# Proteome Landscape of Epithelial-to-Mesenchymal Transition (EMT) of Retinal Pigment Epithelium Shares Commonalities With Malignancy-Associated EMT

**DOI:** 10.1016/j.mcpro.2021.100131

**Published:** 2021-08-27

**Authors:** Srinivasa R. Sripathi, Ming-Wen Hu, Ravi Chakra Turaga, Joseph Mertz, Melissa M. Liu, Jun Wan, Julien Maruotti, Karl J. Wahlin, Cynthia A. Berlinicke, Jiang Qian, Donald J. Zack

**Affiliations:** 1 Department of Ophthalmology, Stem Cell Ocular Regenerative Medicine Center, Wilmer Eye Institute, The Johns Hopkins University School of Medicine, Baltimore, Maryland, USA; 2 Research & Development, Caris Life Sciences, Tempe, Arizona, USA; 3 Department of Medical and Molecular Genetics, Indiana University School of Medicine, Indianapolis, Indiana, USA; 4 Research & Development, Phenocell, Grasse, France; 5 Shiley Eye Institute, University of California, San Diego, LA Jolla, California, USA; 6 Solomon H. Snyder Department of Neuroscience, Department of Molecular Biology and Genetics, Department of Genetic Medicine, The Johns Hopkins University School of Medicine, Baltimore, Maryland, USA; 7 Institute for NanoBioTechnology, Whiting School of Engineering, Johns Hopkins University, Baltimore, Maryland, USA

**Keywords:** hiPS/ES–RPE, EMT, retina, proteomics, MS, transcriptomics, proteogenomics, ACN, acetonitrile, AMD, age-related macular degeneration, bRPLC, basic reversed-phase LC, DIA, data-independent acquisition, ECM, extracellular matrix, EMT, epithelial-to-mesenchymal transition, FC, fold change, FDR, false discovery rate, FGFR, fibroblast growth factor receptor, GO, Gene Ontology, hESC, human-embryonic stem cell, hiPSC, human-induced pluripotent stem cell, hRPE, human stem cell–derived RPE, IPA, ingenuity pathway analysis, PSM, peptide-spectrum match, qPCR, quantitative PCR, RPE, retinal pigment epithelium, RT, room temperature, SLC, solute carrier, STRING, Search Tool for the Retrieval of Interacting Genes/Proteins, STY, phosphoserine, threonine, and tyrosine, TEABC, triethylammonium bicarbonate, TMT, tandem mass tag, UR, upstream regulator, URA, upstream regulator analysis

## Abstract

Stress and injury to the retinal pigment epithelium (RPE) often lead to dedifferentiation and epithelial-to-mesenchymal transition (EMT). These processes have been implicated in several retinal diseases, including proliferative vitreoretinopathy, diabetic retinopathy, and age-related macular degeneration. Despite the importance of RPE-EMT and the large body of data characterizing malignancy-related EMT, comprehensive proteomic studies to define the protein changes and pathways underlying RPE-EMT have not been reported. This study sought to investigate the temporal protein expression changes that occur in a human-induced pluripotent stem cell–based RPE-EMT model. We utilized multiplexed isobaric tandem mass tag labeling followed by high-resolution tandem MS for precise and in-depth quantification of the RPE-EMT proteome. We have identified and quantified 7937 protein groups in our tandem mass tag–based MS analysis. We observed a total of 532 proteins that are differentially regulated during RPE-EMT. Furthermore, we integrated our proteomic data with prior transcriptomic (RNA-Seq) data to provide additional insights into RPE-EMT mechanisms. To validate these results, we have performed a label-free single-shot data-independent acquisition MS study. Our integrated analysis indicates both the commonality and uniqueness of RPE-EMT compared with malignancy-associated EMT. Our comparative analysis also revealed that multiple age-related macular degeneration–associated risk factors are differentially regulated during RPE-EMT. Together, our integrated dataset provides a comprehensive RPE-EMT atlas and resource for understanding the molecular signaling events and associated biological pathways that underlie RPE-EMT onset. This resource has already facilitated the identification of chemical modulators that could inhibit RPE-EMT, and it will hopefully aid in ongoing efforts to develop EMT inhibition as an approach for the treatment of retinal disease.

## Highlights

• Proteomics data were integrated with prior transcriptomic (RNA-Seq) data on RPE-EMT.
• Dysregulated RPE-EMT proteome shares commonality with malignancy-associated EMT.
• Altered RPE-EMT proteome signatures correlated with known AMD-associated risk factors.
• Protein kinases and phosphatases crosstalk modulate RPE-EMT.

## Graphical Abstract

**Figure:**
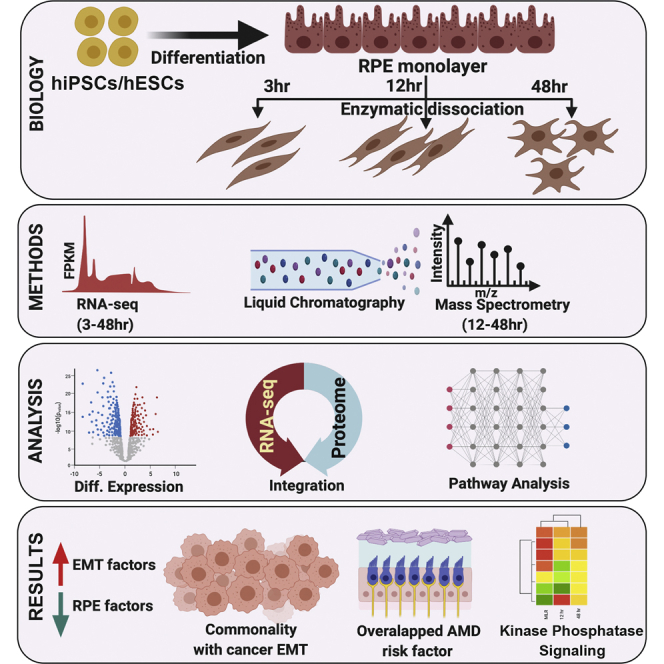

## In Brief

EMT can play a role in retinal diseases. Here, we present a comprehensive proteomic analysis aimed at defining the temporal protein expression changes associated with EMT of stem cell–derived retinal pigment epithelial cells. Tandem mass tag and direct data-independent acquisition MS approaches were performed after inducing RPE-EMT by enzymatic dissociation. We present integration of our proteomic data with prior transcriptomic (RNA-Seq) data to provide additional insights into the RPE-EMT progression.

Epithelial cells can convert into motile mesenchymal-like cells by a process of well-defined transdifferentiation known as epithelial-to-mesenchymal transition (EMT) (1, 2). EMT is a complex biological process that occurs through multiple cellular and molecular events, including disruption of cytoskeletal architecture, loss of cell–cell contact, elevated expression of mesenchymal markers, and decreased expression of epithelial factors (3). Initiation of EMT is one of the critical processes that cause malignant cells to lose their cell–cell junctions, leading to single-cell invasive and migratory behavior (4). Invading and metastasizing tumor cells develop the ability to degrade their underlying basement membrane and extracellular matrix (ECM) by modulating the expression of multiple matrix degradation enzymes, in a process regulated by the core EMT transcription factors SNAIL, SLUG, TWIST1, and ZEB1 (5). Proteases are also implicated in initiating EMT, where in addition to other activities, they disrupt cytoskeletal architecture (6).

Given the importance of EMT in cancer, there have been abundant studies focused upon the mechanisms of cancer cell–associated EMT and its role in malignant transformation, tumor progression, and metastasis (7, 8, 9, 10). More recently, with the realization that EMT plays an essential role in many ocular degenerative diseases, including proliferative vitreoretinopathy (11, 12, 13), neovascular (“wet”) age-related macular degeneration (AMD) (14, 15), atrophic (“dry”) AMD (16, 17), and diabetic retinopathy (18), there has been increasing interest in mechanisms and regulation of EMT of the retinal pigment epithelium (RPE). The RPE, which consists of a monolayer of epithelial cells located between the neural retina and the choriocapillaris, is essential for normal photoreceptor health and function. Degeneration of the RPE plays a critical role in the pathogenesis of the above mentioned and other retinal diseases (19). Environmental factors, including exposure to growth factors, cytokines, hypoxia, and oxidative stress, all of which accumulate with age, can lead to the onset of RPE stress response pathways that ultimately result in the induction of RPE-EMT, leading to RPE dedifferentiation and dysfunction, and potentially vision loss and even blindness.

As part of efforts to better understand RPE-EMT, several model systems for generating RPE-EMT have been reported. Most of these studies have focused on primary, immortalized RPE cell lines, and human fetal, cadaver, and donor specimens (20, 21, 22), all of which imperfectly model *in vivo* human RPE EMT. More recently, in an effort to develop systems that better mimick human RPE biology and pathology, we and others have generated models based upon human stem cell–derived RPE (hRPE) (23, 24). These systems reflect native RPE in terms of gene expression, morphology, and function and are being used for biological discoveries, drug discovery, and as substrates for therapeutic cell transplantation (25, 26, 27).

Despite the increasing appreciation of the importance and use of hRPE, only limited reports have so far focused on the human RPE proteome (28, 29). Using advanced MS–based multiplexed quantitative proteomics, we present here a comprehensive time-course proteomics analysis of hRPE cells undergoing EMT, comparing the proteome of RPE monolayer cells to cells that have been induced to undergo EMT through proteocollagenolytic treatment (30). Multiplex quantitation using isobaric tandem mass tag (TMT-9plex) technology was performed, which allowed us to obtain quantitative comparative data by comparing and processing multiple samples simultaneously (31, 32, 33, 34). In addition to presenting a comprehensive temporal proteomic analysis of hRPE monolayers and enzymatically dissociated single cells by high-resolution MS, we also describe an integrative proteogenomic analysis to identify the proteins and associated biological pathways that drive EMT in RPE. Furthermore, we also validated our TMT labeling approach using a direct data-independent acquisition (DIA) strategy. Together, we hope that our dataset and analysis will provide the vision research community a comprehensive resource for the increasing understanding of RPE-EMT progression mechanisms and point toward potential targets for therapeutic intervention.

## Experimental Procedures

### Human-Induced Pluripotent Stem Cell and Human-Embryonic Stem Cell Culture and RPE Differentiation

Human-induced pluripotent stem cells (hiPSCs) were maintained in culture and differentiated into mature RPE monolayers as previously described (35, 36). Briefly, hiPSCs (EP1 (37) and IMR90.4 [WiCell] and human-embryonic stem cells [hESCs] [H7, WiCell]) were maintained on growth factor-reduced Matrigel (BD Biosciences) in mTeSR1 medium (Stem Cell Technologies), in 10% CO_2_ and 5% O_2_, and amplified by clonal propagation using 5 μM blebbistatin (Sigma). For differentiation, hiPSCs/hESCs were plated at a density of 30,000 cells per cm^2^ and maintained in mTeSR1 to form a confluent monolayer, after which the culture medium was replaced with differentiation medium (Dulbecco's modified Eagle's medium/F12, 15% knockout serum, 2 mM glutamate, 1% nonessential amino acids, 0.1 mM mercaptoethanol, and 1% antibiotic–antimycotic [GIBCO]), supplemented with 10 mM nicotinamide (Sigma), for ∼50 days. Differentiating RPE cells were enzymatically dissociated using 0.25% (w/v) collagenase IV (Gibco) and resuspended in AccuMAX (Sigma) to make a single-cell suspension. Cells were replated onto freshly coated matrigel plates and cultured in RPE medium (70% Dulbecco's modified Eagle's medium, 30% Ham's F12 nutrient mix, 2% B-27 serum-free supplement, and 1% antibiotic–antimycotic) for 2 to 3 months to form mature RPE monolayers.

### RPE-EMT Induction by Enzymatic Dissociation

To induce EMT by enzymatic dissociation, hRPE monolayers were treated with 1× cell detachment solution consisting of proteocollagenolytic enzymes (Accumax; Sigma) for 20 min. Gentle mechanical trituration was performed by pipetting approximately 15 times with a P1000 pipette. Accumax was neutralized with twice the volume of RPE medium, and cells were centrifuged and then resuspended in RPE medium. Cells were plated on matrigel-coated plates at a density of 30,000 cells per cm^2^ and incubated at 37 °C/5% CO_2._ Replated cells were harvested at time points of 12 and 48 h for proteome analysis. Undissociated hRPE monolayers were used as controls.

### RNA Extraction and Quantitative RT-PCR

Total RNA from hRPE monolayers and dissociated cells were extracted using RNeasy Mini Kit (Qiagen), and complementary DNA synthesis was performed using High Capacity cDNA kit (Applied Biosystems). Quantitative PCR (qPCR) analysis was performed with SsoAdvanced Universal SYBR Green Supermix (Bio-Rad). qPCR samples were run in biological triplicate, and expression levels were normalized using the geometric mean of four housekeeping genes (GAPDH, ACTB, SRP72, and CREBBP). Gene-specific primer sequences are provided in supplemental Table S1.

### Immunoblot (Western) Analysis

hiPS-RPE monolayers and dissociated cells were harvested and lysed in radioimmunoprecipitation lysis buffer (0.5 M Tris–HCl, pH 7.4, 1.5 M NaCl, 2.5% deoxycholic acid, 10% NP-40, and 10 mM EDTA) in the presence of protease inhibitor cocktail (P8430; Millipore Sigma), or cells were lysed in ice-cold lysis buffer containing 50 mM Tris–HCl at pH 7.4, 10 mM sodium glycerophosphate, 10 mM sodium pyrophosphate, 150 mM NaCl, 5 mM MgCl_2_, 1 mM EDTA, 1 mM dithiothreitol, 1% (v/v) Triton X-100, 10% glycerol, and 1 mM sodium orthovanadate supplemented with EDTA-free protease and phosphatase inhibitor cocktail (Roche). Whole protein extracts were quantified by bicinchoninic acid protein assay (Thermo) and denatured in Laemmli sample buffer (62.5 mM Tris–HCl, pH 6.8, 25% glycerol, 2% SDS, and 0.01% bromophenol blue). For each sample, 20 to 30 μg of protein was loaded onto a gel, and the samples were resolved using 4 to 20% polyacrylamide criterion TGX precast gels (Bio-Rad), followed by electrotransfer onto a nitrocellulose membrane, which was then blocked in 5% of nonfat milk prepared in Tris-buffered saline (0.1% v/v Tween-20) for 1 h at room temperature (RT). Membranes were probed with primary antibodies at 4 °C overnight, followed by corresponding horseradish peroxidase–conjugated secondary antibodies for 1 h at RT. The following primary antibodies were used for Western blot analysis: SNAIL (3839), N-cadherin (13116), E-cadherin (3195), TWIST1 (46702), JUNB (3753), and FOSL1 (5281) (Cell Signaling Technology); RPE65 (MAB5428; Millipore), BEST1 (NB 300-164; Novus Biologicals), MMP3 (PA5-27936; Thermo), GAPDH (365062; Santa Cruz Biotechnology), and ACTB (664803; BioLegend). The immune reactive bands corresponding to the primary antibodies were visualized using West Pico Chemiluminescent Substrate (Thermo) on an X-ray film or with an iBright imaging system (Invitrogen). Membranes were stained with Ponceau S (P7170; Sigma) to determine sample loading in each lane.

### Protein Extraction and Precipitation

hRPE monolayers and dissociated cells were washed 2× with 1× PBS, lysed in 4% SDS lysis buffer (4% SDS, 0.05 M triethylammonium bicarbonate [TEABC], pH 8.0) and boiled at 95 °C for 5 min. About 500 μg of protein in lysate was reduced with 10 mM DTT for 30 min at 56 °C, and proteins were alkylated with 40 mM iodoacetamide for an additional 30 min in dark. Remaining SDS detergent was removed by methanol:chloroform precipitation. Briefly, a 4:1 ratio of methanol:chloroform was added and vortexed, followed by three volumes of water and centrifugation at 14,000 RPM for 2 min. The top aqueous layer was removed, and another four volumes of methanol were added and centrifuged again at 14,000 RPM for 2 min. The aqueous layer was completely removed and air dried the protein pellet. The protein pellet was solubilized in 6 M urea and incubated on ice for 30 min, and the urea concentration was reduced to 2 M urea by adding 50 mM TEABC buffer. The sample was then subjected to double digestion by adding Lys-C (1:200, enzyme:protein) for 4 h at 32 °C and then trypsinized overnight by adding sequencing grade trypsin (1:50, enzyme:protein). This reaction was quenched by adding 1% TFA (final) and centrifuged at 14,000 RPM for 10 min, and the supernatant was transferred to new collection tubes and subjected to C18 clean up using Sep-PaK cartridges and vacuum dried and stored in −80 °C until needed for TMT labeling.

### TMT 9-plex Labeling

The vacuum dried samples were reconstituted in 100 μl of 100 mM TEABC (pH 8.0) buffer at RT for 15 min. TMT labeling was carried out as per the manufacturer's instructions with slight modifications. The TMT 10-plex kit (Thermo) was brought to RT and reconstituted by adding 41 μl of 100% anhydrous acetonitrile (ACN) at RT for 10 min with brief vortexing. About 400 μg of each reporter tag was added to the sample as follows, 126, 127N, and 127C to RPE monolayer; 128N, 128C, and 129N to dissociated RPE 12 h; 129C, 130N, and 130C to dissociated RPE 48-h time-point samples (Fig. 2*A*). Samples were then vortexed and incubated at RT for 2 h with a brief vortexing for every 30 min. About 10 μl from each reaction was pooled together, vacuum dried, and desalted to check the labeling efficiency. About 2% from each reaction was quenched, pooled, and subjected to LC–MS/MS analysis. Furthermore, 5 μl of 5% hydroxylamine was added to each sample and incubated at RT for 15 min. Following, all the samples were pooled and lyophilized and desalted using C18 Sep-Pak cartridges. The eluted peptides were vacuum dried and subjected to basic reversed-phase LC (bRPLC) fractionation.

**Fig. 1:**
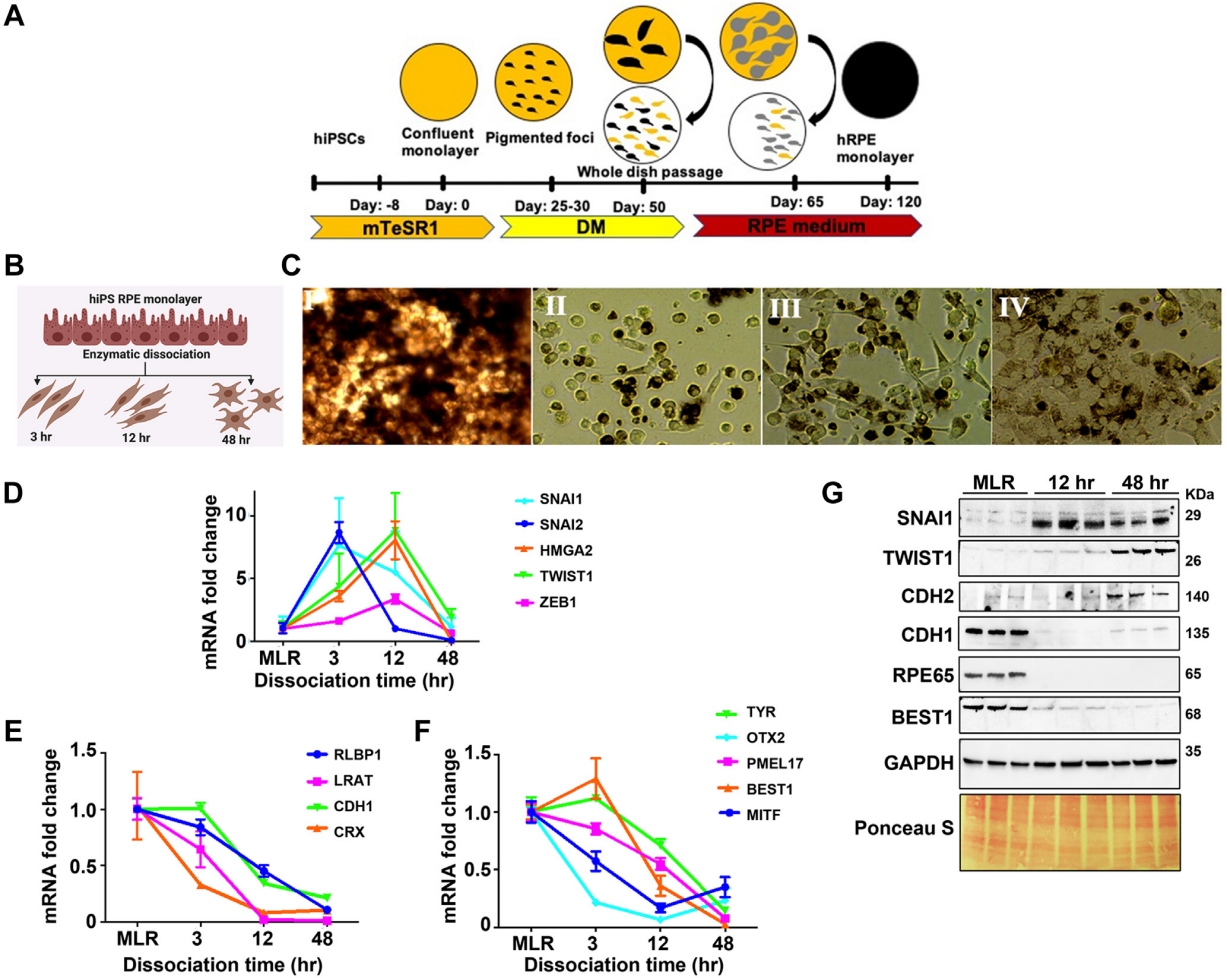
**Enzymatic dissociation-induced EMT in human iPS-derived RPE cells.***A*, schematic representation of hiPS differentiation into RPE monolayers. *B*, schematic representation of RPE-EMT induction in hRPE cells. *C*, bright-field images of 2-month-old hiPS RPE monolayer with cobblestone morphology (I) and fibroblast morphology of hRPE cells after enzymatic dissociation-induced EMT at 3 h (II), 12 h (III), and 48 h (IV). *D*–*F*, differential expression of EMT-associated and RPE-specific genes was measured by quantitative RT-PCR after enzymatic dissociation of monolayers into single cells. Data were normalized by the expression levels in monolayer control conditions. *G*, Western blot analysis revealed the increased expression of SNAI1, TWIST1, and N-cadherin but decreased expression of E-cadherin and no noticeable expression of RPE65 from postdissociated RPE cells compared with undissociated RPE monolayers. EMT, epithelial-to-mesenchymal transition; hiPS, human-induced pluripotent stem cells; hRPE, human stem cell–derived RPE; iPS, induced pluripotent stem cell; RPE, retinal pigment epithelium.

**Fig. 2:**
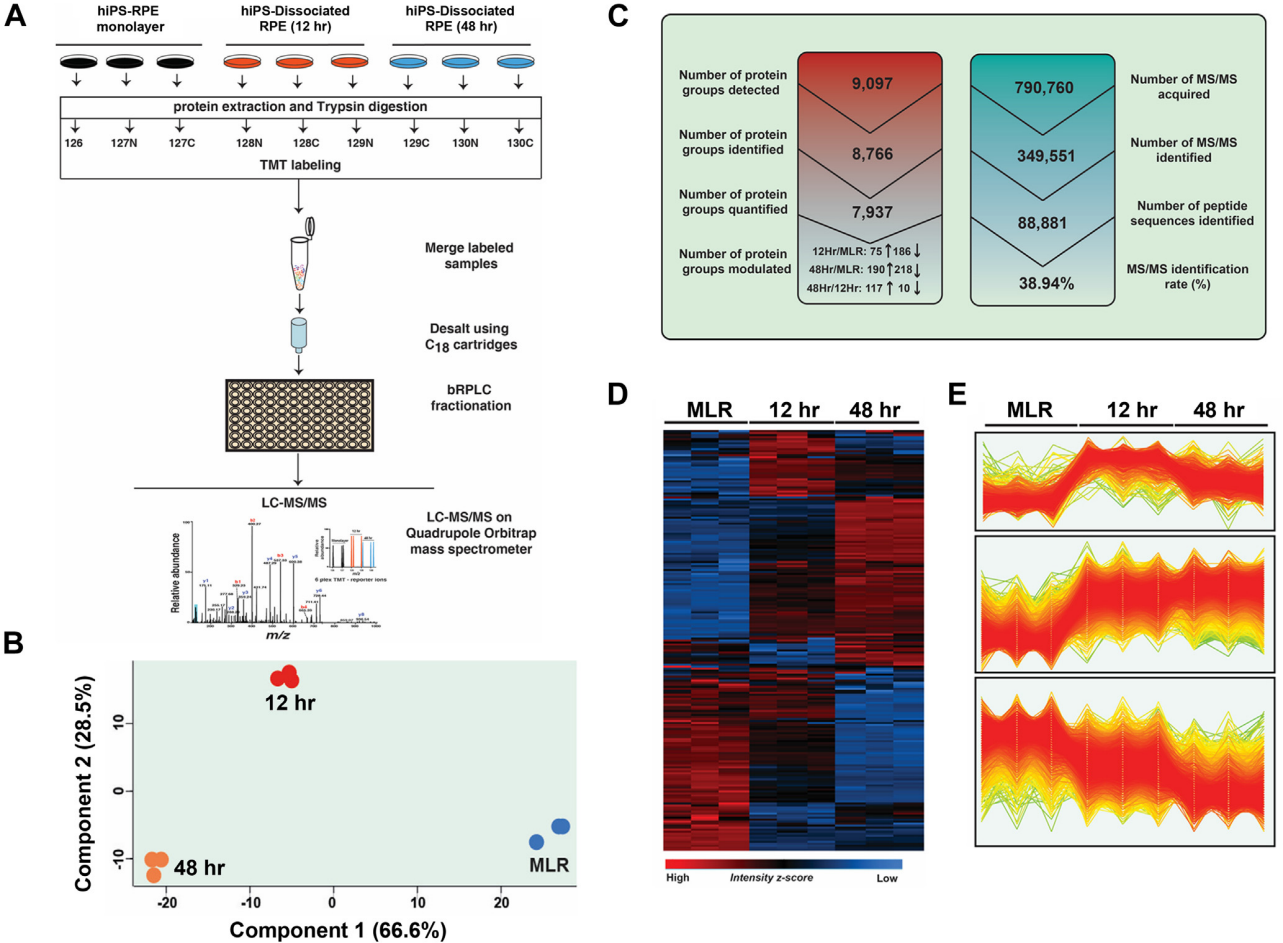
**Temporal quantification of dissociation-induced RPE-EMT global proteome.***A*, schematic workflow of the quantitative MS-based TMT9plex labeling protocol used to quantify proteome change after enzymatic dissociation-induced EMT of hRPE. Following RPE cell lysis, total protein extraction, and trypsin digestion, each sample was labeled with a specific TMT isobaric tag (MLR: 126, 127N, and 127C; 12 h: 128N, 128C, and 129N; 48 h: 129C, 130N, and 130C). The pooled samples were fractionated by basic reversed-phase LC (bRPLC) and subjected to a high-resolution quadrupole Orbitrap Q-Exactive HF mass spectrometer. *B*, principal component analysis (PCA) of protein expression data from dissociation-induced RPE-EMT samples. *C*, summary of results depicting the identified protein groups, unique peptides, MS/MS counts, and identification percentage of the total proteome analysis of dissociation-induced hRPE-EMT proteome. *D*, hierarchical clustering of log2-transformed and *z*-score normalized abundances derived from individual TMT reporter ion intensities of MLR, 12 h, and 48 h samples. *E*, profile plots of three selected clusters showing distinct behavior with respect to each time point: 1. substantial decrease in 12-h and 48-h time points; 2. substantial increase in 12-h and 48-h time points; and 3. strong decrease in 48-h time point only. EMT, epithelial-to-mesenchymal transition; hRPE, human stem cell–derived RPE; RPE, retinal pigment epithelium; TMT, tandem mass tag.

### bRPLC

Peptides were fractionated by bRPLC as described earlier (38). Briefly, TMT-labeled lyophilized peptide mixture was resuspended in 100 μl of buffer A (10 mM TEABC), which was fractionated on an Agilent 1100 LC system using a linear gradient of 8 to 60% buffer B (10 mM TEABC in 90% ACN) for 90 min at a flow rate of 0.5 ml/min. A total of 96 fractions were collected, concatenated to 12 fractions in zig–zag fashion and then vacuum dried and stored in −80 °C until LC–MS/MS analysis.

### LC–MS/MS Analysis

The concatenated 12 bRPLC fractions were analyzed on a Thermo Q-Exactive HF mass spectrometer. Each fraction was injected as a technical replicate. Fractions were reconstituted in 0.1% formic acid (v/v) in 3% ACN (v/v) in 10 μl volume and were loaded onto the autosampler tray of Waters nanoACQUITY LC system, which is in line with the Q-Exactive HF mass spectrometer. The peptides were loaded onto a 25-cm in-house packed (Magic C18 material) analytical column and separated by applying a linear gradient of 110 min, with a total run of 120 min at 300 nl/min flow rate of which the last 10 min were to equilibrate the column for the next injection. The MS data were acquired in a data-dependent acquisition mode by targeting the top 15 precursor ions for fragmentation. These were then surveyed in a 350 to 1800 *m/z* scan range and acquired using Orbitrap mass analyzer at 120,000 resolution at *m/z* 200. The MS2 data were selected by using 1.0 *m/z* with an isolation offset of 0.5 *m/z* using quadrupole and fragmented using stepped normalized higher-energy collisional dissociation between 30 and 35 and acquired using Orbitrap mass analyzer at 30,000 resolution at *m/z* 200. The automatic gain control and ion injection times for both MS1 and MS2 were set as 3E6 and 50 ms and 1E5 and 200 ms, respectively. Dynamic exclusion was set at 30 s, and both MS1 and MS2 were acquired in a profile mode.

### Data Analysis

Data analysis, performed with MaxQuant, version 1.6.0.13 (Max Planck Institute of Biochemistry), was used to search against the human UniProt database (March 2017, containing 42,101 sequences) using the built-in Andromeda search engine with potential contaminants excluded. We used trypsin as a specific protease with a maximum of two missed cleavages and a minimum peptide length of seven amino acids. Parameters used were 20 ppm of first search, 4.5 ppm of second search precursor tolerance, and 20 ppm of MS/MS tolerance. Group-specific parameters: peak picking was carried out by selecting MS2 reporter ion, and the percent of precursor intensity fraction was set at 0.75 for filtering out the coeluting peptide ion ratio compression for an accurate TMT reporter ion quantification. Oxidation (M), acetyl (protein N-term), deamidation (NQ), and phosphorylation (phosphoserine, threonine, and tyrosine [STY]) were set as dynamic modifications and carbamidomethyl (C) as fixed modification. Peptide-spectrum match (PSM) false discovery rate (FDR) of 0.01, protein FDR of 0.01, and site FDR of 0.01 were used. Phosphorylation of (STY) modified peptides was excluded for protein level quantifications. The MaxQuant output tables were processed using Perseus software suite (39, 40), R statistical computation platform, Python data analytics program, and GraphPad Prism (GraphPad Software, Inc) software. Protein copy numbers were estimated using the “Proteomic-Ruler” plugin in the Perseus suite (Max Planck Institute of Biochemistry) (39). TMT-derived intensities for monolayer, 12, and 48 h samples were filtered for contaminants, reverse and proteins identified by site. The remaining protein group raw TMT intensities were used to estimate copy numbers by using histone proteomic ruler as a scaling mode and keeping the ploidy of RPE cell as diploid. These copy numbers were log transformed and used to measure the Pearson correlation between TMT intensities and copy numbers of MLR, 12 h, and 48 h samples. Furthermore, the global correlation matrix was generated using custom-made scripts in Python.

### S-Trap–Assisted On-Column Trypsin Digestion

S-trap–assisted trypsin digestion was performed as described previously (41), with slight modification. Briefly, hiPSC (EP1)-derived RPE cells were lysed in 5% SDS lysis buffer in 100 mM TEABC buffer (pH 7.5) and placed at 95 °C for 5 min. Lysates were sonicated at high energy for 15 cycles (30 s-ON/30 s-OFF) using a Bioruptor (Diagenode) and then centrifuged at 20,000*g* for 20 min. Total protein was quantified using the bicinchoninic acid protein assay (Thermo). A total amount of 300 μg of protein was reduced in 10 mM Tris (2-carboxyethyl) phosphine by incubating at 60 °C for 30 min on a Thermomixer with gentle agitation (1000 RPM). Samples were then allowed to cool at RT and alkylated in 40 mM iodoacetamide by incubating at RT in the dark for 30 min on a Thermomixer with gentle agitation (1000 RPM). Samples were quenched with 5 mM Tris (2-carboxyethyl) phosphine and acidified with 1.2% phosphoric acid in 5% SDS. Seven times the volume of lysate, ∼400 μl of S-Trap wash buffer (90% [v/v] methanol in 100 mM TEABC at pH 7.1) was added to the samples. The columns were then centrifuged at 1000*g* for 1 min and washed four times with S-Trap Wash buffer. An “in-solution on-column” tryptic digestion was performed by adding sequencing grade modified trypsin (Promega) in 50 mM TEABC at pH 8.0 containing a ratio of enzyme:substrate (1:15) onto an S-Trap column. Tryptic peptides were further extracted using 0.15% (v/v) formic acid and eluted in elution buffer (80% [v/v] ACN and 0.15% formic acid). Peptides were quantified by nanoDrop spectrophotometer (A224). Eluted peptides were combined and vacuum dried using a speed vac and stored at −80 °C until MS analysis.

### LC–MS/MS Analysis of DIA

About 500 ng of vacuum dried peptide digest from RPE monolayers and dissociated cells (12 and 48 h) was injected for LC–MS/MS analysis on an Orbitrap Lumos Tribrid mass spectrometer in line with an EasynLC 1000. Samples were dissolved in LC buffer (3% [v/v] ACN in 0.1% [v/v] formic acid). Peptides were loaded onto a trap column at 4 μl/min flow rate and subsequently resolved on a 50 cm analytical column at 300 nl/min flow rate and directly electrosprayed into the mass spectrometer using Easy nano source. A gradient of 5% to 20% of solvent B (80% ACN in 0.1% formic acid) was maintained for 65 min and a gradient of 20% to 30% for another 60 min followed by increase to 50% B for 10 min and 100% B for another 10 min and maintained at 100% A for 5 min, with a total run time of 145 min. The data were acquired in a variable DIA strategy. MS1 scan was acquired at 120,000 resolution at 200 *m/z* in a scan range between 375 and 1500 *m/z*. A total of 45 variable windows covering the scan range between 375 and 1500 *m/z* were used and isolated using quadrupole and fragmented using 35% stepped collision energy and measured using Orbitrap mass analyzer at a resolution of 30,000. The automatic gain control targets for both MS1 and MS2 were set at 100% with a maximum ion injection time of 54 ms for MS2 scans. Duty cycle was set at 3 s with loop count set at 23 to record an MS1 scan. The variable DIA MS2 isolation window list is provided in supplemental Table S3.

### Data Analysis of DIA

Raw data acquired from the DIA strategy were analyzed using the Spectronaut 14.0 software suite (Biognosys). The same human Uniprot database was used for both TMT and DIA analyses. The default search parameters in Spectronaut 14.0 were used, namely, trypsin as a specific protease with a maximum of two missed cleavages allowed. Minimum peptide length of 7 and maximum peptide length of 52 was set. Carbamidomethyl of Cys as fixed modification and acetyl protein N-ter, oxidation of Met, deamidation of Asn and Gln, and phosphorylation of STY were set as variable modifications. 1% FDR at PSM, peptide, and protein level were used with iDpicker inference. MS2 area was used for the quantification and global median-based normalization. The protein group quantities table was exported and analyzed using the Perseus software suite.

### Pathways and Upstream Regulator Analysis

Biological pathway analysis was performed using ingenuity pathway analysis (IPA) (QIAGEN) (42), Search Tool for the Retrieval of Interacting Genes/Proteins (STRING) (43) (https://string-db.org/), and the functional enrichment analysis (FUNrich) tool (44). Proteins that were identified as differentially expressed (greater than two fold change [FC] and *p* < 0.05) during dissociation-induced RPE-EMT compared with untreated monolayers were input into IPA for bioinformatics pathway analysis using protein IDs. Protein datasets were assessed for prediction of canonical pathways and upstream regulators (URs) (42). The kinome trees were generated using Kinome viewer software (45).

### Experimental Design and Statistical Rationale

TMT and DIA experiments were performed on three biological replicates. TMT-bRPLC fractions were analyzed in technical duplicates, and the database search was carried out as a single set by specifying two sets as set I and set II in MaxQuant. Combined intensities from two technical replicates computed by MaxQuant were used for further analysis. All qRT PCR and Western blot experiments were performed with at least three biological replicates as described previously.

## Results

### Proteolytic–Collagenolytic Enzyme Treatment Induces EMT in hiPS-RPE Monolayers

To obtain insight into the proteomic pathways involved in RPE-EMT, we utilized our previously described system for generating RPE monolayers from hiPSCs and hESCs (35, 36) and induced EMT by proteocollagenolytic treatment (46). In brief, hiPSCs (37) and hESCs were differentiated into mature RPE monolayers (hRPE) (Fig. 1*A*). Monolayer cultures were treated with Accumax to detach the cells from their culture substrate and dissociate them into single-cell suspensions, and the dissociated cells were then replated on Matrigel-coated culture plates (Fig. 1*B*). The enzymatically treated RPE cells showed loss of pigmentation and loss of their RPE-like morphological characteristics and exhibited EMT-related phenotypic changes, including elongated-fibroblast cell-like morphology (Fig. 1*C*). We confirmed upregulation of key EMT transcriptional factors (*SNAI1* 7-fold, *SNAI2* 8-fold, *TWIST1* 8-fold, *ZEB1* 3-fold, and *HMGA2* 8-fold) by qPCR at 3 to 12 h postdissociation (Fig. 1*D*). These changes were accompanied by decreased expression of both epithelial cell morphology–related genes (*CDH1* [E-cadherin] fourfold) and RPE-specific genes (*LRAT* 84-fold, *BEST1* 26-fold, *OTX2* 15-fold, *PMEL17* 12-fold, *CRX* 12-fold, *RLBP1* 9-fold, *TYR* 7-fold, and *MITF* 6-fold) (Fig. 1, *E* and *F*).

Next, to confirm the qPCR mRNA expression data at the protein level, we performed Western blotting for EMT and RPE markers that have been previously associated with EMT and malignancy-associated metastasis. Consistent with the gene expression results, our Western blotting analysis revealed RPE-EMT–associated increased expression of the EMT factors SNAI1, TWIST1, and N-cadherin (CDH2). Conversely, we found decreased expression of the epithelial/RPE factors CDH1 and RPE65 (Fig. 1*G*). Overall, consistent with prior work, this analysis showed that dissociation of hRPE monolayers leads to various morphological, mRNA, and protein expression changes typical of RPE cells undergoing EMT.

### Quantitative Global Proteomics Reveals Temporal Changes in RPE-EMT Progression

Untreated hRPE monolayer cultures (control) and dissociated cells at 12 and 48 h post EMT induction (which will be referred to as “MLR,” “12 h,” and “48 h”) were harvested for proteome analysis. Total protein was subjected to tryptic digestion followed by bRPLC chromatographic separation, with samples concatenated into 12 fractions, which were analyzed as technical replicates on a high-resolution quadrupole Orbitrap Q-Exactive HF mass spectrometer. We utilized the MaxQuant pipeline for database searches, keeping the precursor intensity fraction to 0.75 for accurate reporter ion-based quantification to mitigate the quantification accuracy caused by peptide coelution interference. Using this workflow, the MS analysis generated up to 0.8 million MS/MS spectra, resulting in 0.34 million MS/MS events identified at 1% PSM level FDR, which corresponds to a 38.94% identification rate of high-resolution MS data, revealing an in-depth RPE-EMT proteome.

Principal component analysis revealed consistent spatial clustering of the MLR, 12 h, and 48 h dissociation samples, demonstrating a high degree of similarity and reproducibility between replicates (Fig. 2*B*). In total, we identified 88,881 unique peptide sequences at 1% FDR, leading to 9097 protein groups at this level of stringency. We applied a number of stringent filtering criteria to this dataset—we removed potential contaminants, reverse decoy hits, and proteins that were identified by only site, and we considered only protein groups that were identified with at least two unique peptide sequences. After application of these filters, we were able to identify and quantify 7937 protein groups (Fig. 2*C*). The accuracy and reproducibility of the MS/MS data (supplemental Fig. S1*A*) revealed a median Pearson correlation of 0.99. We employed a downstream bioinformatics and statistical analysis using the Perseus (40) and R statistical computation platforms. Unsupervised hierarchical clustering of differentially regulated global proteome dynamics, depicted as a heat map (Fig. 2*D*), showed three major unique clusters, with high consistency of replicates (Fig. 2*E*). Gene Ontology (GO) analysis revealed good subcellular coverage of cellular compartment proteins, showing greater than 50% representation of cytoplasm, 20% each of lysosomal and exosomes, and 10% of mitochondria and nucleolus proteins. We used permutation-based FDR correction for *t* test to identify differentially regulated protein groups between 12 h/MLR and 48 h/MLR comparisons and considered a 2-fold cutoff for further analysis. In total, and as described in more detail later, we identified approximately 8000 proteins and found significantly altered expression of 255 proteins at 12 h (75 upregulated and 180 downregulated) and 431 proteins with altered expression at 48 h (198 upregulated and 233 downregulated).

### Many EMT-Associated Factors Showed Increased Expression, Whereas RPE-Specific Proteins Showed Decreased Expression With EMT

**Fig. 3:**
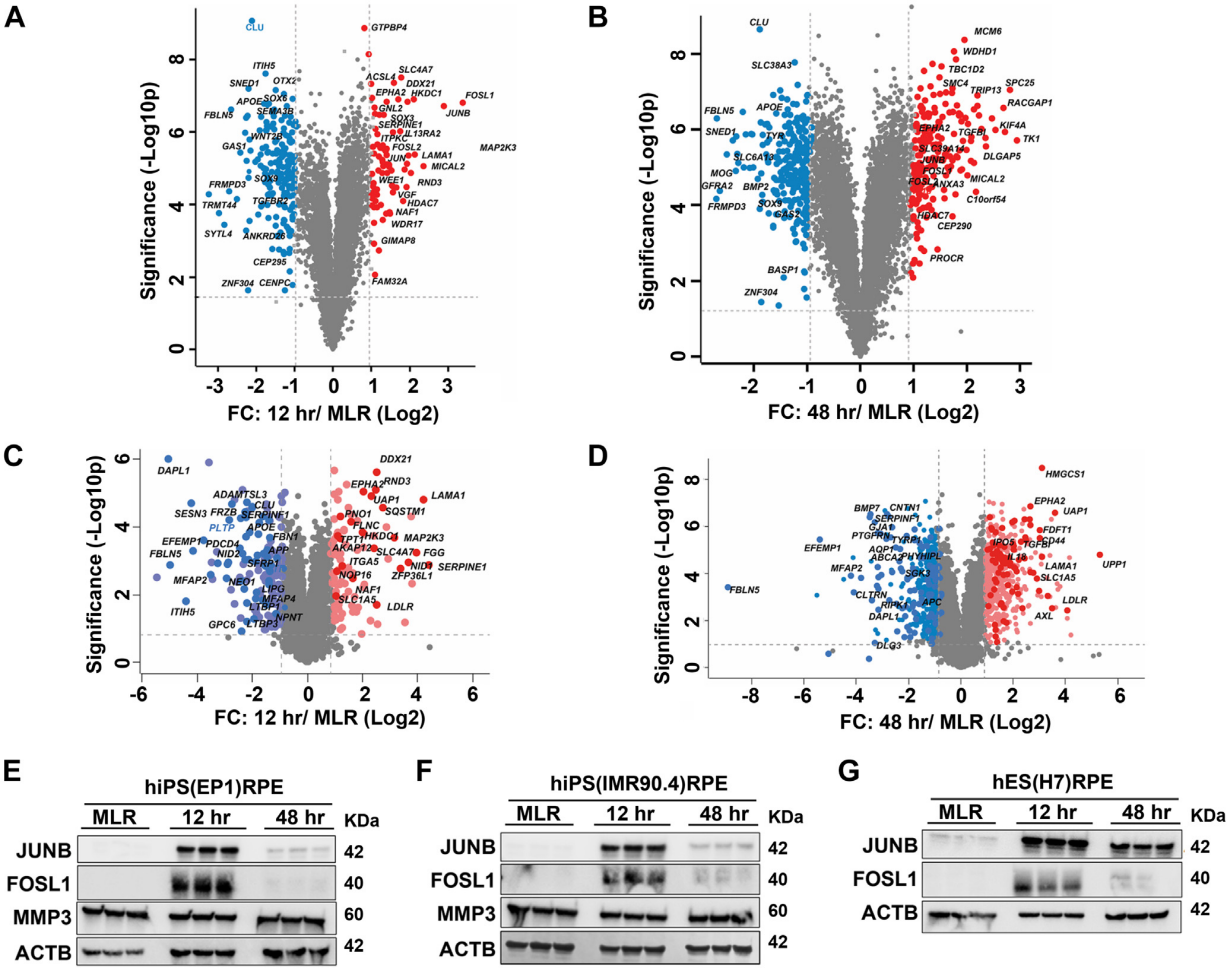
**Dissociation-induced EMT alters malignancy-associated mesenchymal proteins and RPE signature proteins.***A* and *B*, volcano plot illustration of differentially expressed proteins during RPE-EMT that were identified by TMT labeling approach. *C* and *D*, volcano plot illustration of differentially expressed proteins during RPE-EMT were validated by direct-DIA approach. Pairwise comparison between (*A*) group I (12 h/MLR) and (*B*) group II (48 h/MLR). Expression fold changes (*t* test difference log 2, permutation-based FDR: 0.01, S0: 0.001) were calculated and plotted against permutation-based FDR of 0.01 cP-valued −log10 *p* value. Pairwise comparison between 12 h/MLR (*C*) and 48 h/MLR (*D*). *E*–*G*, Western blot validation of differentially regulated proteins identified from TMT labeling. Differentially expressed selective proteins identified from TMT labeling approach were validated in (*E*) hiPS-RPE (EP1), (*F*) hiPS-RPE (IMR90.4), and (*G*) hES (H7) that undergo EMT (12 and 48 h). DIA, data-independent acquisition; EMT, epithelial-to-mesenchymal transition; FDR, false discovery rate; hES, human-embryonic stem cell; hiPS, human-induce pluripotent stem cell; RPE, retinal pigment epithelium; TMT, tandem mass tag.

To identify differentially regulated proteins between intact mature RPE monolayers and RPE cells undergoing dissociation-induced EMT (time points of 12 and 48 h), we performed differential protein expression analysis using the Perseus software suite (39). Using differential expression criteria of at least a two FC with a 1% permutation-based FDR, and a *t* test *p* value <0.05, we identified 75 proteins with increased expression and 180 proteins with decreased expression at the time point of 12 h. At 48 h, 198 proteins were upregulated and 233 proteins were downregulated (supplemental Table S4). These changes are shown as volcano plots in Figure 3, *A* and *B*. Comparative analysis between the 12 h and 48 h time points (supplemental Fig. S1*C*) revealed 116 proteins that showed increased abundance at 48 h compared with 12 h, but only 20 proteins showed decreased expression at 48 h compared with 12 h.

In order to validate the differentially regulated proteins that were quantified by the TMT labeling method, we performed a label-free single shot DIA MS analysis on hRPE monolayers and RPE cells undergoing EMT (12 and 48 h) and analyzed the data using a direct DIA strategy with Spectronaut 14.0 (41). Using this approach, we identified 57,728 precursor and 5276 protein groups at 1% protein level FDR (supplemental Table S5). We achieved a high reproducibility between replicates (supplemental Fig. S1*B*) and a median protein coefficient of variation % of 7.2, 7.6, and 7.0 for MLR, 12 h, and 48 h, respectively (supplemental Fig. S2*B*). Of the protein groups that were identified and quantified from the DIA analysis, >95% matched well with our RPE-TMT data. Next, we compared the differentially regulated proteins that showed similar changes between the MLR/12 h and MLR/48 h comparisons. The volcano plots in Figure 3, *C* and *D* illustrate the patterns of differential protein expression, with proteins that were upregulated or downregulated compared with TMT labeling labeled in red or blue, respectively. We found the overlap between the TMT and DIA analyses to be 17.2% and 13.3% for the 12 h/MLR and 48 h/MLR comparisons, respectively (supplemental Fig. S2*A*). It should be noted that the orthogonal DIA analysis was performed on an independently generated set of hiPS-derived RPE cells (EP1), providing validation for the protein groups that were identified in our TMT analysis.

Besides the DIA analysis, we have also validated a subset of proteins that were significantly altered from our TMT labeling approach by qPCR and Western blotting analysis in three different stem cell–derived RPE lines (hiPSCs [EP1 and IMR90.4] and hESCs [H7]). Consistent with the MS data from the TMT and DIA approaches, our qPCR data show that a number of malignancy-associated EMT factors, including *FOSL1*, *JUNB*, *TUFT1*, *FGF1*, *ITGA5*, *MICAL2*, *LAMA1*, *SLC1A5*, *MMP1*, and *MMP*3, were significantly increased, whereas *TK1* and *VTN1* were significantly decreased in both hiPSC- and hESC-derived RPE cells undergoing EMT (supplemental Figs. S3, S4*A*, and S5*A*). Next, we performed Western blotting validation on several differentially expressed EMT factors, for which high-quality antibodies were commercially available. The Western analysis showed good correlation with our MS data, for example, showing that the malignancy-associated EMT factors JUNB and FOSL1 were upregulated at 12 h RPE-EMT time point (Fig. 3, *E*–*G*).

To determine the absolute protein copy number per cell, we applied the “proteomic-ruler” algorithm method, which uses the MS signal of any protein to estimate protein copies per cell based on the histone content as an internal standard (47). Using this approach, we calculated the absolute protein copies of the RPE-EMT proteome (supplemental Table S14), which enabled us to compare the relative and absolute protein amounts in the MLR, 12 h, and 48 h samples. We observed a high correlation between our copy number analysis and the TMT intensities (supplemental Figs. S9 and S10). Our data show corresponding fold increase changes in malignancy-associated EMT factors, such as FOSL1, JUNB, TK1, MICAL2, LAMA1, TUFT1, FGF1, and ERRFI1 during RPE-EMT (supplemental Fig. S11). RPE differentiation and function-associated proteins, including visual cycle (OTX2, SOX6, and SOX9), cell adhesion molecules (CDH1, CDH3, and ITGB8), pigment synthesis/melanosome biogenesis (TYR and TYRP), pigment epithelial growth factor (SERPINE3), ion transport (BEST1), and tight junction proteins (CLDN3 and CLDN10), were significantly downregulated during RPE-EMT (supplemental Fig. S12). Regulation of transepithelial resistance by active ion channels and transporters, which requires tight junctions between RPE cells, is one of the critical functions of the RPE. hRPE expresses two major transporter families, the ABC and the Na^+^ transporter solute carrier (SLC) super families (48). Differential expression analysis showed that while some SLC family members (SLC2A1, SLC6A13, SLC16A3, SLC23A2, SLC38A3, and SLC39A12) showed decreased expression with EMT, others (SLC1A5, SLC4A7, SLC39A14, and SLC43A3) showed increased expression (supplemental Fig. S13). Together, our proteomic analysis identified differential regulation of malignancy-associated EMT factors and RPE makers during enzymatic dissociation-induced RPE-EMT.

### Integrated Transcriptomic and Proteomic Analysis of hRPE-EMT

Investigating protein levels in each tissue type and how observed protein levels compare with corresponding RNA levels is key to understanding the biology and regulatory processes that control protein expression of disease states. However, proteogenomic studies often show significant discordance between RNA and protein expression levels (49). To determine whether EMT-related protein abundance changes correlate with changes in the corresponding mRNA expression levels, we compared our proteomic RPE-EMT results with data that we previously generated through an RNA-Seq study (46, 50). We compared the significantly altered 7647 genes that we identified from our transcriptome analysis with the current proteome analysis. We found a maximum Pearson correlation of 0.38 between 3-h RNA and 12-h protein level changes from dissociation-induced RPE EMT (supplemental Table S6) (we compared the earlier RNA dataset to the later protein time point to account for the time lag between RNA and protein levels).

**Fig. 4:**
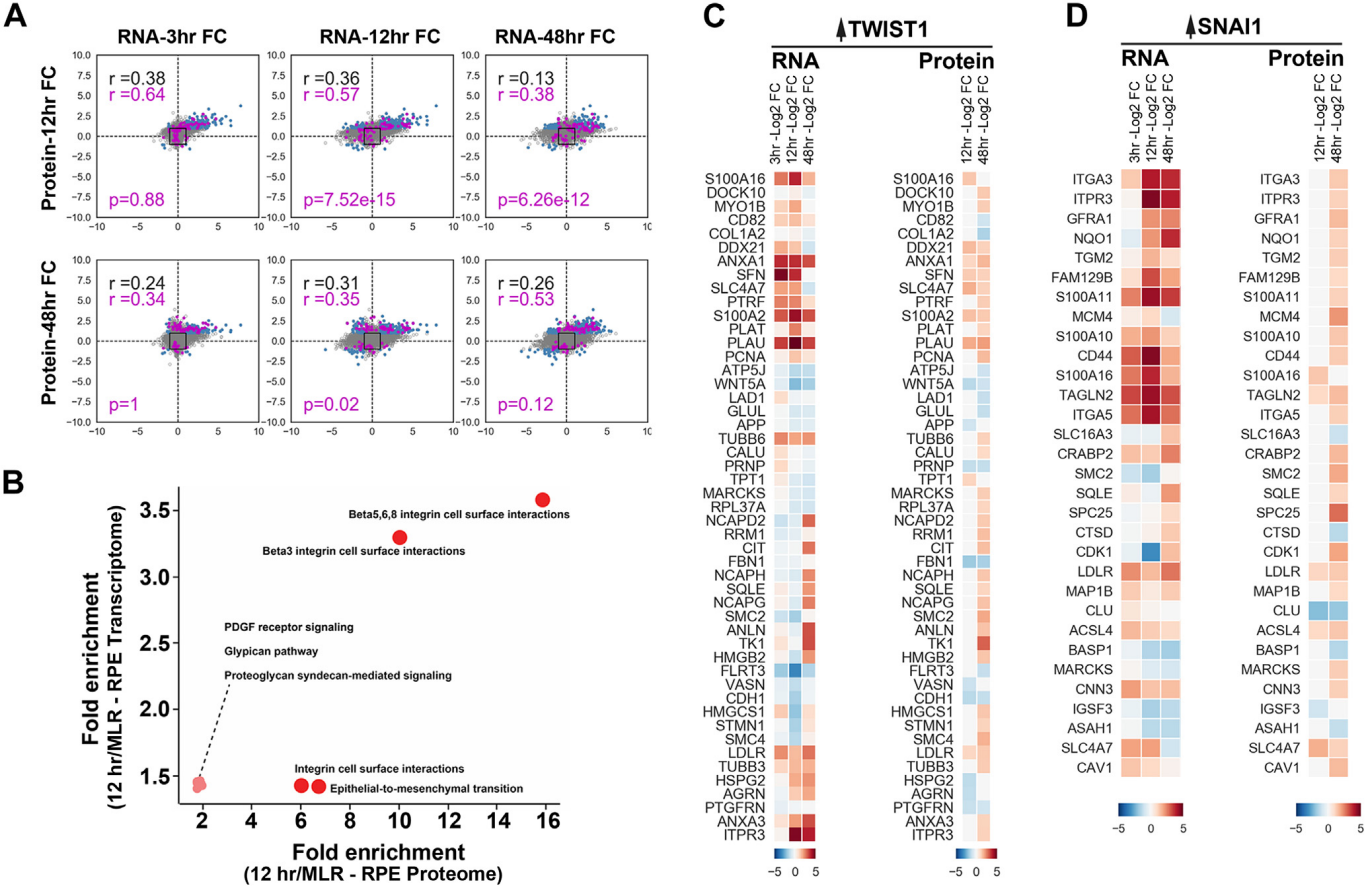
**Integrated transcriptome and proteome changes show the correlation of malignancy-associated EMT and RPE-EMT.***A*, scatter plot showing the level of correlation between RNA (RNA-Seq; 3, 12, and 48 h) and protein (TMT-labeled proteomics; 12 and 48 h) expression changes during RPE-EMT. *Gray dots* represent shared genes/proteins between RNA-Seq and proteomics datasets. *Black squares* represent the threshold of log2FC, and *blue dots* were genes/proteins with log2FC larger than 1. Each *dot* in *magenta* represents a gene/protein that were altered from both hRPE EMT and malignancy-associated cells that were overexpressed with TWIST1 and SNAI1. *B*, scatter plot representation of pathway enrichment analysis that performed using functional enrichment tool (FUNrich). *C*, heat map representing the factors that were significantly altered from both dissociation-induced hRPE EMT model and TWIST1 overexpression-associated cancer EMT model at both RNA and protein levels. *D*, heat map showed the significantly altered factors from both dissociation-induced hRPE EMT model and SNAI1 overexpression-associated cancer EMT model at both RNA and protein levels. EMT, epithelial-to-mesenchymal transition; FC, fold change; RPE, retinal pigment epithelium; TMT, tandem mass tag.

**Fig. 5:**
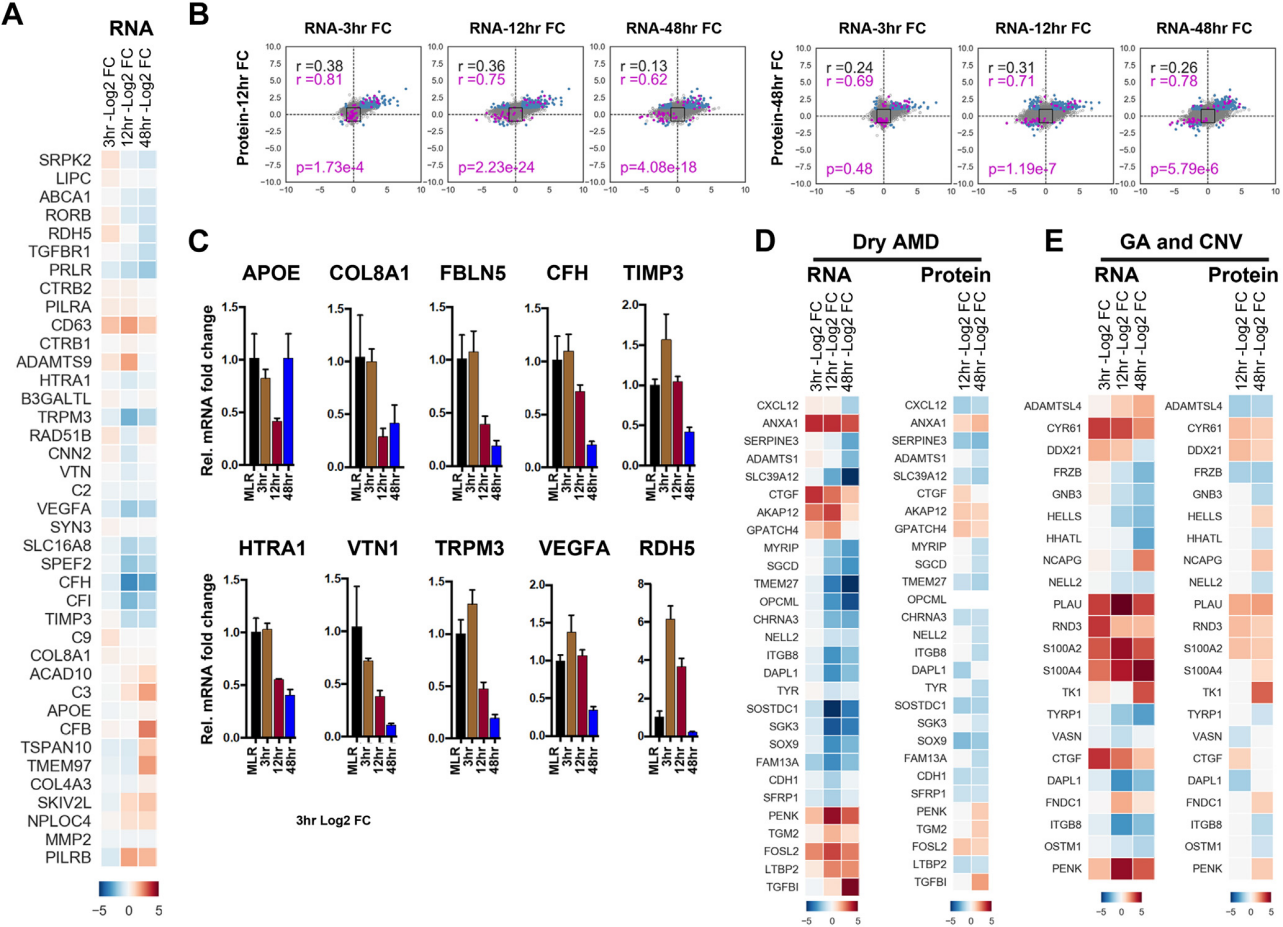
**Integrative RPE-EMT transcriptome and proteome signature correlates with known AMD-associated risk factors.***A*, heat map representing the log2 fold change of AMD-associated risk variants that were significantly altered in RPE-EMT transcriptome study. *B*, scatter plot represents the correlation between changes in RNA (RNA-Seq; 3, 12, and 48 h) and protein (TMT-labeled proteomics; 12 and 48 h) levels following EMT induction. *Dots* in *gray* represent shared genes/proteins between RNA-Seq and proteomics datasets. *Black squares* represent the threshold of log2FC, and *blue dots* were genes/proteins with log2FC larger than 1. *Dots* in *magenta* represent the AMD-associated risk factors that were found in our RNA-Seq and proteomics study. *C*, differential expression of altered AMD-associated risk factors in RPE-EMT was measured by quantitative RT-PCR after enzymatic dissociation of monolayers into single cells. Data were normalized by the expression levels in monolayer control conditions. *D* and *E*, heat maps represent the list of common RPE-EMT genes and proteins that were enriched in a previously reported transcriptome study of AMD donor tissues. AMD, age-related macular degeneration; EMT, epithelial-to-mesenchymal transition; FC, fold change; RPE, retinal pigment epithelium; TMT, tandem mass tag.

Our integrated analysis shows the correlation between RNA and protein expression changes at various time point comparisons, including 3 h RNA *versus* 12 h protein, 3 h RNA *versus* 48 h protein, 12 h RNA *versus* 12 h protein, 12 h RNA *versus* 48 h protein, 48 h RNA *versus* 48 h protein, and 48 h RNA *versus* 12 h protein. Based on the RNA-Seq data, the number of genes that met the log2FC threshold for differential expression was 890 genes at 3 h, 3118 genes at 12 h, and 2798 genes at 48 h. The number of proteins that met the log2FC threshold was 1062 proteins at 12 h and 664 proteins at 48 h (Figs. 4*A* and 5*B*). Magenta points, which are malignancy-associated EMT factors (Fig. 4*A*) that are altered by SNAI1/TWIST1 overexpression (51, 52, 53, 54), were also identified as being altered following the induction of RPE-EMT. We used the same approach to evaluate and compare the datasets of AMD-associated risk factors (55) that are altered in our RPE-EMT study, and these factors were shown as magenta points (Fig. 5*B*). We also compared the RNA- *versus* protein-pathway enrichment analysis using FUNrich (44). We identified several enriched biological pathways, including integrin cell surface signaling, platelet-derived growth factor receptor signaling, glypican pathway, proteoglycan syndecan-mediated signaling, and the EMT pathway (*p* < 0.05) (Fig. 4*B*). Overall, our data indicate that the molecular signatures that coordinate with EMT are well correlated at the transcriptome and proteome levels, suggesting a controlled interplay of gene expression and protein synthesis in RPE-EMT.

### Dysregulated RPE-EMT Proteome Shares Commonality With Malignancy-Associated EMT

EMT plays a critical role in malignancy-associated metastatic dissemination of mammary epithelial cancer cells (5, 56). Lu *et al.* (51) used stable isotope labeling by amino acids in cell culture to quantify proteins of normal human mammary epithelial cells and cells that were experimentally induced to undergo EMT by TWIST1 overexpression. Their study identified over 2600 proteins, with 460 proteins significantly upregulated (277) or downregulated (183). As mentioned previously, the current study demonstrates that TWIST1 is significantly upregulated during dissociation-induced RPE-EMT (Fig. 1, *D* and *G*). Changes in other EMT-associated proteins, such as E-cadherin (CDH1), P-cadherin (CDH3), vimentin (VIM), and fibronectin (FN1), also showed similar changes in mammary epithelial cells compared with hRPE cells undergoing EMT. We compared the TWIST1 overexpression study with our RPE-EMT dataset.

In addition, we extended our RPE-EMT proteomic comparison with another independent proteomic analysis in which TWIST1-induced EMT changes were studied in human mammary epithelial cells (54). As a result, we identified a total number of 50 proteins that were significantly altered in both cancer and RPE-EMT models (supplemental Table S7). We have plotted these protein abundances along with their gene expression changes (Log2 fragments per kilobase per million) from our RNA-Seq study (Fig. 4*C*). Furthermore, we also performed GO enrichment analysis on these subsets of proteins and identified enrichment of various biological processes (epithelial cell differentiation and glucocorticoid signaling). Reactome pathways with *p* value (<0.05) were integrin cell surface interactions, hemostasis, ECM proteoglycans, and retinoid metabolism and transport. Pathway analysis also identified multiple mitotic cell cycle and chromosome condensation-associated pathways, which we felt were of less interest because one might expect to see an altered expression of cell cycle–related genes in dividing RPE cells.

Next, we sought to compare our RPE-EMT proteome with the SNAI1-induced cancer EMT proteome. SNAI1 is a master regulator of cancer EMT, whose overexpression correlates to tumor aggressiveness. Our qPCR and RNA-Seq analysis indicate that SNAI1 mRNA levels are significantly upregulated (7-fold) during dissociation-induced RPE-EMT (Fig. 1*D*). We crossreferenced and compared the significantly altered RPE-EMT genes with the SNAI1 transduced cancer proteome of breast adenocarcinoma MCF7 cells (53) and identified 31 proteins that were significantly altered in both RPE and cancer EMT (supplemental Table S7). We plotted subsets of these genes (Log2 fragments per kilobase per million) and protein abundance as a heat map (Fig. 4*D*). Furthermore, we performed GO enrichment analysis on these 31 proteins and identified significant changes in multiple biological pathways (Kyoto Encyclopedia of Genes and Genomes), including cancer proteoglycans, hematopoietic cell lineage, ECM receptor interactions, and cancer-associated miRNAs. Together, these comparative cancer EMT and RPE-EMT studies show commonality at both the transcriptomic and proteomic levels.

### Altered RPE-EMT Transcriptome and Proteome Signatures Correlate With Known AMD-Associated Risk Factors

A comprehensive Genome Wide Association Studies have identified several genetic loci and genes associated with AMD risk (57, 58). The majority of AMD risk factors are attributed to variants of complement components, transporters, ECM organization, and assembly factors. Since, as noted previously, EMT has been implicated in AMD pathogenesis (16, 17, 59), we examined our transcriptomic and proteomics data to explore whether any of the AMD-associated genes showed EMT-related changes in expression of their corresponding mRNAs/proteins (supplemental Table S8). We particularly focused on 52 well-defined independent common and rare AMD risk variants, which themselves are distributed across 34 genetic loci (57). This analysis identified genes associated with 42 of the AMD-associated variants whose expression, at least one time point (from 3 to 48 h post dissociation), was significantly modulated by dissociation-induced RPE-EMT (Fig. 5*A*). However, of those genes showing transcriptional modulation, only seven showed statistically significantly altered expression at the protein level (APOE, TIMP3, HTRA1, VTN, MMP2, and SLC16A8). We have validated these factors by qPCR in two hiPSCs (EP1 and IMR90.4) and hESCs (H7) and found that AMD-associated risk factors, such as *APOE*, *CFH*, *COL8A1*, *FBLN5*, *HTRA1*, *RDH5*, *SLC16A10*, *TIMP3*, *TRPM3*, *VEGFA*, and *VTN*, are all significantly downregulated during RPE-EMT (Fig. 5*C* and supplemental Figs. S4*B* and S5*B*). Our protein copy number analysis showed that the number of AMD-associated risk factors was decreased (supplemental Fig. S14) during RPE-EMT. To gain further insight into the 42 EMT-modulated AMD risk-associated genes, we analyzed the gene set with STRING, which predicts protein–protein interactions and network analysis. This analysis highlighted enrichment of the complement-coagulation cascades, AGE-RACE signaling pathways in diabetes, cholesterol metabolism, and PI3K–Akt signaling pathways.

Next, we compared our RPE-EMT proteome data with a previously reported transcriptome study on ocular tissues from 68 human donor eyes with phenotypes including early atrophic (“dry”) AMD, dry AMD with geographic atrophy, and neovascular (“wet”) AMD (55). This analysis identified 28 of the EMT-associated factors as being altered in AMD (Fig. 5, *D* and *E*). Furthermore, we performed GO enrichment analysis as another approach to identify common pathways and cellular activities associated with RPE-EMT. This analysis suggested the importance of integrin cell signaling, l-dopachrome biosynthesis, peptide–ligand interactions, ataxia–telangiectasia serine/threonine kinase signaling, and EMT regulation. Together, our comparative analysis indicates that multiple AMD-associated risk factors are differentially regulated during RPE-EMT, further supporting the role of EMT in AMD pathogenesis.

### Global RPE-EMT Proteomics Predicted Biological Pathways, and Upstream Transcriptional Regulators Highly Overlap With Those Implicated in Cancer-Related EMT

**Fig. 6:**
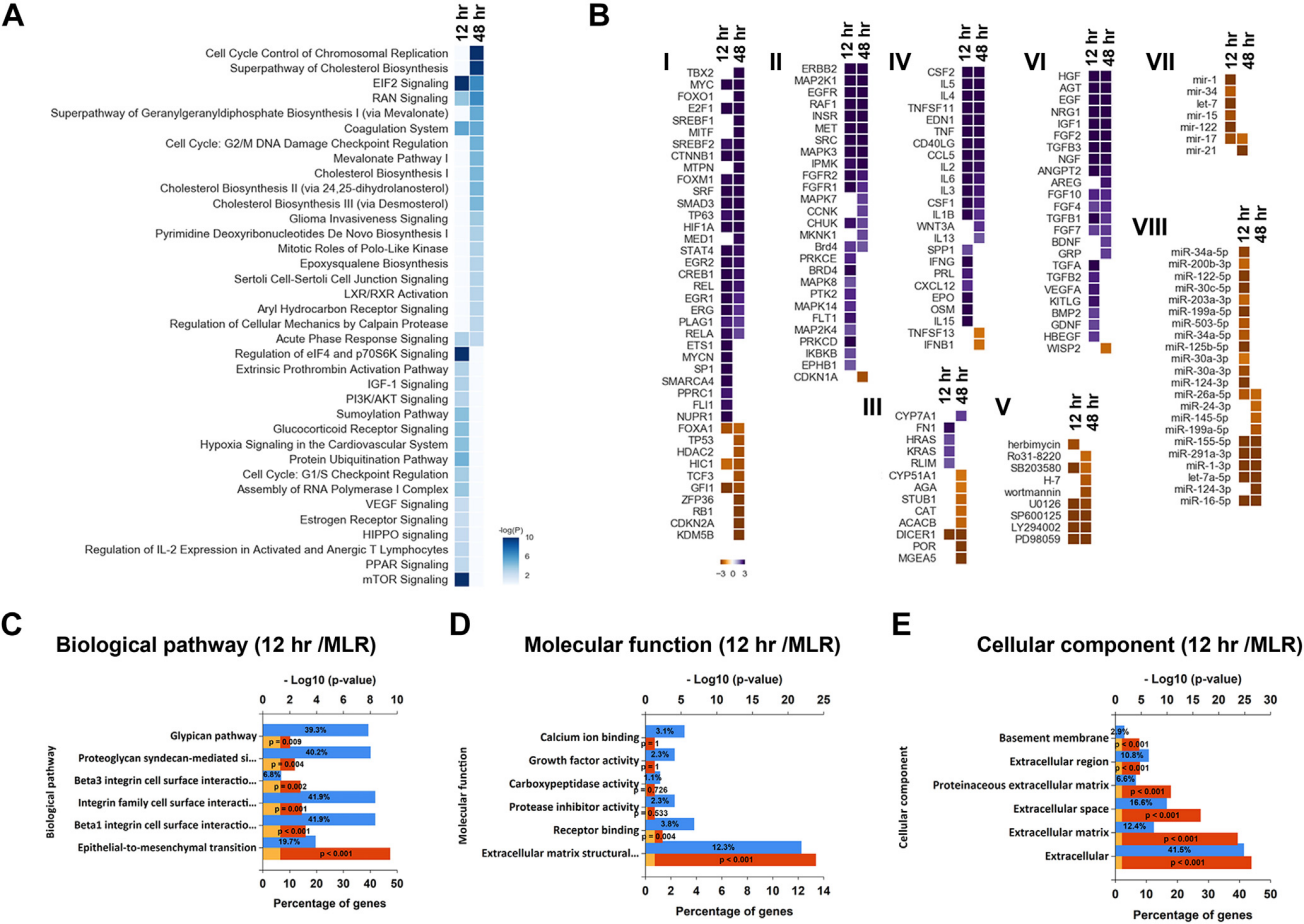
**Biological pathways and upstream transcription regulators altered by RPE-EMT.***A*, top canonical pathways predicted based on RPE-EMT induced gene expression changes, plotted based on relative *p* value. *B*, heat maps of IPA predicted RPE-EMT upstream regulators. IPA uses activation *Z* score to measure the match between expected relationship direction and observed changes in gene expression regulators. Predicted regulators are shown grouped by functional class: (I) transcription factors (TFs), (II) kinases, (III) enzymes, (IV) cytokines, (V) kinase inhibitors, (VI) growth factors, (VII) mature miRNAs, and (VIII) premature miRNAs, with predicted activators shown in *violet* and inhibitors shown in *yellow*. GO enrichment analysis for 12-h time point is shown for biological pathways (*C*), molecular function (*D*), and cellular components (*E*) (*Blue*: percentage of genes; *yellow*: 0.05 reference; *red*: *p* value). EMT, epithelial-to-mesenchymal transition; GO, Gene Ontology; IPA, ingenuity pathway analysis; RPE, retinal pigment epithelium.

To obtain a broader understanding of the essential biological processes and EMT-associated pathways involved with RPE-EMT progression, we performed bioinformatics analysis using IPA. IPA identifies and ranks the significantly altered pathways based on enriched associated proteins from each pathway (Fig. 6*A*; supplemental Table S9). Interestingly, many of the canonical pathways identified as being enriched by the IPA analysis turned out to be pathways already known to modulate EMT during tumor cell invasion, migration, and metastasis. Among the pathways enriched at 12 h postdissociation are cholesterol biosynthesis (60, 61, 62), eukaryotic initiation factor 2 signaling (63), mevalonate pathway (64), and glioma invasiveness signaling (65).

To investigate the cascade of factors that are either activated or inhibited in response to dissociation-induced EMT and act upstream of the observed changes in proteome abundance, we analyzed the dissociation-induced RPE-EMT proteins using the “upstream regulator analysis” (URA) module in IPA. The URA algorithm uses an overlap of *p* values and activation *z* scores to predict experimentally validated “transcription regulators” that may be responsible for observed changes in protein expression. URA highlighted a number of potential URs, including transcription factors, kinases, miRNAs, growth factors, cytokines, and kinase inhibitors (42, 66). We filtered for URs that were predicted by IPA to be activated or inhibited during at least a one-time point during EMT progression (supplemental Table S10). Furthermore, we clustered the resulting URs based on the IPA activation score's absolute value at any dissociation time to identify temporal regulated patterns absolute value of their IPA activation score at the various dissociation time points to identify temporally regulated patterns. This analysis resulted in the identification of several transcription factors that have been previously reported to be involved in malignancy-associated EMT (Fig. 6*B* and supplemental Table S2).

Furthermore, we performed a bioinformatics analysis using the GO annotation and STRING databases to predict associations between the 532 proteins that were differentially regulated at the time points of 12 and 48 h to extract protein-interactions networks based on functional annotations (43). Significantly altered proteins were categorized into three major classes—biological processes, cellular components, and molecular processes. The GO and STRING pathway enrichment analysis indicated that dissociation-induced RPE-EMT is regulated by multiple pathways including integrin signaling, complement, coagulation cascades, axon guidance, proteoglycans in cancer, Wnt signaling, ECM receptor interactions, cell adhesion molecules, and cholesterol metabolism (Fig. 6*C*). Next, our analysis identified candidate cellular components (basement membrane, ECM) (Fig. 6*D*) and molecular process (calcium iron binding, growth factor activity) (Fig. 6*E*). Our enrichment analysis also implicated cell cycle regulatory components such as DNA replication, chromosome segregation, spindle assembly, and kinetochore assembly as being implicated in RPE-EMT (supplemental Fig. S6, *A*–*C*). Together, these findings indicate that RPE-EMT is a complex process regulated by multiple signaling events and network pathways during the enzymatic dissociation of RPE monolayers.

### Expression Patterns of RPE-EMT–Altered Protein Kinases and Phosphatases Are Inversely Related

**Fig. 7:**
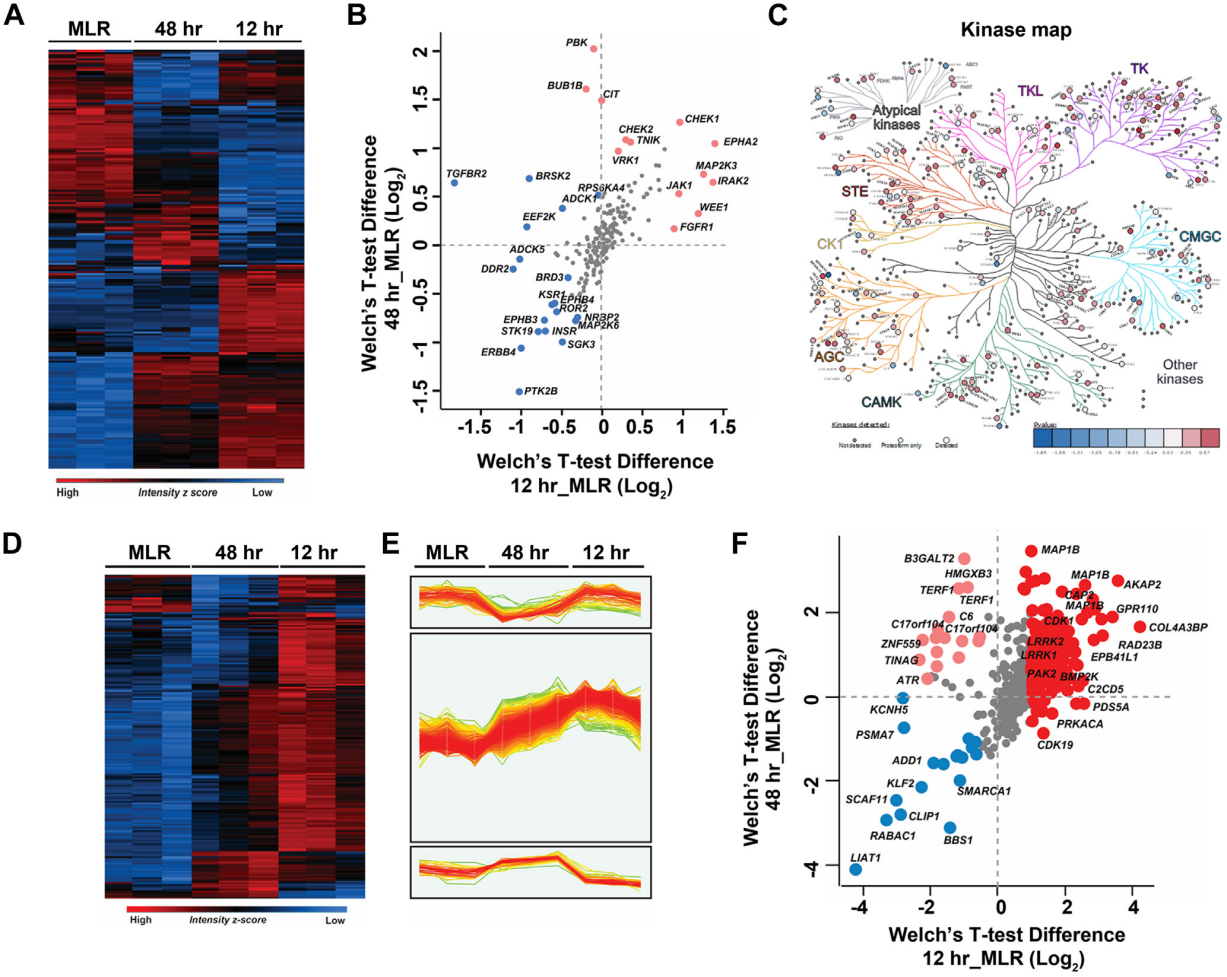
**Temporal enriched kinase profiling of dissociation-induced RPE-EMT.***A*, heat map showing pattern of altered expression of protein kinases during dissociation-induced EMT. *B*, scatter plot of identified kinase changes across the 12- and 48-h time points. *C*, kinome tree representation of the altered kinase profiling in RPE-EMT. *D*, heat map representing the expression of phosphosites across MLR, 12 h, and 48 h samples. *E*, profile plots of three selected clusters showing distinct phosphosite patterns with EMT induction. *F*, scatter plot showing relative quantification of identified phosphosites across the 12- and 48-h time points. EMT, epithelial-to-mesenchymal transition; RPE, retinal pigment epithelium.

Initiating RPE-EMT by detaching hRPE monolayer cultures from their underlying substrate, followed by dissociation into a single-cell population, is a rapid process. Since the molecular events occurring during this induction process are partially regulated by protein phosphorylation, to gain further insight into the mechanisms and pathways involved, we mapped our proteome data to an established kinome resource (67, 68). We identified a total of 202 known protein kinases from the established human kinome map. Furthermore, we performed Welch's *t* test analysis and performed clustering to identify the differentially regulated kinases between monolayers and dissociated RPE cells (Fig. 7, *A* and *B* and supplemental Table S11). We also plotted each of these significantly alerted kinases by protein copy number per cell (supplemental Fig. S15). The 202 potentially interesting kinases that were identified by this analysis are presented as a kinome tree organized based on their *t* test significance (Fig. 7*C* and supplemental Fig. S8*B*), depicting the different classes of kinases that appear to play a crucial role during RPE differentiation and EMT. Ephrins modulate cytoskeletal architecture by coordinating cell adhesion, proliferation, and invasion (69). EPHA2 modulates tumor invasion and metastasis by negatively regulating CDH1 (E-cadherin), while also having a positive correlation with vimentin and β-catenin expression (70). Next to the p53 and KRAS pathways, fibroblast growth factor receptor (FGFR) is the most frequently dysregulated pathway in a wide variety of cancer progression models (71). FGFR signaling regulates SNAI1, which further controls CDH1 expression (72). FGFR1 induces cell proliferation and metastasis *via* ERK11/2–SOX2 signaling in lung cancer (73). JAK1 modulates oncogenic activation of STAT3 in mammary cancer cells driven by ERBB2 receptor tyrosine kinase signaling (74).

We next addressed the question of whether differential enrichment kinases in RPE-EMT have any association with phosphatase expression. We extracted the known phosphatases from the DEPOD (Human DEPhOsphorylation Database) (75) and mapped those against 260 curated phosphatases. We identified 135 phosphatases, of which the majority are tyrosine phosphatase and PPM family members. The differential enrichment of these 135 phosphatases is represented in a heat map (supplemental Fig. S7*A*), which mainly fall into three clusters. Cluster I comprises 49 phosphatases (supplemental Fig. S7*B* and supplemental Table S12) that show an increased abundance at 12 and 48 h. Cluster II comprises 17 phosphatases that show an increased abundance in 12 h but not at 48 h. Cluster III comprises 69 phosphatases that show a reduced abundance at both 12 and 48 h. In order to identify the differential expression of kinases and phosphatases, we applied Welch's *t* test; this analysis revealed that the kinases, mostly the tyrosine kinase family members who are enriched at the 12-h time point, show an opposite pattern compared with the tyrosine phosphatase family members. As shown in supplemental Fig. S7*C*, the majority of the phosphatase family members are unchanged, and a few of the phosphatases exhibited an increase in abundance in the monolayer state. Many of the tyrosine family phosphatases, such as PTP4A3, PTPRZ1, PTPRS, PTPRU, PTPTRD, PTPTRM, and PTPRF, are enriched in monolayers, suggesting a possible regulatory role of these phosphatases on their substrates in the pre-EMT state. In the hierarchical clustering of the identified phosphatases in cluster 2 identified 17 phosphatases (*e.g.*, PTPN1, PTEN, SYNJ1, SYNJ2, PHLPP2, PPM1A, PPM1B, and PTPN14) that showed an increase in abundance at 12 h, suggesting that their substrates could be downregulated. Together, the enriched kinase phosphatase crosstalk suggests candidate molecules and pathways that may modulate and regulate RPE-EMT.

### Phosphosite Analysis of RPE-EMT

We next performed phosphosite analysis on the global proteome dataset to define phosphorylation events potentially involved in RPE-EMT. To identify altered sites, we analyzed the STY site data from MaxQuant search output tables to identify altered sites and processed the data using the Perseus computational software suite. The analysis identified 764 phosphosites (>0.75 phosphosite probability), of which 71.8% were phosphoserine (549), 20.7% were phosphothreonine (158), and 7.5% were phosphotyrosine (57). The summary and expression of phosphosites between MLR, 12 h, and 48 h are represented as a heat map (Fig. 7*D*). The phosphosites identified in each condition between replicates were well clustered, suggesting data accuracy and reproducibility. Interestingly, we identified three unique clusters of phosphosites (Fig. 7*E*), which covered two-thirds of the identified phosphosites that are altered during RPE-EMT. Analysis of the data to look for enriched phosphosites between MLR, 12 h, and 48 h using Welch's *t* test analysis at 1% FDR revealed the enrichment of 496 phosphosites that are increased in abundance more than or equal to 1.5-fold and 30 sites that are decreased in abundance 0.6-fold in 12 h in comparison to MLR. Similarly, 274 phosphosites are increased in abundance more than or equal to 1.5-fold, and 29 sites are decreased in abundance 0.6-fold at 48 h in comparison to MLR. A volcano plot comparing phosphosites at 48 and 12 h revealed 160 phosphosites showing increased abundance and 170 phosphosites showing decreased abundance (supplemental Fig. S8*A*). The phosphosites that are enriched at both 12 and 48 h, compared with MLR, includes AKAP10, GPR110, MAP1B, RAD23B, PAK2, LRRK1, LRRK2, COL4A3BP, EPB41L1, and BMPK2. The phosphosites that are enriched only at 48 h include B3GALT2, HMGX3B, TERF1, C17orf104, ZNF559, and ATR. The phosphosites that are exclusively enriched in the MLR state include LIAT1, RABAC1, CLIP1, SCAF11, KLF2, ADD1, and PSMA7. The summary of the data is depicted as a scatter plot in Figure 7*F*, and the complete list of phosphosites that are identified and enriched in MLR, 12 h, and 48 h is provided in supplemental Table S13. Our ability to identify major RPE regulatory phosphosites without employing any phosphopeptide enrichment technique is consistent with prior work that has previously shown that direct deep proteomic analysis can enable the identification of phosphosites that exhibit high phosphorylation stoichiometry (76). Of course, such an approach has limited power to identify less abundant phosphosites.

## Discussion

RPE injury induces a variety of pathological changes, including transdifferentiation and EMT (24). EMT and associated RPE dysfunction has been implicated in a variety of potentially blinding ocular diseases. Here, we report in-depth temporal global protein profiling of human stem cell–derived RPE monolayers (hRPE) that were induced to undergo EMT and also provide integration of this new dataset with a previously reported temporal mRNA expression RPE-EMT dataset (46). Our integrative proteogenomic analysis showed significant, but far from complete, correlation between the observed transcriptome and proteome expression changes brought about by the induction of EMTs.

Immunoblot analysis confirmed many of our proteomic findings, demonstrating increased expression of SNAI1, TWIST1, CDH2, JUNB, and FOSL1 and decreased expression of CDH1, RPE65, and BEST1, during dissociation-induced RPE-EMT. Induction of RPE-EMT in hiPS RPE cells by combinatorial treatment with transforming growth factor-β and tumor necrosis factor-α has been shown to significantly elevate mRNA levels of the EMT-activating transcription factors SNAI1, SNAI2, and TWIST1 (23). These factors are known to negatively regulate CDH1 expression (77). Decreased CDH1 expression, a crucial initial step in EMT, is associated with disruption of tight junctions and reinforces EMT progression by inducing TWIST1 and ZEB1 in a feed-forward loop (78). SNAI1-induced EMT in cancer cells promotes a more fibroblast-like appearance and increases the invasive behavior by suppressing CDH1 and β-catenin expression through elevated vimentin expression (79).

Supporting a role of RPE-EMT in AMD, a recent study of AMD donor eyes demonstrated elevated SNAI1 and vimentin levels accompanied by reduced CDH1 expression compared with age-matched controls (17). Further supporting an important role of RPE-EMT in AMD, the RPE-EMT factors identified by our comparative proteogenomic analysis are significantly differentially enriched in the corresponding set of genes that have been identified by Genome Wide Association Studies as being associated with increased risk for developing AMD (57, 58).

Other proteins highlighted by our proteomic analysis to show increased expression upon RPE-EMT induction are FOSL1, JUNB, MICAL2, and TUFT1. Notably, differential expression of AP-1, FOS, JUN, and ATF (80) has also been implicated in malignancy-associated EMT. Studies have shown that FOSL1 (FRA-1) modulates tumor heterogeneity and EMT plasticity during lung (81) and pancreatic cancer progression (82). JUNB plays a critical role in tumor cell invasion, migration, and metastasis, both through EMT-dependent and EMT-independent pathways (83, 84). We similarly found that the oncogenic protein TUFT1, whose expression is increased in breast cancer tissues (85), is also upregulated during RPE-EMT. Elevated TUFT1 expression regulates CDH1 and vimentin expression through the hypoxia-inducible factor 1–SNAI1 signaling pathway in pancreatic (86) and hepatocellular cancer metastasis (87). Our data also show increased expression of MICAL2, a tumor-promoting factor regulating cellular growth and axon guidance signaling that has been implicated in ovarian cancer through Wnt/β-catenin pathway (88).

The proteomic analysis also confirmed downregulation of RPE differentiation-related proteins. We observed decreased expression of the transcription factors SOX9 and OTX2, whose synergistic action is essential for the expression of a number of key RPE-specific factors, such as RPE65 and RLBP1 (89). In addition, there was decreased expression of various transporters, including SLC16A8, SLC38A3, and SLC2A1, that are crucial for regulating amino acid and glucose levels in the retina/RPE (90). Interestingly, depletion of SLC16A8 has been demonstrated to lead to visual dysfunction in mice (91). Our pathway enrichment analysis identified dysregulated cholesterol biosynthesis and alterations in the mevalonate cascade during RPE-EMT. It has been reported that cholesterol homeostasis plays a pivotal role in maintaining RPE health, and compromised cholesterol metabolism can lead to RPE dysfunction (92).

The proteomic analysis also implicated modulation of protein phosphorylation pathways as contributing to dissociation-induced RPE-EMT. For example, the kinase enrichment analysis identified increased expression of EPHA2, CHEK1, MAP2K3, IRAK2, and Traf2- and Nck-interacting kinase. These results are consistent with findings in cancer-associated EMT. EPHA2 has been shown to promote EMT in gastric cancer cells by modulating Wnt/β-catenin signaling (93, 94). CHEK1 is a serine–threonine protein kinase that regulates EMT through ZEB1-mediated signaling in breast cancer cells (95). MAP2K3 (MKK3) specifically phosphorylates p38MAPK and activates tumor cell invasion and migration by a JNK-dependent pathway (96, 97). IRAK2 promotes the EMT phenotype through miR-373 regulation in non–small cell lung cancer cells (98). Traf2- and Nck-interacting kinase is a regulatory component of the β-catenin transcription factor that modulates EMT during colorectal cancer metastasis (99); and the soluble form of EPHB4 plays a key role in inhibiting platelet-derived growth factor–induced RPE cell attachment, proliferation, and attachment (69). In addition, our phosphatase/kinase enrichment analysis revealed that tyrosine kinase family members were enriched at the 12 h time point and had a reciprocal relationship with the tyrosine phosphatases, suggesting a regulatory role for both kinases and phosphatases in orchestrating RPE-EMT.

Overall, the global proteome datasets that we generated by TMT labeling and direct-DIA and integrated transcriptomic analyses described here provide a rich resource that will hopefully assist the research community in deepening our understanding of the mechanisms underlying the development and progression of RPE-EMT. Given the increasing appreciation of RPE-EMT in the pathogenesis of a number of blinding retinal diseases, we hope this new database will serve as a resource for aiding in the understanding of retinal disease mechanisms in the context of EMT. We in addition hope that our proteomic data will provide targets and insights that will aid in the development of pharmacologic approaches to modulate RPE-EMT and thereby lead to improvements in the treatment of retinal diseases that involve the development of RPE-EMT.

## Data Availability

All MS files and search results generated from this study have been deposited to the ProteomeXchange Consortium (https://www.ebi.ac.uk/pride/) *via* PRIDE (PRoteomic identification database (100) with the identification number PXD019600) and project name “Epithelial to mesenchymal transition (EMT) of human stem cell–derived RPE shares commonalities with malignancy-associated EMT: a proteomic analysis.” We have included the annotated MS2 spectra for single peptide identifications and phosphorylated STY (supplemental Figs. S16 and S17).

## Supplemental data

This article contains supplemental data.

## Conflict of interest

The authors declare no competing interests.
