# Supplementary material for: Proteome Landscape of Epithelial-to-Mesenchymal Transition (EMT) of Retinal Pigment Epithelium Shares Commonalities With Malignancy-Associated EMT
Source: Mol Cell Proteomics. 2021 Aug 27;20:100131. doi: 10.1016/j.mcpro.2021.100131 (PMC8482521; doi:10.1016/j.mcpro.2021.100131)
Supplement: Supplemental Table S1 [file mmc1.docx]

| **Quantitative Real-Time PCR Primers** | | | | |
| --- | --- | --- | --- | --- |
| **Gene** |  | **Primer Sequence (5' to 3')** | **Annealing Temp (°C).** | **Source** |
| *SNAI1* | F | TCGGAAGCCTAACTACAGCGA | 60 | Sripathi, 2021 |
|  | R | AGATGAGCATTGGCAGCGAG |  |  |
| *CDH1* | F | ATTTTTCCCTCGACACCCGAT | 60 | Sripathi, 2021 |
|  | R | TCCCAGGCGTAGACCAAGA |  |  |
| *BEST1* | F | CTGGGCTTCTACGTGACGC | 60 | Sripathi, 2021 |
|  | R | TTGCTCGTCCTTGCCTTCG |  |  |
| *CREBBP* | F | GAGAGCAAGCAAACGGAGAG | 60 | Maruotti, 2013 |
|  | R | AAGGGAGGCAAACAGGACA |  |  |
| *ACTA2* | F | GTGTTGCCCCTGAAGAGCAT | 60 | Maruotti, 2013 |
|  | R | GCTGGGACATTGAAAGTCTCA |  |  |
| *GAPDH* | F | TAG CCAAATTCGTTGTCATACC | 60 | Sripathi, 2021 |
|  | R | CTGACTTCAACAGCGACACC |  |  |
| *RPE65* | F | TGCGTATGGACTTGGCTTGAATC | 60 | Maruotti, 2013 |
|  | R | TCCTGCTCCTGGGCTCACC |  |  |
| *HMGA2* | F | GGGCCAGGAGGTAGTTTCTC | 60 | Sripathi, 2021 |
|  | R | CCTCGGTGCACCATGTTTGGC |  |  |
| *TWIST1* | F | GTCCGCAGTCTTACGAGGAG | 60 | Sripathi, 2021 |
|  | R | GCTTGAGGGTCTGAATCTTGCT |  |  |
| *SNAI2* | F | TGTGACAAGGAATATGTGAGCC | 60 | Sripathi, 2021 |
|  | R | TGAGCCCTCAGATTTGACCTG |  |  |
| *ZEB1* | F | TTACACCTTTGCATACAGAACCC | 60 | Sripathi, 2021 |
|  | R | TTTACGATTACACCCAGACTGC |  |  |
| *RLBP1* | F | GCTGCTGGAGAATGAGGAAAC | 60 | Maruotti, 2013 |
|  | R | TGGCTGGTGGATGAAGTGG |  |  |
| *MITF* | F | TTCACGAGCGTCCTGTATGCAGAT | 60 | Meyer, 2009 |
|  | R | TTGCAAAGCAGGATCCATCAAGCC |  |  |
| *TYR* | F | ATTGGGACTGGCGGGATG | 60 | Maruotti, 2013 |
|  | R | GCATAAAGACTGATGGCTGTTG |  |  |
| *OTX2* | F | ACCTTGAACTCCACCTCT | 56 | Meyer, 2009 |
|  | R | GCTTCTCTTCTCTGACTCTCTTTG |  |  |
| *FOSL1* | F | CAGTGGATGGTACAGCCTCA | 60 | Sripathi, 2021 |
|  | R | CTGCAGGAAGTCGGTCAGTT |  |  |
| *JUNB* | F | ACGACTCATACACAGCTACGG | 60 | Sripathi, 2021 |
|  | R | GCTCGGTTTCAGGAGTTTGTAGT |  |  |
| *TUFT1* | F | GGAAAGTCCGGCAAATGATAGA | 60 | Sripathi, 2021 |
|  | R | TCCAGATAGGCGATTTTCTCCTT |  |  |
| *FGF1* | F | TTCACAGCCCTGACCGAGAA | 60 | Sripathi, 2021 |
|  | R | TGGCCAGTCTCGGTACTCTT |  |  |
| *ITGA5* | F | GCCTGTGGAGTACAAGTCCTT | 60 | Sripathi, 2021 |
|  | R | AATTCGGGTGAAGTTATCTGTGG |  |  |
| *MICAL2* | F | GGTCAAACCGGAGAGAATGA | 60 | Sripathi, 2021 |
|  | R | GCTACGGCTGGAAAAGTTTG |  |  |
| *LAMA1* | F | GTCAGCGACTCAGAGTGTTTG | 60 | Sripathi, 2021 |
|  | R | CTTGGGTGAAAGATCGTCAGC |  |  |
| *SLC1A5* | F | CATCATCCTCGAAGCAGTCA | 60 | Sripathi, 2021 |
|  | R | CTCCGTACGGTCCACGTAAT |  |  |
| *MMP1* | F | GGGGCTTTGATGTACCCTAGC | 60 | Sripathi, 2021 |
|  | R | TGTCACACGCTTTTGGGGTTT |  |  |
| *MMP3* | F | GCAGTTTGCTCAGCCTATCC | 60 | Sripathi, 2021 |
|  | R | GAGTGTCGGAGTCCAGCTTC |  |  |
| *APOE* | F | CCAATCACAGGCAGGAAGAT | 60 | Sripathi, 2021 |
|  | R | TCCAGTTCCGATTTGTAGGC |  |  |
| *COL8A1* | F | GCTGCCACCTCAAATTCCTC | 60 | Sripathi, 2021 |
|  | R | CTGGTTGCCCTGGTAACCC |  |  |
| *FBLN5* | F | AGCTCTGTGCGAATGTTCCT | 60 | Sripathi, 2021 |
|  | R | GGCAGATGAAAGAGCCGTAG |  |  |
| *HTRA1* | F | TCCCAACAGTTTGCGCCATAA | 60 | Sripathi, 2021 |
|  | R | CCGGCACCTCTCGTTTAGAAA |  |  |
| *TK1* | F | ATTAACCTGCCCACTGTGCT | 60 | Sripathi, 2021 |
|  | R | TGCTGCTGTAGCGAGTGTCT |  |  |
| *VTN1* | F | CGGGGATGTGTTCACTATGCC | 60 | Sripathi, 2021 |
|  | R | CCCCTGACAGTTGATGCGG |  |  |
| CFH | F | CACACAAGATGGATGGTCGC | 60 | This study |
|  | R | GGATGGCAGGCAACGTCTAT |  |  |
| TIMP3 | F | CCTGCTACTACCTGCCTTGC | 60 | This study |
|  | R | GGTCTGTGGCATTGATGATG |  |  |
| TRPM3 | F | GGAGCAGAGGTGAAACTTCG | 60 | This study |
|  | R | CCCATCACAGACAACCACTG |  |  |
| RDH5 | F | TCATTTTGGGATACGAGTCTCCA | 60 | This study |
|  | R | CGGGTCACAGATCAGGTTCA |  |  |
| SLC16A10 | F | ATGCTGGAAACCTTCGGCTC | 60 | This study |
|  | R | TGAAGACGCTGACTATTGGGC |  |  |
| VEGFA | F | CTGCTGTCTTGGGTGCATTGG | 60 | RR Greb 1999 |
|  | R | CACCGCCTCGGCTTGTCACAT |  |  |
