# Supplementary material for: Proteome Landscape of Epithelial-to-Mesenchymal Transition (EMT) of Retinal Pigment Epithelium Shares Commonalities With Malignancy-Associated EMT
Source: Mol Cell Proteomics. 2021 Aug 27;20:100131. doi: 10.1016/j.mcpro.2021.100131 (PMC8482521; doi:10.1016/j.mcpro.2021.100131)

Supplemental Figure. S16: MS2 spectra for single peptide identifications and phospho STY

Raw File	Scan	Method	Score	m/z	Gene names
RO-QC-160121_ZackD_SS_Set_01_Fractior	5062	FTMS; HCI	44.64	484.26	C19orf71

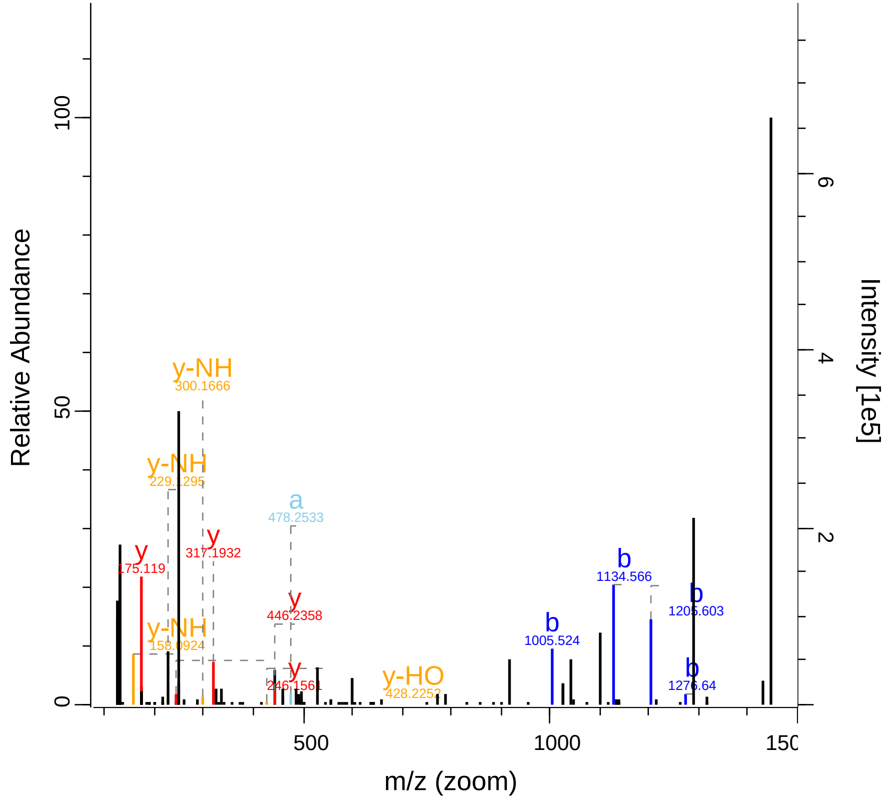

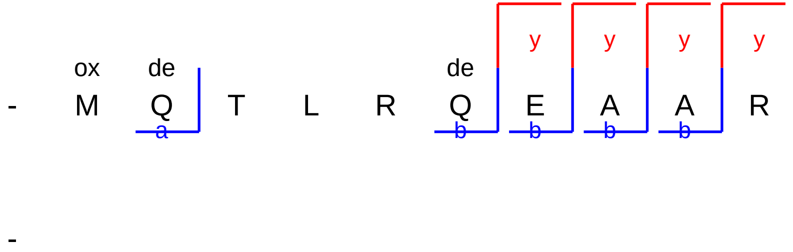

Raw File	Scan	Method	Score	m/z	Gene names
RO-QC-160121_ZackD_SS_Set_01_Fractior	5331	FTMS; HCI	47.26	382.19	KCNE5

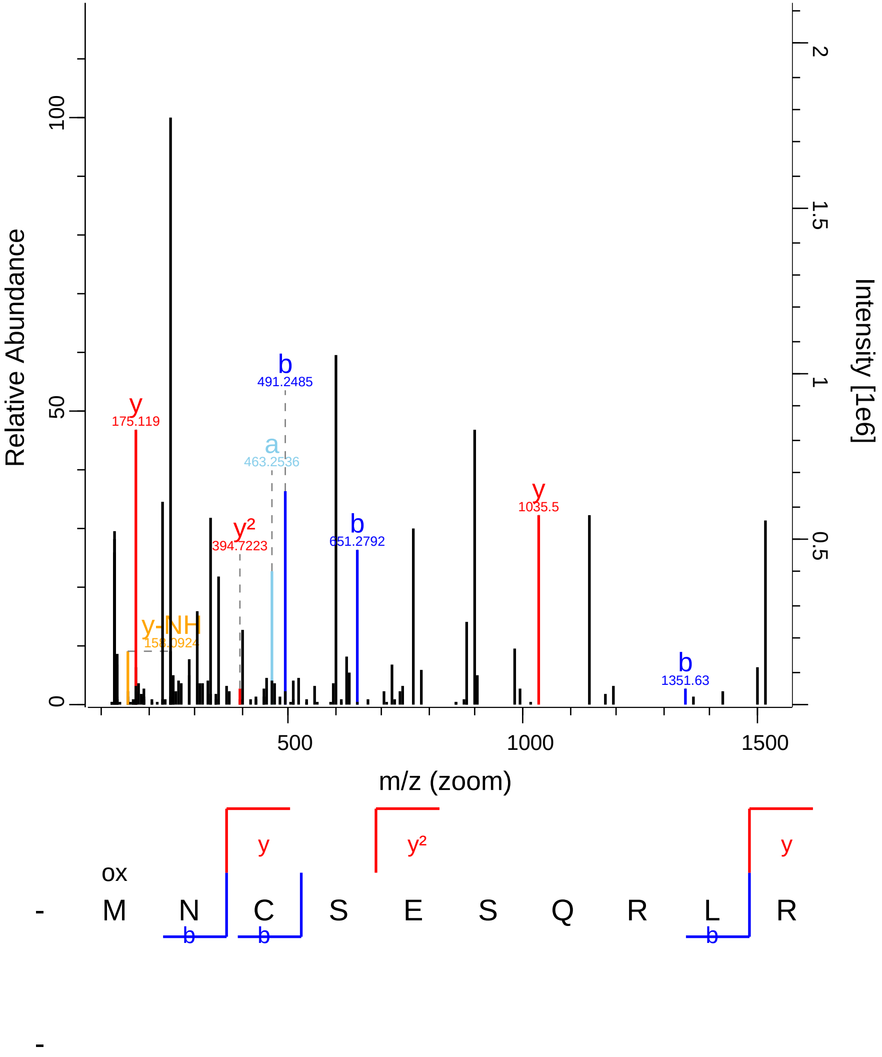

Raw File	Scan	Method	Score	m/z	Gene names
RO-QC-160121_ZackD_SS_Set_01_Fraction	5335	FTMS; HCI	61.34	490.9	SPRED2

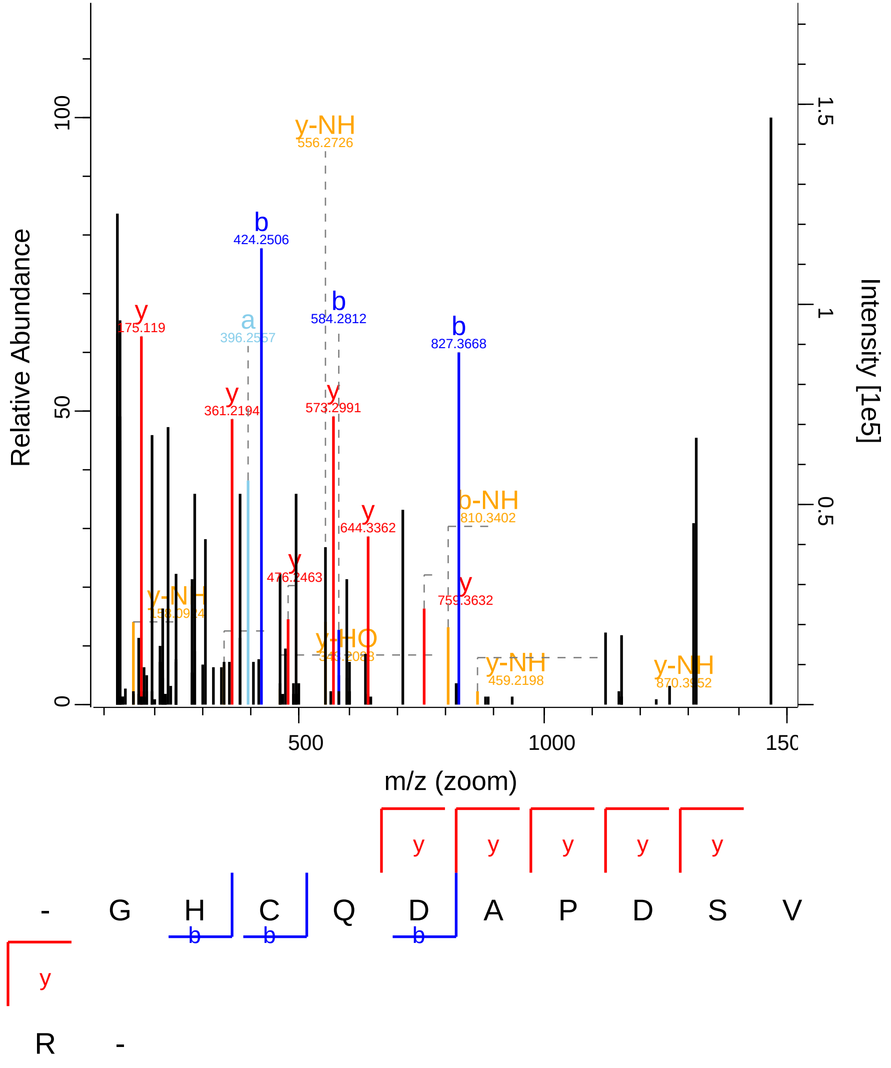

Raw File	Scan	Method	Score	m/z	Gene names
RO-QC-160121_ZackD_SS_Set_01_Fractior	6040	FTMS; HCI	51.53	488.56	WDR25

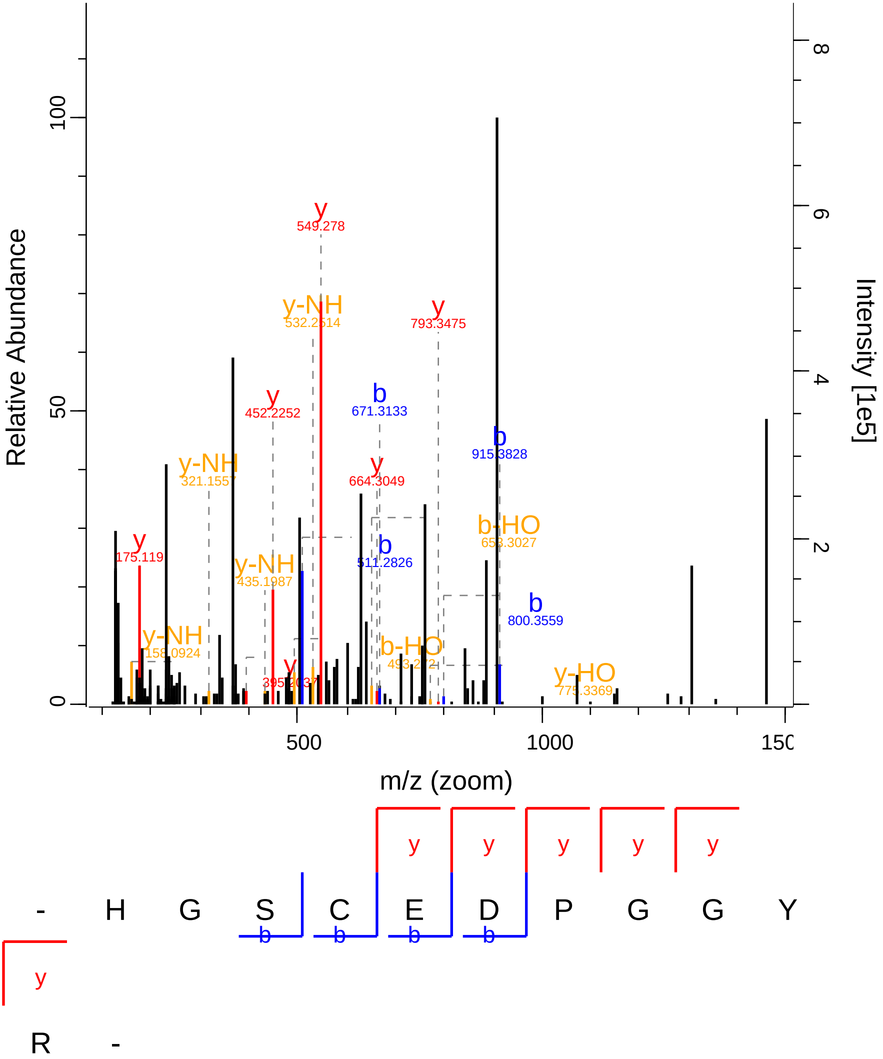

Raw File	Scan	Method	Score	m/z	Gene names
RO-QC-160121_ZackD_SS_Set_01_Fractior	6119	FTMS; HCI	91.09	669.83	TMEM47

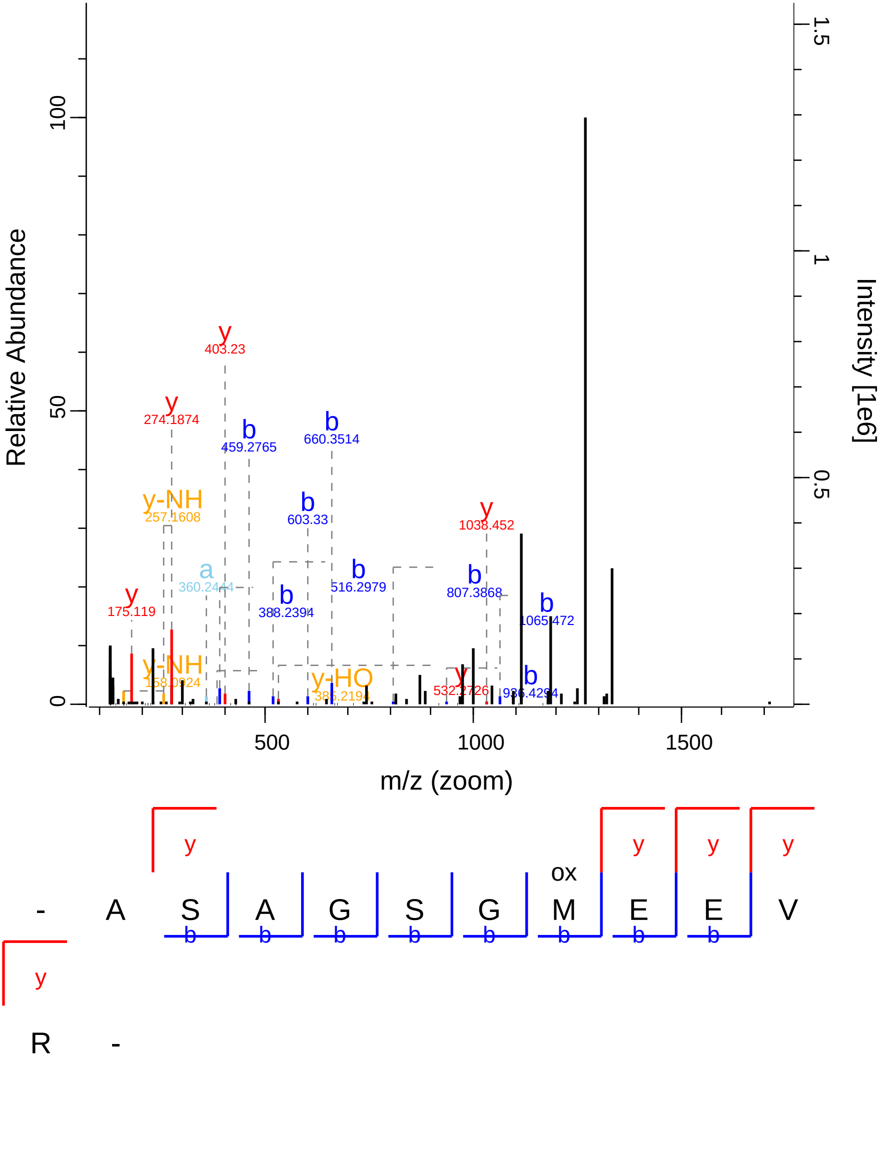

Raw File	Scan	Method	Score	m/z	Gene names
RO-QC-160121_ZackD_SS_Set_01_Fraction	6262	FTMS; HCI	111.86	513.3	LRIF1

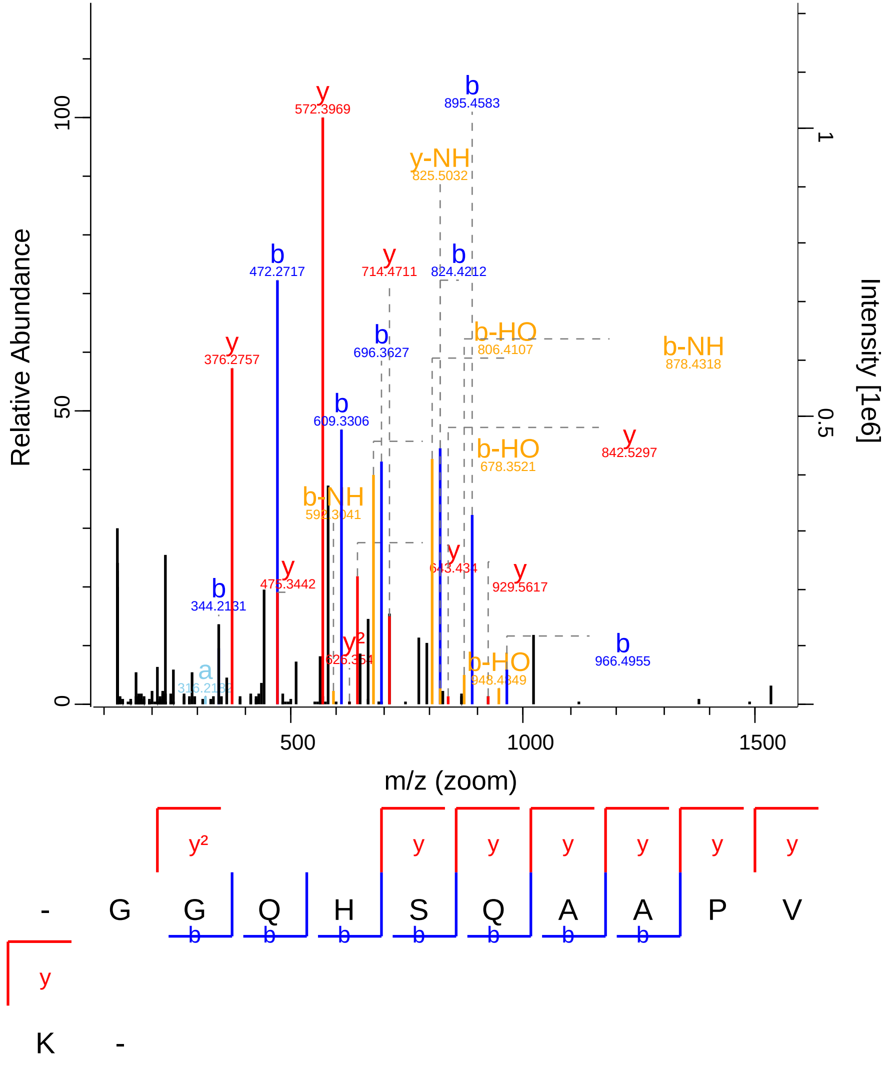

Raw File	Scan	Method	Score	m/z	Gene names
RO-QC-160121_ZackD_SS_Set_01_Fractioı	6314	FTMS; HC	152.85	519.04	ZNF518B

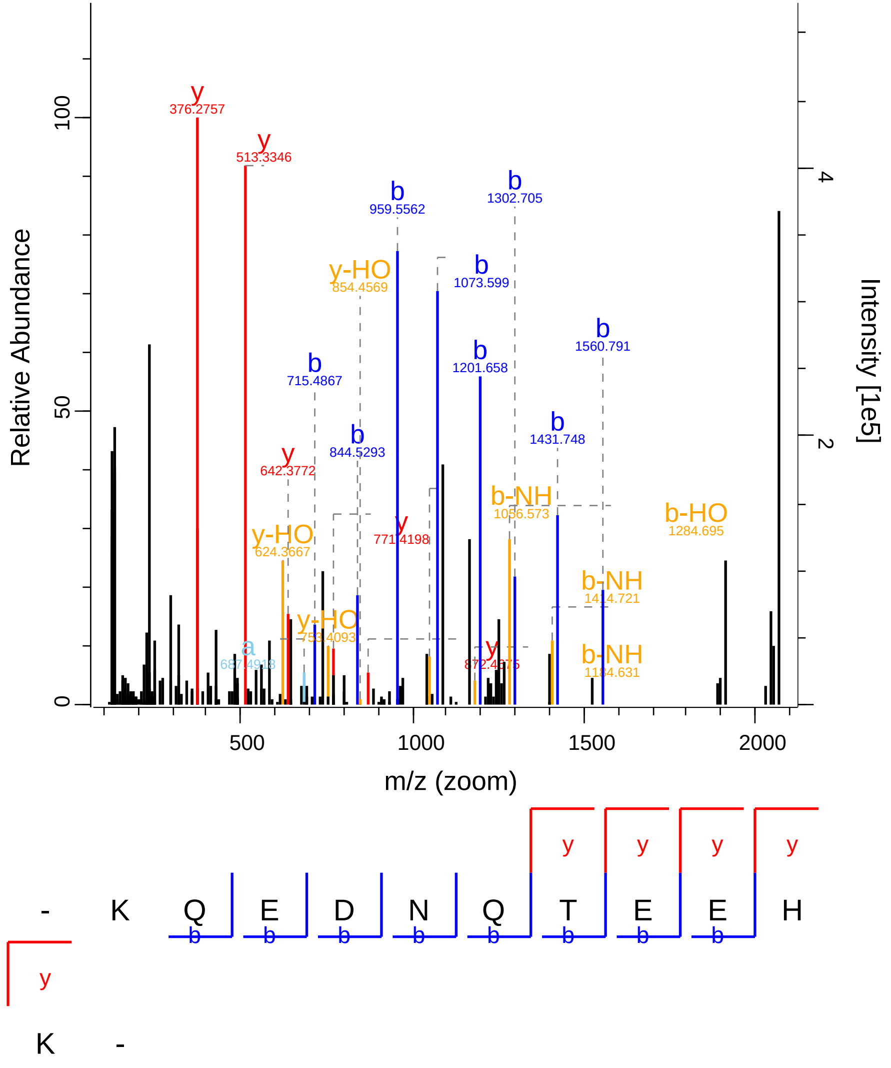

Raw File	Scan	Method	Score	m/z	Gene names
RO-QC-160121_ZackD_SS_Set_01_Fraction	6740	FTMS; HCI	105.14	575.8	GPAM

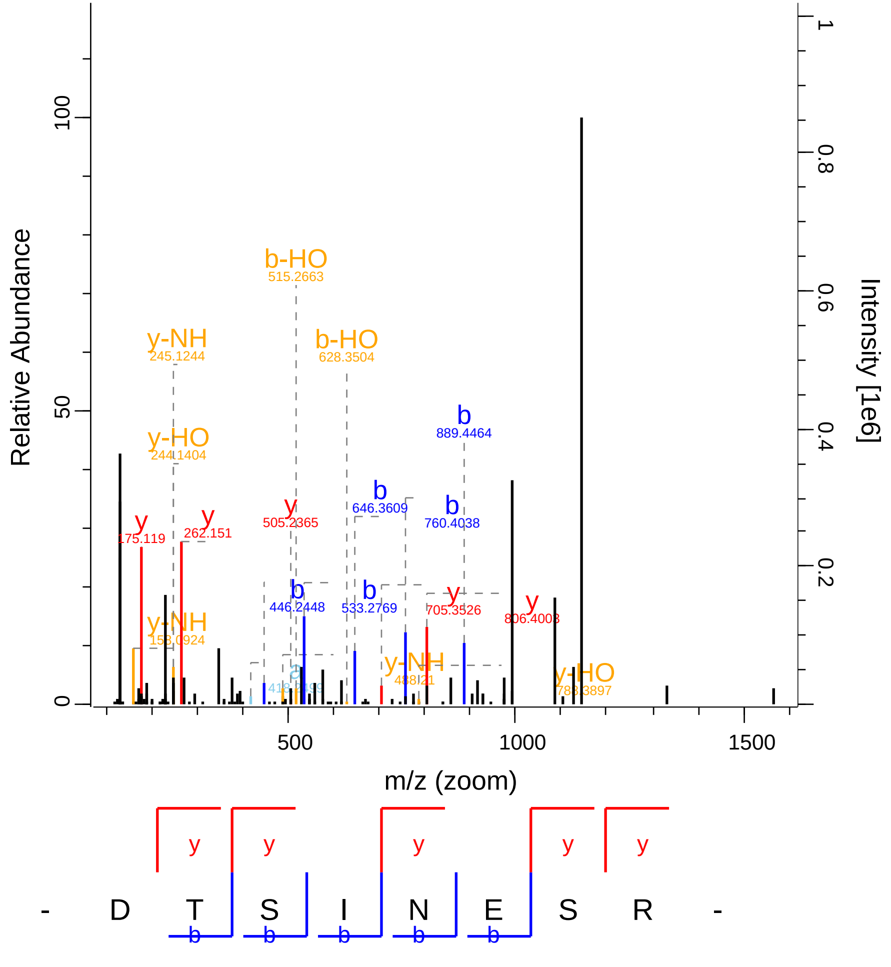

Raw File	Scan	Method	Score	m/z	Gene names
RO-QC-160121_ZackD_SS_Set_01_Fractior	7602	FTMS; HC	196.44	778.93	TTC39B

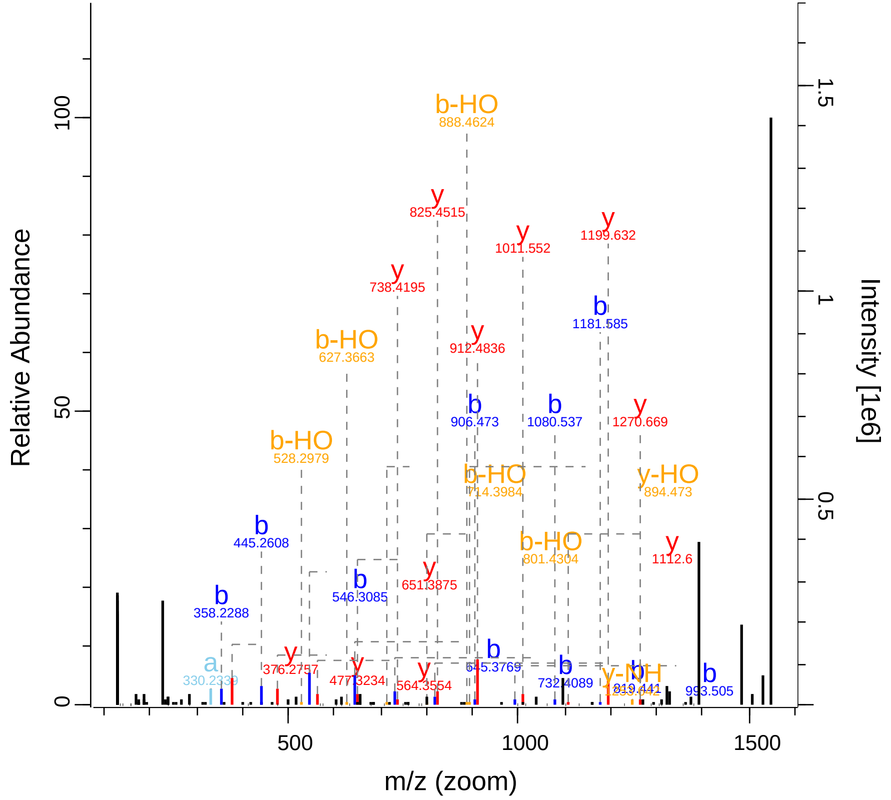

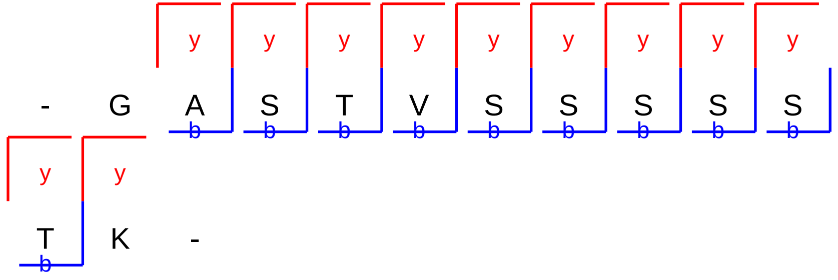

Raw File	Scan	Method	Score	m/z	Gene names
RO-QC-160121_ZackD_SS_Set_01_Fractior	7976	FTMS; HCI	80.24	709.86	SLCO3A1

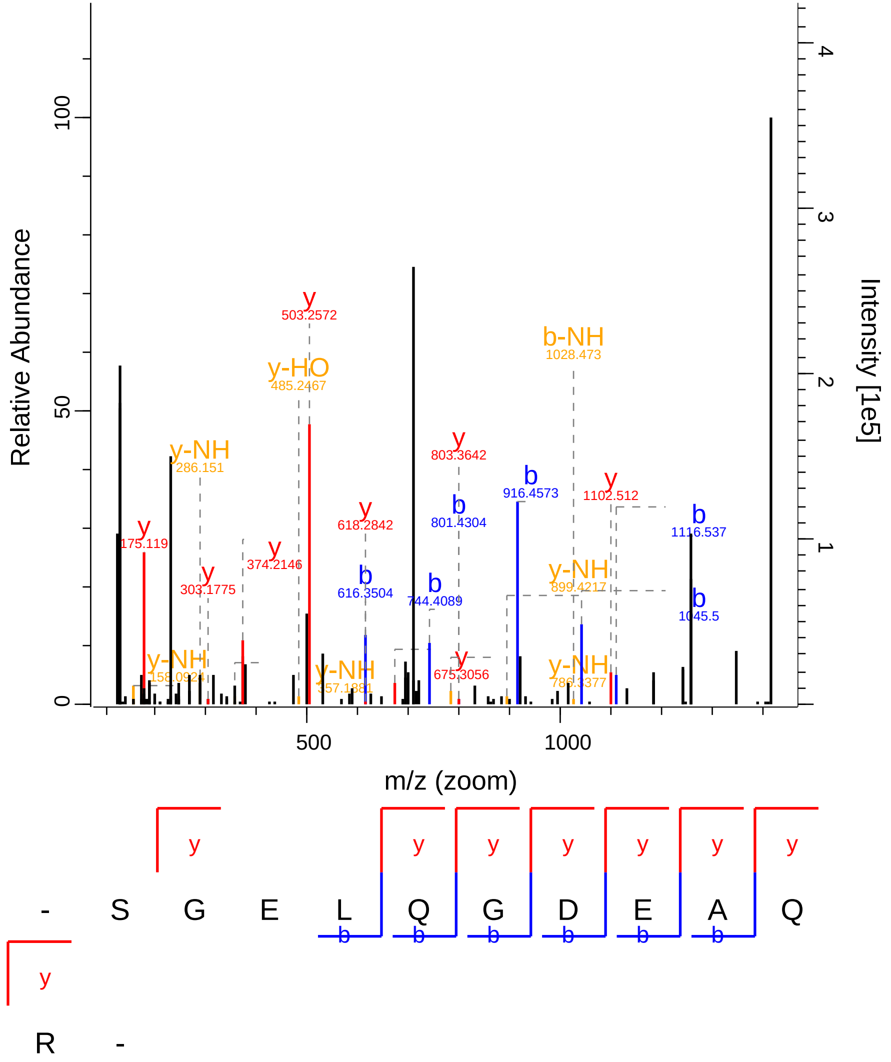

Raw File	Scan	Method	Score	m/z	Gene names
RO-QC-160121_ZackD_SS_Set_01_Fractior	8020	FTMS; HCI	60.68	467.94	SLCO4A1

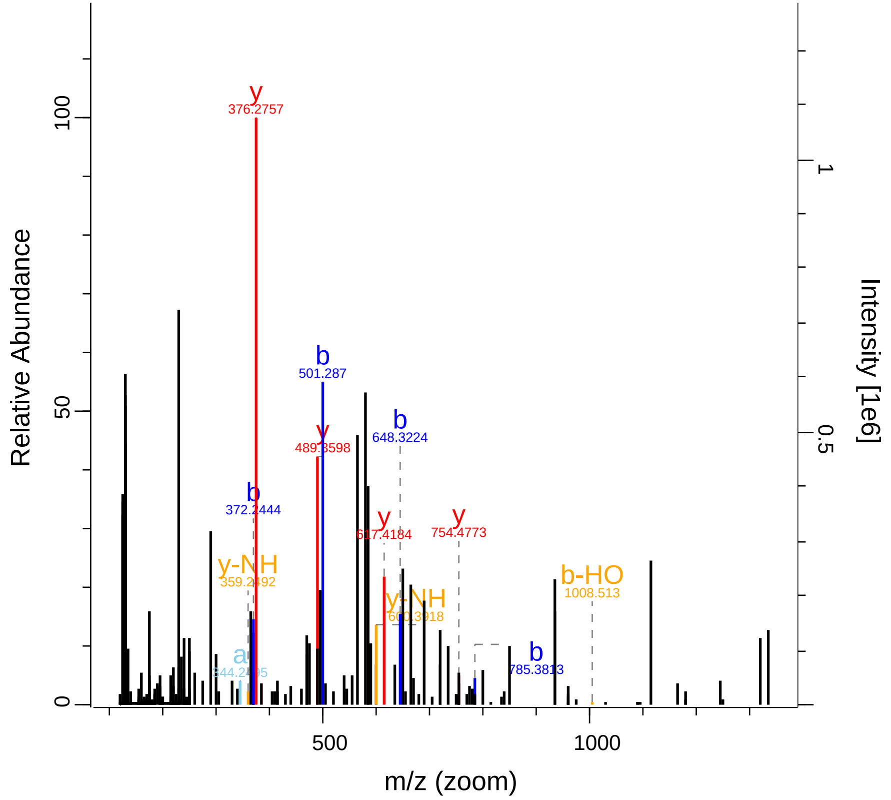

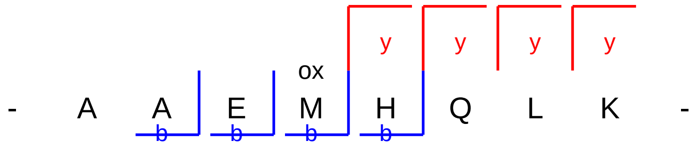

Raw File	Scan	Method	Score	m/z
RO-QC-160121_ZackD_SS_Set_01_Fraction_1	8100	FTMS; HCD	48.76	399.23

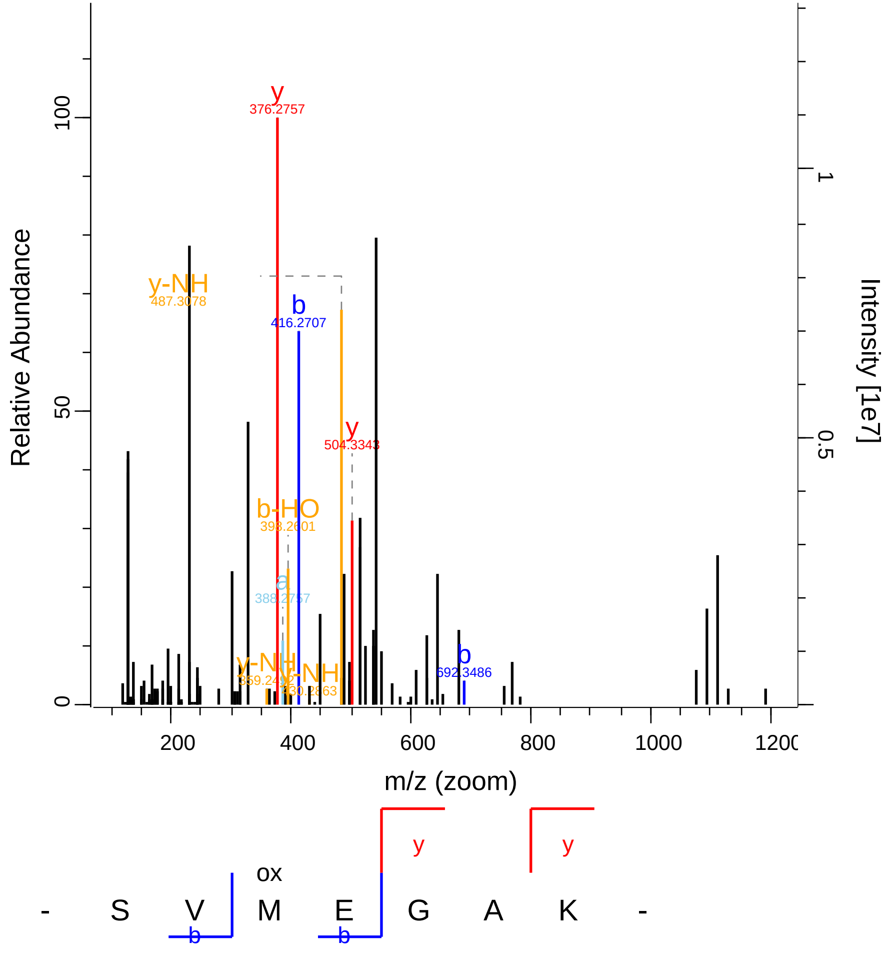

Raw File	Scan	Method	Score	m/z	Gene names
RO-QC-160121_ZackD_SS_Set_01_Fractio[illegible]	8274	FTMS; HC	121.99	717.05	PCGF5

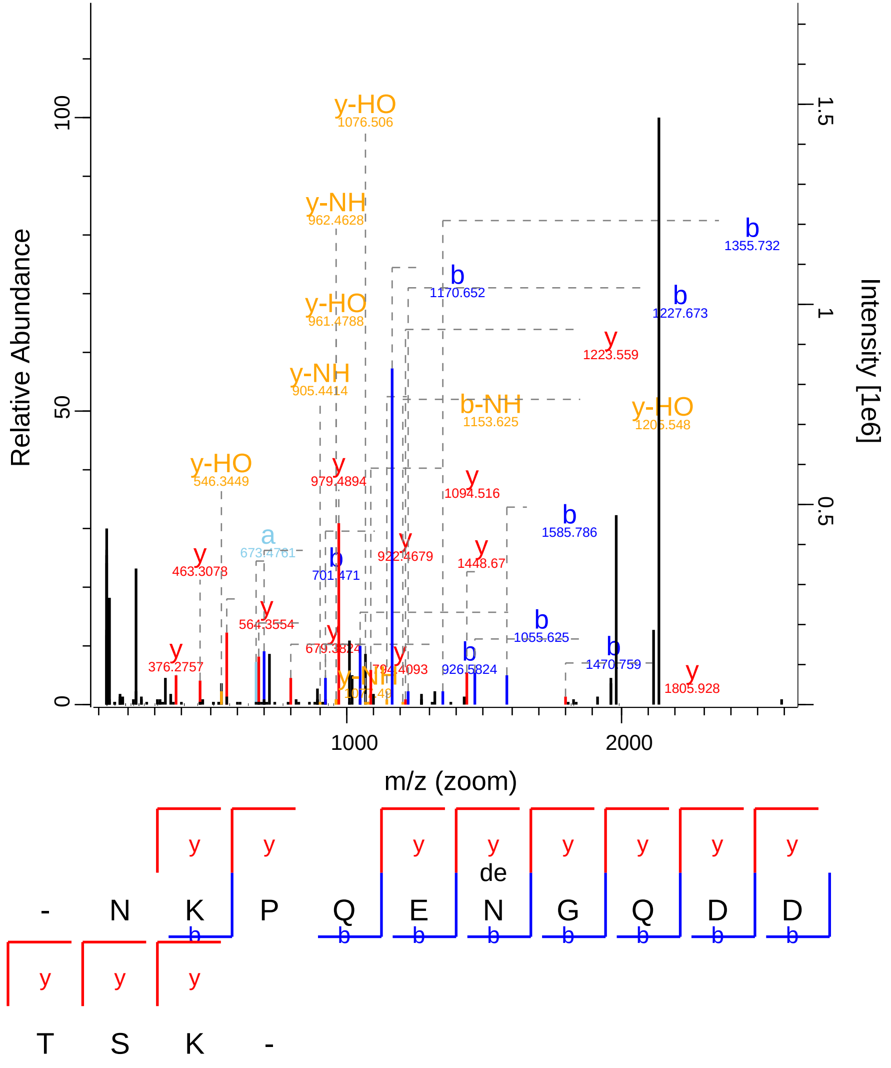

Raw File	Scan	Method	Score	m/z	Gene names
RO-QC-160121_ZackD_SS_Set_01_Fractior	8295	FTMS; HCI	53.97	456.94	TCTN2

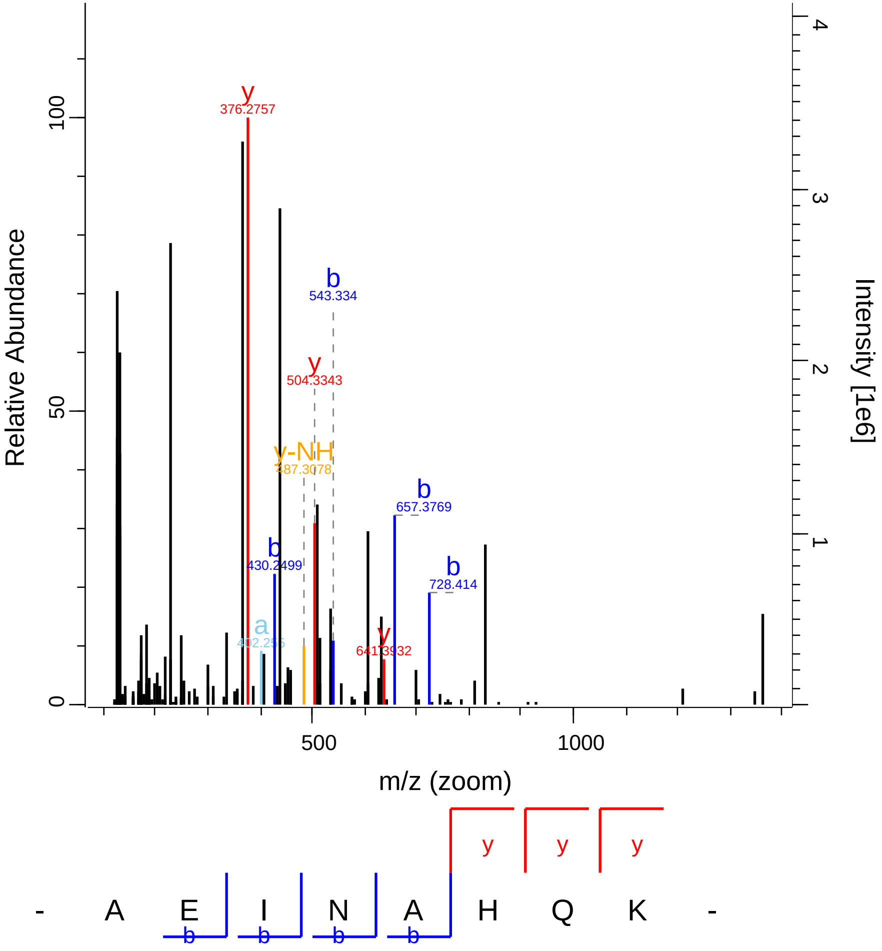

Raw File	Scan	Method	Score	m/z	Gene names
RO-QC-160121_ZackD_SS_Set_01_Fractior	8404	FTMS; HCI	91.58	440.01	EMP2

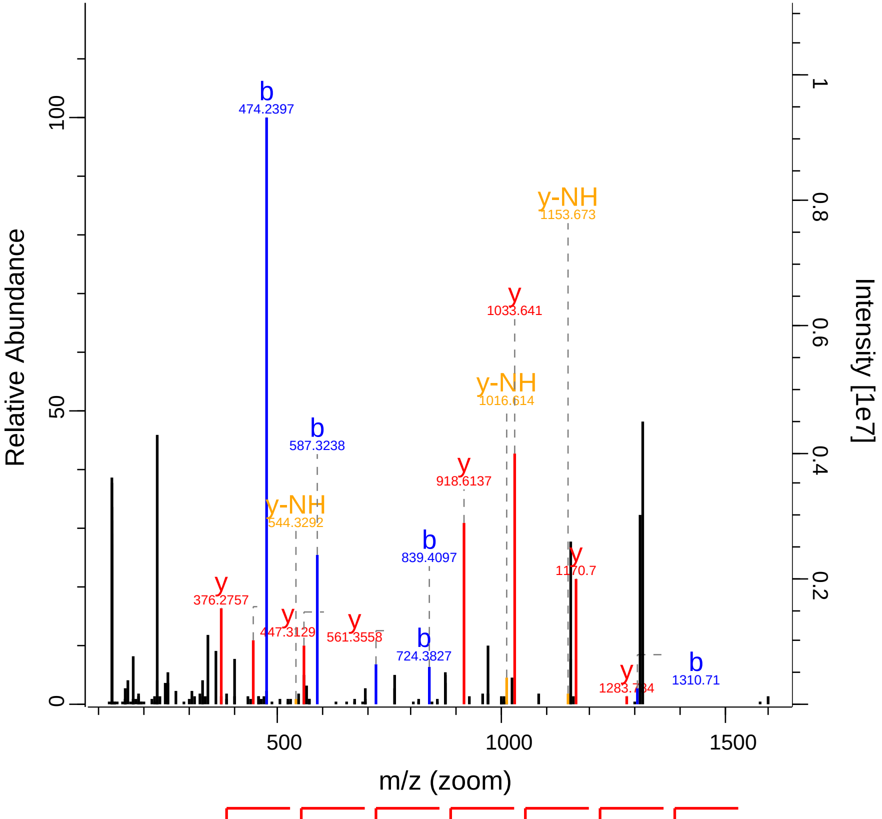

Raw File	Scan	Method	Score	m/z	Gene names
RO-QC-160121_ZackD_SS_Set_01_Fractior	8432	FTMS; HCI	73.3	730.08	CDC20

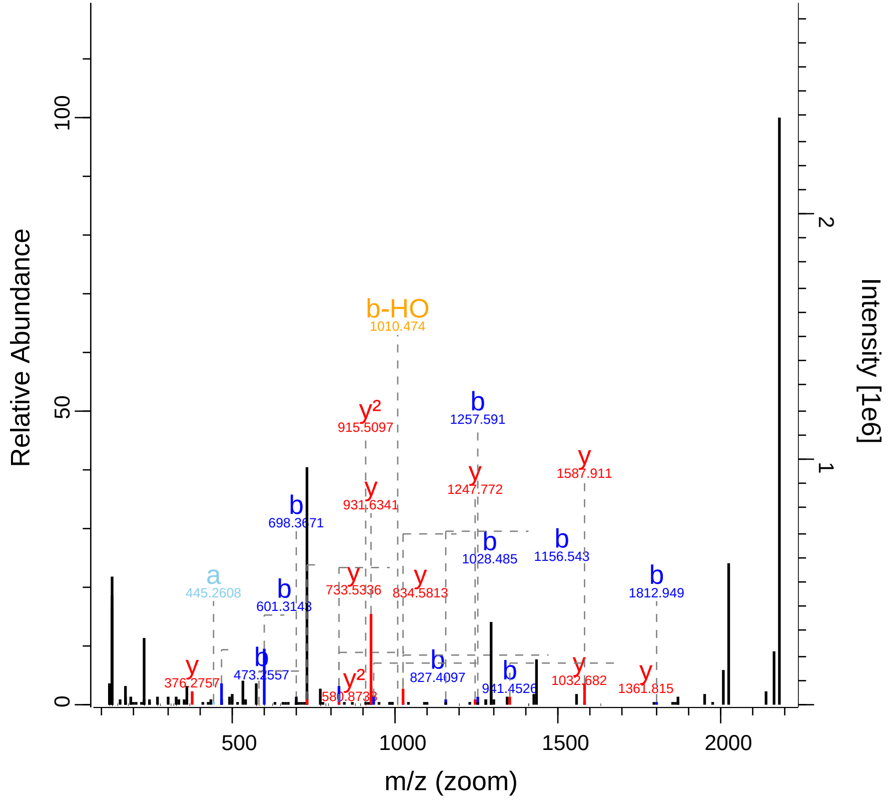

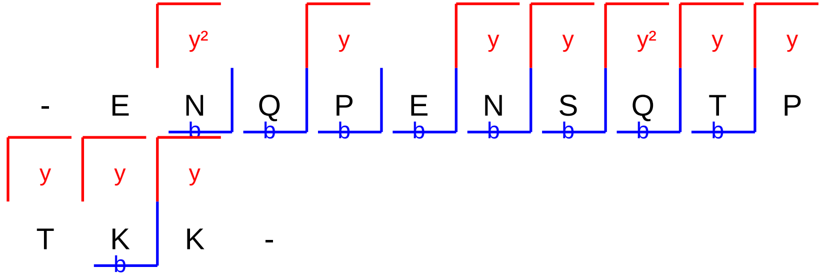

Raw File	Scan	Method	Score	m/z	Gene names
RO-QC-160121_ZackD_SS_Set_01_Fraction	8666	FTMS; HCI	116.12	786.9	SLC46A1

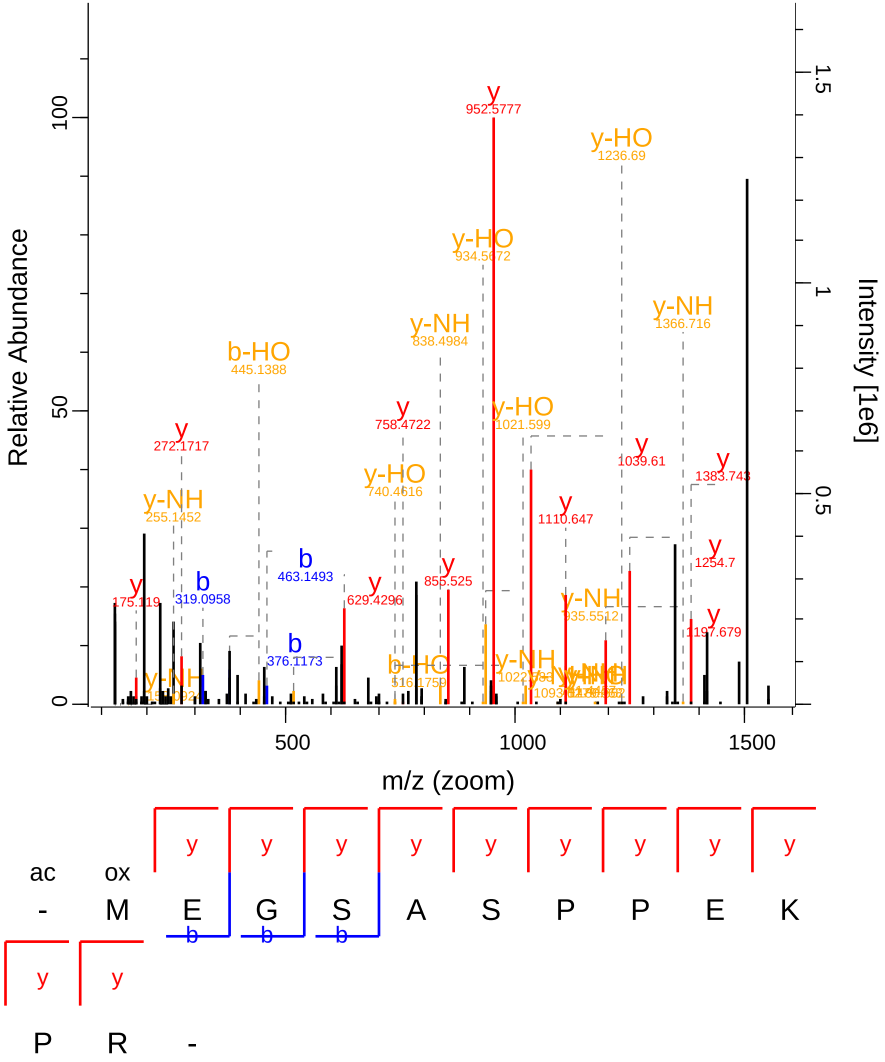

Raw File	Scan	Method	Score	m/z
RO-QC-160121_ZackD_SS_Set_01_Fraction_1	8673	FTMS; HCD	75.74	758.93

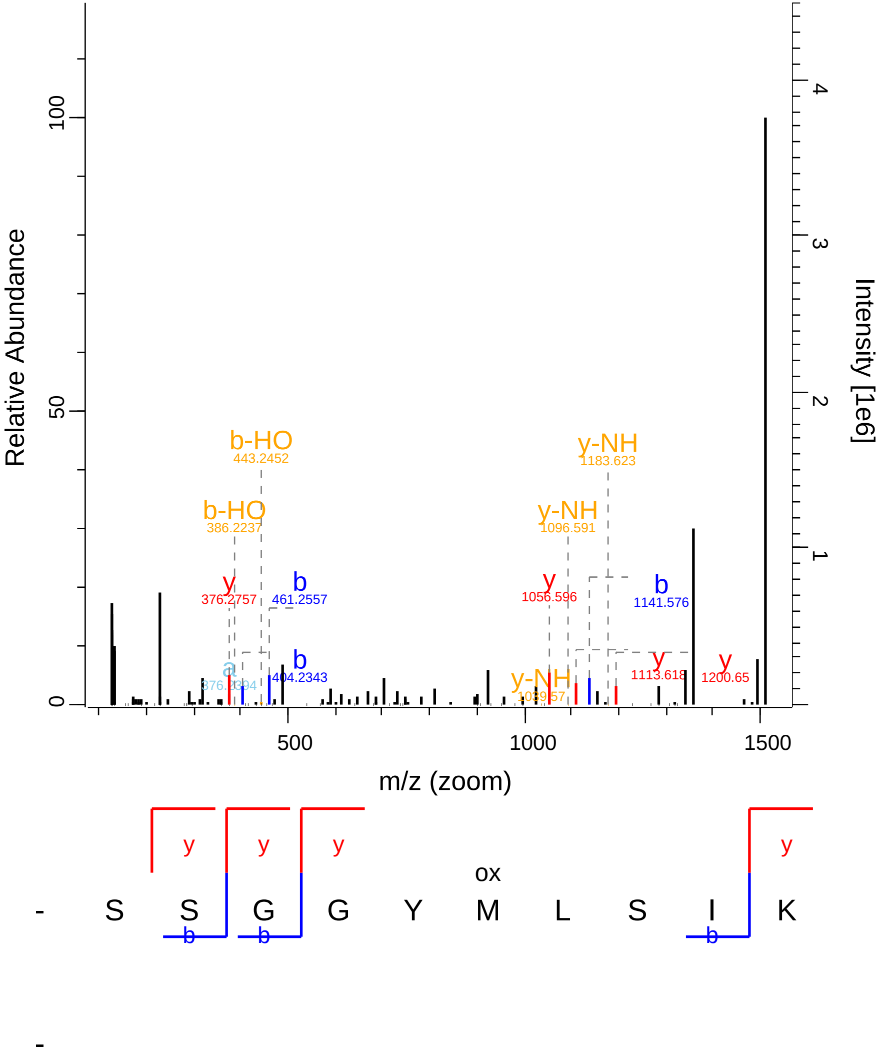

Raw File	Scan	Method	Score	m/z	Gene names
RO-QC-160121_ZackD_SS_Set_01_Fractio[illegible]	8776	FTMS; HC	155.97	866.42	PDGFA

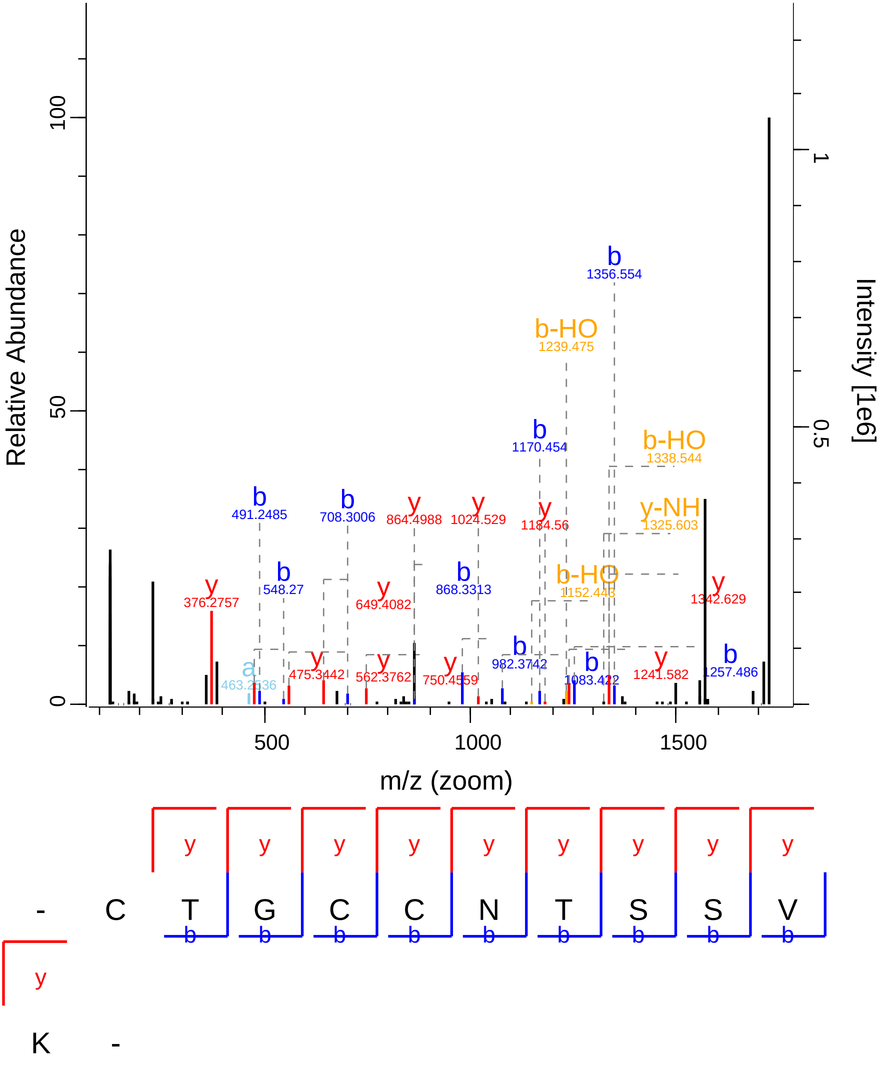

Raw File	Scan	Method	Score	m/z	Gene names
RO-QC-160121_ZackD_SS_Set_01_Fraction	8868	FTMS; HCI	121.73	786.9	SLC46A1

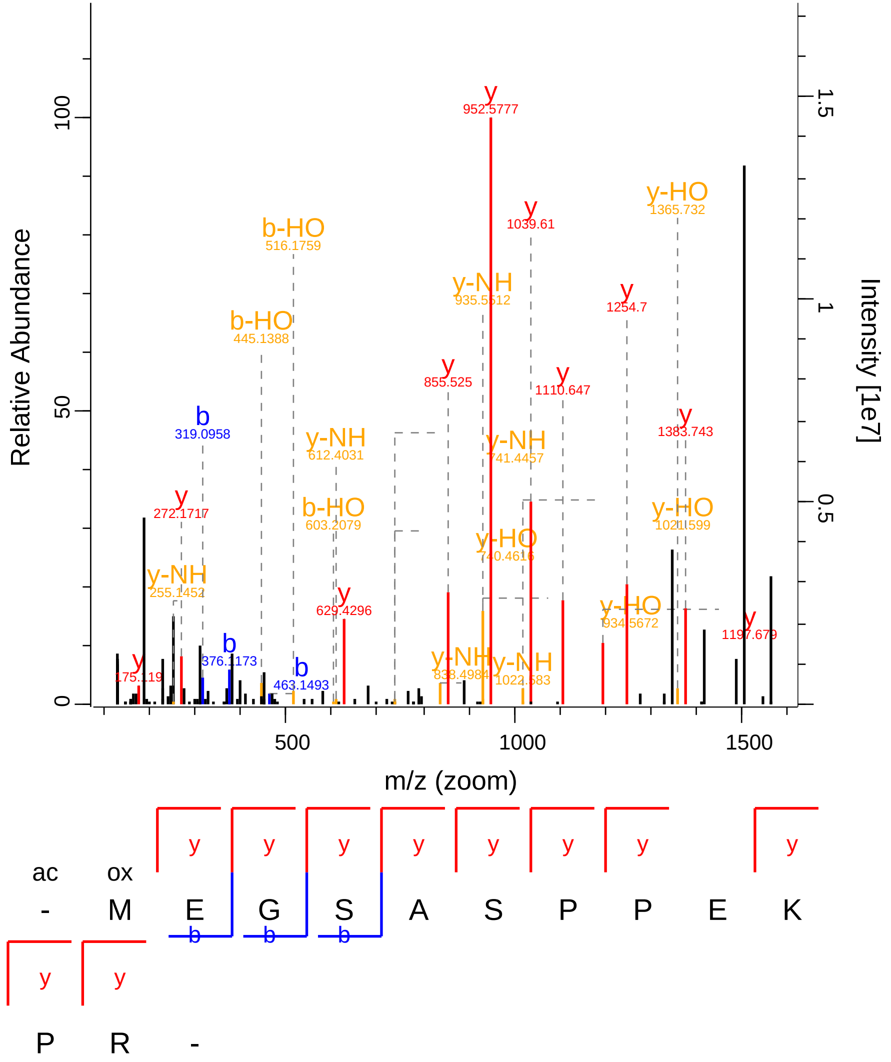

Raw File	Scan	Method	Score	m/z	Gene names
RO-QC-160121_ZackD_SS_Set_01_Fractio	9768	FTMS; HC	109.92	605.95	CHCHD5

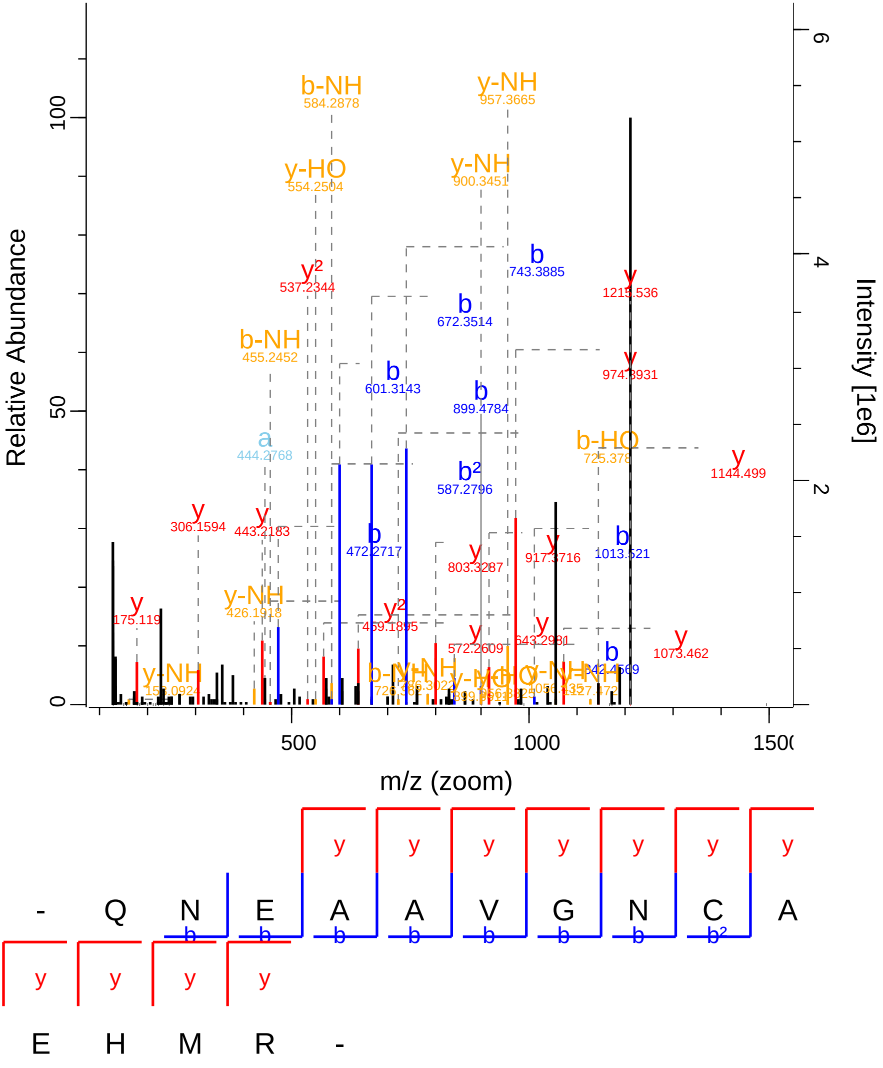

Raw File	Scan	Method	Score	m/z	Gene names
RO-QC-160121_ZackD_SS_Set_01_Fractioı	9840	FTMS; HC	127.76	378.21	GGT7

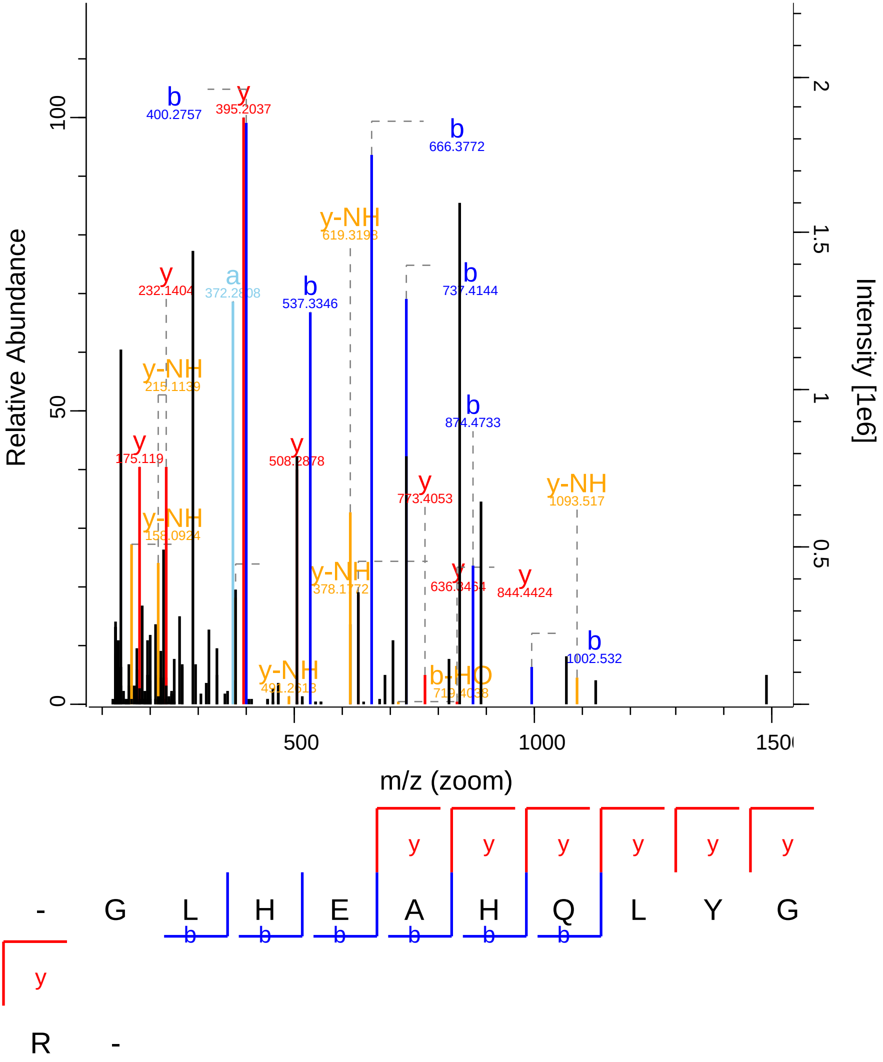

Raw File	Scan	Method	Score	m/z	Gene names
RO-QC-160121_ZackD_SS_Set_01_Fractioı	9945	FTMS; HC	120.46	577.29	ST6GALNAC3

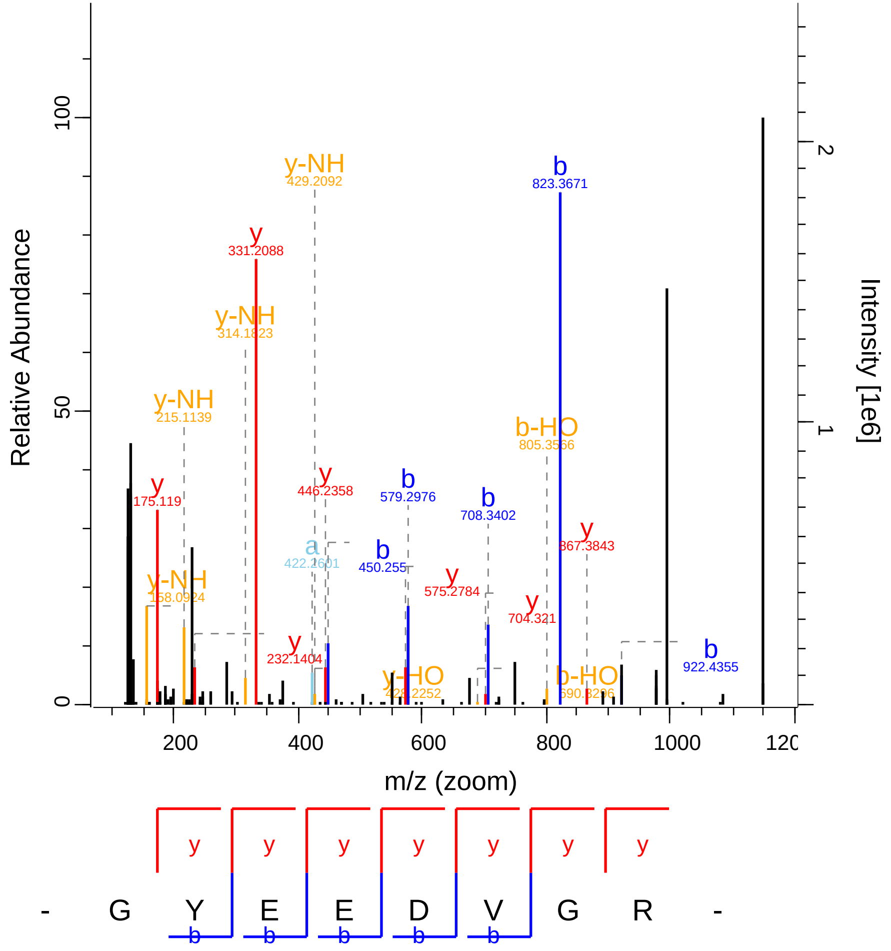

Raw File	Scan	Method	Score	m/z	Gene names
RO-QC-160121_ZackD_SS_Set_01_Fractio	10460	FTMS; HC	108.67	620.95	PLA2G2A

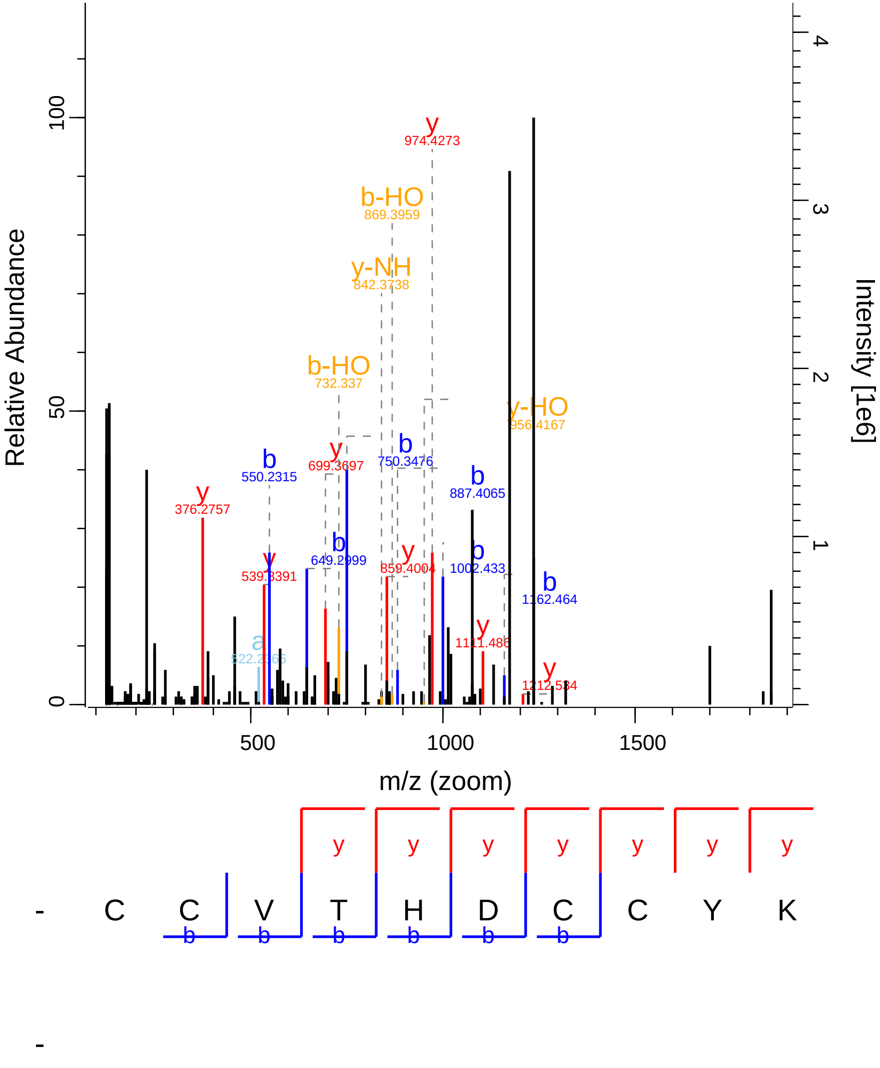

Raw File	Scan	Method	Score	m/z
RO-QC-160121_ZackD_SS_Set_01_Fraction_1	10924	FTMS; HCD	88.19	825.96

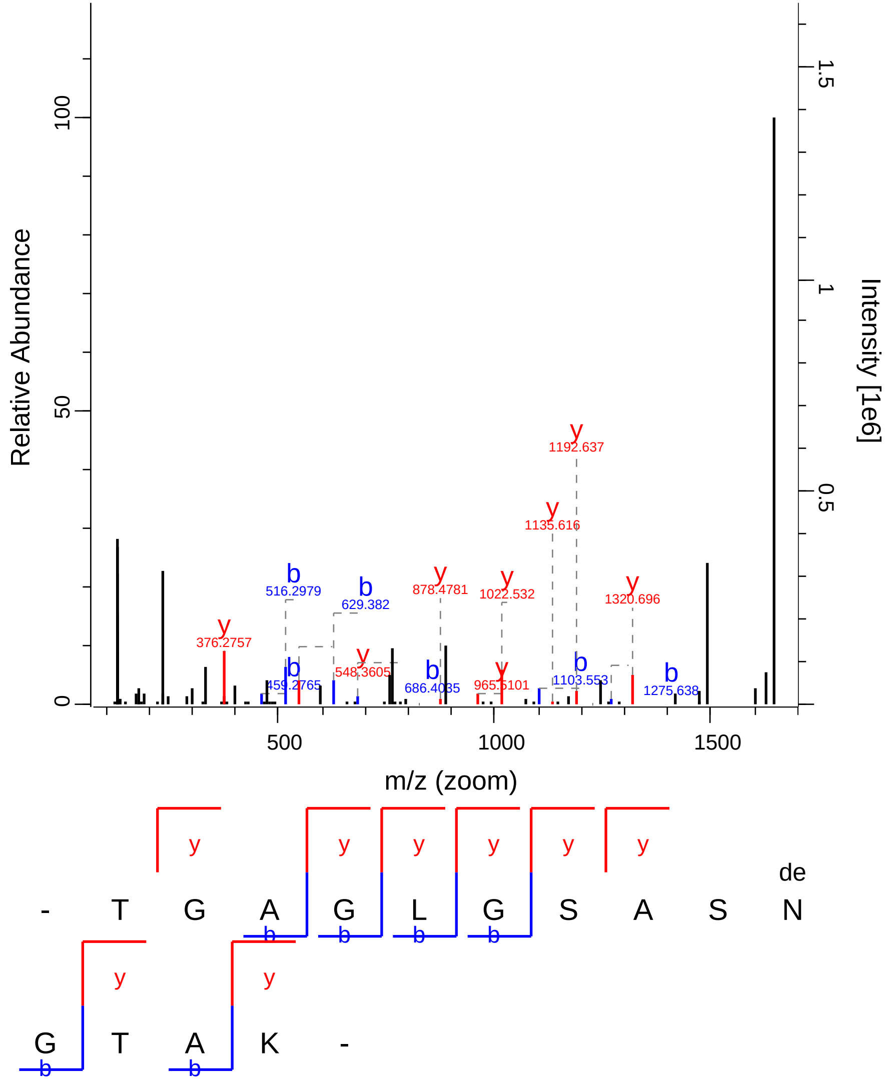

Raw File	Scan	Method	Score	m/z	Gene names
RO-QC-160121_ZackD_SS_Set_01_Fractio	11063	FTMS; HC	120.12	666.9	FAM151B

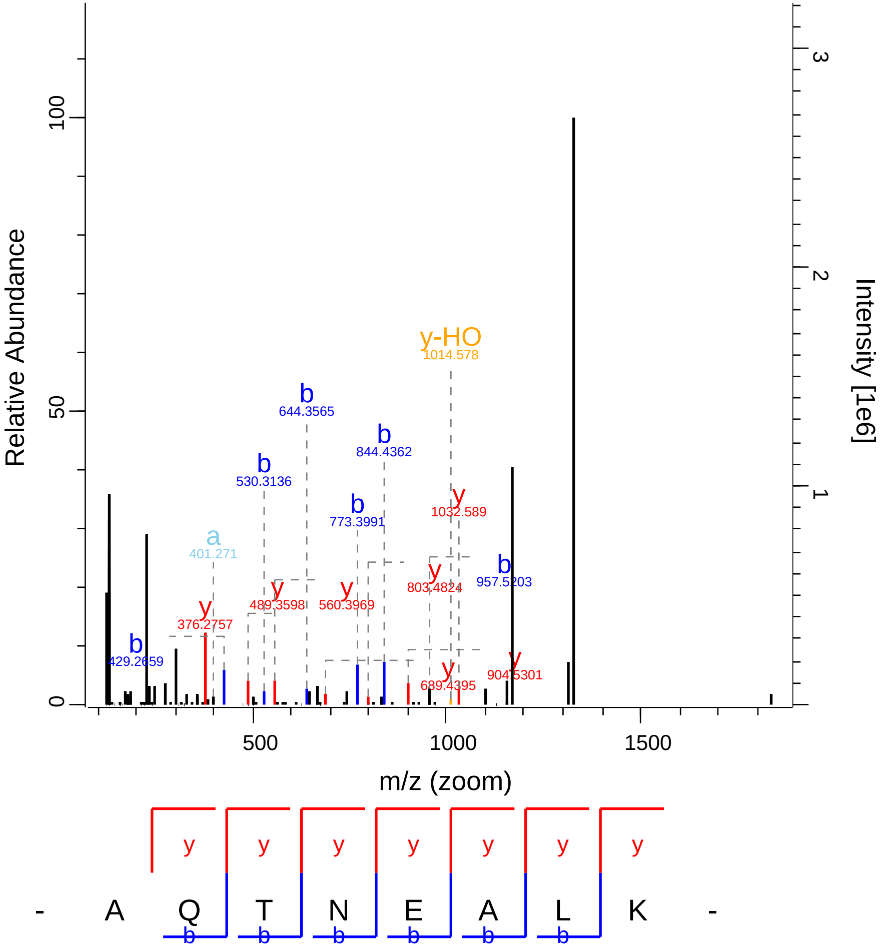

Raw File	Scan	Method	Score	m/z	Gene names
RO-QC-160121_ZackD_SS_Set_01_Fractio	11314	FTMS; HC	50.28	906.92	CC2D2B

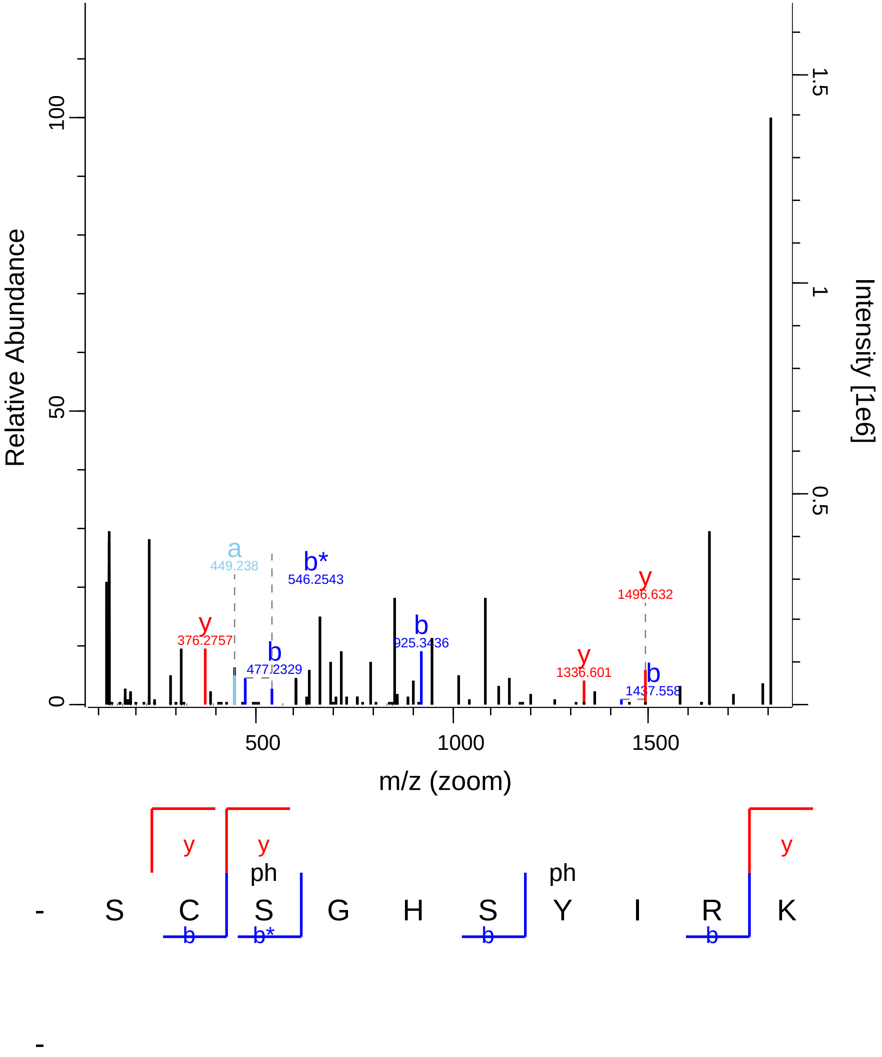

Raw File	Scan	Method	Score	m/z	Gene names
RO-QC-160121_ZackD_SS_Set_01_Fractio	11393	FTMS; HC	47.26	996.96	ANO3

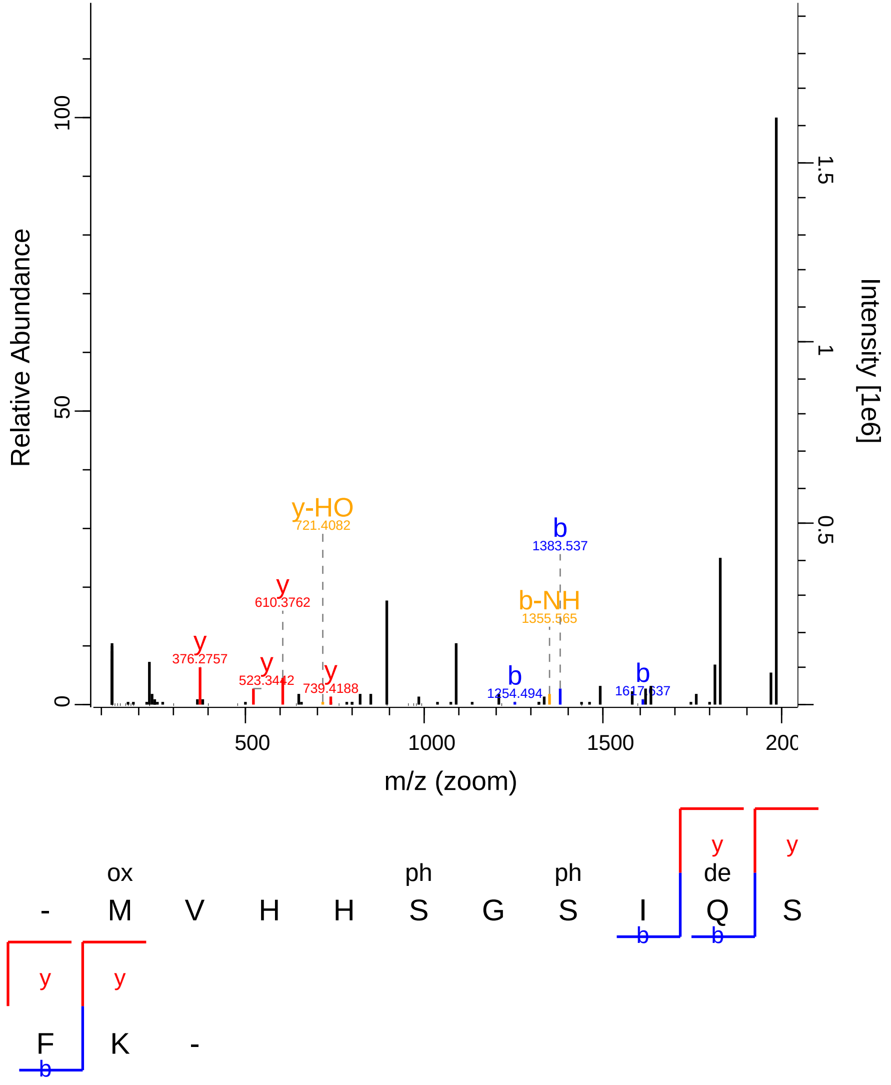

Raw File	Scan	Method	Score	m/z	Gene names
RO-QC-160121_ZackD_SS_Set_01_	1190	FTMS; H	88.95	378.7	GOLGA6L1;GOLGA6L6;GOL

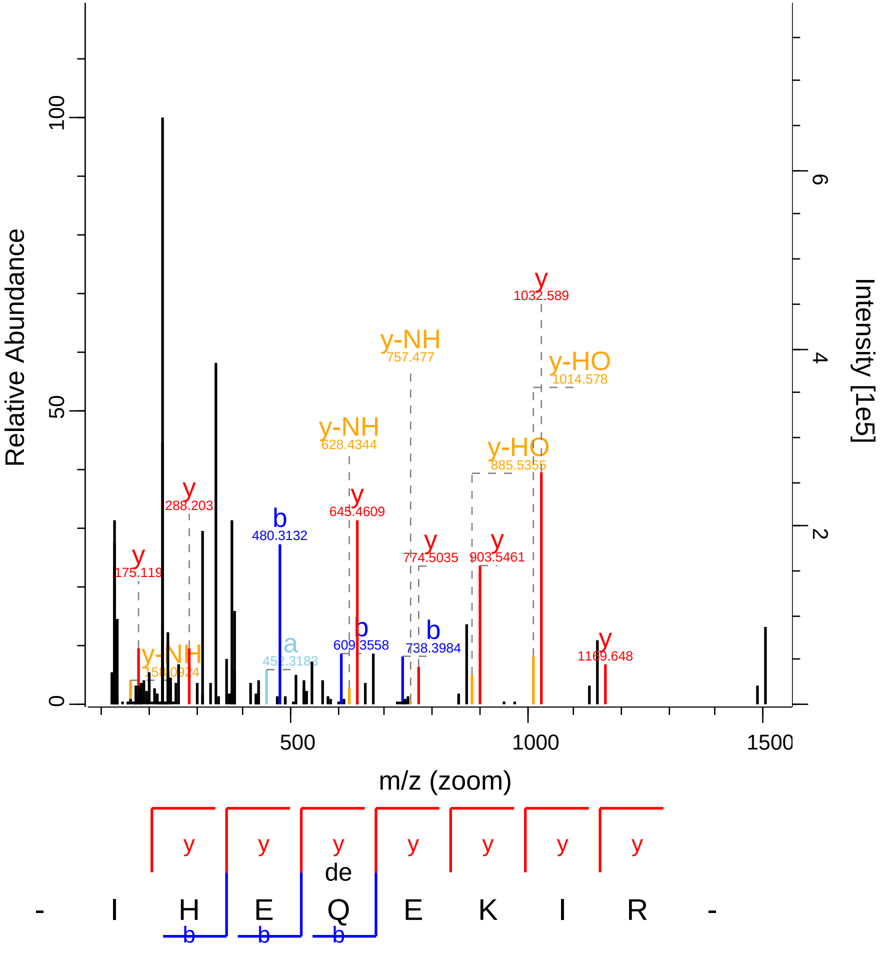

Raw File	Scan	Method	Score	m/z	Gene names
RO-QC-160121_ZackD_SS_Set_01_Fractio	12269	FTMS; HC	175.73	734.92	AHR

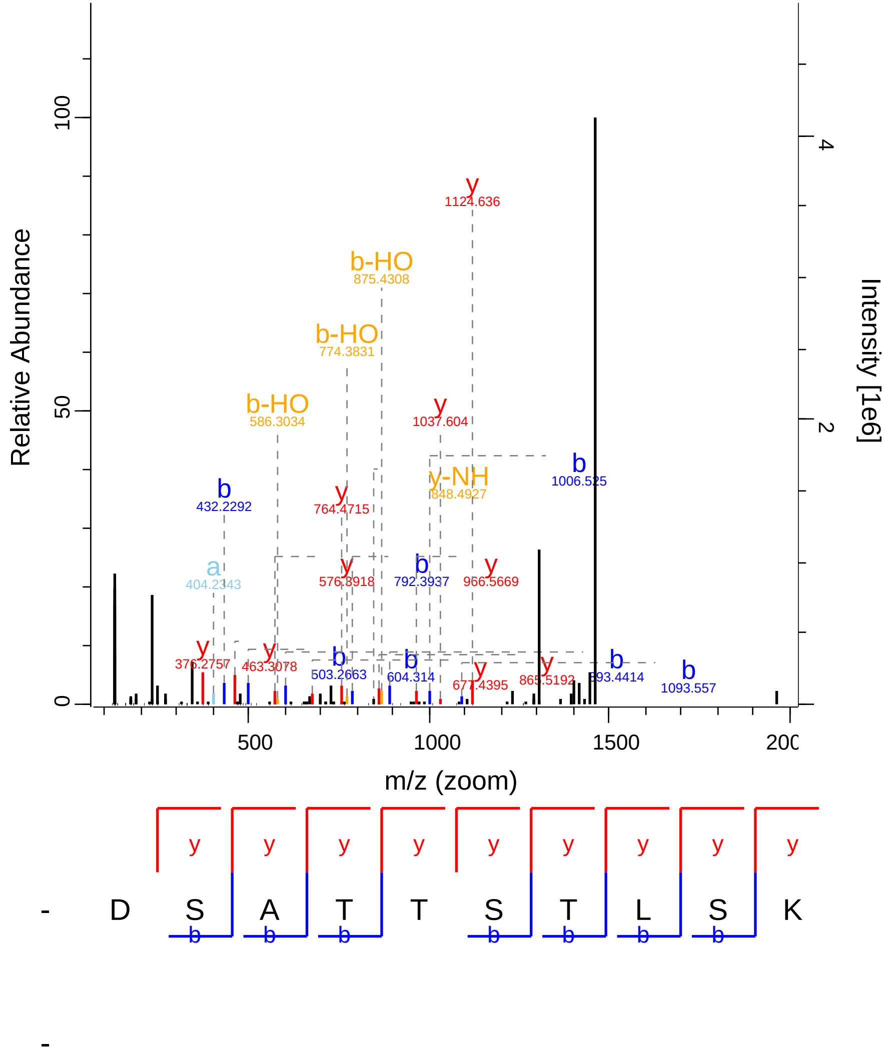

Raw File	Scan	Method	Score	m/z	Gene names
RO-QC-160121_ZackD_SS_Set_01_Fra	12407	FTMS; HC	113.7	596.35	ATP5G1;ATP5G2;ATP

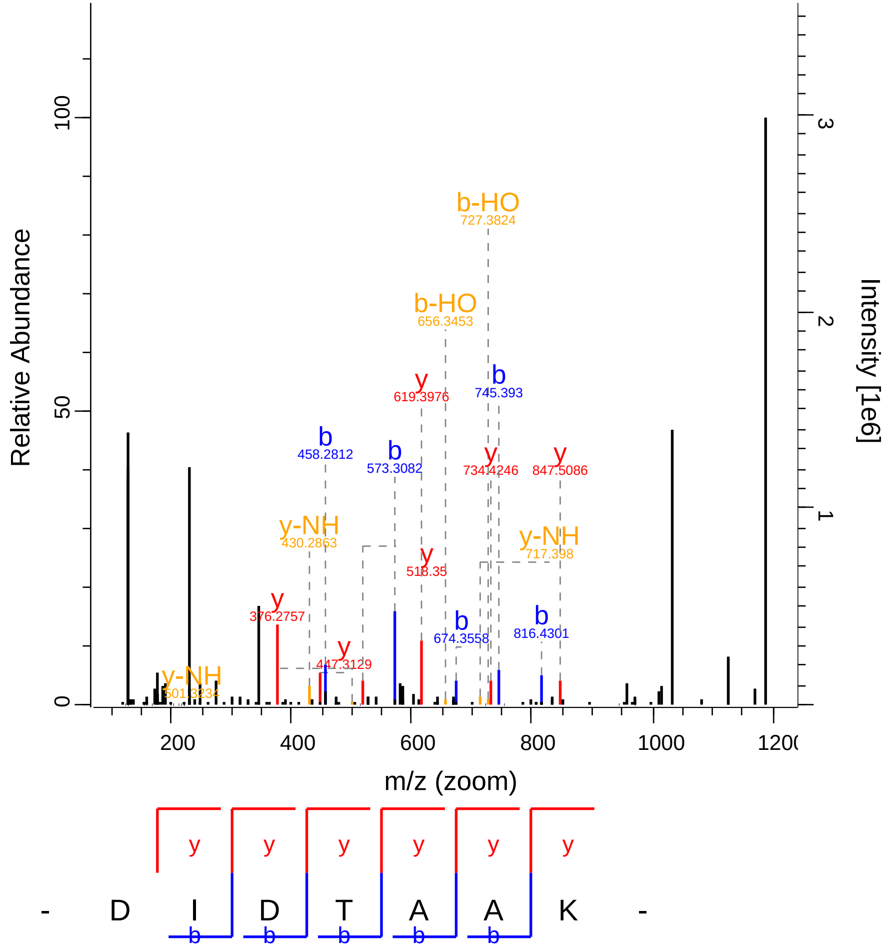

Raw File	Scan	Method	Score	m/z	Gene names
RO-QC-160121_ZackD_SS_Set_01_Fractio	12706	FTMS; HC	167.12	786.44	DGKZ

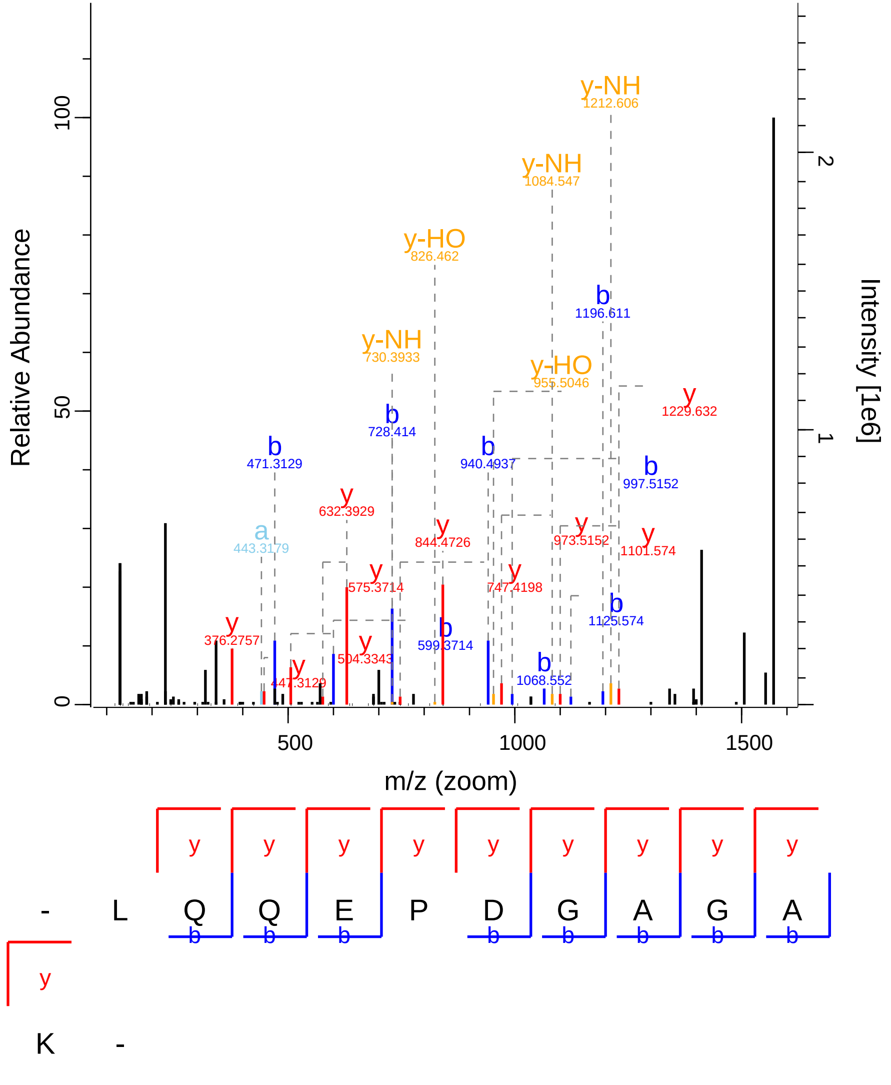

Raw File	Scan	Method	Score	m/z
RO-QC-160121_ZackD_SS_Set_01_Fraction_1	12825	FTMS; HCD	98.63	687.9

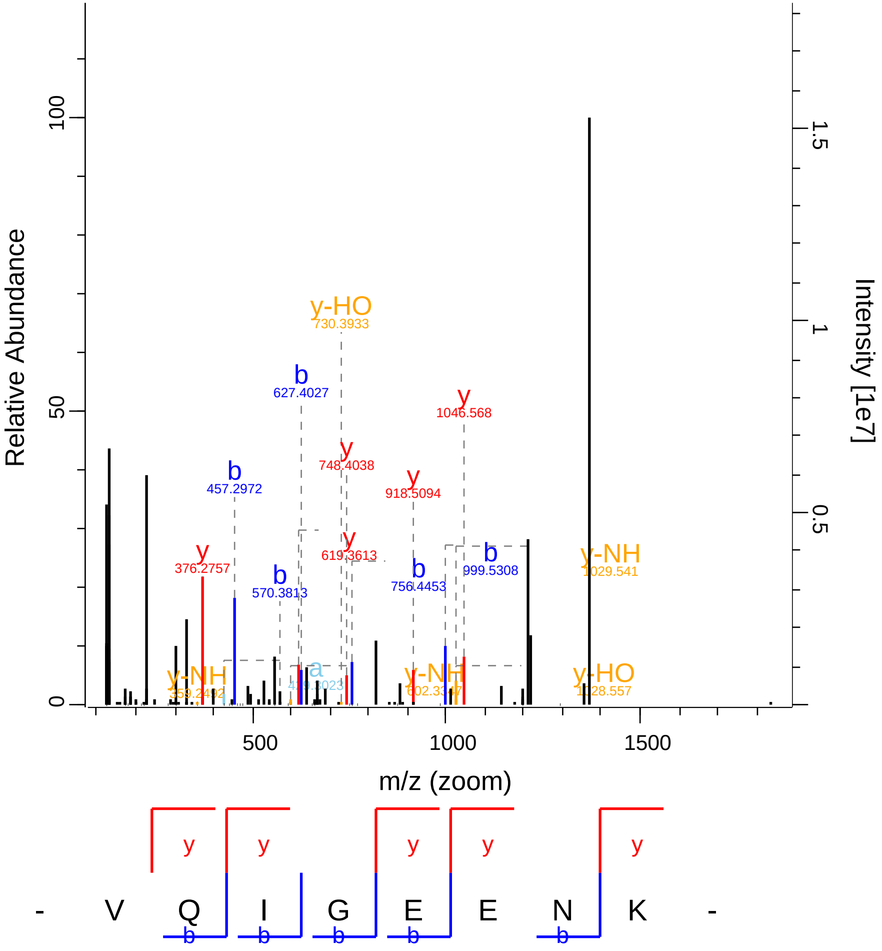

Raw File	Scan	Method	Score	m/z
RO-QC-160121_ZackD_SS_Set_01_Fraction_1	12828	FTMS; HCD	57.05	458.94

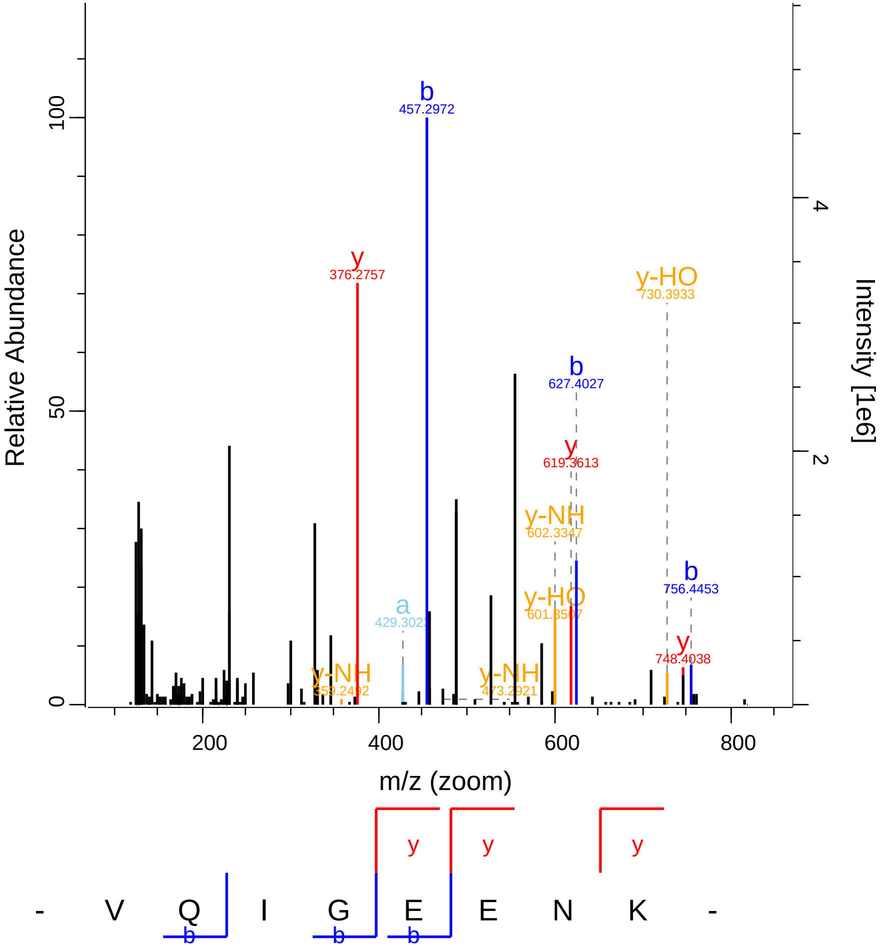

Raw File	Scan	Method	Score	m/z	Gene names
RO-QC-160121_ZackD_SS_Set_01_Fractic	12850	FTMS; HC	93.56	403.22	KCTD15;KCTD1

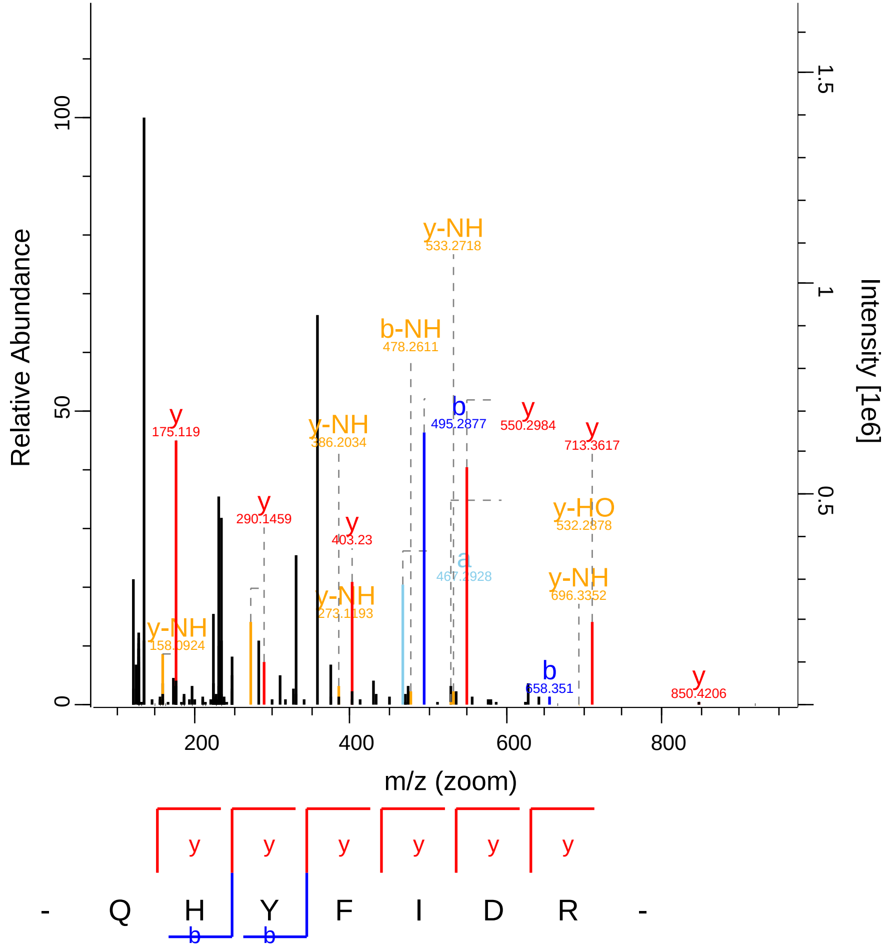

Raw File	Scan	Method	Score	m/z
RO-QC-160121_ZackD_SS_Set_01_Fraction_1	13045	FTMS; HCD	106.36	687.9

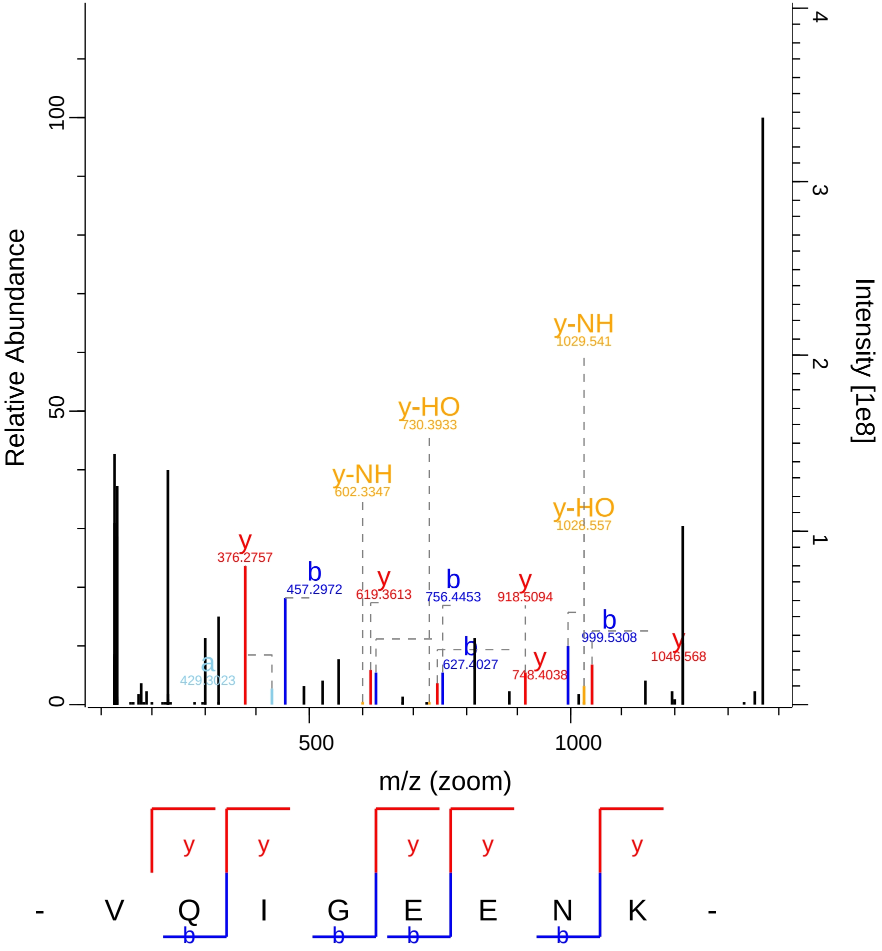

Raw File	Scan	Method	Score	m/z
RO-QC-160121_ZackD_SS_Set_01_Fraction_1	13046	FTMS; HCD	64.26	458.94

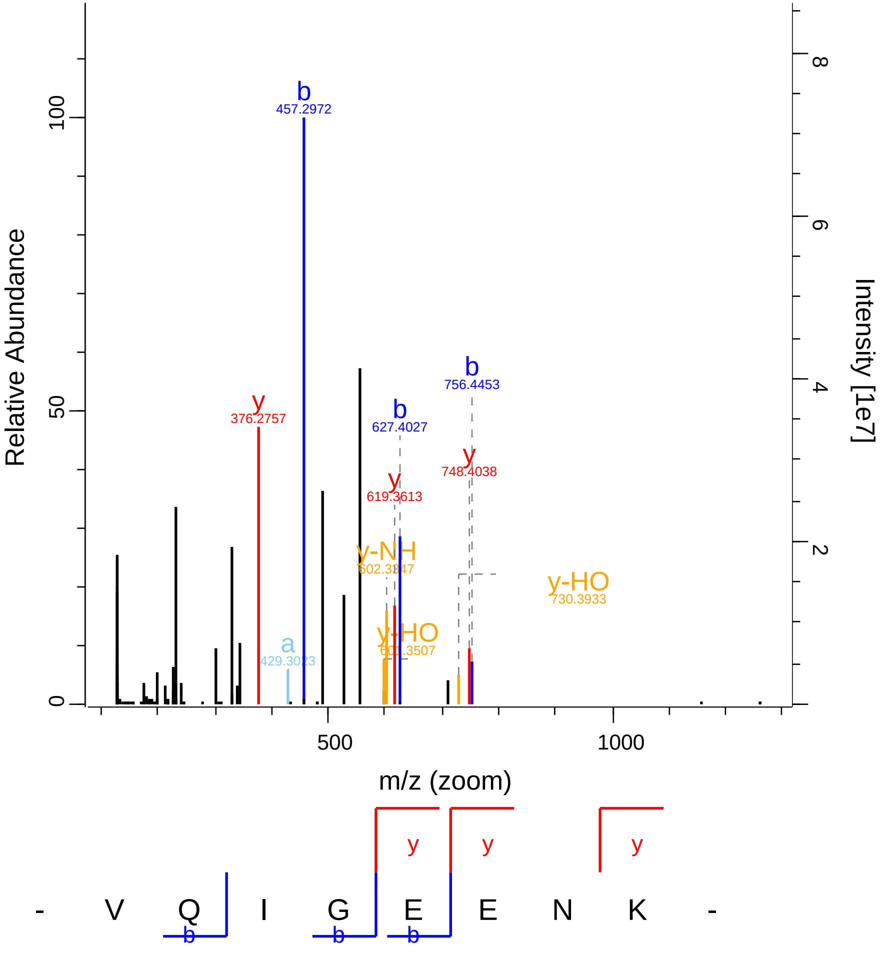

Raw File	Scan	Method	Score	m/z
RO-QC-160121_ZackD_SS_Set_01_Fraction_1	13493	FTMS; HCD	146.01	480.79

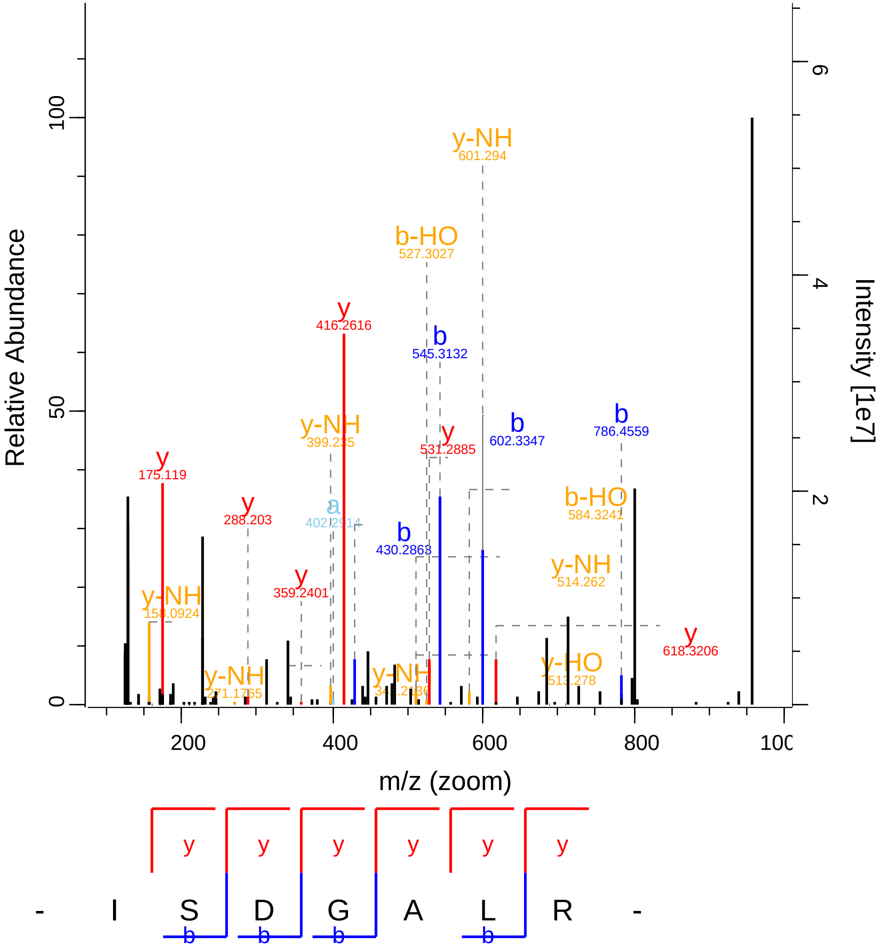

Raw File	Scan	Method	Score	m/z
RO-QC-160121_ZackD_SS_Set_01_Fraction_1	13701	FTMS; HCD	98.5	480.79

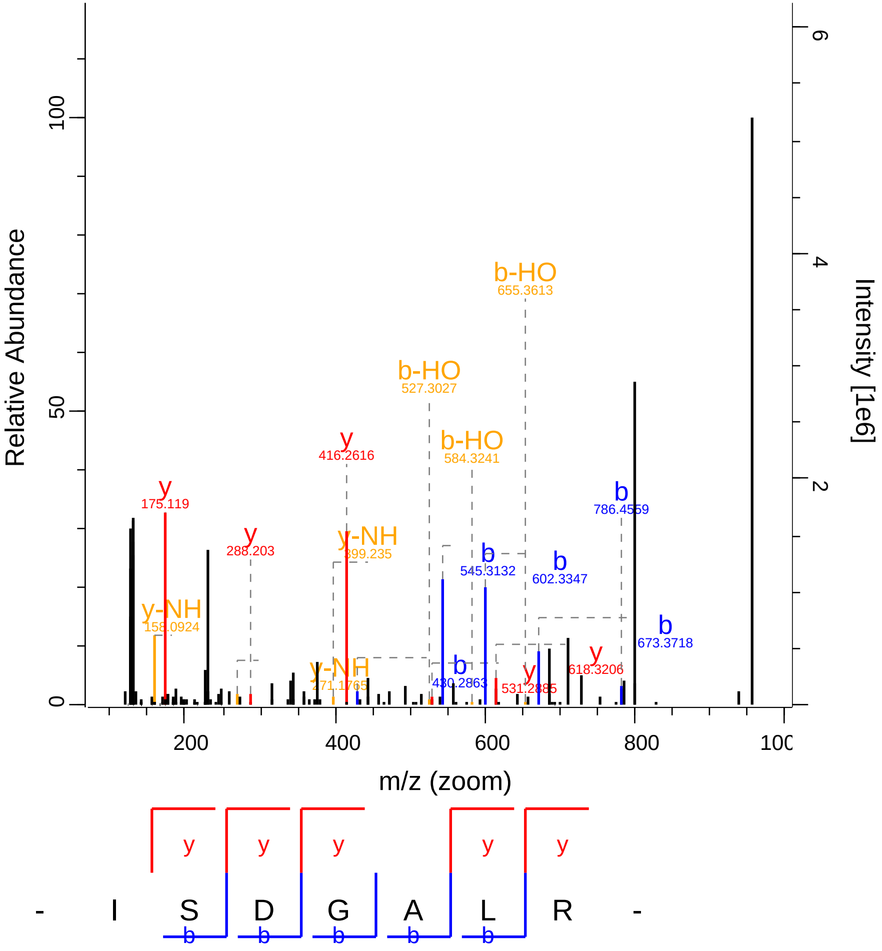

Raw File	Scan	Method	Score	m/z	Gene names
RO-QC-160121_ZackD_SS_Set_01_Fractior	14561	FTMS; HCI	107.21	799.4	KLHDC3

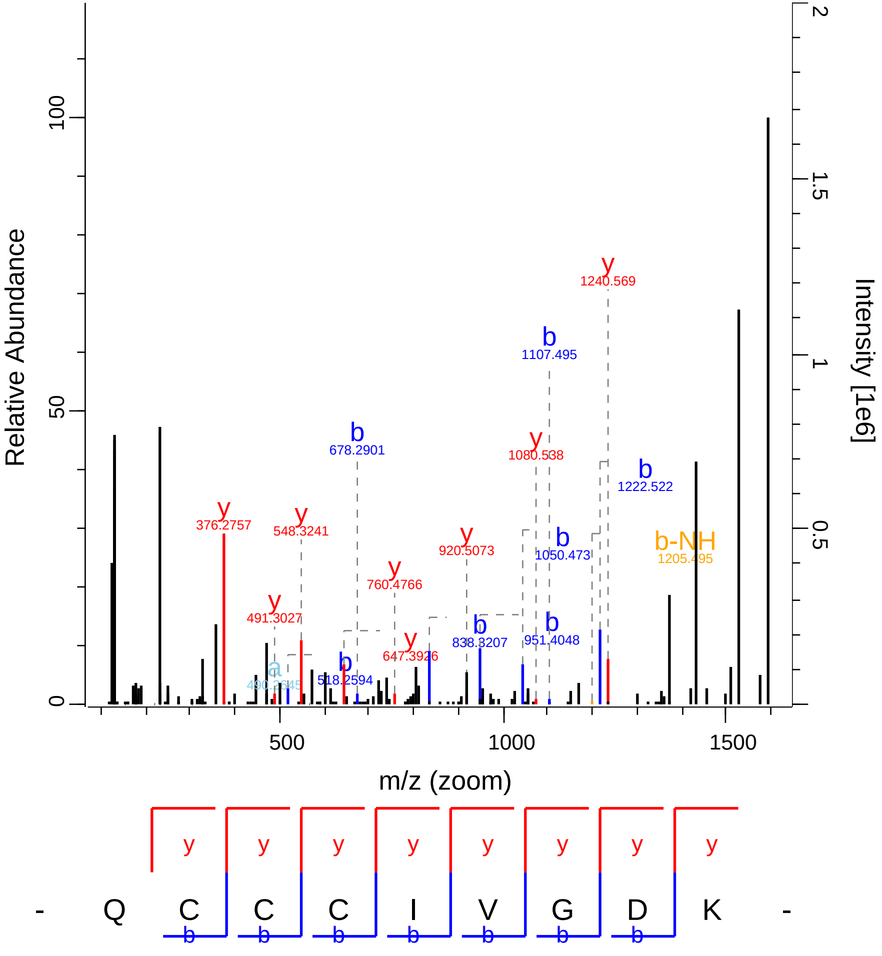

Raw File	Scan	Method	Score	m/z
RO-QC-160121_ZackD_SS_Set_01_Fraction_1	14795	FTMS; HCD	78.15	552.83

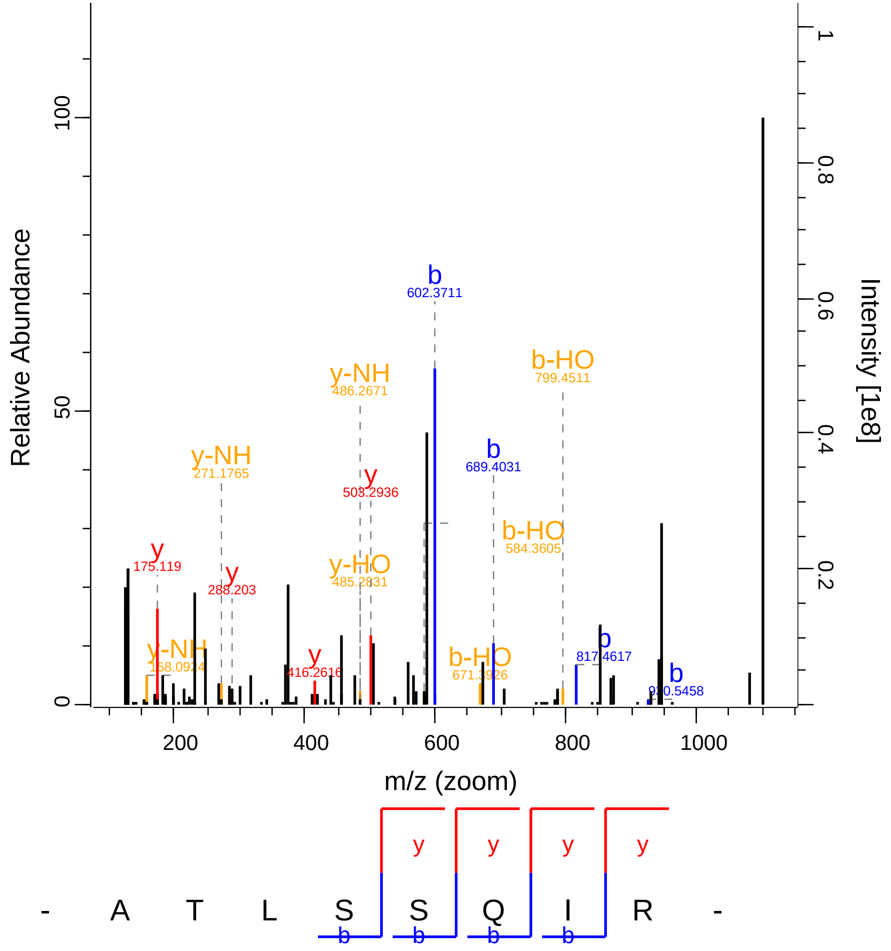

Raw File	Scan	Method	Score	m/z	Gene names
RO-QC-160121_ZackD_SS_Set_01_Fractic	14894	FTMS; HC	138.25	1018.58	SP2

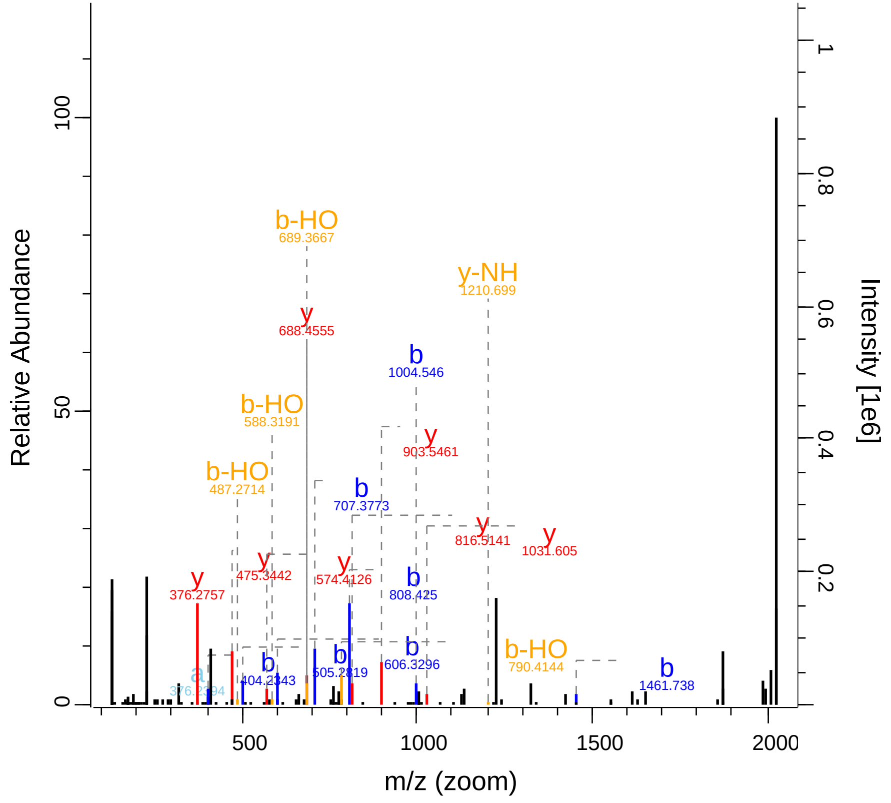

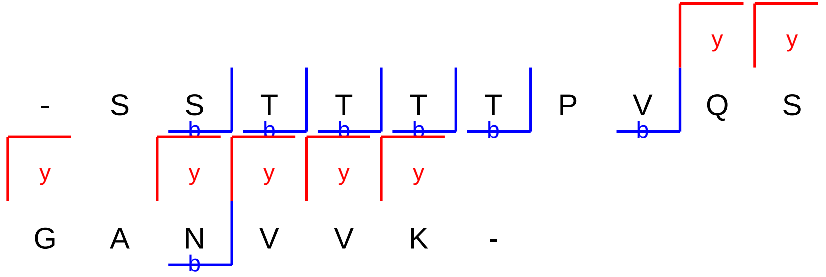

Raw File	Scan	Method	Score	m/z
RO-QC-160121_ZackD_SS_Set_01_Fraction_1	14899	FTMS; HCD	44.3	584.99

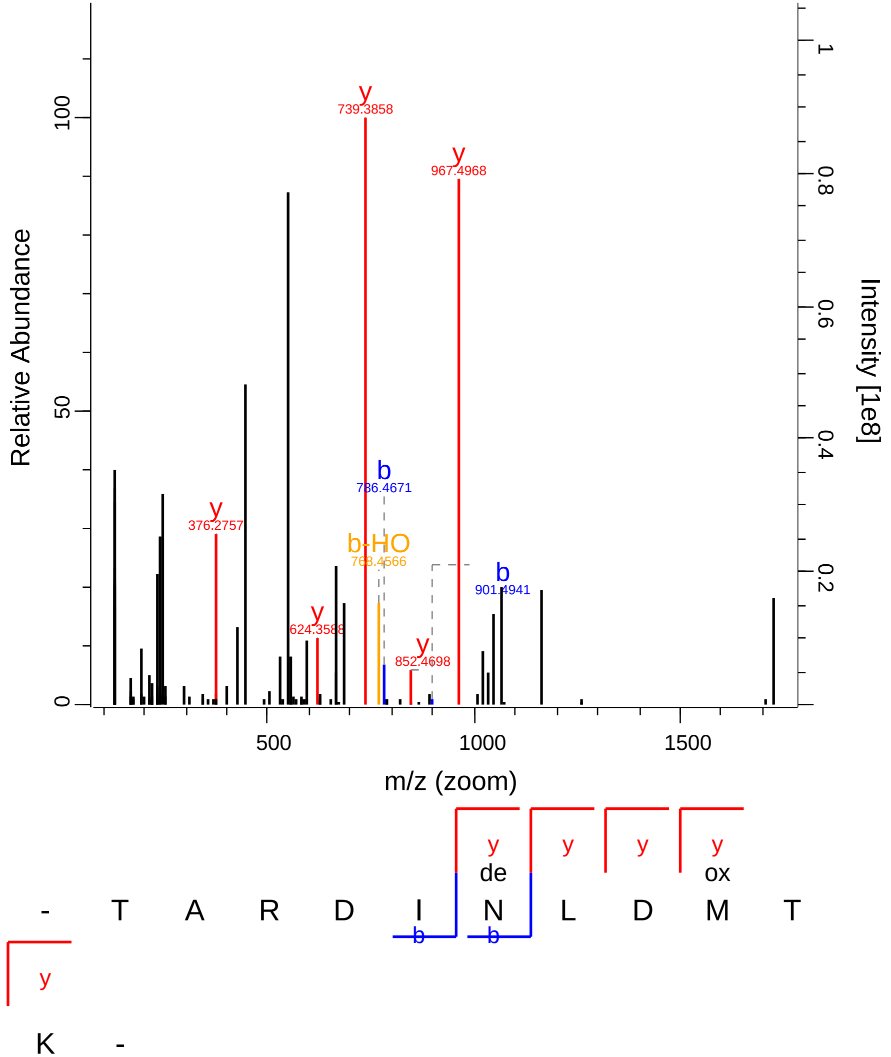

Raw File	Scan	Method	Score	m/z	Gene names
RO-QC-160121_ZackD_SS_Set_01_Fractio	15233	FTMS; HC	109.79	739.93	APITD1

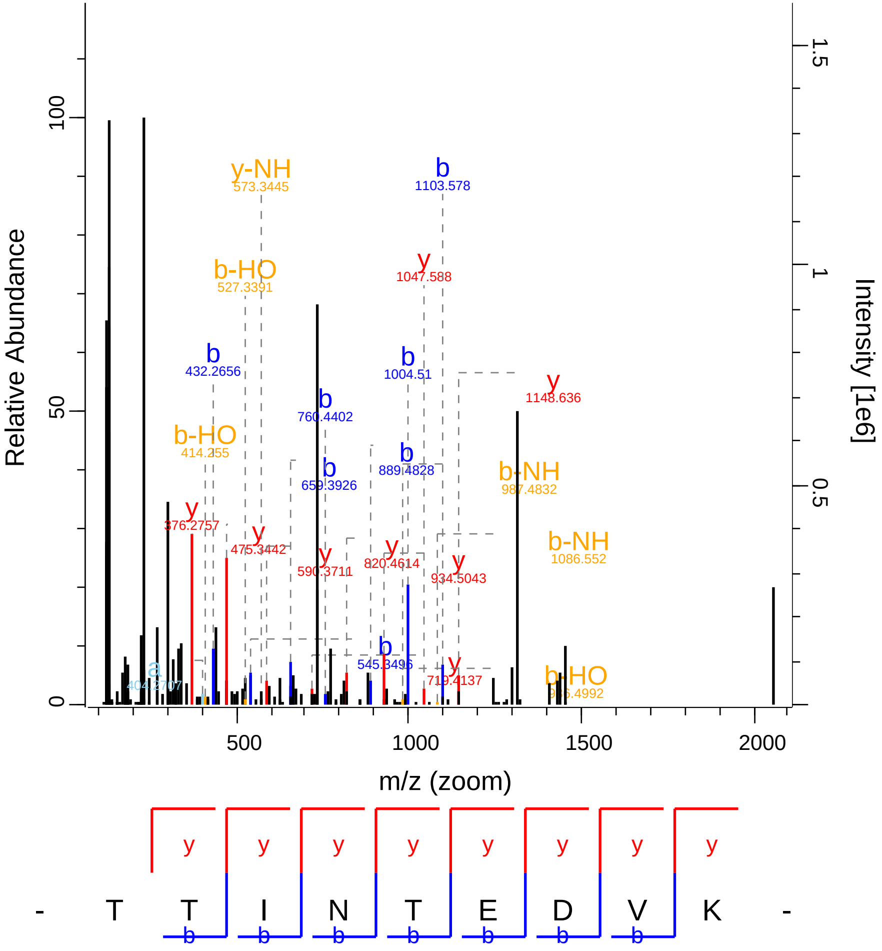

Raw File	Scan	Method	Score	m/z	Gene names
RO-QC-160121_ZackD_SS_Set_01_Fractio	17148	FTMS; HC	88.26	745.66	BSCL2

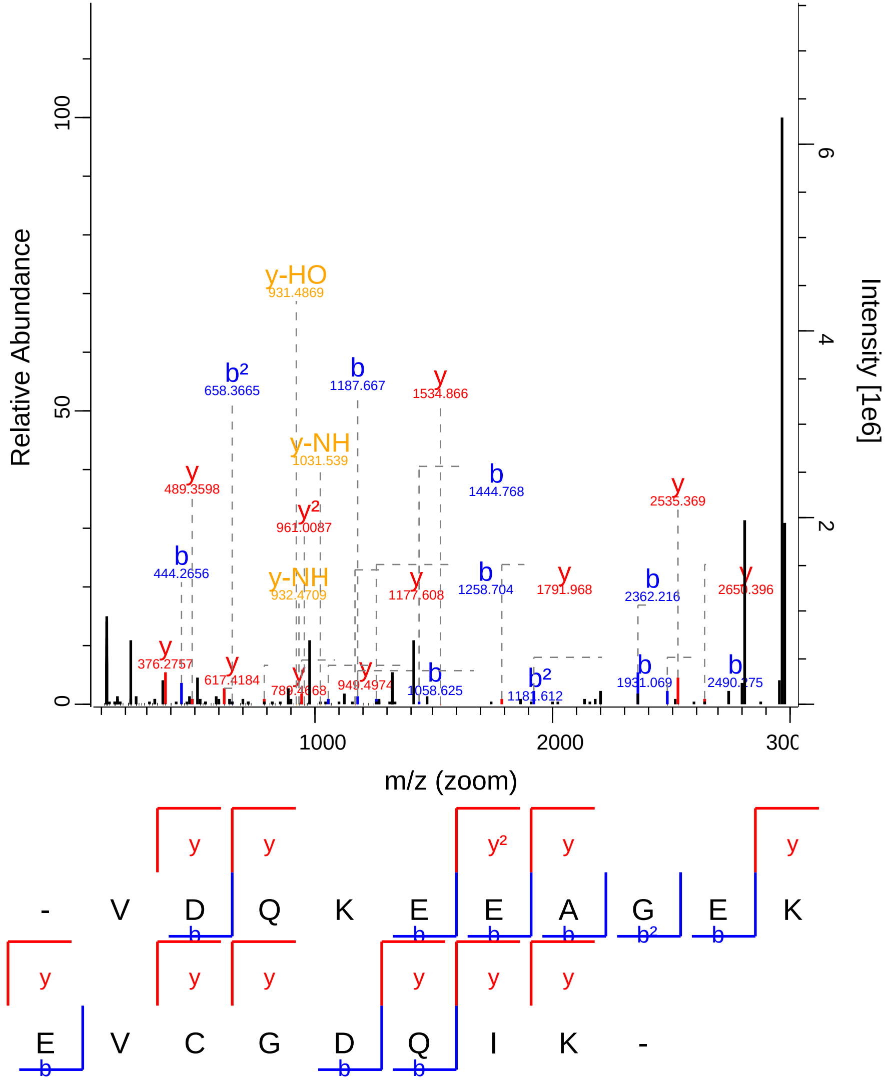

Raw File	Scan	Method	Score	m/z	Gene names
RO-QC-160121_ZackD_SS_Set_01_Fractio	17181	FTMS; HC	121.71	949.46	FOXJ3

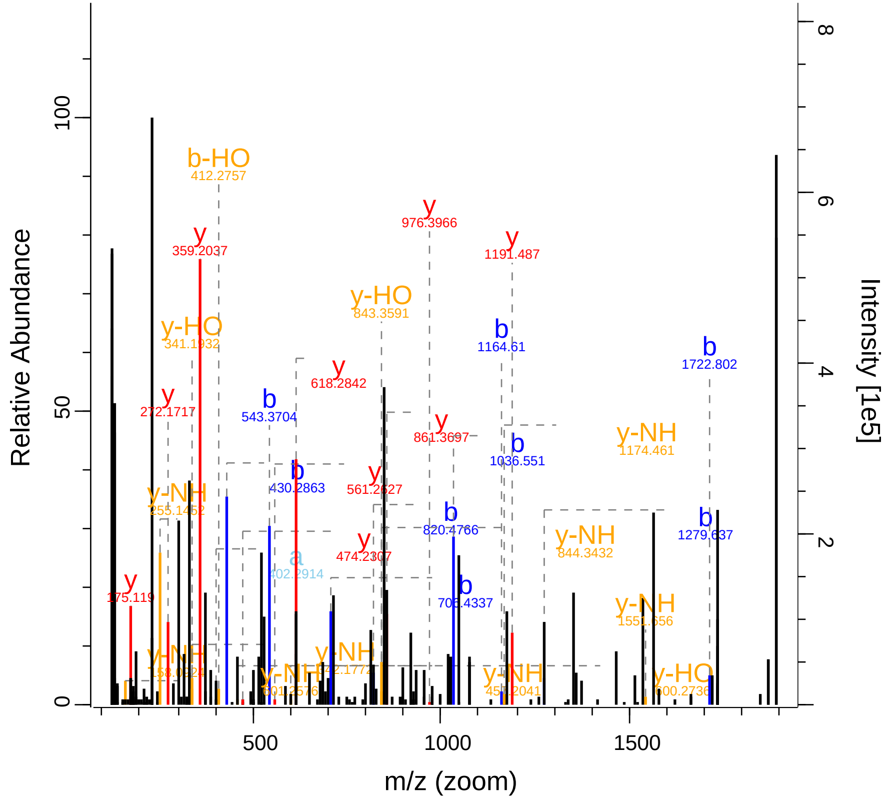

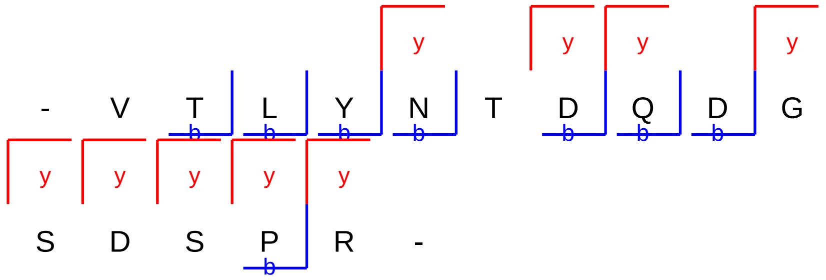

Raw File	Scan	Method	Score	m/z	Gene names
RO-QC-160121_ZackD_SS_Set_01_Fr	17555	FTMS; H(	88.06	545.63	ORMDL3;ORMDL1;OR

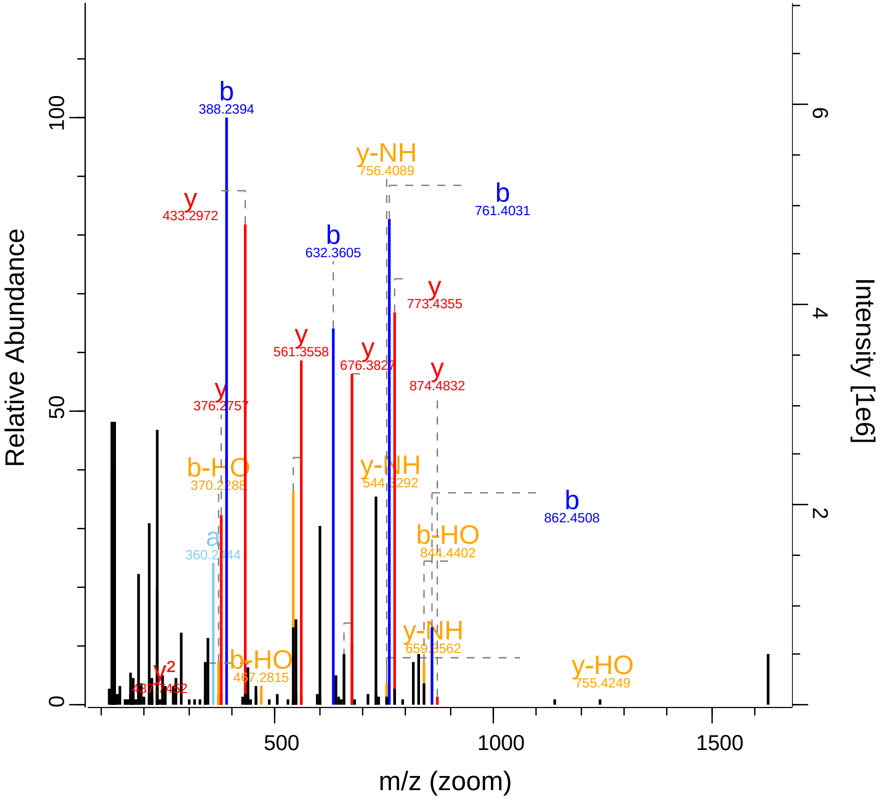

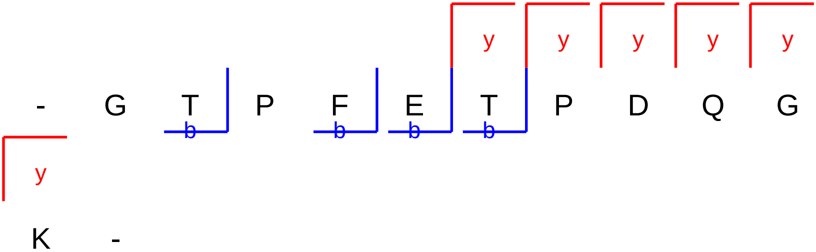

Raw File	Scan	Method	Score	m/z	Gene names
RO-QC-160121_ZackD_SS_Set_01_F	1759[illegible]	FTMS; H	198.2	817.94	ORMDL3;ORMDL1;ORM

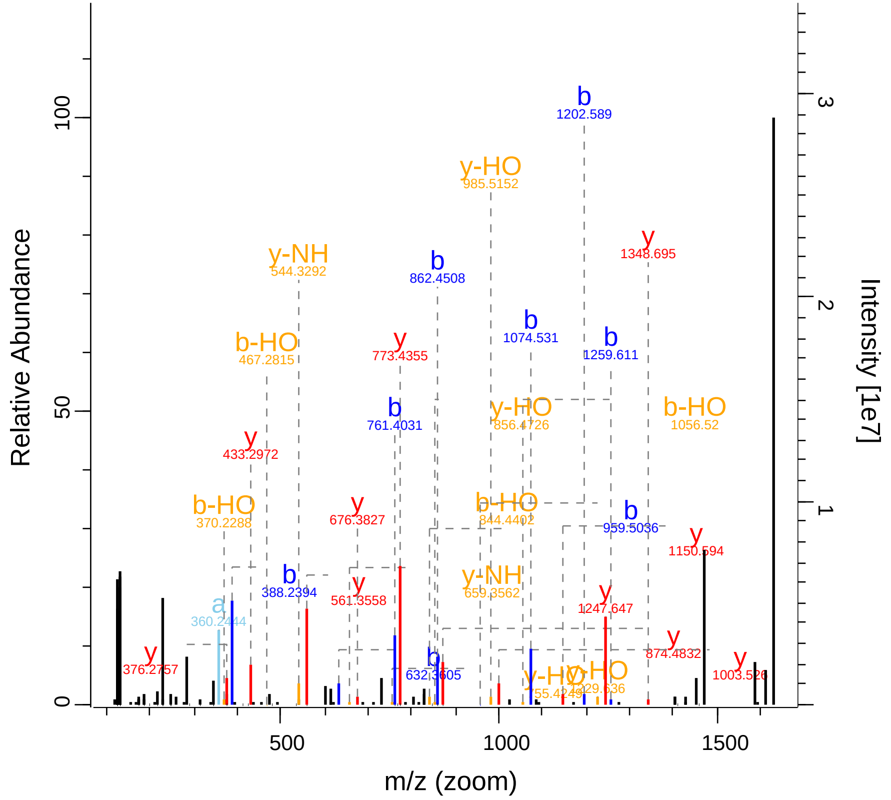

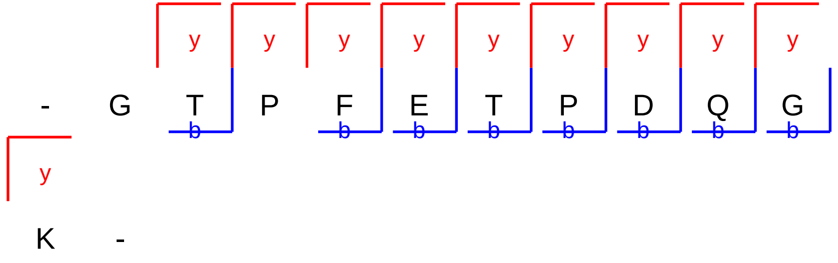

Raw File	Scan	Method	Score	m/z
RO-QC-160121_ZackD_SS_Set_01_Fraction_1	17860	FTMS; HCD	48.17	496.79

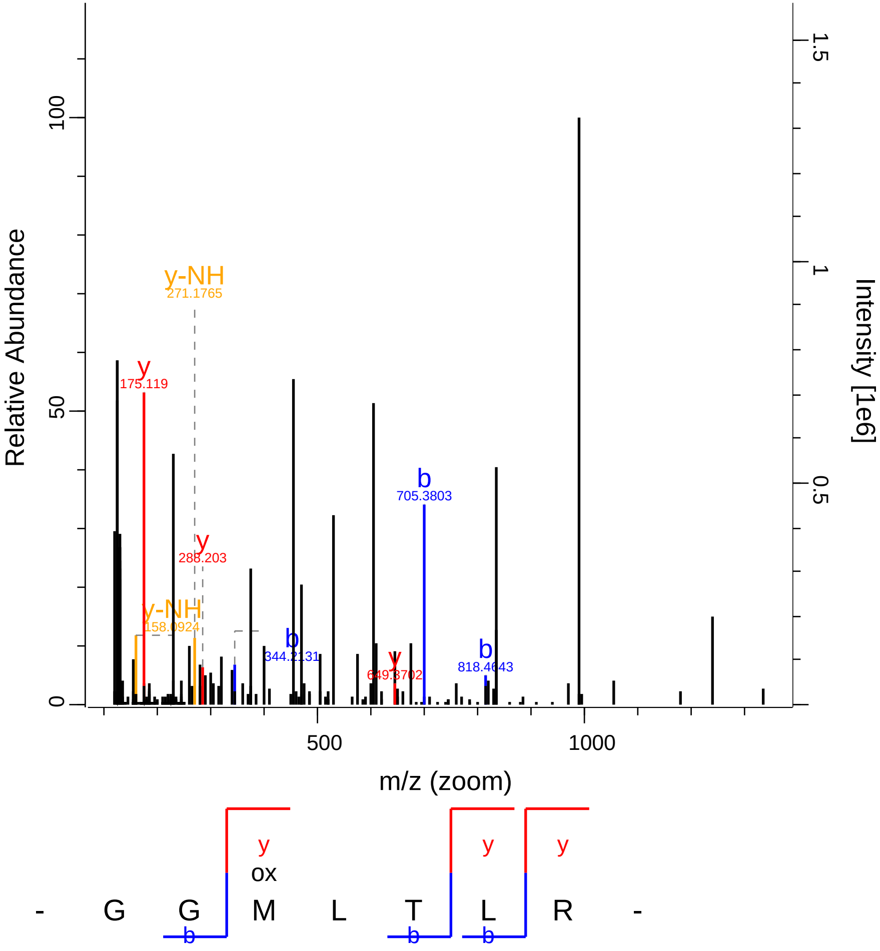

Raw File	Scan	Method	Score	m/z	Gene names
RO-QC-160121_ZackD_SS_Set_01_Fractio	17913	FTMS; HC	152.97	806.92	TRAM2

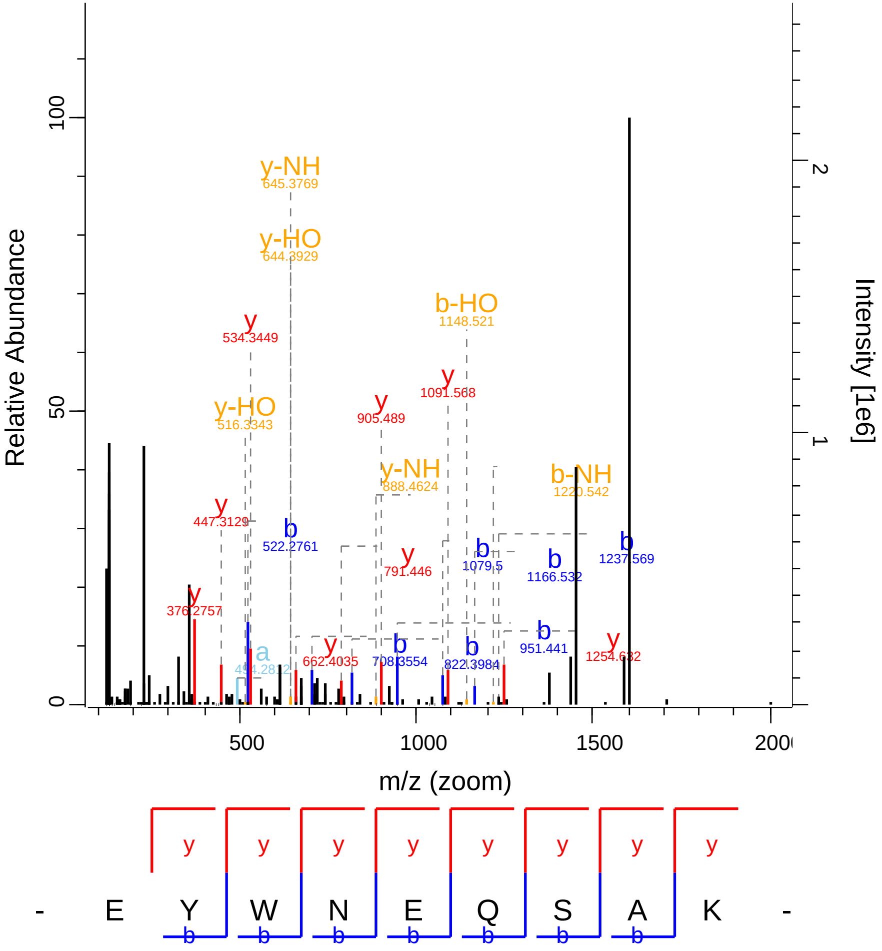

Raw File	Scan	Method	Score	m/z	Gene names
RO-QC-160121_ZackD_SS_Set_01_Fractio	18055	FTMS; HC	66.6	525.64	SESTD1

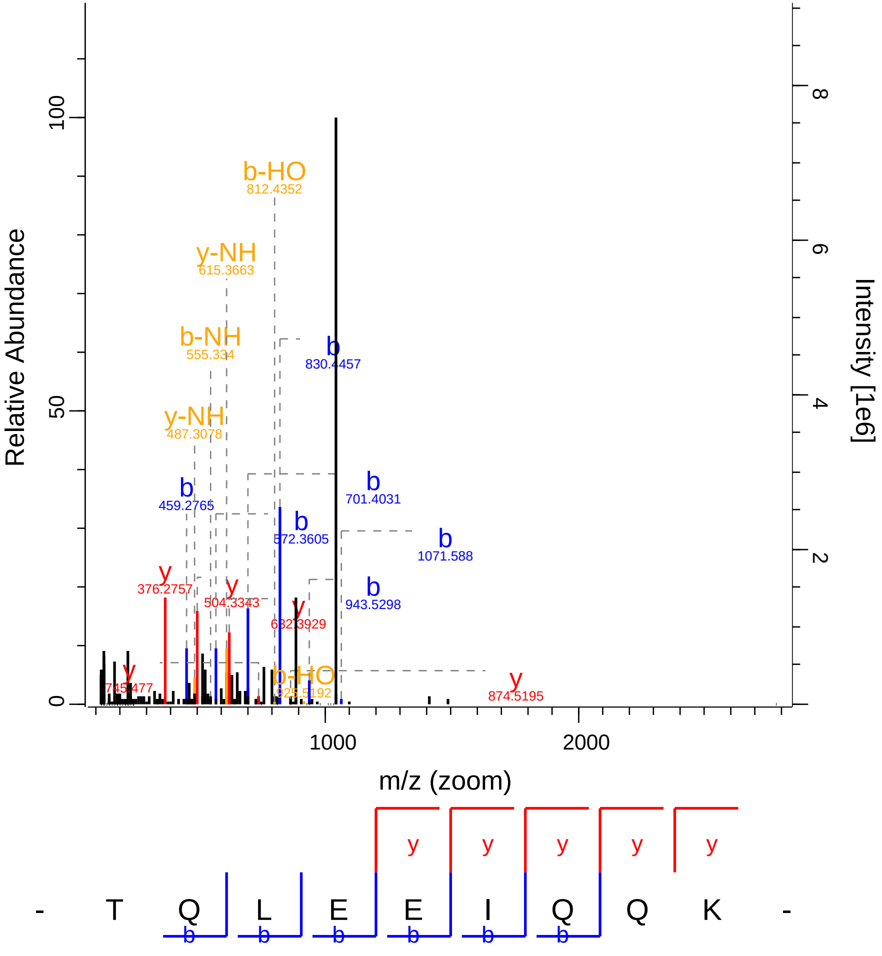

Raw File	Scan	Method	Score	m/z	Gene names
RO-QC-160121_ZackD_SS_Set_01_Fractio	18058	FTMS; HC	97.46	787.96	SESTD1

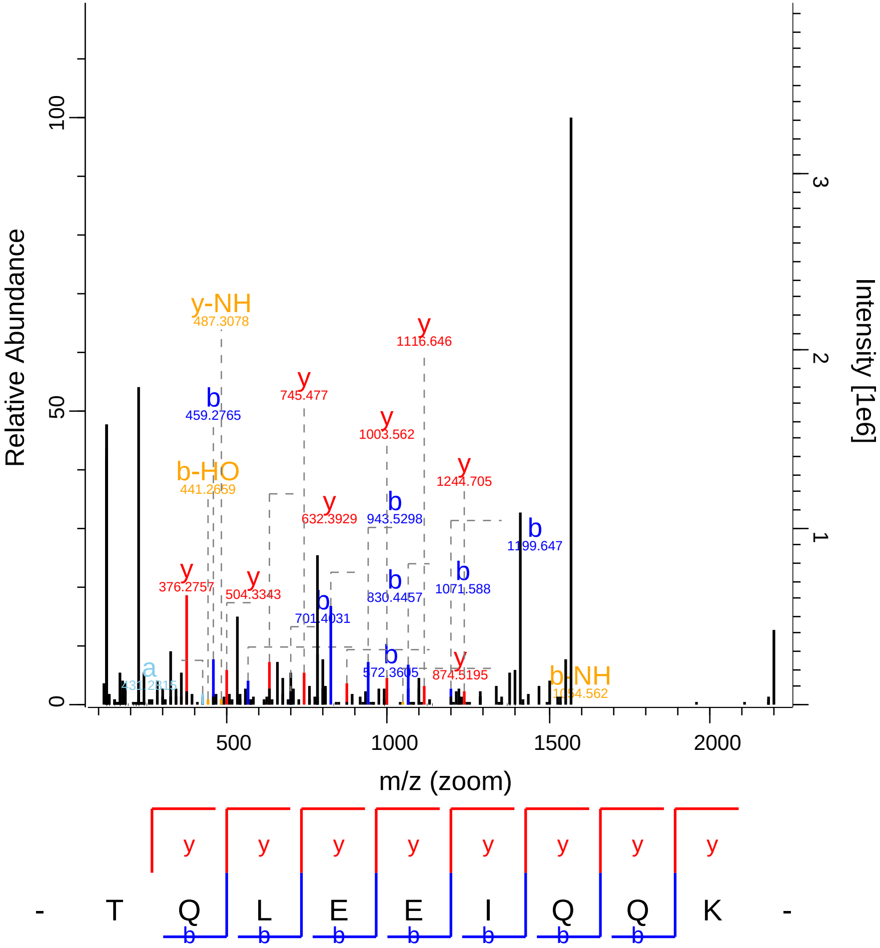

Raw File	Scan	Method	Score	m/z	Gene names
RO-QC-160121_ZackD_SS_Set_01_Fractio	18754	FTMS; HC	126.39	495.95	WBP1L

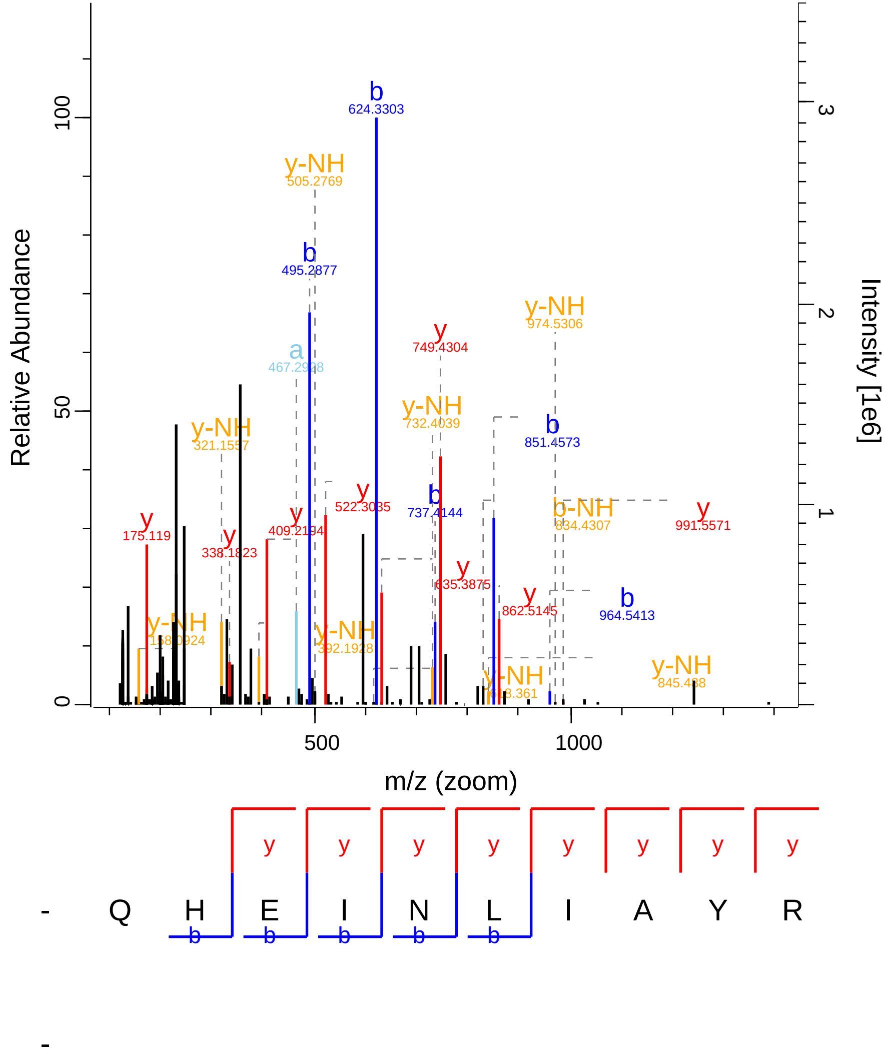

Raw File	Scan	Method	Score	m/z	Gene names
RO-QC-160121_ZackD_SS_Set_01_Fractio	18941	FTMS; HC	41.24	515.78	MMRN1

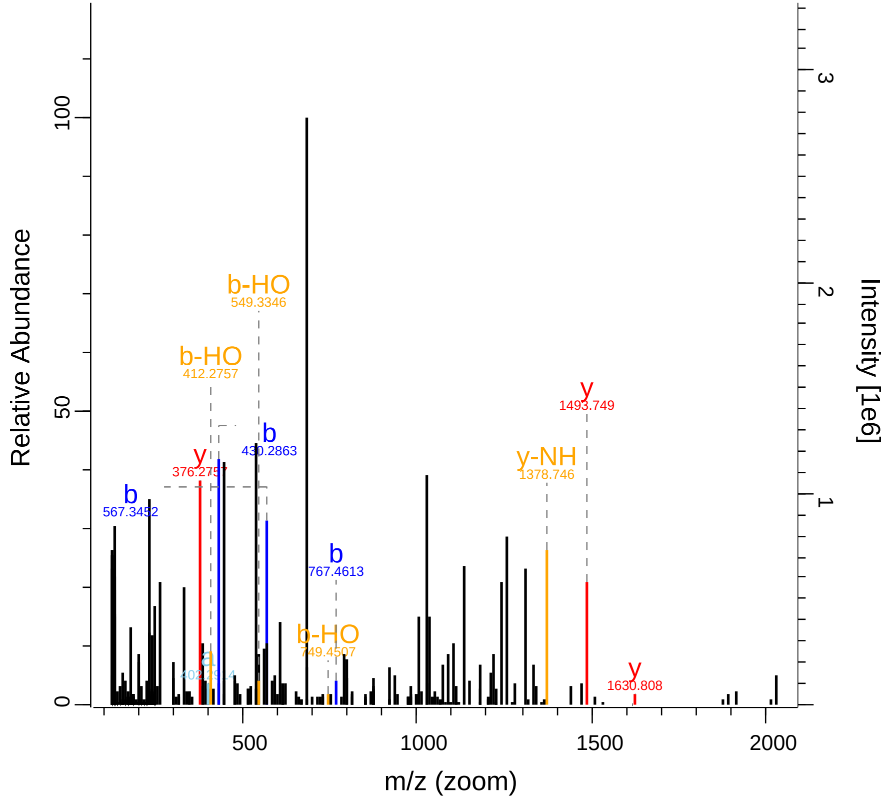

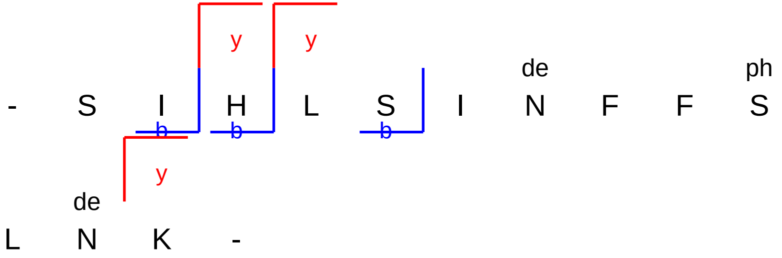

Raw File	Scan	Method	Score	m/z
RO-QC-160121_ZackD_SS_Set_01_Fraction_1	19519	FTMS; HCD	99.69	515.99

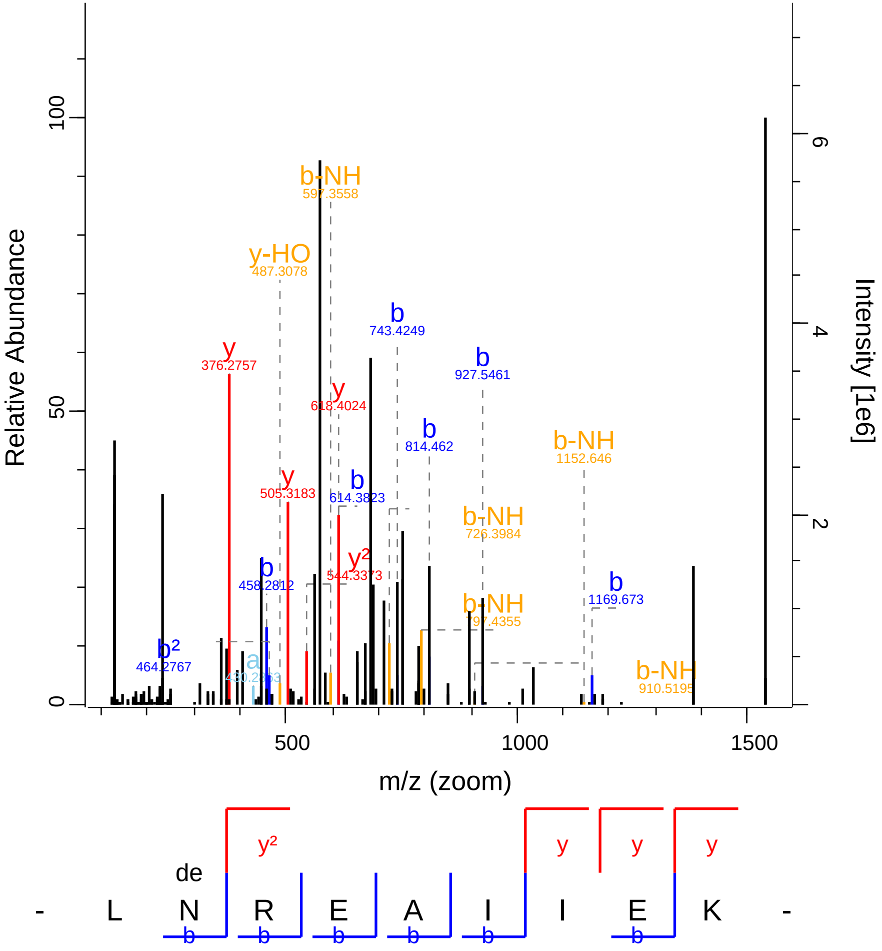

Raw File	Scan	Method	Score	m/z	Gene names
RO-QC-160121_ZackD_SS_Set_01_Fractio	20143	FTMS; HC	61.48	582.68	WWC3

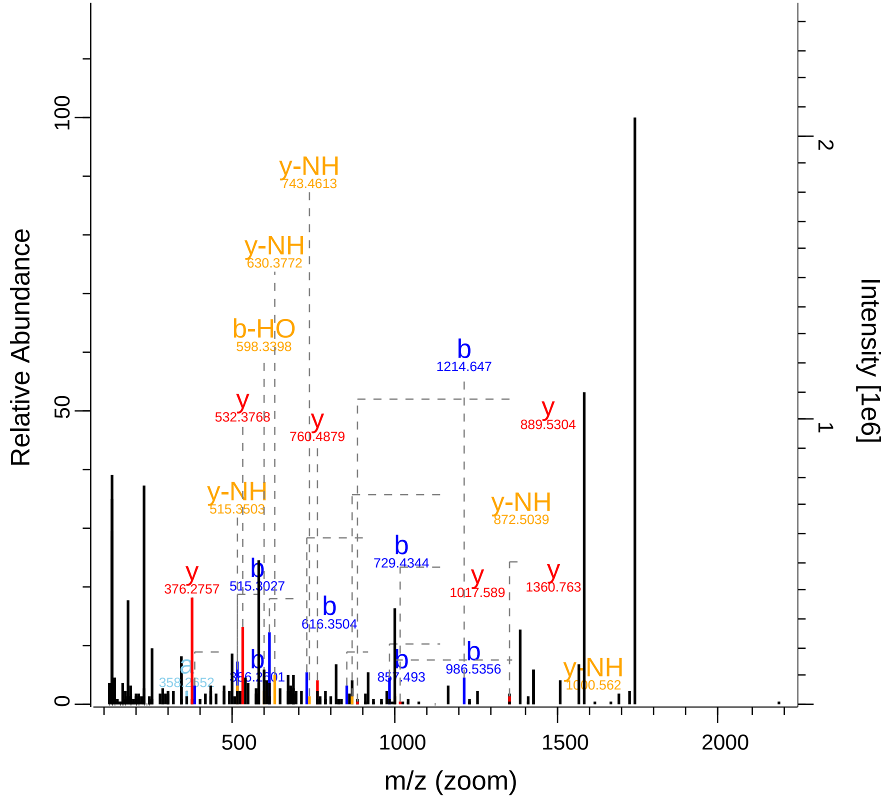

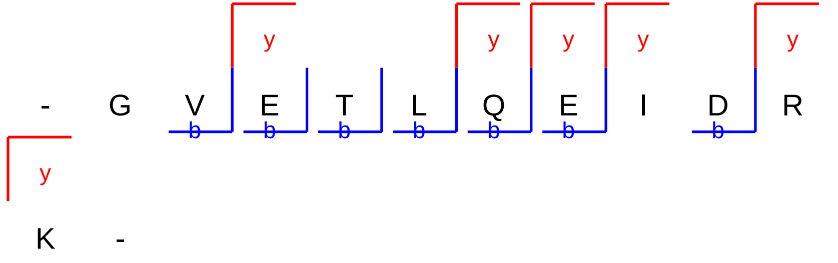

Raw File	Scan	Method	Score	m/z	Gene names
RO-QC-160121_ZackD_SS_Set_01_Fractio	20239	FTMS; HC	75.39	493.93	FRMD6

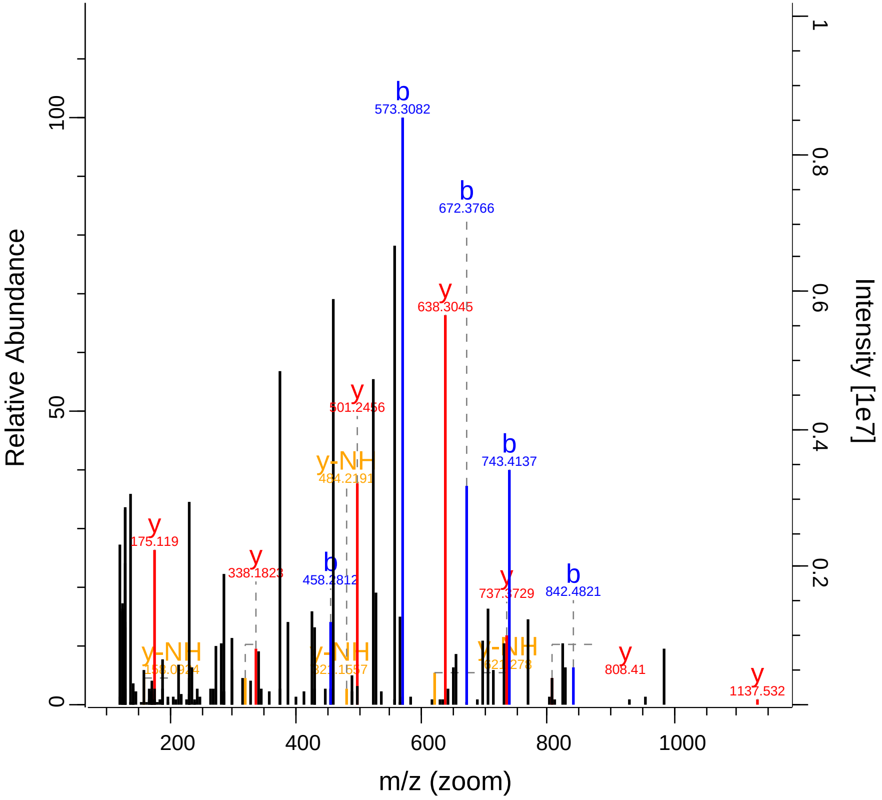

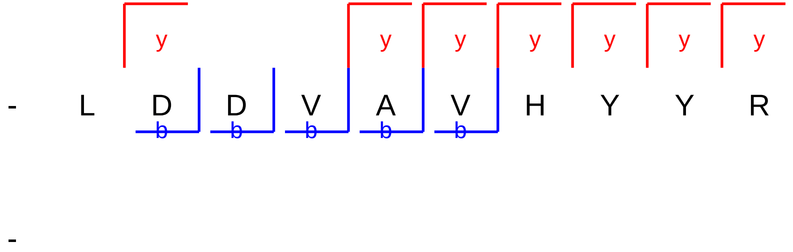

Raw File	Scan	Method	Score	m/z	Gene names
RO-QC-160121_ZackD_SS_Set_01_Fractio	20270	FTMS; HC	111.91	534.56	REEP1

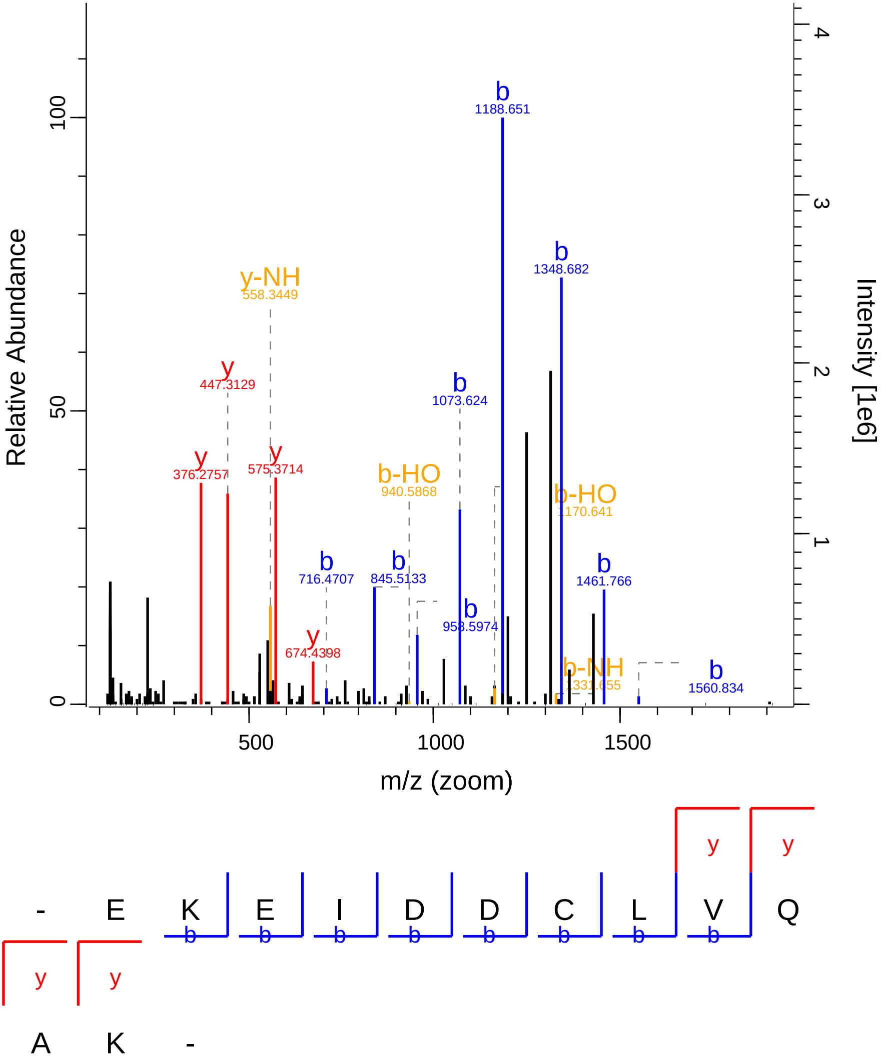

Raw File	Scan	Method	Score	m/z	Gene names
RO-QC-160121_ZackD_SS_Set_01_Fractio	20288	FTMS; HC	126.95	712.41	REEP1

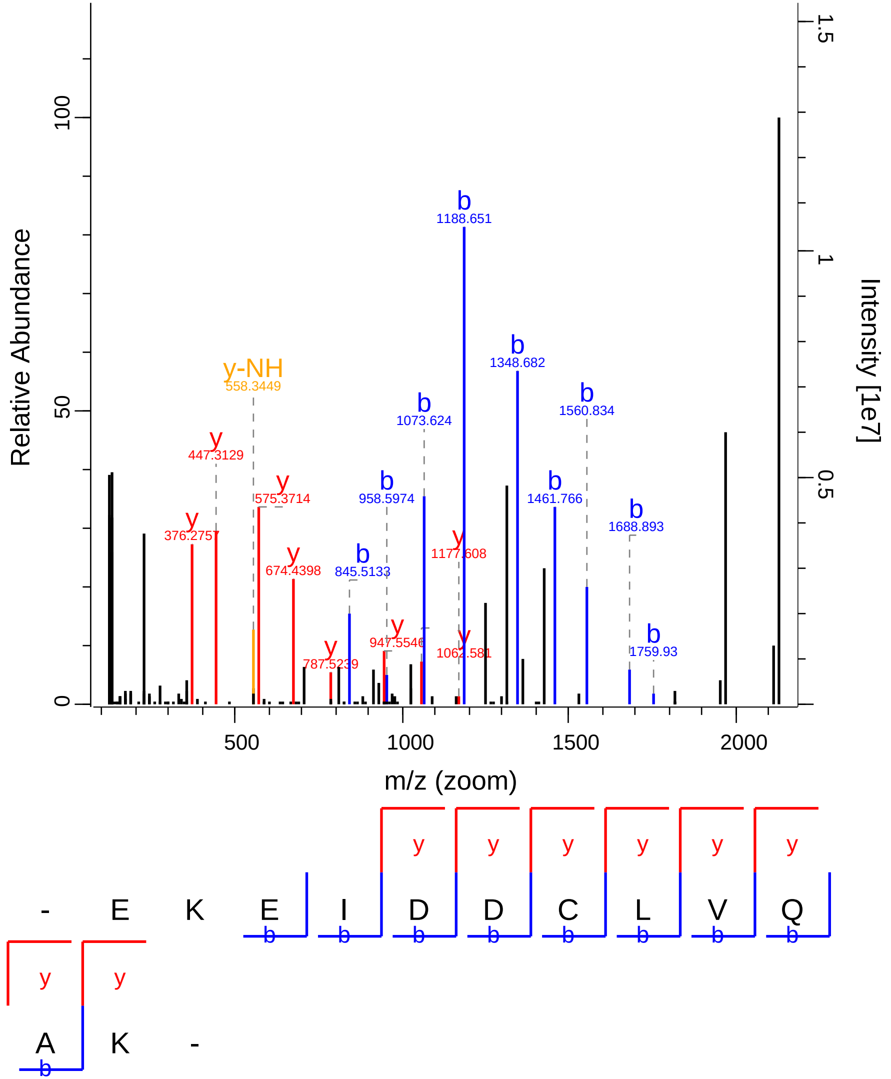

Raw File	Scan	Method	Score	m/z	Gene names
RO-QC-160121_ZackD_SS_Set_01_Fractio	20313	FTMS; HC	125.75	439.49	MED10

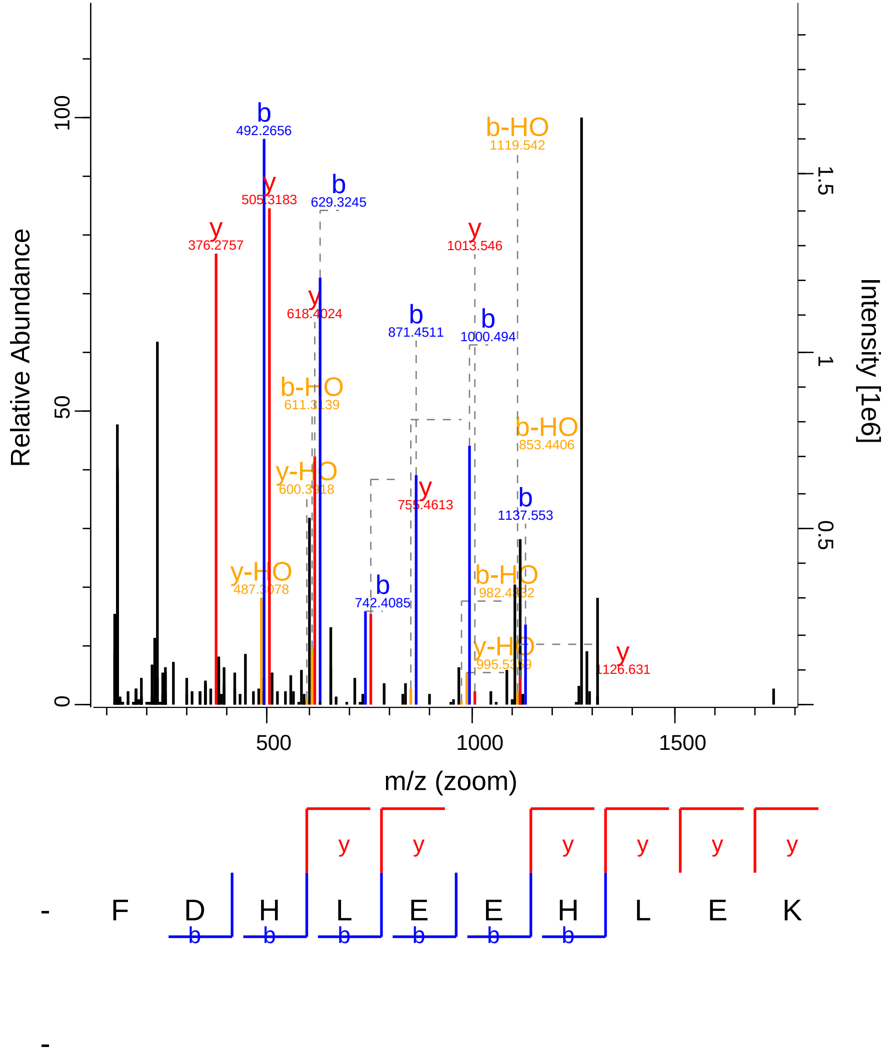

Raw File	Scan	Method	Score	m/z	Gene names
RO-QC-160121_ZackD_SS_Set_01_Fractio	20403	FTMS; HC	57.75	723.36	SVEP1

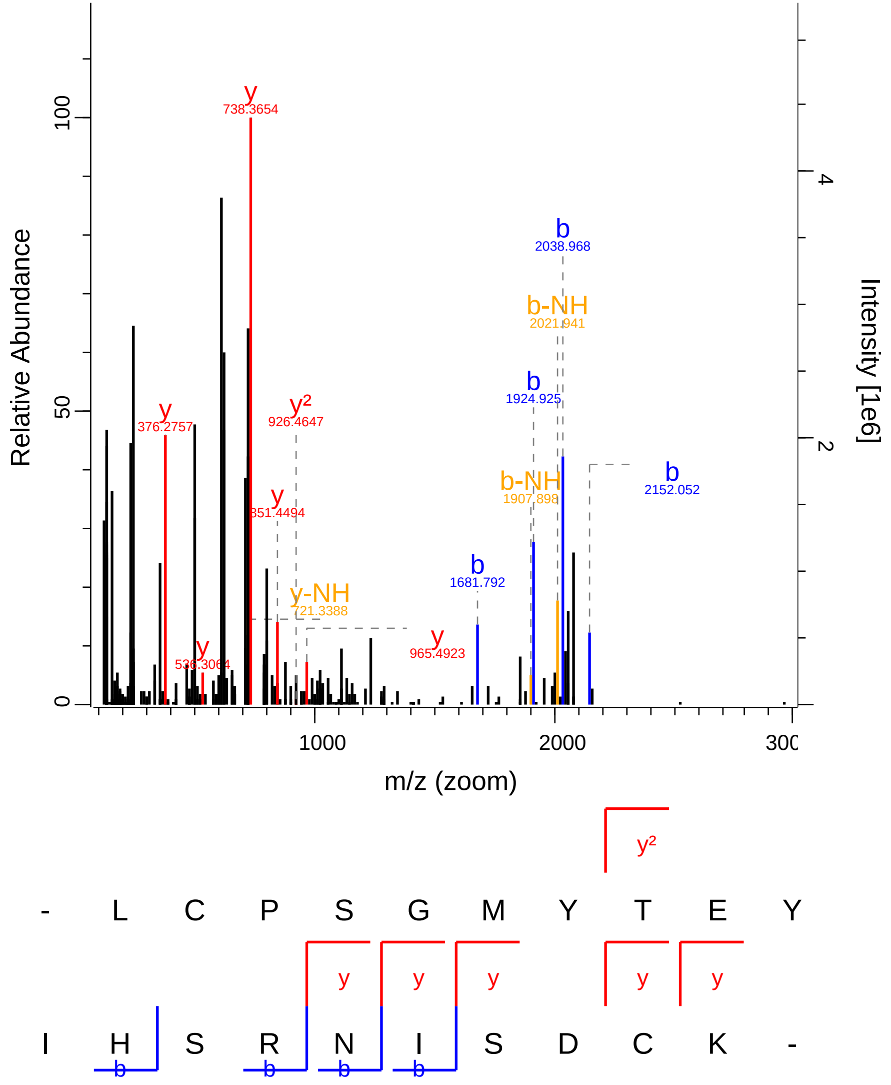

Raw File	Scan	Method	Score	m/z	Gene names
RO-QC-160121_ZackD_SS_Set_01_Fractio	20565	FTMS; HC	100.79	840.44	CDC42EP3

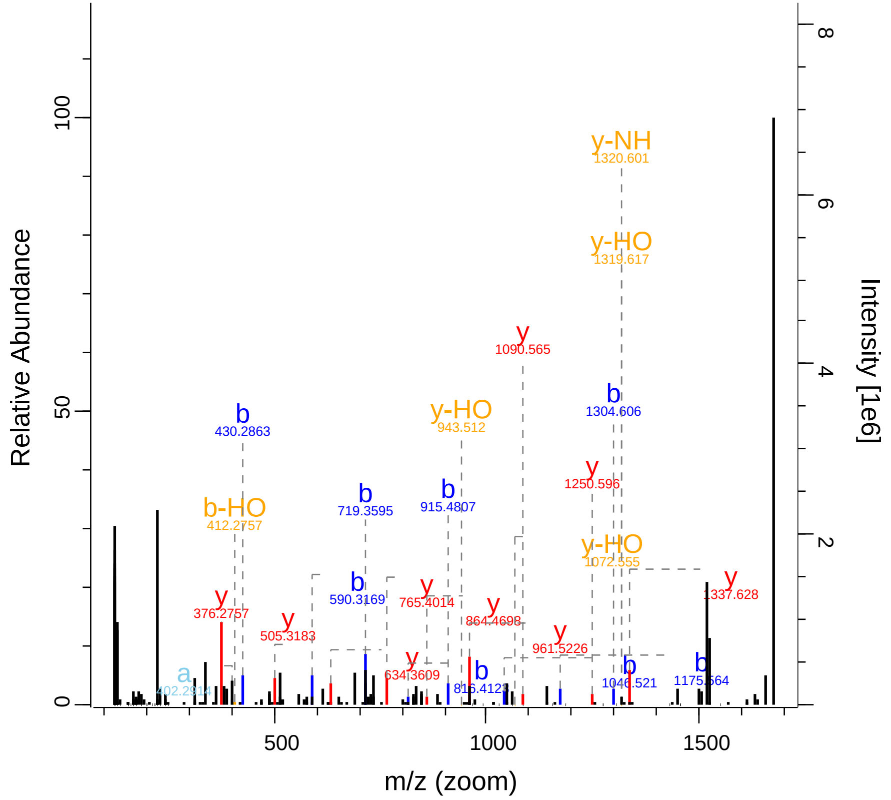

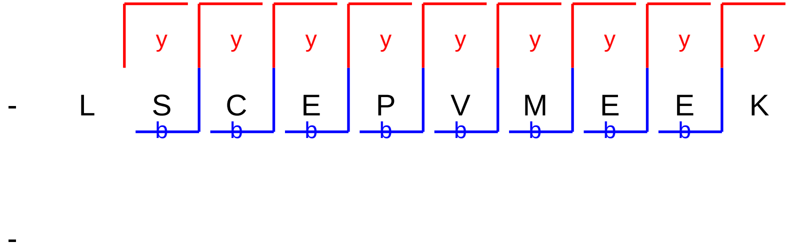

Raw File	Scan	Method	Score	m/z	Gene names
RO-QC-160121_ZackD_SS_Set_01_Fractio	20744	FTMS; HC	80.24	423.25	ZNF536

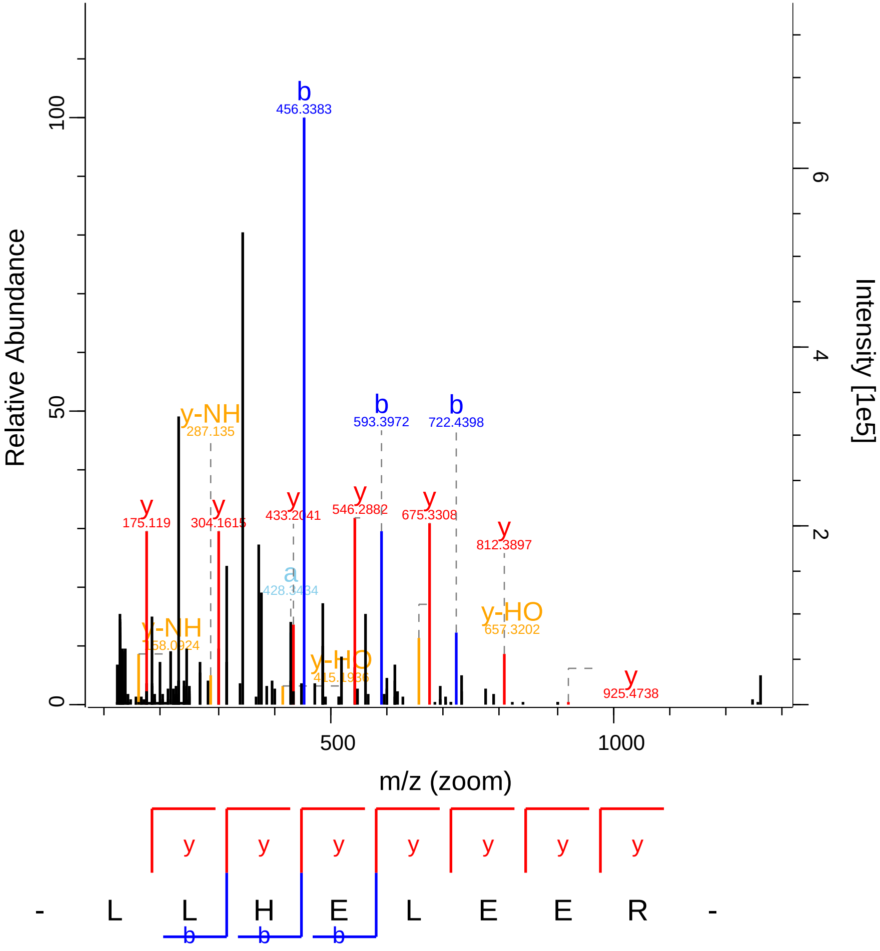

Raw File	Scan	Method	Score	m/z	Gene names
RO-QC-160121_ZackD_SS_Set_01_Fractic	21930	FTMS; HC	97.46	1035.06	TRPV2

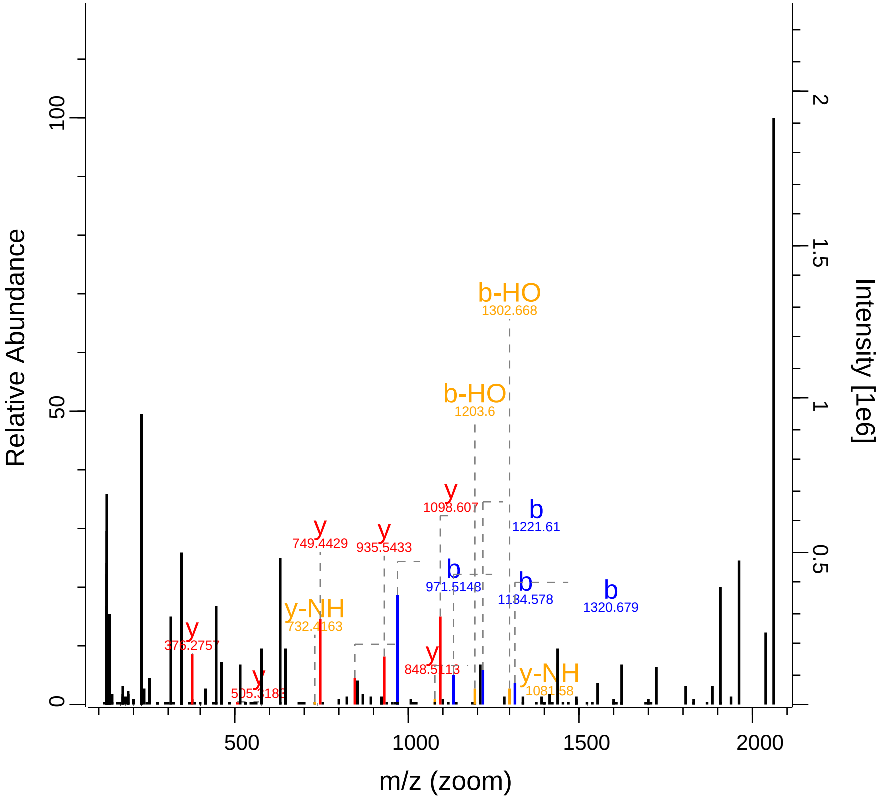

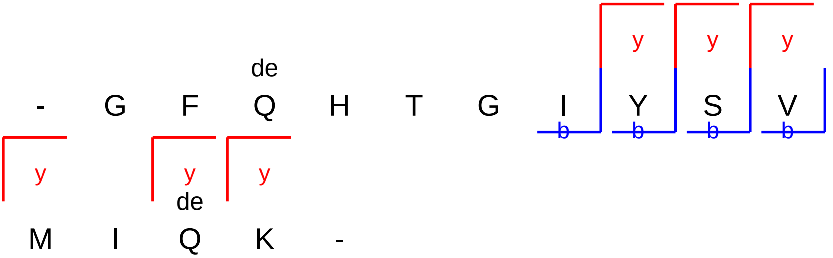

Raw File	Scan	Method	Score	m/z	Gene names
RO-QC-160121_ZackD_SS_Set_01_Fractio	22513	FTMS; HC	81.16	708.36	SLC25A15

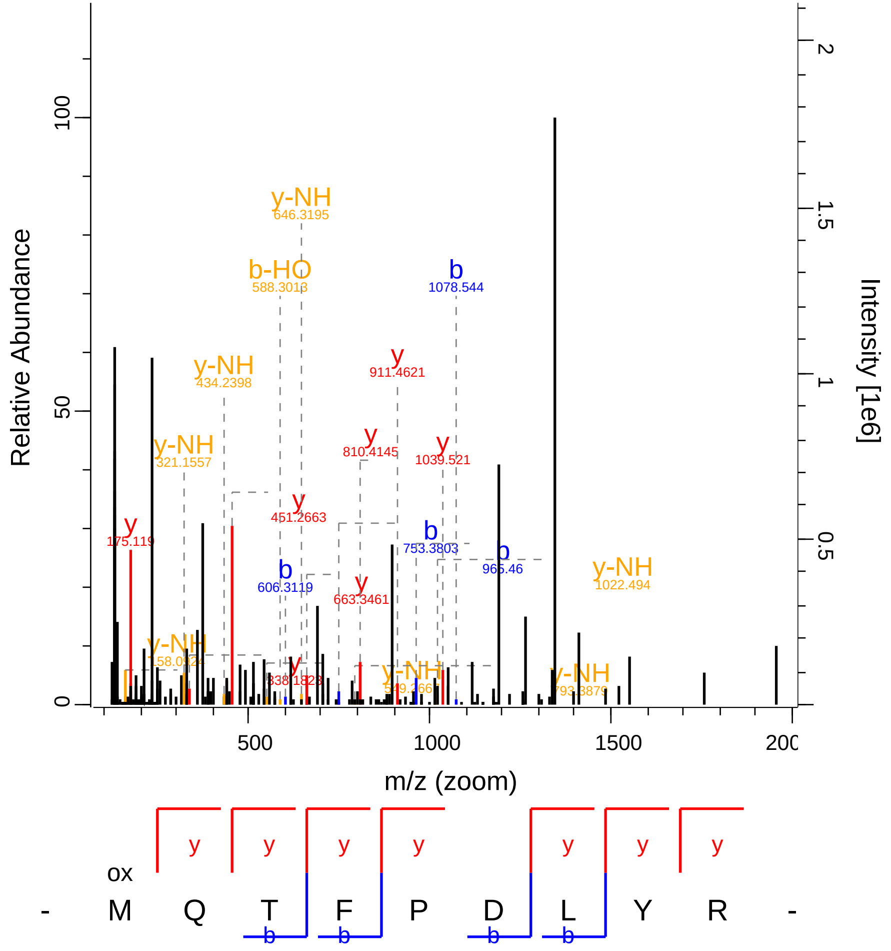

Raw File	Scan	Method	Score	m/z
RO-QC-160121_ZackD_SS_Set_01_Fraction_1	23119	FTMS; HCD	103.29	844.69

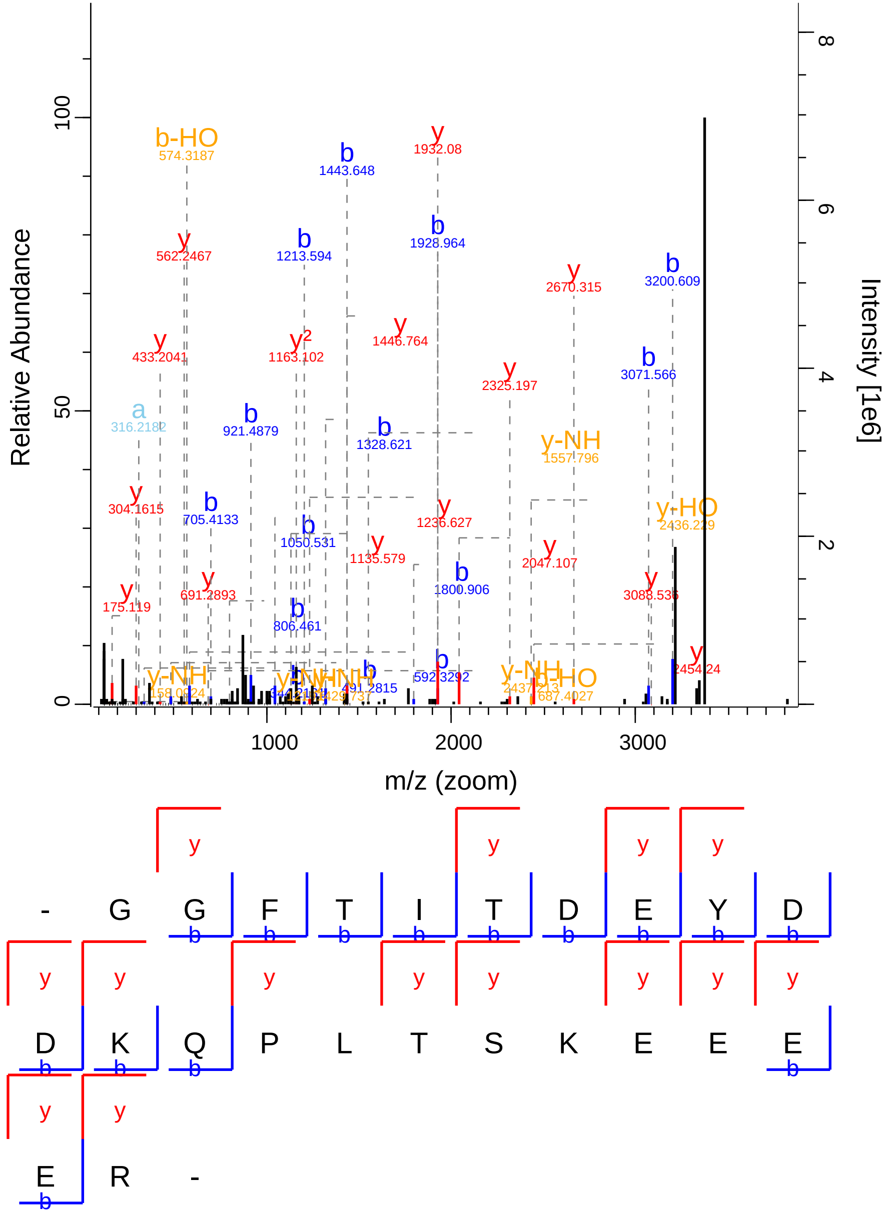

Raw File	Scan	Method	Score	m/z	Gene names
RO-QC-160121_ZackD_SS_Set_01_Fractio	23810	FTMS; HC	105.14	637.34	FBXW2

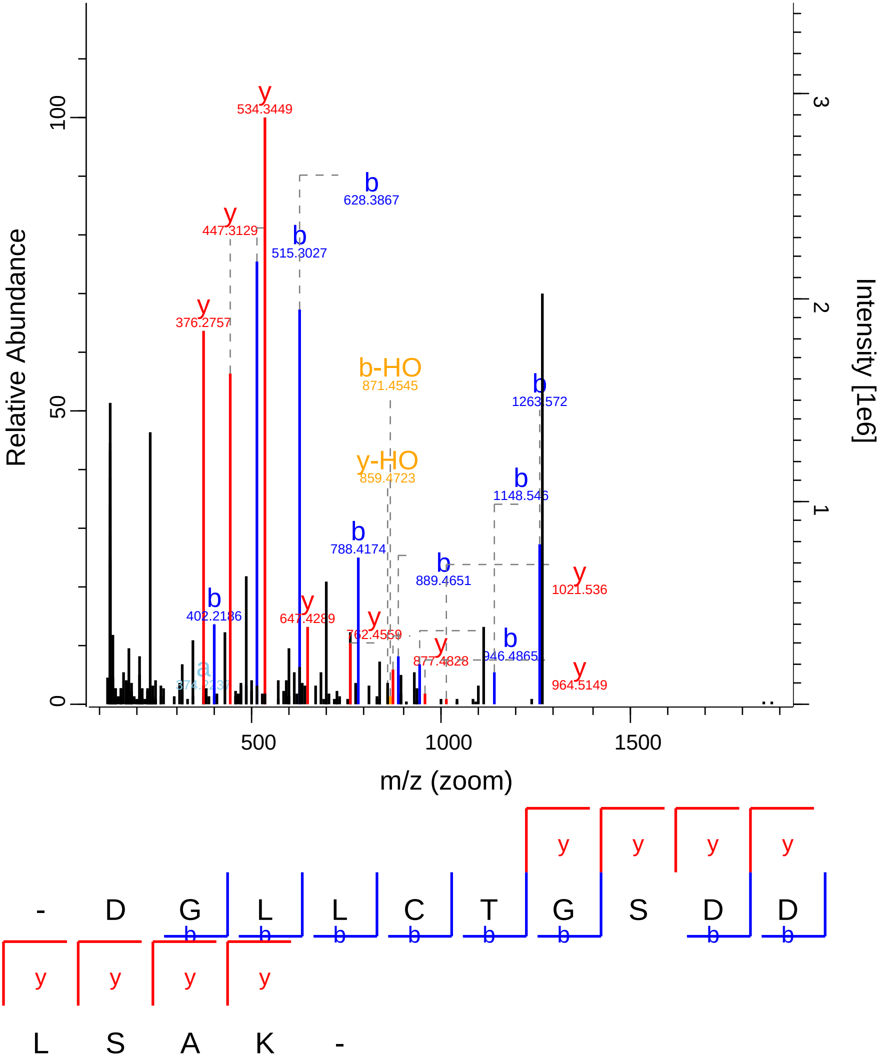

Raw File	Scan	Method	Score	m/z	Gene names
RO-QC-160121_ZackD_SS_Set_01_Fractio	24091	FTMS; HC	45.33	603.34	KLHL13;KLHL9

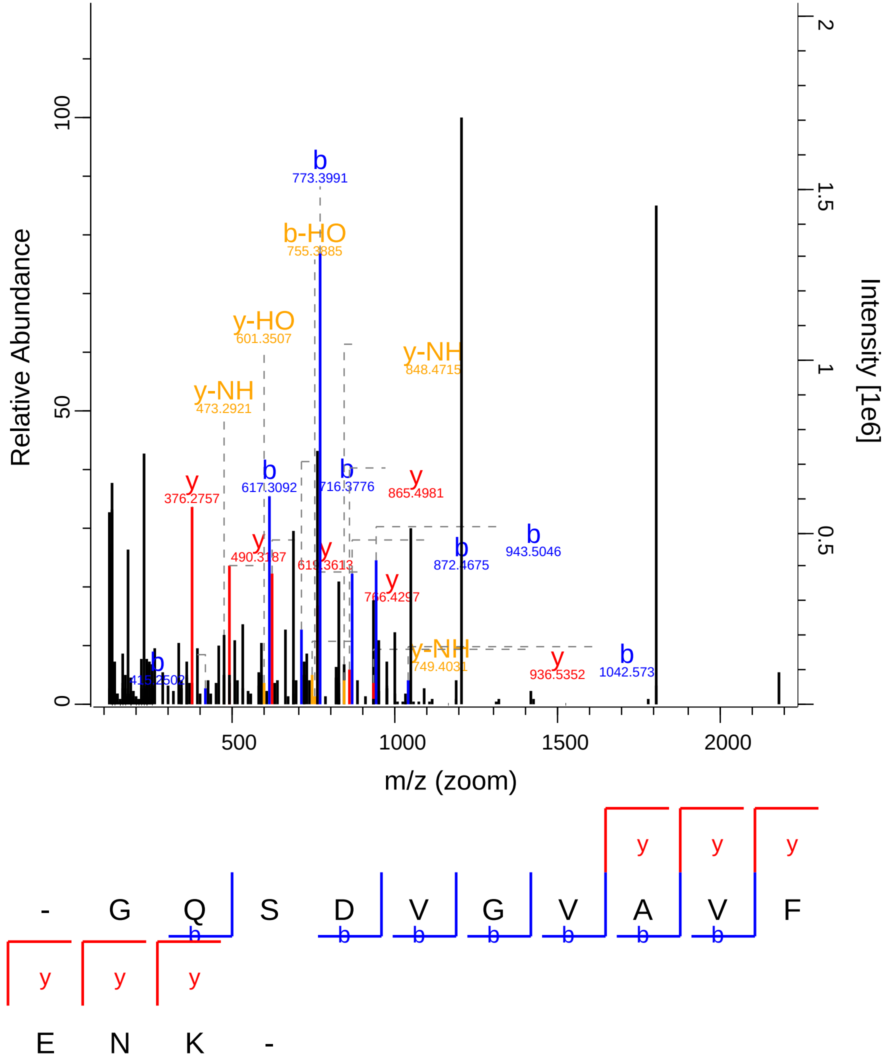

Raw File	Scan	Method	Score	m/z	Gene names
RO-QC-160121_ZackD_SS_Set_01_Fractior	24148	FTMS; HC	155.42	904.5	KLHL13;KLHL9

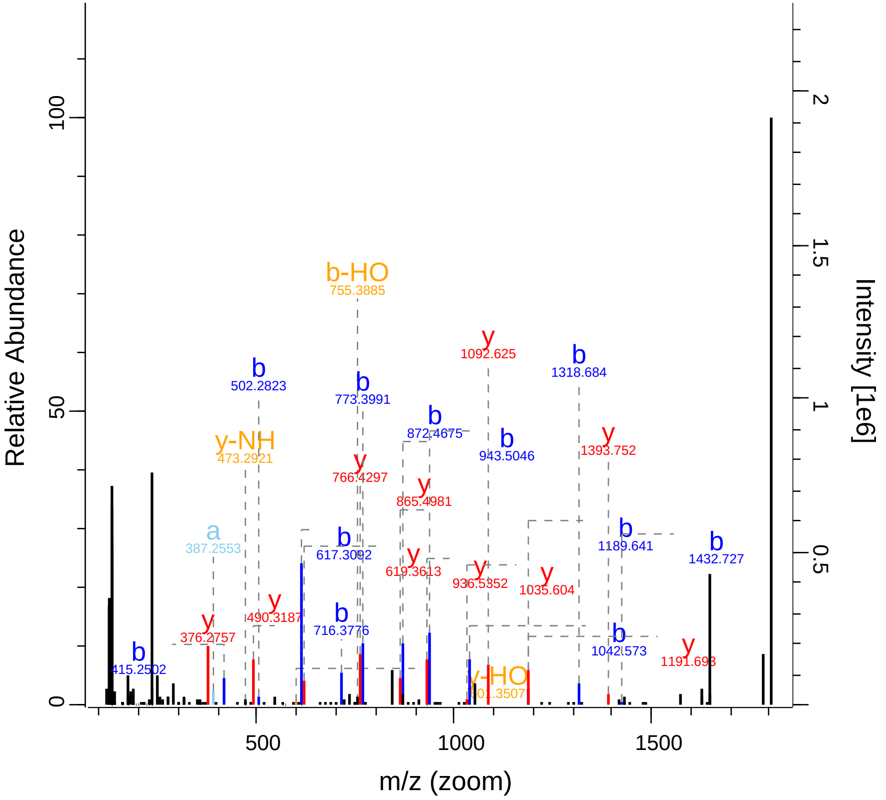

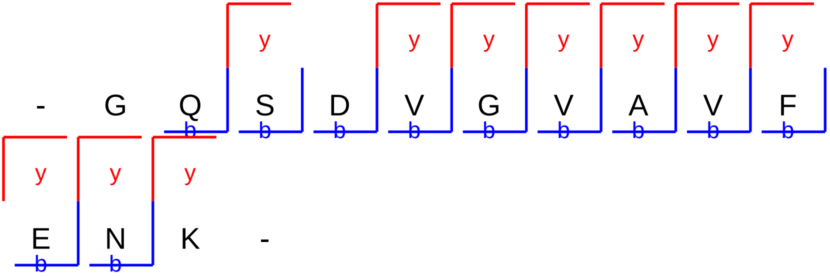

Raw File	Scan	Method	Score	m/z
RO-QC-160121_ZackD_SS_Set_01_Fraction_1	24316	FTMS; HCD	118.71	874.99

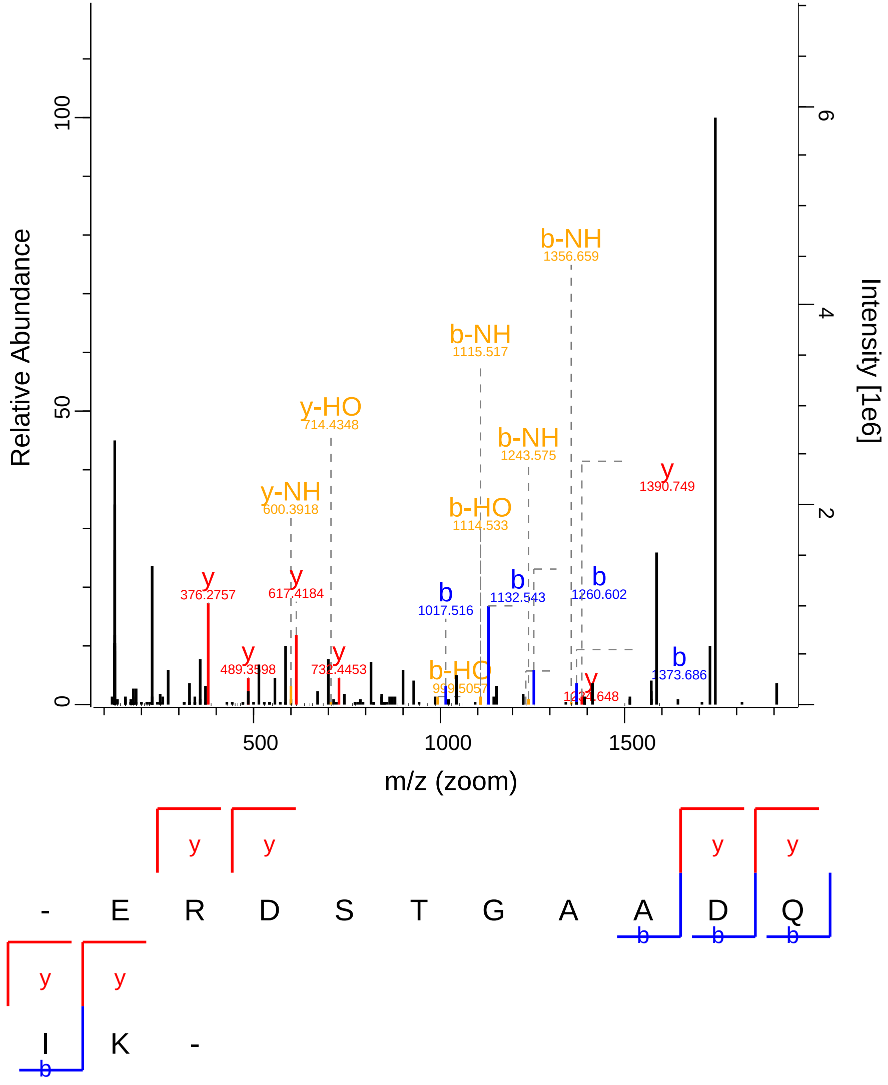

Raw File	Scan	Method	Score	m/z	Gene names
RO-QC-160121_ZackD_SS_Set_01_Fractio	24437	FTMS; HC	120.45	531.32	MAP4K2

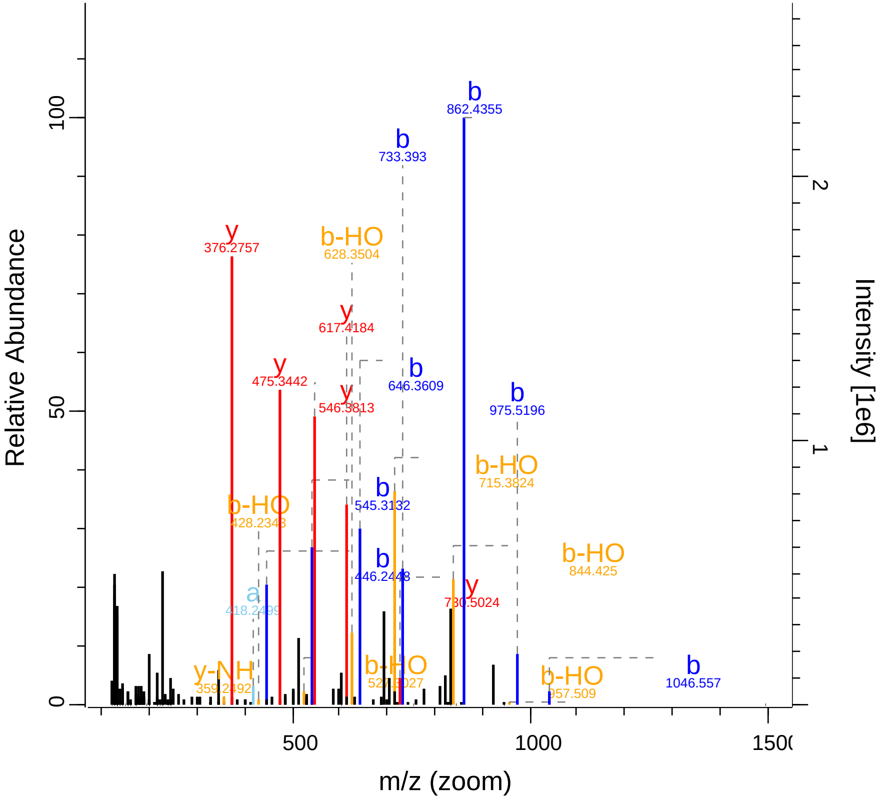

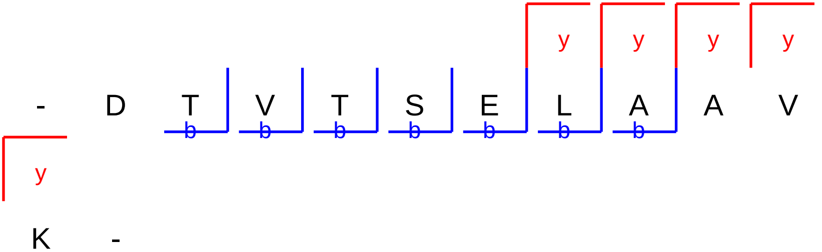

Raw File	Scan	Method	Score	m/z
RO-QC-160121_ZackD_SS_Set_01_Fraction_1	24667	FTMS; HCD	108.74	687.92

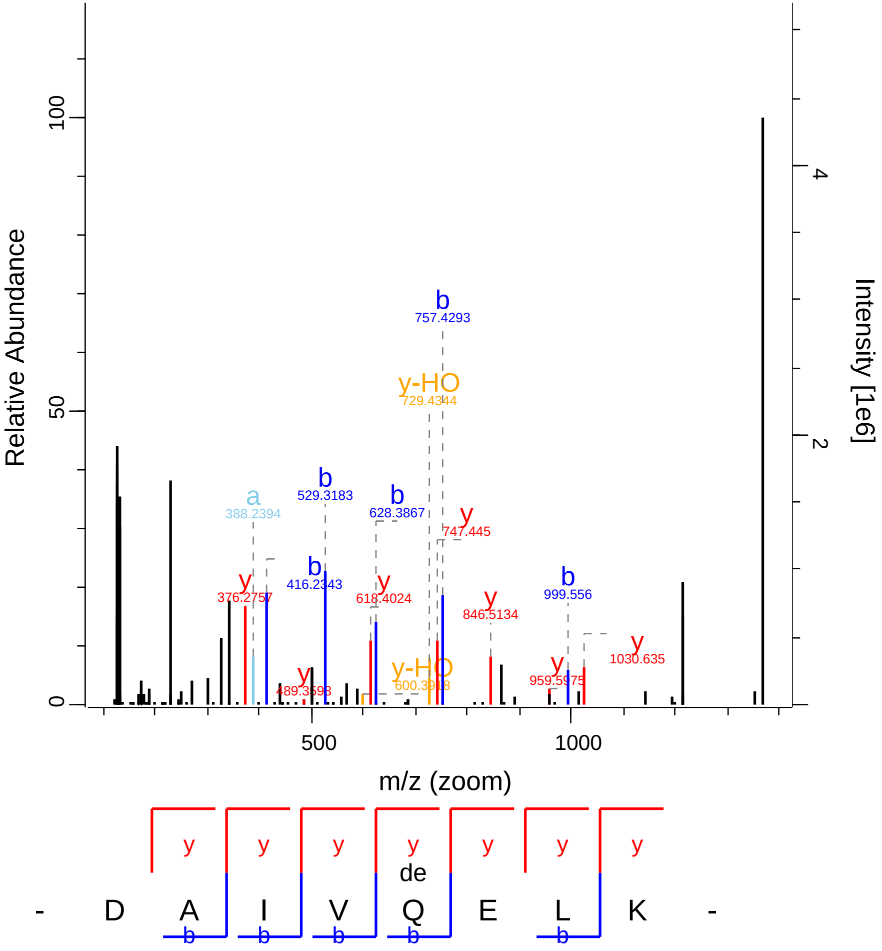

Raw File	Scan	Method	Score	m/z	Gene names
RO-QC-160121_ZackD_SS_Set_01_Fractio	25509	FTMS; HC	67.43	855.11	SLC31A1

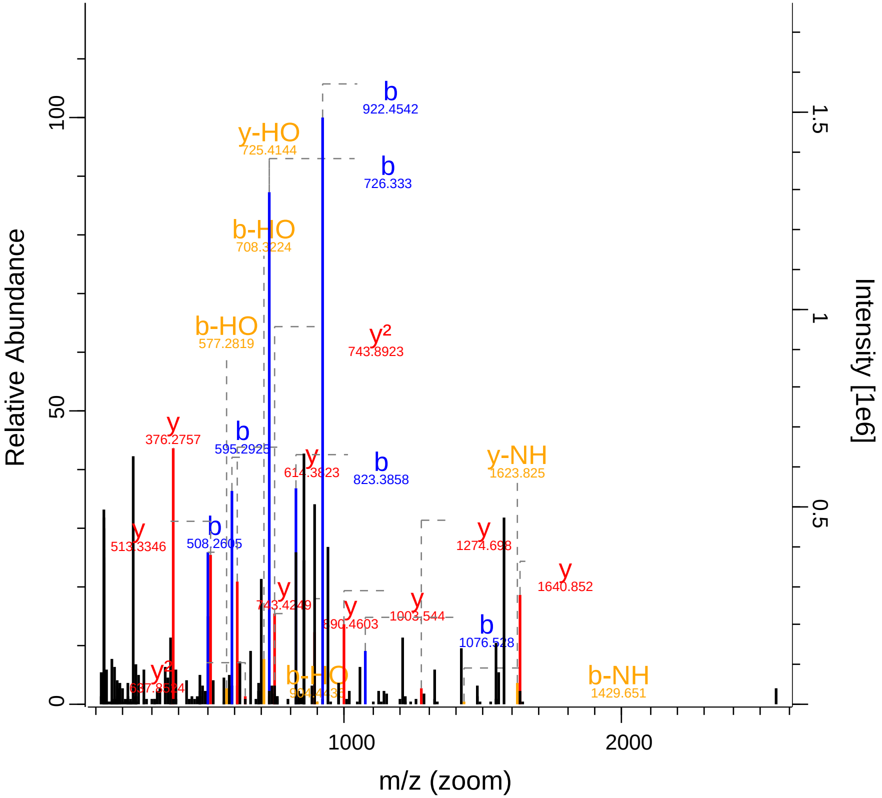

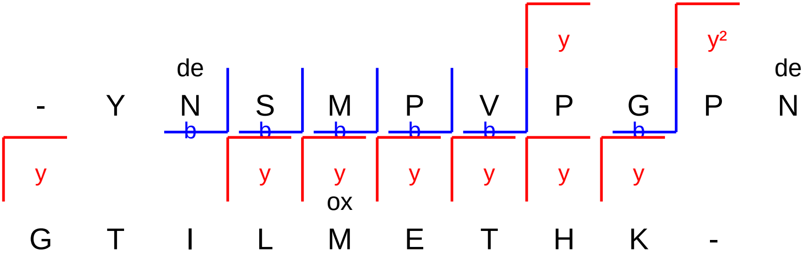

Raw File	Scan	Method	Score	m/z
RO-QC-160121_ZackD_SS_Set_01_Fraction_1	26166	FTMS; HCD	42.04	613.66

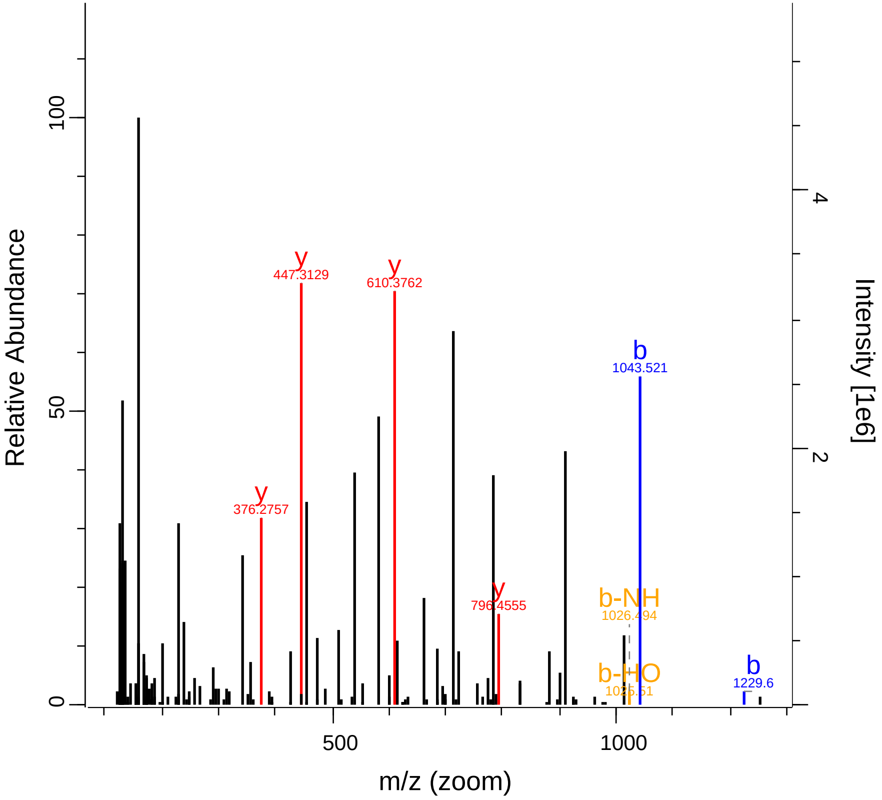

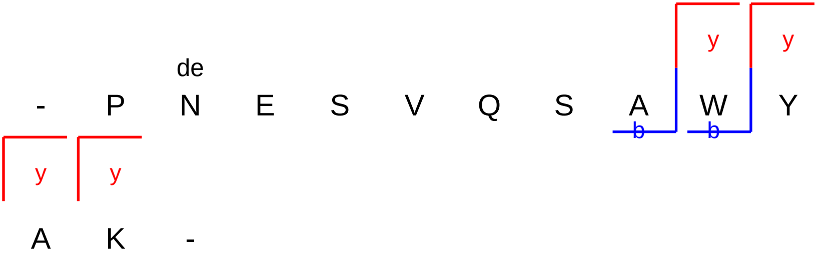

Raw File	Scan	Method	Score	m/z	Gene names
RO-QC-160121_ZackD_SS_Set_01_Fractio	26336	FTMS; HC	112.94	702.92	CHRDL1

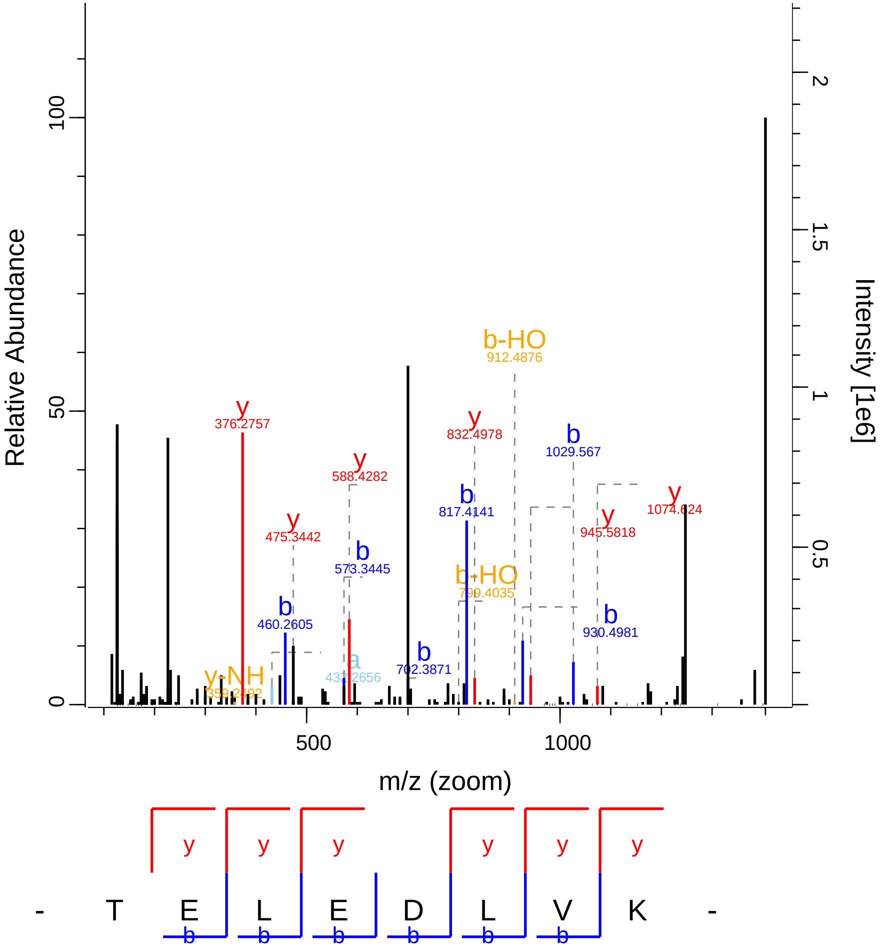

Raw File	Scan	Method	Score	m/z	Gene names
RO-QC-160121_ZackD_SS_Set_01_Fractio	26386	FTMS; HC	74.94	687.88	FIZ1

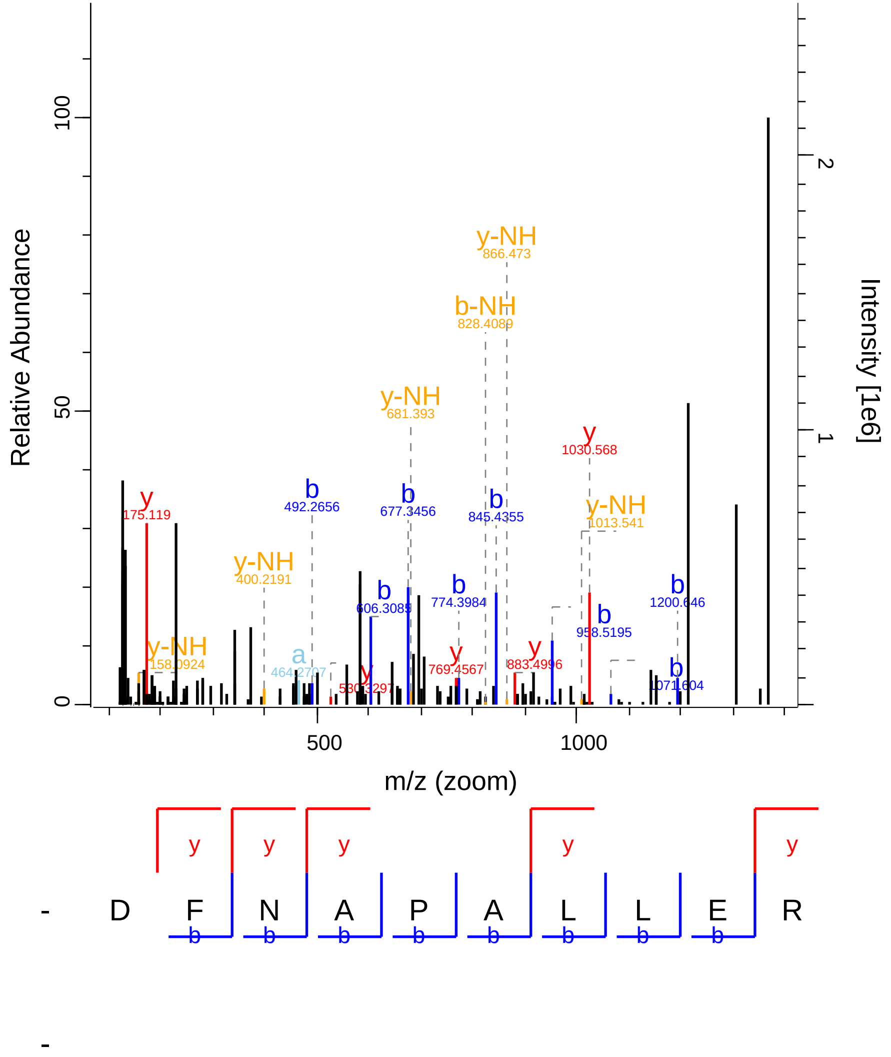

Raw File	Scan	Method	Score	m/z	Gene names
RO-QC-160121_ZackD_SS_Set_01_Fractioı	26742	FTMS; HCI	133.95	909.8	DRAM2

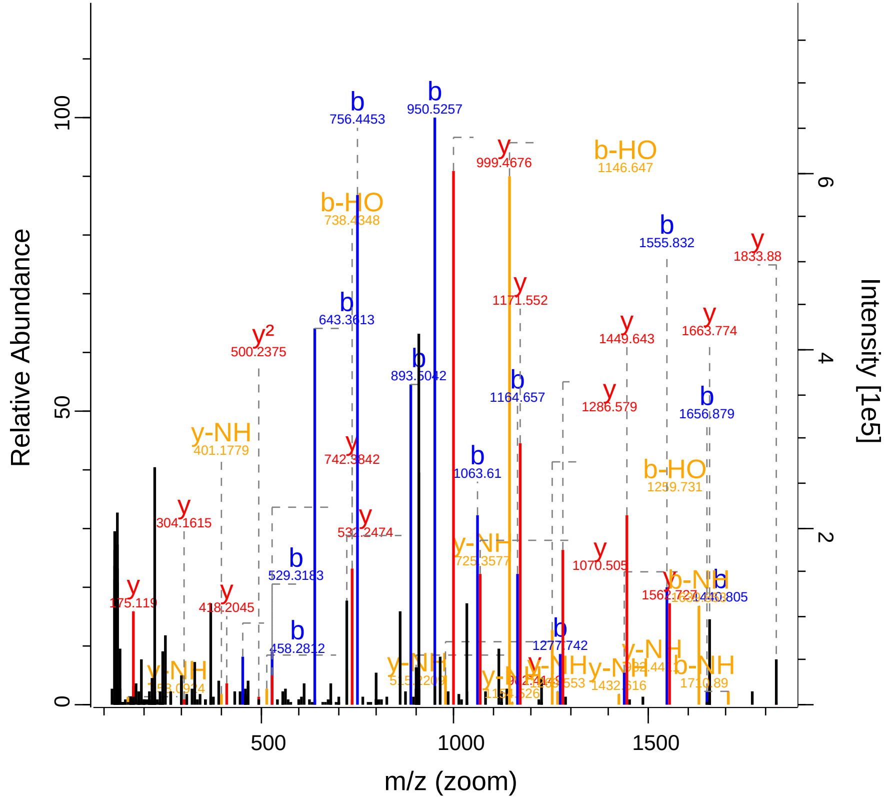

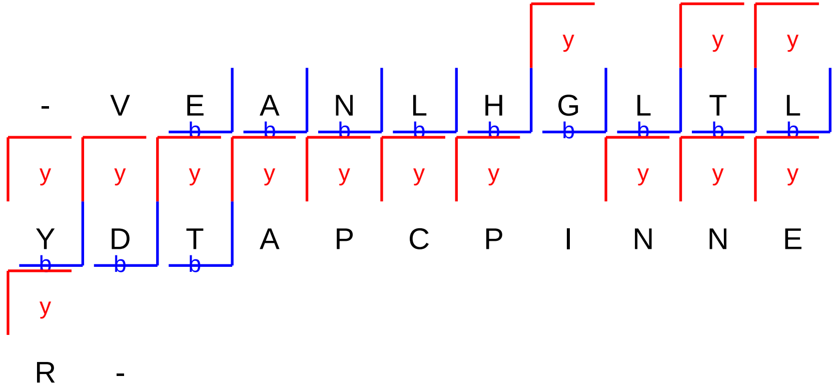

Raw File	Scan	Method	Score	m/z	Gene names
RO-QC-160121_ZackD_SS_Set_01_Fractio	27123	FTMS; HC	148.2	910.13	DRAM2

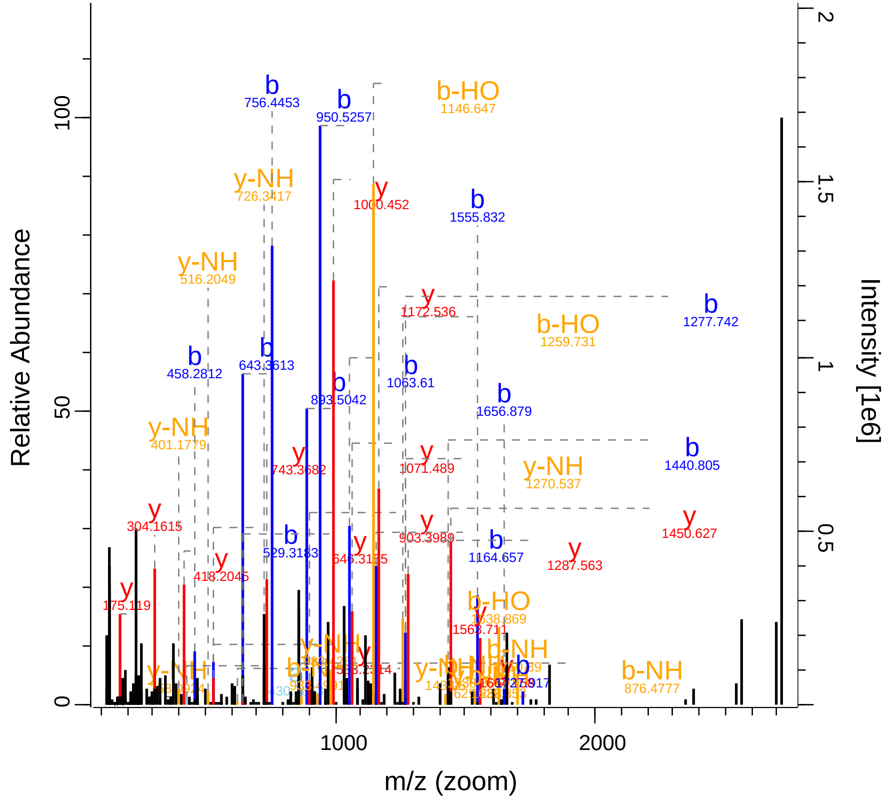

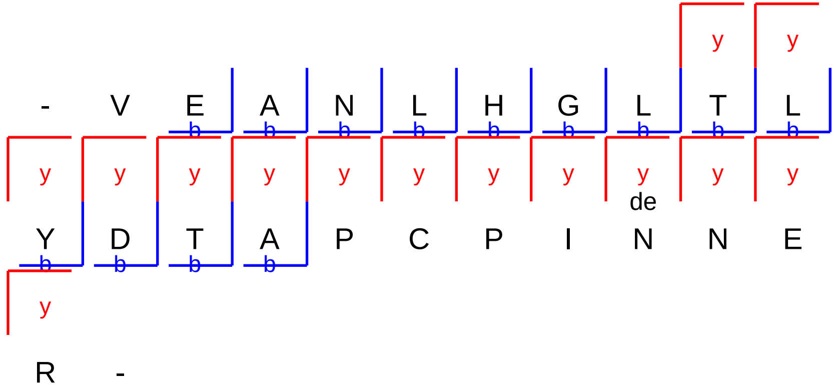

Raw File	Scan	Method	Score	m/z	Gene names
RO-QC-160121_ZackD_SS_Set_01_Fractio	27138	FTMS; HC	141.58	926.97	FAM58A

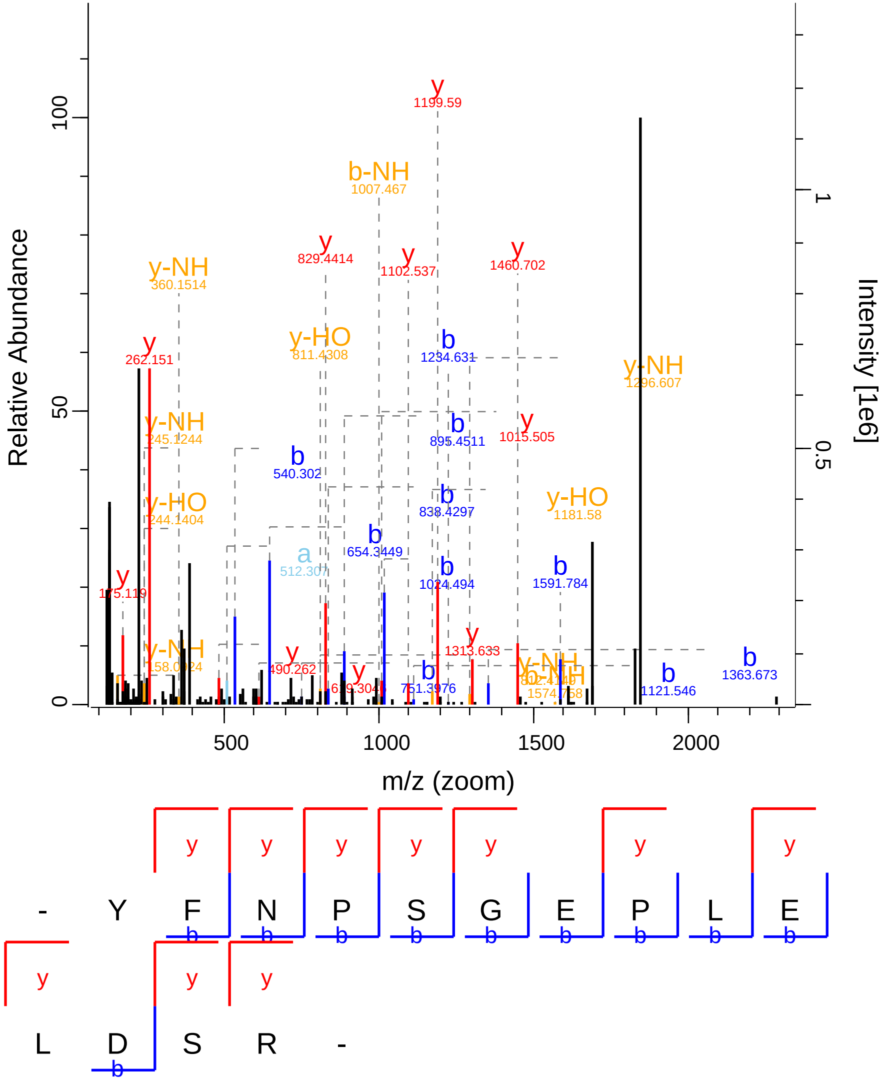

Raw File	Scan	Method	Score	m/z	Gene names
RO-QC-160121_ZackD_SS_Set_01_Fractio	27150	FTMS; HC	49.63	696.03	RASEF

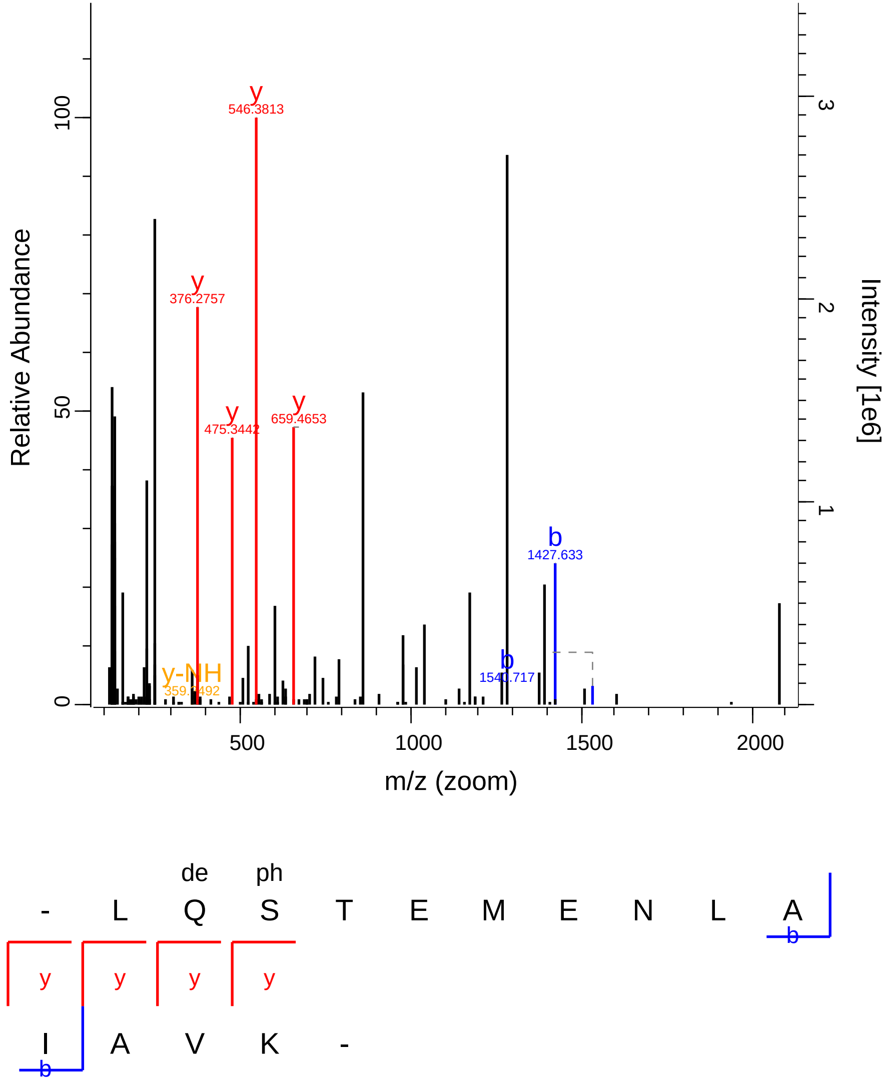

Raw File	Scan	Method	Score	m/z	Gene names
RO-QC-160121_ZackD_SS_Set_01_Fractio	27206	FTMS; HC	57.58	682.85	DRAM2

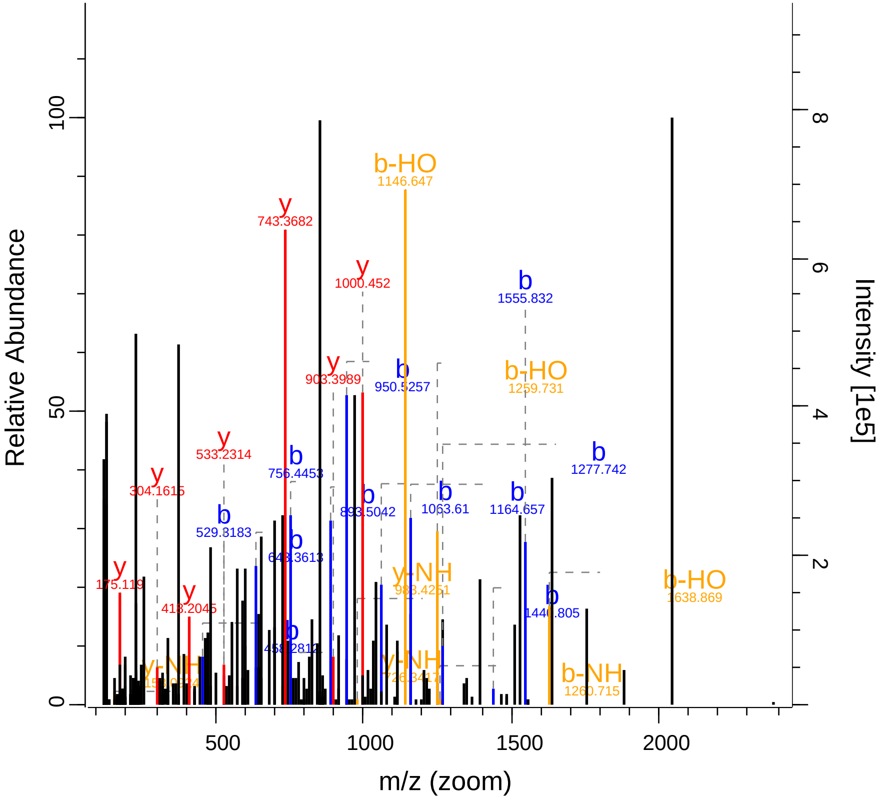

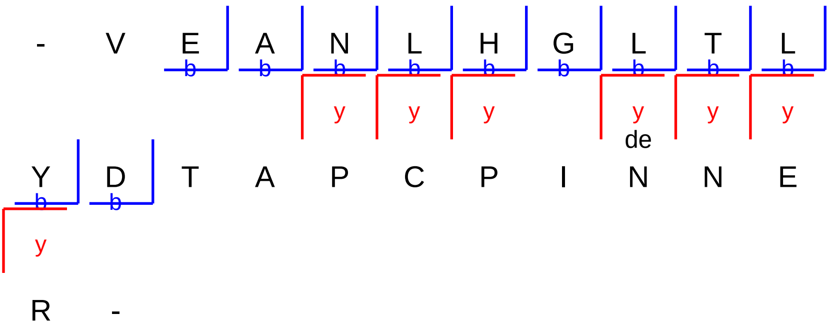

Raw File	Scan	Method	Score	m/z	Gene names
RO-QC-160121_ZackD_SS_Set_01_Fractio	27308	FTMS; HC	116.24	612.69	PLA2G16

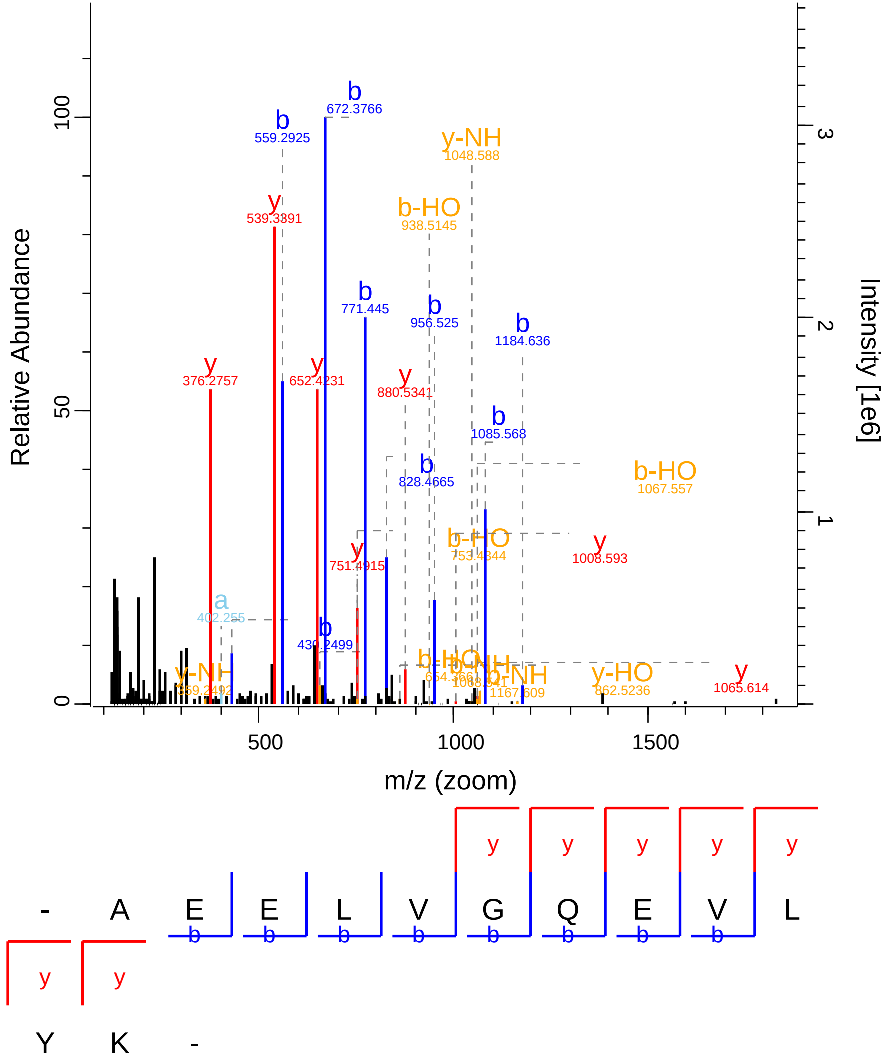

Raw File	Scan	Method	Score	m/z	Gene names
RO-QC-160121_ZackD_SS_Set_01_Fractio	27487	FTMS; HC	92.87	984.05	BAP1

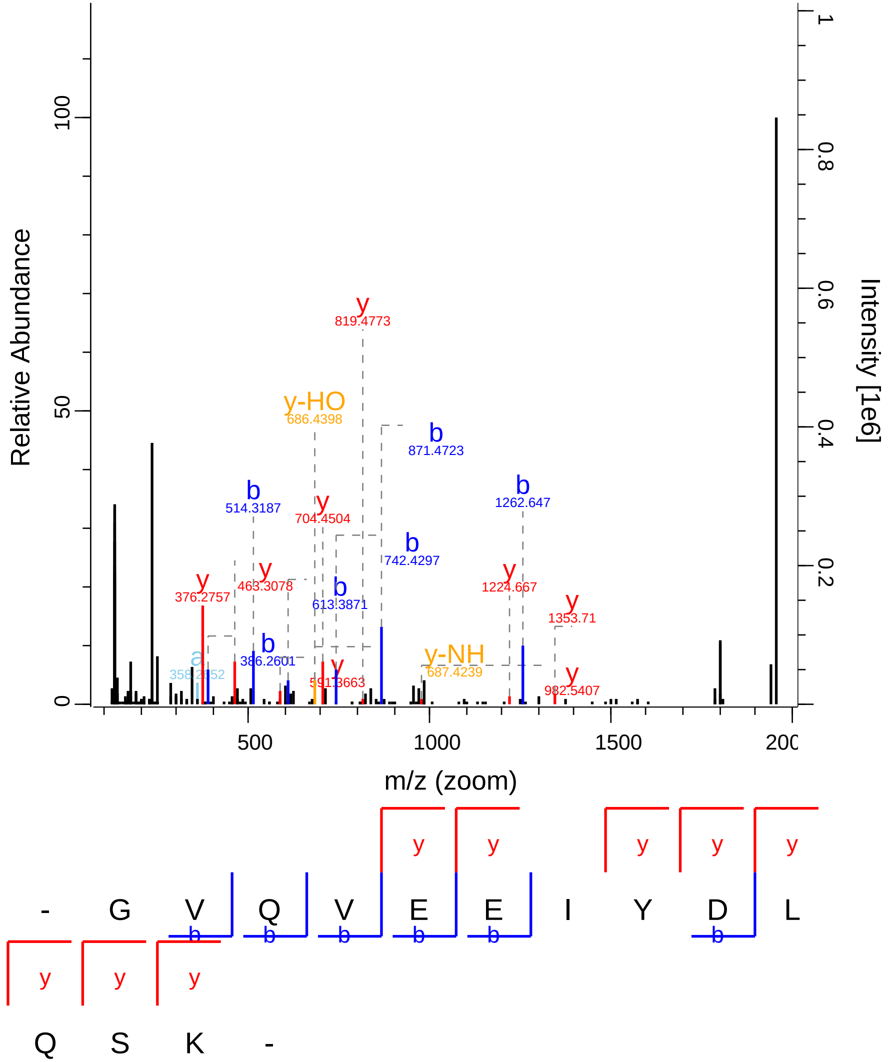

Raw File	Scan	Method	Score	m/z	Gene names
RO-QC-160121_ZackD_SS_Set_01_Fractio	28467	FTMS; HC	151.68	916.98	LOXL4

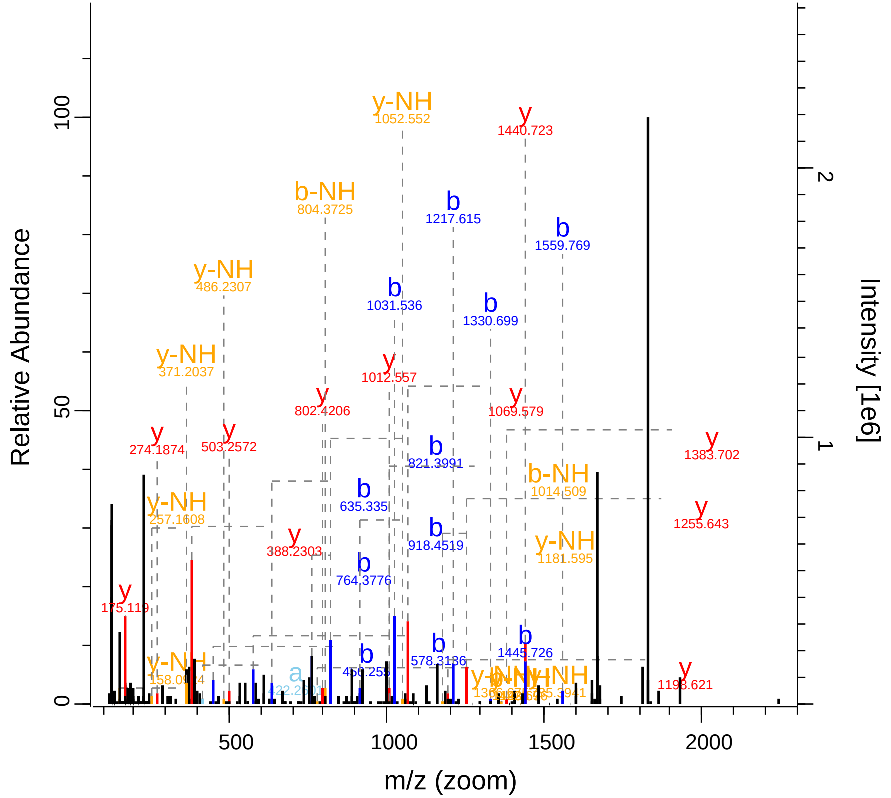

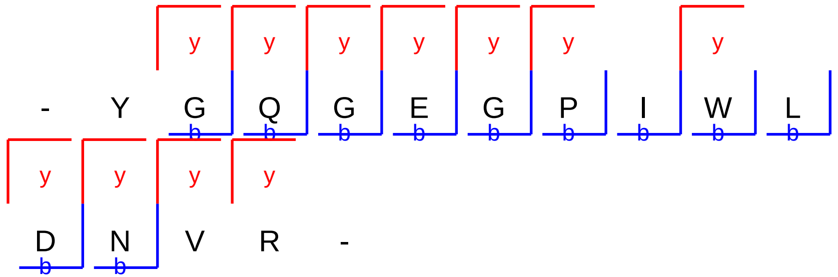

Raw File	Scan	Method	Score	m/z	Gene names
RO-QC-160121_ZackD_SS_Set_01_Fractio	29253	FTMS; HC	93.21	863.48	FAU

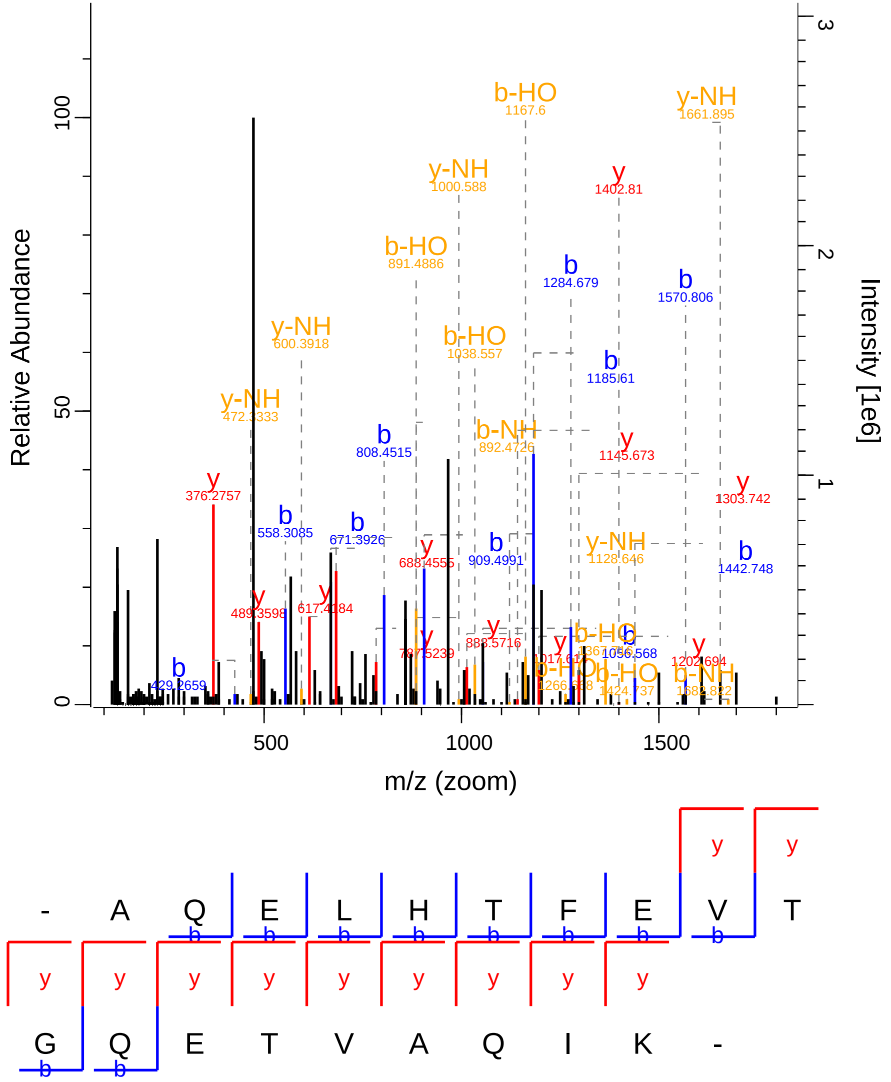

Raw File	Scan	Method	Score	m/z	Gene names
RO-QC-160121_ZackD_SS_Set_01_Fractio	29268	FTMS; HC	87.16	647.86	FAU

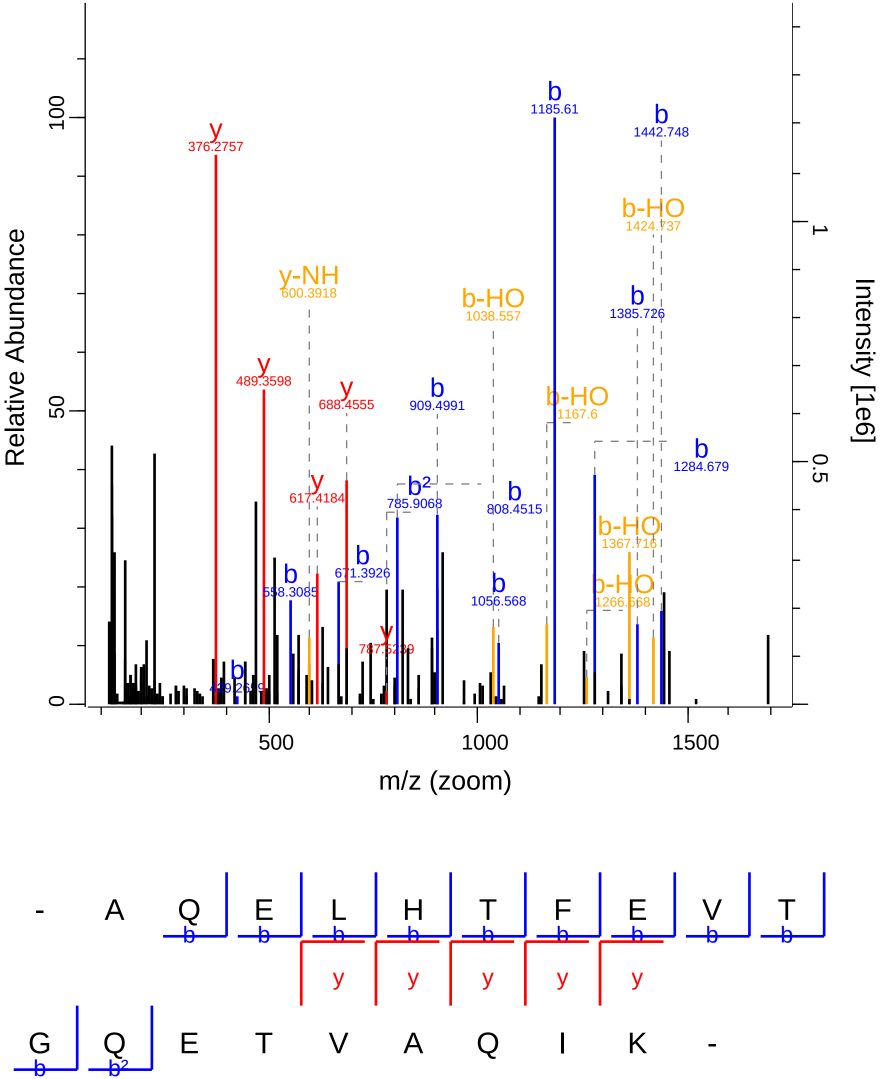

Raw File	Scan	Method	Score	m/z	Gene names
RO-QC-160121_ZackD_SS_Set_01_Fractio	30116	FTMS; HC	69.92	678.04	SERPINF2

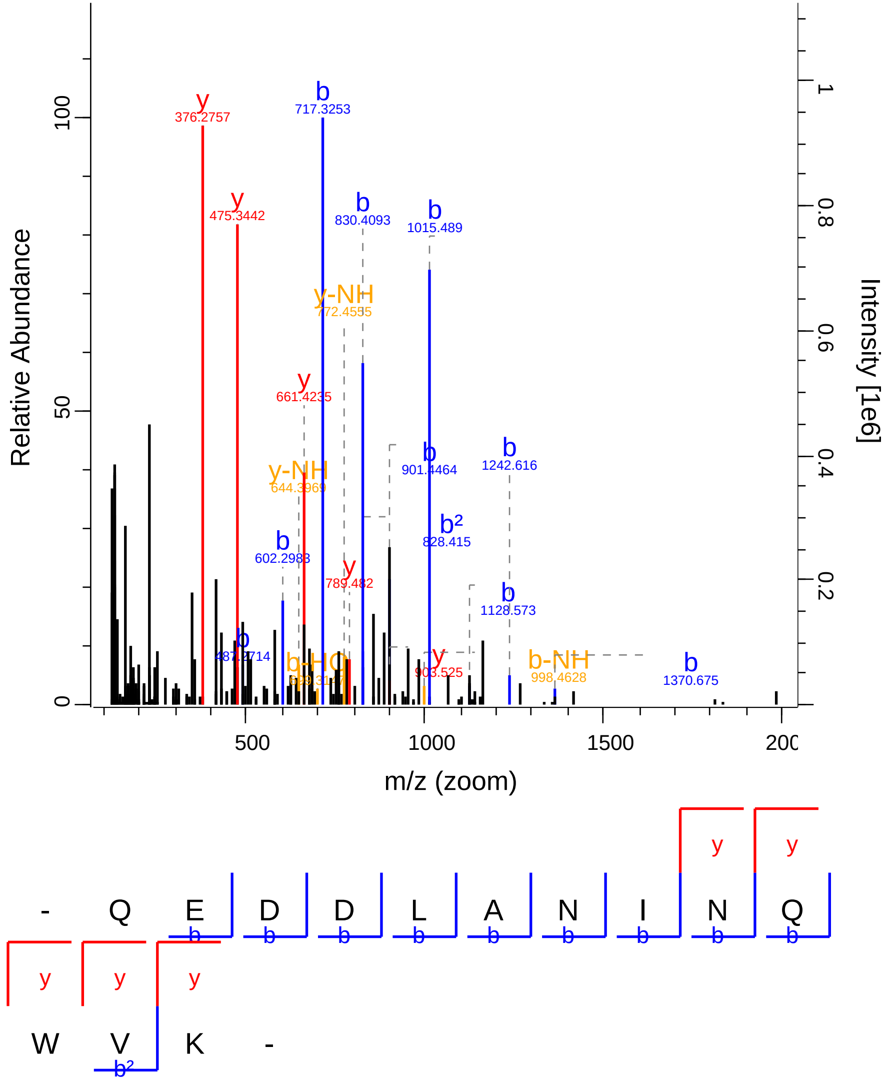

Raw File	Scan	Method	Score	m/z
RO-QC-160121_ZackD_SS_Set_01_Fraction_1	30220	FTMS; HCD	47.29	1027.03

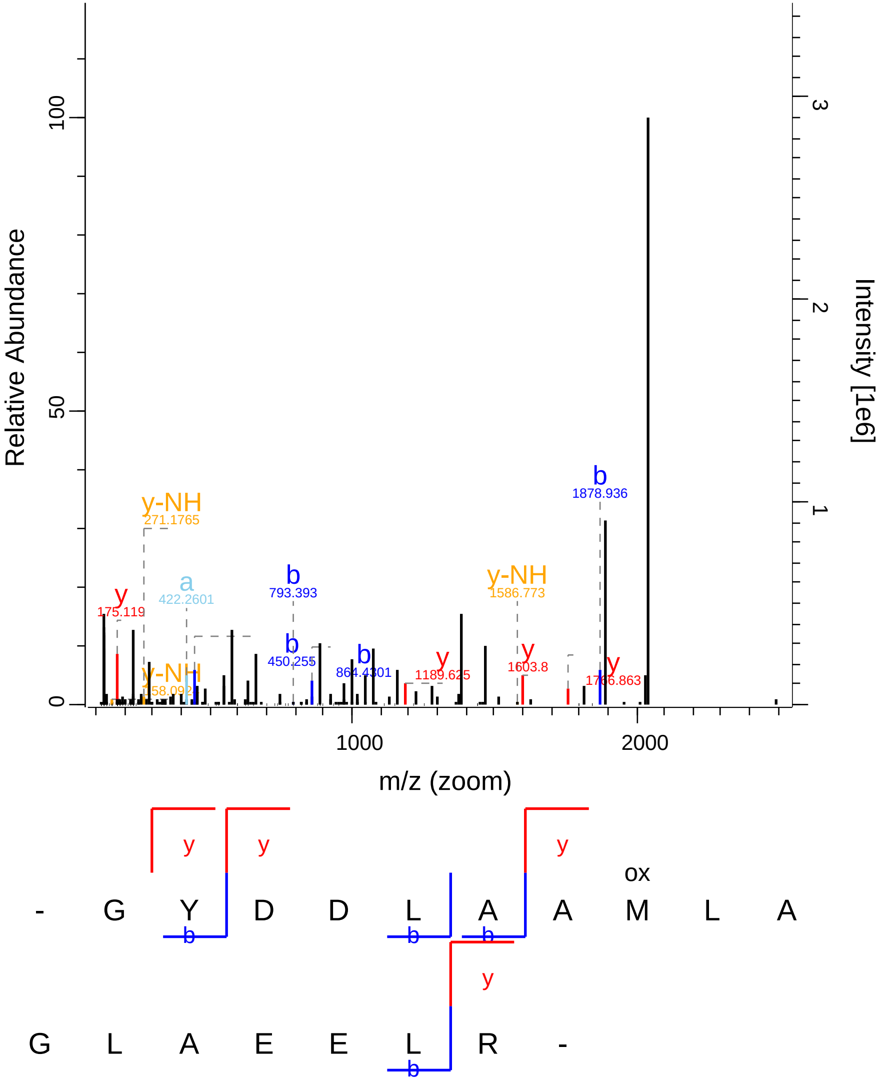

Raw File	Scan	Method	Score	m/z	Gene names
RO-QC-160121_ZackD_SS_Set_01_Fractio	30592	FTMS; HC	113.47	744.37	GANC

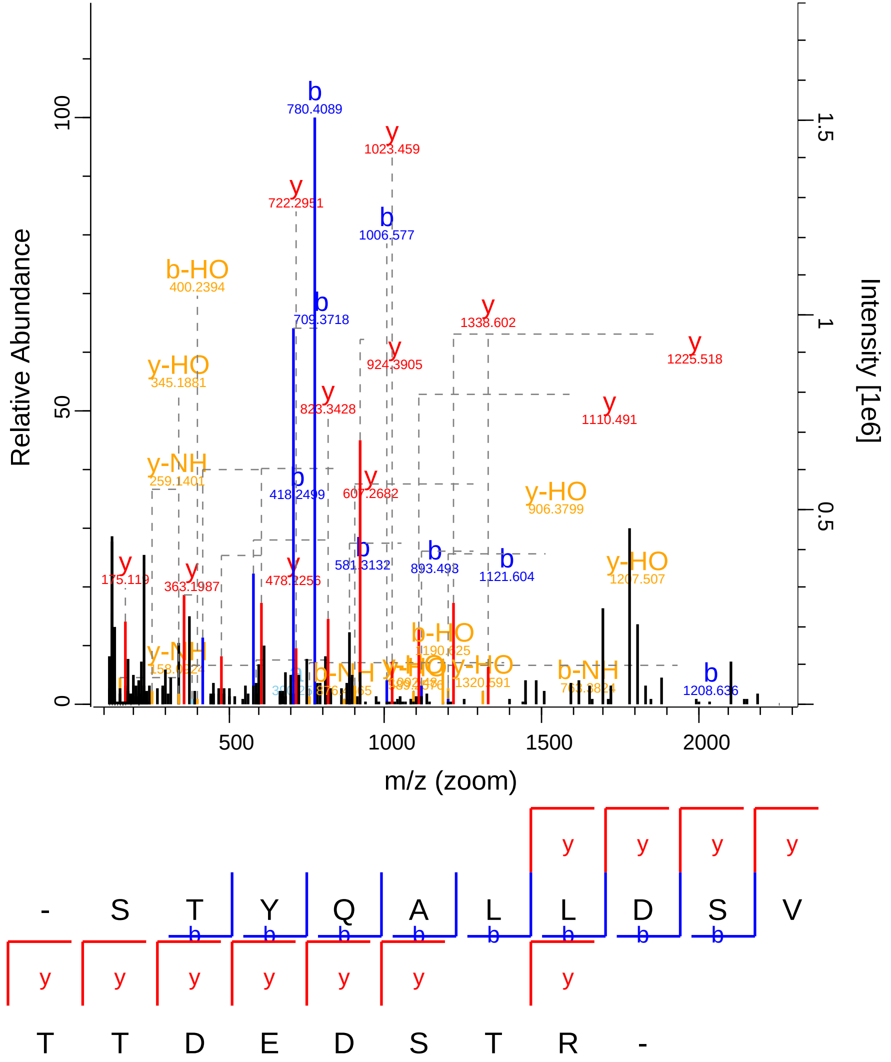

Raw File	Scan	Method	Score	m/z	Gene names
RO-QC-160121_ZackD_SS_Set_01_Fractio	30674	FTMS; HC	109.79	707.34	CLEC19A

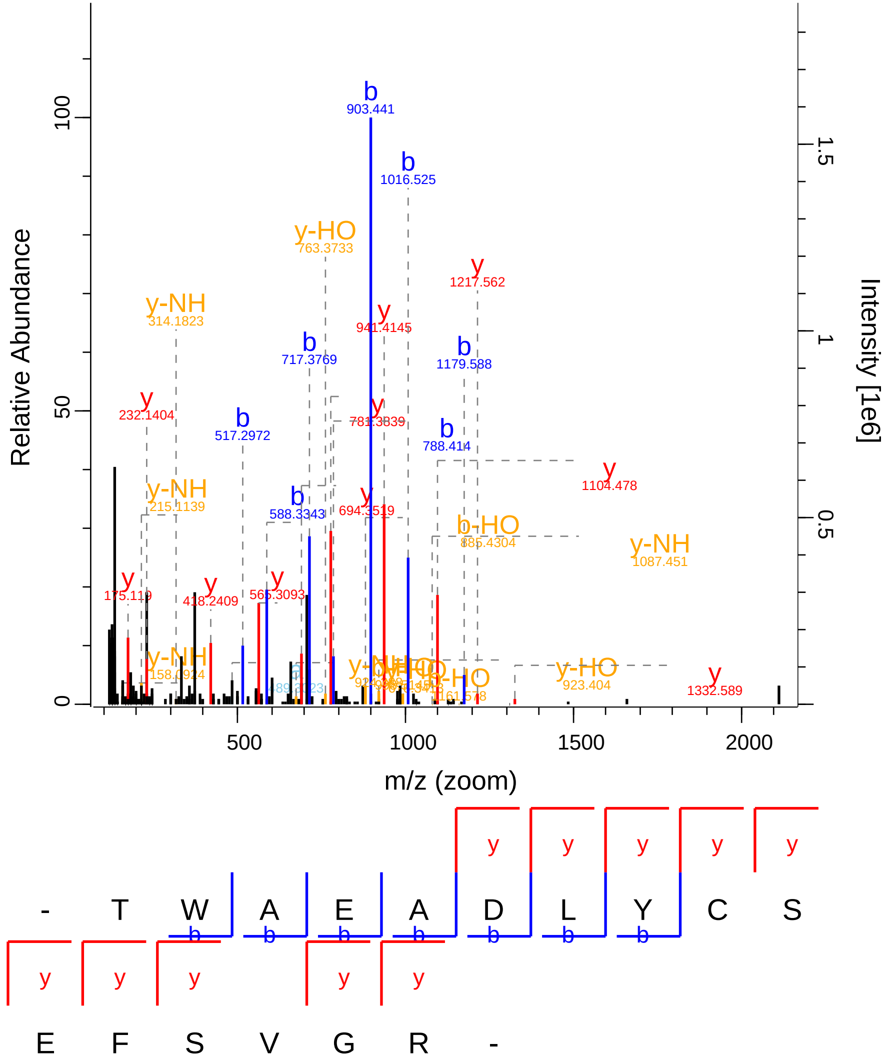

Raw File	Scan	Method	Score	m/z	Gene names
RO-QC-160121_ZackD_SS_Set_01_Fractio	30701	FTMS; HC	131.13	626.69	TMEM184C

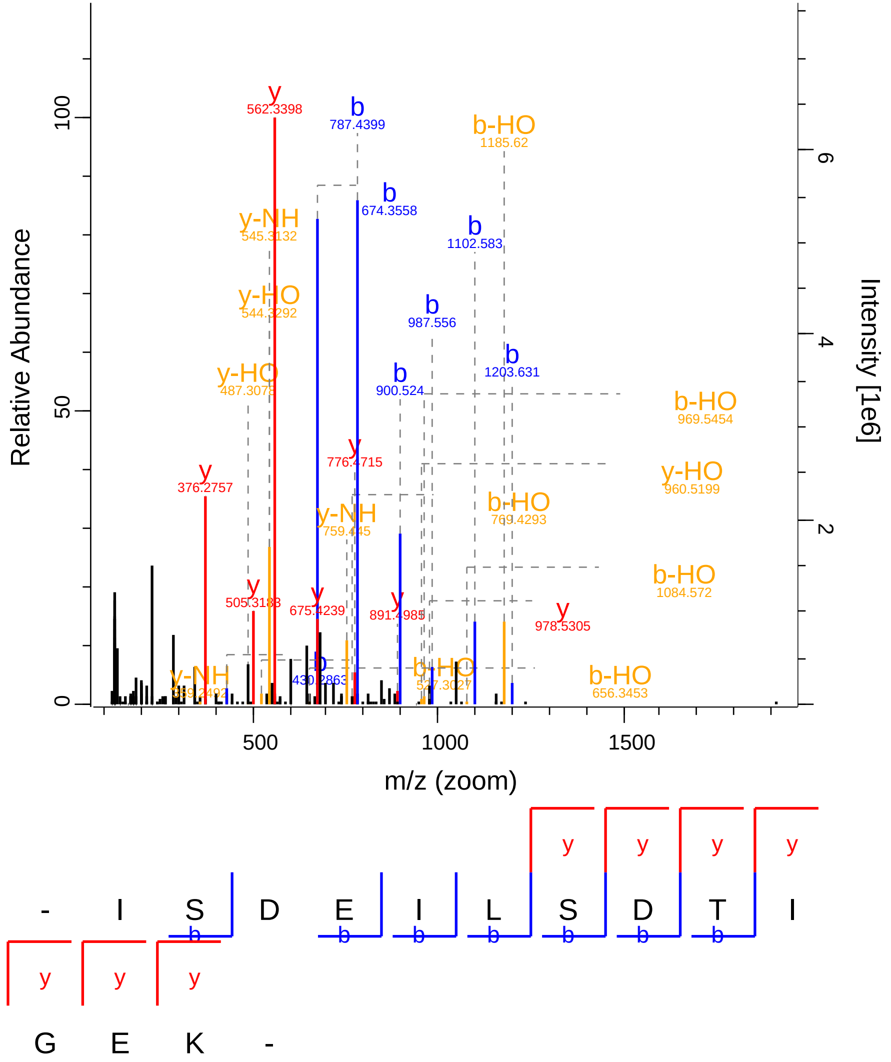

Raw File	Scan	Method	Score	m/z	Gene names
RO-QC-160121_ZackD_SS_Set_01_Fractio	30719	FTMS; HC	112.13	940.03	TMEM184C

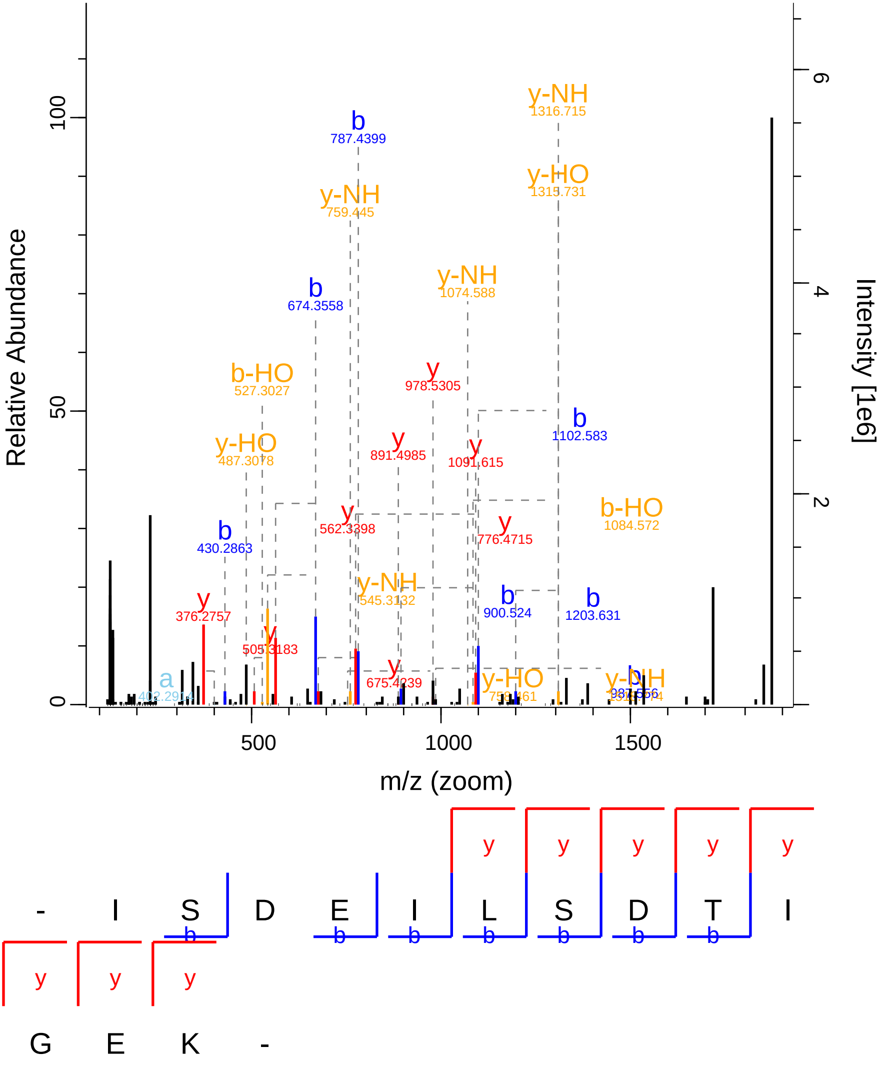

Raw File	Scan	Method	Score	m/z	Gene names
RO-QC-160121_ZackD_SS_Set_01_Fractio	30874	FTMS; HC	118.76	626.69	TMEM184C

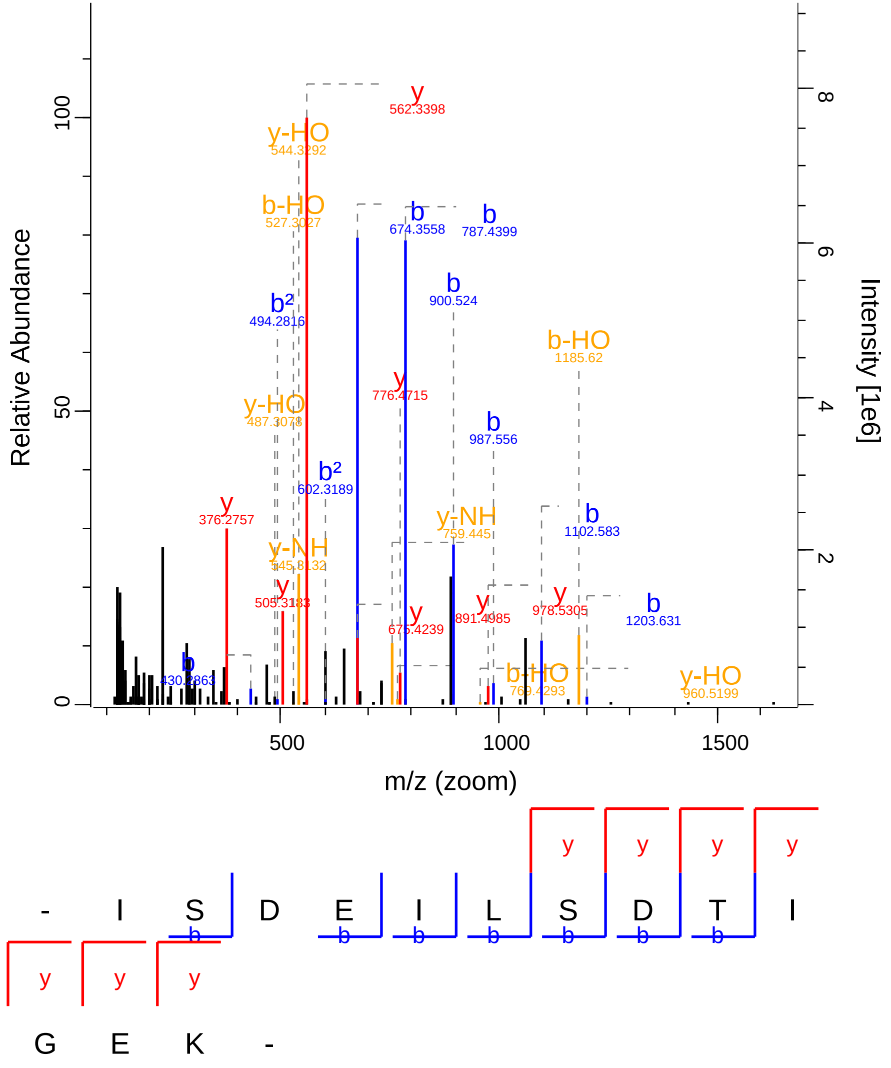

Raw File	Scan	Method	Score	m/z	Gene names
RO-QC-160121_ZackD_SS_Set_01_Fractio	30919	FTMS; HC	144.57	939.53	TMEM184C

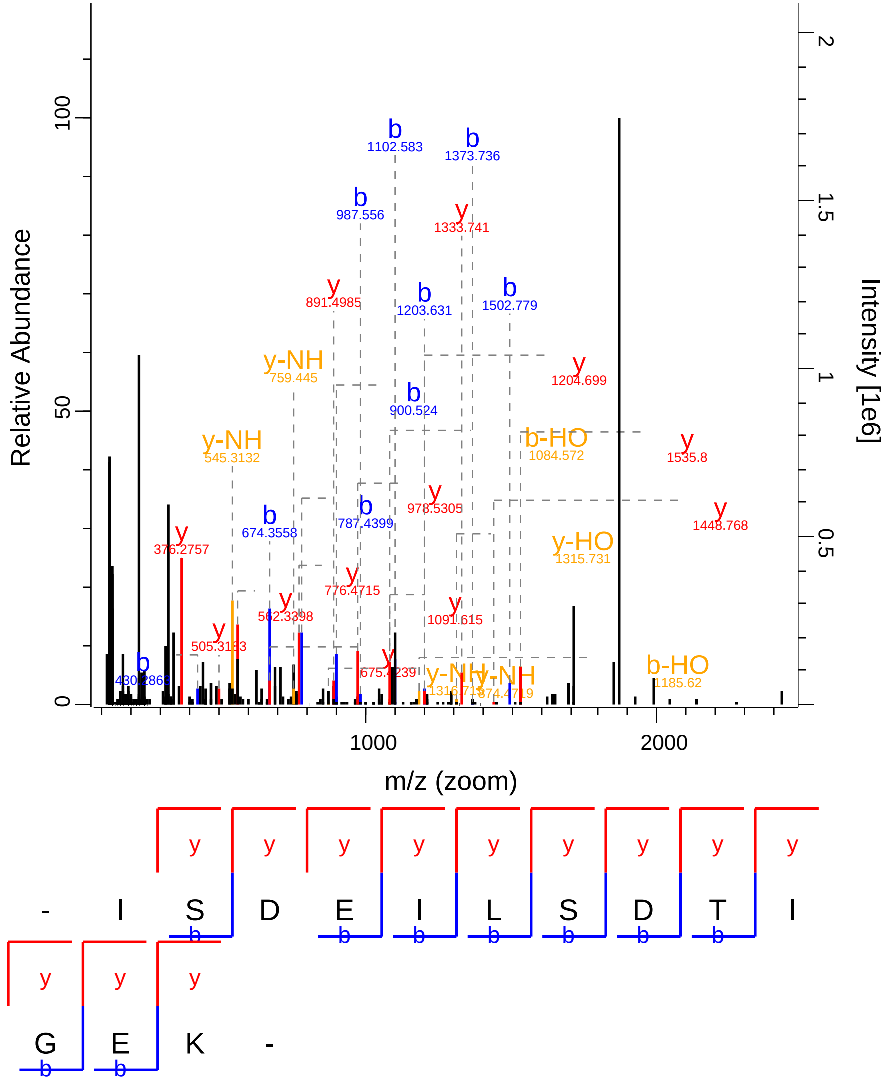

Raw File	Scan	Method	Score	m/z	Gene names
RO-QC-160121_ZackD_SS_Set_01_Fractio	31423	FTMS; HC	98.69	740.73	SMYD2

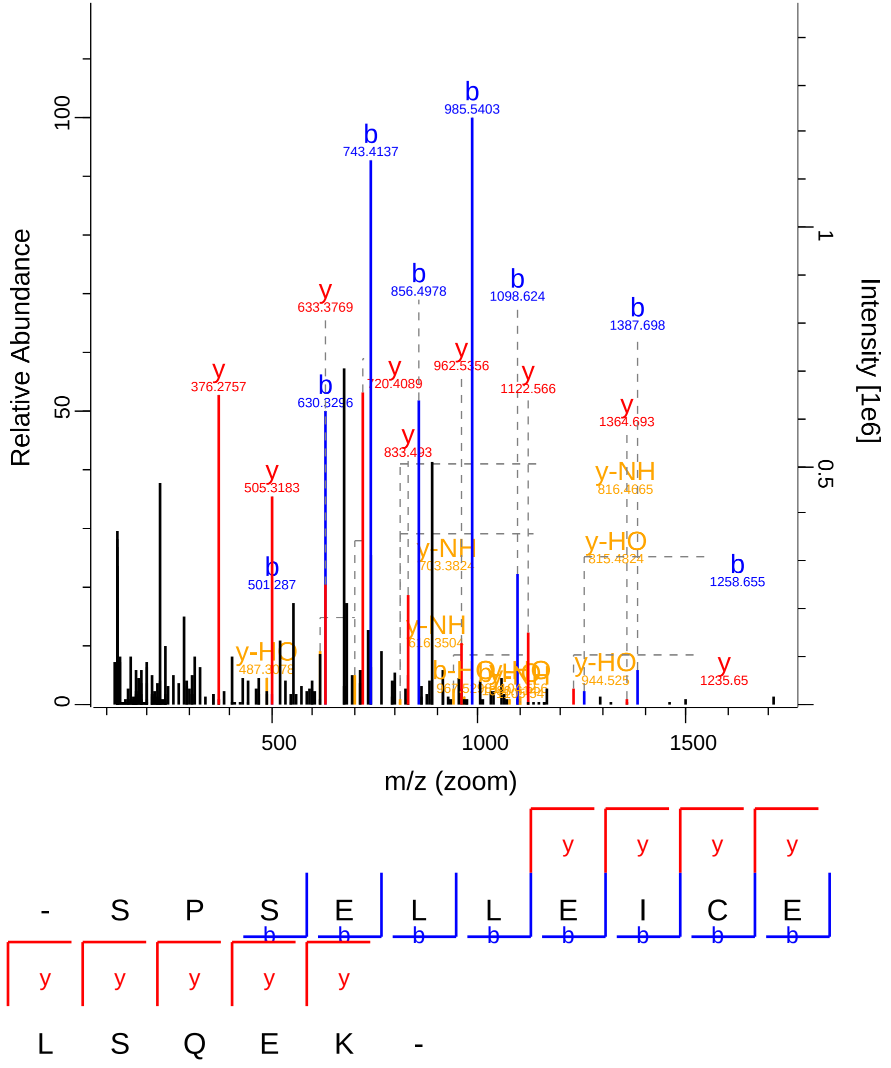

Raw File	Scan	Method	Score	m/z	Gene names
RO-QC-160121_ZackD_SS_Set_01_Fractio	31568	FTMS; HC	151.56	765.08	SCLT1

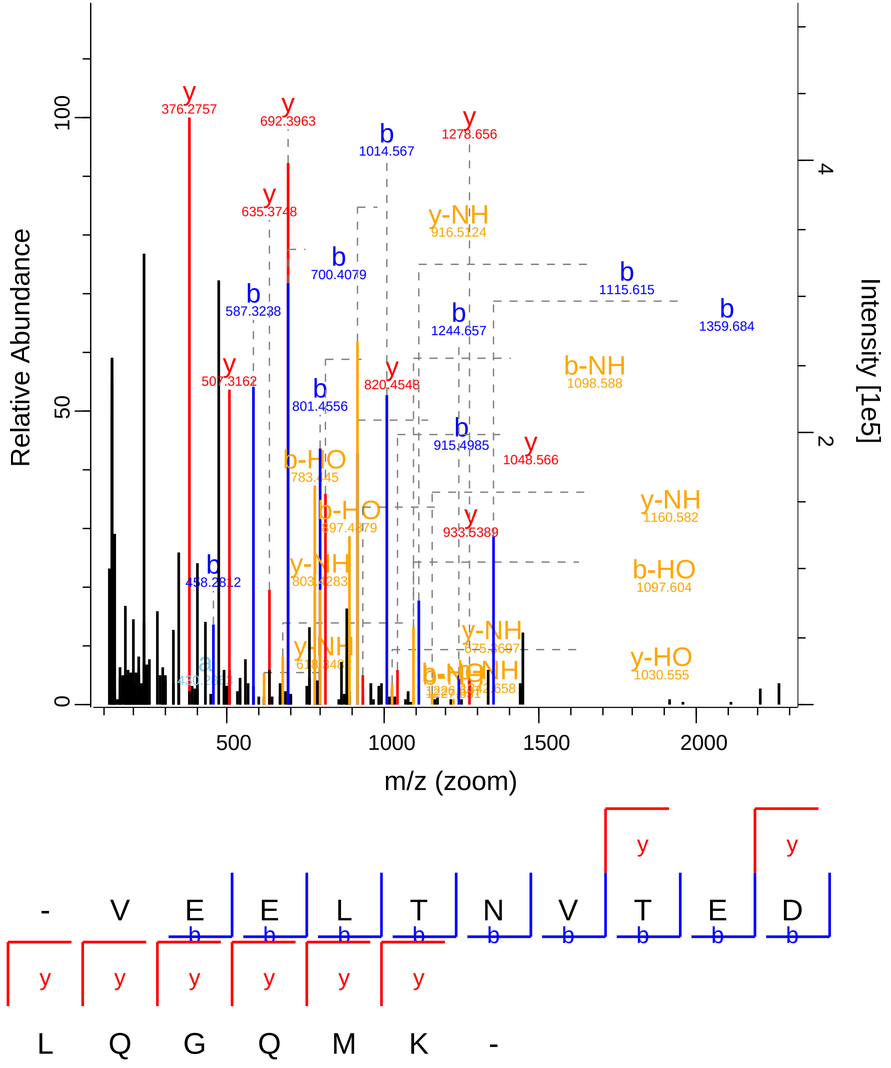

Raw File	Scan	Method	Score	m/z	Gene names
RO-QC-160121_ZackD_SS_Set_01_Fractio	32160	FTMS; HC	138.99	881.95	R3HDM4

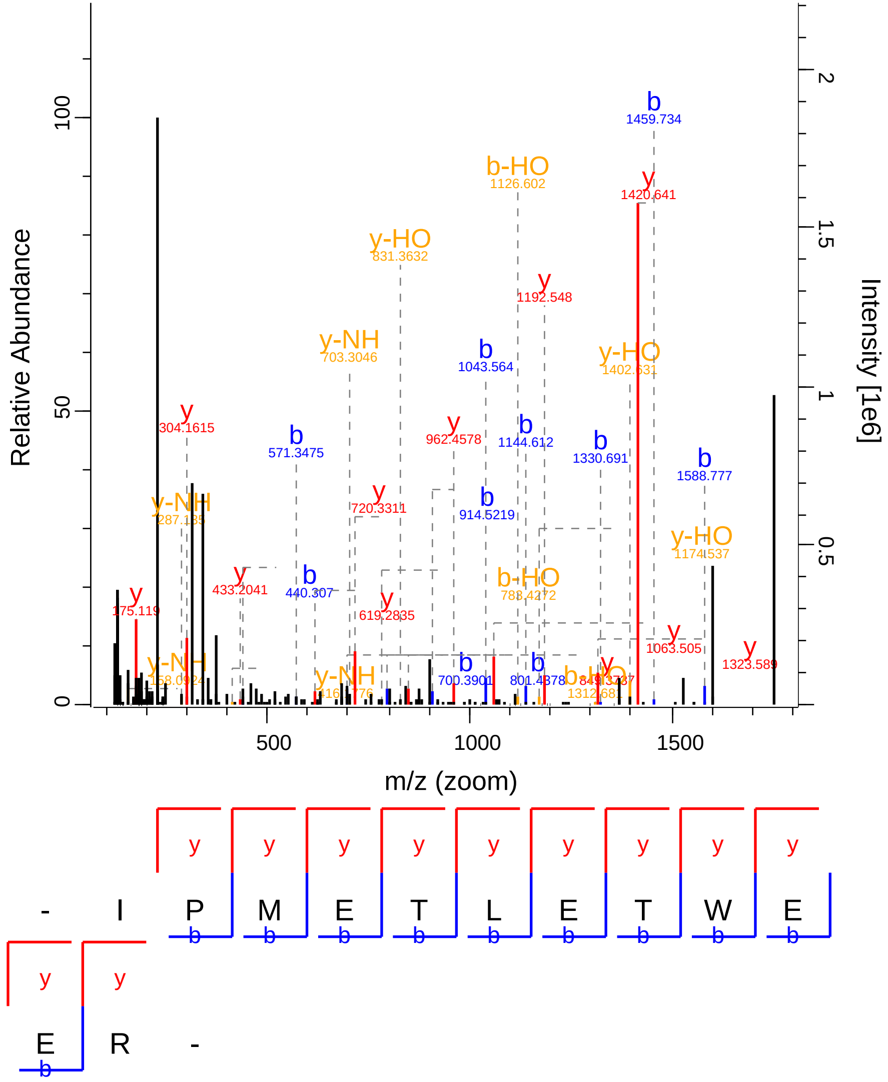

Raw File	Scan	Method	Score	m/z	Gene names
RO-QC-160121_ZackD_SS_Set_01_Fractio	32526	FTMS; HC	78.69	761.35	VCPKMT

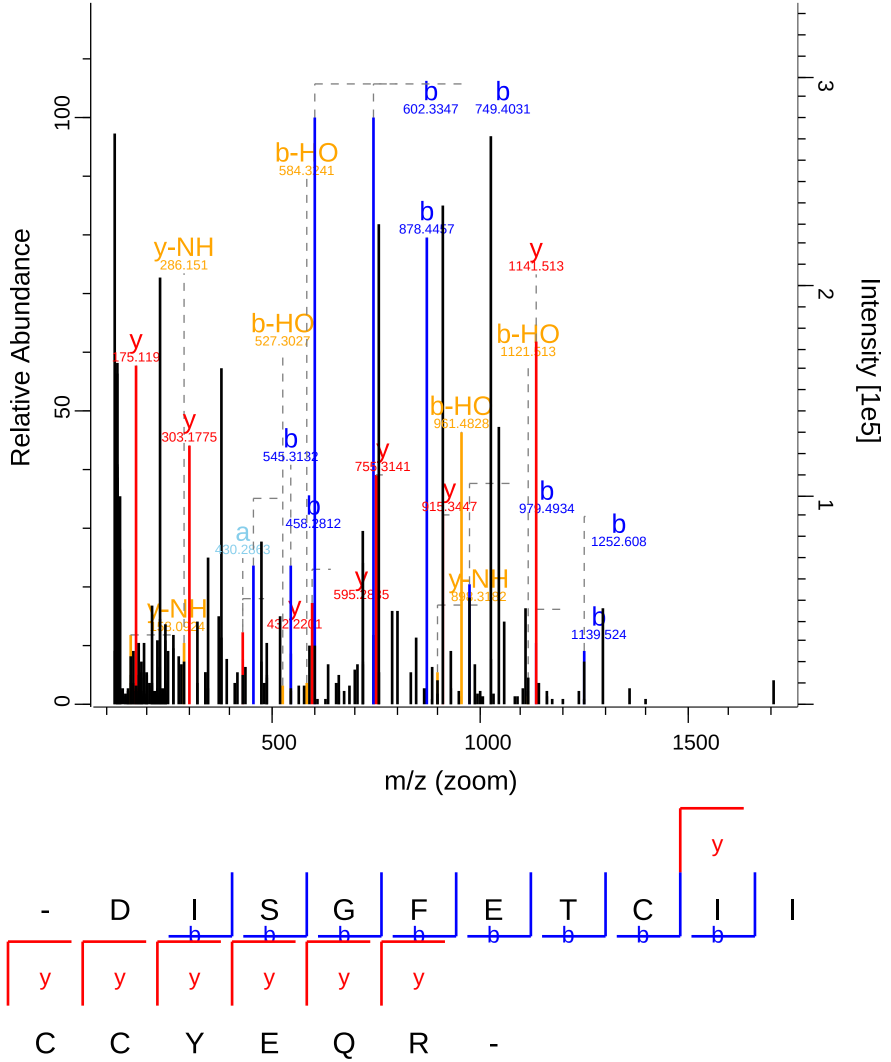

Raw File	Scan	Method	Score	m/z	Gene names
RO-QC-160121_ZackD_SS_Set_01_Fractioı	32786	FTMS; HCI	130.24	679.7	CCDC61

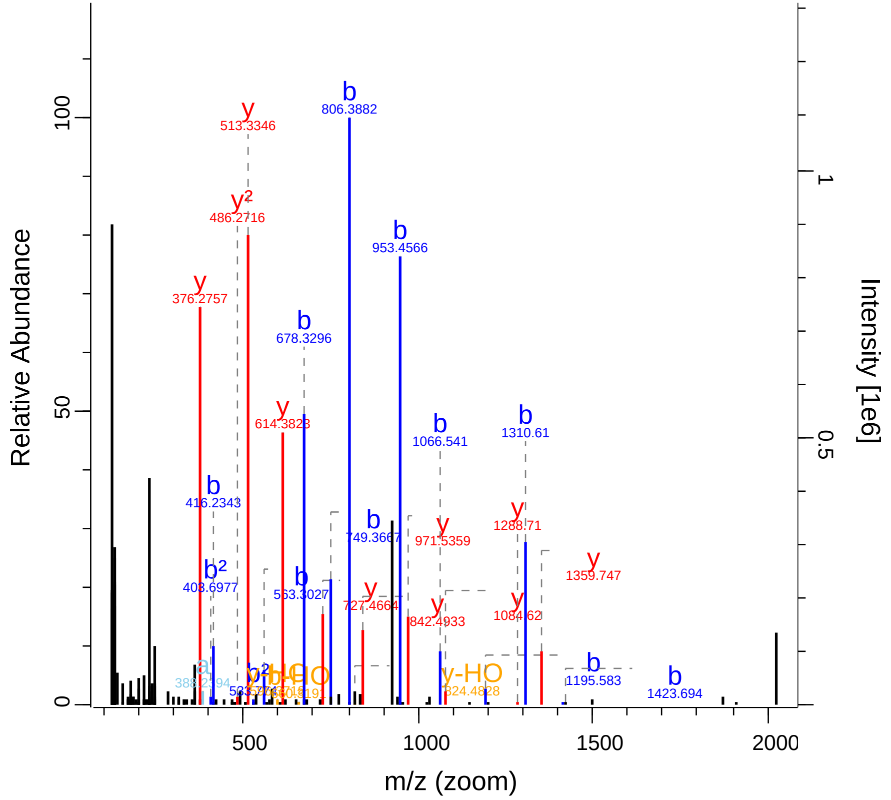

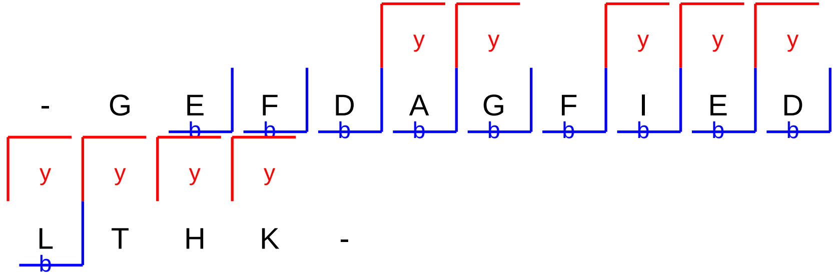

Raw File	Scan	Method	Score	m/z	Gene names
RO-QC-160121_ZackD_SS_Set_01_Fractio	33301	FTMS; HC	77.02	810.42	RGMA

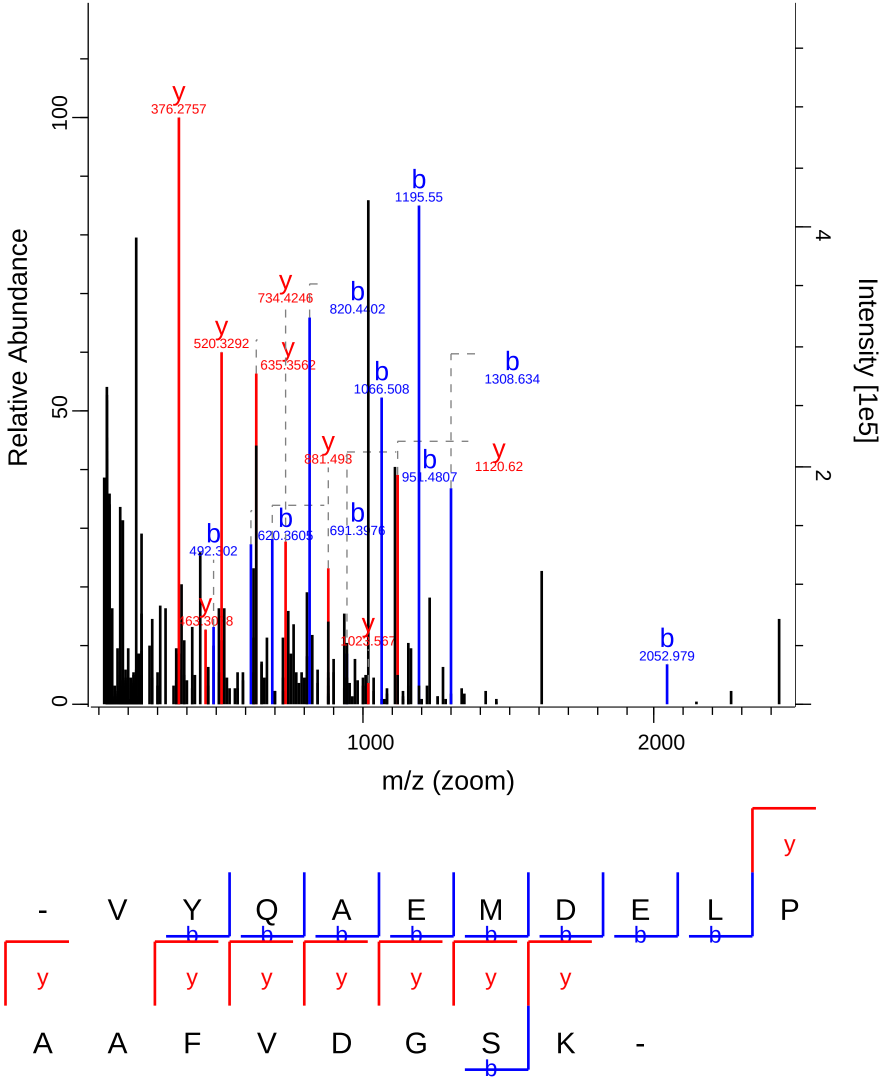

Raw File	Scan	Method	Score	m/z	Gene names
RO-QC-160121_ZackD_SS_Set_01_Fractio	33488	FTMS; HC	161.48	952.52	NRSN2

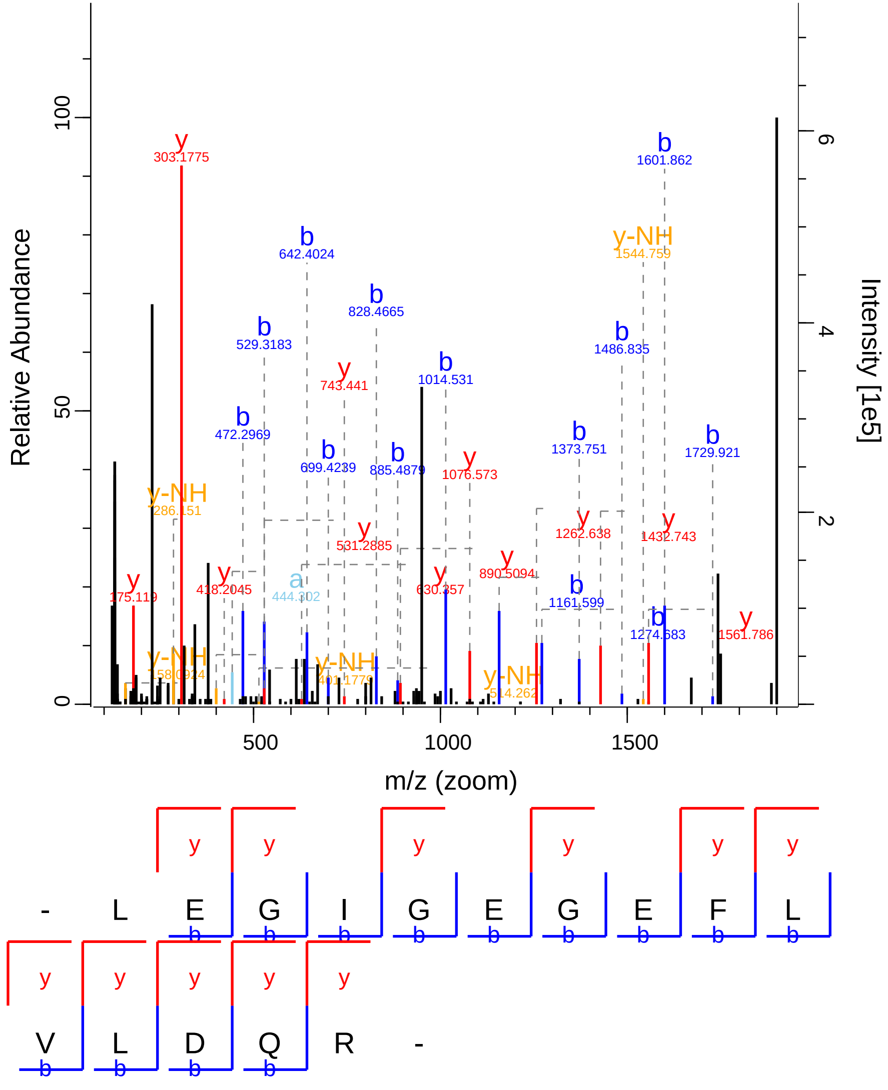

Raw File	Scan	Method	Score	m/z	Gene names
RO-QC-160121_ZackD_SS_Set_01_Fractic	33876	FTMS; HC	76.29	1037.86	SFT2D1

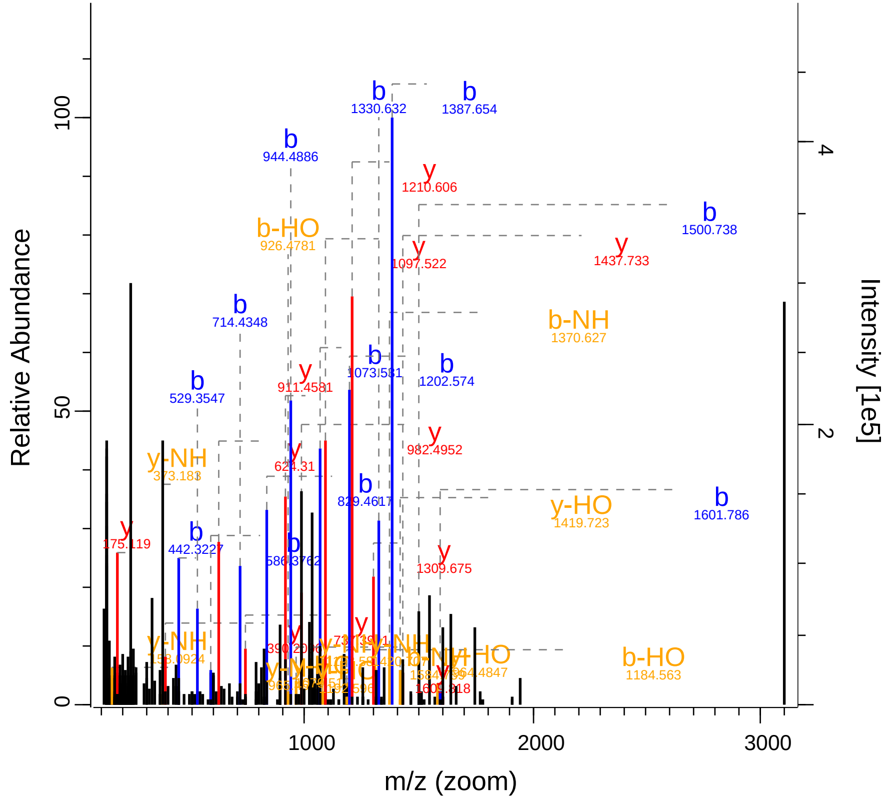

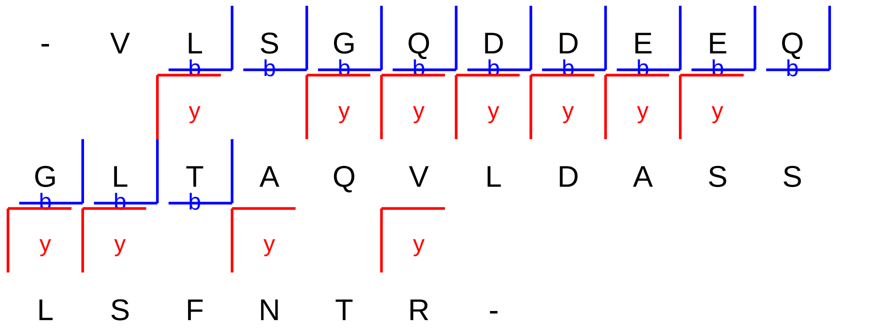

Raw File	Scan	Method	Score	m/z
RO-QC-160121_ZackD_SS_Set_01_Fraction_1	34336	FTMS; HCD	88.68	766.43

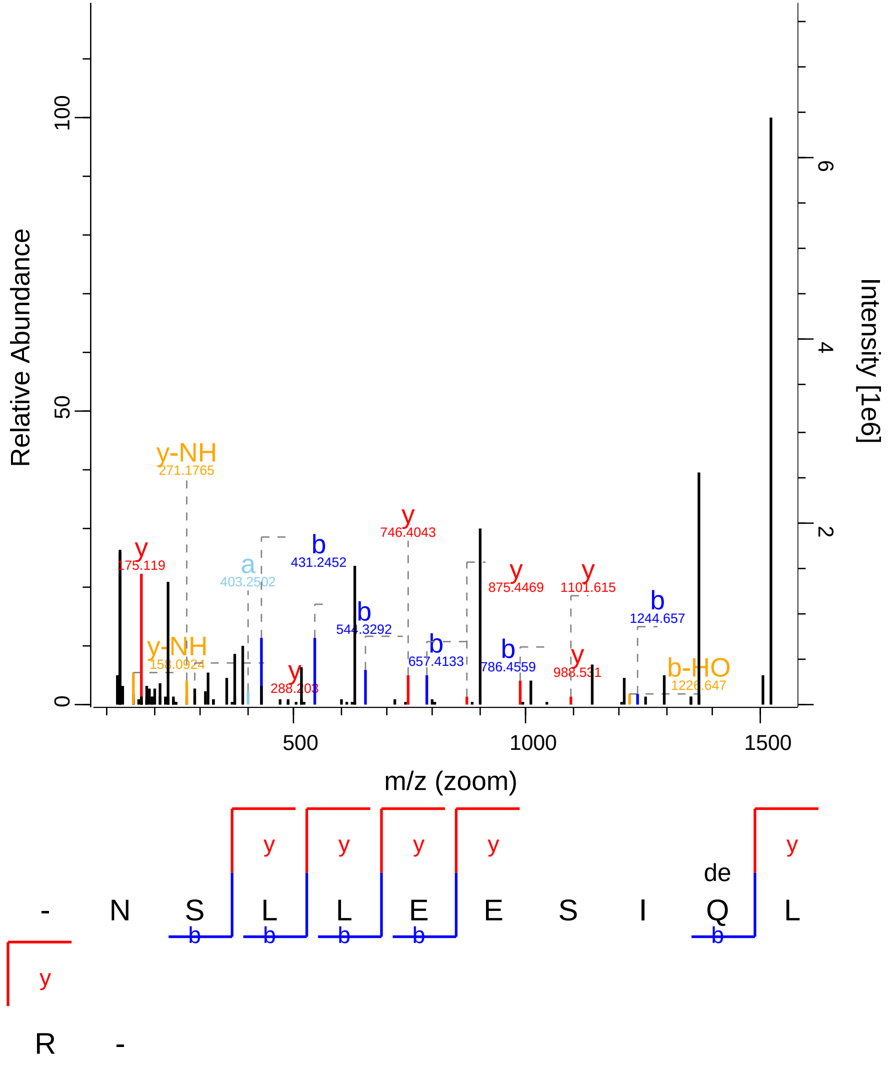

Raw File	Scan	Method	Score	m/z	Gene names
RO-QC-160121_ZackD_SS_Set_01_Fractior	4758	FTMS; HCI	66.27	412.22	ZSCAN26

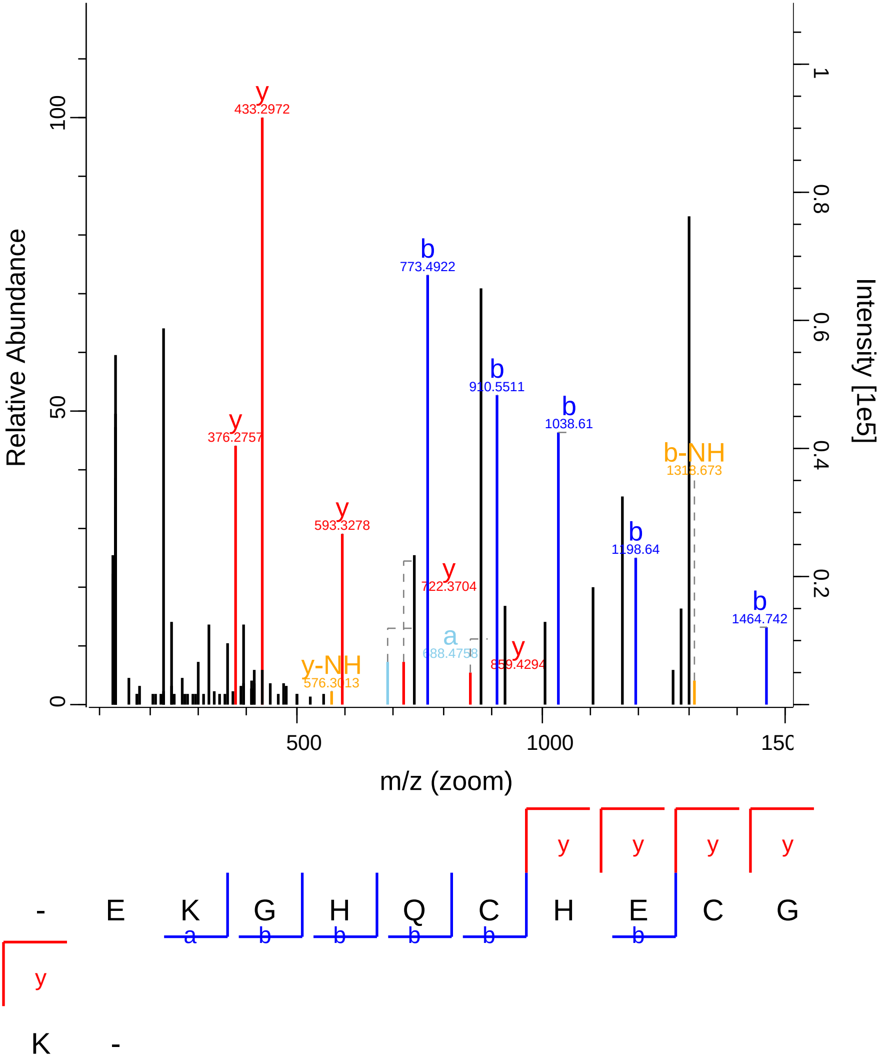

Raw File	Scan	Method	Score	m/z	Gene names
RO-QC-160121_ZackD_SS_Set_01_Fractior	4775	FTMS; HCI	43.08	515.02	ZSCAN26

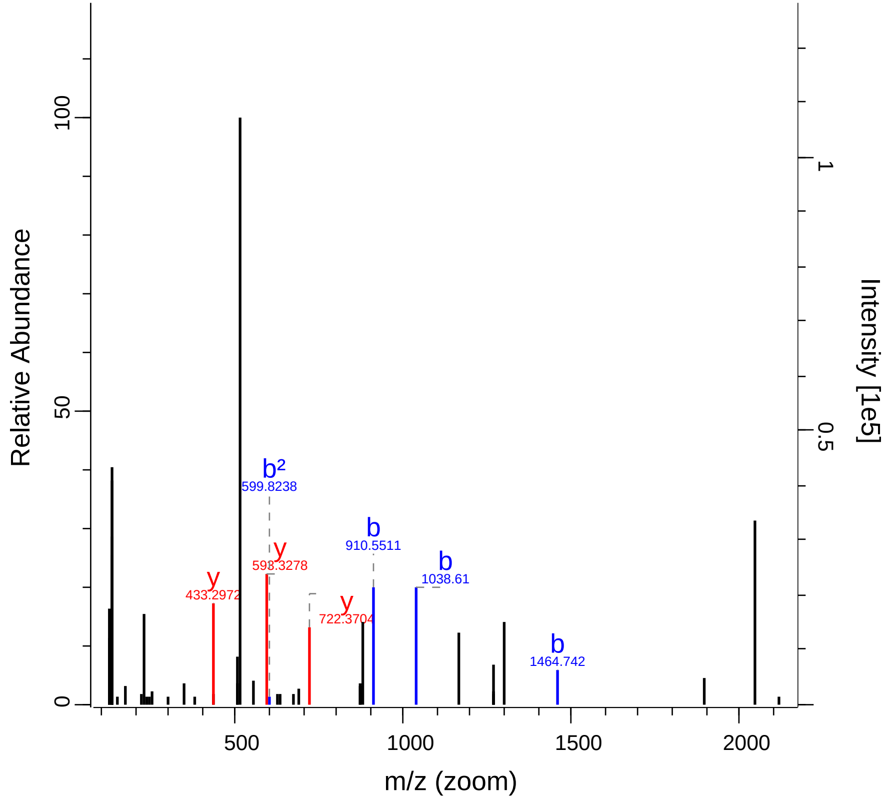

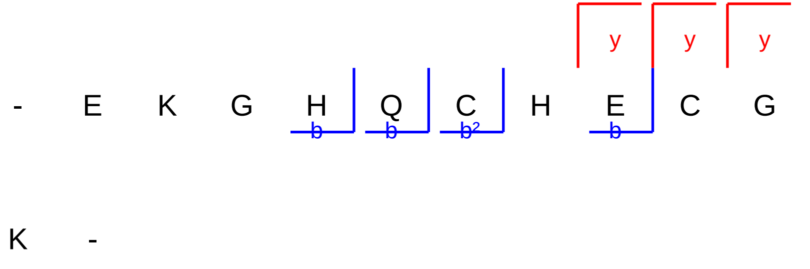

Raw File	Scan	Method	Score	m/z	Gene names
RO-QC-160121_ZackD_SS_Set_01_Fractior	4789	FTMS; HCI	94.69	443.76	C16orf87

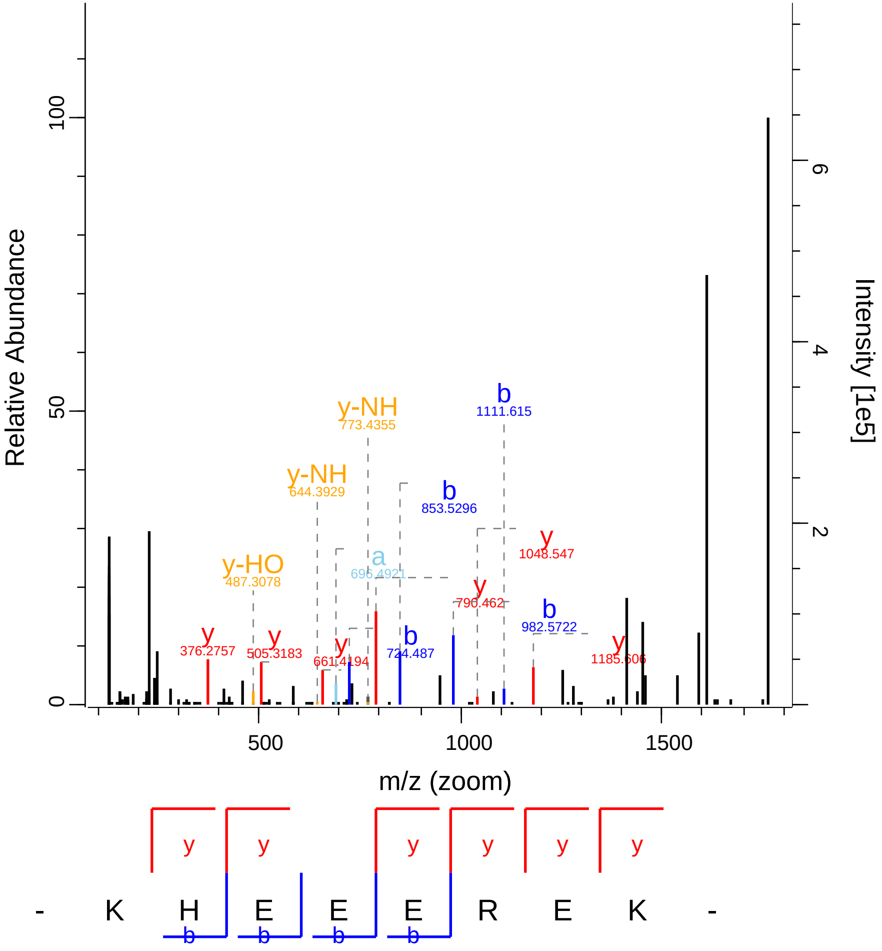

Raw File	Scan	Method	Score	m/z	Gene names
RO-QC-160121_ZackD_SS_Set_01_Fractior	4795	FTMS; HC	166.33	355.21	C16orf87

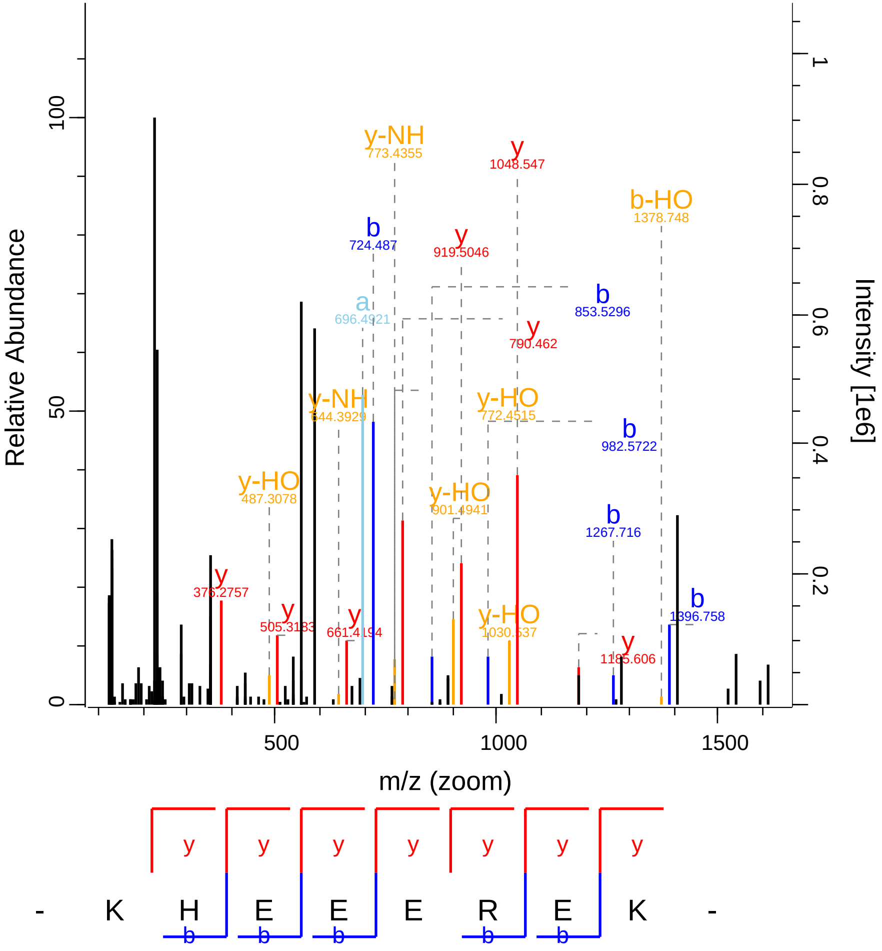

Raw File	Scan	Method	Score	m/z	Gene names
RO-QC-160121_ZackD_SS_Set_01_Fractior	5154	FTMS; HCI	69.82	515.24	DOT1L

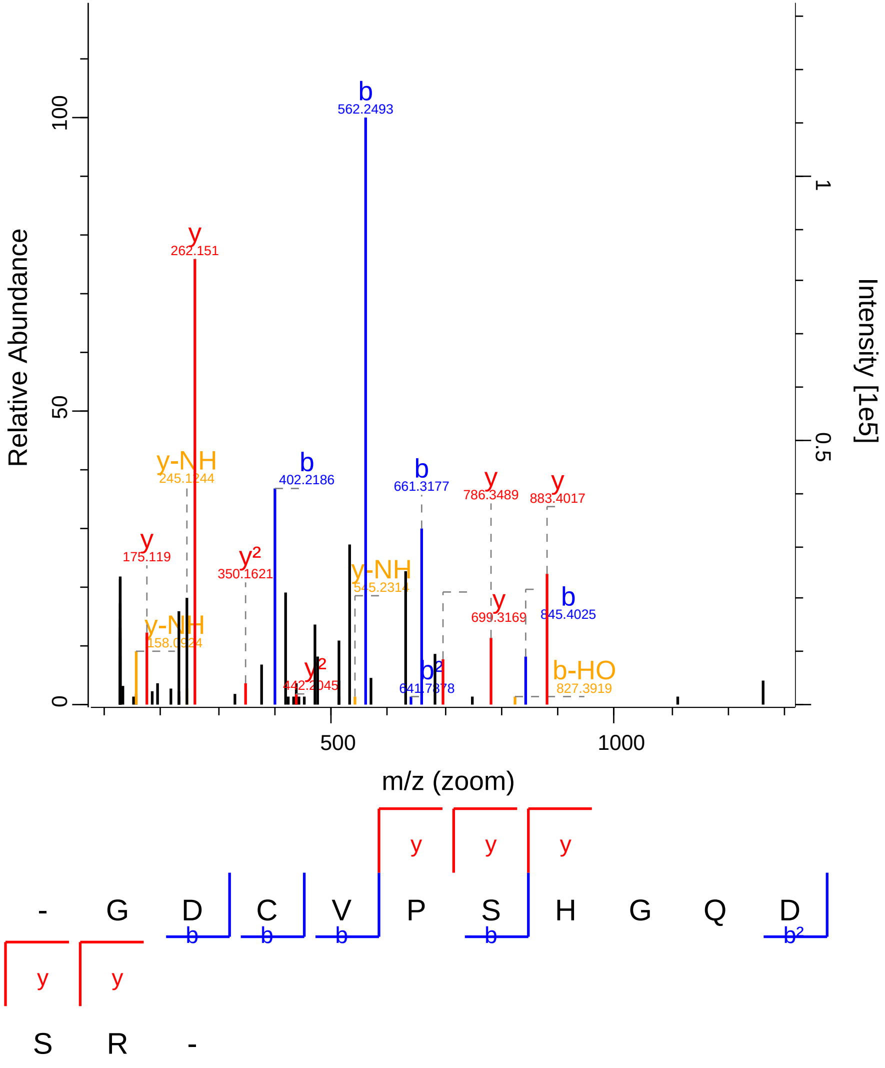

Raw File	Scan	Method	Score	m/z	Gene names
RO-QC-160121_ZackD_SS_Set_01_Fractior	5482	FTMS; HCI	41.54	520.91	GREM1

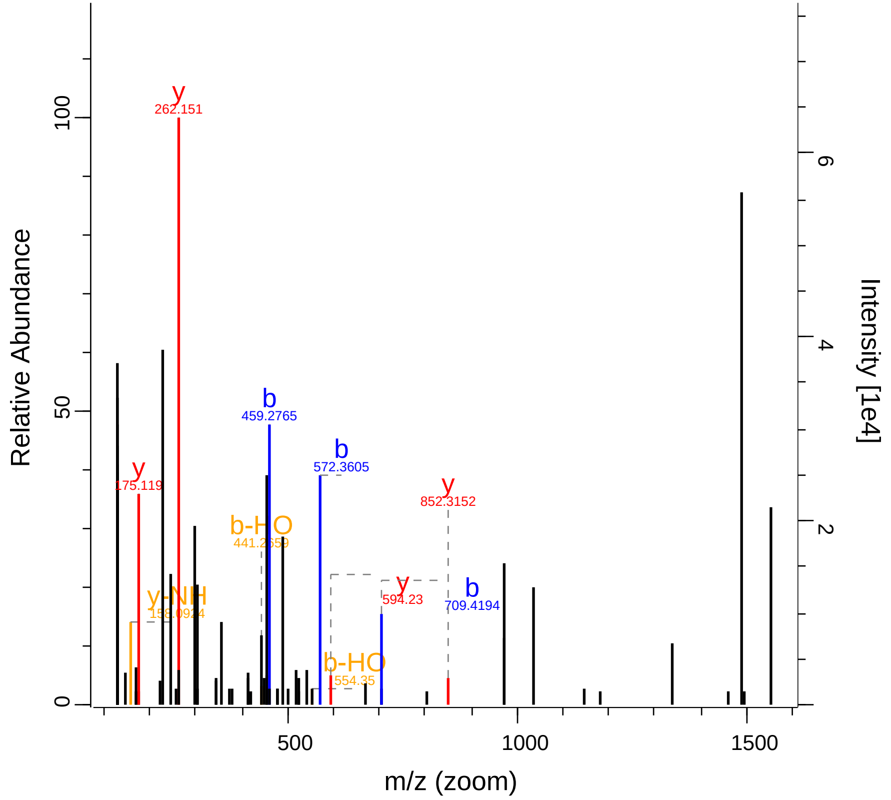

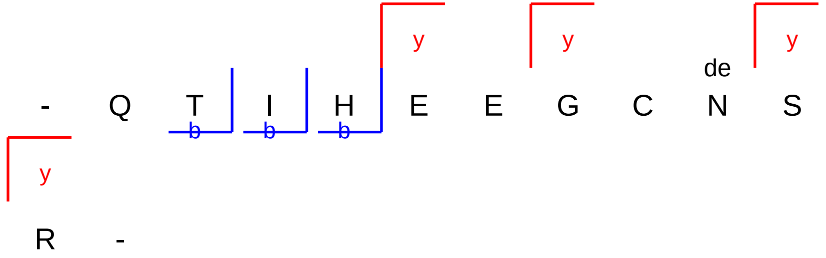

Raw File	Scan	Method	Score	m/z	Gene names
RO-QC-160121_ZackD_SS_Set_01_Fractio	5667	FTMS; HC	127.76	640.99	RUNDC1

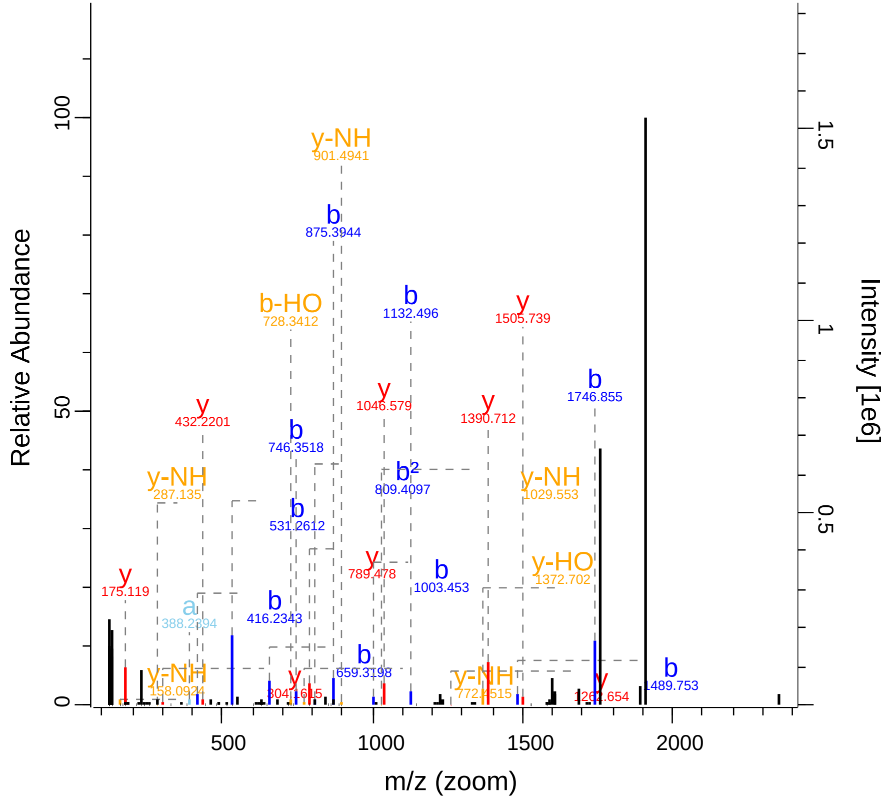

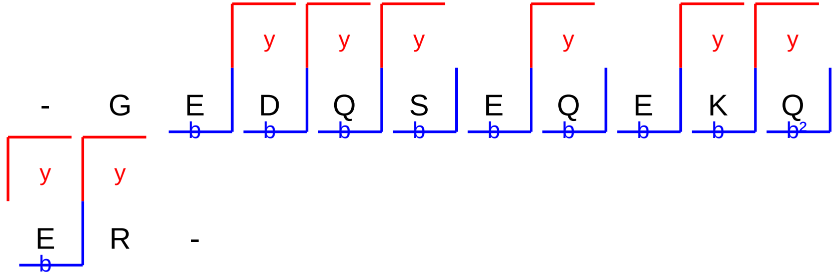

Raw File	Scan	Method	Score	m/z	Gene names
RO-QC-160121_ZackD_SS_Set_01_Fract	5688 2	FTMS; HC	88.73	643.68	ARHGEF35;ARHGE

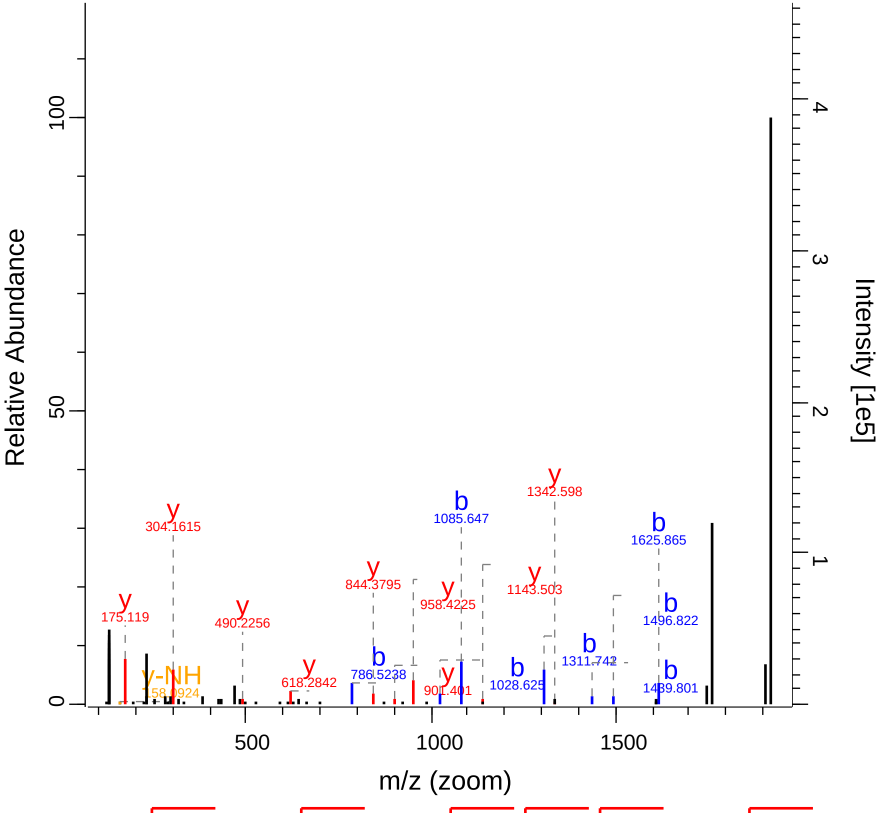

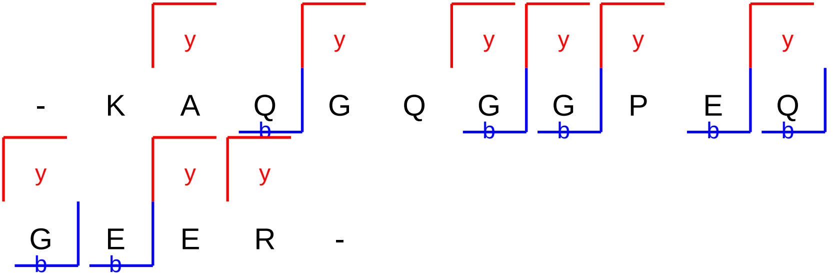

Raw File	Scan	Method	Score	m/z	Gene names
RO-QC-160121_ZackD_SS_Set_01_Fractior	5750	FTMS; HCI	77.53	573.65	FAM110C

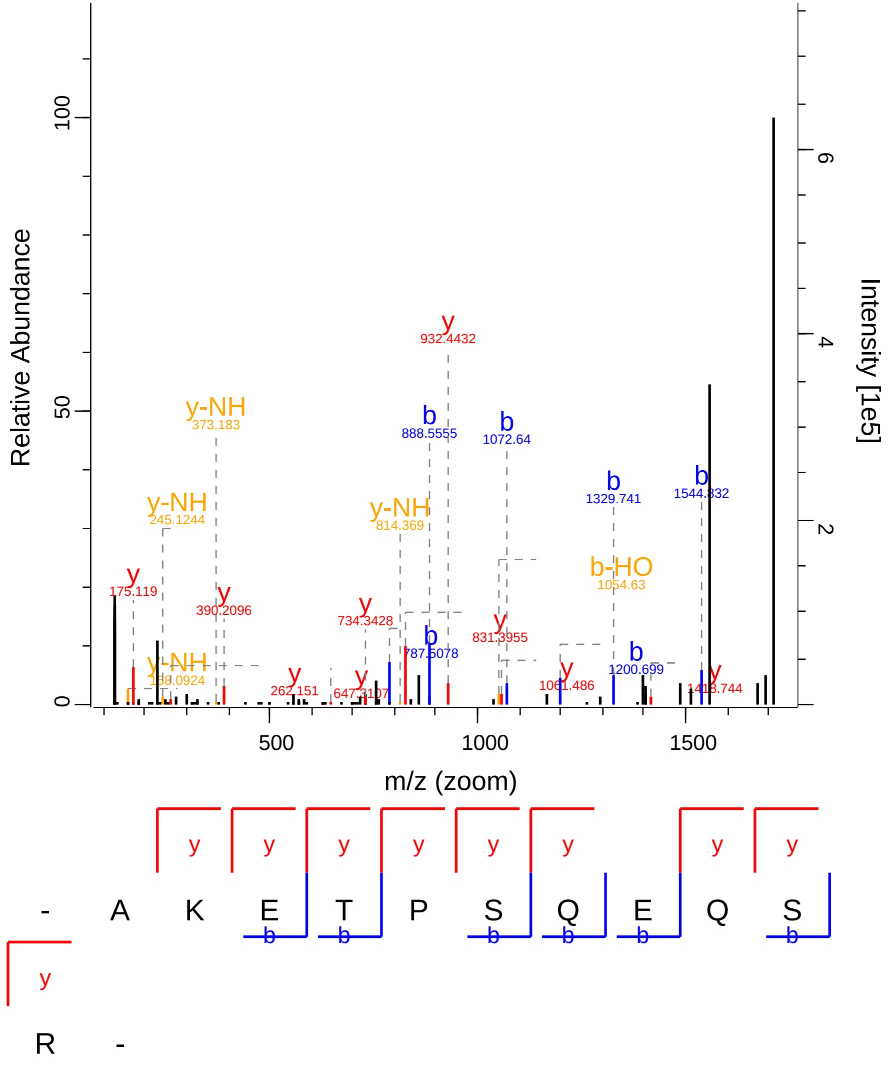

Raw File	Scan	Method	Score	m/z	Gene names
RO-QC-160121_ZackD_SS_Set_01_Fractior	5879	FTMS; HCI	92.84	669.83	TMEM47

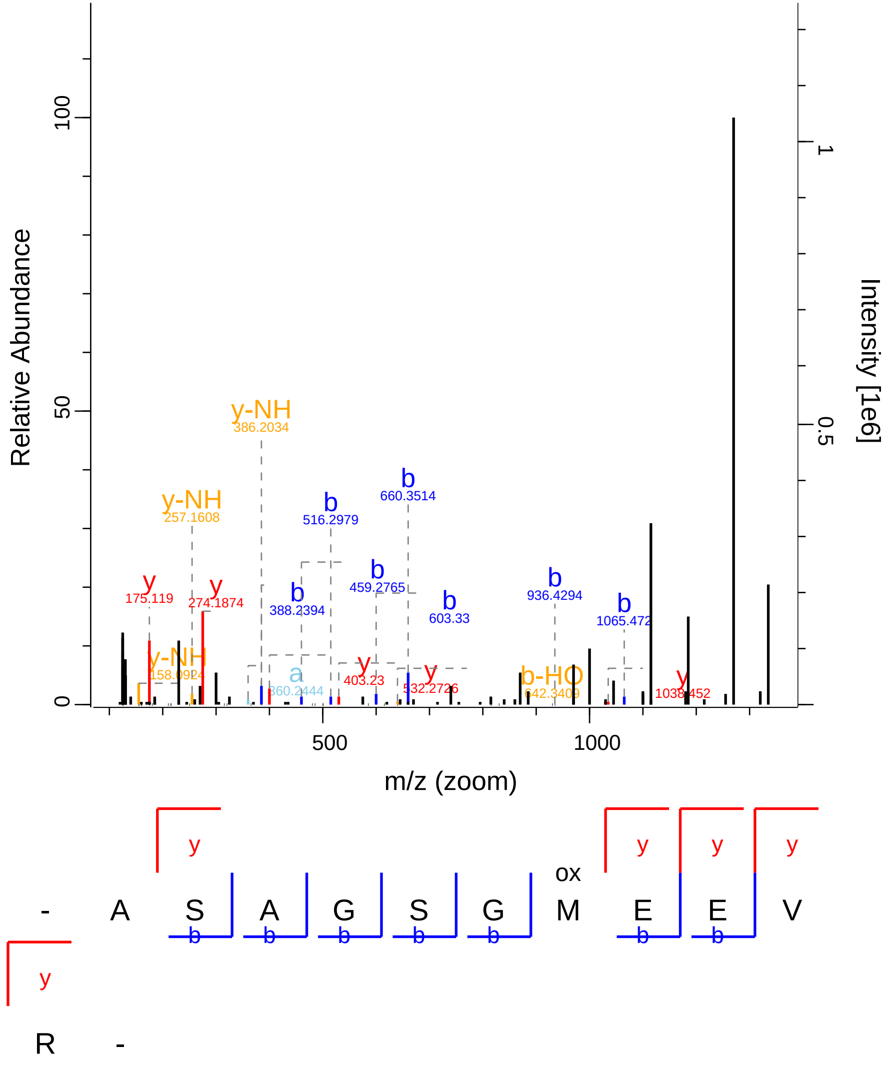

Raw File	Scan	Method	Score	m/z	Gene names
RO-QC-160121_ZackD_SS_Set_01_Fractio	6018	FTMS; HC	170.89	690.35	SLC2A12

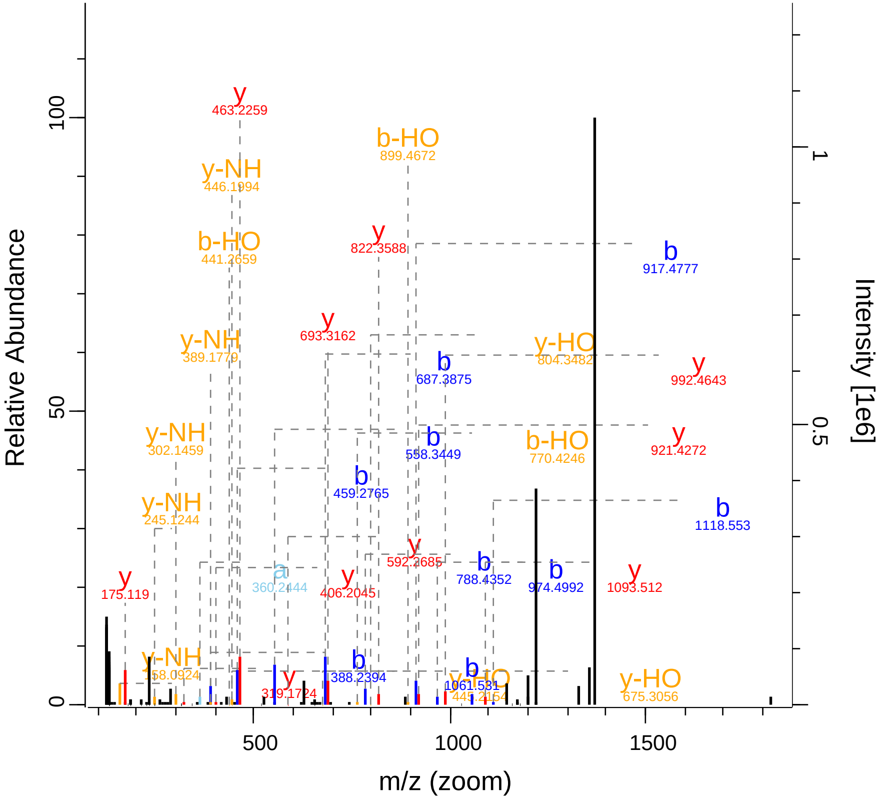

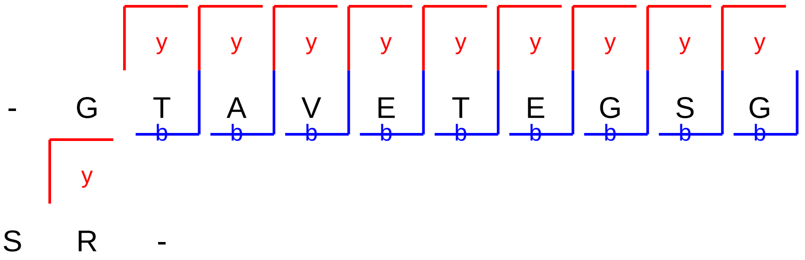

Raw File	Scan	Method	Score	m/z
RO-QC-160121_ZackD_SS_Set_01_Fraction_2	6054	FTMS; HCD	44.19	507.79

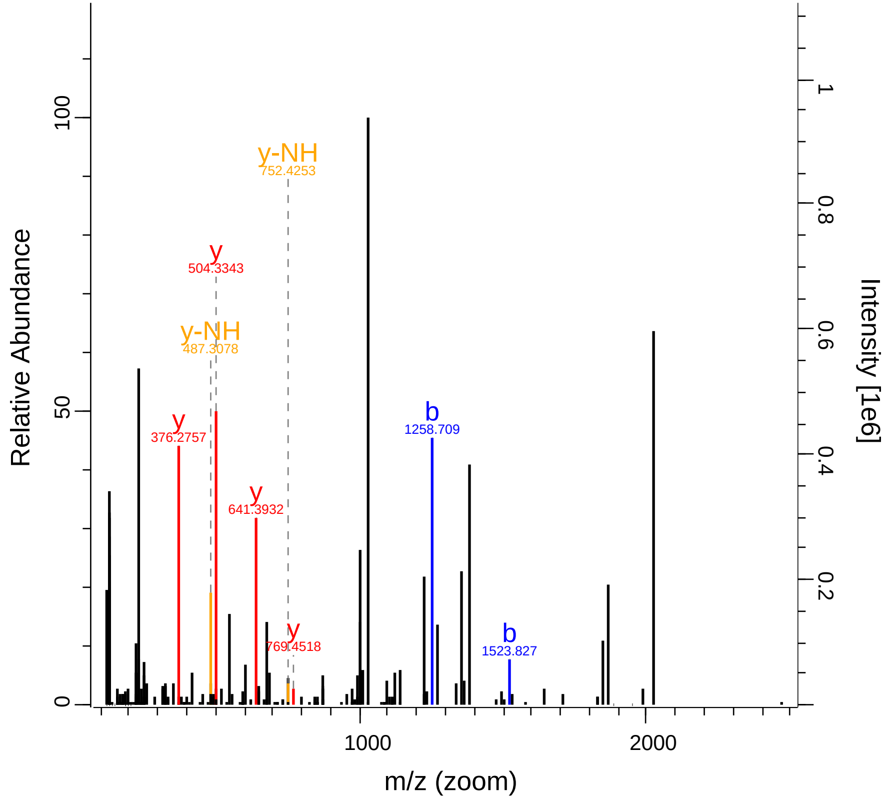

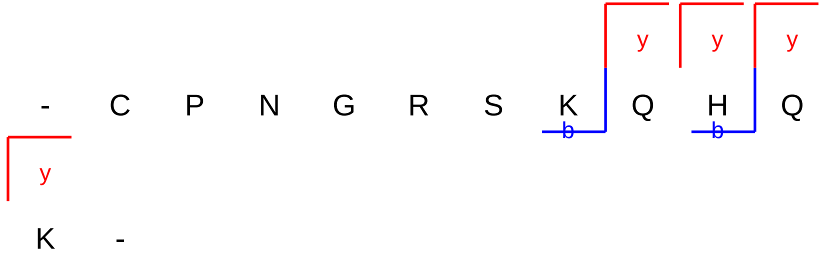

Raw File	Scan	Method	Score	m/z	Gene names
RO-QC-160121_ZackD_SS_Set_01_Fractior	6154	FTMS; HCI	76.84	755.85	RCN3

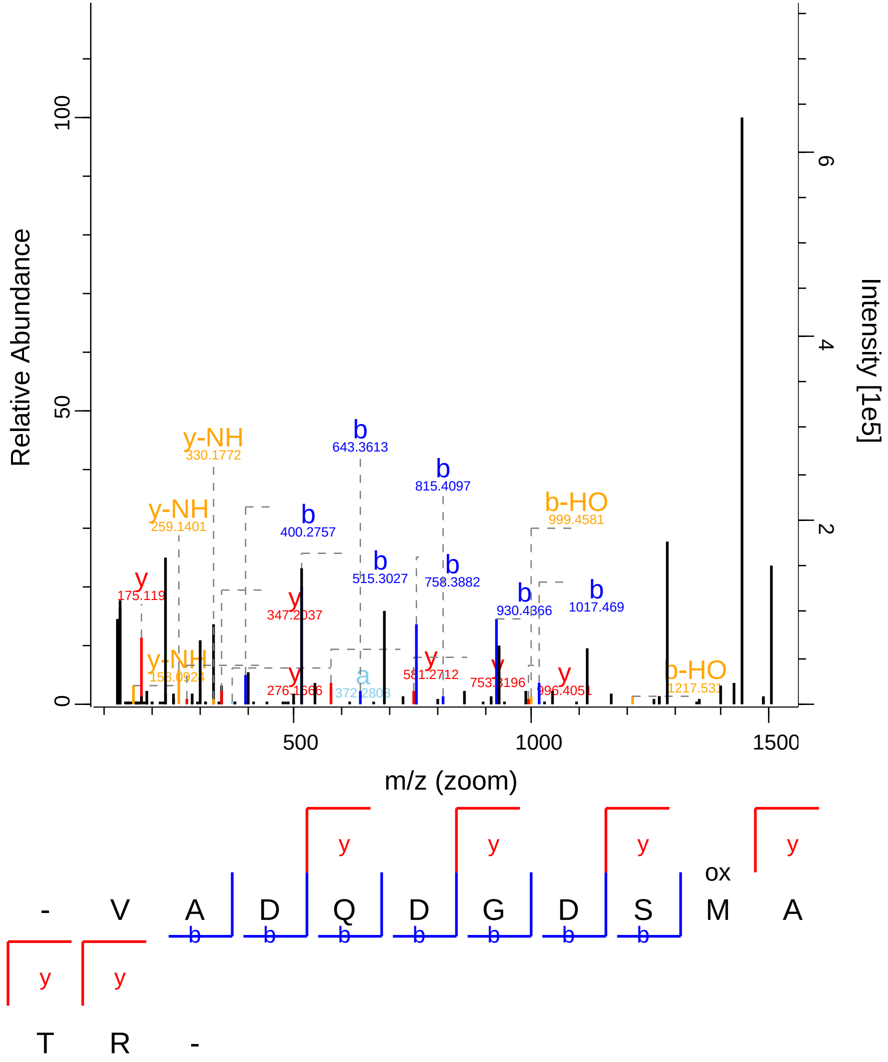

Raw File	Scan	Method	Score	m/z
RO-QC-160121_ZackD_SS_Set_01_Fraction_2	6221	FTMS; HCD	57.49	507.79

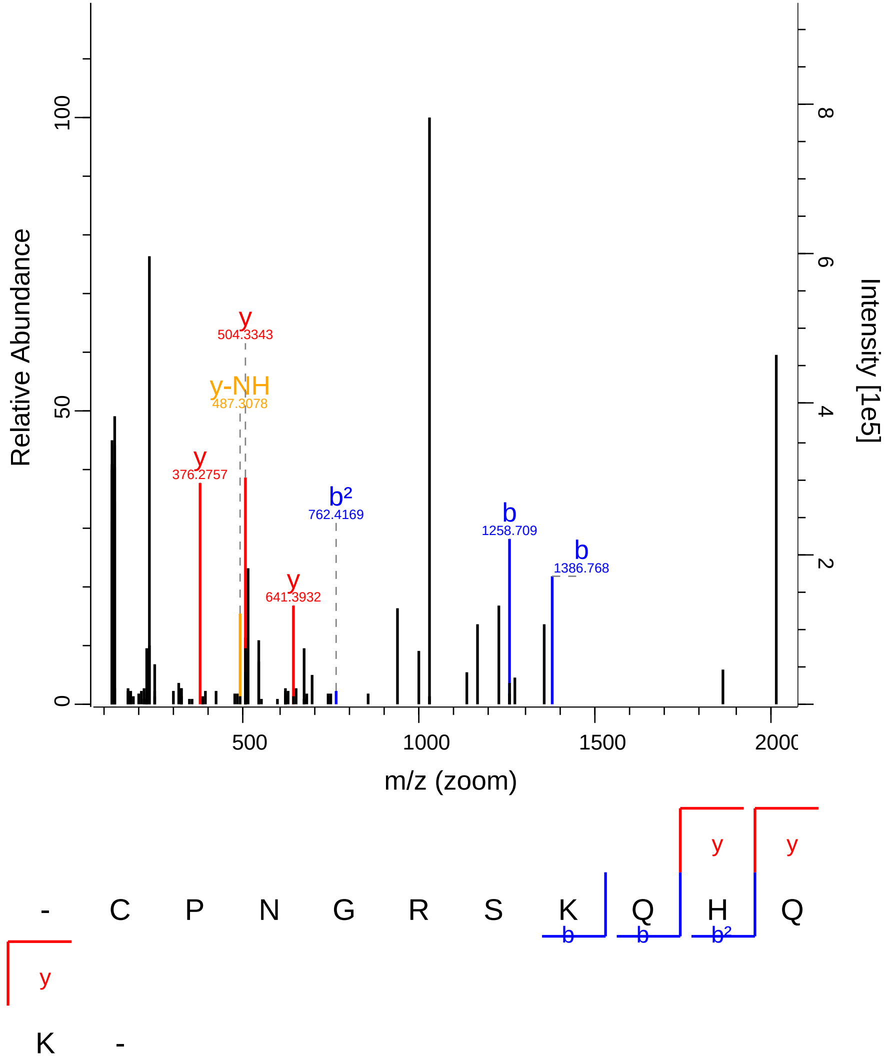

Raw File	Scan	Method	Score	m/z	Gene names
RO-QC-160121_ZackD_SS_Set_01_Fractio	6423	FTMS; HC	191.45	649.85	CD99

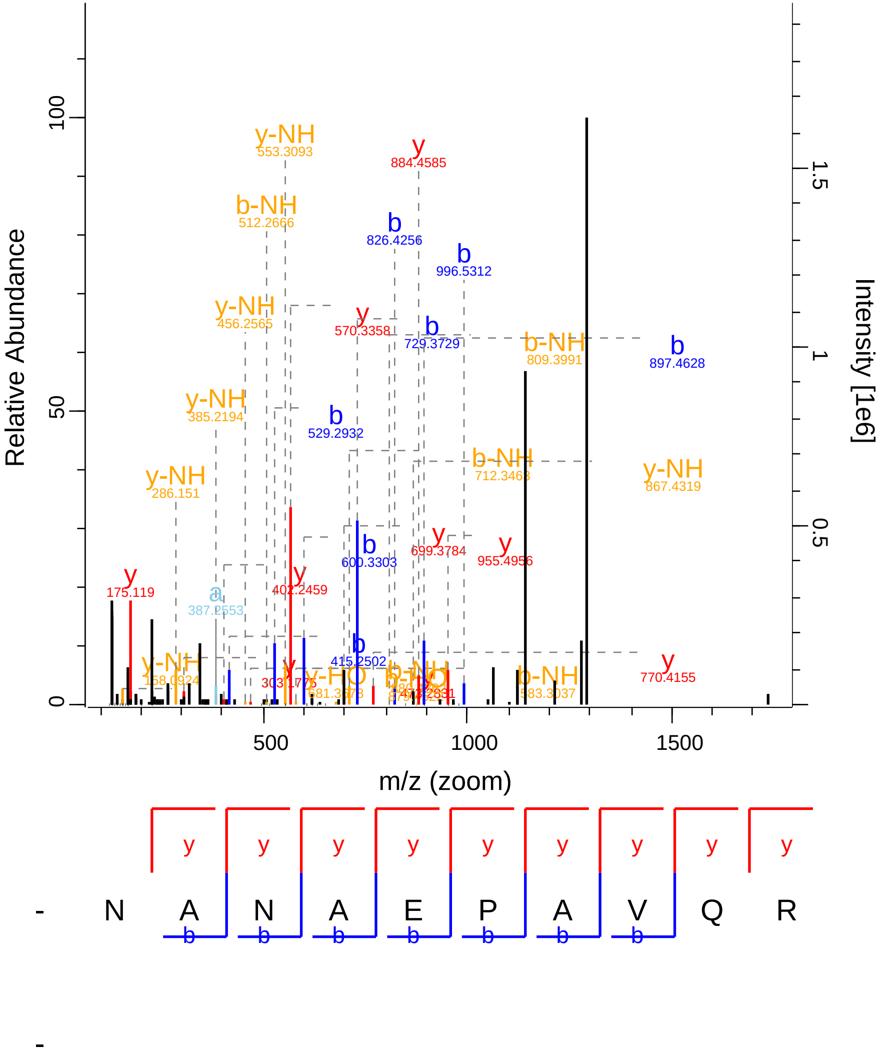

Raw File	Scan	Method	Score	m/z	Gene names
RO-QC-160121_ZackD_SS_Set_01_Fractior	6473	FTMS; HCI	51.45	713.39	LARP6

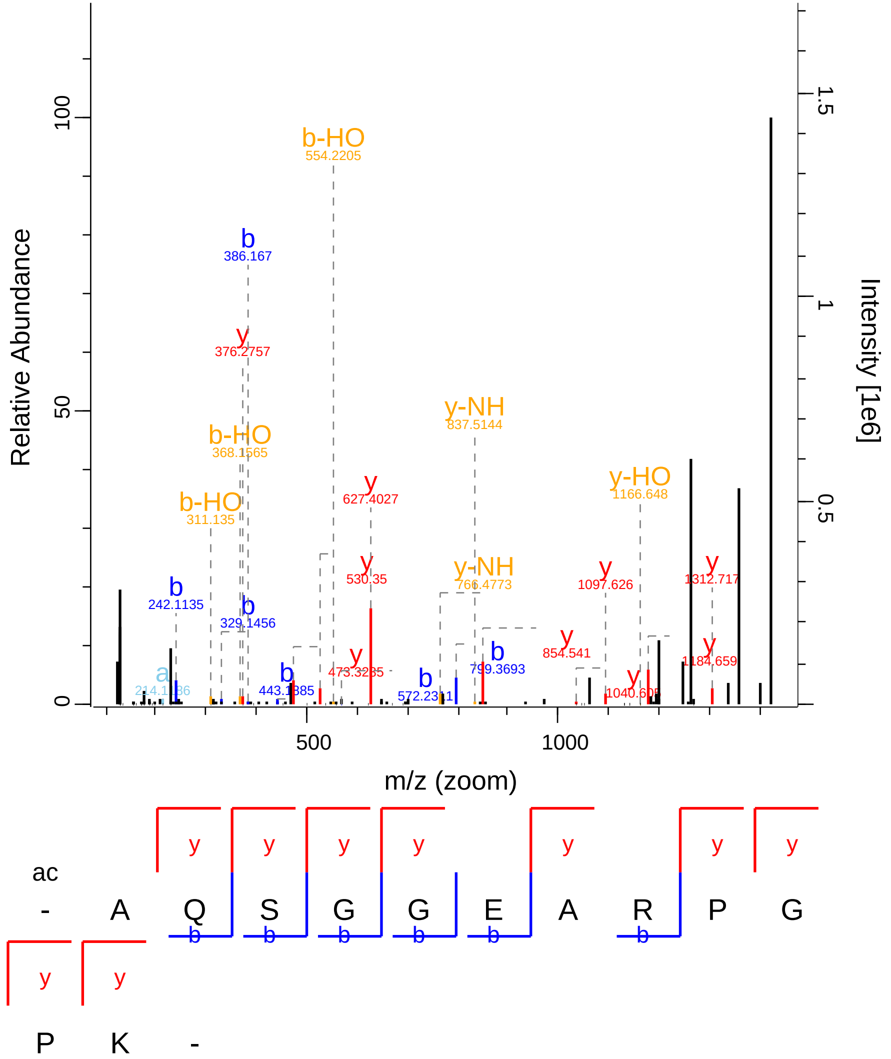

Raw File	Scan	Method	Score	m/z	Gene names
RO-QC-160121_ZackD_SS_Set_01_Fractio	6504	FTMS; HC	140.84	681.79	VOPP1

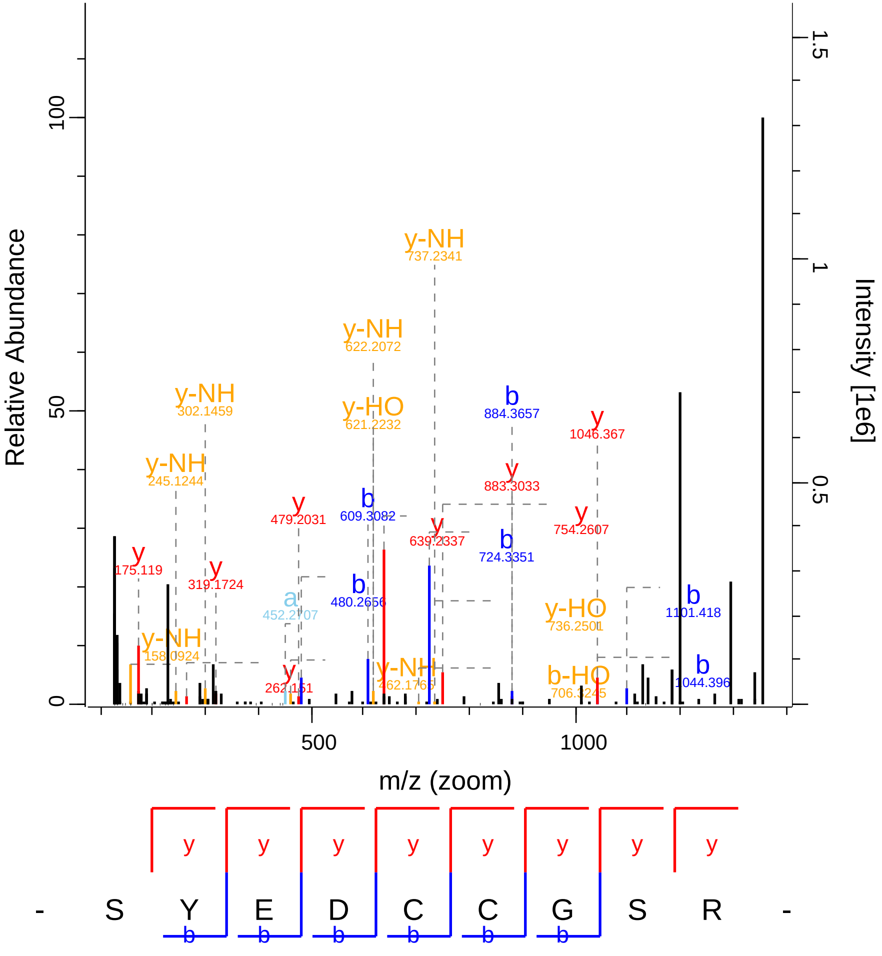

Raw File	Scan	Method	Score	m/z	Gene names
RO-QC-160121_ZackD_SS_Set_01_Fraction	6670	FTMS; HCI	95.31	565.8	KIAA1161

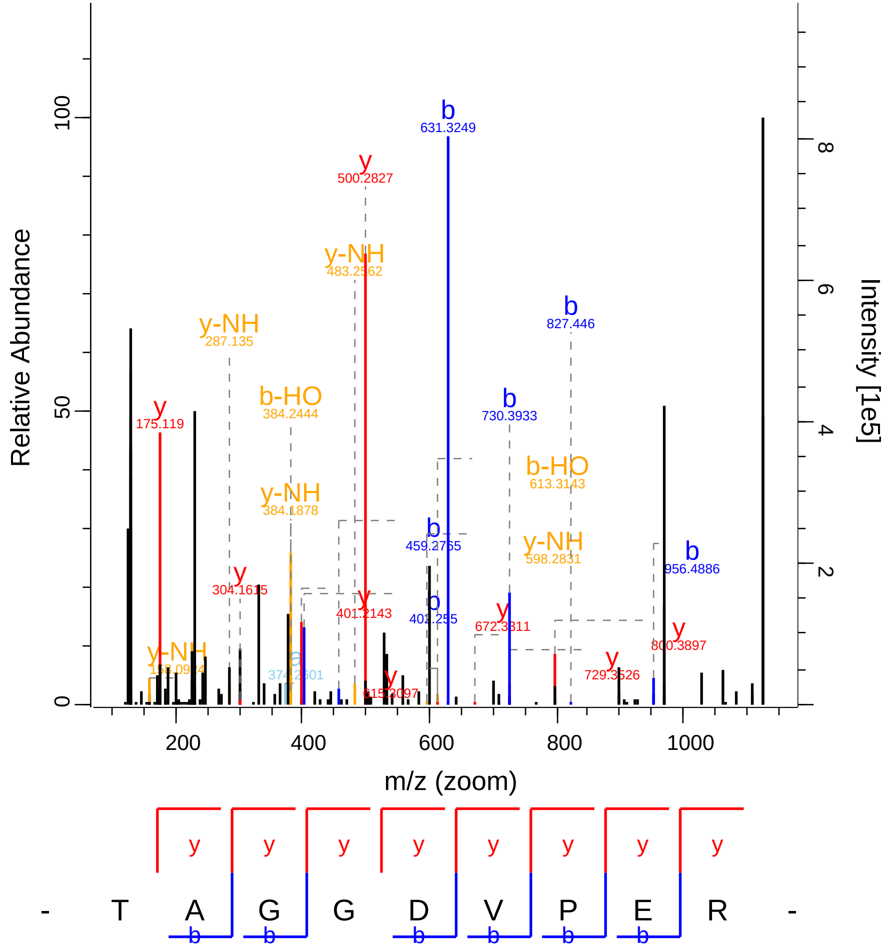

Raw File	Scan	Method	Score	m/z
RO-QC-160121_ZackD_SS_Set_01_Fraction_2	6712	FTMS; HCD	50.61	638.86

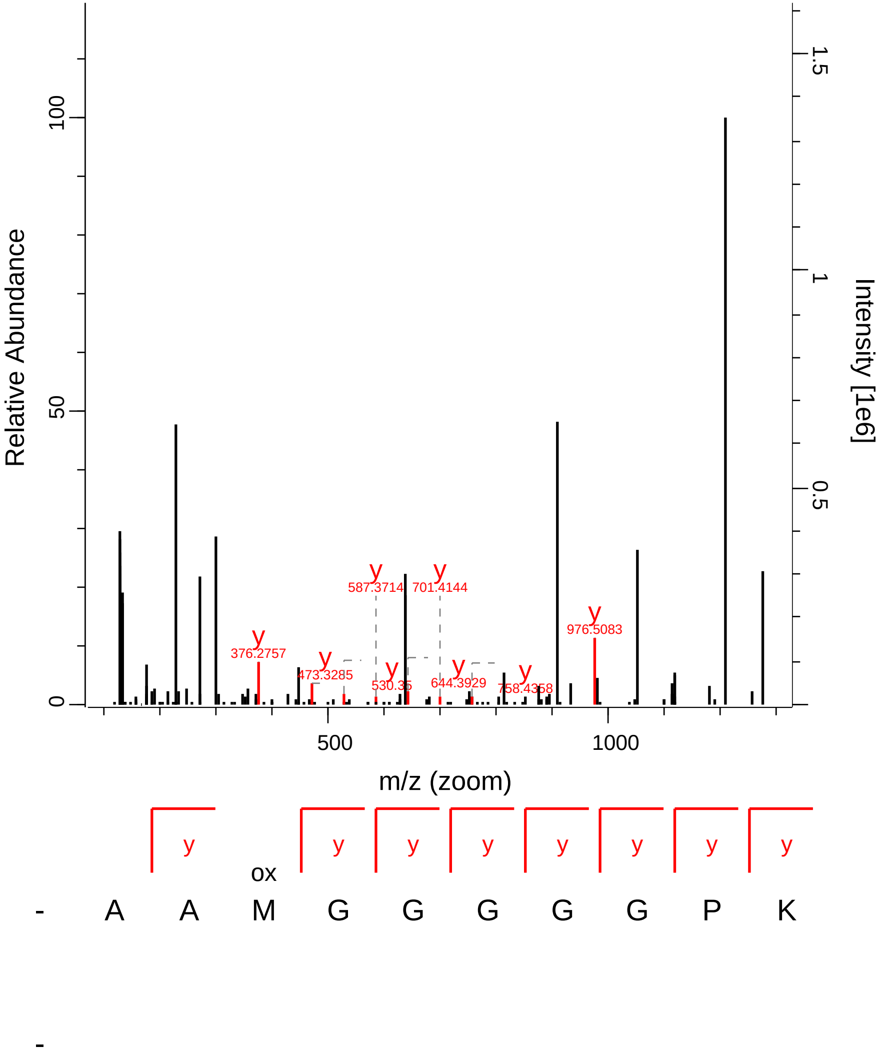

Raw File	Scan	Method	Score	m/z	Gene names
RO-QC-160121_ZackD_SS_Set_01_Fractio	7083	FTMS; HC	186.02	780.38	ZNF511

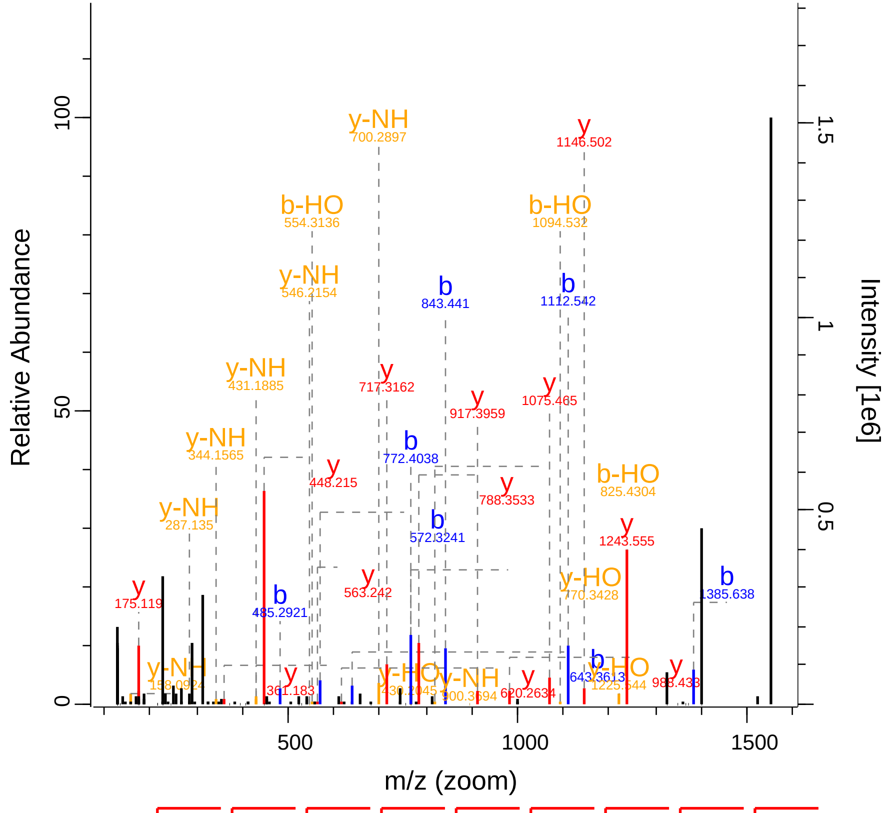

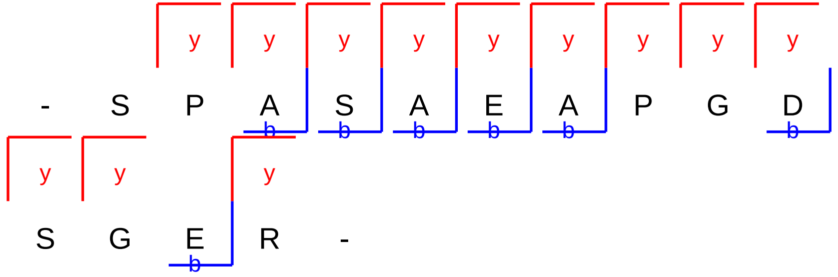

Raw File	Scan	Method	Score	m/z	Gene names
RO-QC-160121_ZackD_SS_Set_01_Fractior	7119	FTMS; HCI	29.73	520.92	ZNF511

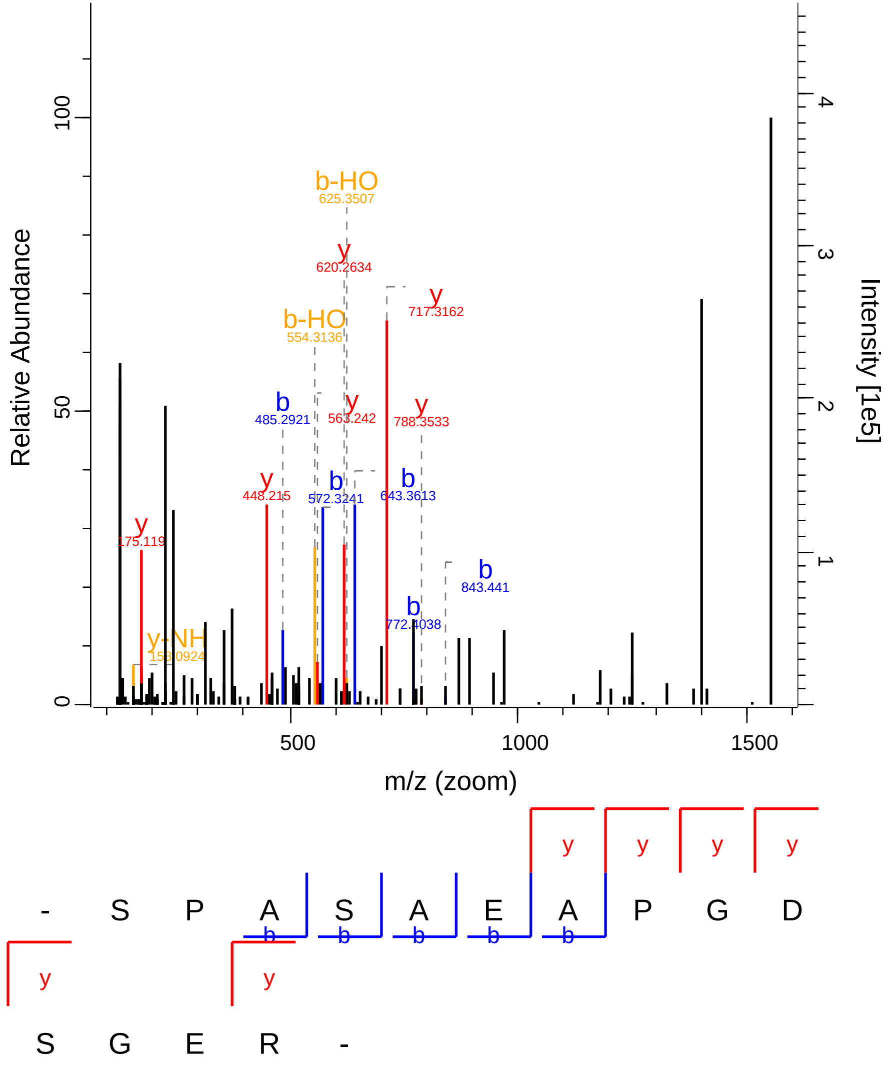

Raw File	Scan	Method	Score	m/z	Gene names
RO-QC-160121_ZackD_SS_Set_01_Fractio	7241	FTMS; HC	133.81	798.88	PROSER1

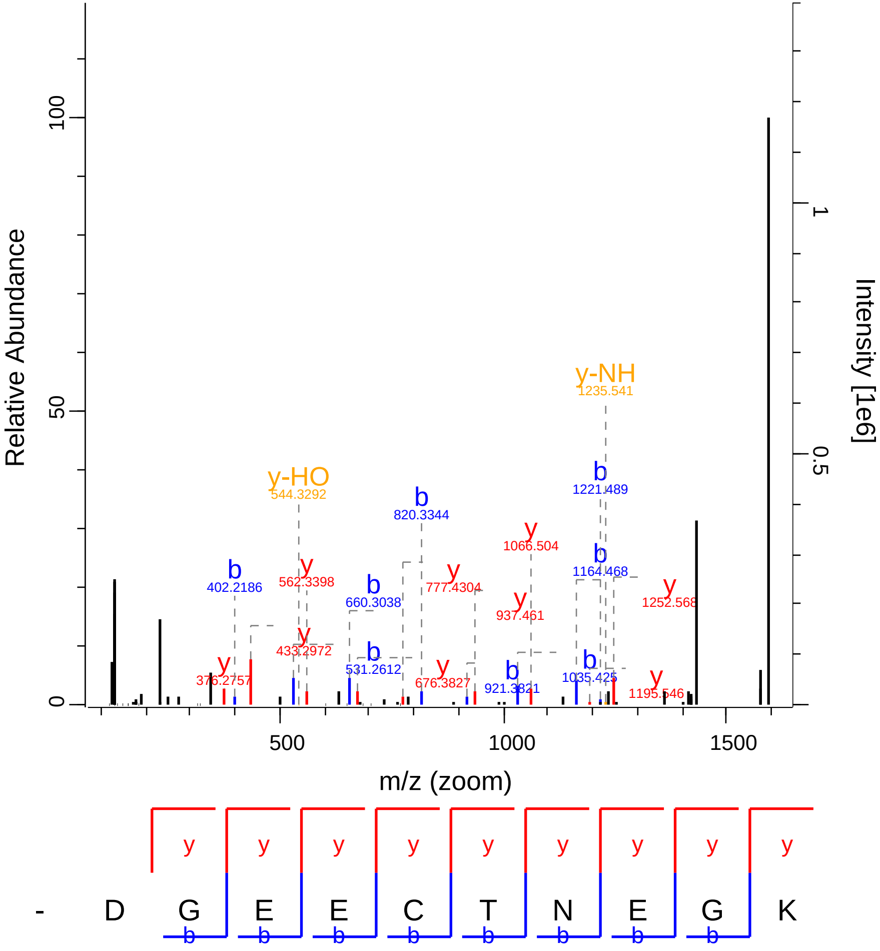

-

Raw File	Scan	Method	Score	m/z	Gene names
RO-QC-160121_ZackD_SS_Set_01_Fractior	7512	FTMS; HCI	83.86	489.51	DCP2

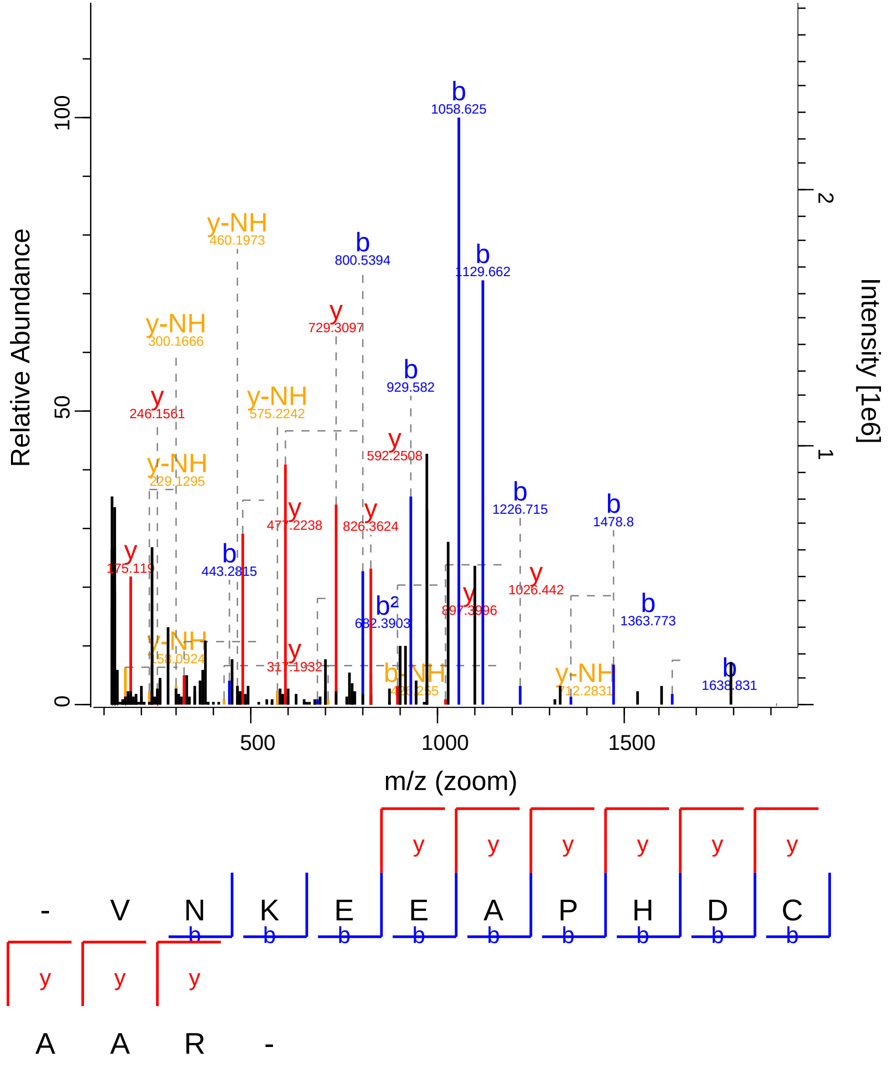

Raw File	Scan	Method	Score	m/z	Gene names
RO-QC-160121_ZackD_SS_Set_01_Fractioi	7863	FTMS; HC	116.96	716.72	PCGF5

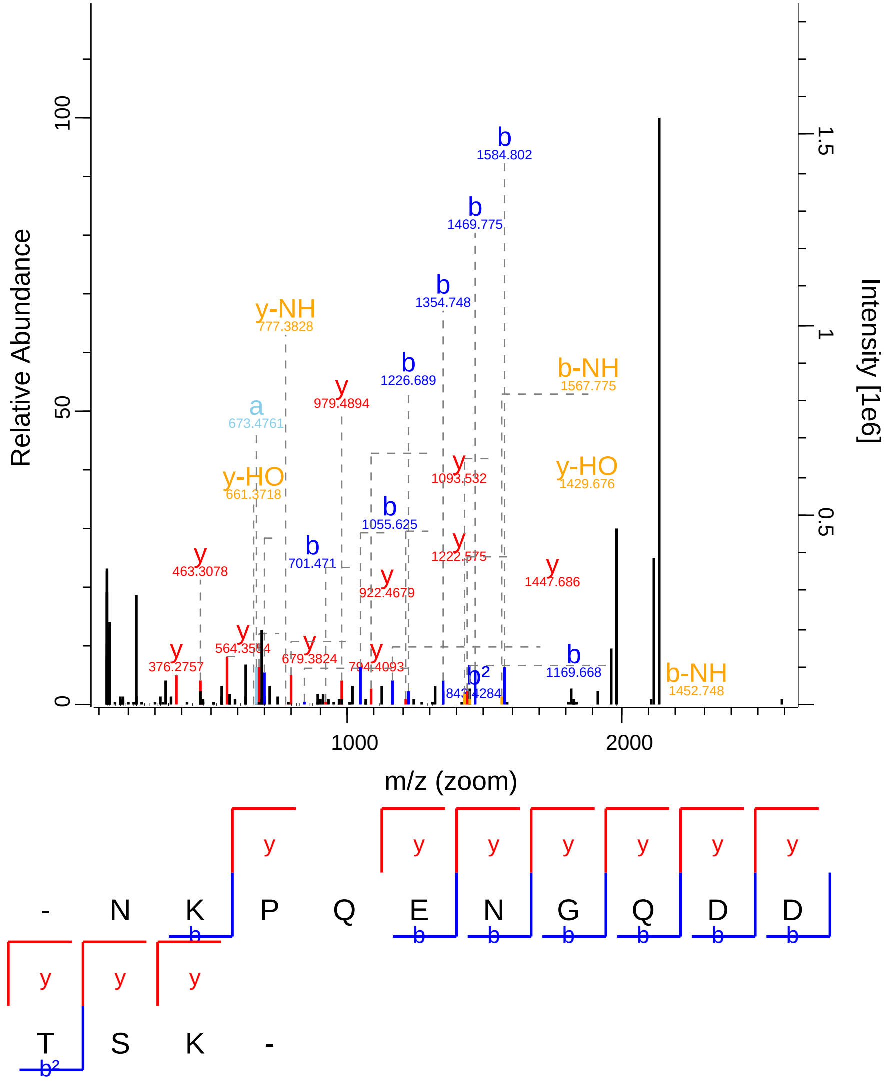

Raw File	Scan	Method	Score	m/z	Gene names
RO-QC-160121_ZackD_SS_Set_01_Fractior	7928	FTMS; HC	148.05	743.86	CCBE1

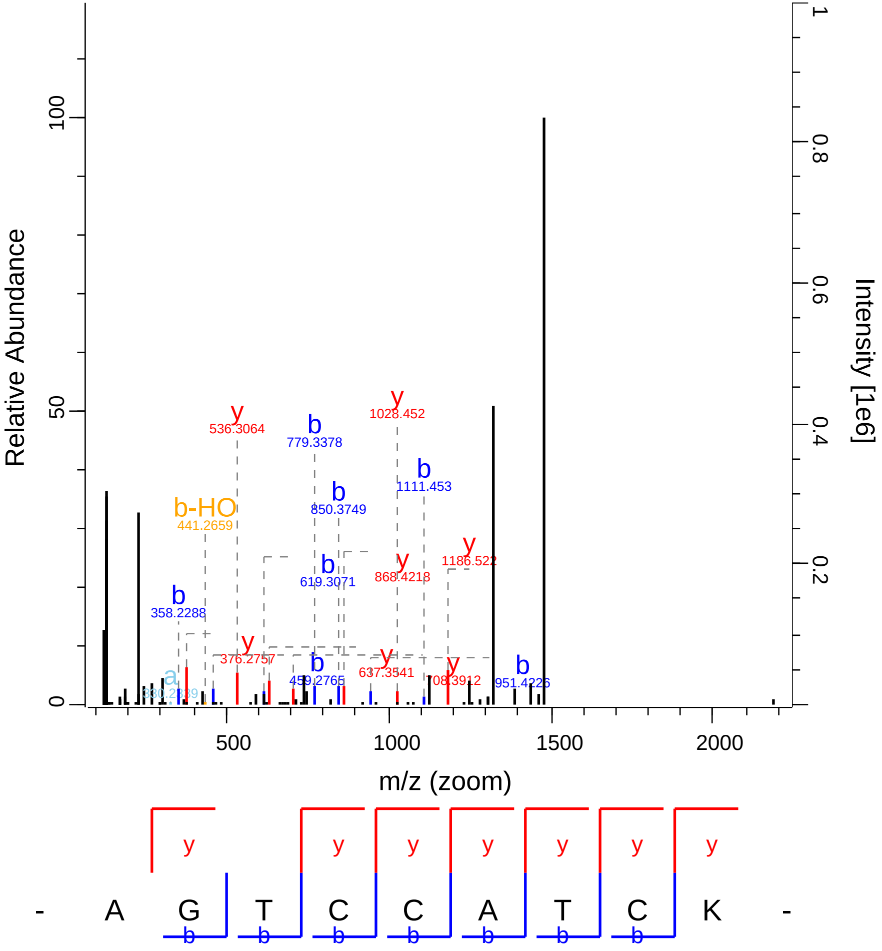

Raw File	Scan	Method	Score	m/z	Gene names
RO-QC-160121_ZackD_SS_Set_01_Fractior	8038	FTMS; HCI	44.61	395.48	SHKBP1

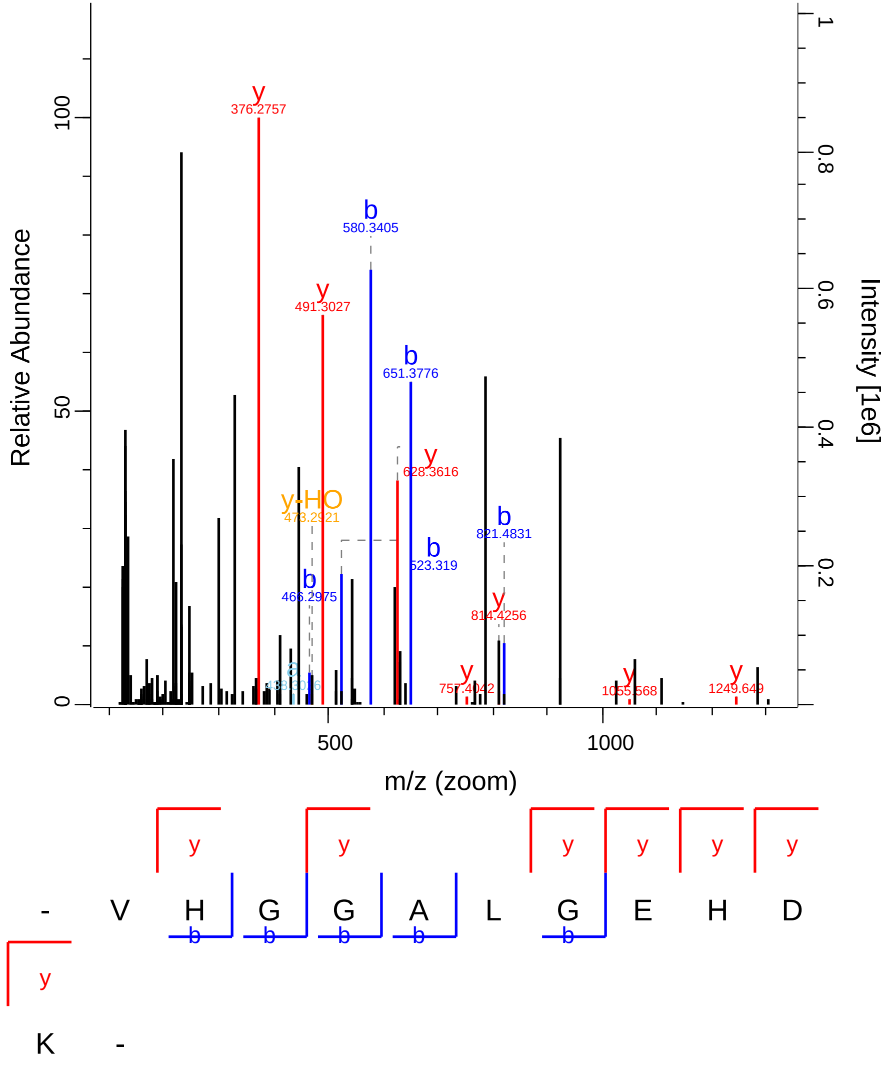

Raw File	Scan	Method	Score	m/z	Gene names
RO-QC-160121_ZackD_SS_Set_01_Fractior	8118	FTMS; HCI	63.76	548.26	A2M

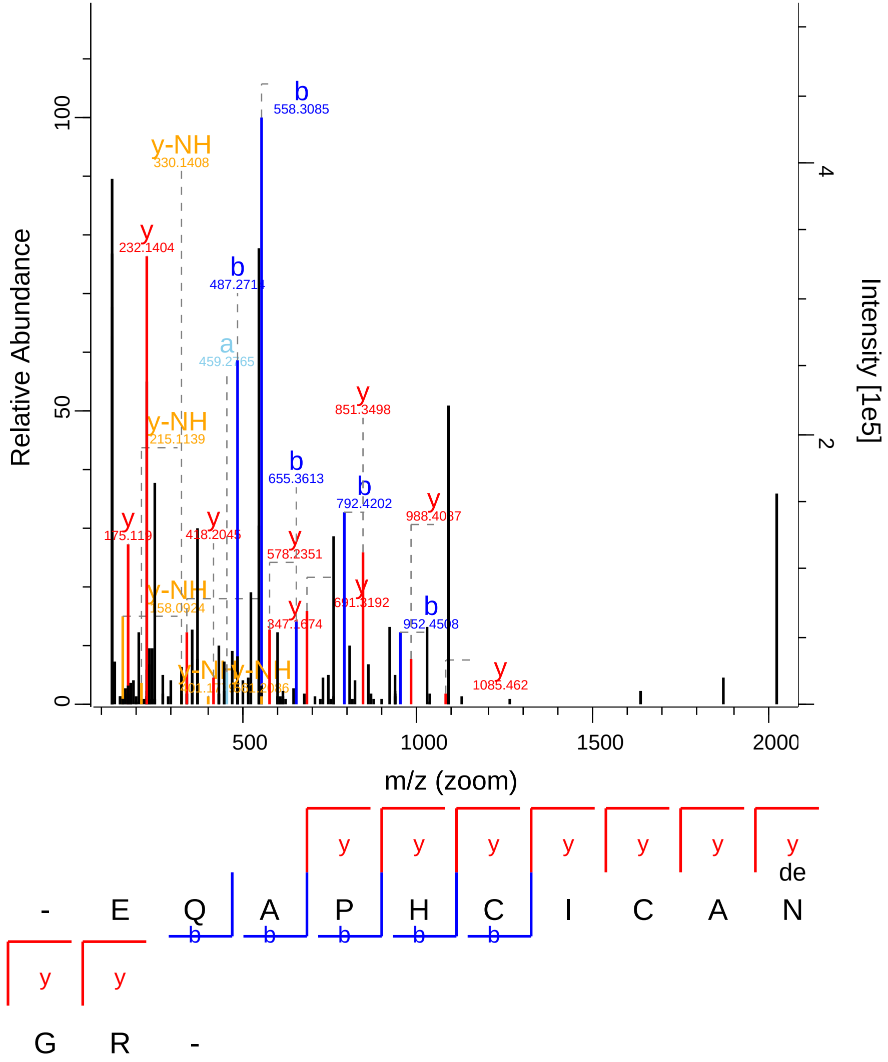

Raw File	Scan	Method	Score	m/z	Gene names
RO-QC-160121_ZackD_SS_Set_01_Fractio	8496	FTMS; HC	216.12	815.47	NCKAP5

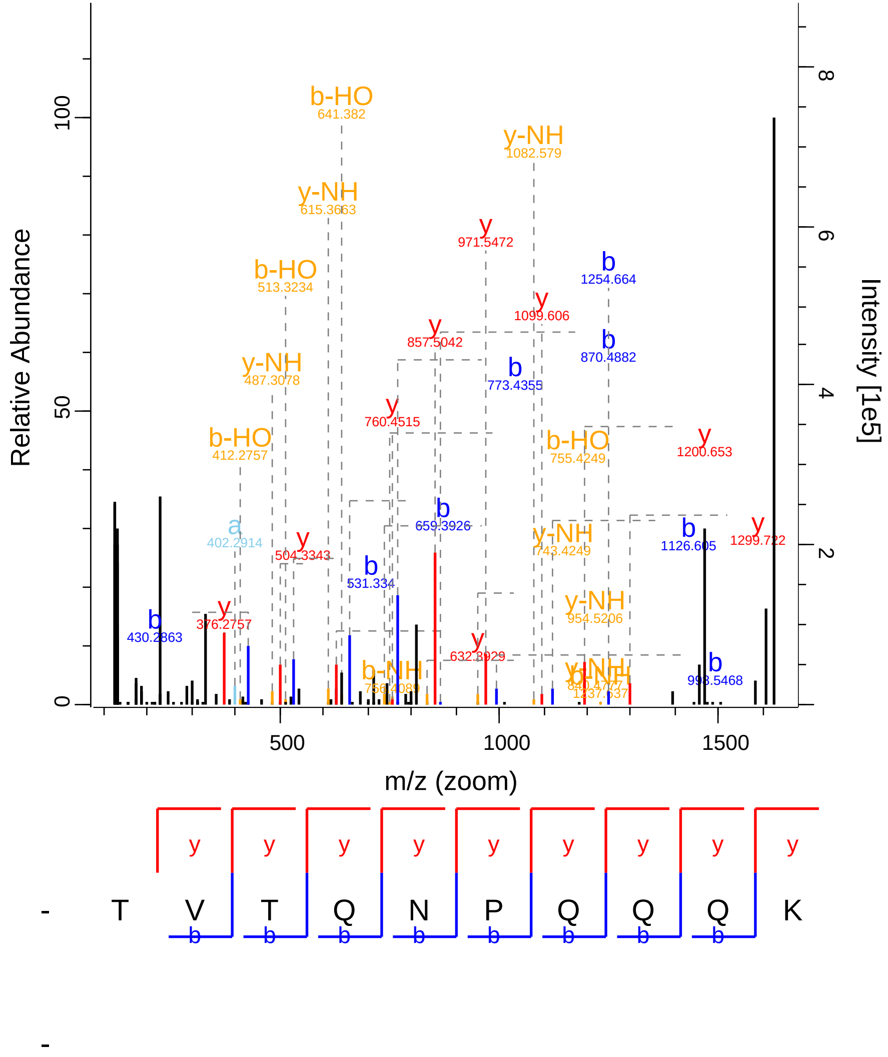

Raw File	Scan	Method	Score	m/z	Gene names
RO-QC-160121_ZackD_SS_Set_01_Fraction	8509	FTMS; HCI	116.52	808.9	ZMYND19

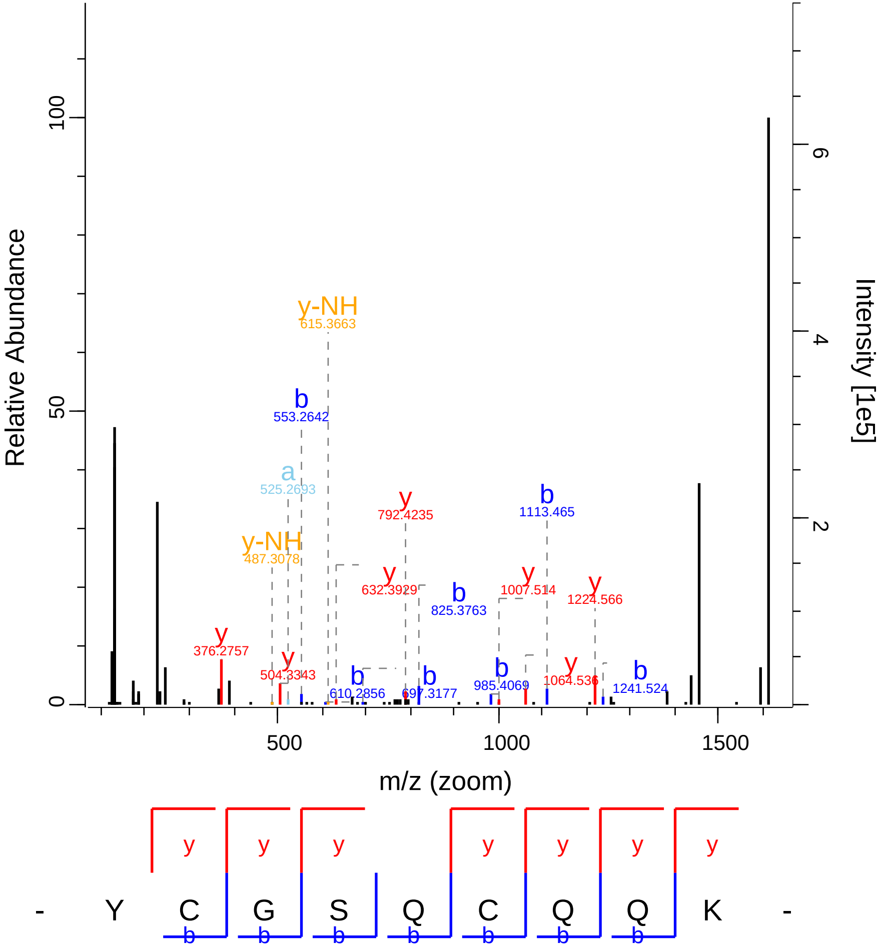

Raw File	Scan	Method	Score	m/z	Gene names
RO-QC-160121_ZackD_SS_Set_01_Fractior	8554	FTMS; HCI	67.17	803.96	VSIG1

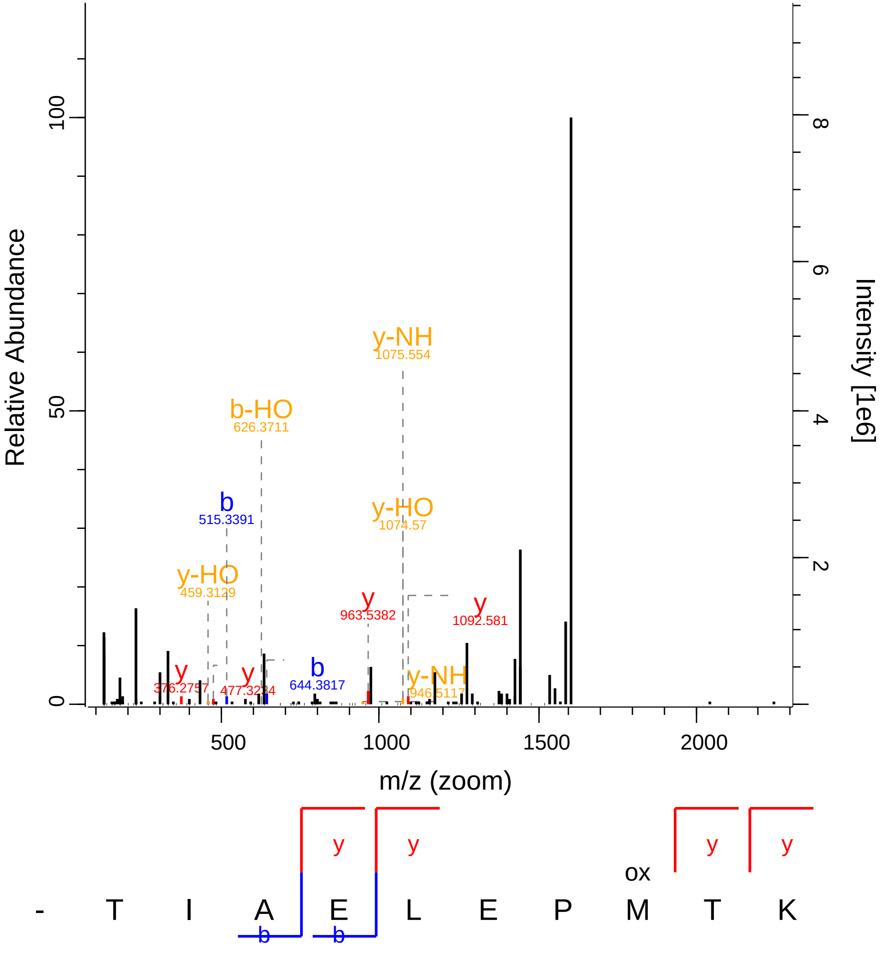

-

Raw File	Scan	Method	Score	m/z	Gene names
RO-QC-160121_ZackD_SS_Set_01_Fractior	9038	FTMS; HCI	85.06	453.26	RNASEH1

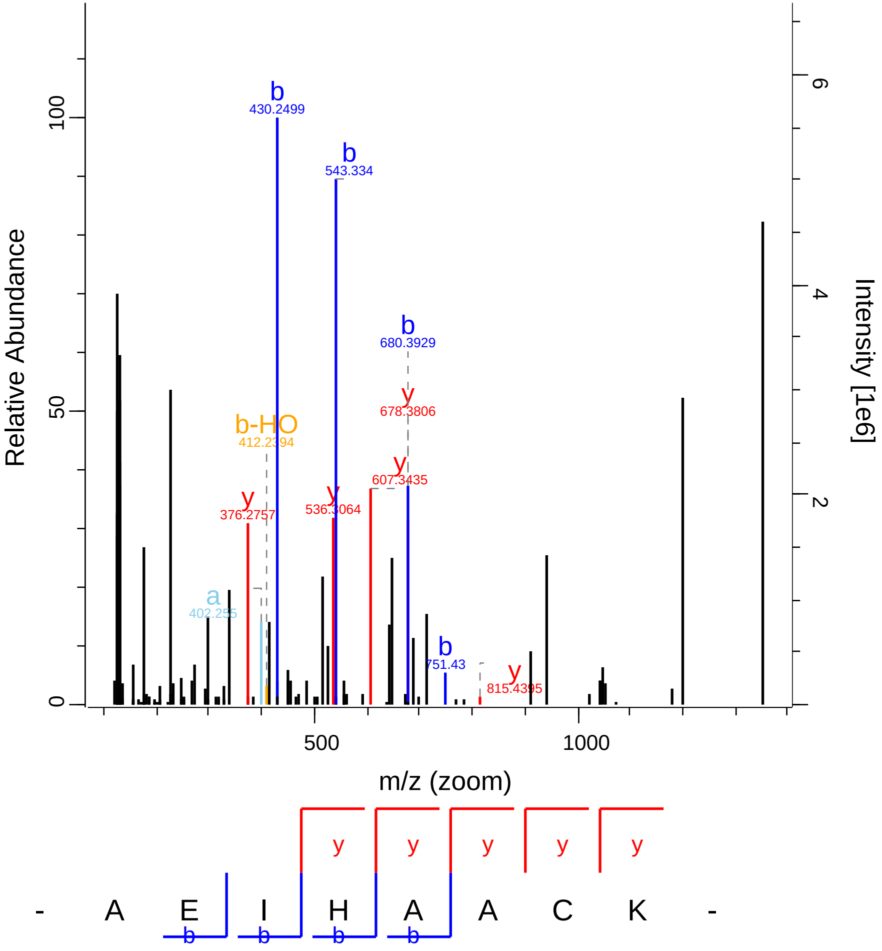

Raw File	Scan	Method	Score	m/z	Gene names
RO-QC-160121_ZackD_SS_Set_01_Fractior	10262	FTMS; HCI	55.11	841.9	GLRX2

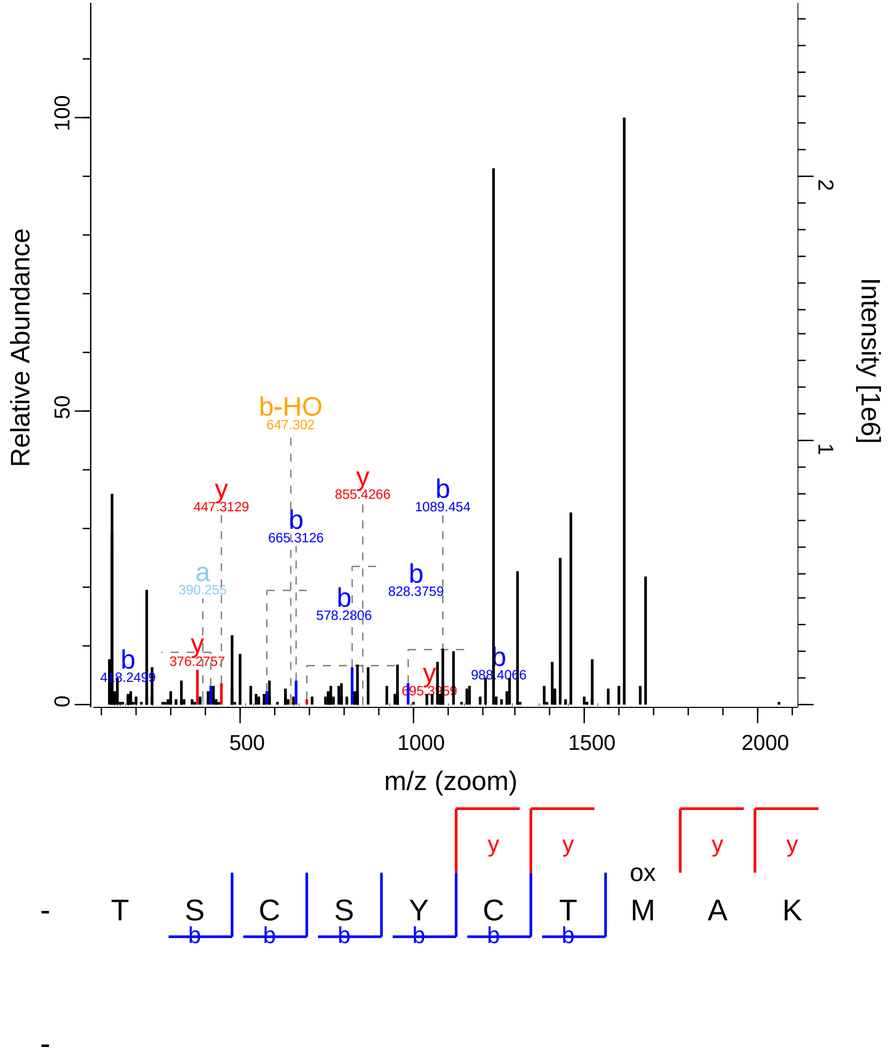

Raw File	Scan	Method	Score	m/z	Gene names
RO-QC-160121_ZackD_SS_Set_01_Fractio	10335	FTMS; HC	76.76	571.31	SMIM7

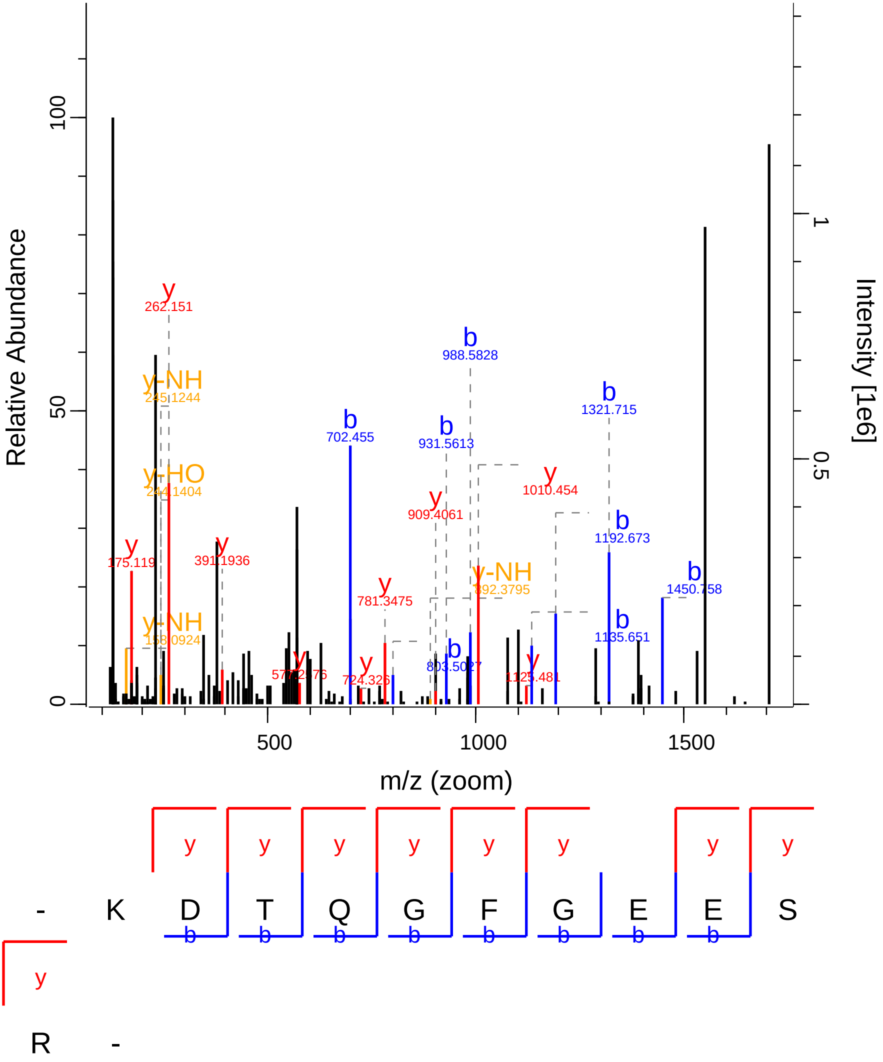

Raw File	Scan	Method	Score	m/z	Gene names
RO-QC-160121_ZackD_SS_Set_01_Fractio	10481	FTMS; HC	154.35	637.88	G6PC3

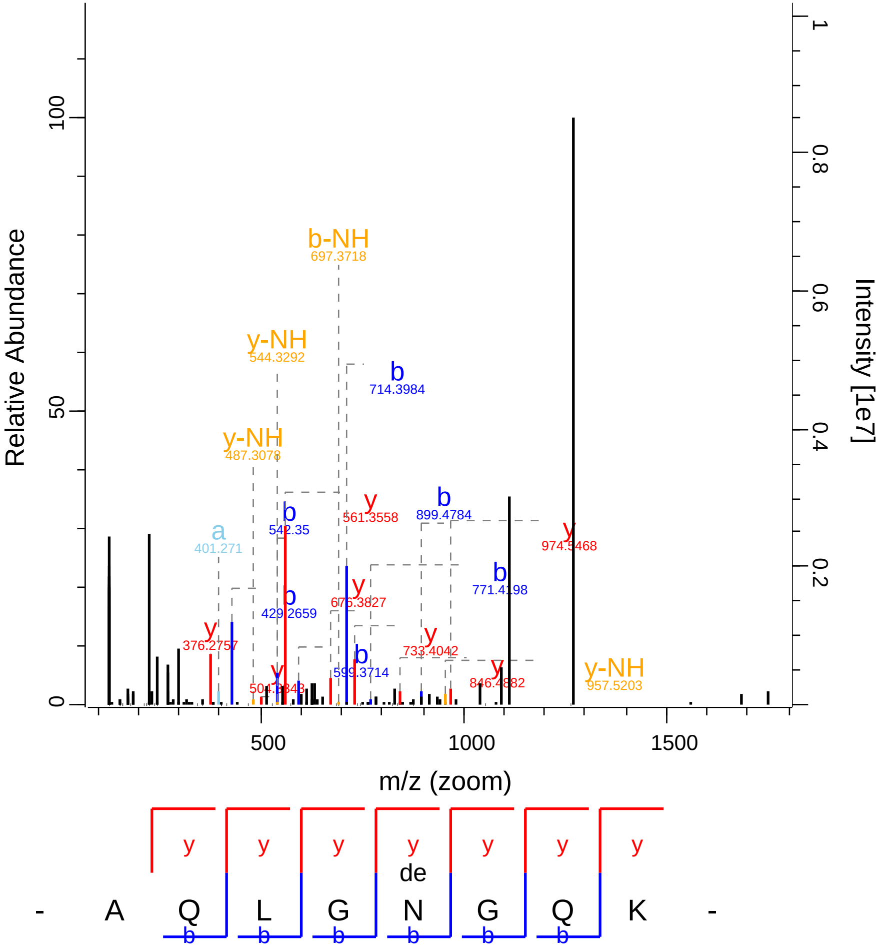

Raw File	Scan	Method	Score	m/z	Gene names
RO-QC-160121_ZackD_SS_Set_01_Fractio	10926	FTMS; HC	190.85	674.72	PRKRIR

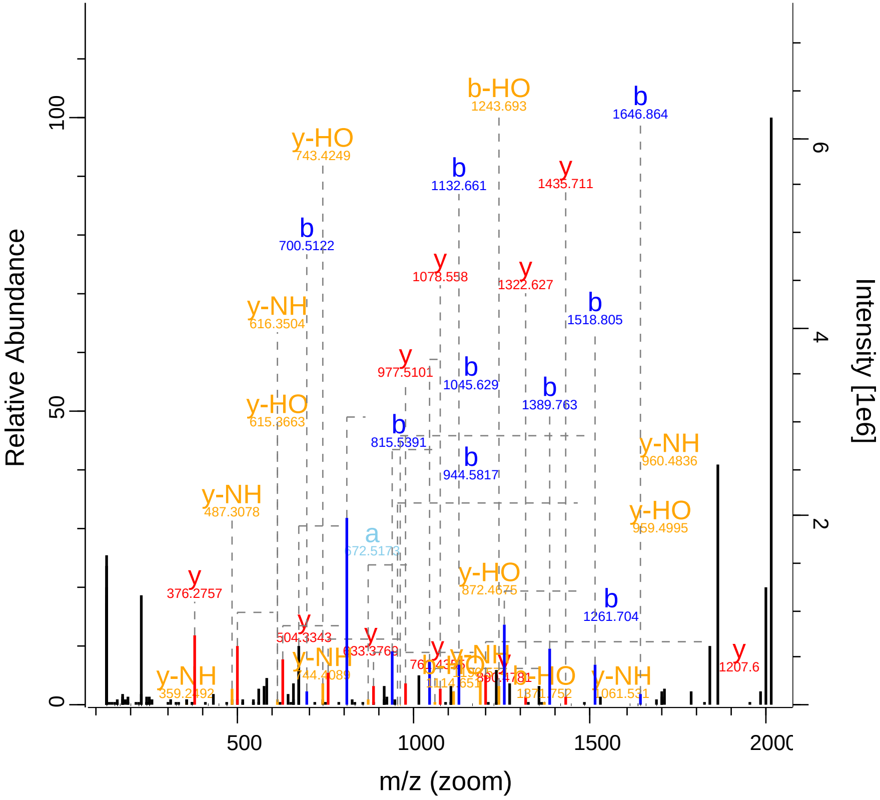

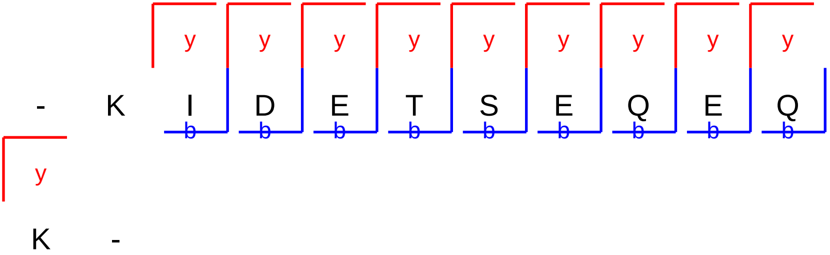

Raw File	Scan	Method	Score	m/z
RO-QC-160121_ZackD_SS_Set_01_Fraction_2	11248	FTMS; HCD	82.67	673.4

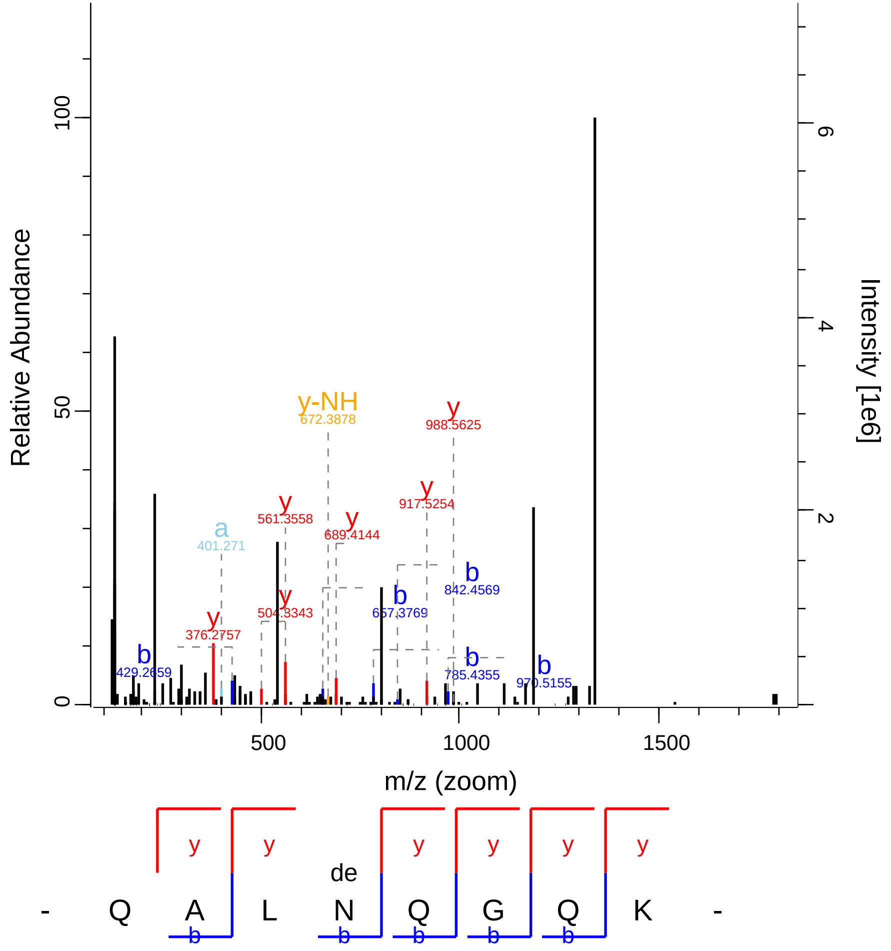

Raw File	Scan	Method	Score	m/z	Gene names
RO-QC-160121_ZackD_SS_Set_01_Fractio	11563	FTMS; HC	100.45	464.61	KCNV2

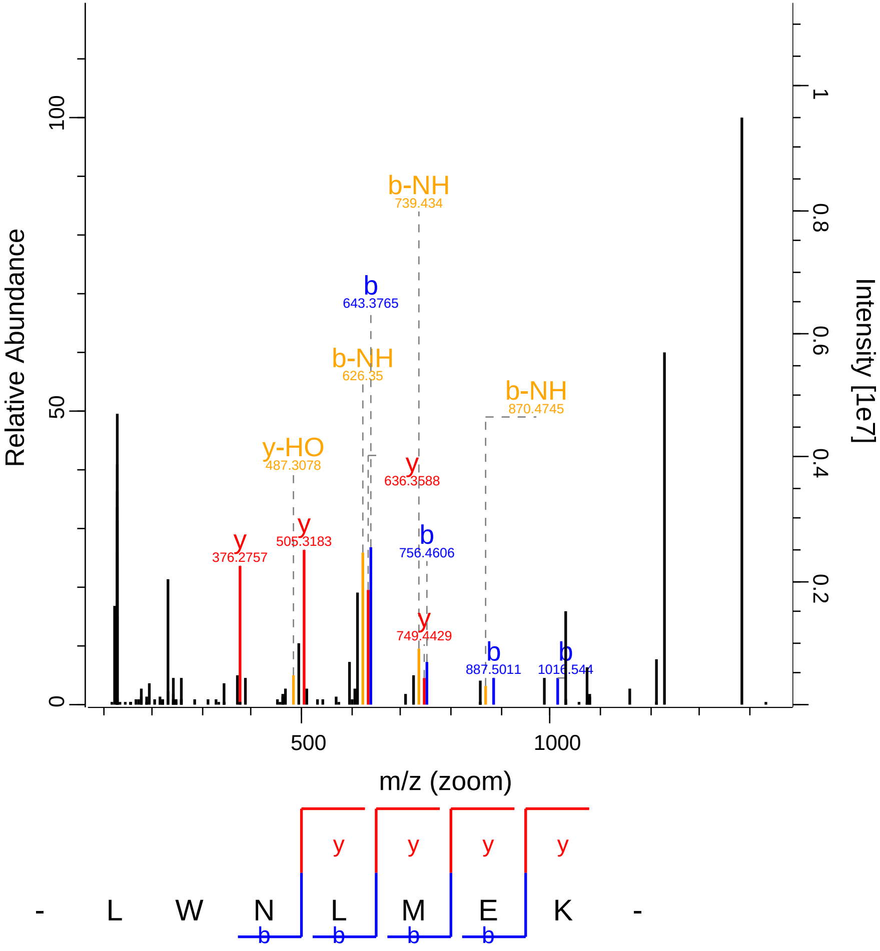

Raw File	Scan	Method	Score	m/z	Gene names
RO-QC-160121_ZackD_SS_Set_01_Fractic	12017	FTMS; HC	75.51	1035.99	MRC1

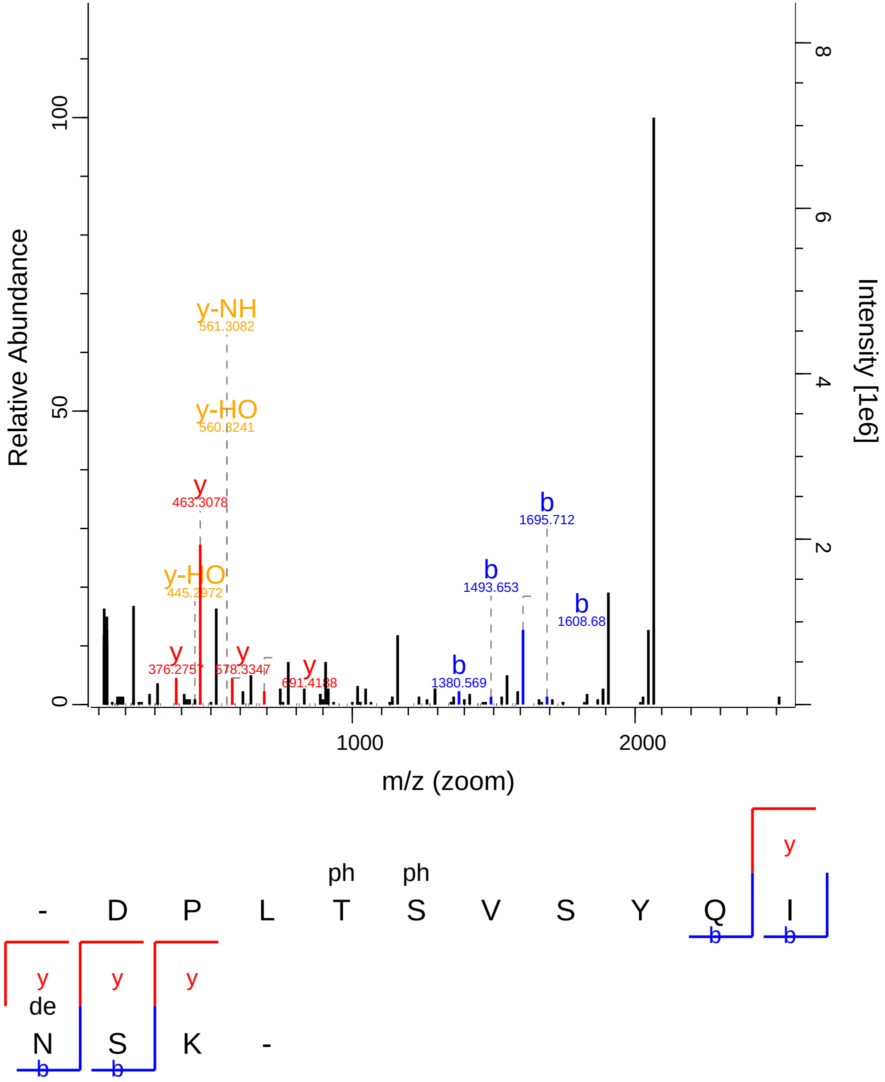

Raw File	Scan	Method	Score	m/z	Gene names
RO-QC-160121_ZackD_SS_Set_01_Fractio	12308	FTMS; HC	136.64	765.91	SLC29A1

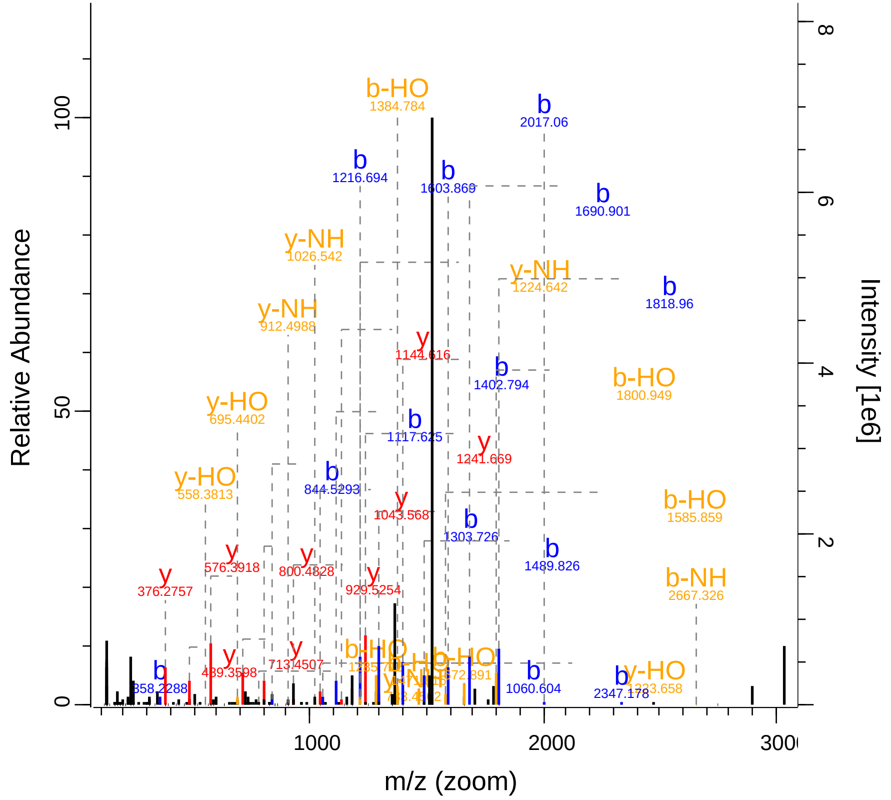

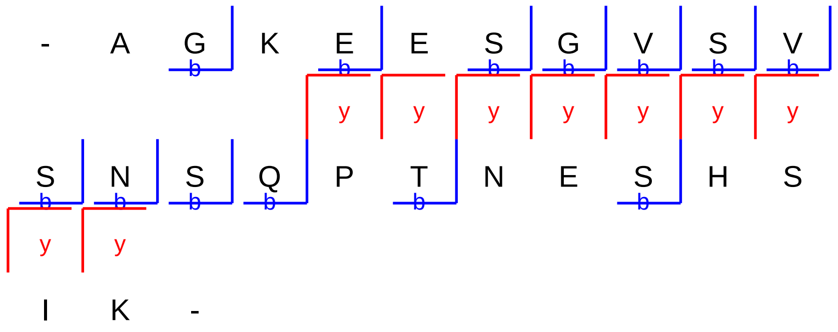

Raw File	Scan	Method	Score	m/z	Gene names
RO-QC-160121_ZackD_SS_Set_01_Fr	1232(	FTMS; H(	128.57	596.35	ATP5G1;ATP5G2;ATP

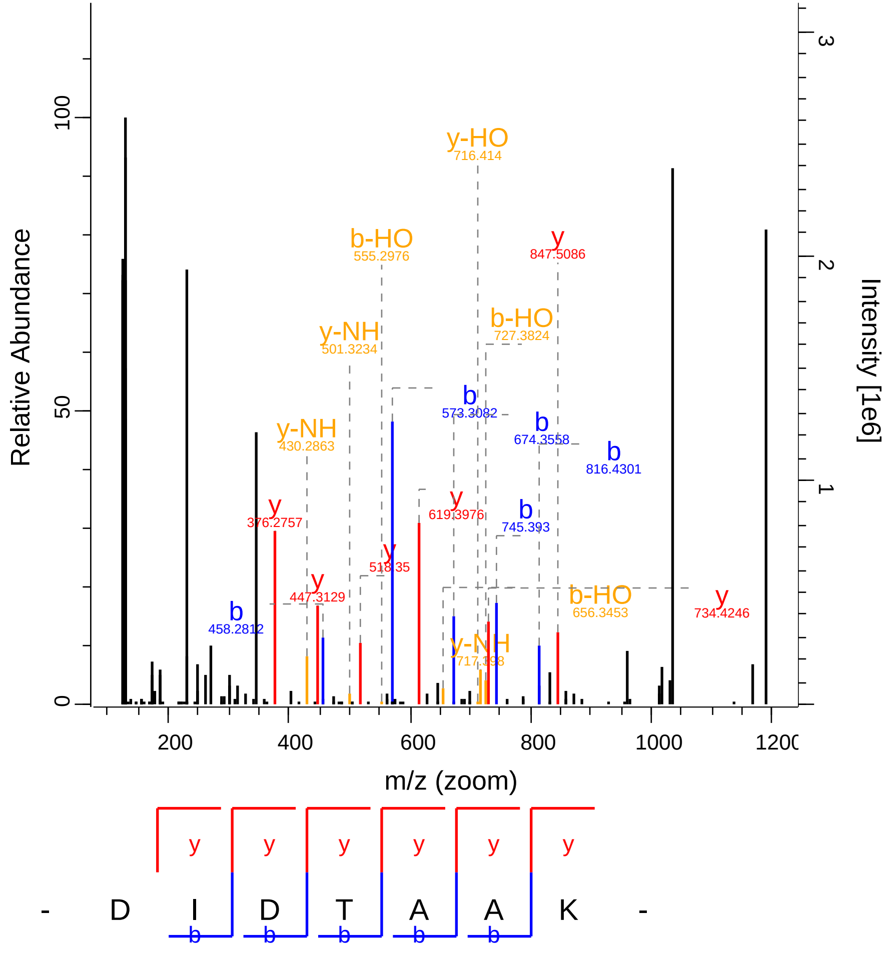

Raw File	Scan	Method	Score	m/z
RO-QC-160121_ZackD_SS_Set_01_Fraction_2	12569	FTMS; HCD	106.36	638.39

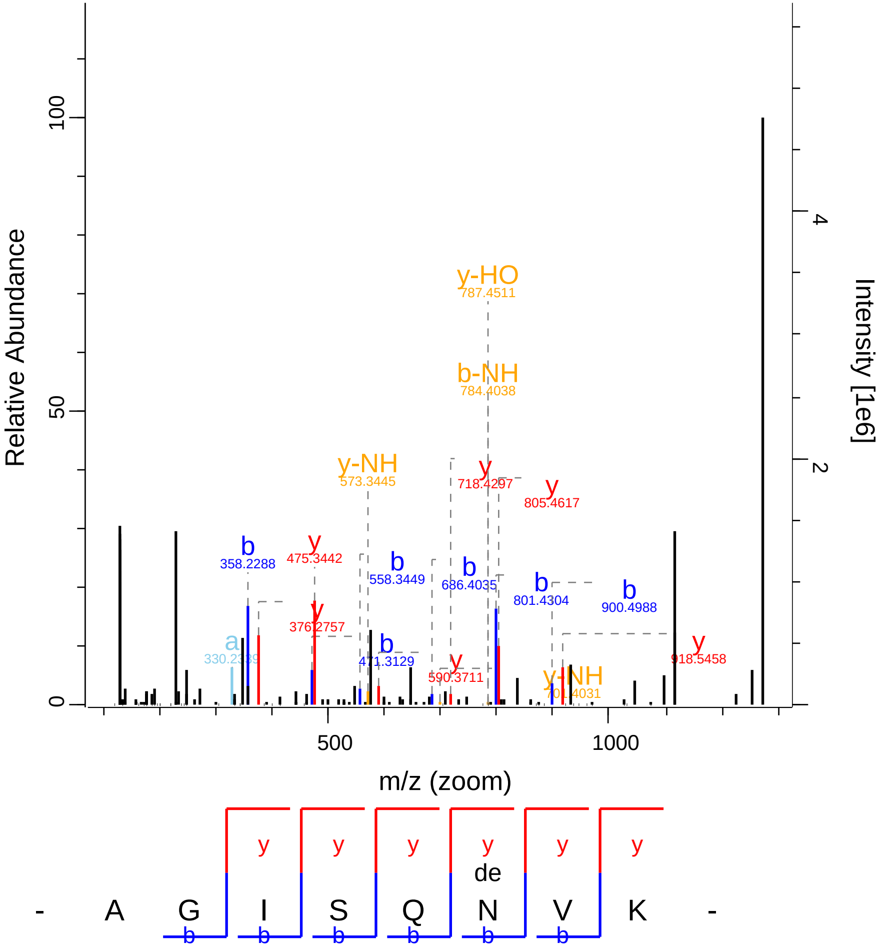

Raw File	Scan	Method	Score	m/z	Gene names
RO-QC-160121_ZackD_SS_Set_01_Fractio	12787	FTMS; HC	59.71	818.77	SLC6A17

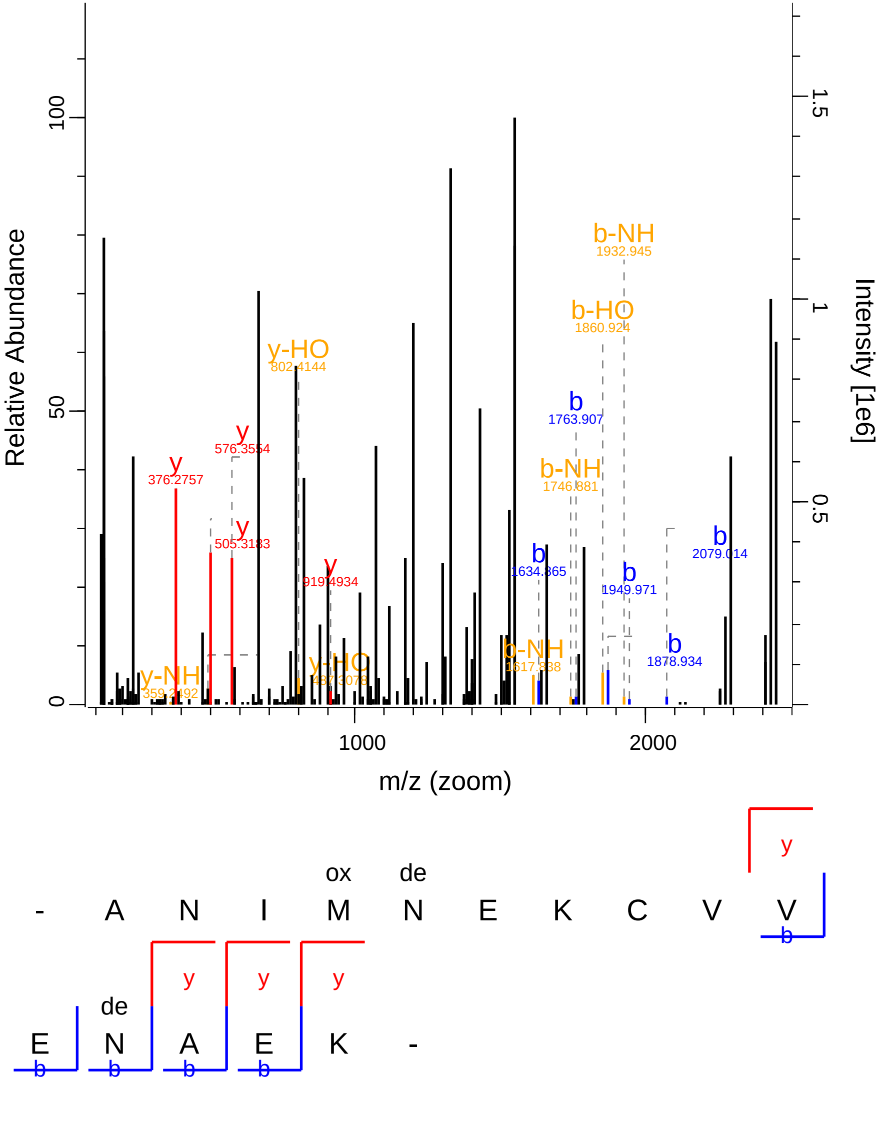

Raw File	Scan	Method	Score	m/z	Gene names
RO-QC-160121_ZackD_SS_Set_01_Fractio	13378	FTMS; HC	62.55	637.86	ZNF706

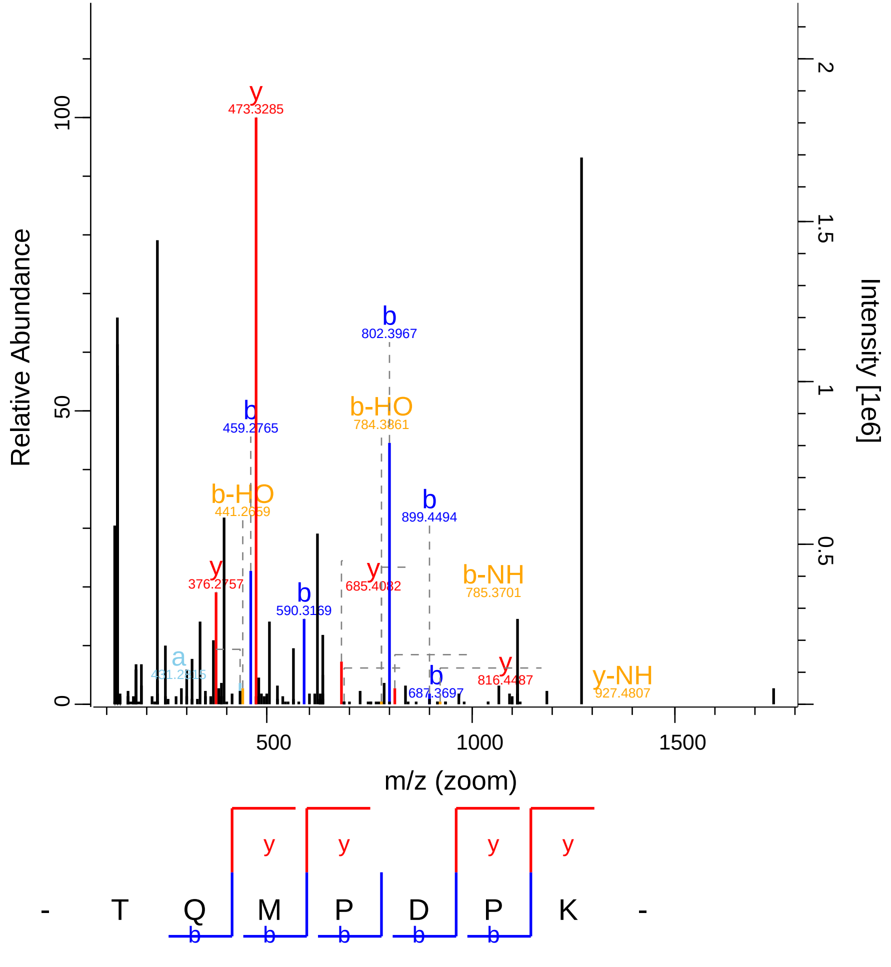

Raw File	Scan	Method	Score	m/z
RO-QC-160121_ZackD_SS_Set_01_Fraction_2	13605	FTMS; HCD	105.12	594.87

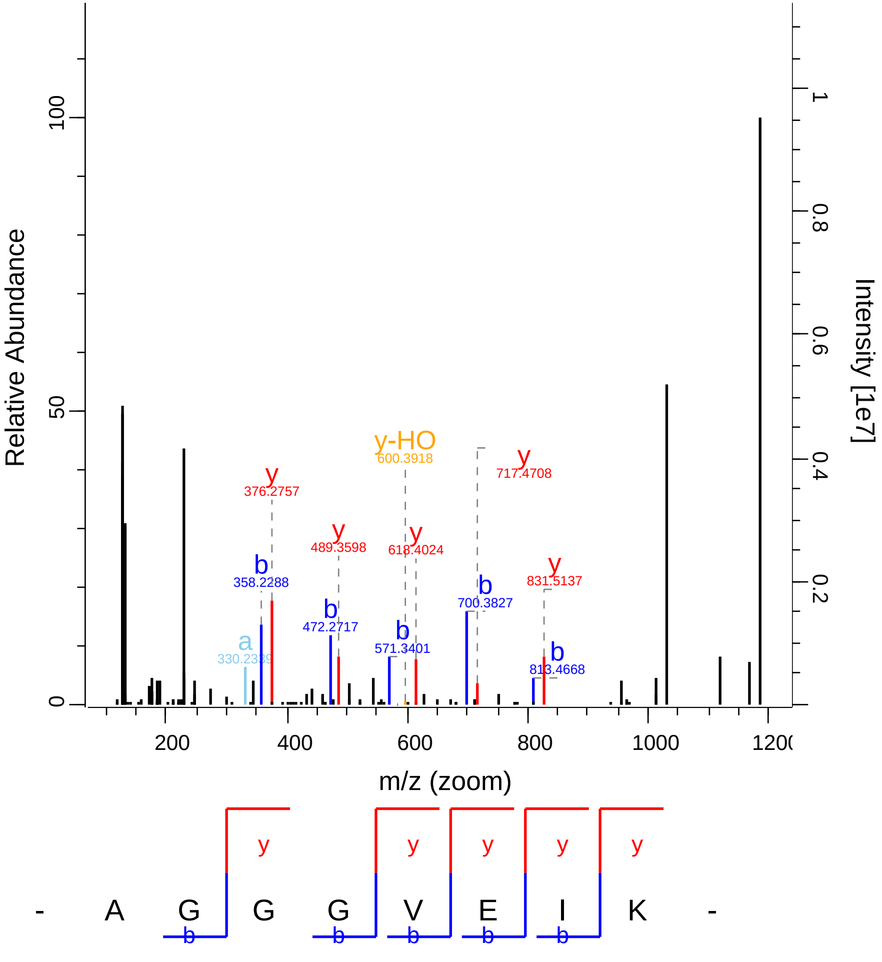

Raw File	Scan	Method	Score	m/z	Gene names
RO-QC-160121_ZackD_SS_Set_01_Fractio	13815	FTMS; HC	60.02	780.45	WRAP73

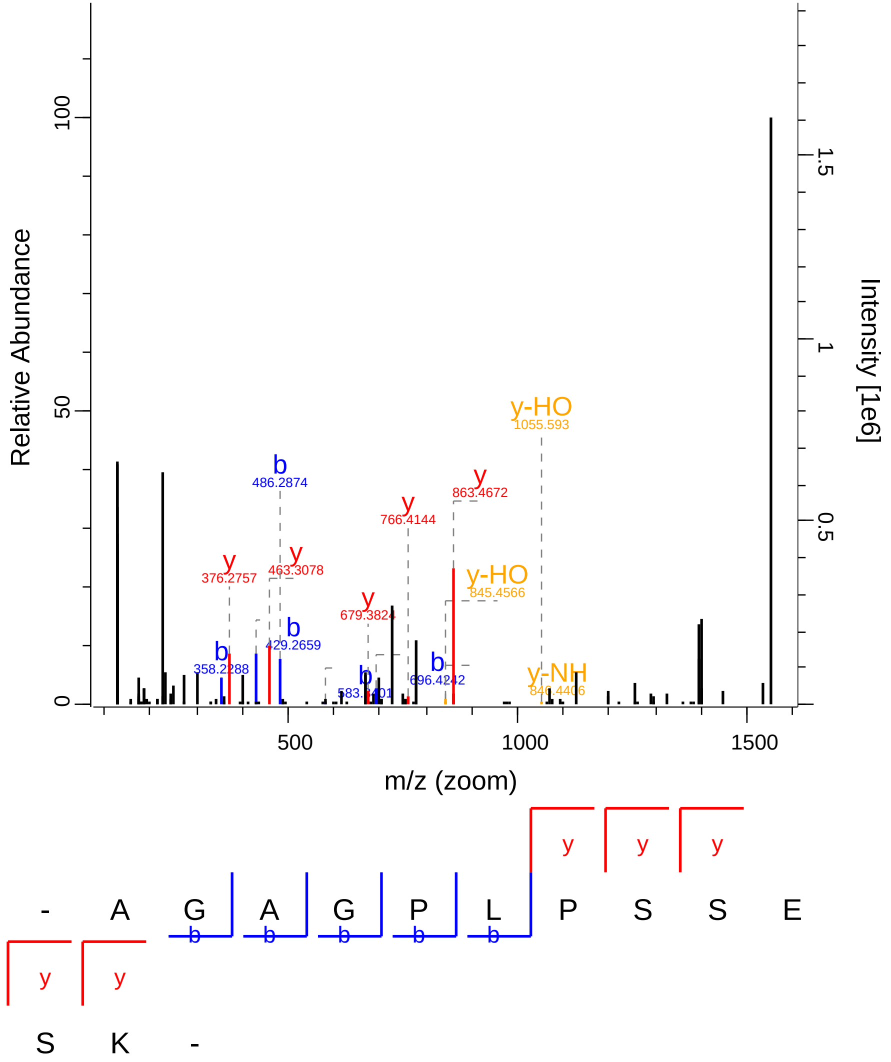

Raw File	Scan	Method	Score	m/z	Gene names
RO-QC-160121_ZackD_SS_Set_01_Fractio	13932	FTMS; HC	94.19	548.99	STRADB

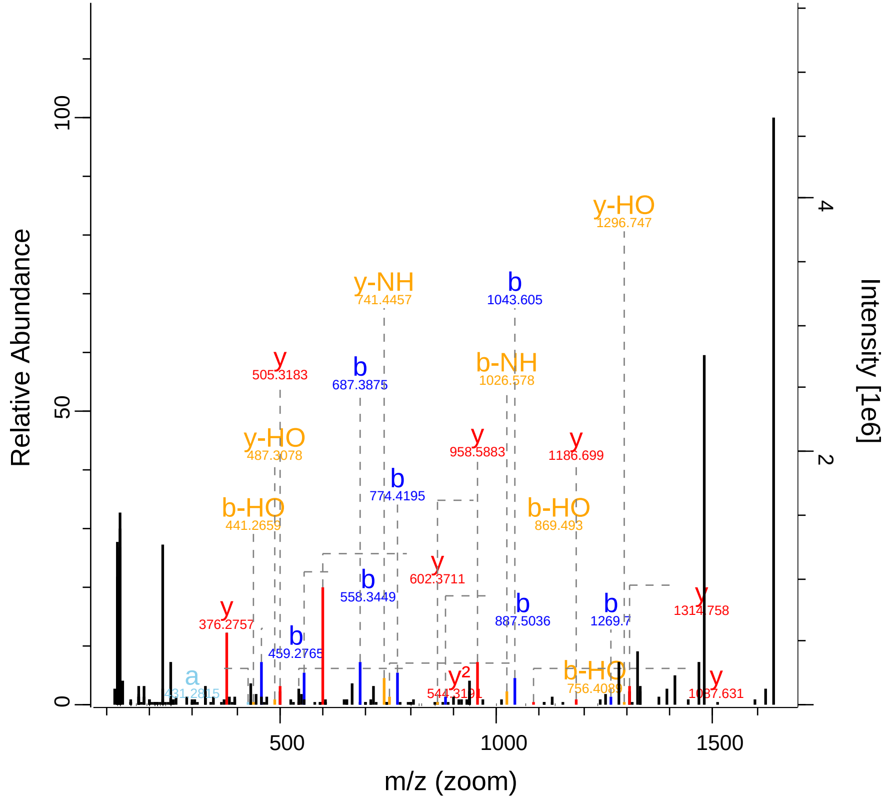

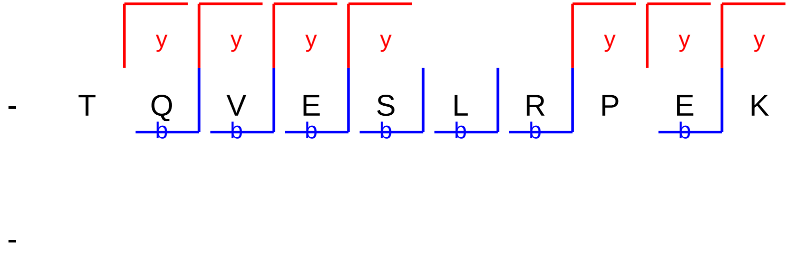

Raw File	Scan	Method	Score	m/z	Gene names
RO-QC-160121_ZackD_SS_Set_01_Fractio	14080	FTMS; HC	141.27	740.35	ANAPC16

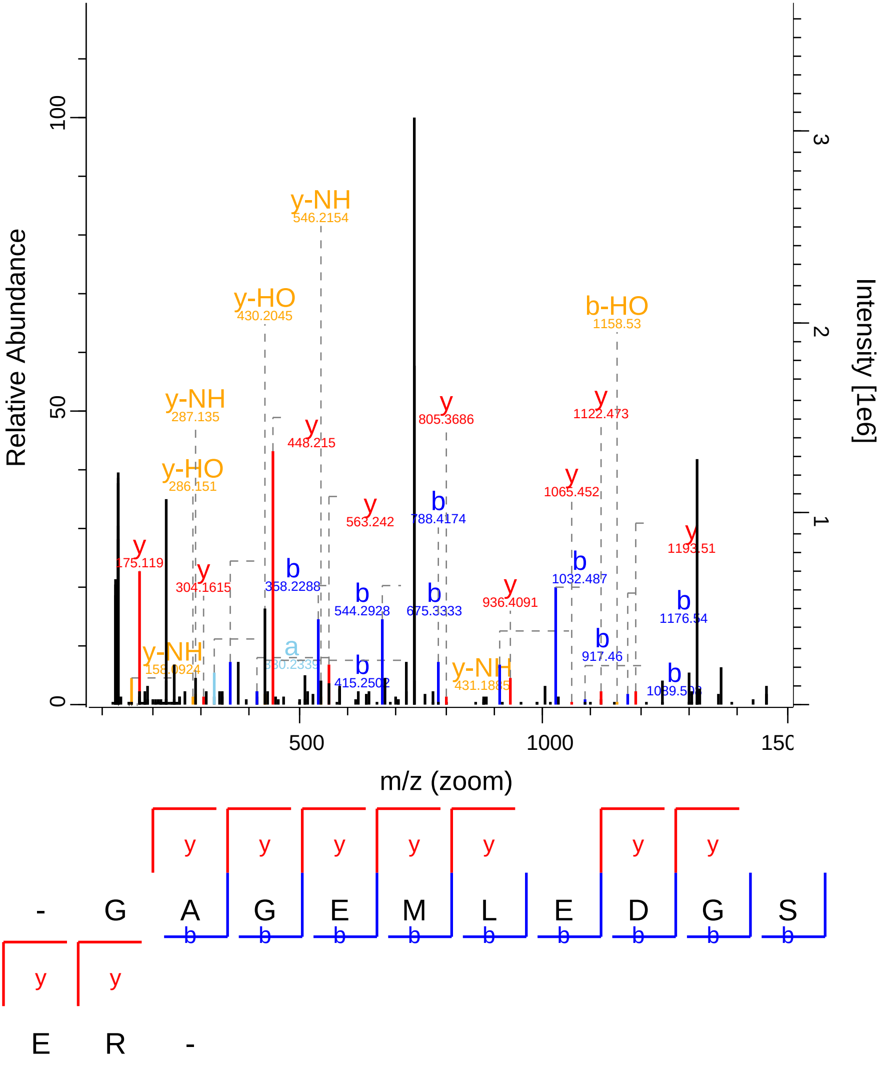

Raw File	Scan	Method	Score	m/z	Gene names
RO-QC-160121_ZackD_SS_Set_01_Fractio	14107	FTMS; HC	129.38	753.92	MED28

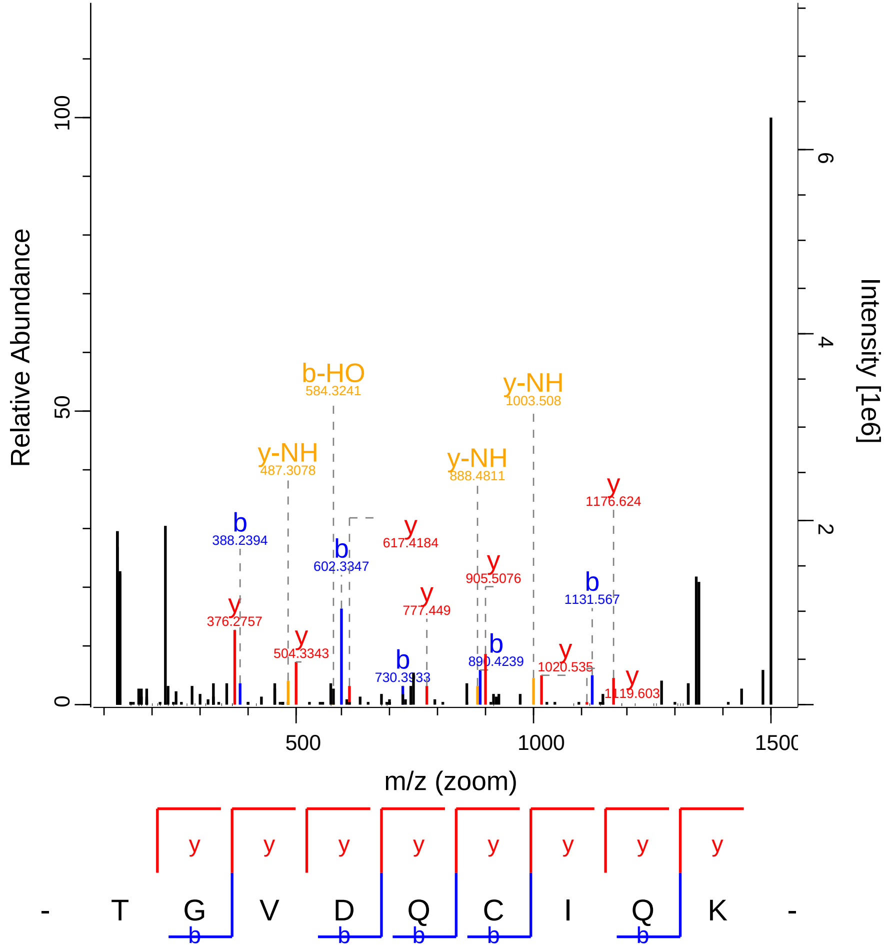

Raw File	Scan	Method	Score	m/z	Gene names
RO-QC-160121_ZackD_SS_Set_01_Fractio	14201	FTMS; HC	84.66	956.01	GPR110

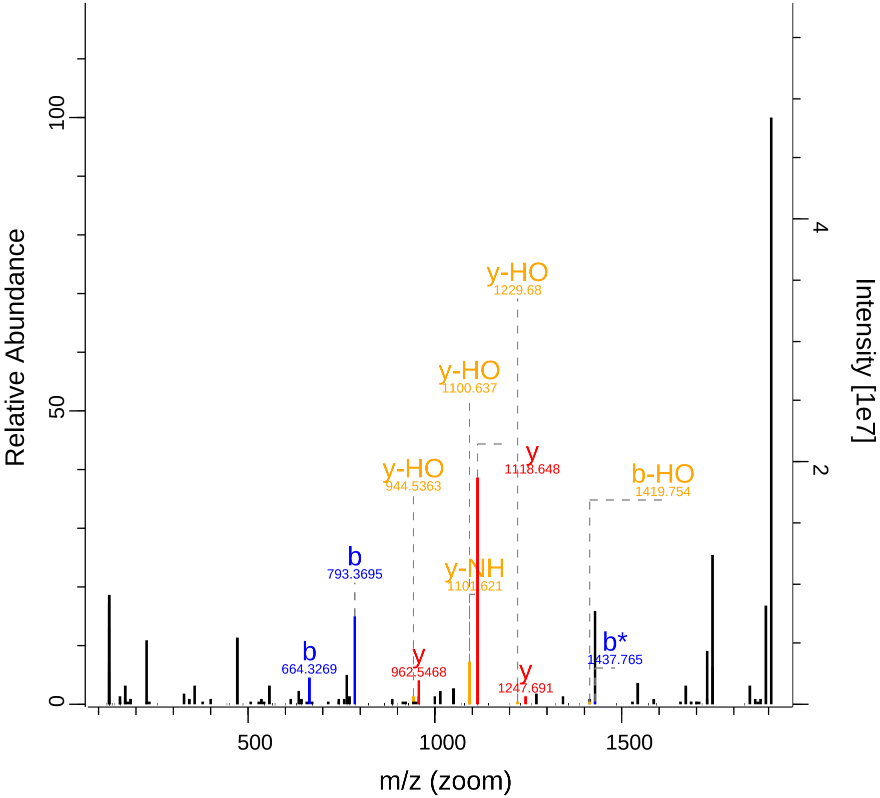

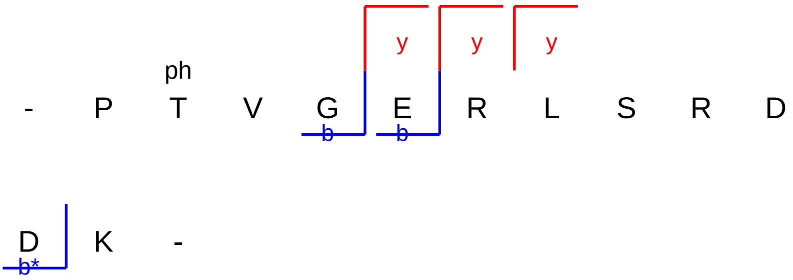

Raw File	Scan	Method	Score	m/z	Gene names
RO-QC-160121_ZackD_SS_Set_01_Fractio	14882	FTMS; HC	49.81	677.87	TIGD1

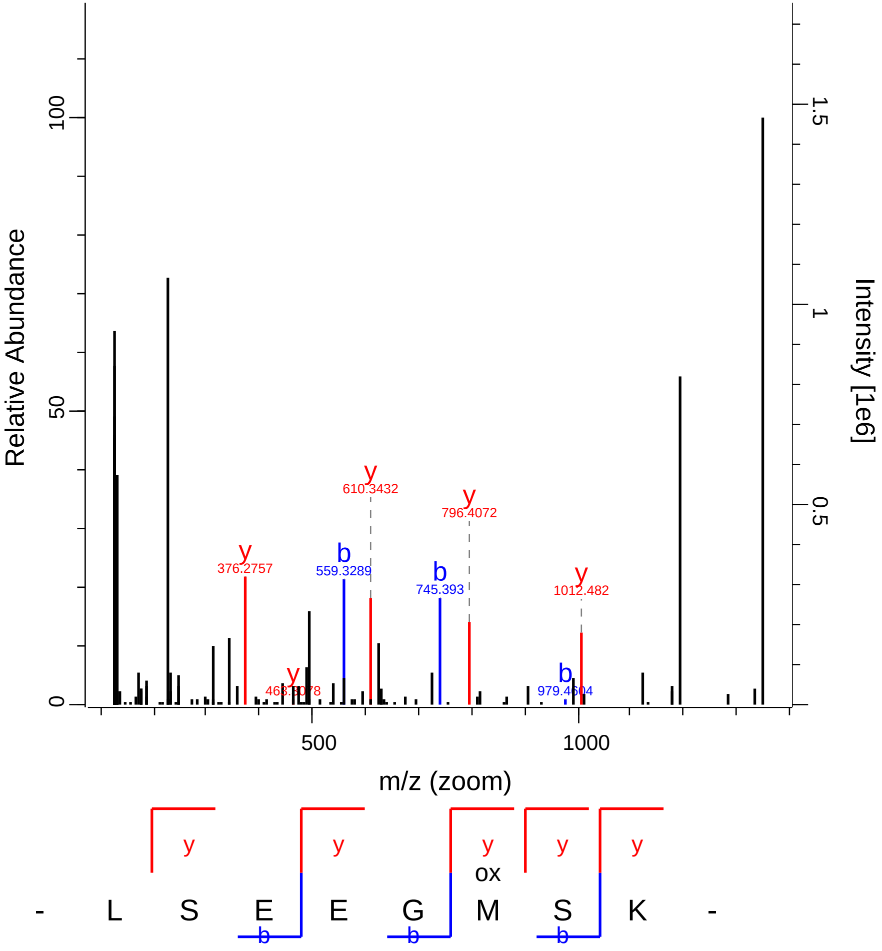

Raw File	Scan	Method	Score	m/z	Gene names
RO-QC-160121_ZackD_SS_Set_01_Fractio	14902	FTMS; HC	98.58	596.32	TMEM41B

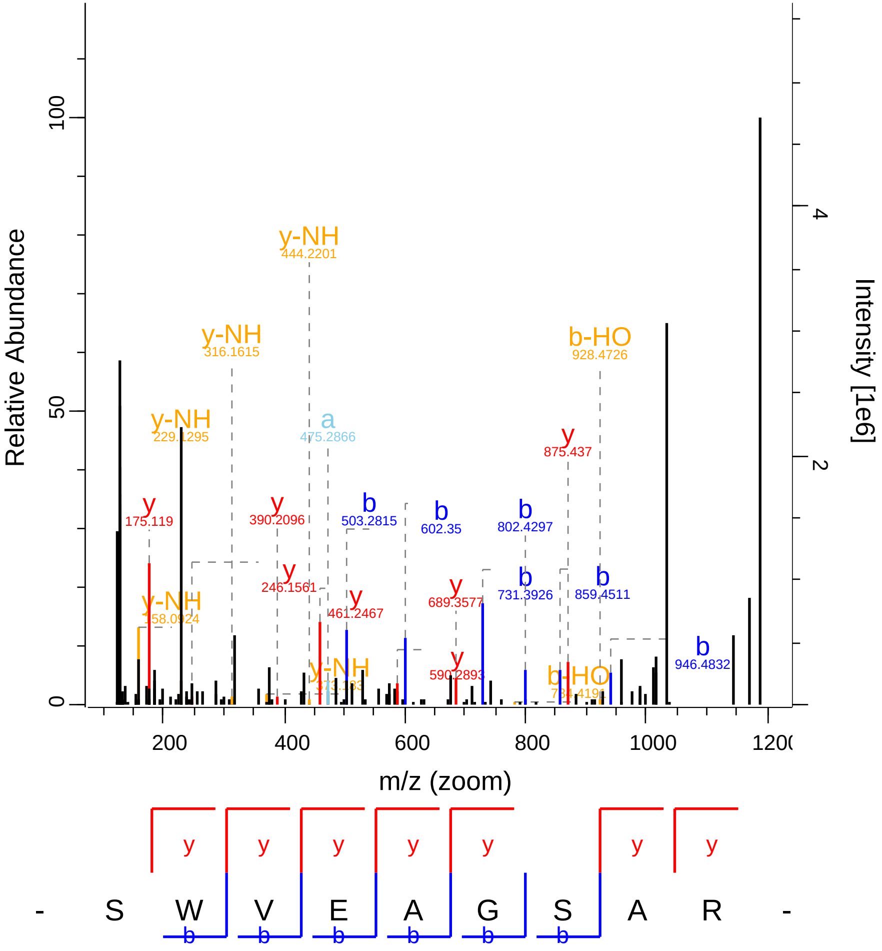

Raw File	Scan	Method	Score	m/z	Gene names
RO-QC-160121_ZackD_SS_Set_01_Fractio	15364	FTMS; HC	109.65	770.43	SAYSD1

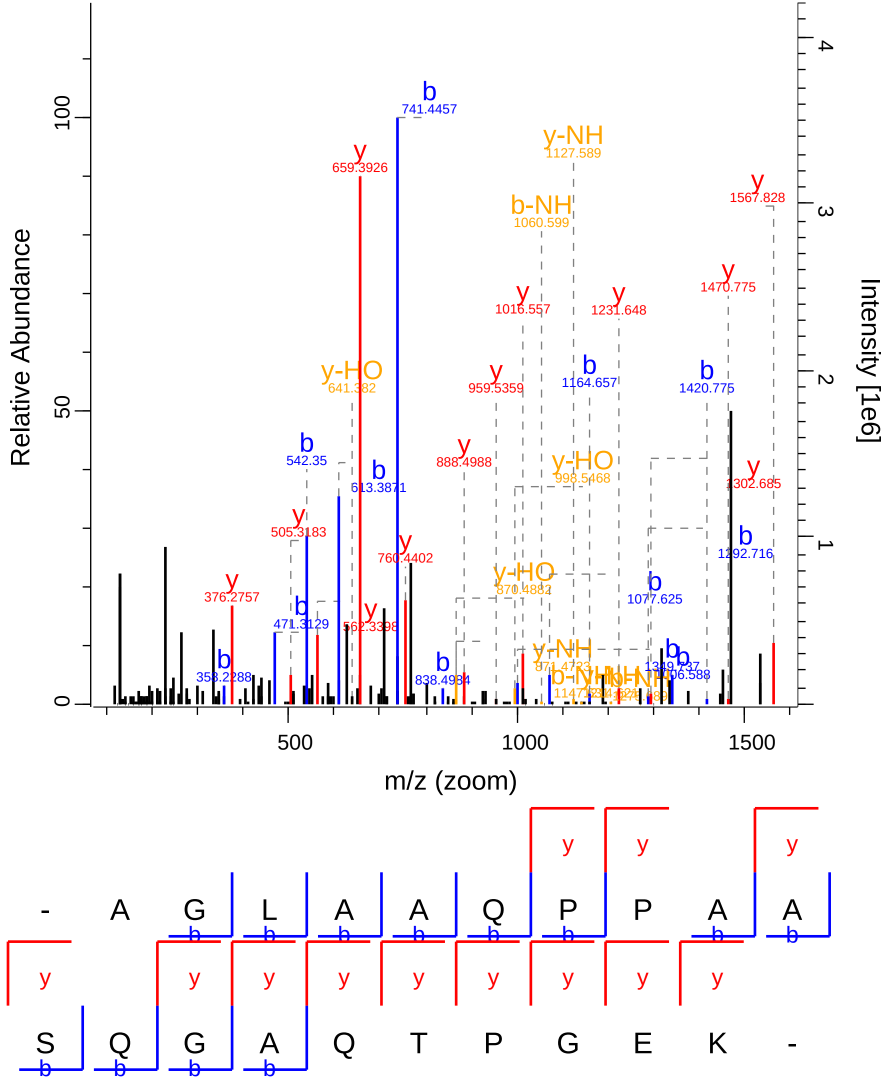

Raw File	Scan	Method	Score	m/z
RO-QC-160121_ZackD_SS_Set_01_Fraction_2	15762	FTMS; HCD	76.06	495.96

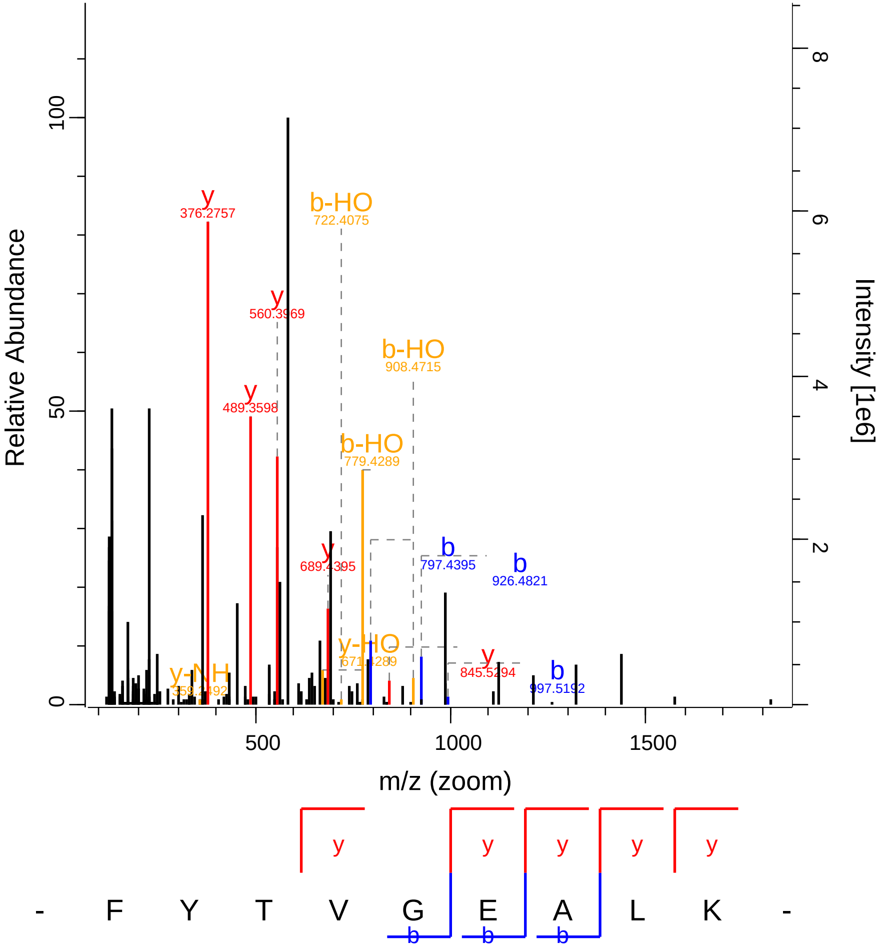

Raw File	Scan	Method	Score	m/z	Gene names
RO-QC-160121_ZackD_SS_Set_01_Fractio	15894	FTMS; HC	135.43	722.41	SLC25A14

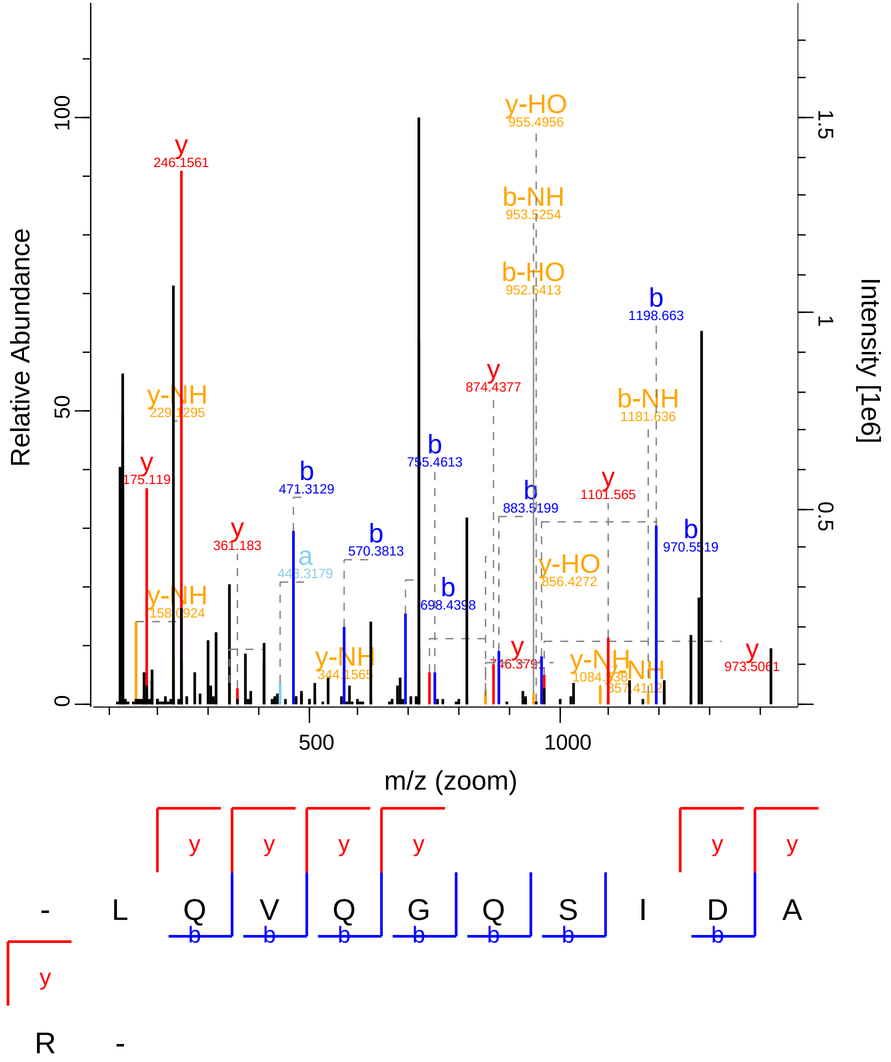

Raw File	Scan	Method	Score	m/z
RO-QC-160121_ZackD_SS_Set_01_Fraction_2	15981	FTMS; HCD	111.82	702.91

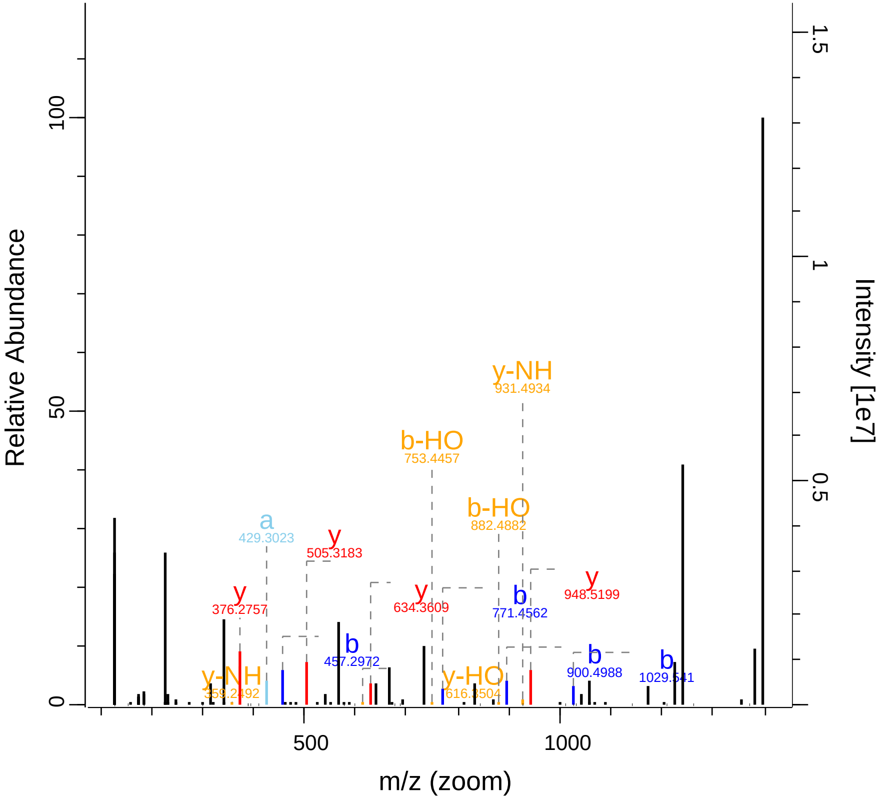

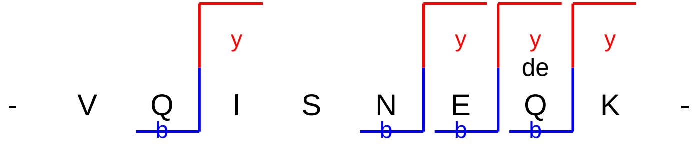

Raw File	Scan	Method	Score	m/z	Gene names
RO-QC-160121_ZackD_SS_Set_01_Fractio	16225	FTMS; HC	103.22	745.39	HS6ST1

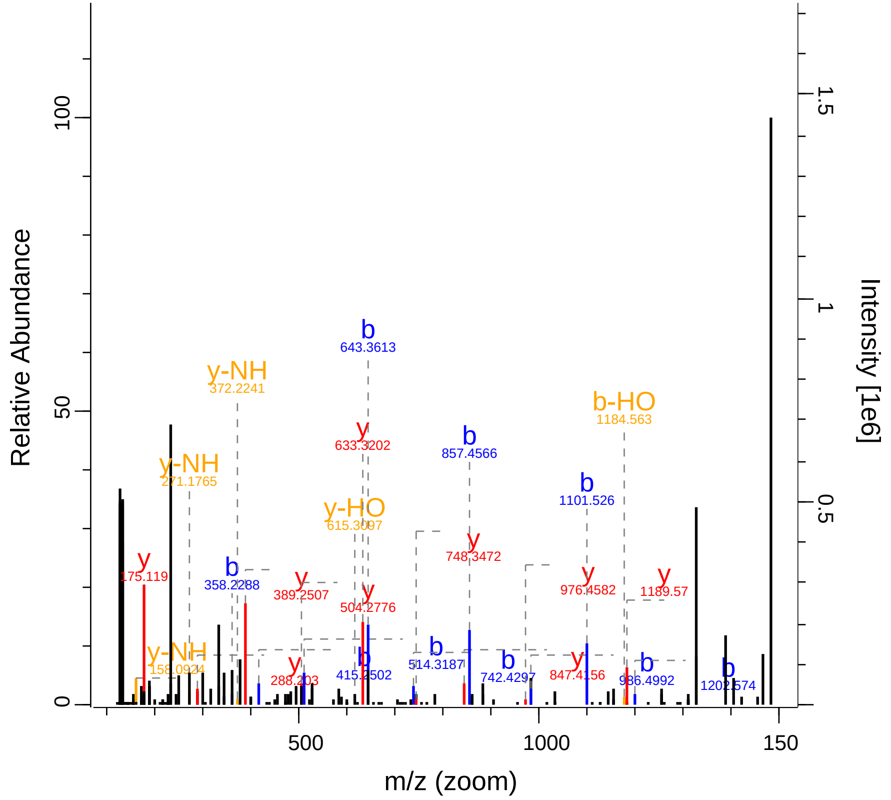

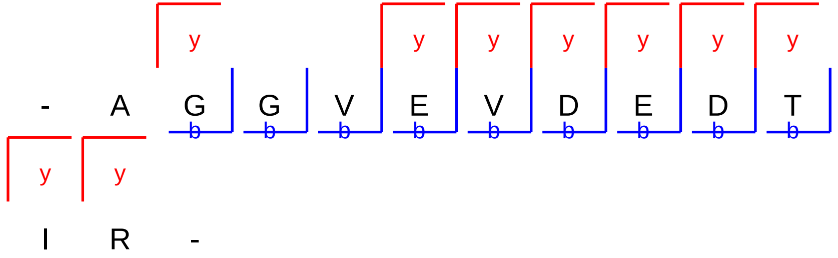

Raw File	Scan	Method	Score	m/z
RO-QC-160121_ZackD_SS_Set_01_Fraction_2	17069	FTMS; HCD	46.35	967.95

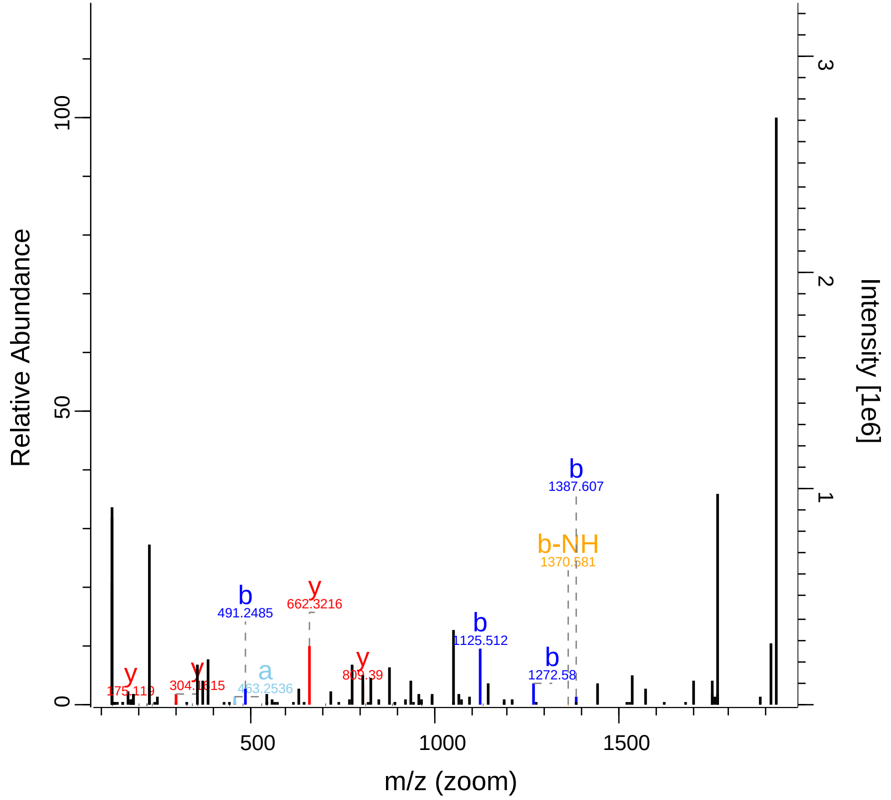

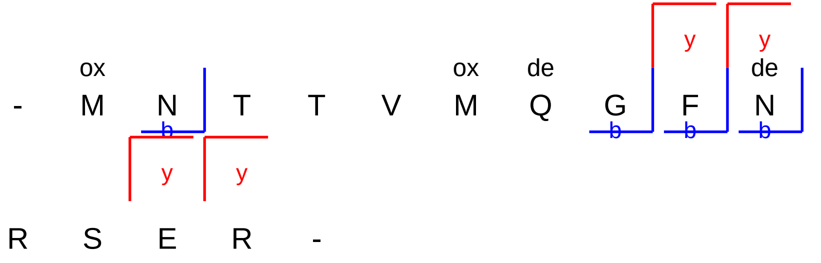

Raw File	Scan	Method	Score	m/z	Gene names
RO-QC-160121_ZackD_SS_Set_01_Fractio	17170	FTMS; HC	103.34	763.95	FAAP100

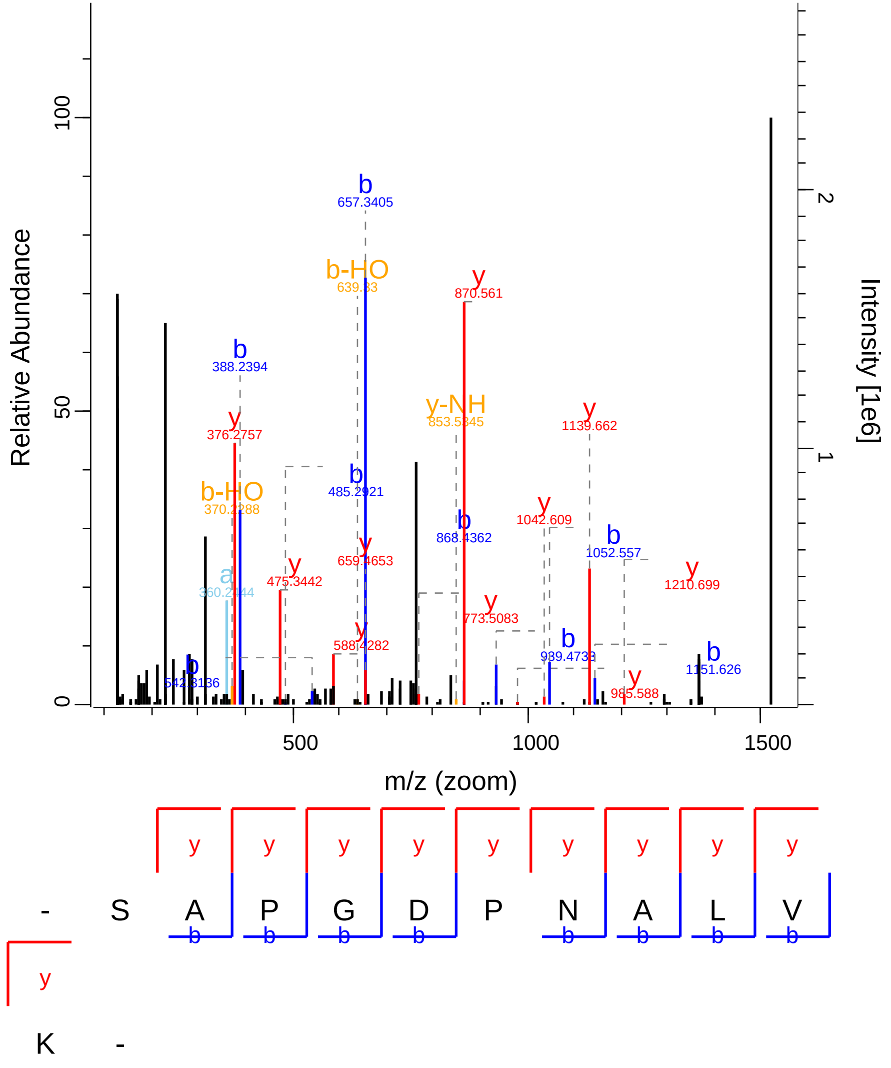

Raw File	Scan	Method	Score	m/z	Gene names
RO-QC-160121_ZackD_SS_Set_01_Fractio	17801	FTMS; HC	143.42	827.42	IQCB1

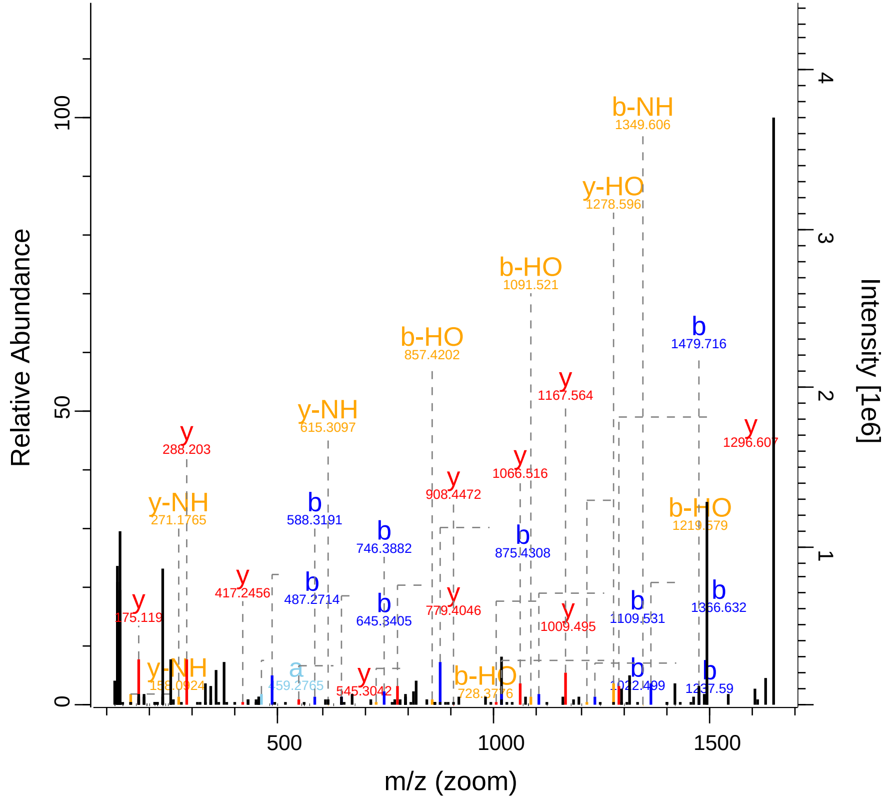

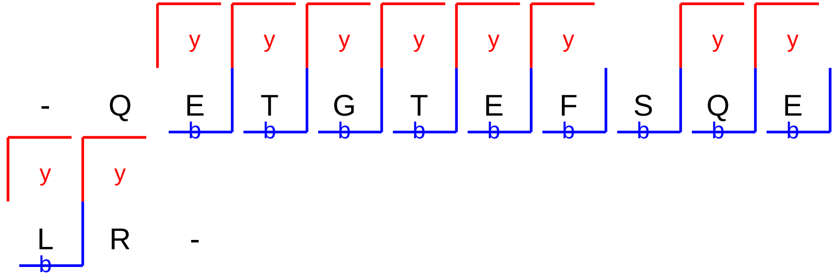

Raw File	Scan	Method	Score	m/z
RO-QC-160121_ZackD_SS_Set_01_Fraction_2	18154	FTMS; HCD	57.49	967.45

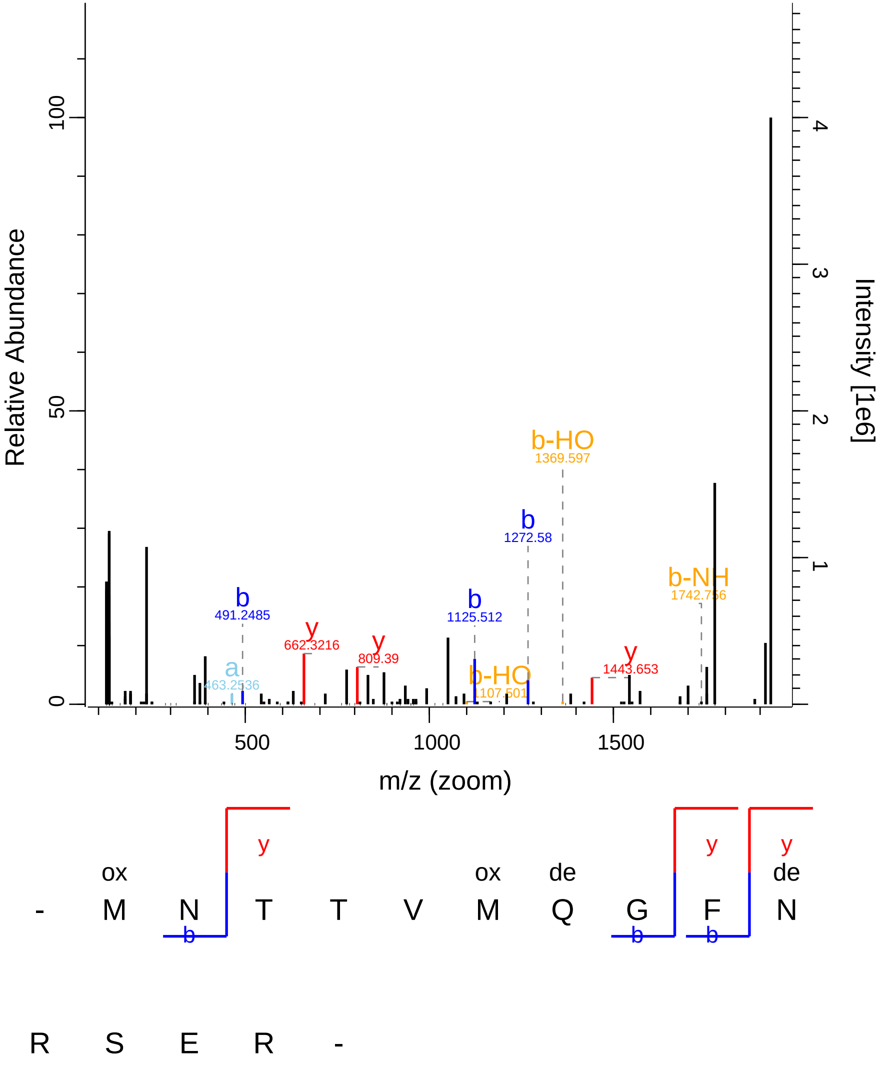

Raw File	Scan	Method	Score	m/z	Gene names
RO-QC-160121_ZackD_SS_Set_01_Fractio	18367	FTMS; HC	114.89	688.41	PTPN21

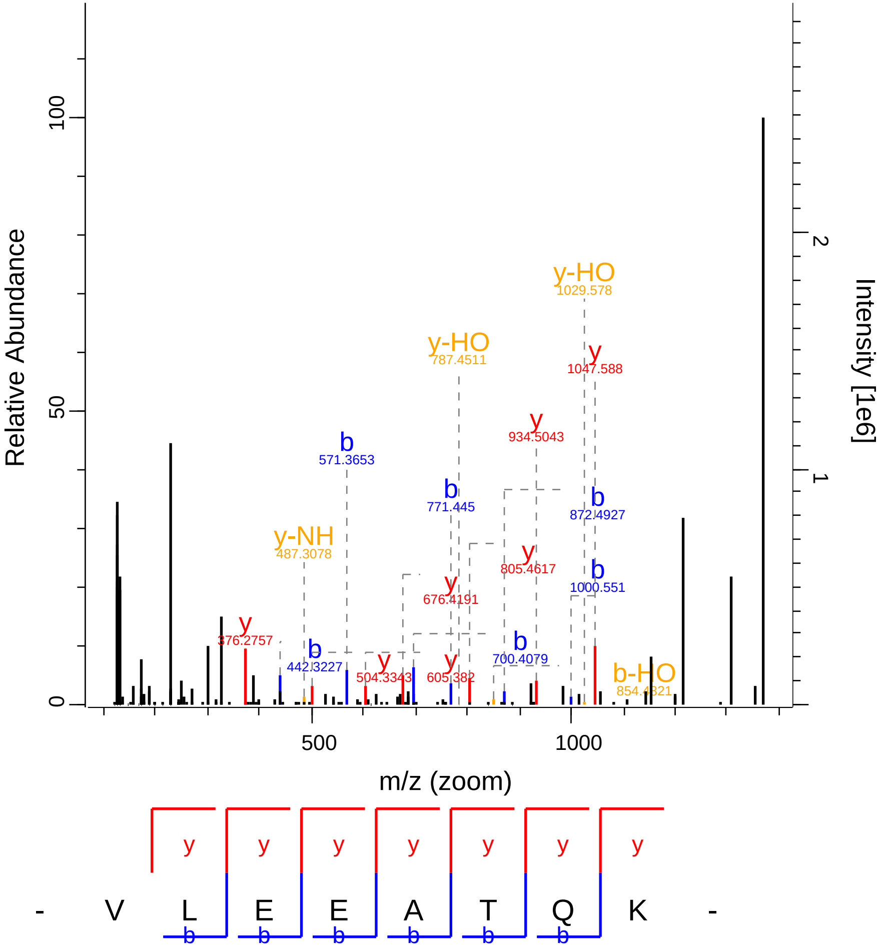

Raw File	Scan	Method	Score	m/z
RO-QC-160121_ZackD_SS_Set_01_Fraction_2	18380	FTMS; HCD	56.78	967.45

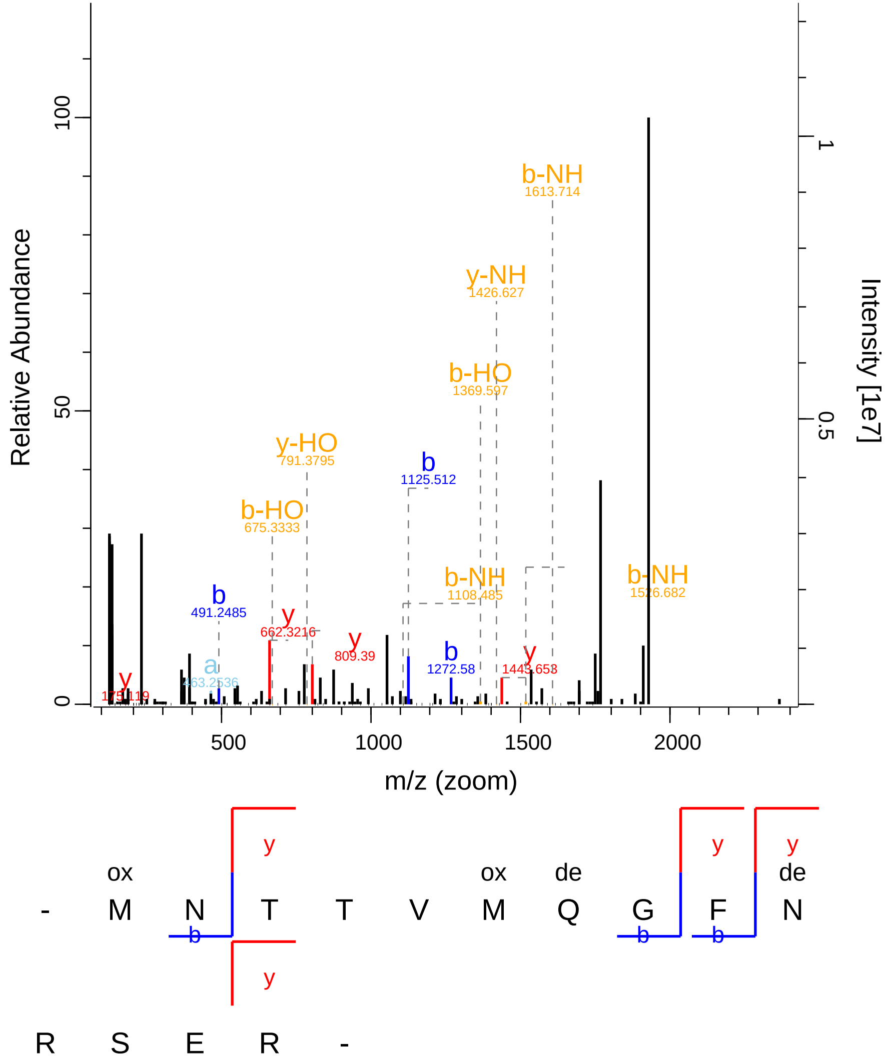

Raw File	Scan	Method	Score	m/z	Gene names
RO-QC-160121_ZackD_SS_Set_01_Fractio	19014	FTMS; HC	77.19	799.77	ATP6V0C

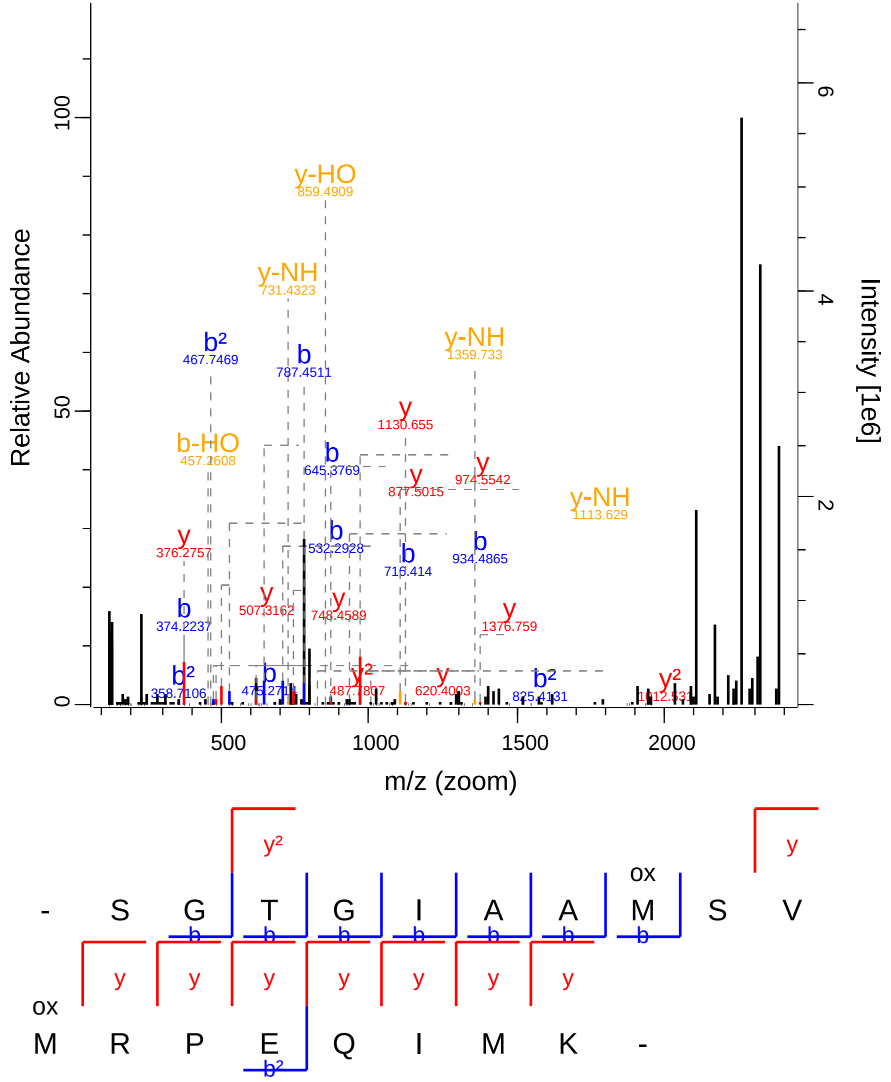

Raw File	Scan	Method	Score	m/z	Gene names
RO-QC-160121_ZackD_SS_Set_01_Fractior	19264	FTMS; HCI	54.77	800.1	ATP6V0C

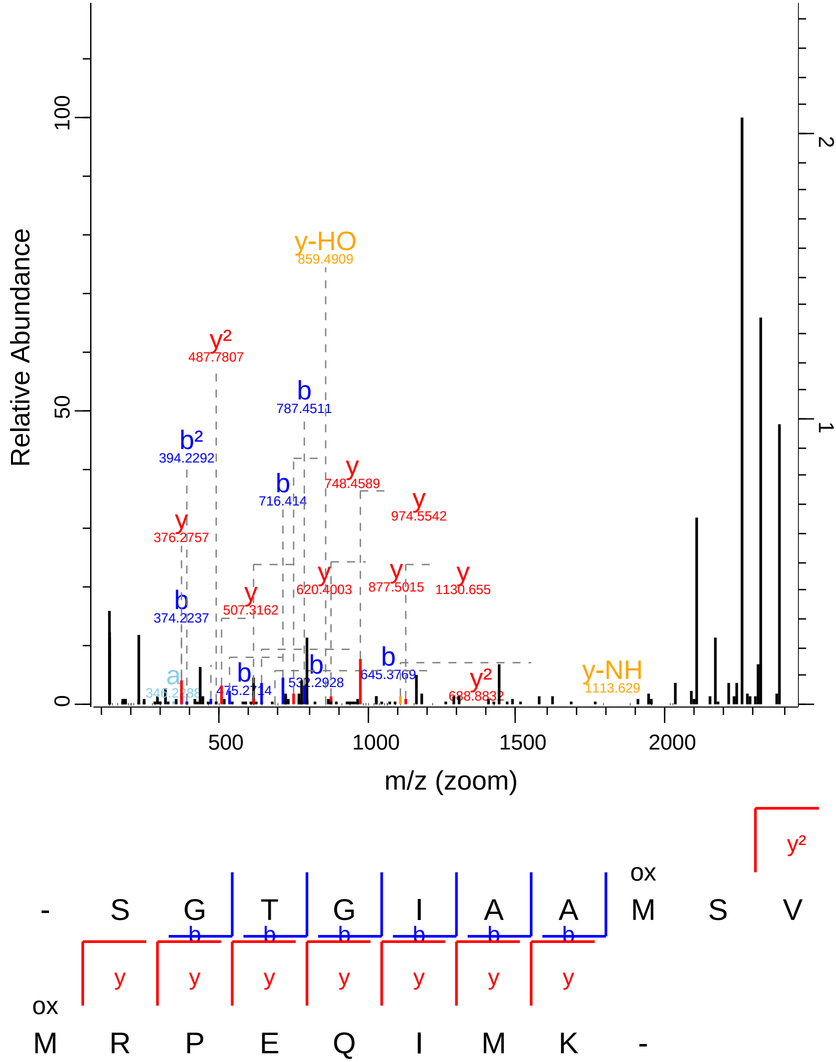

Raw File	Scan	Method	Score	m/z	Gene names
RO-QC-160121_ZackD_SS_Set_01_Fractio	19358	FTMS; HC	89.08	661.37	ABHD13

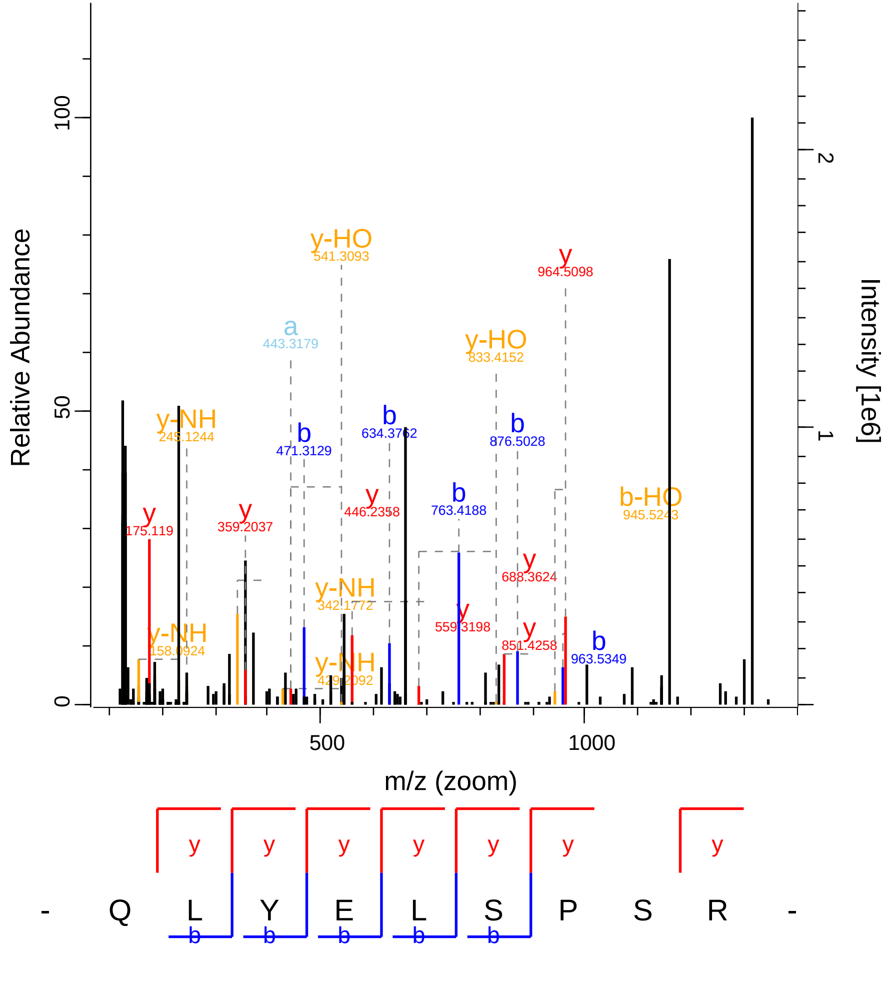

Raw File	Scan	Method	Score	m/z	Gene names
RO-QC-160121_ZackD_SS_Set_01_Fractio	19686	FTMS; HC	110.61	578.31	BNIP3L

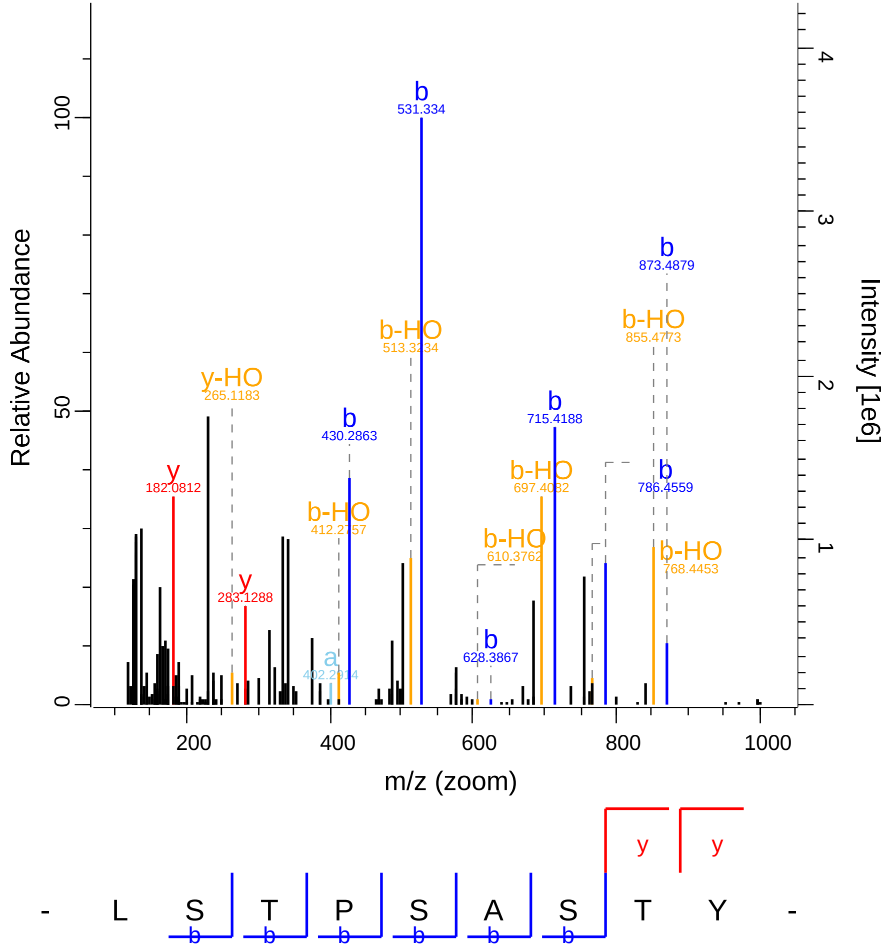

Raw File	Scan	Method	Score	m/z
RO-QC-160121_ZackD_SS_Set_01_Fraction_2	20166	FTMS; HCD	117.16	430.27

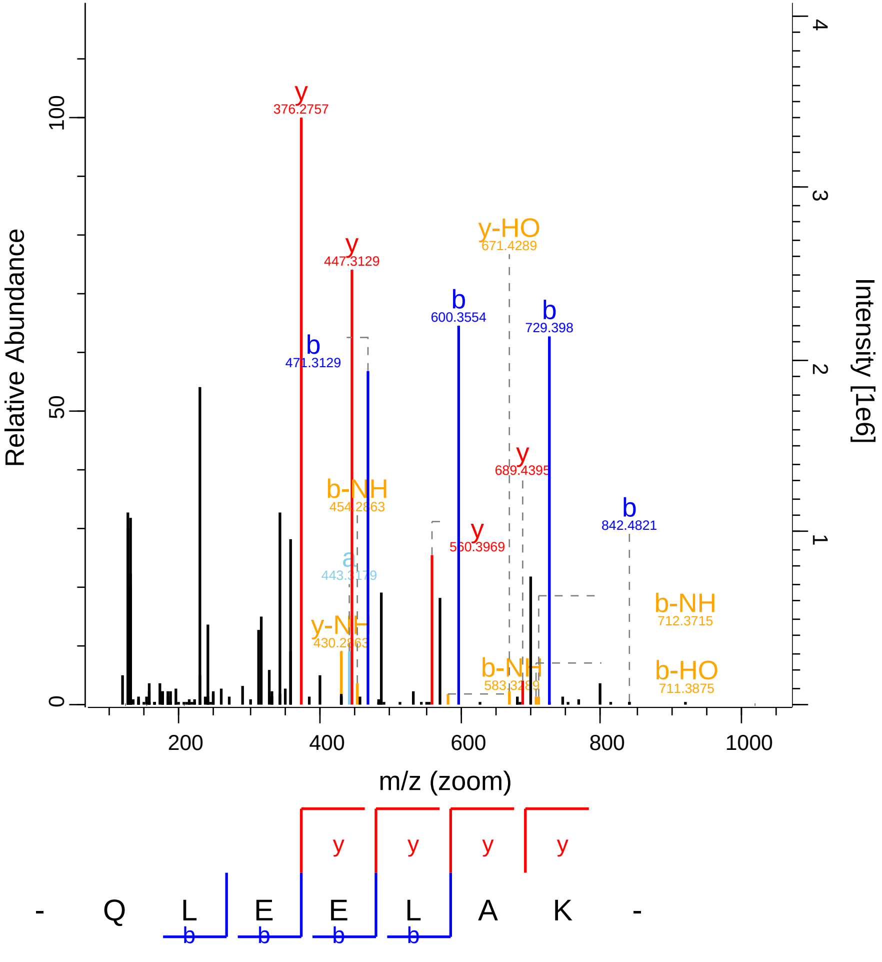

Raw File	Scan	Method	Score	m/z	Gene names
RO-QC-160121_ZackD_SS_Set_01_Fractic	20785	FTMS; HC	40.49	1015.84	DCST1

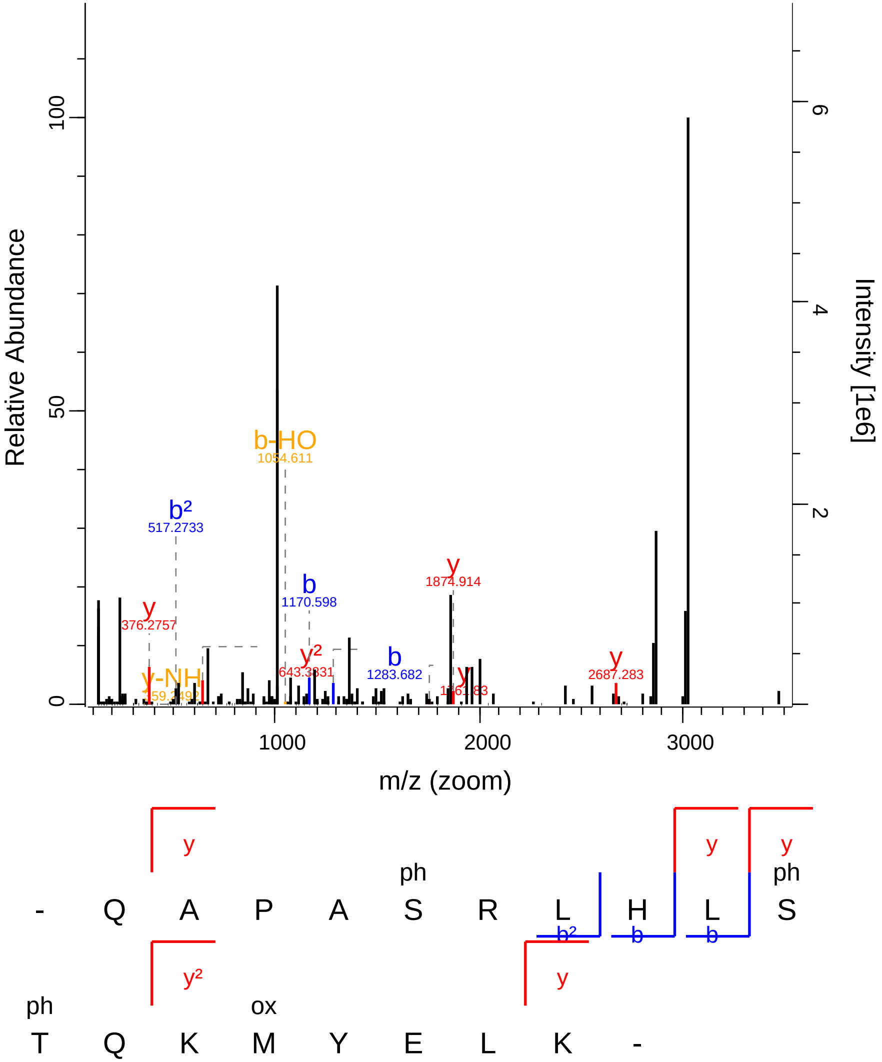

Raw File	Scan	Method	Score	m/z	Gene names
RO-QC-160121_ZackD_SS_Set_01_Fractio	22837	FTMS; HC	69.1	551.33	YBEY

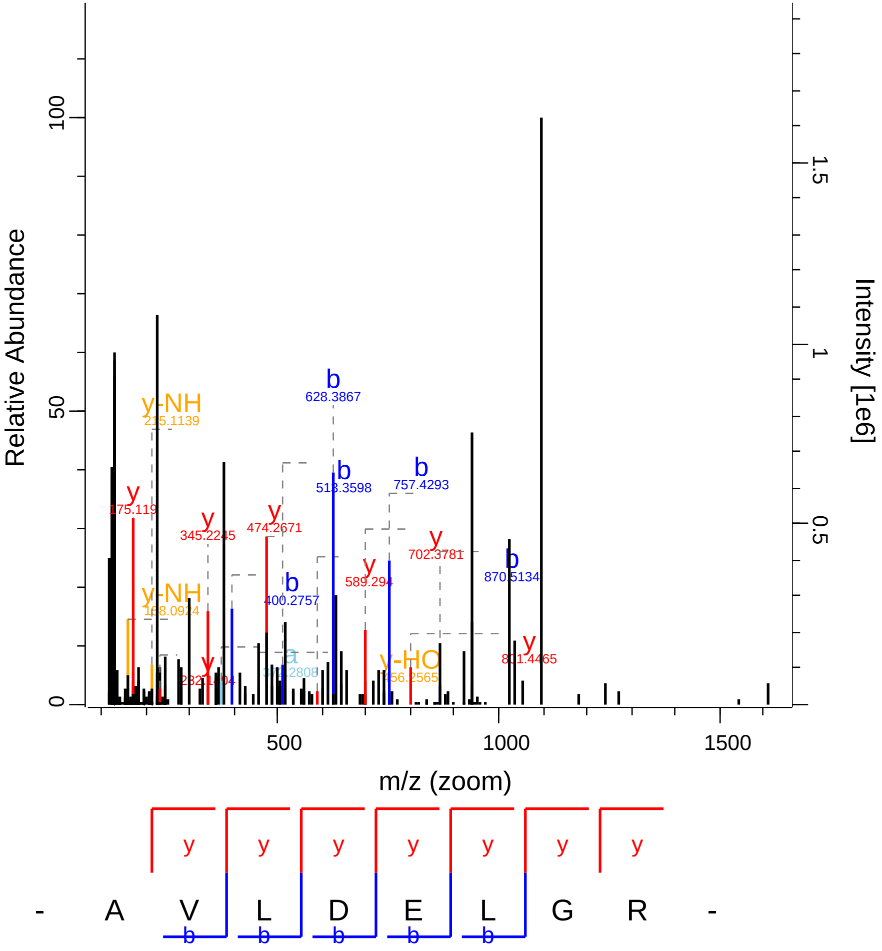

Raw File	Scan	Method	Score	m/z	Gene names
RO-QC-160121_ZackD_SS_Set_01_Fractic	23074	FTMS; HC	124.86	1086.52	HIGD1A

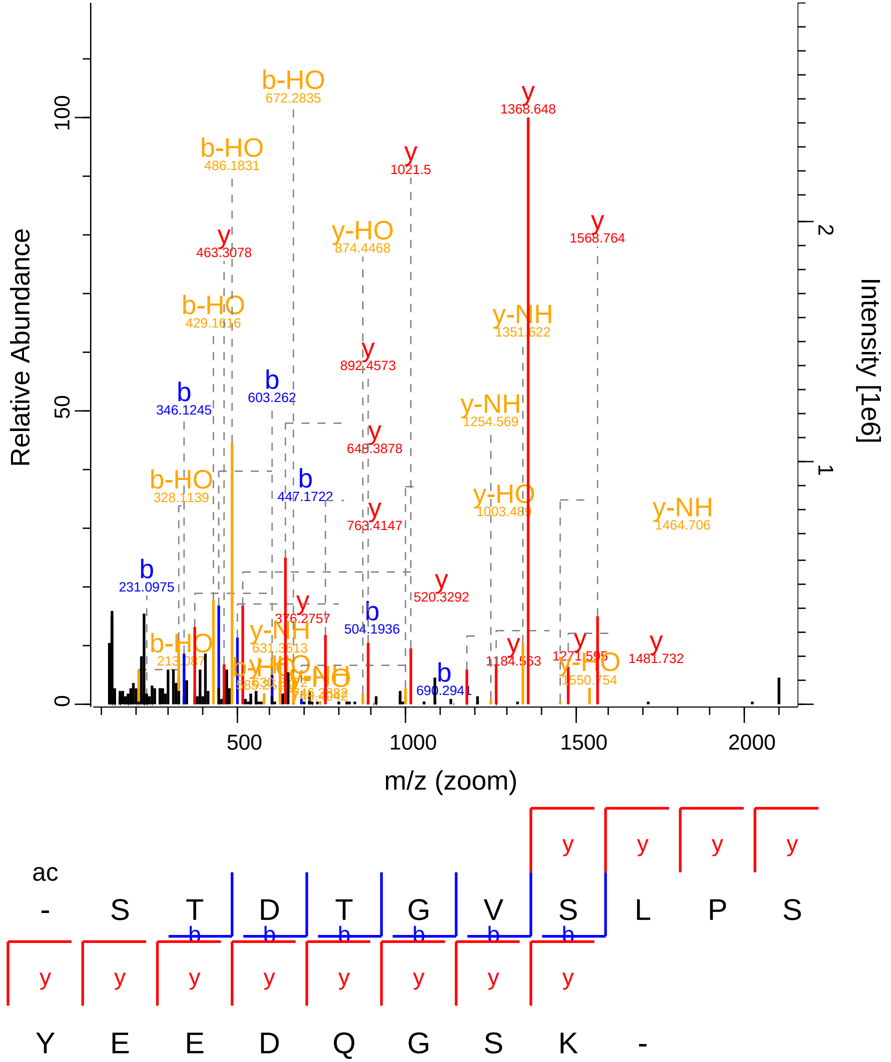

Raw File	Scan	Method	Score	m/z	Gene names
RO-QC-160121_ZackD_SS_Set_01_Fractio	23118	FTMS; HC	93.75	724.68	HIGD1A

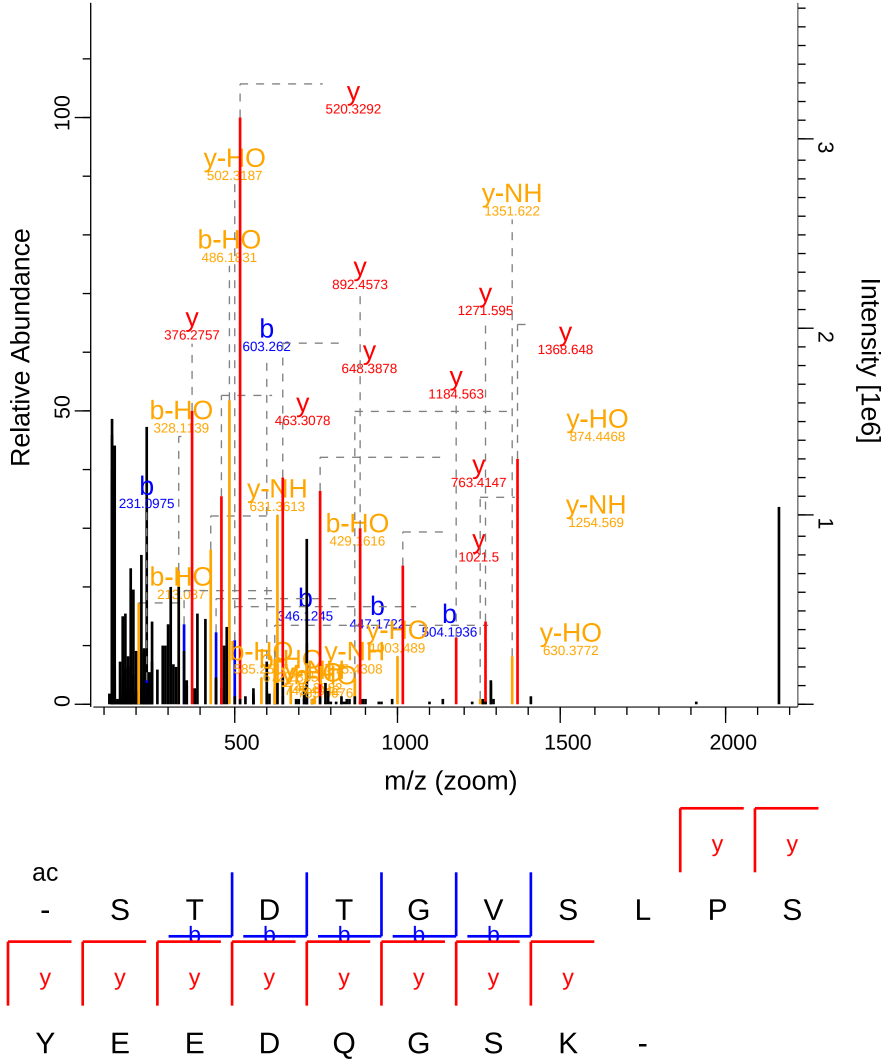

Raw File	Scan	Method	Score	m/z	Gene names
RO-QC-160121_ZackD_SS_Set_01_Fractio	23283	FTMS; HC	105.94	611.84	ALG14

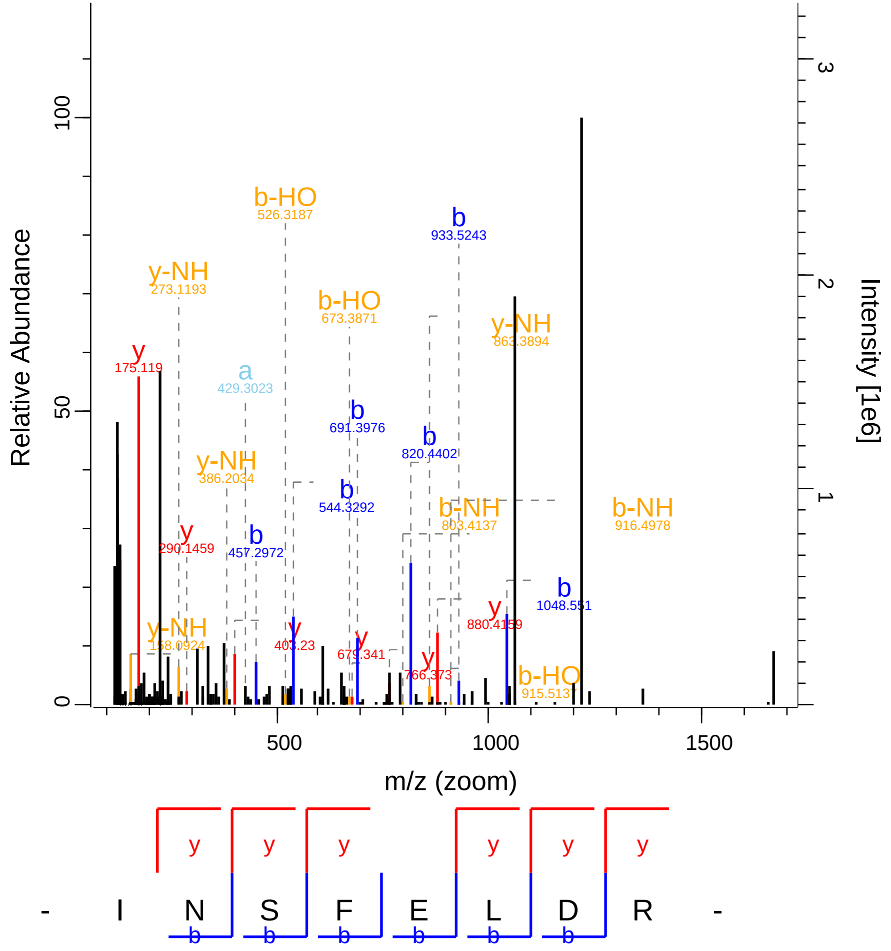

Raw File	Scan	Method	Score	m/z	Gene names
RO-QC-160121_ZackD_SS_Set_01_Fractic	23308	FTMS; HC	125.28	1086.52	HIGD1A

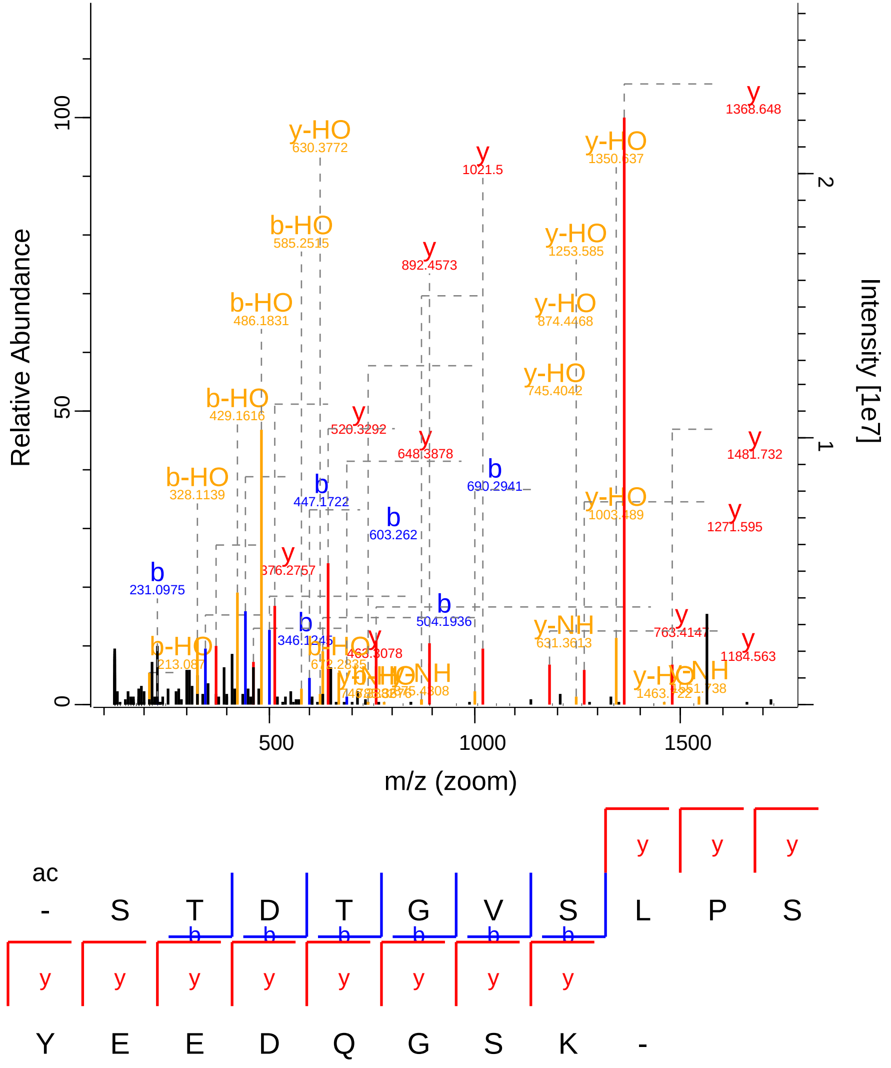

Raw File	Scan	Method	Score	m/z	Gene names
RO-QC-160121_ZackD_SS_Set_01_Fractio	23365	FTMS; HC	99.45	724.68	HIGD1A

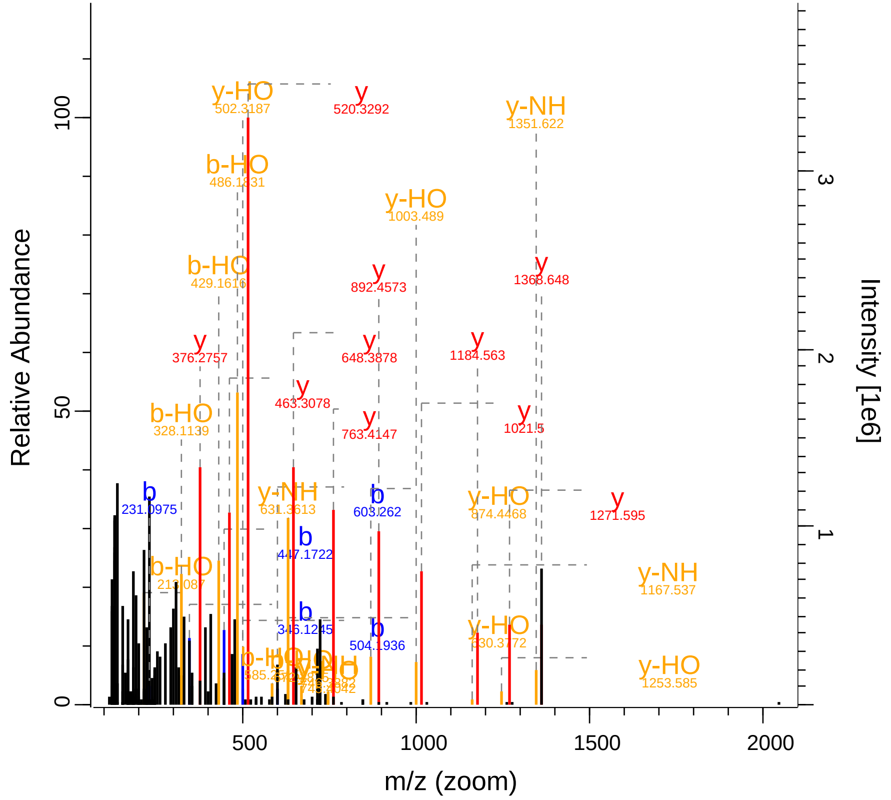

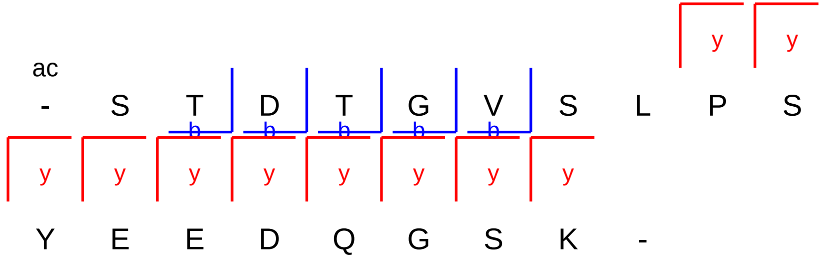

Raw File	Scan	Method	Score	m/z	Gene names
RO-QC-160121_ZackD_SS_Set_01_Fractic	23487	FTMS; HC	55.23	1086.19	SLX4

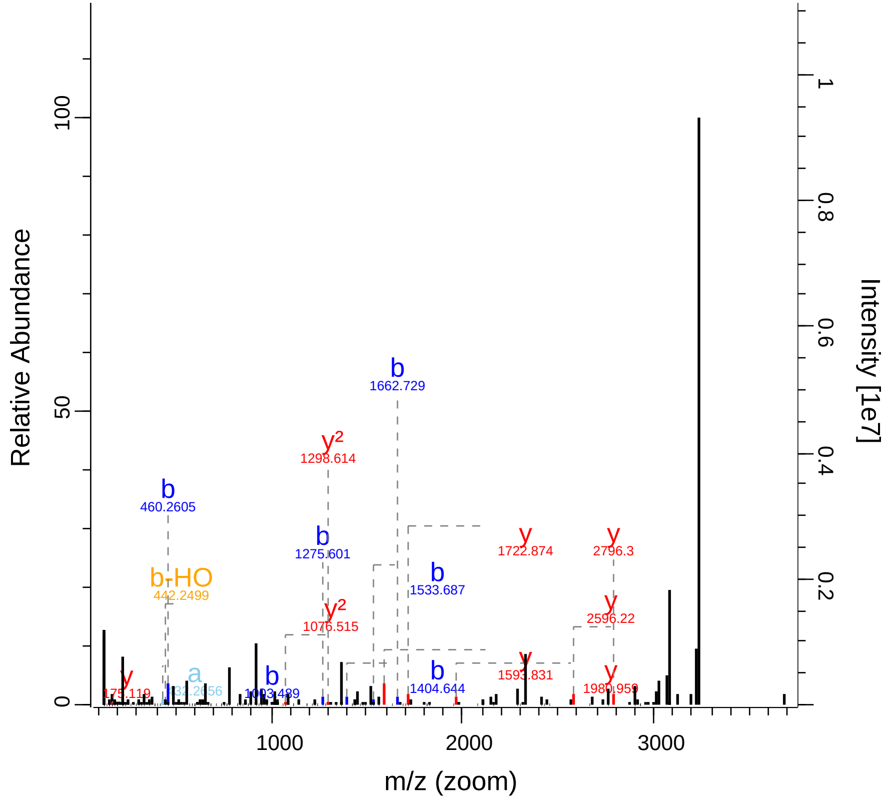

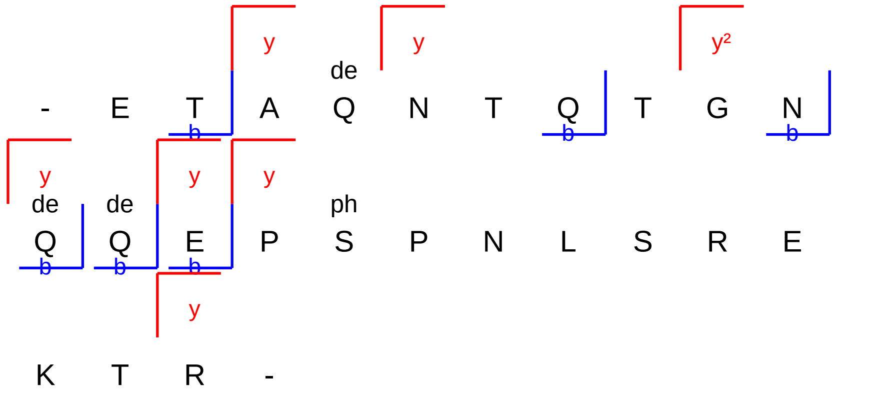

Raw File	Scan	Method	Score	m/z	Gene names
RO-QC-160121_ZackD_SS_Set_01_Fractio[illegible]	23839	FTMS; HC[illegible]	133.71	858.5	TINF2

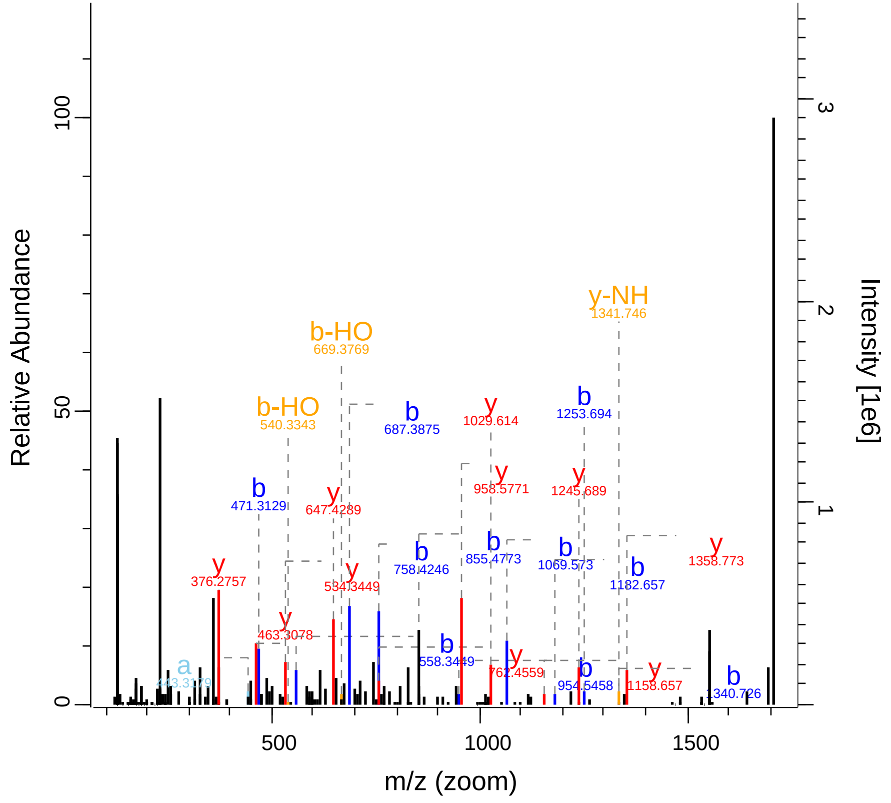

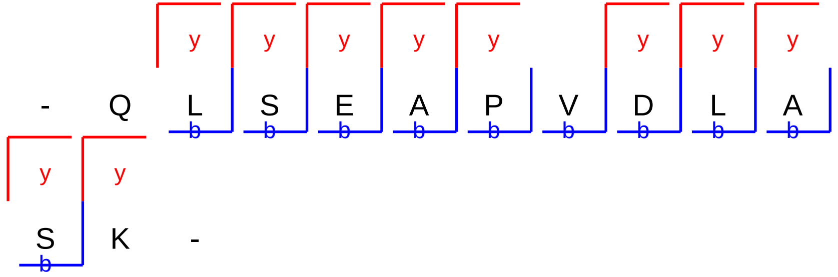

Raw File	Scan	Method	Score	m/z	Gene names
RO-QC-160121_ZackD_SS_Set_01_Fractio	24136	FTMS; HC	136.48	803.45	ATXN7L3B

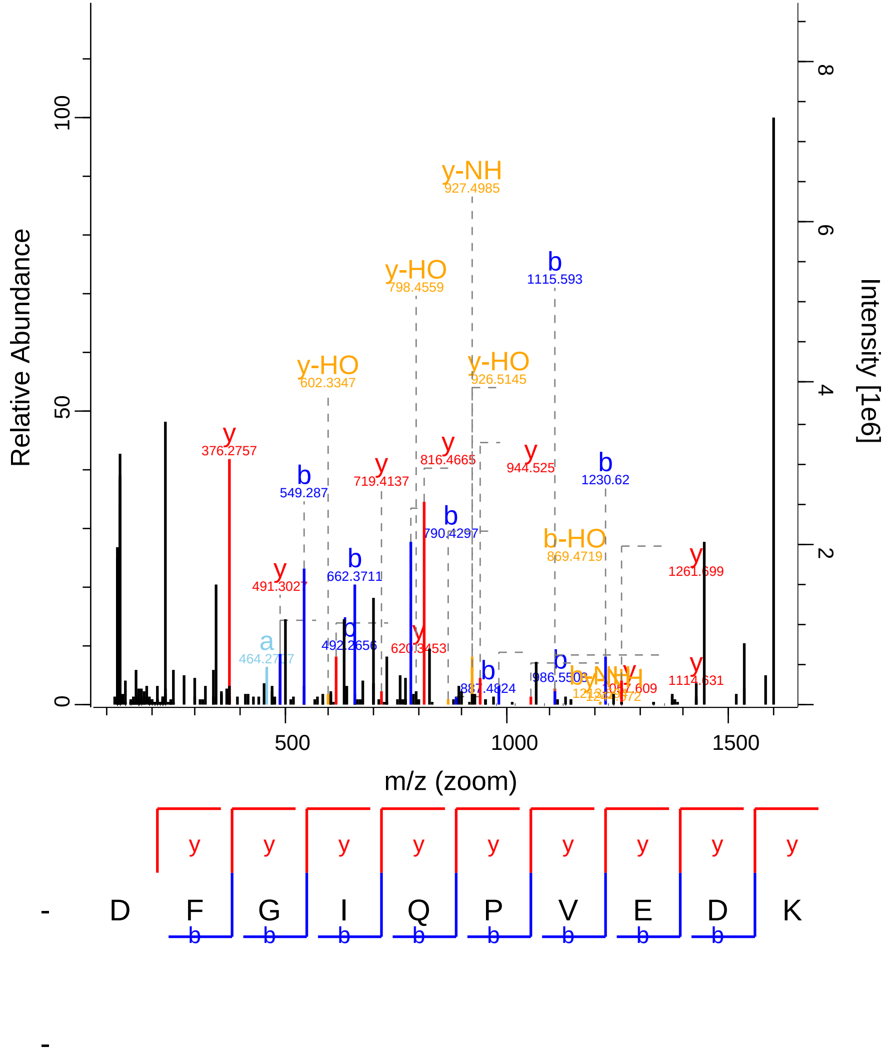

Raw File	Scan	Method	Score	m/z	Gene names
RO-QC-160121_ZackD_SS_Set_01_Fract	24430	FTMS; HC	127.56	697.9	FAM133B;FAM133

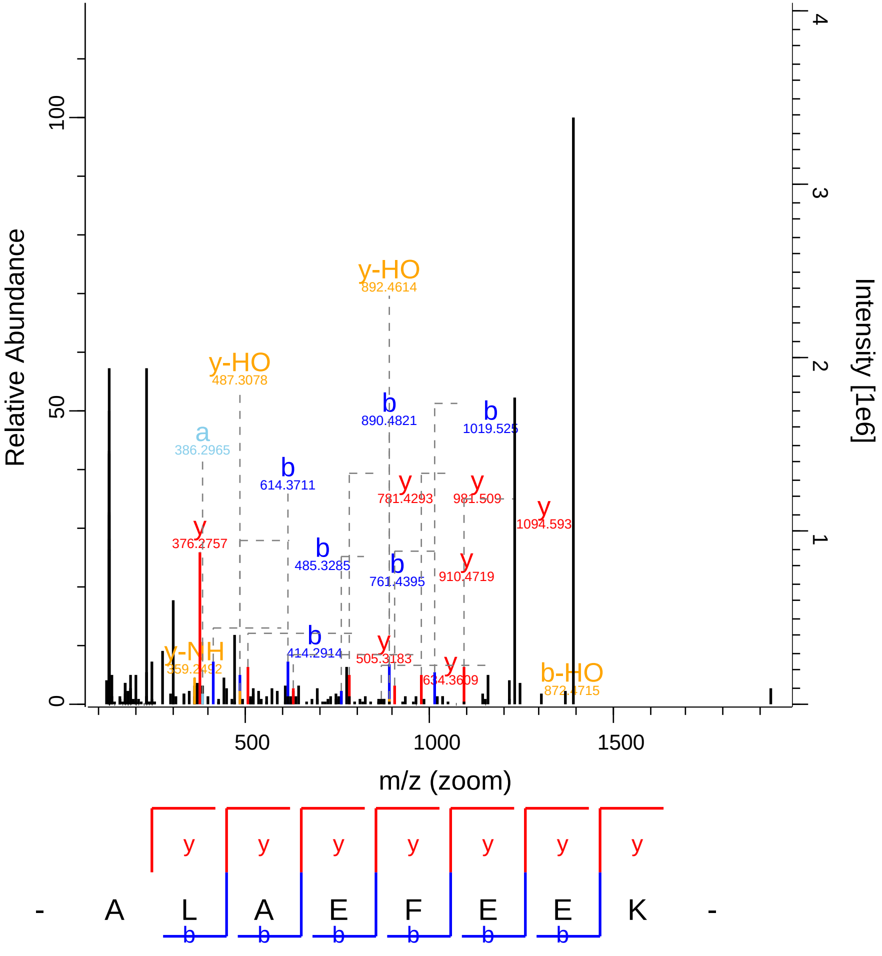

Raw File	Scan	Method	Score	m/z
RO-QC-160121_ZackD_SS_Set_01_Fraction_2	24903	FTMS; HCD	43.25	470.94

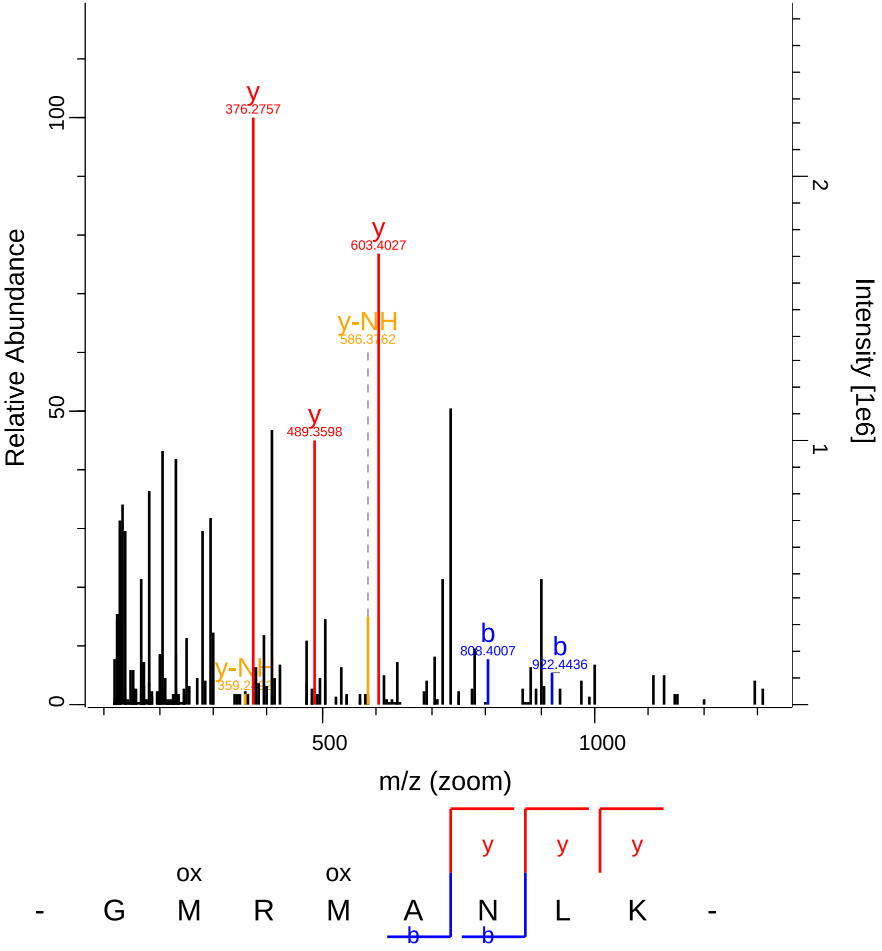

Raw File	Scan	Method	Score	m/z	Gene names
RO-QC-160121_ZackD_SS_Set_01_Fractio	26075	FTMS; HC	112.5	756.06	DCAF16

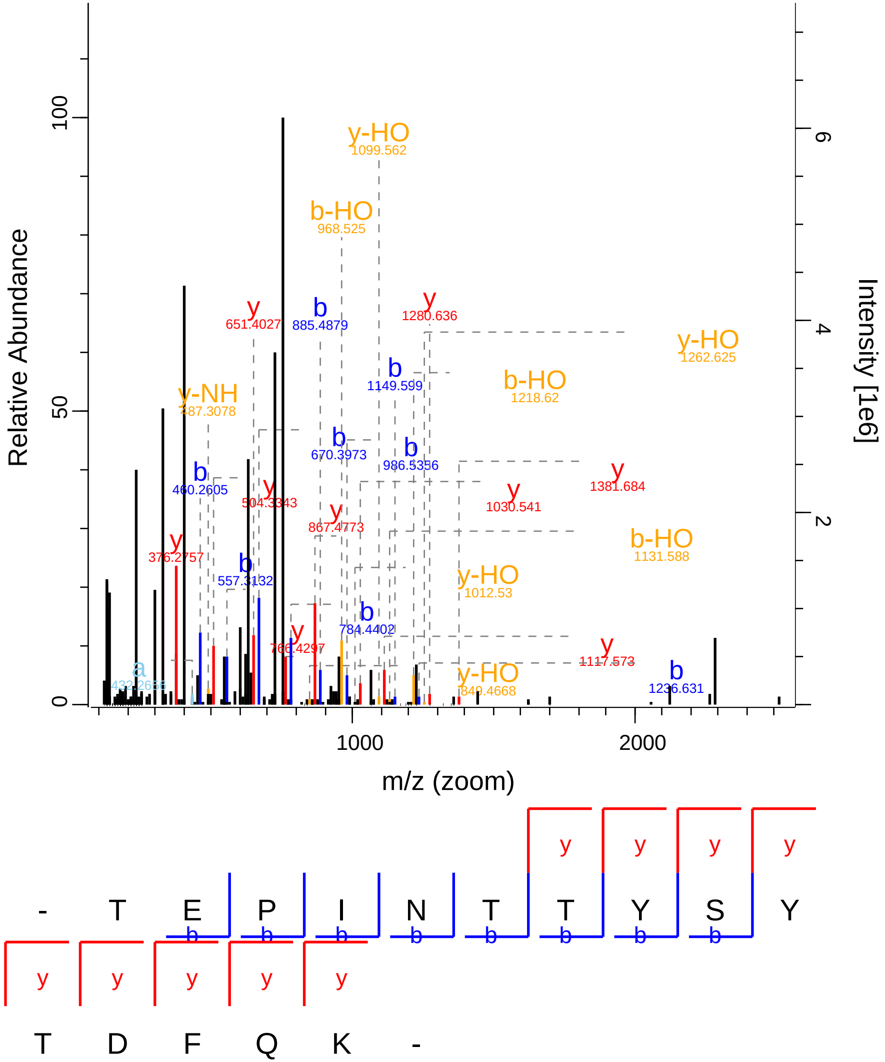

Raw File	Scan	Method	Score	m/z	Gene names
RO-QC-160121_ZackD_SS_Set_01_Fractio	26838	FTMS; HC	114.4	692.41	PMF1

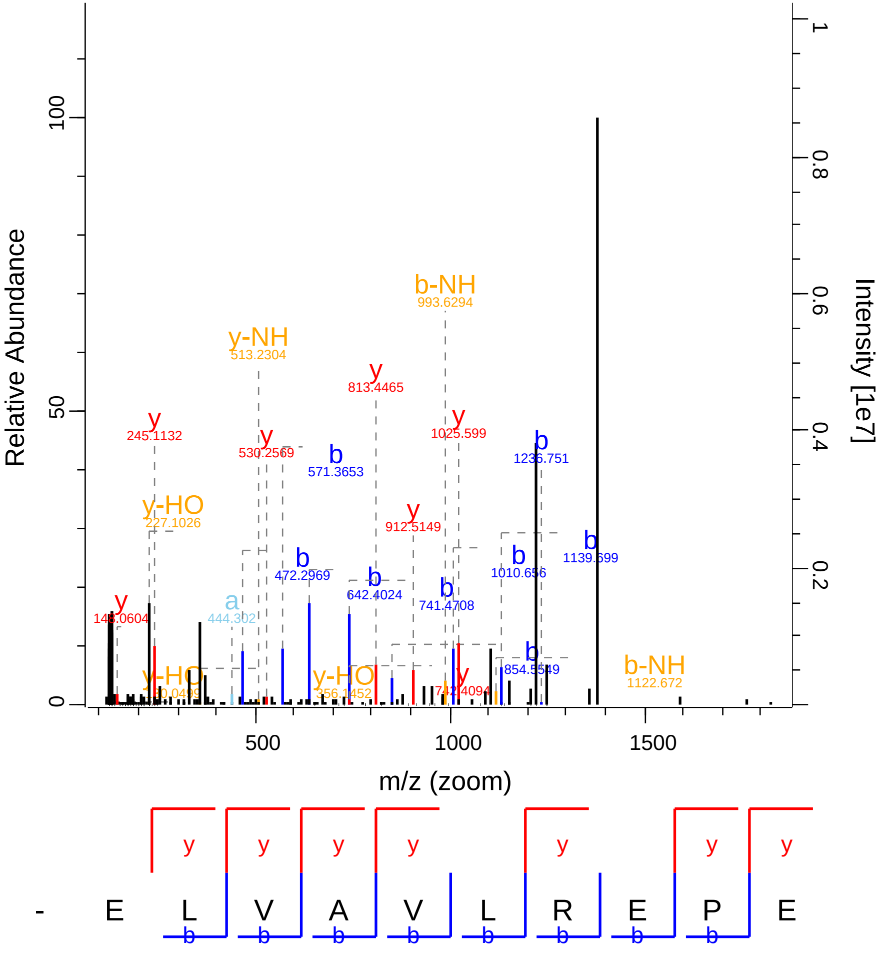

-

Raw File	Scan	Method	Score	m/z	Gene names
RO-QC-160121_ZackD_SS_Set_01_Fractio	26946	FTMS; HC	130.87	882.79	ERCC6L

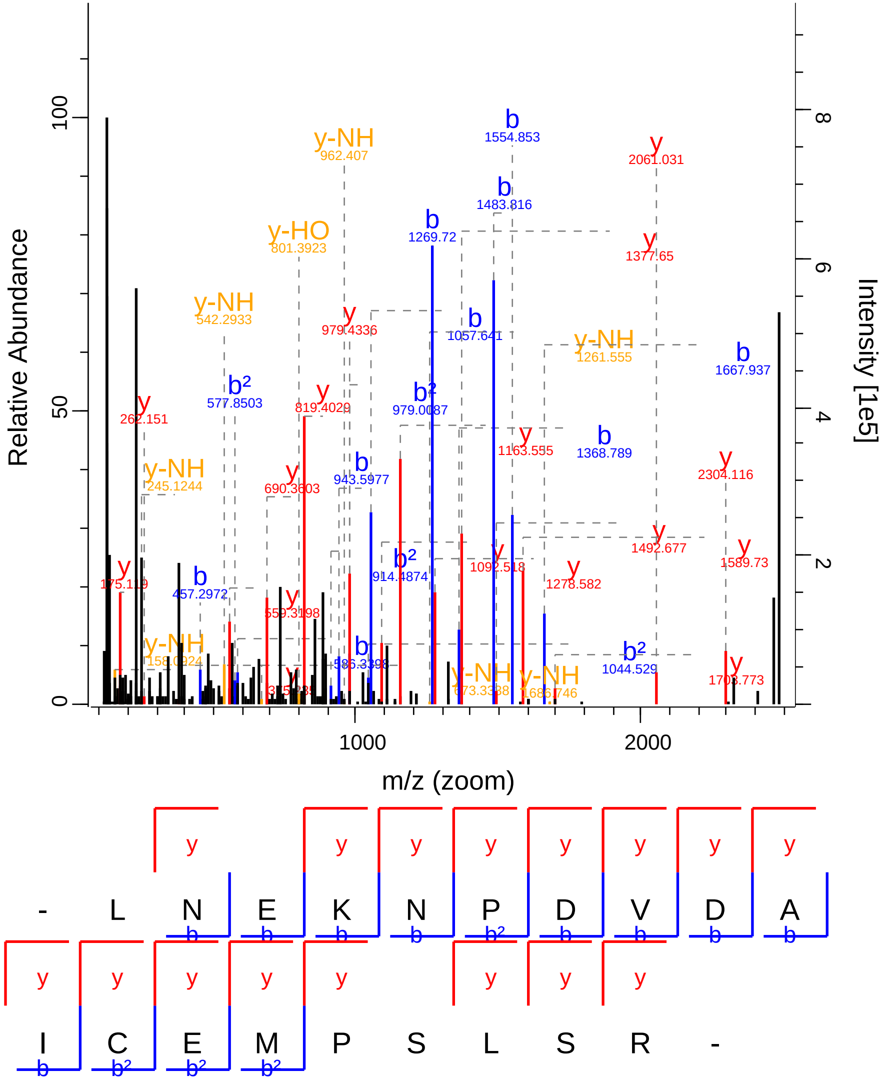

Raw File	Scan	Method	Score	m/z	Gene names
RO-QC-160121_ZackD_SS_Set_01_Fractio	27497	FTMS; HC	54.78	554.81	ADAMDEC1

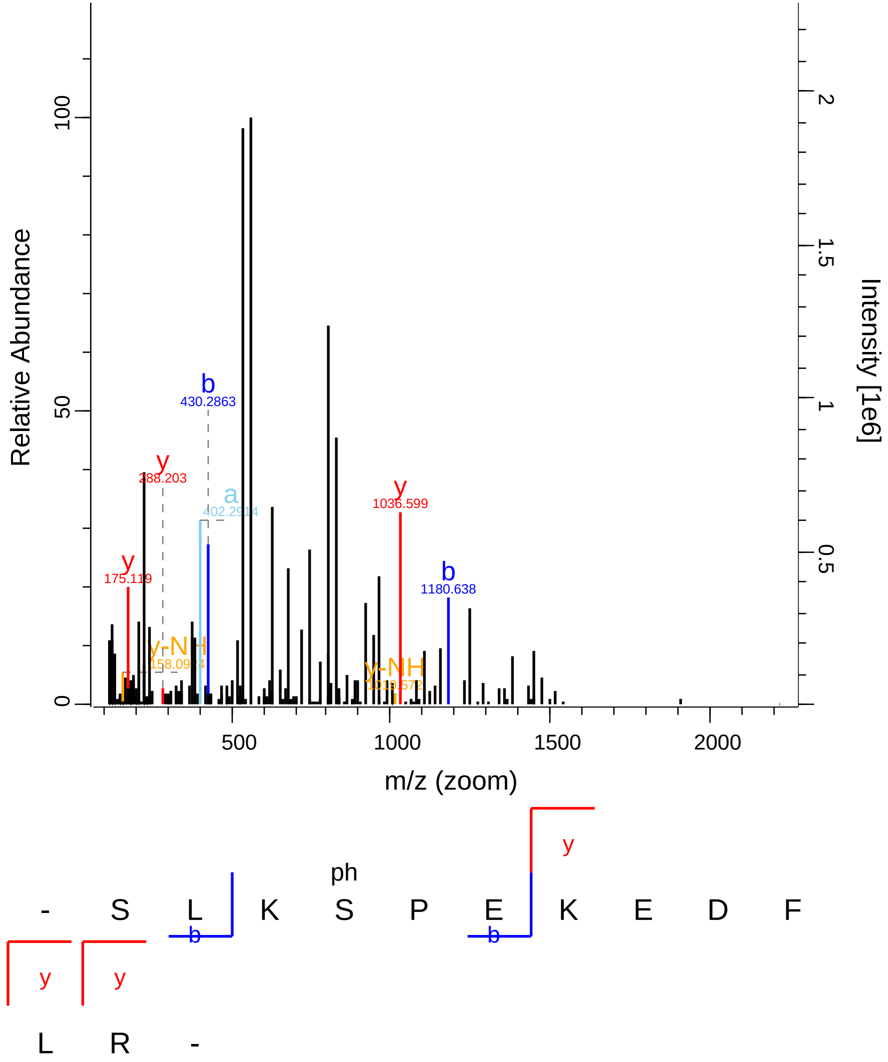

Raw File	Scan	Method	Score	m/z	Gene names
RO-QC-160121_ZackD_SS_Set_01_Fracti	27934	FTMS; HC	293.35	1249.68	EVA1B

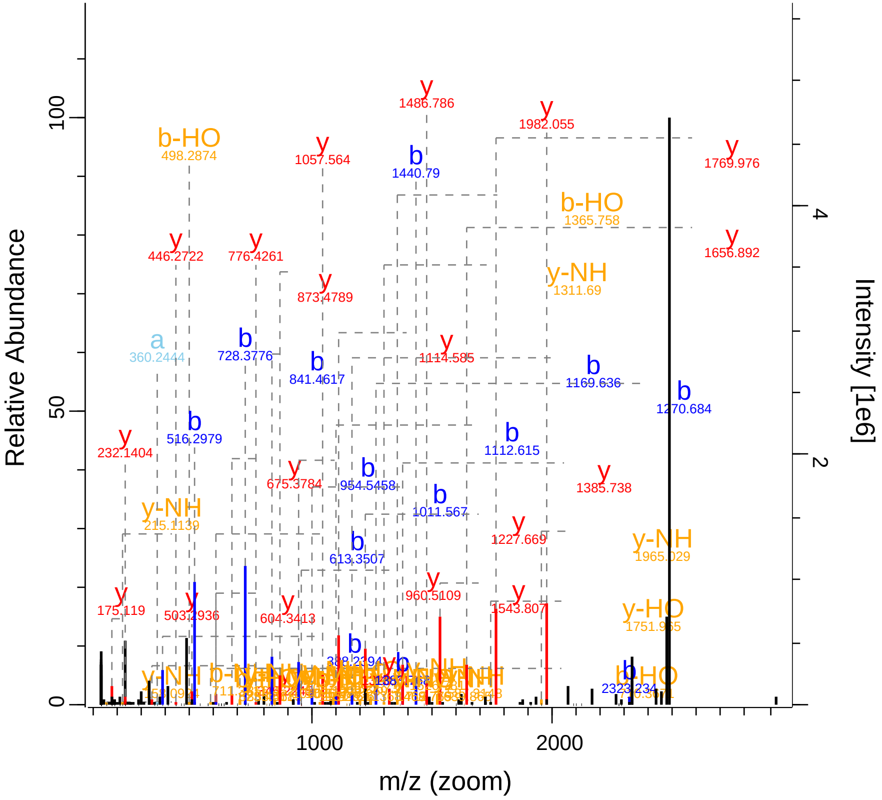

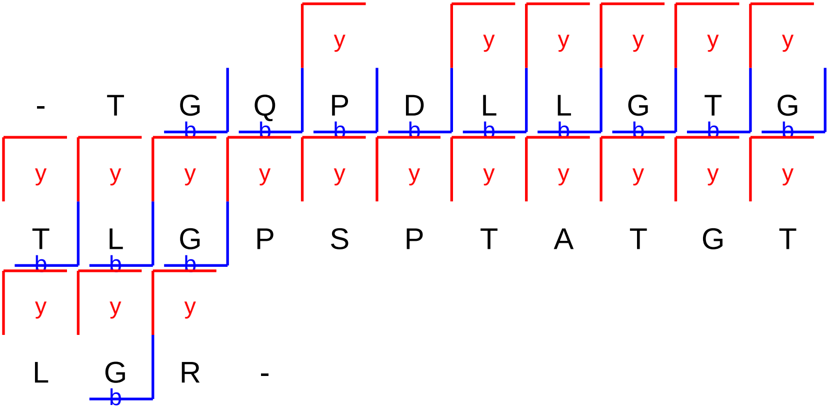

Raw File	Scan	Method	Score	m/z	Gene names
RO-QC-160121_ZackD_SS_Set_01_Fractic	28045	FTMS; HC	220.53	1037.59	NACA2

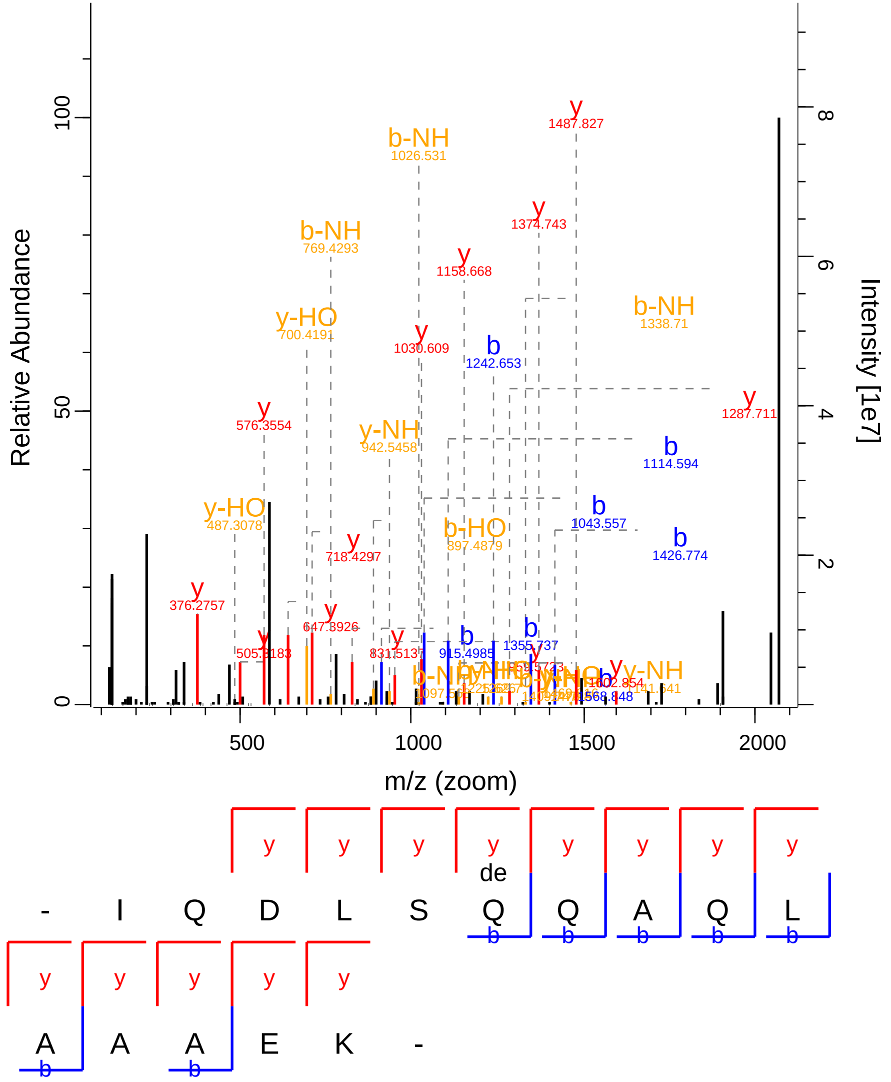

Raw File	Scan	Method	Score	m/z	Gene names
RO-QC-160121_ZackD_SS_Set_01_Fractio	28276	FTMS; HC	122.77	935.48	SOCS6

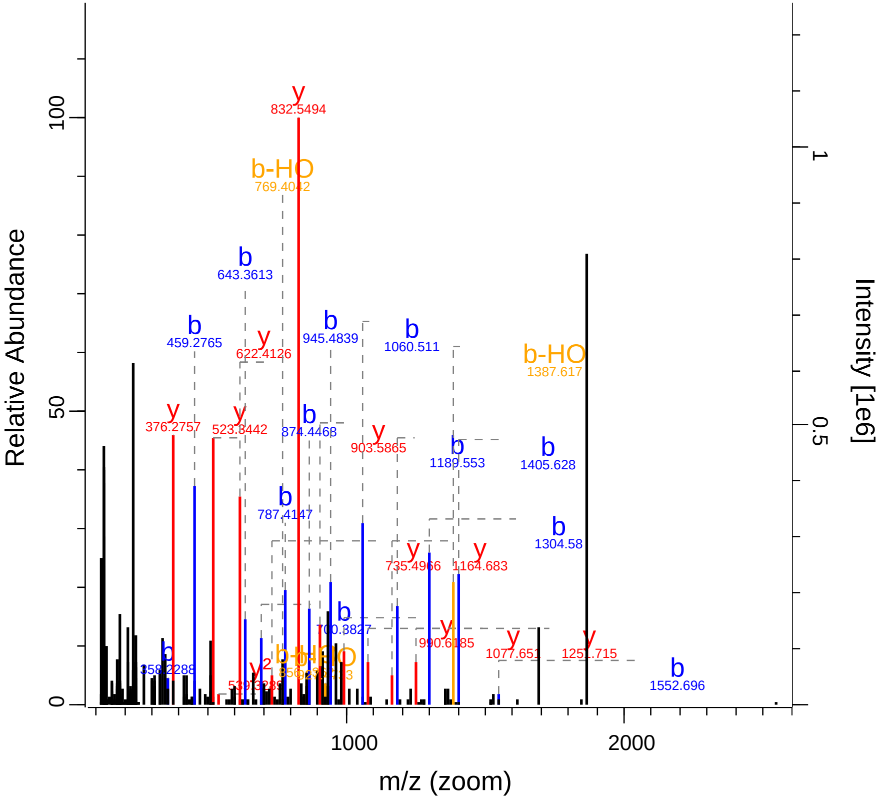

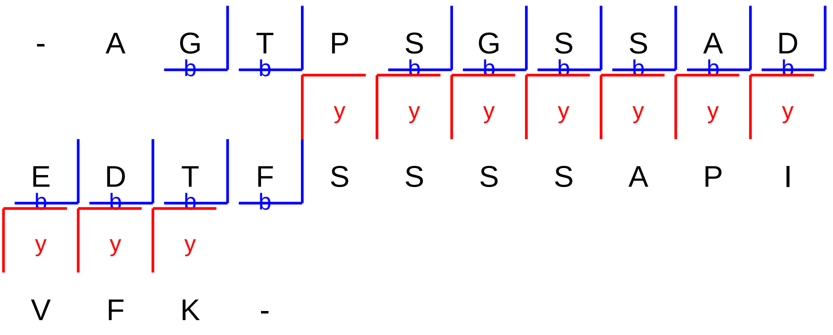

Raw File	Scan	Method	Score	m/z	Gene names
RO-QC-160121_ZackD_SS_Set_01_Fractio	28439	FTMS; HC	155.97	500.62	C1orf122

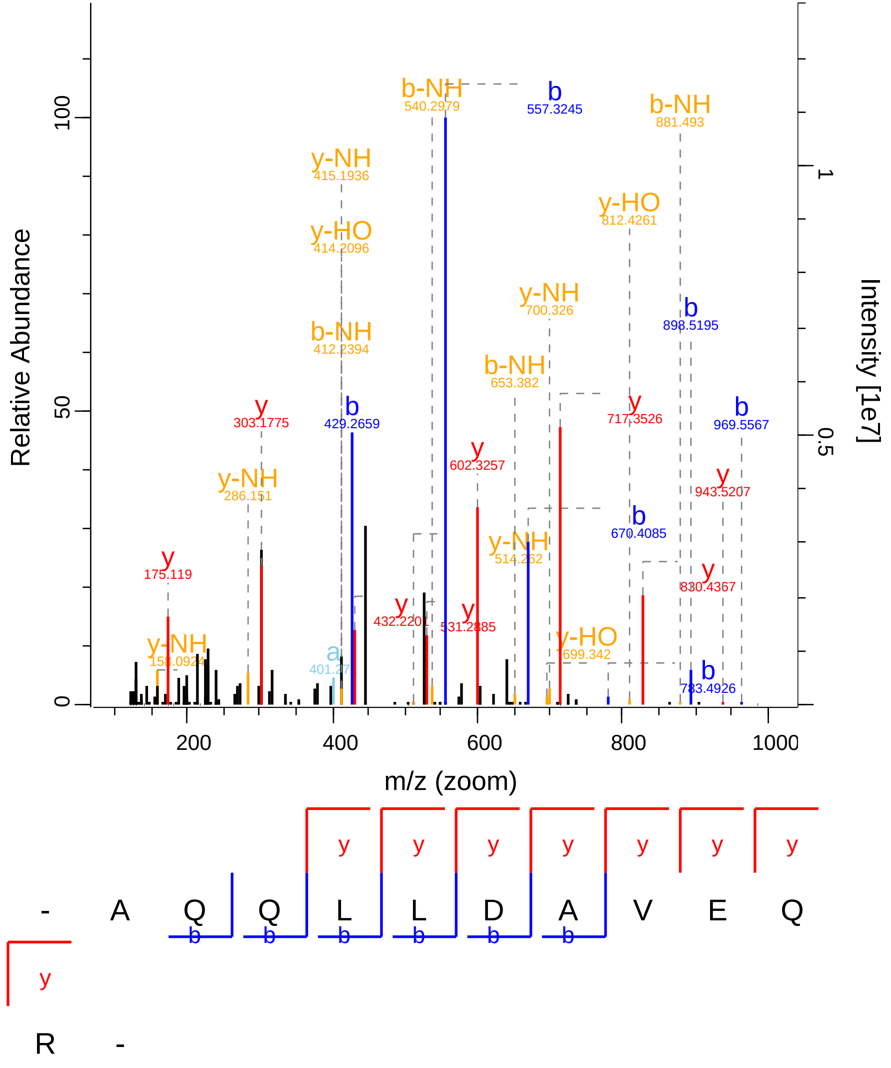

Raw File	Scan	Method	Score	m/z	Gene names
RO-QC-160121_ZackD_SS_Set_01_Fractio	28539	FTMS; HC	189.58	750.42	C1orf122

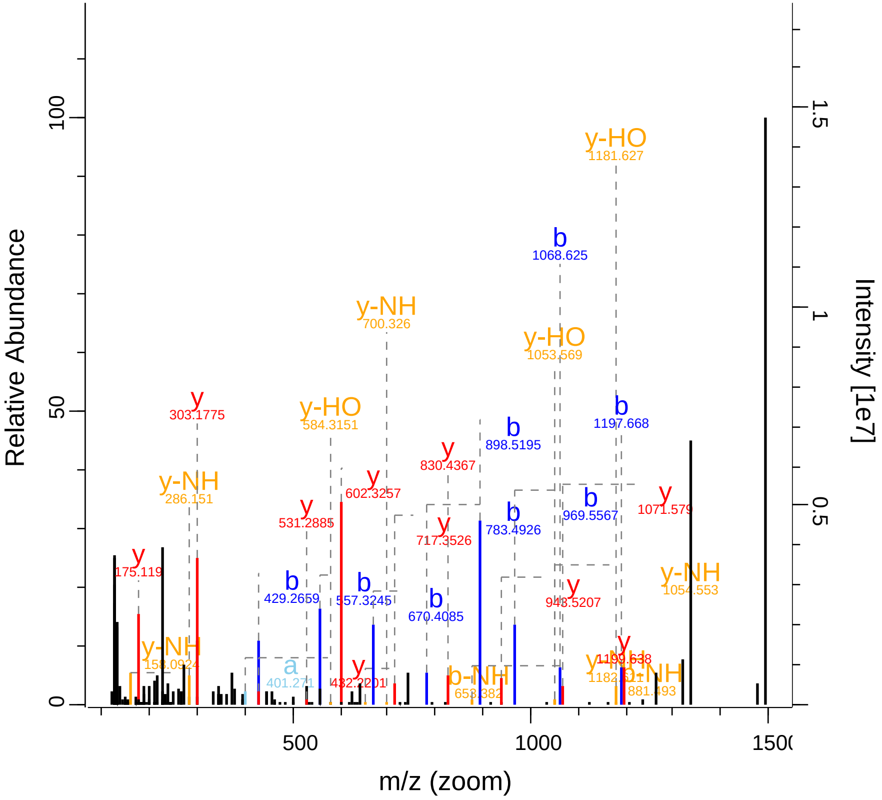

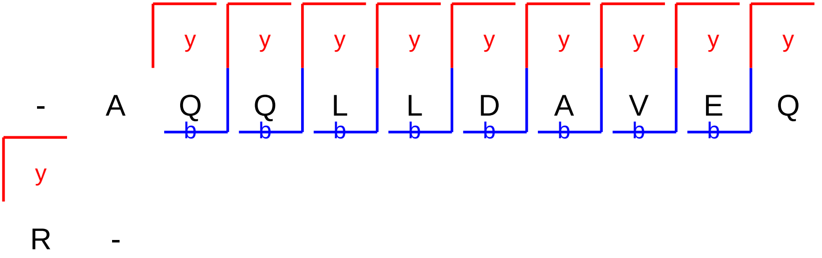

Raw File	Scan	Method	Score	m/z	Gene names
RO-QC-160121_ZackD_SS_Set_01_Fractic	28599	FTMS; HC	97.49	1027.19	PTOV1

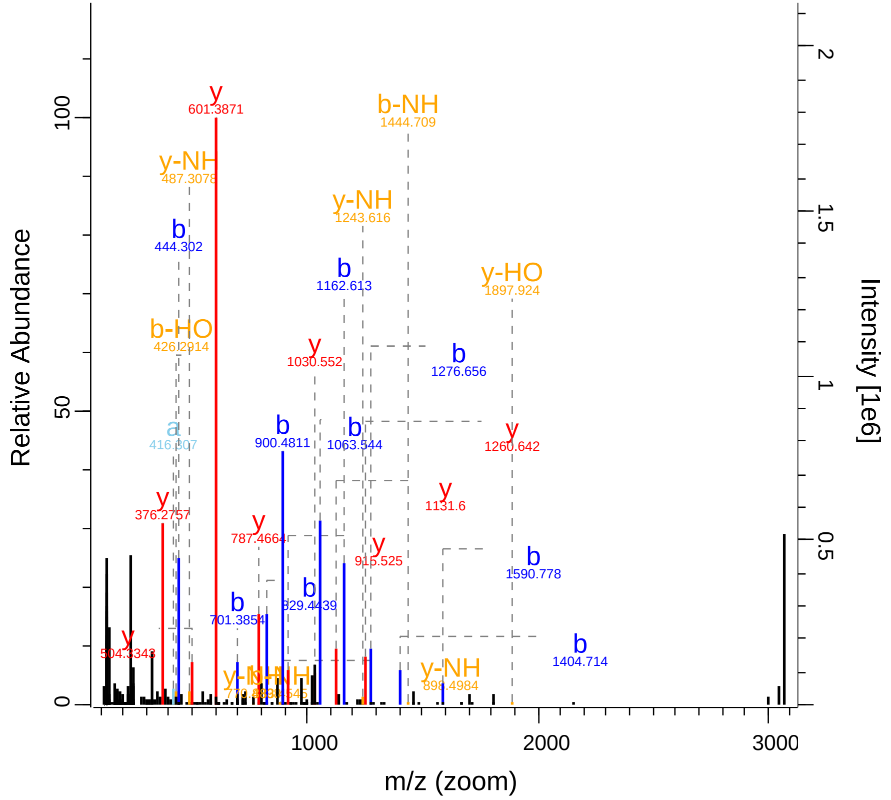

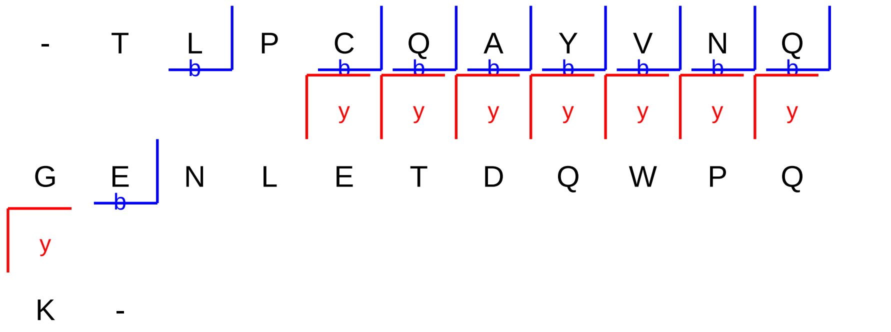

Raw File	Scan	Method	Score	m/z	Gene names
RO-QC-160121_ZackD_SS_Set_01_Fractio	28845	FTMS; HC	100.22	688.69	PVRL3

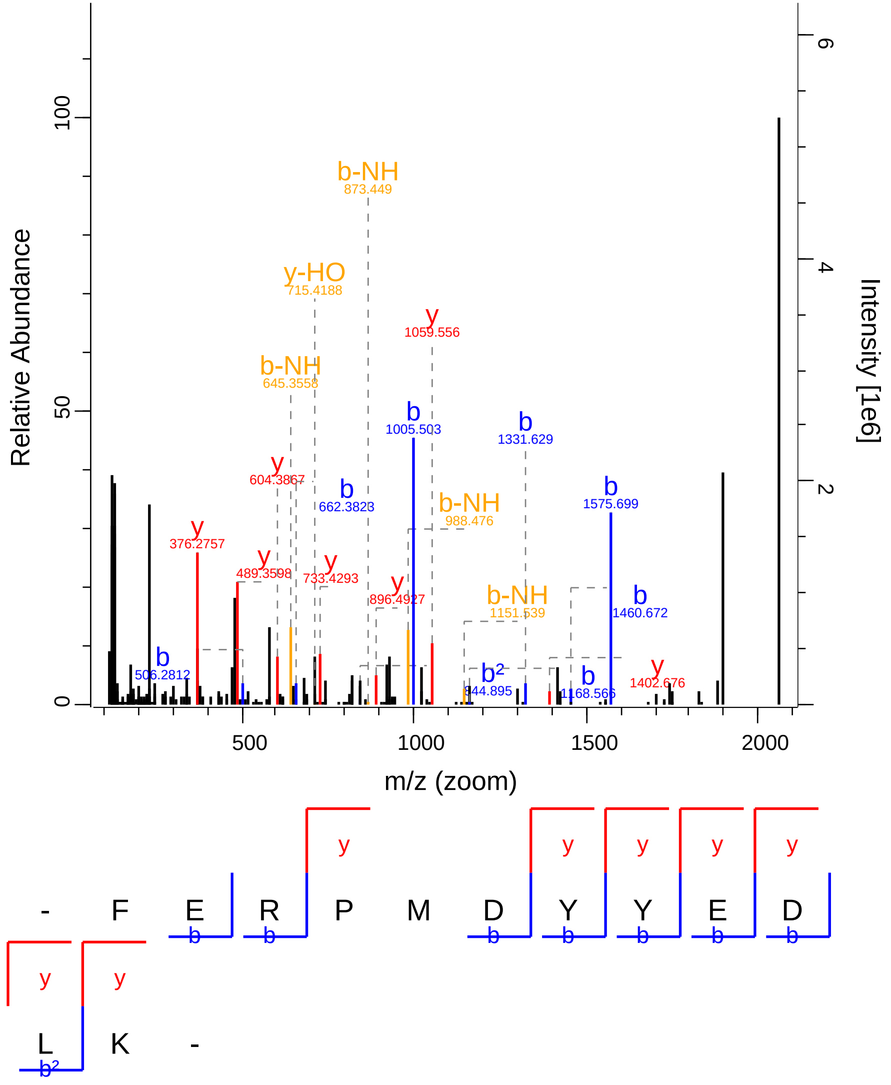

Raw File	Scan	Method	Score	m/z	Gene names
RO-QC-160121_ZackD_SS_Set_01_Fractic	28926	FTMS; HC	62.47	1036.09	TMPRSS11B

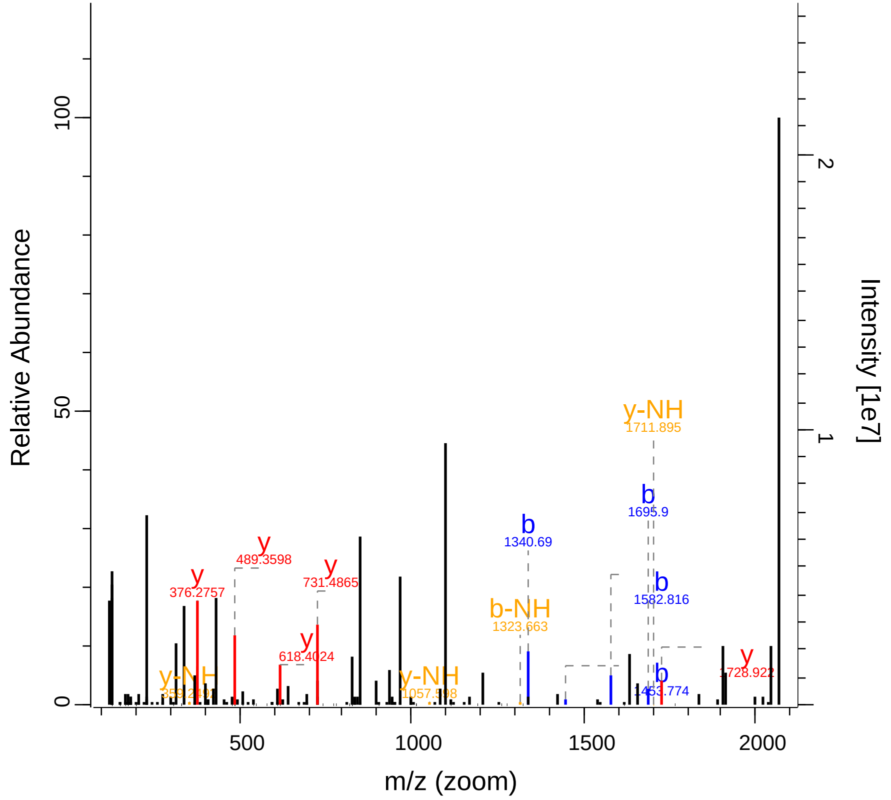

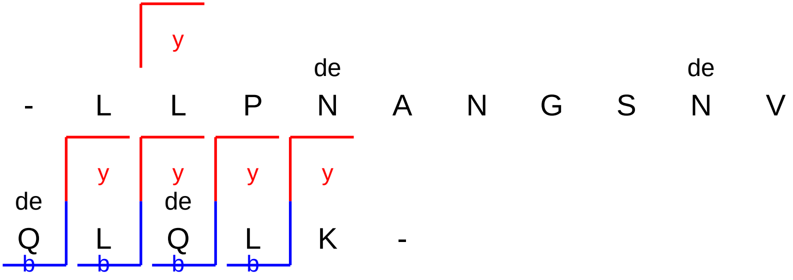

Raw File	Scan	Method	Score	m/z	Gene names
RO-QC-160121_ZackD_SS_Set_01_Fractio	28947	FTMS; HC	75.9	933.81	VAMP4

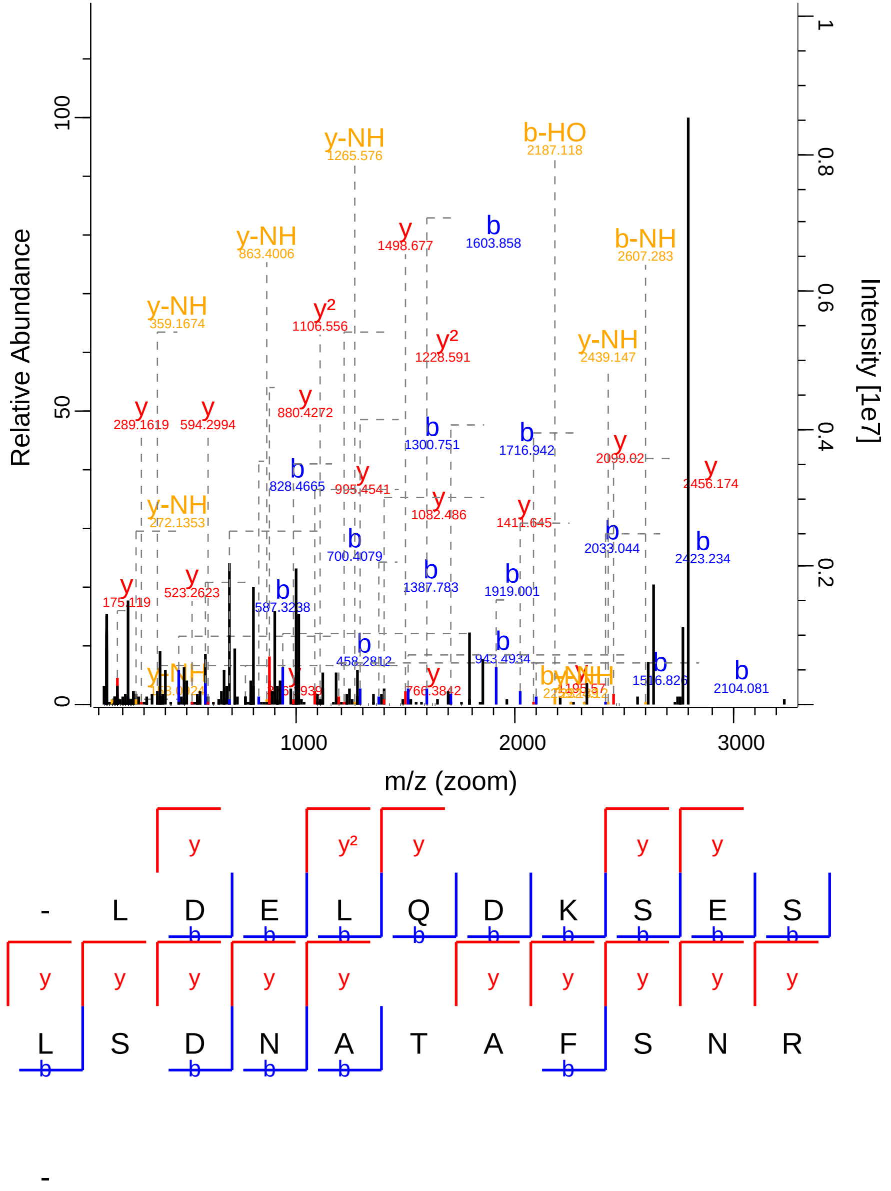

Raw File	Scan	Method	Score	m/z	Gene names
RO-QC-160121_ZackD_SS_Set_01_Fractio	28952	FTMS; HC	139.11	849.44	SLC31A1

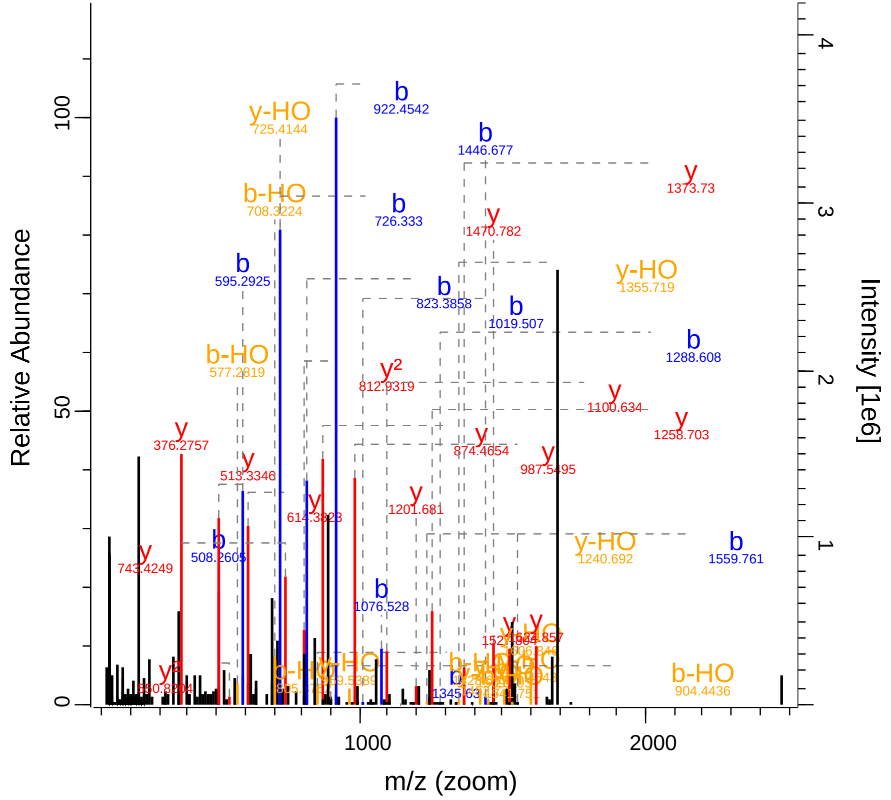

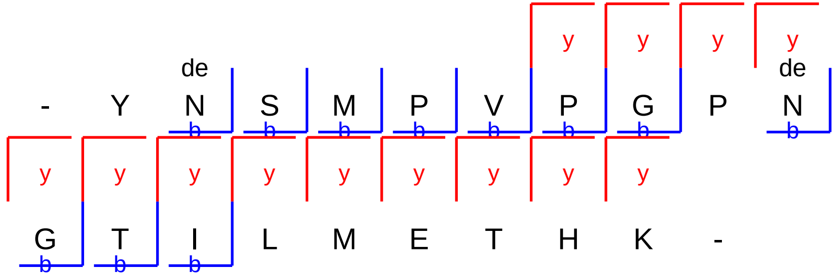

Raw File	Scan	Method	Score	m/z	Gene names
RO-QC-160121_ZackD_SS_Set_01_Fractio	29064	FTMS; HC	70.62	619.03	SYT7

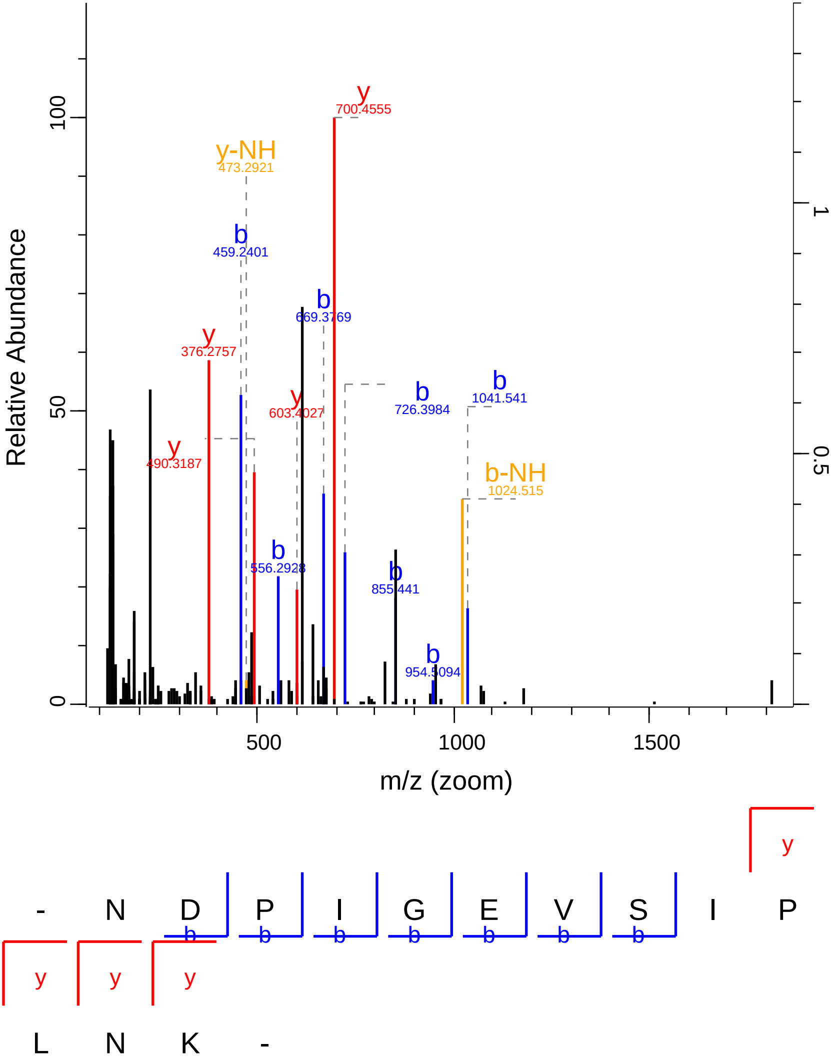

Raw File	Scan	Method	Score	m/z	Gene names
RO-QC-160121_ZackD_SS_Set_01_Fractio	29178	FTMS; HC	97.49	849.78	SLC31A1

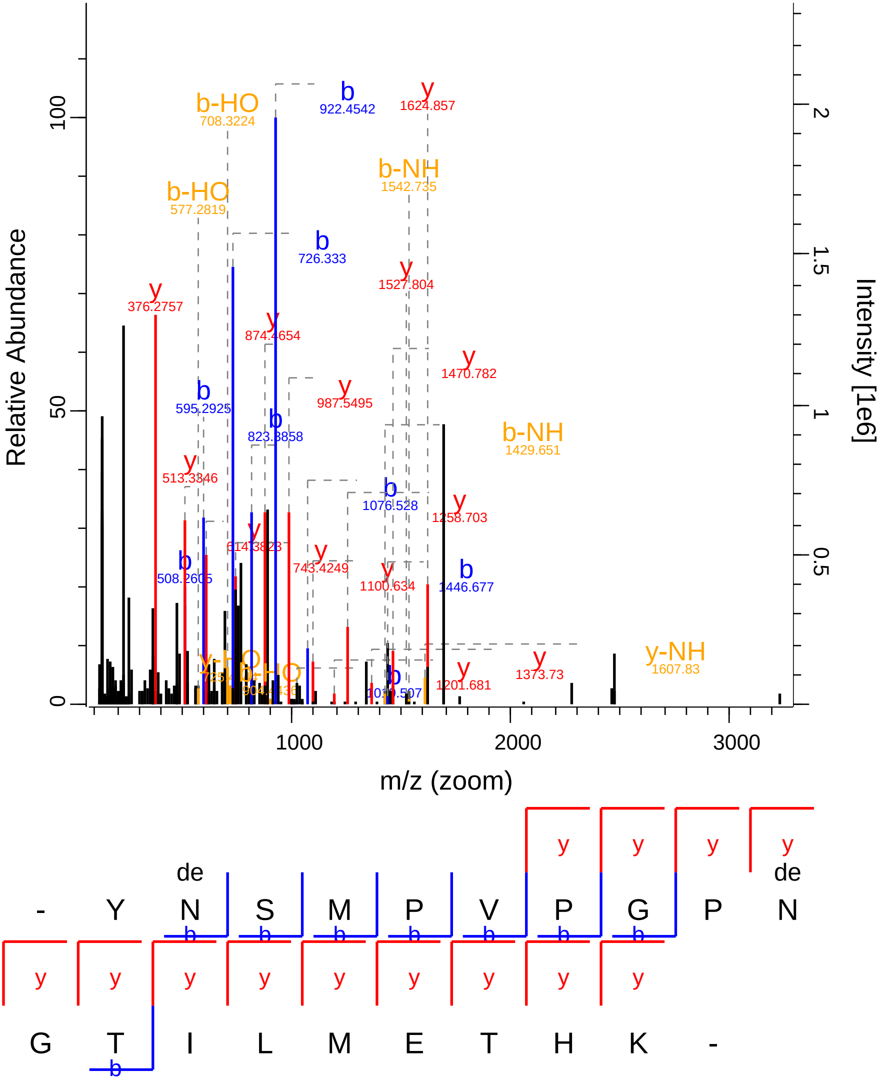

Raw File	Scan	Method	Score	m/z	Gene names
RO-QC-160121_ZackD_SS_Set_01_Fractio	29652	FTMS; HC	105.32	849.44	SLC31A1

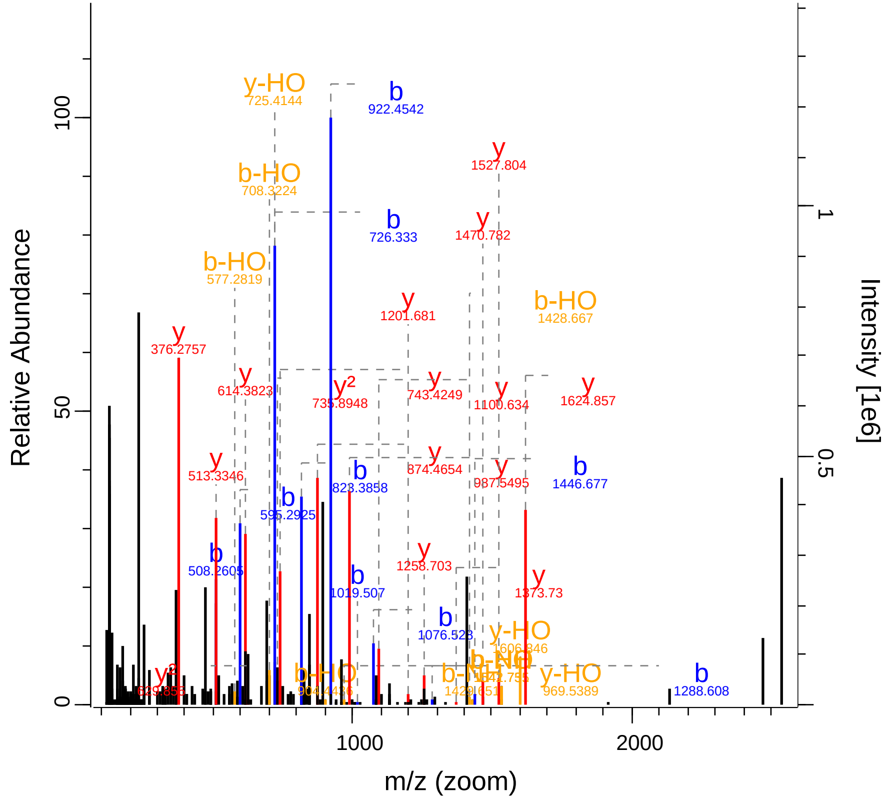

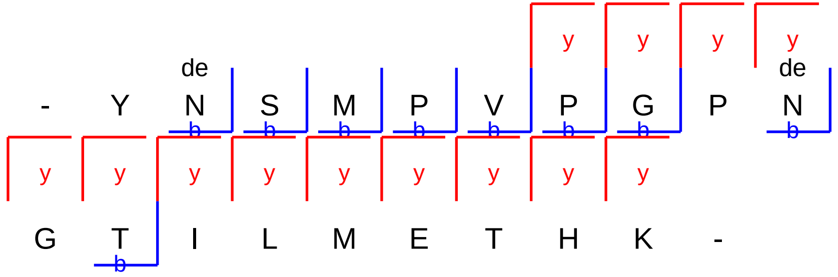

Raw File	Scan	Method	Score	m/z	Gene names
RO-QC-160121_ZackD_SS_Set_01_Fractic	30067	FTMS; HC	73.63	1001.53	DUSP6

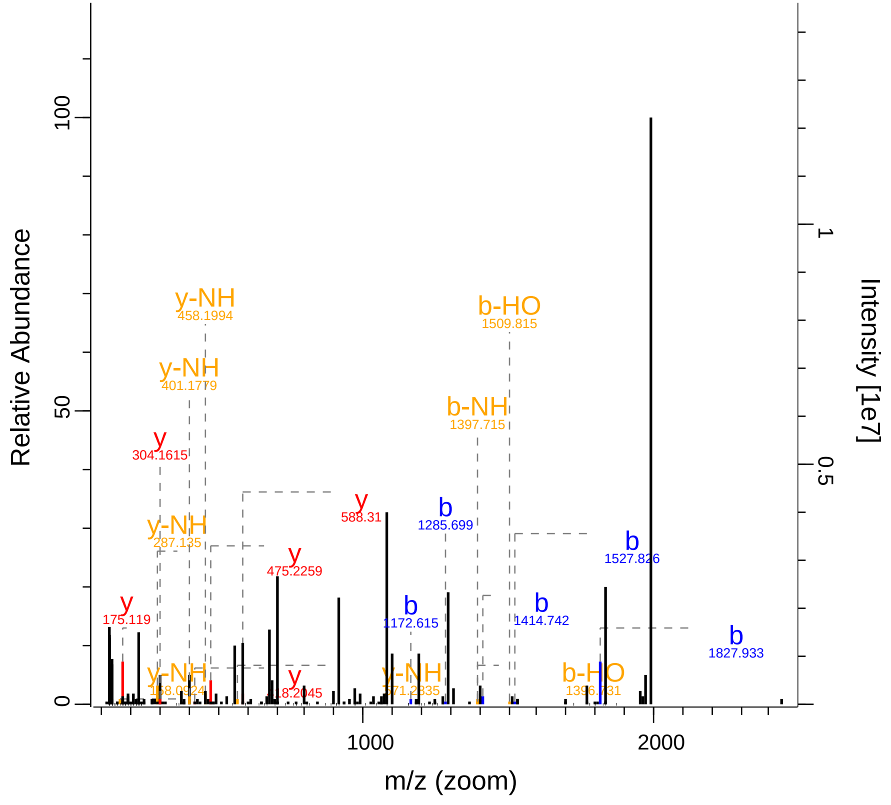

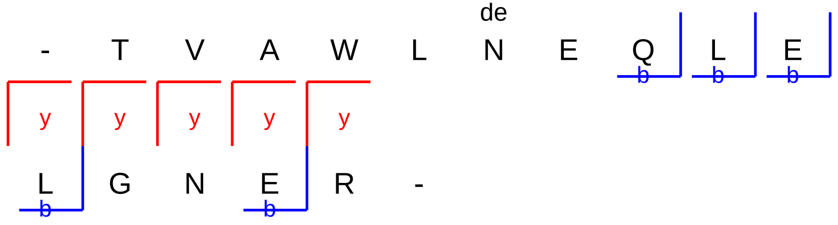

Raw File	Scan	Method	Score	m/z	Gene names
RO-QC-160121_ZackD_SS_Set_01_Fractio	30413	FTMS; HC	203.11	934.52	C14orf119

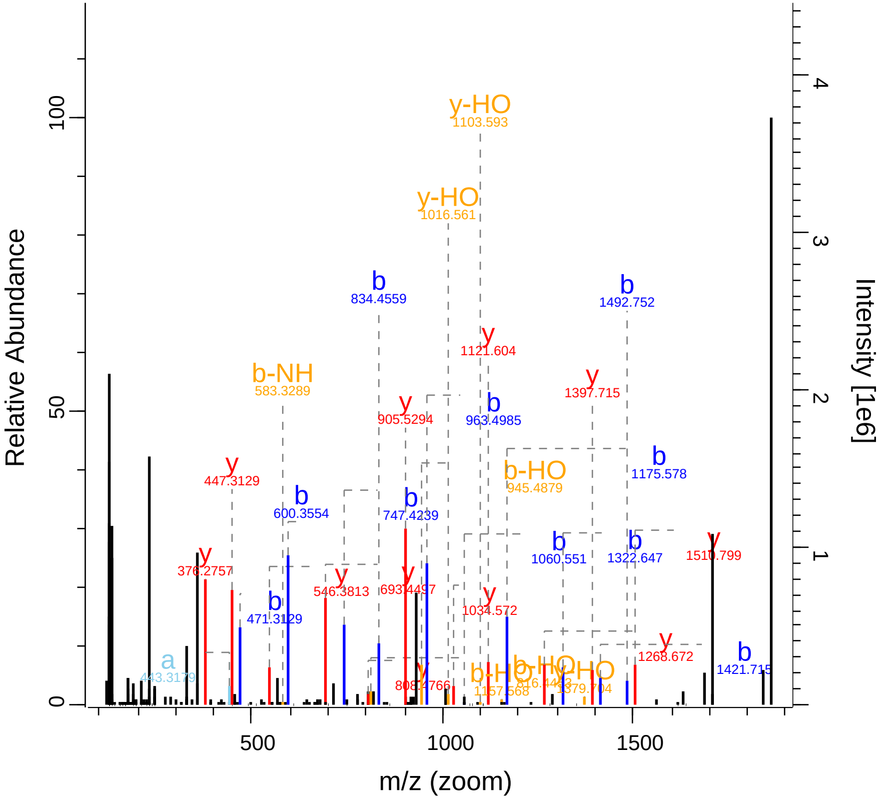

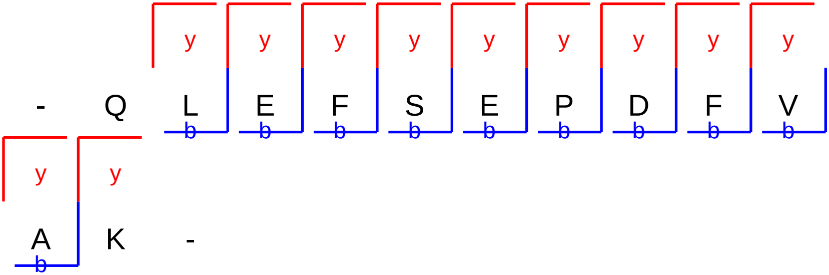

Raw File	Scan	Method	Score	m/z	Gene names
RO-QC-160121_ZackD_SS_Set_01_Fractio	30425	FTMS; HC	79.28	623.68	C14orf119

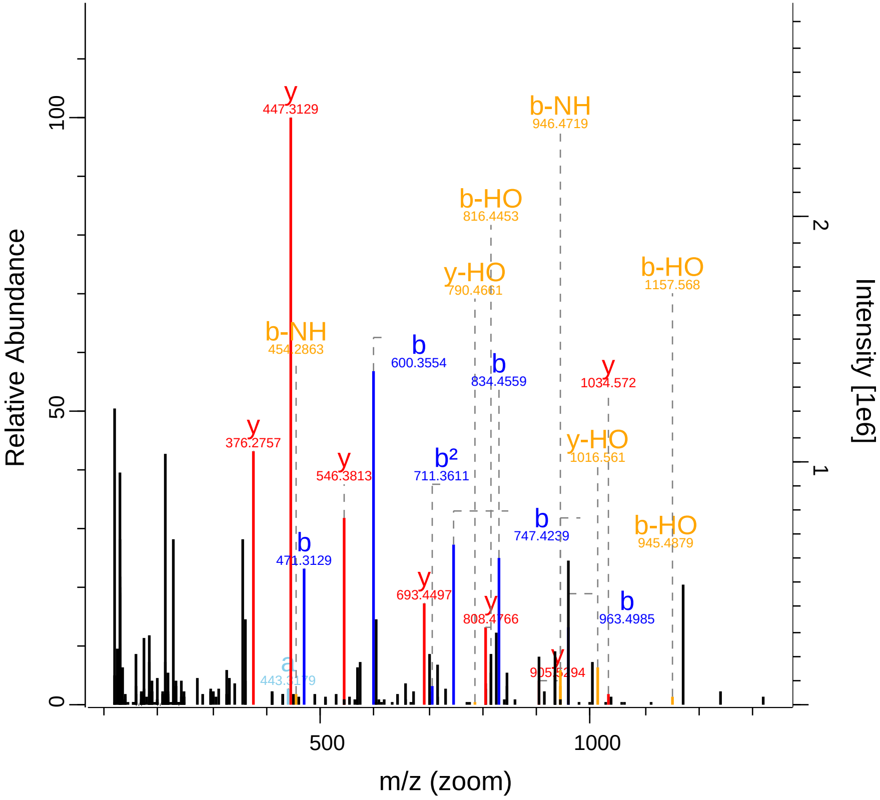

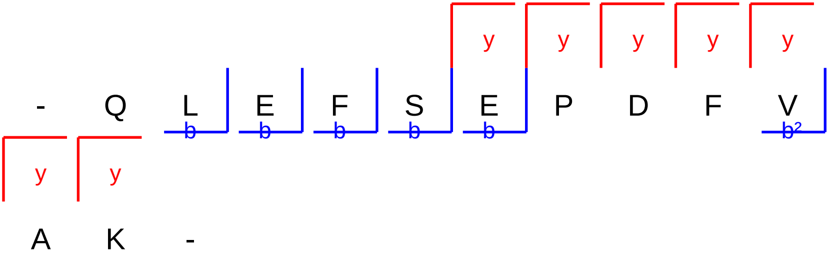

Raw File	Scan	Method	Score	m/z	Gene names
RO-QC-160121_ZackD_SS_Set_01_Fractio	30456	FTMS; HC	200.68	812.91	FOXRED2

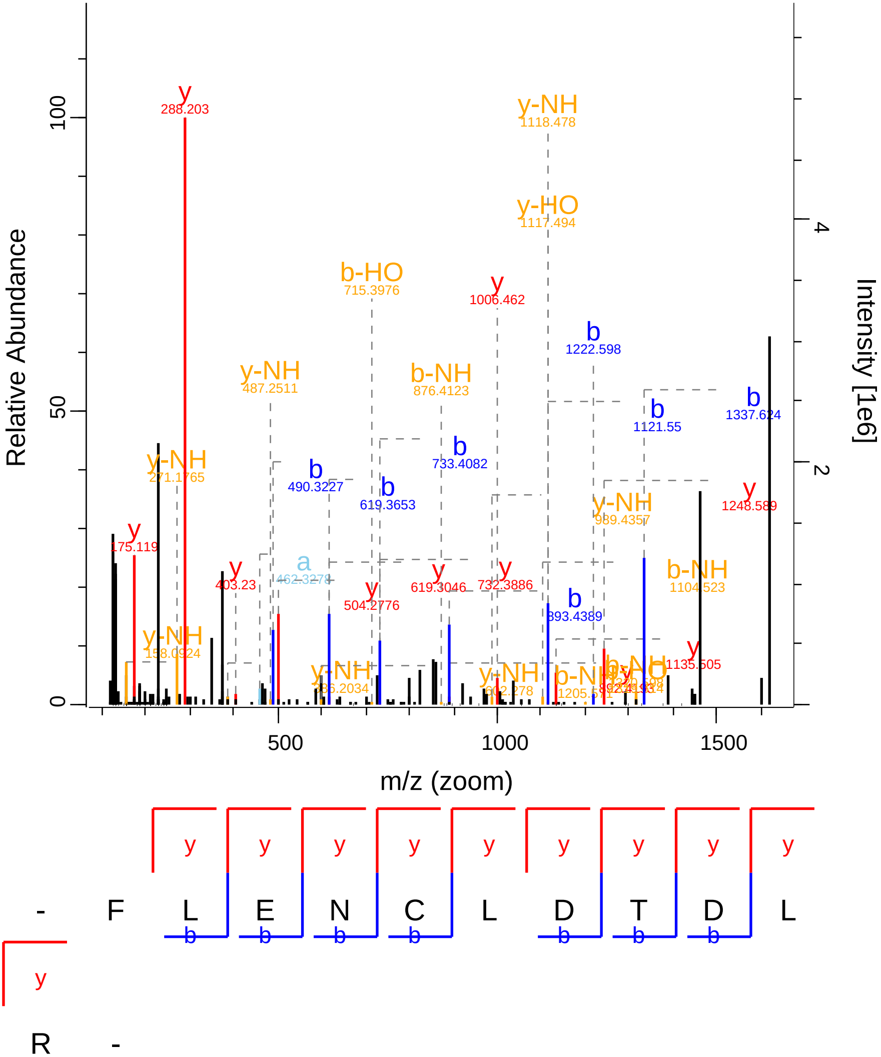

Raw File	Scan	Method	Score	m/z	Gene names
RO-QC-160121_ZackD_SS_Set_01_Fractio	35240	FTMS; HC	42.34	548.65	OR10G3

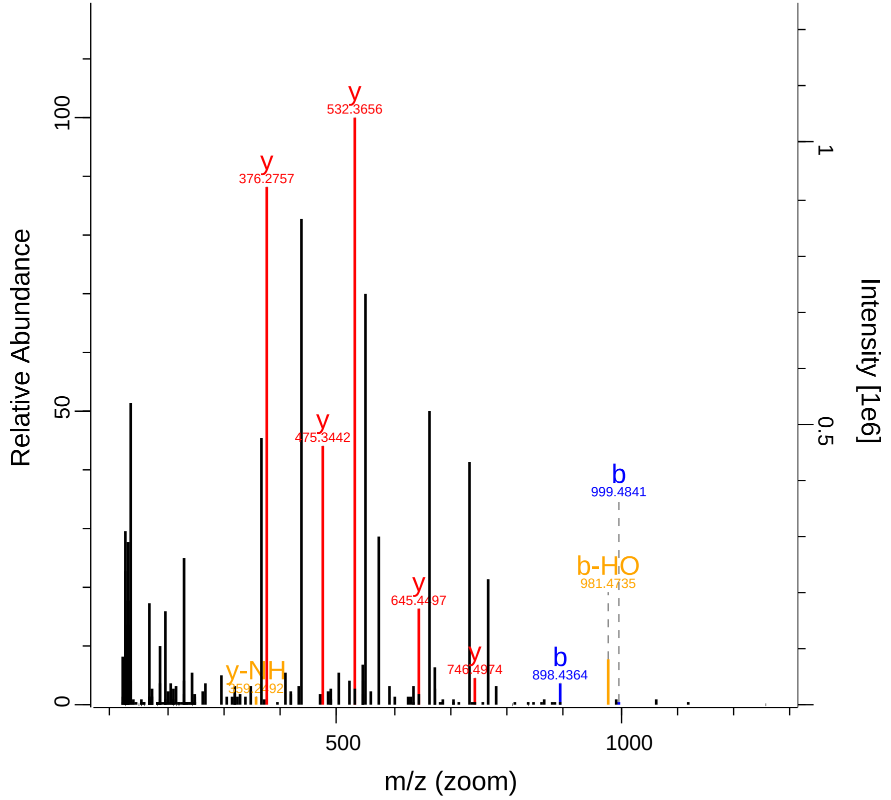

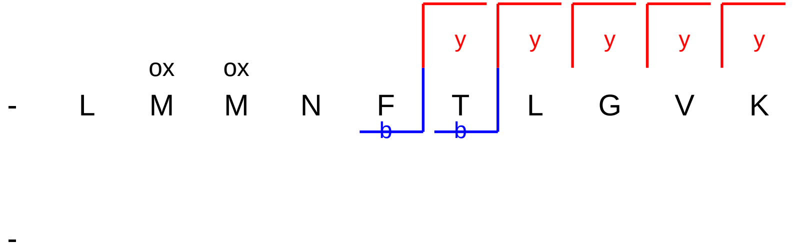

Raw File	Scan	Method	Score	m/z	Gene names
RO-QC-160121_ZackD_SS_Set_01_Fractio	35303	FTMS; HC	47.55	779.08	VAV1

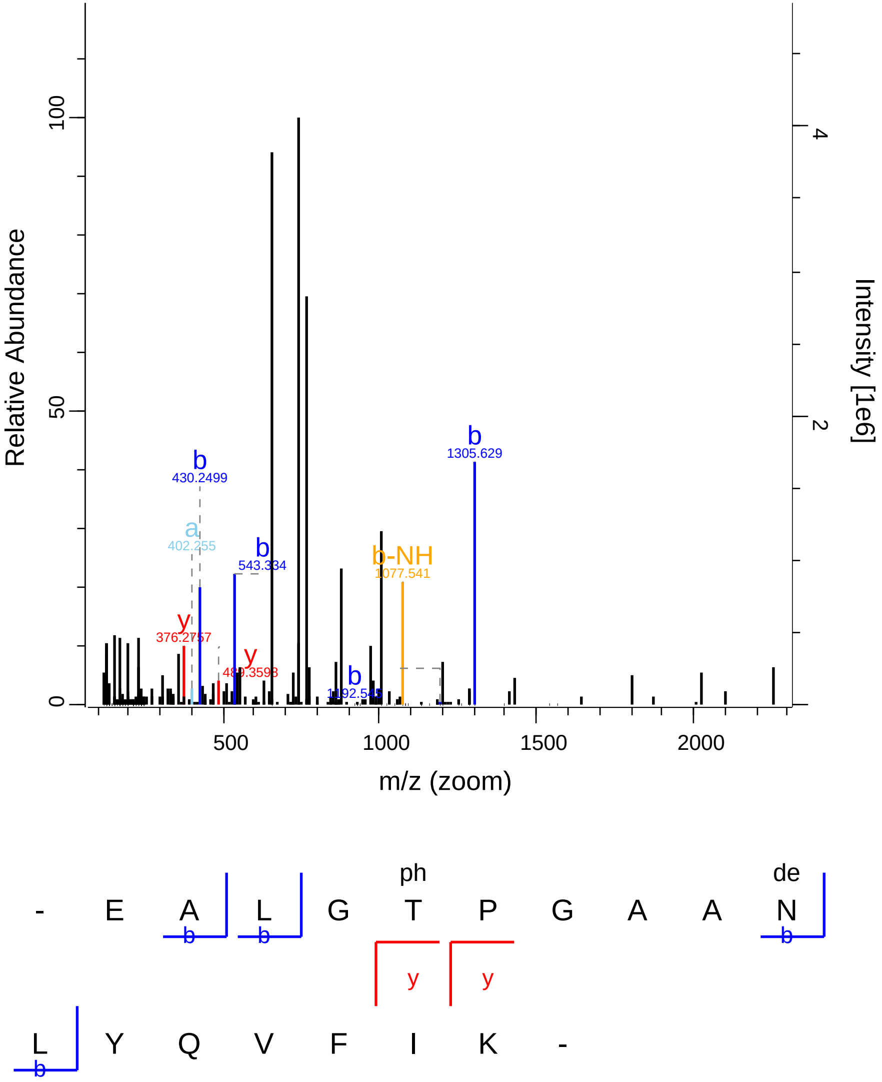

Raw File	Scan	Method	Score	m/z	Gene names
RO-QC-160121_ZackD_SS_Set_01_Fractio	35313	FTMS; HC	40.04	822.46	OR10G3

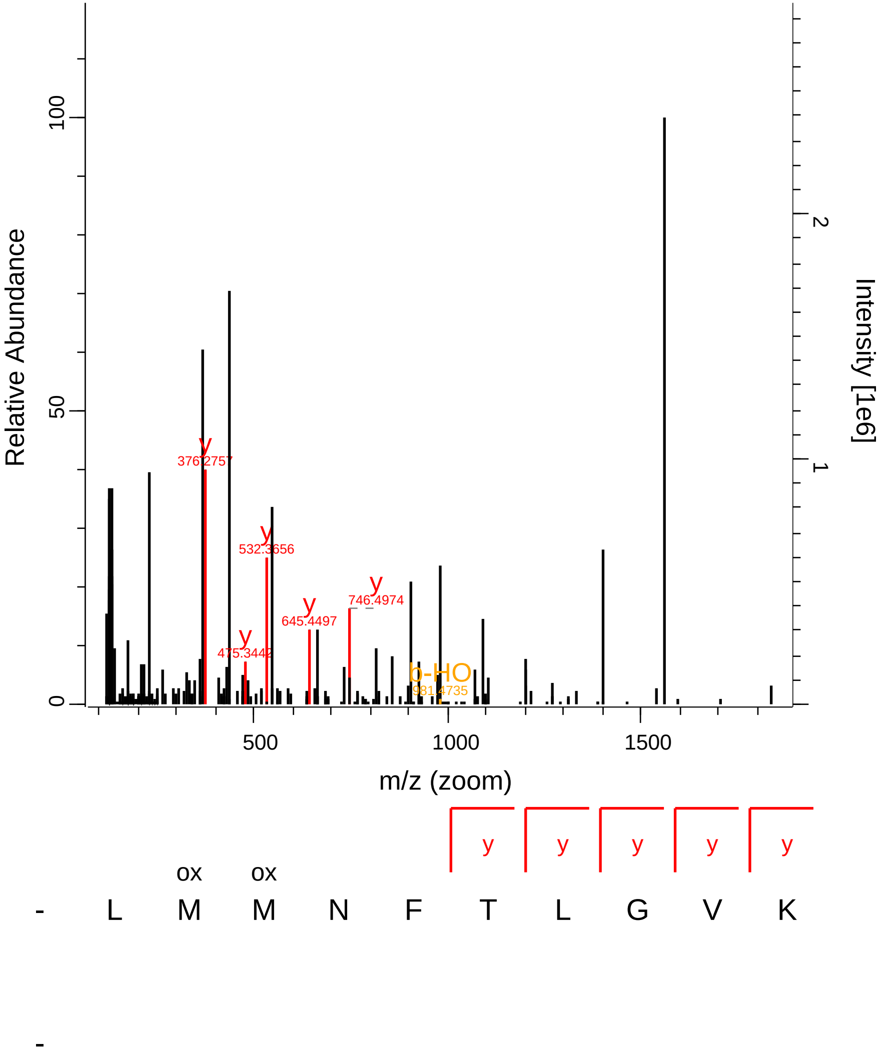

Raw File	Scan	Method	Score	m/z	Gene names
RO-QC-160121_ZackD_SS_Set_01_Fractio	36064	FTMS; HC	44.1	994.85	DAPP1

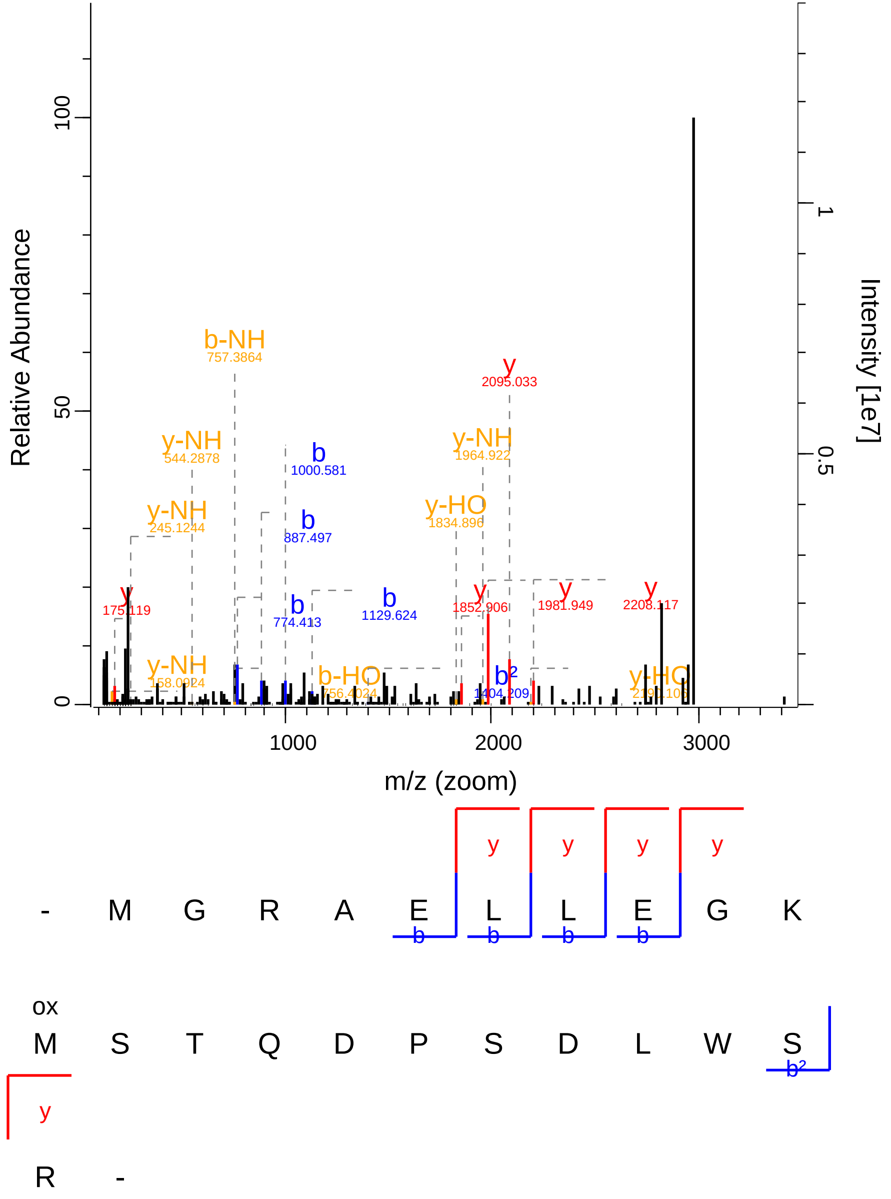

Raw File	Scan	Method	Score	m/z	Gene names
RO-QC-160121_ZackD_SS_Set_01_Fractic	36842	FTMS; HC	53.4	1323.69	SNCAIP

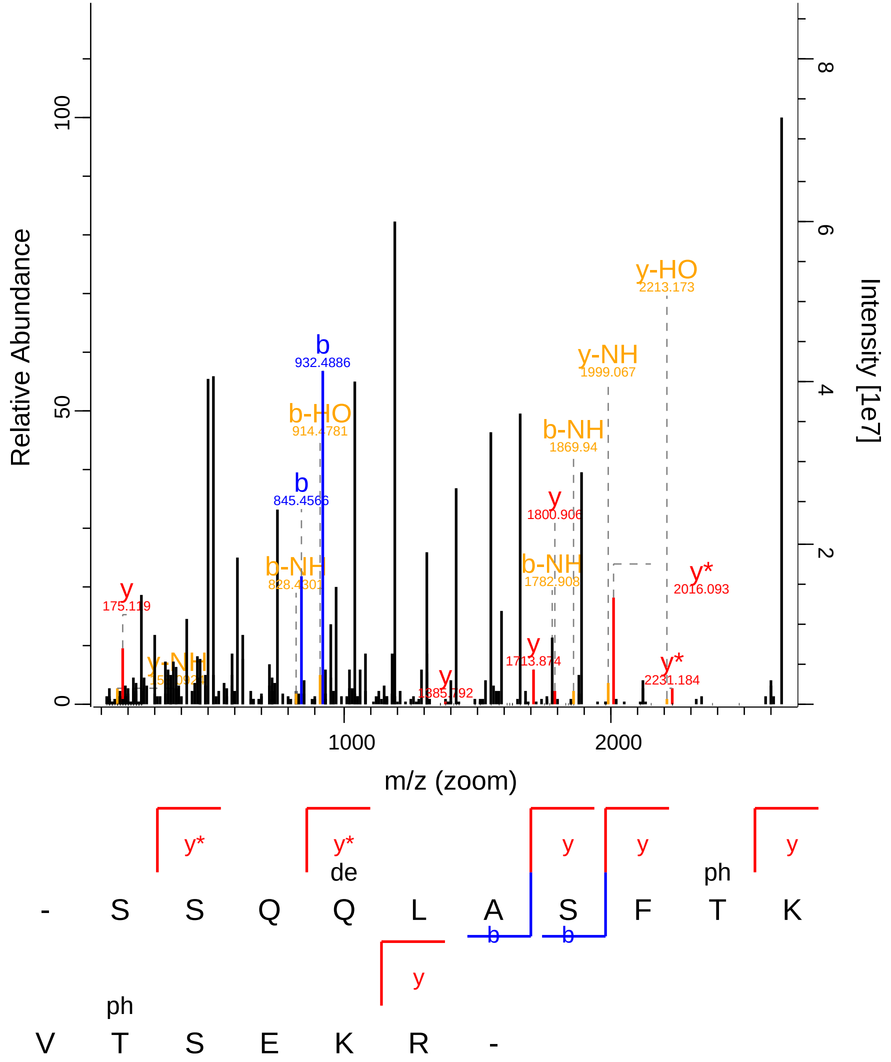

Raw File	Scan	Method	Score	m/z	Gene names
RO-QC-160121_ZackD_SS_Set_01_Fractic	37102	FTMS; HC	46.16	1097.57	HHIP

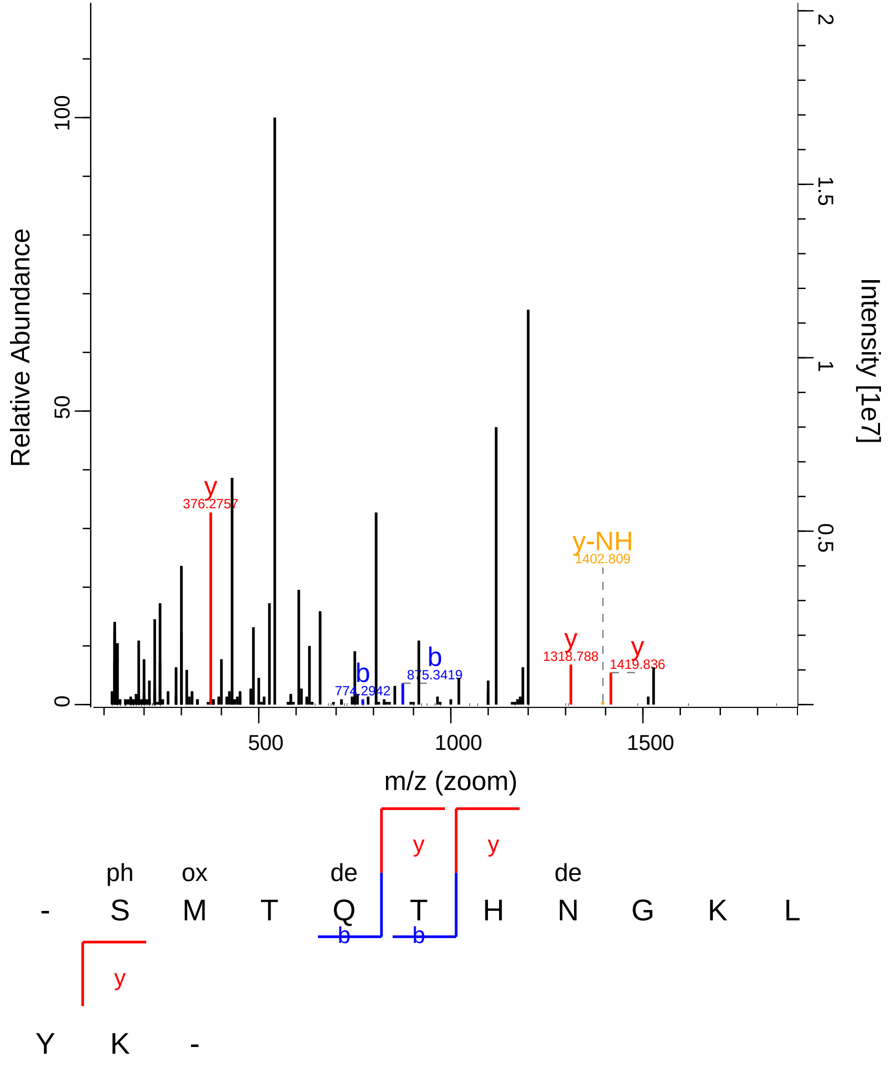

Raw File	Scan	Method	Score	m/z	Gene names
RO-QC-160121_ZackD_SS_Set_01_Fractio[illegible]	37738	FTMS; HC[illegible]	172.21	771.9	SLC48A1

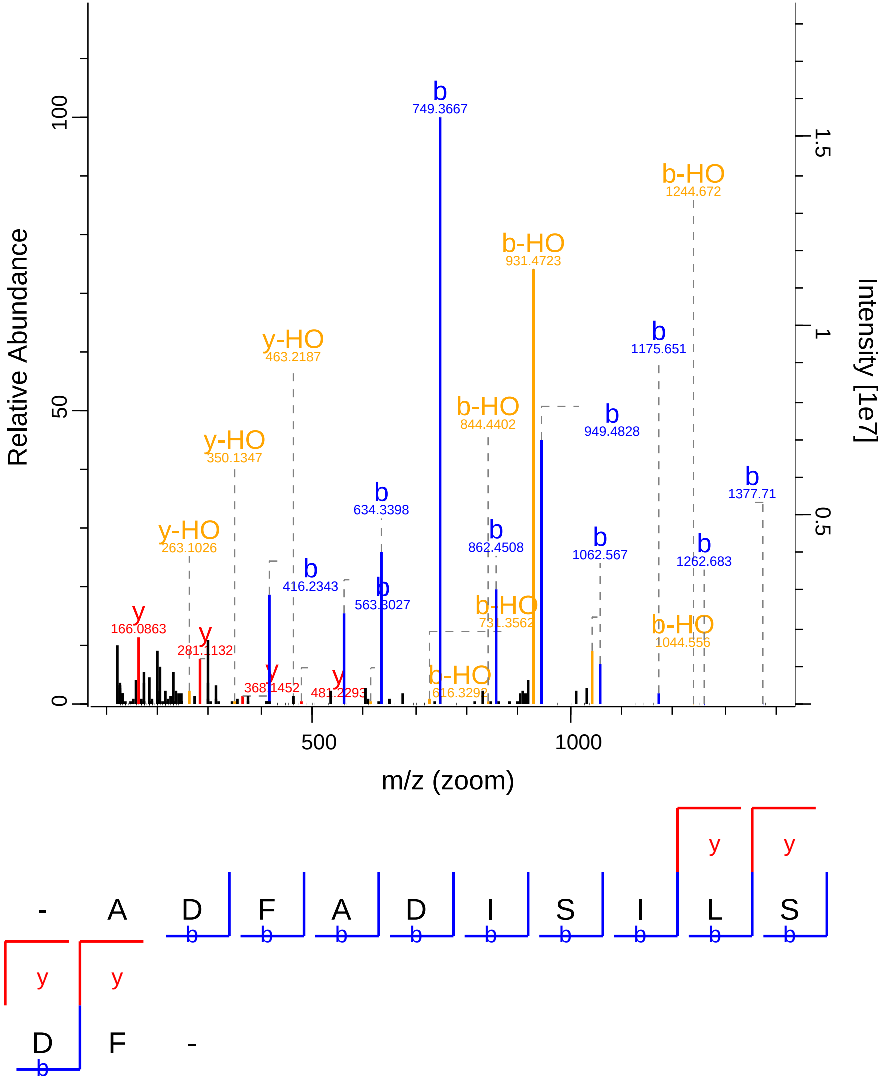

Raw File	Scan	Method	Score	m/z	Gene names
RO-QC-160121_ZackD_SS_Set_01_Fractic	37739	FTMS; HC	132.79	1056.53	LRRC8D

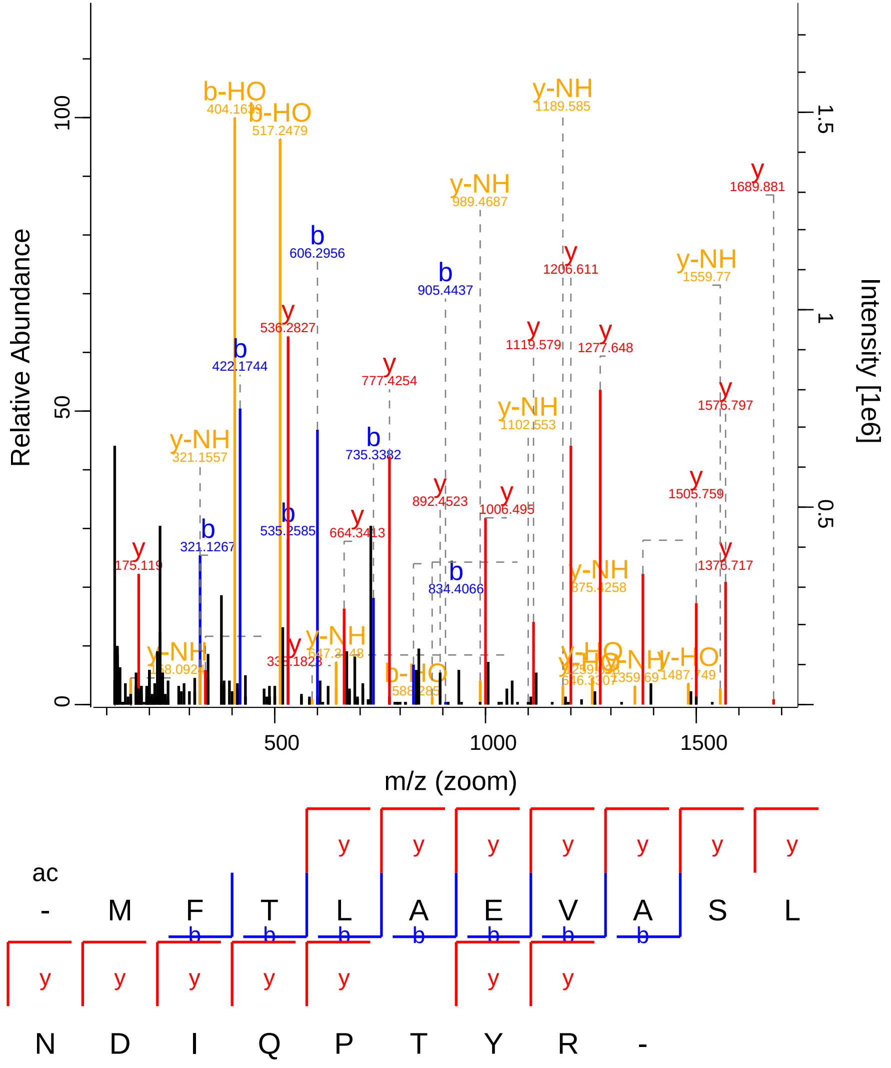

Raw File	Scan	Method	Score	m/z	Gene names
RO-QC-160121_ZackD_SS_Set_01_Fractio	38148	FTMS; HC	41.87	872.14	LRRK2

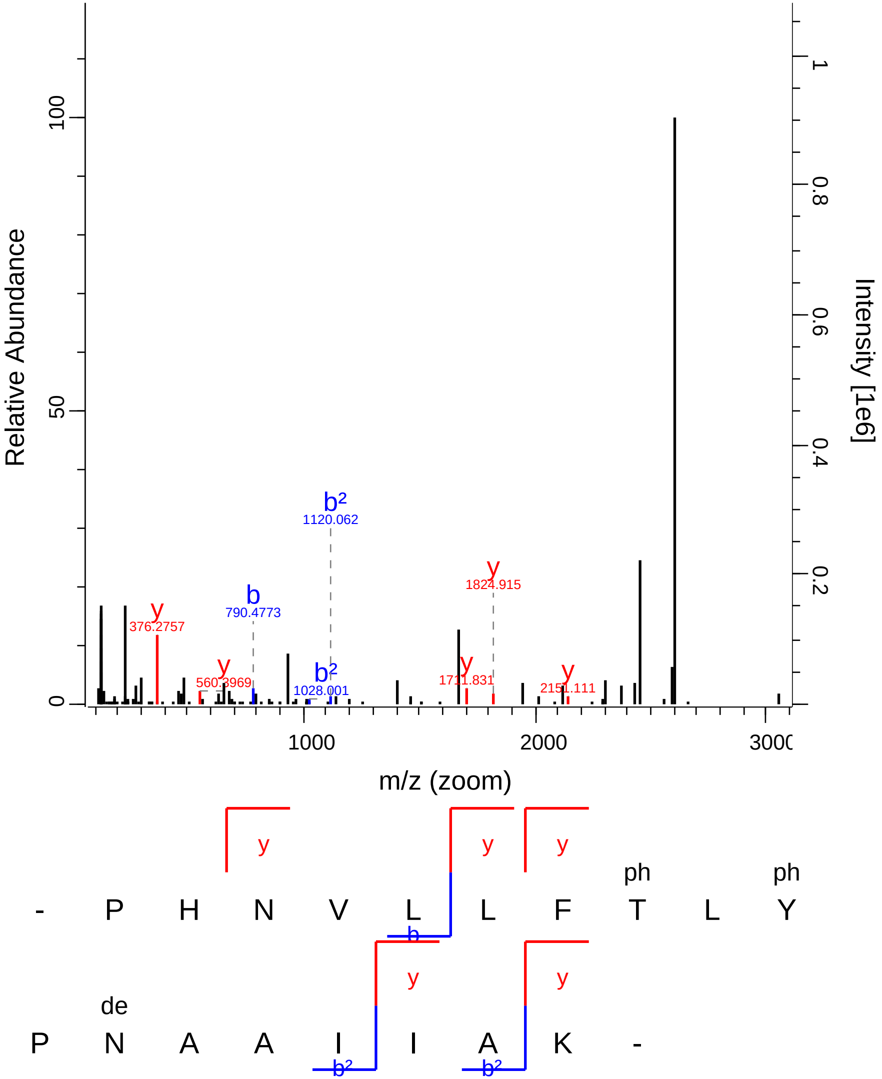

Raw File	Scan	Method	Score	m/z	Gene names
RO-QC-160121_ZackD_SS_Set_01_Fractio	5569	FTMS; HC	151.98	522.94	TRAF3IP1

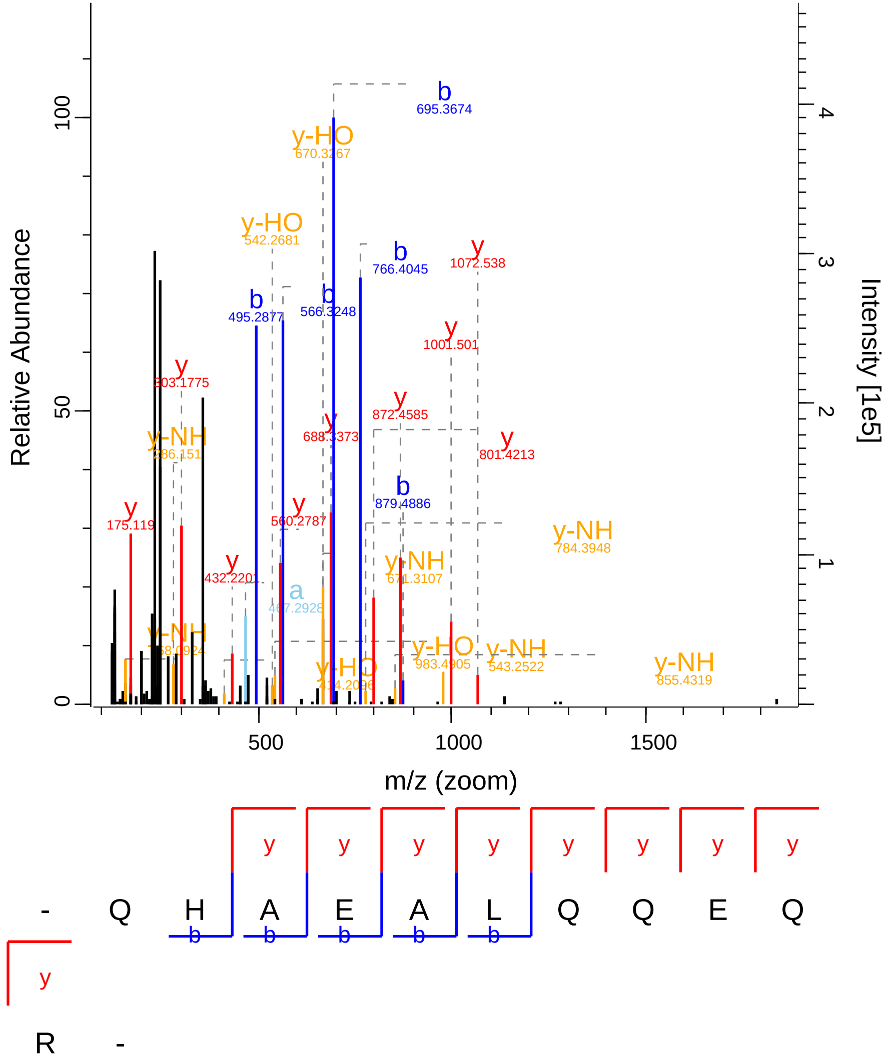

Raw File	Scan	Method	Score	m/z	Gene names
RO-QC-160121_ZackD_SS_Set_01_Fractior	5615	FTMS; HC	106.29	783.91	TRAF3IP1

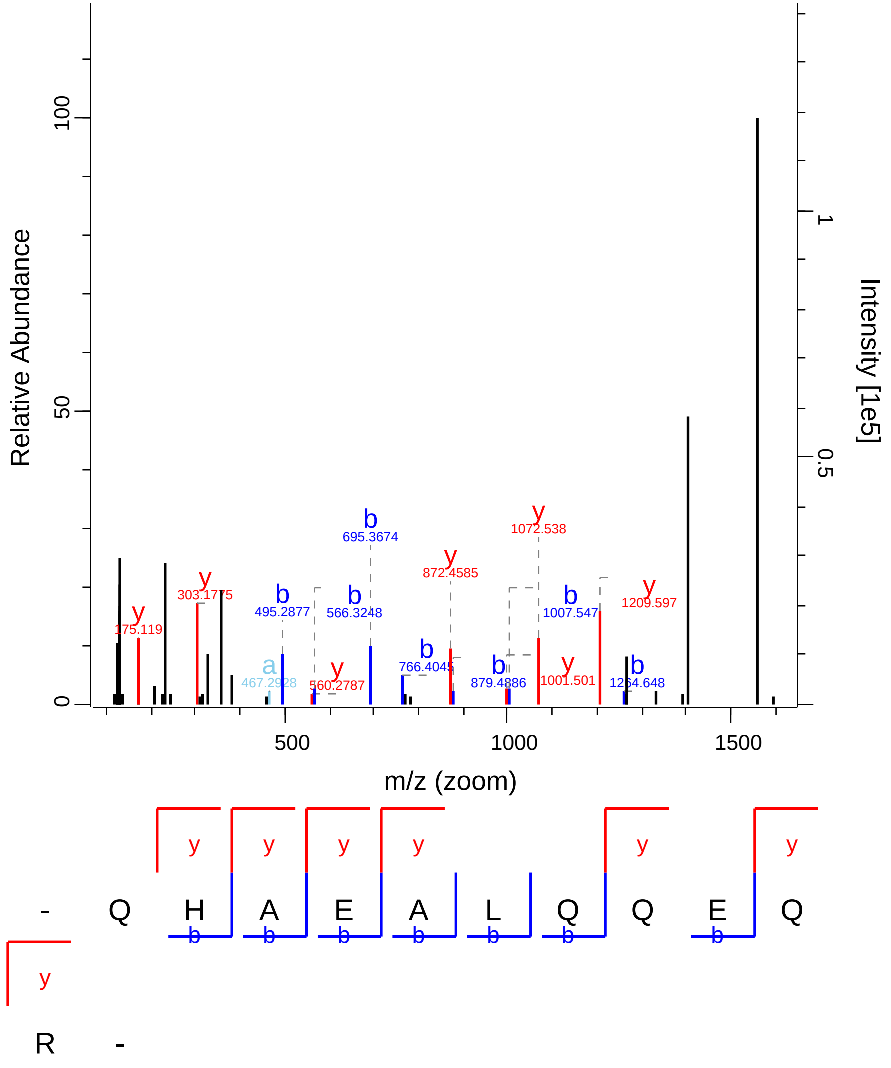

Raw File	Scan	Method	Score	m/z	Gene names
RO-QC-160121_ZackD_SS_Set_01_Fractior	5790	FTMS; HCI	56.09	496.28	MYC

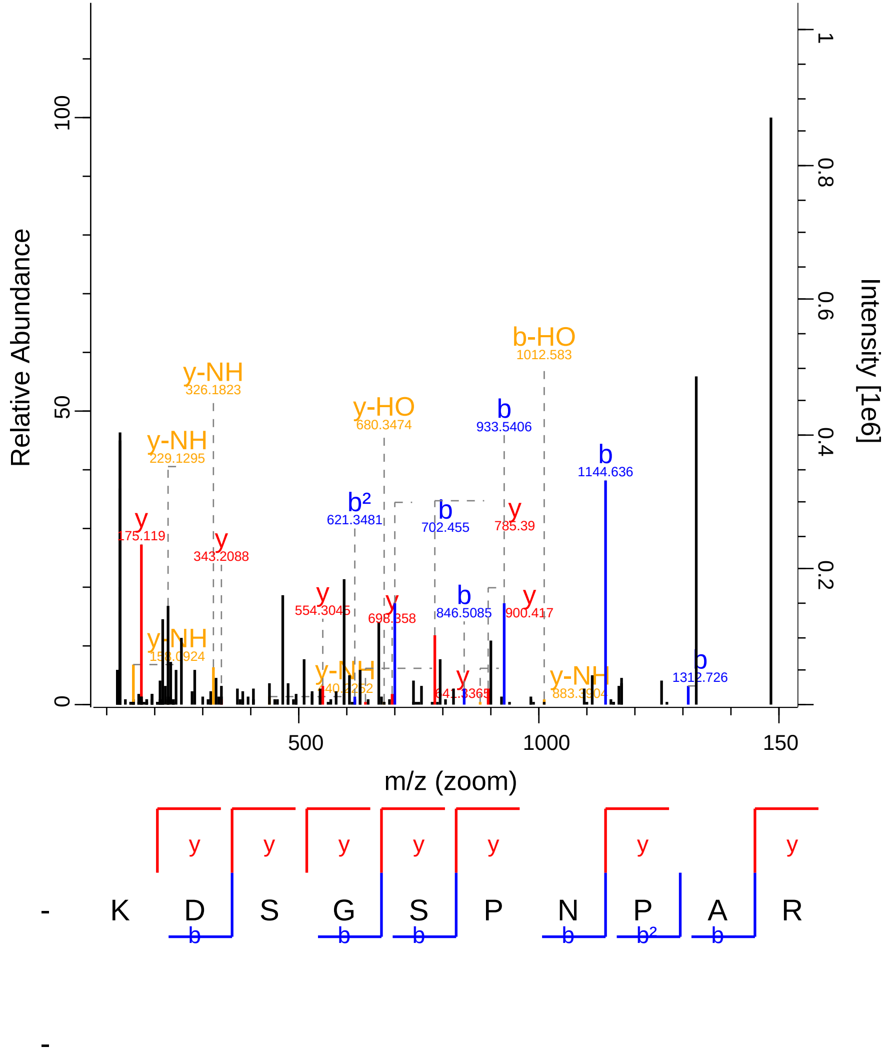

Raw File	Scan	Method	Score	m/z	Gene names
RO-QC-160121_ZackD_SS_Set_01_Fraction	5949	FTMS; HCI	104.75	548.8	WIPF1

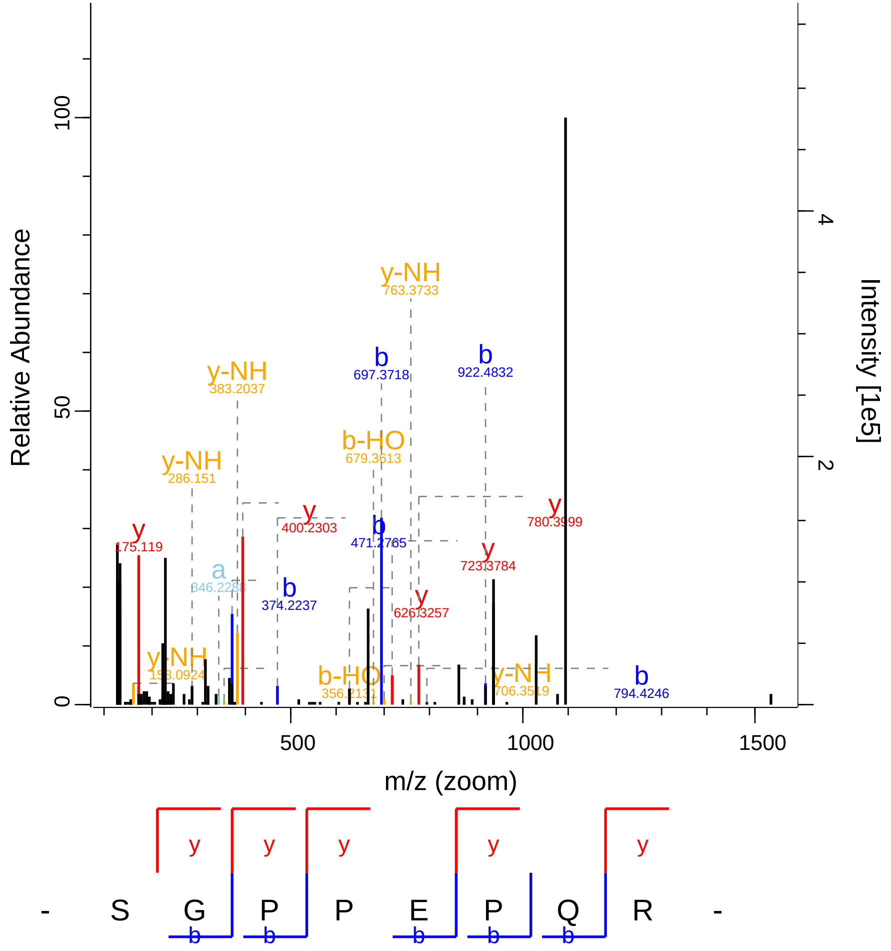

Raw File	Scan	Method	Score	m/z	Gene names
RO-QC-160121_ZackD_SS_Set_01_Fraction	6656	FTMS; HCI	75.69	575.8	GPAM

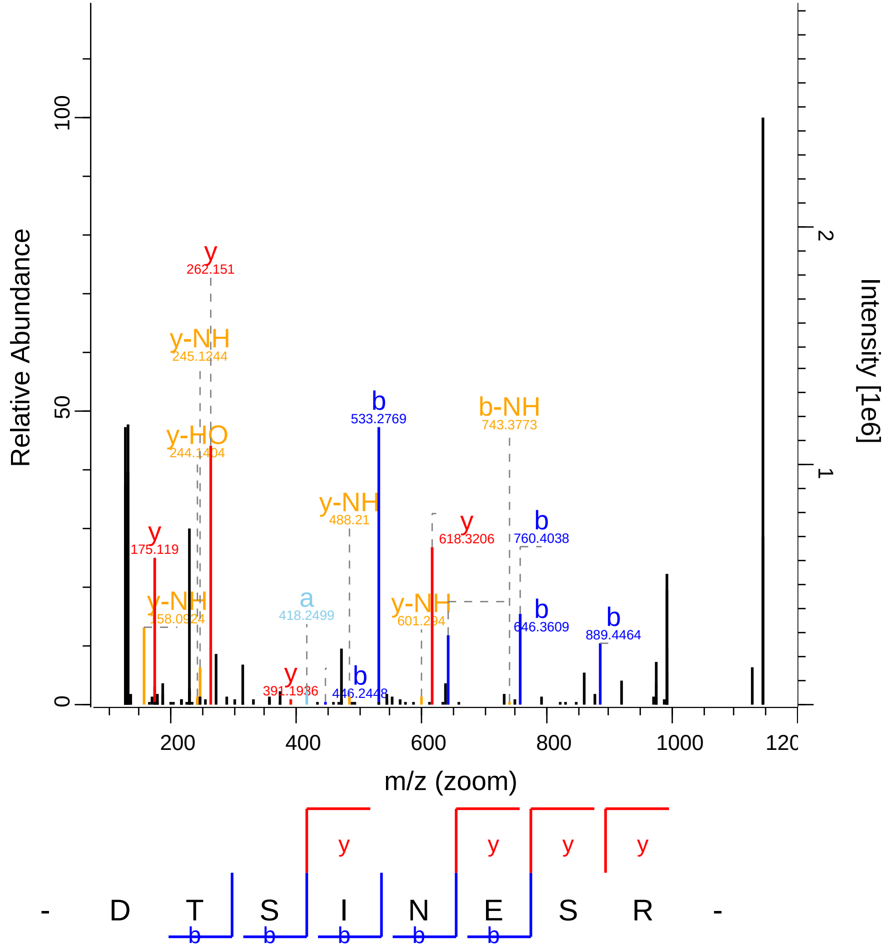

Raw File	Scan	Method	Score	m/z	Gene names
RO-QC-160121_ZackD_SS_Set_01_Fractioı	6787	FTMS; HC	154.75	686.31	C5orf45

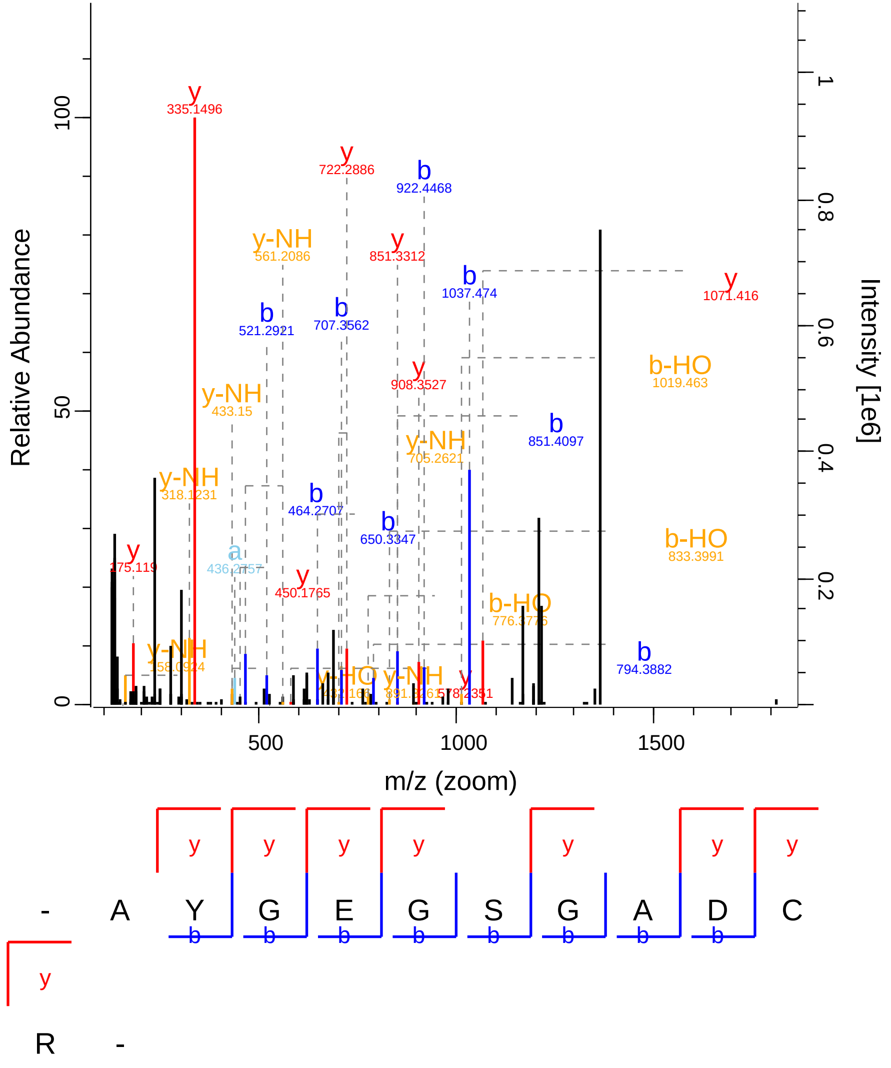

Raw File	Scan	Method	Score	m/z	Gene names
RO-QC-160121_ZackD_SS_Set_01_Fraction	7320	FTMS; HCI	99.26	604.8	RNF115

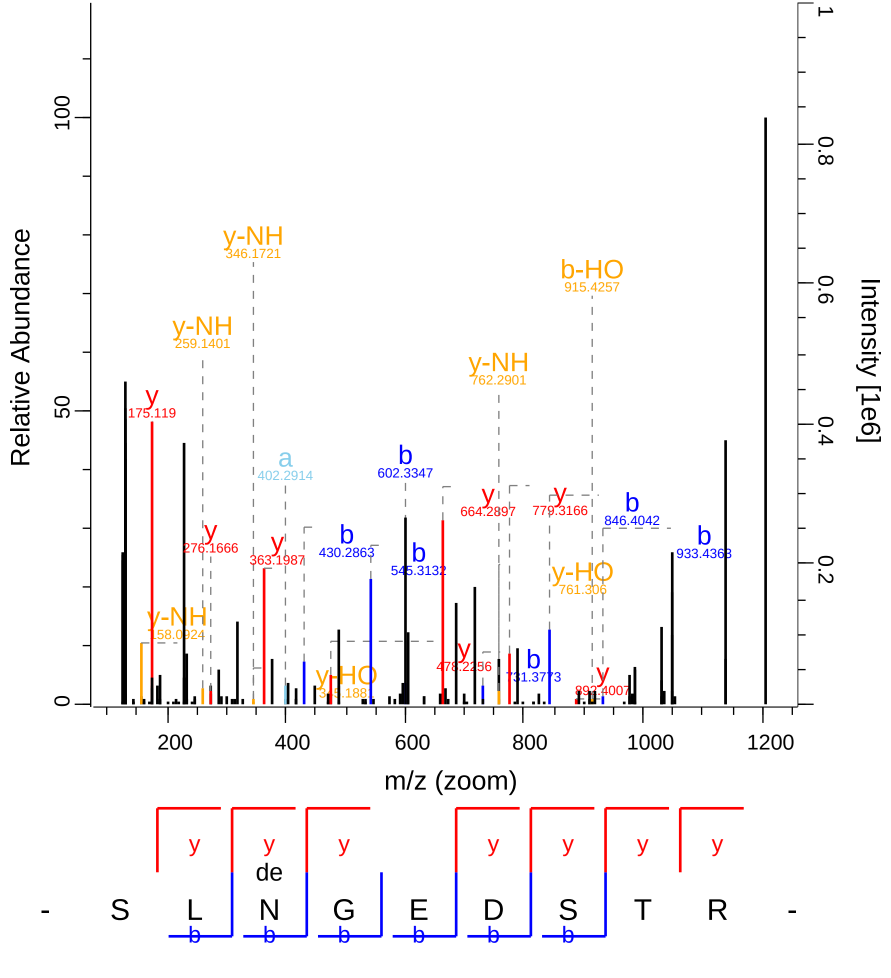

Raw File	Scan	Method	Score	m/z	Gene names
RO-QC-160121_ZackD_SS_Set_01_Fractio	7612	FTMS; HC	121.99	608.38	HERC5

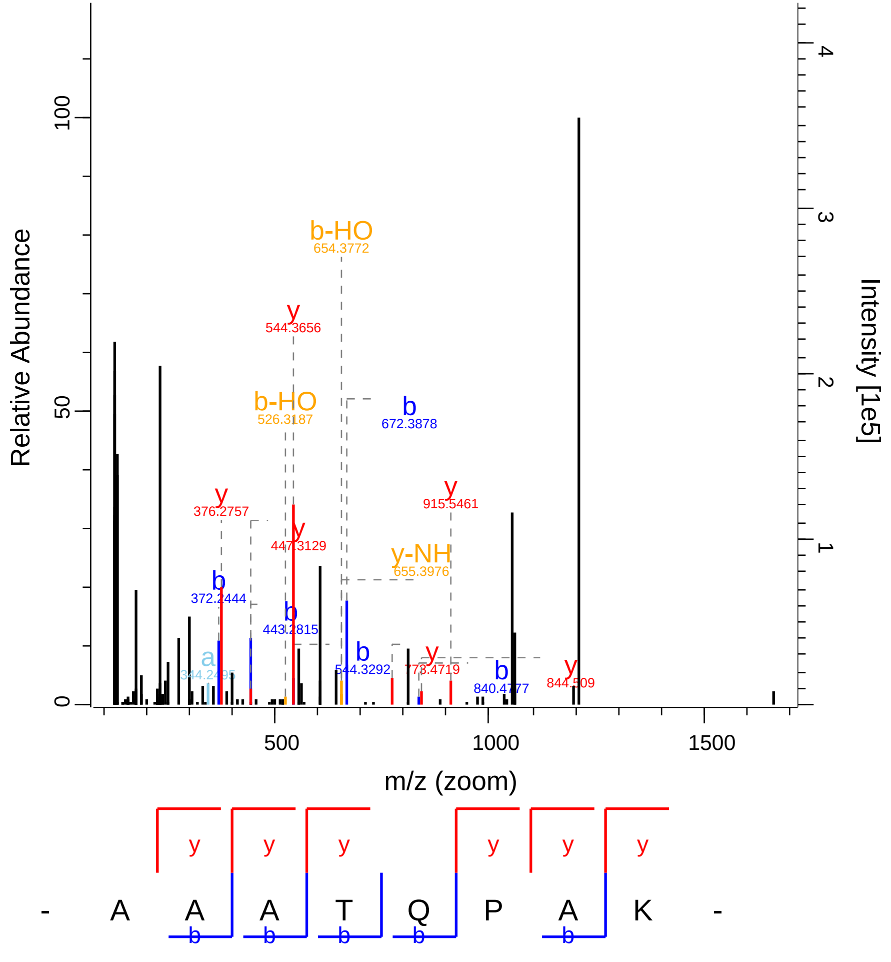

Raw File	Scan	Method	Score	m/z	Gene names
RO-QC-160121_ZackD_SS_Set_01_Fraction_	7643	FTMS; HCI	178.23	660	CASP2

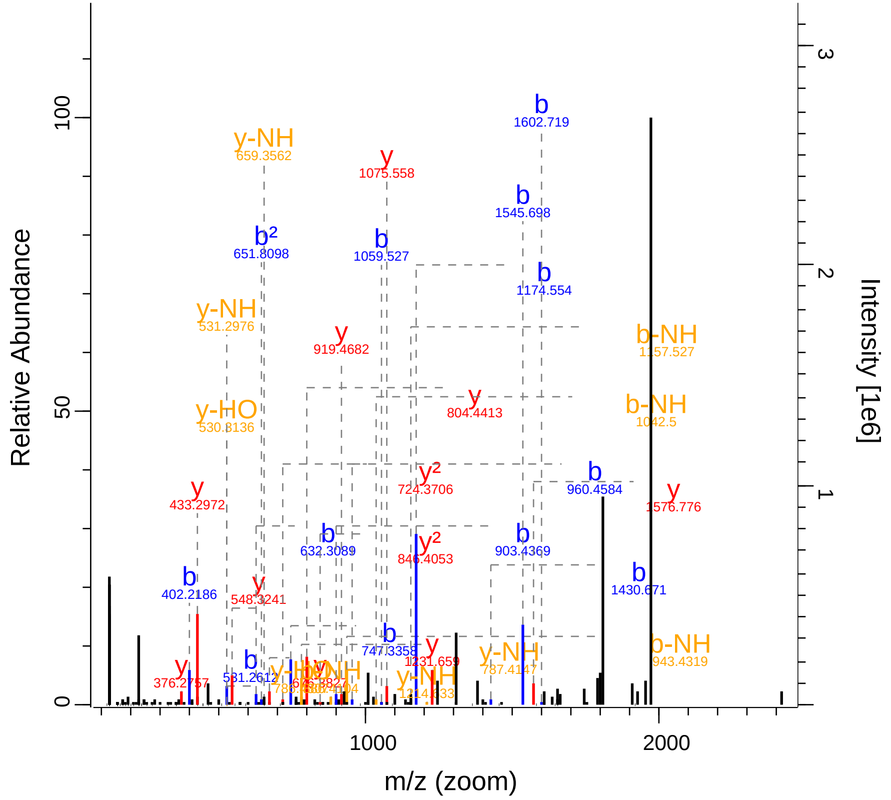

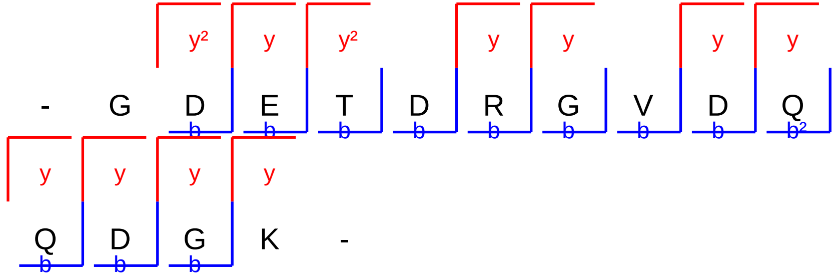

Raw File	Scan	Method	Score	m/z
RO-QC-160121_ZackD_SS_Set_01_Fraction_3	8470	FTMS; HCD	71.3	451.25

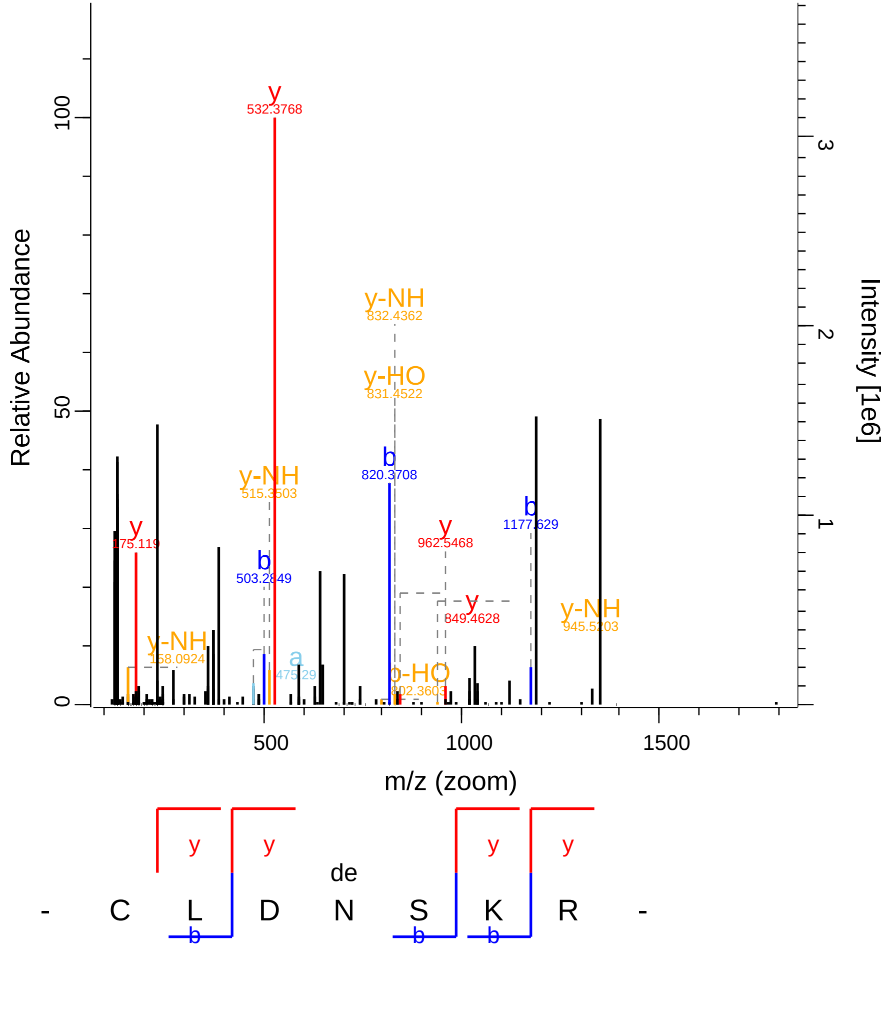

Raw File	Scan	Method	Score	m/z	Gene names
RO-QC-160121_ZackD_SS_Set_01_Fractio	9100	FTMS; HC	140.24	860.94	CNOT8

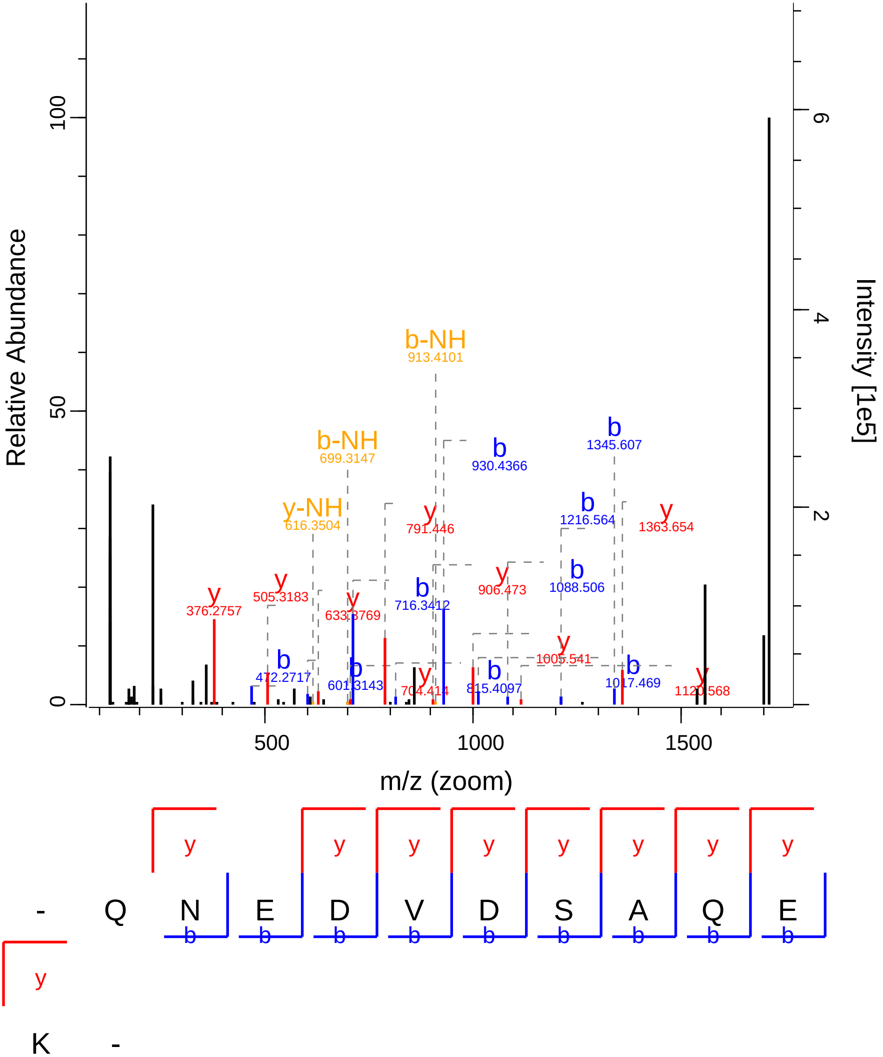

Raw File	Scan	Method	Score	m/z
RO-QC-160121_ZackD_SS_Set_01_Fraction_3	9751	FTMS; HCD	48.24	502.81

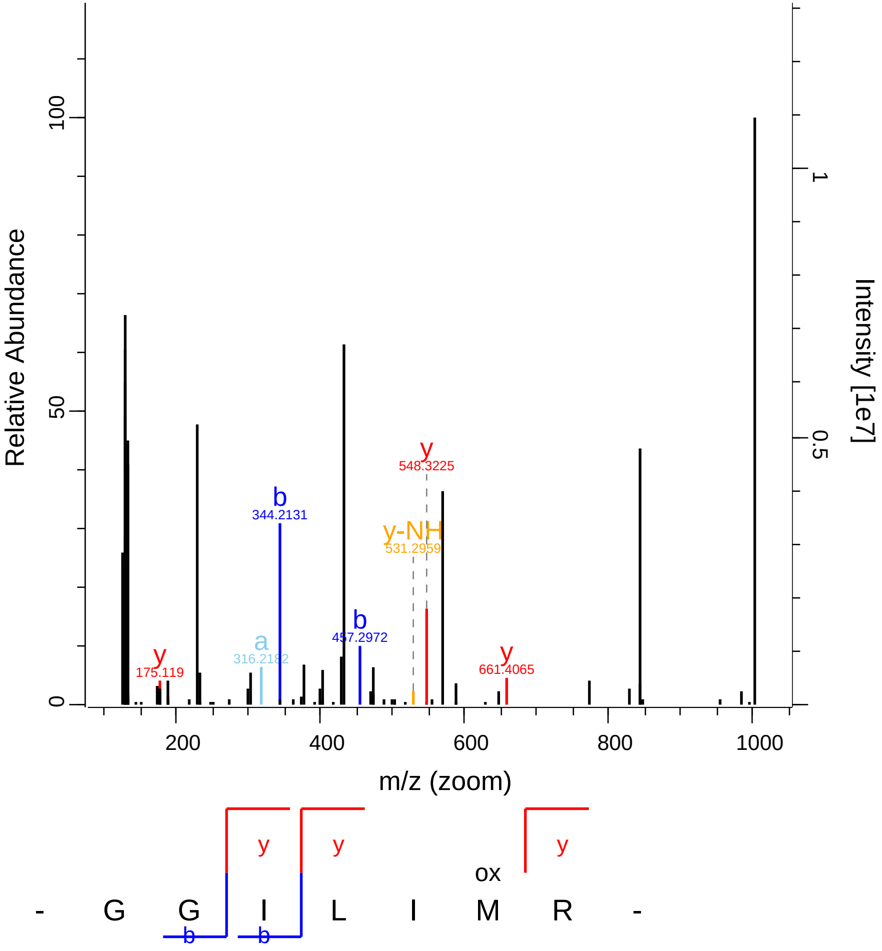

Raw File	Scan	Method	Score	m/z	Gene names
RO-QC-160121_ZackD_SS_Set_01_Fractio	9985	FTMS; HC	107.56	623.83	TXNDC11

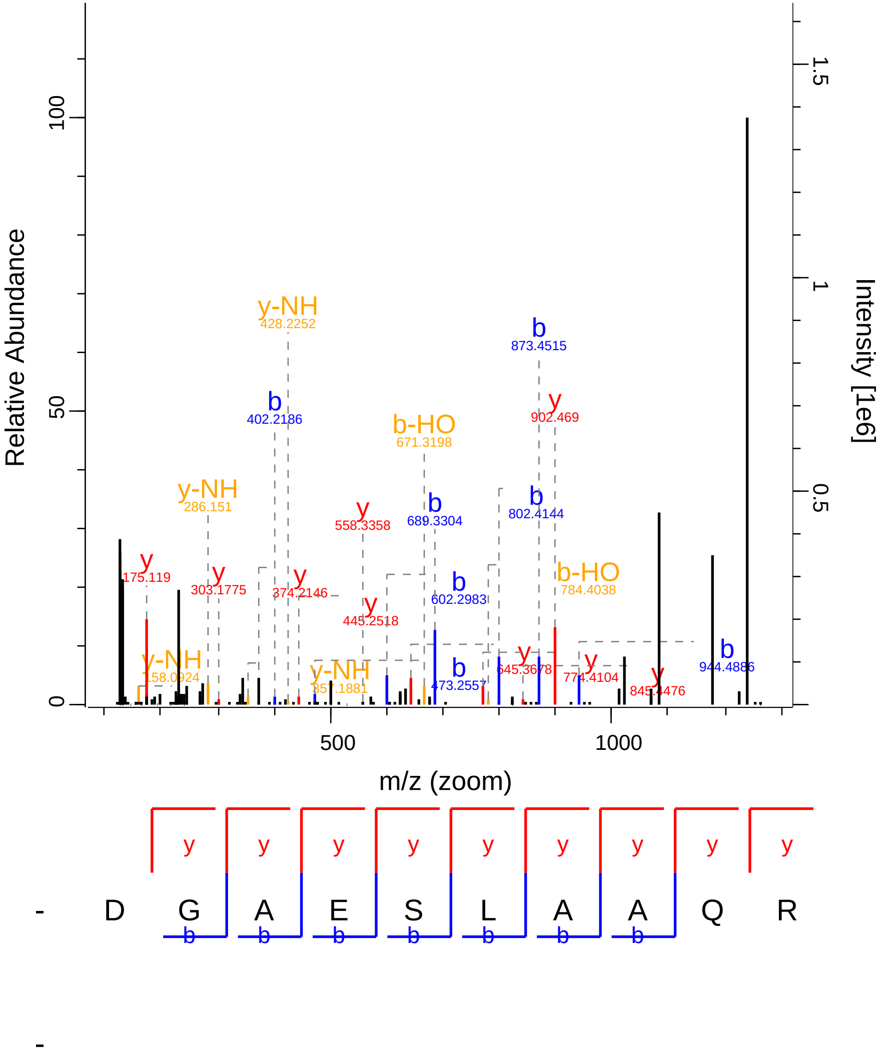

Raw File	Scan	Method	Score	m/z
RO-QC-160121_ZackD_SS_Set_01_Fraction_3	10440	FTMS; HCD	41.3	403.9

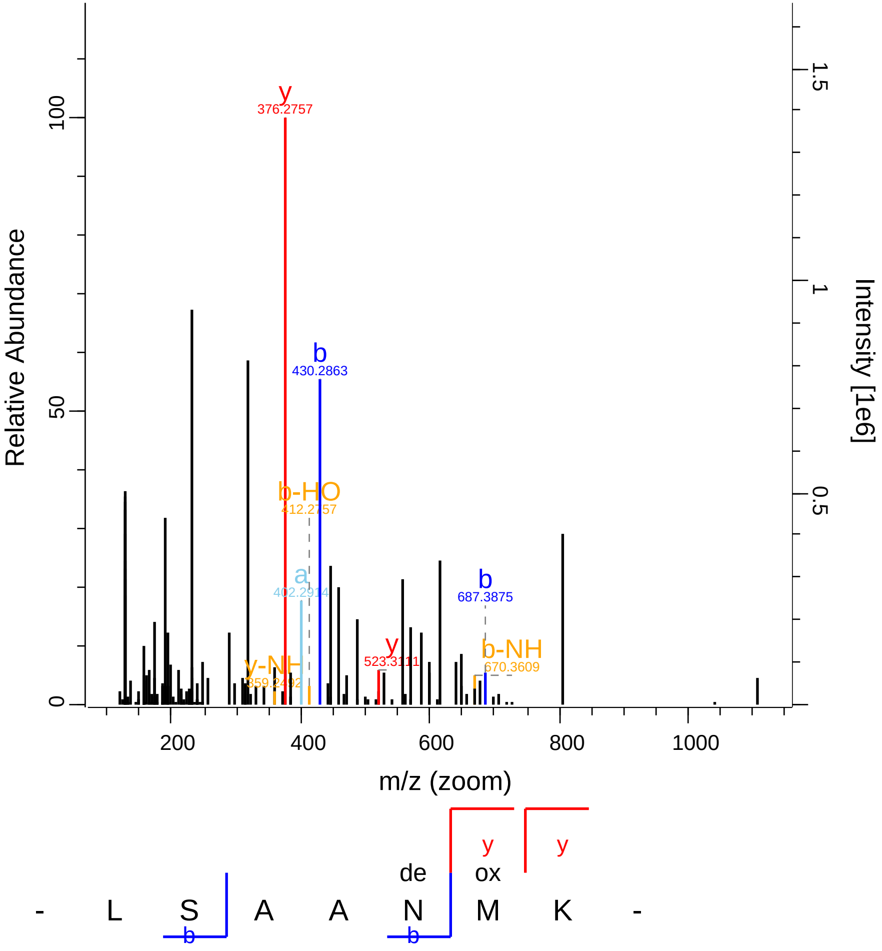

Raw File	Scan	Method	Score	m/z	Gene names
RO-QC-160121_ZackD_SS_Set_01_Fractio	10536	FTMS; HC	77.73	493.29	CADM3

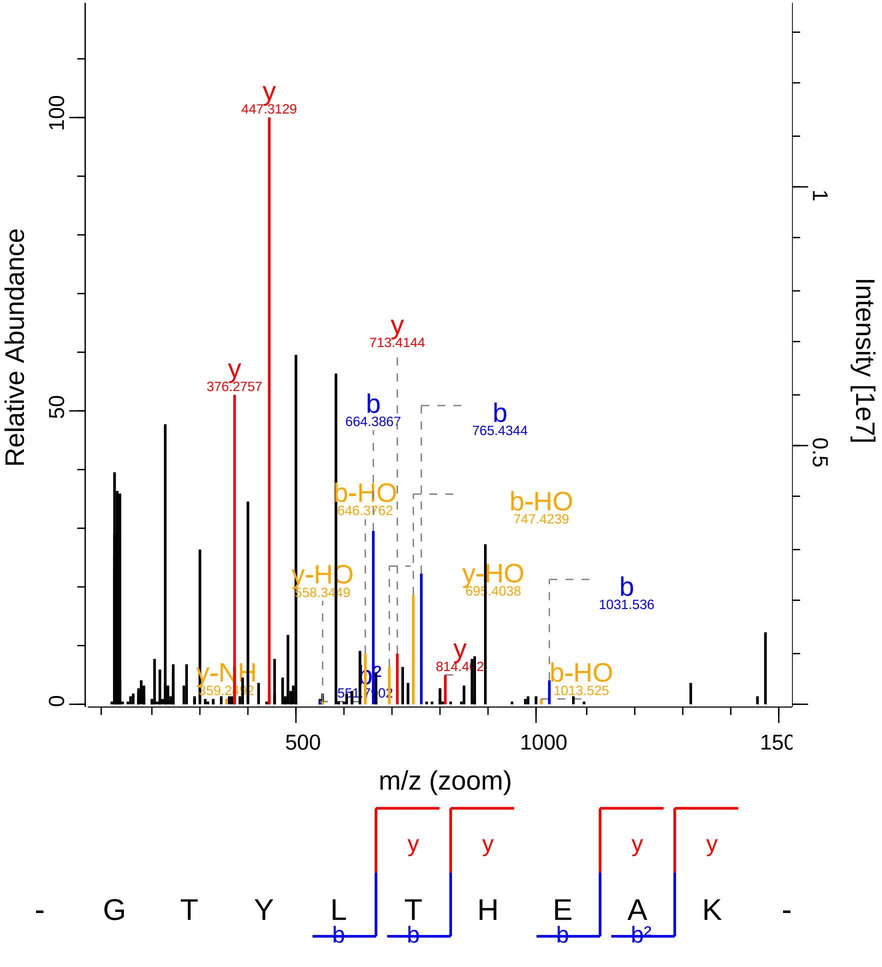

Raw File	Scan	Method	Score	m/z	Gene names
RO-QC-160121_ZackD_SS_Set_01_Fractio	10616	FTMS; HC	96.25	425.59	G6PC3

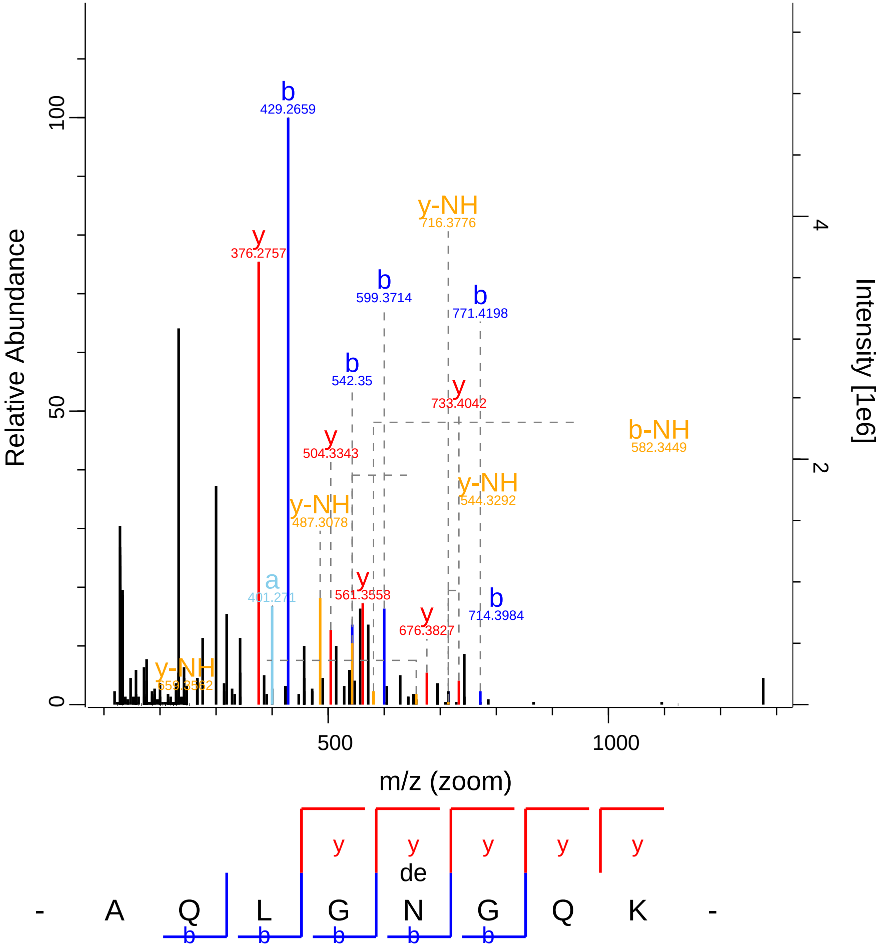

Raw File	Scan	Method	Score	m/z	Gene names
RO-QC-160121_ZackD_SS_Set_01_Fractio	10679	FTMS; HC	93.26	469.92	EFHC2

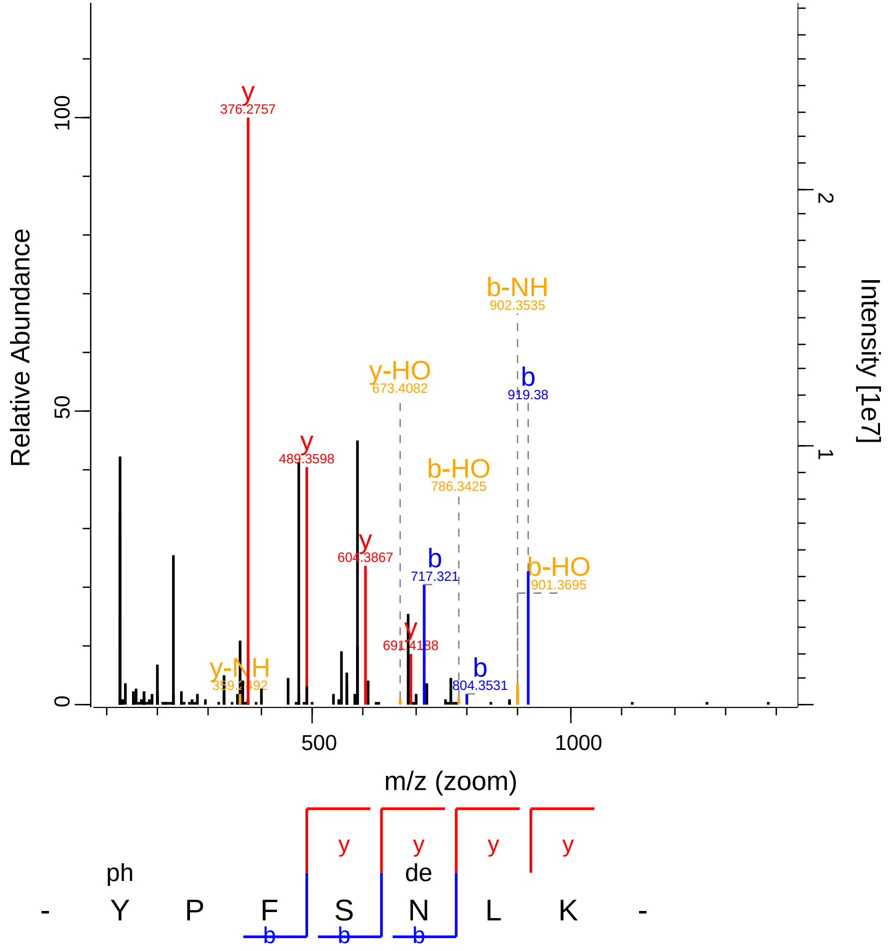

Raw File	Scan	Method	Score	m/z	Gene names
RO-QC-160121_ZackD_SS_Set_01_Fractio	10855	FTMS; HC	139.28	637.88	G6PC3

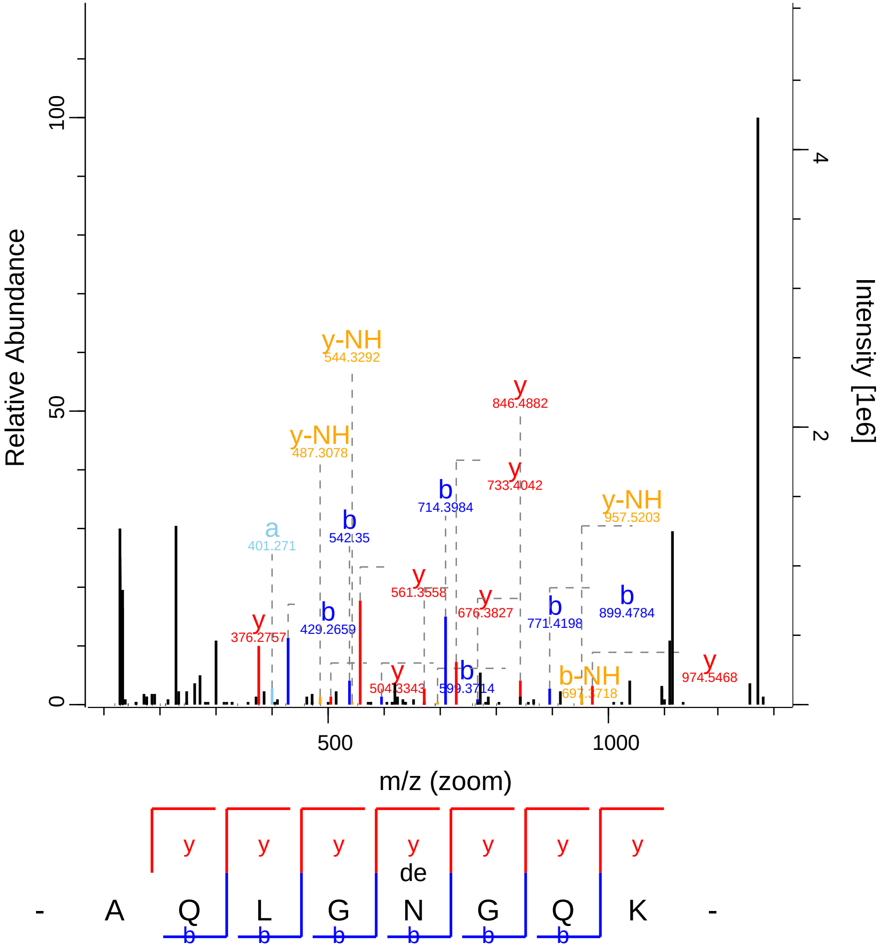

Raw File	Scan	Method	Score	m/z	Gene names
RO-QC-160121_ZackD_SS_Set_01_Fractio	11488	FTMS; HC	182.29	739.38	PLP2

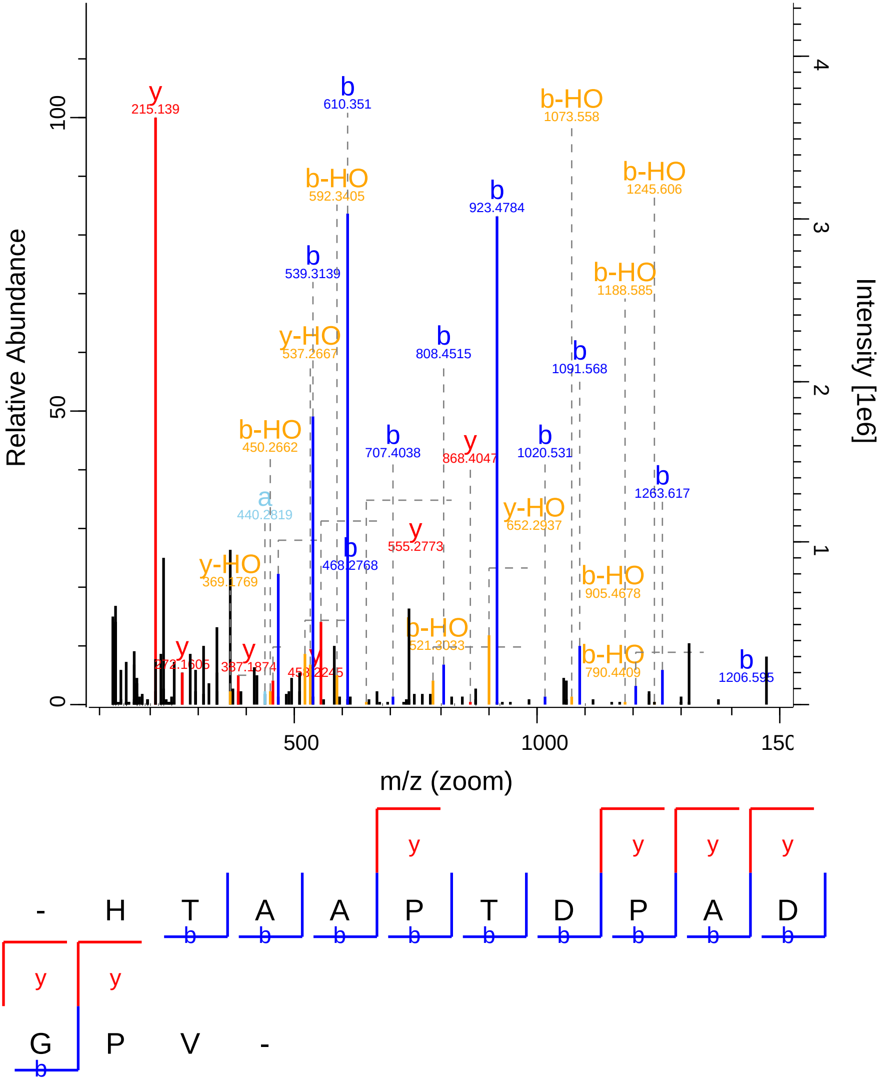

Raw File	Scan	Method	Score	m/z	Gene names
RO-QC-160121_ZackD_SS_Set_01_Fractio	11695	FTMS; HC	157.91	739.38	PLP2

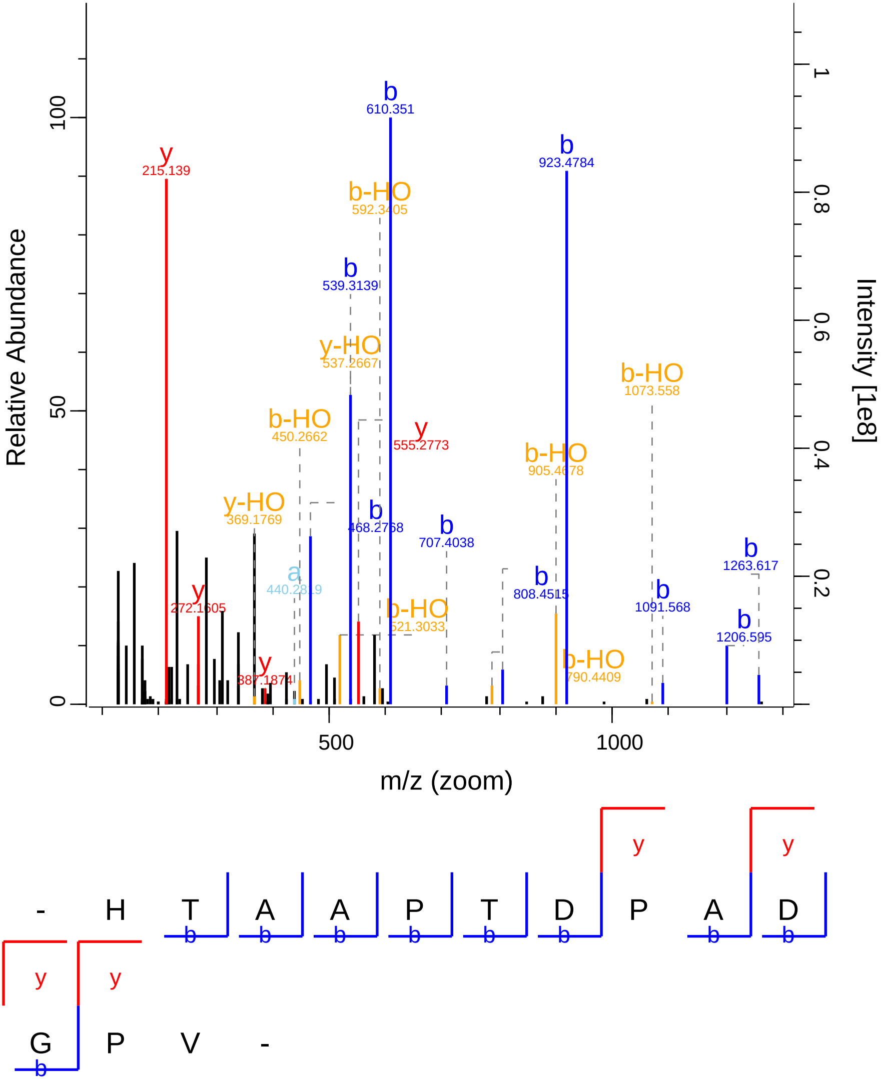

Raw File	Scan	Method	Score	m/z	Gene names
RO-QC-160121_ZackD_SS_Set_01_Fractio	11937	FTMS; HC	105.1	739.38	PLP2

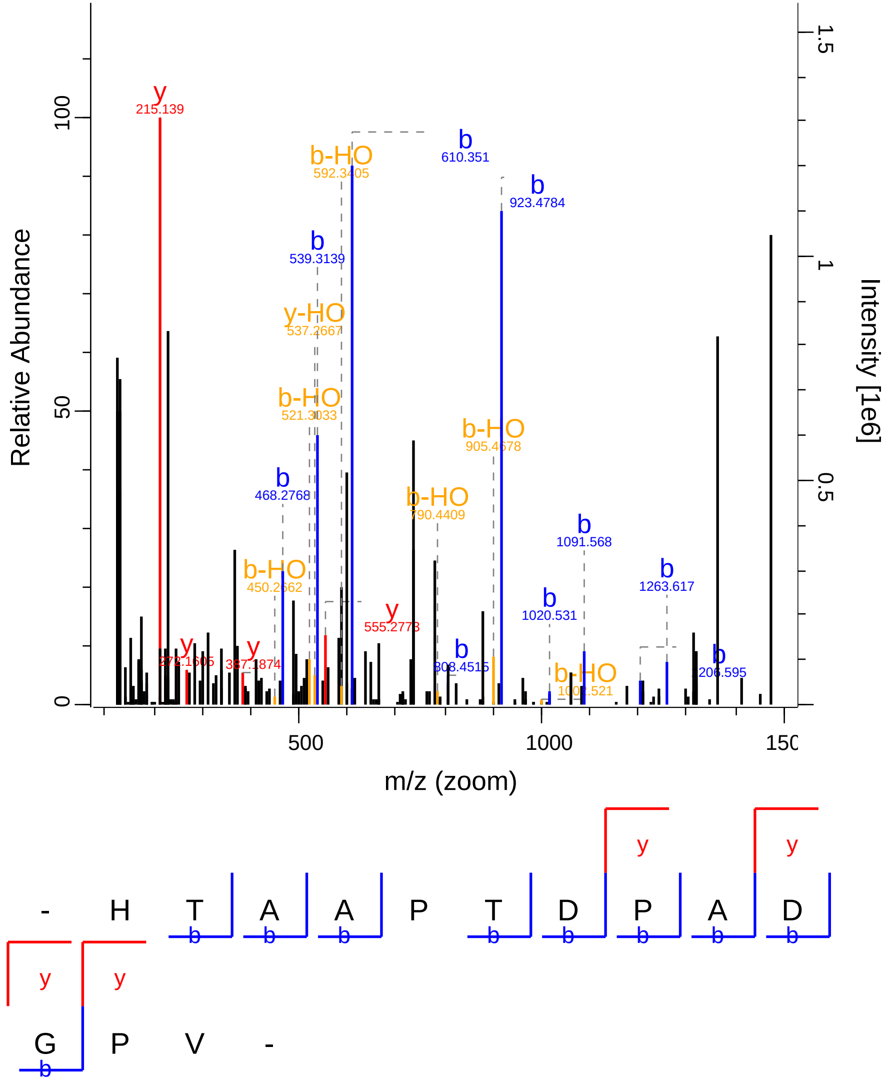

Raw File	Scan	Method	Score	m/z	Gene names
RO-QC-160121_ZackD_SS_Set_01_Fractior	12664	FTMS; HCI	105.98	778.9	SLC46A1

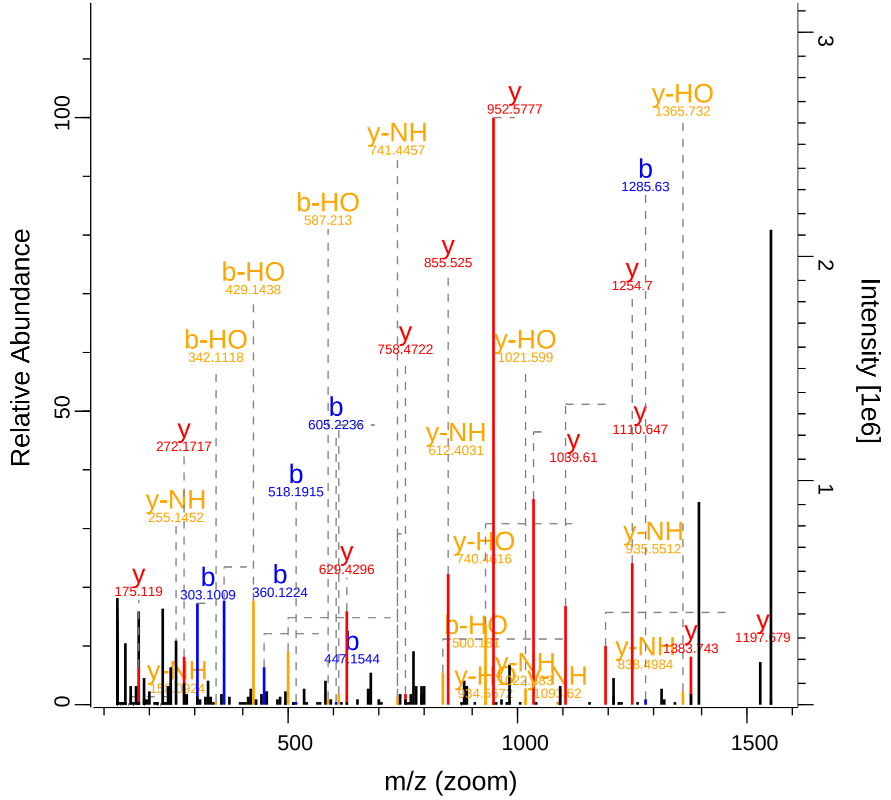

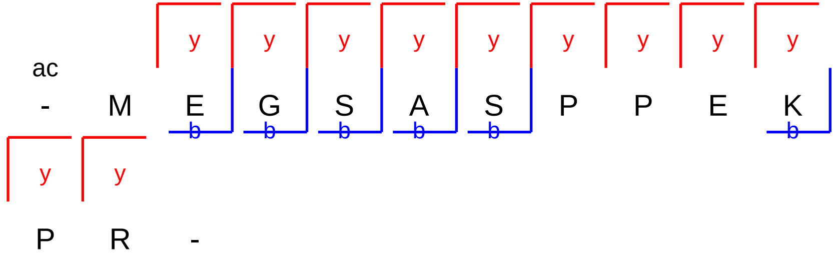

Raw File	Scan	Method	Score	m/z
RO-QC-160121_ZackD_SS_Set_01_Fraction_3	12769	FTMS; HCD	162.5	638.39

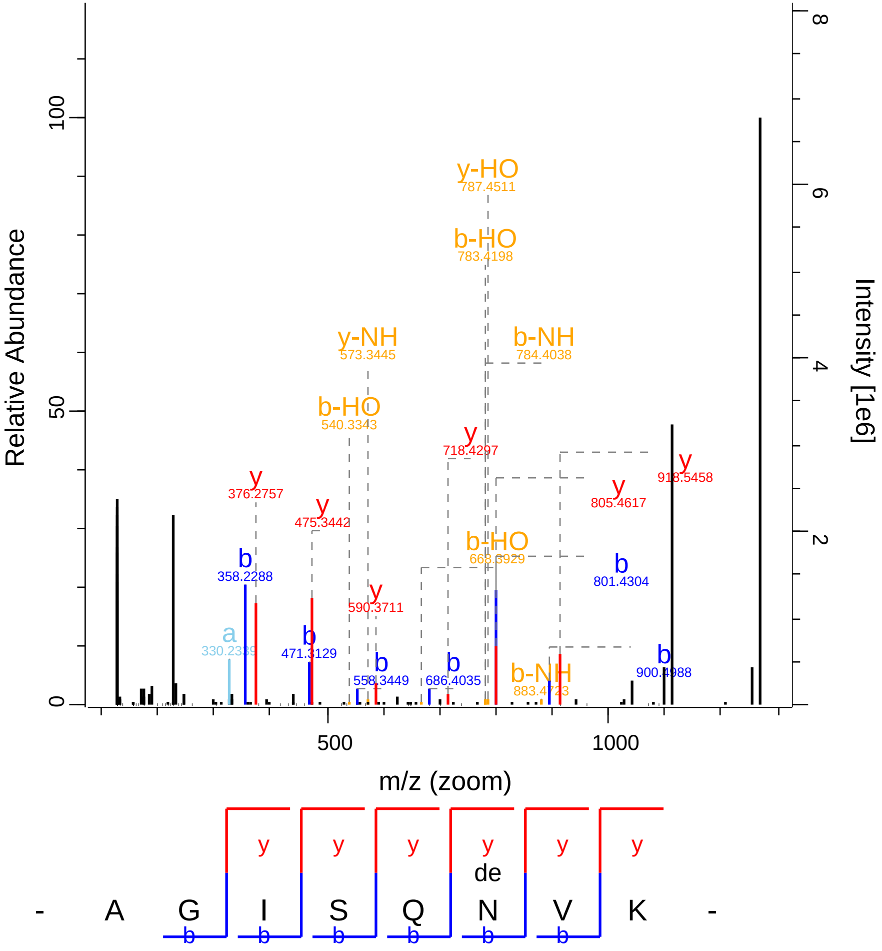

Raw File	Scan	Method	Score	m/z	Gene names
RO-QC-160121_ZackD_SS_Set_01_Fractio	13309	FTMS; HC	61.38	600.62	CCHCR1

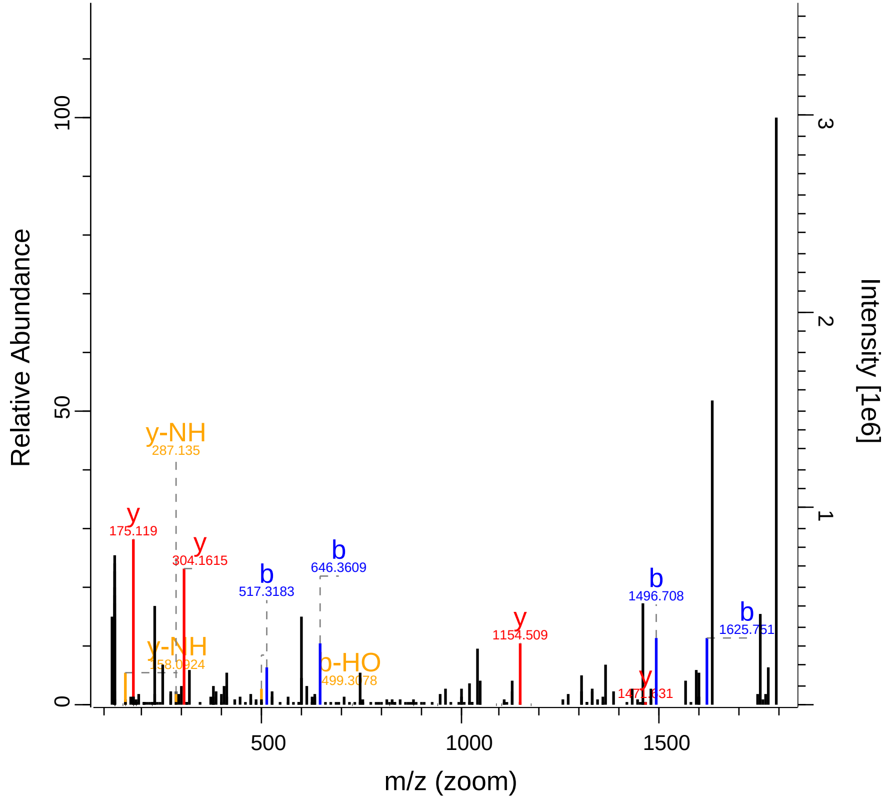

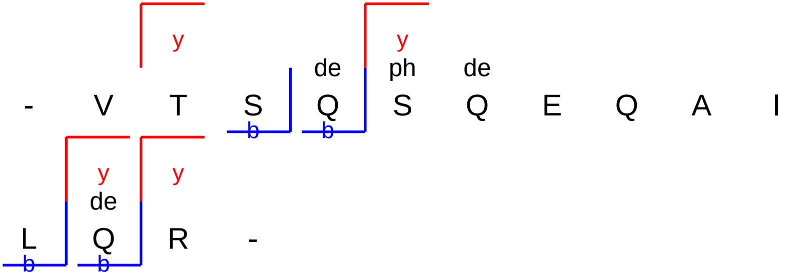

Raw File	Scan	Method	Score	m/z	Gene names
RO-QC-160121_ZackD_SS_Set_01_Fractio	13584	FTMS; HC	82.27	716.35	CHTF18

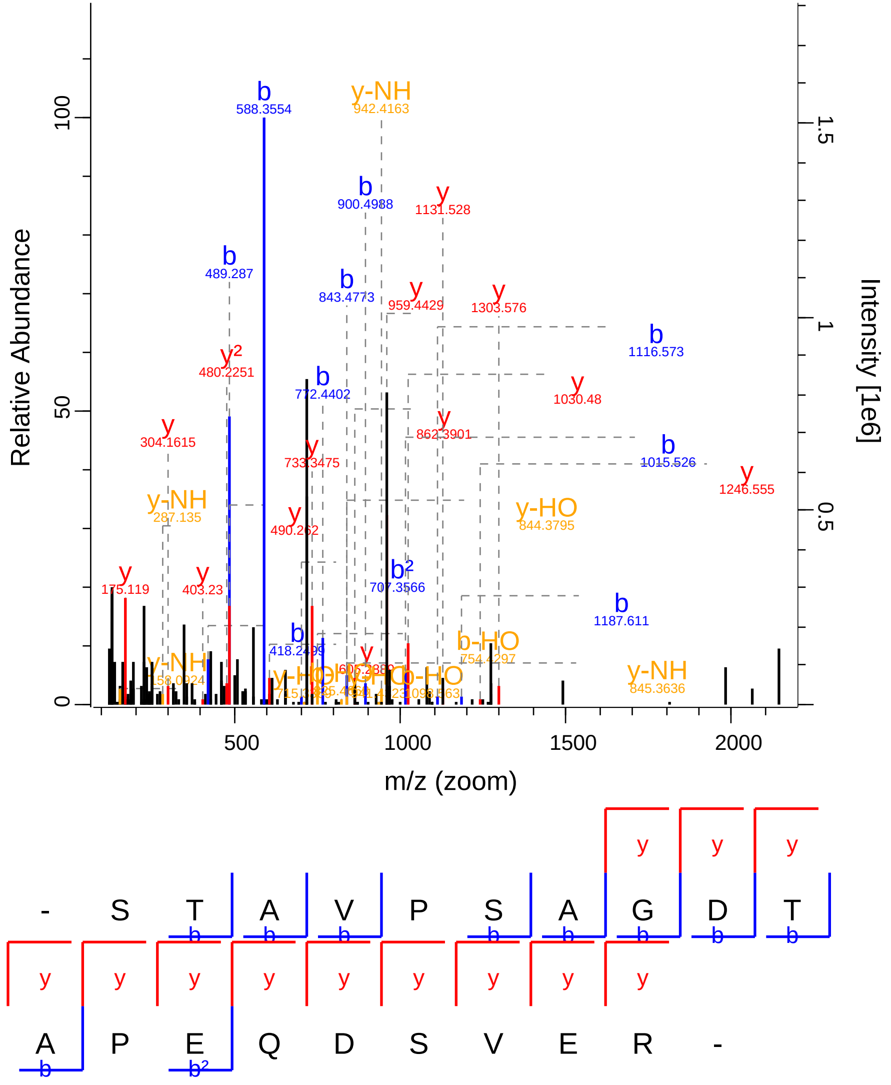

Raw File	Scan	Method	Score	m/z
RO-QC-160121_ZackD_SS_Set_01_Fraction_3	15067	FTMS; HCD	44.14	457.58

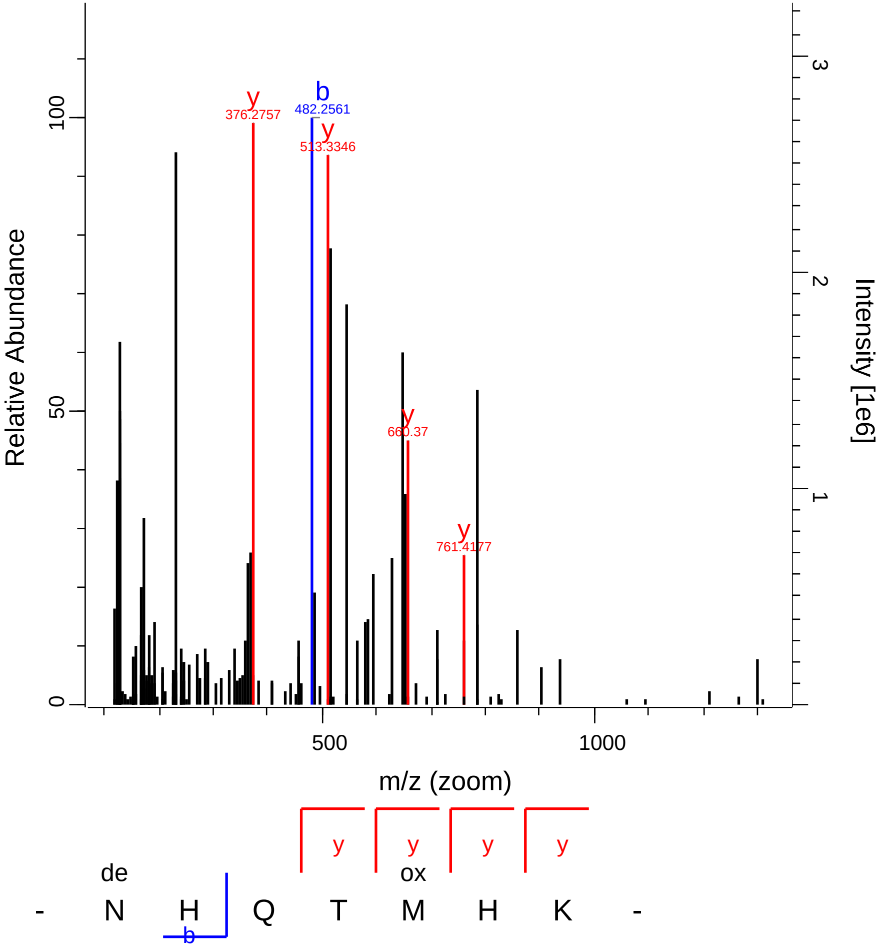

Raw File	Scan	Method	Score	m/z
RO-QC-160121_ZackD_SS_Set_01_Fraction_3	15216	FTMS; HCD	45.08	702.36

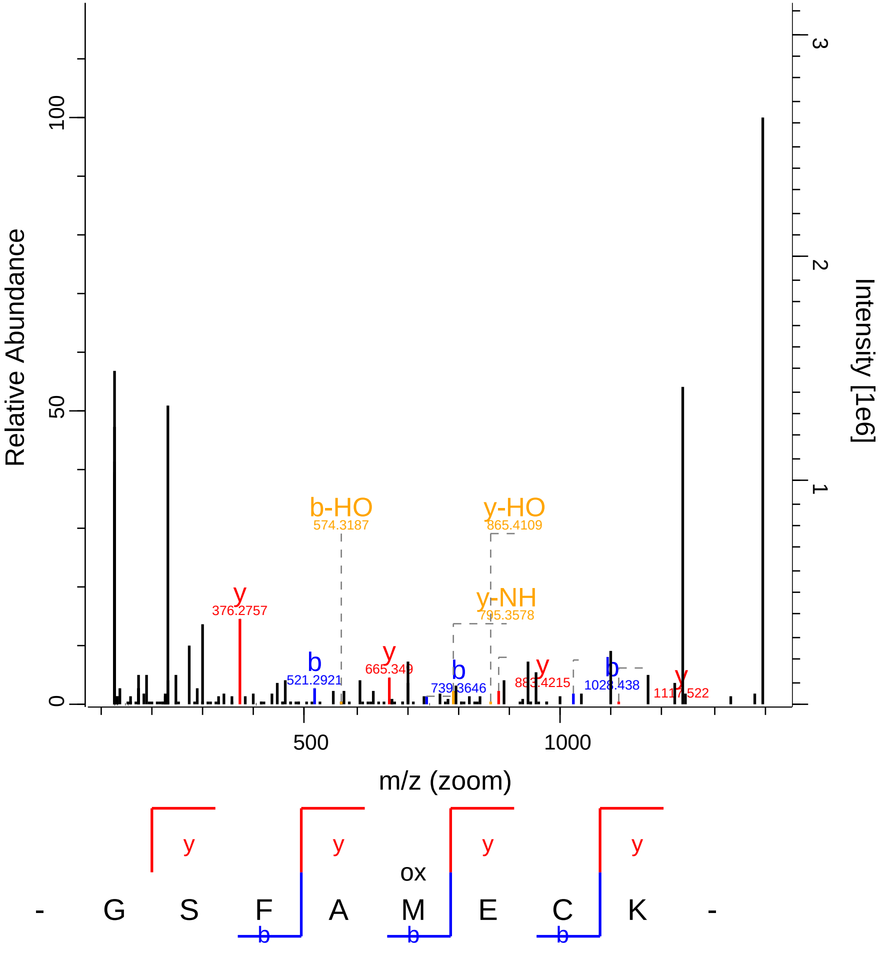

Raw File	Scan	Method	Score	m/z	Gene names
RO-QC-160121_ZackD_SS_Set_01_Fractio	15248	FTMS; HC	59.35	403.98	BPIFB4

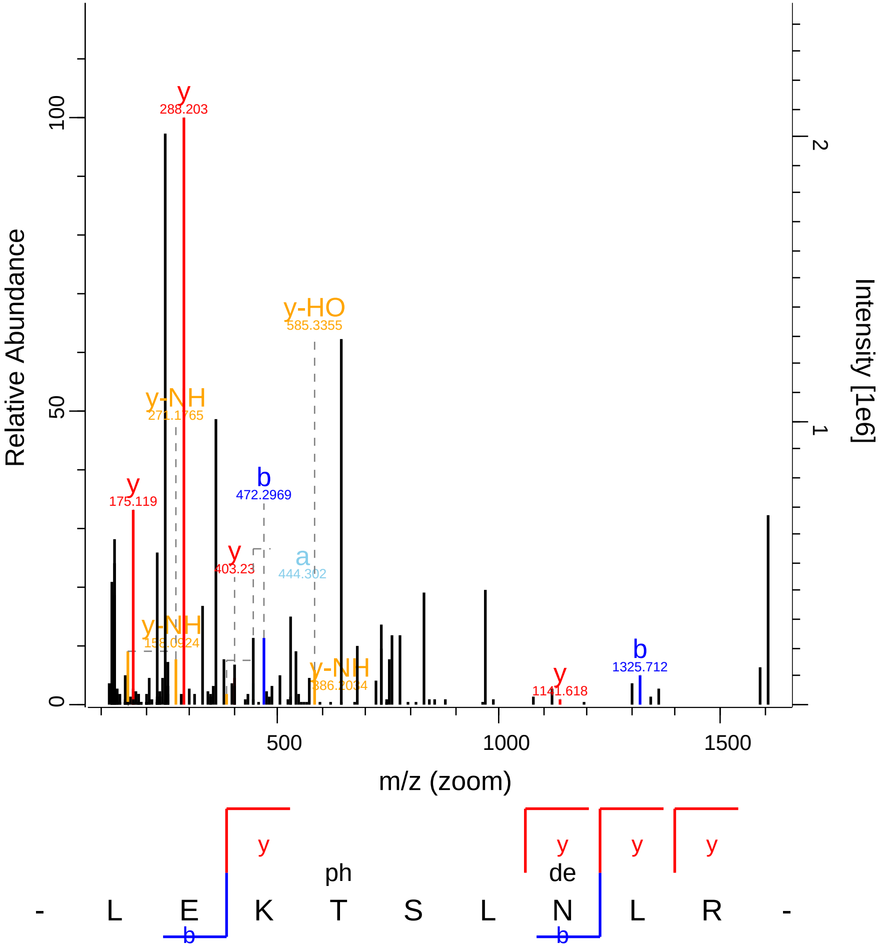

Raw File	Scan	Method	Score	m/z	Gene names
RO-QC-160121_ZackD_SS_Set_01_Fractio	15930	FTMS; HC	119.18	719.74	ARMCX5

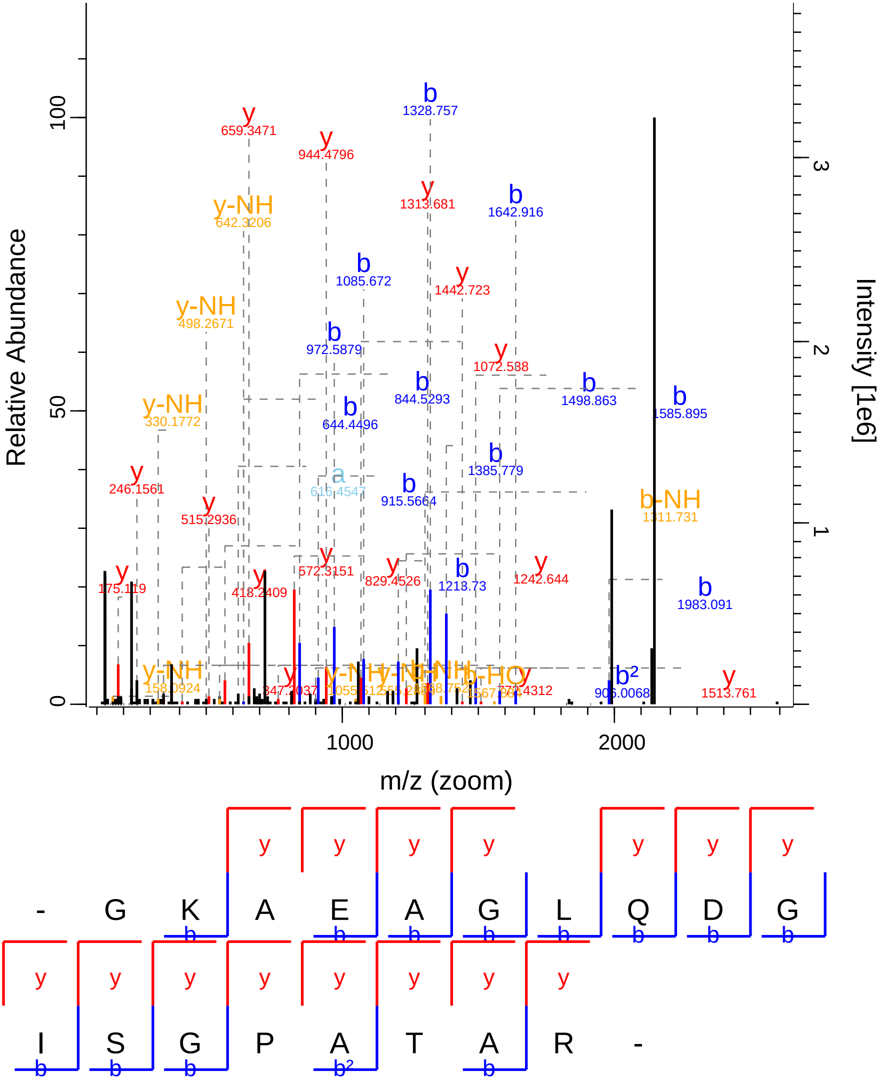

Raw File	Scan	Method	Score	m/z	Gene names
RO-QC-160121_ZackD_SS_Set_01_Fractio	16108	FTMS; HC	93.14	703.41	ATG12

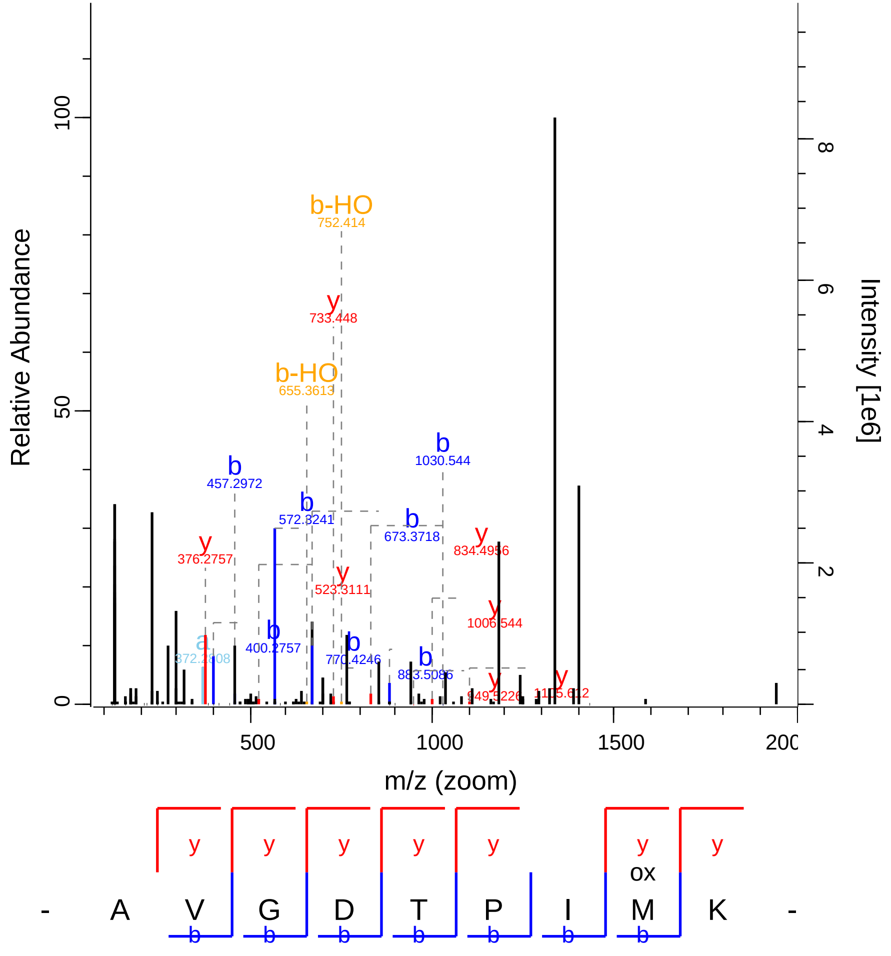

Raw File	Scan	Method	Score	m/z	Gene names
RO-QC-160121_ZackD_SS_Set_01_Fractio	16153	FTMS; HC	69.82	469.28	ATG12

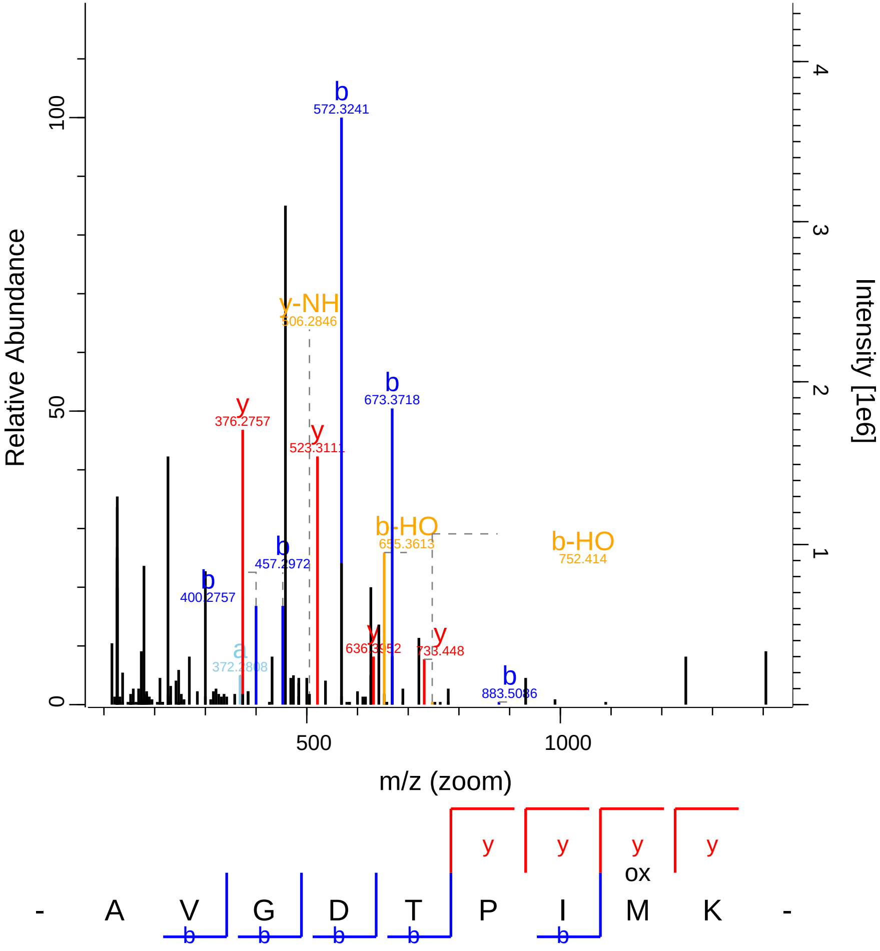

Raw File	Scan	Method	Score	m/z	Gene names
RO-QC-160121_ZackD_SS_Set_01_Fractior	16367	FTMS; HCI	57.41	691.3	TOR1AIP2

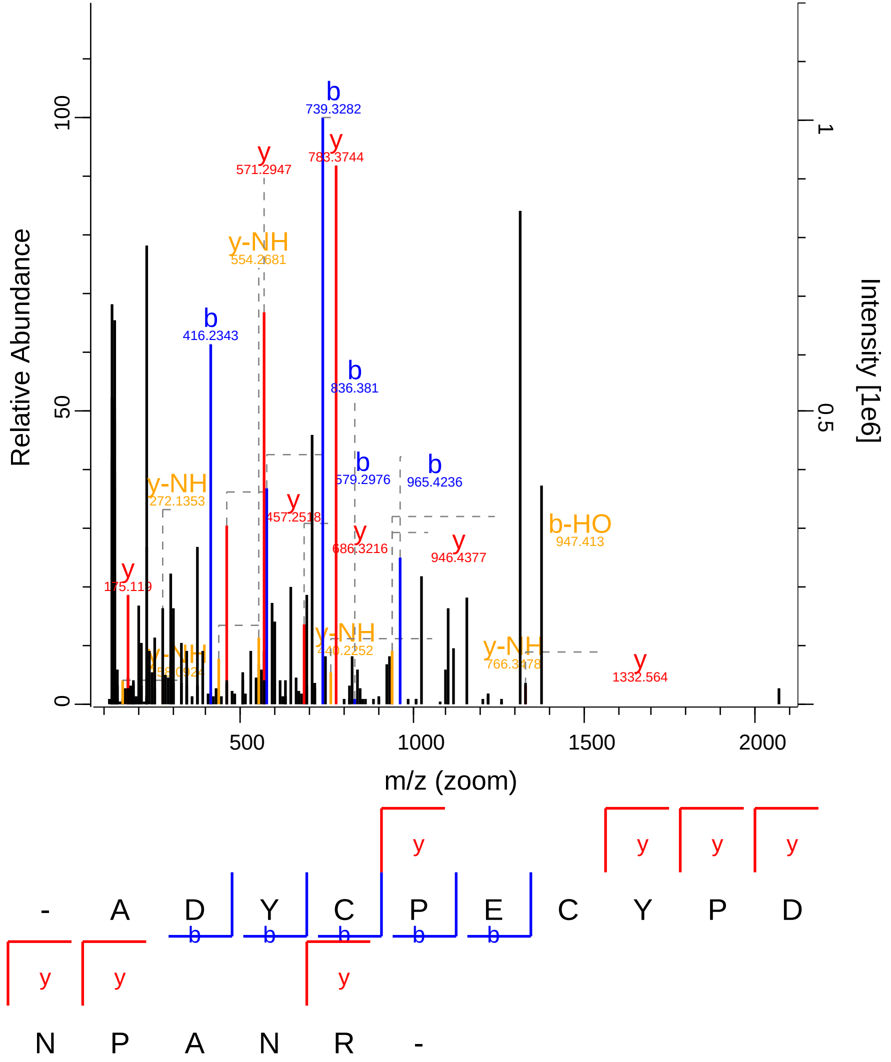

Raw File	Scan	Method	Score	m/z	Gene names
RO-QC-160121_ZackD_SS_Set_01_Fractic	16381	FTMS; HC	126.19	1036.45	TOR1AIP2

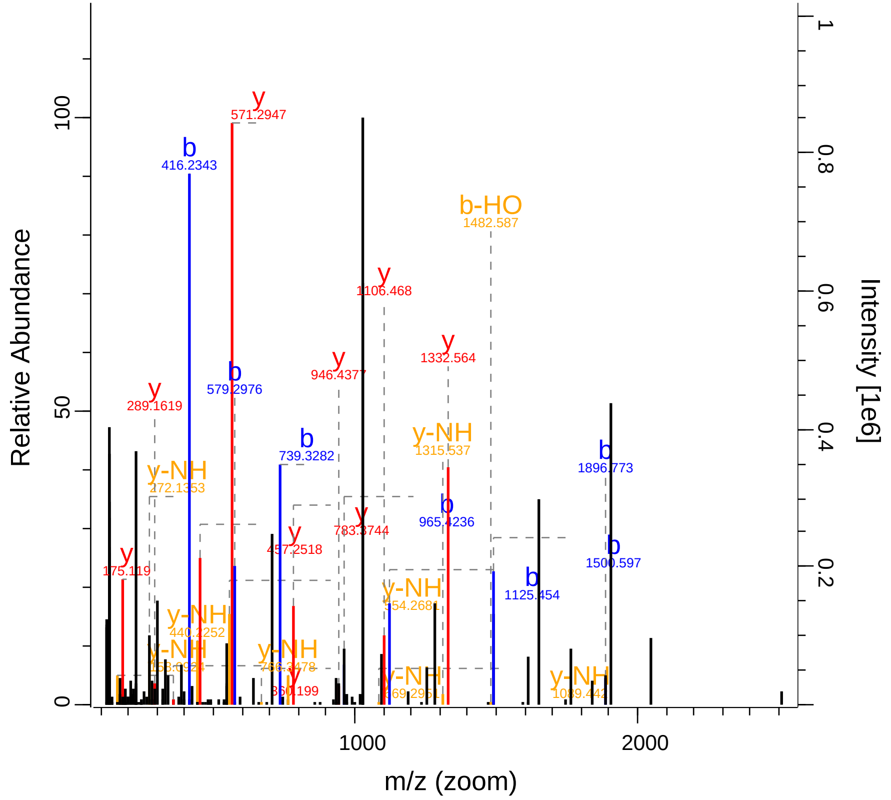

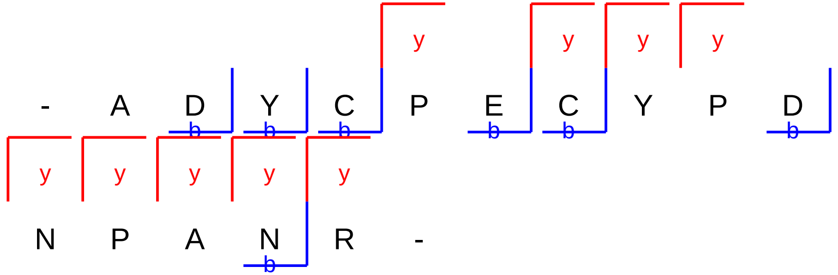

Raw File	Scan	Method	Score	m/z	Gene names
RO-QC-160121_ZackD_SS_Set_01_Fractio	16784	FTMS; HC	138.77	705.39	PQLC1

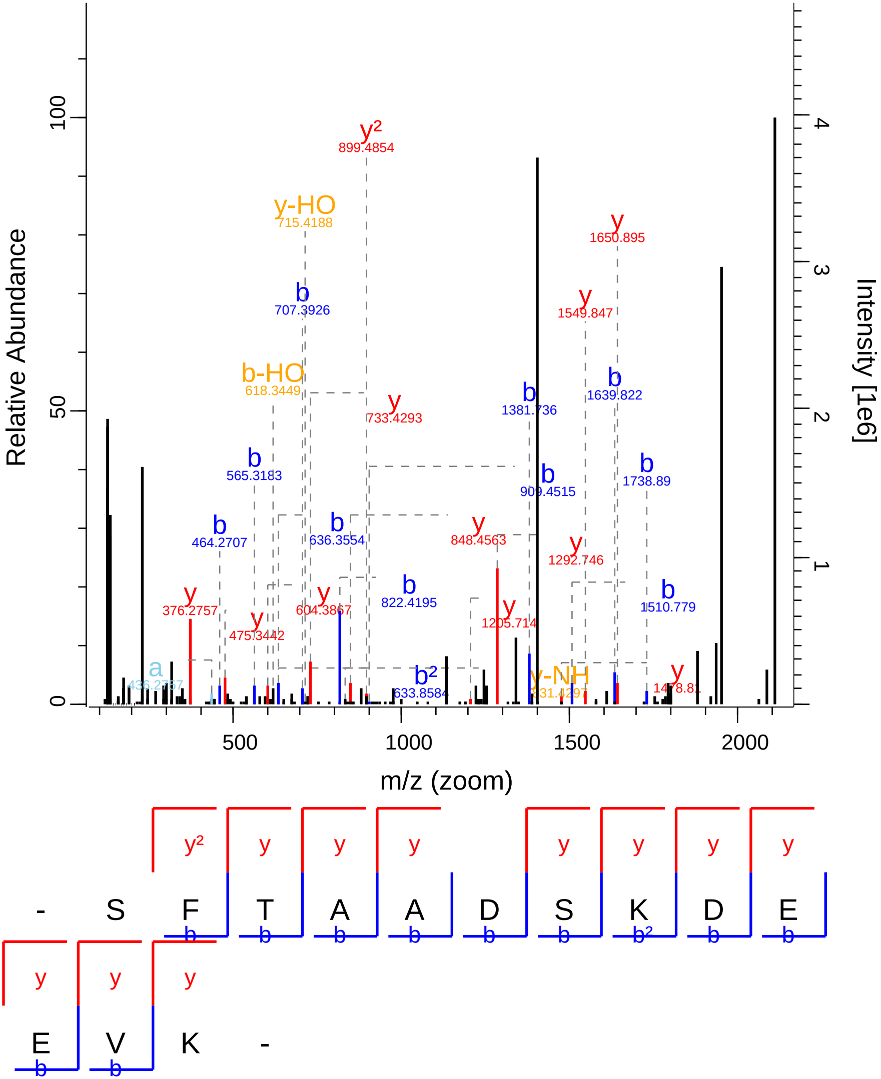

Raw File	Scan	Method	Score	m/z	Gene names
RO-QC-160121_ZackD_SS_Set_01_Fractio	17782	FTMS; HC	188.04	707.38	MFSD1

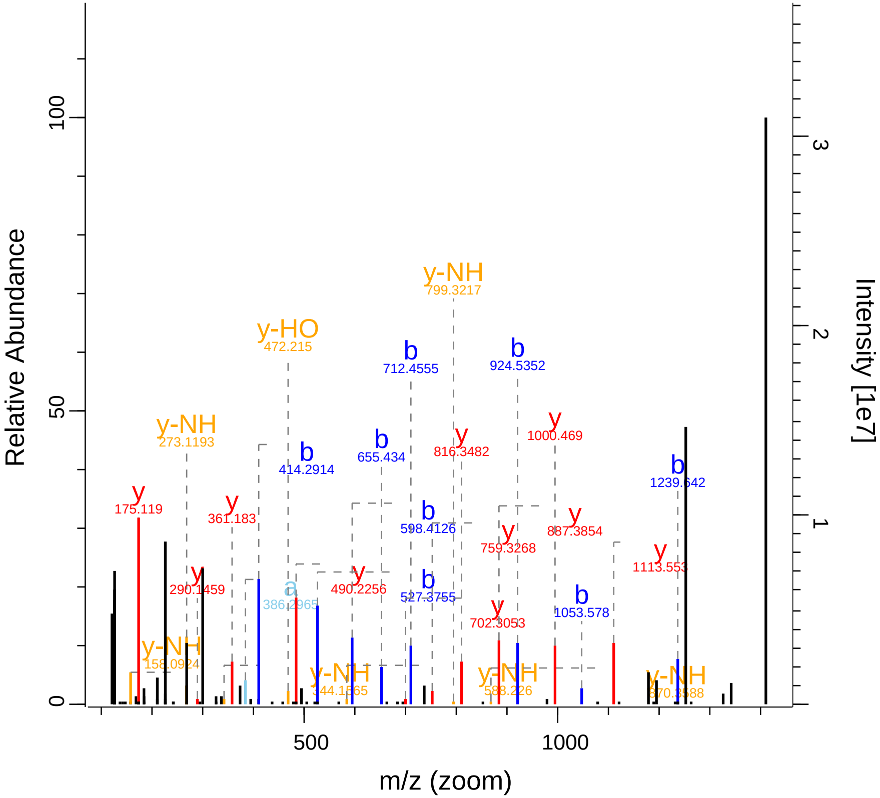

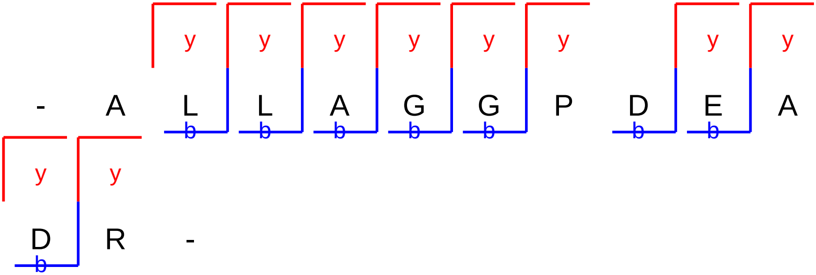

Raw File	Scan	Method	Score	m/z
RO-QC-160121_ZackD_SS_Set_01_Fraction_3	17874	FTMS; HCD	78.52	440.6

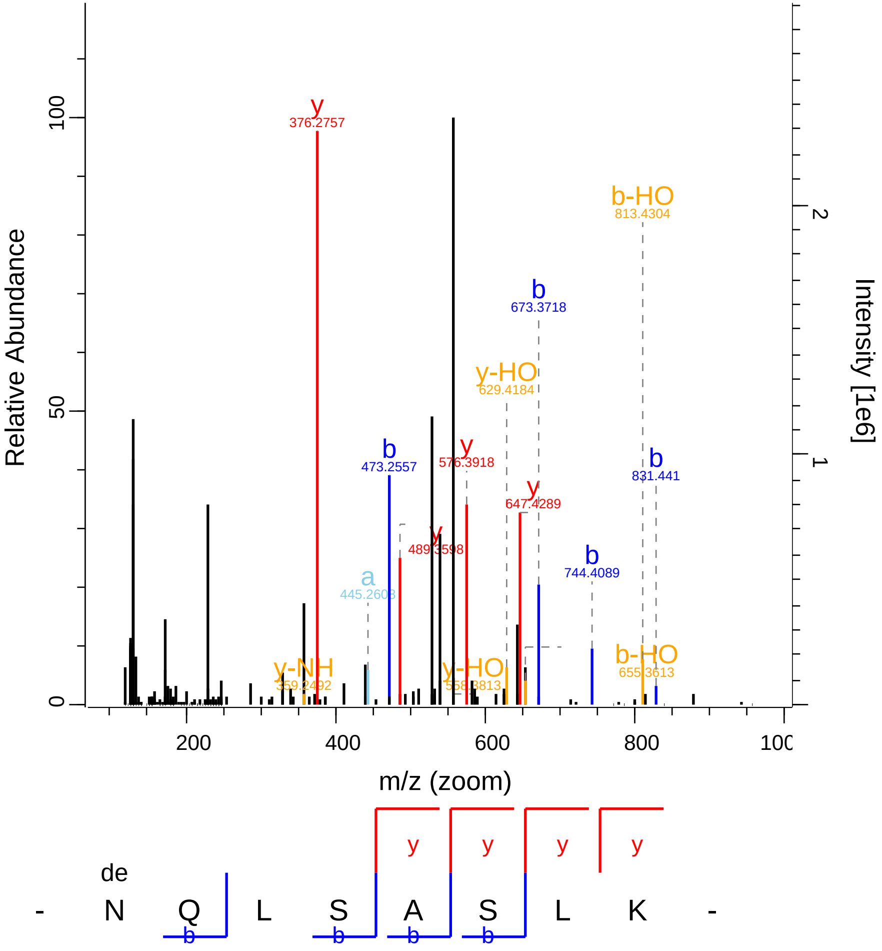

Raw File	Scan	Method	Score	m/z	Gene names
RO-QC-160121_ZackD_SS_Set_01_Fractio	18051	FTMS; HC	107.75	752.09	CERS1

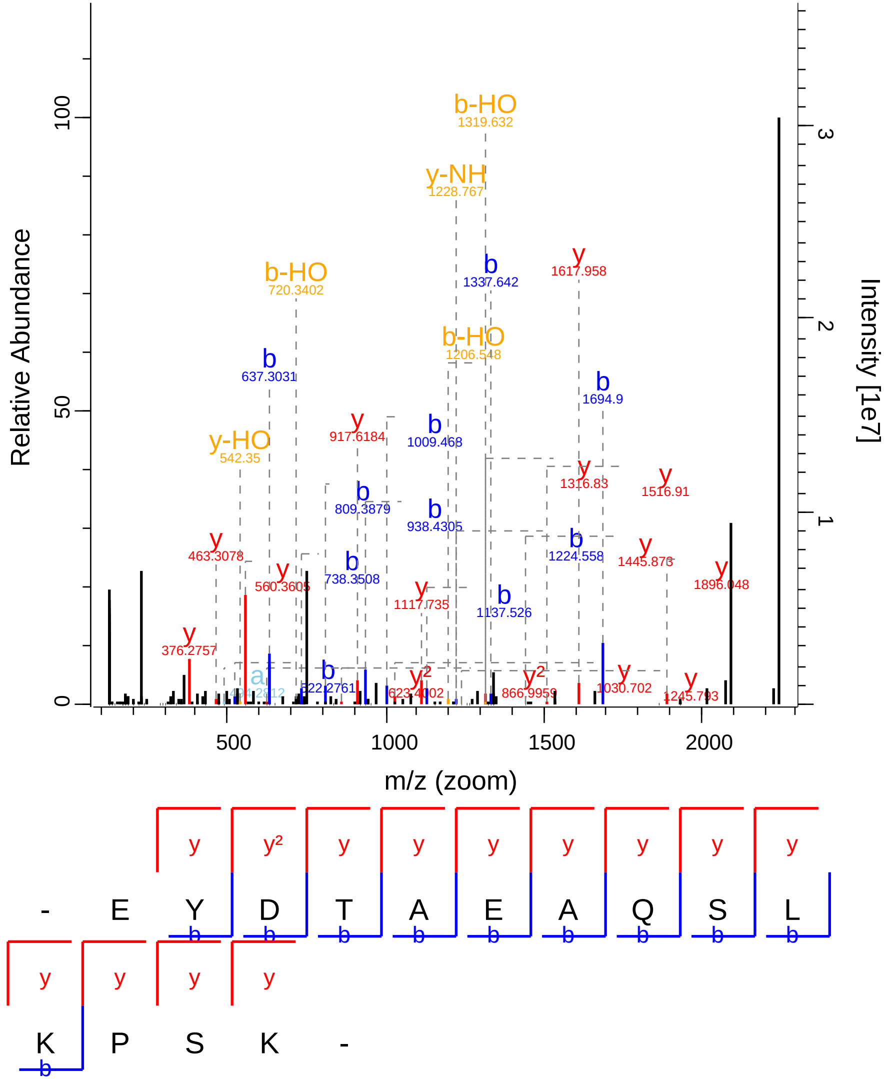

Raw File	Scan	Method	Score	m/z
RO-QC-160121_ZackD_SS_Set_01_Fraction_3	18077	FTMS; HCD	111.29	590.85

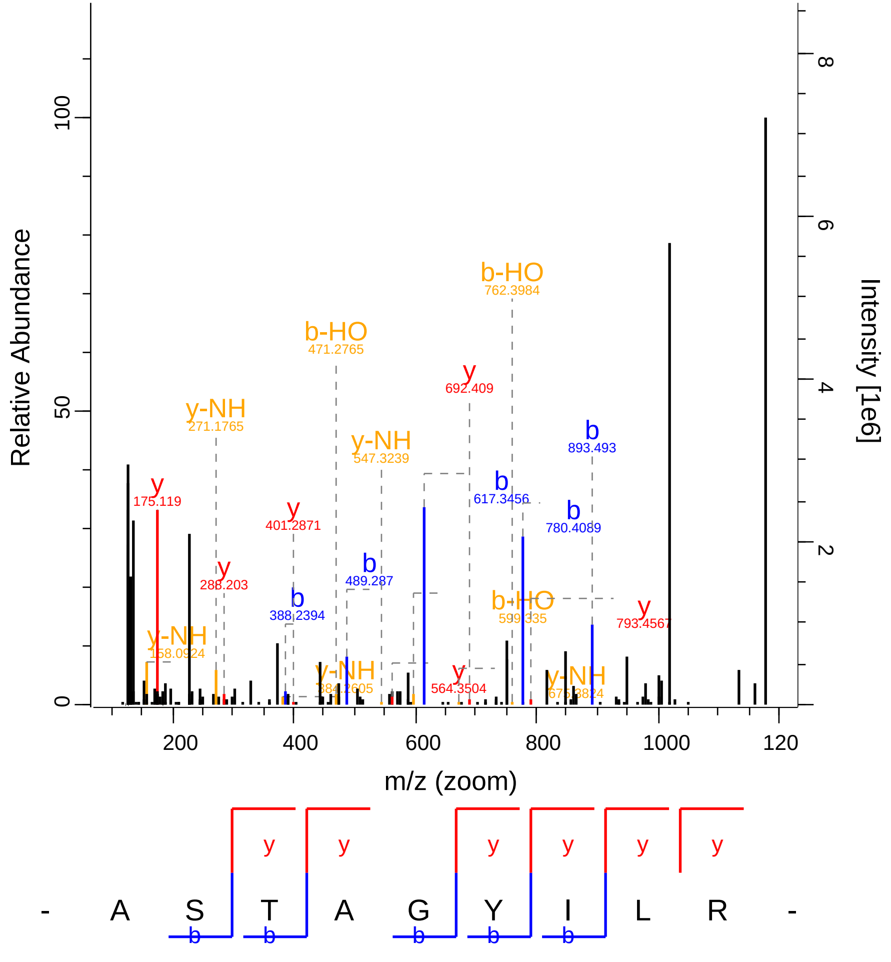

Raw File	Scan	Method	Score	m/z	Gene names
RO-QC-160121_ZackD_SS_Set_01_Fractio	18080	FTMS; HC	66.53	564.57	CERS1

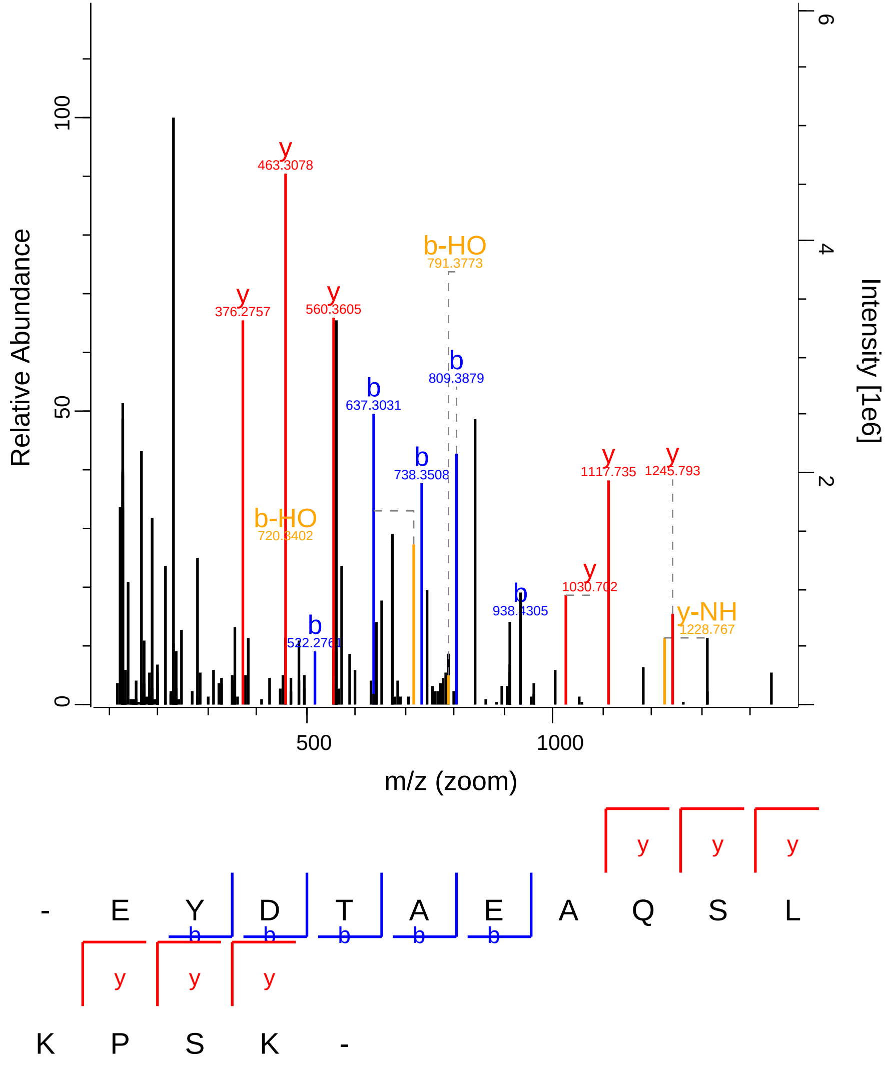

Raw File	Scan	Method	Score	m/z
RO-QC-160121_ZackD_SS_Set_01_Fraction_3	18725	FTMS; HCD	61.78	454.25

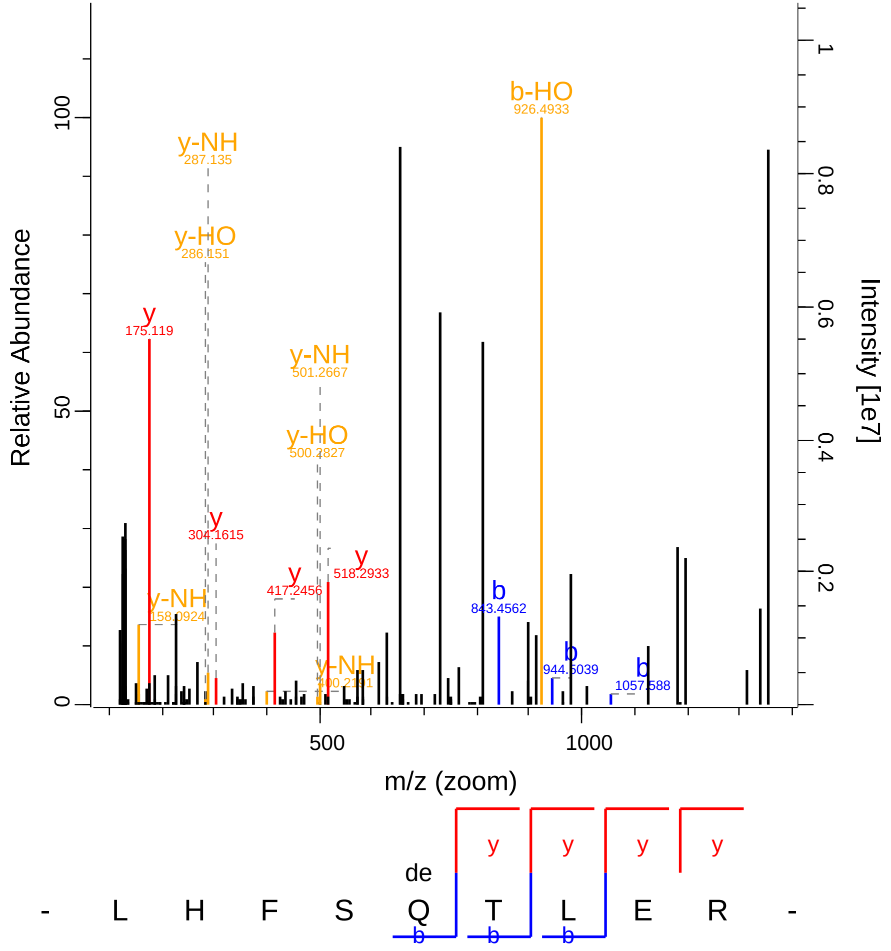

Raw File	Scan	Method	Score	m/z	Gene names
RO-QC-160121_ZackD_SS_Set_01_Fractio	18916	FTMS; HC	95.5	452.58	HBB;HBD

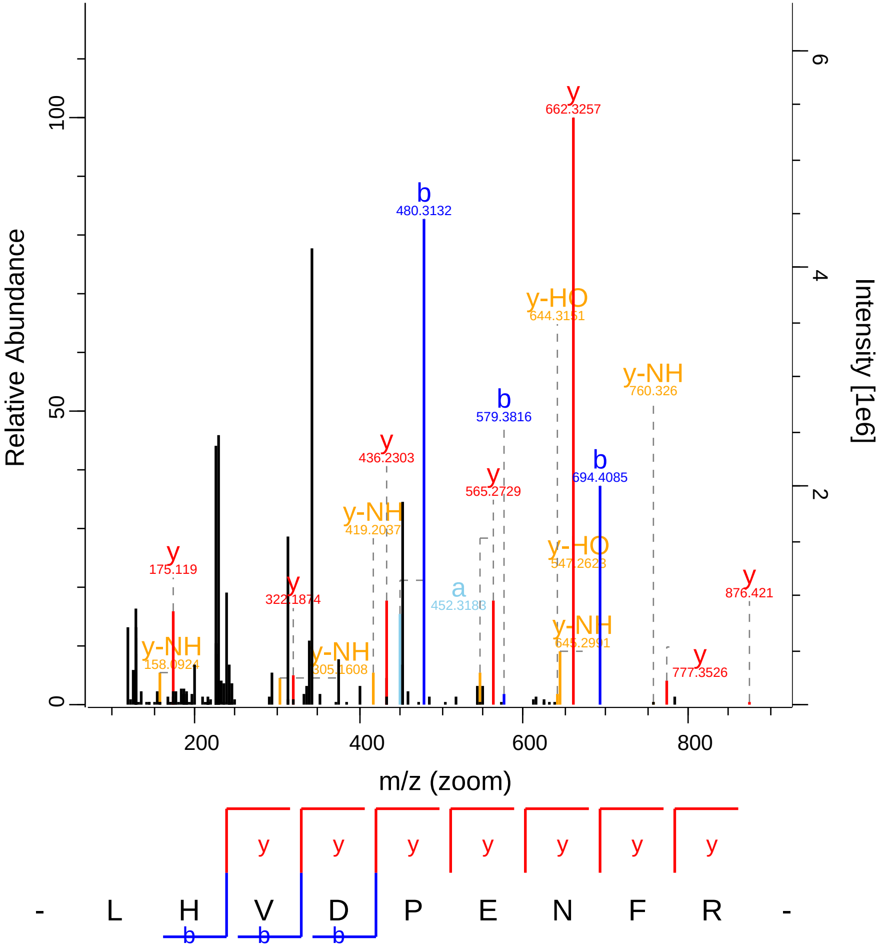

Raw File	Scan	Method	Score	m/z	Gene names
RO-QC-160121_ZackD_SS_Set_01_Fractior	19123	FTMS; HCI	47.09	800.1	ATP6V0C

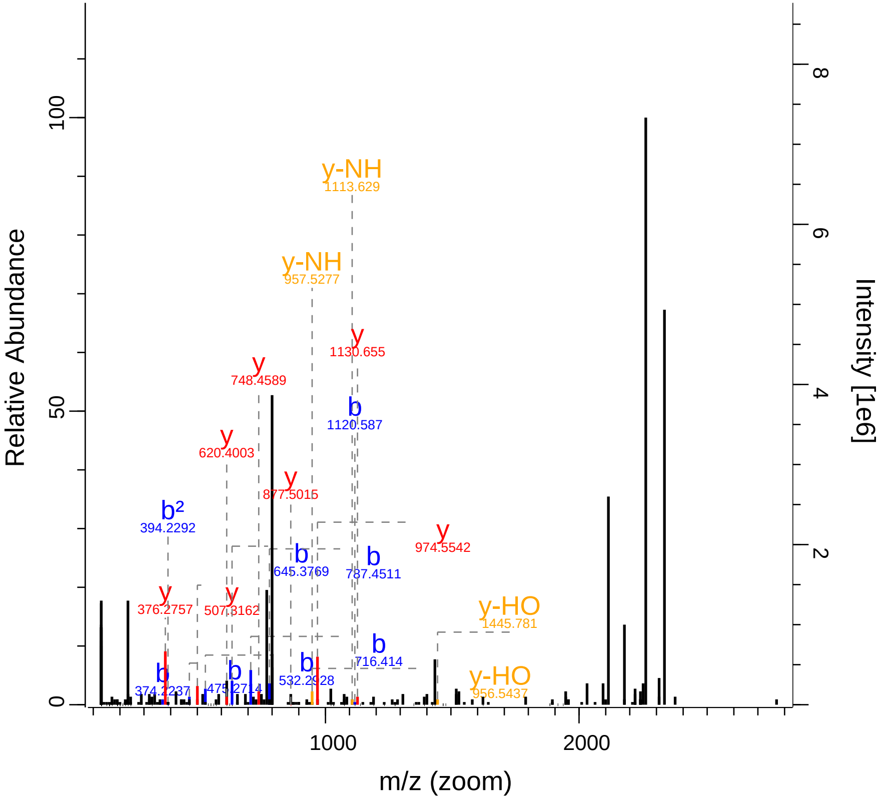

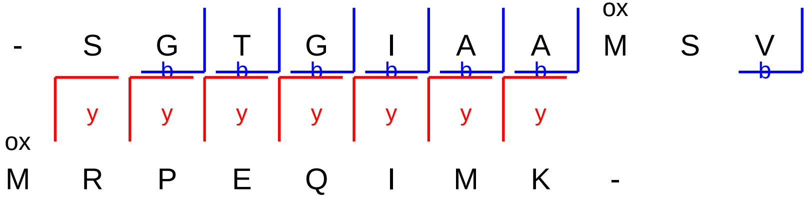

Raw File	Scan	Method	Score	m/z	Gene names
RO-QC-160121_ZackD_SS_Set_01_Fractio	19471	FTMS; HC	122.45	657.02	FGF1

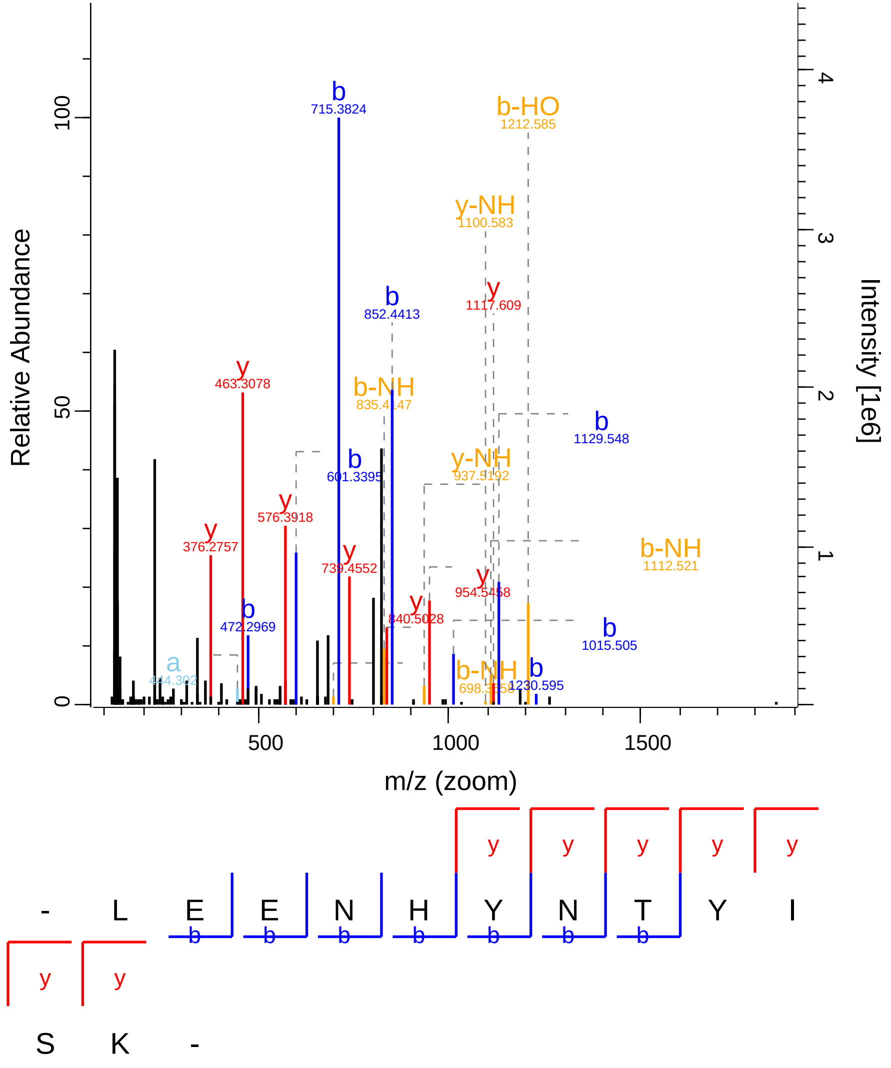

Raw File	Scan	Method	Score	m/z	Gene names
RO-QC-160121_ZackD_SS_Set_01_Fractio	19658	FTMS; HC	150.09	913.11	SOD3

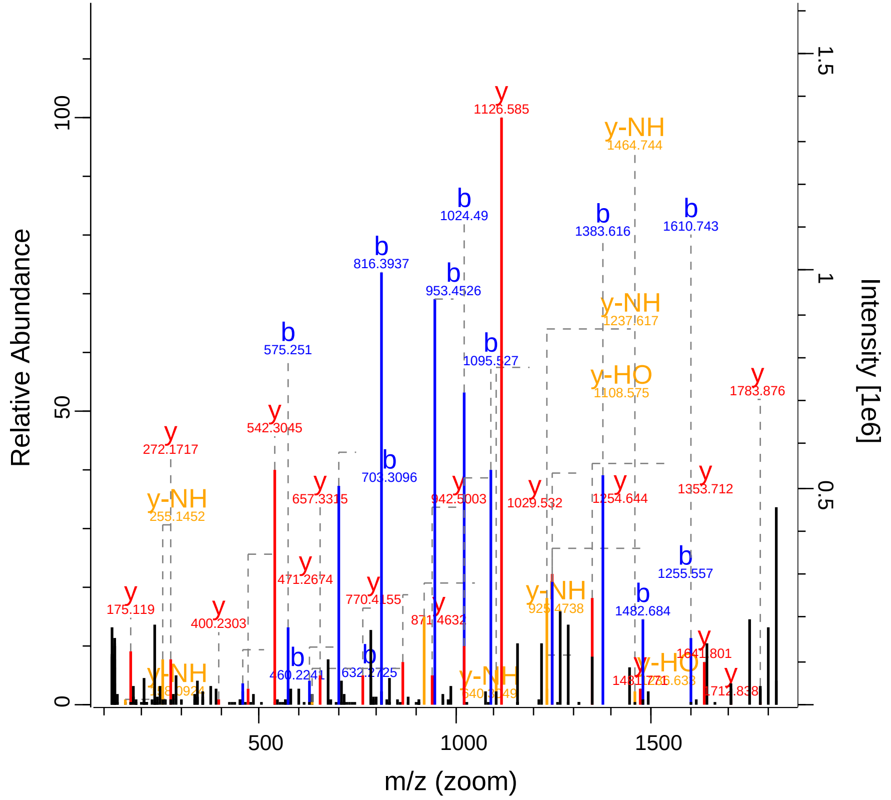

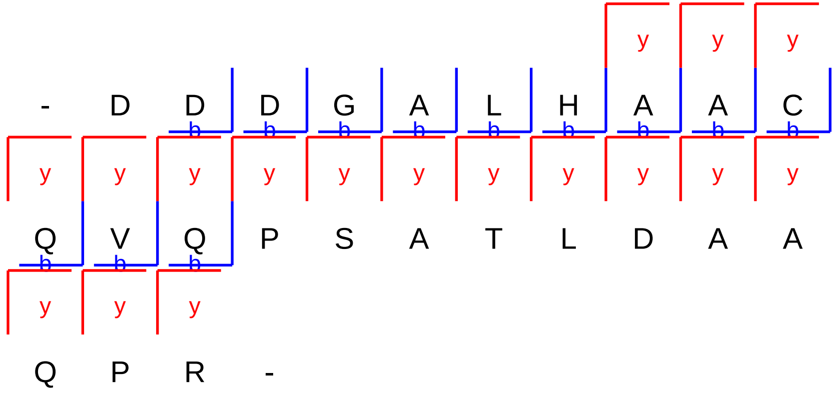

Raw File	Scan	Method	Score	m/z	Gene names
RO-QC-160121_ZackD_SS_Set_01_Fractioı	20356	FTMS; HCI	137.89	498.3	SIPA1L2

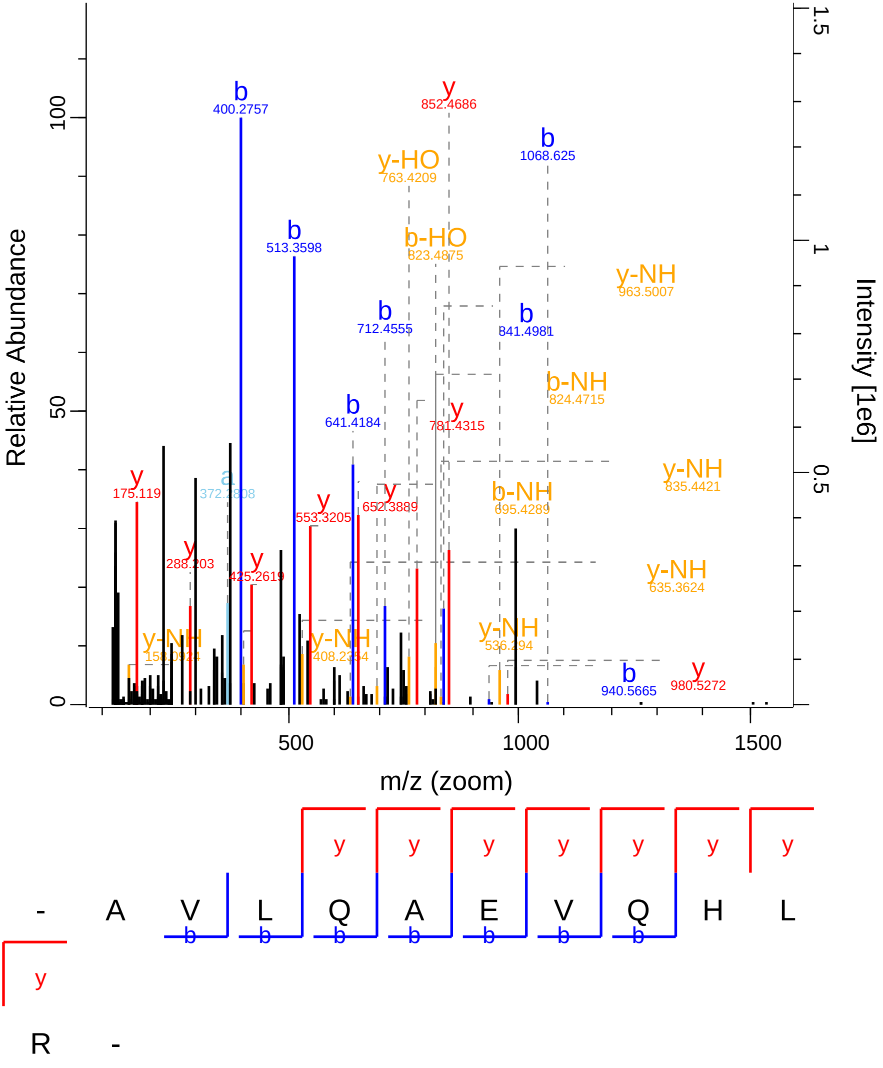

Raw File	Scan	Method	Score	m/z	Gene names
RO-QC-160121_ZackD_SS_Set_01_Fractio	20433	FTMS; HC	113.44	781.46	PASK

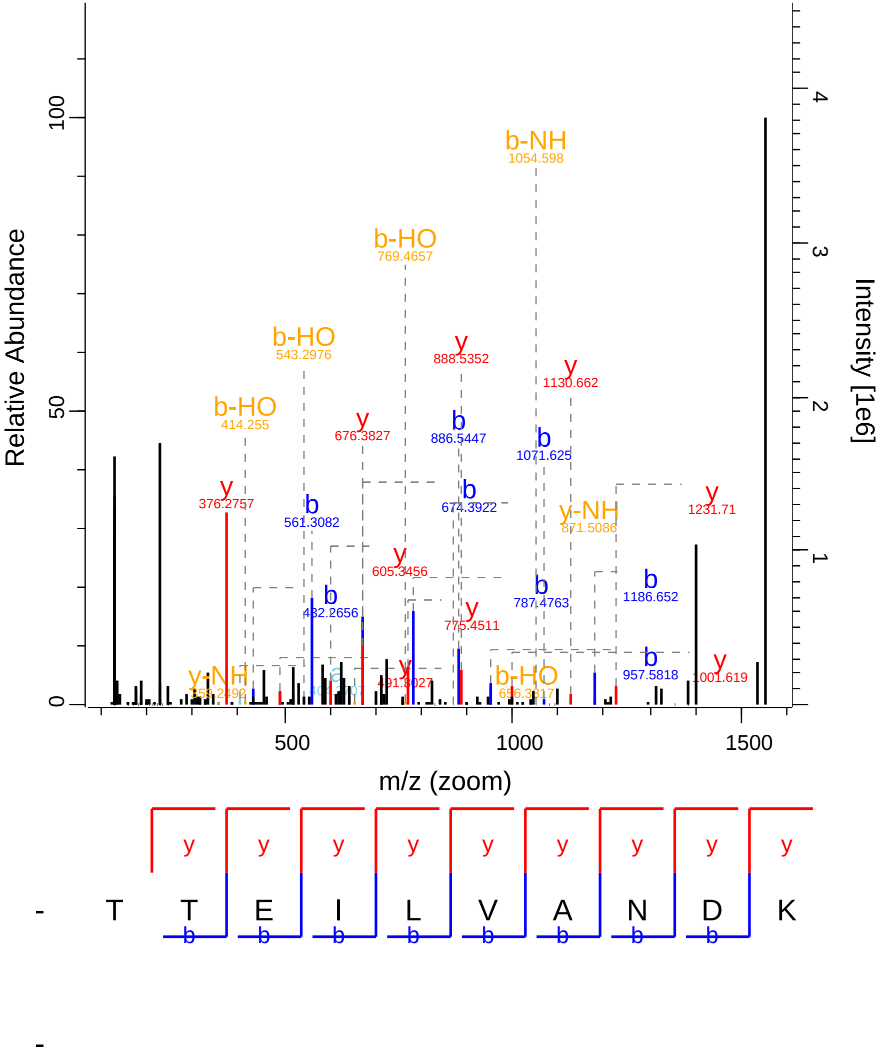

Raw File	Scan	Method	Score	m/z	Gene names
RO-QC-160121_ZackD_SS_Set_01_Fractio	21092	FTMS; HC	108.98	741.44	KRT80

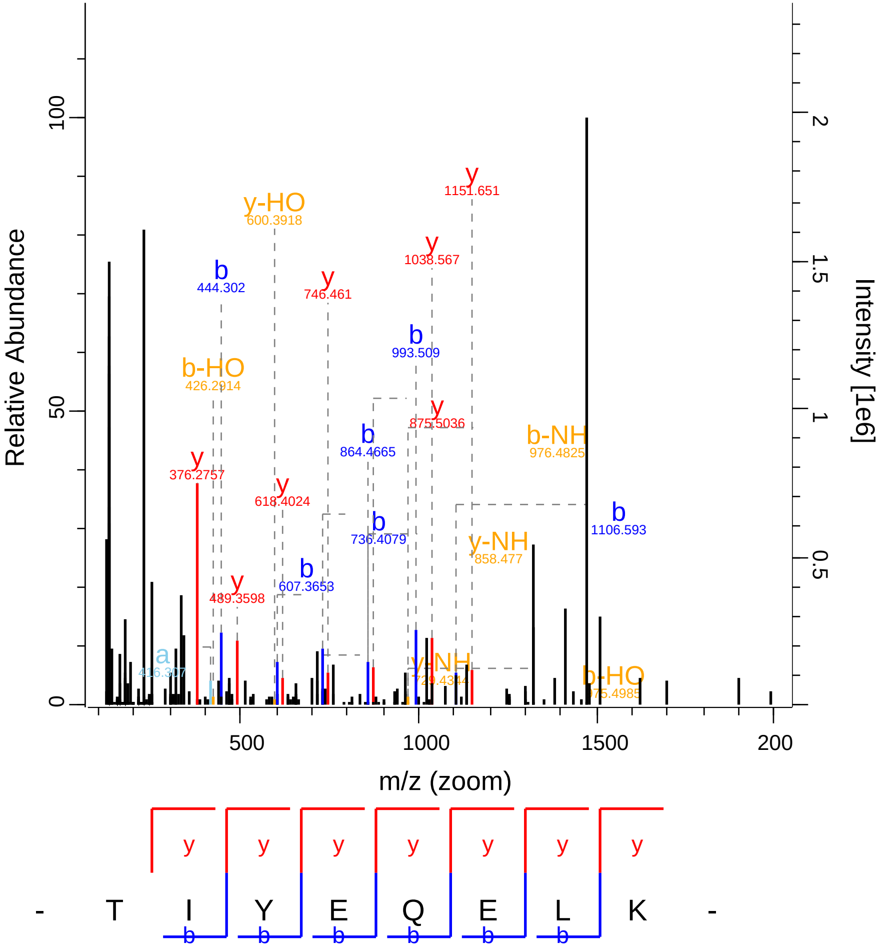

Raw File	Scan	Method	Score	m/z	Gene names
RO-QC-160121_ZackD_SS_Set_01_Fractio	21112	FTMS; HC	73.93	637.36	RHOBTB3

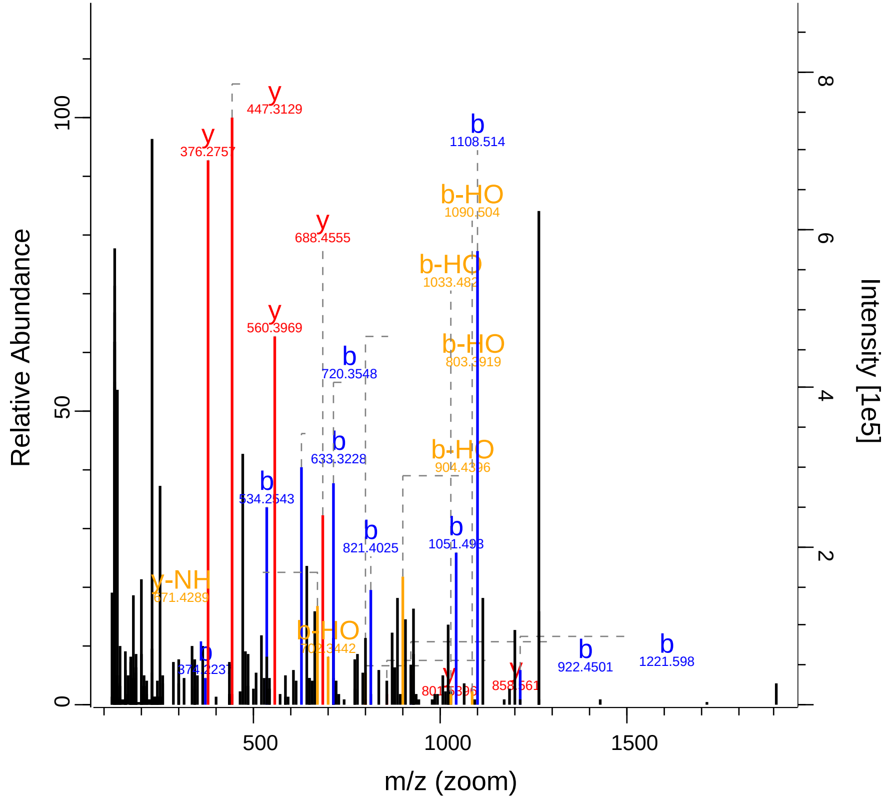

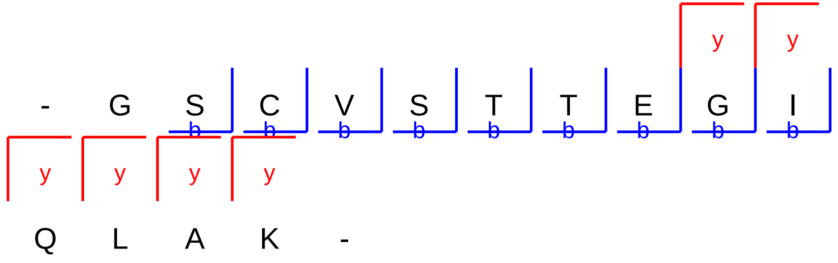

Raw File	Scan	Method	Score	m/z	Gene names
RO-QC-160121_ZackD_SS_Set_01_Fractio	21140	FTMS; HC	101.71	955.03	RHOBTB3

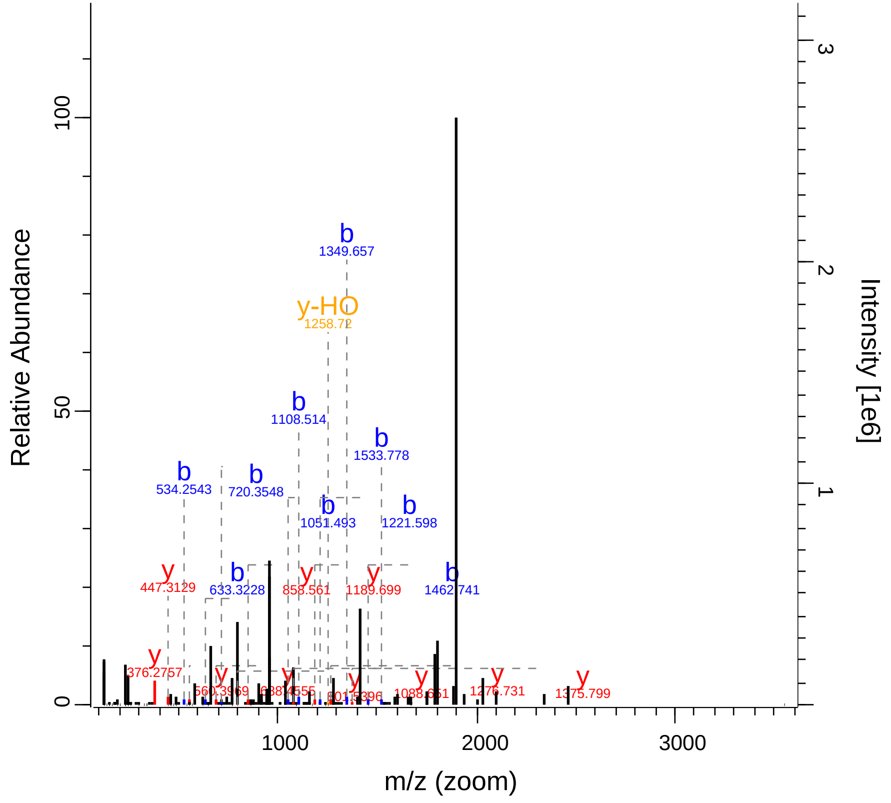

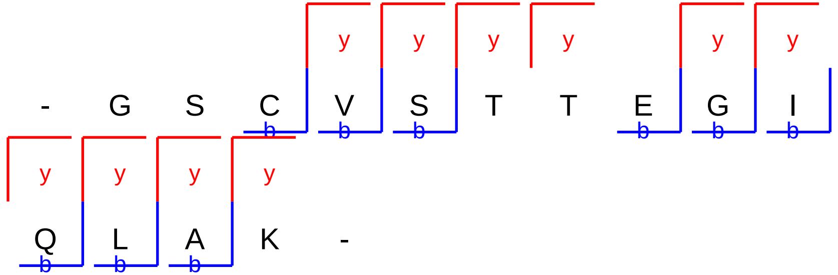

Raw File	Scan	Method	Score	m/z
RO-QC-160121_ZackD_SS_Set_01_Fraction_3	21382	FTMS; HCD	49.42	874.5

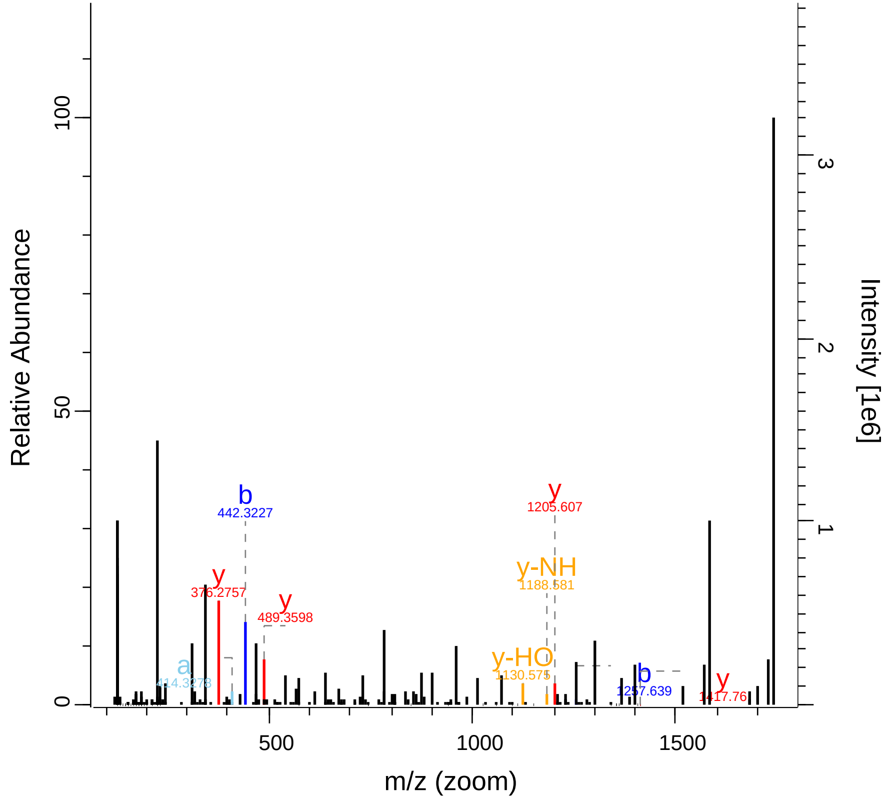

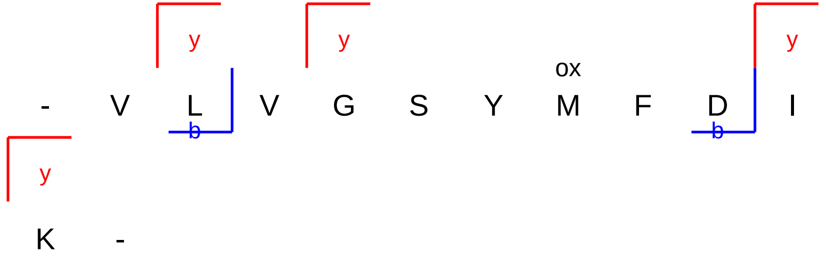

Raw File	Scan	Method	Score	m/z	Gene names
RO-QC-160121_ZackD_SS_Set_01_Fractio	22036	FTMS; HC	82.17	927.02	EPN2

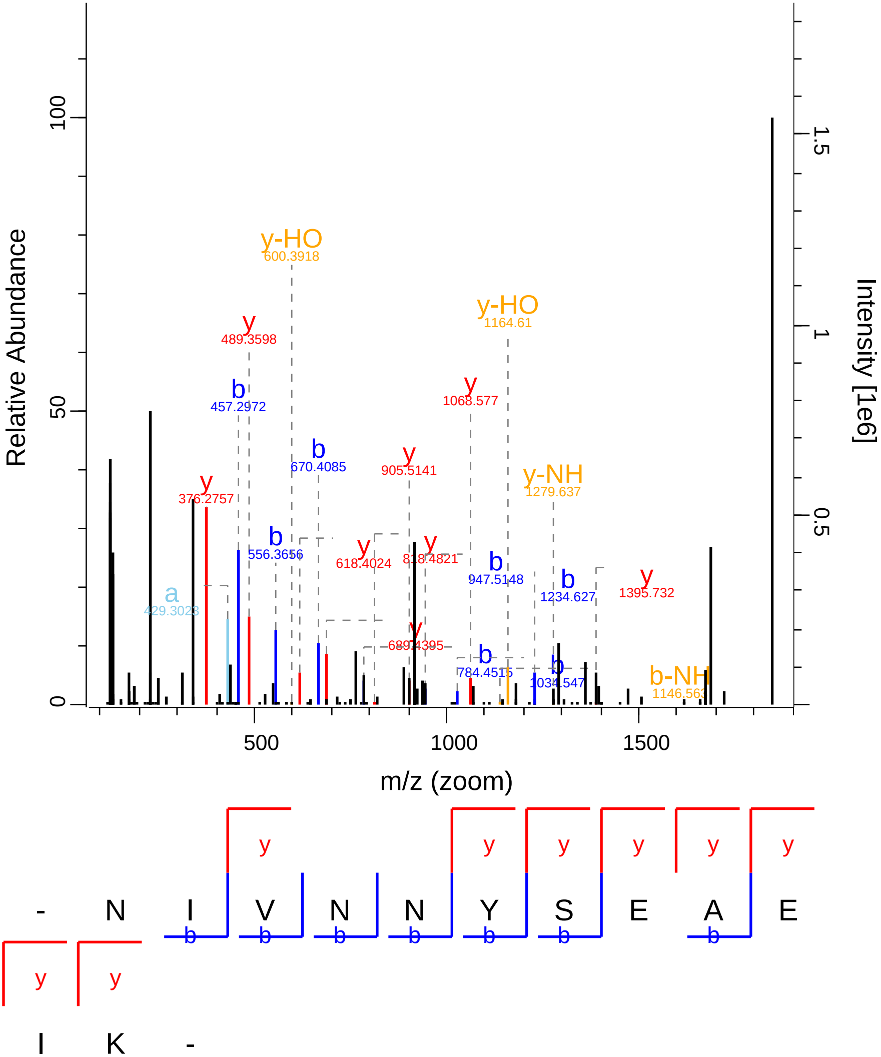

Raw File	Scan	Method	Score	m/z
RO-QC-160121_ZackD_SS_Set_01_Fraction_3	22705	FTMS; HCD	83.26	470.95

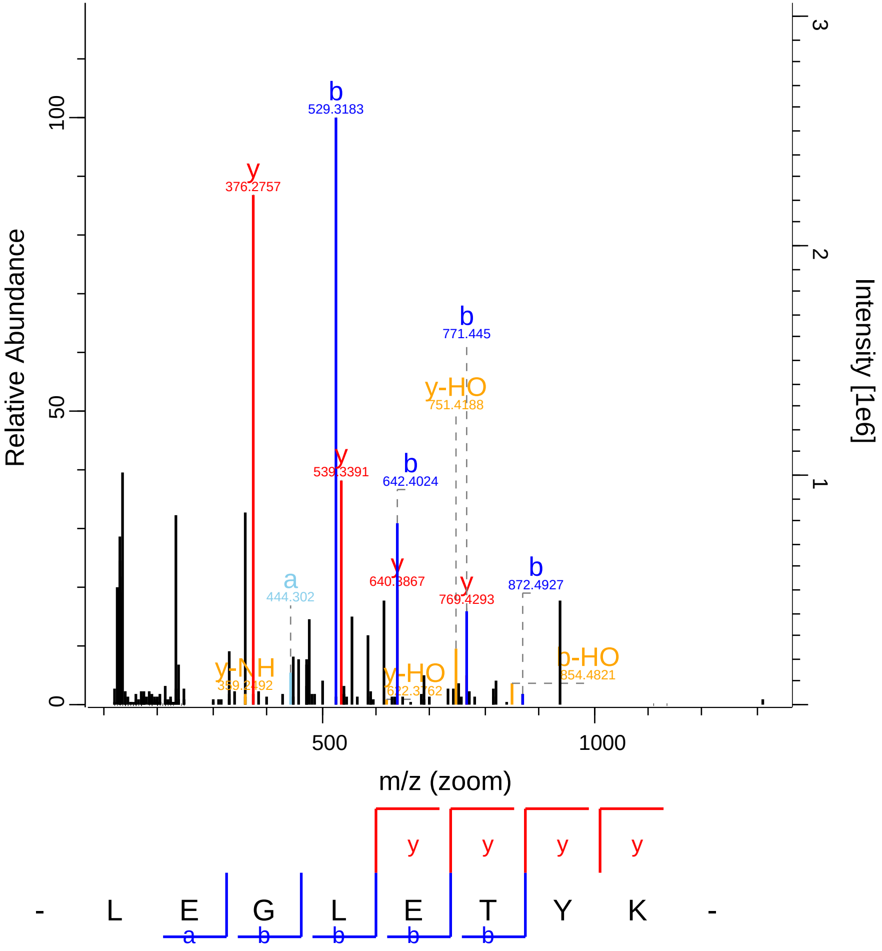

Raw File	Scan	Method	Score	m/z	Gene names
RO-QC-160121_ZackD_SS_Set_01_Fractio	23230	FTMS; HC	94.85	810.96	CASC5

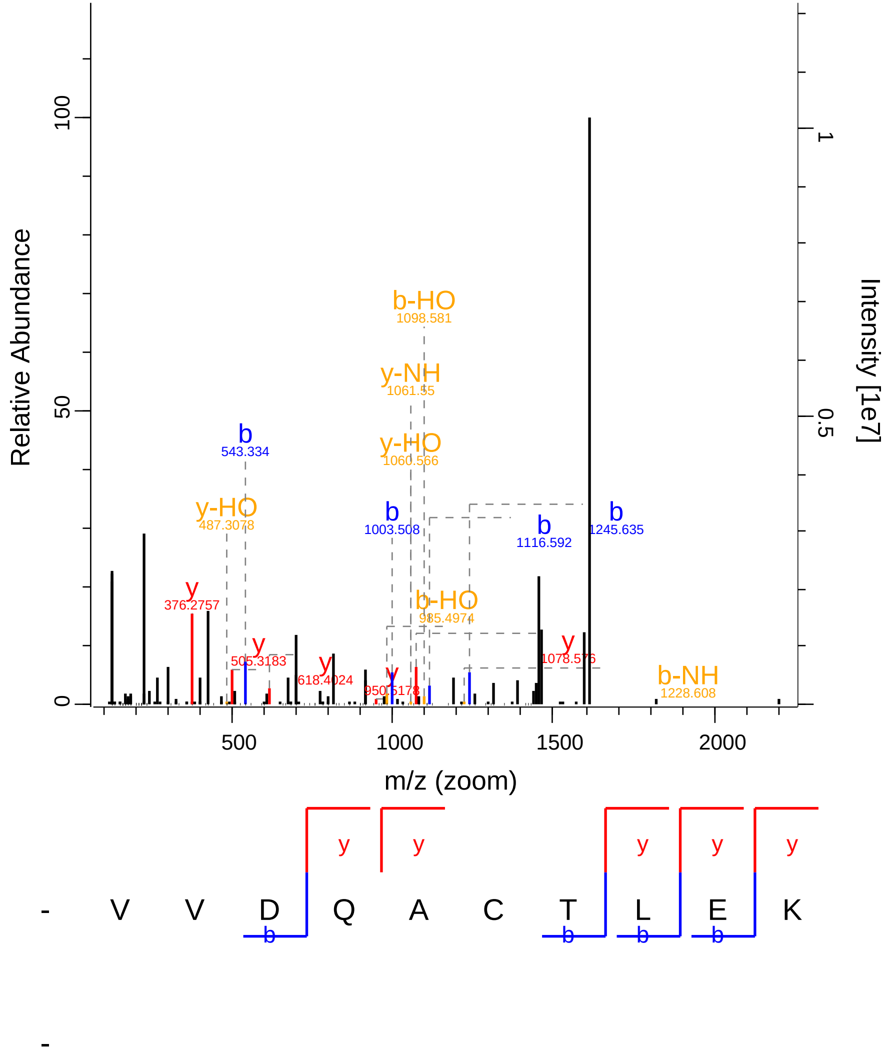

Raw File	Scan	Method	Score	m/z	Gene names
RO-QC-160121_ZackD_SS_Set_01_Fractio	23285	FTMS; HC	104.17	650.37	TRDMT1

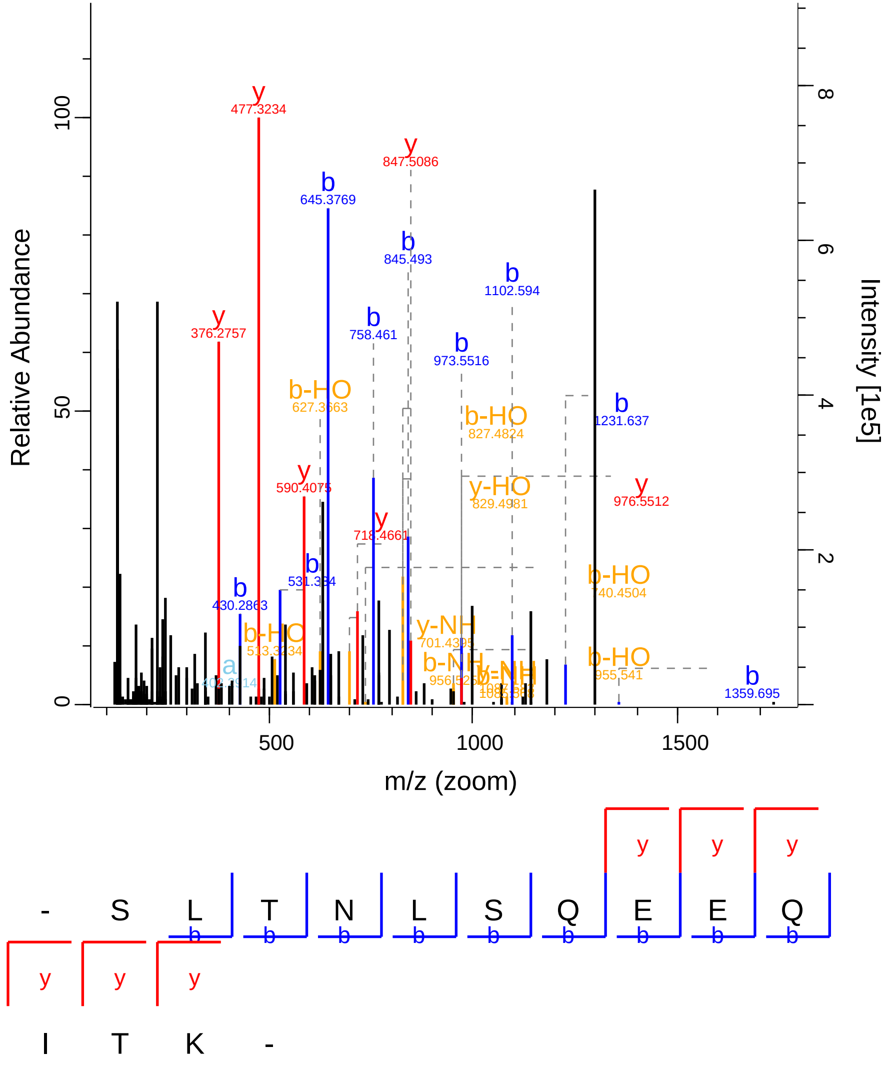

Raw File	Scan	Method	Score	m/z	Gene names
RO-QC-160121_ZackD_SS_Set_01_Fraction	23490	FTMS; HCI	89.3	829	TRDC

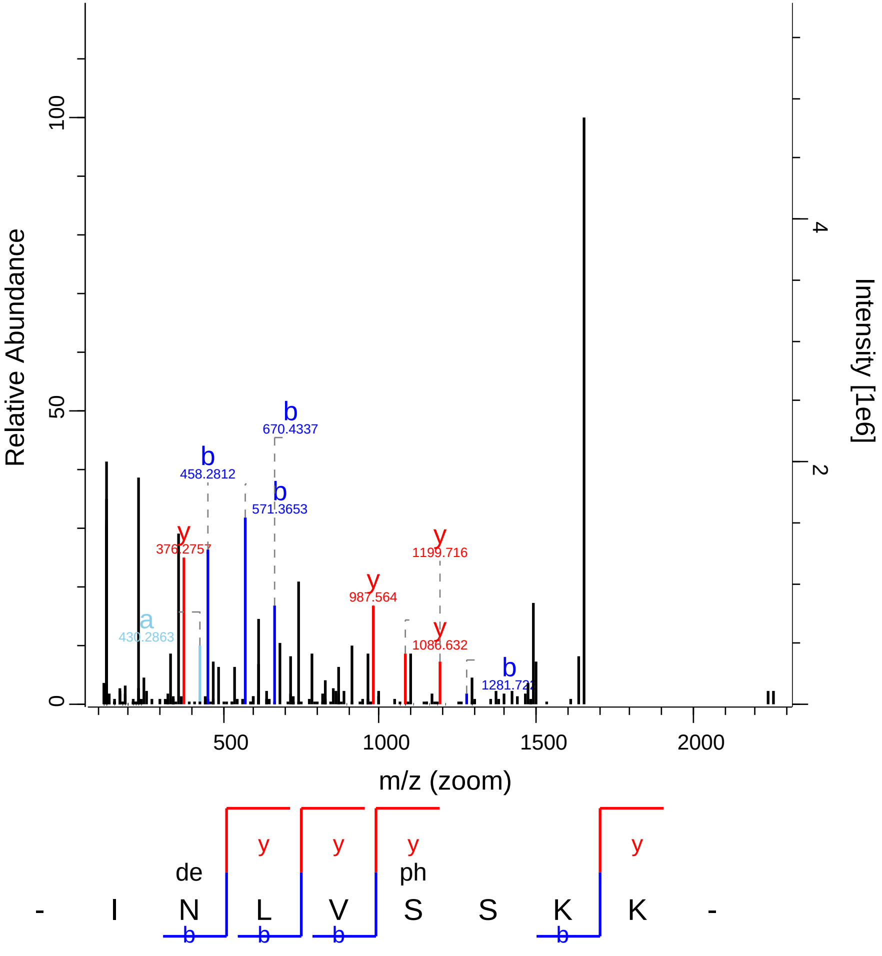

Raw File	Scan	Method	Score	m/z	Gene names
RO-QC-160121_ZackD_SS_Set_01_Fractio	23573	FTMS; HC	88.5	873.51	PGAP2

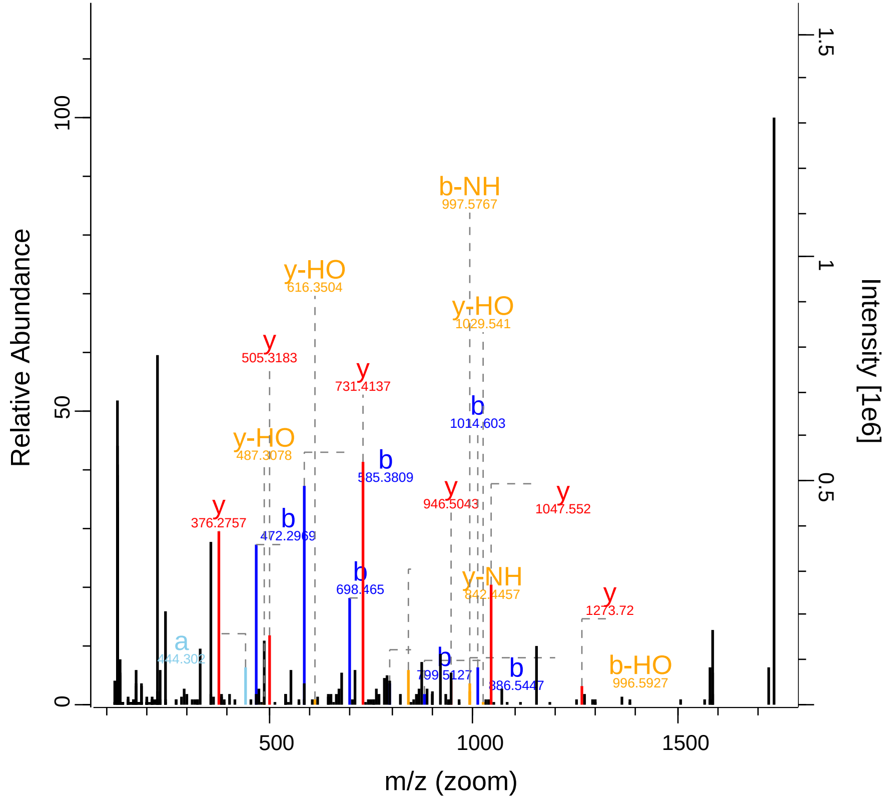

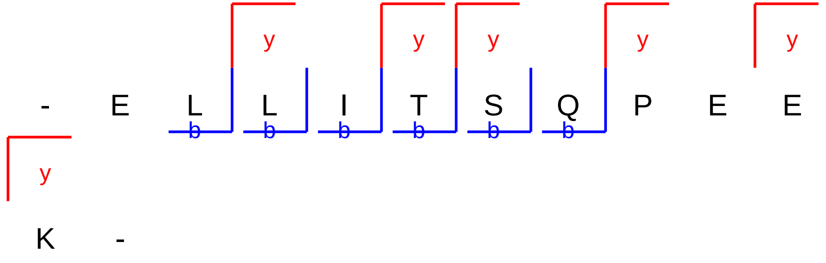

Raw File	Scan	Method	Score	m/z	Gene names
RO-QC-160121_ZackD_SS_Set_01_Fractio	23691	FTMS; HC	98.16	552.65	FKBPL

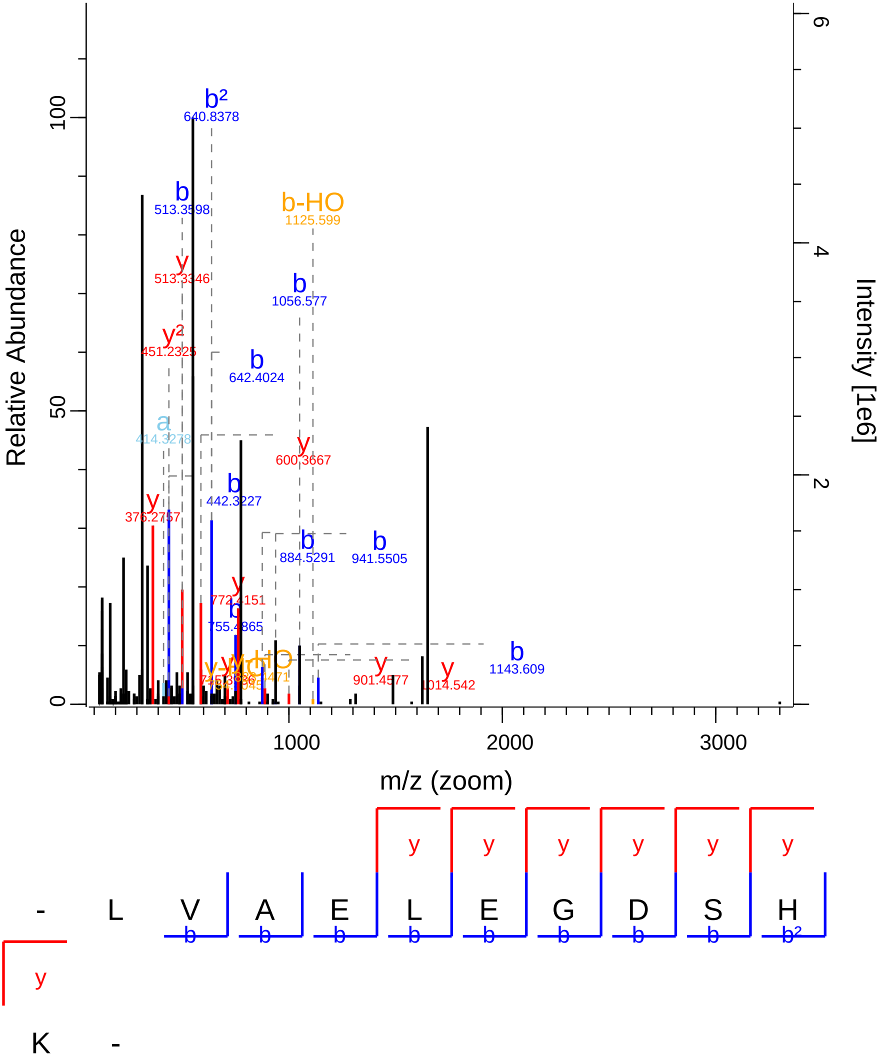

Raw File	Scan	Method	Score	m/z	Gene names
RO-QC-160121_ZackD_SS_Set_01_Fractio	23701	FTMS; HC	115.18	582.34	PGAP2

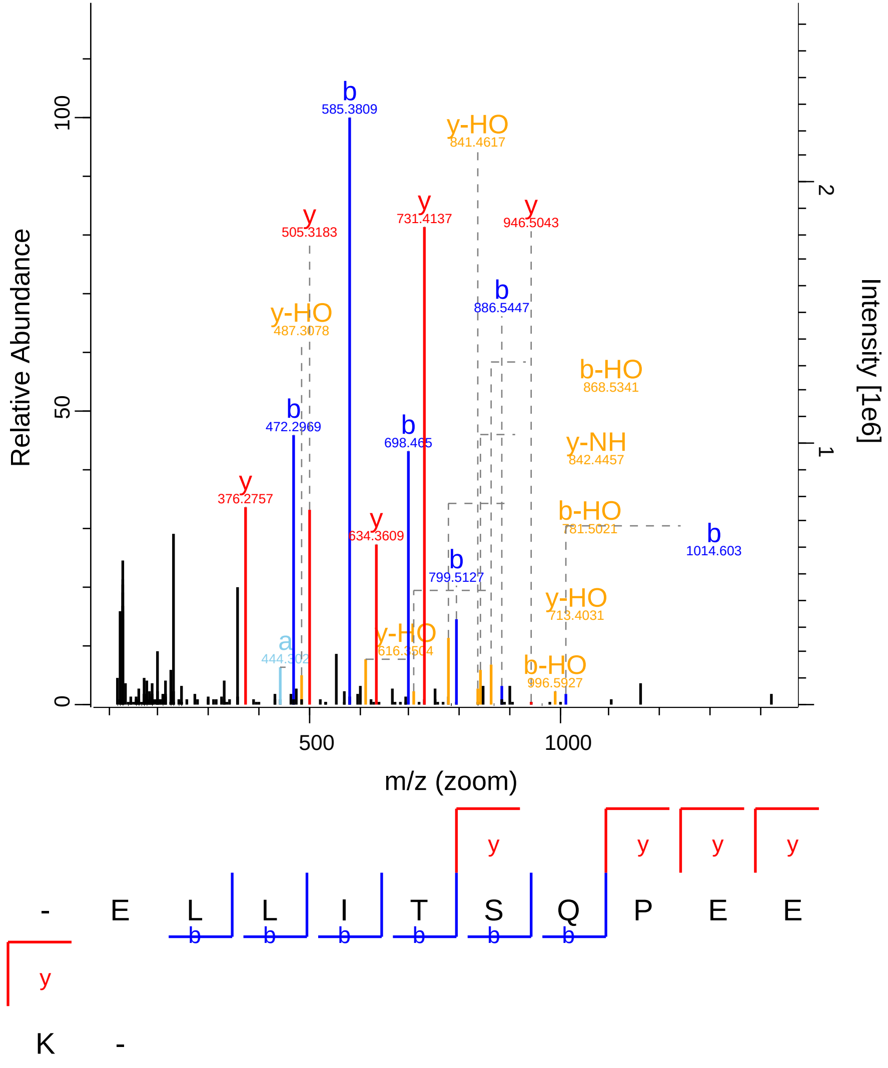

Raw File	Scan	Method	Score	m/z	Gene names
RO-QC-160121_ZackD_SS_Set_01_Fractio	24236	FTMS; HC	100.79	514.26	APOBEC3B

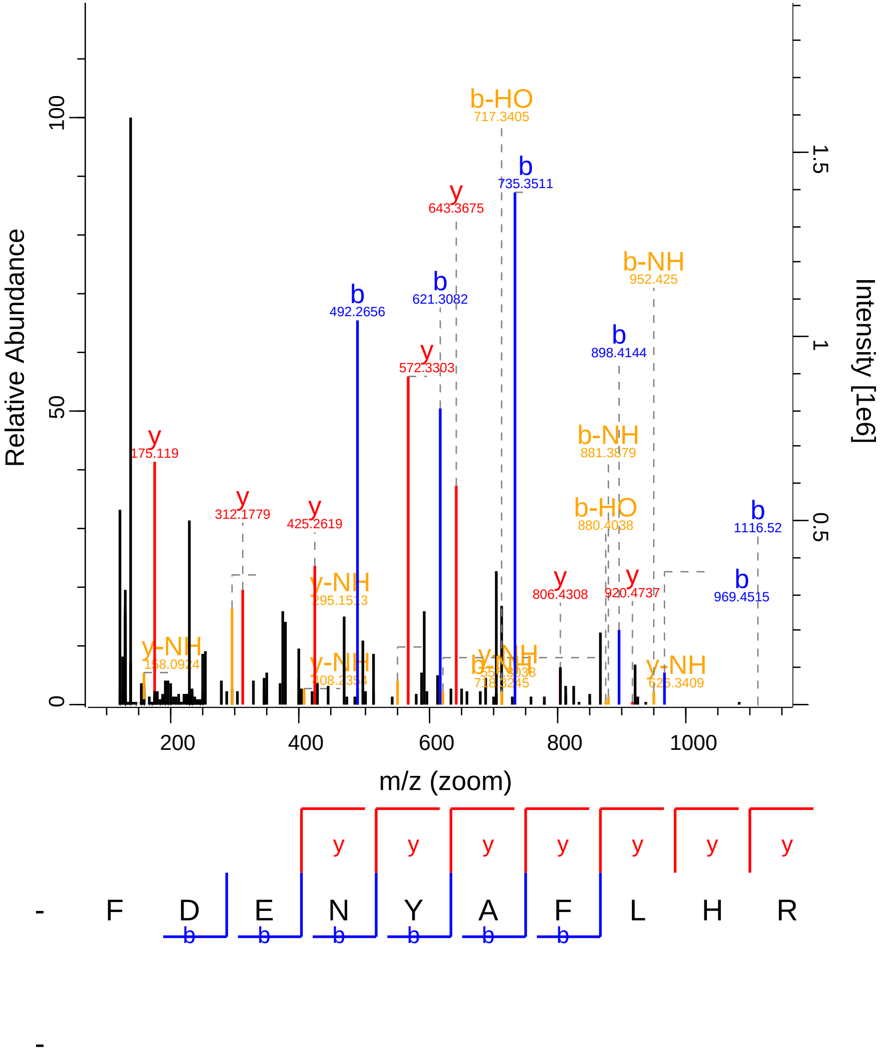

Raw File	Scan	Method	Score	m/z	Gene names
RO-QC-160121_ZackD_SS_Set_01_Fractio	24386	FTMS; HC	99	473.62	CD14

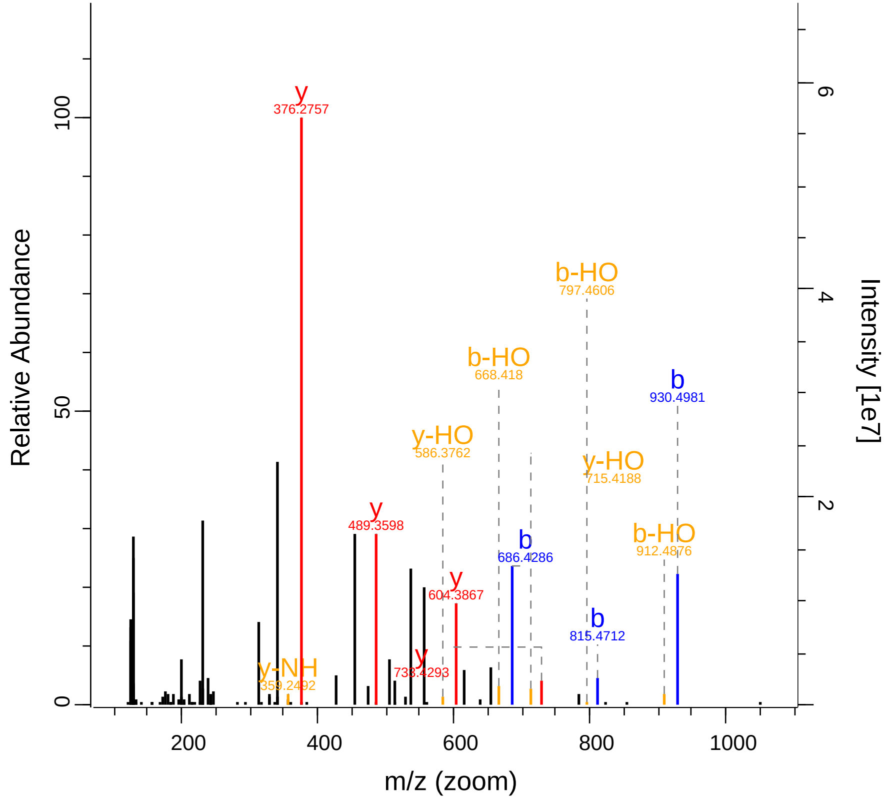

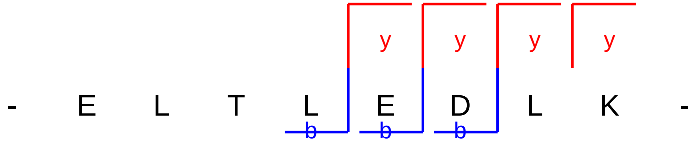

Raw File	Scan	Method	Score	m/z	Gene names
RO-QC-160121_ZackD_SS_Set_01_Fractio	24473	FTMS; HC	42.56	739.04	TRPM6

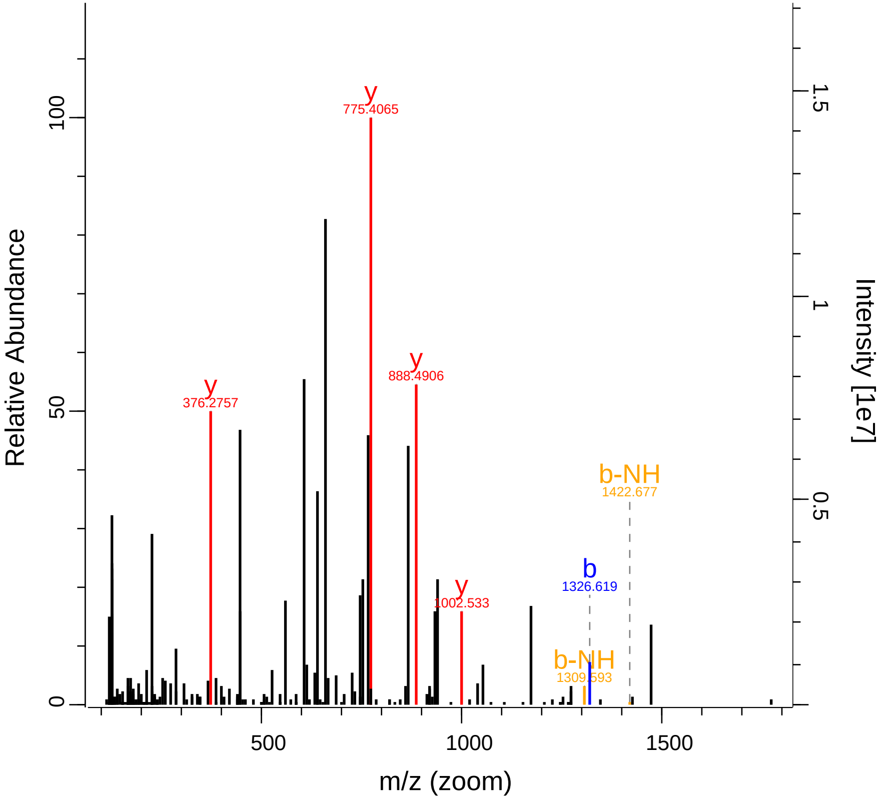

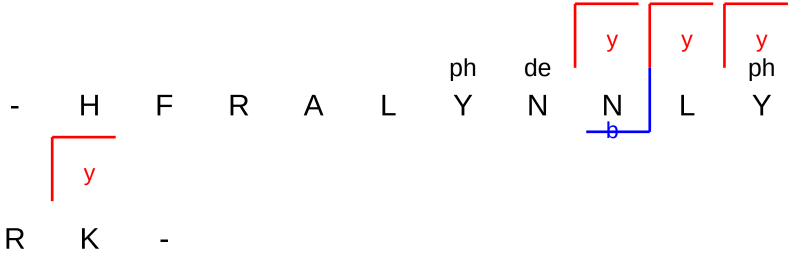

Raw File	Scan	Method	Score	m/z	Gene names
RO-QC-160121_ZackD_SS_Set_01_Fractio	24571	FTMS; HC	141.58	915.52	PRRX1

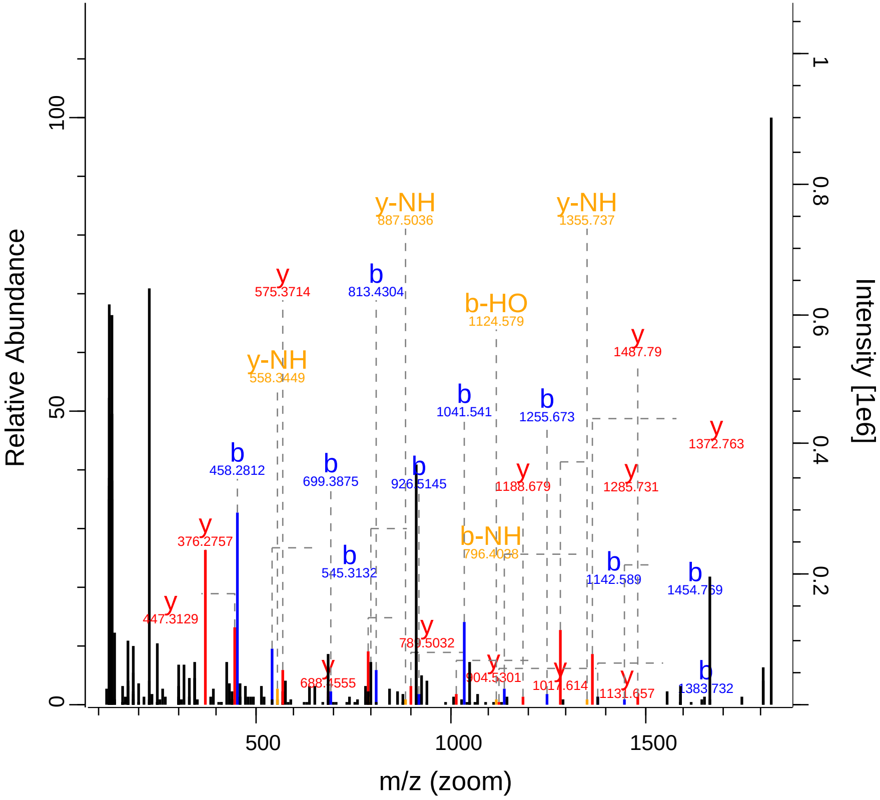

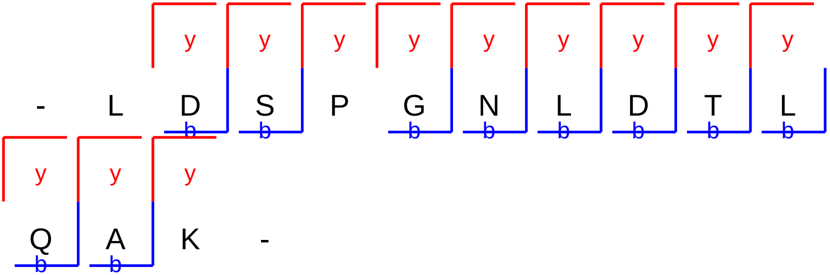

Raw File	Scan	Method	Score	m/z
RO-QC-160121_ZackD_SS_Set_01_Fraction_3	24670	FTMS; HCD	75.38	911.02

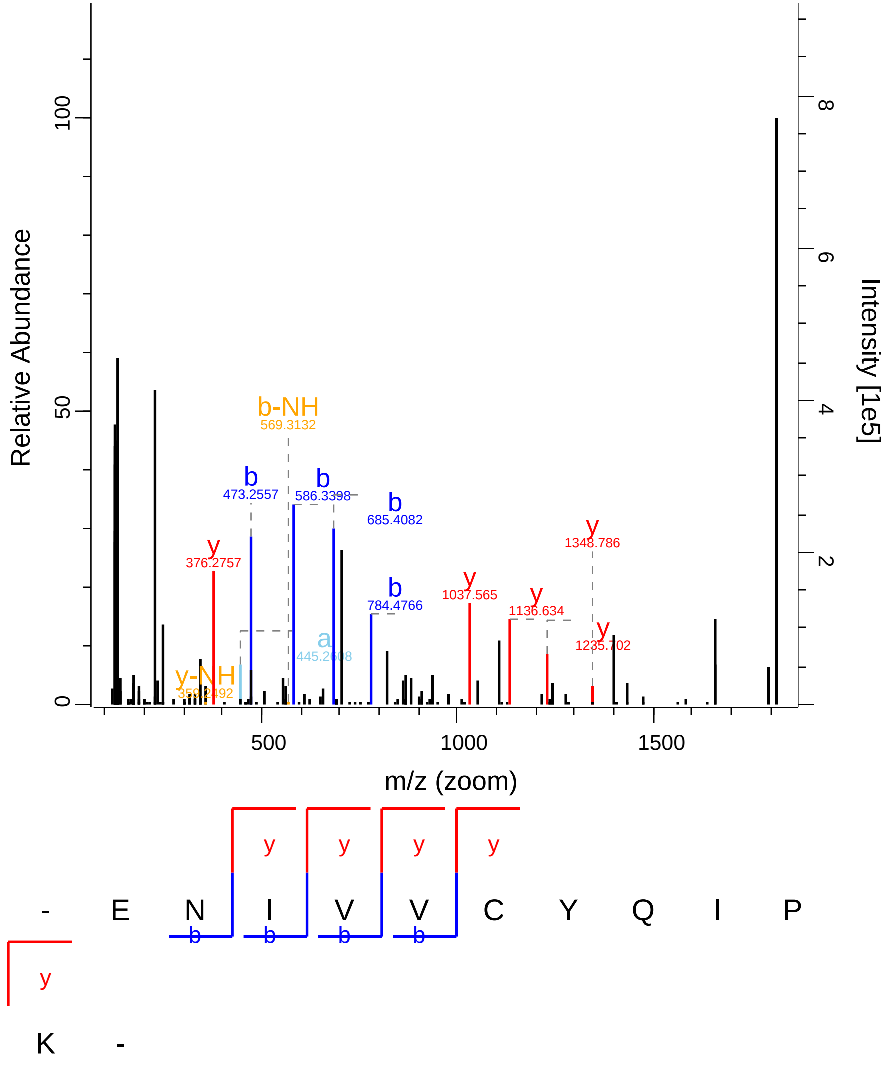

Raw File	Scan	Method	Score	m/z	Gene names
RO-QC-160121_ZackD_SS_Set_01_Fractic	25151	FTMS; HC	186.56	1210.56	CASP9

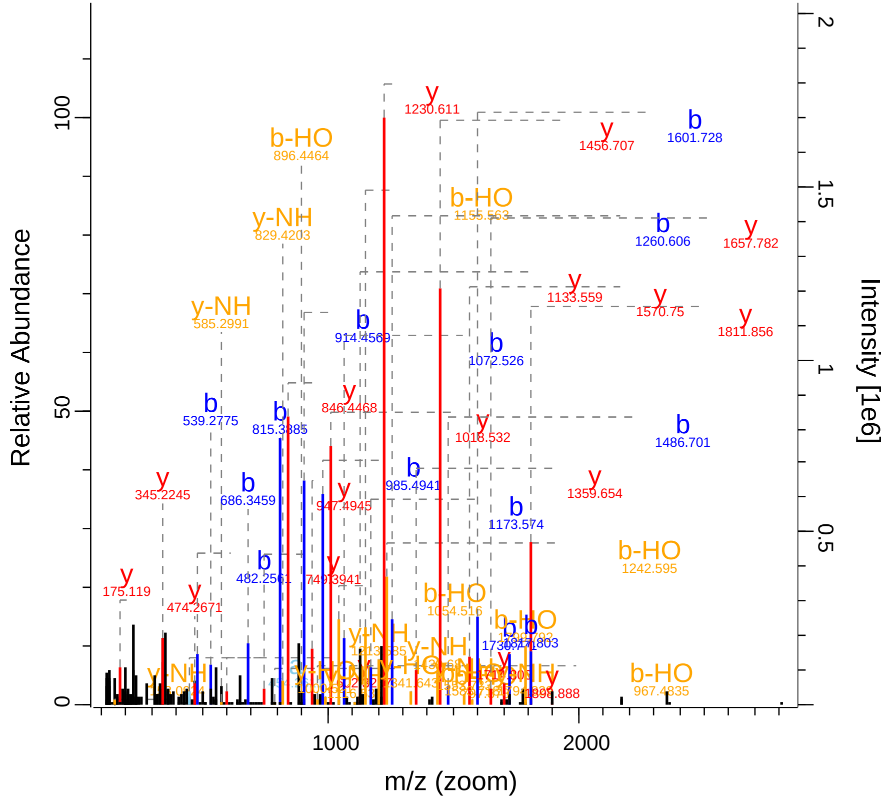

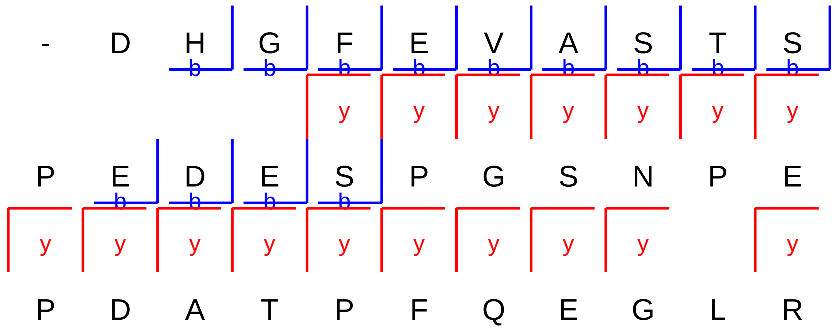

Raw File	Scan	Method	Score	m/z	Gene names
RO-QC-160121_ZackD_SS_Set_01_Fractio	25434	FTMS; HC	141.08	831.93	GPRC5B

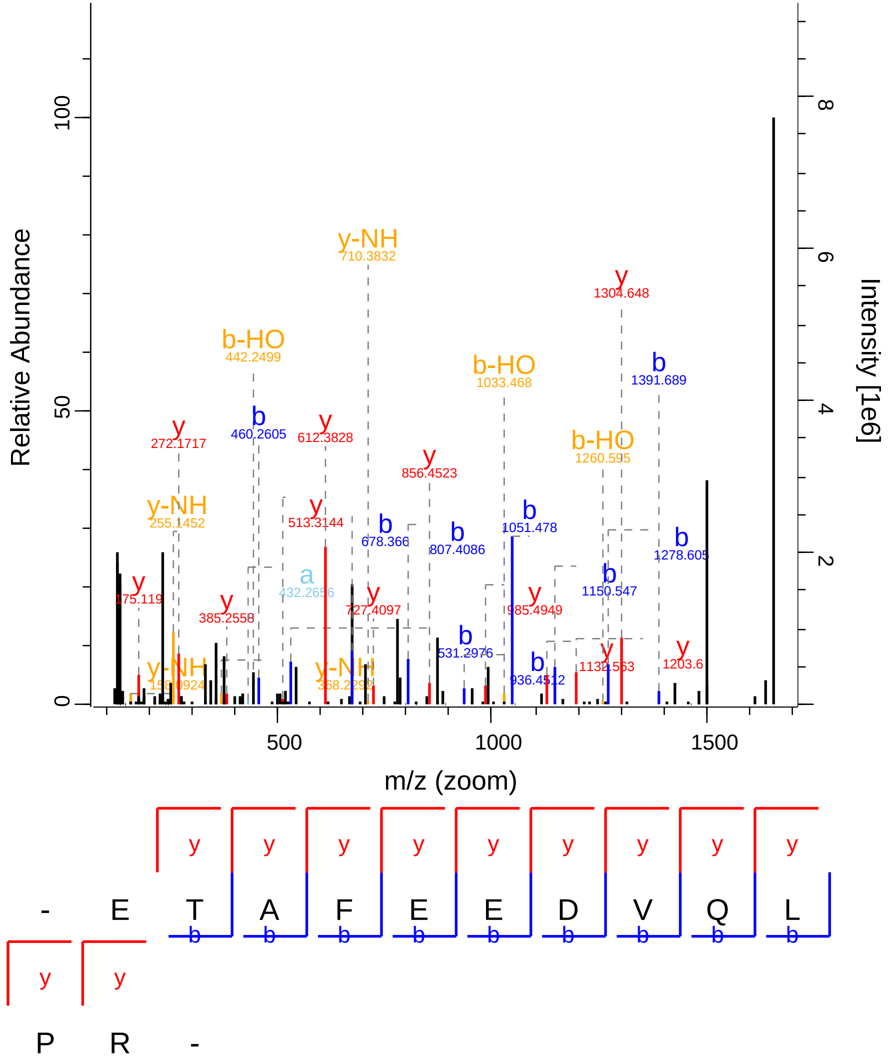

Raw File	Scan	Method	Score	m/z	Gene names
RO-QC-160121_ZackD_SS_Set_01_Fractio	25487	FTMS; HC	66.5	554.96	GPRC5B

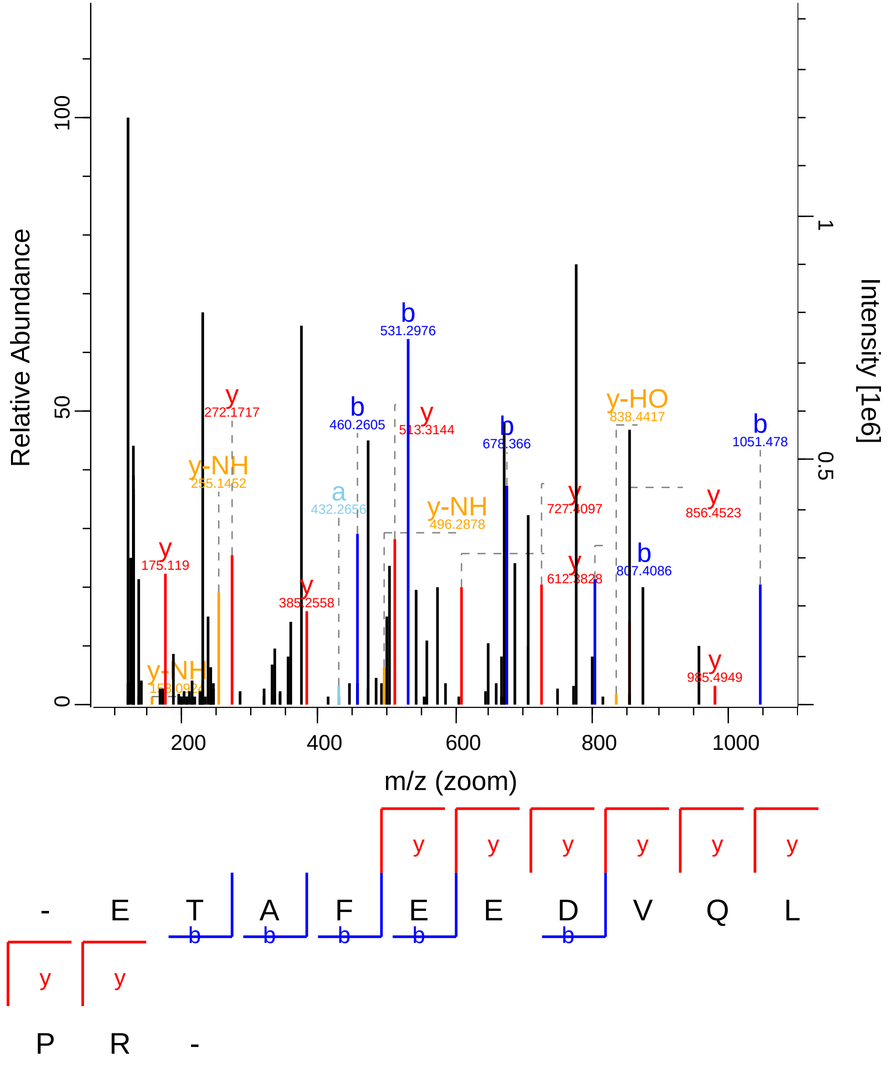

Raw File	Scan	Method	Score	m/z	Gene names
RO-QC-160121_ZackD_SS_Set_01_Fractio	25501	FTMS; HC	104.45	854.78	SLC31A1

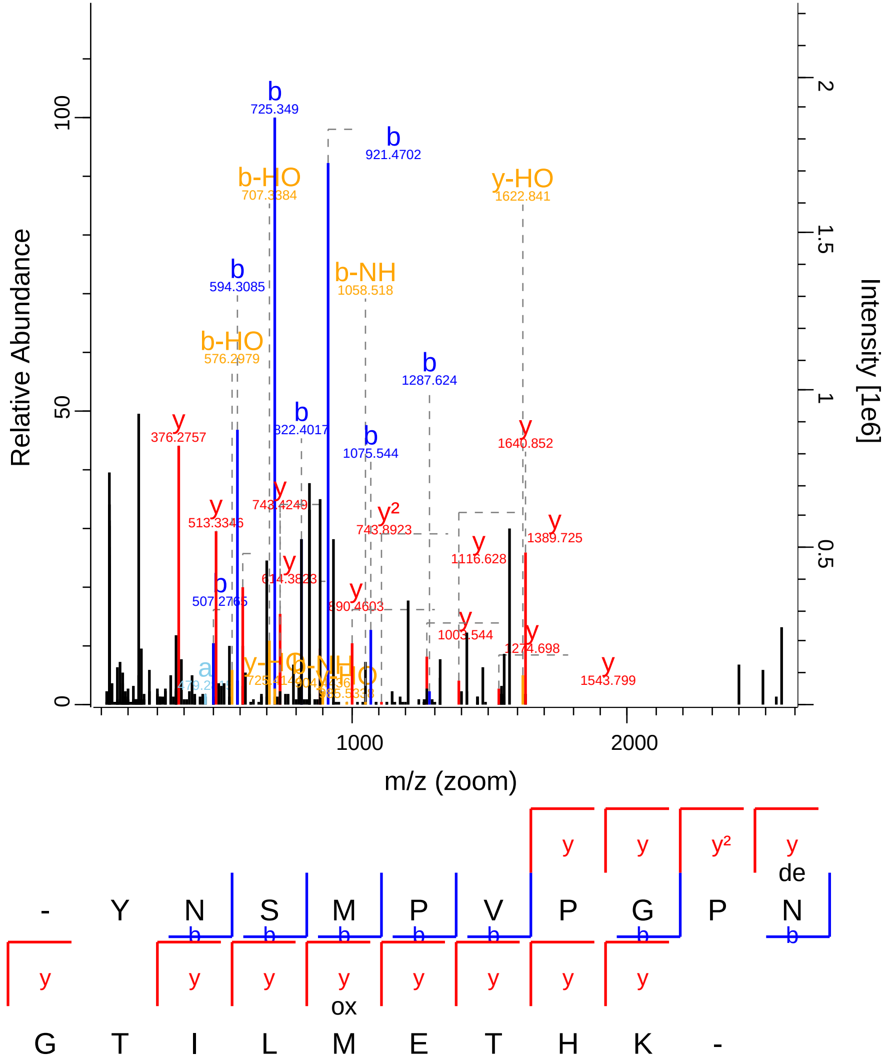

Raw File	Scan	Method	Score	m/z	Gene names
RO-QC-160121_ZackD_SS_Set_01_Fractio	26129	FTMS; HC	93.32	465.94	PTRH1

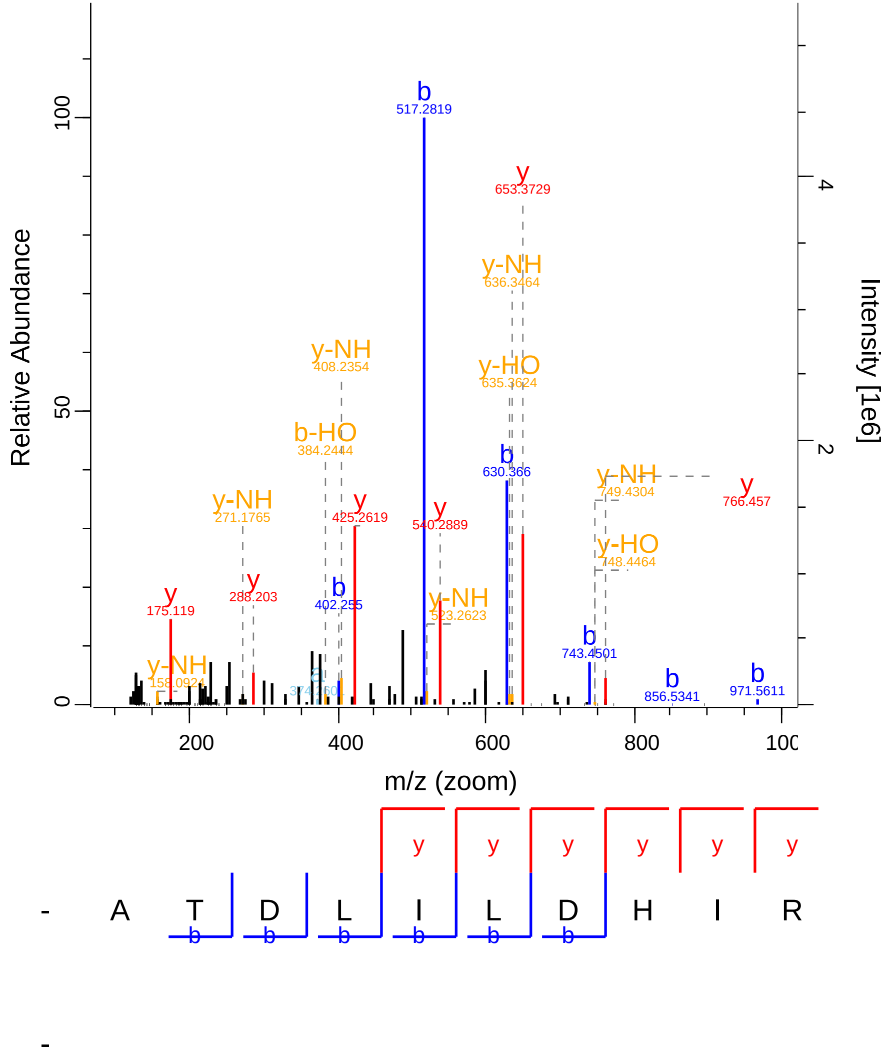

Raw File	Scan	Method	Score	m/z	Gene names
RO-QC-160121_ZackD_SS_Set_01_Fraction	26188	FTMS; HCI	61.21	1010	FTMT

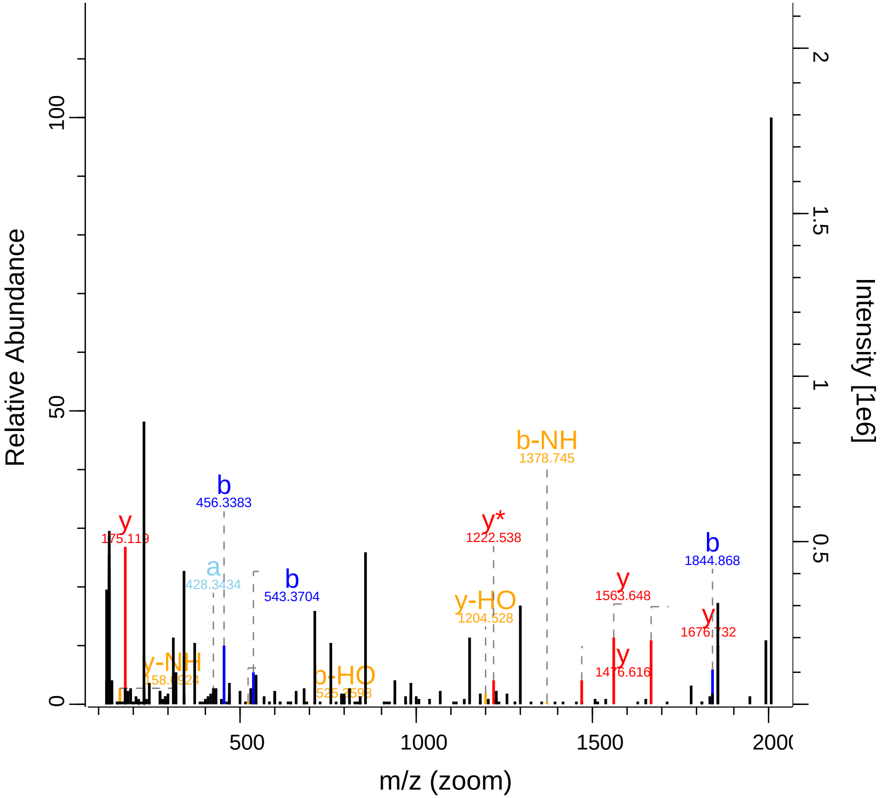

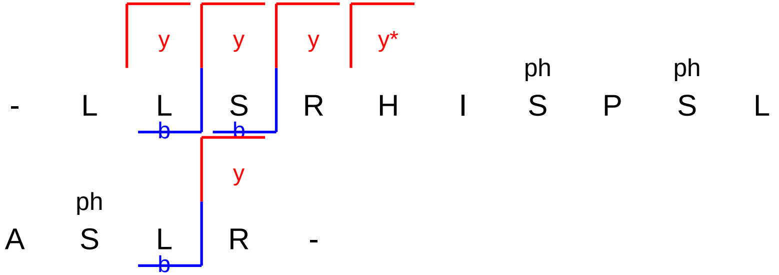

Raw File	Scan	Method	Score	m/z	Gene names
RO-QC-160121_ZackD_SS_Set_01_Fractio	26339	FTMS; HC	89.91	465.94	PTRH1

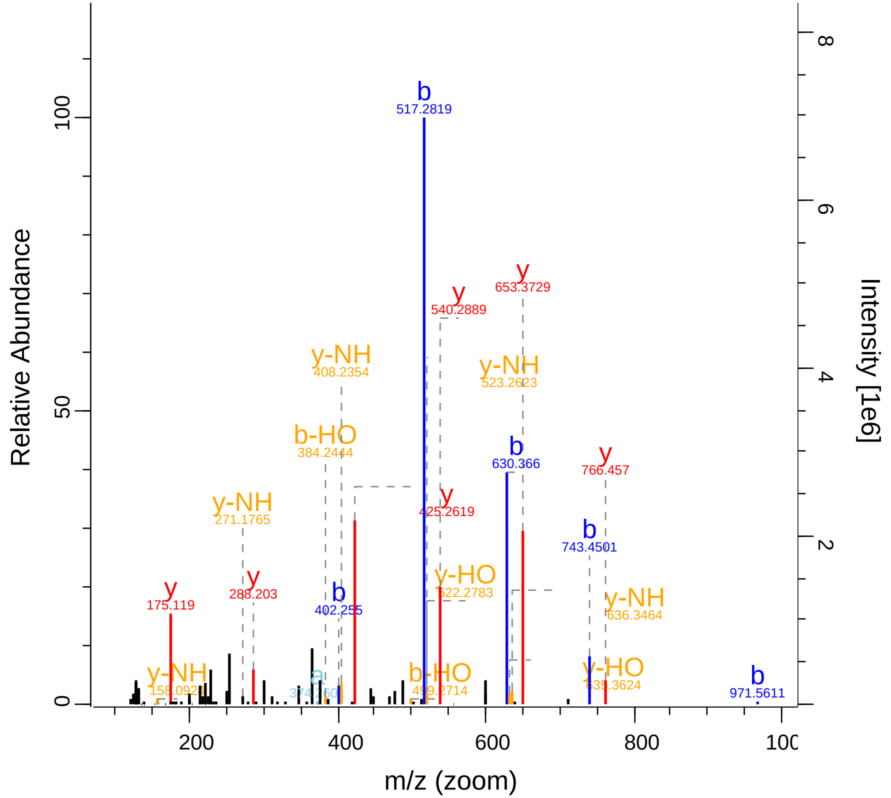

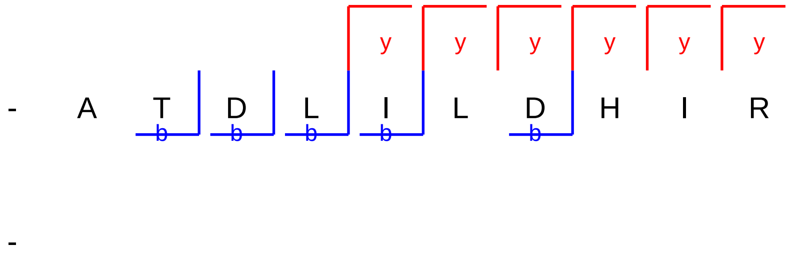

Raw File	Scan	Method	Score	m/z	Gene names
RO-QC-160121_ZackD_SS_Set_01_Fractio	26403	FTMS; HC	46.61	1010.5	FTMT

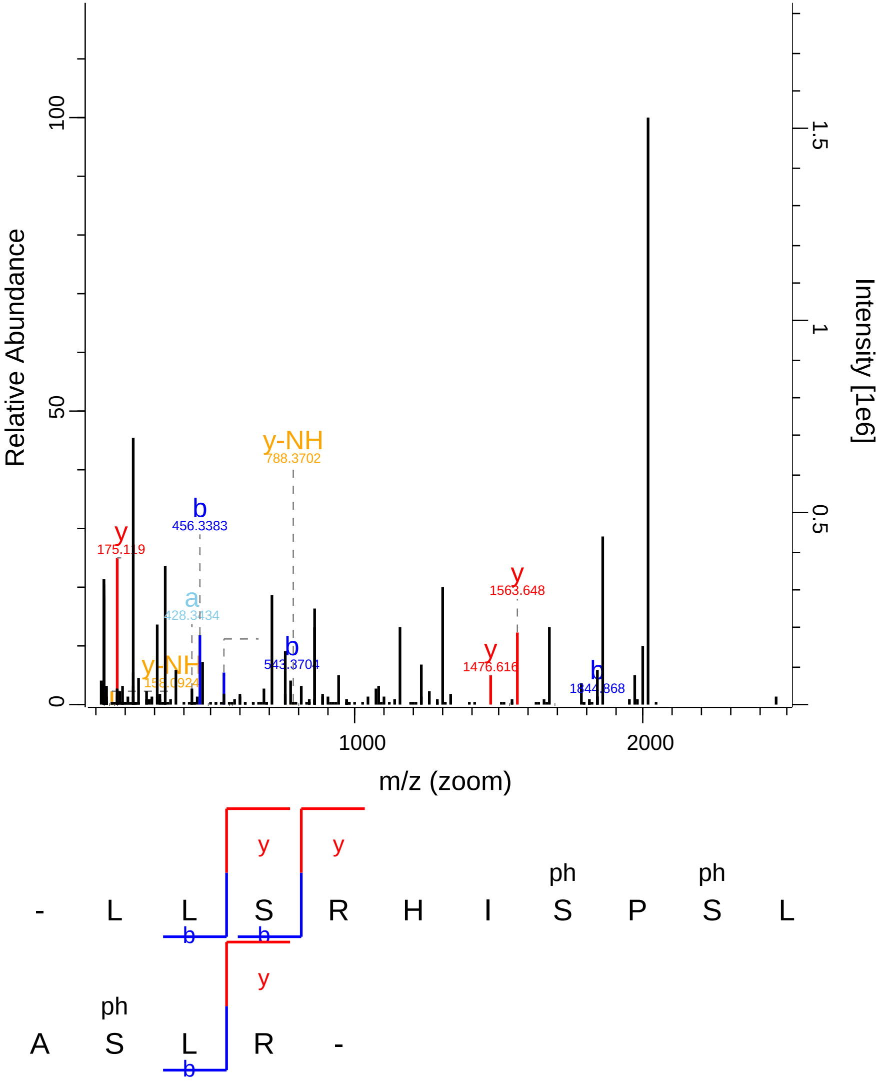

Raw File	Scan	Method	Score	m/z	Gene names
RO-QC-160121_ZackD_SS_Set_01_Fractior	26904	FTMS; HCI	46.49	830.4	BTC

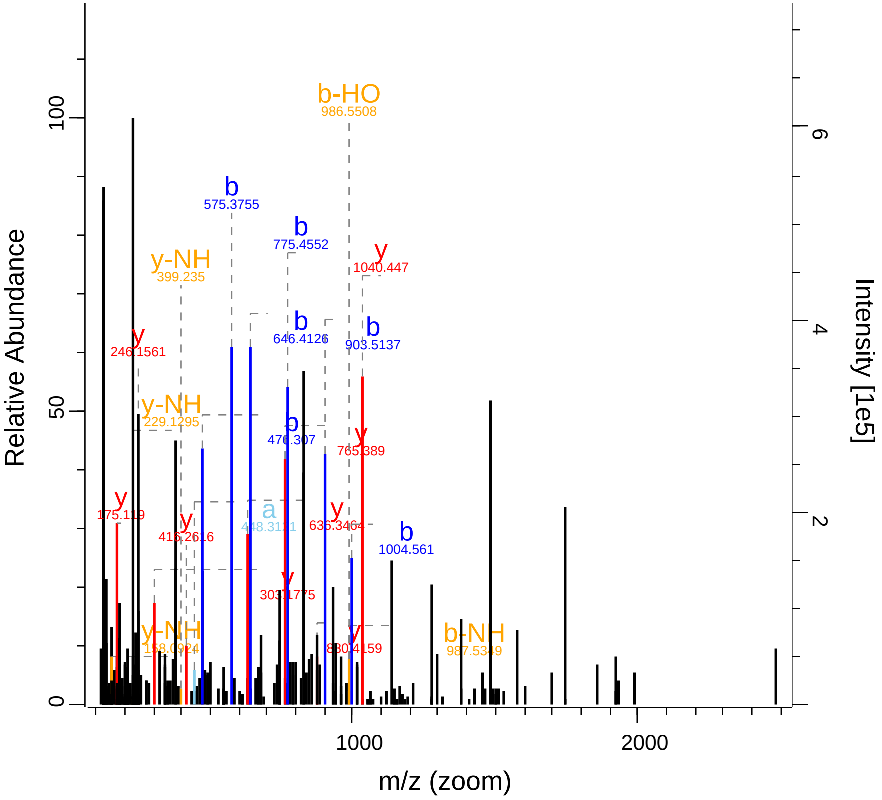

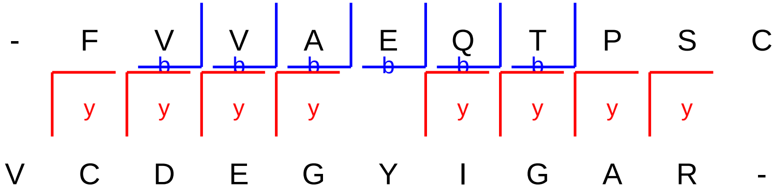

Raw File	Scan	Method	Score	m/z
RO-QC-160121_ZackD_SS_Set_01_Fraction_3	27507	FTMS; HCD	40.99	898.27

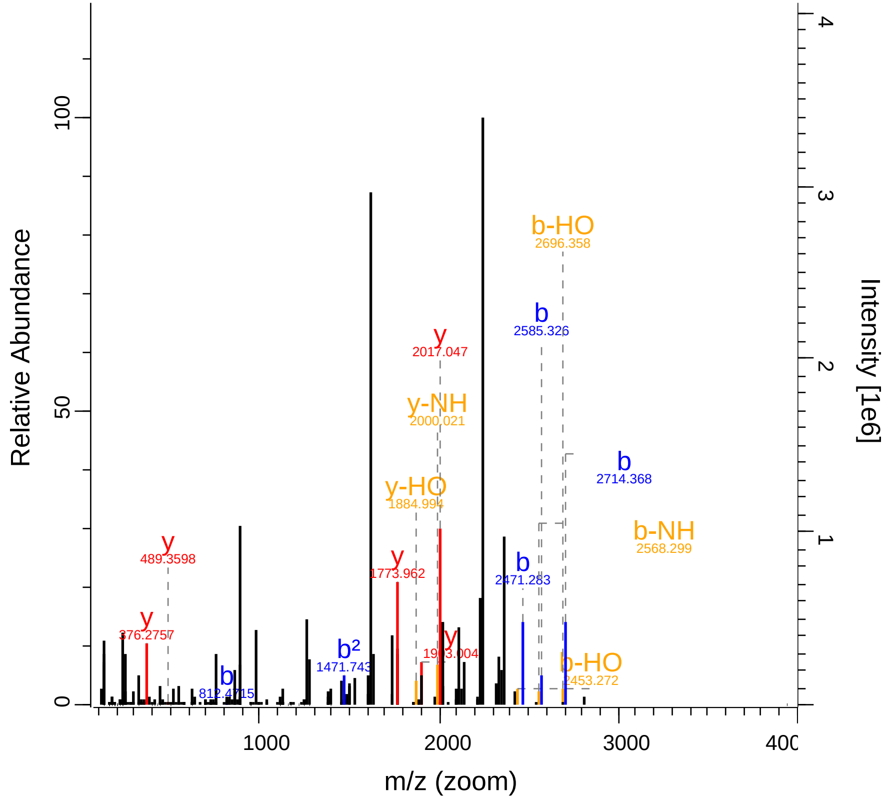

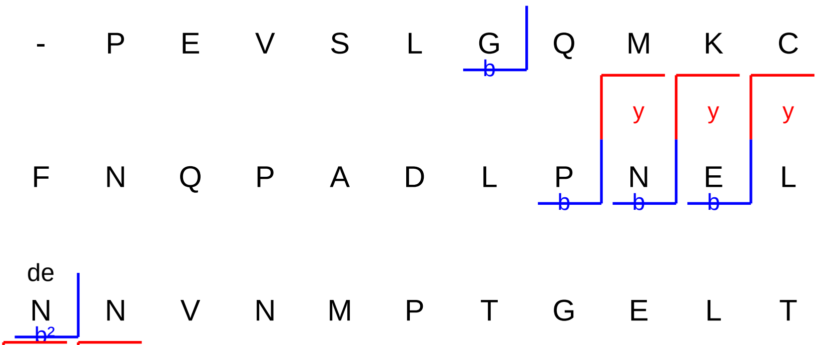

Raw File	Scan	Method	Score	m/z	Gene names
RO-QC-160121_ZackD_SS_Set_01_Fractio	28048	FTMS; HC	110.66	563.68	IFNGR1

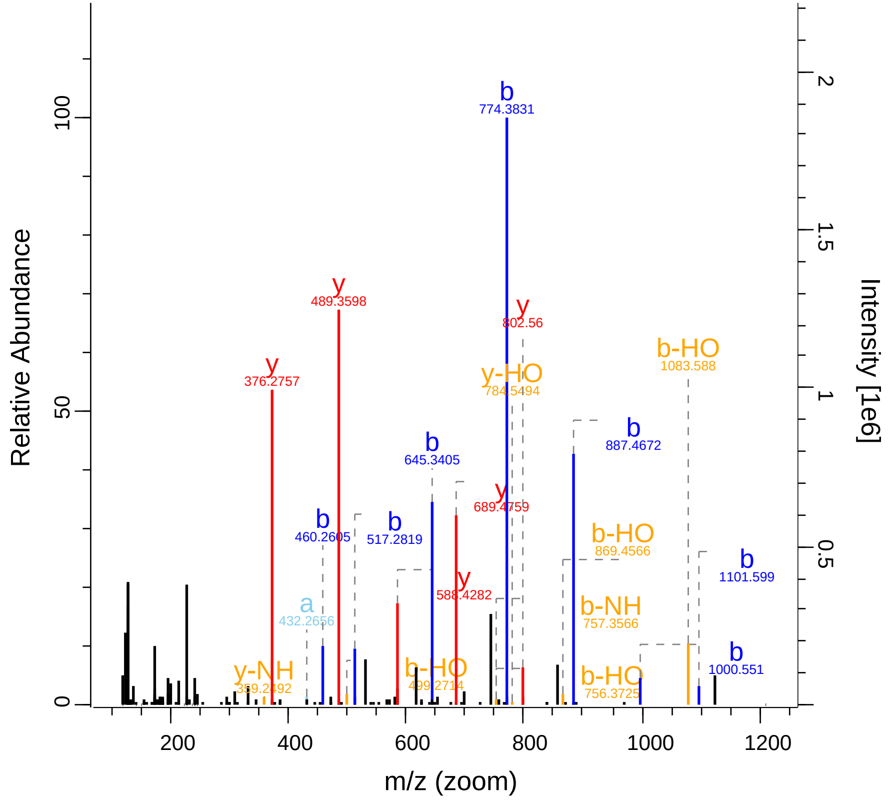

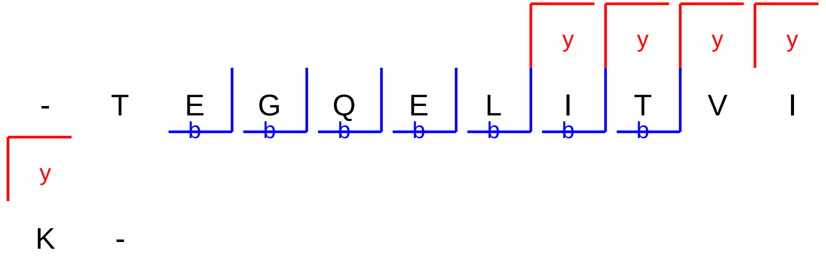

Raw File	Scan	Method	Score	m/z	Gene names
RO-QC-160121_ZackD_SS_Set_01_Fractio	29280	FTMS; HC	94.69	771.36	EIF4EBP1

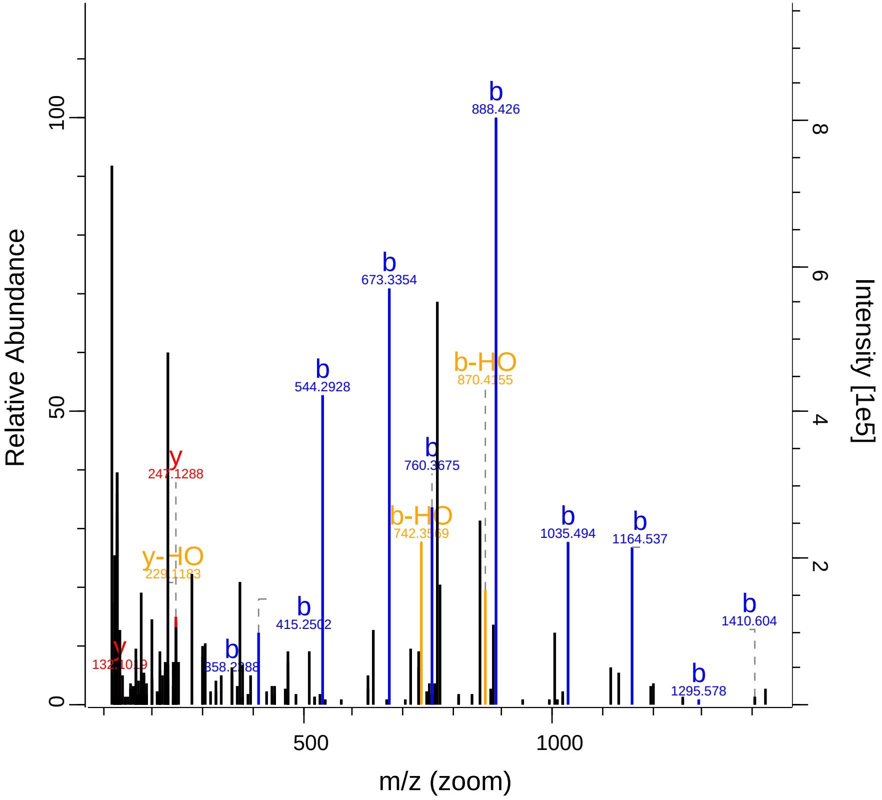

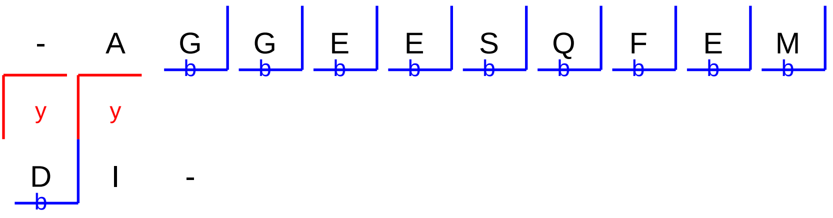

Raw File	Scan	Method	Score	m/z	Gene names
RO-QC-160121_ZackD_SS_Set_01_Fractic	30078	FTMS; HC	89.51	1008.88	TMEM206

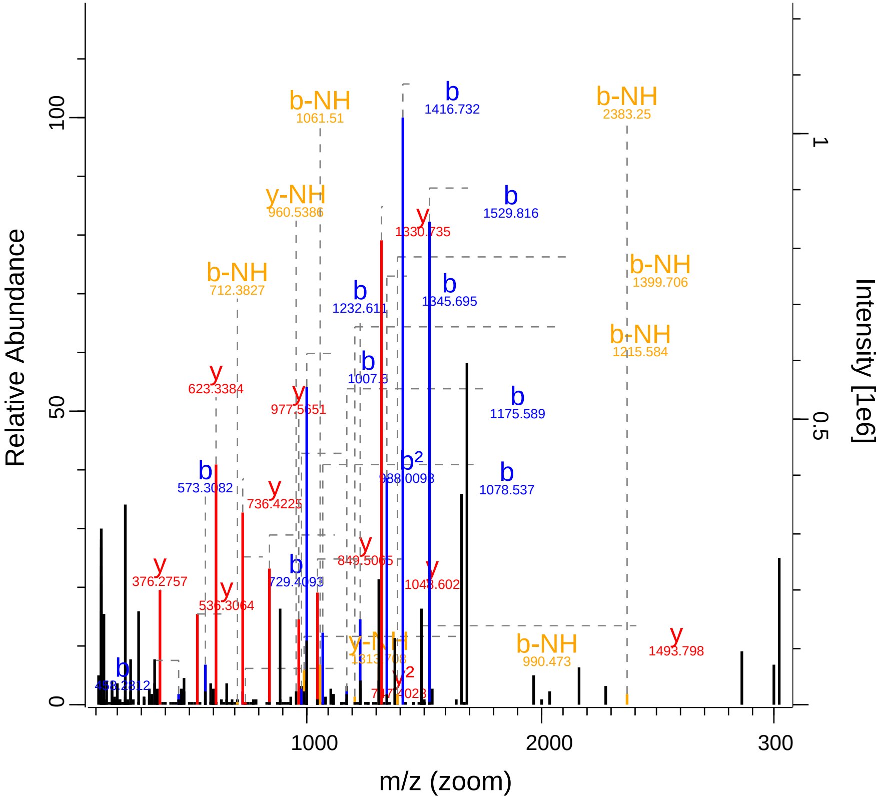

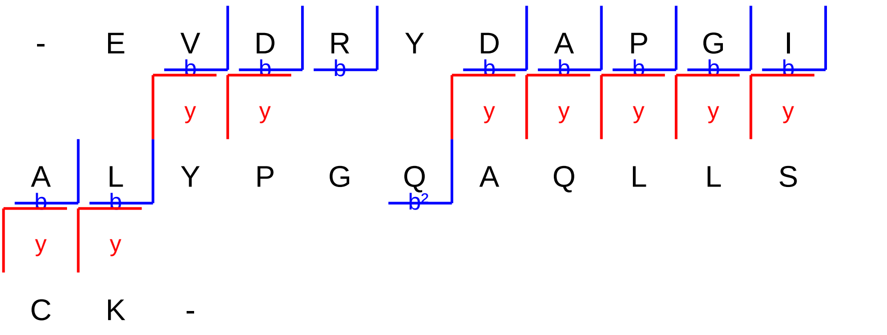

Raw File	Scan	Method	Score	m/z	Gene names
RO-QC-160121_ZackD_SS_Set_01_Fractio	30909	FTMS; HC	100.55	628.32	TADA2B

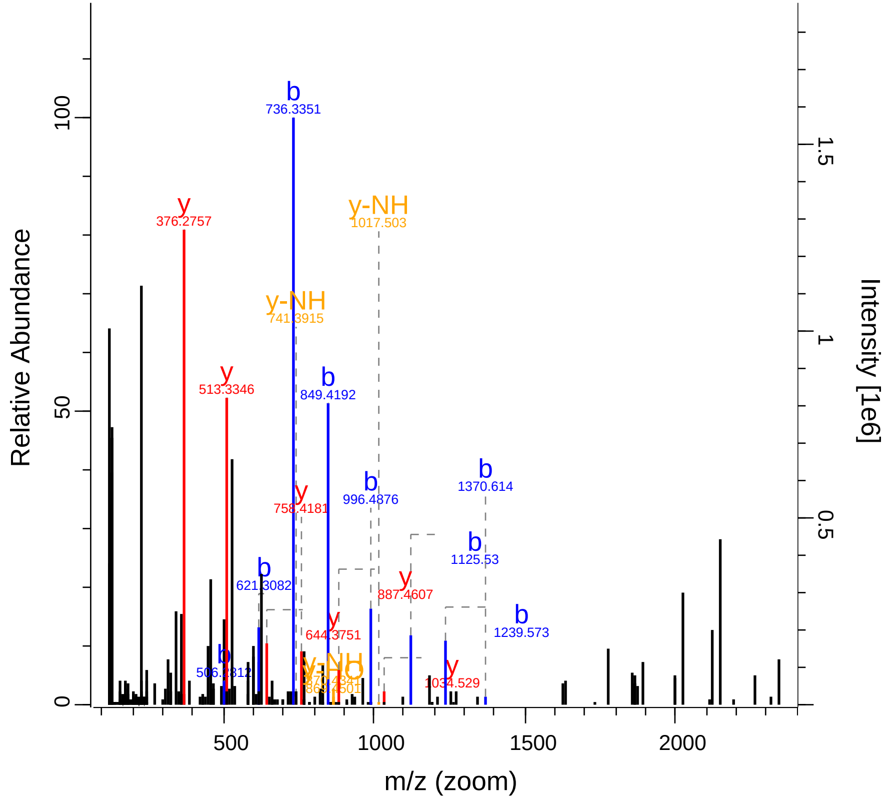

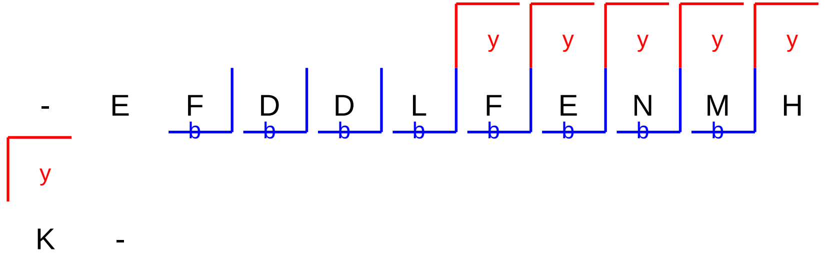

Raw File	Scan	Method	Score	m/z	Gene names
RO-QC-160121_ZackD_SS_Set_01_Fractio	32184	FTMS; HC	123.53	995.19	NDUFAF6

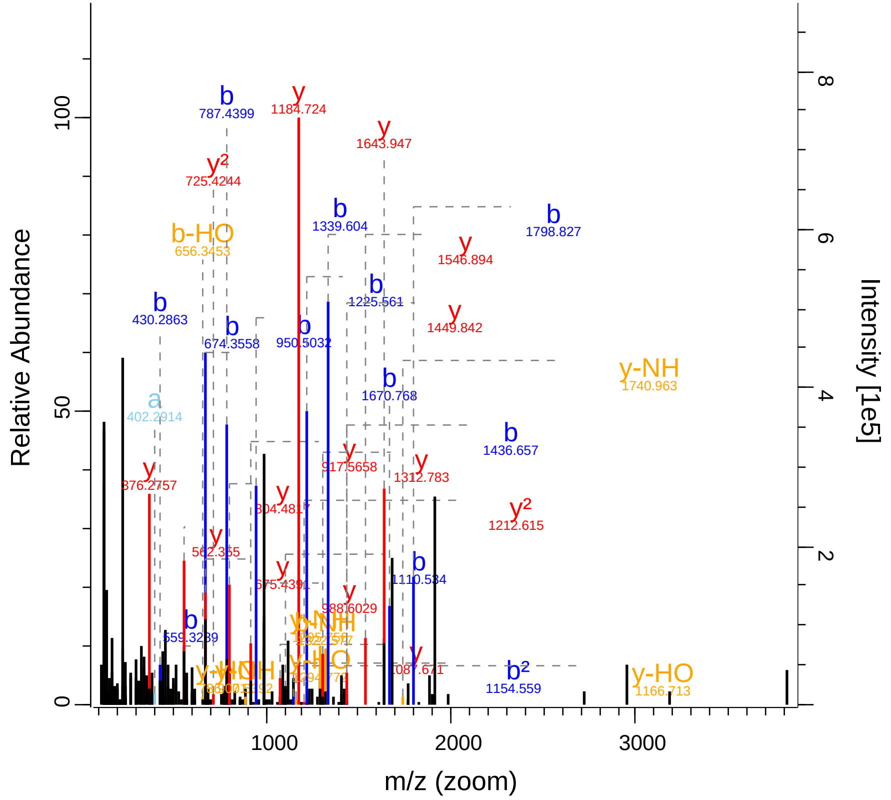

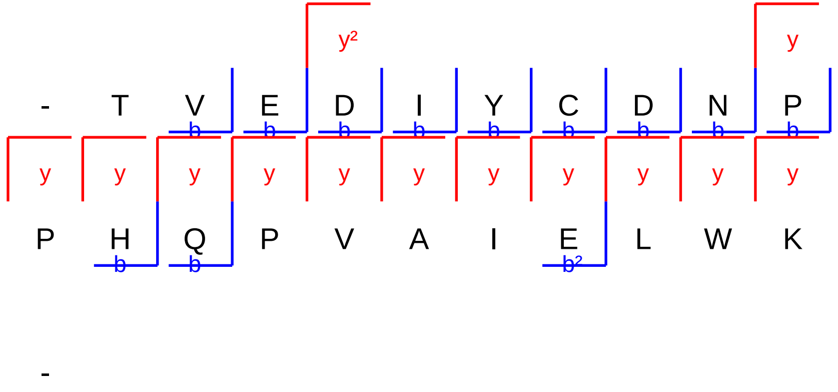

Raw File	Scan	Method	Score	m/z
RO-QC-160121_ZackD_SS_Set_01_Fraction_3	33457	FTMS; HCD	40.51	950.83

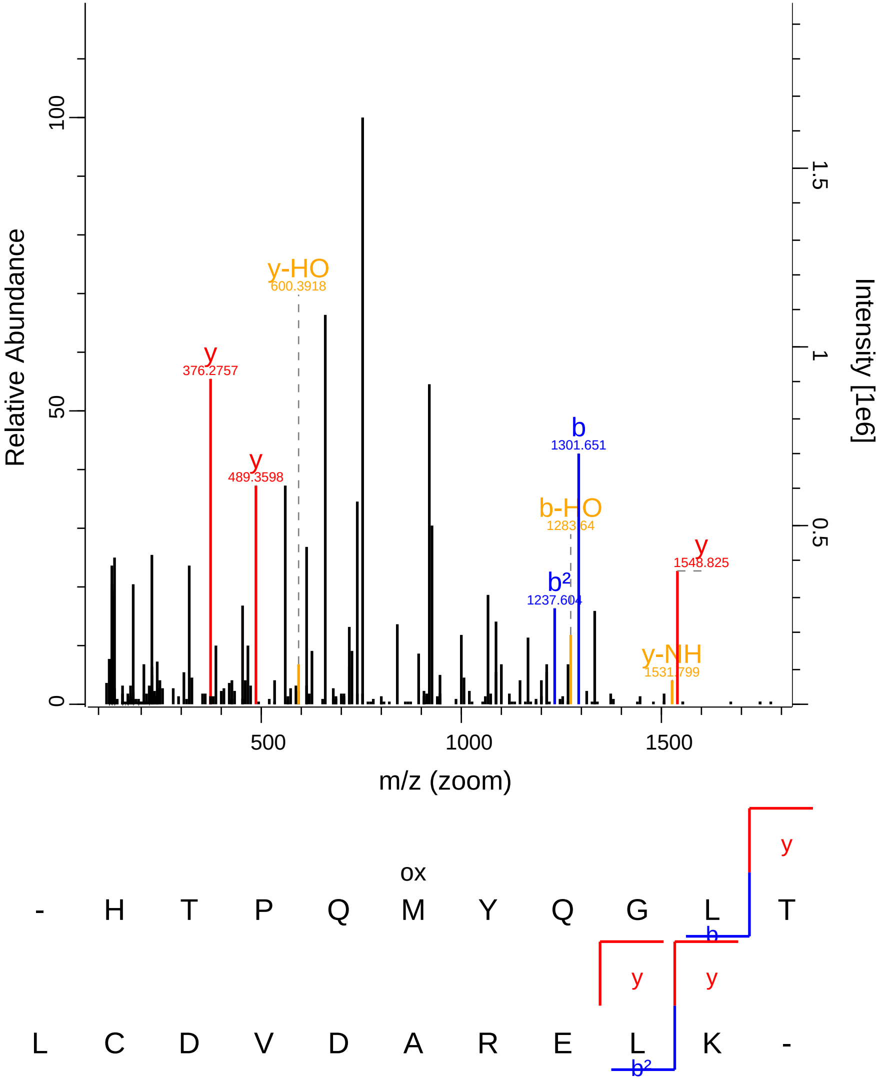

Raw File	Scan	Method	Score	m/z	Gene names
RO-QC-160121_ZackD_SS_Set_01_Fractio	34180	FTMS; HC	121.21	555.97	IRAK1

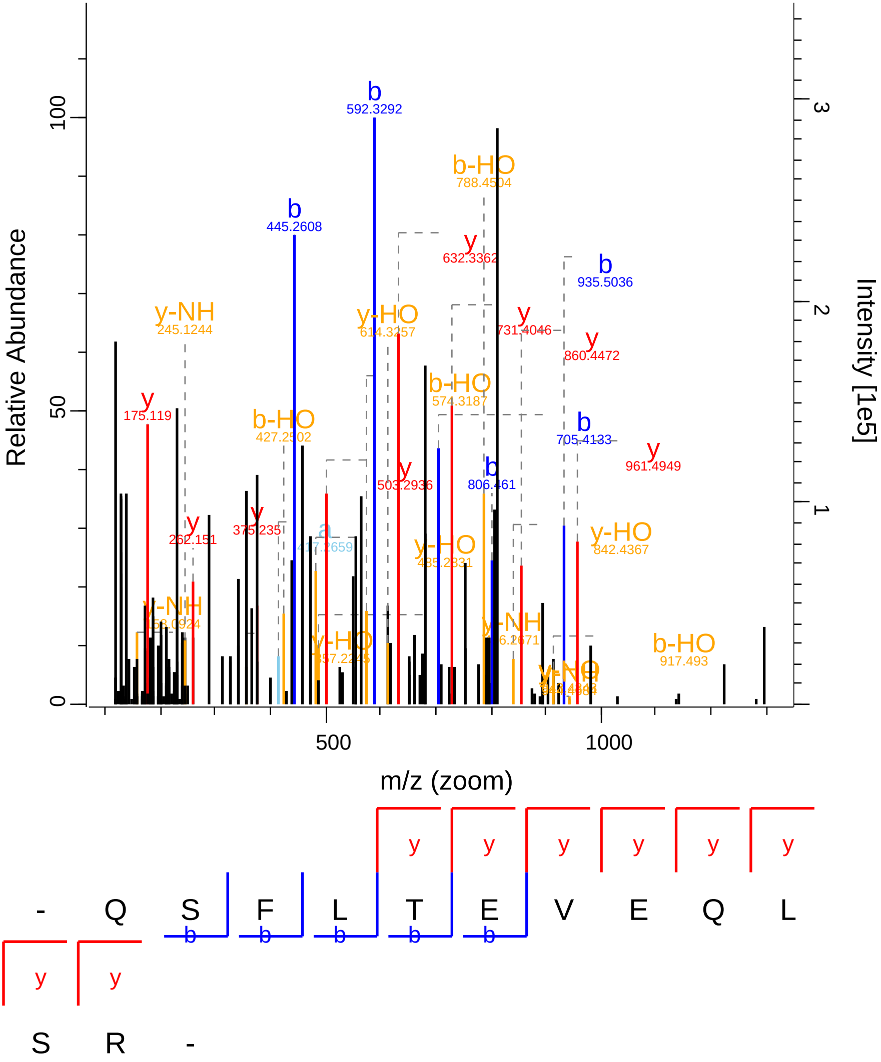

Raw File	Scan	Method	Score	m/z	Gene names
RO-QC-160121_ZackD_SS_Set_01_Fractio	34190	FTMS; HC	40.04	822.47	OR10G3

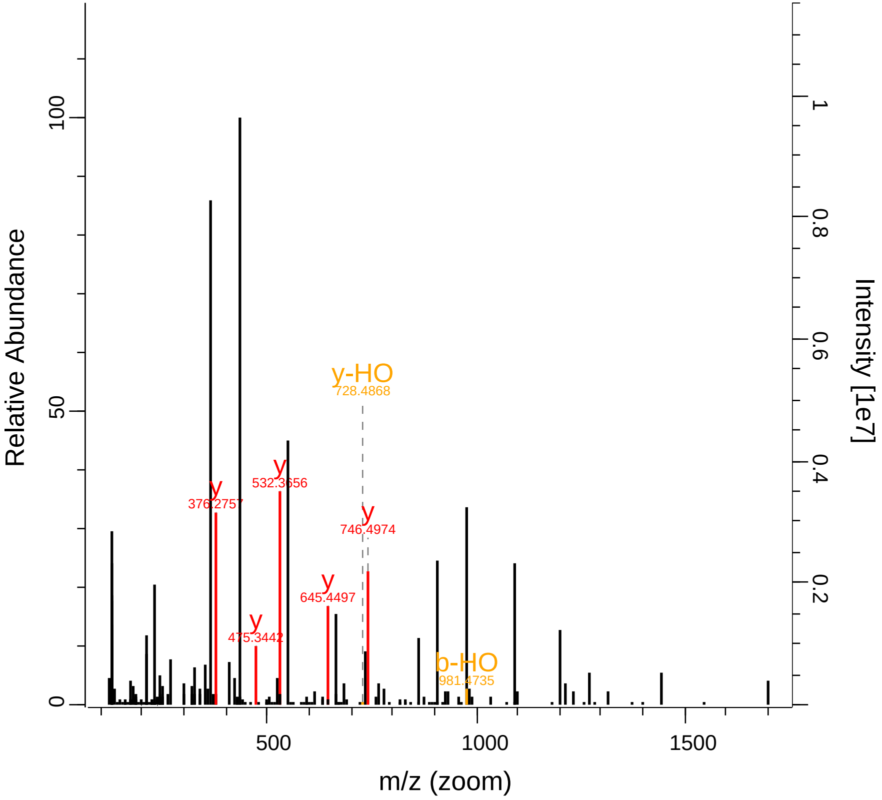

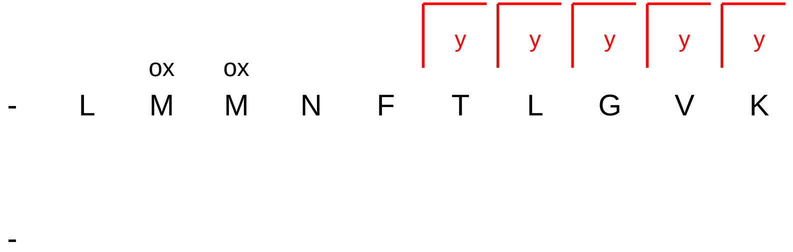

Raw File	Scan	Method	Score	m/z	Gene names
RO-QC-160121_ZackD_SS_Set_01_Fractic	34840	FTMS; HC	216.44	1032.51	CHCHD10

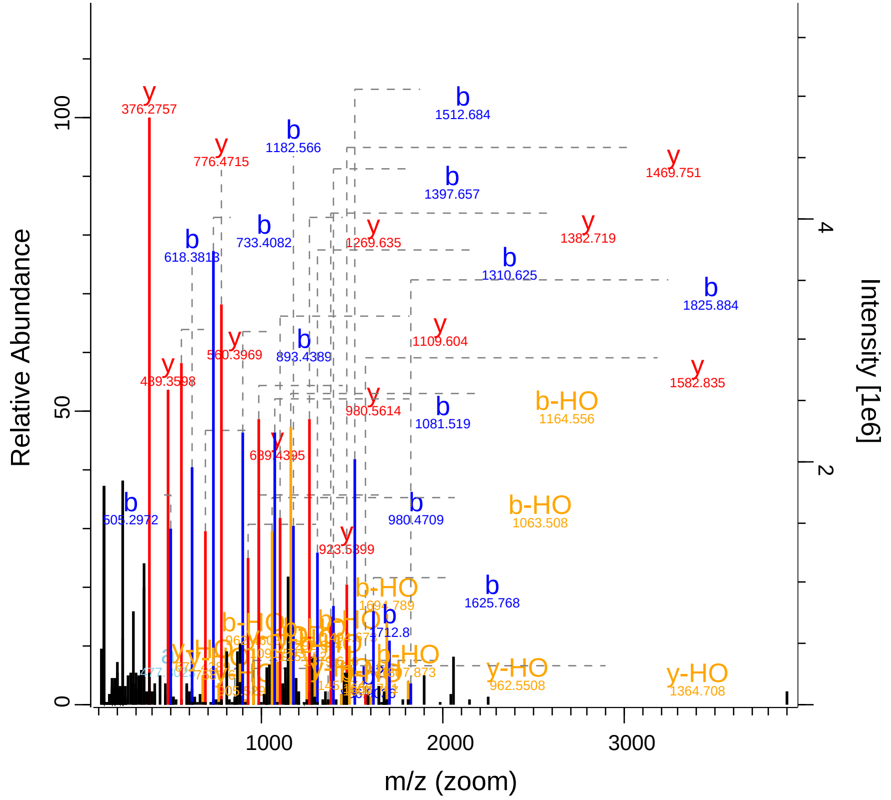

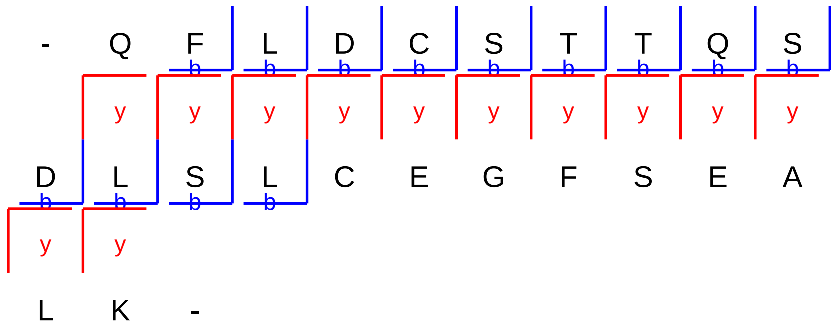

Raw File	Scan	Method	Score	m/z	Gene names
RO-QC-160121_ZackD_SS_Set_01_Fractio	35383	FTMS; HC	114.56	605.68	MRGBP

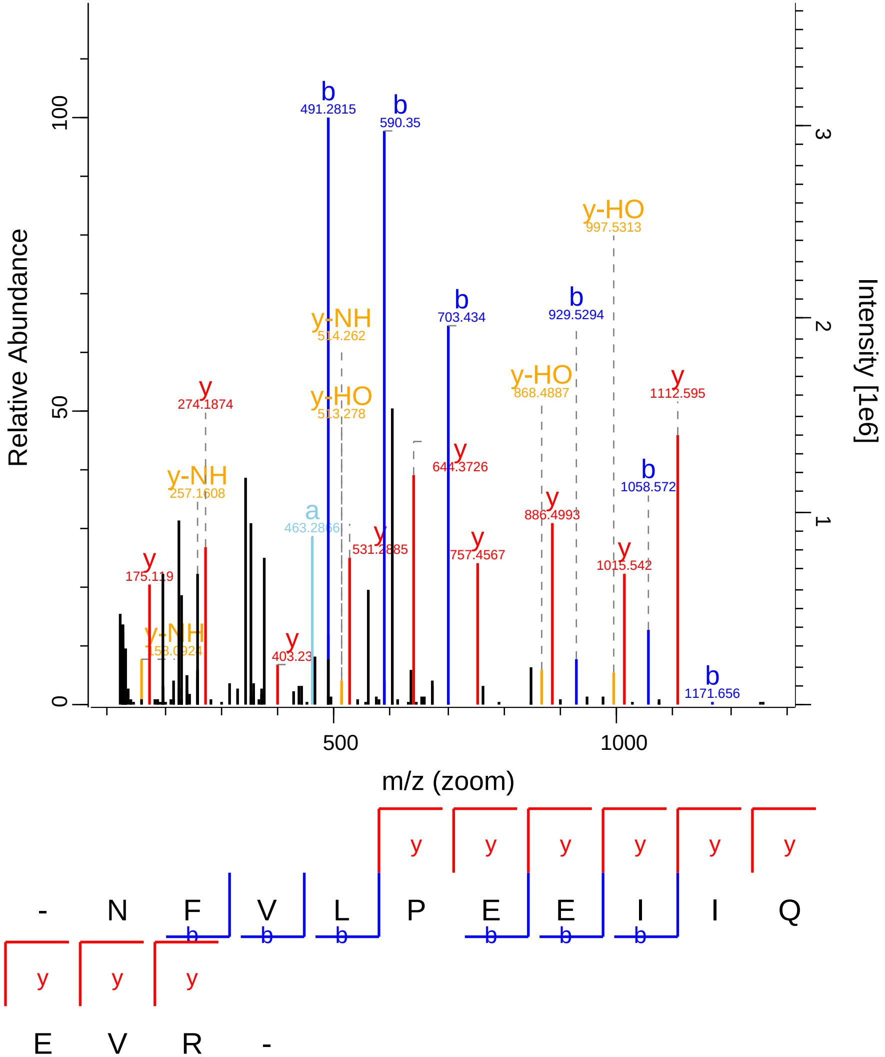

Raw File	Scan	Method	Score	m/z	Gene names
RO-QC-160121_ZackD_SS_Set_01_Fractic	36026	FTMS; HC	71.02	1001.96	C5orf45

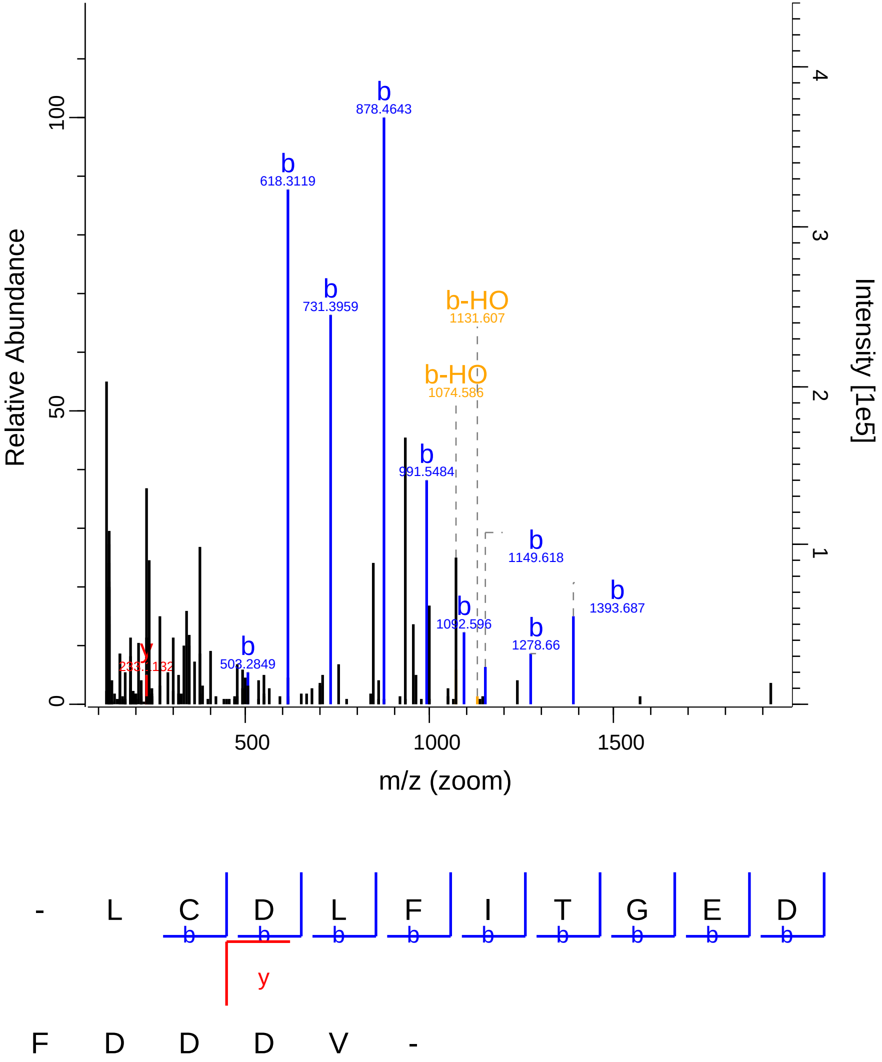

Raw File	Scan	Method	Score	m/z	Gene names
RO-QC-160121_ZackD_SS_Set_01_Fractio	36148	FTMS; HC	149	787.78	FUNDC1

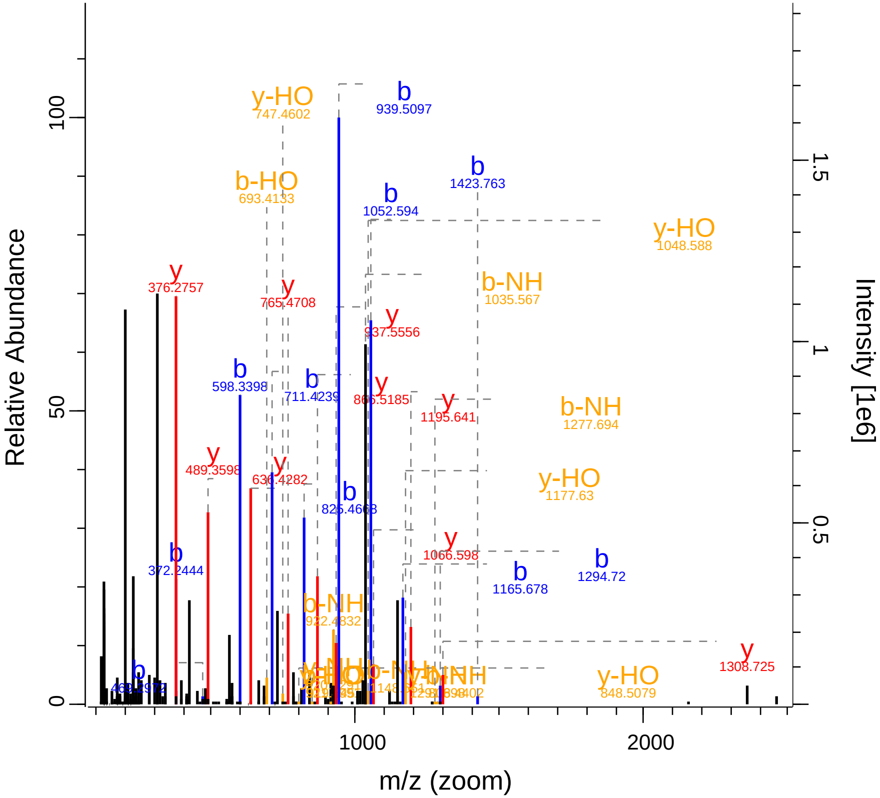

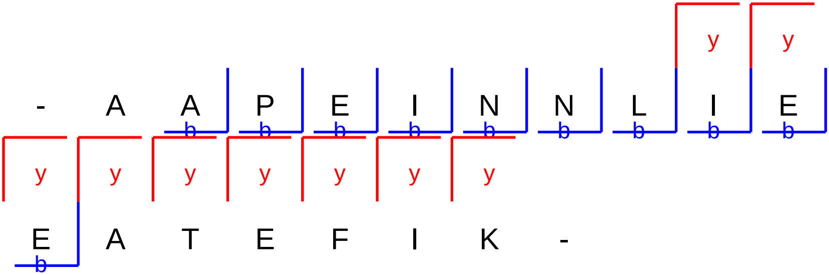

Raw File	Scan	Method	Score	m/z
RO-QC-160121_ZackD_SS_Set_01_Fraction_4	5289	FTMS; HCD	59.54	577.97

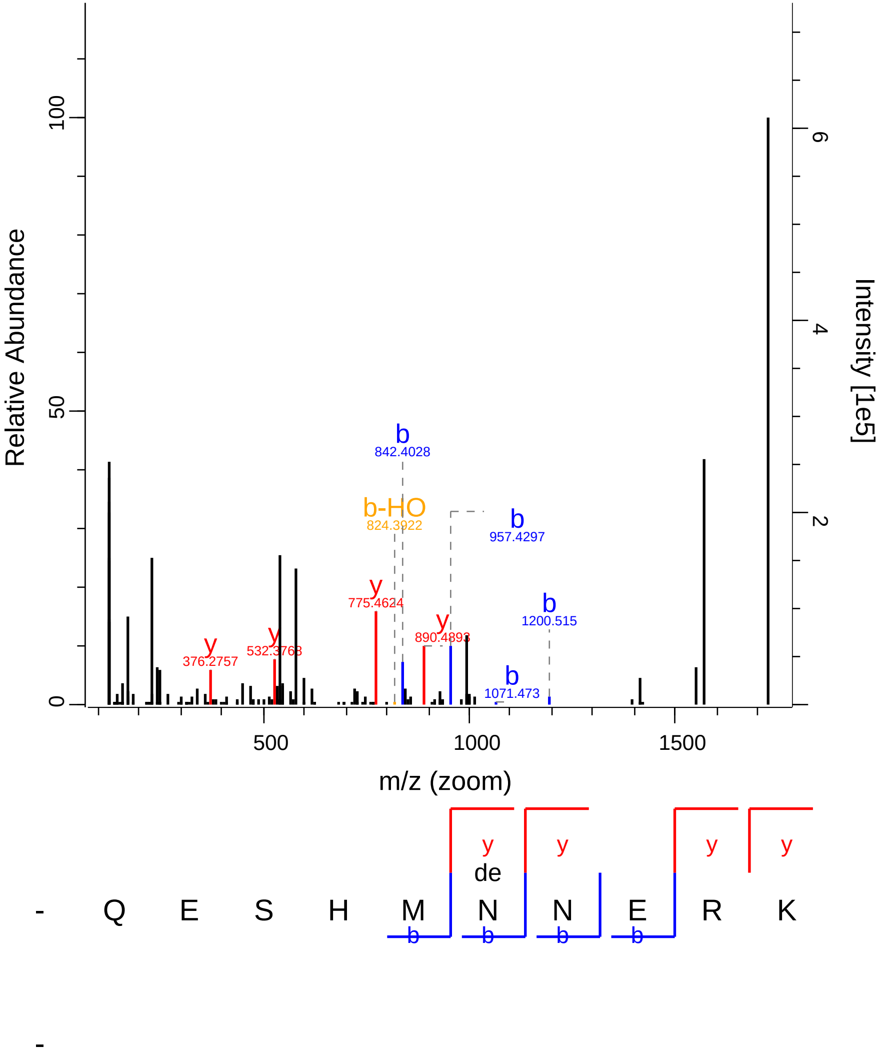

Raw File	Scan	Method	Score	m/z	Gene names
RO-QC-160121_ZackD_SS_Set_01_Fractio	5727	FTMS; HC	107.01	566.79	NXNL1

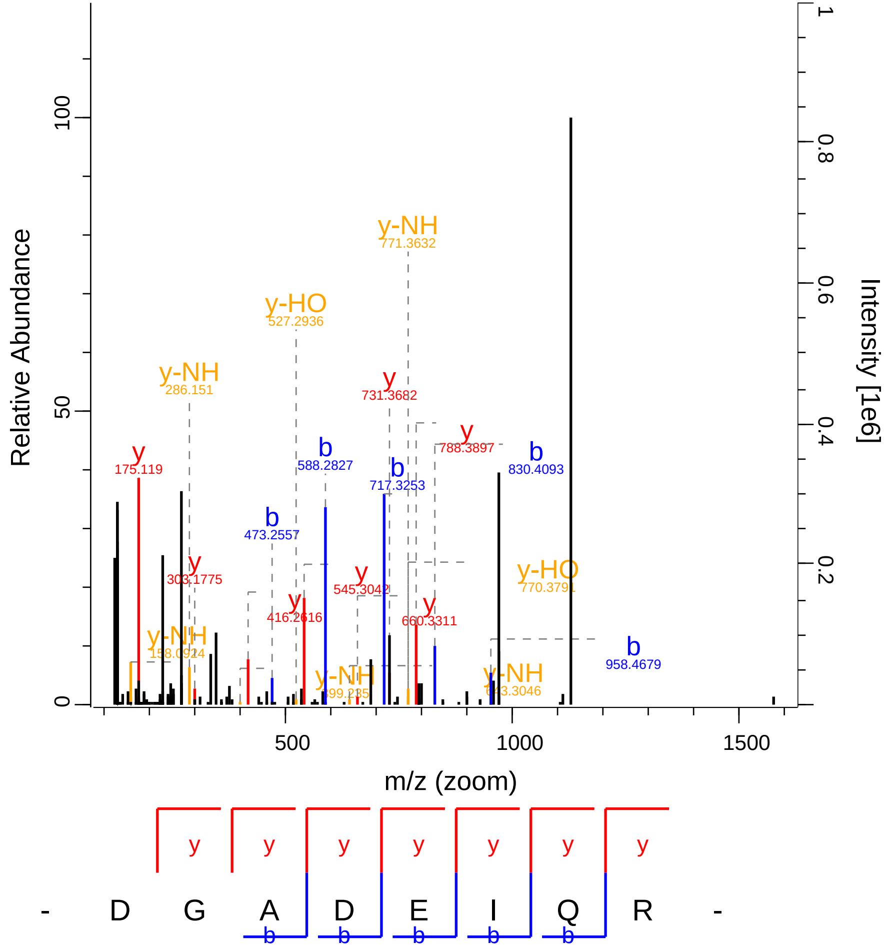

Raw File	Scan	Method	Score	m/z	Gene names
RO-QC-160121_ZackD_SS_Set_01_Fractior	6072	FTMS; HCI	62.3	586.99	FAM111B

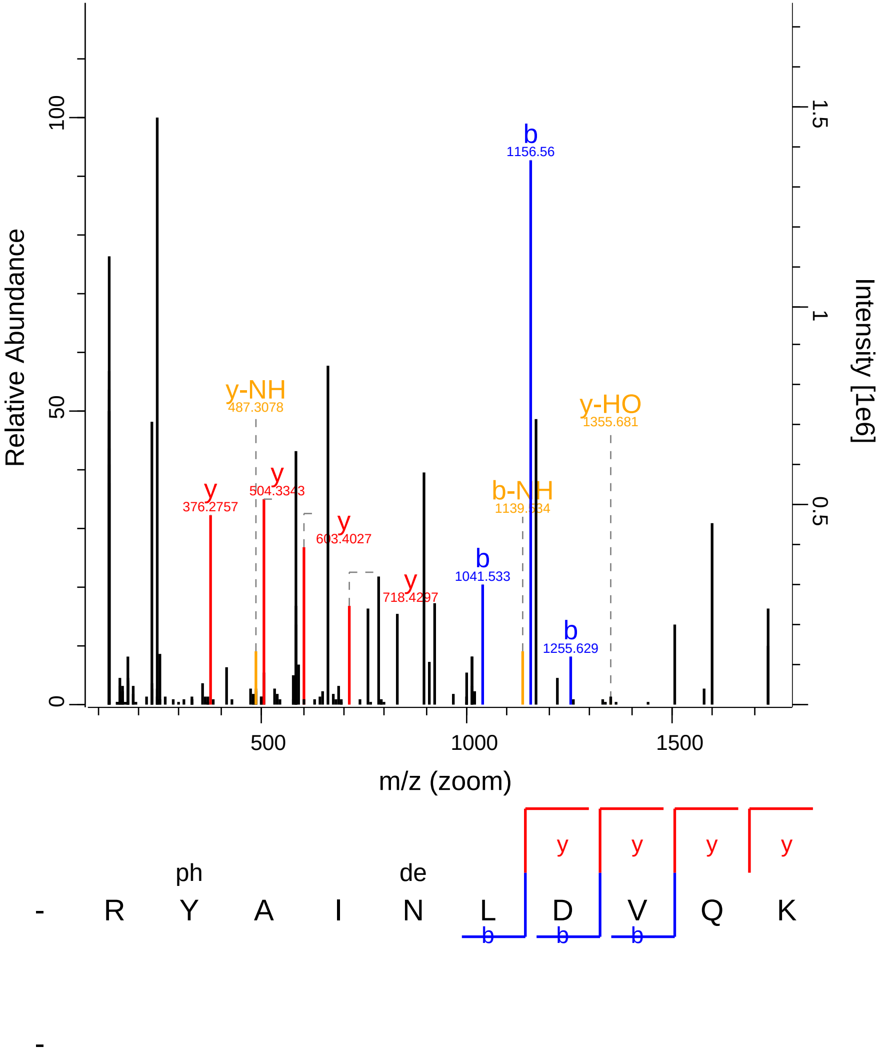

Raw File	Scan	Method	Score	m/z	Gene names
RO-QC-160121_ZackD_SS_Set_01_Fractior	6150	FTMS; HCl	41.03	476.94	POLR2F

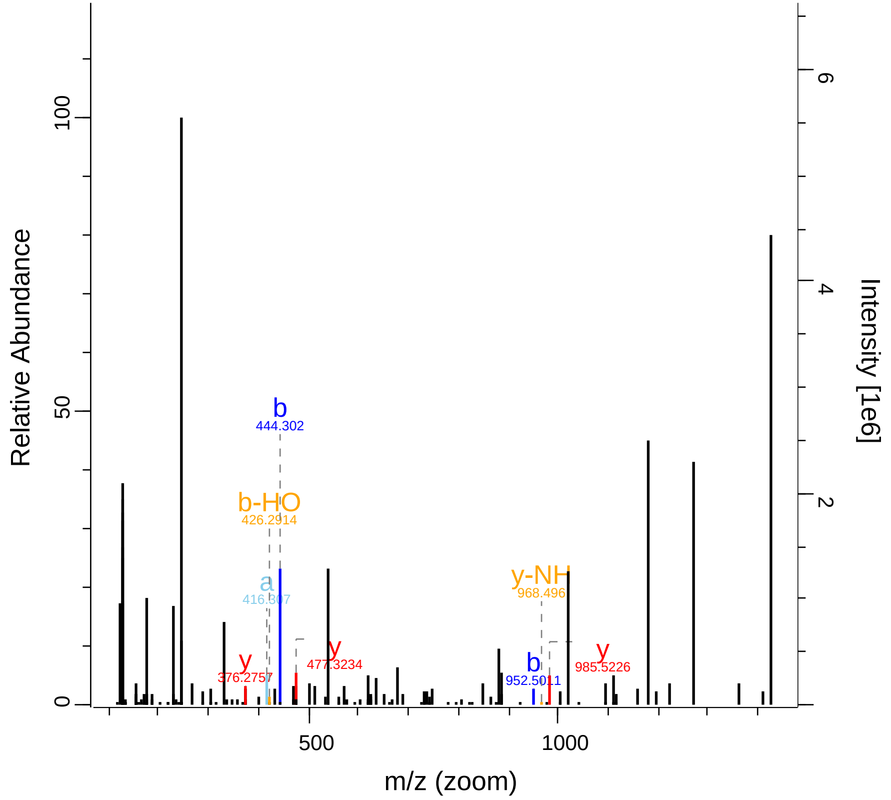

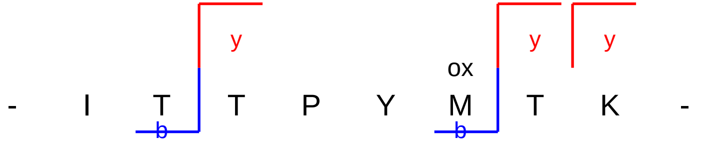

Raw File	Scan	Method	Score	m/z	Gene names
RO-QC-160121_ZackD_SS_Set_01_Fractior	6275	FTMS; HCI	79.66	438.23	ZNF581

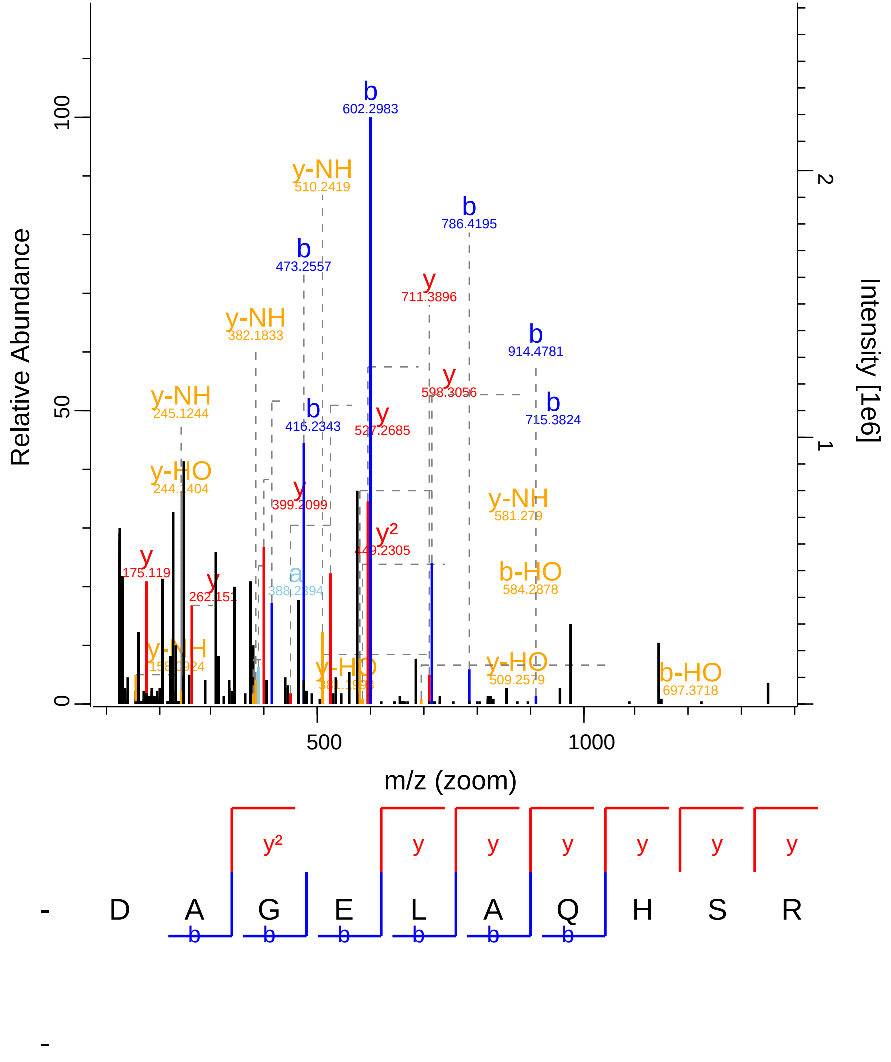

Raw File	Scan	Method	Score	m/z	Gene names
RO-QC-160121_ZackD_SS_Set_01_Fractior	6734	FTMS; HCI	96.6	464.92	SMIM13

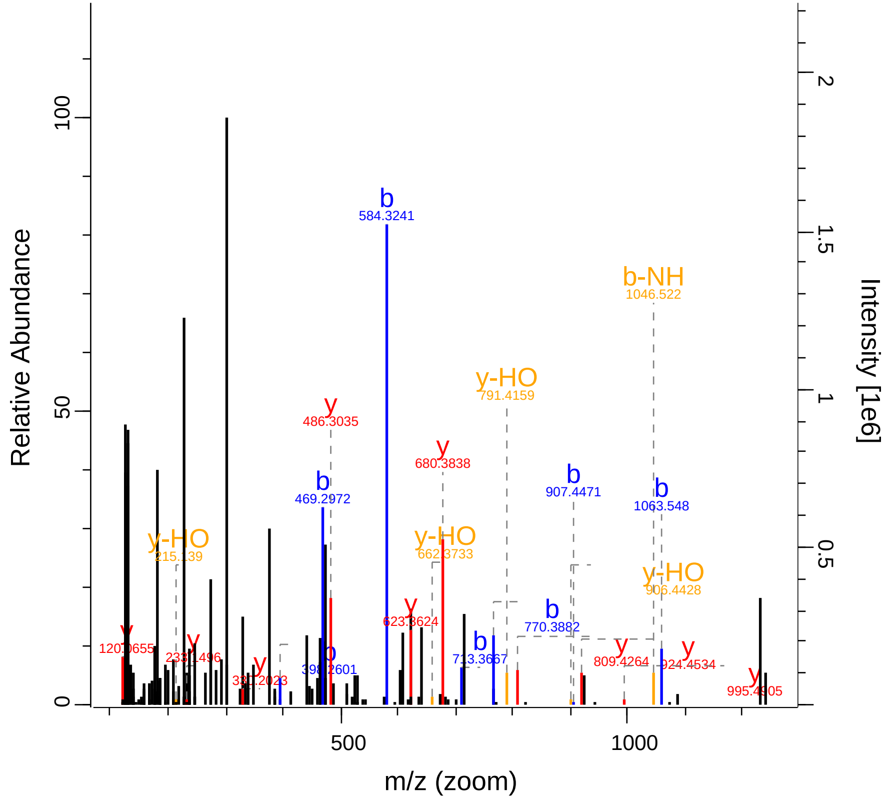

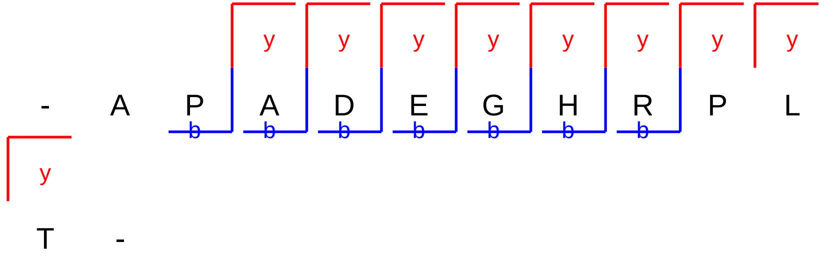

Raw File	Scan	Method	Score	m/z	Gene names
RO-QC-160121_ZackD_SS_Set_01_Fractior	7054	FTMS; HCI	56.92	660.39	PEX26

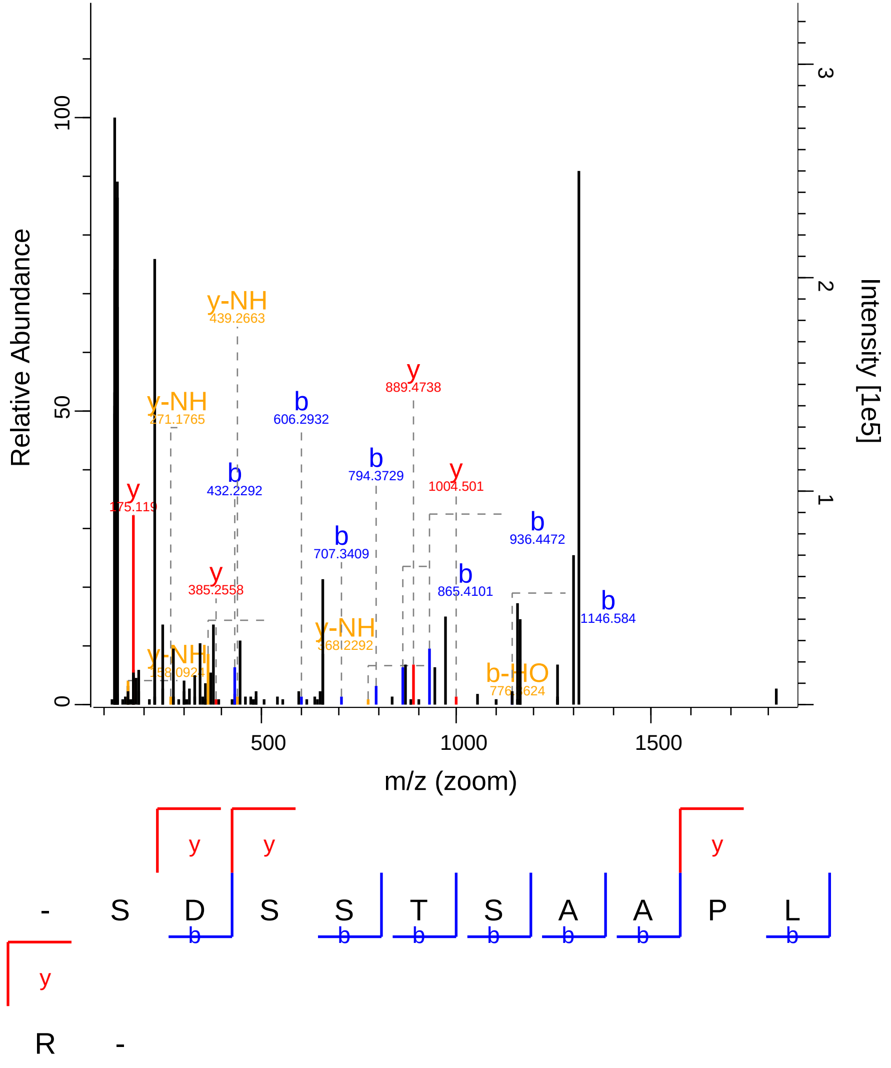

Raw File	Scan	Method	Score	m/z	Gene names
RO-QC-160121_ZackD_SS_Set_01_Fractio	7099	FTMS; HC	131.52	660.85	PEX26

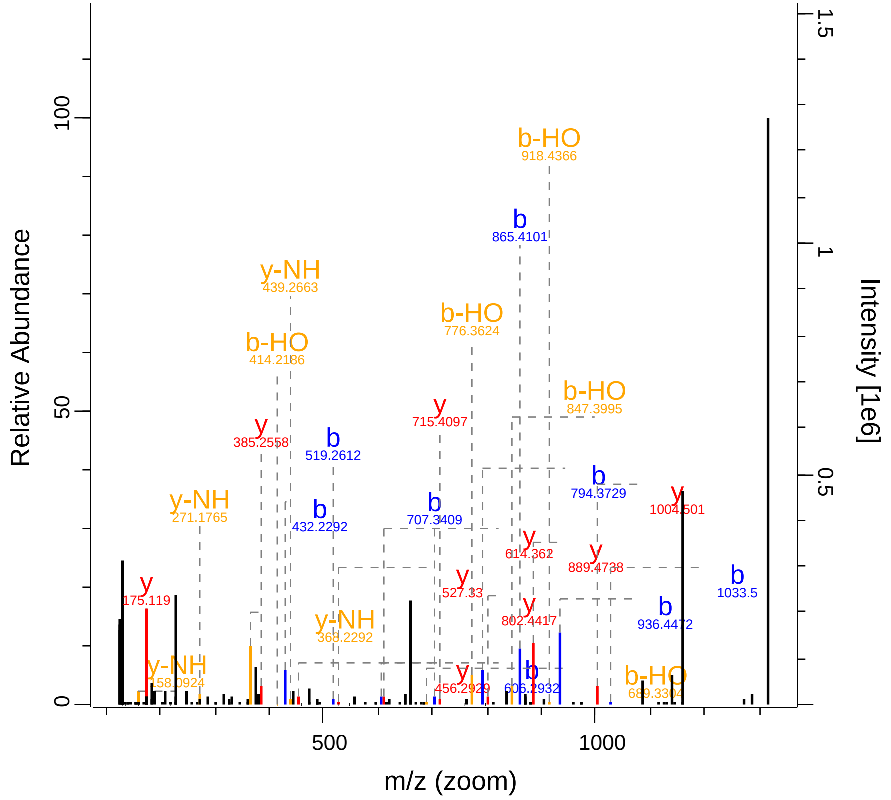

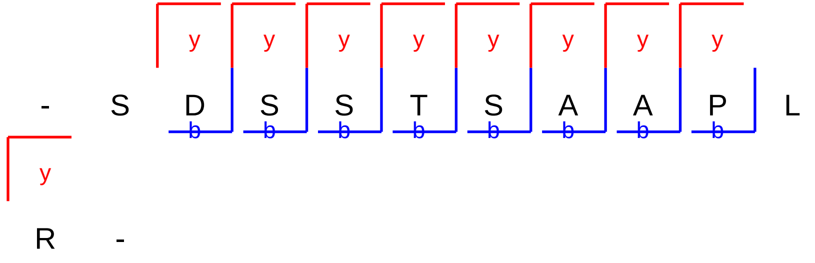

Raw File	Scan	Method	Score	m/z	Gene names
RO-QC-160121_ZackD_SS_Set_01_Fractior	7407	FTMS; HC	140.88	699.34	TK2

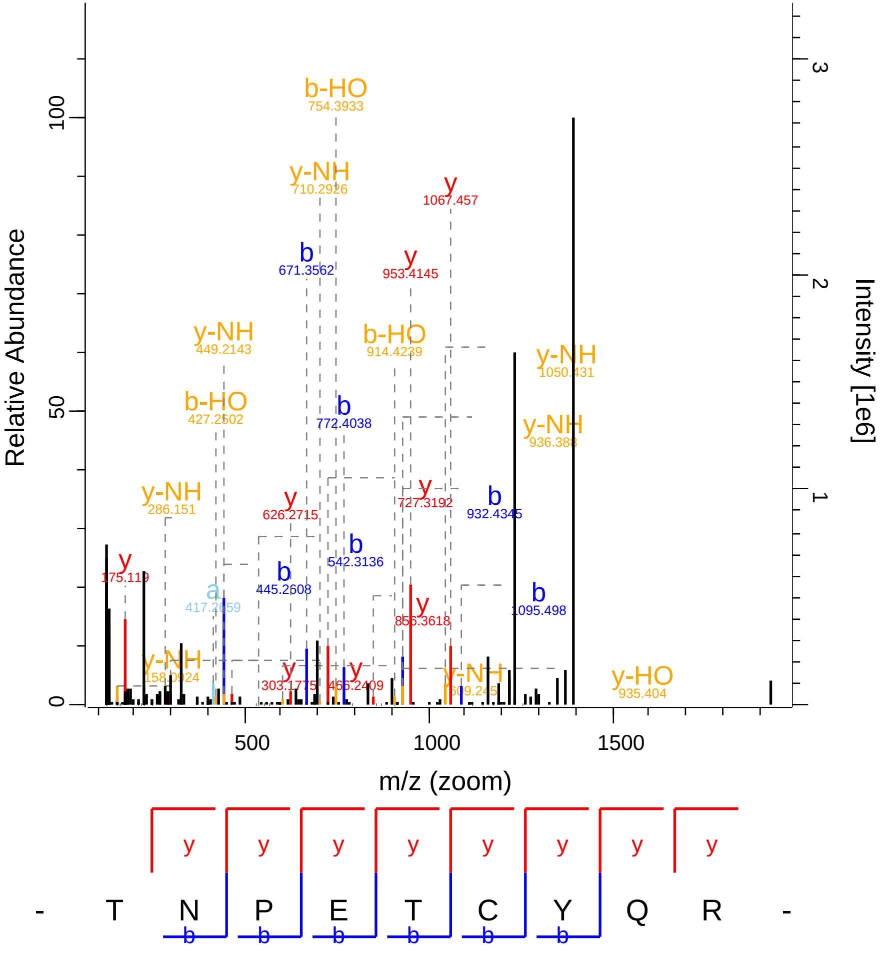

Raw File	Scan	Method	Score	m/z	Gene names
RO-QC-160121_ZackD_SS_Set_01_Fractior	8303	FTMS; HCl	167.2	860.94	CNOT8

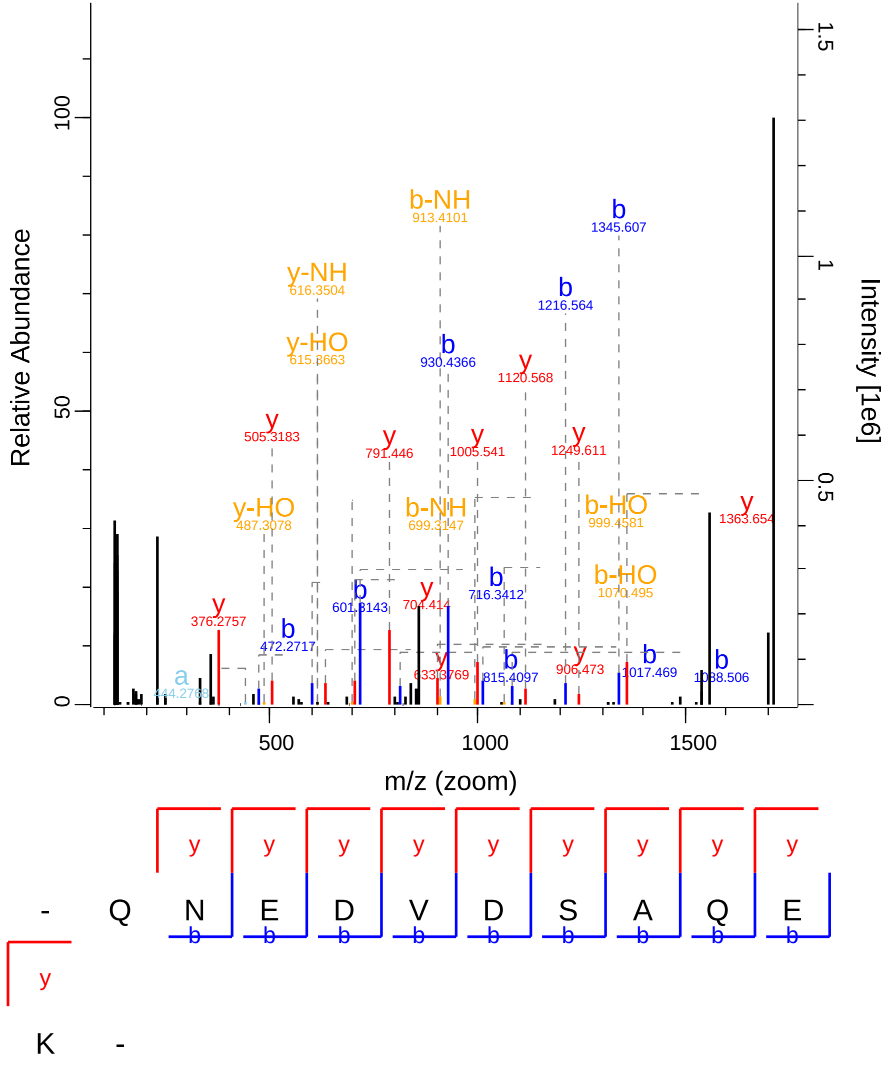

Raw File	Scan	Method	Score	m/z	Gene names
RO-QC-160121_ZackD_SS_Set_01_Fractior	8575	FTMS; HCI	94.77	761.91	UBXN2B

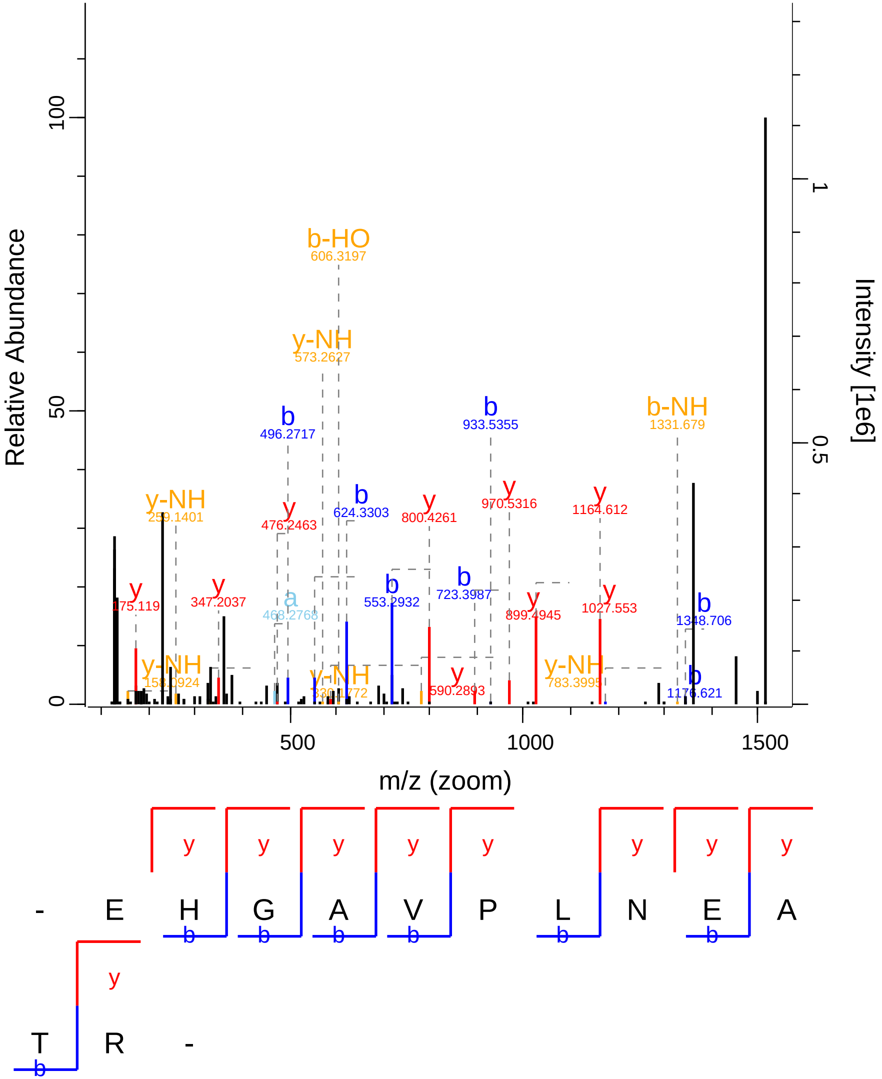

Raw File	Scan	Method	Score	m/z	Gene names
RO-QC-160121_ZackD_SS_Set_01_Fractior	9009	FTMS; HCI	91.21	685.06	AMER1

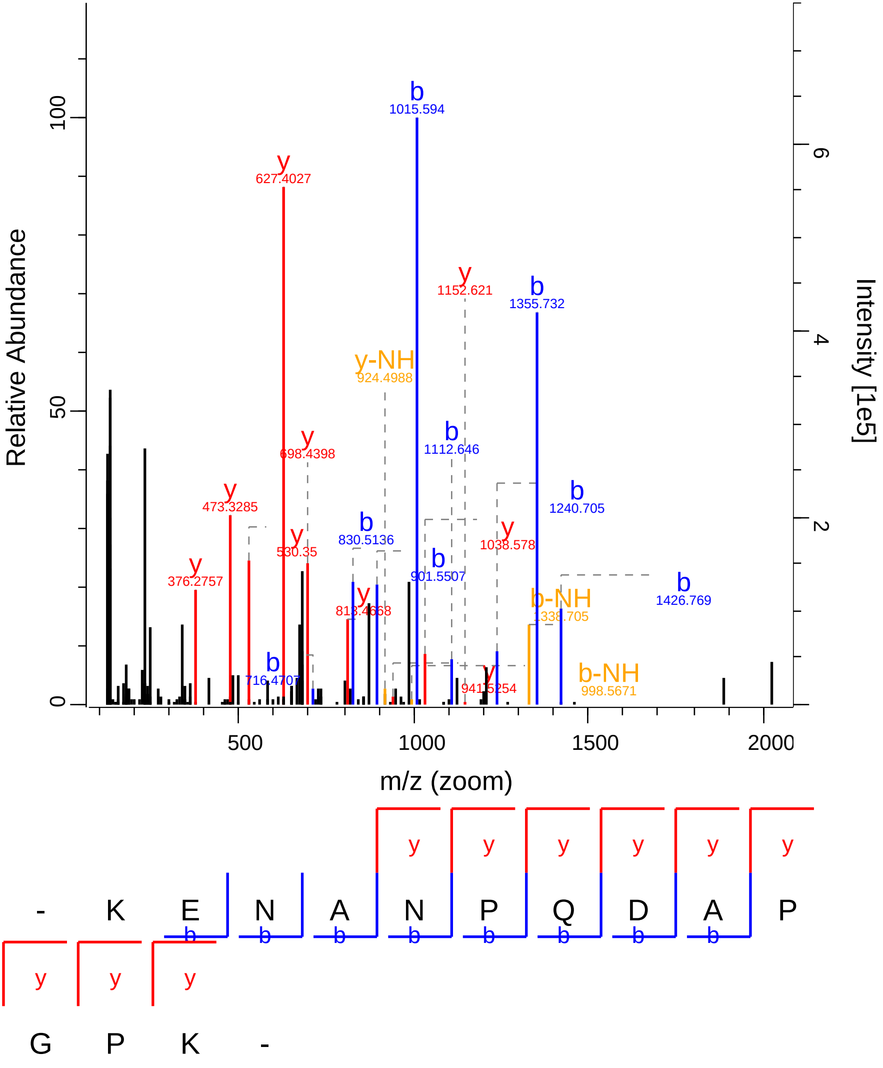

Raw File	Scan	Method	Score	m/z	Gene names
RO-QC-160121_ZackD_SS_Set_01_Fractior	9107	FTMS; HCl	84.74	661.83	TMEM47

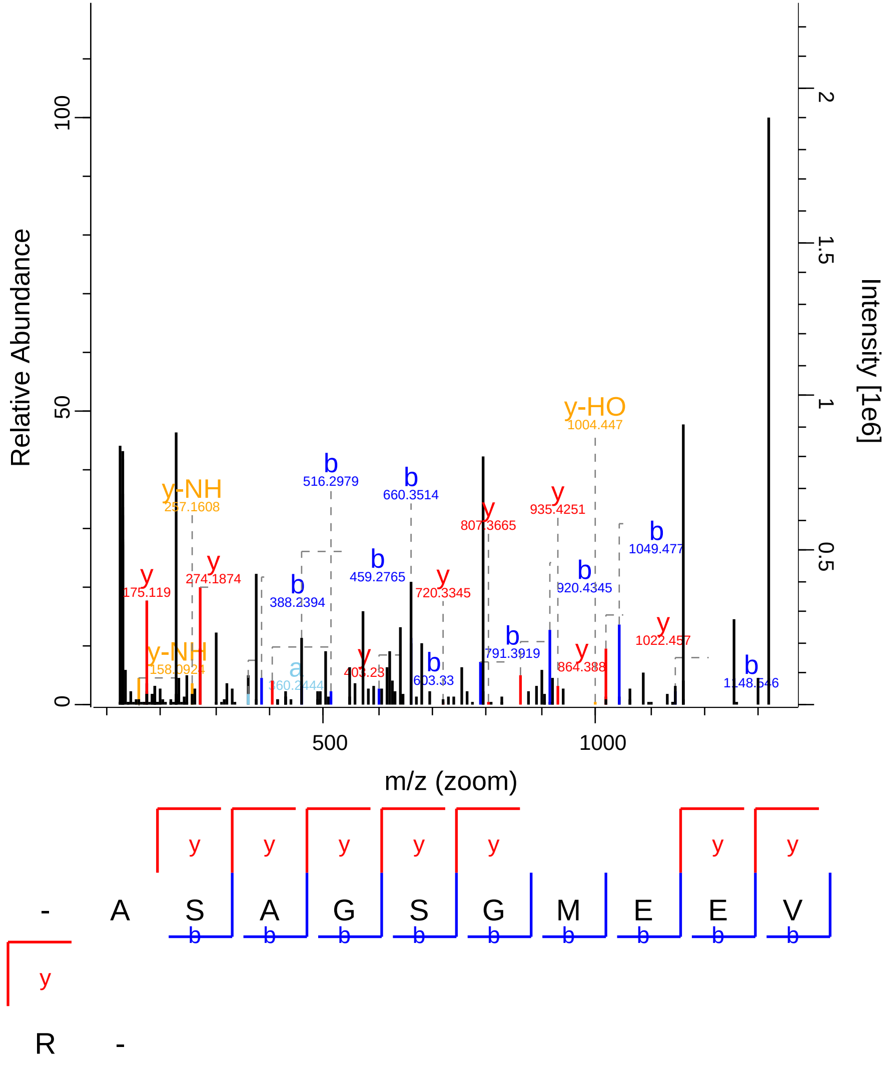

Raw File	Scan	Method	Score	m/z	Gene names
RO-QC-160121_ZackD_SS_Set_01_Fractio	11328	FTMS; HC	111.06	818.98	TRPT1

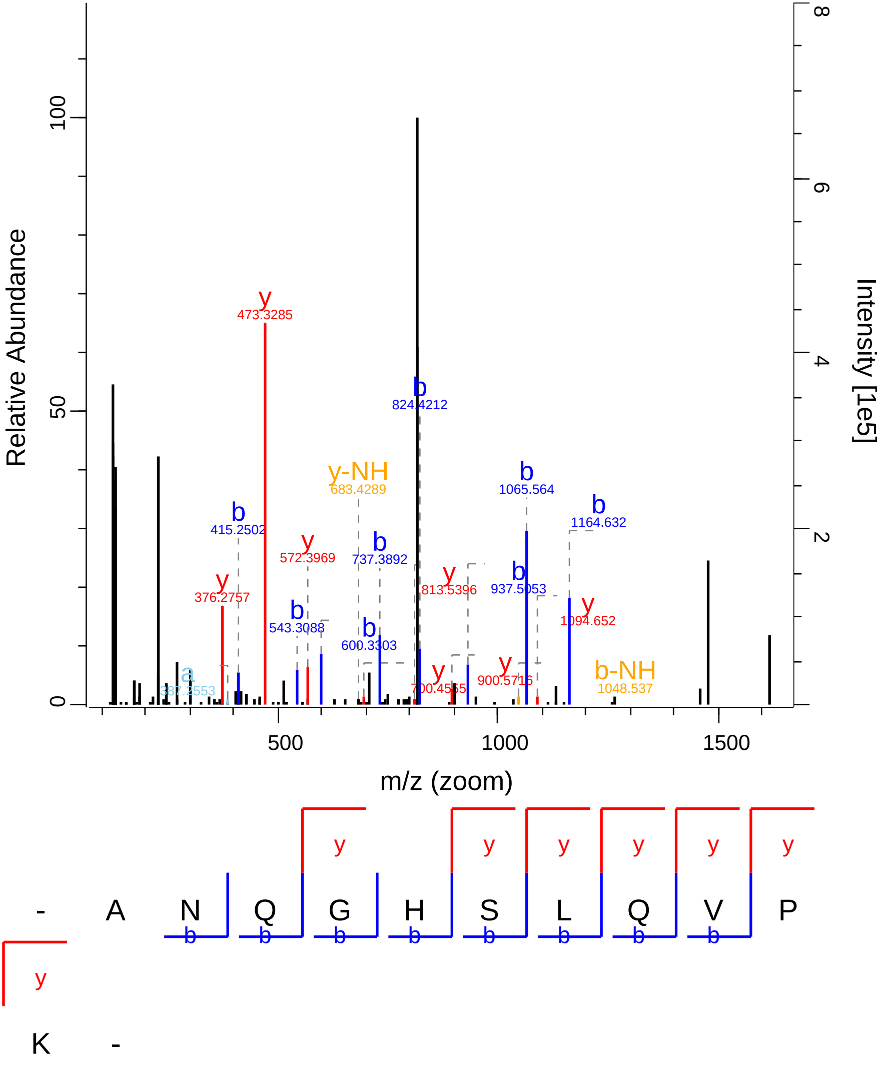

Raw File	Scan	Method	Score	m/z
RO-QC-160121_ZackD_SS_Set_01_Fraction_4	11866	FTMS; HCD	57.28	413.25

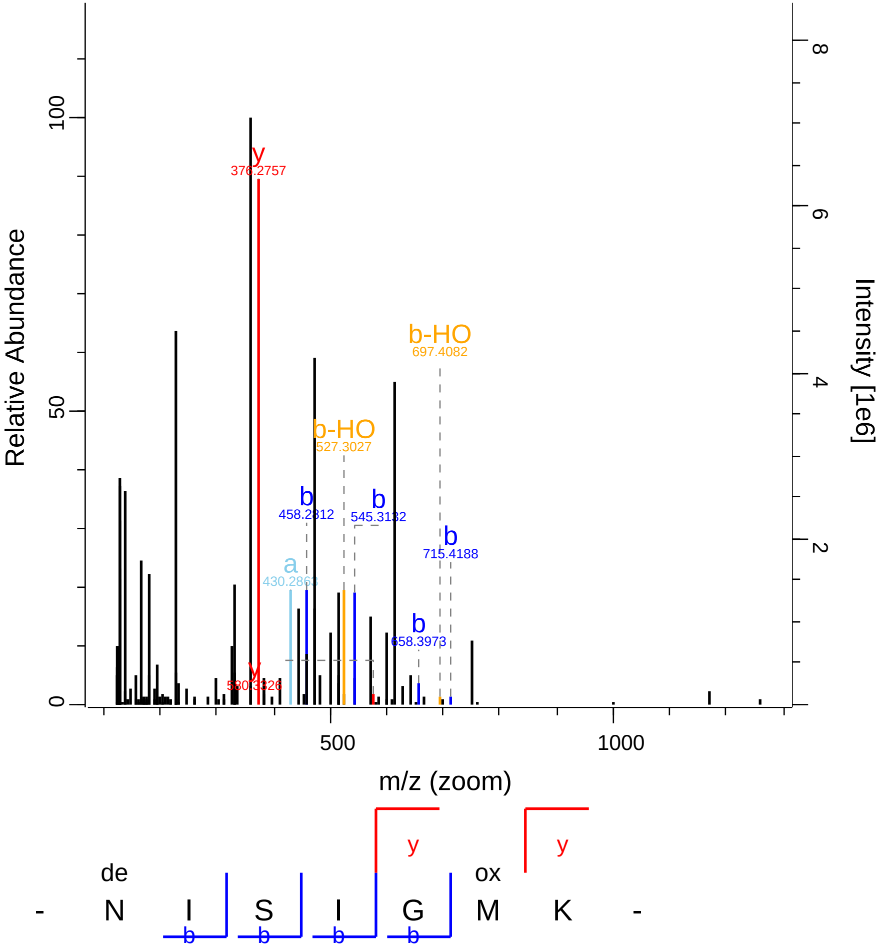

Raw File	Scan	Method	Score	m/z	Gene names
RO-QC-160121_ZackD_SS_Set_01_Fractio	12071	FTMS; HC	47.08	943.51	CDCA3

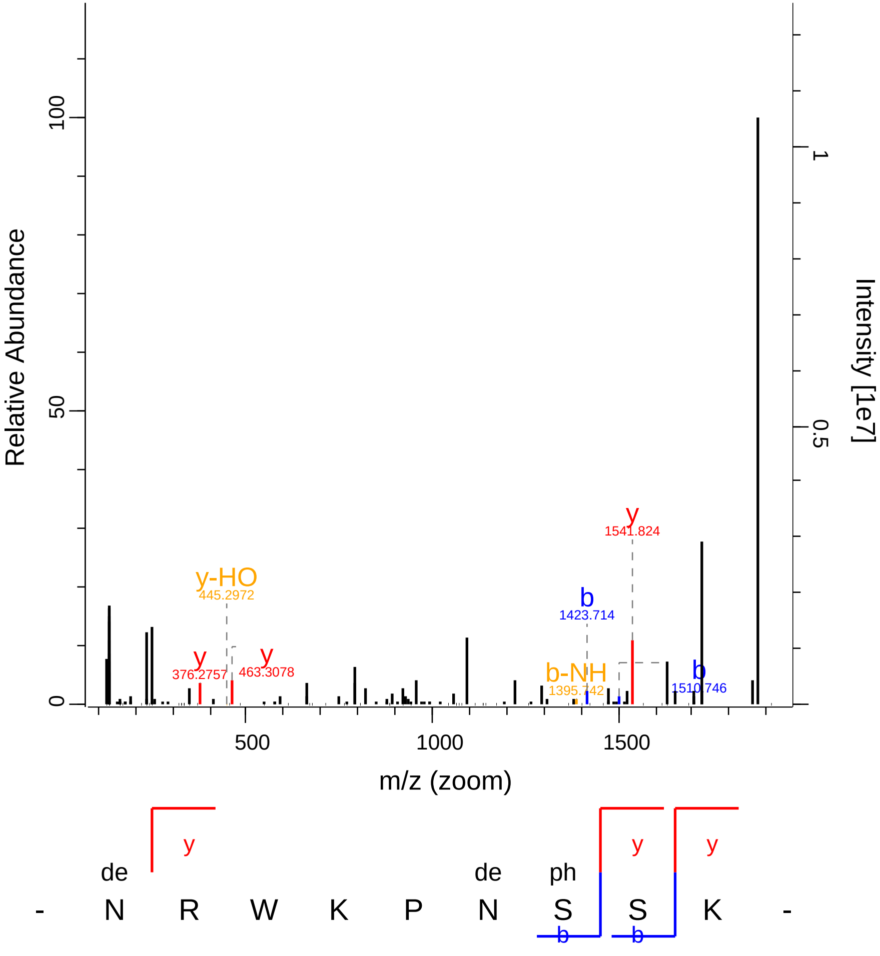

Raw File	Scan	Method	Score	m/z	Gene names
RO-QC-160121_ZackD_SS_Set_01_Fractio	12127	FTMS; HC	146.79	644.39	C6orf47

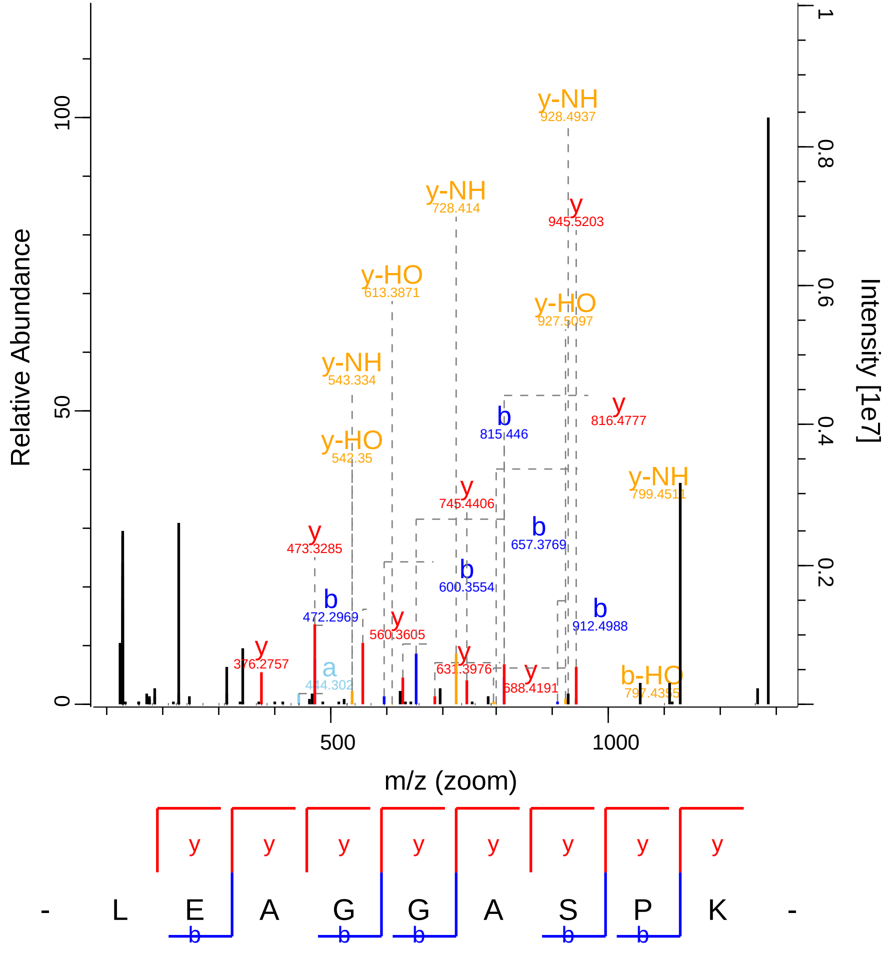

Raw File	Scan	Method	Score	m/z	Gene names
RO-QC-160121_ZackD_SS_Set_01_Fractior	12153	FTMS; HCI	69.98	778.9	SLC46A1

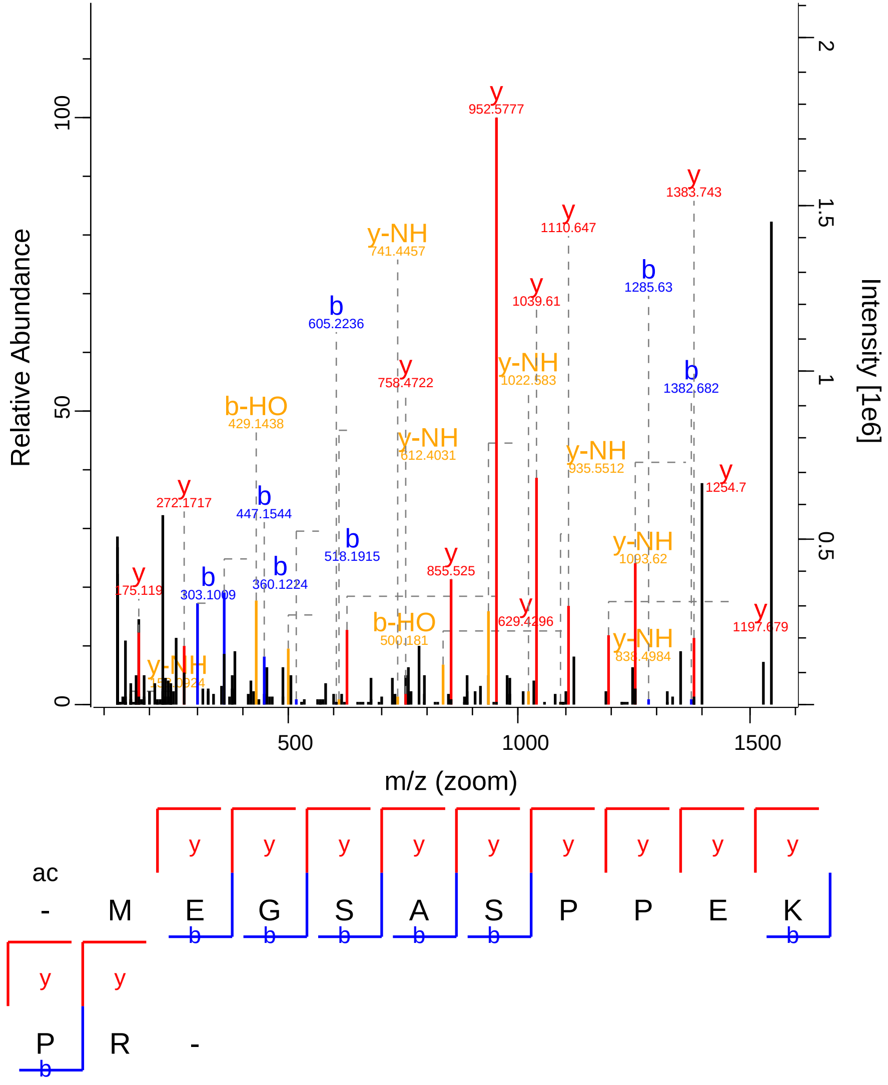

Raw File	Scan	Method	Score	m/z
RO-QC-160121_ZackD_SS_Set_01_Fraction_4	12483	FTMS; HCD	59.64	393.9

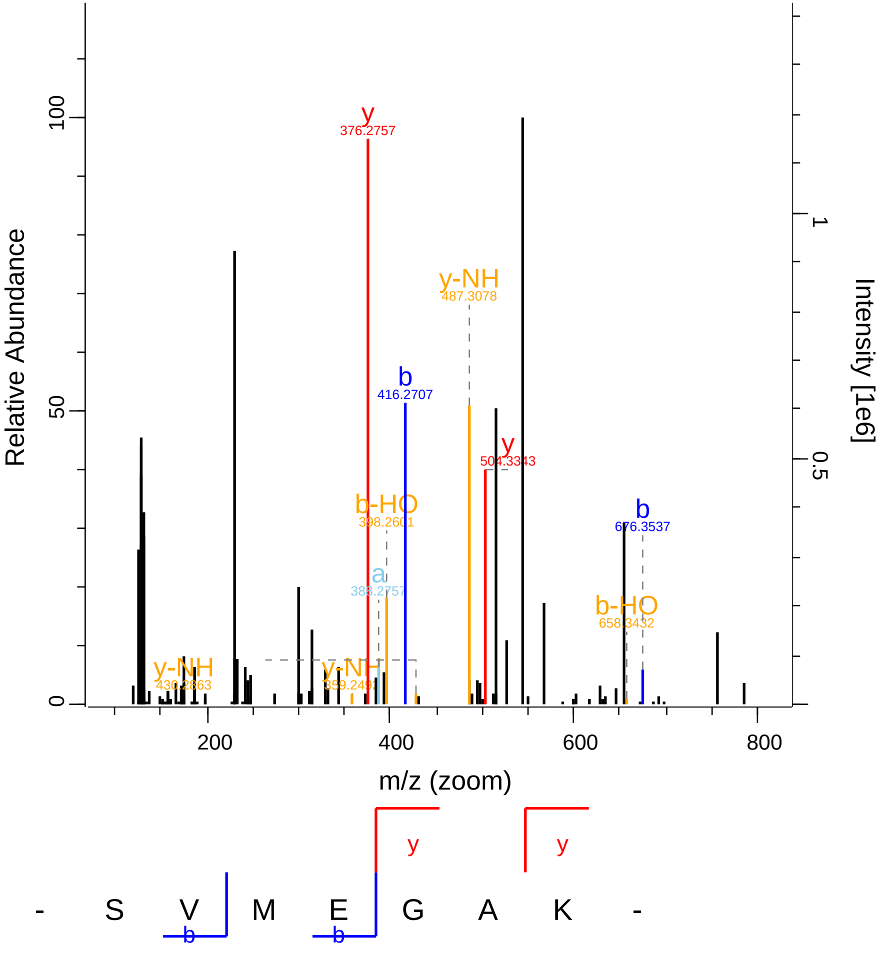

Raw File	Scan	Method	Score	m/z	Gene names
RO-QC-160121_ZackD_SS_Set_01_Fractio	12706	FTMS; HC	126.76	779.92	COL26A1

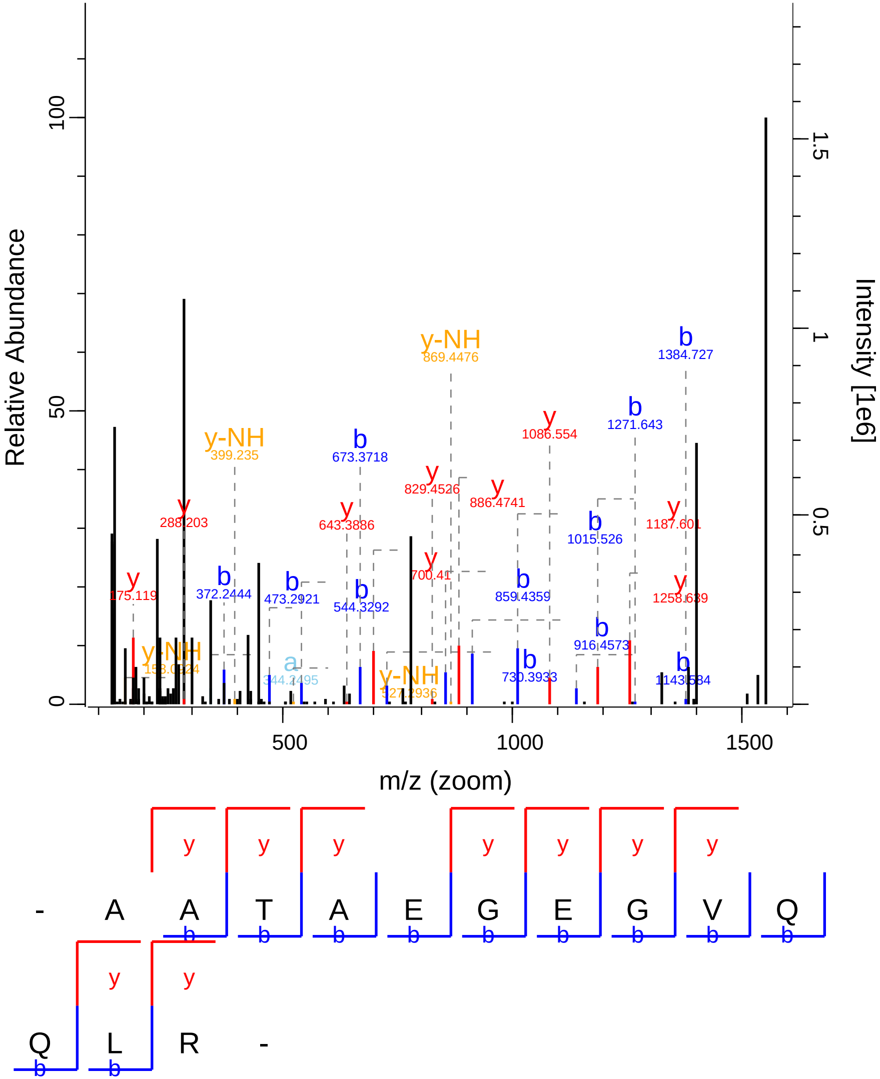

Raw File	Scan	Method	Score	m/z
RO-QC-160121_ZackD_SS_Set_01_Fraction_4	13003	FTMS; HCD	94.16	364.86

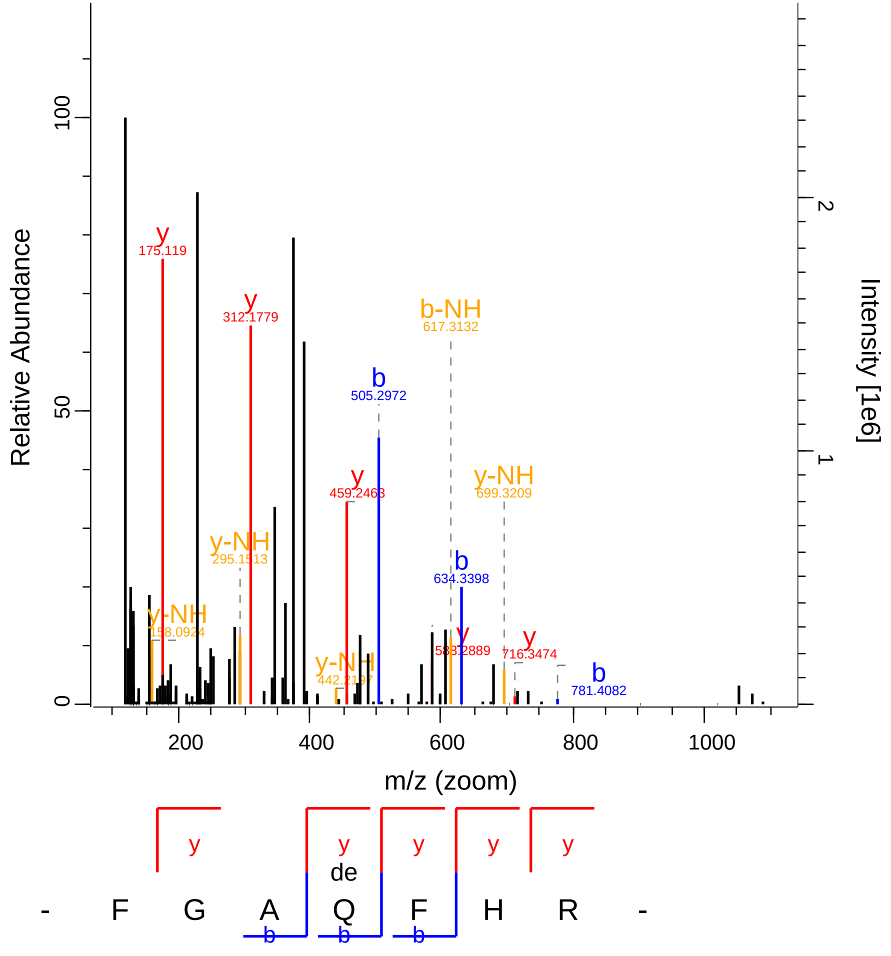

Raw File	Scan	Method	Score	m/z	Gene names
RO-QC-160121_ZackD_SS_Set_01_Fractior	14276	FTMS; HCI	86.88	833.9	GLRX2

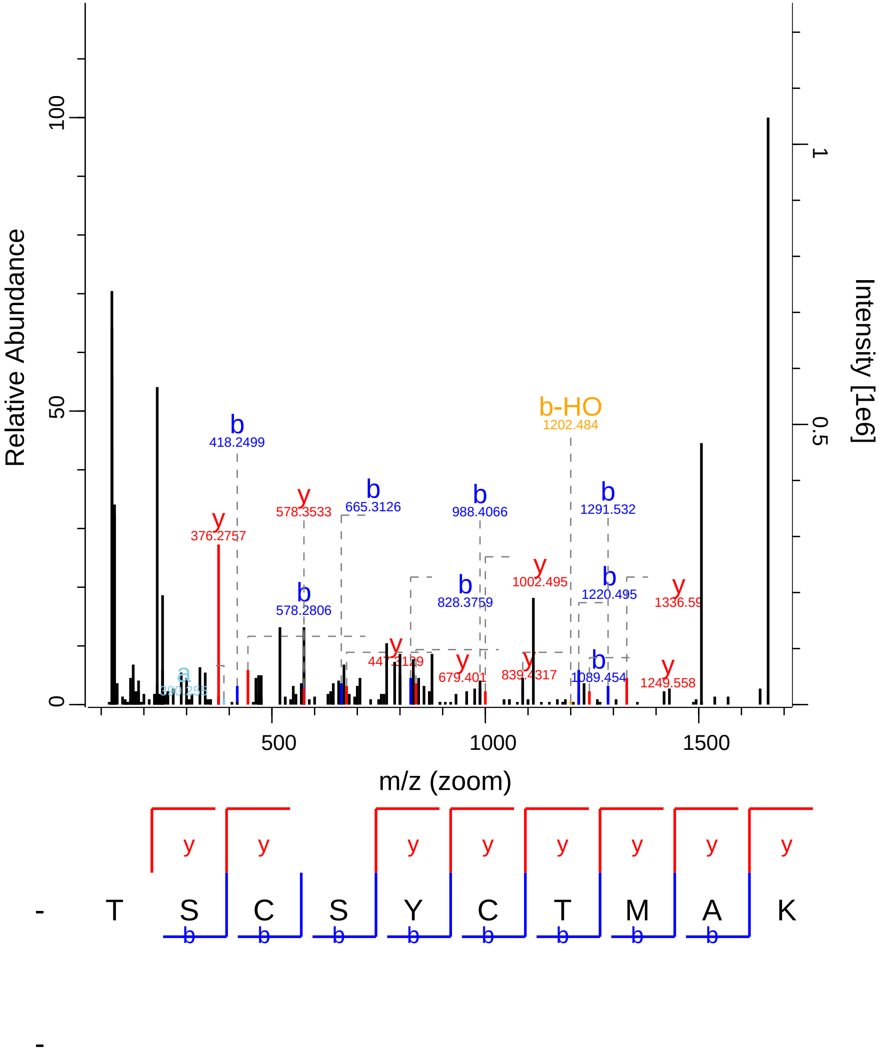

Raw File	Scan	Method	Score	m/z	Gene names
RO-QC-160121_ZackD_SS_Set_01_Fractio	14706	FTMS; HC	60.68	458.95	FAM117A

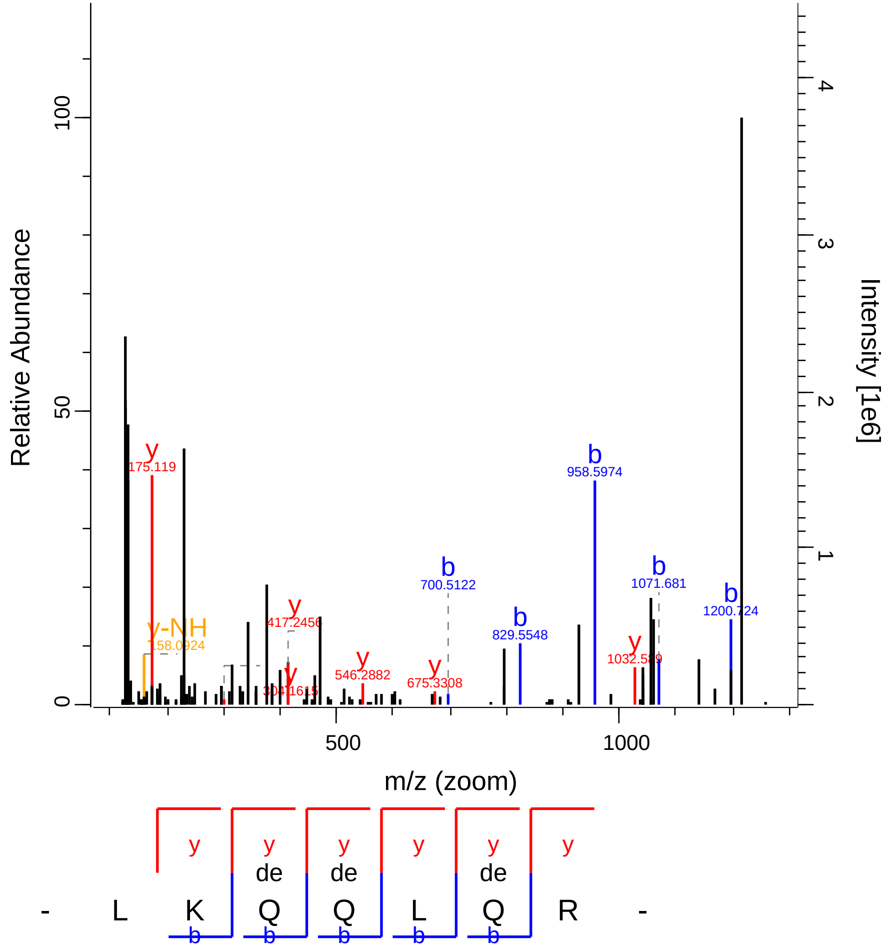

Raw File	Scan	Method	Score	m/z	Gene names
RO-QC-160121_ZackD_SS_Set_01_Fractio	14924	FTMS; HC	62.66	458.95	FAM117A

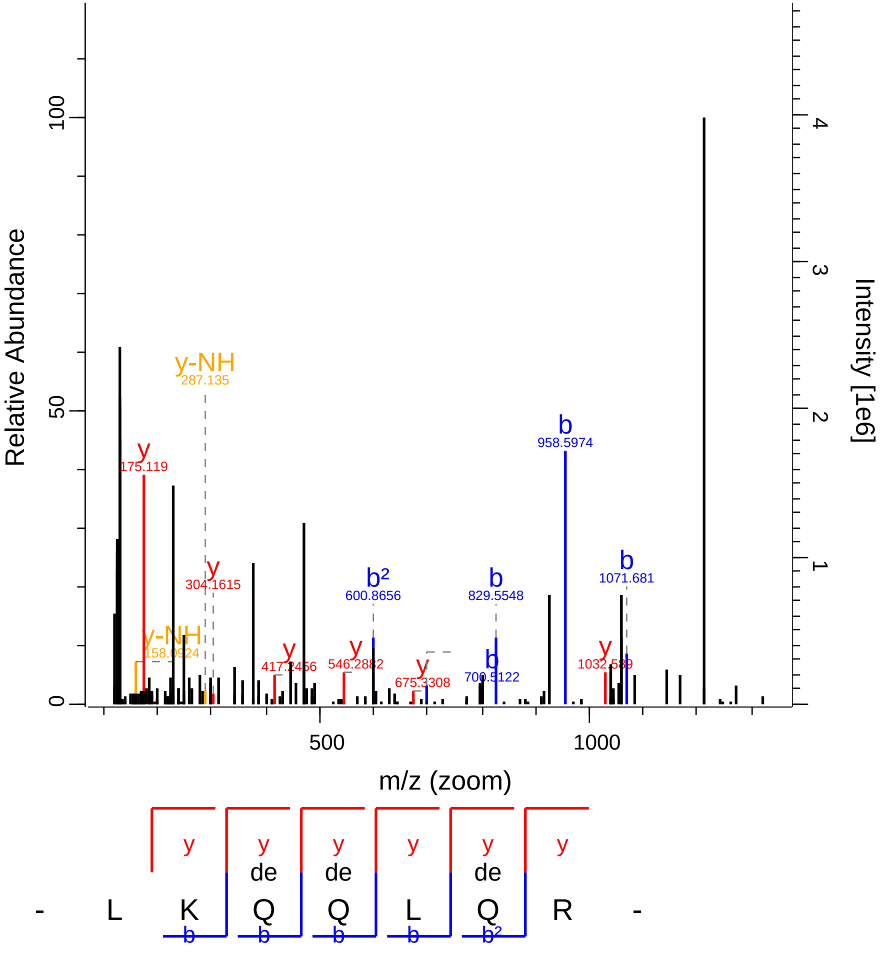

Raw File	Scan	Method	Score	m/z	Gene names
RO-QC-160121_ZackD_SS_Set_01_Fractio	15497	FTMS; HC	113.62	679.91	CLDN19

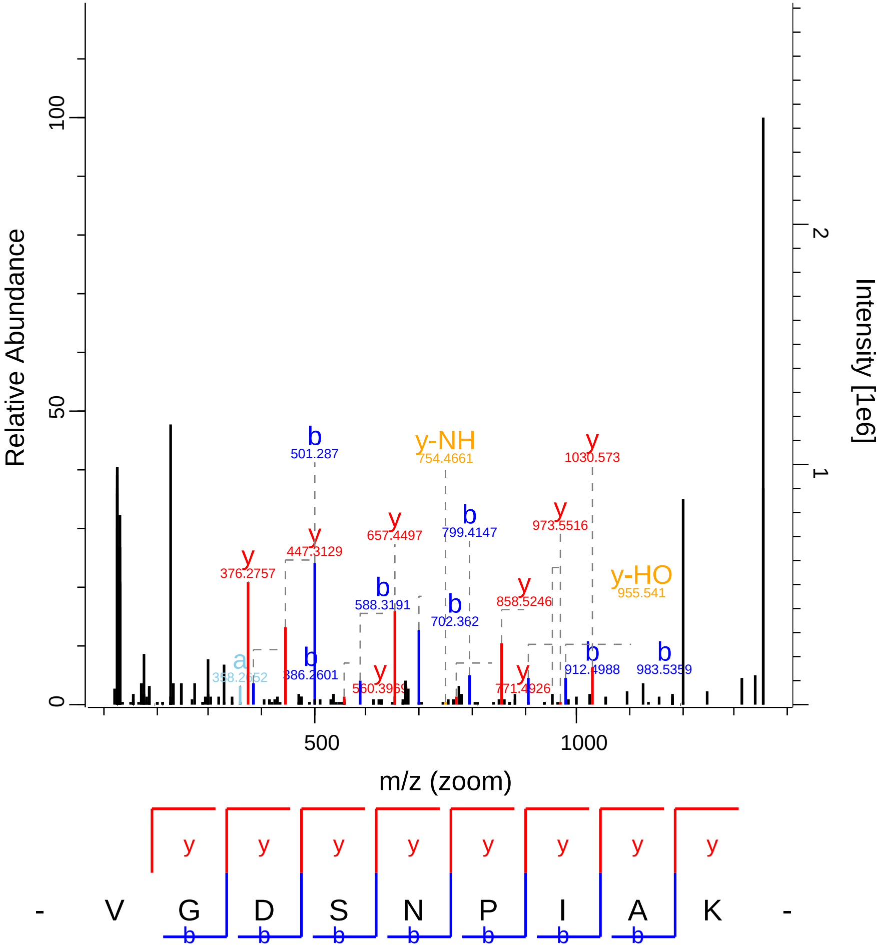

Raw File	Scan	Method	Score	m/z	Gene names
RO-QC-160121_ZackD_SS_Set_01_Fractio	15541	FTMS; HC	73.26	824.47	AIPL1

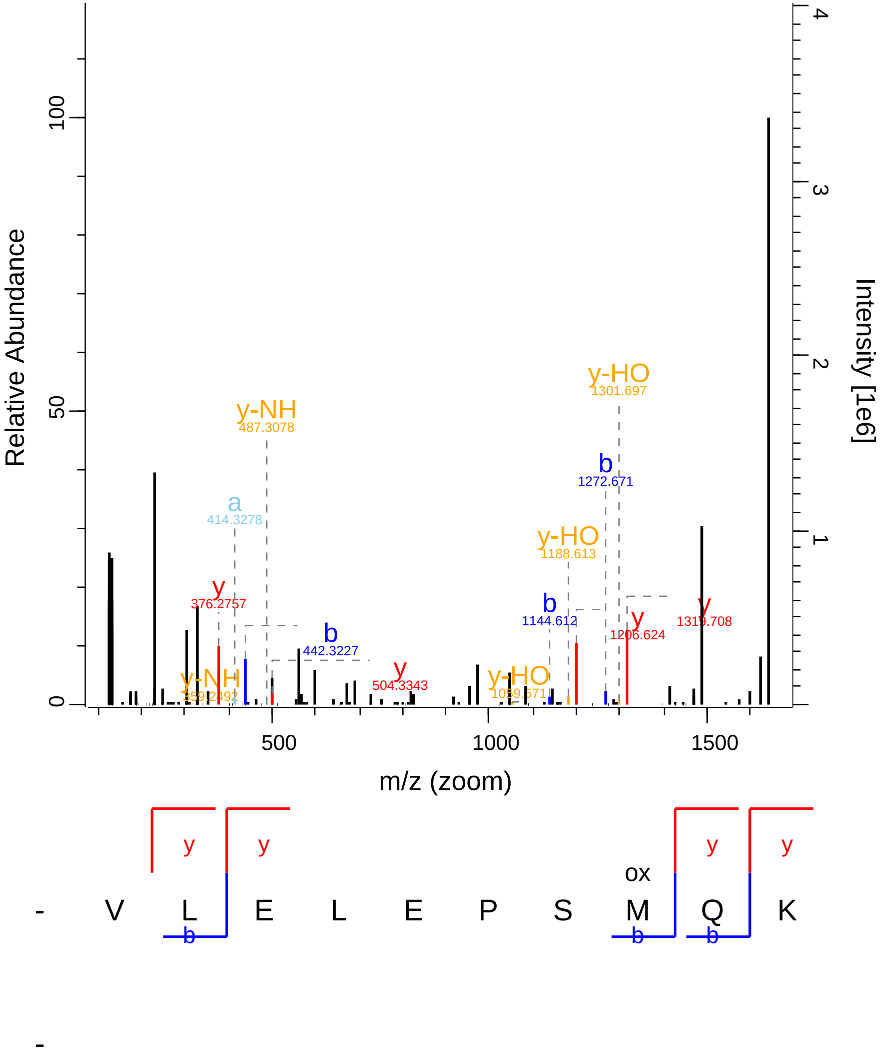

Raw File	Scan	Method	Score	m/z	Gene names
RO-QC-160121_ZackD_SS_Set_01_Fractio	15559	FTMS; HC	96.71	691.39	SLC7A6

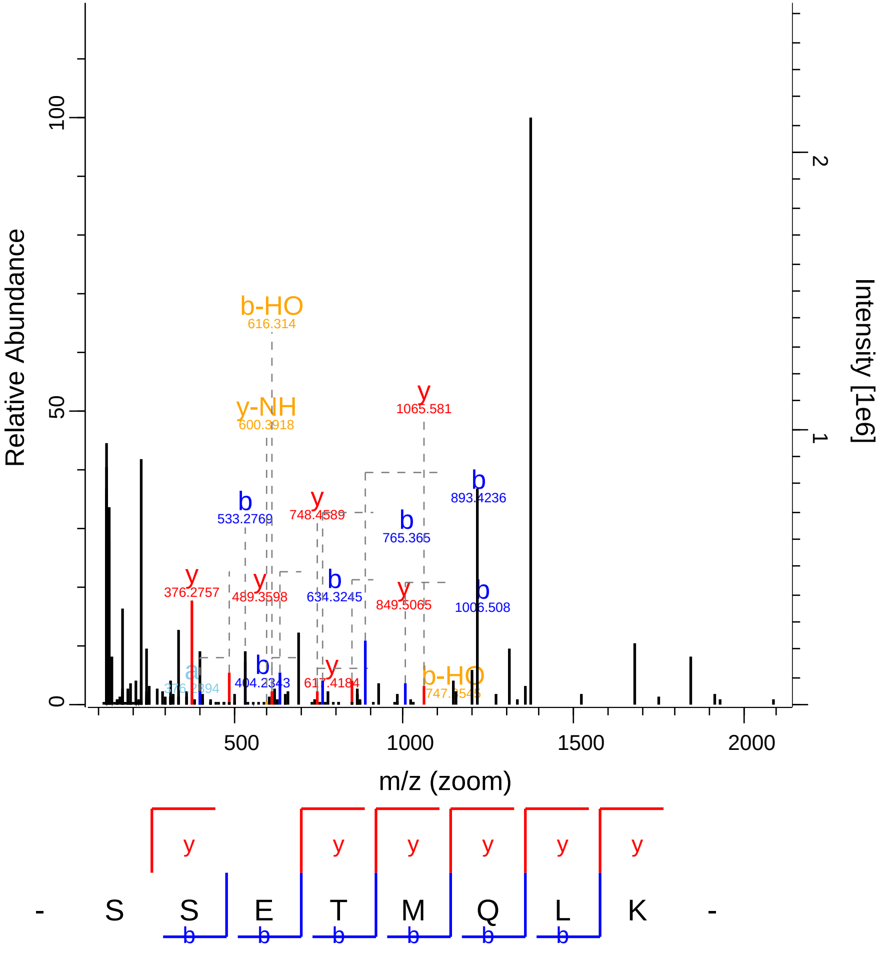

Raw File	Scan	Method	Score	m/z	Gene names
RO-QC-160121_ZackD_SS_Set_01_Fractio	15743	FTMS; HC	85.21	824.47	AIPL1

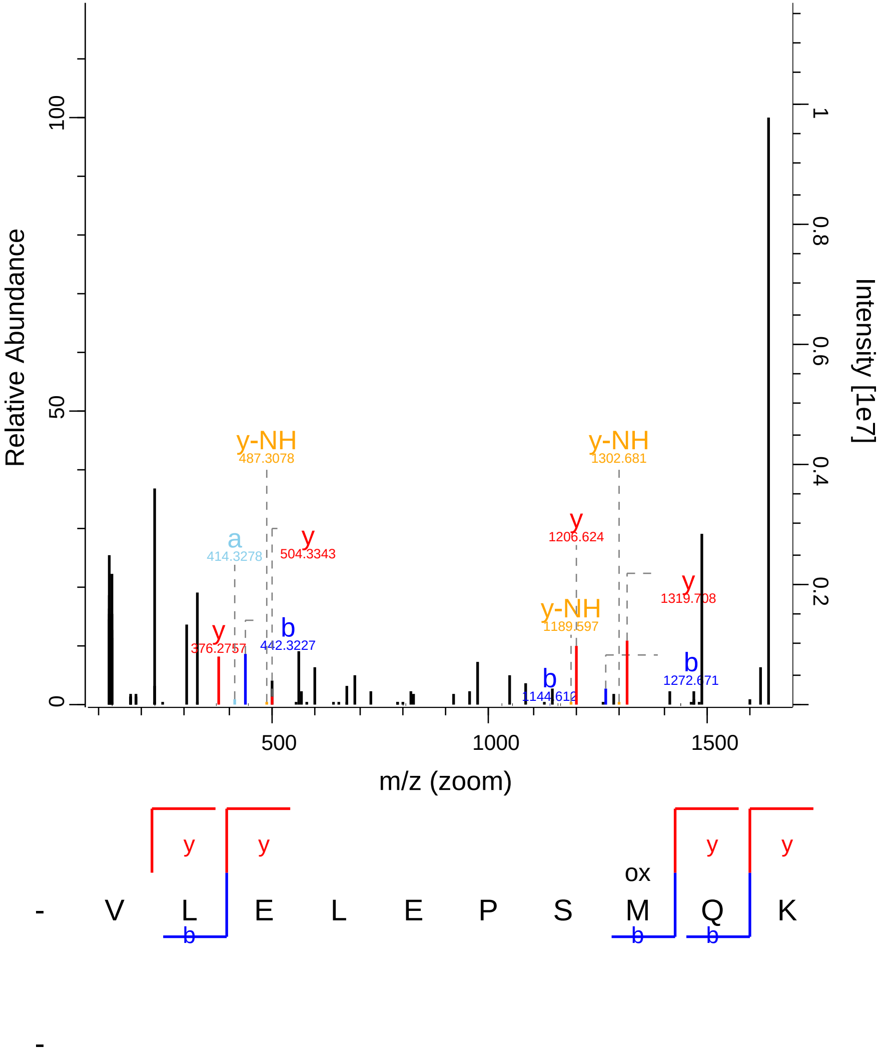

Raw File	Scan	Method	Score	m/z	Gene names
RO-QC-160121_ZackD_SS_Set_01_Fractio	15757	FTMS; HC	107.47	691.39	SLC7A6

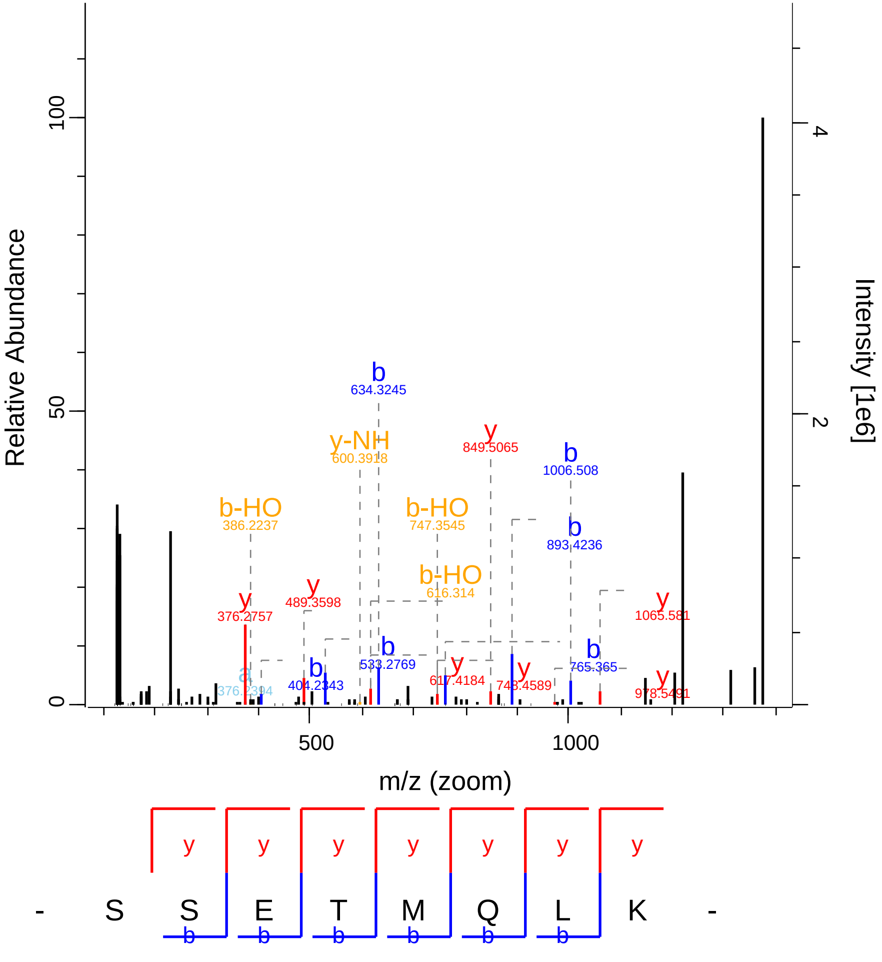

Raw File	Scan	Method	Score	m/z	Gene names
RO-QC-160121_ZackD_SS_Set_01_Fractio	16322	FTMS; HC	97.97	714.85	EID2

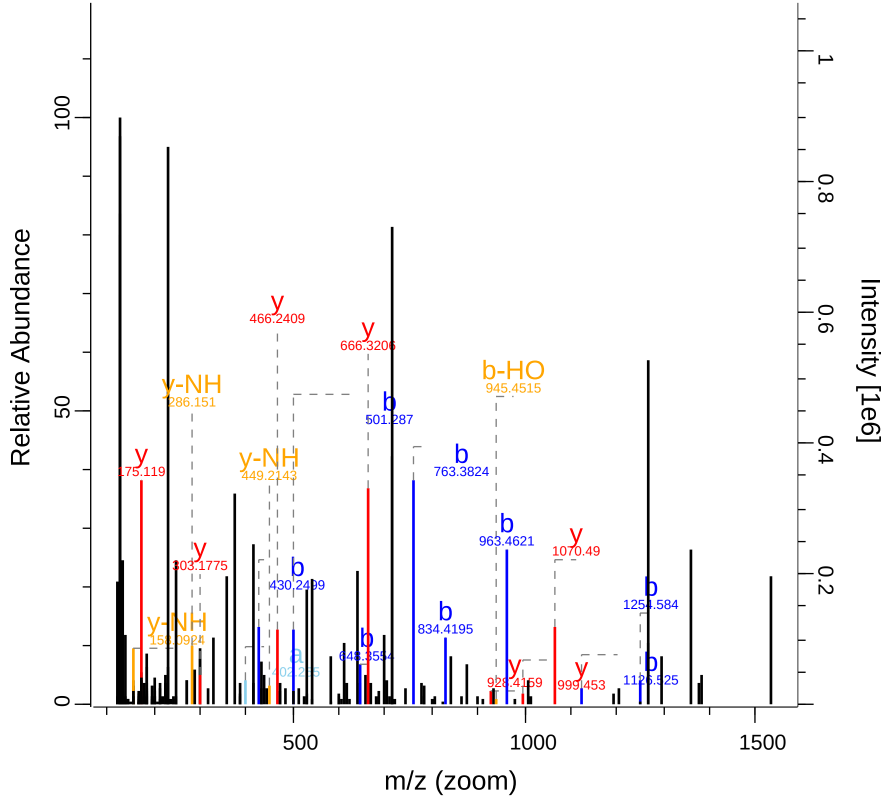

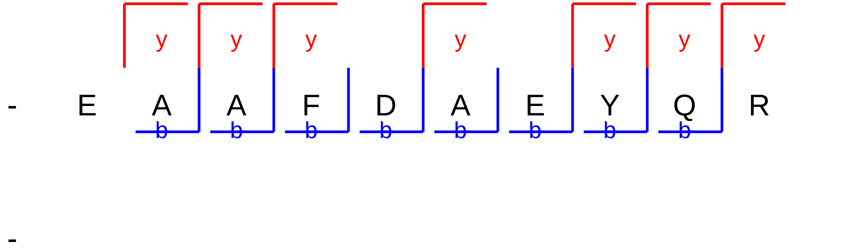

Raw File	Scan	Method	Score	m/z	Gene names
RO-QC-160121_ZackD_SS_Set_01_Fractio	16328	FTMS; HC	104.52	823.48	KDM4D

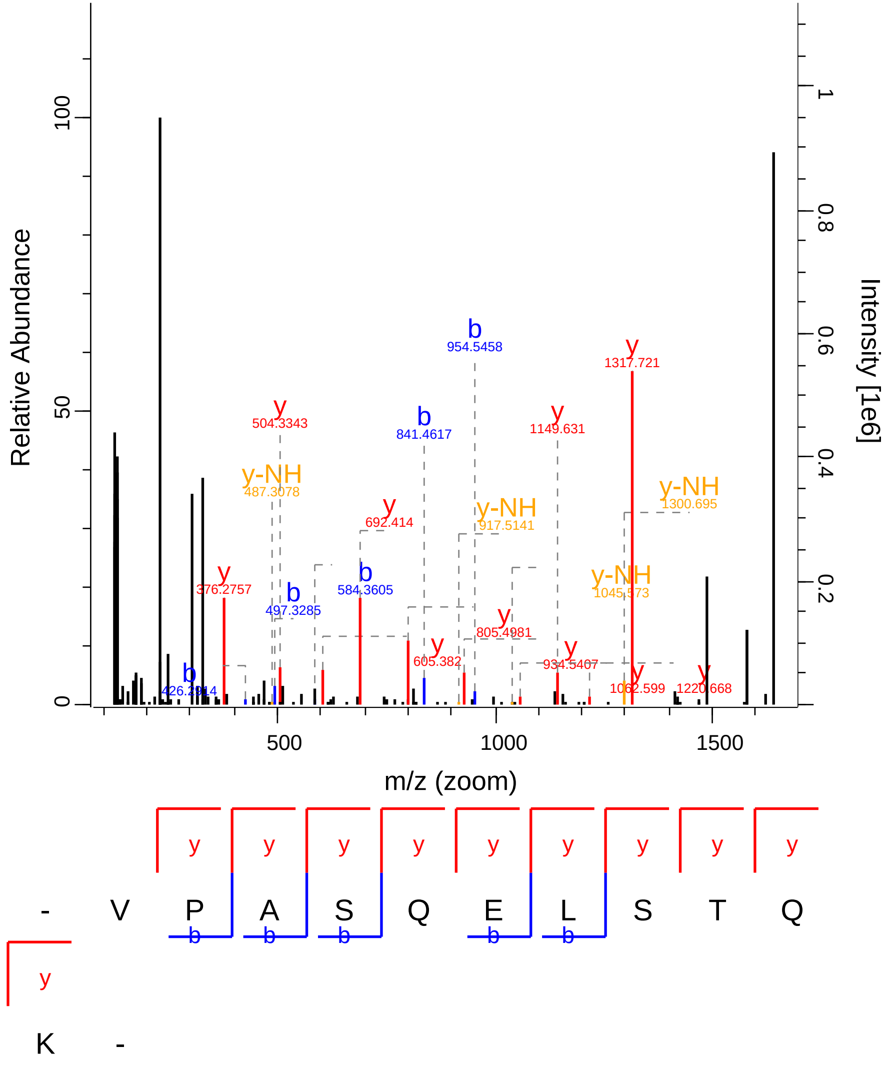

Raw File	Scan	Method	Score	m/z
RO-QC-160121_ZackD_SS_Set_01_Fraction_4	17480	FTMS; HCD	85.16	767.43

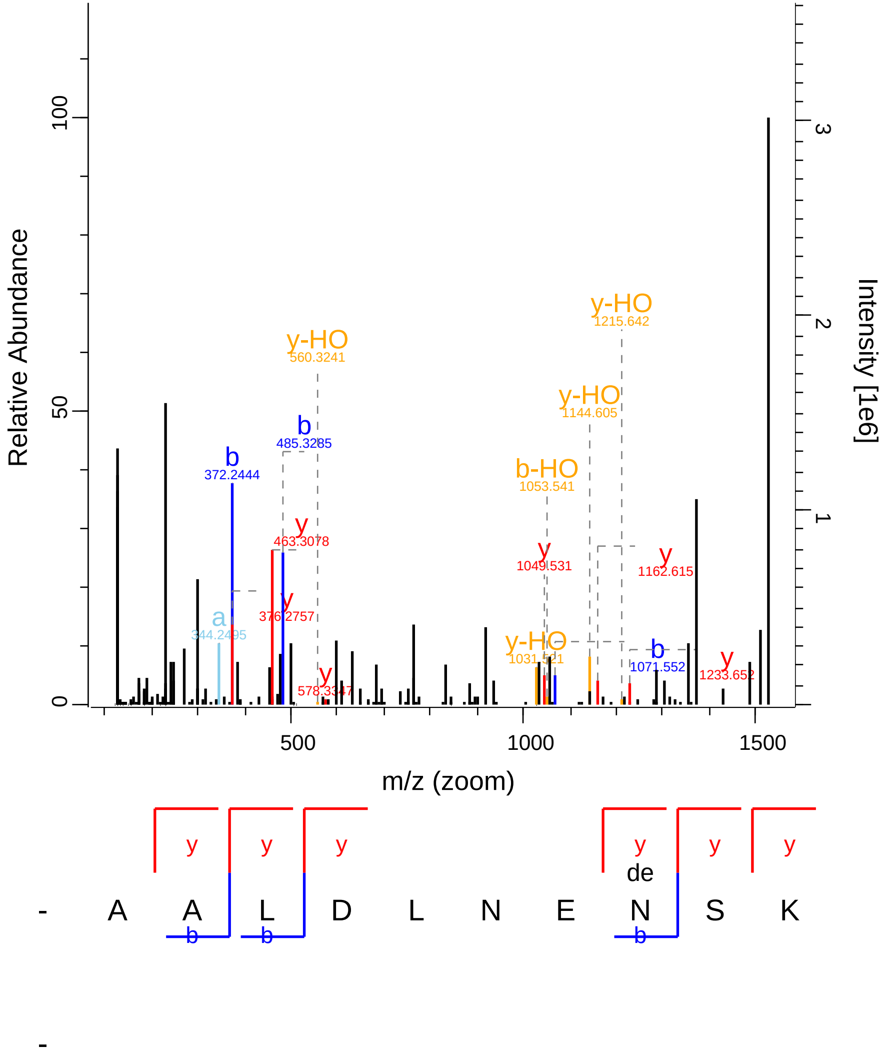

Raw File	Scan	Method	Score	m/z	Gene names
RO-QC-160121_ZackD_SS_Set_01_Fractio	17526	FTMS; HC	143.04	719.36	GEMIN6

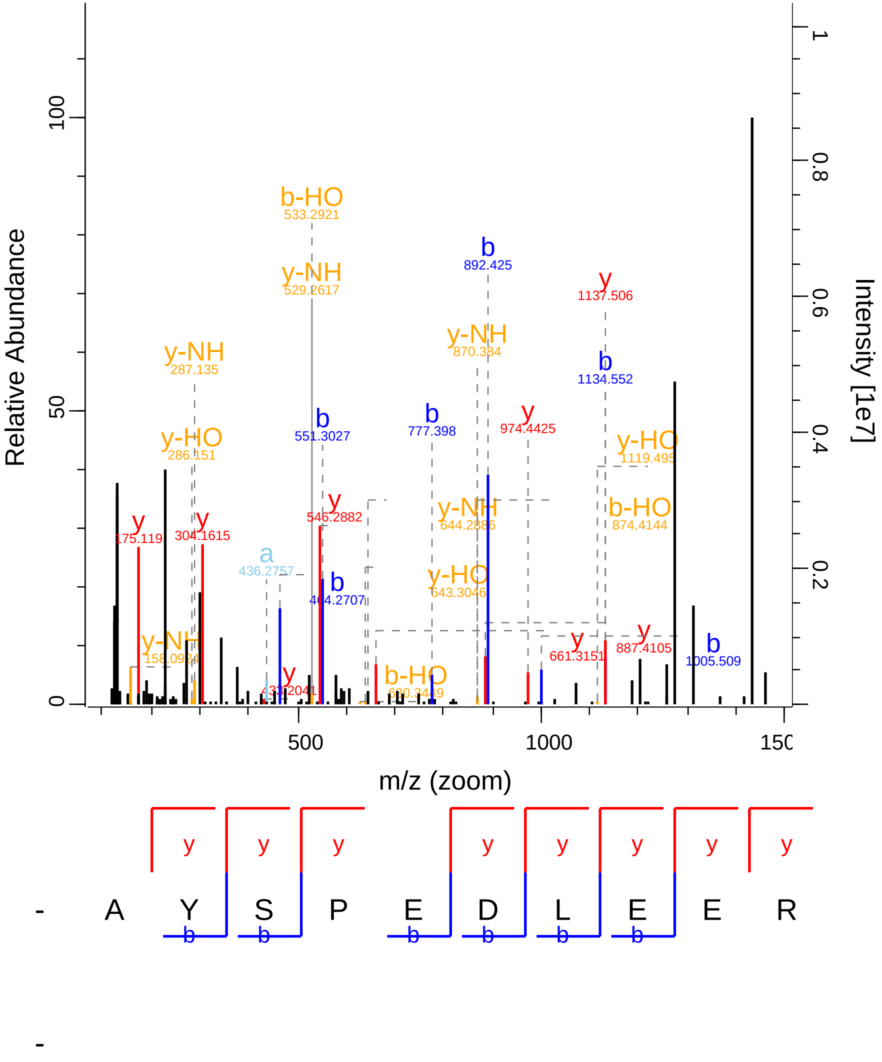

Raw File	Scan	Method	Score	m/z	Gene names
RO-QC-160121_ZackD_SS_Set_01_Fractio	17975	FTMS; HC	46.71	648.06	DAGLA

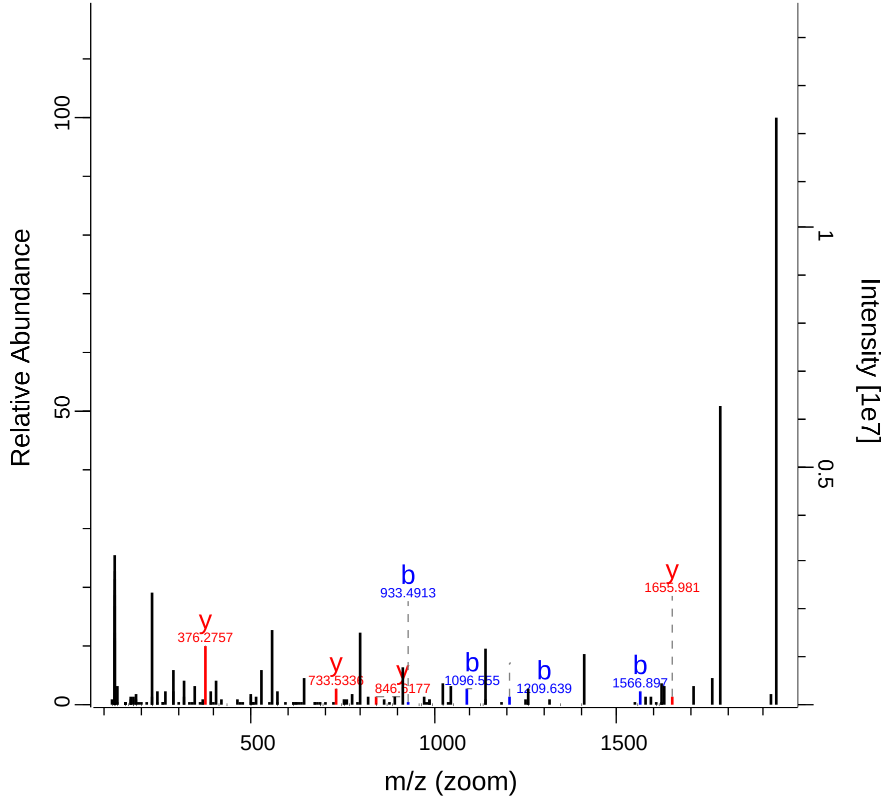

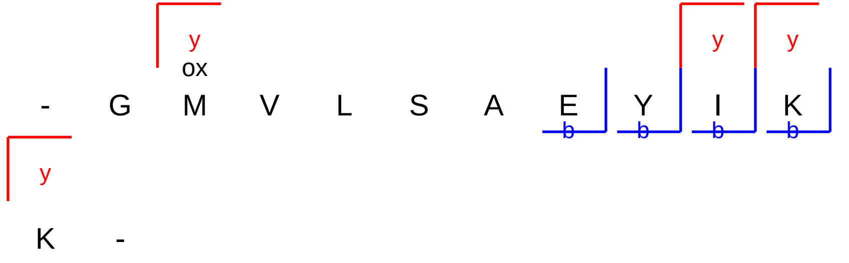

Raw File	Scan	Method	Score	m/z	Gene names
RO-QC-160121_ZackD_SS_Set_01_Fractior	18287	FTMS; HCI	121.9	934.5	TBPL1

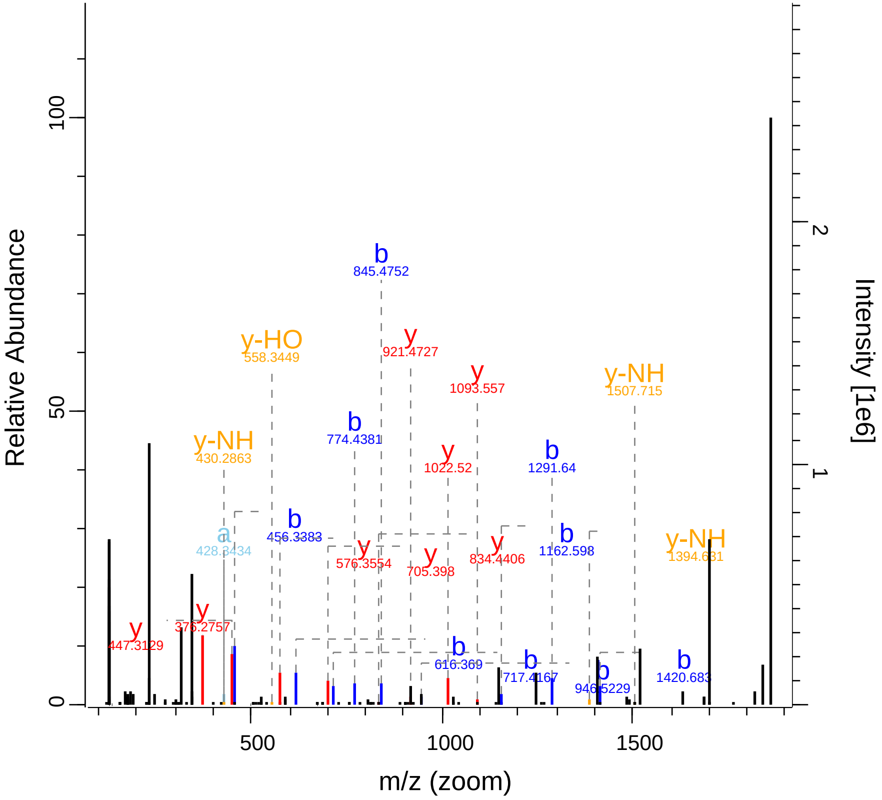

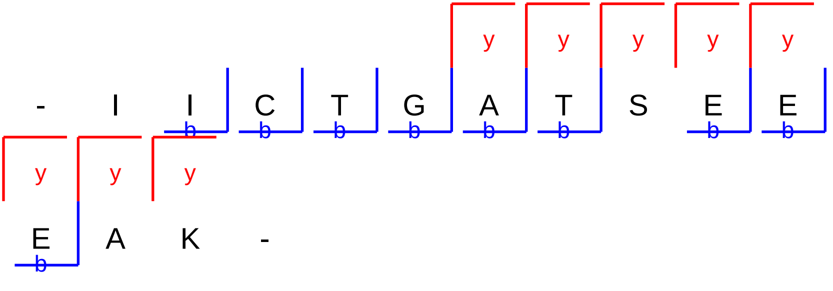

Raw File	Scan	Method	Score	m/z	Gene names
RO-QC-160121_ZackD_SS_Set_01_Fractio	18606	FTMS; HC	109.44	713.94	SYS1

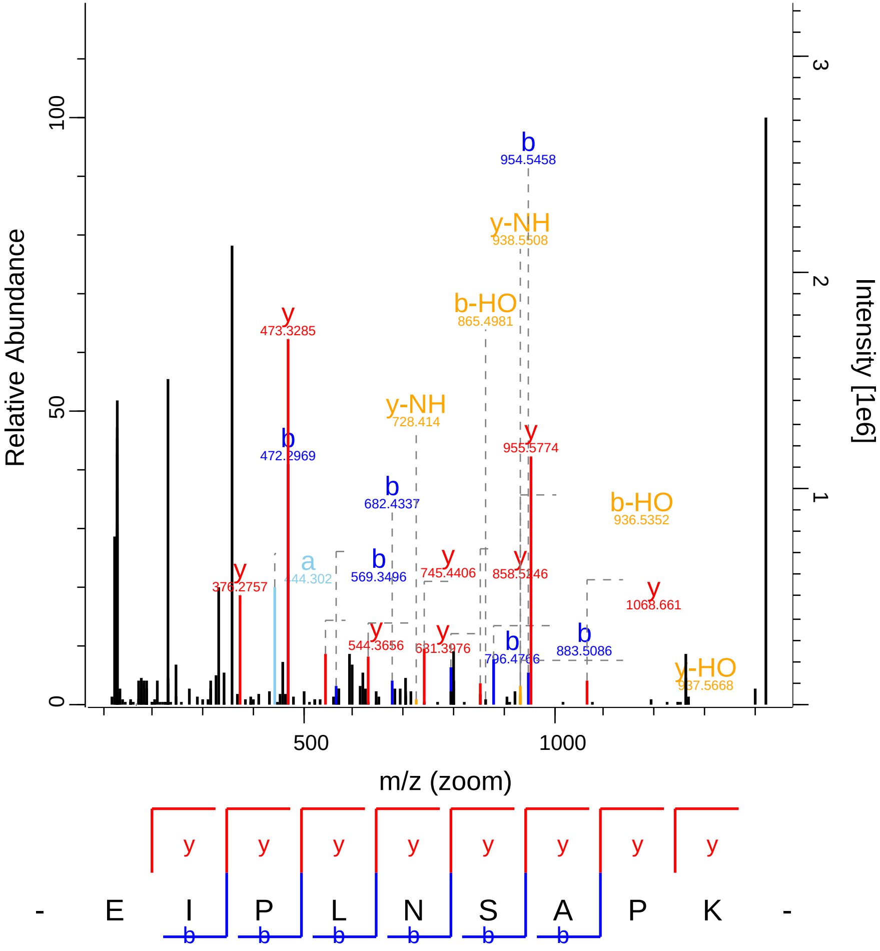

Raw File	Scan	Method	Score	m/z	Gene names
RO-QC-160121_ZackD_SS_Set_01_Fractior	18628	FTMS; HCI	51.61	638.6	MCTP2

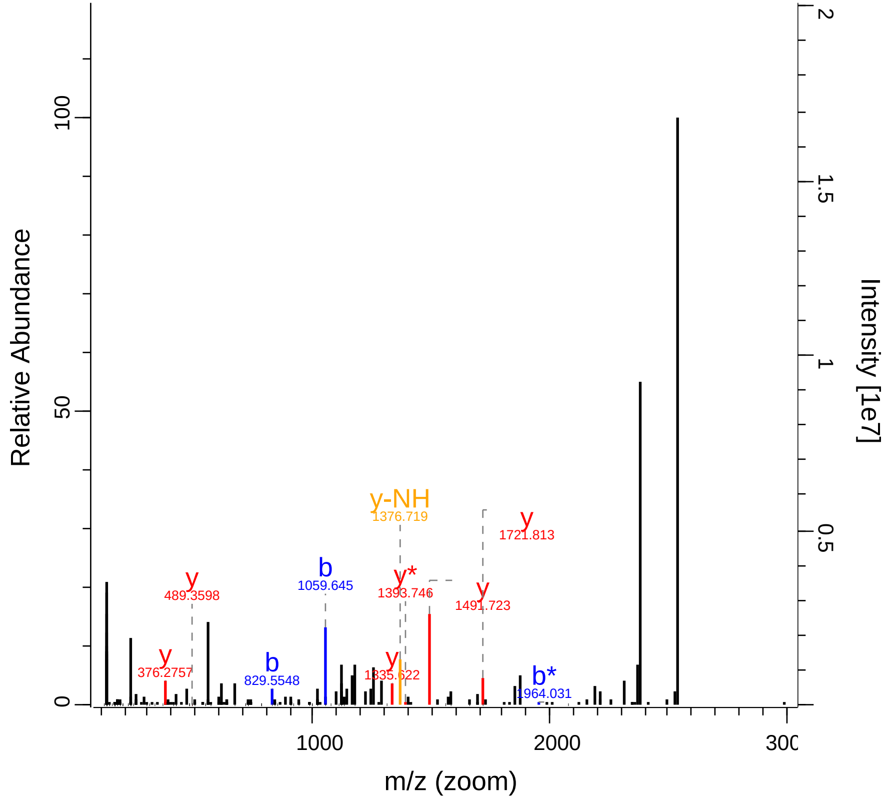

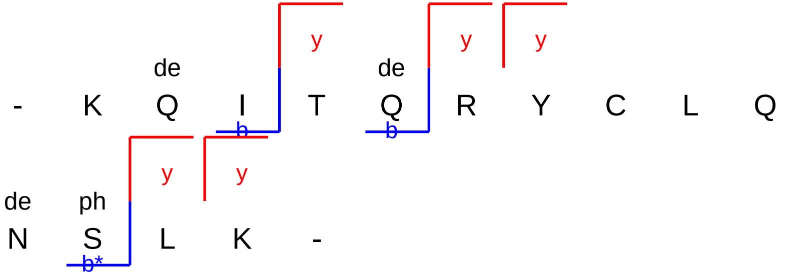

Raw File	Scan	Method	Score	m/z	Gene names
RO-QC-160121_ZackD_SS_Set_01_Fractio	18643	FTMS; HC	63.28	476.29	SYS1

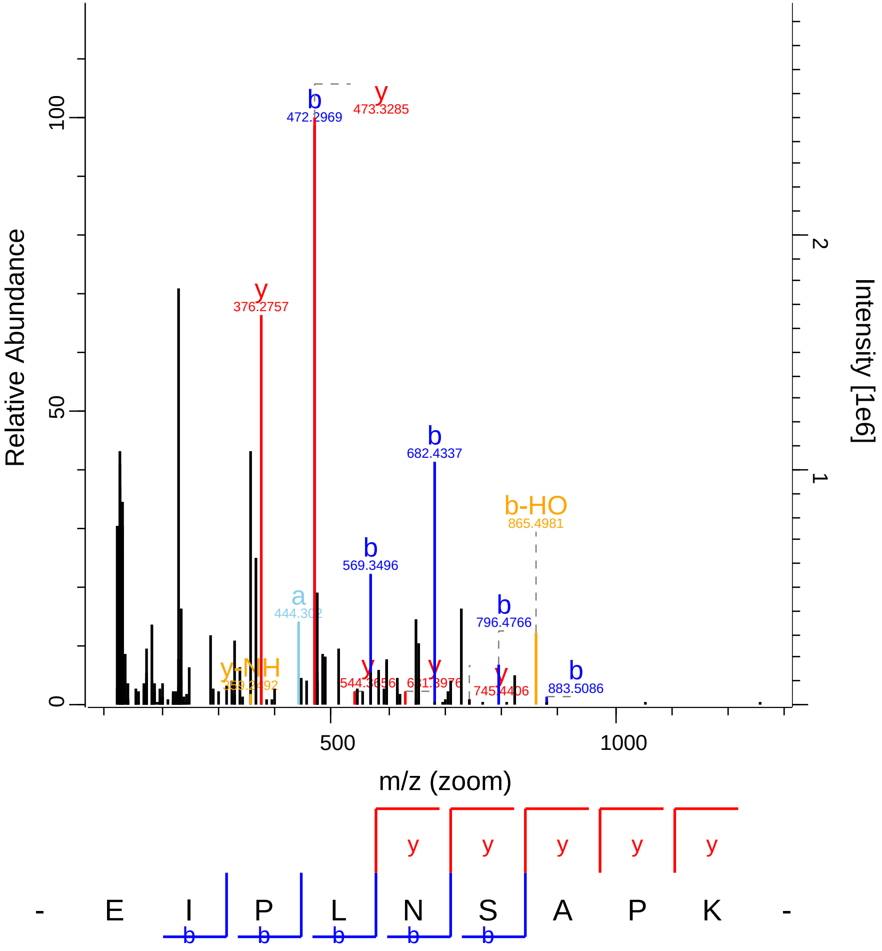

Raw File	Scan	Method	Score	m/z	Gene names
RO-QC-160121_ZackD_SS_Set_01_Fractio	18662	FTMS; HC	102.98	804.72	TTF1

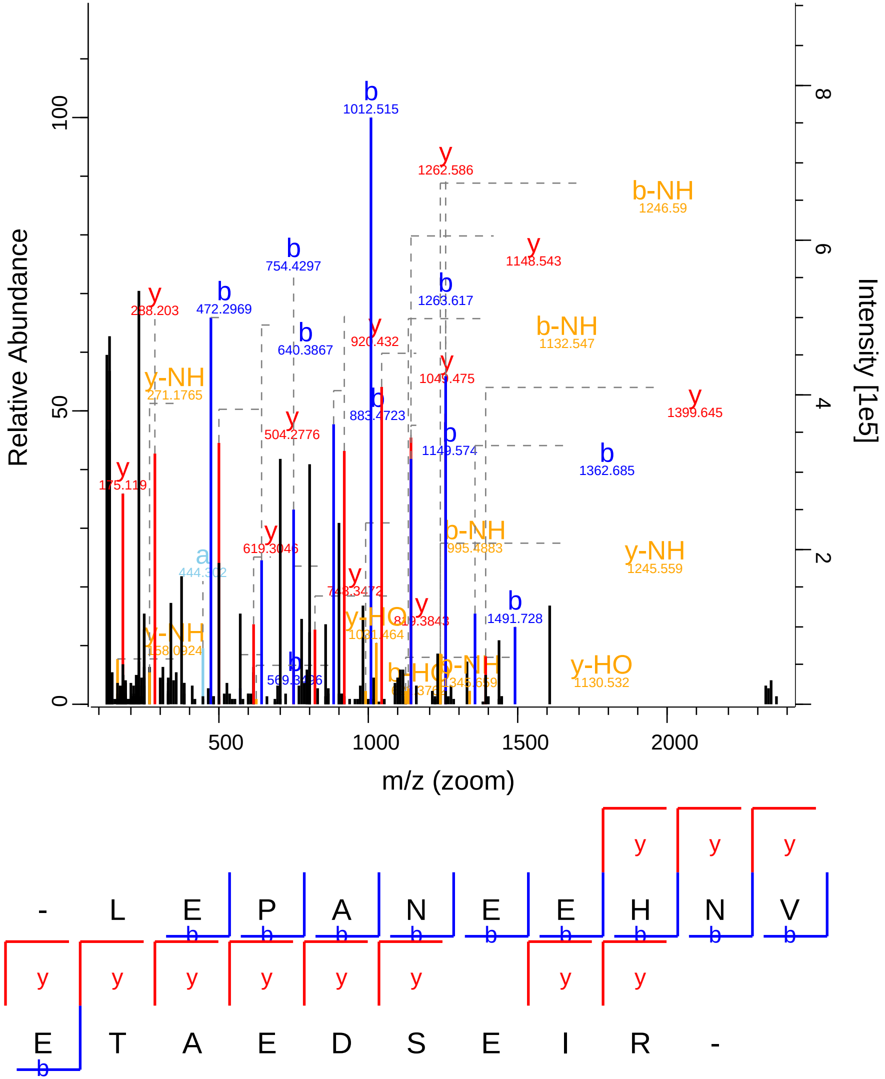

Raw File	Scan	Method	Score	m/z	Gene names
RO-QC-160121_ZackD_SS_Set_01_Fractio	19879	FTMS; HC	194.54	933.03	TMEM208

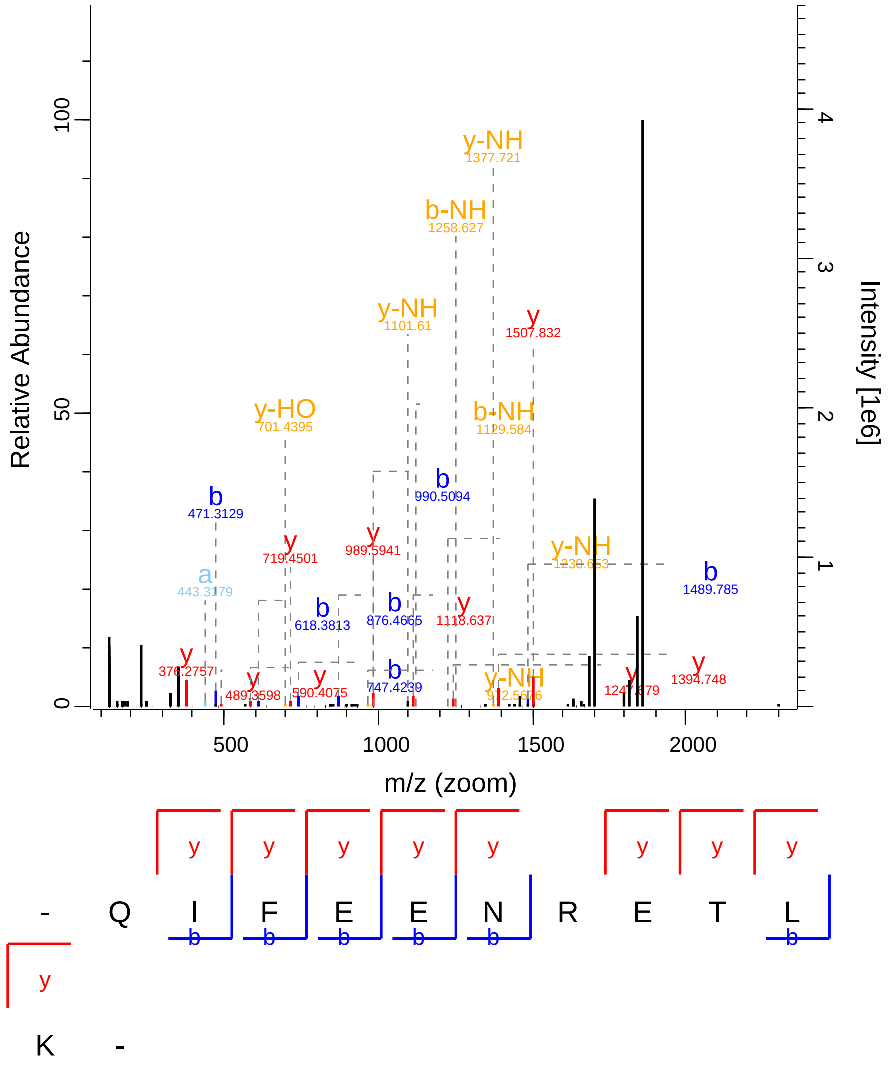

Raw File	Scan	Method	Score	m/z	Gene names
RO-QC-160121_ZackD_SS_Set_01_Fractio	19916	FTMS; HC	316.83	864.96	CBY1

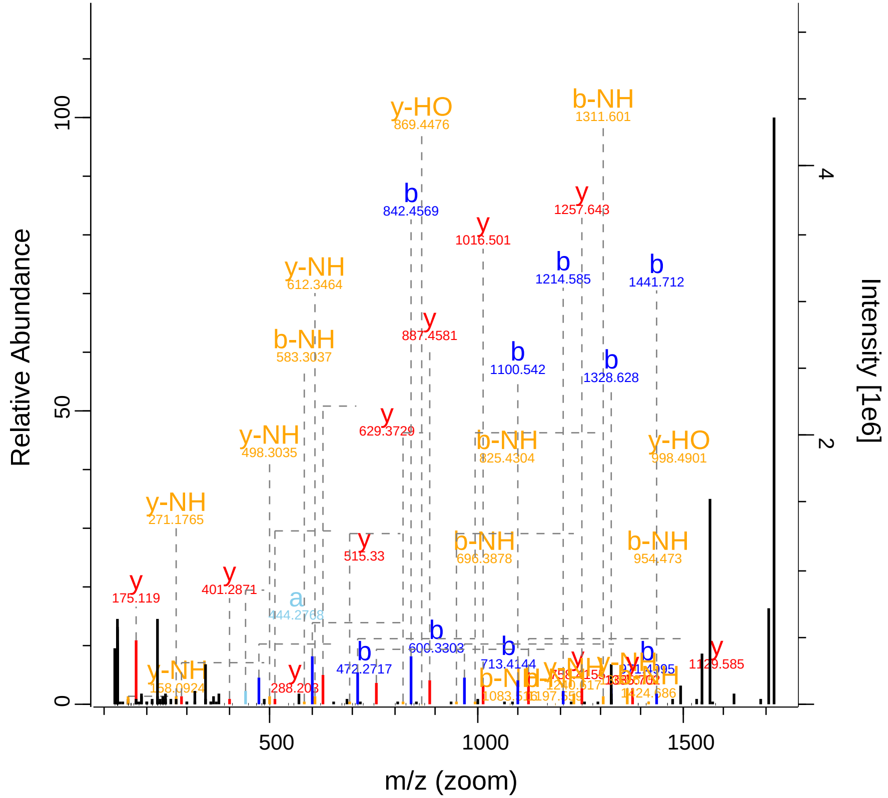

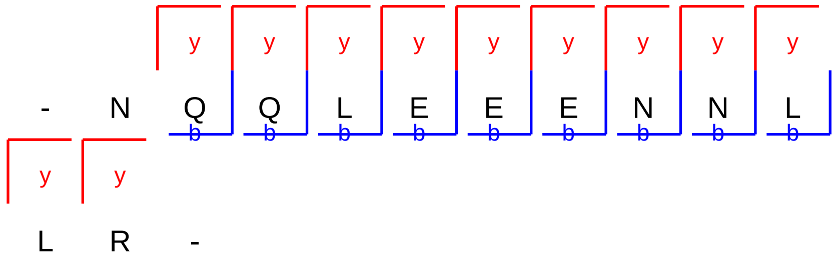

Raw File	Scan	Method	Score	m/z	Gene names
RO-QC-160121_ZackD_SS_Set_01_Fractio	19952	FTMS; HC	108.47	909.91	ANAPC11

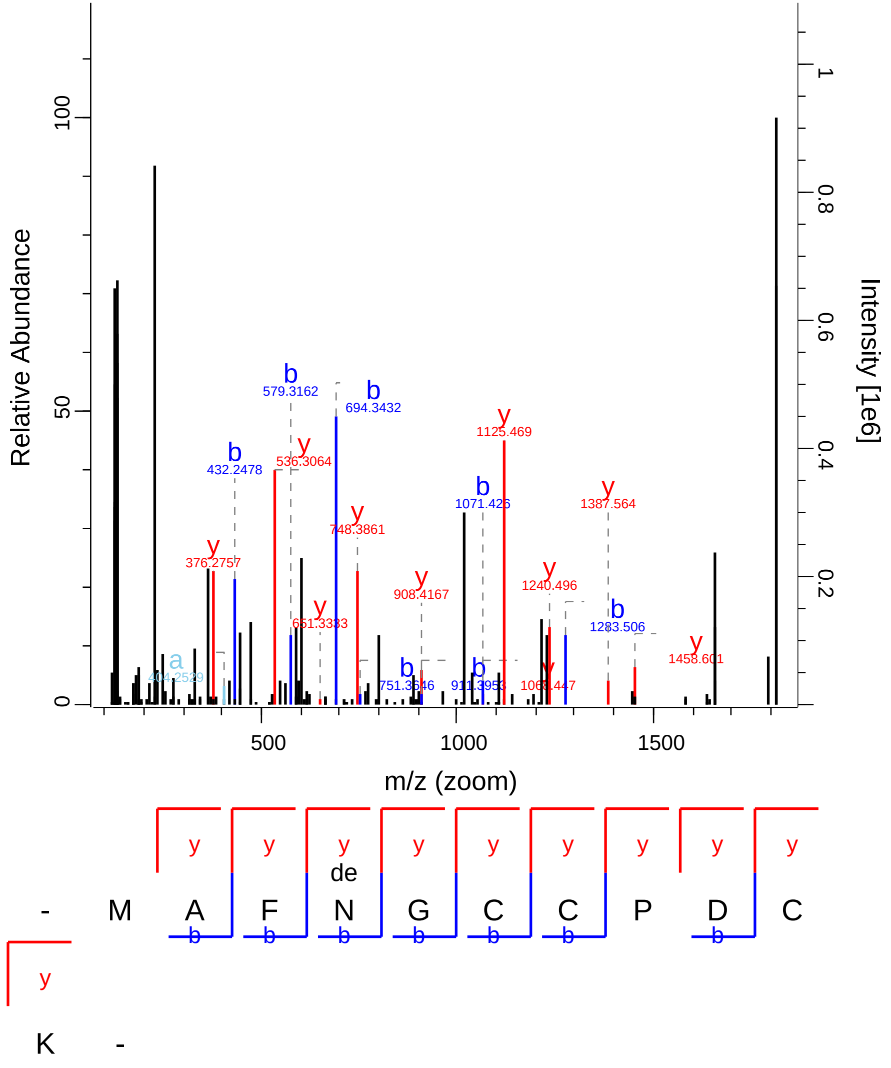

Raw File	Scan	Method	Score	m/z	Gene names
RO-QC-160121_ZackD_SS_Set_01_Fractio	19964	FTMS; HC	52.52	600.07	ATP6V0C

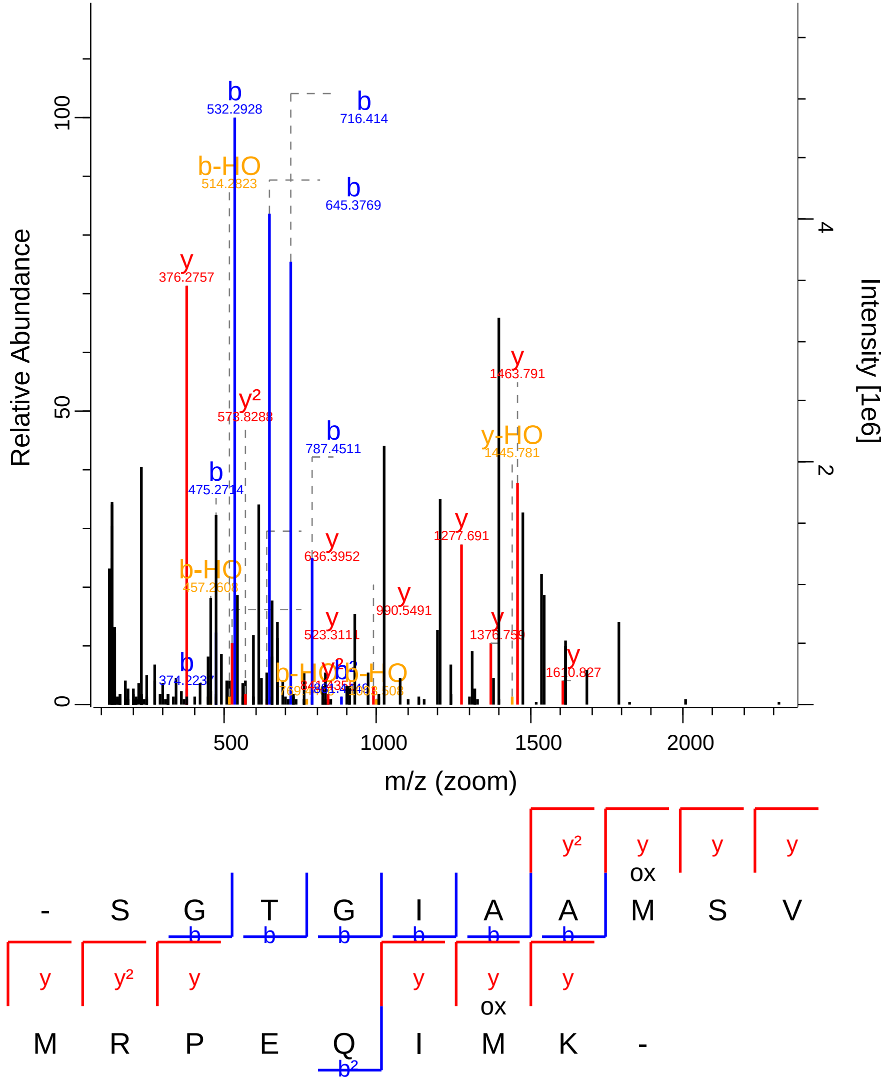

Raw File	Scan	Method	Score	m/z	Gene names
RO-QC-160121_ZackD_SS_Set_01_Fractio	20336	FTMS; HC	47.22	799.76	ATP6V0C

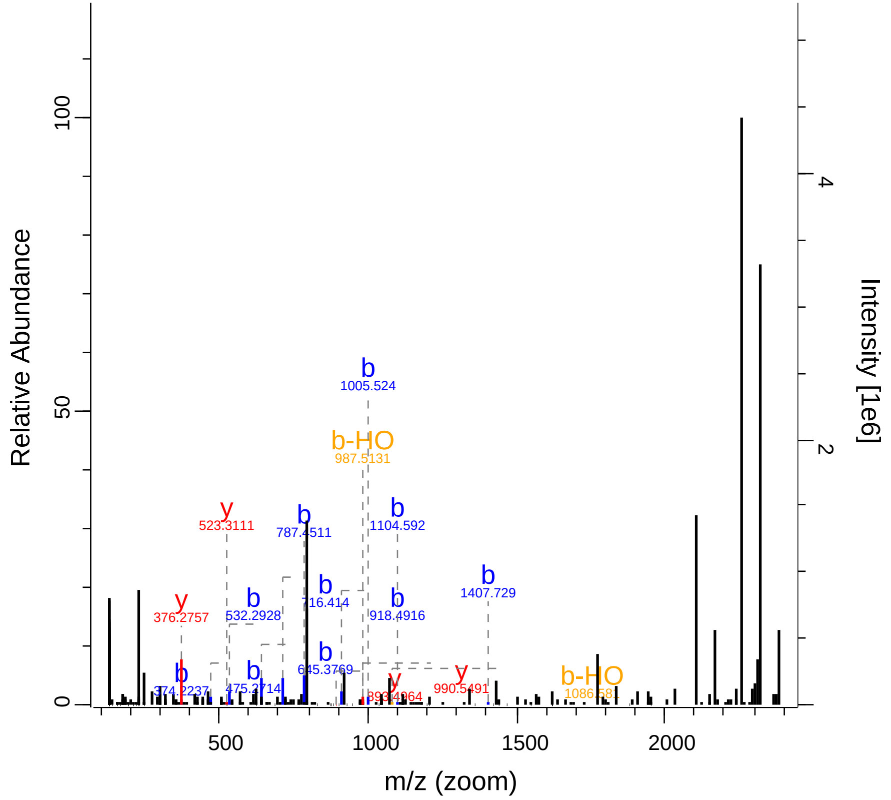

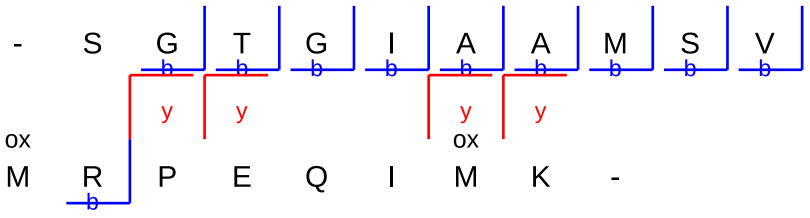

Raw File	Scan	Method	Score	m/z	Gene names
RO-QC-160121_ZackD_SS_Set_01_Fractio	20620	FTMS; HC	67.56	810.96	FSD2

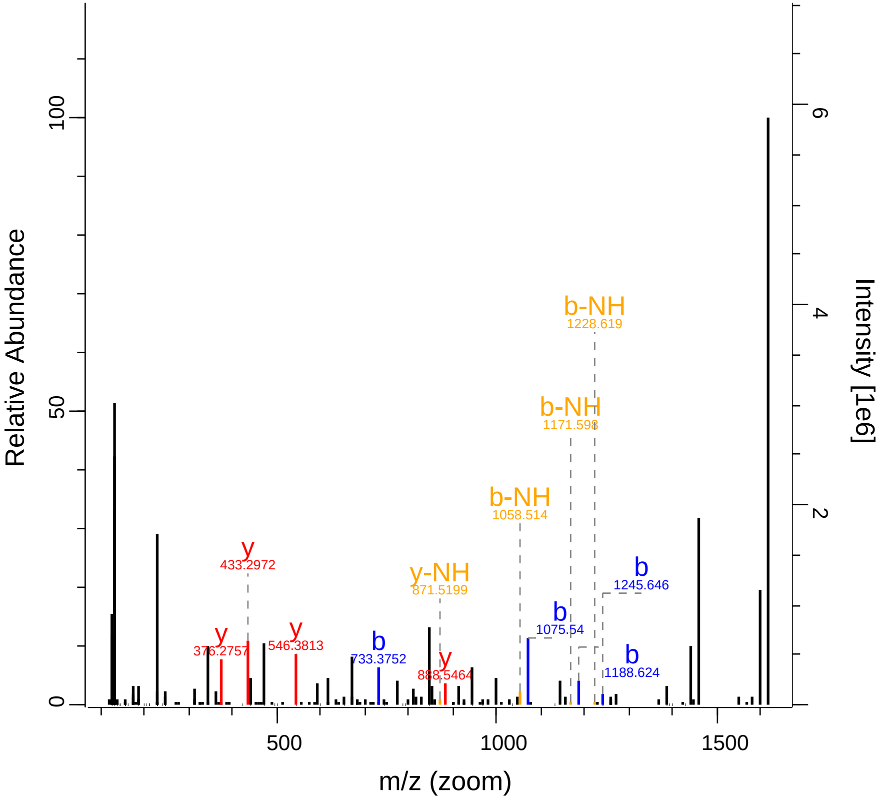

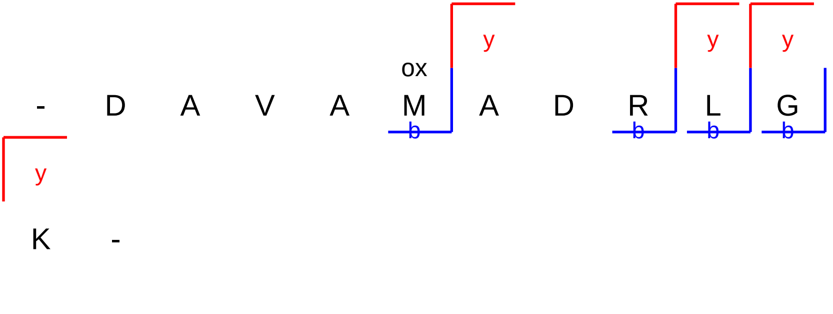

Raw File	Scan	Method	Score	m/z	Gene names
RO-QC-160121_ZackD_SS_Set_01_Fractior	20676	FTMS; HCI	99.22	930.5	PMS2P11

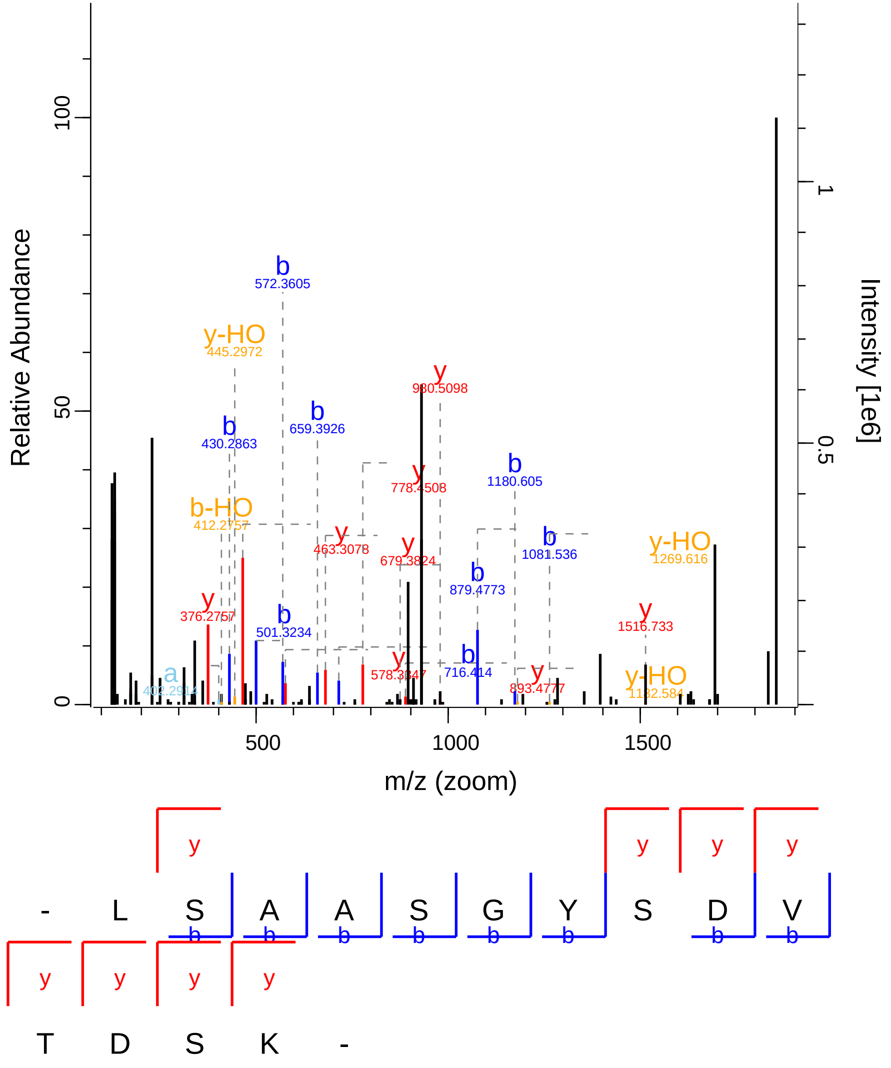

Raw File	Scan	Method	Score	m/z	Gene names
RO-QC-160121_ZackD_SS_Set_01_Fractio	21501	FTMS; HC	170.91	951.53	AURKA

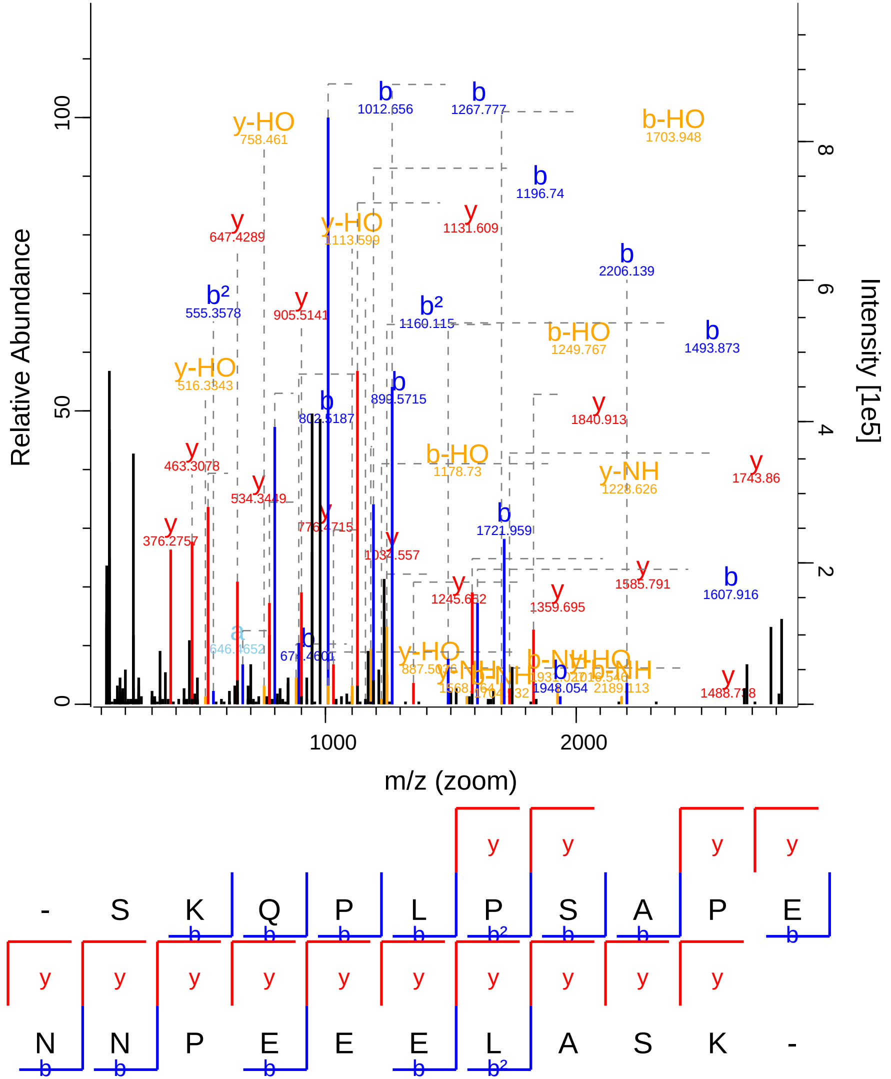

Raw File	Scan	Method	Score	m/z	Gene names
RO-QC-160121_ZackD_SS_Set_01_Fractio	21535	FTMS; HC	74.46	594.01	ORAI3

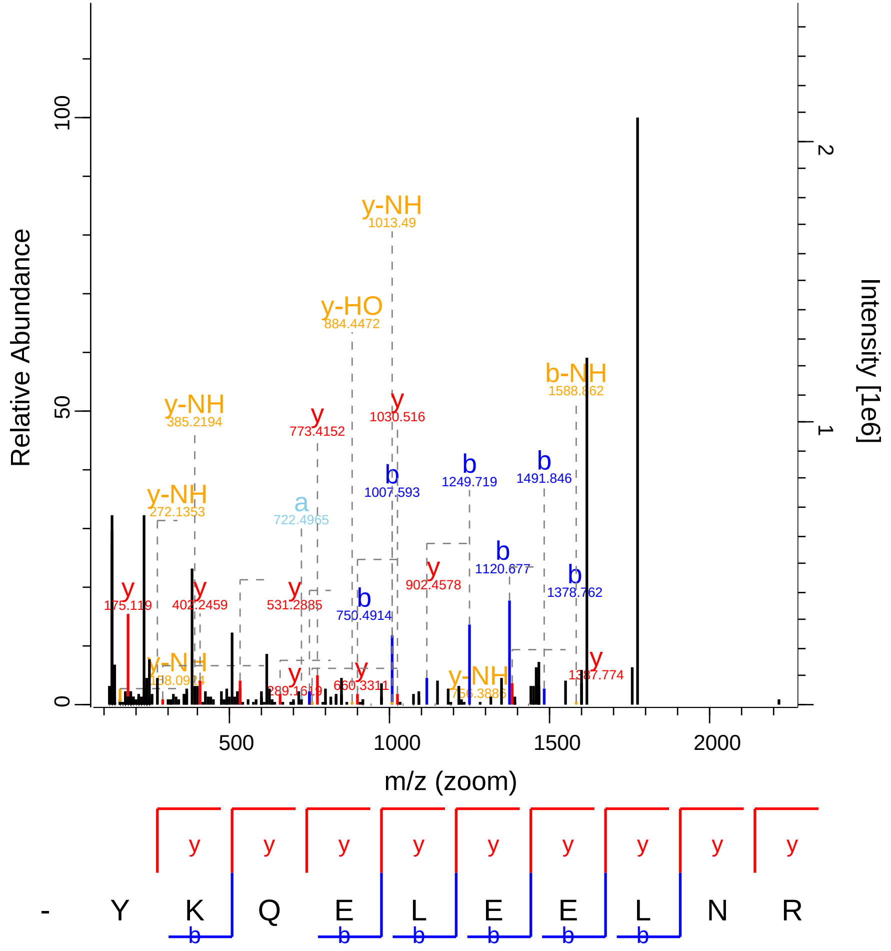

-

Raw File	Scan	Method	Score	m/z	Gene names
RO-QC-160121_ZackD_SS_Set_01_Fractio	21655	FTMS; HC	50.28	942.49	DYRK4

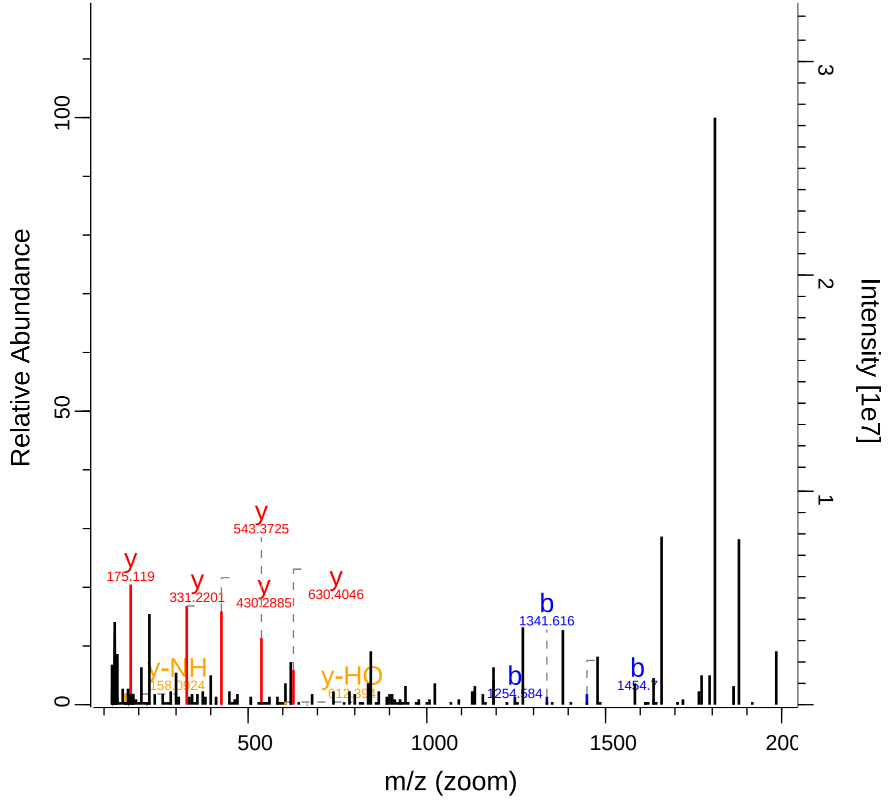

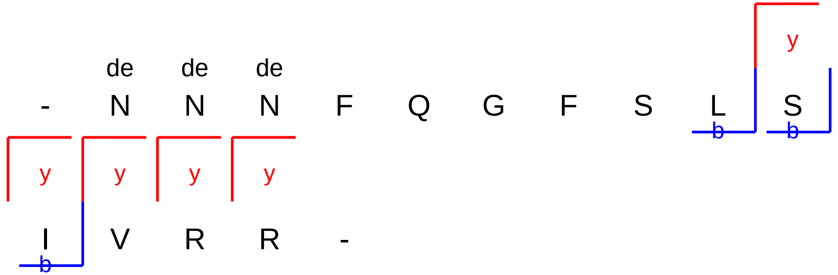

Raw File	Scan	Method	Score	m/z	Gene names
RO-QC-160121_ZackD_SS_Set_01_Fractio	21705	FTMS; HC	40.5	521.53	PRSS55

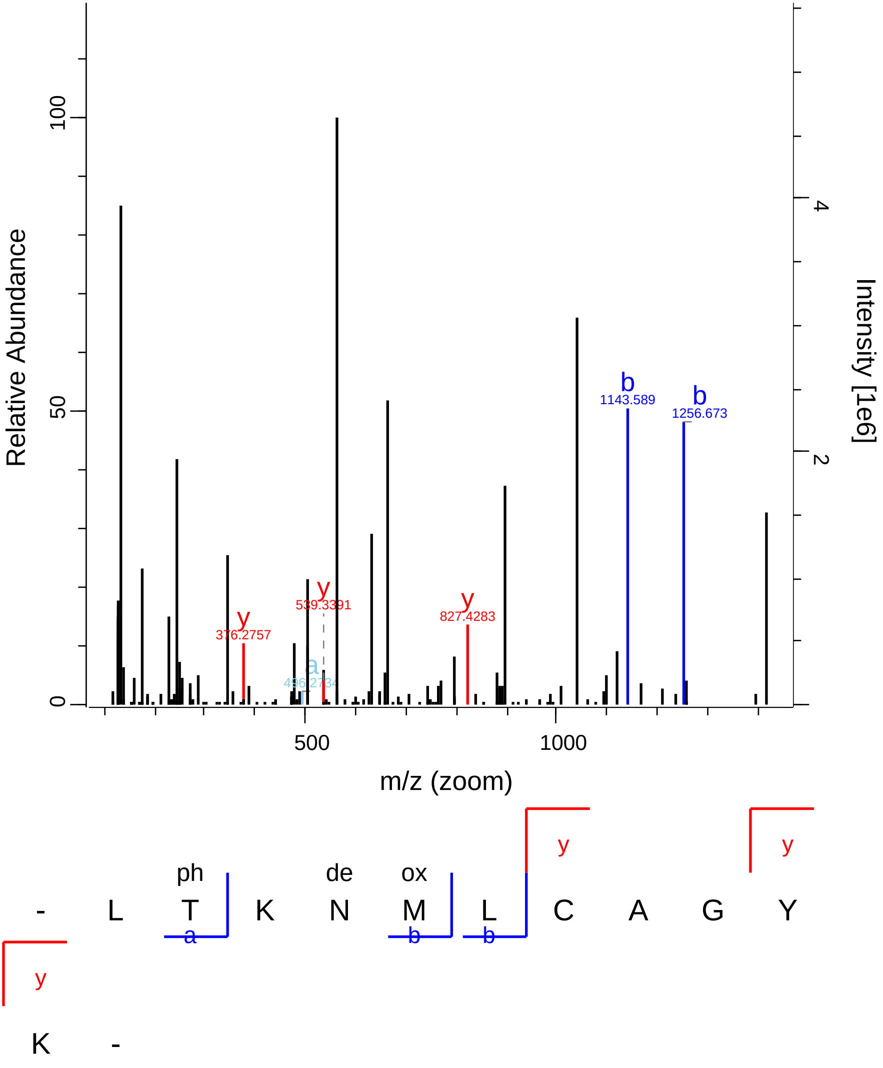

Raw File	Scan	Method	Score	m/z
RO-QC-160121_ZackD_SS_Set_01_Fraction_4	21772	FTMS; HCD	74.3	710.4

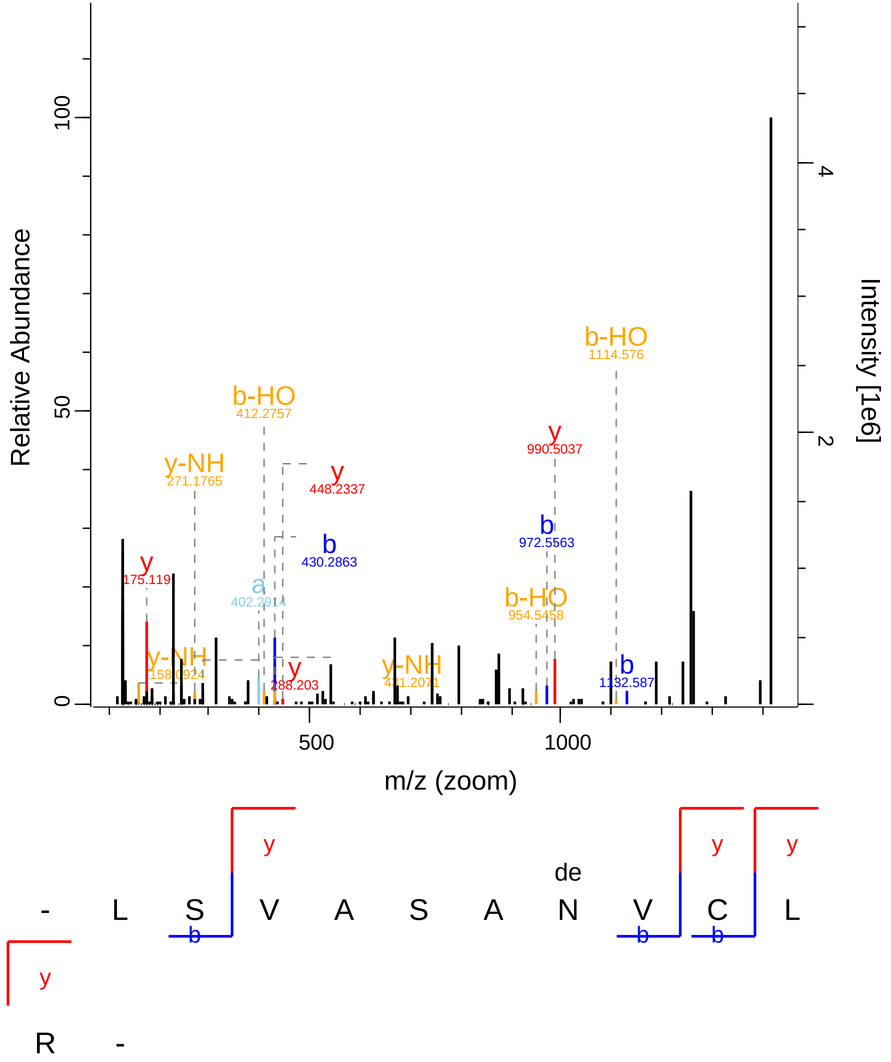

Raw File	Scan	Method	Score	m/z	Gene names
RO-QC-160121_ZackD_SS_Set_01_Fractio	23043	FTMS; HC	45.26	854.78	SLC31A1

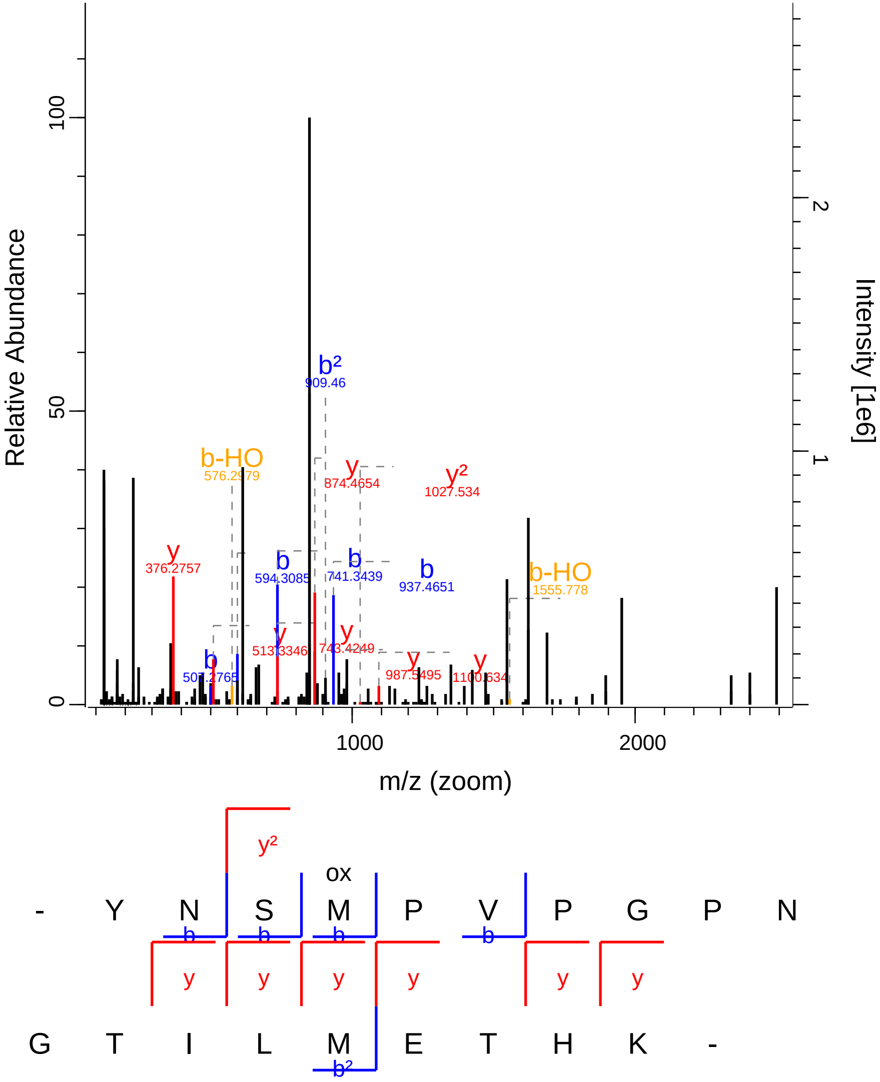

Raw File	Scan	Method	Score	m/z
RO-QC-160121_ZackD_SS_Set_01_Fraction_4	23260	FTMS; HCD	84.48	470.95

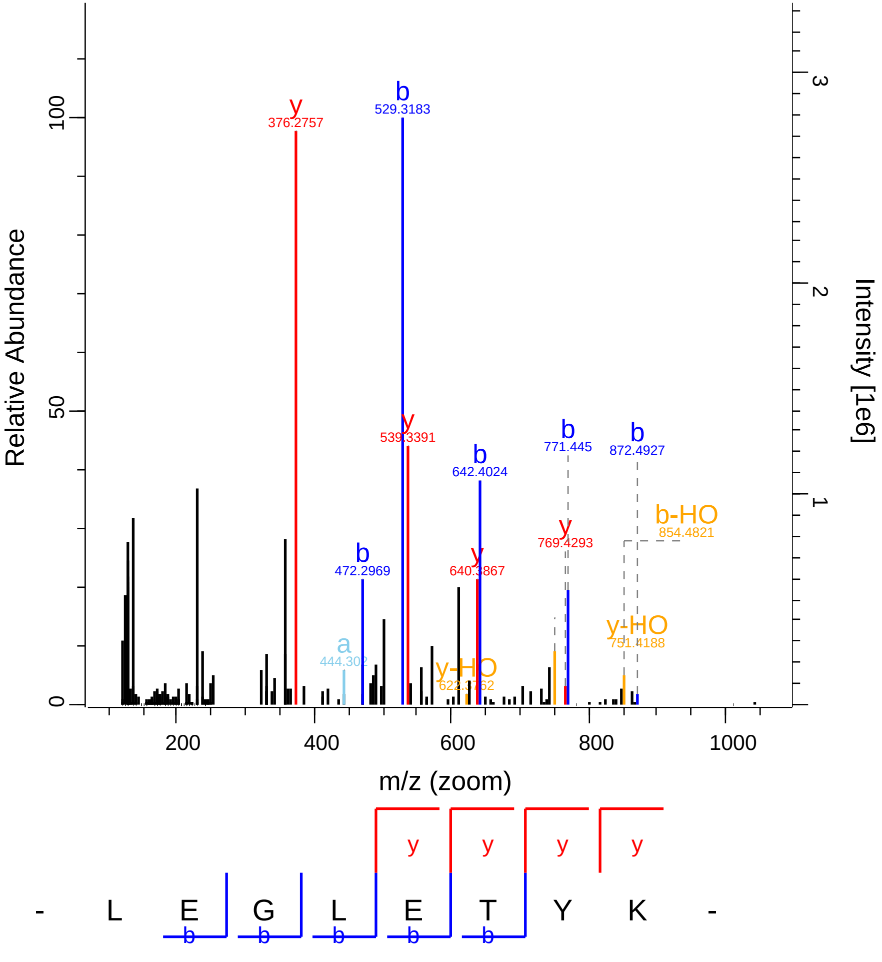

Raw File	Scan	Method	Score	m/z	Gene names
RO-QC-160121_ZackD_SS_Set_01_Fractio	23281	FTMS; HC	68.18	692.71	CEP78

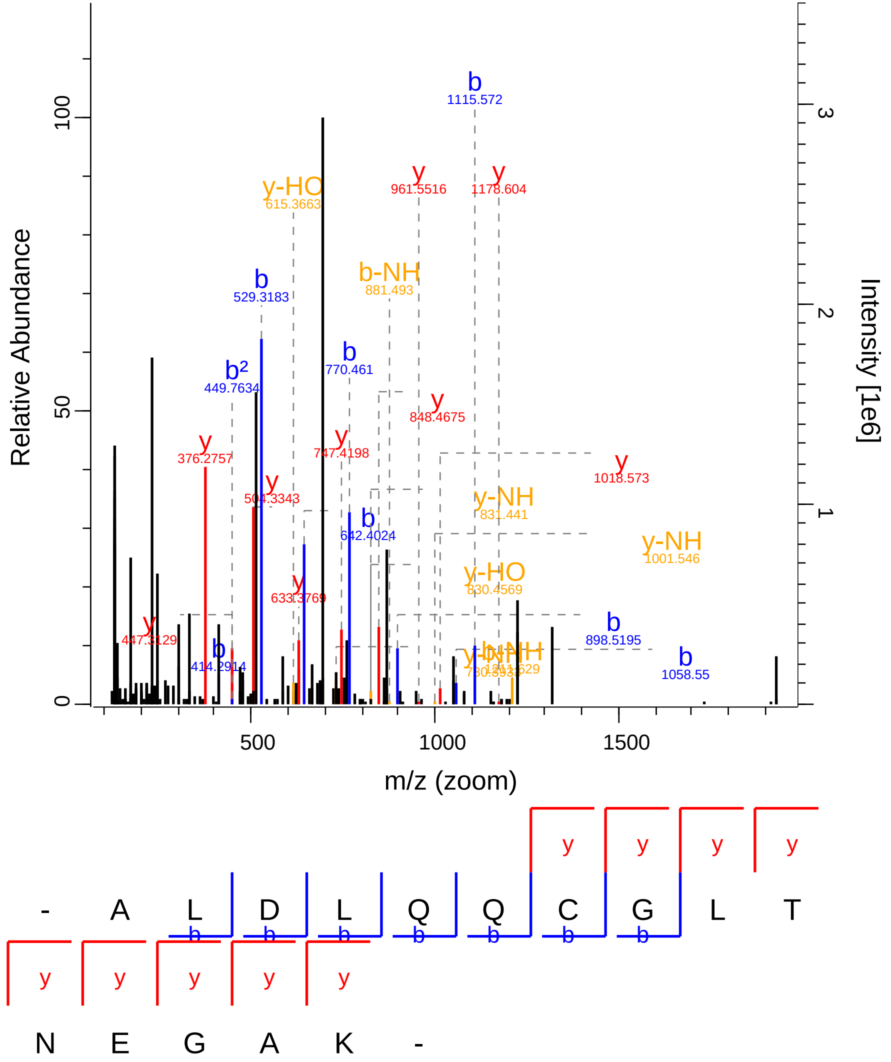

Raw File	Scan	Method	Score	m/z
RO-QC-160121_ZackD_SS_Set_01_Fraction_4	23581	FTMS; HCD	99.14	450.6

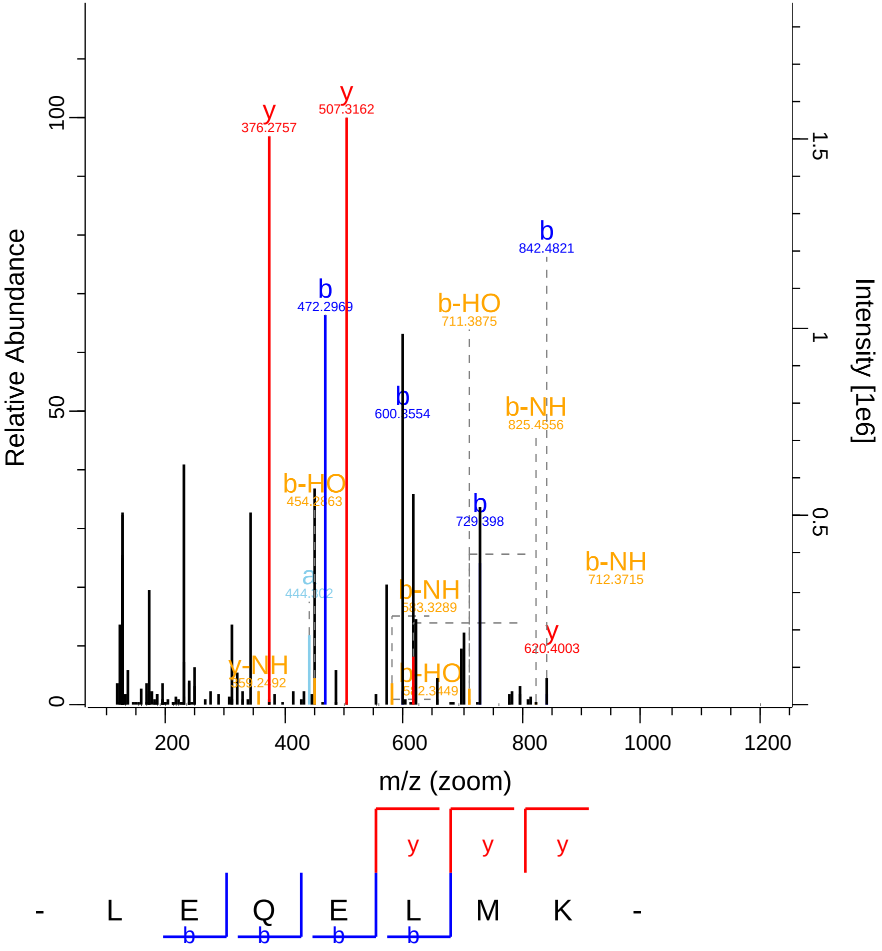

Raw File	Scan	Method	Score	m/z	Gene names
RO-QC-160121_ZackD_SS_Set_01_Fractio	24037	FTMS; HC	60.48	473.62	CD14

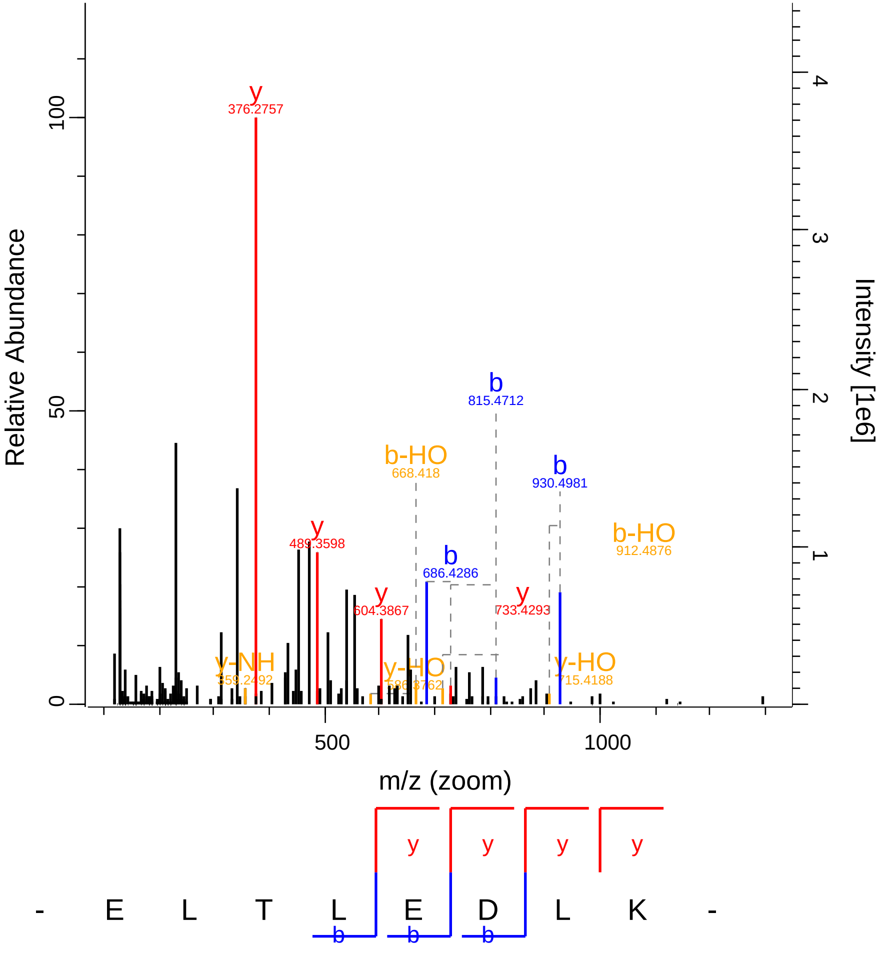

Raw File	Scan	Method	Score	m/z
RO-QC-160121_ZackD_SS_Set_01_Fraction_4	24063	FTMS; HCD	57.1	618.36

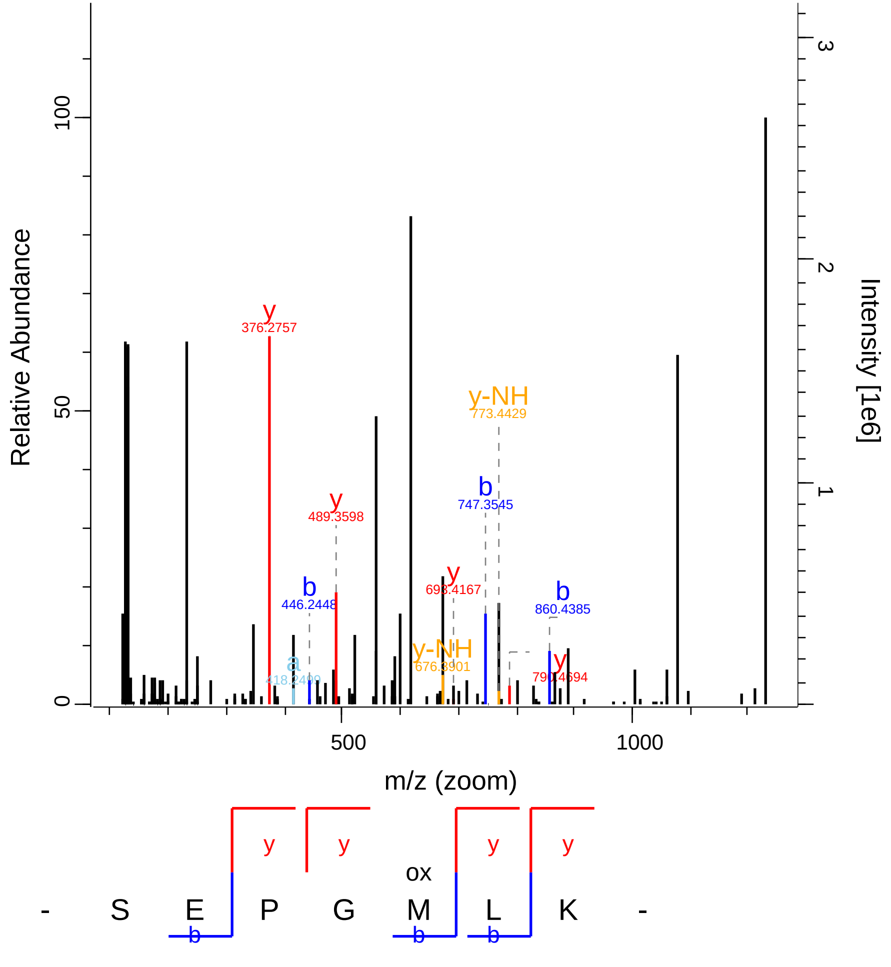

Raw File	Scan	Method	Score	m/z	Gene names
RO-QC-160121_ZackD_SS_Set_01_Fractio	24254	FTMS; HC	77.92	473.62	CD14

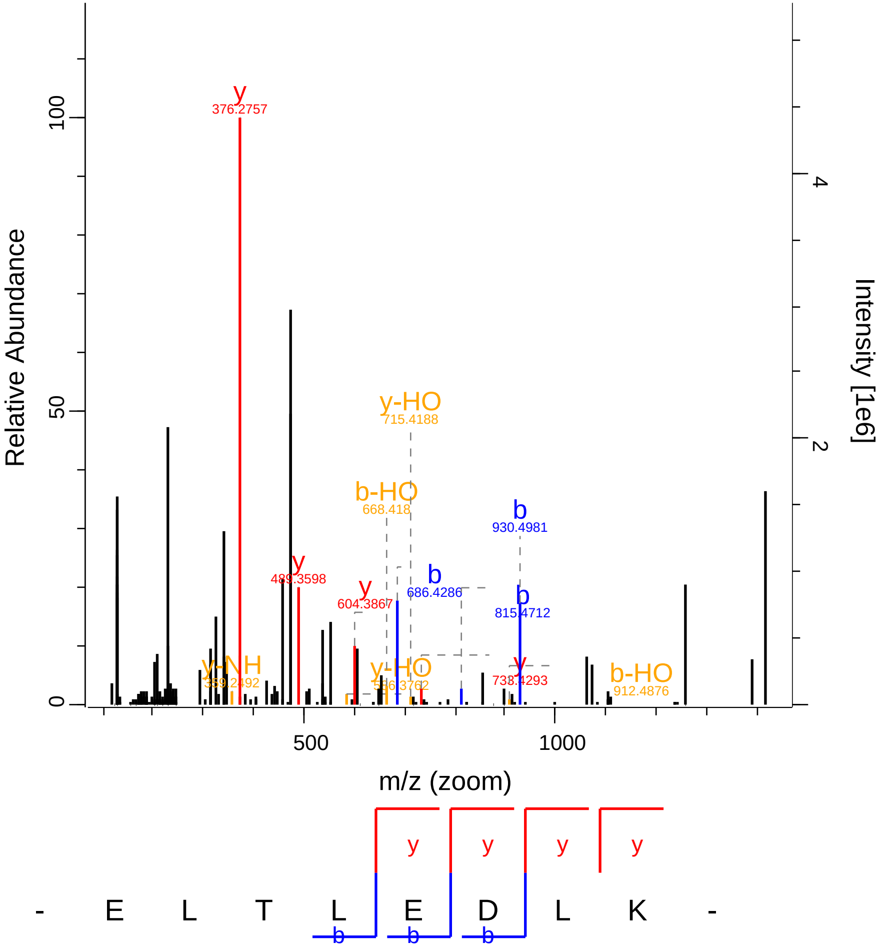

Raw File	Scan	Method	Score	m/z
RO-QC-160121_ZackD_SS_Set_01_Fraction_4	24634	FTMS; HCD	192.1	913.96

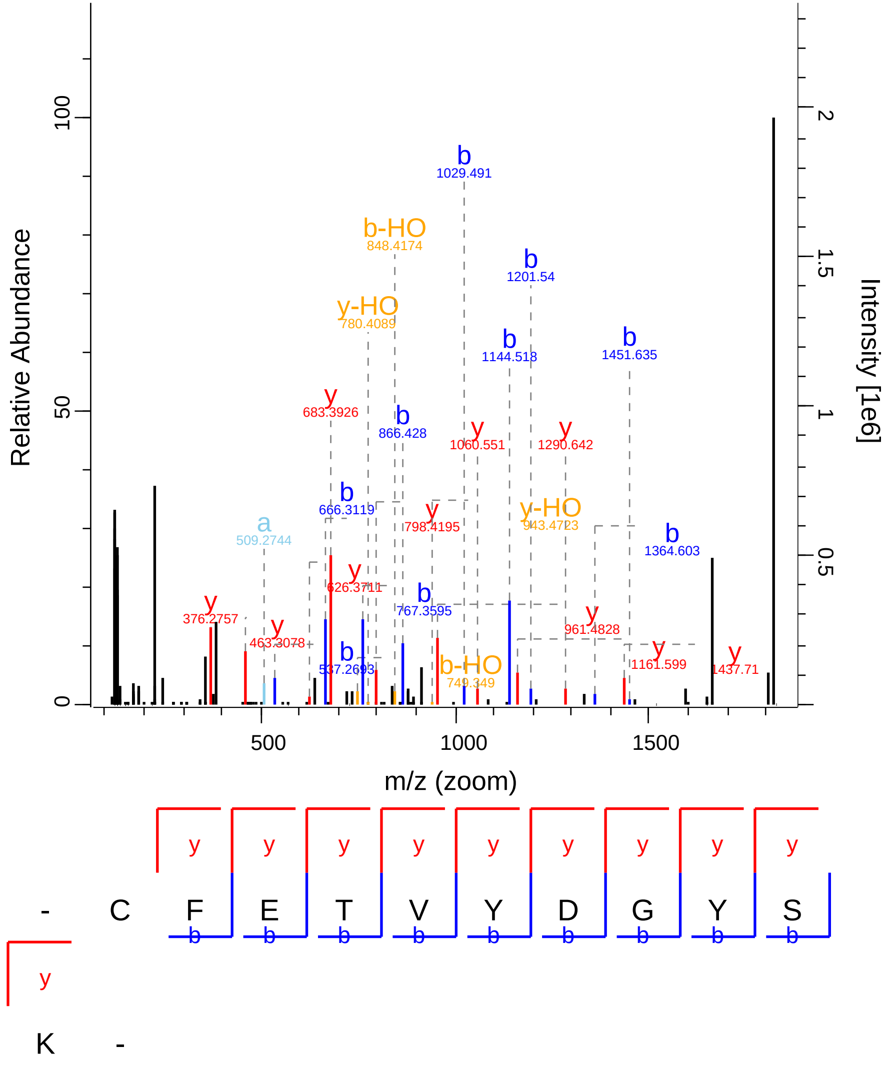

Raw File	Scan	Method	Score	m/z	Gene names
RO-QC-160121_ZackD_SS_Set_01_Fractio	24894	FTMS; HC	130.56	551.97	MMP23A

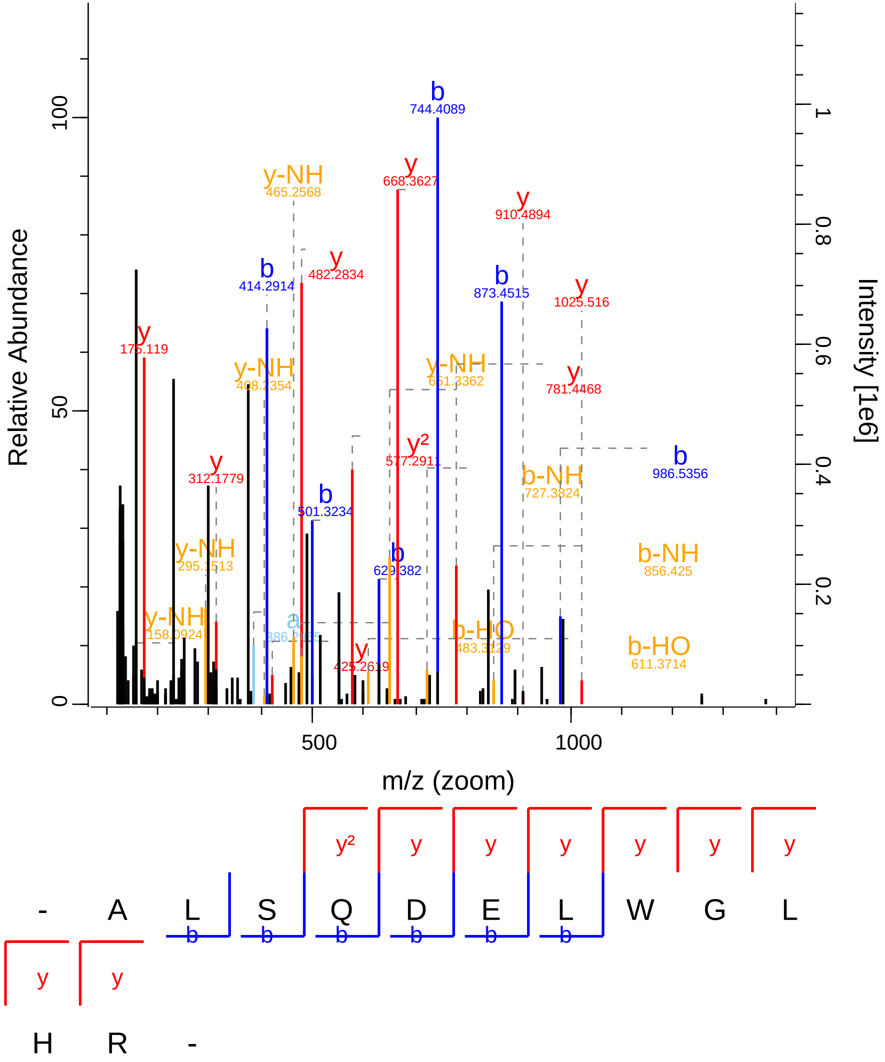

Raw File	Scan	Method	Score	m/z	Gene names
RO-QC-160121_ZackD_SS_Set_01_Fractio	25277	FTMS; HC	142.97	798.76	FAM103A1

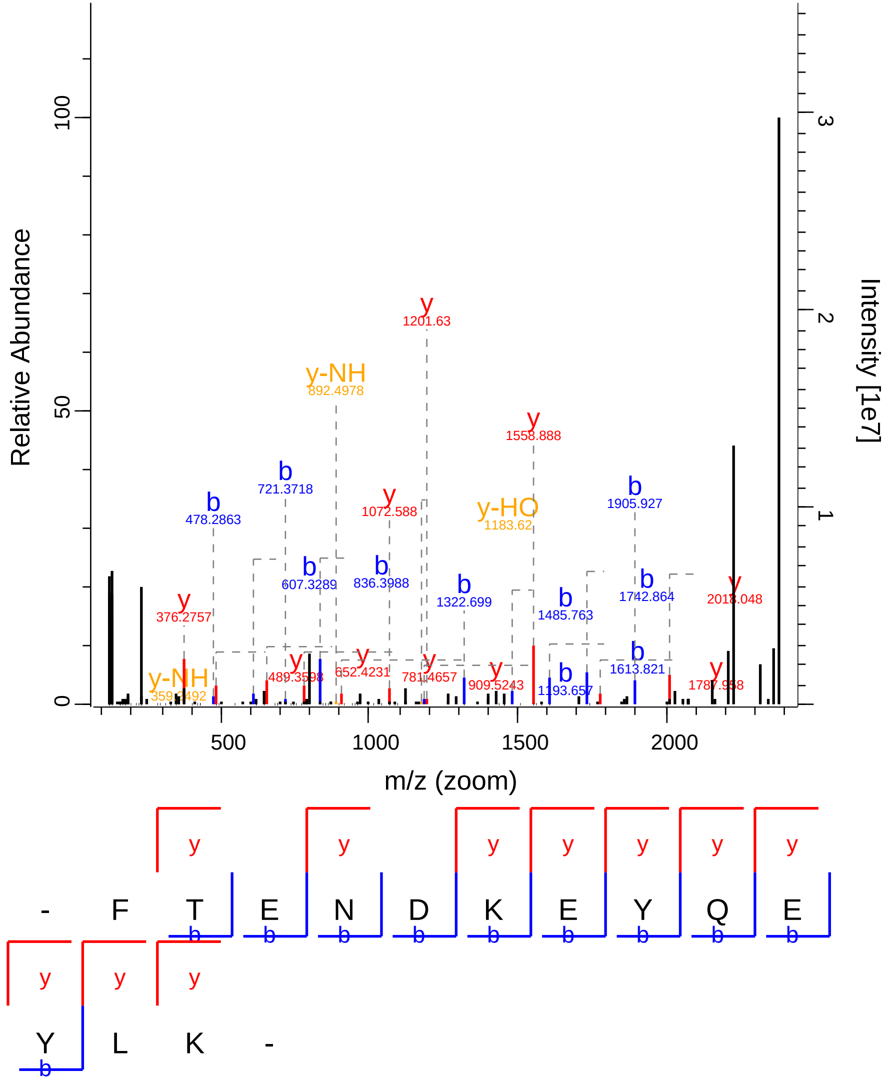

Raw File	Scan	Method	Score	m/z	Gene names
RO-QC-160121_ZackD_SS_Set_01_Fractio	26465	FTMS; HC	77.9	847.08	CNIH4

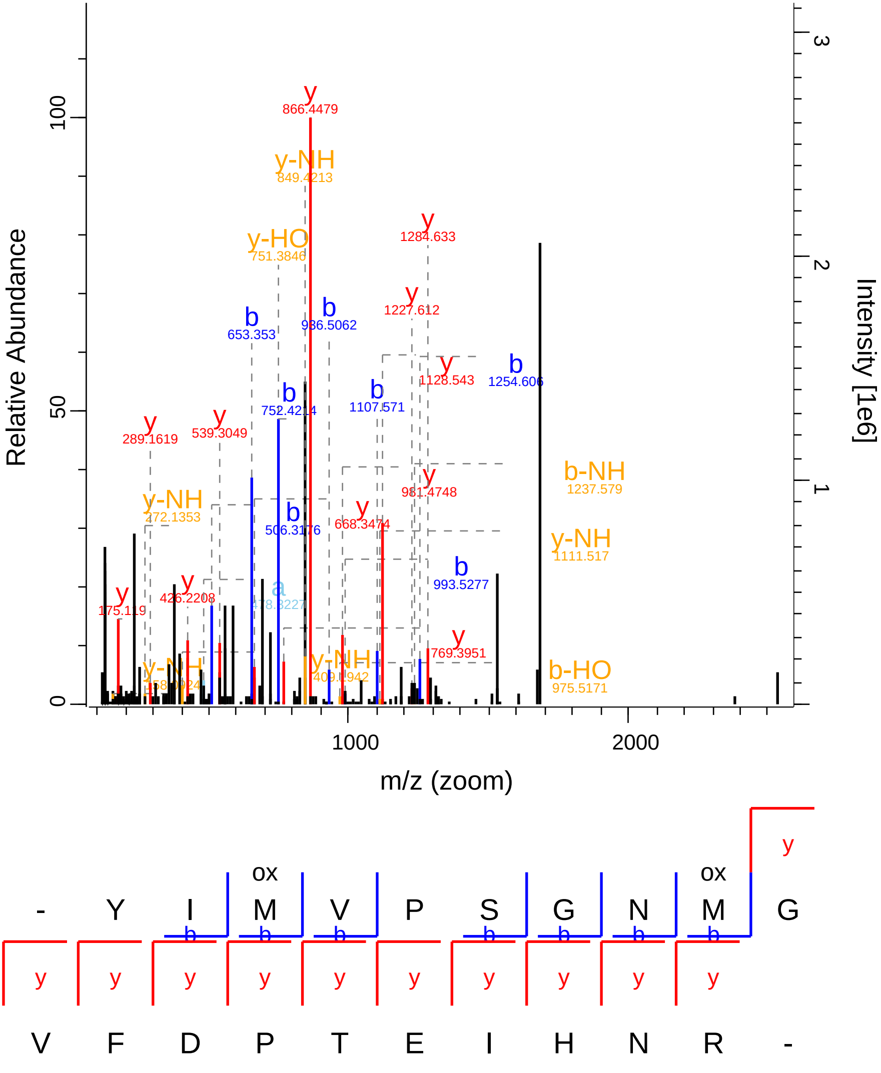

Raw File	Scan	Method	Score	m/z	Gene names
RO-QC-160121_ZackD_SS_Set_01_Fractio	26595	FTMS; HC	59.9	635.56	CNIH4

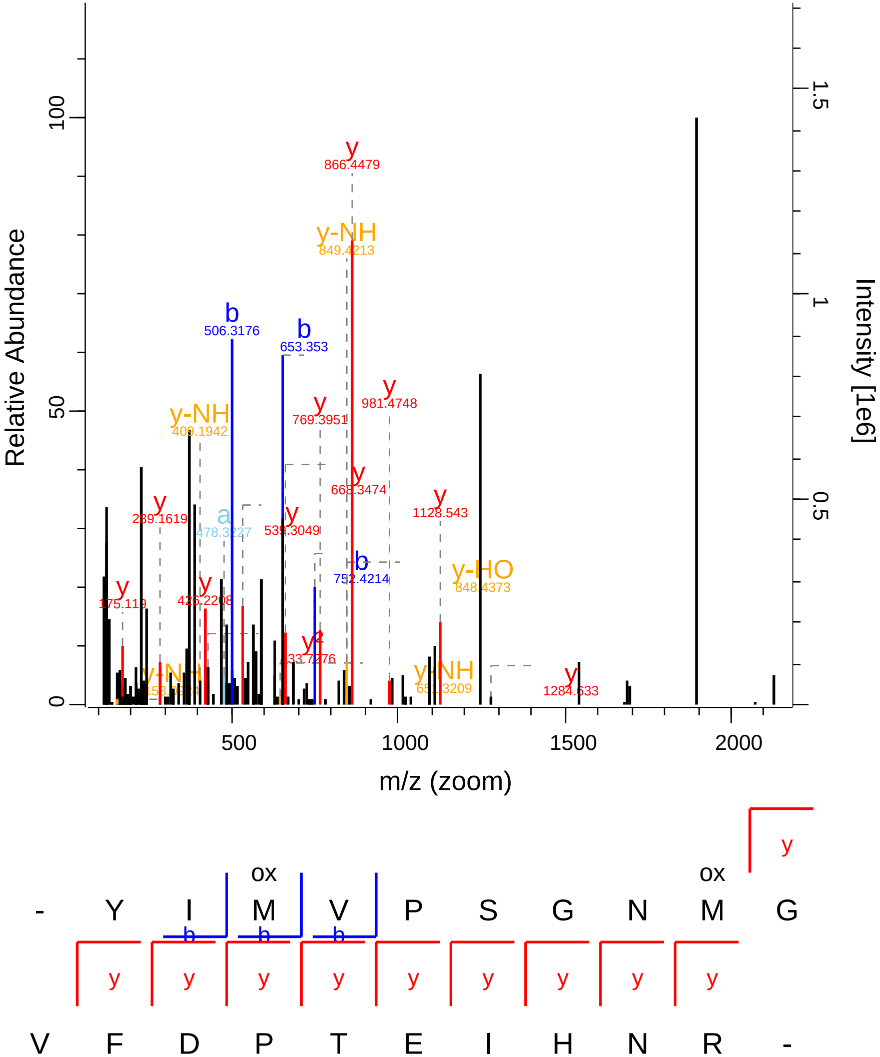

Raw File	Scan	Method	Score	m/z	Gene names
RO-QC-160121_ZackD_SS_Set_01_Fractio	26677	FTMS; HC	111.77	847.08	CNIH4

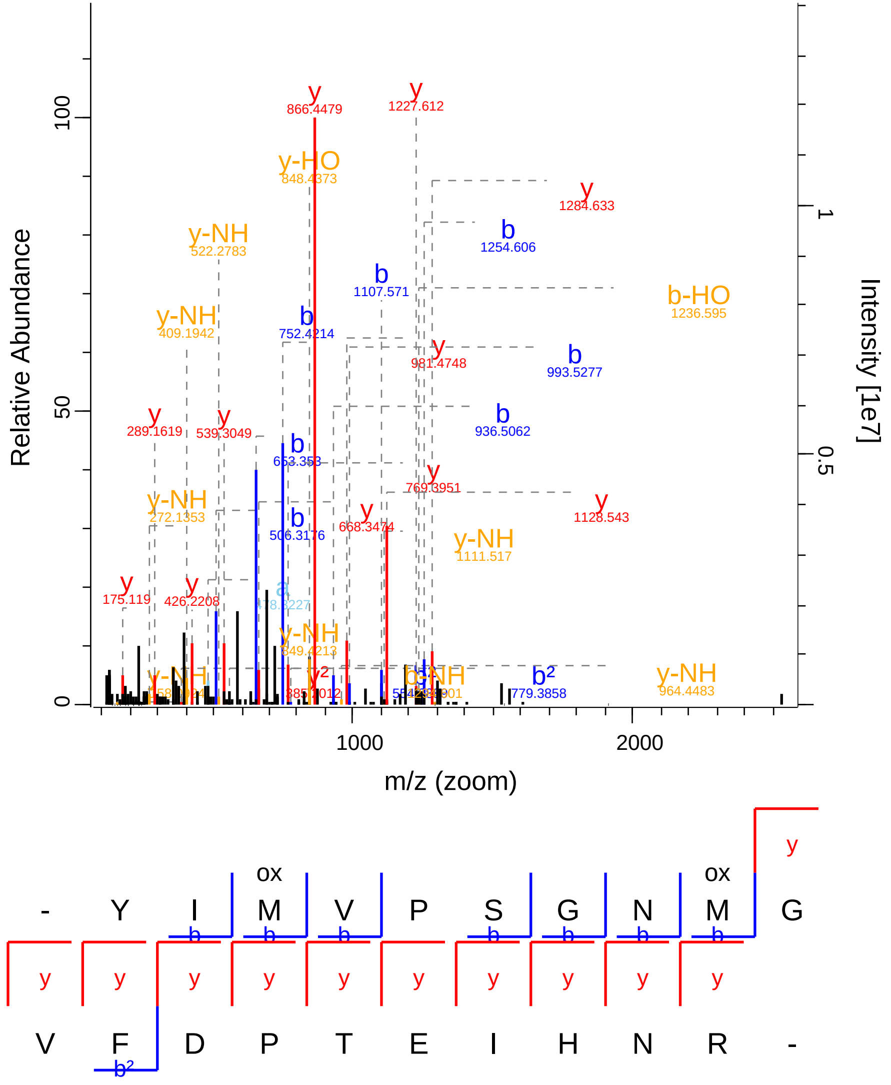

Raw File	Scan	Method	Score	m/z	Gene names
RO-QC-160121_ZackD_SS_Set_01_Fractio	27063	FTMS; HC	95.53	634.89	PKP1

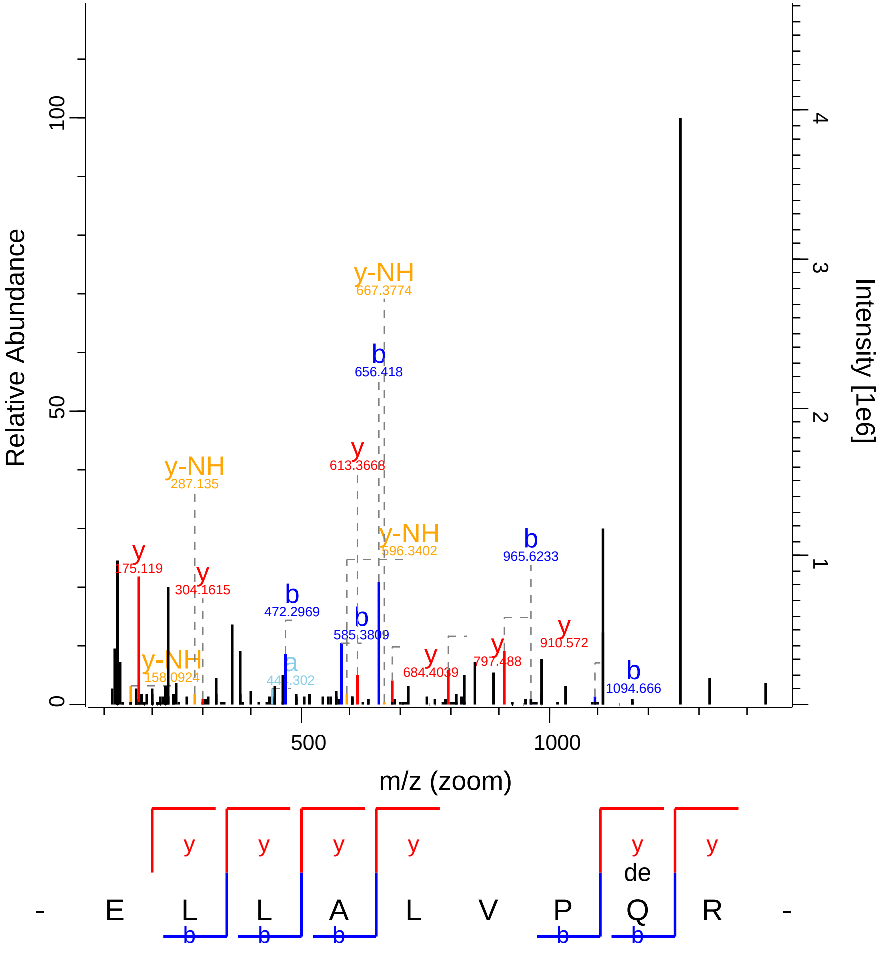

Raw File	Scan	Method	Score	m/z	Gene names
RO-QC-160121_ZackD_SS_Set_01_Fractic	27507	FTMS; HC	43.8	1042.25	CASP12

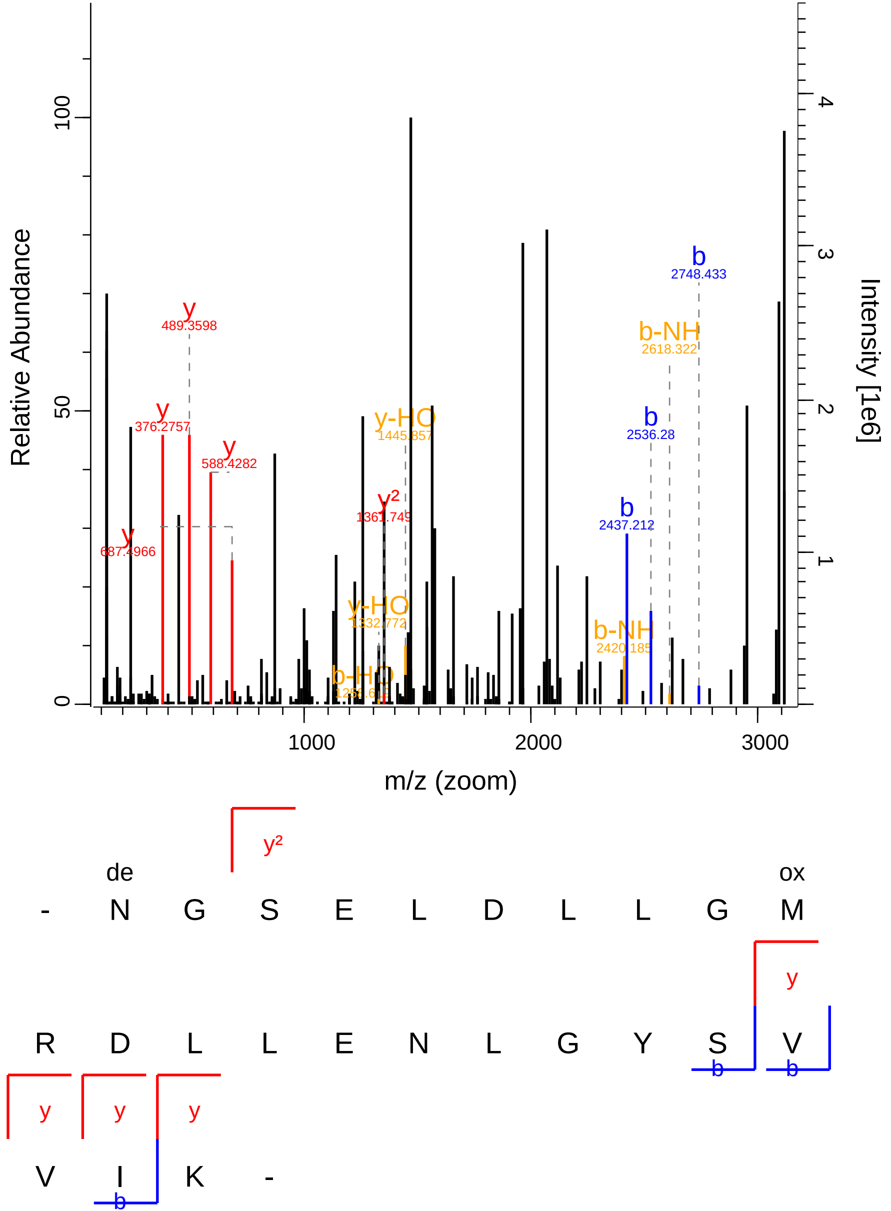

Raw File	Scan	Method	Score	m/z	Gene names
RO-QC-160121_ZackD_SS_Set_01_Fractic	27548	FTMS; HC	140.31	1191.62	CDC42SE2

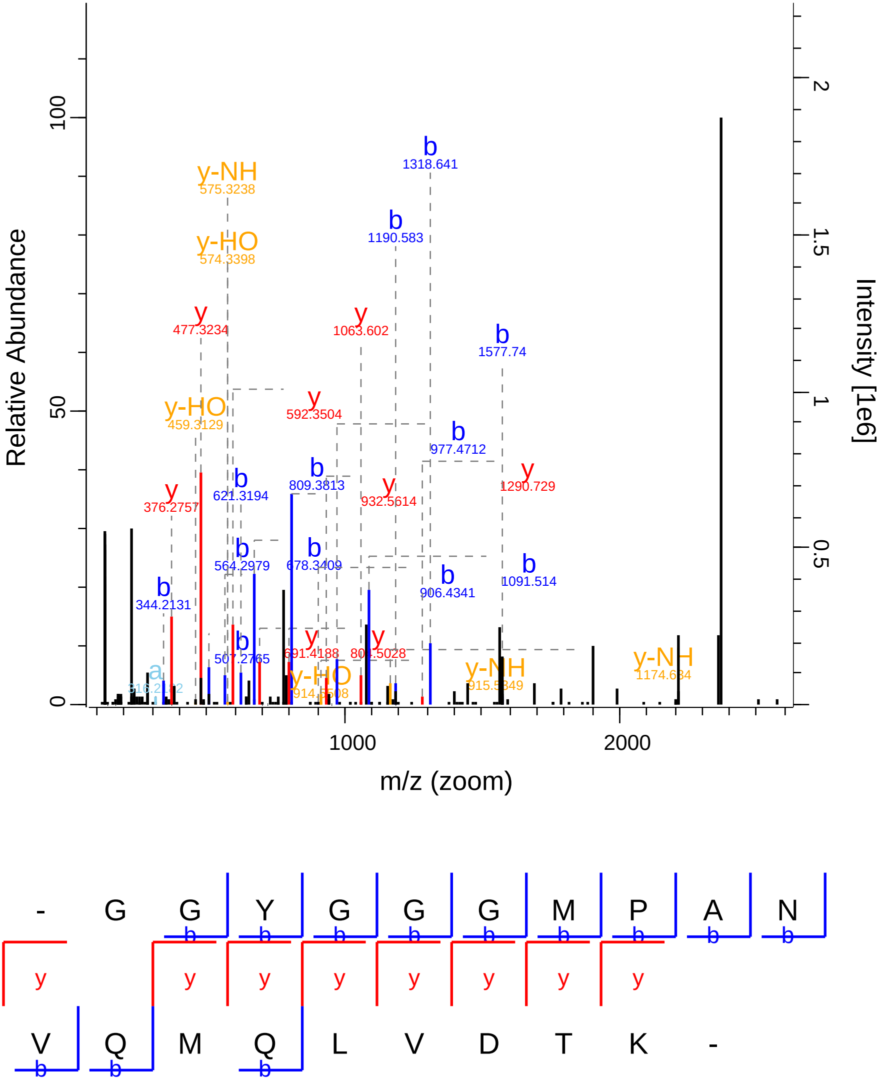

Raw File	Scan	Method	Score	m/z	Gene names
RO-QC-160121_ZackD_SS_Set_01_Fractic	27612	FTMS; HC	237.32	1191.12	CDC42SE2

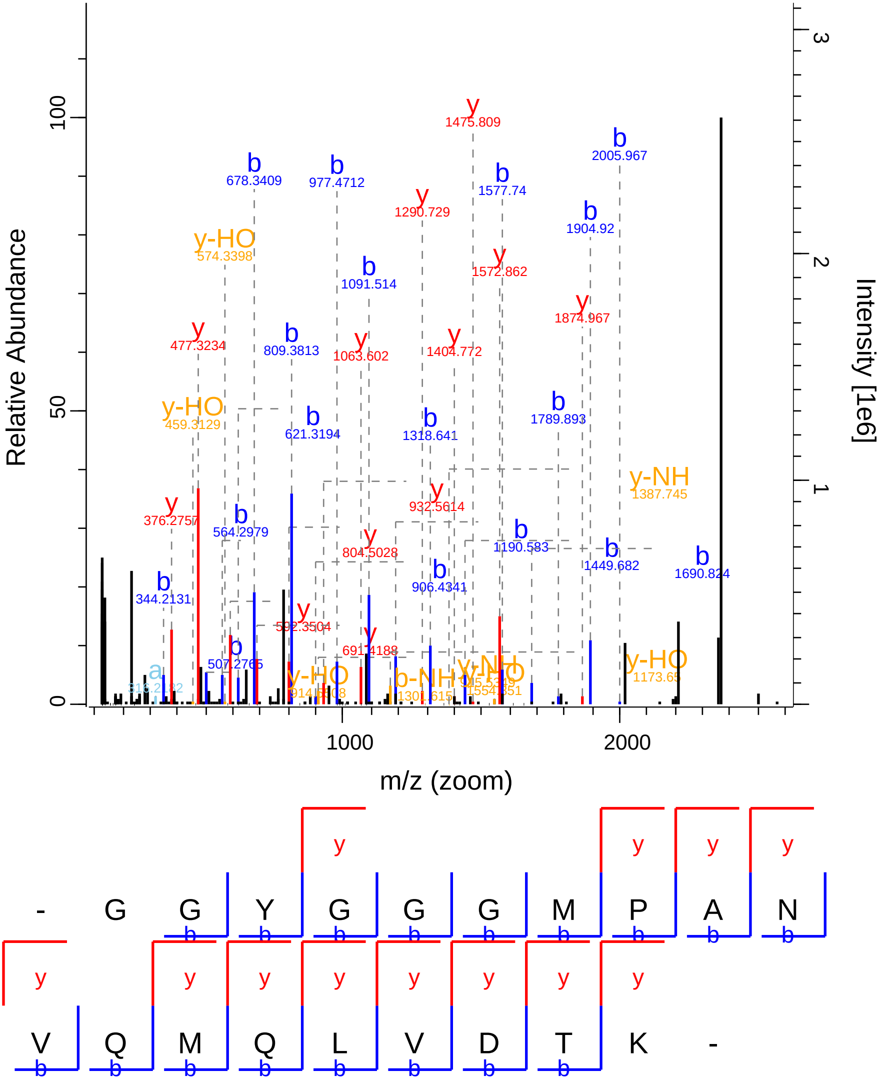

Raw File	Scan	Method	Score	m/z	Gene names
RO-QC-160121_ZackD_SS_Set_01_Fractio	27653	FTMS; HC	124.2	794.42	CDC42SE2

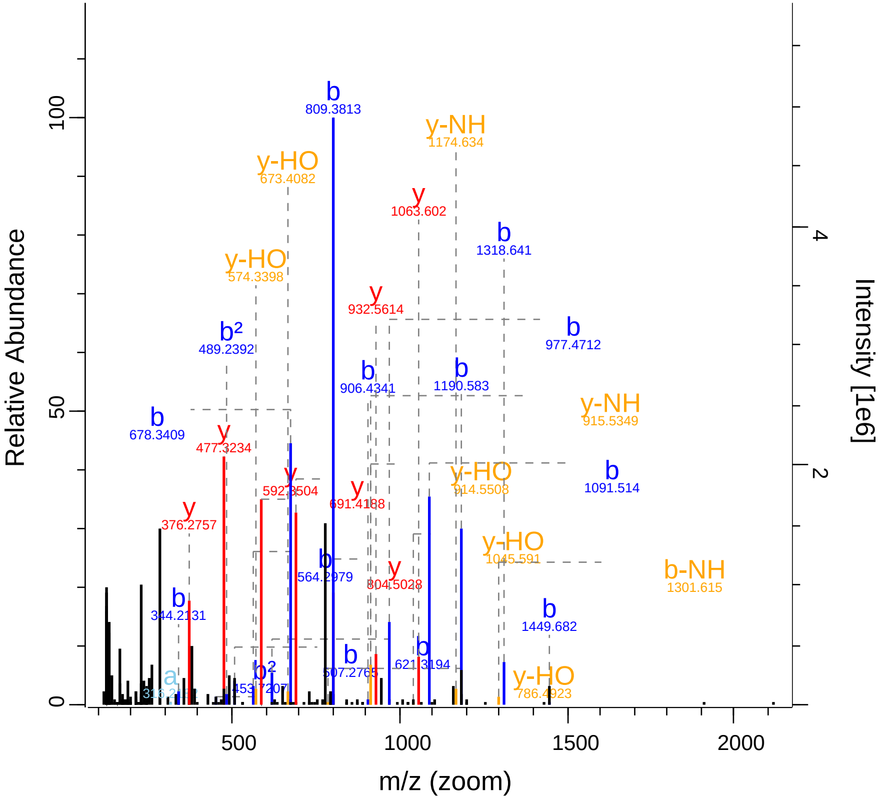

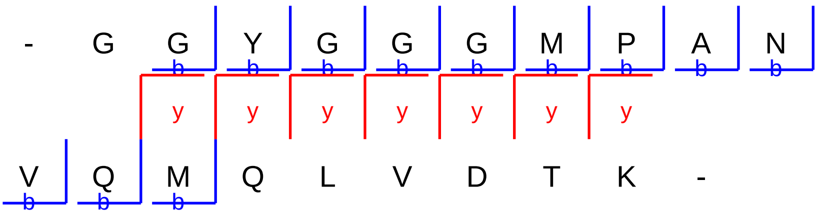

Raw File	Scan	Method	Score	m/z	Gene names
RO-QC-160121_ZackD_SS_Set_01_Fractio	27686	FTMS; HC	88.4	849.45	SLC31A1

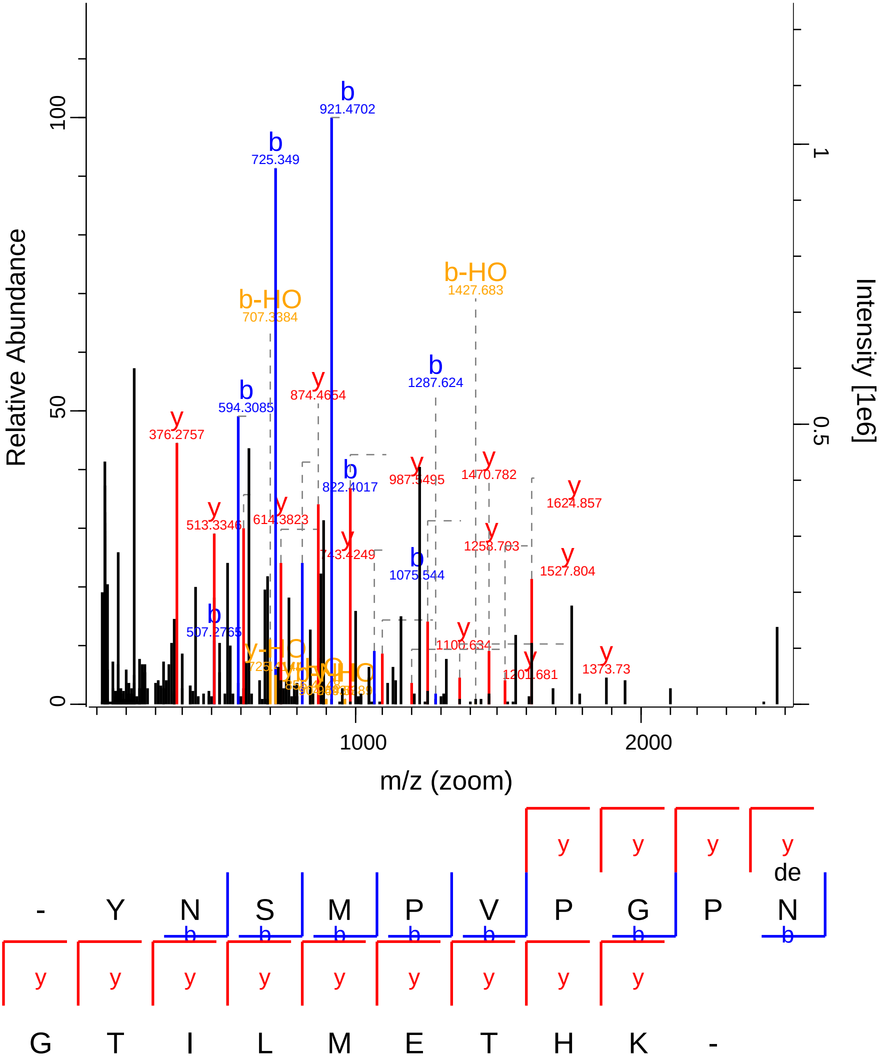

Raw File	Scan	Method	Score	m/z	Gene names
RO-QC-160121_ZackD_SS_Set_01_Fractio	27705	FTMS; HC	116.77	797.47	KBTBD8

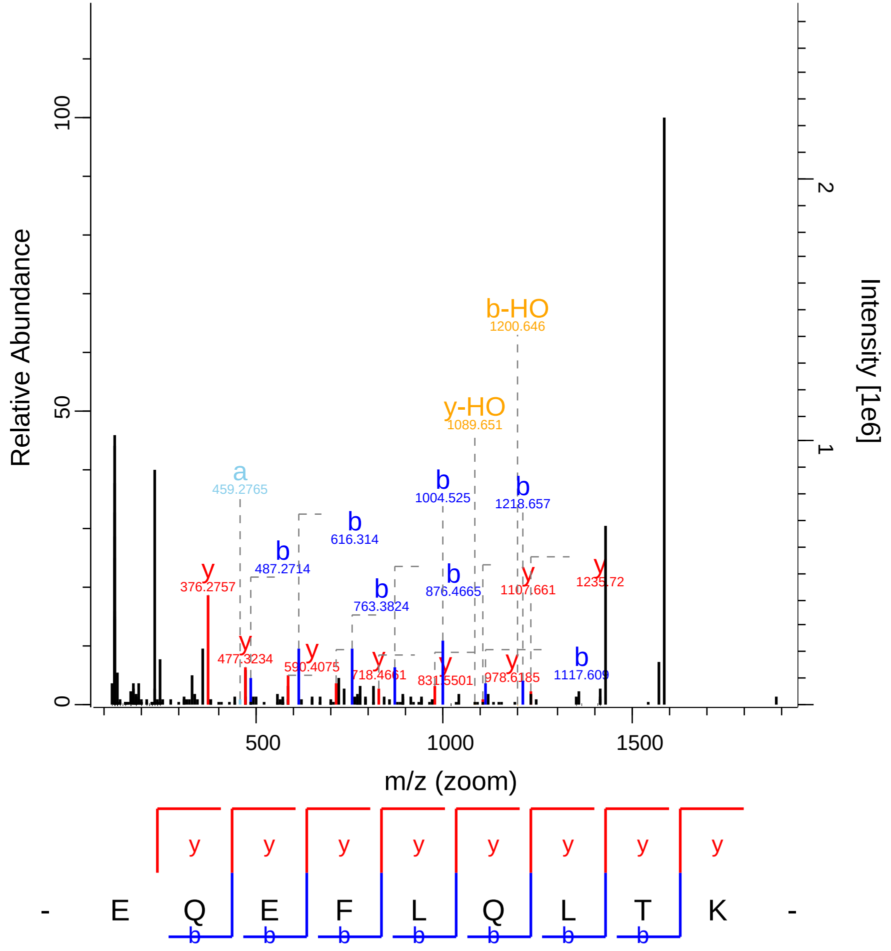

Raw File	Scan	Method	Score	m/z	Gene names
RO-QC-160121_ZackD_SS_Set_01_Fractio	27769	FTMS; HC	174.41	666.39	CDNF

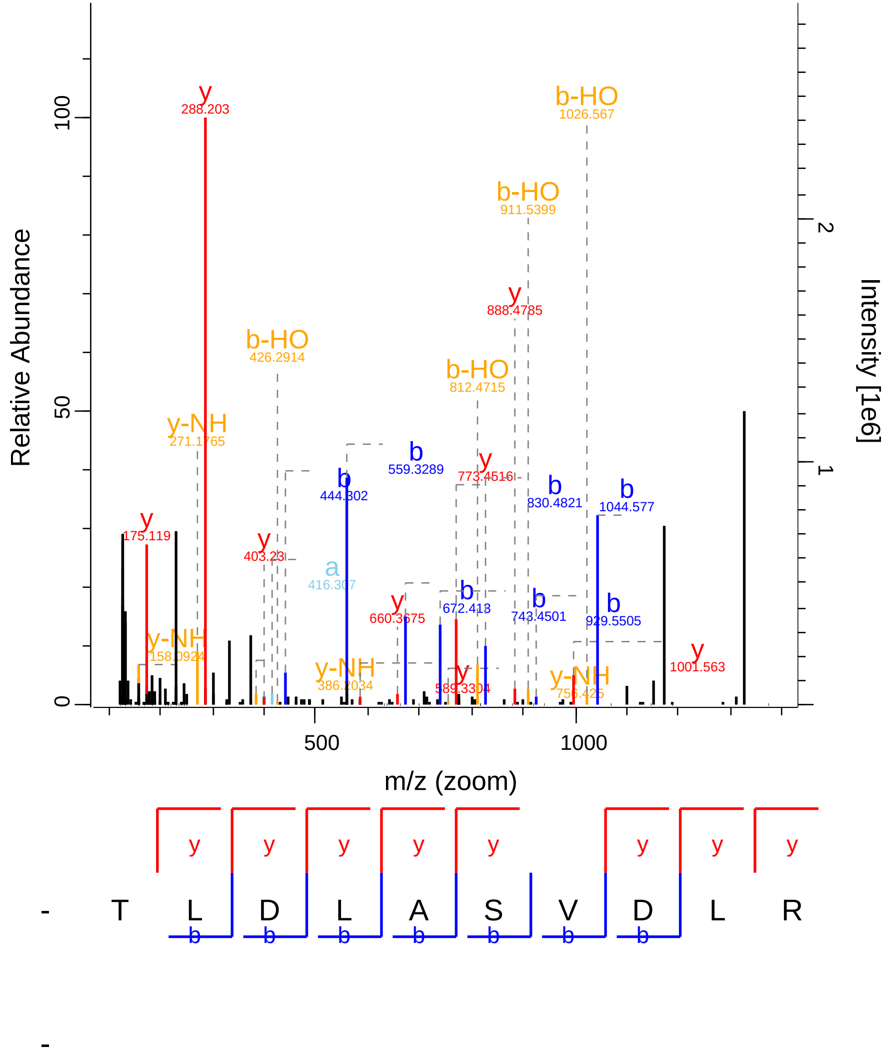

Raw File	Scan	Method	Score	m/z	Gene names
RO-QC-160121_ZackD_SS_Set_01_Fractior	27862	FTMS; HCI	126.24	657.4	IMMP2L

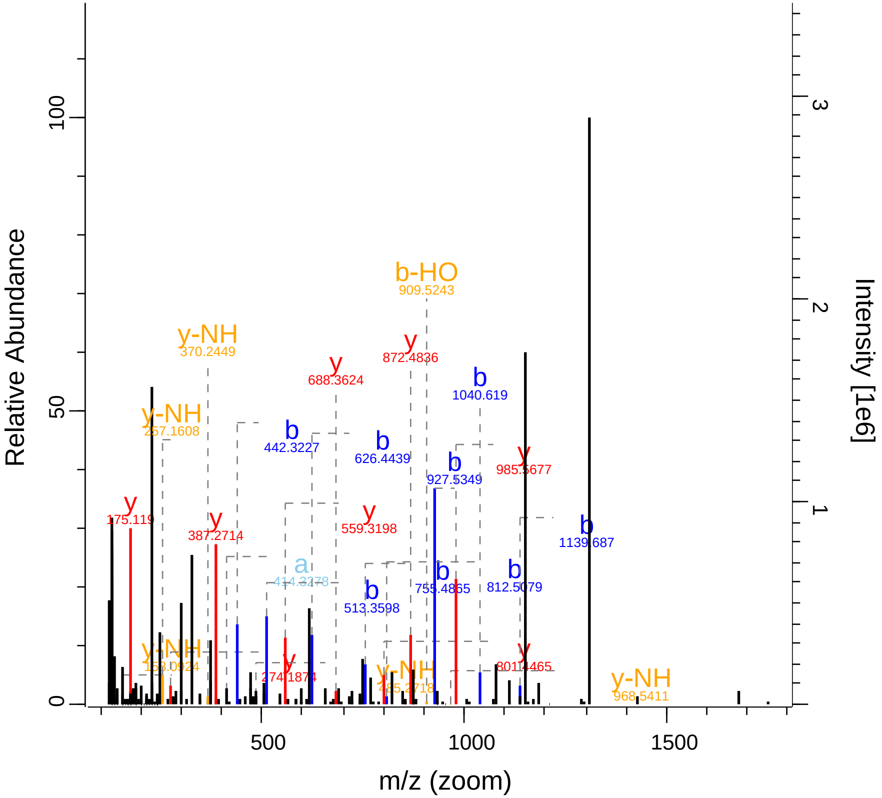

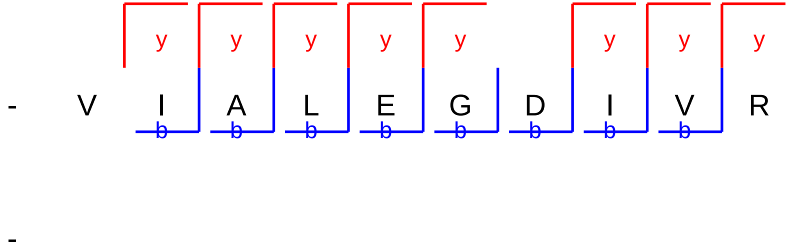

Raw File	Scan	Method	Score	m/z	Gene names
RO-QC-160121_ZackD_SS_Set_01_Fractio	27895	FTMS; HC	118.52	849.45	SLC31A1

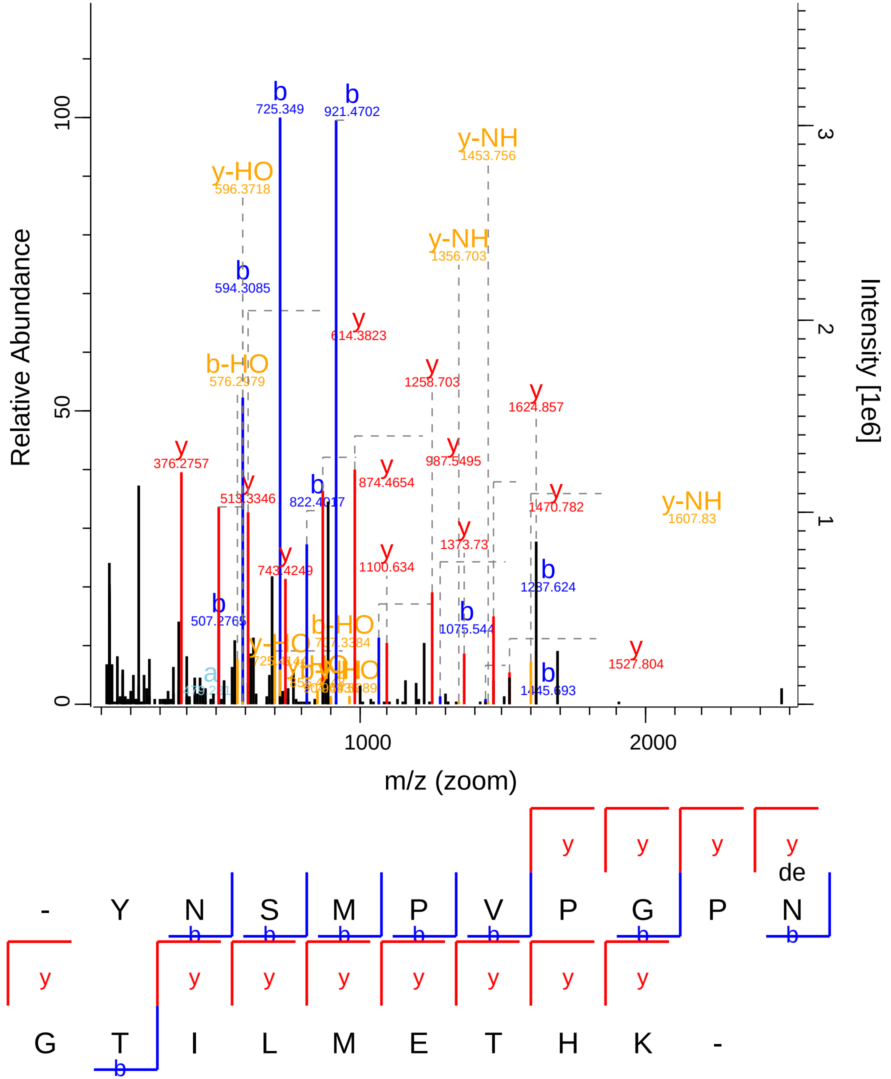

Raw File	Scan	Method	Score	m/z	Gene names
RO-QC-160121_ZackD_SS_Set_01_Fractio	27927	FTMS; HC	121.74	637.09	SLC31A1

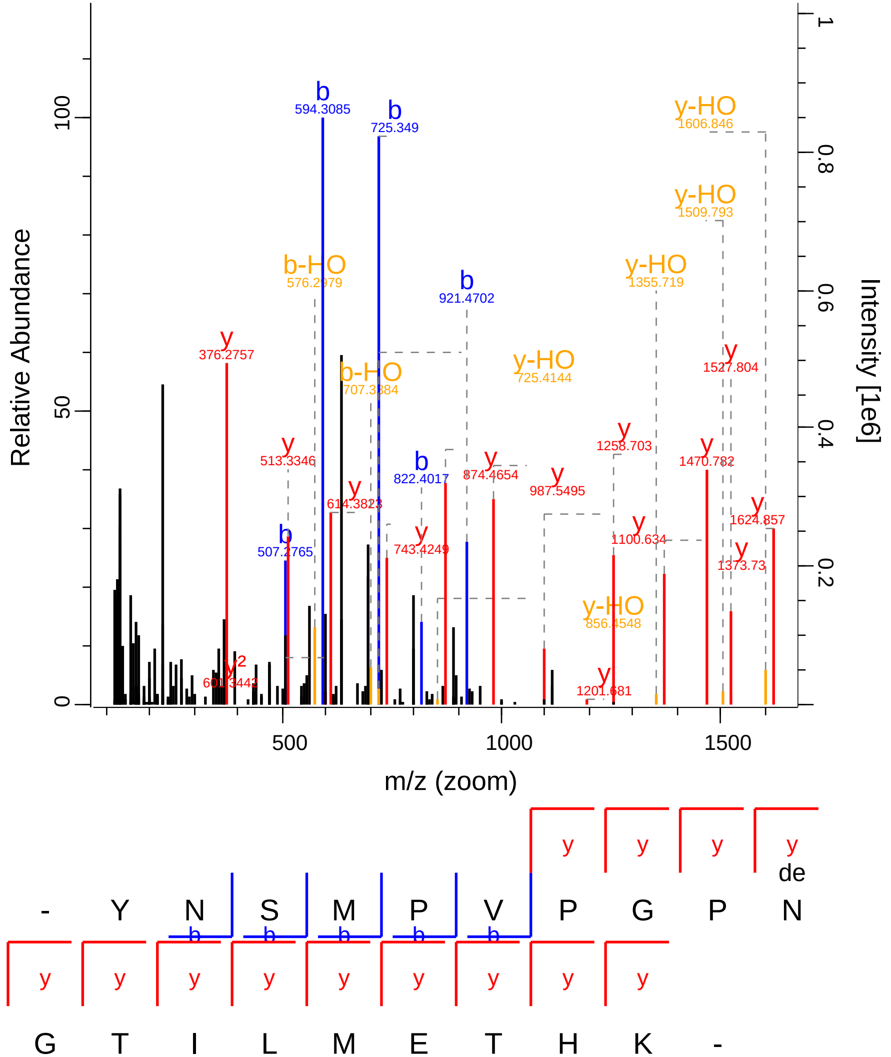

Raw File	Scan	Method	Score	m/z	Gene names
RO-QC-160121_ZackD_SS_Set_01_Fractio	28411	FTMS; HC	144.76	937.11	GYPC

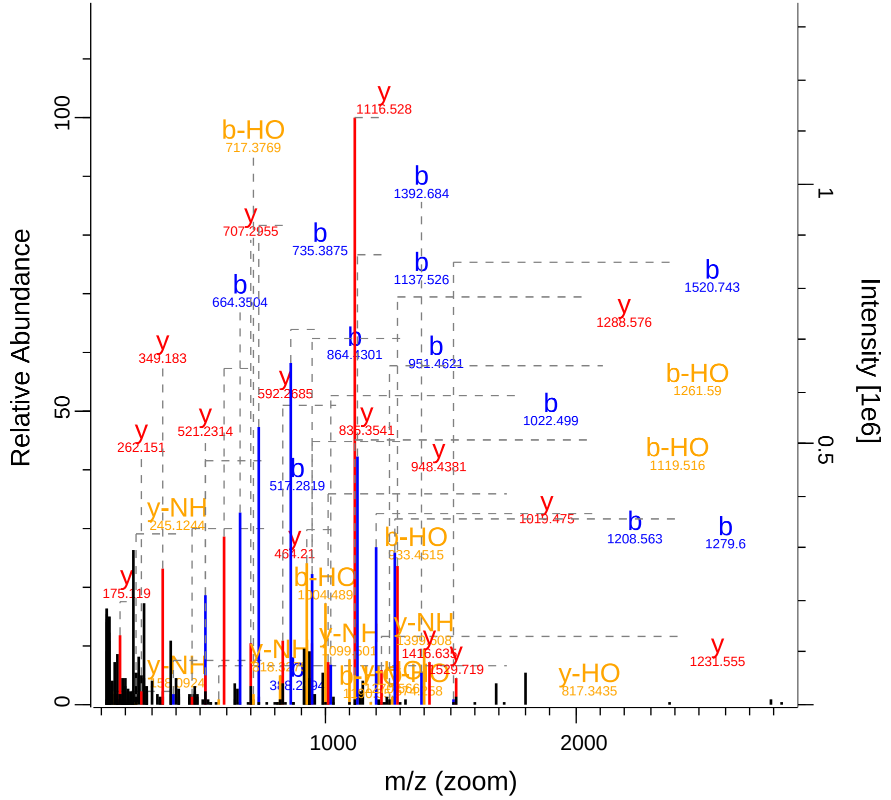

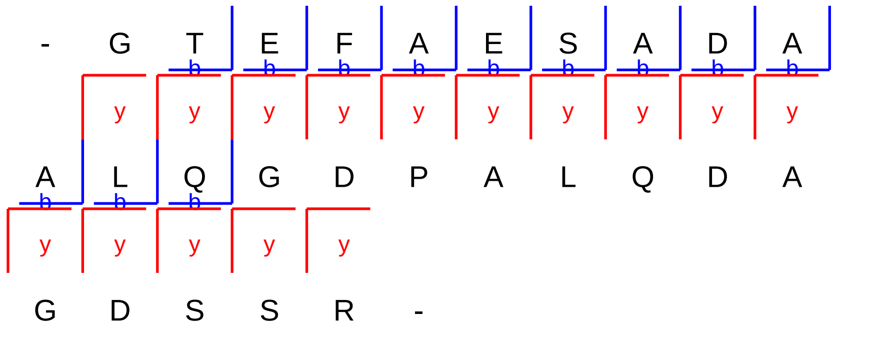

Raw File	Scan	Method	Score	m/z	Gene names
RO-QC-160121_ZackD_SS_Set_01_Fractio	28667	FTMS; HC	88.55	874.46	SMUG1

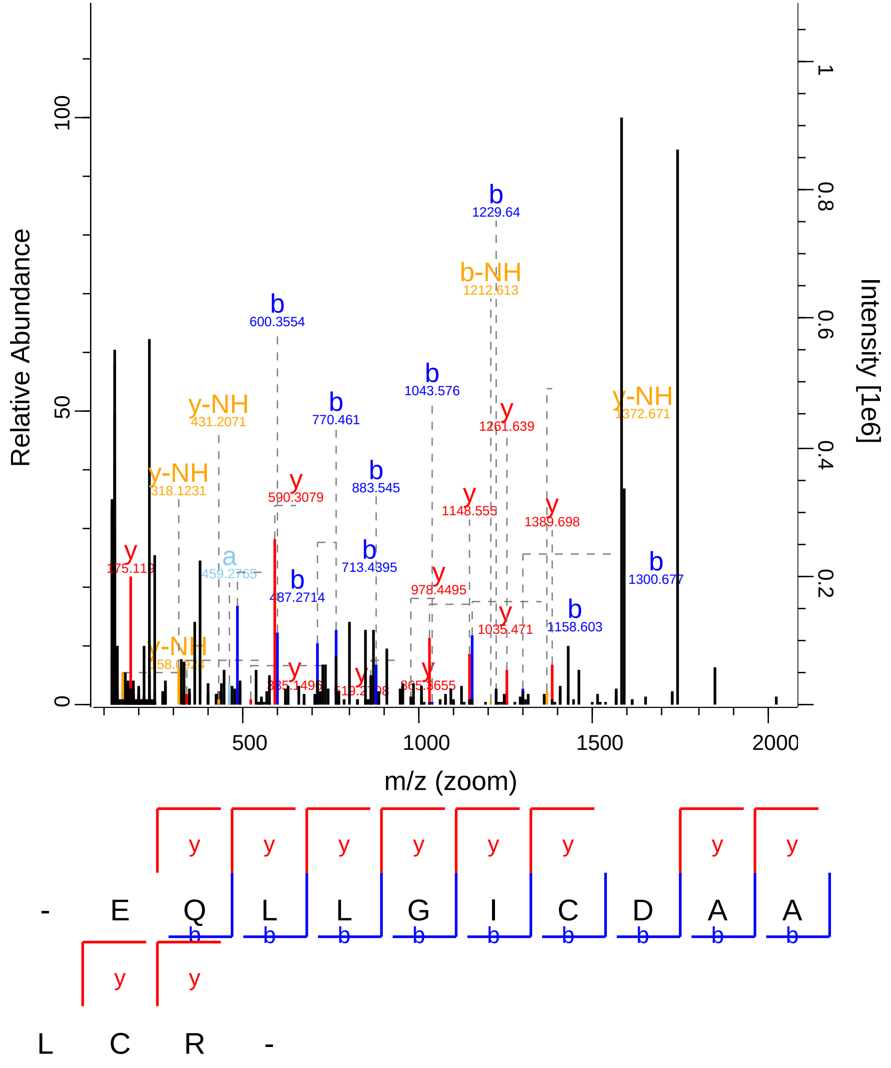

Raw File	Scan	Method	Score	m/z	Gene names
RO-QC-160121_ZackD_SS_Set_01_Fractic	28717	FTMS; HC	56.01	1026.49	SIRPB2

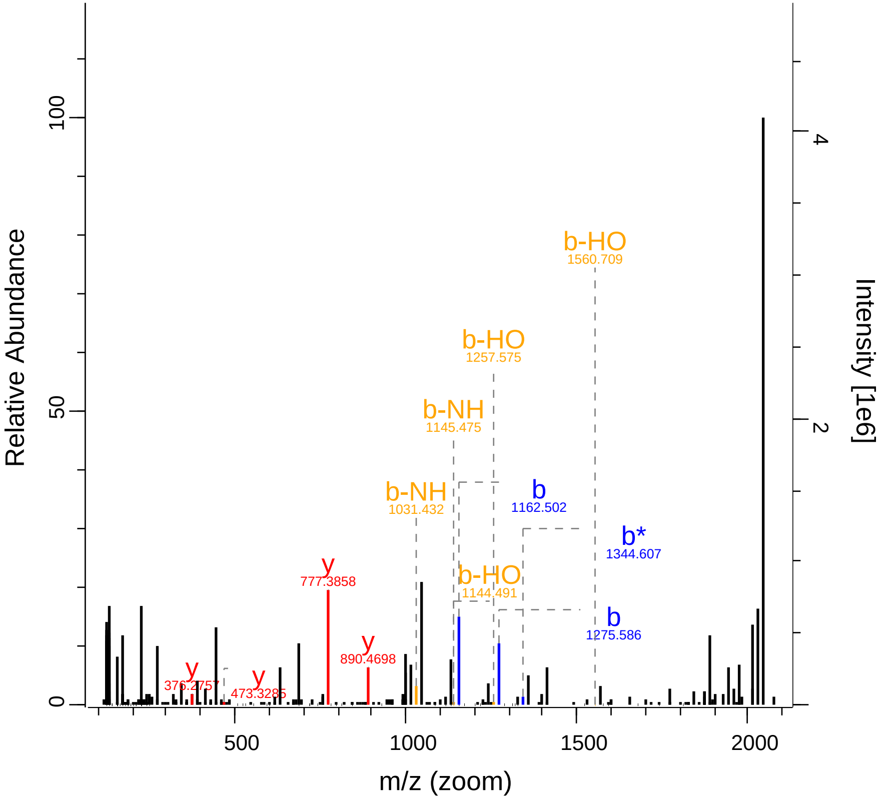

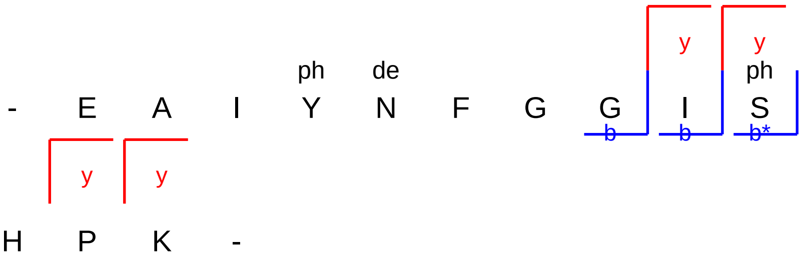

Raw File	Scan	Method	Score	m/z	Gene names
RO-QC-160121_ZackD_SS_Set_01_Fractio	28988	FTMS; HC	78.36	829.75	TMEM117

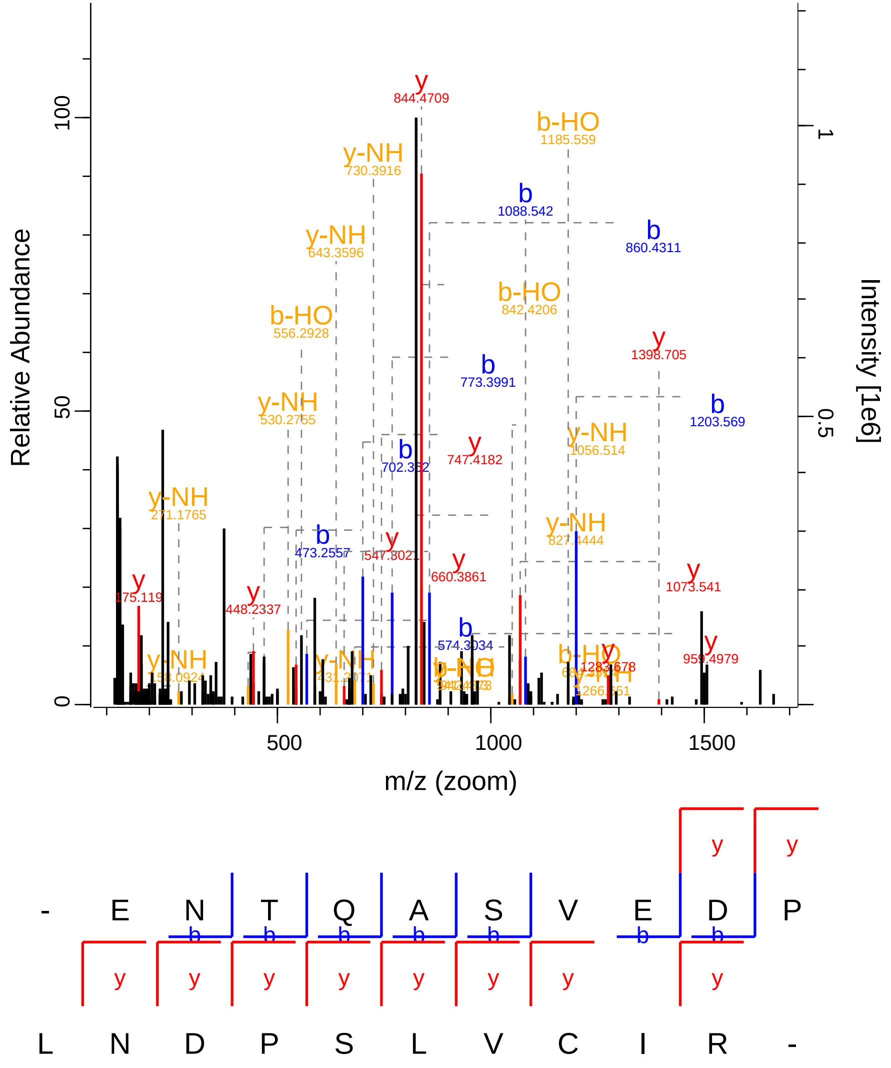

Raw File	Scan	Method	Score	m/z	Gene names
RO-QC-160121_ZackD_SS_Set_01_Fractio	29066	FTMS; HC	74.58	993.88	C1orf43

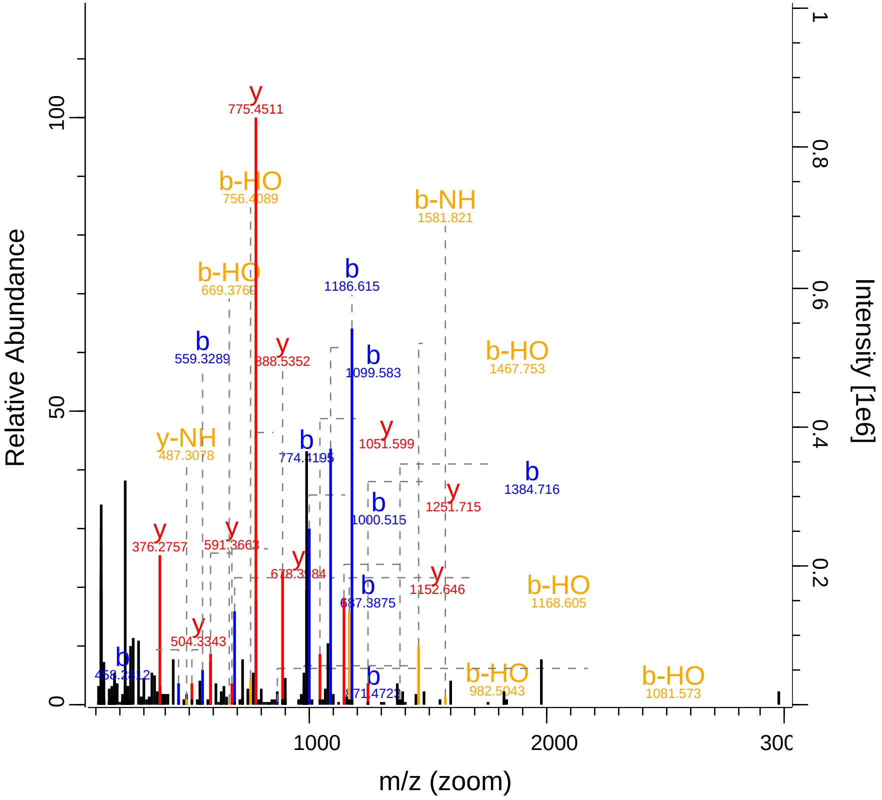

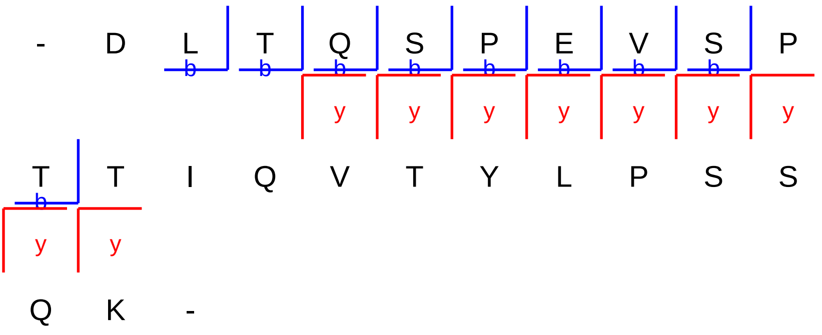

Raw File	Scan	Method	Score	m/z	Gene names
RO-QC-160121_ZackD_SS_Set_01_Fractio	29446	FTMS; HC	42.6	737.86	TRIL

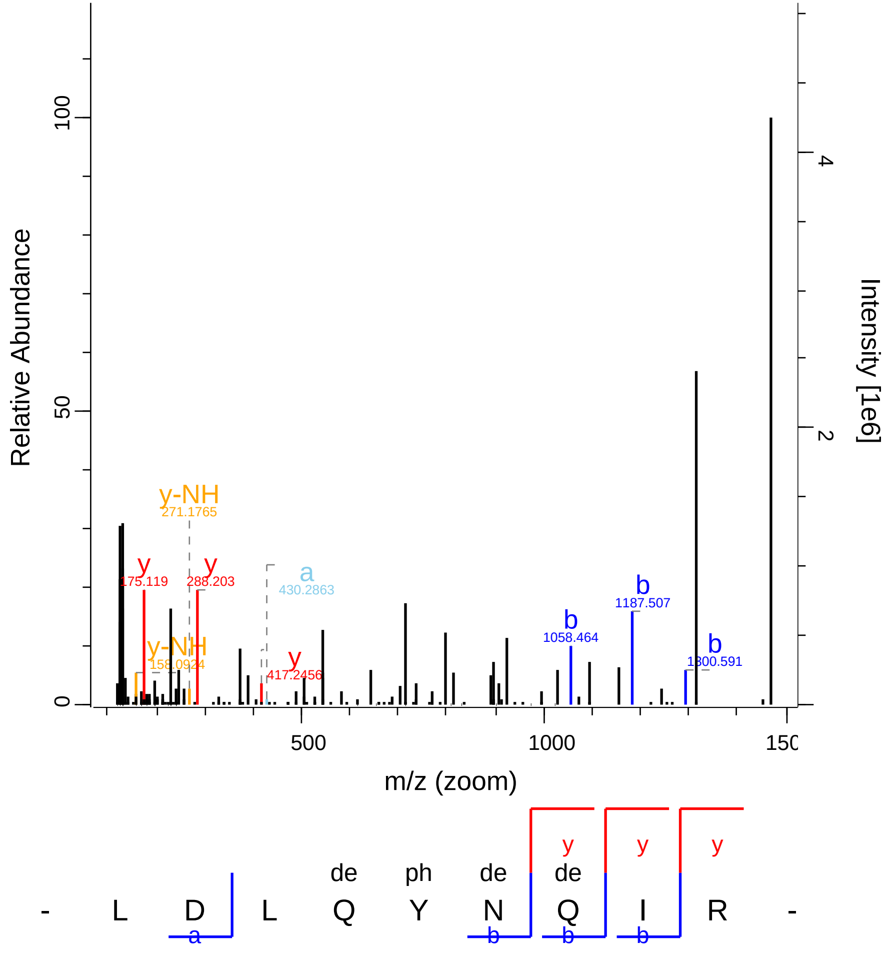

Raw File	Scan	Method	Score	m/z	Gene names
RO-QC-160121_ZackD_SS_Set_01_Fractic	30021	FTMS; HC	75.62	1008.54	TMEM206

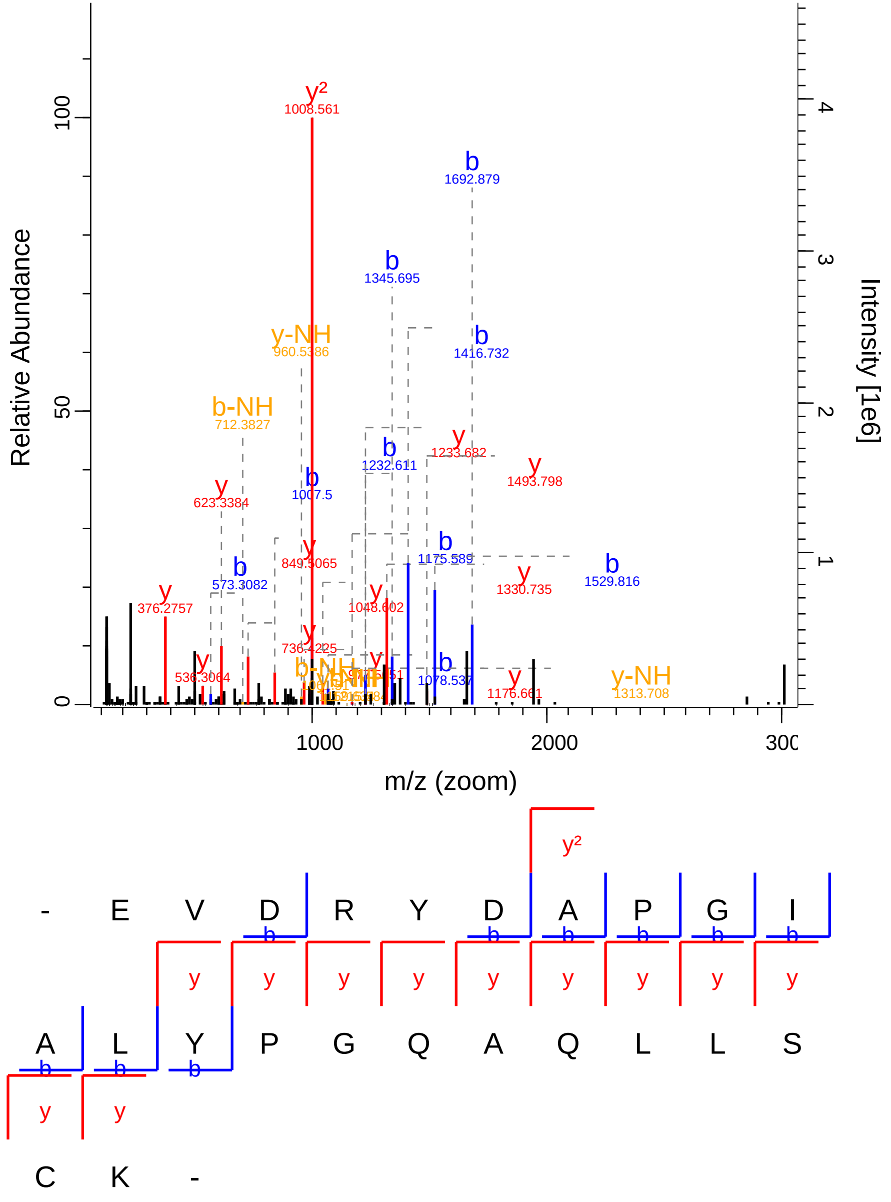

Raw File	Scan	Method	Score	m/z	Gene names
RO-QC-160121_ZackD_SS_Set_01_Fractio	30176	FTMS; HC	101.39	800.47	PRTFDC1

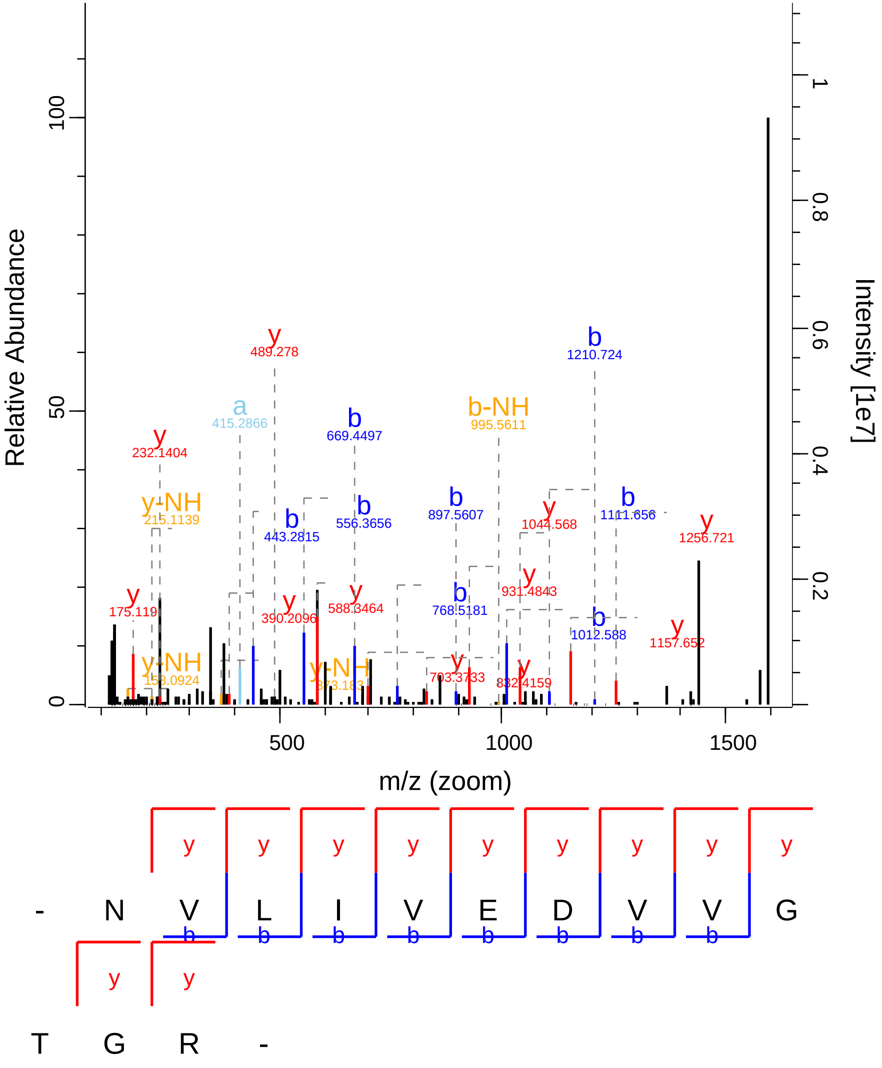

Raw File	Scan	Method	Score	m/z	Gene names
RO-QC-160121_ZackD_SS_Set_01_Fractio	31103	FTMS; HC	113.22	577.34	MIS12

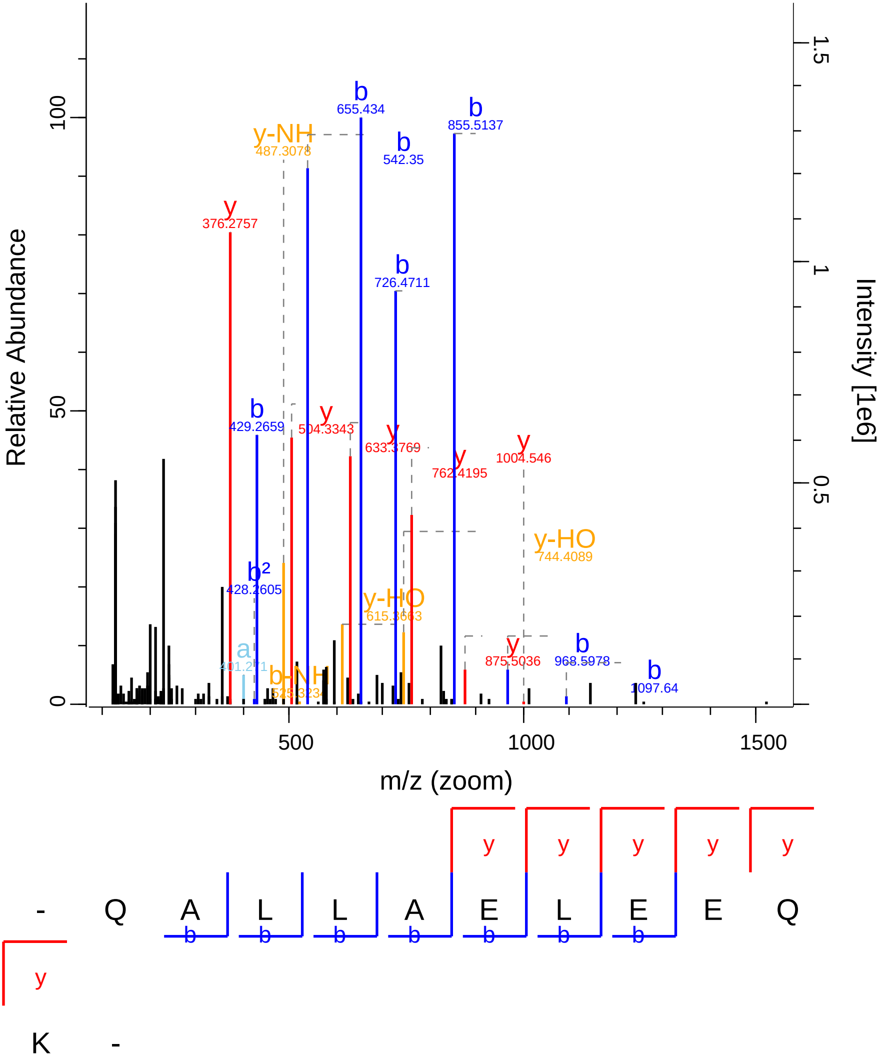

Raw File	Scan	Method	Score	m/z	Gene names
RO-QC-160121_ZackD_SS_Set_01_Fractio	31128	FTMS; HC	110.08	865.51	MIS12

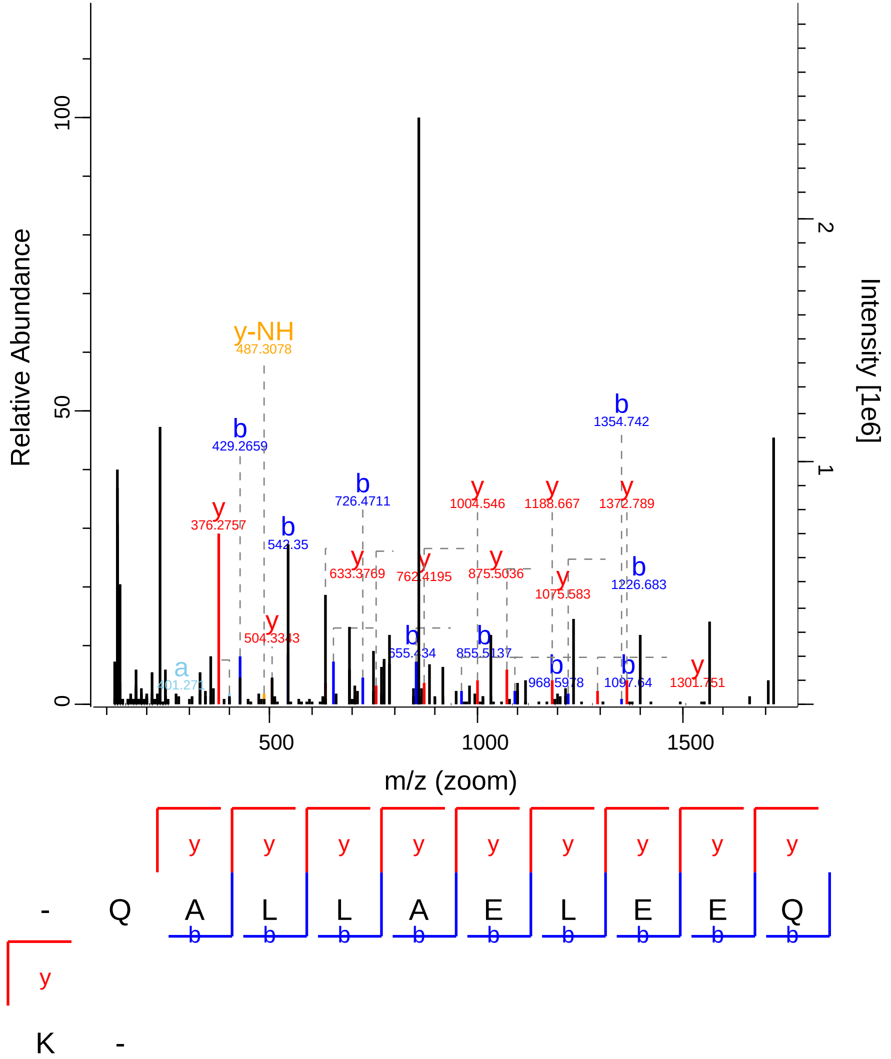

Raw File	Scan	Method	Score	m/z	Gene names
RO-QC-160121_ZackD_SS_Set_01_Fractio	31213	FTMS; HC	57.34	911.82	MXD3

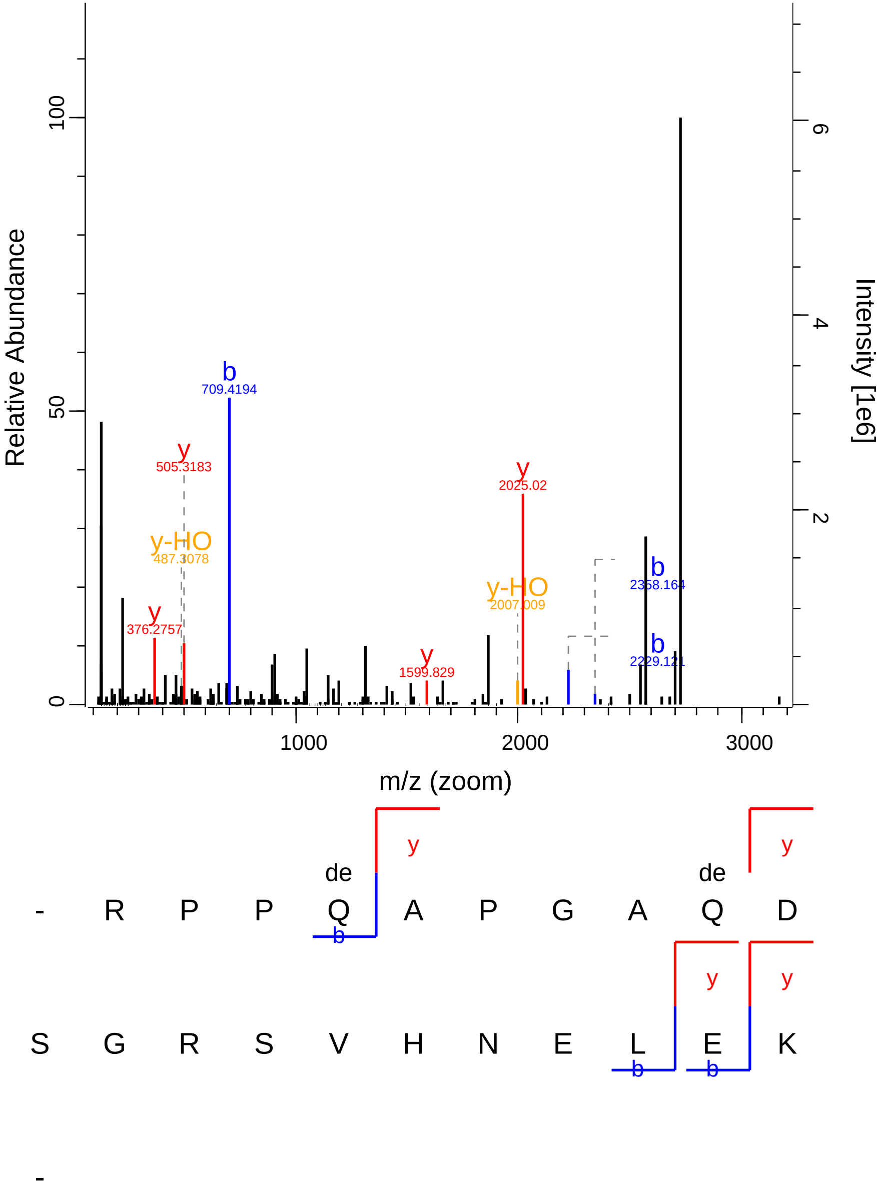

Raw File	Scan	Method	Score	m/z
RO-QC-160121_ZackD_SS_Set_01_Fraction_4	31714	FTMS; HCD	67.9	506.65

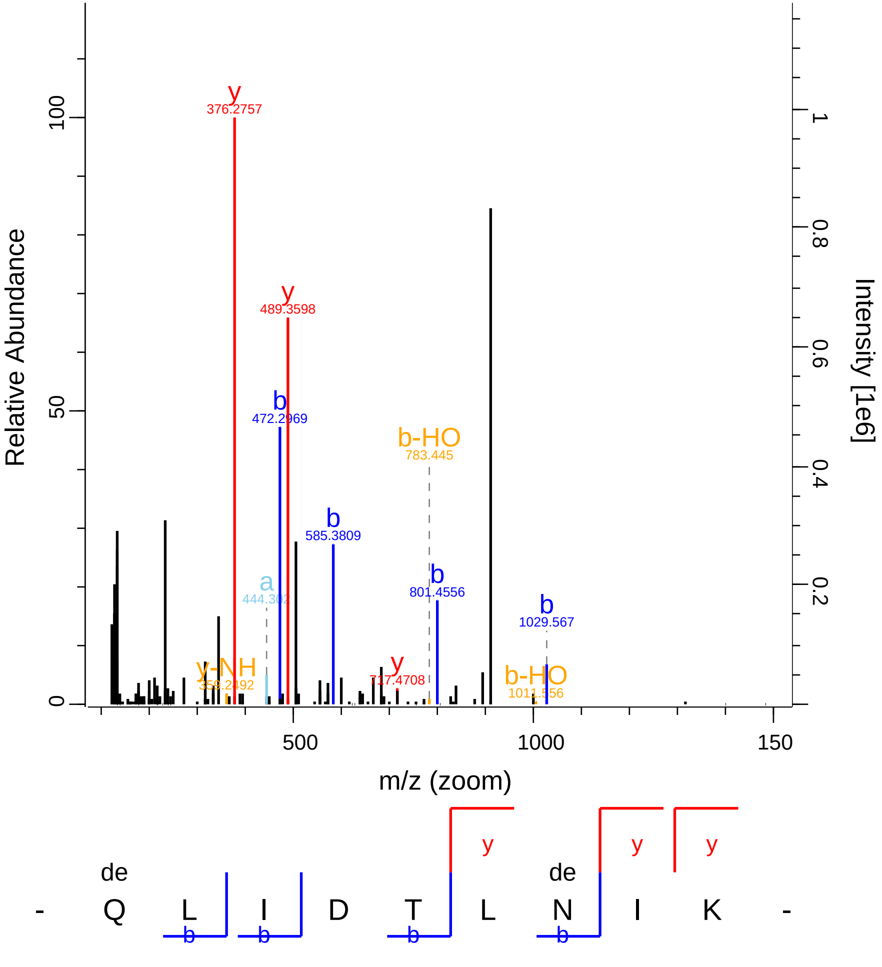

Raw File	Scan	Method	Score	m/z	Gene names
RO-QC-160121_ZackD_SS_Set_01_Fractio	31723	FTMS; HC	115.7	501.61	ATF6B

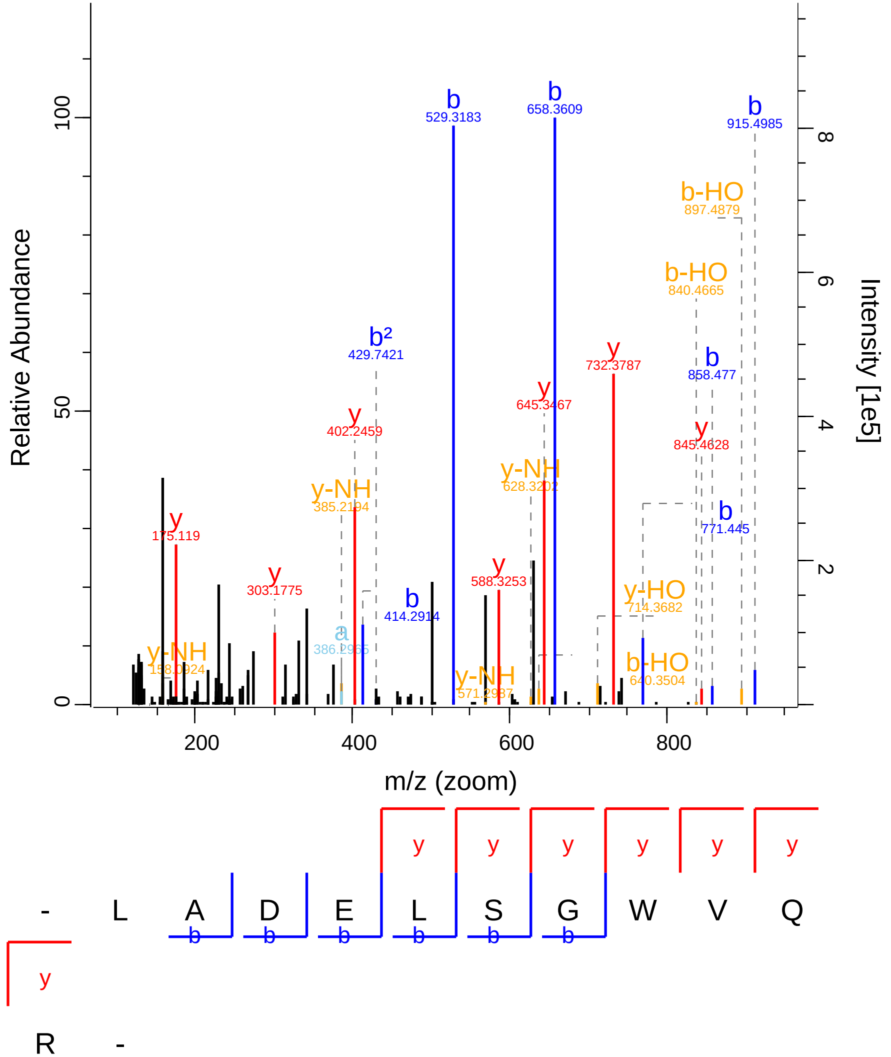

Raw File	Scan	Method	Score	m/z	Gene names
RO-QC-160121_ZackD_SS_Set_01_Fractio	32163	FTMS; HC	156.29	916.51	TMUB2

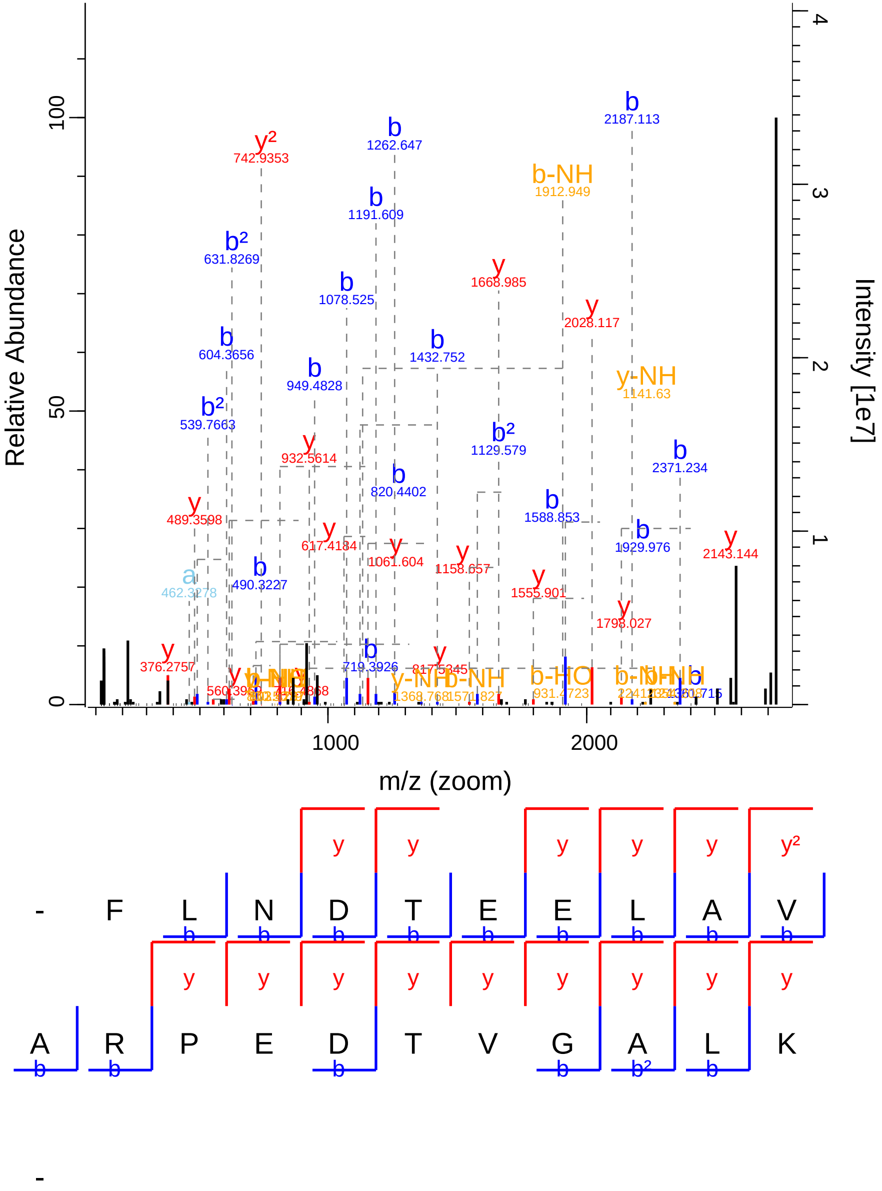

Raw File	Scan	Method	Score	m/z	Gene names
RO-QC-160121_ZackD_SS_Set_01_Fractio	32176	FTMS; HC	149.85	687.63	TMUB2

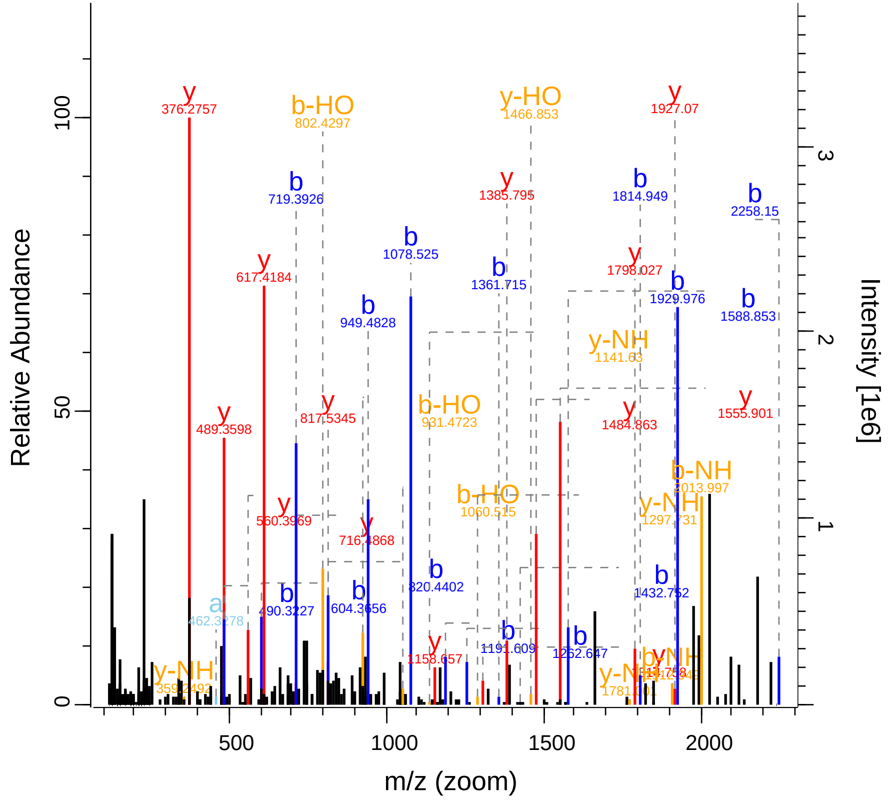

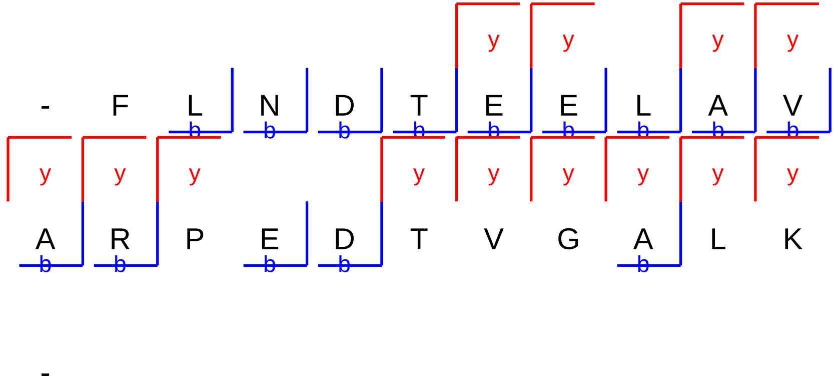

Raw File	Scan	Method	Score	m/z	Gene names
RO-QC-160121_ZackD_SS_Set_01_Fractio	33209	FTMS; HC	103.71	935.85	ALKBH1

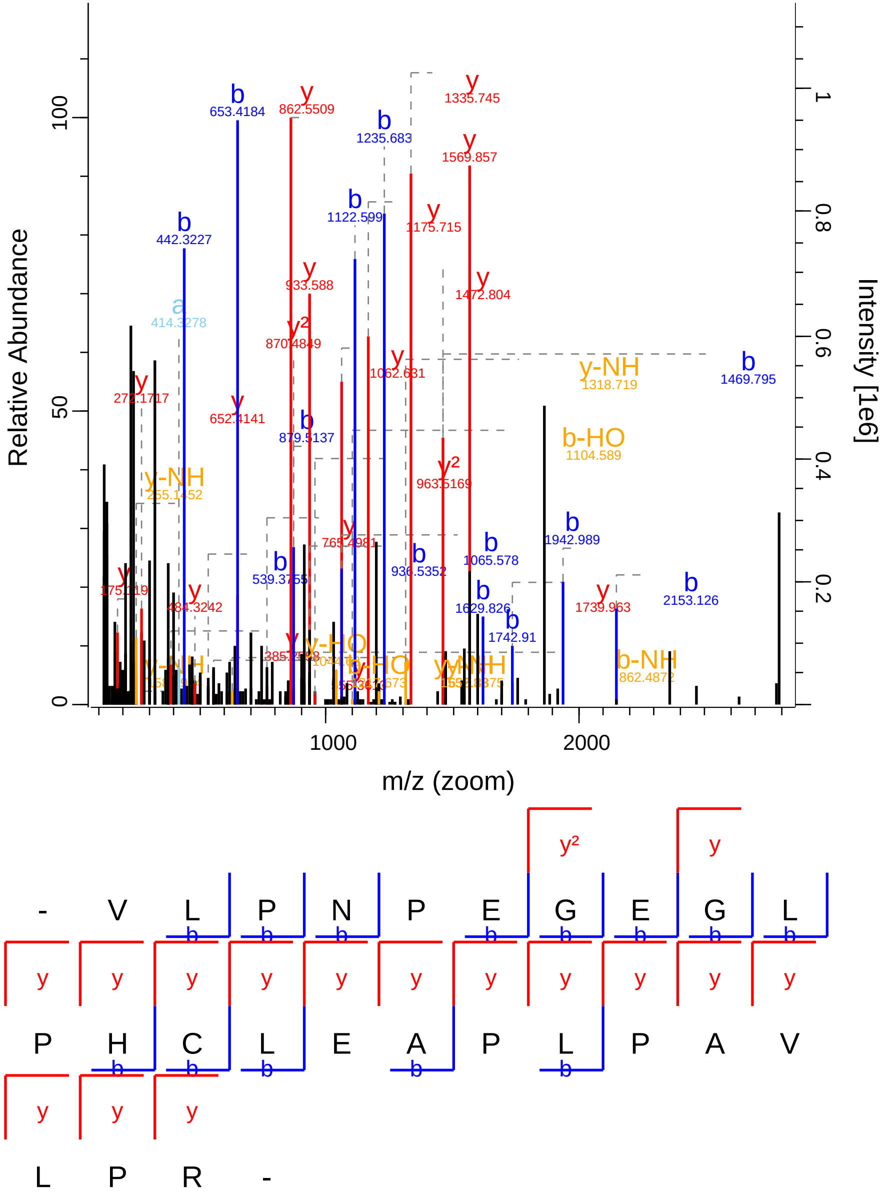

Raw File	Scan	Method	Score	m/z	Gene names
RO-QC-160121_ZackD_SS_Set_01_Fractio	33488	FTMS; HC	40.15	581.92	NLRC5

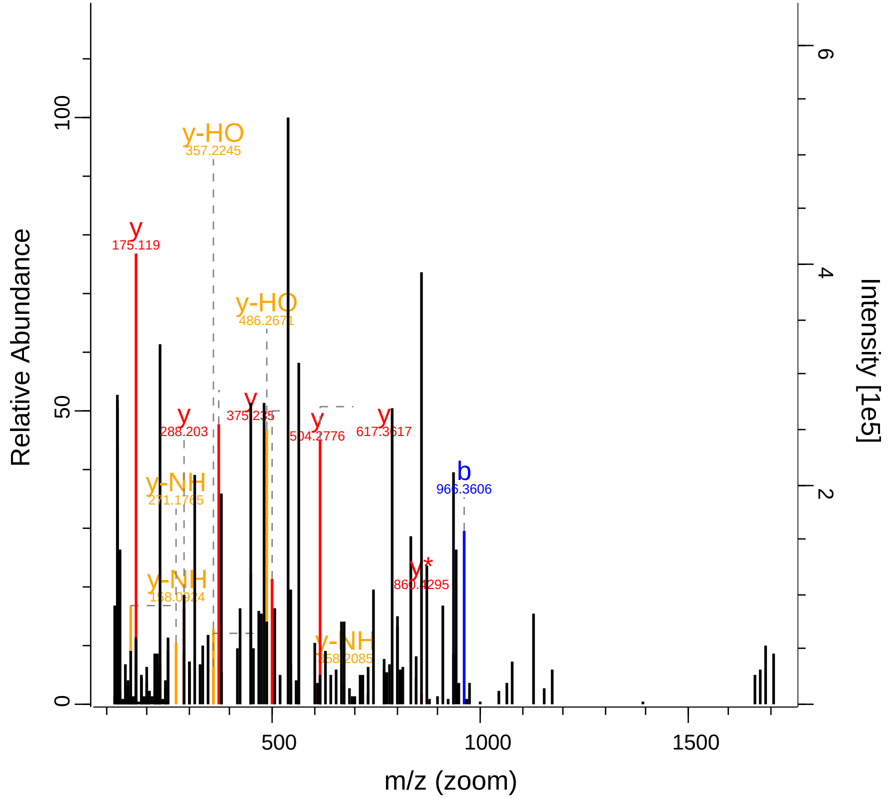

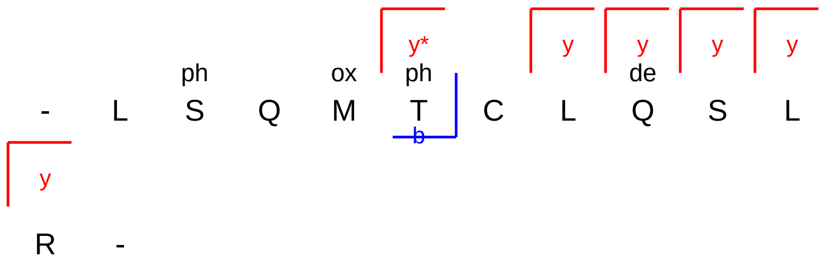

Raw File	Scan	Method	Score	m/z	Gene names
RO-QC-160121_ZackD_SS_Set_01_Fractic	35232	FTMS; HC	178.69	1068.91	FAM122A

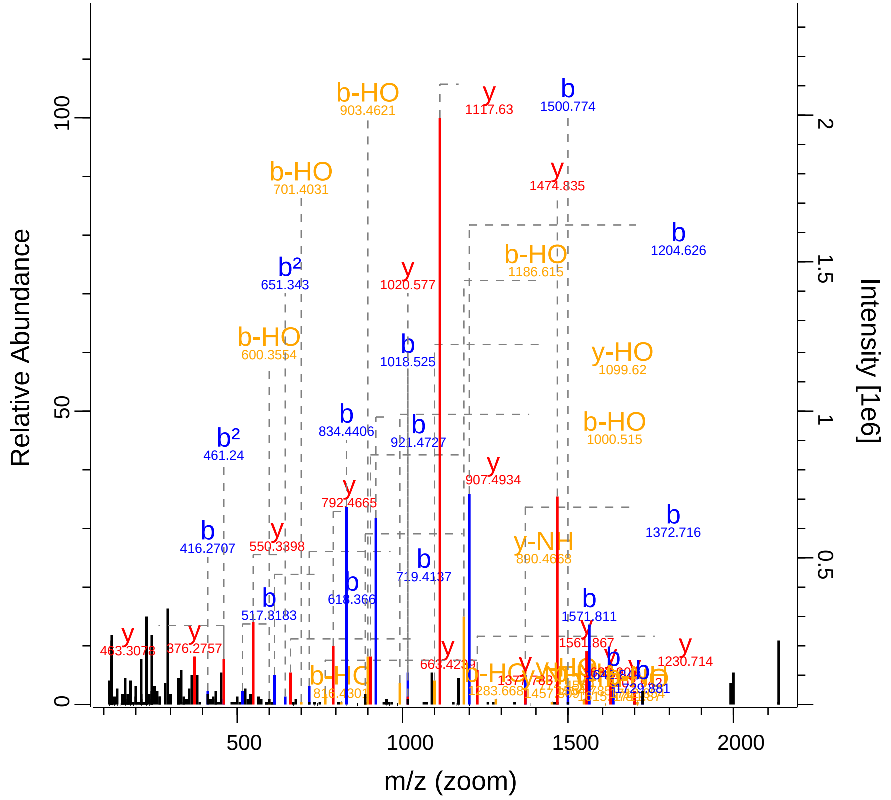

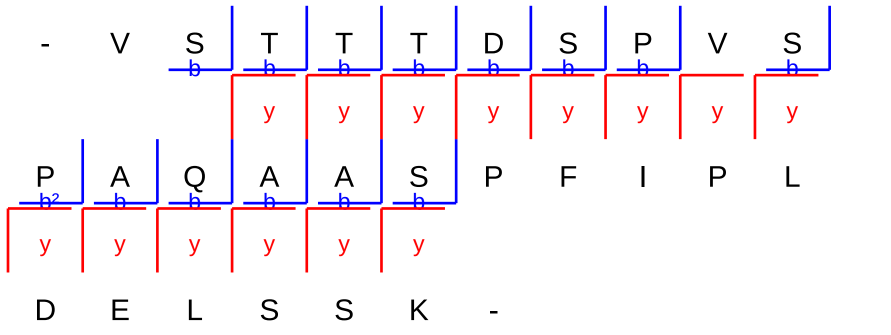

Raw File	Scan	Method	Score	m/z	Gene names
RO-QC-160121_ZackD_SS_Set_01_Fractio	35267	FTMS; HC	39.99	801.94	FAM122A

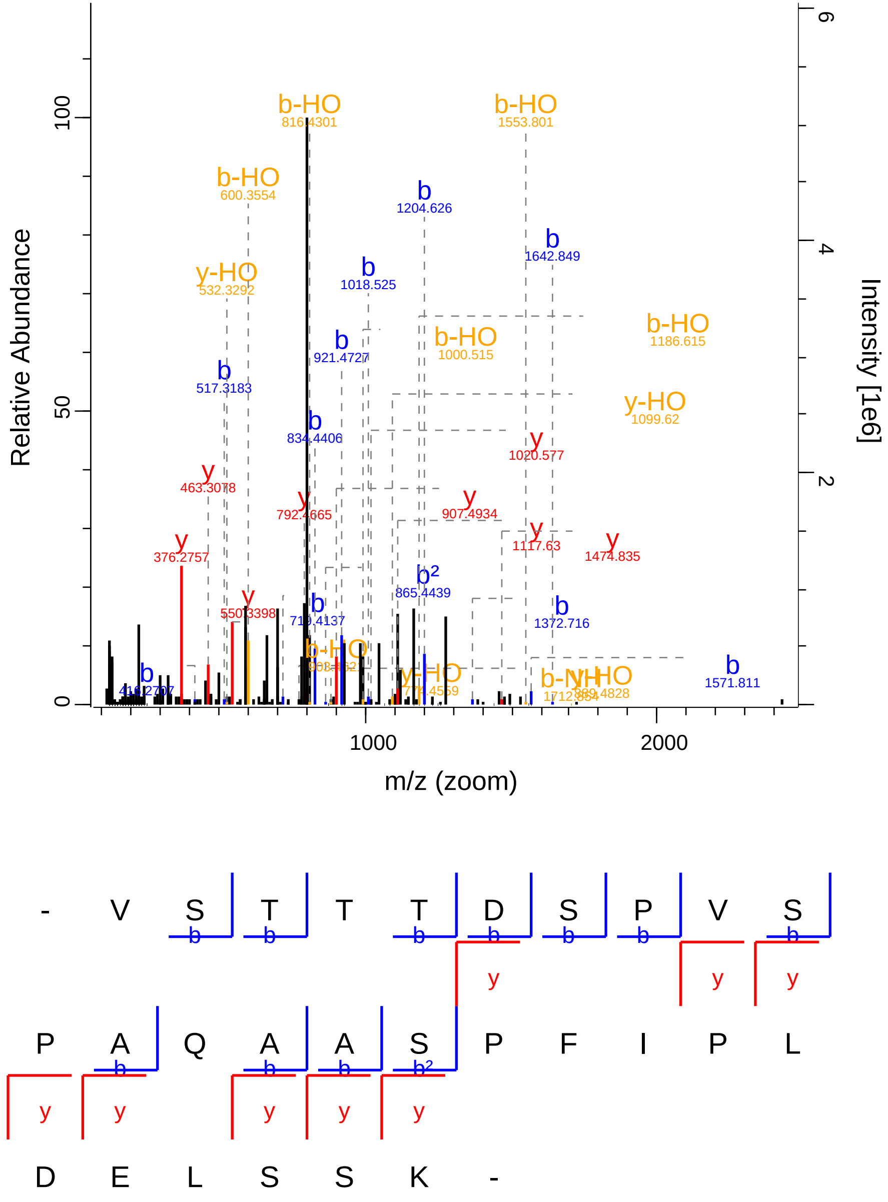

Raw File	Scan	Method	Score	m/z	Gene names
RO-QC-160121_ZackD_SS_Set_01_Fractio	35297	FTMS; HC	67.96	620.36	WNT11

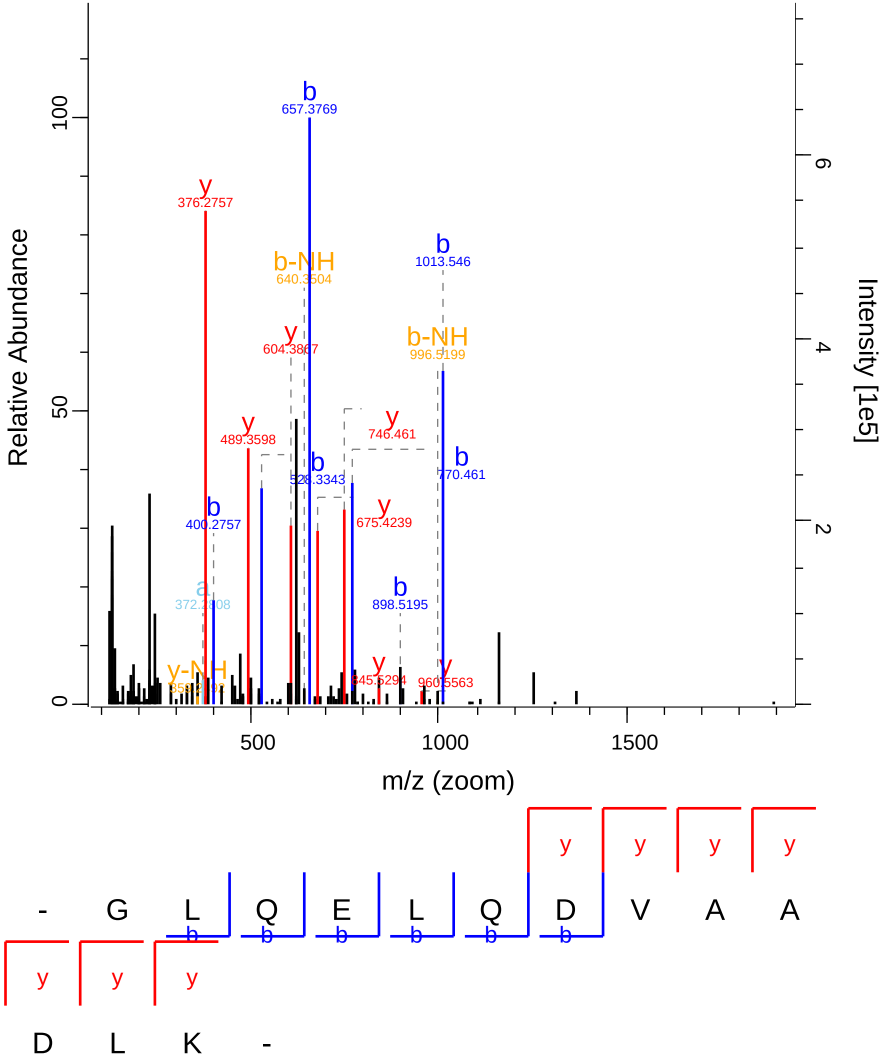

Raw File	Scan	Method	Score	m/z
RO-QC-160121_ZackD_SS_Set_01_Fraction_4	35481	FTMS; HCD	54.41	771.41

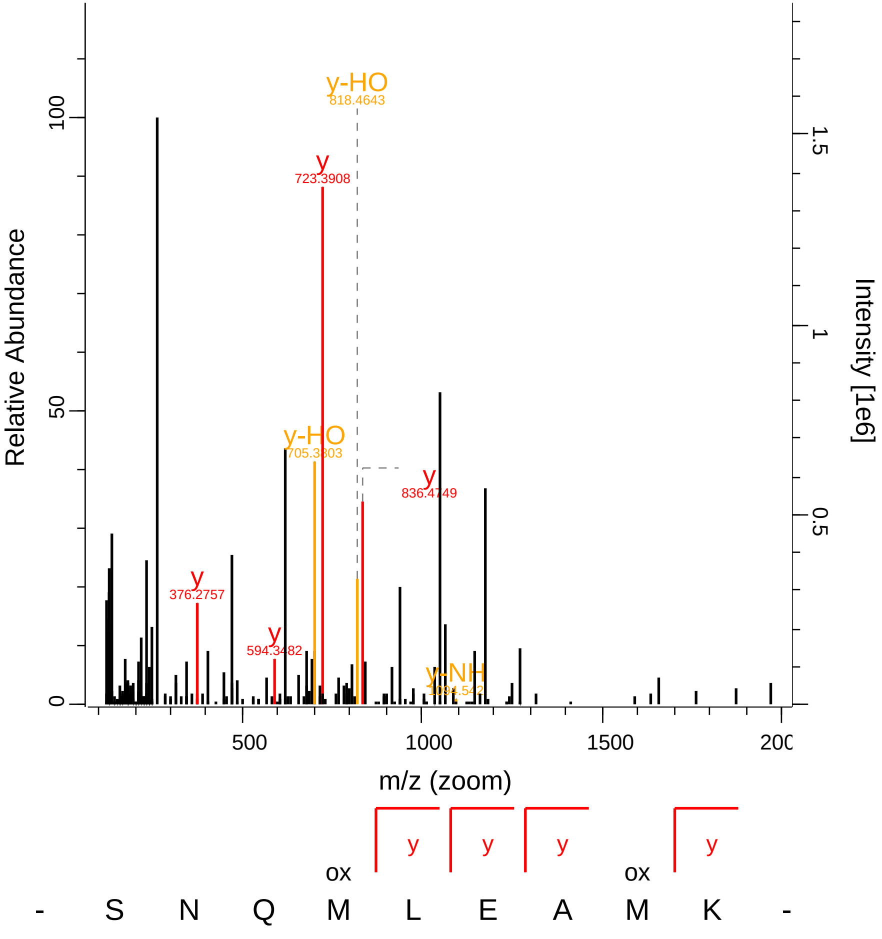

Raw File	Scan	Method	Score	m/z	Gene names
RO-QC-160121_ZackD_SS_Set_01_Fractio	35769	FTMS; HC	71.08	663.67	ABRACL

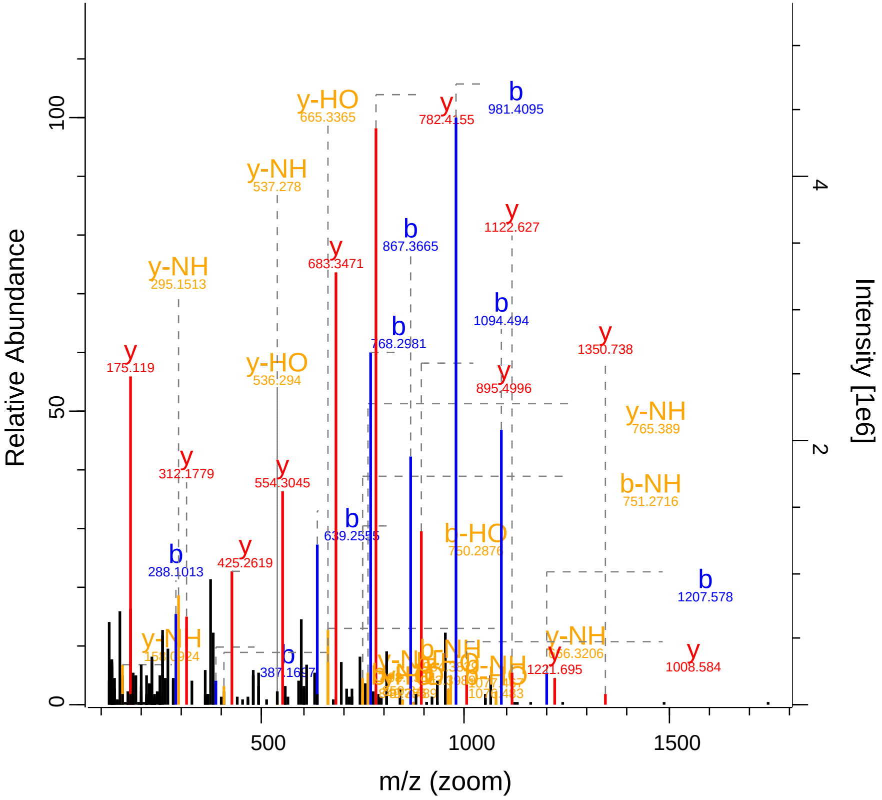

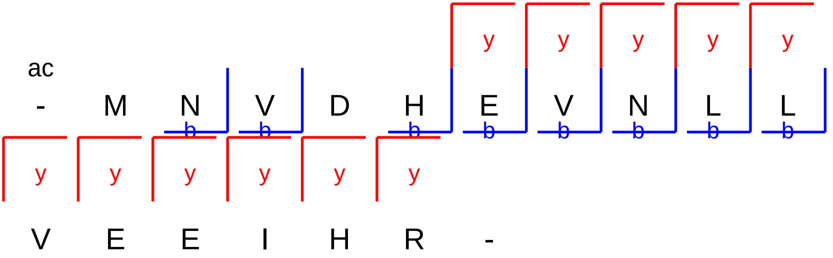

Raw File	Scan	Method	Score	m/z	Gene names
RO-QC-160121_ZackD_SS_Set_01_Fractic	36357	FTMS; HC	89.55	1230.64	NEK10

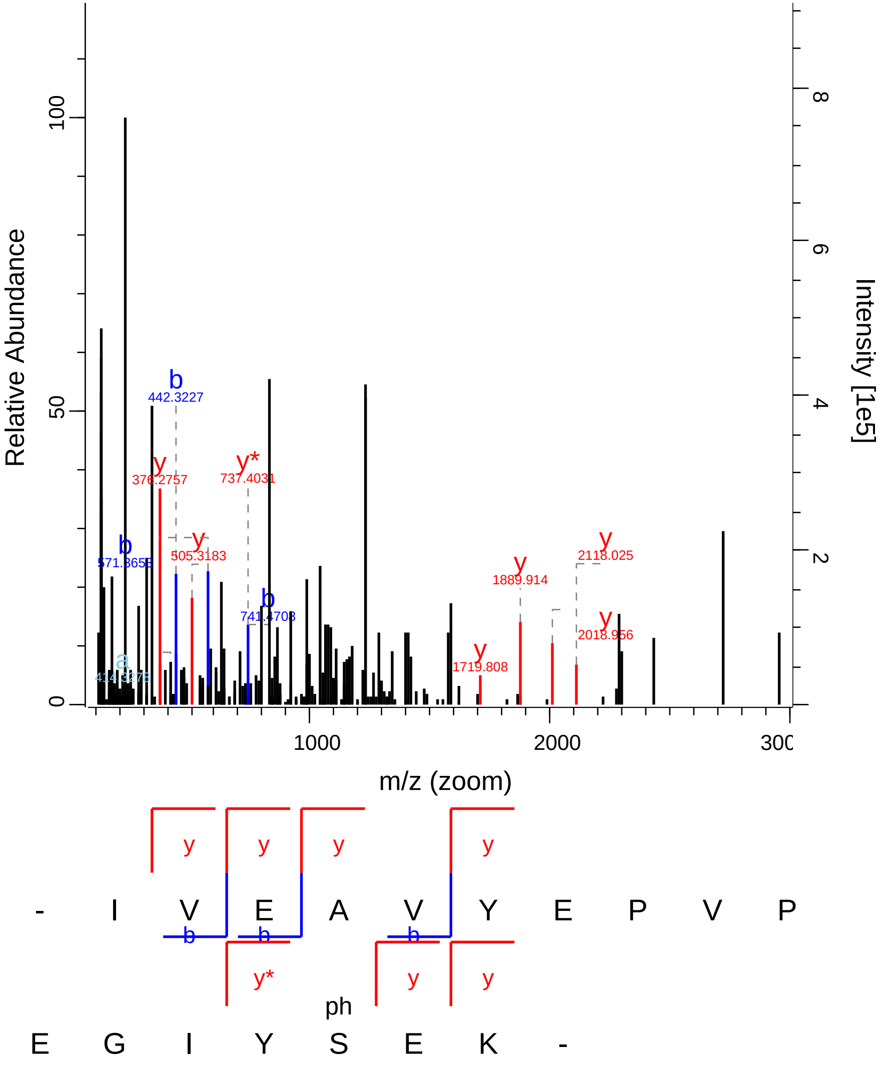

Raw File	Scan	Method	Score	m/z
RO-QC-160121_ZackD_SS_Set_01_Fraction_4	36833	FTMS; HCD	96.25	548.68

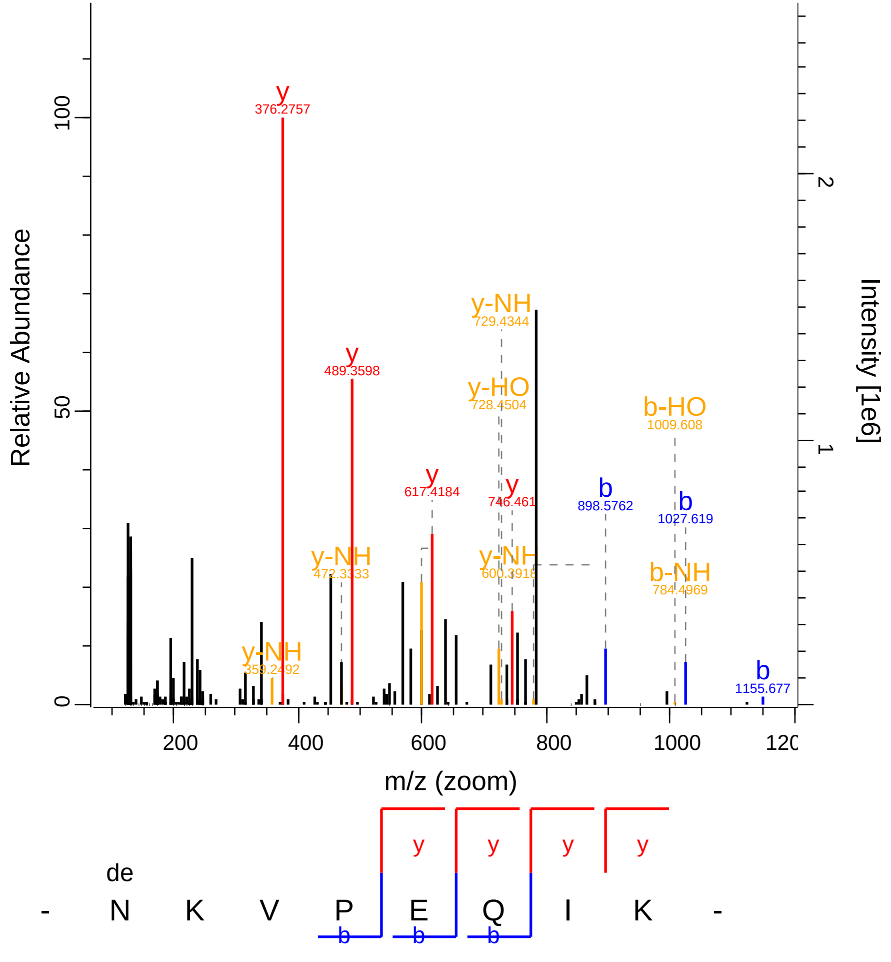

Raw File	Scan	Method	Score	m/z
RO-QC-160121_ZackD_SS_Set_01_Fraction_5	5164	FTMS; HCD	52.48	363.95

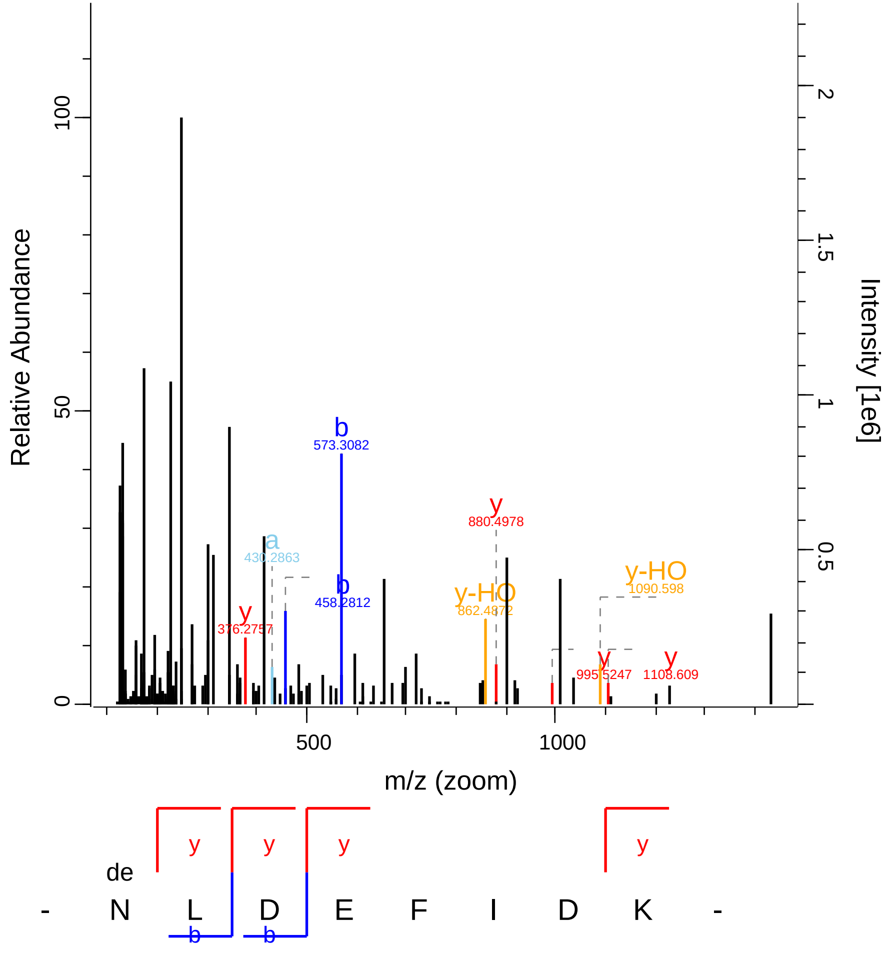

Raw File	Scan	Method	Score	m/z	Gene names
RO-QC-160121_ZackD_SS_Set_01_Fractior	5422	FTMS; HCI	41.98	786.39	HAS1

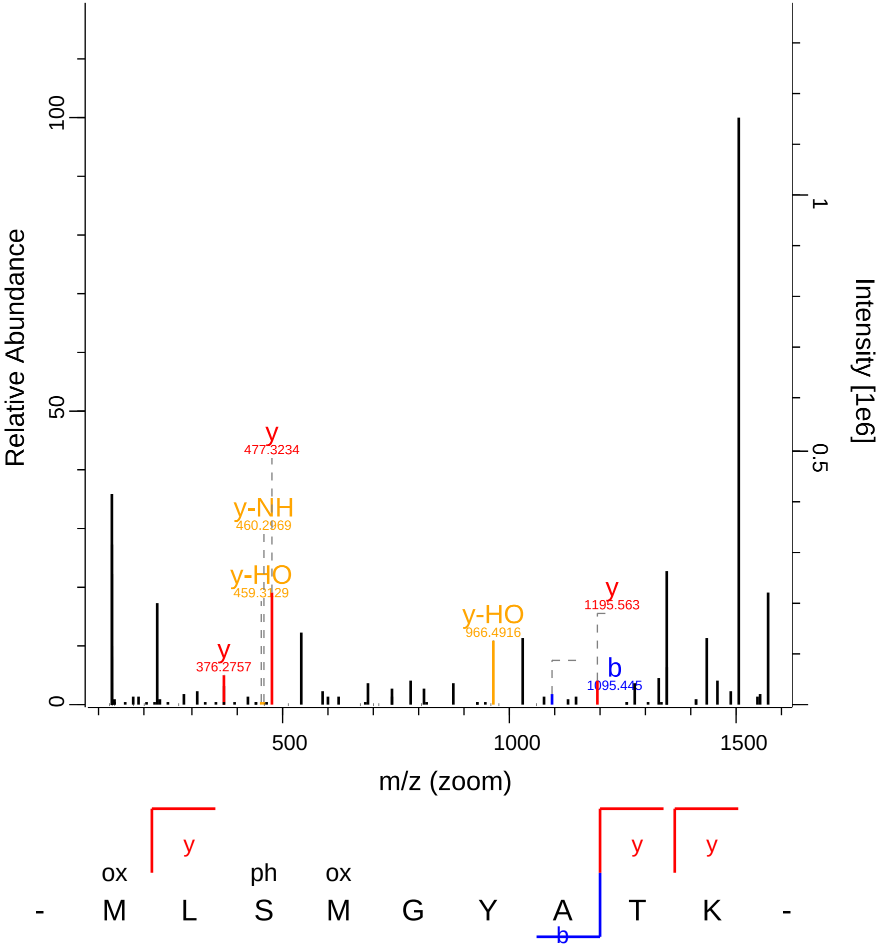

Raw File	Scan	Method	Score	m/z	Gene names
RO-QC-160121_ZackD_SS_Set_01_Fractio[illegible]	5806	FTMS; HC	191.85	621.34	FAM89A

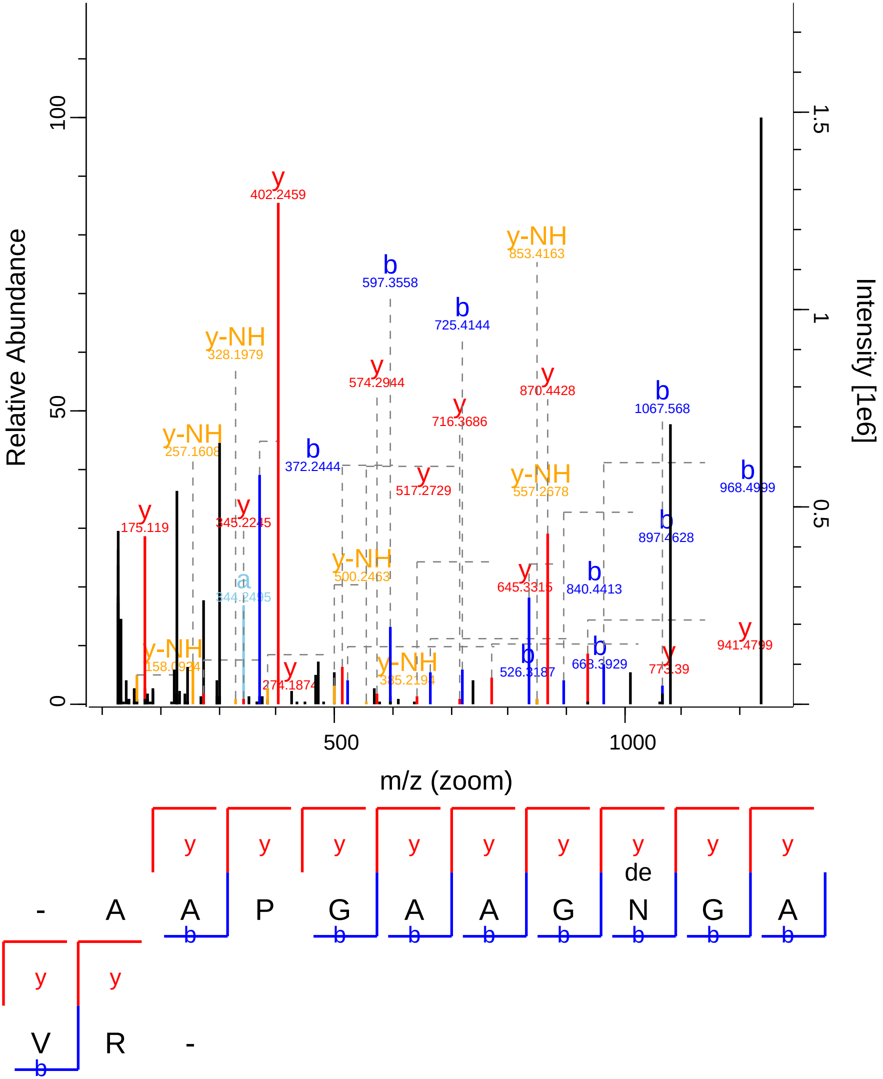

Raw File	Scan	Method	Score	m/z	Gene names
RO-QC-160121_ZackD_SS_Set_01_Fractior	5861	FTMS; HCI	32.41	459.28	GTF3A

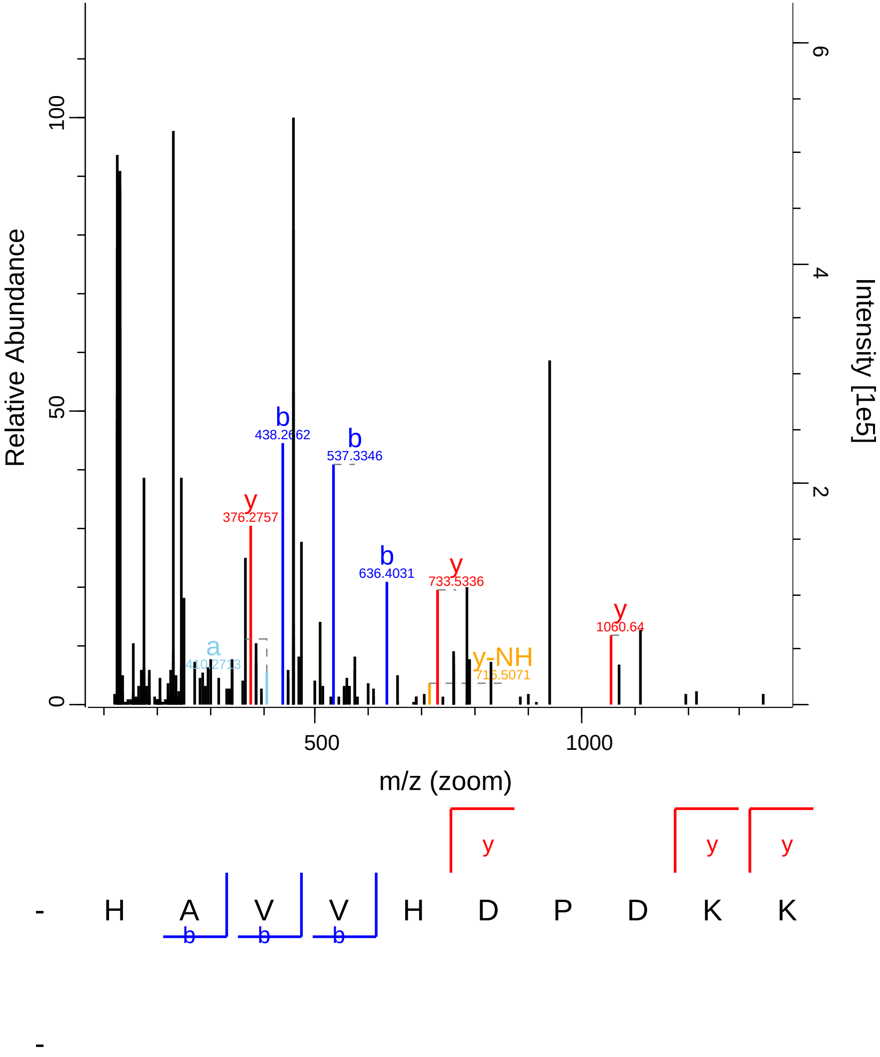

Raw File	Scan	Method	Score	m/z	Gene names
RO-QC-160121_ZackD_SS_Set_01_Fractior	6811	FTMS; HCI	87.52	658.01	KIF26B

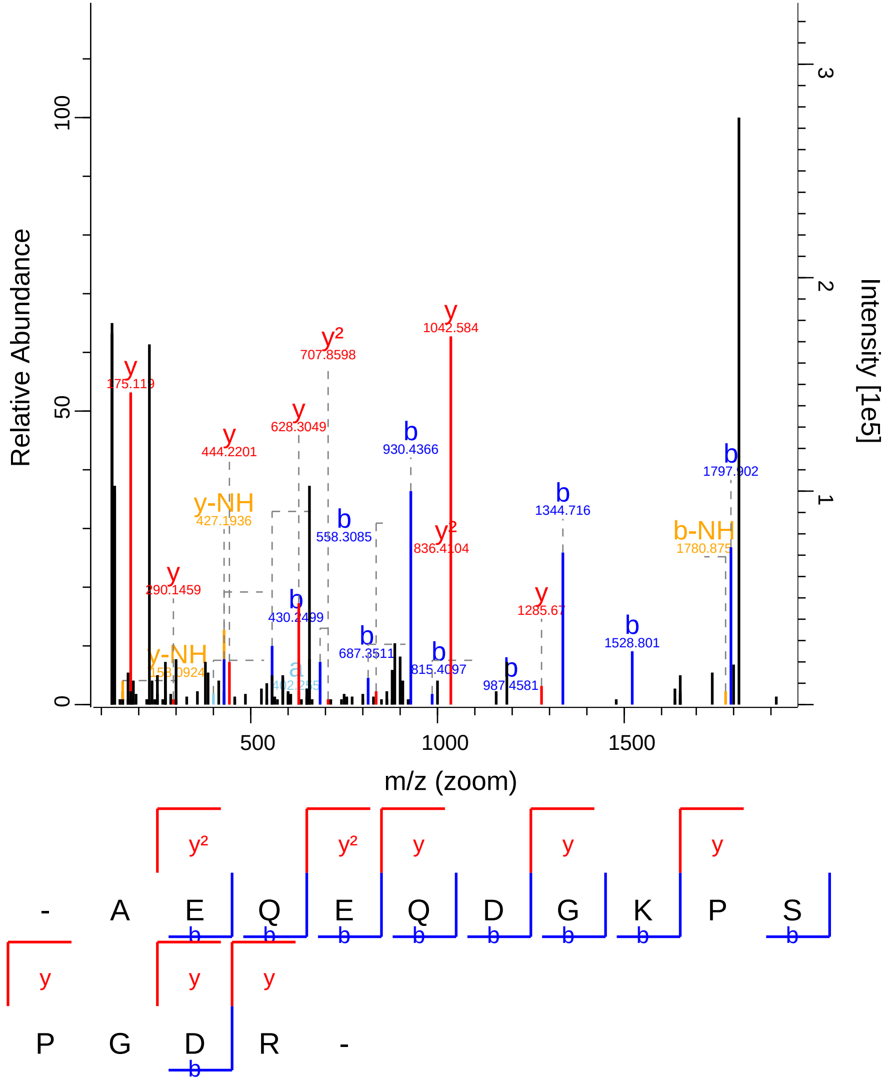

Raw File	Scan	Method	Score	m/z	Gene names
RO-QC-160121_ZackD_SS_Set_01_Fractior	7165	FTMS; HCl	93.35	646.37	UTP23

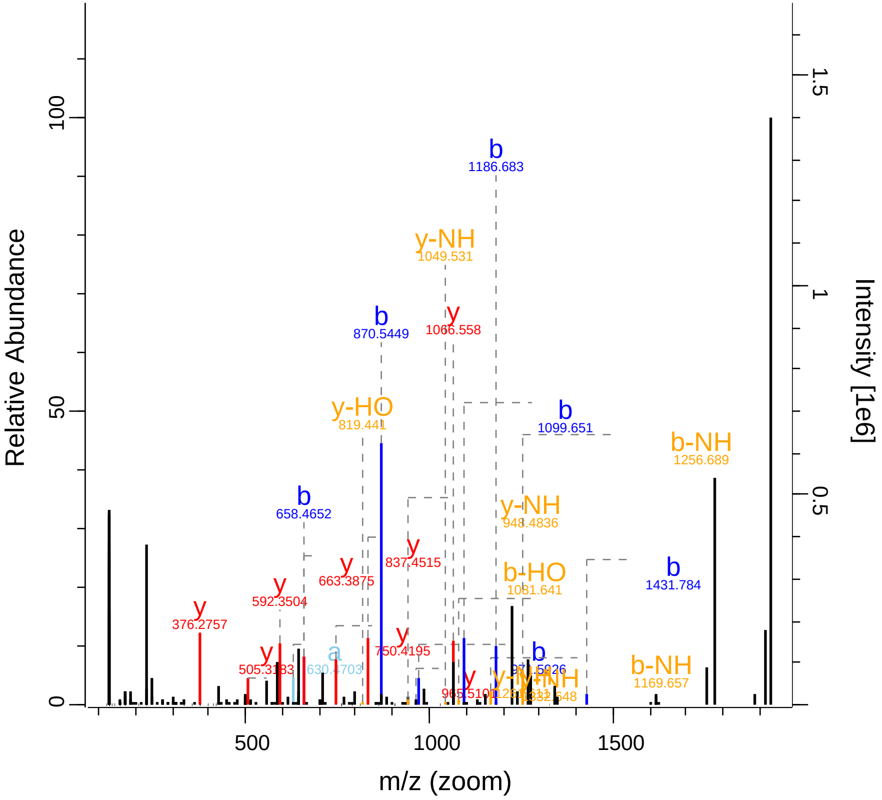

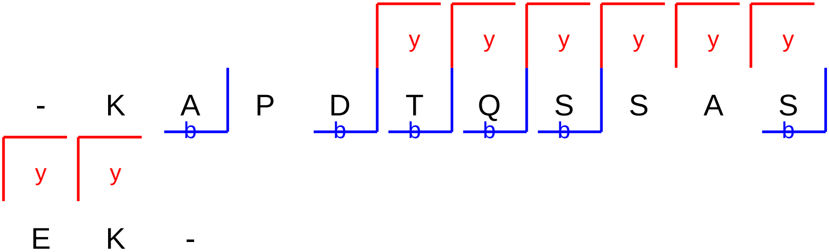

Raw File	Scan	Method	Score	m/z	Gene names
RO-QC-160121_ZackD_SS_Set_01_Fractior	7463	FTMS; HCI	57.6	386.97	DESI2

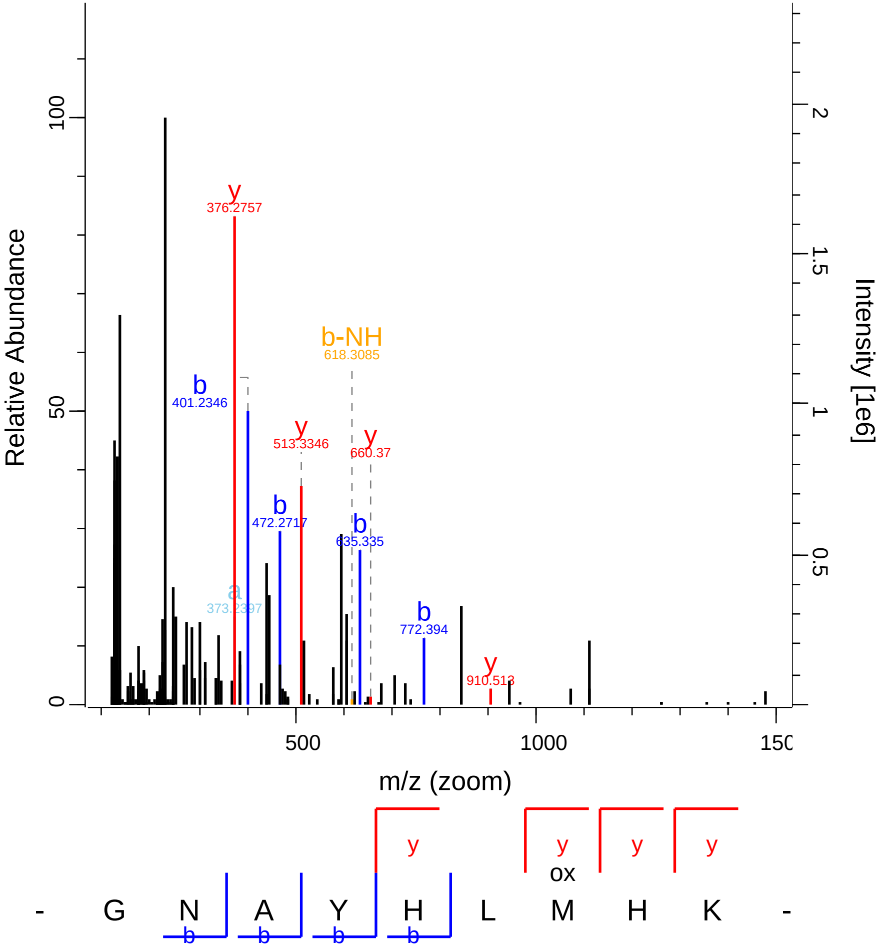

Raw File	Scan	Method	Score	m/z	Gene names
RO-QC-160121_ZackD_SS_Set_01_Fraction	7679	FTMS; HCI	106.38	552.8	ASB3

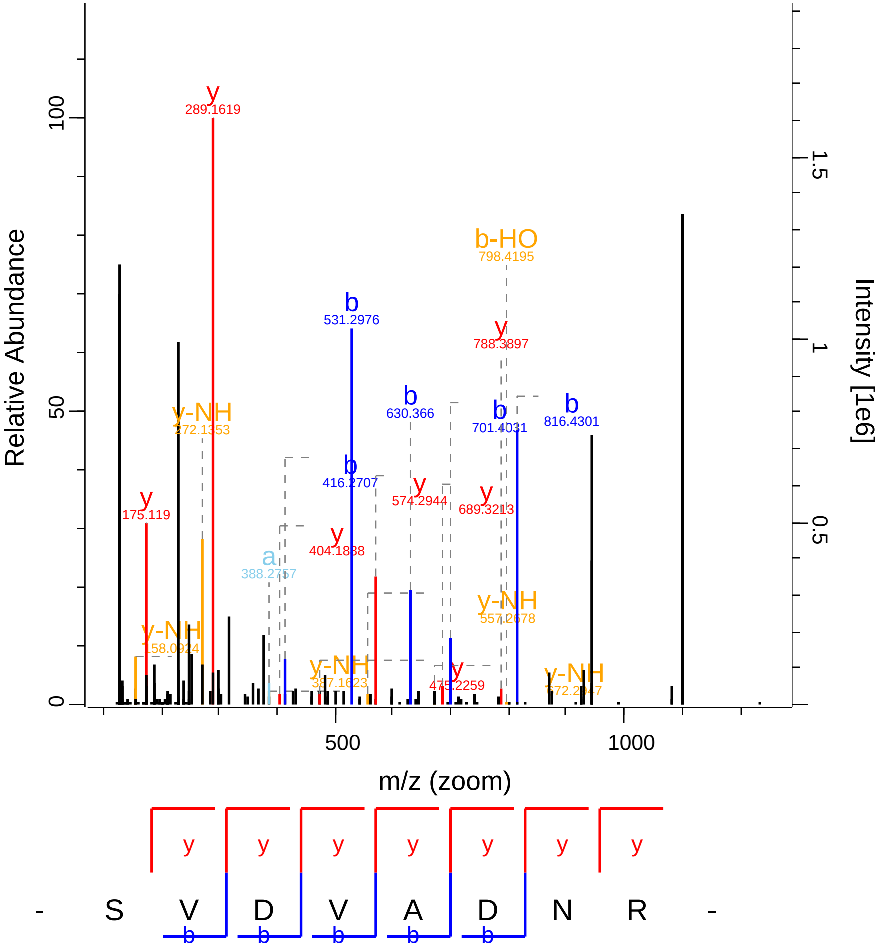

Raw File	Scan	Method	Score	m/z
RO-QC-160121_ZackD_SS_Set_01_Fraction_5	8040	FTMS; HCD	102.62	437.77

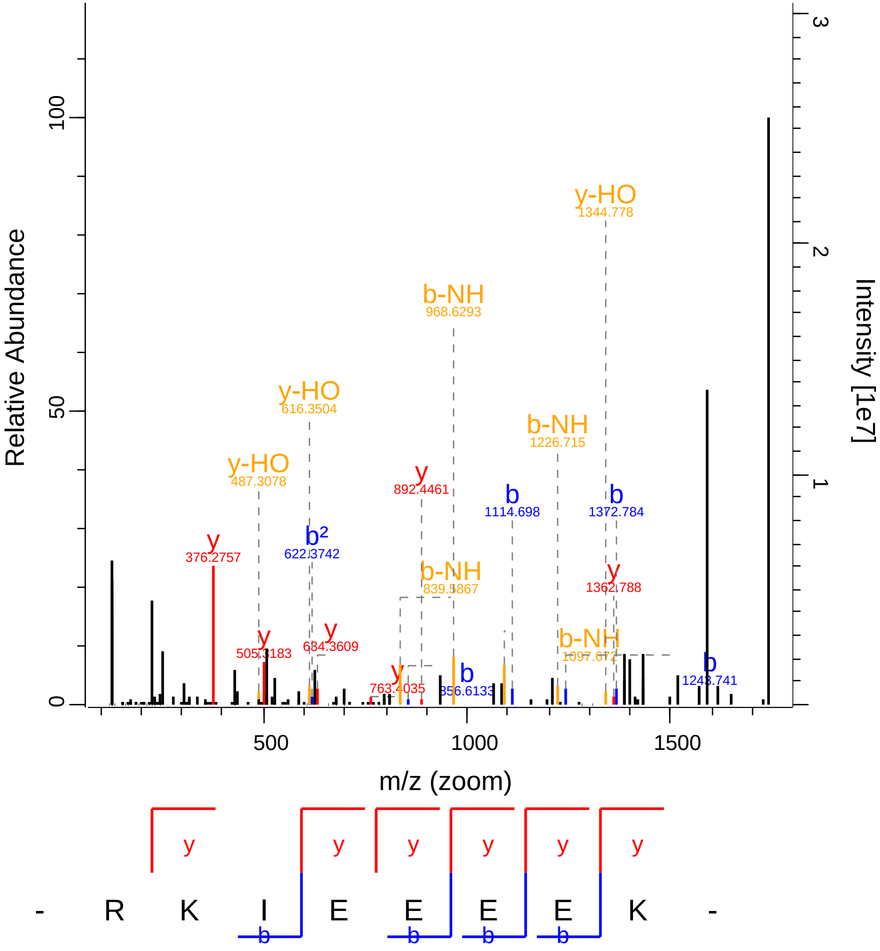

Raw File	Scan	Method	Score	m/z	Gene names
RO-QC-160121_ZackD_SS_Set_01_Fractior	8927	FTMS; HCI	75.38	499.62	KLHL8

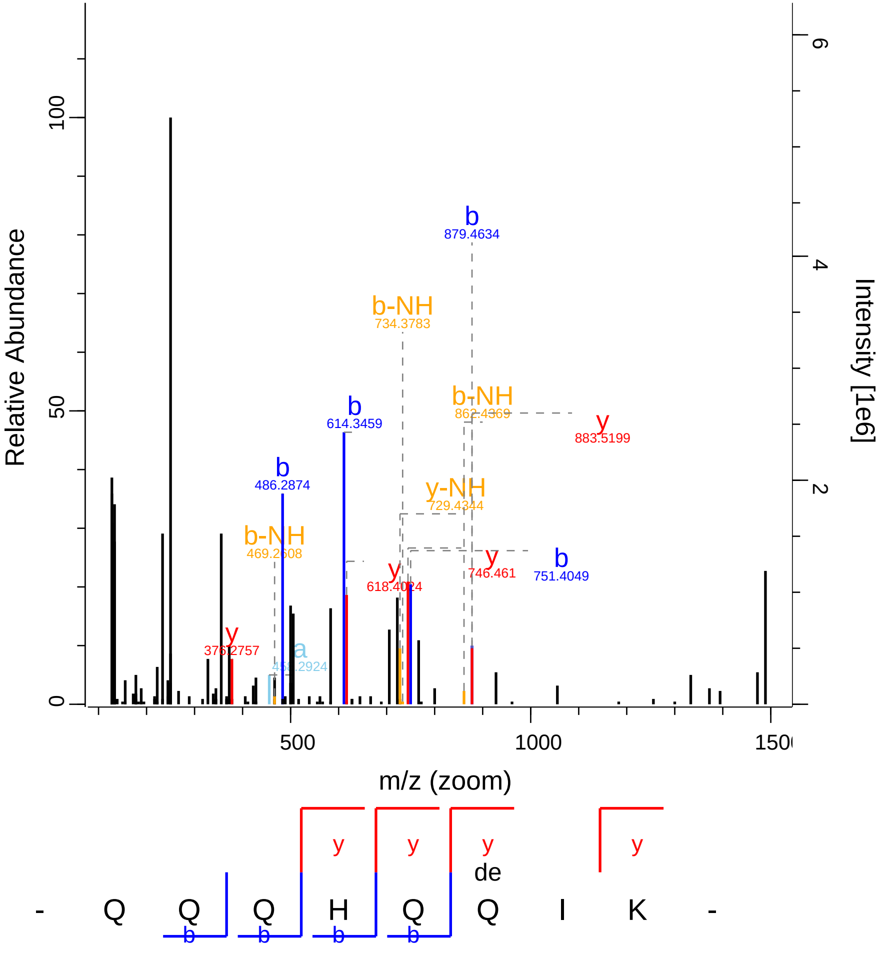

Raw File	Scan	Method	Score	m/z
RO-QC-160121_ZackD_SS_Set_01_Fraction_5	10541	FTMS; HCD	69.34	706.4

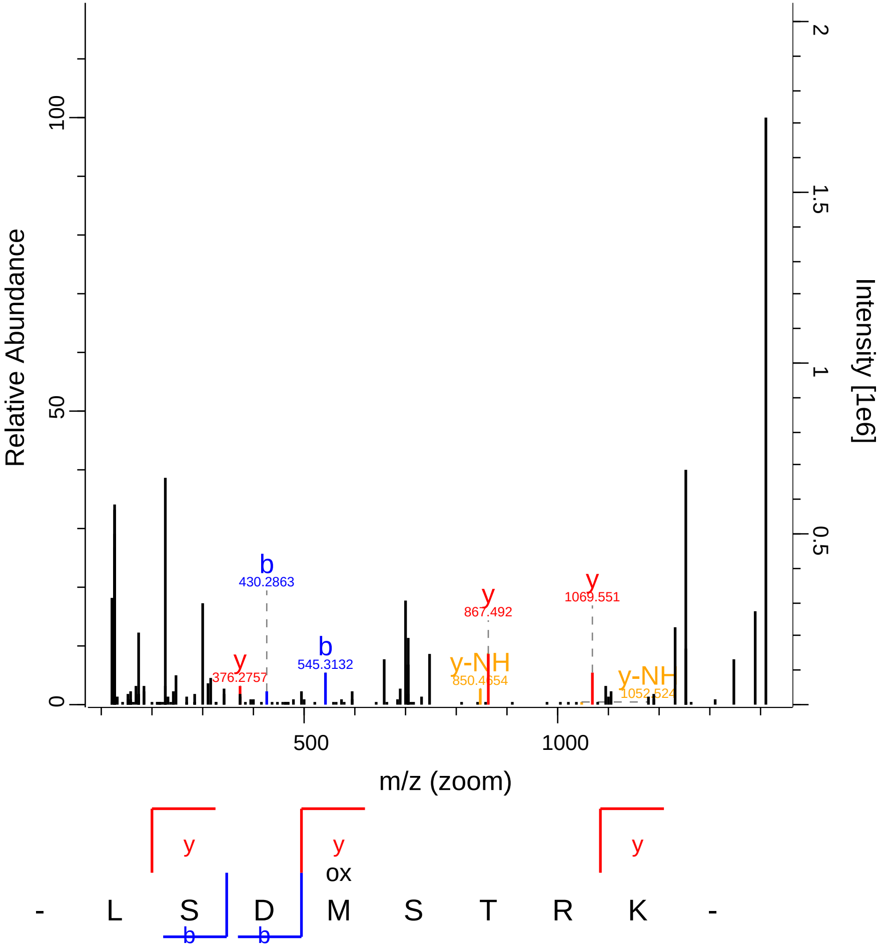

Raw File	Scan	Method	Score	m/z	Gene names
RO-QC-160121_ZackD_SS_Set_01_Fractior	11036	FTMS; HCI	51.79	691.4	KIAA0586

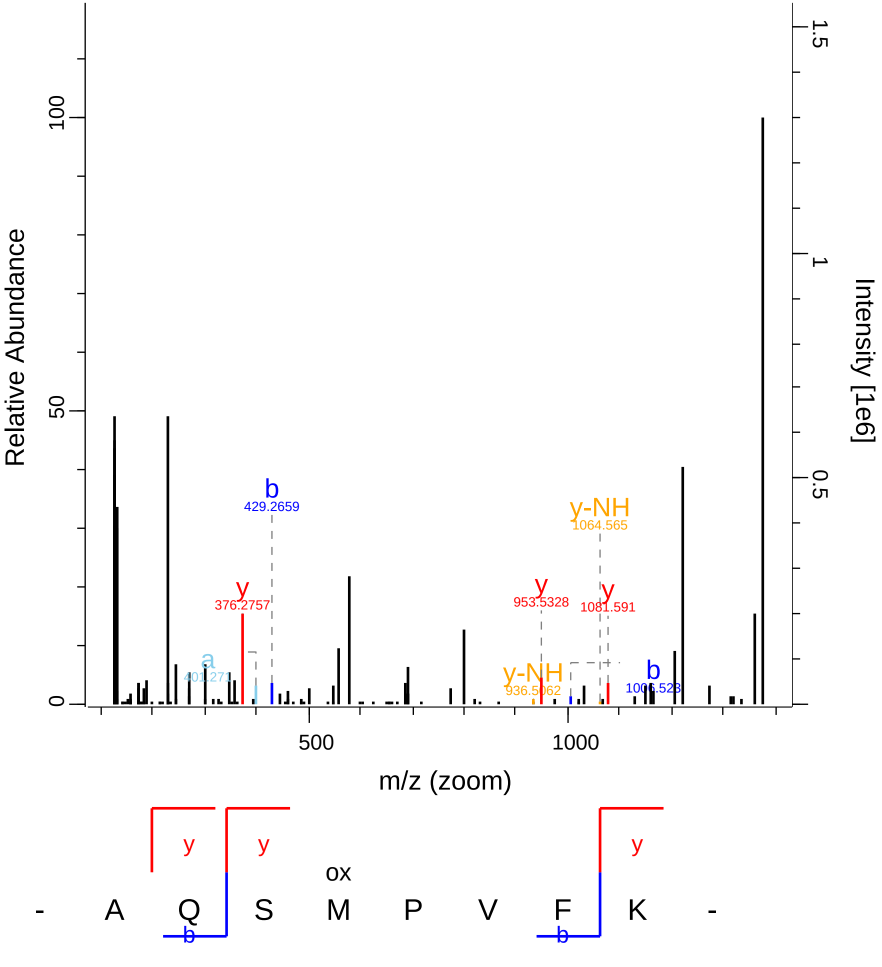

Raw File	Scan	Method	Score	m/z
RO-QC-160121_ZackD_SS_Set_01_Fraction_5	13515	FTMS; HCD	51.69	568.33

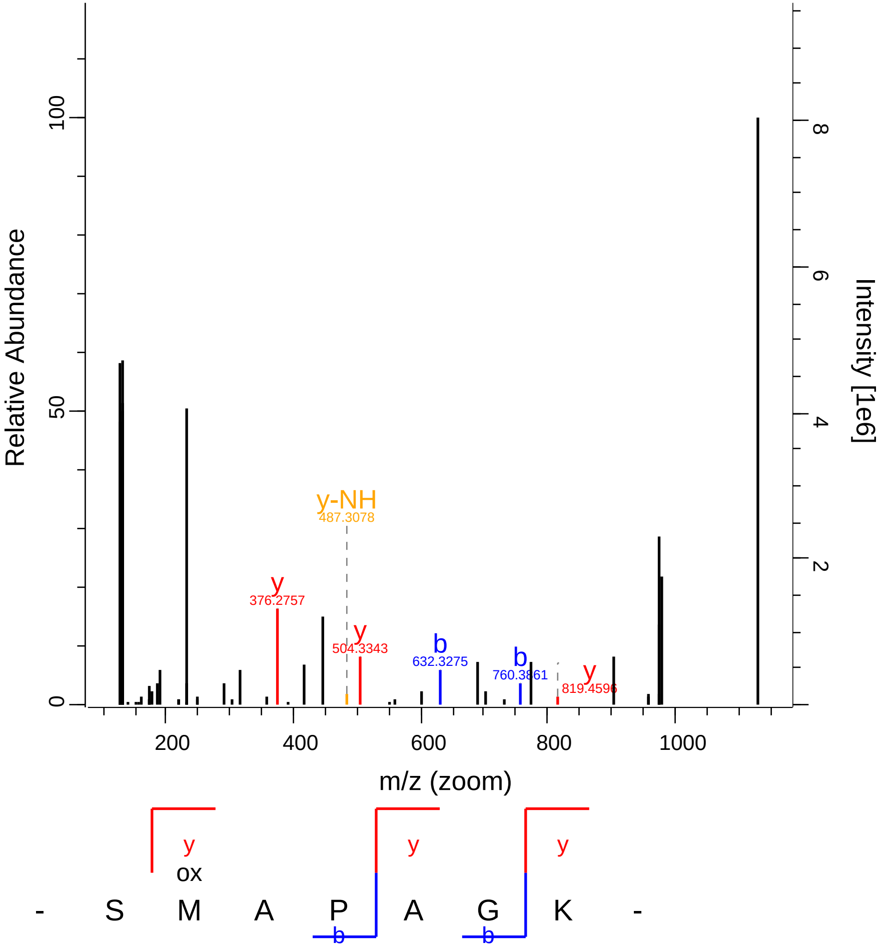

Raw File	Scan	Method	Score	m/z
RO-QC-160121_ZackD_SS_Set_01_Fraction_5	13541	FTMS; HCD	57.56	387.91

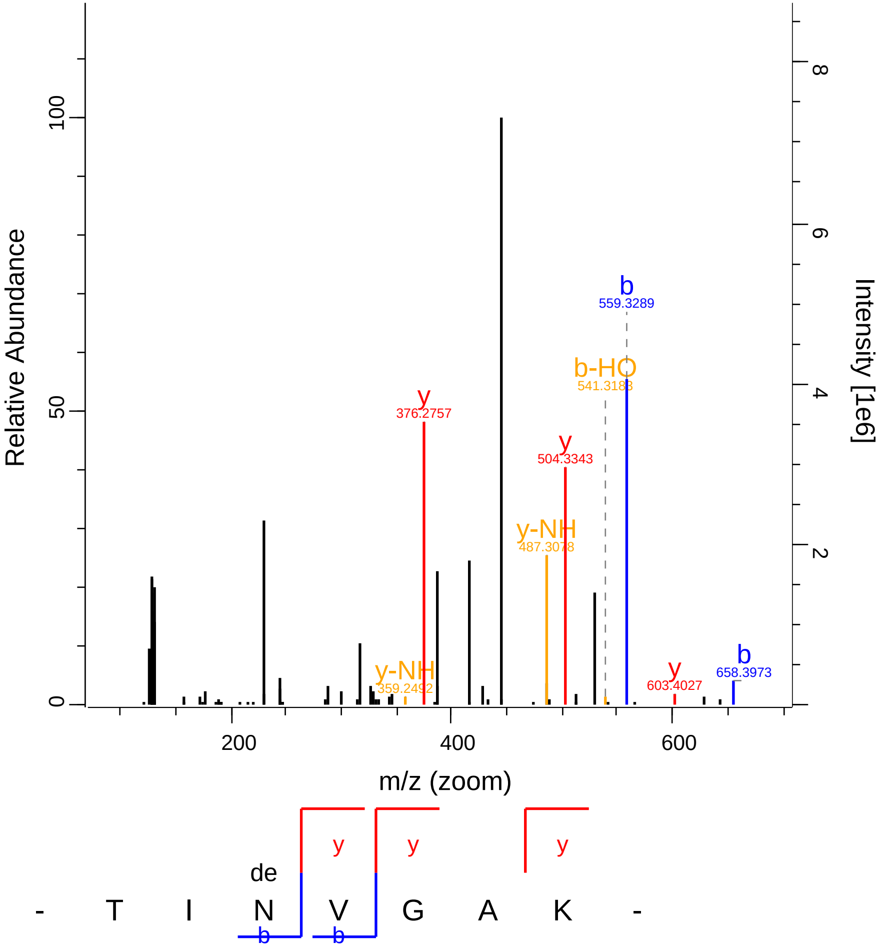

Raw File	Scan	Method	Score	m/z	Gene names
RO-QC-160121_ZackD_SS_Set_01_Fractio	14902	FTMS; HC	84.24	687.92	FAM117A

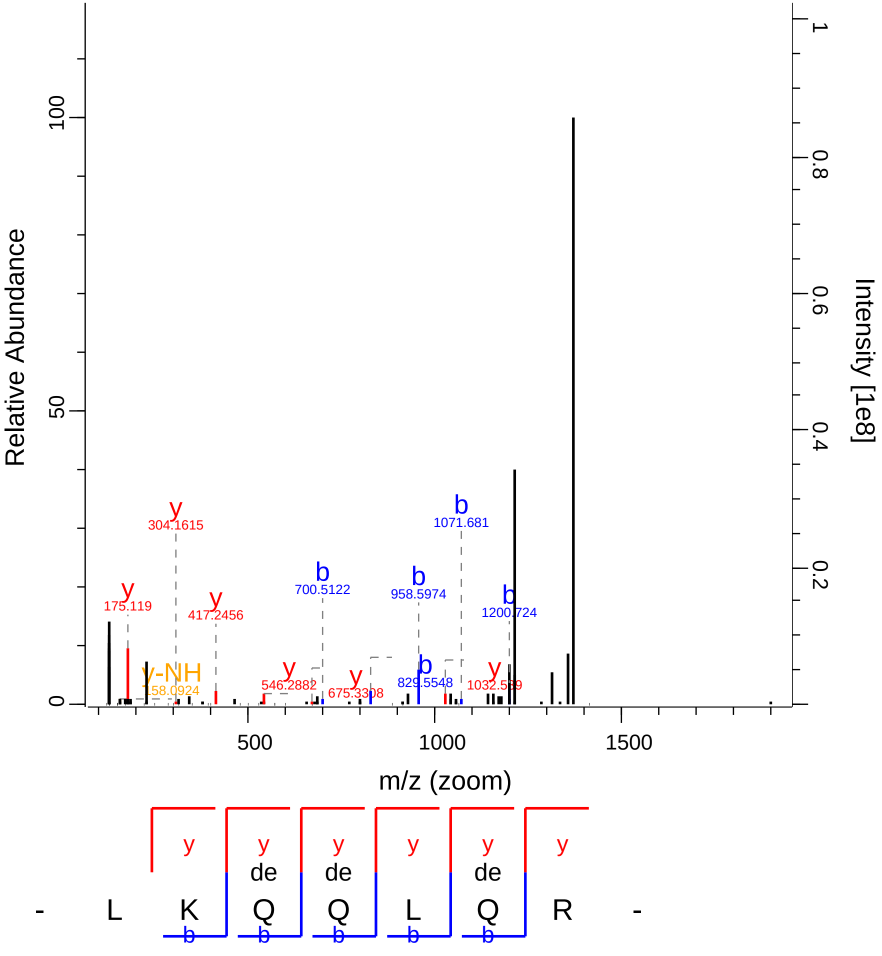

Raw File	Scan	Method	Score	m/z
RO-QC-160121_ZackD_SS_Set_01_Fraction_5	15193	FTMS; HCD	65.9	597.35

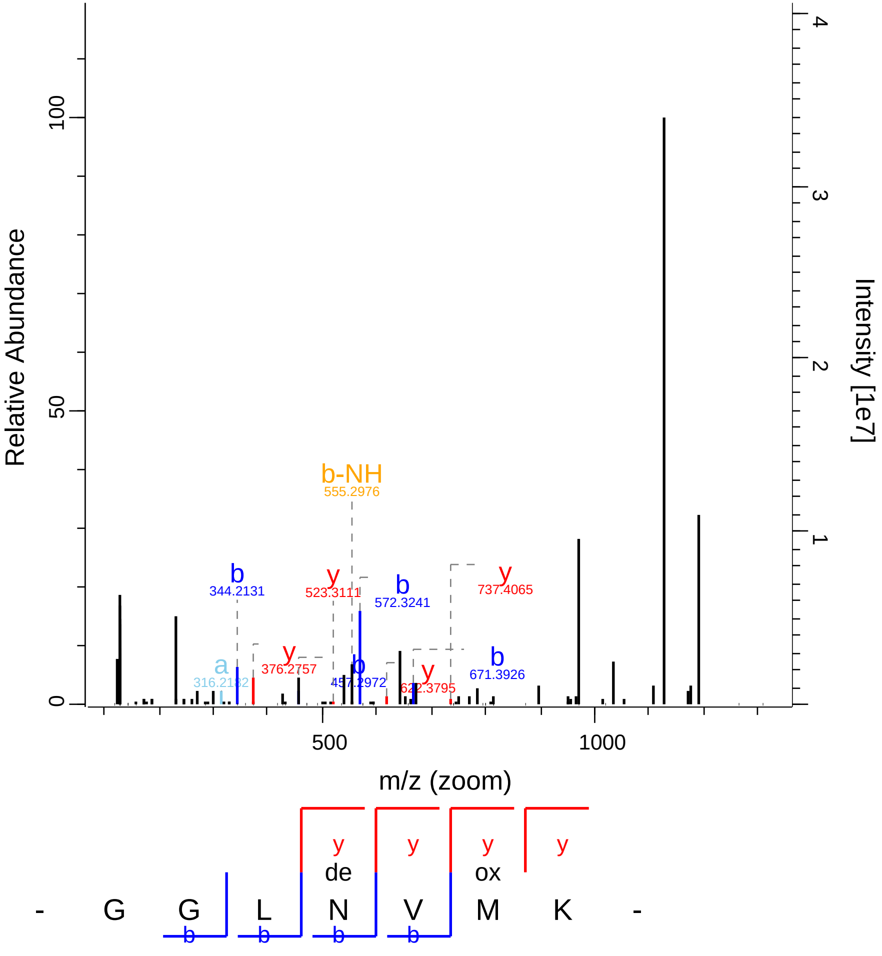

Raw File	Scan	Method	Score	m/z
RO-QC-160121_ZackD_SS_Set_01_Fraction_5	15242	FTMS; HCD	64.78	398.57

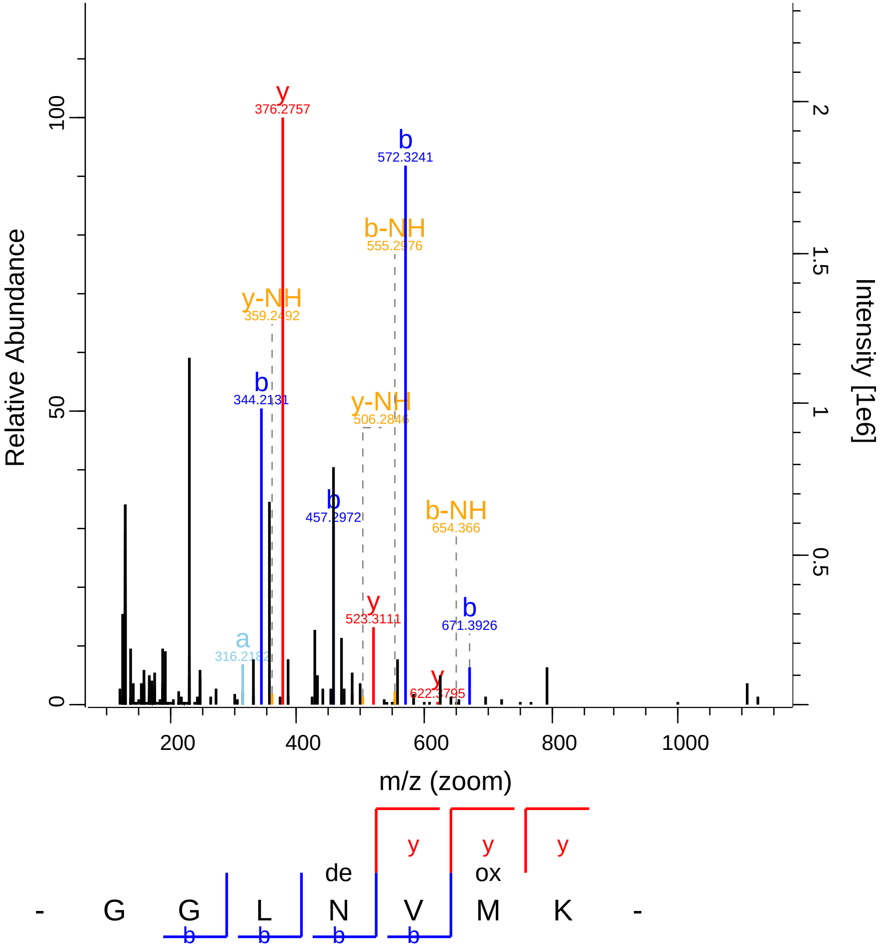

Raw File	Scan	Method	Score	m/z	Gene names
RO-QC-160121_ZackD_SS_Set_01_Fractio	15923	FTMS; HC	85.21	824.47	AIPL1

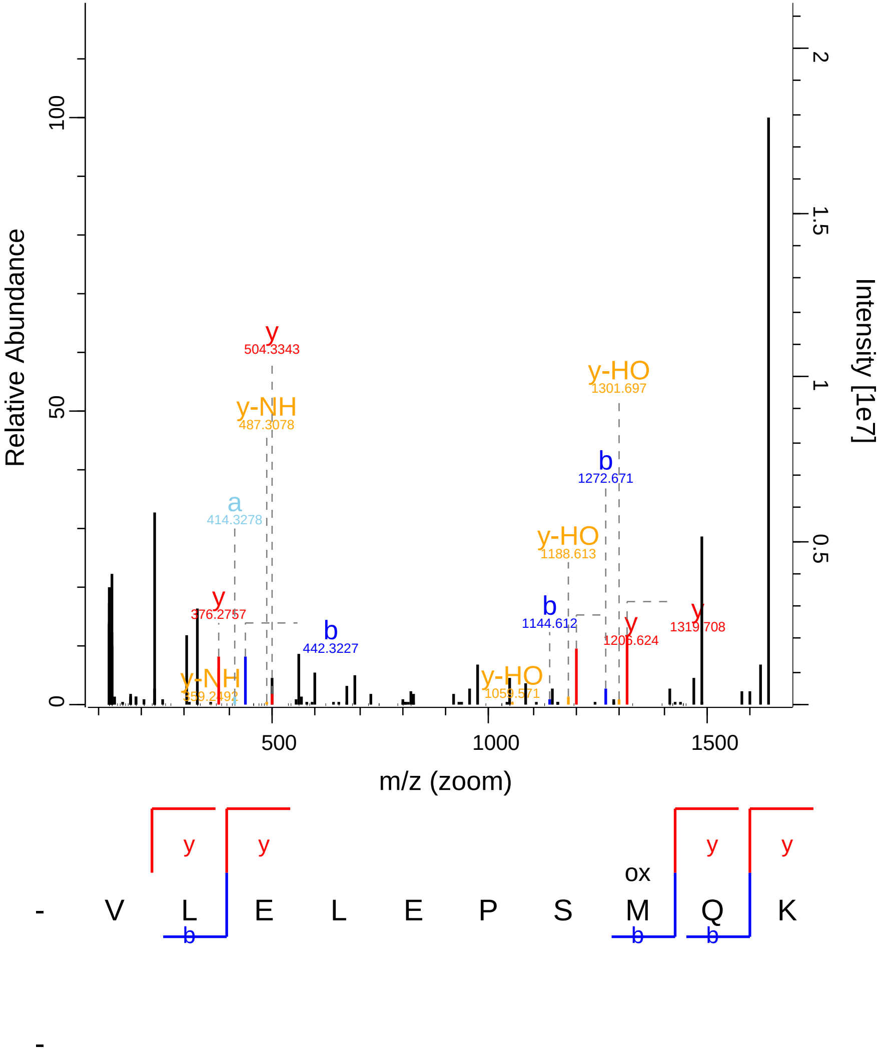

Raw File	Scan	Method	Score	m/z	Gene names
RO-QC-160121_ZackD_SS_Set_01_Fractio	16534	FTMS; HC	60.4	663.57	TMEM8B

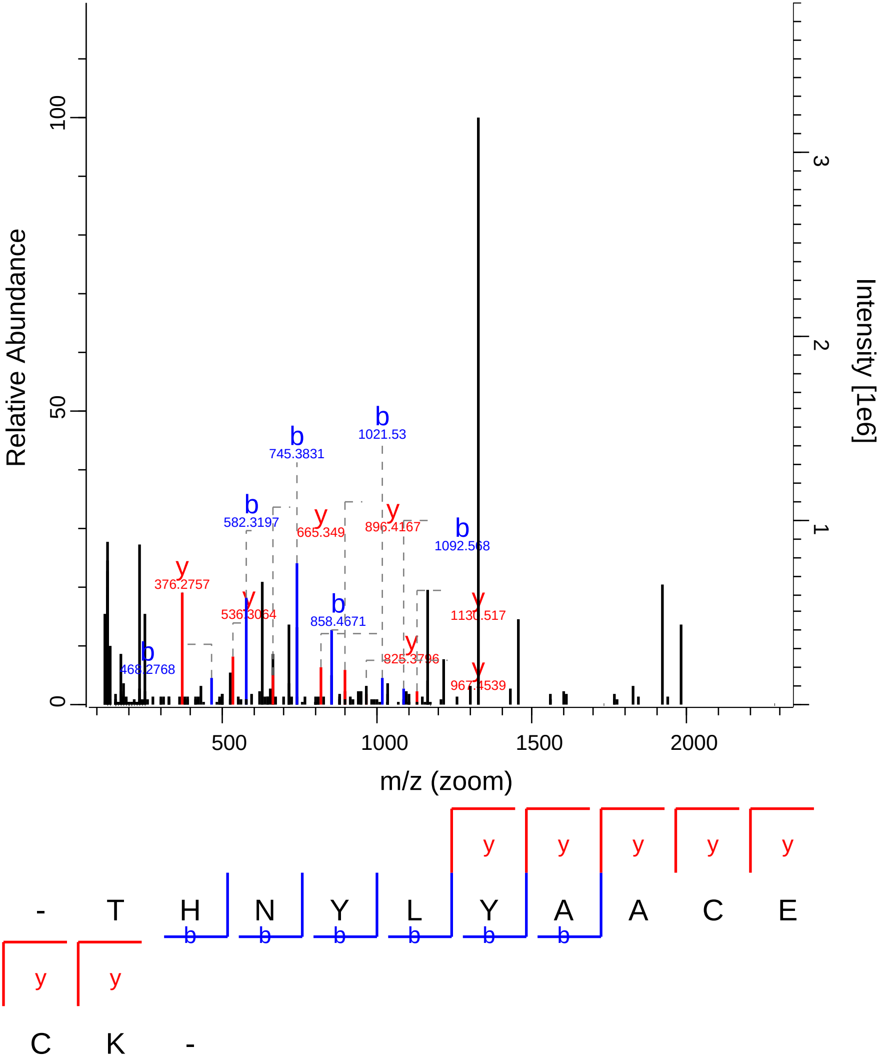

Raw File	Scan	Method	Score	m/z	Gene names
RO-QC-160121_ZackD_SS_Set_01_Fractio	16575	FTMS; HC	143.61	836.49	CIZ1

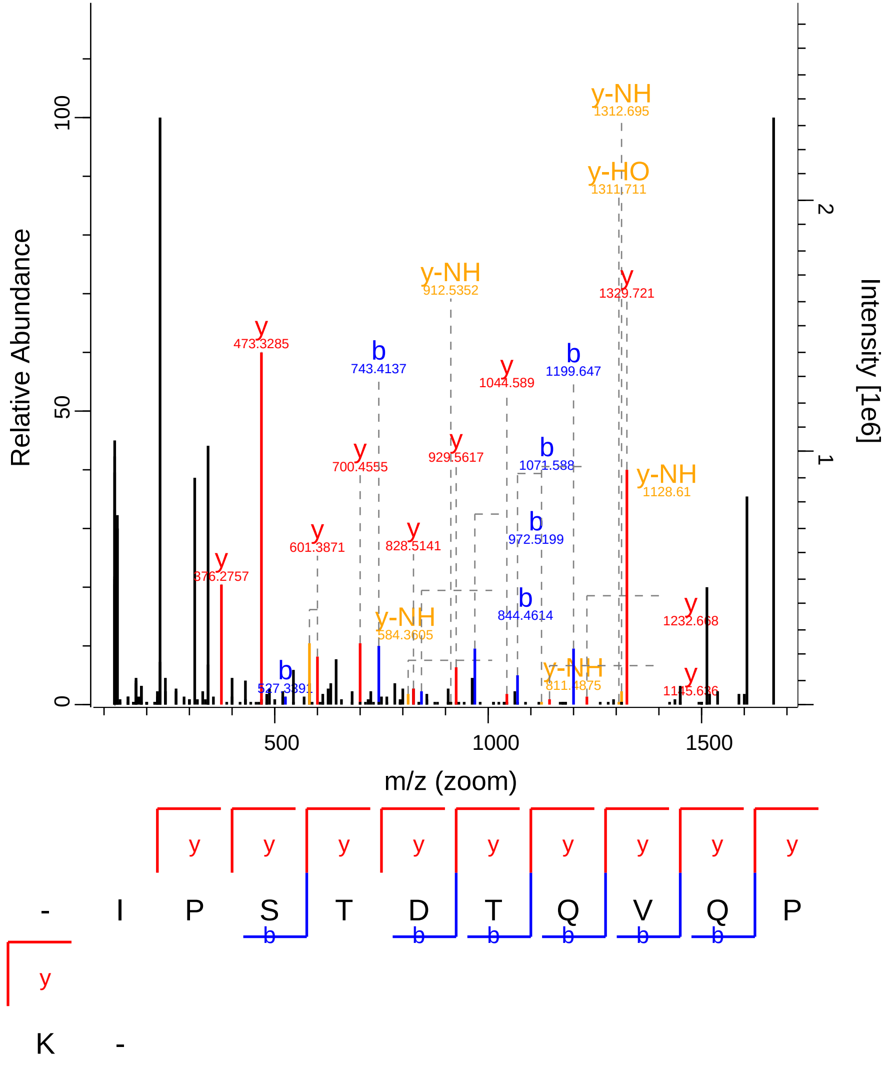

Raw File	Scan	Method	Score	m/z	Gene names
RO-QC-160121_ZackD_SS_Set_01_Fractio	16591	FTMS; HC	85.46	663.33	TMEM8B

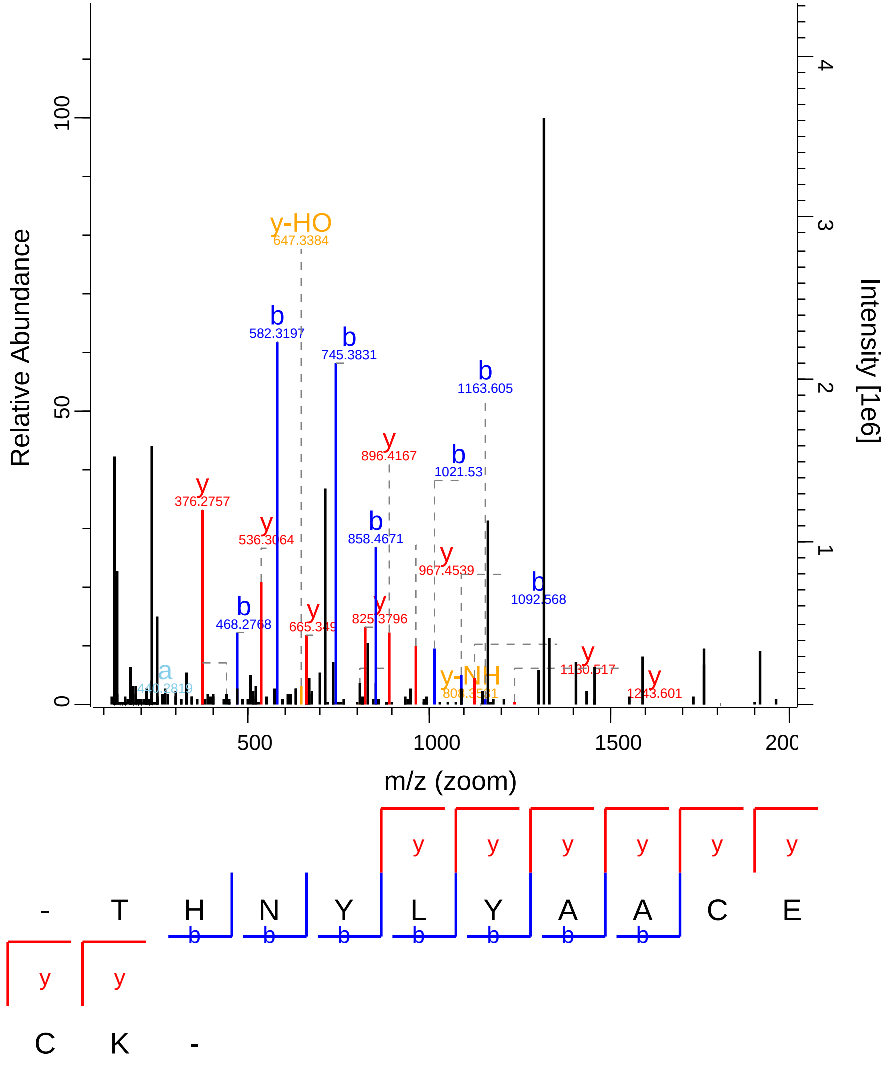

Raw File	Scan	Method	Score	m/z	Gene names
RO-QC-160121_ZackD_SS_Set_01_Fractio	16626	FTMS; HC	143.01	697.88	NAA40

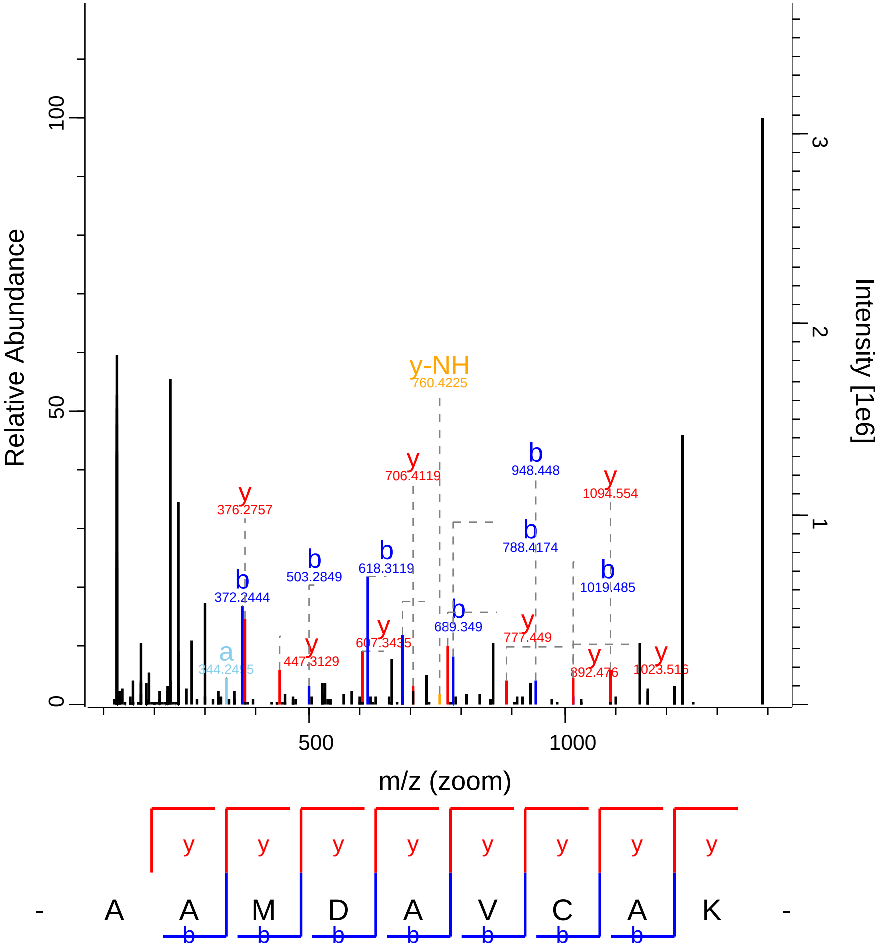

Raw File	Scan	Method	Score	m/z	Gene names
RO-QC-160121_ZackD_SS_Set_01_Fractio	16801	FTMS; HC	95.53	602.82	NPC1L1

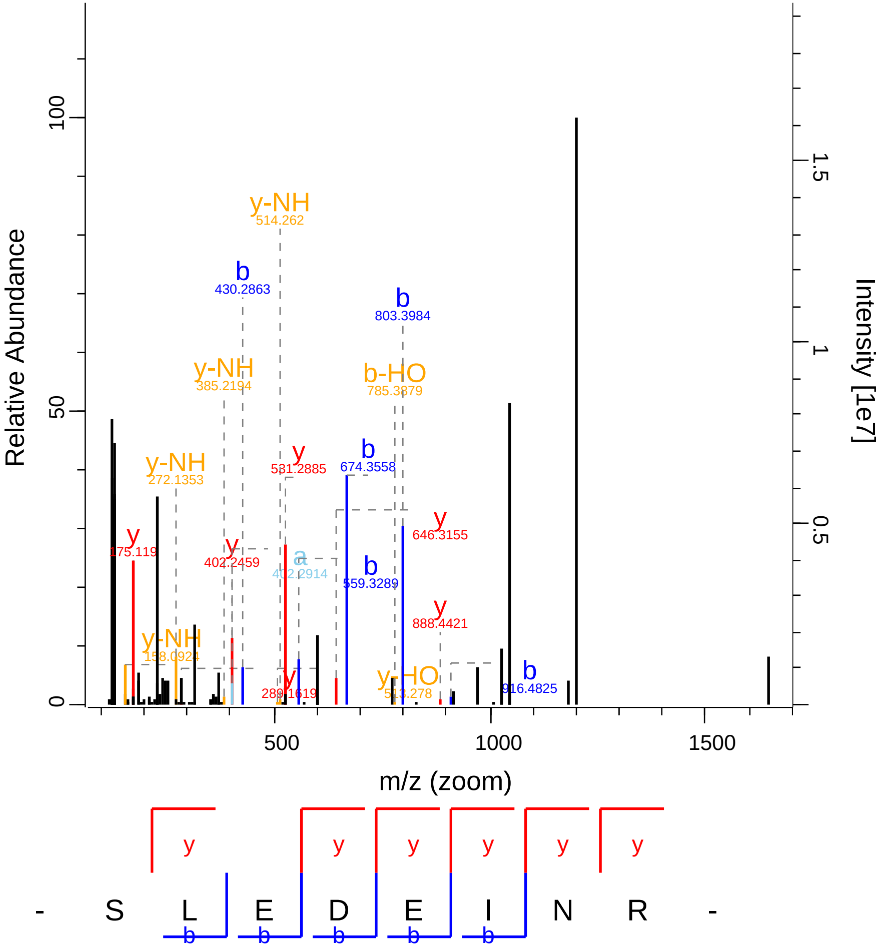

Raw File	Scan	Method	Score	m/z	Gene names
RO-QC-160121_ZackD_SS_Set_01_Fractio	17048	FTMS; HC	63.49	602.82	NPC1L1

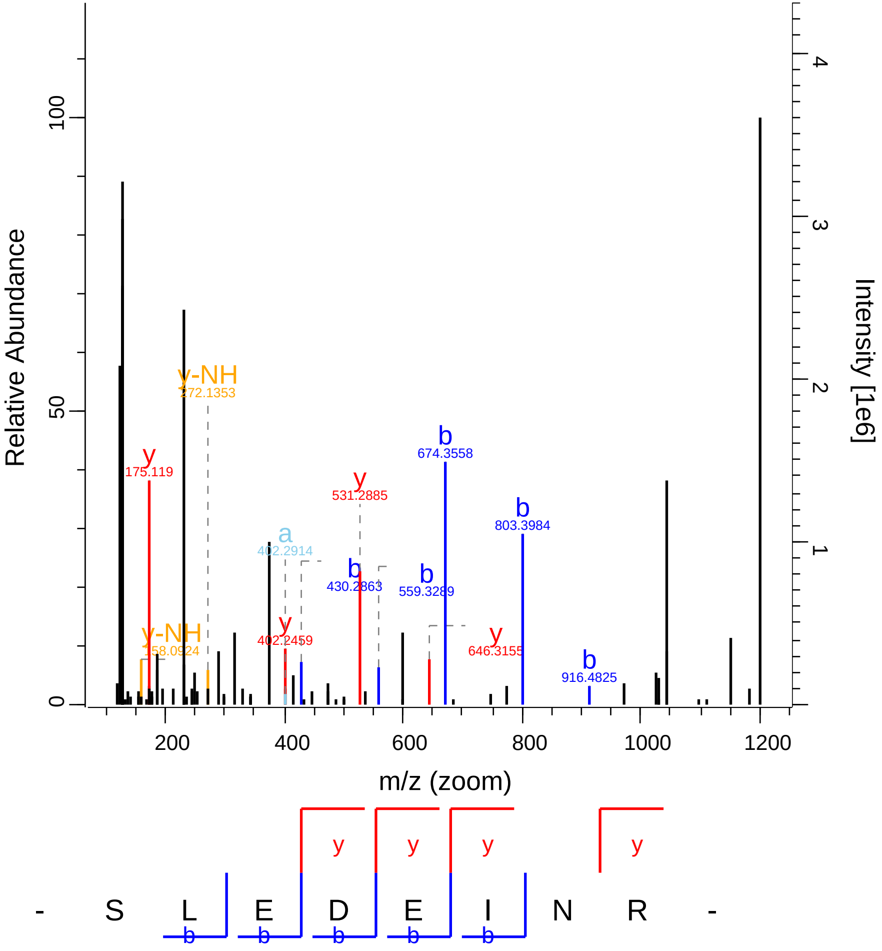

Raw File	Scan	Method	Score	m/z	Gene names
RO-QC-160121_ZackD_SS_Set_01_Fractio	18112	FTMS; HC	107.09	611.37	SCOC

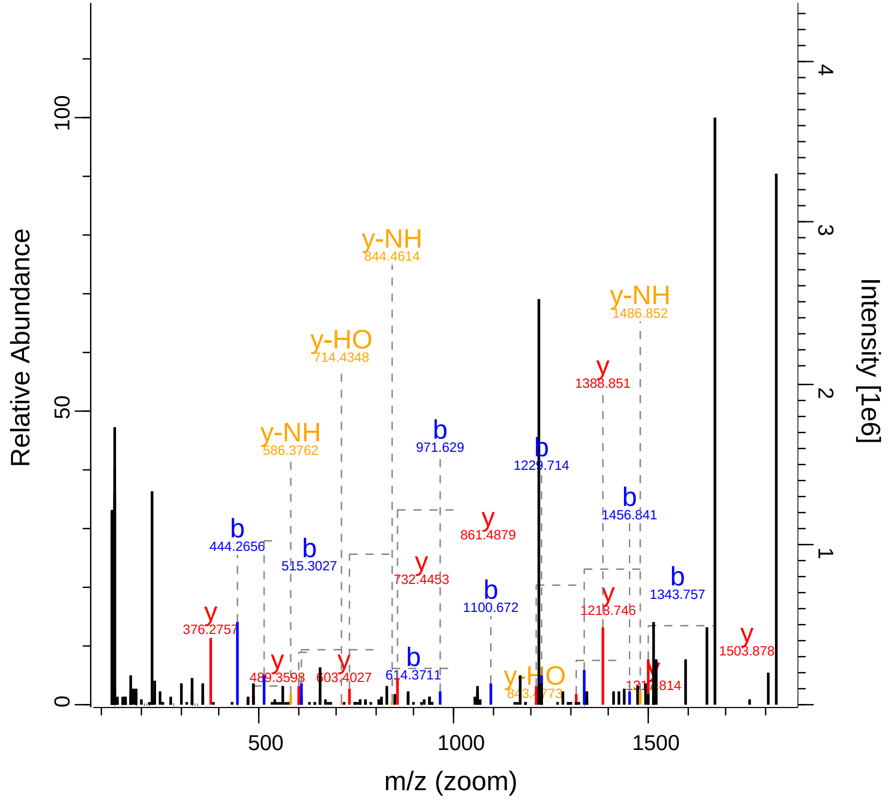

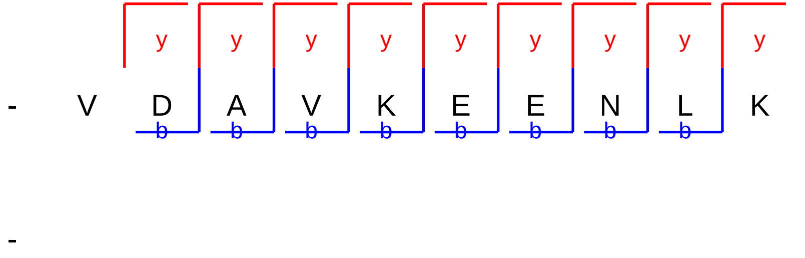

Raw File	Scan	Method	Score	m/z	Gene names
RO-QC-160121_ZackD_SS_Set_01_Fractio	18794	FTMS; HC	85.53	713.94	SYS1

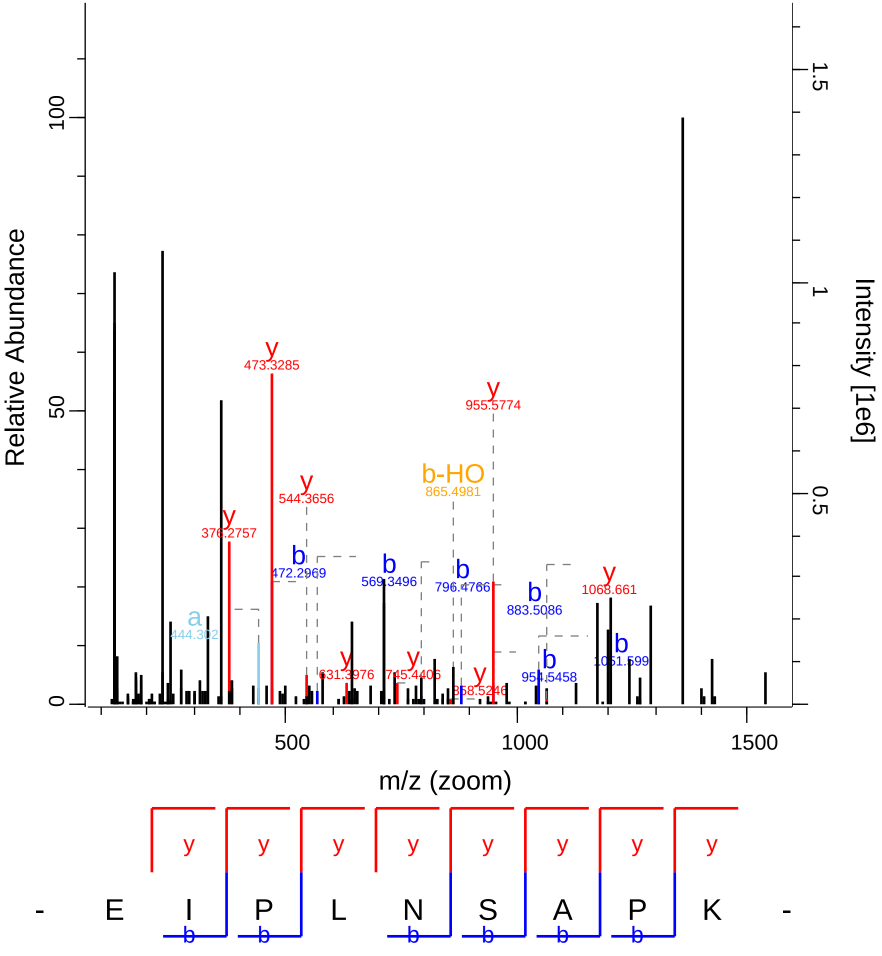

Raw File	Scan	Method	Score	m/z	Gene names
RO-QC-160121_ZackD_SS_Set_01_Fractic	19103	FTMS; HC	45.66	1173.57	LRRC28

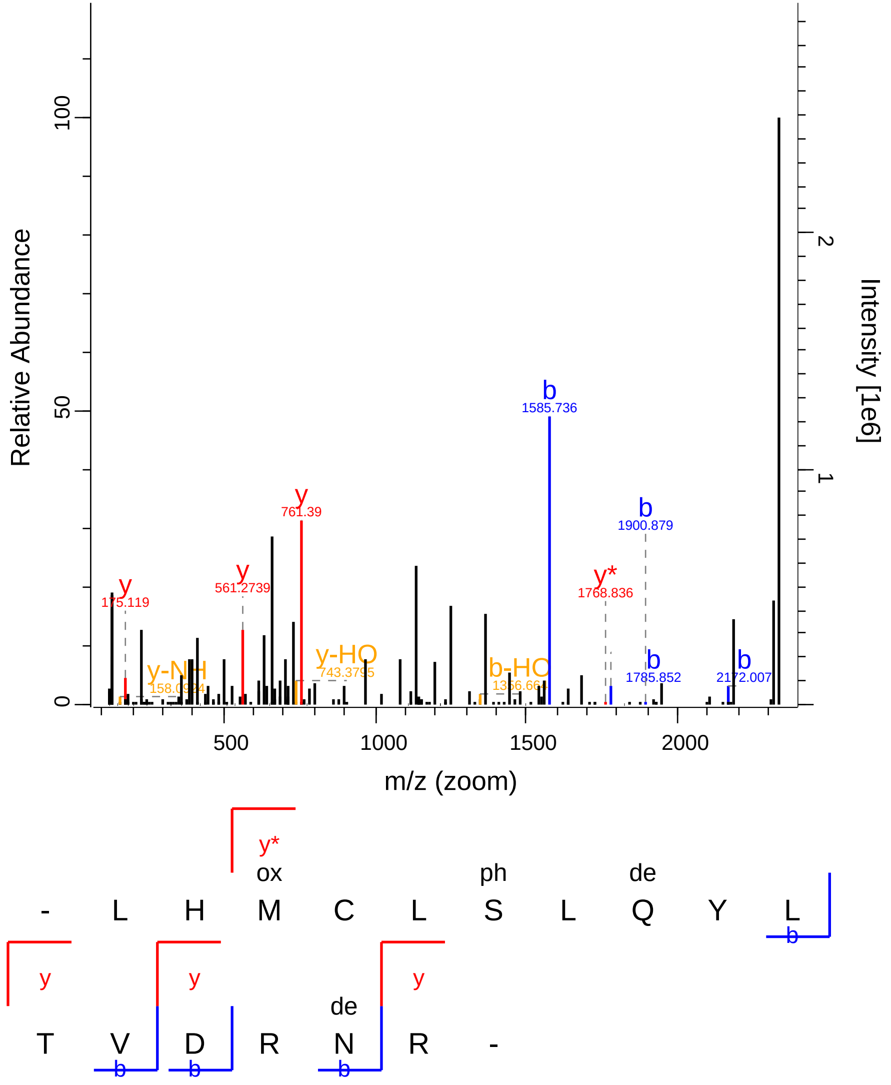

Raw File	Scan	Method	Score	m/z
RO-QC-160121_ZackD_SS_Set_01_Fraction_5	19469	FTMS; HCD	64.44	1095.62

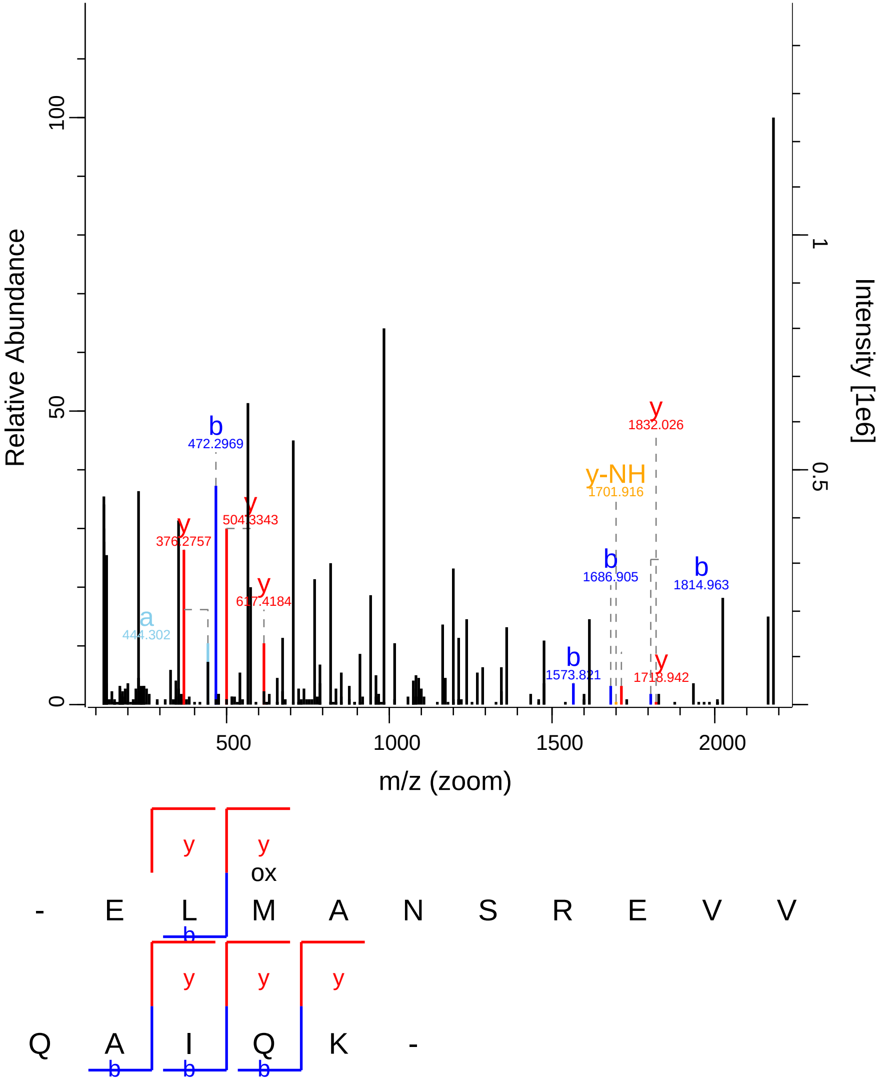

Raw File	Scan	Method	Score	m/z	Gene names
RO-QC-160121_ZackD_SS_Set_01_Fractio	20287	FTMS; HC	44.58	600.07	ATP6V0C

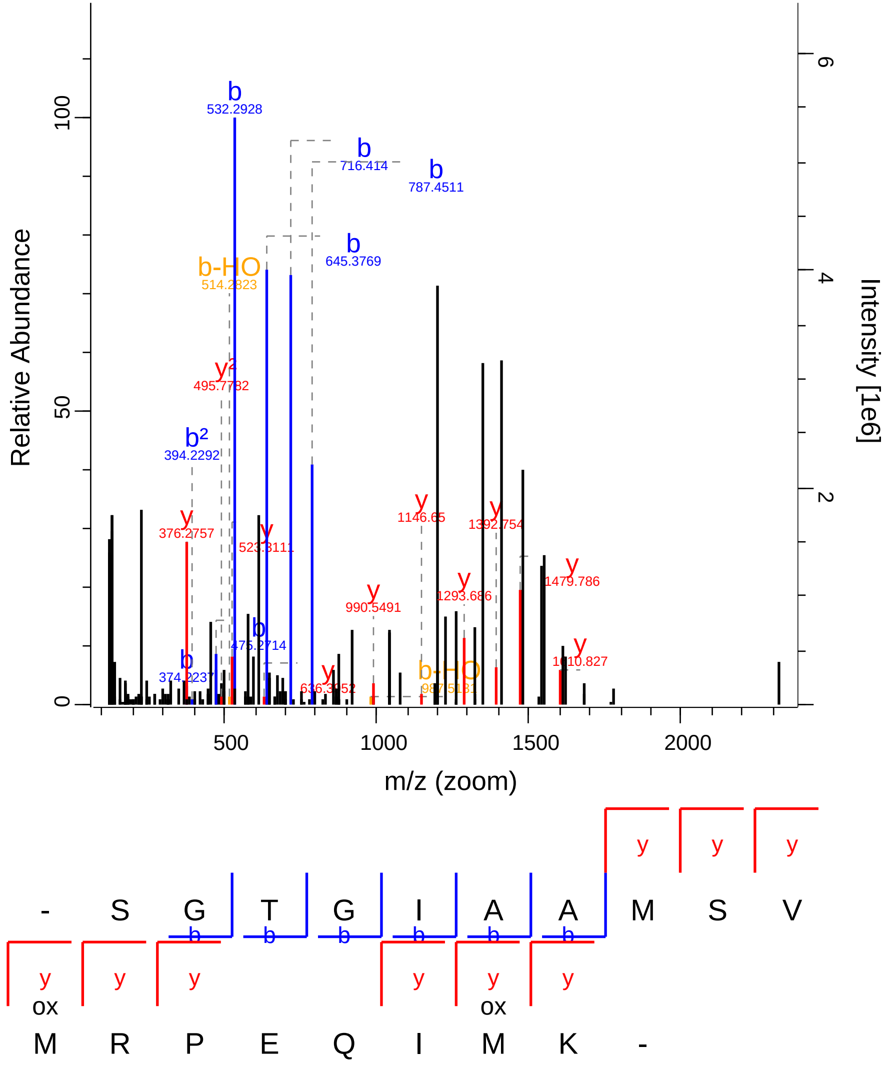

Raw File	Scan	Method	Score	m/z	Gene names
RO-QC-160121_ZackD_SS_Set_01_Fractio	20831	FTMS; HC	66.06	1235.1	HELT

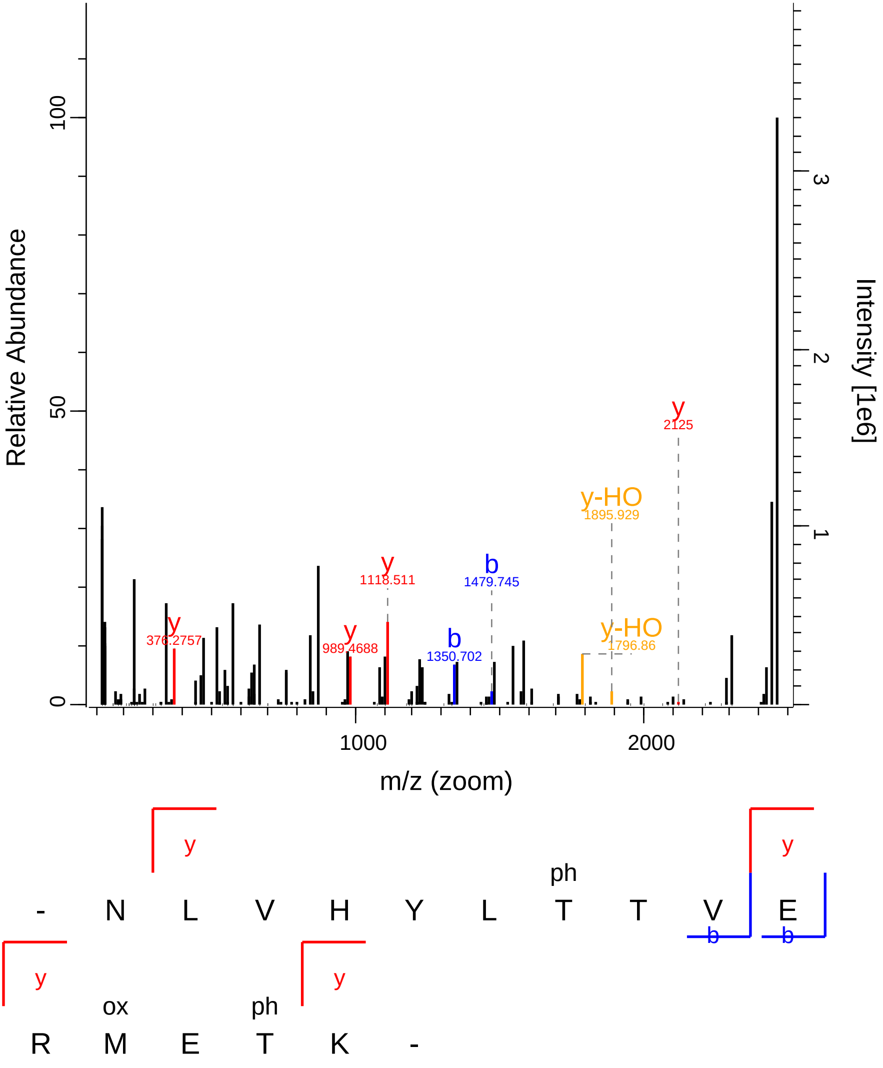

Raw File	Scan	Method	Score	m/z	Gene names
RO-QC-160121_ZackD_SS_Set_01_Fractio	20872	FTMS; HC	47.07	689.86	CNKSR1

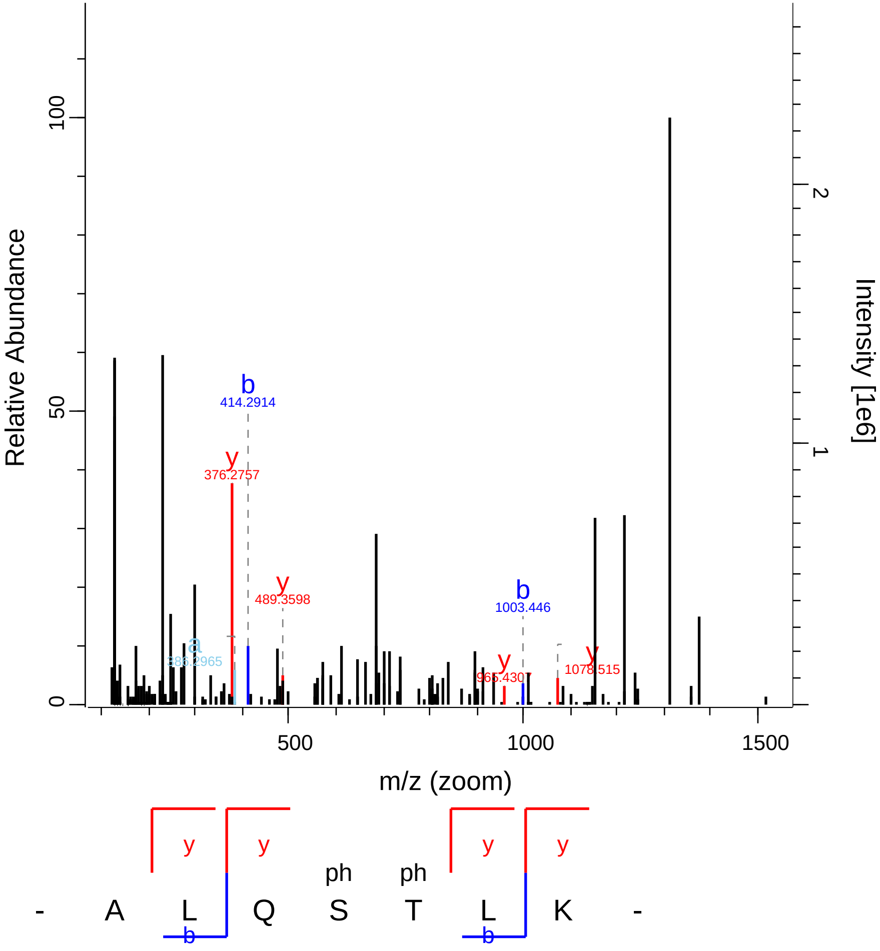

Raw File	Scan	Method	Score	m/z	Gene names
RO-QC-160121_ZackD_SS_Set_01_Fractio	21184	FTMS; HC	43	471.91	NKPD1

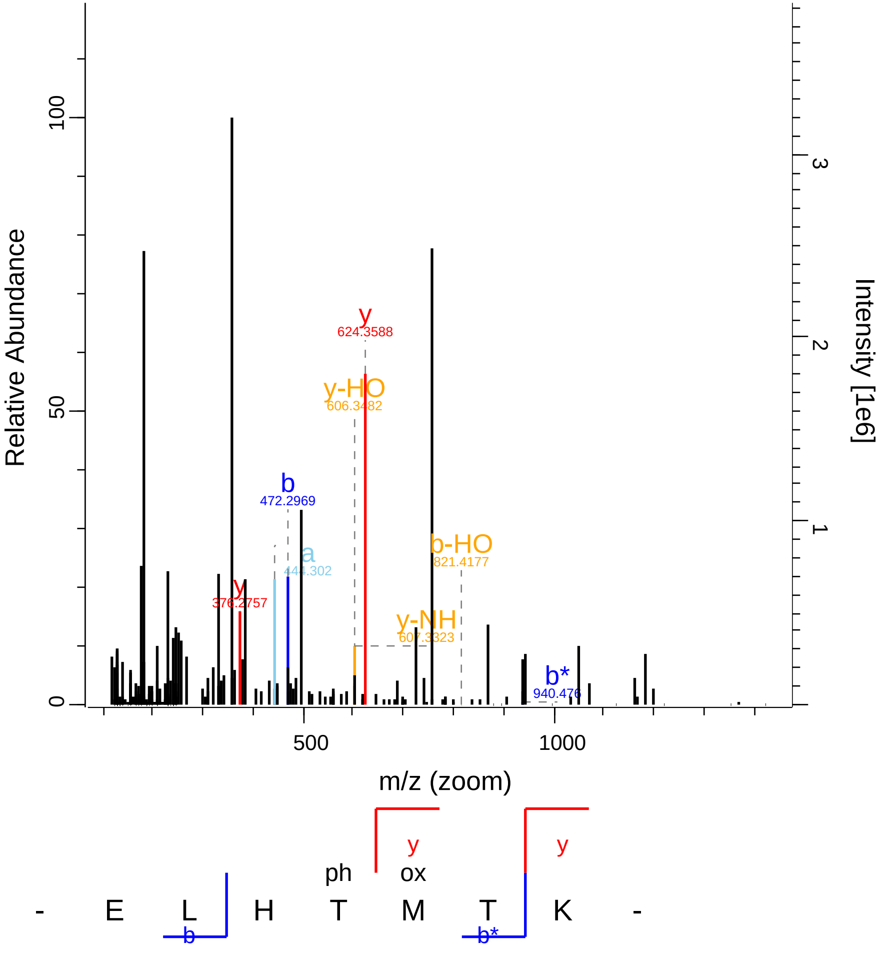

Raw File	Scan	Method	Score	m/z	Gene names
RO-QC-160121_ZackD_SS_Set_01_Fractio	21676	FTMS; HC	49.31	487.78	HPDL

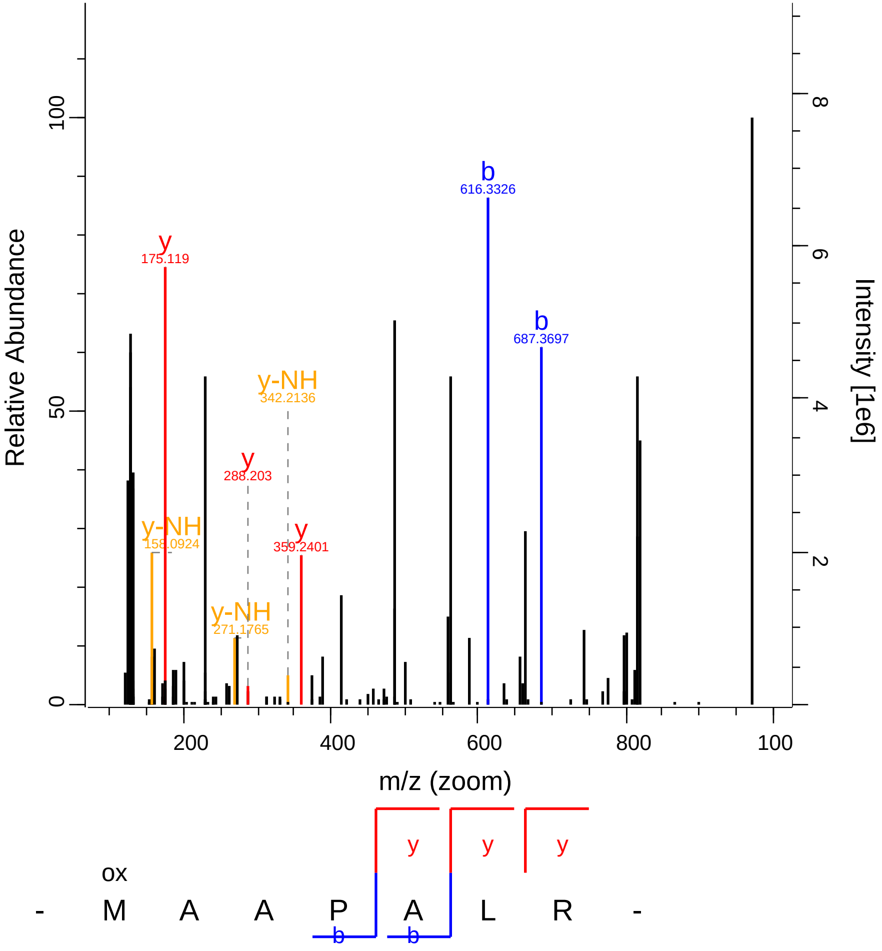

Raw File	Scan	Method	Score	m/z	Gene names
RO-QC-160121_ZackD_SS_Set_01_Fractio	22116	FTMS; HC	119.68	818.44	GPATCH2

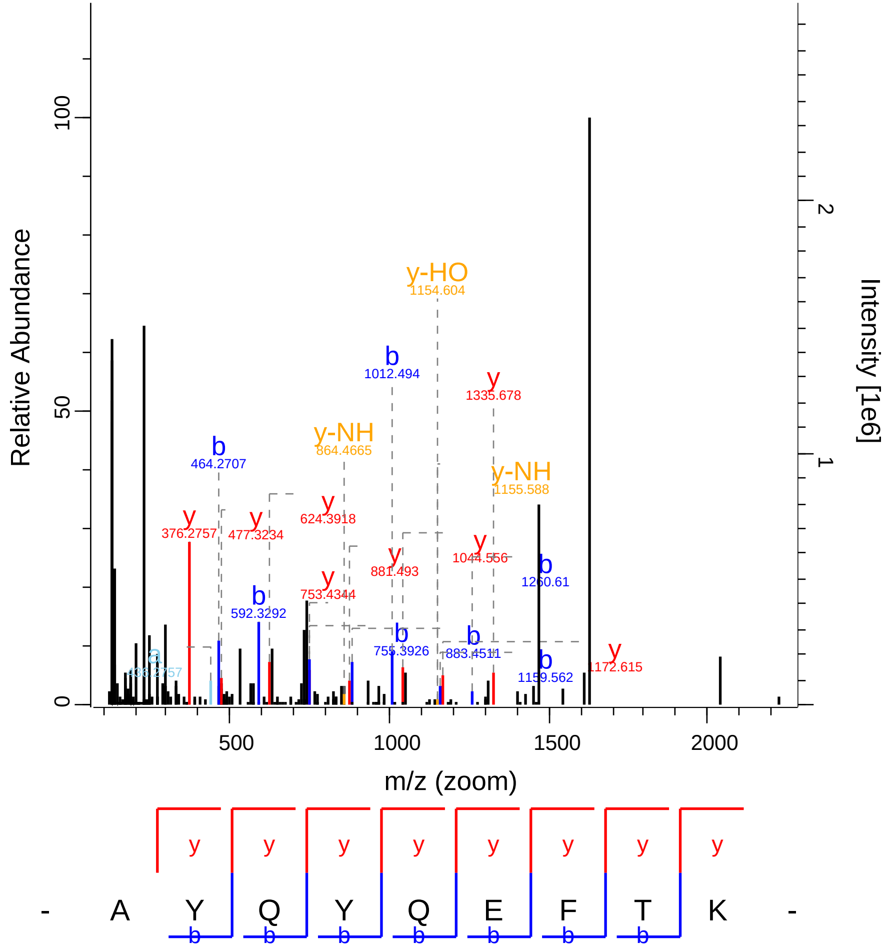

Raw File	Scan	Method	Score	m/z	Gene names
RO-QC-160121_ZackD_SS_Set_01_Fractio	22630	FTMS; HC	62.63	883.49	SEC22A

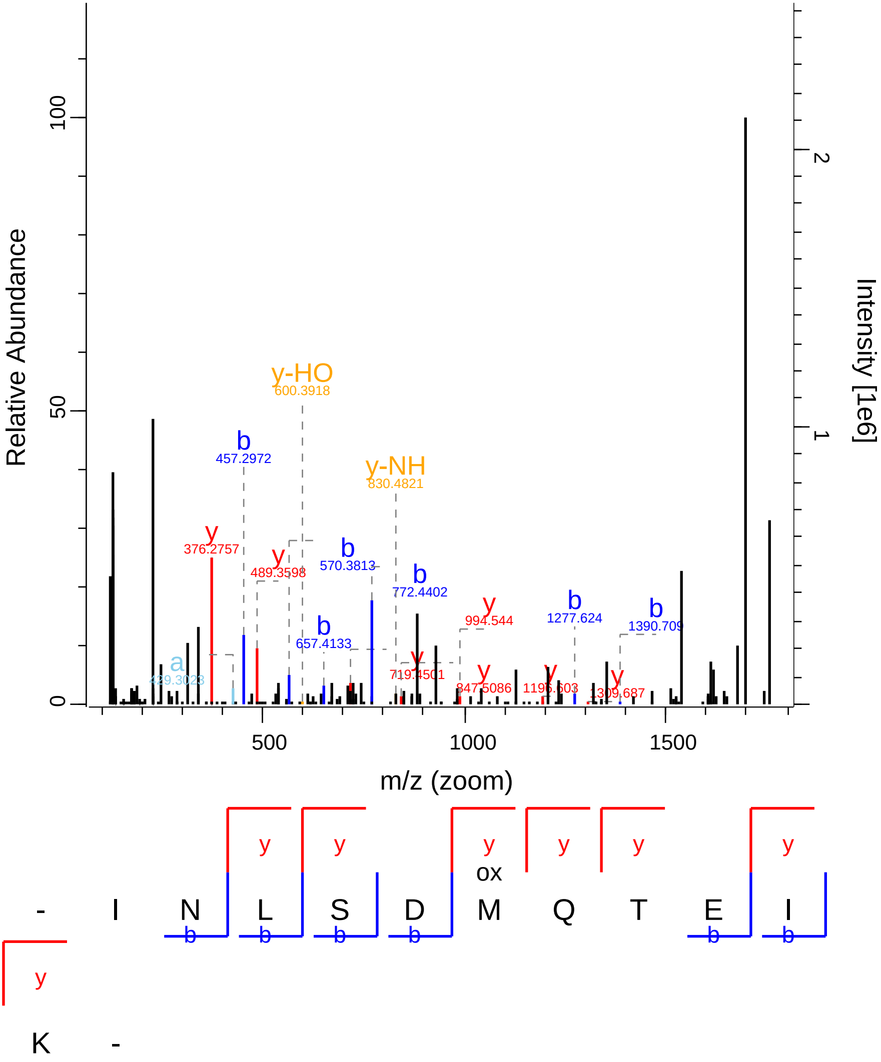

Raw File	Scan	Method	Score	m/z	Gene names
RO-QC-160121_ZackD_SS_Set_01_Fractio	23290	FTMS; HC	70.96	794.77	ATP6V0C

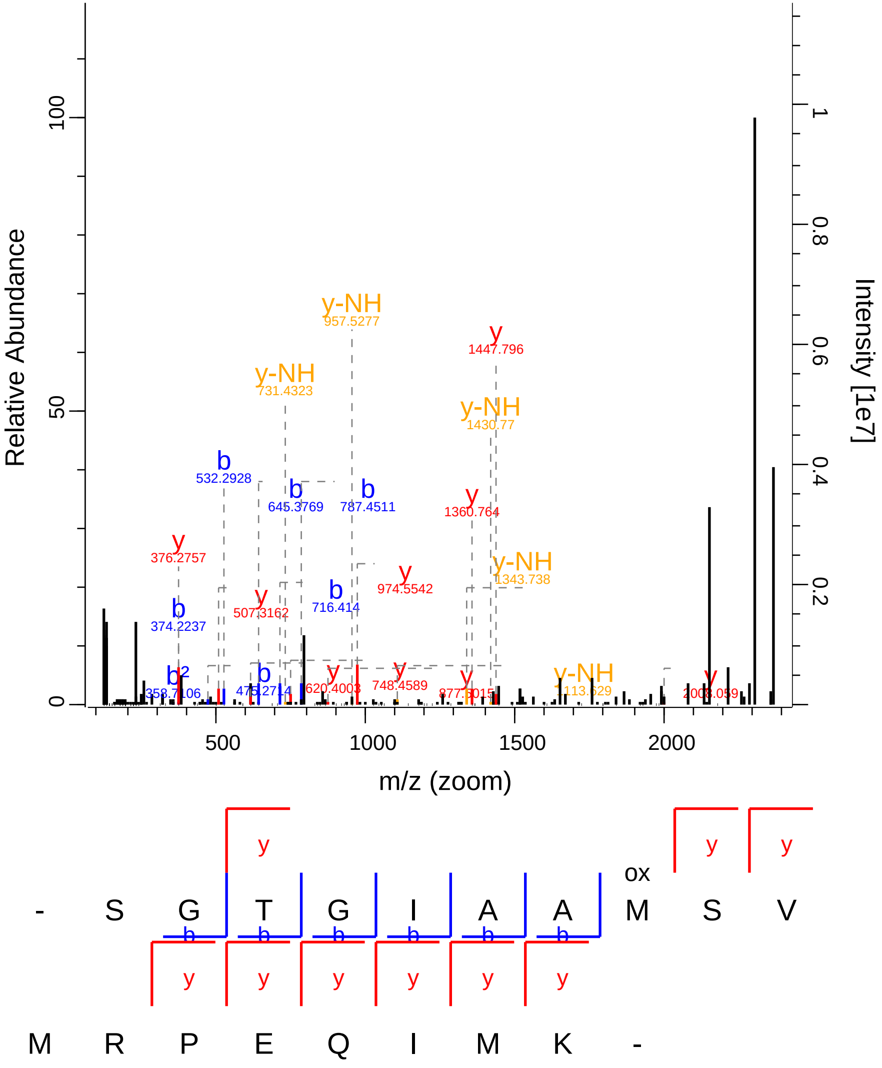

Raw File	Scan	Method	Score	m/z	Gene names
RO-QC-160121_ZackD_SS_Set_01_Fractic	23482	FTMS; HC	147.96	1191.14	ATP6V0C

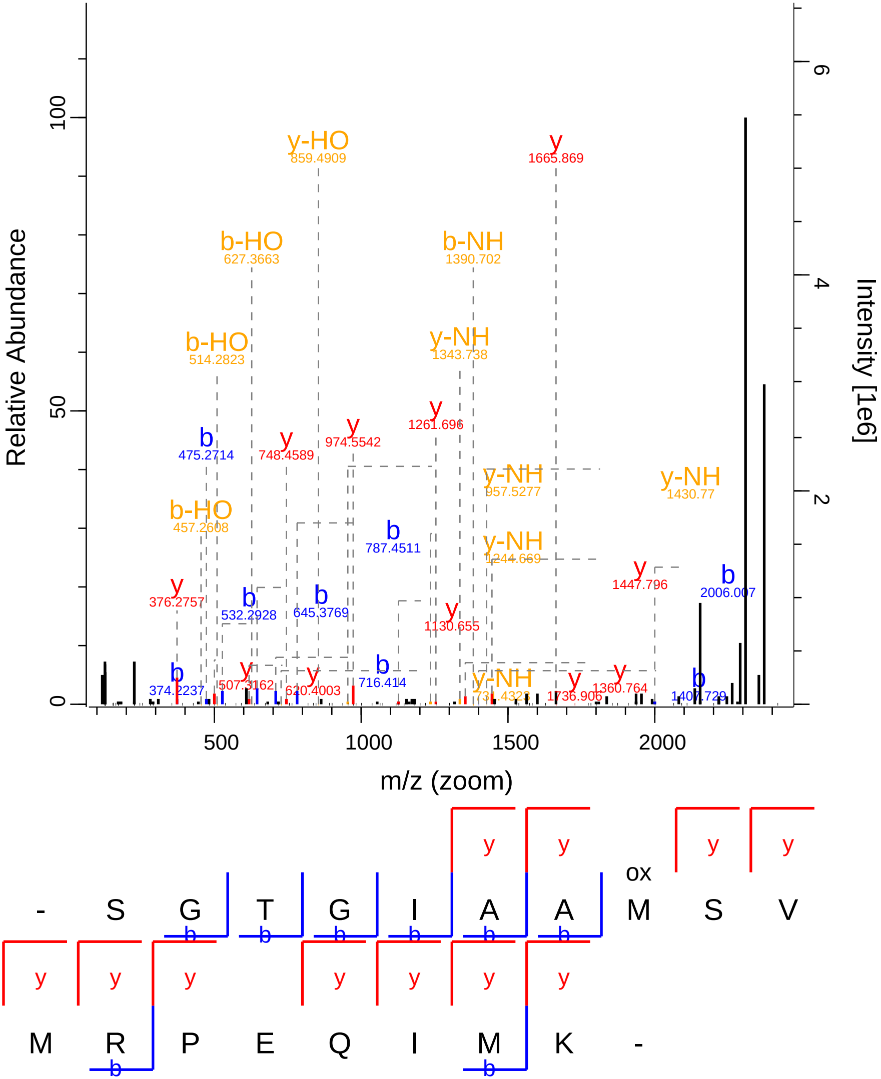

Raw File	Scan	Method	Score	m/z	Gene names
RO-QC-160121_ZackD_SS_Set_01_Fractio	23487	FTMS; HC	64.7	825.11	TMEM243

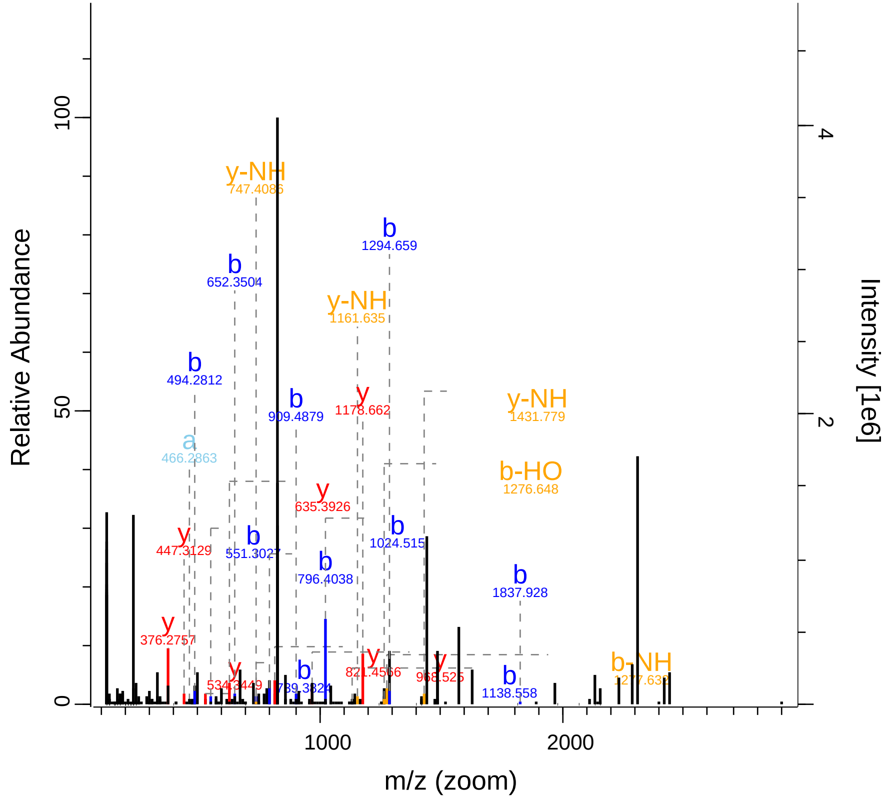

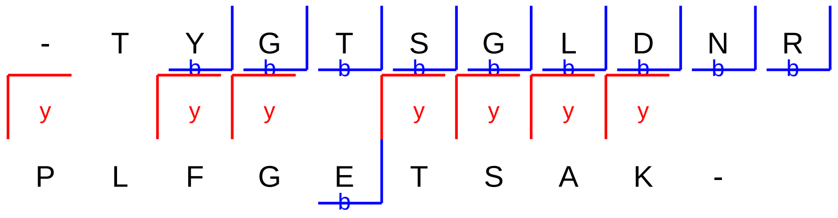

Raw File	Scan	Method	Score	m/z	Gene names
RO-QC-160121_ZackD_SS_Set_01_Fractio	23539	FTMS; HC	99.04	794.77	ATP6V0C

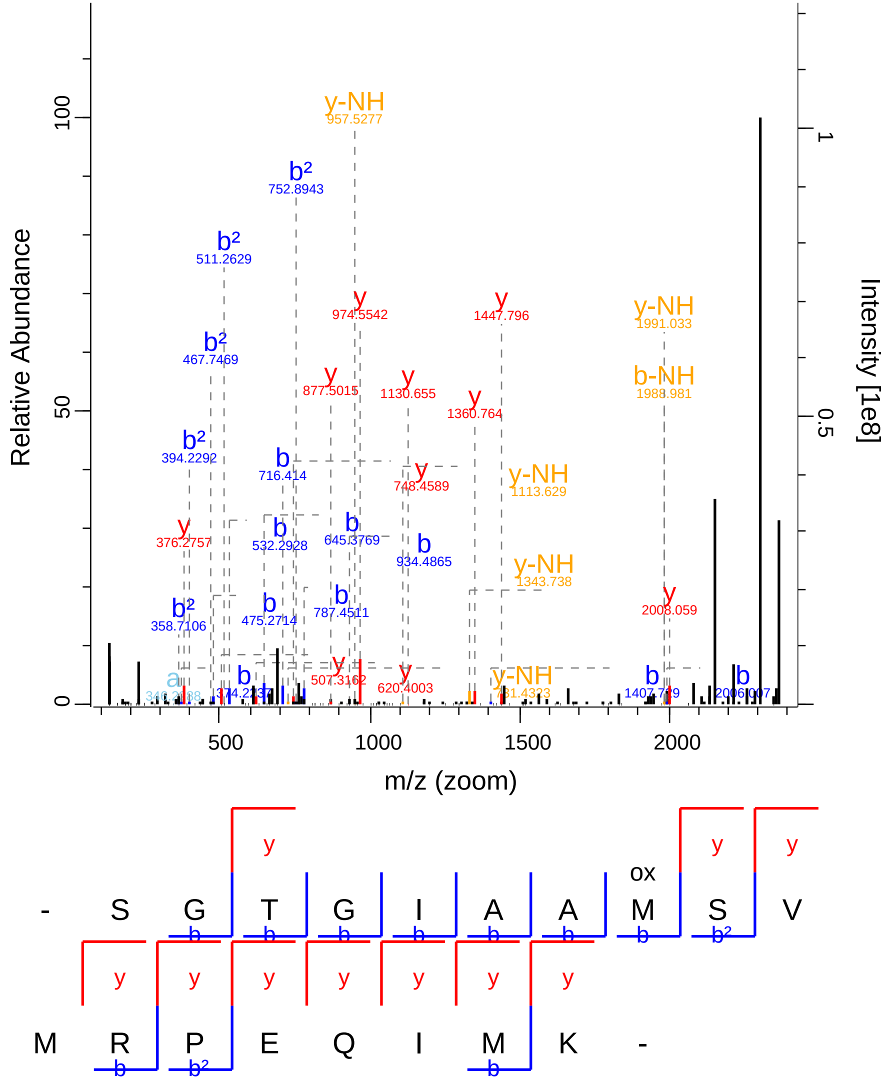

Raw File	Scan	Method	Score	m/z	Gene names
RO-QC-160121_ZackD_SS_Set_01_Fractio	23622	FTMS; HC	117.81	600.86	TMEM167A

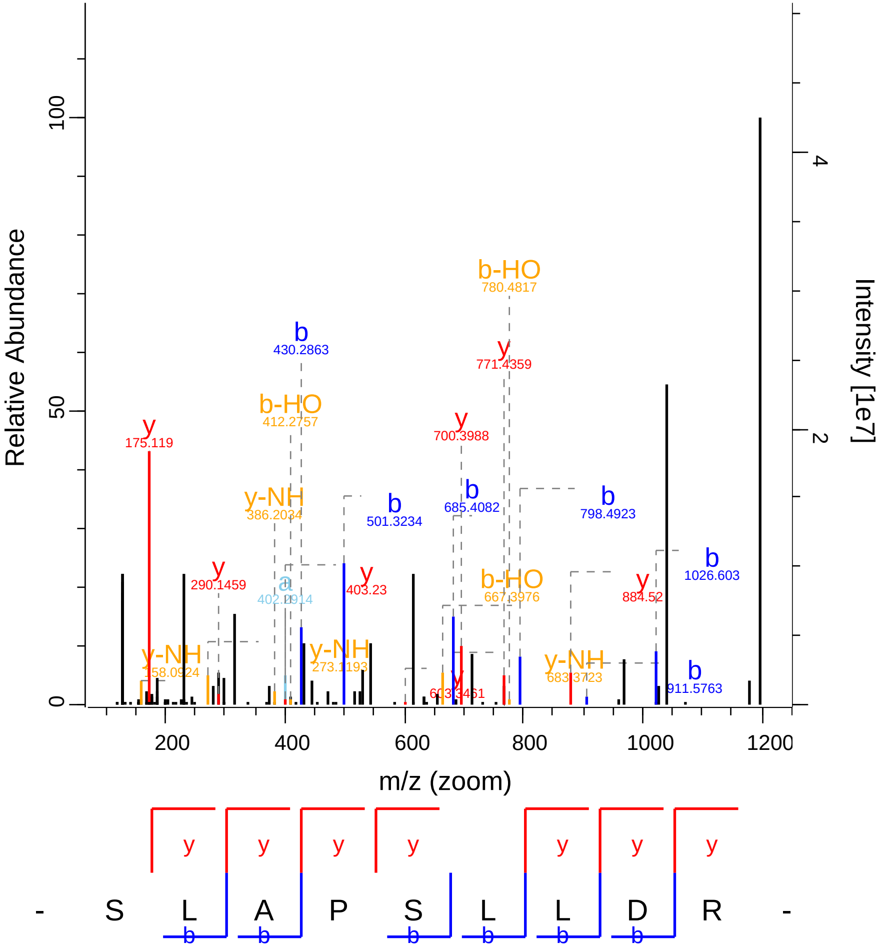

Raw File	Scan	Method	Score	m/z	Gene names
RO-QC-160121_ZackD_SS_Set_01_Fractio	24368	FTMS; HC	156.35	722.45	ARL4C

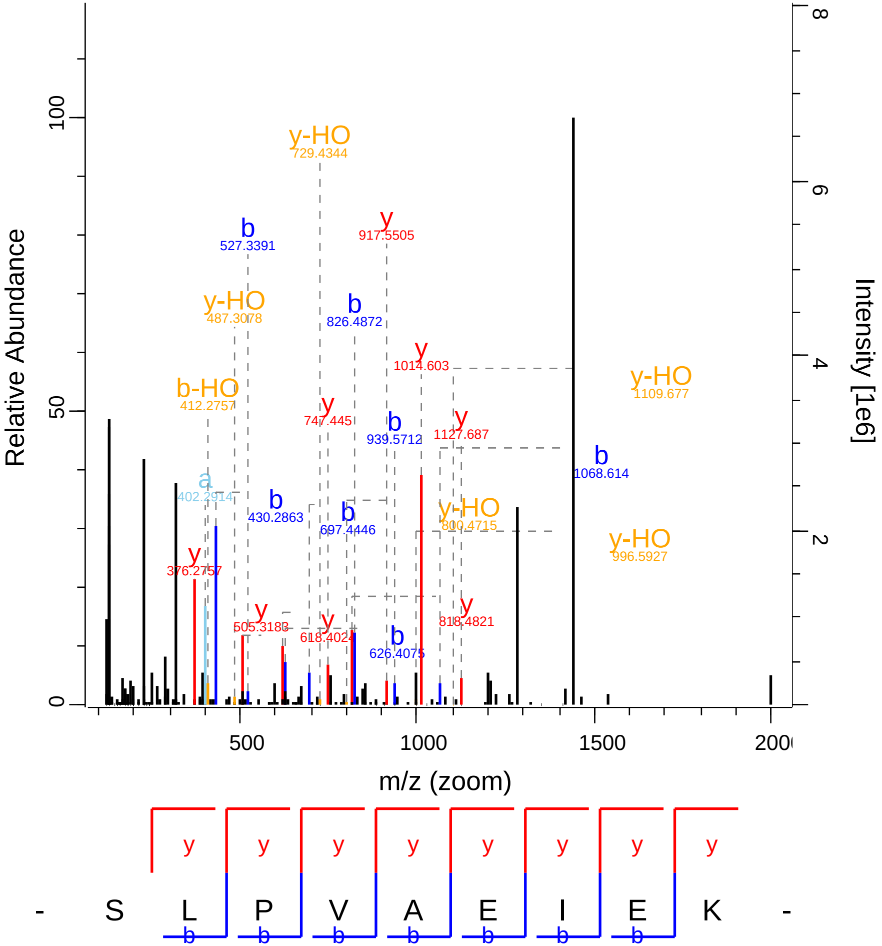

Raw File	Scan	Method	Score	m/z	Gene names
RO-QC-160121_ZackD_SS_Set_01_Fractio	25299	FTMS; HC	230.26	1124.54	RHBDD1

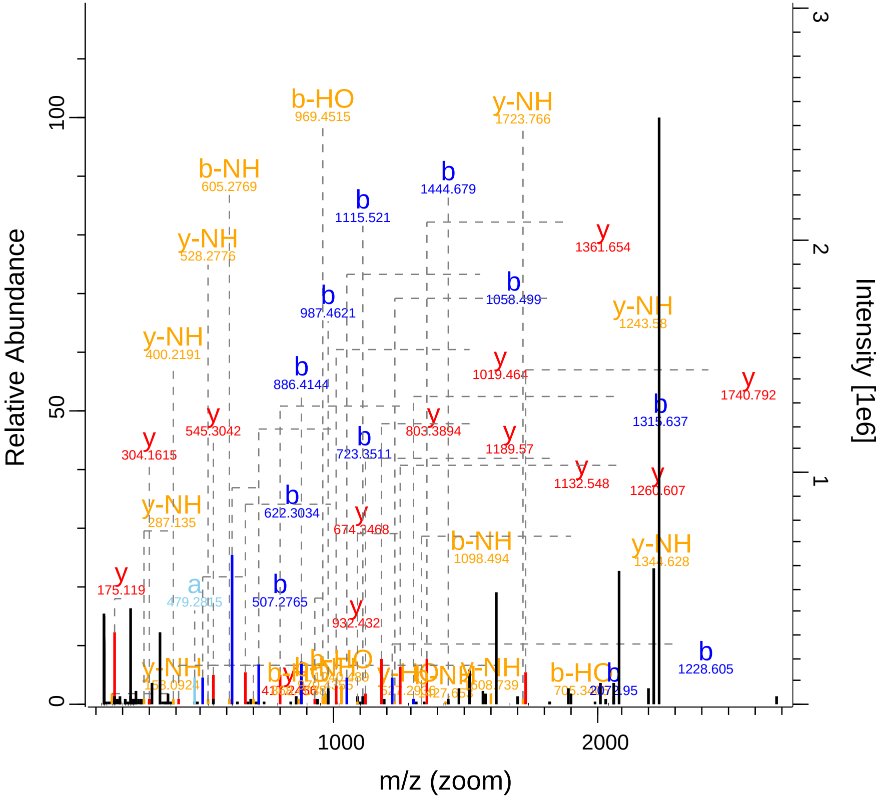

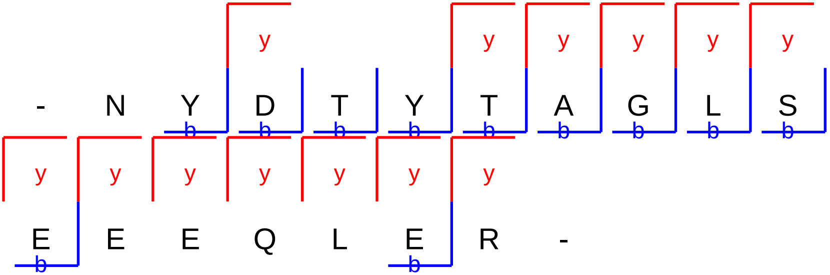

Raw File	Scan	Method	Score	m/z	Gene names
RO-QC-160121_ZackD_SS_Set_01_Fractic	25332	FTMS; HC	369.51	1124.03	RHBDD1

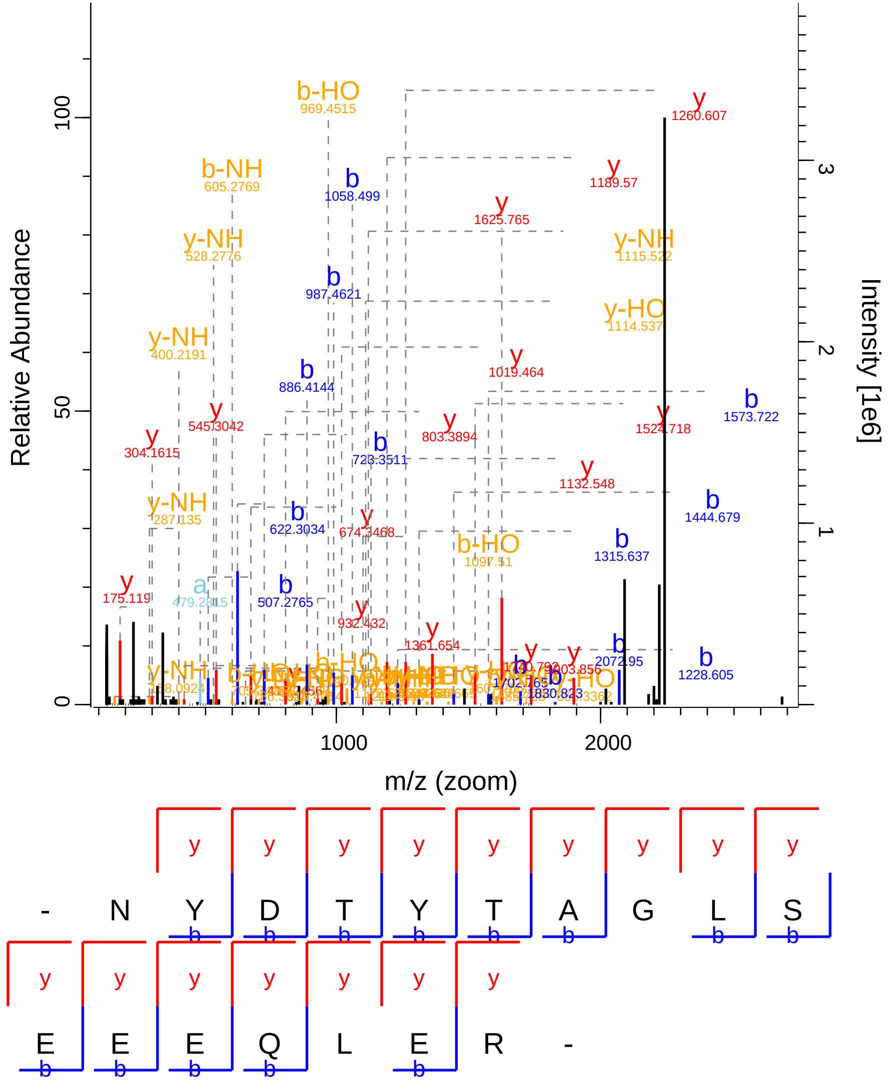

Raw File	Scan	Method	Score	m/z	Gene names
RO-QC-160121_ZackD_SS_Set_01_Fractio	25719	FTMS; HC	48.28	511.96	ASB7

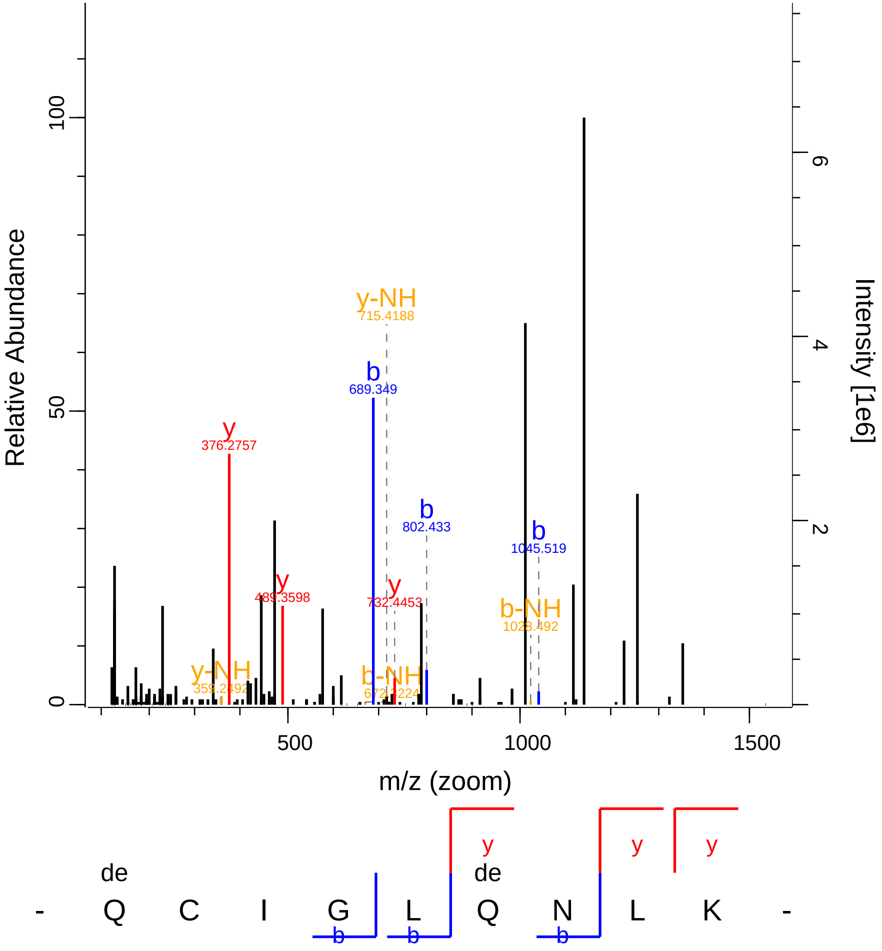

Raw File	Scan	Method	Score	m/z	Gene names
RO-QC-160121_ZackD_SS_Set_01_Fractio	26235	FTMS; HC	122.66	955.03	MPV17L2

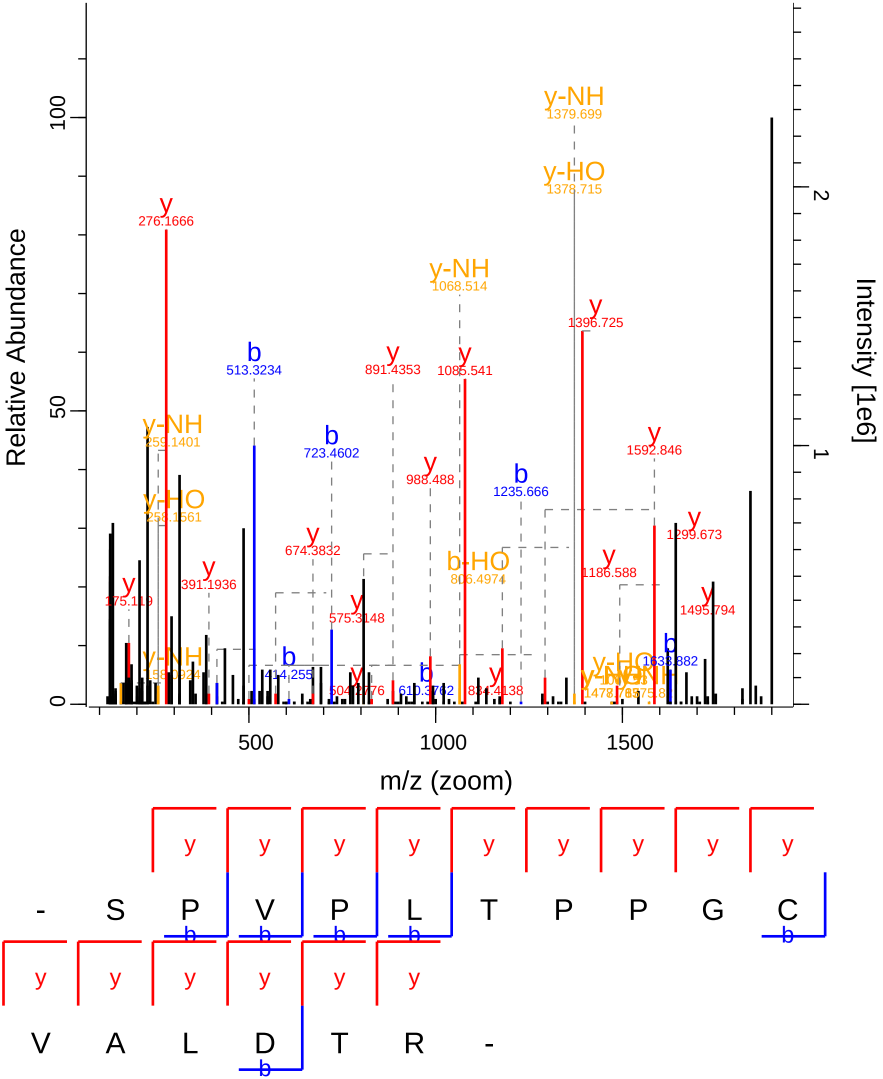

Raw File	Scan	Method	Score	m/z	Gene names
RO-QC-160121_ZackD_SS_Set_01_Fractio	26282	FTMS; HC	63.83	637.02	MPV17L2

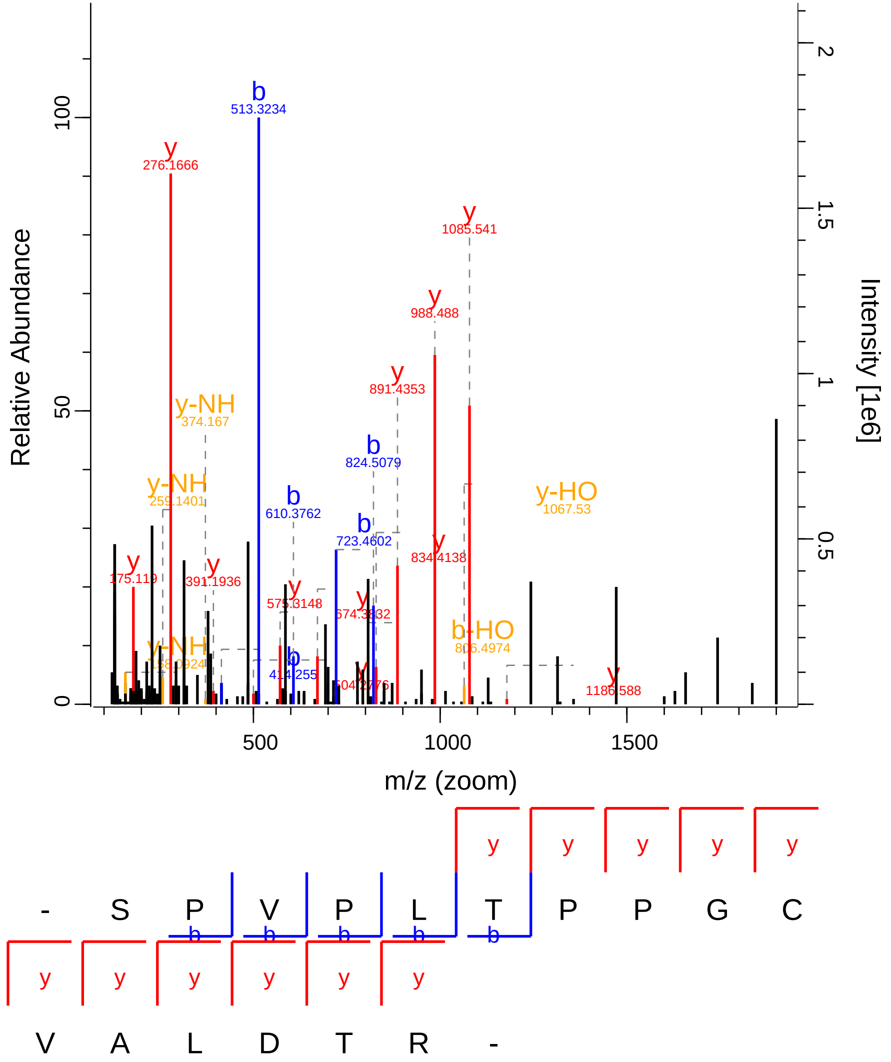

Raw File	Scan	Method	Score	m/z	Gene names
RO-QC-160121_ZackD_SS_Set_01_Fractio	26881	FTMS; HC	61.84	717.44	AIM2

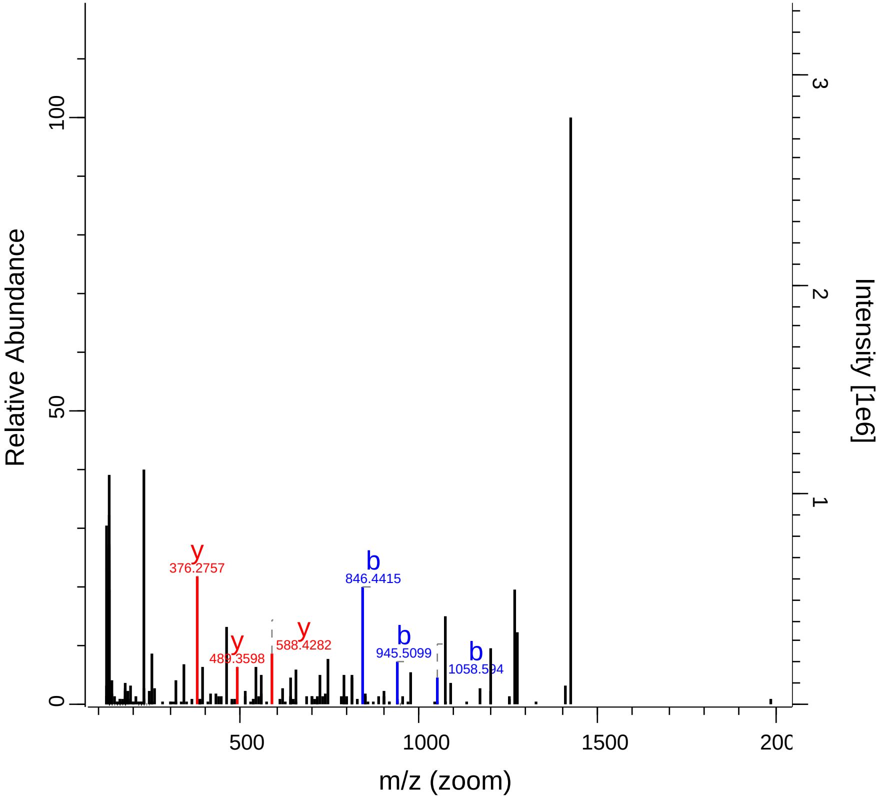

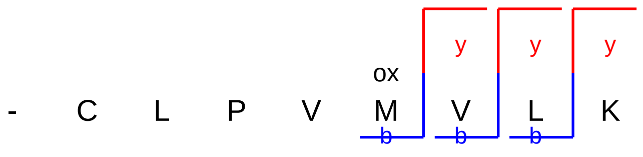

Raw File	Scan	Method	Score	m/z	Gene names
RO-QC-160121_ZackD_SS_Set_01_Fractior	27542	FTMS; HCI	45.33	801.8	RASSF3

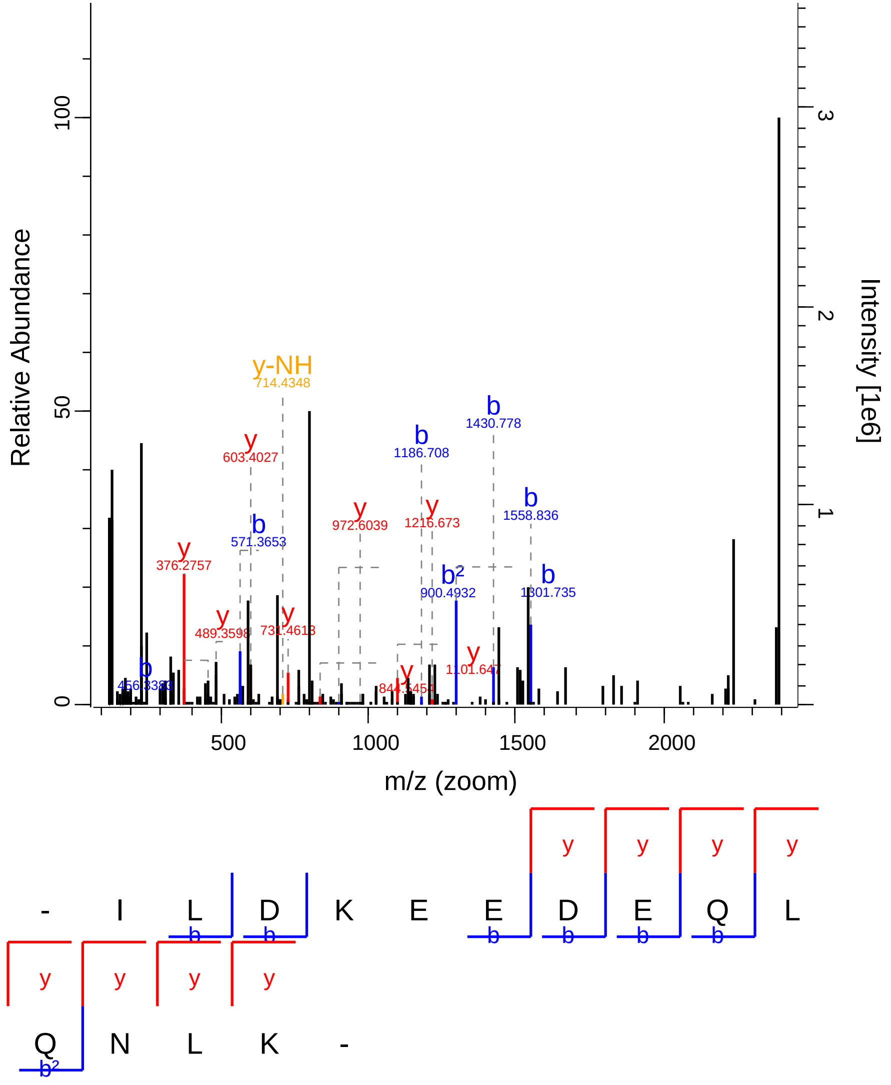

Raw File	Scan	Method	Score	m/z	Gene names
RO-QC-160121_ZackD_SS_Set_01_Fractio	28220	FTMS; HC	77.6	494.63	GATC

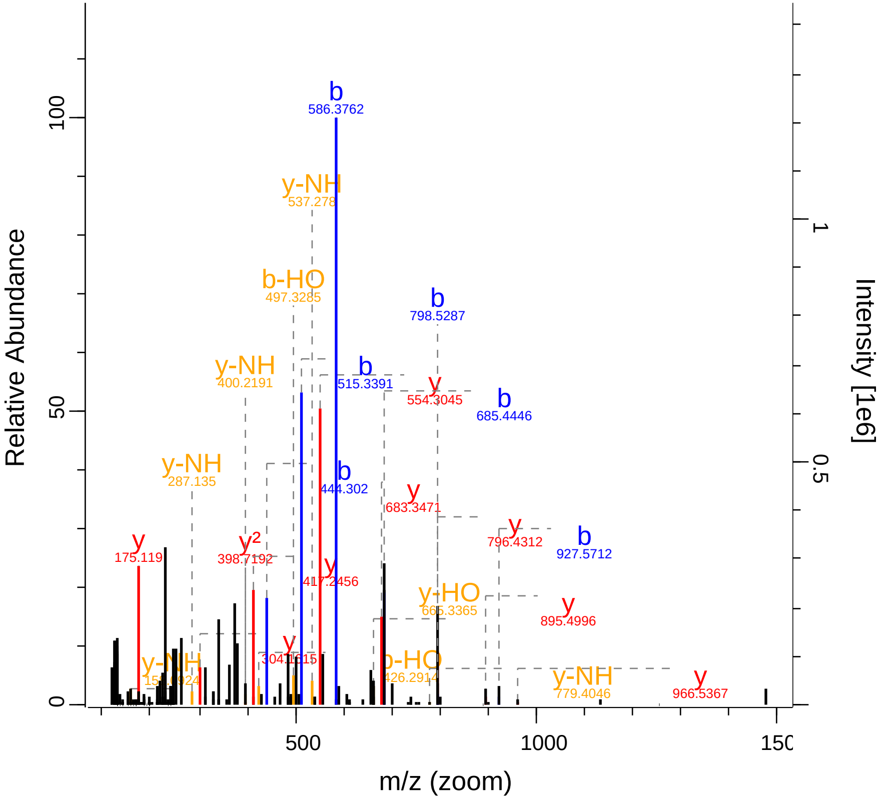

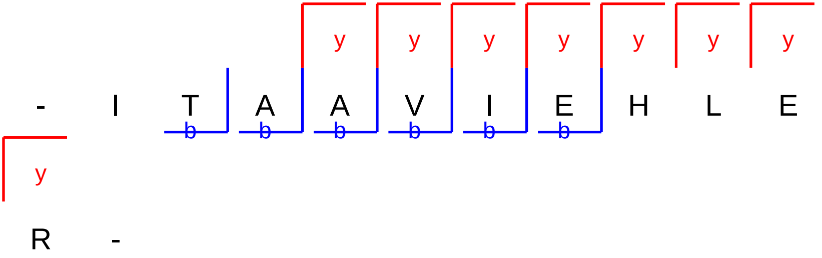

Raw File	Scan	Method	Score	m/z	Gene names
RO-QC-160121_ZackD_SS_Set_01_Fractio	28519	FTMS; HC	60.03	819.42	HN1

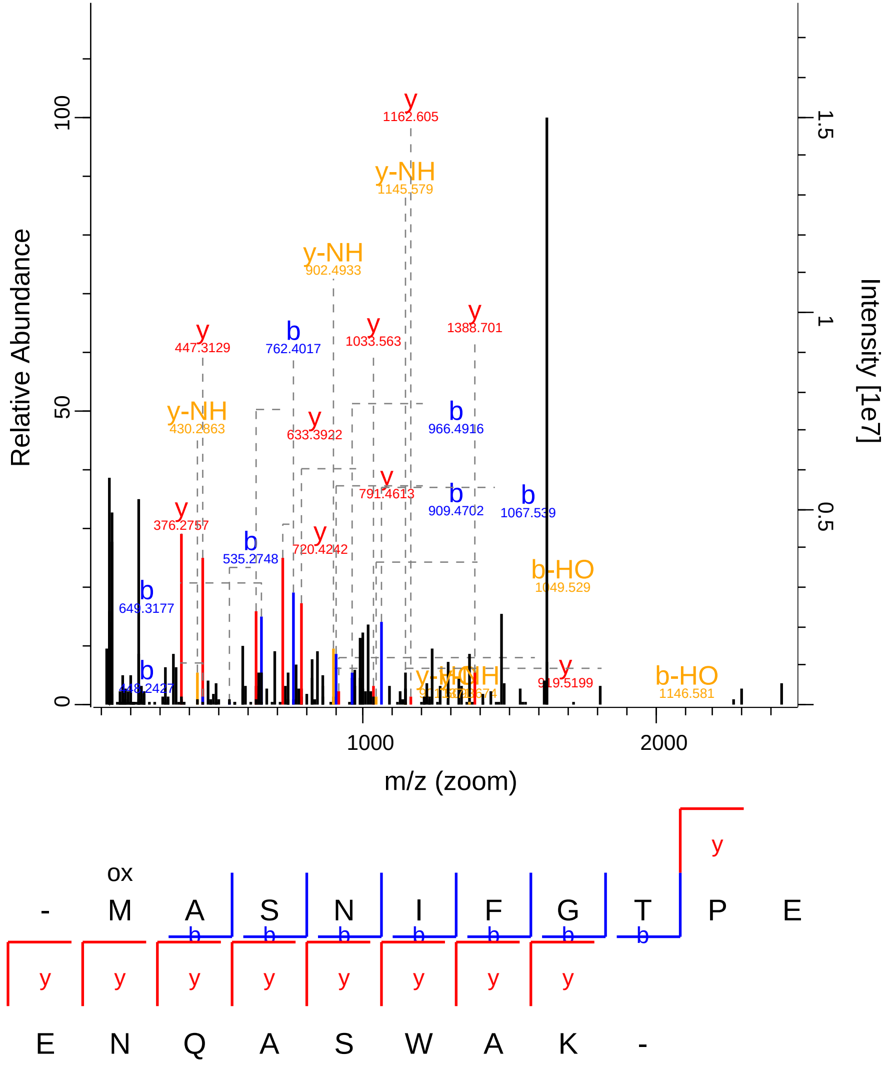

Raw File	Scan	Method	Score	m/z	Gene names
RO-QC-160121_ZackD_SS_Set_01_Fractio	28579	FTMS; HC	132.24	1228.62	HN1

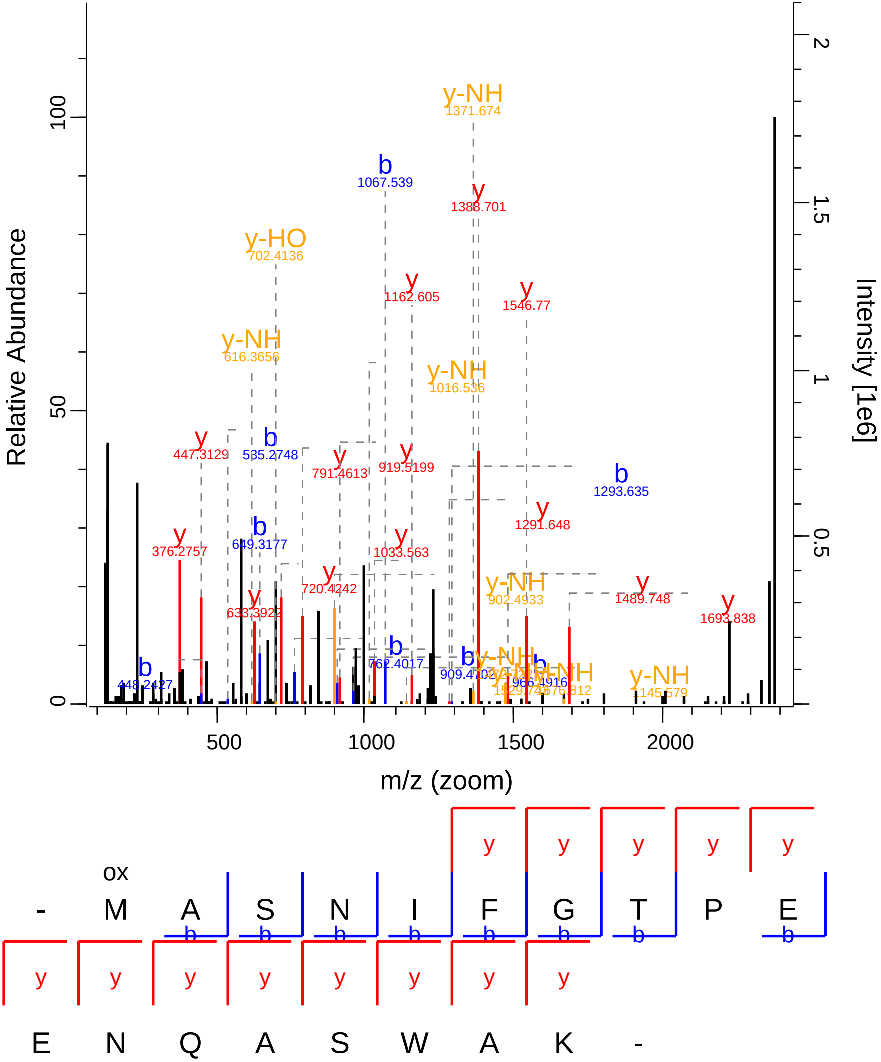

Raw File	Scan	Method	Score	m/z	Gene names
RO-QC-160121_ZackD_SS_Set_01_Fractio	28770	FTMS; HC	41.22	594.99	IFI44

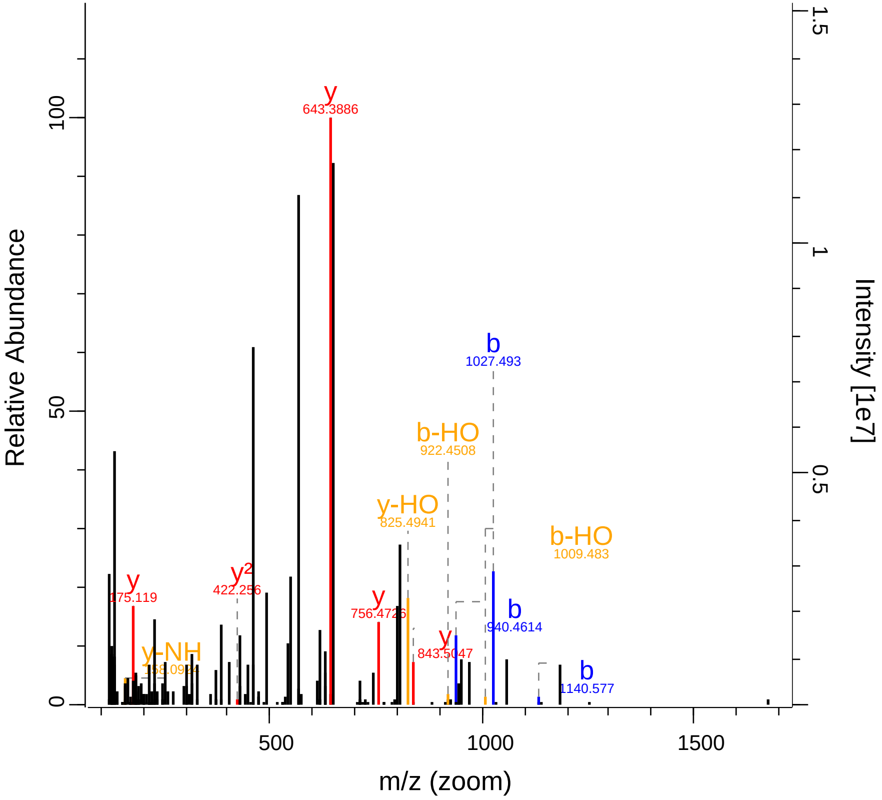

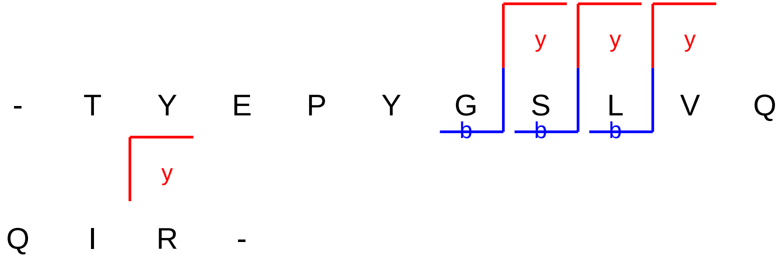

Raw File	Scan	Method	Score	m/z	Gene names
RO-QC-160121_ZackD_SS_Set_01_Fractio	29802	FTMS; HC	172.84	738.14	C1GALT1C1

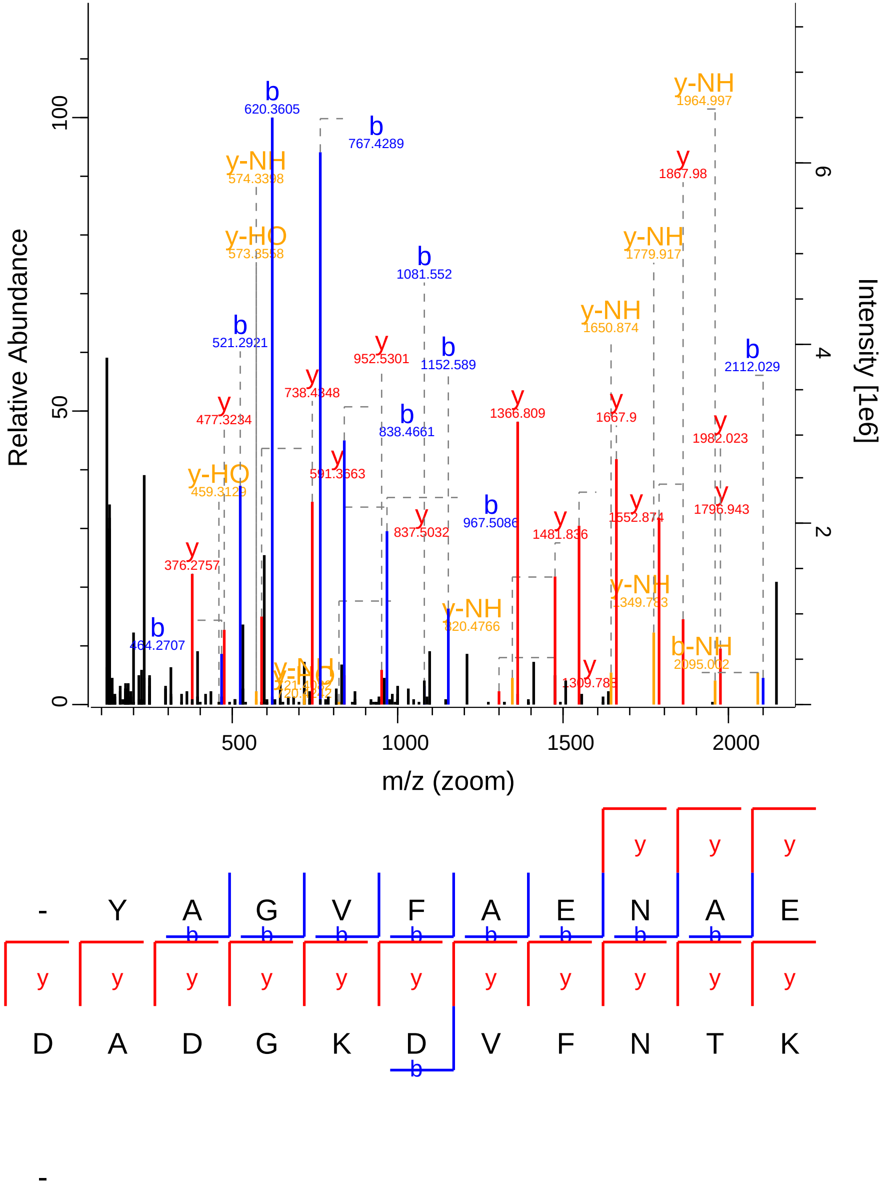

Raw File	Scan	Method	Score	m/z	Gene names
RO-QC-160121_ZackD_SS_Set_01_Fractio	29808	FTMS; HC	174.2	983.85	C1GALT1C1

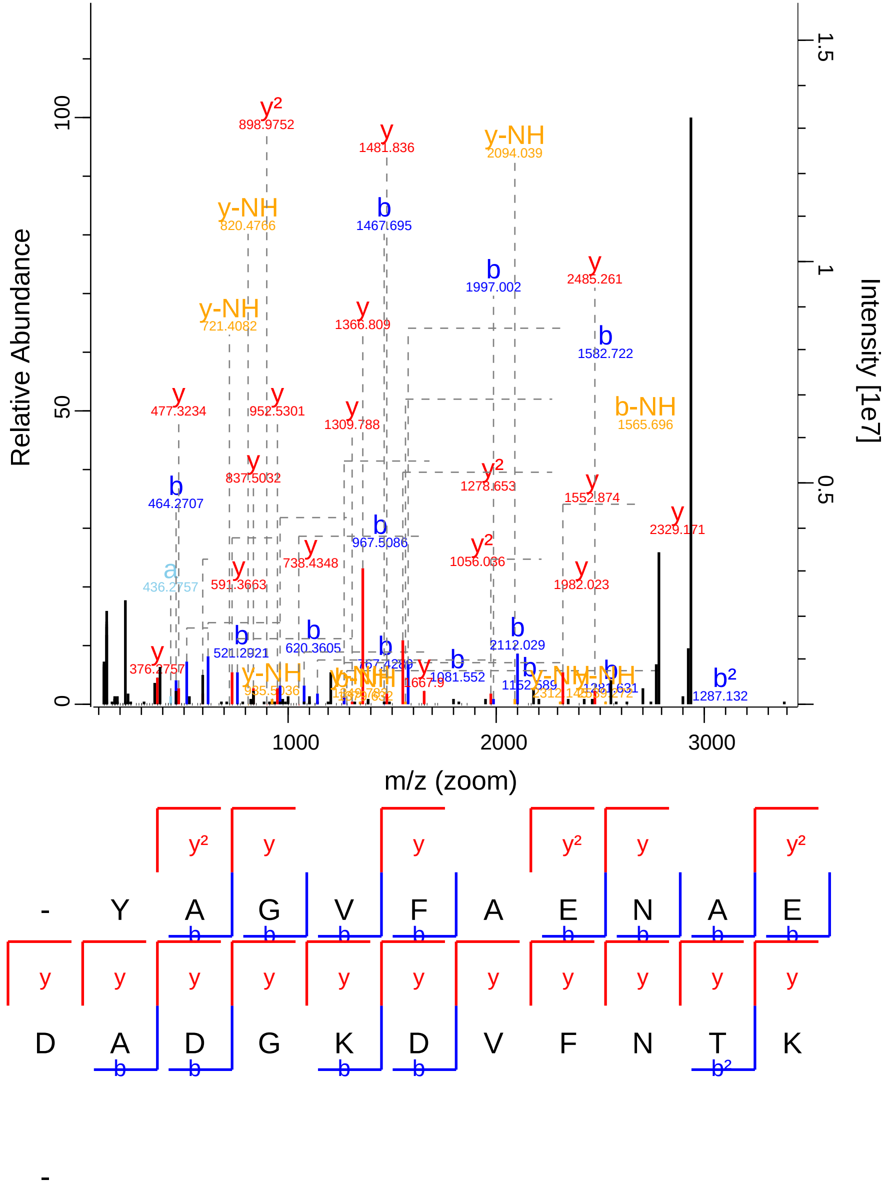

Raw File	Scan	Method	Score	m/z	Gene names
RO-QC-160121_ZackD_SS_Set_01_Fractio	29886	FTMS; HC	79.16	644.38	DNLZ

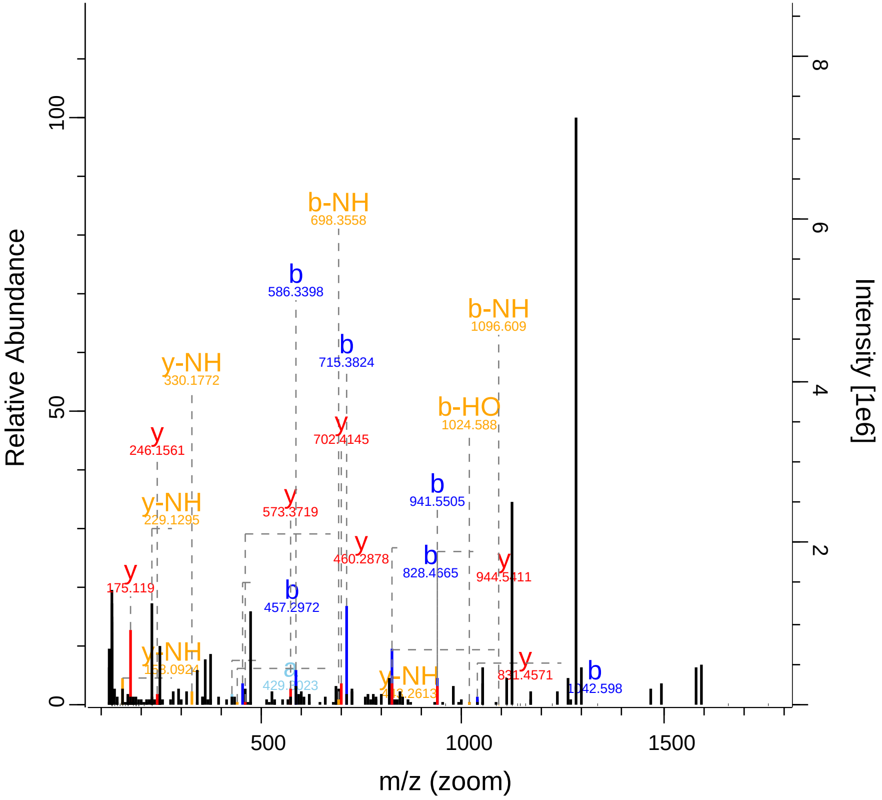

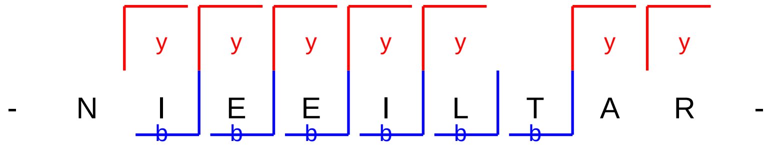

Raw File	Scan	Method	Score	m/z	Gene names
RO-QC-160121_ZackD_SS_Set_01_Fractio	30451	FTMS; HC	168.43	998.06	B3GALNT1

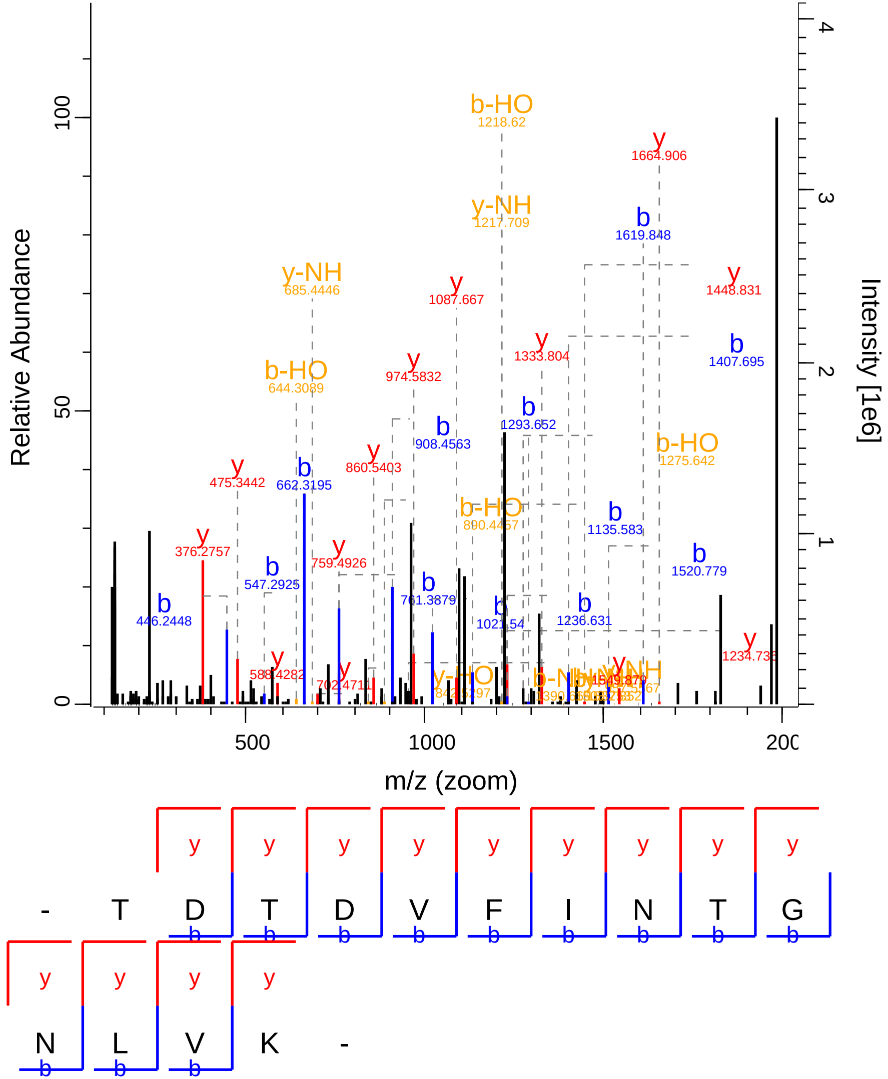

Raw File	Scan	Method	Score	m/z	Gene names
RO-QC-160121_ZackD_SS_Set_01_Fractio	30745	FTMS; HC	61.44	662.35	TMEM87B

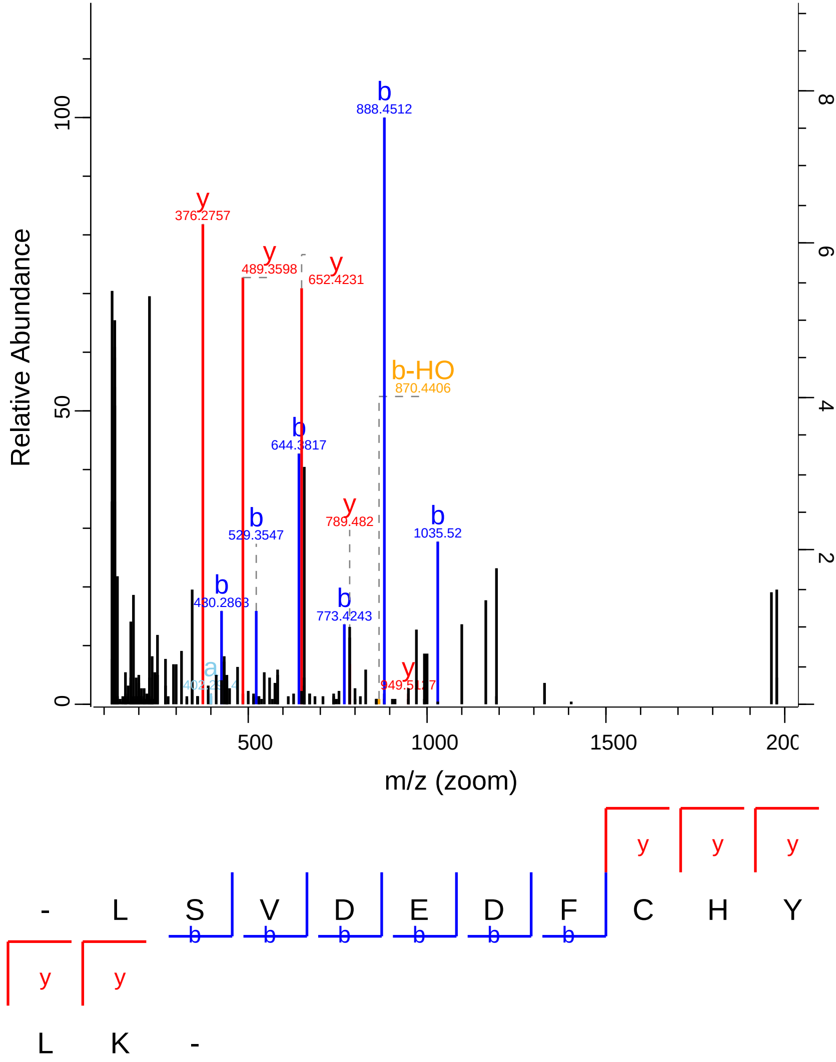

Raw File	Scan	Method	Score	m/z	Gene names
RO-QC-160121_ZackD_SS_Set_01_Fractic	32023	FTMS; HC	41.73	1303.26	DMKN

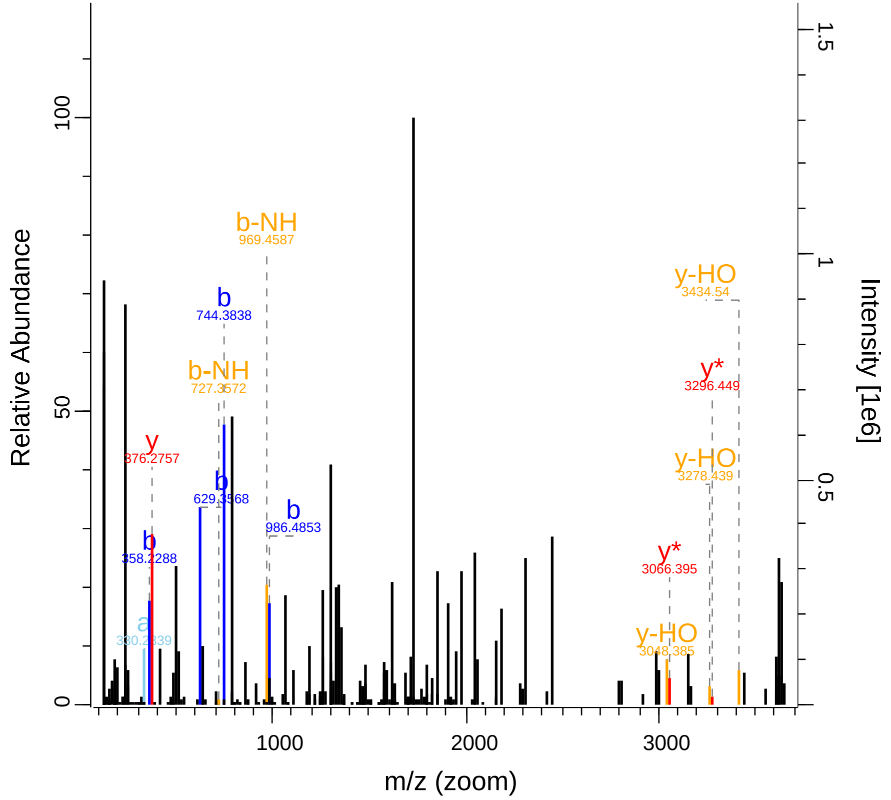

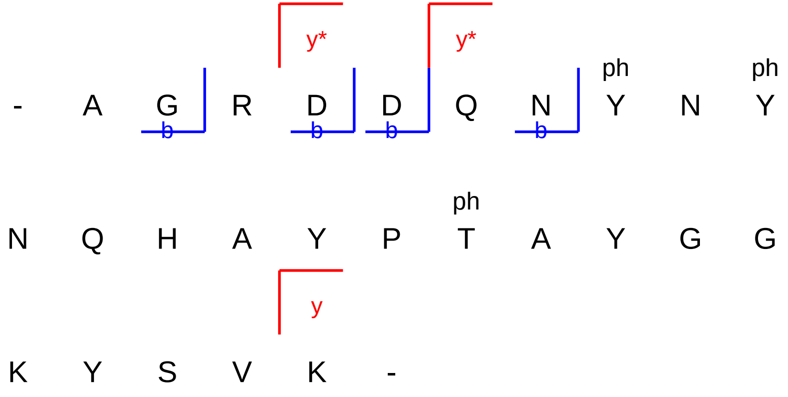

Raw File	Scan	Method	Score	m/z	Gene names
RO-QC-160121_ZackD_SS_Set_01_Fractic	32818	FTMS; HC	313.92	1245.66	IER3IP1

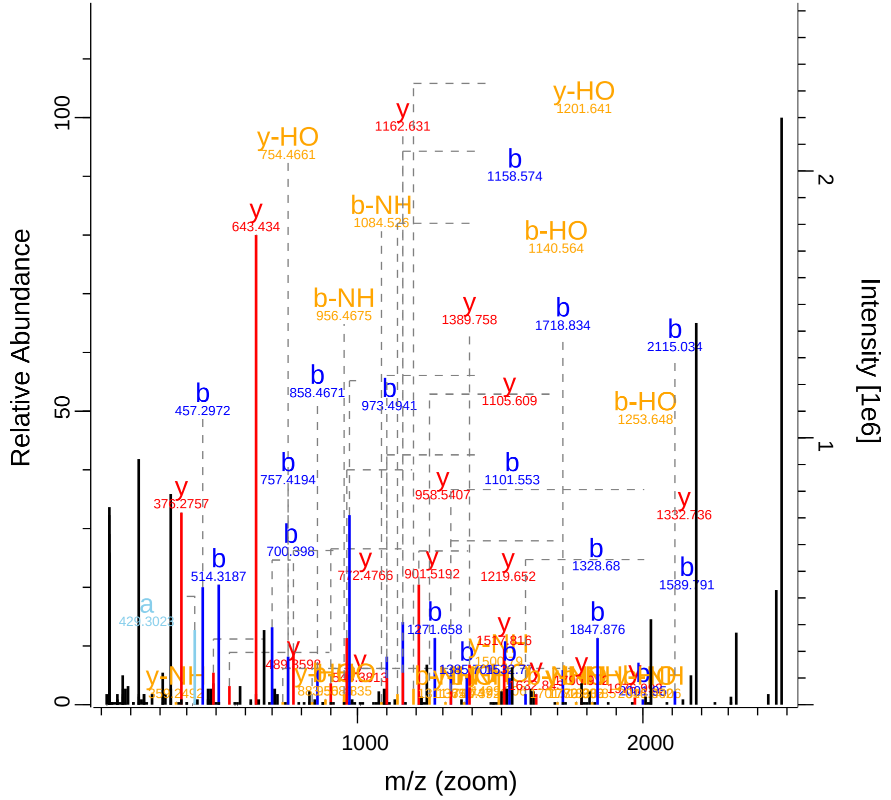

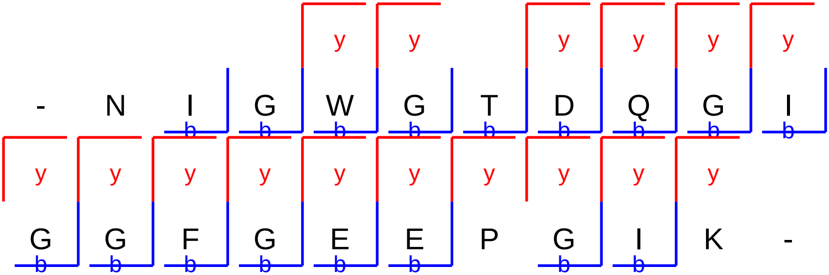

Raw File	Scan	Method	Score	m/z	Gene names
RO-QC-160121_ZackD_SS_Set_01_Fractio	32832	FTMS; HC	137.43	831.11	IER3IP1

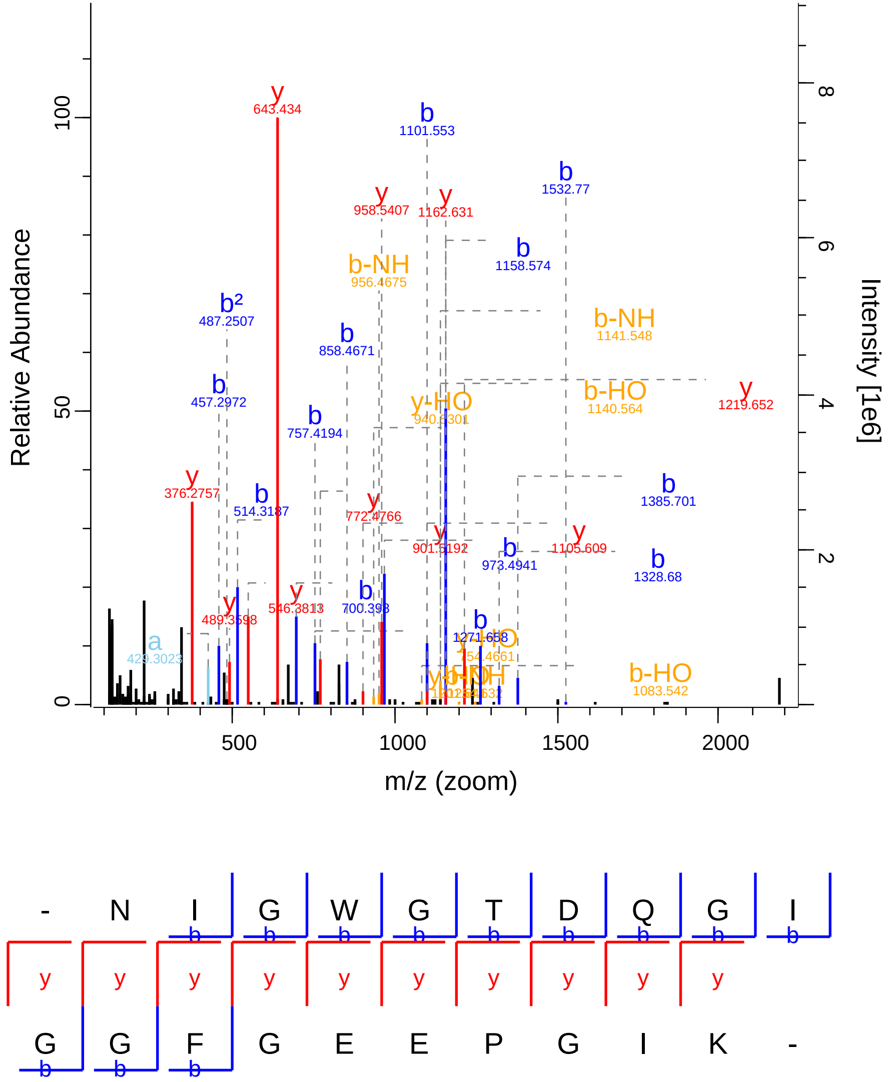

Raw File	Scan	Method	Score	m/z	Gene names
RO-QC-160121_ZackD_SS_Set_01_Fractic	32837	FTMS; HC	93.35	1246.66	IER3IP1

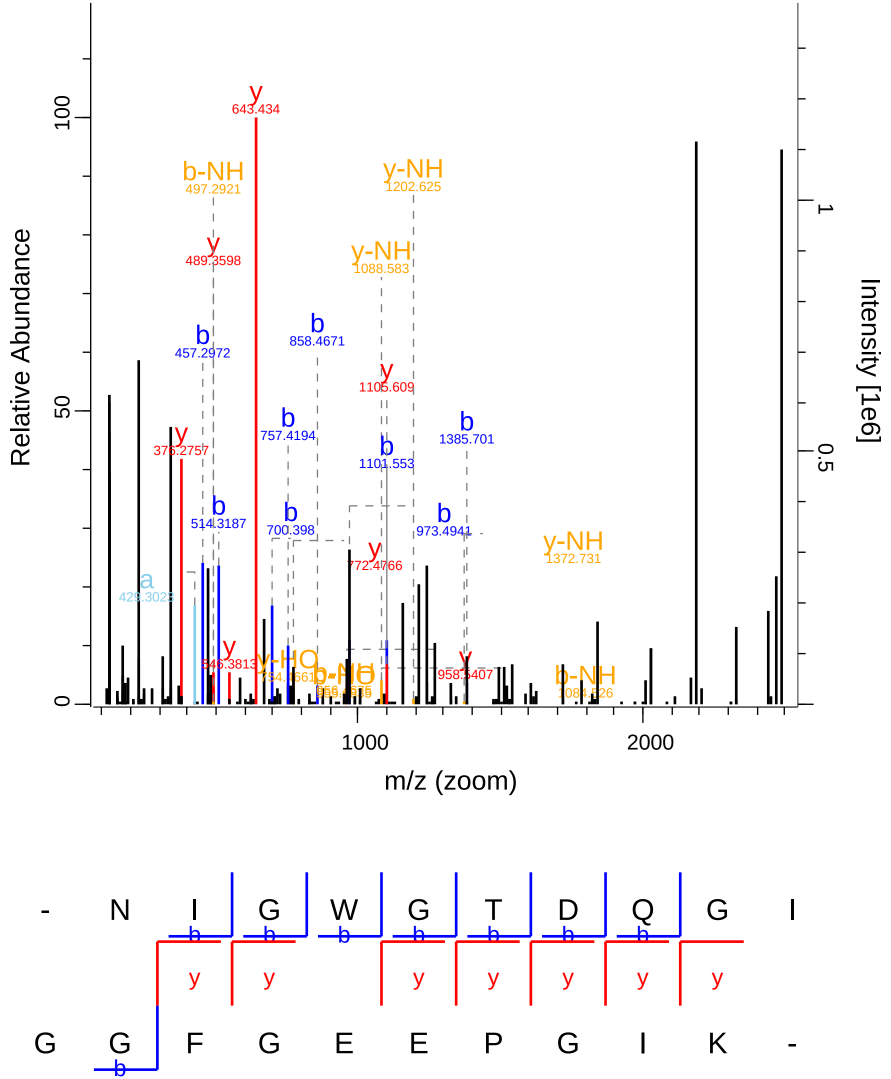

Raw File	Scan	Method	Score	m/z	Gene names
RO-QC-160121_ZackD_SS_Set_01_Fractio	32870	FTMS; HC	85.81	623.58	IER3IP1

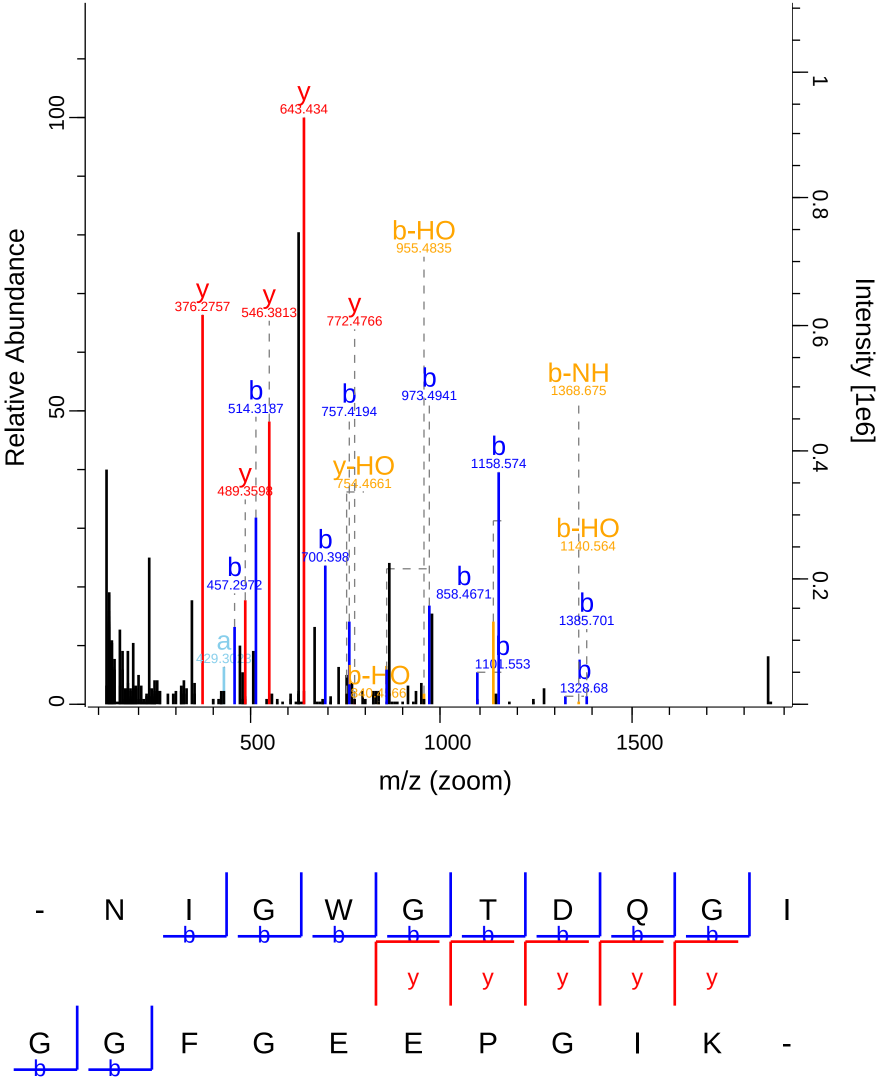

Raw File	Scan	Method	Score	m/z	Gene names
RO-QC-160121_ZackD_SS_Set_01_Fracti	33028	FTMS; HC	324.38	1246.16	IER3IP1

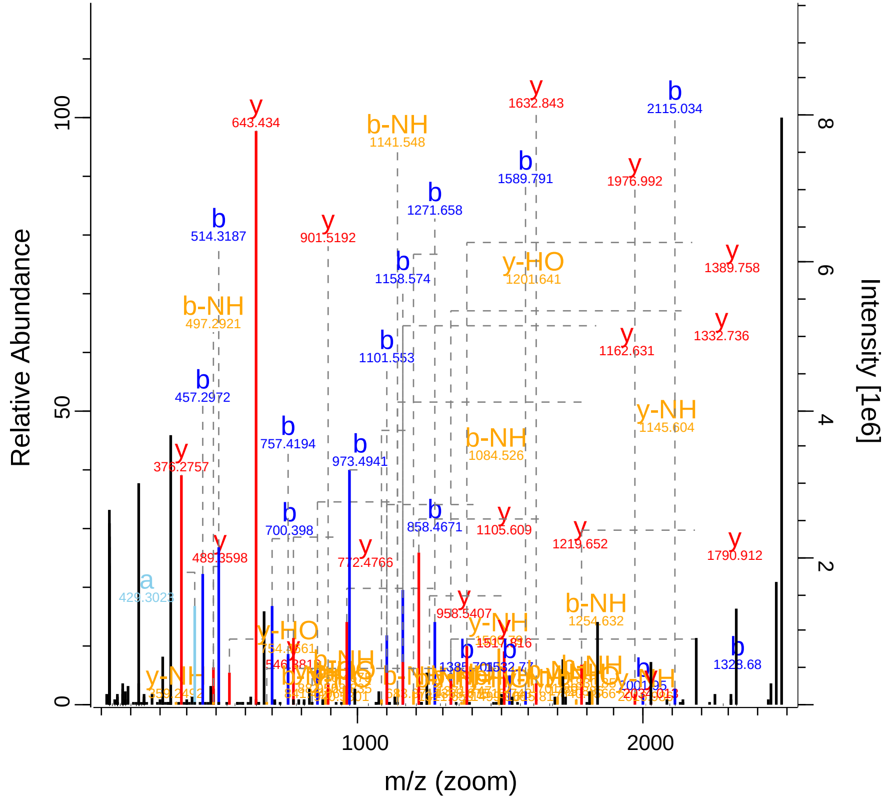

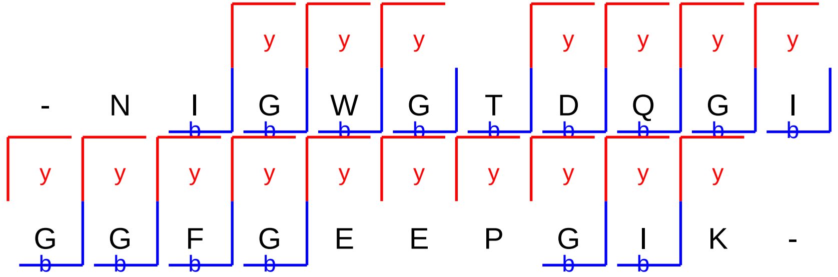

Raw File	Scan	Method	Score	m/z	Gene names
RO-QC-160121_ZackD_SS_Set_01_Fractio	33031	FTMS; HC	163.79	831.11	IER3IP1

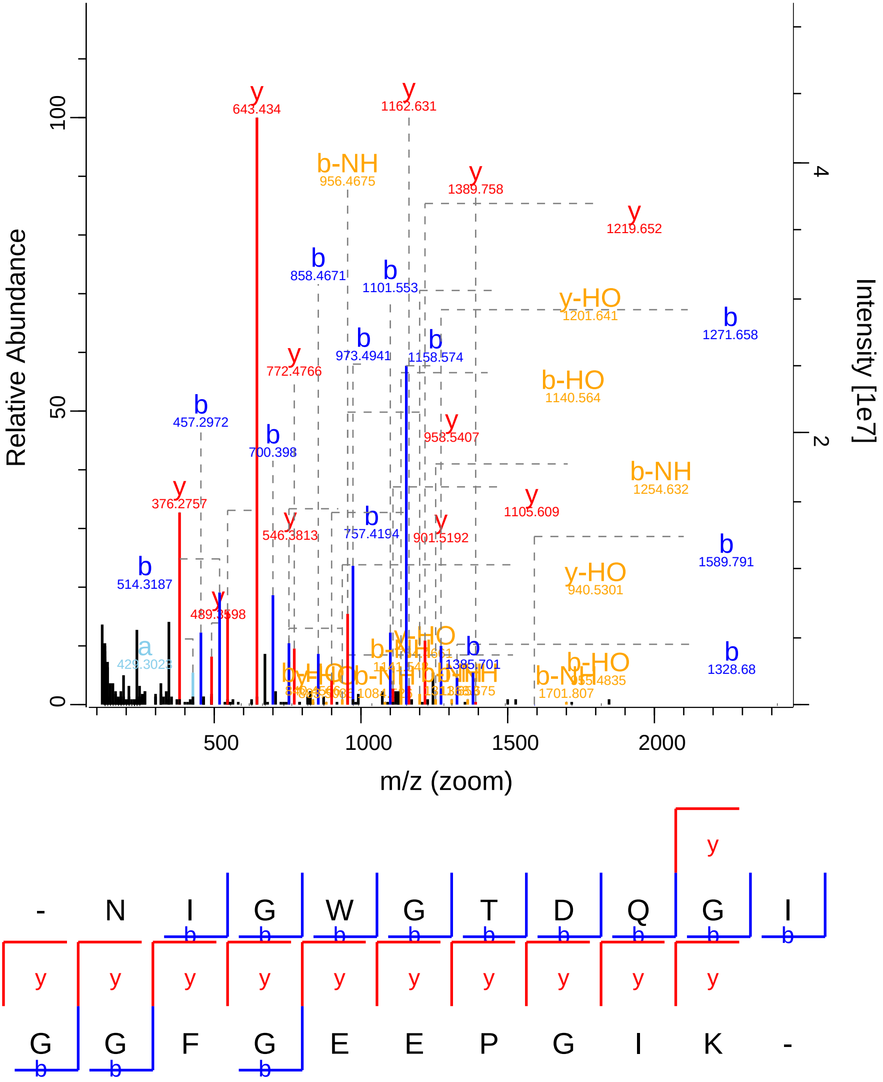

Raw File	Scan	Method	Score	m/z	Gene names
RO-QC-160121_ZackD_SS_Set_01_Fractio	33077	FTMS; HC	84.25	623.58	IER3IP1

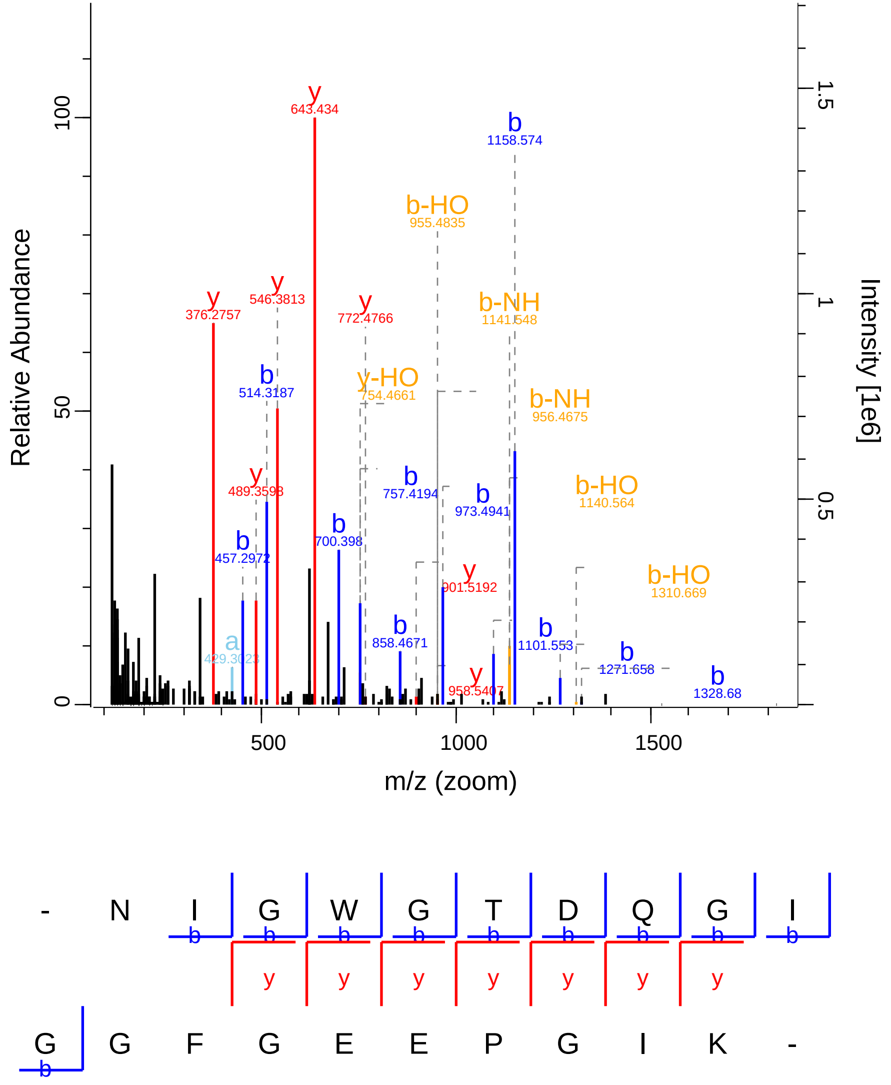

Raw File	Scan	Method	Score	m/z	Gene names
RO-QC-160121_ZackD_SS_Set_01_Fractio	33901	FTMS; HC	85.36	716.72	GPT2;GPT

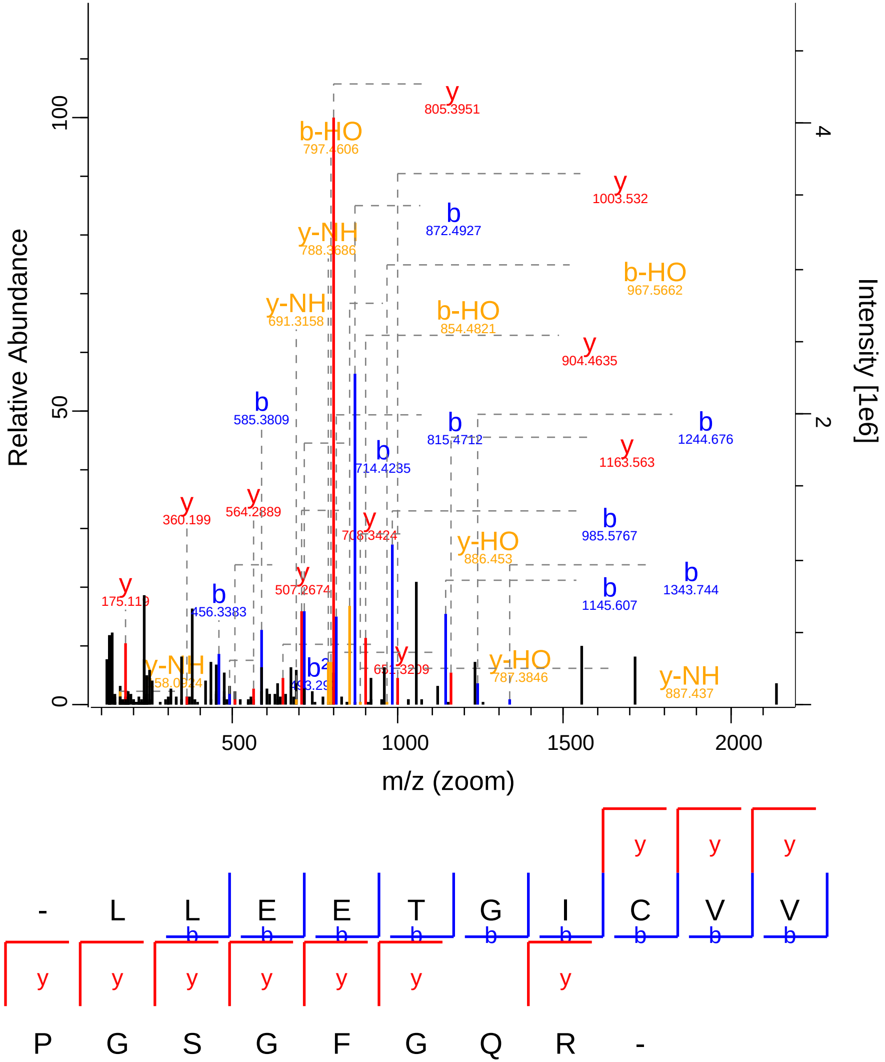

Raw File	Scan	Method	Score	m/z	Gene names
RO-QC-160121_ZackD_SS_Set_01_Fractio	33922	FTMS; HC	157.52	1075.07	GPT2;GPT

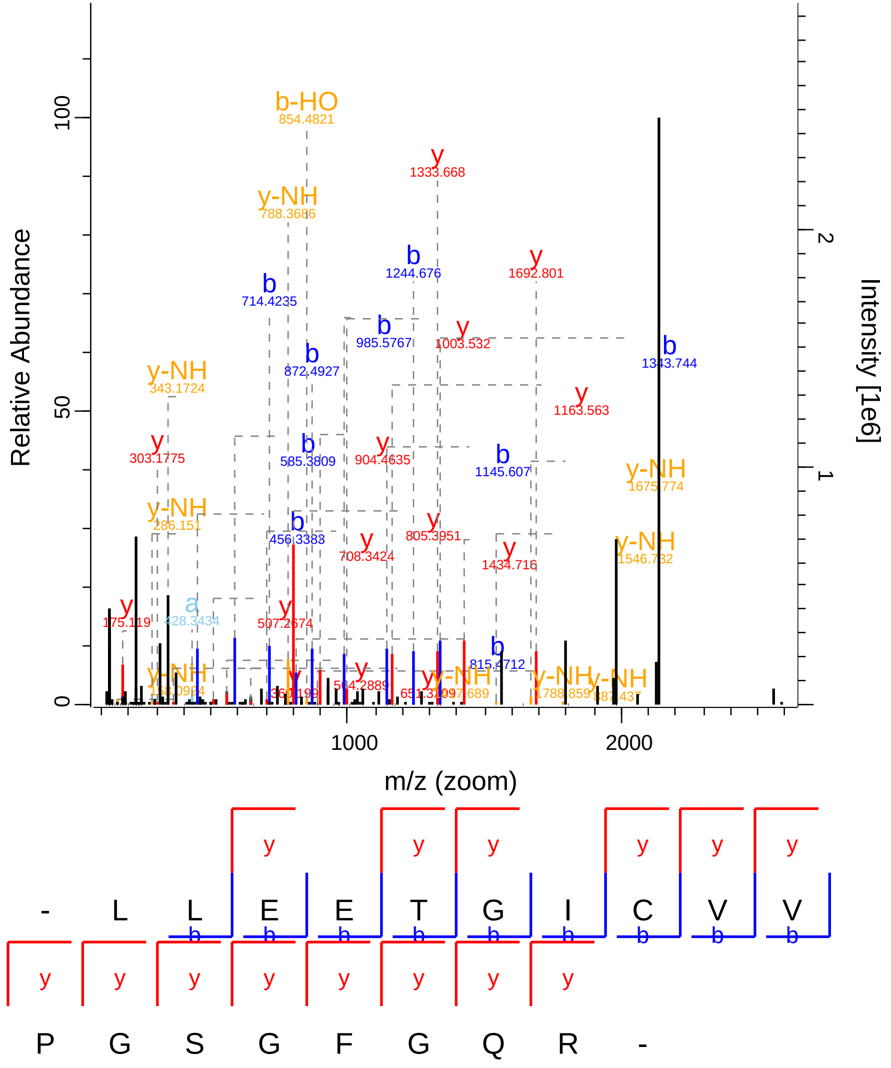

Raw File	Scan	Method	Score	m/z	Gene names
RO-QC-160121_ZackD_SS_Set_01_Fractio	35156	FTMS; HC	99.89	757.74	FJX1

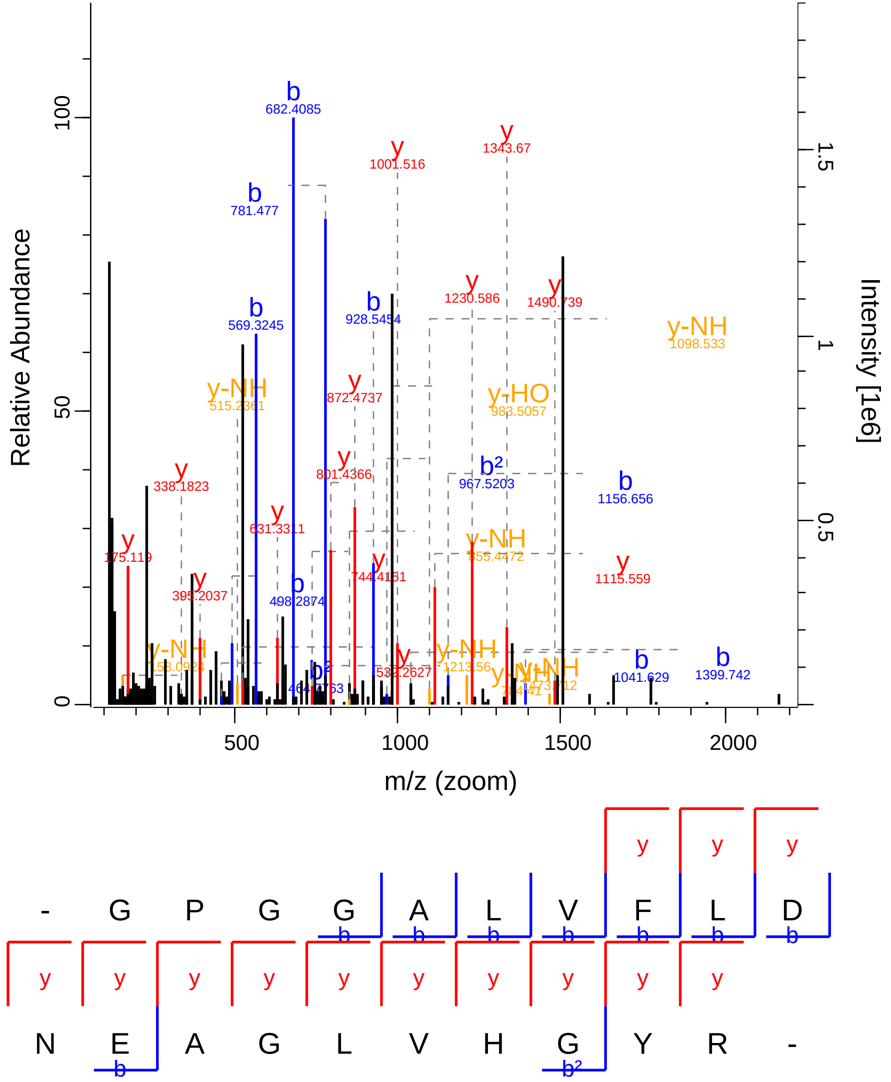

Raw File	Scan	Method	Score	m/z	Gene names
RO-QC-160121_ZackD_SS_Set_01_Fractio	35330	FTMS; HC	75.57	692.07	PPP4R4

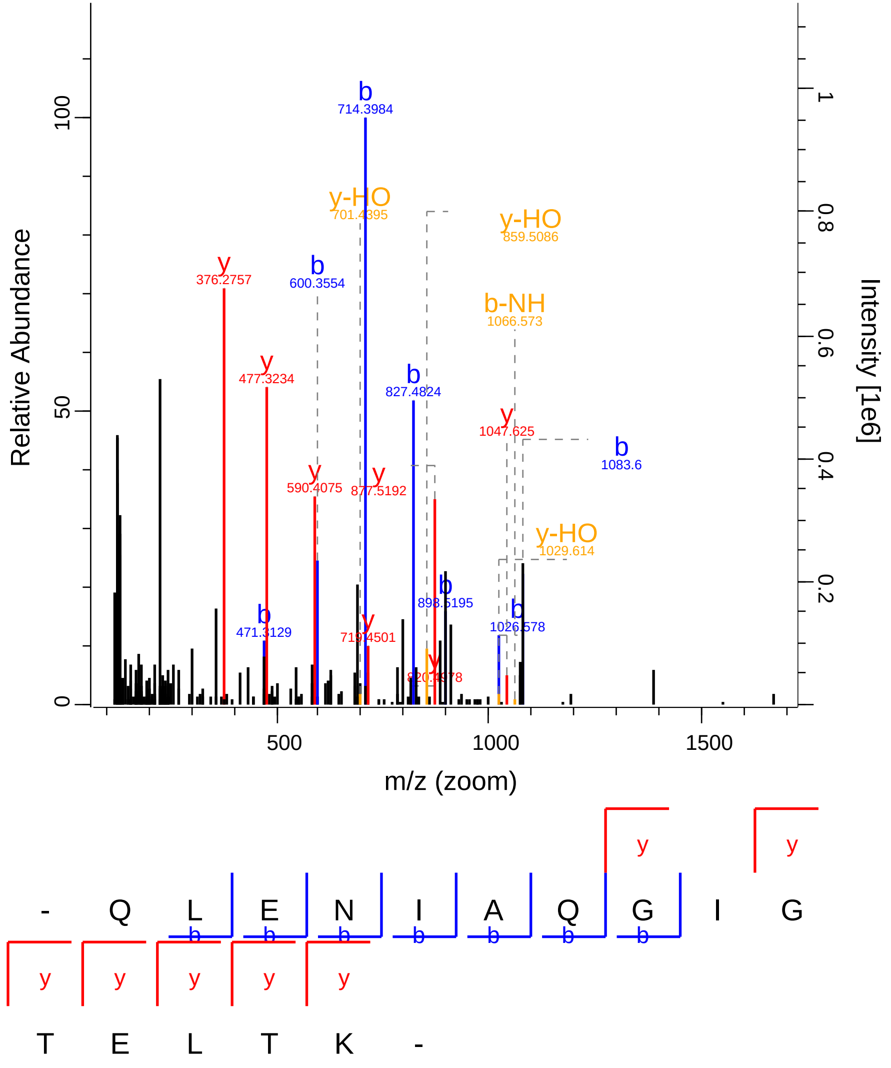

Raw File	Scan	Method	Score	m/z	Gene names
RO-QC-160121_ZackD_SS_Set_01_Fractio	36372	FTMS; HC	73.11	722.39	BMP8B;BMP8A

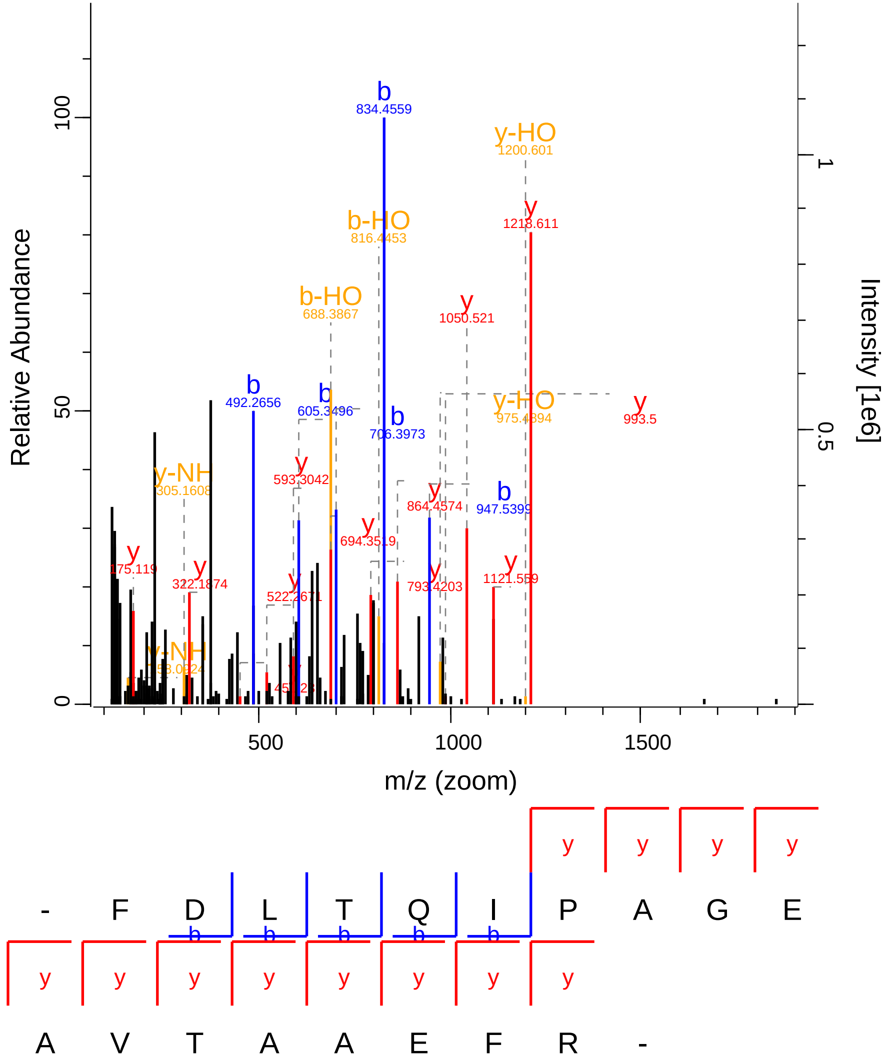

Raw File	Scan	Method	Score	m/z	Gene names
RO-QC-160121_ZackD_SS_Set_01_Fractio	37650	FTMS; HC	82.41	723.03	DEGS1

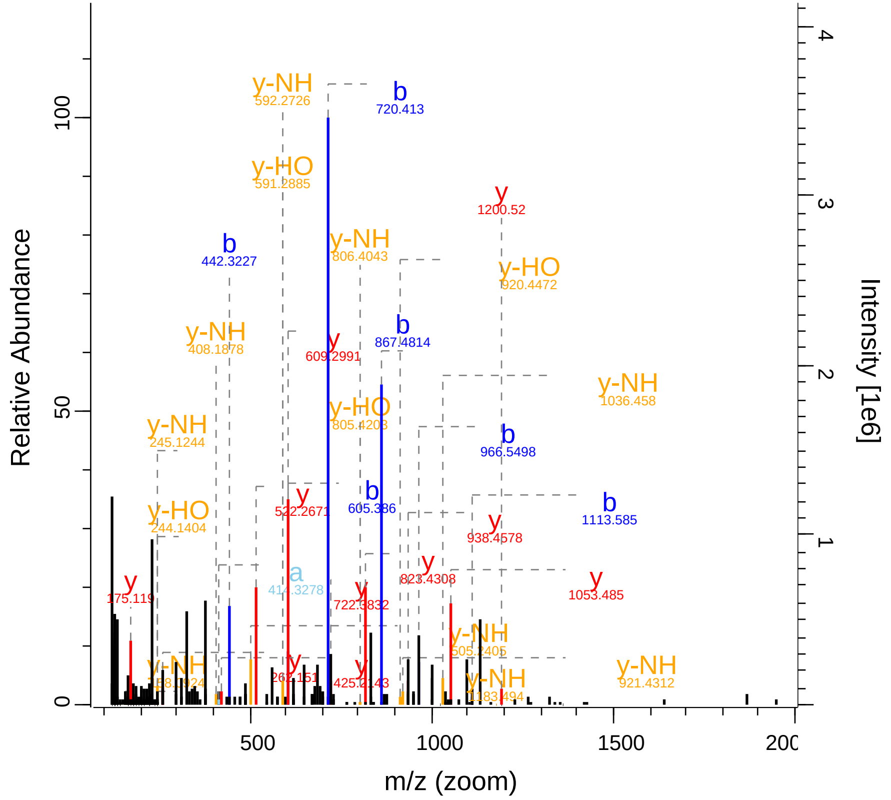

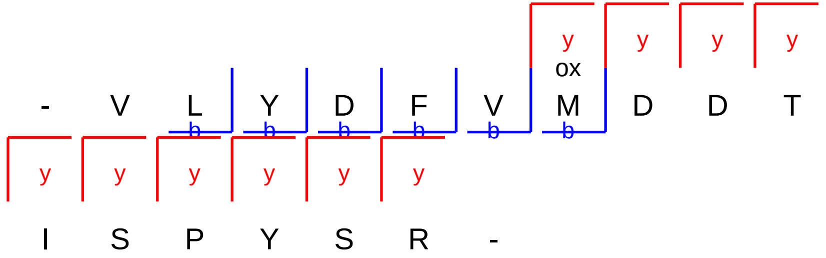

Raw File	Scan	Method	Score	m/z	Gene names
RO-QC-160121_ZackD_SS_Set_01_Fractic	37786	FTMS; HC	41.05	1196.66	LRRC49

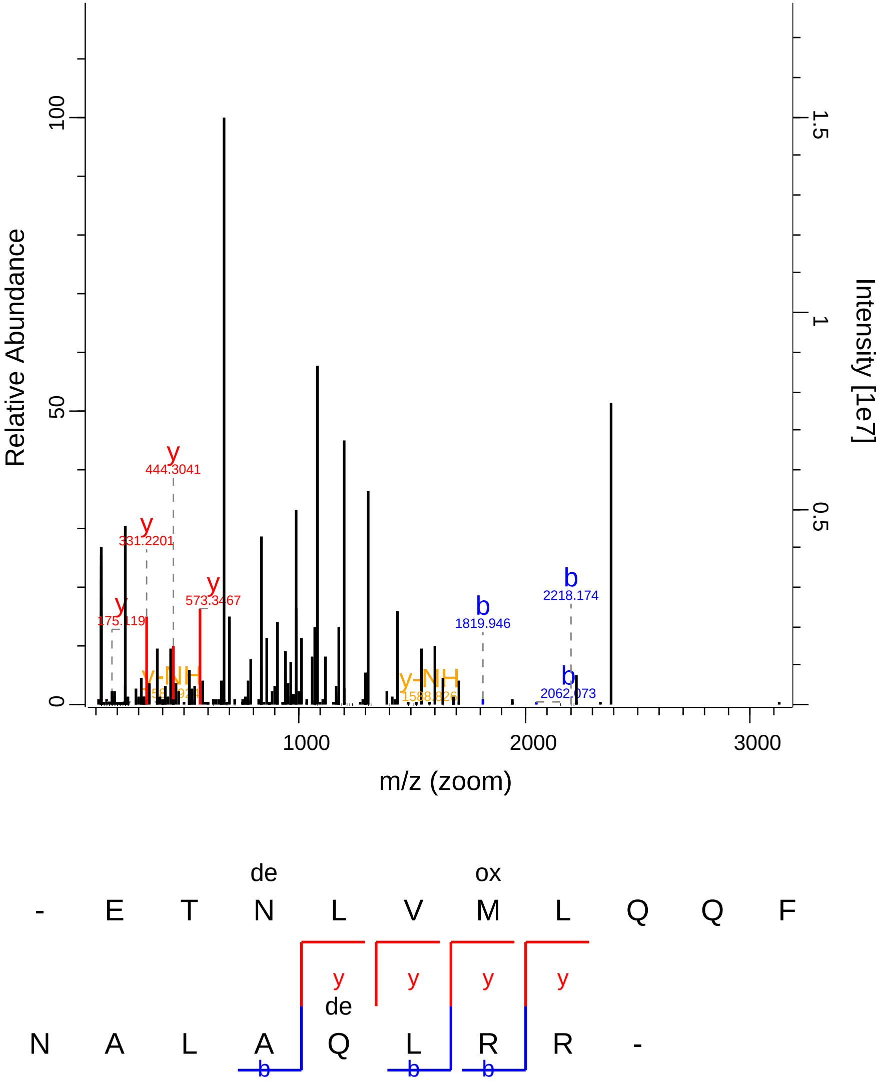

Raw File	Scan	Method	Score	m/z	Gene names
RO-QC-160121_ZackD_SS_Set_01_Fractio	38762	FTMS; HC	41.09	762.45	SYT5

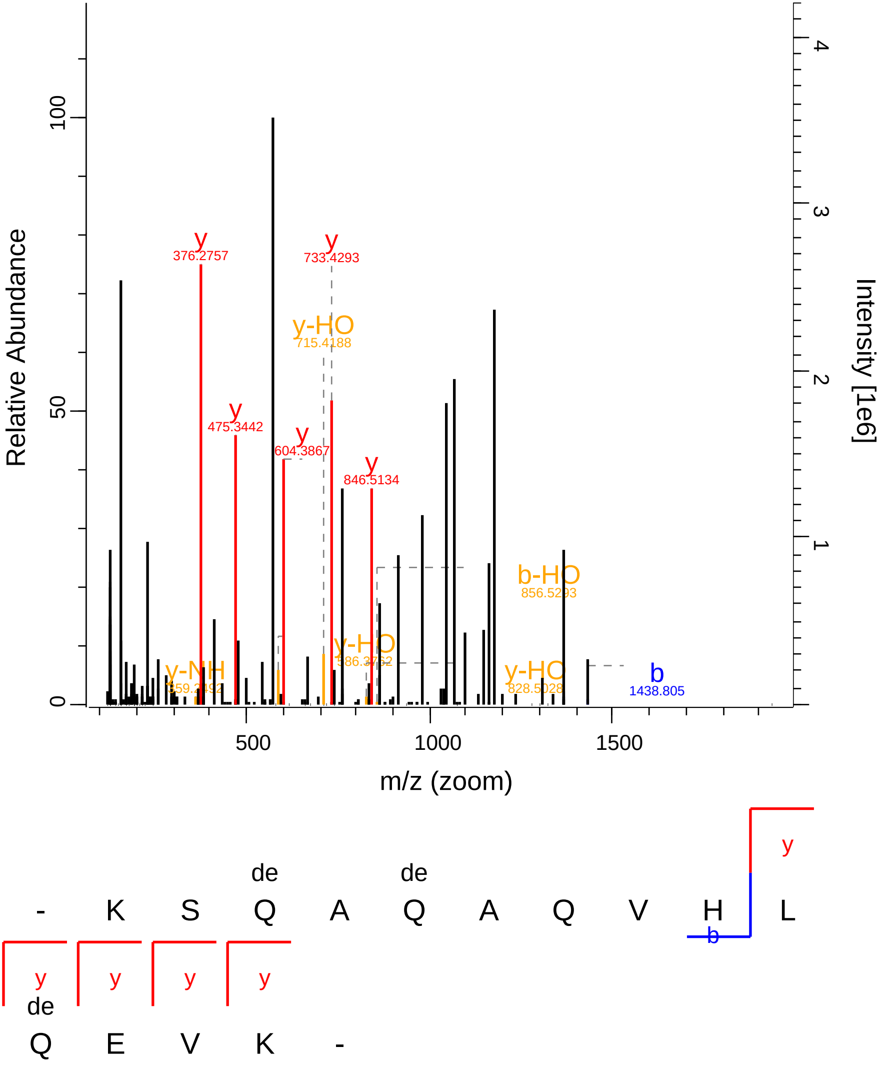

Raw File	Scan	Method	Score	m/z	Gene names
RO-QC-160121_ZackD_SS_Set_01_Fractio	38775	FTMS; HC	85.26	796.93	KLHDC9

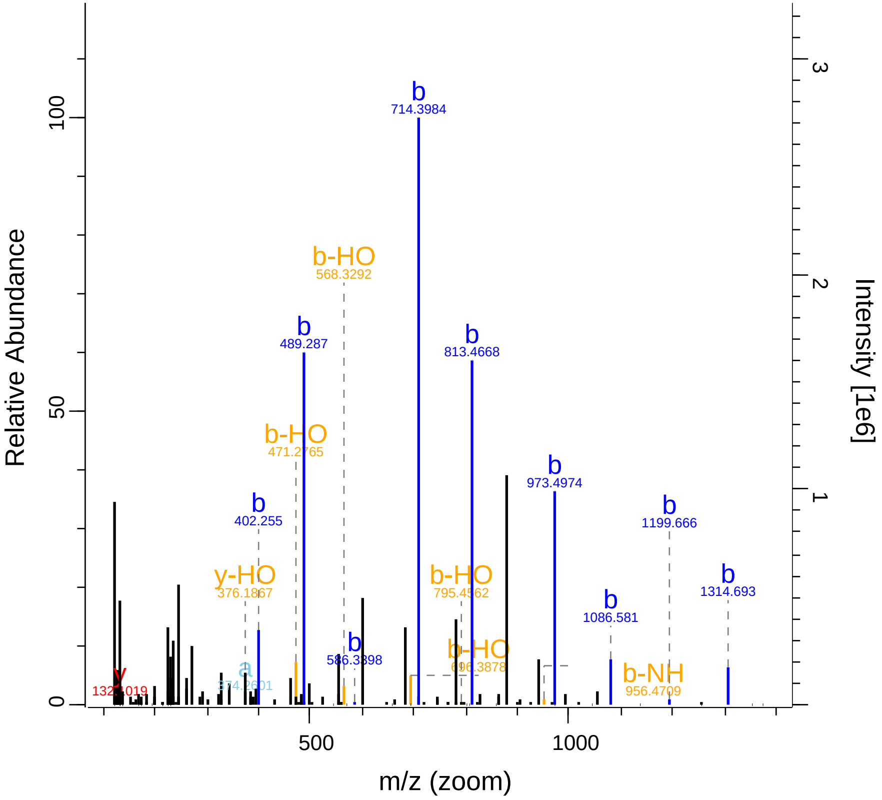

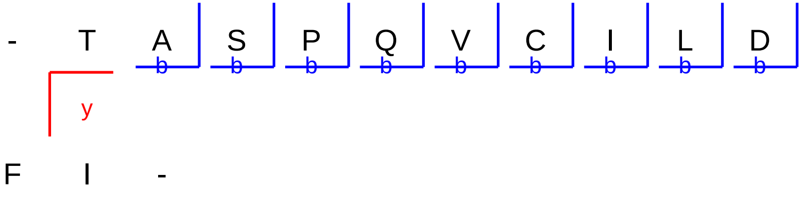

Raw File	Scan	Method	Score	m/z	Gene names
RO-QC-160121_ZackD_SS_Set_01_Fractio	38796	FTMS; HC	141.34	706.04	GTPBP8

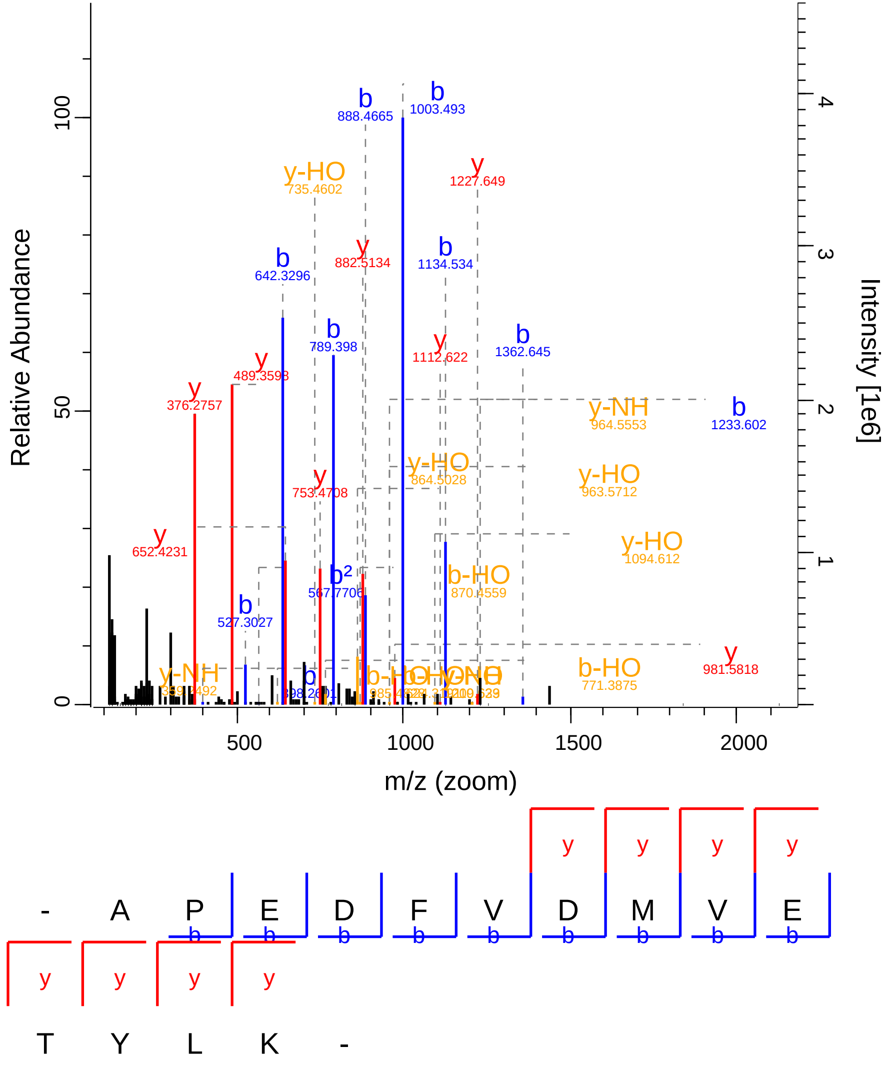

Raw File	Scan	Method	Score	m/z	Gene names
RO-QC-160121_ZackD_SS_Set_01_Fractio	39079	FTMS; HC	161.67	840.92	SENP8

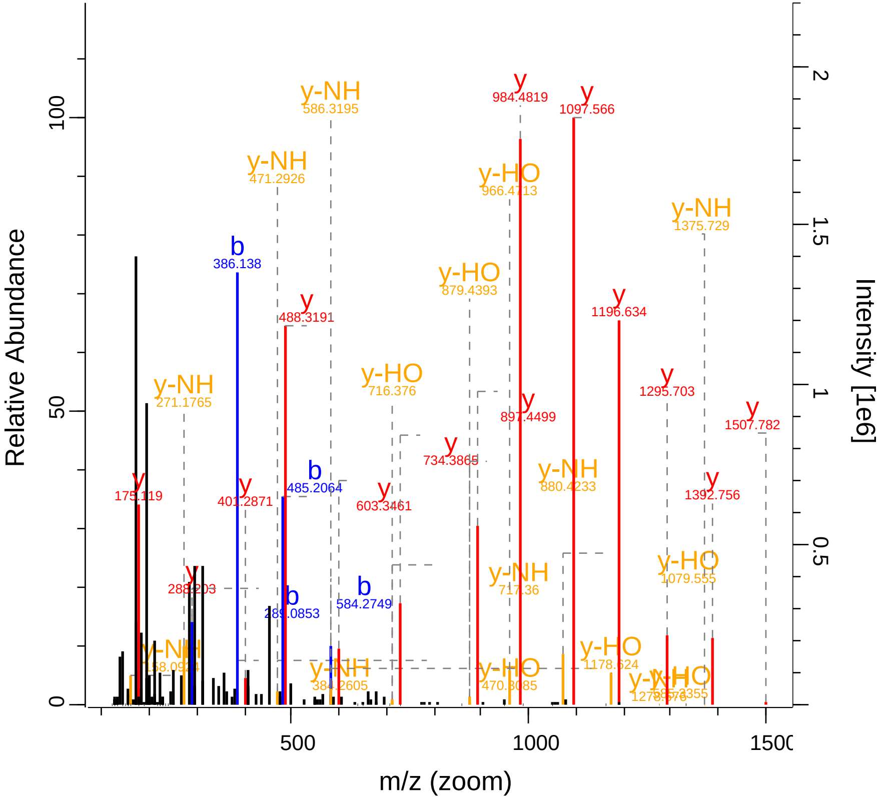

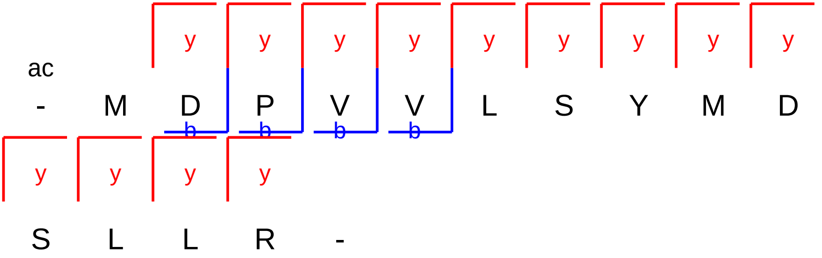

Raw File	Scan	Method	Score	m/z	Gene names
RO-QC-160121_ZackD_SS_Set_01_Fractior	4517	FTMS; HCI	84.05	507.95	METTL25

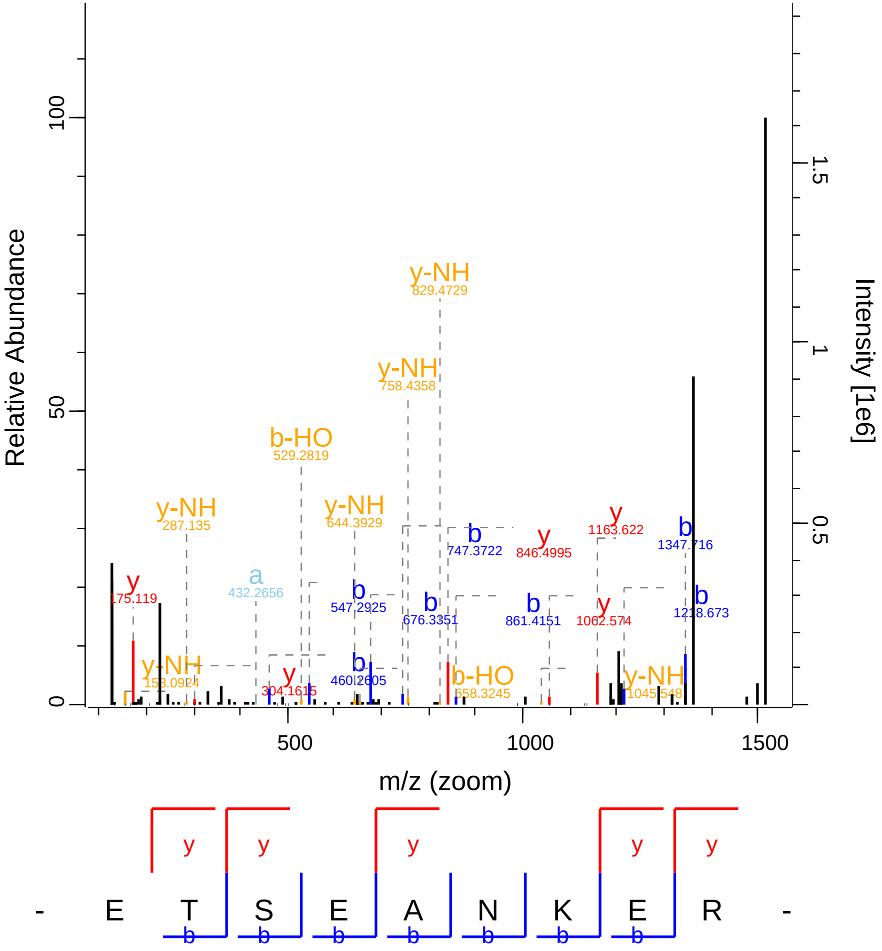

Raw File	Scan	Method	Score	m/z	Gene names
RO-QC-160121_ZackD_SS_Set_01_Fractior	4984	FTMS; HCI	57.14	638.97	SUV420H1

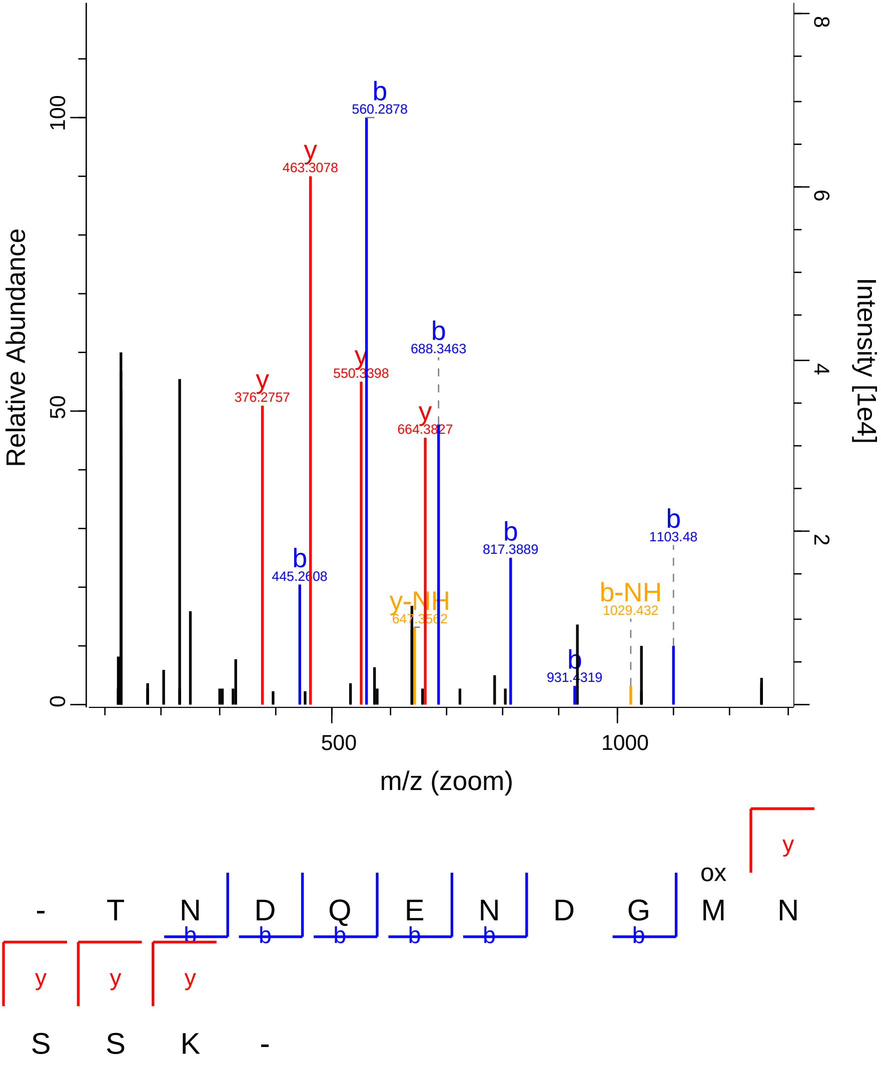

Raw File	Scan	Method	Score	m/z	Gene names
RO-QC-160121_ZackD_SS_Set_01_Fractio[illegible]	5172	FTMS; HC	150.94	854.73	ATXN7

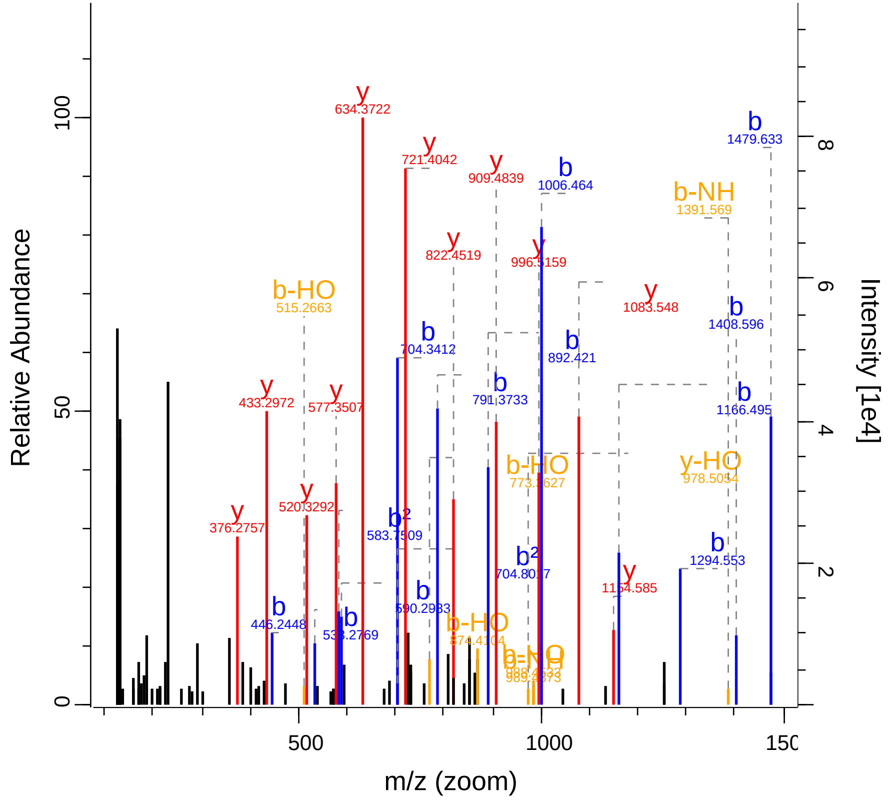

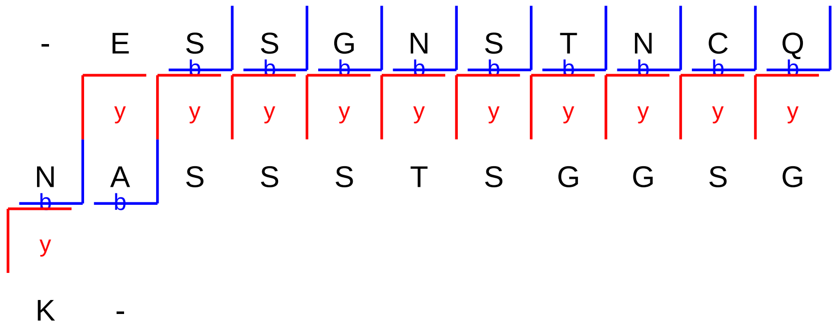

Raw File	Scan	Method	Score	m/z	Gene names
RO-QC-160121_ZackD_SS_Set_01_Fraction	5282	FTMS; HCI	107.37	573.3	CMTM3

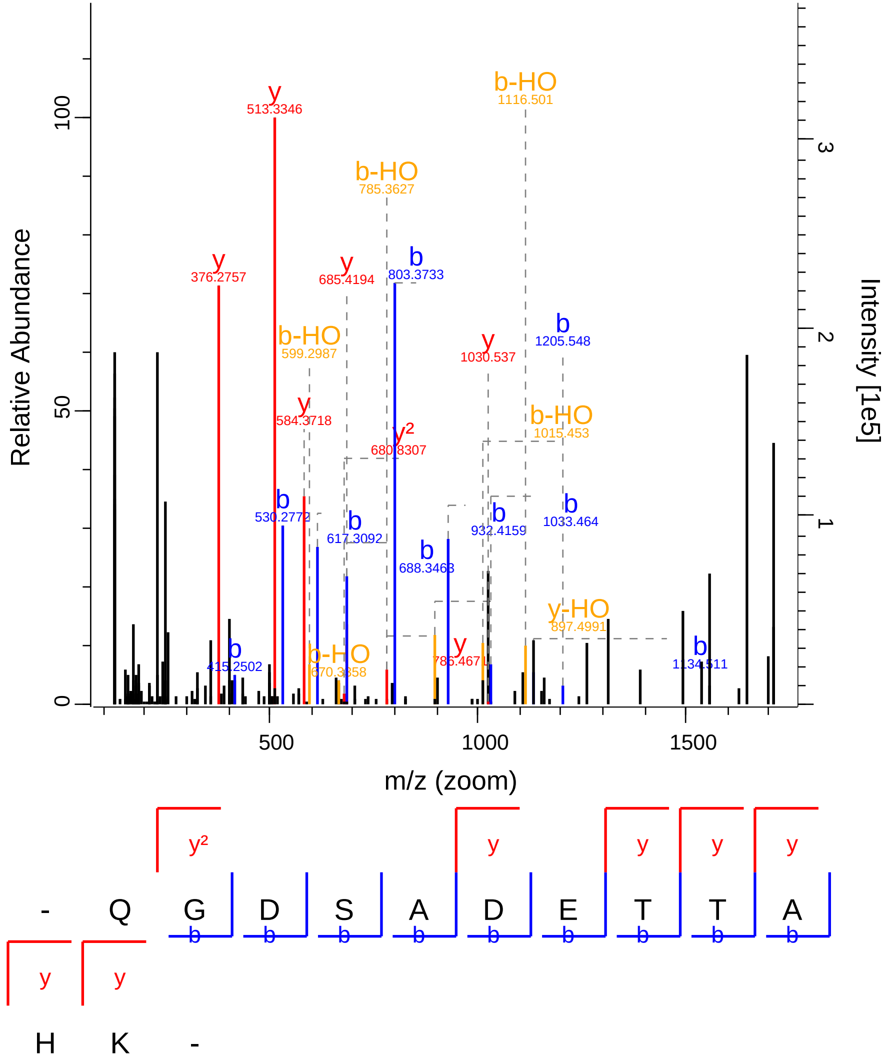

Raw File	Scan	Method	Score	m/z	Gene names
RO-QC-160121_ZackD_SS_Set_01_Fractior	5357	FTMS; HCI	70.93	683.83	LMBR1

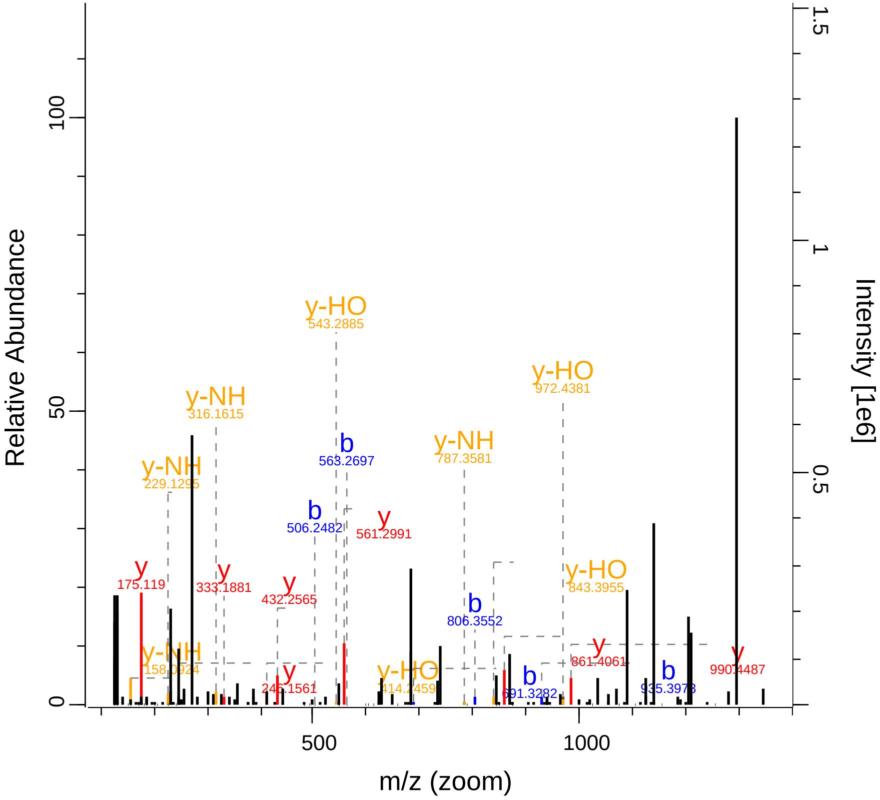

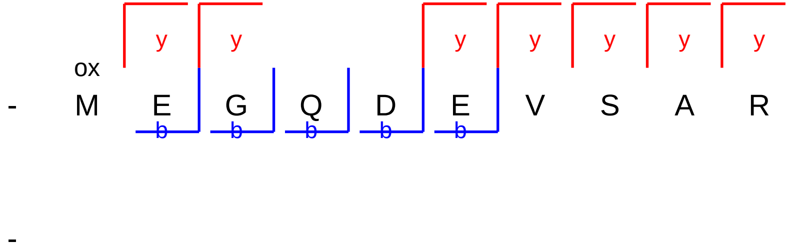

Raw File	Scan	Method	Score	m/z	Gene names
RO-QC-160121_ZackD_SS_Set_01_Fractior	5531	FTMS; HC	116.88	566.79	NXNL1

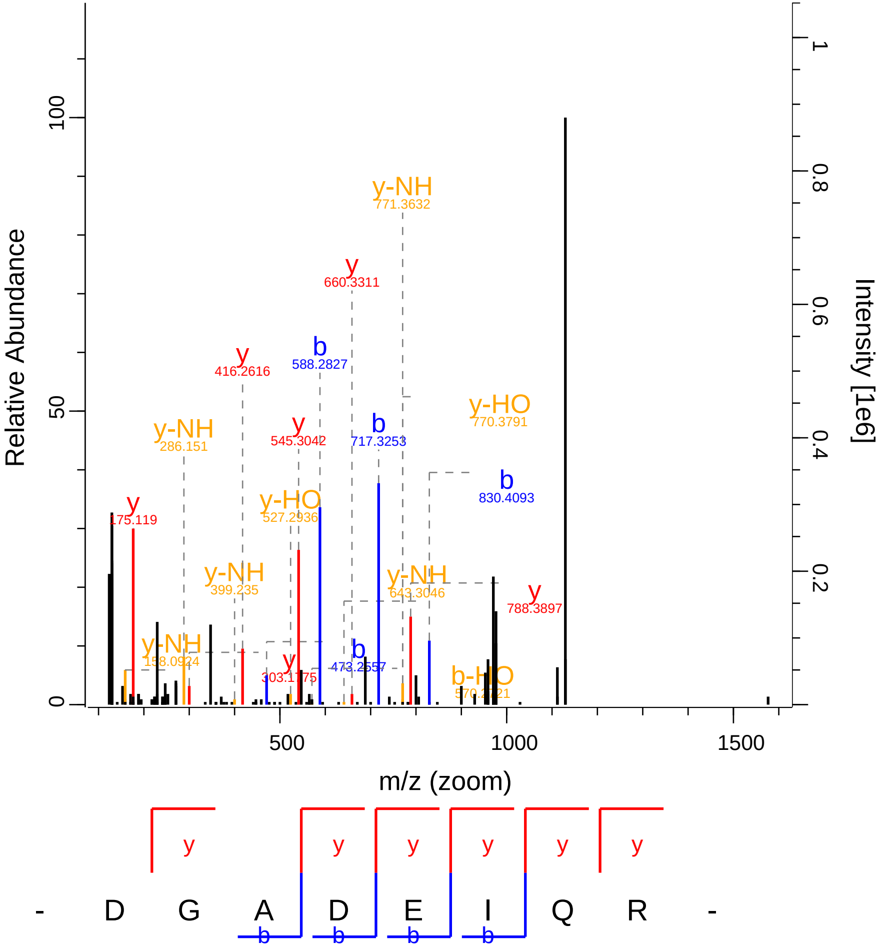

Raw File	Scan	Method	Score	m/z	Gene names
RO-QC-160121_ZackD_SS_Set_01_Fractior	6188	FTMS; HCI	56.65	672.34	ADAMTS3

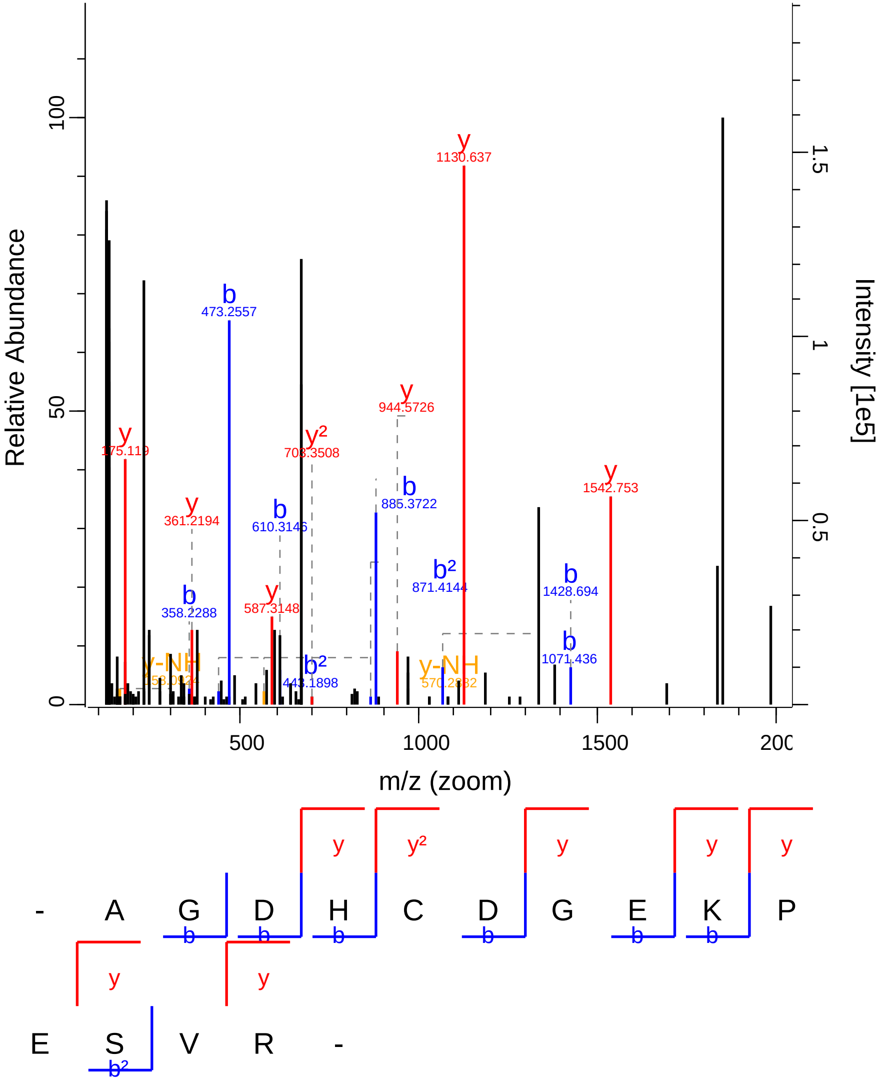

Raw File	Scan	Method	Score	m/z	Gene names
RO-QC-160121_ZackD_SS_Set_01_Fractior	6343	FTMS; HC	107.83	385.24	MGP

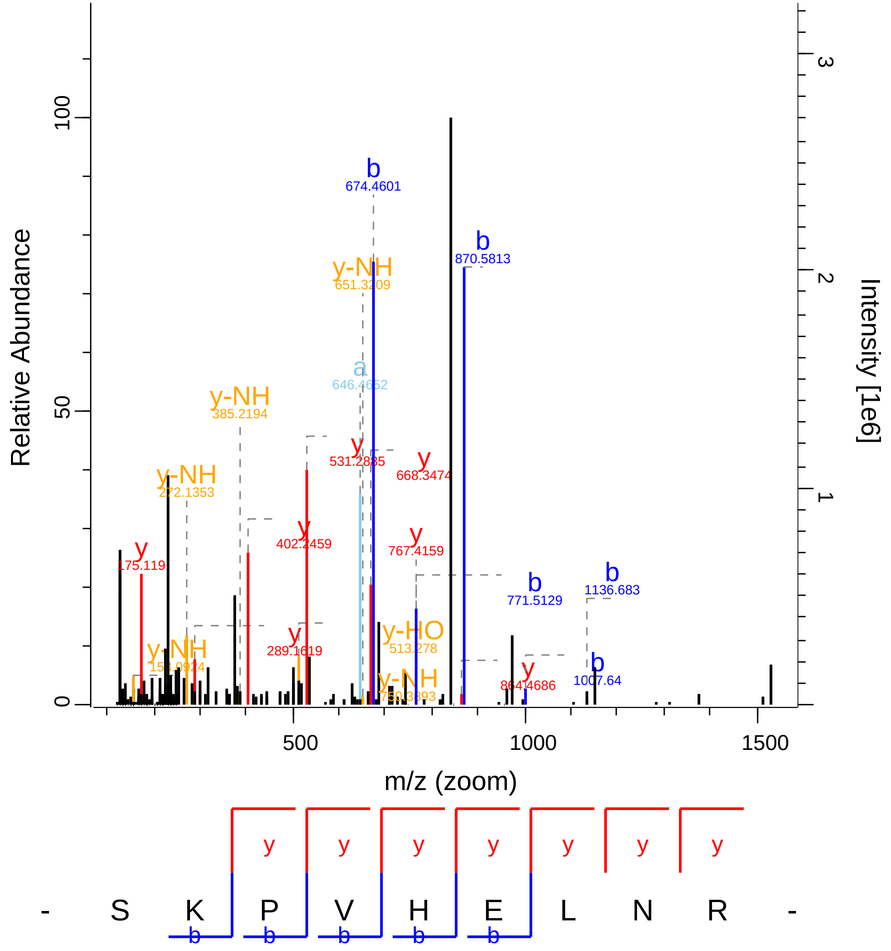

Raw File	Scan	Method	Score	m/z	Gene names
RO-QC-160121_ZackD_SS_Set_01_Fractior	6662	FTMS; HCI	67.11	551.66	HAND2

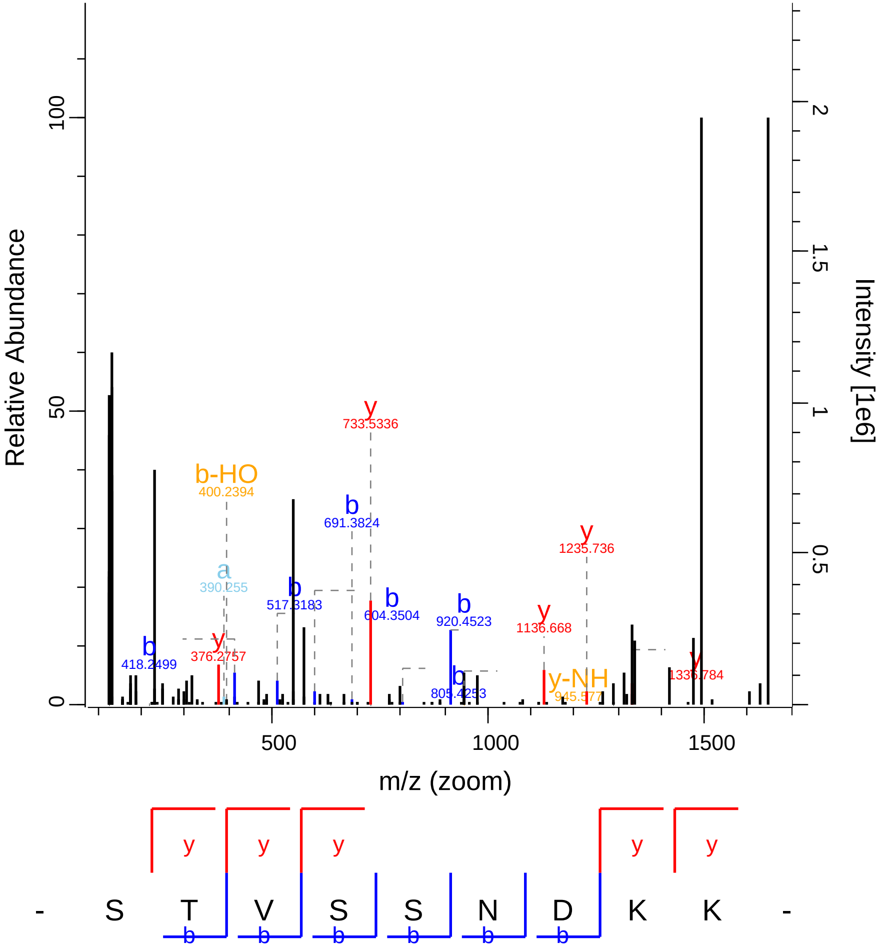

Raw File	Scan	Method	Score	m/z	Gene names
RO-QC-160121_ZackD_SS_Set_01_Fractio	6763	FTMS; HC	111.06	674.36	TIPIN

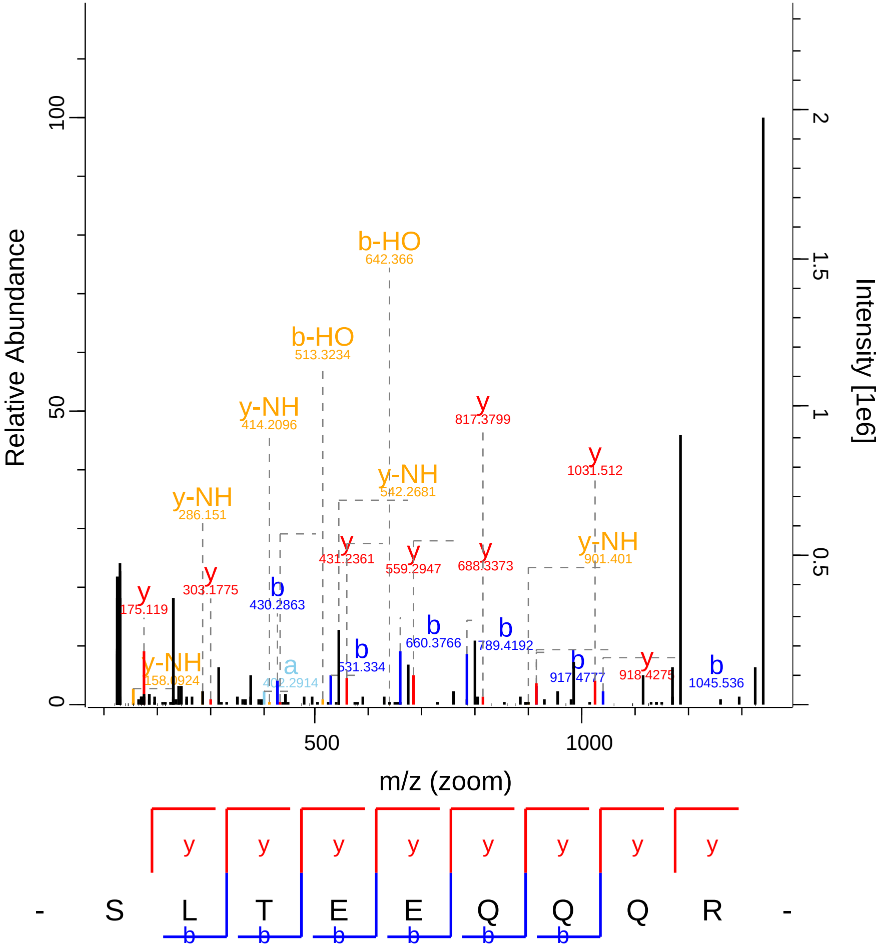

Raw File	Scan	Method	Score	m/z	Gene names
RO-QC-160121_ZackD_SS_Set_01_Fractior	6996	FTMS; HC	120.46	682.87	ING5

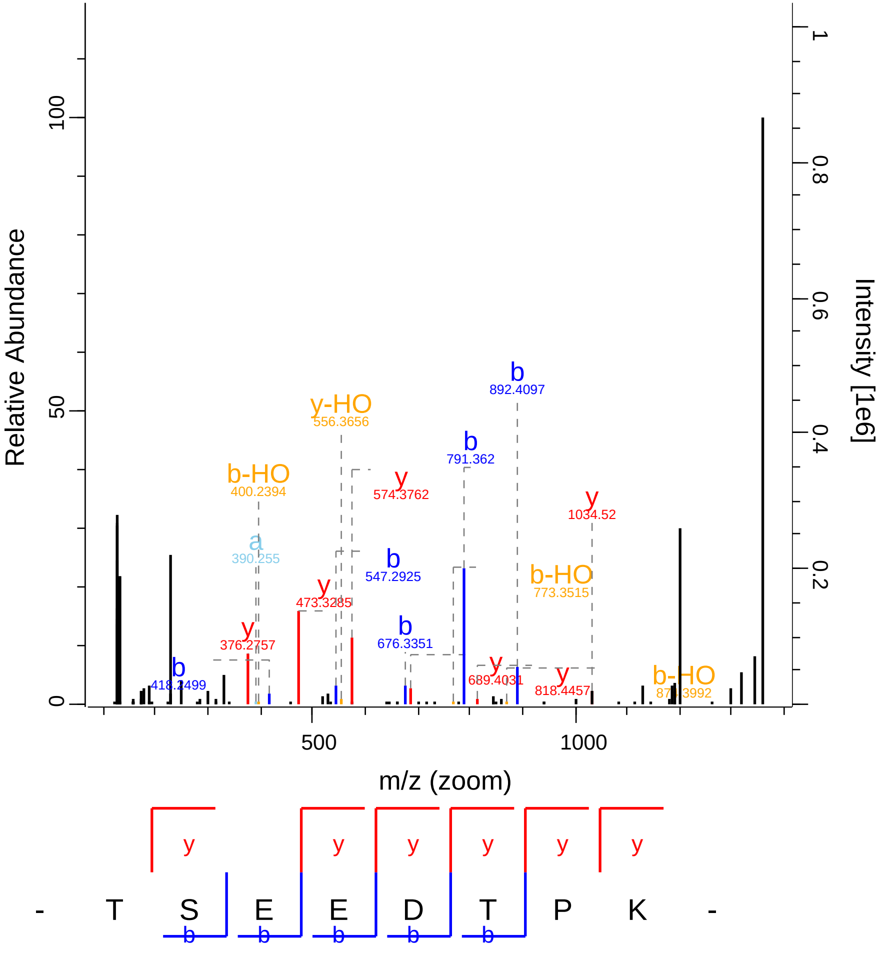

Raw File	Scan	Method	Score	m/z	Gene names
RO-QC-160121_ZackD_SS_Set_01_Fractio	7181	FTMS; HC	102.89	761.04	ADRA2B

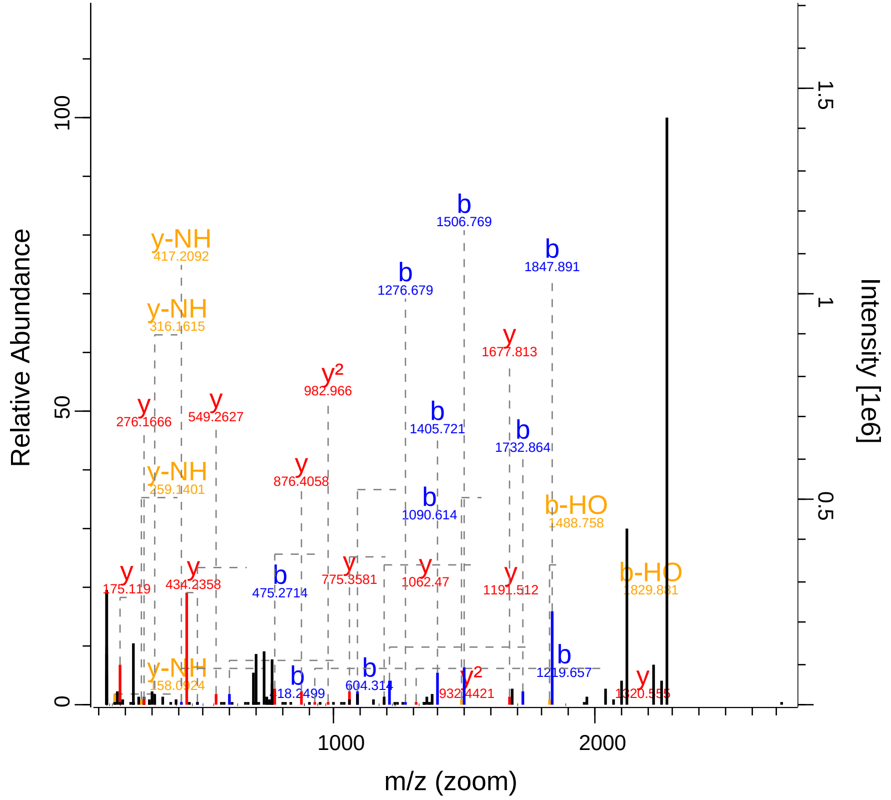

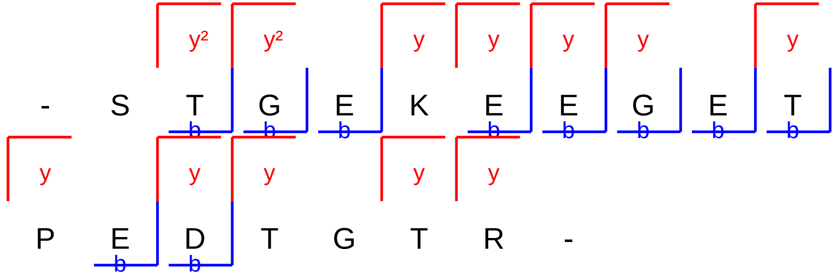

Raw File	Scan	Method	Score	m/z
RO-QC-160121_ZackD_SS_Set_01_Fraction_6	7241	FTMS; HCD	74.42	370.22

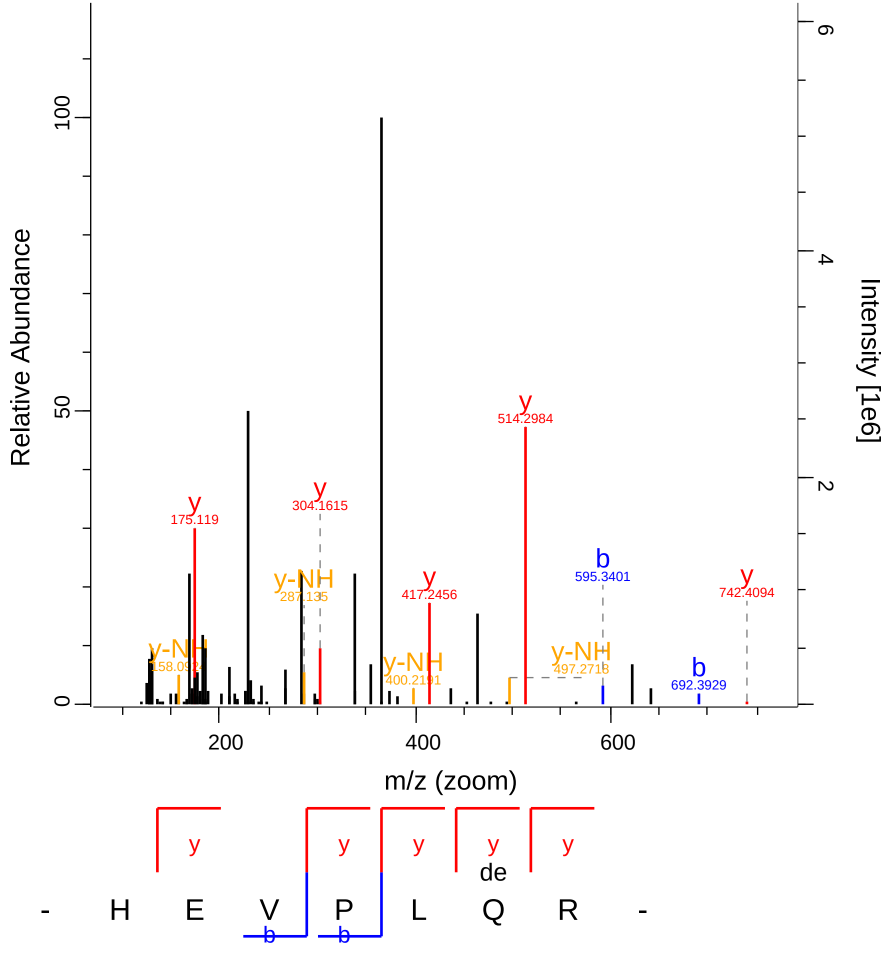

Raw File	Scan	Method	Score	m/z	Gene names
RO-QC-160121_ZackD_SS_Set_01_Fractior	7808	FTMS; HCI	45.88	433.75	KCNA10

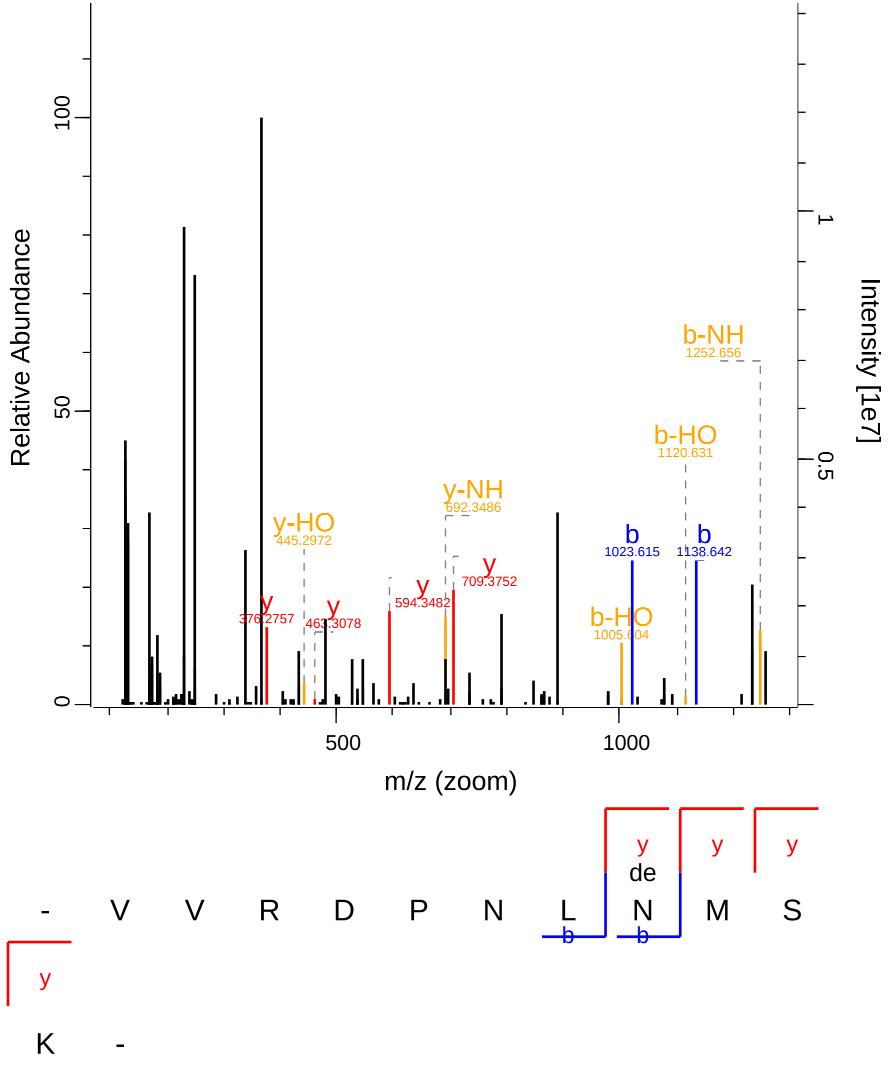

Raw File	Scan	Method	Score	m/z	Gene names
RO-QC-160121_ZackD_SS_Set_01_Fraction	7926	FTMS; HCl	142.07	768.4	PLEKHH2

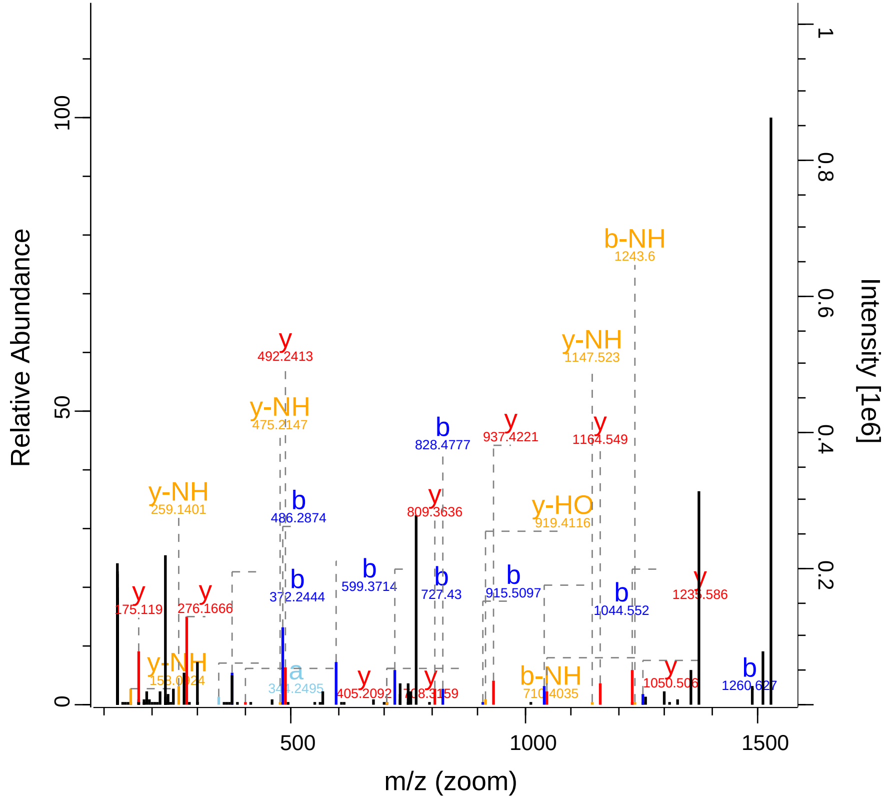

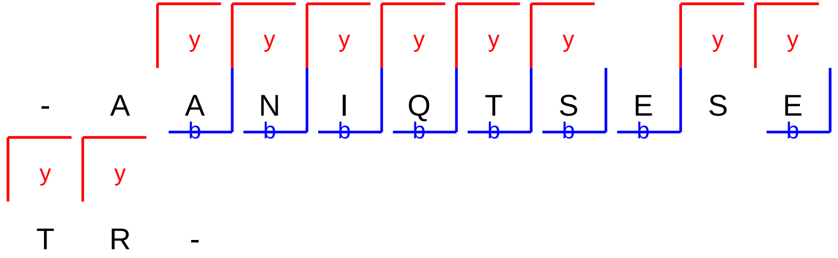

Raw File	Scan	Method	Score	m/z	Gene names
RO-QC-160121_ZackD_SS_Set_01_Fractior	8102	FTMS; HCI	95.5	780.92	INSIG2

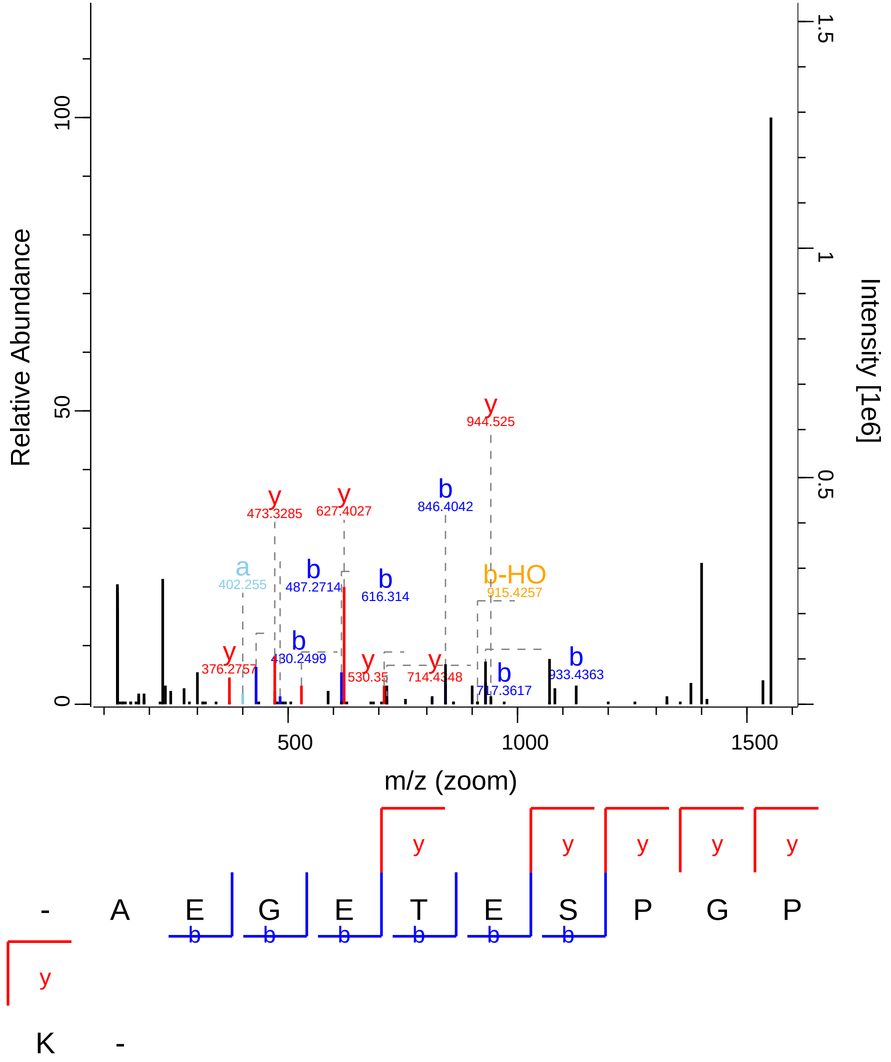

Raw File	Scan	Method	Score	m/z	Gene names
RO-QC-160121_ZackD_SS_Set_01_Fractio	8820	FTMS; HC	139.31	637.39	G6PC3

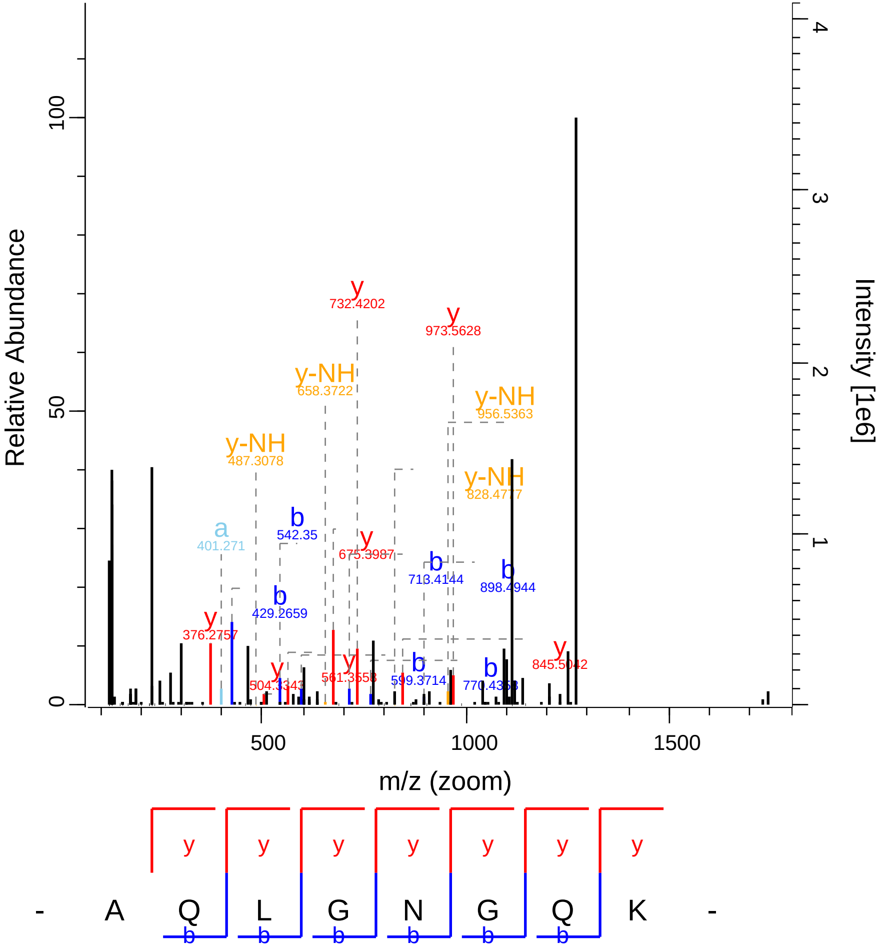

Raw File	Scan	Method	Score	m/z	Gene names
RO-QC-160121_ZackD_SS_Set_01_Fractior	8994	FTMS; HCI	90.05	567.34	SLC35C1

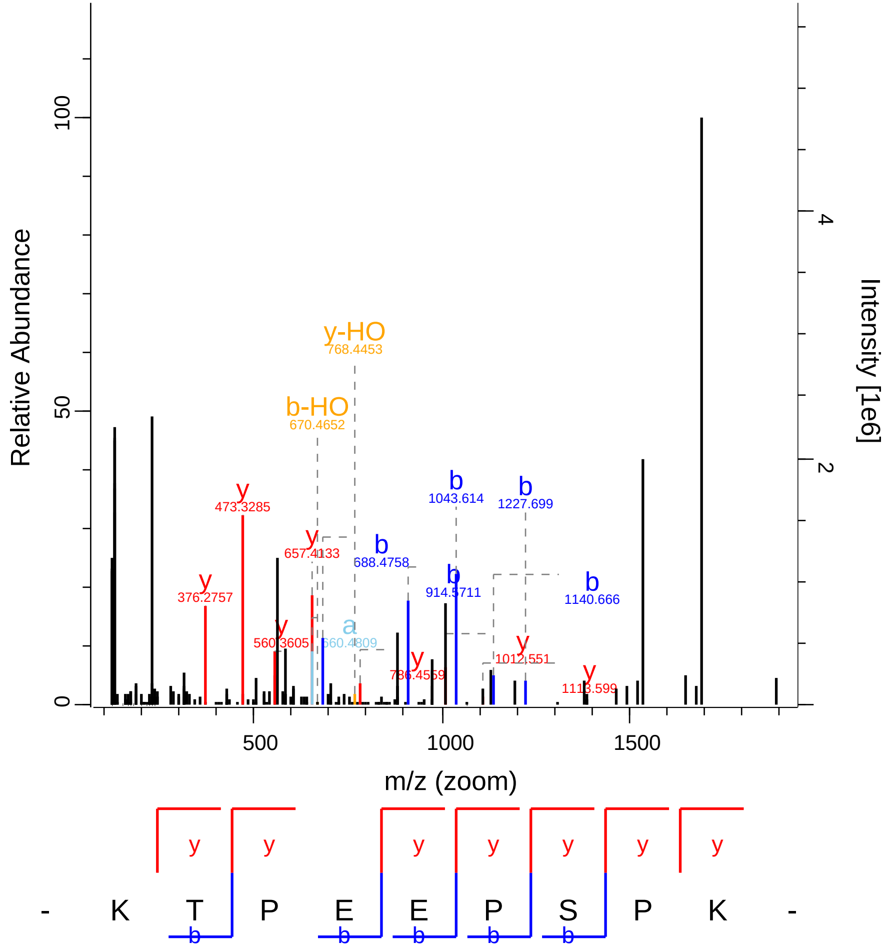

Raw File	Scan	Method	Score	m/z	Gene names
RO-QC-160121_ZackD_SS_Set_01_Fractior	9177	FTMS; HCI	79.12	428.27	AZIN2

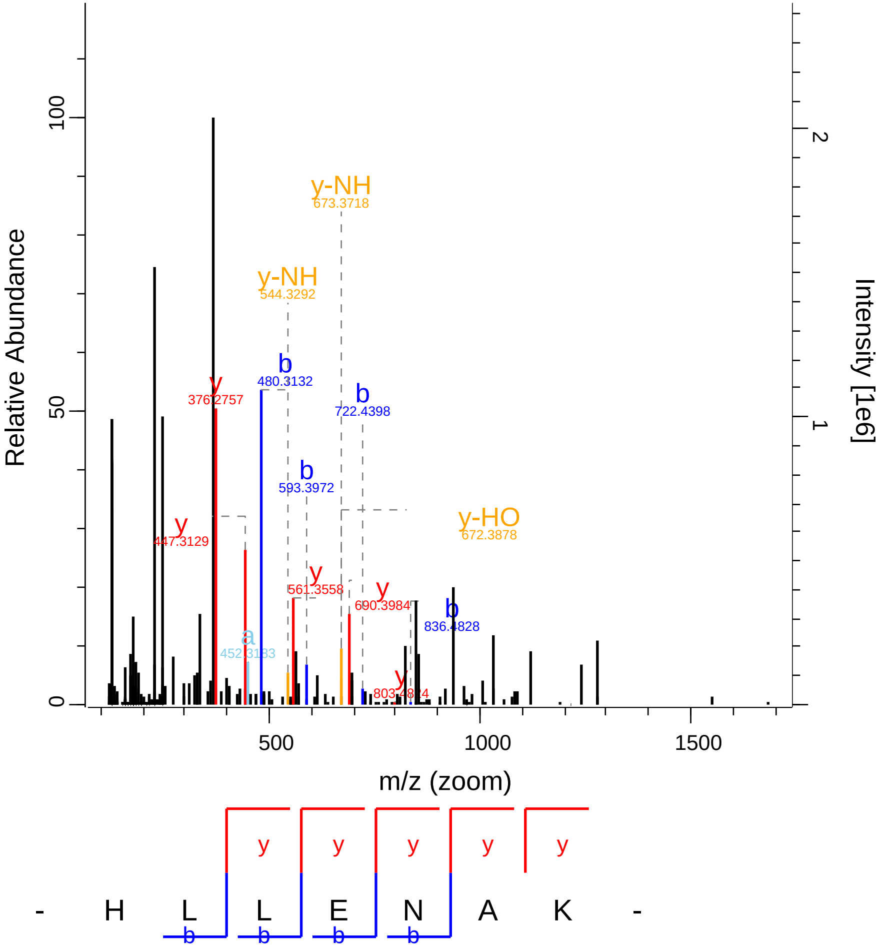

Raw File	Scan	Method	Score	m/z
RO-QC-160121_ZackD_SS_Set_01_Fraction_6	9732	FTMS; HCD	44.61	423.59

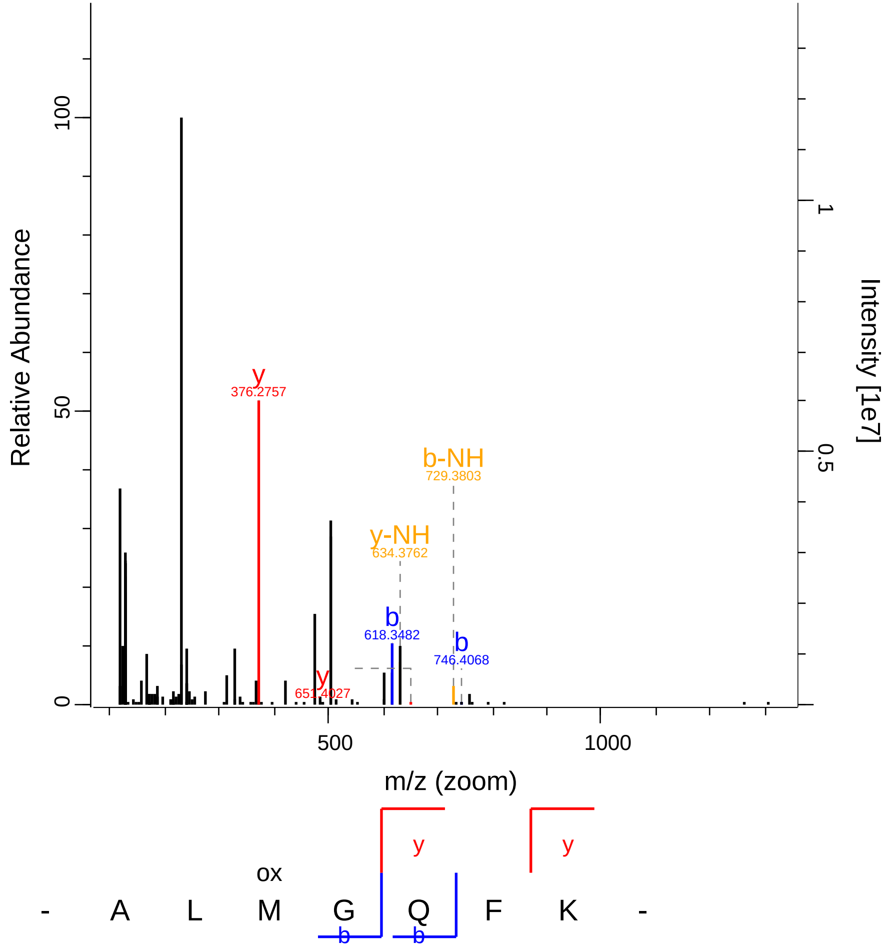

Raw File	Scan	Method	Score	m/z	Gene names
RO-QC-160121_ZackD_SS_Set_01_Fractio	10043	FTMS; HC	53.71	685.85	CRIPT

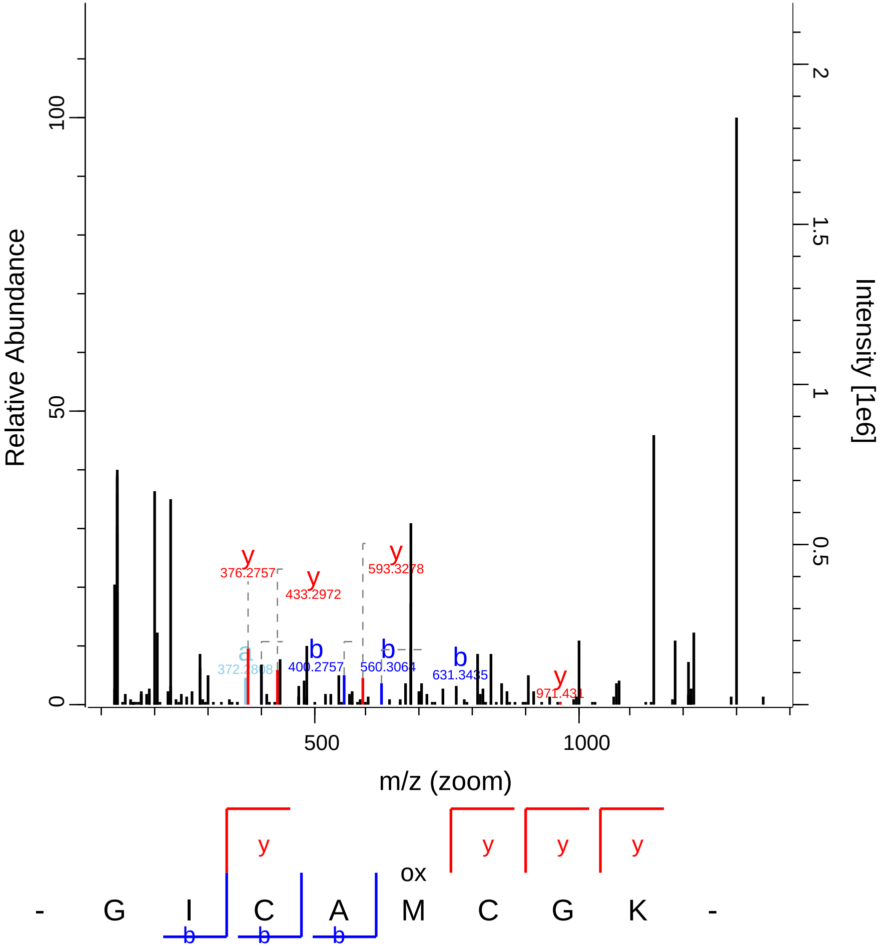

Raw File	Scan	Method	Score	m/z	Gene names
RO-QC-160121_ZackD_SS_Set_01_Fractio	10049	FTMS; HC	179.42	680.38	ANKRD16

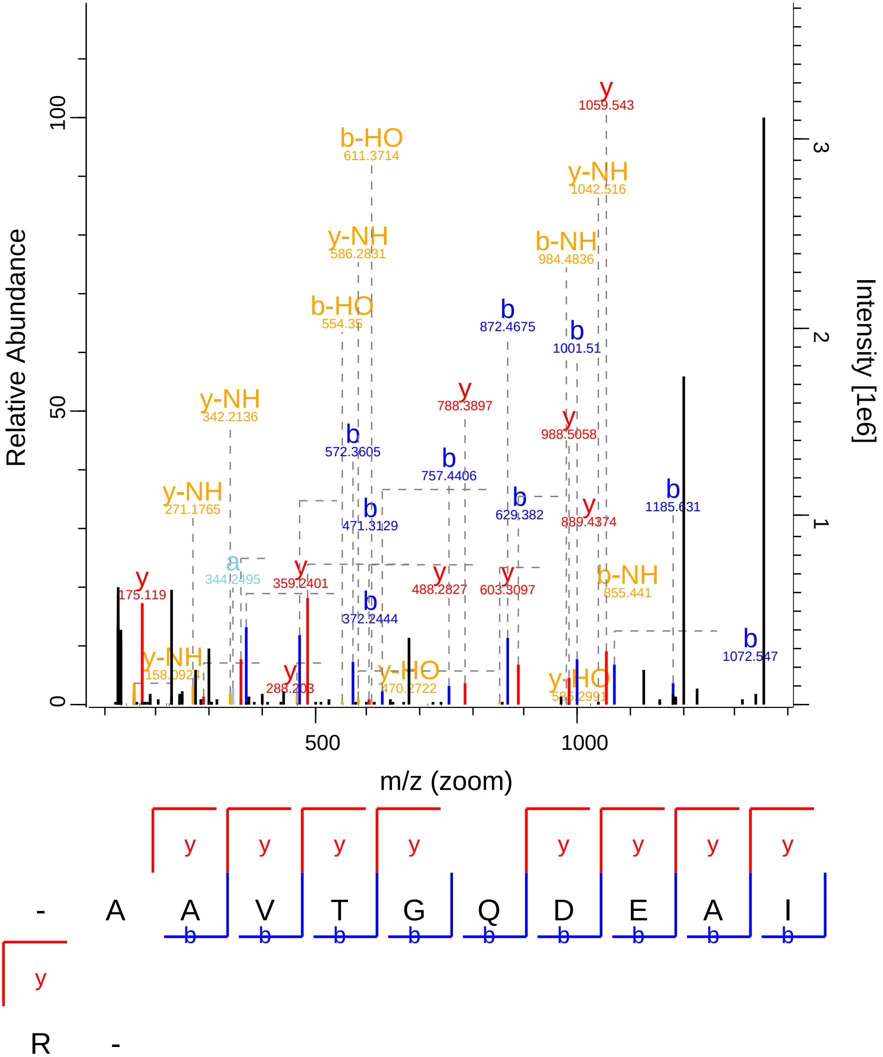

Raw File	Scan	Method	Score	m/z	Gene names
RO-QC-160121_ZackD_SS_Set_01_Fractio	10823	FTMS; HC	80.61	721.68	C16orf70

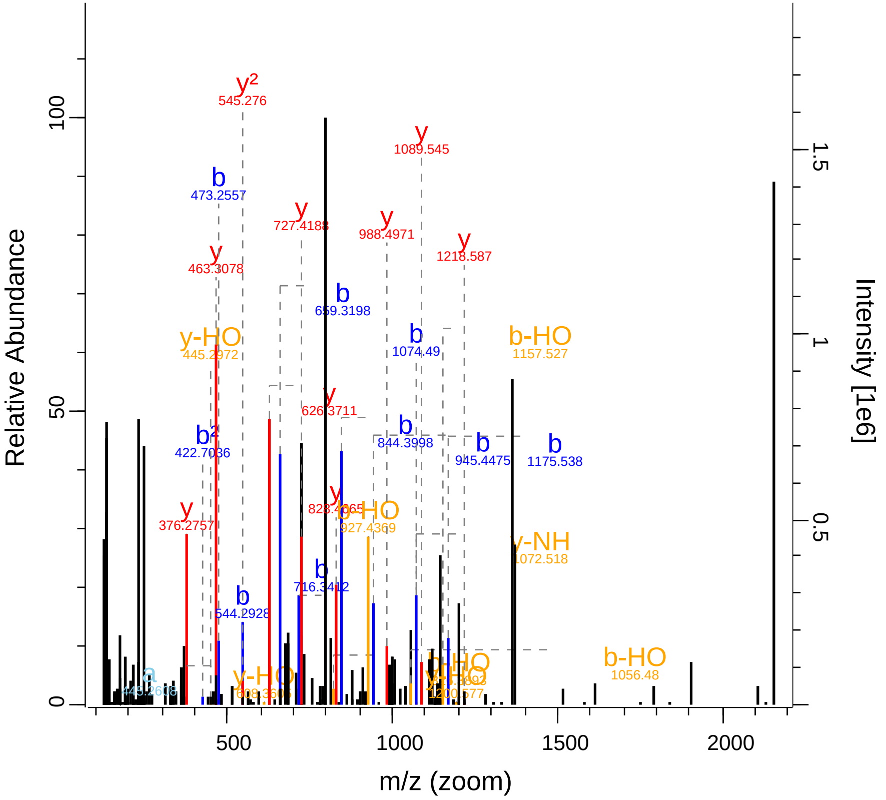

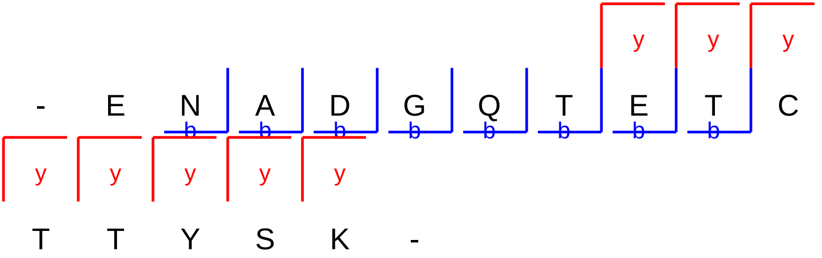

Raw File	Scan	Method	Score	m/z	Gene names
RO-QC-160121_ZackD_SS_Set_01_Fractio	11033	FTMS; HCI	105.39	356.9	CENPA

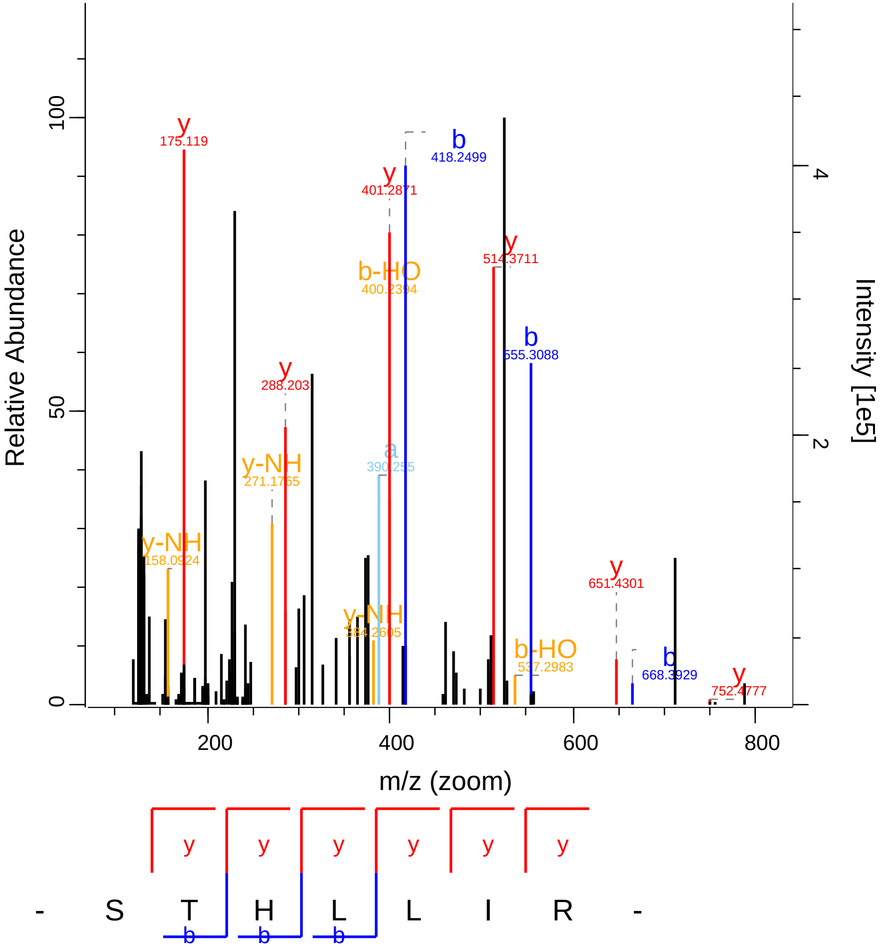

Raw File	Scan	Method	Score	m/z
RO-QC-160121_ZackD_SS_Set_01_Fraction_6	11374	FTMS; HCD	66.68	449.28

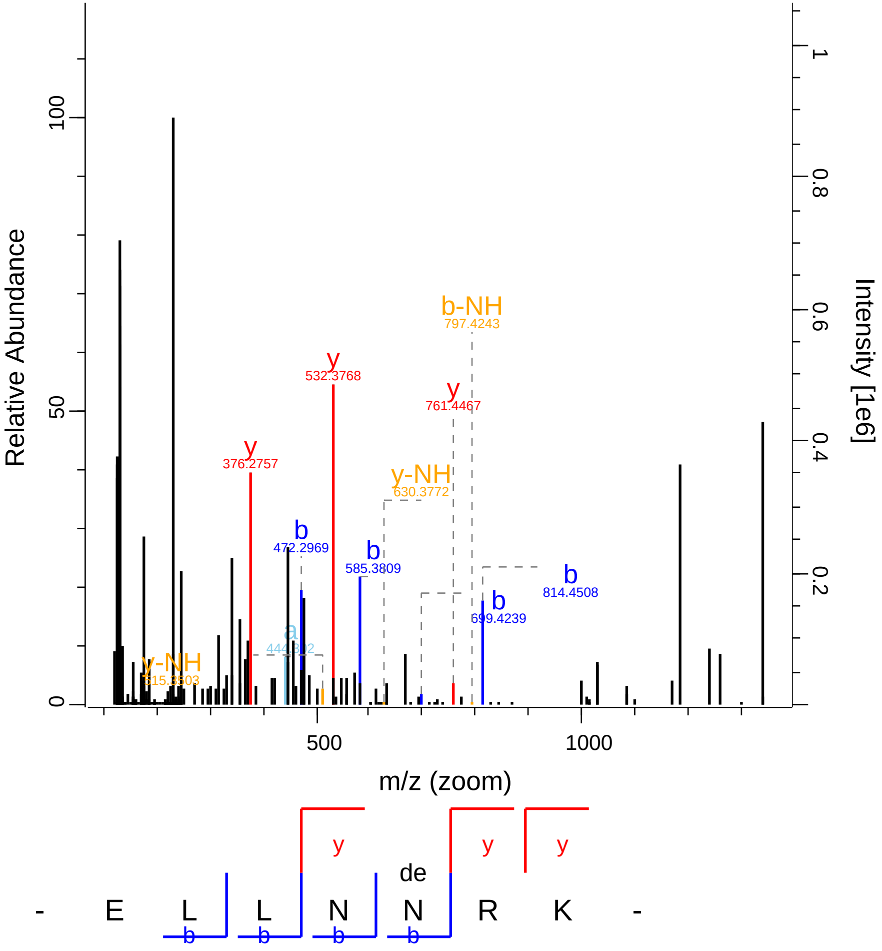

Raw File	Scan	Method	Score	m/z	Gene names
RO-QC-160121_ZackD_SS_Set_01_Fra	11549	FTMS; HC	81.12	596.35	ATP5G1;ATP5G2;ATP

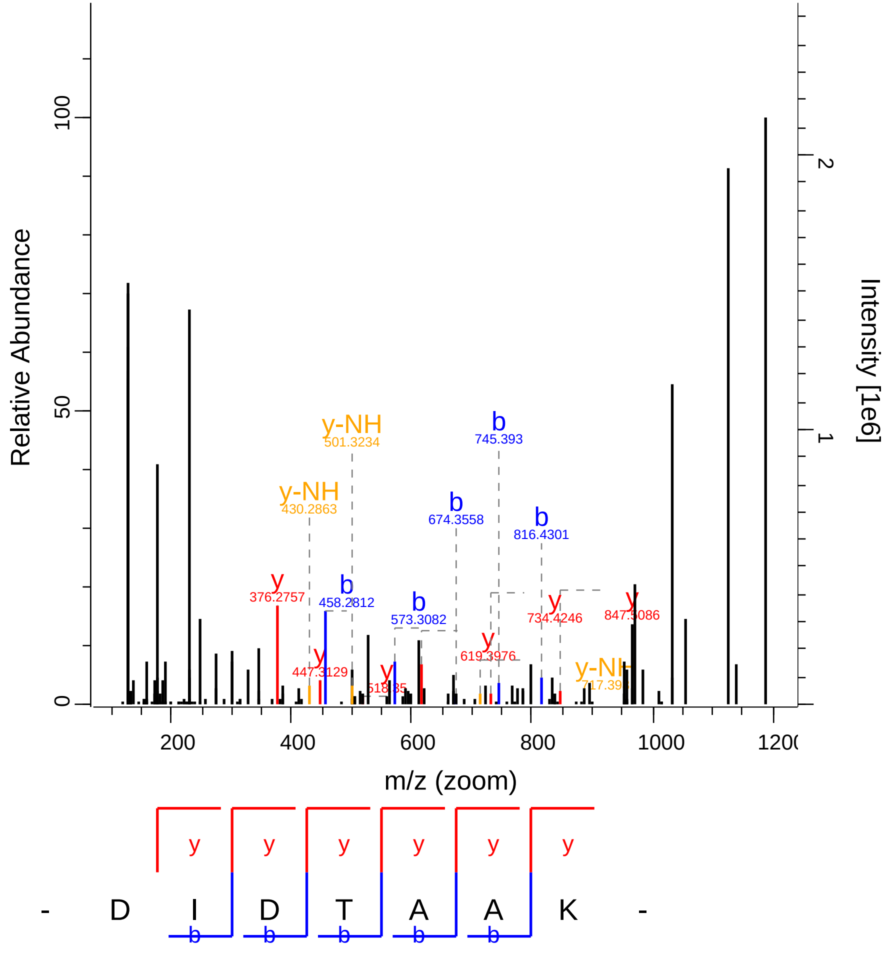

Raw File	Scan	Method	Score	m/z	Gene names
RO-QC-160121_ZackD_SS_Set_01_Fractio	12398	FTMS; HC	96.41	718.35	R3HCC1L

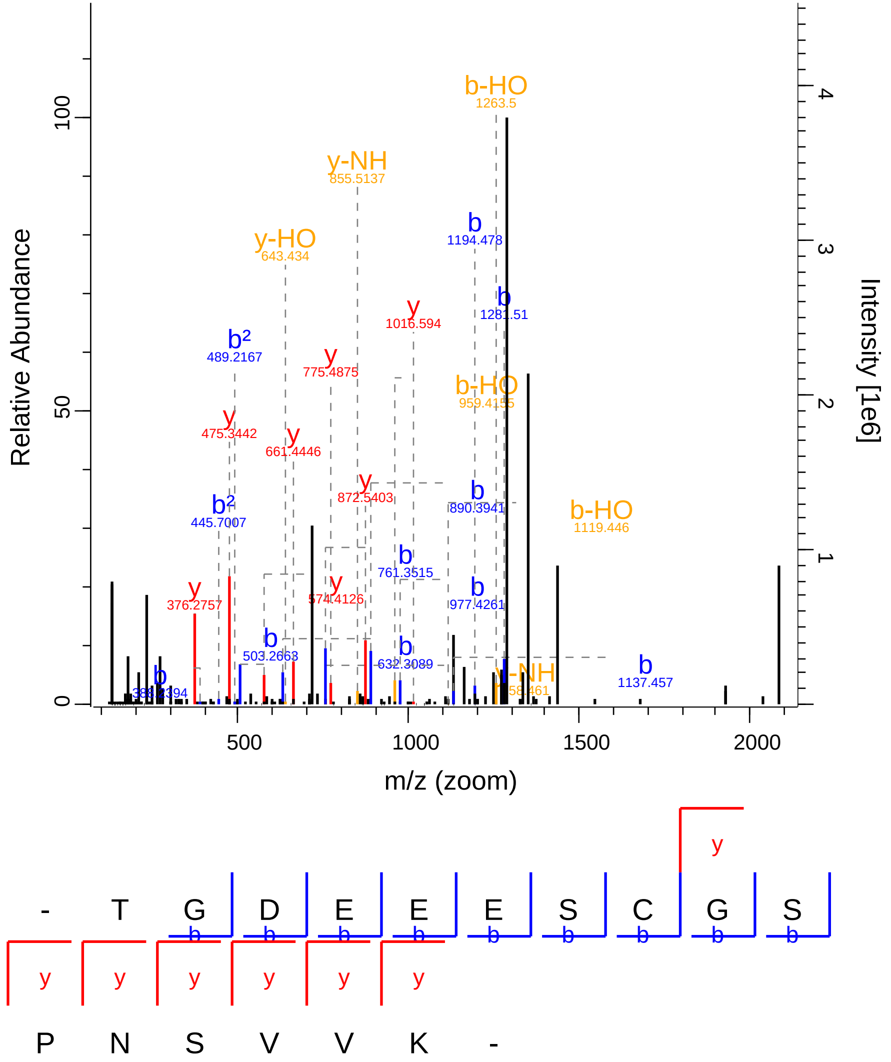

Raw File	Scan	Method	Score	m/z	Gene names
RO-QC-160121_ZackD_SS_Set_01_Fractior	12572	FTMS; HCI	96.25	379.9	FRMD4B

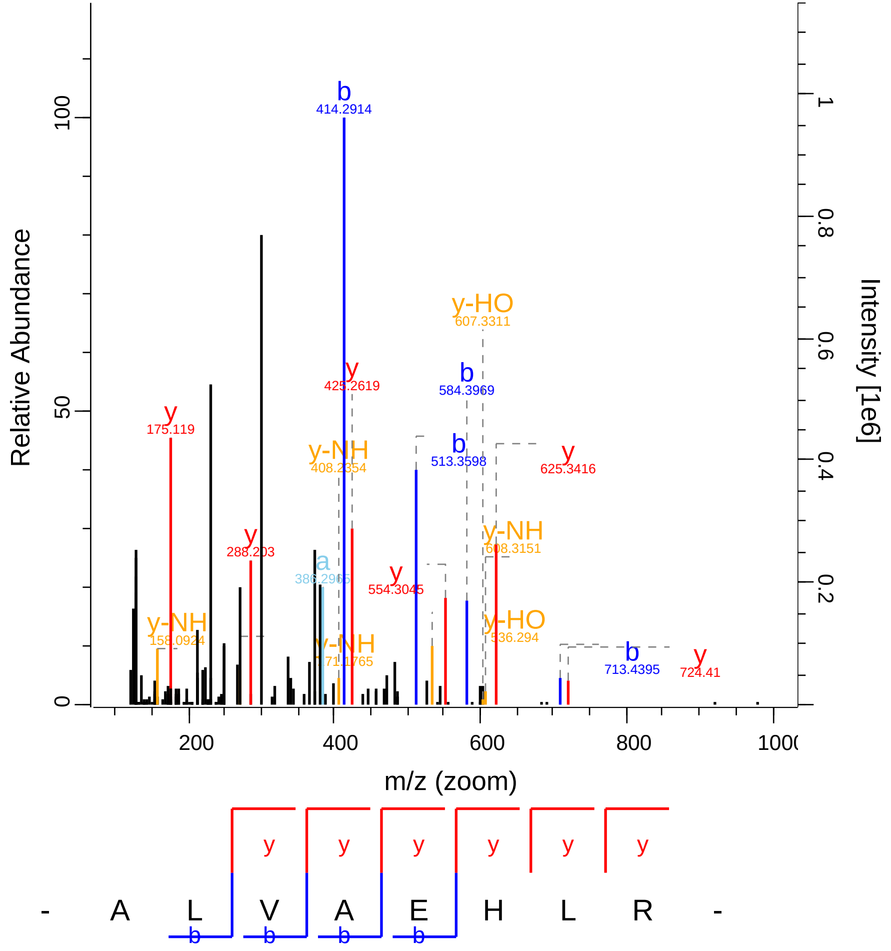

Raw File	Scan	Method	Score	m/z	Gene names
RO-QC-160121_ZackD_SS_Set_01_Fractio	13029	FTMS; HC	74.46	403.74	CCDC146

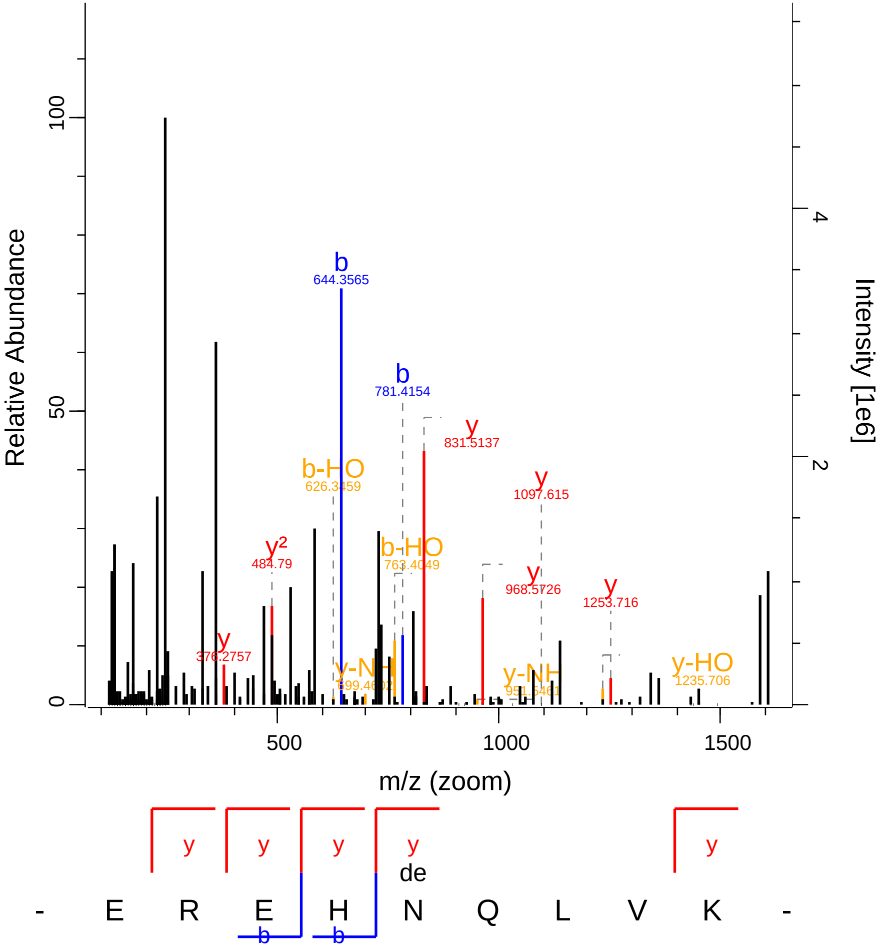

Raw File	Scan	Method	Score	m/z	Gene names
RO-QC-160121_ZackD_SS_Set_01_Fractio	13243	FTMS; HC	97.74	714.86	FRAS1

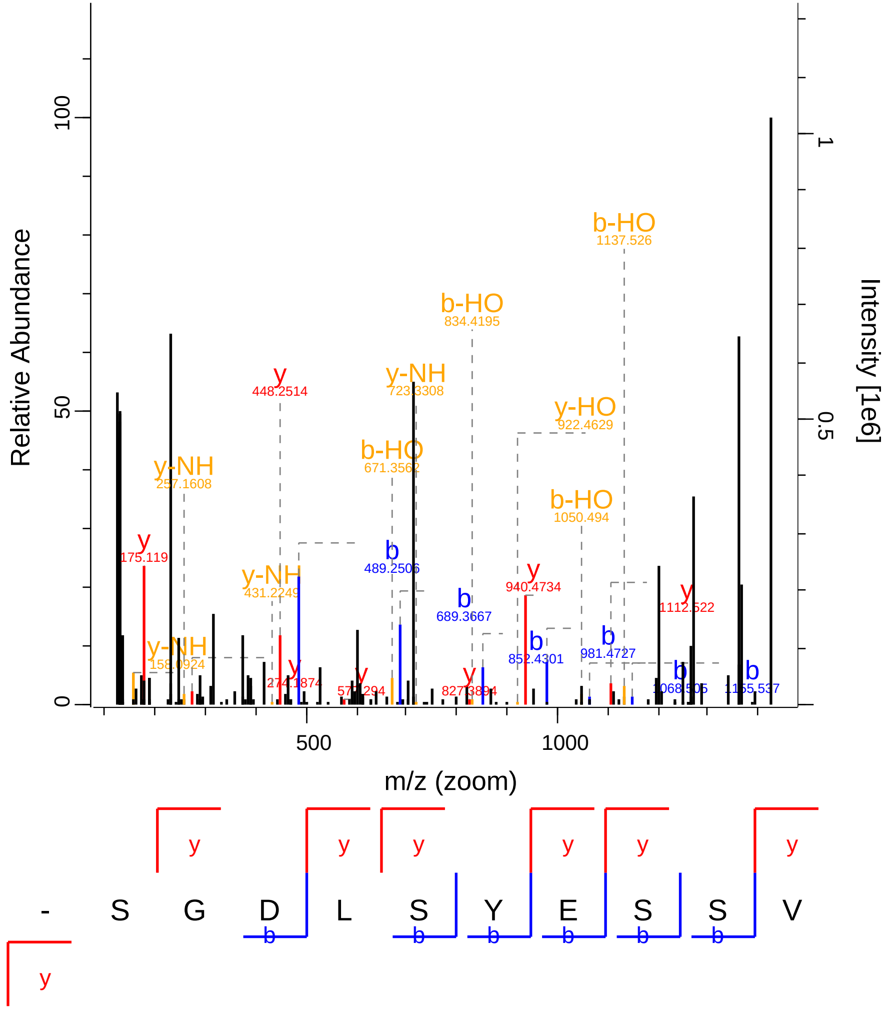

Raw File	Scan	Method	Score	m/z	Gene names
RO-QC-160121_ZackD_SS_Set_01_Fractio	13733	FTMS; HC	99.28	569.36	RIC8B

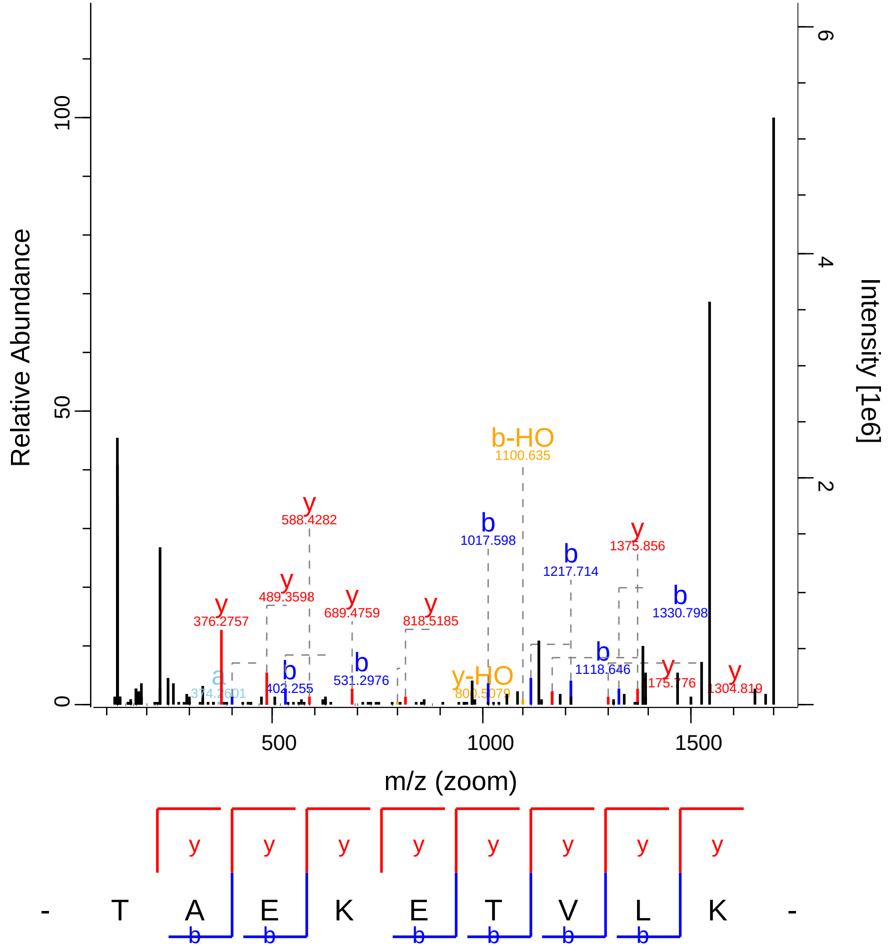

Raw File	Scan	Method	Score	m/z	Gene names
RO-QC-160121_ZackD_SS_Set_01_Fractio	14924	FTMS; HC	121.93	763.88	FCF1

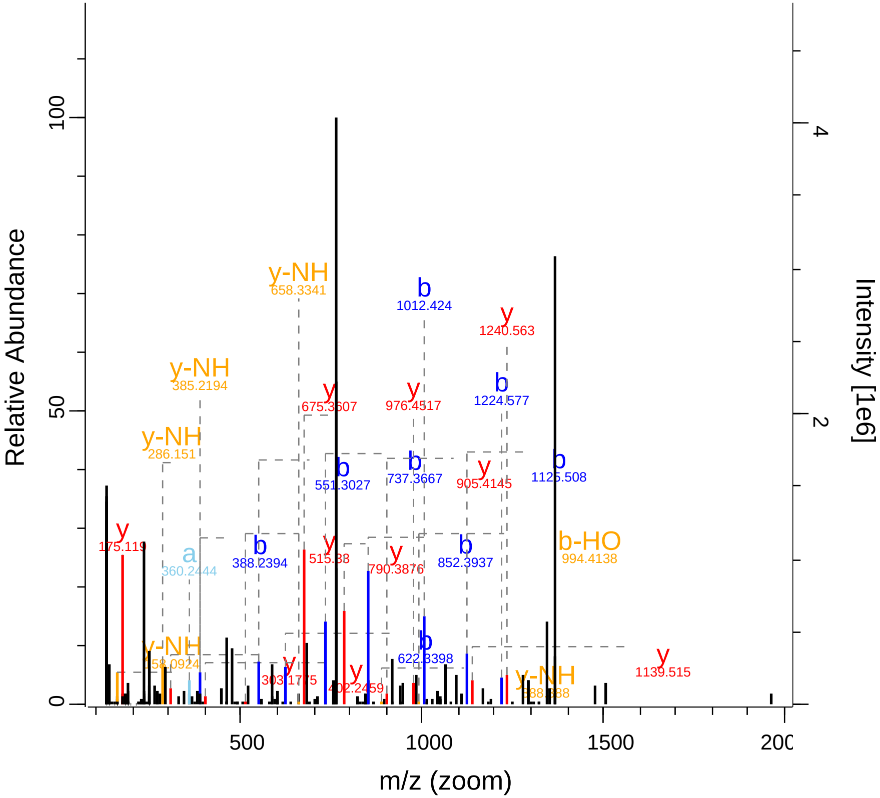

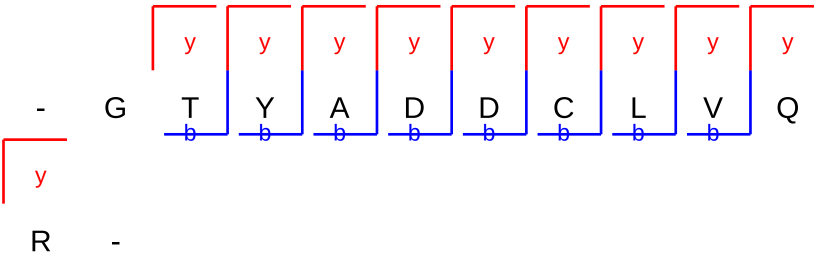

Raw File	Scan	Method	Score	m/z	Gene names
RO-QC-160121_ZackD_SS_Set_01_Fractio	15160	FTMS; HC	40.02	466.51	GPR124

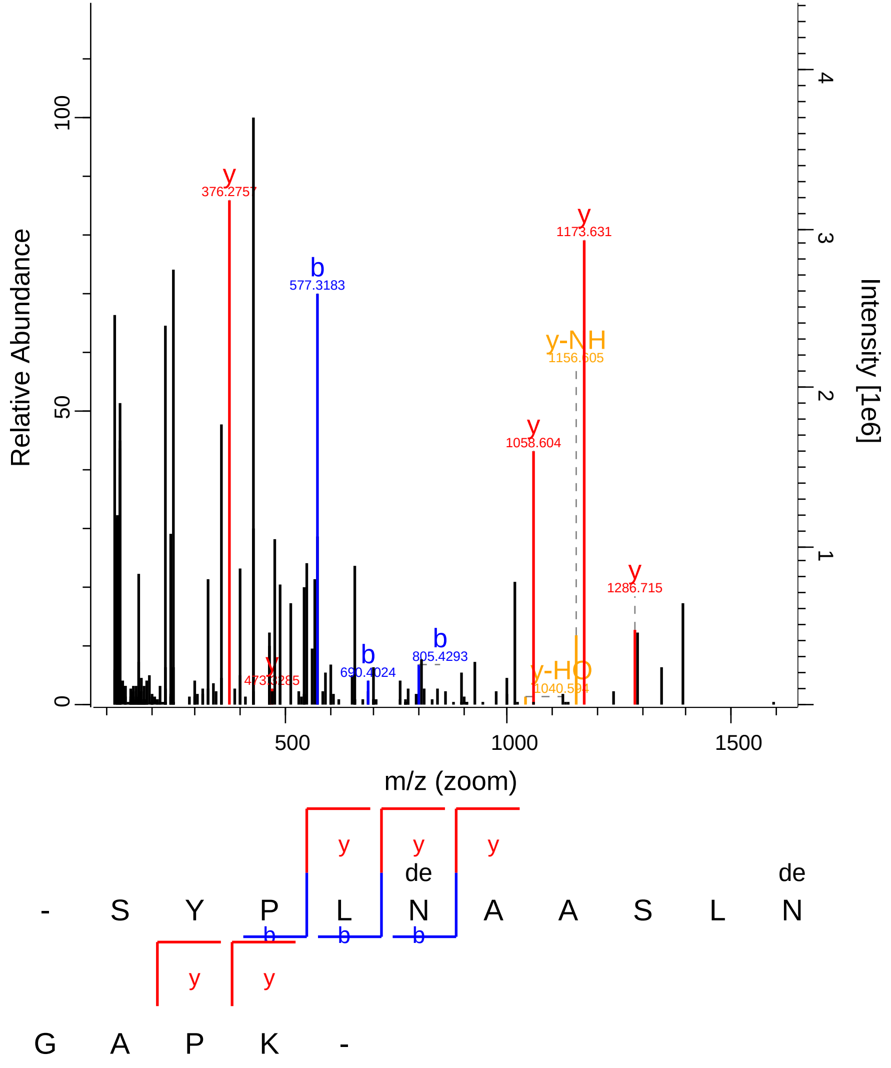

Raw File	Scan	Method	Score	m/z	Gene names
RO-QC-160121_ZackD_SS_Set_01_Fractio	15470	FTMS; HC	103.69	441.94	DALRD3

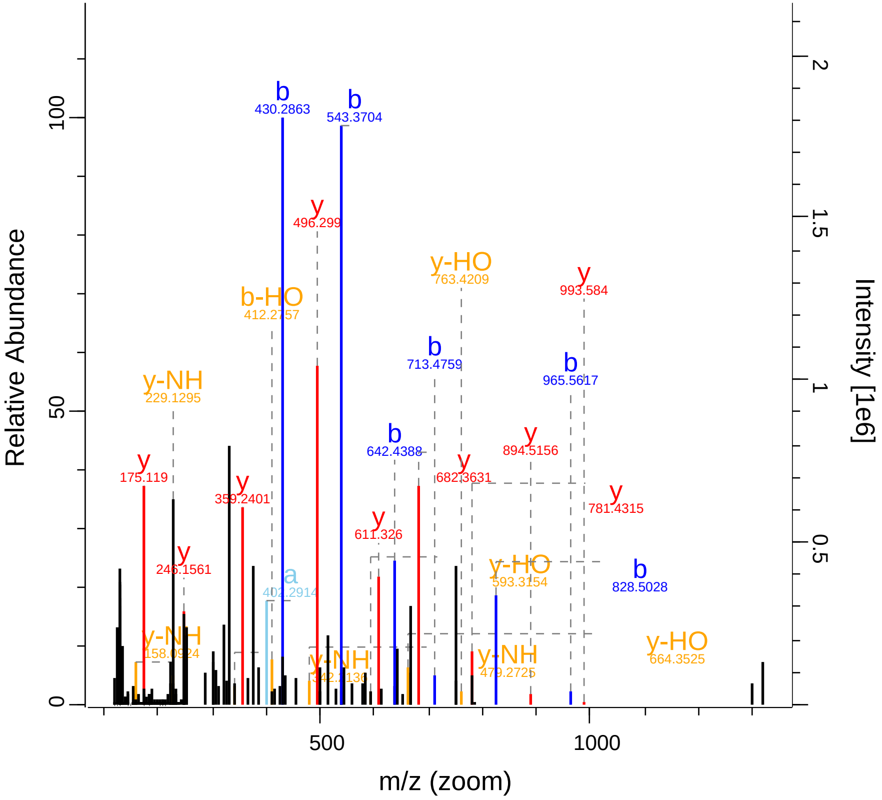

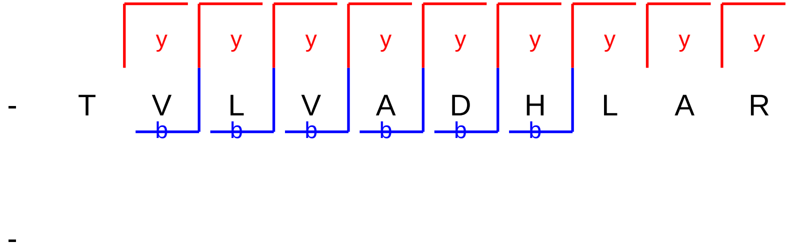

Raw File	Scan	Method	Score	m/z	Gene names
RO-QC-160121_ZackD_SS_Set_01_Fractio	15513	FTMS; HC	93.1	538.32	ATP5SL

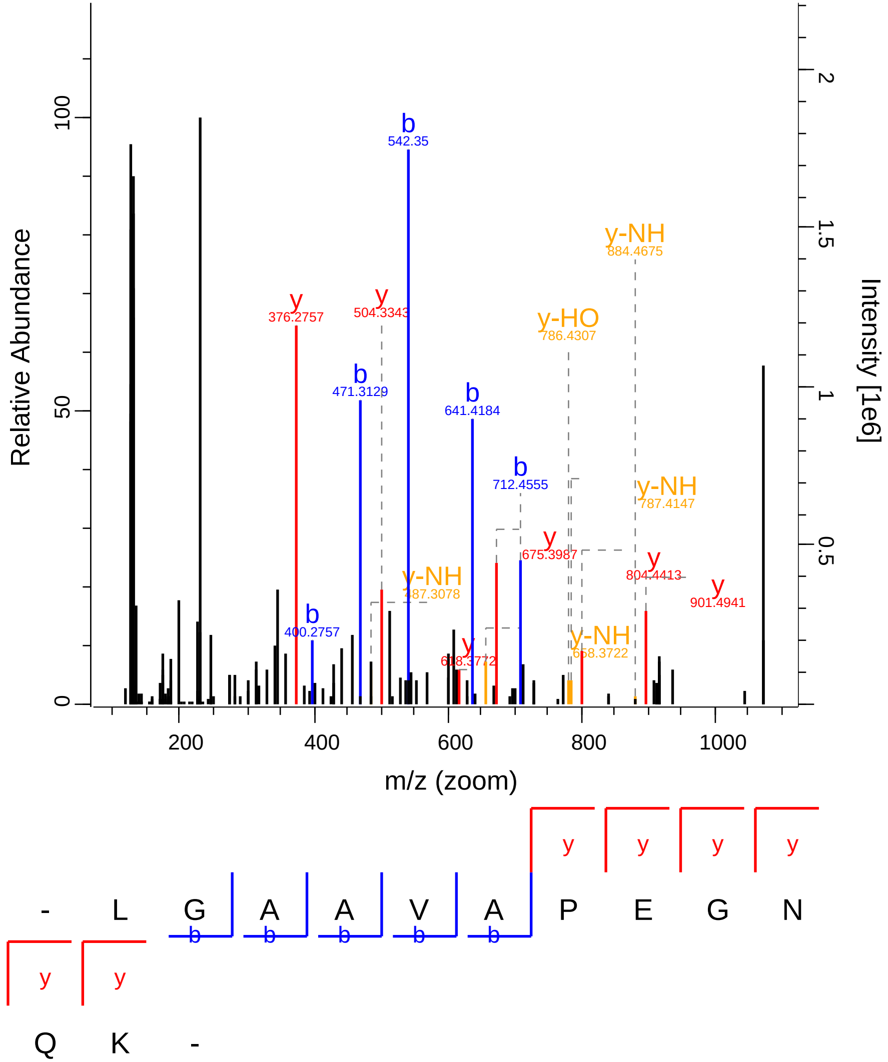

Raw File	Scan	Method	Score	m/z	Gene names
RO-QC-160121_ZackD_SS_Set_01_Fractio	15576	FTMS; HC	72.2	538.65	ATP5SL

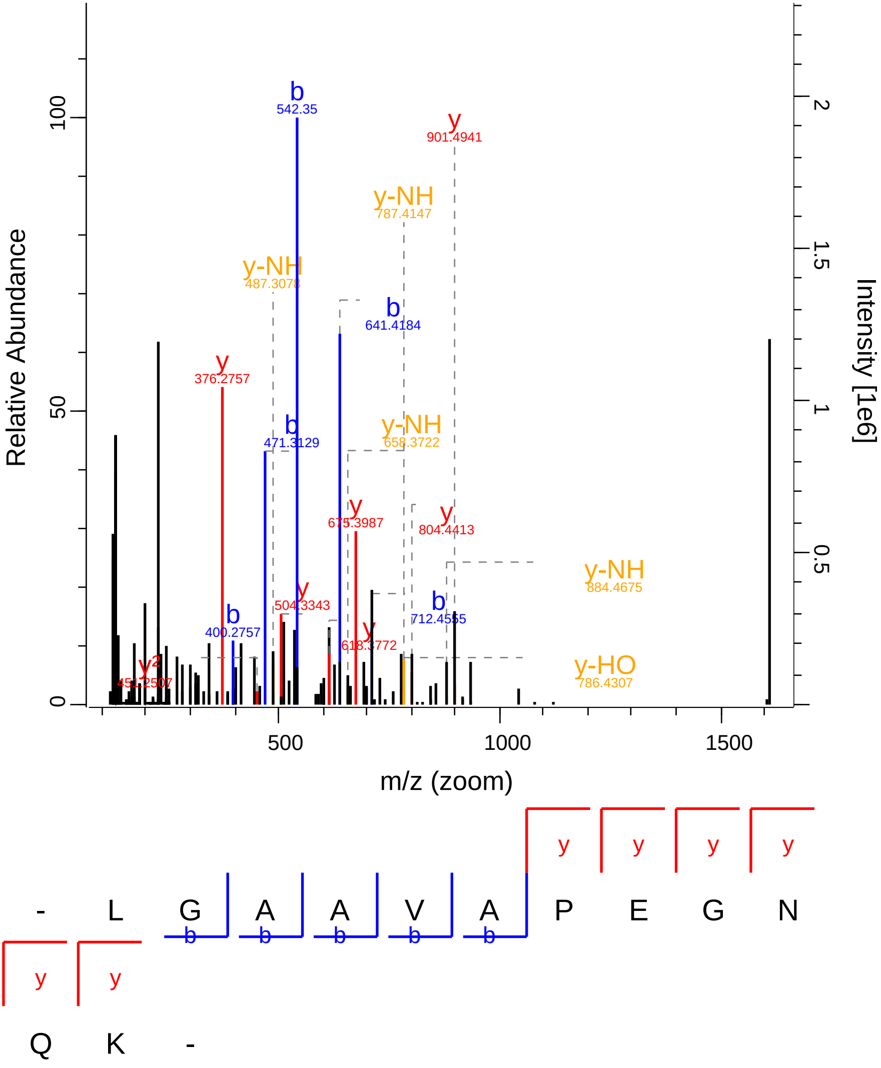

Raw File	Scan	Method	Score	m/z
RO-QC-160121_ZackD_SS_Set_01_Fraction_6	15578	FTMS; HCD	50.7	680.34

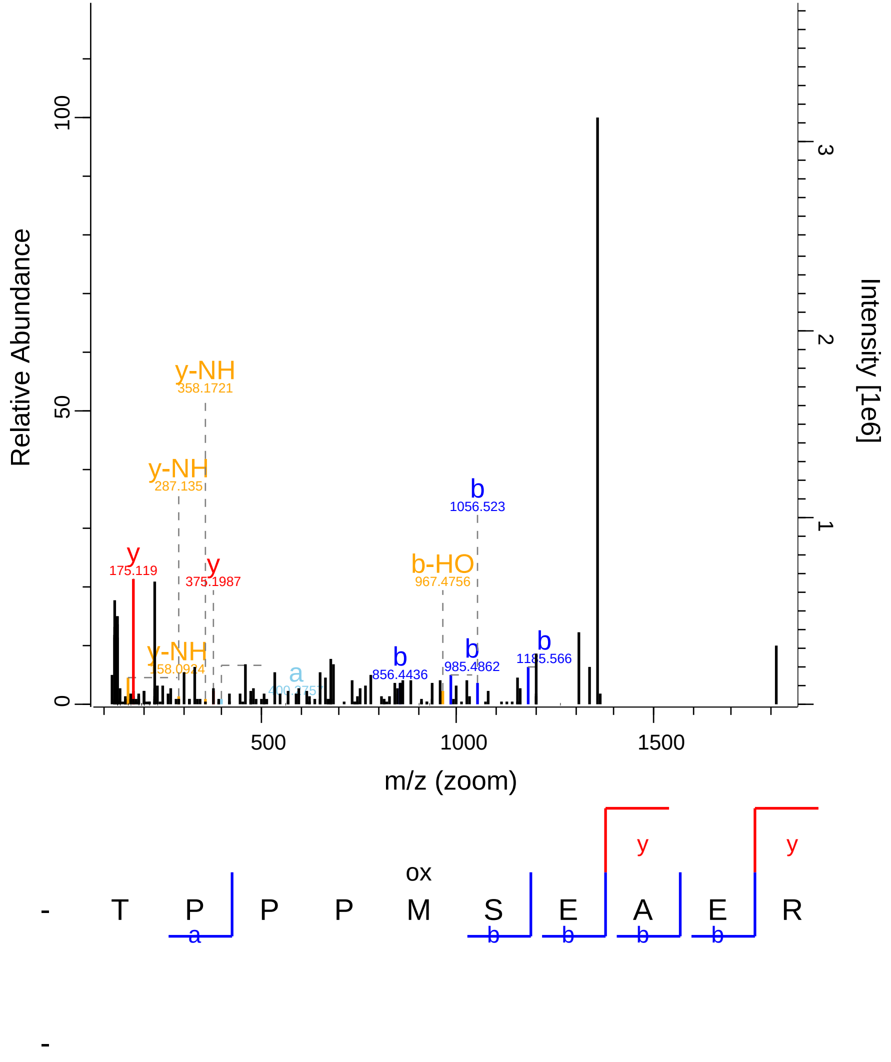

Raw File	Scan	Method	Score	m/z
RO-QC-160121_ZackD_SS_Set_01_Fraction_6	16571	FTMS; HCD	58.82	667.91

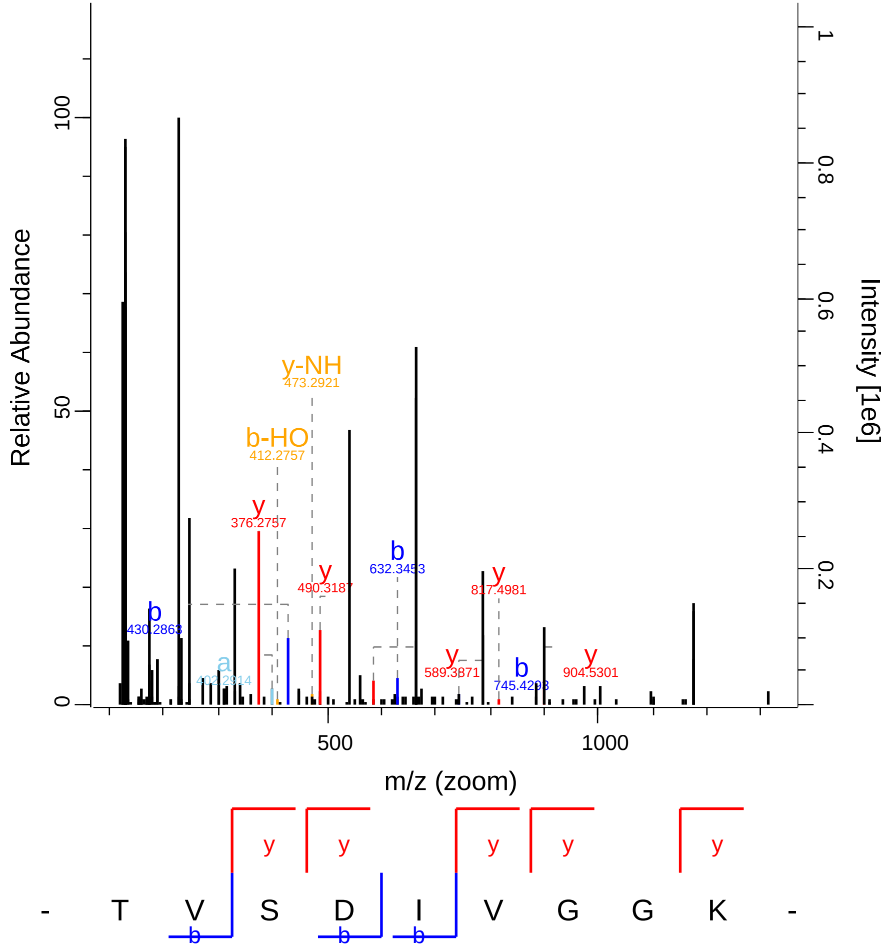

Raw File	Scan	Method	Score	m/z	Gene names
RO-QC-160121_ZackD_SS_Set_01_Fractio	16650	FTMS; HC	100.77	760.73	ZFYVE27

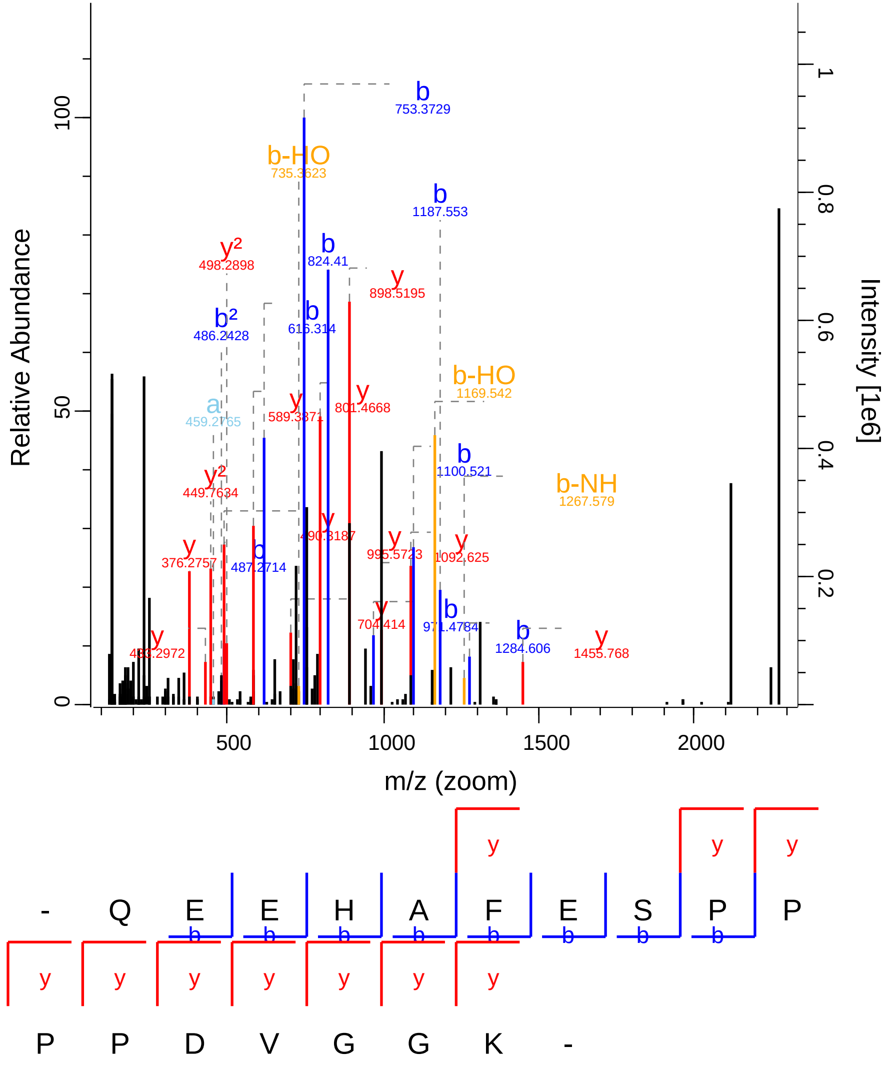

Raw File	Scan	Method	Score	m/z	Gene names
RO-QC-160121_ZackD_SS_Set_01_Fractio	16914	FTMS; HC	141.55	484.83	CLIP4

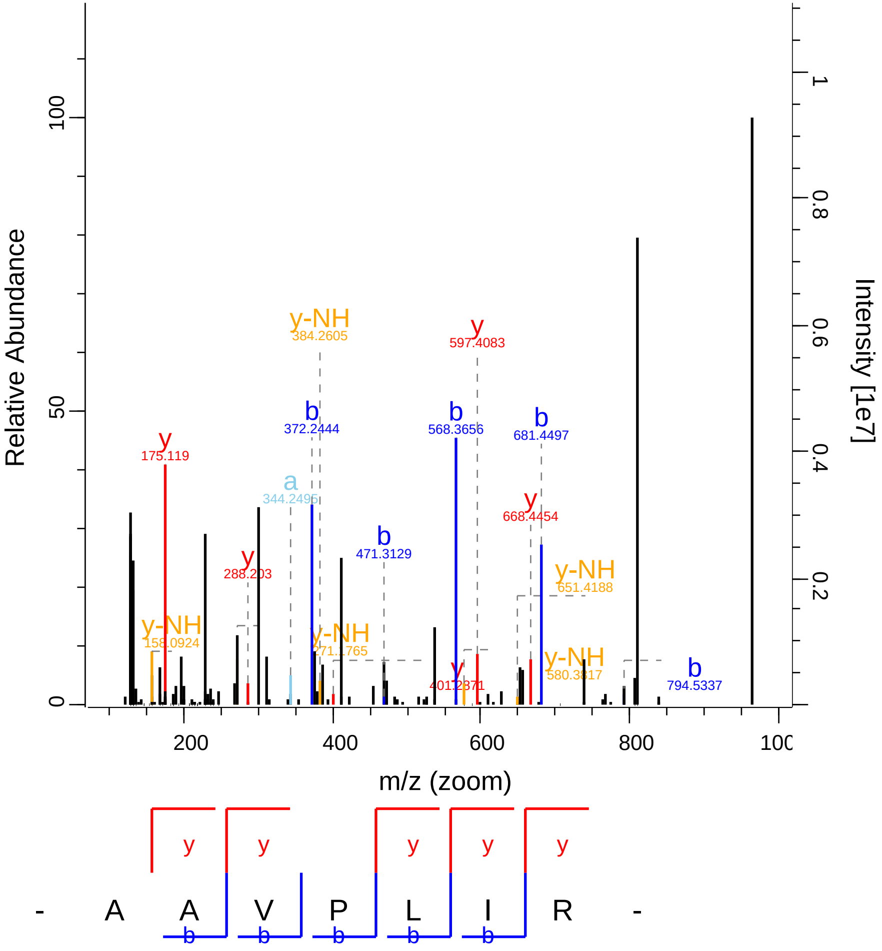

Raw File	Scan	Method	Score	m/z	Gene names
RO-QC-160121_ZackD_SS_Set_01_Fractic	16943	FTMS; HC	89.25	1063.53	LRRC8B

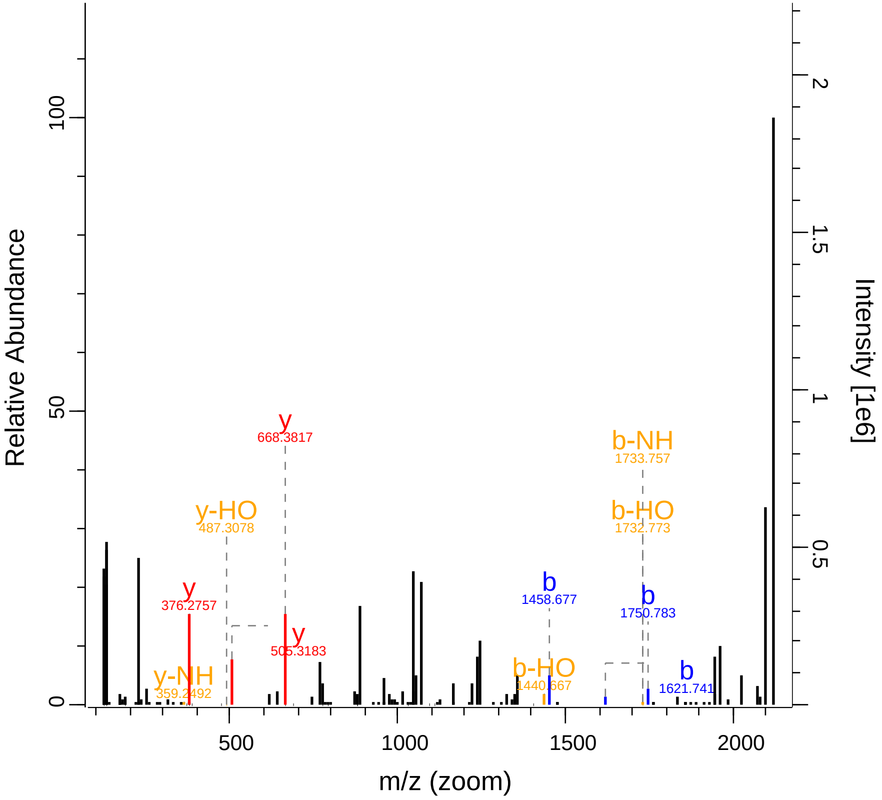

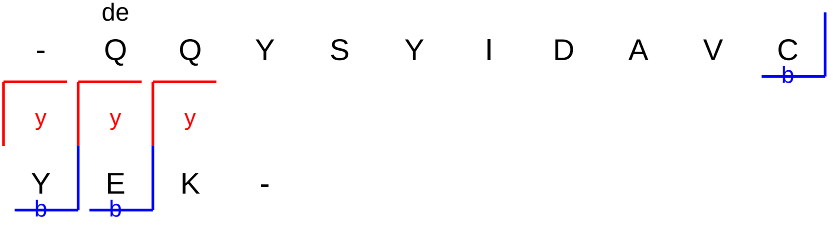

Raw File	Scan	Method	Score	m/z	Gene names
RO-QC-160121_ZackD_SS_Set_01_Fractio	17577	FTMS; HC	87.31	461.29	IL33

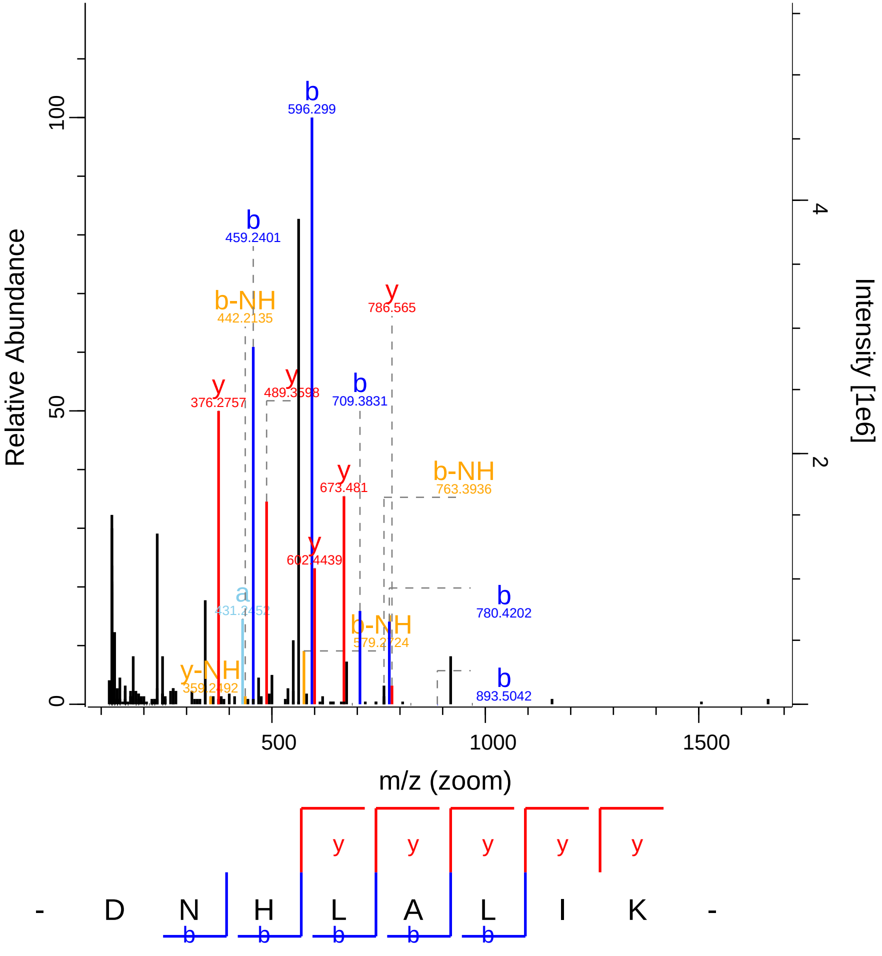

Raw File Sc Met Sc m/ Gene names

RO-QC-160121_Z 1& FT 3! 1{ 6! USP17L1;USP17L24;USP17L20;USP17L19;USP17L17;USP1

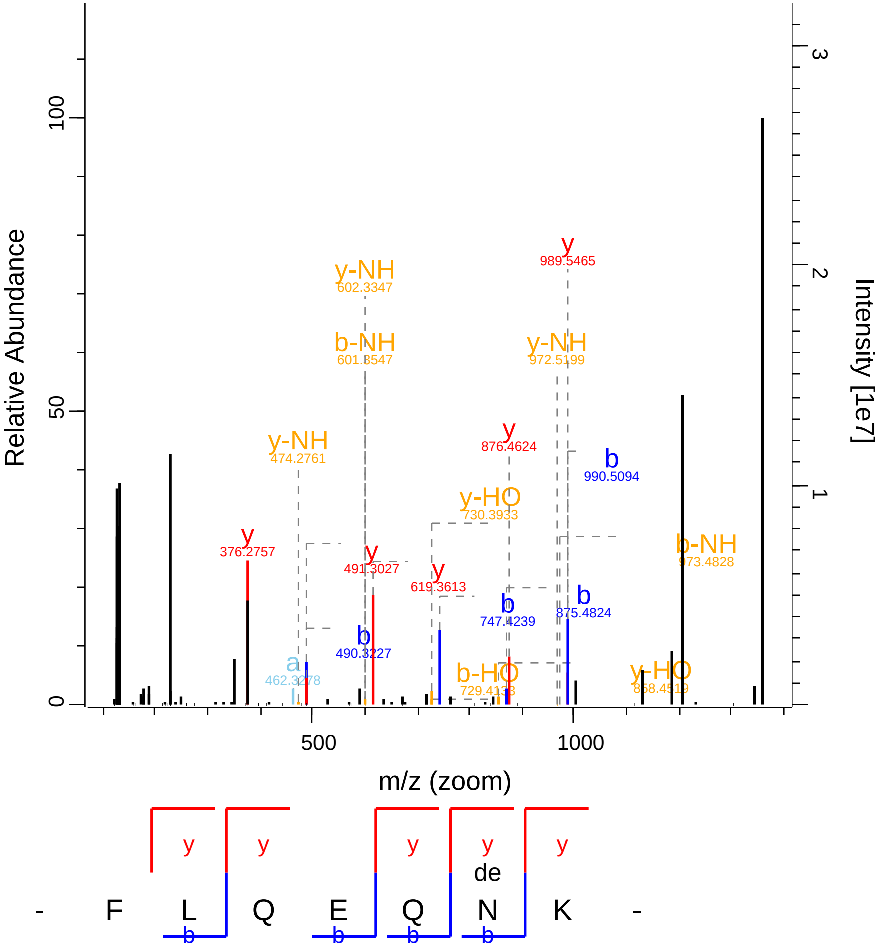

Raw File Sc Met Sc m/ Gene names

RO-QC-160121_Z 16 FT 9 8 4 6 USP17L1;USP17L24;USP17L20;USP17L19;USP17L17;USP1

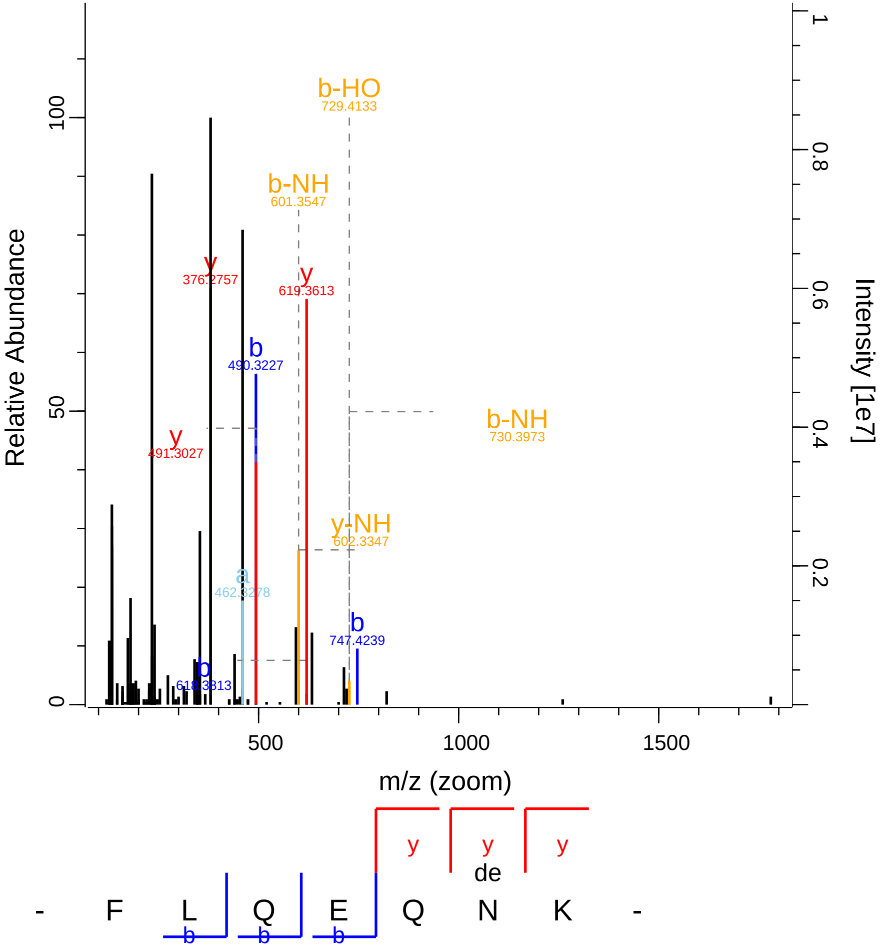

Raw File Sc Met Sc m/ Gene names

RO-QC-160121_Z 1€ FT 4 9 S 4 USP17L1;USP17L24;USP17L20;USP17L19;USP17L17;USP1

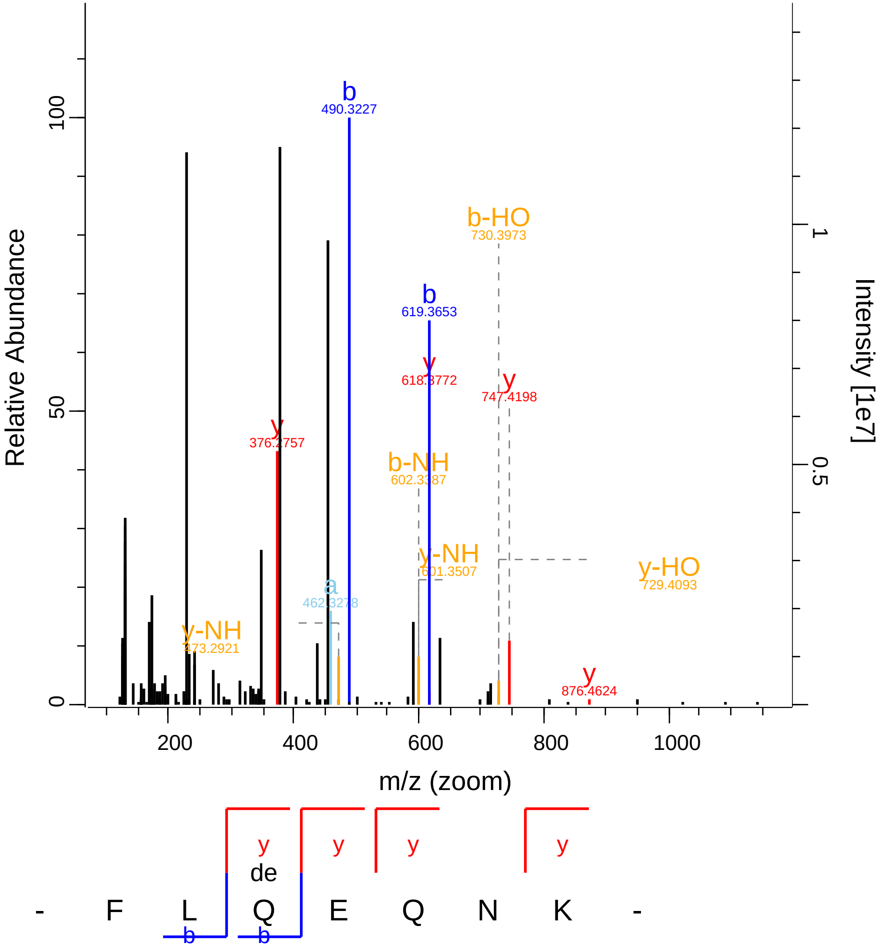

Raw File	Scan	Method	Score	m/z	Gene names
RO-QC-160121_ZackD_SS_Set_01_Fractio	18647	FTMS; HC	102.53	980.44	MT-ND3

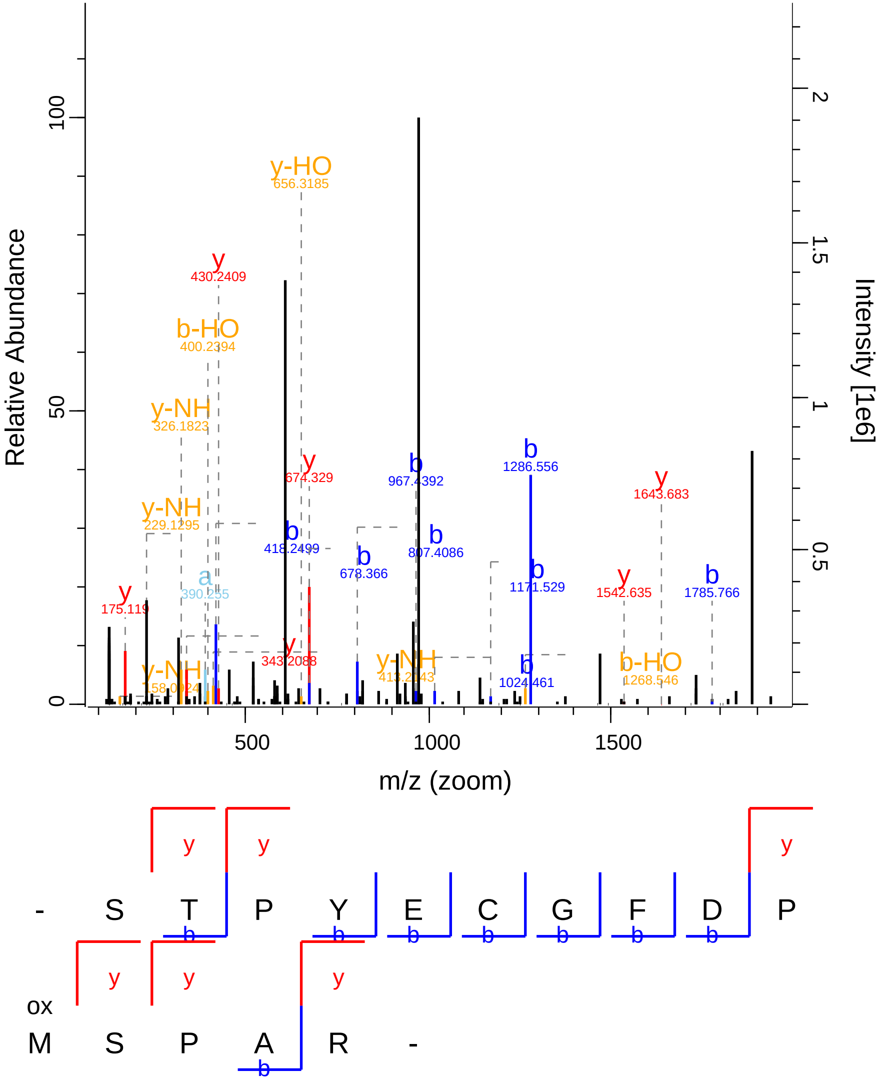

Raw File	Scan	Method	Score	m/z	Gene names
RO-QC-160121_ZackD_SS_Set_01_Fractio	18704	FTMS; HC	57.97	653.97	MT-ND3

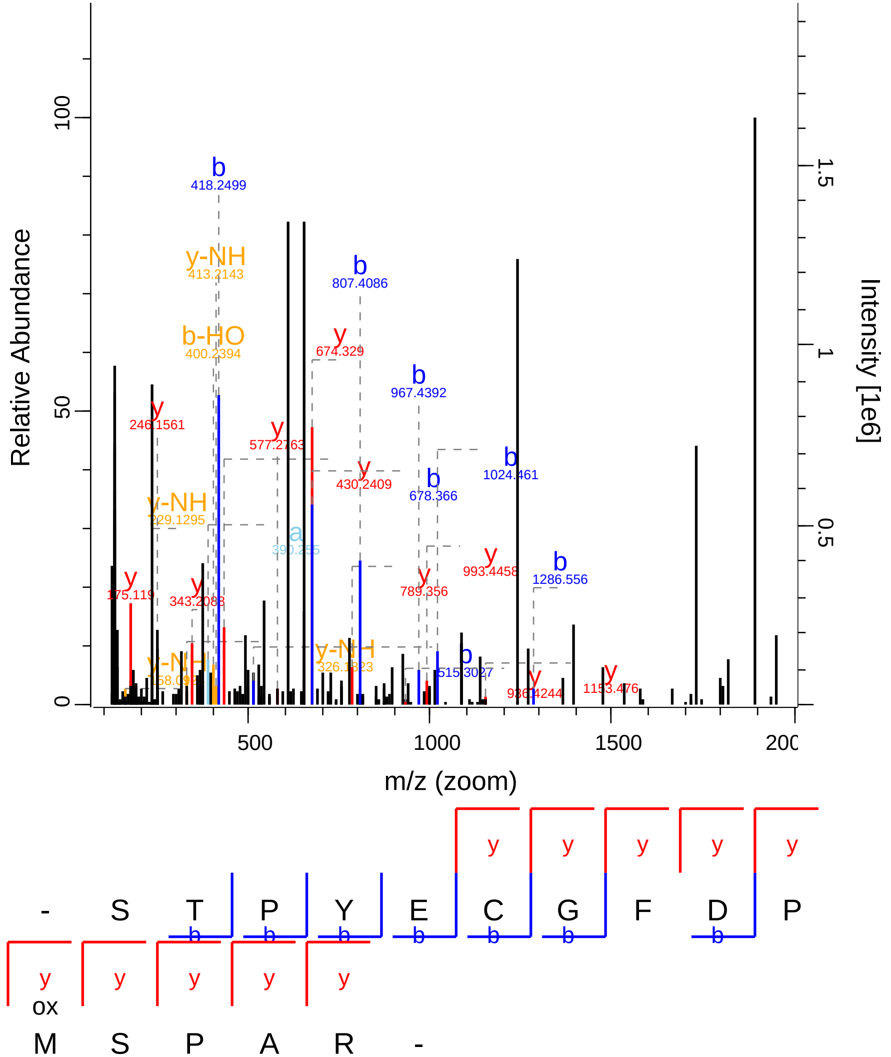

Raw File	Sc	Met	Sc	m/	Gene names
RO-QC-160121_Z	1c	FT	8	4	USP17L1;USP17L24;USP17L20;USP17L19;USP17L17;USP1

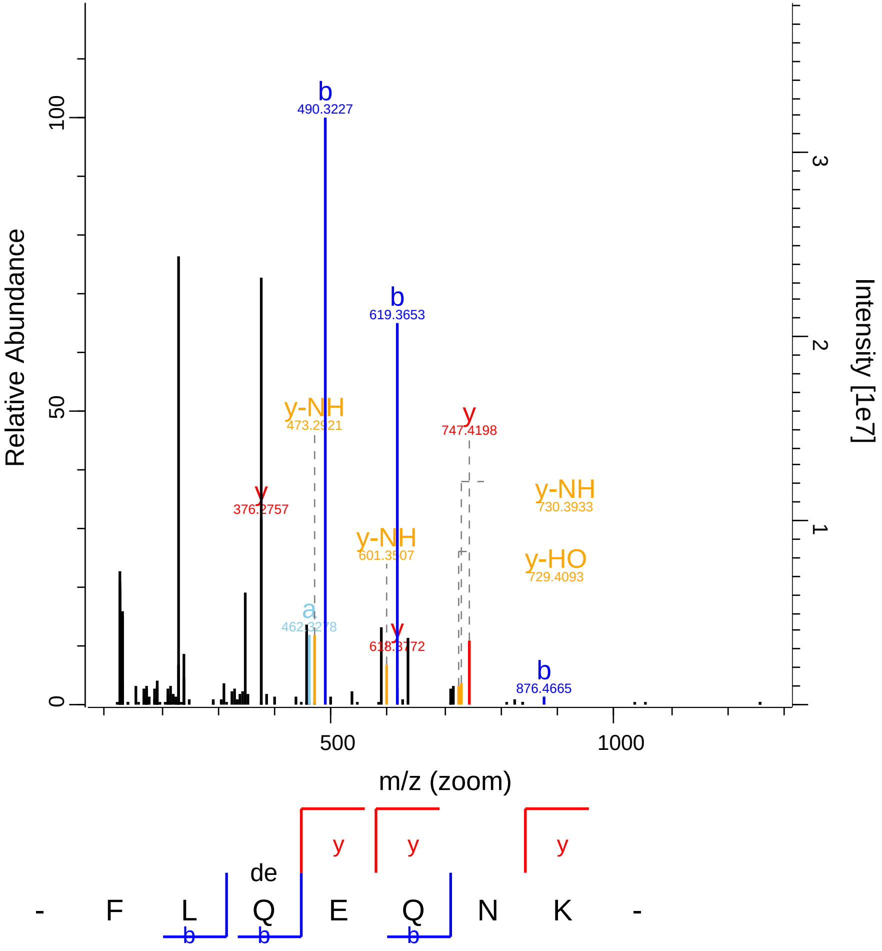

Raw File Sc Met Sc m/ Gene names

RO-QC-160121_Z 1 FT 0 S 4 USP17L1;USP17L24;USP17L20;USP17L19;USP17L17;USP1

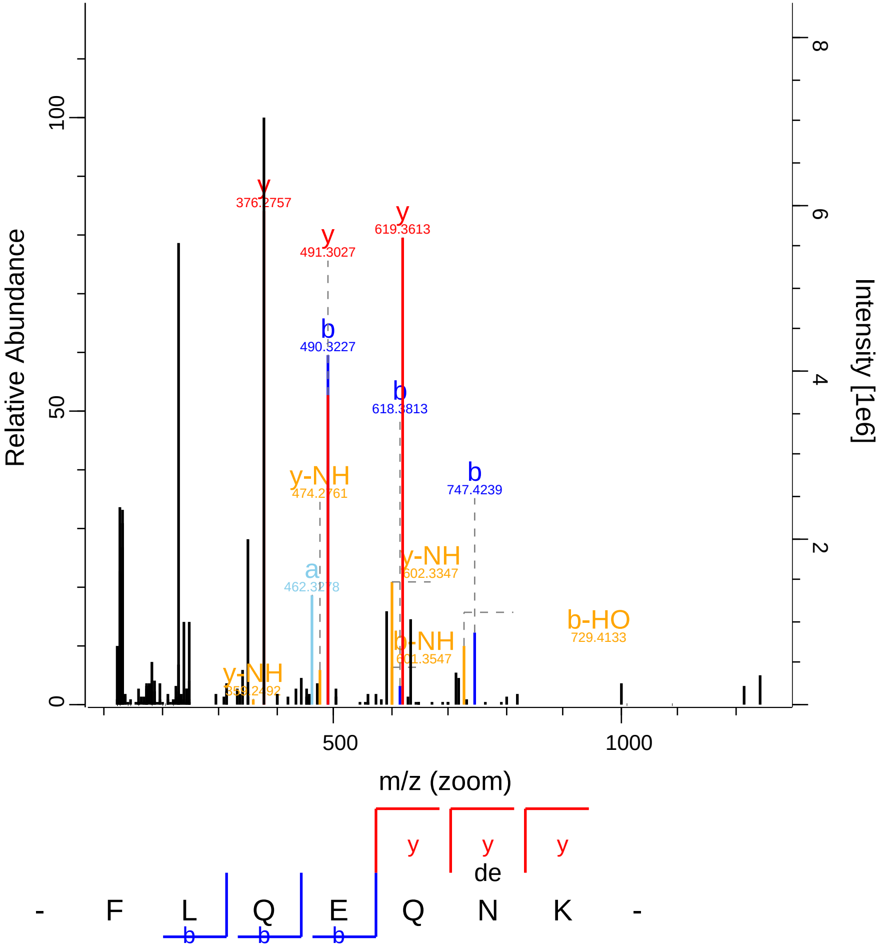

Raw File	Scan	Method	Score	m/z	Gene names
RO-QC-160121_ZackD_SS_Set_01_Fractio	19166	FTMS; HC	83.31	807.41	YIPF6

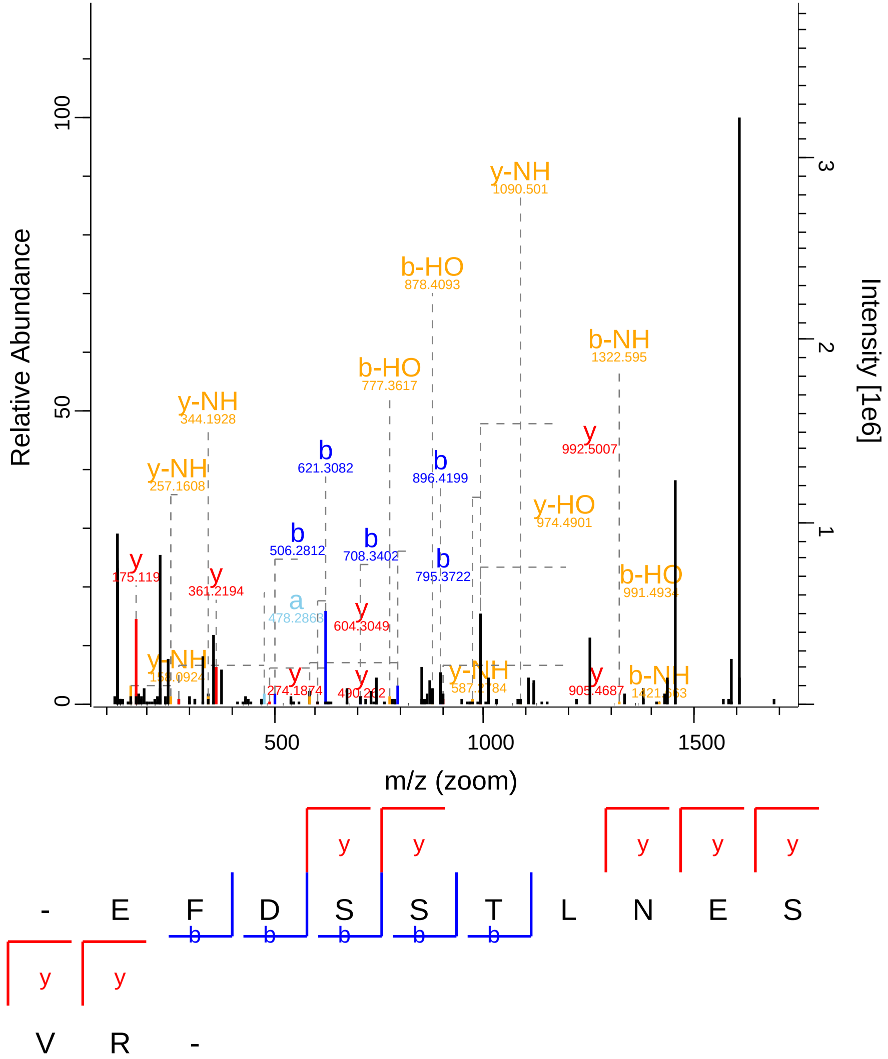

Raw File	Scan	Method	Score	m/z
RO-QC-160121_ZackD_SS_Set_01_Fraction_6	19335	FTMS; HCD	45.14	608.7

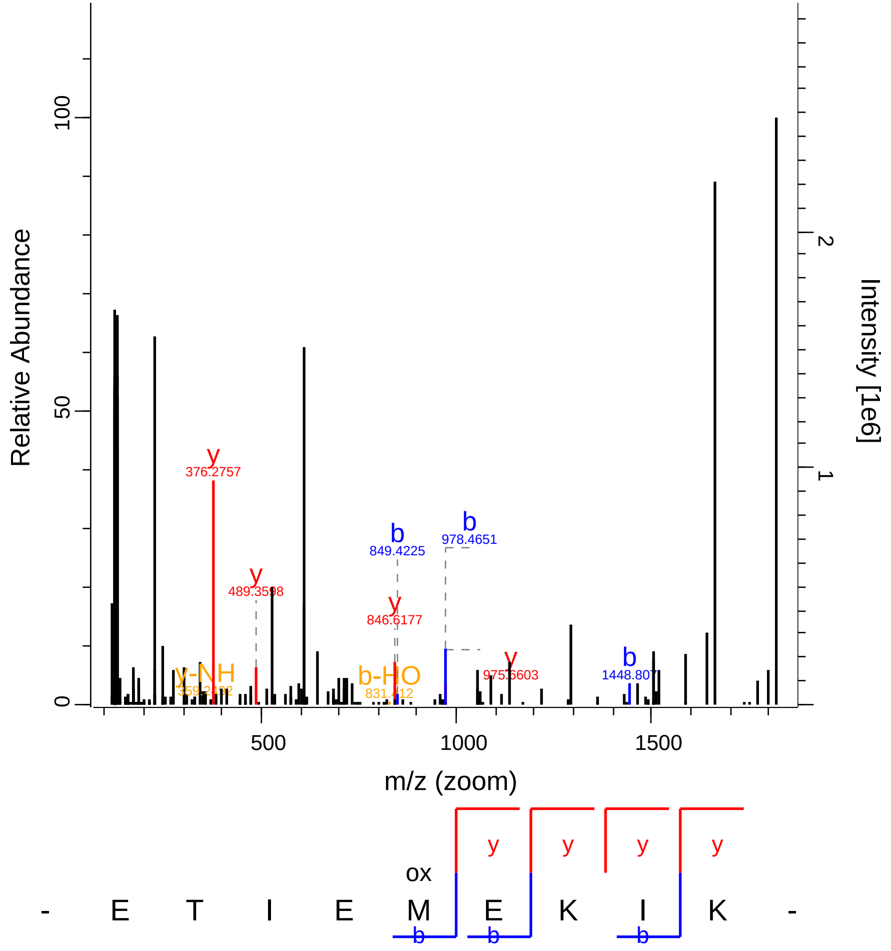

Raw File	Scan	Method	Score	m/z	Gene names
RO-QC-160121_ZackD_SS_Set_01_Fractio	19470	FTMS; HC	115.06	748.88	CES2

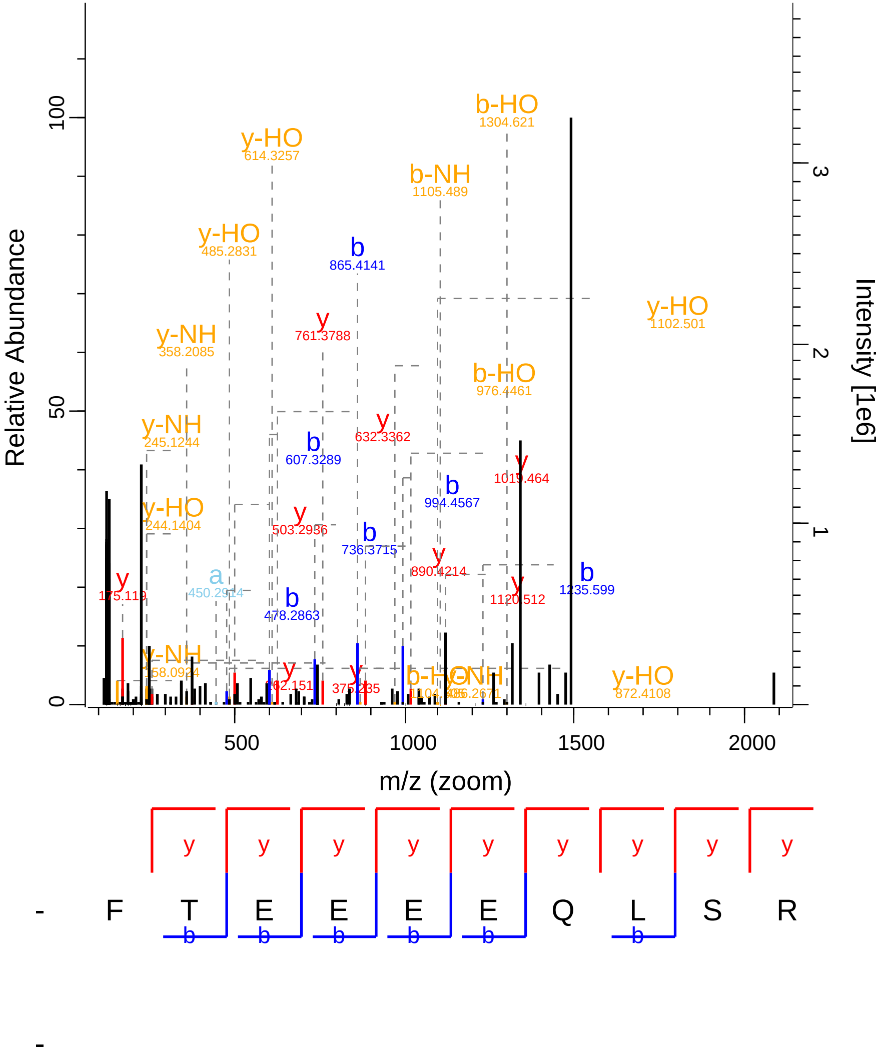

Raw File	Scan	Method	Score	m/z
RO-QC-160121_ZackD_SS_Set_01_Fraction_6	20606	FTMS; HCD	103.56	924.46

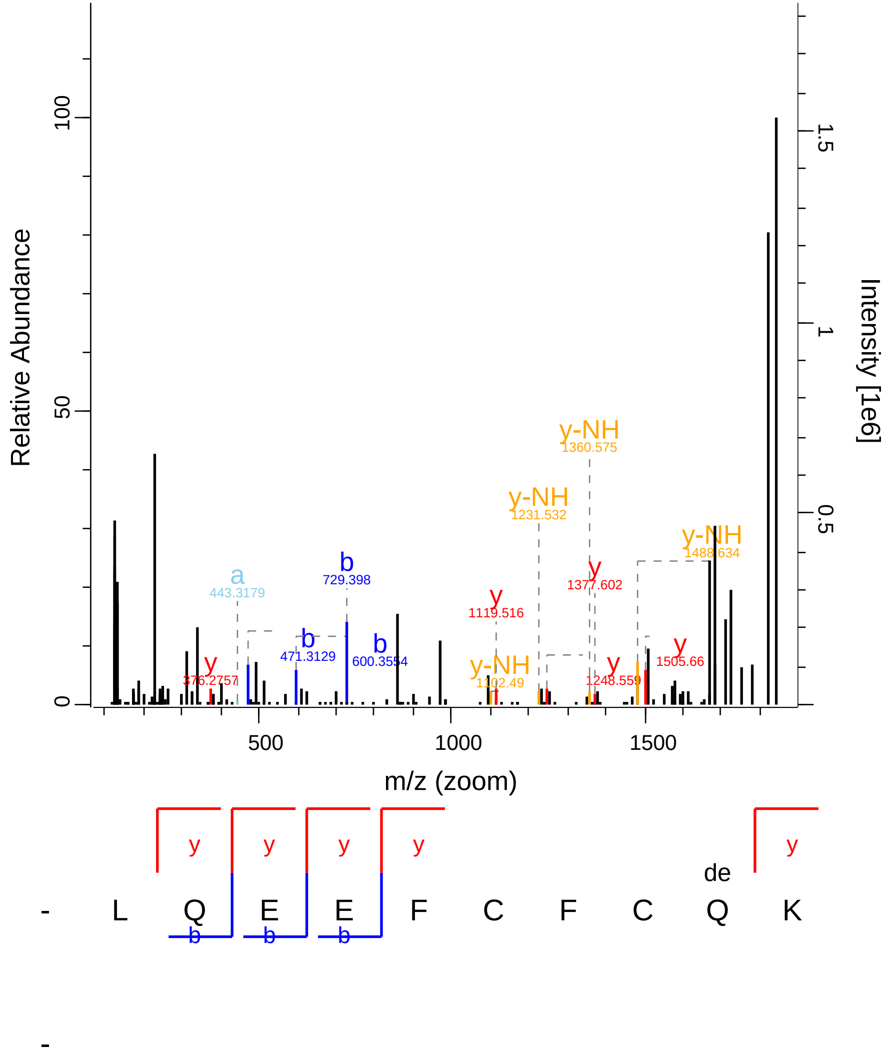

Raw File	Scan	Method	Score	m/z	Gene names
RO-QC-160121_ZackD_SS_Set_01_Fractic	21099	FTMS; HC	45.26	1264.15	FSCN3

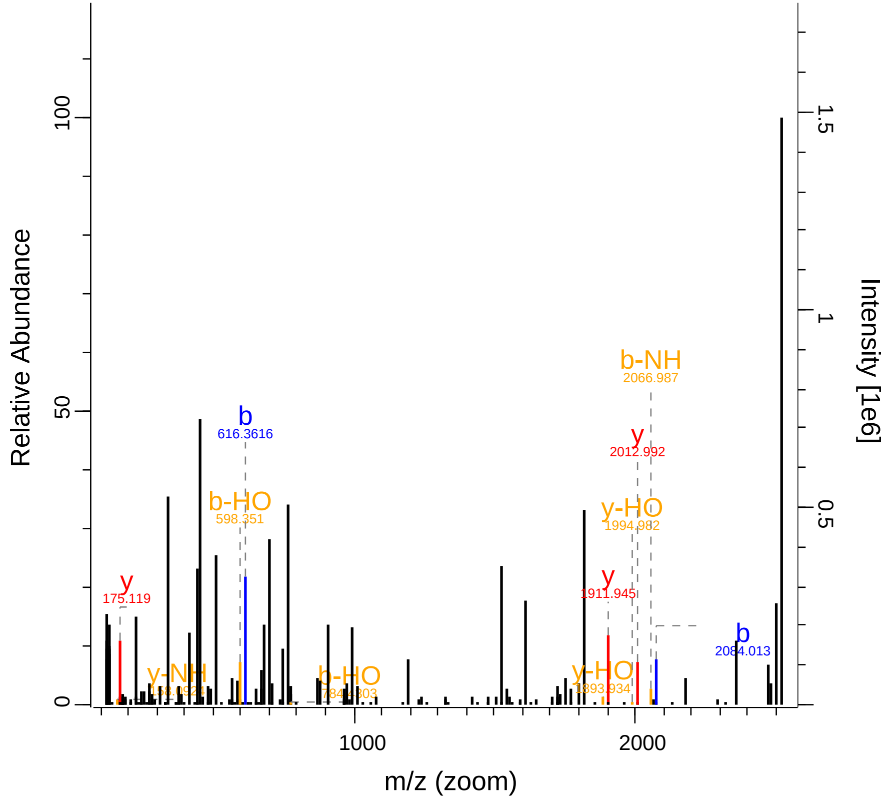

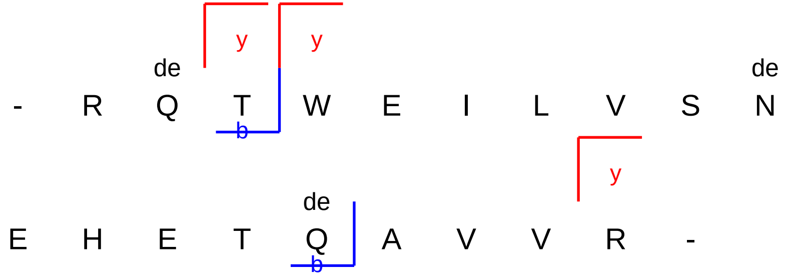

Raw File	Scan	Method	Score	m/z	Gene names
RO-QC-160121_ZackD_SS_Set_01_Fractio	21161	FTMS; HC	88.1	652.03	SLC25A17

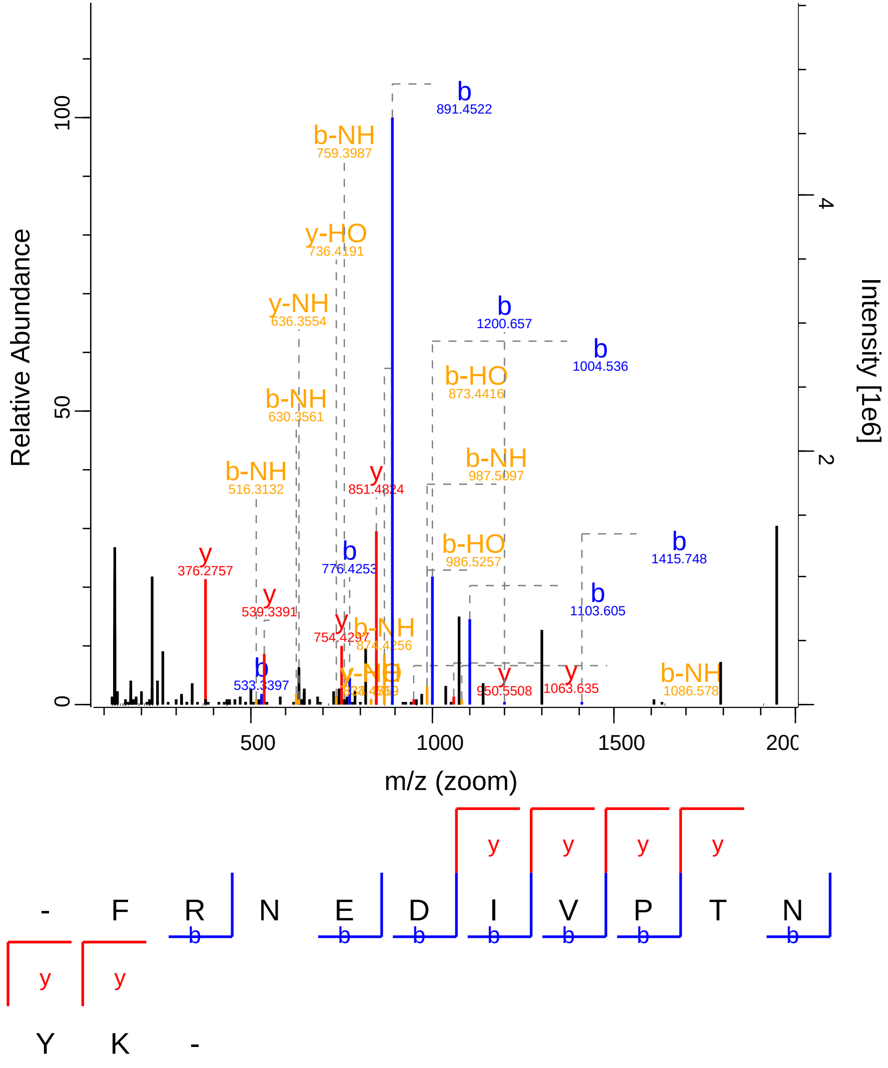

Raw File	Scan	Method	Score	m/z	Gene names
RO-QC-160121_ZackD_SS_Set_01_Fractio	21501	FTMS; HC	132.24	935.47	NUDT6

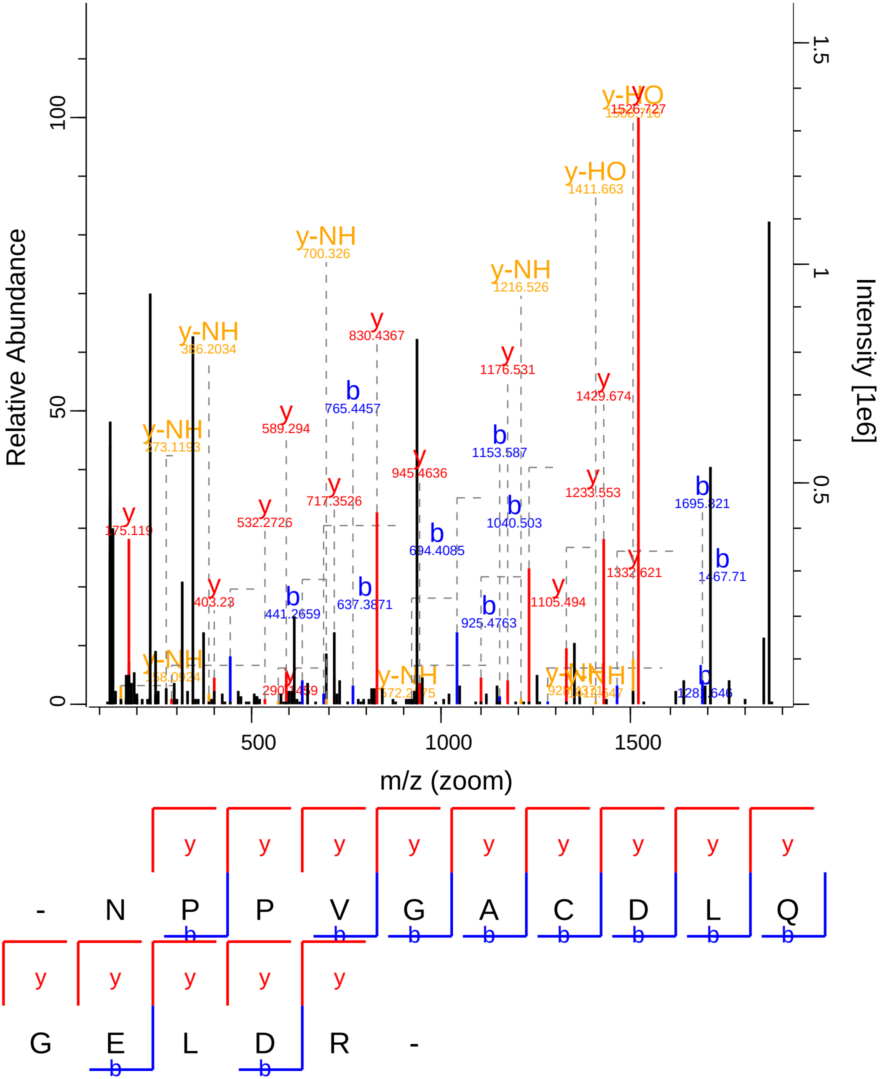

Raw File	Scan	Method	Score	m/z	Gene names
RO-QC-160121_ZackD_SS_Set_01_Fractio	21899	FTMS; HC	137	705.41	CLMN

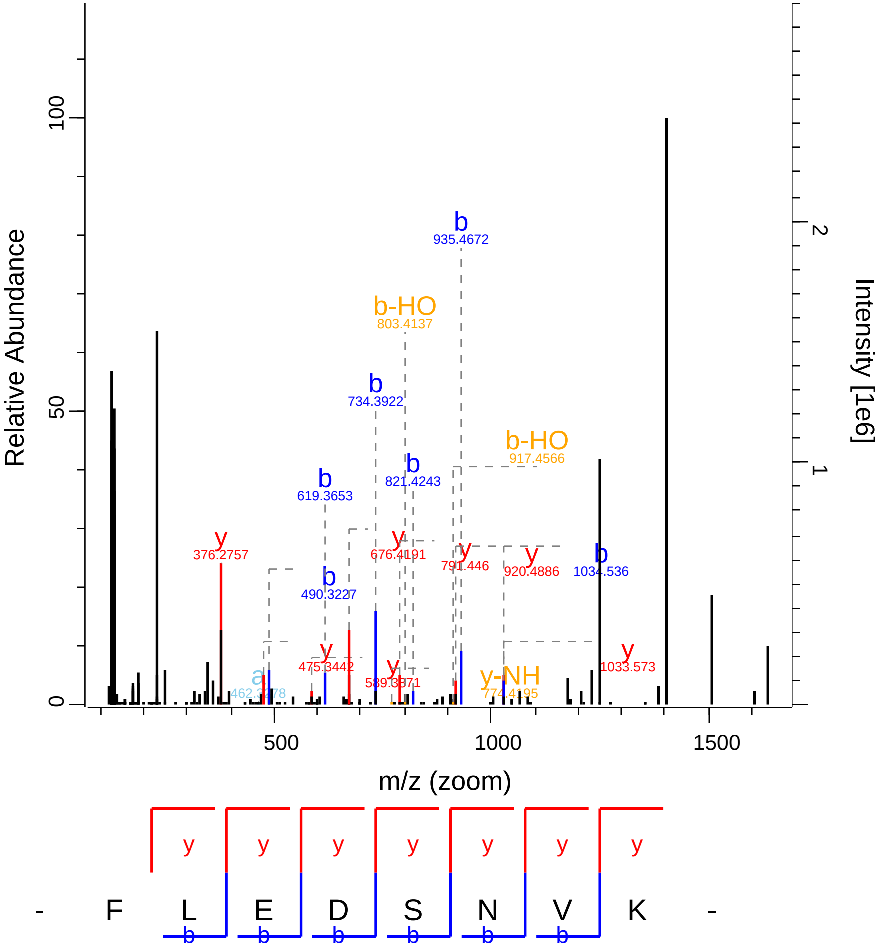

Raw File	Scan	Method	Score	m/z	Gene names
RO-QC-160121_ZackD_SS_Set_01_Fractio	22824	FTMS; HC	125.47	600.86	TMEM167A

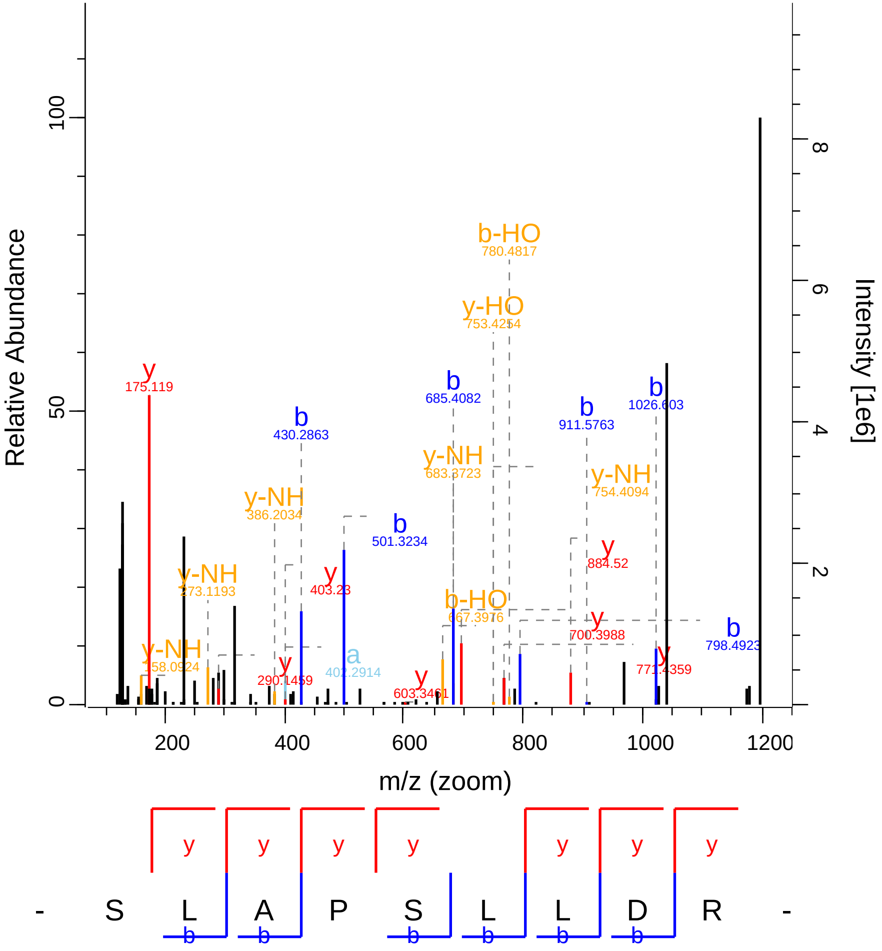

Raw File	Scan	Method	Score	m/z	Gene names
RO-QC-160121_ZackD_SS_Set_01_Fractic	22859	FTMS; HC	84.29	1191.64	ATP6V0C

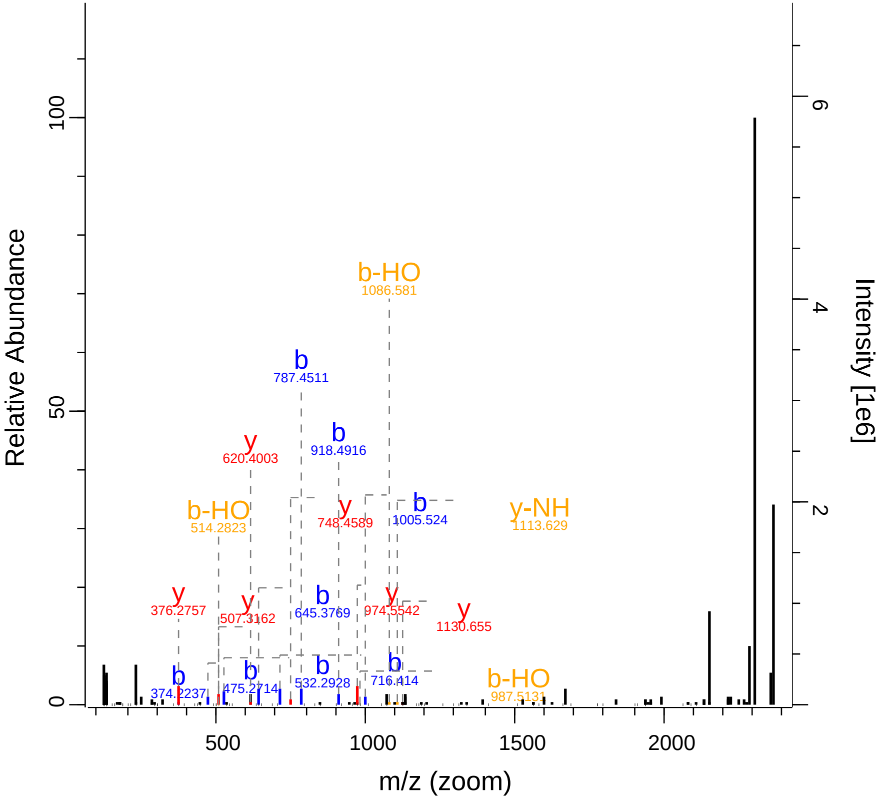

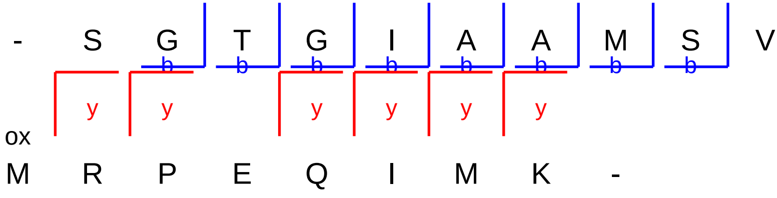

Raw File	Scan	Method	Score	m/z	Gene names
RO-QC-160121_ZackD_SS_Set_01_Fractio	22925	FTMS; HC	86.55	794.43	ATP6V0C

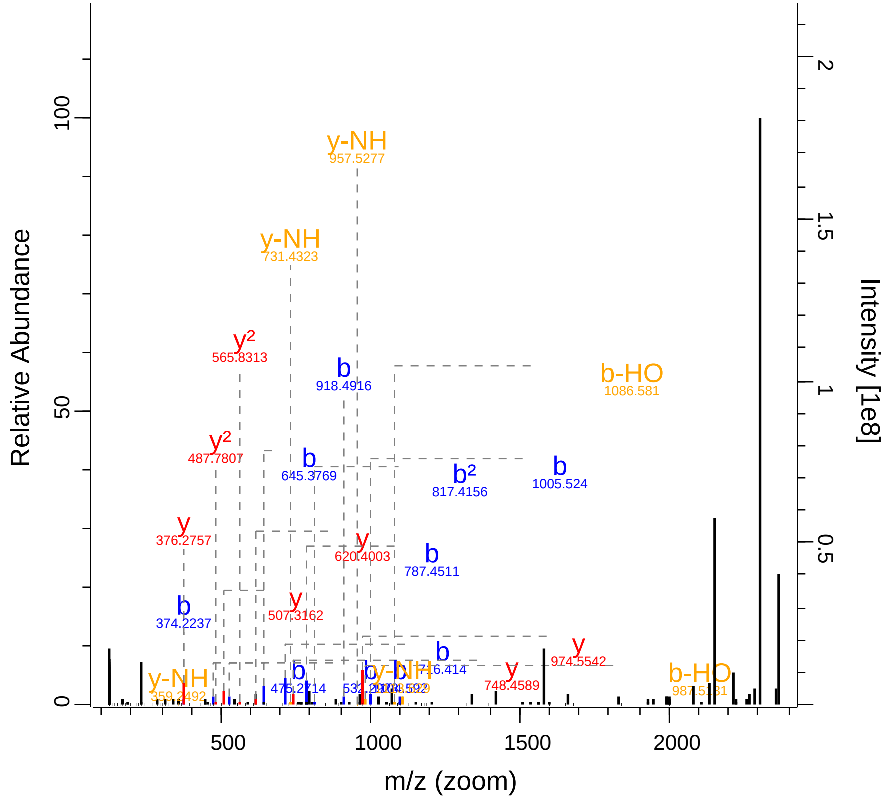

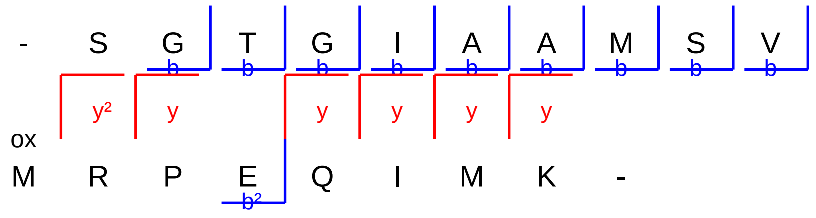

Raw File	Scan	Method	Score	m/z	Gene names
RO-QC-160121_ZackD_SS_Set_01_Fractic	22964	FTMS; HC	84.48	1192.14	ATP6V0C

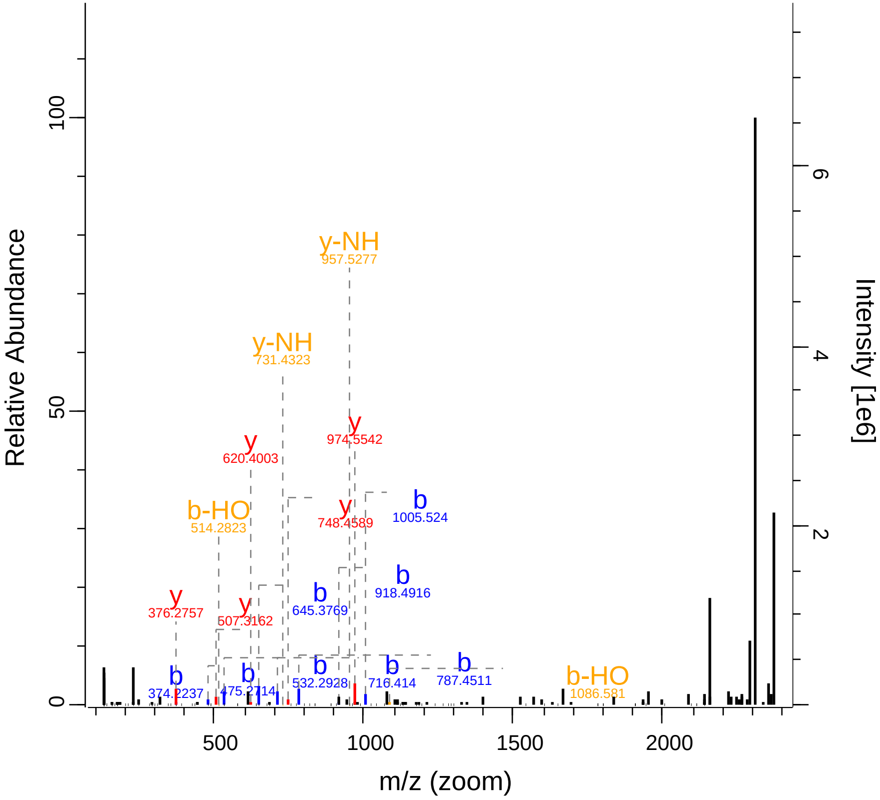

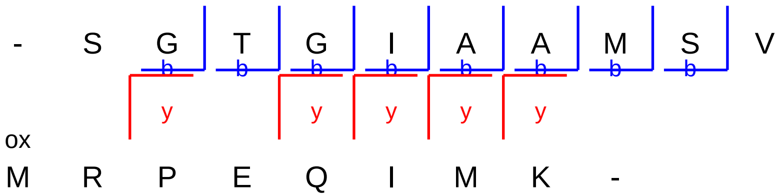

Raw File	Scan	Method	Score	m/z	Gene names
RO-QC-160121_ZackD_SS_Set_01_Fractio	23146	FTMS; HC	60.95	794.77	ATP6V0C

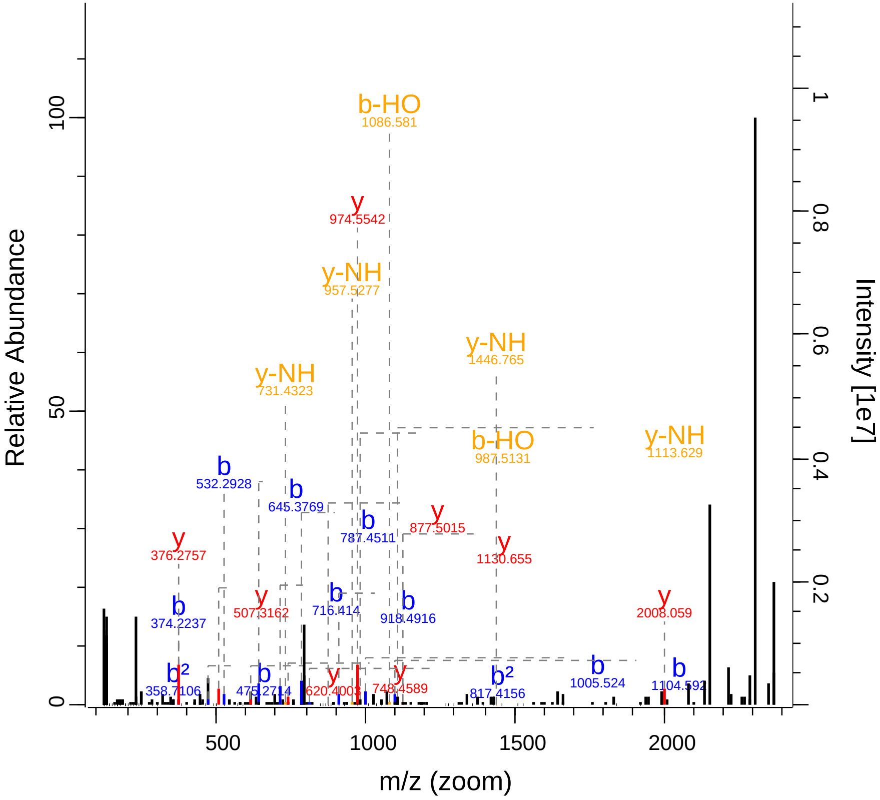

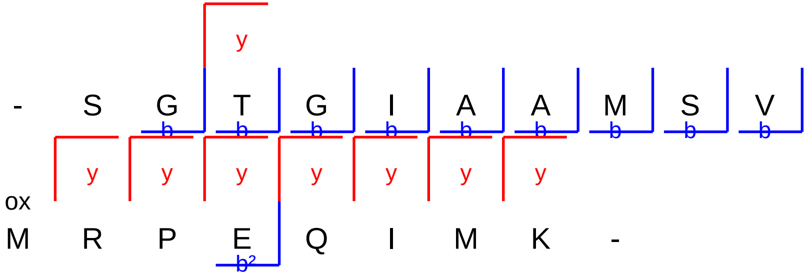

Raw File	Scan	Method	Score	m/z	Gene names
RO-QC-160121_ZackD_SS_Set_01_Fractior	23440	FTMS; HCI	96.19	638.4	CABP7

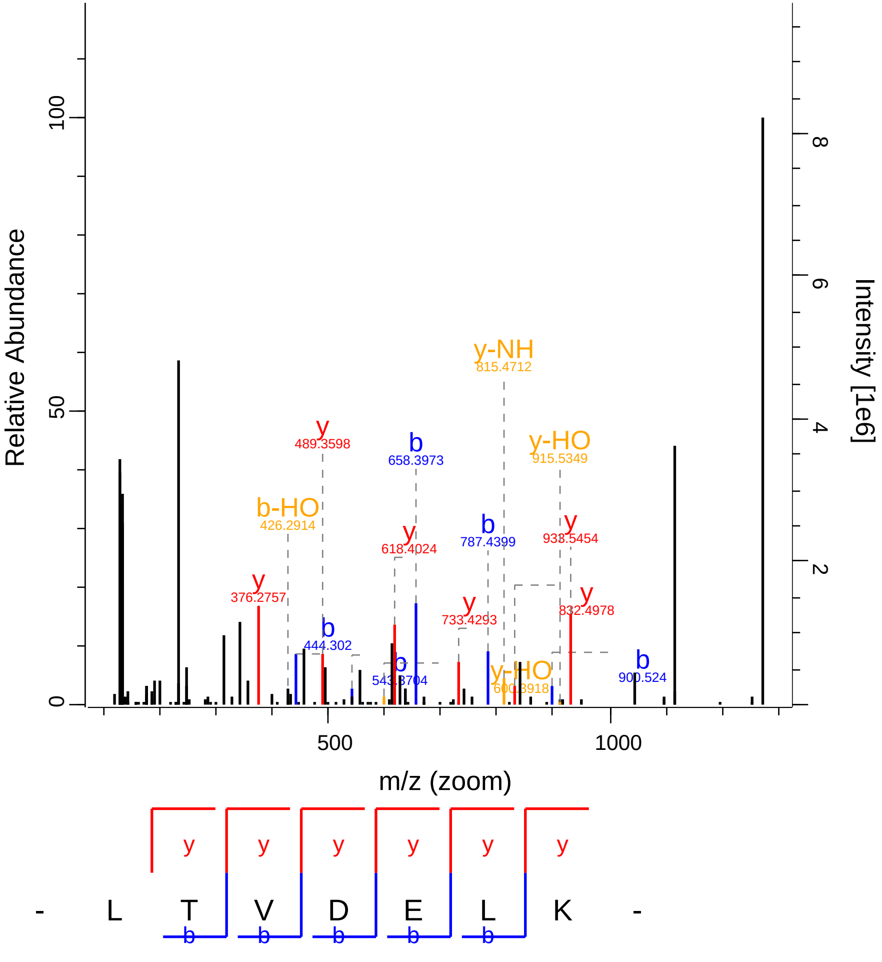

Raw File	Scan	Method	Score	m/z	Gene names
RO-QC-160121_ZackD_SS_Set_01_Fractio	23617	FTMS; HC	114.72	425.94	CABP7

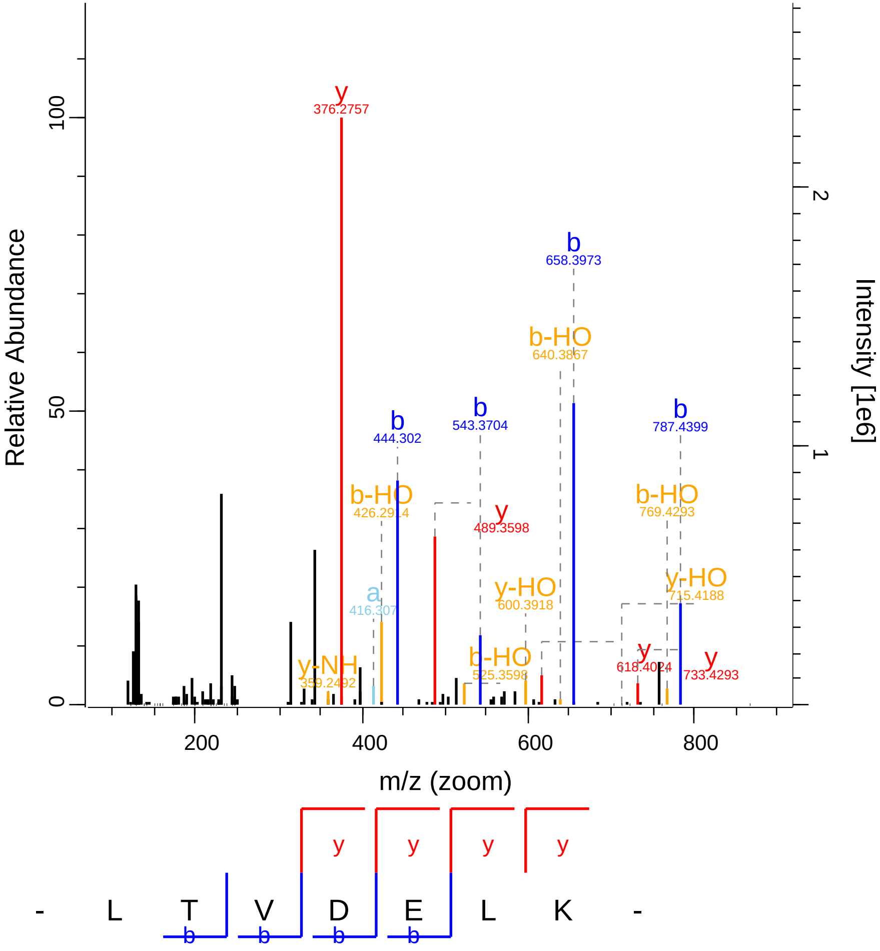

Raw File	Scan	Method	Score	m/z	Gene names
RO-QC-160121_ZackD_SS_Set_01_Fractioı	23653	FTMS; HCI	116.88	638.4	CABP7

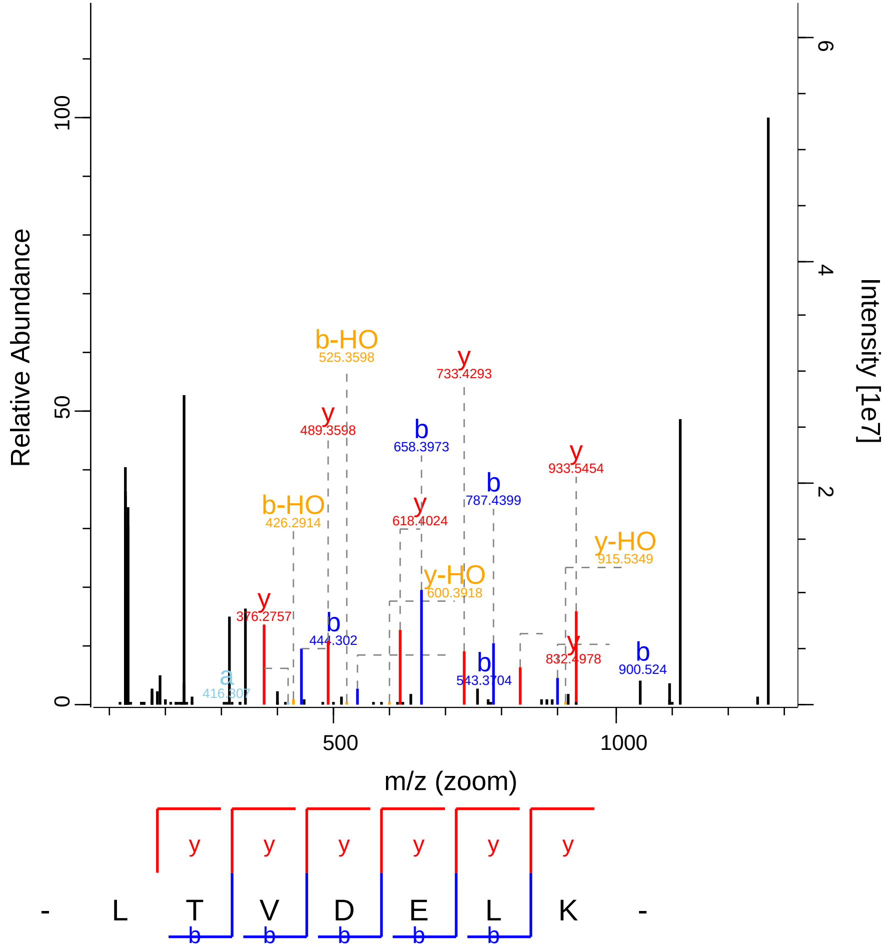

Raw File	Scan	Method	Score	m/z
RO-QC-160121_ZackD_SS_Set_01_Fraction_6	24066	FTMS; HCD	44.2	1031.55

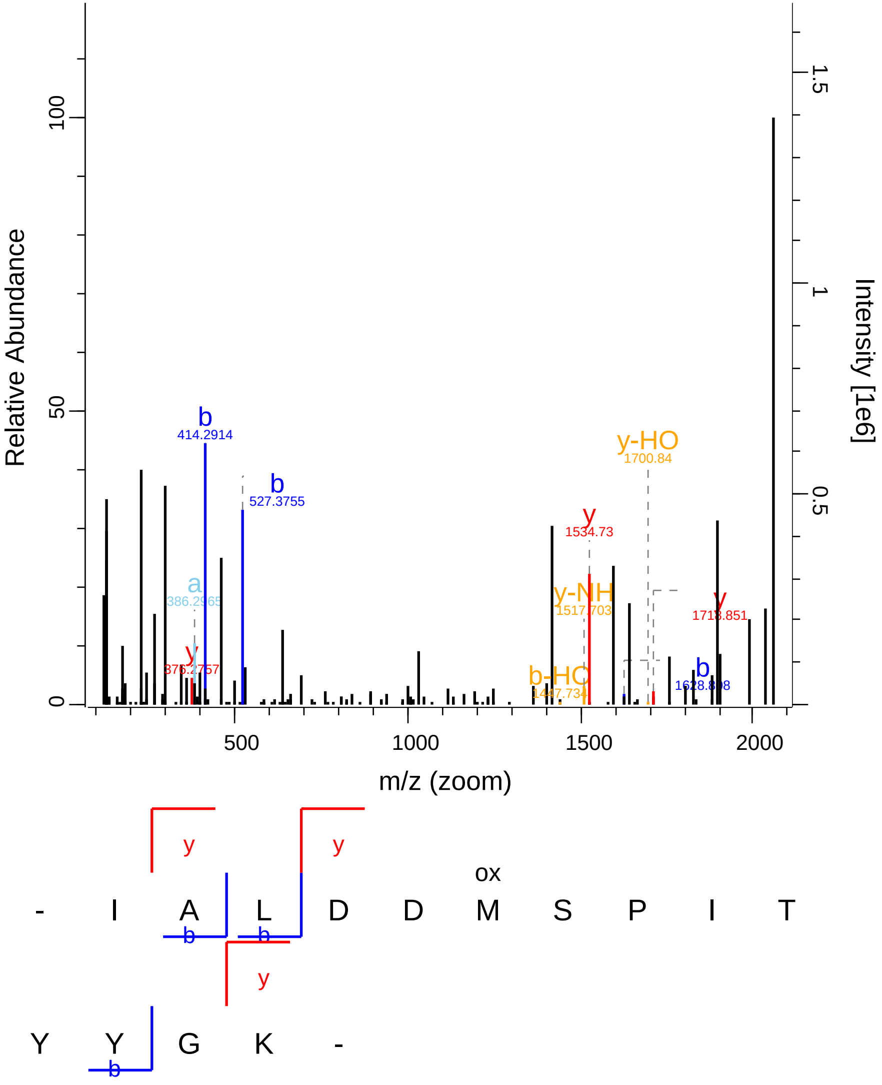

Raw File	Scan	Method	Score	m/z	Gene names
RO-QC-160121_ZackD_SS_Set_01_Fractio	24285	FTMS; HC	105.19	618.71	REC8

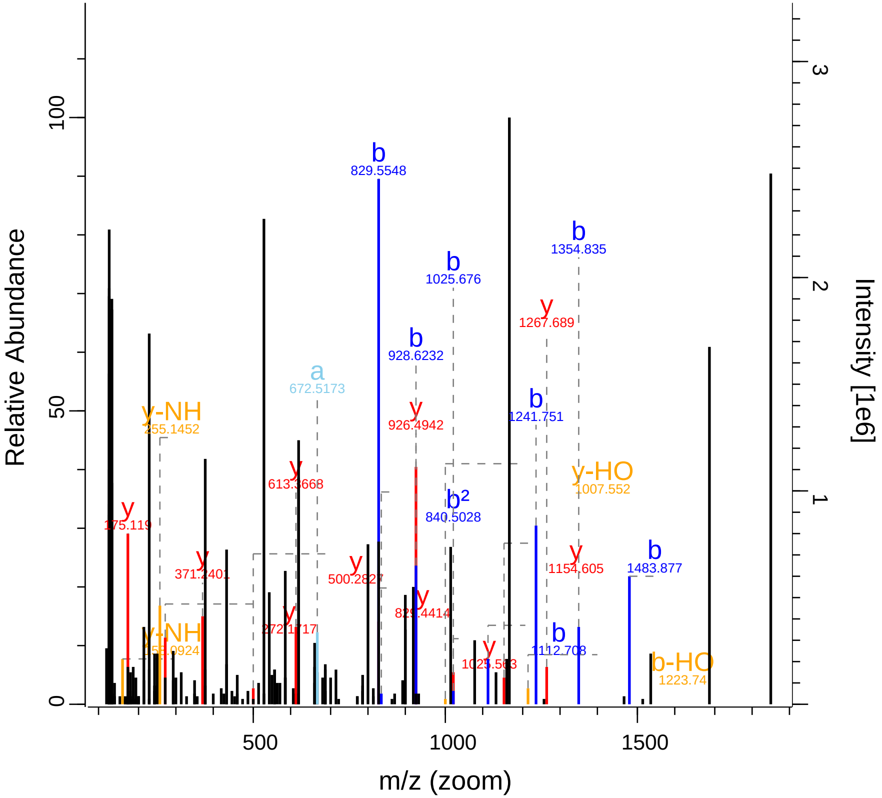

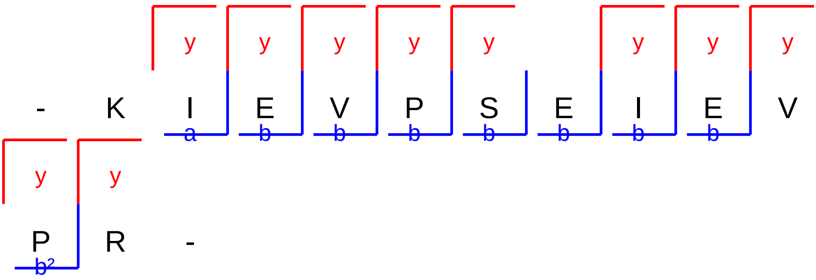

Raw File	Scan	Method	Score	m/z	Gene names
RO-QC-160121_ZackD_SS_Set_01_Fractio	26278	FTMS; HC	145.89	893.47	MRPL35

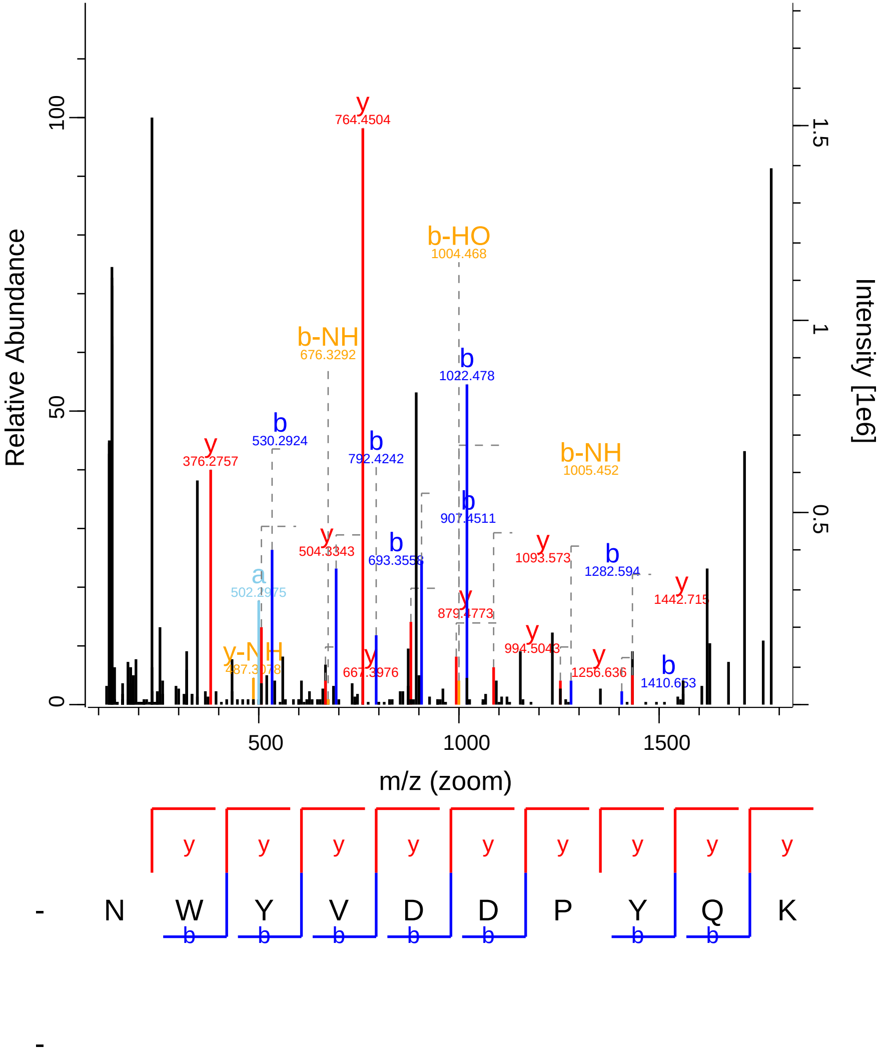

Raw File	Scan	Method	Score	m/z
RO-QC-160121_ZackD_SS_Set_01_Fraction_6	26625	FTMS; HCD	106.04	454.95

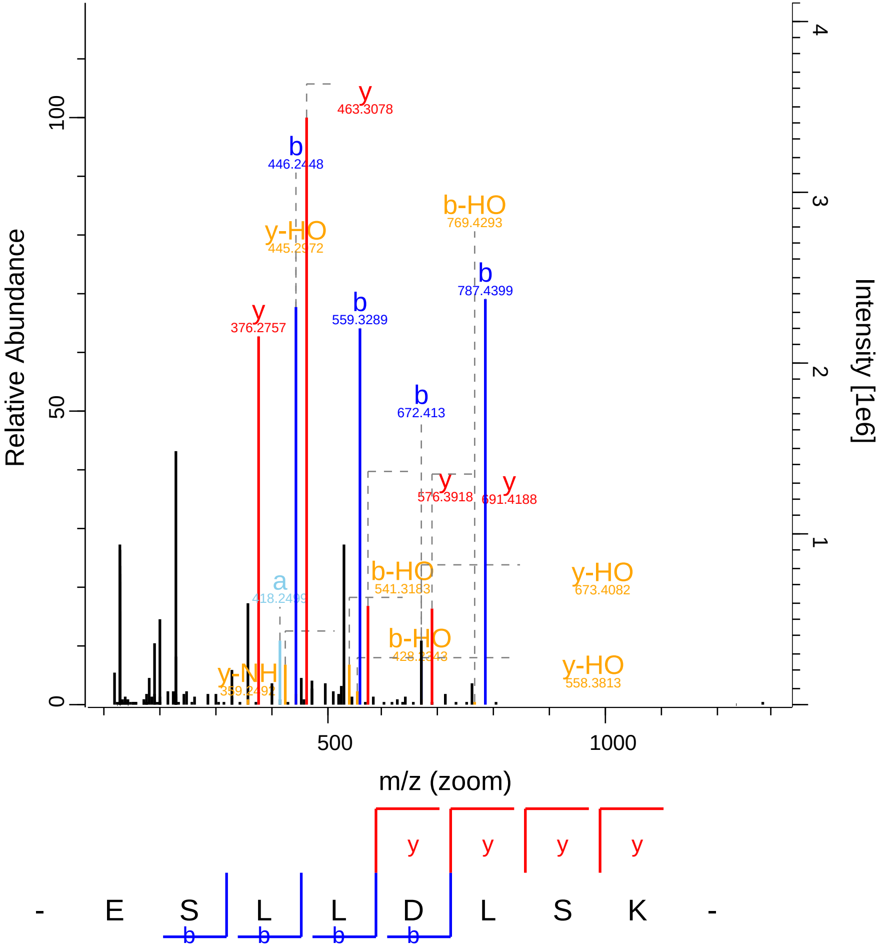

Raw File	Scan	Method	Score	m/z	Gene names
RO-QC-160121_ZackD_SS_Set_01_Fractio	26676	FTMS; HC	86.09	405.21	SCTR

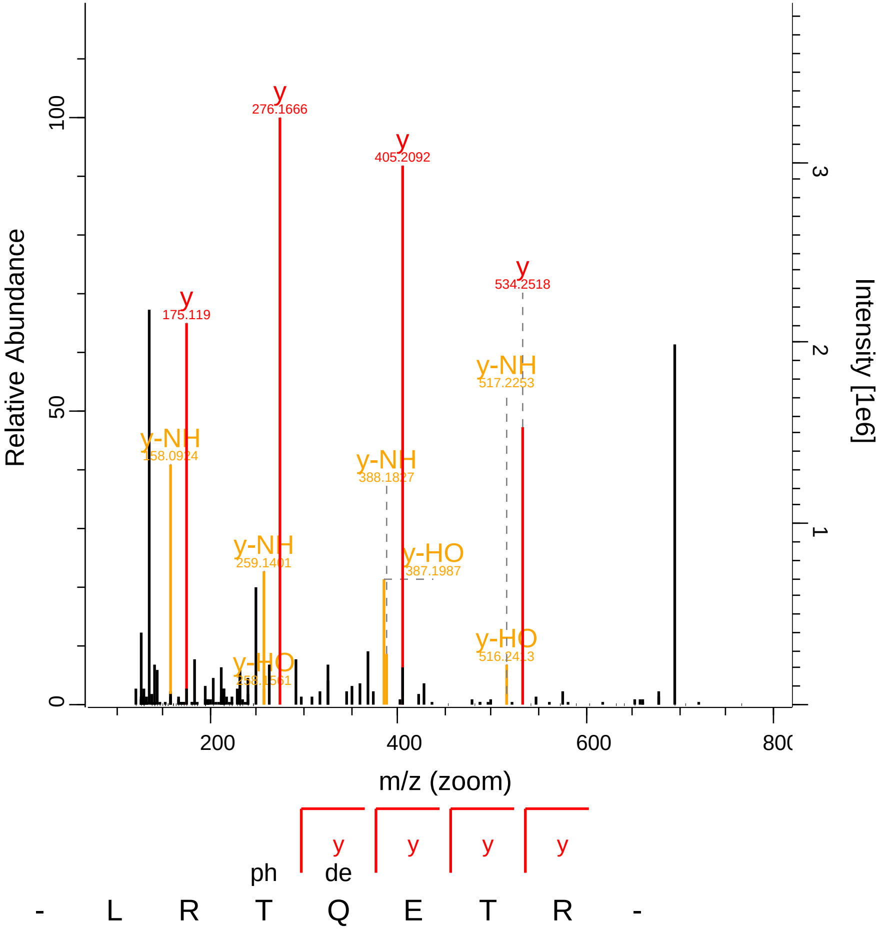

Raw File	Scan	Method	Score	m/z
RO-QC-160121_ZackD_SS_Set_01_Fraction_6	26756	FTMS; HCD	122.52	681.92

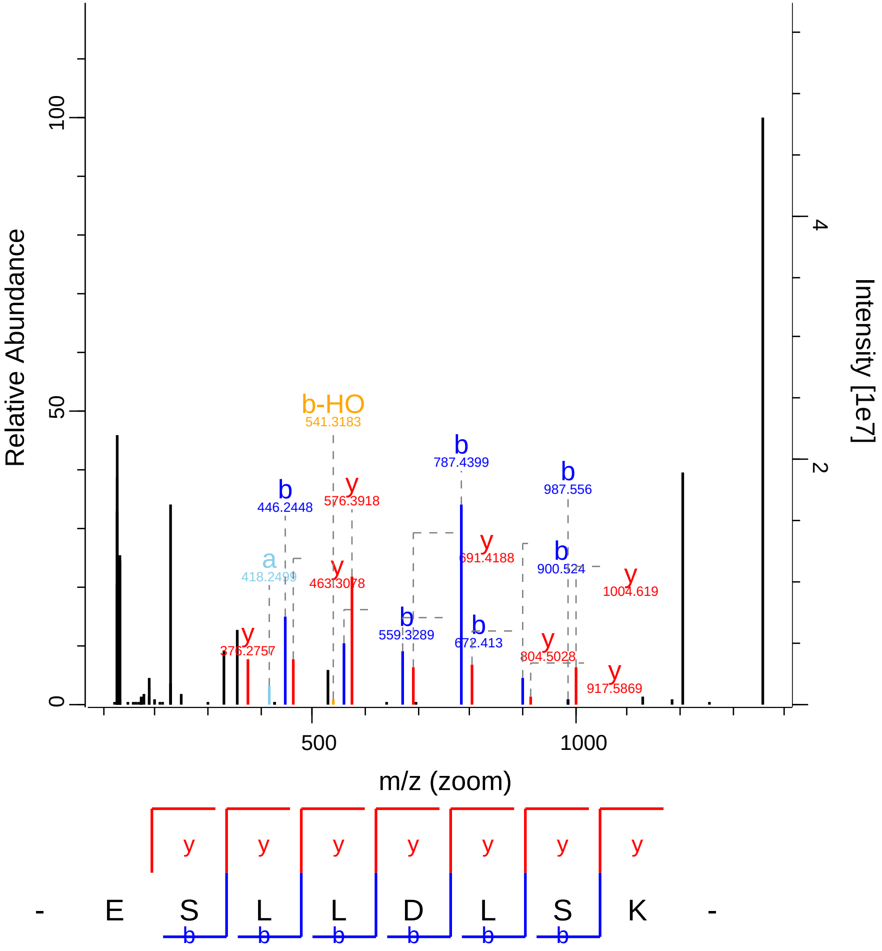

Raw File	Scan	Method	Score	m/z
RO-QC-160121_ZackD_SS_Set_01_Fraction_6	26841	FTMS; HCD	93.37	454.95

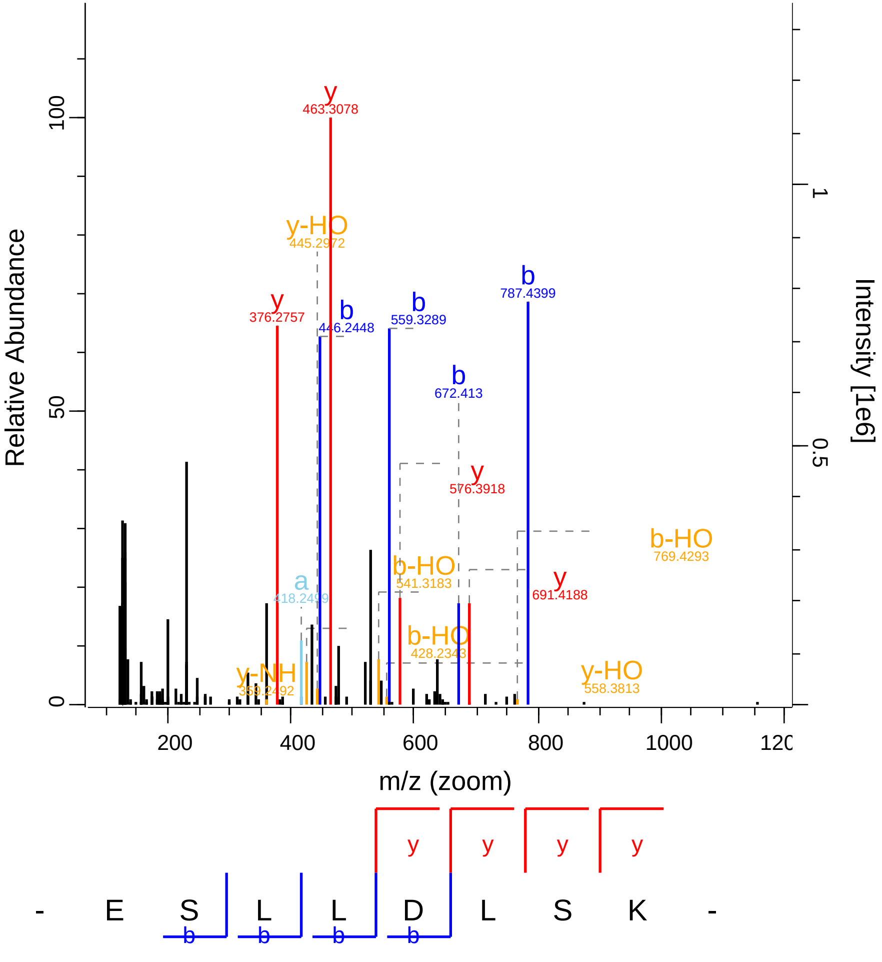

Raw File	Scan	Method	Score	m/z	Gene names
RO-QC-160121_ZackD_SS_Set_01_Fractio	26892	FTMS; HC	86.09	405.21	SCTR

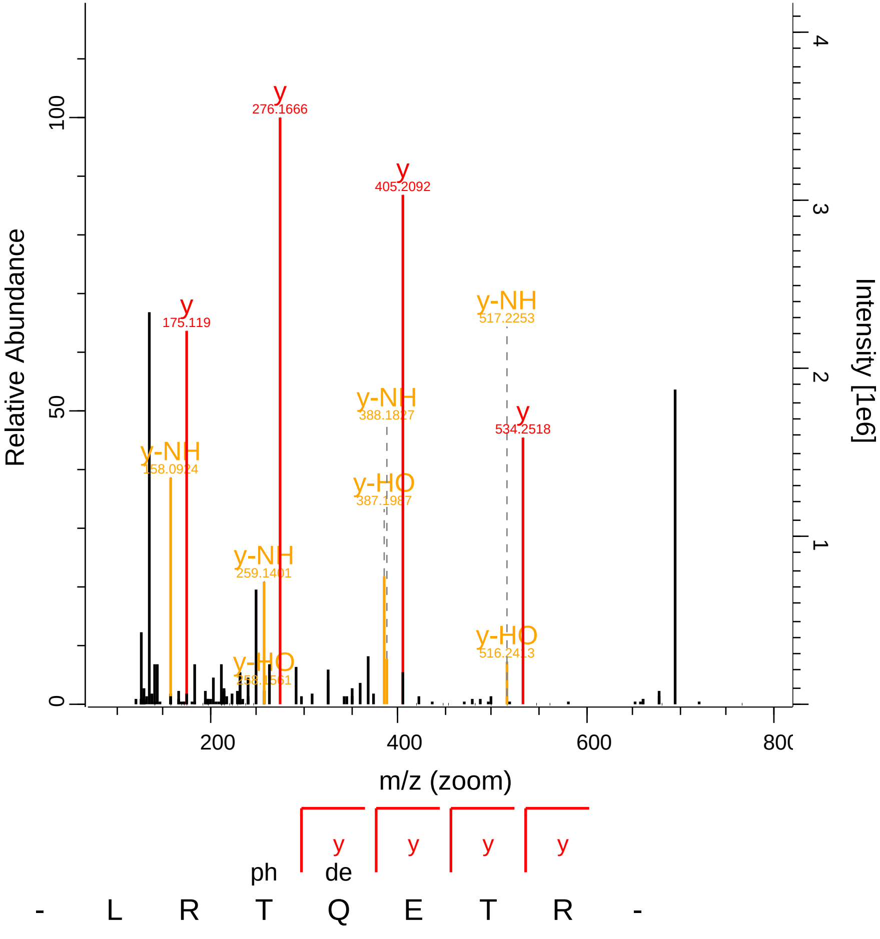

Raw File	Scan	Method	Score	m/z	Gene names
RO-QC-160121_ZackD_SS_Set_01_Fractio	27133	FTMS; HC	86.09	405.21	SCTR

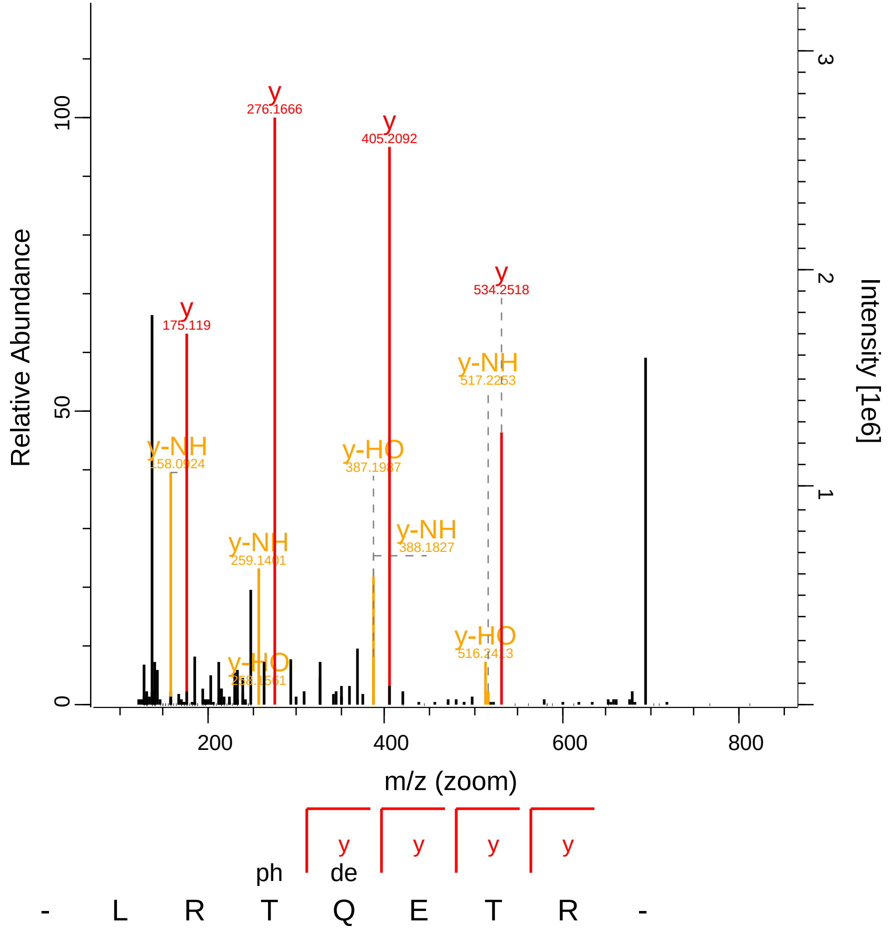

Raw File	Scan	Method	Score	m/z	Gene names
RO-QC-160121_ZackD_SS_Set_01_Fractio	28736	FTMS; HC	63.28	968.53	OPALIN

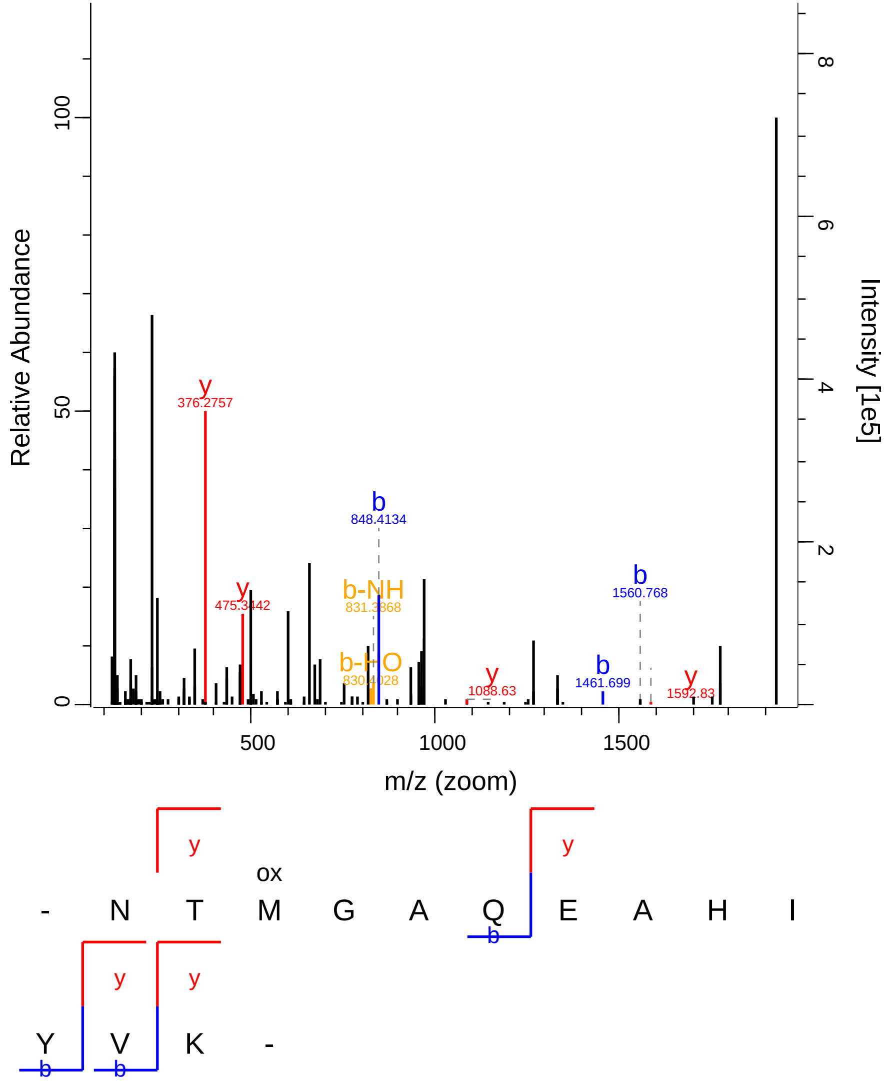

Raw File	Scan	Method	Score	m/z	Gene names
RO-QC-160121_ZackD_SS_Set_01_Fractior	28876	FTMS; HCI	55.17	613.1	MELK

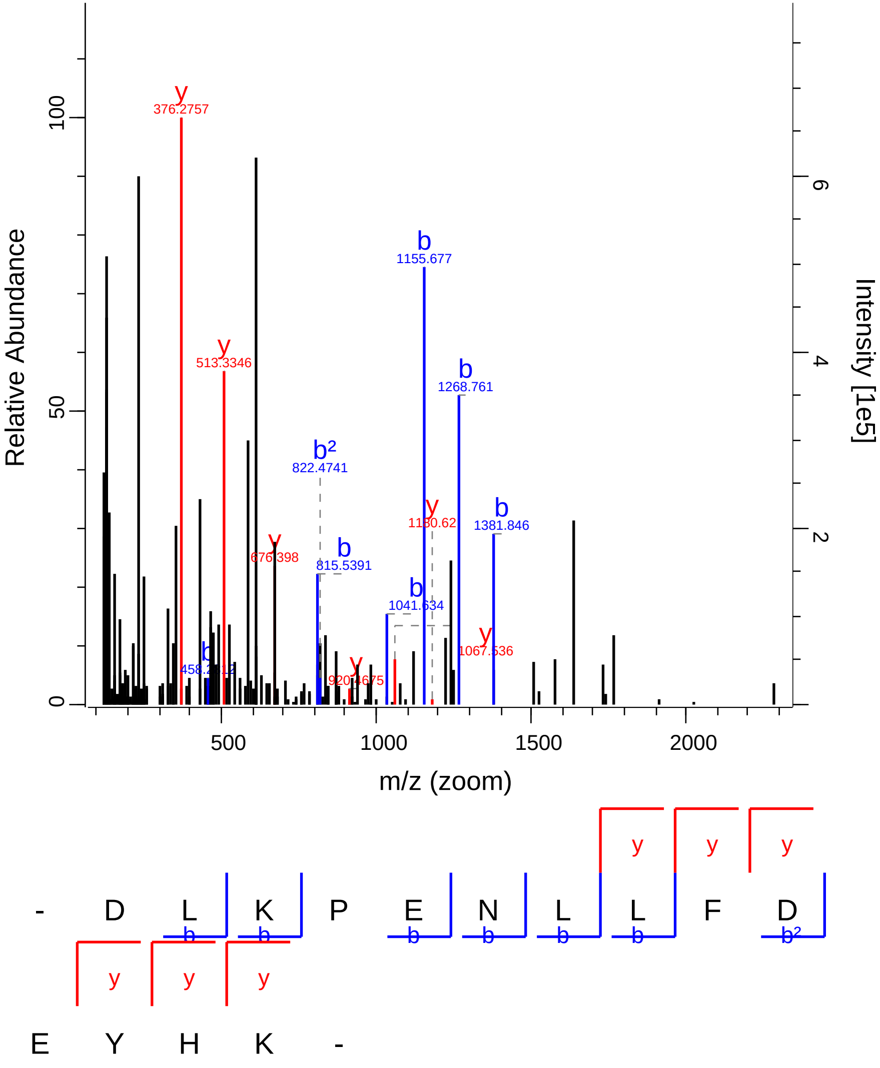

Raw File	Scan	Method	Score	m/z	Gene names
RO-QC-160121_ZackD_SS_Set_01_Fractio	29436	FTMS; HC	41.64	732.38	SLC22A7

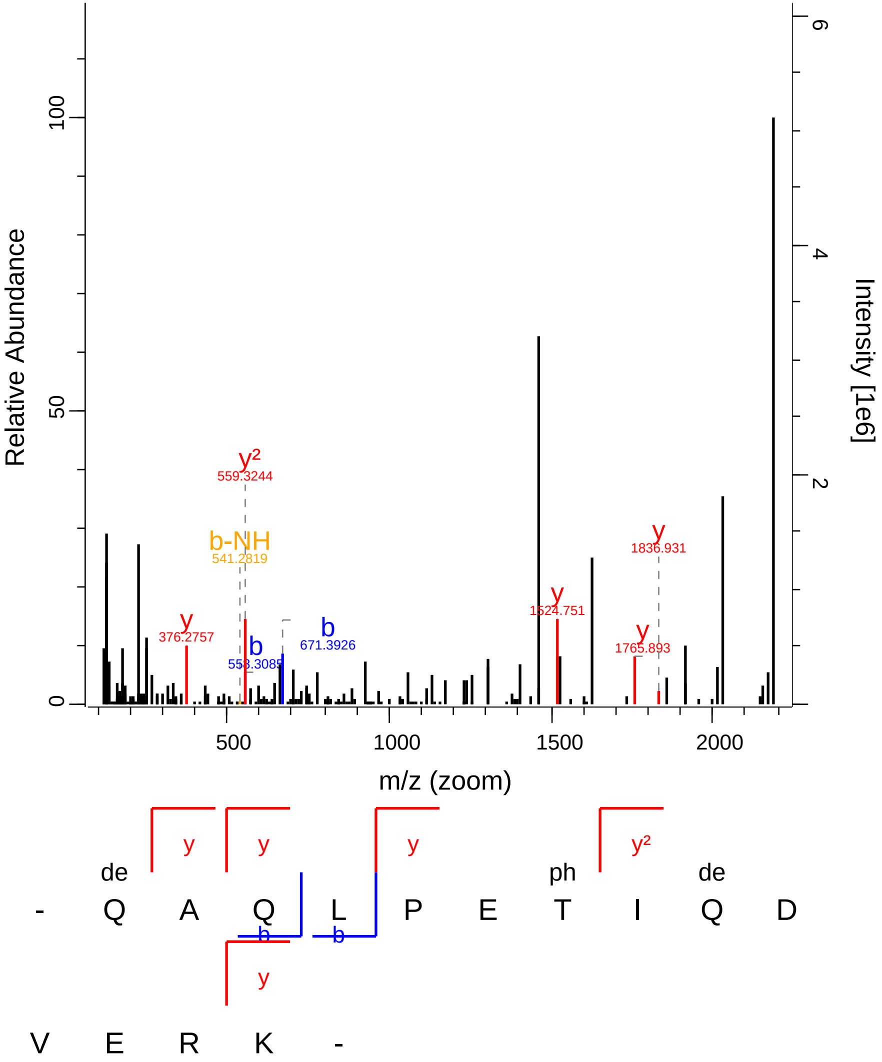

Raw File	Scan	Method	Score	m/z	Gene names
RO-QC-160121_ZackD_SS_Set_01_Fractio	30066	FTMS; HC	82.42	585.66	GSKIP

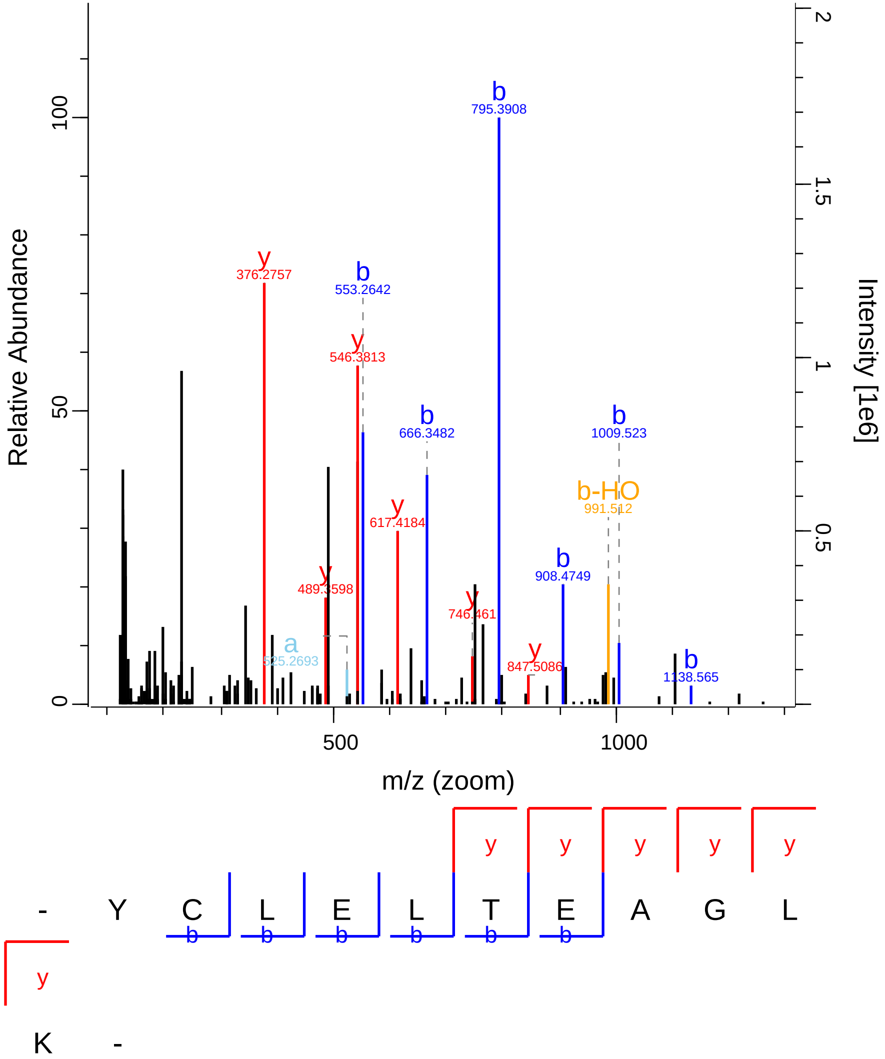

Raw File	Scan	Method	Score	m/z	Gene names
RO-QC-160121_ZackD_SS_Set_01_Fractio	30114	FTMS; HC	123.35	682.88	MT-CYB

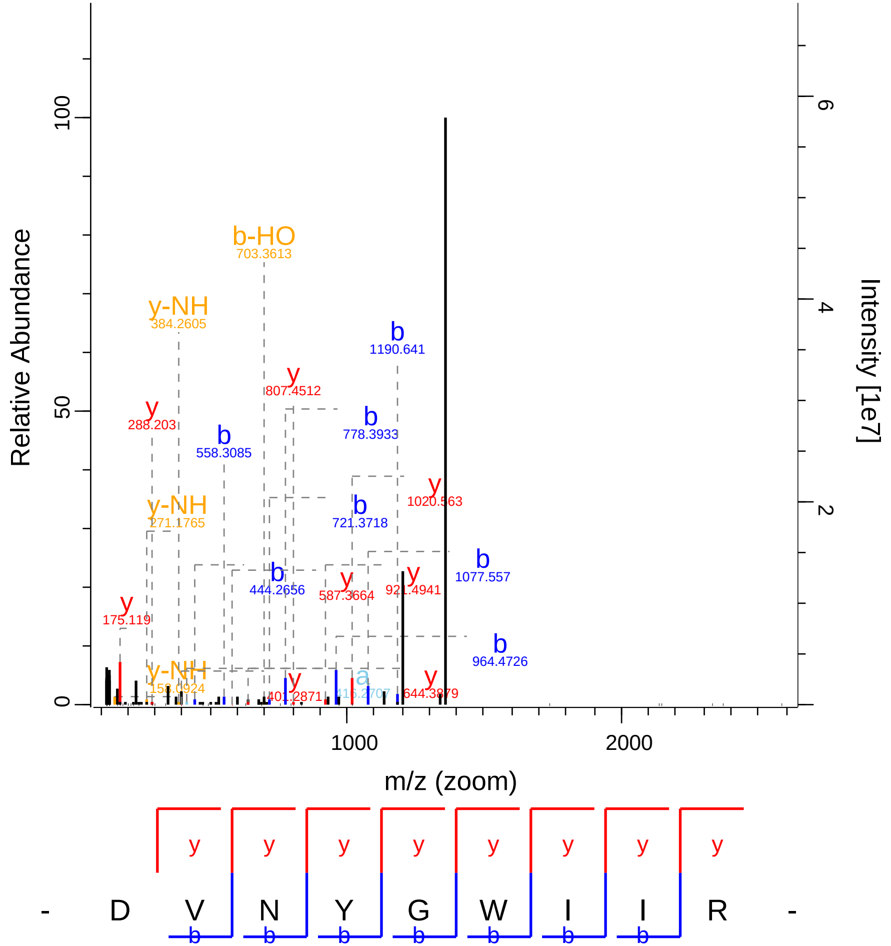

Raw File	Scan	Method	Score	m/z	Gene names
RO-QC-160121_ZackD_SS_Set_01_Fractio	30119	FTMS; HC	179.1	877.99	GSKIP

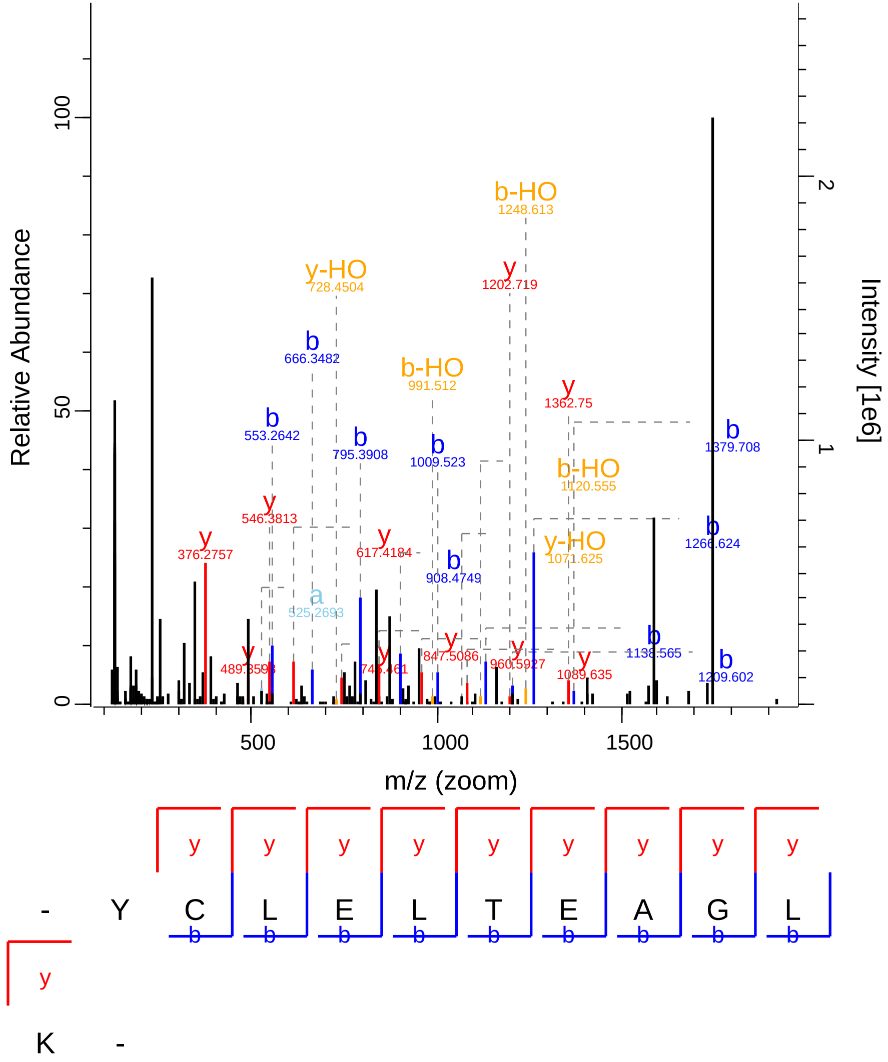

Raw File	Scan	Method	Score	m/z	Gene names
RO-QC-160121_ZackD_SS_Set_01_Fractio	30509	FTMS; HC	136.7	841.75	CNIH4

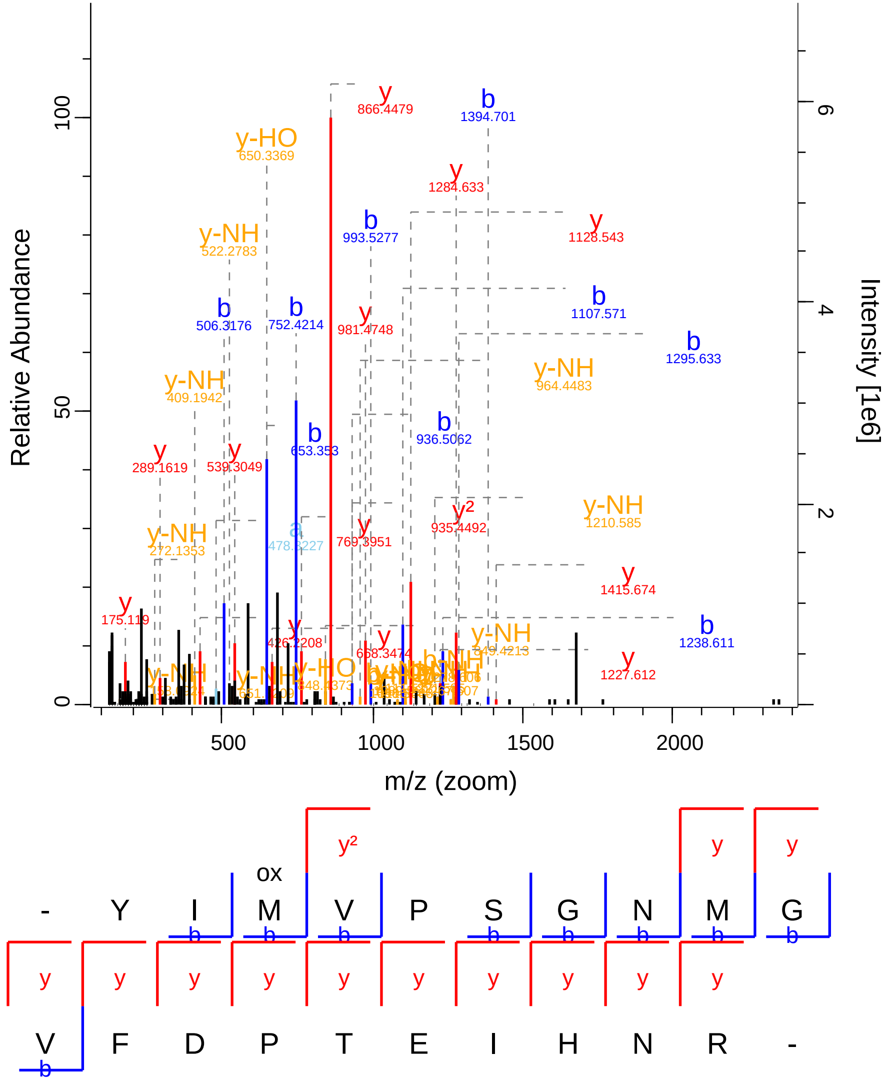

Raw File	Scan	Method	Score	m/z	Gene names
RO-QC-160121_ZackD_SS_Set_01_Fractio	30611	FTMS; HC	48.82	631.57	CNIH4

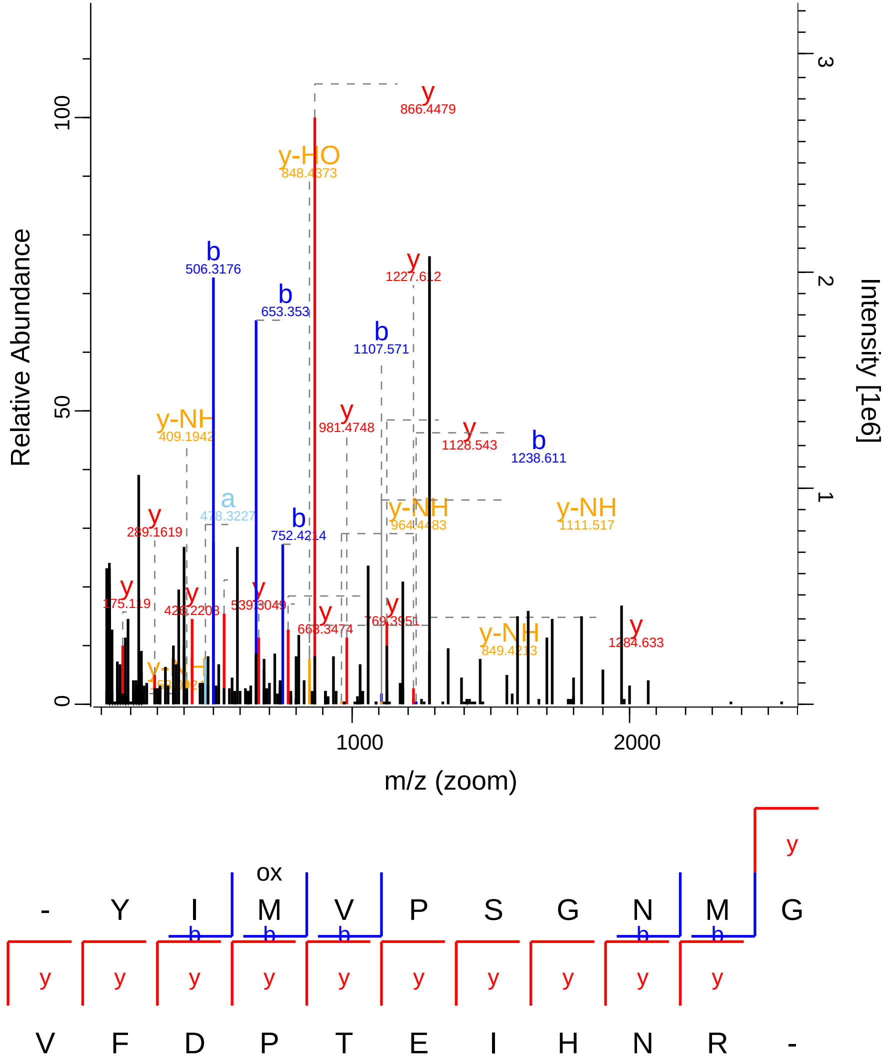

Raw File	Scan	Method	Score	m/z	Gene names
RO-QC-160121_ZackD_SS_Set_01_Fractio	30714	FTMS; HC	142.26	841.42	CNIH4

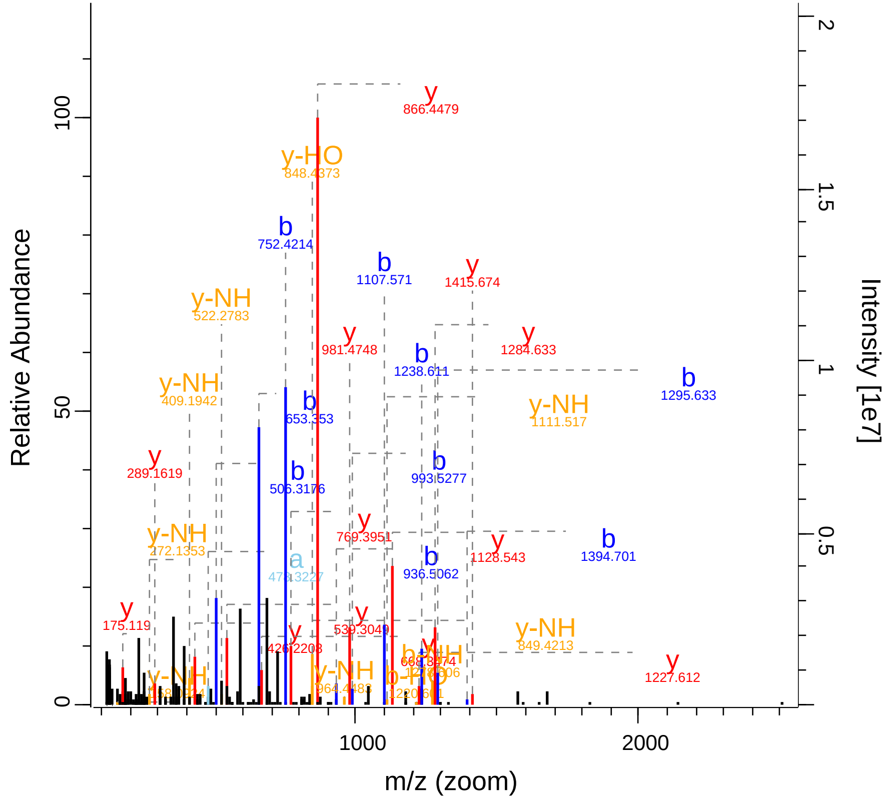

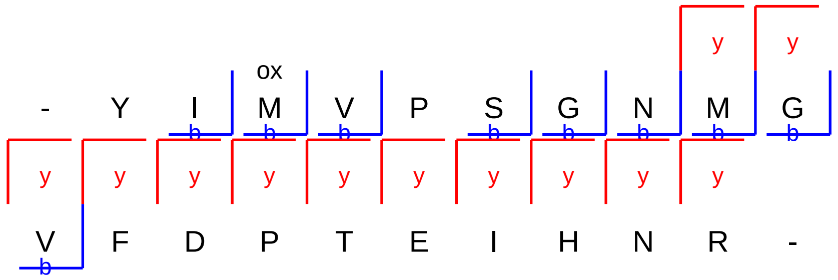

Raw File	Scan	Method	Score	m/z	Gene names
RO-QC-160121_ZackD_SS_Set_01_Fractio	30839	FTMS; HC	130.2	655.38	ALKBH8

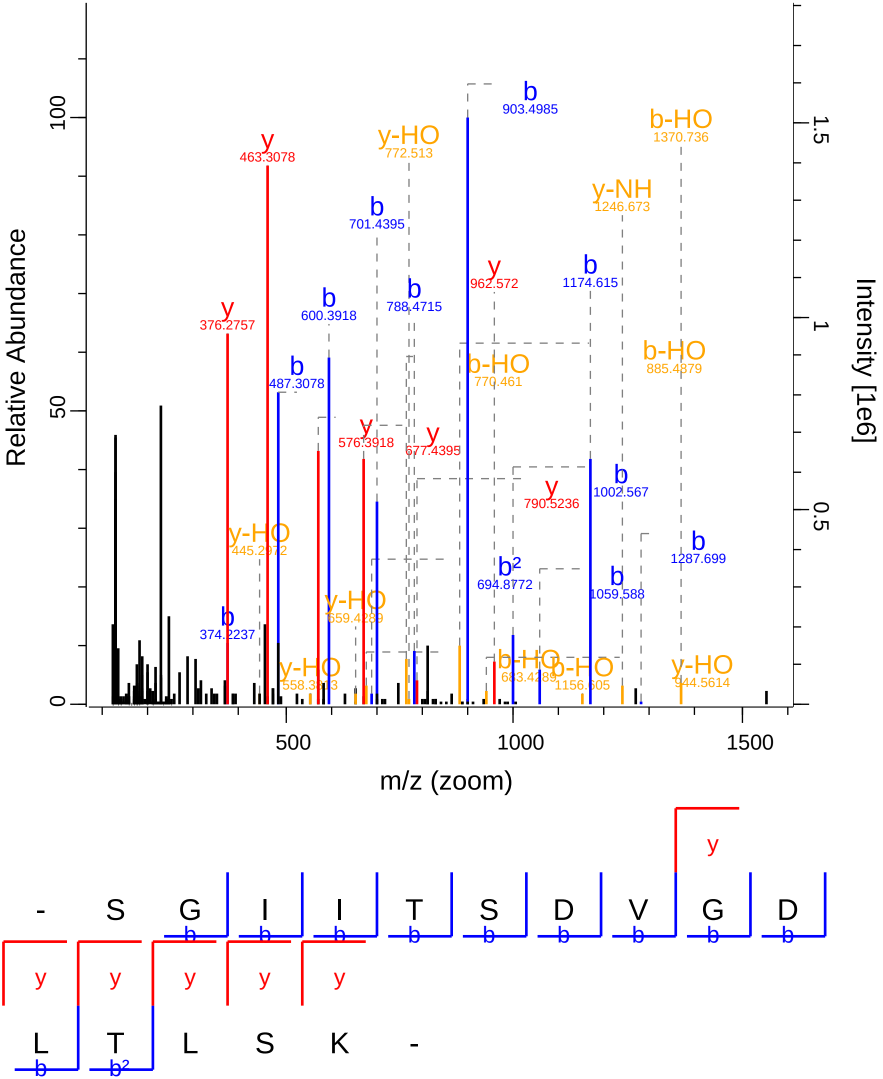

Raw File	Scan	Method	Score	m/z	Gene names
RO-QC-160121_ZackD_SS_Set_01_Fractio	30867	FTMS; HC	125.18	839.48	GTF3C6

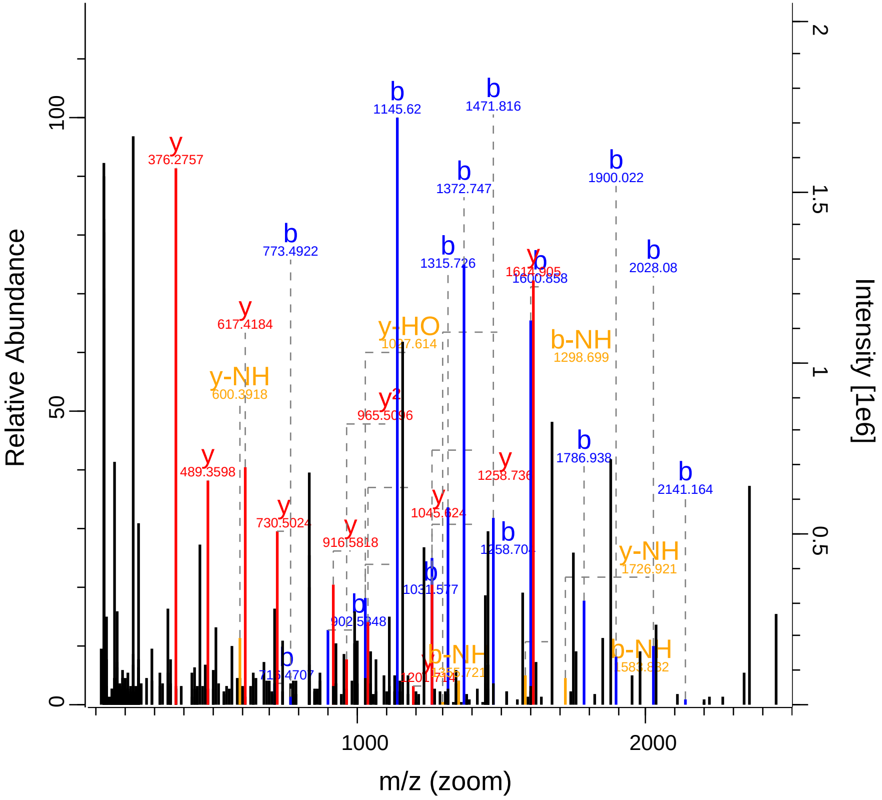

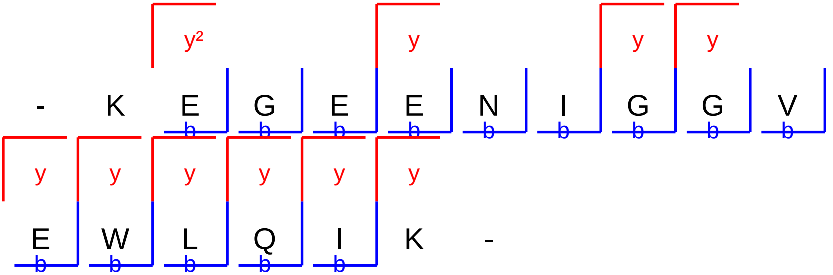

Raw File	Scan	Method	Score	m/z	Gene names
RO-QC-160121_ZackD_SS_Set_01_Fractio	30900	FTMS; HC	89.26	631.34	POP7

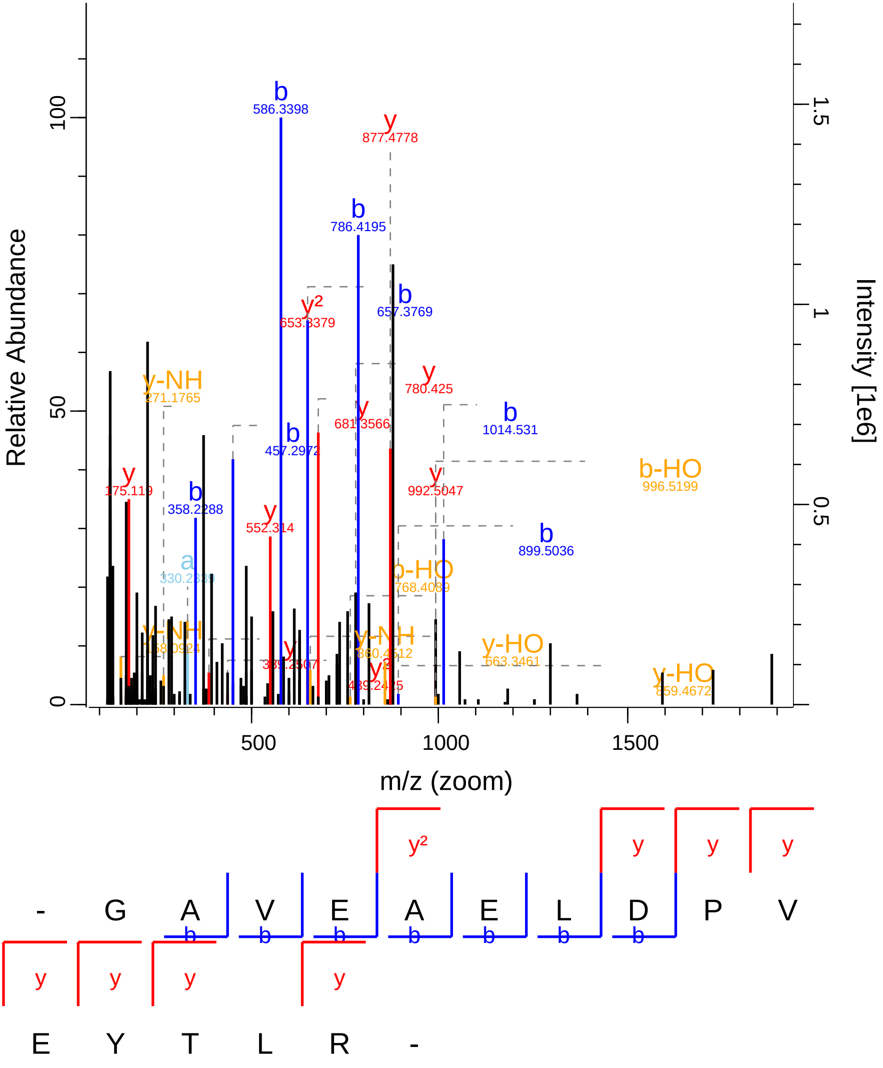

Raw File	Scan	Method	Score	m/z	Gene names
RO-QC-160121_ZackD_SS_Set_01_Fractio	30901	FTMS; HC	208.95	946.01	POP7

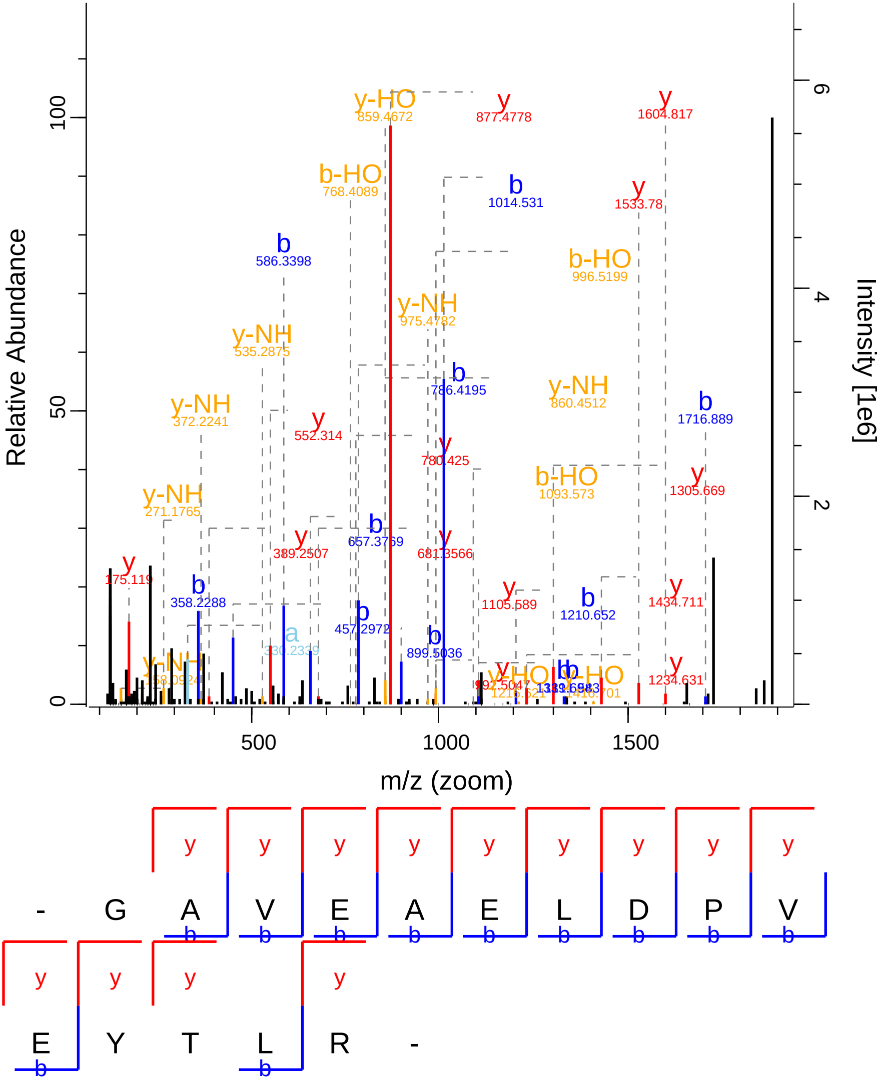

Raw File	Scan	Method	Score	m/z	Gene names
RO-QC-160121_ZackD_SS_Set_01_Fractio	31027	FTMS; HC	72.43	829.03	C4orf33

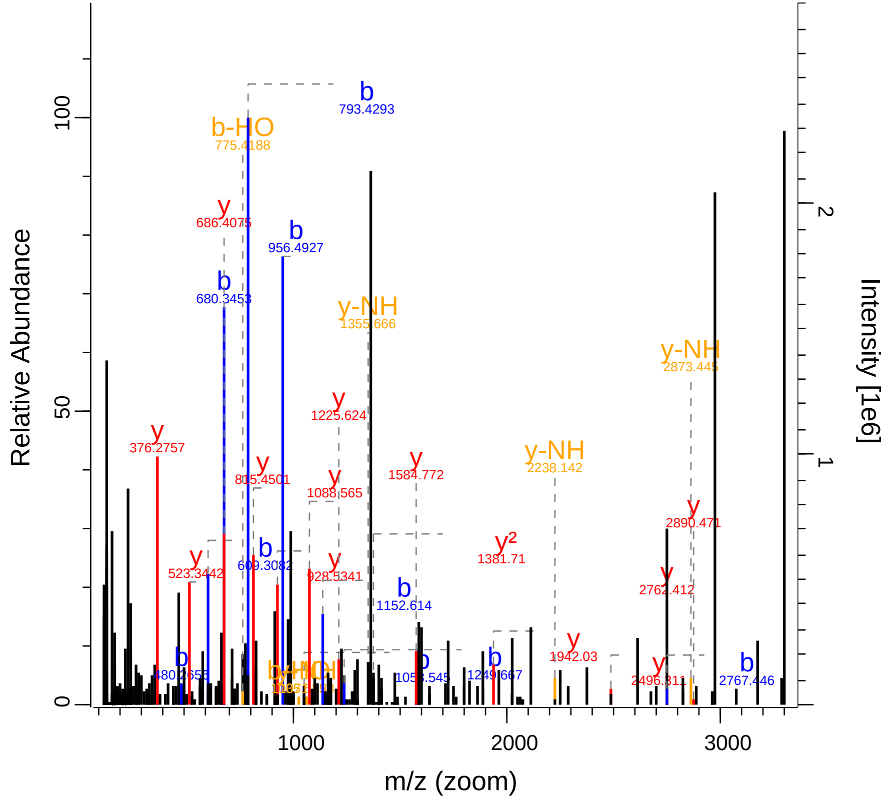

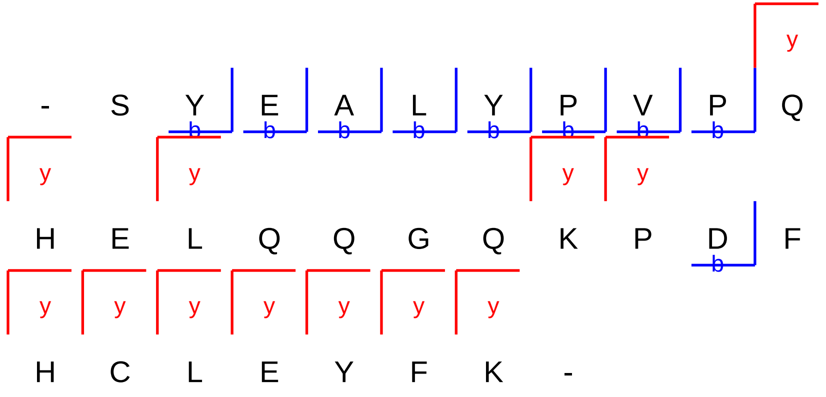

Raw File	Scan	Method	Score	m/z	Gene names
RO-QC-160121_ZackD_SS_Set_01_Fractior	31460	FTMS; HCI	72.21	872.5	KBTBD2

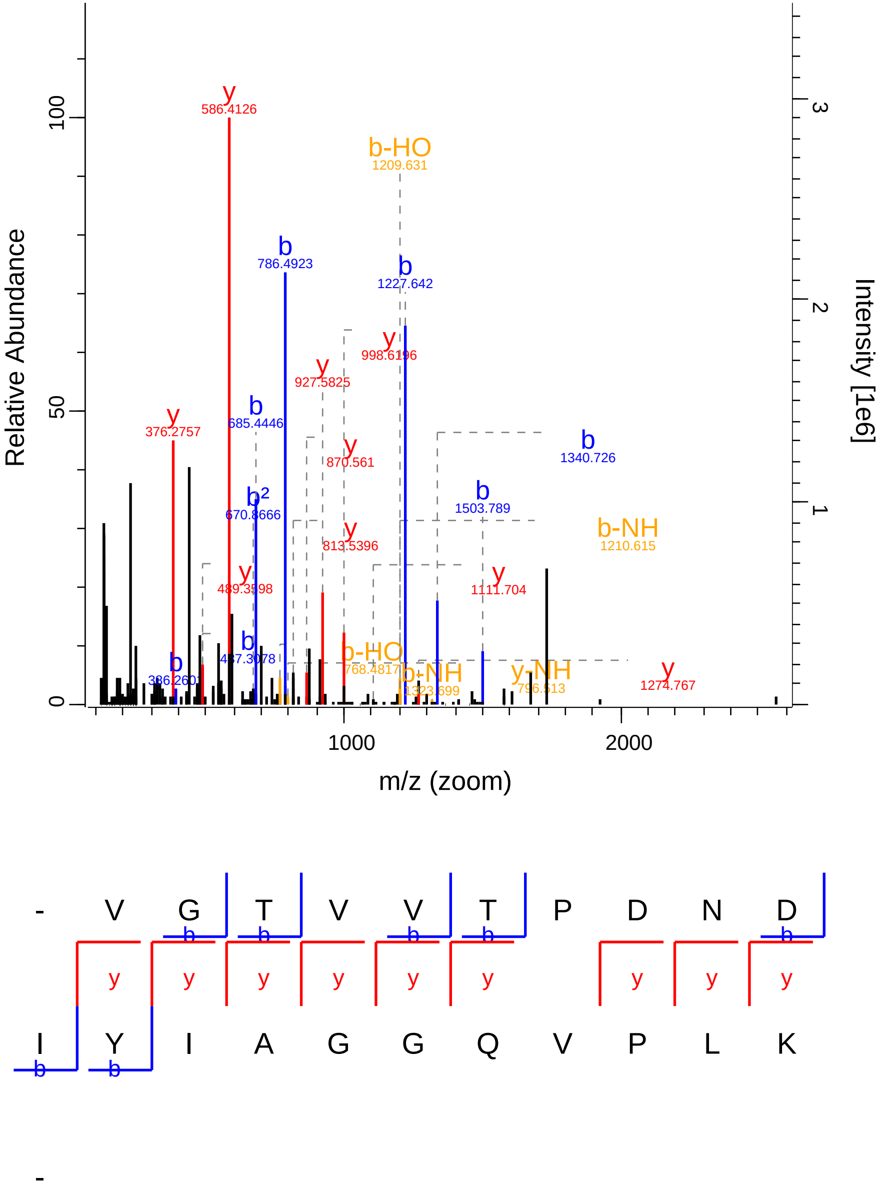

Raw File	Scan	Method	Score	m/z
RO-QC-160121_ZackD_SS_Set_01_Fraction_6	31834	FTMS; HCD	78.33	736.96

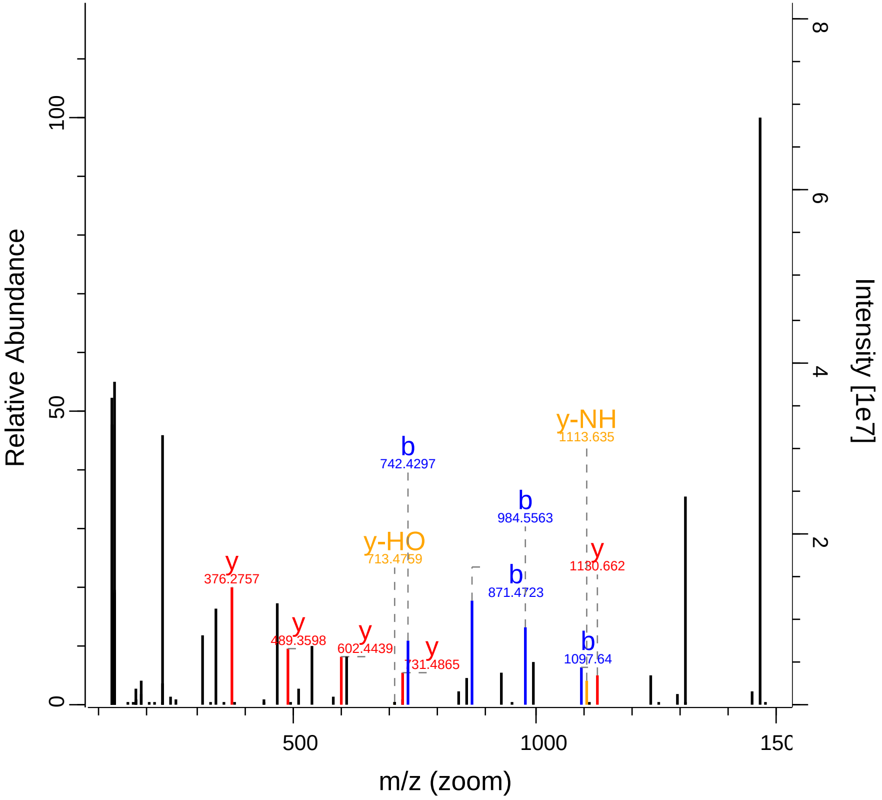

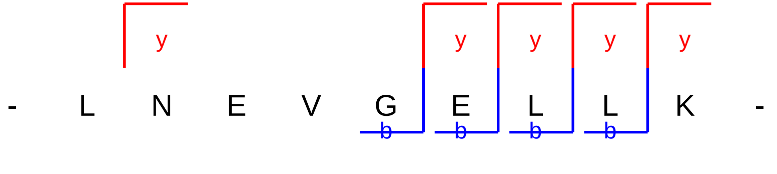

Raw File	Scan	Method	Score	m/z	Gene names
RO-QC-160121_ZackD_SS_Set_01_Fractio	32157	FTMS; HC	70.44	857.46	UBE2S

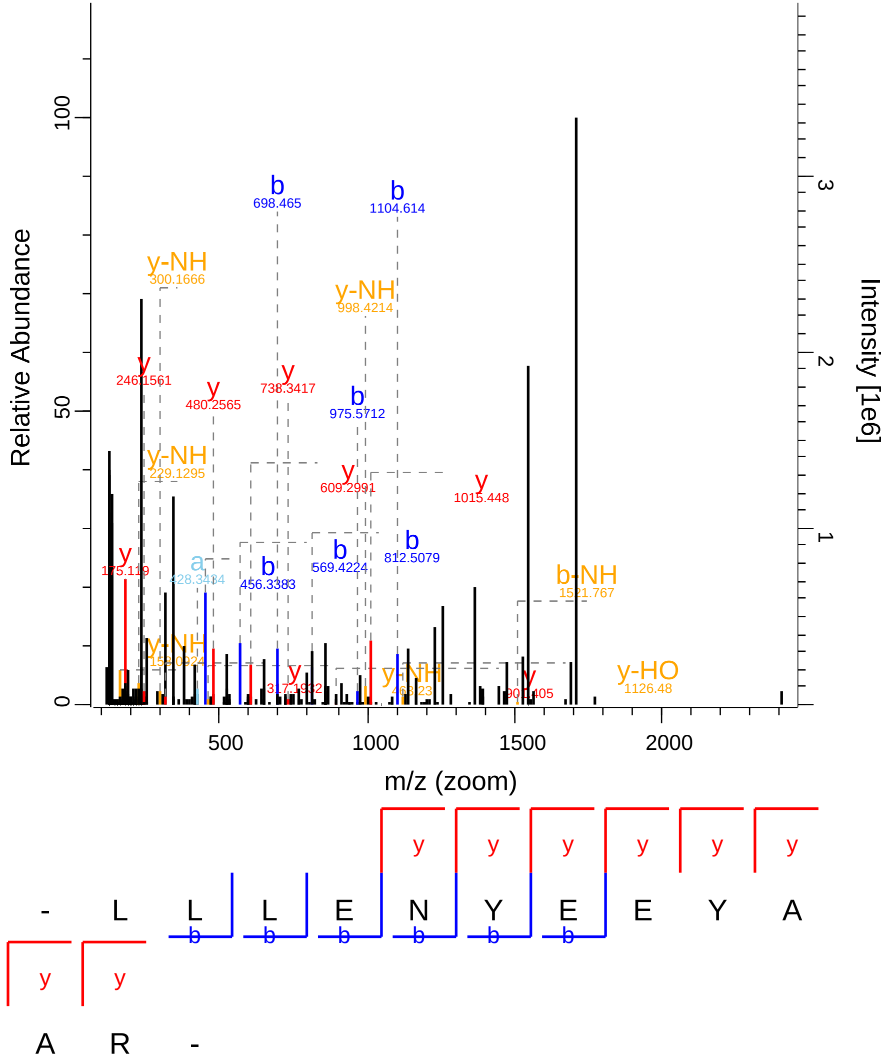

Raw File	Scan	Method	Score	m/z	Gene names
RO-QC-160121_ZackD_SS_Set_01_Fractior	32676	FTMS; HCI	102.89	729.1	BOLA1

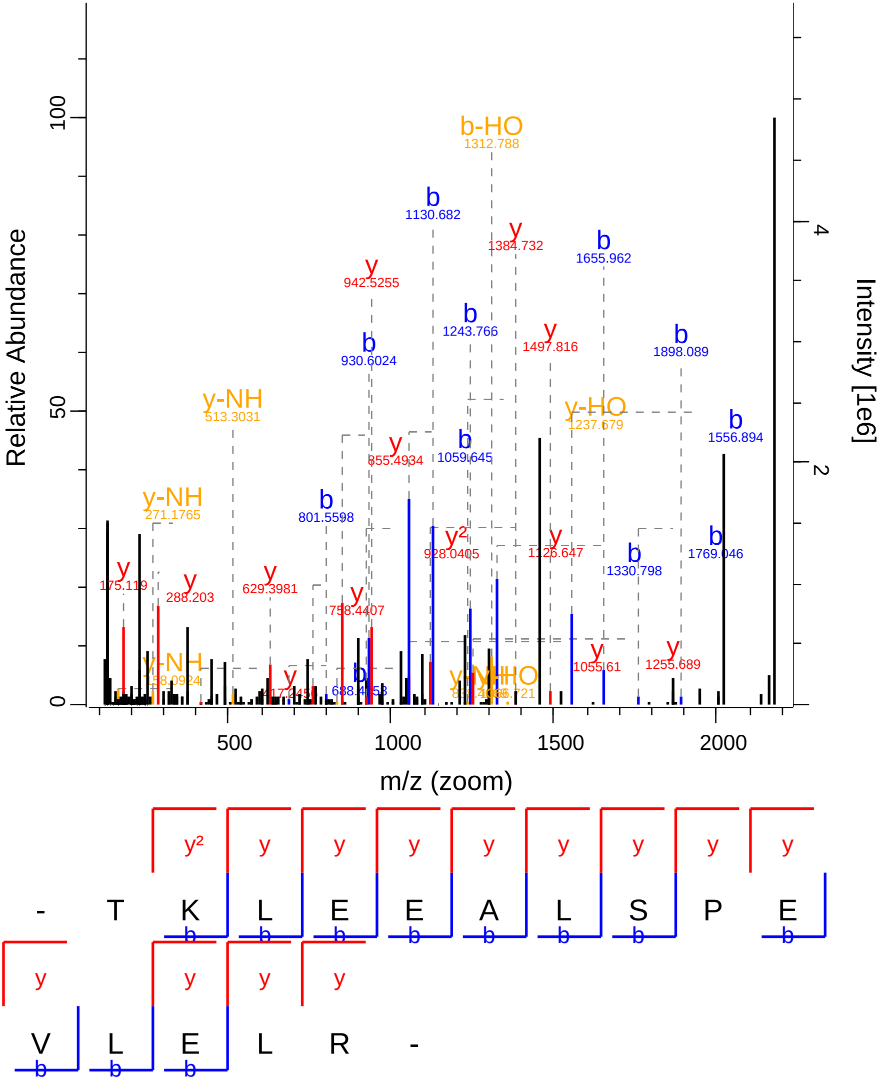

Raw File	Scan	Method	Score	m/z
RO-QC-160121_ZackD_SS_Set_01_Fraction_6	32685	FTMS; HCD	67.99	800.96

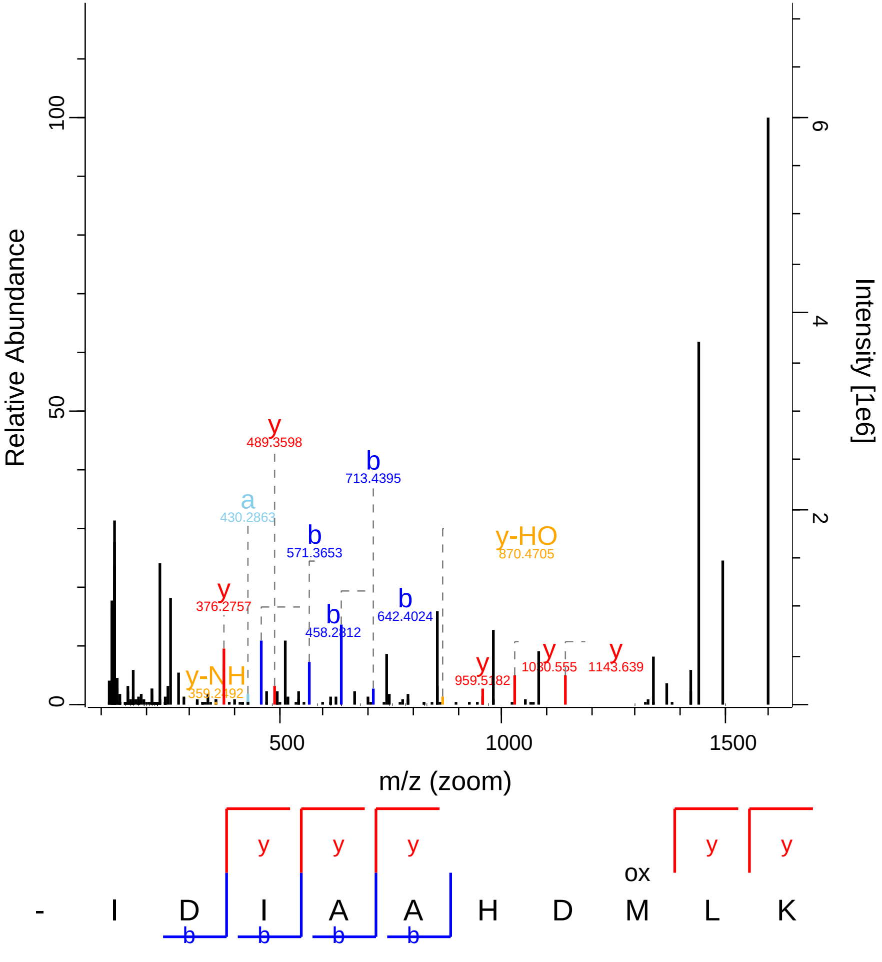

Raw File	Scan	Method	Score	m/z	Gene names
RO-QC-160121_ZackD_SS_Set_01_Fractio	33207	FTMS; HC	45.1	911.98	C12orf29

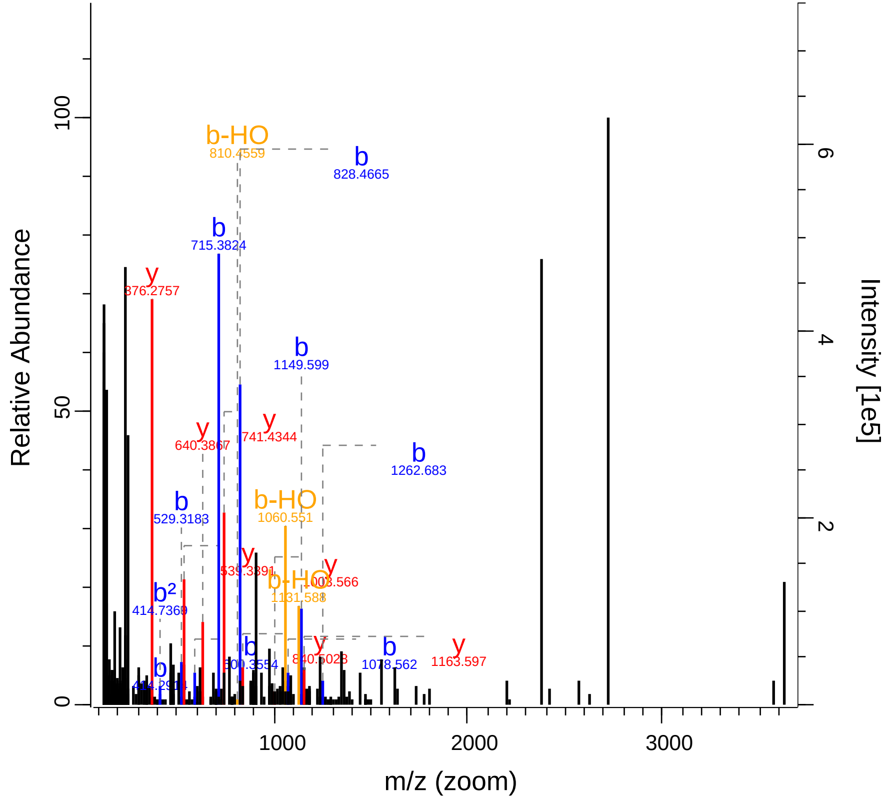

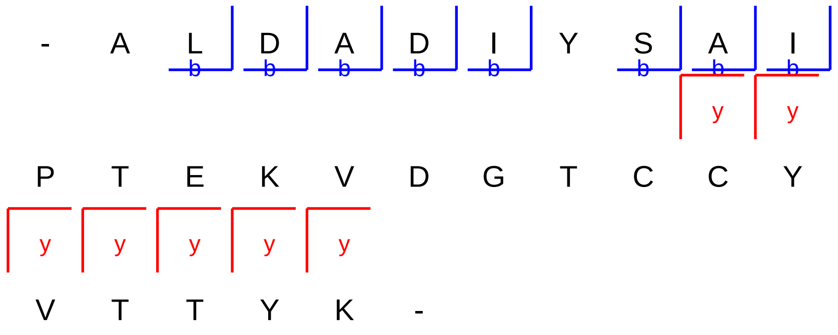

Raw File	Scan	Method	Score	m/z	Gene names
RO-QC-160121_ZackD_SS_Set_01_Fractio	33396	FTMS; HC	95.2	739.42	MZT1

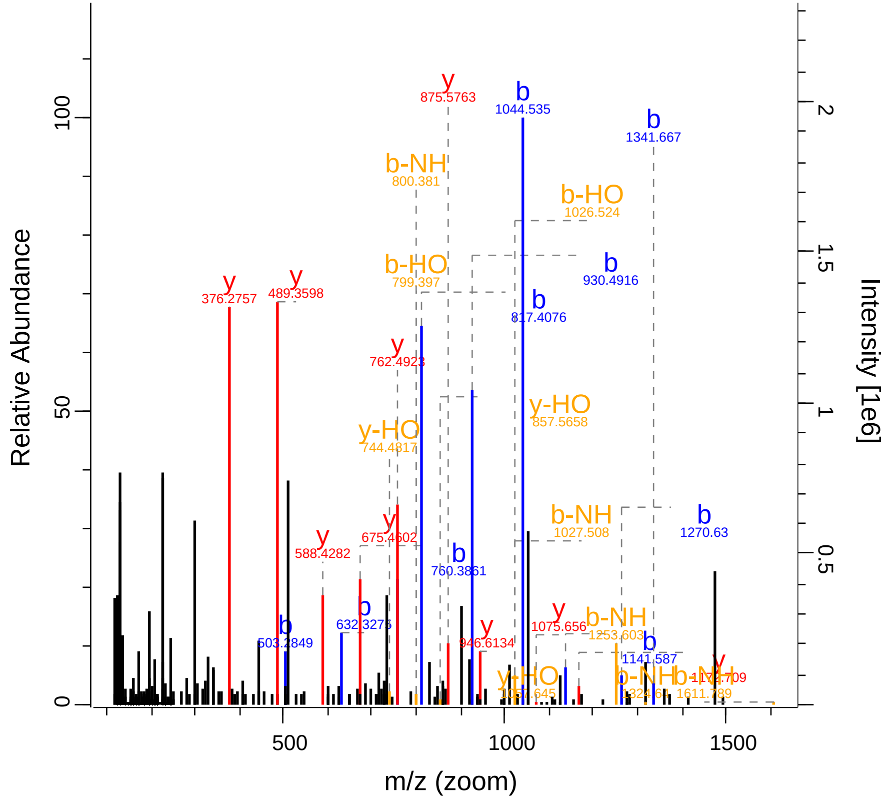

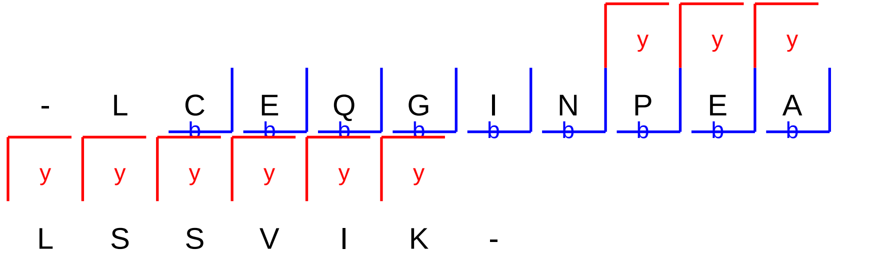

Raw File	Scan	Method	Score	m/z	Gene names
RO-QC-160121_ZackD_SS_Set_01_Fractio	33438	FTMS; HC	52.33	616.02	ATRIP

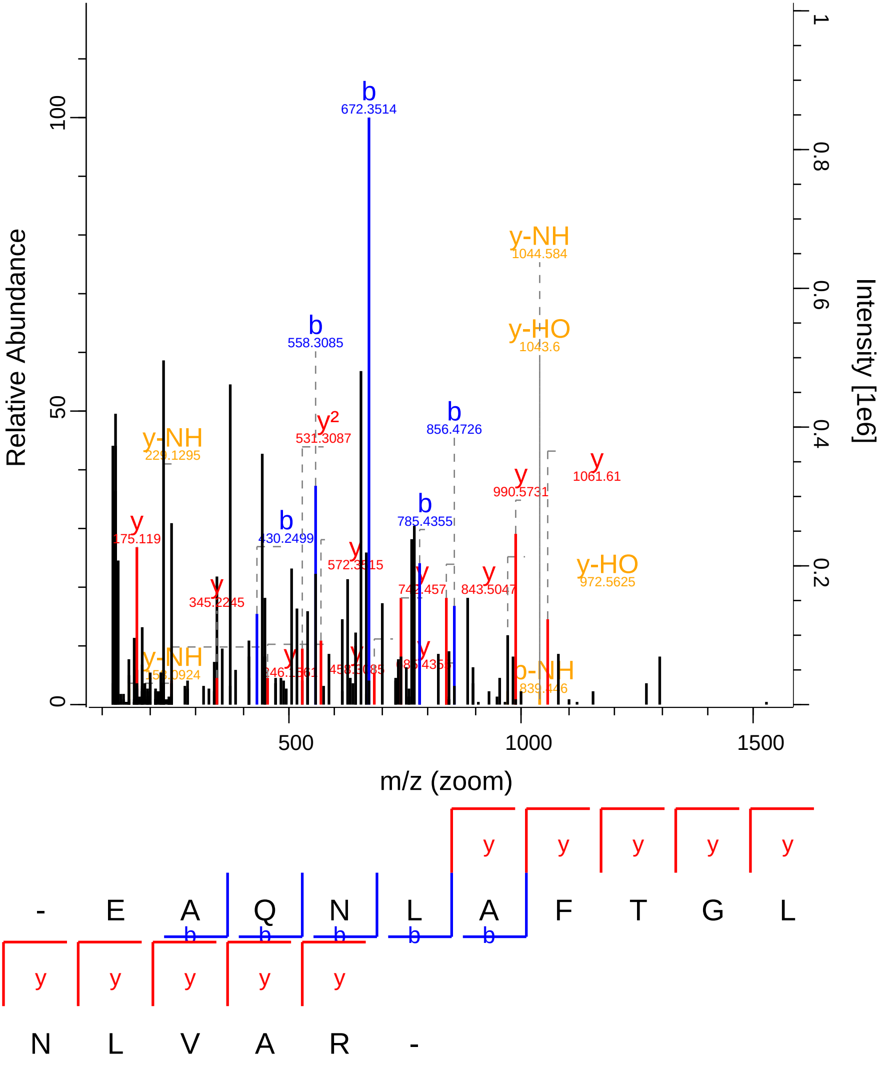

Raw File	Scan	Method	Score	m/z	Gene names
RO-QC-160121_ZackD_SS_Set_01_Fractio	35975	FTMS; HC	45.1	1030.8	DENND6A

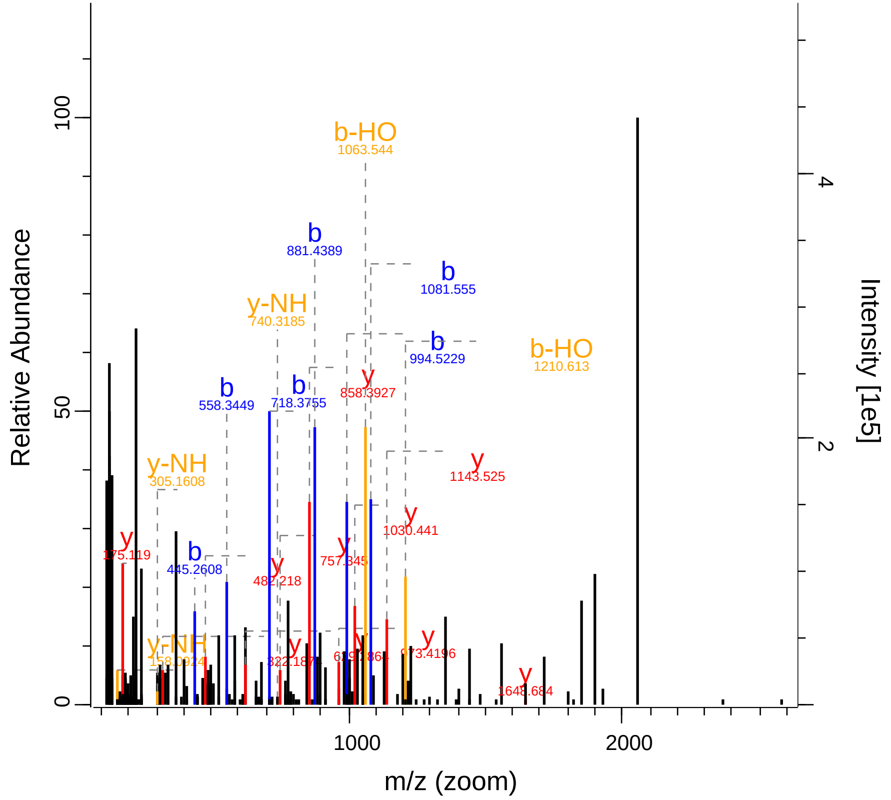

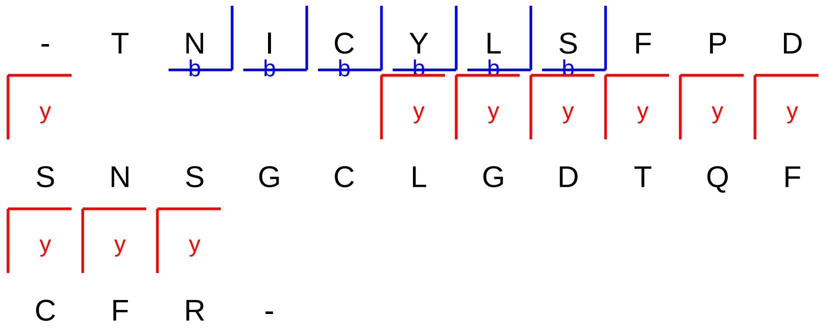

Raw File	Scan	Method	Score	m/z	Gene names
RO-QC-160121_ZackD_SS_Set_01_Fractic	37572	FTMS; HC	232	1066.02	MT-CO1

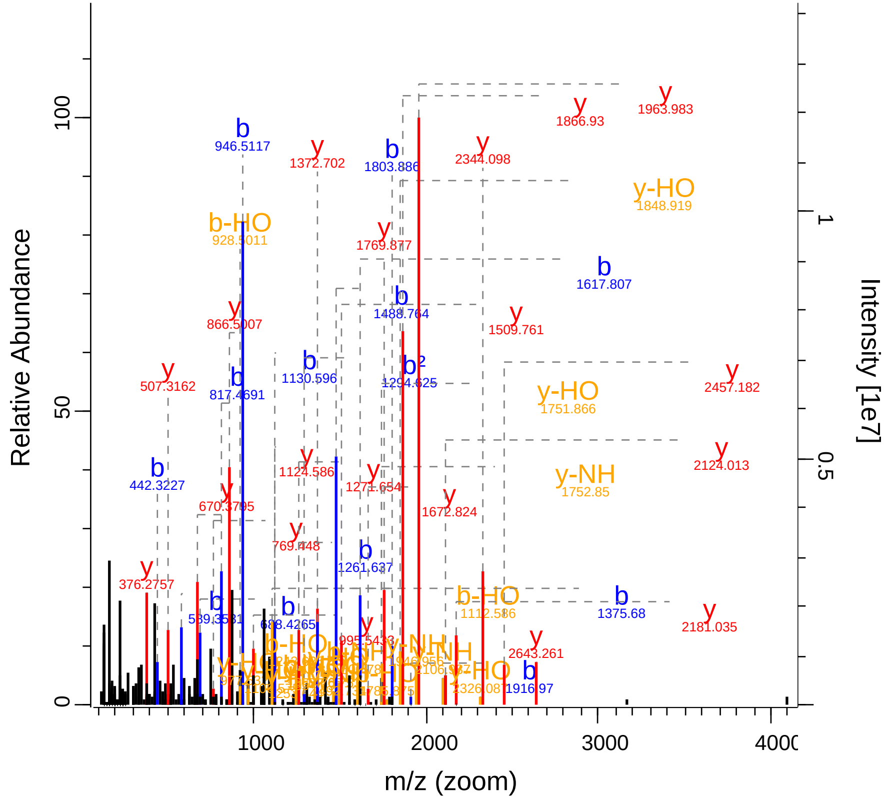

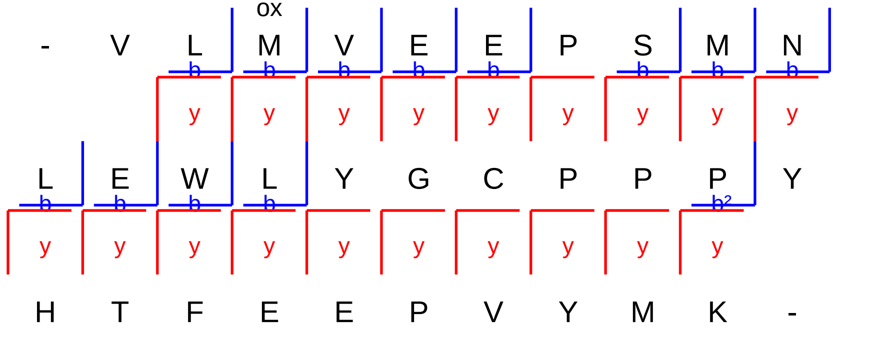

Raw File	Scan	Method	Score	m/z	Gene names
RO-QC-160121_ZackD_SS_Set_01_Fractio	38027	FTMS; HC	91.85	860.44	PRSS58

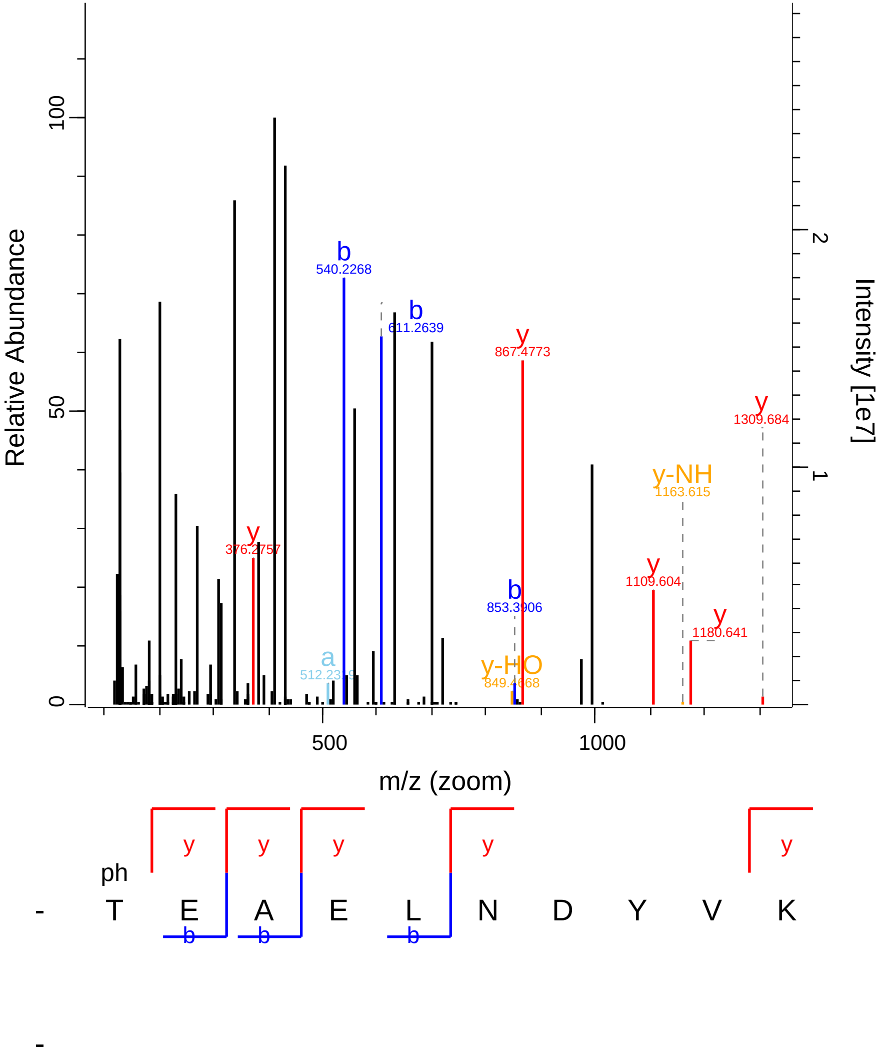

Raw File	Scan	Method	Score	m/z
RO-QC-160121_ZackD_SS_Set_01_Fraction_6	38363	FTMS; HCD	64.8	682.35

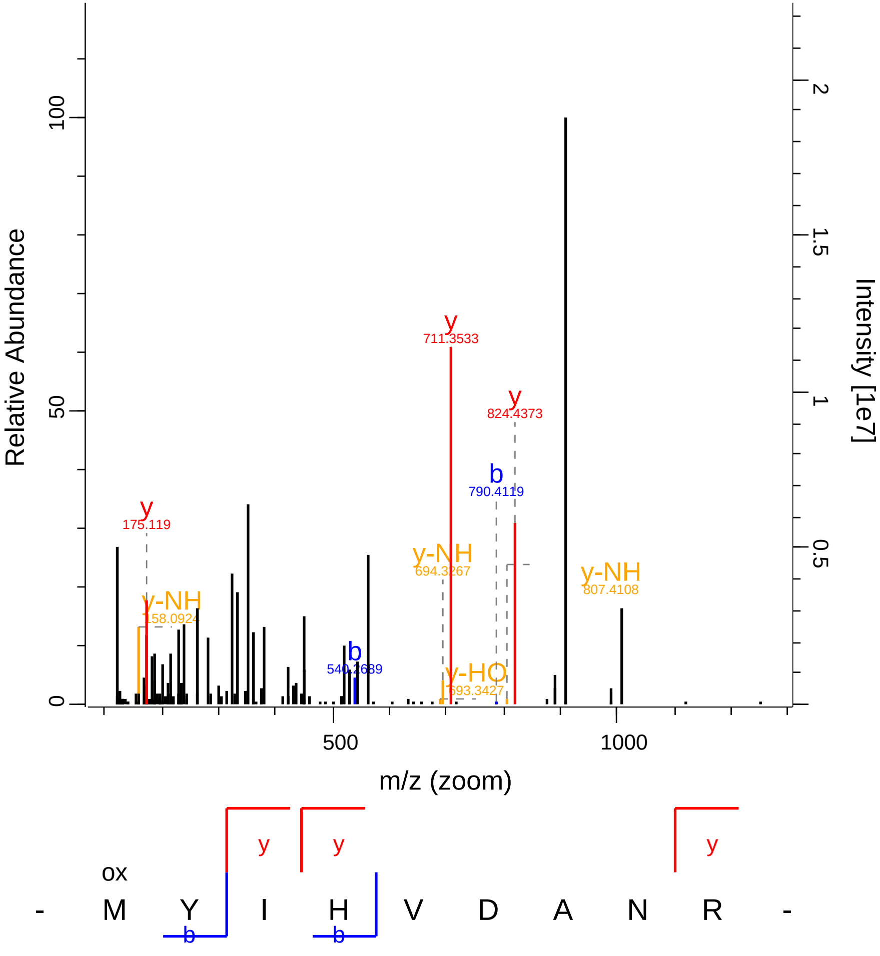

Raw File	Scan	Method	Score	m/z	Gene names
RO-QC-160121_ZackD_SS_Set_01_Fractio	38649	FTMS; HC	122.51	885.45	PXDC1

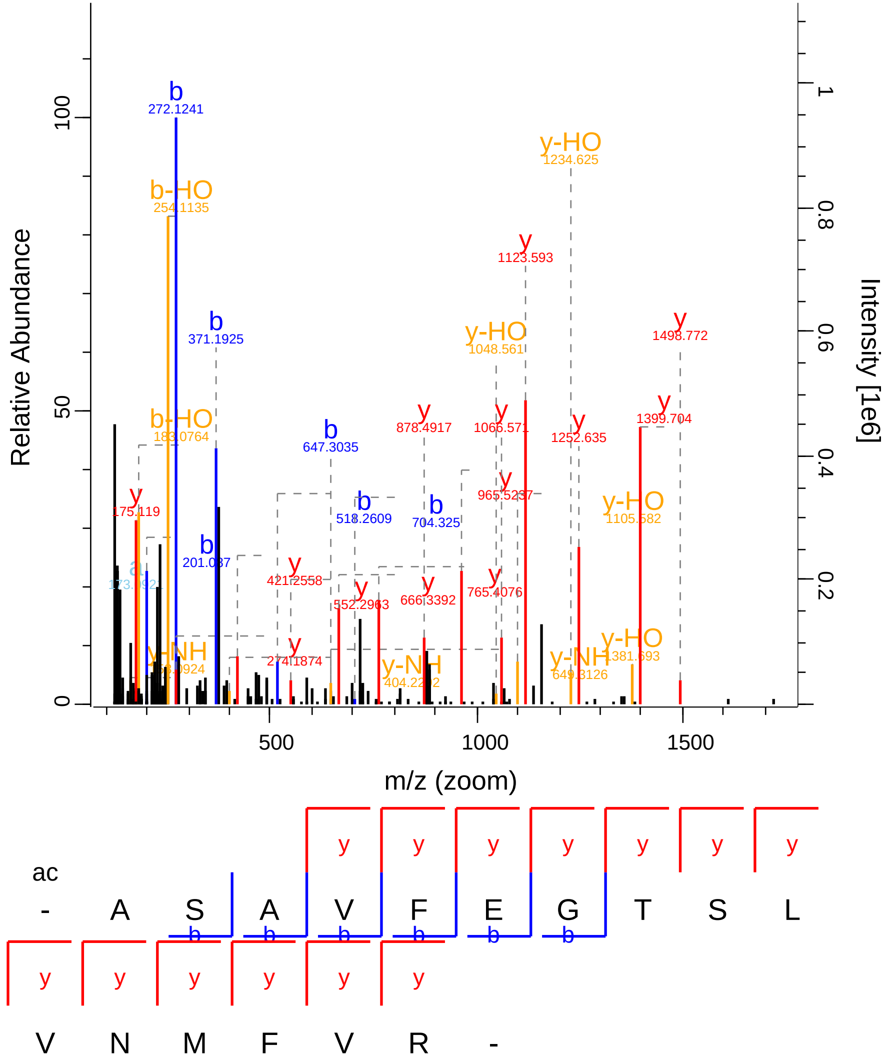

Raw File	Scan	Method	Score	m/z	Gene names
RO-QC-160121_ZackD_SS_Set_01_Fractio	38798	FTMS; HC	105.86	930.03	GRK6;GRK5

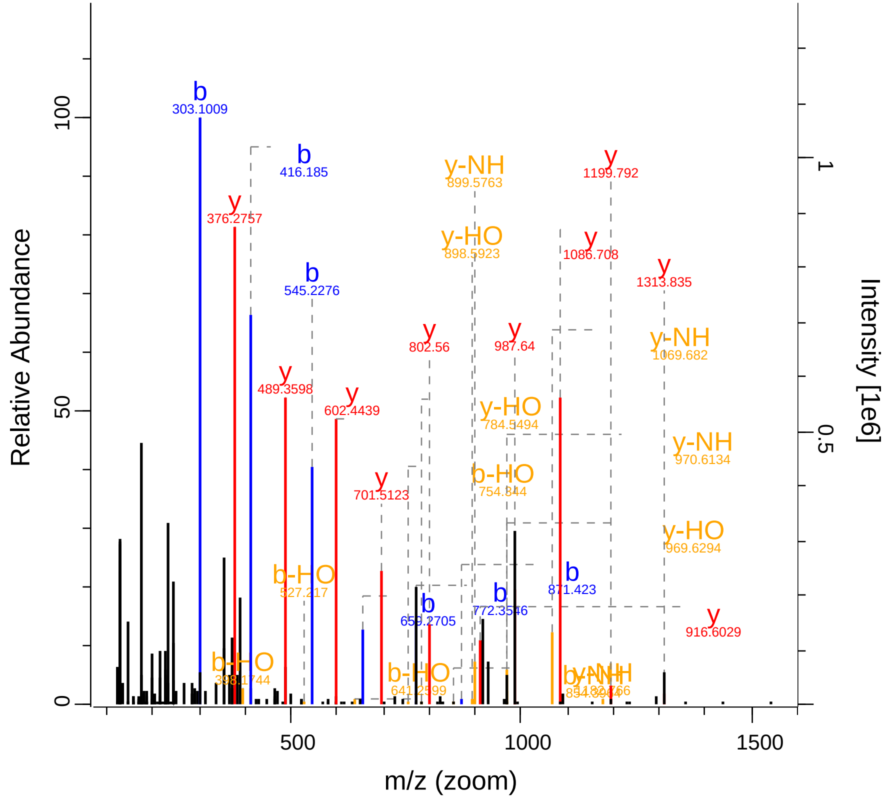

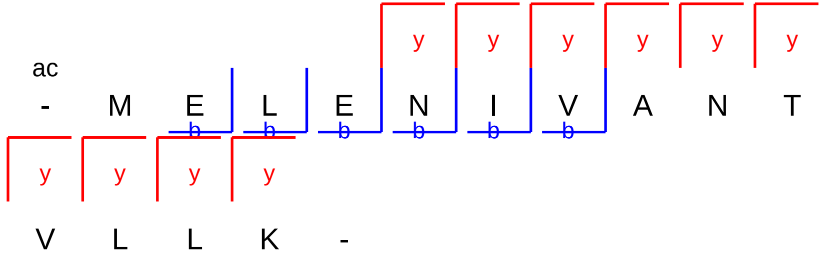

Raw File	Scan	Method	Score	m/z
RO-QC-160121_ZackD_SS_Set_01_Fraction_7	5134	FTMS; HCD	85.29	815.4

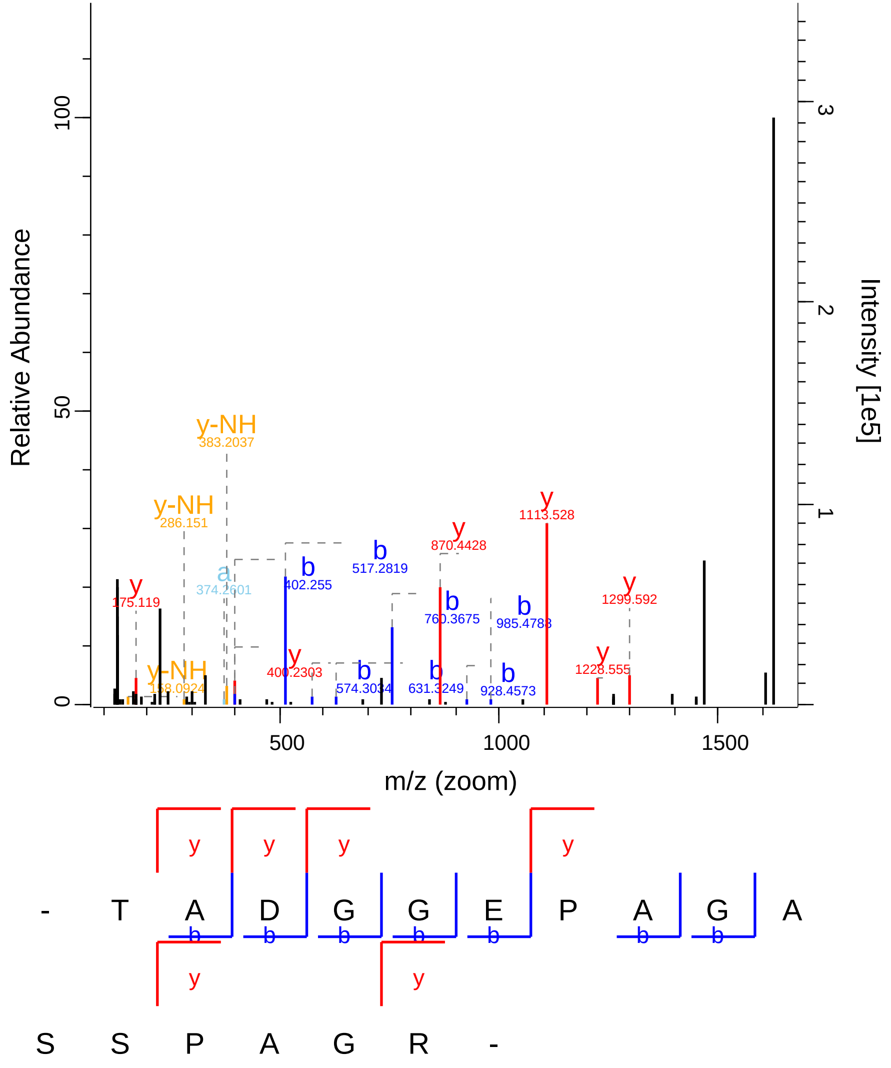

Raw File	Scan	Method	Score	m/z
RO-QC-160121_ZackD_SS_Set_01_Fraction_7	5260	FTMS; HCD	58.81	543.94

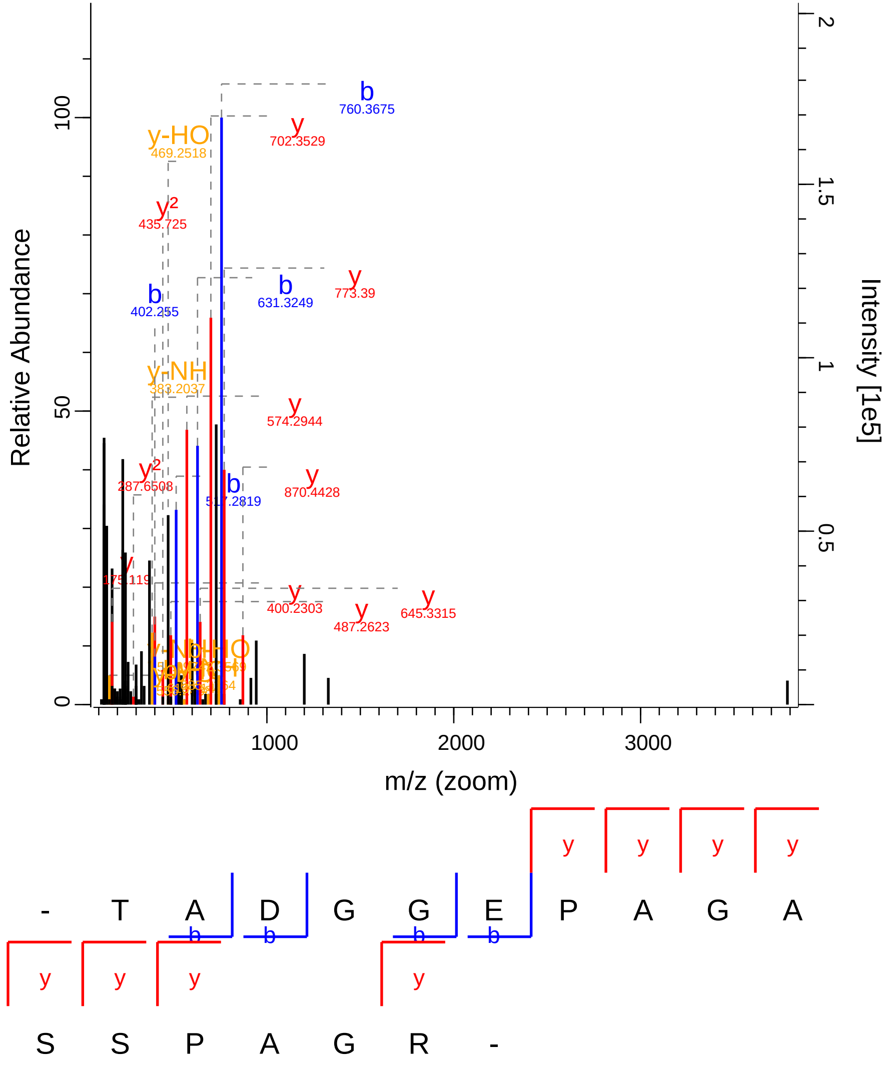

Raw File	Scan	Method	Score	m/z
RO-QC-160121_ZackD_SS_Set_01_Fraction_7	5283	FTMS; HCD	120.87	815.41

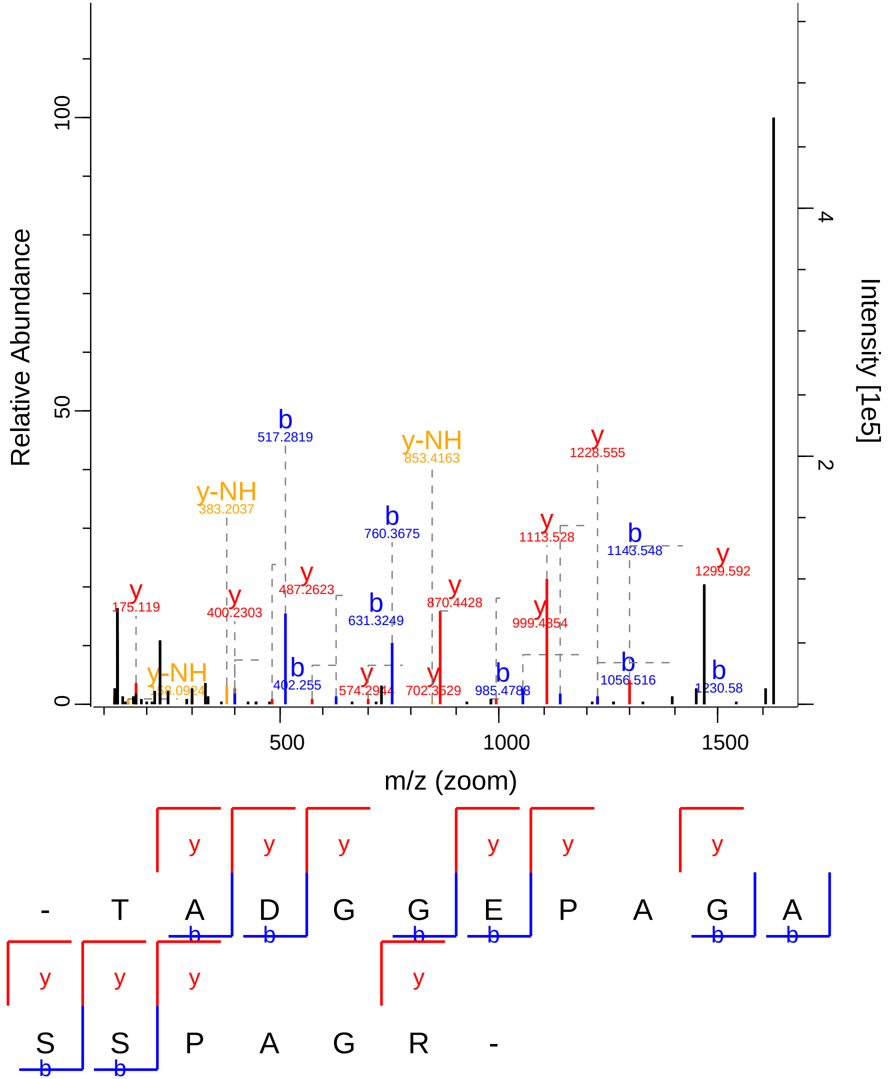

Raw File	Scan	Method	Score	m/z	Gene names
RO-QC-160121_ZackD_SS_Set_01_Fractior	6188	FTMS; HCI	105.9	574.28	SECISBP2

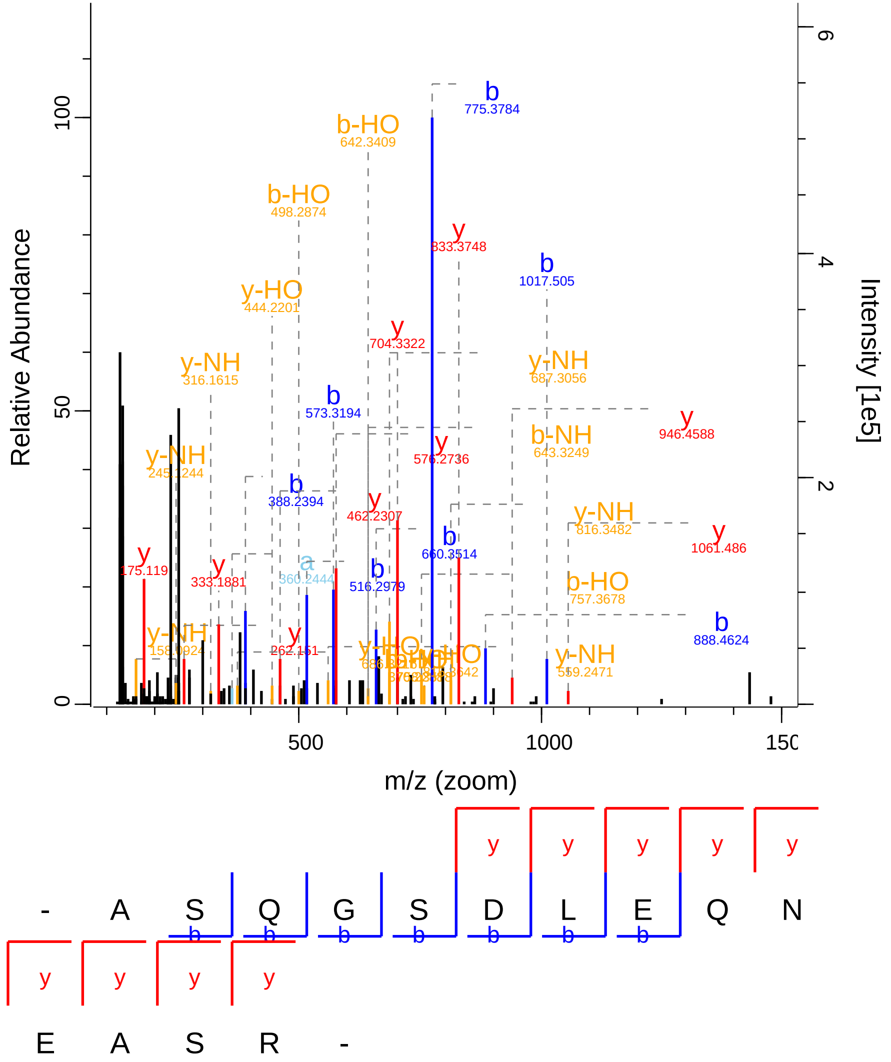

Raw File	Scan	Method	Score	m/z	Gene names
RO-QC-160121_ZackD_SS_Set_01_Fractior	6200	FTMS; HCI	58.17	861.42	SECISBP2

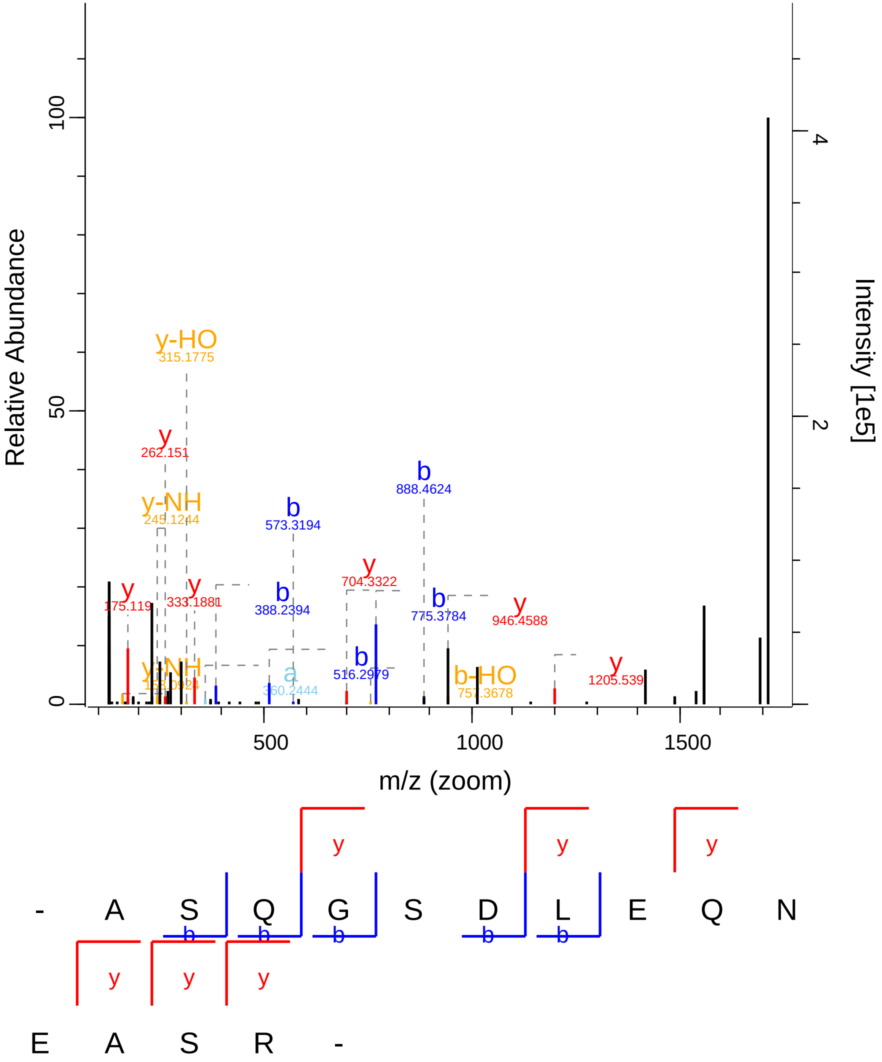

Raw File	Scan	Method	Score	m/z
RO-QC-160121_ZackD_SS_Set_01_Fraction_7	6377	FTMS; HCD	51.35	459.27

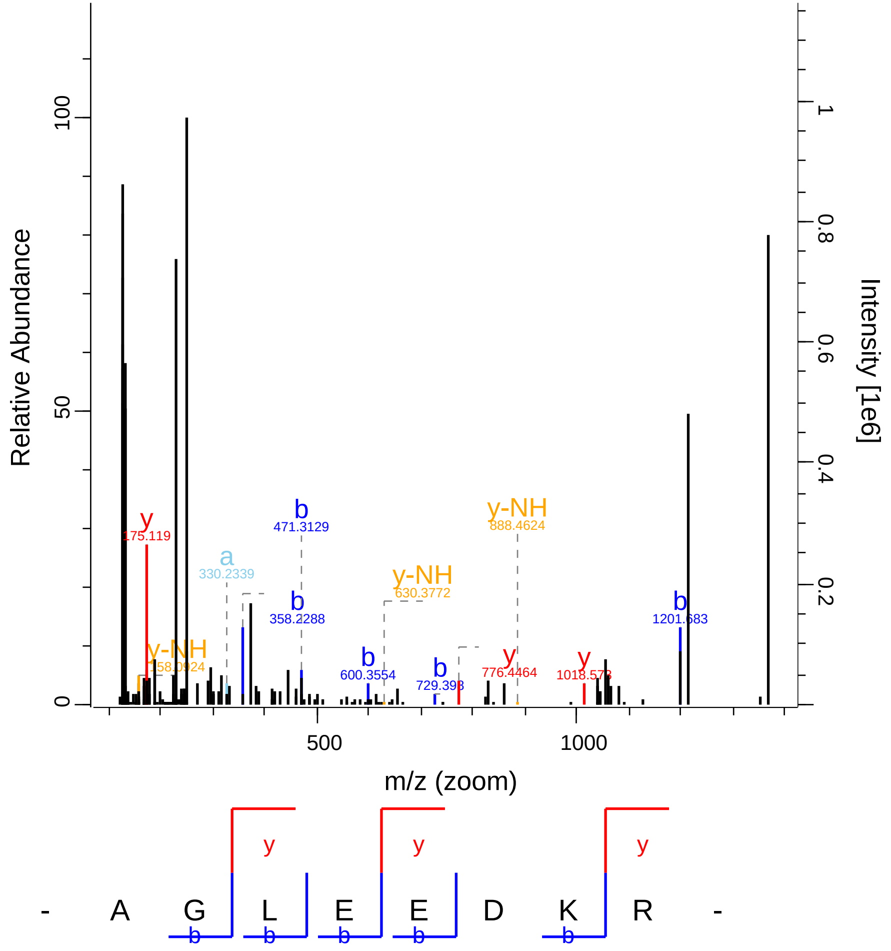

Raw File	Scan	Method	Score	m/z	Gene names
RO-QC-160121_ZackD_SS_Set_01_Fractior	6648	FTMS; HCI	89.25	356.22	CNKSR2

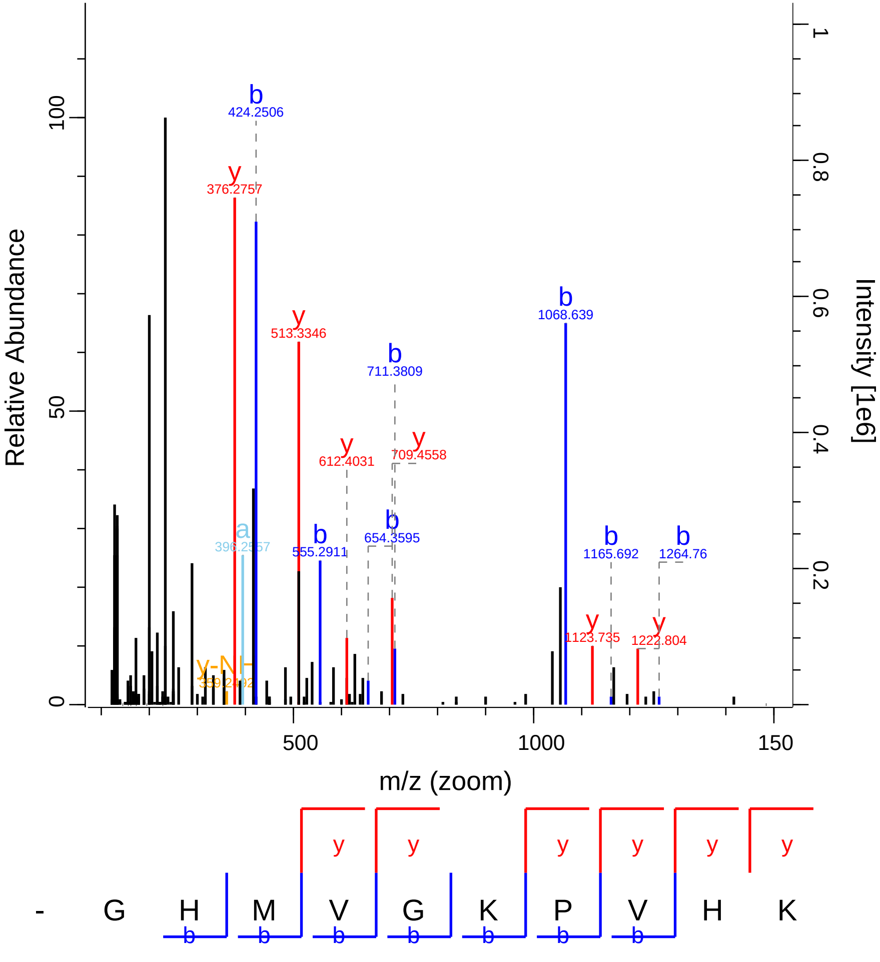

Raw File	Scan	Method	Score	m/z
RO-QC-160121_ZackD_SS_Set_01_Fraction_7	6793	FTMS; HCD	72.26	692.05

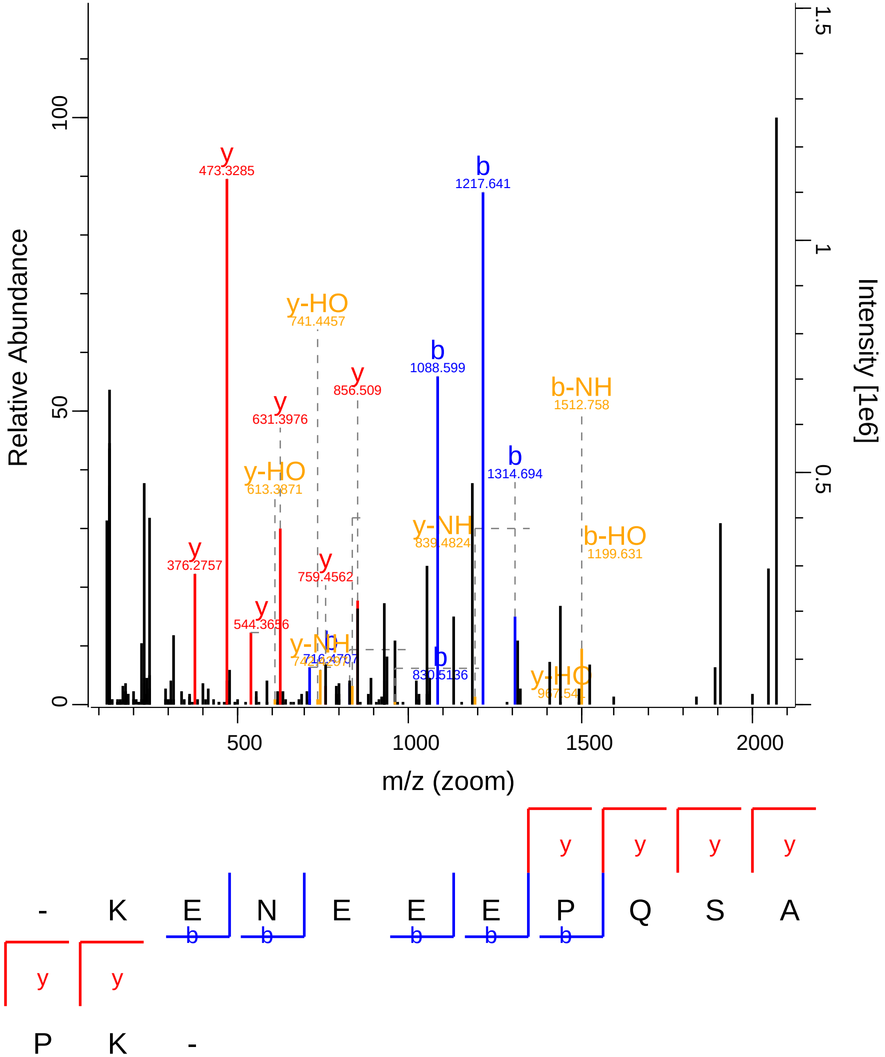

Raw File	Scan	Method	Score	m/z	Gene names
RO-QC-160121_ZackD_SS_Set_01_Fractio[illegible]	6940	FTMS; HC	124.42	739.42	BBX

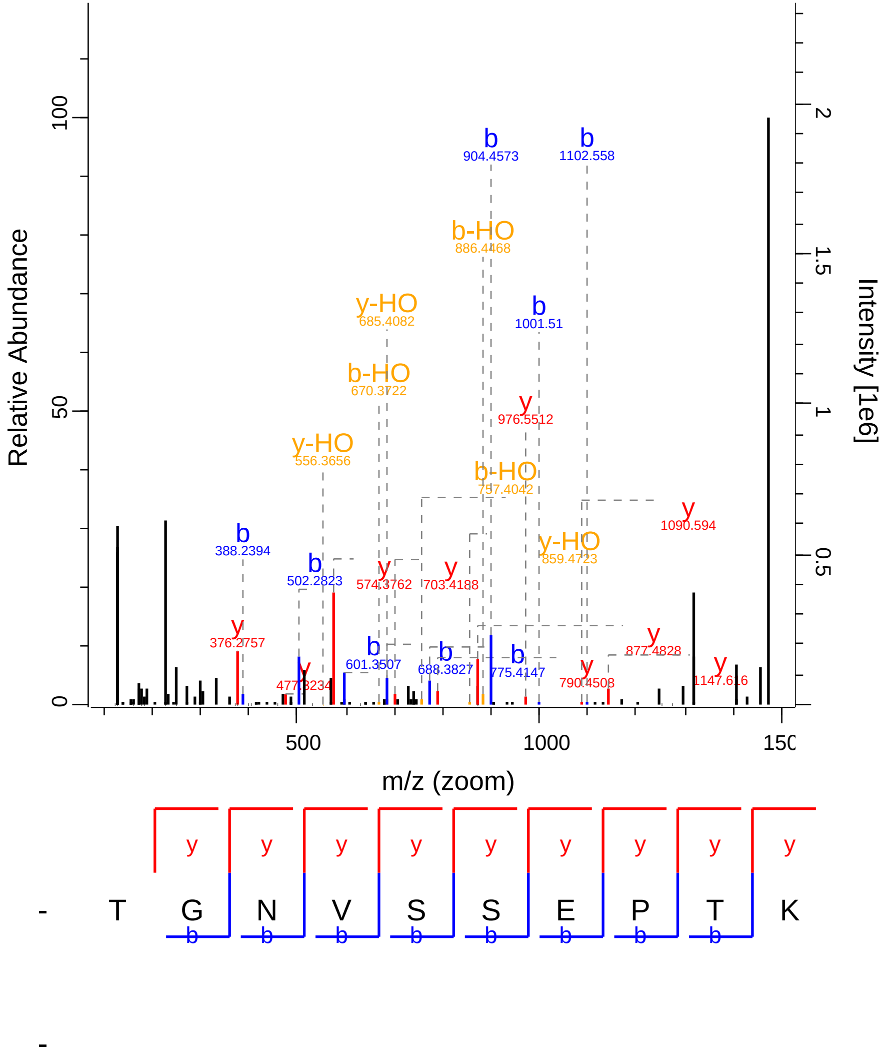

Raw File	Scan	Method	Score	m/z	Gene names
RO-QC-160121_ZackD_SS_Set_01_Fractio	7128	FTMS; HC	162.93	777.39	TMEM51

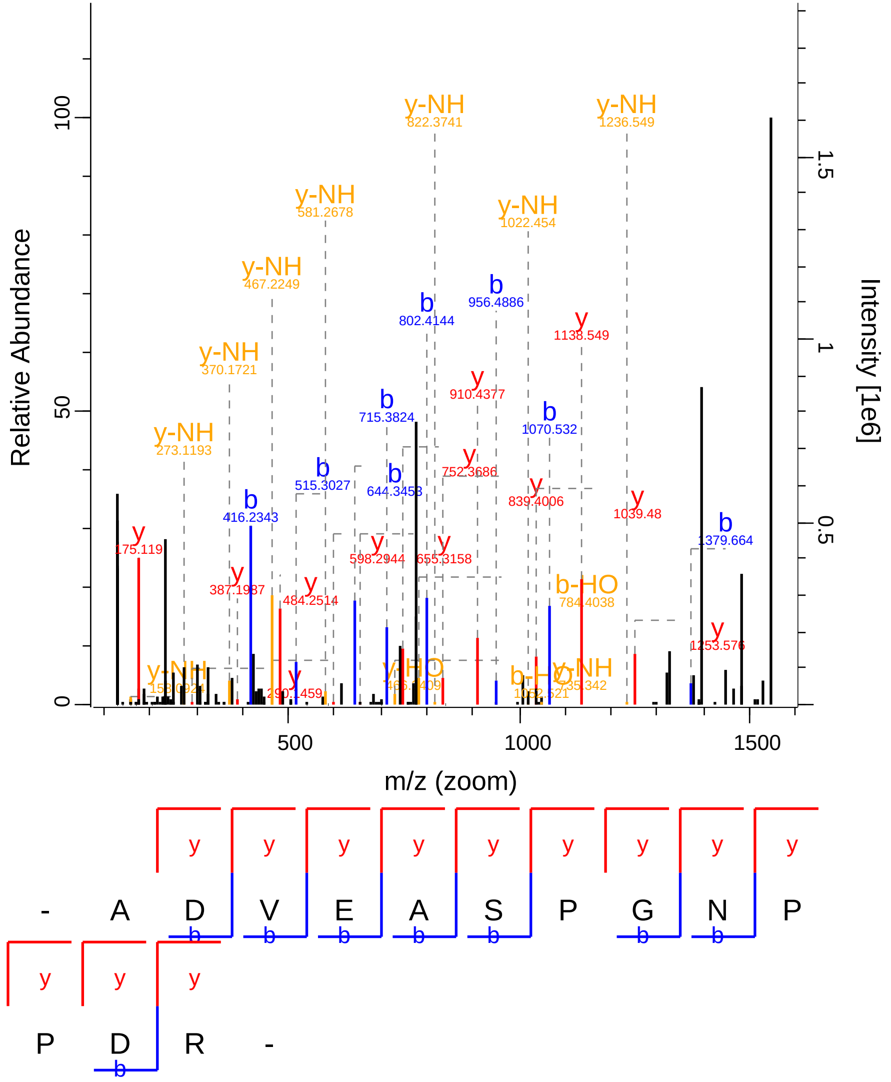

Raw File	Scan	Method	Score	m/z	Gene names
RO-QC-160121_ZackD_SS_Set_01_Fractio	7399	FTMS; HC	140.17	659.84	FKRP

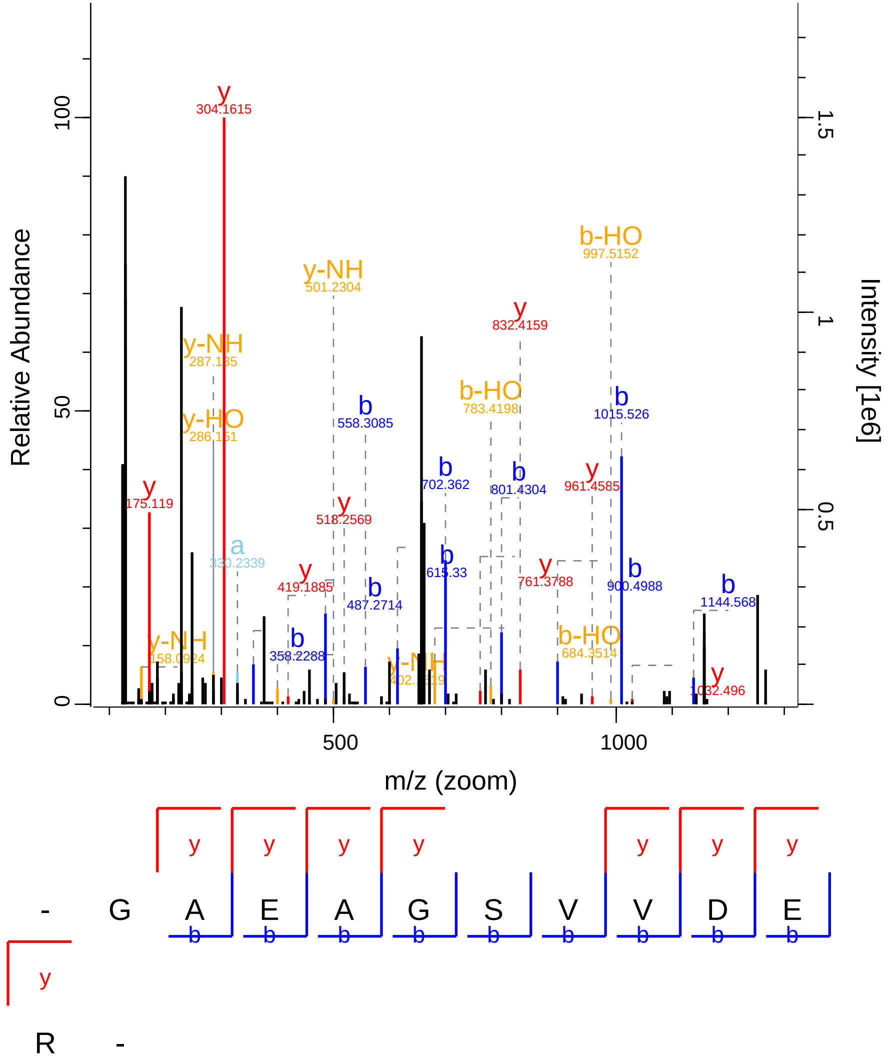

Raw File	Scan	Method	Score	m/z	Gene names
RO-QC-160121_ZackD_SS_Set_01_Fractior	7820	FTMS; HCI	56.12	699.39	SLC7A6

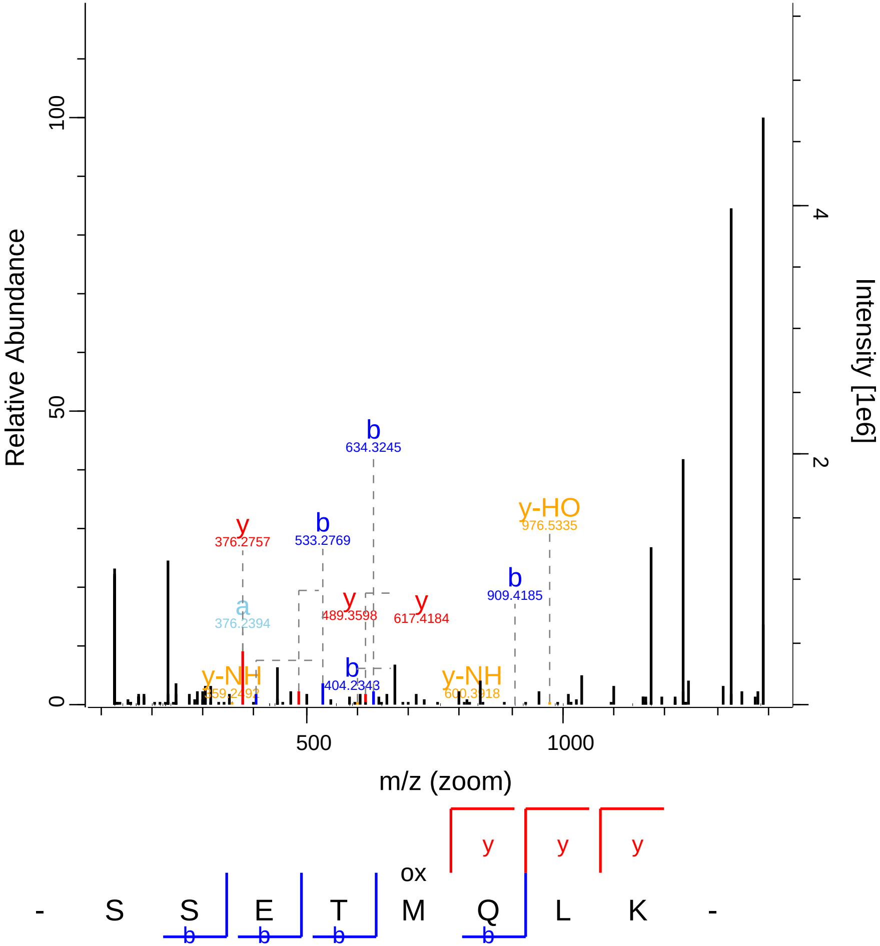

Raw File	Scan	Method	Score	m/z	Gene names
RO-QC-160121_ZackD_SS_Set_01_Fraction	9128	FTMS; HCI	153.54	779.9	SLC39A1

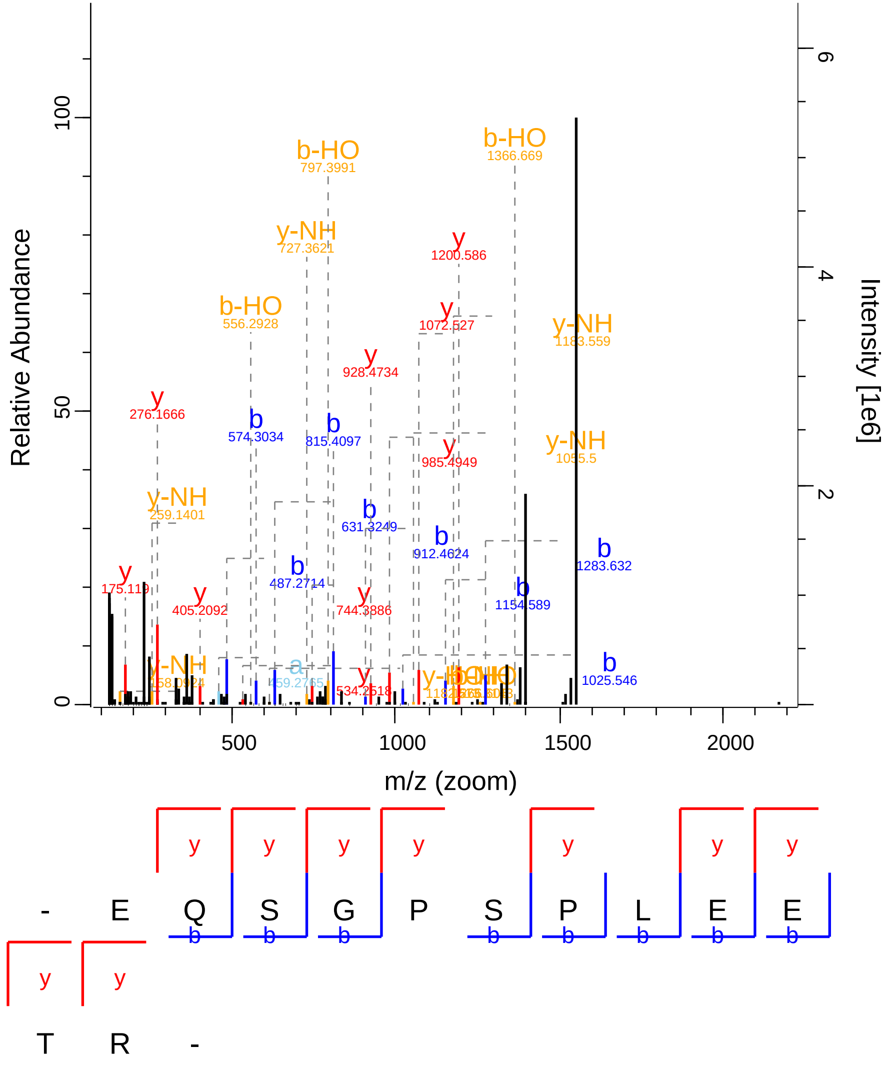

Raw File	Scan	Method	Score	m/z	Gene names
RO-QC-160121_ZackD_SS_Set_01_Fra	9482	FTMS; HC	75.11	596.35	ATP5G1;ATP5G2;ATP5

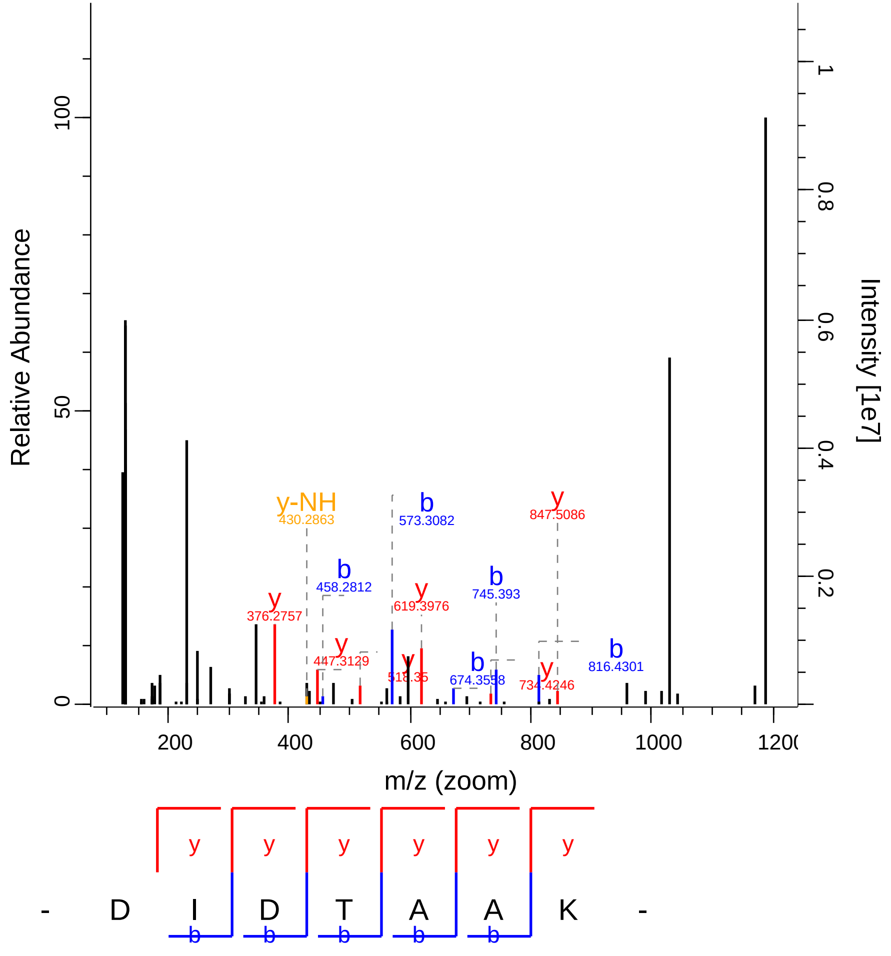

Raw File	Scan	Method	Score	m/z
RO-QC-160121_ZackD_SS_Set_01_Fraction_7	10232	FTMS; HCD	48.28	724.91

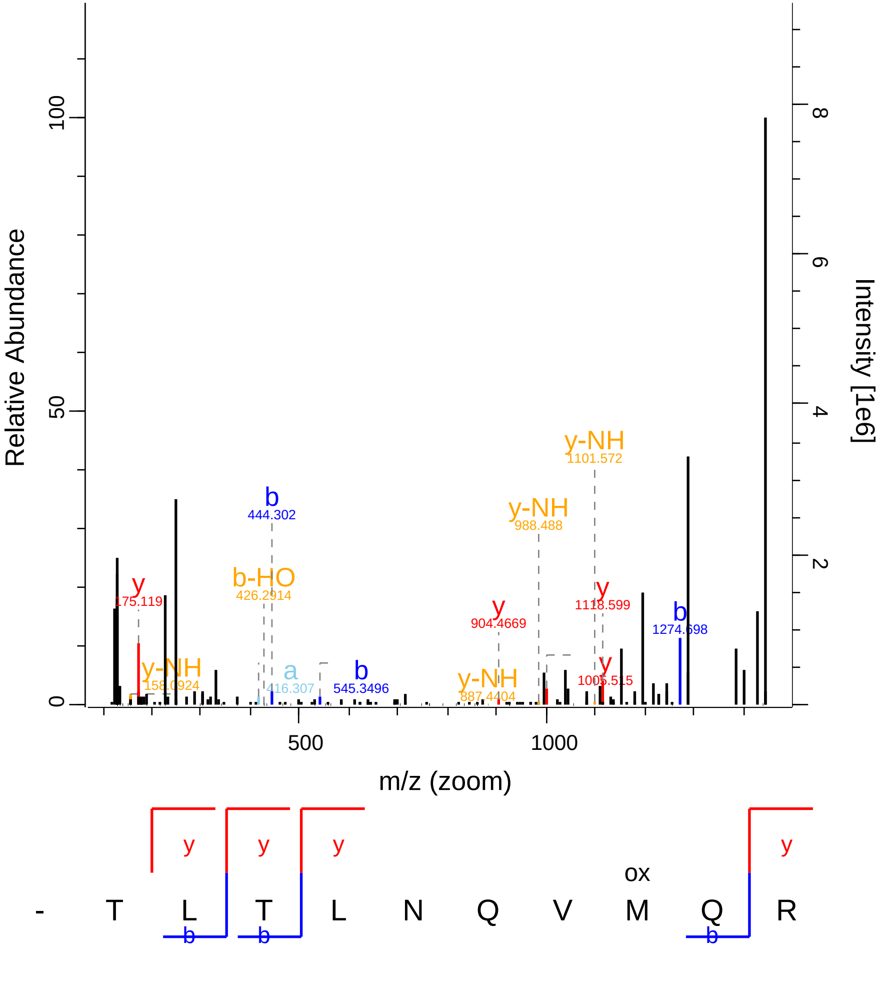

Raw File	Scan	Method	Score	m/z	Gene names
RO-QC-160121_ZackD_SS_Set_01_Fractio	11312	FTMS; HC	83.26	623.87	TMEM141

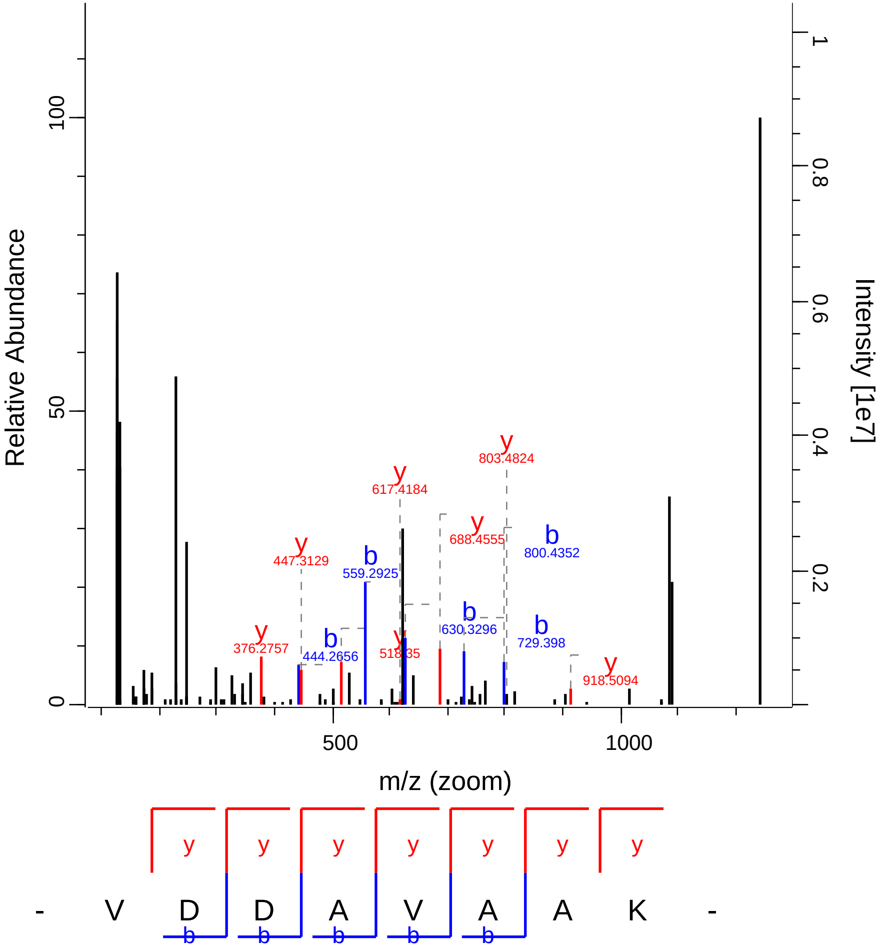

Raw File	Scan	Method	Score	m/z
RO-QC-160121_ZackD_SS_Set_01_Fraction_7	11422	FTMS; HCD	107.32	552.83

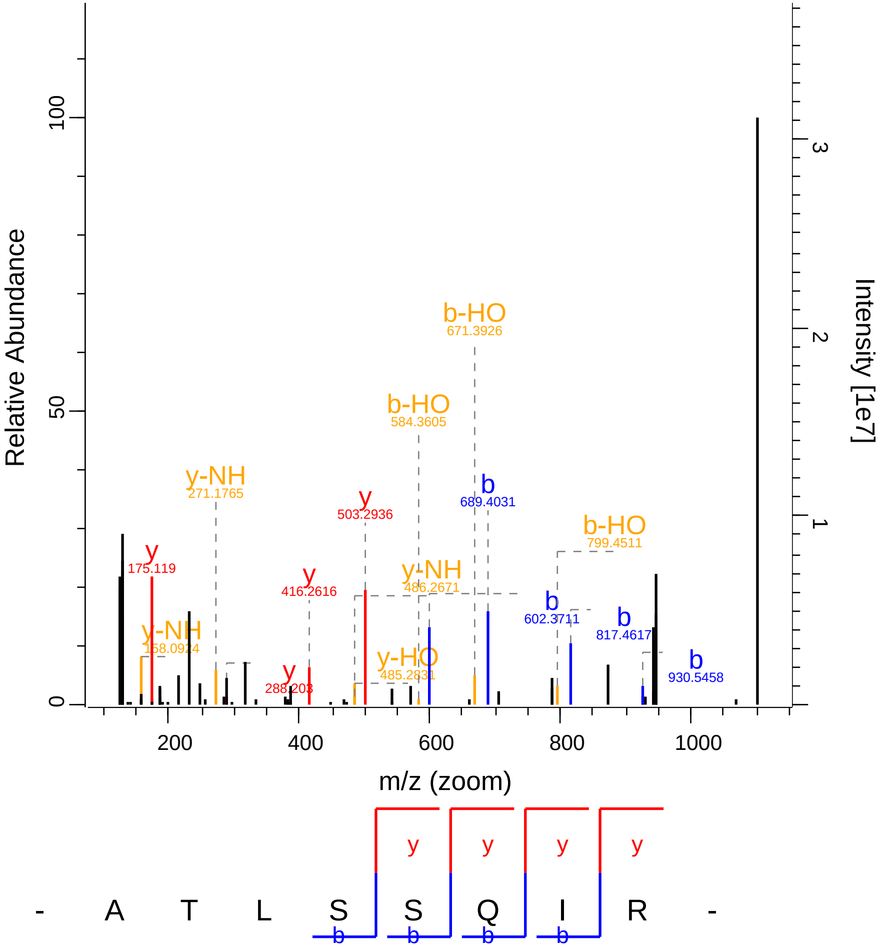

Raw File	Scan	Method	Score	m/z	Gene names
RO-QC-160121_ZackD_SS_Set_01_Fractio	11570	FTMS; HC	107.66	623.87	TMEM141

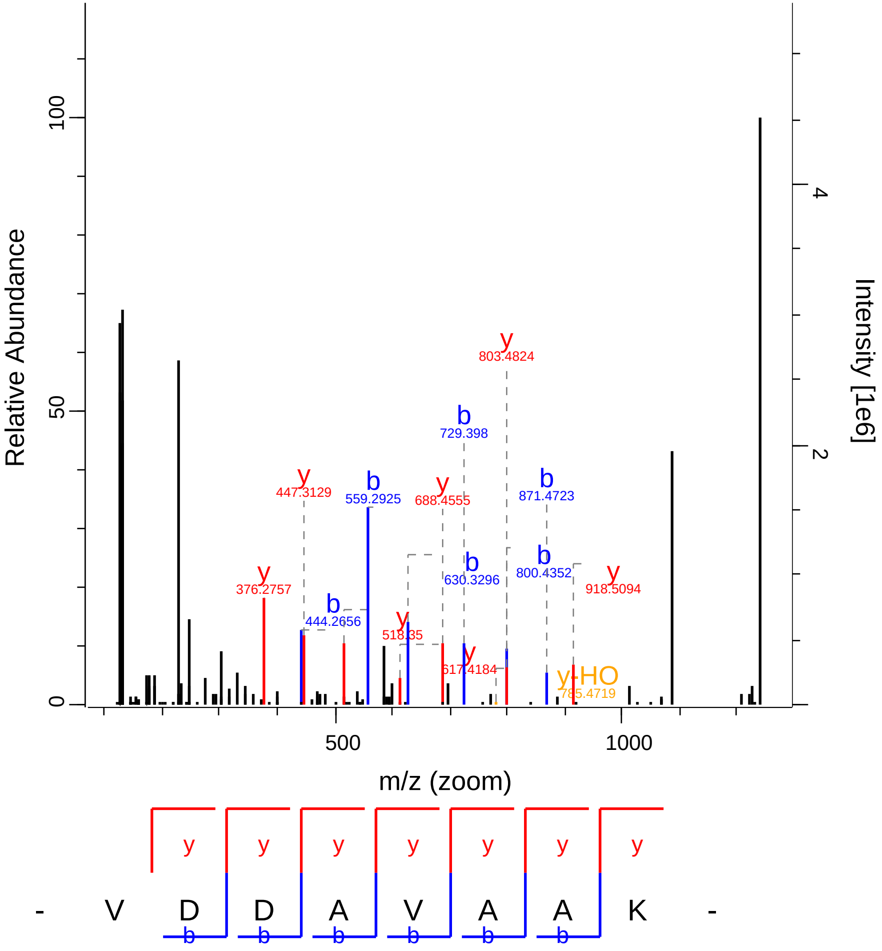

Raw File	Scan	Method	Score	m/z	Gene names
RO-QC-160121_ZackD_SS_Set_01_Fractio	11647	FTMS; HC	130.98	804.44	MRPL34

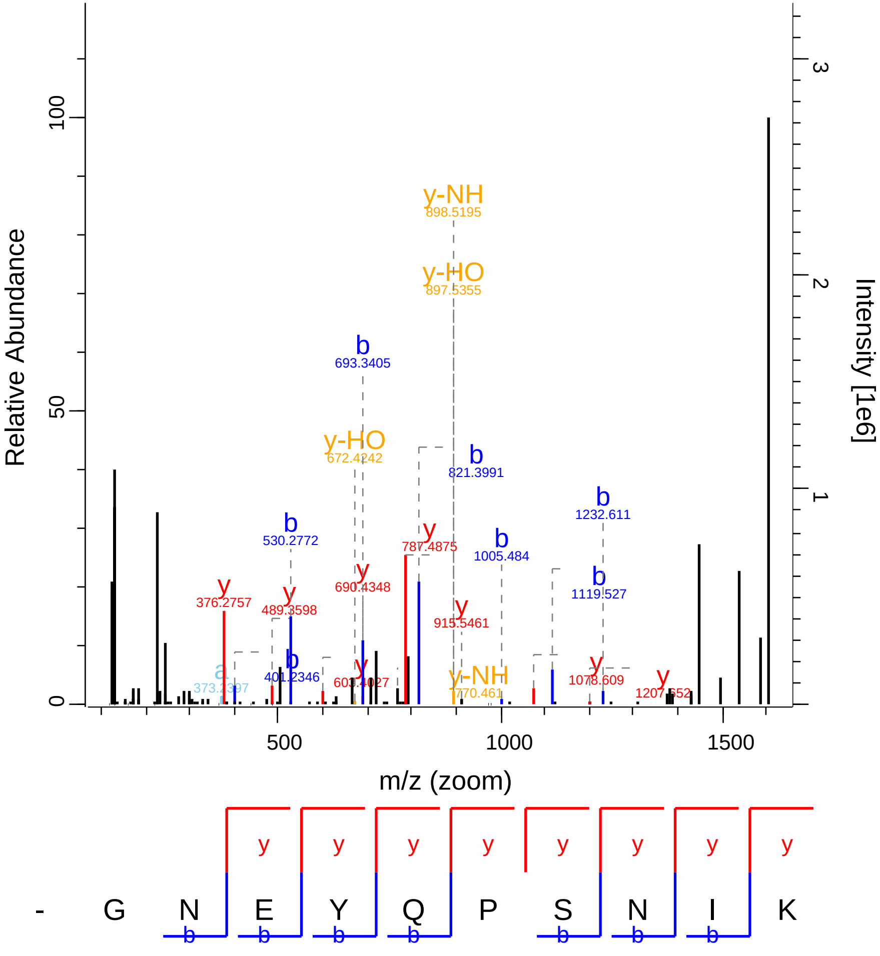

Raw File	Scan	Method	Score	m/z
RO-QC-160121_ZackD_SS_Set_01_Fraction_7	12214	FTMS; HCD	53.47	755.37

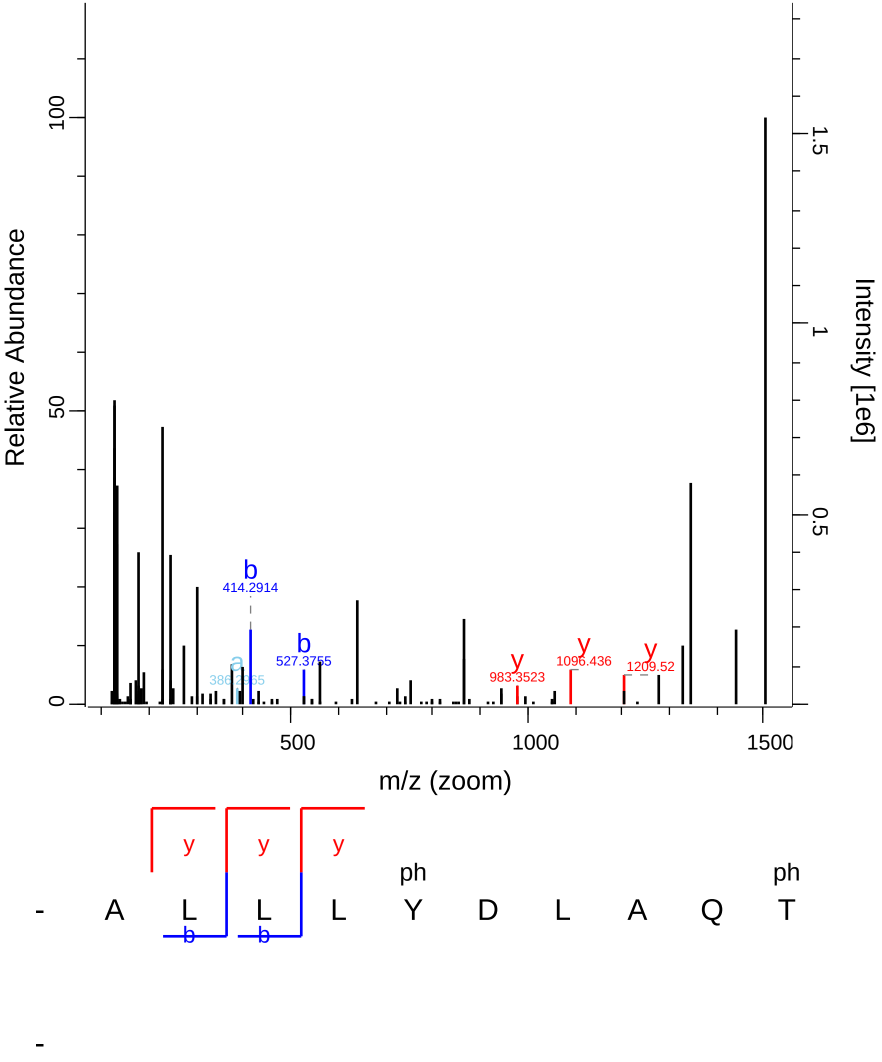

Raw File	Scan	Method	Score	m/z	Gene names
RO-QC-160121_ZackD_SS_Set_01_Fractio	13426	FTMS; HC	123.42	724.88	WDFY2

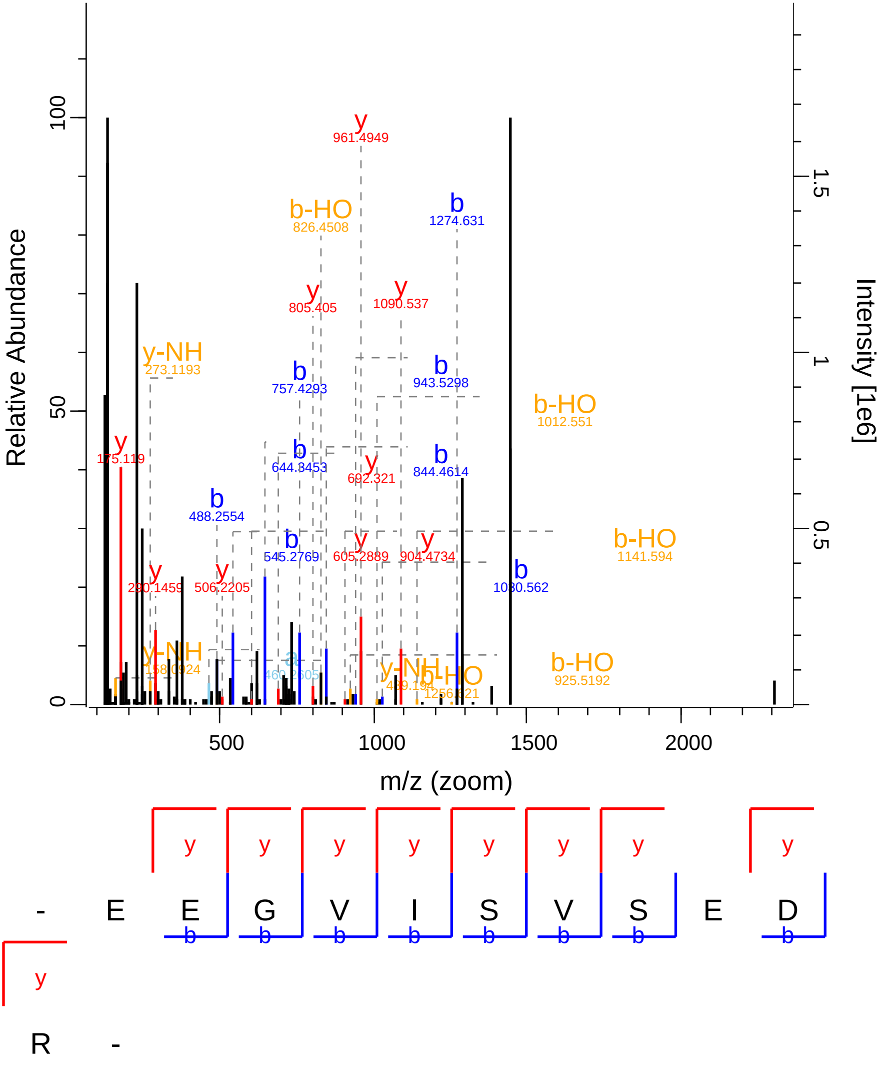

Raw File	Scan	Method	Score	m/z
RO-QC-160121_ZackD_SS_Set_01_Fraction_7	13920	FTMS; HCD	69.45	1057.58

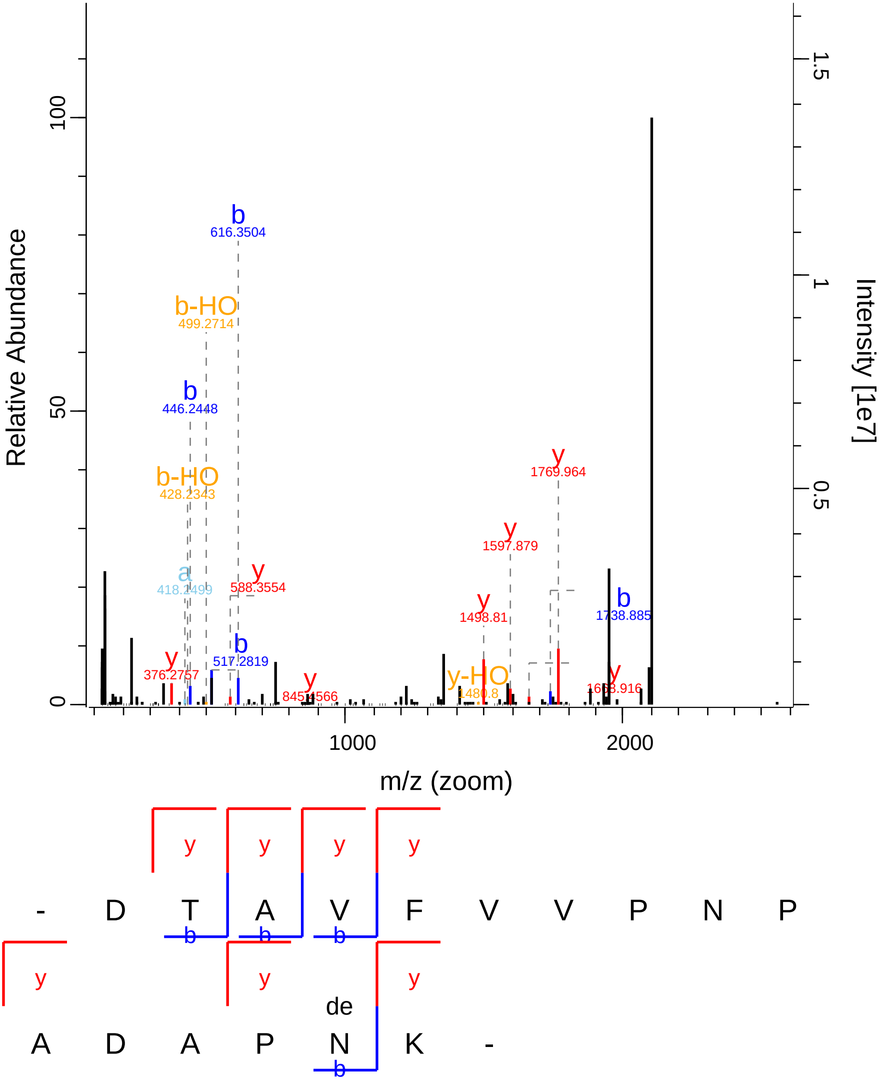

Raw File	Scan	Method	Score	m/z	Gene names
RO-QC-160121_ZackD_SS_Set_01_Fractio	14460	FTMS; HC	41.65	621.42	OFCC1

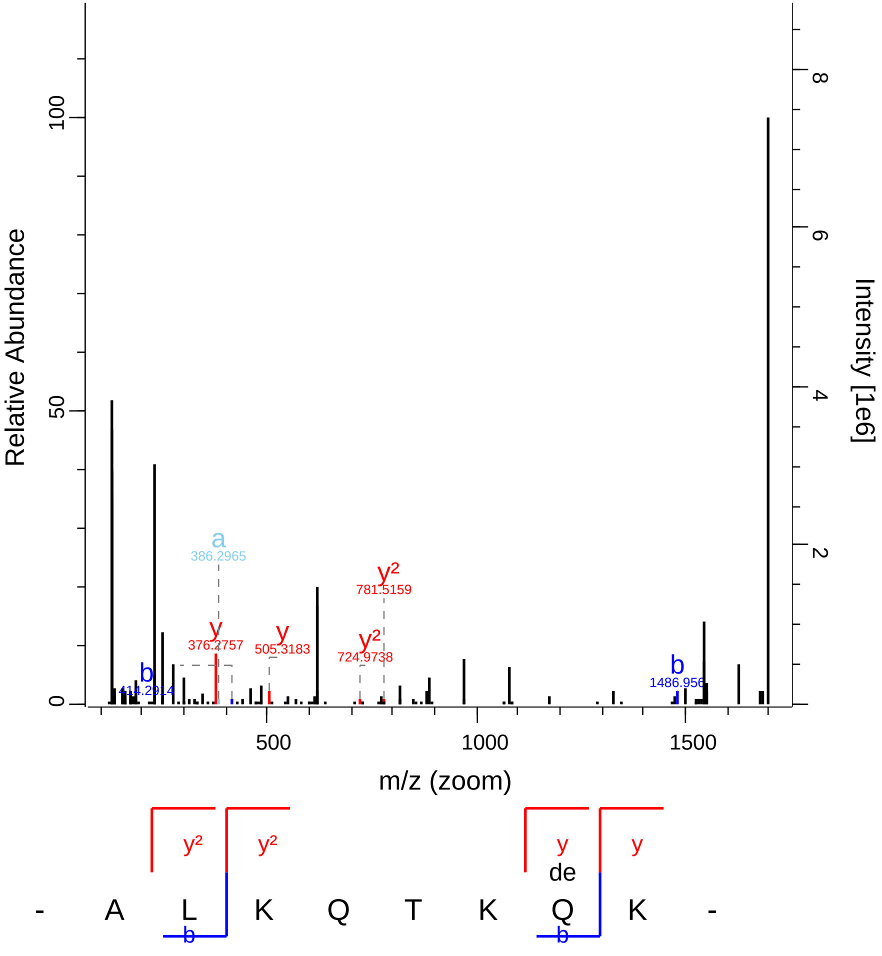

Raw File	Scan	Method	Score	m/z	Gene names
RO-QC-160121_ZackD_SS_Set_01_Fractio	18691	FTMS; HC	43.99	683.41	KCTD19

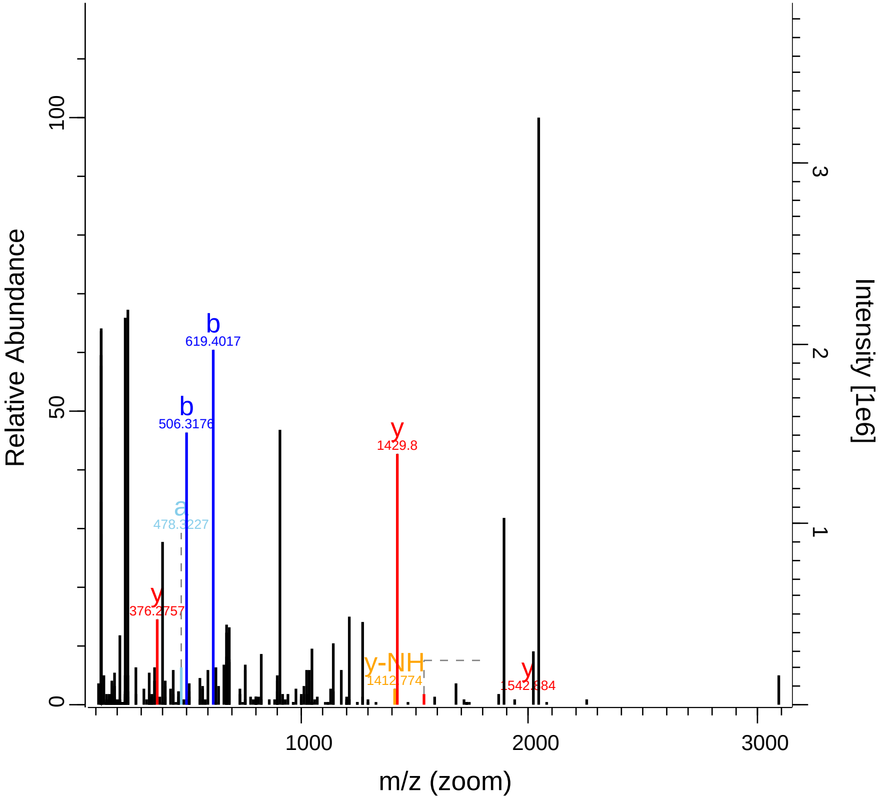

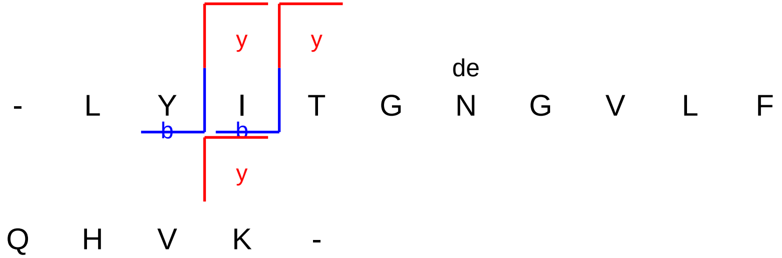

Raw File	Scan	Method	Score	m/z	Gene names
RO-QC-160121_ZackD_SS_Set_01_Fractic	20382	FTMS; HC	76.14	1134.08	SPTLC3

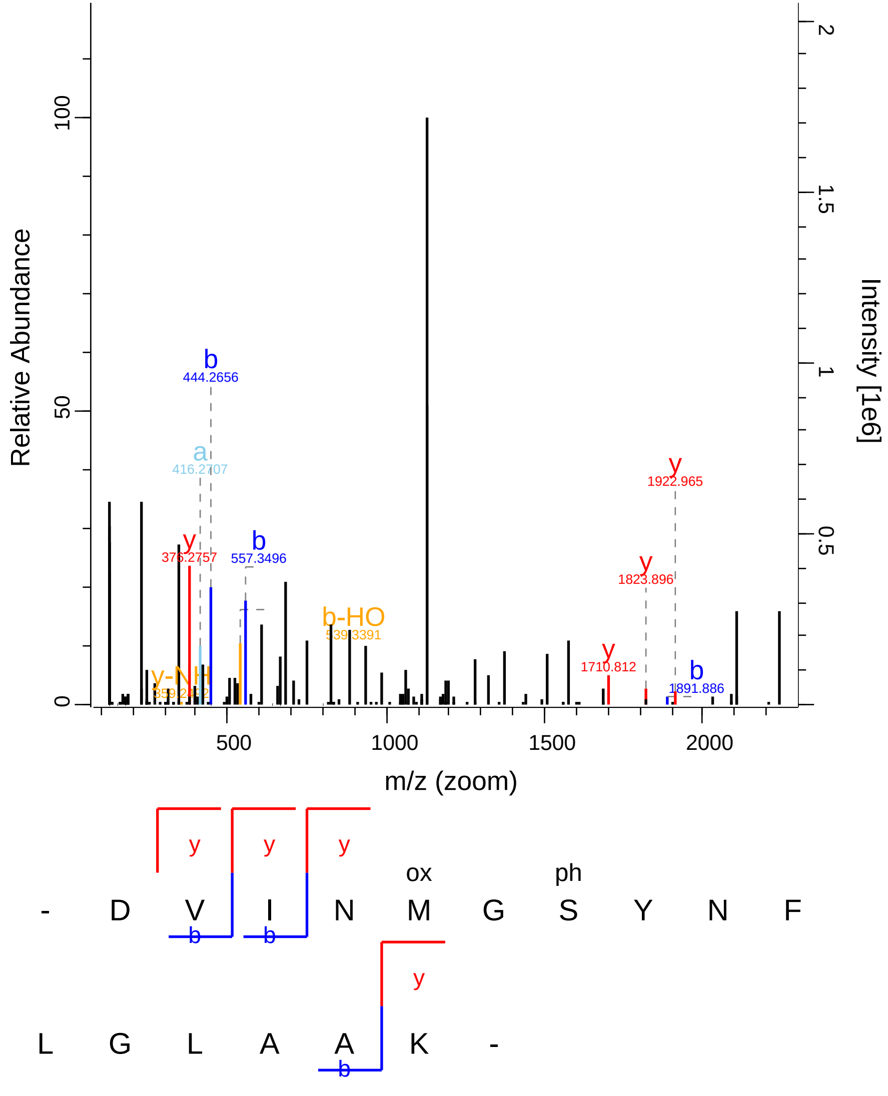

Raw File	Scan	Method	Score	m/z	Gene names
RO-QC-160121_ZackD_SS_Set_01_Fractio	20401	FTMS; HC	50.35	543.67	FKBP11

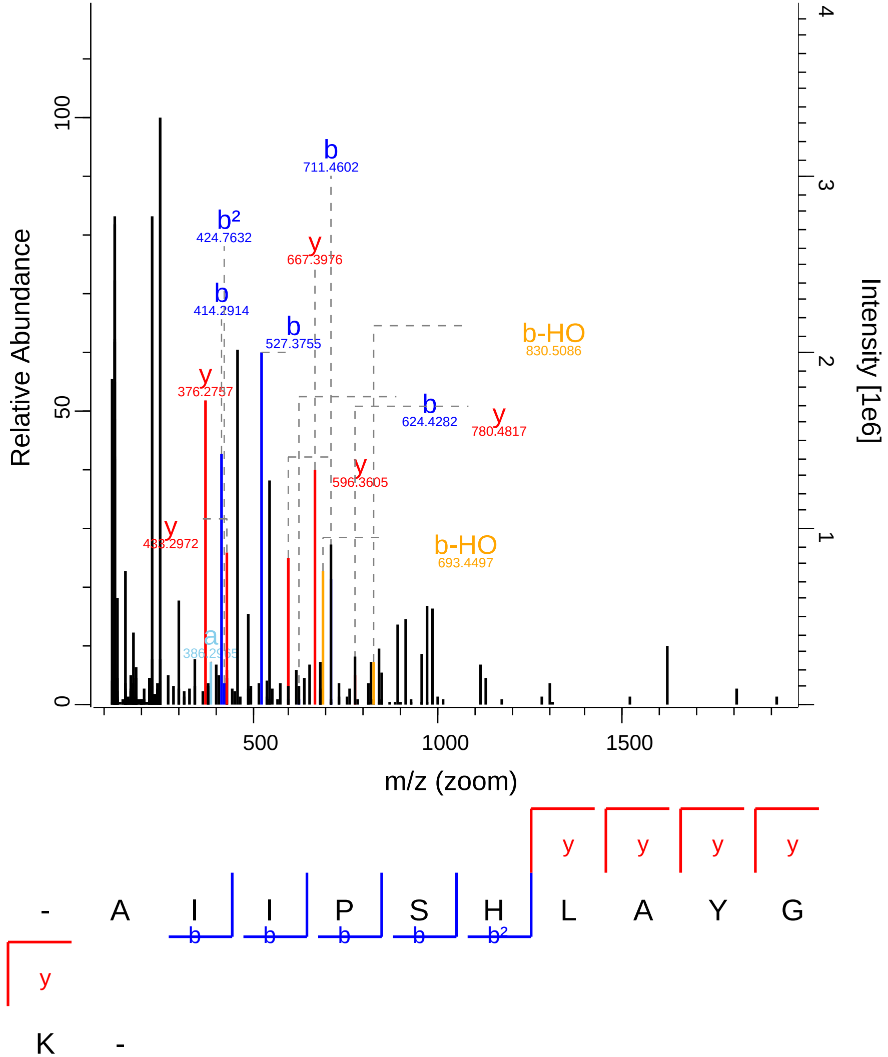

Raw File	Scan	Method	Score	m/z	Gene names
RO-QC-160121_ZackD_SS_Set_01_Fractioı	20507	FTMS; HCI	123.35	814.5	FKBP11

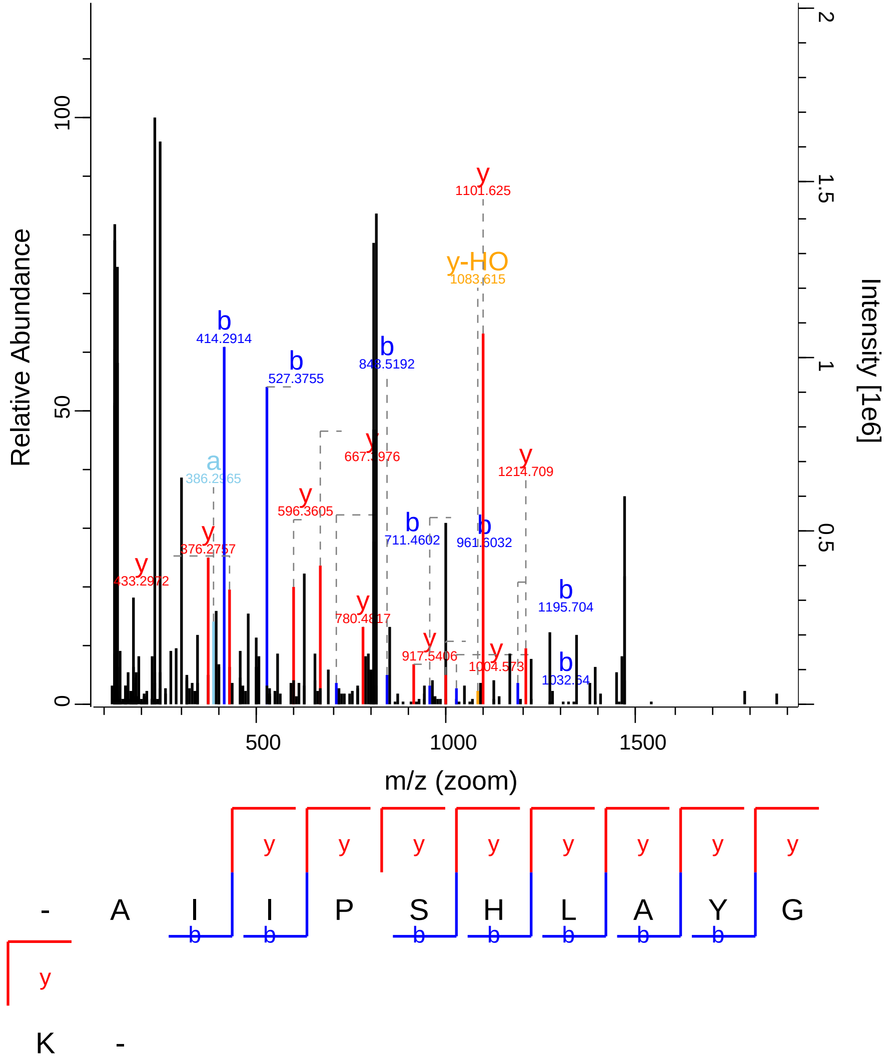

Raw File	Scan	Method	Score	m/z	Gene names
RO-QC-160121_ZackD_SS_Set_01_Fractio	20762	FTMS; HC	41.38	749.11	ACMSD

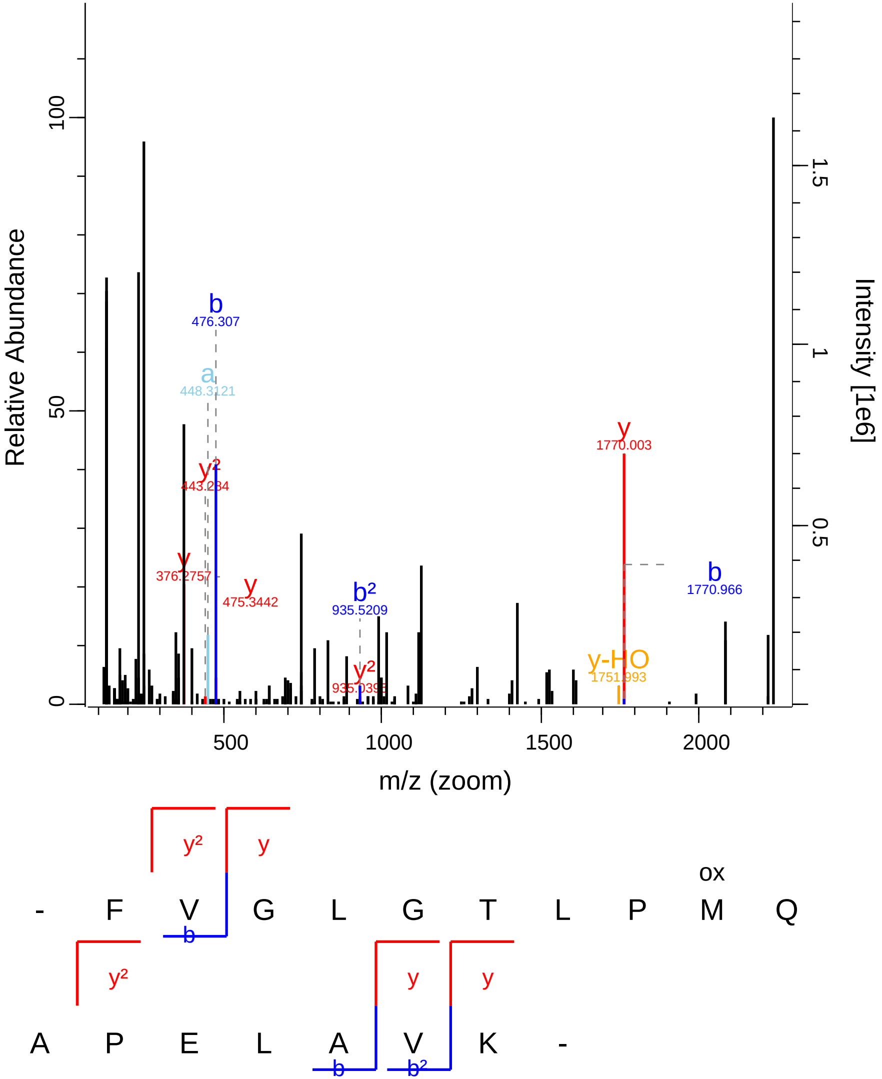

Raw File	Scan	Method	Score	m/z
RO-QC-160121_ZackD_SS_Set_01_Fraction_7	21760	FTMS; HCD	113.22	732.42

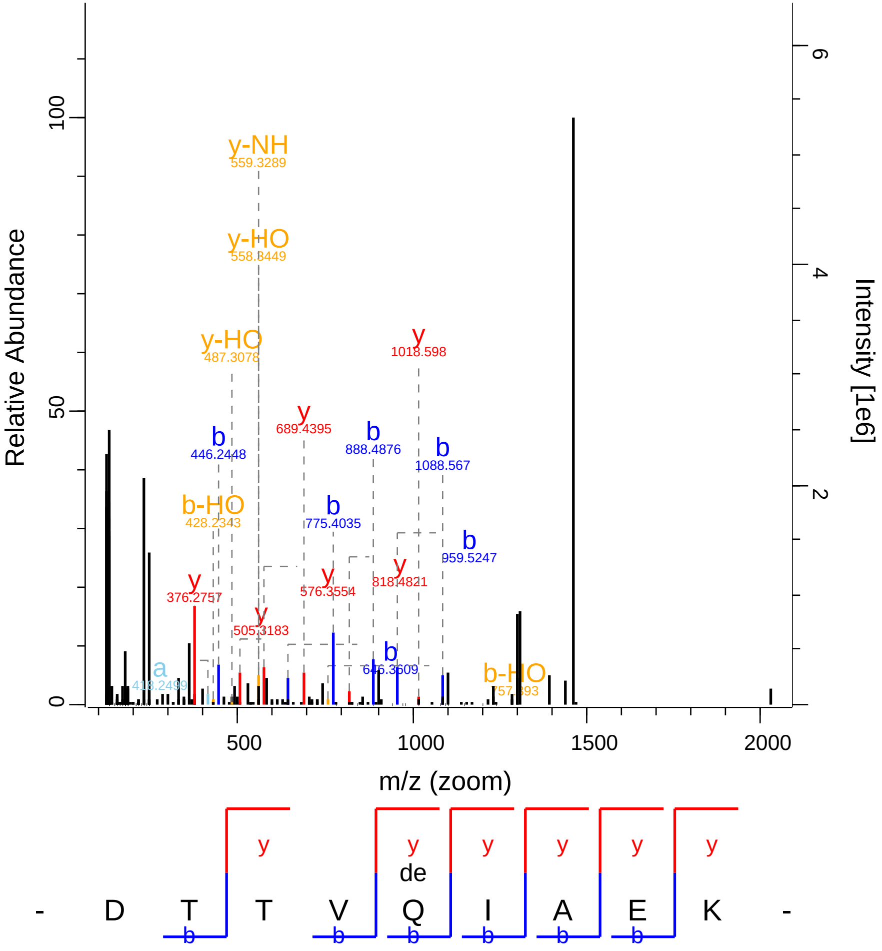

Raw File	Scan	Method	Score	m/z	Gene names
RO-QC-160121_ZackD_SS_Set_01_Fractio	22281	FTMS; HC	79.69	453.26	YPEL5

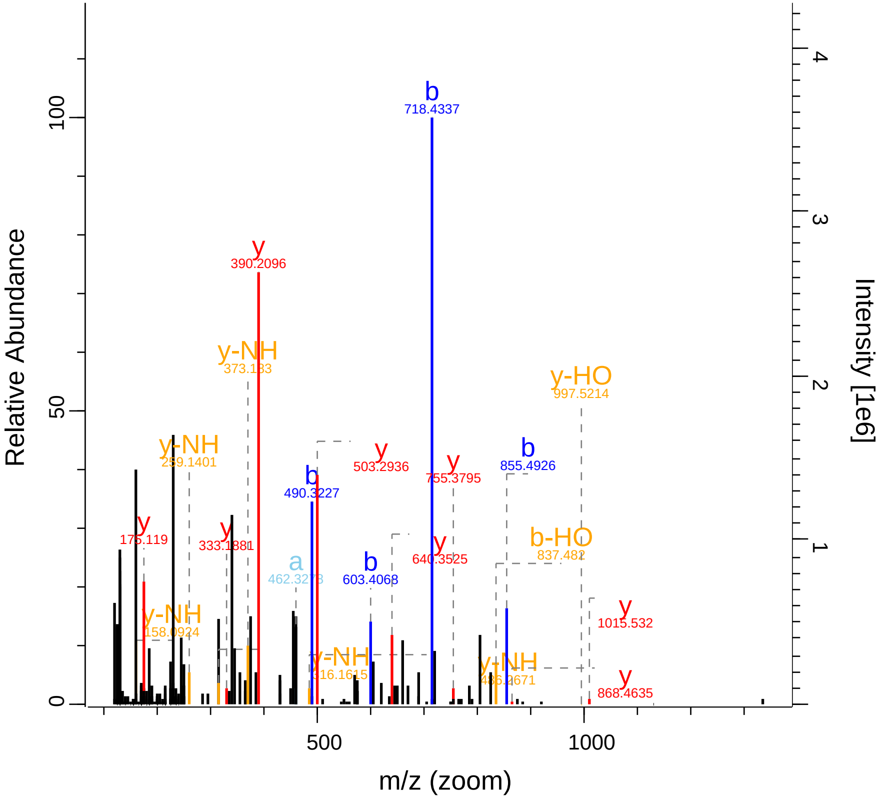

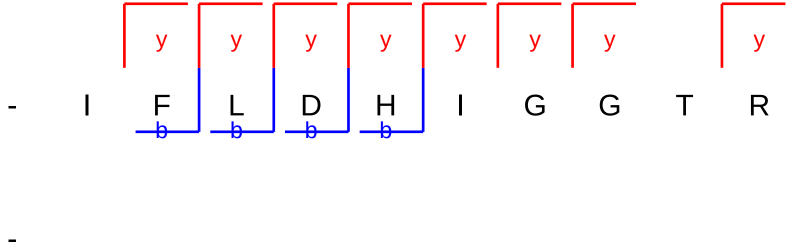

Raw File	Scan	Method	Score	m/z	Gene names
RO-QC-160121_ZackD_SS_Set_01_Fra	23531	FTMS; HC	72.93	960.06	ANKRD18A;ANKRD1

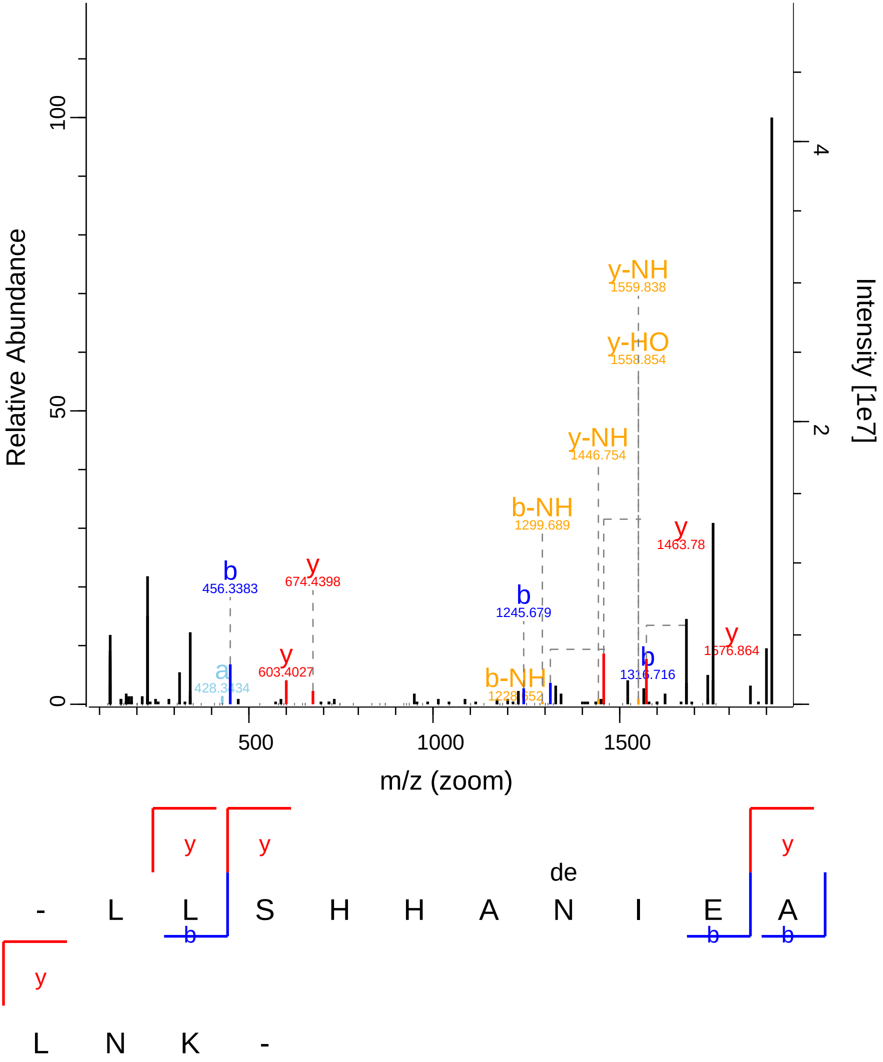

Raw File	Scan	Method	Score	m/z	Gene names
RO-QC-160121_ZackD_SS_Set_01_Fractio	24445	FTMS; HC	144.17	803.41	SMIM4

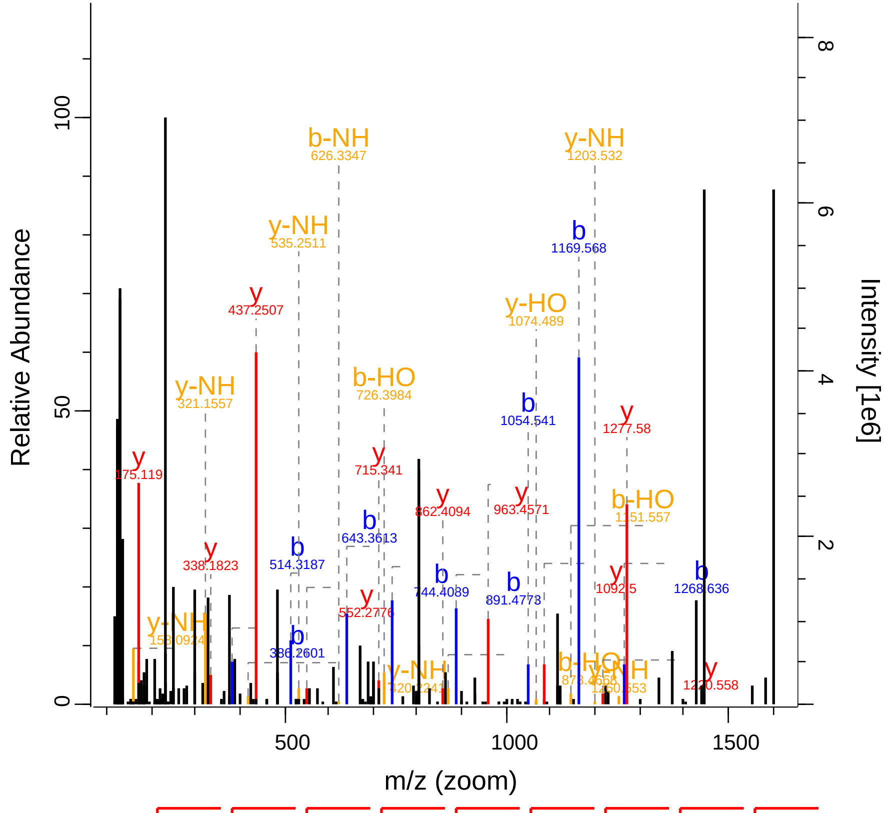

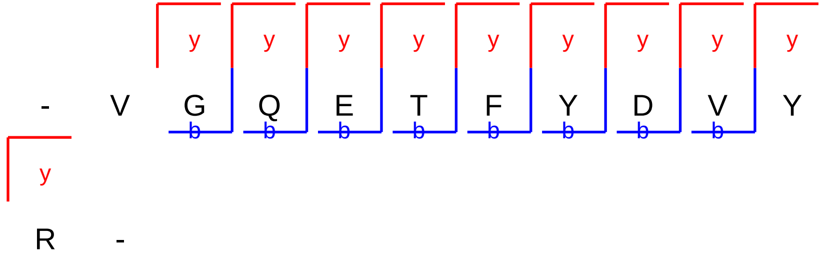

Raw File	Scan	Method	Score	m/z	Gene names
RO-QC-160121_ZackD_SS_Set_01_Fractio	26834	FTMS; HC	54.41	902.59	FHAD1

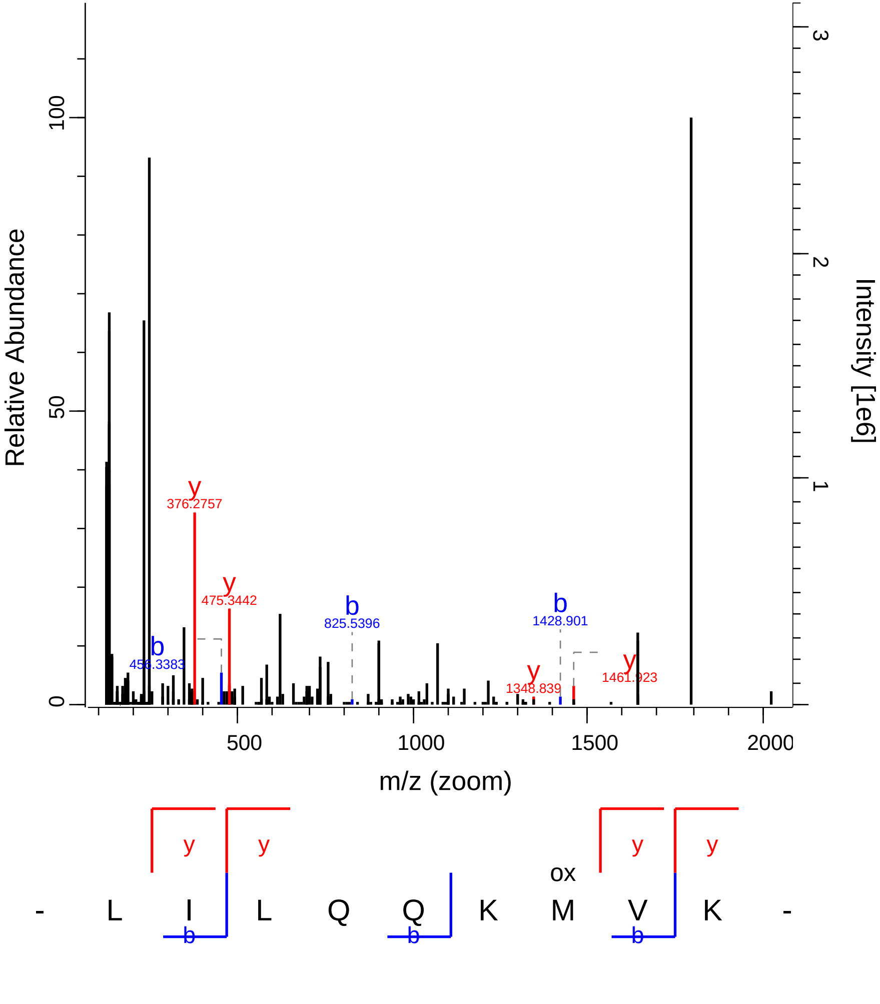

Raw File	Scan	Method	Score	m/z
RO-QC-160121_ZackD_SS_Set_01_Fraction_7	27428	FTMS; HCD	62.82	969.04

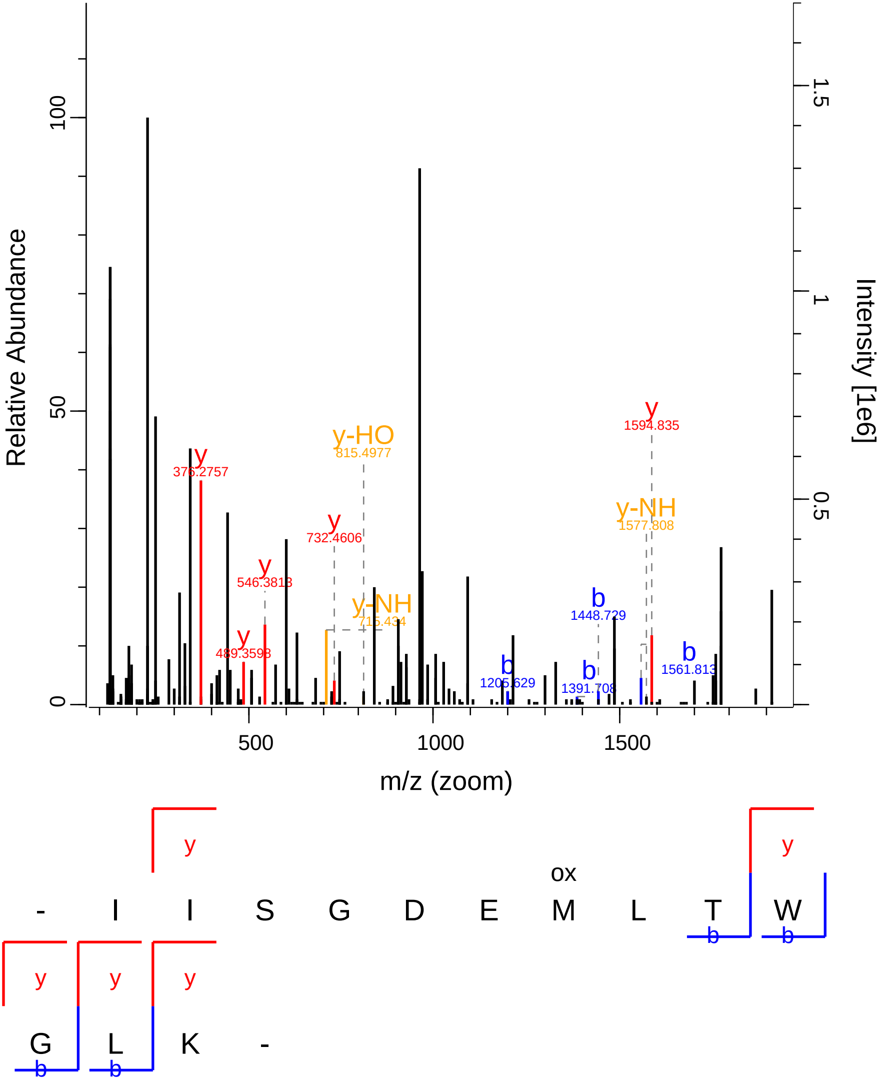

Raw File	Scan	Method	Score	m/z	Gene names
RO-QC-160121_ZackD_SS_Set_01_Fractio	27526	FTMS; HC	165.83	837.97	RGR

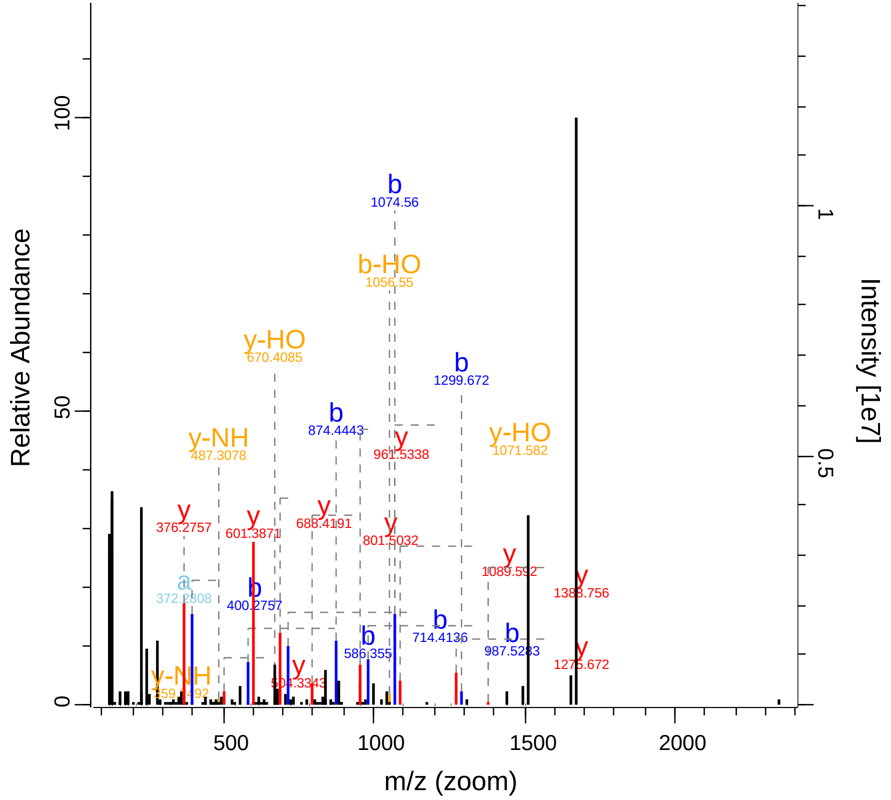

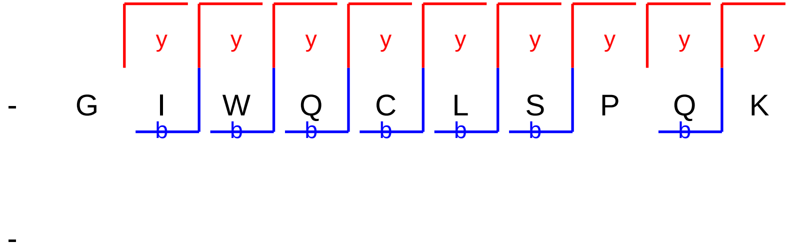

Raw File	Scan	Method	Score	m/z	Gene names
RO-QC-160121_ZackD_SS_Set_01_Fractic	27986	FTMS; HC	54.61	1075.59	DRAXIN

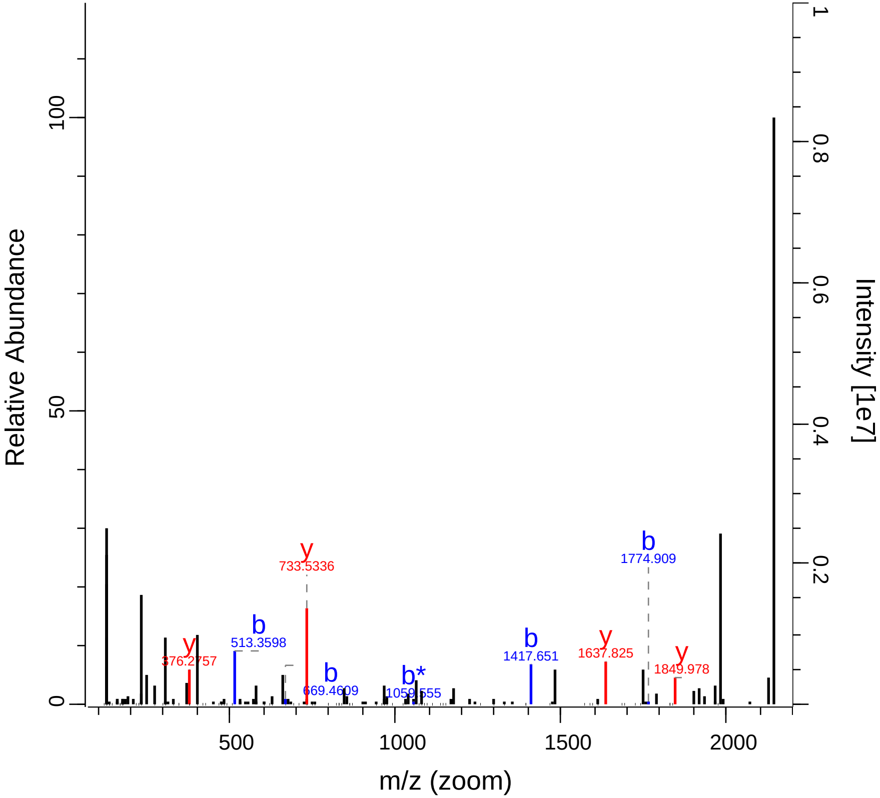

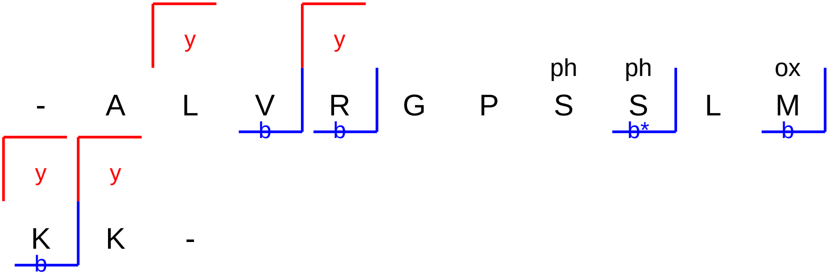

Raw File	Scan	Method	Score	m/z	Gene names
RO-QC-160121_ZackD_SS_Set_01_Fractio	28306	FTMS; HC	87.57	692.05	NACAP1

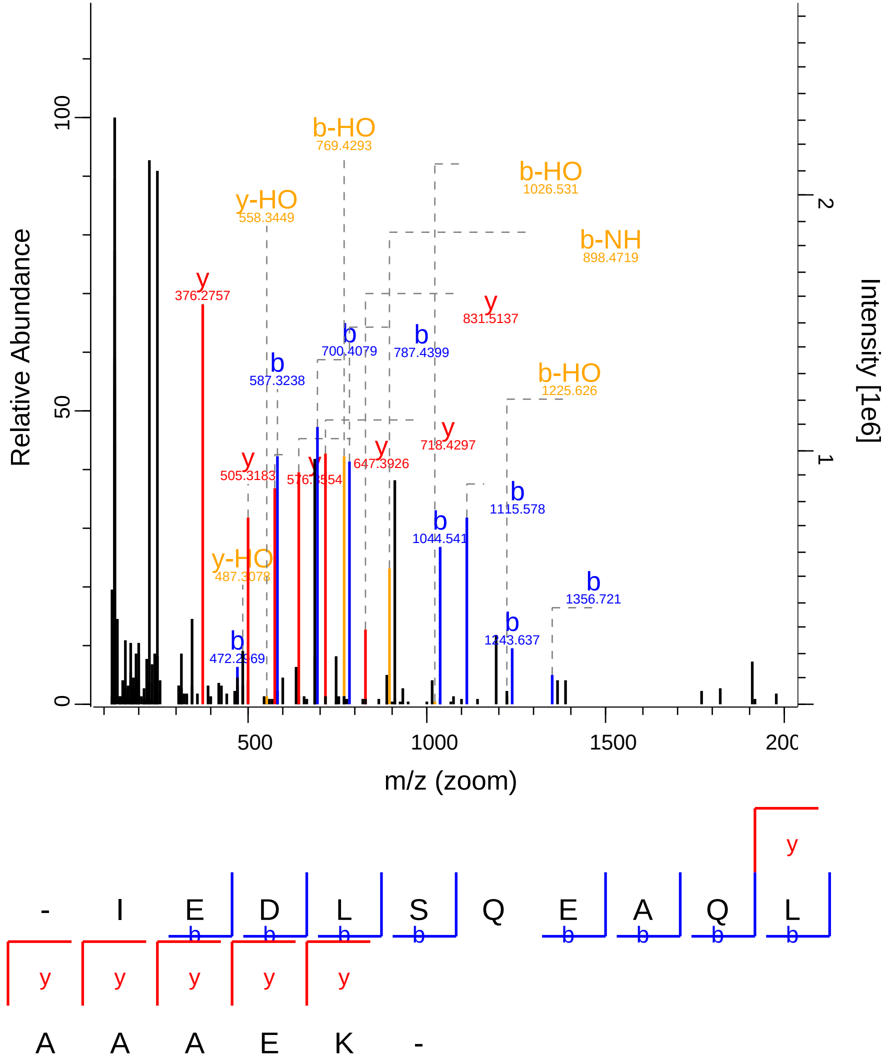

Raw File	Scan	Method	Score	m/z
RO-QC-160121_ZackD_SS_Set_01_Fraction_7	28564	FTMS; HCD	63.28	1137.17

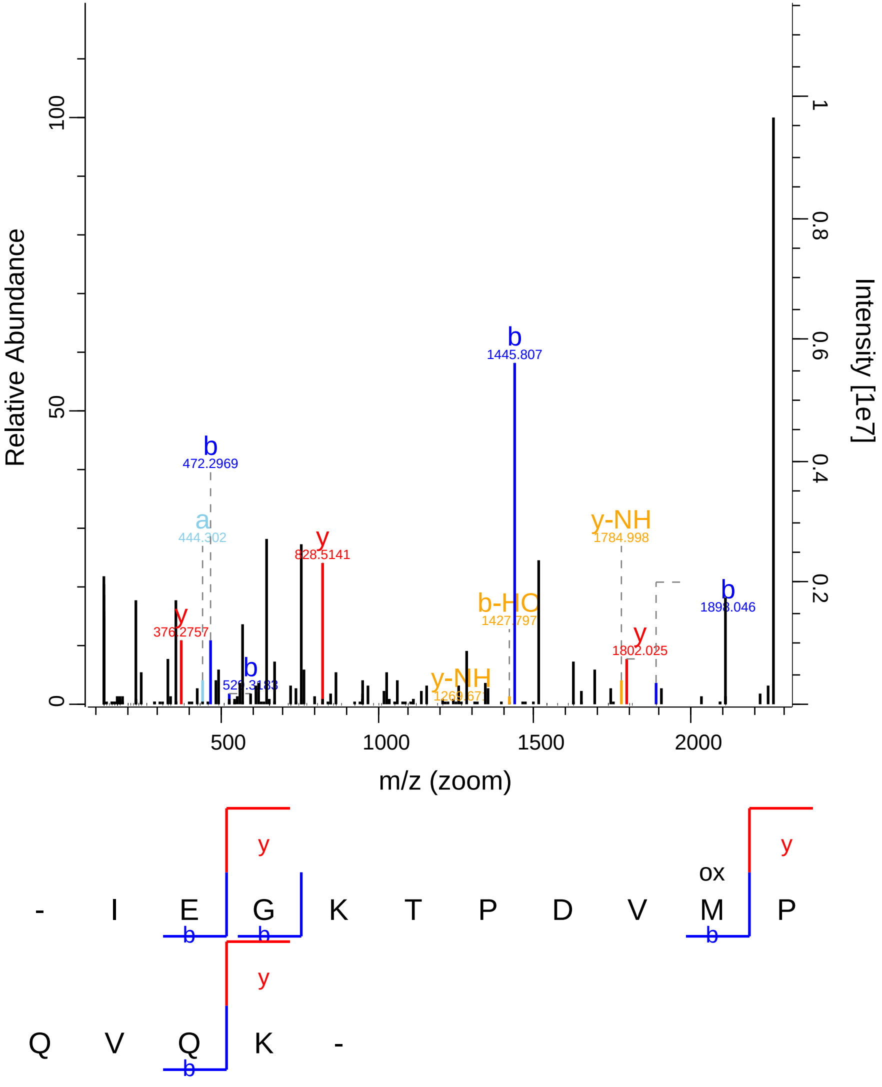

Raw File	Scan	Method	Score	m/z	Gene names
RO-QC-160121_ZackD_SS_Set_01_Fractic	28874	FTMS; HC	50.3	1740.91	POU6F2

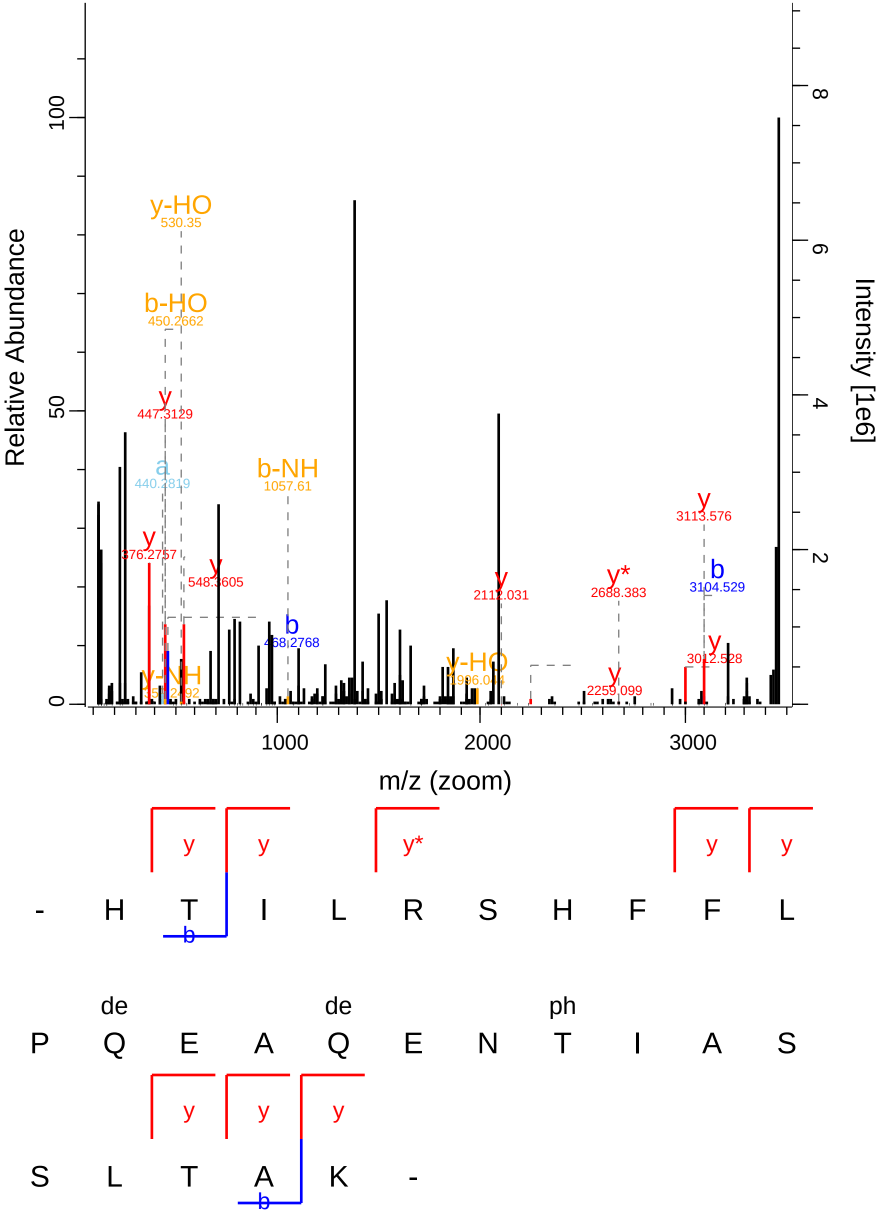

Raw File	Scan	Method	Score	m/z	Gene names
RO-QC-160121_ZackD_SS_Set_01_Fractio	28980	FTMS; HC	88.73	429.26	KRT82

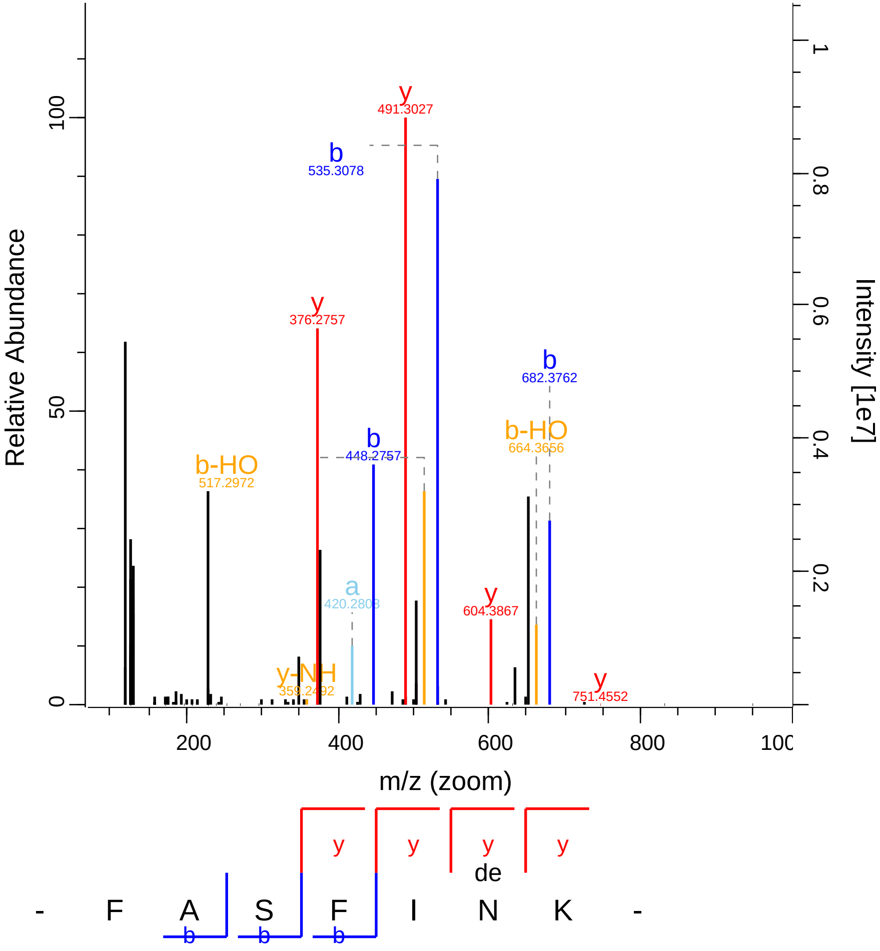

Raw File	Scan	Method	Score	m/z	Gene names
RO-QC-160121_ZackD_SS_Set_01_Fractio	29088	FTMS; HC	117.93	643.38	KRT82

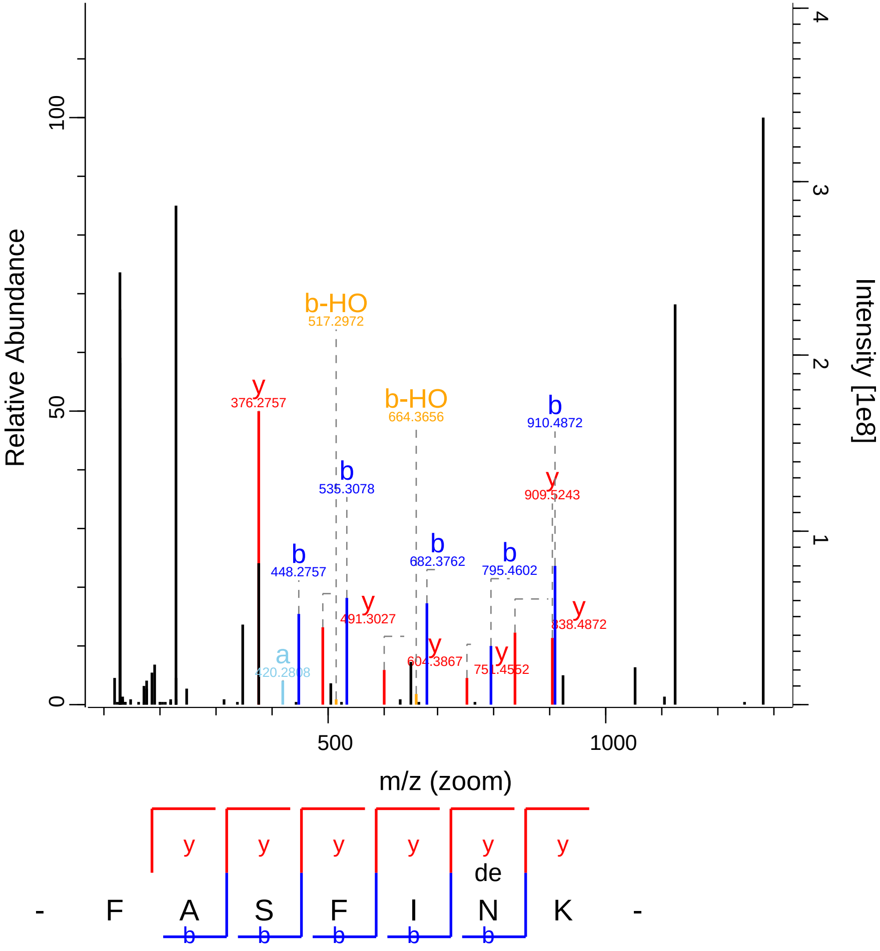

Raw File	Scan	Method	Score	m/z	Gene names
RO-QC-160121_ZackD_SS_Set_01_Fractio	29249	FTMS; HC	88.73	429.26	KRT82

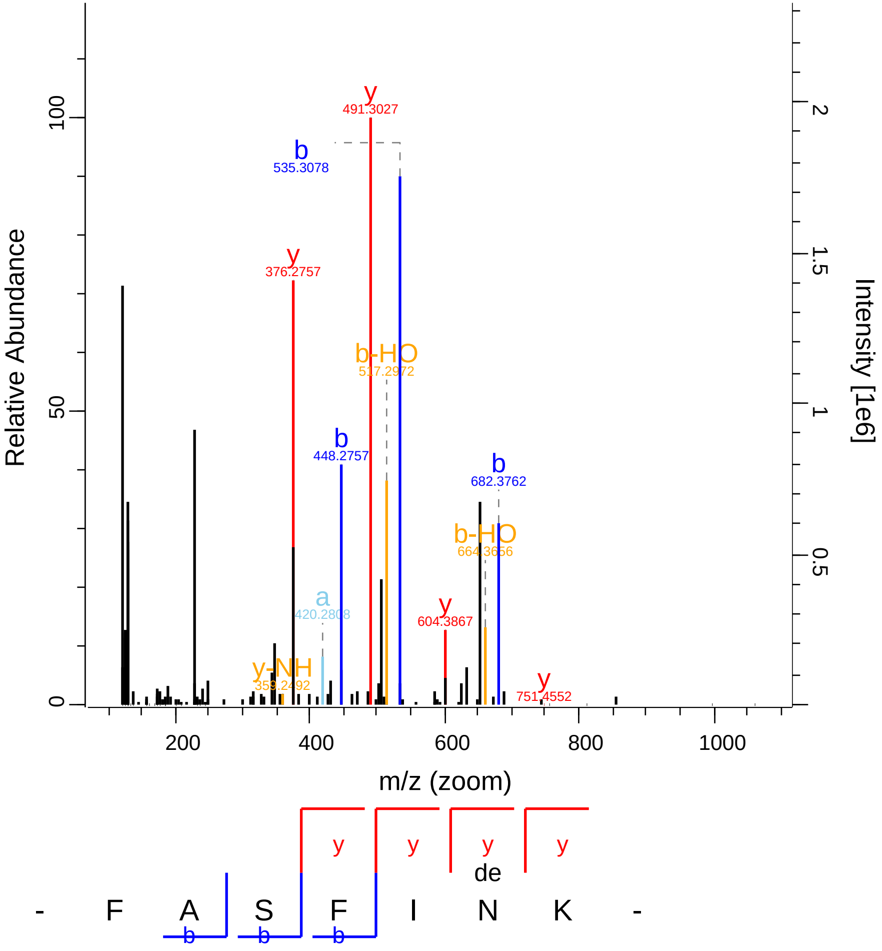

Raw File	Scan	Method	Score	m/z	Gene names
RO-QC-160121_ZackD_SS_Set_01_Fractic	29871	FTMS; HC	55.45	1136.05	LGI4

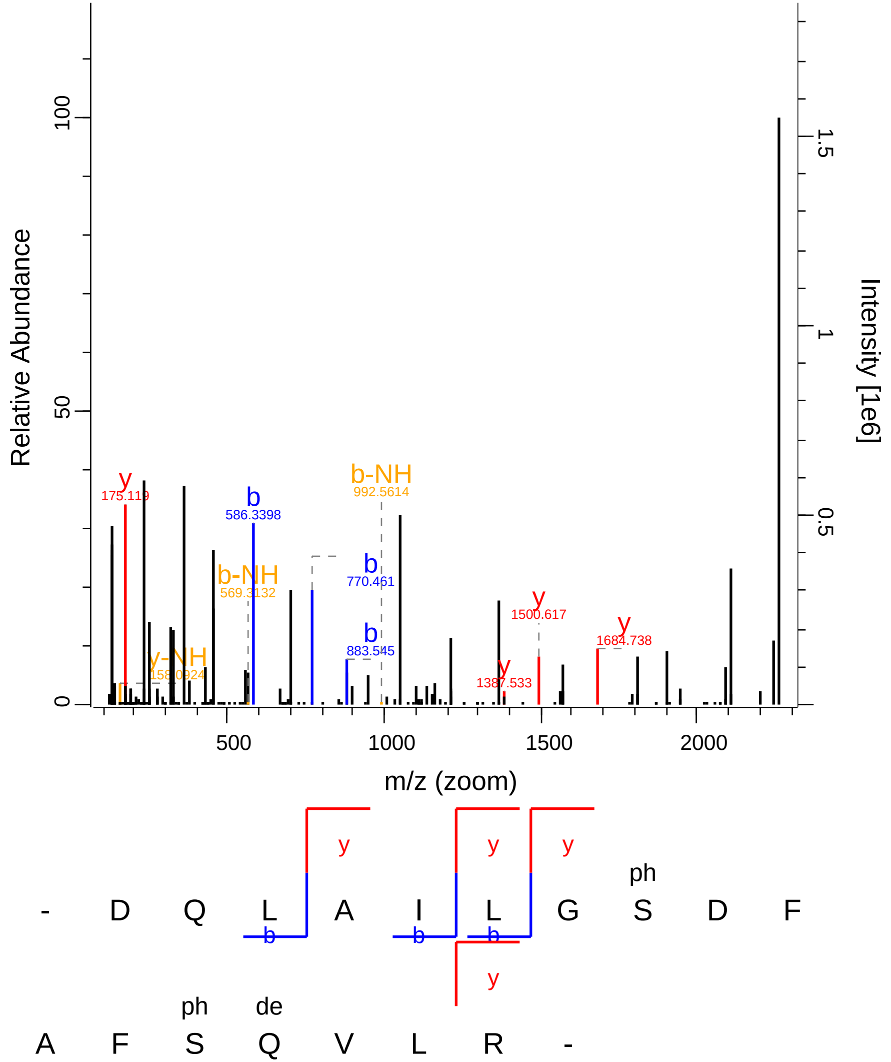

Raw File	Scan	Method	Score	m/z	Gene names
RO-QC-160121_ZackD_SS_Set_01_Fractio	31295	FTMS; HC	119.27	701.46	UNC79

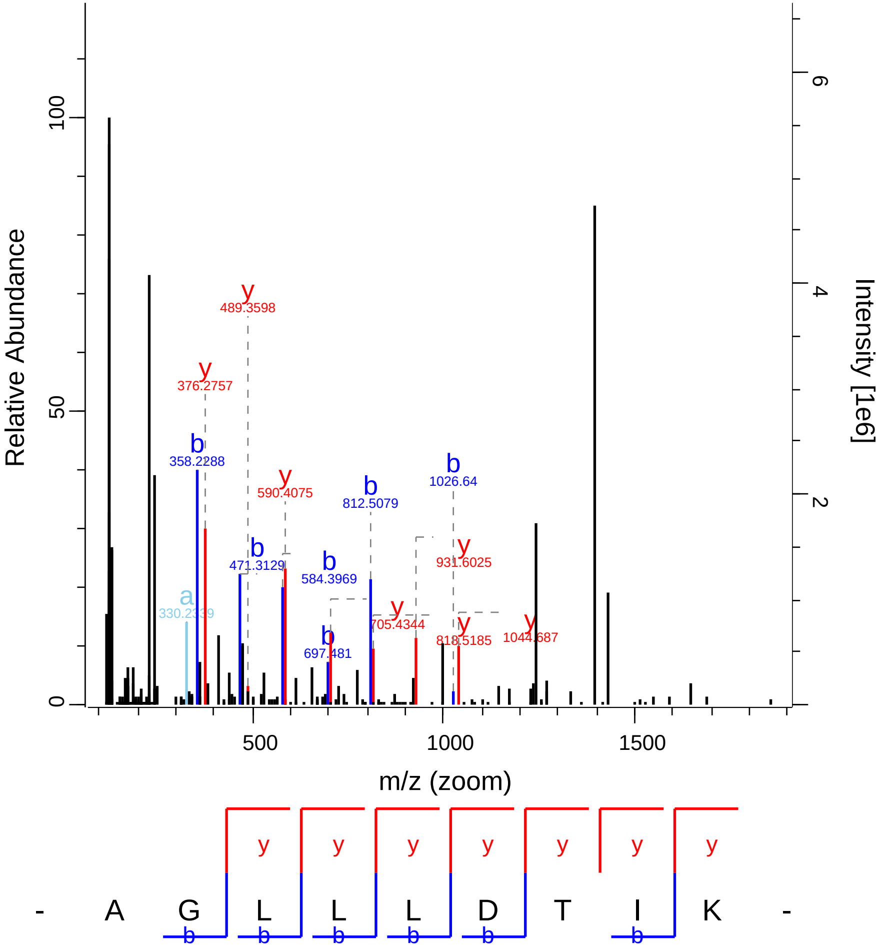

Raw File	Scan	Method	Score	m/z	Gene names
RO-QC-160121_ZackD_SS_Set_01_Fractic	32362	FTMS; HC	70.94	1318.66	NEUROD4

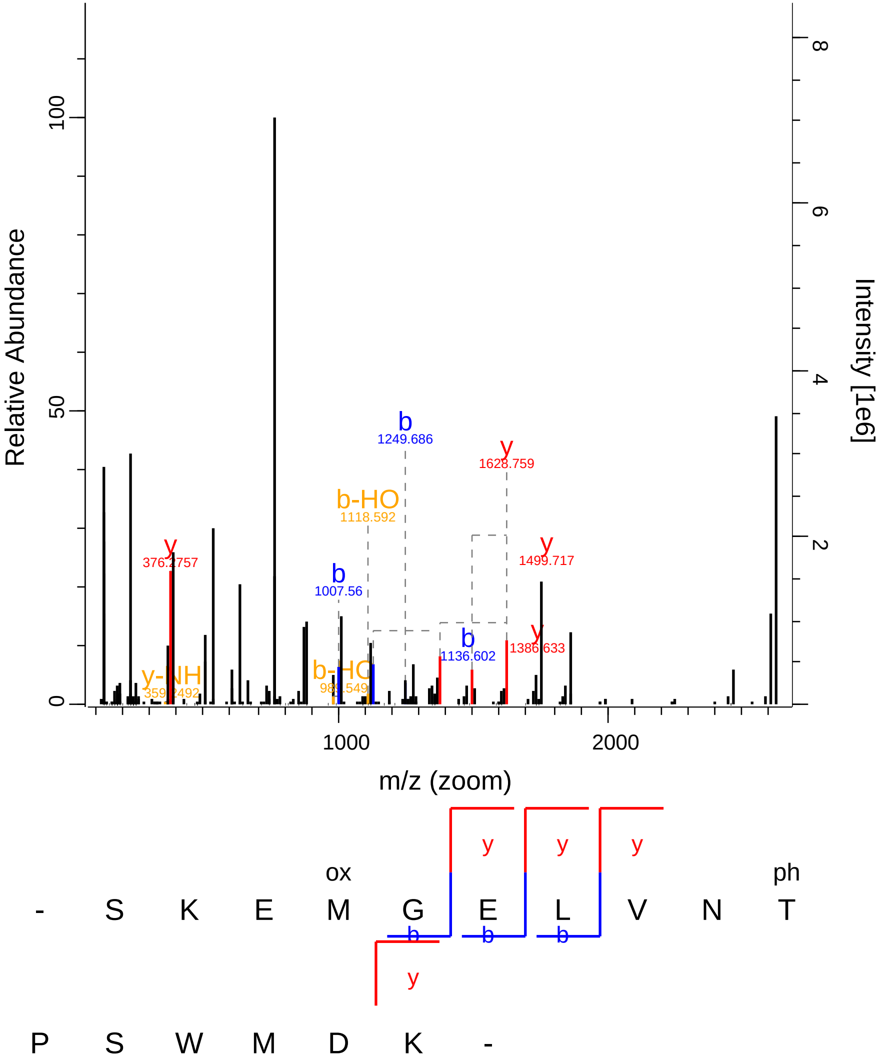

Raw File	Scan	Method	Score	m/z	Gene names
RO-QC-160121_ZackD_SS_Set_01_Fractic	32769	FTMS; HC	54.98	1192.24	CEP70

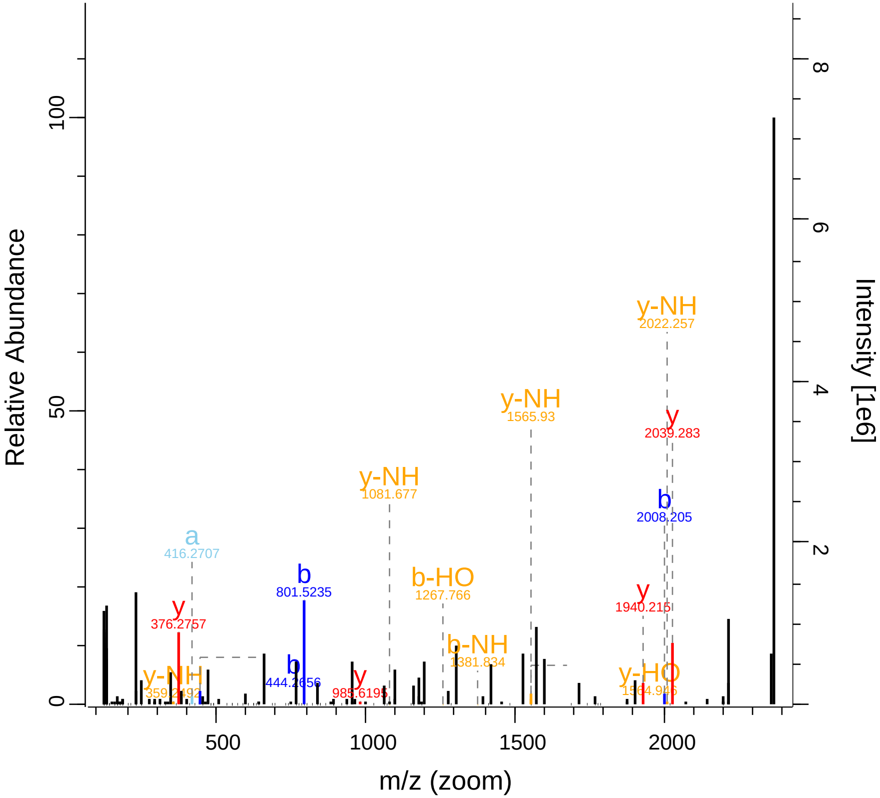

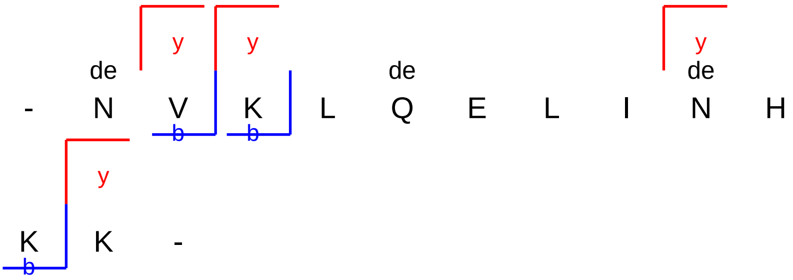

Raw File	Scan	Method	Score	m/z	Gene names
RO-QC-160121_ZackD_SS_Set_01_Fractio	32858	FTMS; HC	200.16	1095.65	KRTCAP2

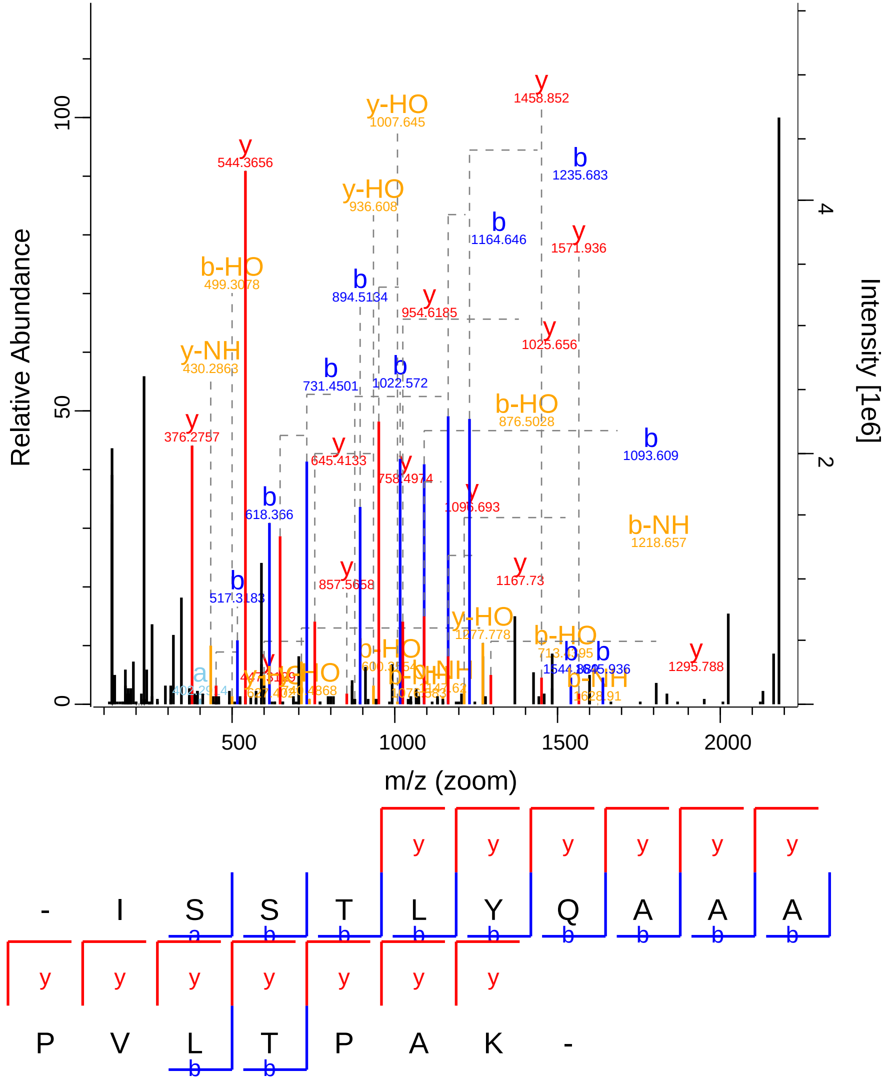

Raw File	Scan	Method	Score	m/z	Gene names
RO-QC-160121_ZackD_SS_Set_01_Fractio	32912	FTMS; HC	63.69	548.33	KRTCAP2

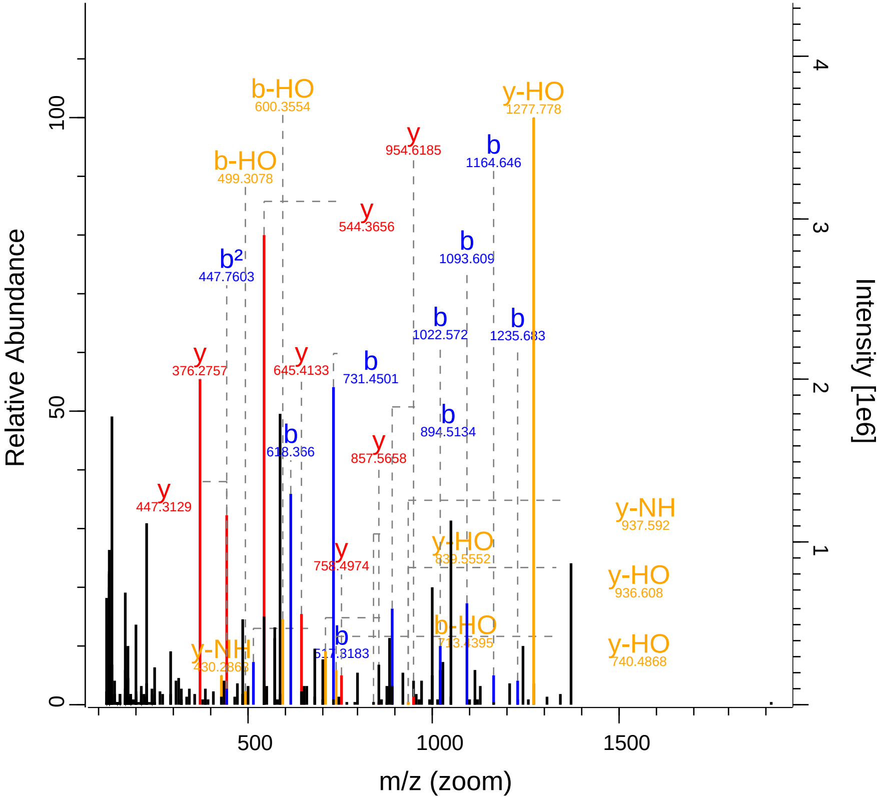

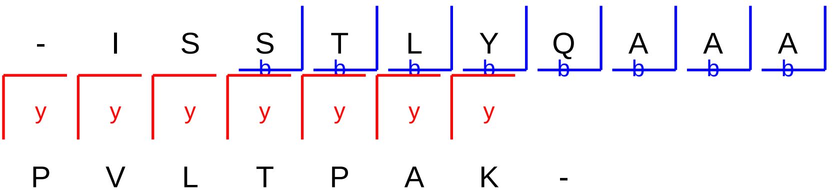

Raw File	Scan	Method	Score	m/z	Gene names
RO-QC-160121_ZackD_SS_Set_01_Fractio	33013	FTMS; HC	95.2	730.77	KRTCAP2

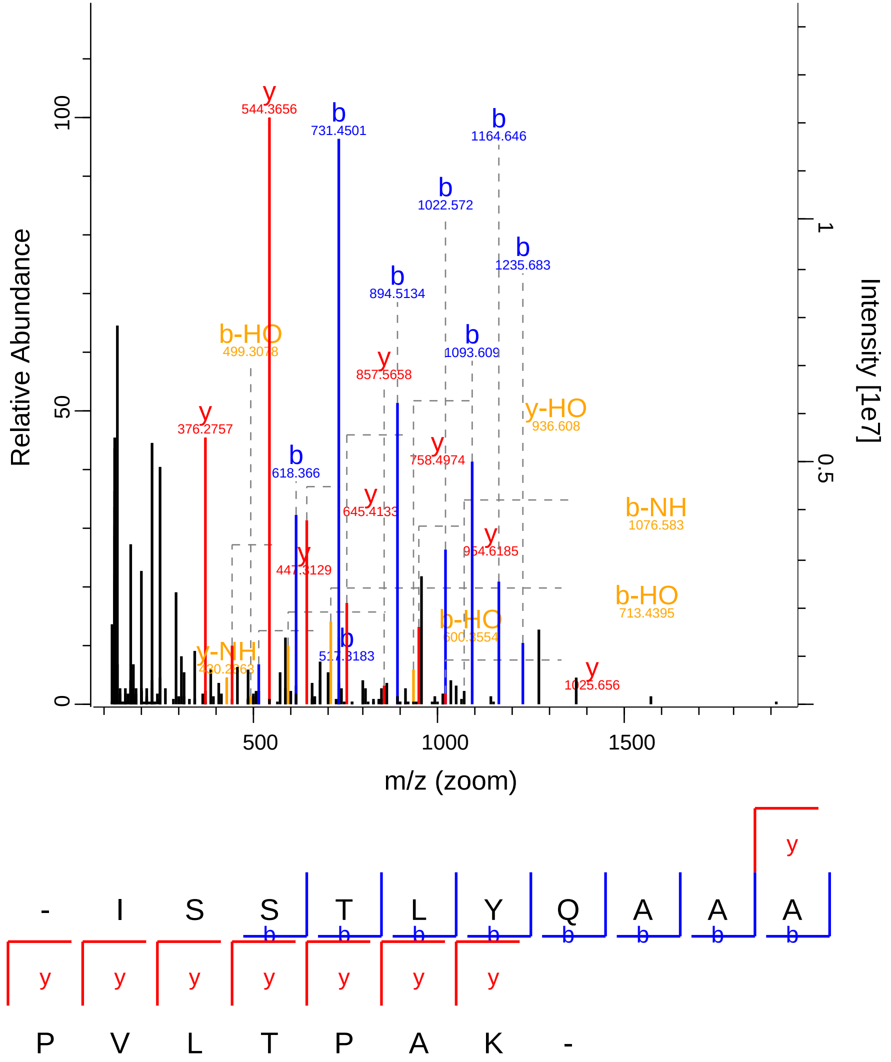

Raw File	Scan	Method	Score	m/z	Gene names
RO-QC-160121_ZackD_SS_Set_01_Fractic	33107	FTMS; HC	236.3	1095.15	KRTCAP2

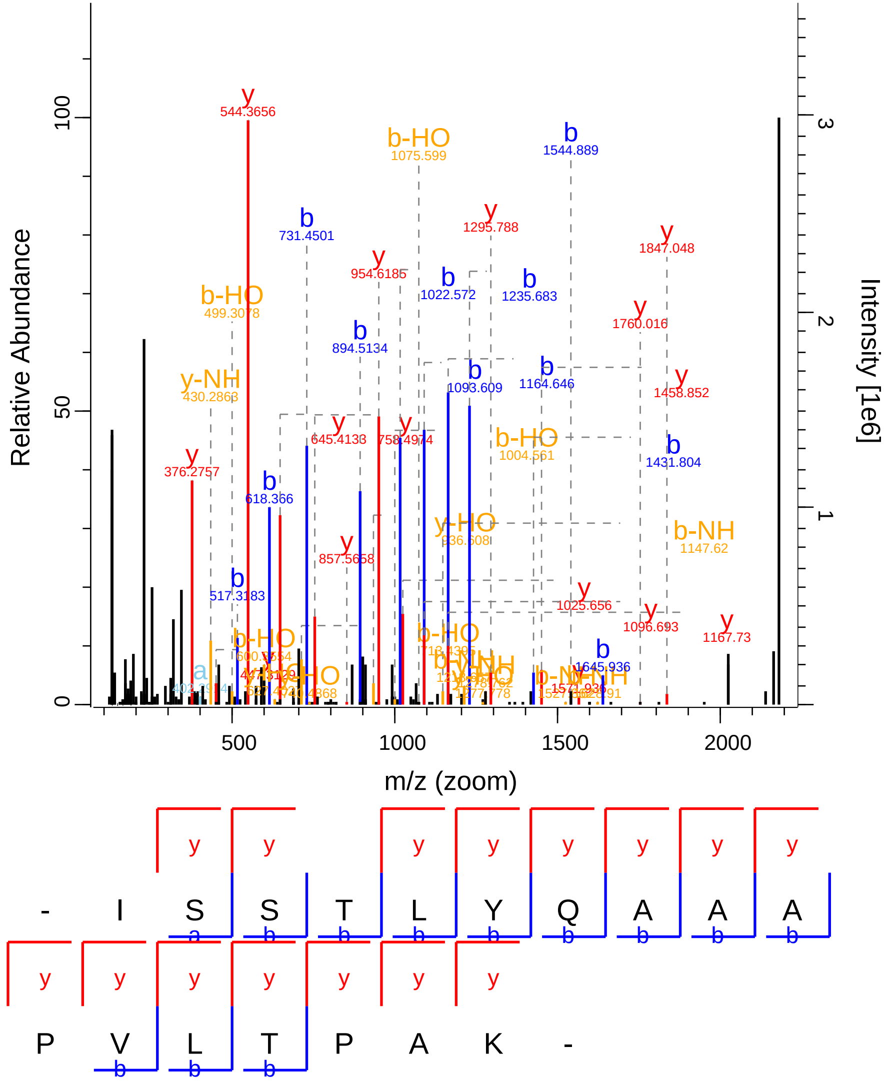

Raw File	Scan	Method	Score	m/z
RO-QC-160121_ZackD_SS_Set_01_Fraction_7	33192	FTMS; HCD	50.42	1002.56

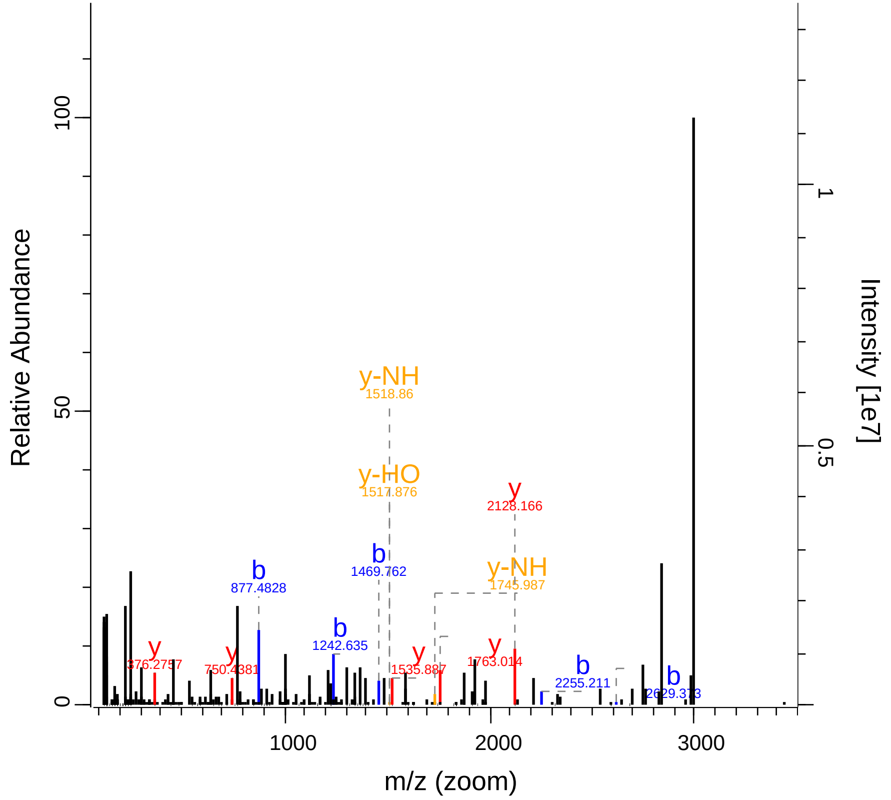

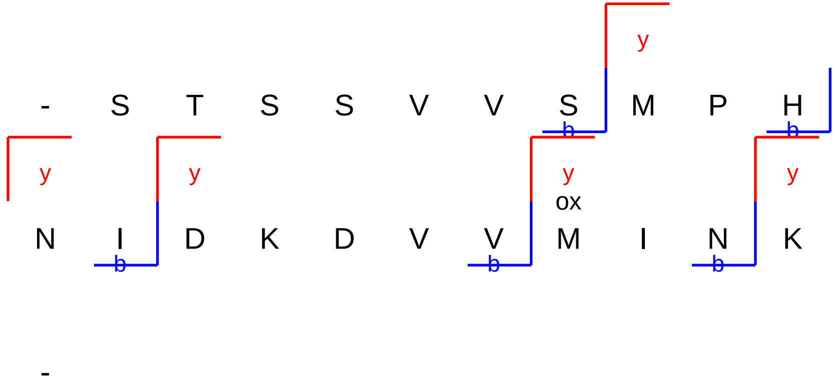

Raw File	Scan	Method	Score	m/z
RO-QC-160121_ZackD_SS_Set_01_Fraction_7	33784	FTMS; HCD	47.07	465.28

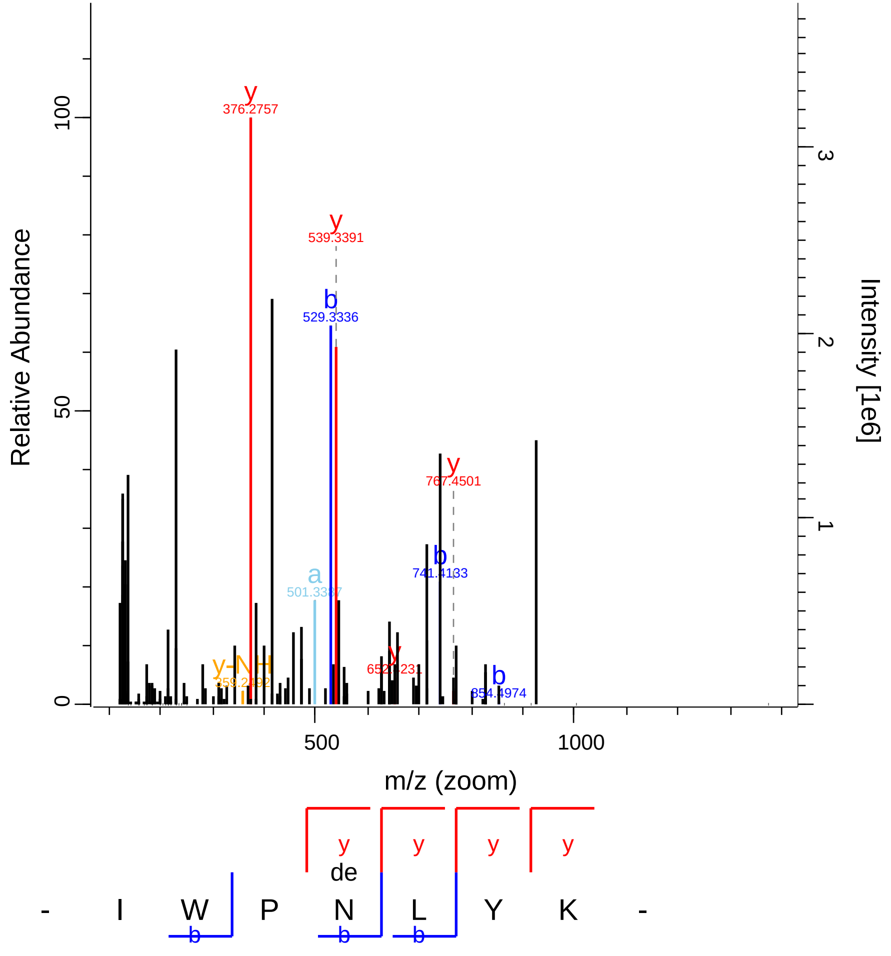

Raw File	Scan	Method	Score	m/z
RO-QC-160121_ZackD_SS_Set_01_Fraction_7	34008	FTMS; HCD	99.17	464.95

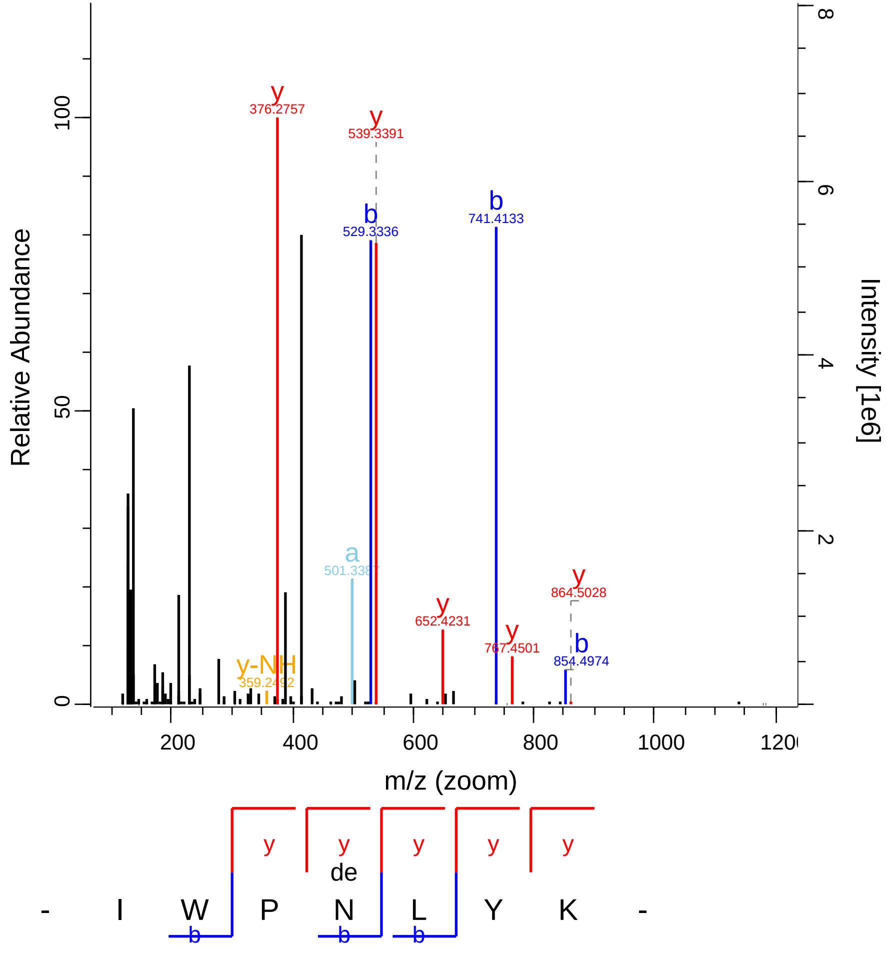

Raw File	Scan	Method	Score	m/z	Gene names
RO-QC-160121_ZackD_SS_Set_01_Fractio	34098	FTMS; HC	74.94	552.01	KLHDC4

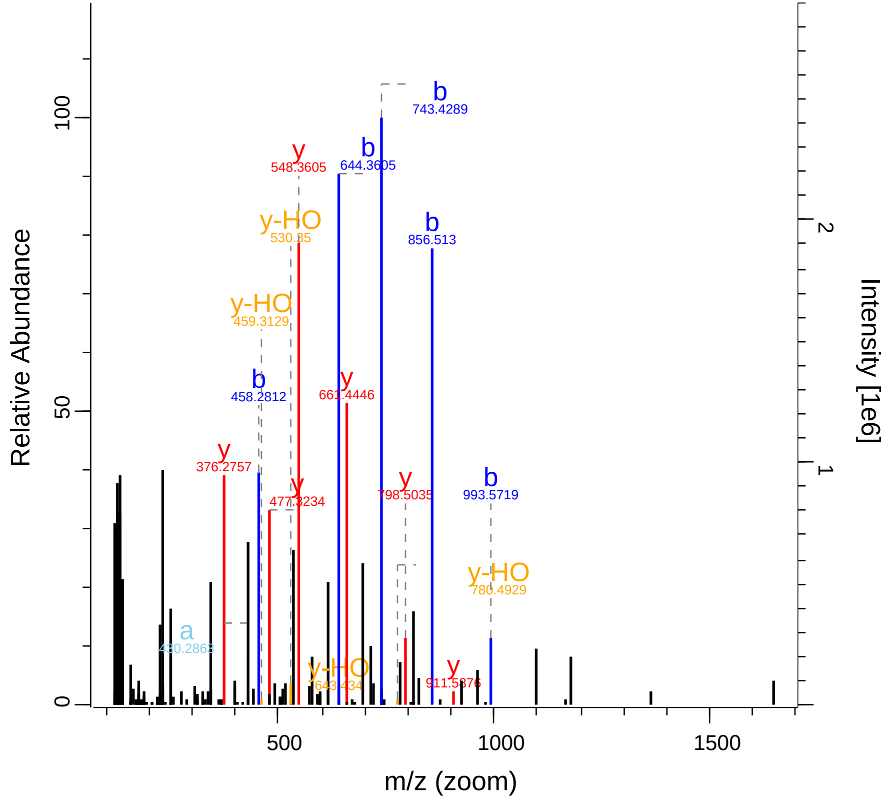

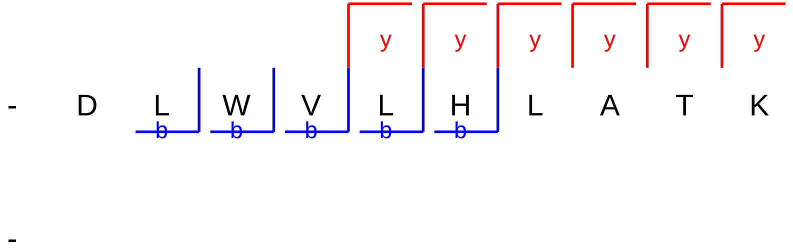

Raw File	Scan	Method	Score	m/z	Gene names
RO-QC-160121_ZackD_SS_Set_01_Fractic	34832	FTMS; HC	50.03	1155.63	EBF2

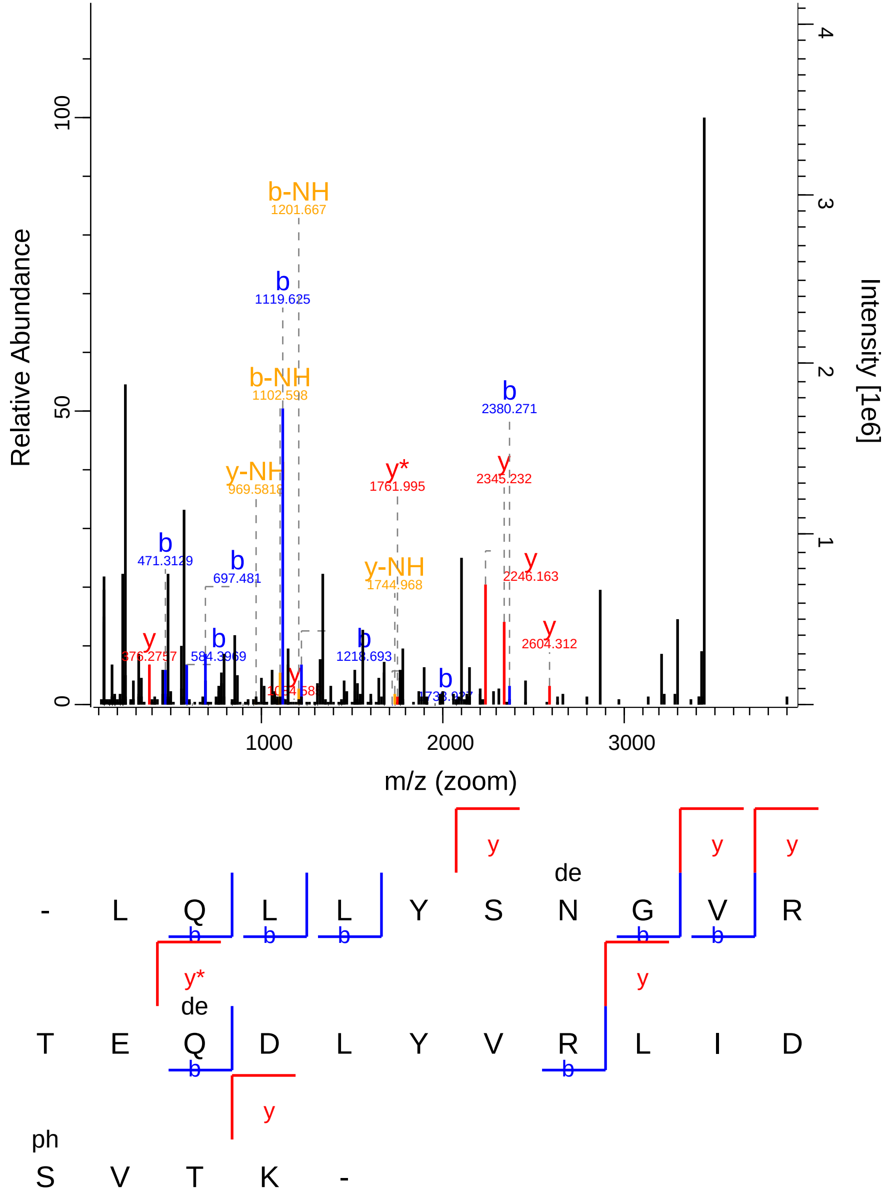

Raw File	Scan	Method	Score	m/z	Gene names
RO-QC-160121_ZackD_SS_Set_01_Fractio	36251	FTMS; HC	87.16	901.73	PARM1

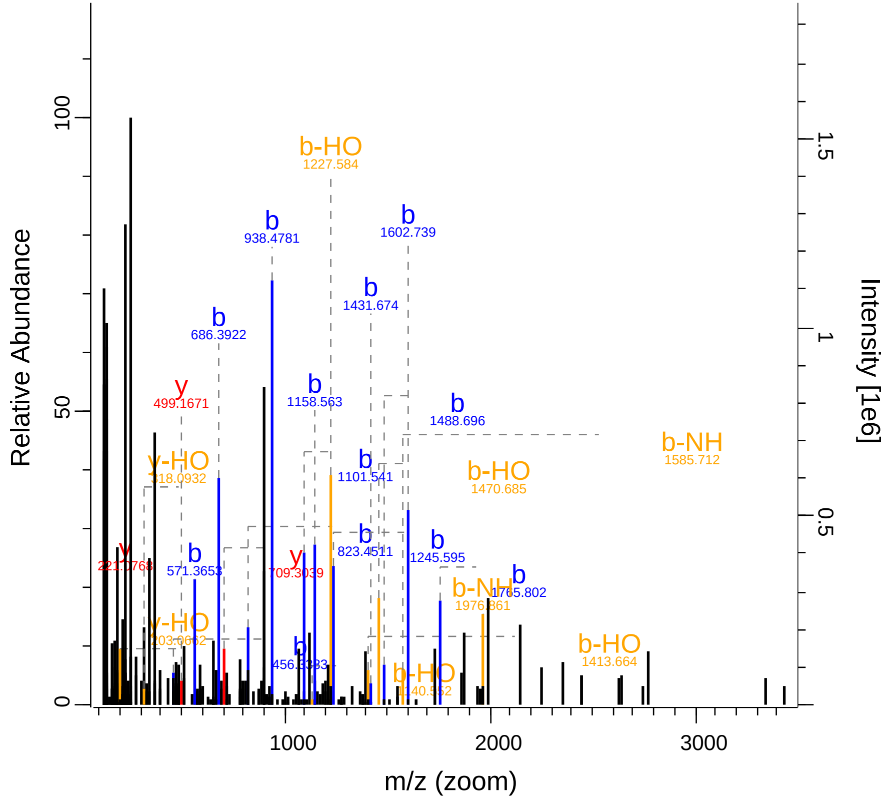

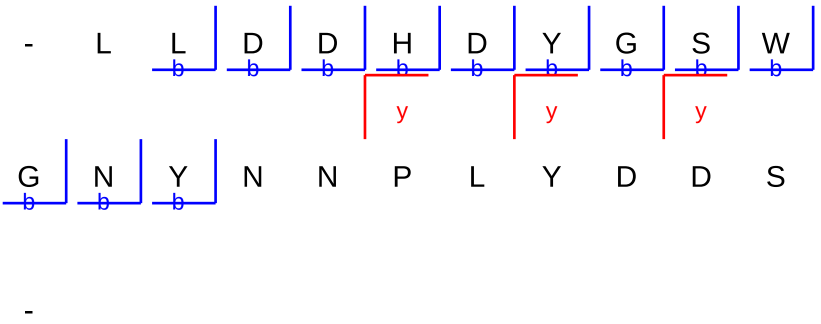

Raw File	Scan	Method	Score	m/z	Gene names
RO-QC-160121_ZackD_SS_Set_01_Fractio	36373	FTMS; HC	104.14	496.61	GTF2A1

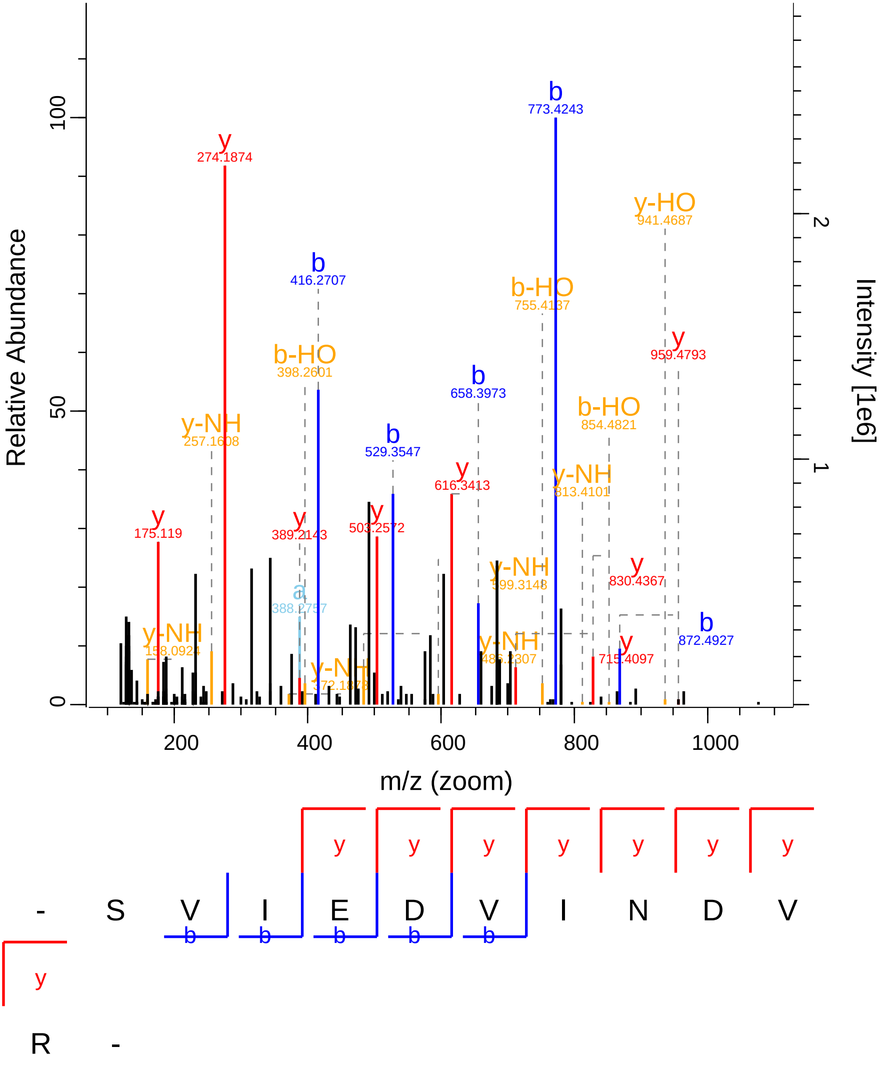

Raw File	Scan	Method	Score	m/z	Gene names
RO-QC-160121_ZackD_SS_Set_01_Fractio	36866	FTMS; HC	180.78	874.77	KCNK1

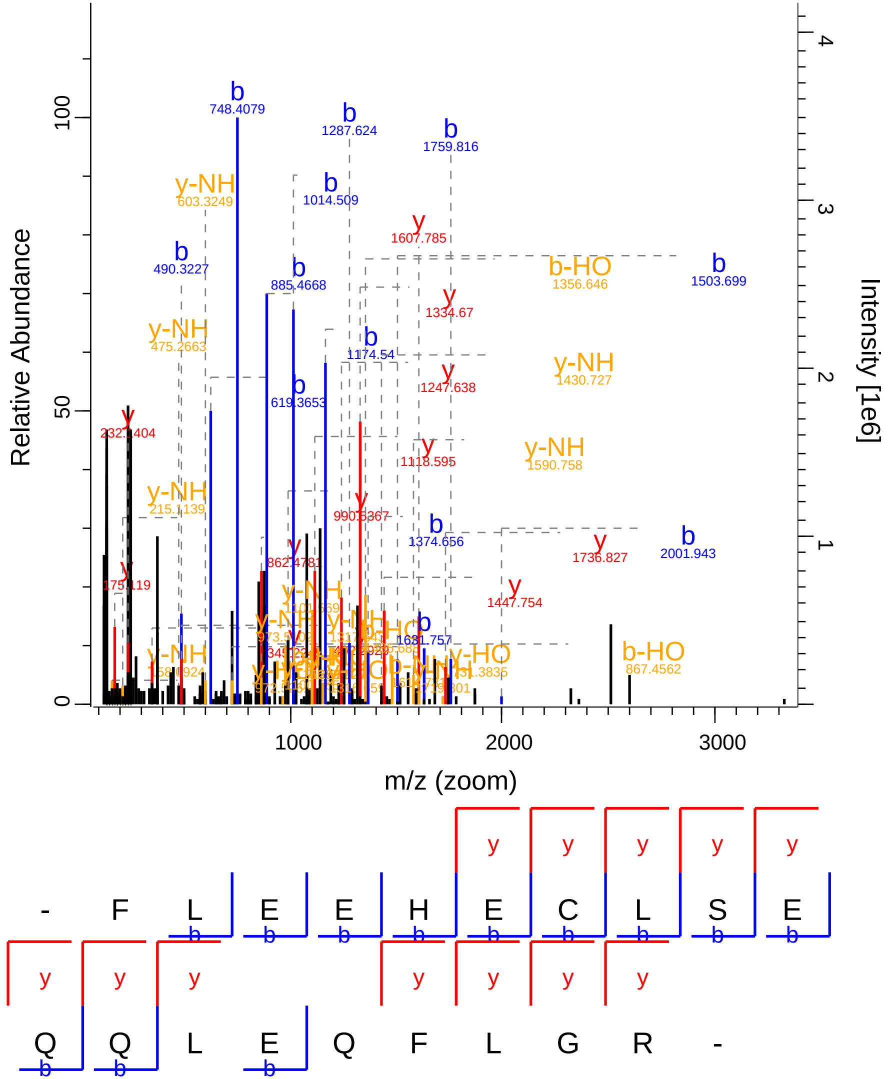

Raw File	Scan	Method	Score	m/z	Gene names
RO-QC-160121_ZackD_SS_Set_01_Fractio	36881	FTMS; HC	80.22	695.94	TMBIM6

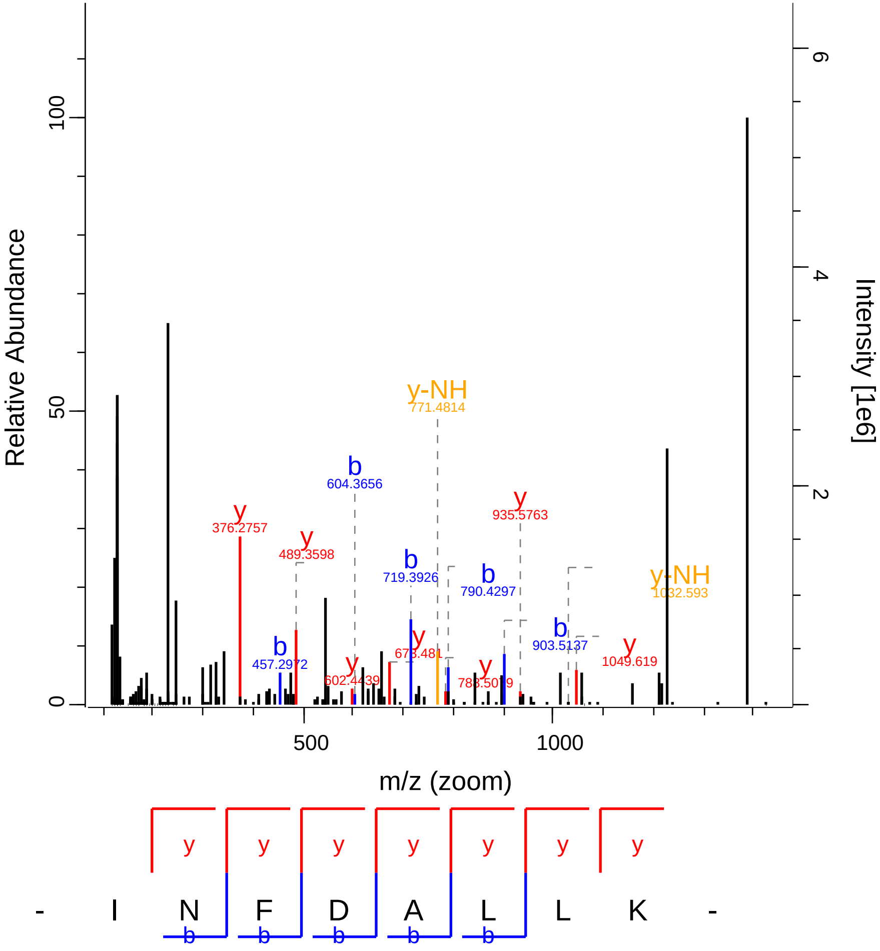

Raw File	Scan	Method	Score	m/z	Gene names
RO-QC-160121_ZackD_SS_Set_01_Fractio	37840	FTMS; HC	55.65	515.99	FBXO4

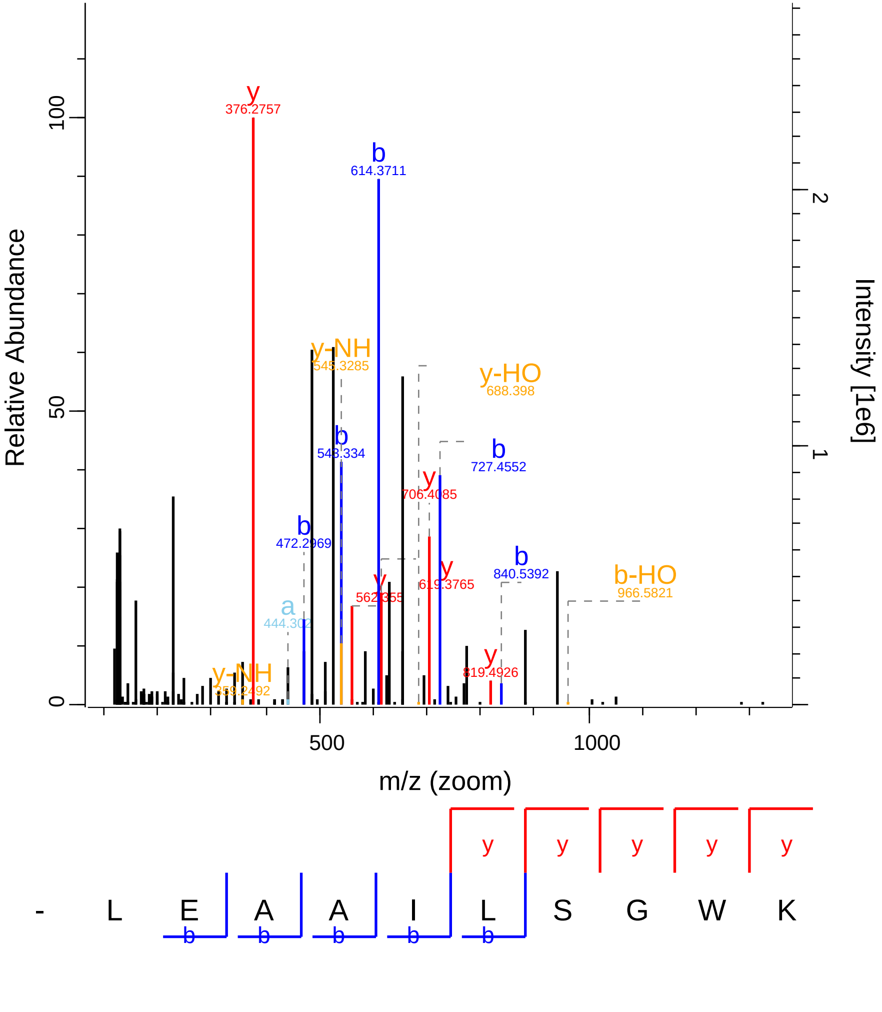

Raw File	Scan	Method	Score	m/z
RO-QC-160121_ZackD_SS_Set_01_Fraction_7	37902	FTMS; HCD	52.48	773.48

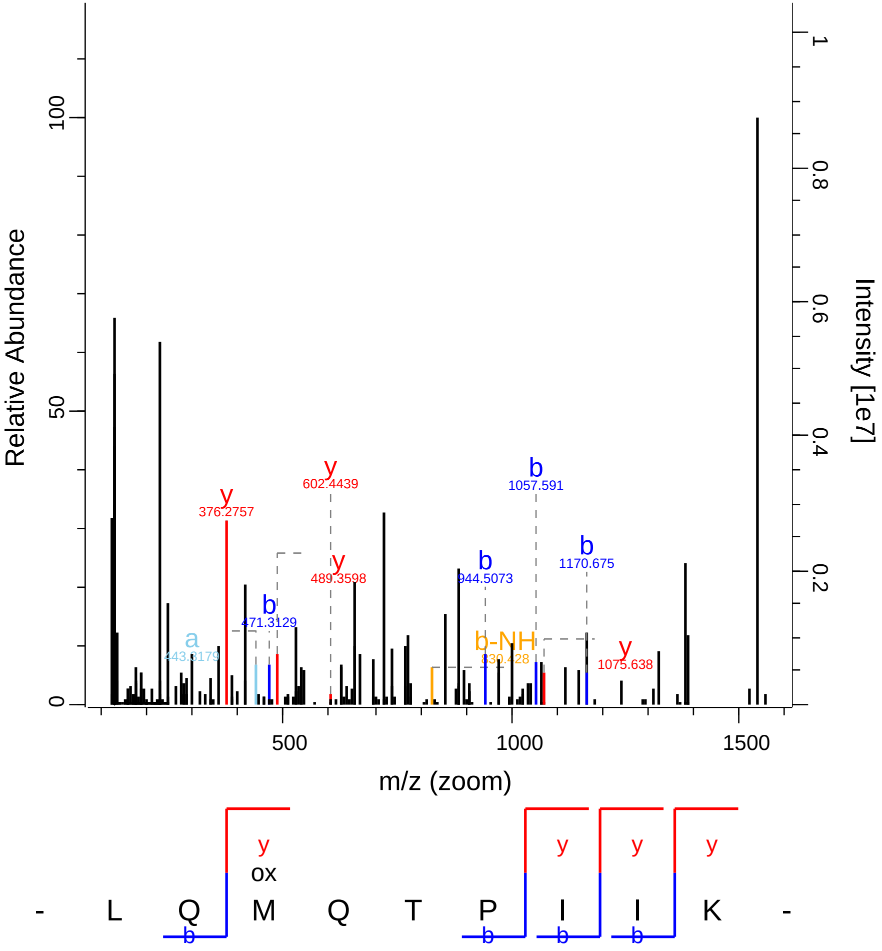

Raw File	Scan	Method	Score	m/z	Gene names
RO-QC-160121_ZackD_SS_Set_01_Fractic	38391	FTMS; HC	144.18	1037.52	SFT2D1

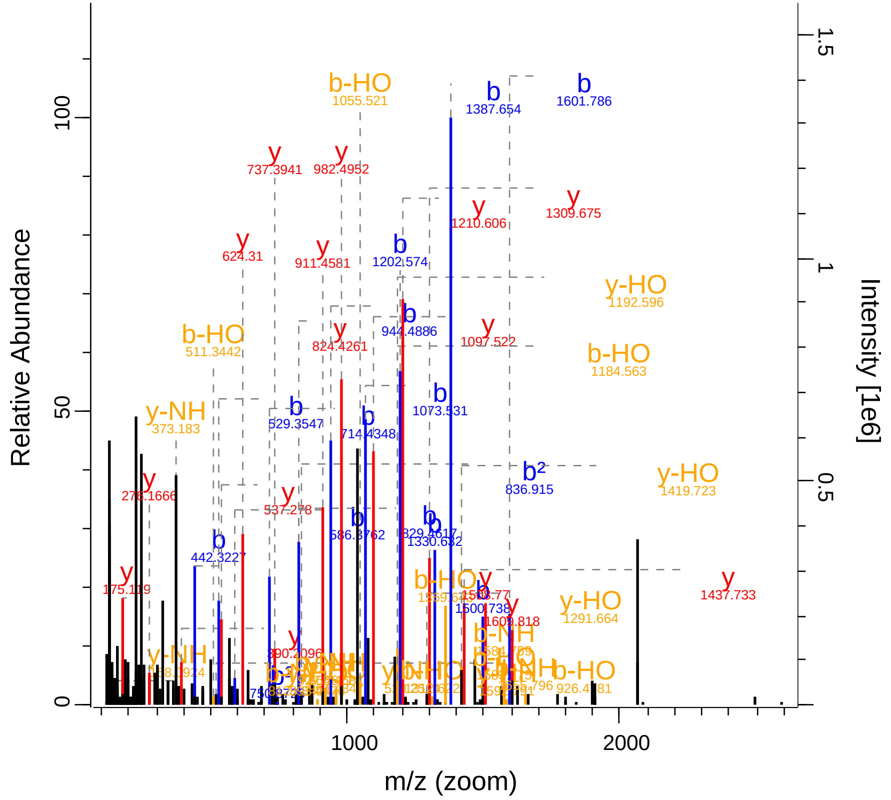

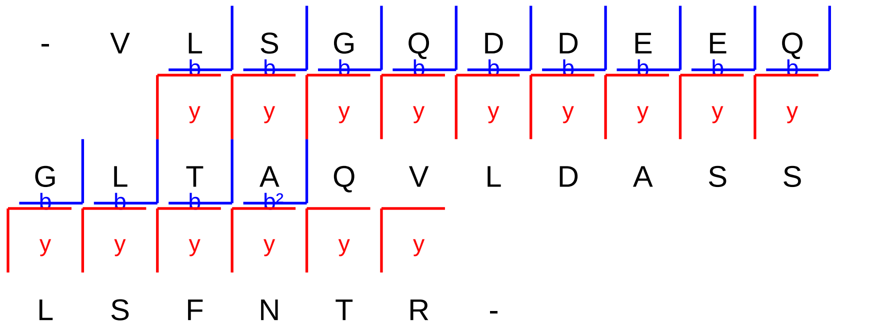

Raw File	Scan	Method	Score	m/z
RO-QC-160121_ZackD_SS_Set_01_Fraction_7	38418	FTMS; HCD	51.55	776.8

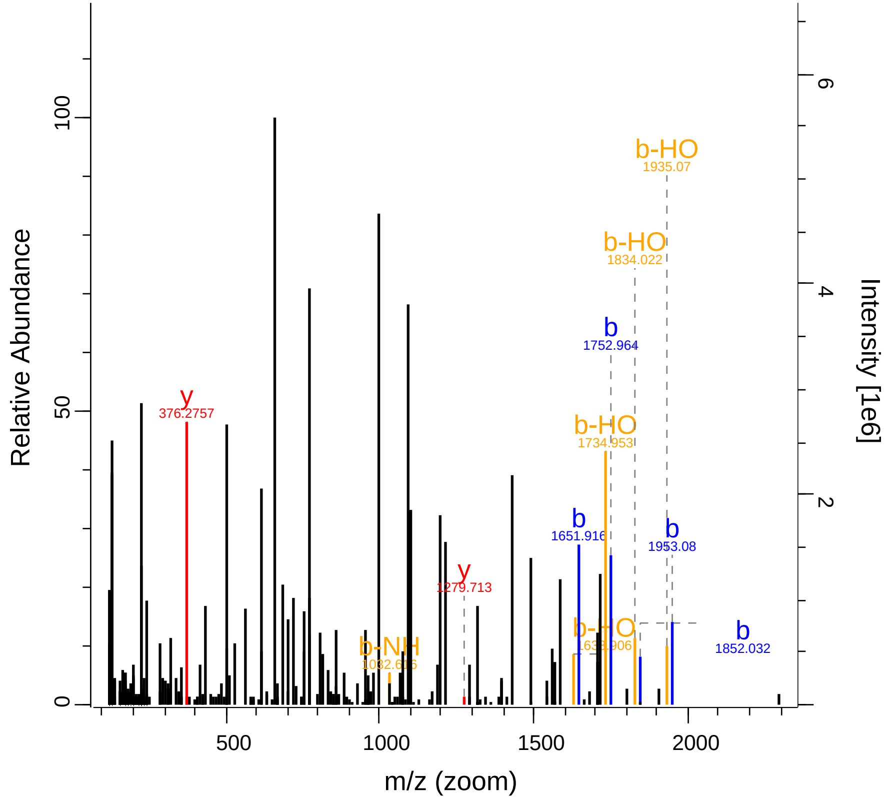

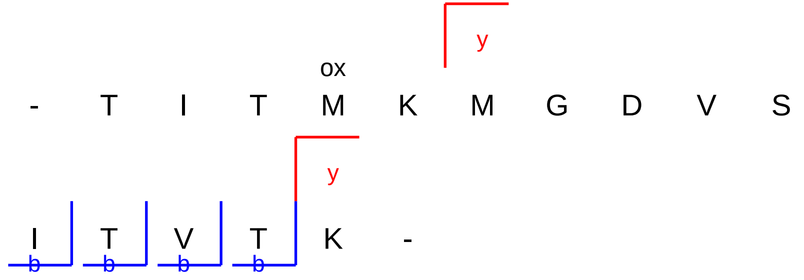

Raw File	Scan	Method	Score	m/z
RO-QC-160121_ZackD_SS_Set_01_Fraction_7	38647	FTMS; HCD	61.35	481.3

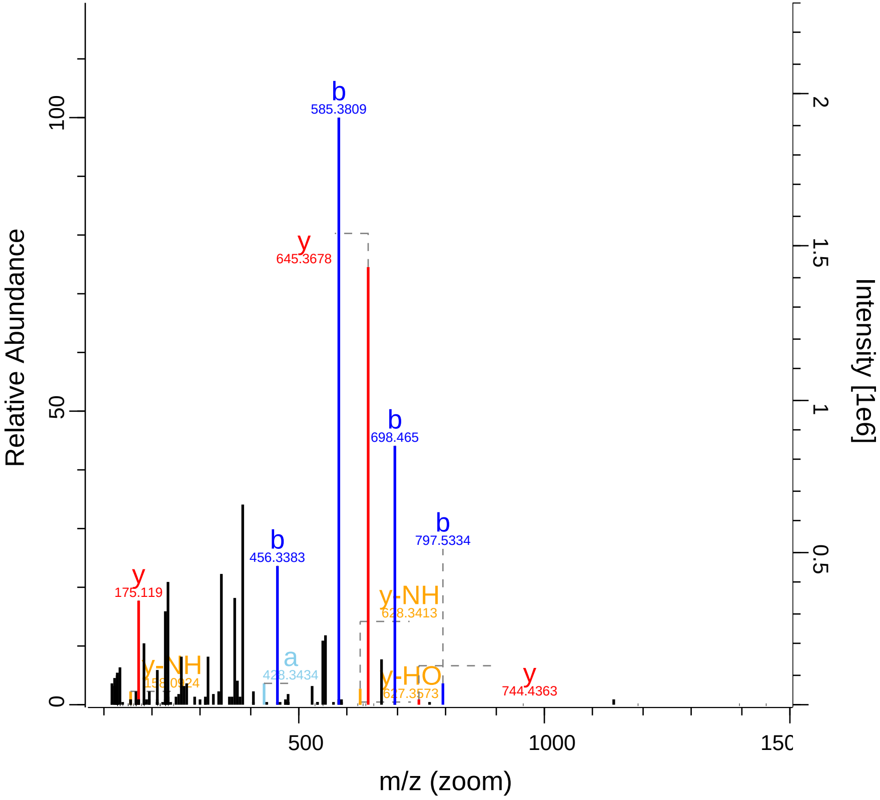

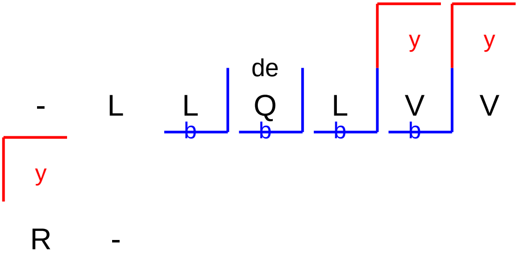

Raw File	Scan	Method	Score	m/z	Gene names
RO-QC-160121_ZackD_SS_Set_01_Fractio	40882	FTMS; HC	188.04	945.56	UQCR11

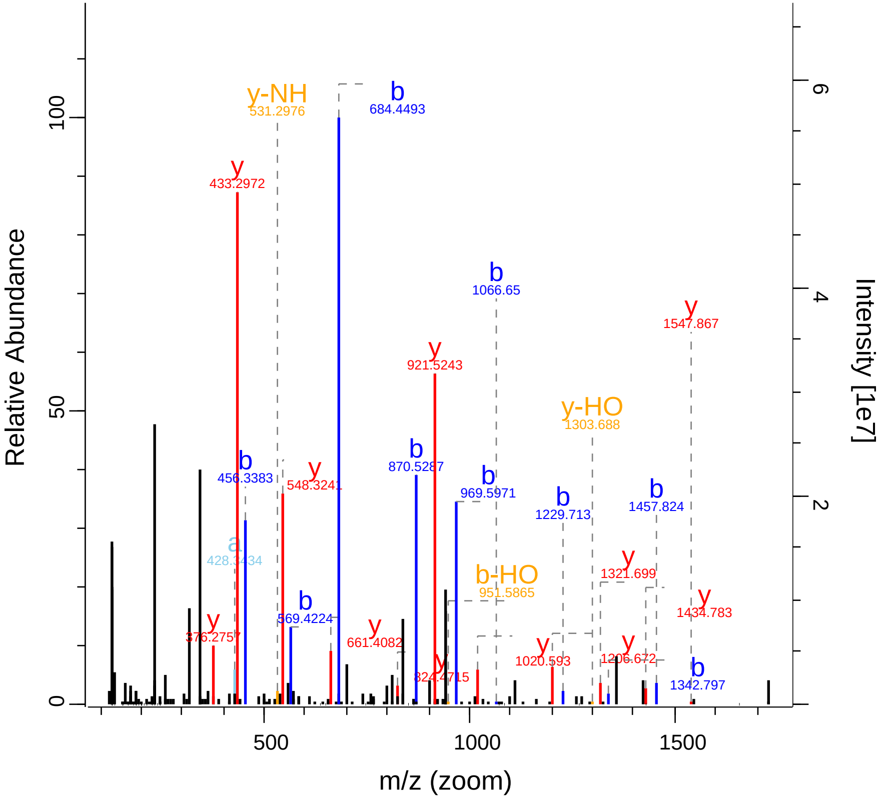

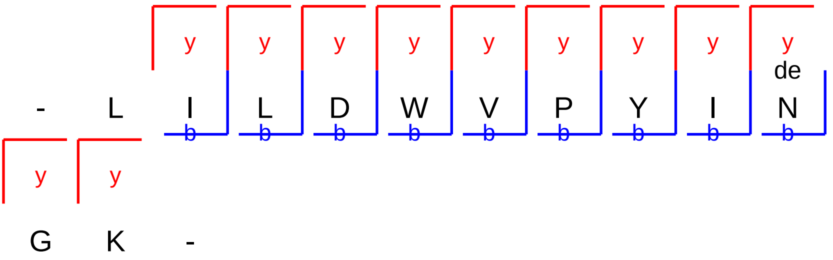

Raw File	Scan	Method	Score	m/z	Gene names
RO-QC-160121_ZackD_SS_Set_01_Fractio	40885	FTMS; HC	106.29	630.71	UQCR11

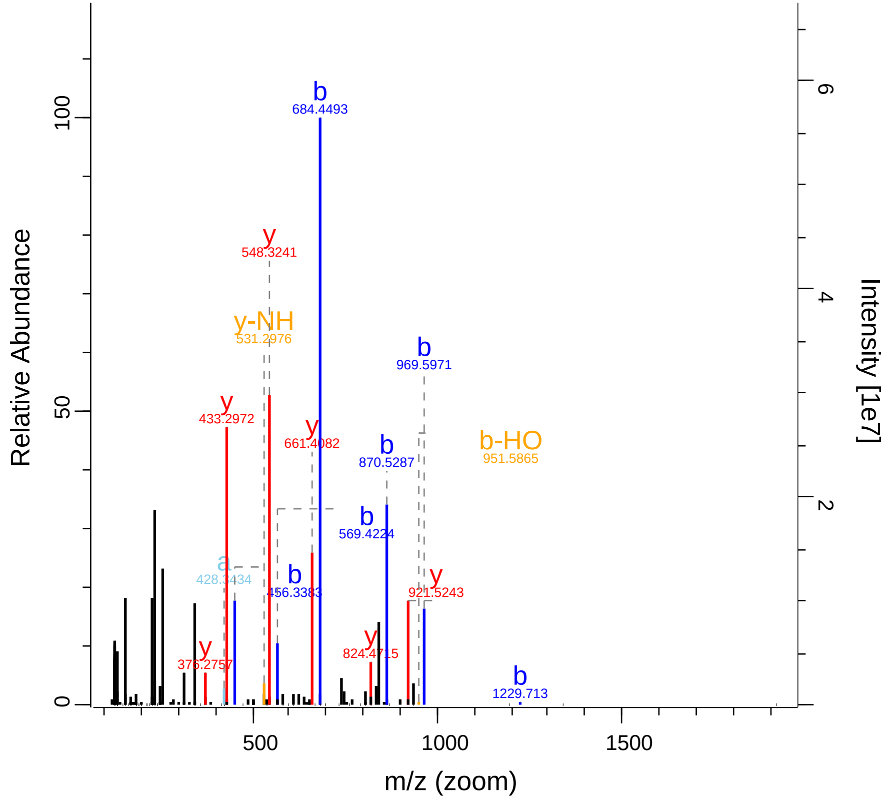

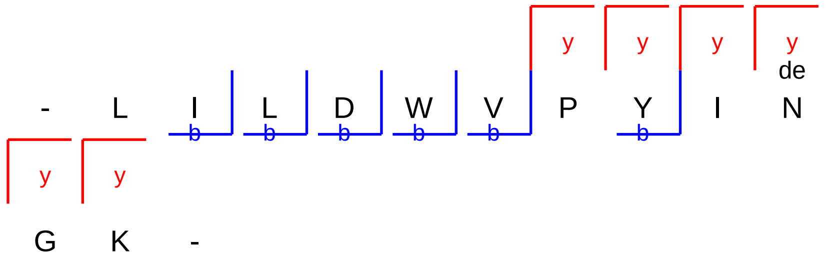

Raw File	Scan	Method	Score	m/z	Gene names
RO-QC-160121_ZackD_SS_Set_01_Fractior	41823	FTMS; HCI	83.61	643.9	STRA13

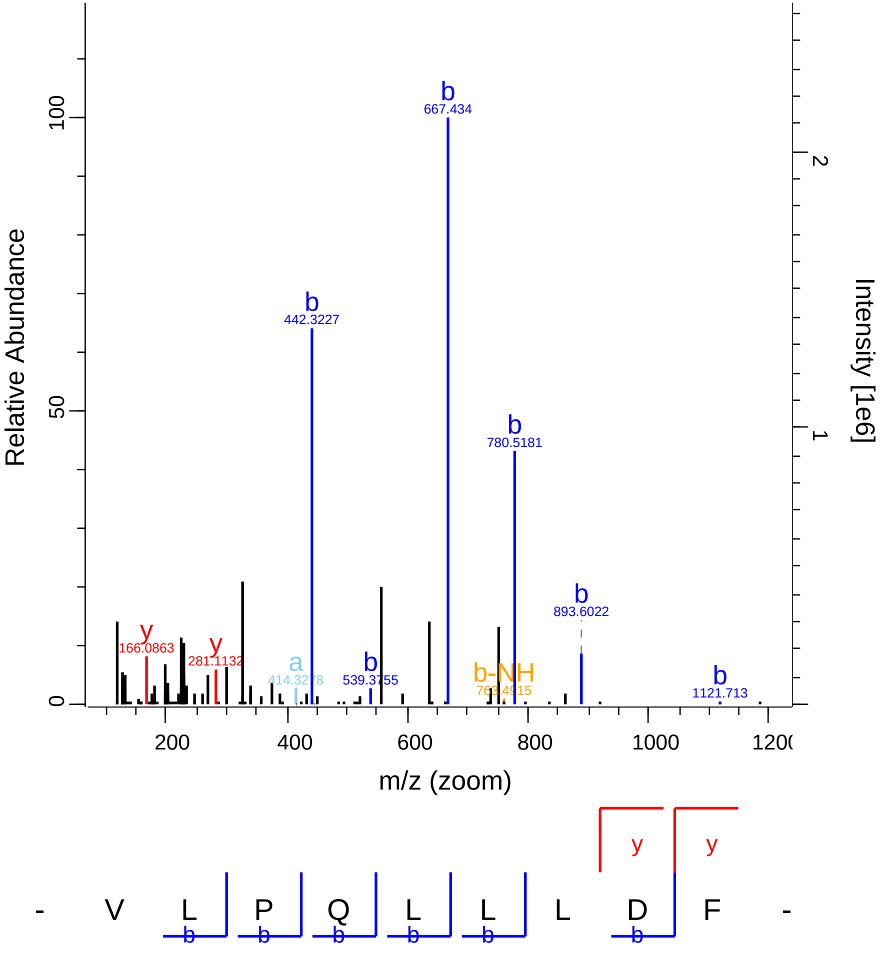

Raw File	Scan	Method	Score	m/z	Gene names
RO-QC-160121_ZackD_SS_Set_01_Fractio	5948	FTMS; HC	108.43	752.92	CDK10

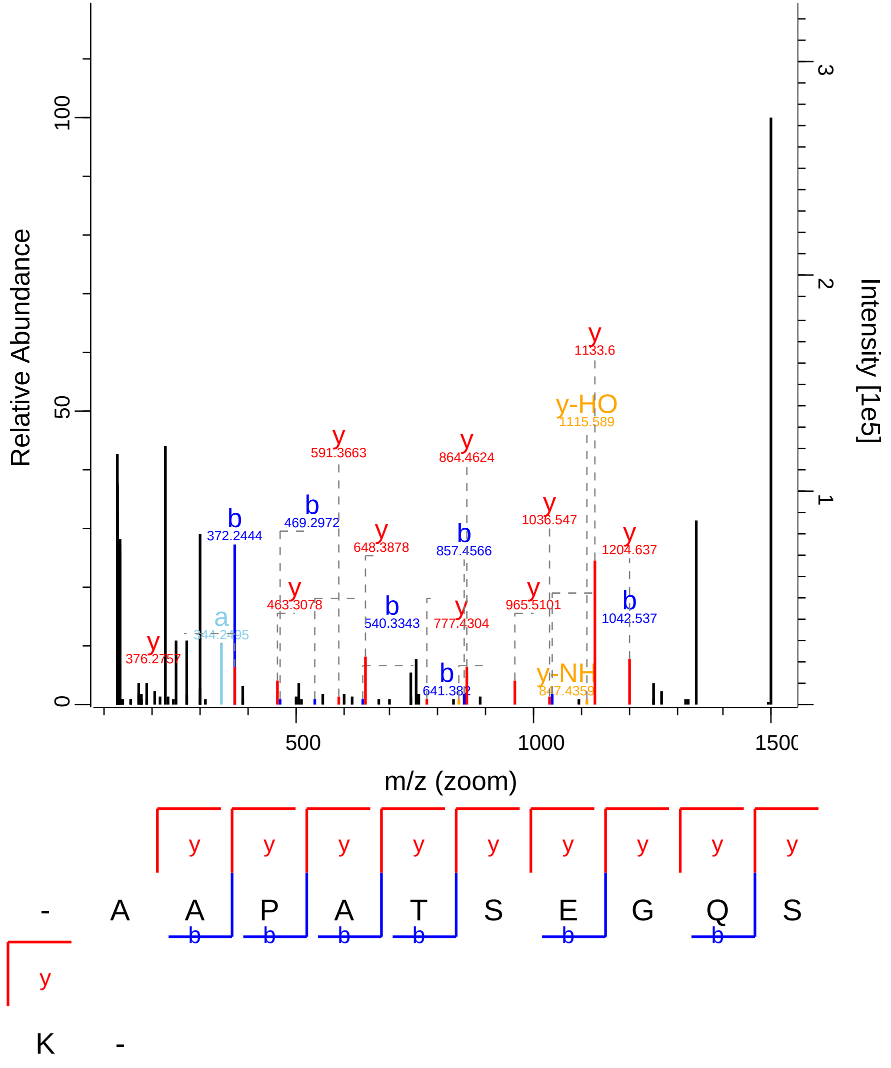

Raw File	Scan	Method	Score	m/z	Gene names
RO-QC-160121_ZackD_SS_Set_01_Fractior	6069	FTMS; HCI	71.98	579.64	TATDN2

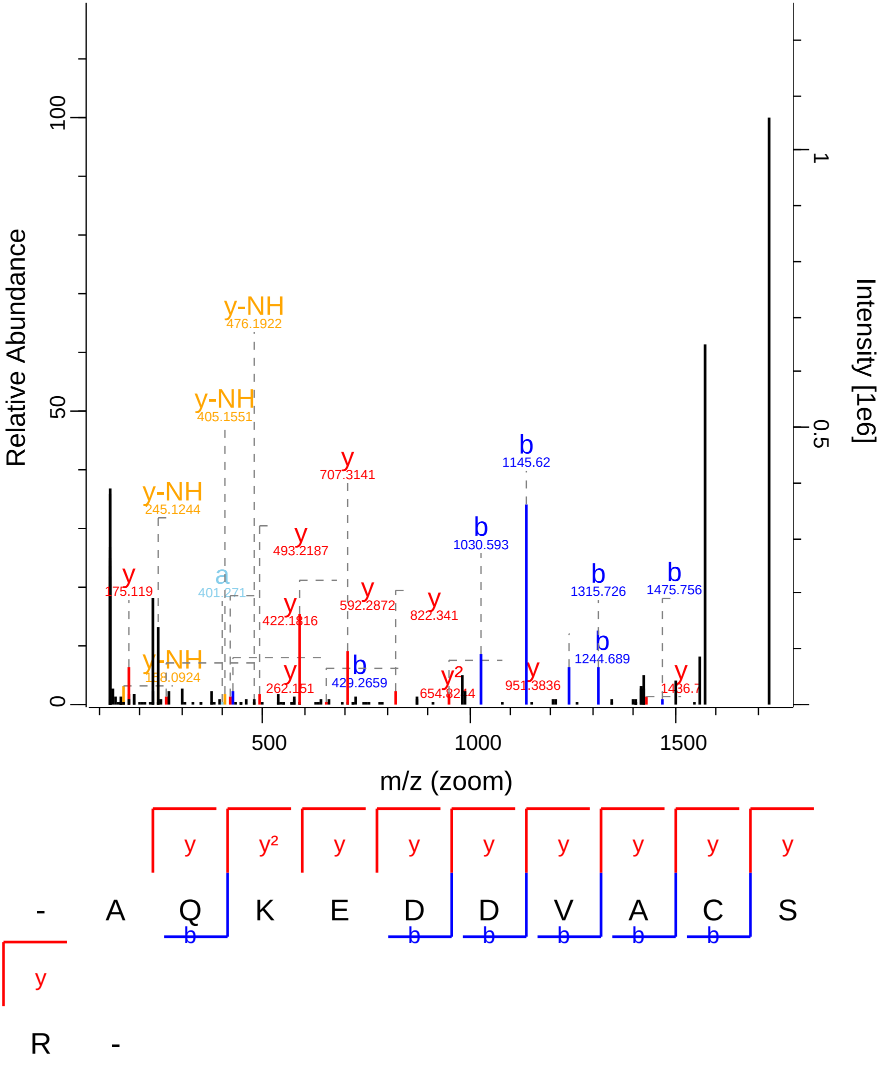

Raw File	Scan	Method	Score	m/z	Gene names
RO-QC-160121_ZackD_SS_Set_01_Fractio	6216	FTMS; HC	182.28	767.06	EPC1

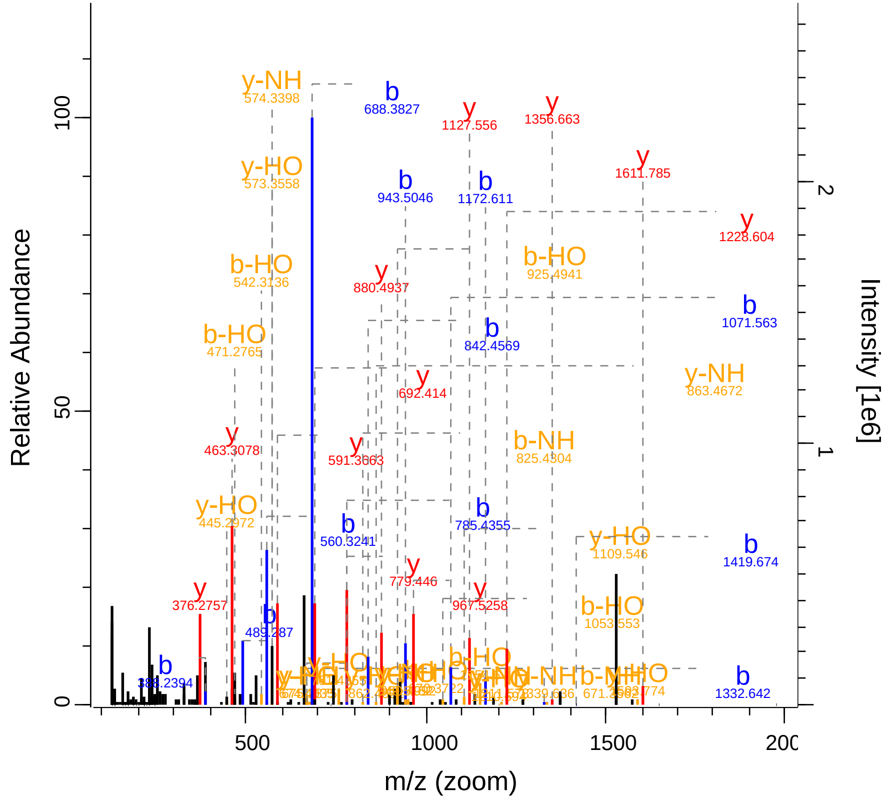

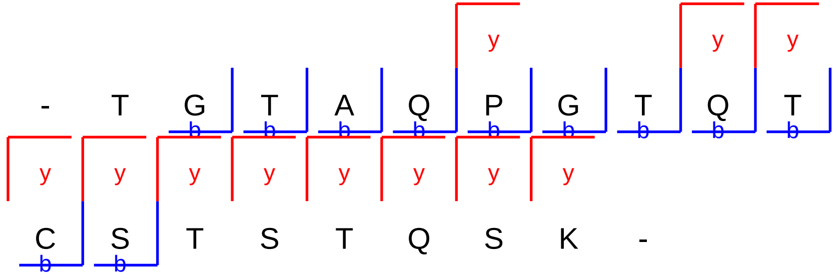

Raw File	Scan	Method	Score	m/z	Gene names
RO-QC-160121_ZackD_SS_Set_01_Fractior	6241	FTMS; HCI	50.35	622.32	PECAM1

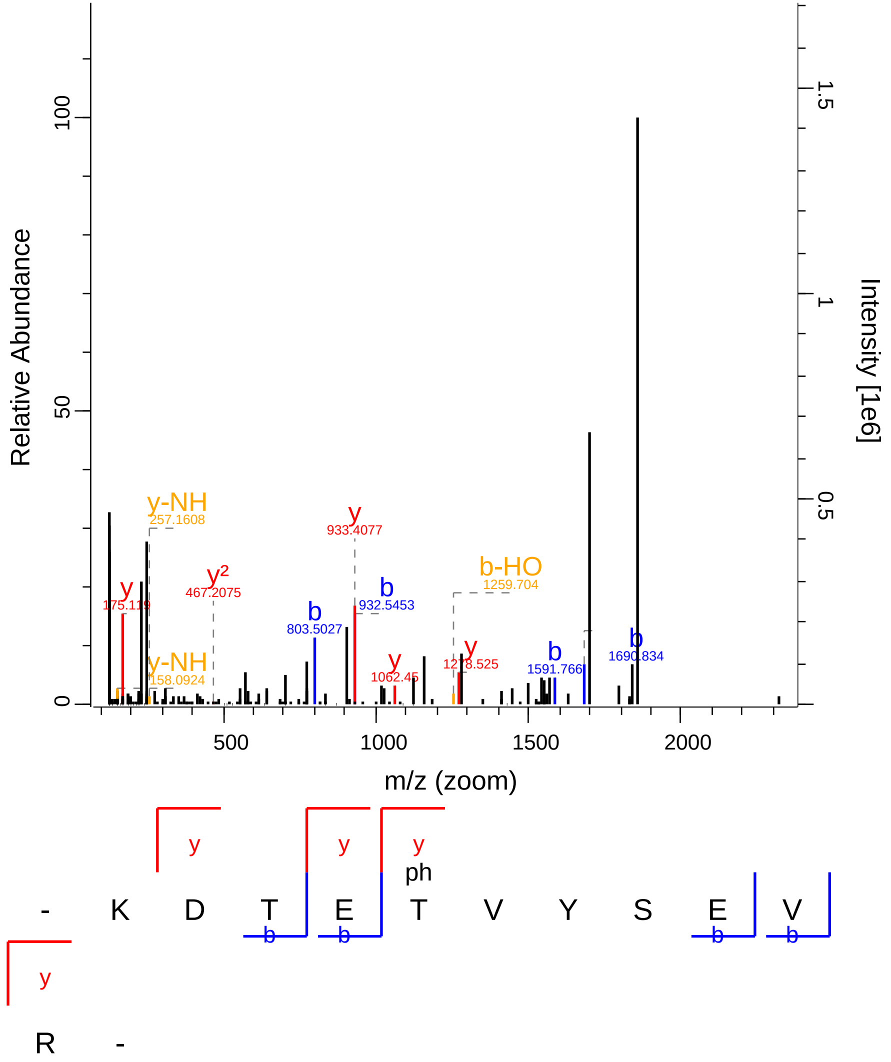

Raw File	Scan	Method	Score	m/z	Gene names
RO-QC-160121_ZackD_SS_Set_01_Fractior	6389	FTMS; HCI	55.45	598.34	USP31

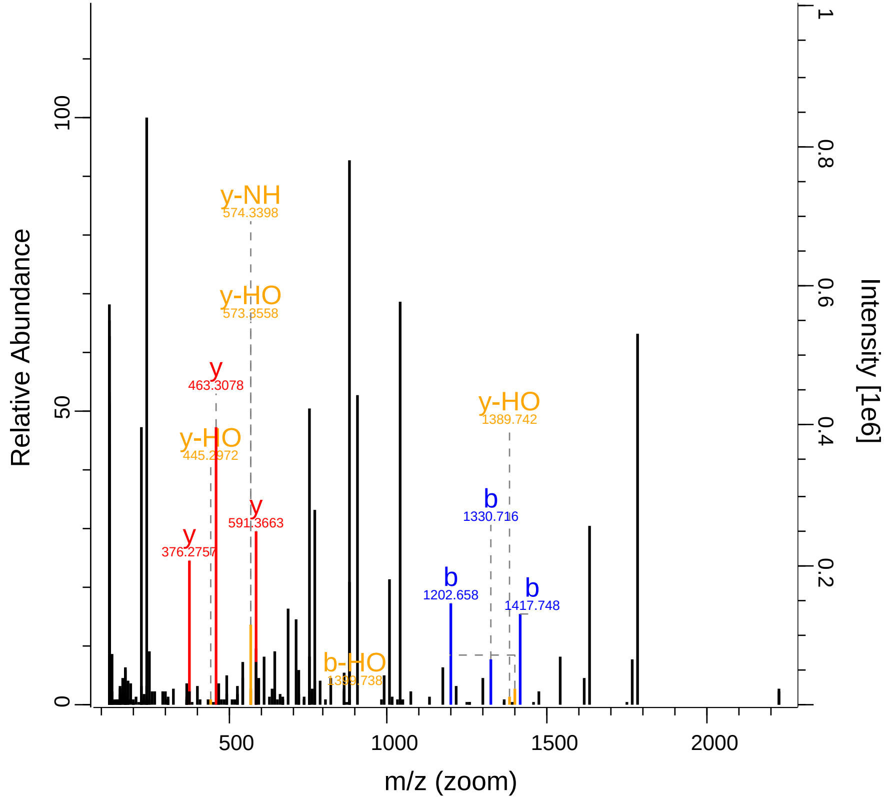

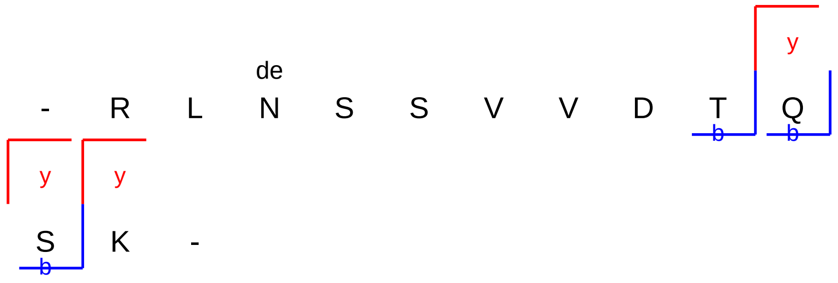

Raw File	Scan	Method	Score	m/z	Gene names
RO-QC-160121_ZackD_SS_Set_01_Fractior	6606	FTMS; HC	132.32	549.28	MACROD2

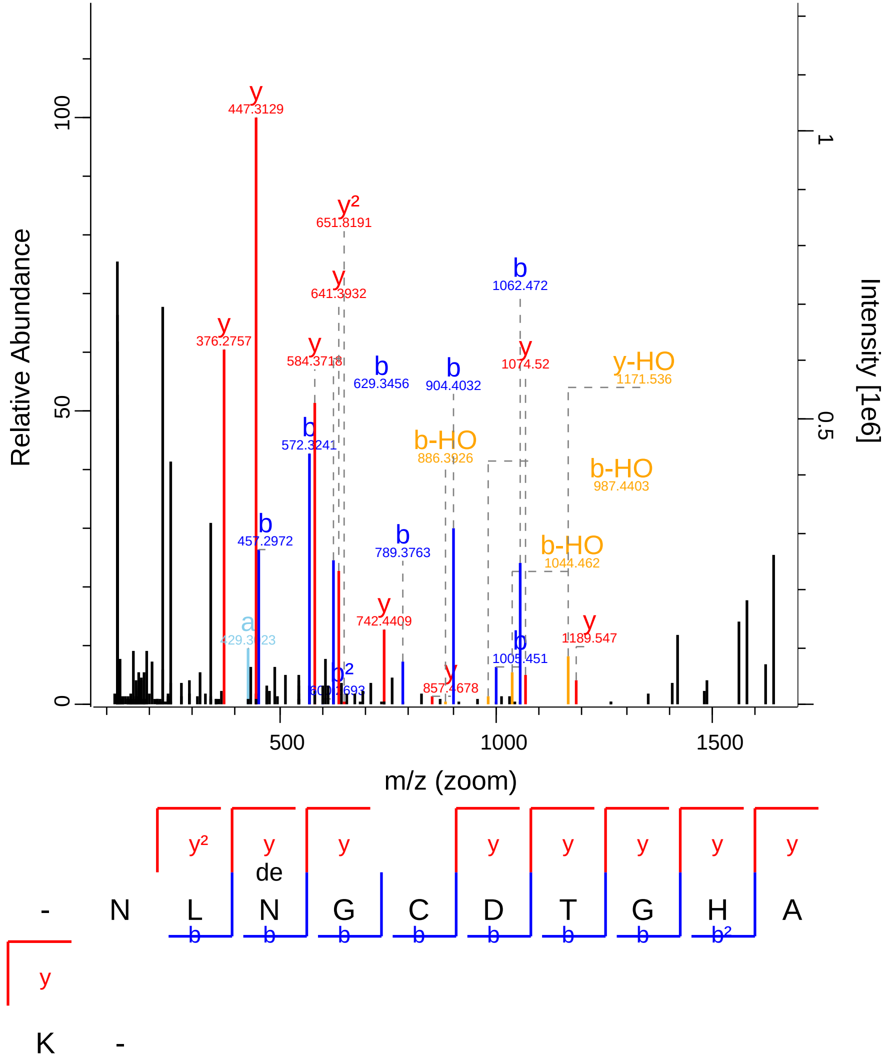

Raw File	Scan	Method	Score	m/z	Gene names
RO-QC-160121_ZackD_SS_Set_01_Fractio[illegible]	6902	FTMS; HC	128.61	721.86	TMEFF1

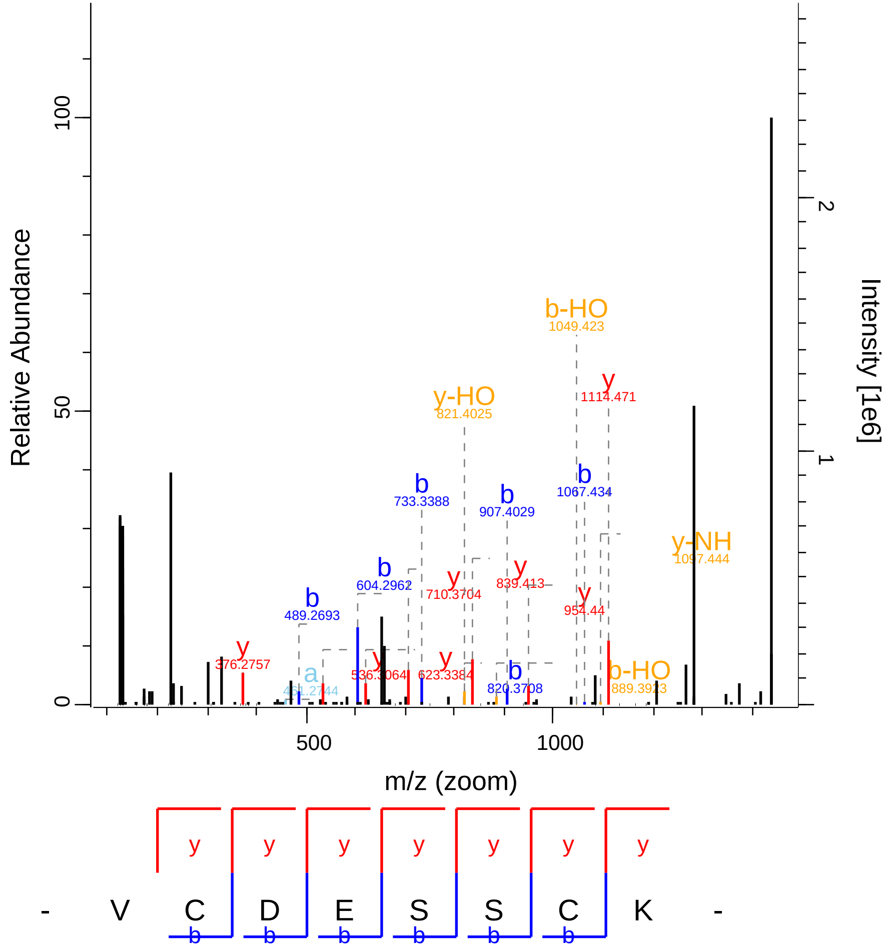

Raw File	Scan	Method	Score	m/z
RO-QC-160121_ZackD_SS_Set_01_Fraction_8	7144	FTMS; HCD	55.4	400.73

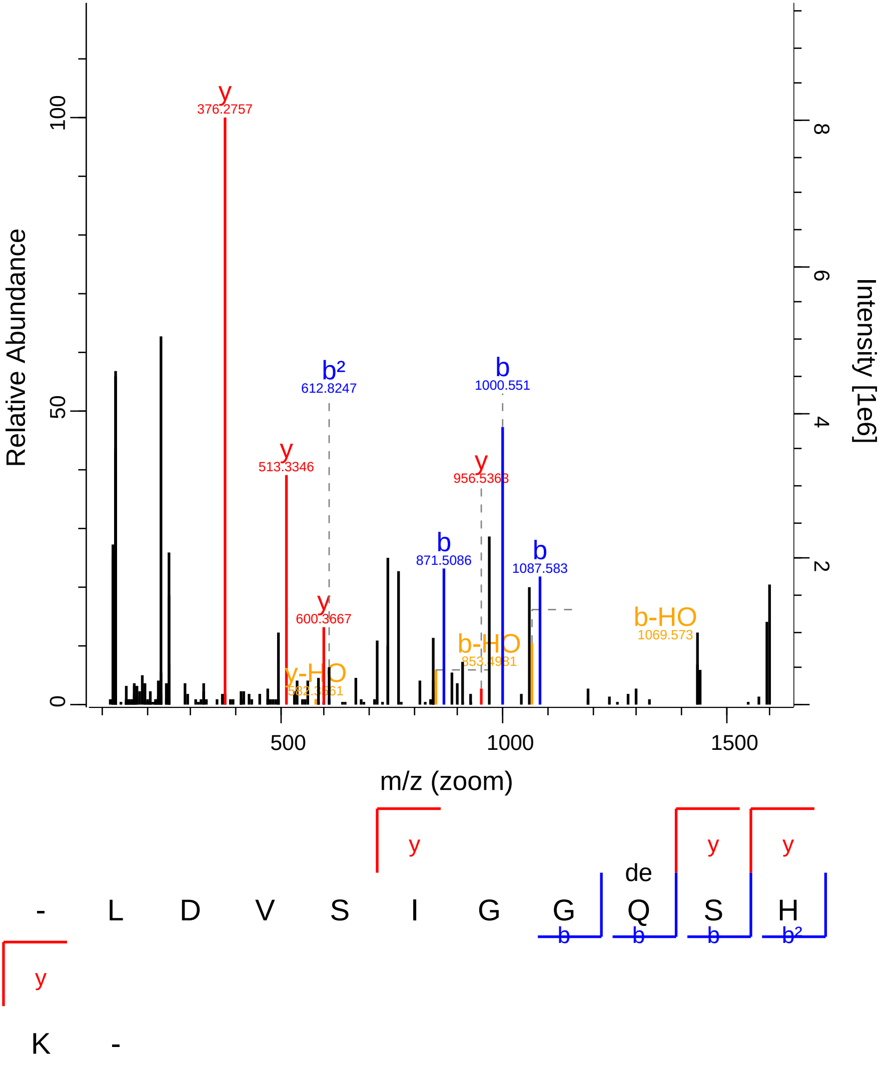

Raw File	Scan	Method	Score	m/z	Gene names
RO-QC-160121_ZackD_SS_Set_01_Fractior	7313	FTMS; HCI	41.28	562.68	GLOD5

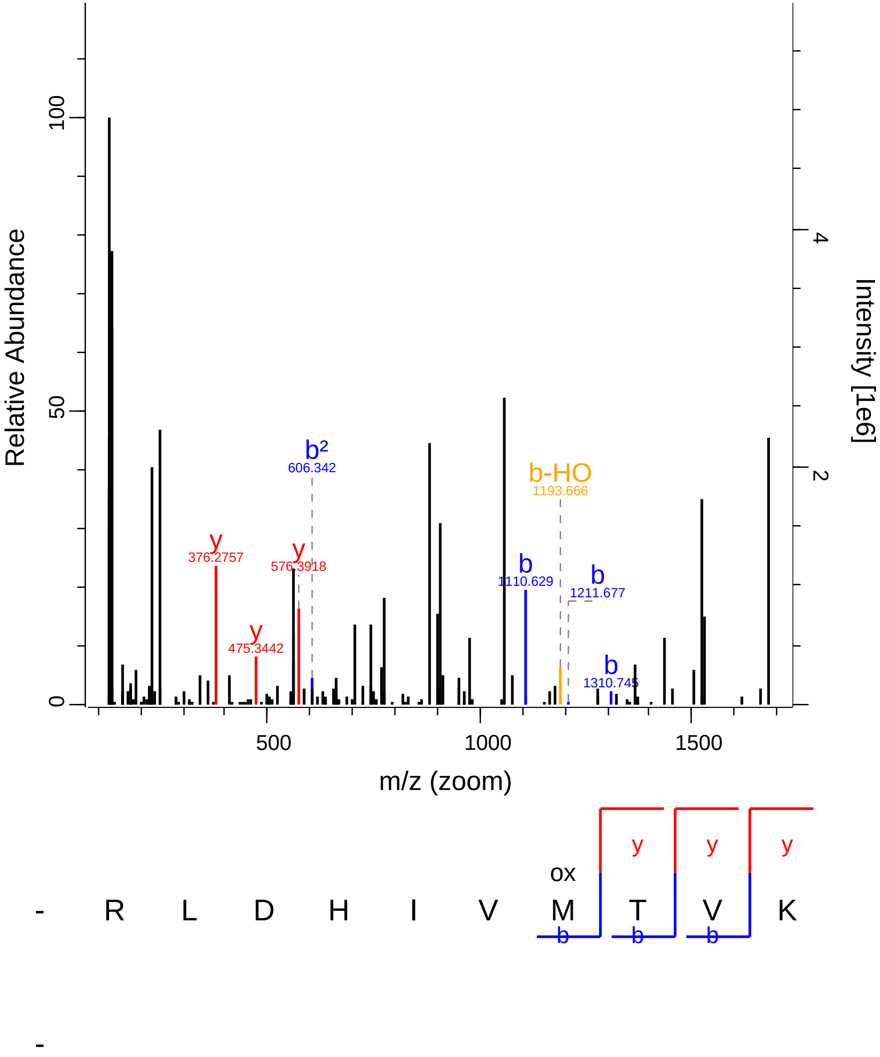

Raw File	Scan	Method	Score	m/z	Gene names
RO-QC-160121_ZackD_SS_Set_01_Fractior	7335	FTMS; HCI	65.06	843.51	GLOD5

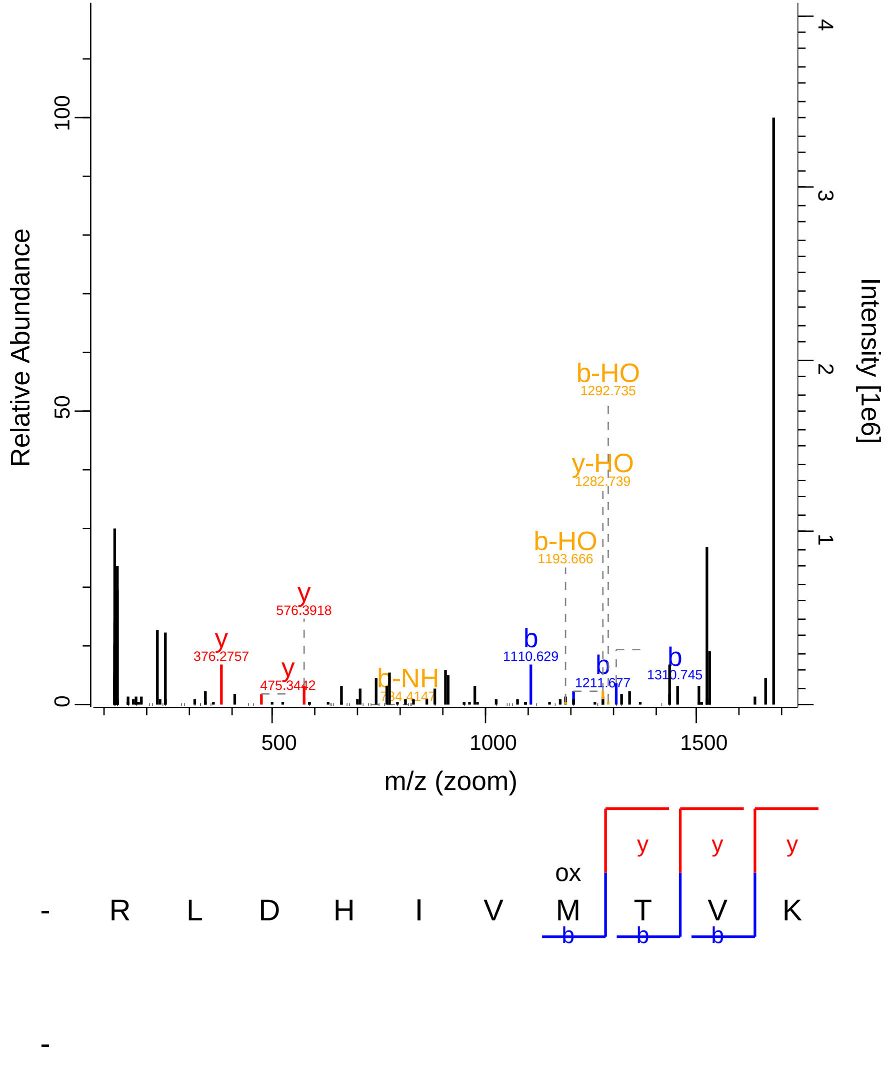

Raw File	Scan Method	Score	m/z	Gene names
RO-QC-160121_ZackD_SS_Set_01_Fra	8768 FTMS; HC	102.72	596.35	ATP5G1;ATP5G2;ATP

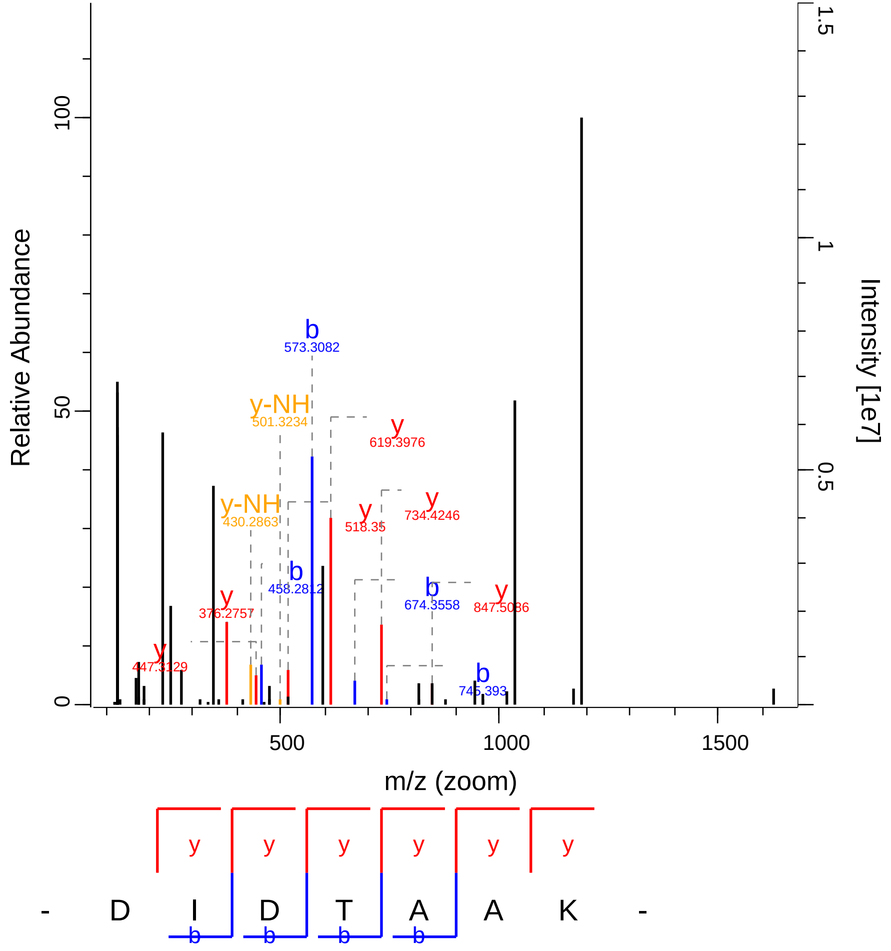

Raw File	Scan	Method	Score	m/z	Gene names
RO-QC-160121_ZackD_SS_Set_01_Fraction	9167	FTMS; HCI	223.08	779.9	SLC39A1

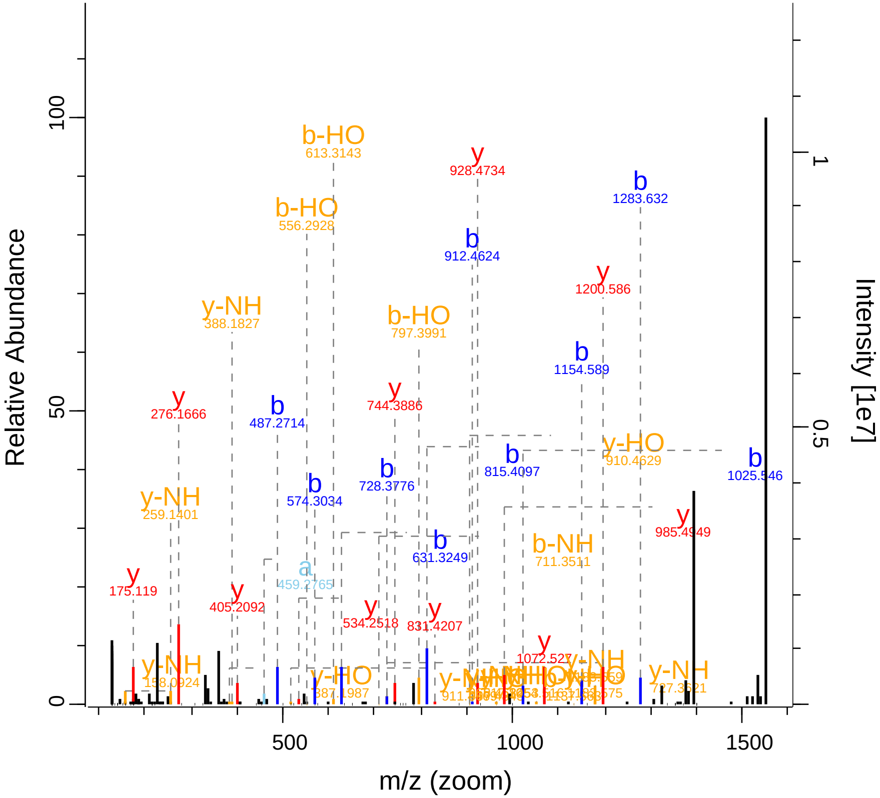

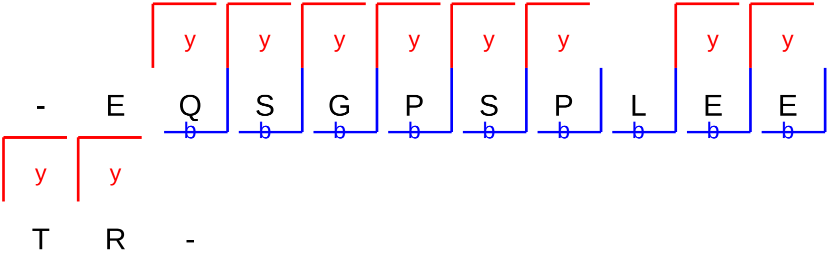

Raw File	Scan	Method	Score	m/z	Gene names
RO-QC-160121_ZackD_SS_Set_01_Fractior	9311	FTMS; HCI	69.95	663.38	RBM20

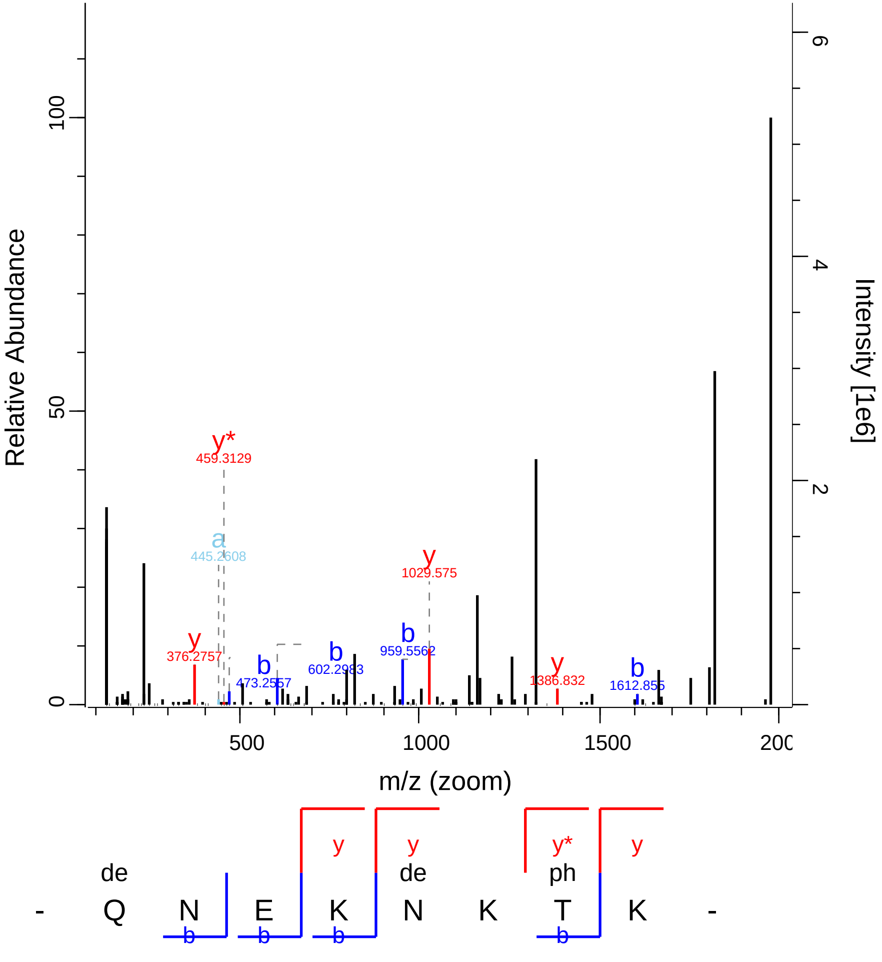

Raw File	Scan Method	Score	m/z	Gene names
RO-QC-160121_ZackD_SS_Set_01_Fra 9327	FTMS; HC	143	596.35	ATP5G1;ATP5G2;ATP5

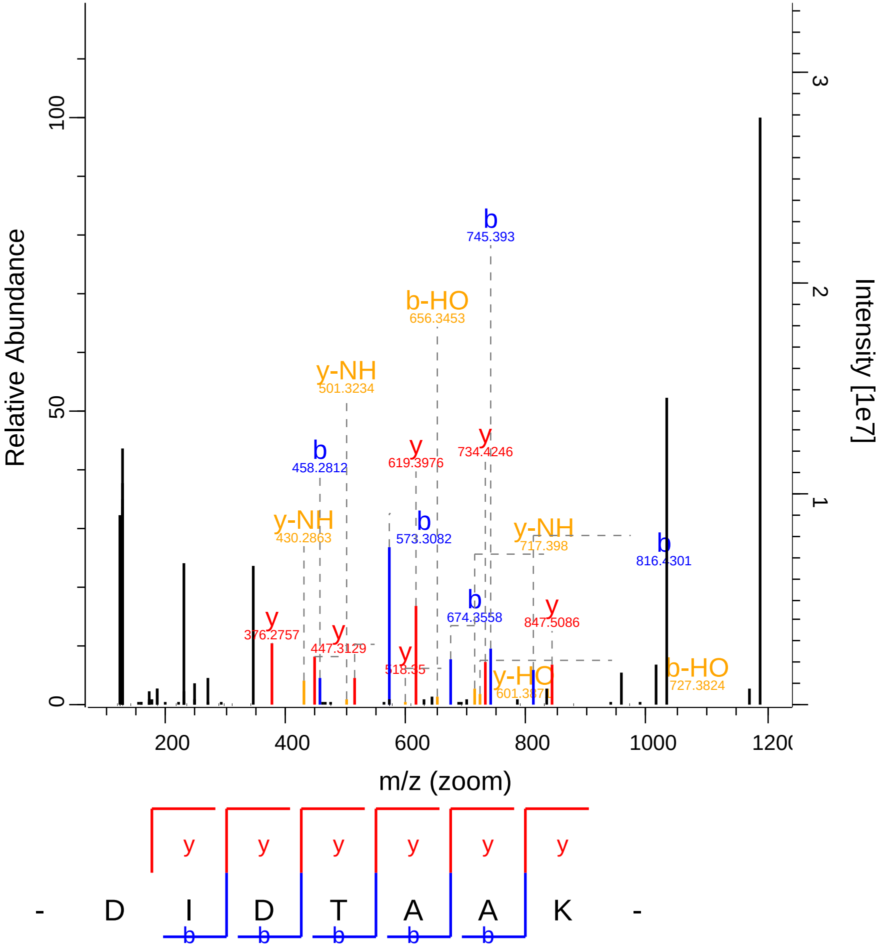

Raw File	Scan	Method	Score	m/z	Gene names
RO-QC-160121_ZackD_SS_Set_01_Fra	9482	FTMS; HC	88.03	397.9	ATP5G1;ATP5G2;ATP5

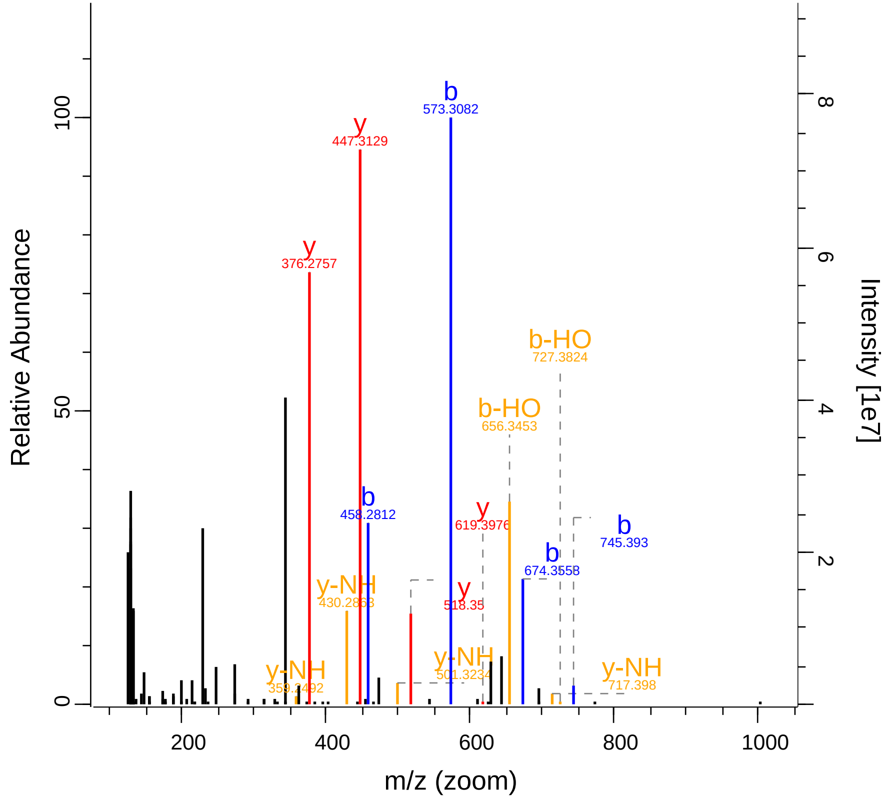

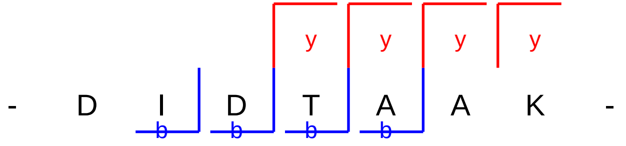

Raw File	Scan Method	Score	m/z	Gene names
RO-QC-160121_ZackD_SS_Set_01_Fra	9578 FTMS; HC	143	596.35	ATP5G1;ATP5G2;ATP5

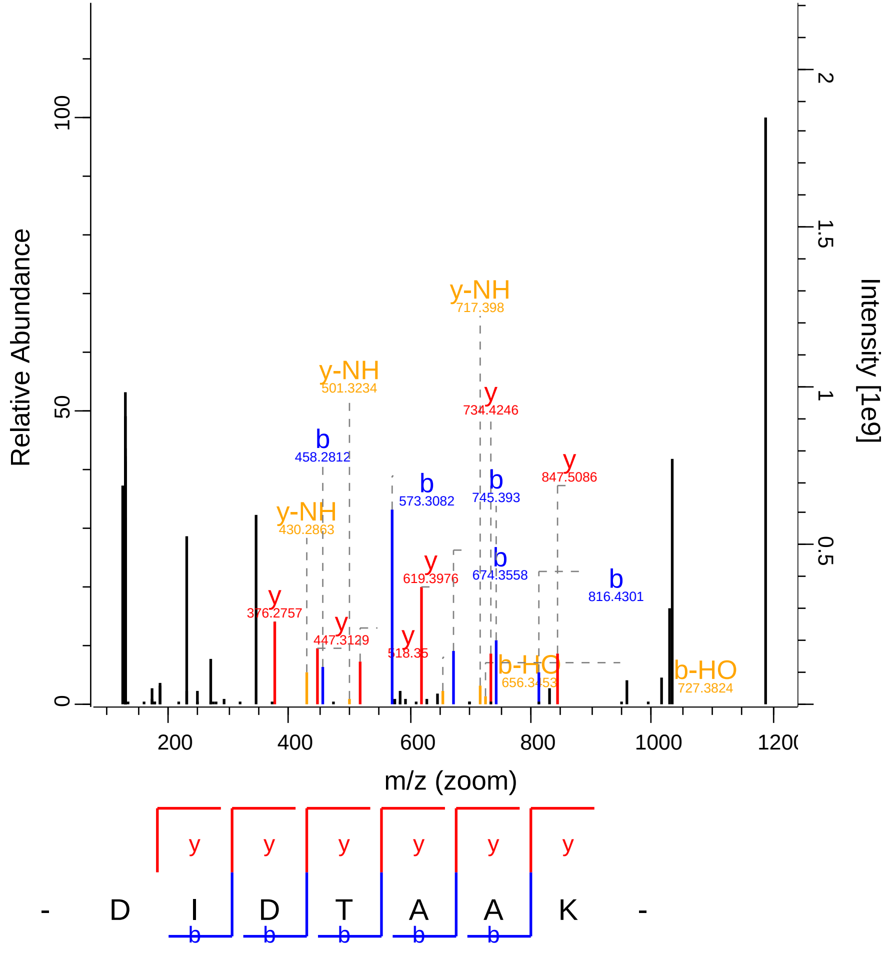

Raw File	Scan	Method	Score	m/z
RO-QC-160121_ZackD_SS_Set_01_Fraction_8	11024	FTMS; HCD	92.44	688.4

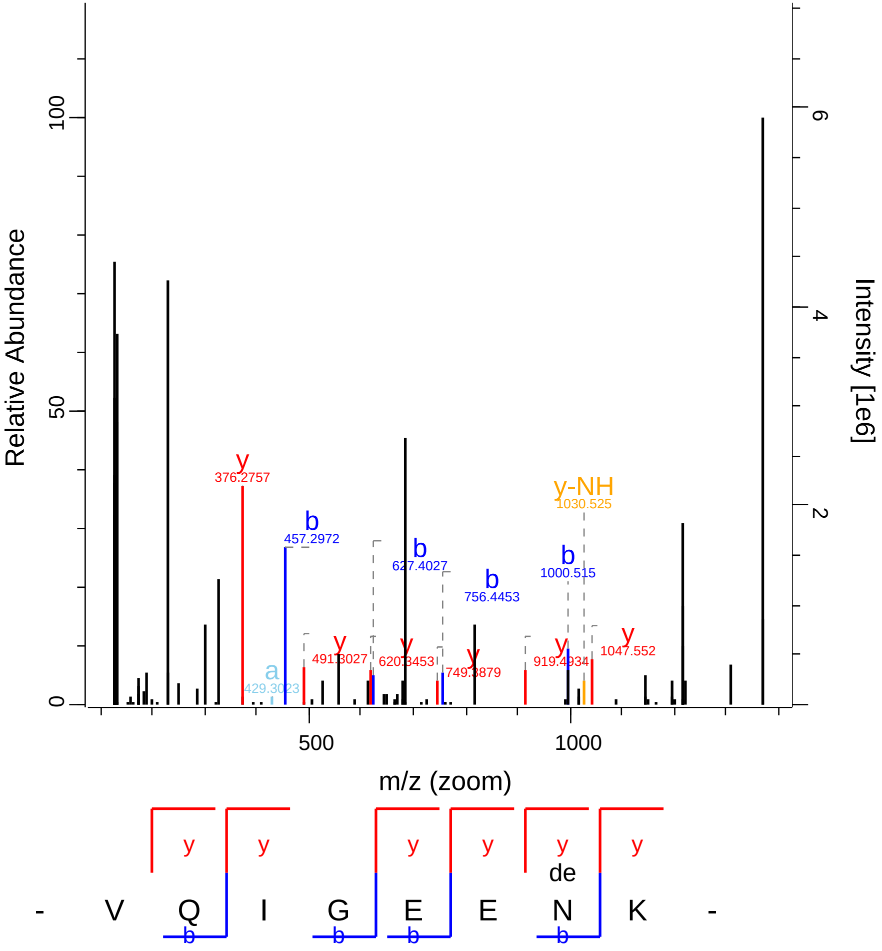

Raw File	Scan	Method	Score	m/z
RO-QC-160121_ZackD_SS_Set_01_Fraction_8	11164	FTMS; HCD	60.59	824.49

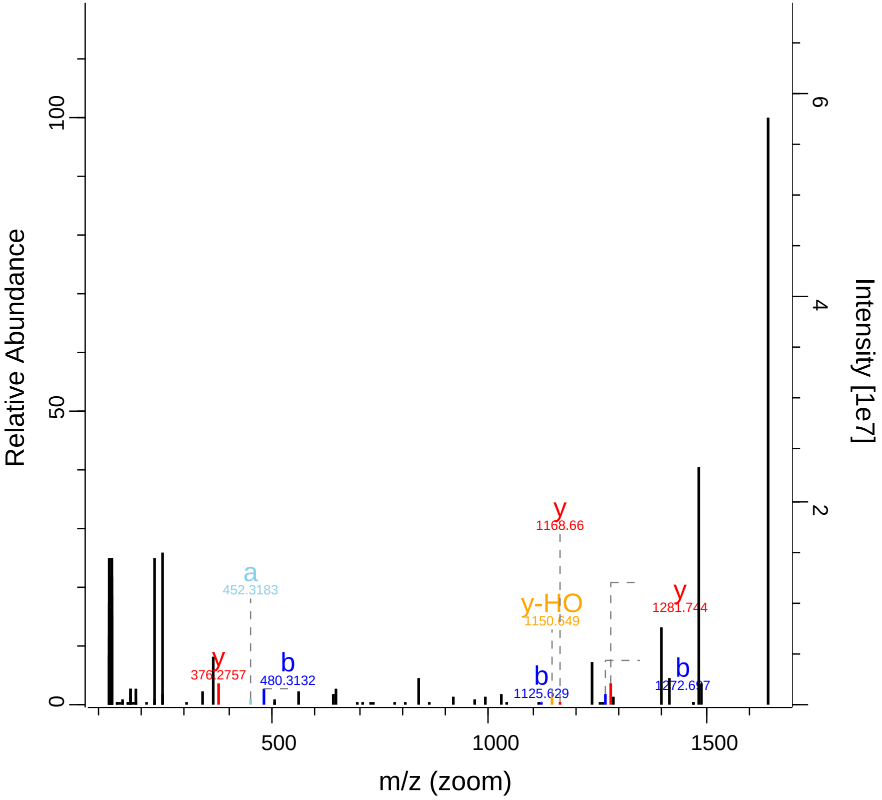

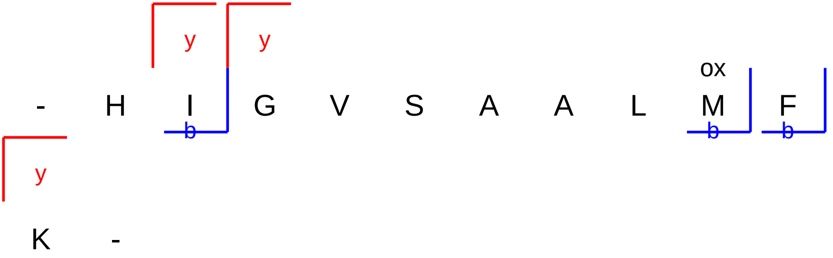

Raw File	Scan	Method	Score	m/z
RO-QC-160121_ZackD_SS_Set_01_Fraction_8	11592	FTMS; HCD	42.6	835.41

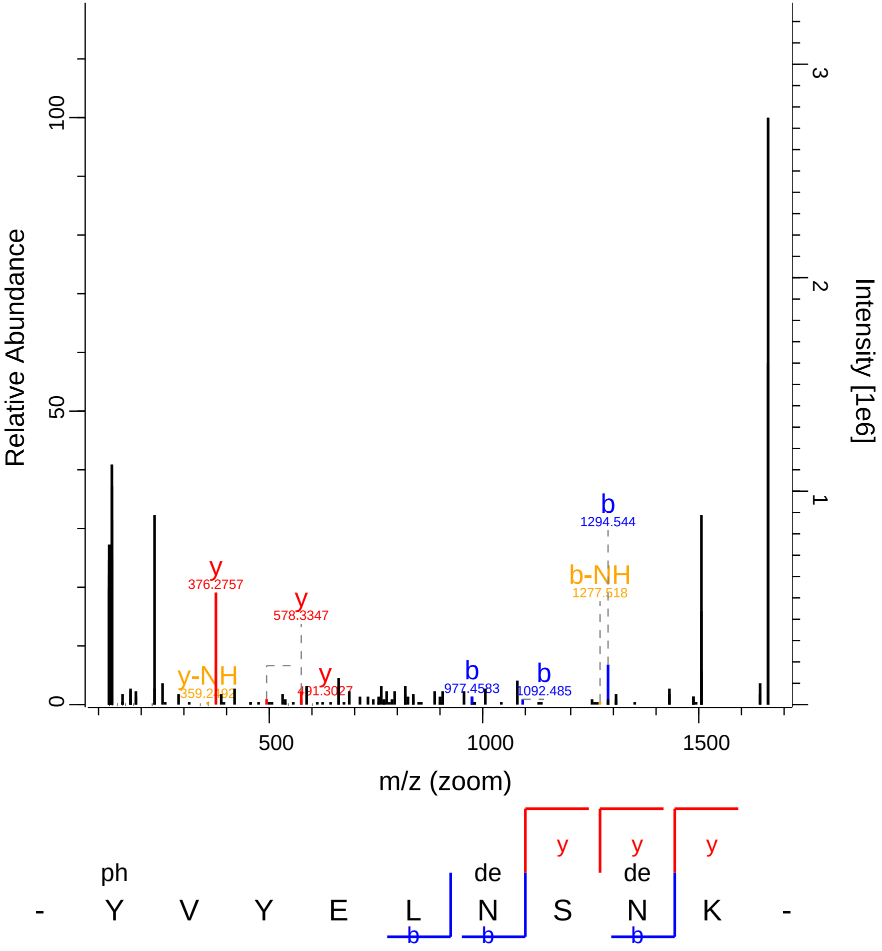

Raw File	Scan	Method	Score	m/z
RO-QC-160121_ZackD_SS_Set_01_Fraction_8	12262	FTMS; HCD	66.99	575.36

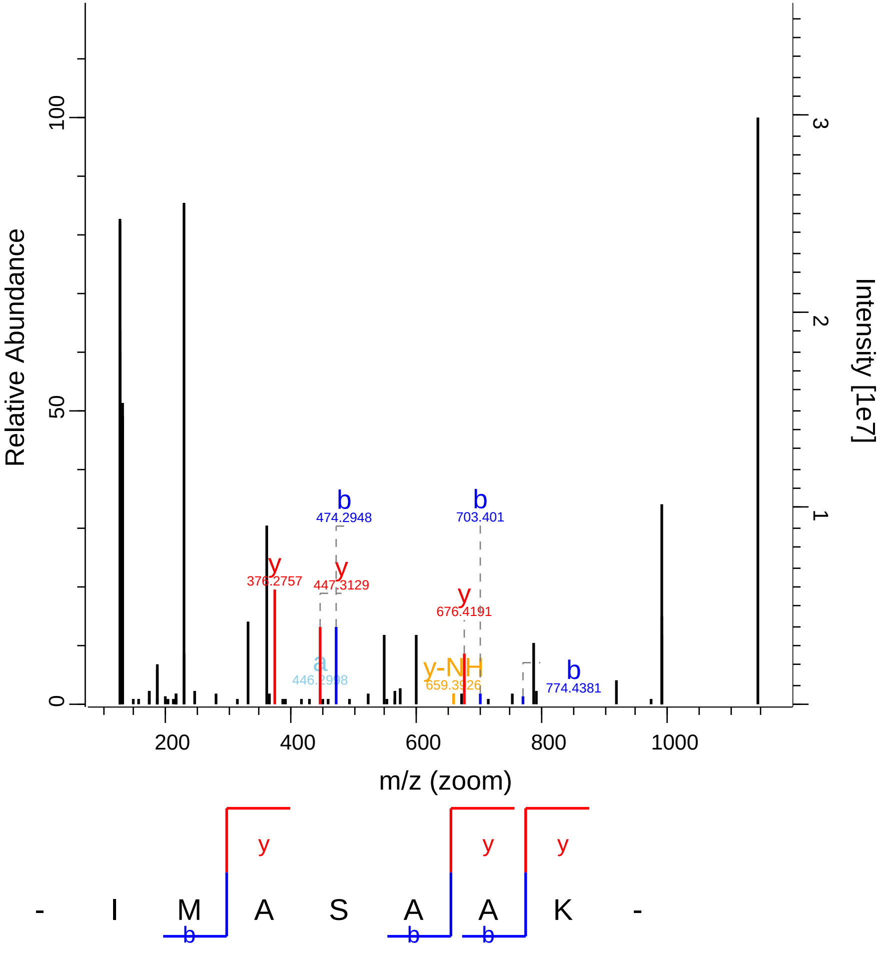

Raw File	Scan	Method	Score	m/z	Gene names
RO-QC-160121_ZackD_SS_Set_01_Fractio	12907	FTMS; HC	101.04	801.18	ALKBH2

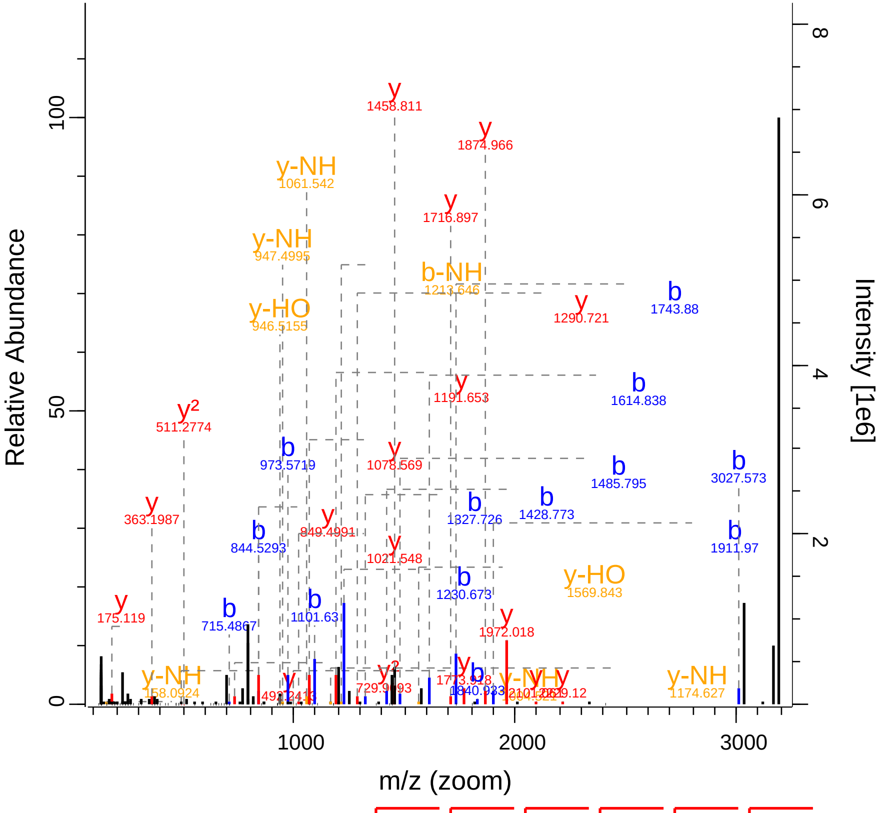

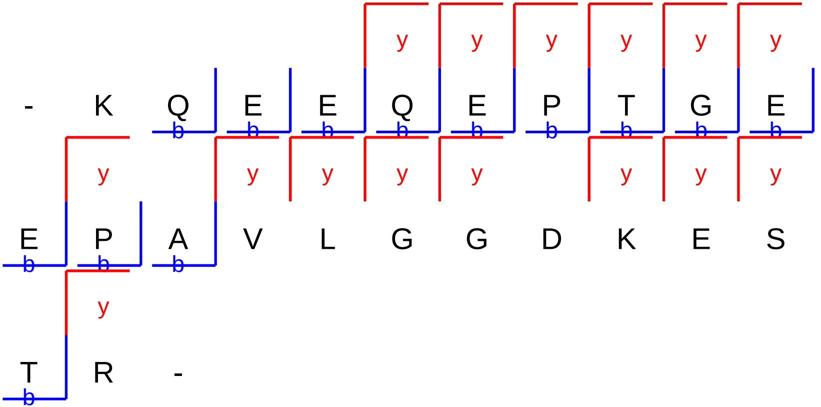

Raw File	Scan	Method	Score	m/z	Gene names
RO-QC-160121_ZackD_SS_Set_01_Fractio	12989	FTMS; HC	94.85	767.93	ZNF251

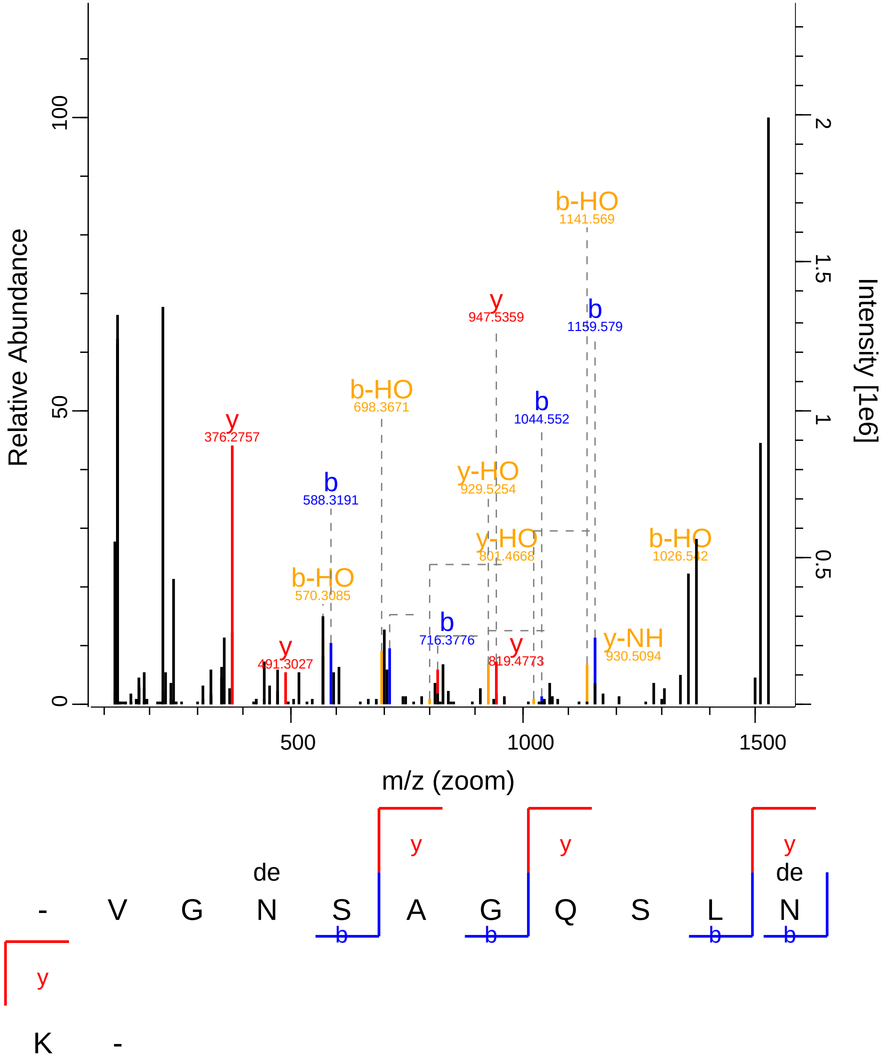

Raw File	Scan	Method	Score	m/z	Gene names
RO-QC-160121_ZackD_SS_Set_01_Fractio	13919	FTMS; HC	70.92	854.46	CARD14

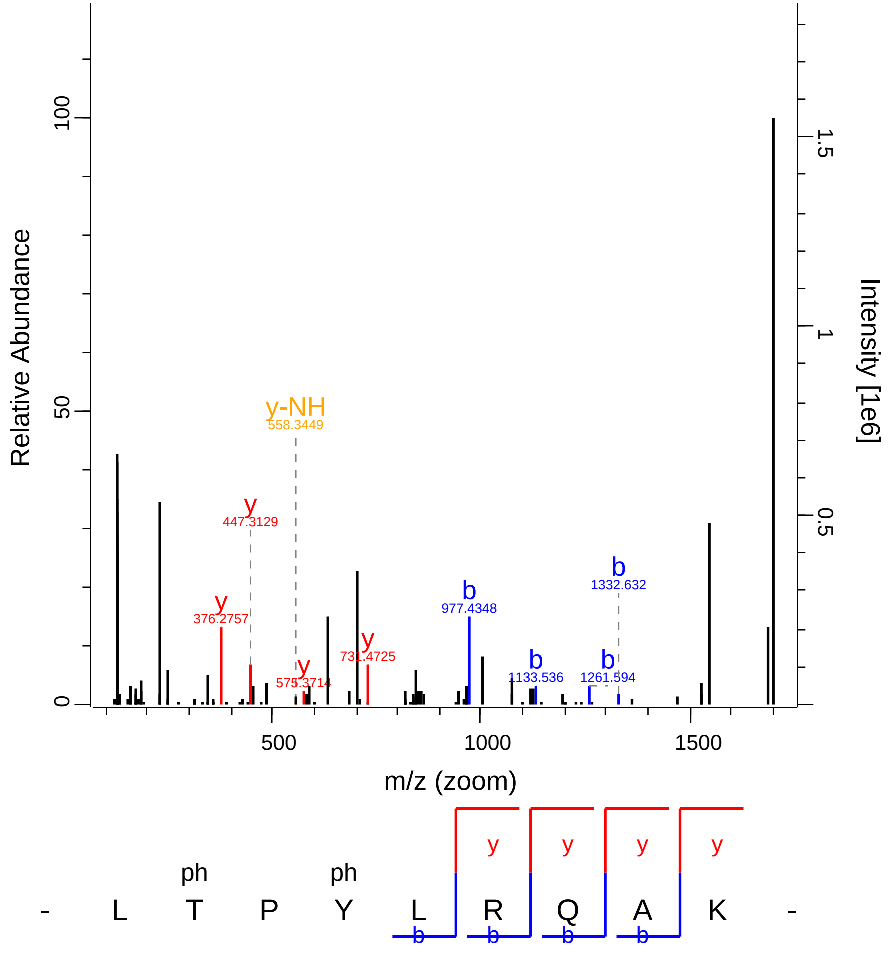

Raw File	Scan	Method	Score	m/z
RO-QC-160121_ZackD_SS_Set_01_Fraction_8	14363	FTMS; HCD	55.39	582.36

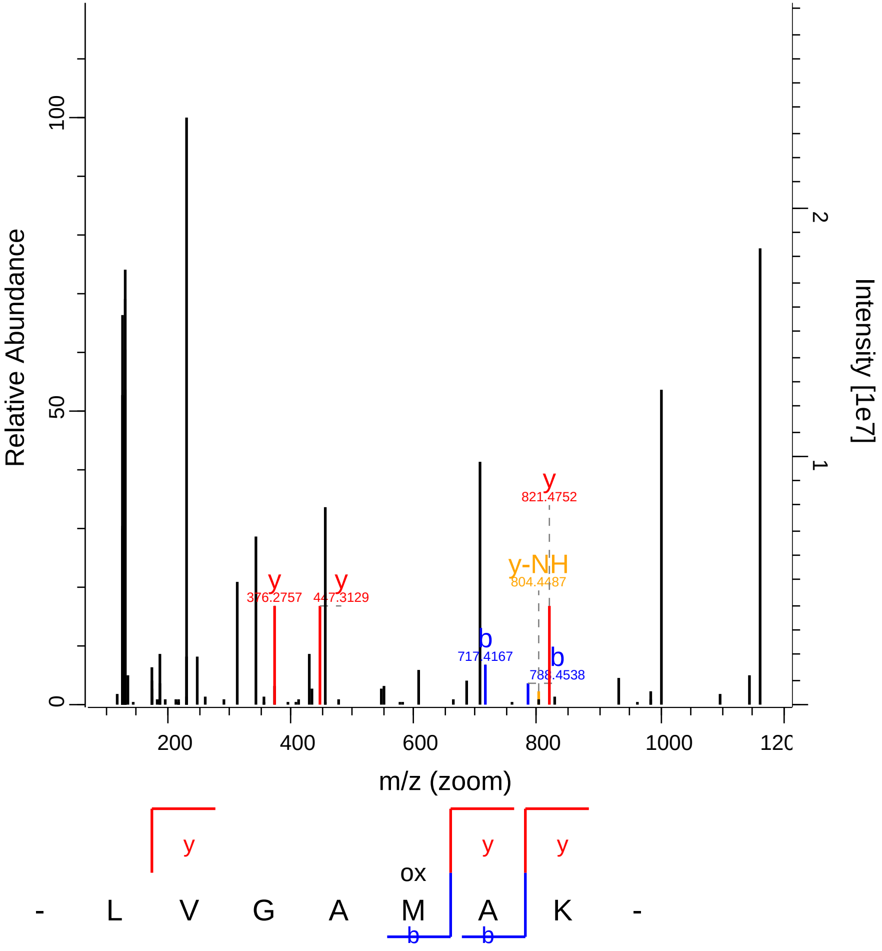

Raw File	Scan	Method	Score	m/z
RO-QC-160121_ZackD_SS_Set_01_Fraction_8	14442	FTMS; HCD	40.58	716.08

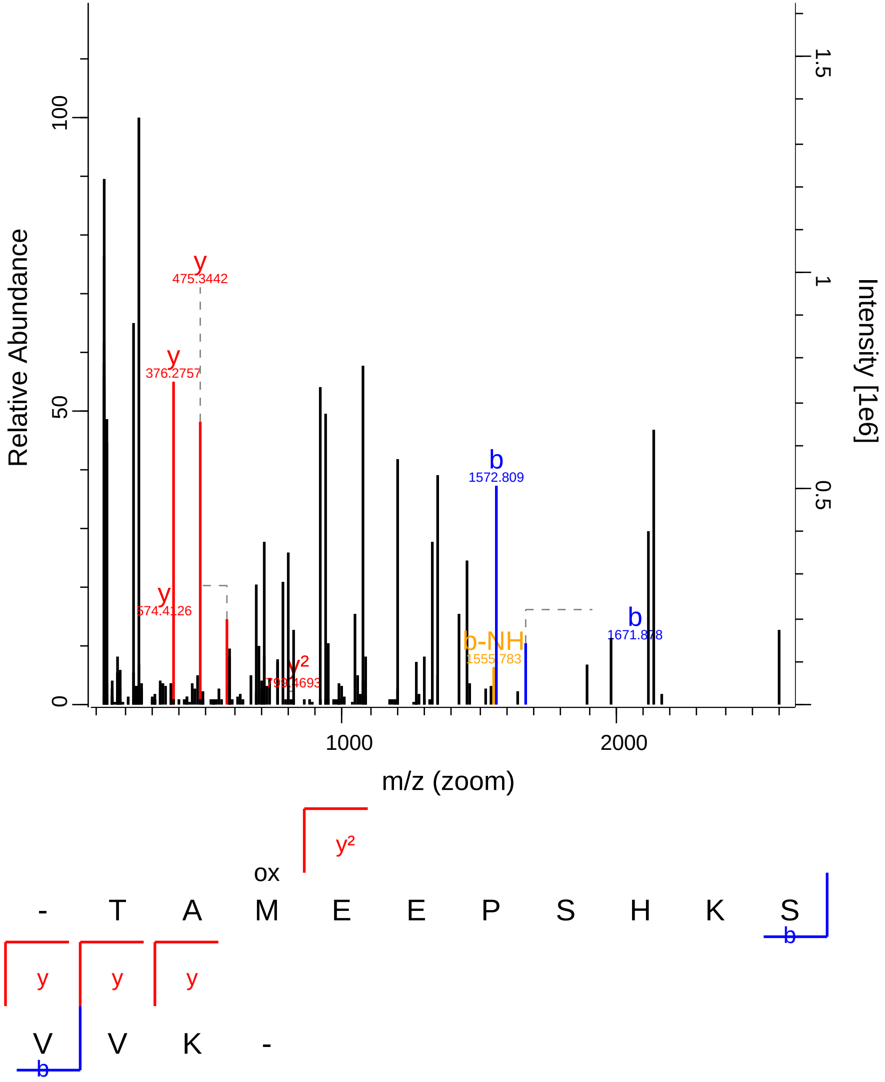

Raw File	Scan	Method	Score	m/z	Gene names
RO-QC-160121_ZackD_SS_Set_01_Fractio	14674	FTMS; HC	59.9	470.07	WDR31

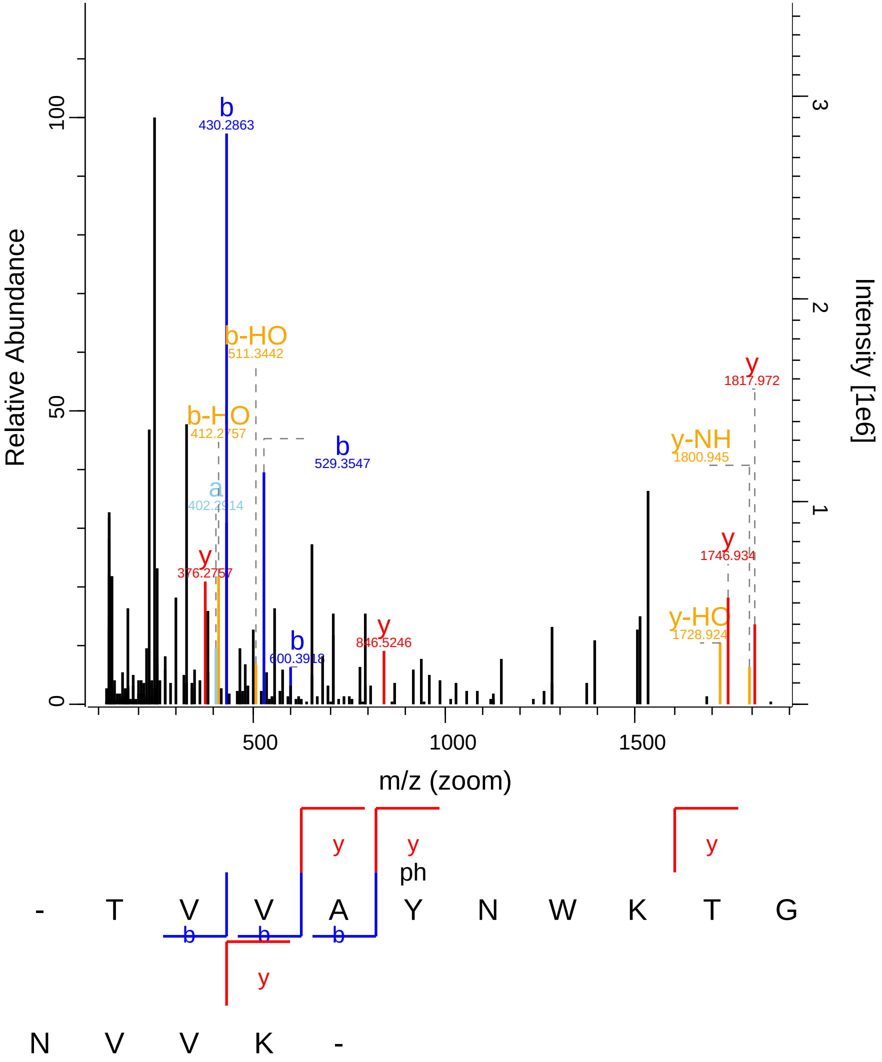

Raw File	Scan	Method	Score	m/z	Gene names
RO-QC-160121_ZackD_SS_Set_01_Fractio	14985	FTMS; HC	45.92	683.04	OSBPL7

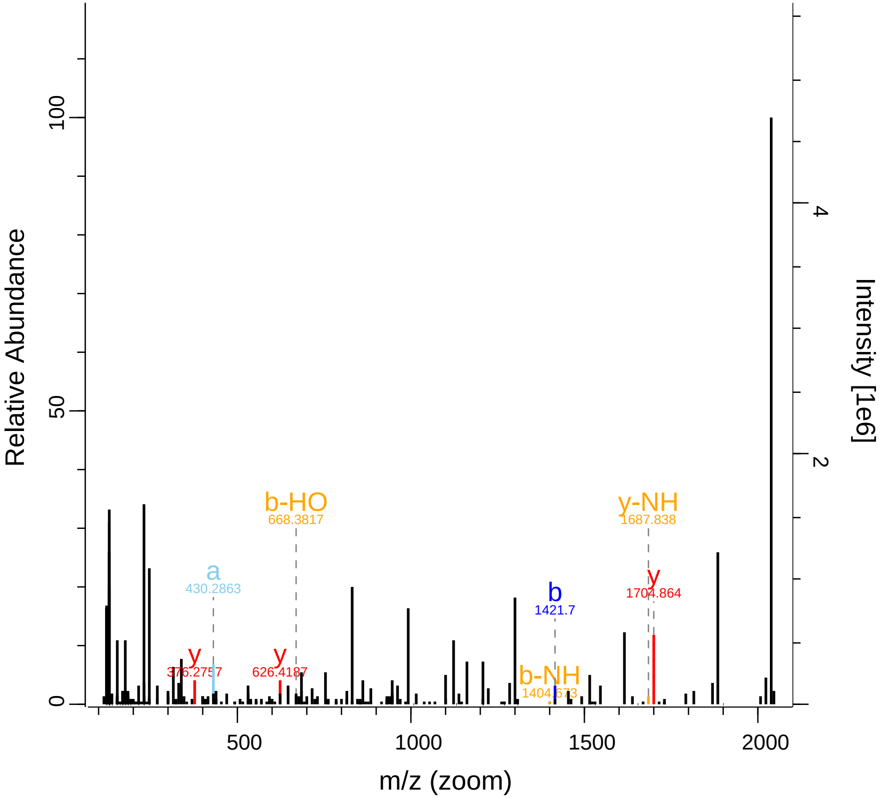

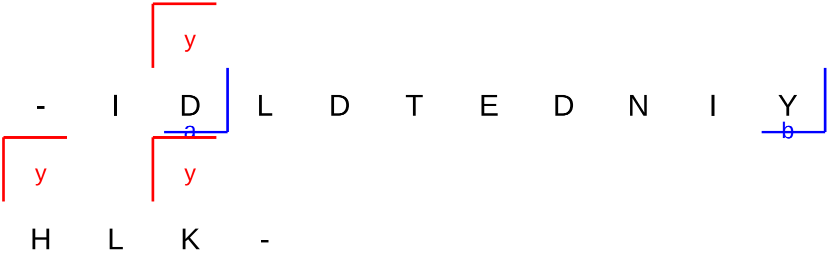

Raw File	Scan	Method	Score	m/z	Gene names
RO-QC-160121_ZackD_SS_Set_01_Fractior	15009	FTMS; HCI	46.73	676.4	ZNF425

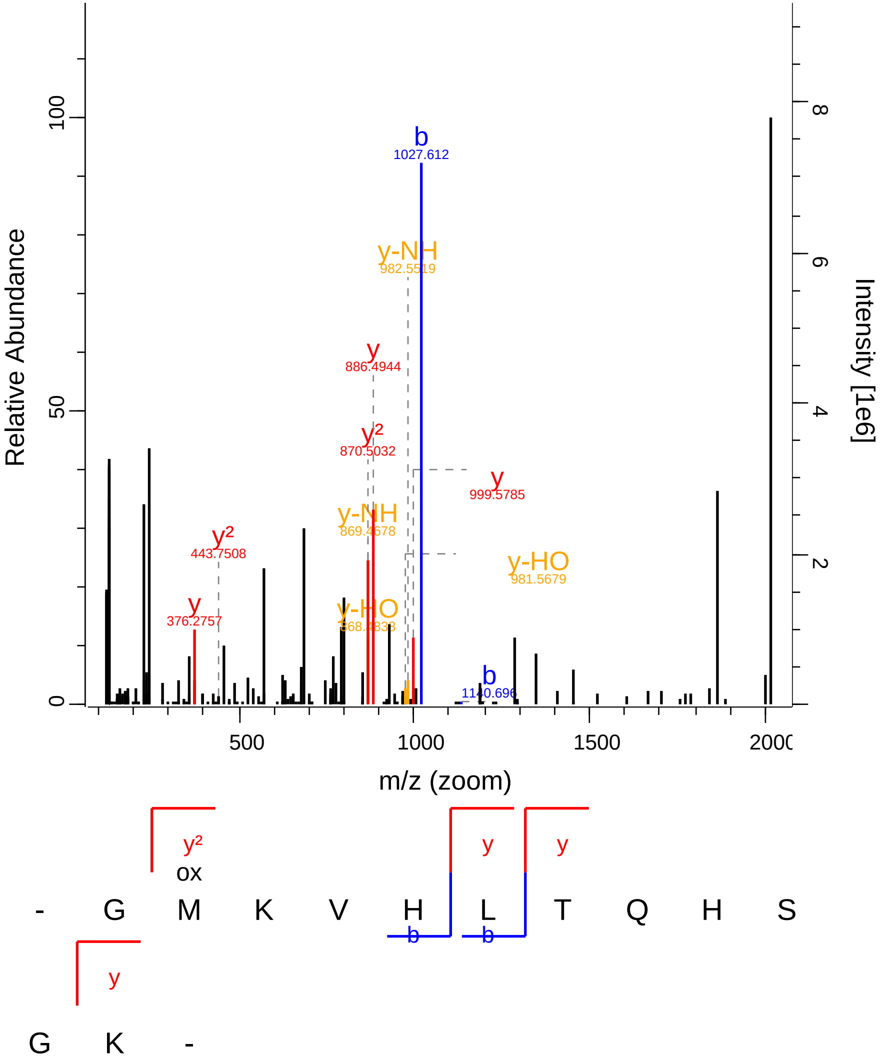

Raw File	Scan	Method	Score	m/z	Gene names
RO-QC-160121_ZackD_SS_Set_01_Fractio	15864	FTMS; HC	56.48	684.73	GGNBP1

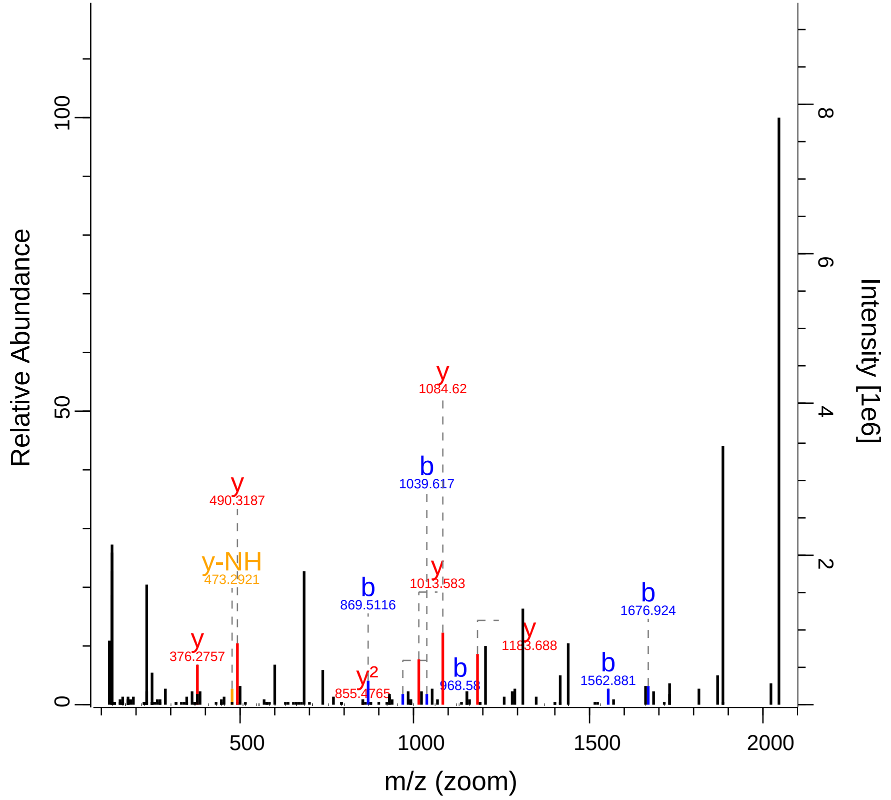

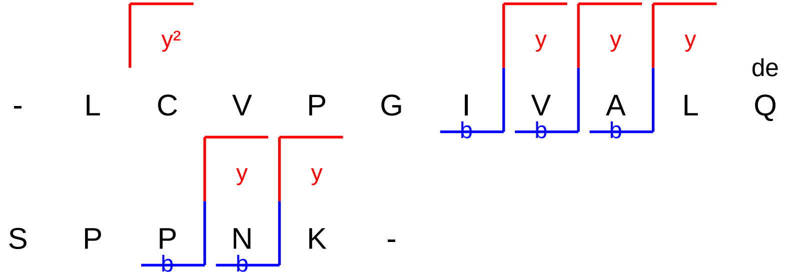

Raw File	Scan	Method	Score	m/z
RO-QC-160121_ZackD_SS_Set_01_Fraction_8	17070	FTMS; HCD	84.74	772.44

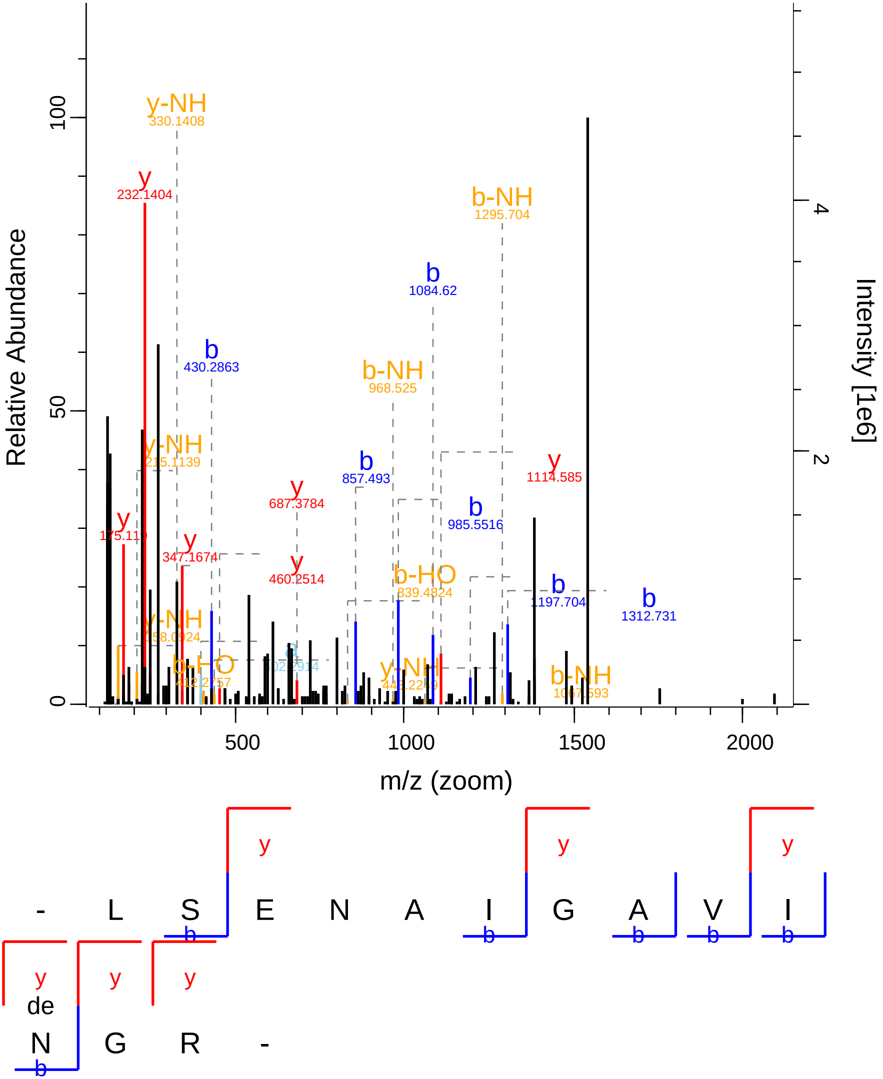

Raw File	Scan	Method	Score	m/z	Gene names
RO-QC-160121_ZackD_SS_Set_01_Fractio	18533	FTMS; HC	51.35	964.13	SVEP1

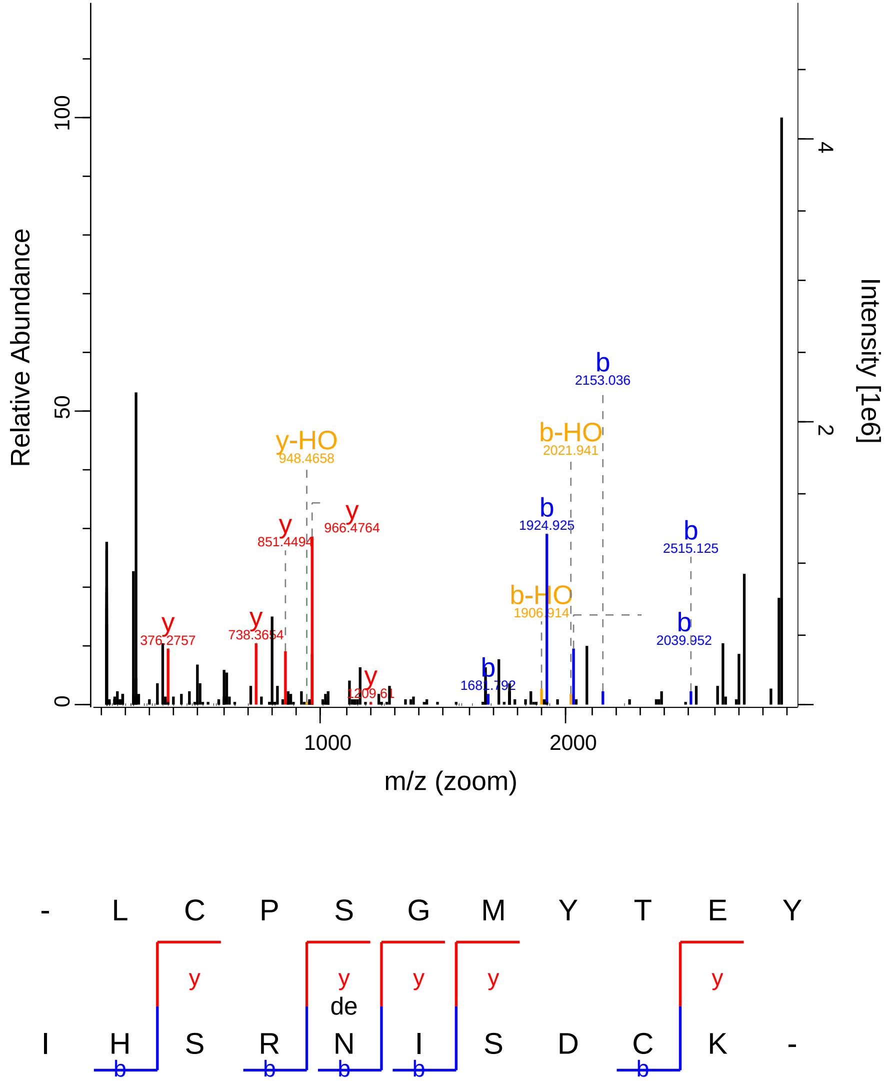

Raw File	Scan	Method	Score	m/z	Gene names
RO-QC-160121_ZackD_SS_Set_01_Fractio	18614	FTMS; HC	80.52	1445.7	SVEP1

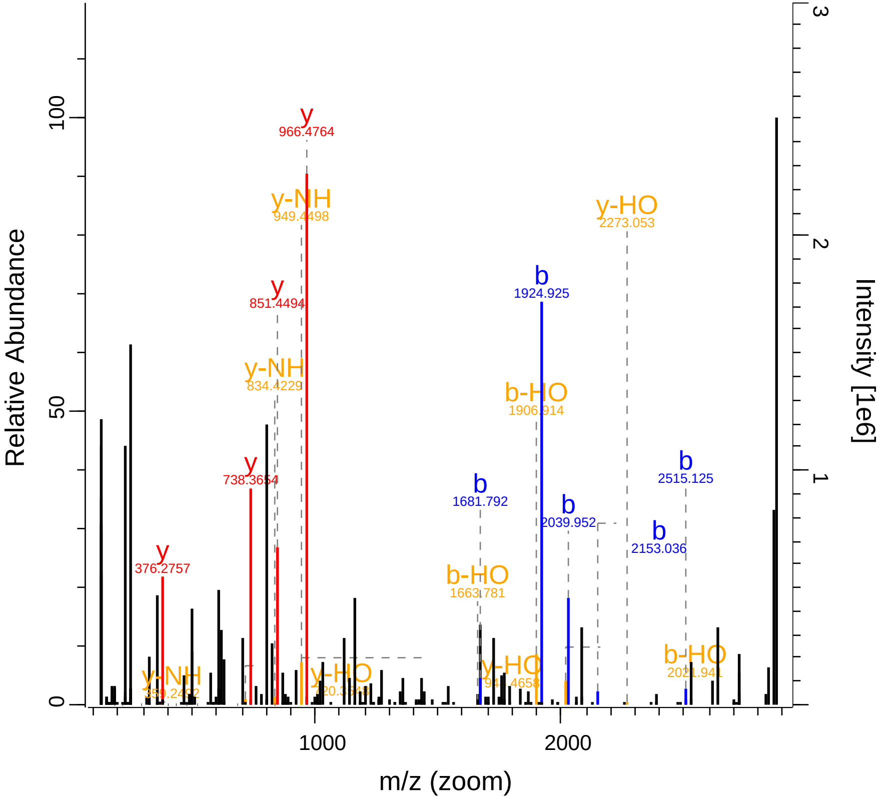

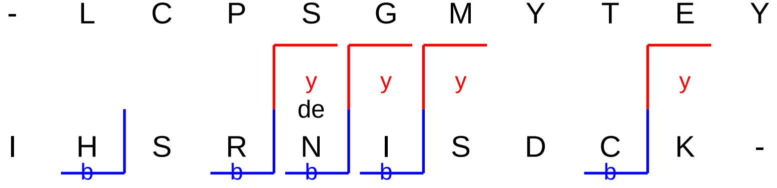

Raw File	Scan	Method	Score	m/z	Gene names
RO-QC-160121_ZackD_SS_Set_01_Fractior	19032	FTMS; HCI	62.2	723.6	SVEP1

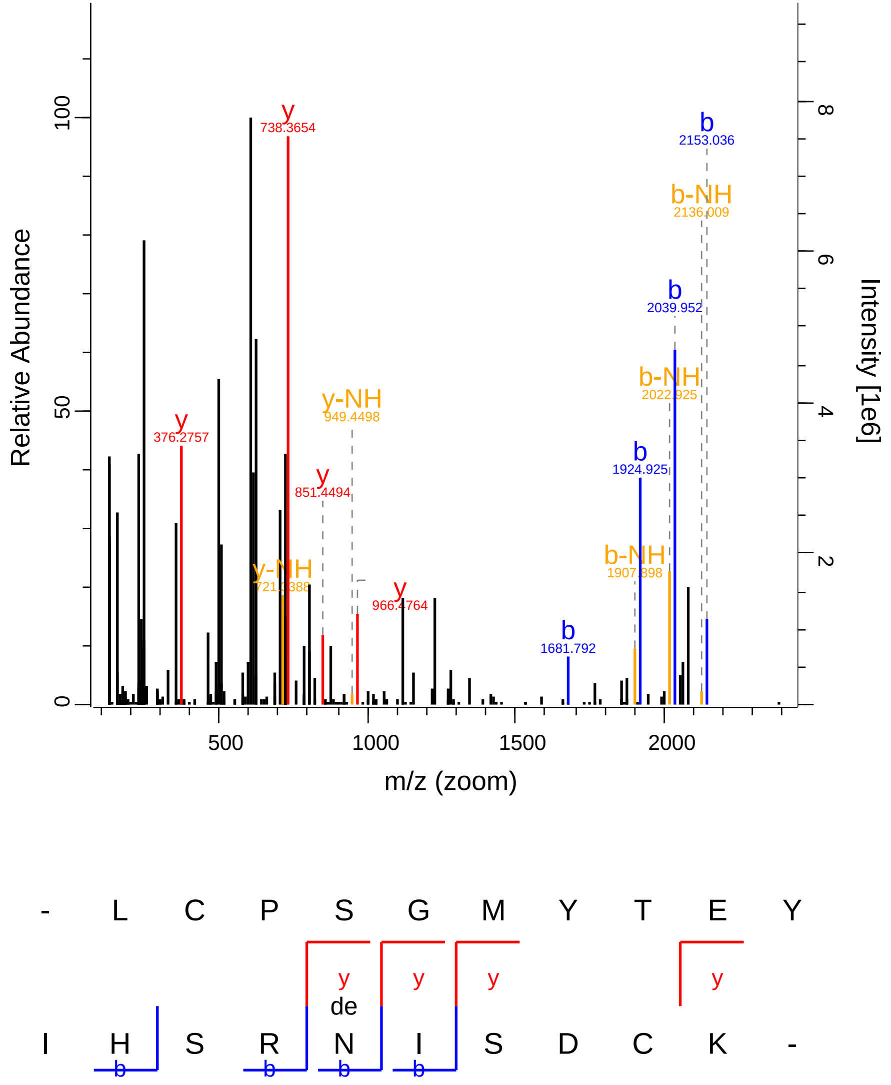

Raw File	Scan	Method	Score	m/z	Gene names
RO-QC-160121_ZackD_SS_Set_01_Fractio	19286	FTMS; HC	71.88	746.44	GRIN2A

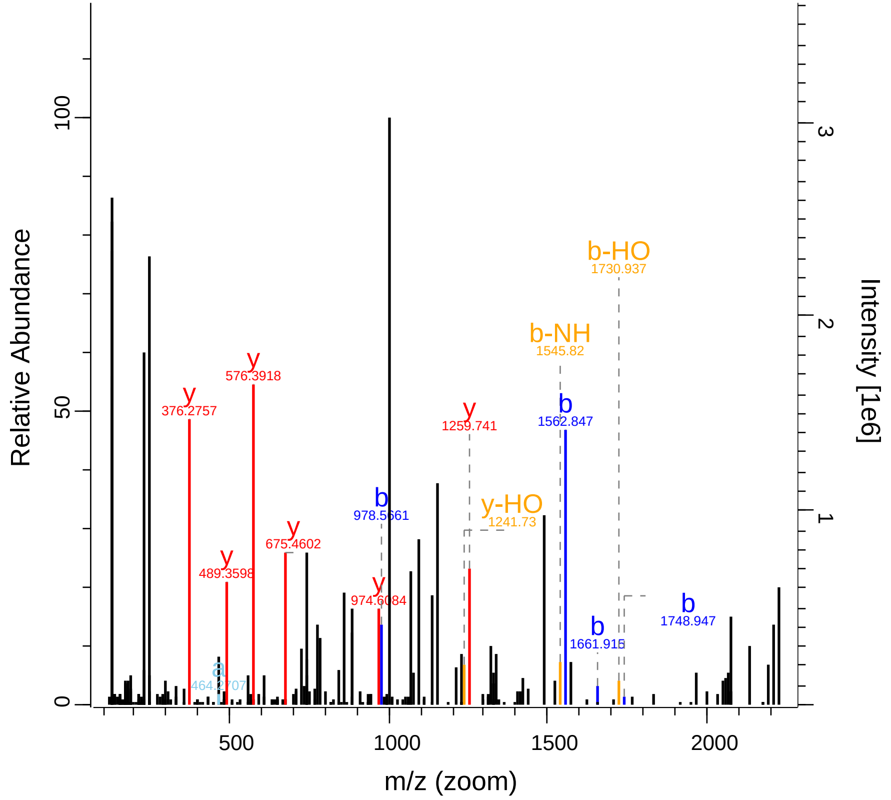

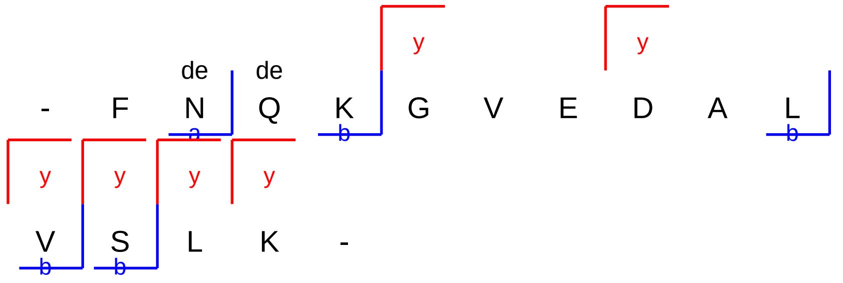

Raw File	Scan	Method	Score	m/z
RO-QC-160121_ZackD_SS_Set_01_Fraction_8	19999	FTMS; HCD	52.48	779.93

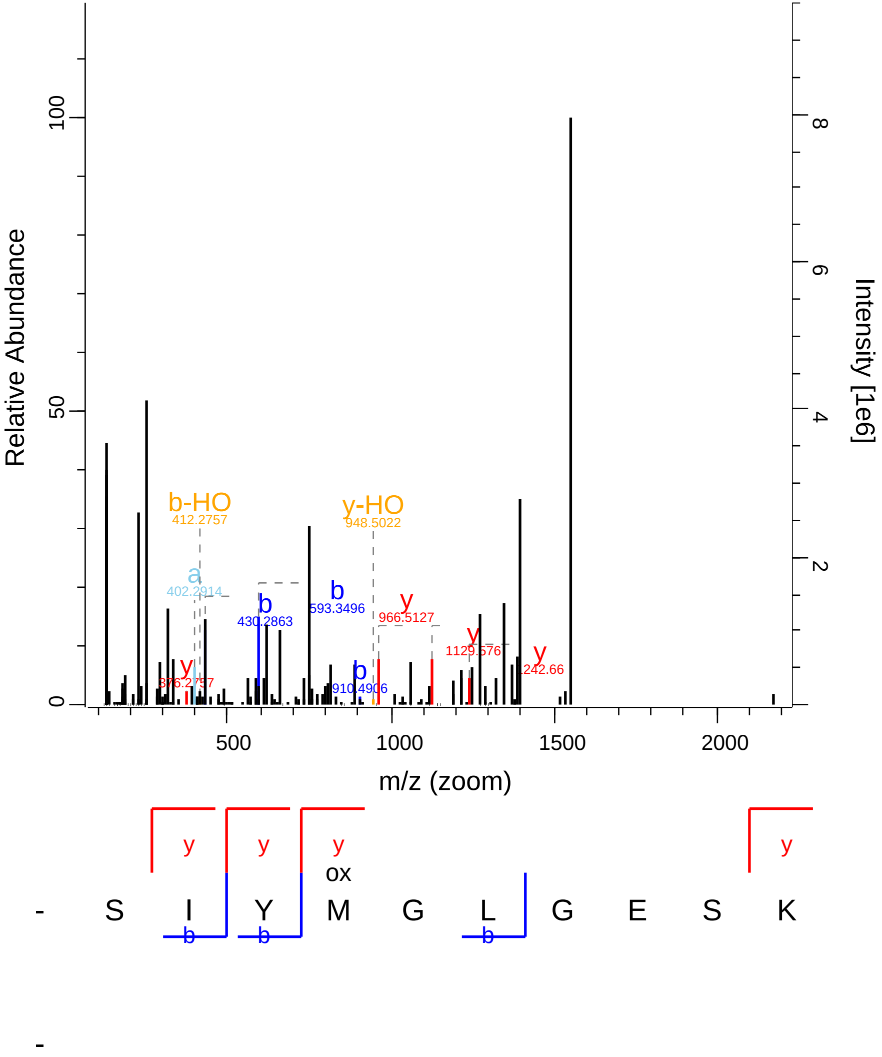

Raw File	Scan	Method	Score	m/z	Gene names
RO-QC-160121_ZackD_SS_Set_01_Fractio	20152	FTMS; HC	55.53	825.42	CELSR3

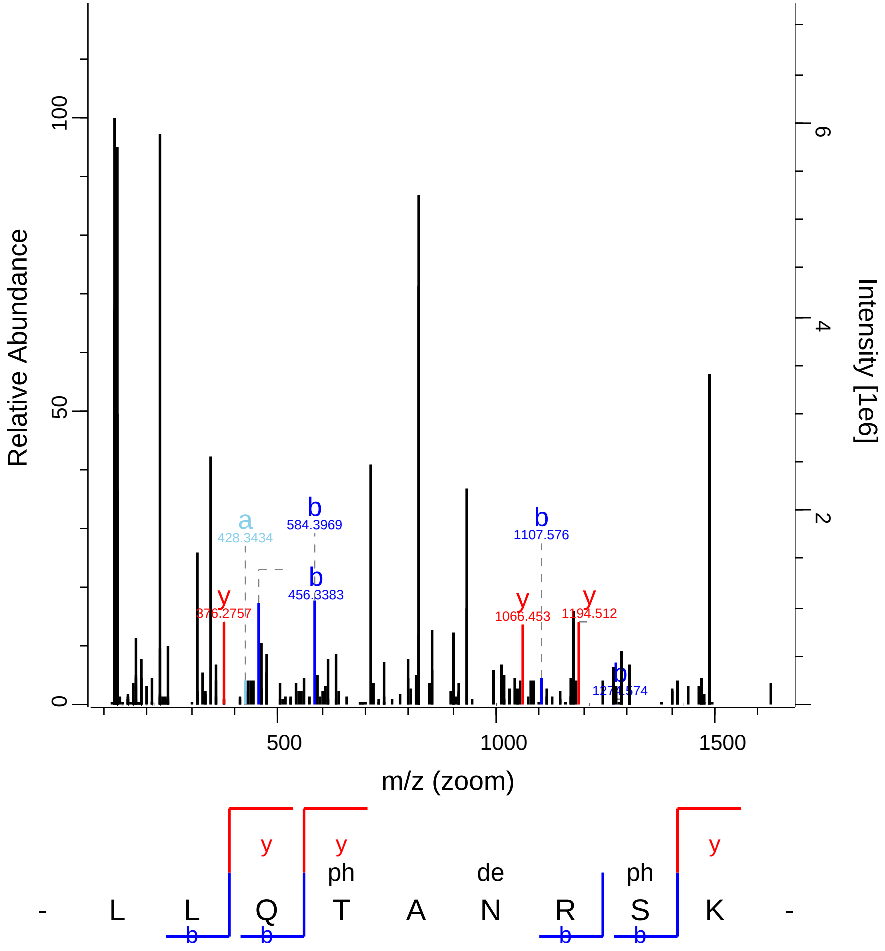

Raw File	Scan	Method	Score	m/z
RO-QC-160121_ZackD_SS_Set_01_Fraction_8	21652	FTMS; HCD	48.28	496.3

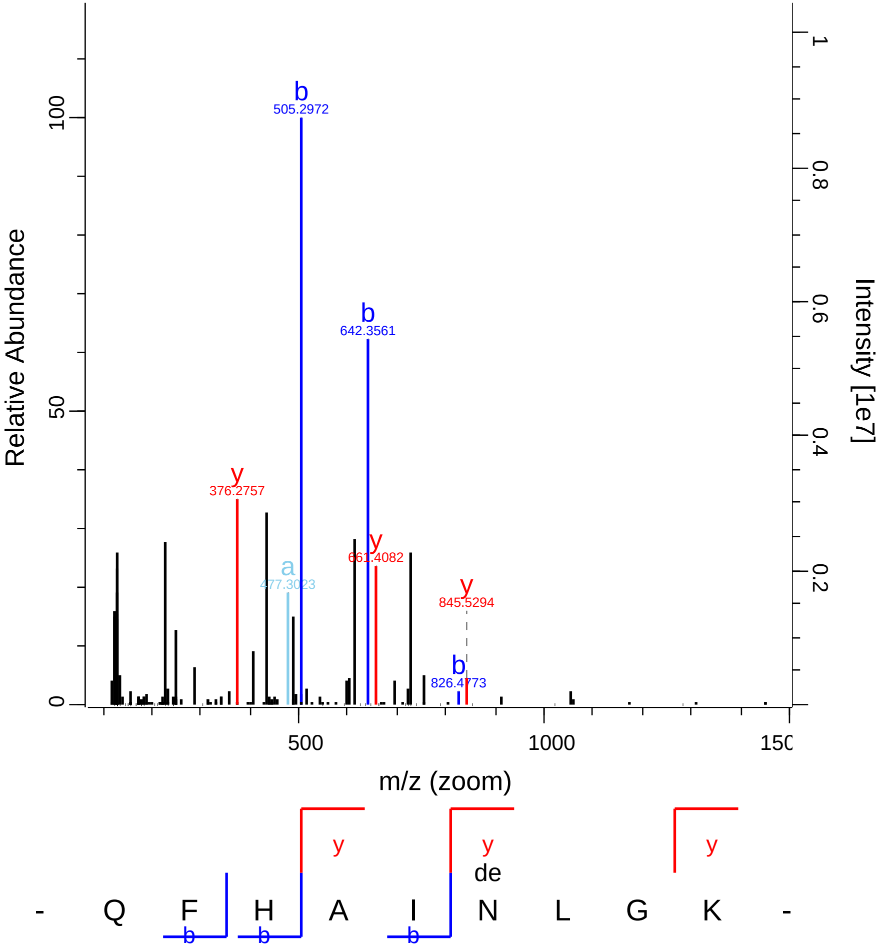

Raw File	Scan	Method	Score	m/z	Gene names
RO-QC-160121_ZackD_SS_Set_01_Fractio	22559	FTMS; HC	168.8	780.46	CAGE1

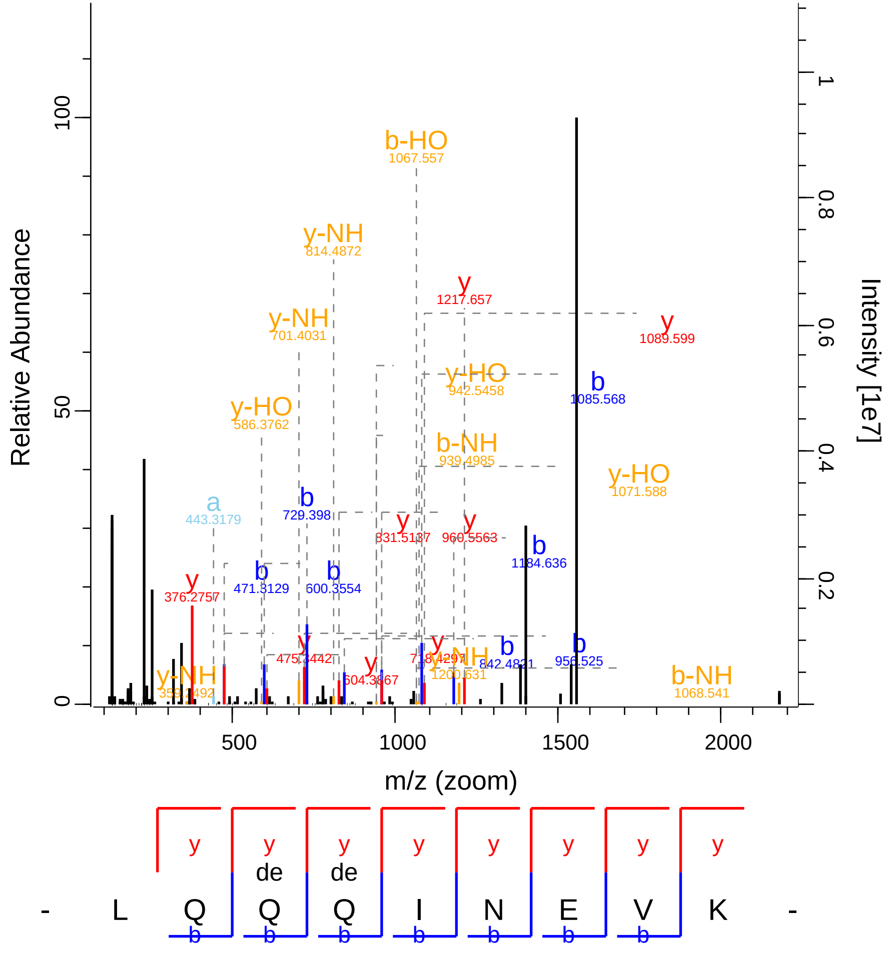

Raw File	Scan	Method	Score	m/z
RO-QC-160121_ZackD_SS_Set_01_Fraction_8	22744	FTMS; HCD	75.42	1311.72

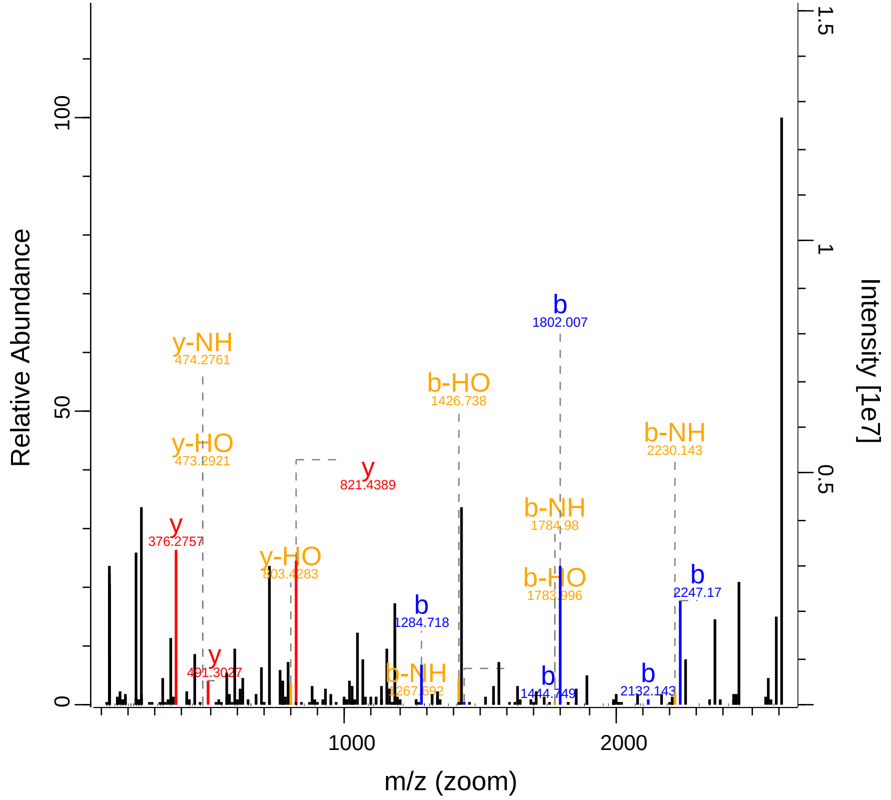

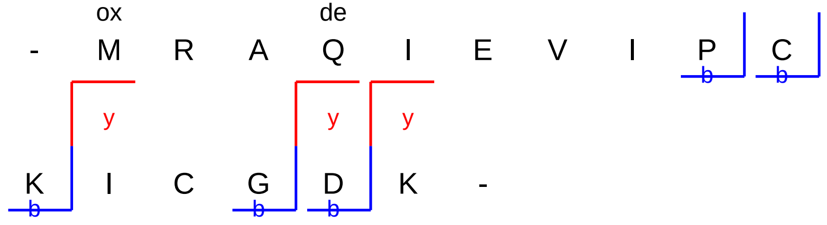

Raw File	Scan	Method	Score	m/z
RO-QC-160121_ZackD_SS_Set_01_Fraction_8	22871	FTMS; HCD	77.75	396.6

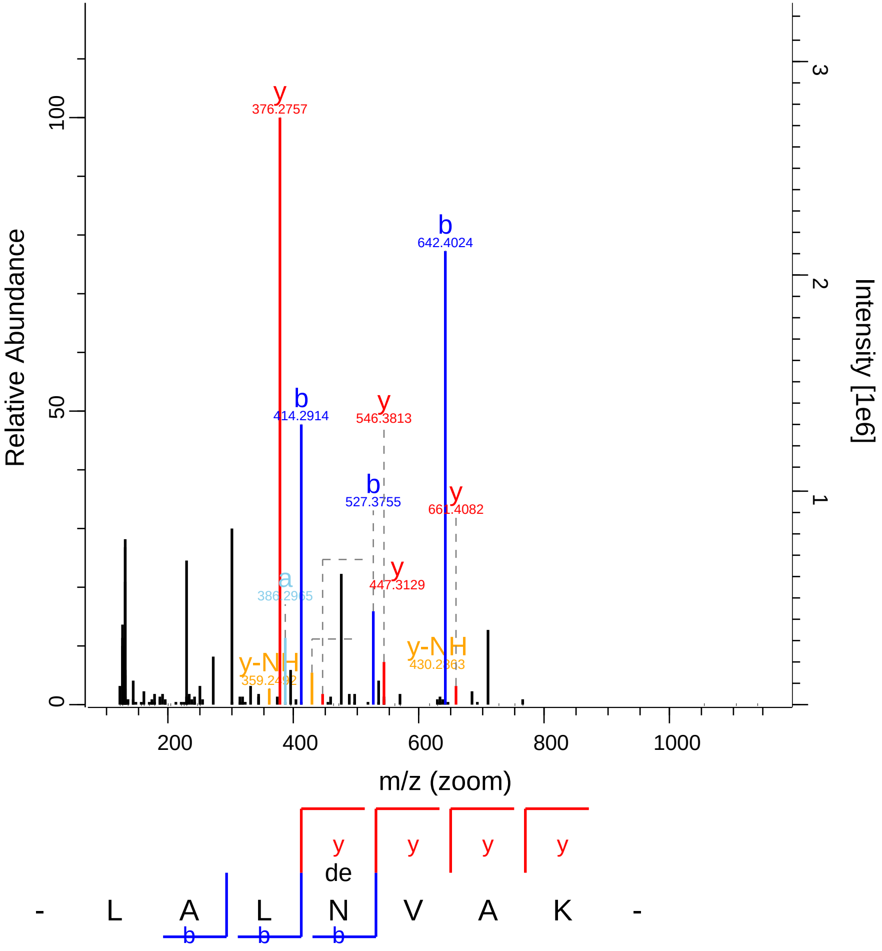

Raw File	Scan	Method	Score	m/z
RO-QC-160121_ZackD_SS_Set_01_Fraction_8	23502	FTMS; HCD	86.95	609.4

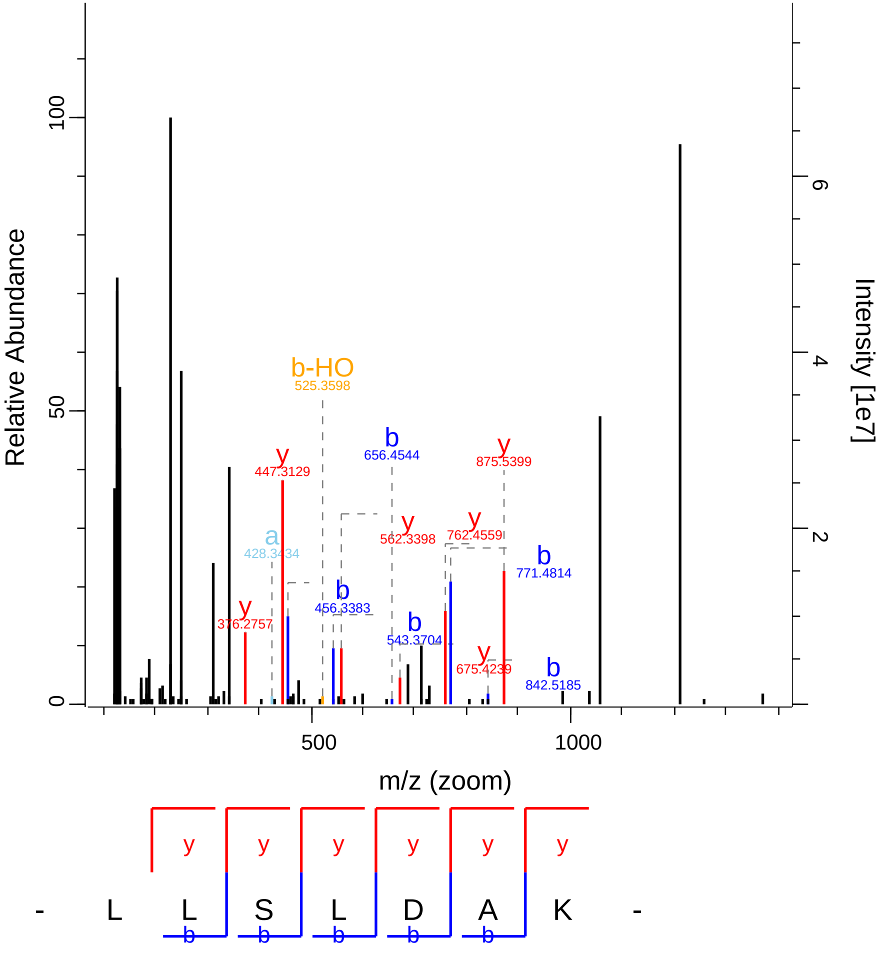

Raw File	Scan	Method	Score	m/z
RO-QC-160121_ZackD_SS_Set_01_Fraction_8	23541	FTMS; HCD	110.61	406.6

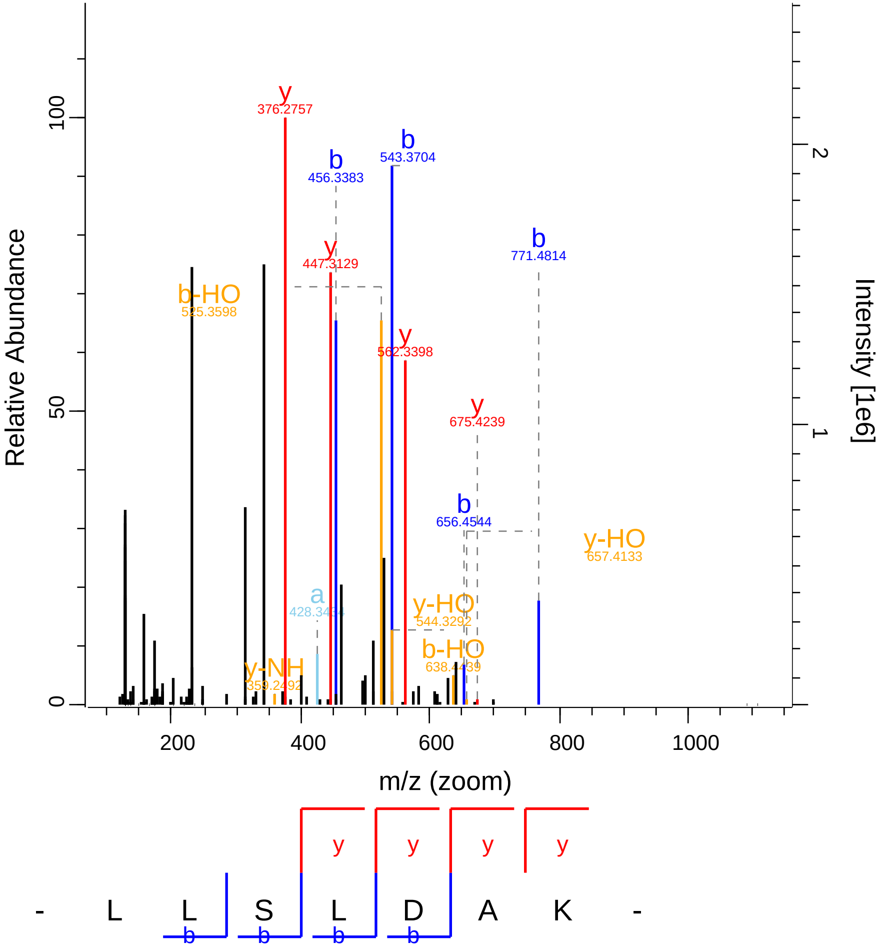

Raw File	Scan	Method	Score	m/z	Gene names
RO-QC-160121_ZackD_SS_Set_01_Fractio	23867	FTMS; HC	56.42	829.91	C6orf120

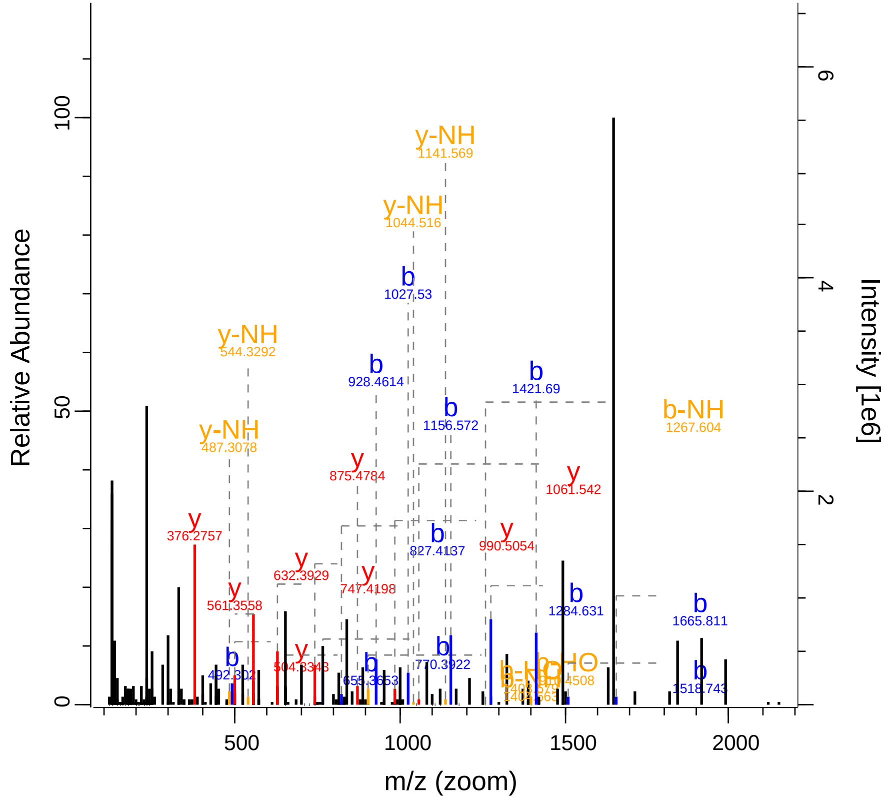

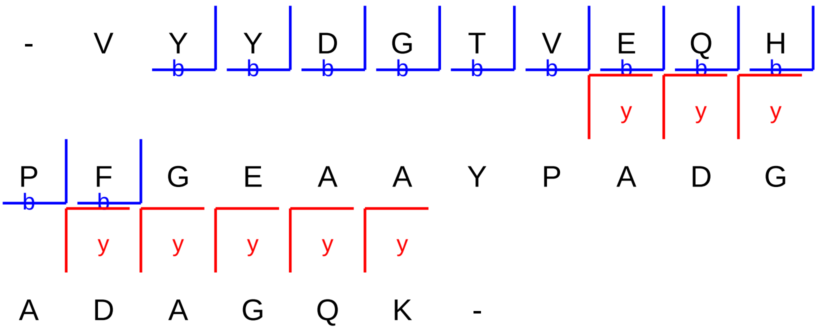

Raw File	Scan	Method	Score	m/z	Gene names
RO-QC-160121_ZackD_SS_Set_01_Fractio	23885	FTMS; HC	81.94	829.66	C6orf120

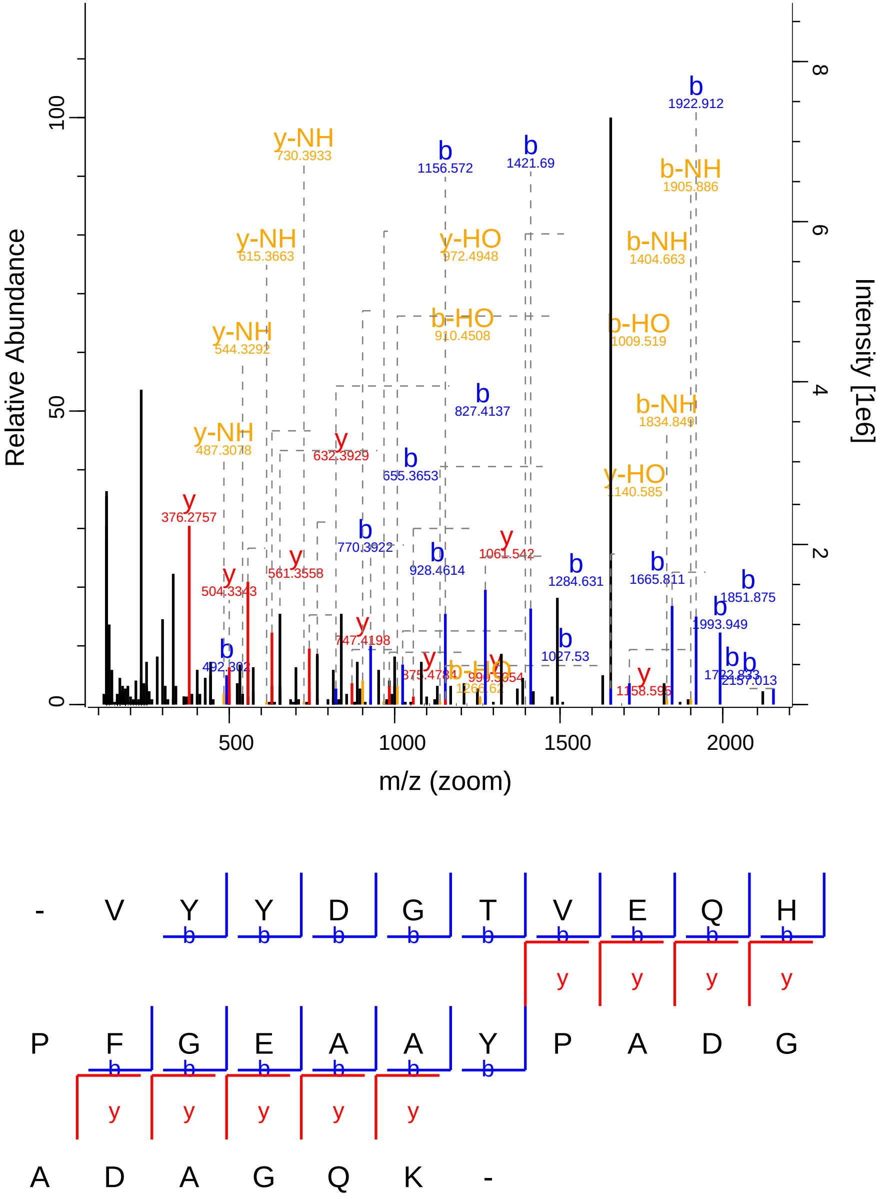

Raw File	Scan	Method	Score	m/z	Gene names
RO-QC-160121_ZackD_SS_Set_01_Fractic	23895	FTMS; HC	161.39	1105.87	C6orf120

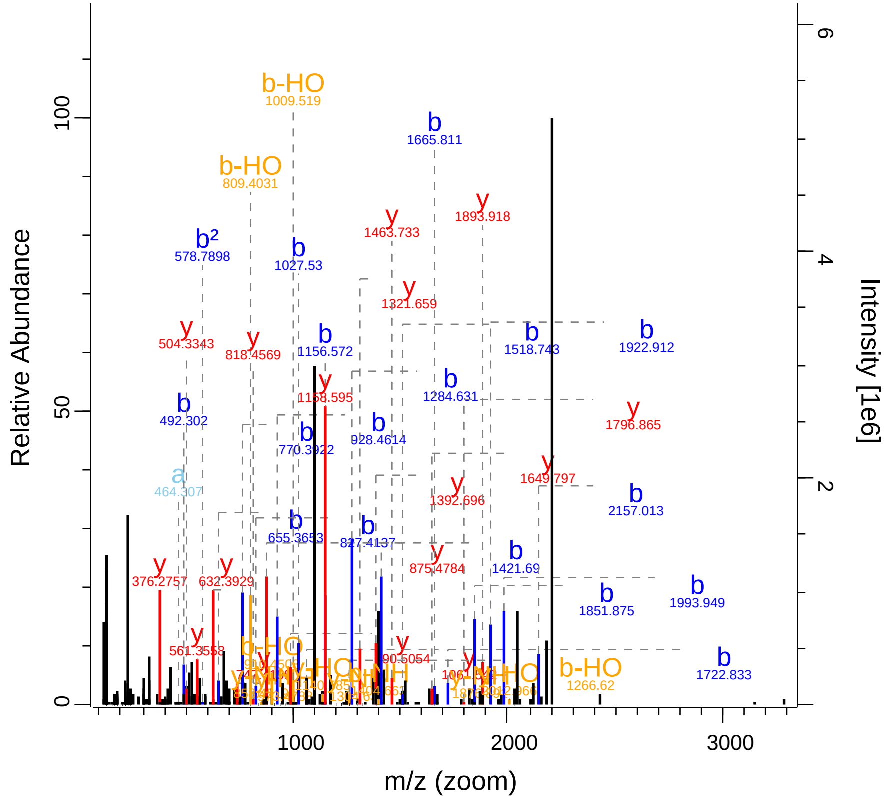

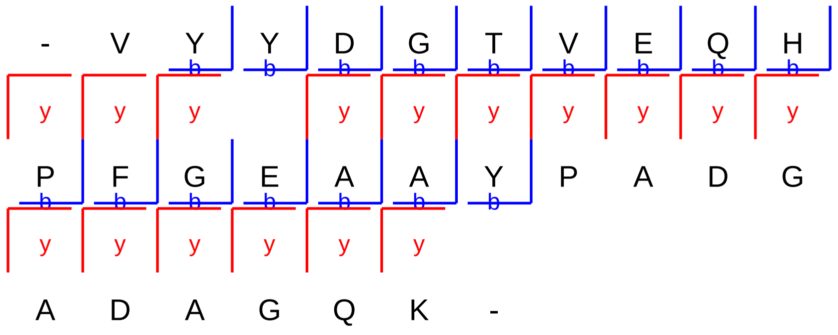

Raw File	Scan	Method	Score	m/z
RO-QC-160121_ZackD_SS_Set_01_Fraction_8	24572	FTMS; HCD	46.71	598.99

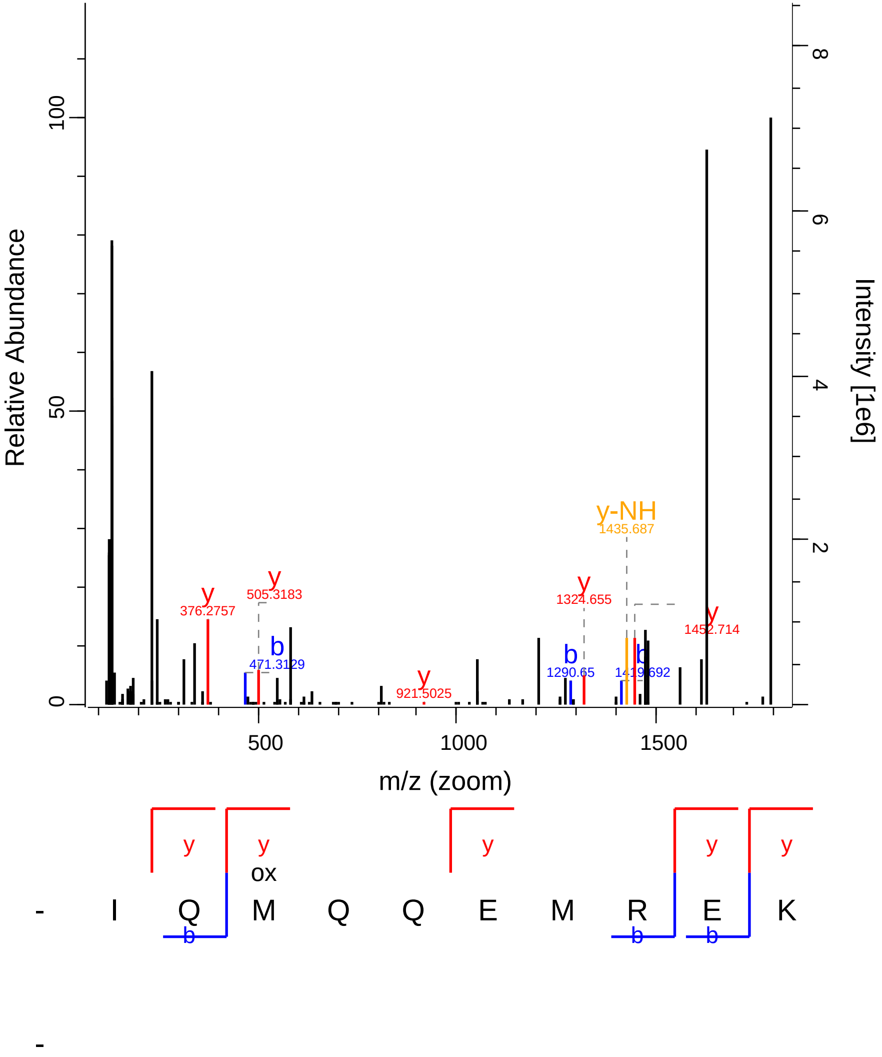

Raw File	Scan	Method	Score	m/z	Gene names
RO-QC-160121_ZackD_SS_Set_01_Fractio	25672	FTMS; HC	124.08	736.91	HIC1

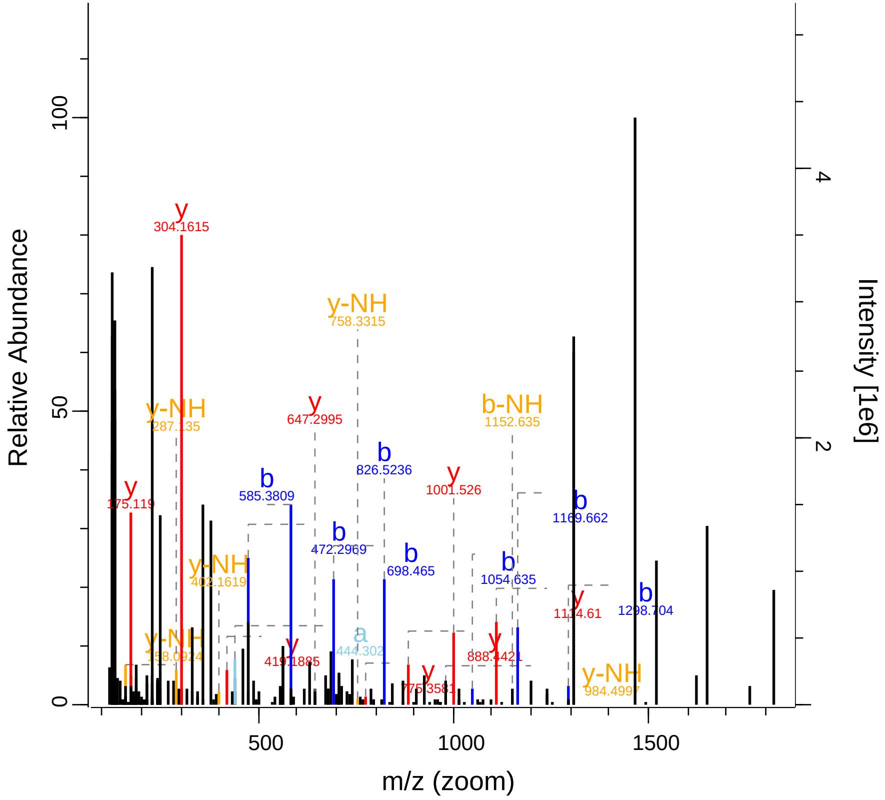

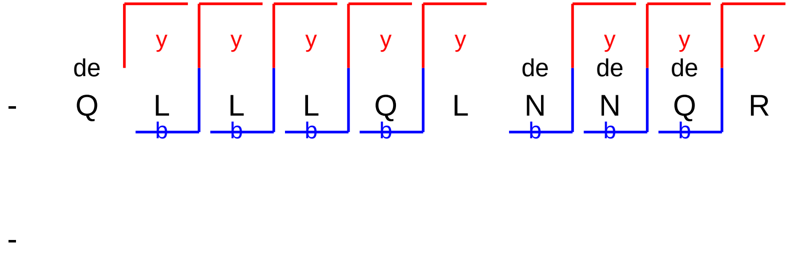

Raw File	Scan	Method	Score	m/z	Gene names
RO-QC-160121_ZackD_SS_Set_01_Fractio	26321	FTMS; HC	124.67	766.48	UBIAD1

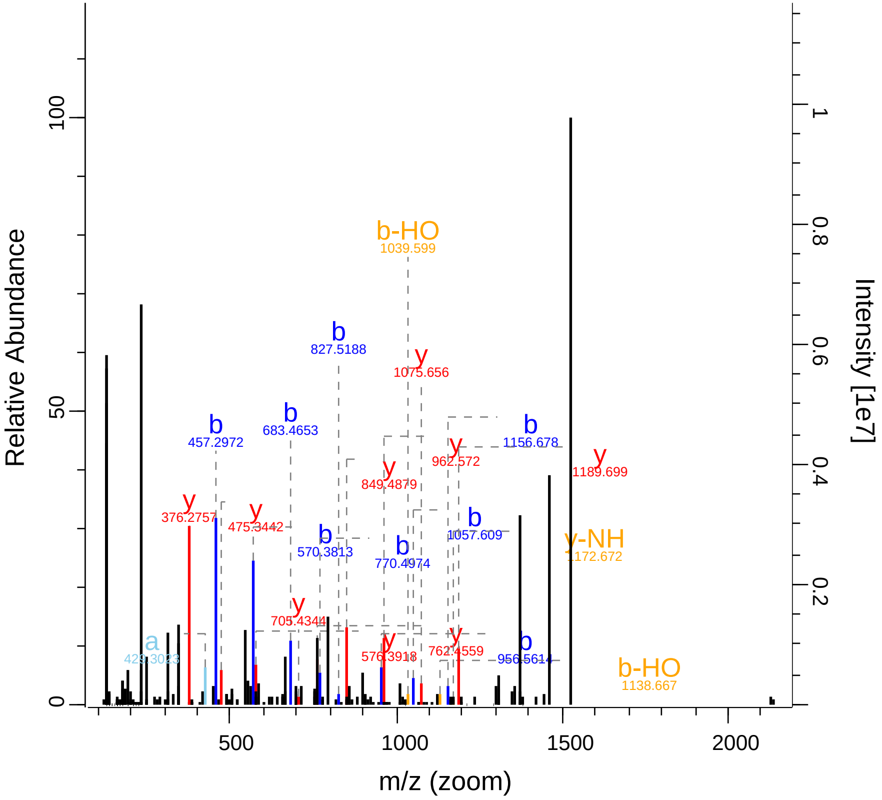

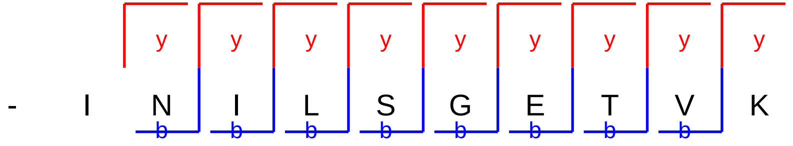

-

Raw File	Scan	Method	Score	m/z	Gene names
RO-QC-160121_ZackD_SS_Set_01_Fractic	26990	FTMS; HC	56.2	1116.63	CNGA2

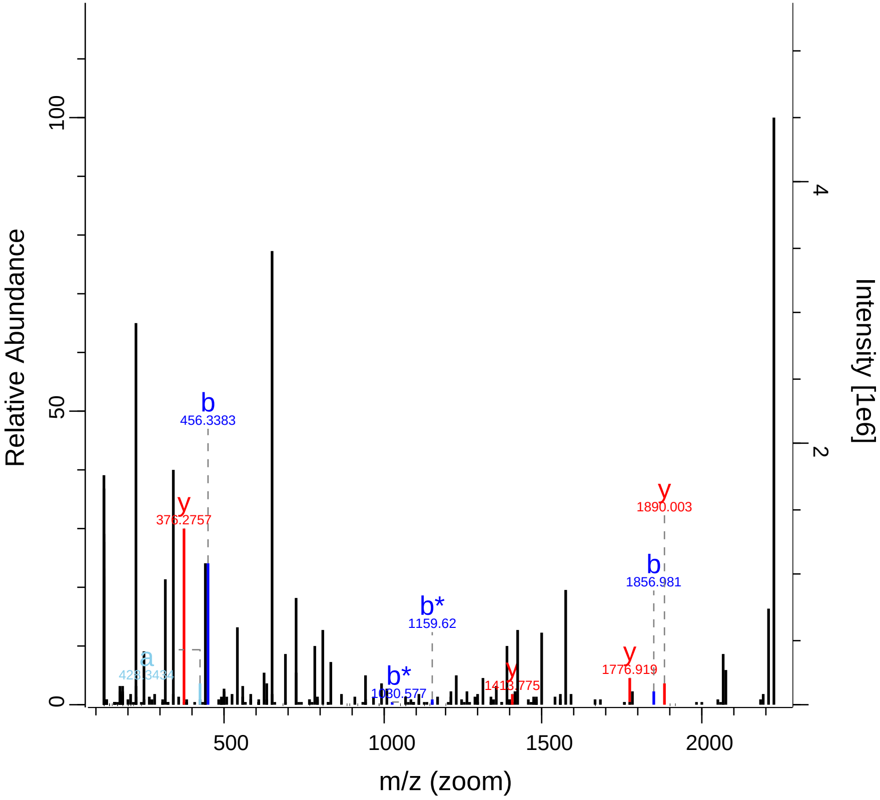

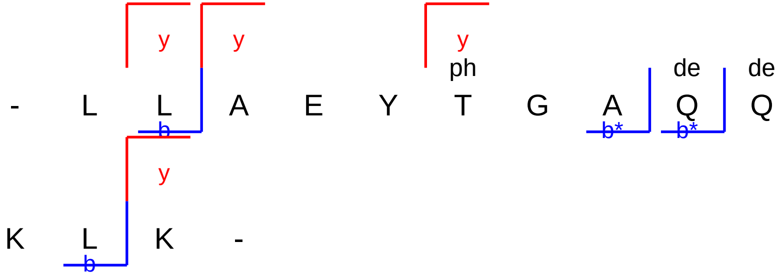

Raw File	Scan	Method	Score	m/z	Gene names
RO-QC-160121_ZackD_SS_Set_01_Fractio	29768	FTMS; HC	66.5	577.65	FICD

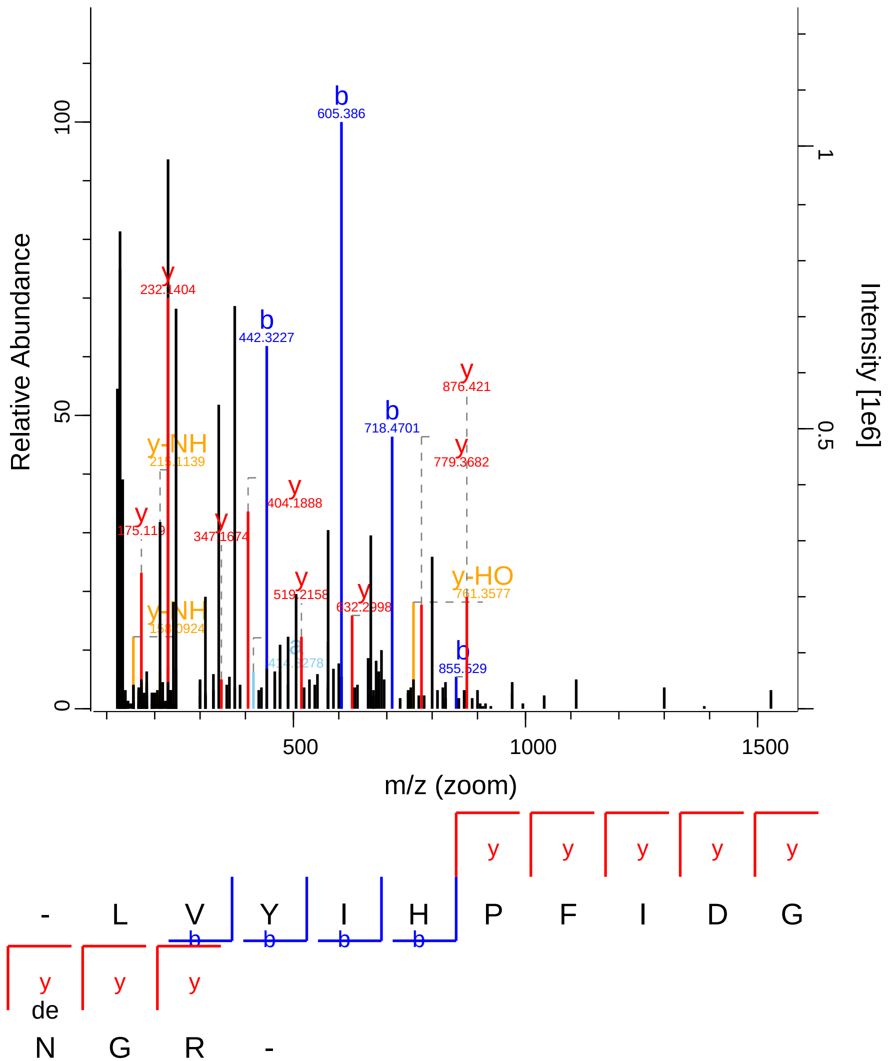

Raw File	Scan	Method	Score	m/z
RO-QC-160121_ZackD_SS_Set_01_Fraction_8	31114	FTMS; HCD	96.71	457.64

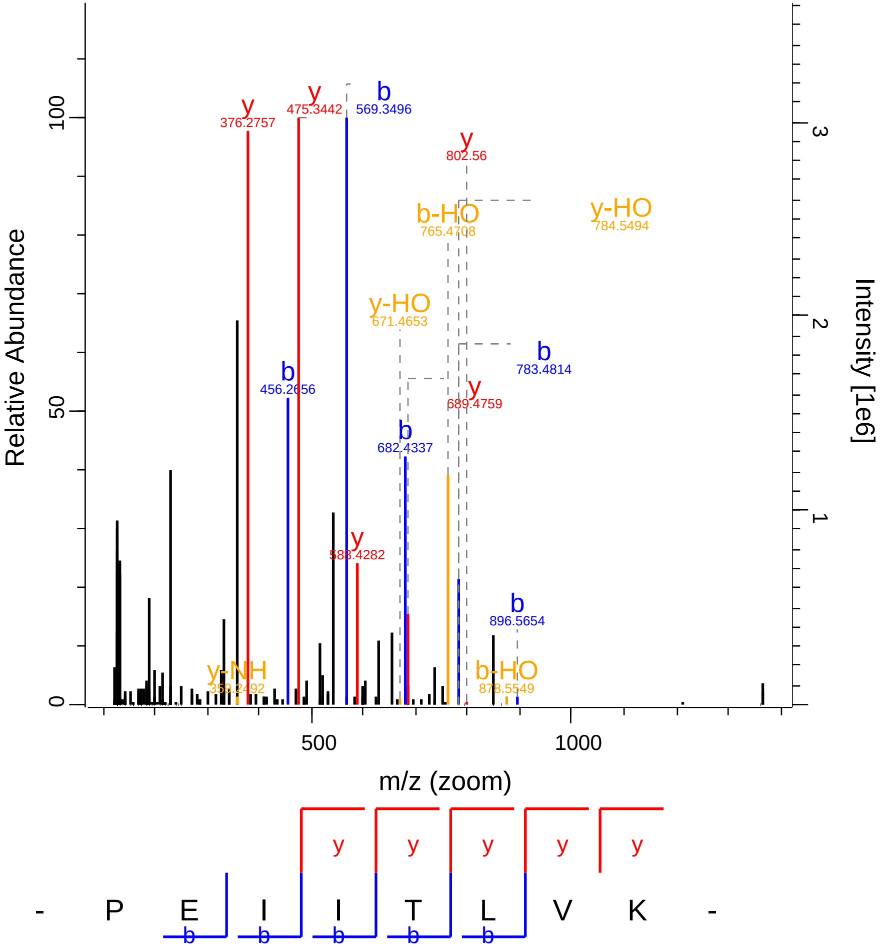

Raw File	Scan	Method	Score	m/z	Gene names
RO-QC-160121_ZackD_SS_Set_01_Fractic	33181	FTMS; HC	47.67	1463.77	ST6GALNAC5

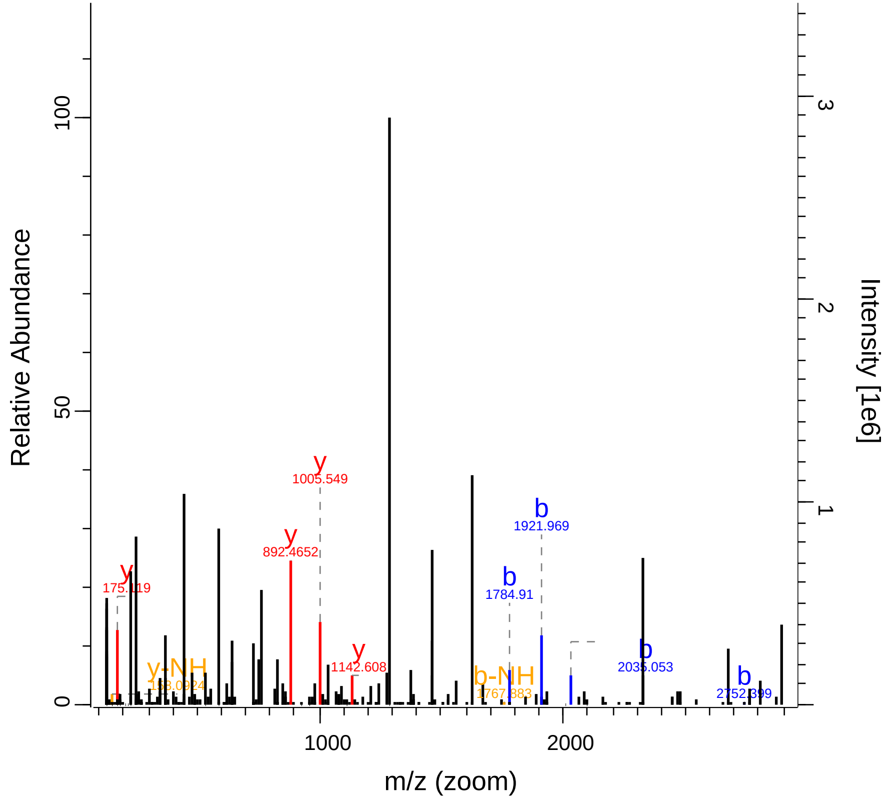

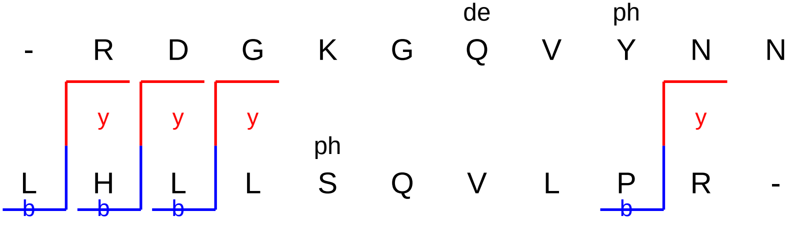

Raw File	Scan	Method	Score	m/z	Gene names
RO-QC-160121_ZackD_SS_Set_01_Fractic	33217	FTMS; HC	91.94	1315.12	ZNF540

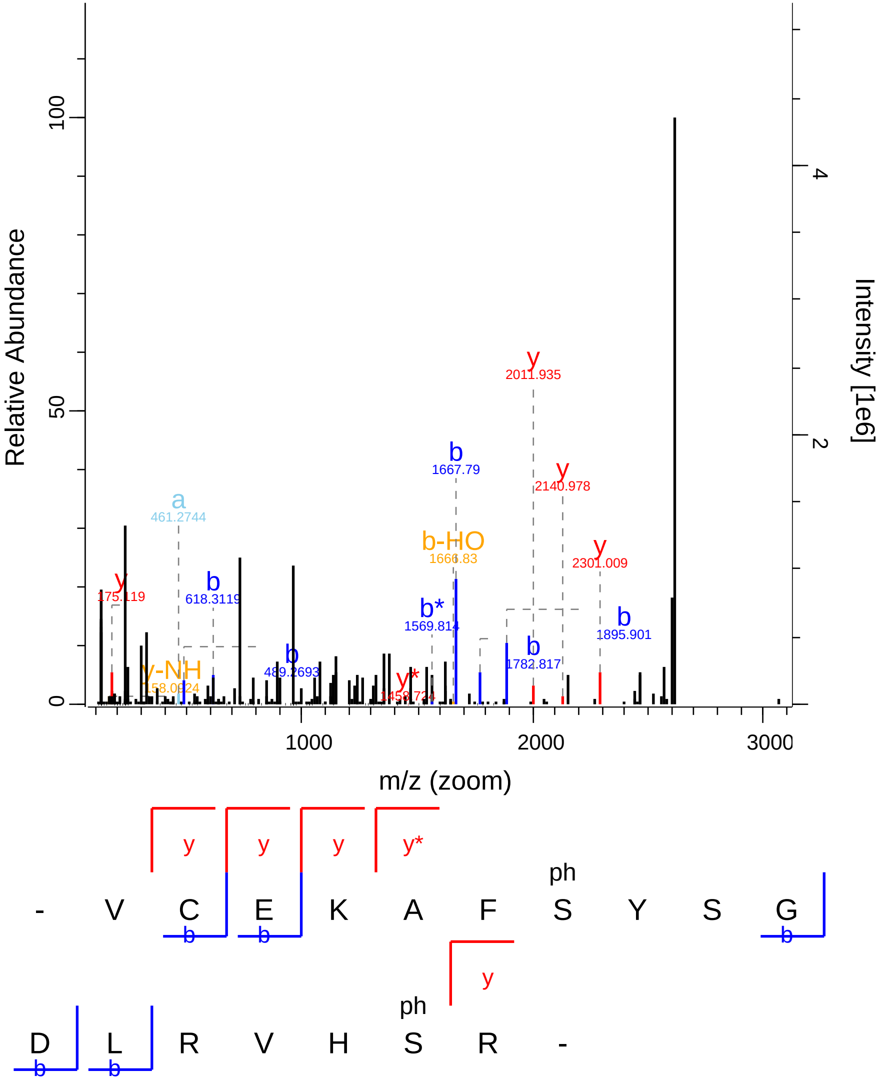

Raw File	Scan	Method	Score	m/z
RO-QC-160121_ZackD_SS_Set_01_Fraction_8	33481	FTMS; HCD	44.64	798.45

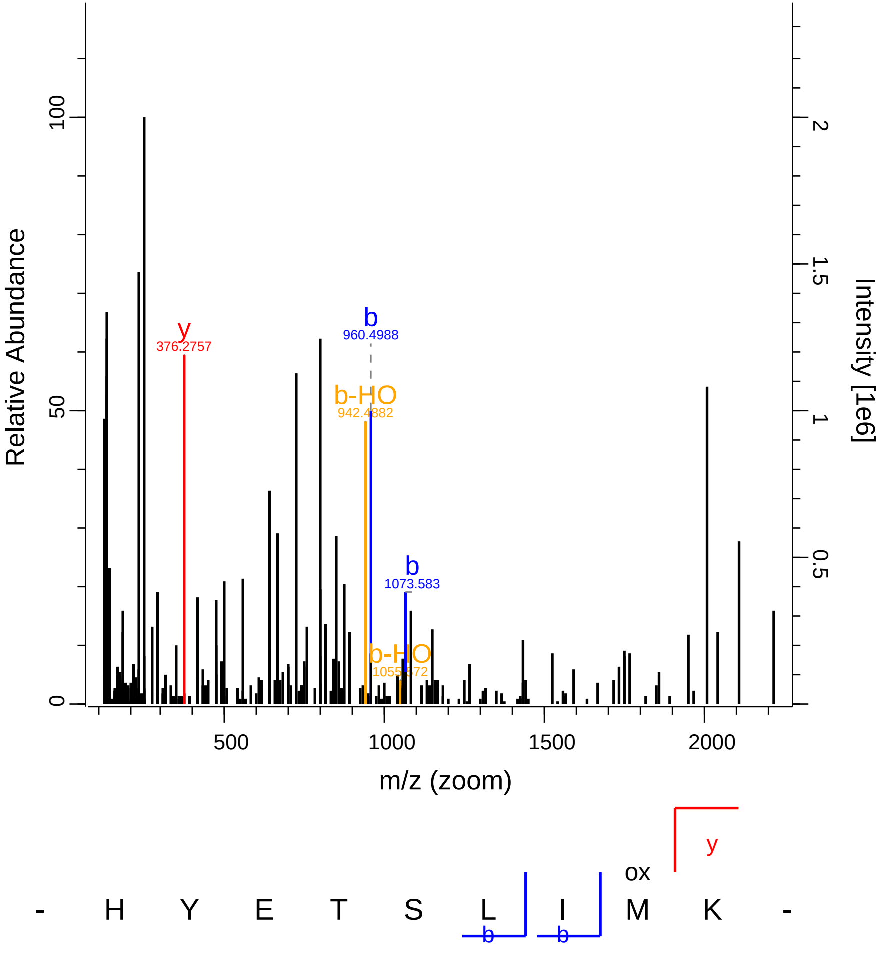

Raw File	Scan	Method	Score	m/z	Gene names
RO-QC-160121_ZackD_SS_Set_01_Fractic	33931	FTMS; HC	56.23	1315.12	ZNF540

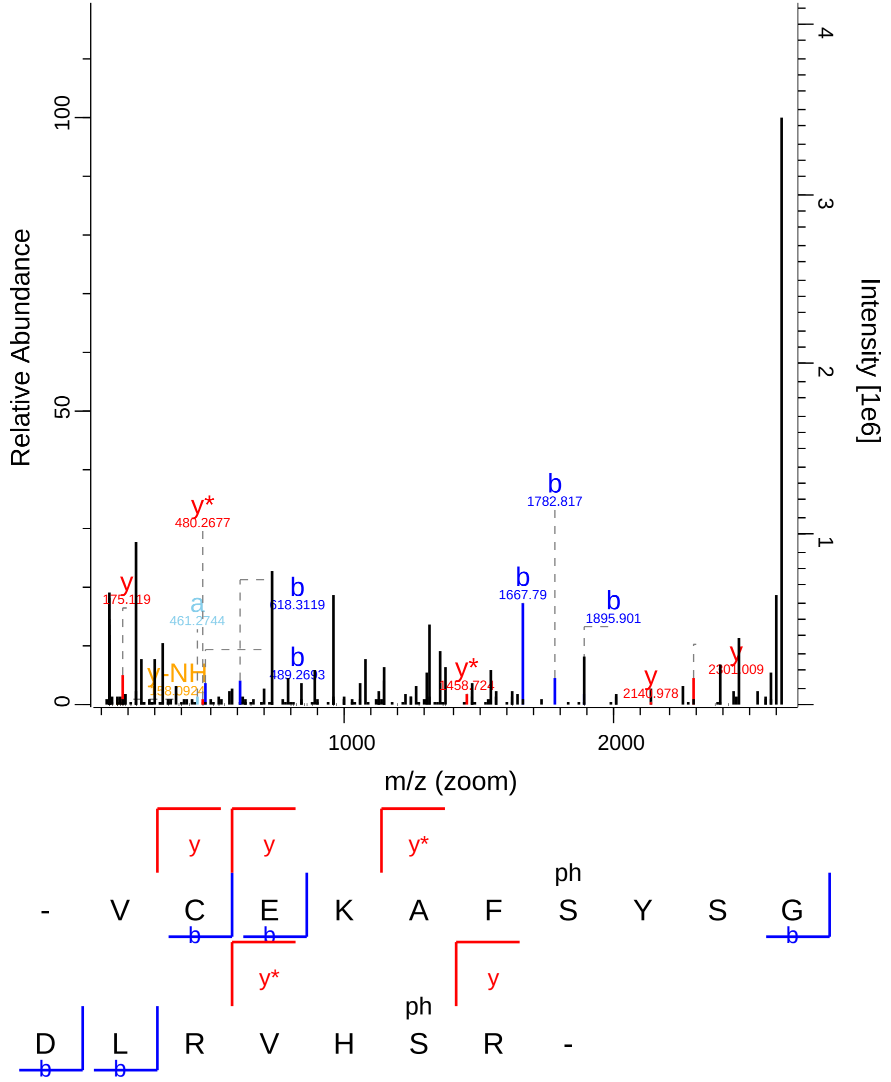

Raw File	Scan	Method	Score	m/z	Gene names
RO-QC-160121_ZackD_SS_Set_01_Fractio	34863	FTMS; HC	72.61	890.03	ASH1L

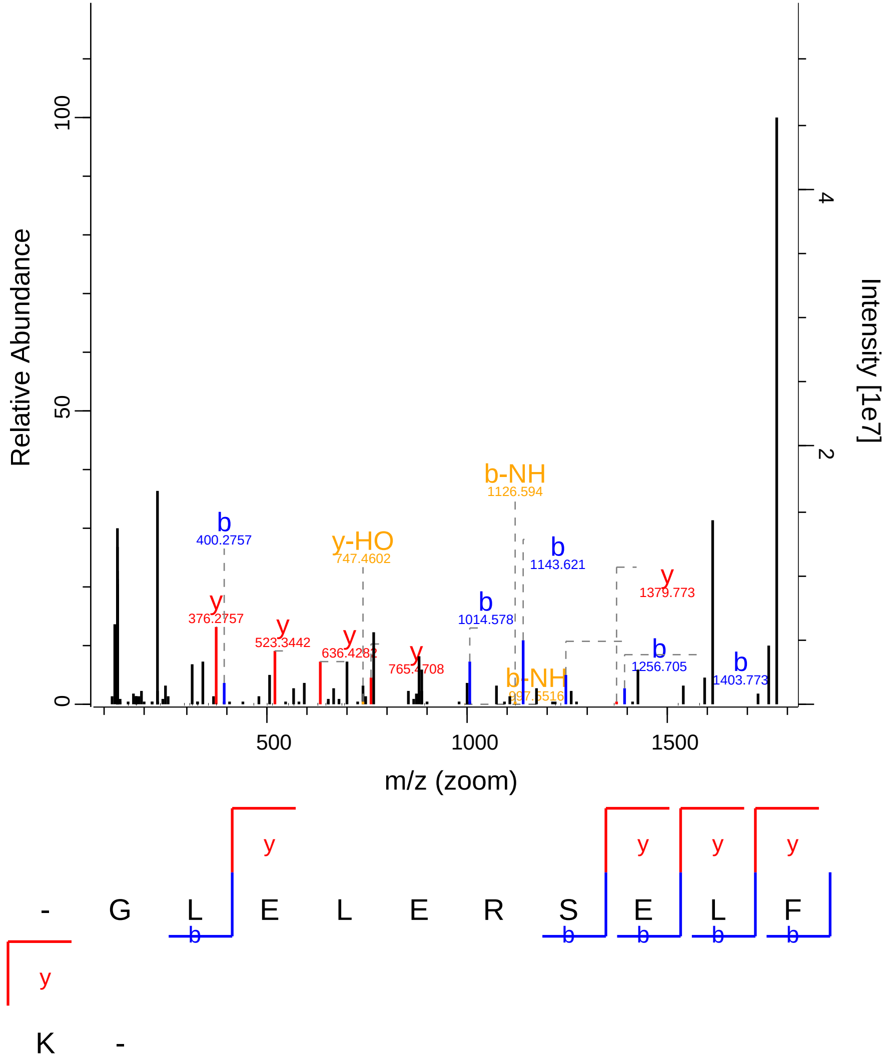

Raw File	Scan	Method	Score	m/z	Gene names
RO-QC-160121_ZackD_SS_Set_01_Fractio	34870	FTMS; HC	69.98	703.89	BLOC1S6

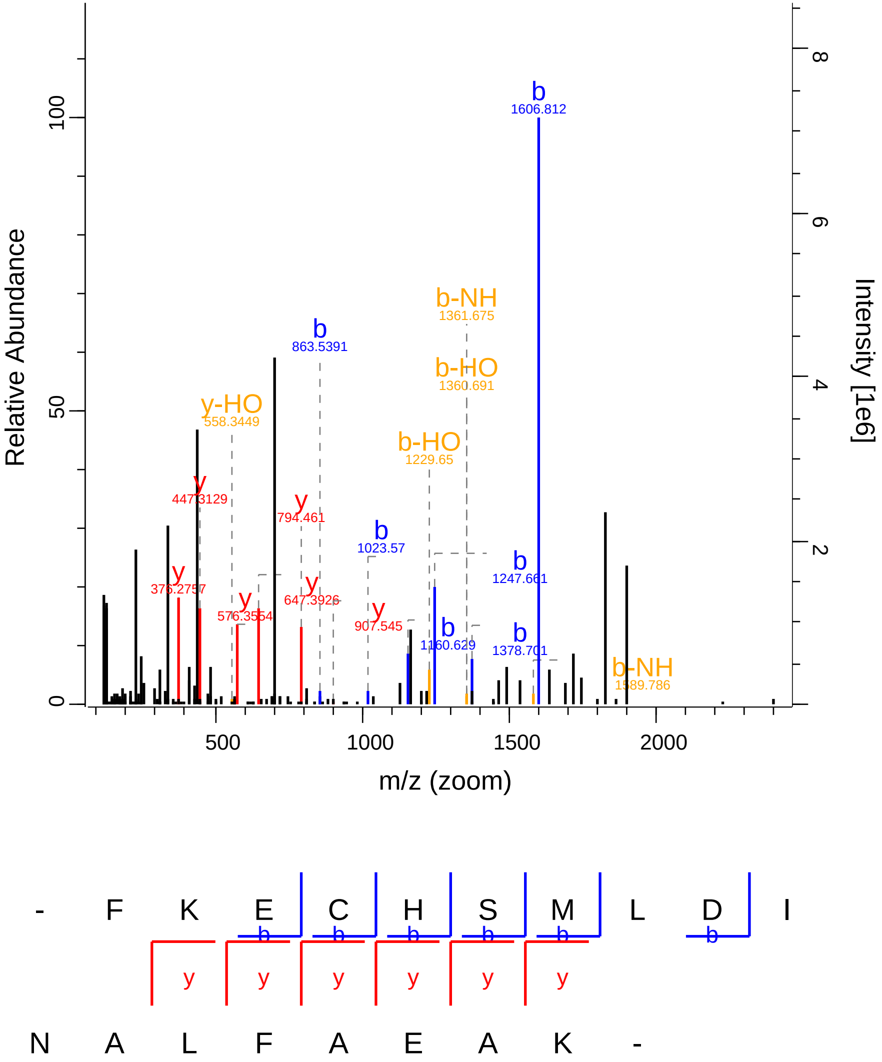

Raw File	Scan	Method	Score	m/z	Gene names
RO-QC-160121_ZackD_SS_Set_01_Fractio	35997	FTMS; HC	44.2	921.21	CCDC176

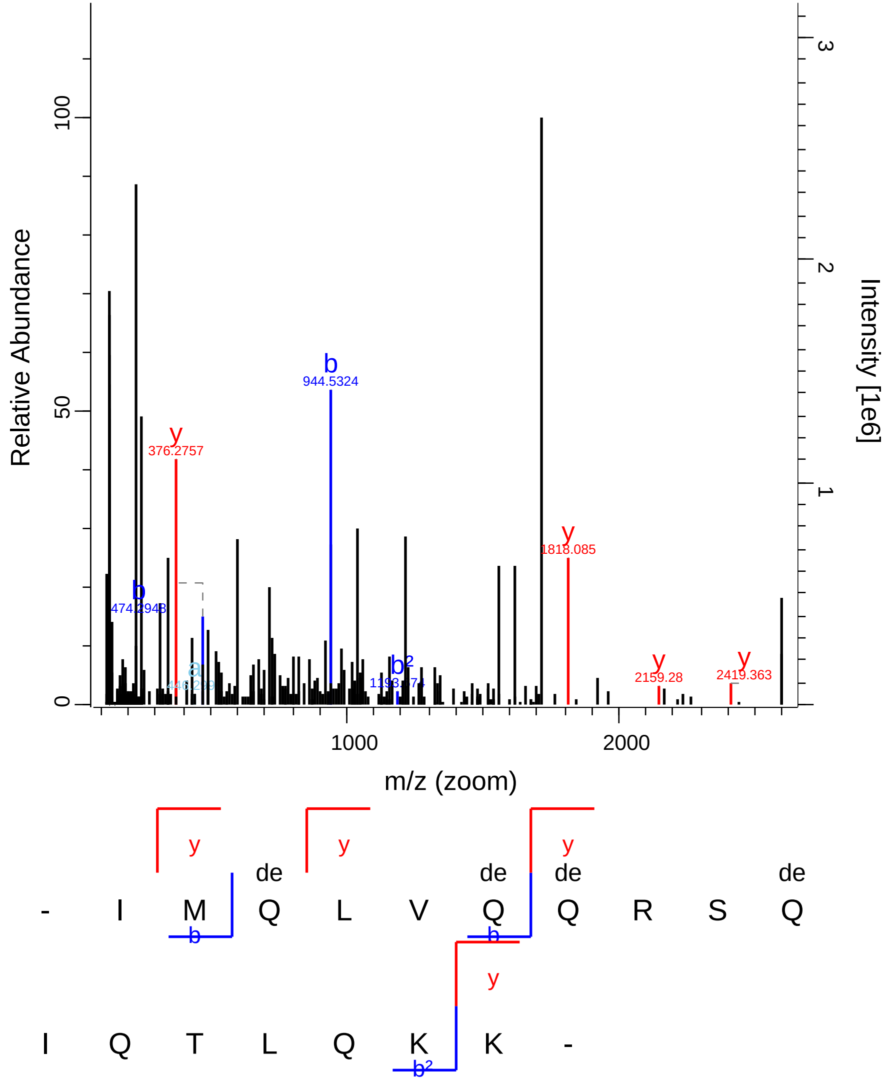

Raw File	Scan	Method	Score	m/z	Gene names
RO-QC-160121_ZackD_SS_Set_01_Fractic	36041	FTMS; HC	64.65	1504.35	UNC80

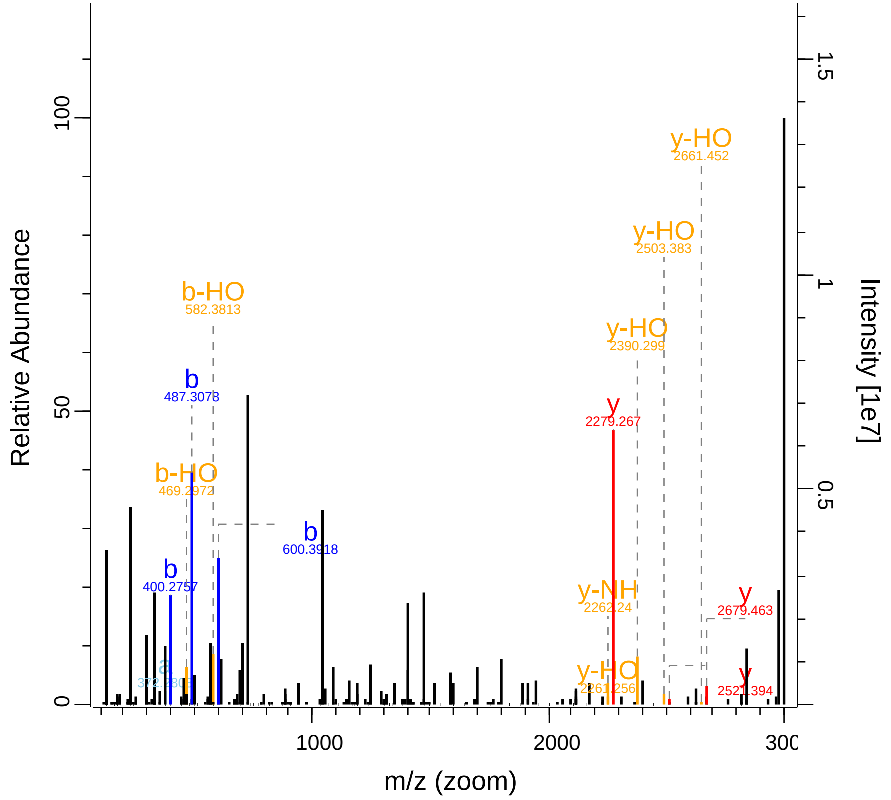

y y y

de de

- V A S I Q S E P G Q

b b b

de

Q N L L V Q Q P L G R

K R -

Raw File	Scan	Method	Score	m/z	Gene names
RO-QC-160121_ZackD_SS_Set_01_Fractio	36493	FTMS; HC	111.49	932.15	DKK3

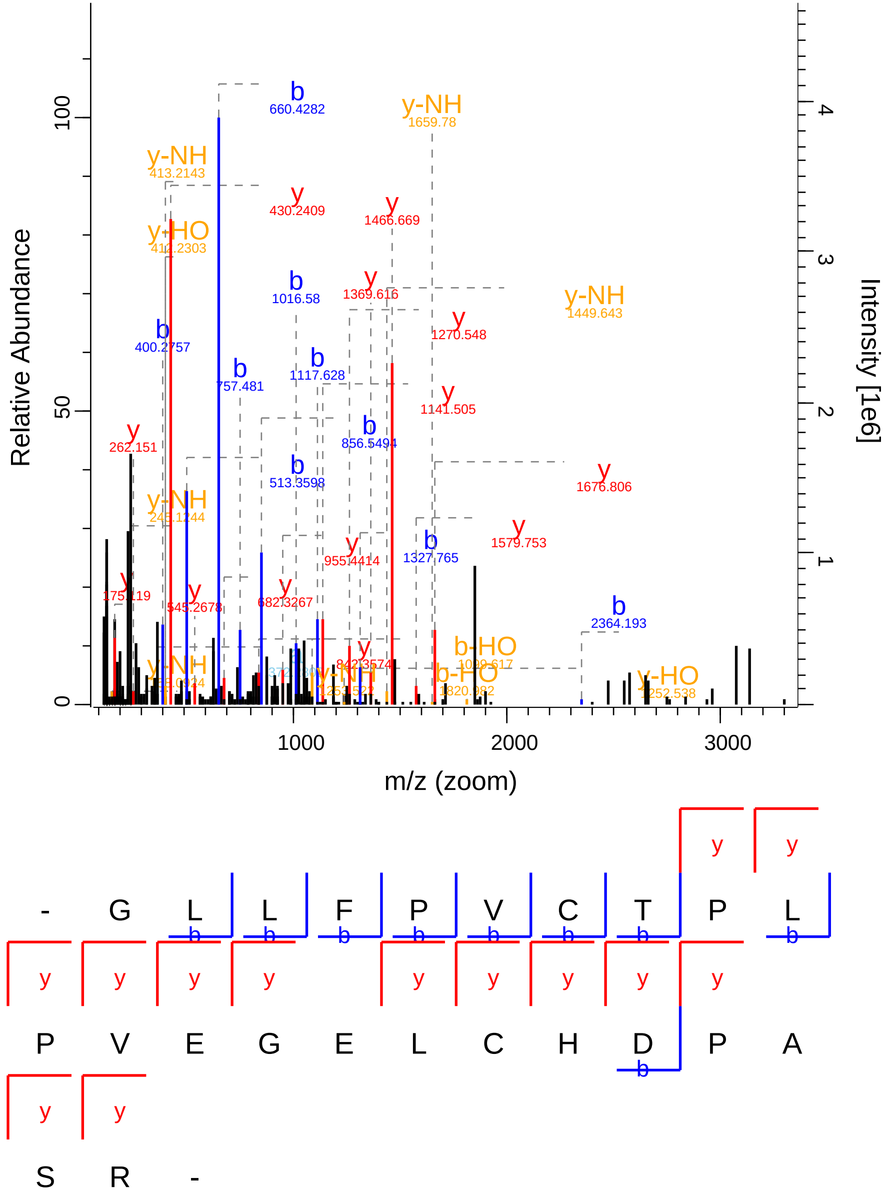

Raw File	Scan	Method	Score	m/z
RO-QC-160121_ZackD_SS_Set_01_Fraction_8	36937	FTMS; HCD	43.25	817.47

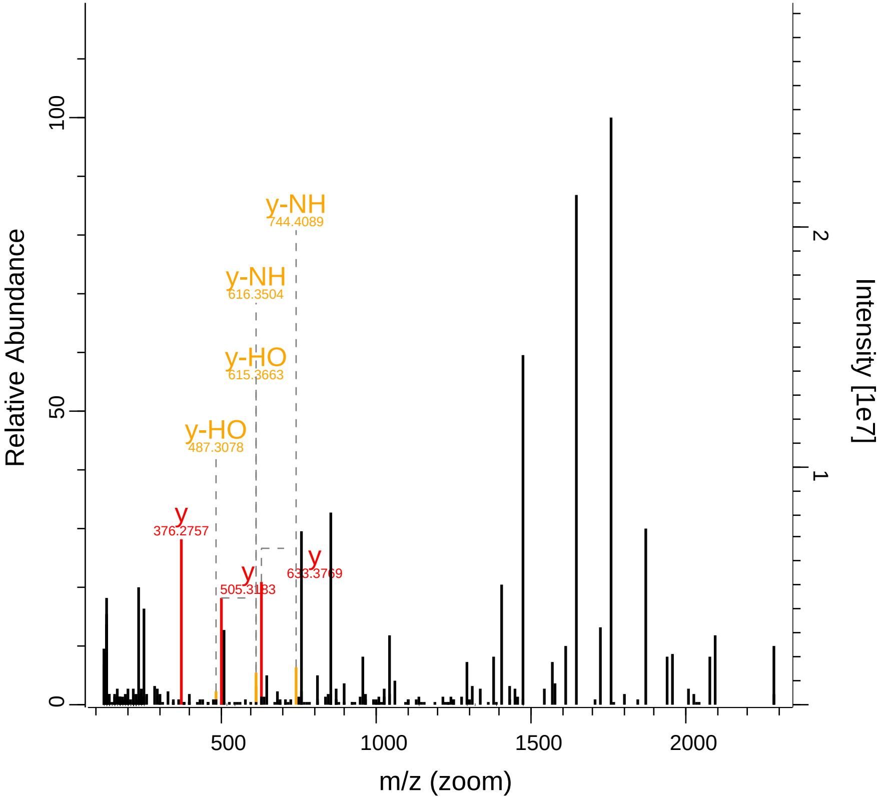

ac ox

y y y

- A A K I A M Q Q E K

-

Raw File	Scan	Method	Score	m/z	Gene names
RO-QC-160121_ZackD_SS_Set_01_Fractio	38067	FTMS; HC	101.17	627.05	ALKBH7

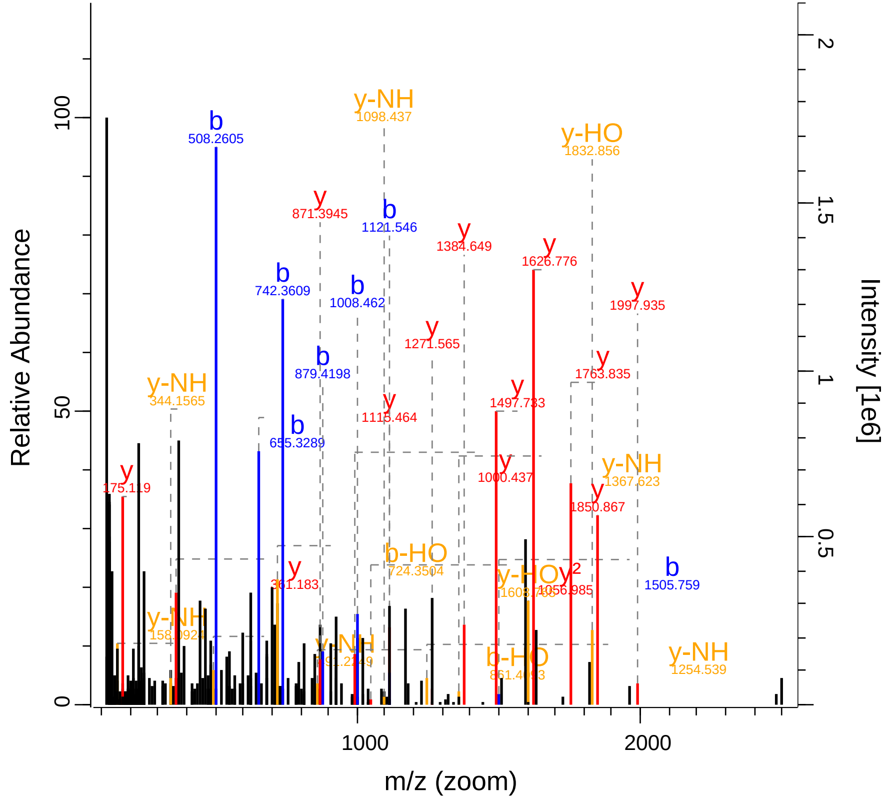

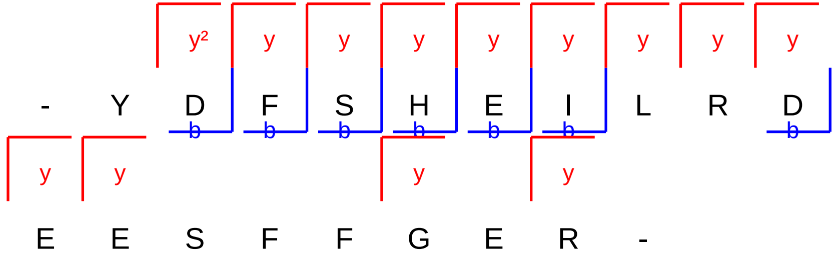

Raw File	Scan	Method	Score	m/z	Gene names
RO-QC-160121_ZackD_SS_Set_01_Fractio	38264	FTMS; HC	118.26	785.95	SNX7

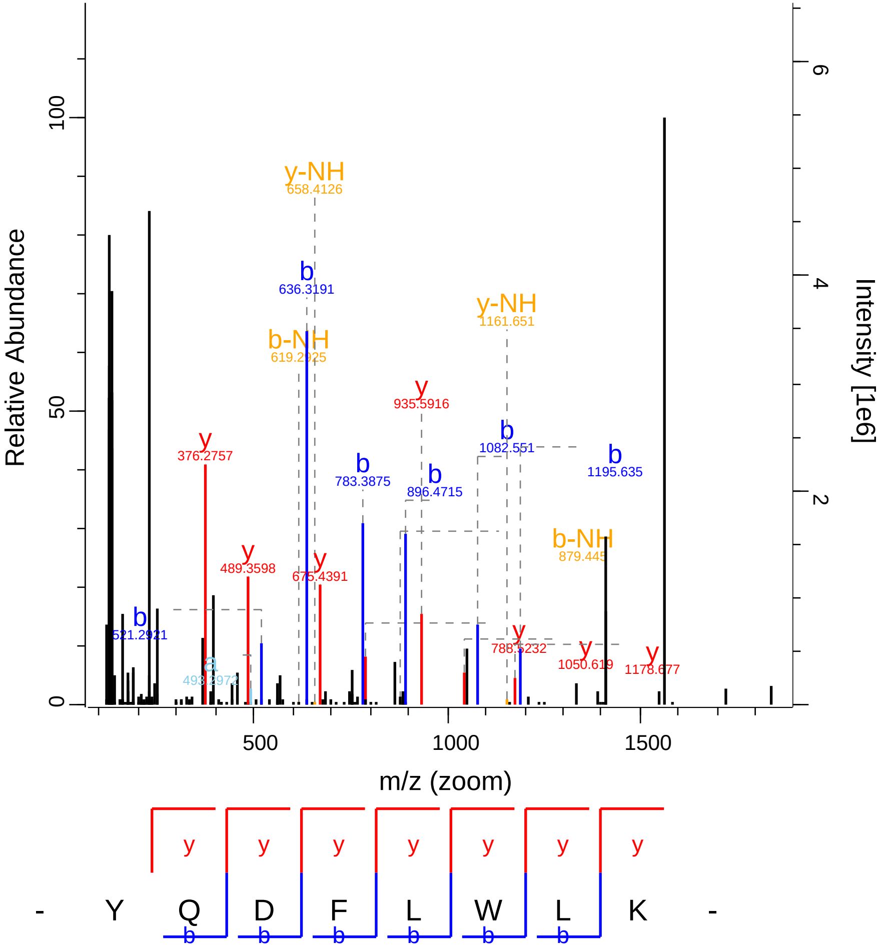

Raw File	Scan	Method	Score	m/z	Gene names
RO-QC-160121_ZackD_SS_Set_01_Fractioı	41432	FTMS; HCI	104.61	873.5	N6AMT2

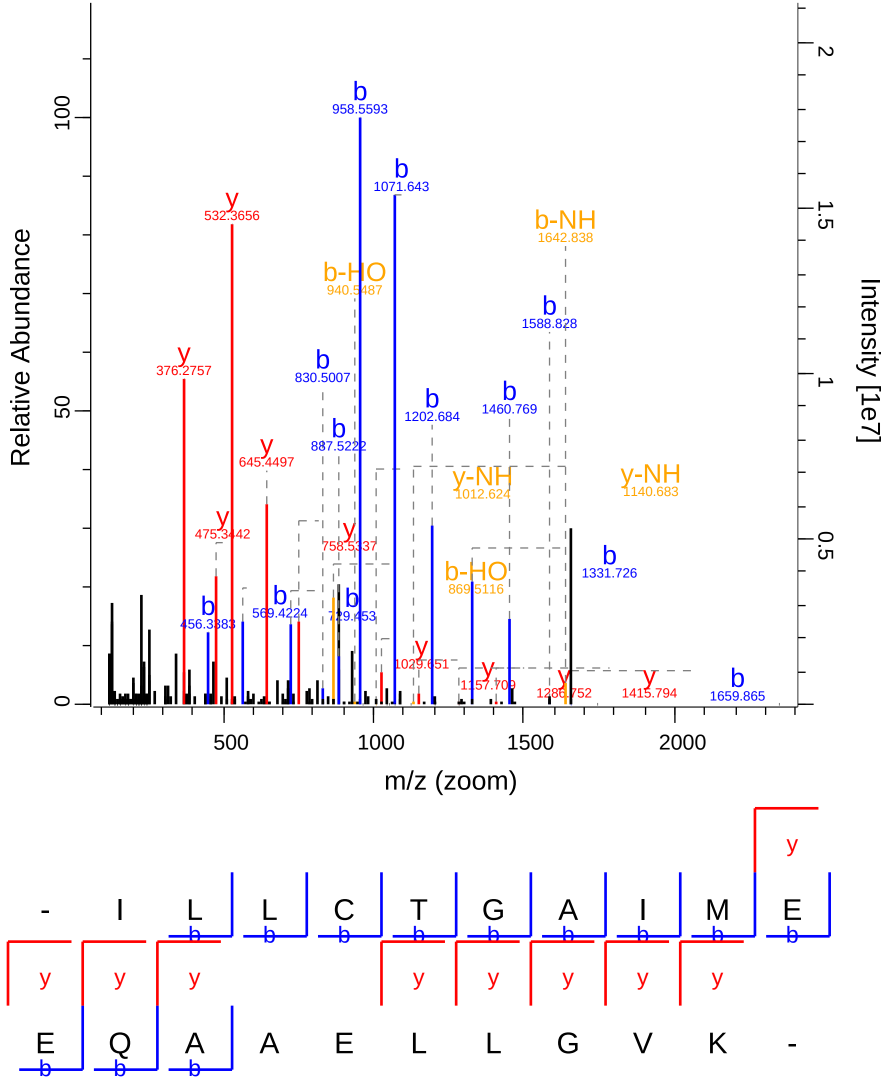

Raw File	Scan	Method	Score	m/z	Gene names
RO-QC-160121_ZackD_SS_Set_01_Fr	4150[illegible]	FTMS; H[illegible]	148.9[illegible]	596.3[illegible]	ATP5G1;ATP5G2;ATP

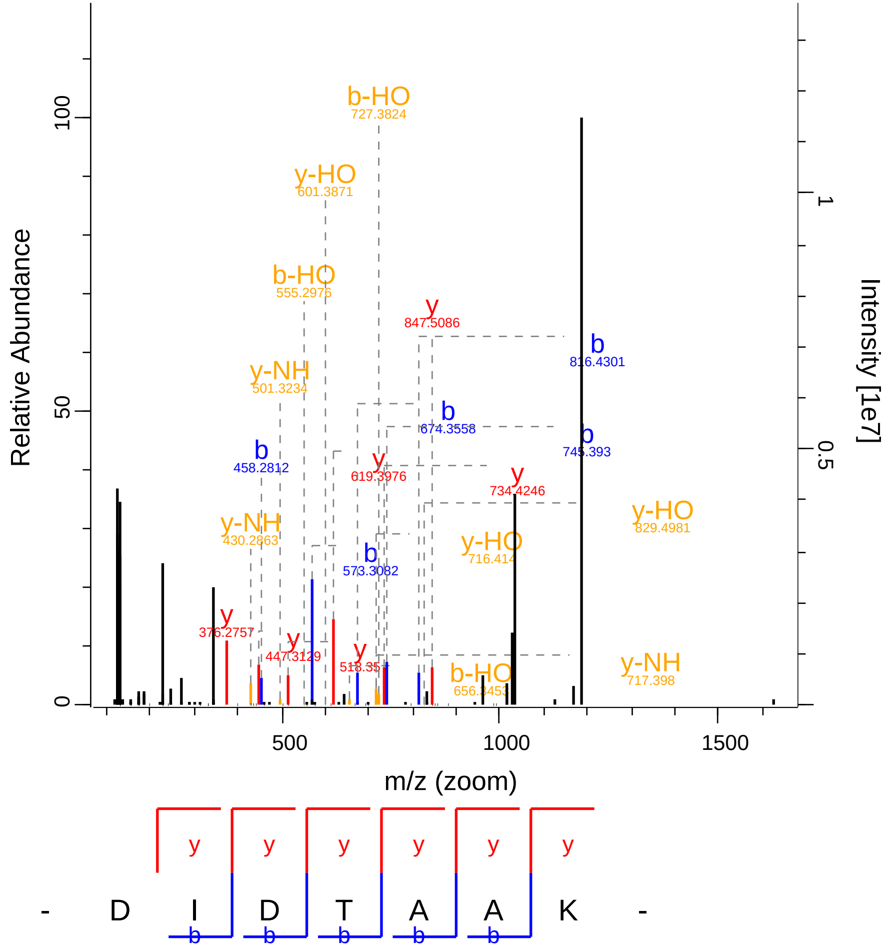

Raw File	Scan	Method	Score	m/z	Gene names
RO-QC-160121_ZackD_SS_Set_01_Fractio	5885	FTMS; HC	149.33	876.92	GLIPR1

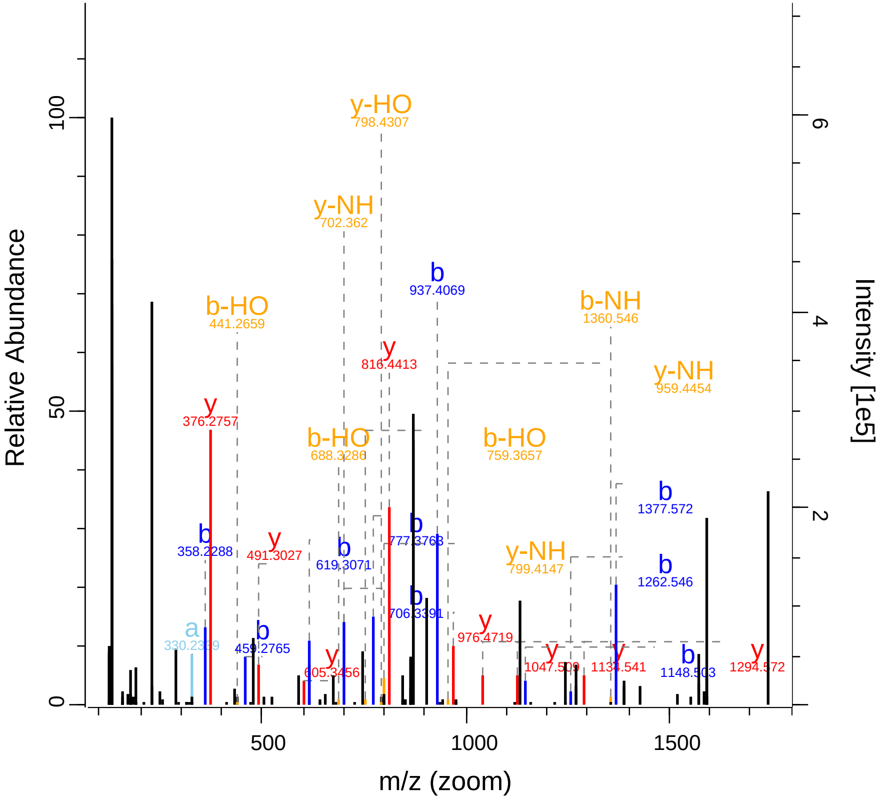

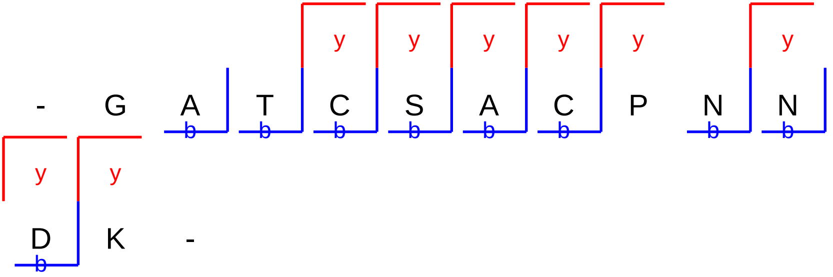

Raw File	Scan	Method	Score	m/z	Gene names
RO-QC-160121_ZackD_SS_Set_01_Fraction	6066	FTMS; HCI	101.32	634.3	GPR125

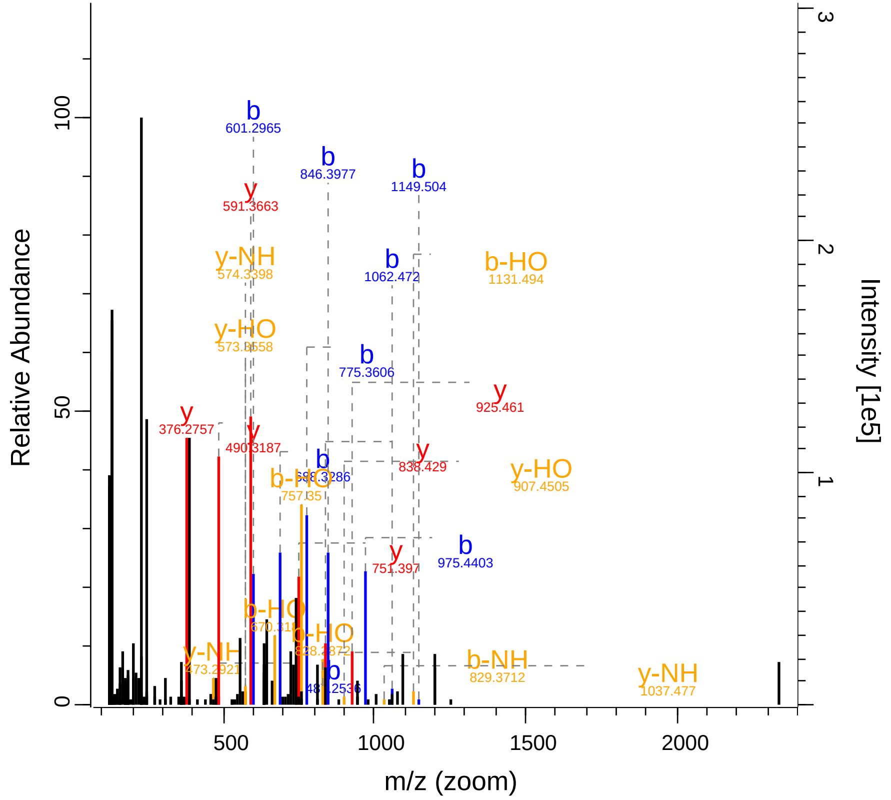

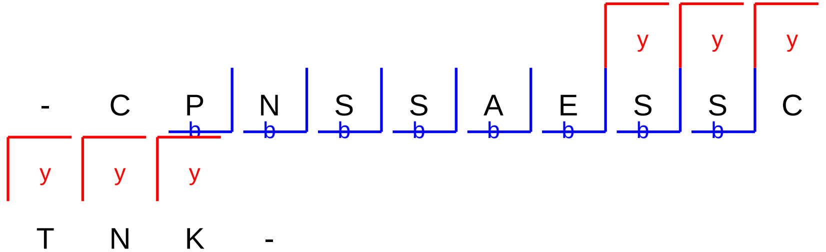

Raw File	Scan	Method	Score	m/z
RO-QC-160121_ZackD_SS_Set_01_Fraction_9	7200	FTMS; HCD	28.62	352.79

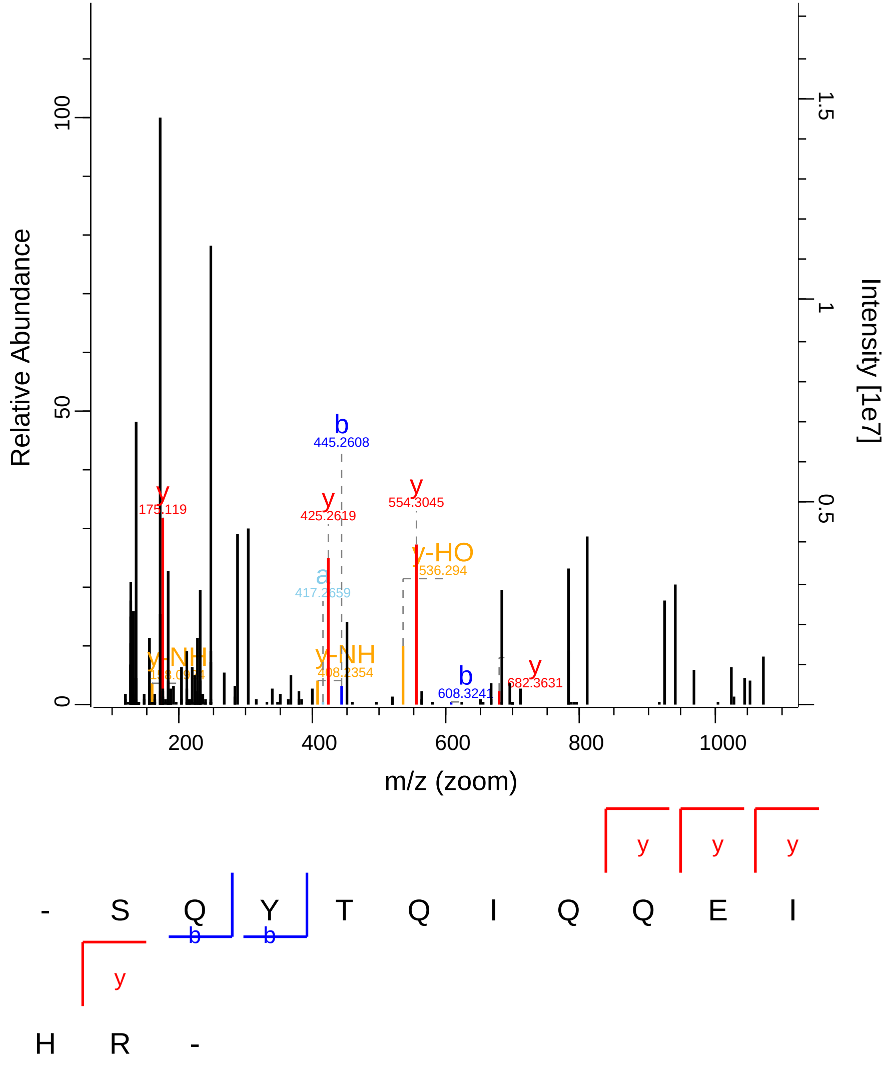

Raw File	Scan	Method	Score	m/z	Gene names
RO-QC-160121_ZackD_SS_Set_01_Fractio	7703	FTMS; HC	202.16	796.94	ZMYM1

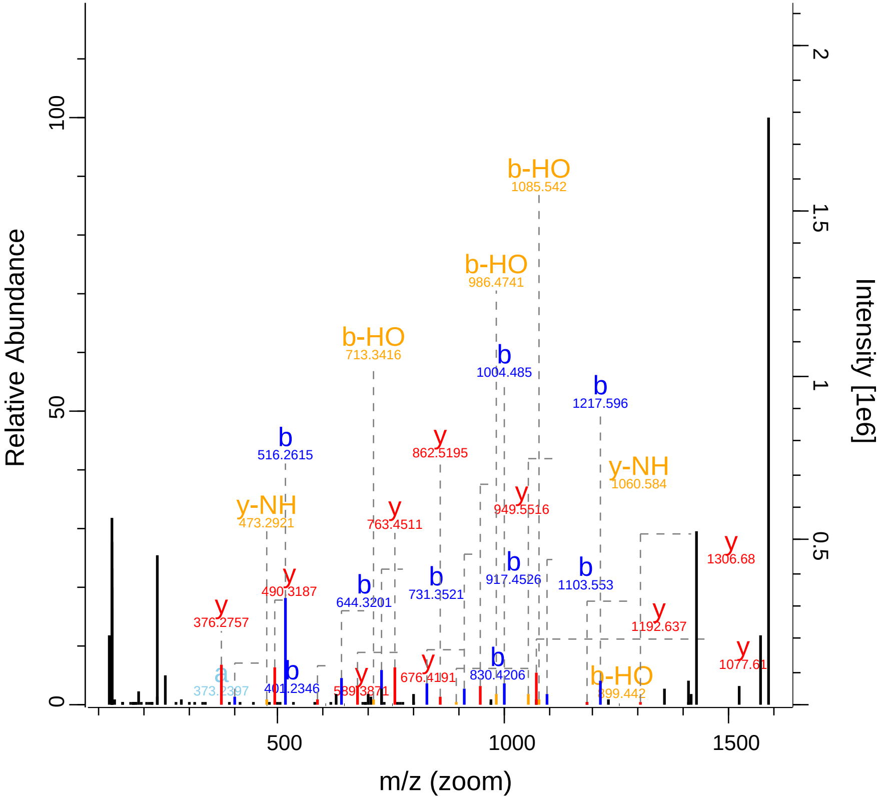

		y	y	y	y	y	y	y	y	y
-	G	N	D	Q	S	V	S	S	V	N
		b	b	b	b	b	b	b	b	b
y										
K	-									

Raw File	Scan	Method	Score	m/z	Gene names
RO-QC-160121_ZackD_SS_Set_01_Fractior	7729	FTMS; HCI	66.43	496.32	SLC35D1

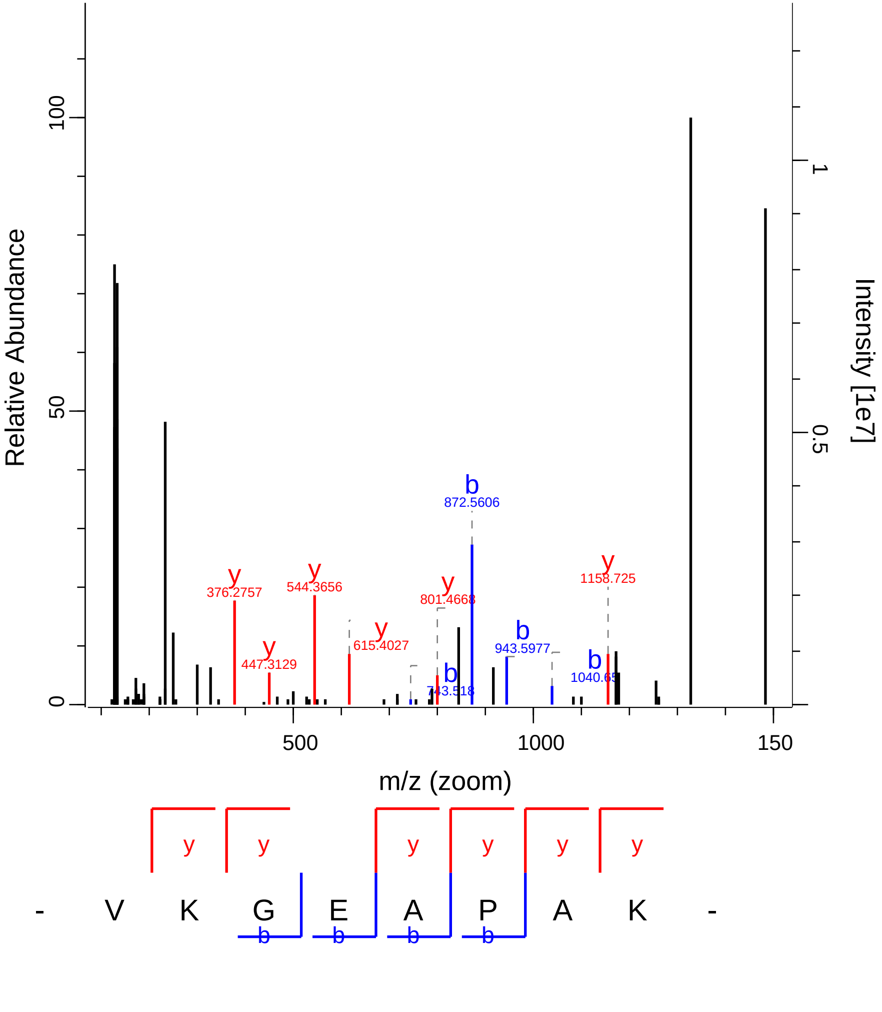

Raw File	Scan	Method	Score	m/z	Gene names
RO-QC-160121_ZackD_SS_Set_01_Fra	9936	FTMS; HC	80.23	596.35	ATP5G1;ATP5G2;ATP5

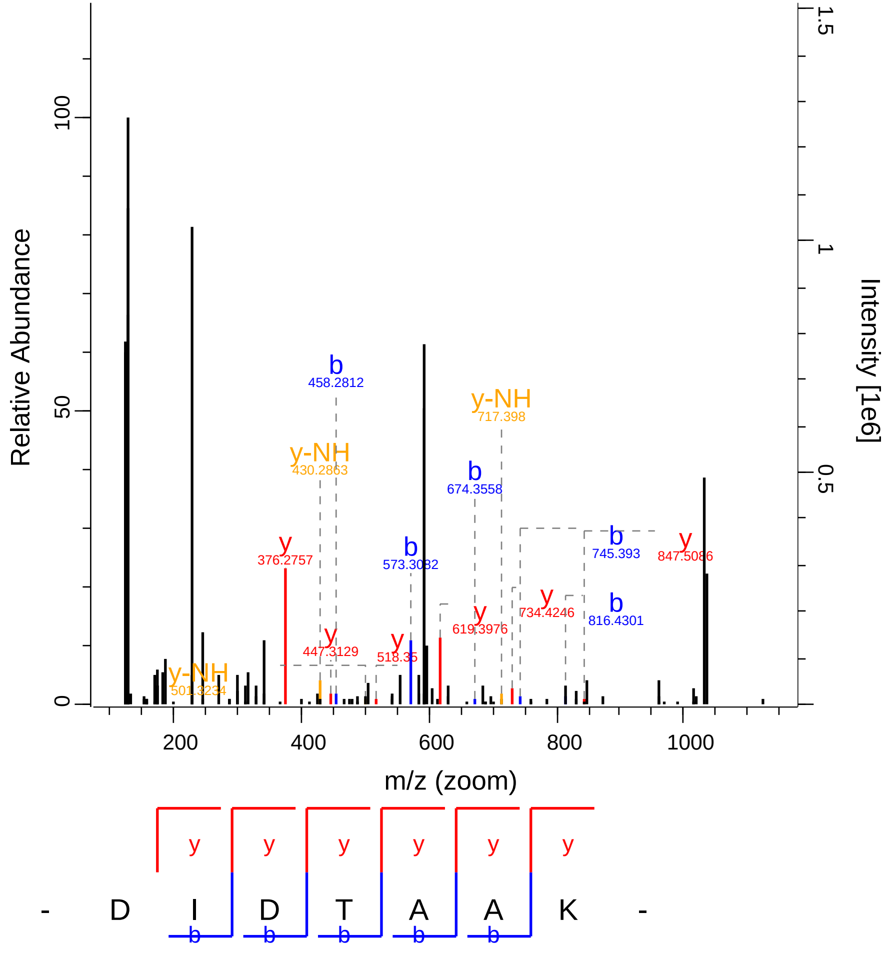

Raw File	Scan	Method	Score	m/z	Gene names
RO-QC-160121_ZackD_SS_Set_01_Fra	10153	FTMS; HC	114.6	596.35	ATP5G1;ATP5G2;ATP

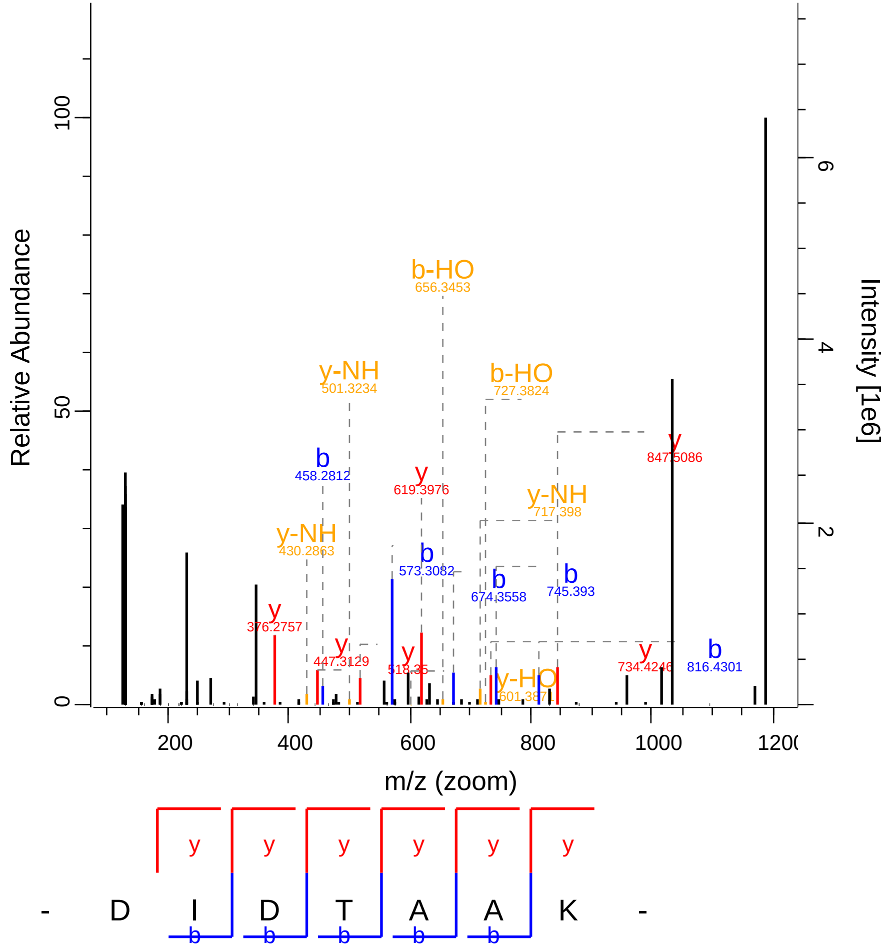

Raw File	Scan	Method	Score	m/z	Gene names
RO-QC-160121_ZackD_SS_Set_01_Fr	10372	FTMS; H(	158.12	596.35	ATP5G1;ATP5G2;ATP

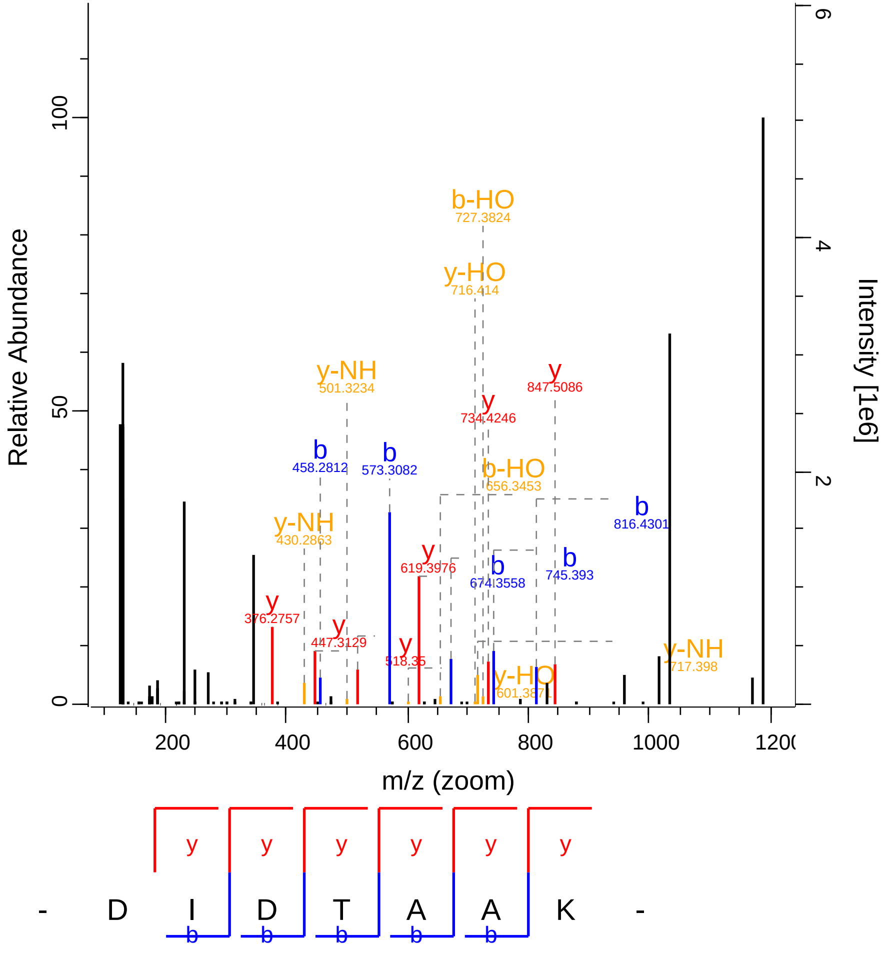

Raw File	Scan	Method	Score	m/z	Gene names
RO-QC-160121_ZackD_SS_Set_01_Fractio	13068	FTMS; HC	117.98	744.89	APH1B

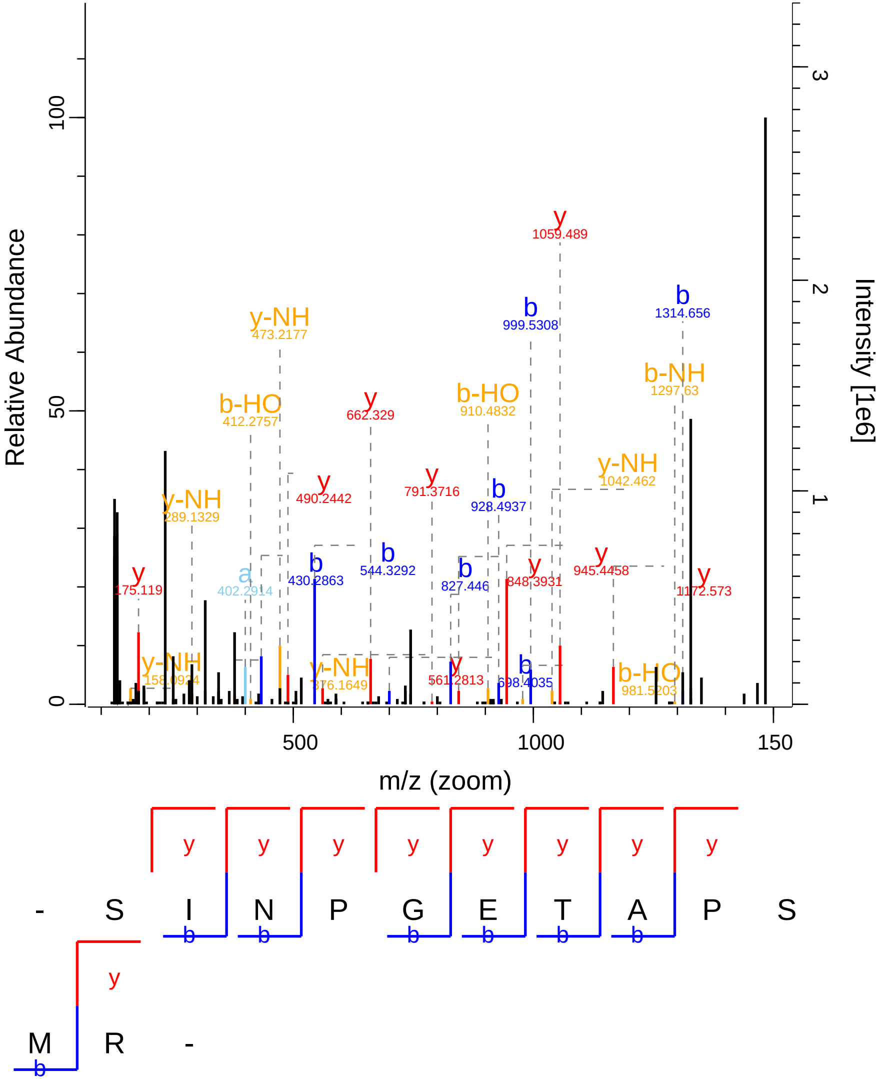

Raw File	Scan	Method	Score	m/z	Gene names
RO-QC-160121_ZackD_SS_Set_01_Fractio	14039	FTMS; HC	133.99	719.89	CYSTM1

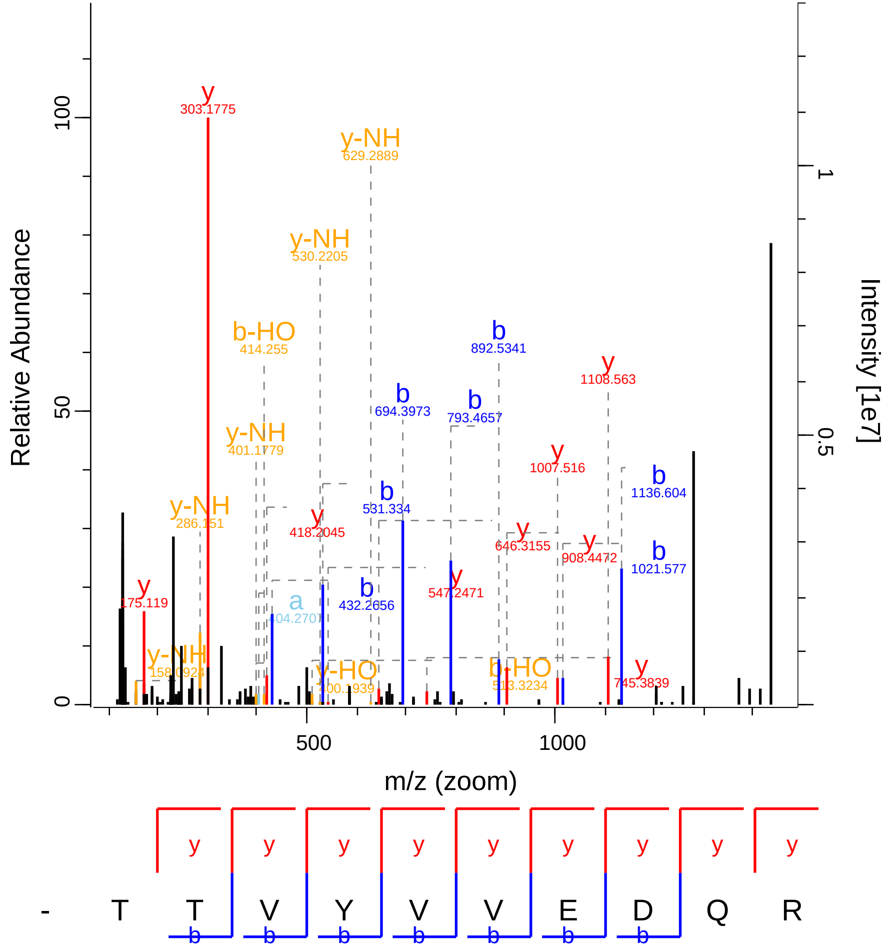

Raw File	Scan	Method	Score	m/z	Gene names
RO-QC-160121_ZackD_SS_Set_01_Fractio	14308	FTMS; HC	170.5	719.89	CYSTM1

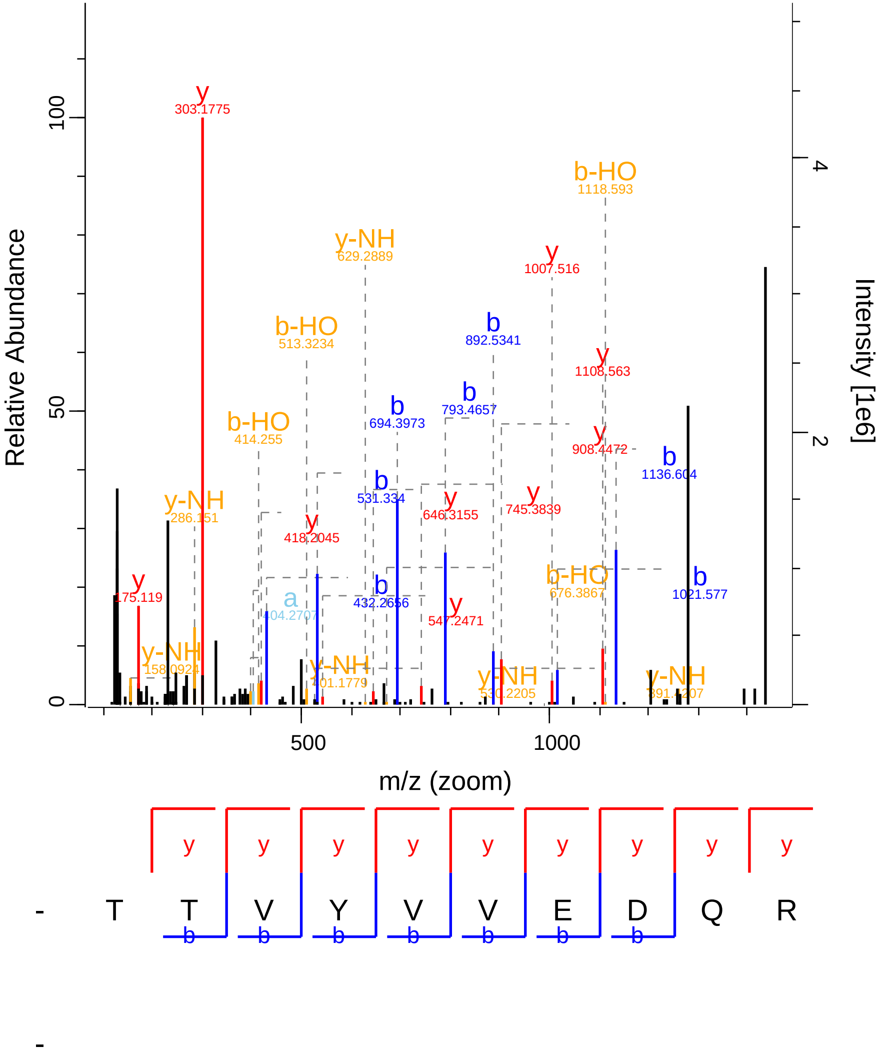

Raw File	Scan	Method	Score	m/z	Gene names
RO-QC-160121_ZackD_SS_Set_01_Fractio	14396	FTMS; HC	89.75	709.89	FBXL19

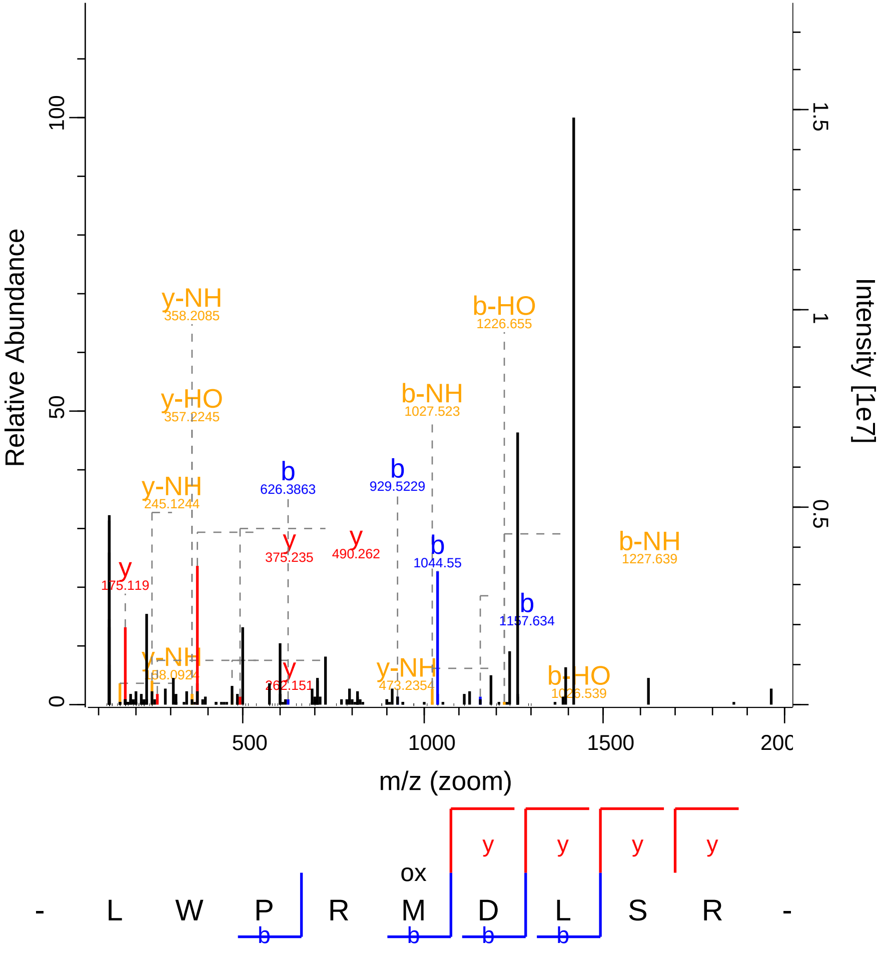

Raw File	Scan	Method	Score	m/z	Gene names
RO-QC-160121_ZackD_SS_Set_01_Fractio	14460	FTMS; HC	126.07	403.48	MED7

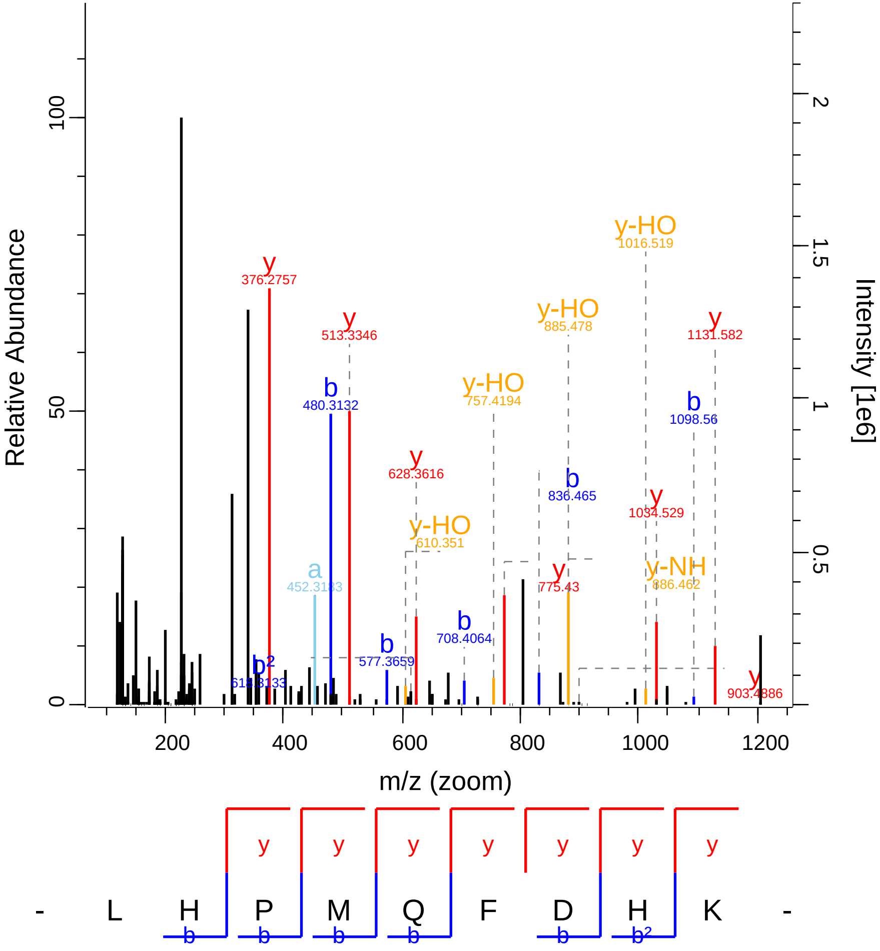

Raw File	Scan	Method	Score	m/z	Gene names
RO-QC-160121_ZackD_SS_Set_01_Fractio	15794	FTMS; HC	45.08	714.91	POLR2F

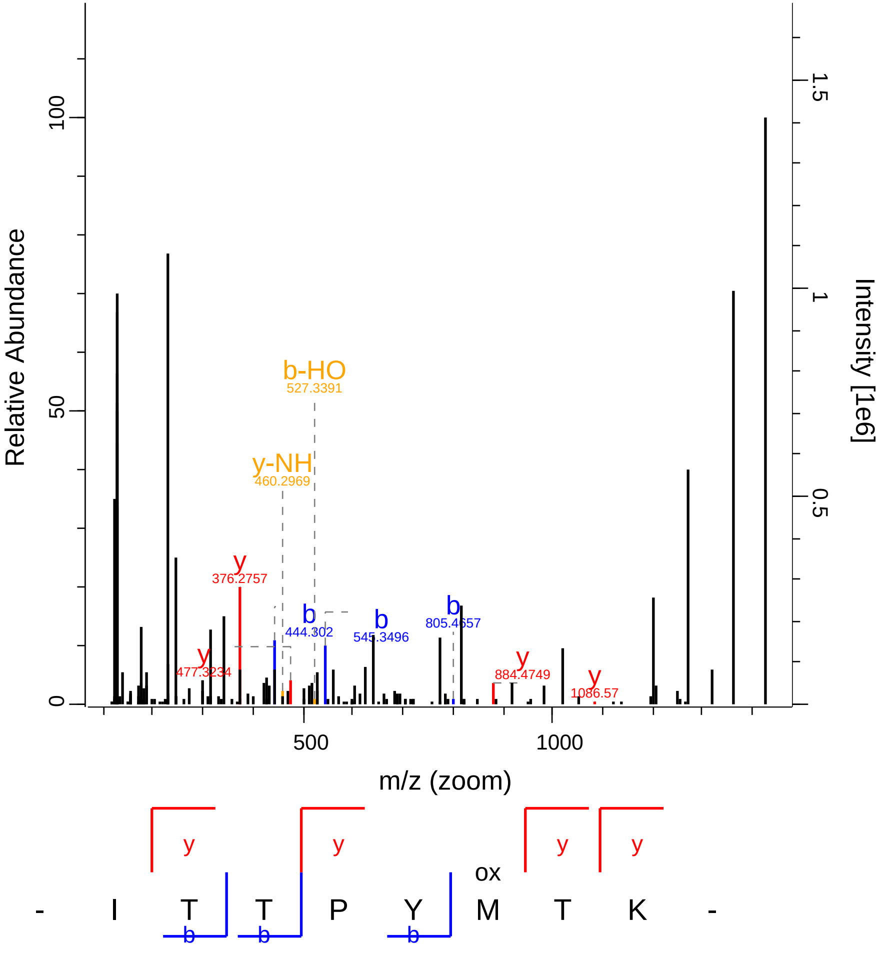

Raw File	Scan	Method	Score	m/z	Gene names
RO-QC-160121_ZackD_SS_Set_01_Fractio	16146	FTMS; HC	136.57	463.31	TMC1

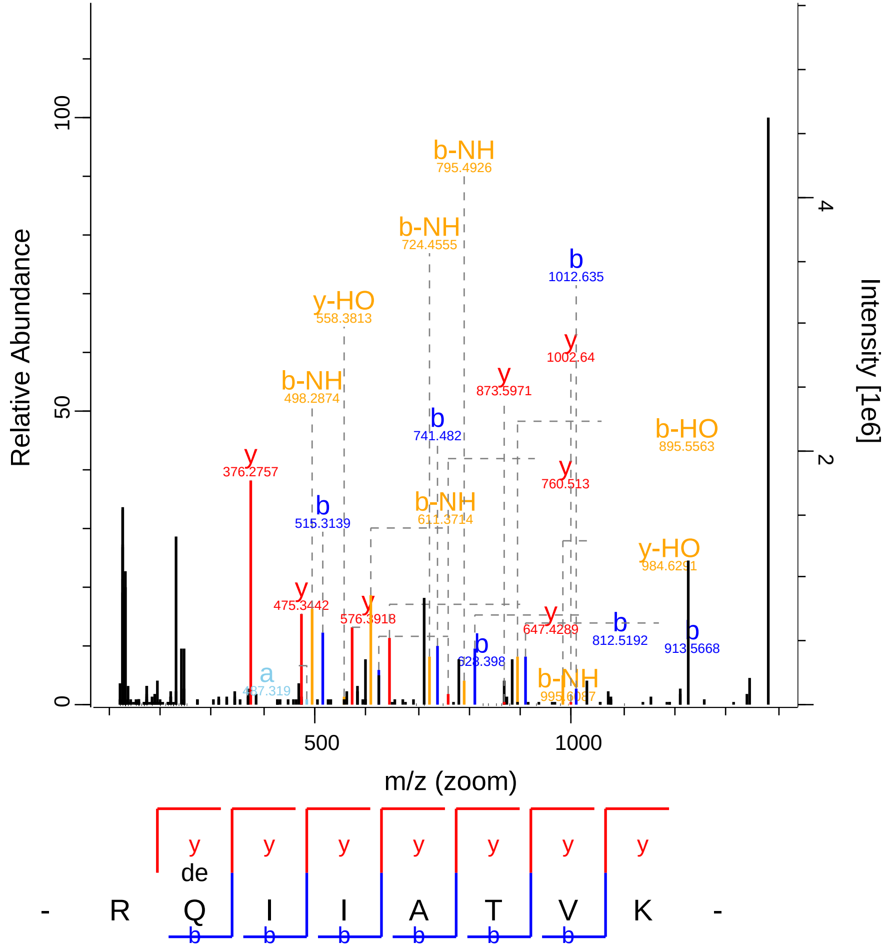

Raw File	Scan	Method	Score	m/z
RO-QC-160121_ZackD_SS_Set_01_Fraction_9	16248	FTMS; HCD	118.06	580.38

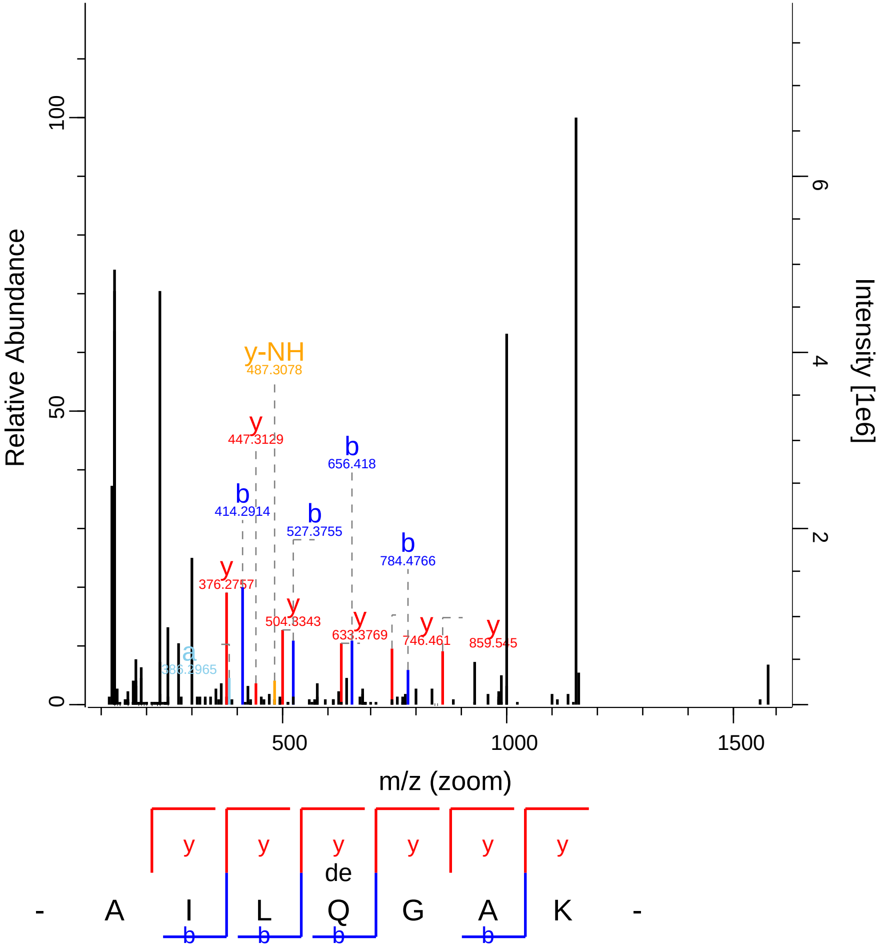

Raw File	Scan	Method	Score	m/z	Gene names
RO-QC-160121_ZackD_SS_Set_01_Fractio	17301	FTMS; HC	113.88	778.72	F2R

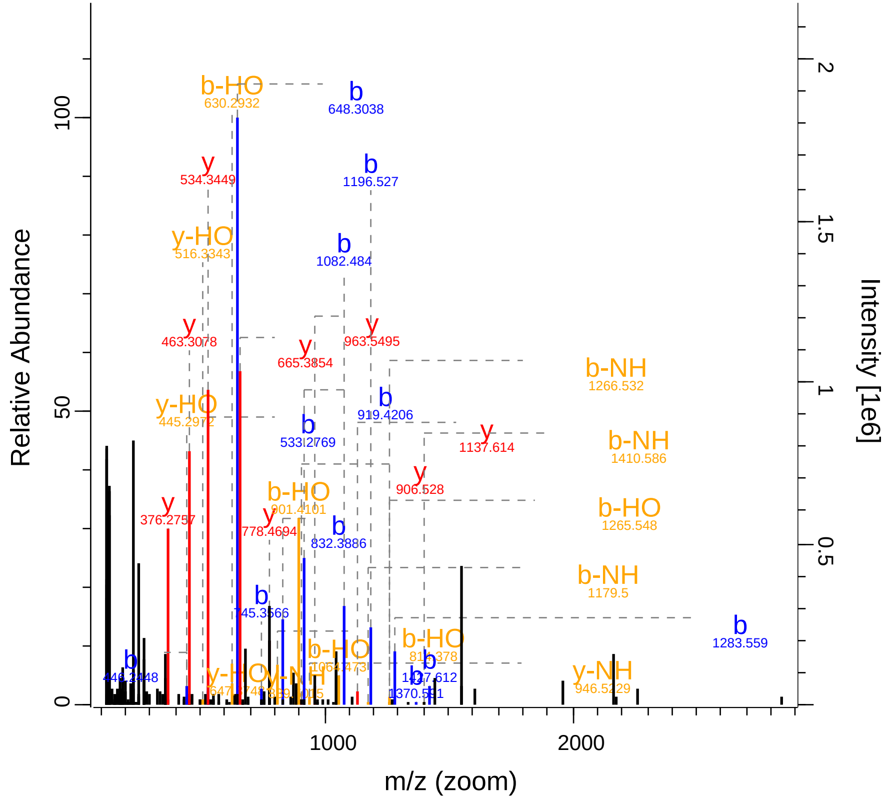

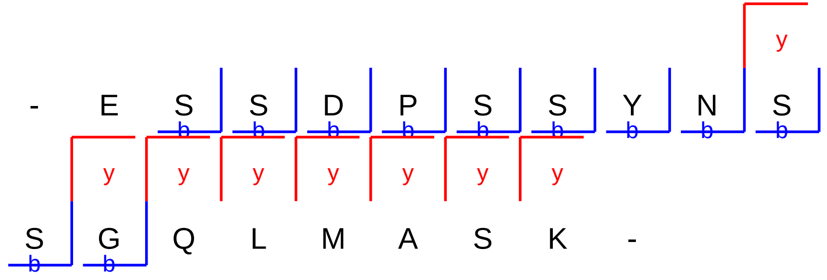

Raw File	Scan	Method	Score	m/z	Gene names
RO-QC-160121_ZackD_SS_Set_01_Fractio	18587	FTMS; HC	50.04	640.86	PROK2

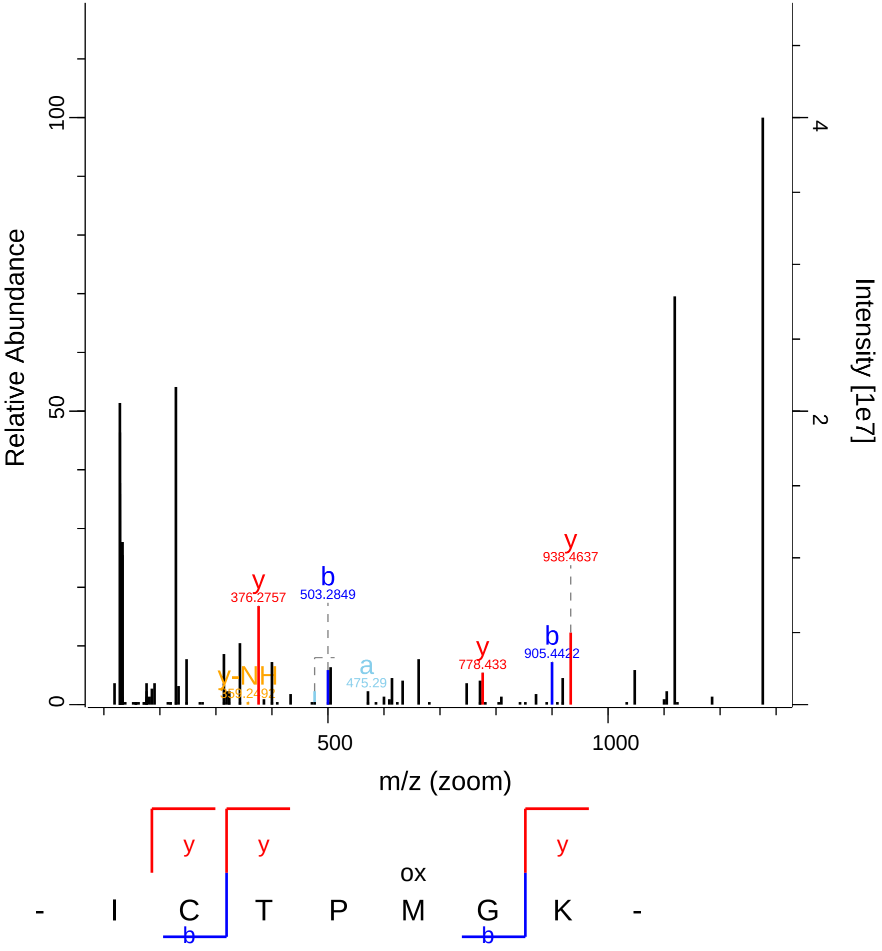

Raw File	Scan	Method	Score	m/z	Gene names
RO-QC-160121_ZackD_SS_Set_01_Fractio	18840	FTMS; HC	50.04	640.86	PROK2

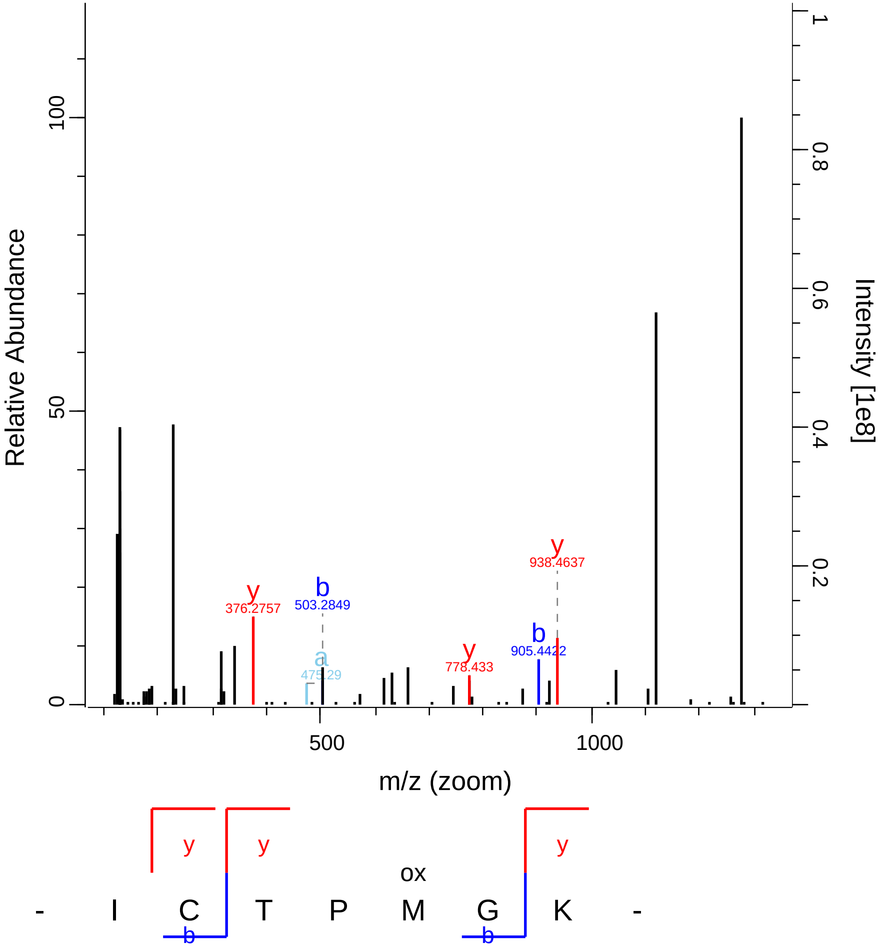

Raw File	Scan	Method	Score	m/z	Gene names
RO-QC-160121_ZackD_SS_Set_01_Fractio	18972	FTMS; HC	130.94	737.93	SLC29A3

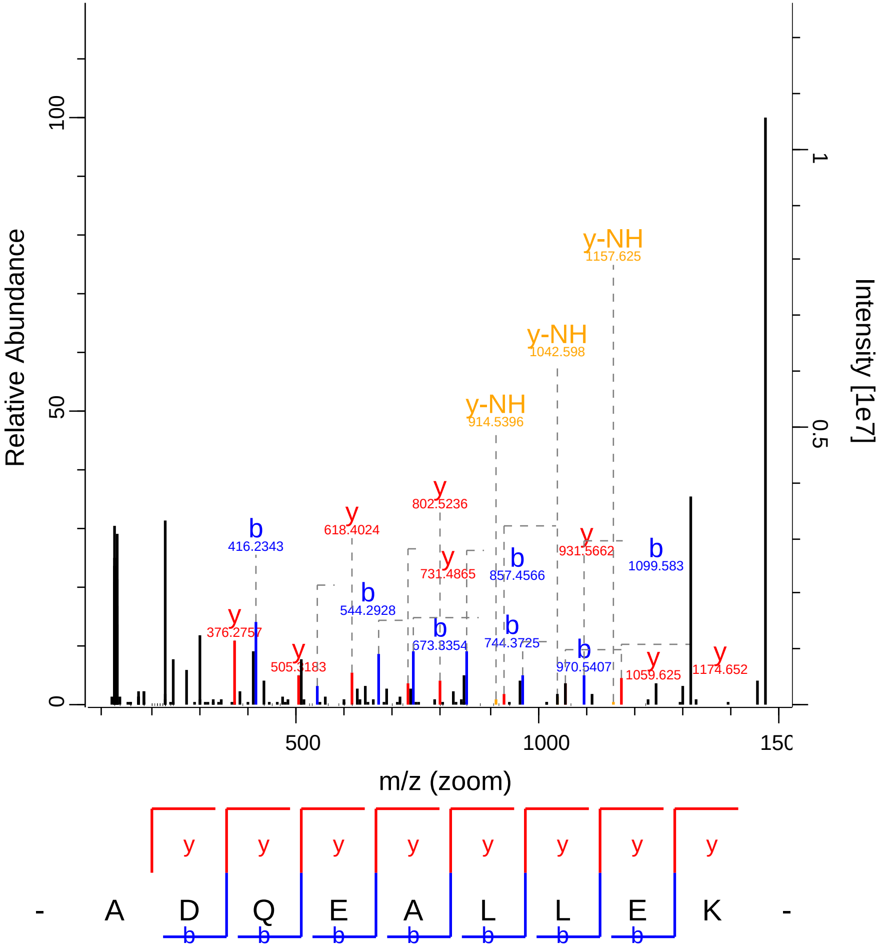

Raw File	Scan	Method	Score	m/z
RO-QC-160121_ZackD_SS_Set_01_Fraction_9	19902	FTMS; HCD	132.48	496.79

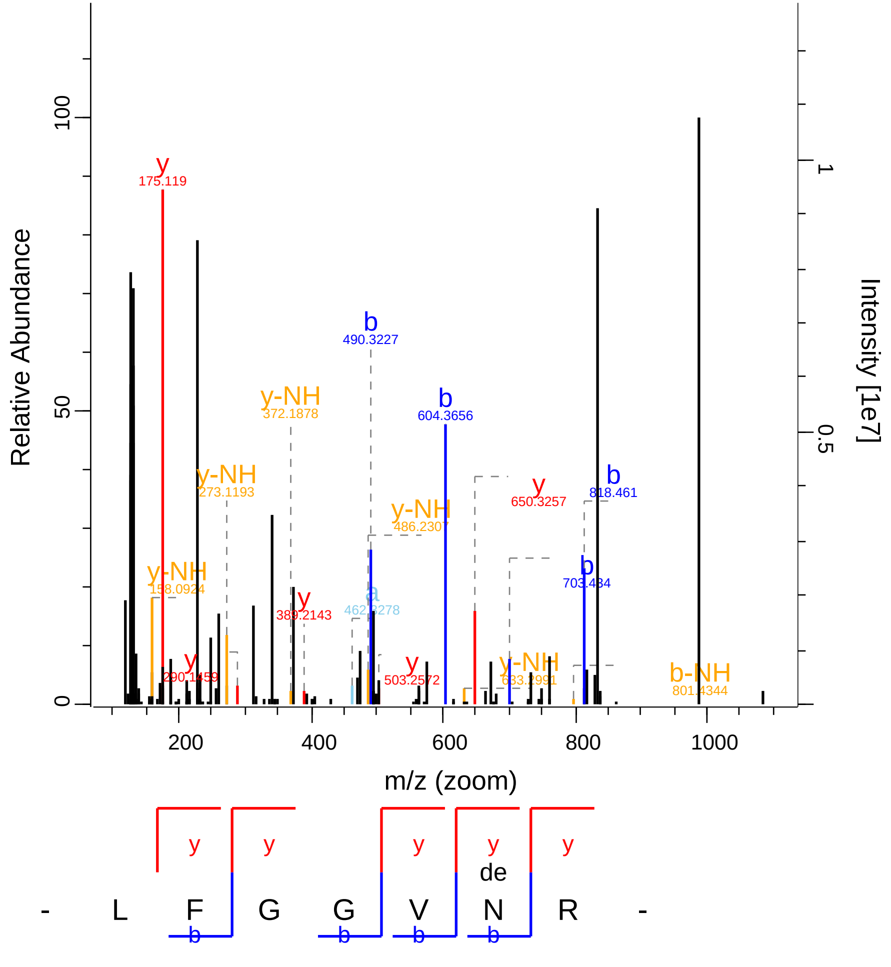

Raw File	Scan	Method	Score	m/z
RO-QC-160121_ZackD_SS_Set_01_Fraction_9	20183	FTMS; HCD	51.93	500.6

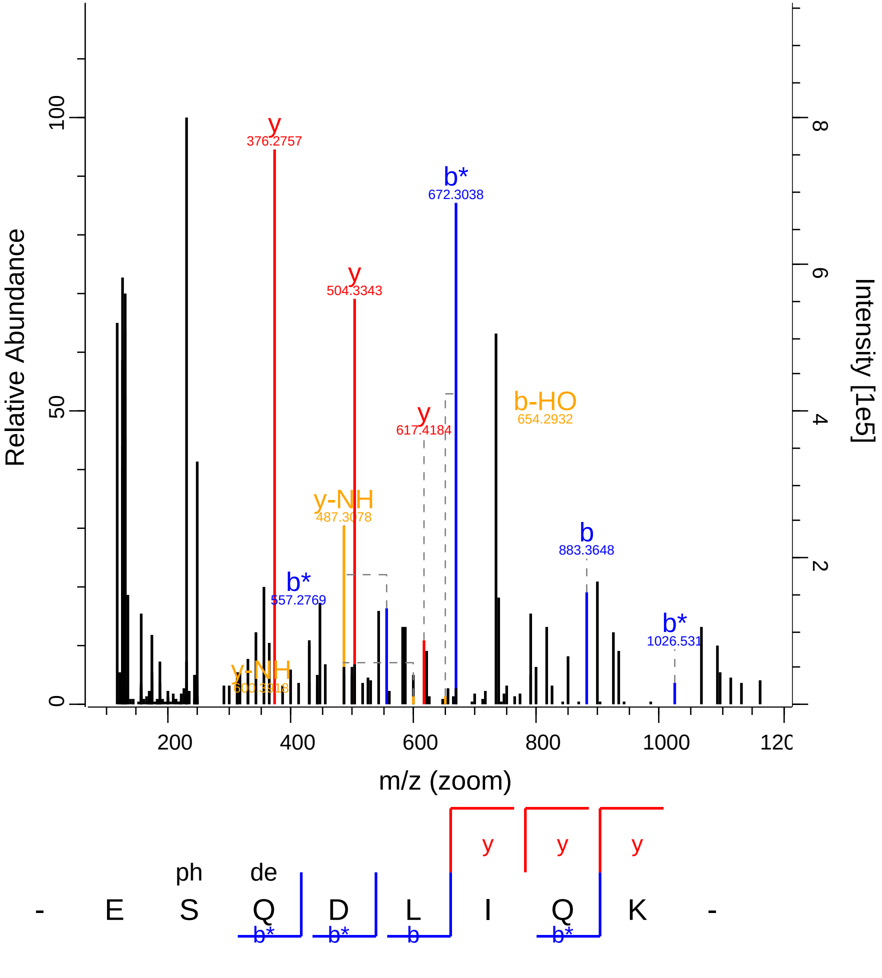

Raw File	Scan	Method	Score	m/z	Gene names
RO-QC-160121_ZackD_SS_Set_01_Fractio	20386	FTMS; HC	67.15	451.51	TMCC2

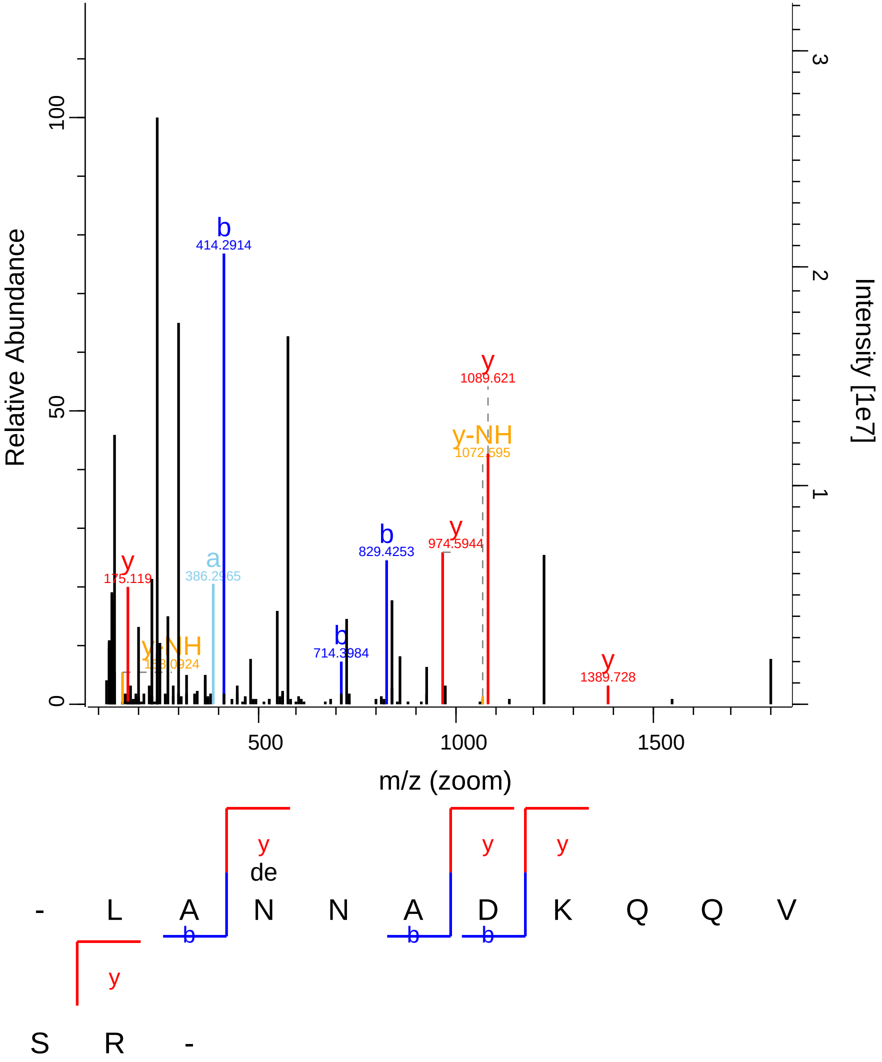

Raw File	Scan	Method	Score	m/z	Gene names
RO-QC-160121_ZackD_SS_Set_01_Fractio	21049	FTMS; HC	139.26	952.16	C6orf106

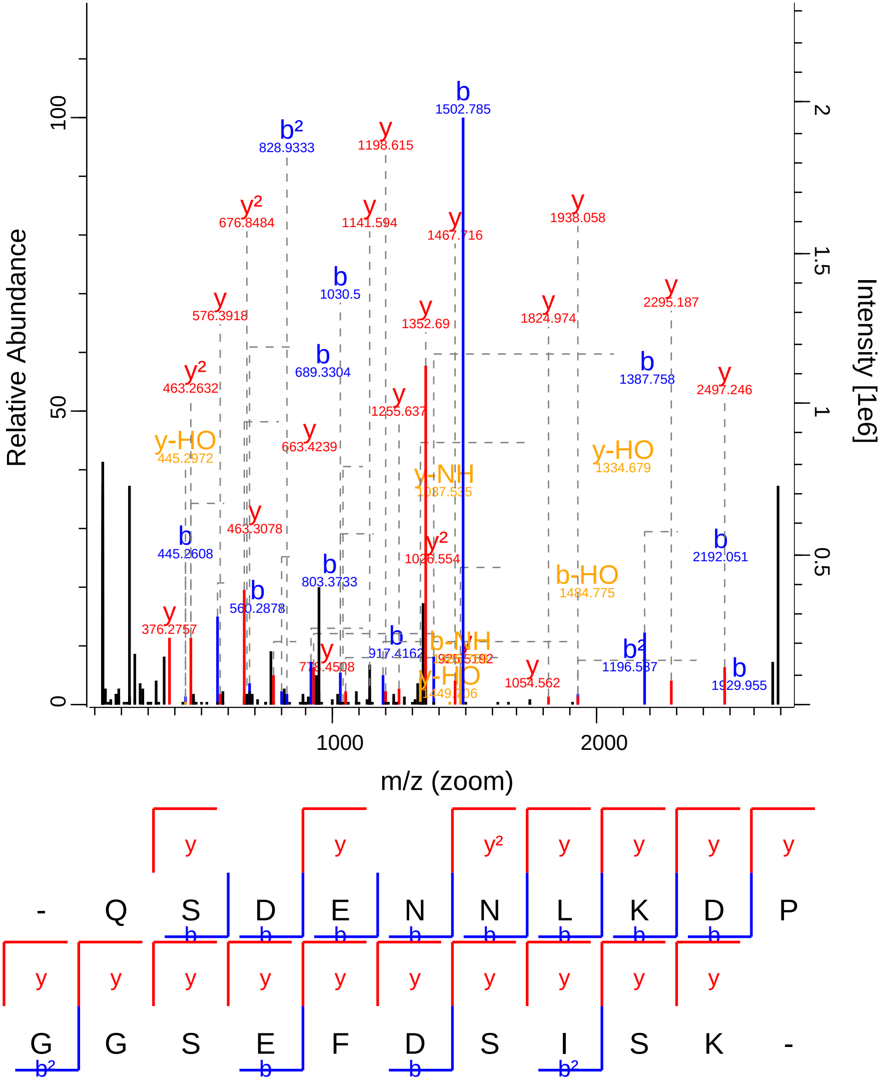

Raw File	Scan	Method	Score	m/z	Gene names
RO-QC-160121_ZackD_SS_Set_01_Fractio	21591	FTMS; HC	81.87	516.65	FAM53C

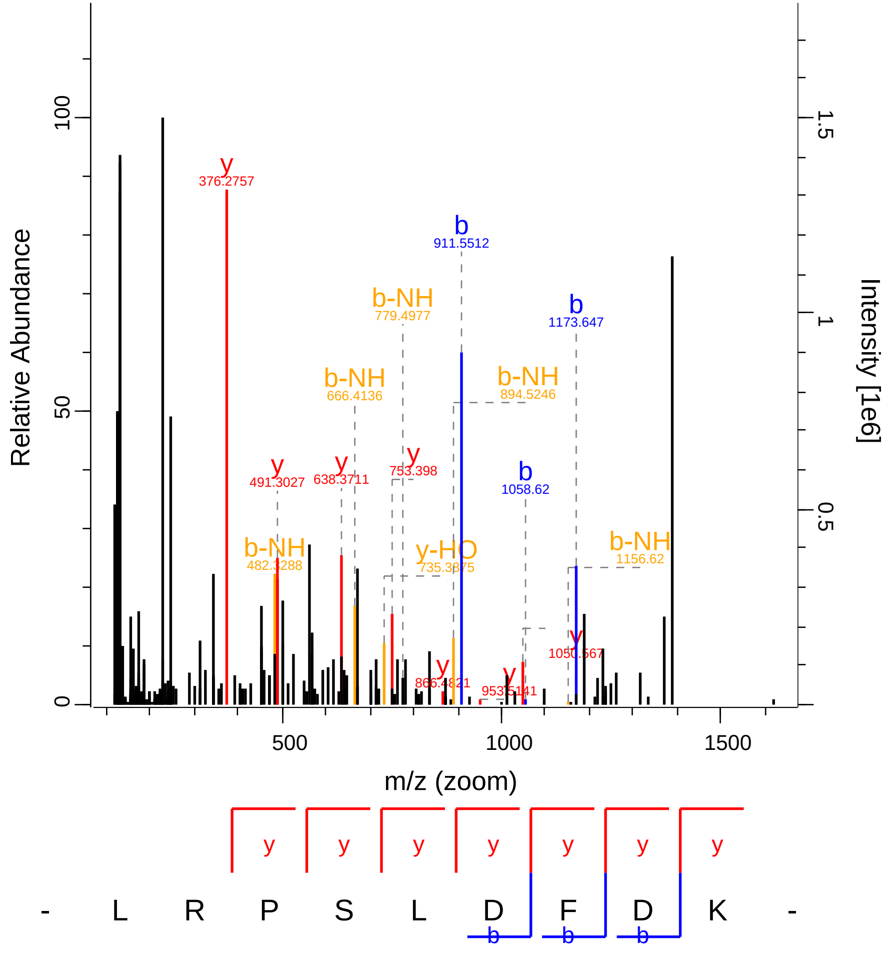

Raw File	Scan	Method	Score	m/z	Gene names
RO-QC-160121_ZackD_SS_Set_01_Fractio	22593	FTMS; HC	43.08	498.54	FLG2

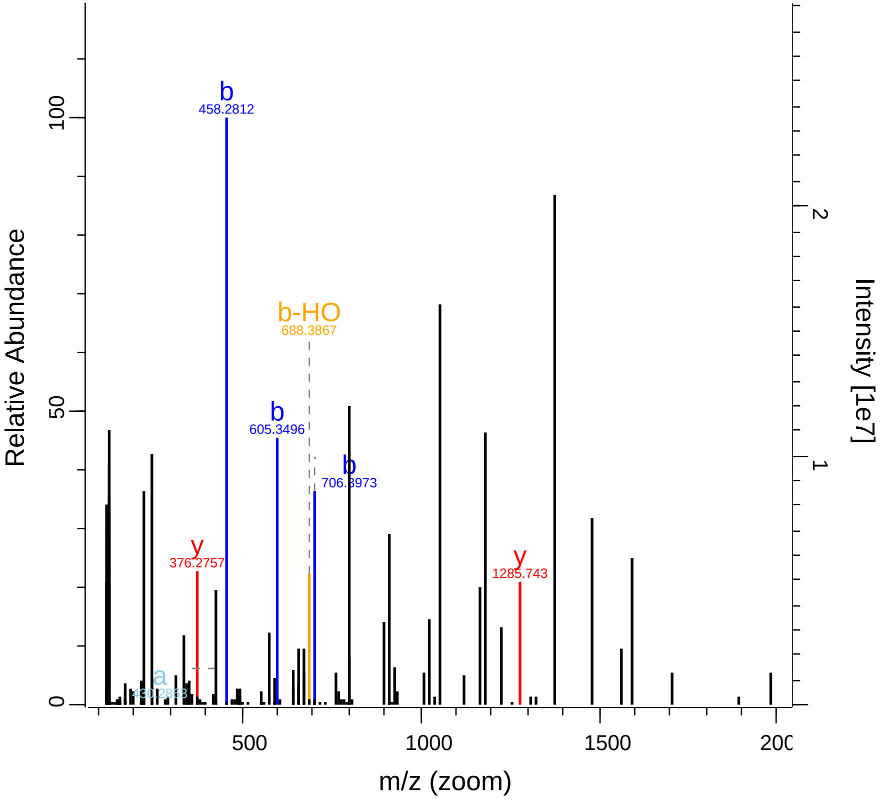

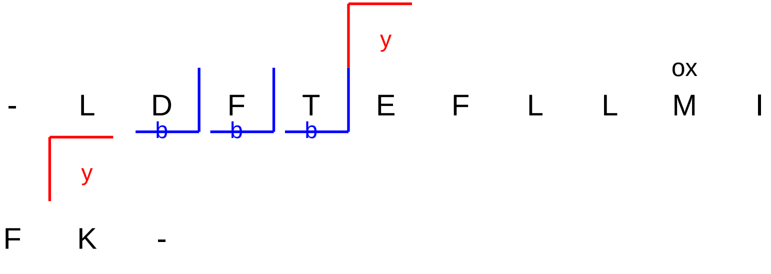

Raw File	Scan	Method	Score	m/z	Gene names
RO-QC-160121_ZackD_SS_Set_01_Fractio	22764	FTMS; HC	65.88	751.42	EMC6

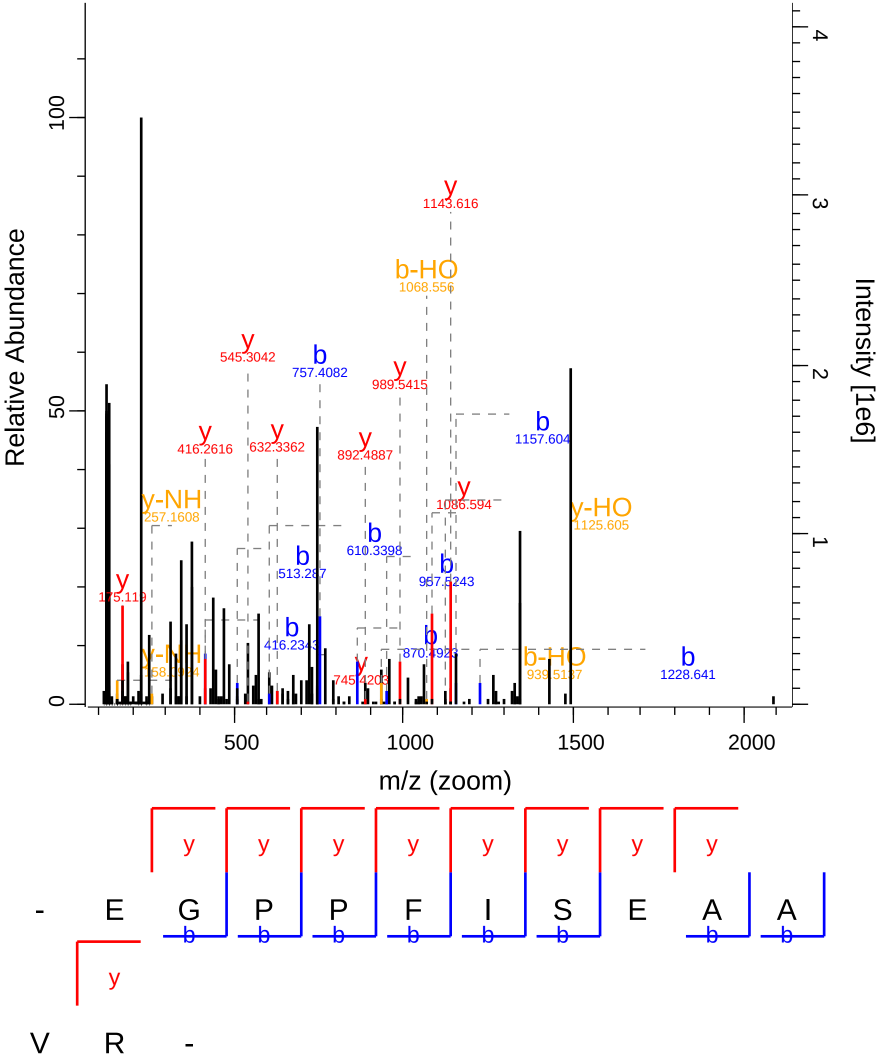

Raw File	Scan	Method	Score	m/z
RO-QC-160121_ZackD_SS_Set_01_Fraction_9	23174	FTMS; HCD	47.07	410.6

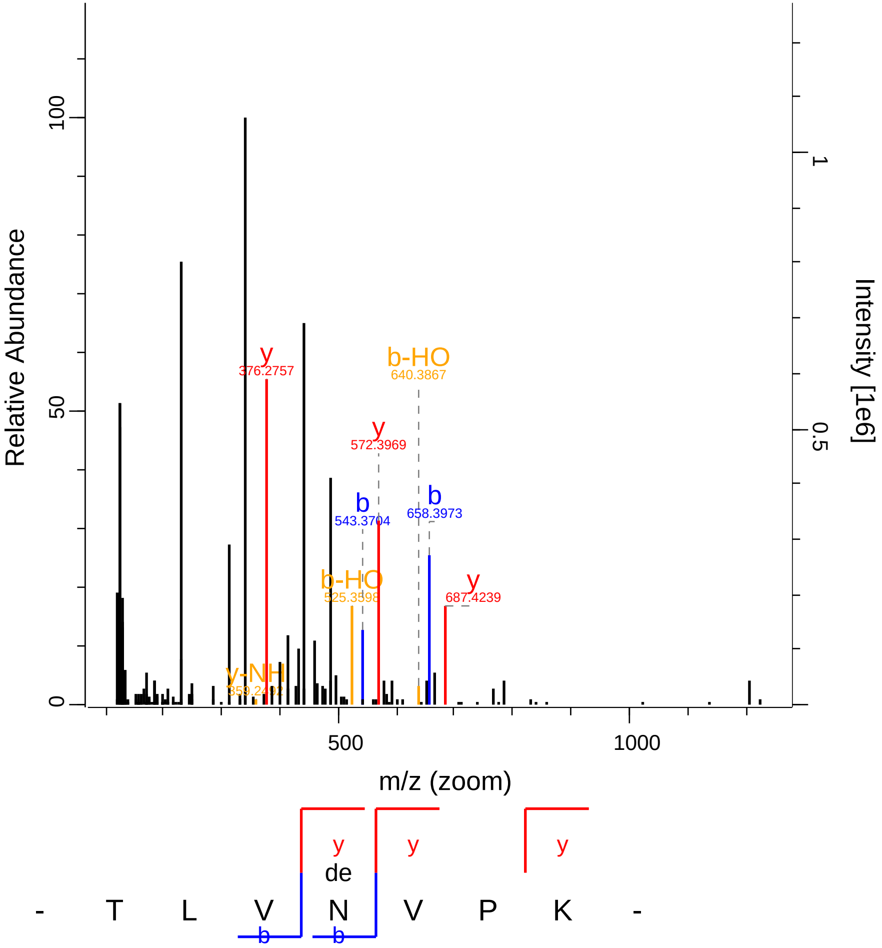

Raw File	Scan	Method	Score	m/z	Gene names
RO-QC-160121_ZackD_SS_Set_01_Fractic	23306	FTMS; HC	54.78	1219.65	CELA2A

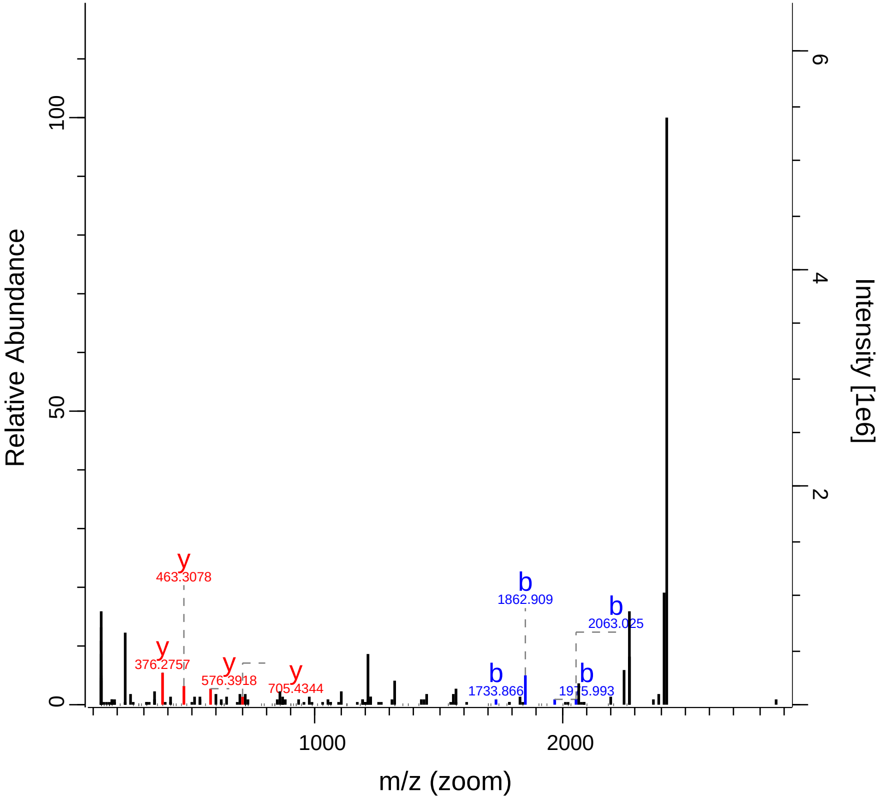

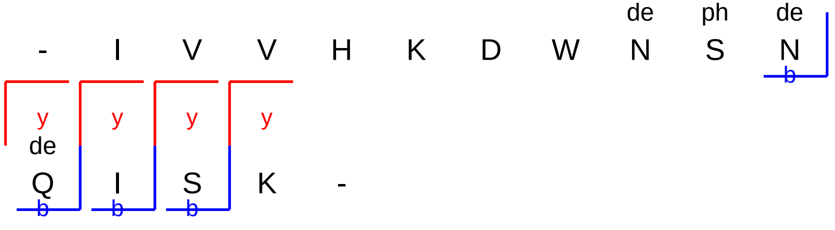

Raw File	Scan	Method	Score	m/z	Gene names
RO-QC-160121_ZackD_SS_Set_01_Fractio	23321	FTMS; HC	69.82	829.49	RTTN

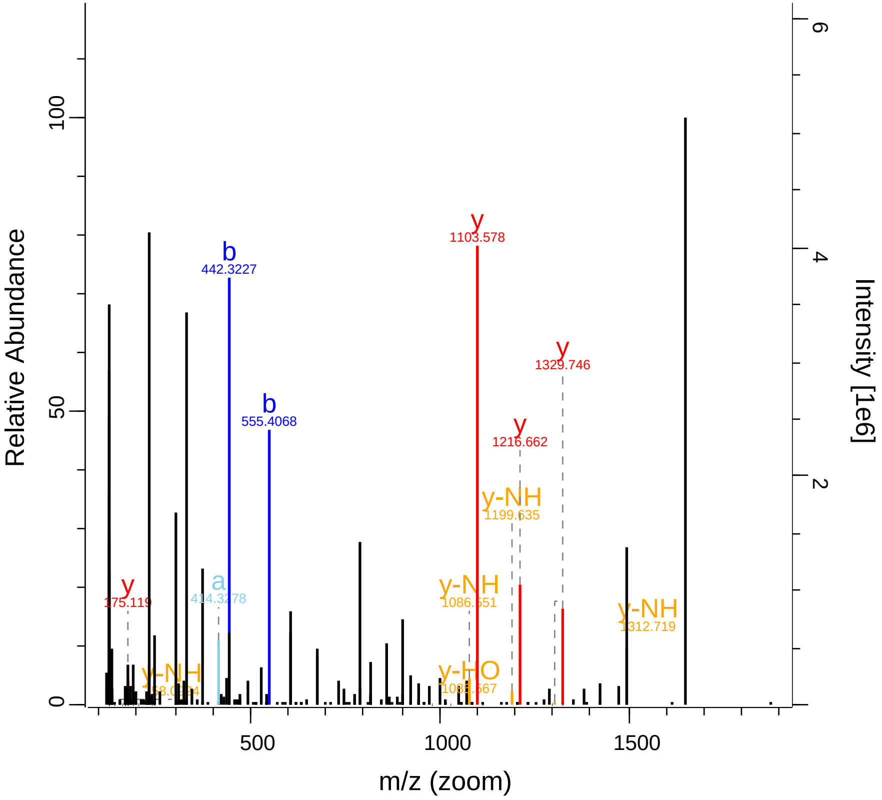

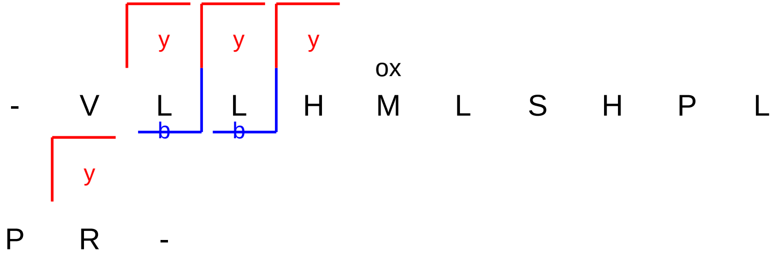

Raw File	Scan	Method	Score	m/z	Gene names
RO-QC-160121_ZackD_SS_Set_01_Fractic	24192	FTMS; HC	53.25	1460.32	NPAT

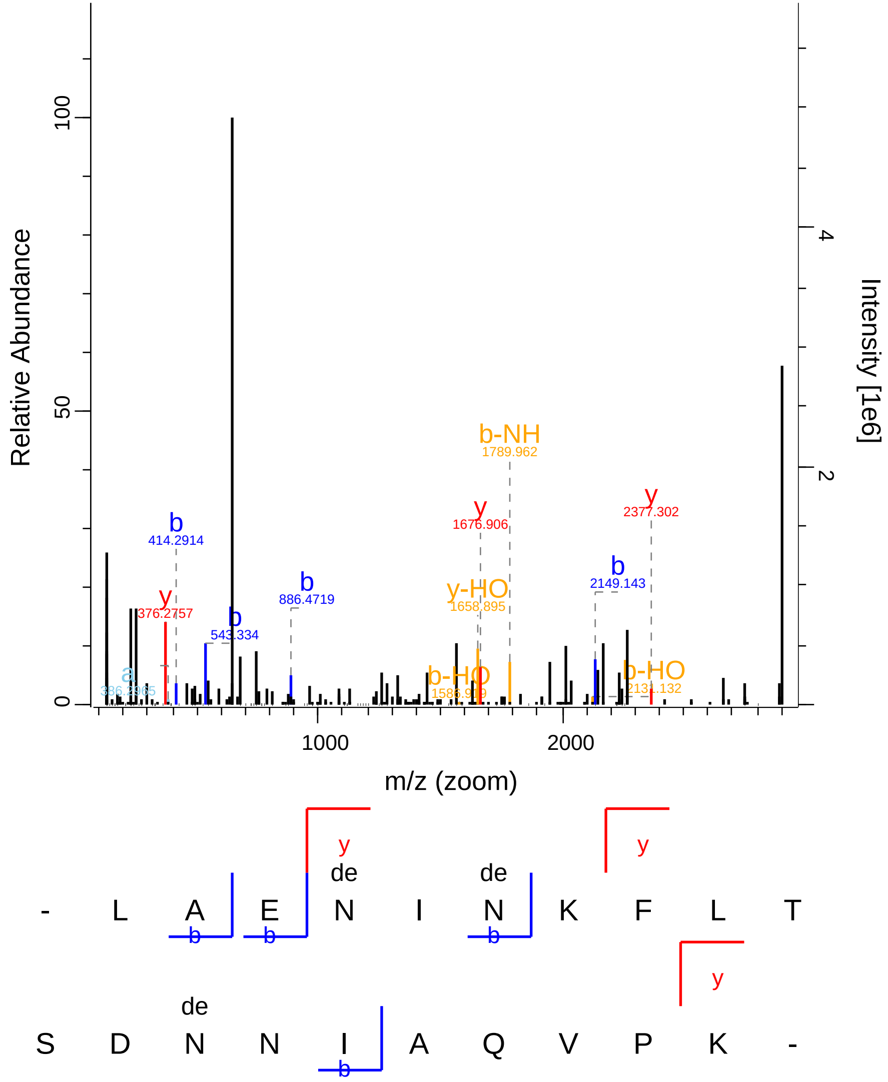

Raw File	Scan	Method	Score	m/z
RO-QC-160121_ZackD_SS_Set_01_Fraction_9	24527	FTMS; HCD	66.68	428.59

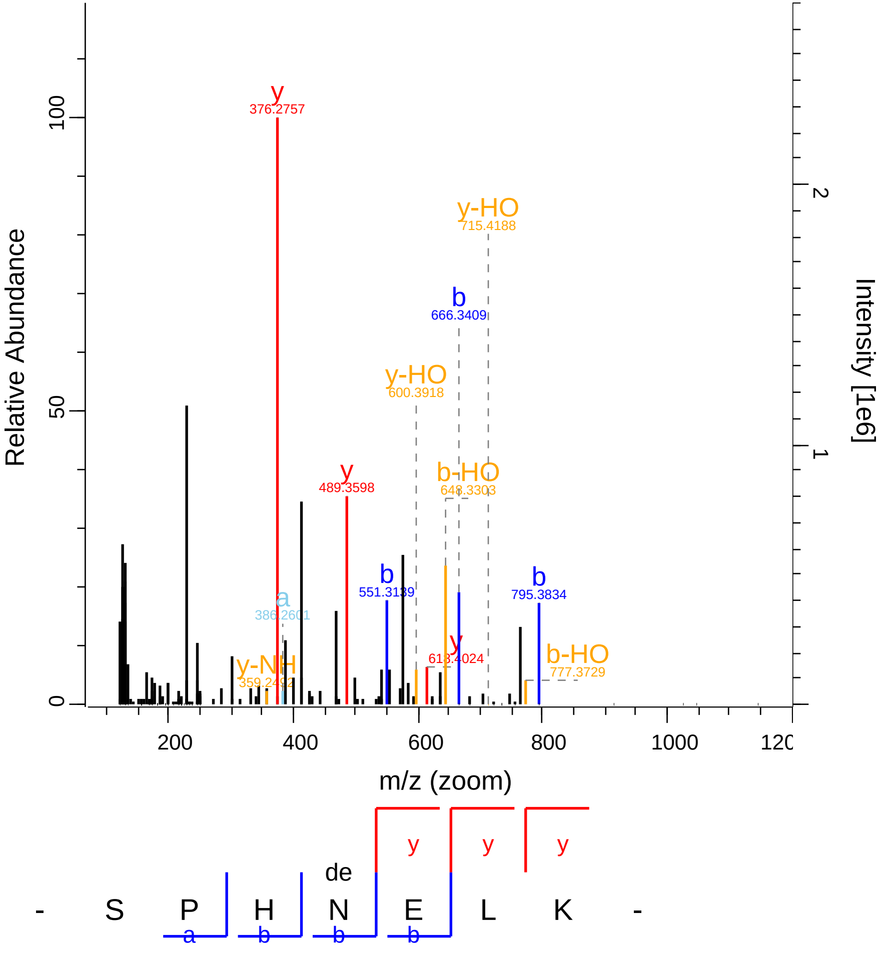

Raw File	Scan	Method	Score	m/z	Gene names
RO-QC-160121_ZackD_SS_Set_01_Fractio	25444	FTMS; HC	121.05	649.89	EBP

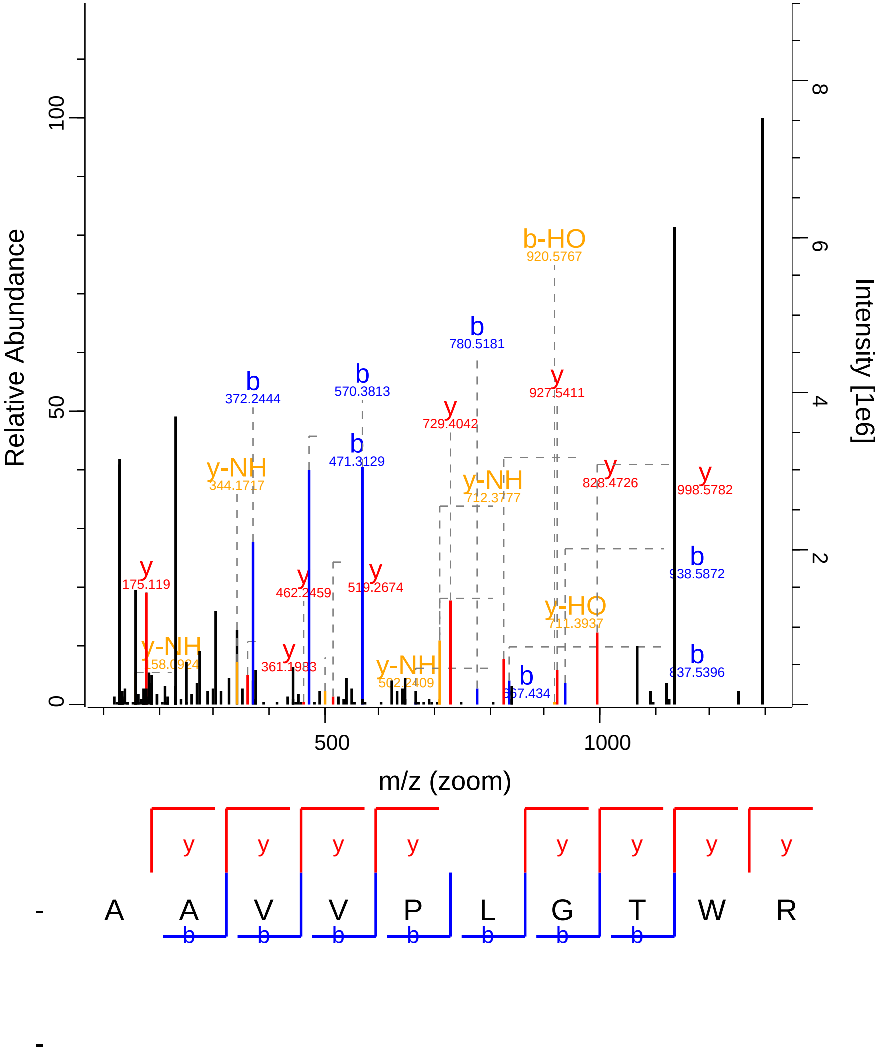

Raw File	Scan	Method	Score	m/z	Gene names
RO-QC-160121_ZackD_SS_Set_01_Fractio	25446	FTMS; HC	93.11	984.04	CLEC4M

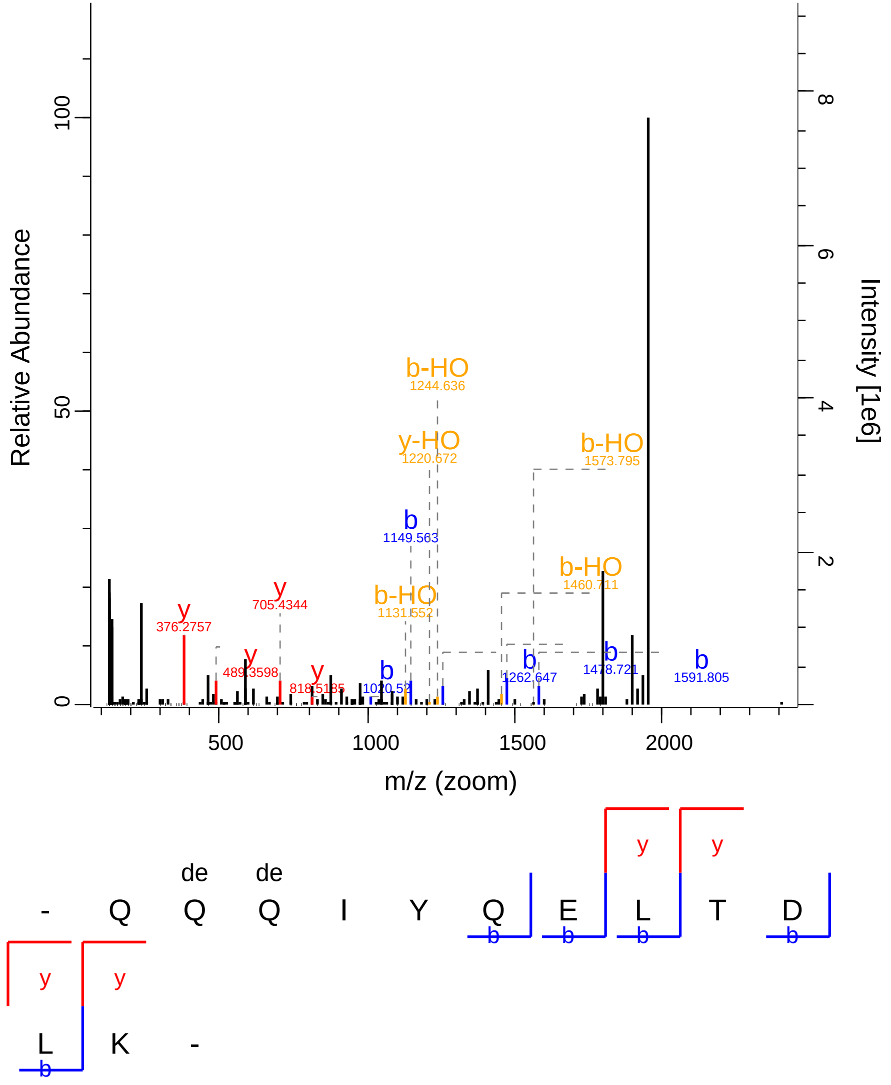

Raw File	Scan	Method	Score	m/z	Gene names
RO-QC-160121_ZackD_SS_Set_01_Fractio	26615	FTMS; HC	86.54	892.82	CASP6

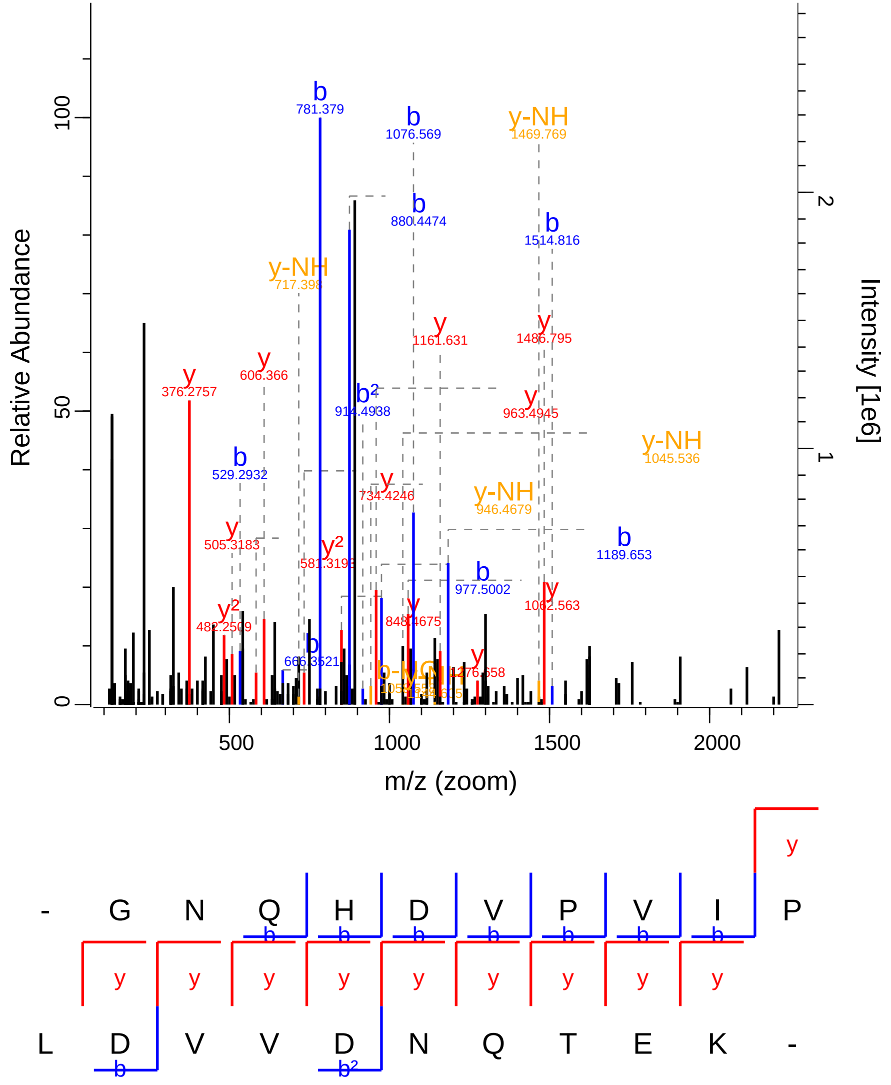

Raw File	Scan	Method	Score	m/z	Gene names
RO-QC-160121_ZackD_SS_Set_01_Fractic	26666	FTMS; HC	48.64	1237.69	LRRC66

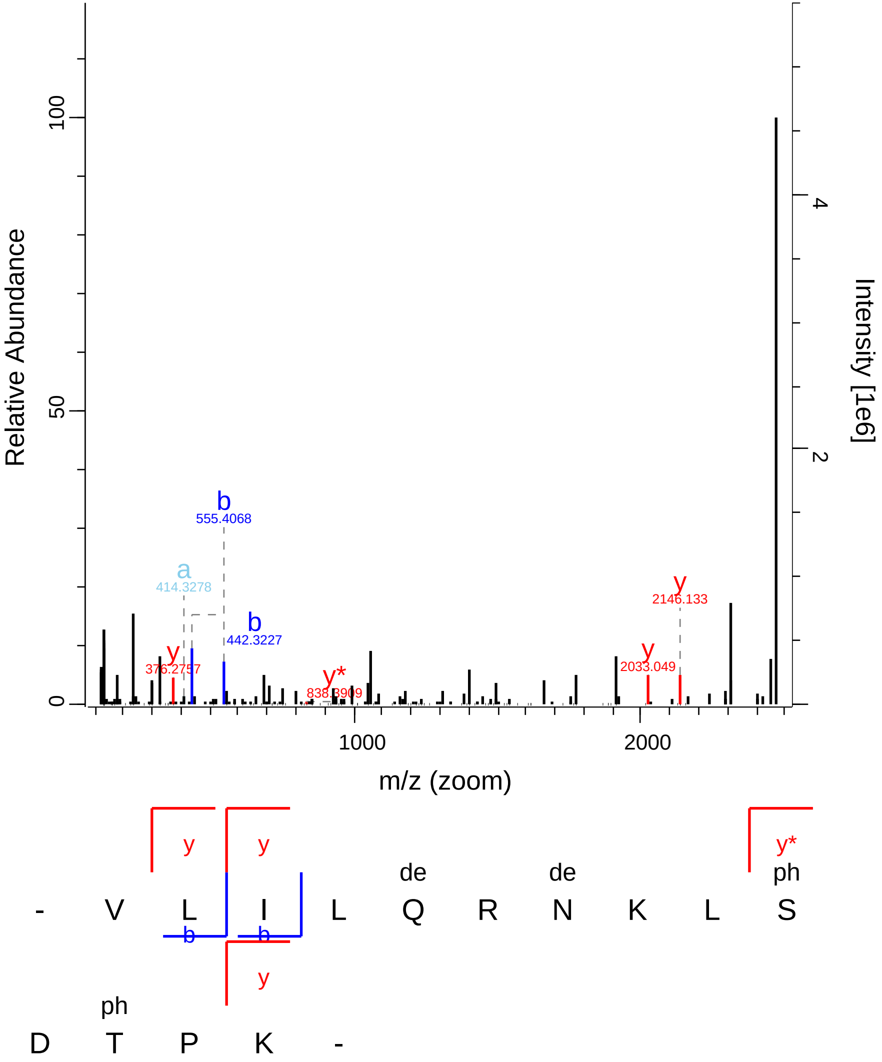

Raw File	Scan	Method	Score	m/z
RO-QC-160121_ZackD_SS_Set_01_Fraction_9	27129	FTMS; HCD	137.77	656.93

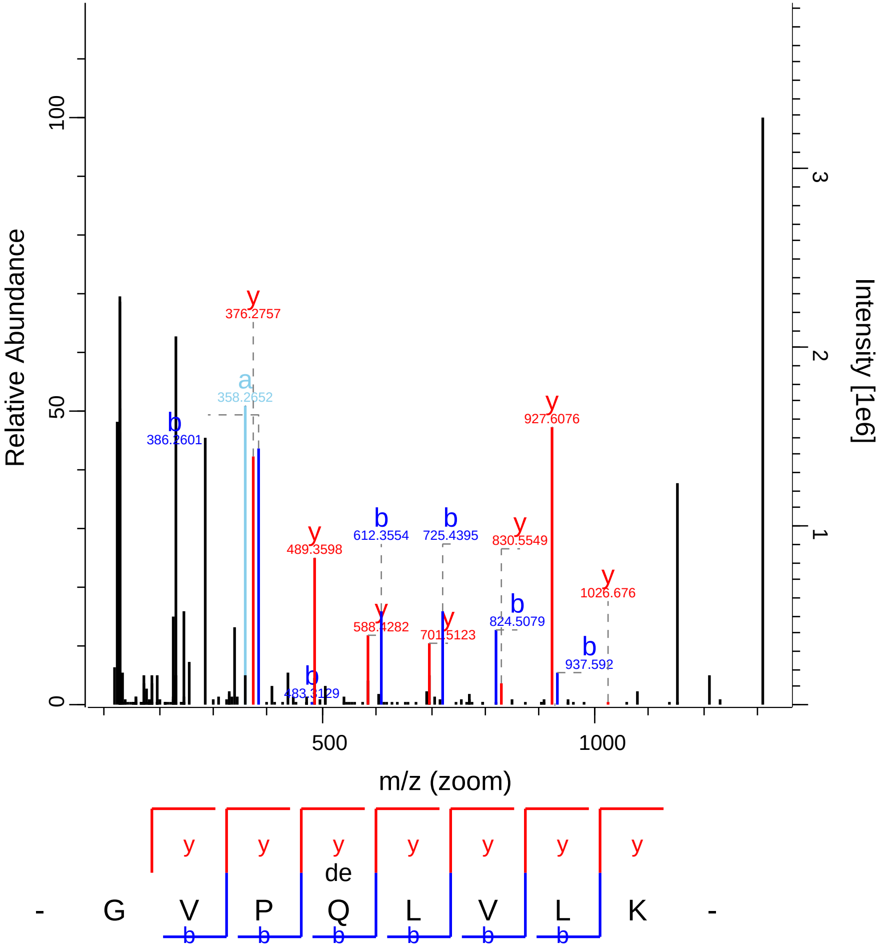

Raw File	Scan	Method	Score	m/z	Gene names
RO-QC-160121_ZackD_SS_Set_01_Fractic	27234	FTMS; HC	46.61	1237.69	LRRC66

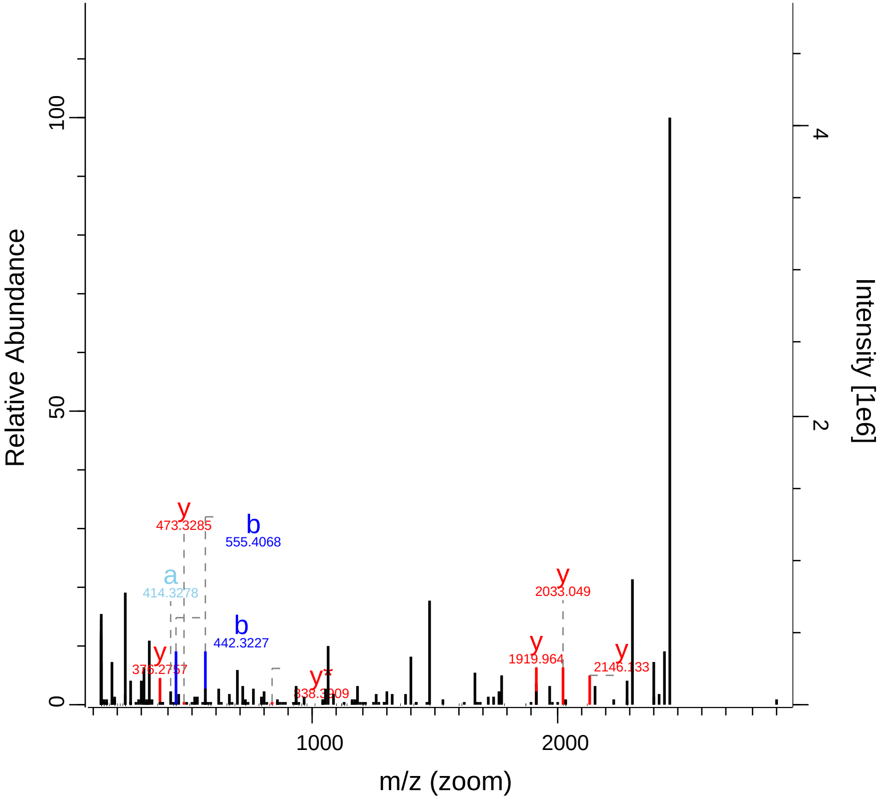

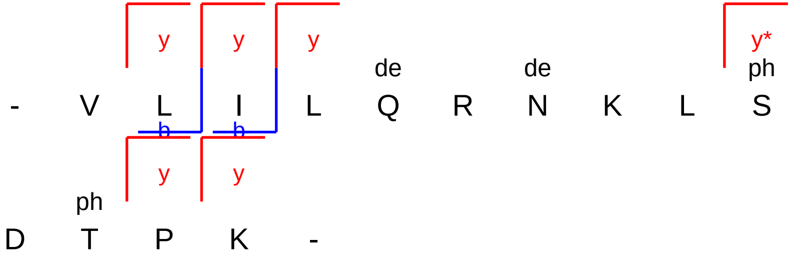

Raw File	Scan	Method	Score	m/z	Gene names
RO-QC-160121_ZackD_SS_Set_01_Fractio	27772	FTMS; HC	110.26	789.1	ATP6V0C

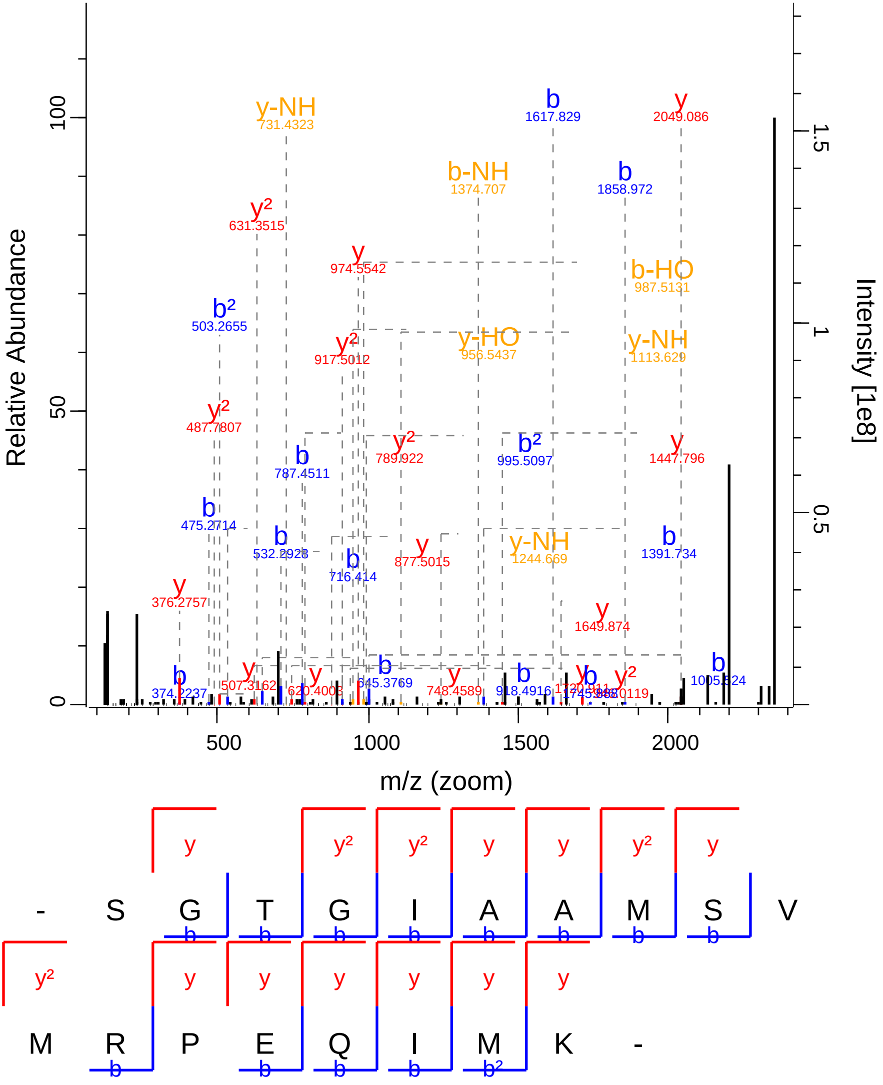

Raw File	Scan	Method	Score	m/z	Gene names
RO-QC-160121_ZackD_SS_Set_01_Fractic	27857	FTMS; HC	194.23	1183.15	ATP6V0C

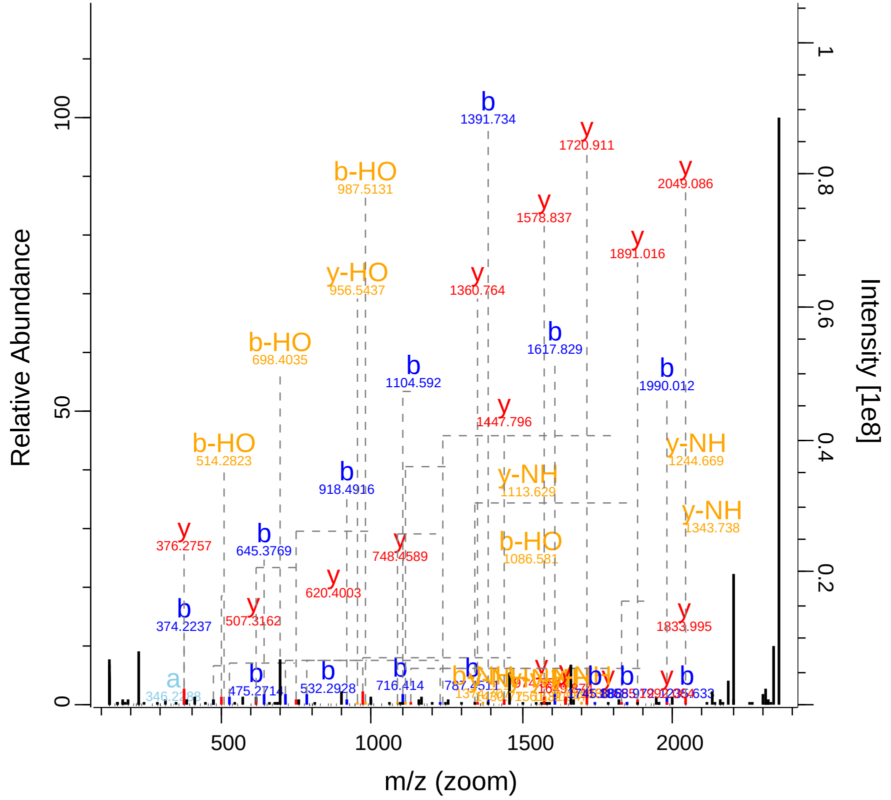

- S G T G I A A M S V

M R P E Q I M K -

Raw File	Scan	Method	Score	m/z
RO-QC-160121_ZackD_SS_Set_01_Fraction_9	27872	FTMS; HCD	44.14	515.99

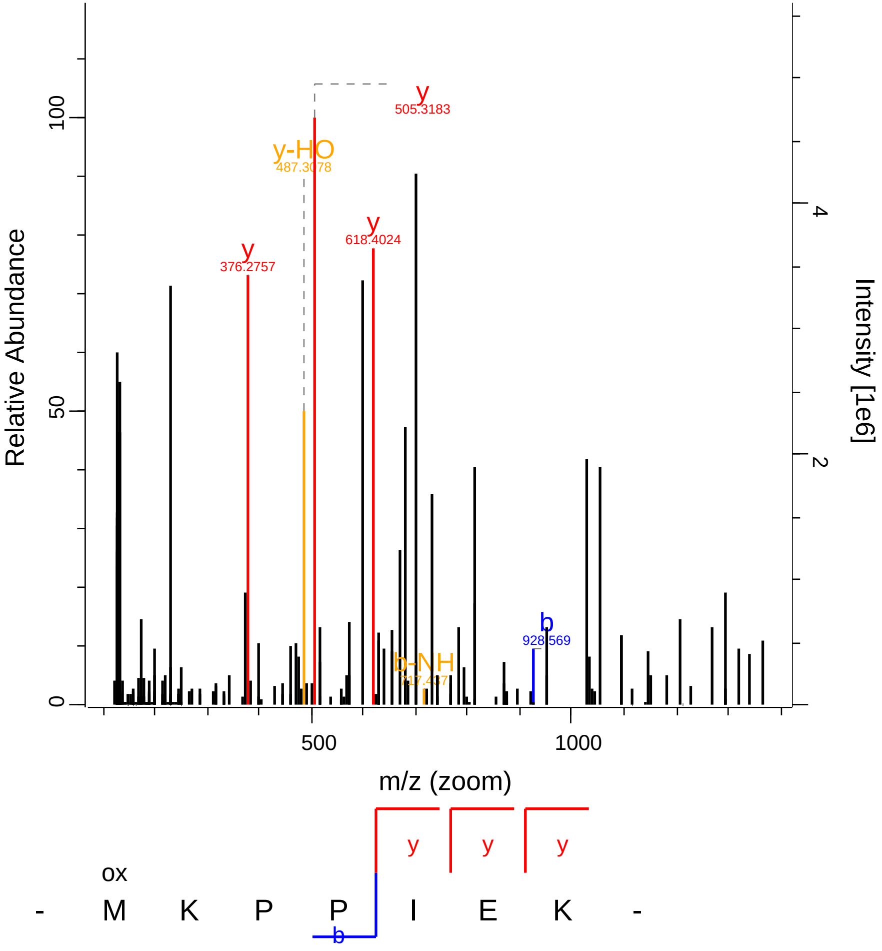

Raw File	Scan	Method	Score	m/z	Gene names
RO-QC-160121_ZackD_SS_Set_01_Fractio	27960	FTMS; HC	59.87	592.33	ATP6V0C

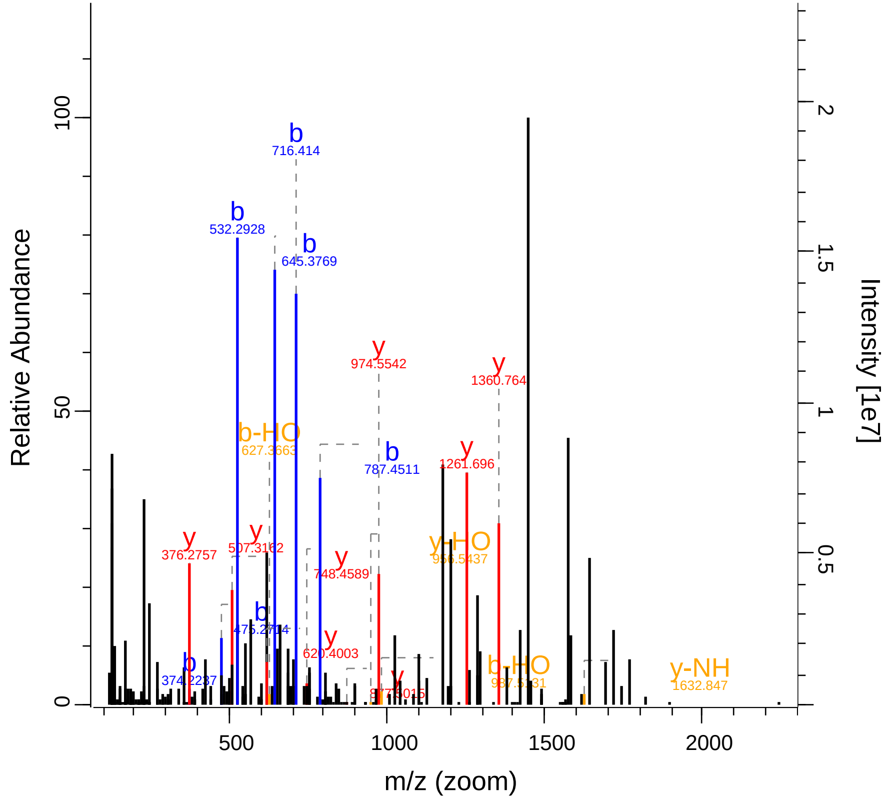

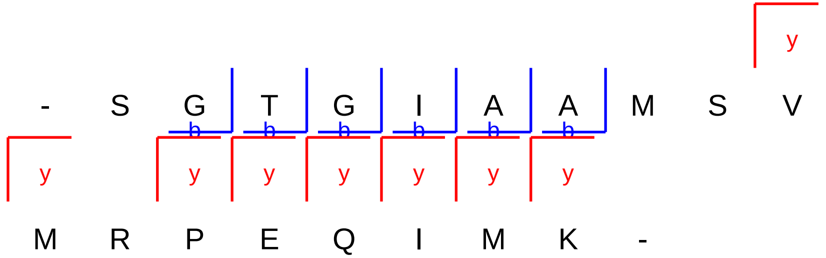

Raw File	Scan	Method	Score	m/z	Gene names
RO-QC-160121_ZackD_SS_Set_01_Fractio	28029	FTMS; HC	111.77	789.44	ATP6V0C

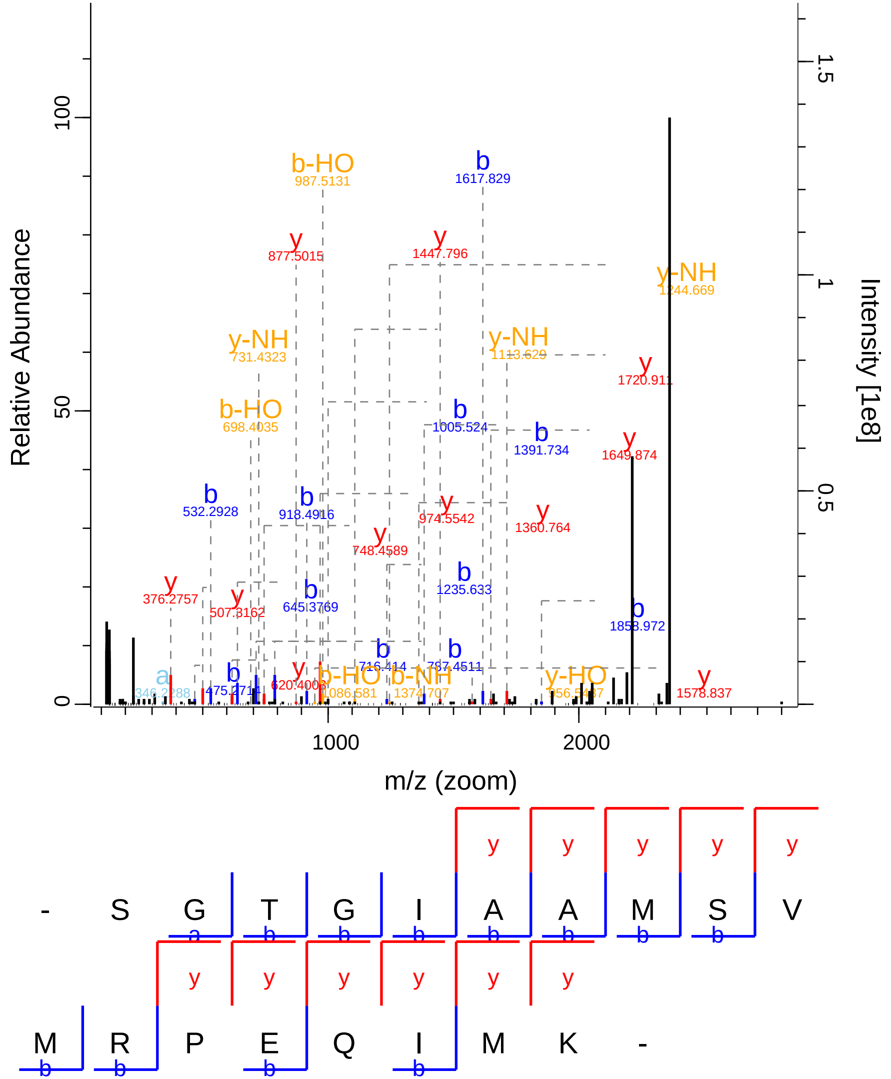

Raw File	Scan	Method	Score	m/z
RO-QC-160121_ZackD_SS_Set_01_Fraction_9	28275	FTMS; HCD	65.81	791.46

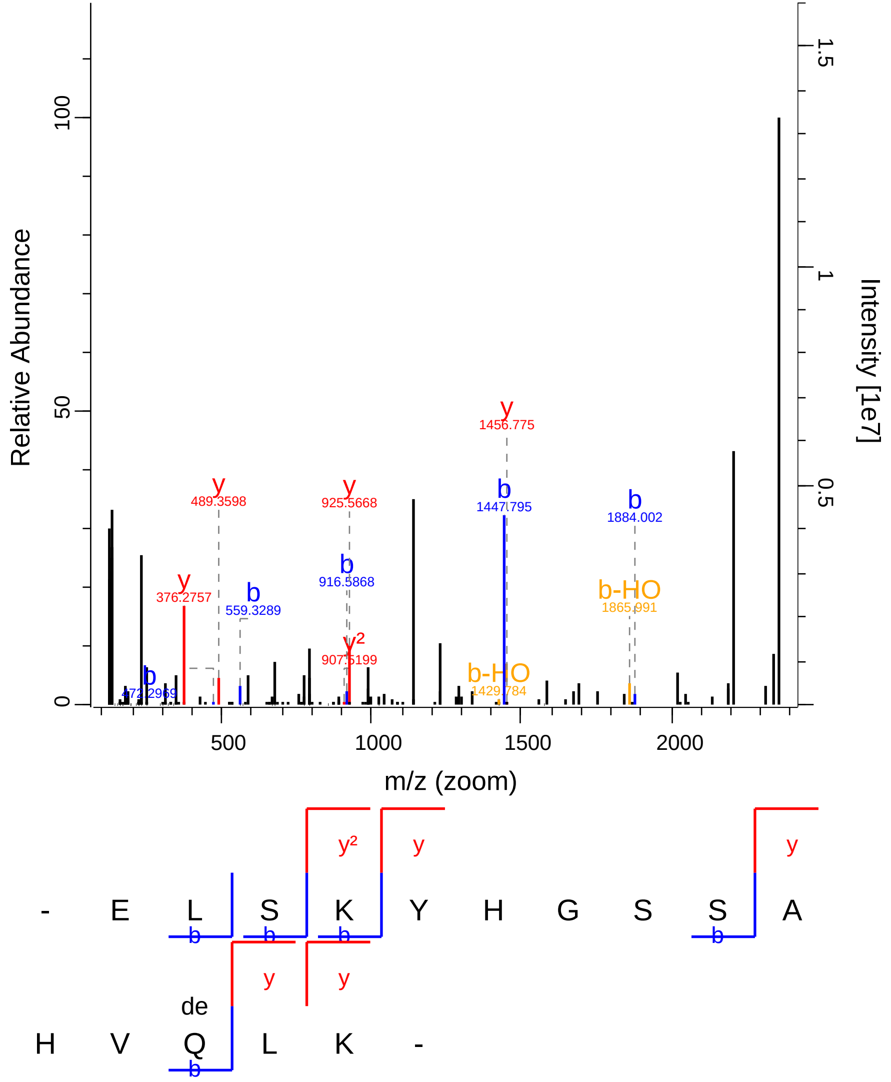

Raw File	Scan	Method	Score	m/z	Gene names
RO-QC-160121_ZackD_SS_Set_01_Fractio	28437	FTMS; HC	90.4	944.98	COX6A1

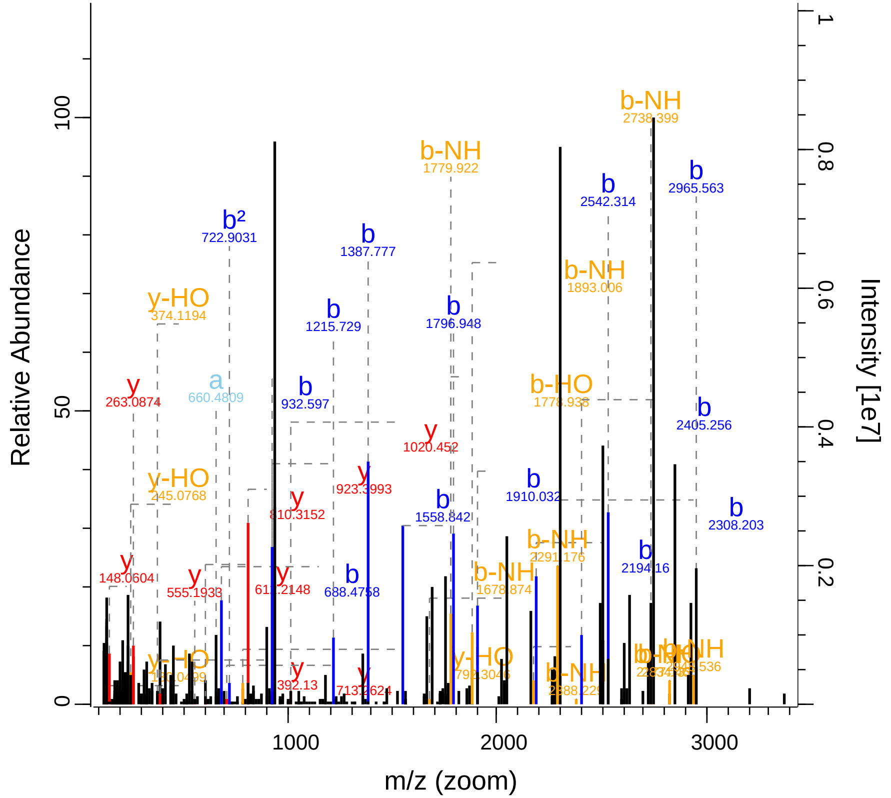

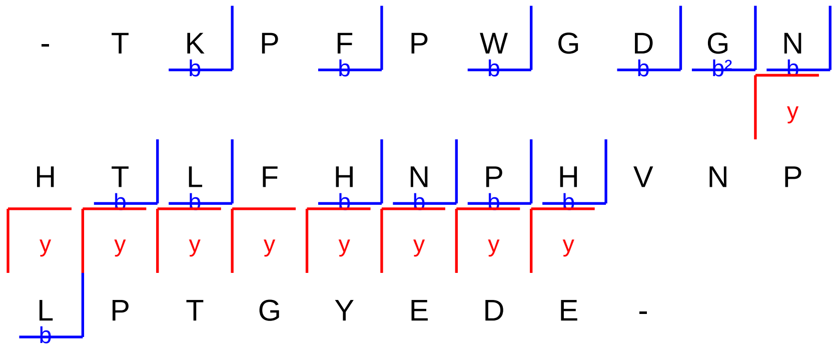

Raw File	Scan	Method	Score	m/z	Gene names
RO-QC-160121_ZackD_SS_Set_01_Fractic	28440	FTMS; HC	66.98	1259.63	COX6A1

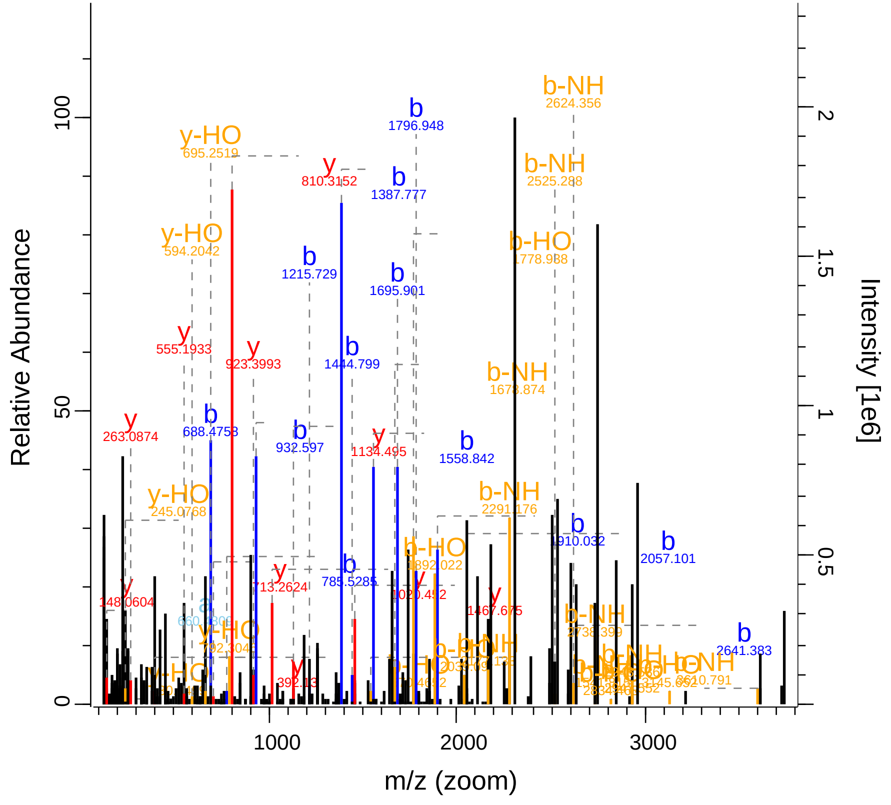

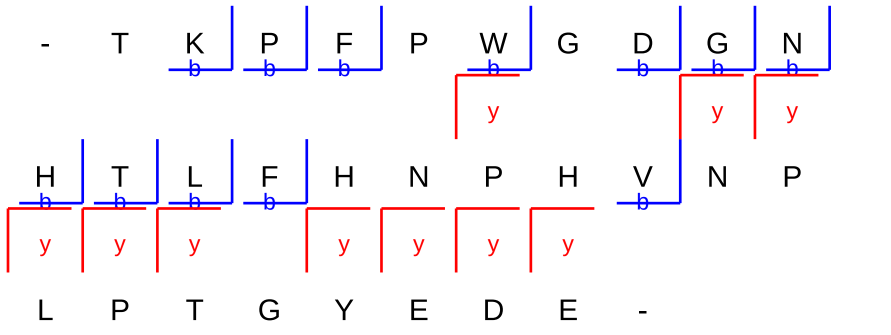

Raw File	Scan	Method	Score	m/z	Gene names
RO-QC-160121_ZackD_SS_Set_01_Fractio	28452	FTMS; HC	73.81	756.18	COX6A1

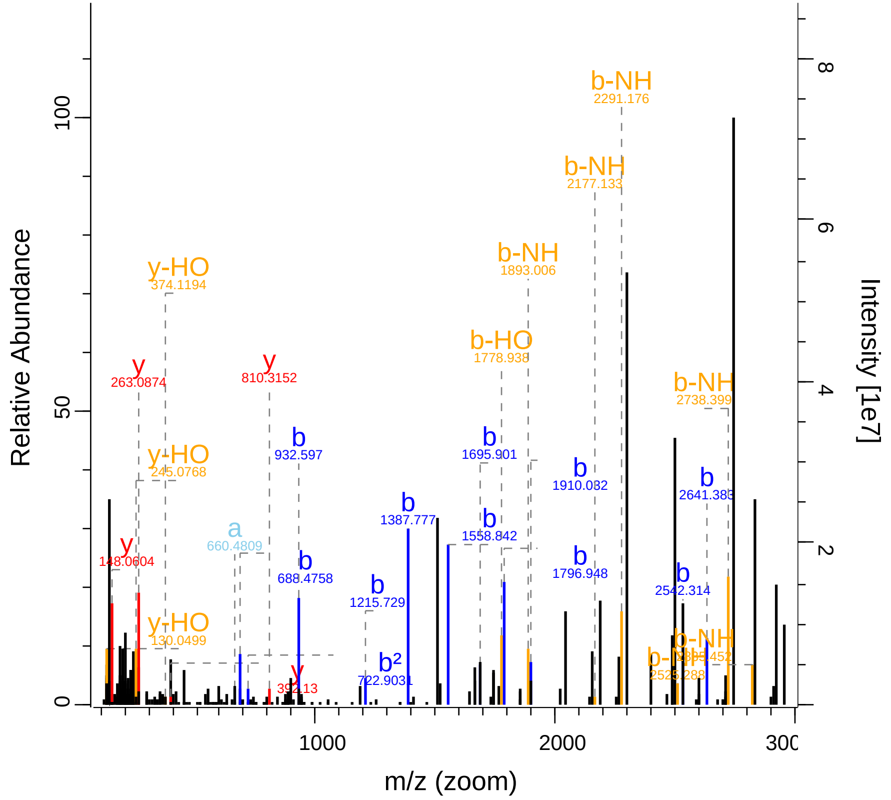

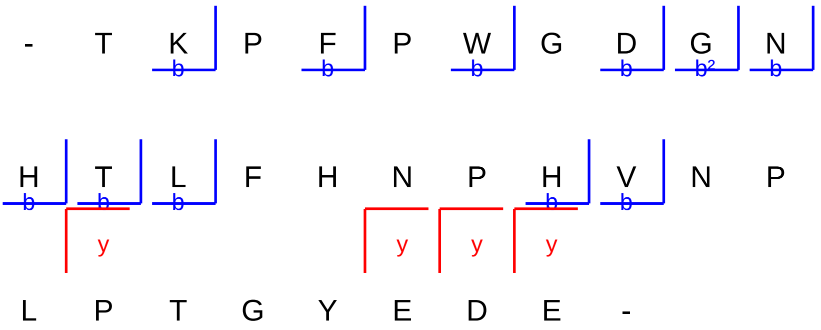

Raw File	Scan	Method	Score	m/z	Gene names
RO-QC-160121_ZackD_SS_Set_01_Fractic	29863	FTMS; HC	56.09	1041.06	TICRR

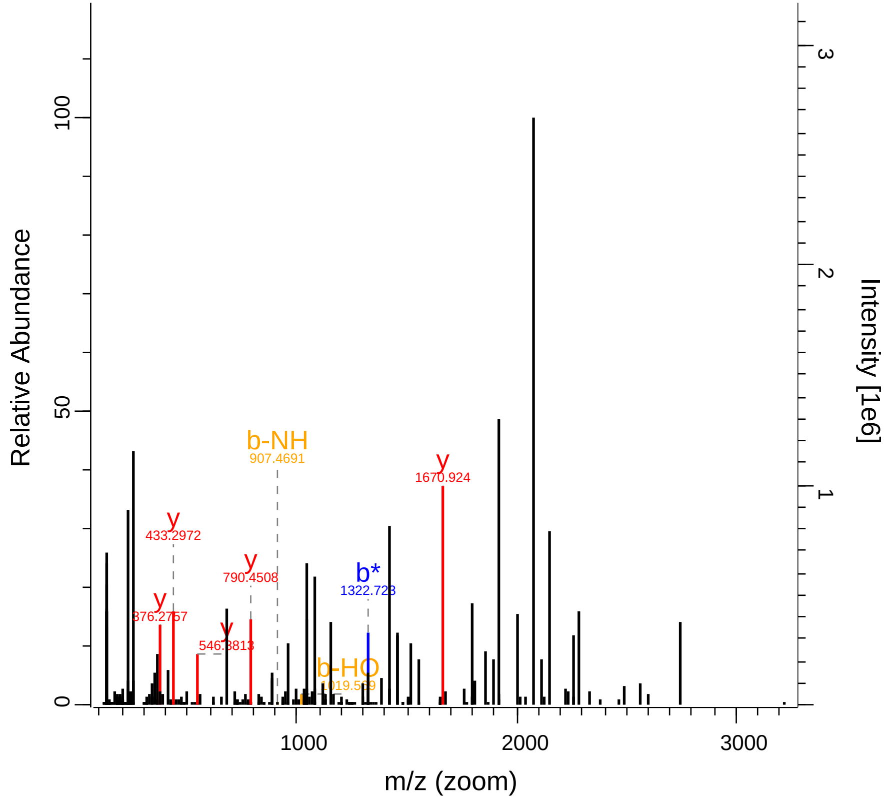

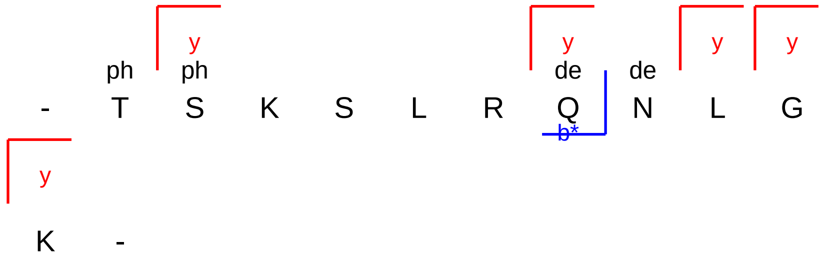

Raw File	Scan	Method	Score	m/z	Gene names
RO-QC-160121_ZackD_SS_Set_01_Fractio	30567	FTMS; HC	52.61	899.13	CEMIP

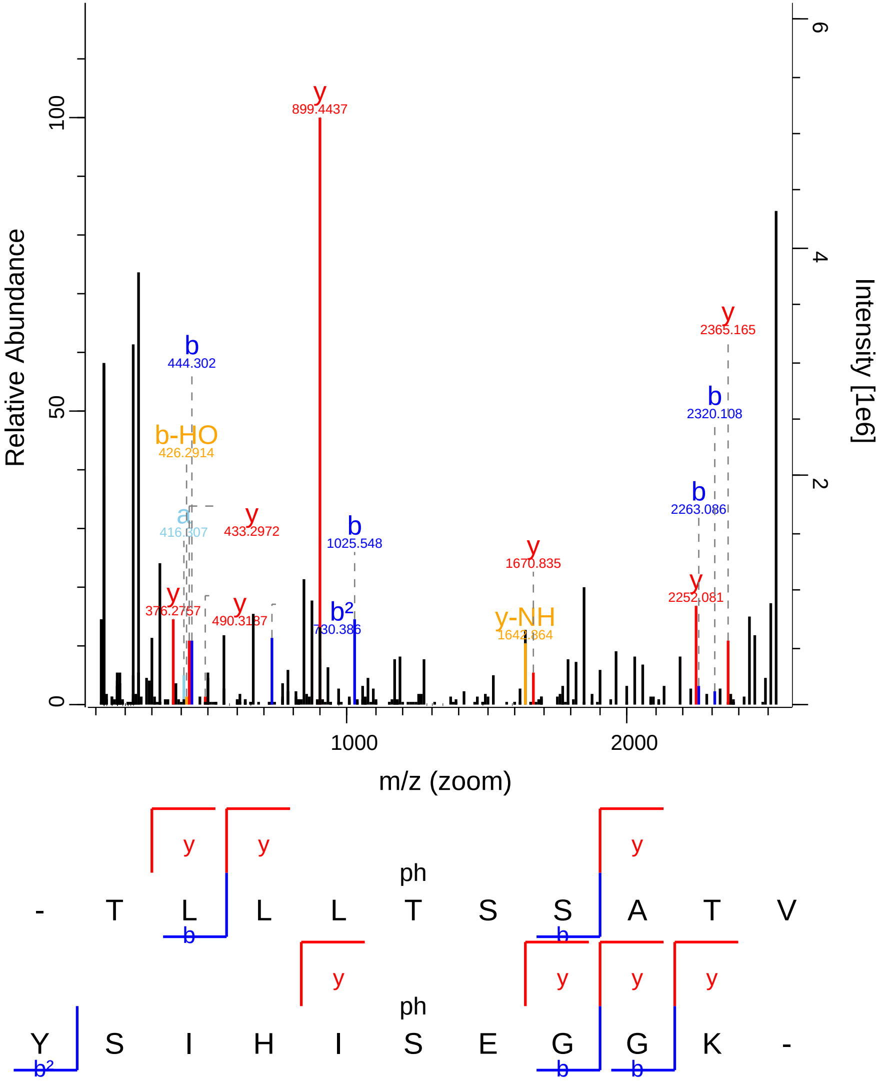

Raw File	Scan	Method	Score	m/z	Gene names
RO-QC-160121_ZackD_SS_Set_01_Fractio	31584	FTMS; HC	119.27	961.47	NOP10

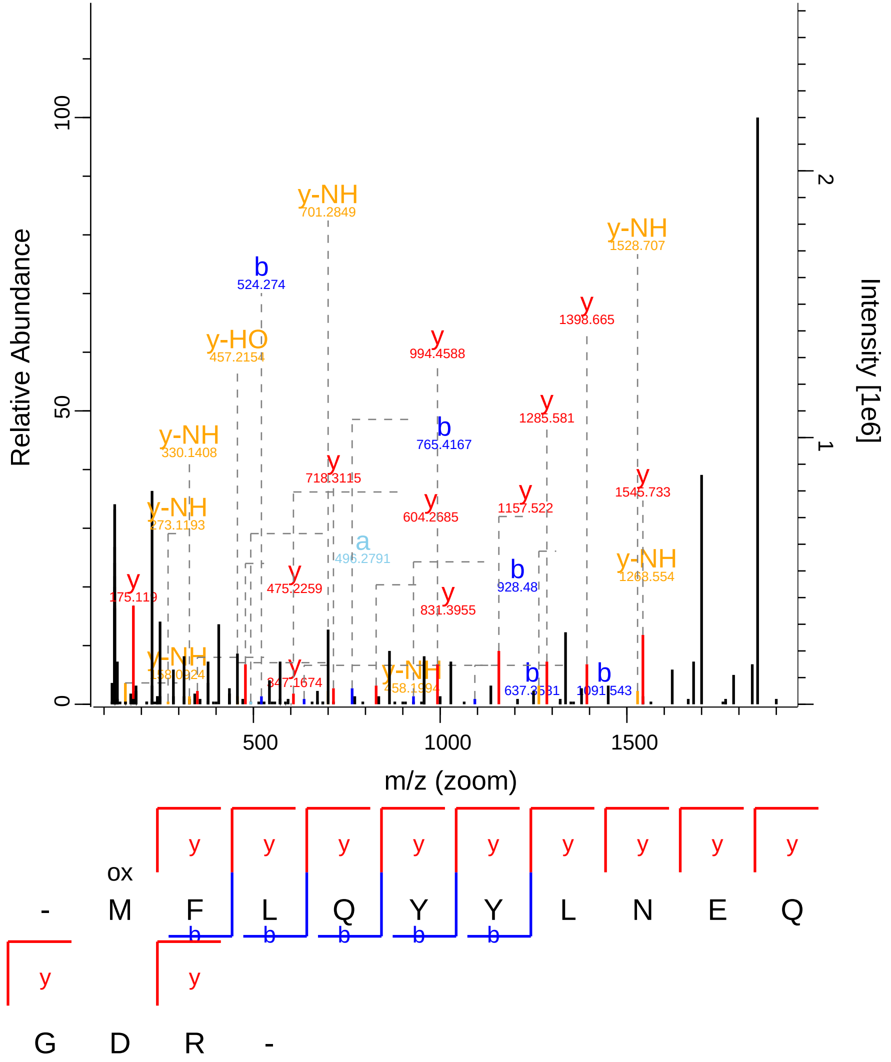

Raw File	Scan	Method	Score	m/z
RO-QC-160121_ZackD_SS_Set_01_Fraction_9	31589	FTMS; HCD	91.07	719.91

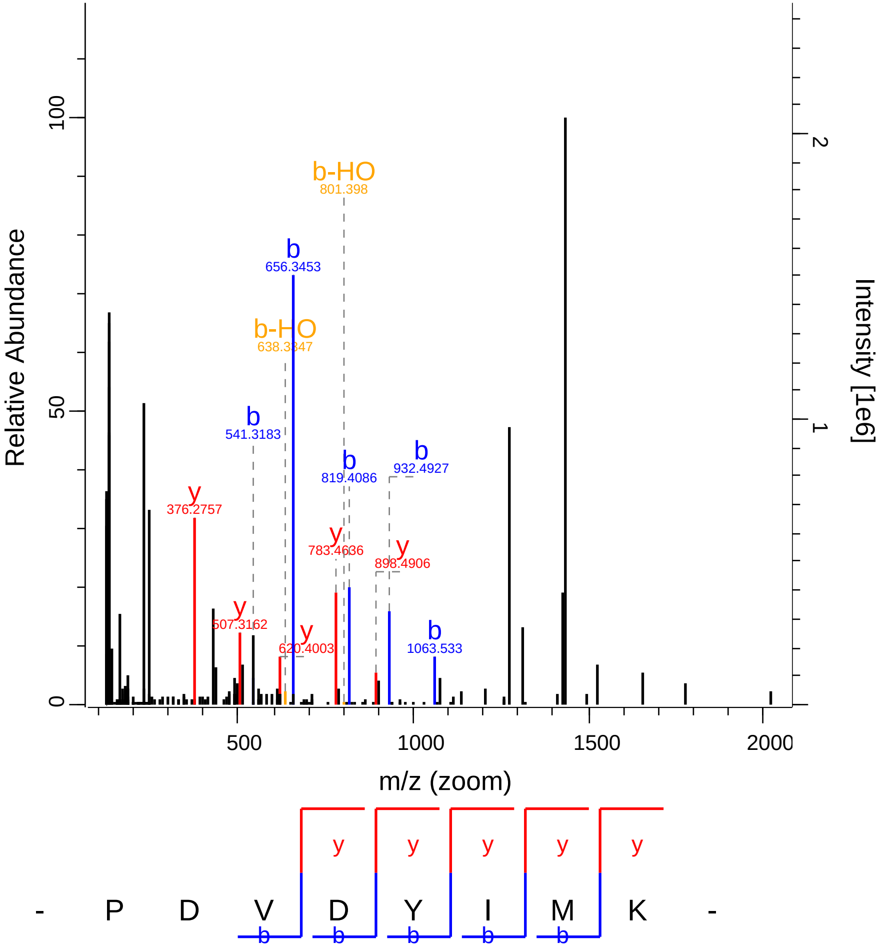

Raw File	Scan	Method	Score	m/z
RO-QC-160121_ZackD_SS_Set_01_Fraction_9	31659	FTMS; HCD	59.54	1166.6

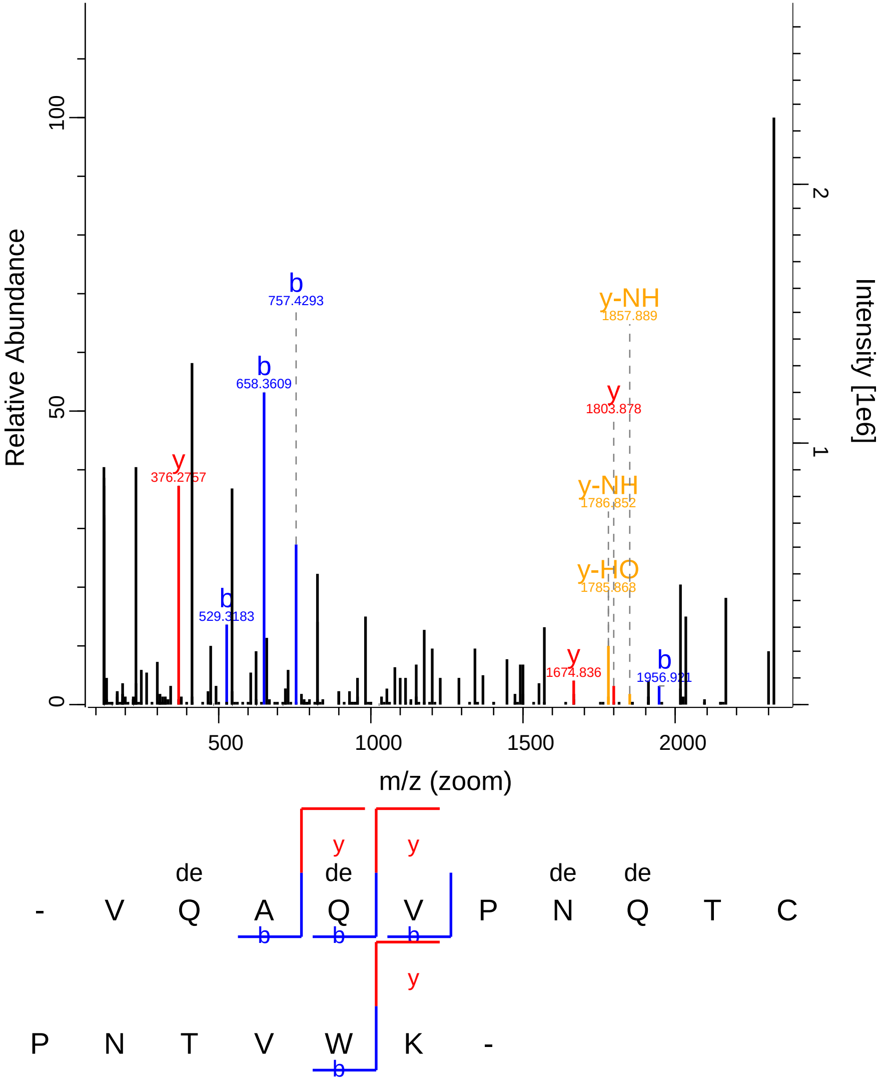

Raw File	Scan	Method	Score	m/z
RO-QC-160121_ZackD_SS_Set_01_Fraction_9	31695	FTMS; HCD	47.08	480.61

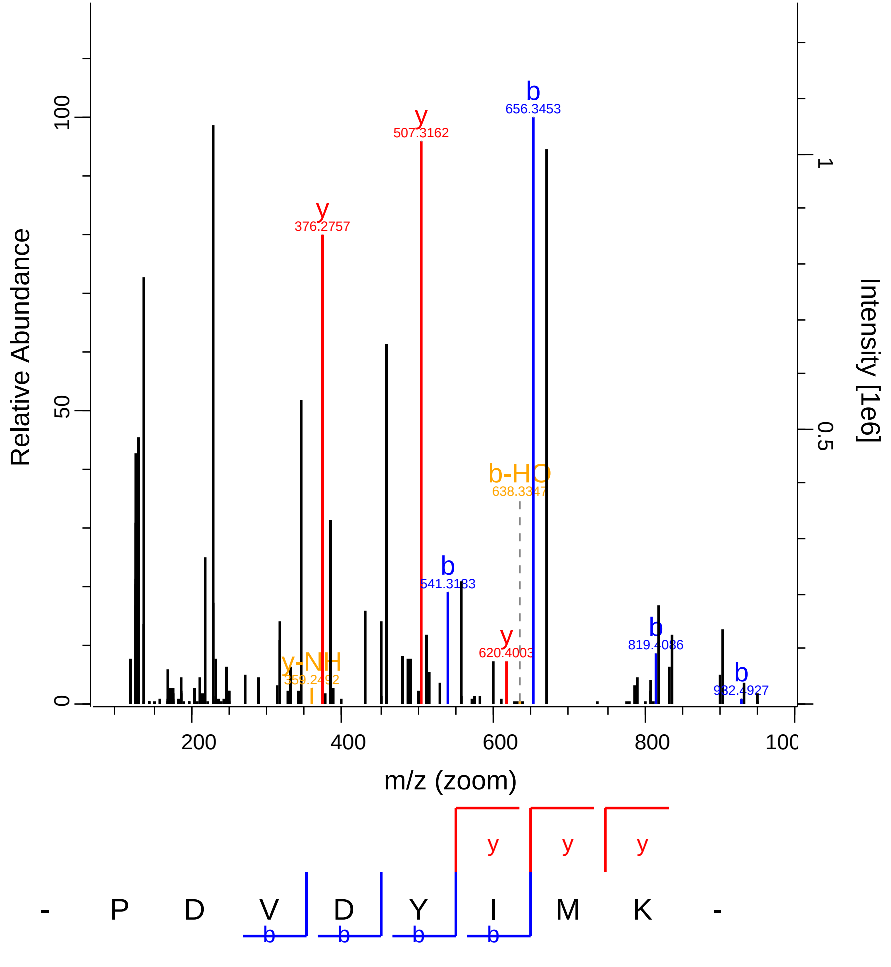

Raw File	Scan	Method	Score	m/z
RO-QC-160121_ZackD_SS_Set_01_Fraction_9	31791	FTMS; HCD	91.07	719.91

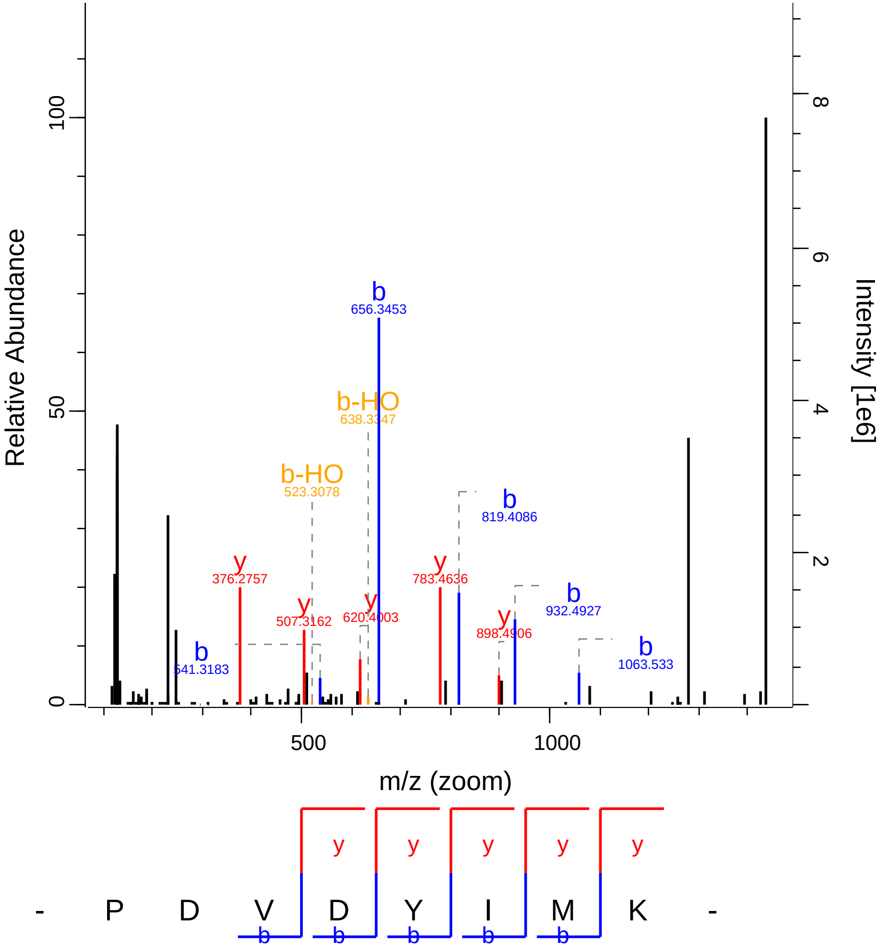

Raw File	Scan	Method	Score	m/z
RO-QC-160121_ZackD_SS_Set_01_Fraction_9	31836	FTMS; HCD	49.5	544

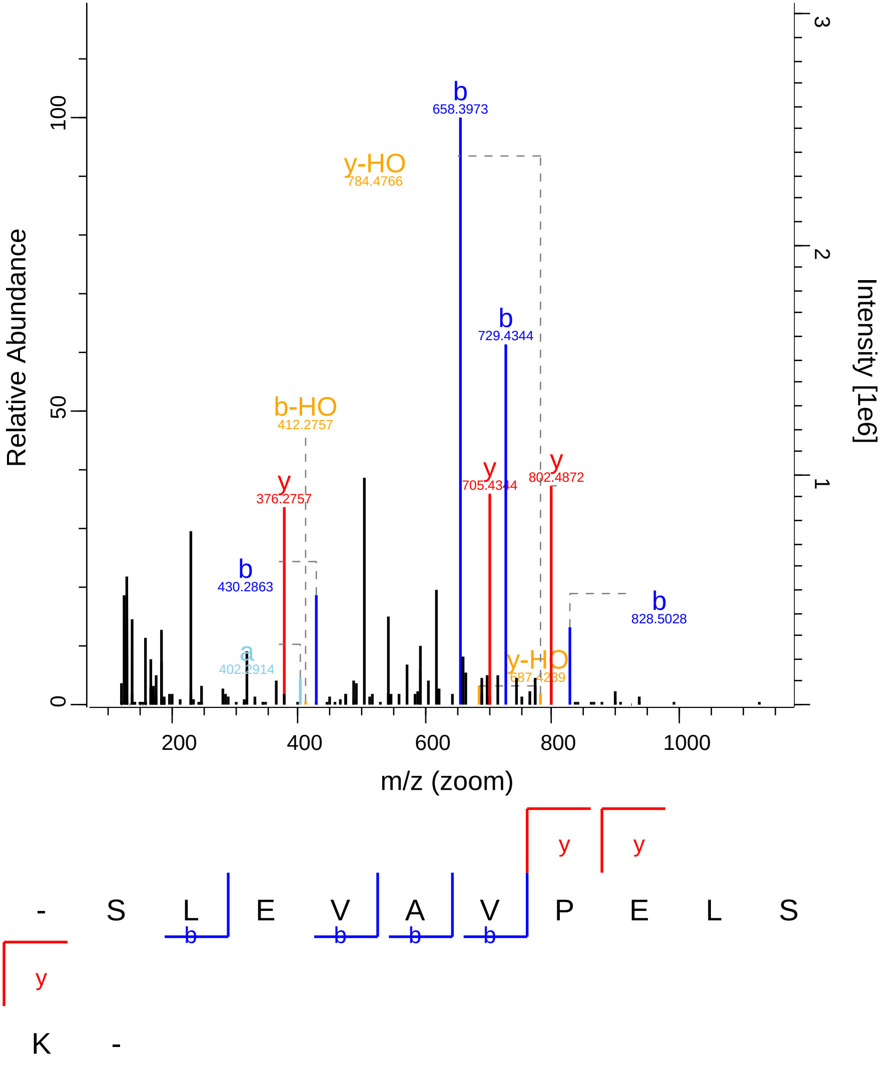

Raw File	Scan	Method	Score	m/z
RO-QC-160121_ZackD_SS_Set_01_Fraction_9	31898	FTMS; HCD	91.58	815.5

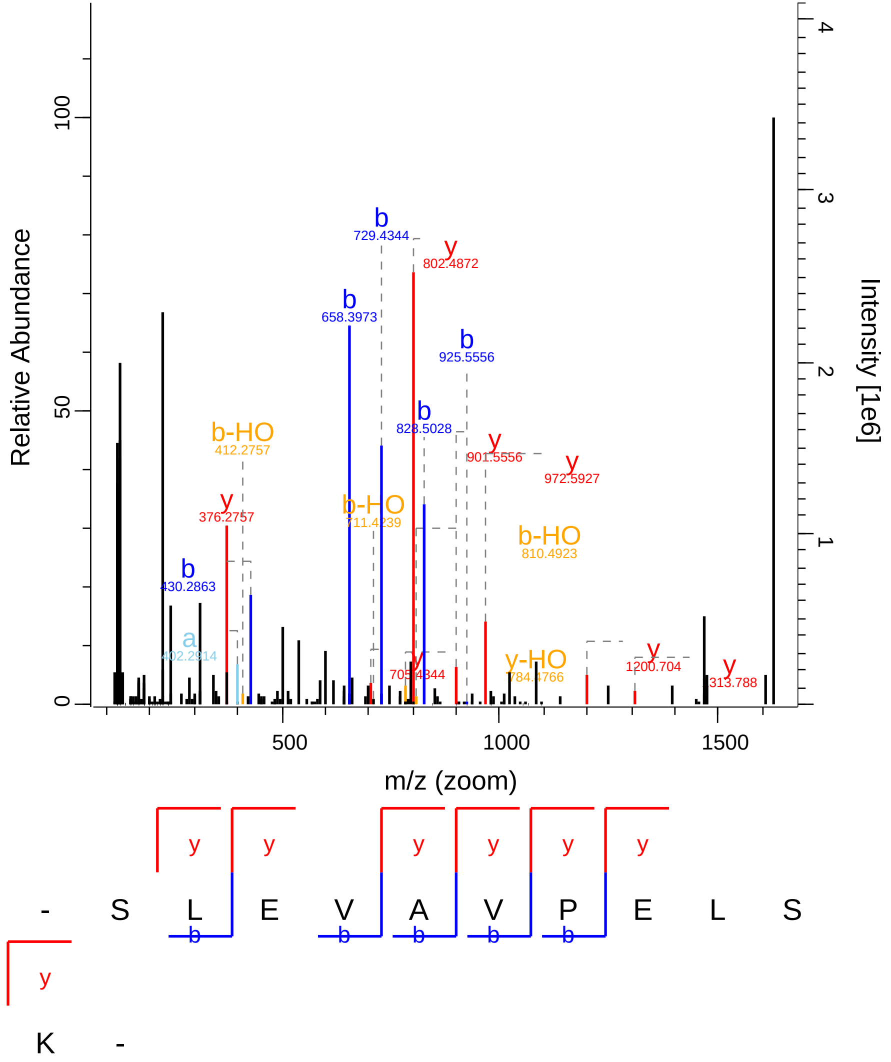

Raw File	Scan	Method	Score	m/z
RO-QC-160121_ZackD_SS_Set_01_Fraction_9	32054	FTMS; HCD	69.67	544

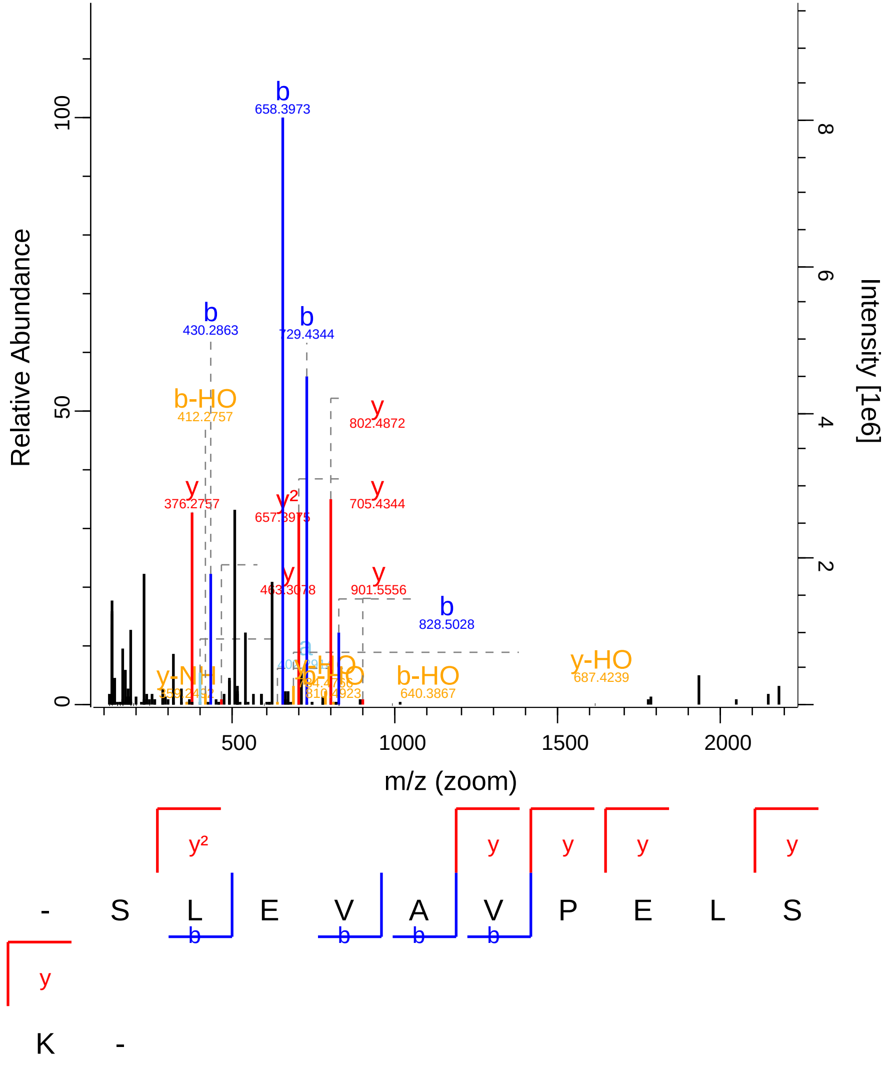

Raw File	Scan	Method	Score	m/z	Gene names
RO-QC-160121_ZackD_SS_Set_01_Fractio	32823	FTMS; HC	65.02	836.76	CNIH4

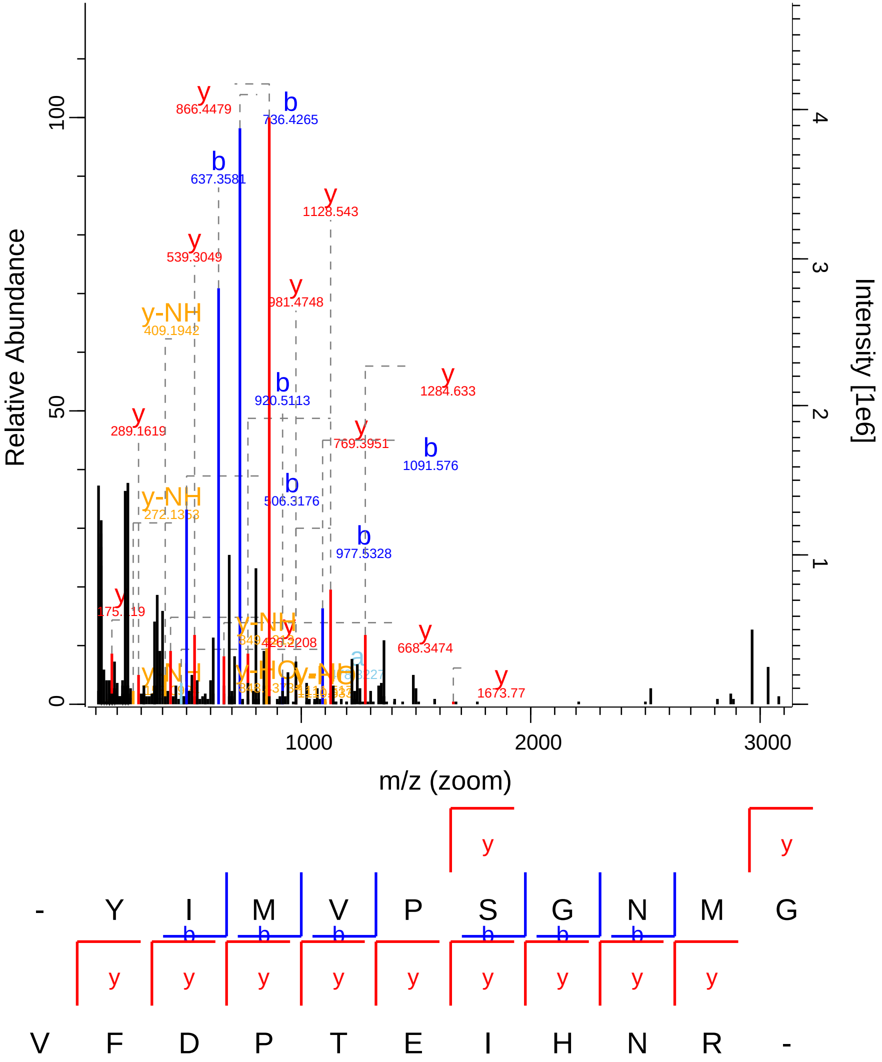

Raw File	Scan	Method	Score	m/z	Gene names
RO-QC-160121_ZackD_SS_Set_01_Fractio	32889	FTMS; HC	105.77	627.32	CNIH4

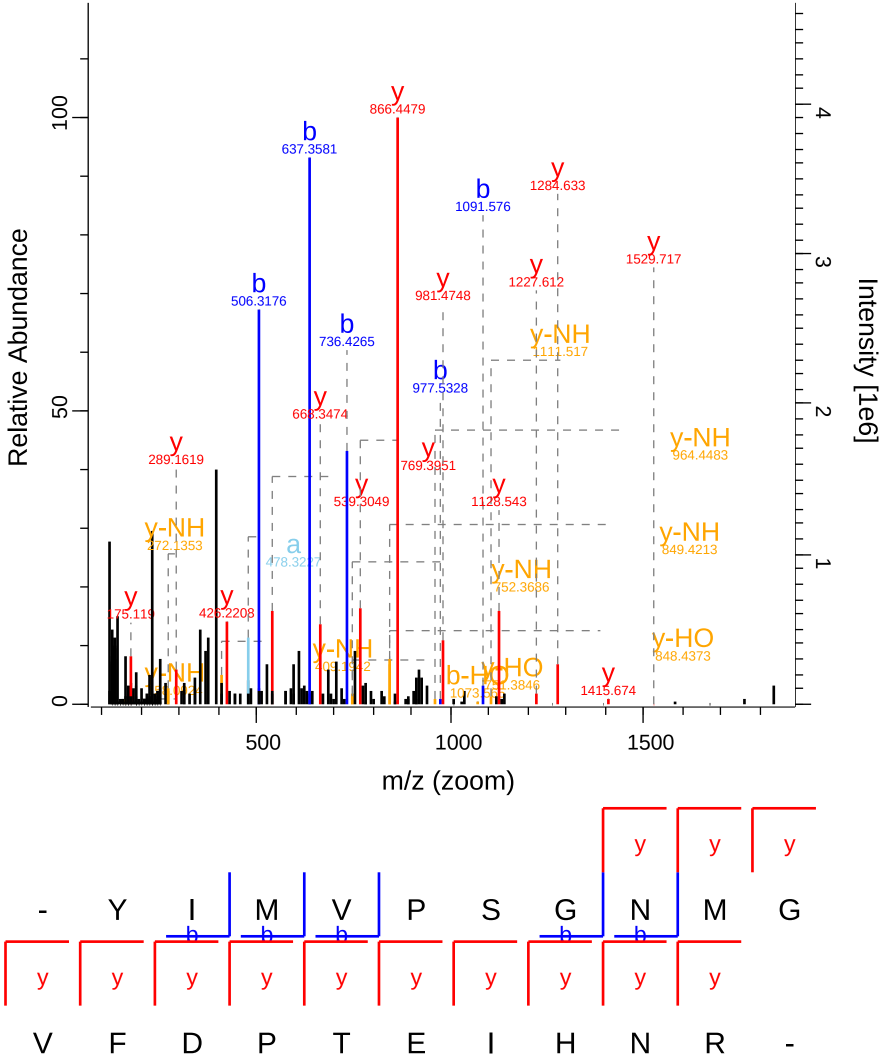

Raw File	Scan	Method	Score	m/z	Gene names
RO-QC-160121_ZackD_SS_Set_01_Fractio	33044	FTMS; HC	147.1	836.42	CNIH4

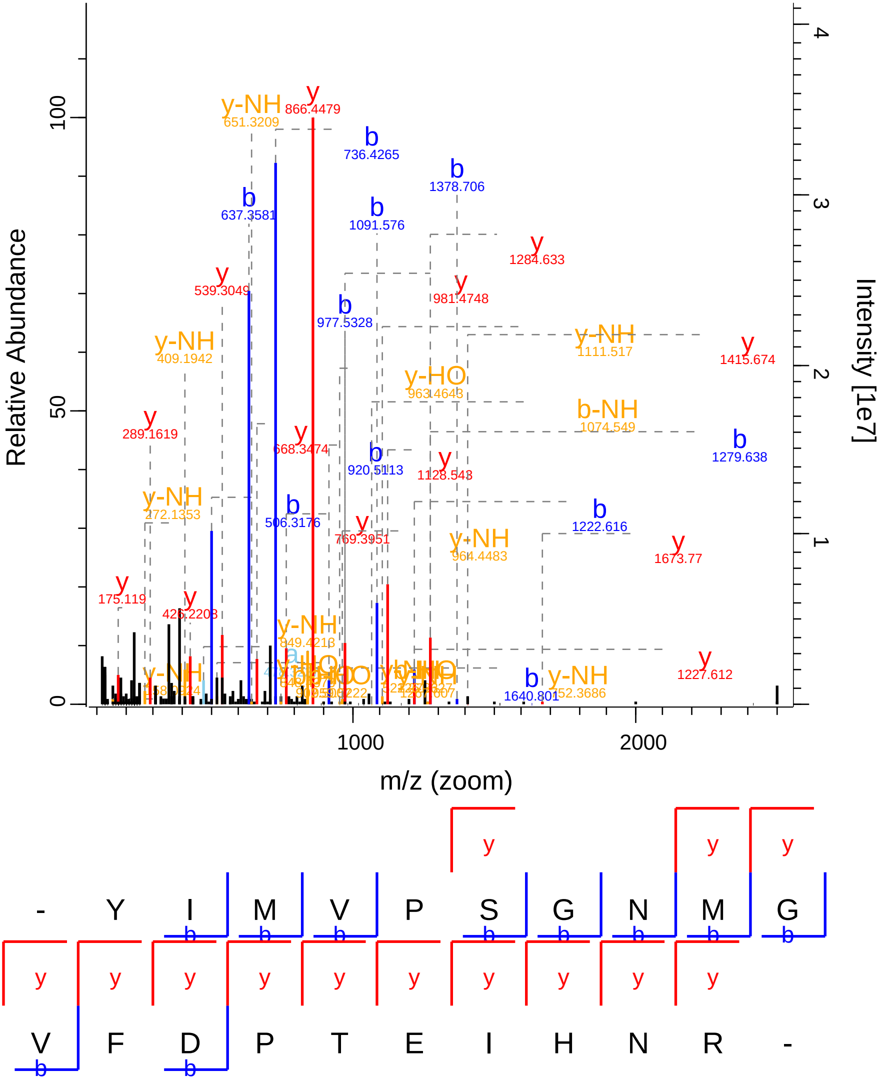

Raw File	Scan	Method	Score	m/z	Gene names
RO-QC-160121_ZackD_SS_Set_01_Fractior	33946	FTMS; HCI	82.26	673.9	C19orf12

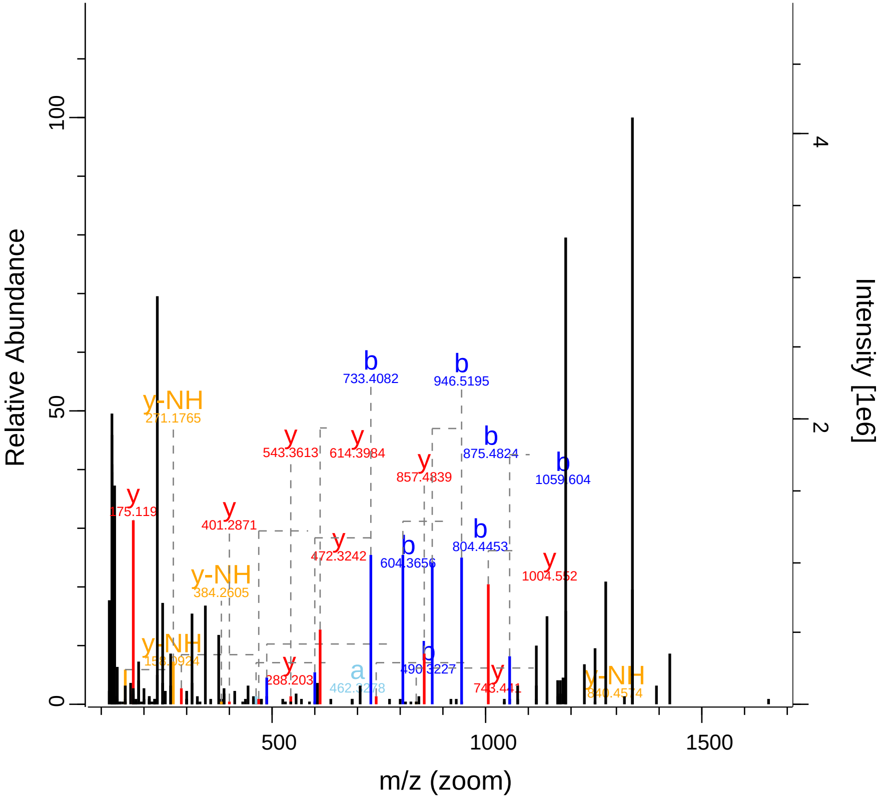

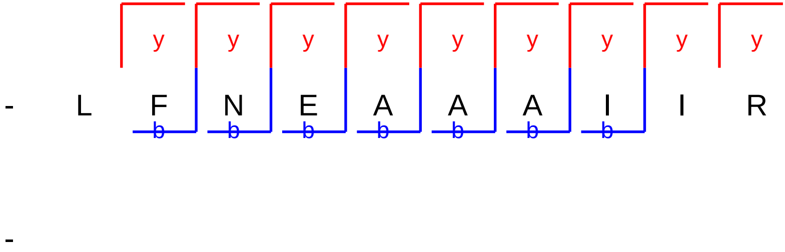

Raw File	Scan	Method	Score	m/z	Gene names
RO-QC-160121_ZackD_SS_Set_01_Fractior	35601	FTMS; HCI	40.18	976.5	ENAM

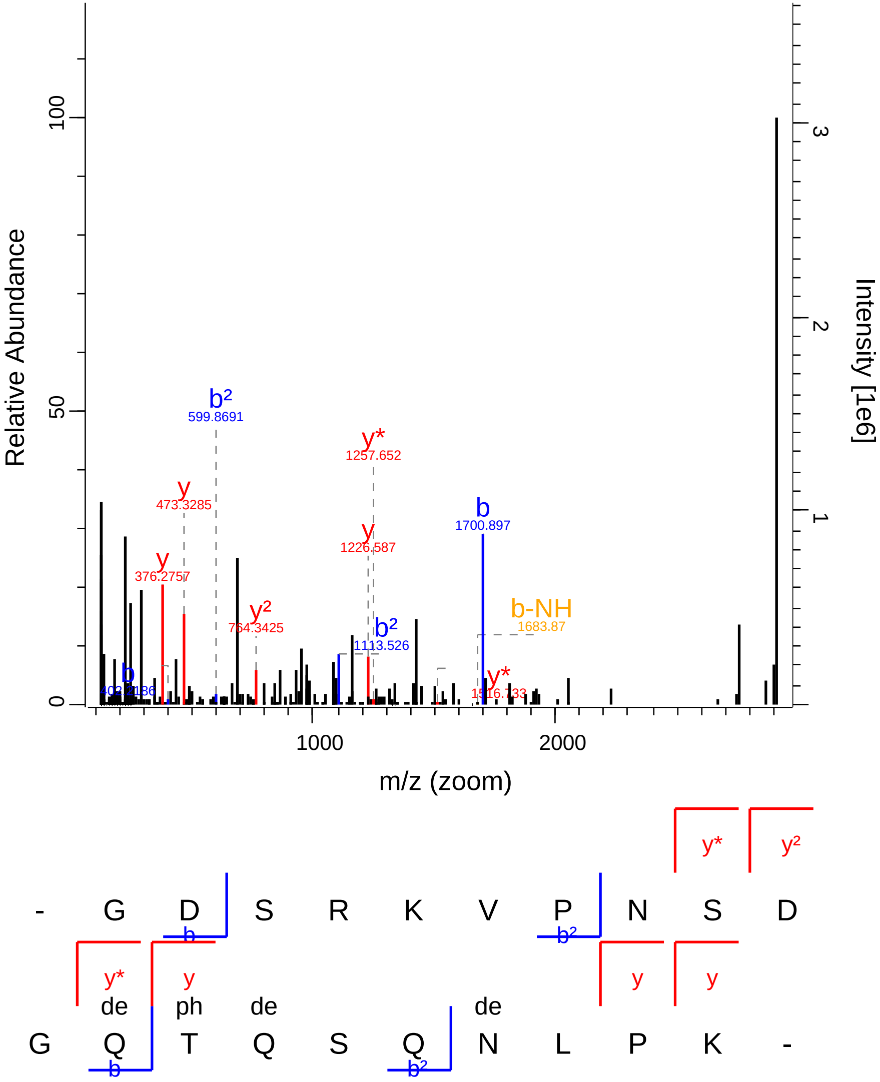

Raw File	Scan	Method	Score	m/z
RO-QC-160121_ZackD_SS_Set_01_Fraction_9	35790	FTMS; HCD	53.33	901.48

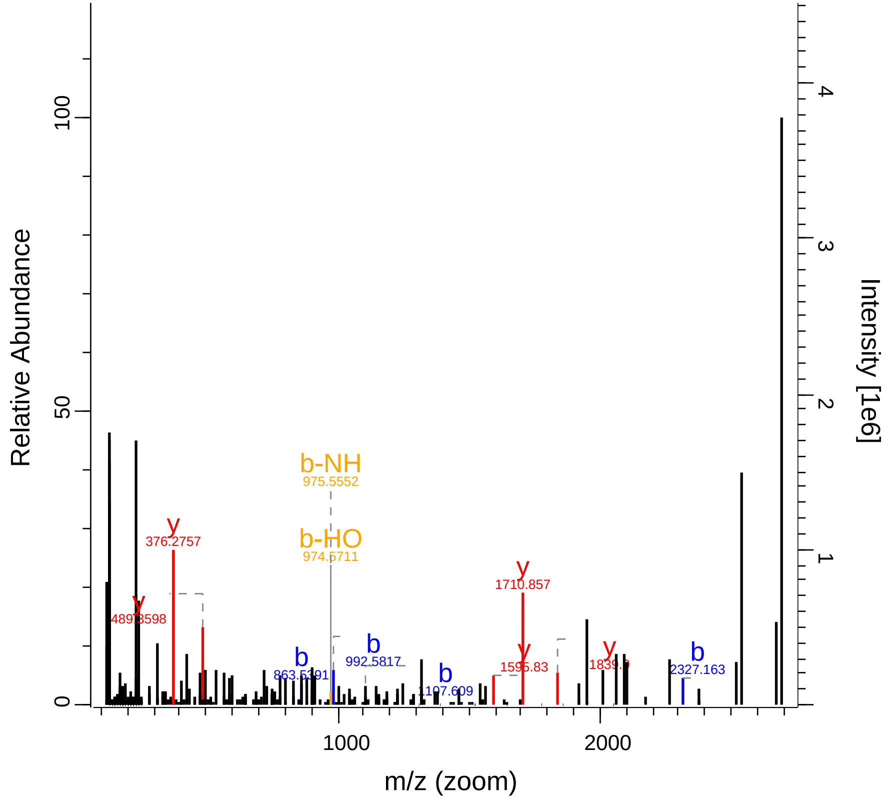

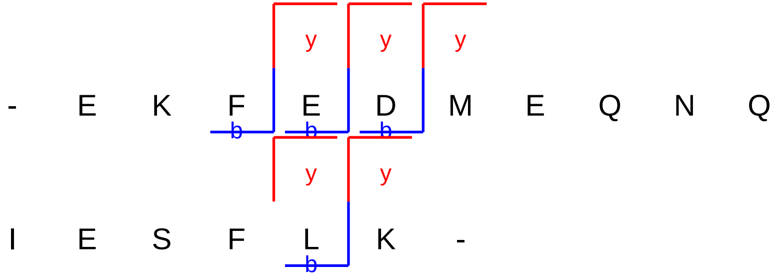

Raw File	Scan	Method	Score	m/z
RO-QC-160121_ZackD_SS_Set_01_Fraction_9	35835	FTMS; HCD	34.61	676.62

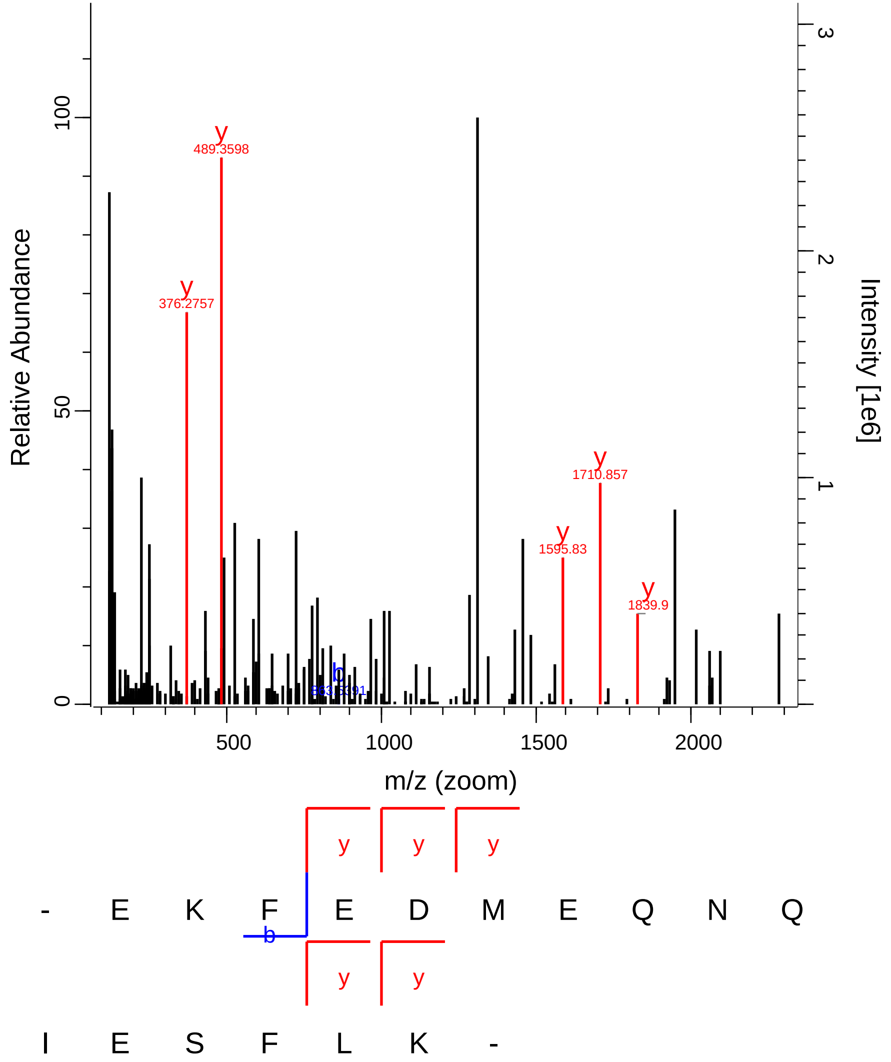

Raw File	Scan	Method	Score	m/z
RO-QC-160121_ZackD_SS_Set_01_Fraction_9	36009	FTMS; HCD	64.65	901.48

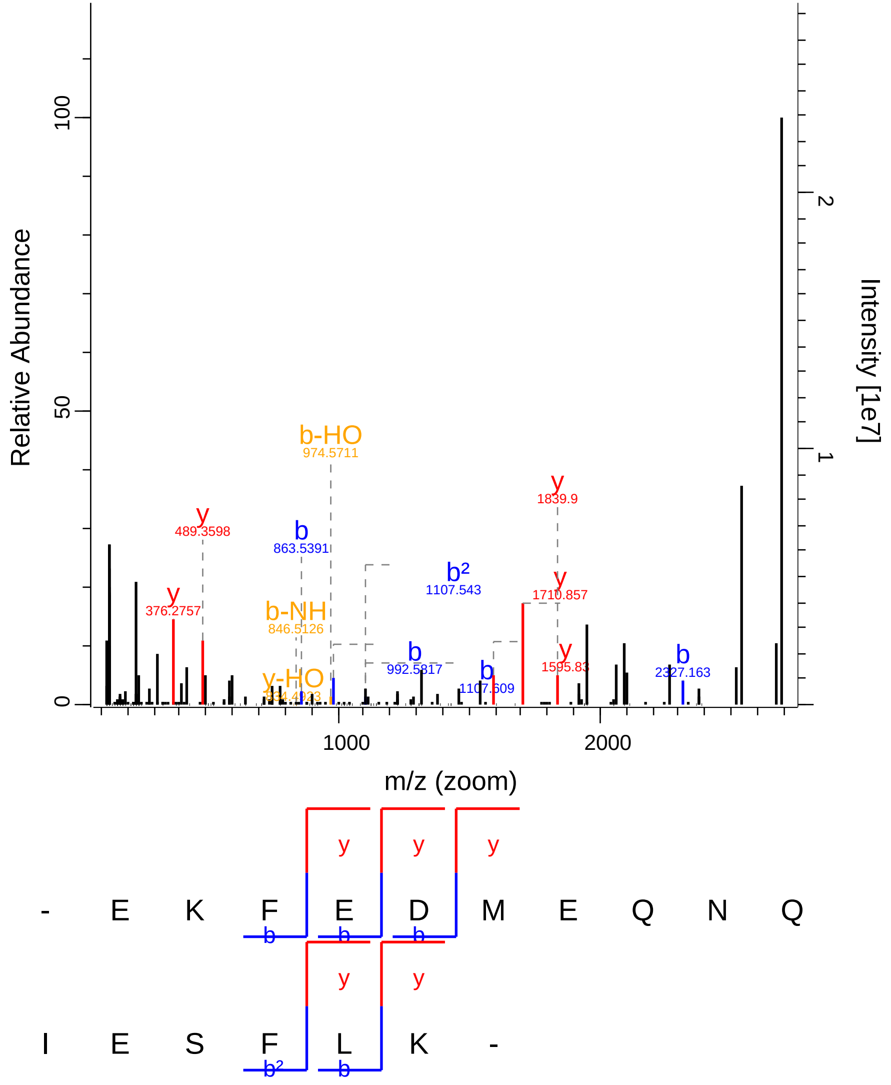

Raw File	Scan	Method	Score	m/z
RO-QC-160121_ZackD_SS_Set_01_Fraction_9	36053	FTMS; HCD	45.01	676.36

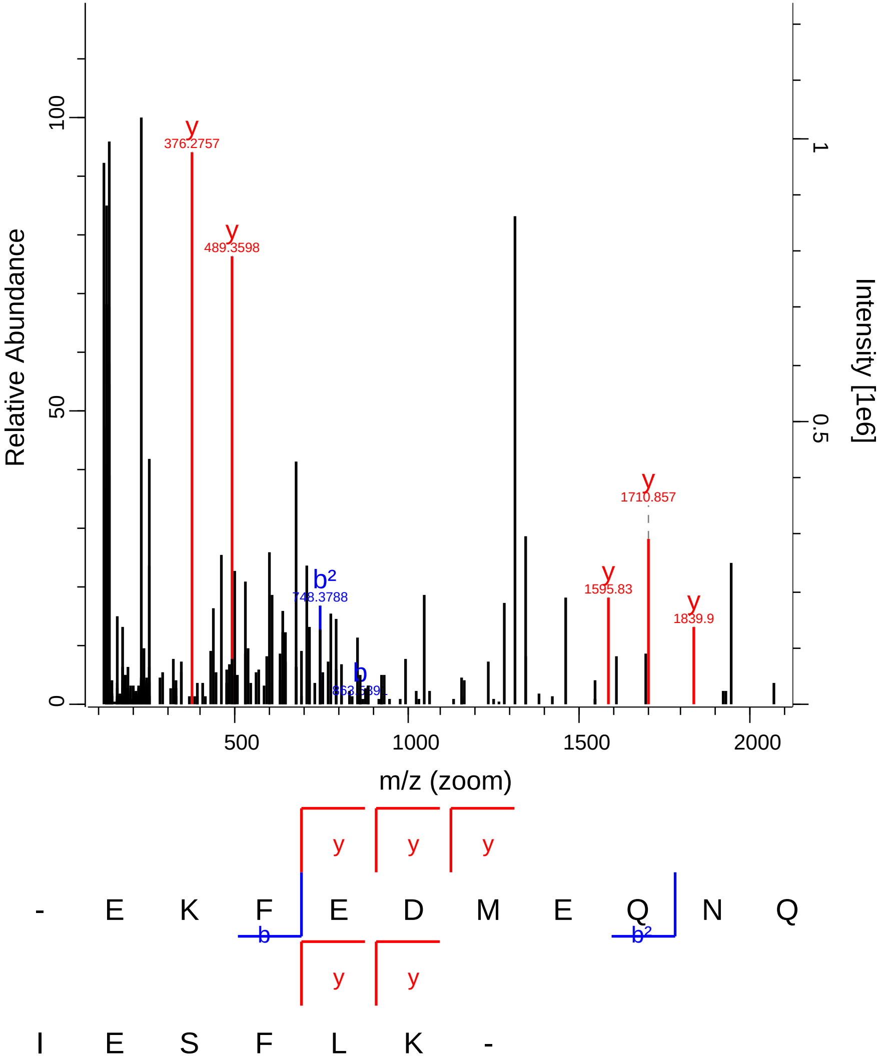

Raw File	Scan	Method	Score	m/z
RO-QC-160121_ZackD_SS_Set_01_Fraction_9	36951	FTMS; HCD	41.4	573.02

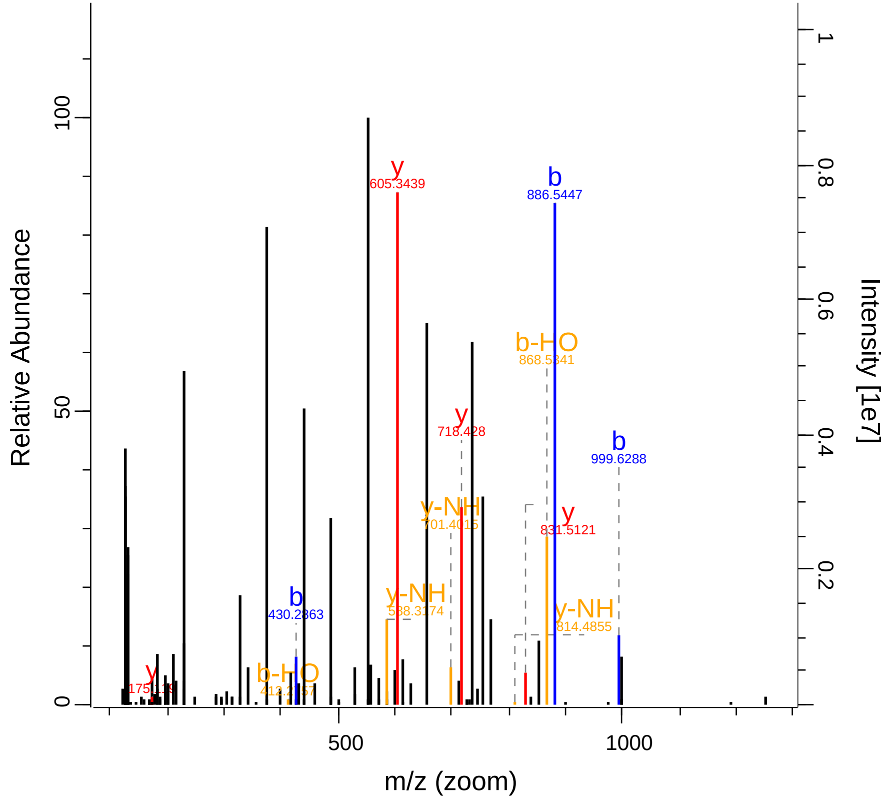

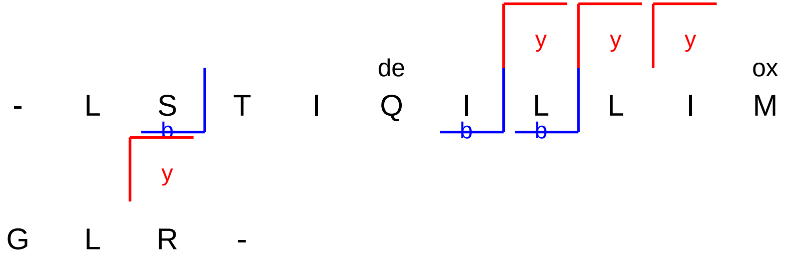

Raw File	Scan	Method	Score	m/z	Gene names
RO-QC-160121_ZackD_SS_Set_01_Fractic	37211	FTMS; HC	64.65	1327.22	KIAA0226L

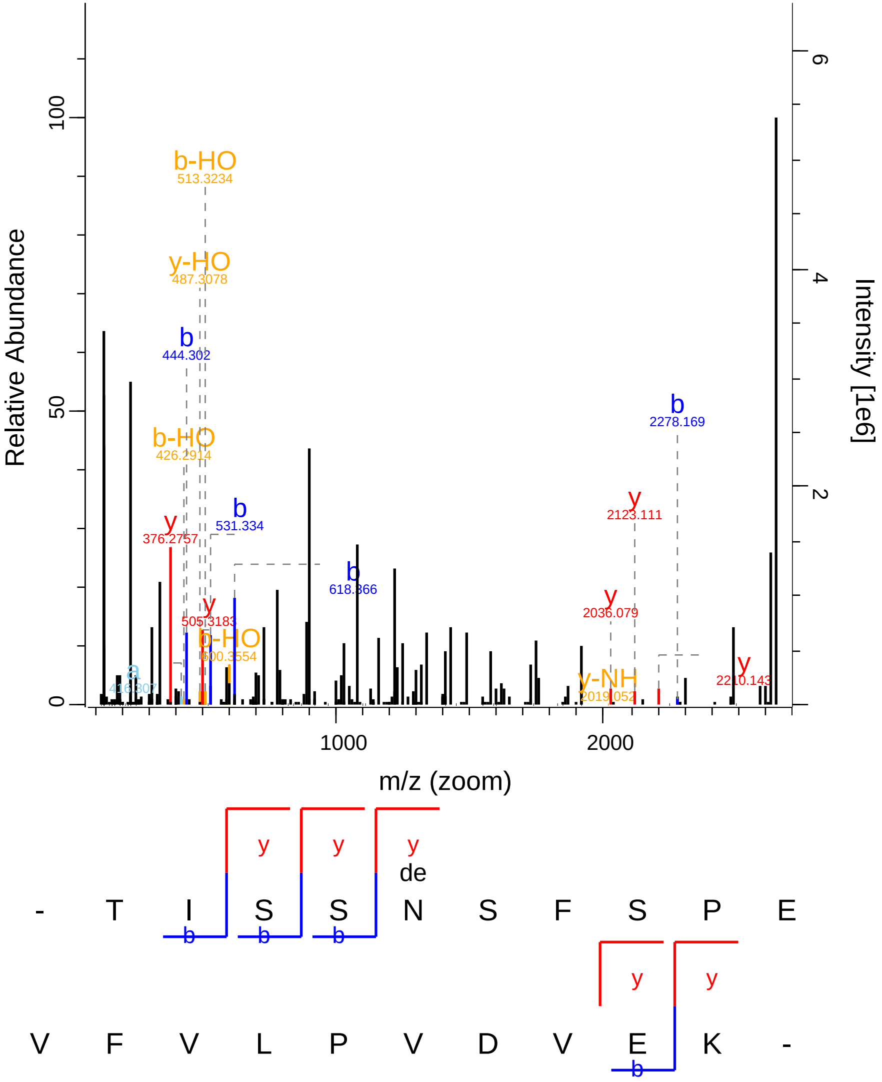

Raw File	Scan	Method	Score	m/z	Gene names
RO-QC-160121_ZackD_SS_Set_01_Fractic	37620	FTMS; HC	85.53	1081.12	C6

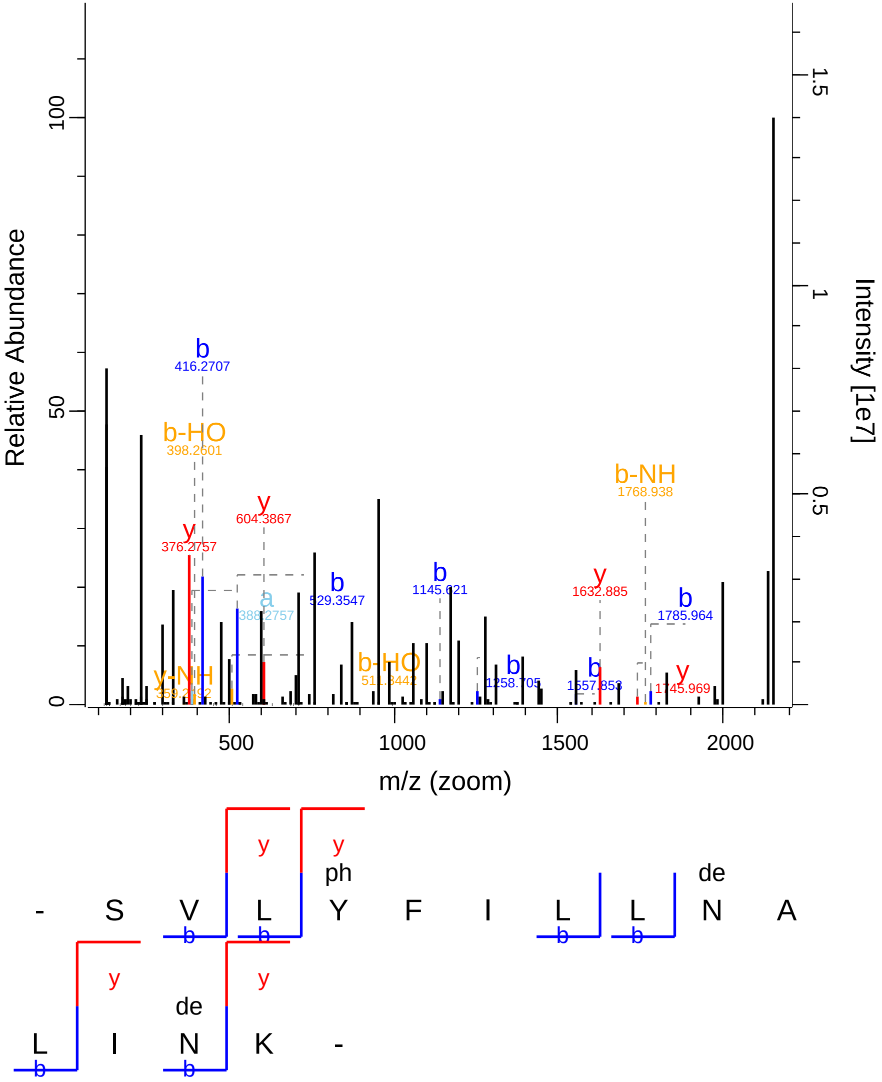

Raw File	Scan	Method	Score	m/z	Gene names
RO-QC-160121_ZackD_SS_Set_01_Fractic	37956	FTMS; HC	97.8	1028.59	ZKSCAN2

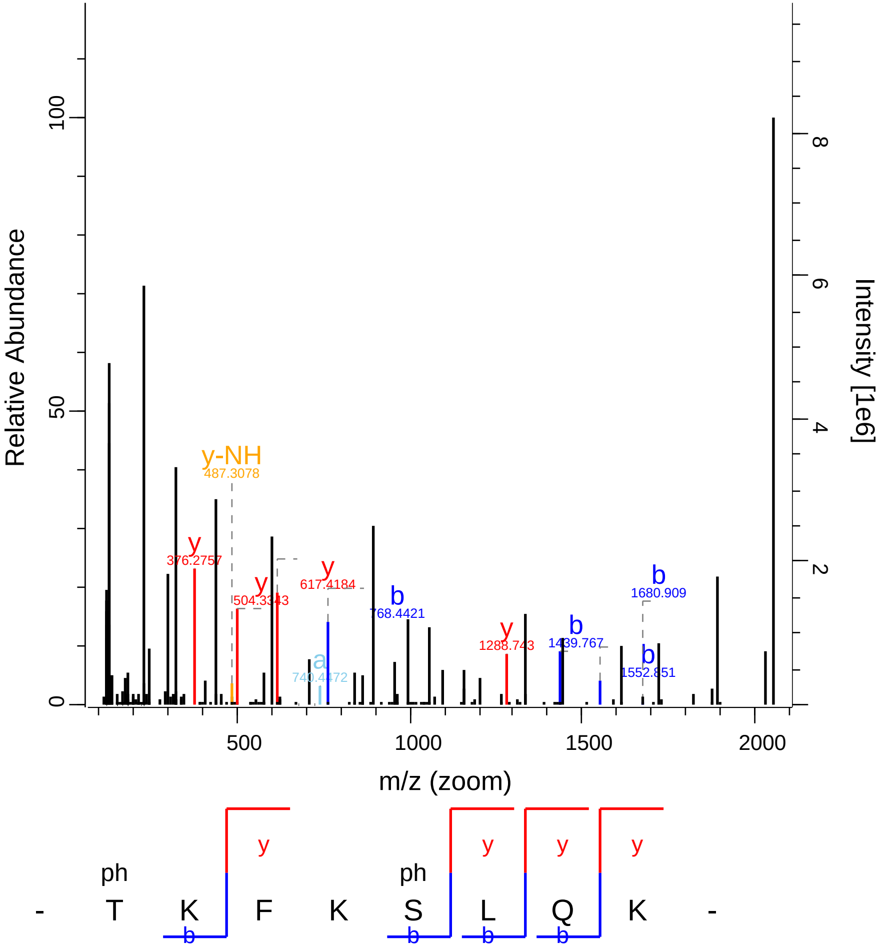

Raw File	Scan	Method	Score	m/z	Gene names
RO-QC-160121_ZackD_SS_Set_01_Fractic	41167	FTMS; HC	145.81	1149.13	NPW

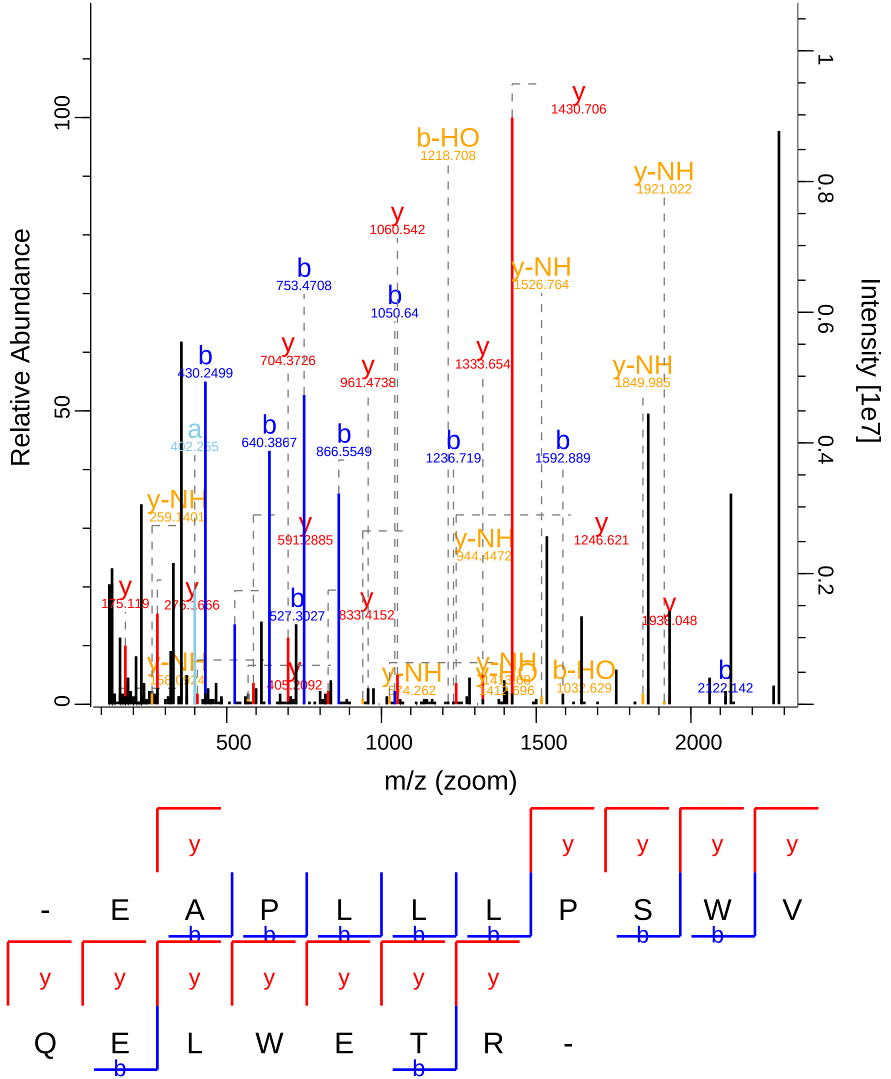

Raw File	Scan	Method	Score	m/z	Gene names
RO-QC-160121_ZackD_SS_Set_01_Fractio	41182	FTMS; HC	77.19	766.42	NPW

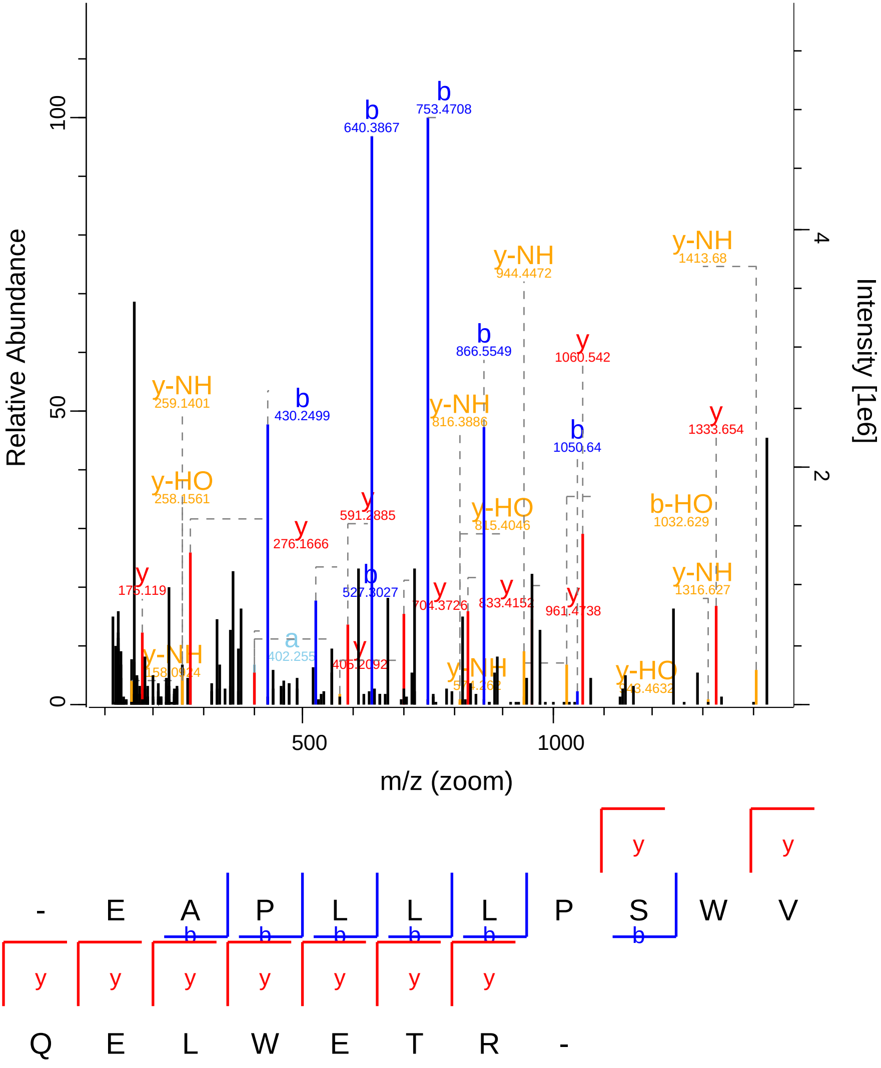

Raw File	Scan	Method	Score	m/z
RO-QC-160121_ZackD_SS_Set_01_Fraction_10	4331	FTMS; HCD	46.57	464.91

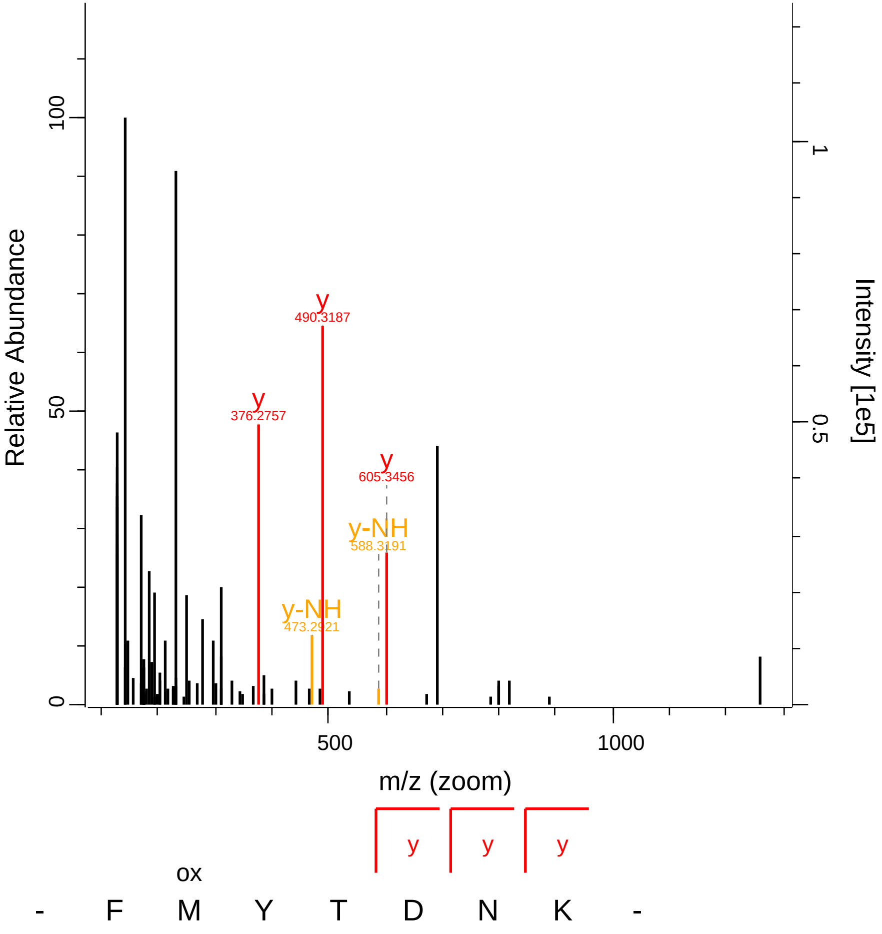

Raw File	Scan	Method	Score	m/z	Gene names
RO-QC-160121_ZackD_SS_Set_01_Fraction	4532	FTMS; HC	72.64	776.86	USP42

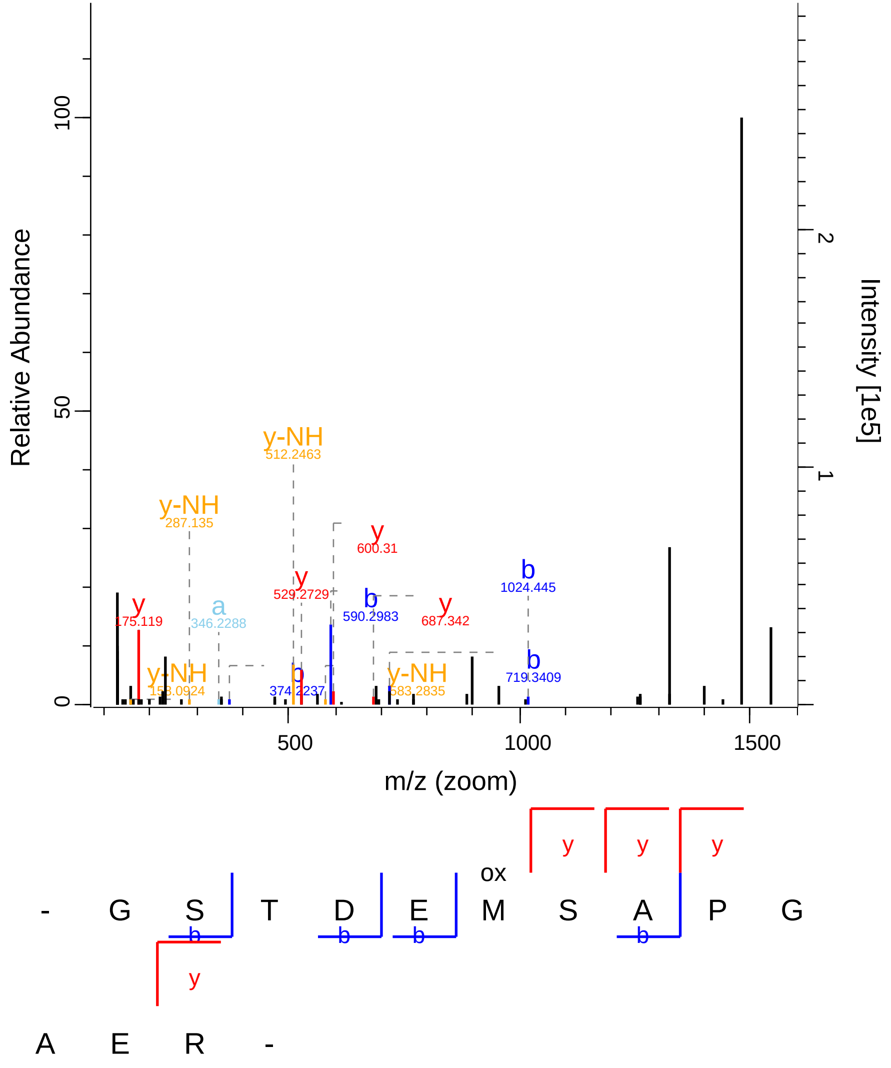

Raw File	Scan	Method	Score	m/z	Gene names
RO-QC-160121_ZackD_SS_Set_01_Fraction	5143	FTMS; HC	102.53	494.92	TMCO6

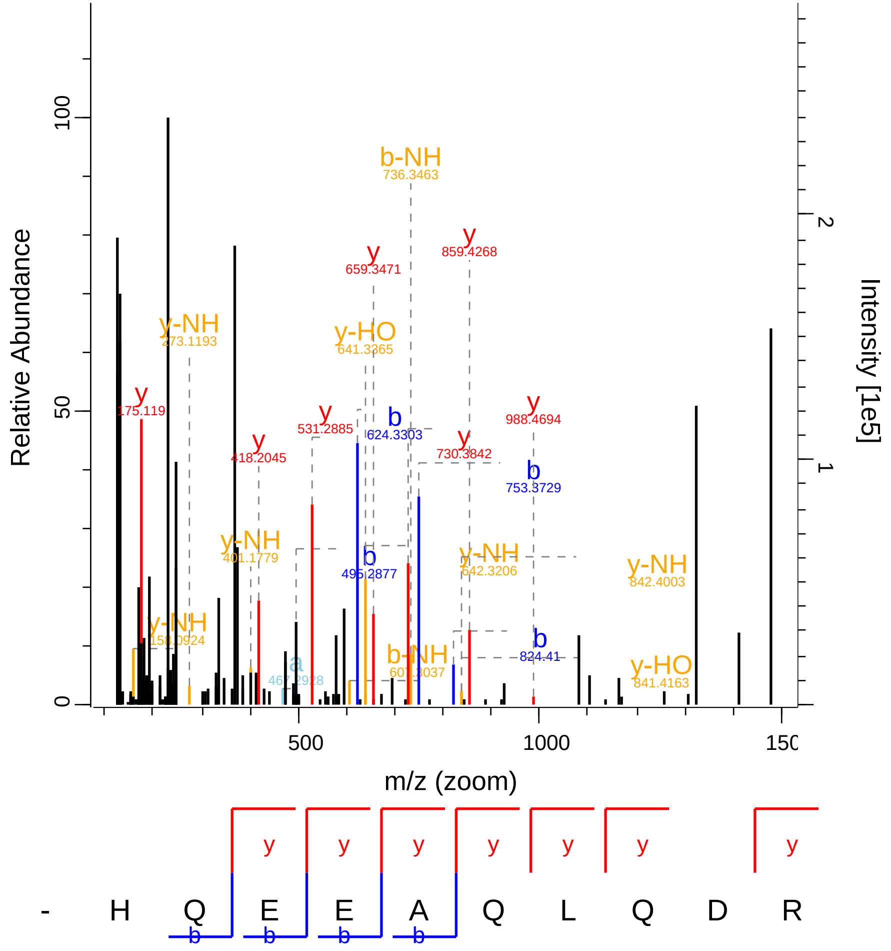

Raw File	Scan	Method	Score	m/z
RO-QC-160121_ZackD_SS_Set_01_Fraction_10	5408	FTMS; HCD	72.55	447.59

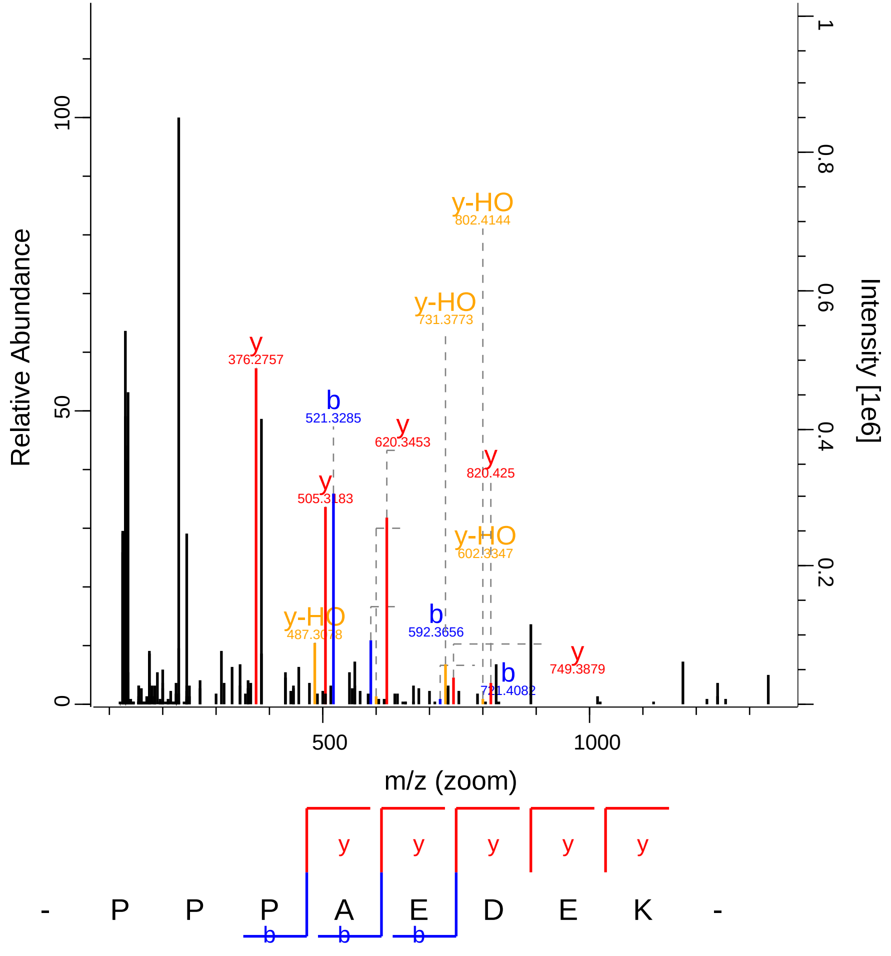

Raw File	Scan	Method	Score	m/z	Gene names
RO-QC-160121_ZackD_SS_Set_01_Fraction	5508	FTMS; HC	44.39	834.41	B3GALT2

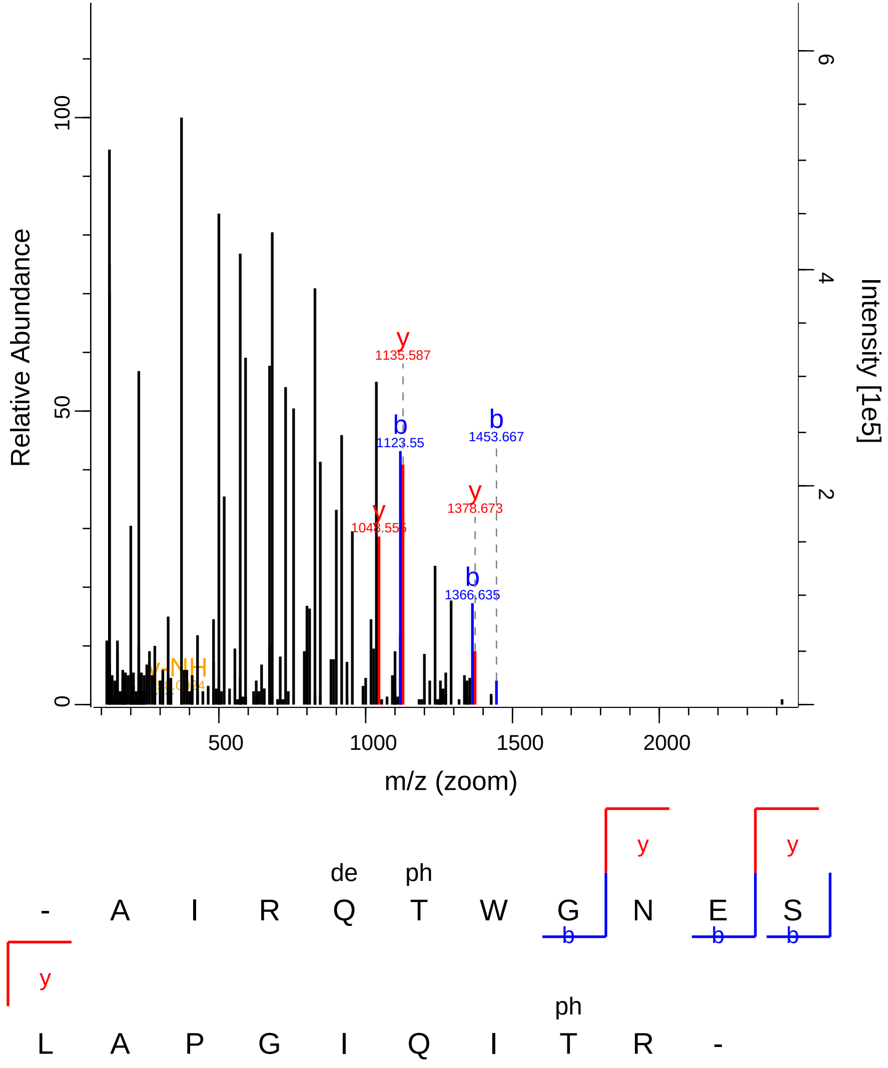

Raw File	Scan	Method	Score	m/z	Gene names
RO-QC-160121_ZackD_SS_Set_01_Fraction	5543	FTMS; HC	168	650.35	CD99

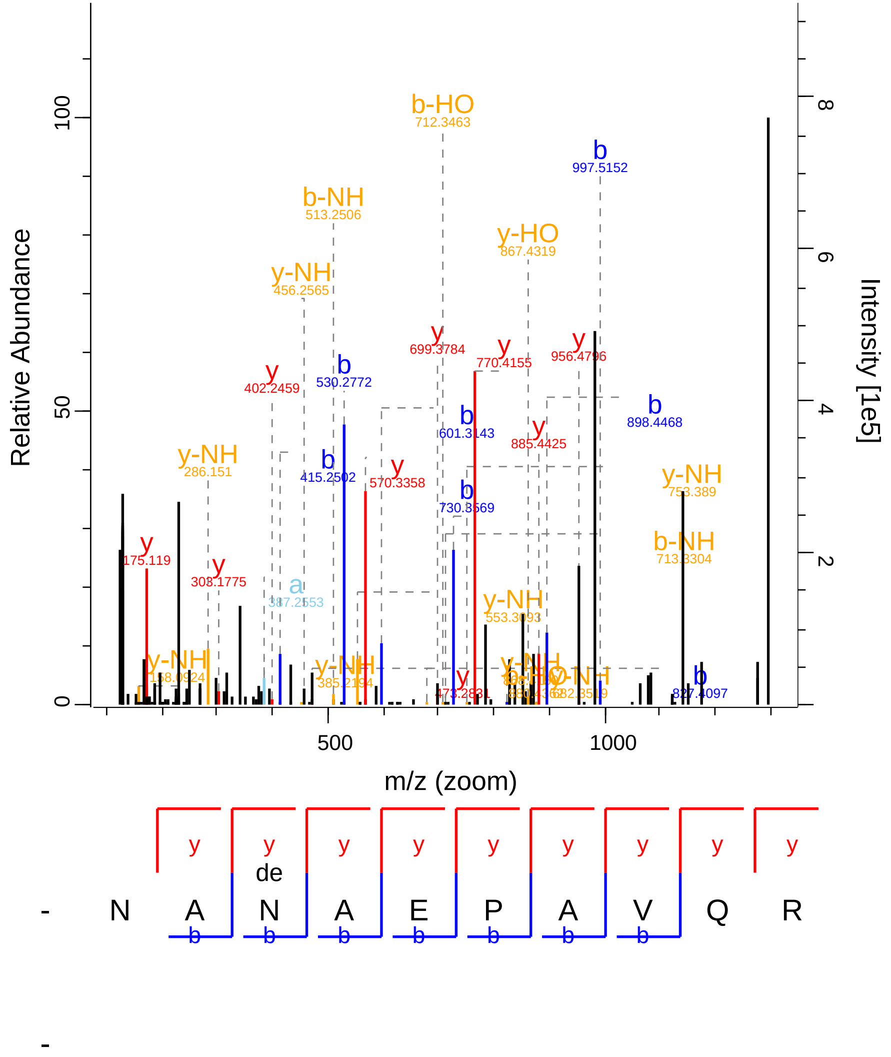

Raw File	Scan	Method	Score	m/z	Gene names
RO-QC-160121_ZackD_SS_Set_01_Fraction_	5613	FTMS; HC	115.71	674.3	FZD7

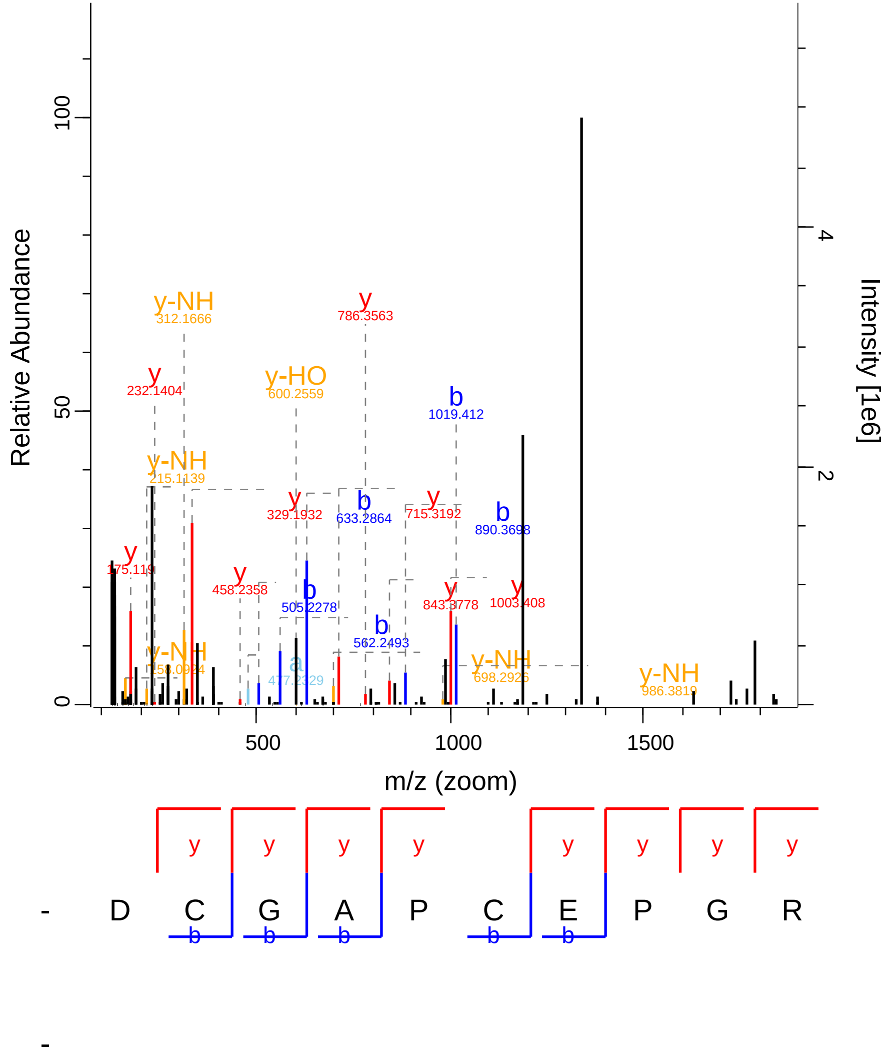

Raw File	Scan	Method	Score	m/z	Gene names
RO-QC-160121_ZackD_SS_Set_01_Fraction	5758	FTMS; HC	161.44	650.35	CD99

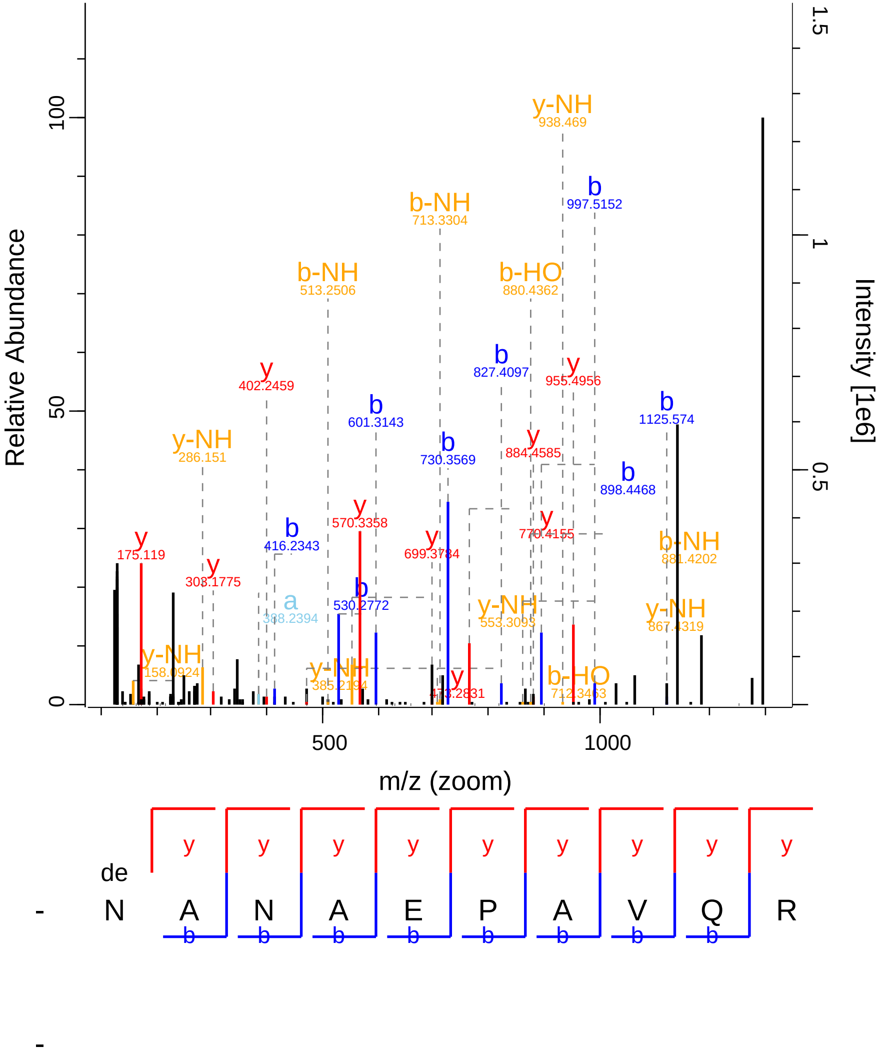

Raw File	Scan	Method	Score	m/z	Gene names
RO-QC-160121_ZackD_SS_Set_01_Fraction	5865	FTMS; HC	54.55	440.52	C15orf41

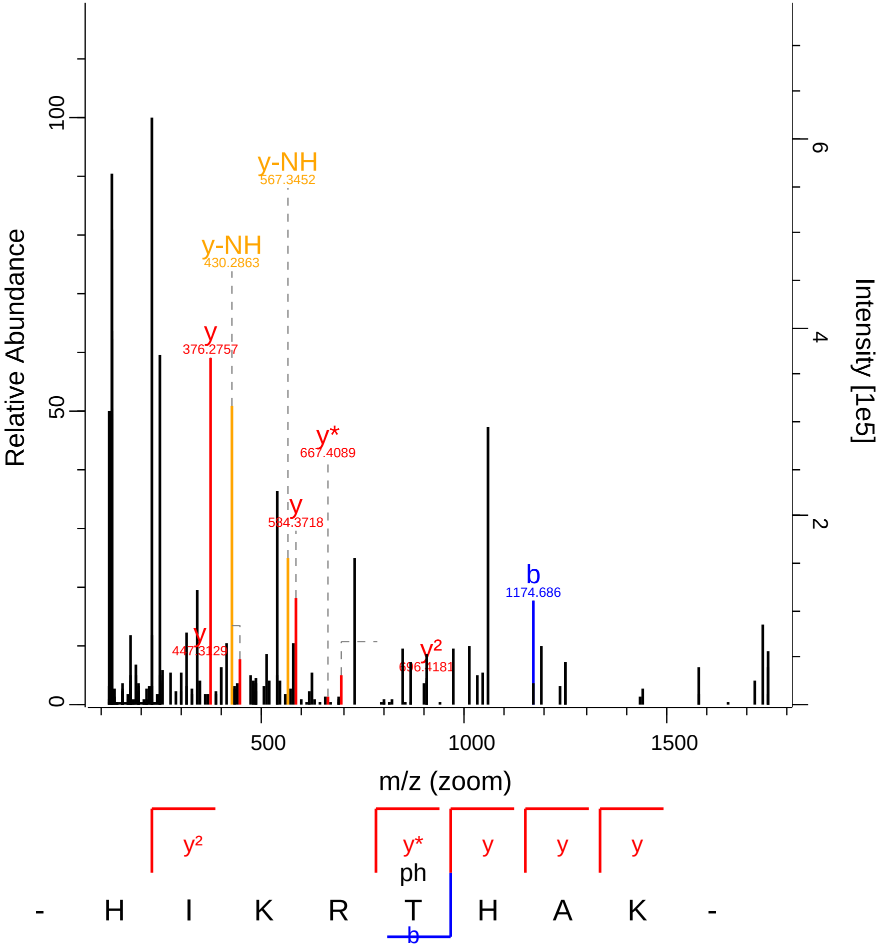

Raw File	Scan	Method	Score	m/z	Gene names
RO-QC-160121_ZackD_SS_Set_01_Fraction	6052	FTMS; HC	53.49	668.82	DSG1

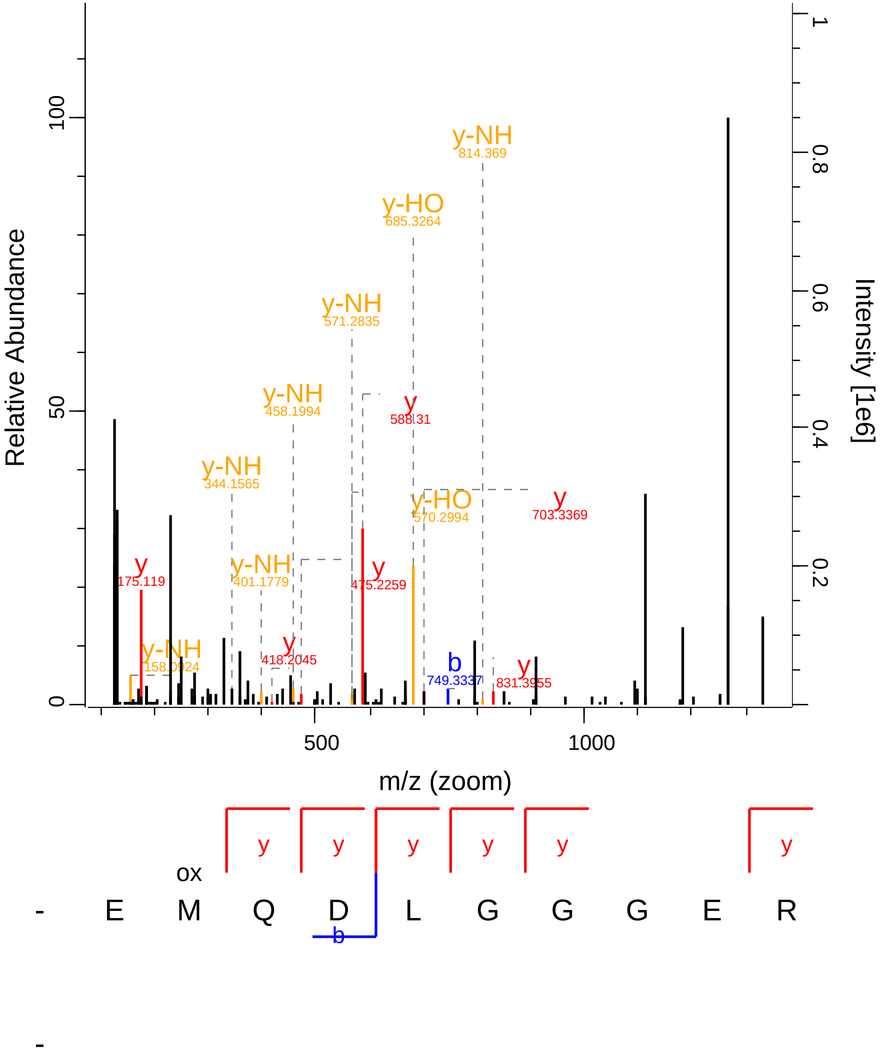

Raw File	Scan	Method	Score	m/z	Gene names
RO-QC-160121_ZackD_SS_Set_01_Fraction	6141	FTMS; HC	131.12	650.35	CD99

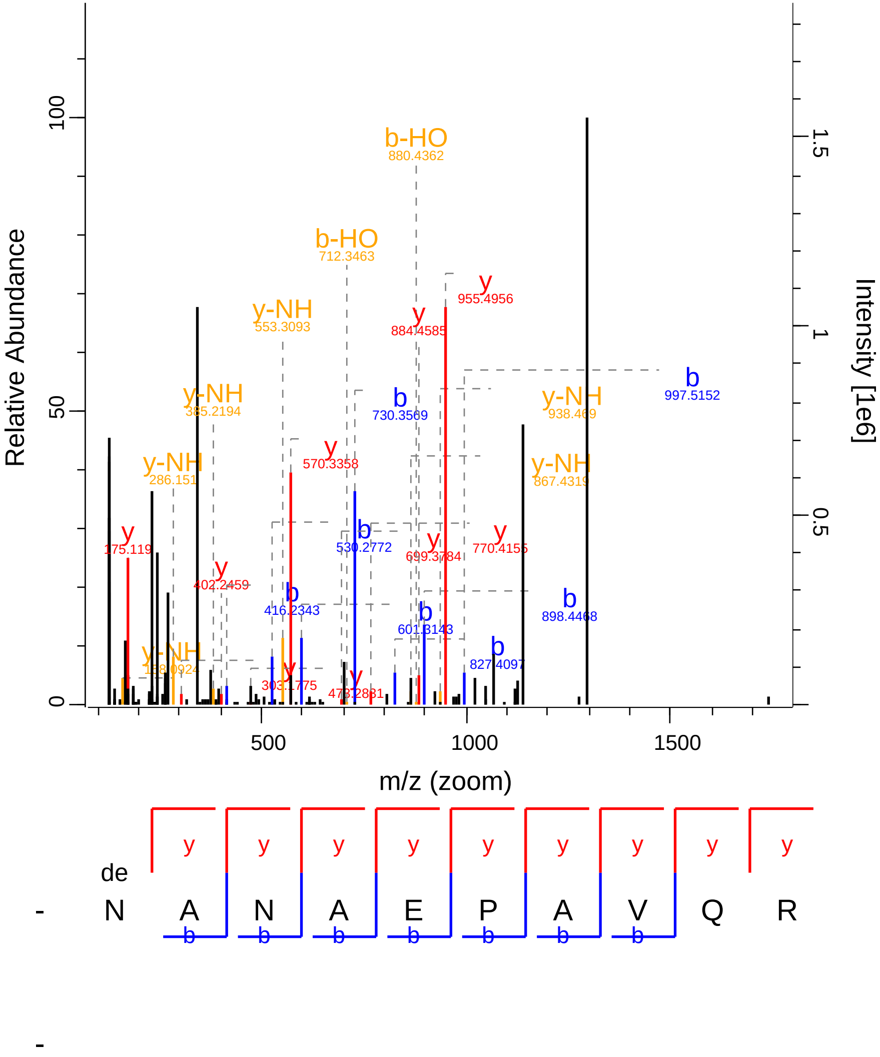

Raw File	Scan	Method	Score	m/z
RO-QC-160121_ZackD_SS_Set_01_Fraction_10	6410	FTMS; HCD	98.52	460.92

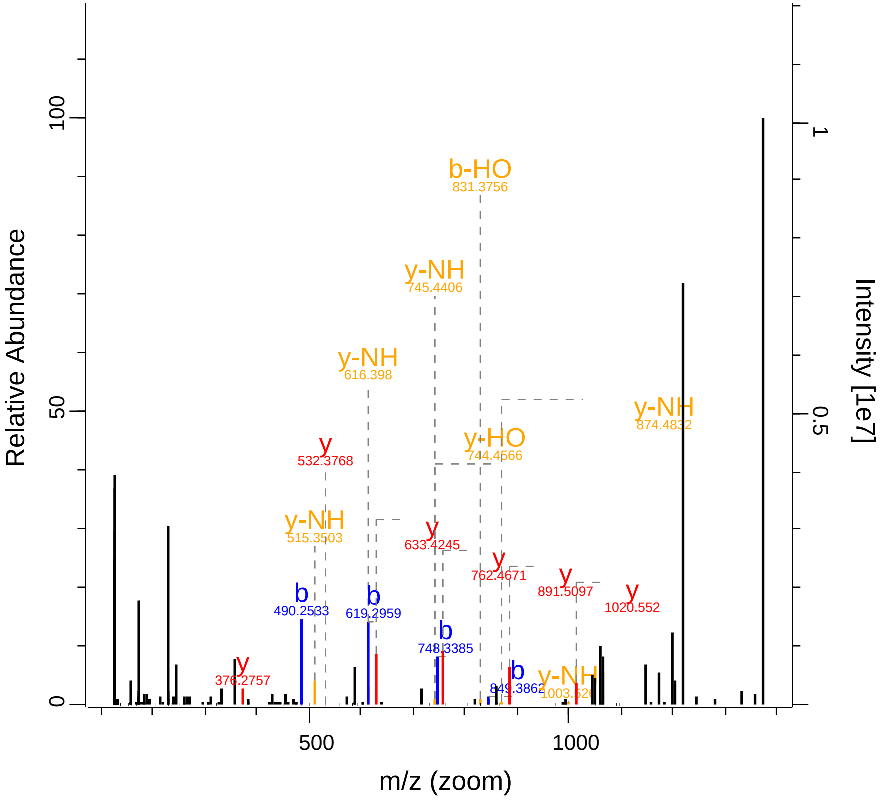

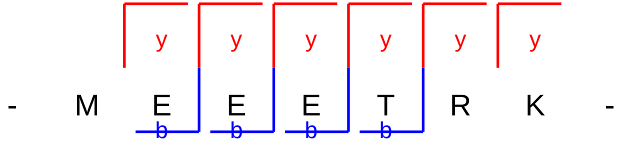

Raw File	Scan	Method	Score	m/z
RO-QC-160121_ZackD_SS_Set_01_Fraction_10	6844	FTMS; HCD	68	352.79

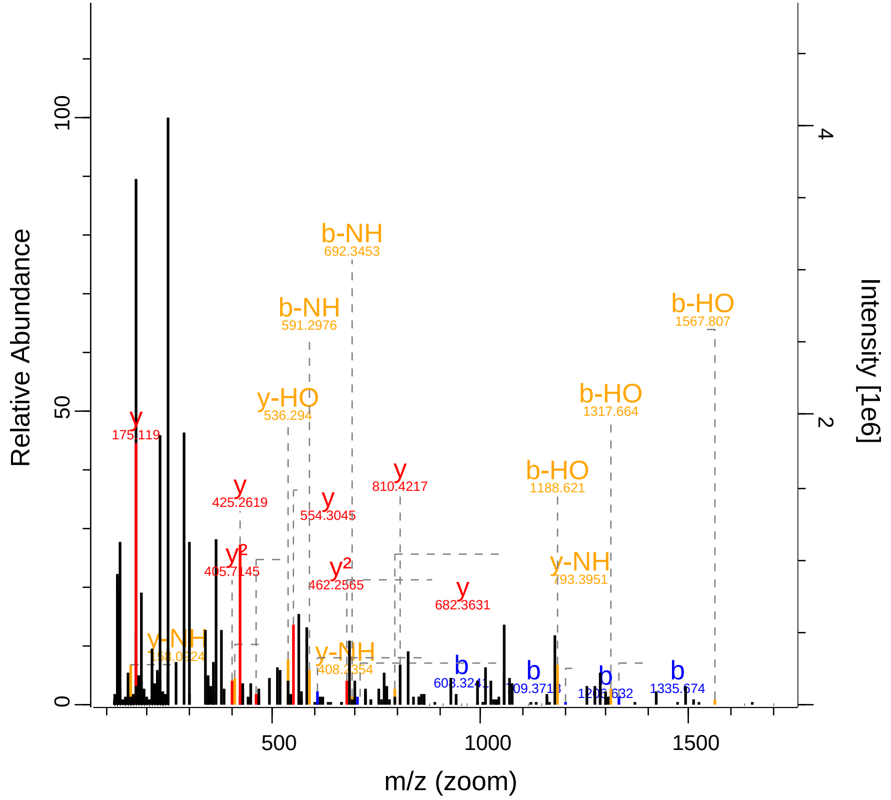

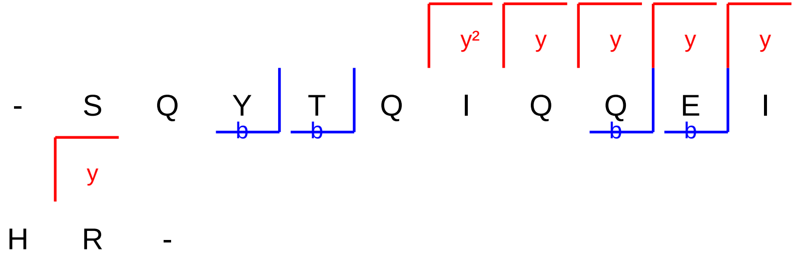

Raw File	Scan	Method	Score	m/z
RO-QC-160121_ZackD_SS_Set_01_Fraction_10	7655	FTMS; HCD	40.45	362.87

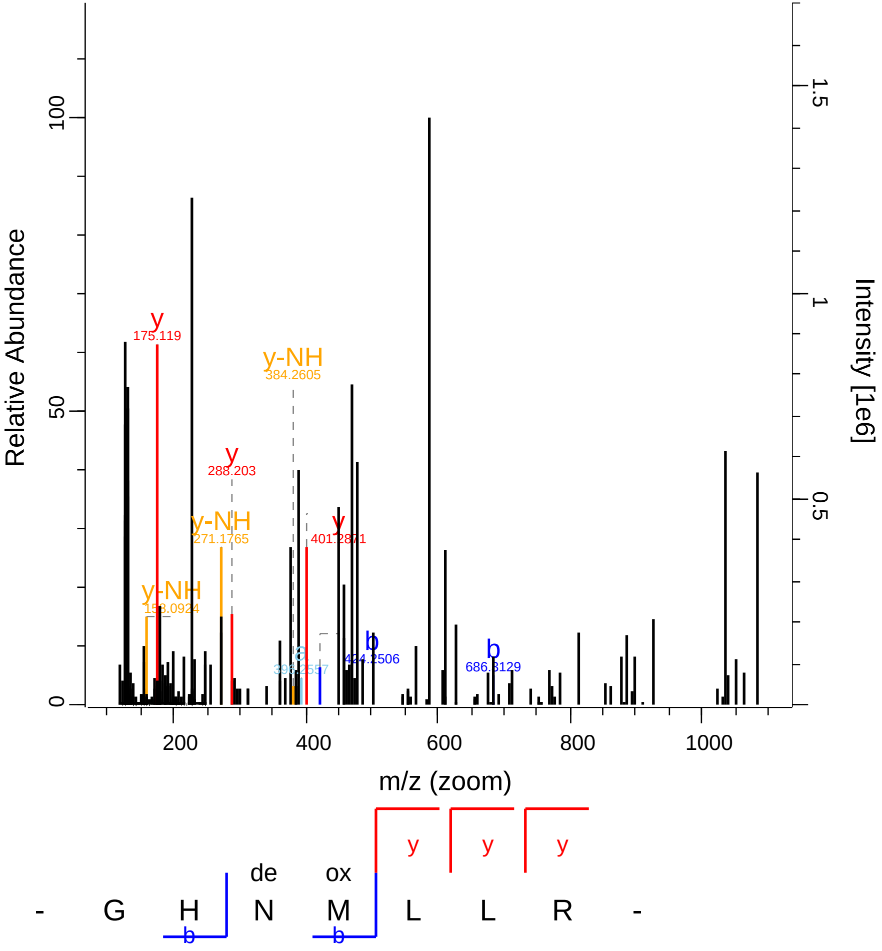

Raw File	Scan	Method	Score	m/z	Gene names
RO-QC-160121_ZackD_SS_Set_01_Fraction	8397	FTMS; HC	44.45	809.89	ERVK-16

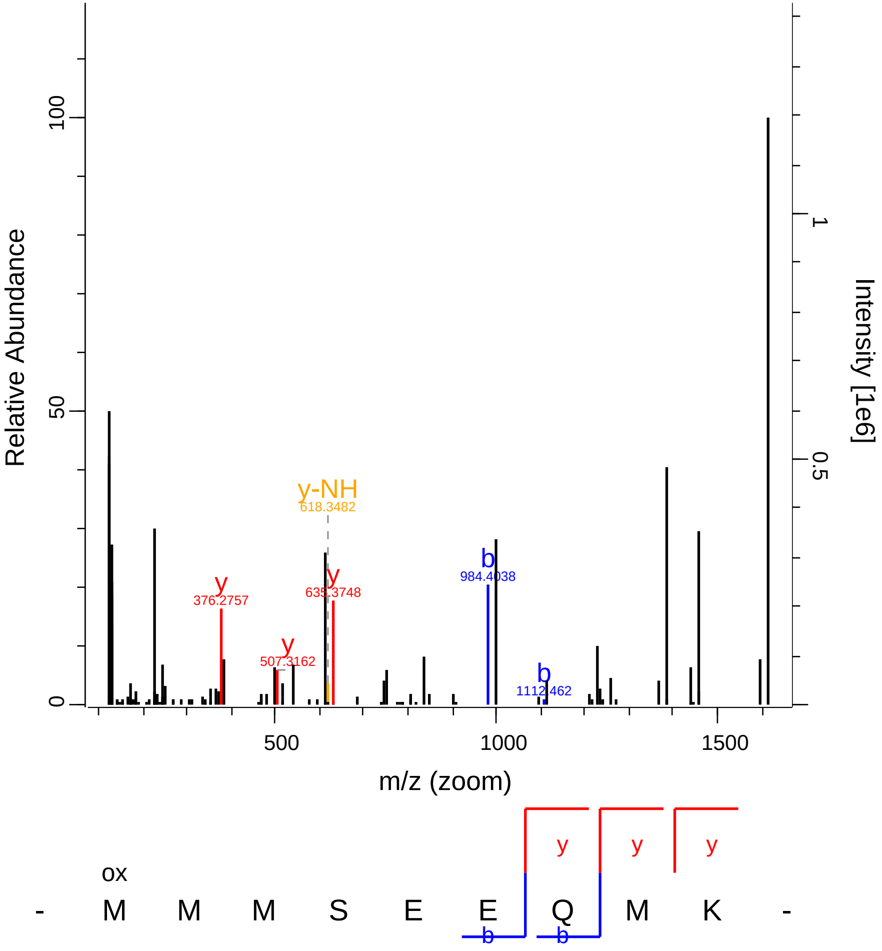

Raw File	Scan	Method	Score	m/z	Gene names
RO-QC-160121_ZackD_SS_Set_01_Fractior	11789	FTMS; HC	96.01	730.38	SLC16A10

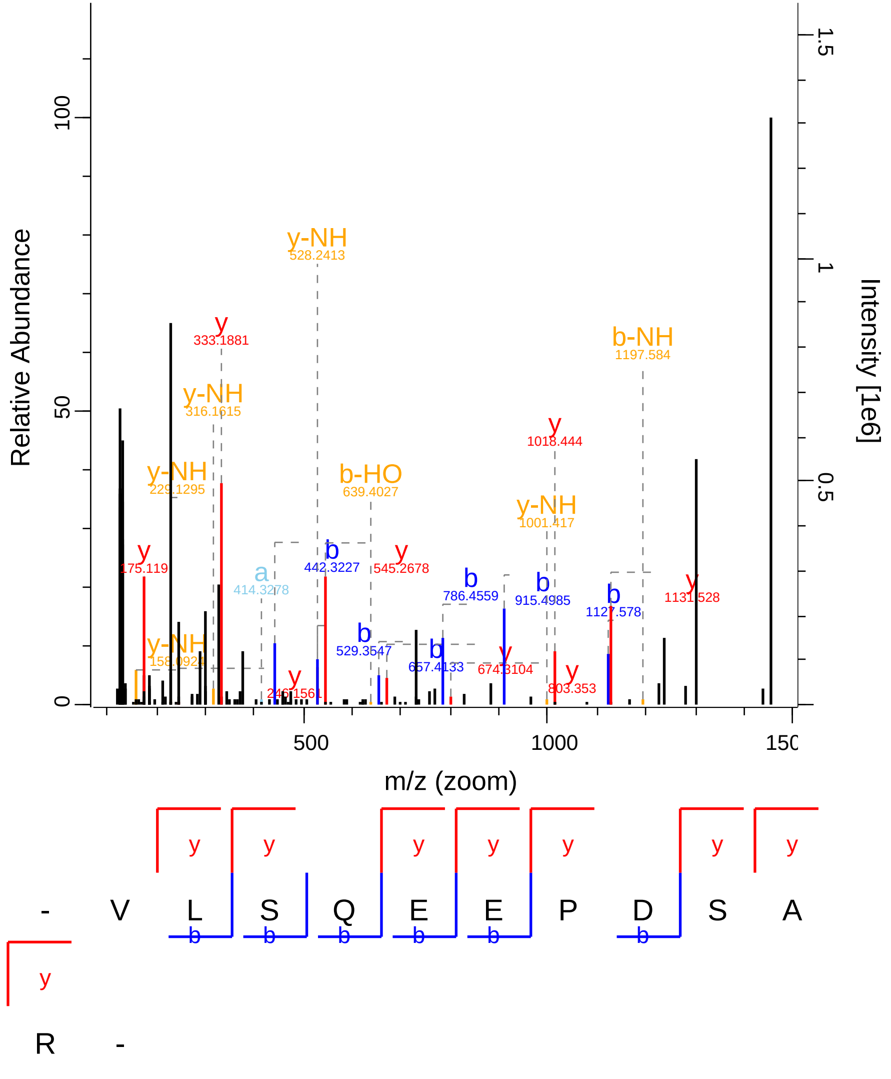

Raw File	Scan	Method	Score	m/z	Gene names
RO-QC-160121_ZackD_SS_Set_01_Fractior	11844	FTMS; HC	44.69	729.45	SFMBT2

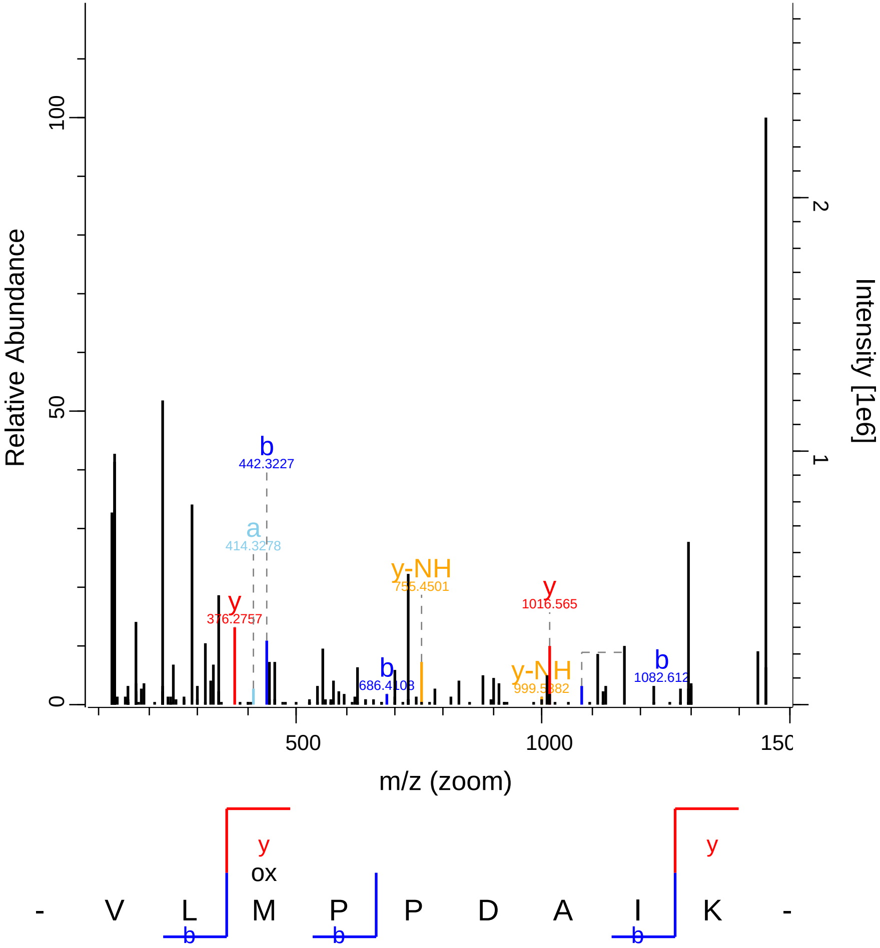

Raw File	Scan	Method	Score	m/z	Gene names
RO-QC-160121_ZackD_SS_Set_01_Fractio	11865	FTMS; HC	202.57	798.37	FXYD1

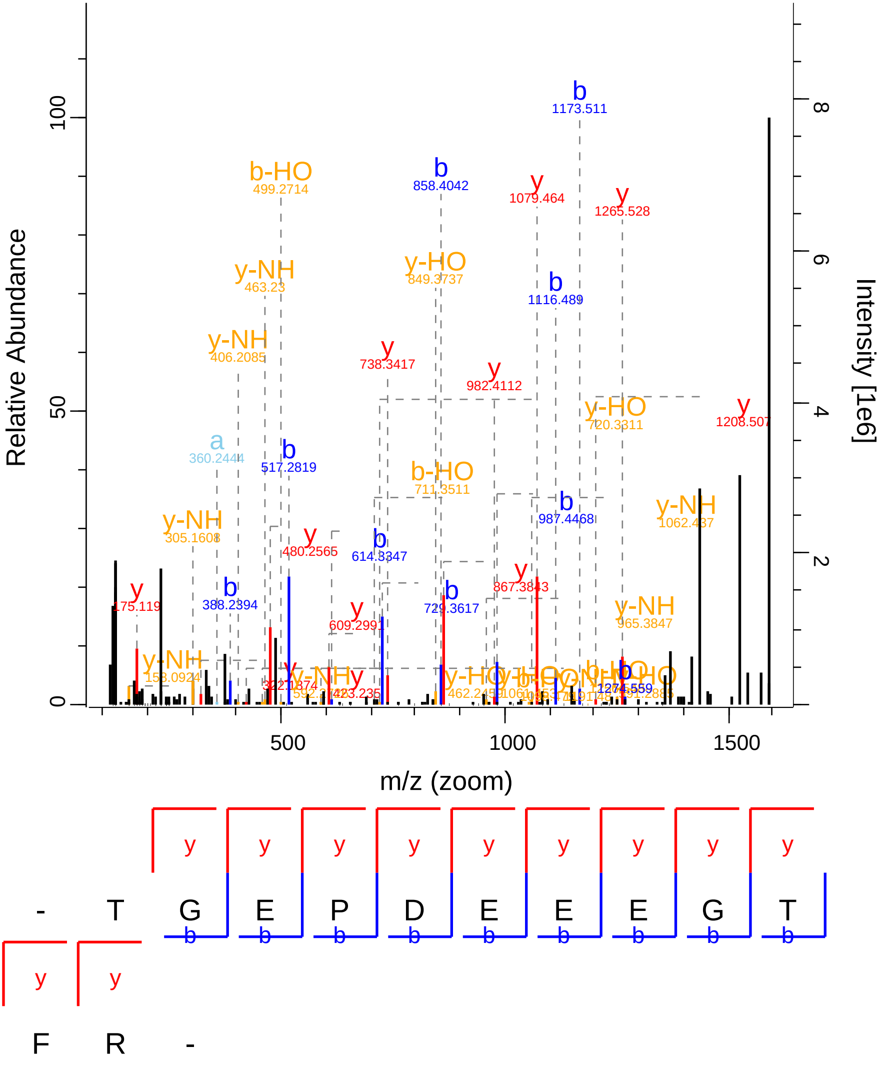

Raw File	Scan	Method	Score	m/z	Gene names
RO-QC-160121_ZackD_SS_Set_01_Fractio[illegible]	12133	FTMS; HC[illegible]	261.87	798.37	FXYD1

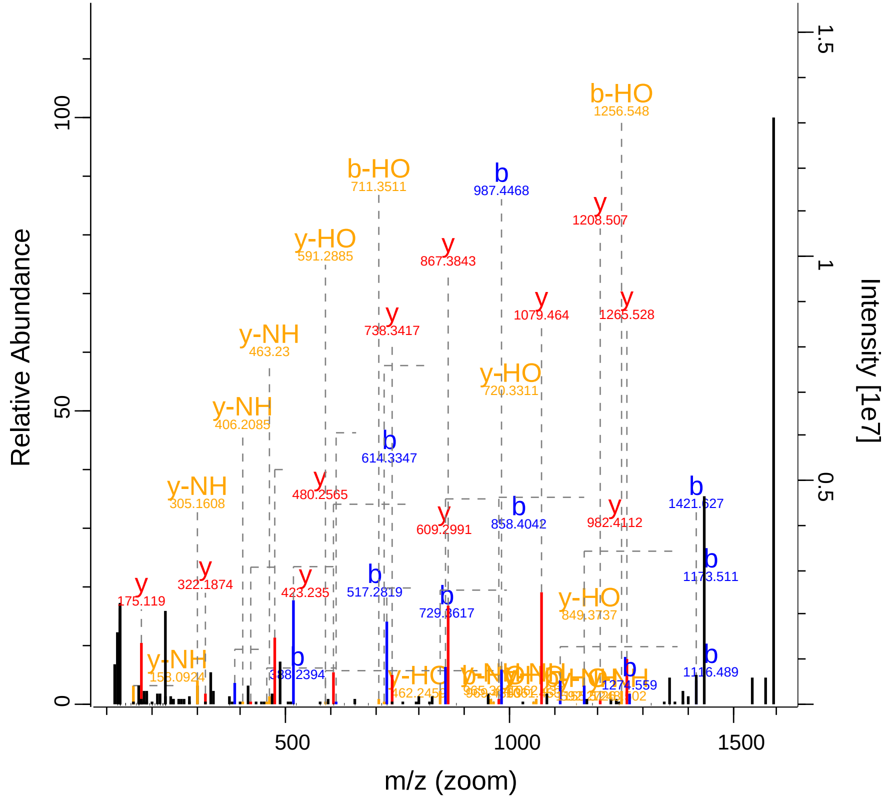

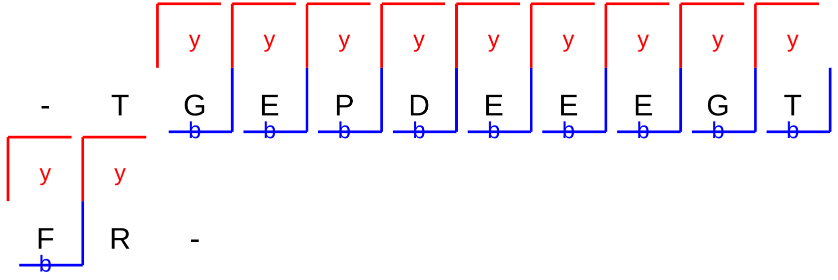

Raw File	Scan	Method	Score	m/z	Gene names
RO-QC-160121_ZackD_SS_Set_01_Fractior	13277	FTMS; HC	111.04	602.89	EXOC3L4

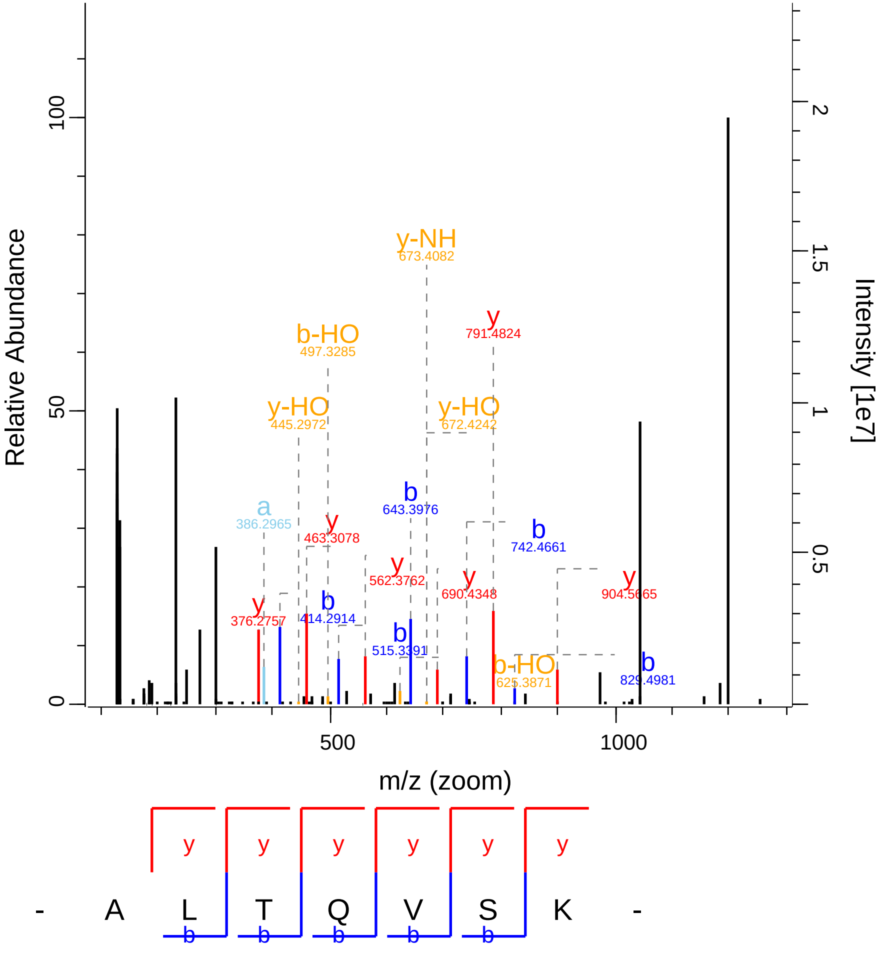

Raw File	Scan	Method	Score	m/z	Gene names
RO-QC-160121_ZackD_SS_Set_01_Fractior	13410	FTMS; HC	58.98	402.26	EXOC3L4

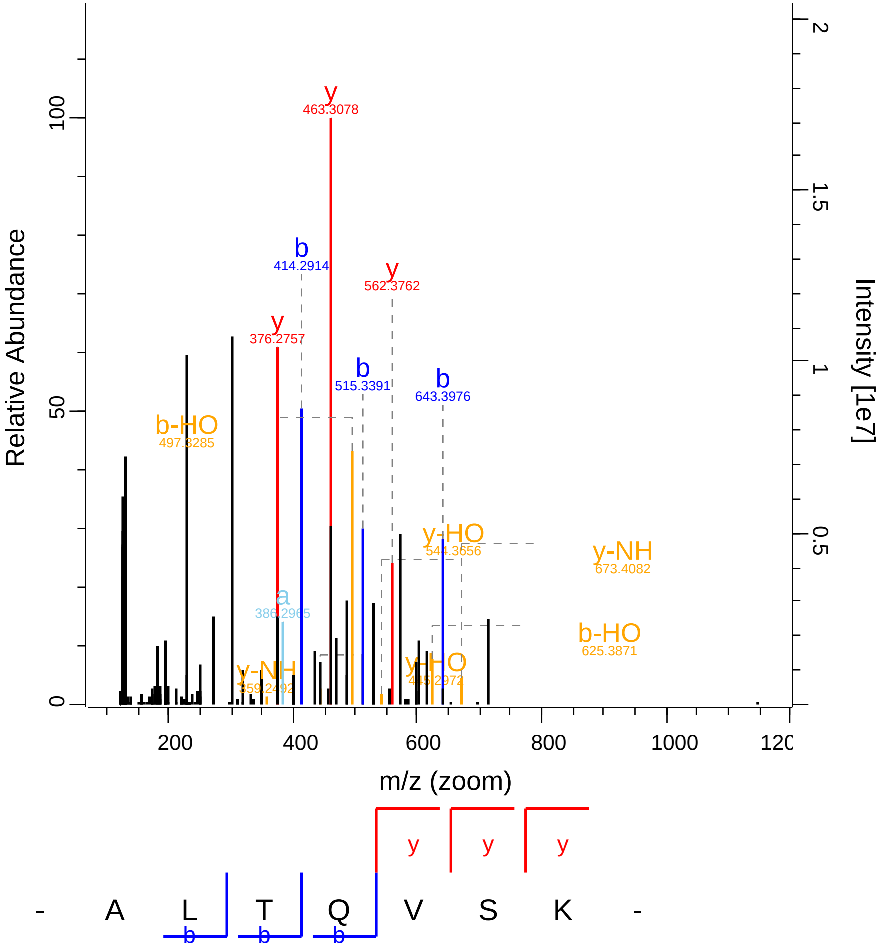

Raw File	Scan	Method	Score	m/z	Gene names
RO-QC-160121_ZackD_SS_Set_01_Fractior	13612	FTMS; HC	65.25	715.38	TMIGD3

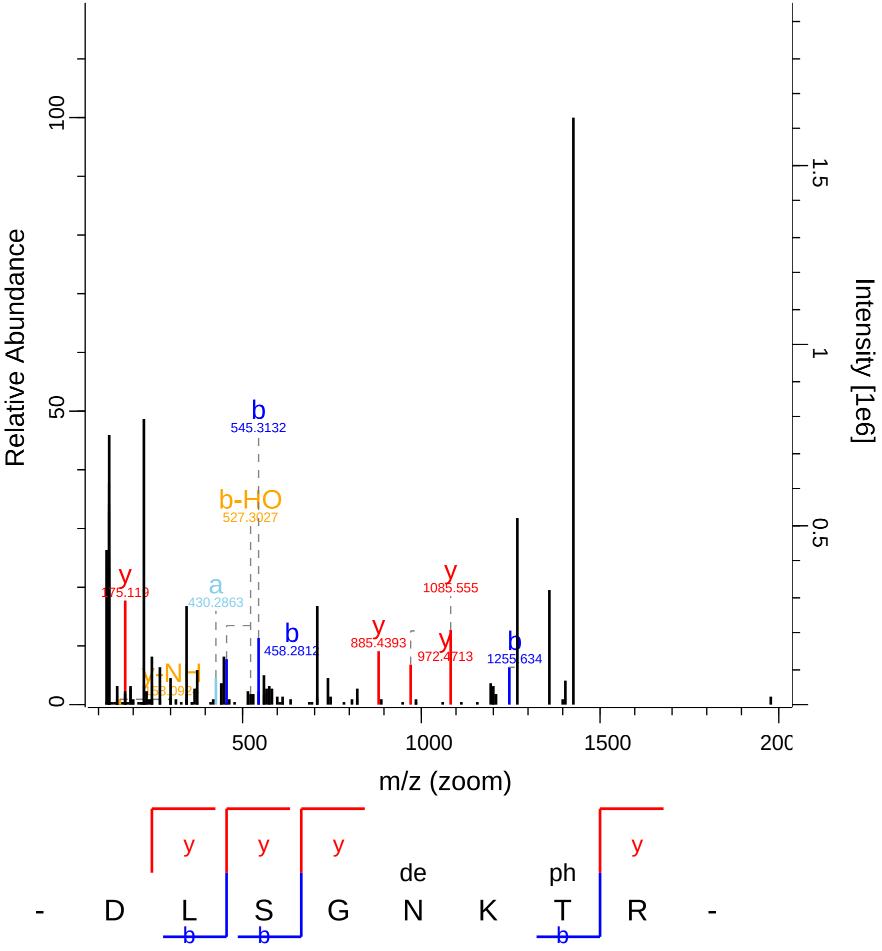

Raw File	Scan	Method	Score	m/z	Gene names
RO-QC-160121_ZackD_SS_Set_01_Fractior	13946	FTMS; HC	83.62	577.69	PEO1

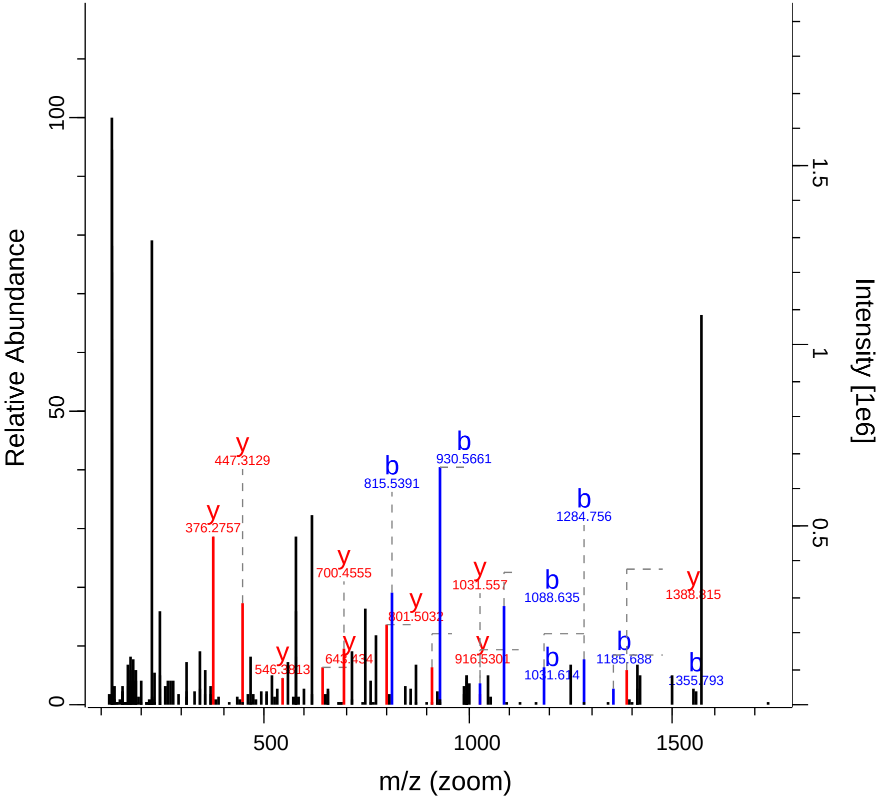

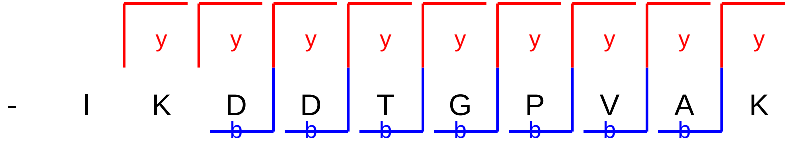

Raw File	Scan	Method	Score	m/z
RO-QC-160121_ZackD_SS_Set_01_Fraction_10	14837	FTMS; HCD	49.36	378.21

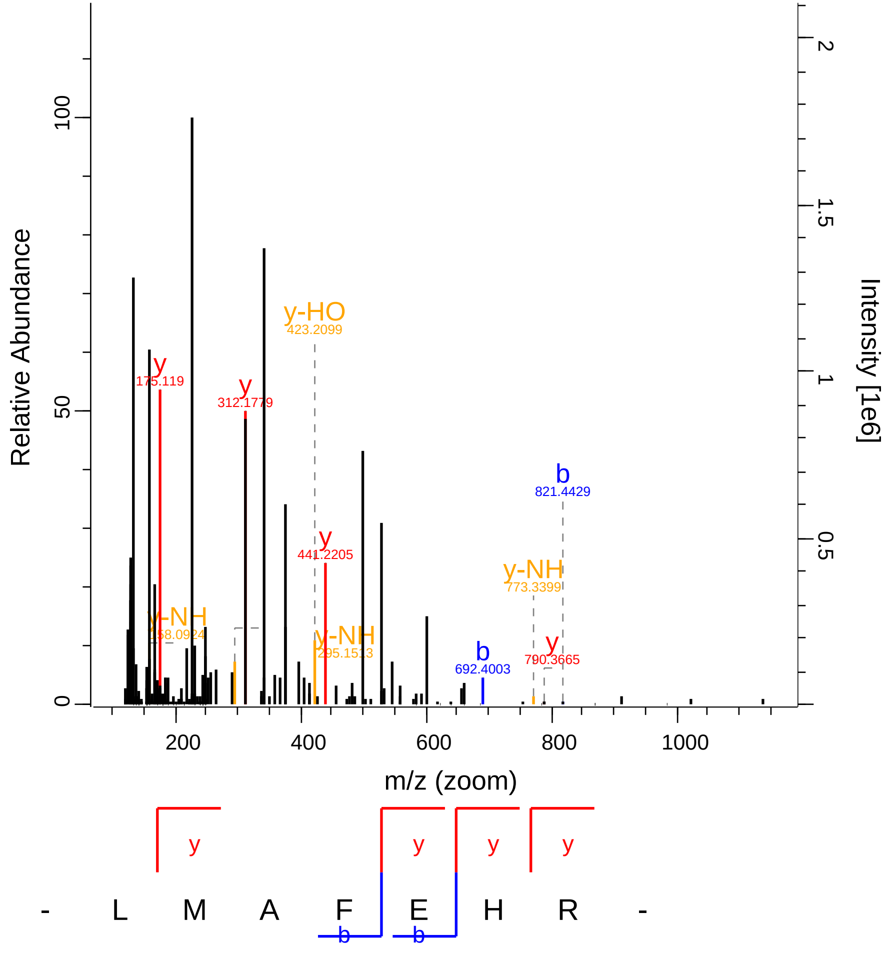

Raw File	Scan	Method	Score	m/z	Gene names
RO-QC-160121_ZackD_SS_Set_01_Fractior	15081	FTMS; HC	77.68	413.57	TAF12

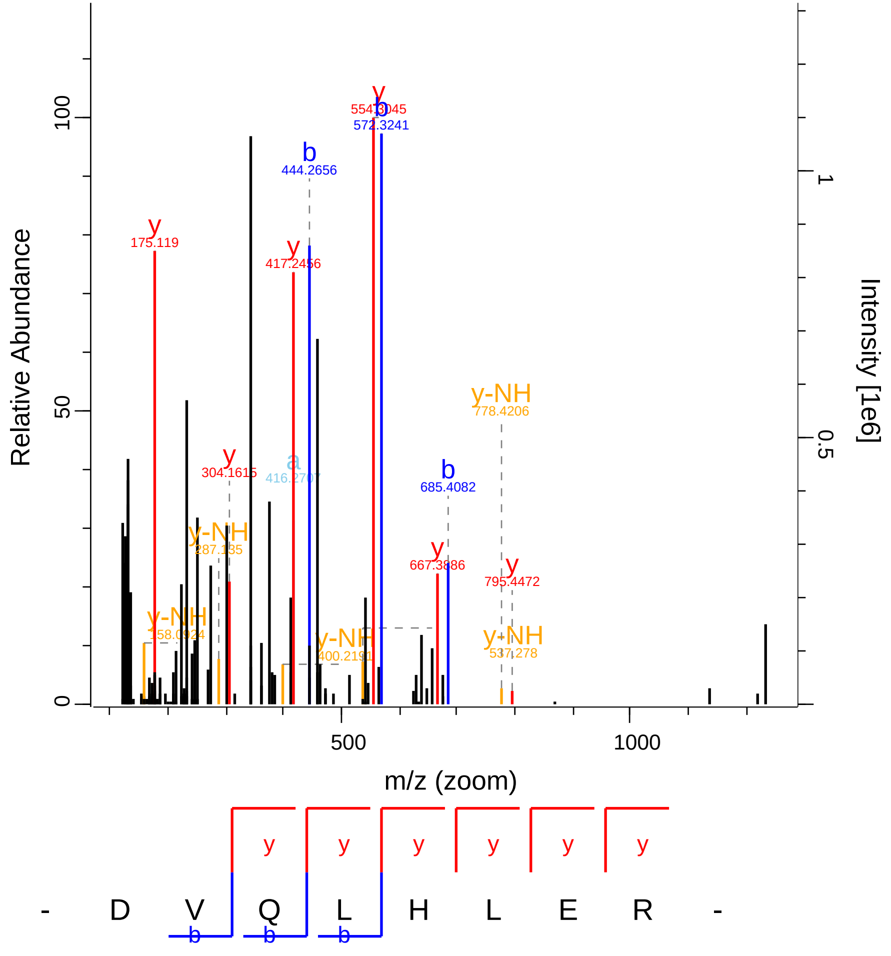

Raw File	Scan	Method	Score	m/z	Gene names
RO-QC-160121_ZackD_SS_Set_01_Fractio[illegible]	15367	FTMS; HC	150.35	719.89	CYSTM1

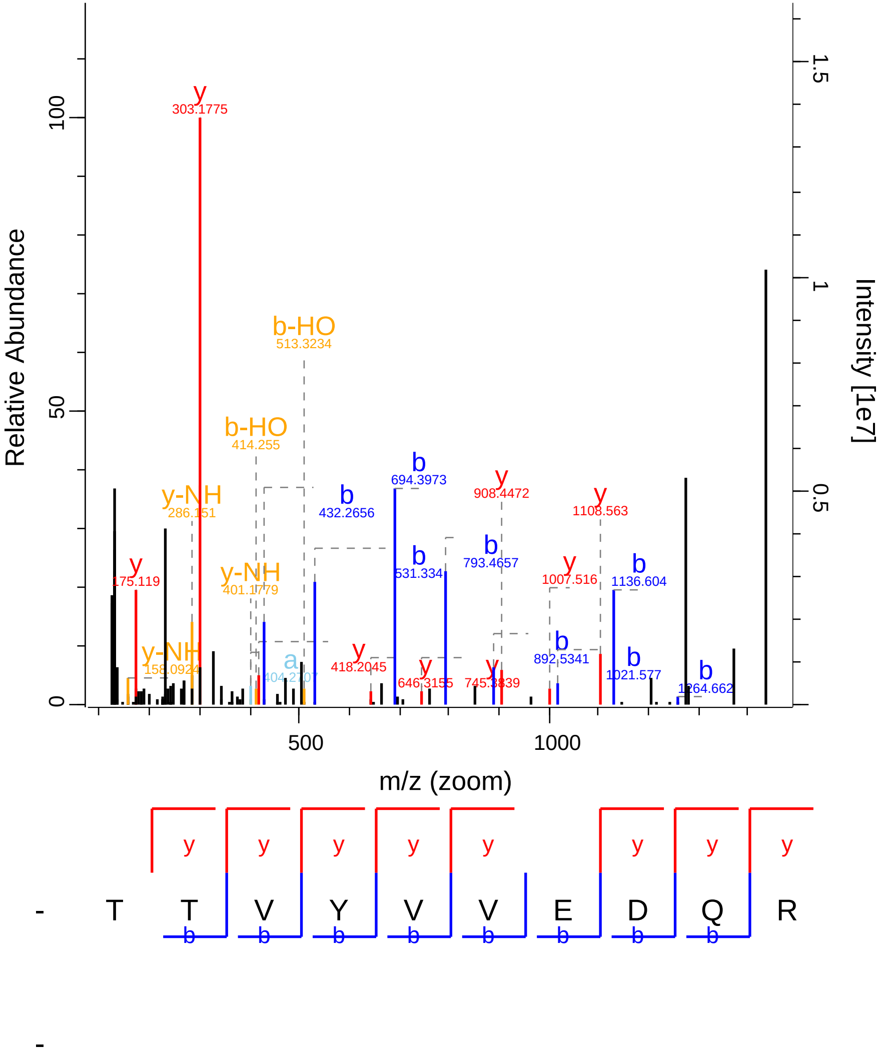

Raw File	Scan	Method	Score	m/z	Gene names
RO-QC-160121_ZackD_SS_Set_01_Fractior	15465	FTMS; HC	43.7	906.16	ZNF385A

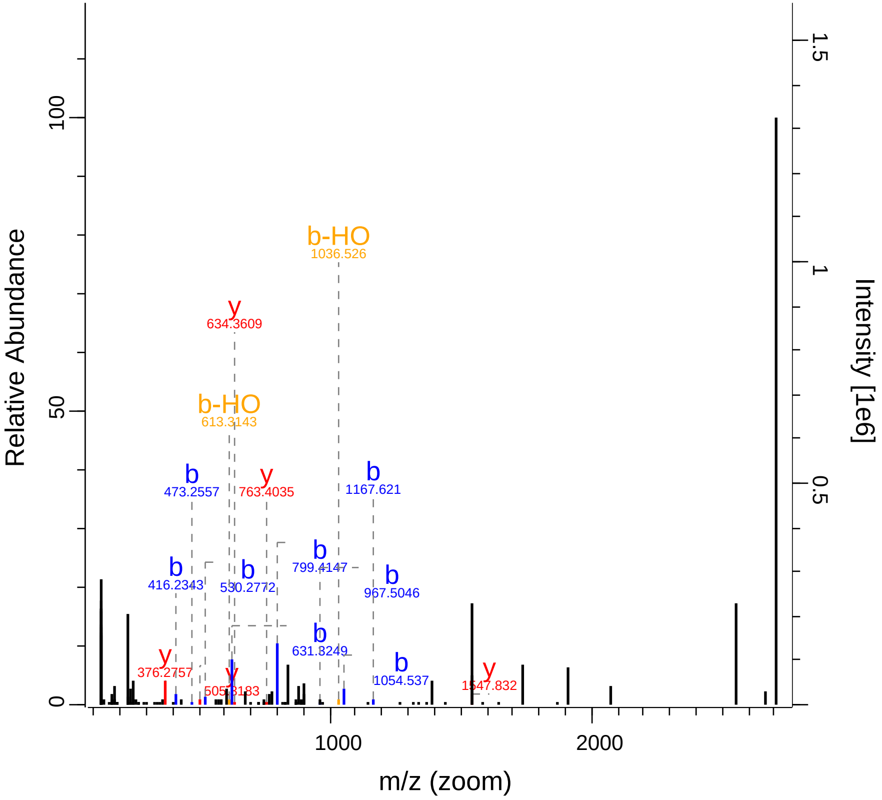

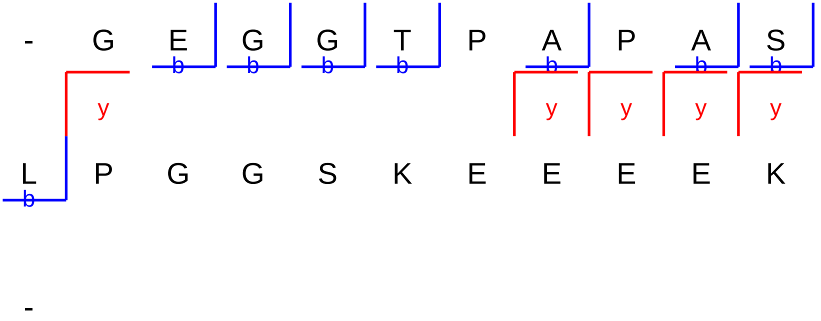

Raw File	Scan	Method	Score	m/z	Gene names
RO-QC-160121_ZackD_SS_Set_01_Fractioı	15497	FTMS; HC	131.54	905.49	ZNF385A

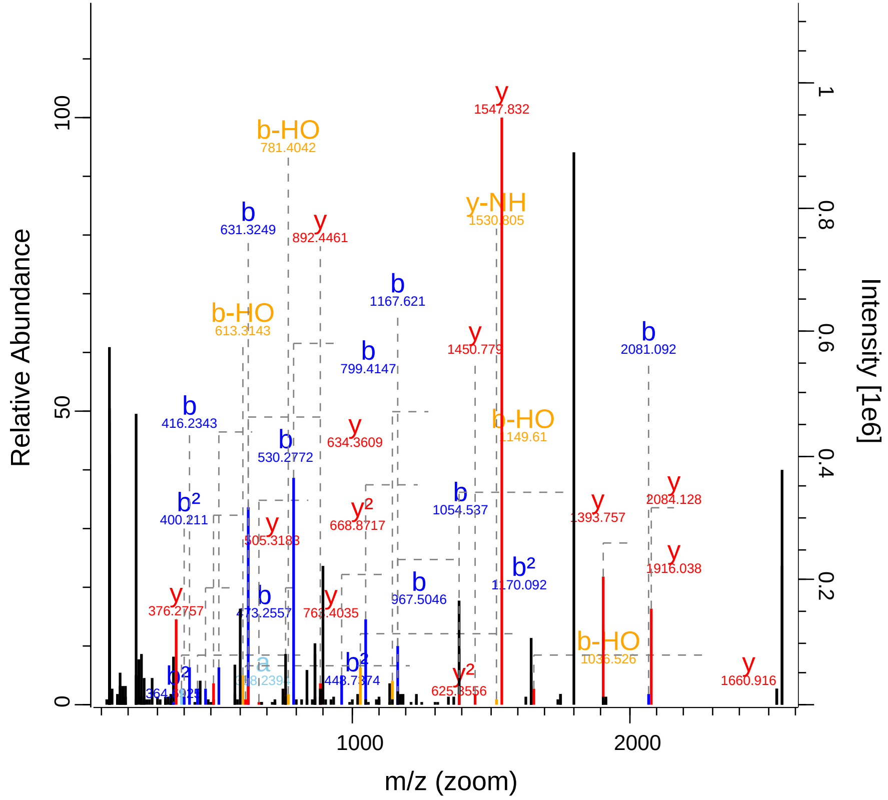

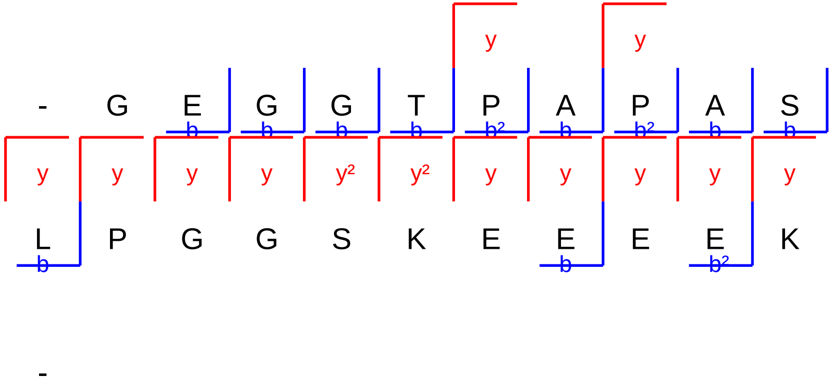

Raw File	Scan	Method	Score	m/z
RO-QC-160121_ZackD_SS_Set_01_Fraction_10	15943	FTMS; HCD	51.84	461.53

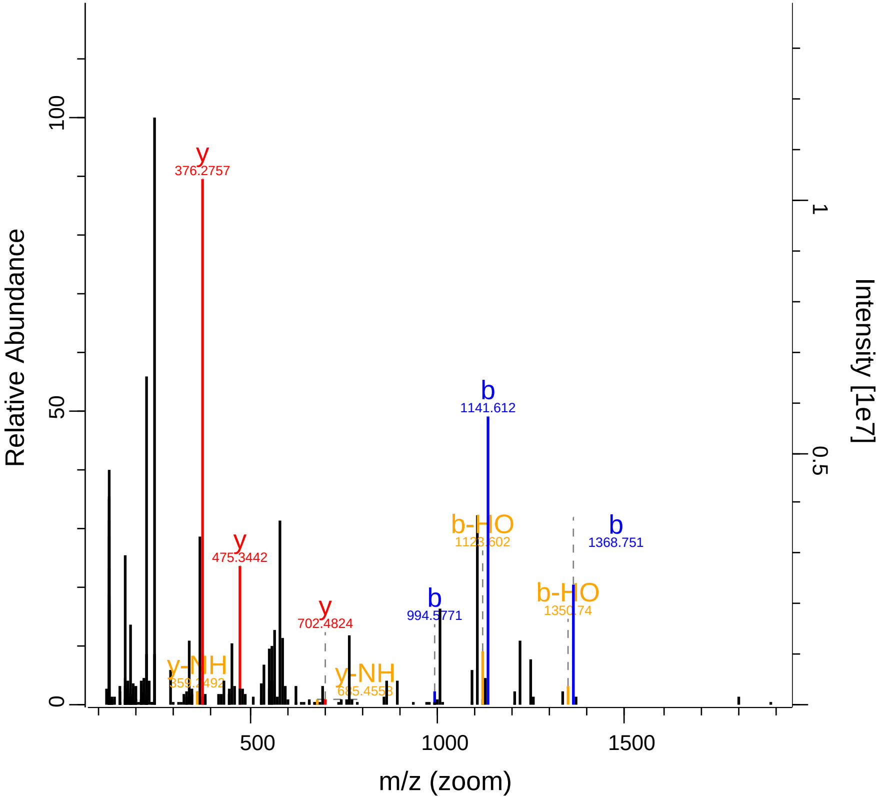

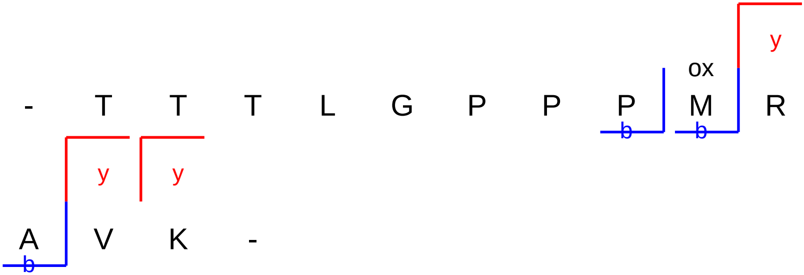

Raw File	Scan	Method	Score	m/z	Gene names
RO-QC-160121_ZackD_SS_Set_01_Fractior	16211	FTMS; HC	53.17	586.97	HBA1

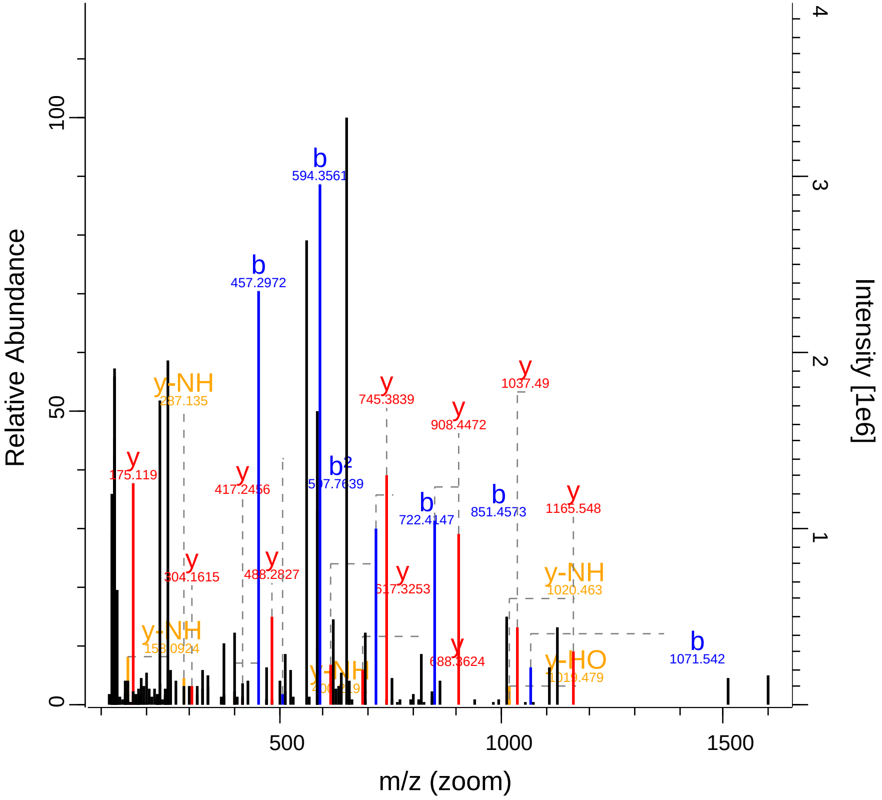

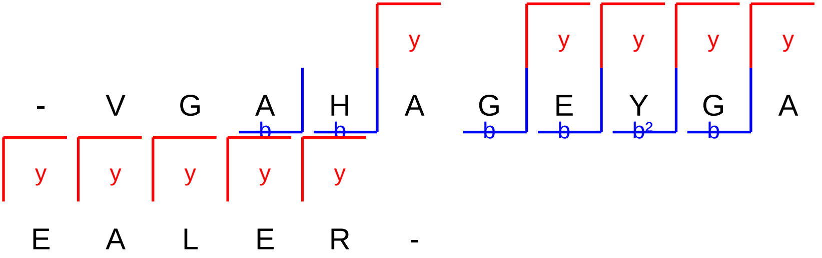

Raw File	Scan	Method	Score	m/z	Gene names
RO-QC-160121_ZackD_SS_Set_01_Fractio	16792	FTMS; HC	131.09	865.44	IGKC

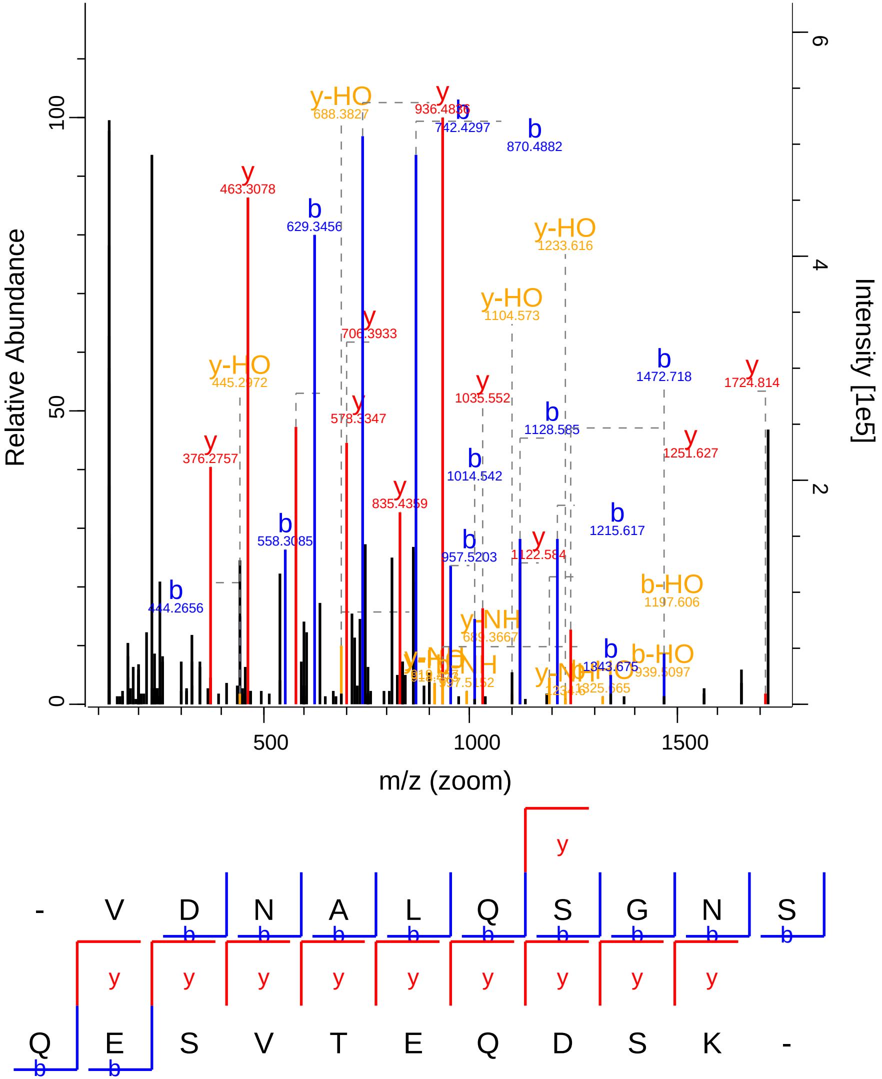

Raw File	Scan	Method	Score	m/z	Gene names
RO-QC-160121_ZackD_SS_Set_01_Fractior	16997	FTMS; HC	71.38	608.34	UTS2

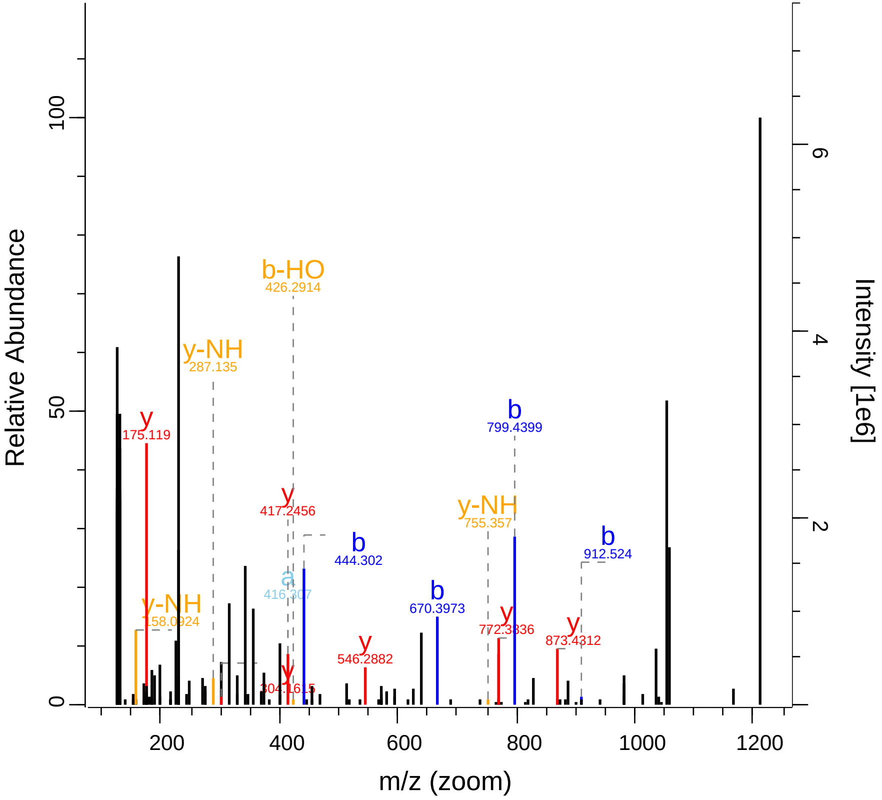

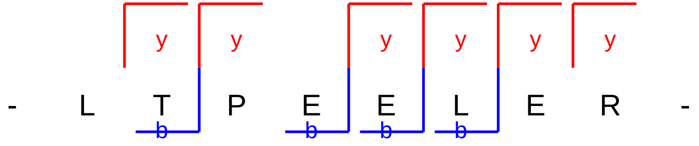

Raw File	Scan	Method	Score	m/z
RO-QC-160121_ZackD_SS_Set_01_Fraction_10	18681	FTMS; HCD	81.78	587.39

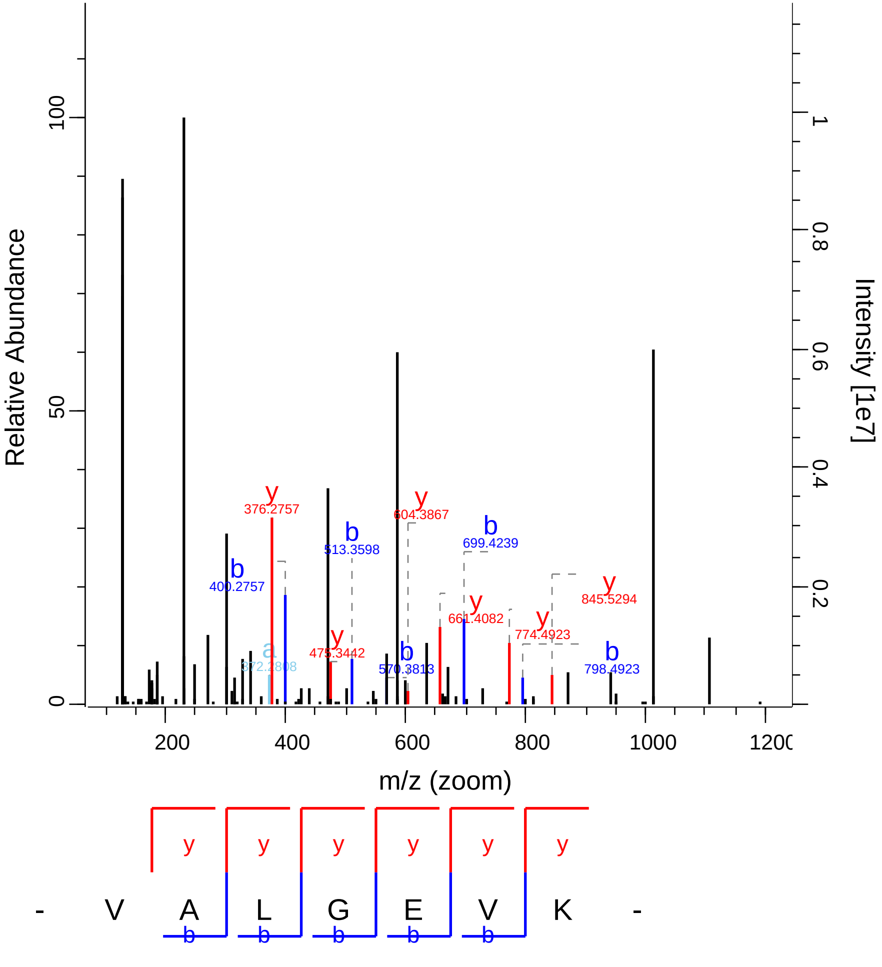

Raw File	Scan	Method	Score	m/z
RO-QC-160121_ZackD_SS_Set_01_Fraction_10	18742	FTMS; HCD	99.92	391.93

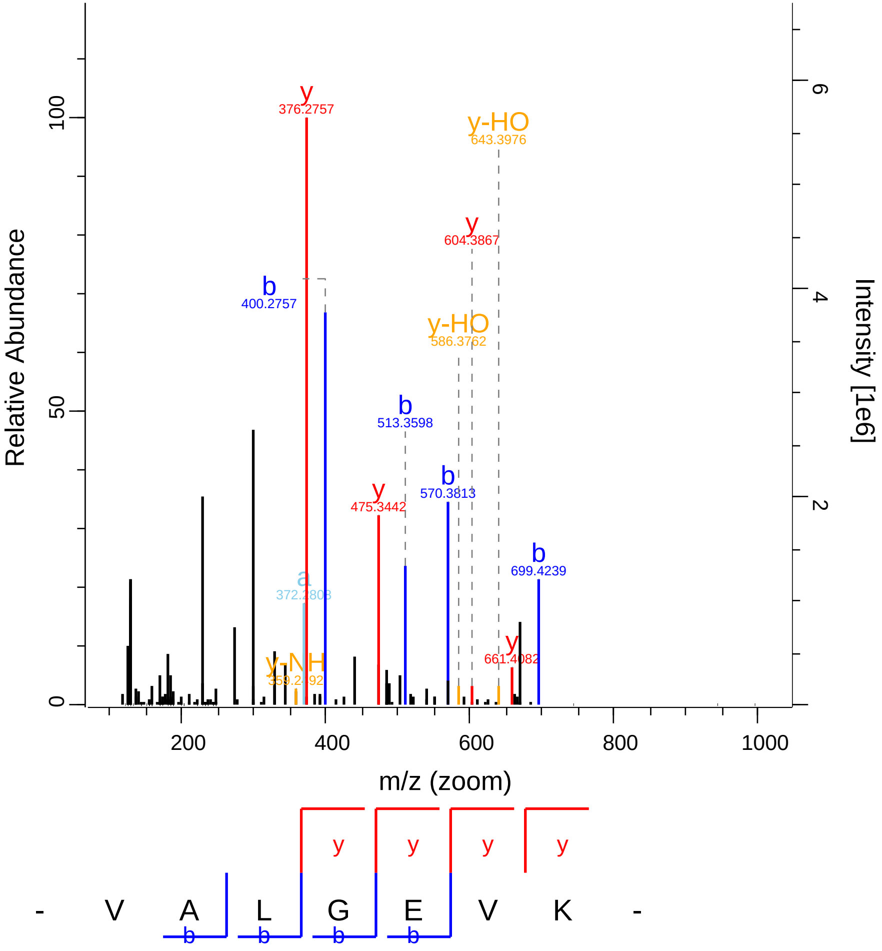

Raw File	Scan	Method	Score	m/z	Gene names
RO-QC-160121_ZackD_SS_Set_01_Fractior	18835	FTMS; HC	68.02	721.92	PPP1R2P1

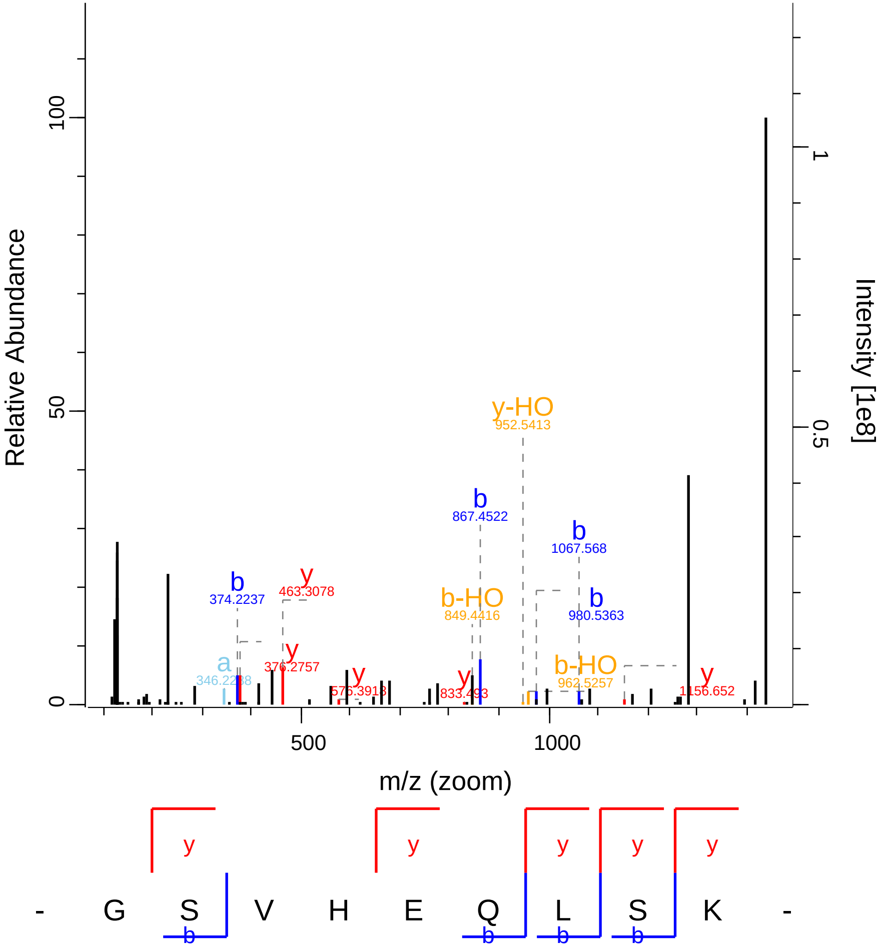

Raw File	Scan	Method	Score	m/z
RO-QC-160121_ZackD_SS_Set_01_Fraction_10	19013	FTMS; HCD	94.16	391.92

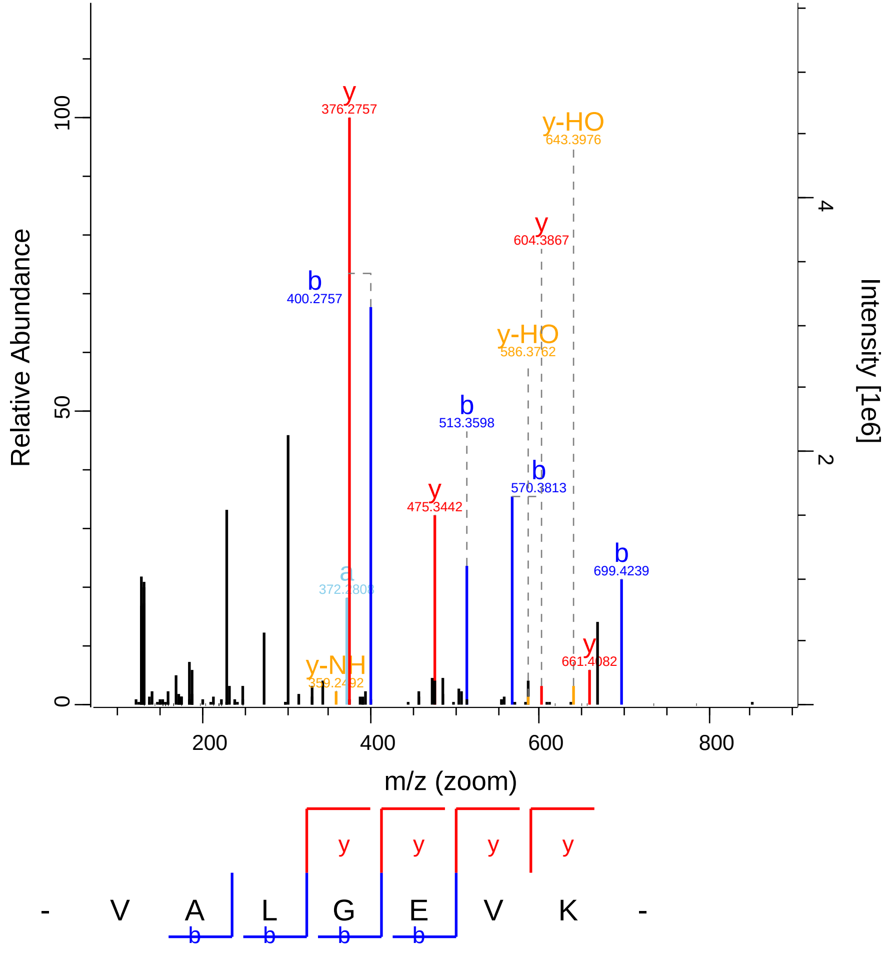

Raw File	Scan	Method	Score	m/z
RO-QC-160121_ZackD_SS_Set_01_Fraction_10	21847	FTMS; HCD	62.84	597.37

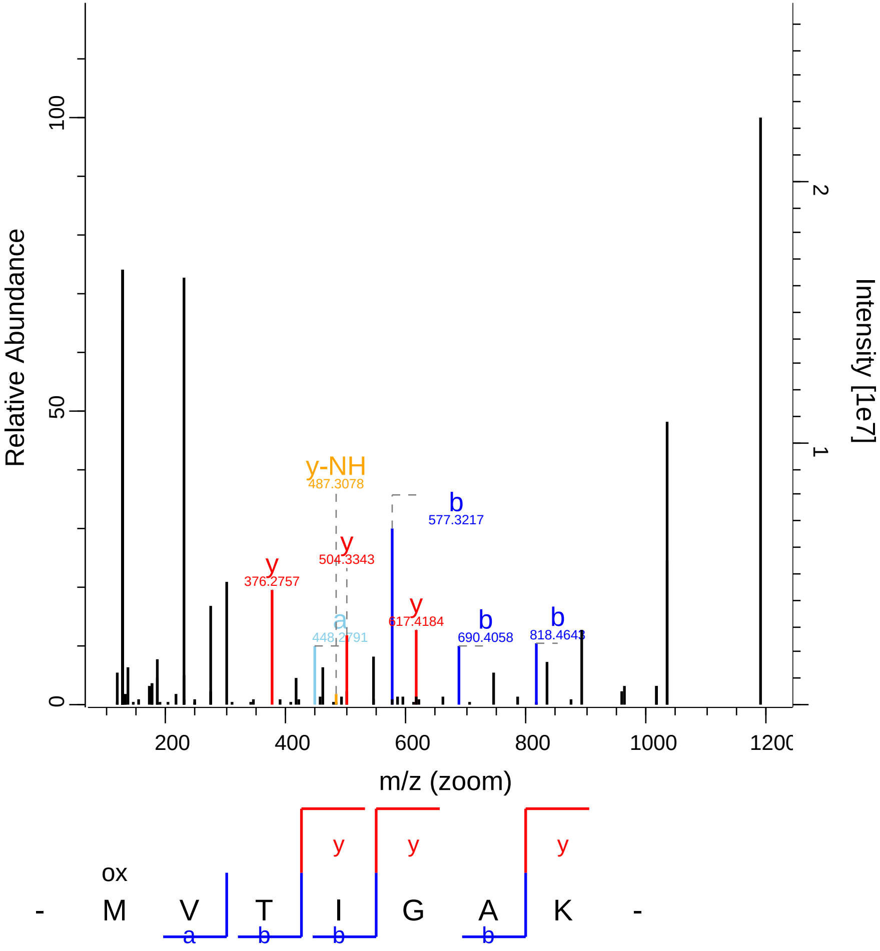

Raw File	Scan	Method	Score	m/z
RO-QC-160121_ZackD_SS_Set_01_Fraction_10	21904	FTMS; HCD	41.5	398.92

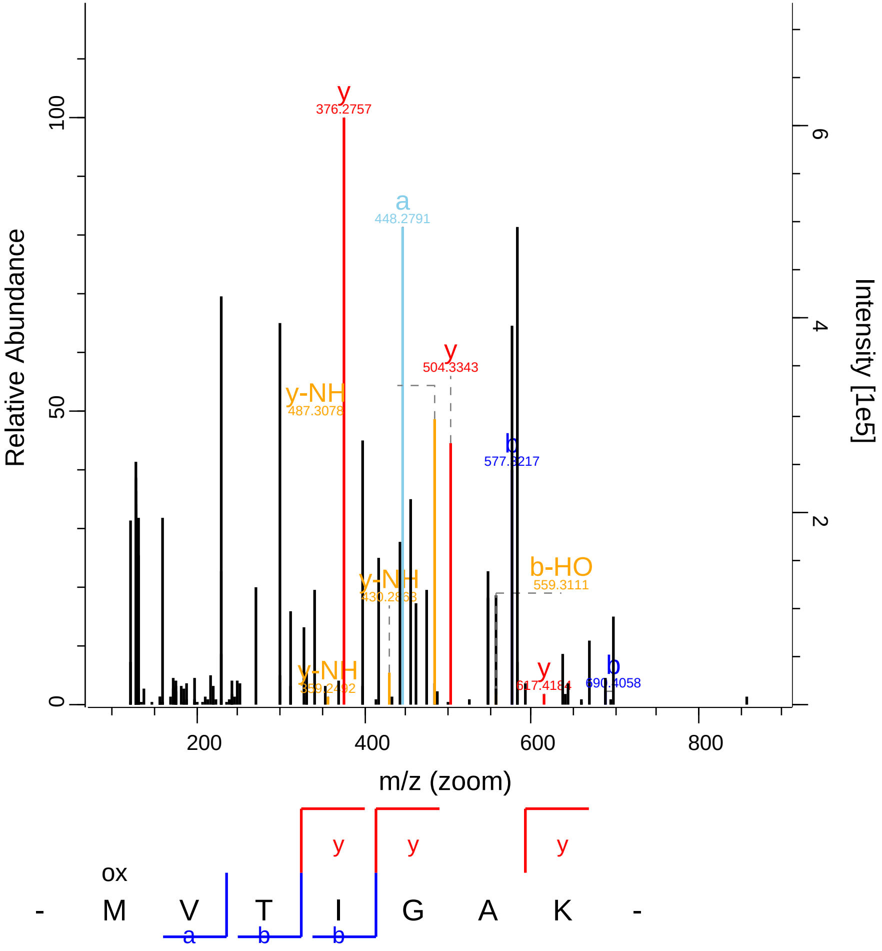

Raw File	Scan	Method	Score	m/z	Gene names
RO-QC-160121_ZackD_SS_Set_01_Fraction	24277	FTMS; HC	140.95	989.2	DPY19L3

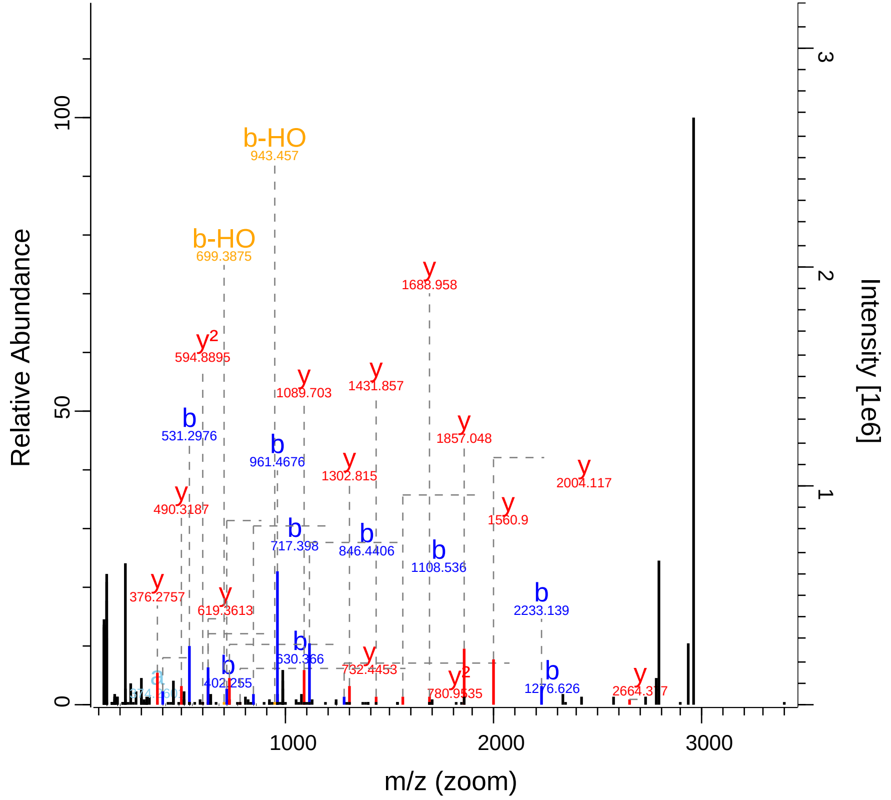

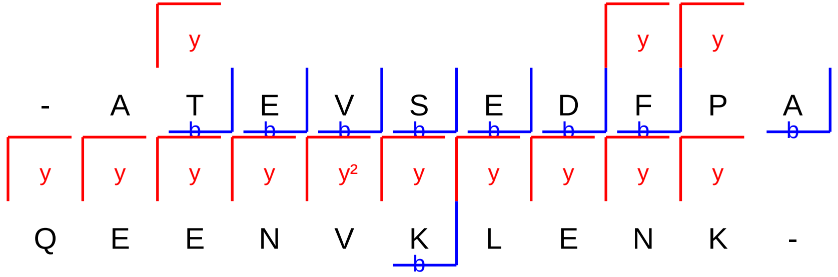

Raw File	Scan	Method	Score	m/z	Gene names
RO-QC-160121_ZackD_SS_Set_01_Fractior	24371	FTMS; HC	141.2	708.92	ALG12

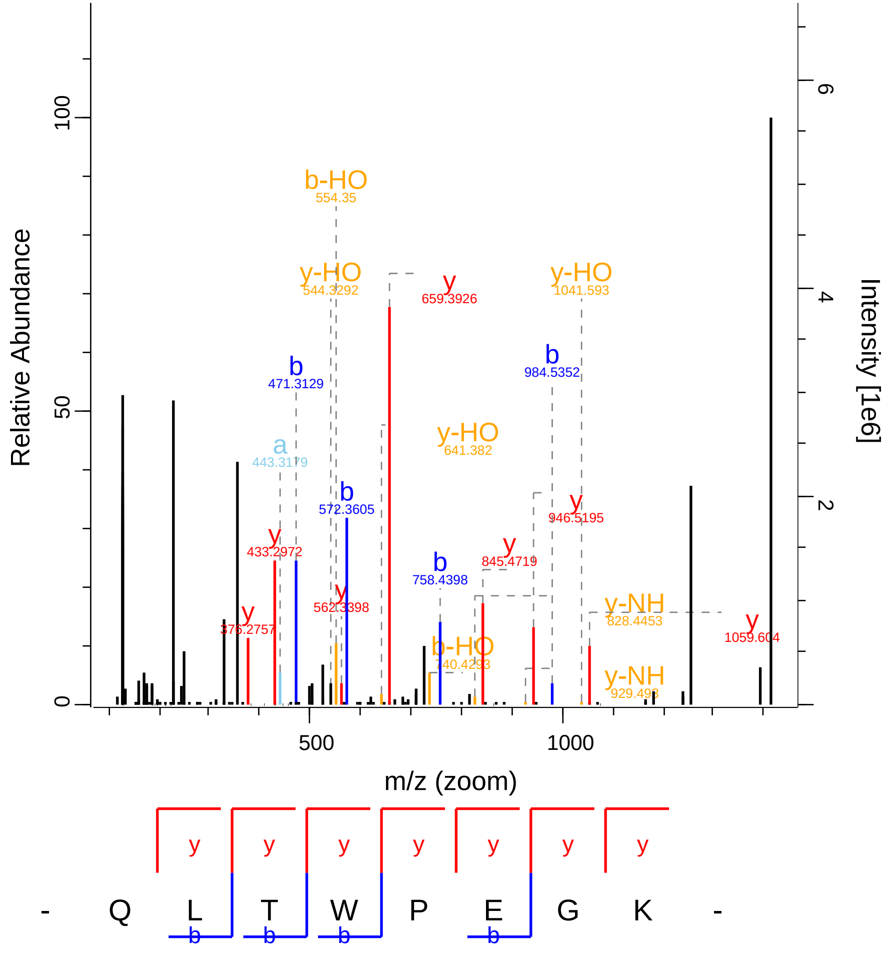

Raw File	Scan	Method	Score	m/z	Gene names
RO-QC-160121_ZackD_SS_Set_01_Fractio	24422	FTMS; HC	174.42	972.45	MT-ND3

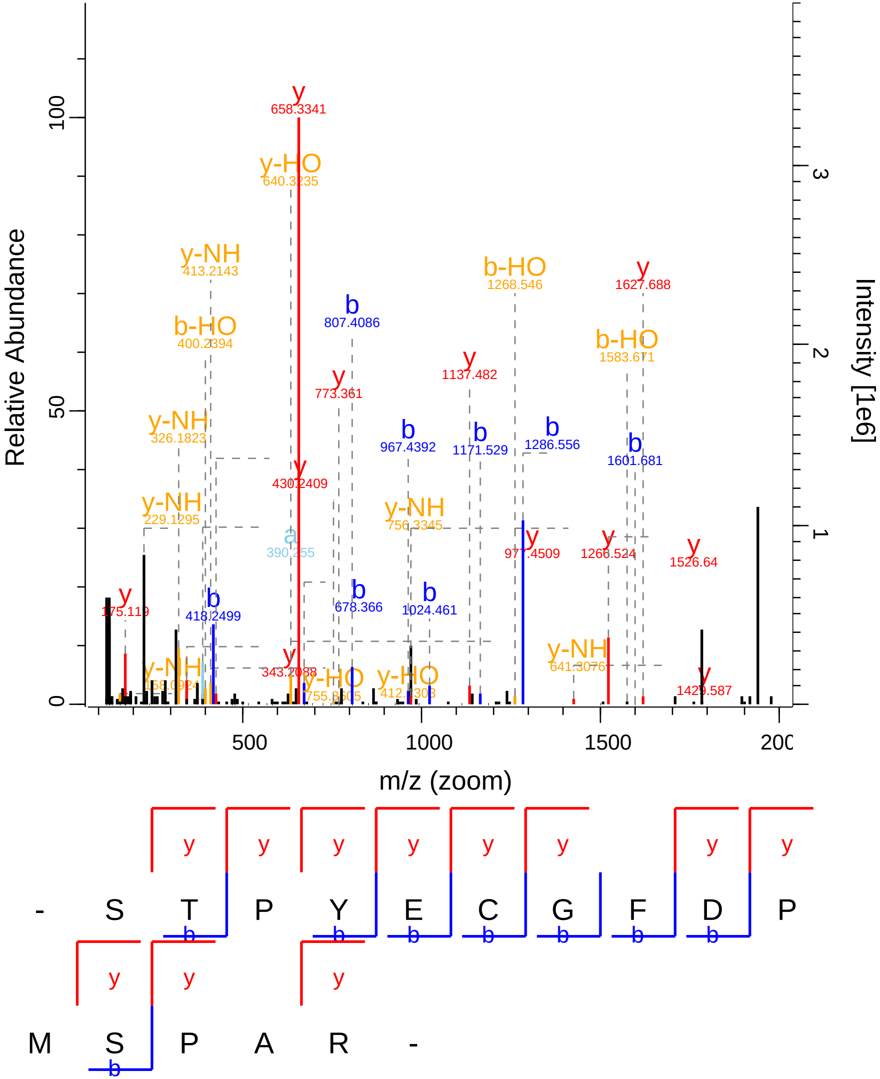

Raw File	Scan	Method	Score	m/z	Gene names
RO-QC-160121_ZackD_SS_Set_01_Fractior	24487	FTMS; HC	48.13	648.97	MT-ND3

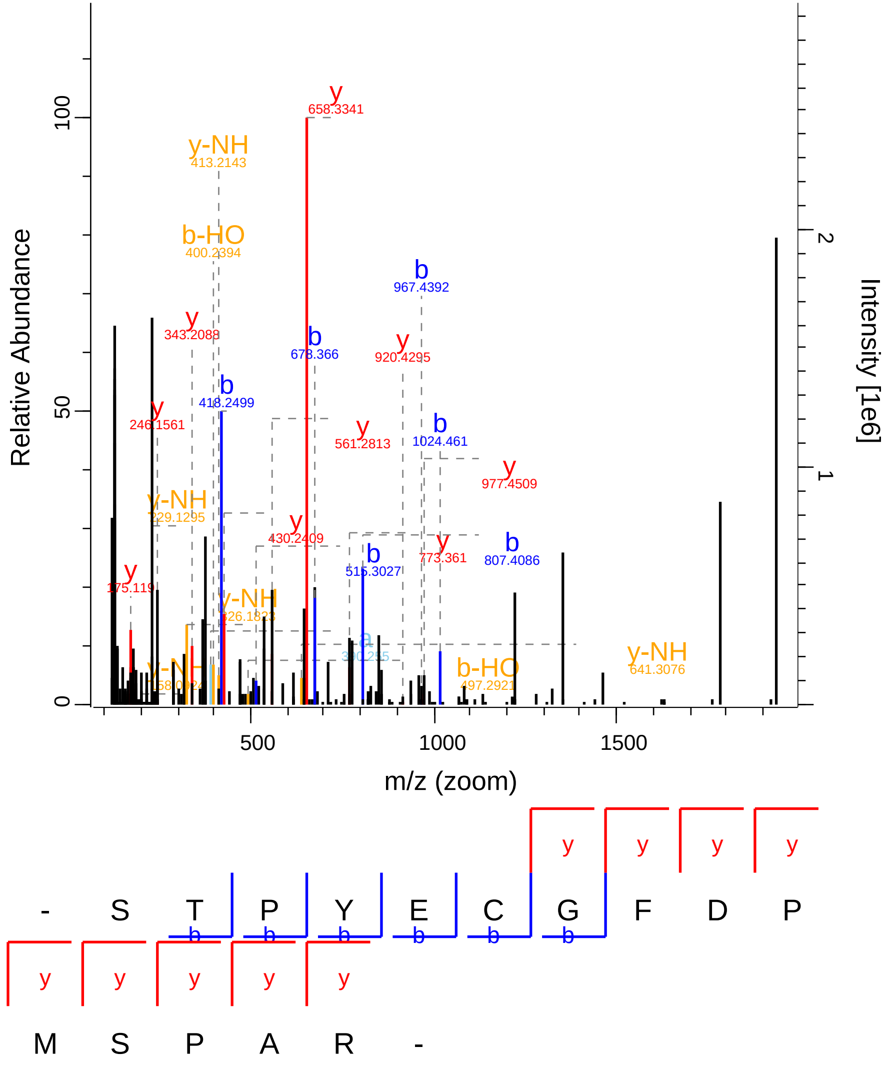

Raw File	Scan	Method	Score	m/z
RO-QC-160121_ZackD_SS_Set_01_Fraction_10	24950	FTMS; HCD	102.72	637.91

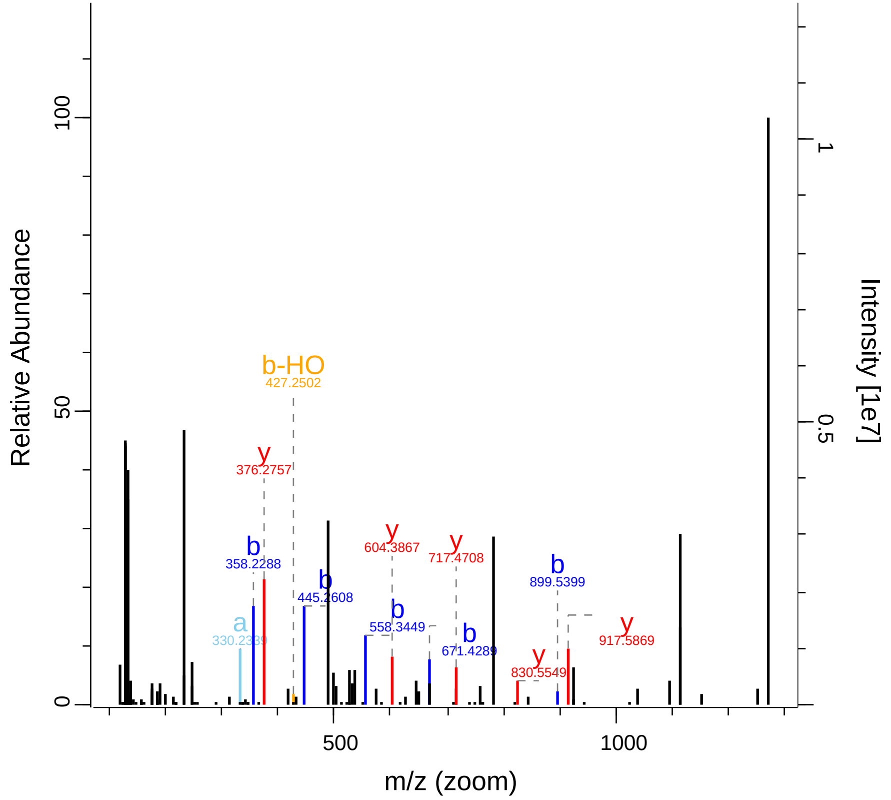

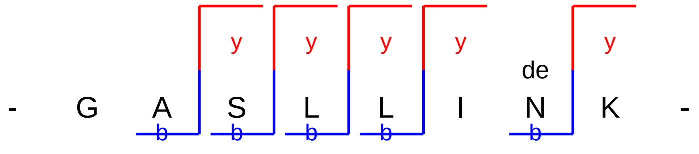

Raw File	Scan	Method	Score	m/z	Gene names
RO-QC-160121_ZackD_SS_Set_01_Fractior	25288	FTMS; HC	45.86	611.56	HOXD8

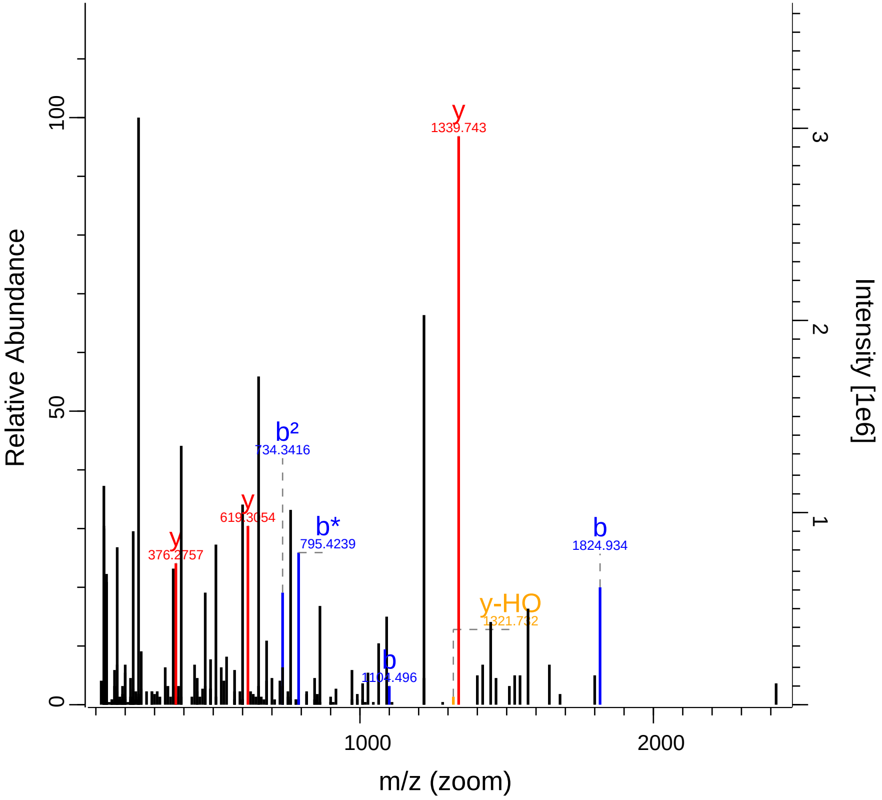

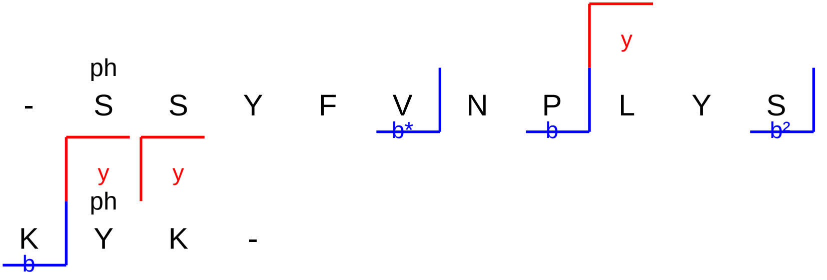

Raw File	Scan	Method	Score	m/z	Gene names
RO-QC-160121_ZackD_SS_Set_01_Fractior	26976	FTMS; HC	93.78	819.12	LMO4

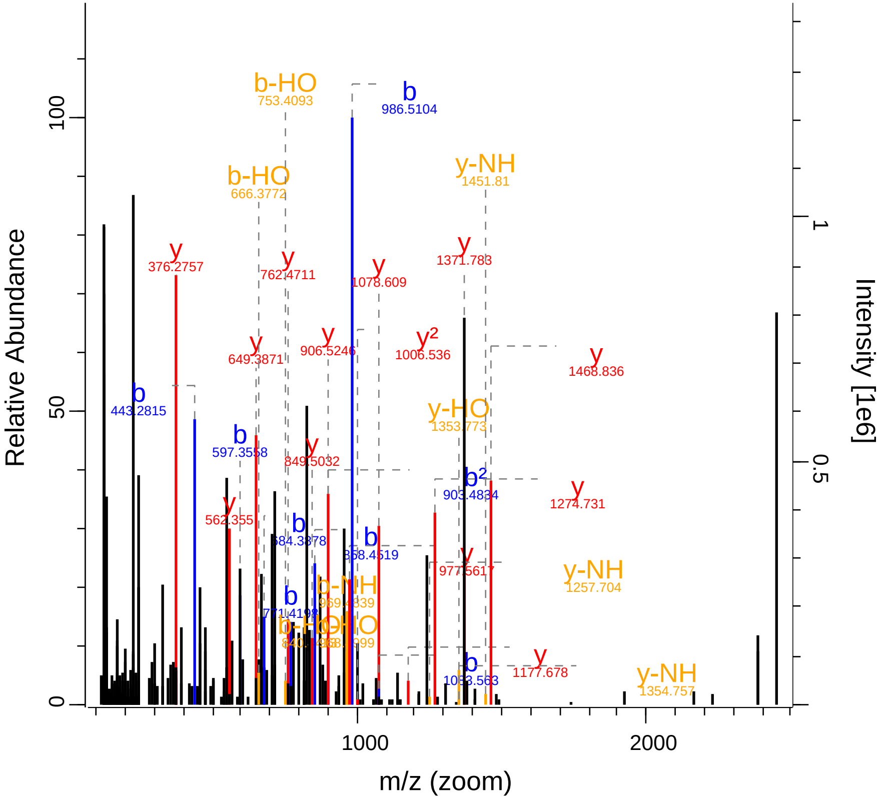

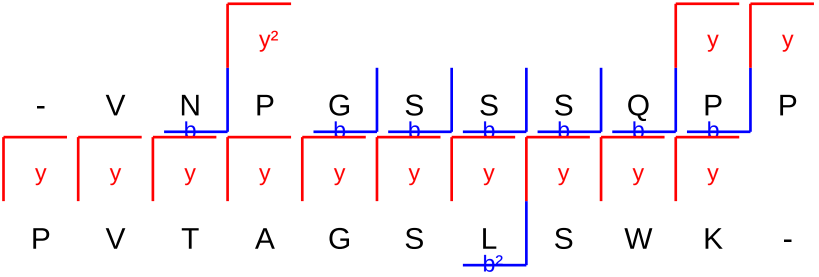

Raw File	Scan	Method	Score	m/z
RO-QC-160121_ZackD_SS_Set_01_Fraction_10	28254	FTMS; HCD	132.29	656.93

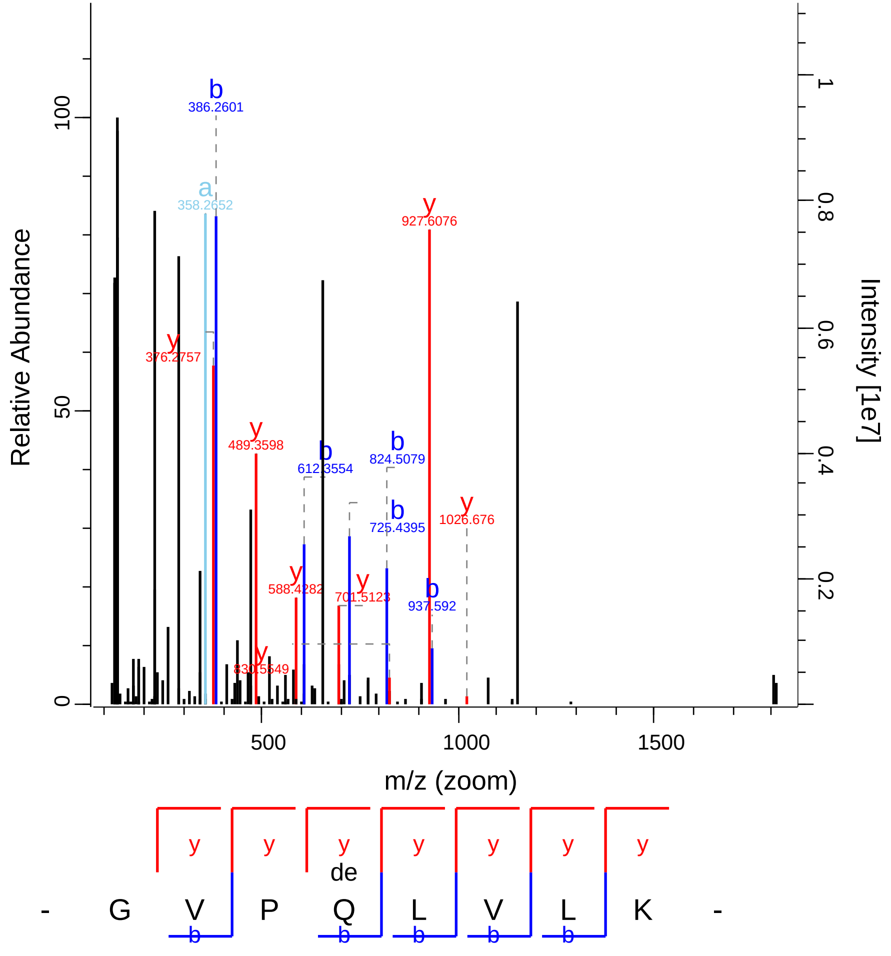

Raw File	Scan	Method	Score	m/z
RO-QC-160121_ZackD_SS_Set_01_Fraction_10	29052	FTMS; HCD	41.03	485.6

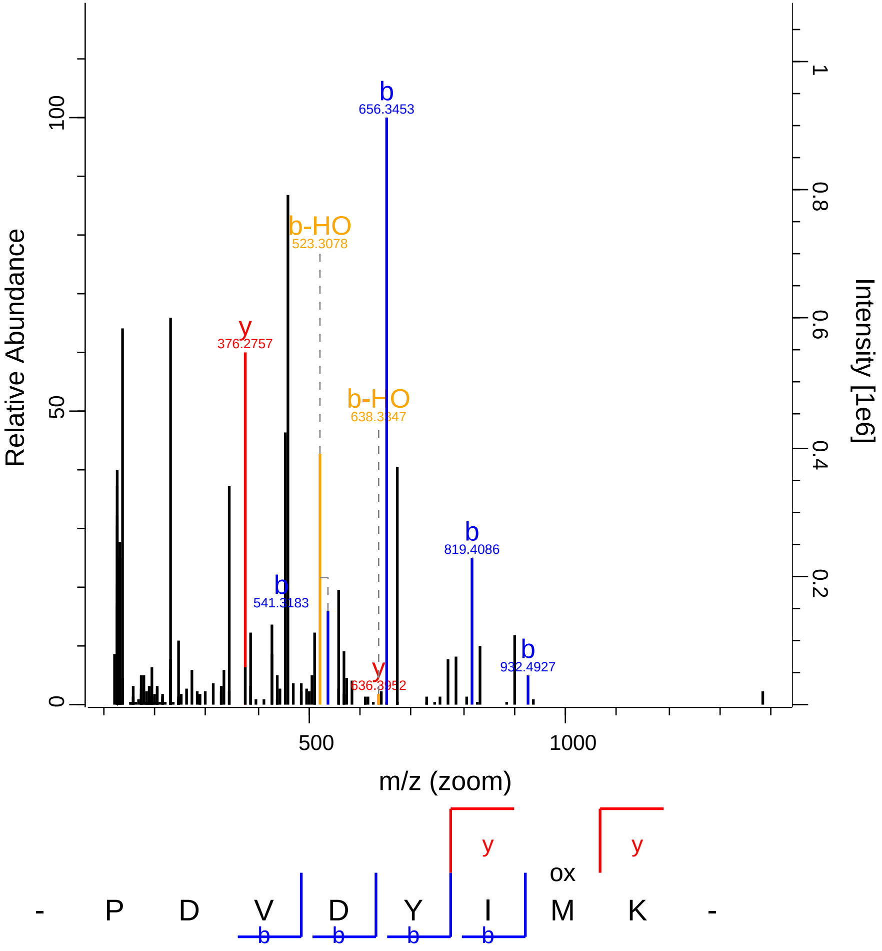

Raw File	Scan	Method	Score	m/z
RO-QC-160121_ZackD_SS_Set_01_Fraction_10	29419	FTMS; HCD	102.06	851.02

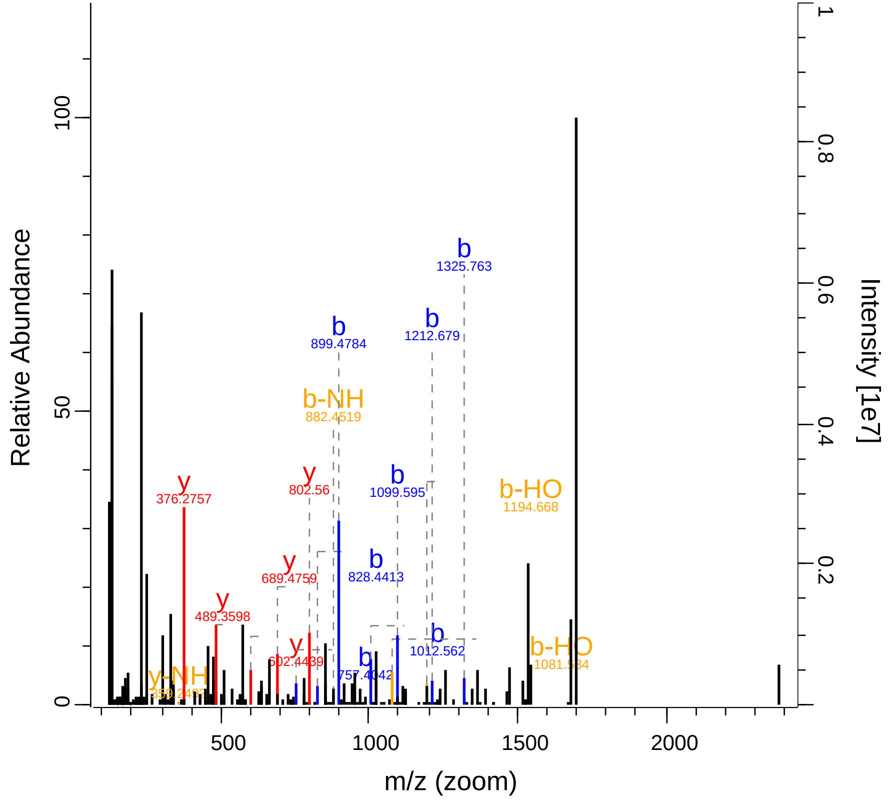

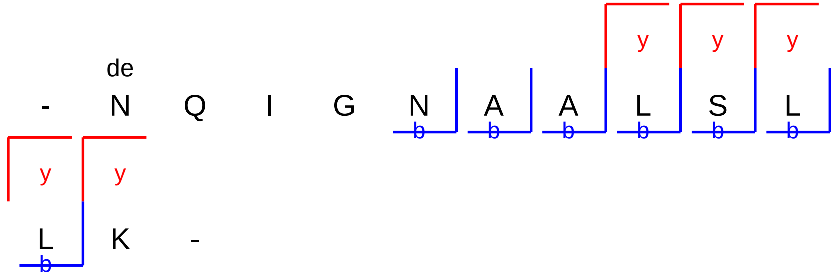

Raw File	Scan	Method	Score	m/z
RO-QC-160121_ZackD_SS_Set_01_Fraction_10	29981	FTMS; HCD	49.3	568.64

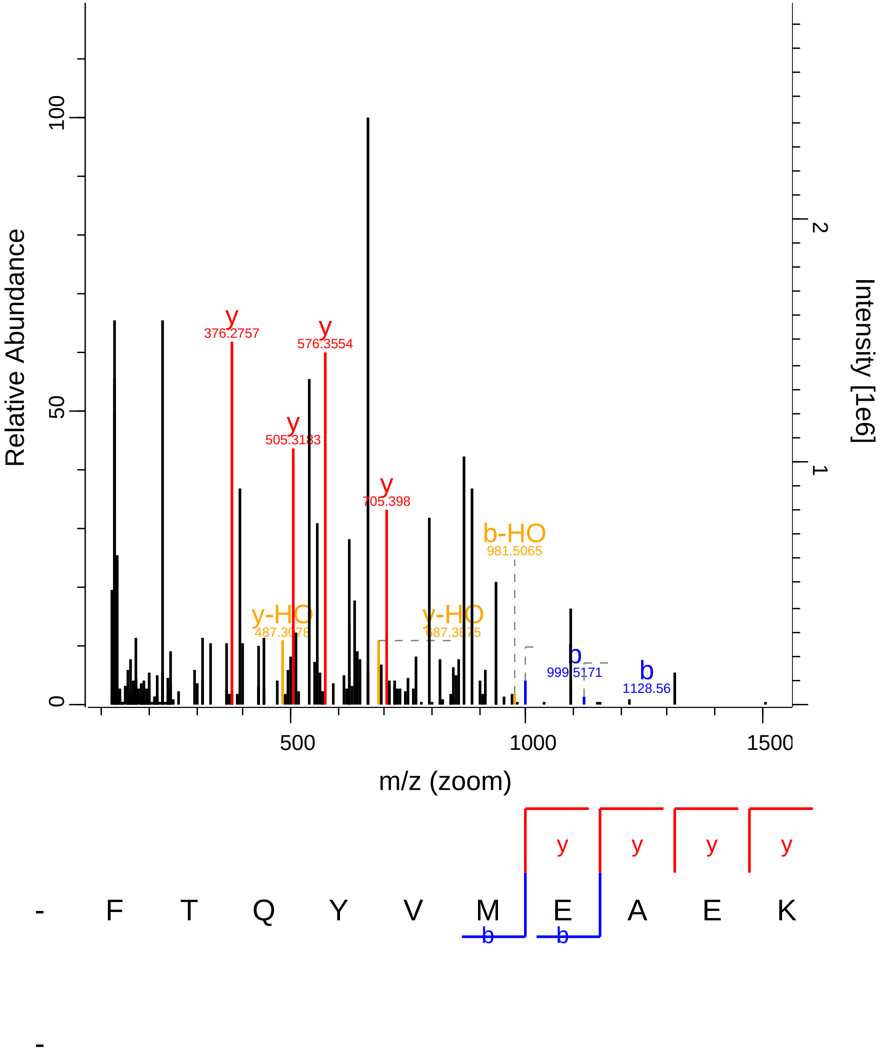

Raw File	Scan	Method	Score	m/z	Gene names
RO-QC-160121_ZackD_SS_Set_01_Fractior	30301	FTMS; HC	96.44	756.18	COX6A1

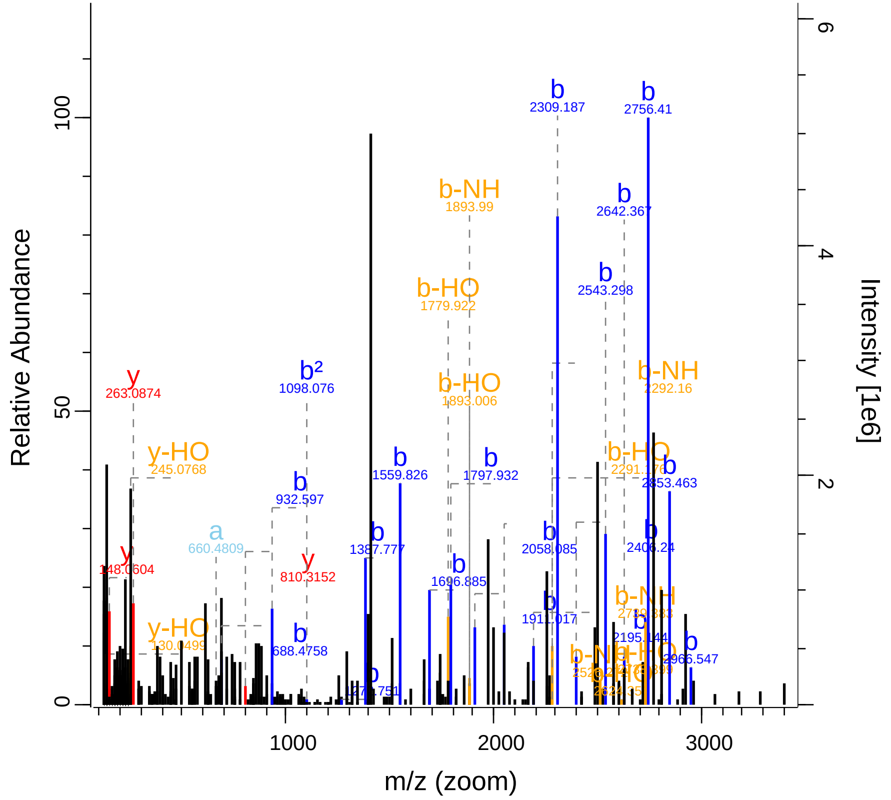

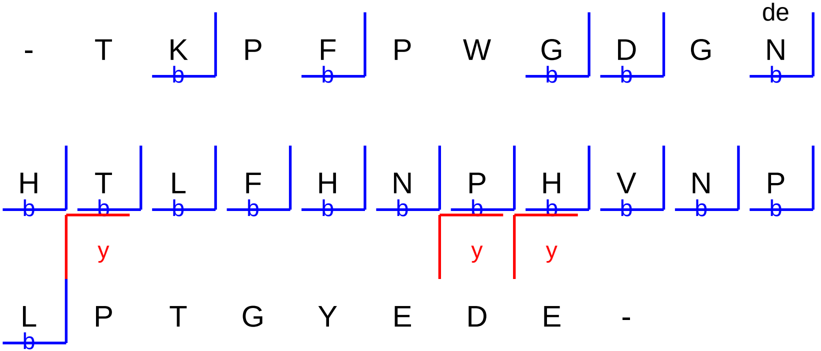

Raw File	Scan	Method	Score	m/z	Gene names
RO-QC-160121_ZackD_SS_Set_01_Fractior	30502	FTMS; HC	50.21	716.77	DRC7

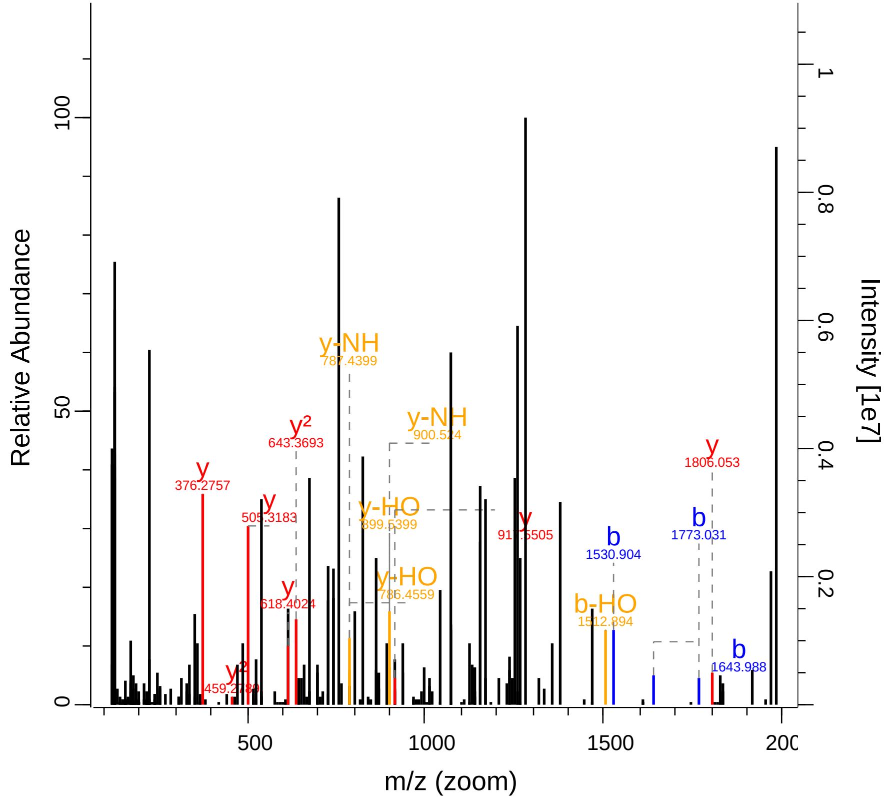

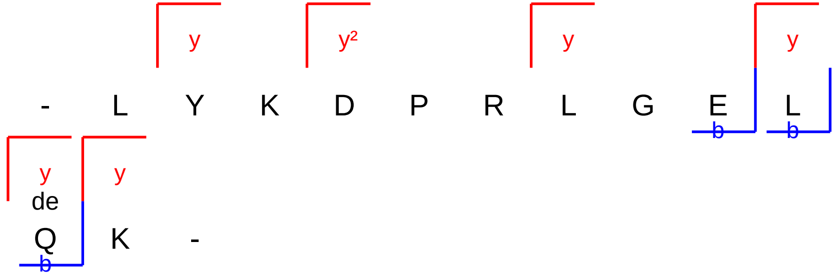

Raw File	Scan	Method	Score	m/z	Gene names
RO-QC-160121_ZackD_SS_Set_01_Fractio	30764	FTMS; HC	139.86	938.99	PHF23

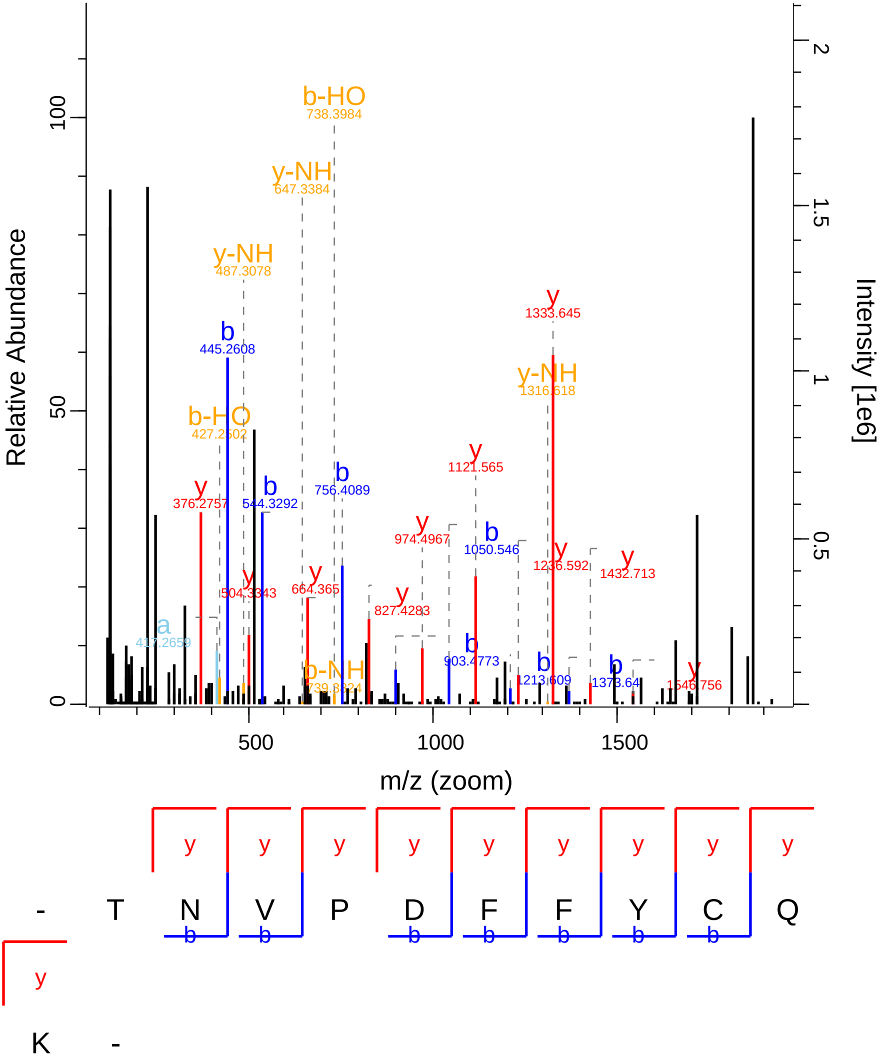

Raw File	Scan	Method	Score	m/z	Gene names
RO-QC-160121_ZackD_SS_Set_01_Fractior	30781	FTMS; HC	71.08	626.33	PHF23

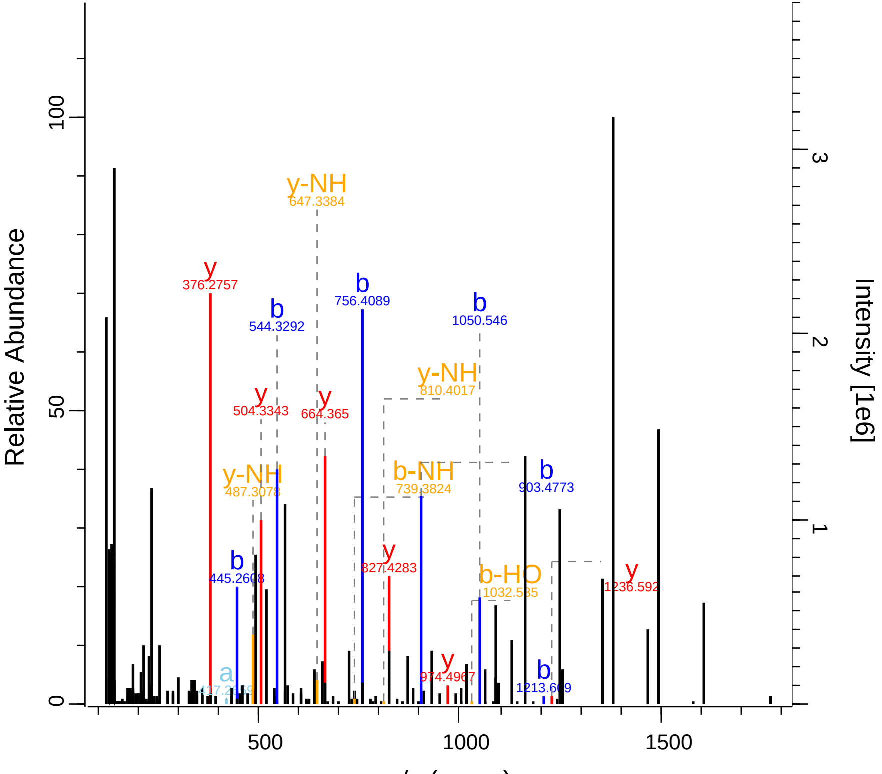

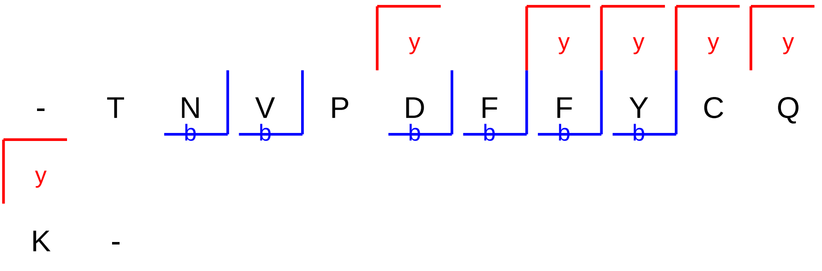

Raw File	Scan	Method	Score	m/z	Gene names
RO-QC-160121_ZackD_SS_Set_01_Fractior	31927	FTMS; HC	93.75	813.75	HN1

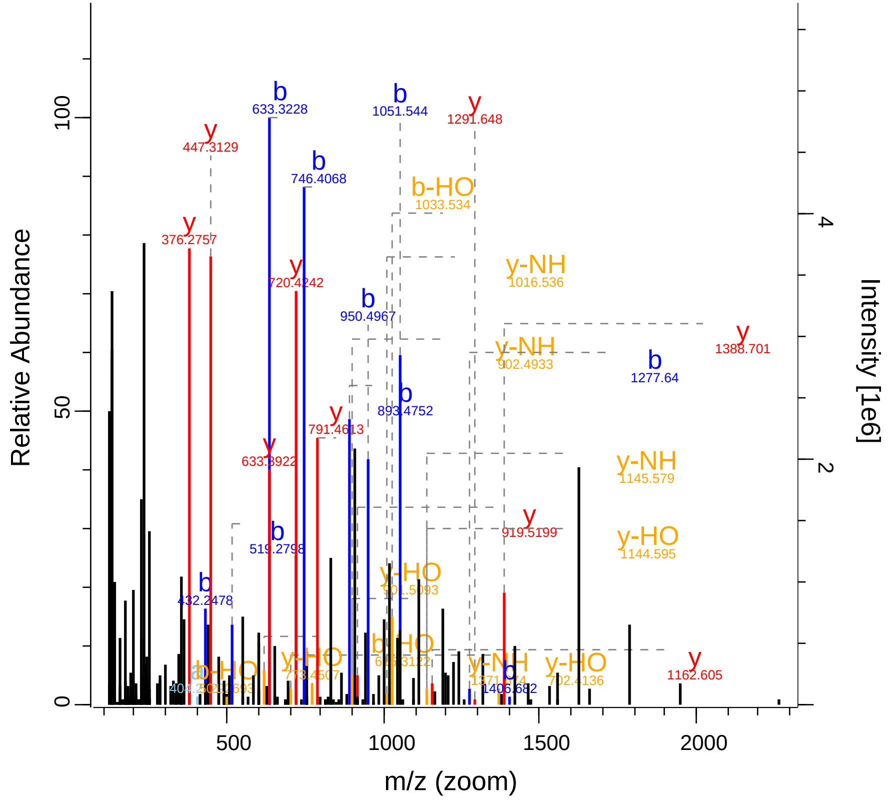

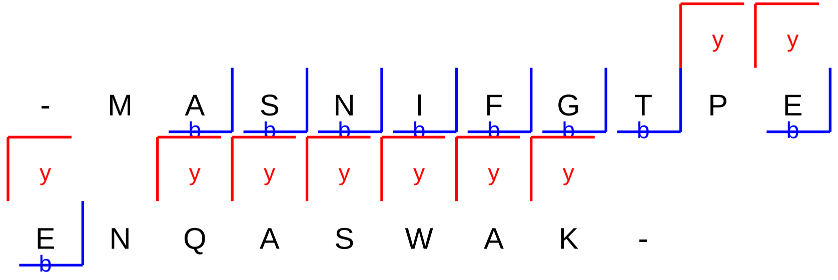

Raw File	Scan	Method	Score	m/z	Gene names
RO-QC-160121_ZackD_SS_Set_01_Fractio	31968	FTMS; HC	243.92	1220.63	HN1

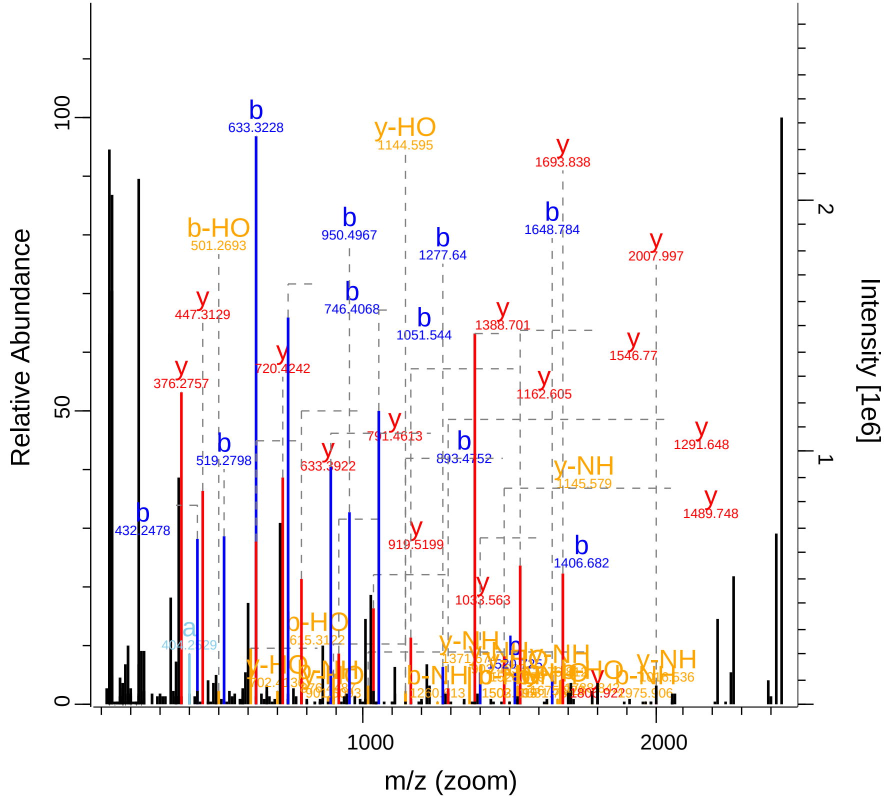

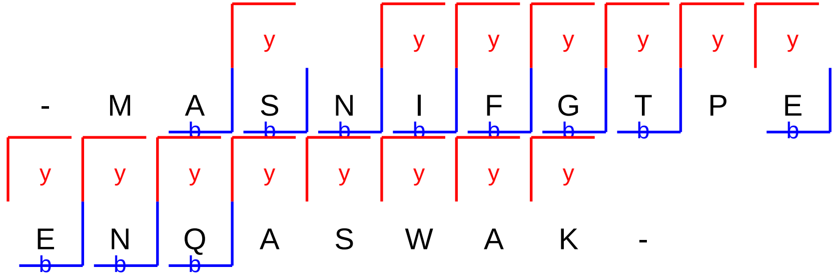

Raw File	Scan	Method	Score	m/z
RO-QC-160121_ZackD_SS_Set_01_Fraction_10	34050	FTMS; HCD	53.55	521.99

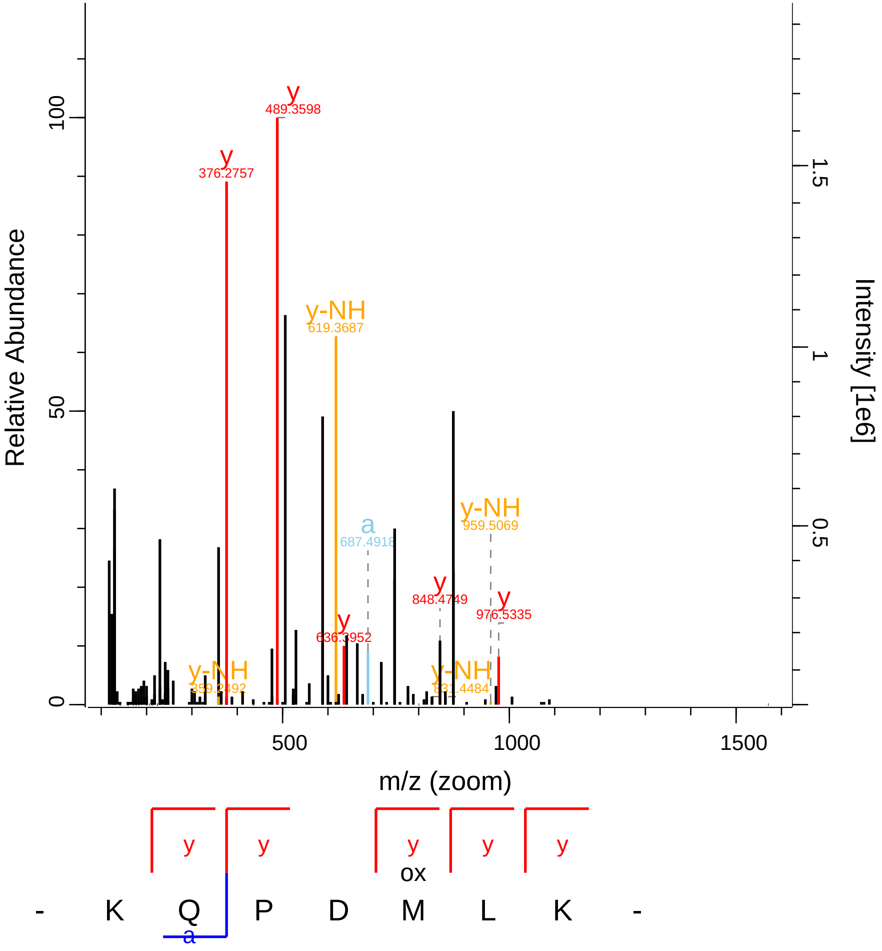

Raw File	Scan	Method	Score	m/z
RO-QC-160121_ZackD_SS_Set_01_Fraction_10	34257	FTMS; HCD	58.17	521.66

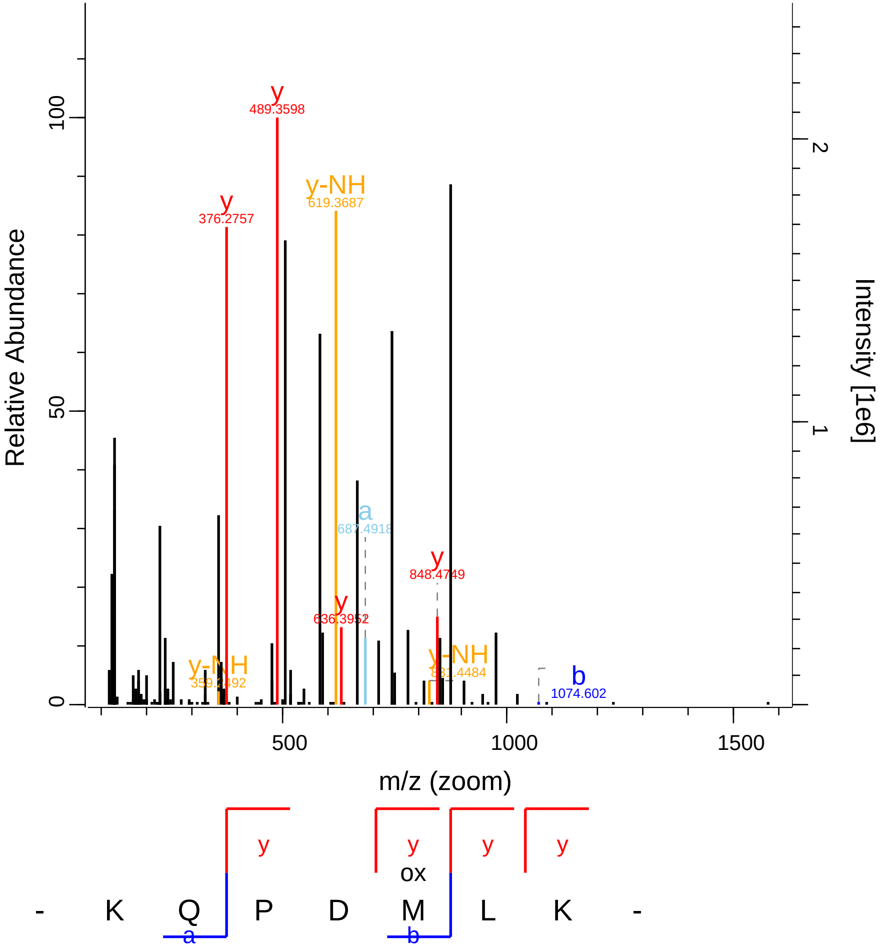

Raw File	Scan	Method	Score	m/z	Gene names
RO-QC-160121_ZackD_SS_Set_01_Fractior	35303	FTMS; HC	77.74	574.31	KCTD7

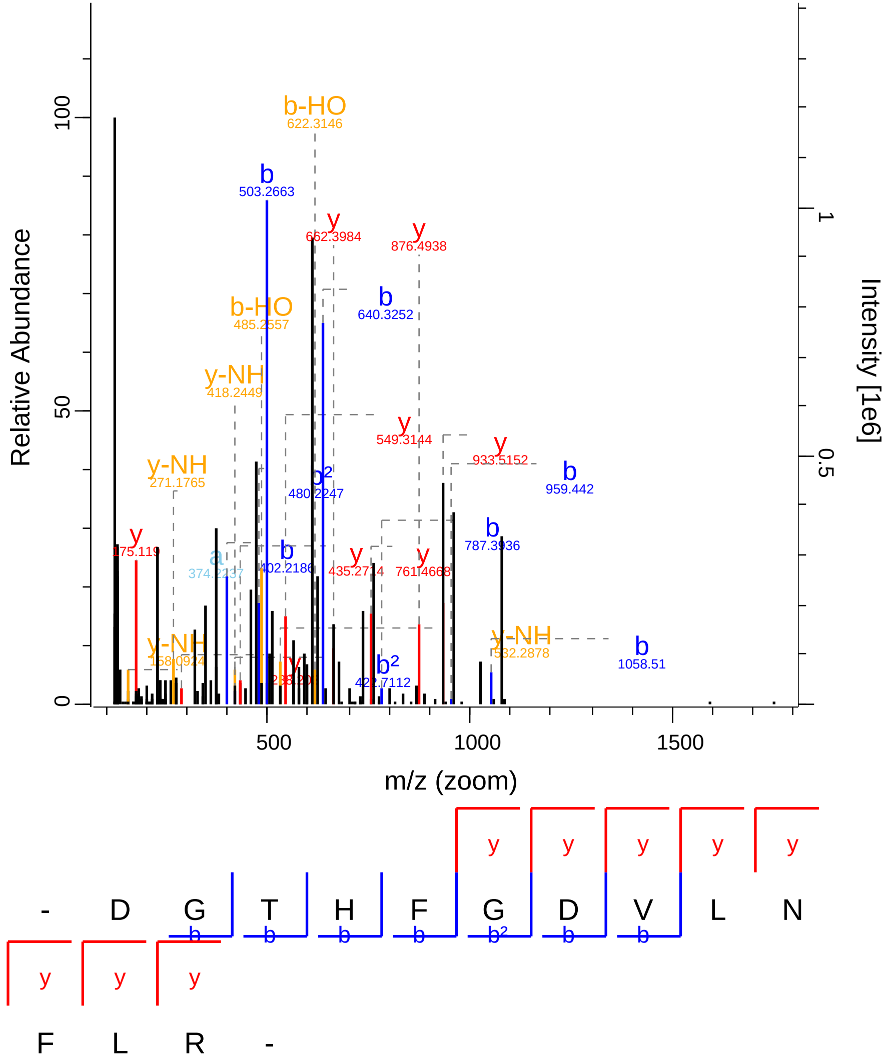

Raw File	Scan	Method	Score	m/z
RO-QC-160121_ZackD_SS_Set_01_Fraction_10	35838	FTMS; HCD	90.11	1222.67

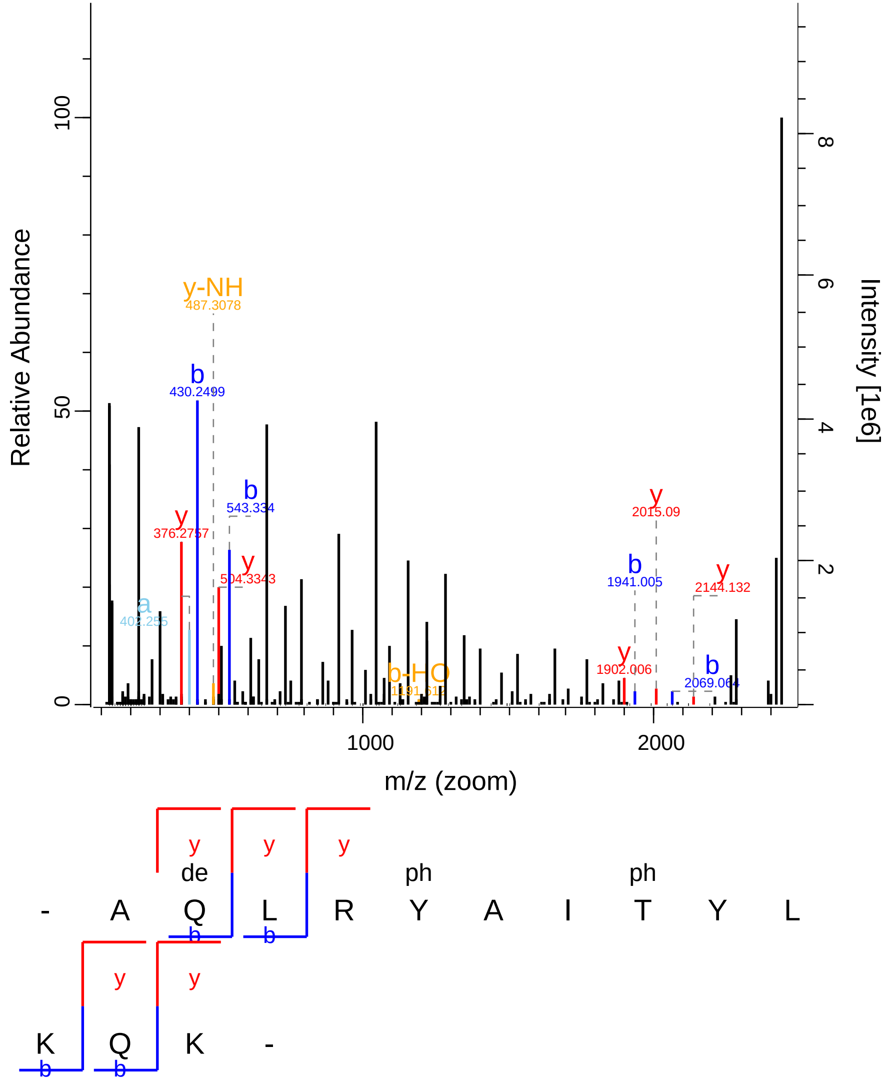

Raw File	Scan	Method	Score	m/z
RO-QC-160121_ZackD_SS_Set_01_Fraction_10	36822	FTMS; HCD	56.43	881.92

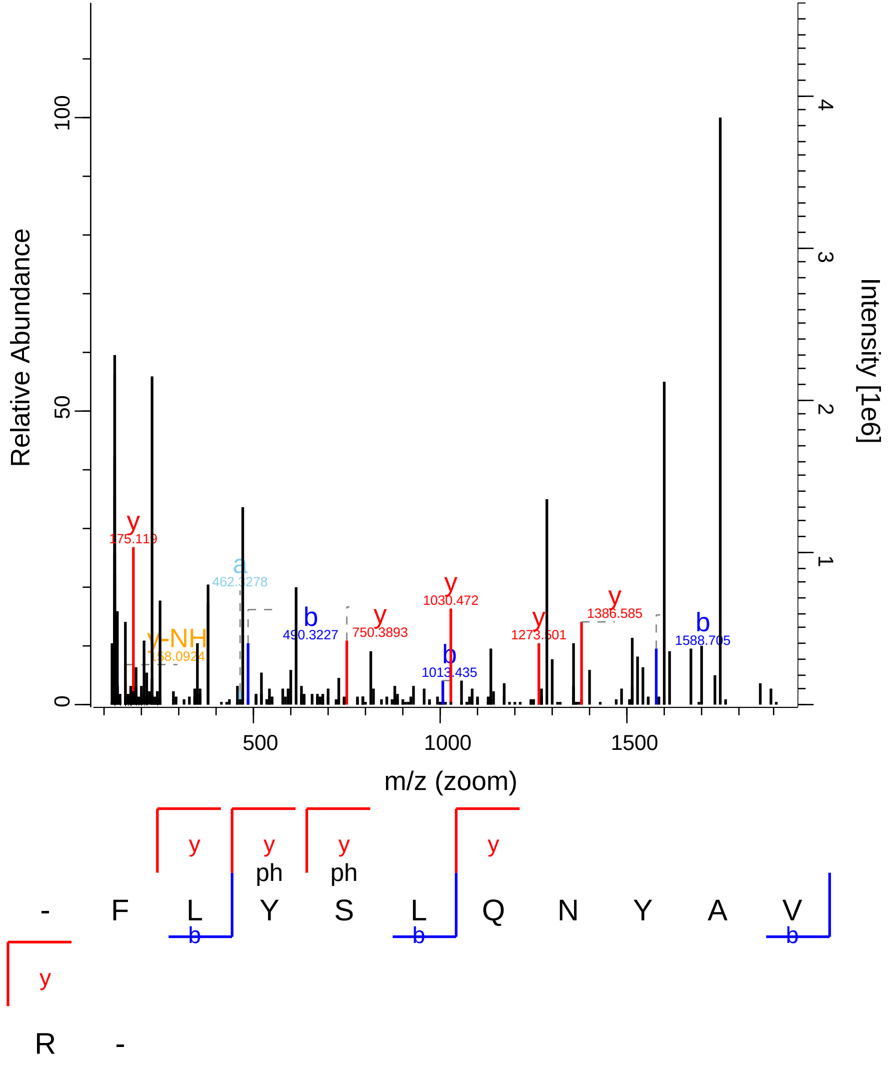

Raw File	Scan	Method	Score	m/z	Gene names
RO-QC-160121_ZackD_SS_Set_01_Fractior	37168	FTMS; HC	173.27	955.47	RABIF

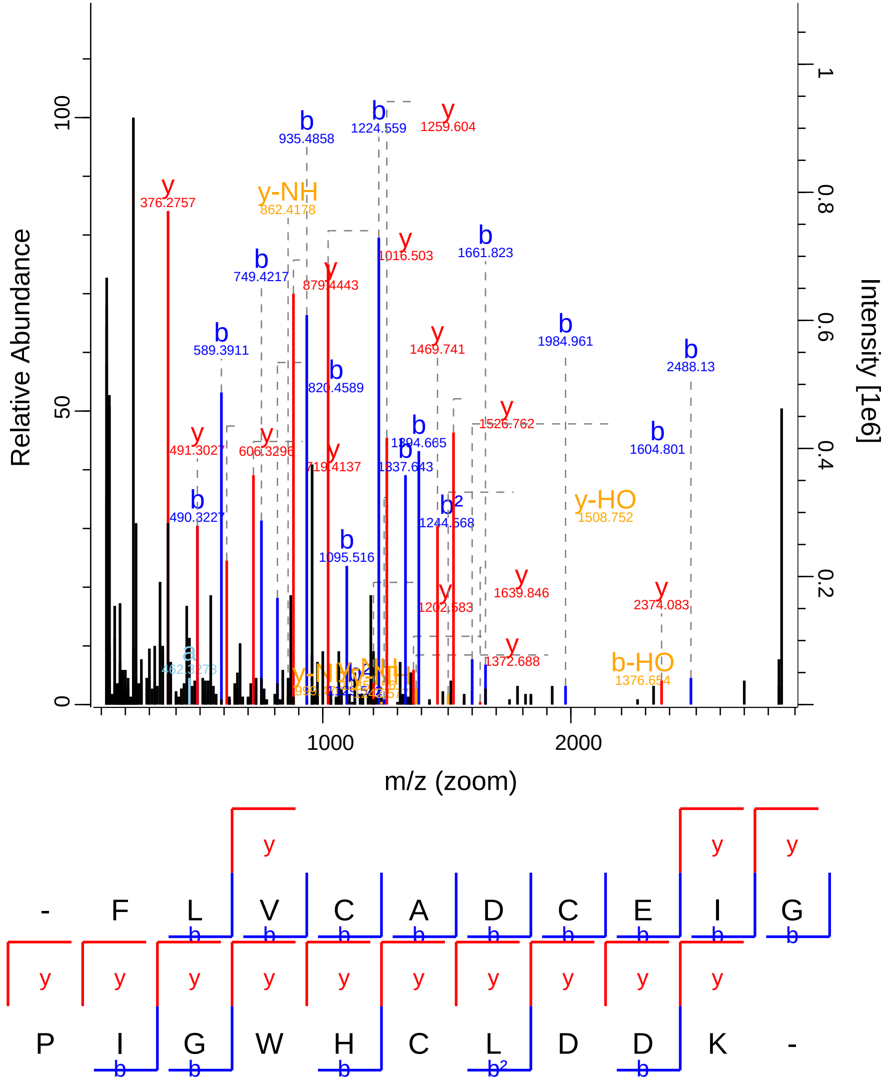

Raw File	Scan	Method	Score	m/z	Gene names
RO-QC-160121_ZackD_SS_Set_01_Fractio	37713	FTMS; HC	142.38	1045.73	FAM174A

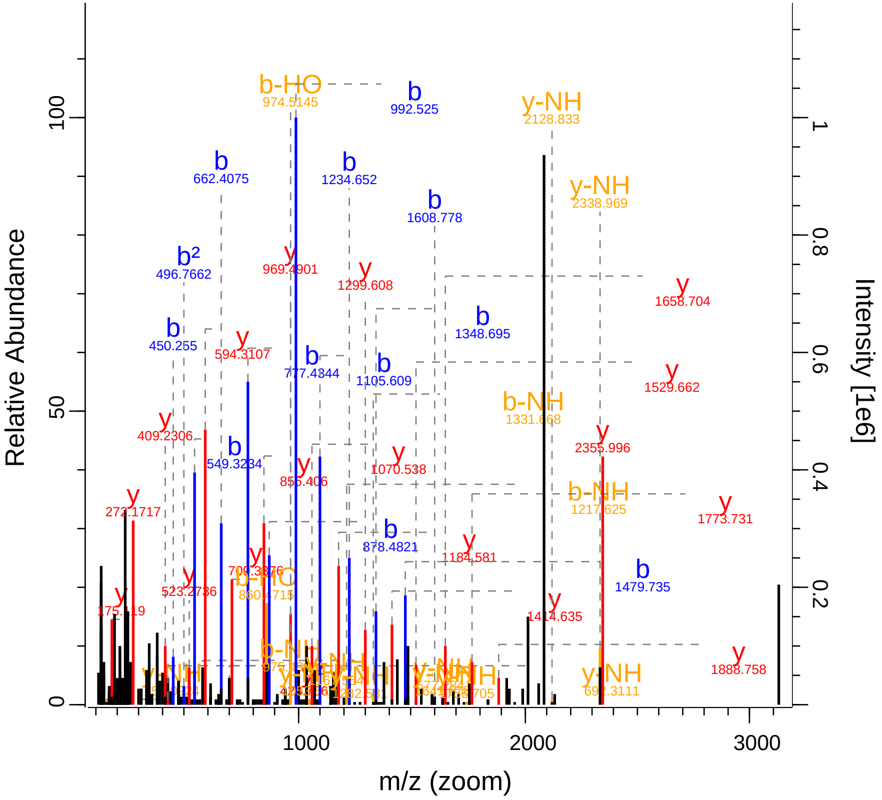

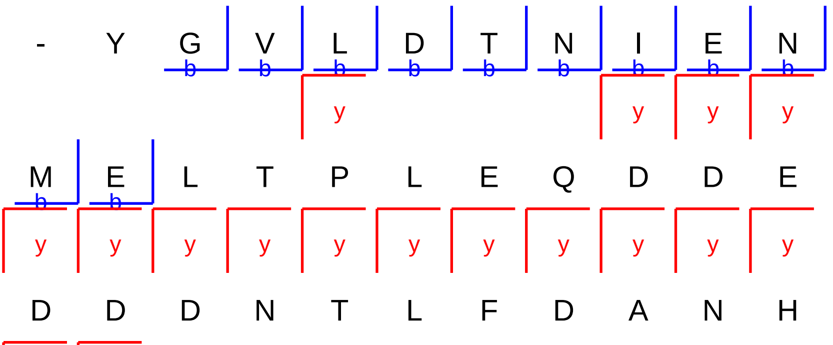

Raw File	Scan	Method	Score	m/z	Gene names
RO-QC-160121_ZackD_SS_Set_01_Fractior	38959	FTMS; HC	79.97	618.02	OR4E1

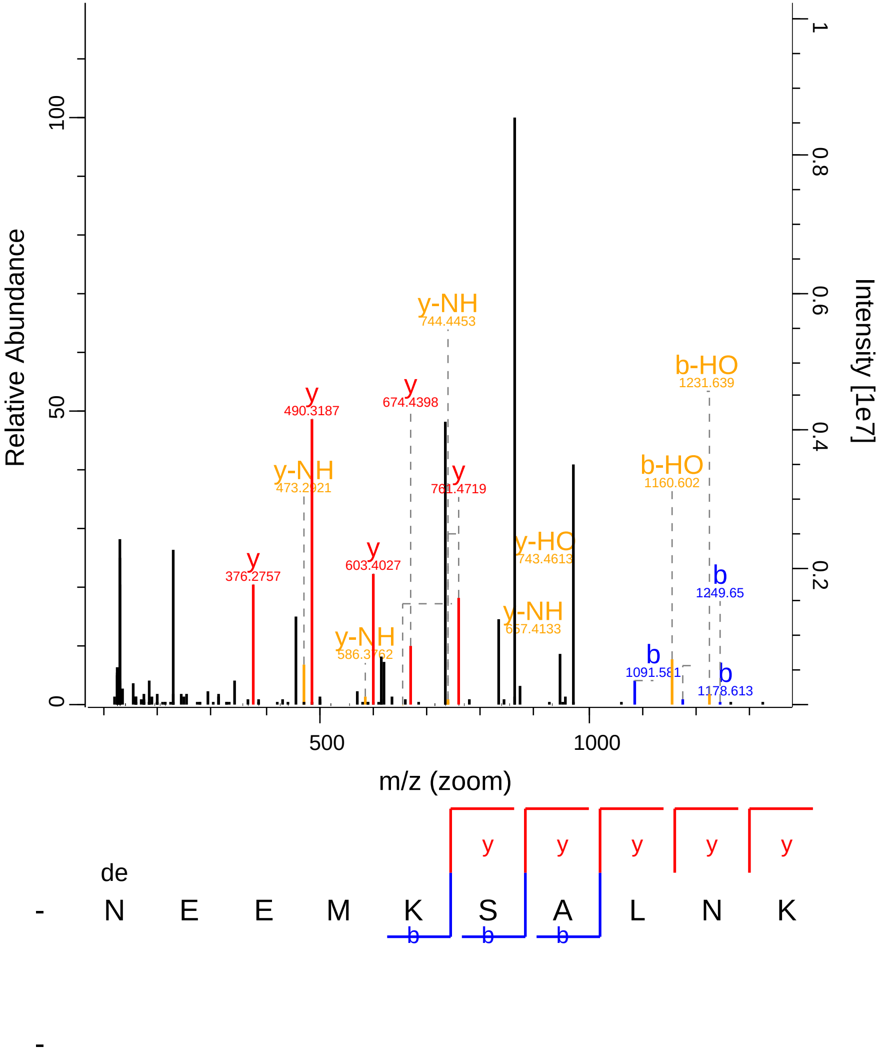

Raw File	Scan	Method	Score	m/z	Gene names
RO-QC-160121_ZackD_SS_Set_01_Fractio	38986	FTMS; HC	120.24	808.43	DXO

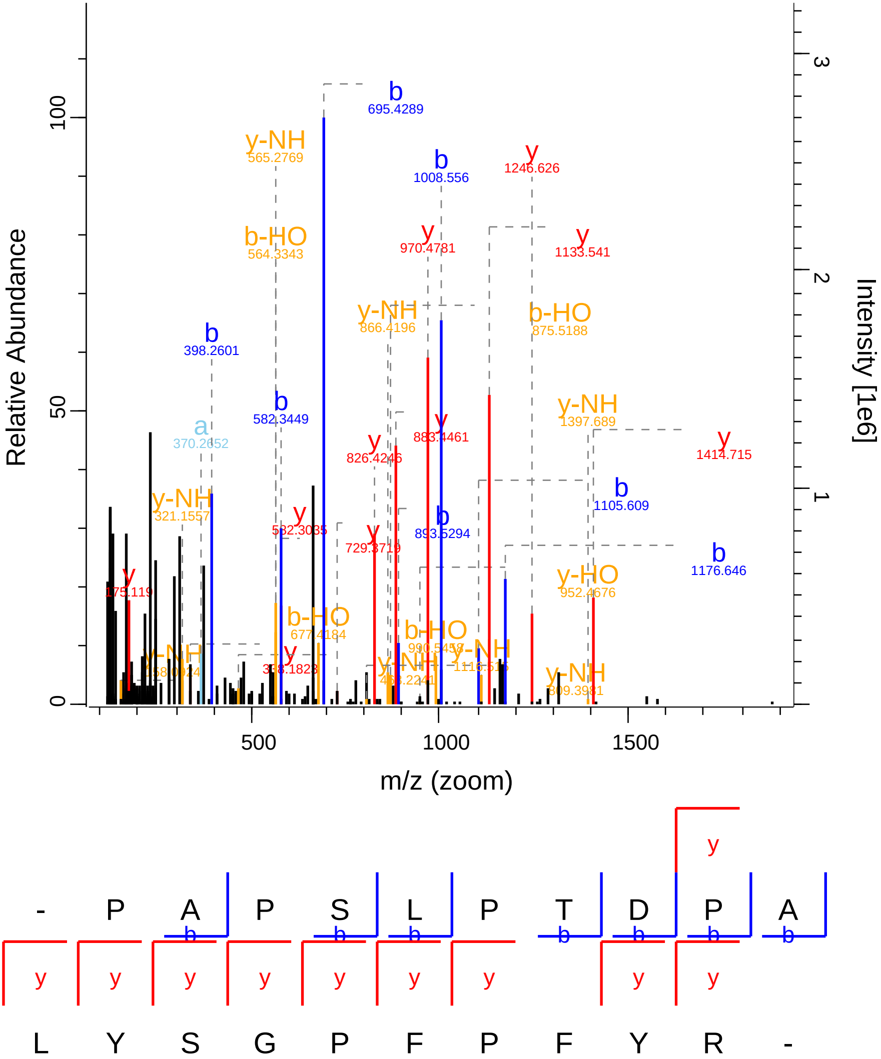

Raw File	Scan	Method	Score	m/z	Gene names
RO-QC-160121_ZackD_SS_Set_01_Fractioı	39489	FTMS; HC	113.37	1091.1	FAM221A

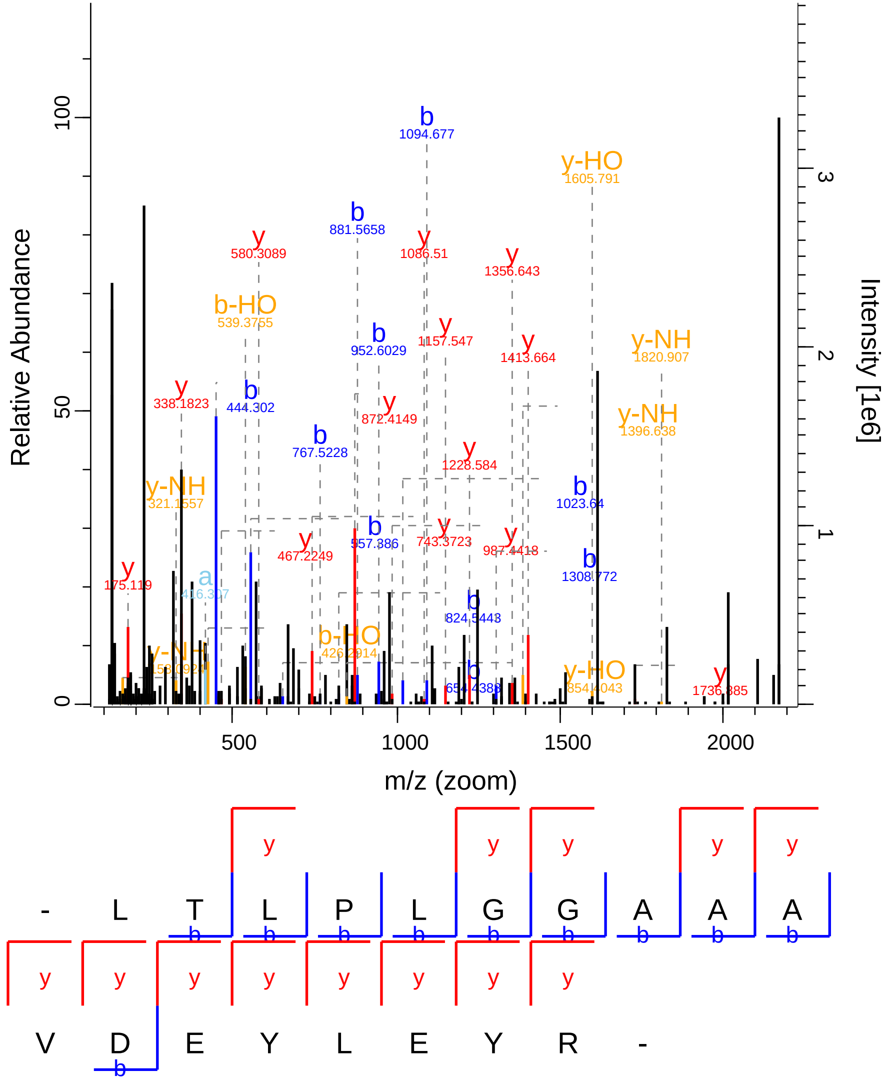

Raw File	Scan	Method	Score	m/z	Gene names
RO-QC-160121_ZackD_SS_Set_01_Fractio	39646	FTMS; HC	204.92	1416.03	MT-CO1

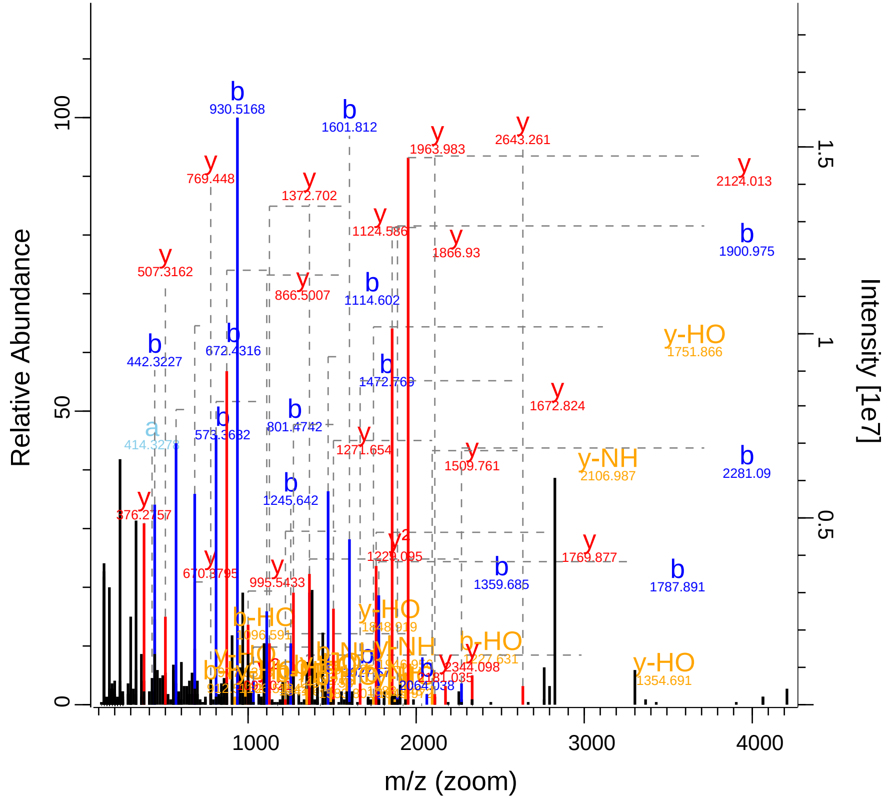

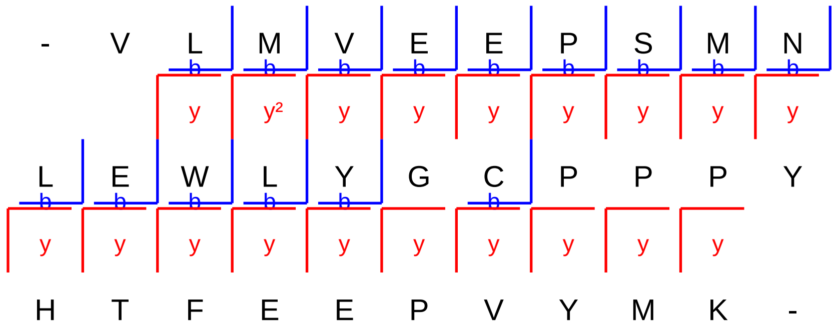

Raw File	Scan	Method	Score	m/z	Gene names
RO-QC-160121_ZackD_SS_Set_01_Fractio	40182	FTMS; HC	94.69	1017.52	FREM1

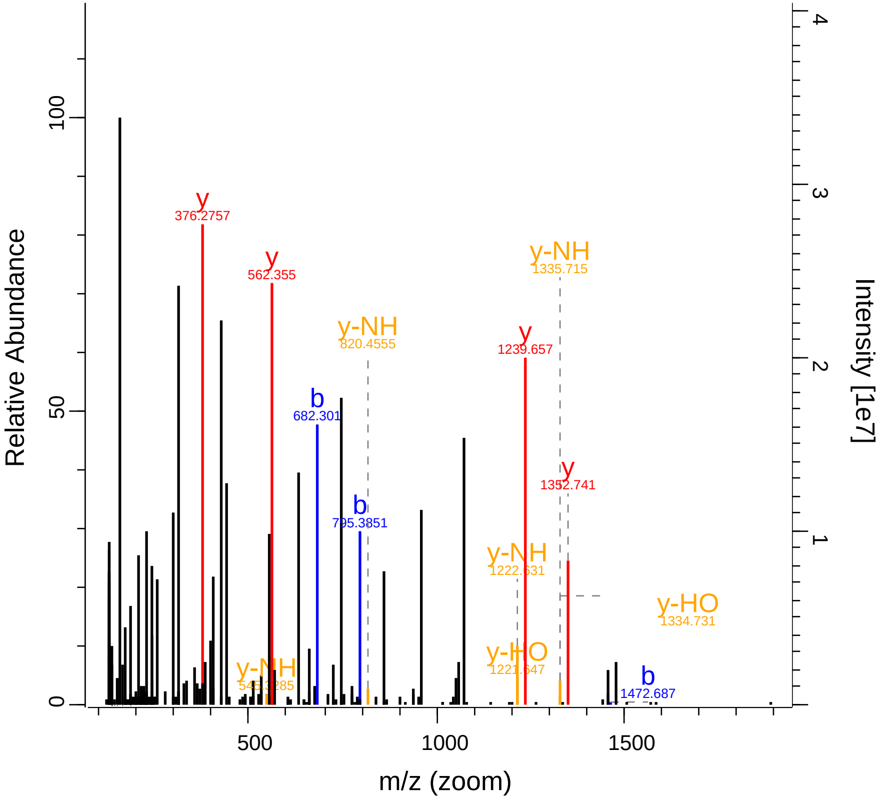

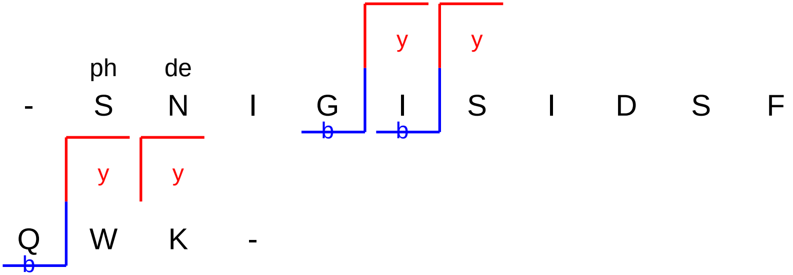

Raw File	Scan	Method	Score	m/z	Gene names
RO-QC-160121_ZackD_SS_Set_01_Fractio	40908	FTMS; HC	61.38	1440.68	TCF7L1

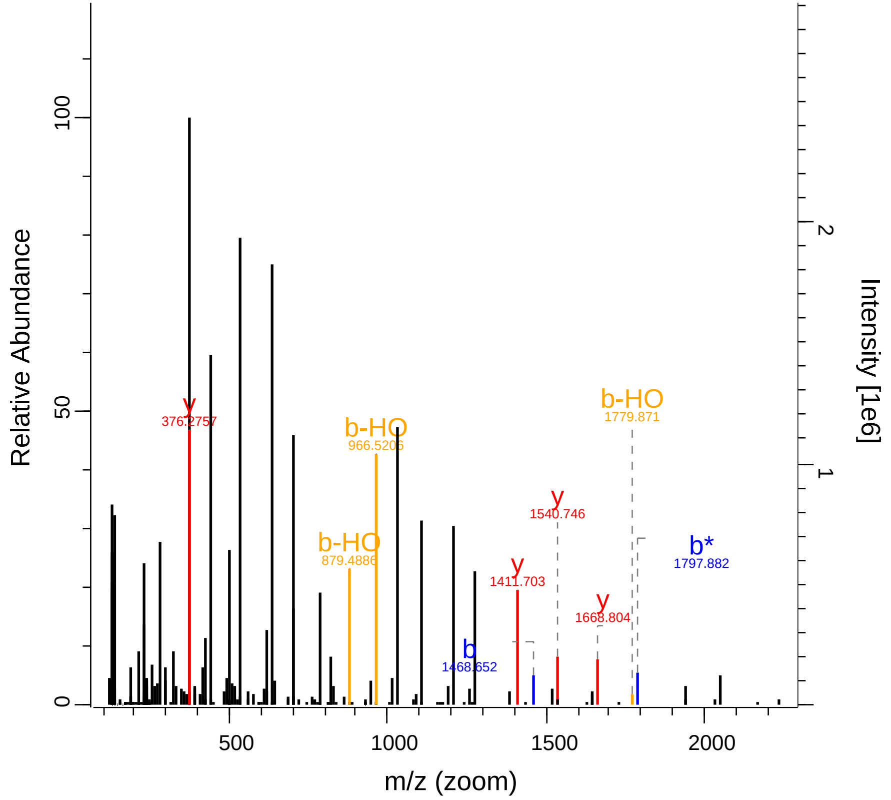

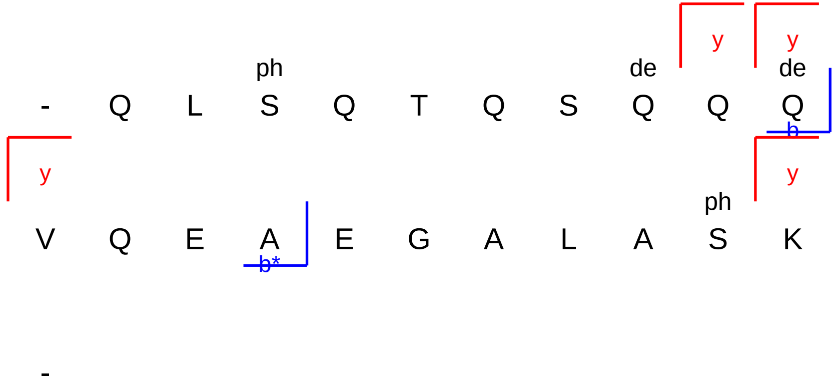

Raw File	Scan	Method	Score	m/z	Gene names
RO-QC-160121_ZackD_SS_Set_01_Fractior	41031	FTMS; HC	90.46	835.11	BSCL2

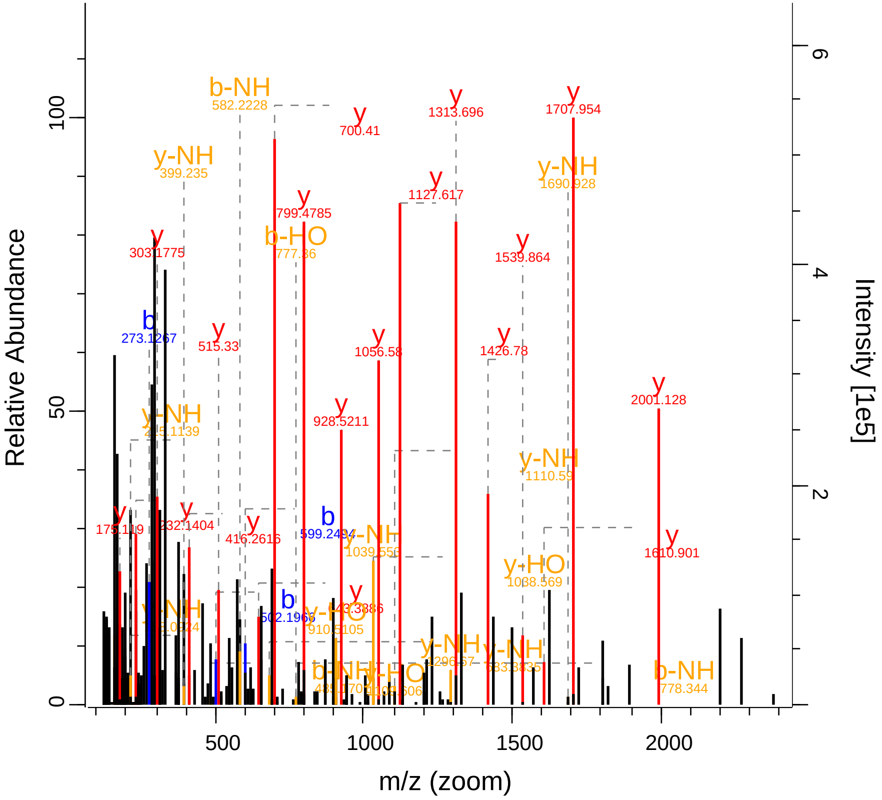

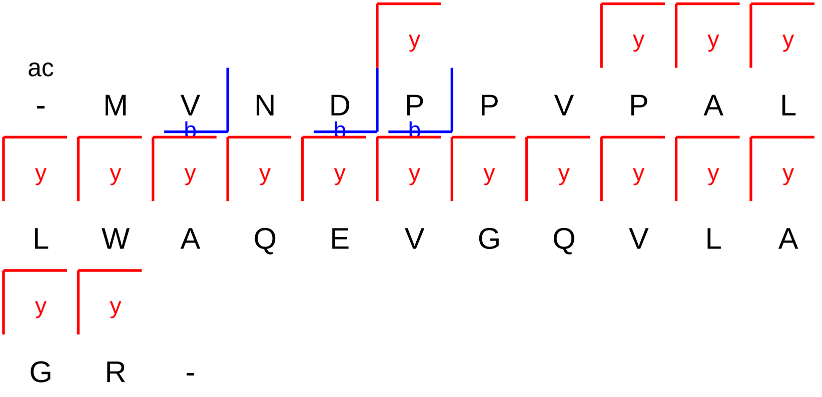

Raw File	Scan	Method	Score	m/z	Gene names
RO-QC-160121_ZackD_SS_Set_01_Fractio	41035	FTMS; HC	96.67	1252.17	BSCL2

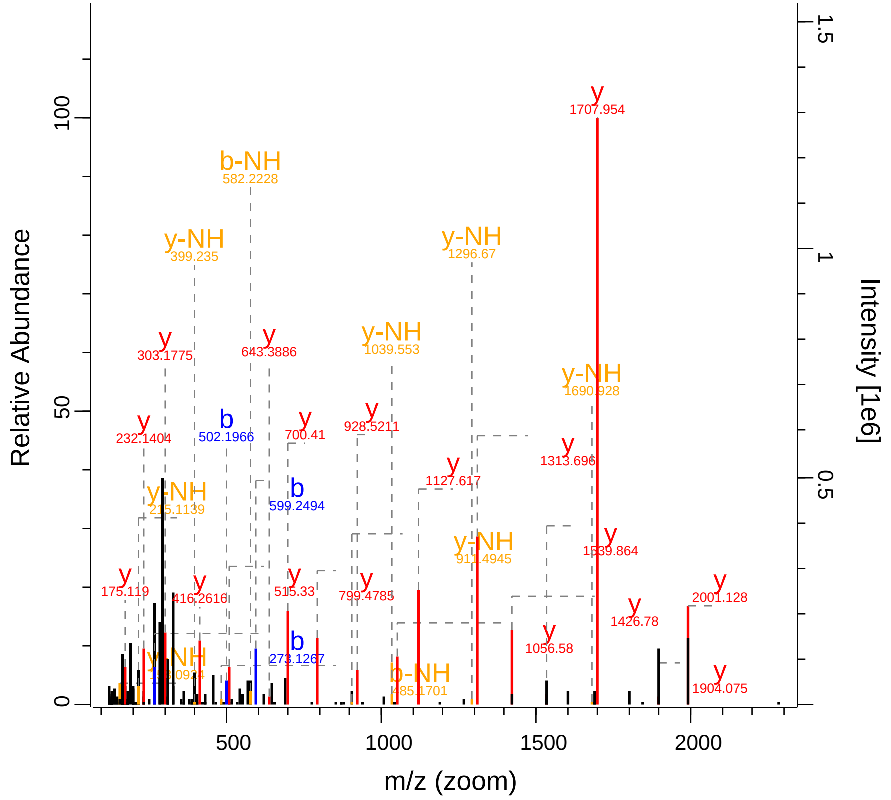

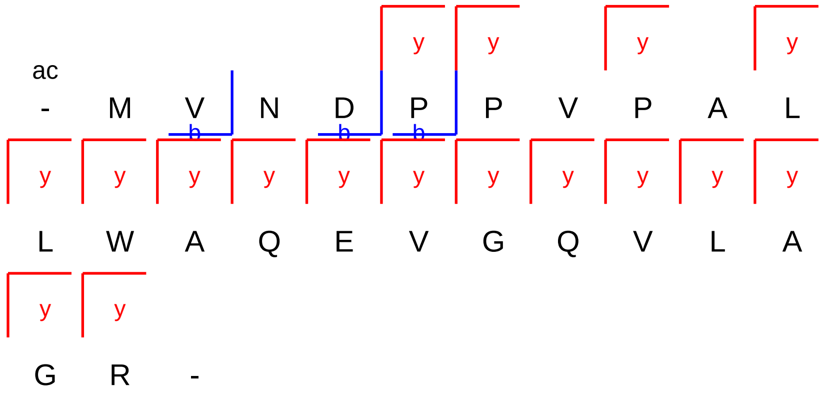

Raw File	Scan	Method	Score	m/z	Gene names
RO-QC-160121_ZackD_SS_Set_01_Fractior	41110	FTMS; HC	92.06	780.47	LACC1

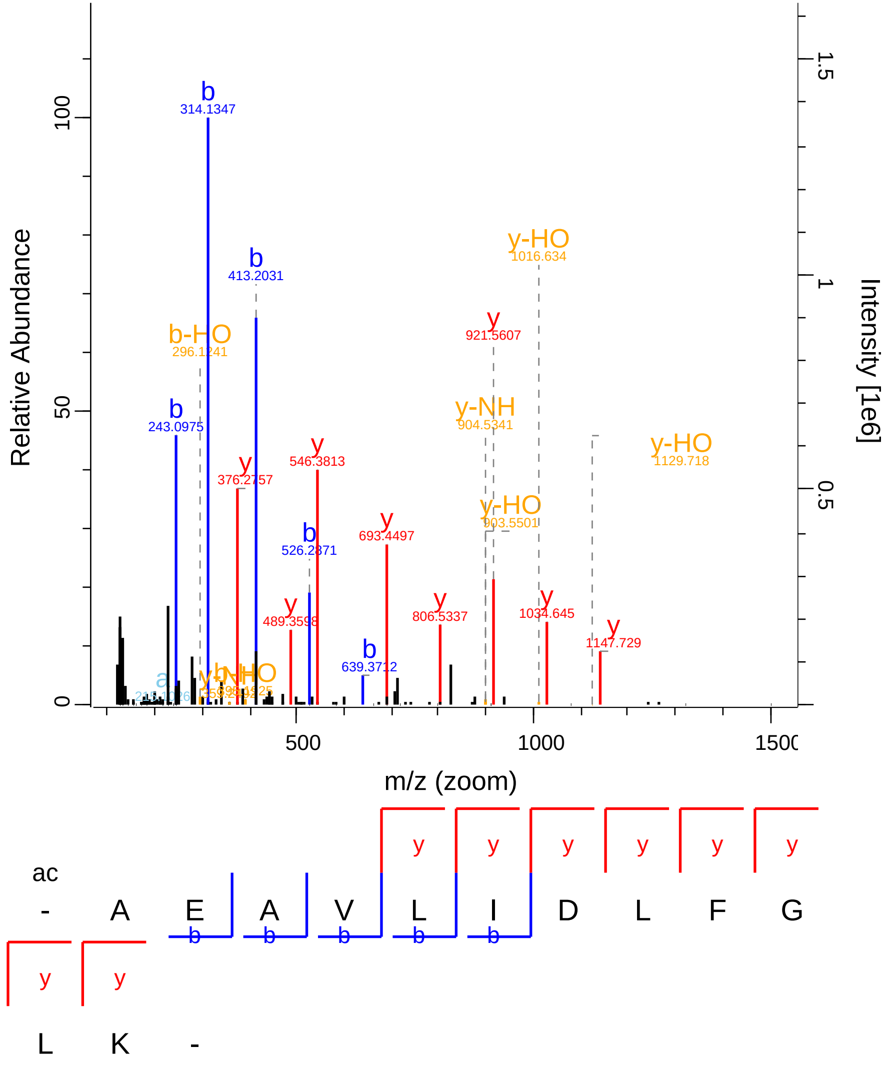

Raw File	Scan	Method	Score	m/z	Gene names
RO-QC-160121_ZackD_SS_Set_01_Fraction_	4156	FTMS; HC	42.91	619.29	ZBTB1

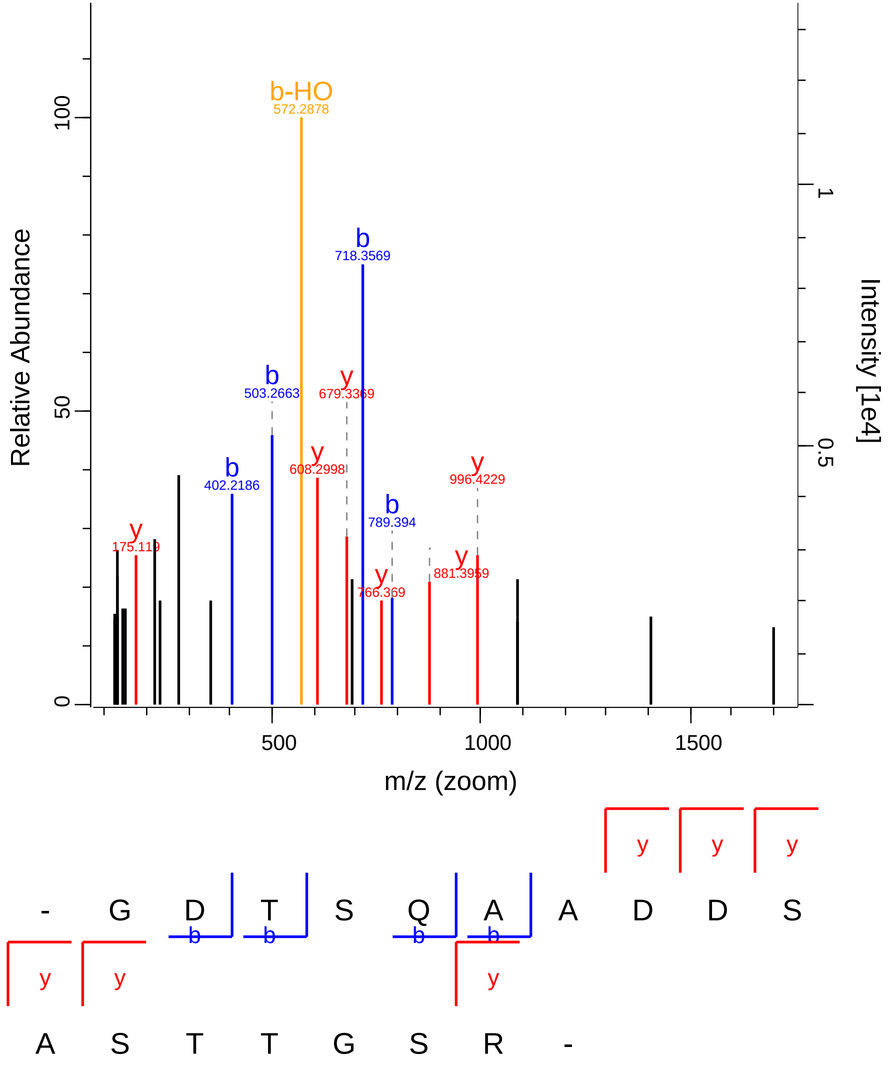

Raw File	Scan	Method	Score	m/z	Gene names
RO-QC-160121_ZackD_SS_Set_01_Fraction	4891	FTMS; HC	43.38	552.31	PADI4

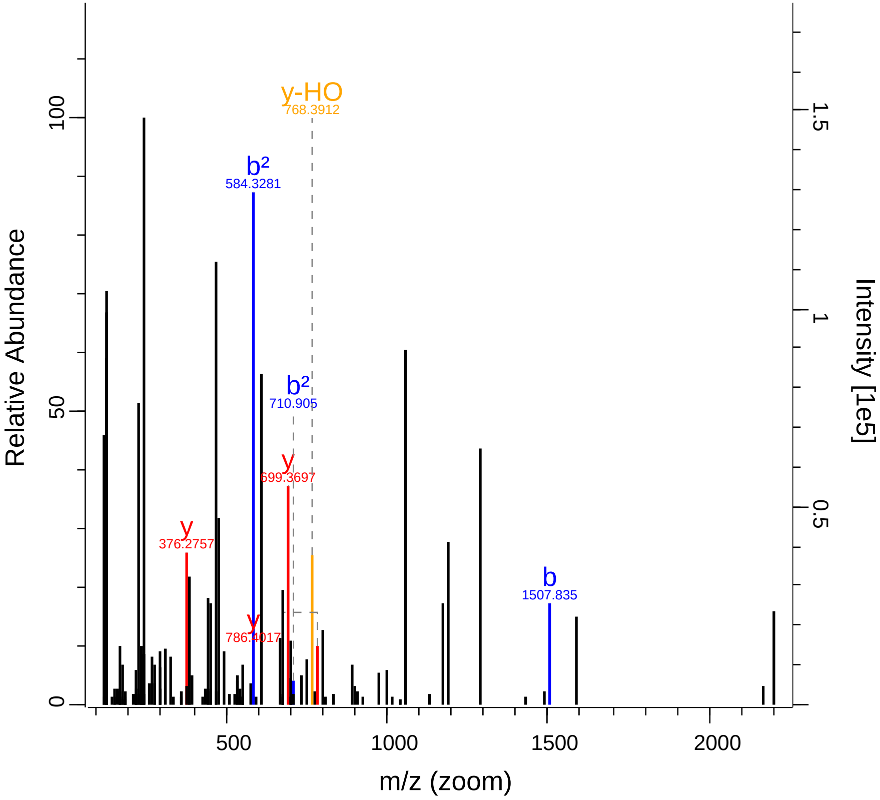

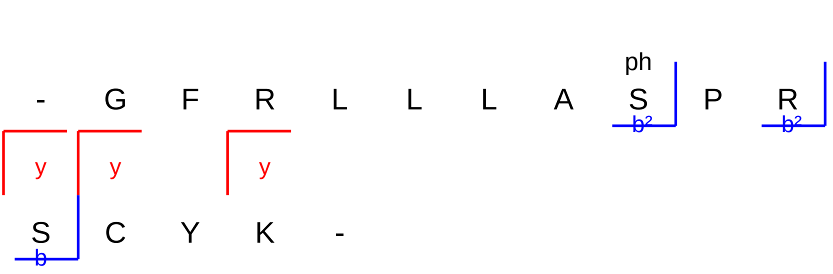

Raw File	Scan	Method	Score	m/z	Gene names
RO-QC-160121_ZackD_SS_Set_01_Fraction	4988	FTMS; HC	117.98	674.05	MOSPD1

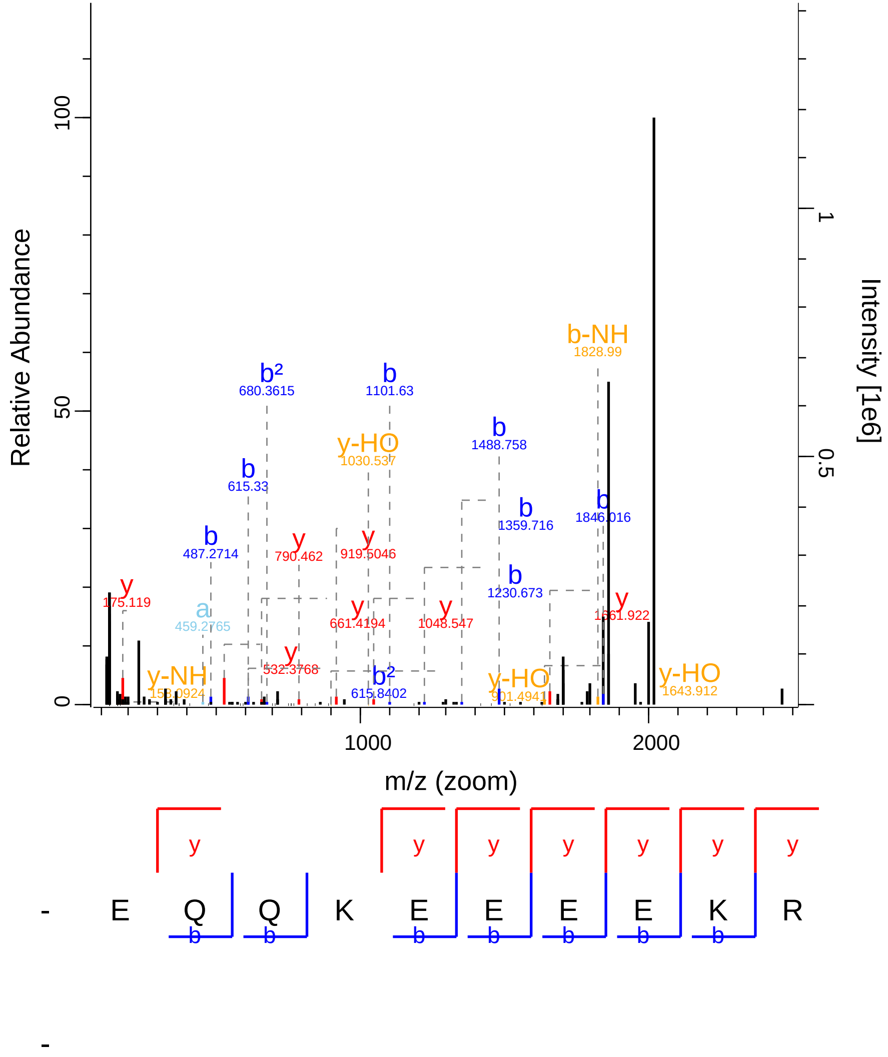

Raw File	Scan	Method	Score	m/z	Gene names
RO-QC-160121_ZackD_SS_Set_01_Fraction	5145	FTMS; HC	70.26	610.26	EGFL7

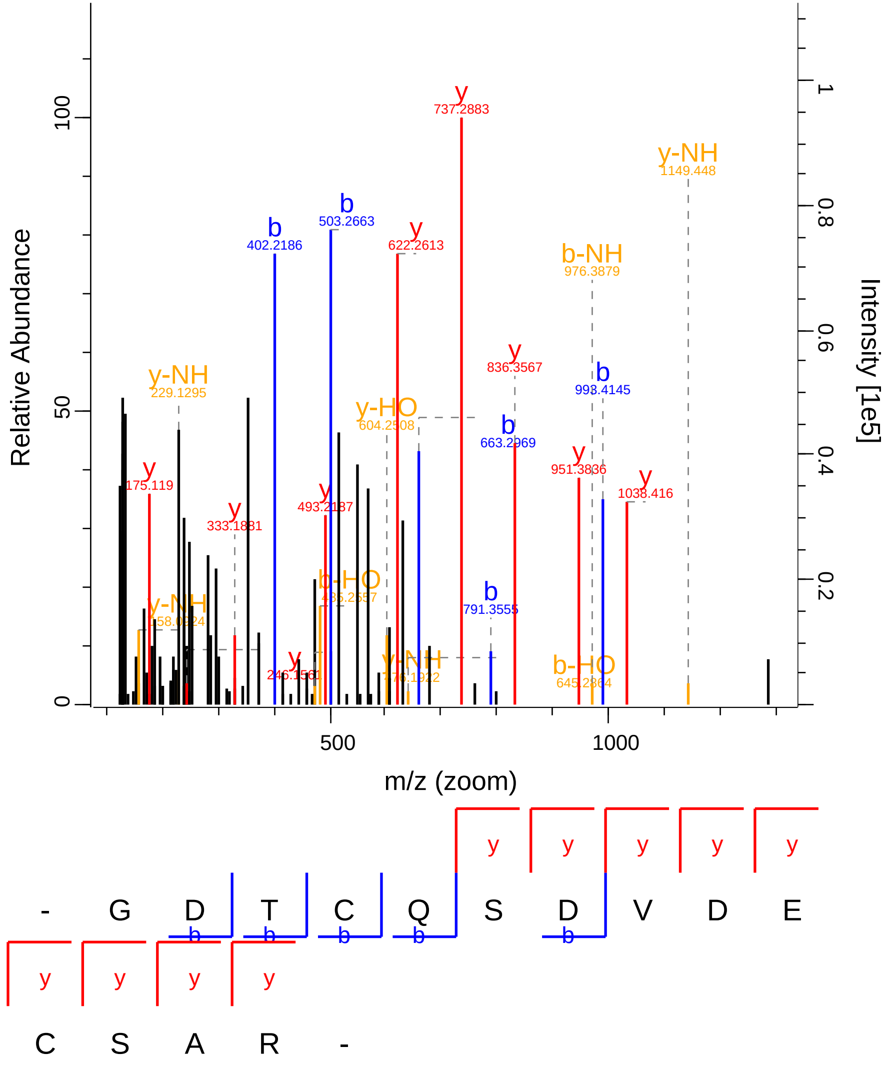

Raw File	Scan	Method	Score	m/z	Gene names
RO-QC-160121_ZackD_SS_Set_01_Fraction	5165	FTMS; HC	89.55	914.89	EGFL7

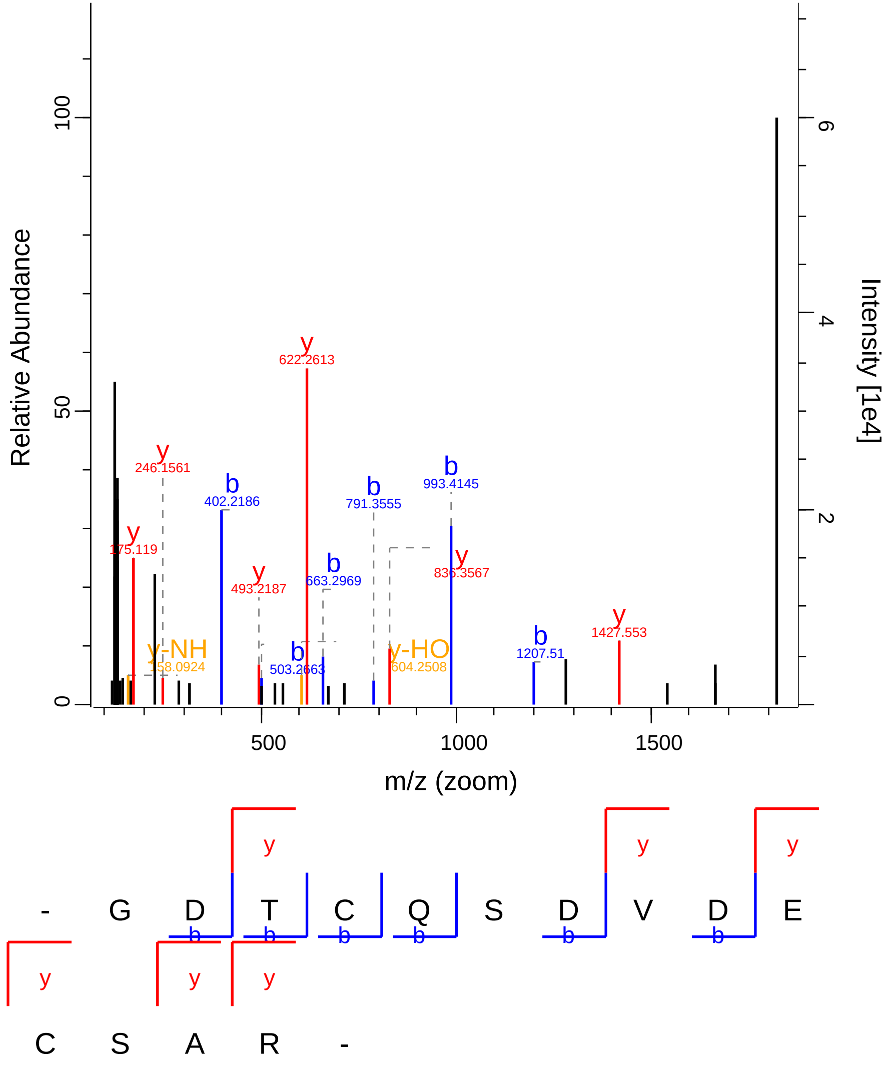

Raw File	Scan	Method	Score	m/z	Gene names
RO-QC-160121_ZackD_SS_Set_01_Fraction_	5489	FTMS; HC	81.3	396.75	EZH1

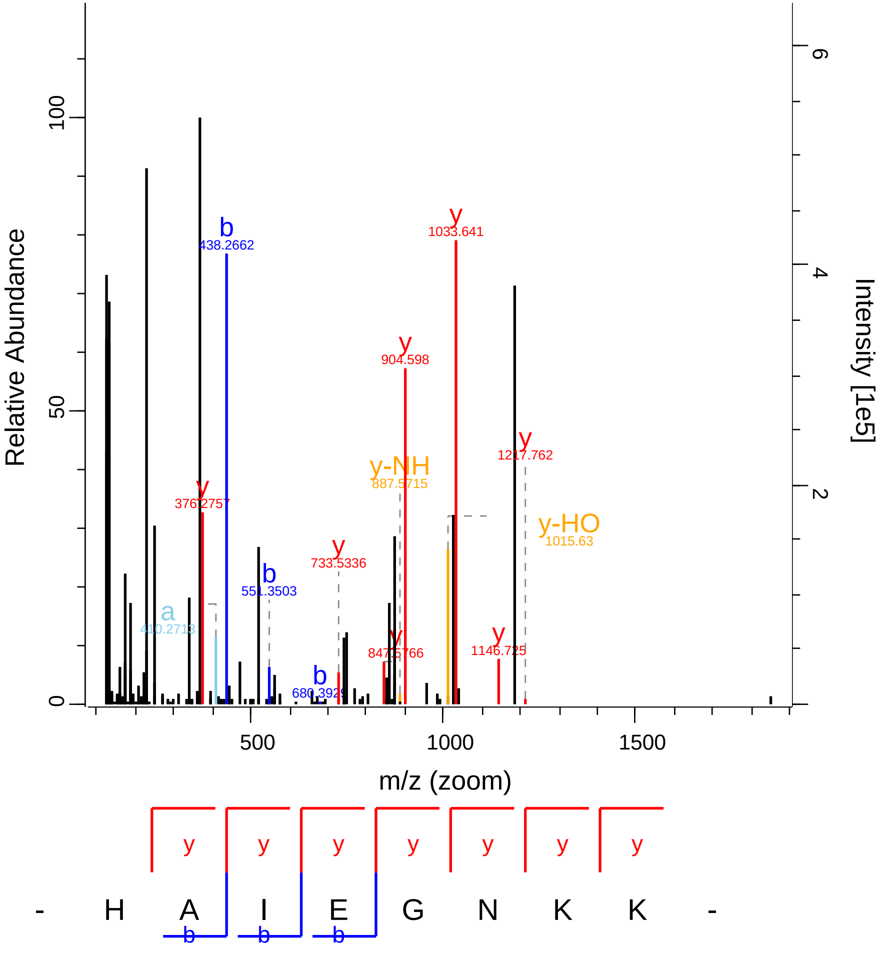

Raw File	Scan	Method	Score	m/z	Gene names
RO-QC-160121_ZackD_SS_Set_01_Fraction	5535	FTMS; HC	55.06	840.92	TASP1

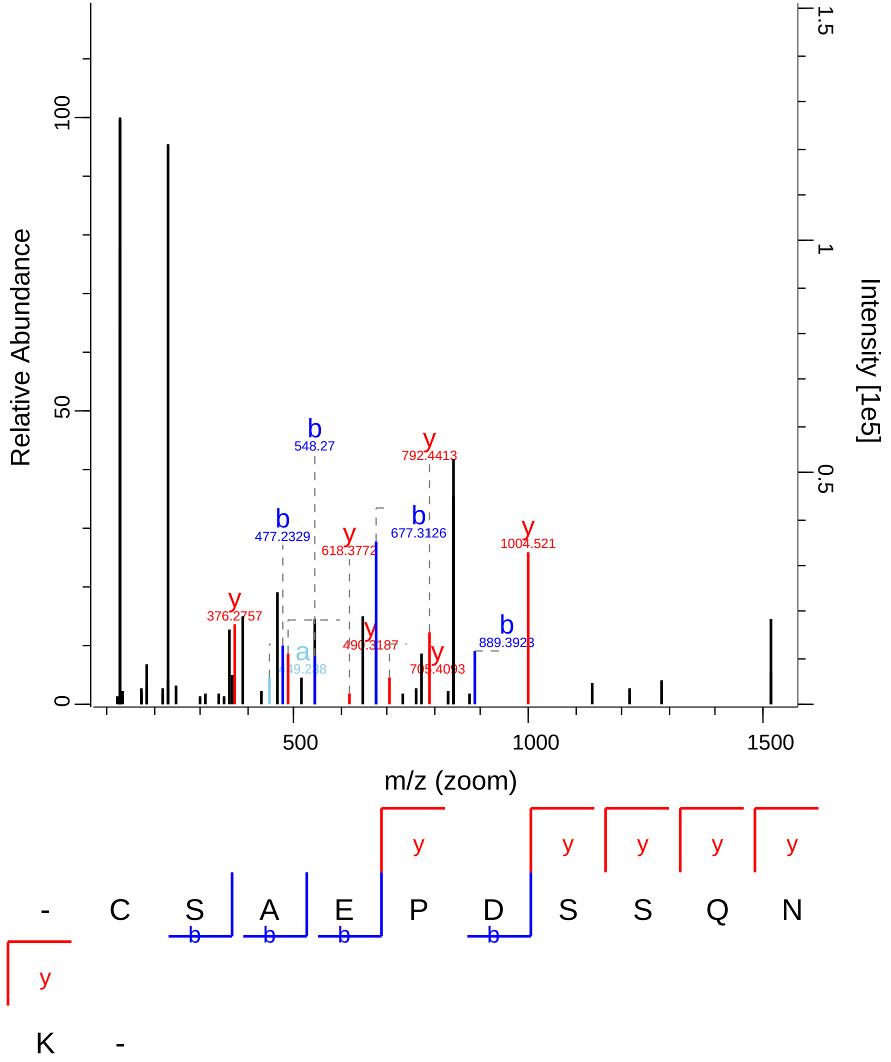

Raw File	Scan	Method	Score	m/z	Gene names
RO-QC-160121_ZackD_SS_Set_01_Fraction_	5745	FTMS; HCI	81.34	797.4	CCDC69

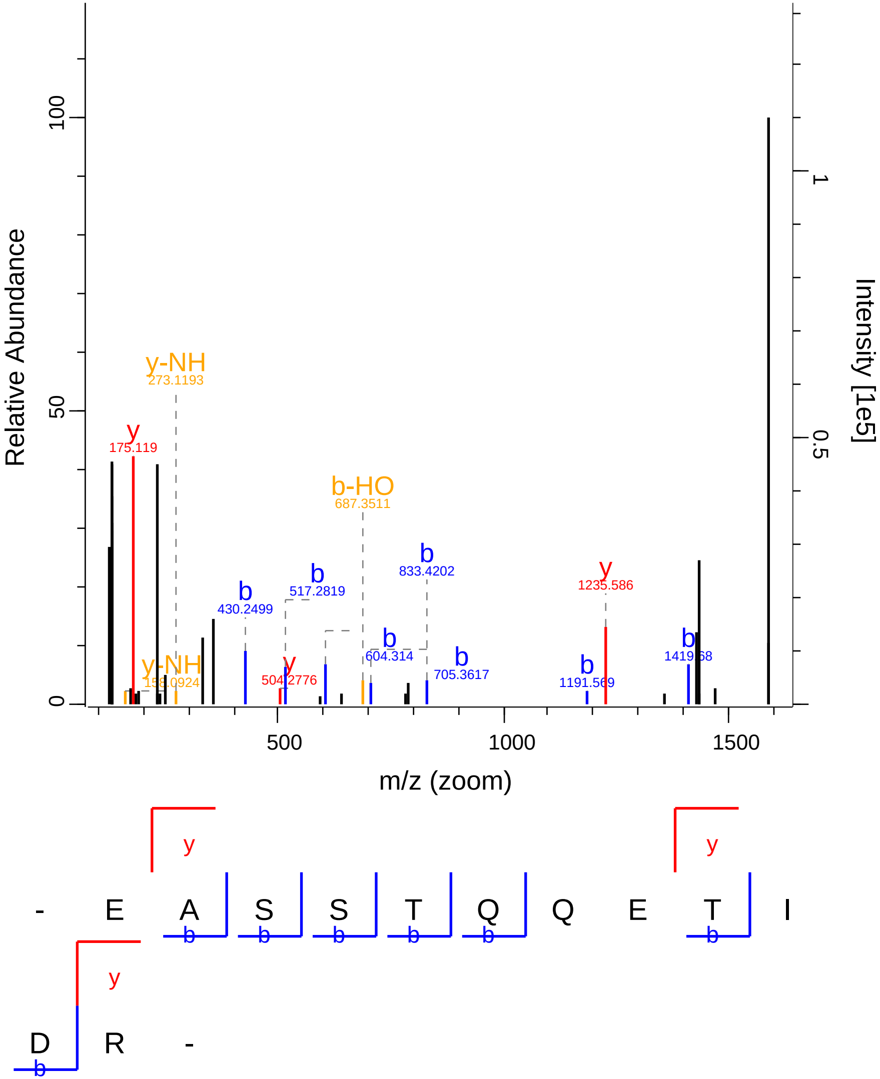

Raw File	Scan	Method	Score	m/z	Gene names
RO-QC-160121_ZackD_SS_Set_01_Fraction	6249	FTMS; HC	208.56	780.89	GKAP1

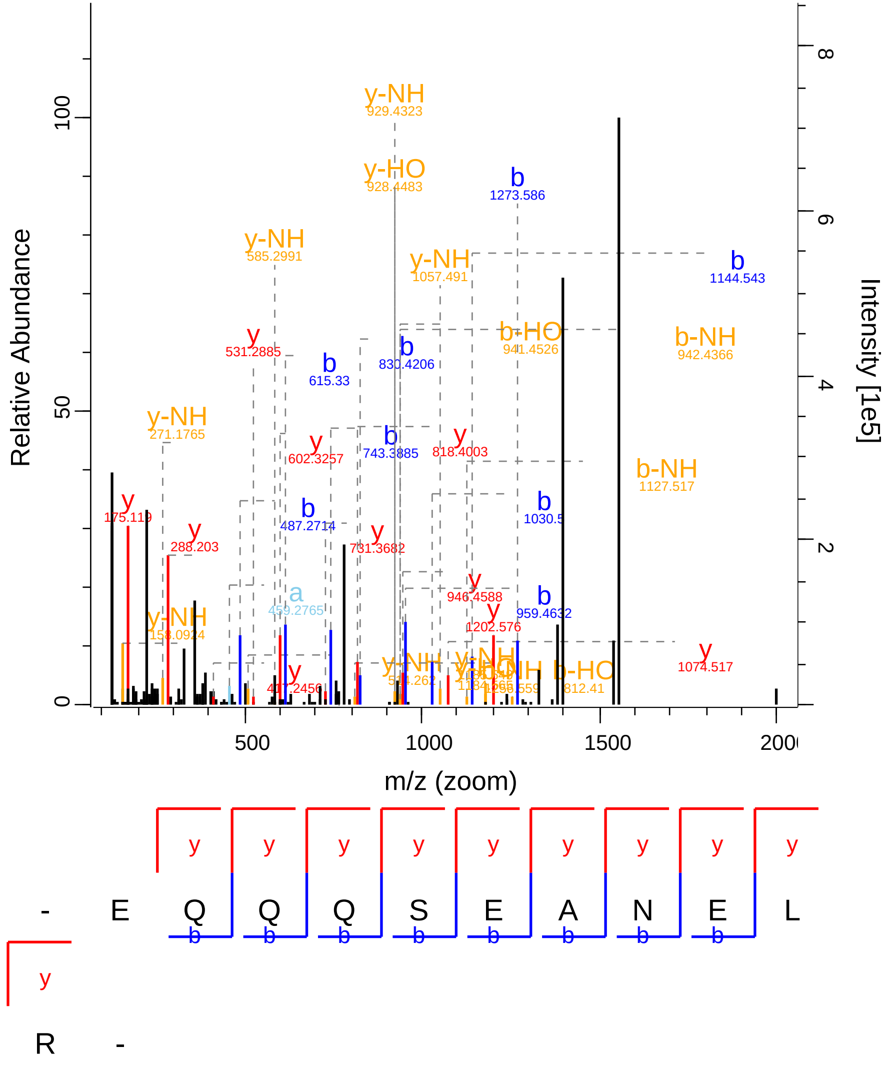

Raw File	Scan	Method	Score	m/z	Gene names
RO-QC-160121_ZackD_SS_Set_01_Fraction	6615	FTMS; HC	70.52	694.34	MLF2

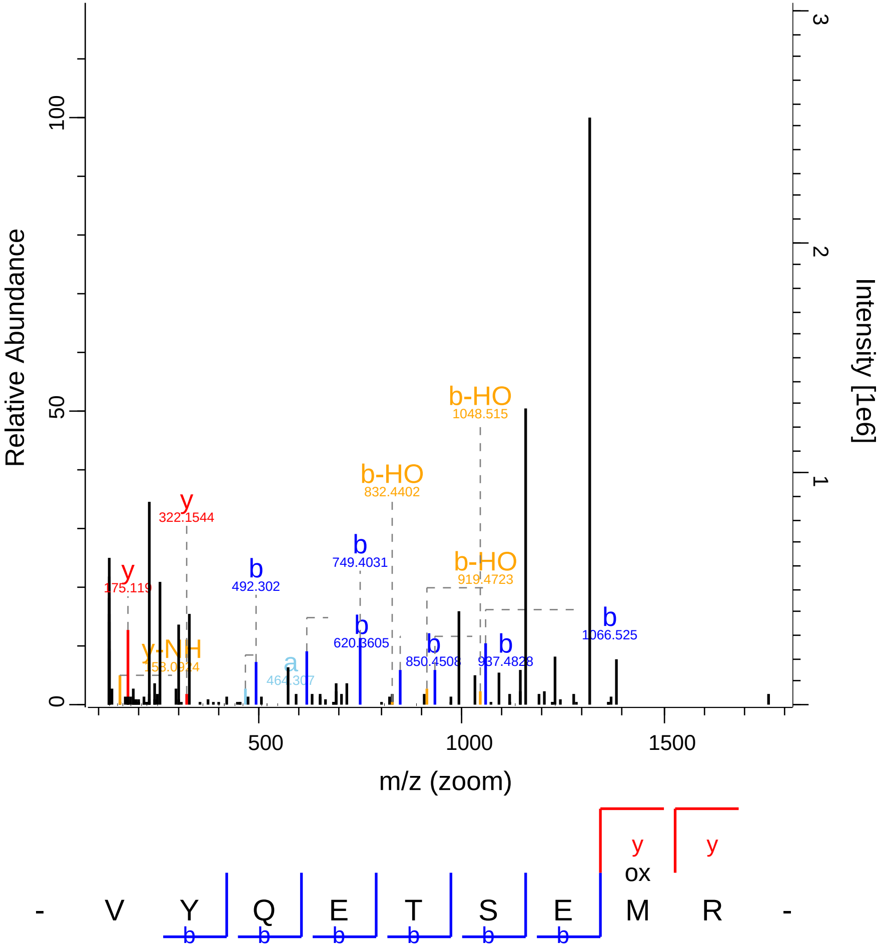

Raw File	Scan	Method	Score	m/z	Gene names
RO-QC-160121_ZackD_SS_Set_01_Fraction	7793	FTMS; HC	109.01	726.89	SERF2

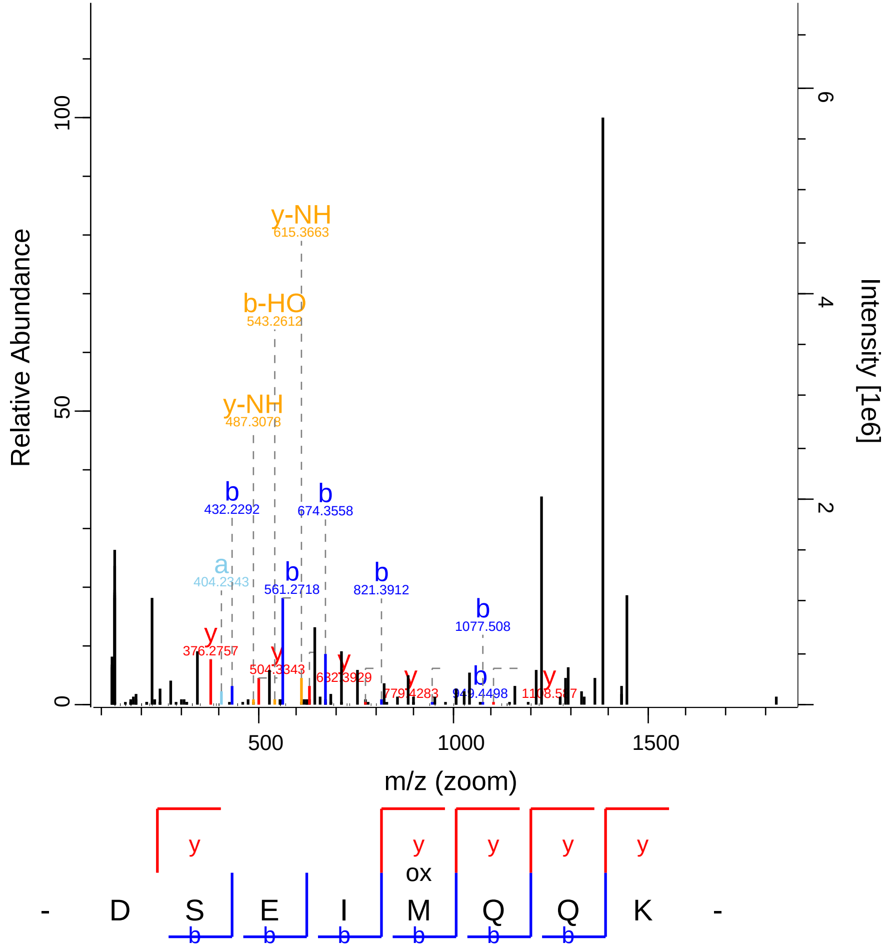

Raw File	Scan	Method	Score	m/z
RO-QC-160121_ZackD_SS_Set_01_Fraction_11	8021	FTMS; HCD	52.4	795.4

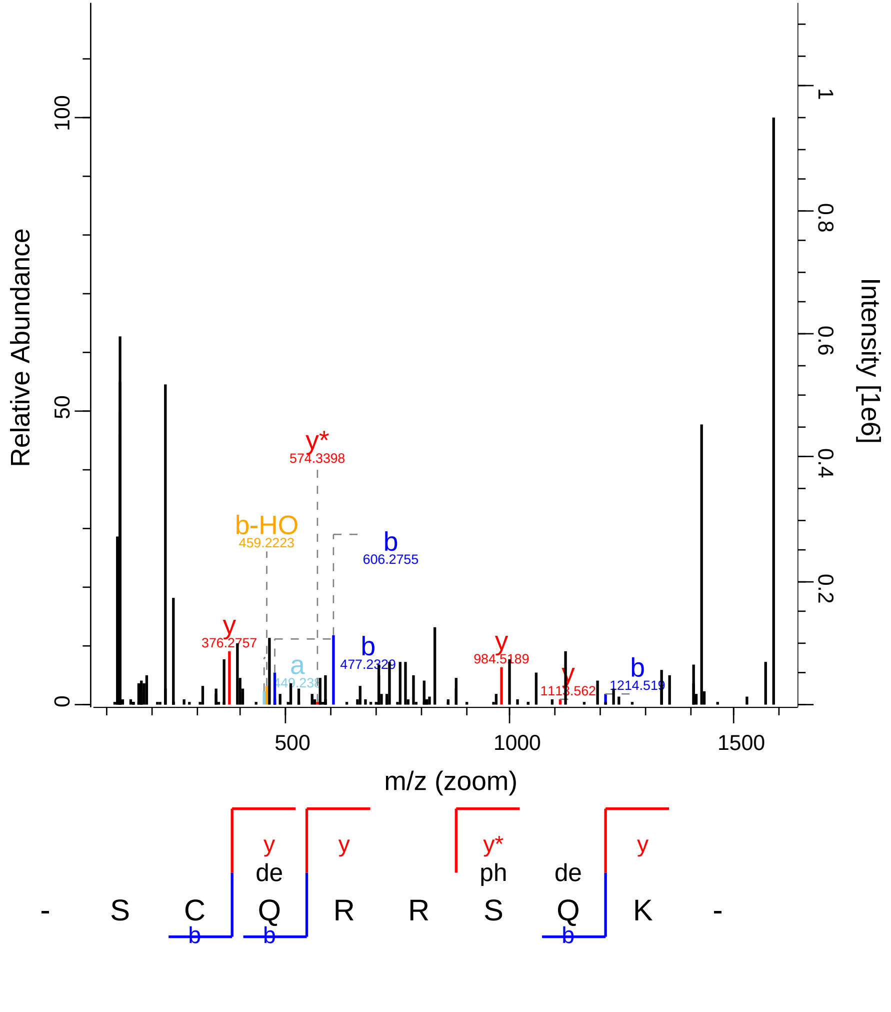

Raw File	Scan	Method	Score	m/z	Gene names
RO-QC-160121_ZackD_SS_Set_01_Fraction	8386	FTMS; HC	45.14	751.89	LRRC74A

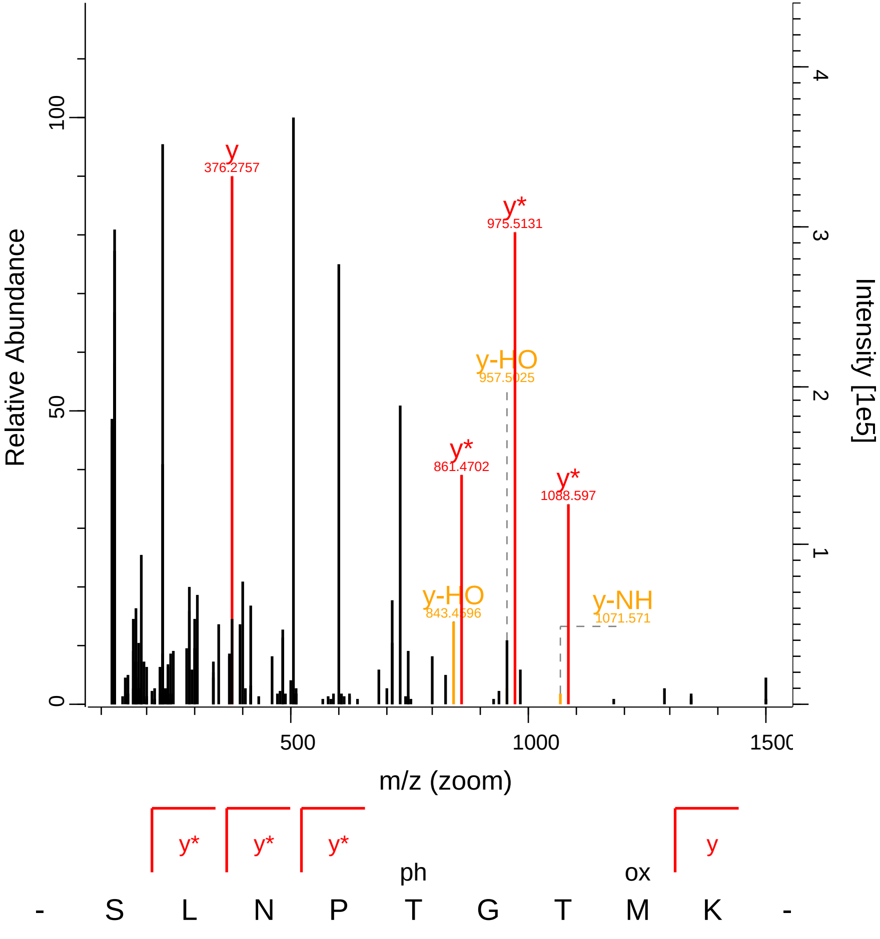

Raw File	Scan	Method	Score	m/z	Gene names
RO-QC-160121_ZackD_SS_Set_01_Fraction	9009	FTMS; HC	104.39	831.89	LEPROT

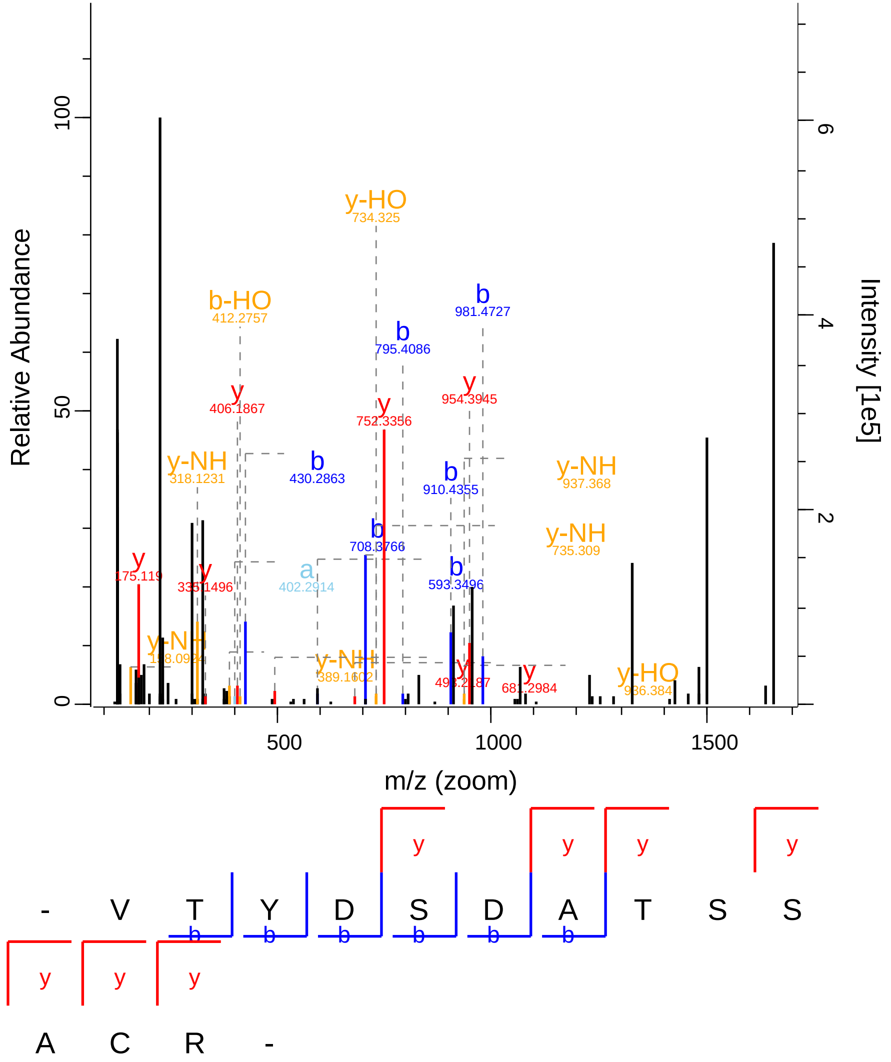

Raw File	Scan	Method	Score	m/z	Gene names
RO-QC-160121_ZackD_SS_Set_01_Fraction	9072	FTMS; HC	112.02	554.59	LEPROT

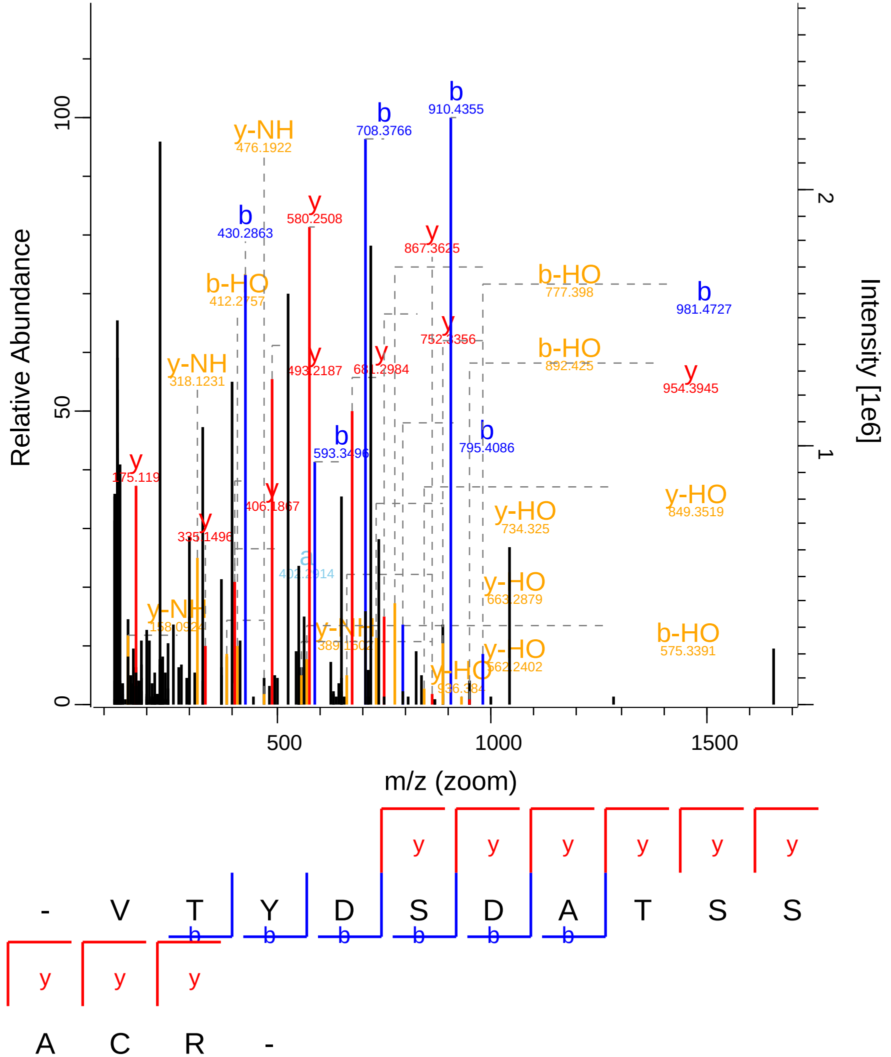

Raw File	Scan	Method	Score	m/z	Gene names
RO-QC-160121_ZackD_SS_Set_01_Fraction	9767	FTMS; HC	104.45	366.22	C2orf15

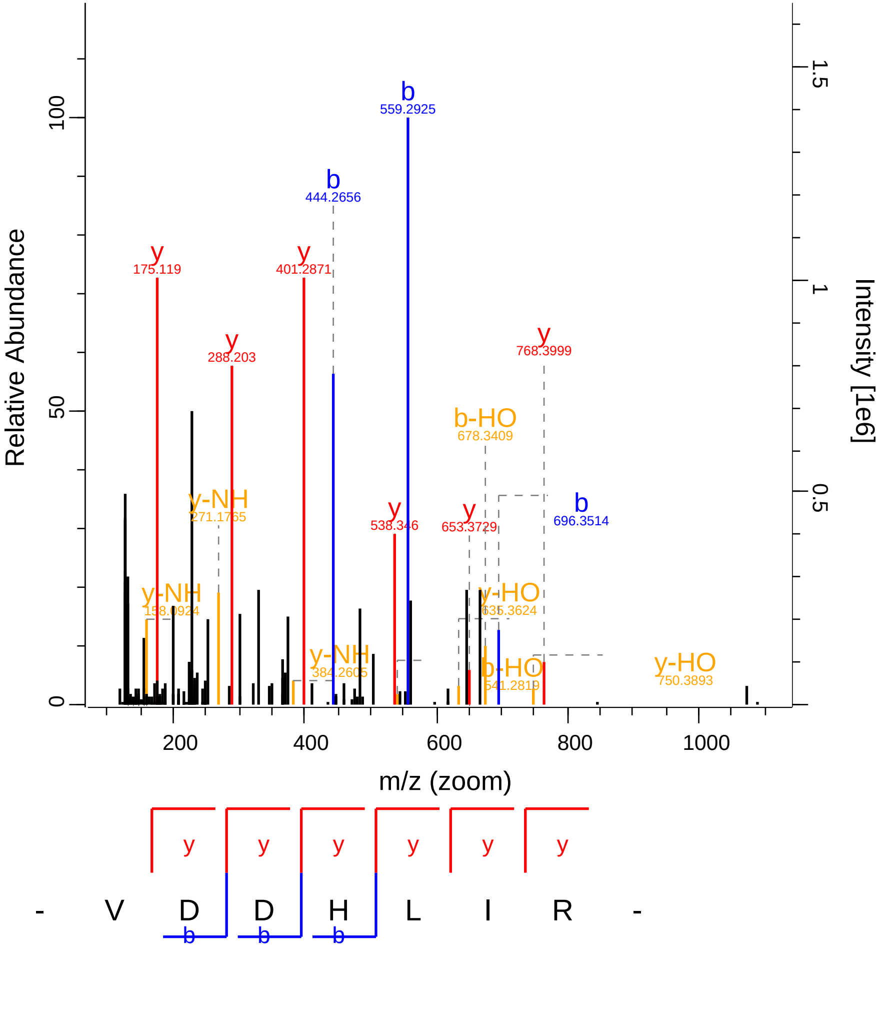

Raw File	Scan	Method	Score	m/z	Gene names
RO-QC-160121_ZackD_SS_Set_01_Fractior	10203	FTMS; HC	38.78	400.49	USH1C

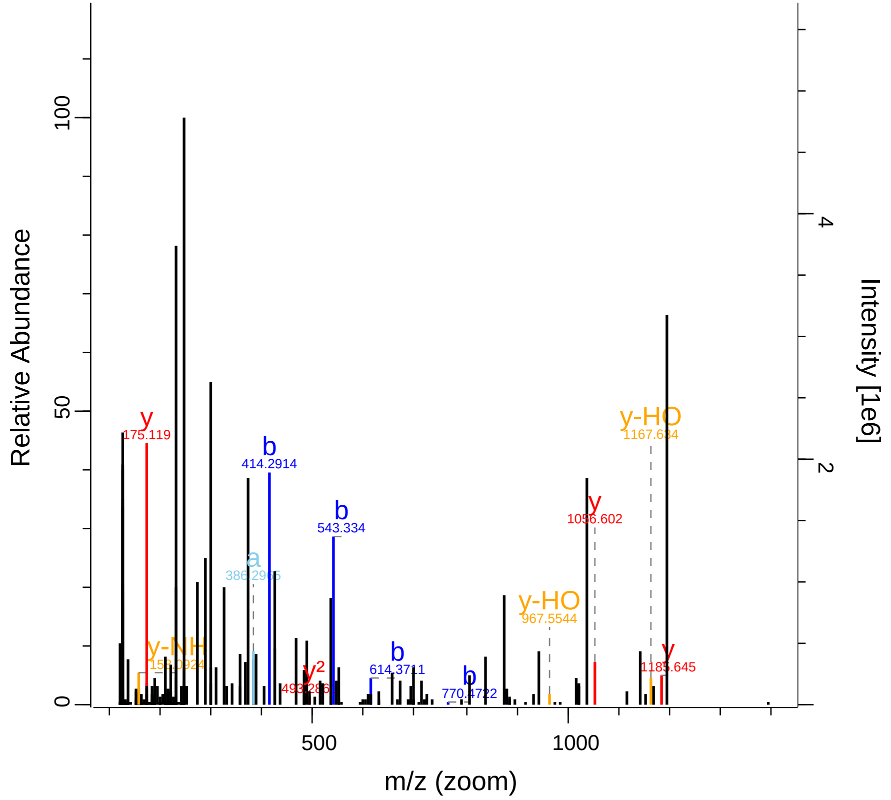

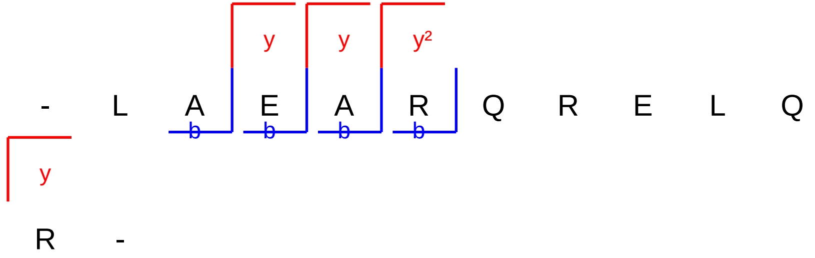

Raw File	Scan	Method	Score	m/z	Gene names
RO-QC-160121_ZackD_SS_Set_01_Fraction	10901	FTMS; HC	141.89	832.9	YIPF5

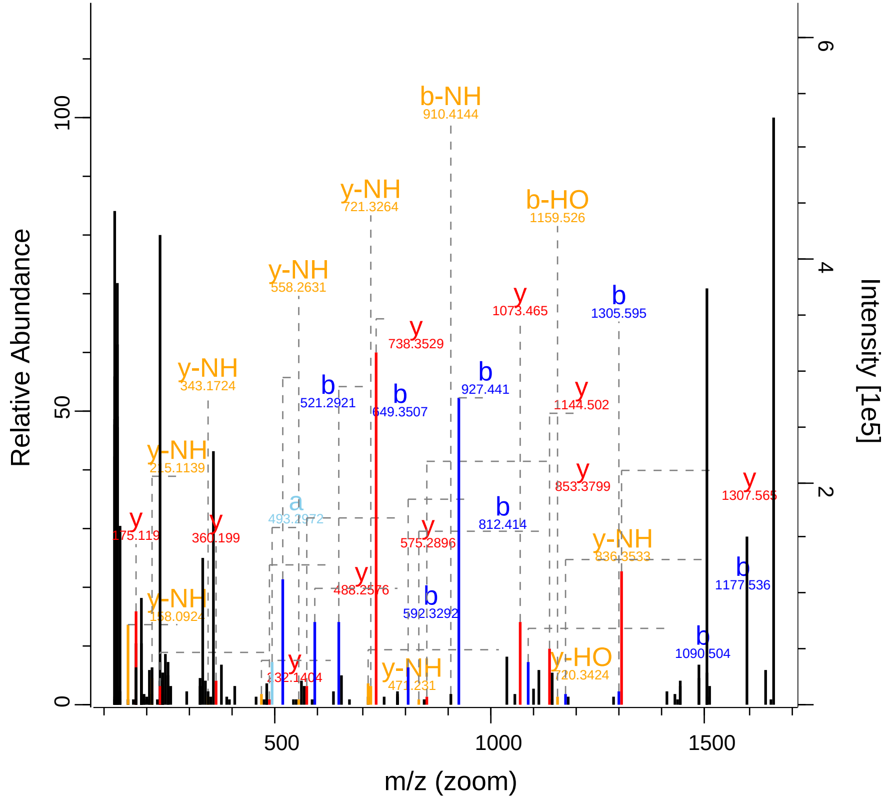

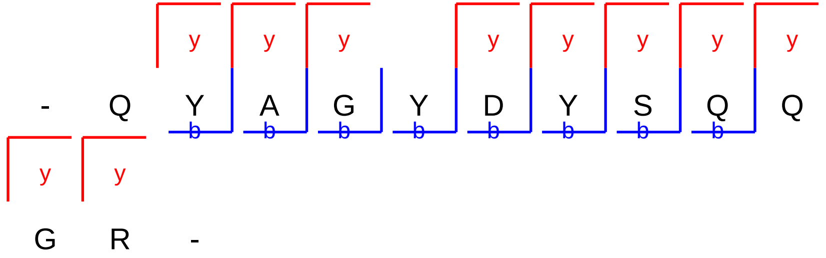

Raw File	Scan	Method	Score	m/z	Gene names
RO-QC-160121_ZackD_SS_Set_01_Fraction	10995	FTMS; HC	107.09	555.6	YIPF5

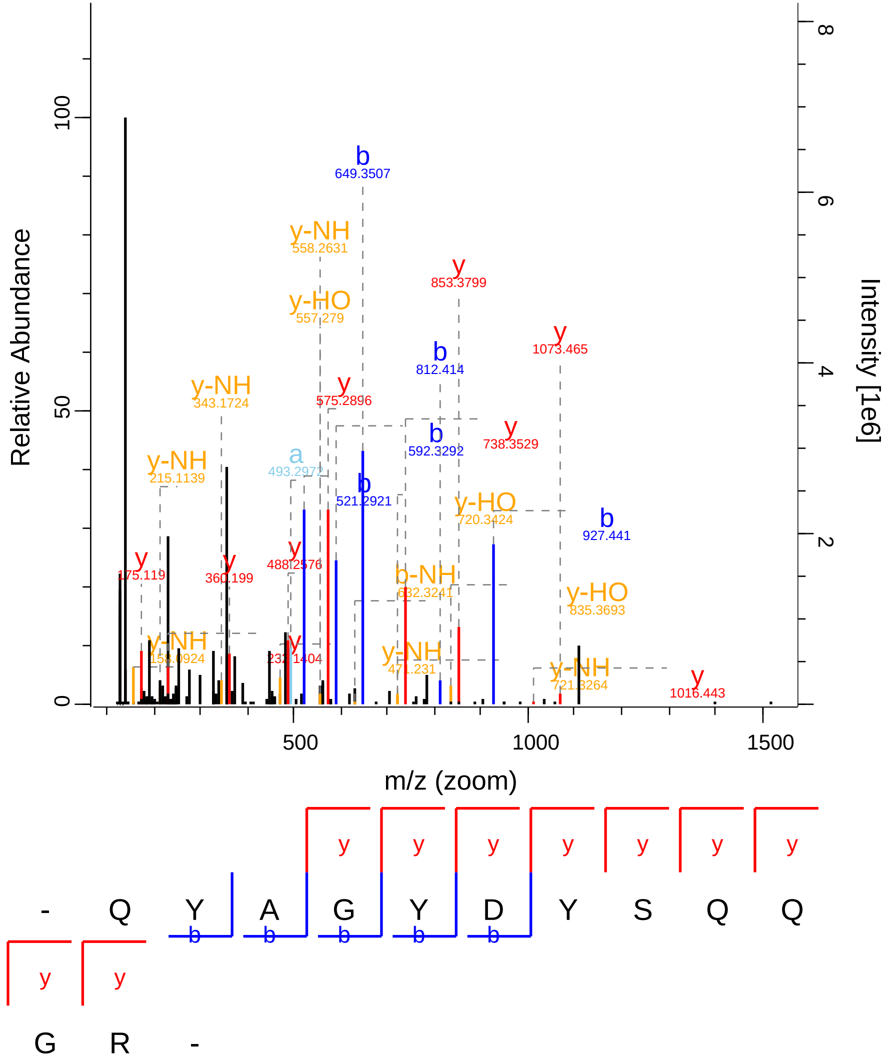

Raw File	Scan	Method	Score	m/z	Gene names
RO-QC-160121_ZackD_SS_Set_01_Fractior	11015	FTMS; HC	42.21	476.47	C6orf165

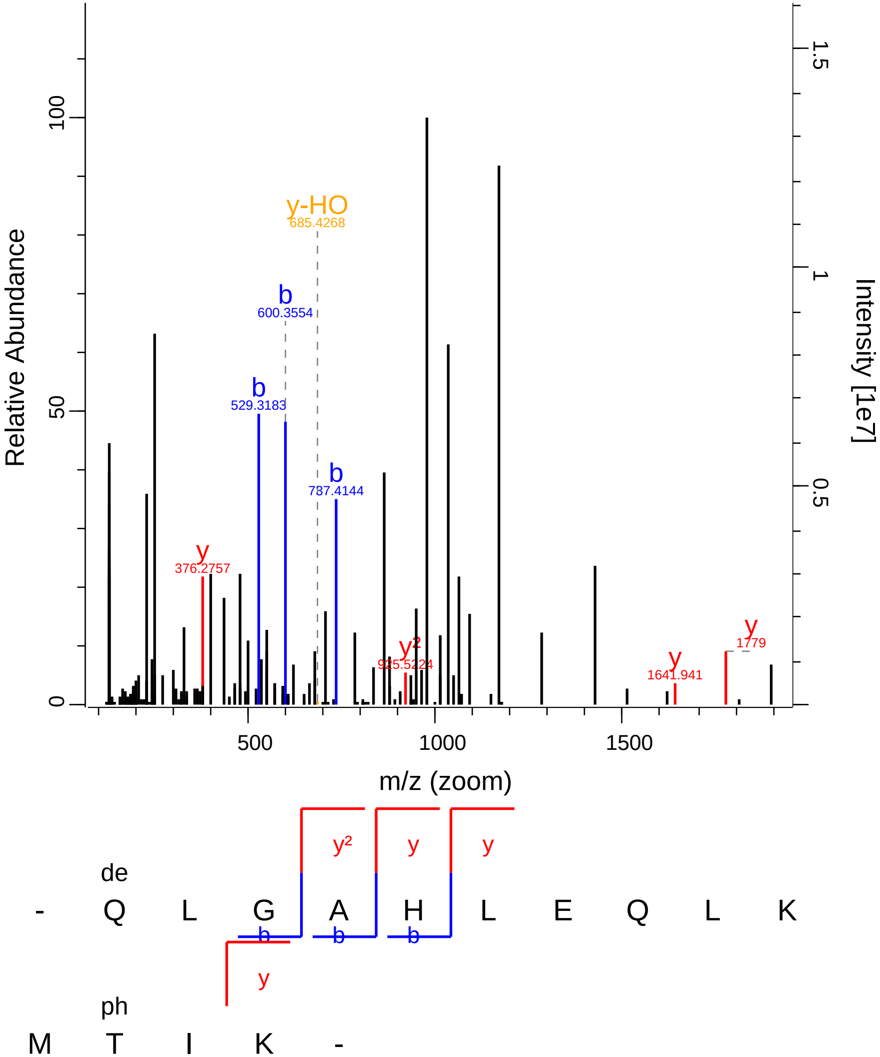

Raw File	Scan	Method	Score	m/z	Gene names
RO-QC-160121_ZackD_SS_Set_01_Fractior	11367	FTMS; HC	135.86	686.34	MLF2

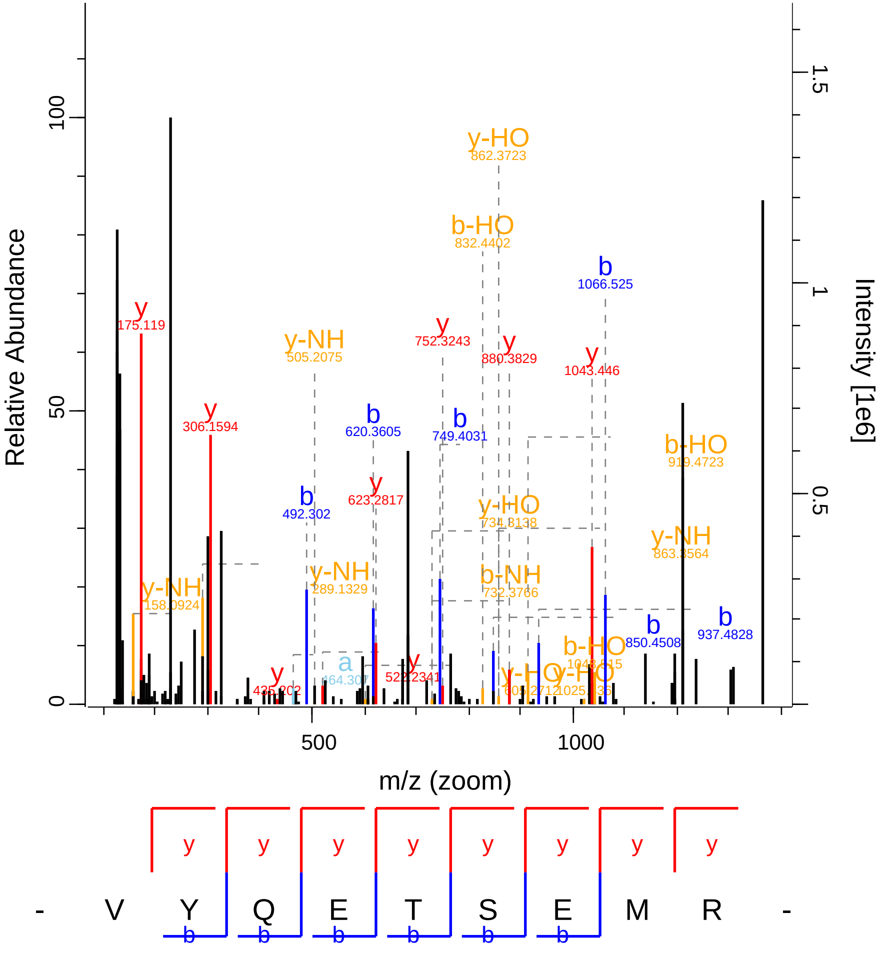

Raw File	Scan	Method	Score	m/z
RO-QC-160121_ZackD_SS_Set_01_Fraction_11	12880	FTMS; HCD	50.97	378.21

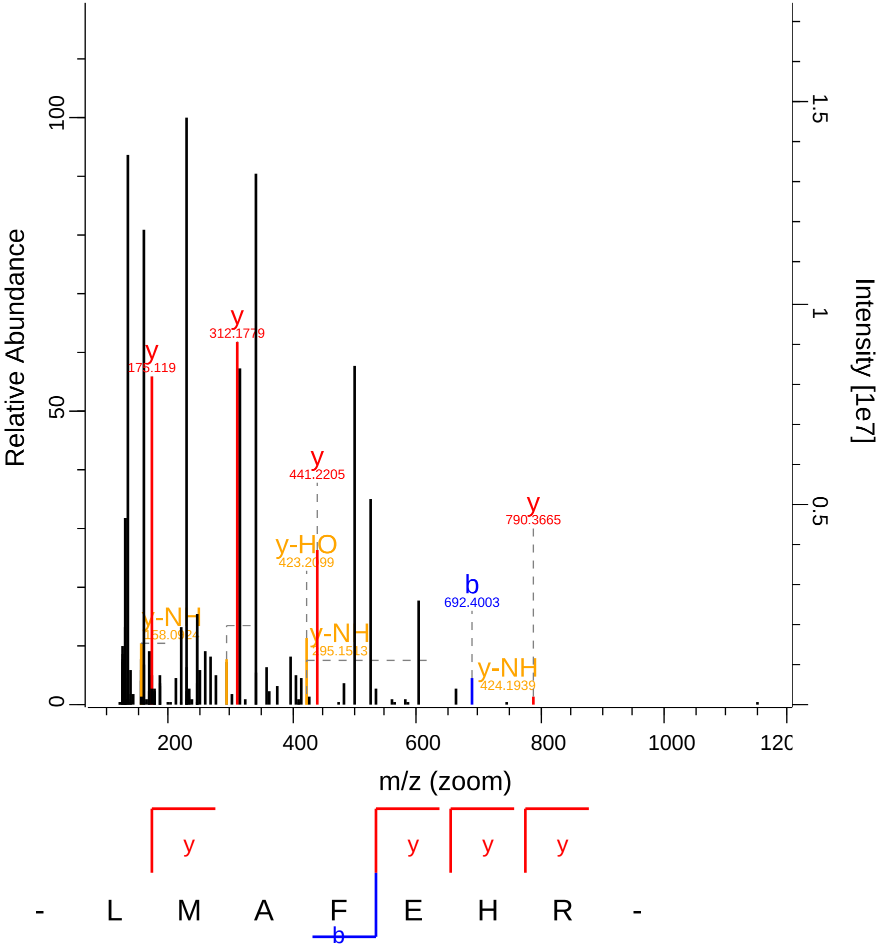

Raw File	Scan	Method	Score	m/z
RO-QC-160121_ZackD_SS_Set_01_Fraction_11	12897	FTMS; HCD	47.92	383.54

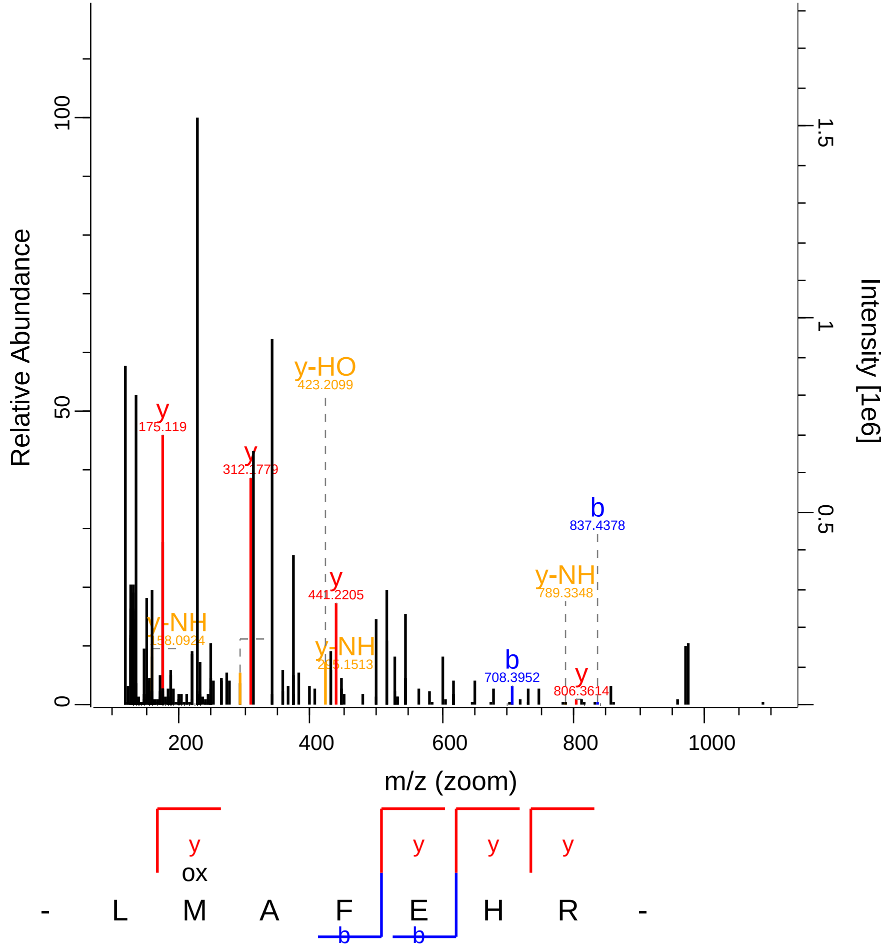

Raw File	Scan	Method	Score	m/z	Gene names
RO-QC-160121_ZackD_SS_Set_01_Fractior	13012	FTMS; HC	82.54	725.07	ZNF350

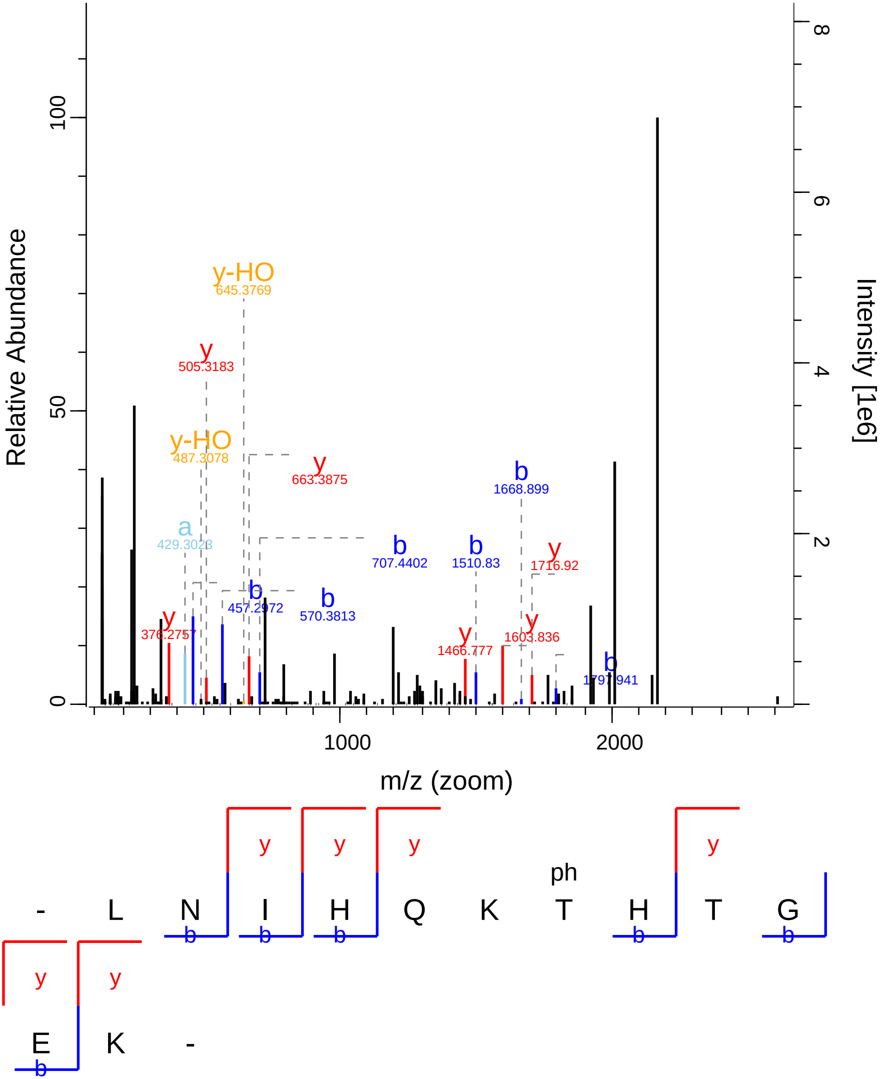

Raw File	Scan	Method	Score	m/z
RO-QC-160121_ZackD_SS_Set_01_Fraction_11	13070	FTMS; HCD	60.36	566.81

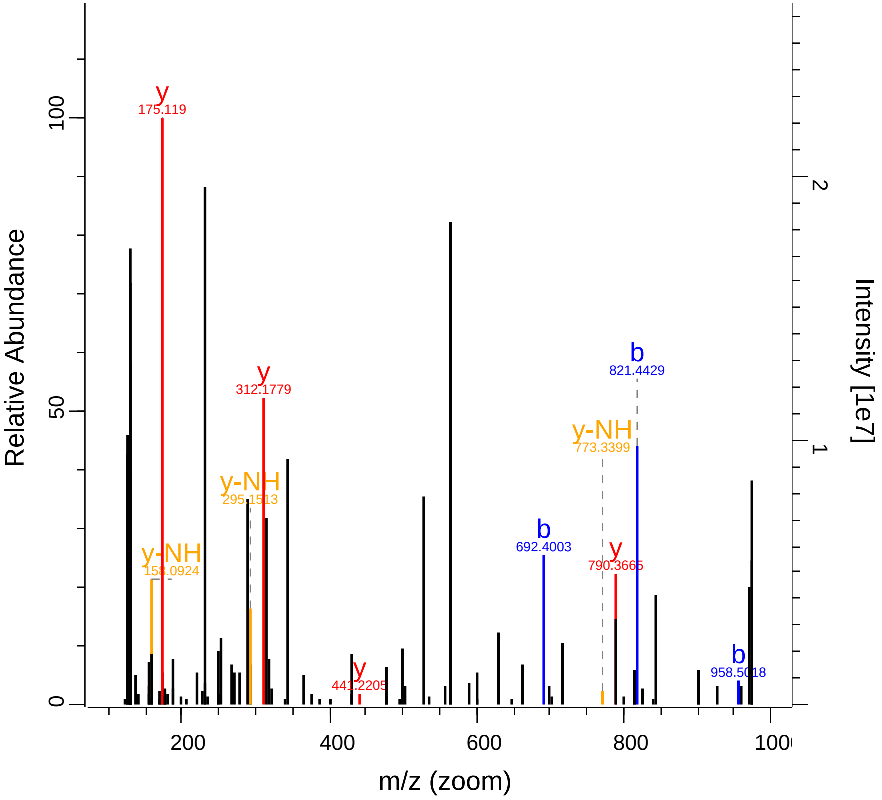

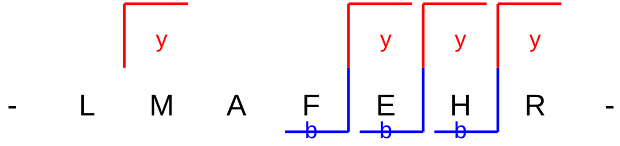

Raw File	Scan	Method	Score	m/z
RO-QC-160121_ZackD_SS_Set_01_Fraction_11	13112	FTMS; HCD	38.82	378.21

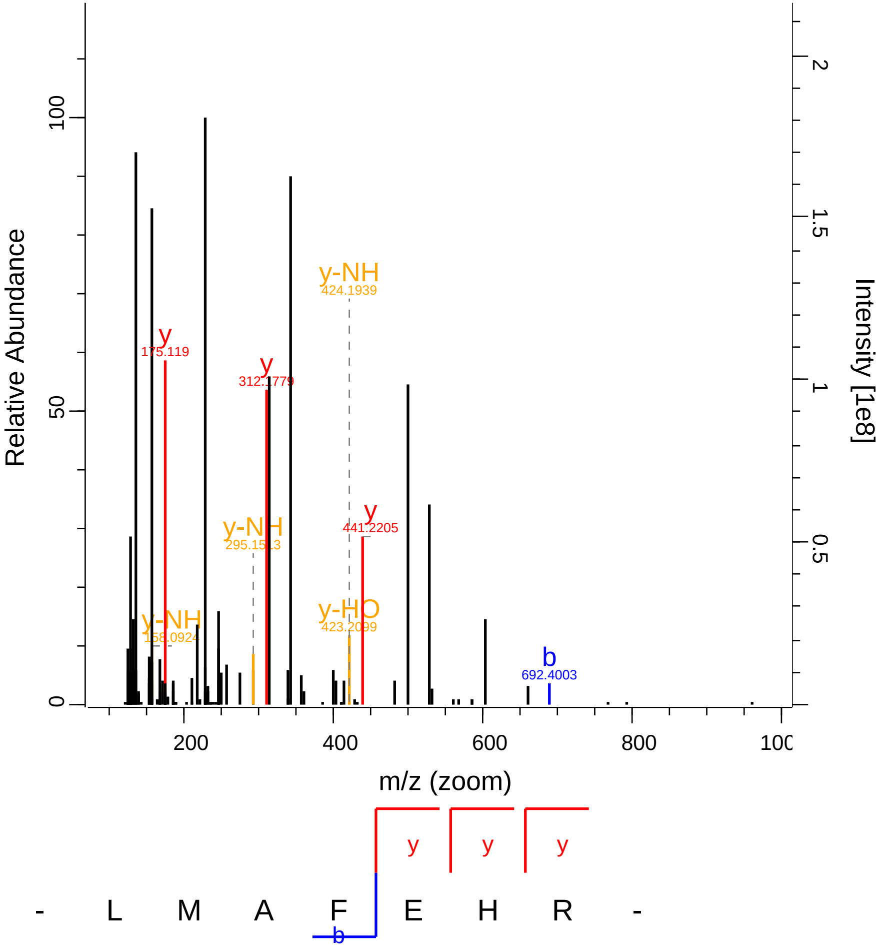

Raw File	Scan	Method	Score	m/z	Gene names
RO-QC-160121_ZackD_SS_Set_01_Fractio[illegible]	13301	FTMS; HC	116.28	783.71	F2R

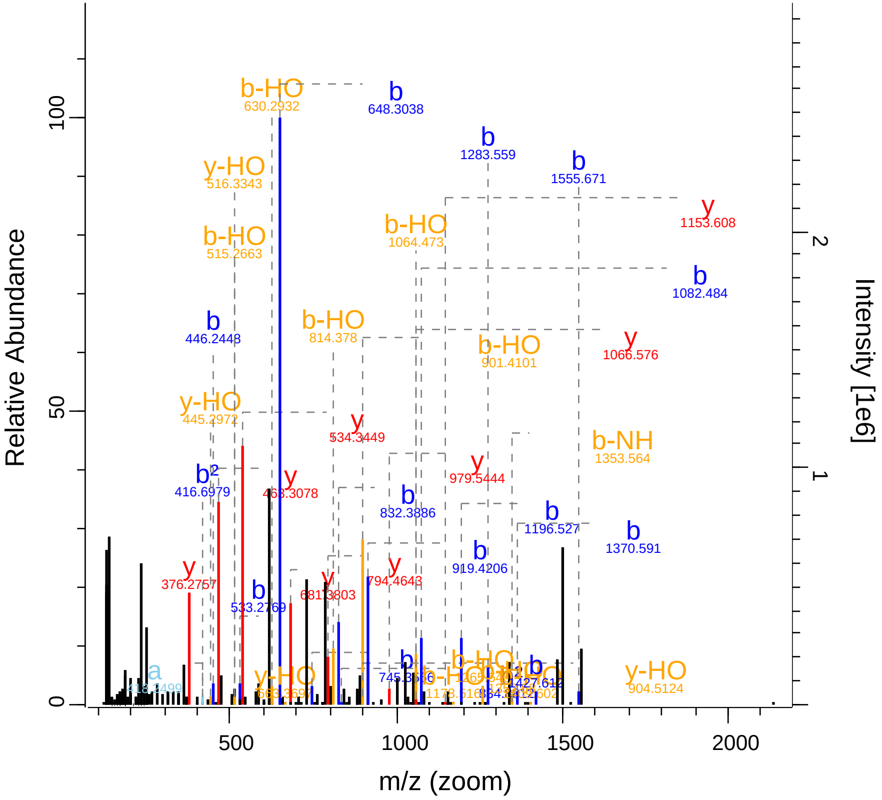

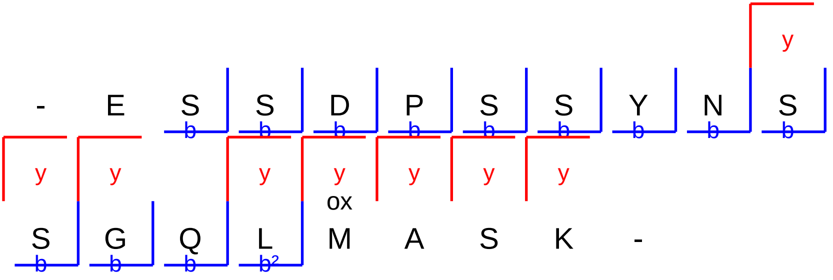

Raw File	Scan	Method	Score	m/z
RO-QC-160121_ZackD_SS_Set_01_Fraction_11	13893	FTMS; HCD	48.24	540.83

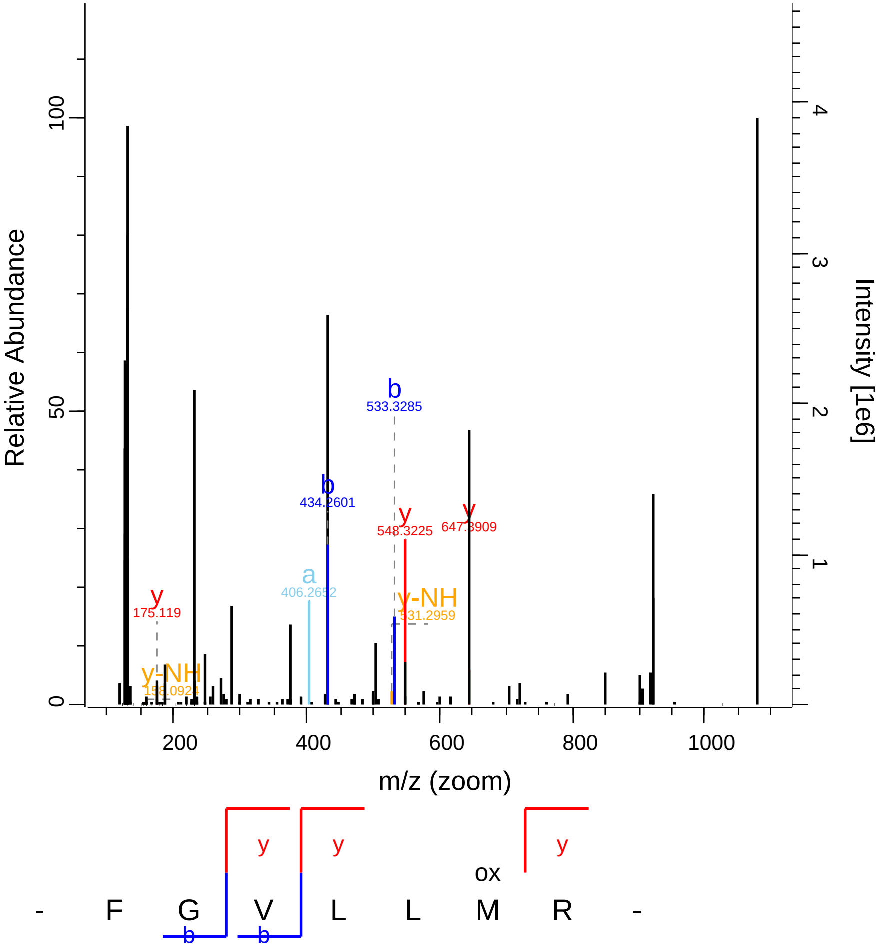

Raw File	Scan	Method	Score	m/z
RO-QC-160121_ZackD_SS_Set_01_Fraction_11	15121	FTMS; HCD	60.79	378.21

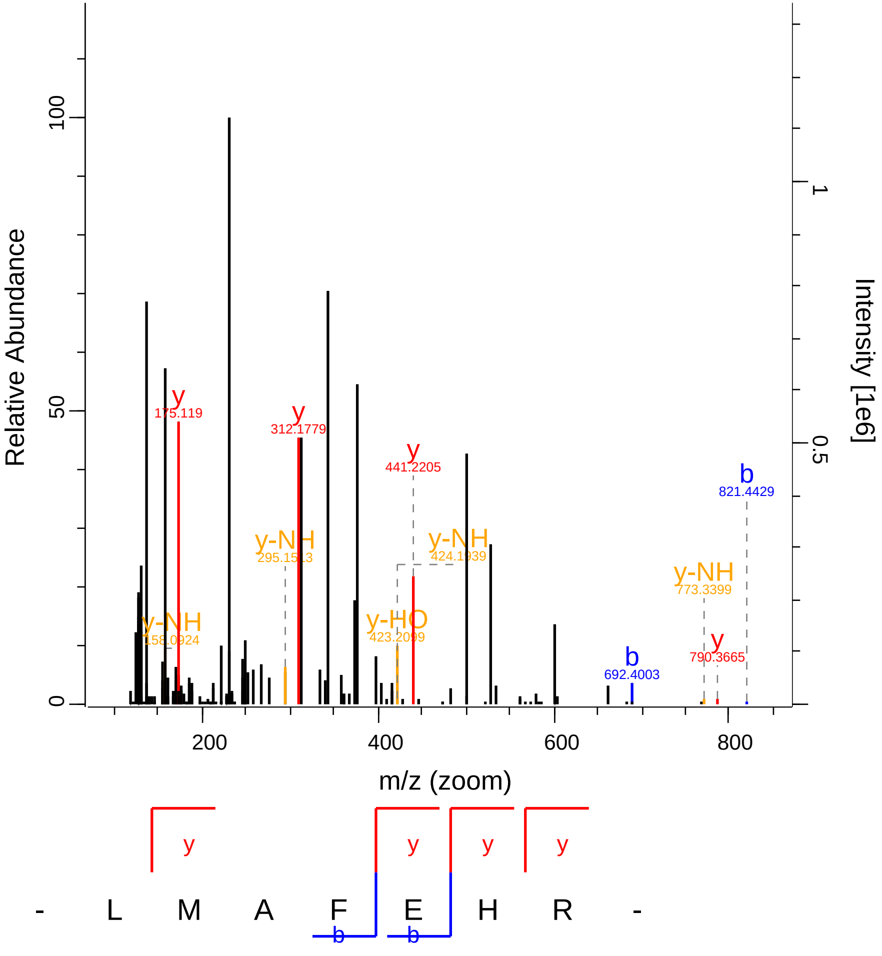

Raw File	Scan	Method	Score	m/z	Gene names
RO-QC-160121_ZackD_SS_Set_01_Fractior	15978	FTMS; HC	70.91	403.91	C7orf43

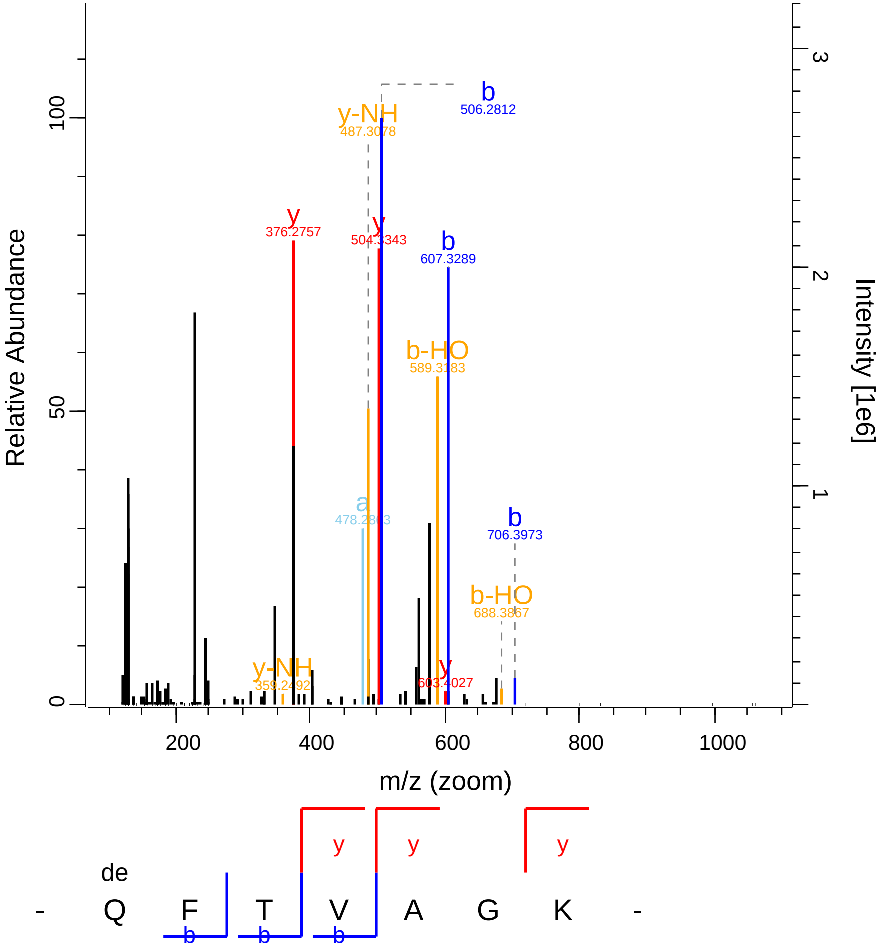

Raw File	Scan	Method	Score	m/z	Gene names
RO-QC-160121_ZackD_SS_Set_01_Fractior	16327	FTMS; HC	98.63	608.34	UTS2

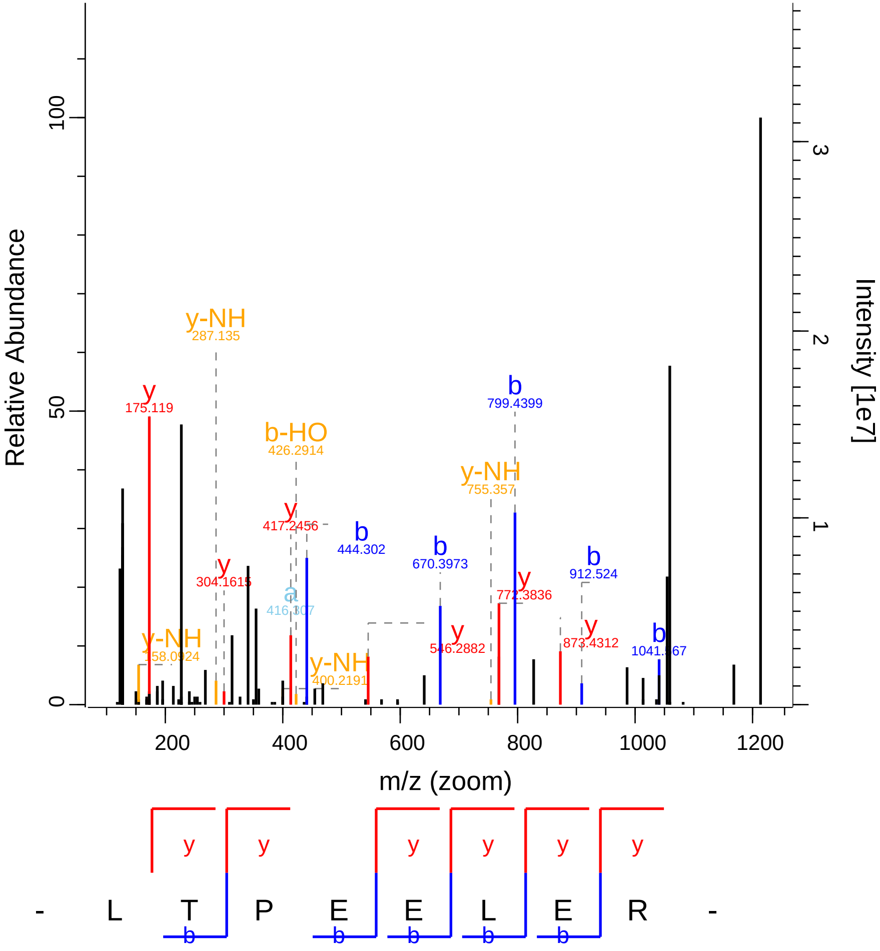

Raw File	Scan	Method	Score	m/z	Gene names
RO-QC-160121_ZackD_SS_Set_01_Fractior	16386	FTMS; HC	117.23	973.51	APOC3

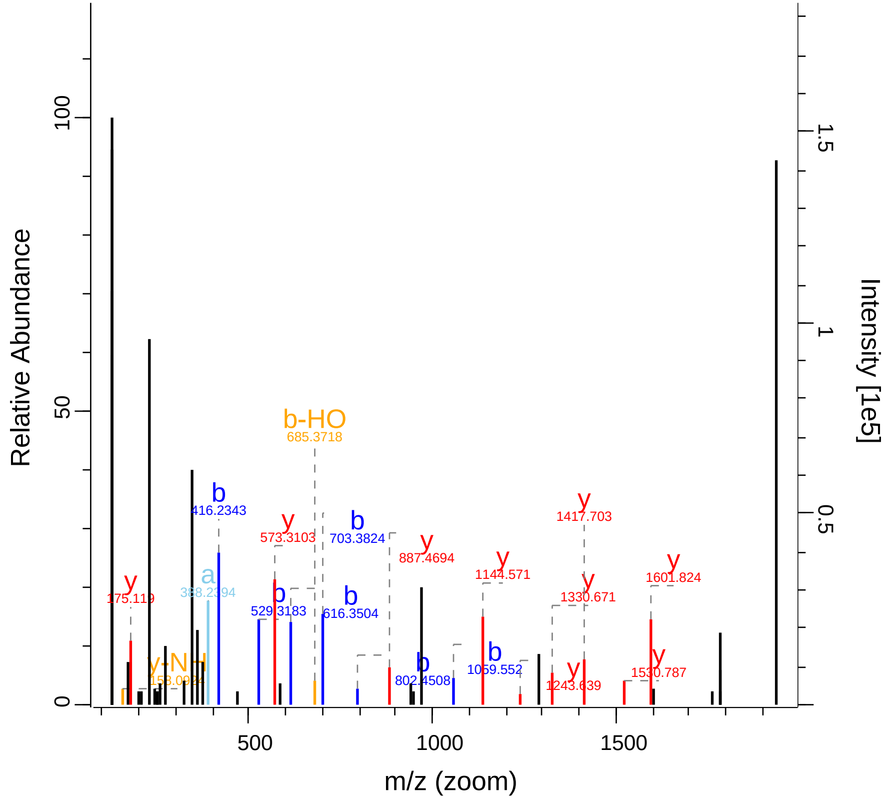

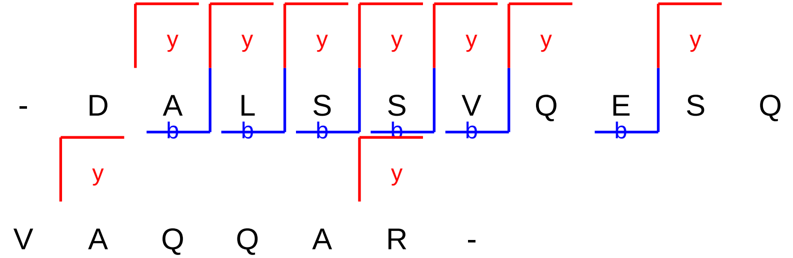

Raw File	Scan	Method	Score	m/z	Gene names
RO-QC-160121_ZackD_SS_Set_01_Fraction	16432	FTMS; HC	111.74	405.9	UTS2

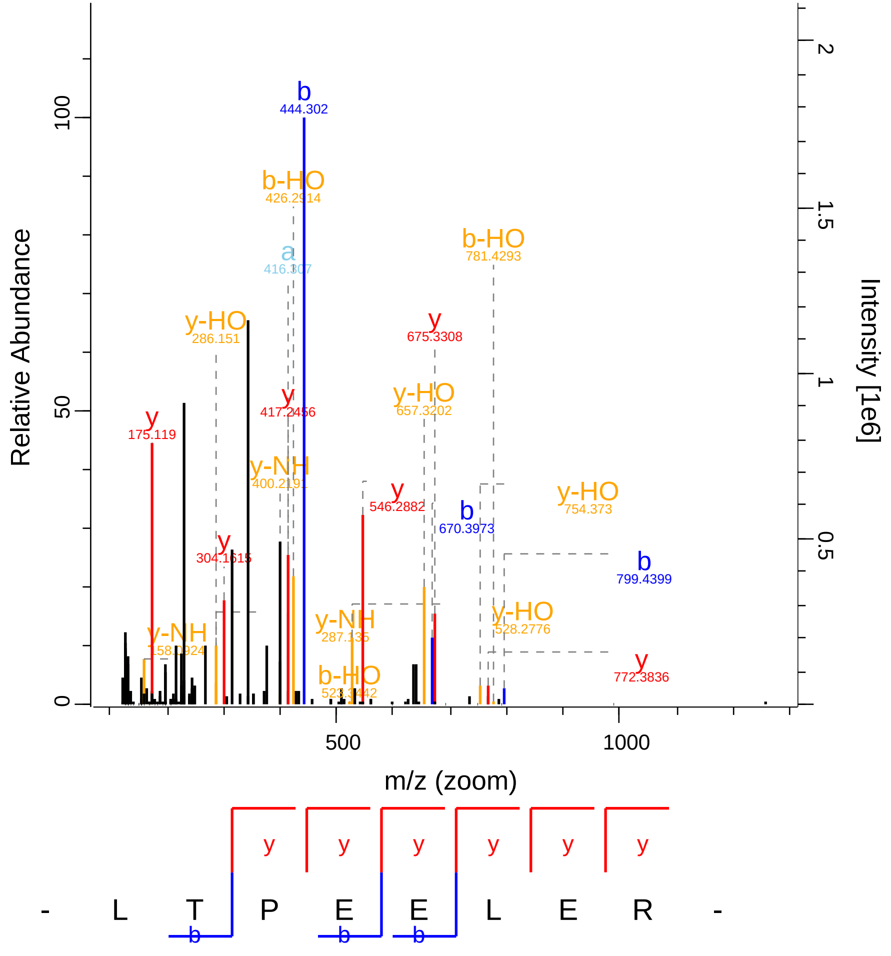

Raw File	Scan	Method	Score	m/z	Gene names
RO-QC-160121_ZackD_SS_Set_01_Fractio[illegible]	16451	FTMS; HC	143.96	897.99	CCDC59

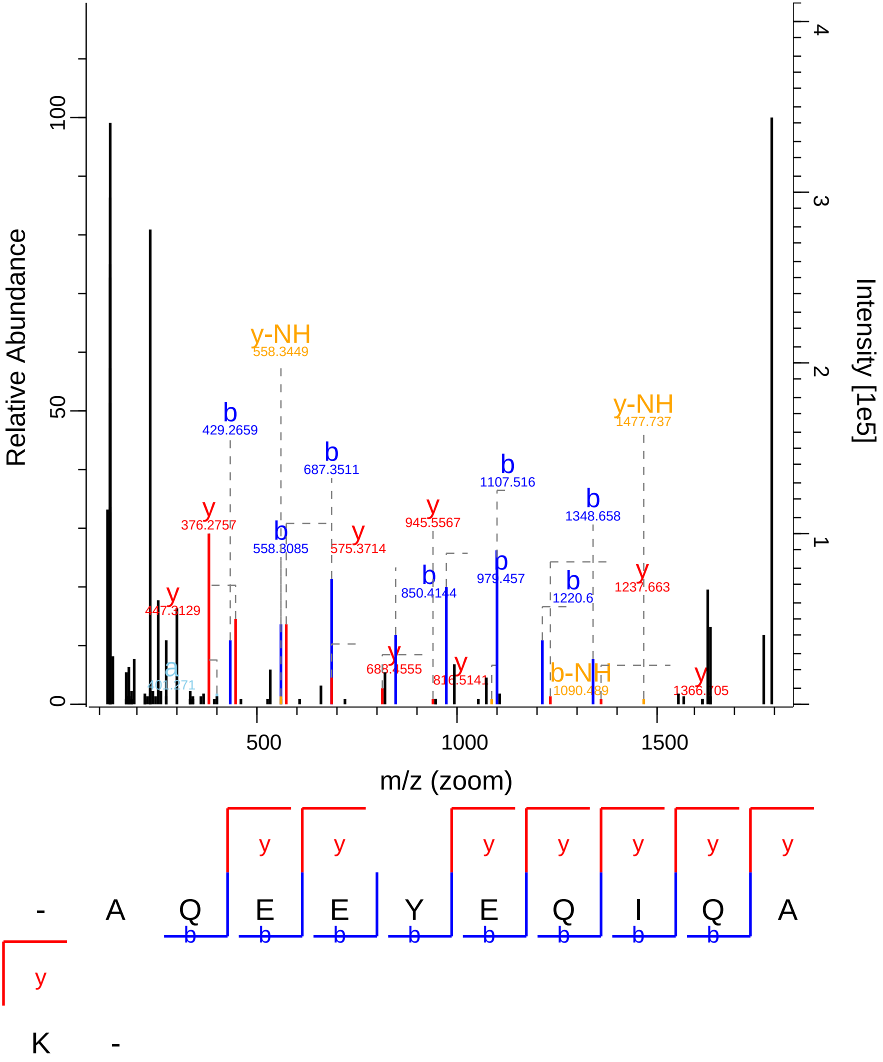

Raw File	Scan	Method	Score	m/z	Gene names
RO-QC-160121_ZackD_SS_Set_01_Fractior	16568	FTMS; HC	98.46	608.34	UTS2

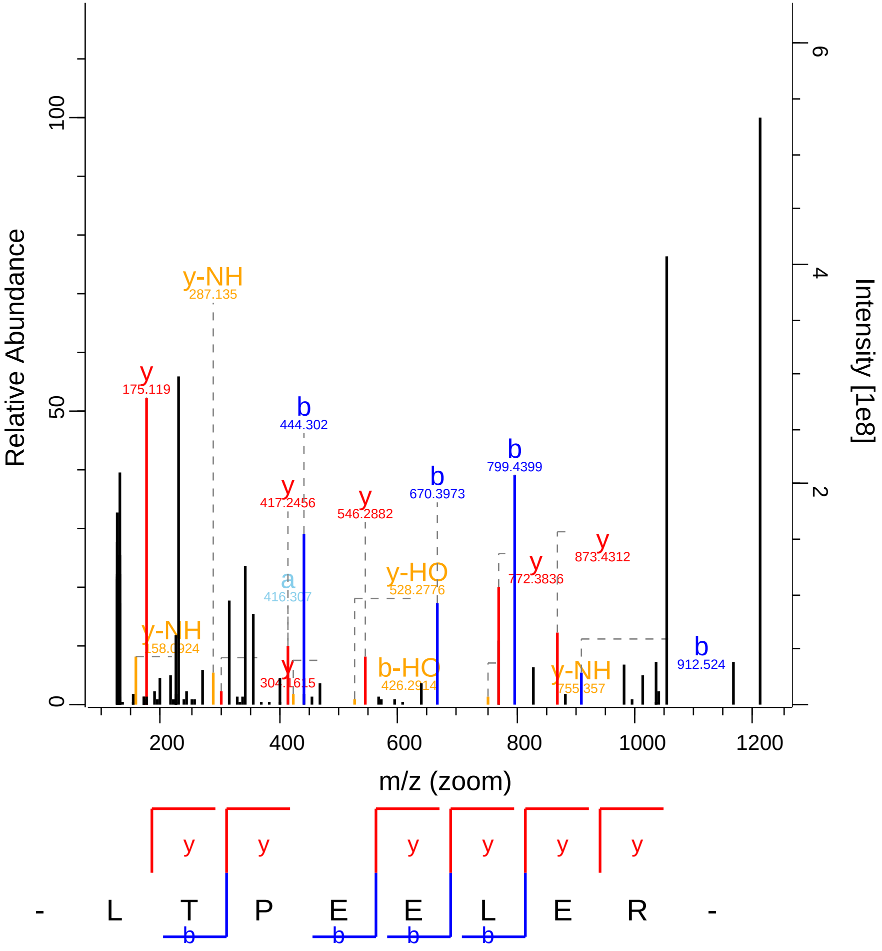

Raw File	Scan	Method	Score	m/z	Gene names
RO-QC-160121_ZackD_SS_Set_01_Fractio	16723	FTMS; HC	110.66	882.48	NTRK2

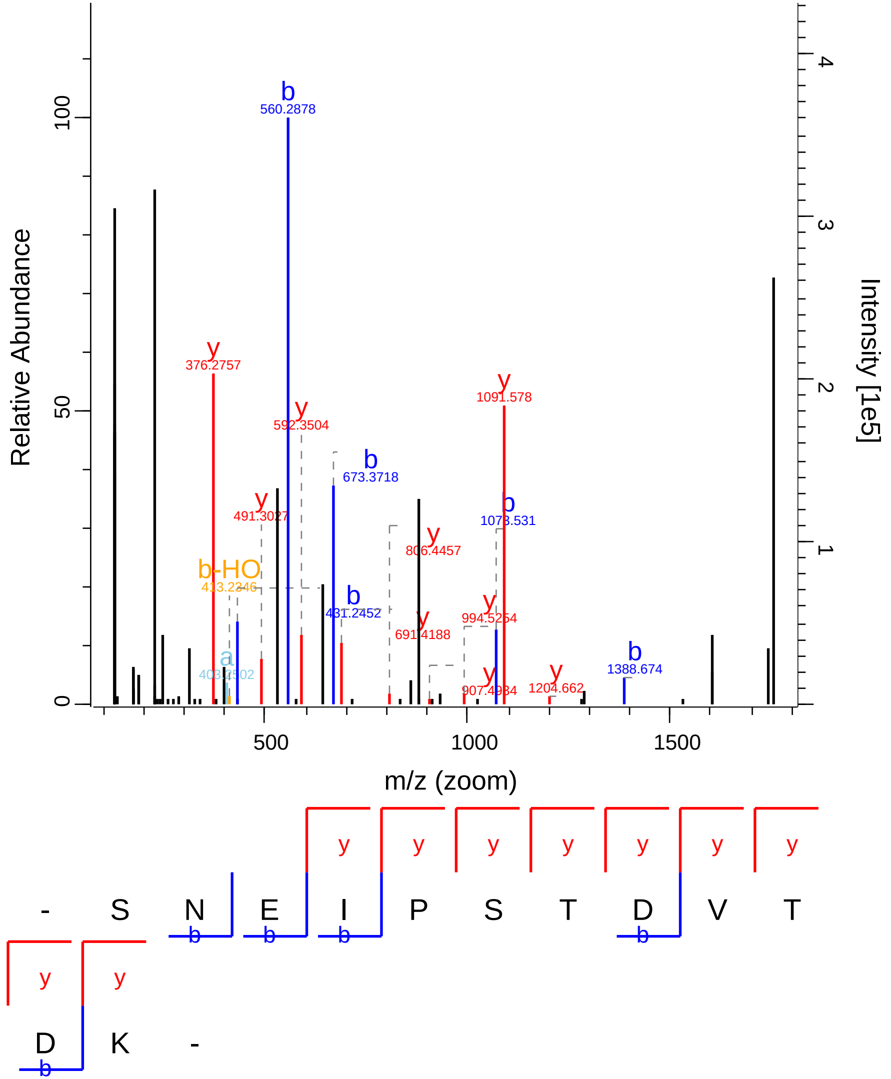

Raw File	Scan	Method	Score	m/z
RO-QC-160121_ZackD_SS_Set_01_Fraction_11	17033	FTMS; HCD	120.07	710.41

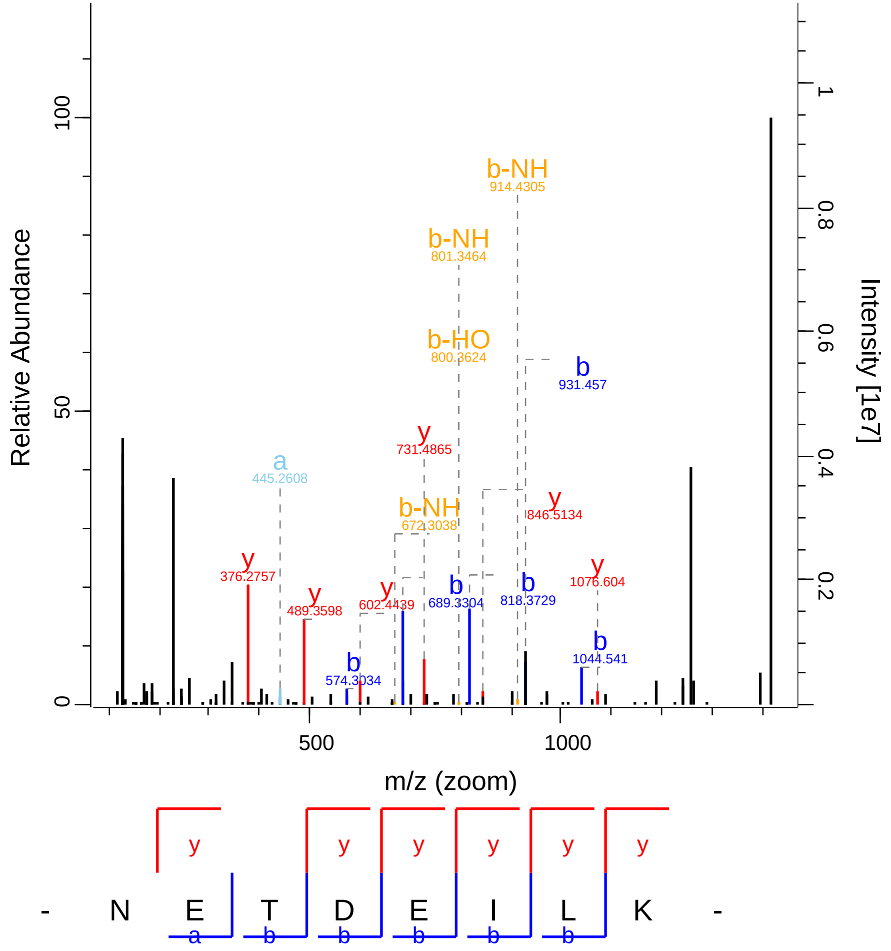

Raw File	Scan	Method	Score	m/z	Gene names
RO-QC-160121_ZackD_SS_Set_01_Fractior	17150	FTMS; HC	92.86	649.05	MAD2L1BP

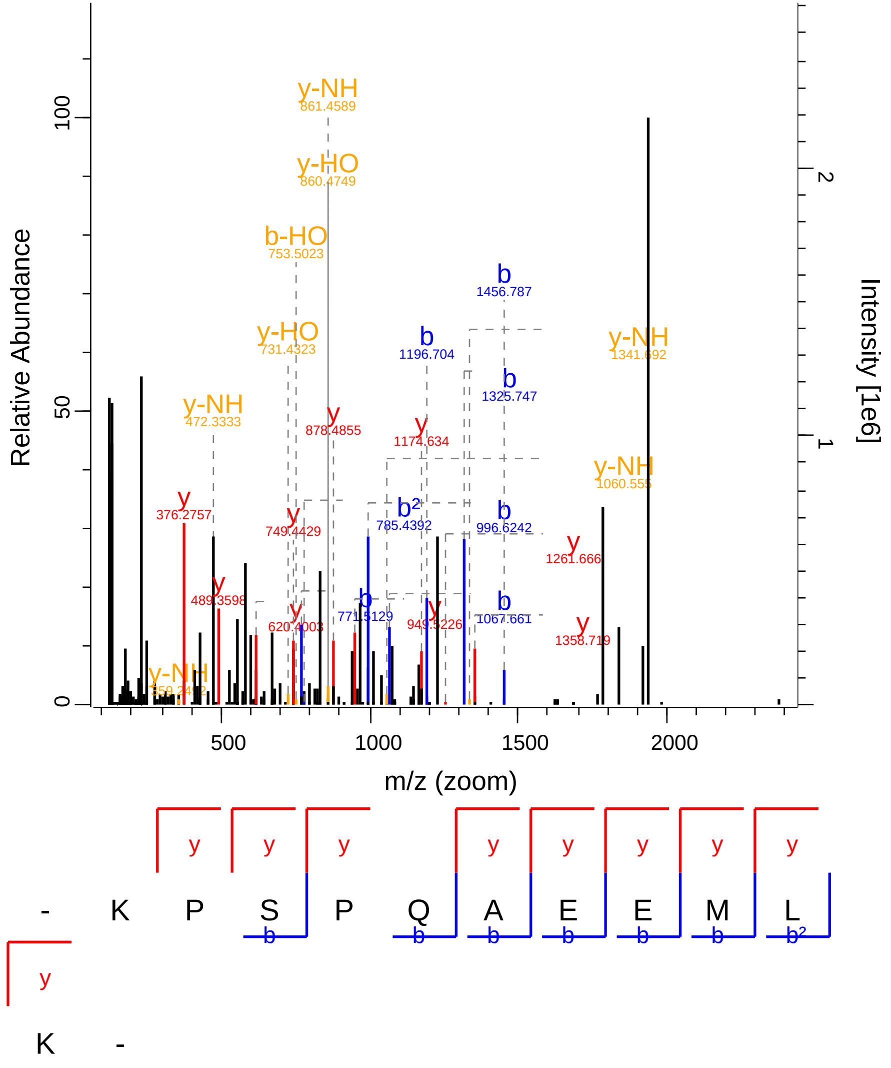

Raw File	Scan	Method	Score	m/z	Gene names
RO-QC-160121_ZackD_SS_Set_01_Fractic	17942	FTMS; HC	44.46	786.76	FCGR3B;FCGR:

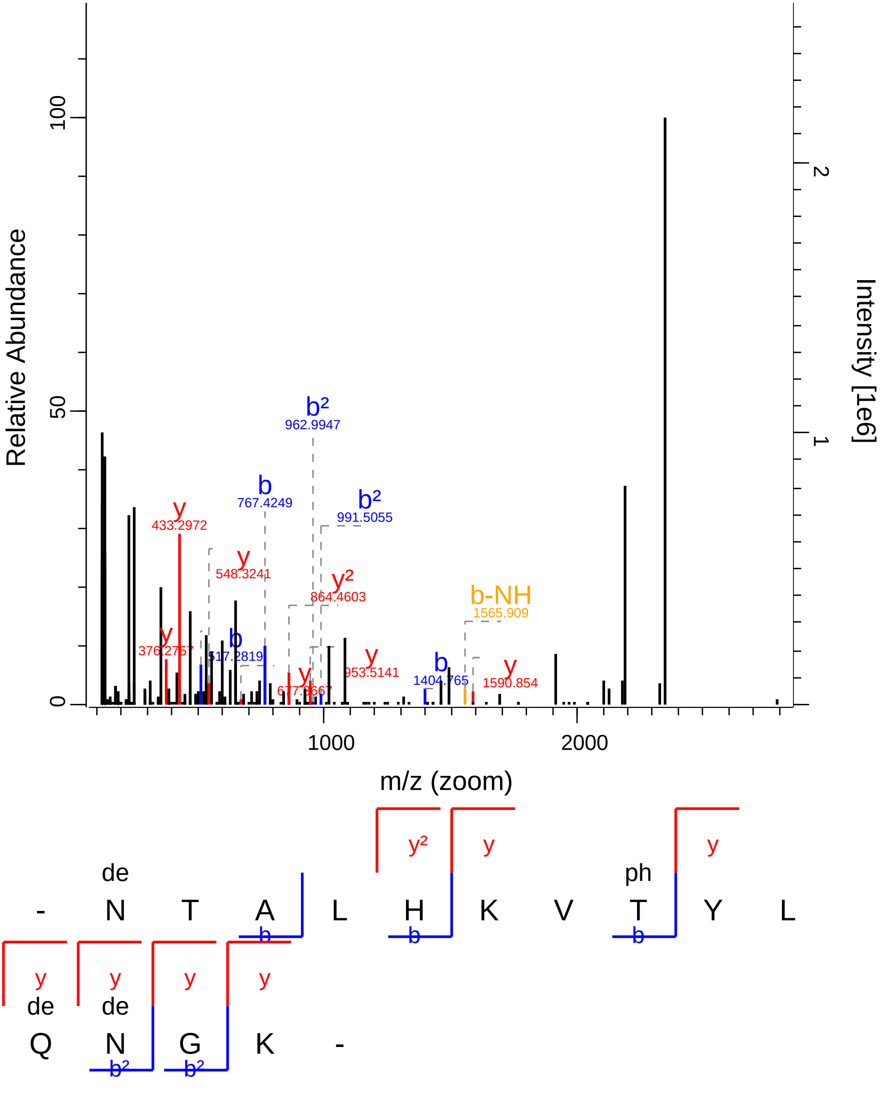

Raw File	Scan	Method	Score	m/z	Gene names
RO-QC-160121_ZackD_SS_Set_01_Fractior	18919	FTMS; HC	49.55	709.44	PCDH17

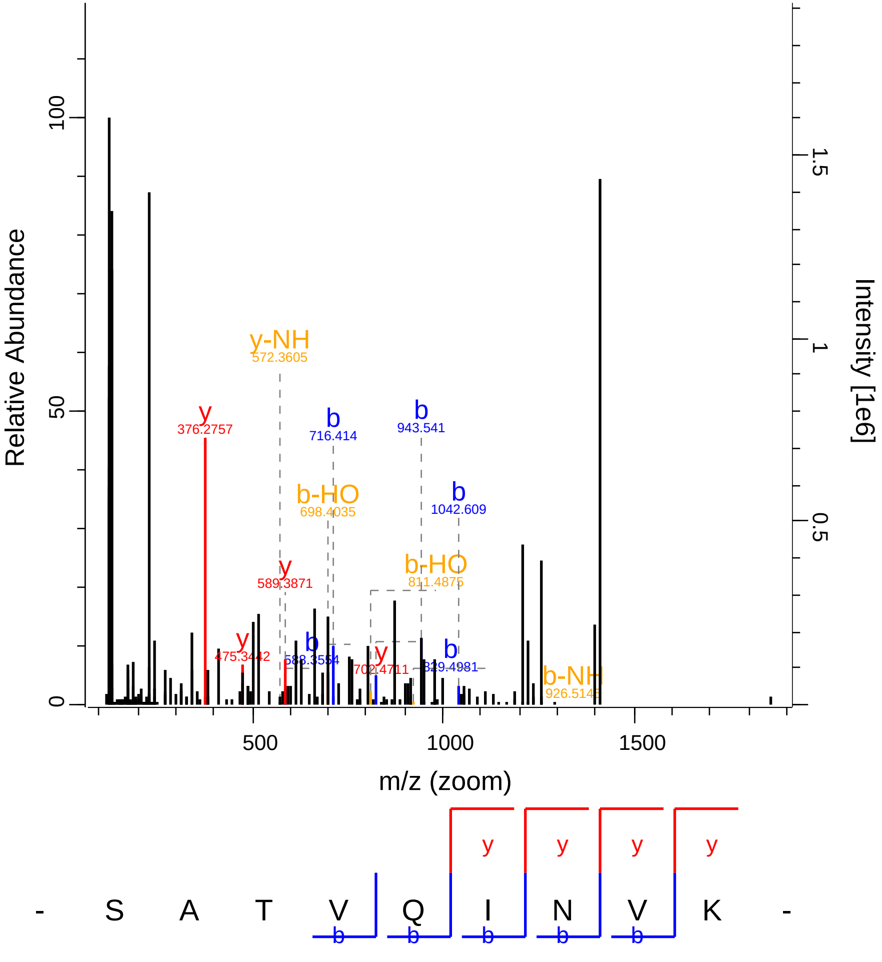

Raw File	Scan	Method	Score	m/z	Gene names
RO-QC-160121_ZackD_SS_Set_01_Fractior	18951	FTMS; HC	40.35	473.63	PCDH17

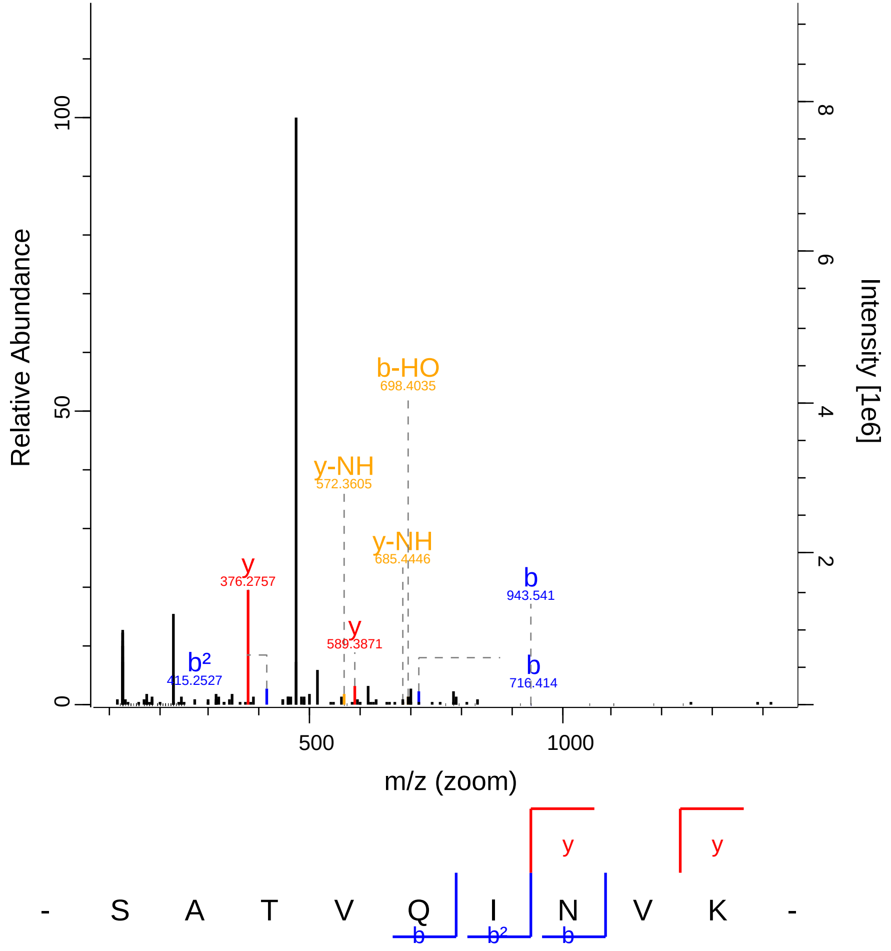

Raw File	Scan	Method	Score	m/z	Gene names
RO-QC-160121_ZackD_SS_Set_01_Fractior	19155	FTMS; HC	75.74	709.44	PCDH17

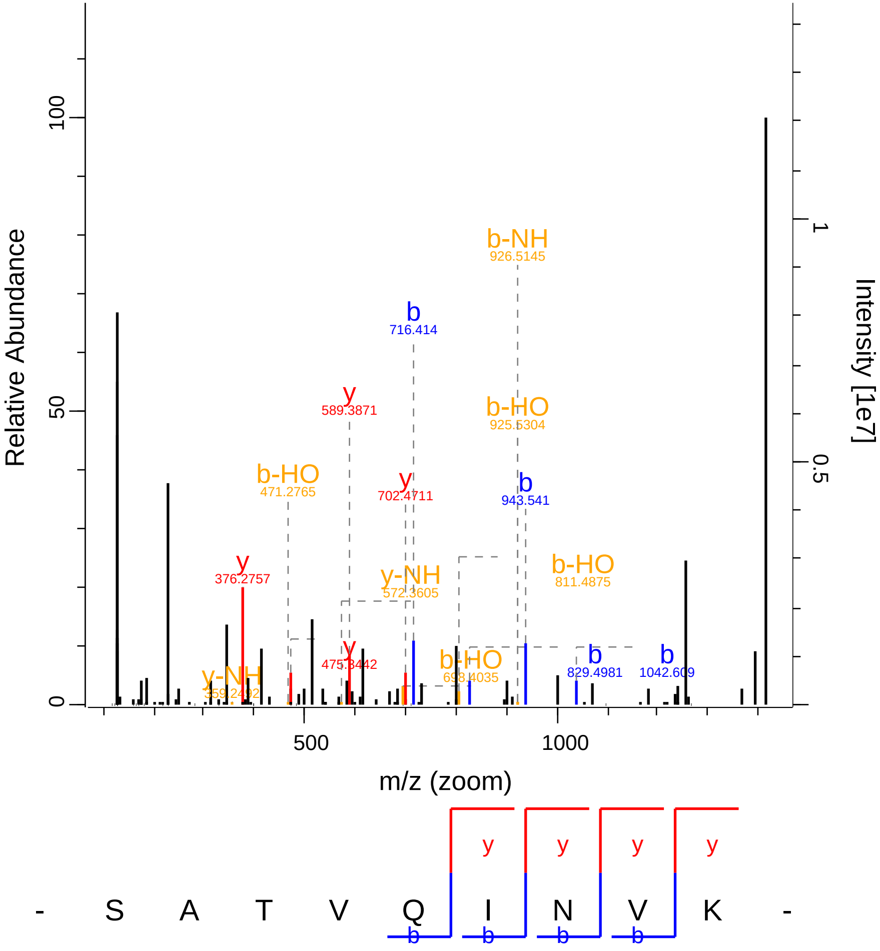

Raw File	Scan	Method	Score	m/z	Gene names
RO-QC-160121_ZackD_SS_Set_01_Fraction	19187	FTMS; HC	68.02	473.3	PCDH17

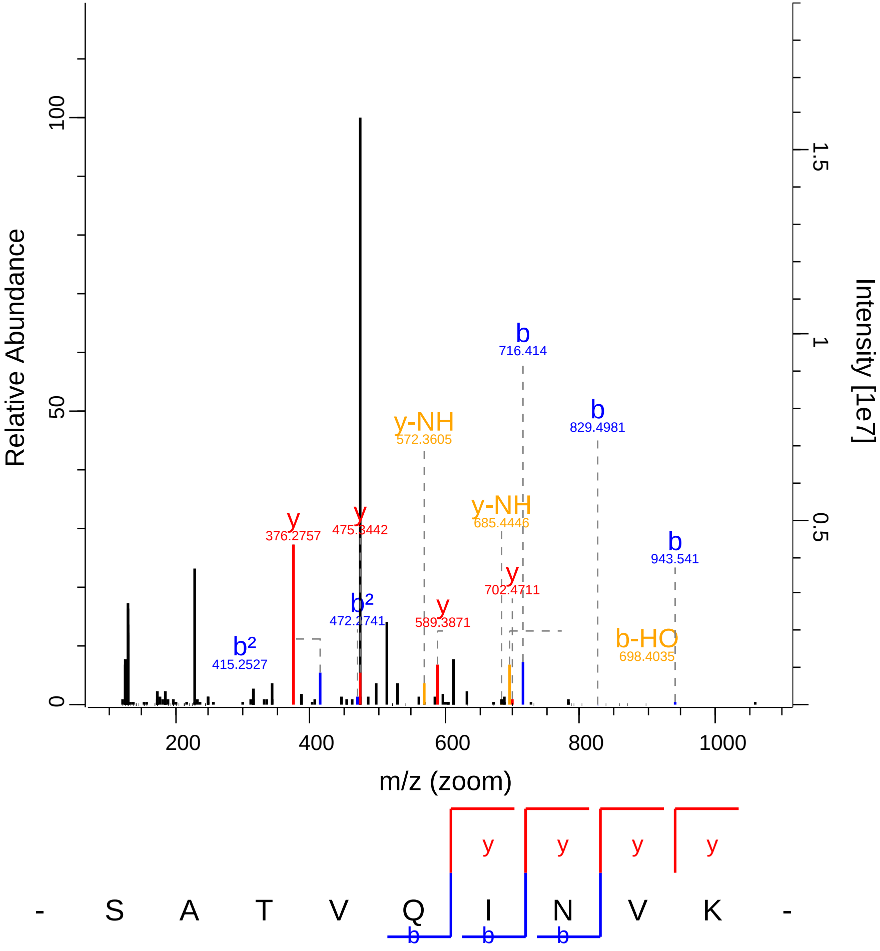

Raw File	Scan	Method	Score	m/z
RO-QC-160121_ZackD_SS_Set_01_Fraction_11	19194	FTMS; HCD	79.47	445.27

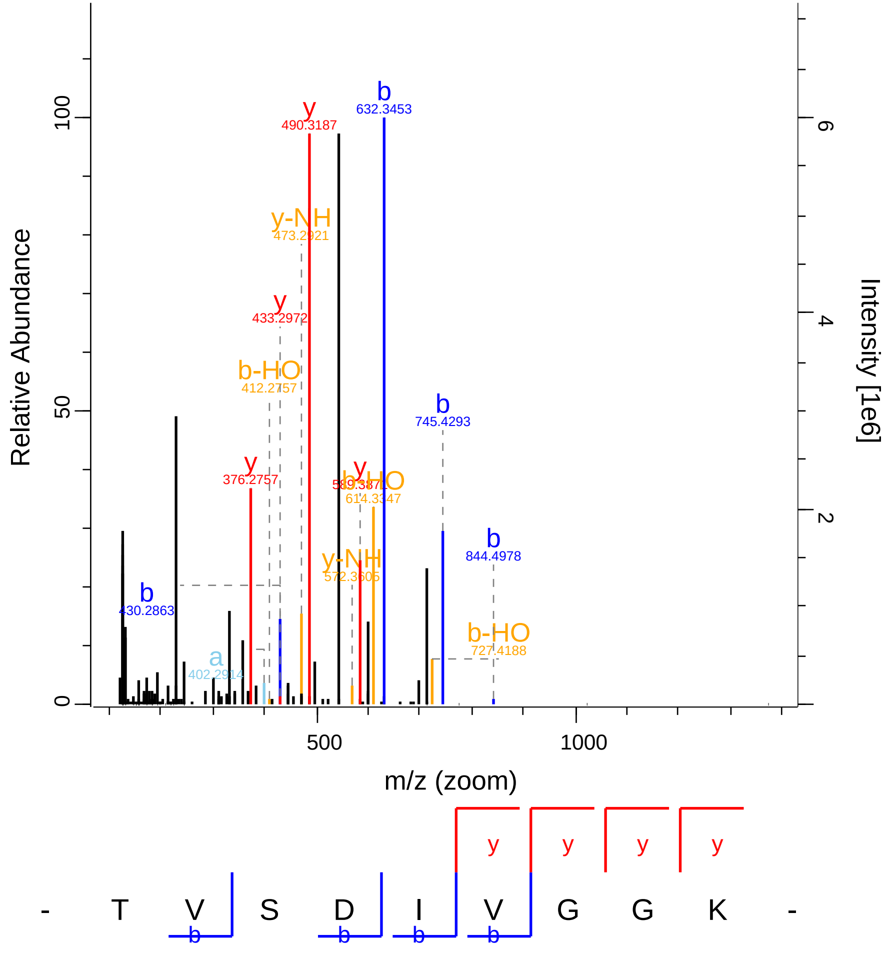

Raw File	Scan	Method	Score	m/z	Gene names
RO-QC-160121_ZackD_SS_Set_01_Fraction	20032	FTMS; HC	40.96	800.1	ATP6V0C

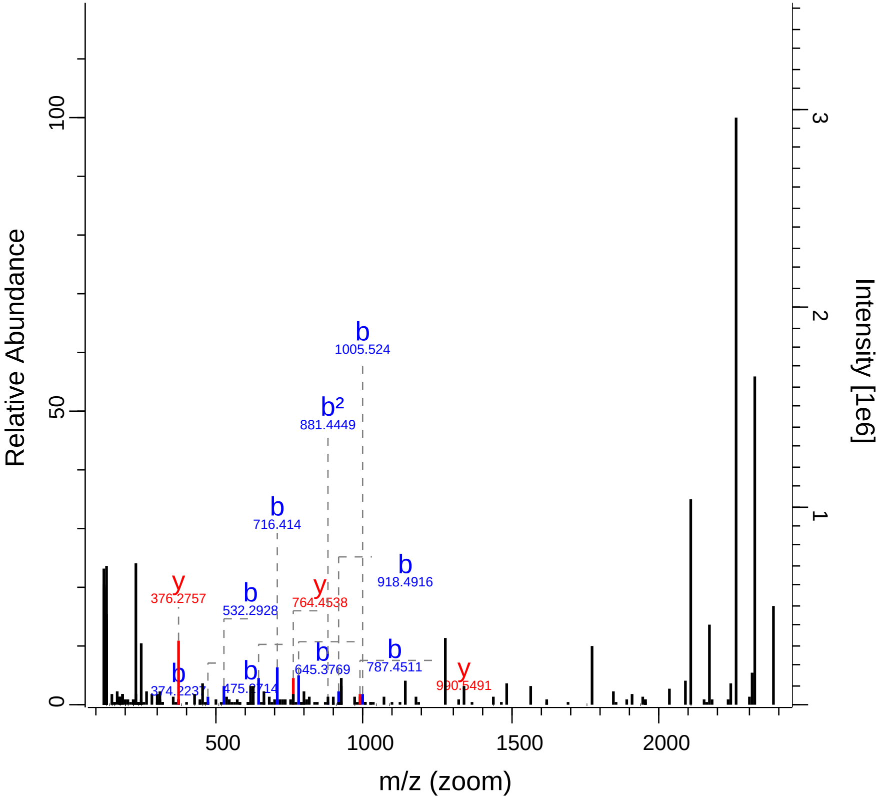

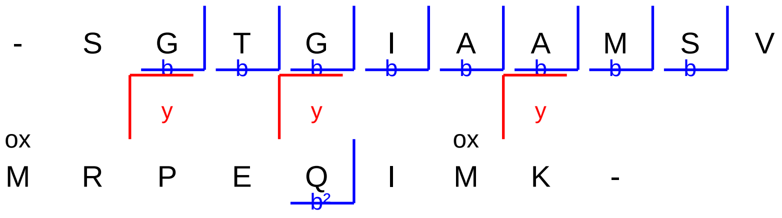

Raw File	Scan	Method	Score	m/z	Gene names
RO-QC-160121_ZackD_SS_Set_01_Fractioı	20111	FTMS; HC	192.44	773.42	ZMYM6NB

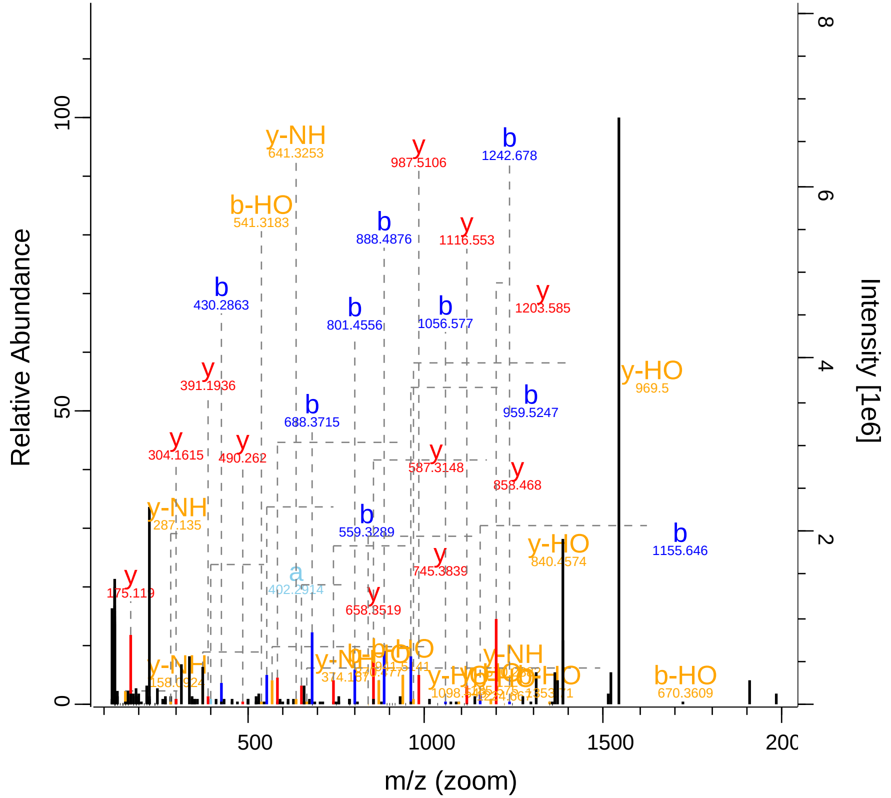

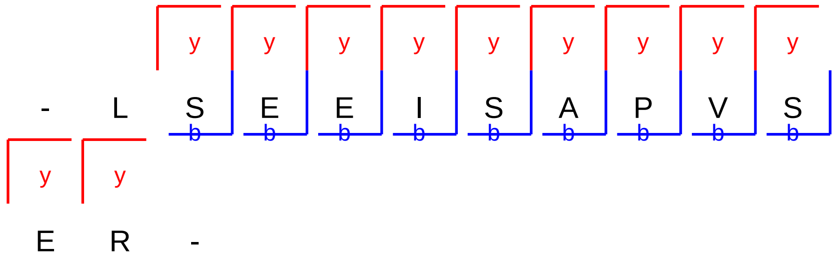

Raw File	Scan	Method	Score	m/z	Gene names
RO-QC-160121_ZackD_SS_Set_01_Fractior	20385	FTMS; HC	90.15	509.61	CPA6

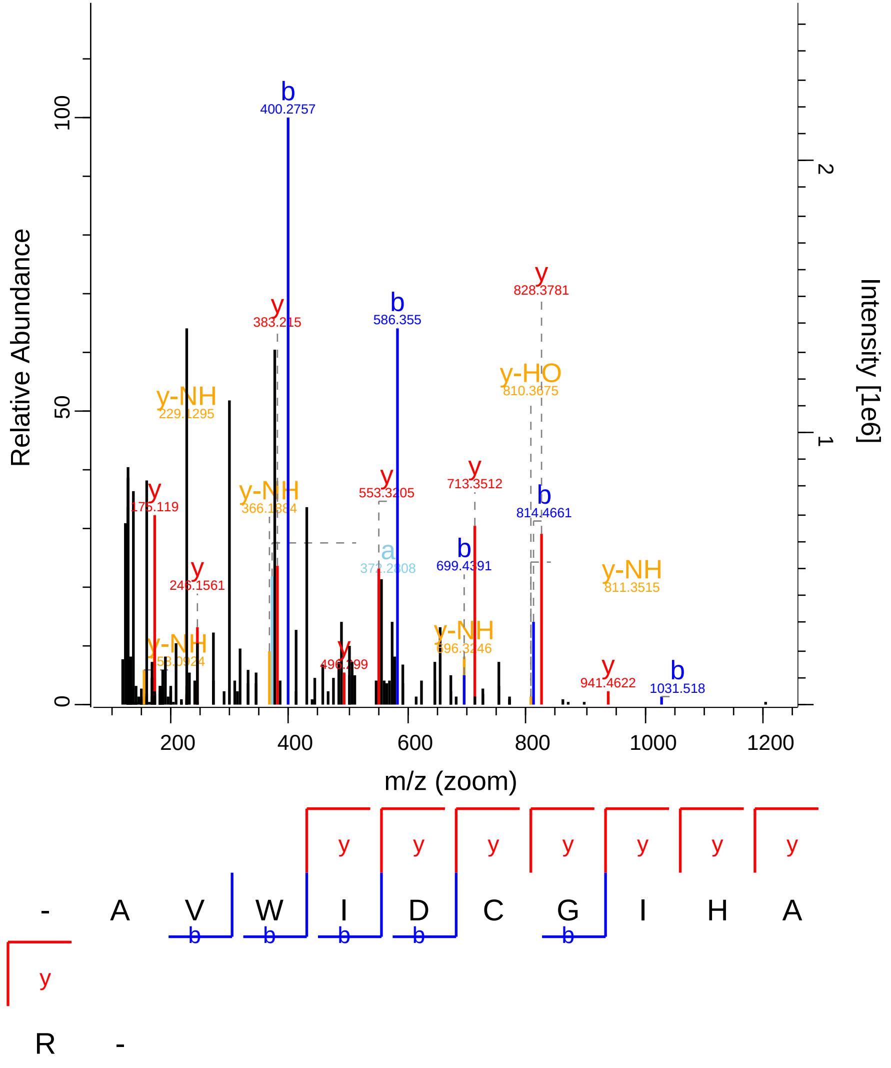

Raw File	Scan	Method	Score	m/z	Gene names
RO-QC-160121_ZackD_SS_Set_01_Fractio	21107	FTMS; HC	169.21	861.95	RCBTB2

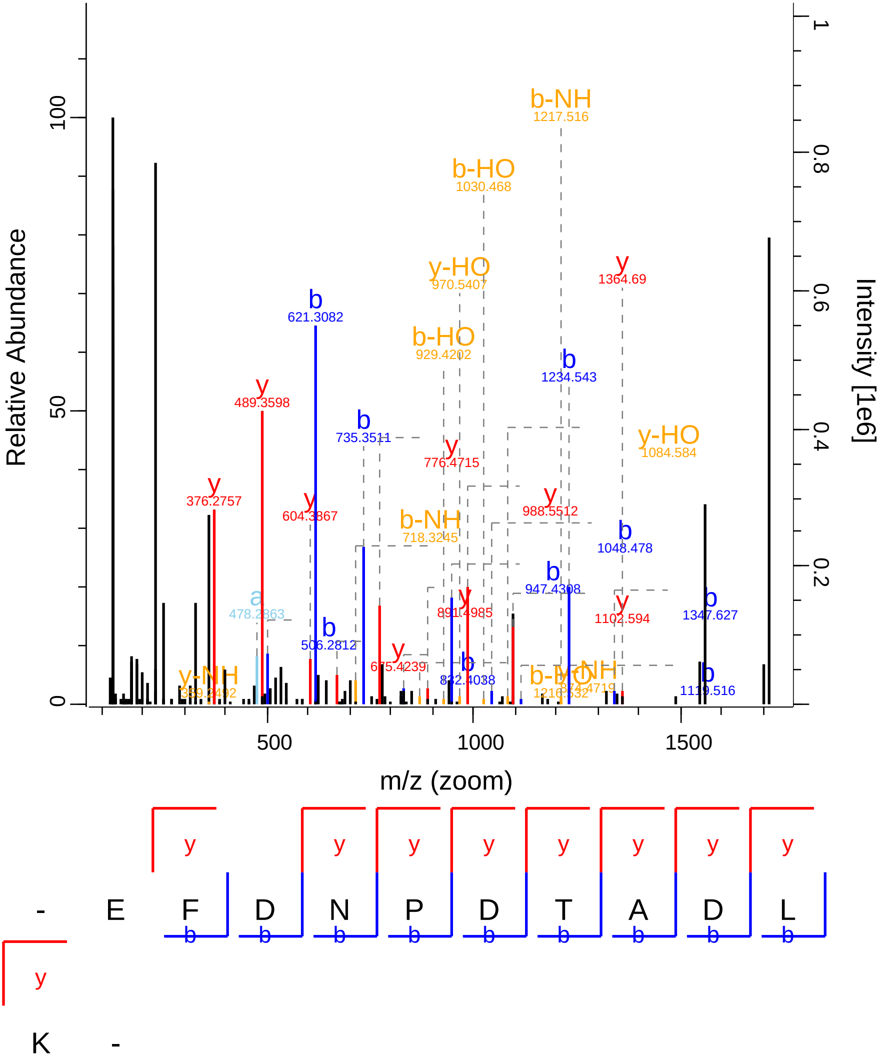

Raw File	Scan	Method	Score	m/z	Gene names
RO-QC-160121_ZackD_SS_Set_01_Fractior	21142	FTMS; HC	58.67	574.97	RCBTB2

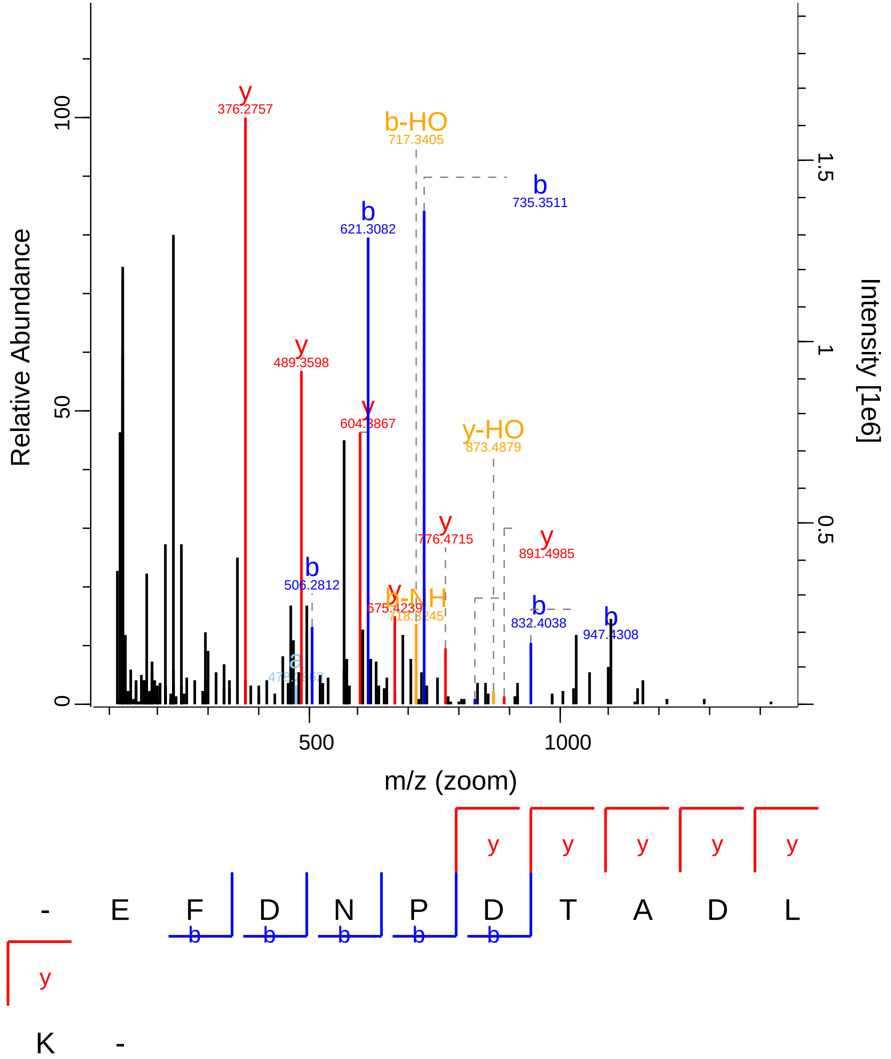

Raw File	Scan	Method	Score	m/z	Gene names
RO-QC-160121_ZackD_SS_Set_01_Fractior	21574	FTMS; HC	106.2	711.88	CD58

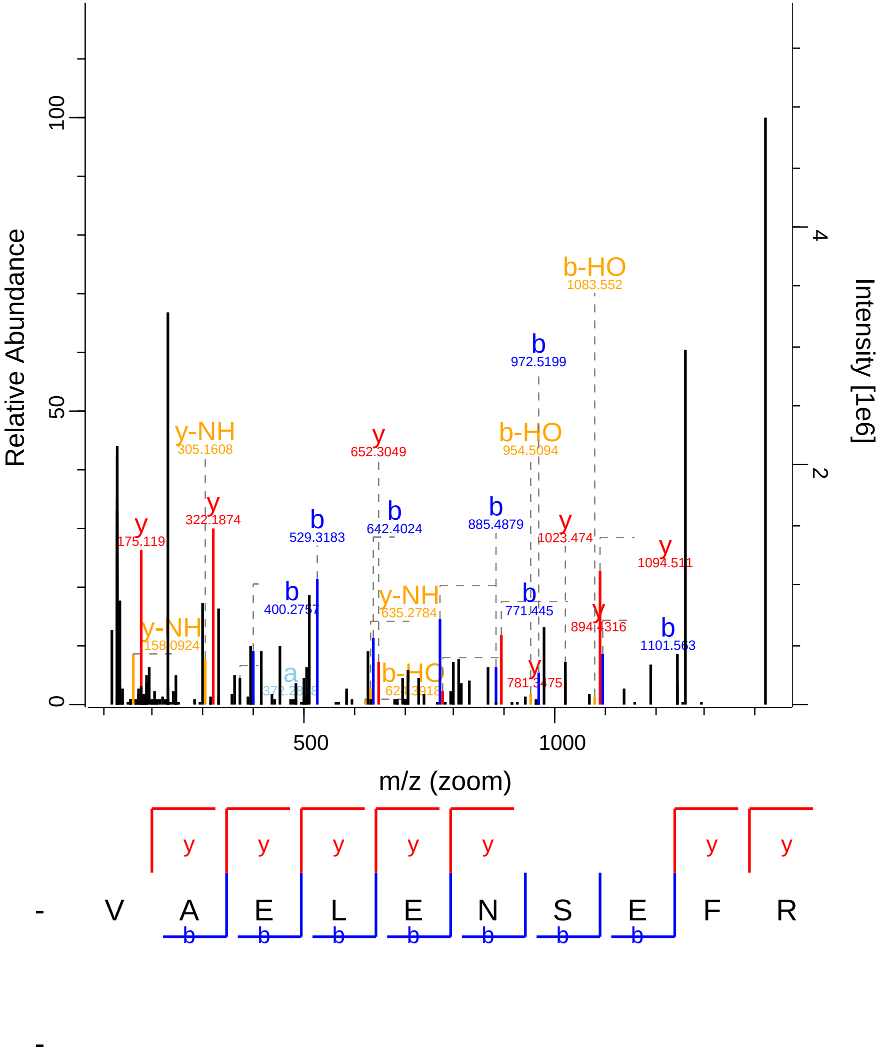

Raw File	Scan	Method	Score	m/z	Gene names
RO-QC-160121_ZackD_SS_Set_01_Fractior	21901	FTMS; HC	147.74	855.46	RCBTB1

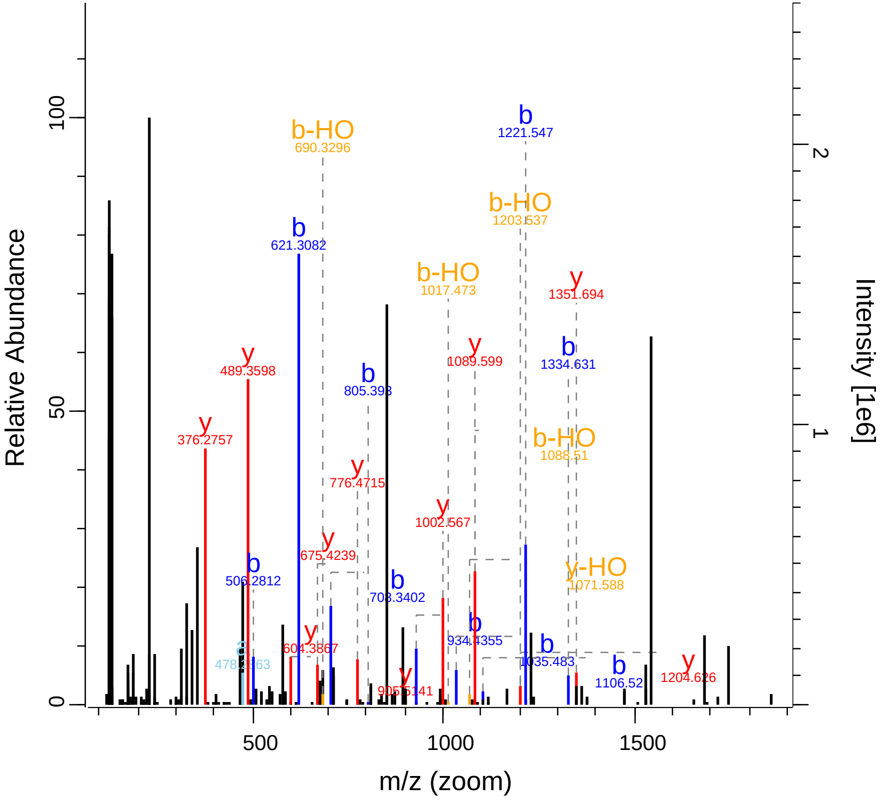

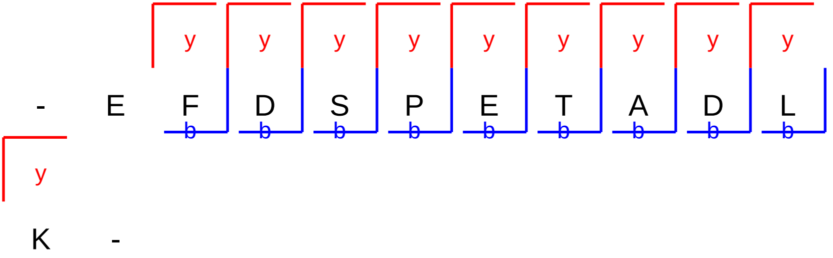

Raw File	Scan	Method	Score	m/z	Gene names
RO-QC-160121_ZackD_SS_Set_01_Fraction	21981	FTMS; HC	92.49	522.3	SAMD8

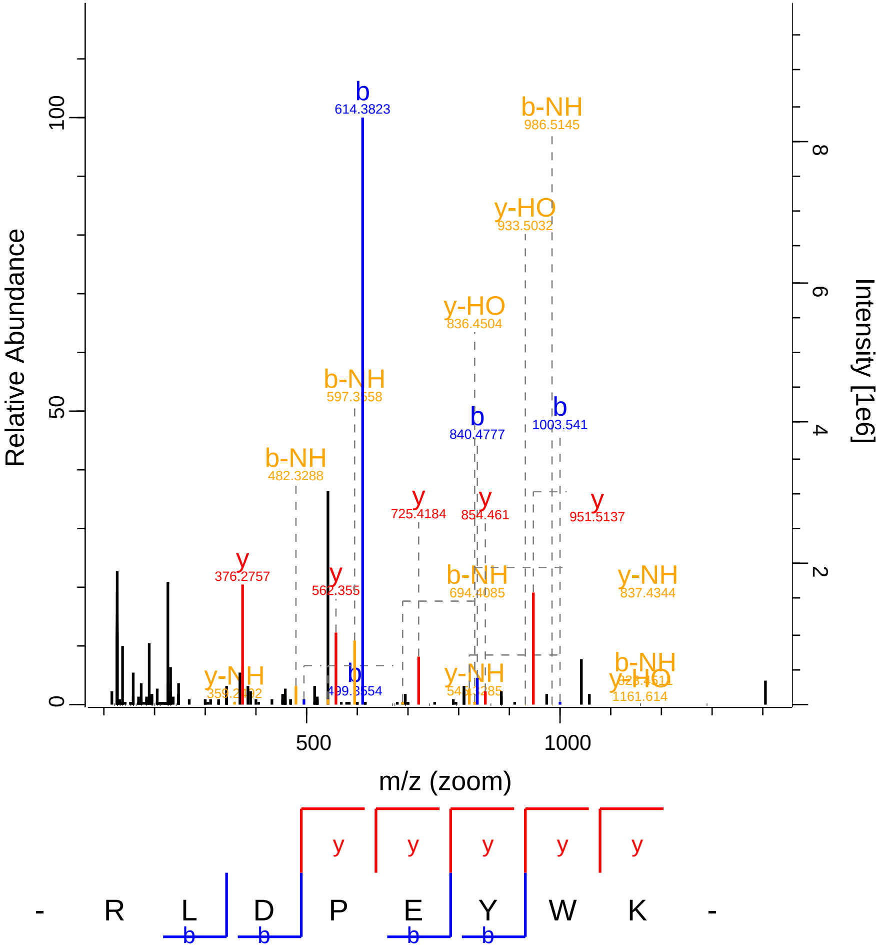

Raw File	Scan	Method	Score	m/z	Gene names
RO-QC-160121_ZackD_SS_Set_01_Fractior	22783	FTMS; HC	77.67	806.45	GSDMA

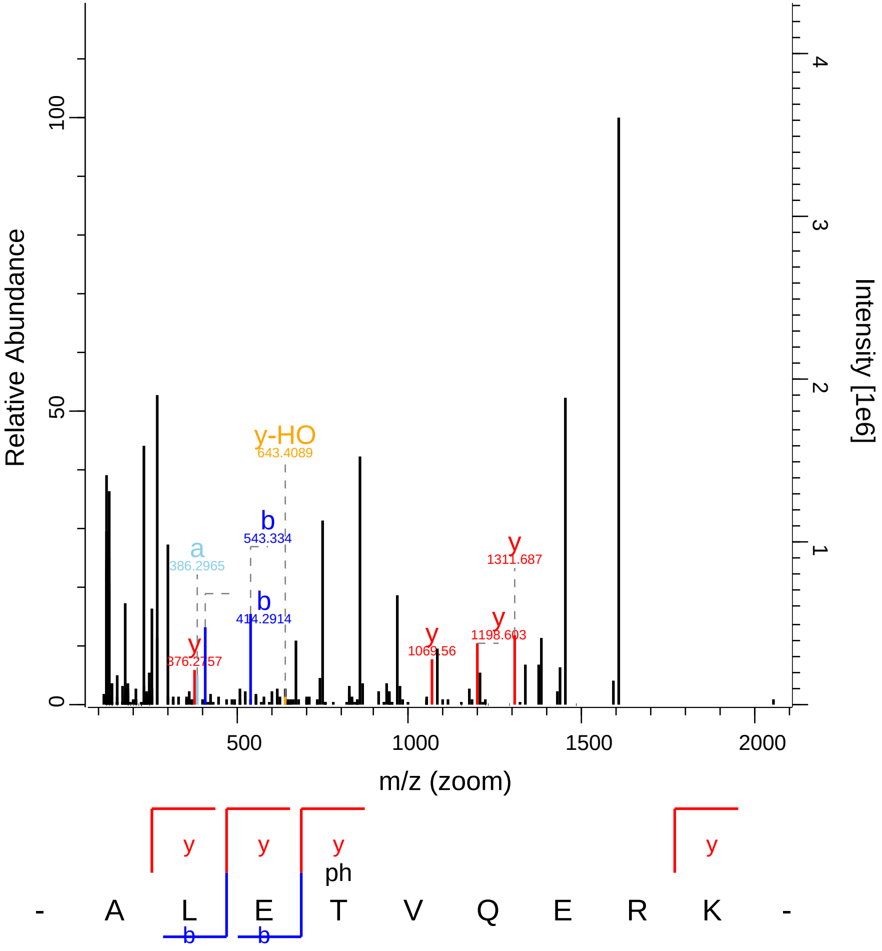

Raw File	Scan	Method	Score	m/z	Gene names
RO-QC-160121_ZackD_SS_Set_01_Fractior	23408	FTMS; HC	86.01	497.63	ZNF487

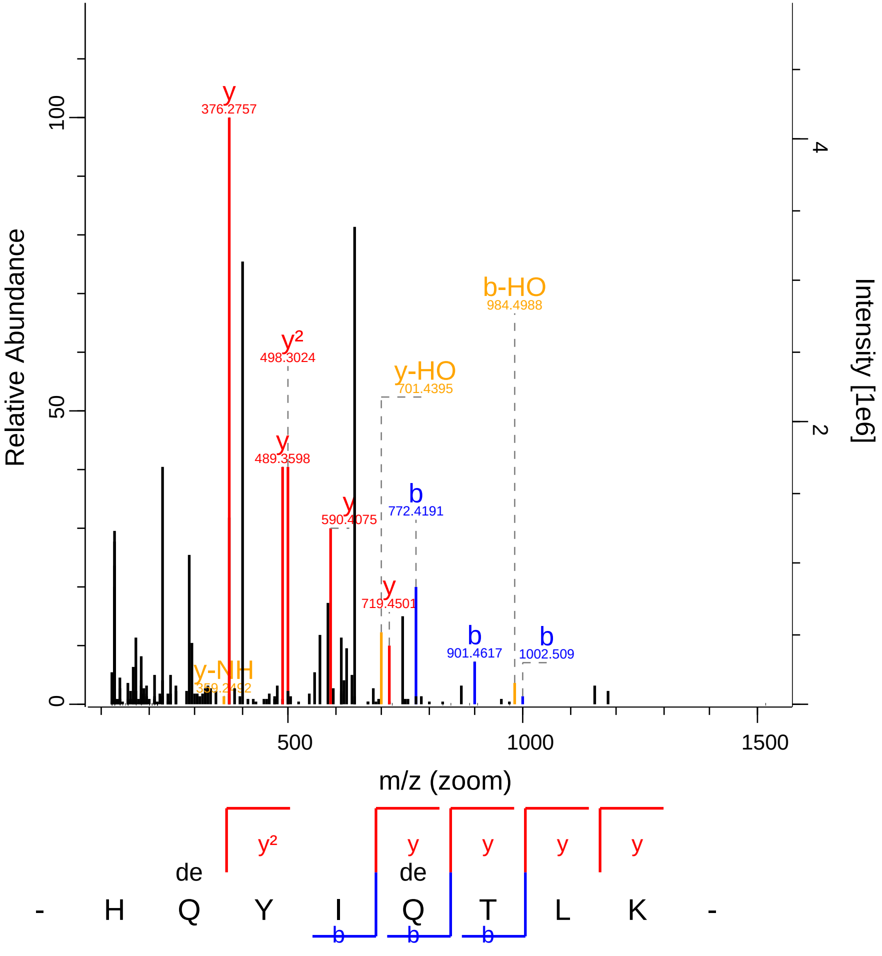

Raw File	Scan	Method	Score	m/z
RO-QC-160121_ZackD_SS_Set_01_Fraction_11	23588	FTMS; HCD	107.66	431.26

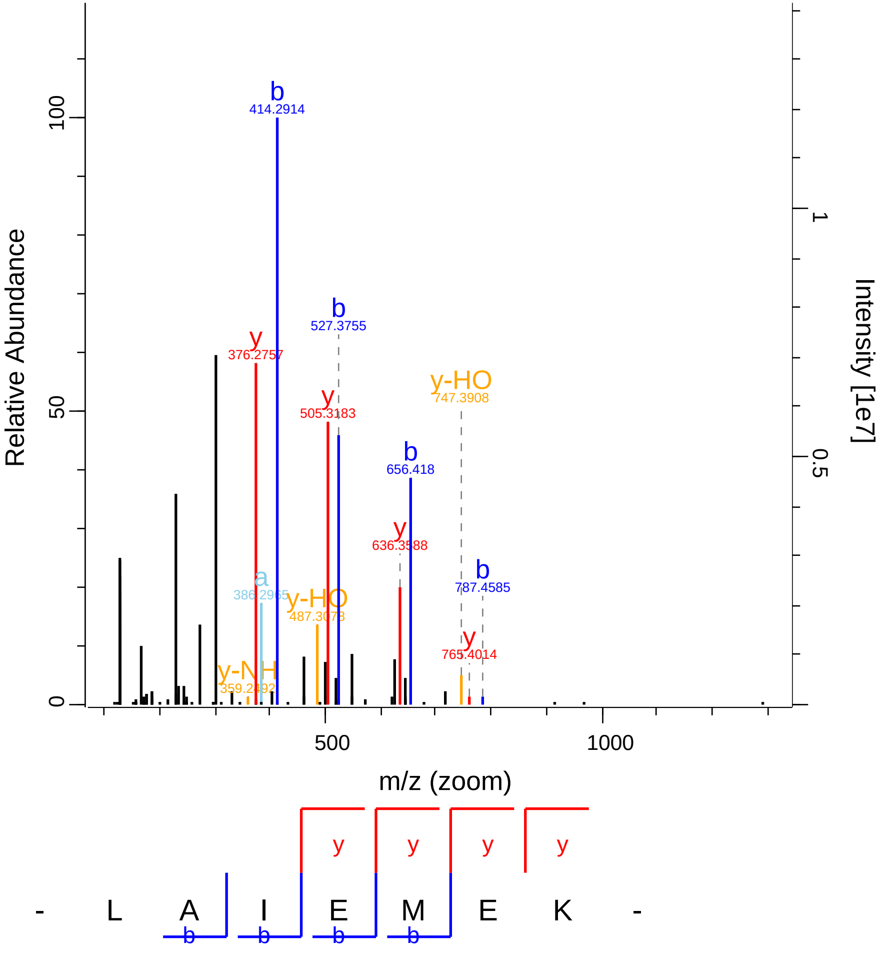

Raw File	Scan	Method	Score	m/z	Gene names
RO-QC-160121_ZackD_SS_Set_01_Fractior	23718	FTMS; HC	136.3	589.61	TMBIM1

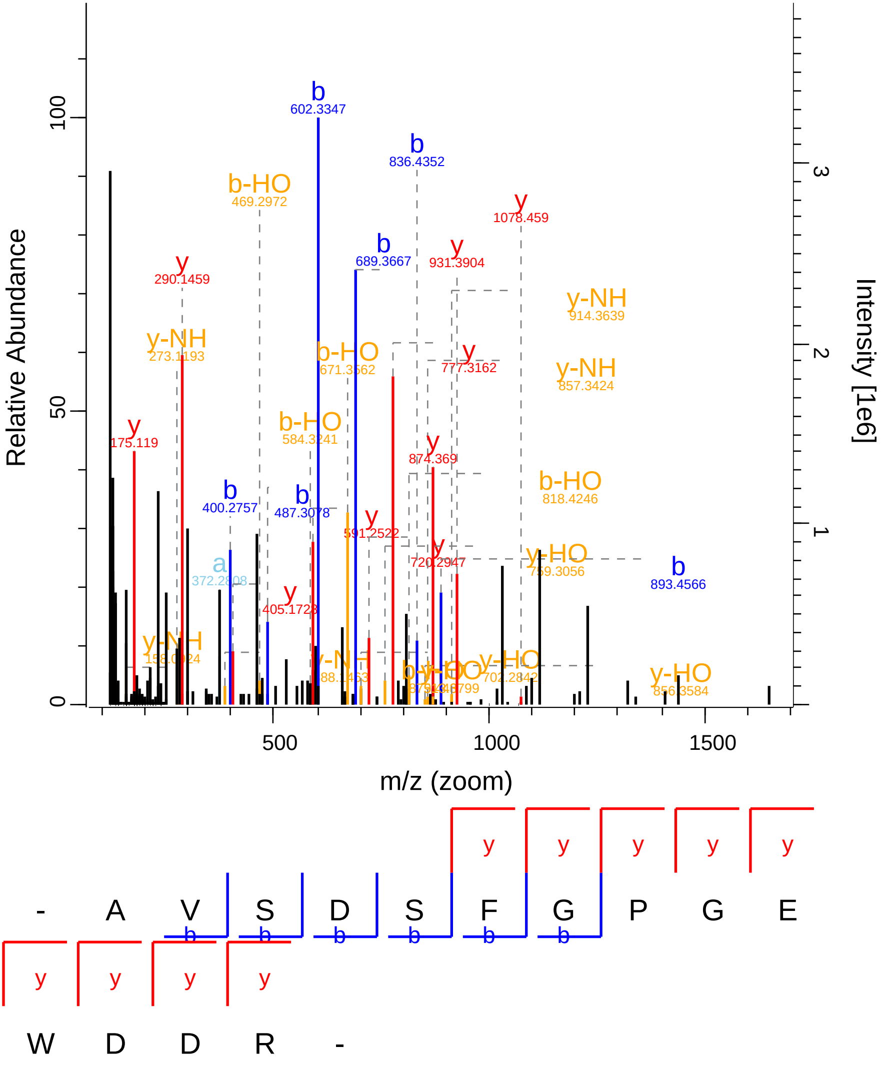

Raw File	Scan	Method	Score	m/z	Gene names
RO-QC-160121_ZackD_SS_Set_01_Fractio[illegible]	23826	FTMS; HC	289.71	883.91	TMBIM1

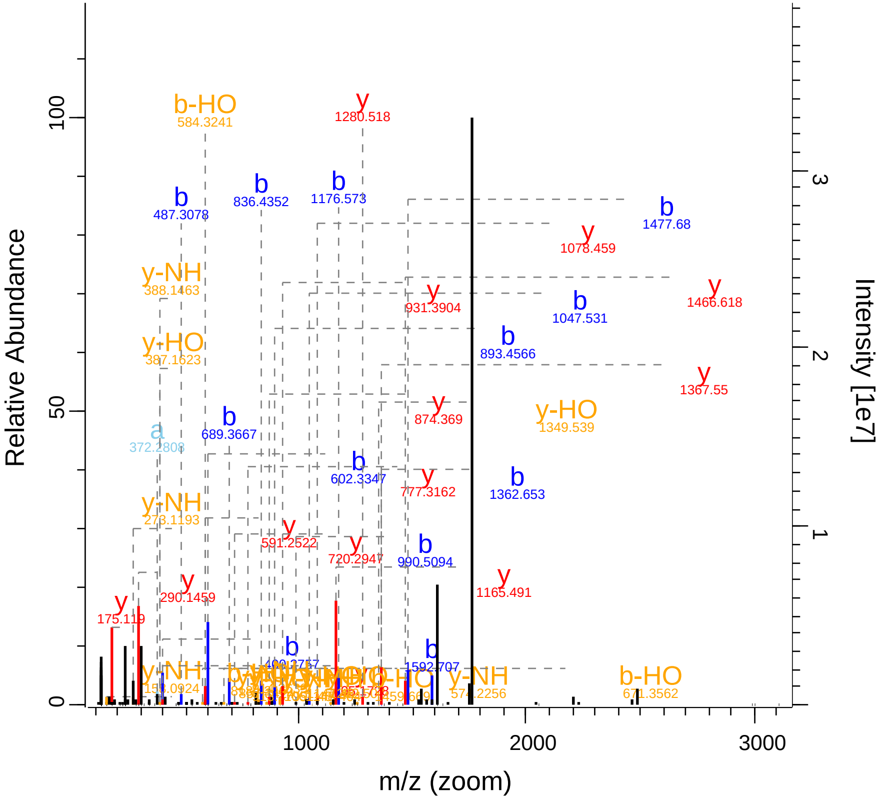

- A V S D S F G P G E
W D D R -

(y and b ion fragment markers shown between residues)

Raw File	Scan	Method	Score	m/z
RO-QC-160121_ZackD_SS_Set_01_Fraction_11	24188	FTMS; HCD	64.76	668.9

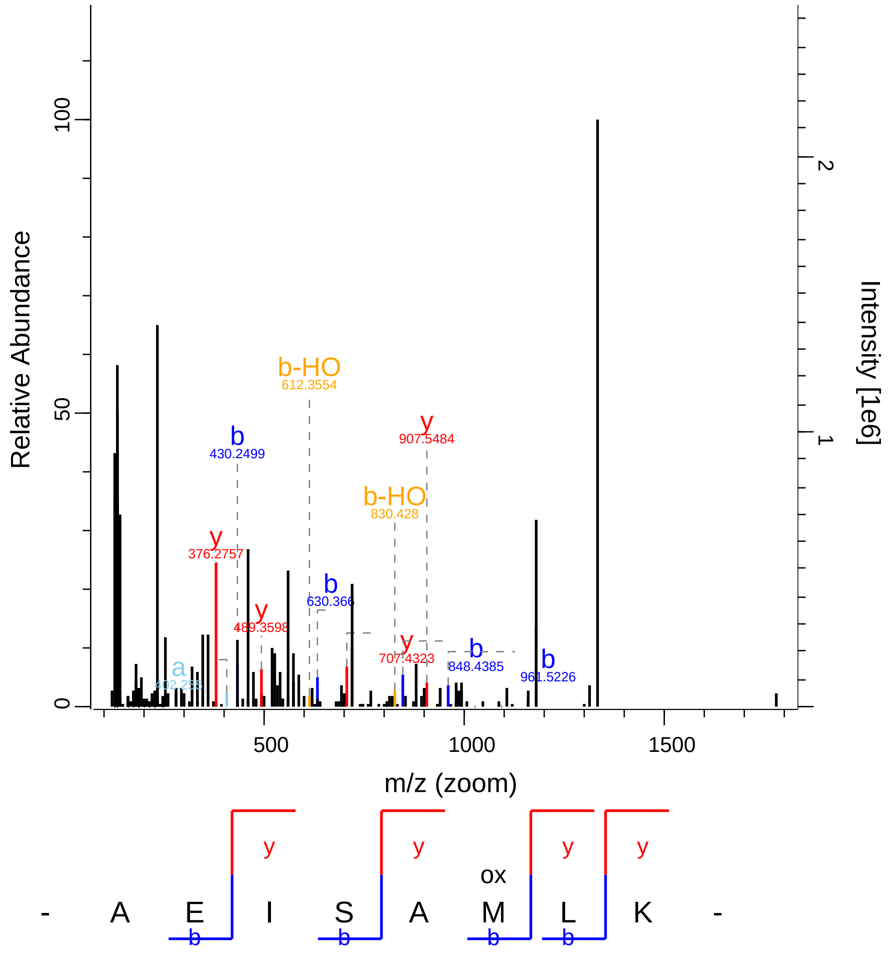

Raw File	Scan	Method	Score	m/z	Gene names
RO-QC-160121_ZackD_SS_Set_01_Fractior	25016	FTMS; HC	58.12	596.07	ATP6V0C

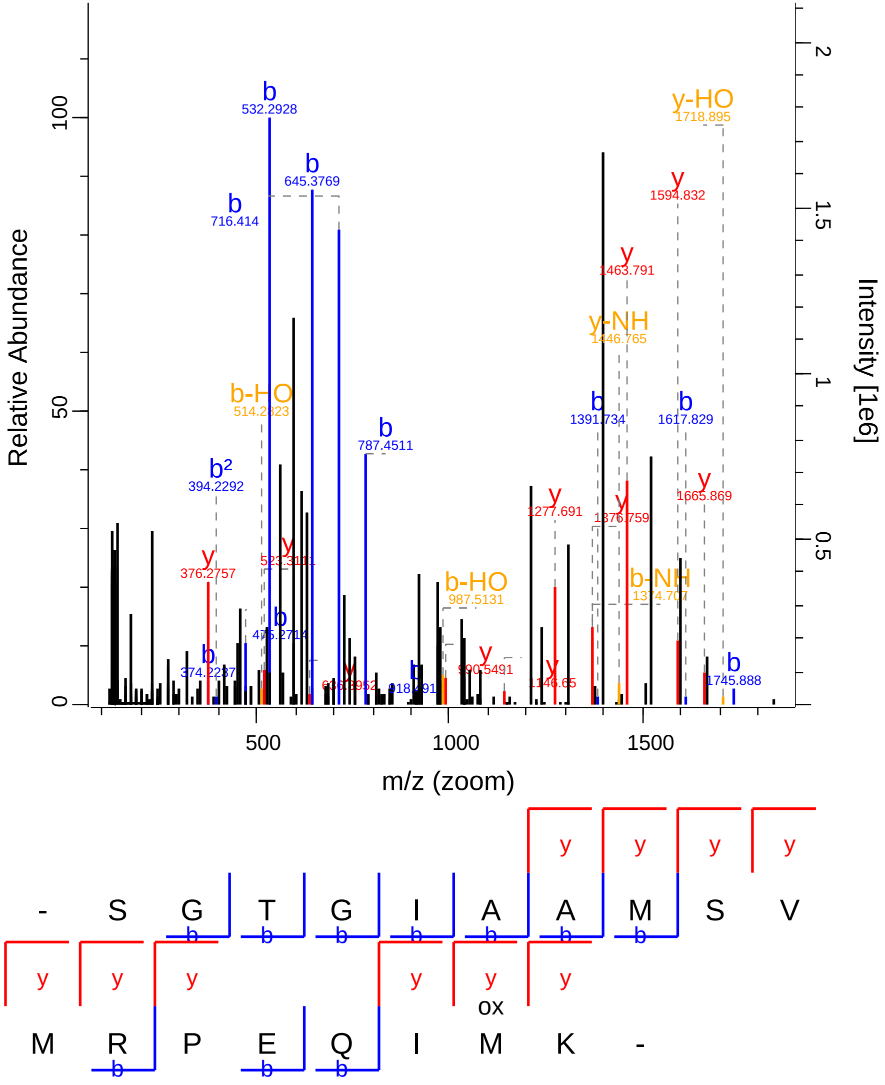

Raw File	Scan	Method	Score	m/z	Gene names
RO-QC-160121_ZackD_SS_Set_01_Fractior	25216	FTMS; HC	95.77	794.77	ATP6V0C

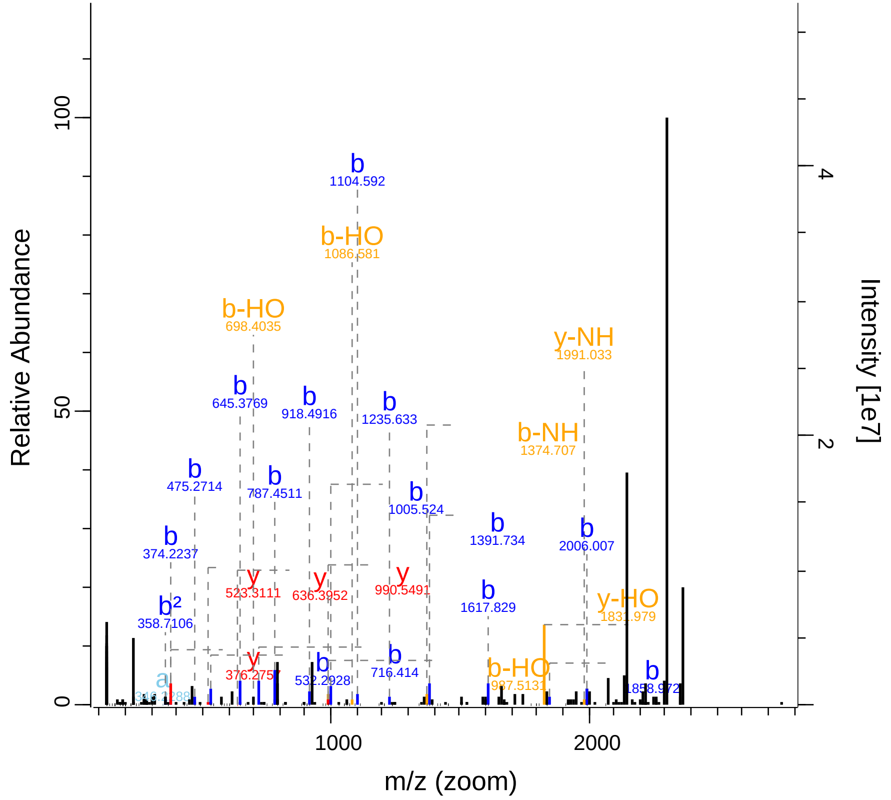

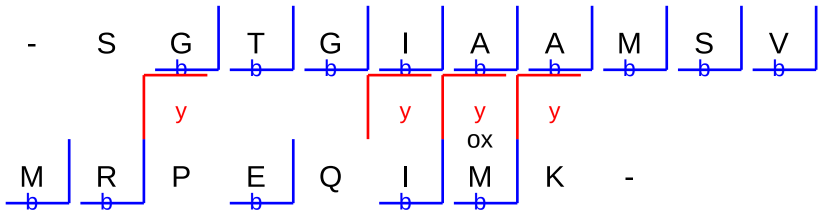

Raw File	Scan	Method	Score	m/z	Gene names
RO-QC-160121_ZackD_SS_Set_01_Fractior	25217	FTMS; HC	56.85	596.33	ATP6V0C

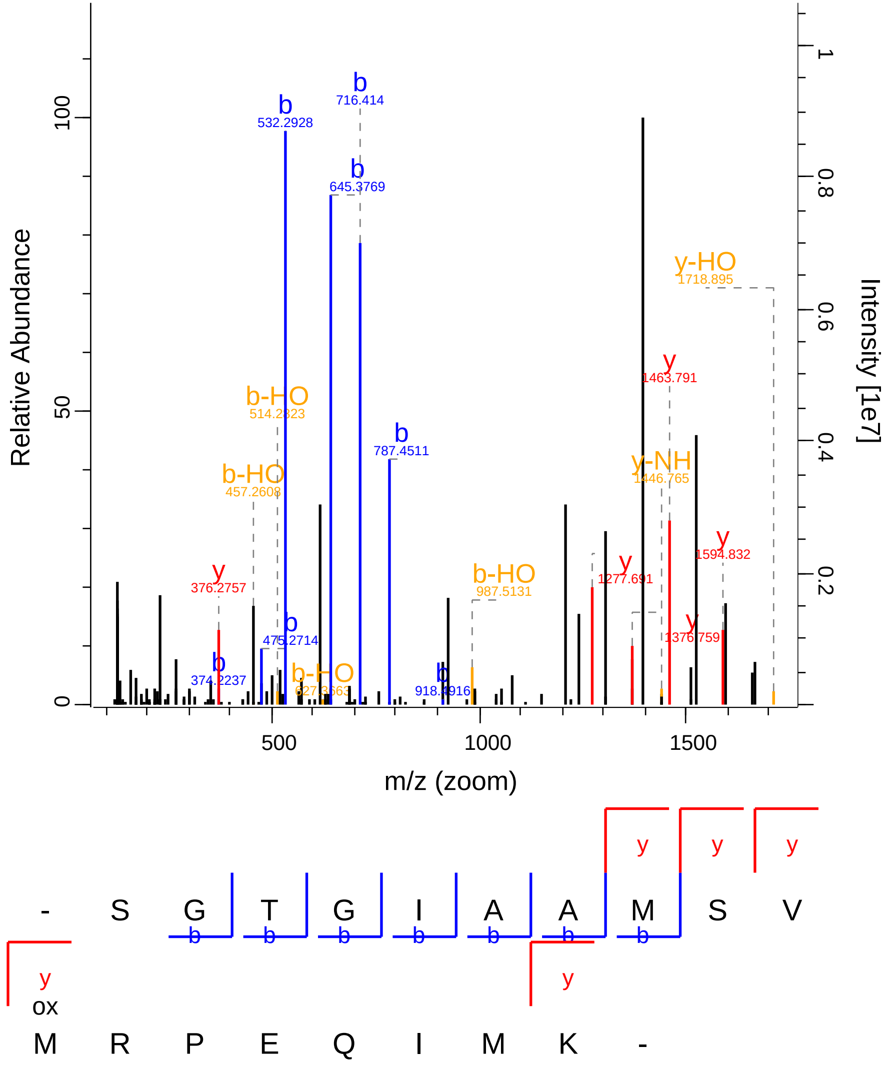

Raw File	Scan	Method	Score	m/z	Gene names
RO-QC-160121_ZackD_SS_Set_01_Fractior	25437	FTMS; HC	79.01	794.76	ATP6V0C

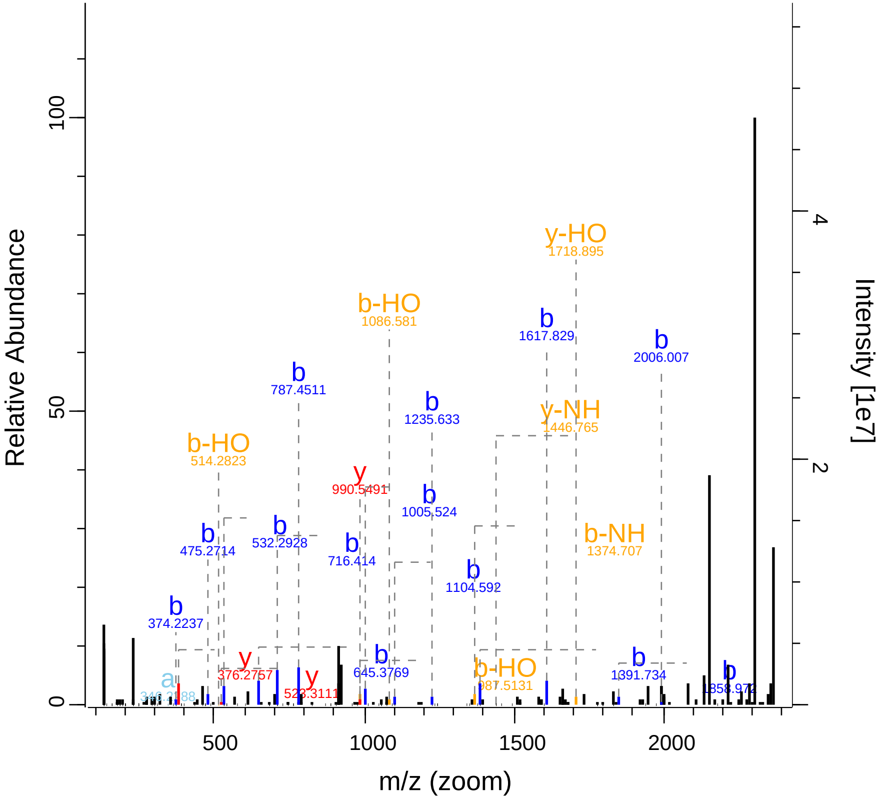

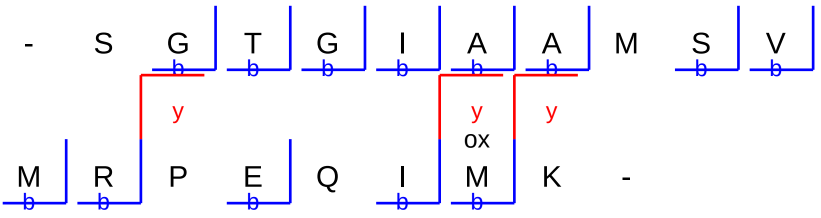

Raw File	Scan	Method	Score	m/z	Gene names
RO-QC-160121_ZackD_SS_Set_01_Fractior	25440	FTMS; HC	47.22	596.33	ATP6V0C

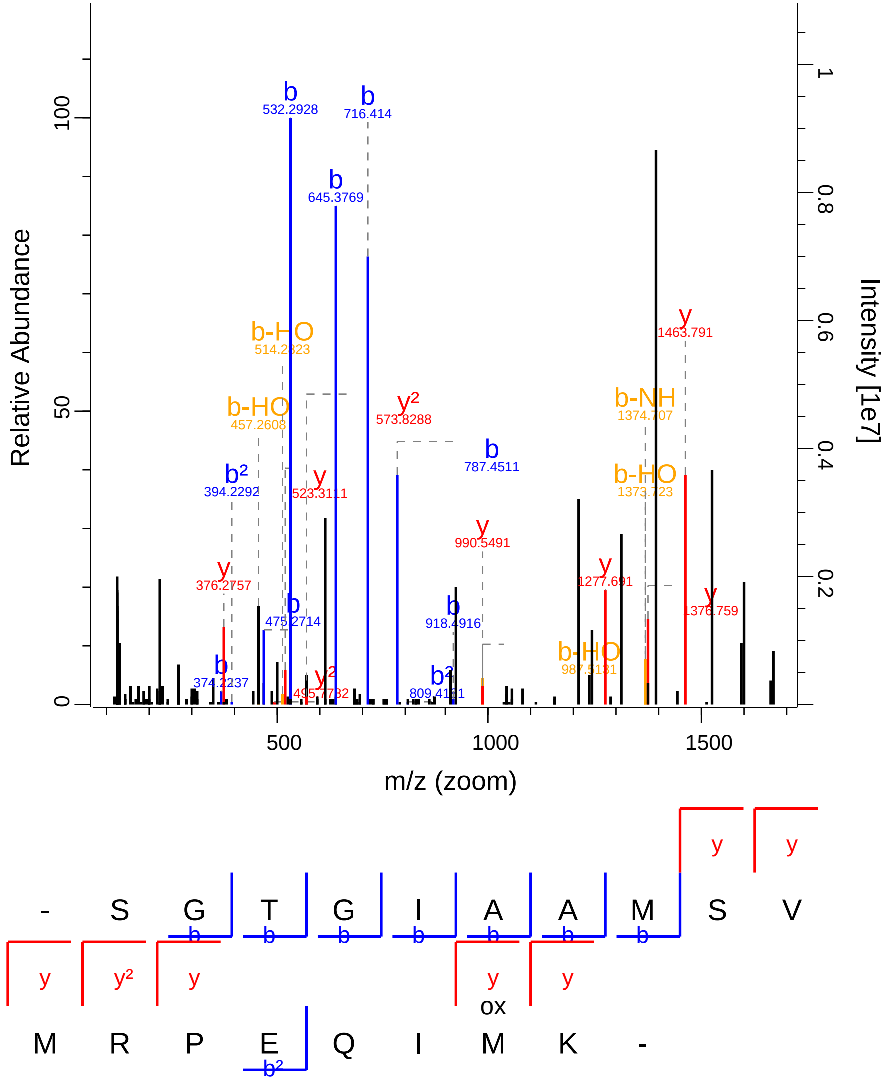

Raw File	Scan	Method	Score	m/z
RO-QC-160121_ZackD_SS_Set_01_Fraction_11	26500	FTMS; HCD	44.3	533.54

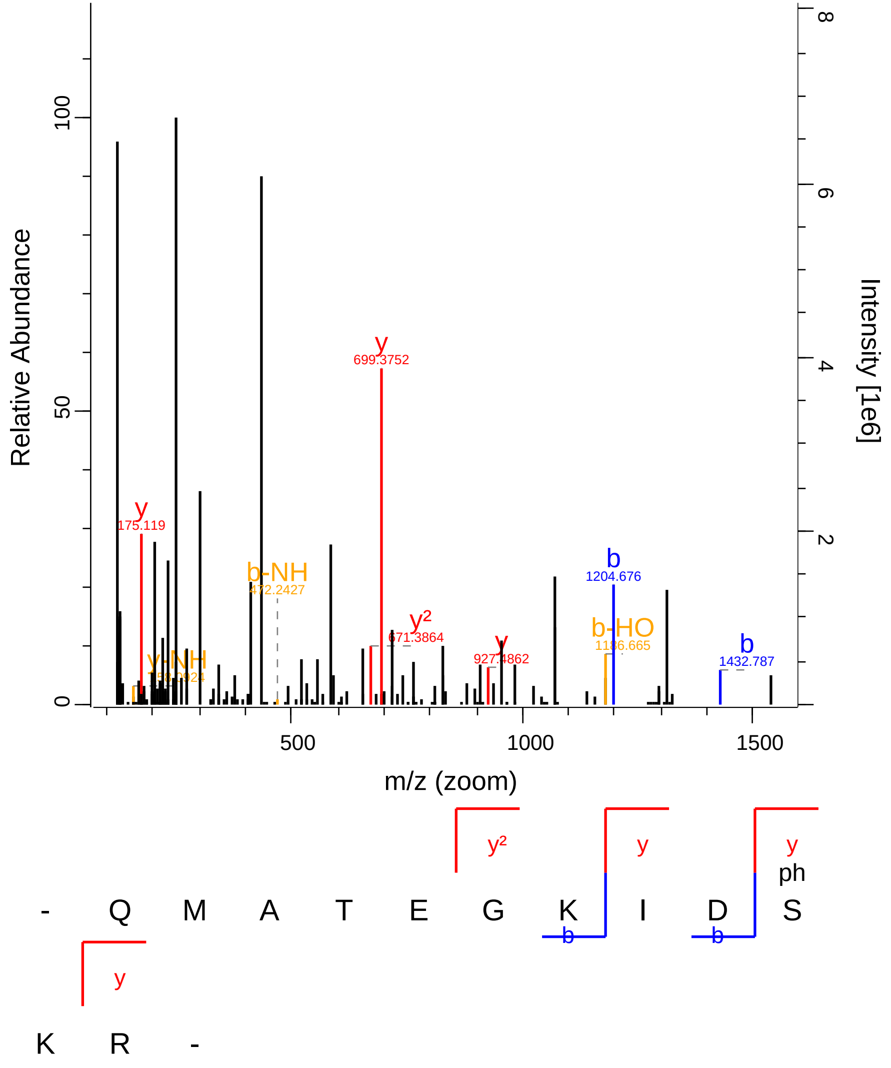

Raw File	Scan	Method	Score	m/z	Gene names
RO-QC-160121_ZackD_SS_Set_01_Fractior	27364	FTMS; HC	93.37	596.32	DRAM1

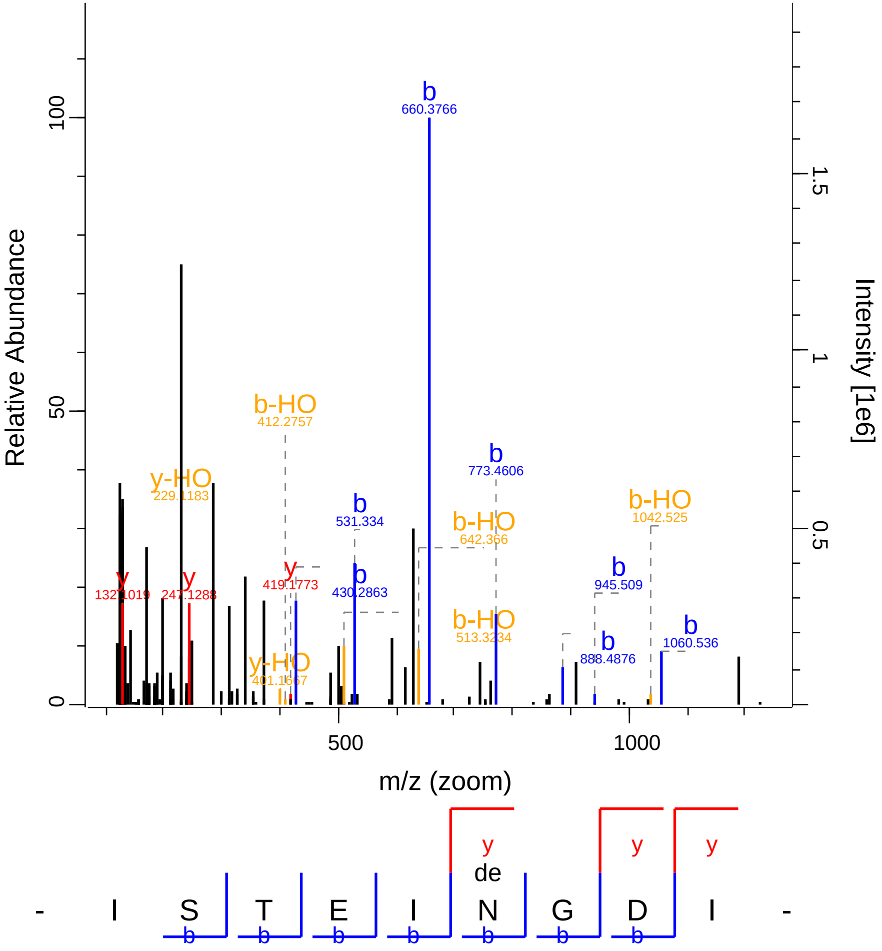

Raw File	Scan	Method	Score	m/z	Gene names
RO-QC-160121_ZackD_SS_Set_01_Fractior	28573	FTMS; HC	77.39	781.11	MDFIC

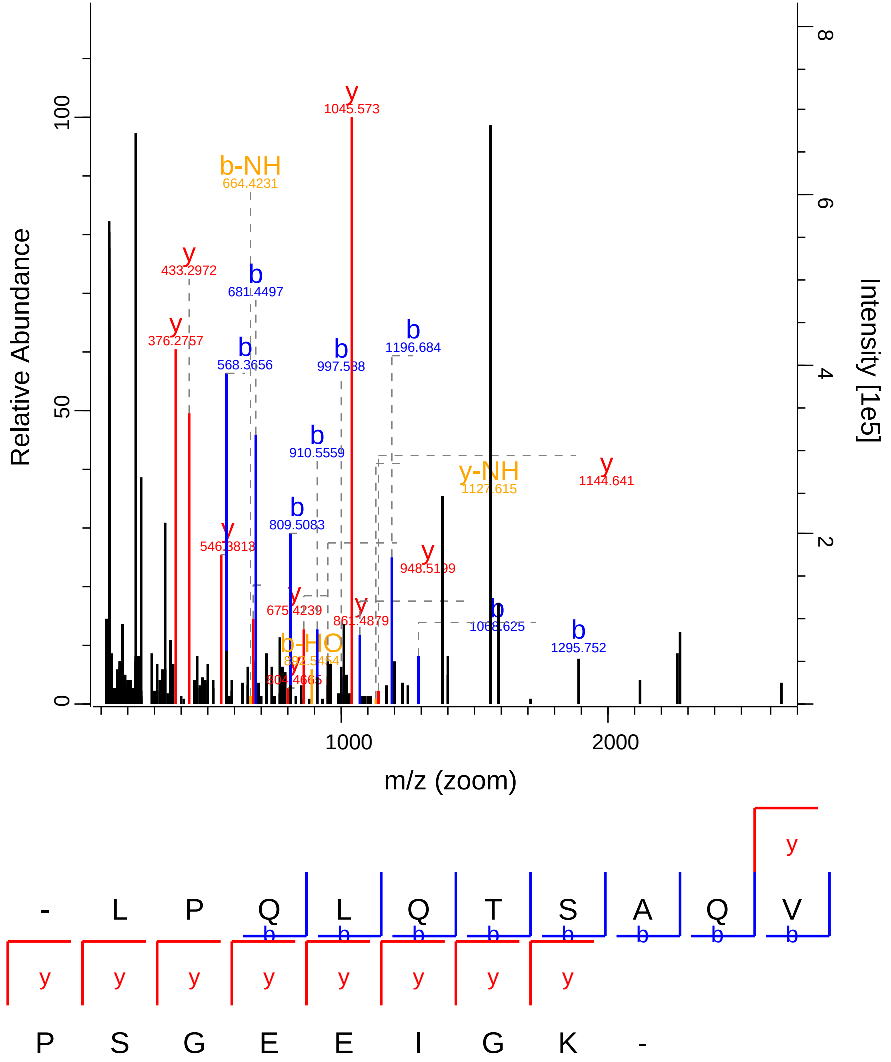

Raw File	Scan	Method	Score	m/z	Gene names
RO-QC-160121_ZackD_SS_Set_01_Fractio[illegible]	28985	FTMS; HC	138.71	811.42	ANKRD49

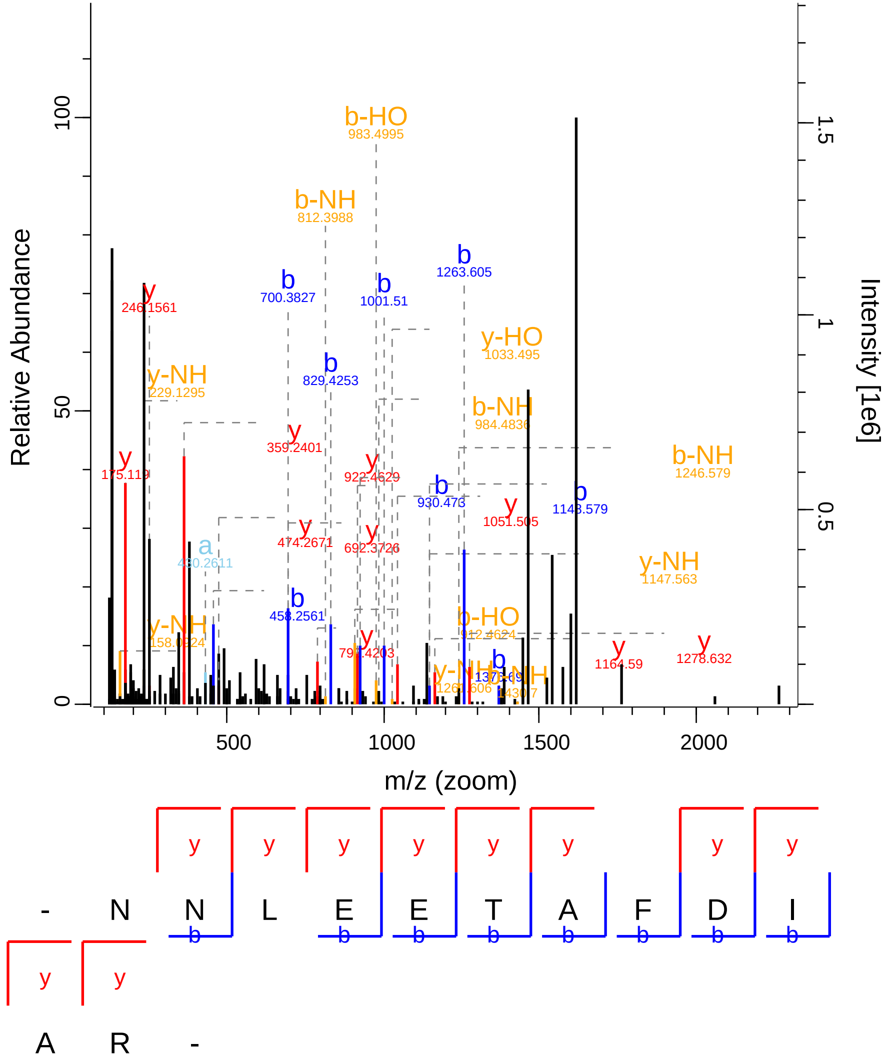

Raw File	Scan	Method	Score	m/z	Gene names
RO-QC-160121_ZackD_SS_Set_01_Fractior	30095	FTMS; HC	107.9	851.02	ST8SIA2

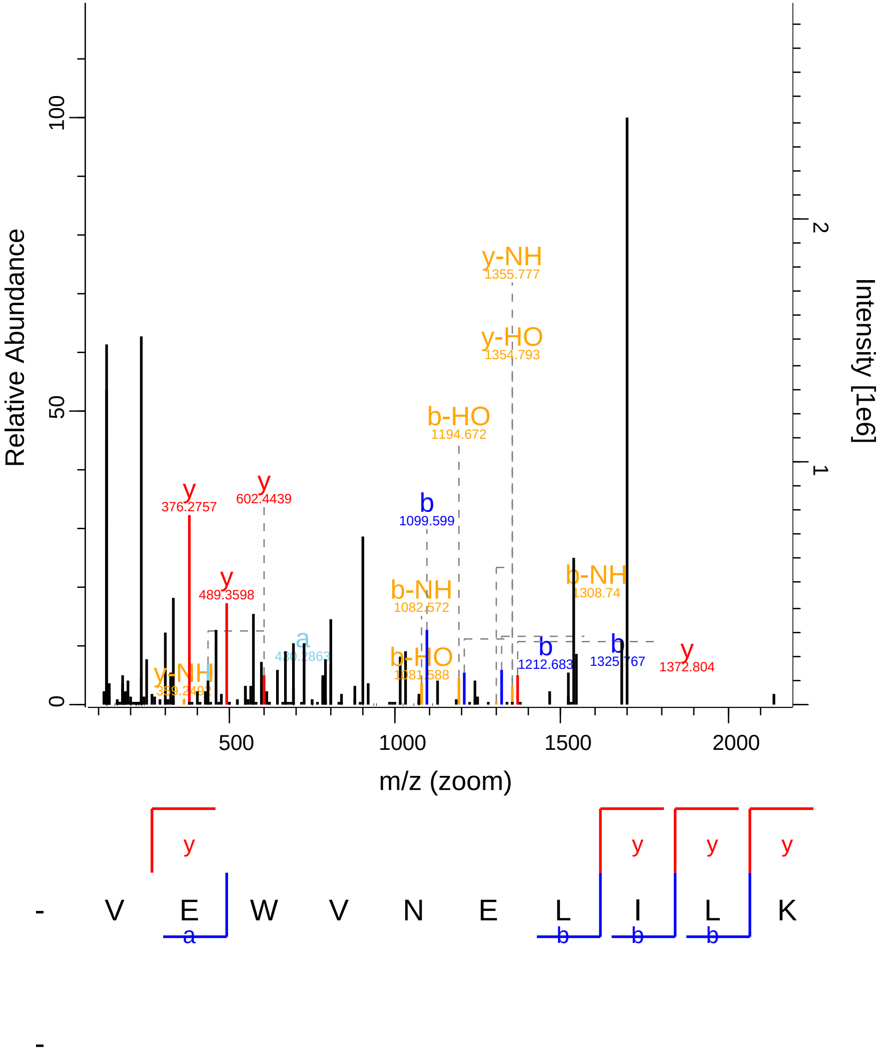

Raw File	Scan	Method	Score	m/z	Gene names
RO-QC-160121_ZackD_SS_Set_01_Fractio	30205	FTMS; HC	111.77	841.75	CNIH4

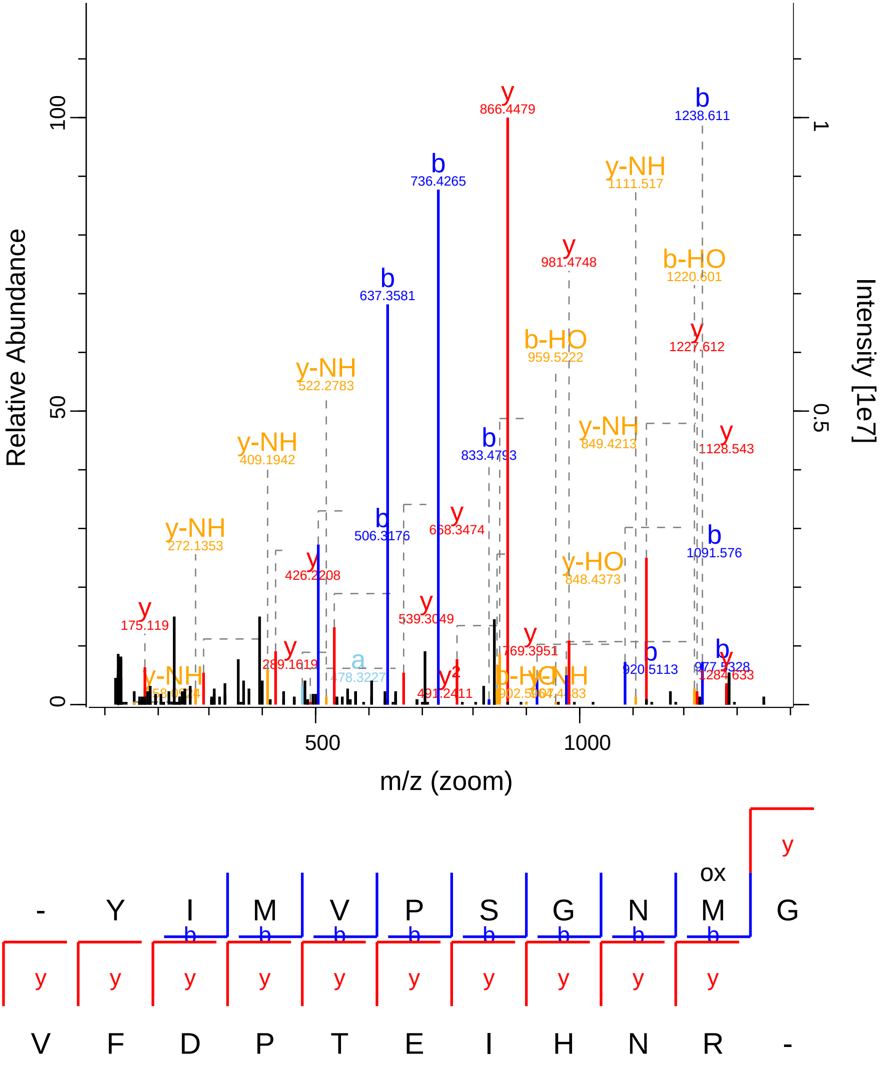

Raw File	Scan	Method	Score	m/z	Gene names
RO-QC-160121_ZackD_SS_Set_01_Fractior	30228	FTMS; HC	52.73	631.57	CNIH4

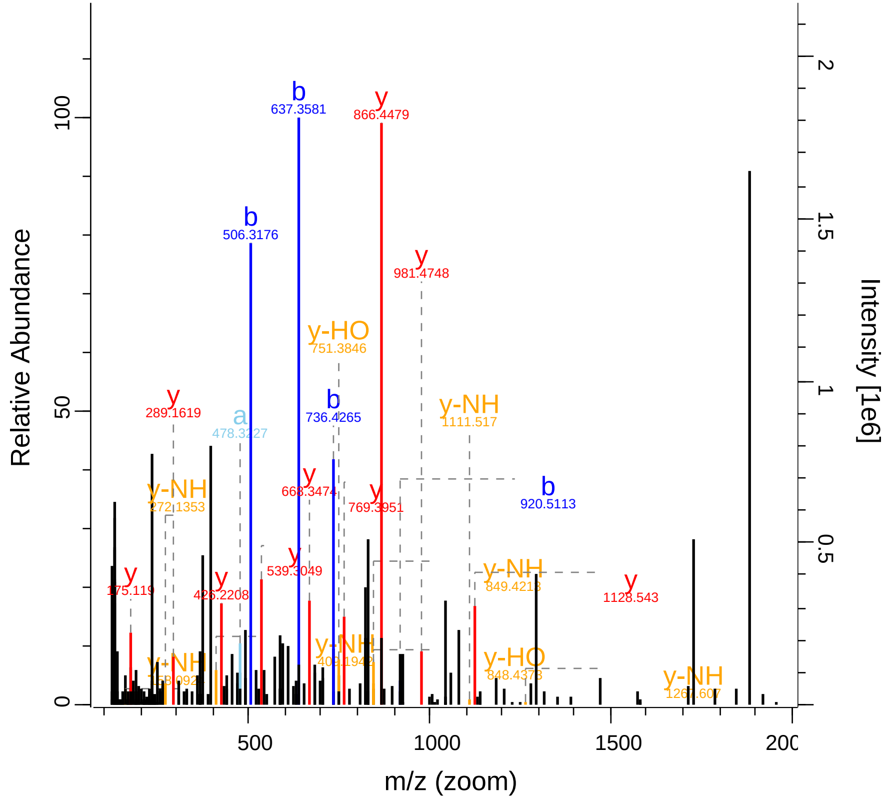

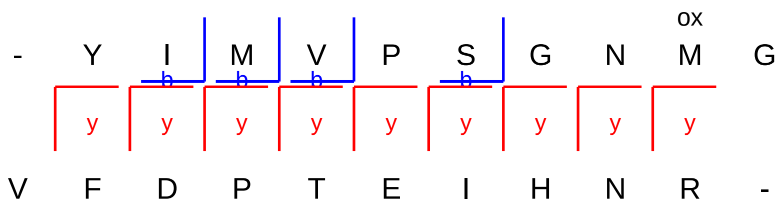

Raw File	Scan	Method	Score	m/z	Gene names
RO-QC-160121_ZackD_SS_Set_01_Fractior	30690	FTMS; HC	75.48	704.69	EMP3

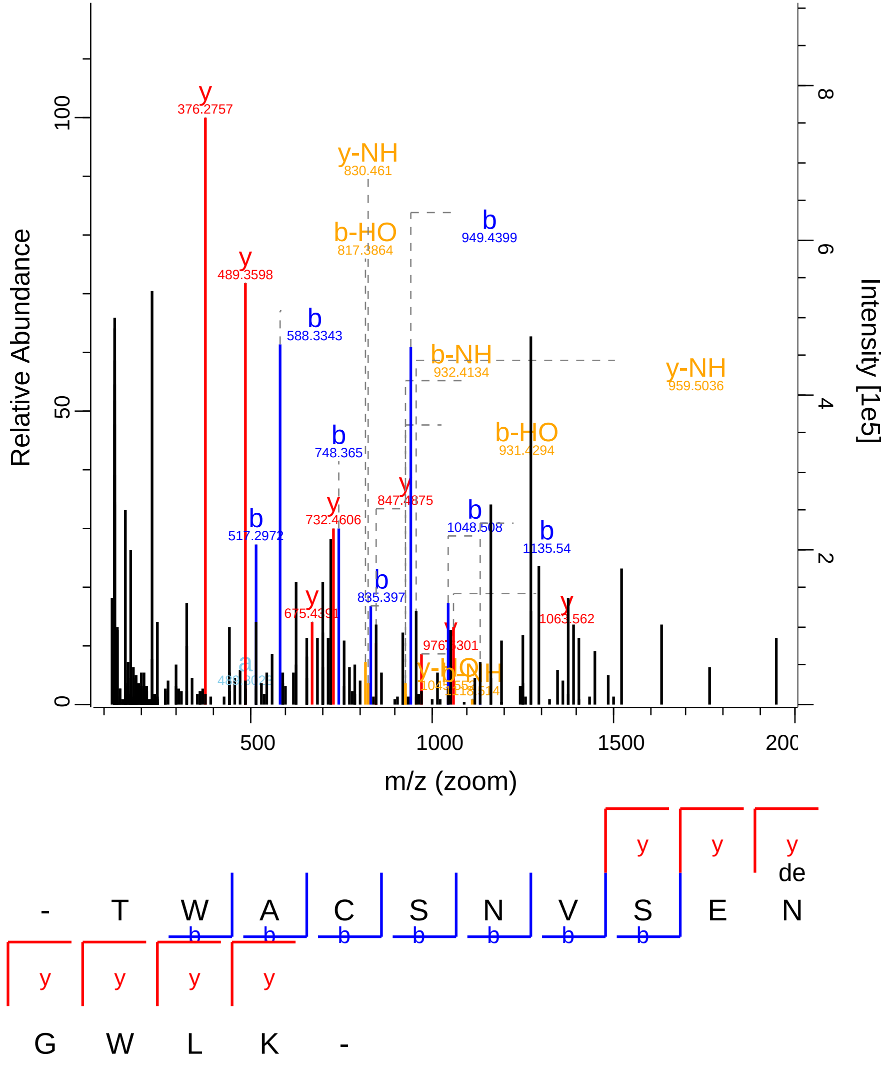

Raw File	Scan	Method	Score	m/z	Gene names
RO-QC-160121_ZackD_SS_Set_01_Fractio	30749	FTMS; HC	184.03	1056.54	EMP3

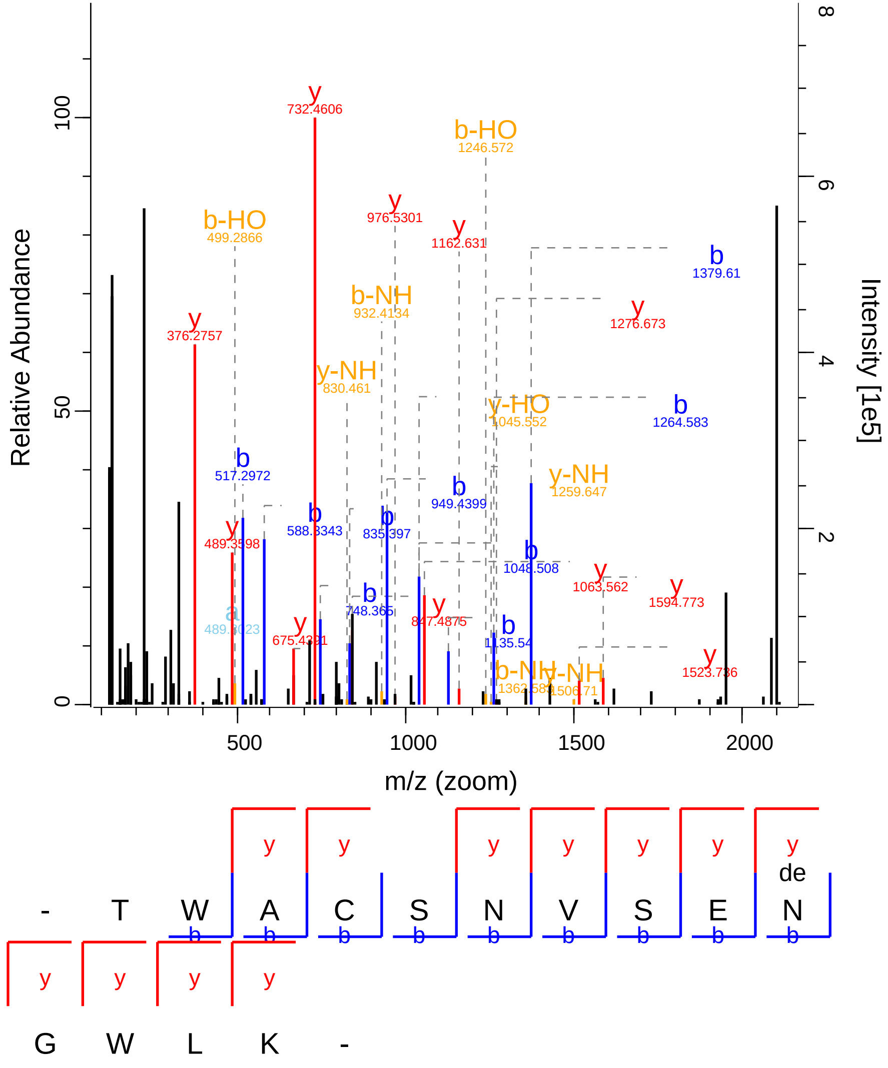

Raw File	Scan	Method	Score	m/z	Gene names
RO-QC-160121_ZackD_SS_Set_01_Fractior	30760	FTMS; HC	78.33	991.06	OGN

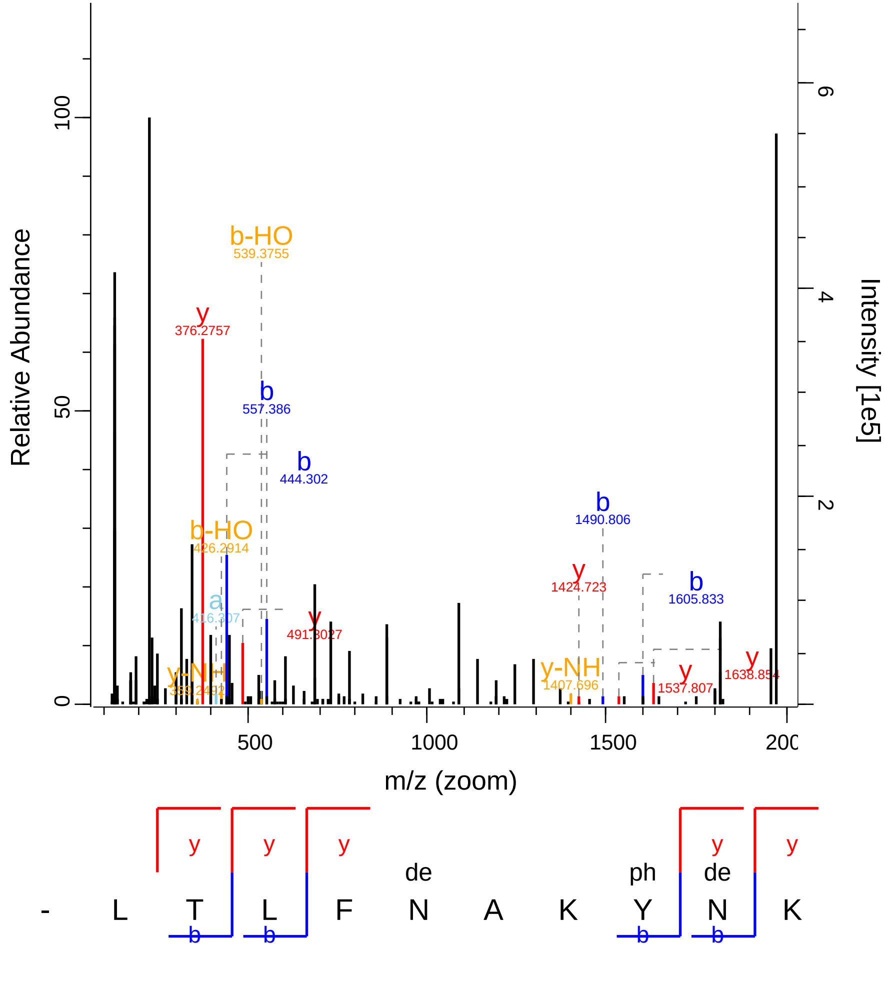

Raw File	Scan	Method	Score	m/z	Gene names
RO-QC-160121_ZackD_SS_Set_01_Fraction	31648	FTMS; HC	58.09	715.4	SEPHS2

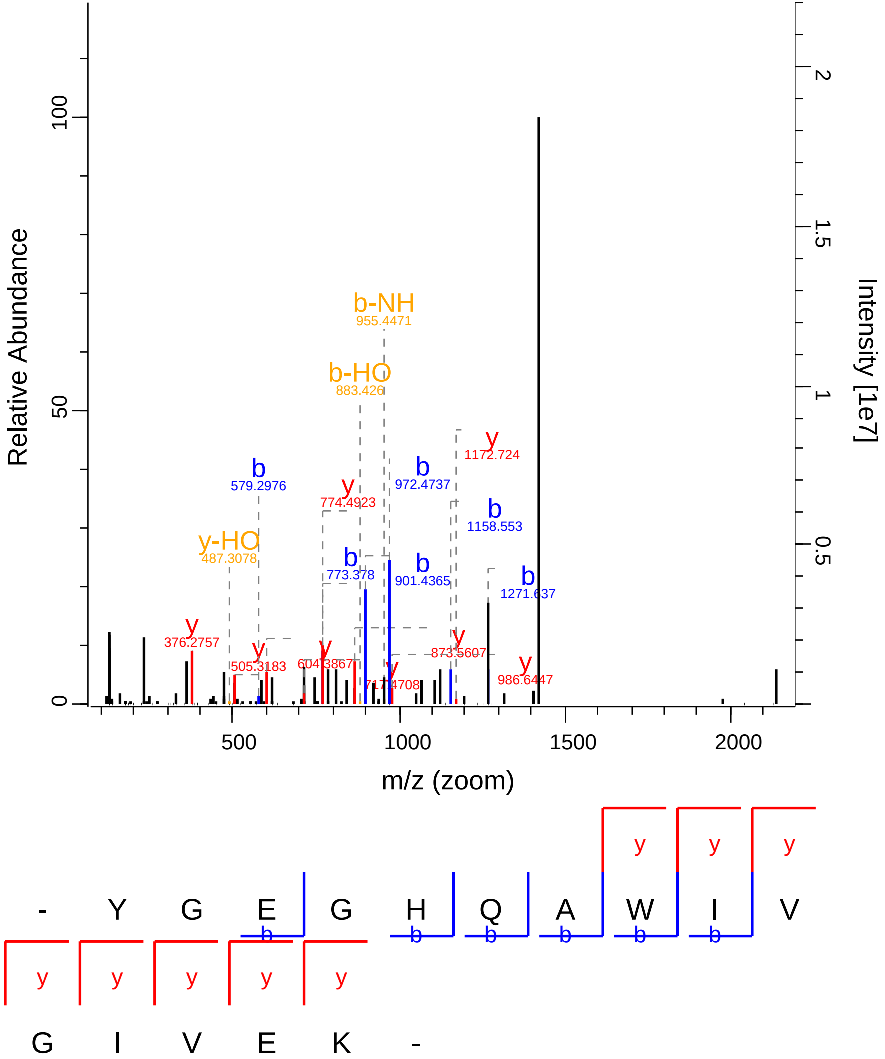

Raw File	Scan	Method	Score	m/z	Gene names
RO-QC-160121_ZackD_SS_Set_01_Fractior	31673	FTMS; HC	90.71	537.05	SEPHS2

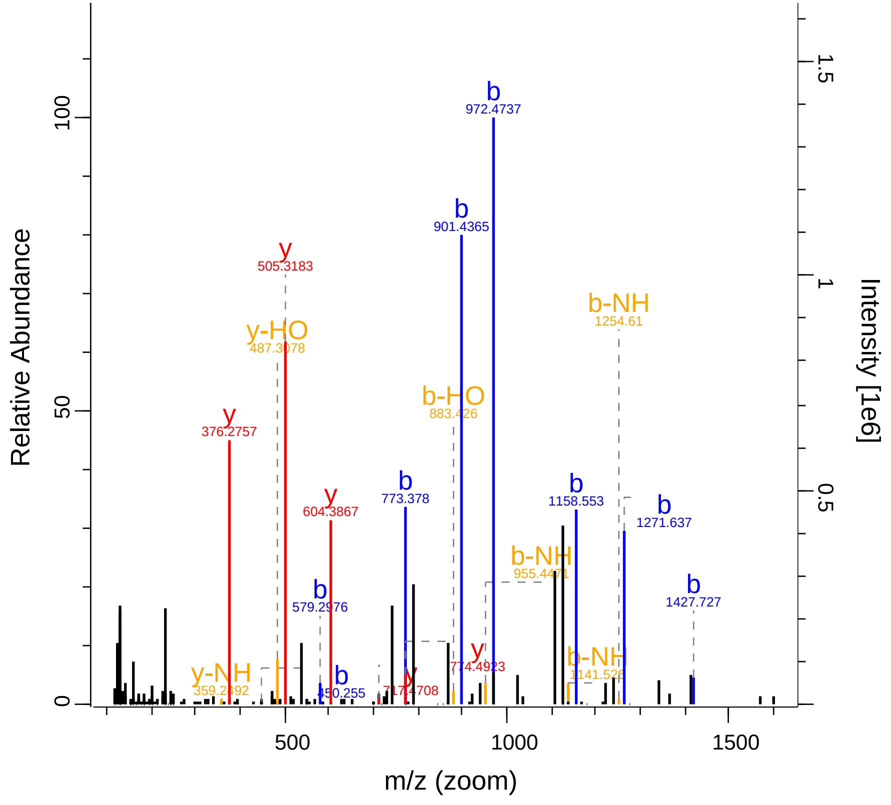

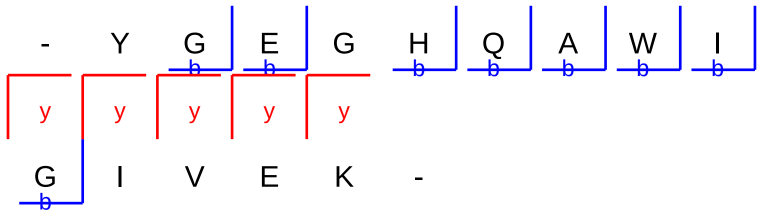

Raw File	Scan	Method	Score	m/z	Gene names
RO-QC-160121_ZackD_SS_Set_01_Fractior	31712	FTMS; HC	55.75	715.74	SEPHS2

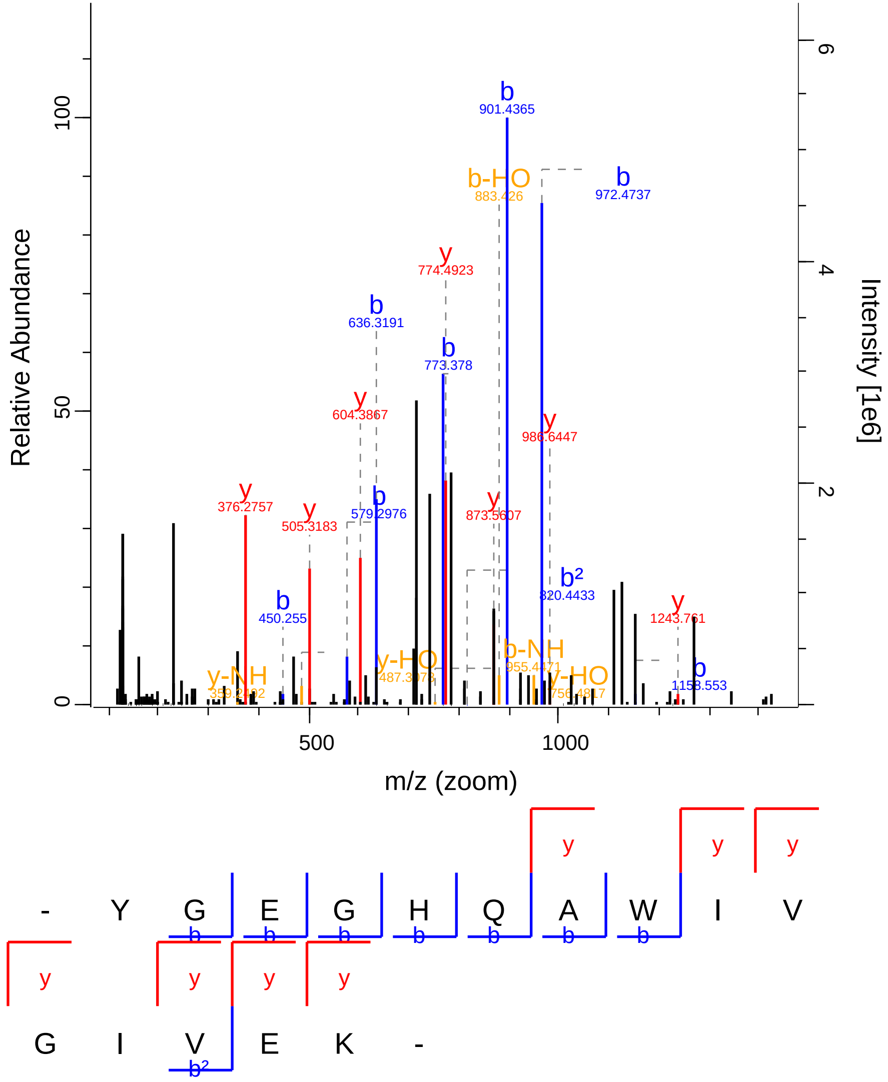

Raw File	Scan	Method	Score	m/z	Gene names
RO-QC-160121_ZackD_SS_Set_01_Fraction_	33189	FTMS; HC	127.83	815	ARL10

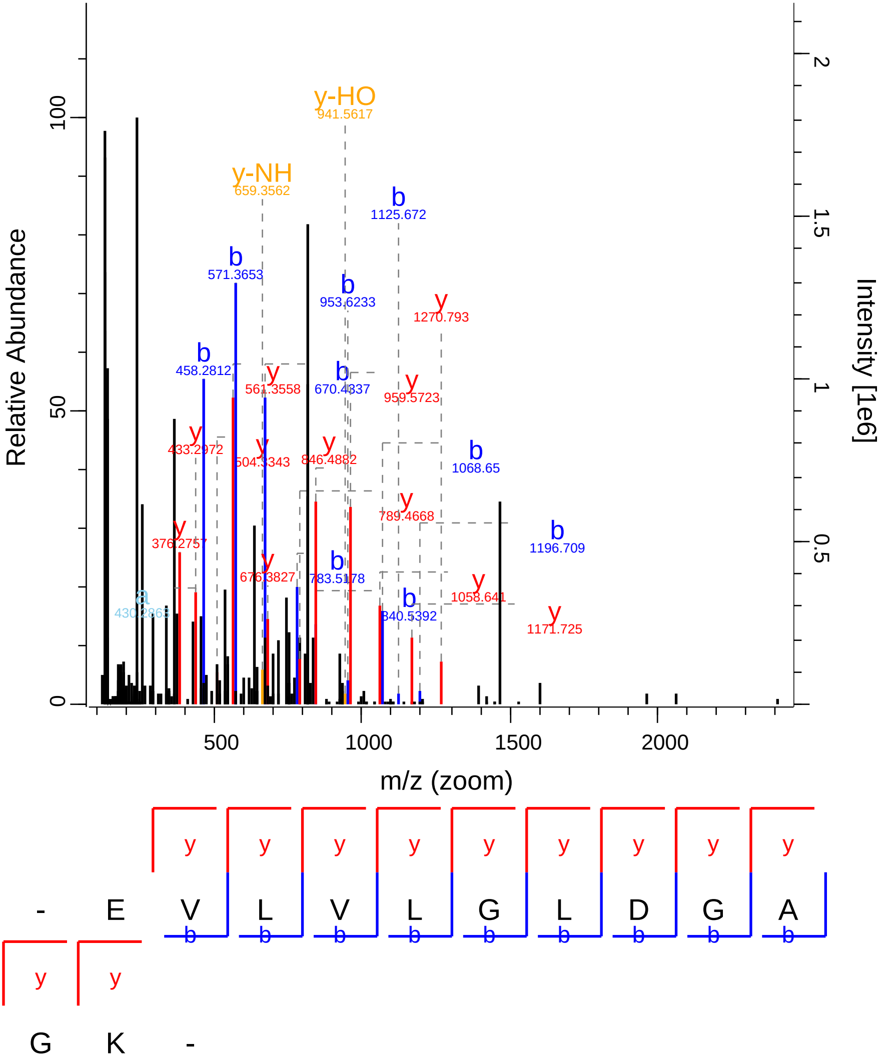

Raw File	Scan	Method	Score	m/z	Gene names
RO-QC-160121_ZackD_SS_Set_01_Fractior	33199	FTMS; HC	93.49	648.36	CLK3

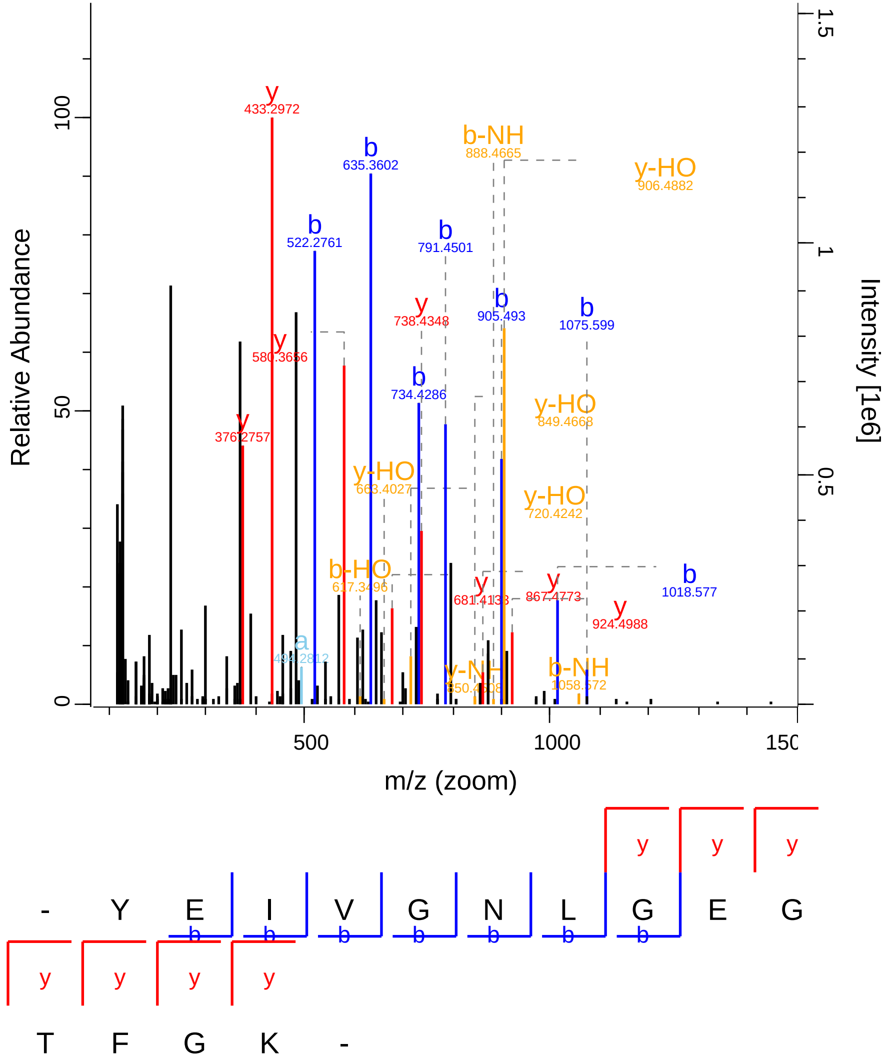

Raw File	Scan	Method	Score	m/z	Gene names
RO-QC-160121_ZackD_SS_Set_01_Fractior	33222	FTMS; HC	63.94	543.67	ARL10

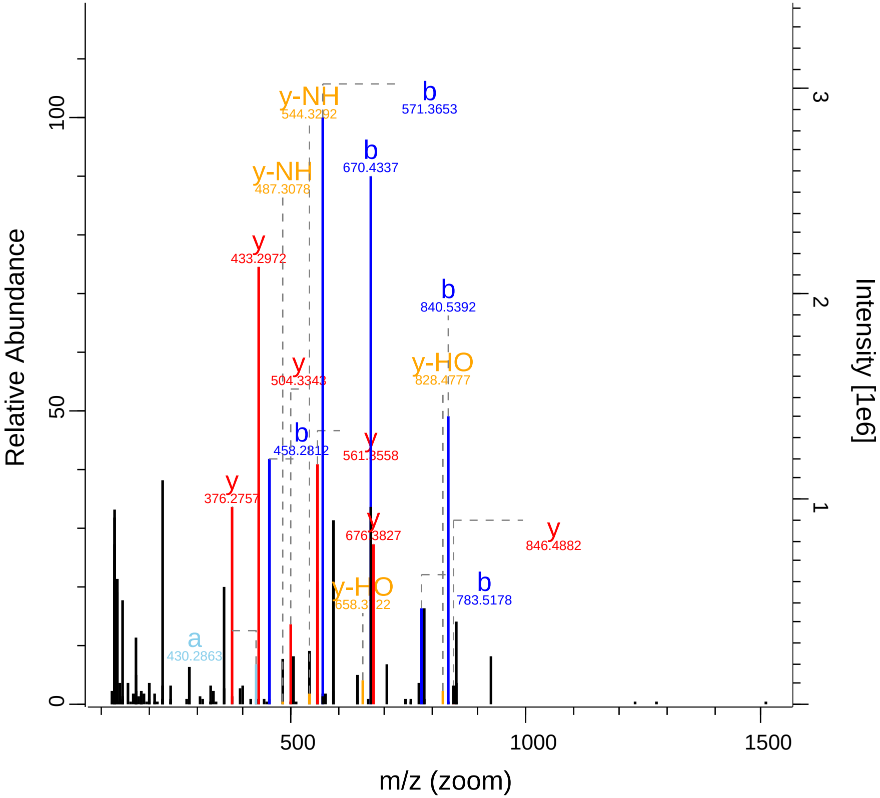

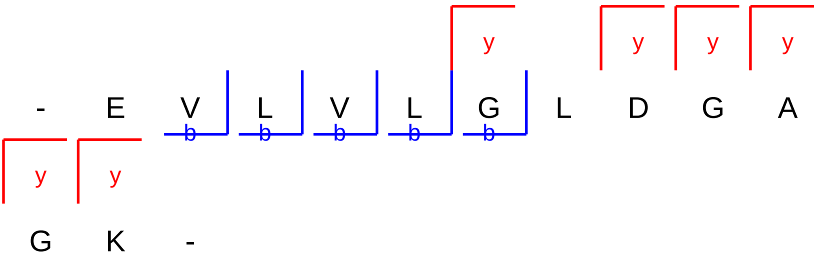

Raw File	Scan	Method	Score	m/z	Gene names
RO-QC-160121_ZackD_SS_Set_01_Fraction	33590	FTMS; HC	54.72	651.9	SHISA4

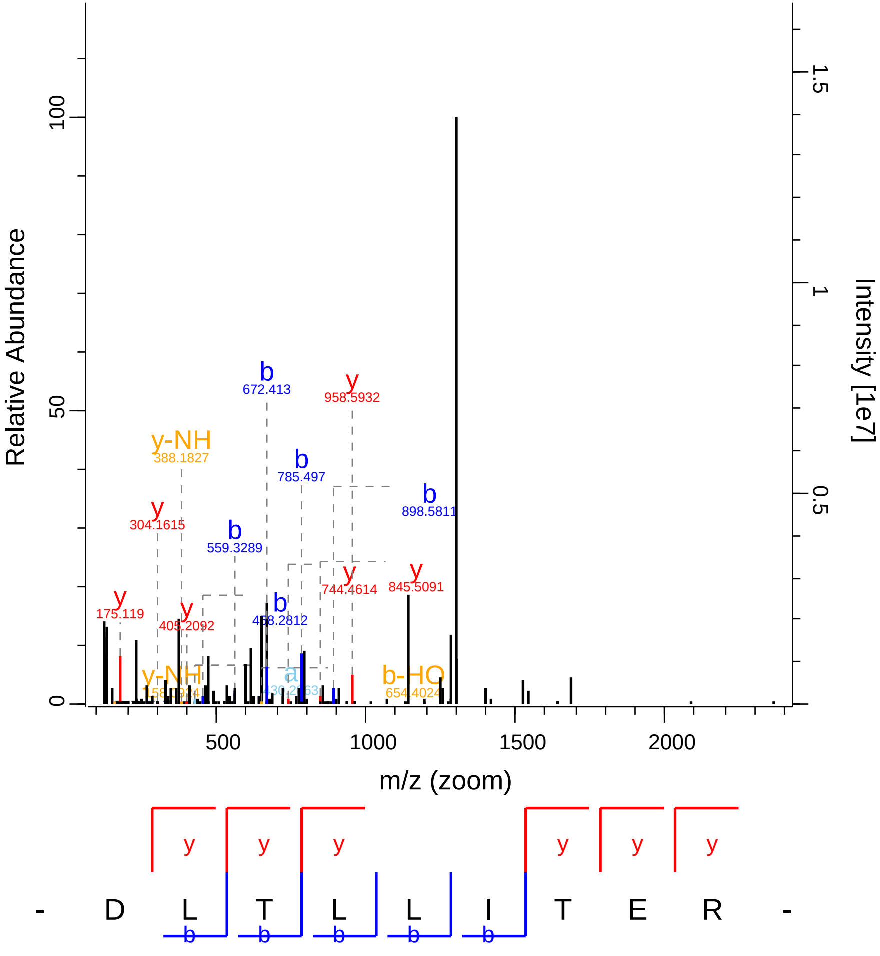

Raw File	Scan	Method	Score	m/z	Gene names
RO-QC-160121_ZackD_SS_Set_01_Fraction	34420	FTMS; HC	107.45	883.5	ZG16B

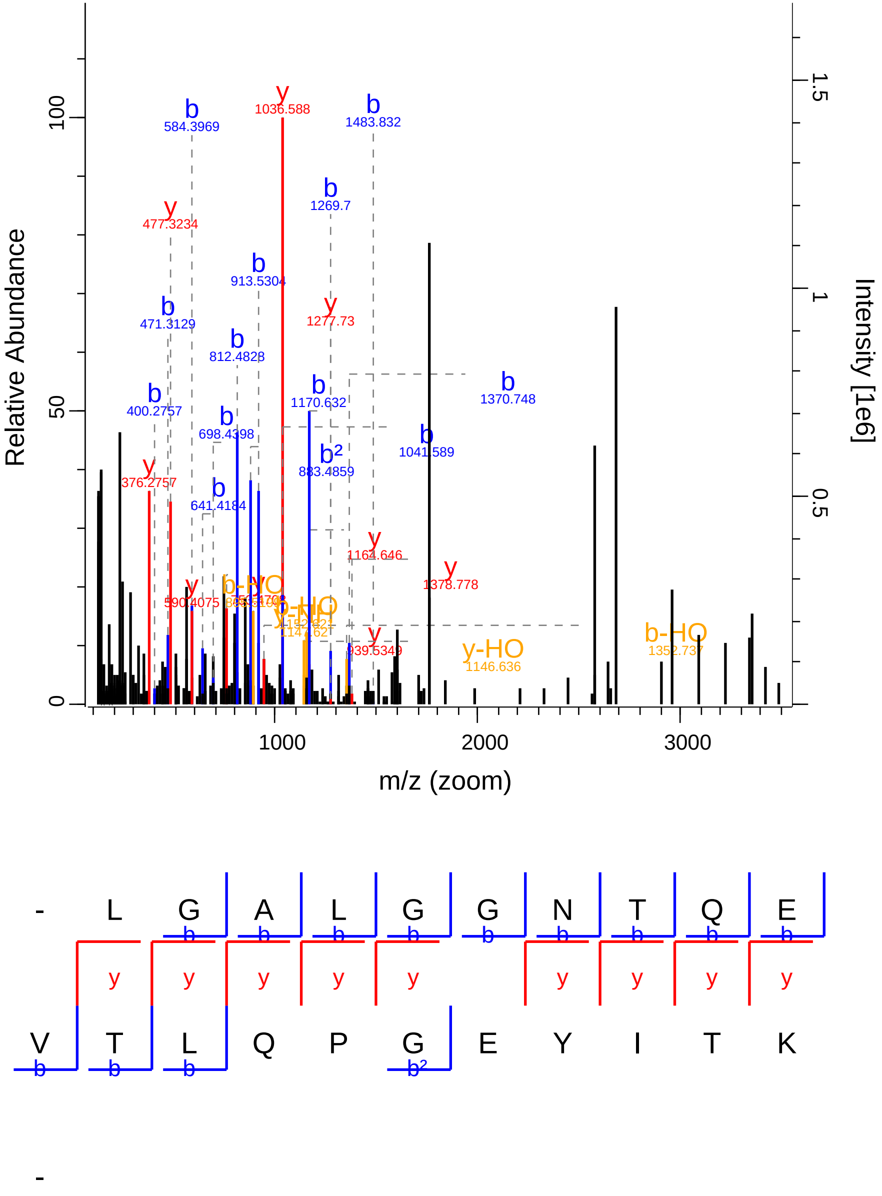

Raw File	Scan	Method	Score	m/z	Gene names
RO-QC-160121_ZackD_SS_Set_01_Fractior	36023	FTMS; HC	43.16	837.23	TDRD10

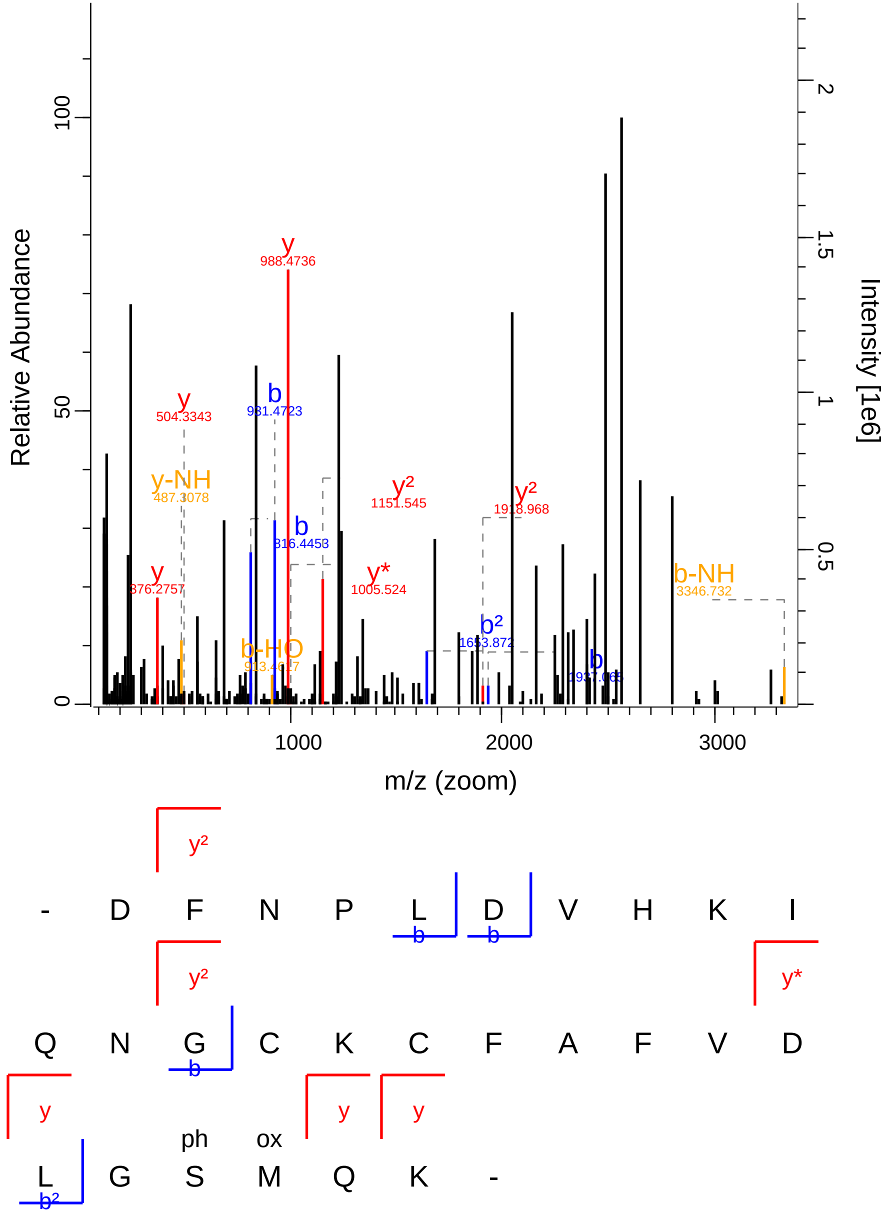

Raw File	Scan	Method	Score	m/z
RO-QC-160121_ZackD_SS_Set_01_Fraction_11	36304	FTMS; HCD	43.53	969.85

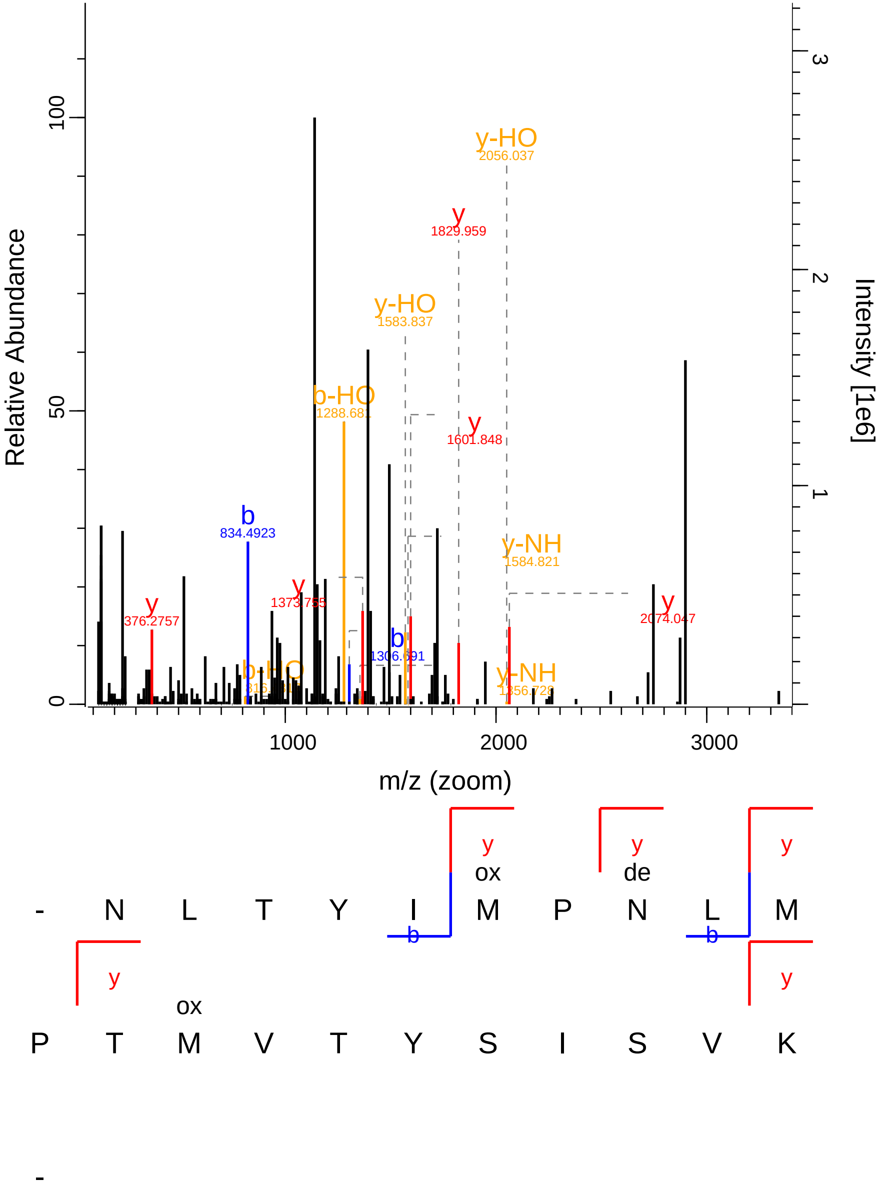

Raw File	Scan	Method	Score	m/z
RO-QC-160121_ZackD_SS_Set_01_Fraction_11	36919	FTMS; HCD	44.05	982.78

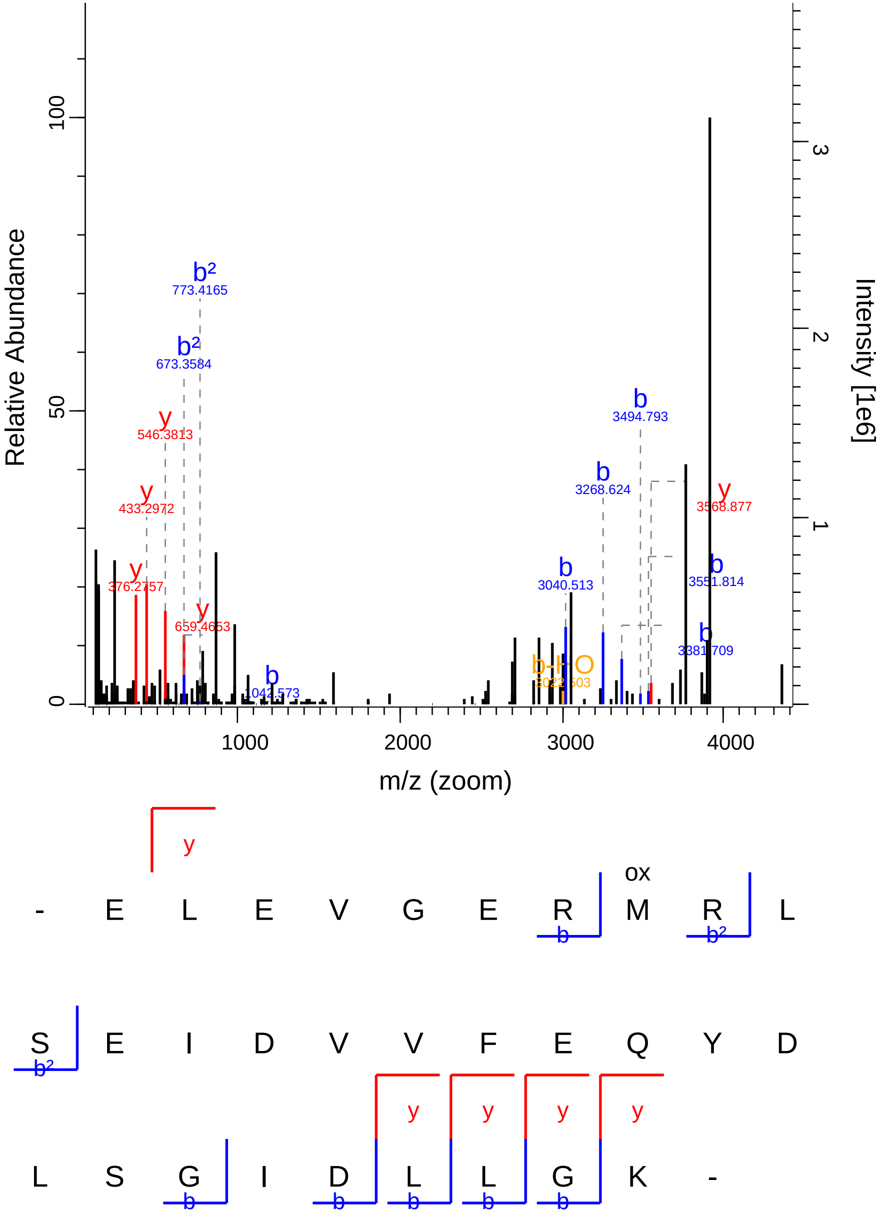

Raw File	Scan	Method	Score	m/z	Gene names
RO-QC-160121_ZackD_SS_Set_01_Fractior	38717	FTMS; HC	79.87	733.07	UBE2J2

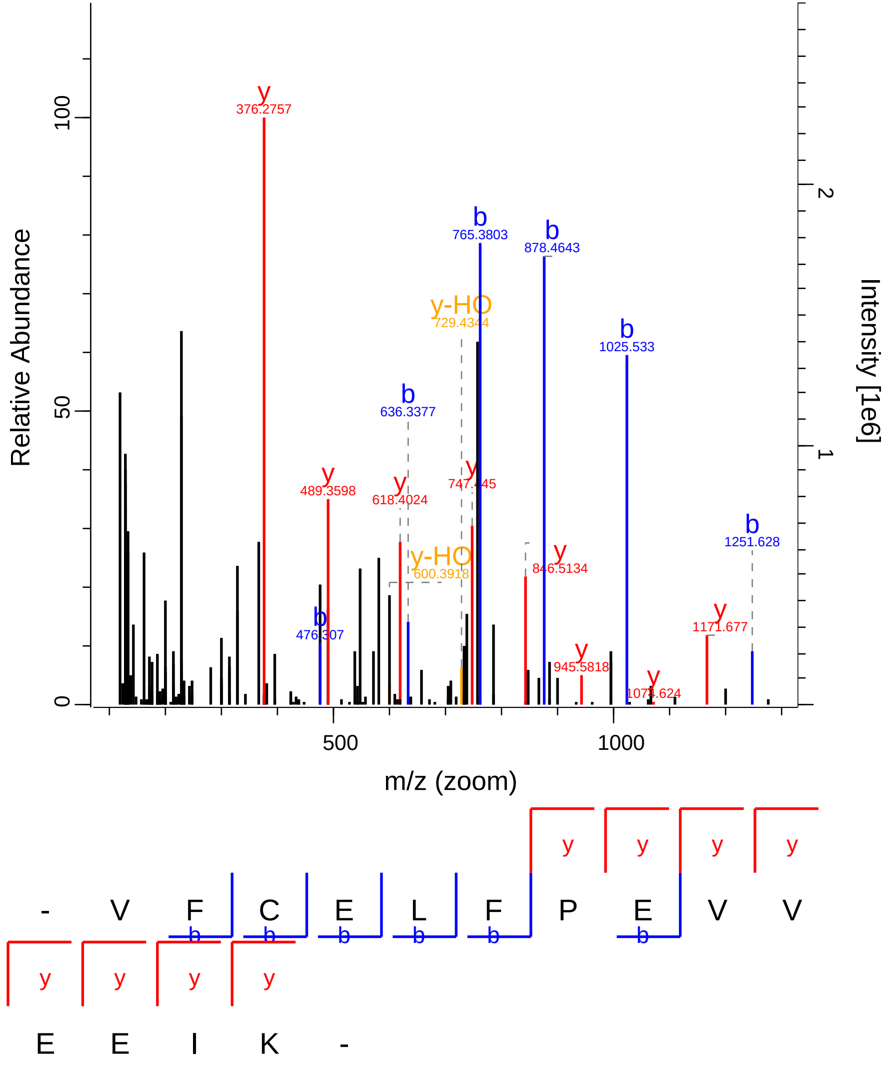

Raw File	Scan	Method	Score	m/z	Gene names
RO-QC-160121_ZackD_SS_Set_01_Fractioı	39123	FTMS; HC	108.43	993.52	TOMM7

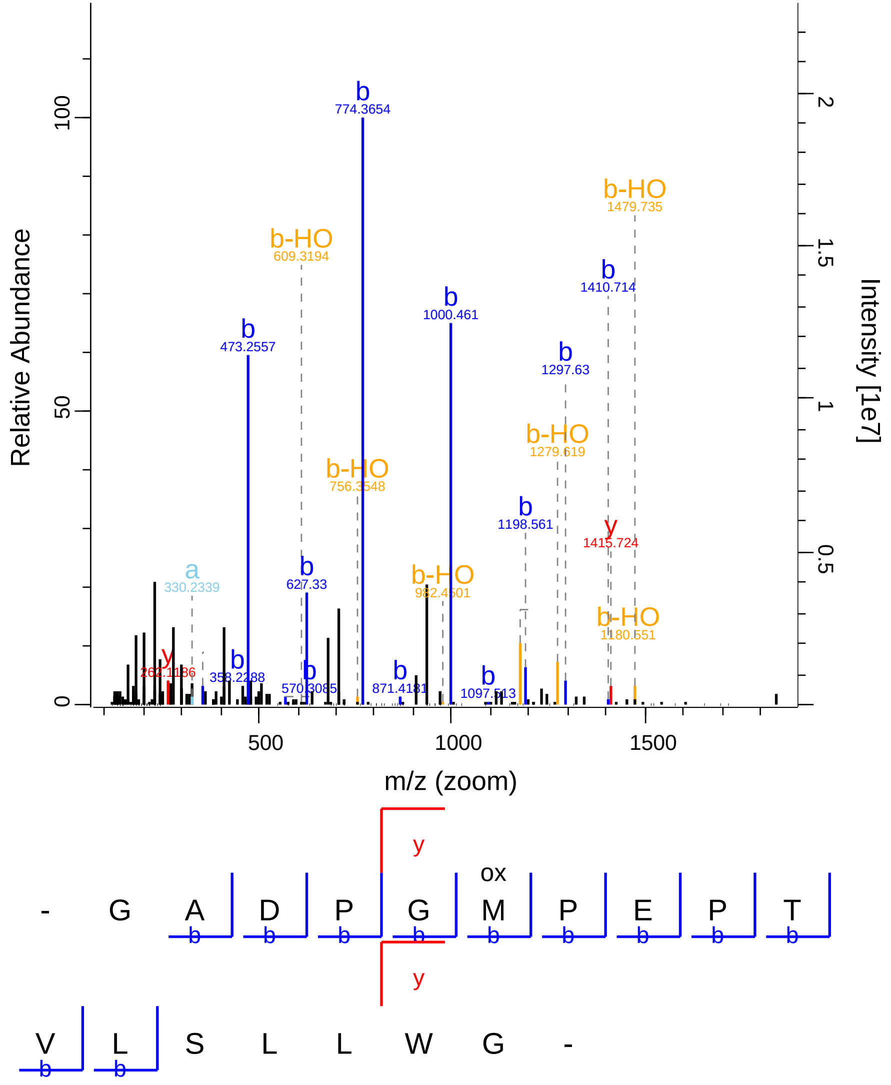

Raw File	Scan	Method	Score	m/z	Gene names
RO-QC-160121_ZackD_SS_Set_01_Fractior	39234	FTMS; HC	82.42	985.52	TOMM7

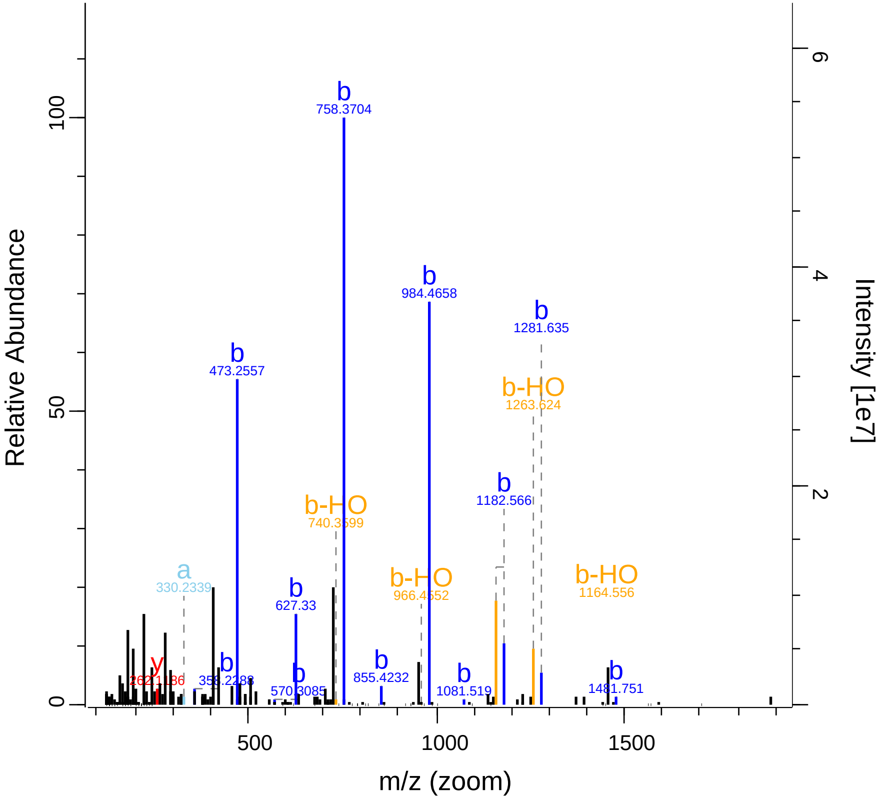

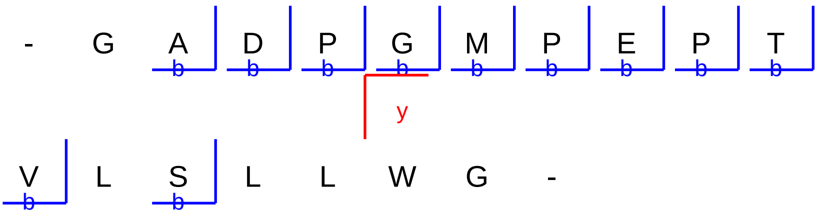

Raw File	Scan	Method	Score	m/z	Gene names
RO-QC-160121_ZackD_SS_Set_01_Fractior	39250	FTMS; HC	38.38	657.35	TOMM7

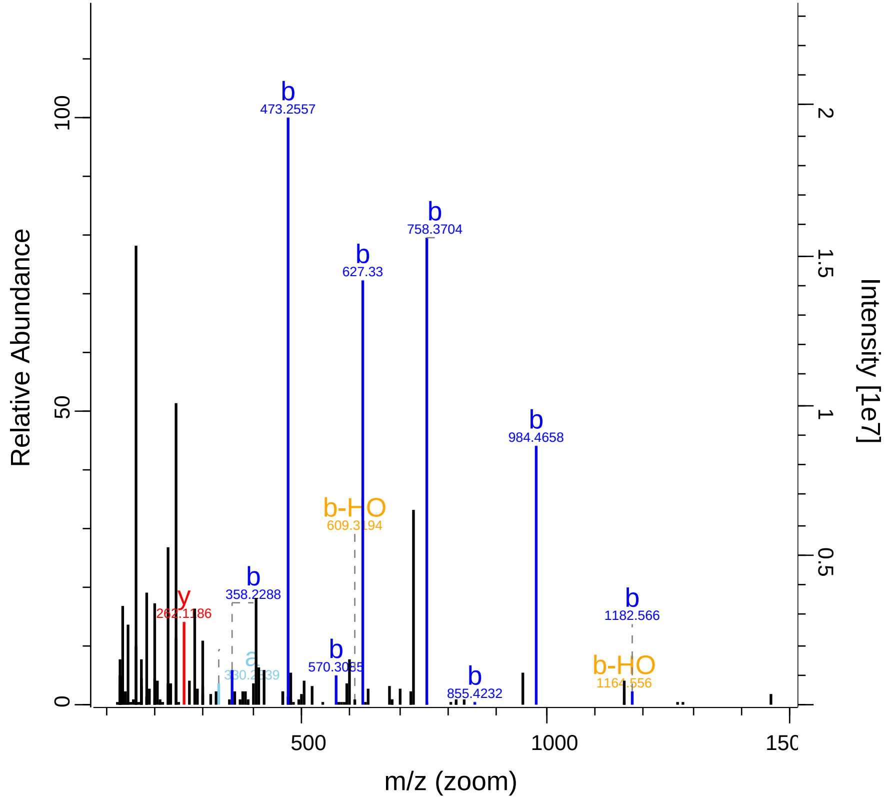

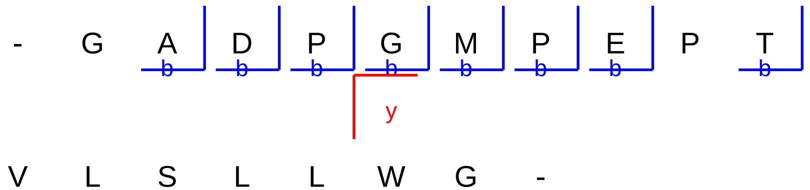

Raw File	Scan	Method	Score	m/z
RO-QC-160121_ZackD_SS_Set_01_Fraction_12	4717	FTMS; HCD	84.66	470.02

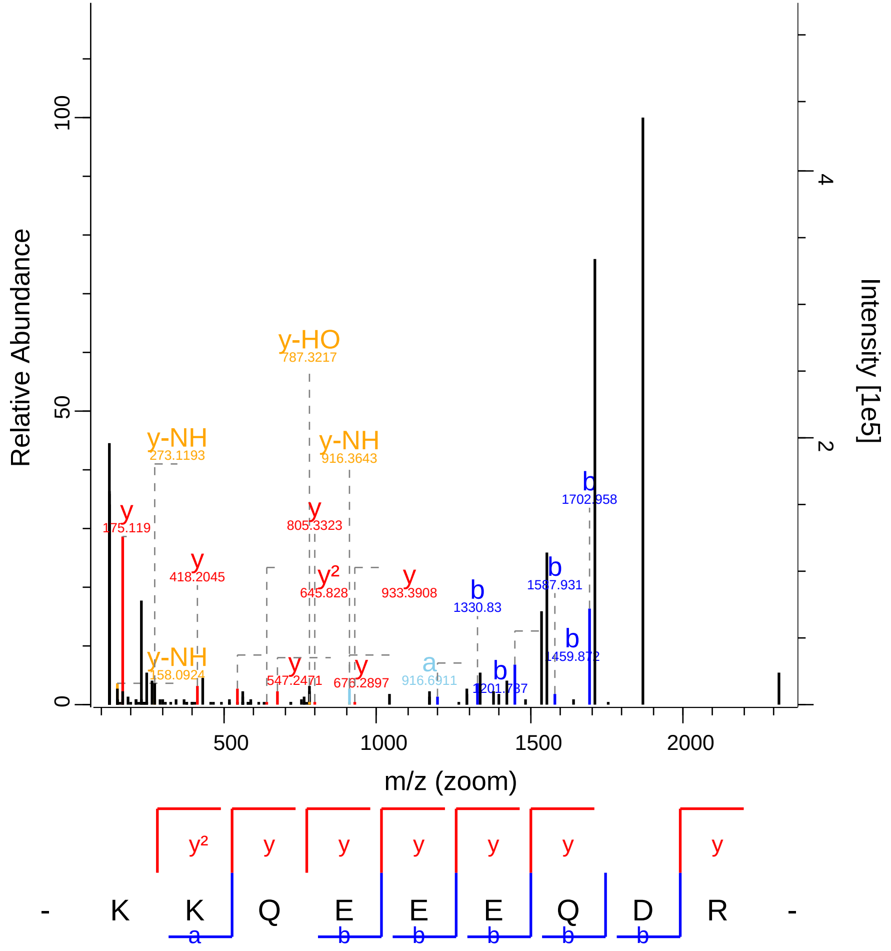

Raw File	Scan	Method	Score	m/z	Gene names
RO-QC-160121_ZackD_SS_Set_01_Fraction_	5409	FTMS; HCI	75.38	390	ODC1

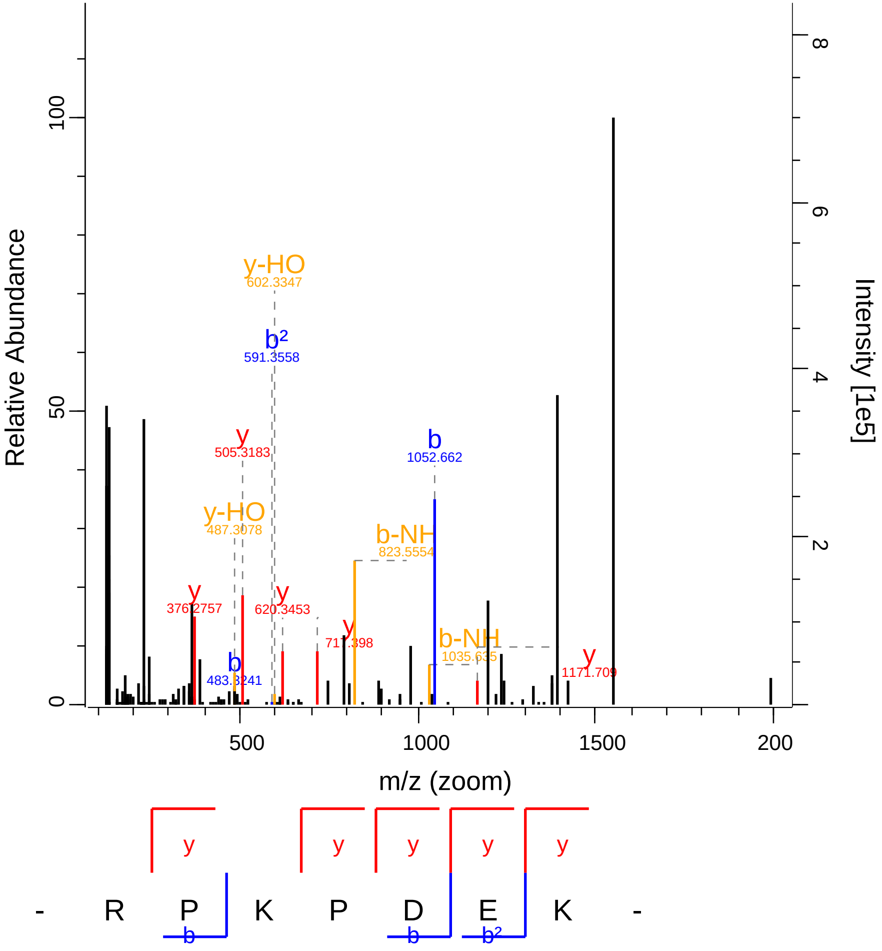

Raw File	Scan	Method	Score	m/z	Gene names
RO-QC-160121_ZackD_SS_Set_01_Fraction_	5494	FTMS; HCI	91.07	397	ADAM28

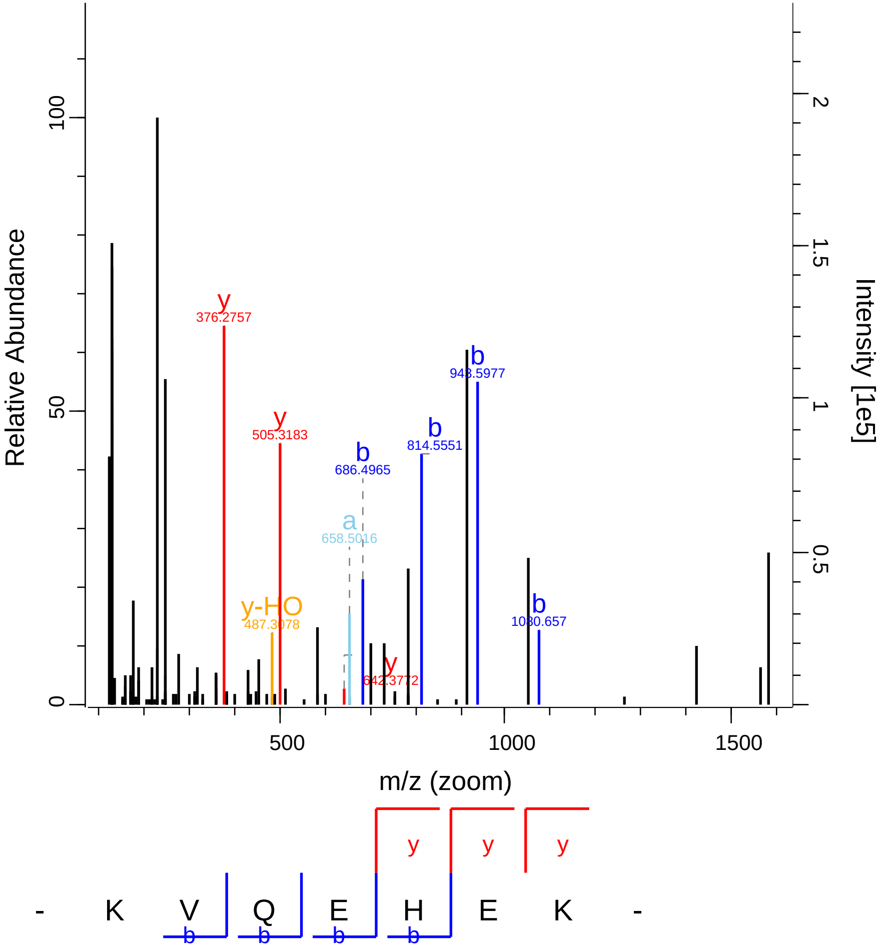

Raw File	Scan	Method	Score	m/z	Gene names
RO-QC-160121_ZackD_SS_Set_01_Fraction	5544	FTMS; HC	66.57	683.83	LMBR1

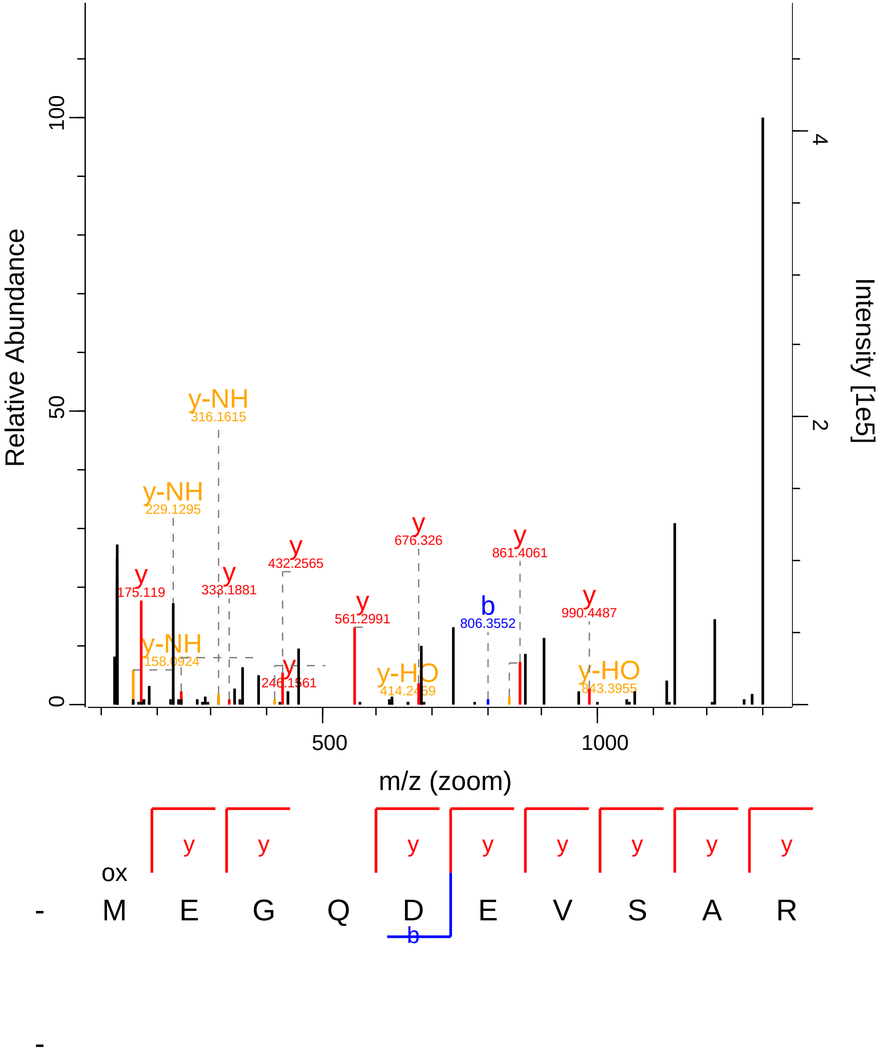

Raw File	Scan	Method	Score	m/z	Gene names
RO-QC-160121_ZackD_SS_Set_01_Fraction	5925	FTMS; HC	117.86	787.88	PCSK1N

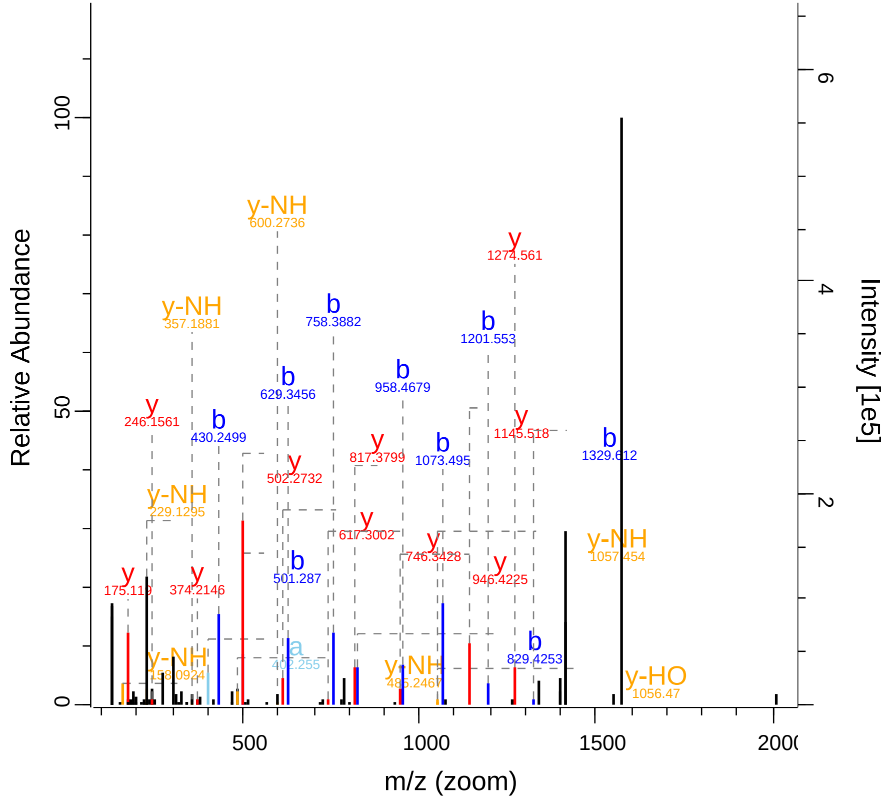

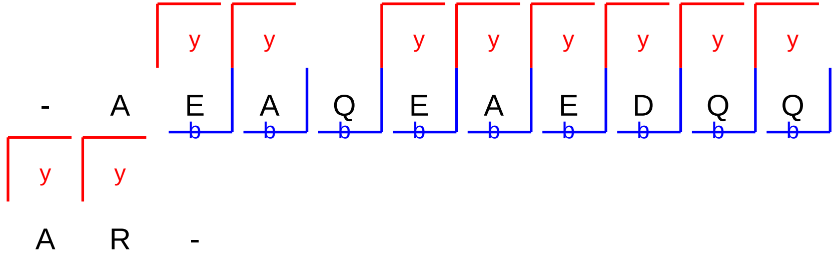

Raw File	Scan	Method	Score	m/z	Gene names
RO-QC-160121_ZackD_SS_Set_01_Fraction	5945	FTMS; HC	122.33	525.59	PCSK1N

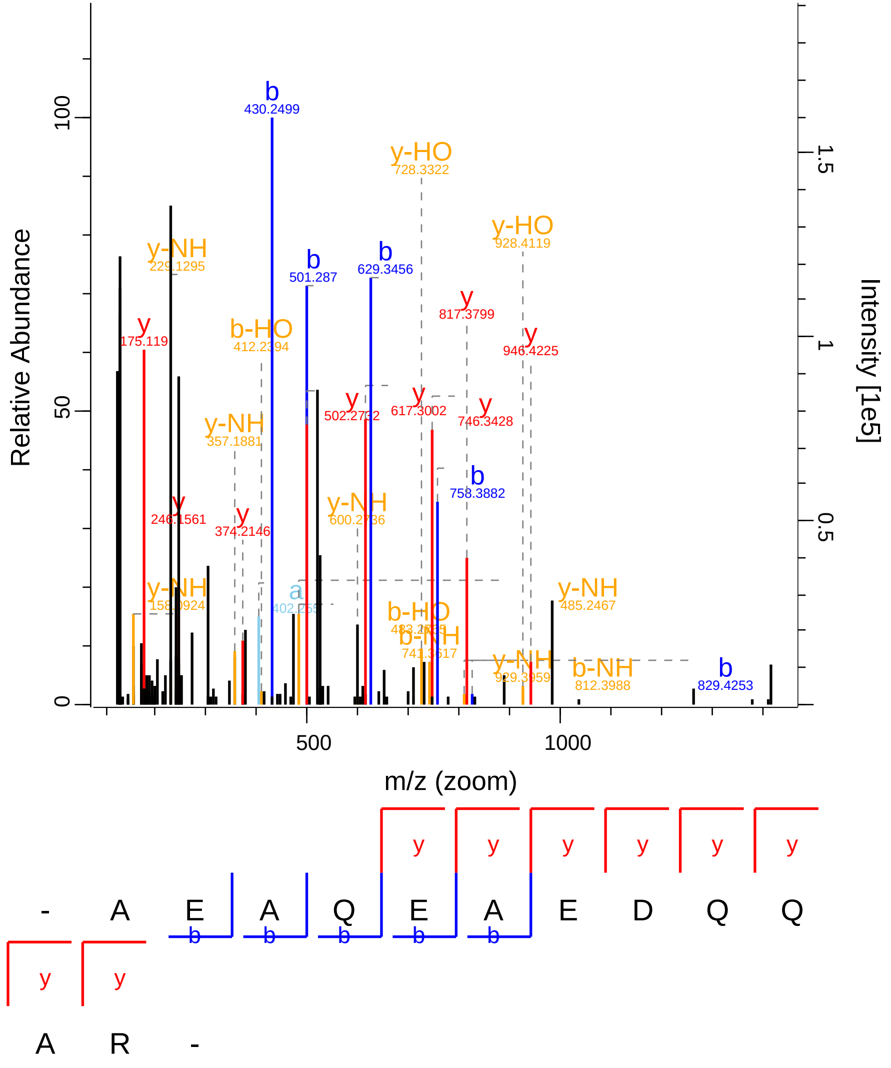

Raw File	Scan	Method	Score	m/z	Gene names
RO-QC-160121_ZackD_SS_Set_01_Fraction	6087	FTMS; HC	82.29	771.35	ABCA7

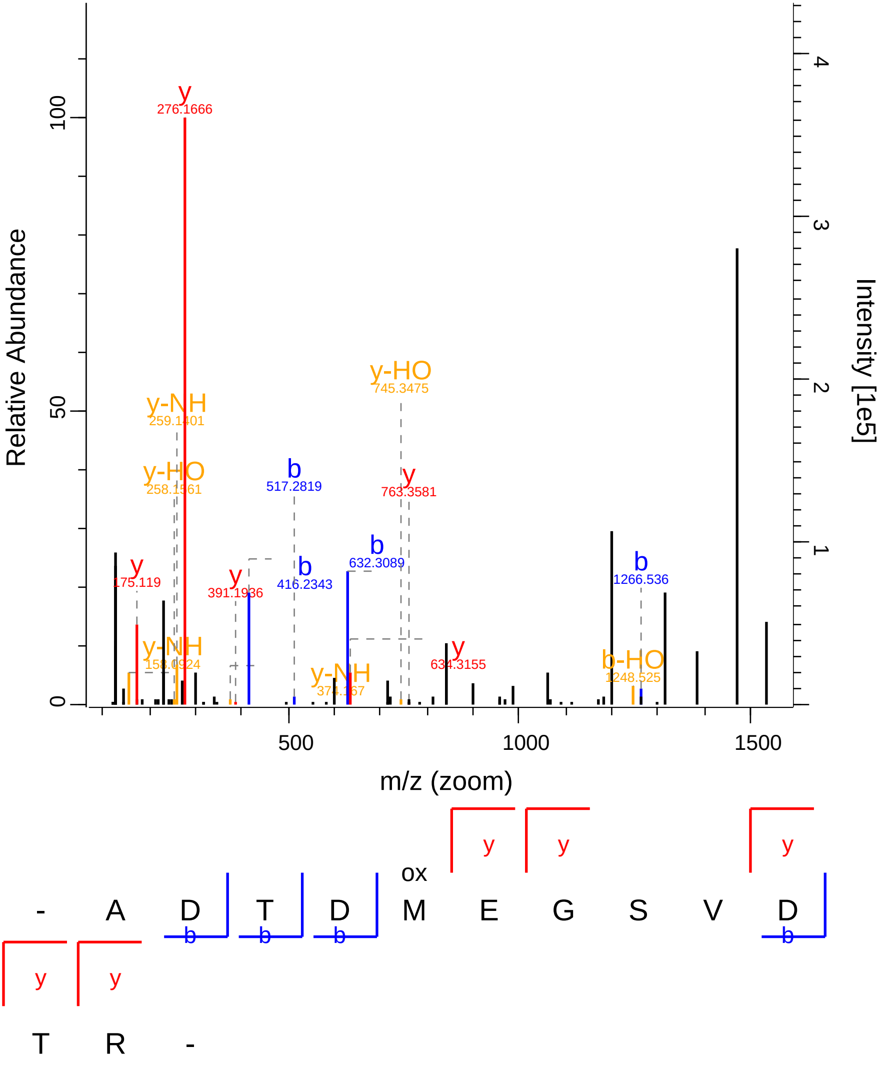

Raw File	Scan	Method	Score	m/z	Gene names
RO-QC-160121_ZackD_SS_Set_01_Fraction	6195	FTMS; HC	60.91	972.99	CDCA7

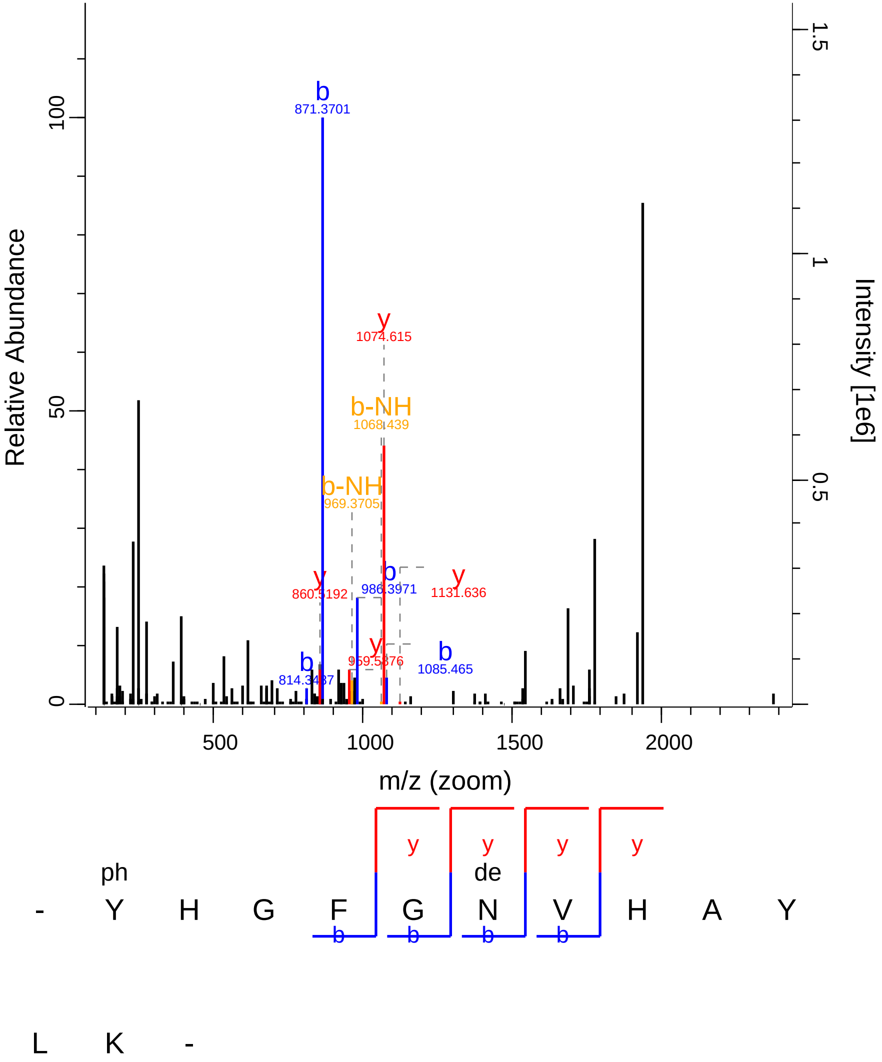

Raw File	Scan	Method	Score	m/z	Gene names
RO-QC-160121_ZackD_SS_Set_01_Fraction	6449	FTMS; HC	49.85	431.51	PIGF

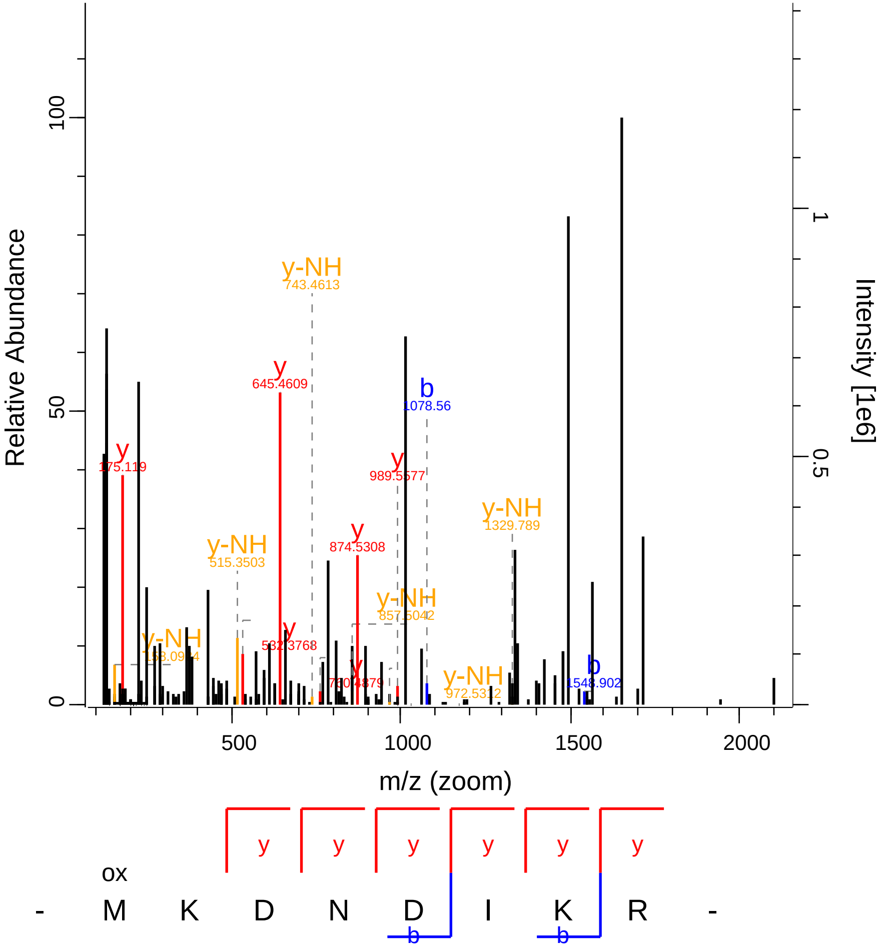

Raw File	Scan	Method	Score	m/z	Gene names
RO-QC-160121_ZackD_SS_Set_01_Fraction	6877	FTMS; HC	126.07	679.89	LENG9

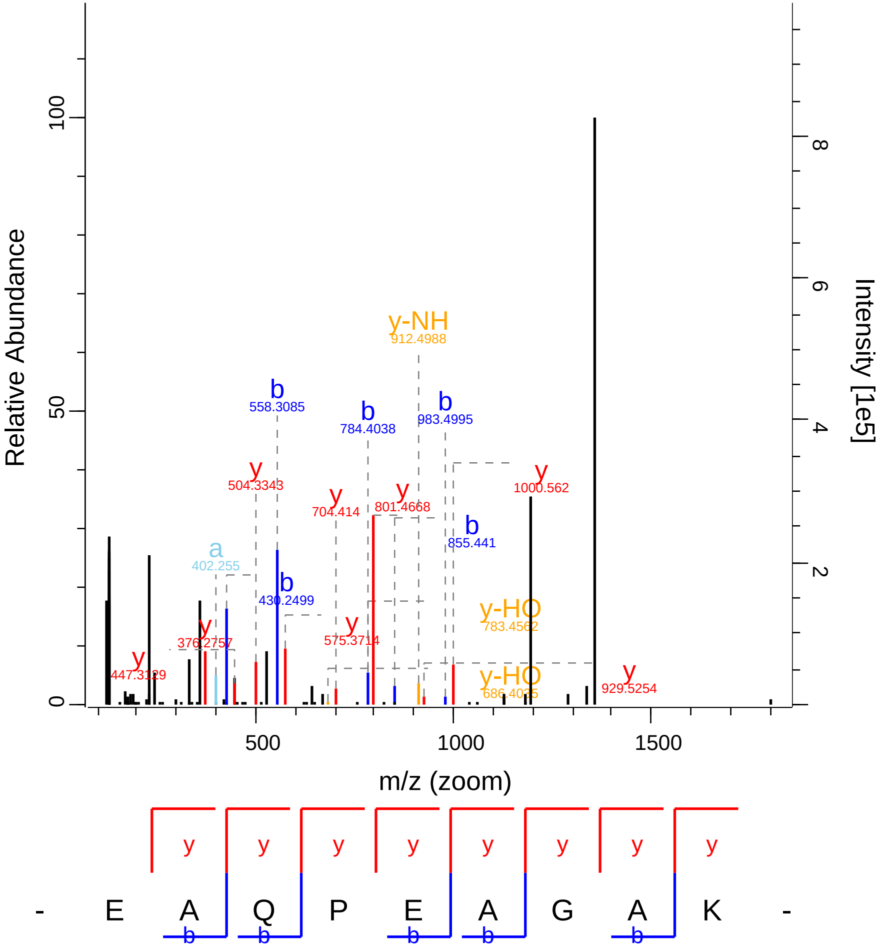

Raw File	Scan	Method	Score	m/z	Gene names
RO-QC-160121_ZackD_SS_Set_01_Fraction	7582	FTMS; HC	215.5	780.89	GKAP1

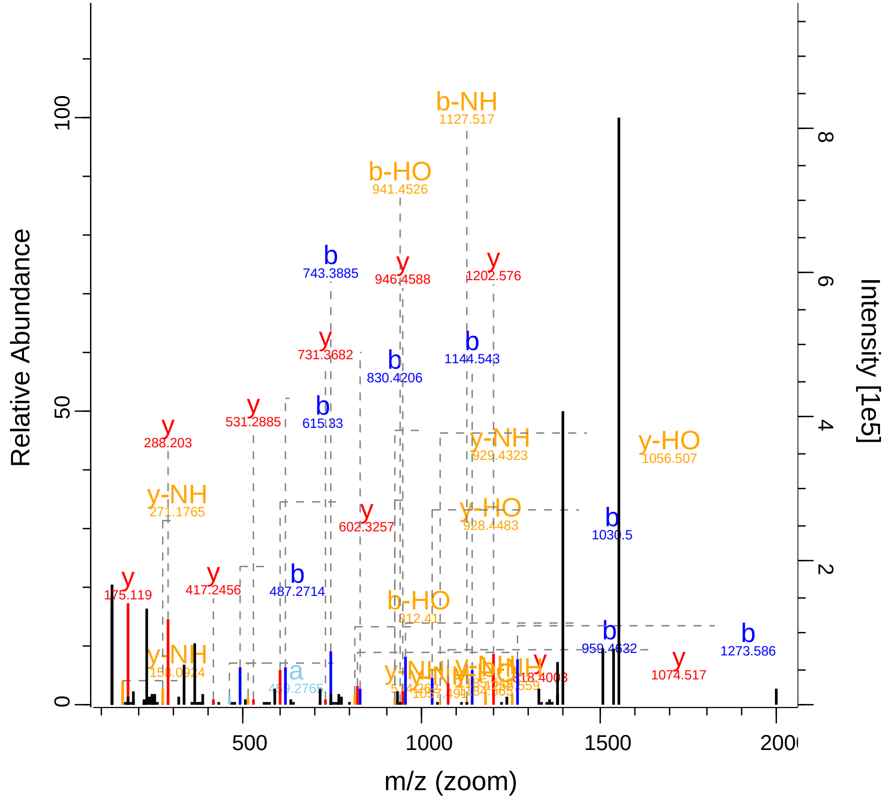

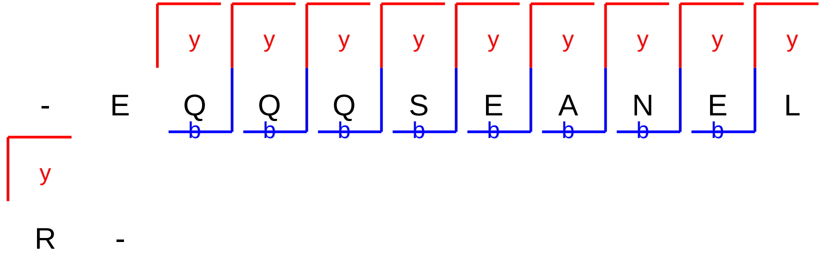

Raw File	Scan	Method	Score	m/z	Gene names
RO-QC-160121_ZackD_SS_Set_01_Fraction	7742	FTMS; HC	117.72	593.32	C17orf100

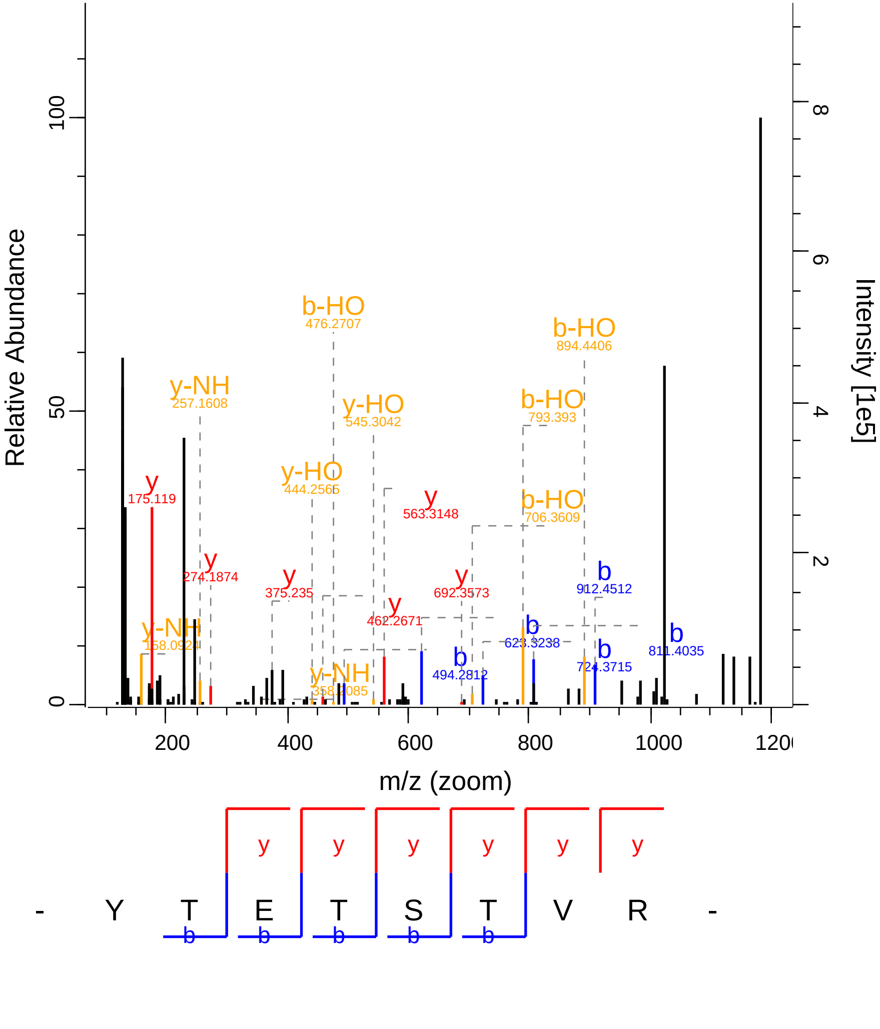

Raw File	Scan	Method	Score	m/z	Gene names
RO-QC-160121_ZackD_SS_Set_01_Fraction	7964	FTMS; HC	58.68	433.75	KCNA10

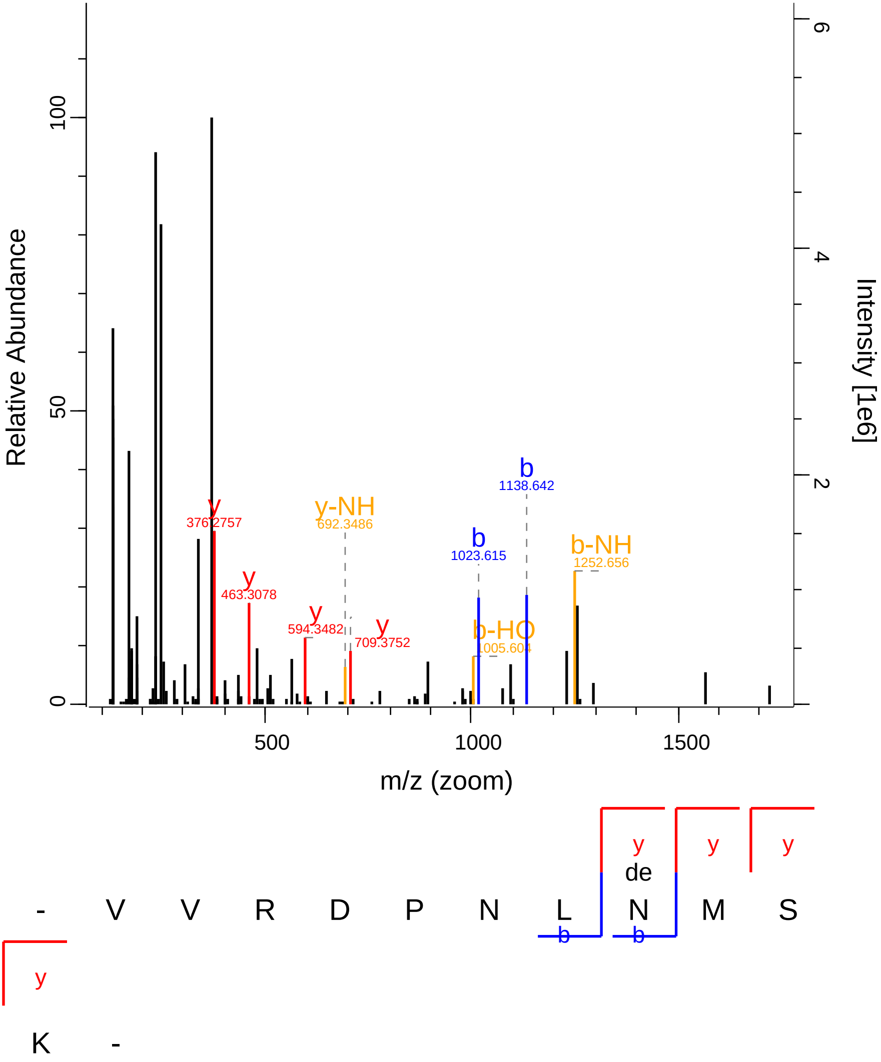

Raw File	Scan	Method	Score	m/z	Gene names
RO-QC-160121_ZackD_SS_Set_01_Fraction	8283	FTMS; HC	45.88	433.75	KCNA10

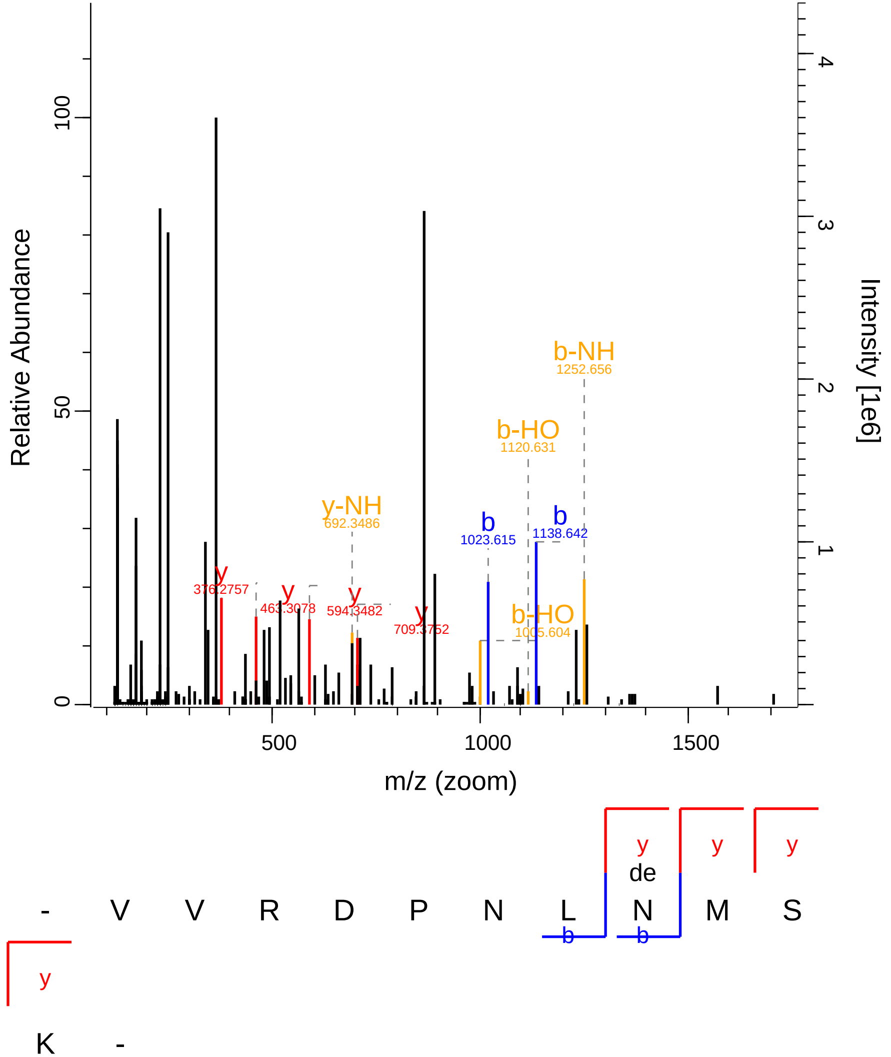

Raw File	Scan	Method	Score	m/z	Gene names
RO-QC-160121_ZackD_SS_Set_01_Fraction_	8287	FTMS; HC	51.69	518.79	RNF187

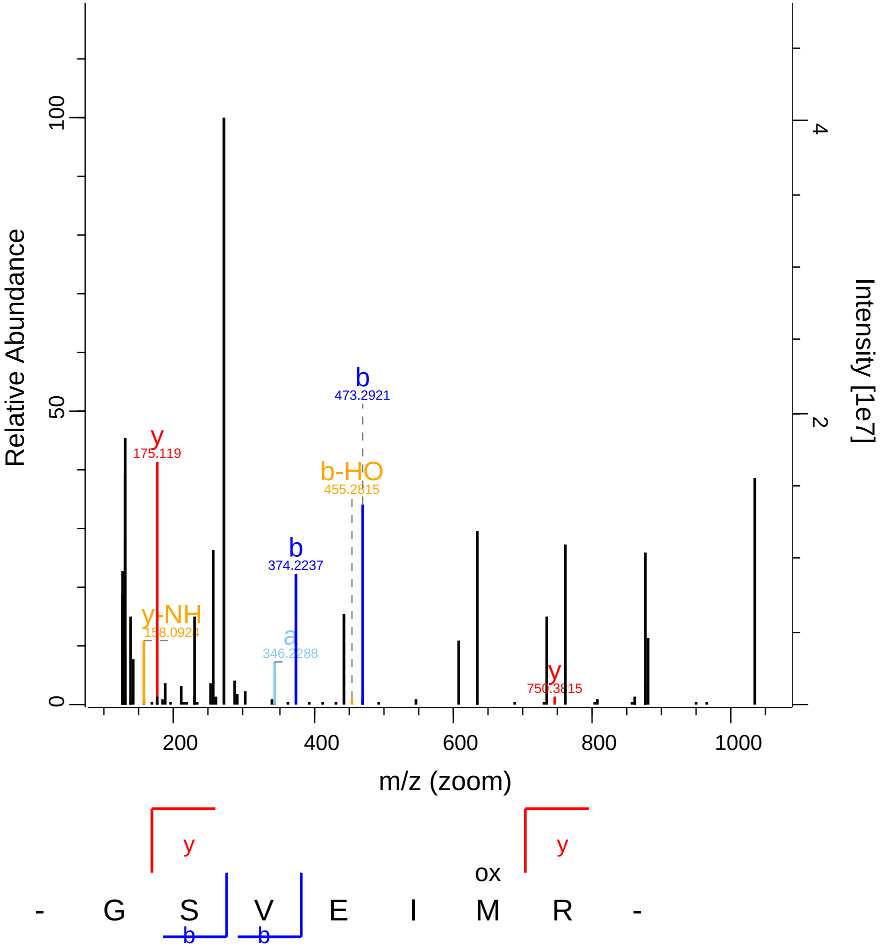

Raw File	Scan	Method	Score	m/z	Gene names
RO-QC-160121_ZackD_SS_Set_01_Fraction	8405	FTMS; HC	149.94	631.84	MBTPS2

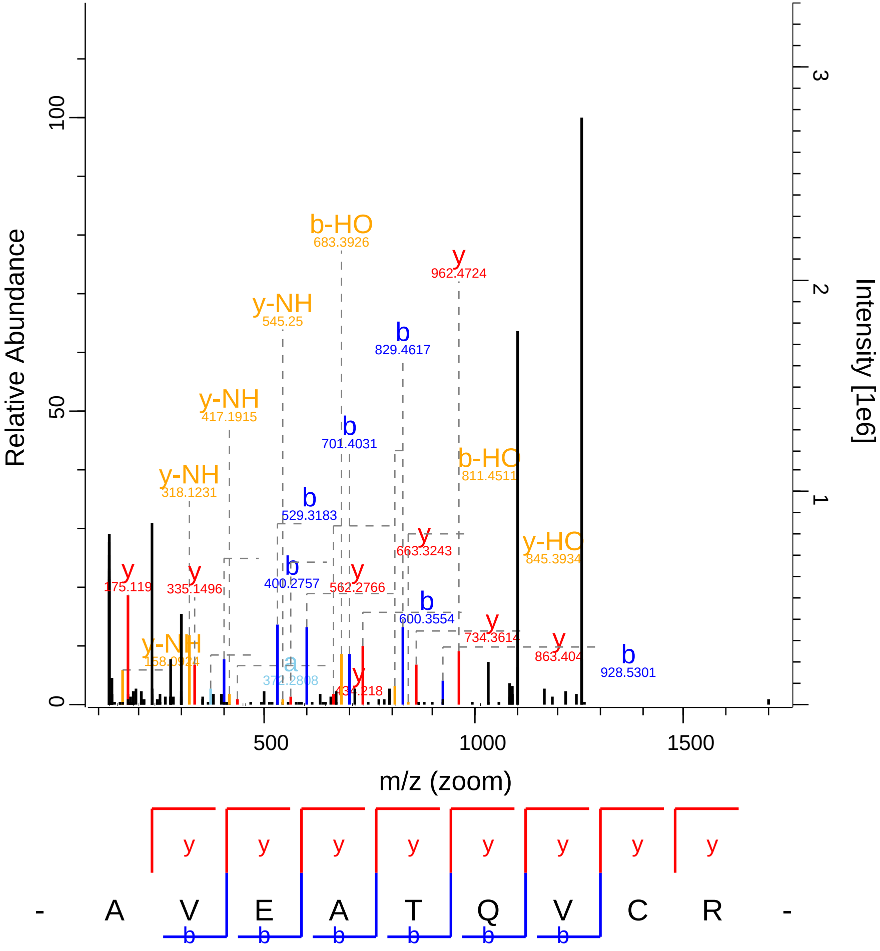

Raw File	Scan	Method	Score	m/z	Gene names
RO-QC-160121_ZackD_SS_Set_01_Fraction	8799	FTMS; HC	42.79	464.66	C6orf10

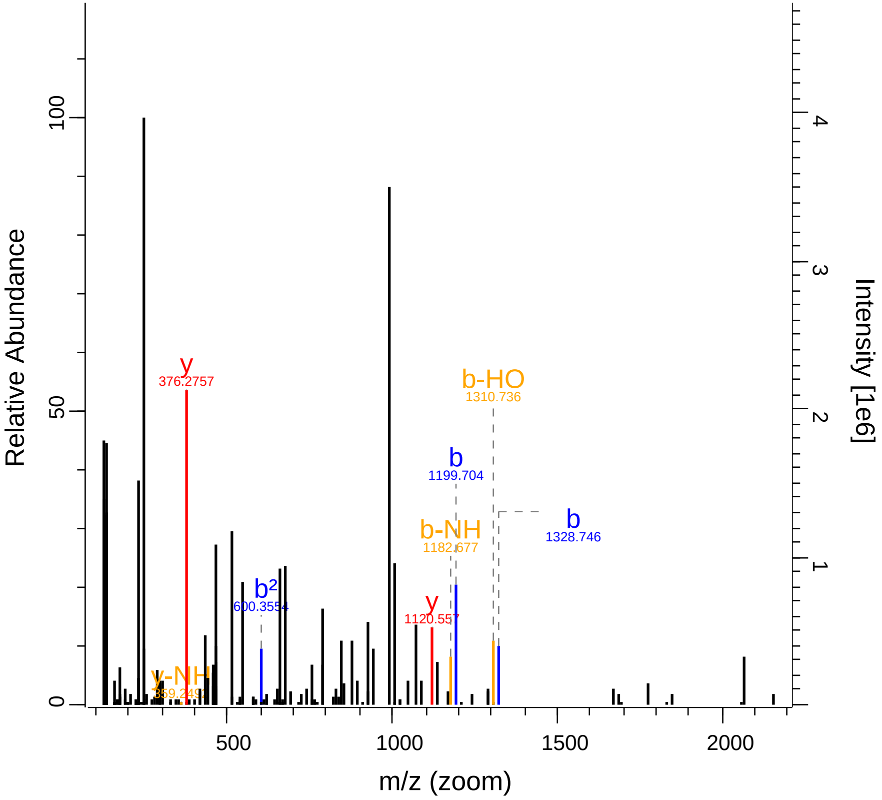

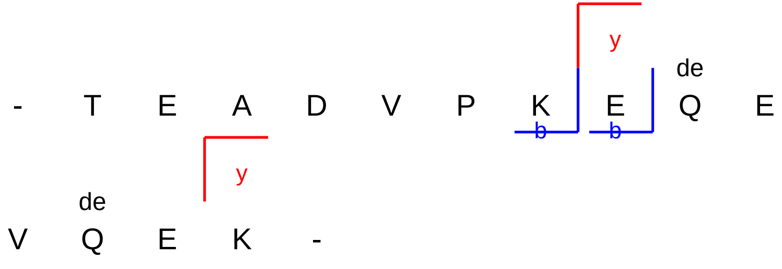

Raw File	Scan	Method	Score	m/z	Gene names
RO-QC-160121_ZackD_SS_Set_01_Fraction	9093	FTMS; HC	125.83	630.34	LIPT2

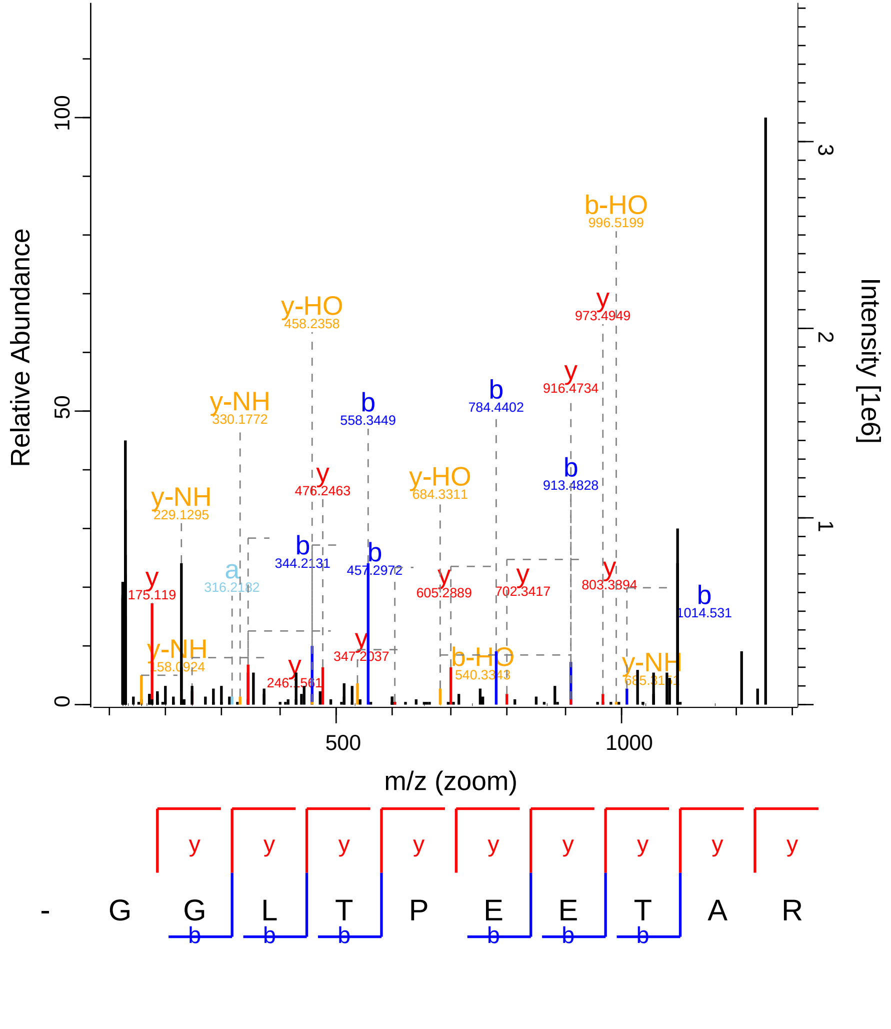

Raw File	Scan	Method	Score	m/z
RO-QC-160121_ZackD_SS_Set_01_Fraction_12	10371	FTMS; HCD	46.35	562.33

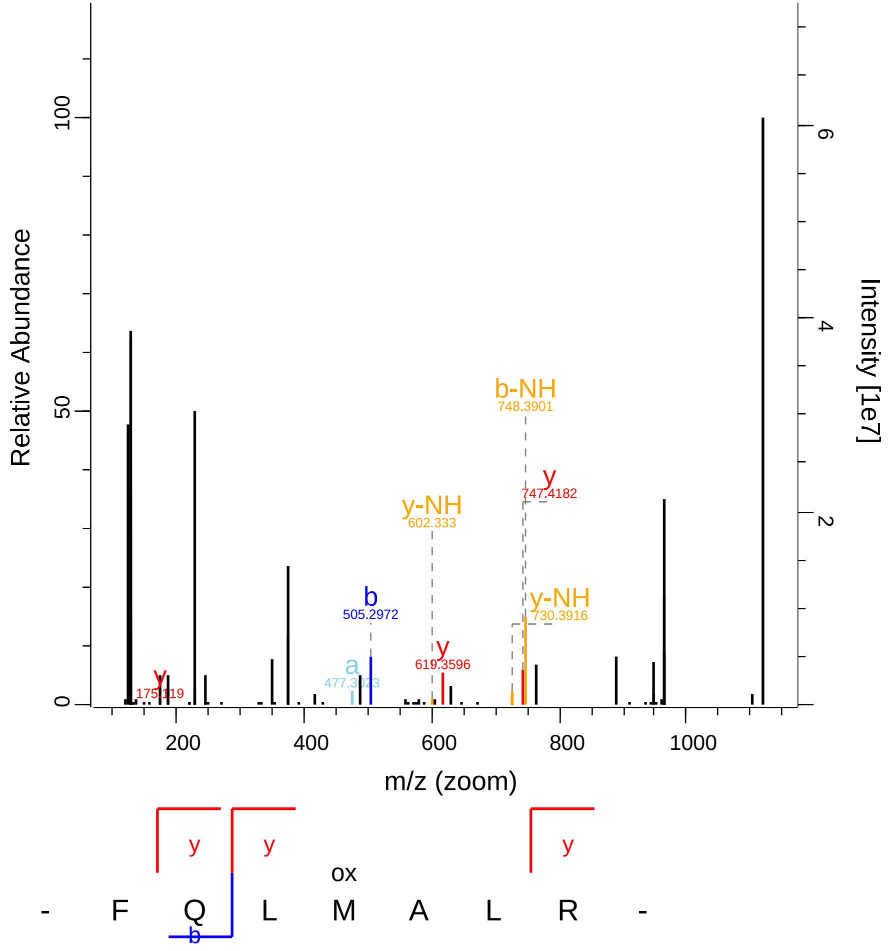

Raw File	Scan	Method	Score	m/z	Gene names
RO-QC-160121_ZackD_SS_Set_01_Fraction	10796	FTMS; HC	158.38	733.9	PIGB

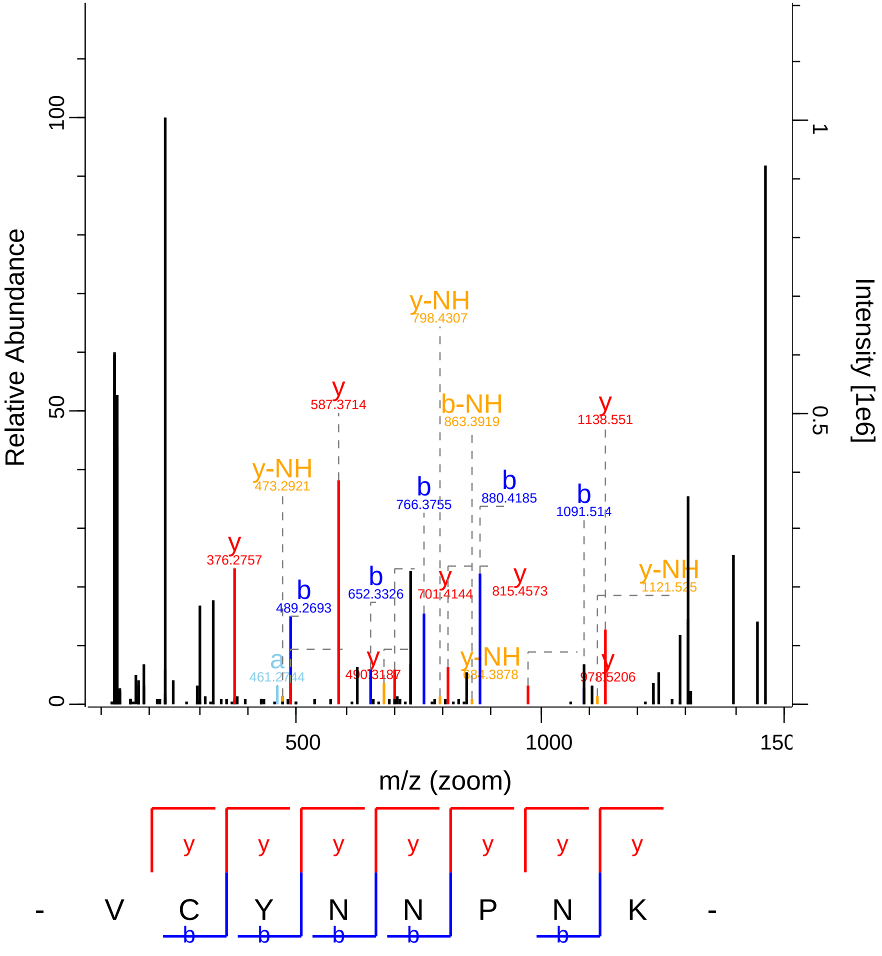

Raw File	Scan	Method	Score	m/z	Gene names
RO-QC-160121_ZackD_SS_Set_01_Fractior	11699	FTMS; HC	112.17	637.42	NOVA2

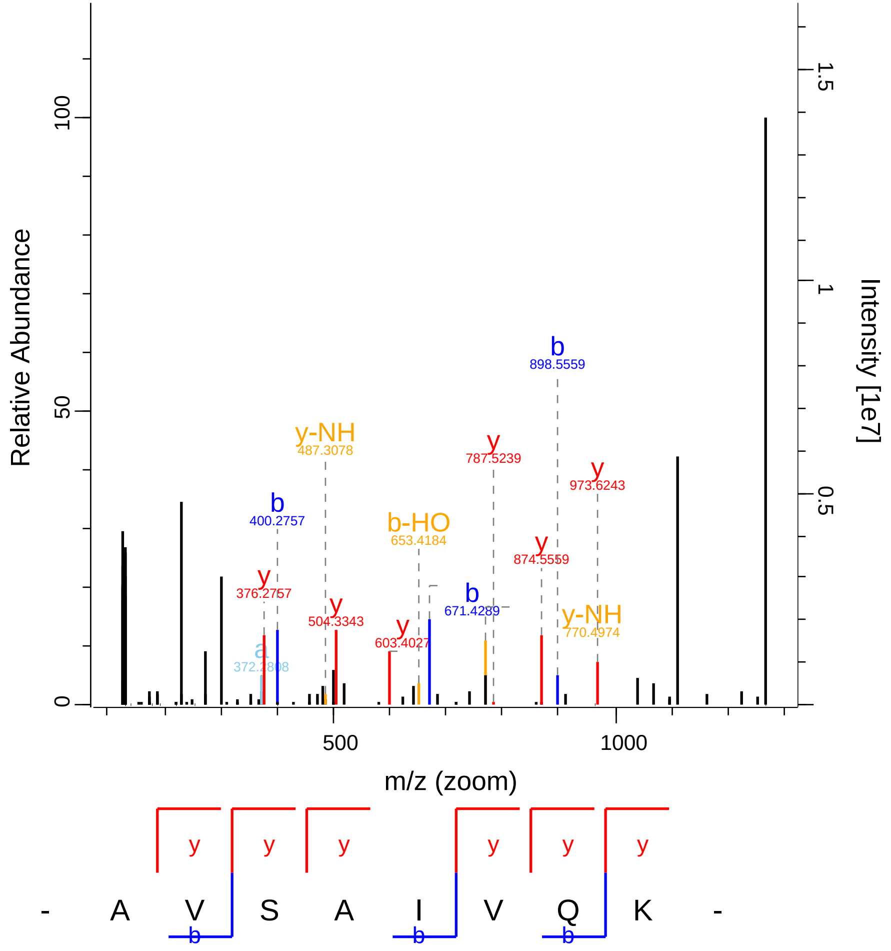

Raw File	Scan	Method	Score	m/z	Gene names
RO-QC-160121_ZackD_SS_Set_01_Fractior	11758	FTMS; HC	58.92	425.28	NOVA2

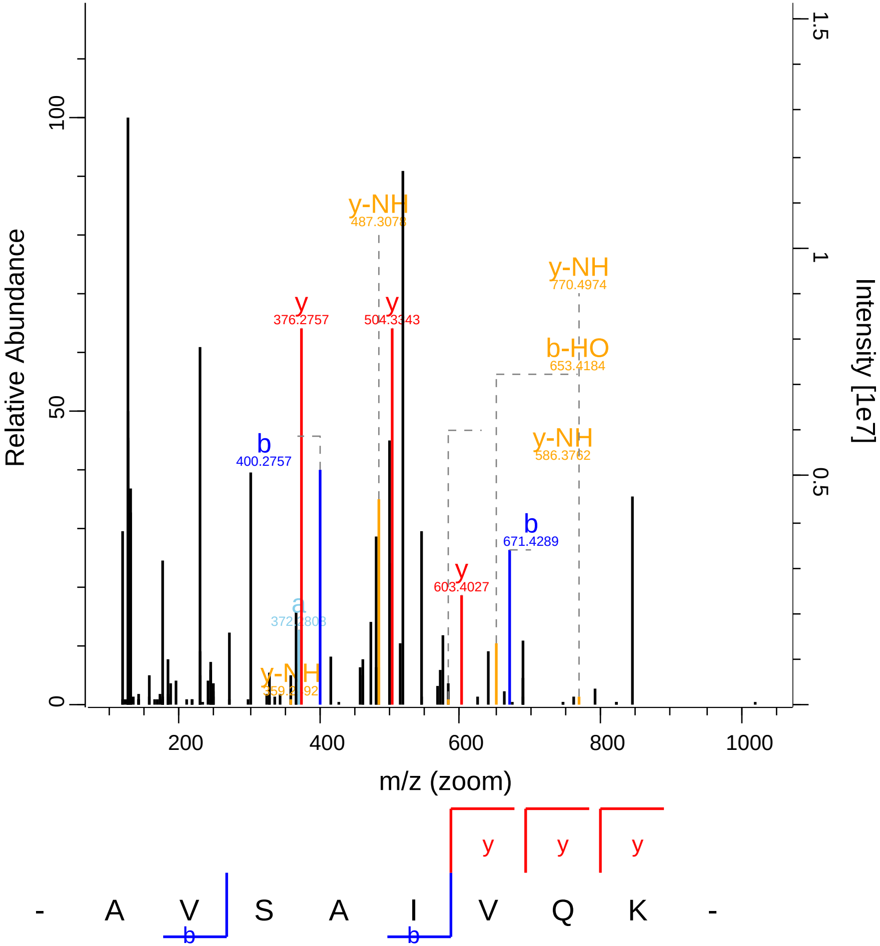

Raw File	Scan	Method	Score	m/z
RO-QC-160121_ZackD_SS_Set_01_Fraction_12	12595	FTMS; HCD	50.9	653.39

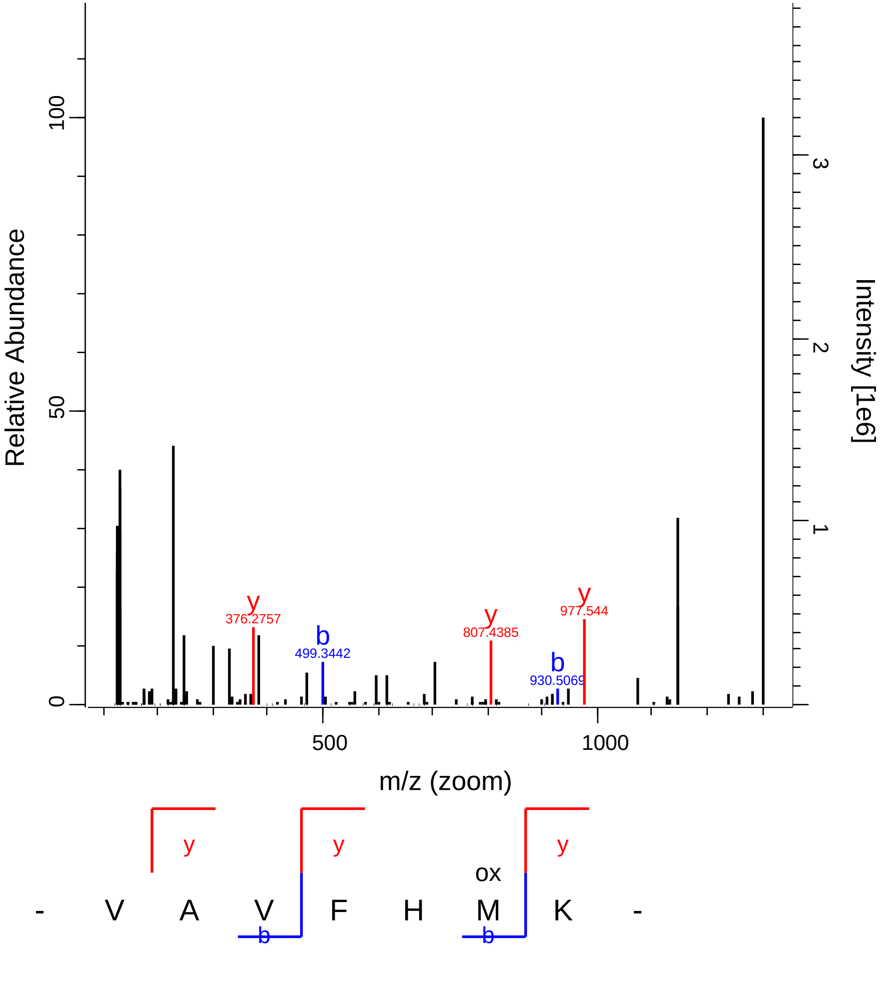

Raw File	Scan	Method	Score	m/z	Gene names
RO-QC-160121_ZackD_SS_Set_01_Fraction	12763	FTMS; HC	44.79	803.9	SCG3

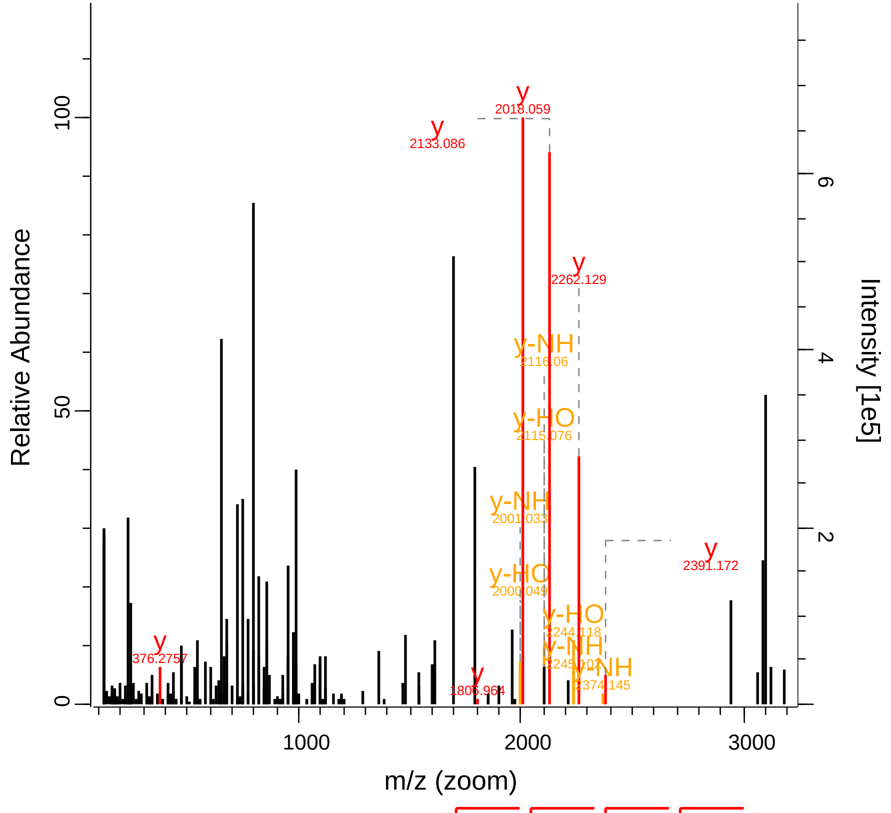

y y y y

de

- E A N N Y E E D P N

y

de de

K P T S W T E N Q A G

y

K -

Raw File	Scan	Method	Score	m/z
RO-QC-160121_ZackD_SS_Set_01_Fraction_12	14120	FTMS; HCD	57.1	423.28

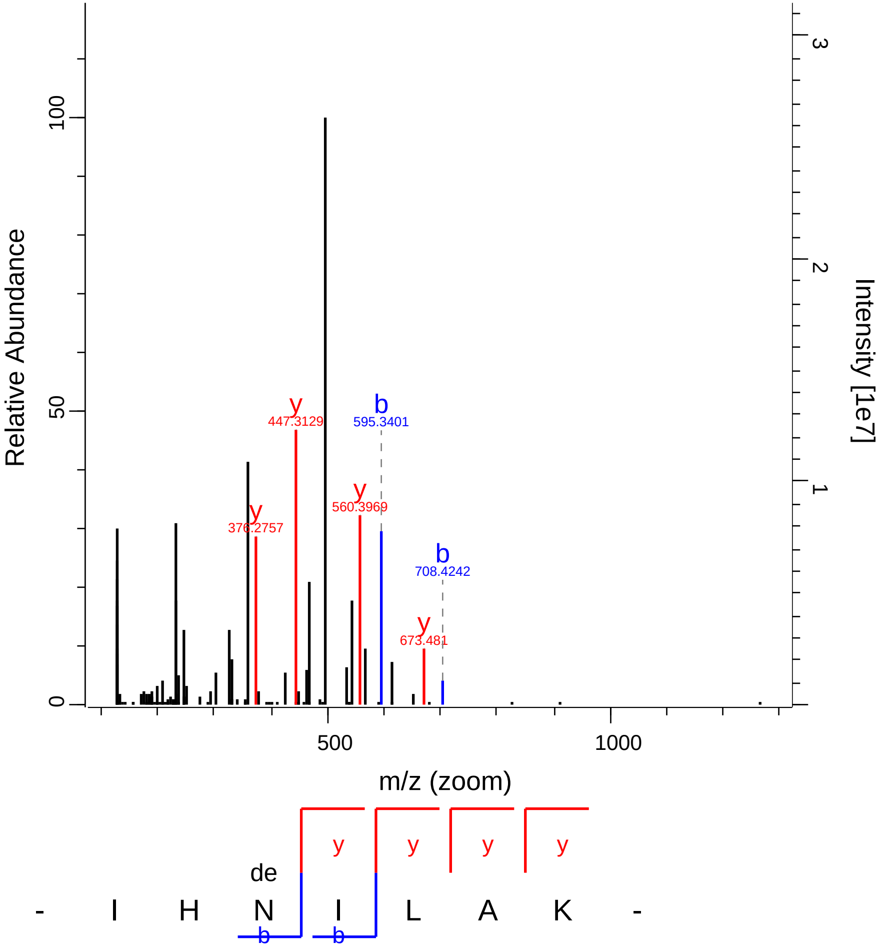

Raw File	Scan	Method	Score	m/z
RO-QC-160121_ZackD_SS_Set_01_Fraction_12	14347	FTMS; HCD	57.1	423.28

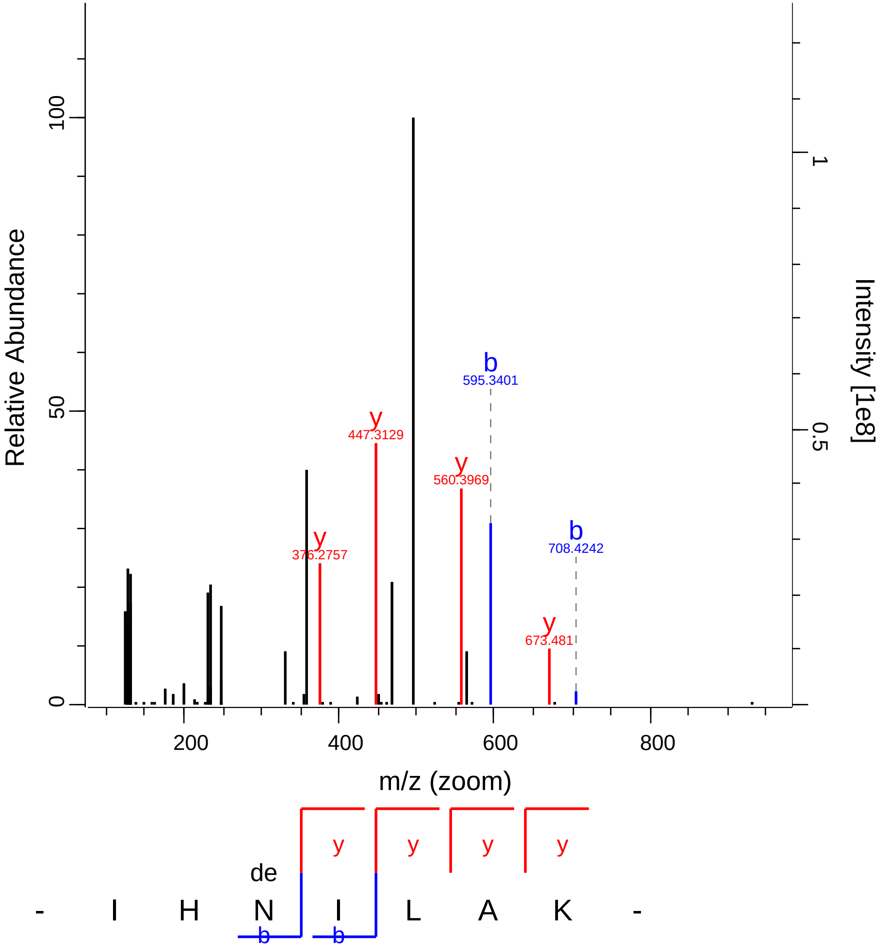

Raw File	Scan	Method	Score	m/z	Gene names
RO-QC-160121_ZackD_SS_Set_01_Fractior	15131	FTMS; HC	151.96	697.35	CCSMST1

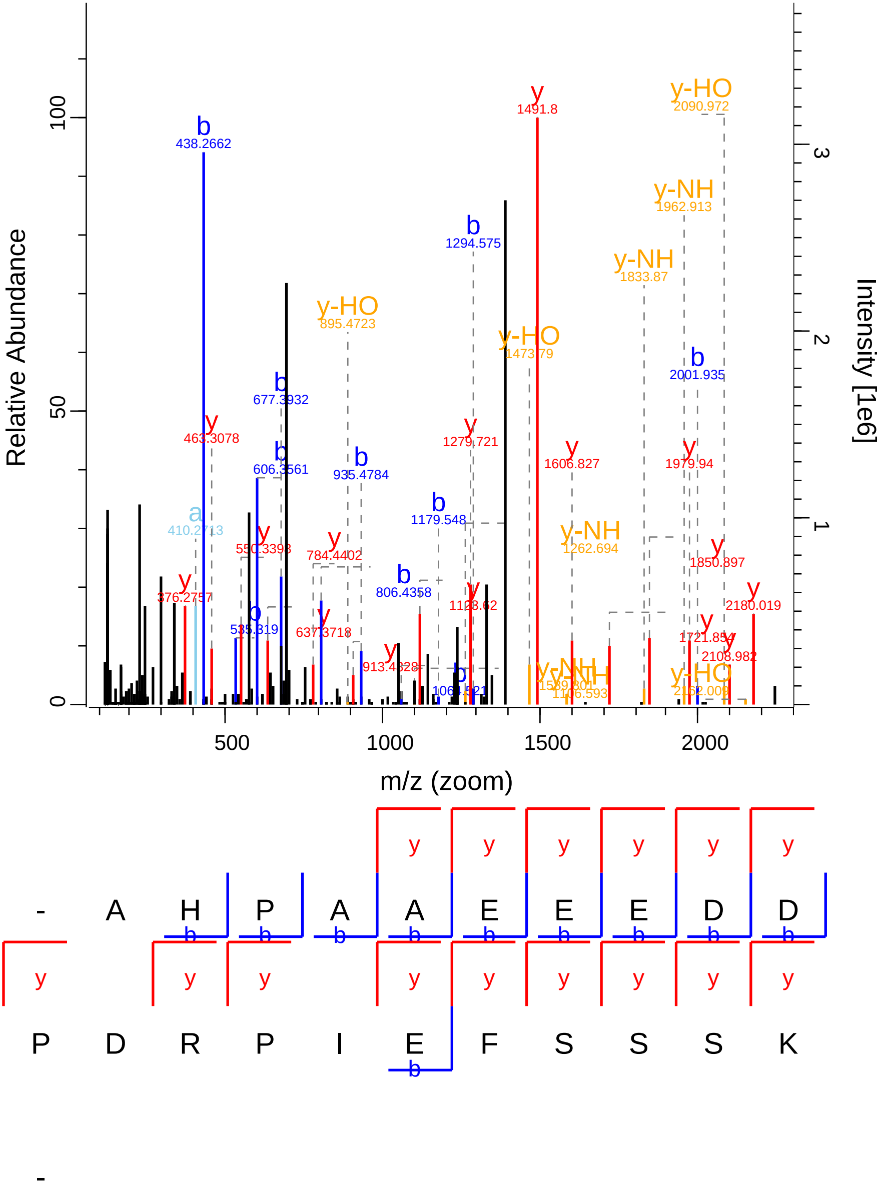

Raw File	Scan	Method	Score	m/z	Gene names
RO-QC-160121_ZackD_SS_Set_01_Fractior	15393	FTMS; HC	176.95	819.98	PLK2

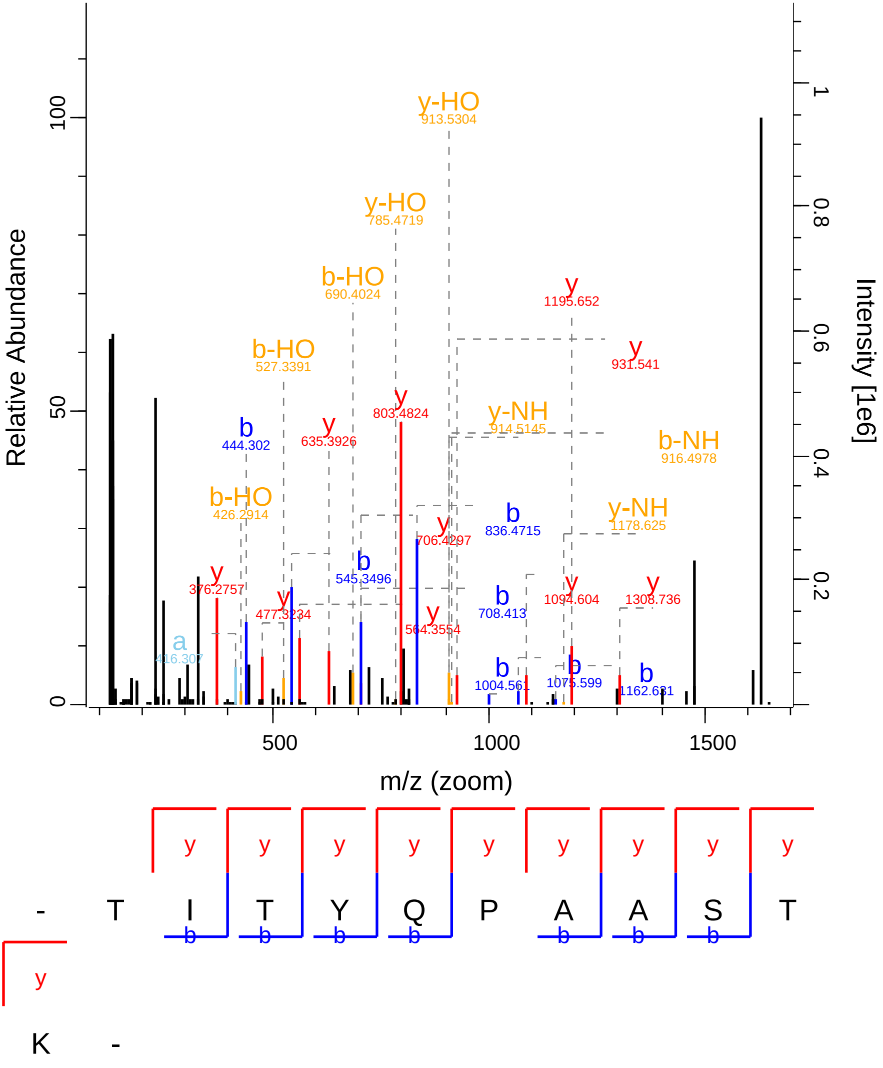

Raw File	Scan	Method	Score	m/z	Gene names
RO-QC-160121_ZackD_SS_Set_01_Fractior	15852	FTMS; HC	94.11	394.89	SSC5D

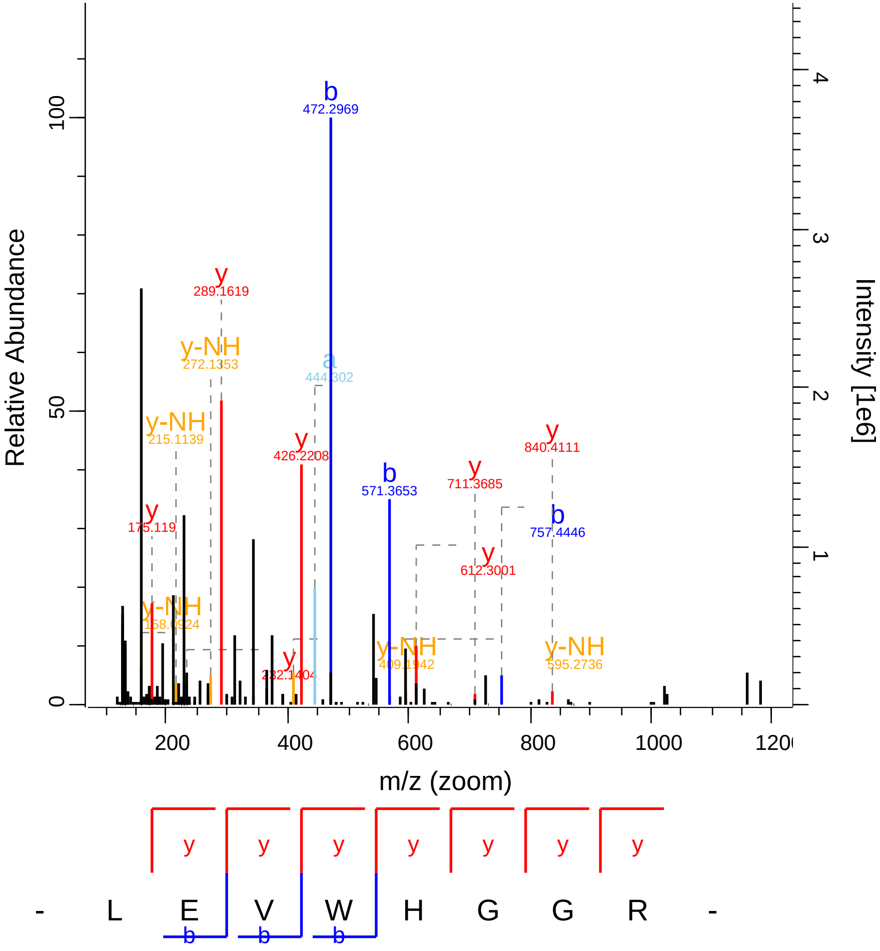

Raw File	Scan	Method	Score	m/z	Gene names
RO-QC-160121_ZackD_SS_Set_01_Fractior	16637	FTMS; HC	93.26	841.44	AIF1L

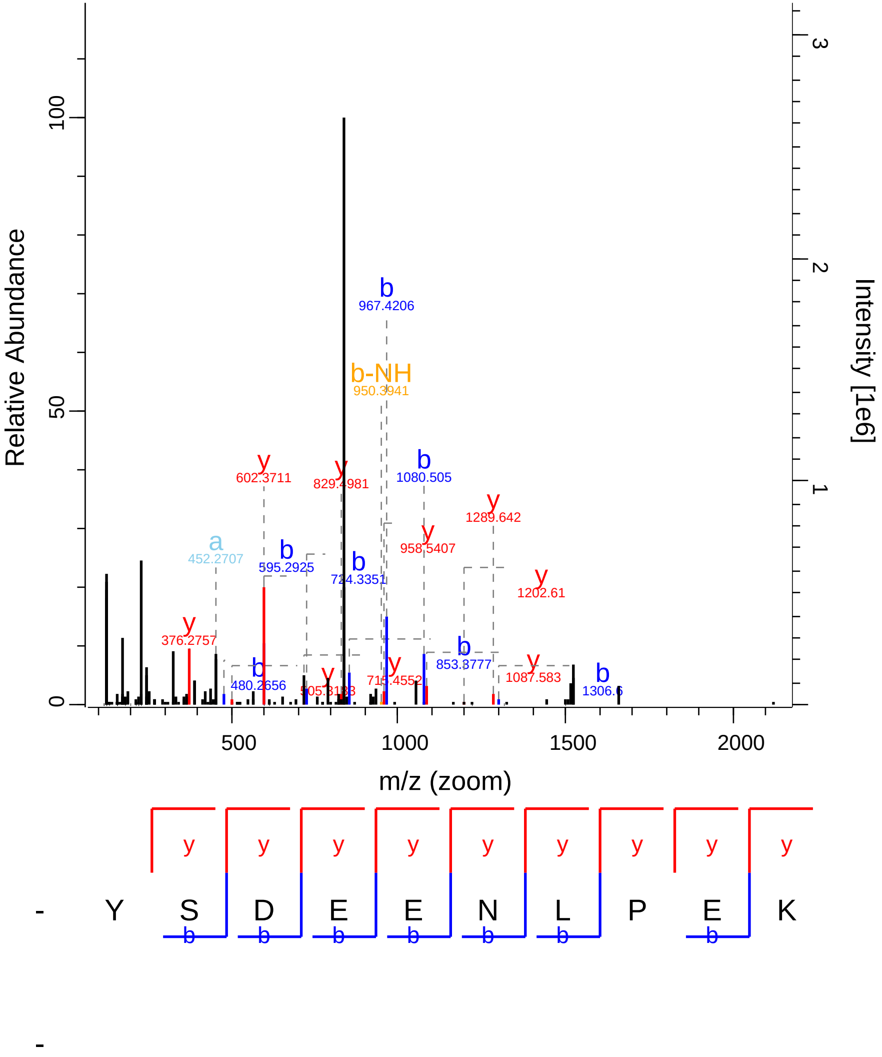

Raw File	Scan	Method	Score	m/z	Gene names
RO-QC-160121_ZackD_SS_Set_01_Fractio	16840	FTMS; HC	107.66	781.49	LRRIQ1

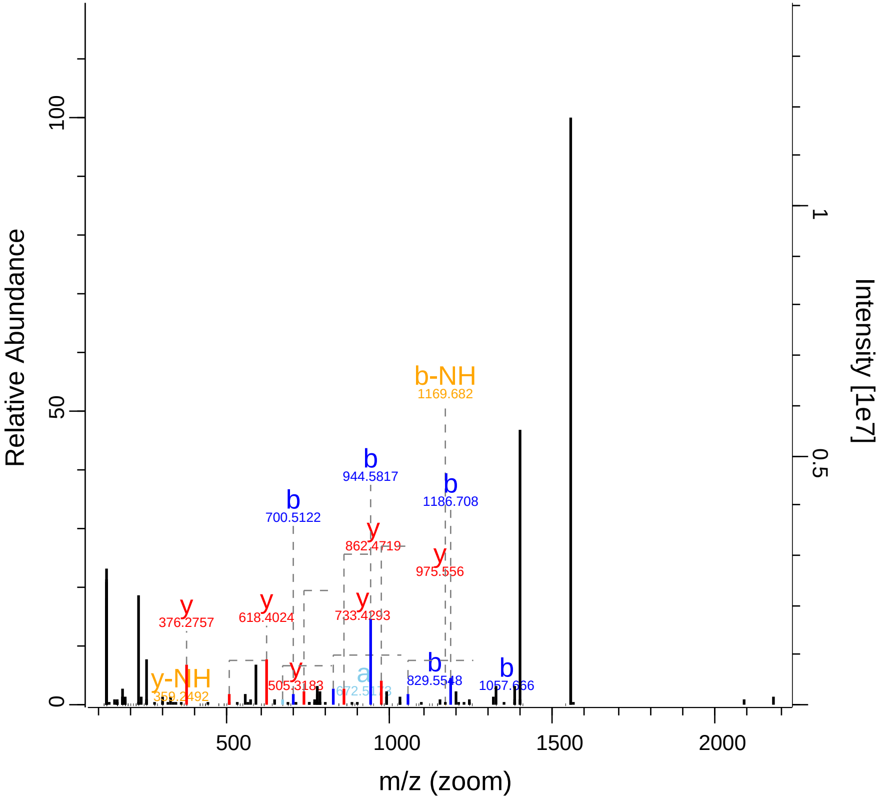

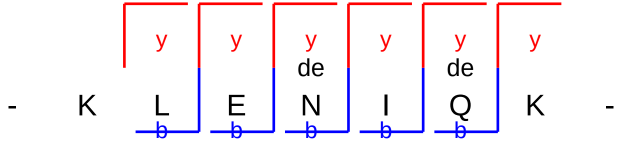

Raw File	Scan	Method	Score	m/z	Gene names
RO-QC-160121_ZackD_SS_Set_01_Fractioi	17625	FTMS; HC	314.29	897.46	SMIM1

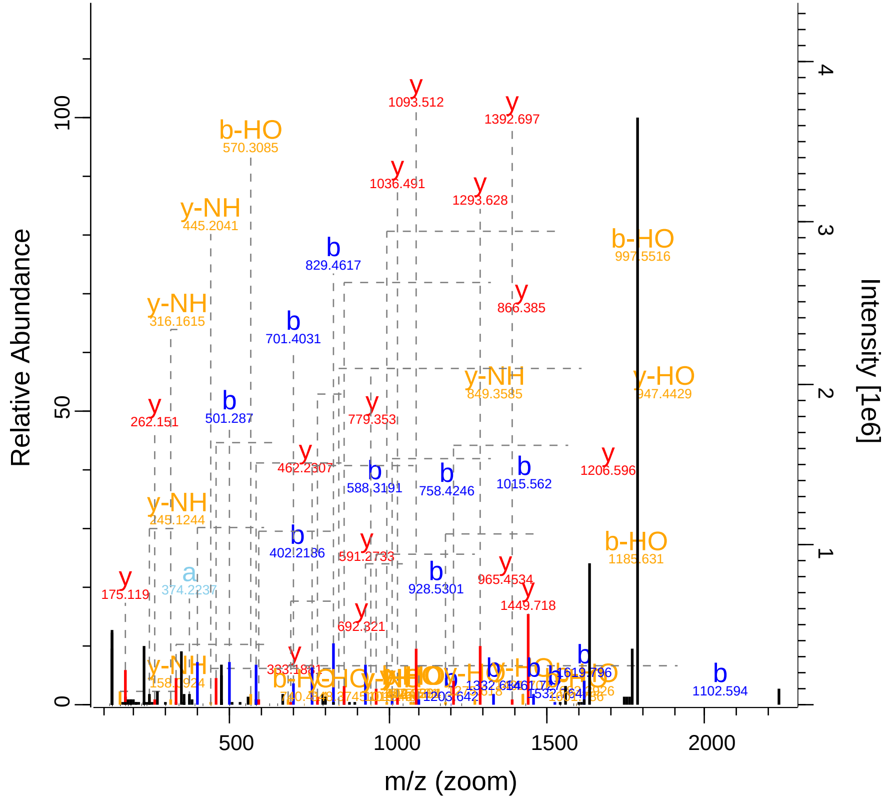

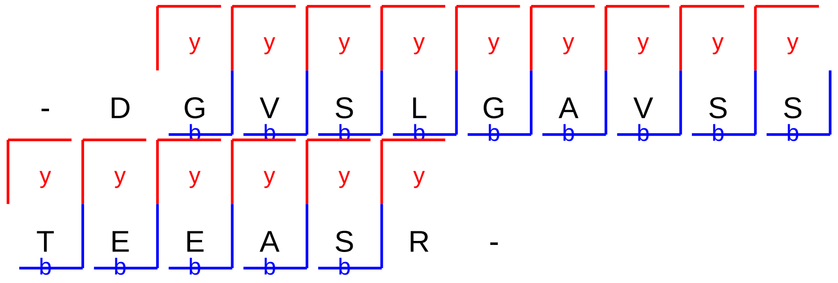

Raw File	Scan	Method	Score	m/z	Gene names
RO-QC-160121_ZackD_SS_Set_01_Fractio[illegible]	17823	FTMS; HC	114.93	732.91	POMP

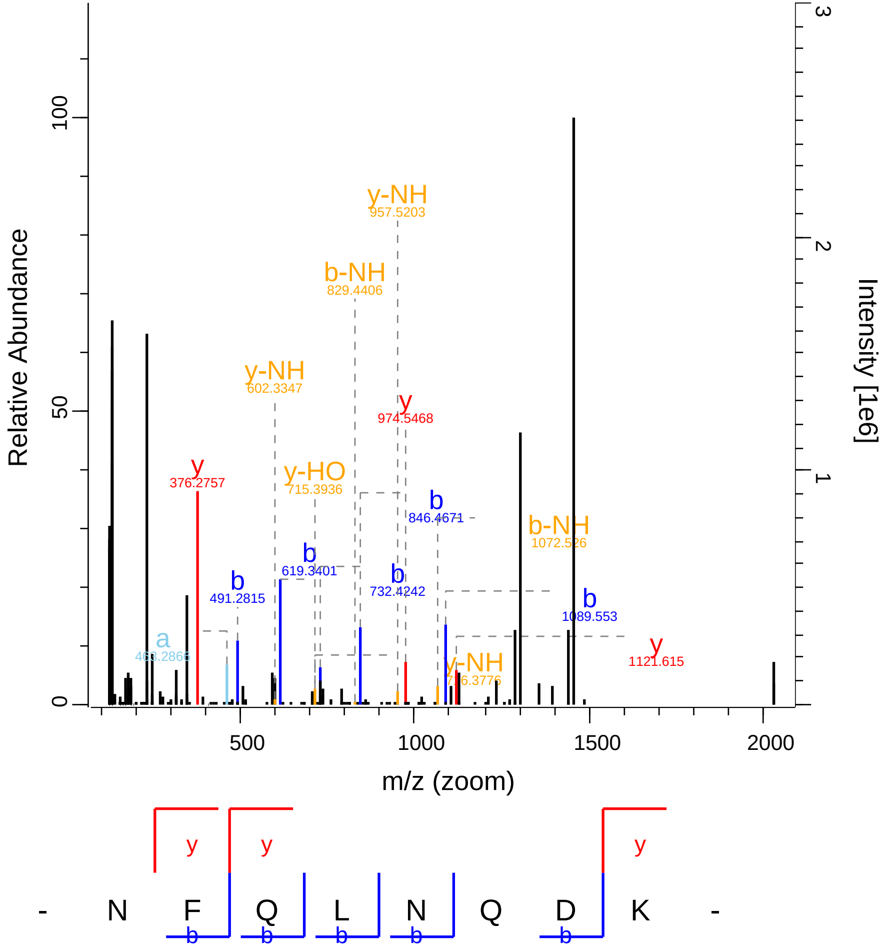

Raw File	Scan	Method	Score	m/z	Gene names
RO-QC-160121_ZackD_SS_Set_01_Fractior	18149	FTMS; HC	96.01	980.44	MT-ND3

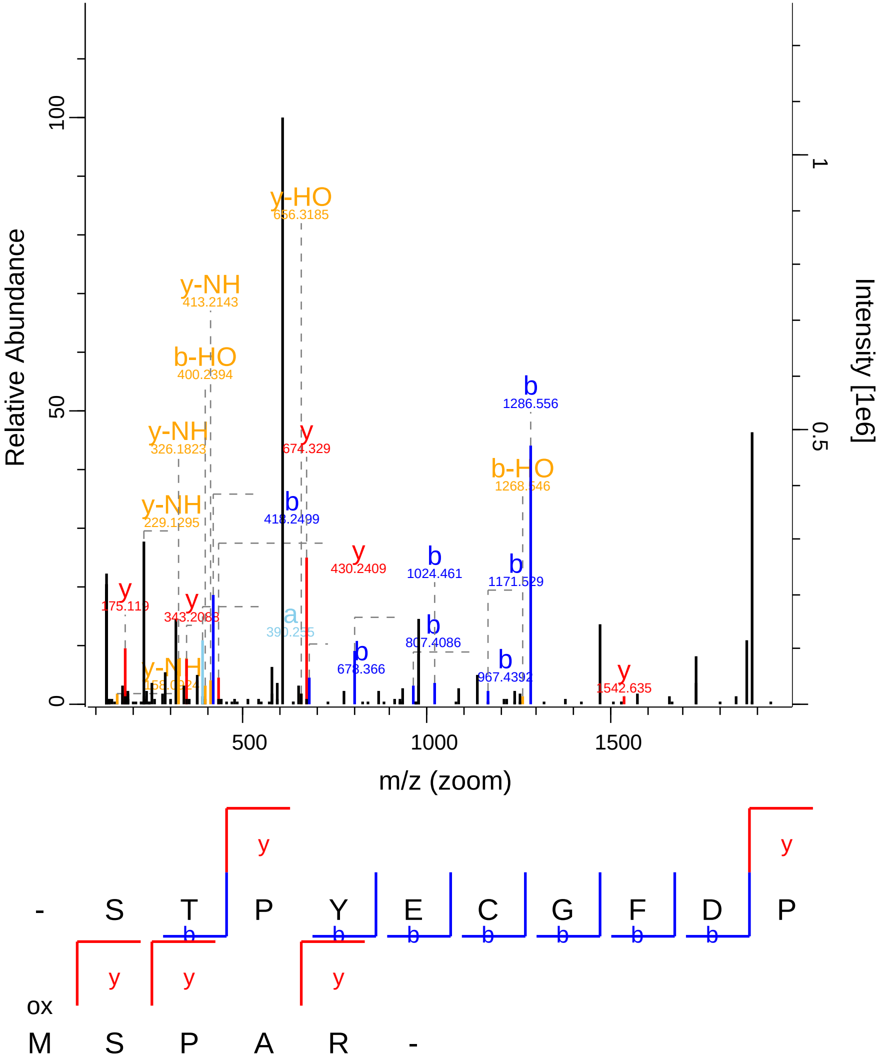

Raw File	Scan	Method	Score	m/z
RO-QC-160121_ZackD_SS_Set_01_Fraction_12	19137	FTMS; HCD	116.35	589.36

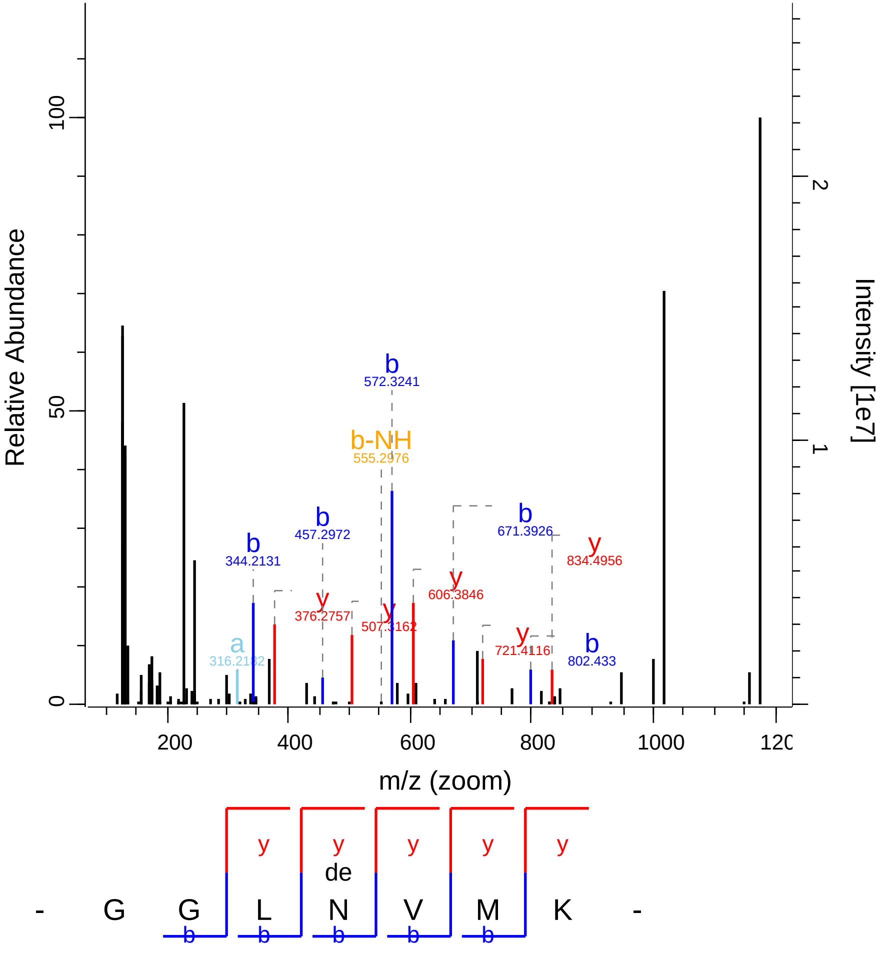

Raw File	Scan	Method	Score	m/z	Gene names
RO-QC-160121_ZackD_SS_Set_01_Fractior	20102	FTMS; HC	50.12	964.47	C3orf49

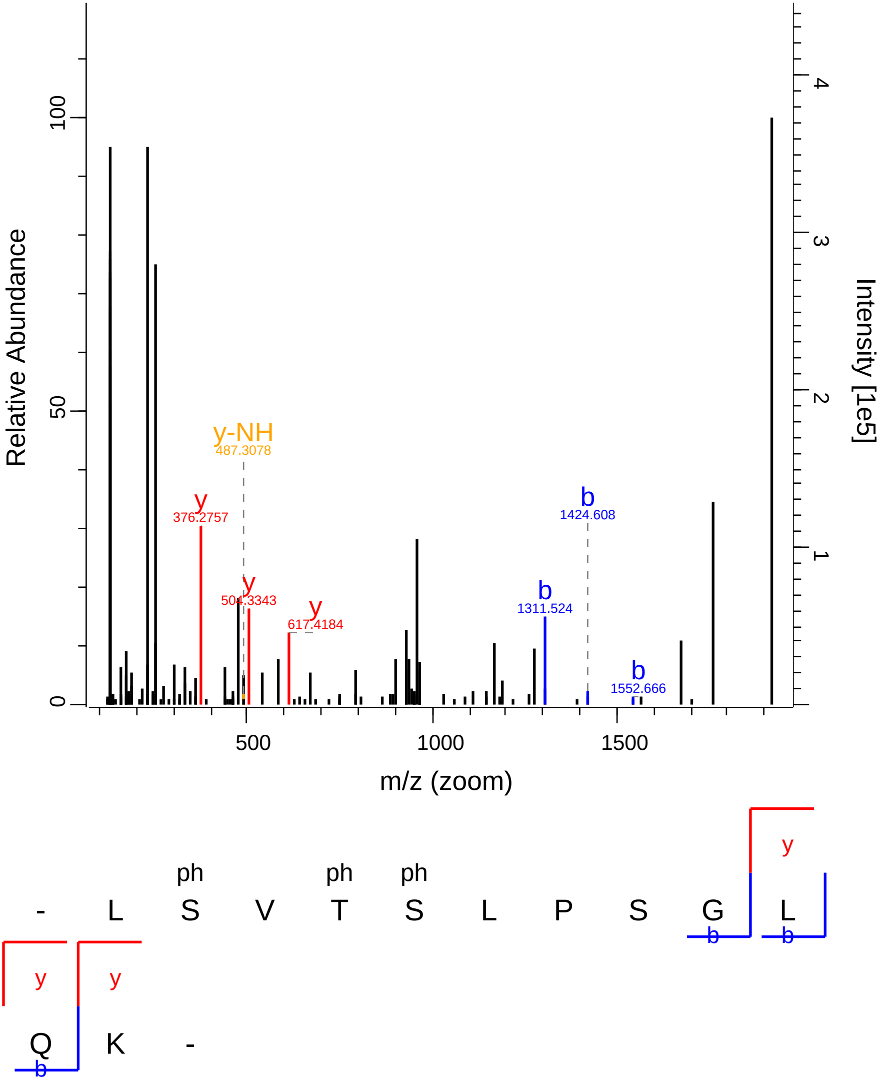

Raw File	Scan	Method	Score	m/z
RO-QC-160121_ZackD_SS_Set_01_Fraction_12	21036	FTMS; HCD	137	724.92

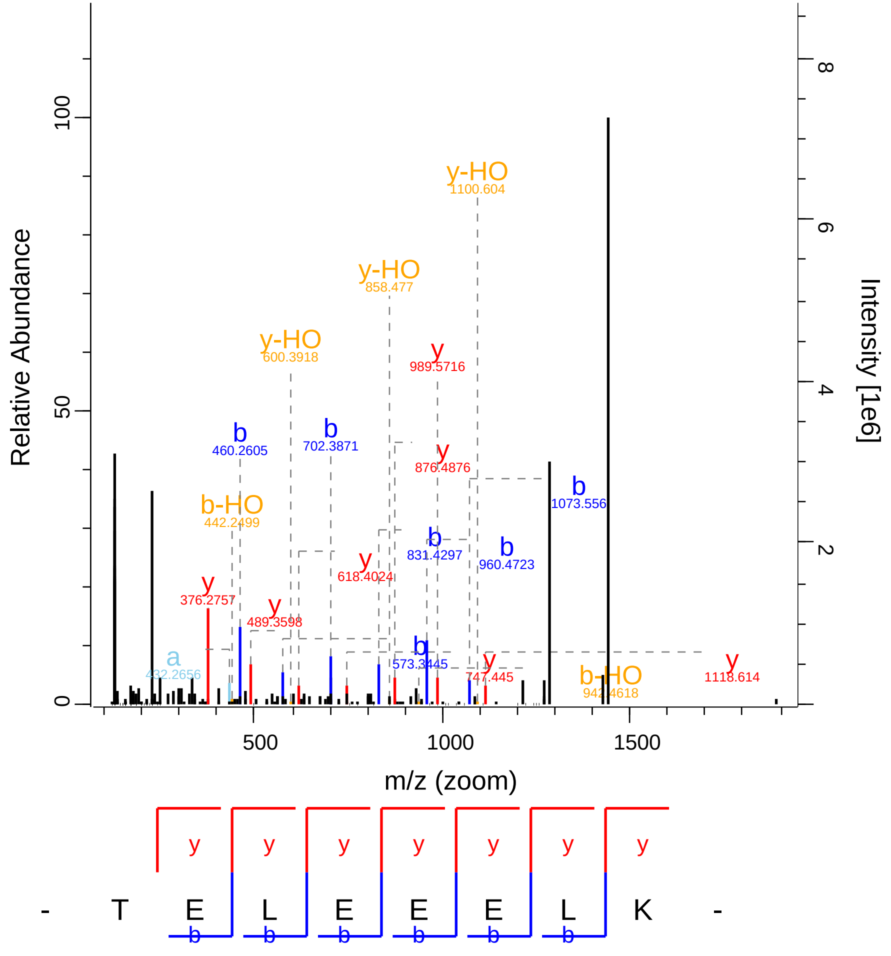

Raw File	Scan	Method	Score	m/z	Gene names
RO-QC-160121_ZackD_SS_Set_01_Fractior	21185	FTMS; HC	109.29	497.31	BLOC1S6

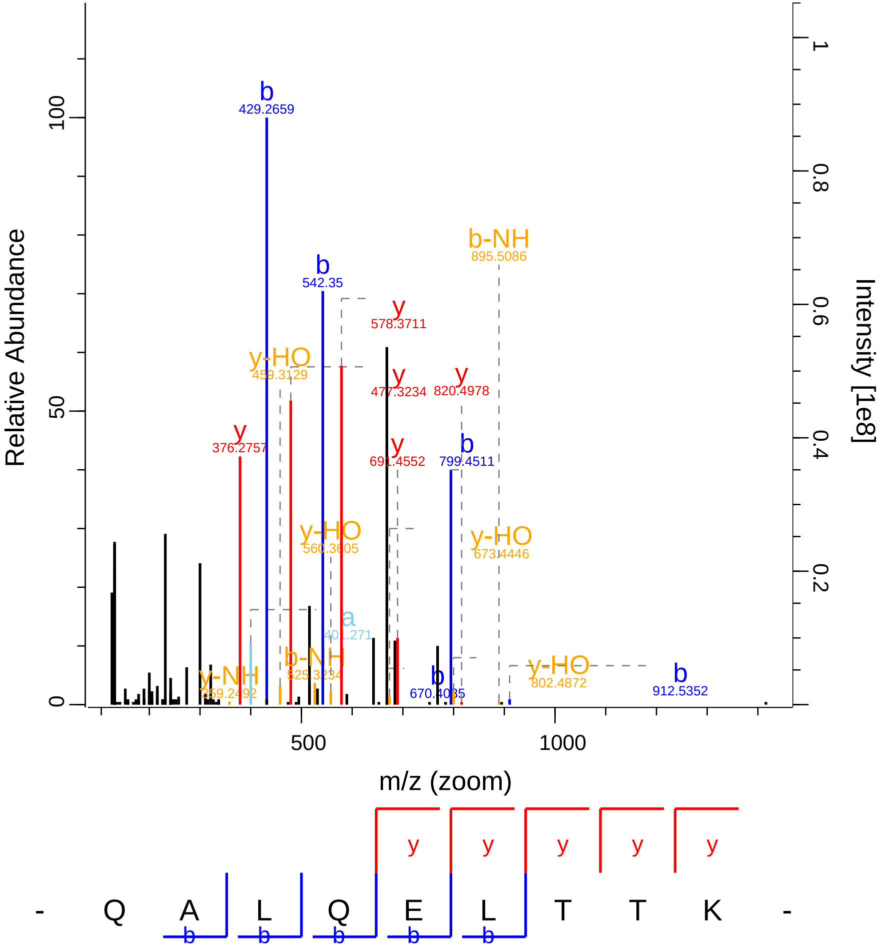

Raw File	Scan	Method	Score	m/z	Gene names
RO-QC-160121_ZackD_SS_Set_01_Fractior	21438	FTMS; HC	54.61	826.47	T

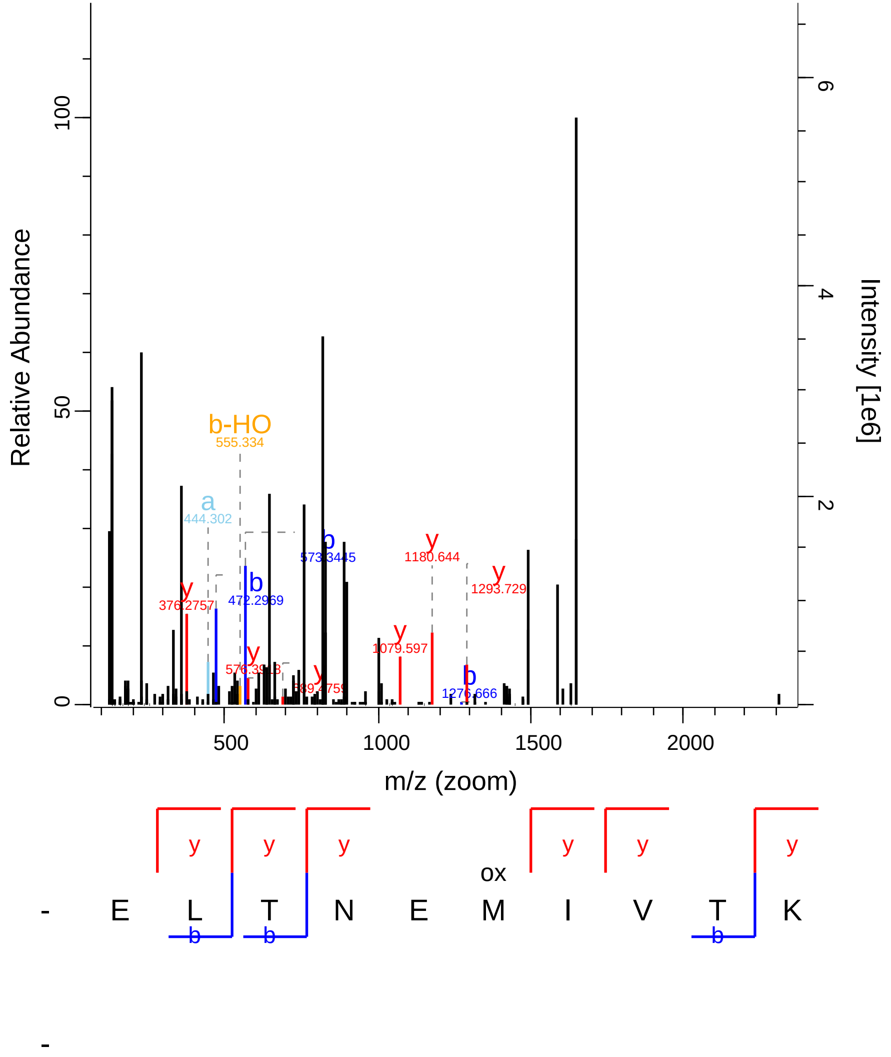

Raw File	Scan	Method	Score	m/z	Gene names
RO-QC-160121_ZackD_SS_Set_01_Fractior	21679	FTMS; HC	60.59	826.47	T

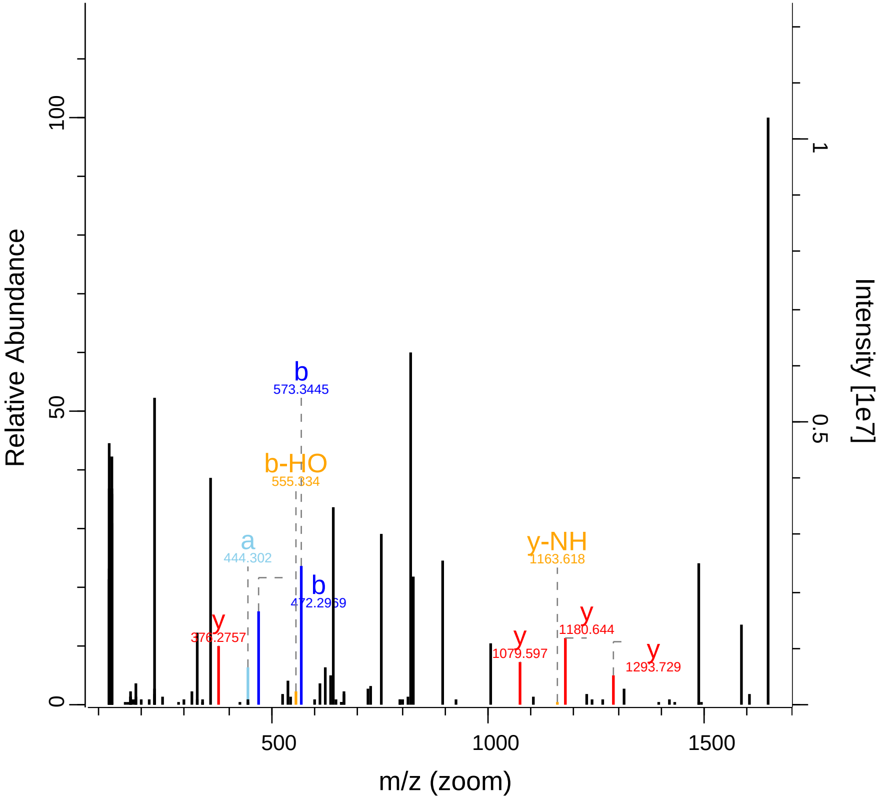

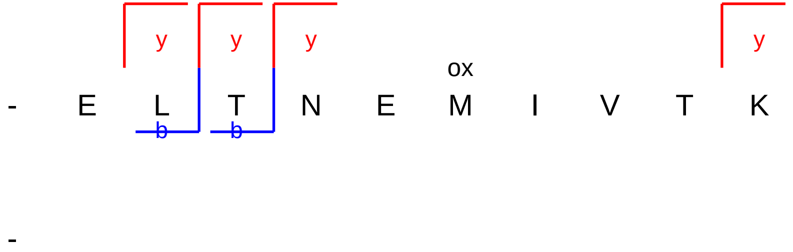

Raw File	Scan	Method	Score	m/z
RO-QC-160121_ZackD_SS_Set_01_Fraction_12	21836	FTMS; HCD	57.11	520.77

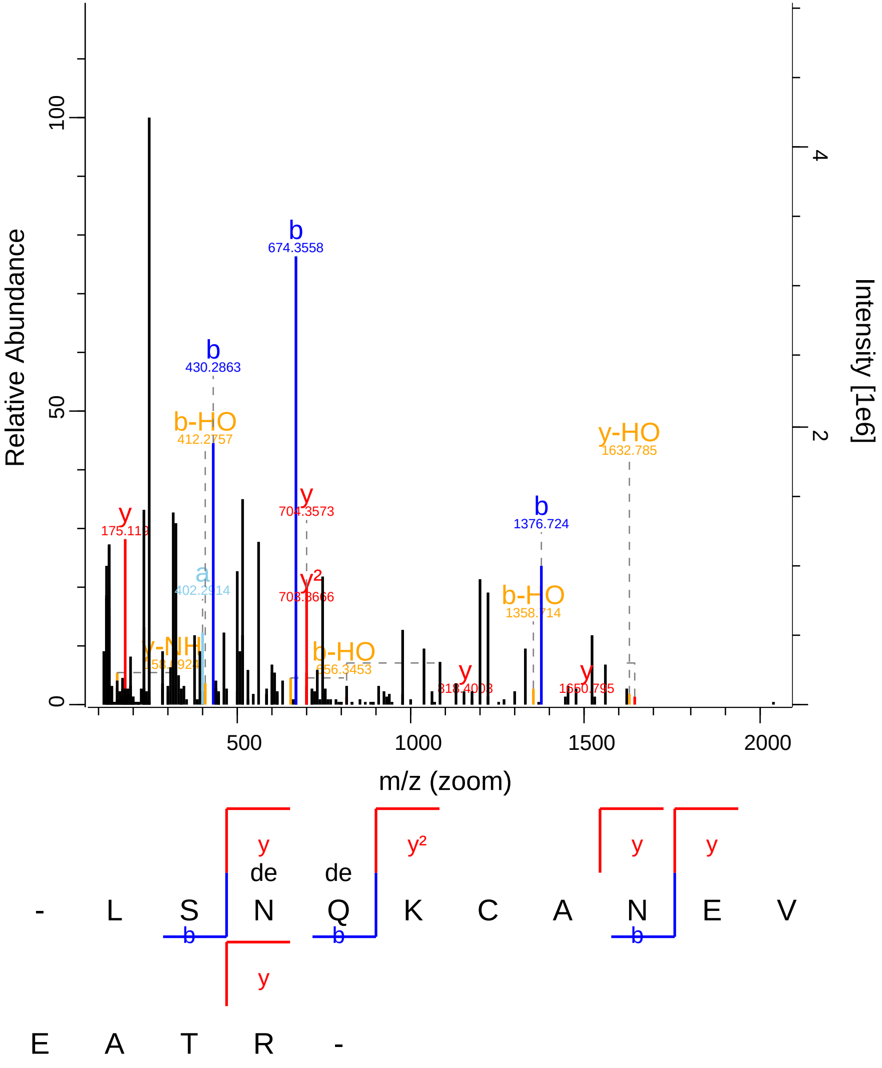

Raw File	Scan	Method	Score	m/z
RO-QC-160121_ZackD_SS_Set_01_Fraction_12	22431	FTMS; HCD	98.42	439.28

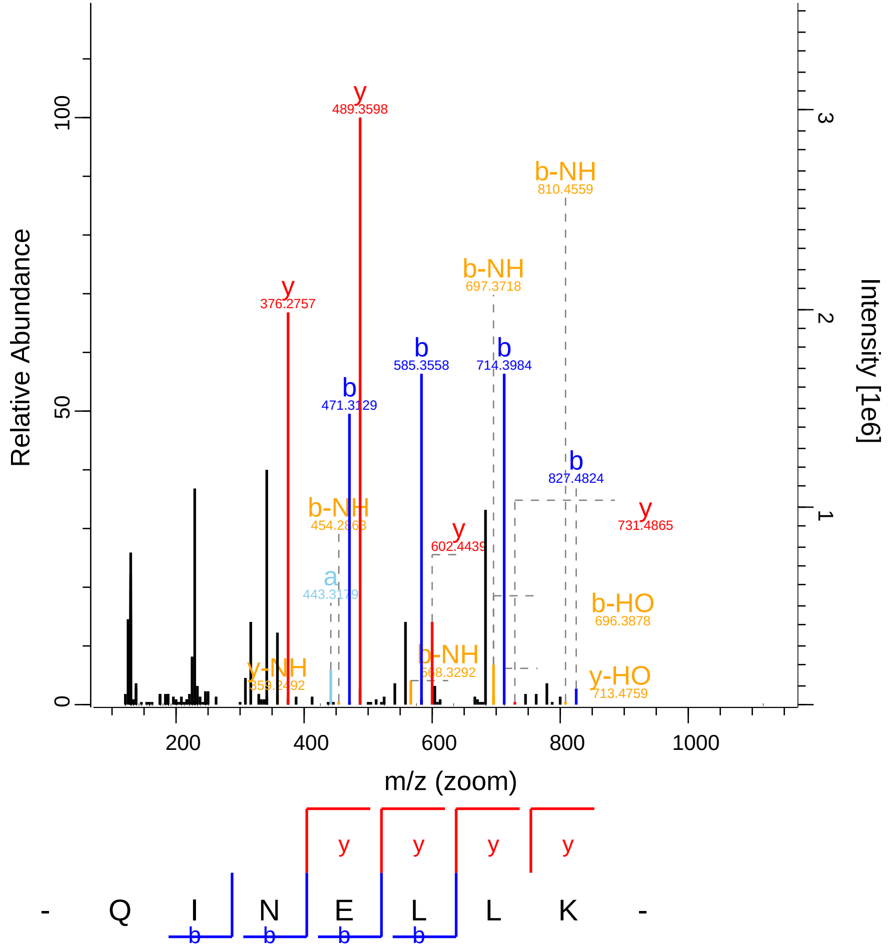

Raw File	Scan	Method	Score	m/z	Gene names
RO-QC-160121_ZackD_SS_Set_01_Fractior	22538	FTMS; HC	64.3	912.51	UGCG

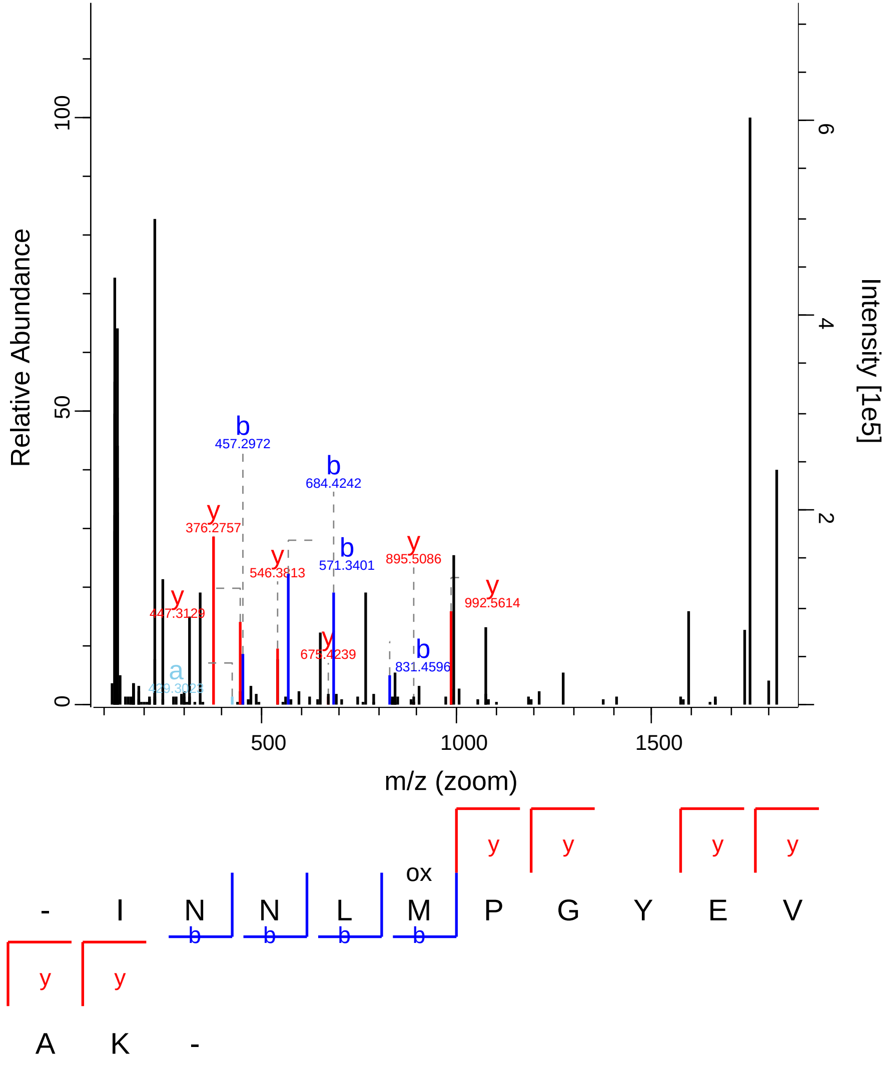

Raw File	Scan	Method	Score	m/z
RO-QC-160121_ZackD_SS_Set_01_Fraction_12	22661	FTMS; HCD	63.31	439.28

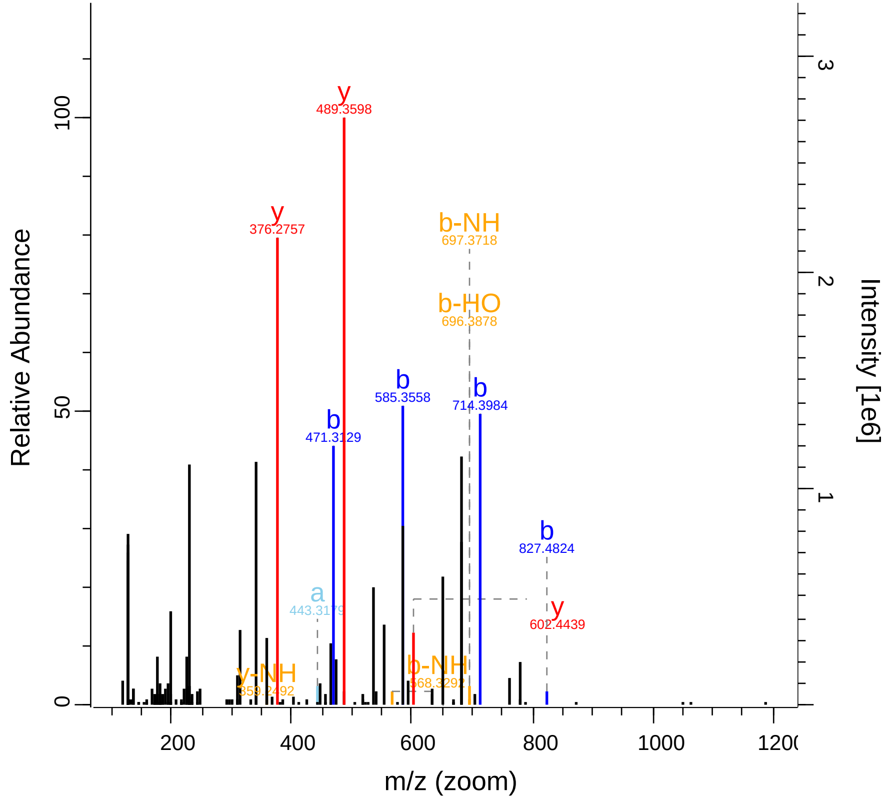

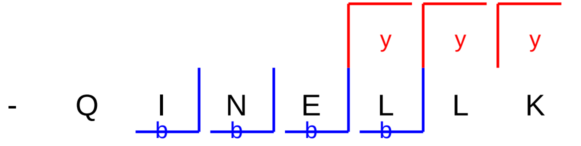

Raw File	Scan	Method	Score	m/z	Gene names
RO-QC-160121_ZackD_SS_Set_01_Fractior	22719	FTMS; HC	104.06	429.59	KANSL2

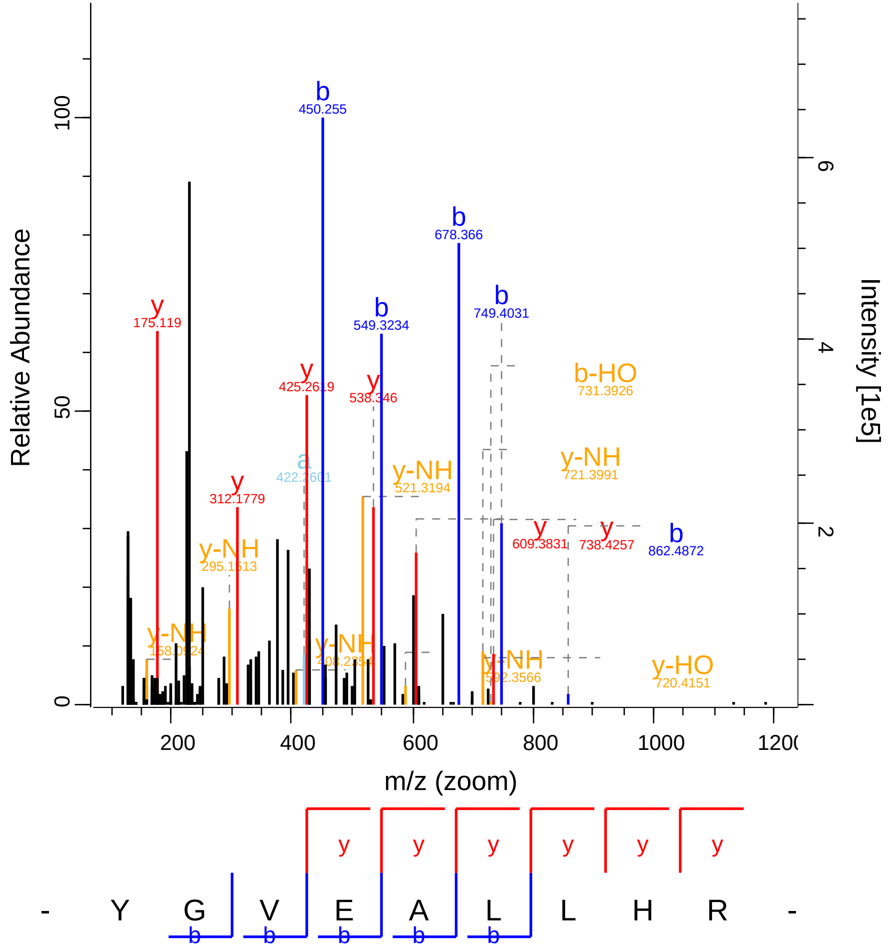

Raw File	Scan	Method	Score	m/z	Gene names
RO-QC-160121_ZackD_SS_Set_01_Fractior	22901	FTMS; HC	111.46	538.97	SEC61B

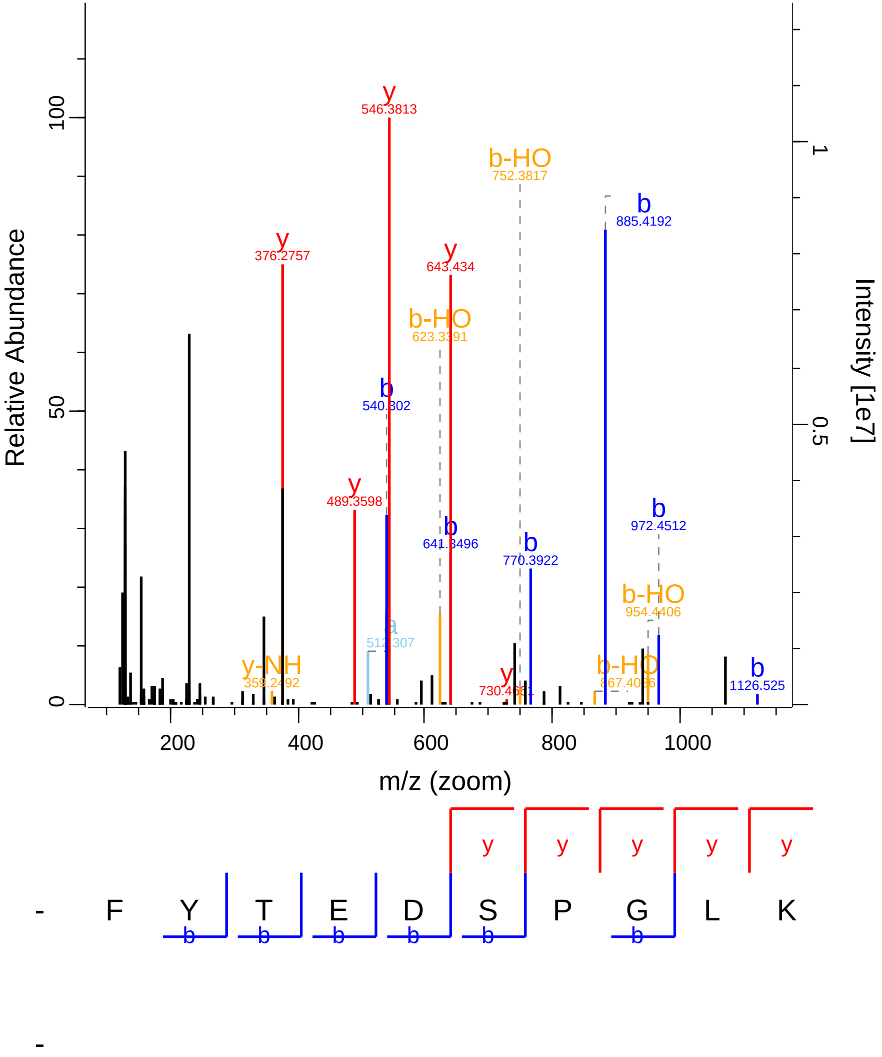

Raw File	Scan	Method	Score	m/z	Gene names
RO-QC-160121_ZackD_SS_Set_01_Fractior	22931	FTMS; HC	163.32	807.94	SEC61B

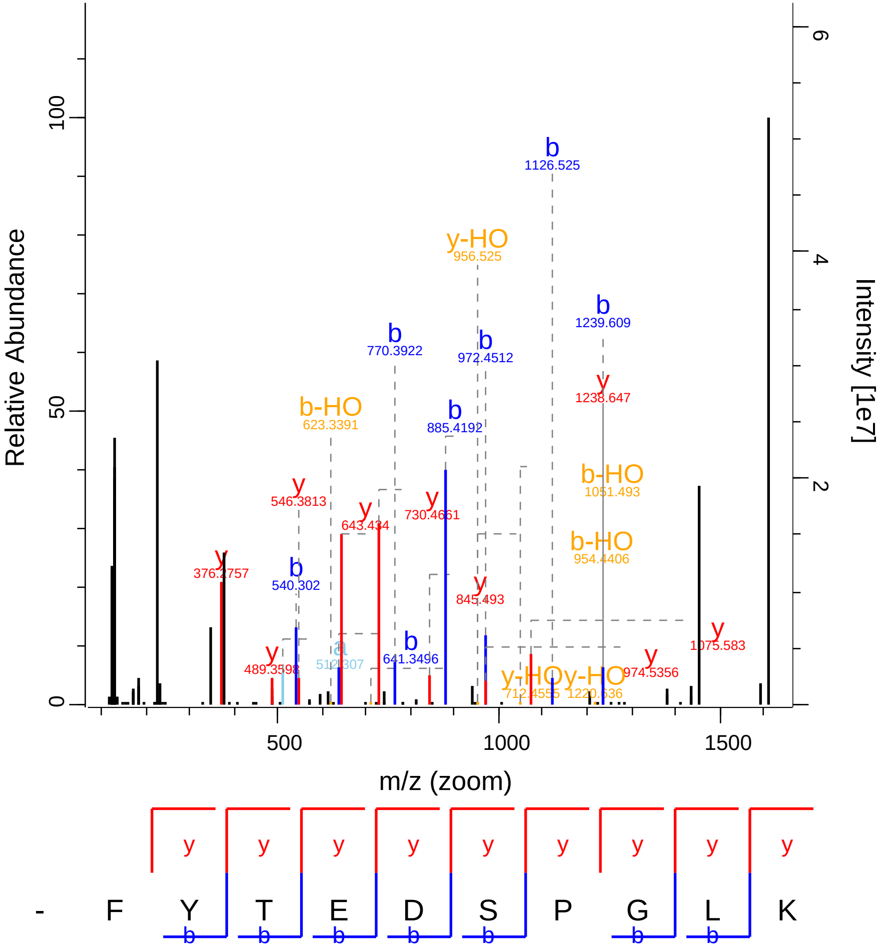

-

Raw File	Scan	Method	Score	m/z	Gene names
RO-QC-160121_ZackD_SS_Set_01_Fractior	23166	FTMS; HC	90.15	807.94	SEC61B

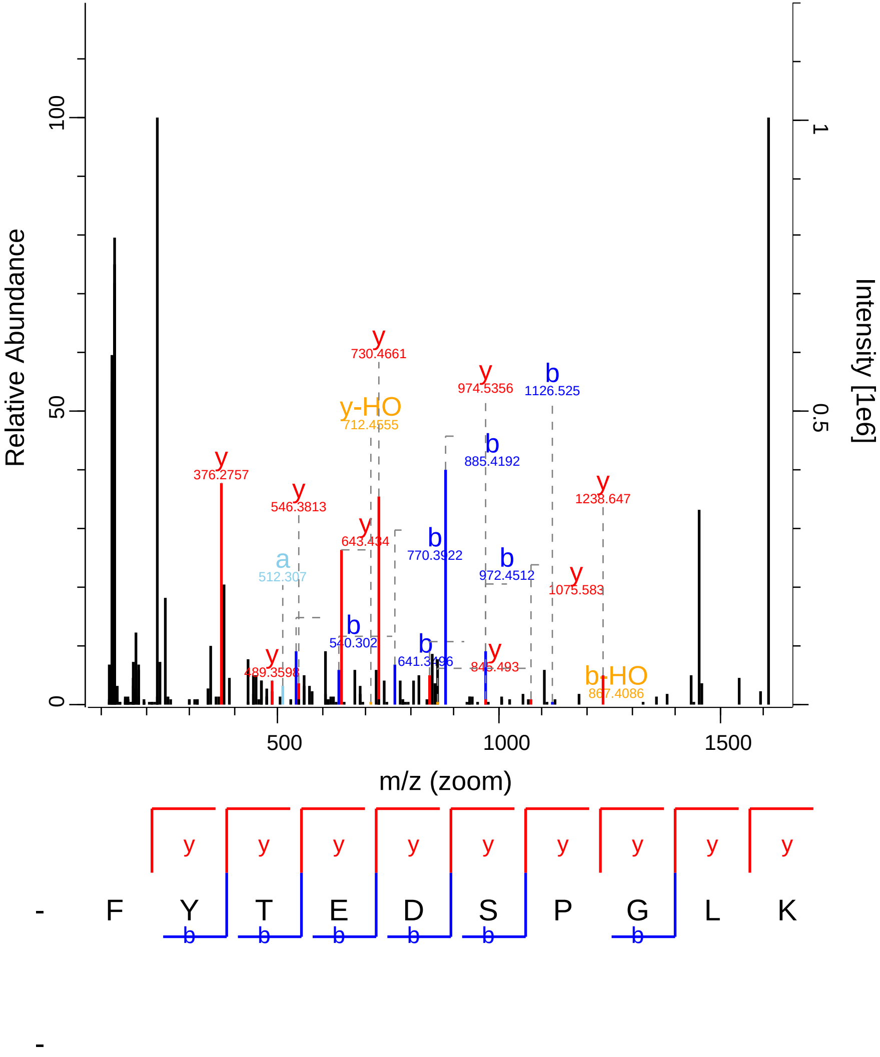

Raw File	Scan	Method	Score	m/z	Gene names
RO-QC-160121_ZackD_SS_Set_01_Fractior	23947	FTMS; HC	51.28	931.53	FAM64A

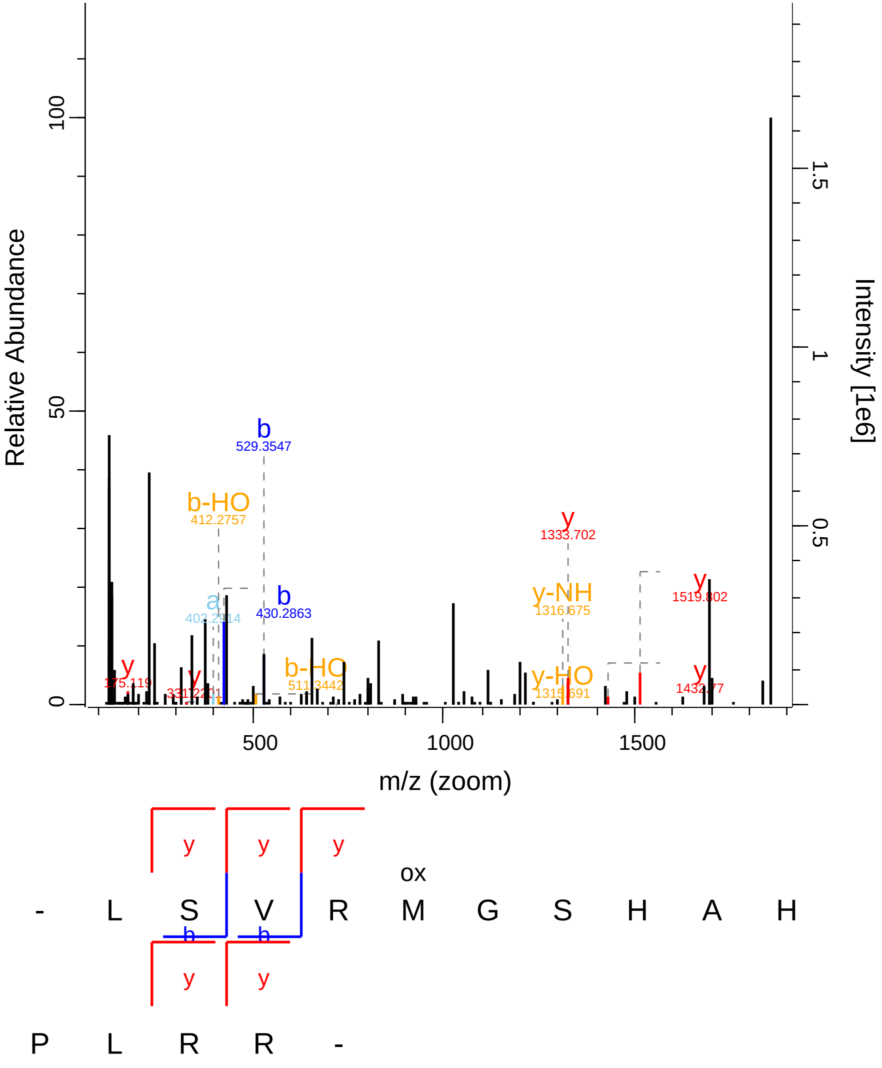

Raw File	Scan	Method	Score	m/z	Gene names
RO-QC-160121_ZackD_SS_Set_01_Fractio	25167	FTMS; HC	130.07	1080.58	CD99L2

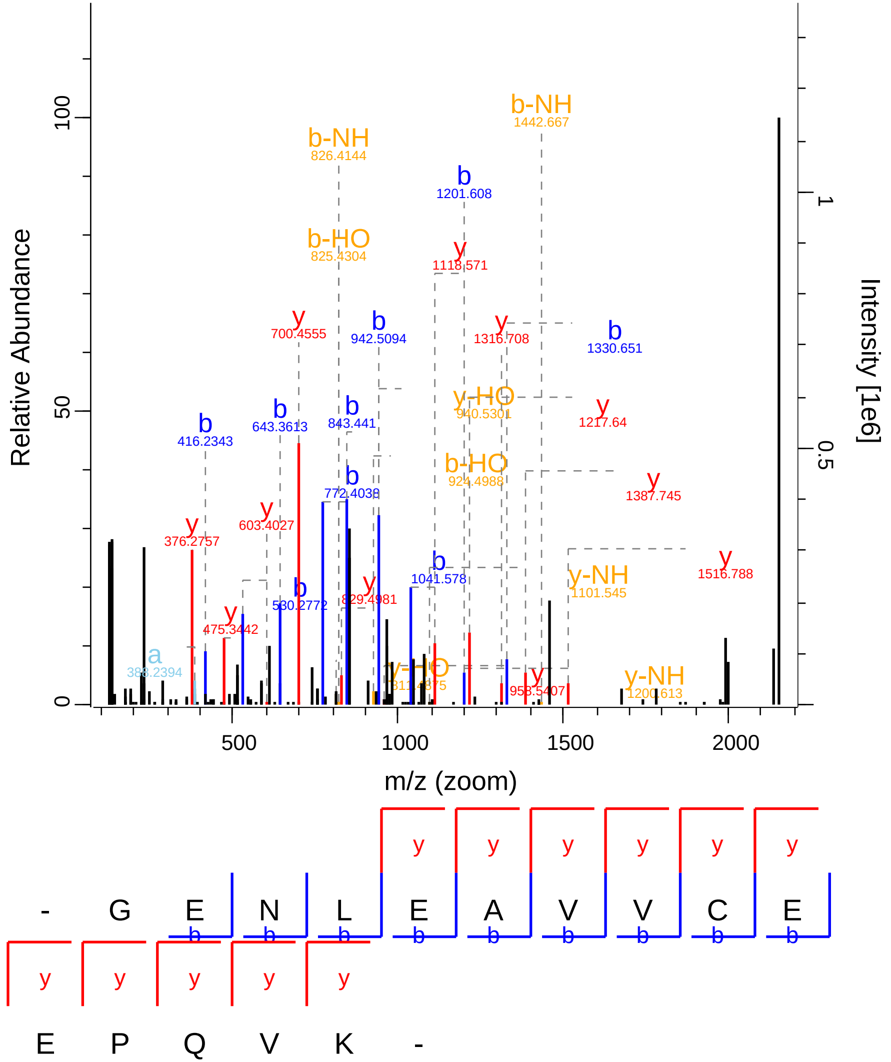

Raw File	Scan	Method	Score	m/z	Gene names
RO-QC-160121_ZackD_SS_Set_01_Fractioı	25657	FTMS; HC	122.33	863.44	MT-ND6

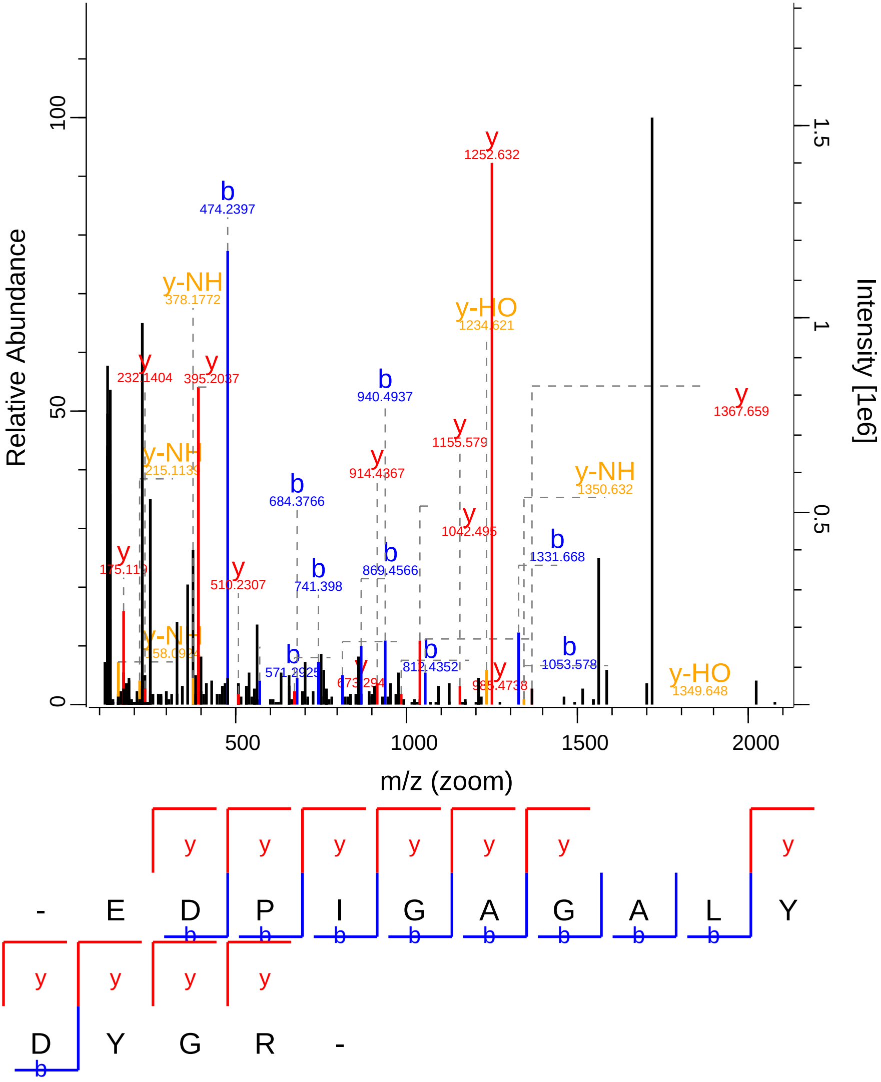

Raw File	Scan	Method	Score	m/z
RO-QC-160121_ZackD_SS_Set_01_Fraction_12	25792	FTMS; HCD	42.74	903.49

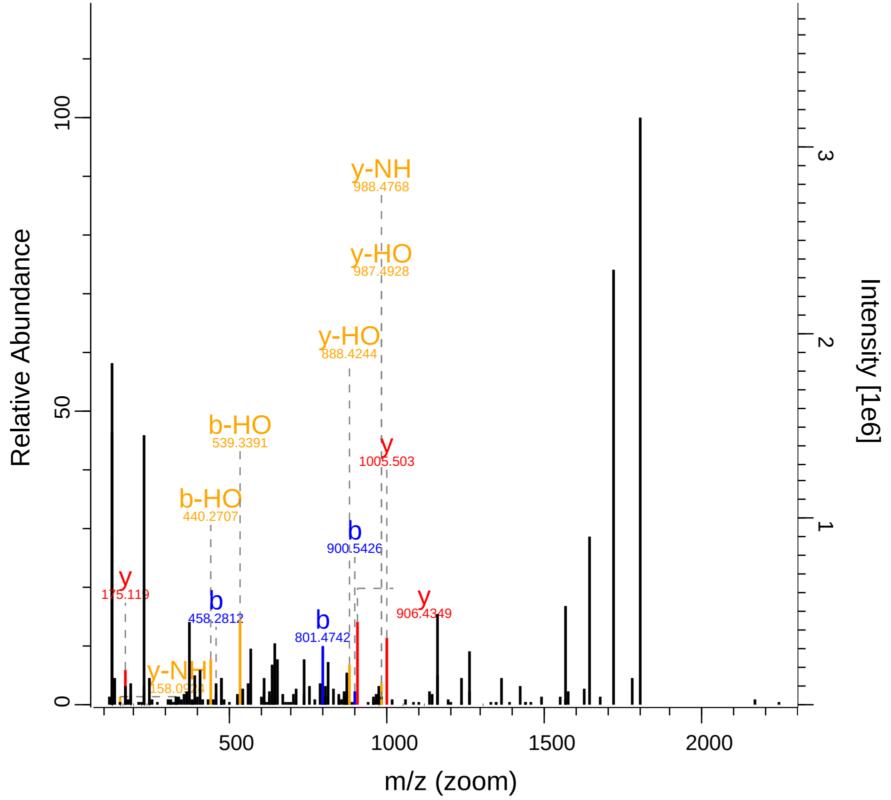

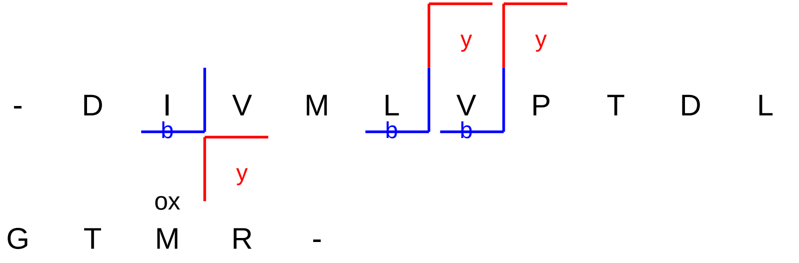

Raw File	Scan	Method	Score	m/z	Gene names
RO-QC-160121_ZackD_SS_Set_01_Fractior	25892	FTMS; HC	34.72	595.98	MRPL35

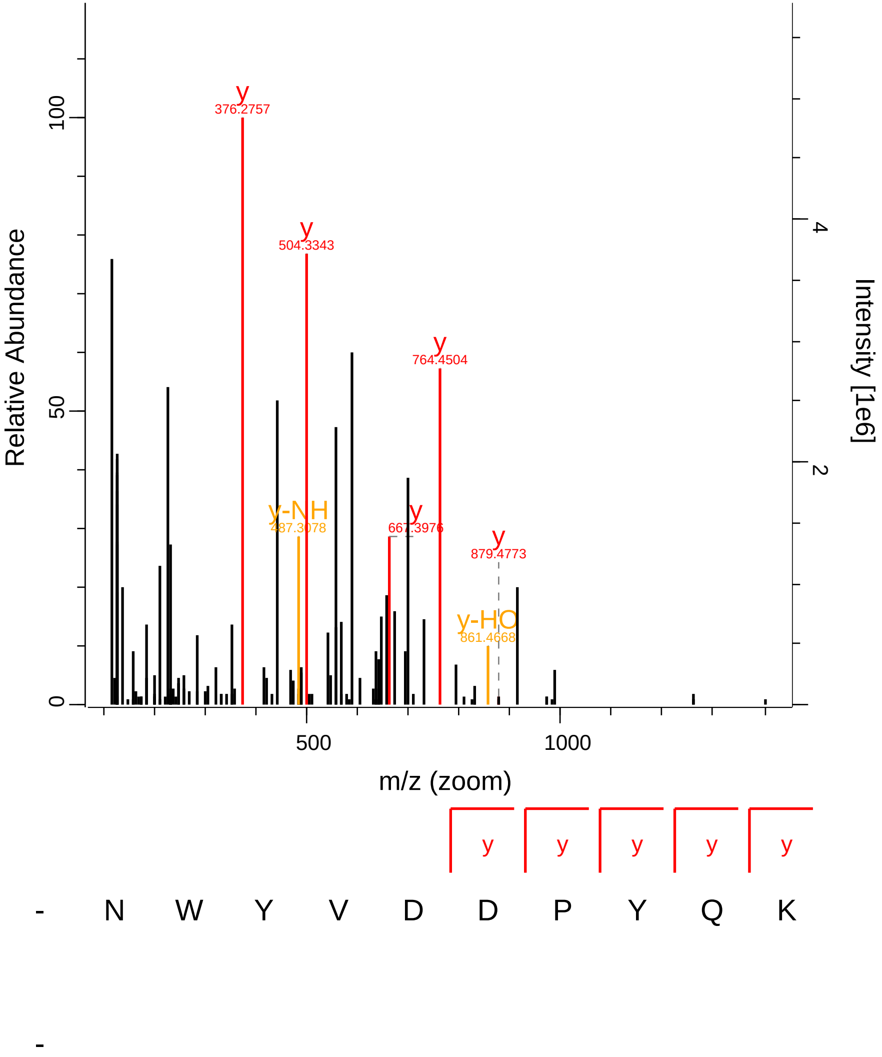

Raw File	Scan	Method	Score	m/z
RO-QC-160121_ZackD_SS_Set_01_Fraction_12	25992	FTMS; HCD	71.03	678.39

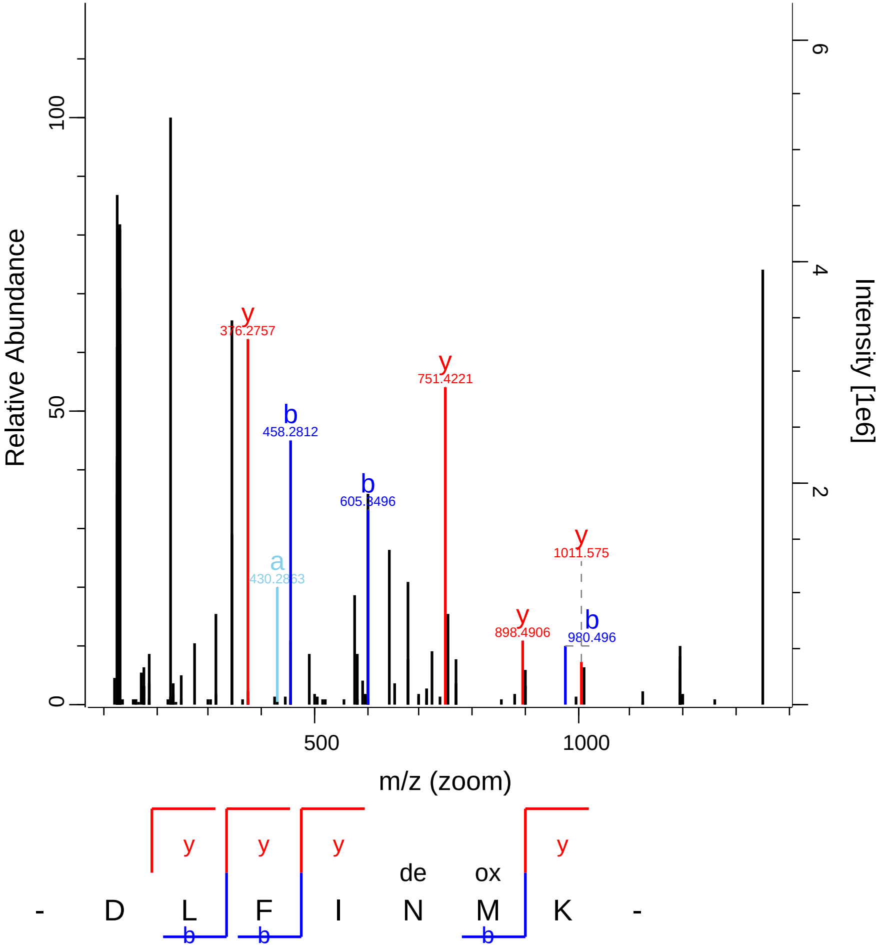

Raw File	Scan	Method	Score	m/z	Gene names
RO-QC-160121_ZackD_SS_Set_01_Fractio	26216	FTMS; HC	48.09	1049.52	ZNF107

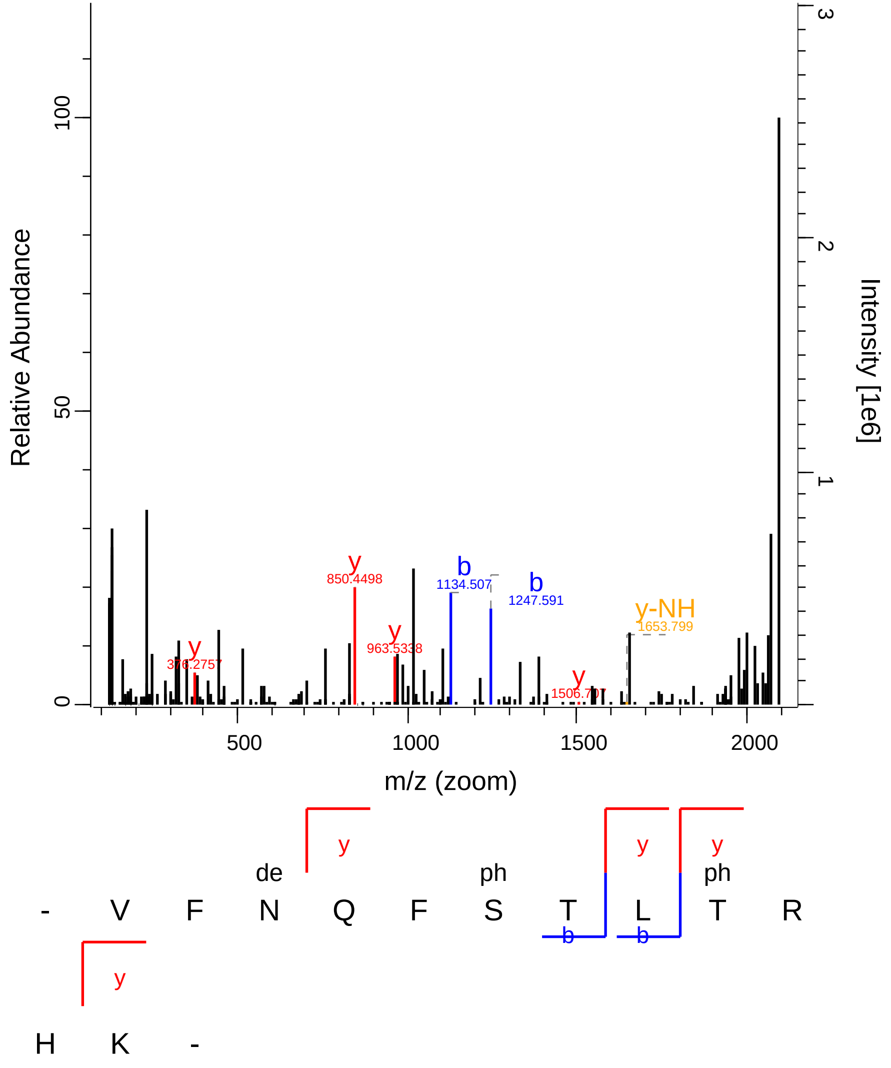

Raw File	Scan	Method	Score	m/z	Gene names
RO-QC-160121_ZackD_SS_Set_01_Fractior	26465	FTMS; HC	45.92	849.45	SLC31A1

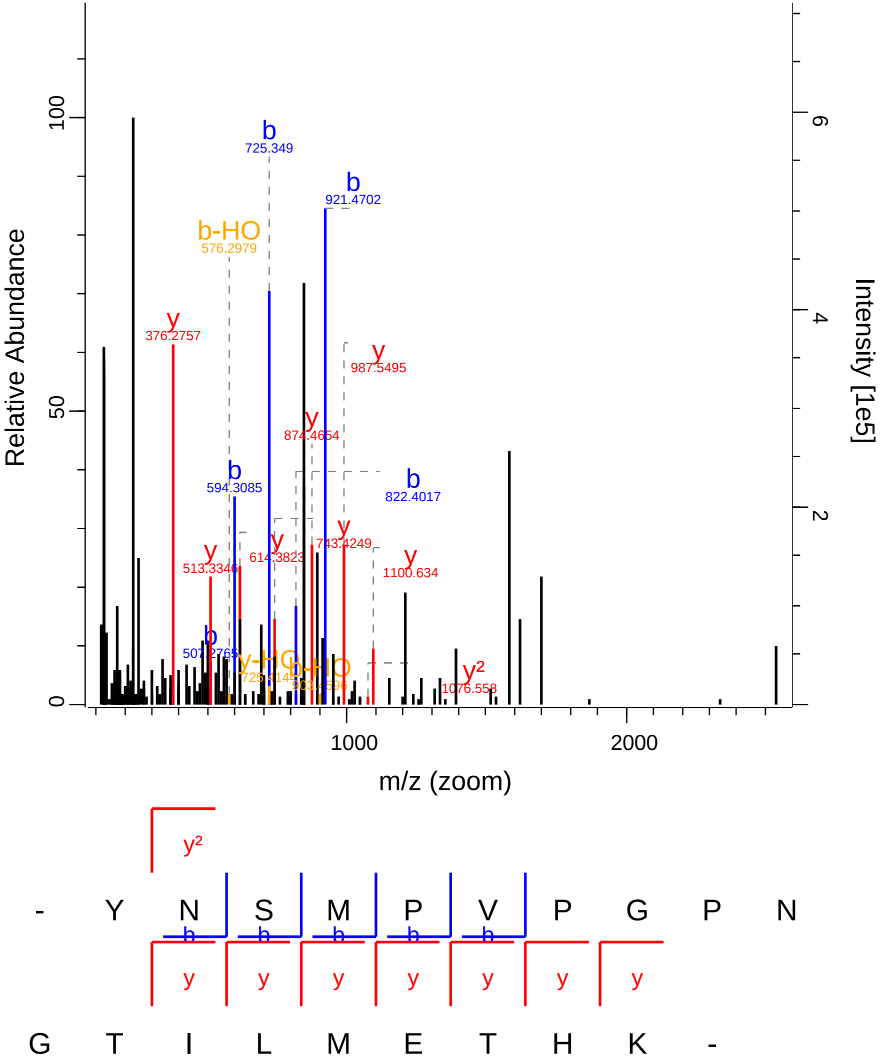

Raw File	Scan	Method	Score	m/z	Gene names
RO-QC-160121_ZackD_SS_Set_01_Fraction	27280	FTMS; HC	112.44	544.3	SLC2A8

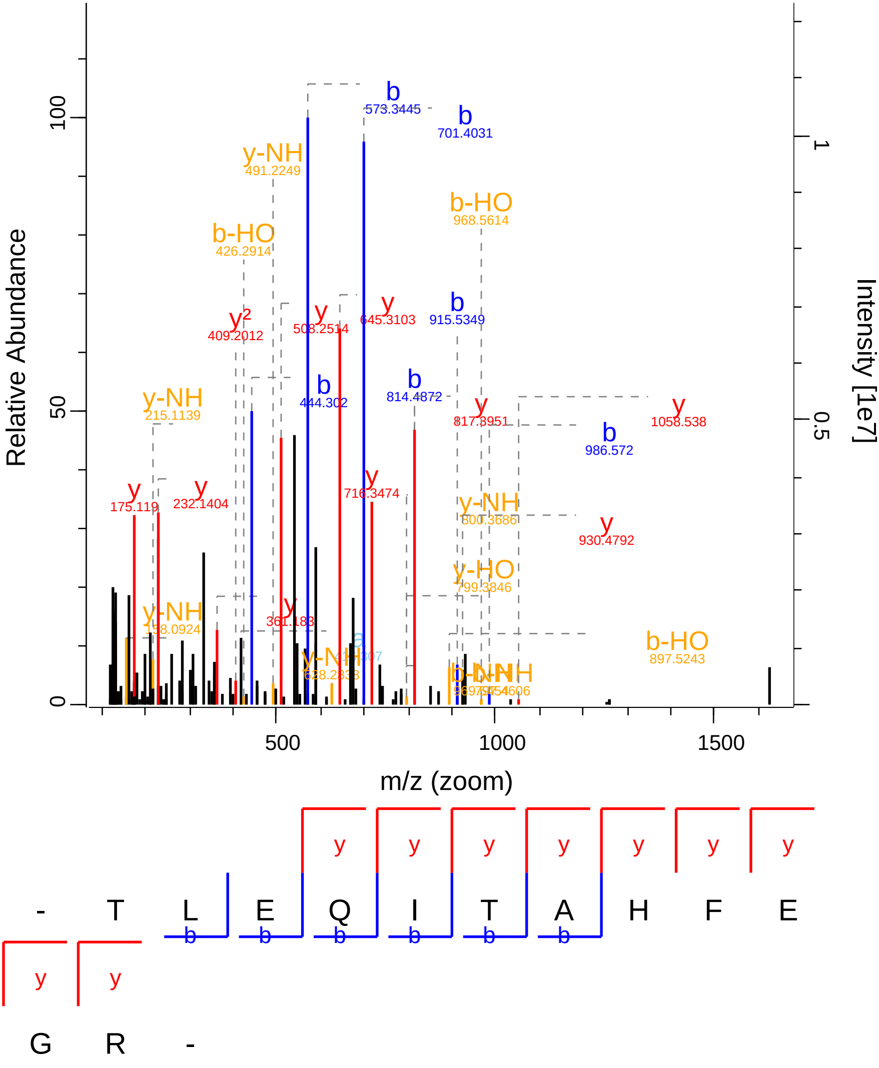

Raw File	Scan	Method	Score	m/z	Gene names
RO-QC-160121_ZackD_SS_Set_01_Fractioı	28542	FTMS; HC	132.88	690.93	MAL2

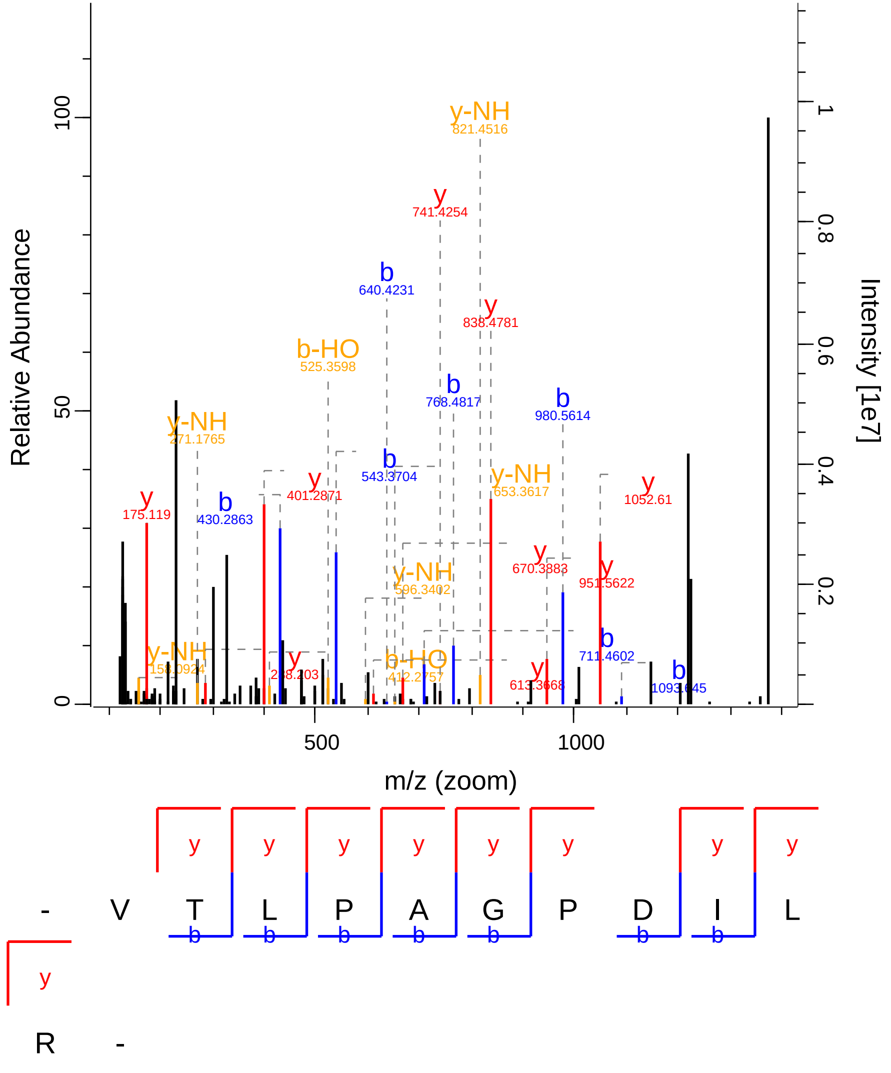

Raw File	Scan	Method	Score	m/z	Gene names
RO-QC-160121_ZackD_SS_Set_01_Fractio	28729	FTMS; HC	52.79	1379.74	MAMDC2

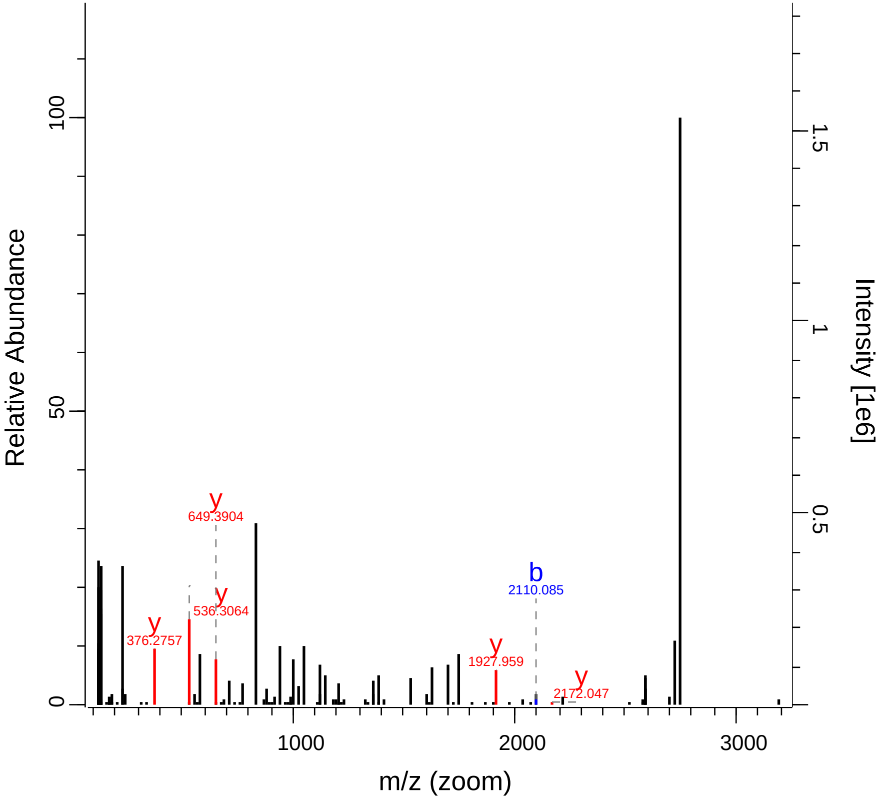

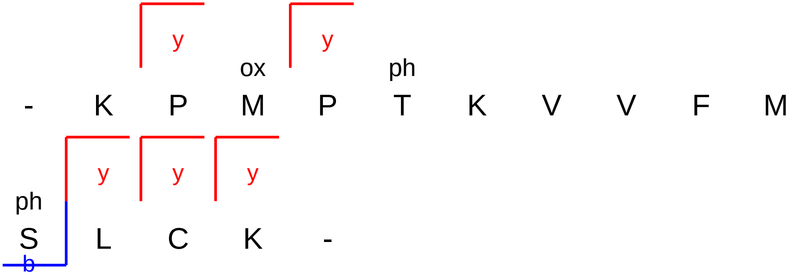

Raw File	Scan	Method	Score	m/z	Gene names
RO-QC-160121_ZackD_SS_Set_01_Fractior	30156	FTMS; HC	59.57	601.03	FAM184B

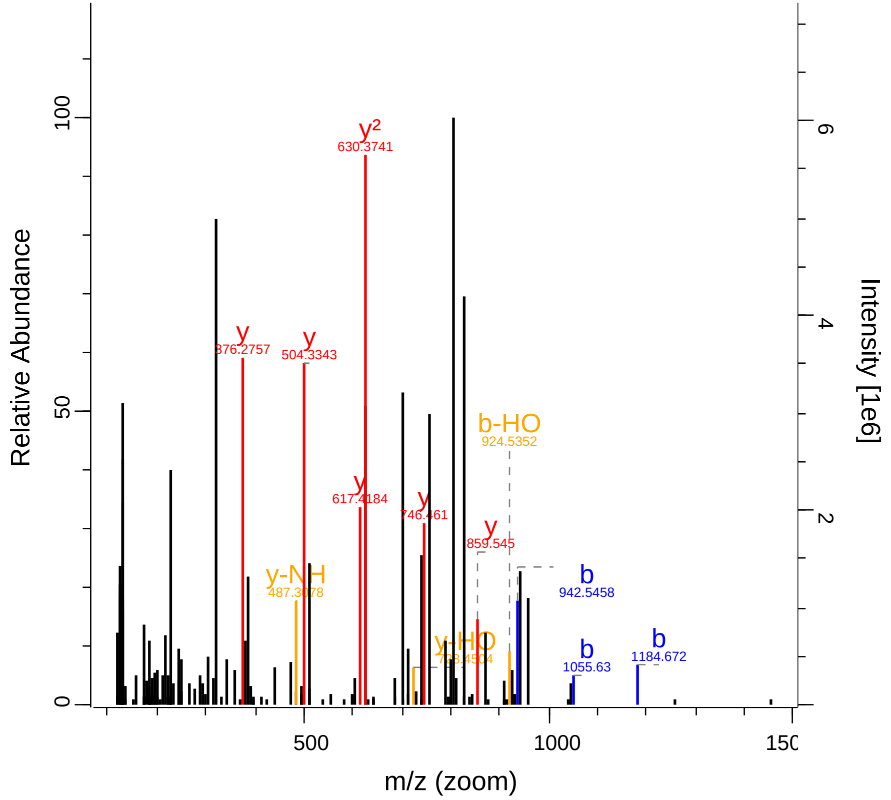

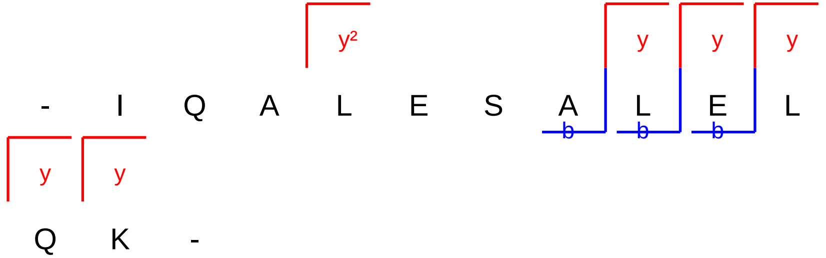

Raw File	Scan	Method	Score	m/z	Gene names
RO-QC-160121_ZackD_SS_Set_01_Fractior	30314	FTMS; HC	49.12	842.16	SIRT7

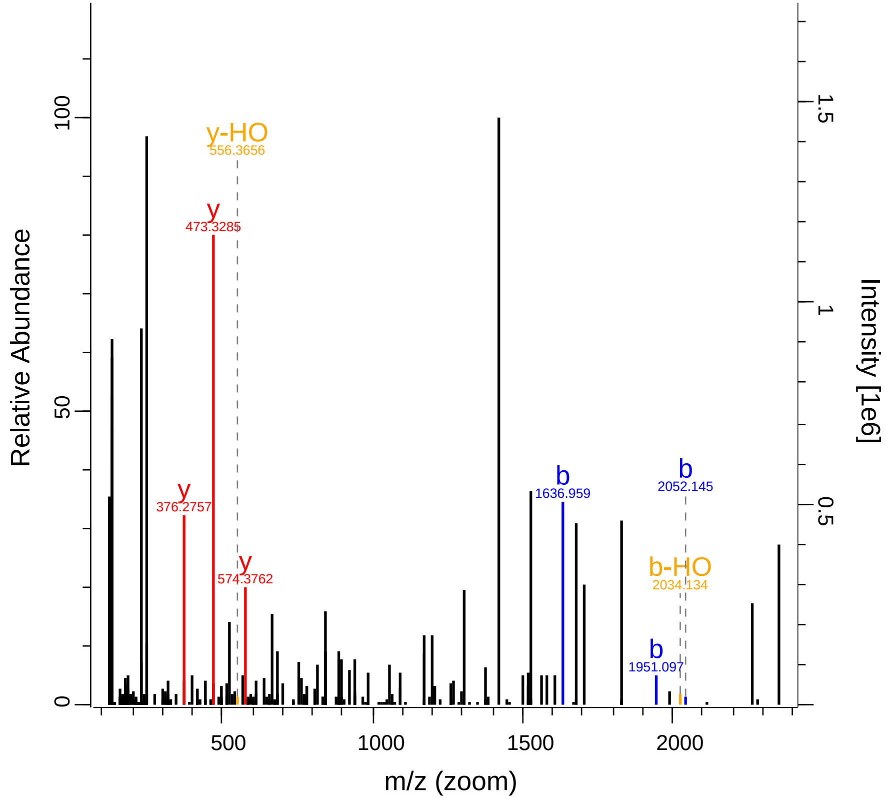

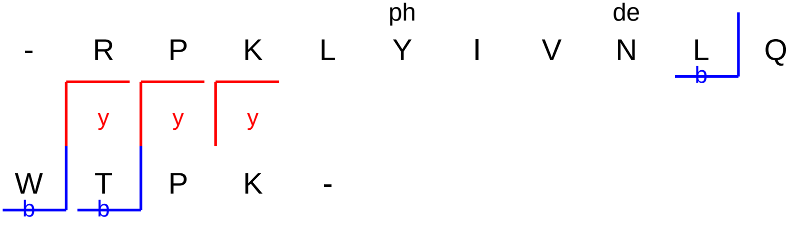

Raw File	Scan	Method	Score	m/z	Gene names
RO-QC-160121_ZackD_SS_Set_01_Fractior	30386	FTMS; HC	59.57	601.03	FAM184B

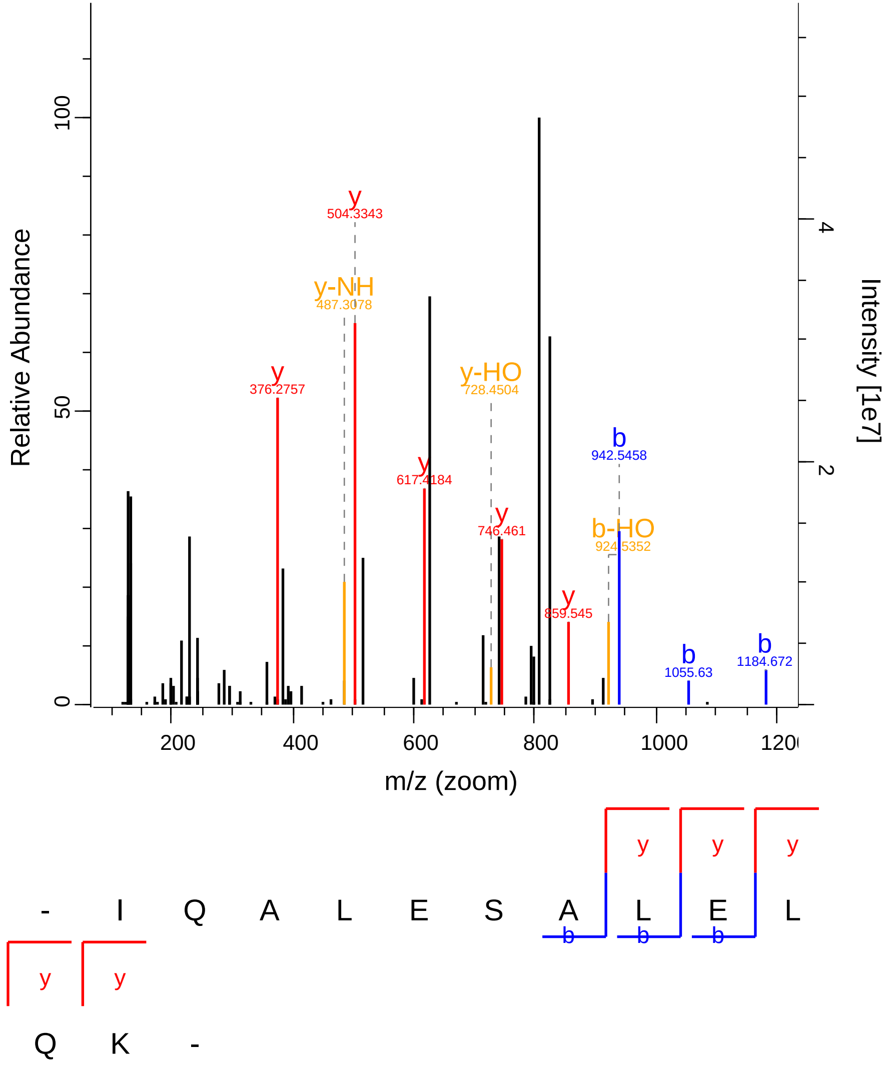

Raw File	Scan	Method	Score	m/z	Gene names
RO-QC-160121_ZackD_SS_Set_01_Fractior	30387	FTMS; HC	90.11	901.05	FAM184B

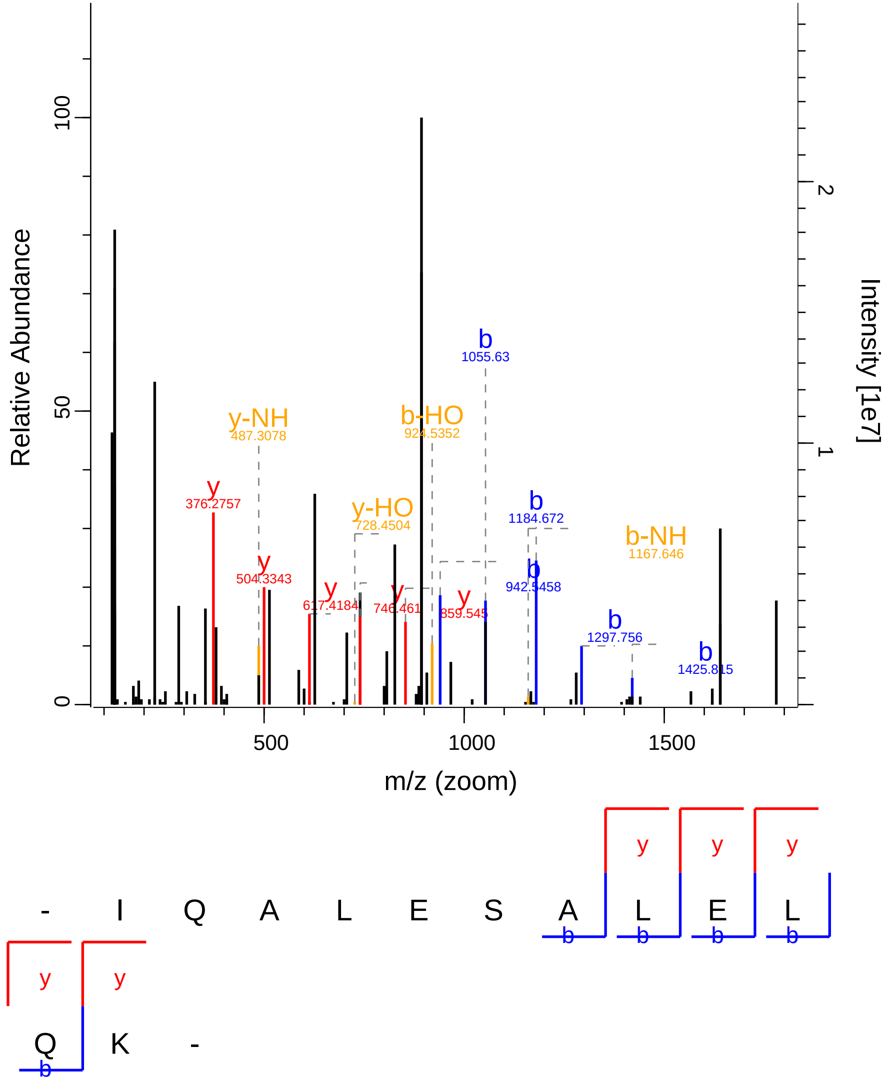

Raw File	Scan	Method	Score	m/z	Gene names
RO-QC-160121_ZackD_SS_Set_01_Fractior	30499	FTMS; HC	72.53	821.45	LRP10

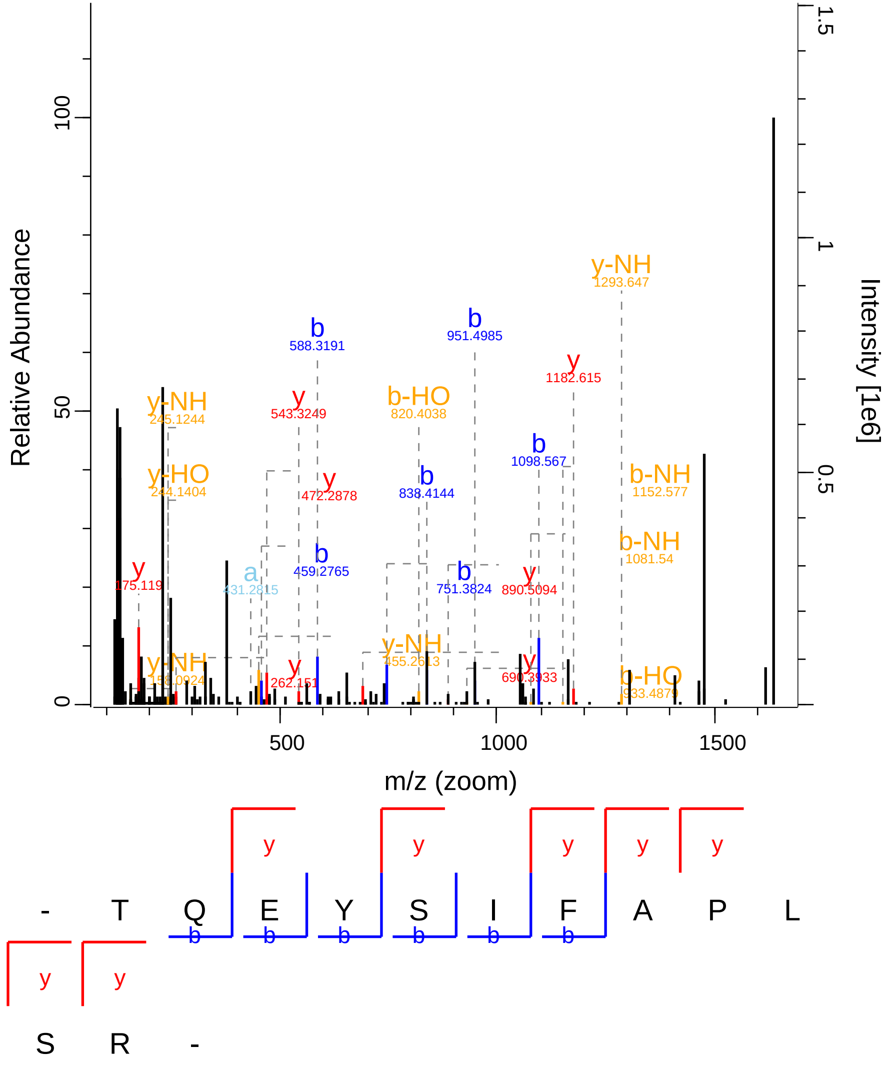

Raw File	Scan	Method	Score	m/z	Gene names
RO-QC-160121_ZackD_SS_Set_01_Fractior	30654	FTMS; HC	64.55	601.03	FAM184B

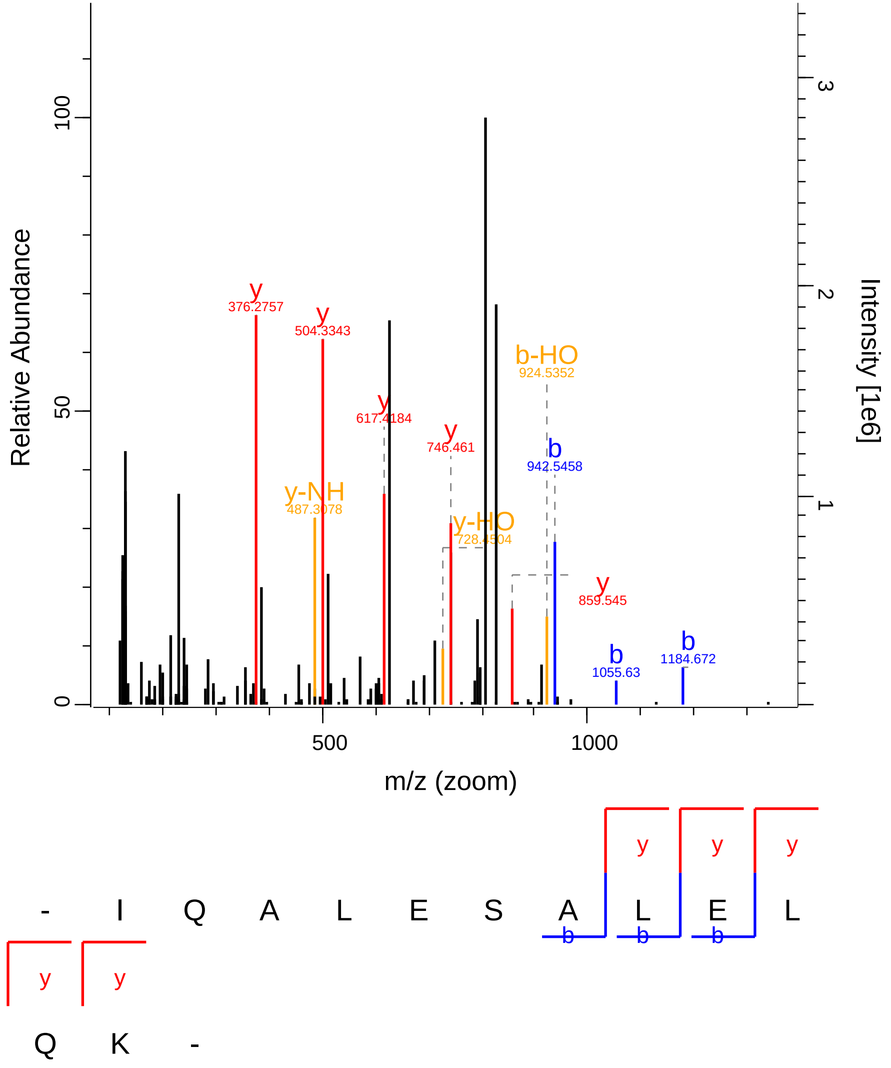

Raw File	Scan	Method	Score	m/z	Gene names
RO-QC-160121_ZackD_SS_Set_01_Fraction	30706	FTMS; HC	48.11	758.4	EFCAB10

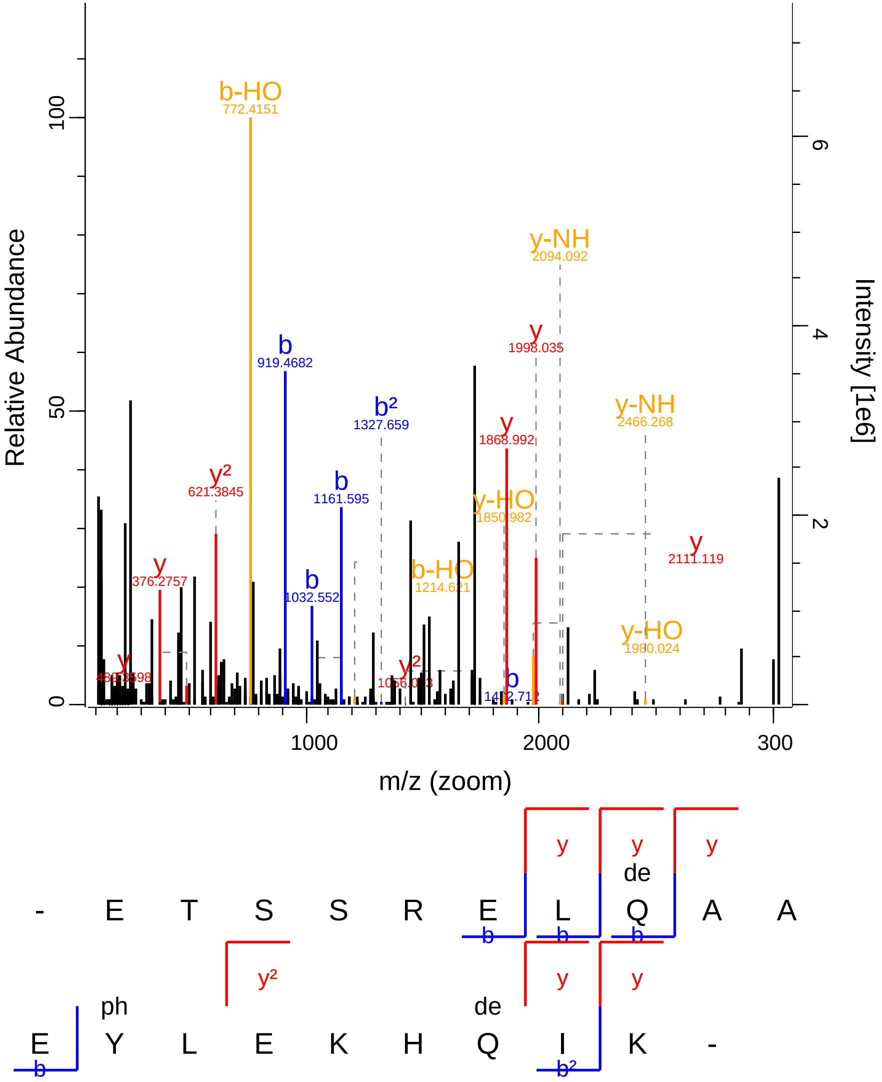

Raw File	Scan	Method	Score	m/z	Gene names
RO-QC-160121_ZackD_SS_Set_01_Fractior	30856	FTMS; HC	57.8	601.03	FAM184B

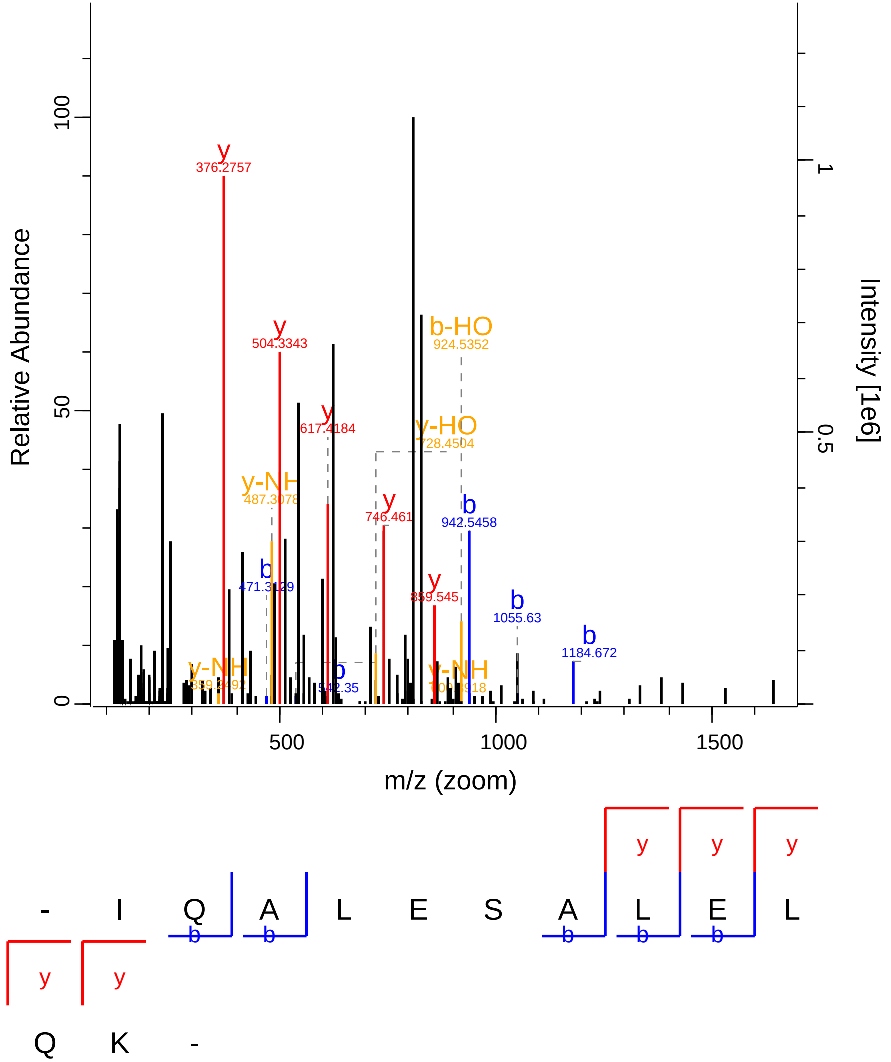

Raw File	Scan	Method	Score	m/z	Gene names
RO-QC-160121_ZackD_SS_Set_01_Fractio	31058	FTMS; HC	181.31	879.47	CLK2

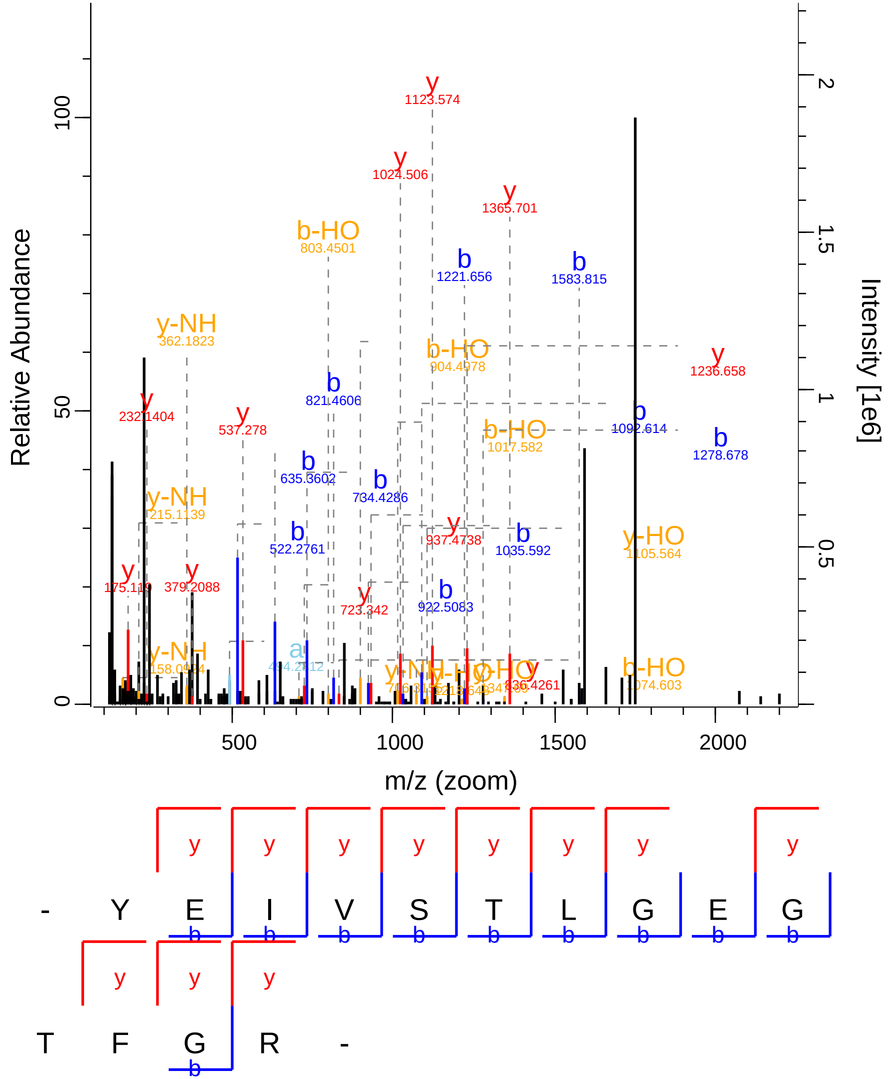

Raw File	Scan	Method	Score	m/z	Gene names
RO-QC-160121_ZackD_SS_Set_01_Fractior	31451	FTMS; HC	66.99	657.04	SLC46A3

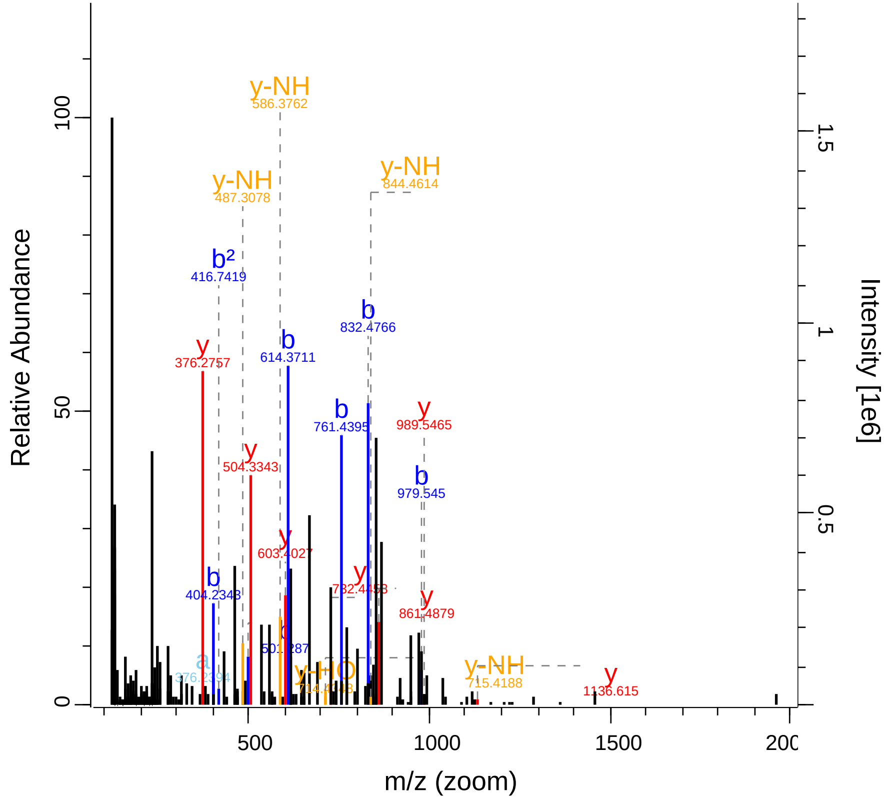

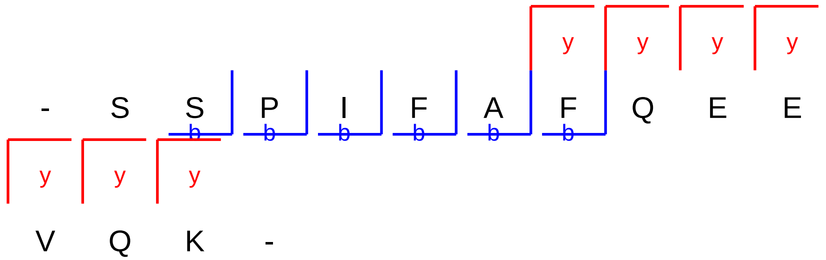

Raw File	Scan	Method	Score	m/z	Gene names
RO-QC-160121_ZackD_SS_Set_01_Fractior	31476	FTMS; HC	110.66	985.05	SLC46A3

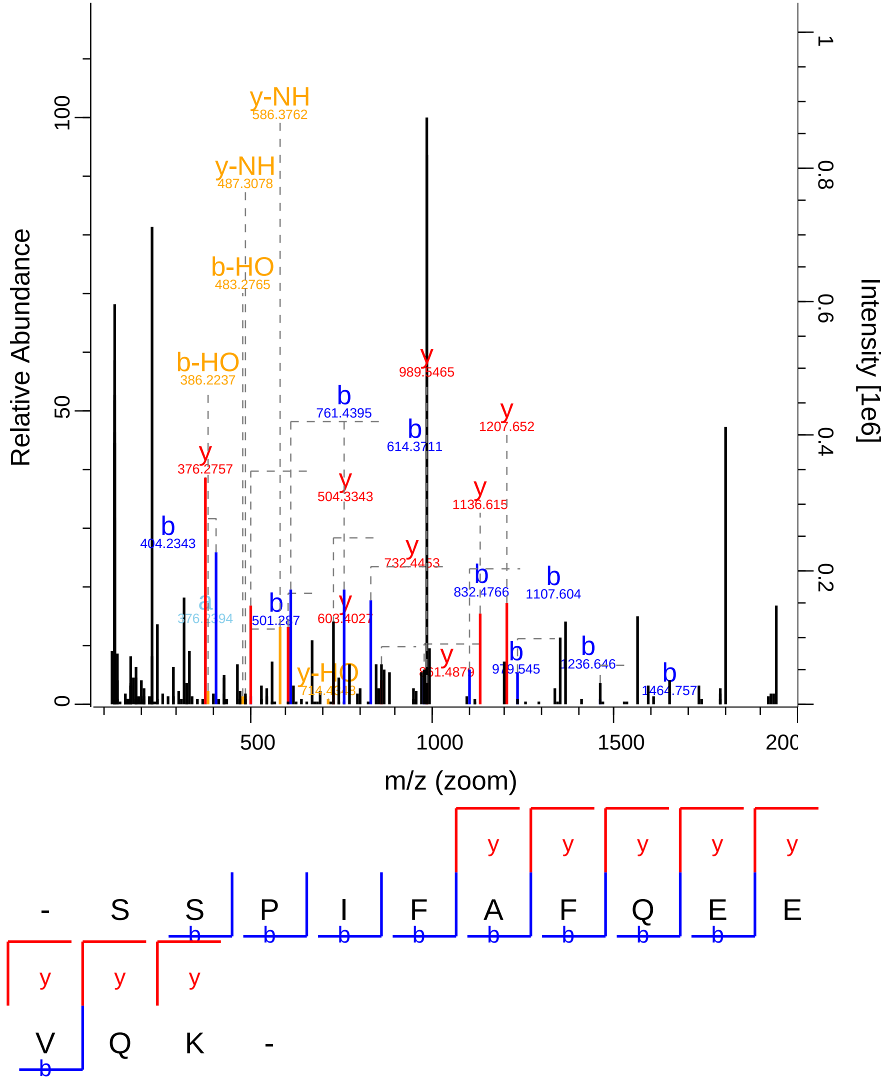

Raw File	Scan	Method	Score	m/z
RO-QC-160121_ZackD_SS_Set_01_Fraction_12	31610	FTMS; HCD	141.47	701.93

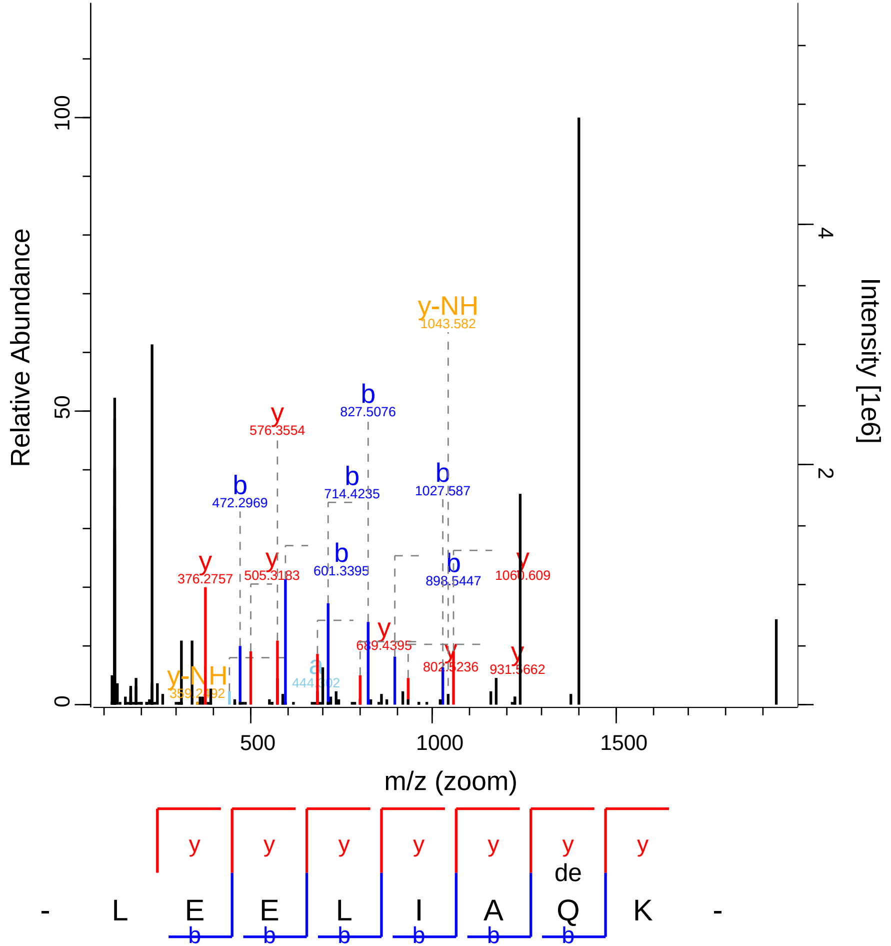

Raw File	Scan	Method	Score	m/z	Gene names
RO-QC-160121_ZackD_SS_Set_01_Fraction	31636	FTMS; HC	108.56	656.7	SLC46A3

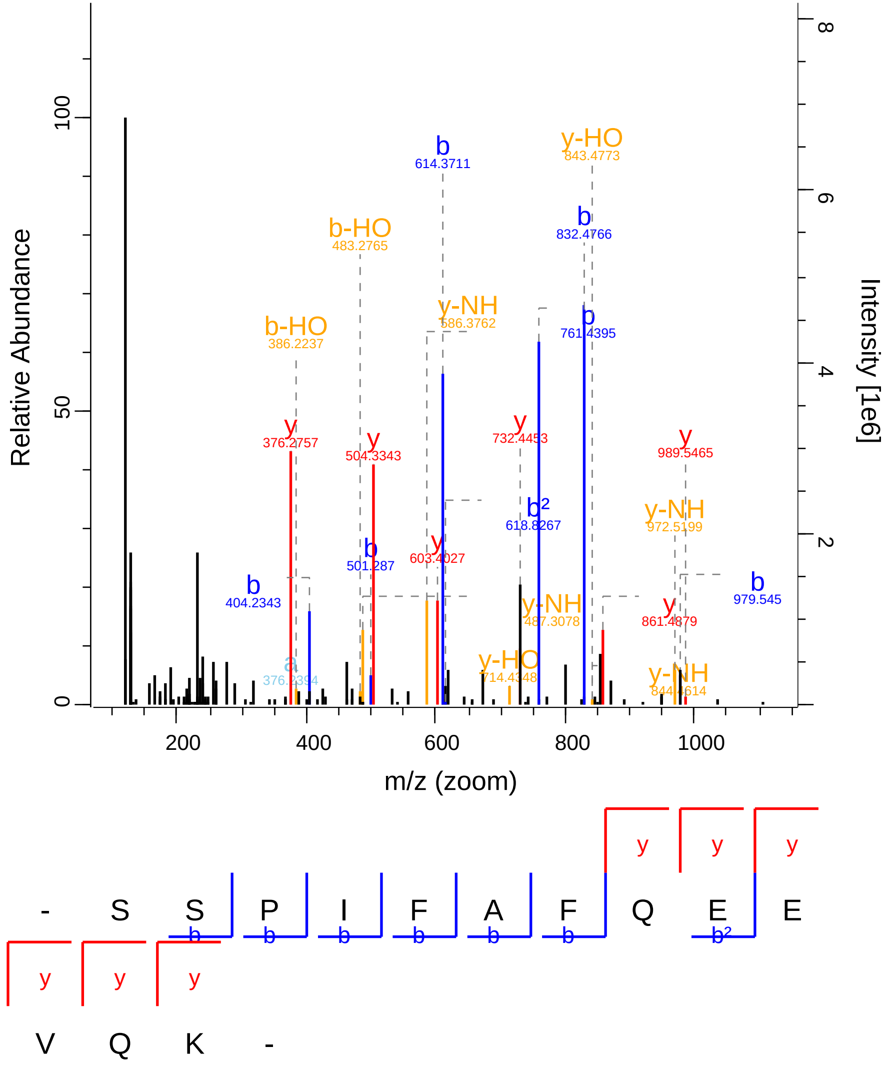

Raw File	Scan	Method	Score	m/z	Gene names
RO-QC-160121_ZackD_SS_Set_01_Fractio	31689	FTMS; HC	123.67	985.05	SLC46A3

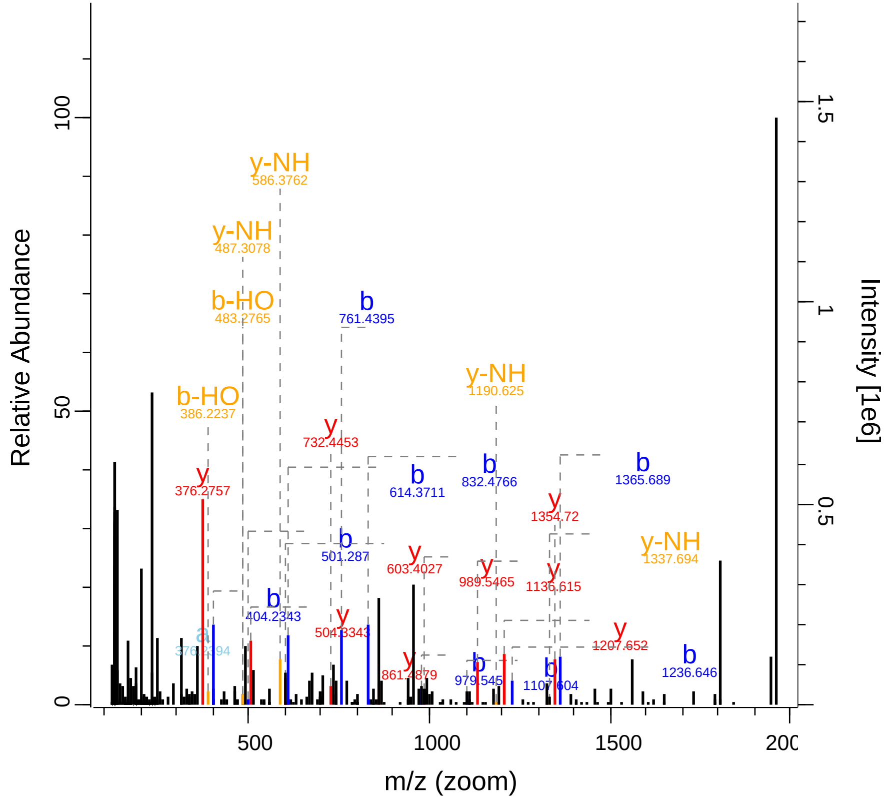

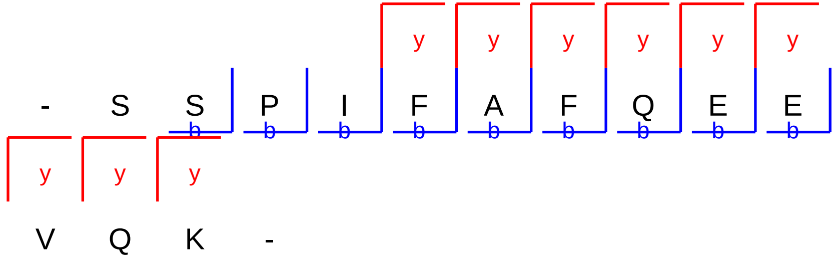

Raw File	Scan	Method	Score	m/z	Gene names
RO-QC-160121_ZackD_SS_Set_01_Fractio	31733	FTMS; HC	114.84	715.46	HAUS2

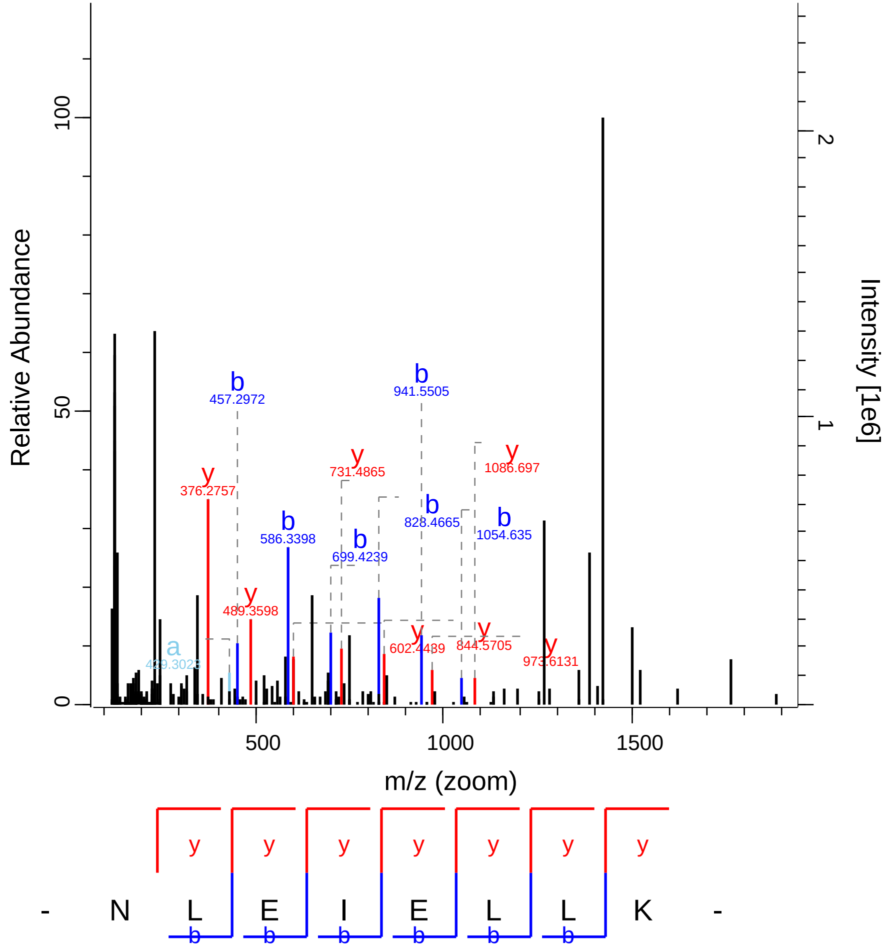

Raw File	Scan	Method	Score	m/z	Gene names
RO-QC-160121_ZackD_SS_Set_01_Fractior	32452	FTMS; HC	70.98	563.67	SLC35A2

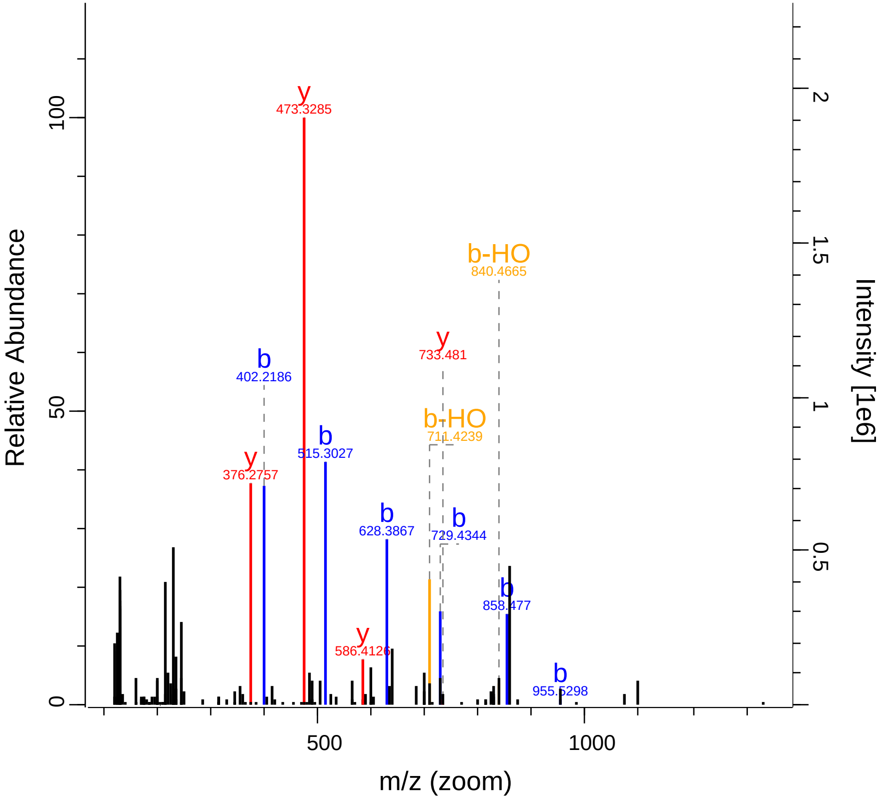

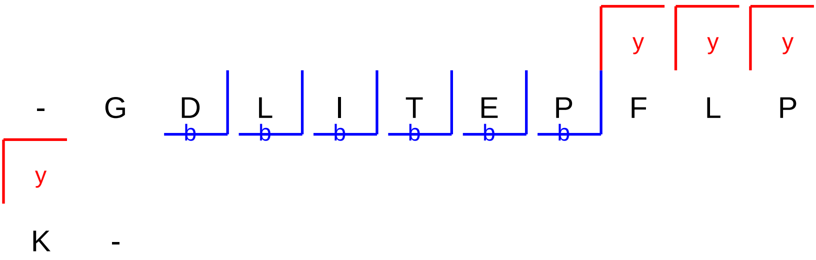

Raw File	Scan	Method	Score	m/z	Gene names
RO-QC-160121_ZackD_SS_Set_01_Fractio	32642	FTMS; HC	105.86	563.34	SLC35A2

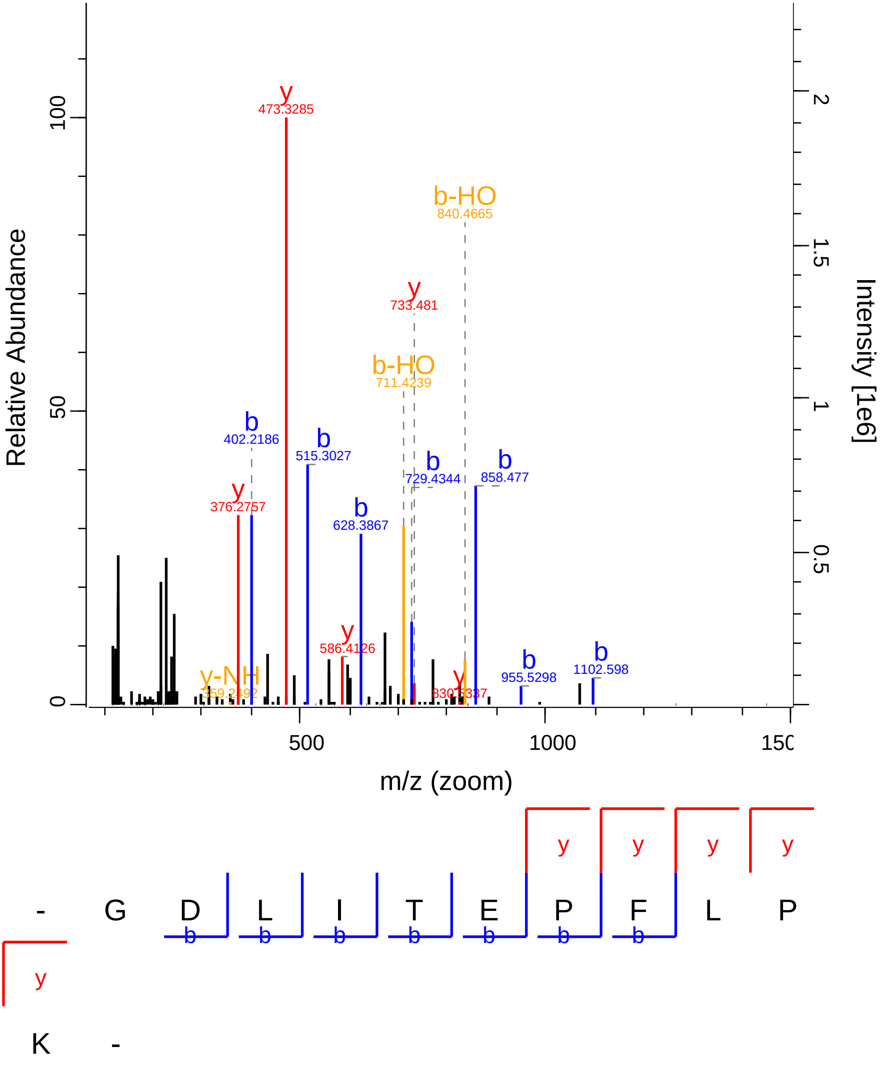

Raw File	Scan	Method	Score	m/z	Gene names
RO-QC-160121_ZackD_SS_Set_01_Fraction	34363	FTMS; HC	51.77	570.3	POP5

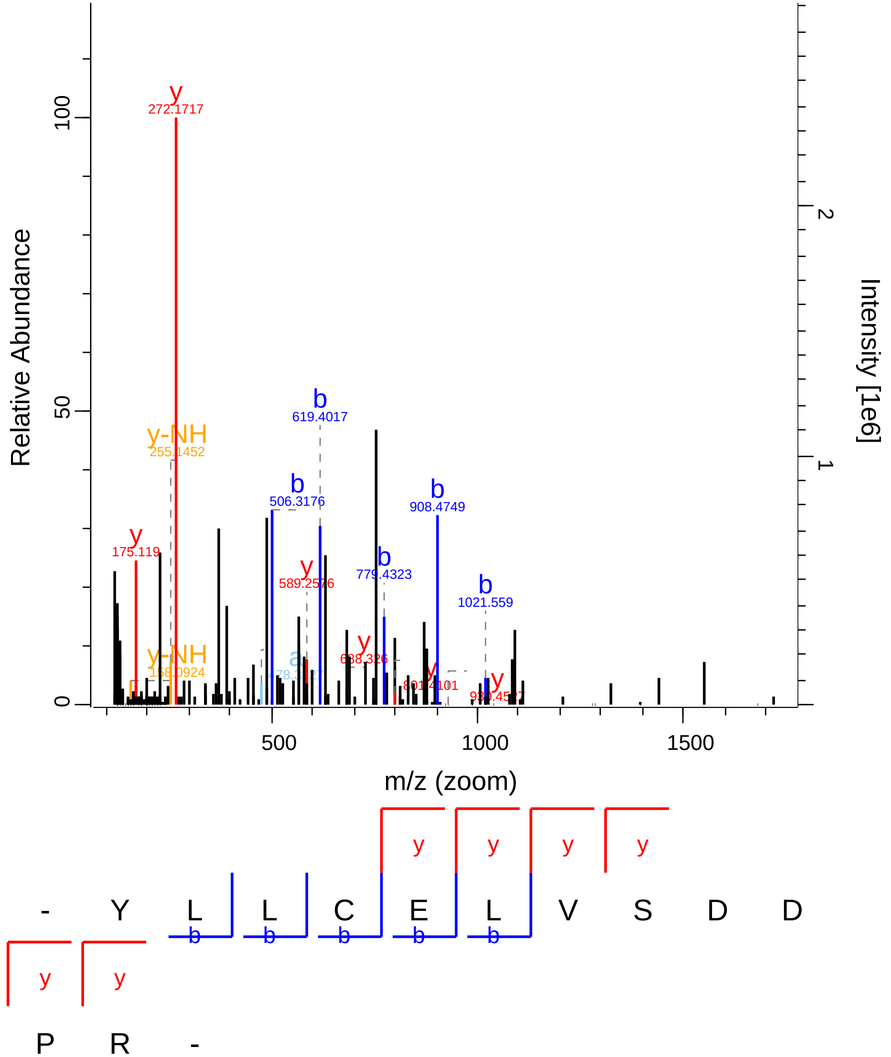

Raw File	Scan	Method	Score	m/z	Gene names
RO-QC-160121_ZackD_SS_Set_01_Fractior	34454	FTMS; HC	91.85	854.94	POP5

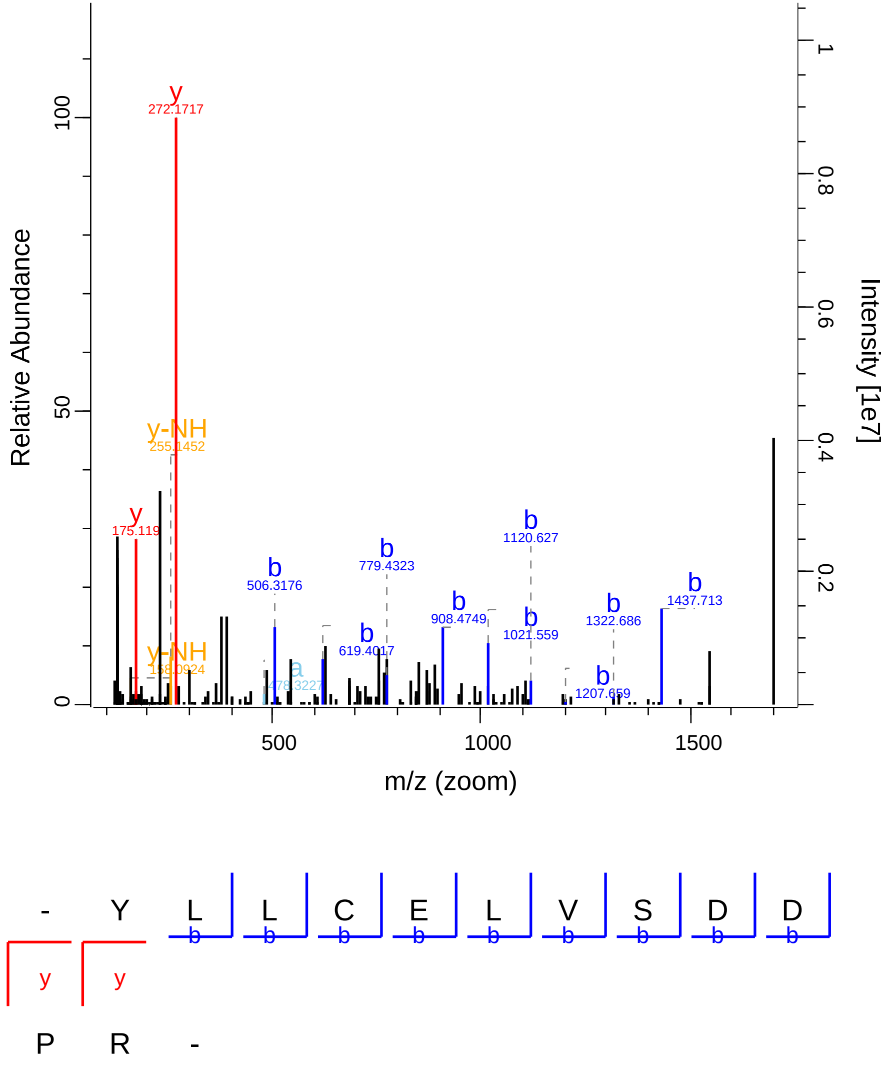

Raw File	Scan	Method	Score	m/z	Gene names
RO-QC-160121_ZackD_SS_Set_01_Fraction	35367	FTMS; HC	113.29	645.7	METRN

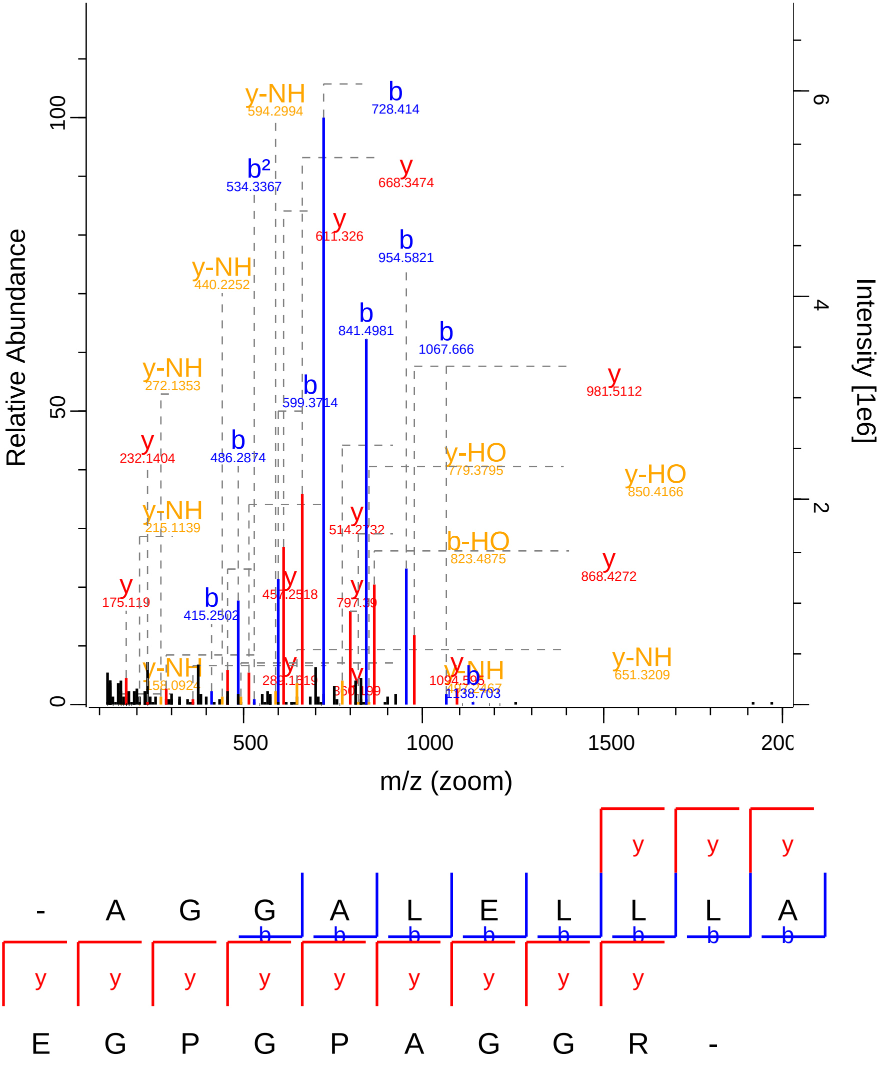

Raw File	Scan	Method	Score	m/z	Gene names
RO-QC-160121_ZackD_SS_Set_01_Fraction	35767	FTMS; HC	54.7	799.8	HMGXB3

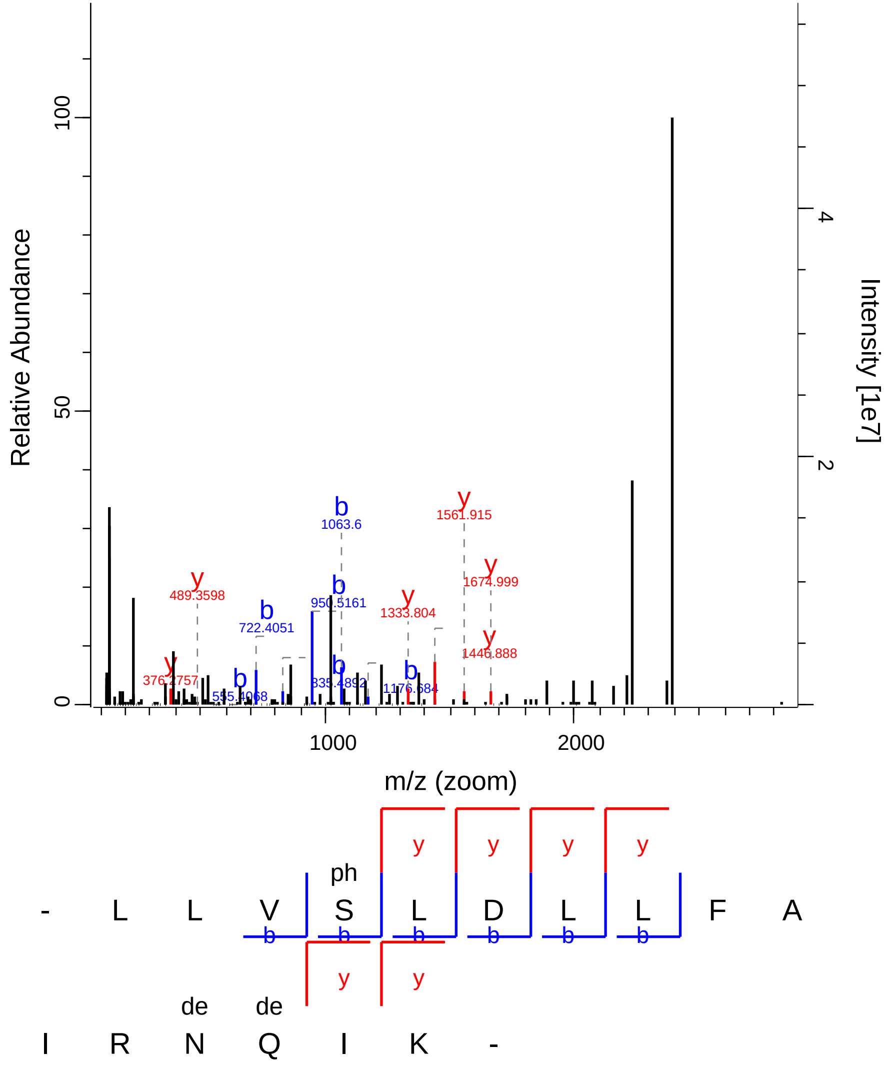

Raw File	Scan	Method	Score	m/z	Gene names
RO-QC-160121_ZackD_SS_Set_01_Fraction	35769	FTMS; HC	48.82	600.1	HMGXB3

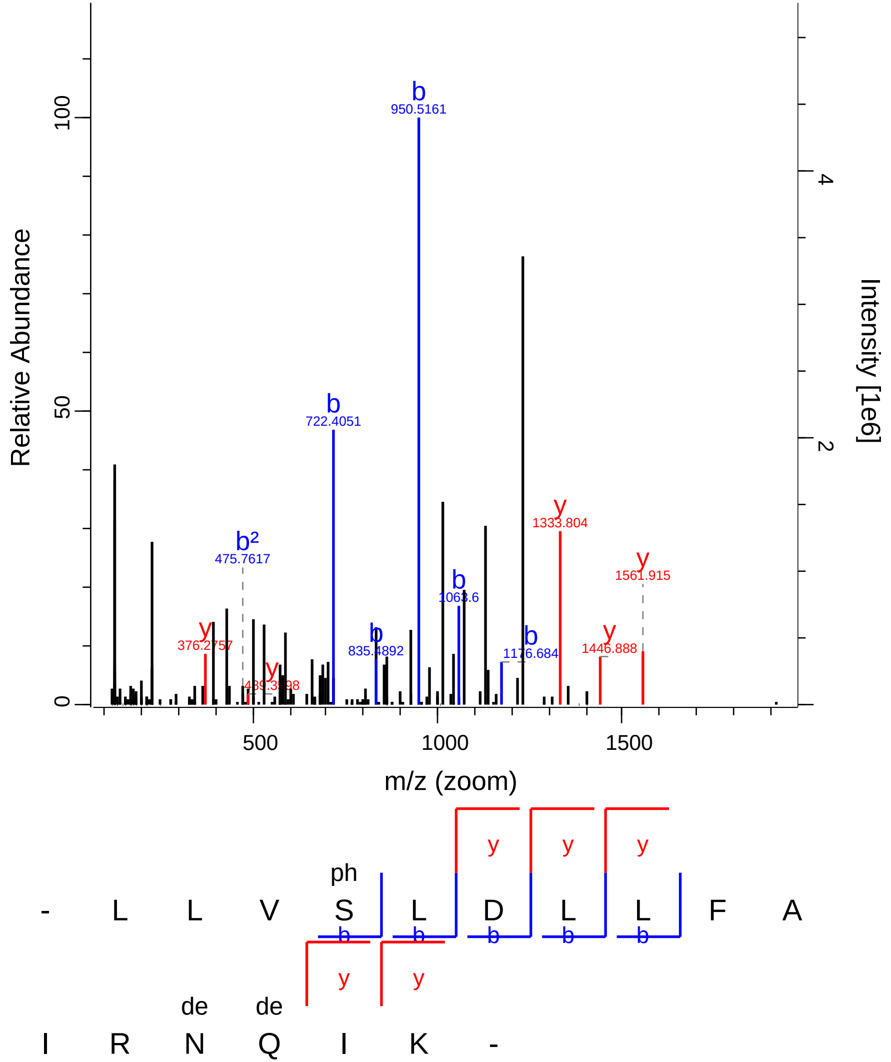

Raw File	Scan	Method	Score	m/z
RO-QC-160121_ZackD_SS_Set_01_Fraction_12	35845	FTMS; HCD	63.57	1070.09

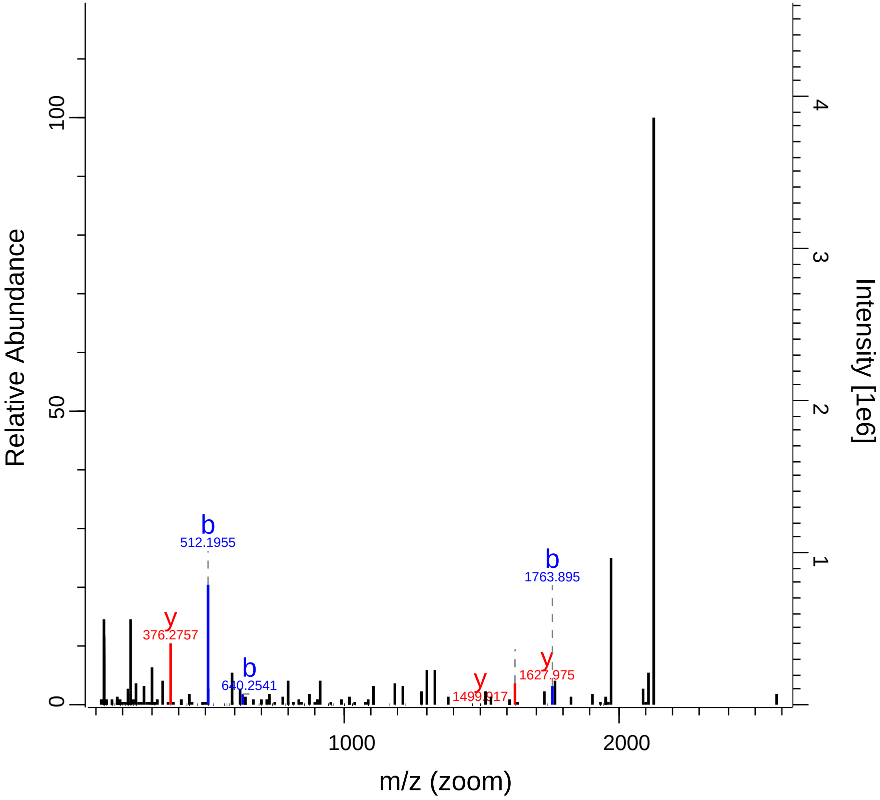

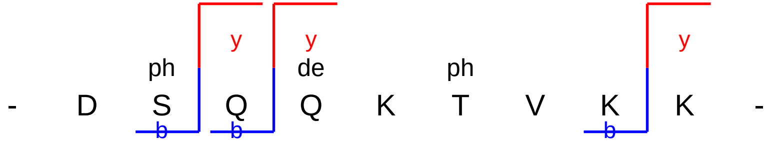

Raw File	Scan	Method	Score	m/z	Gene names
RO-QC-160121_ZackD_SS_Set_01_Fractior	36473	FTMS; HC	48.63	842.76	CCDC108

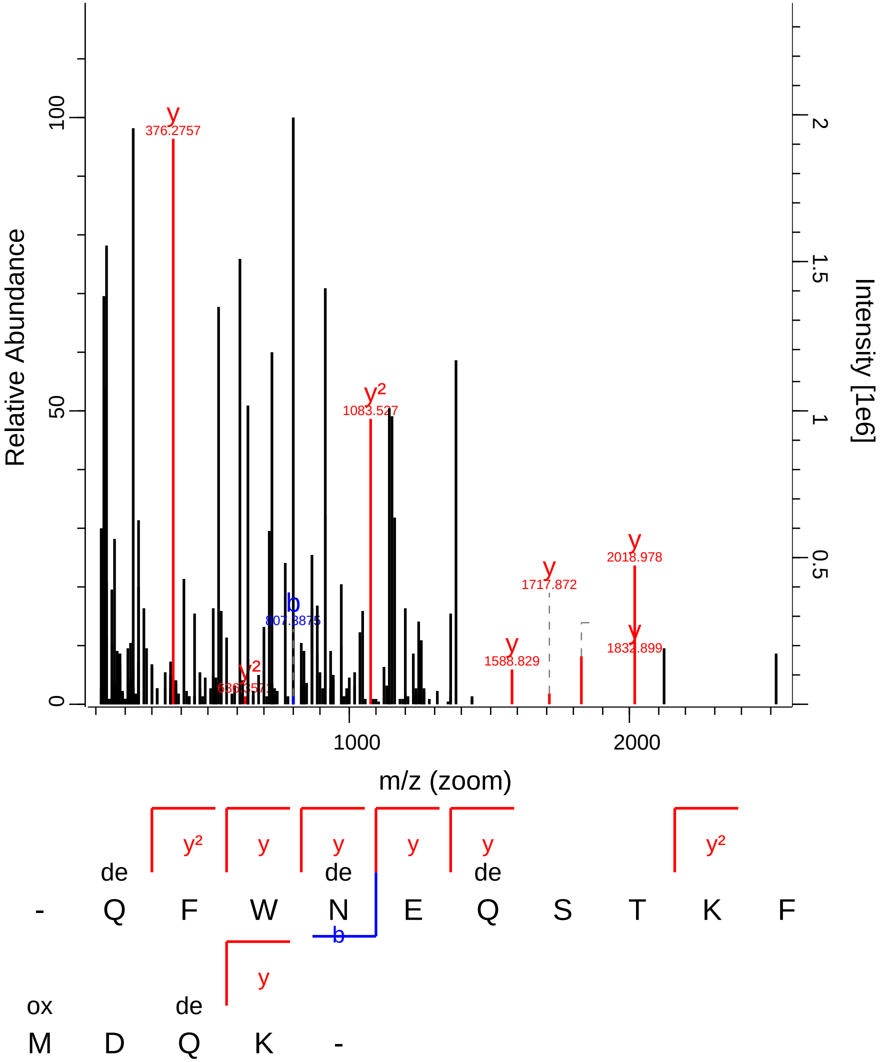

Raw File	Scan	Method	Score	m/z	Gene names
RO-QC-160121_ZackD_SS_Set_01_Fractio	36922	FTMS; HC	97.06	1066.52	MT-CO1

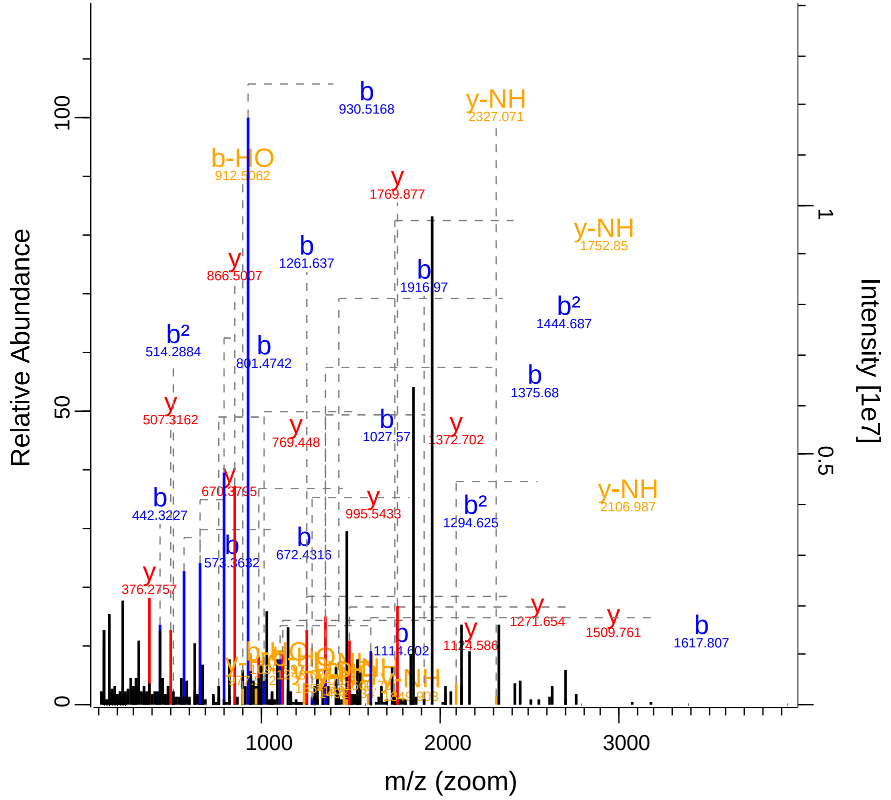

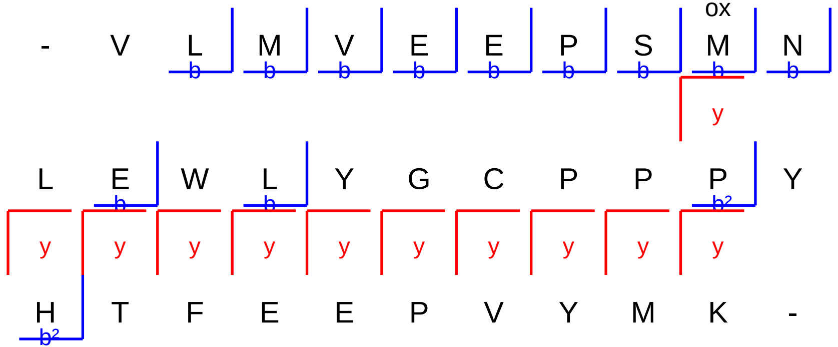

Raw File	Scan	Method	Score	m/z	Gene names
RO-QC-160121_ZackD_SS_Set_01_Fractio	37321	FTMS; HC	238.01	1076.04	DEGS1

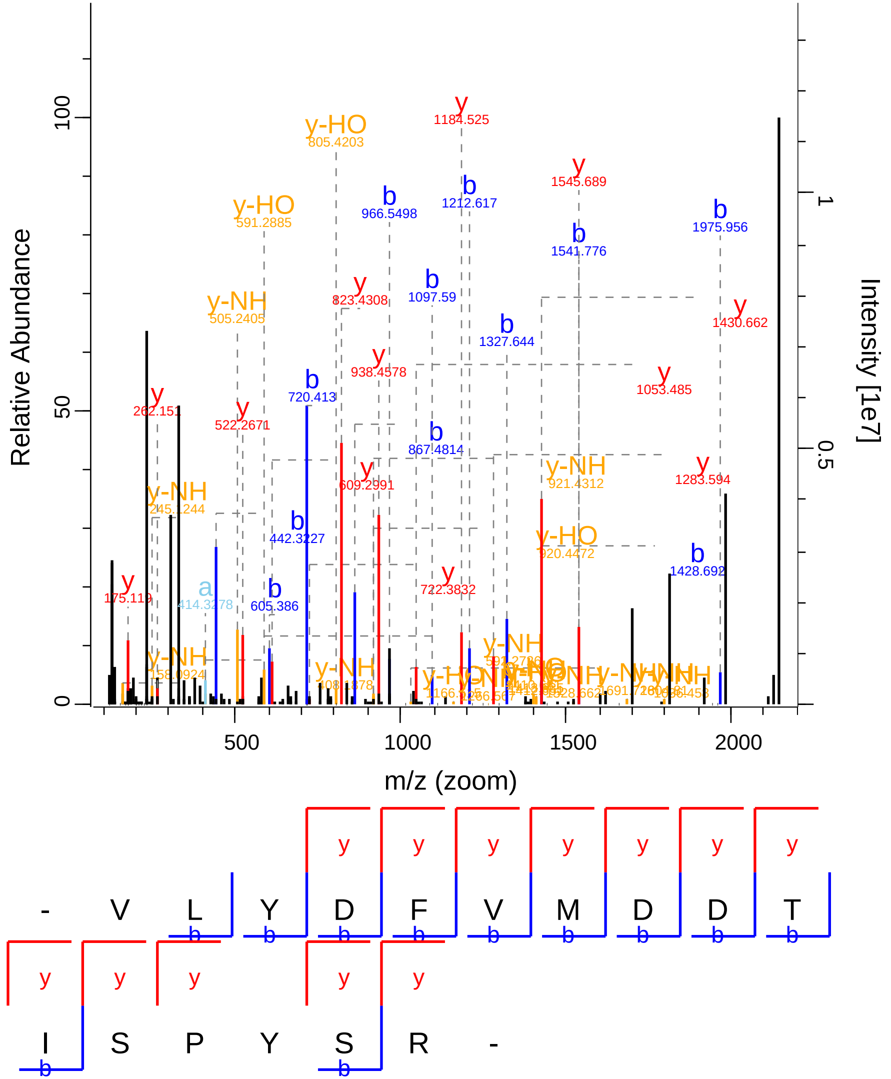

Raw File	Scan	Method	Score	m/z	Gene names
RO-QC-160121_ZackD_SS_Set_01_Fraction	37324	FTMS; HC	120.77	717.7	DEGS1

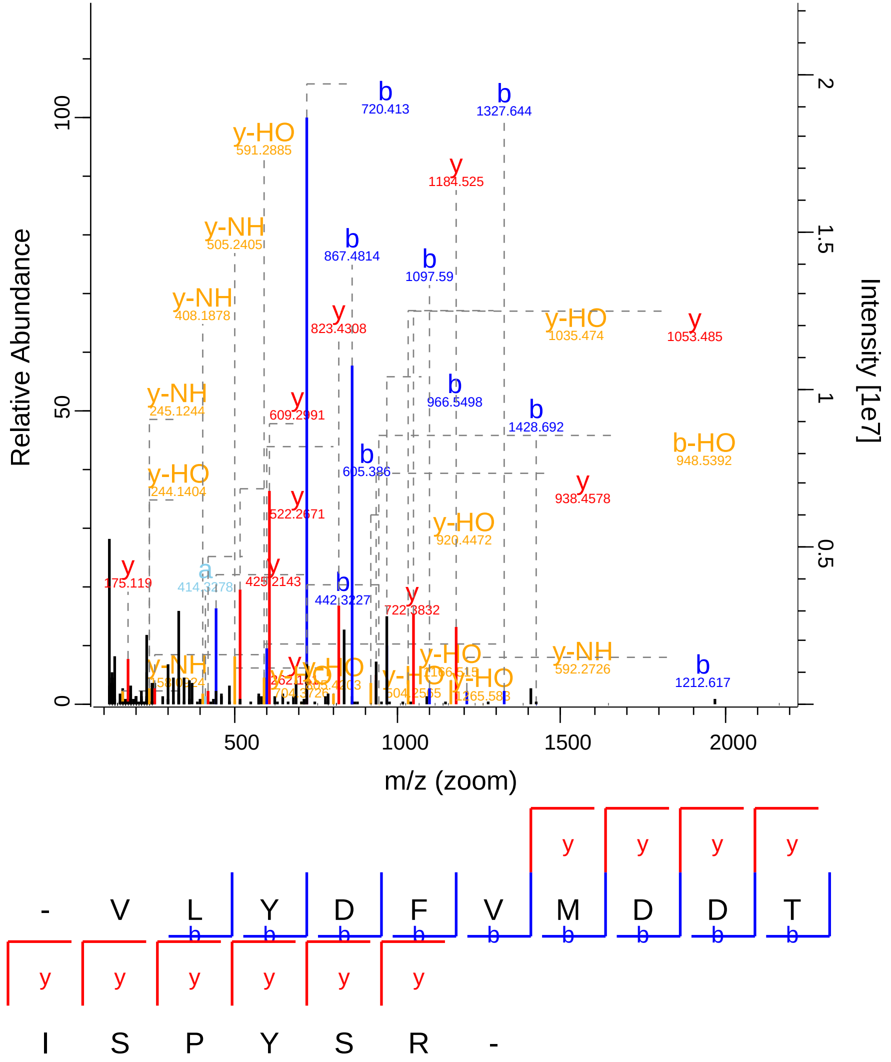

Raw File	Scan	Method	Score	m/z
RO-QC-160121_ZackD_SS_Set_01_Fraction_12	37846	FTMS; HCD	74.34	654.87

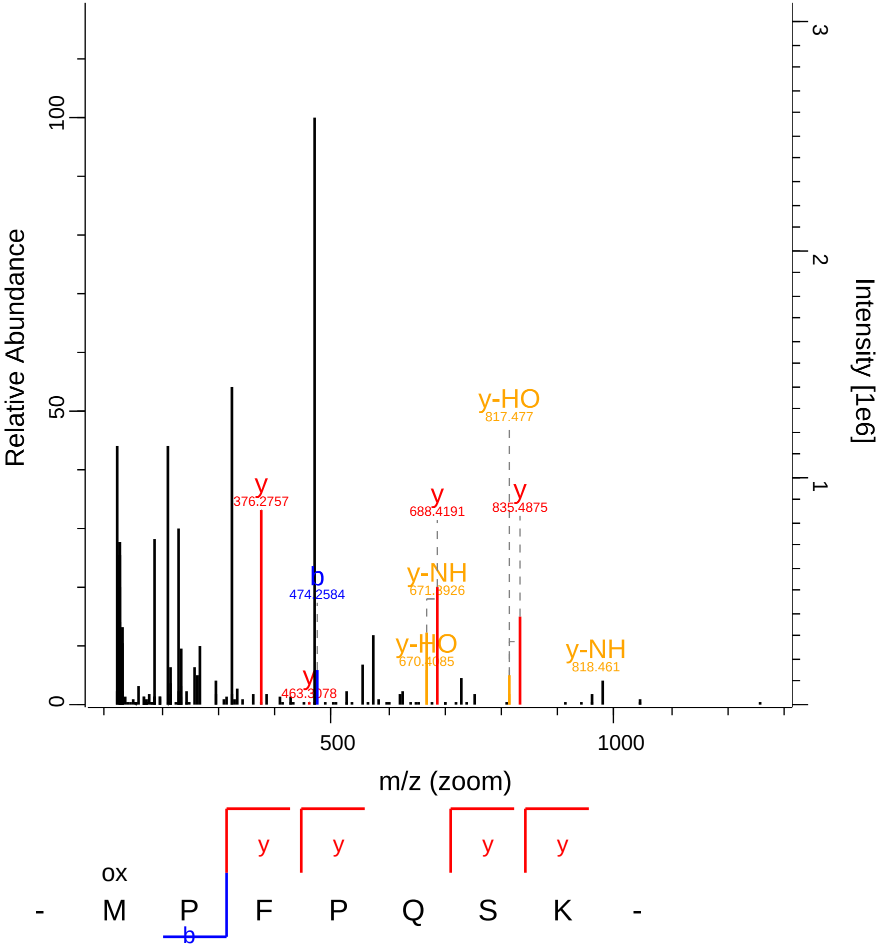

Raw File	Scan	Method	Score	m/z	Gene names
RO-QC-160121_ZackD_SS_Set_01_Fractio	38007	FTMS; HC	158.79	993.02	TOMM7

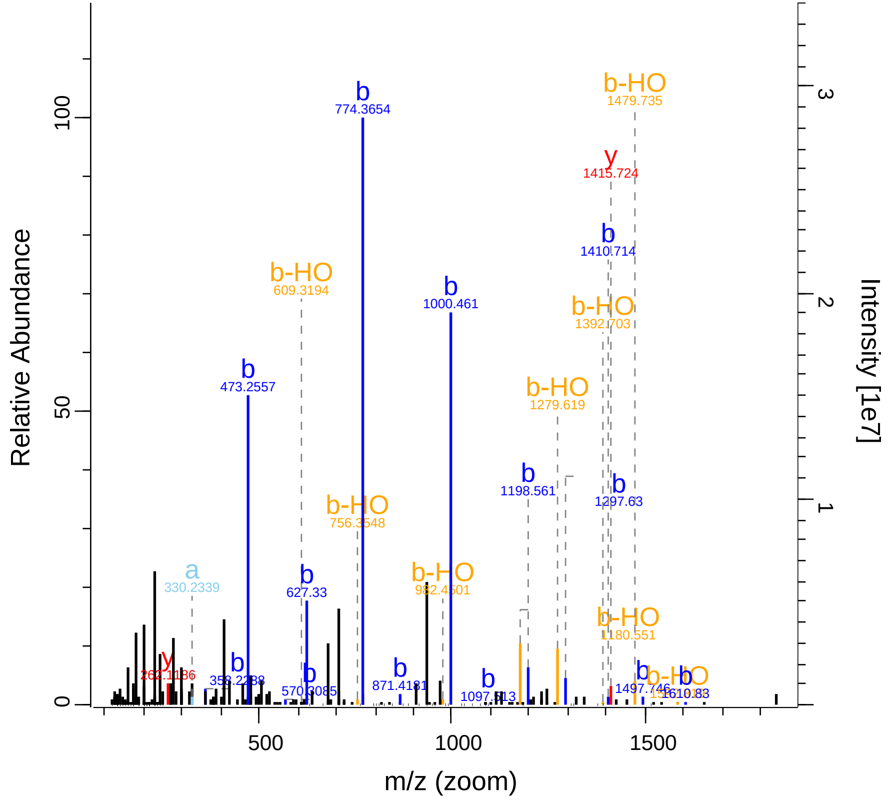

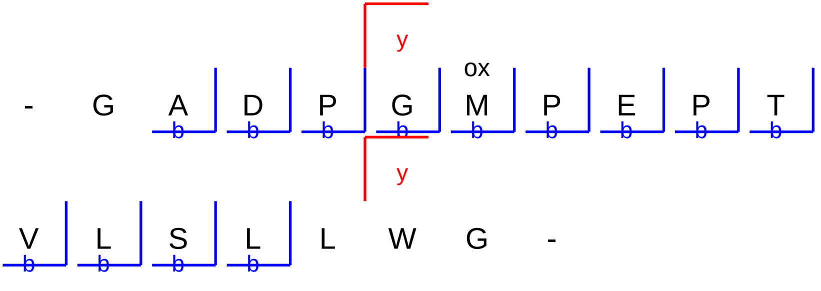

Raw File	Scan	Method	Score	m/z	Gene names
RO-QC-160121_ZackD_SS_Set_01_Fractior	38010	FTMS; HC	54.7	662.35	TOMM7

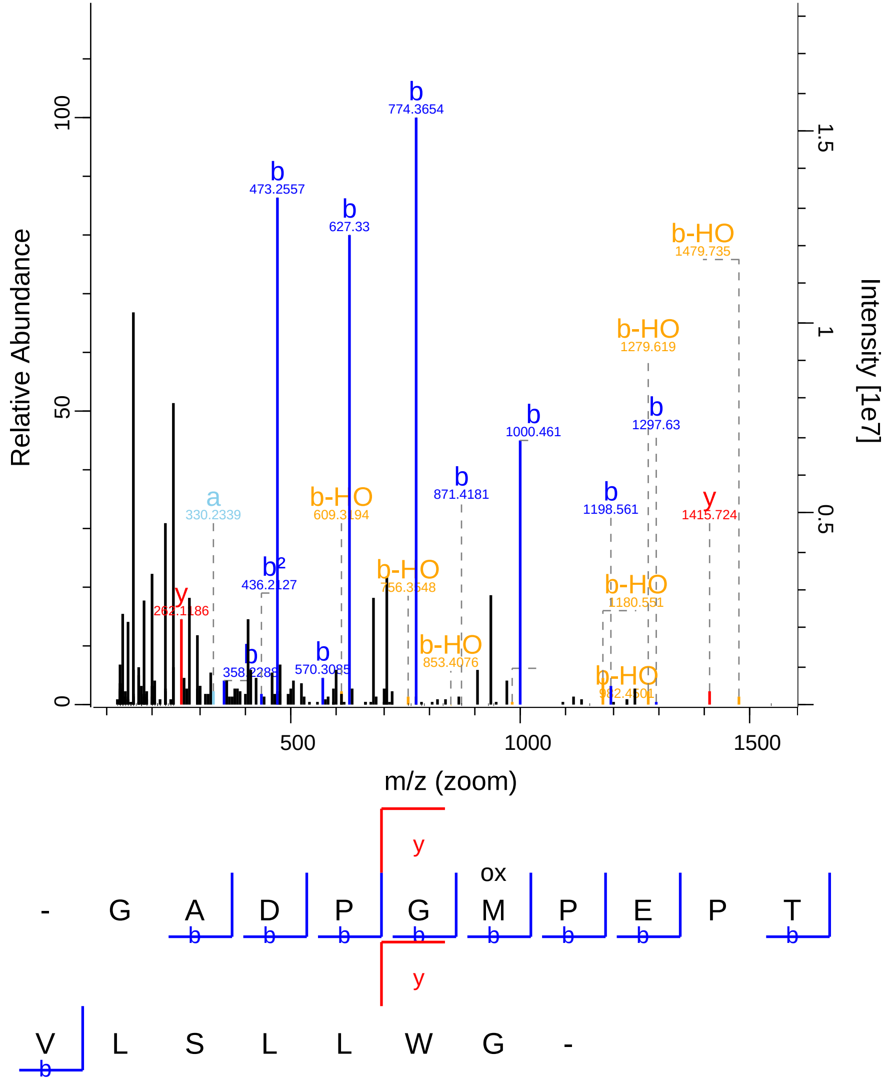

Raw File	Scan	Method	Score	m/z	Gene names
RO-QC-160121_ZackD_SS_Set_01_Fractior	38286	FTMS; HC	84.17	840.44	BSCL2

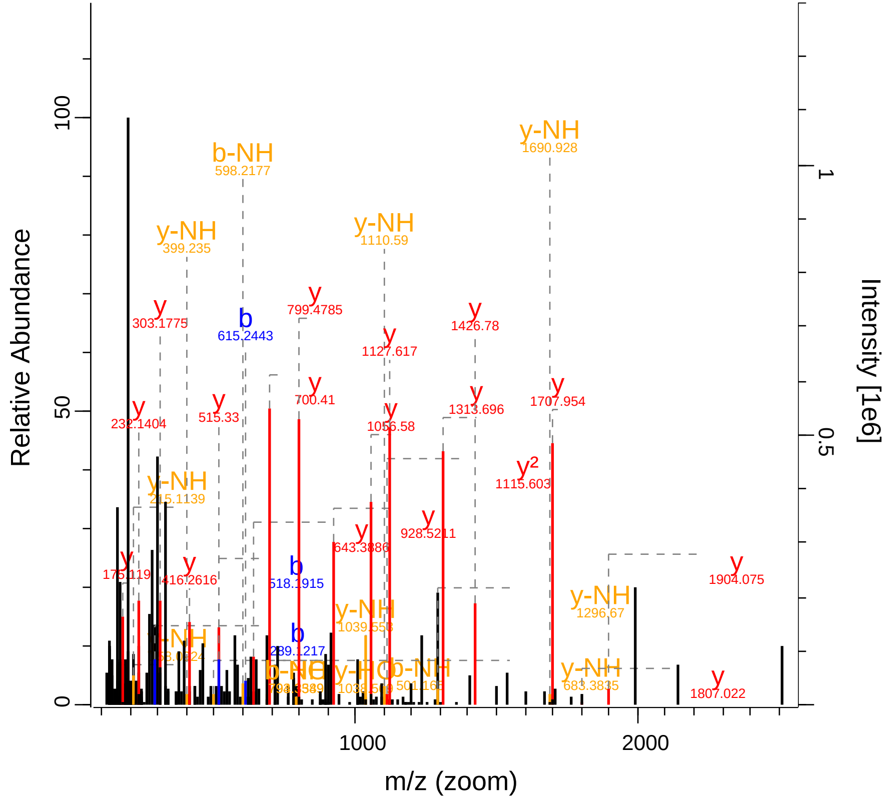

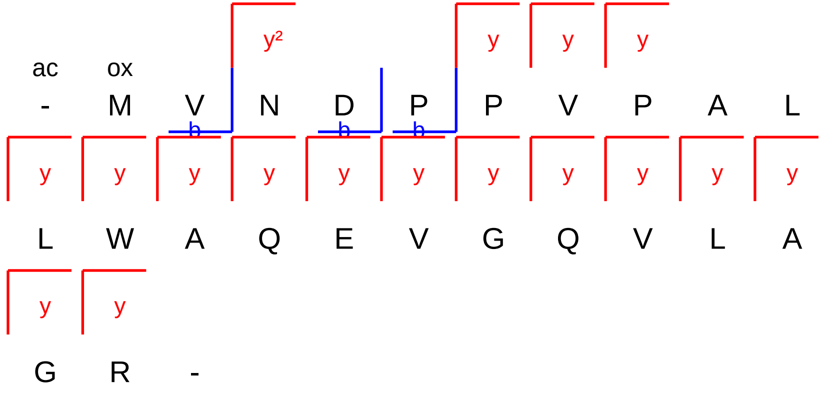

Raw File	Scan	Method	Score	m/z	Gene names
RO-QC-160121_ZackD_SS_Set_01_Fractio	38298	FTMS; HC	77.42	1260.17	BSCL2

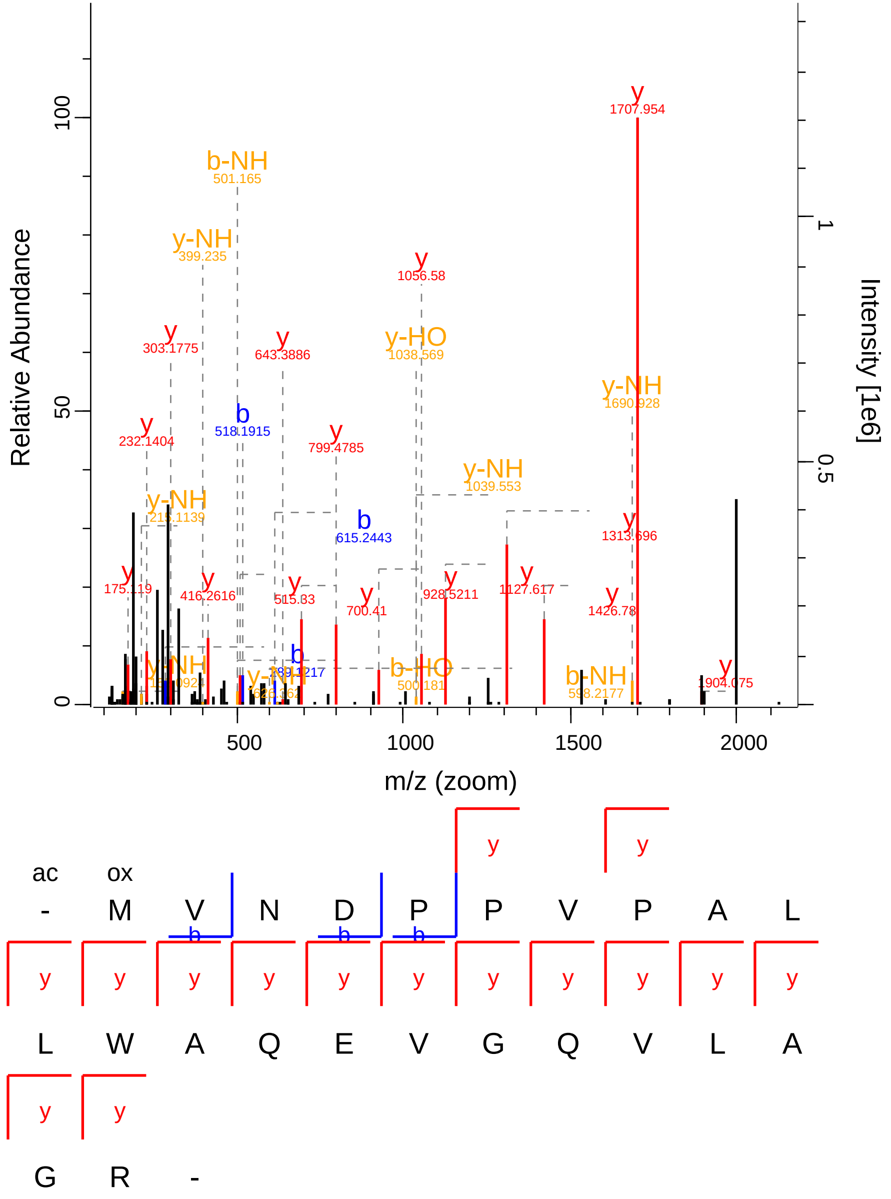

Raw File	Scan	Method	Score	m/z	Gene names
RO-QC-160121_ZackD_SS_Set_02_Fractior	4873	FTMS; HCI	49.42	638.31	BARD1

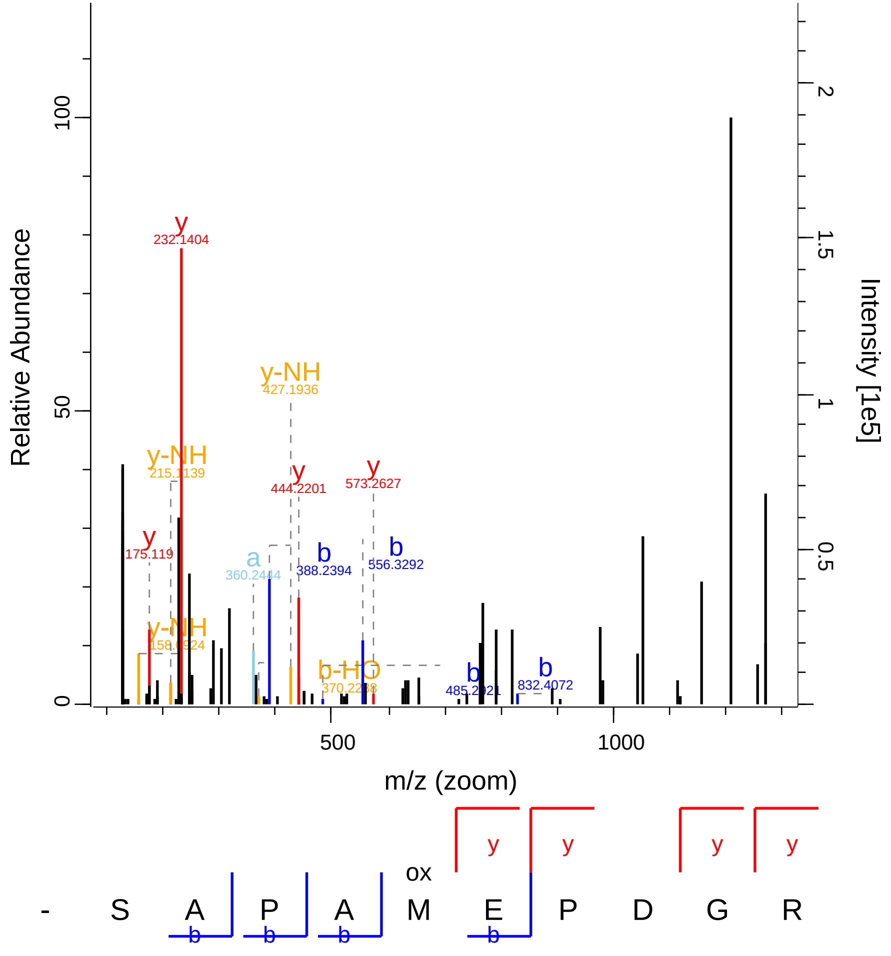

-

Raw File	Scan	Method	Score	m/z	Gene names
RO-QC-160121_ZackD_SS_Set_02_Fractio	5111	FTMS; HC	112.01	669.83	TMEM47

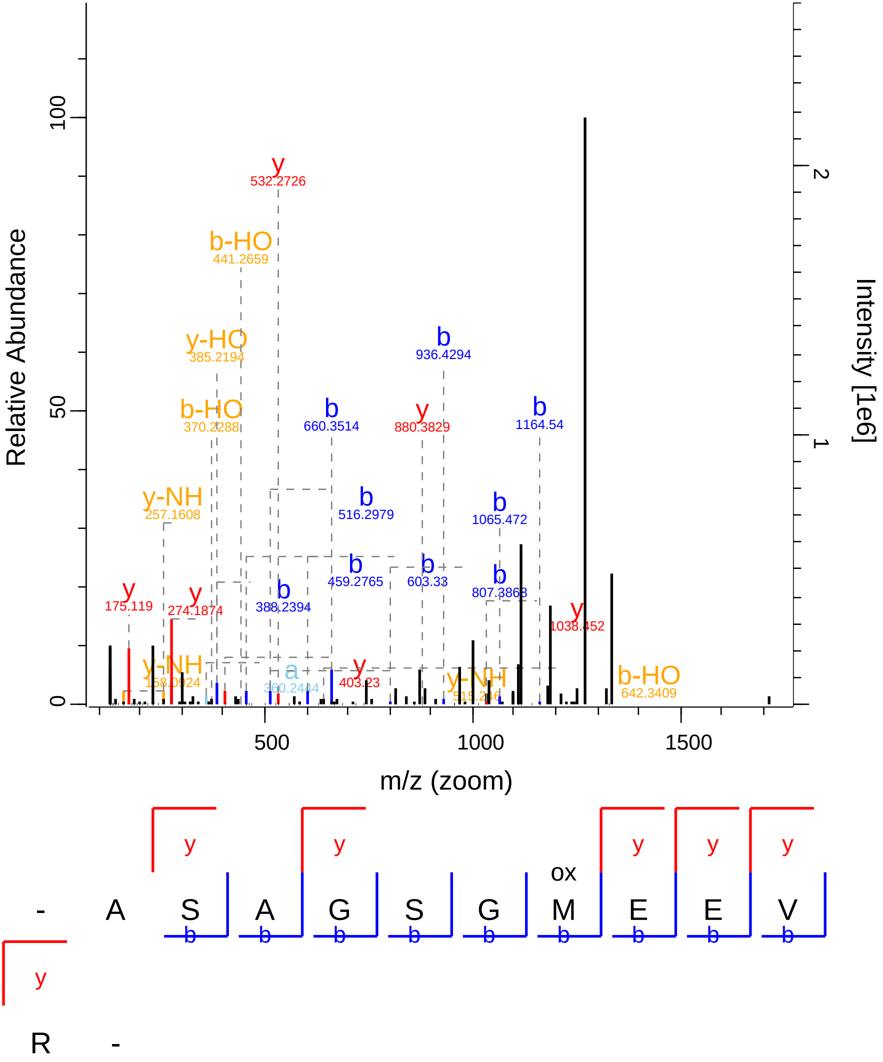

Raw File	Scan	Method	Score	m/z	Gene names
RO-QC-160121_ZackD_SS_Set_02_Fractio	5210	FTMS; HCI	50.7	677.88	SPIN2B;SPIN2

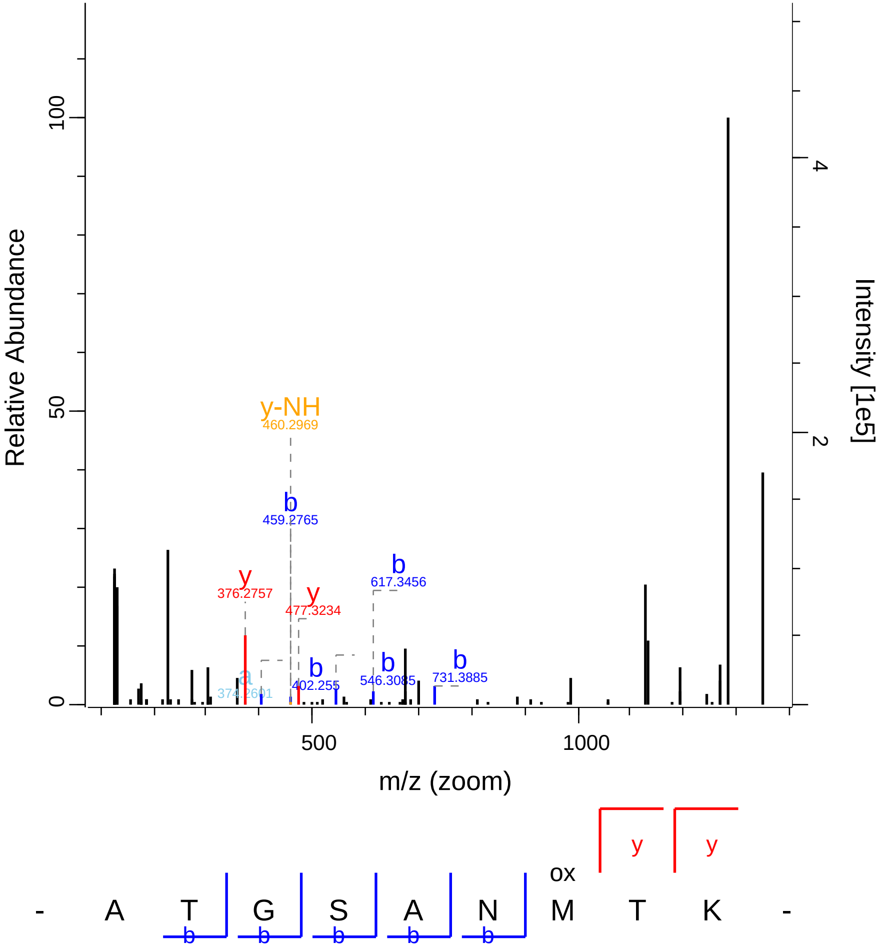

Raw File	Scan	Method	Score	m/z
RO-QC-160121_ZackD_SS_Set_02_Fraction_1	5314	FTMS; HCD	38.63	507.54

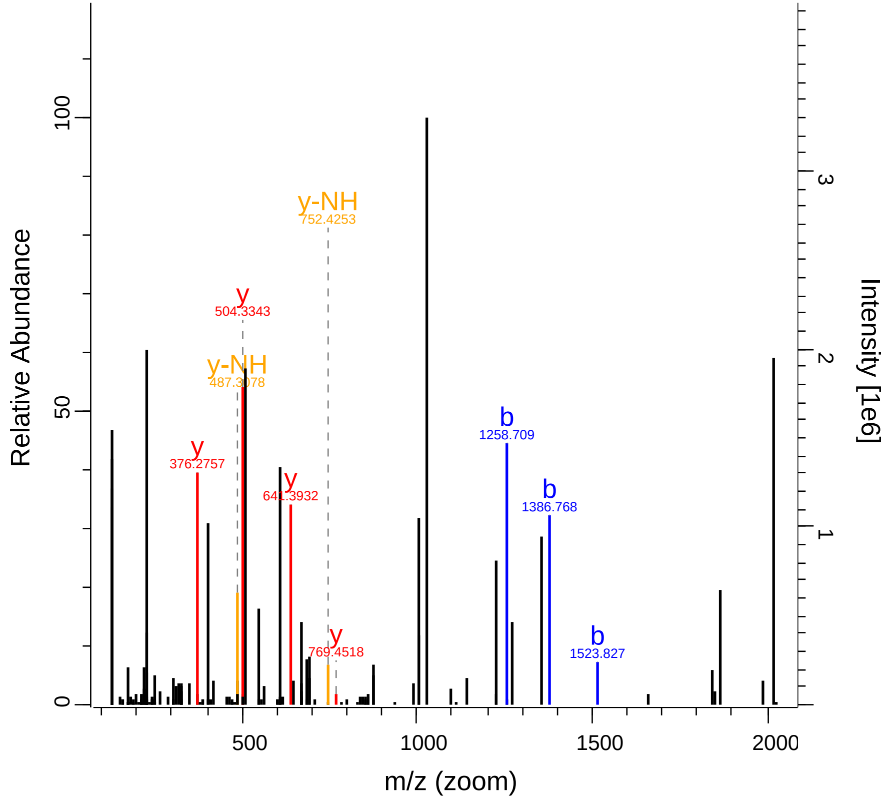

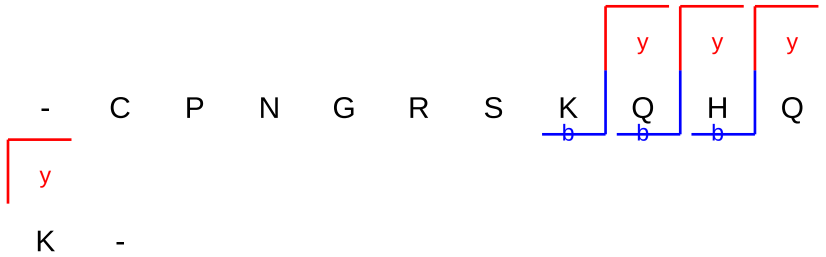

Raw File	Scan	Method	Score	m/z	Gene names
RO-QC-160121_ZackD_SS_Set_02_Fractio[illegible]	5330	FTMS; HC	116.01	519.04	ZNF518B

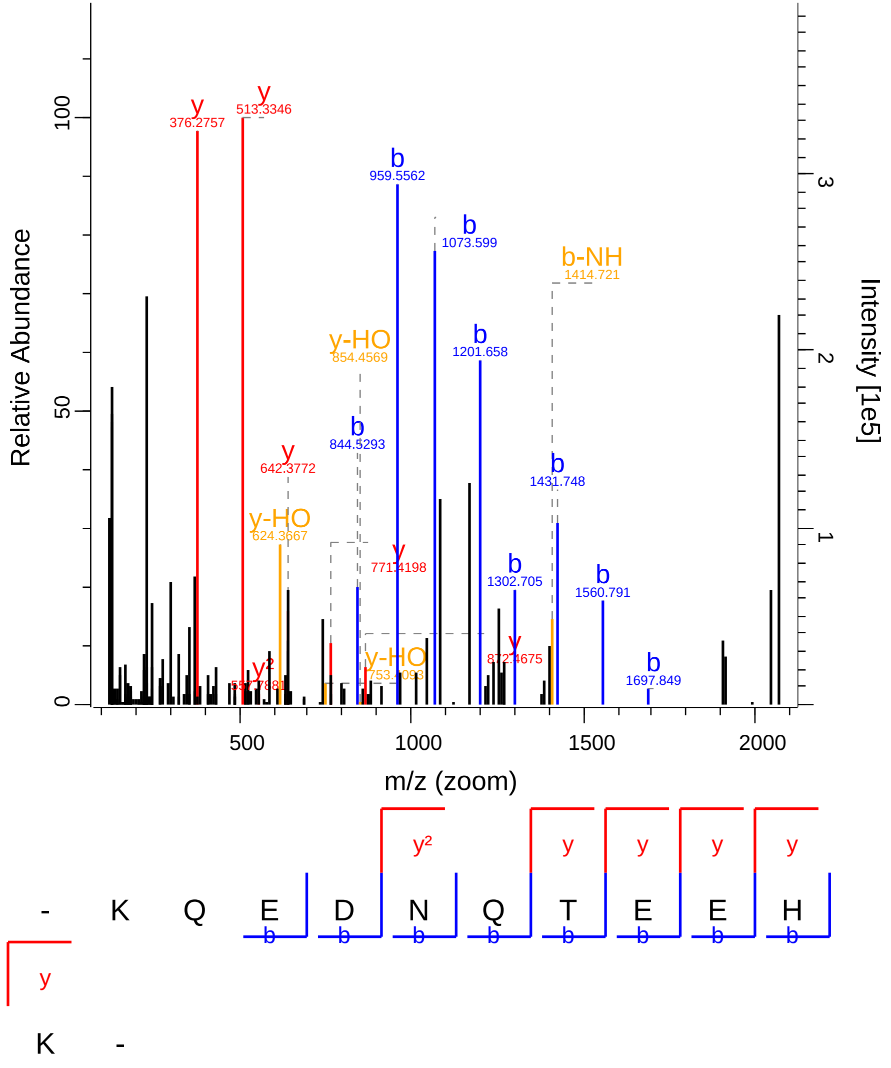

Raw File	Scan	Method	Score	m/z
RO-QC-160121_ZackD_SS_Set_02_Fraction_1	5331	FTMS; HCD	69.01	676.39

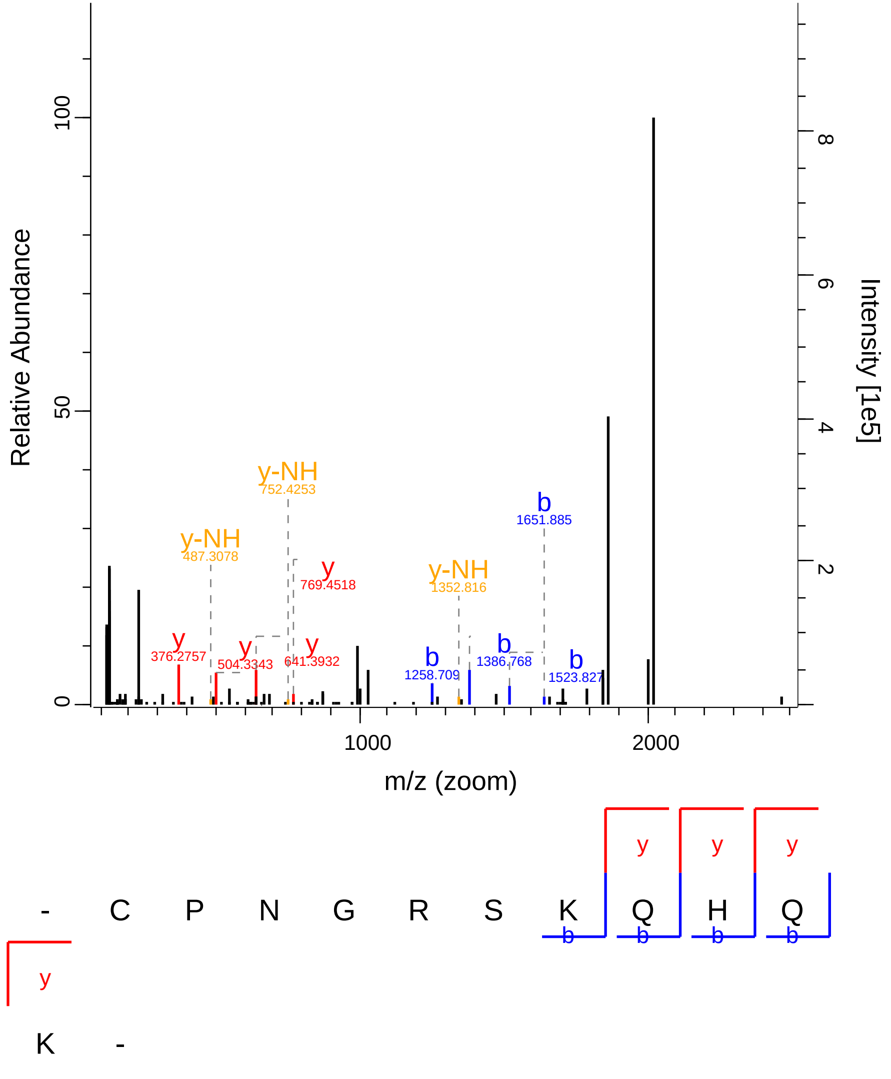

Raw File	Scan	Method	Score	m/z	Gene names
RO-QC-160121_ZackD_SS_Set_02_Fraction	5707	FTMS; HCI	103.83	575.8	GPAM

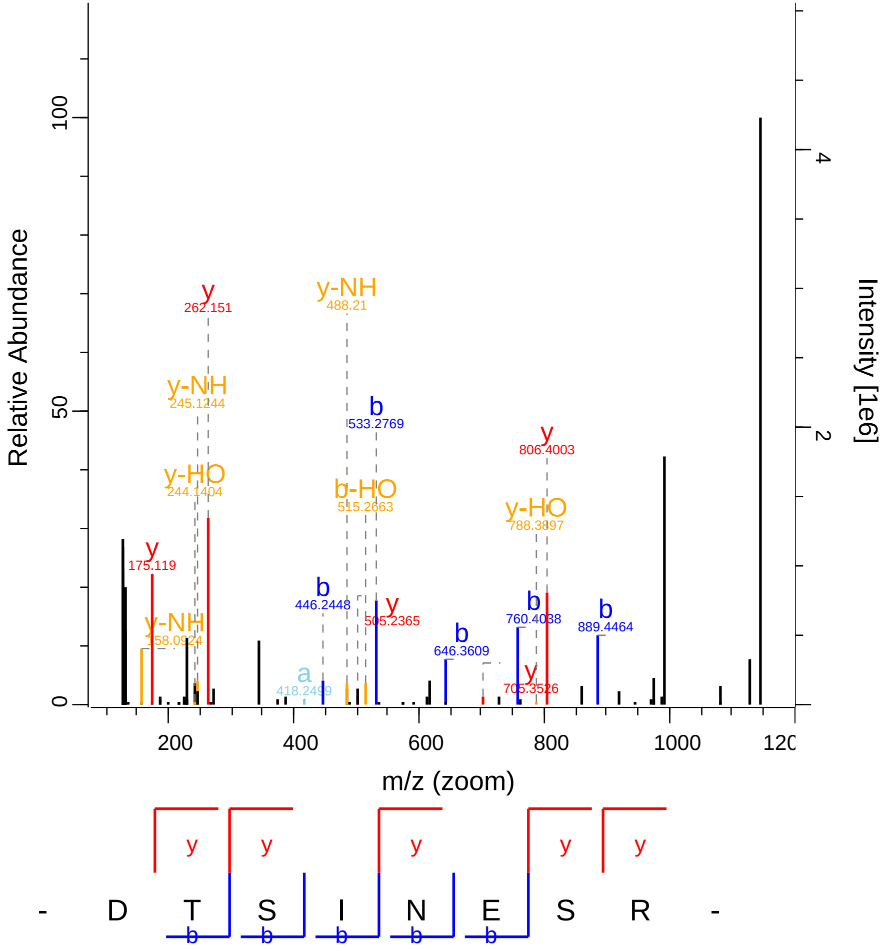

Raw File	Scan	Method	Score	m/z	Gene names
RO-QC-160121_ZackD_SS_Set_02_Fractior	6493	FTMS; HCI	98.03	702.89	TRMT12

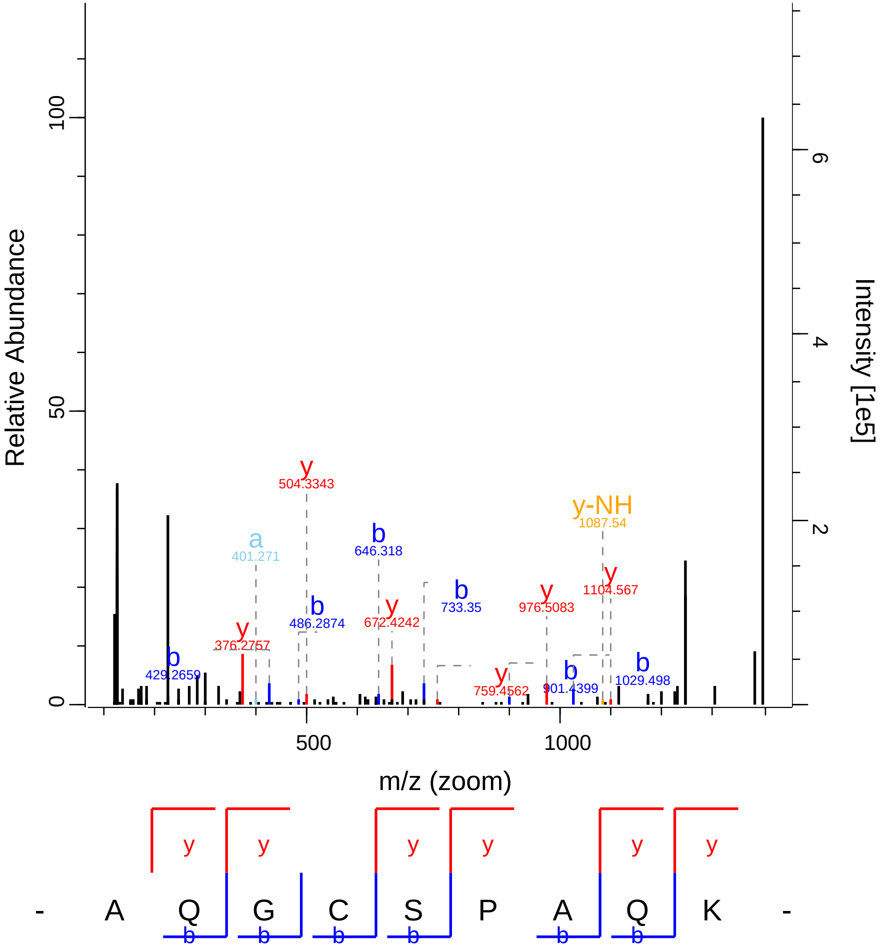

Raw File	Scan	Method	Score	m/z	Gene names
RO-QC-160121_ZackD_SS_Set_02_Fractio	6608	FTMS; HC	223.14	778.93	TTC39B

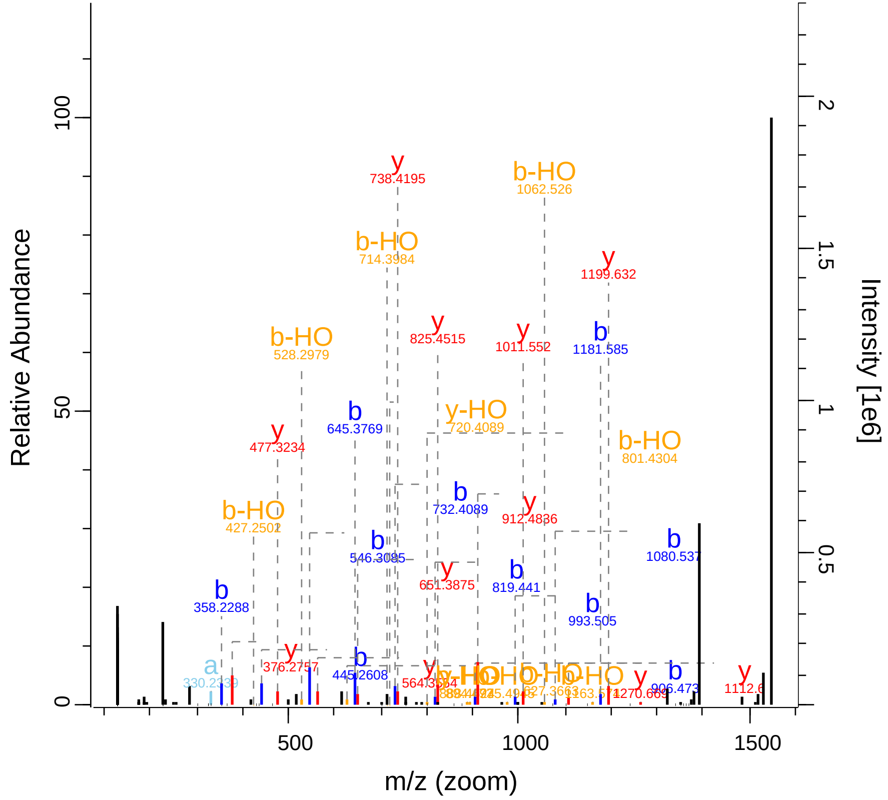

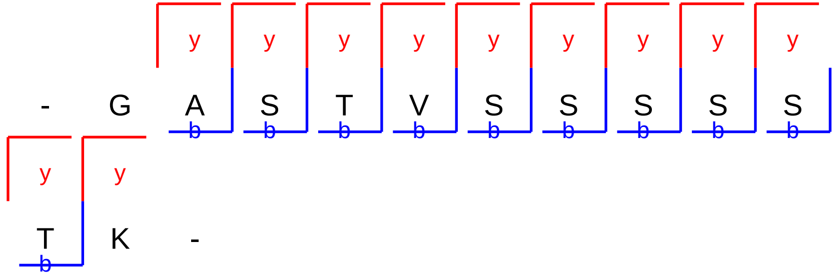

Raw File	Scan	Method	Score	m/z
RO-QC-160121_ZackD_SS_Set_02_Fraction_1	7114	FTMS; HCD	46.35	598.34

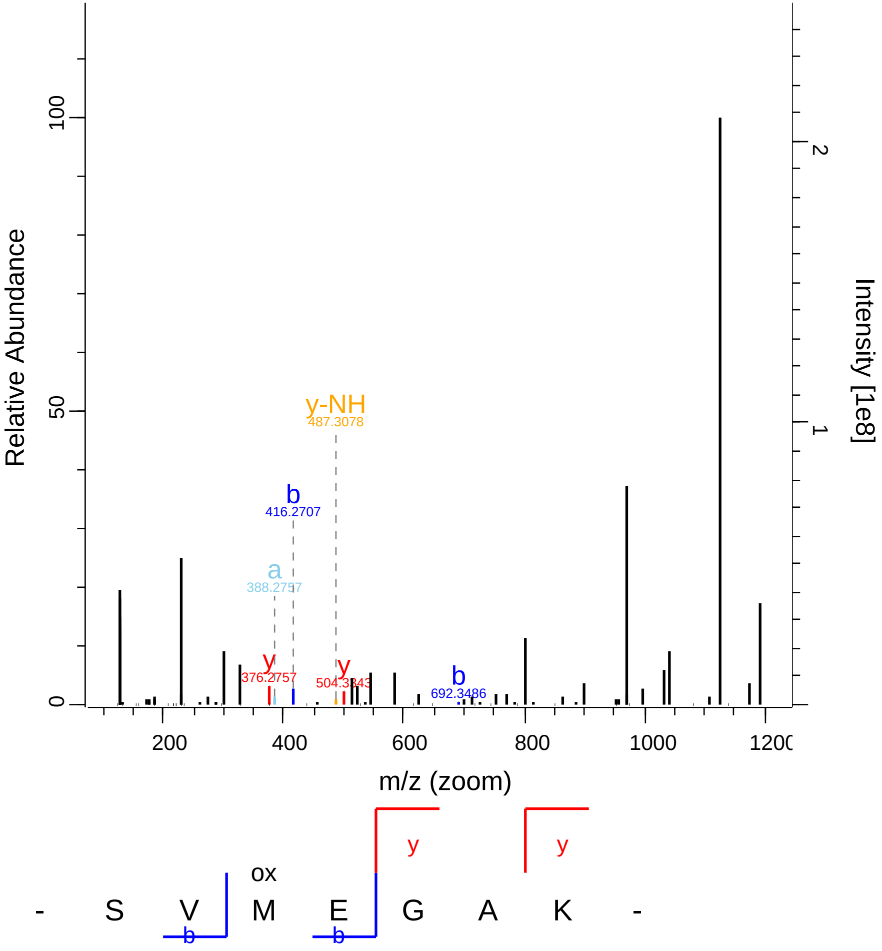

Raw File	Scan	Method	Score	m/z
RO-QC-160121_ZackD_SS_Set_02_Fraction_1	7115	FTMS; HCD	59.64	399.23

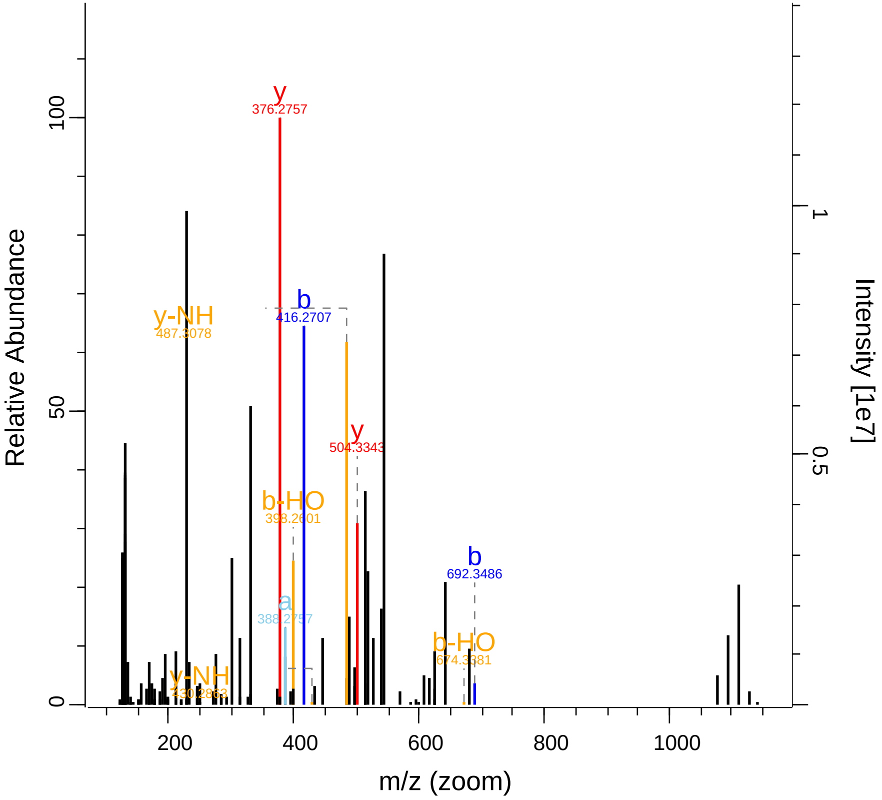

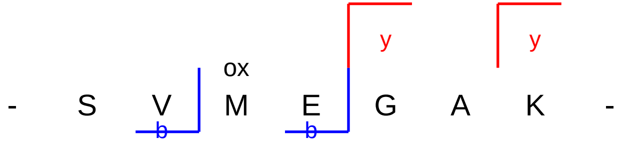

Raw File	Scan	Method	Score	m/z	Gene names
RO-QC-160121_ZackD_SS_Set_02_Fractio	7122	FTMS; HC	104.44	426.53	SHISA8

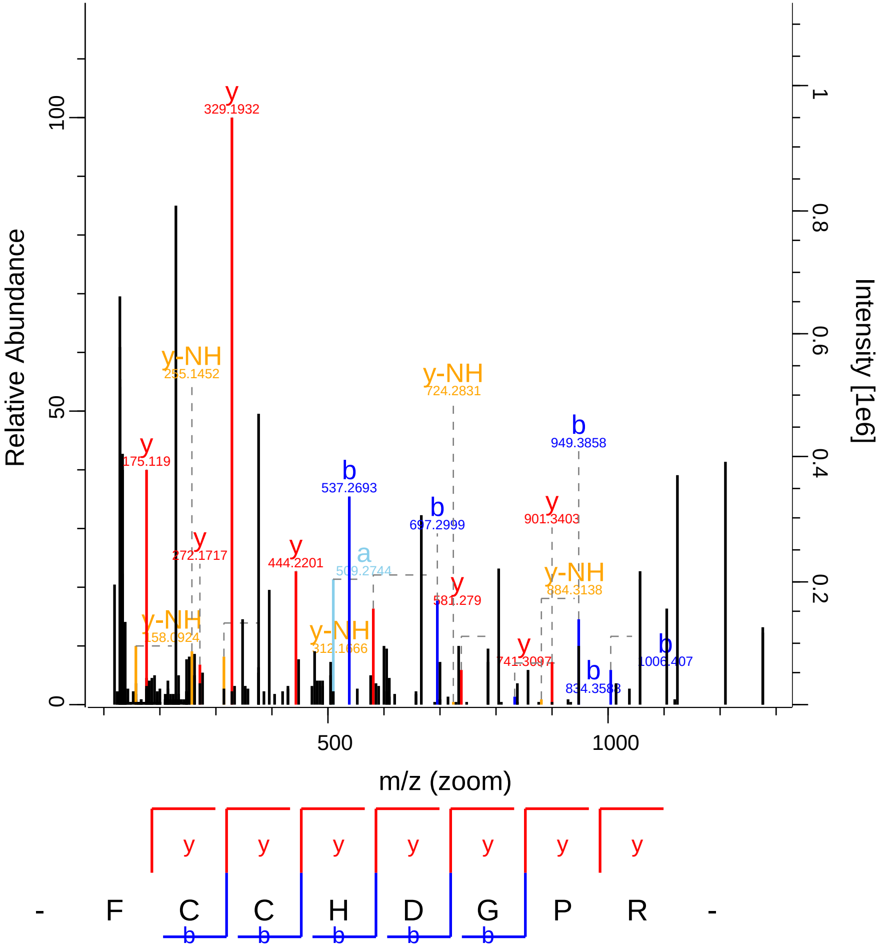

Raw File	Scan	Method	Score	m/z	Gene names
RO-QC-160121_ZackD_SS_Set_02_Fractior	7266	FTMS; HCI	79.91	456.94	TCTN2

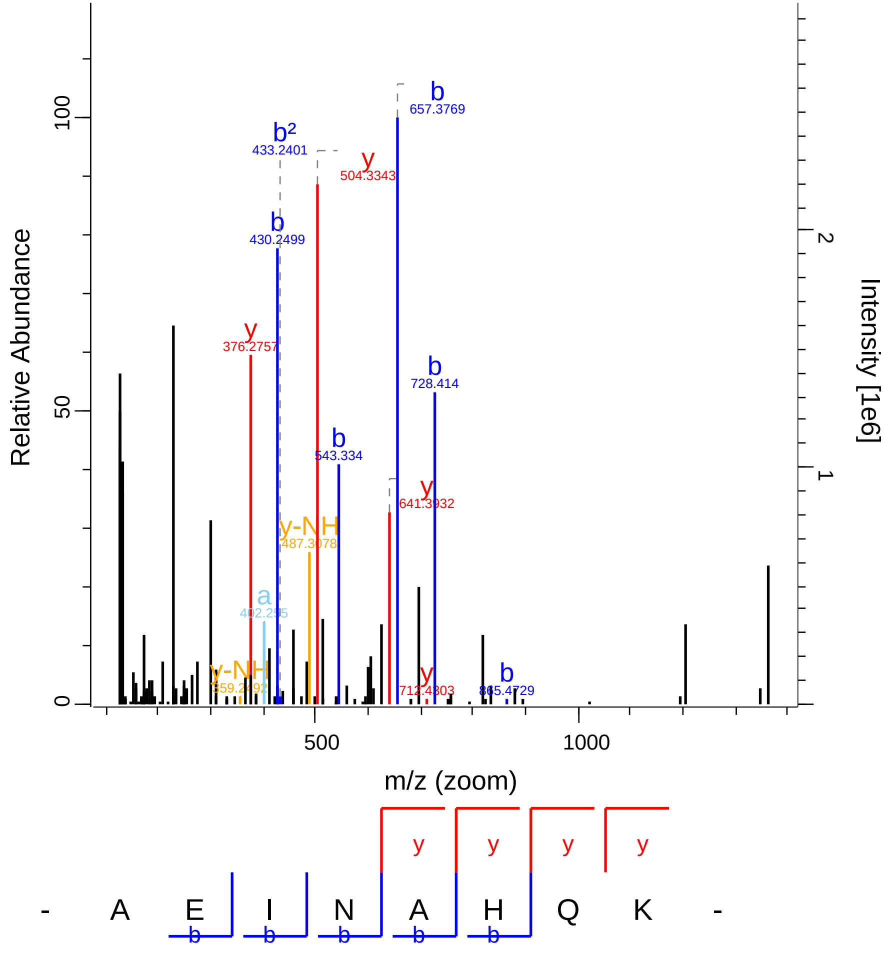

Raw File	Scan	Method	Score	m/z	Gene names
RO-QC-160121_ZackD_SS_Set_02_Fractior	7444	FTMS; HCI	94.09	717.05	PCGF5

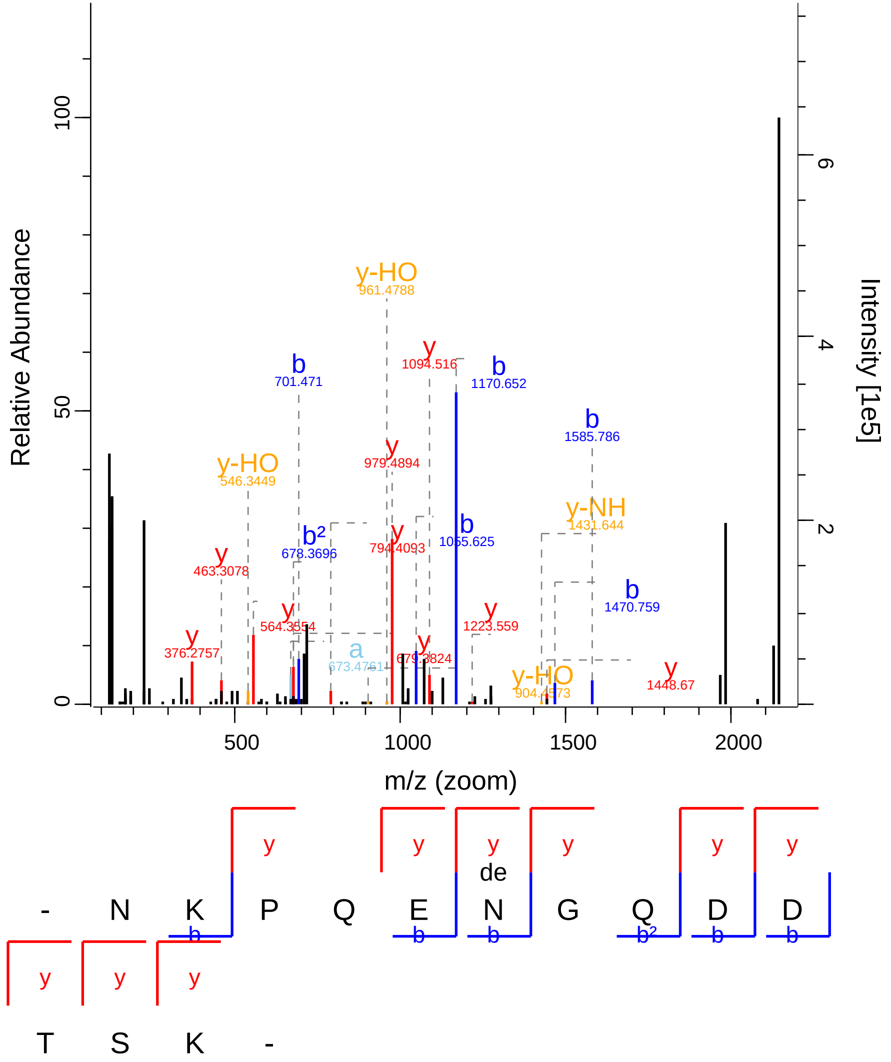

Raw File	Scan	Method	Score	m/z	Gene names
RO-QC-160121_ZackD_SS_Set_02_Fractior	7588	FTMS; HCI	45.86	730.41	CDC20

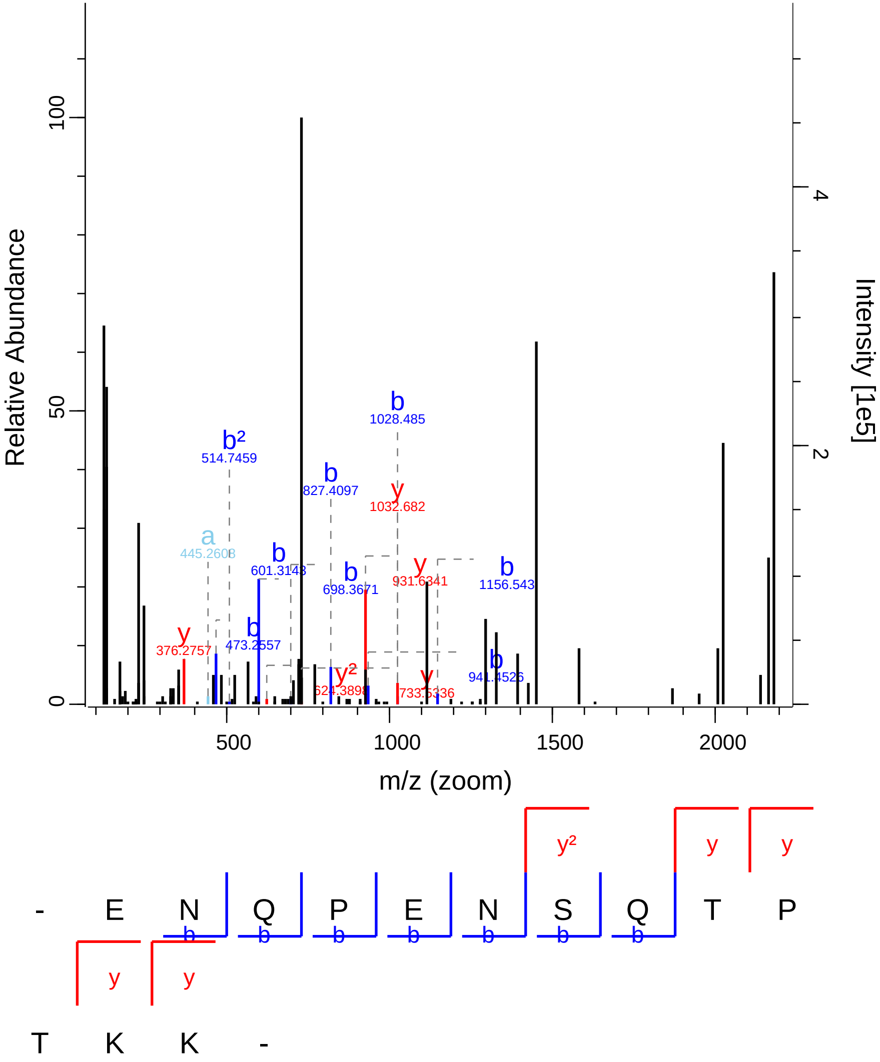

Raw File	Scan	Method	Score	m/z
RO-QC-160121_ZackD_SS_Set_02_Fraction_1	7878	FTMS; HCD	71.88	758.93

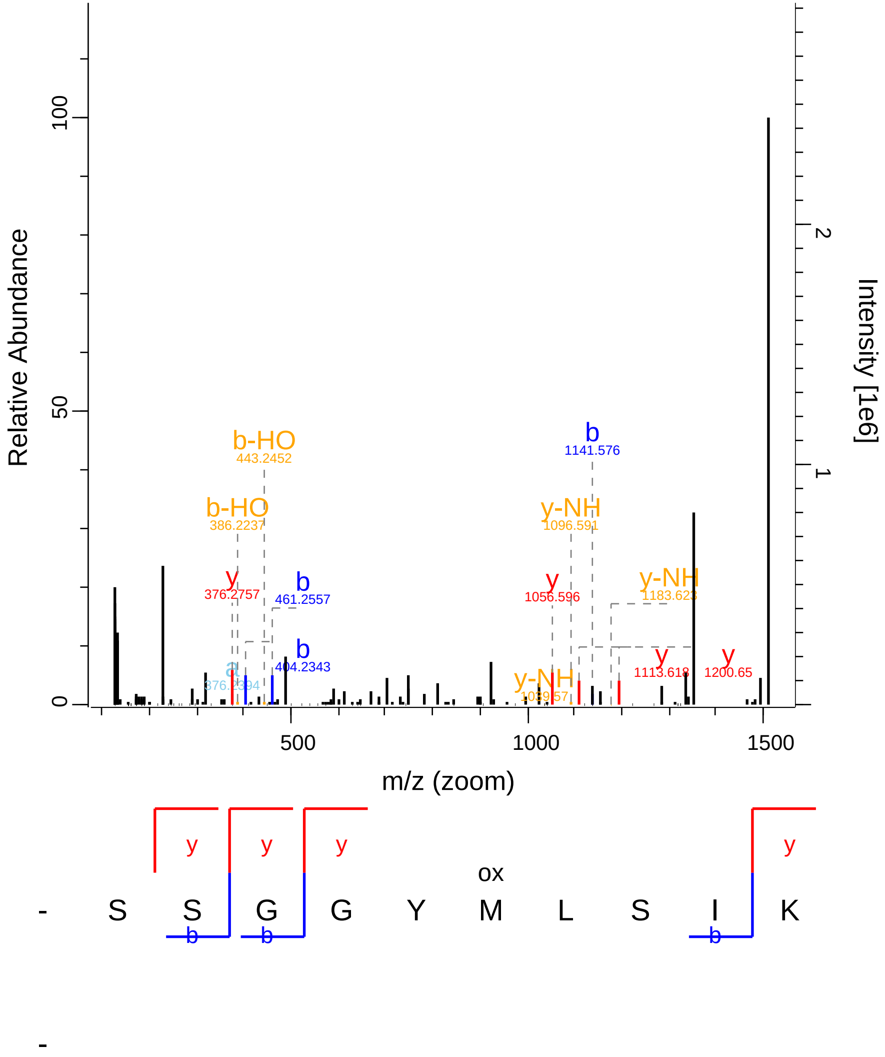

Raw File	Scan	Method	Score	m/z	Gene names
RO-QC-160121_ZackD_SS_Set_02_Fraction	7932	FTMS; HCI	142.43	786.9	SLC46A1

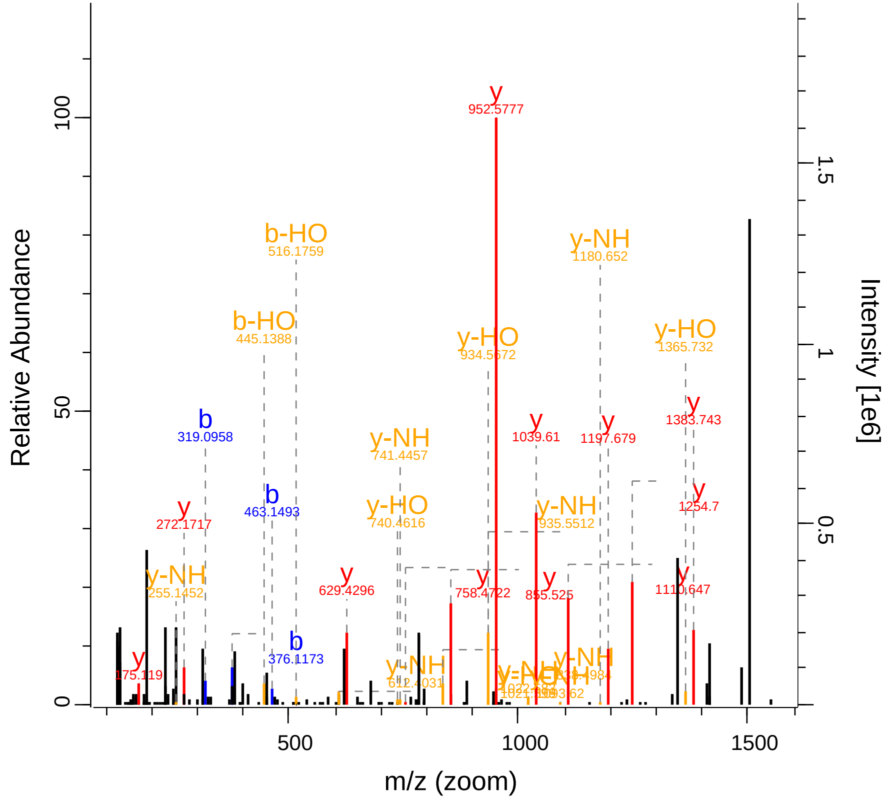

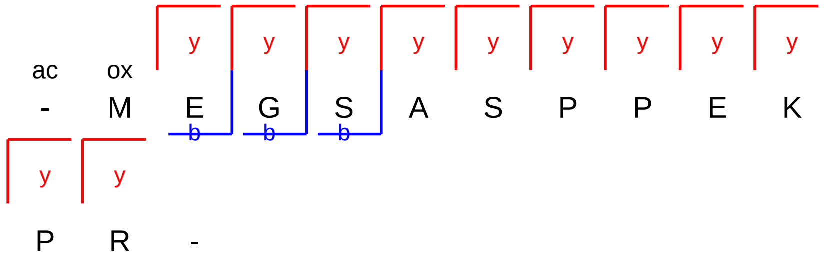

Raw File	Scan	Method	Score	m/z	Gene names
RO-QC-160121_ZackD_SS_Set_02_Fractio	7948	FTMS; HC	148.28	866.42	PDGFA

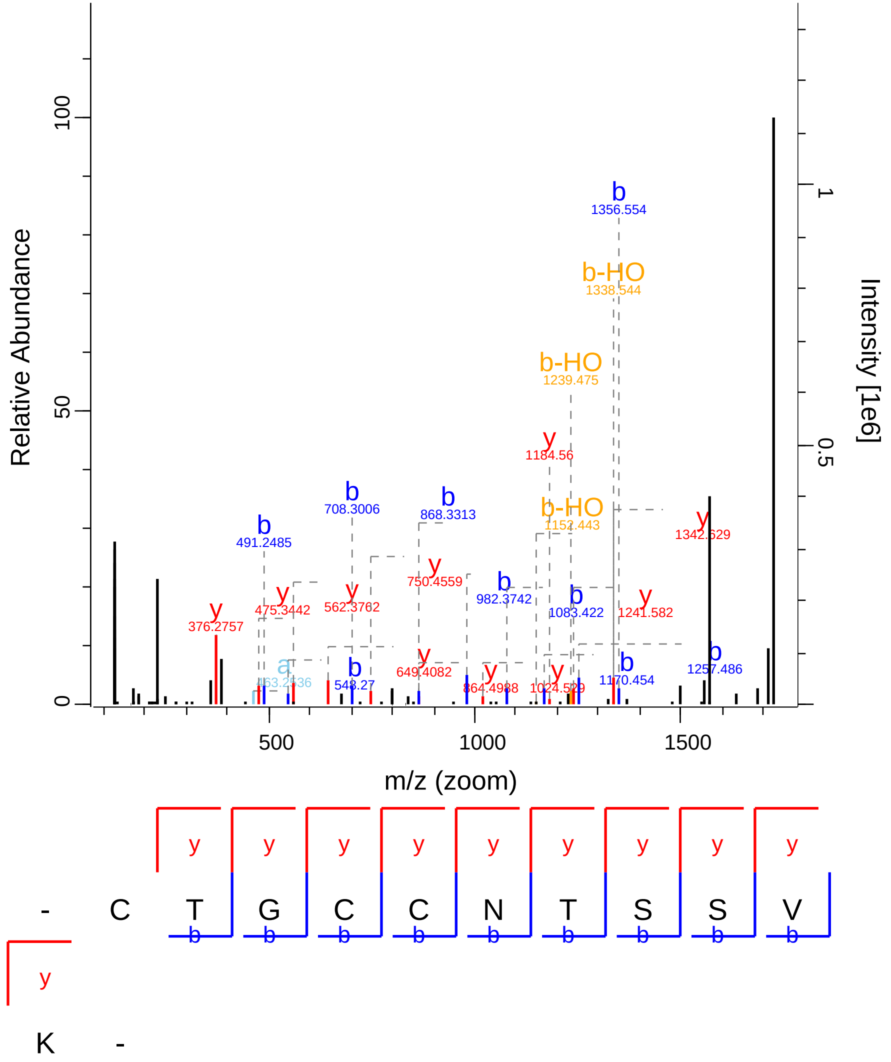

Raw File	Scan	Method	Score	m/z	Gene names
RO-QC-160121_ZackD_SS_Set_02_Fraction	8148	FTMS; HCI	117.7	786.9	SLC46A1

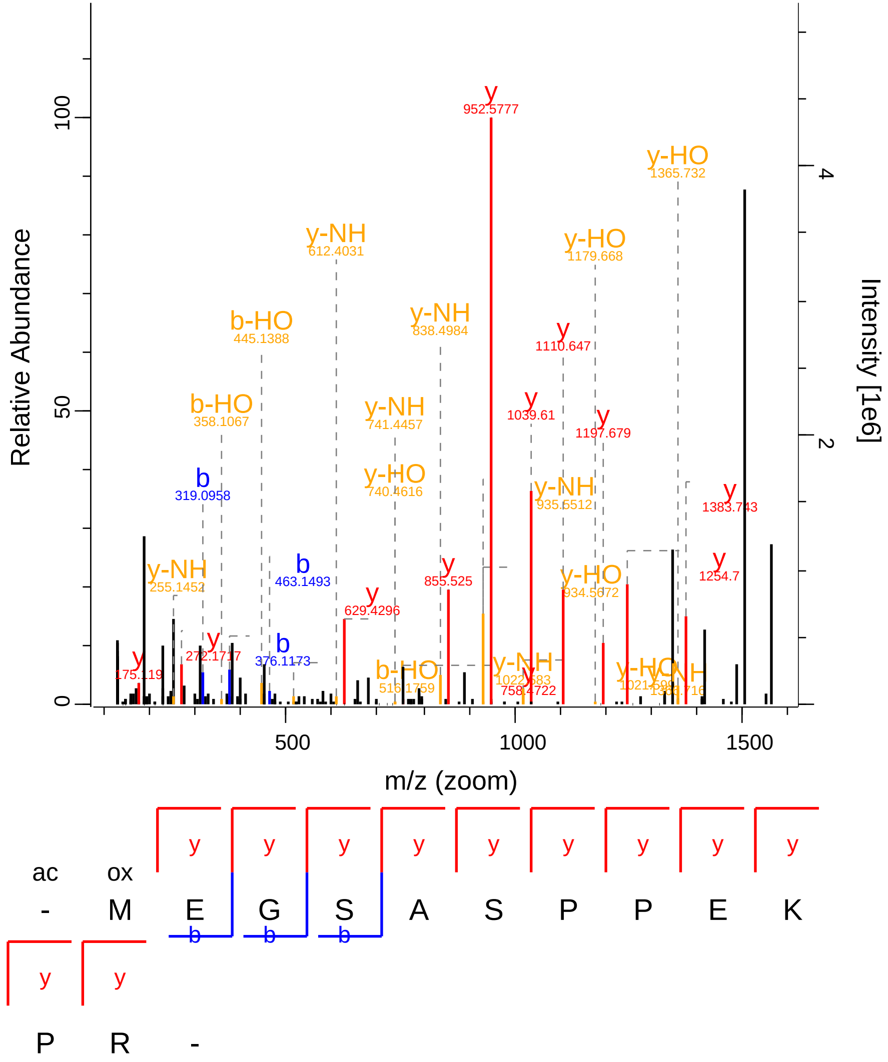

Raw File	Scan	Method	Score	m/z	Gene names
RO-QC-160121_ZackD_SS_Set_02_Fractior	9053	FTMS; HCI	88.31	605.95	CHCHD5

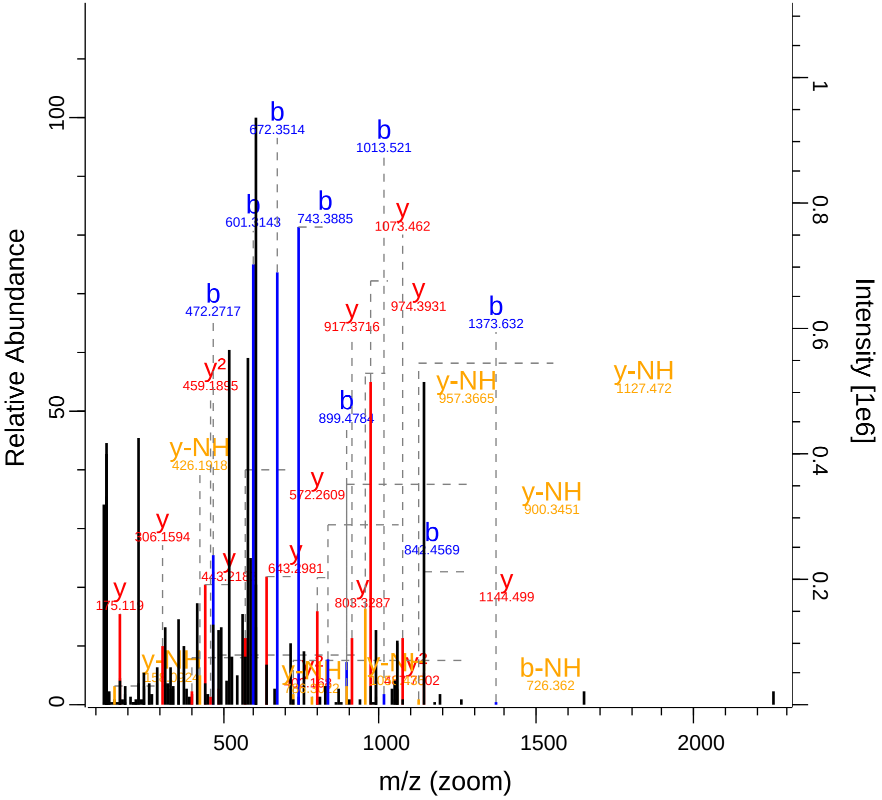

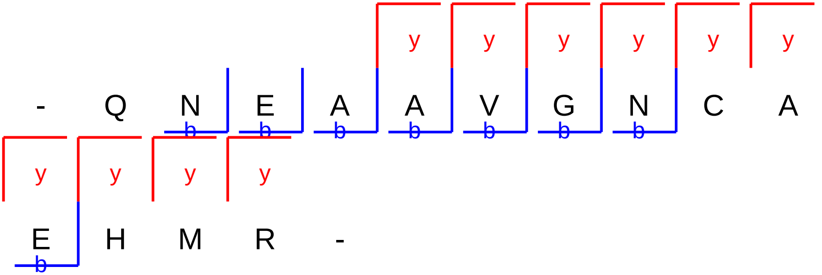

Raw File	Scan	Method	Score	m/z	Gene names
RO-QC-160121_ZackD_SS_Set_02_Fractioı	9076	FTMS; HC	115.78	378.21	GGT7

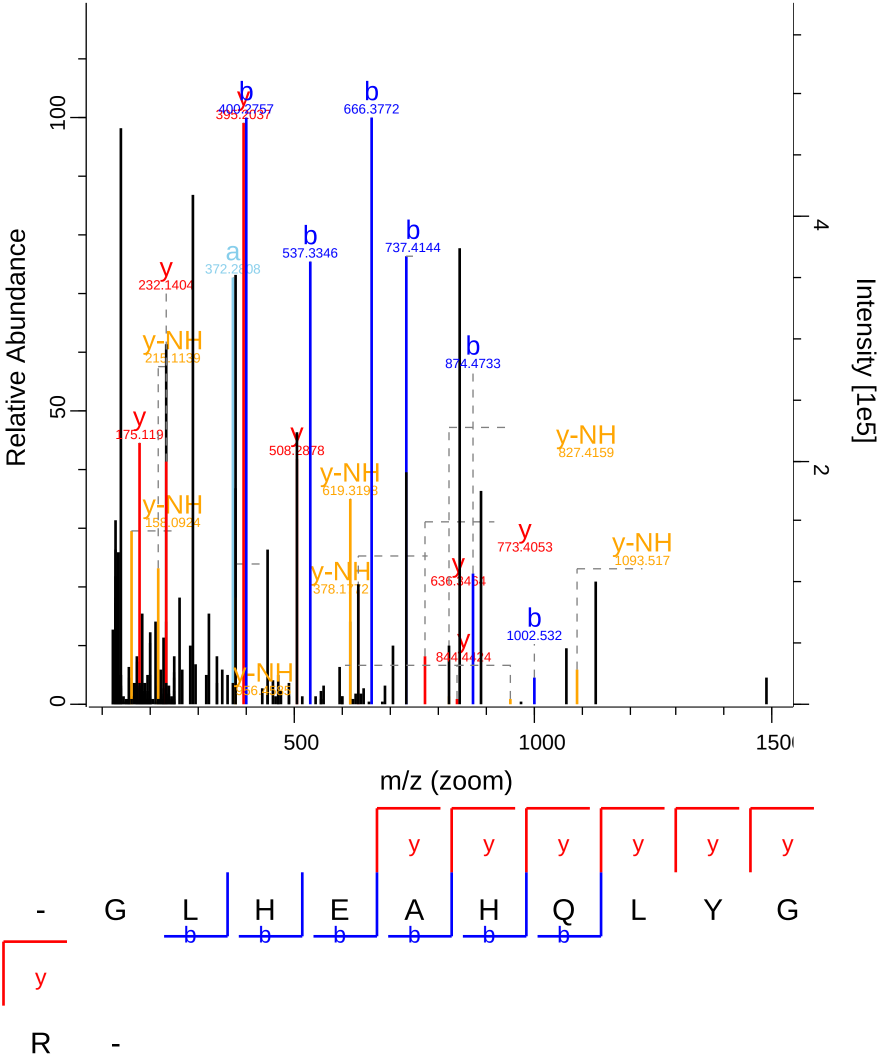

Raw File	Scan	Method	Score	m/z	Gene names
RO-QC-160121_ZackD_SS_Set_02_Fractioı	9466	FTMS; HC	167.67	828.48	AURKB

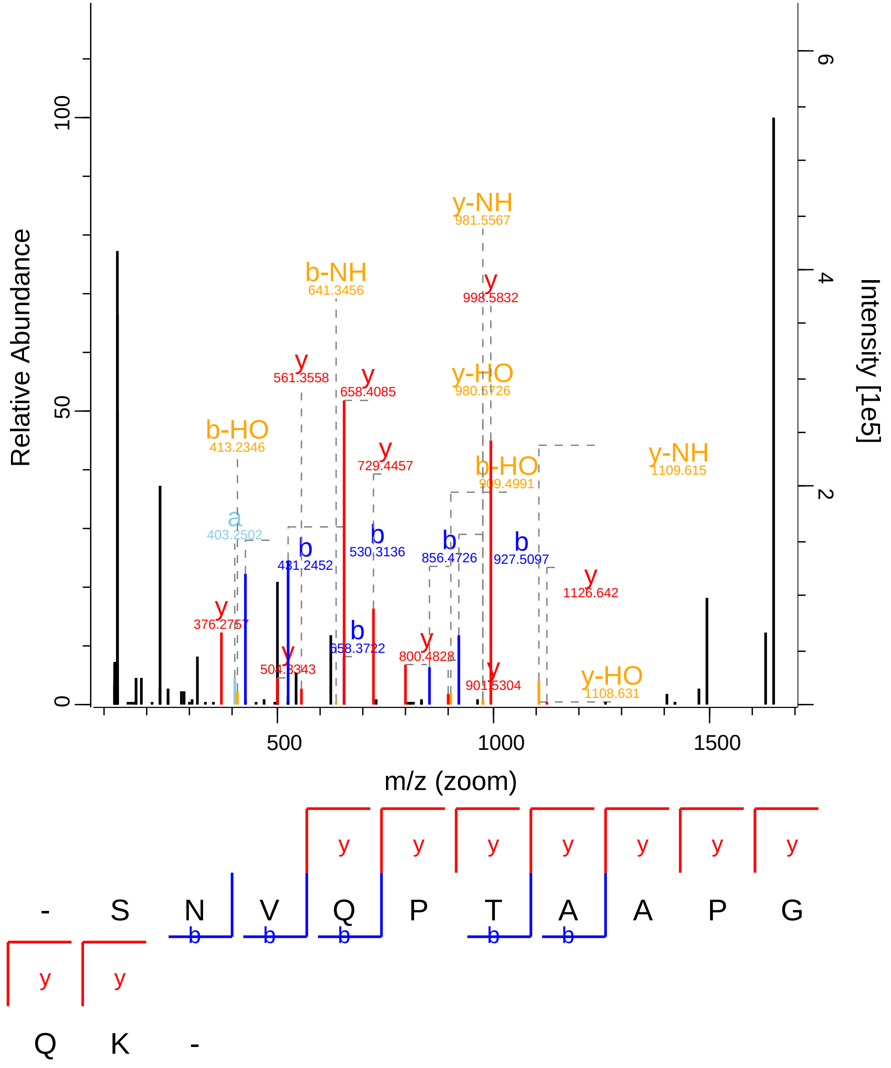

Raw File	Scan	Method	Score	m/z
RO-QC-160121_ZackD_SS_Set_02_Fraction_1	10369	FTMS; HCD	97.91	825.96

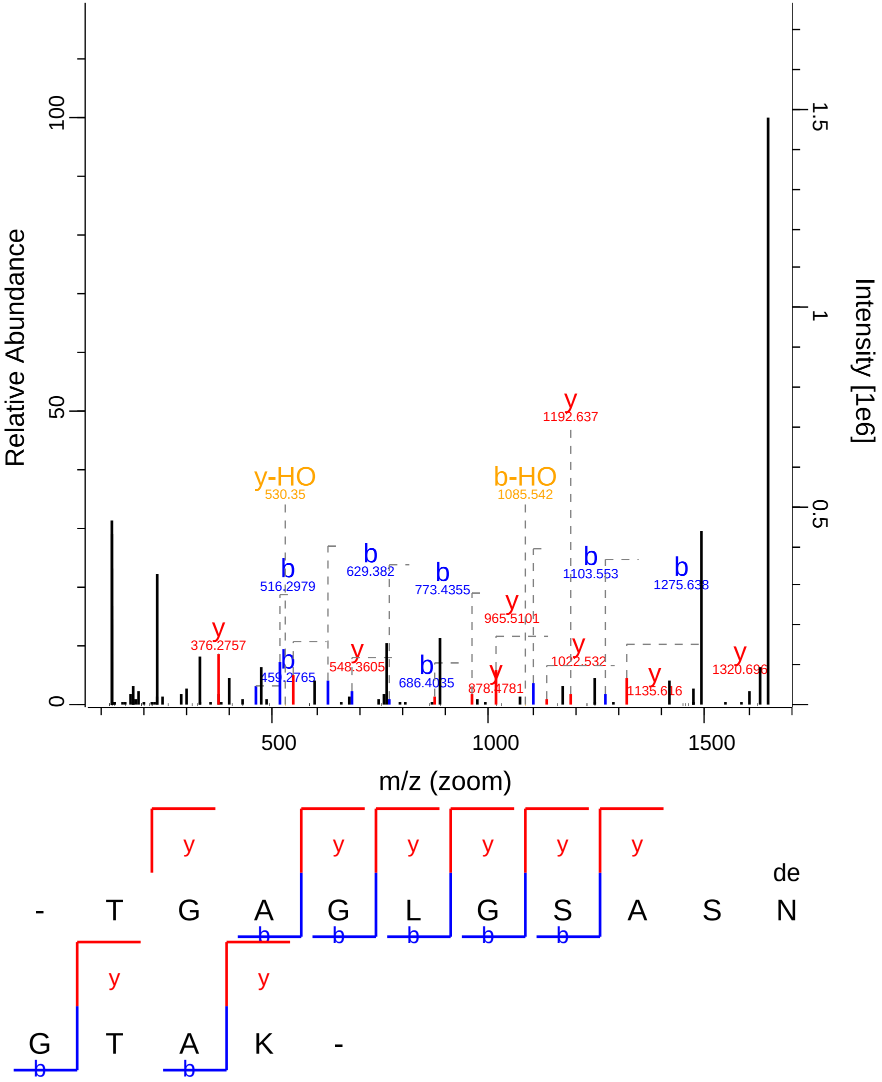

Raw File	Scan	Method	Score	m/z	Gene names
RO-QC-160121_ZackD_SS_Set_02_Fractio	10781	FTMS; HC	171.92	813.95	TRMT13

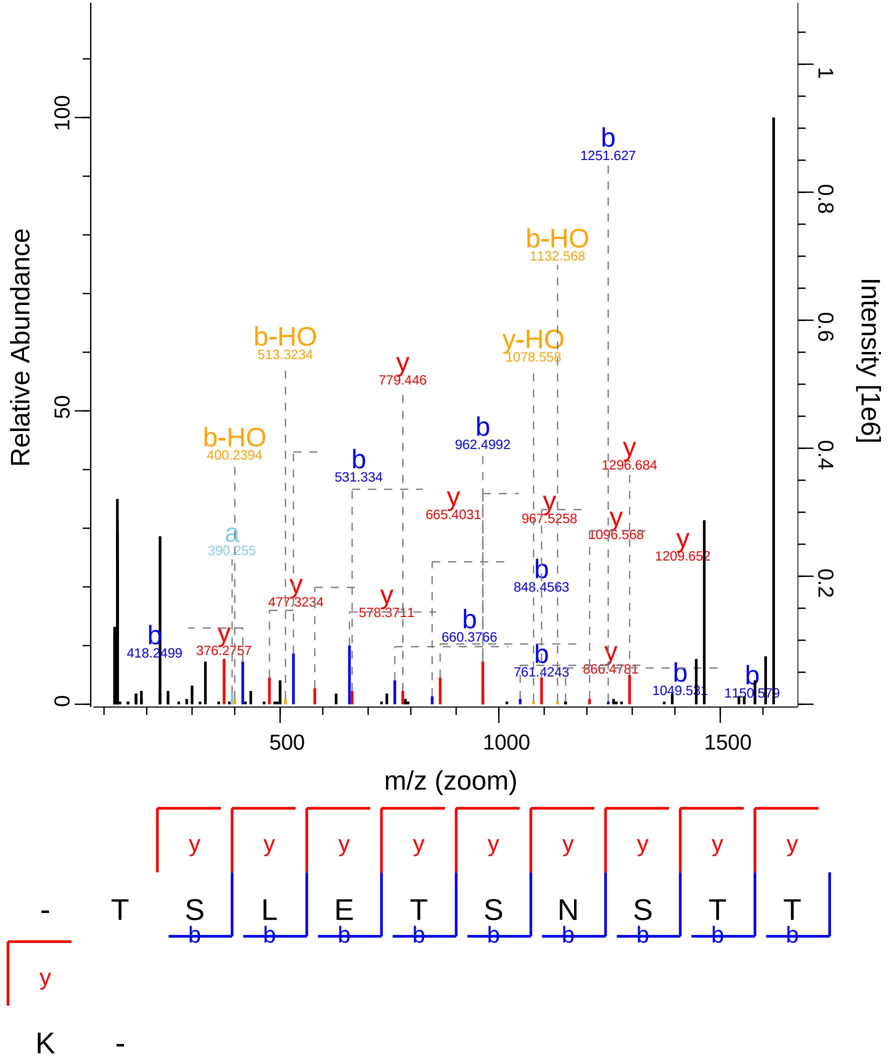

Raw File	Scan	Method	Score	m/z	Gene names
RO-QC-160121_ZackD_SS_Set_02_Fractio	10894	FTMS; HC	47.26	996.96	ANO3

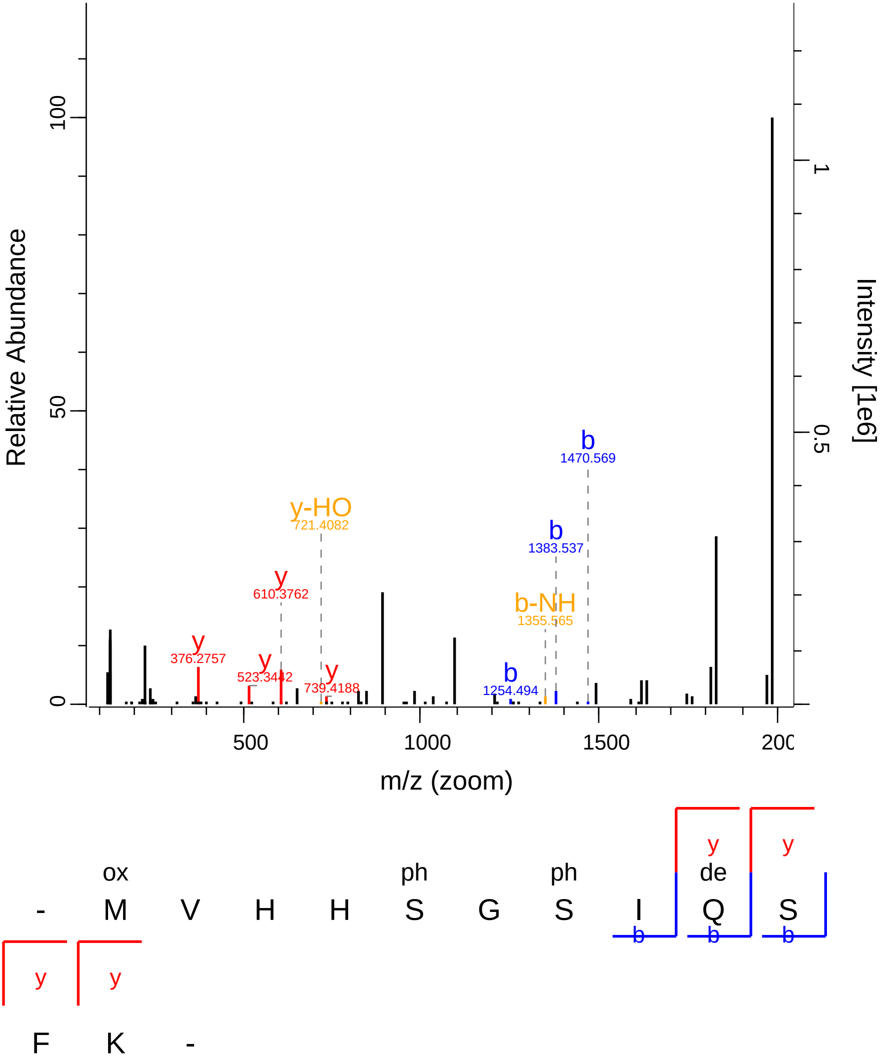

Raw File	Scan	Method	Score	m/z	Gene names
RO-QC-160121_ZackD_SS_Set_02_Fractio	11172	FTMS; HC	89.14	464.61	KCNV2

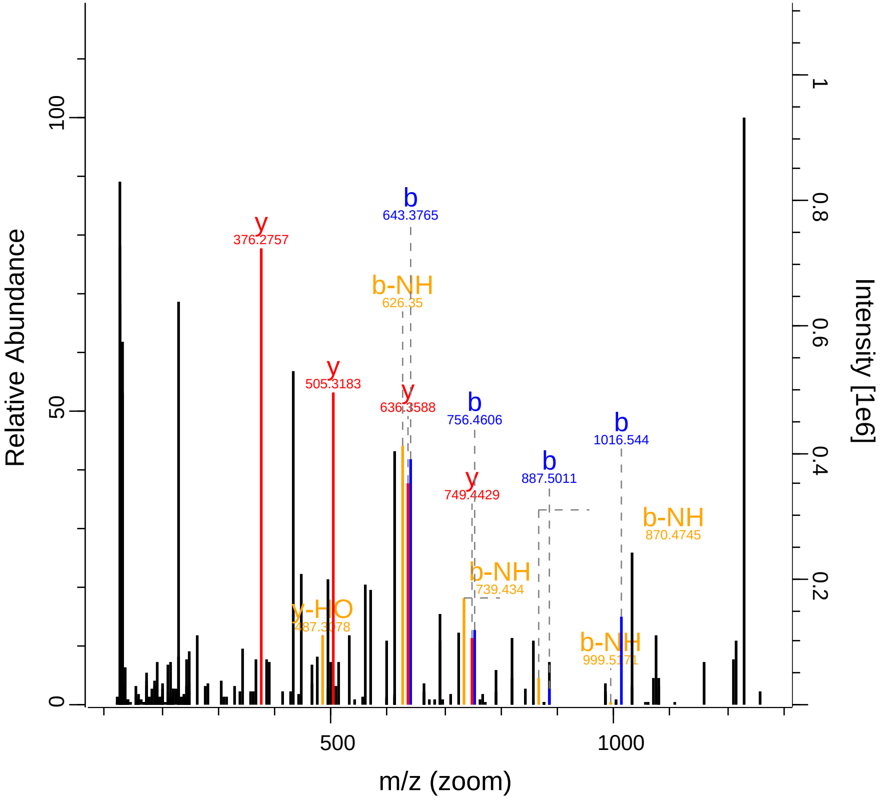

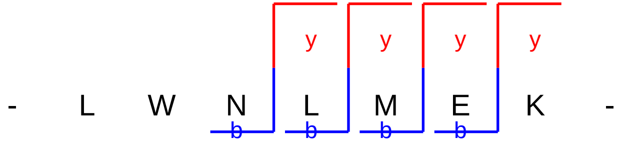

Raw File	Scan	Method	Score	m/z	Gene names
RO-QC-160121_ZackD_SS_Set_02_	1122!	FTMS; H	88.18	378.7:	GOLGA6L1;GOLGA6L6;GOL

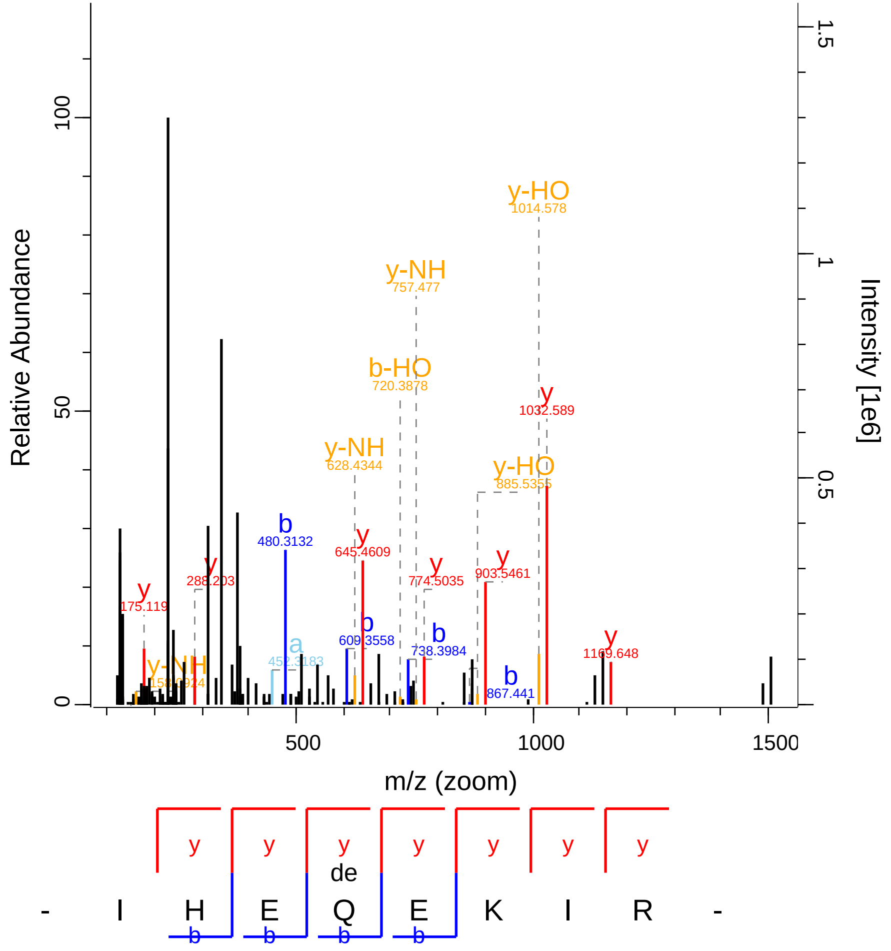

Raw File	Scan	Method	Score	m/z	Gene names
RO-QC-160121_ZackD_SS_Set_02_Fractio	11652	FTMS; HC	65.31	391.71	CCDC82

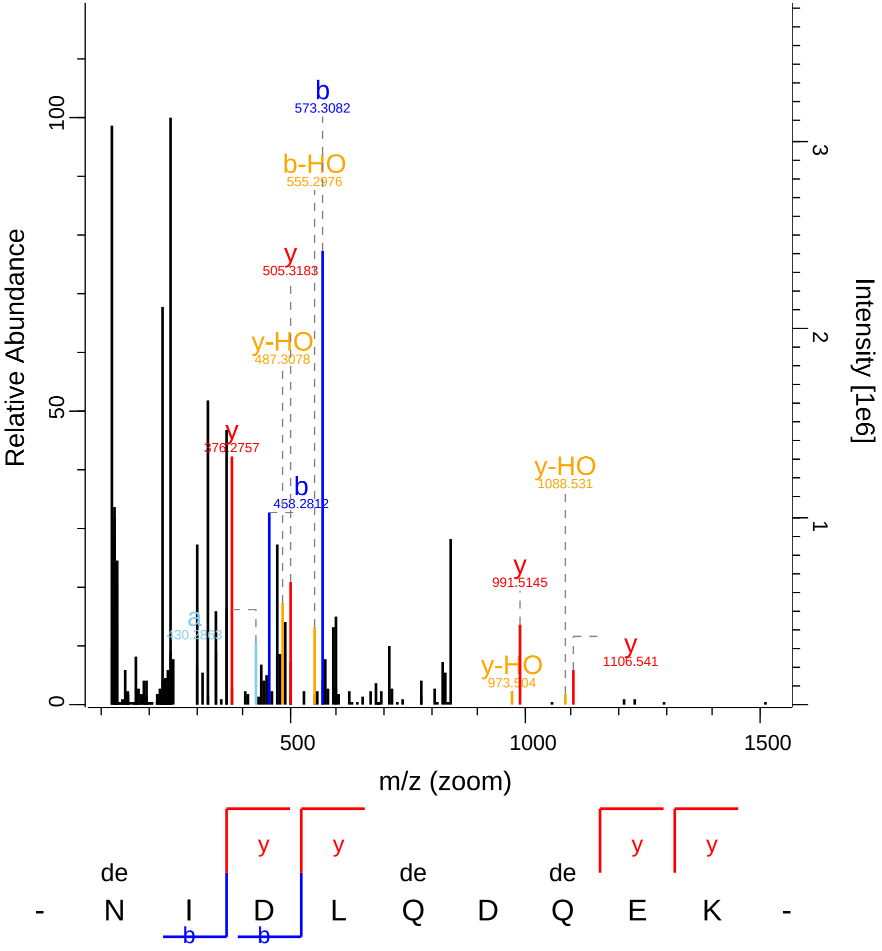

Raw File	Scan	Method	Score	m/z	Gene names
RO-QC-160121_ZackD_SS_Set_02_Fractio	11660	FTMS; HC	33.89	400.49	USH1C

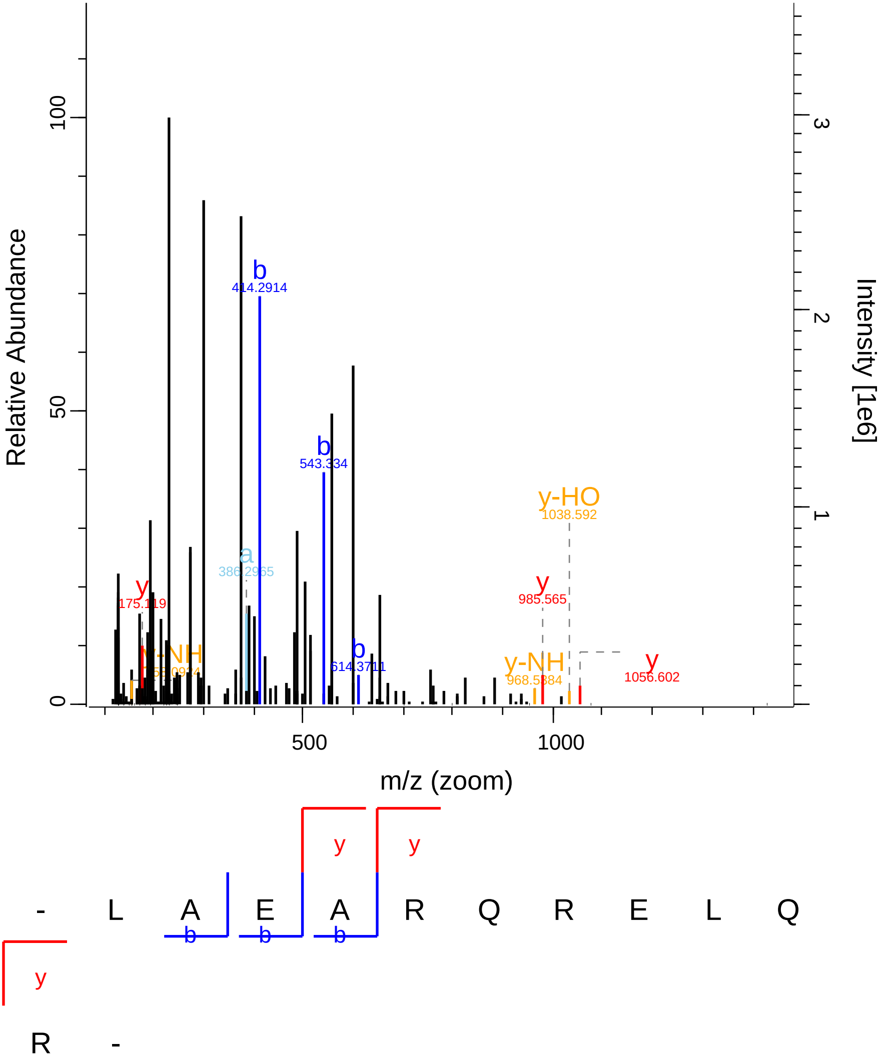

Raw File	Scan	Method	Score	m/z	Gene names
RO-QC-160121_ZackD_SS_Set_02_Fractio	11697	FTMS; HC	211.27	734.92	AHR

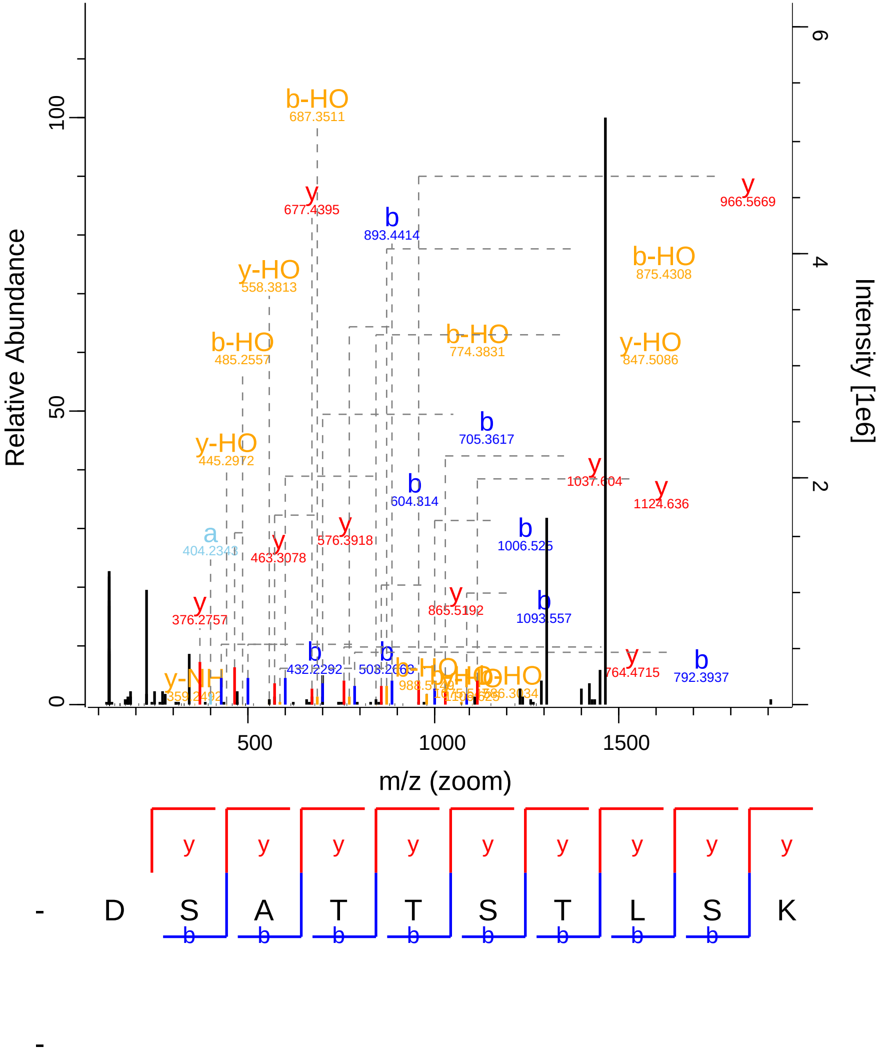

Raw File	Scan	Method	Score	m/z	Gene names
RO-QC-160121_ZackD_SS_Set_02_Fractio	12237	FTMS; HC	151.15	786.44	DGKZ

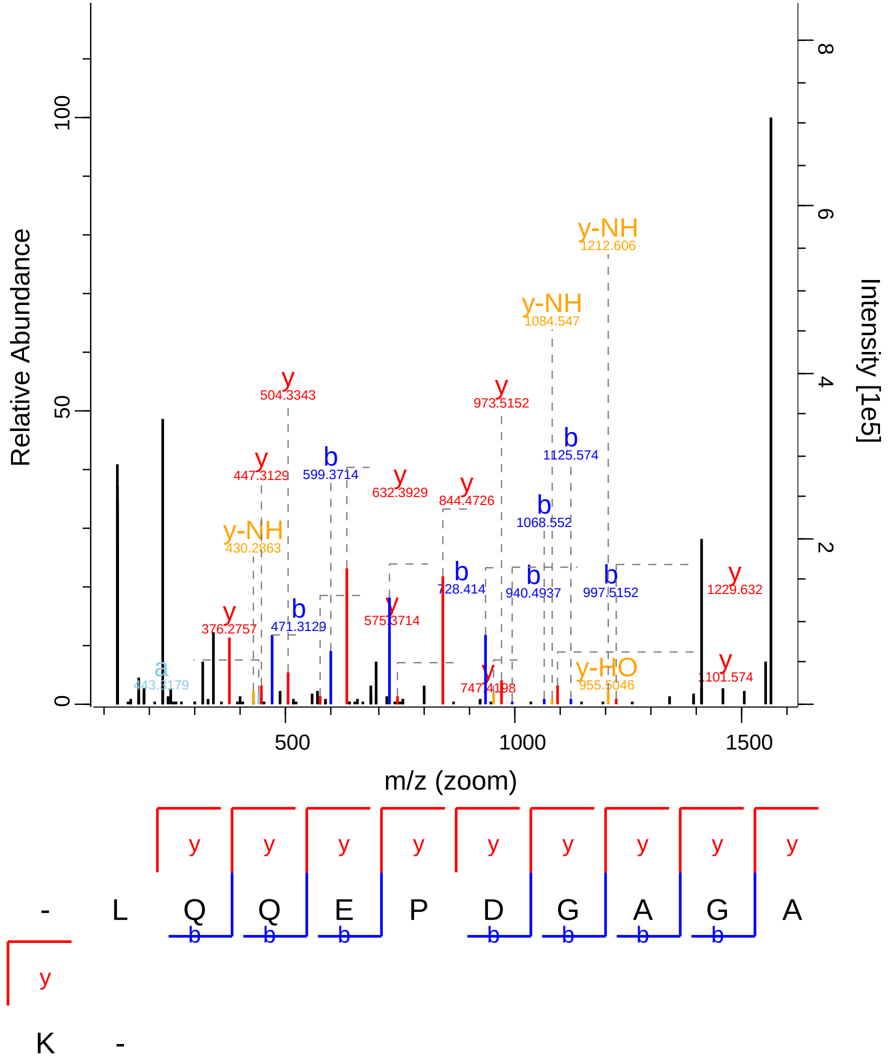

Raw File	Scan	Method	Score	m/z
RO-QC-160121_ZackD_SS_Set_02_Fraction_1	12323	FTMS; HCD	84.5	687.9

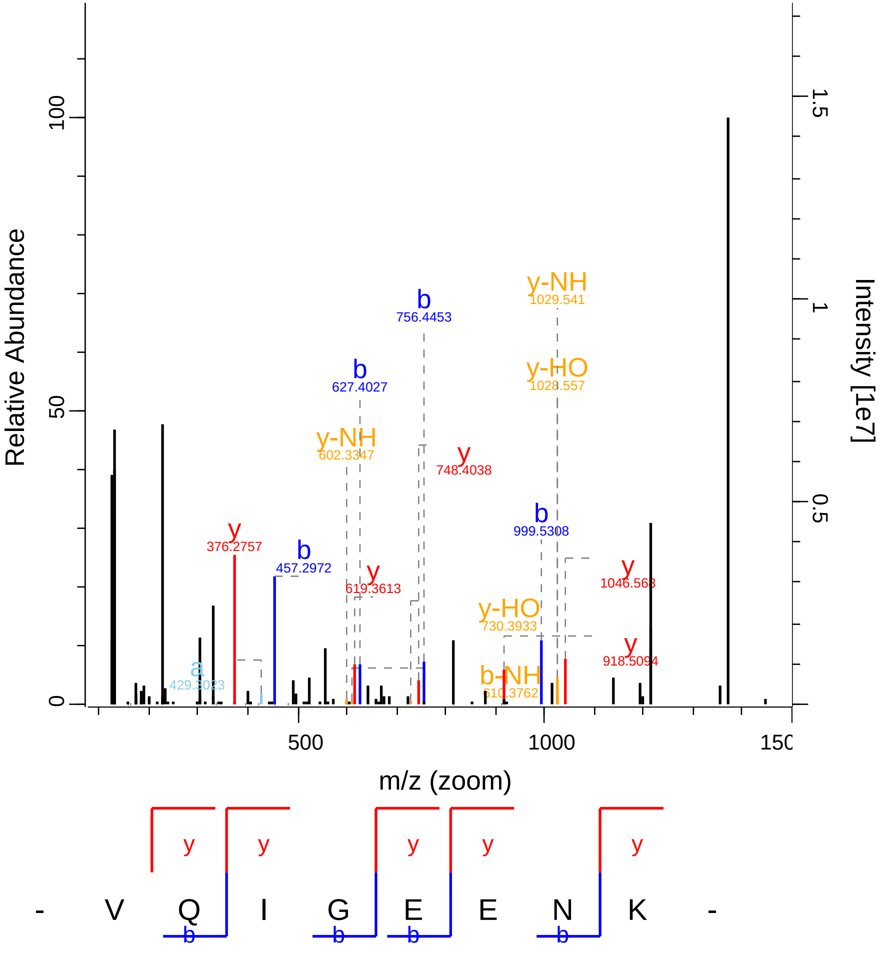

Raw File	Scan	Method	Score	m/z
RO-QC-160121_ZackD_SS_Set_02_Fraction_1	12355	FTMS; HCD	64.26	458.94

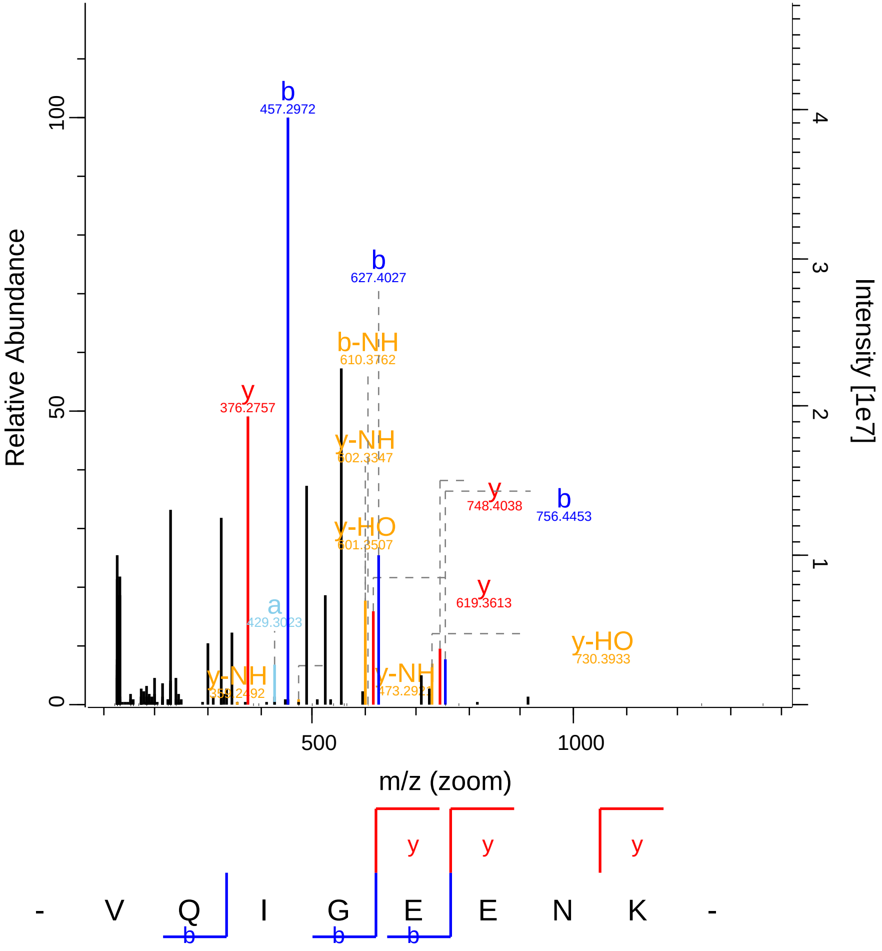

Raw File	Scan	Method	Score	m/z
RO-QC-160121_ZackD_SS_Set_02_Fraction_1	12540	FTMS; HCD	106.04	687.9

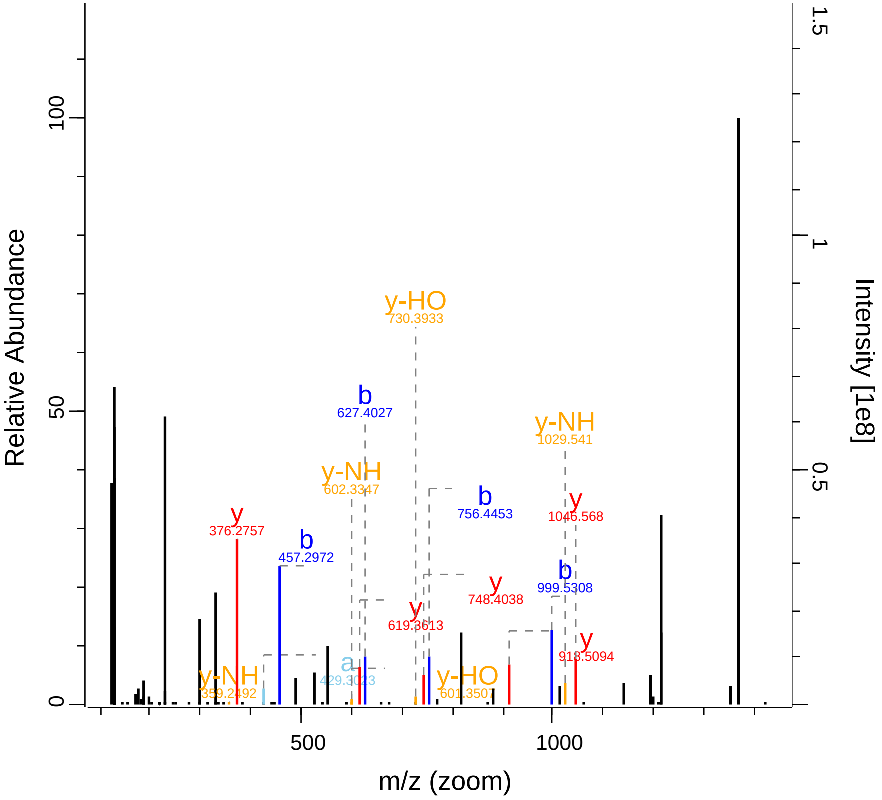

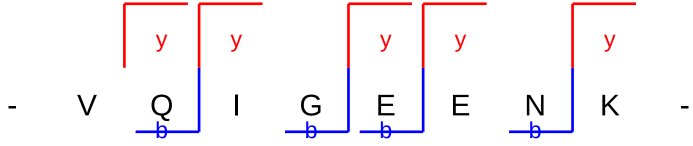

Raw File	Scan	Method	Score	m/z
RO-QC-160121_ZackD_SS_Set_02_Fraction_1	12572	FTMS; HCD	64.26	458.94

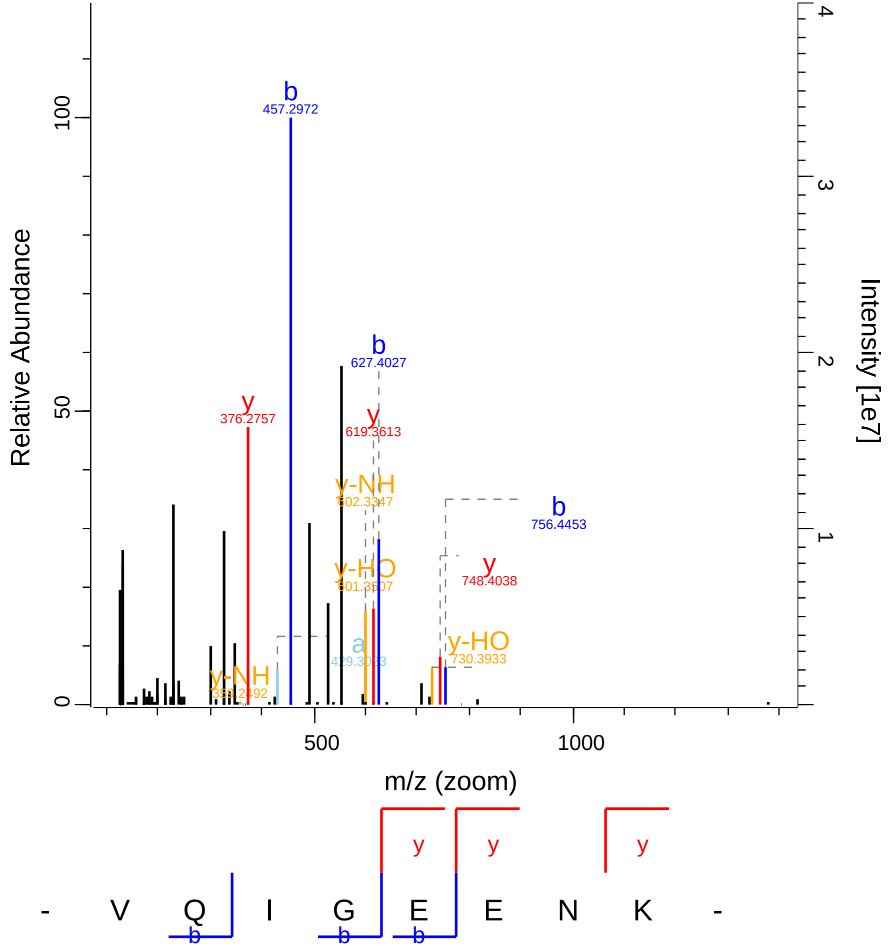

Raw File	Scan	Method	Score	m/z
RO-QC-160121_ZackD_SS_Set_02_Fraction_1	12648	FTMS; HCD	49.1	800.42

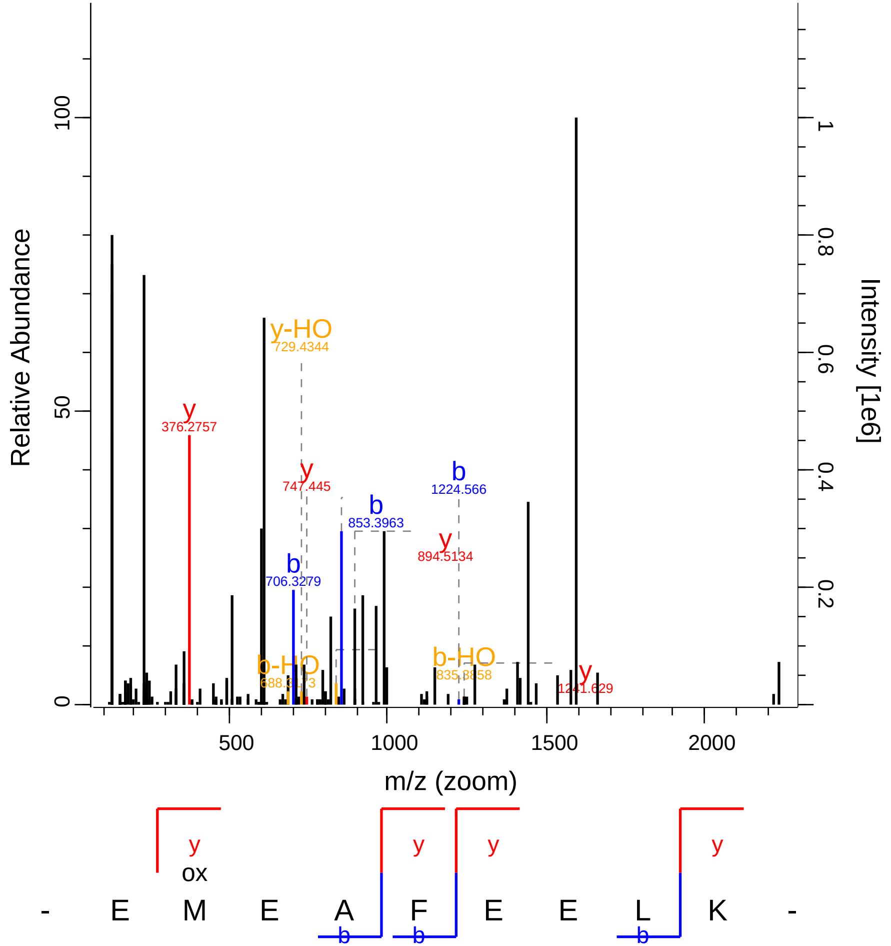

Raw File	Scan	Method	Score	m/z
RO-QC-160121_ZackD_SS_Set_02_Fraction_1	12728	FTMS; HCD	41.45	400.71

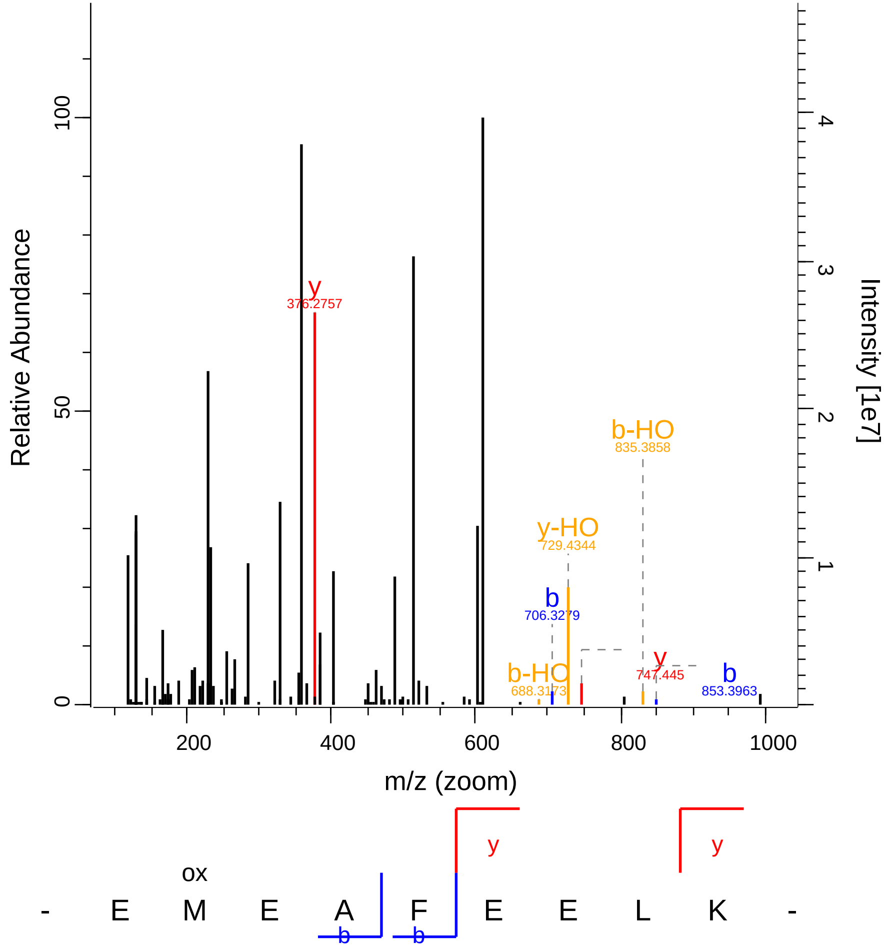

Raw File	Scan	Method	Score	m/z
RO-QC-160121_ZackD_SS_Set_02_Fraction_1	12944	FTMS; HCD	102.8	480.79

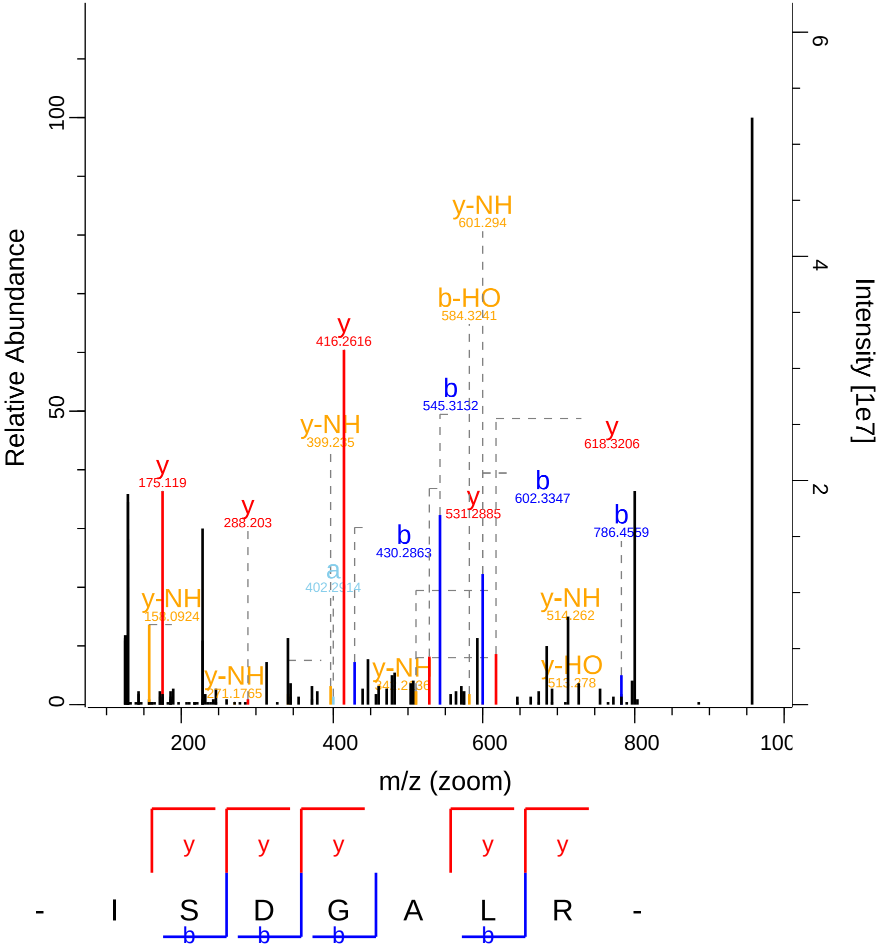

Raw File	Scan	Method	Score	m/z	Gene names
RO-QC-160121_ZackD_SS_Set_02_Fractior	13932	FTMS; HCl	116.3	704.9	DGCR2

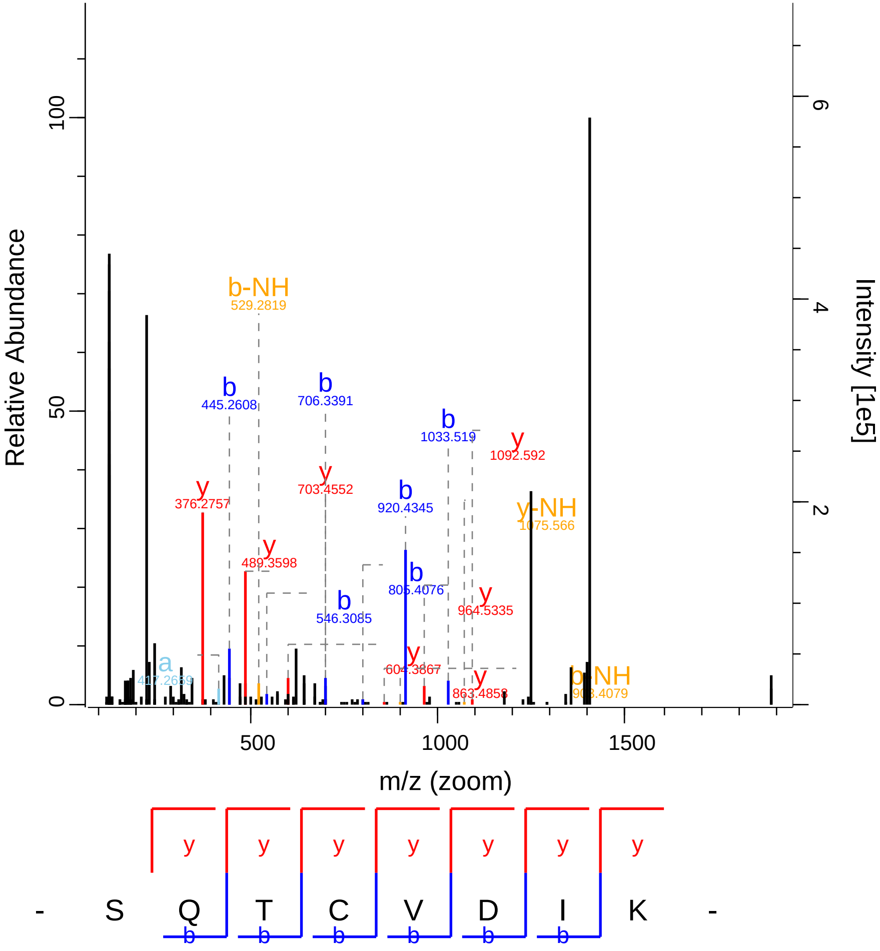

Raw File	Scan	Method	Score	m/z	Gene names
RO-QC-160121_ZackD_SS_Set_02_Fractio	14100	FTMS; HC	184.02	838.68	SLC43A2

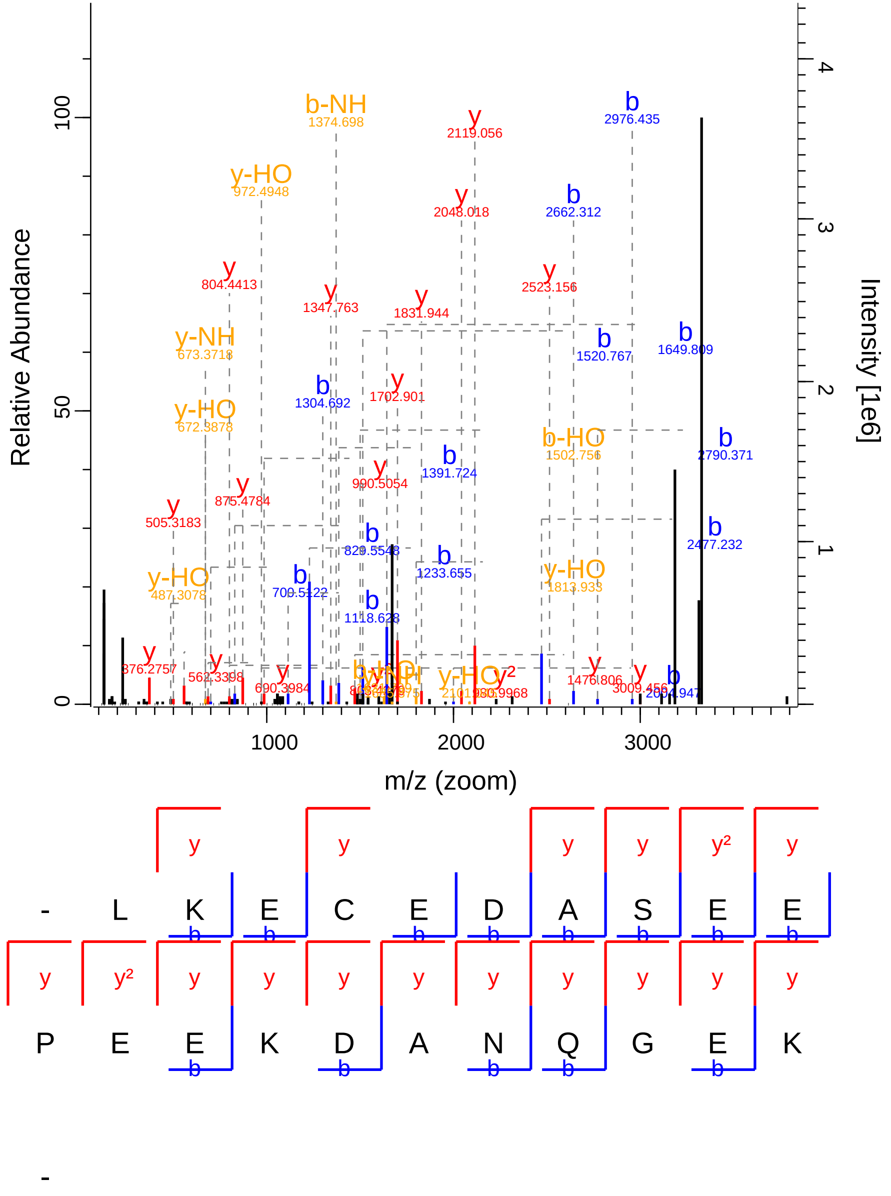

Raw File	Scan	Method	Score	m/z	Gene names
RO-QC-160121_ZackD_SS_Set_02_Fractic	14481	FTMS; HC	140.14	1018.58	SP2

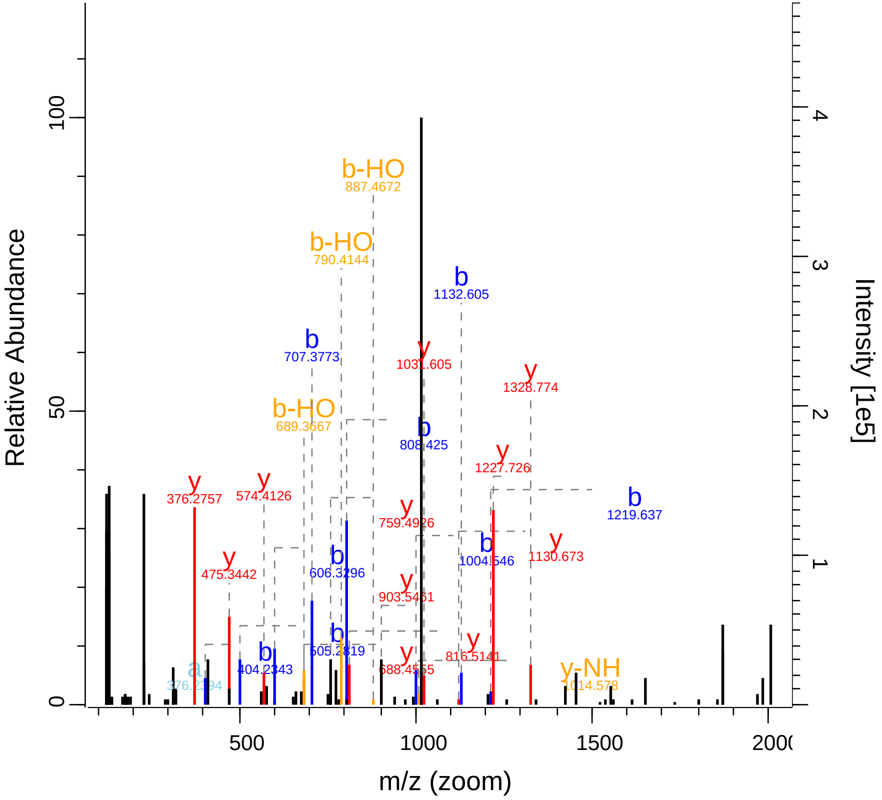

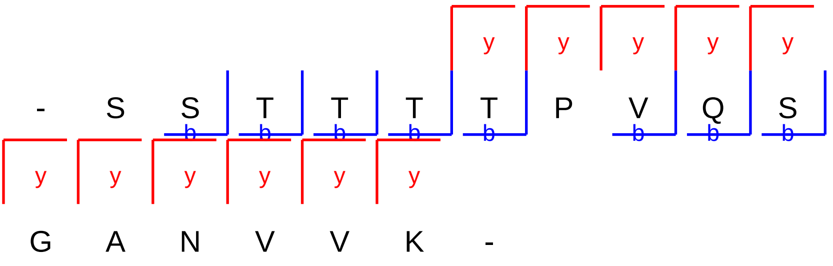

Raw File	Scan	Method	Score	m/z	Gene names
RO-QC-160121_ZackD_SS_Set_02_Fractio	16778	FTMS; HC	63.73	715.41	FANCB

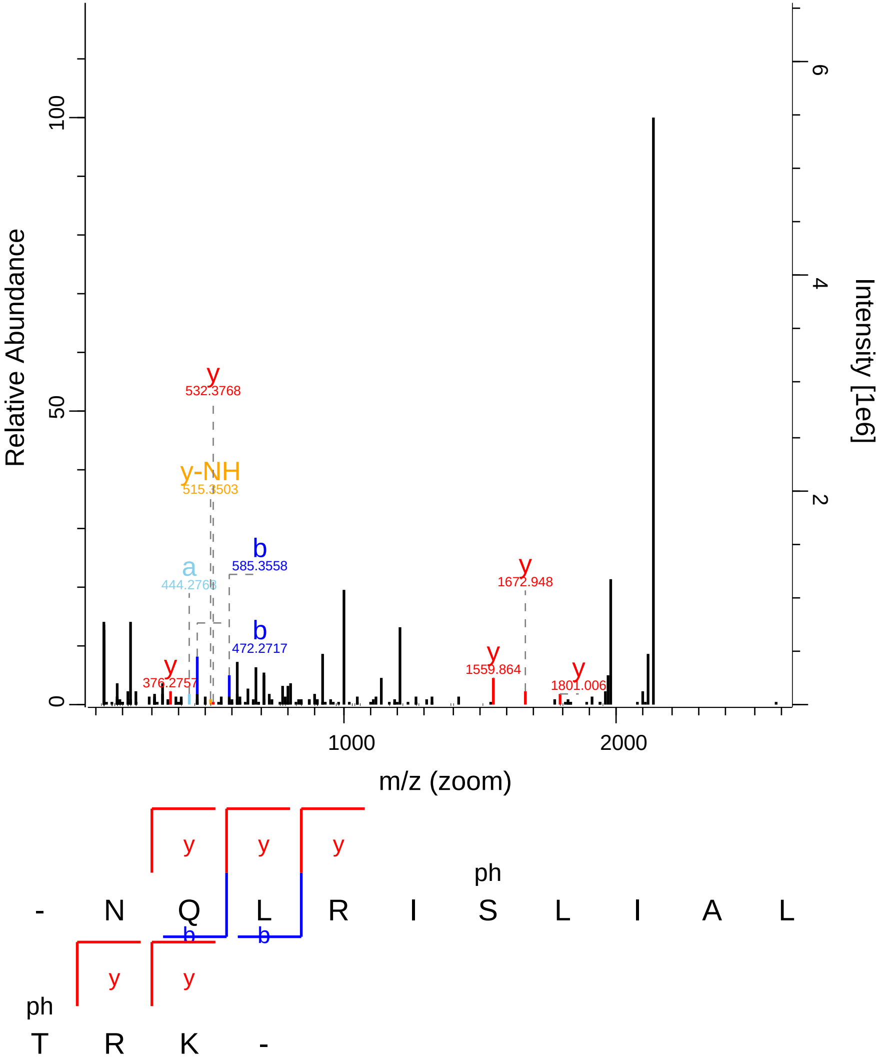

Raw File	Scan	Method	Score	m/z	Gene names
RO-QC-160121_ZackD_SS_Set_02_Fractio	16820	FTMS; HC	222.55	948.96	FOXJ3

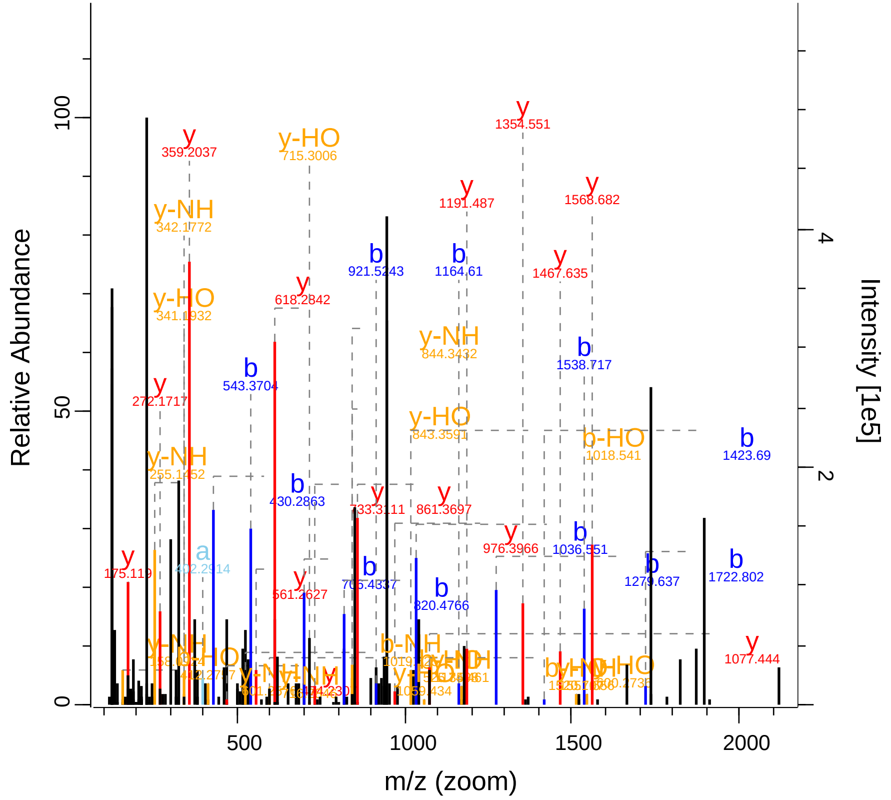

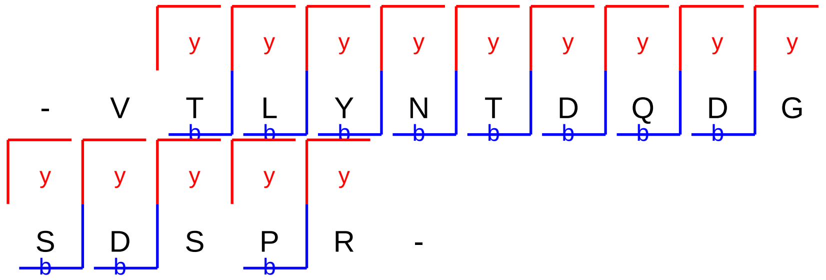

Raw File	Scan	Method	Score	m/z	Gene names
RO-QC-160121_ZackD_SS_Set_02_Fractio	16954	FTMS; HC	97.45	745.71	RGS10

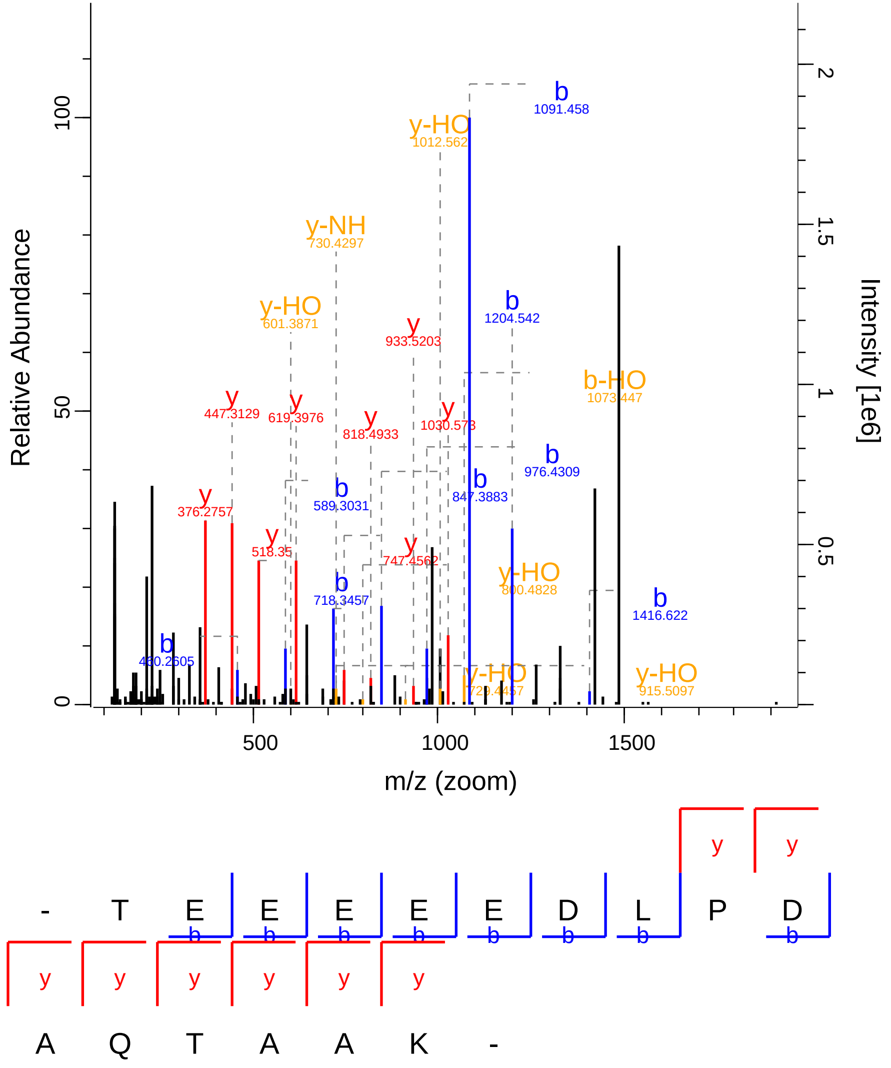

Raw File	Scan	Method	Score	m/z	Gene names
RO-QC-160121_ZackD_SS_Set_02_Fr	17136	FTMS; H(	92.43	545.63	ORMDL3;ORMDL1;OR

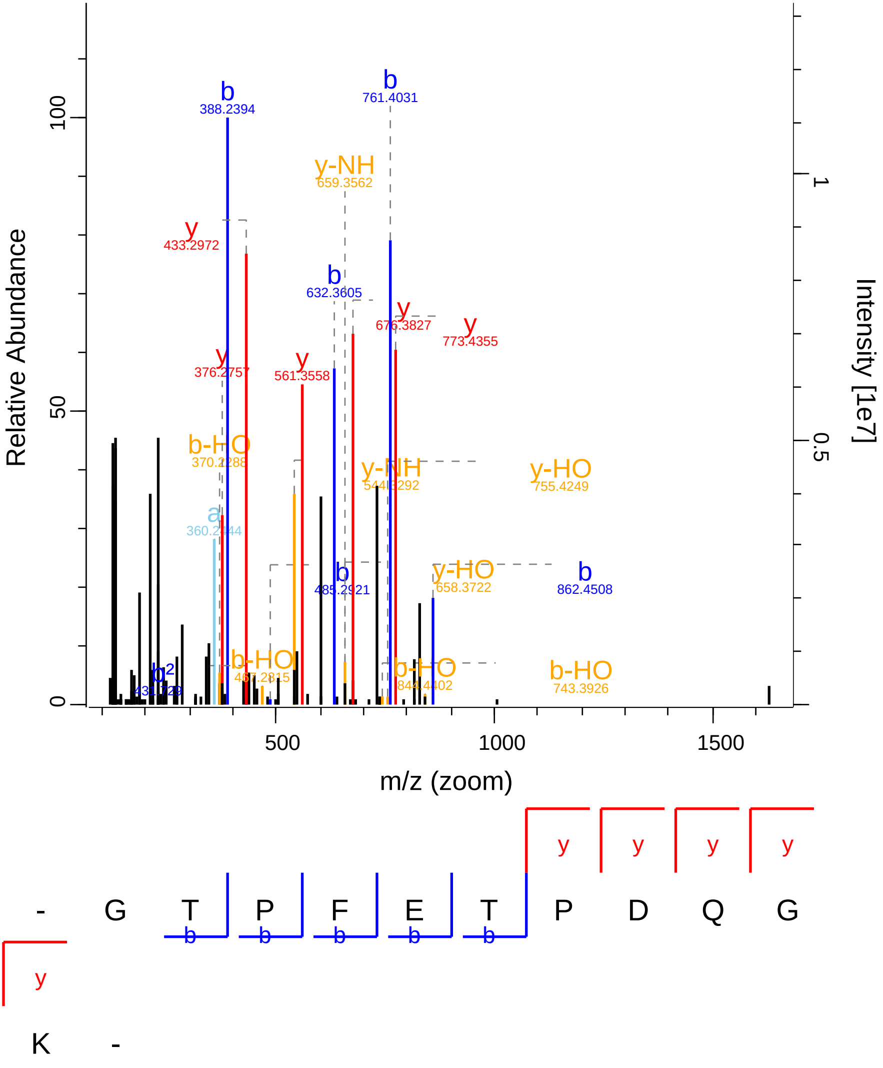

Raw File	Scan	Method	Score	m/z	Gene names
RO-QC-160121_ZackD_SS_Set_02_Fr	1718[illegible]	FTMS; H(	218.6	817.94	ORMDL3;ORMDL1;OR

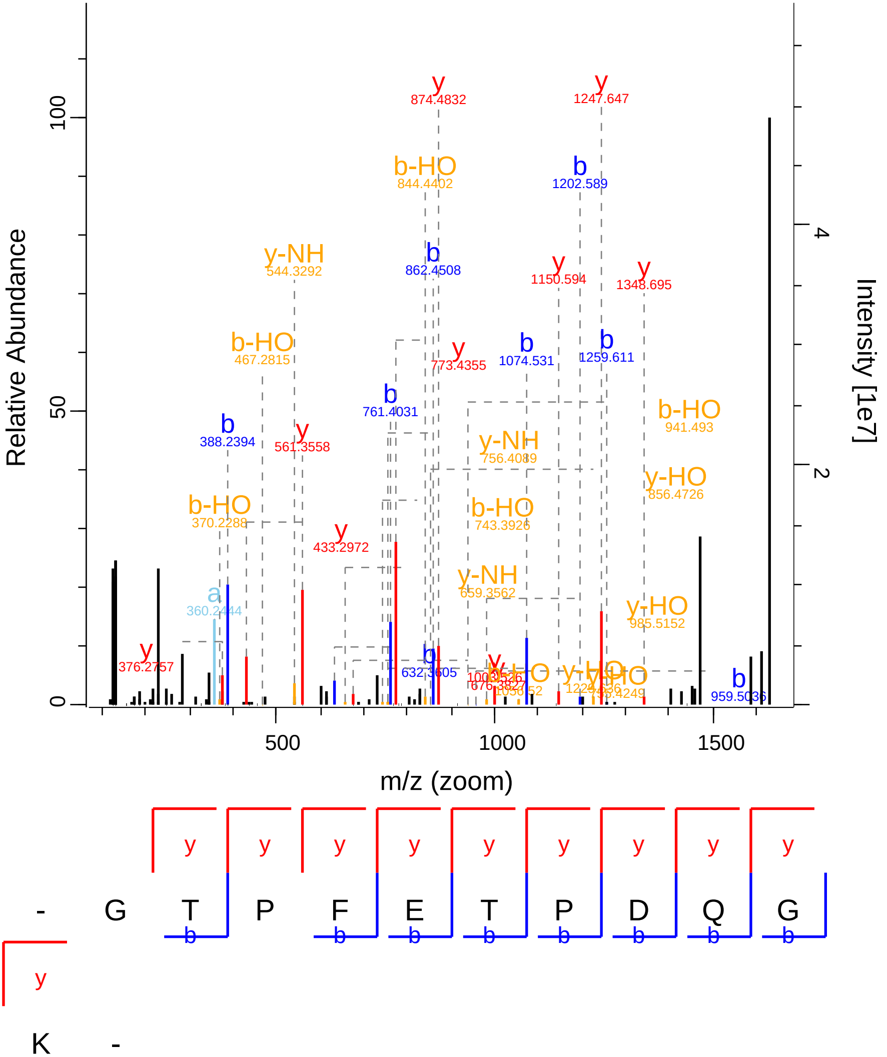

Raw File	Scan	Method	Score	m/z	Gene names
RO-QC-160121_ZackD_SS_Set_02_Fractio	17565	FTMS; HC	179.81	806.92	TRAM2

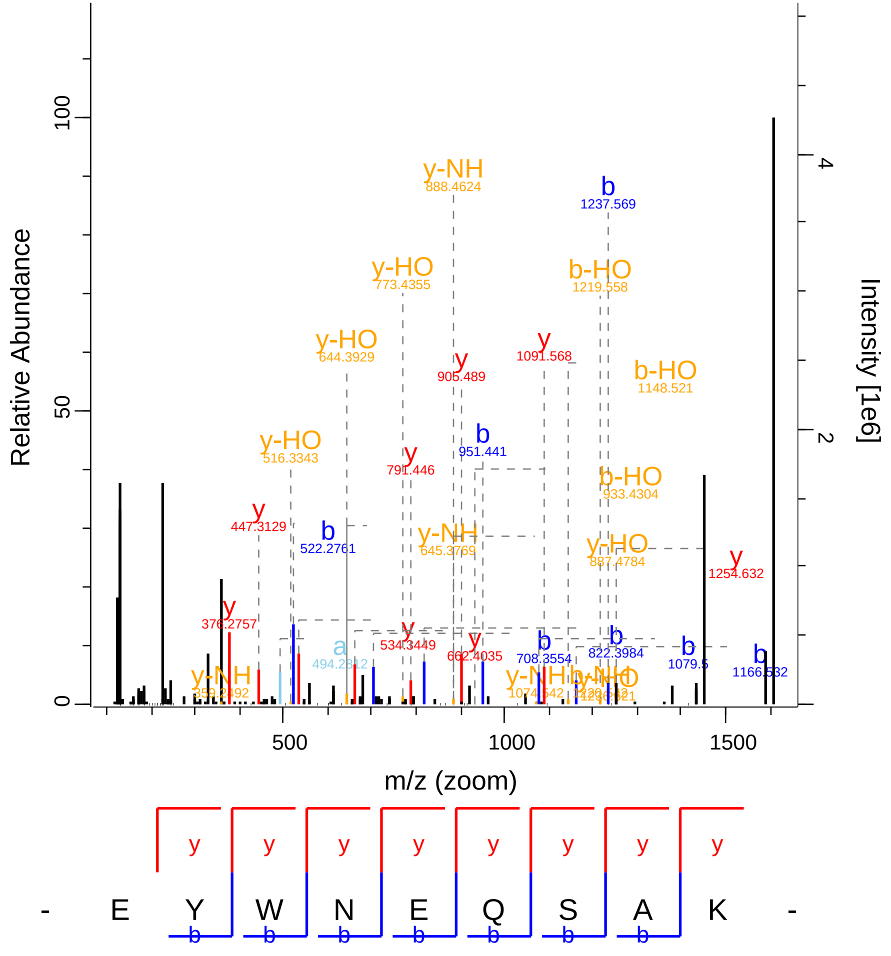

Raw File	Scan	Method	Score	m/z	Gene names
RO-QC-160121_ZackD_SS_Set_02_Fractio	17738	FTMS; HC	60.4	525.64	SESTD1

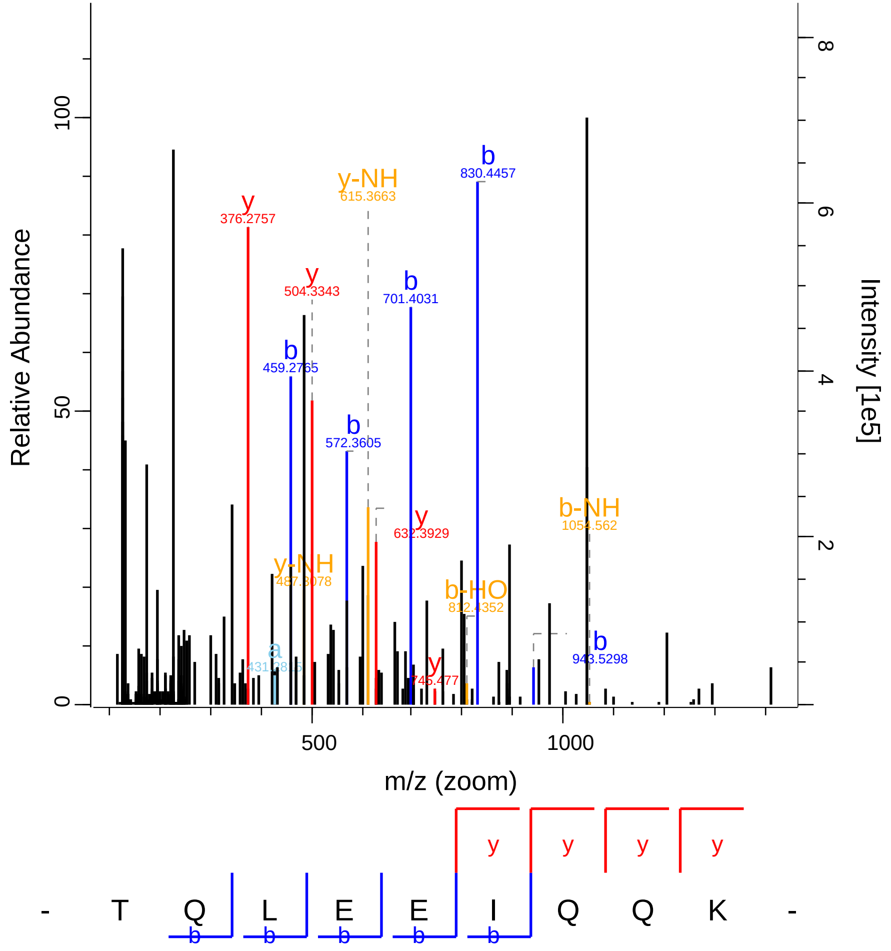

Raw File	Scan	Method	Score	m/z	Gene names
RO-QC-160121_ZackD_SS_Set_02_Fractio	17952	FTMS; HC	106.88	682.36	FBXO30

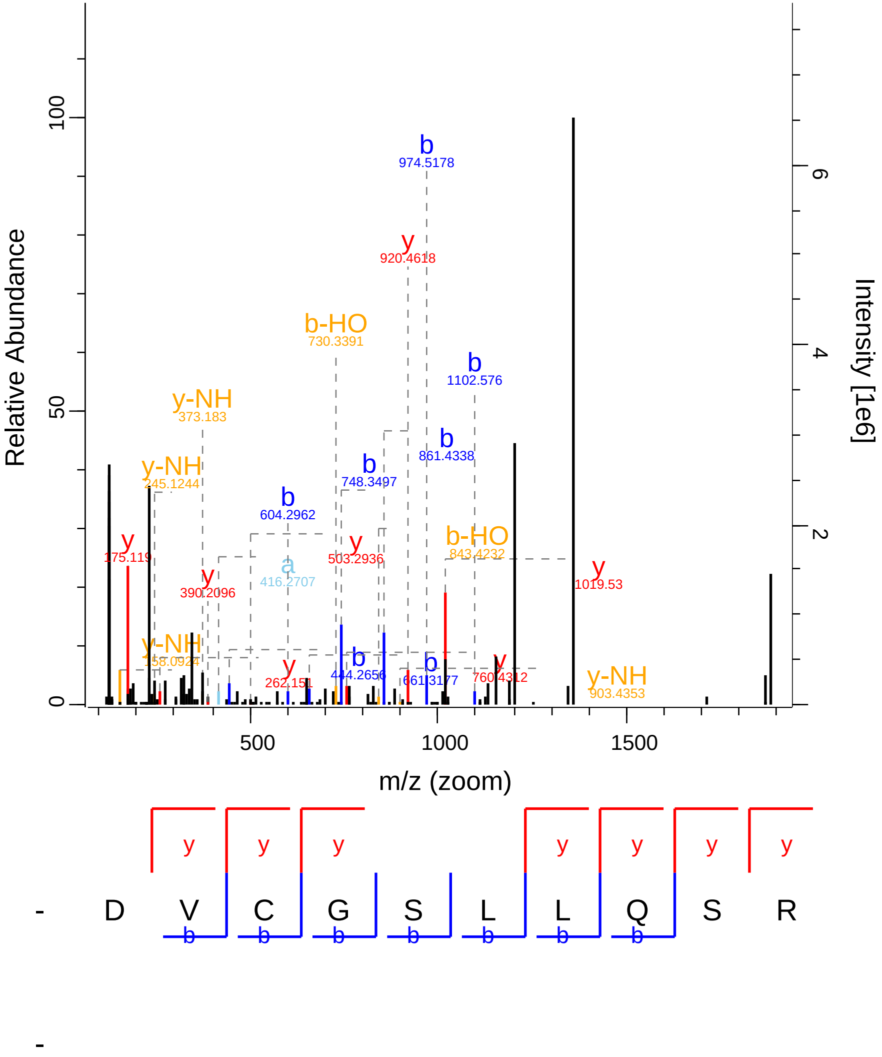

Raw File	Scan	Method	Score	m/z	Gene names
RO-QC-160121_ZackD_SS_Set_02_Fractic	18586	FTMS; HC	46.35	1164.61	PATL2

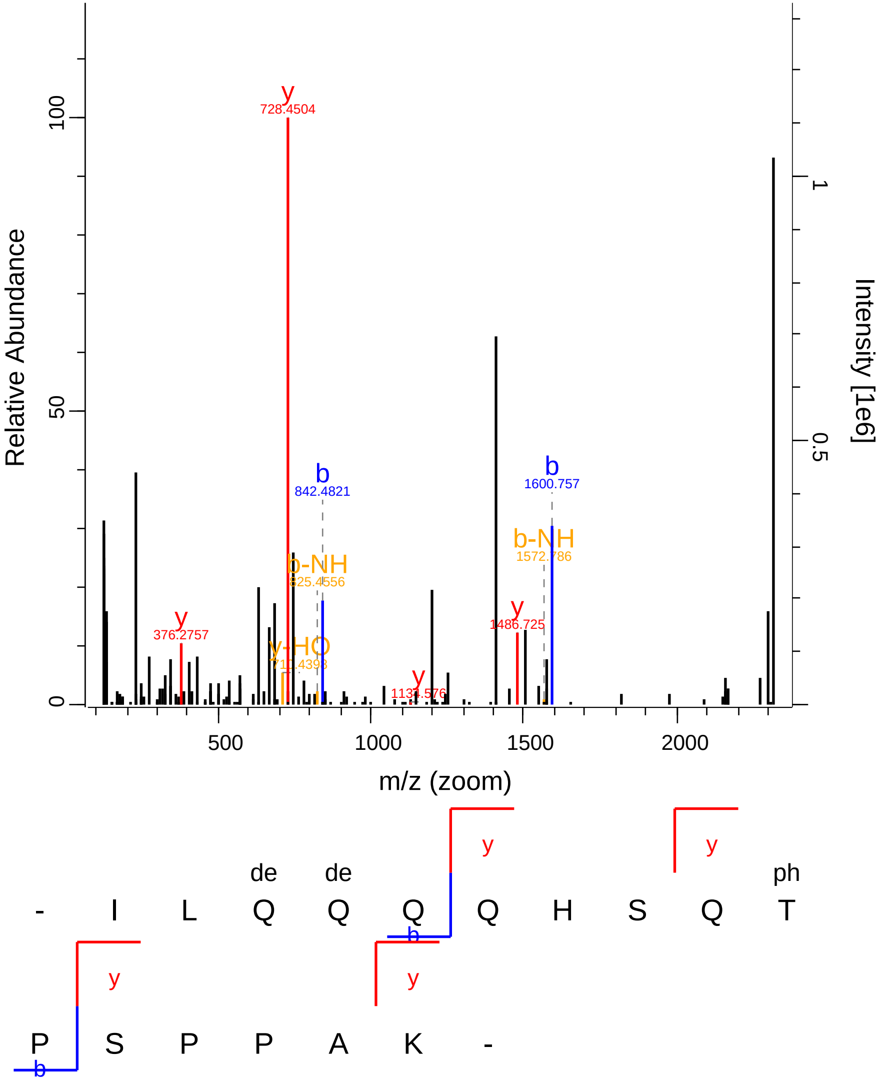

Raw File	Scan	Method	Score	m/z
RO-QC-160121_ZackD_SS_Set_02_Fraction_1	19330	FTMS; HCD	104.43	515.65

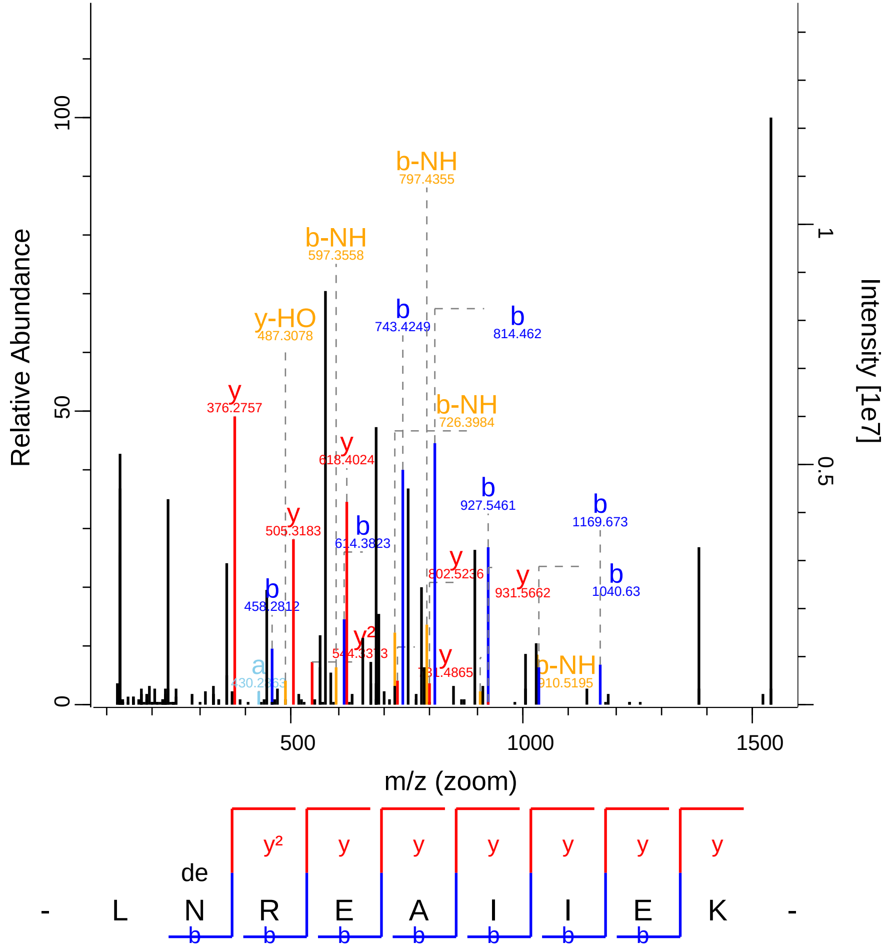

Raw File	Scan	Method	Score	m/z	Gene names
RO-QC-160121_ZackD_SS_Set_02_Fractio	20146	FTMS; HC	73.07	439.74	MED10

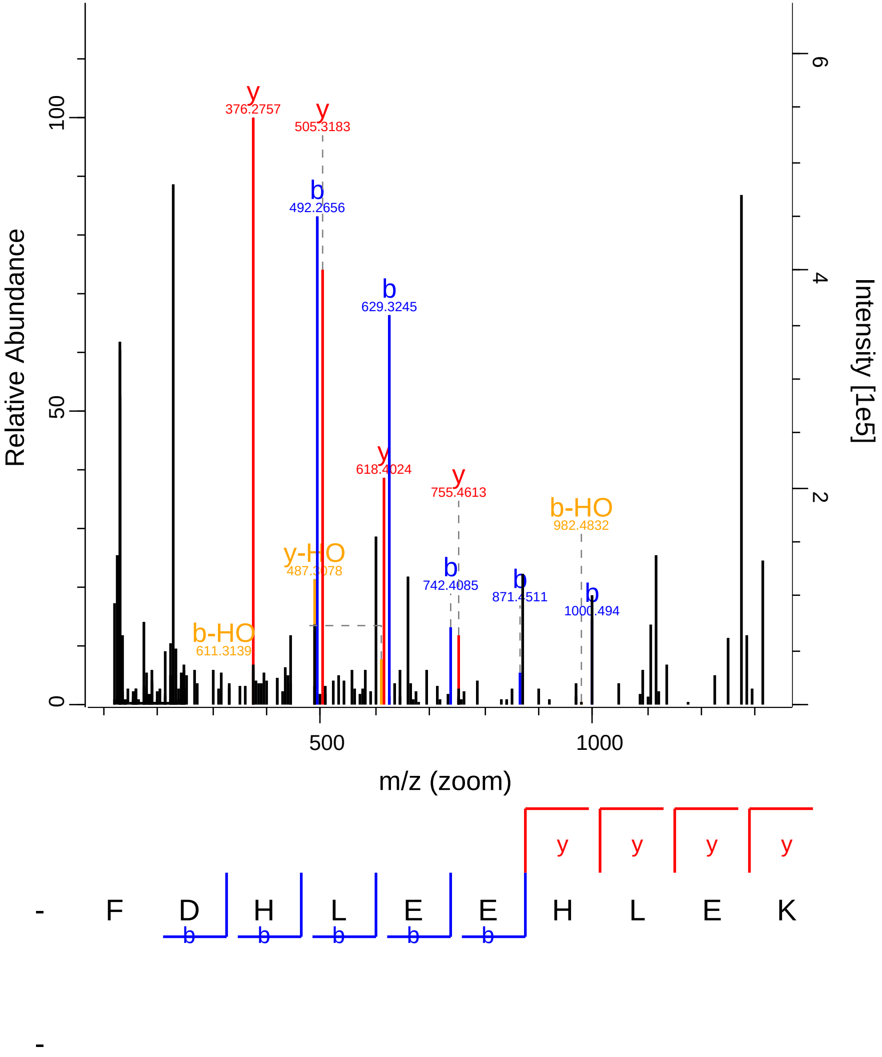

Raw File	Scan	Method	Score	m/z	Gene names
RO-QC-160121_ZackD_SS_Set_02_Fractio	20158	FTMS; HC	97.95	534.56	REEP1

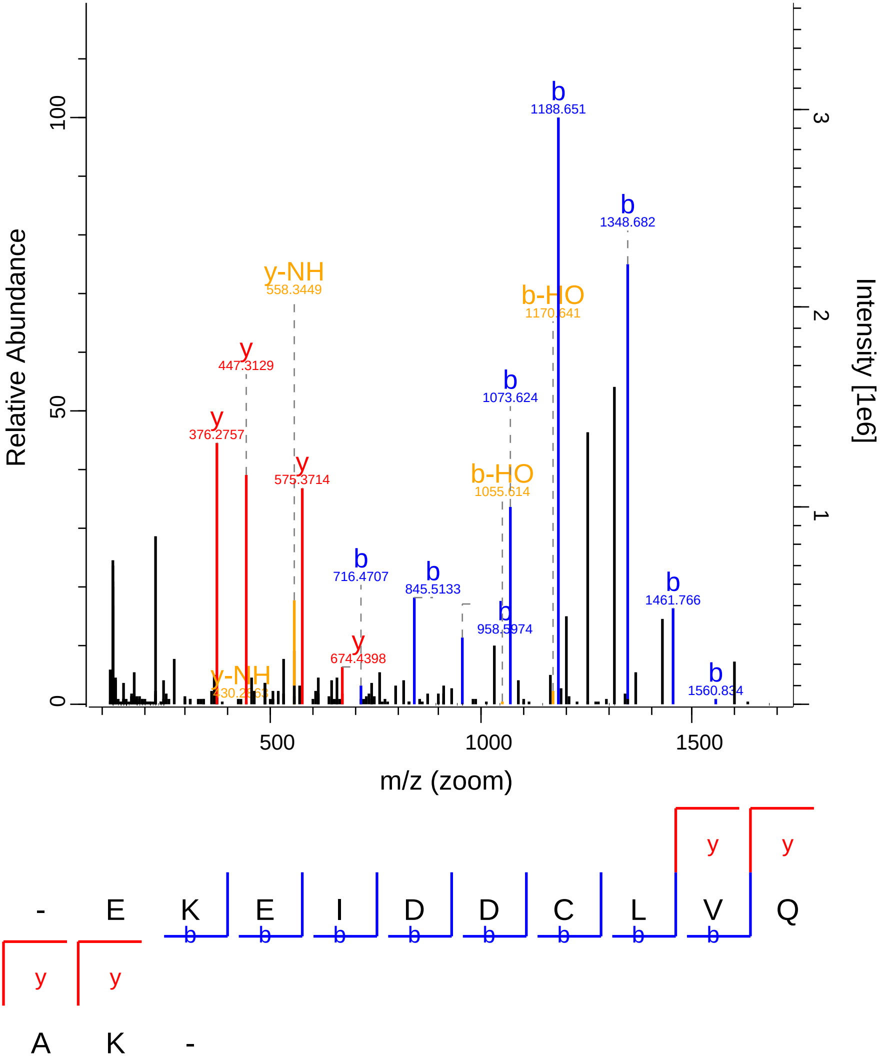

Raw File	Scan	Method	Score	m/z	Gene names
RO-QC-160121_ZackD_SS_Set_02_Fractio	20175	FTMS; HC	118.37	712.41	REEP1

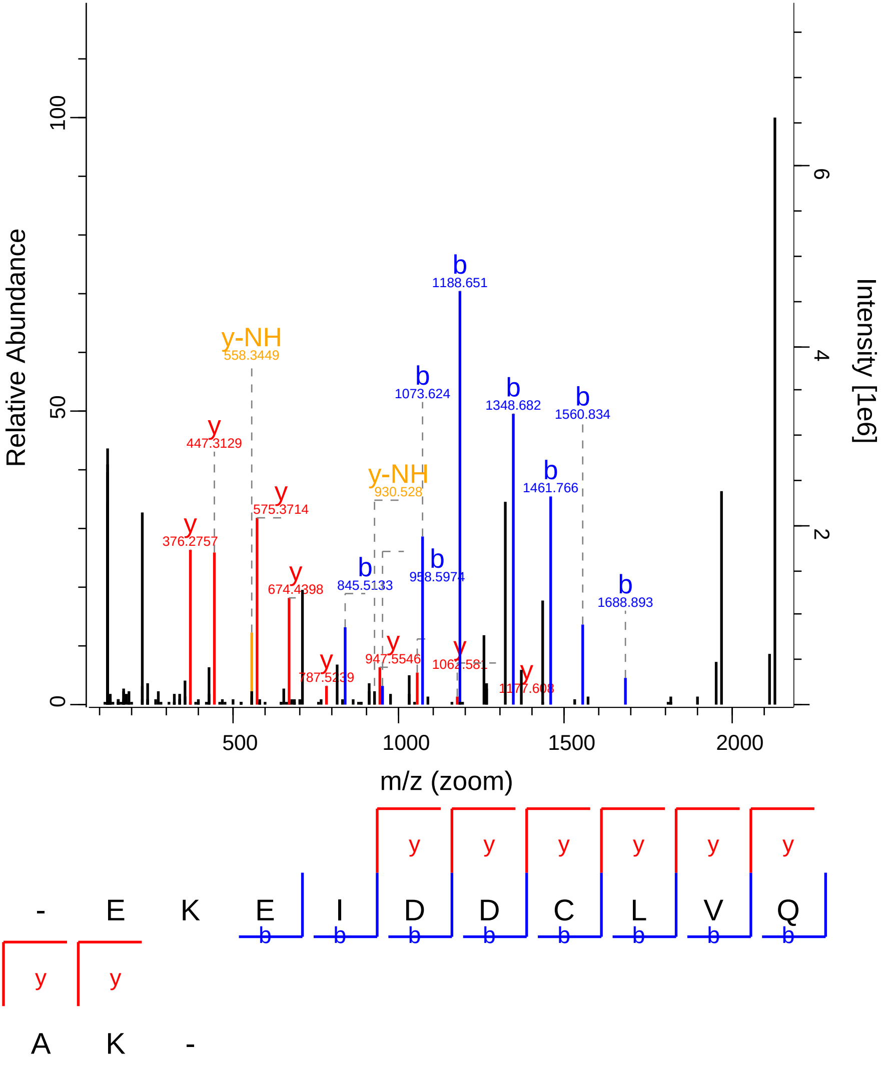

Raw File	Scan	Method	Score	m/z	Gene names
RO-QC-160121_ZackD_SS_Set_02_Fractio	20180	FTMS; HC	76.59	681.73	PPP2R3B

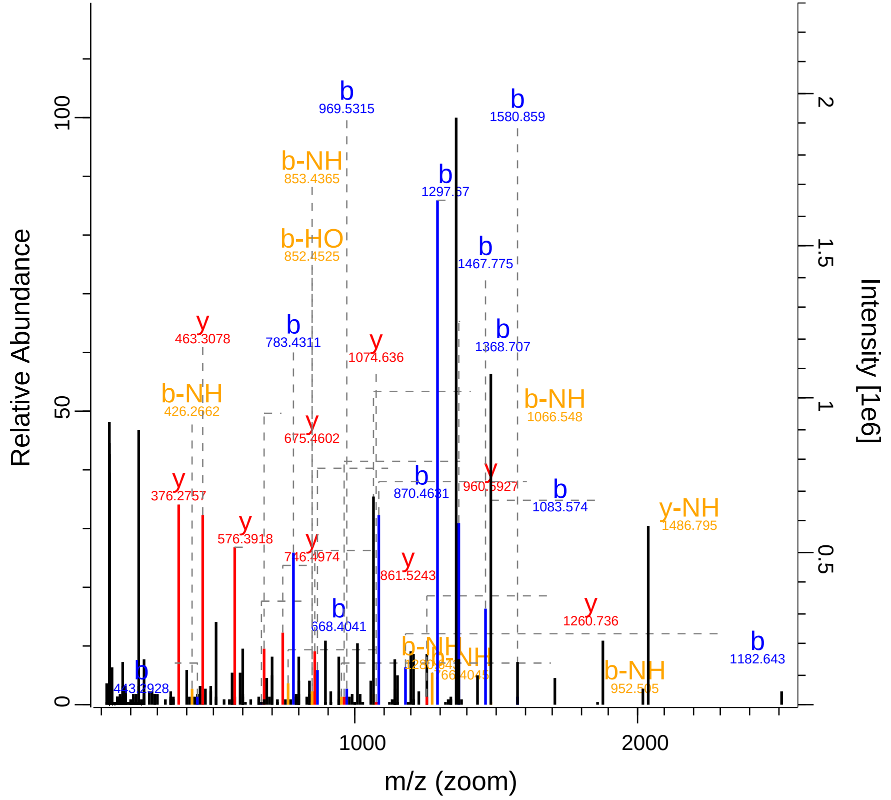

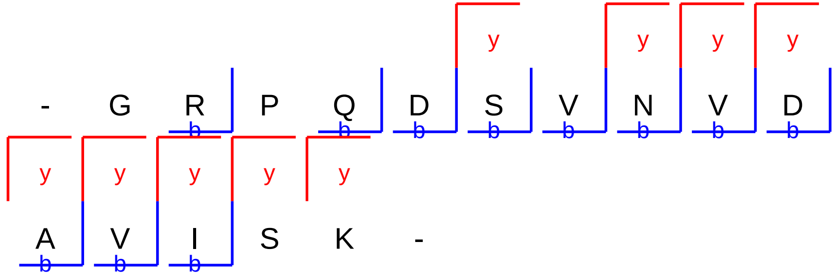

Raw File	Scan	Method	Score	m/z	Gene names
RO-QC-160121_ZackD_SS_Set_02_Fractio	20300	FTMS; HC	38.27	723.36	SVEP1

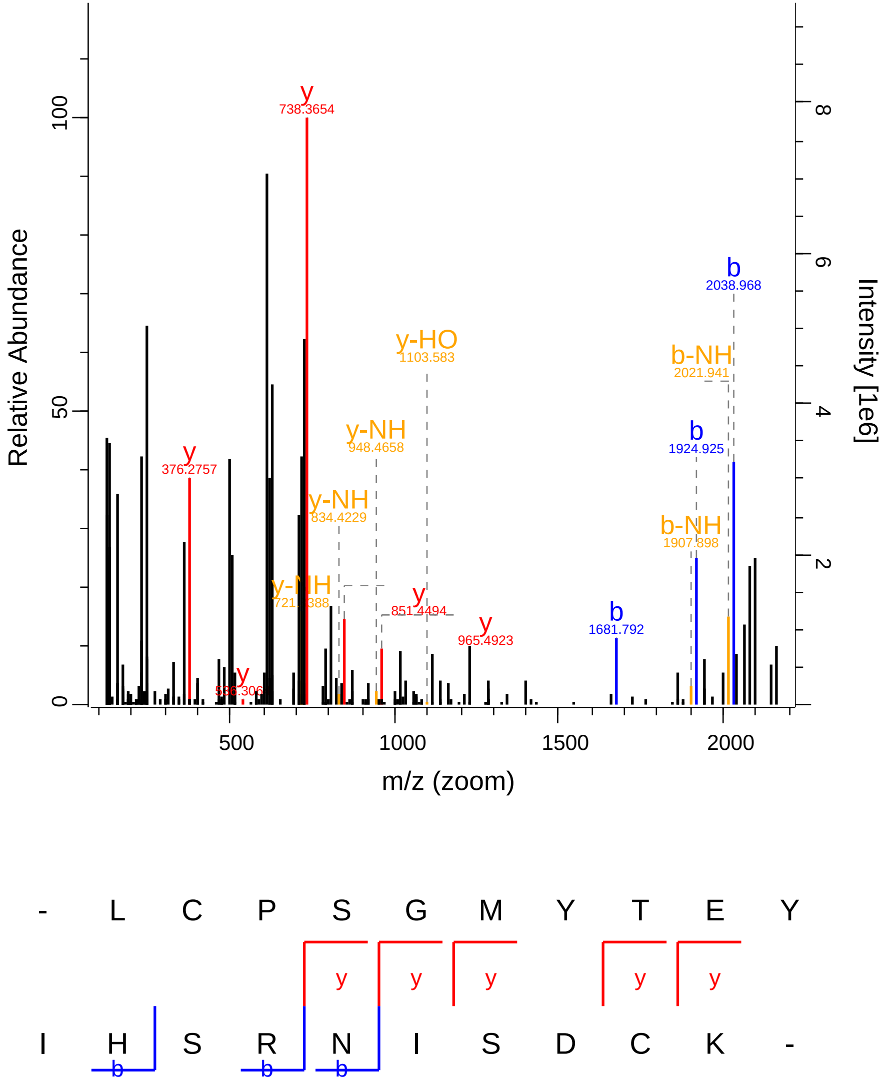

Raw File	Scan	Method	Score	m/z	Gene names
RO-QC-160121_ZackD_SS_Set_02_Fractio	20643	FTMS; HC	112.84	423.25	ZNF536

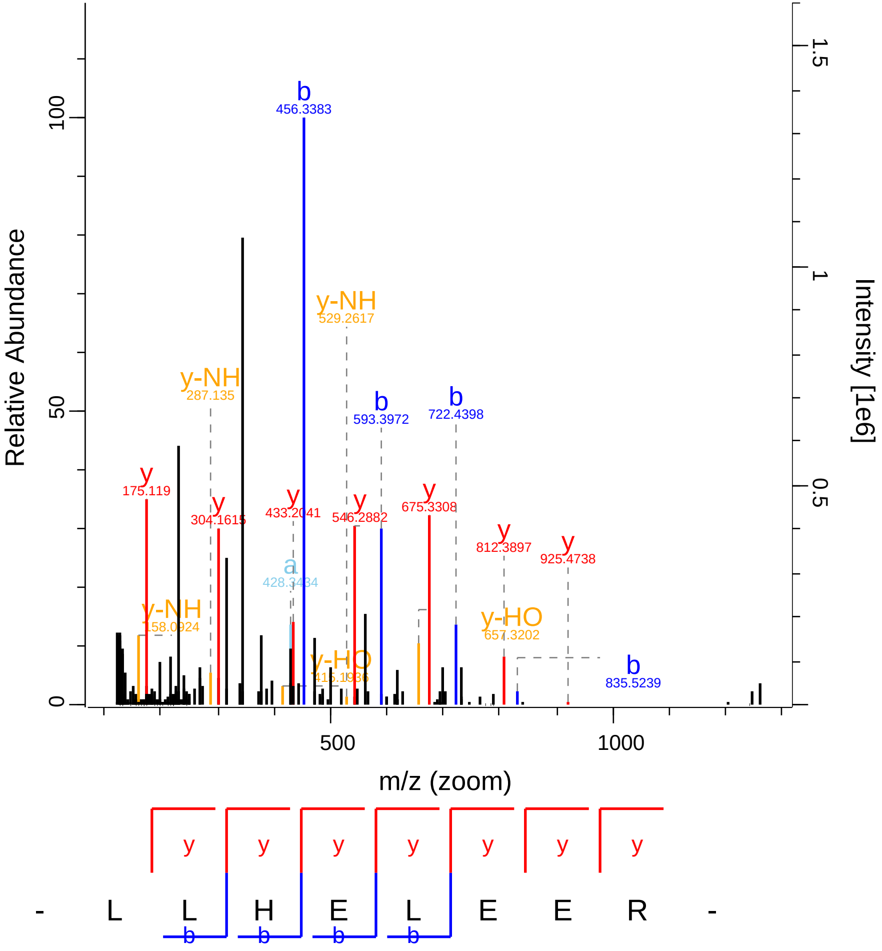

Raw File	Scan	Method	Score	m/z
RO-QC-160121_ZackD_SS_Set_02_Fraction_1	21849	FTMS; HCD	95.1	892.47

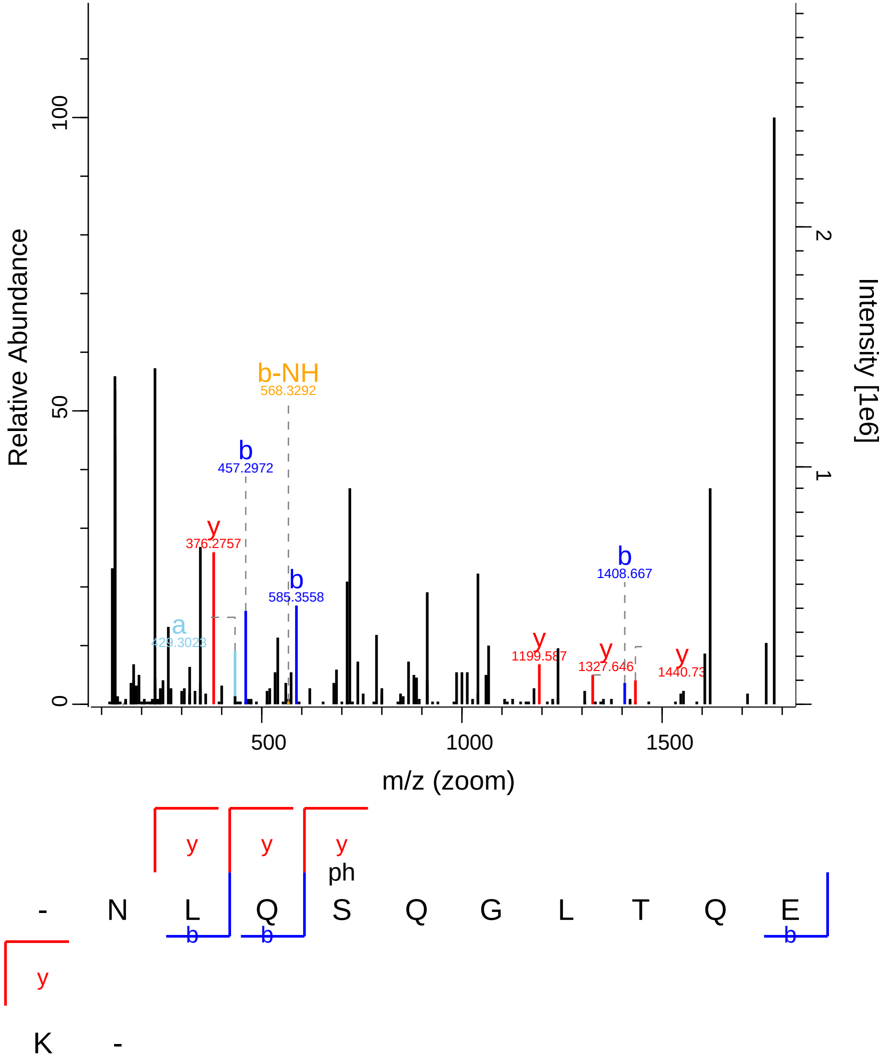

Raw File	Scan	Method	Score	m/z	Gene names
RO-QC-160121_ZackD_SS_Set_02_Fracti	23396	FTMS; HC	87.41	747.05	TRIM16L;TRIM1

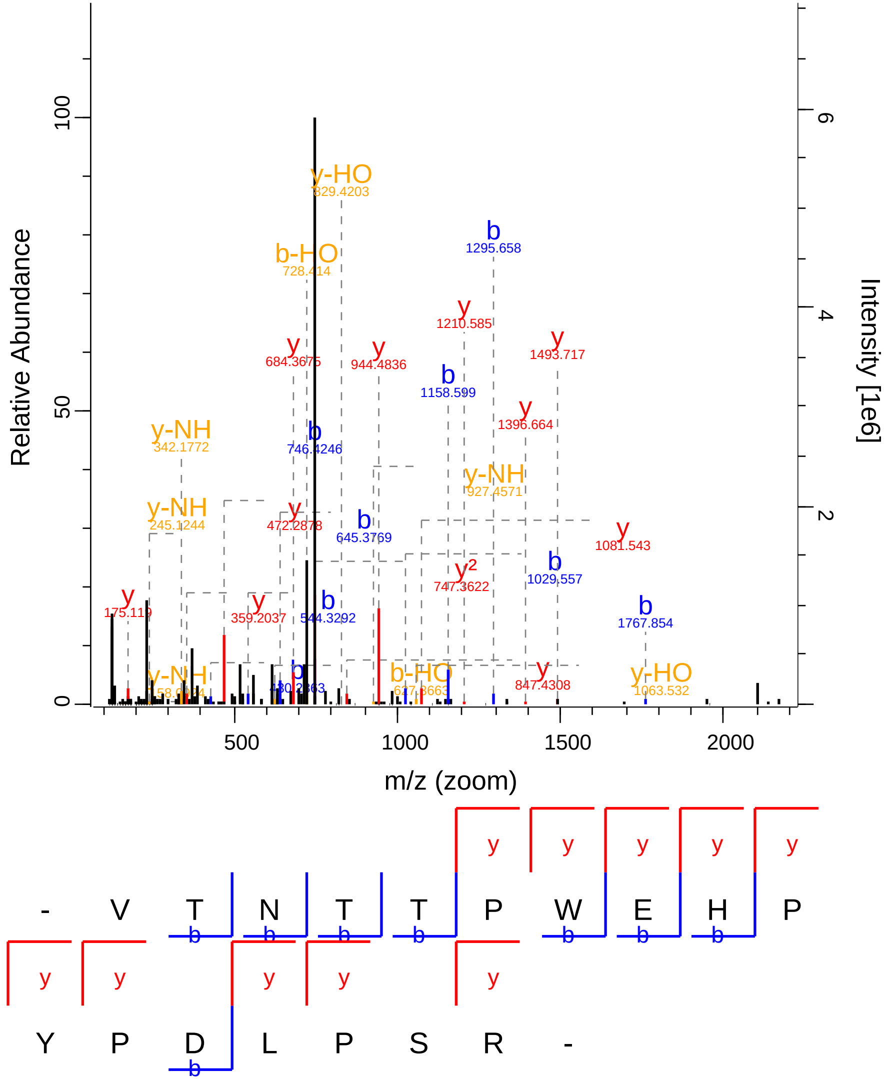

Raw File	Scan	Method	Score	m/z	Gene names
RO-QC-160121_ZackD_SS_Set_02_Fractio	23478	FTMS; HC	76.03	854.78	SLC31A1

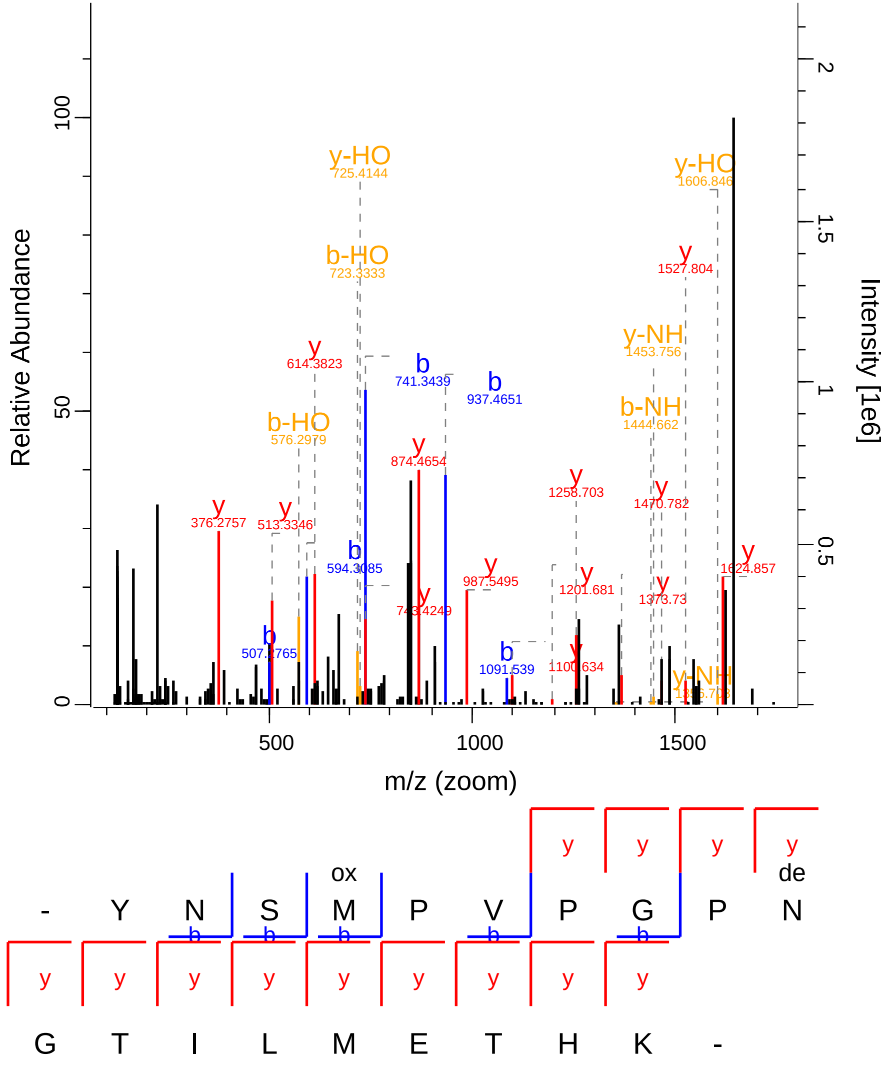

Raw File	Scan	Method	Score	m/z	Gene names
RO-QC-160121_ZackD_SS_Set_02_Fractio	23817	FTMS; HC	97.46	846.47	METTL18

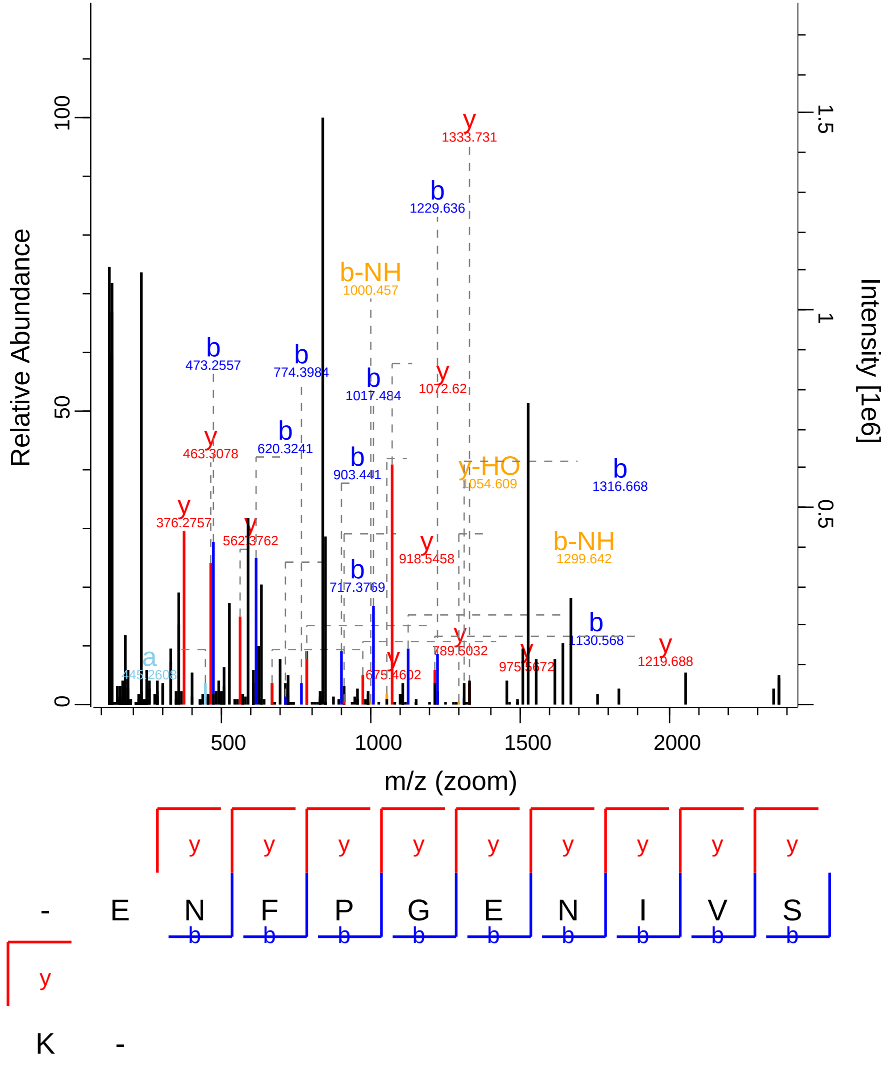

Raw File	Scan	Method	Score	m/z	Gene names
RO-QC-160121_ZackD_SS_Set_02_Fractioı	24065	FTMS; HCI	196.5	904.5	KLHL13;KLHL9

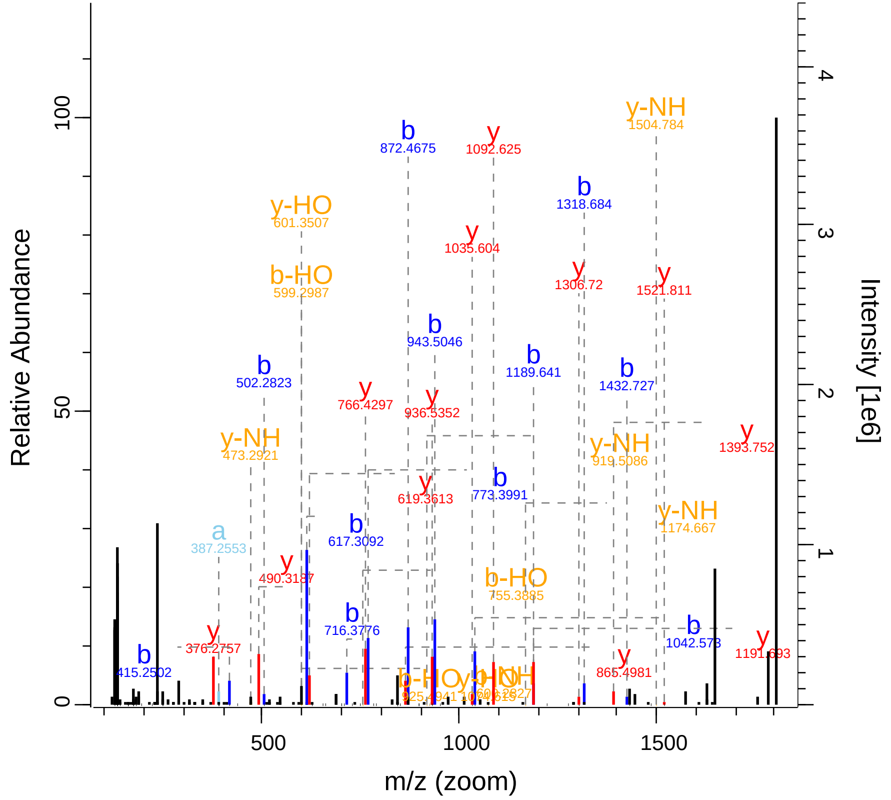

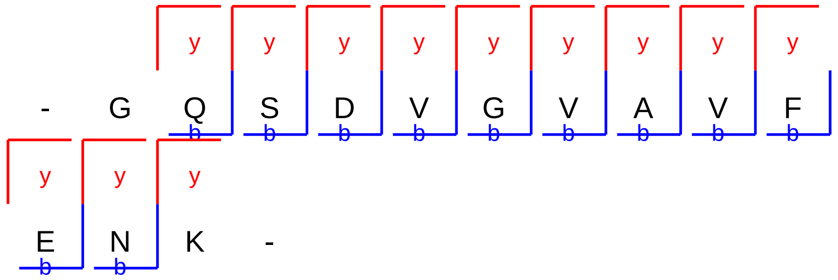

Raw File	Scan	Method	Score	m/z
RO-QC-160121_ZackD_SS_Set_02_Fraction_1	24187	FTMS; HCD	56.26	875.49

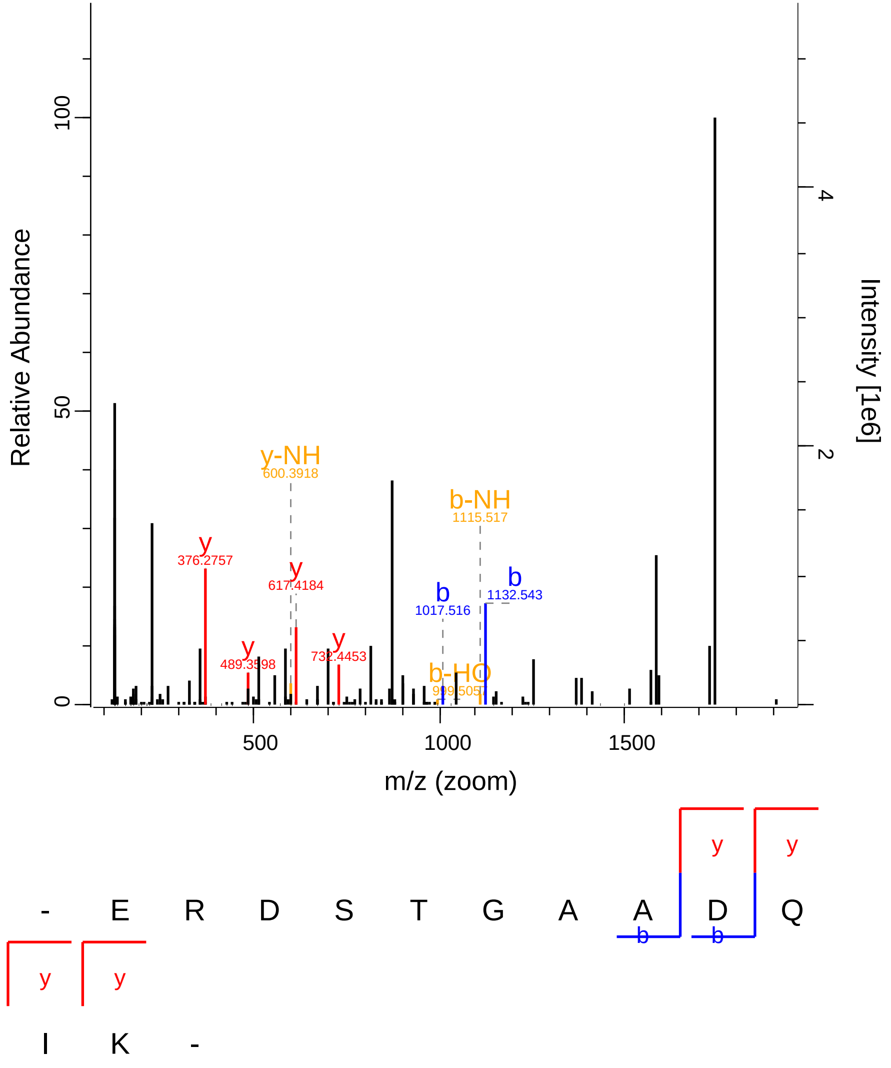

Raw File	Scan	Method	Score	m/z	Gene names
RO-QC-160121_ZackD_SS_Set_02_Fractio	24775	FTMS; HCI	138.45	762.4	EVA1A

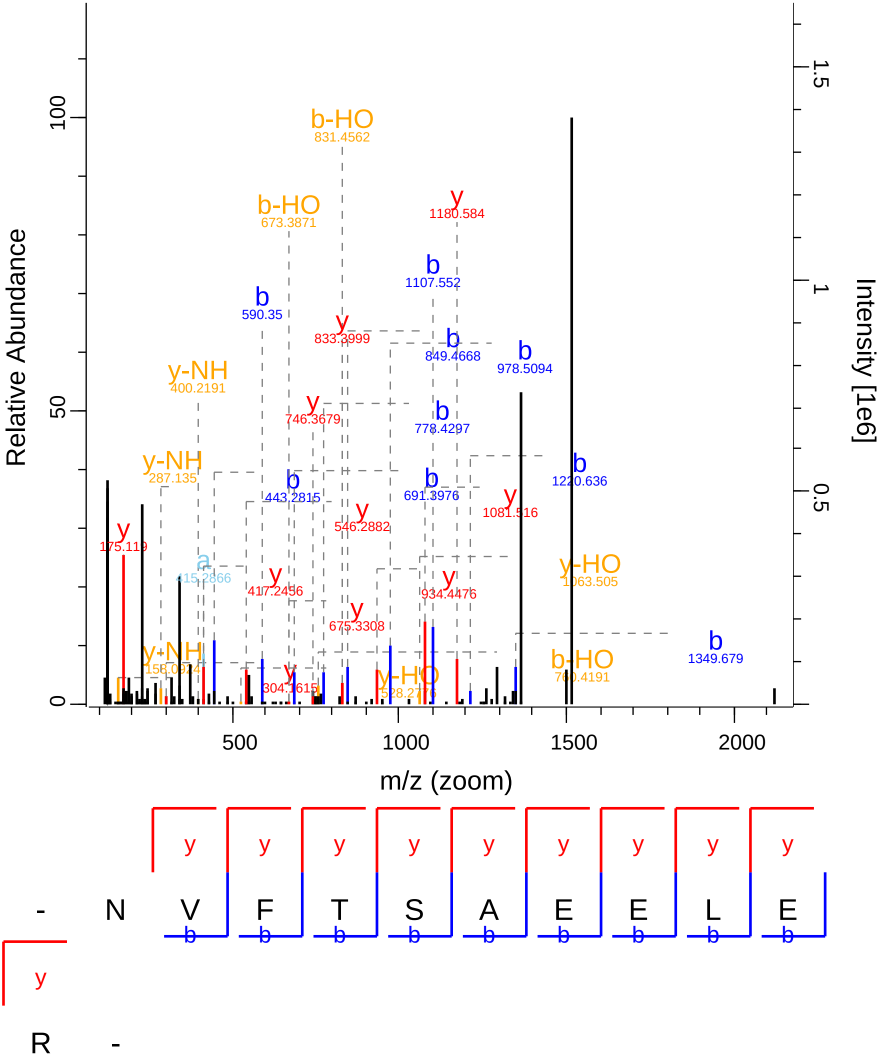

Raw File	Scan	Method	Score	m/z	Gene names
RO-QC-160121_ZackD_SS_Set_02_Fractior	24955	FTMS; HCI	89.8	762.4	EVA1A

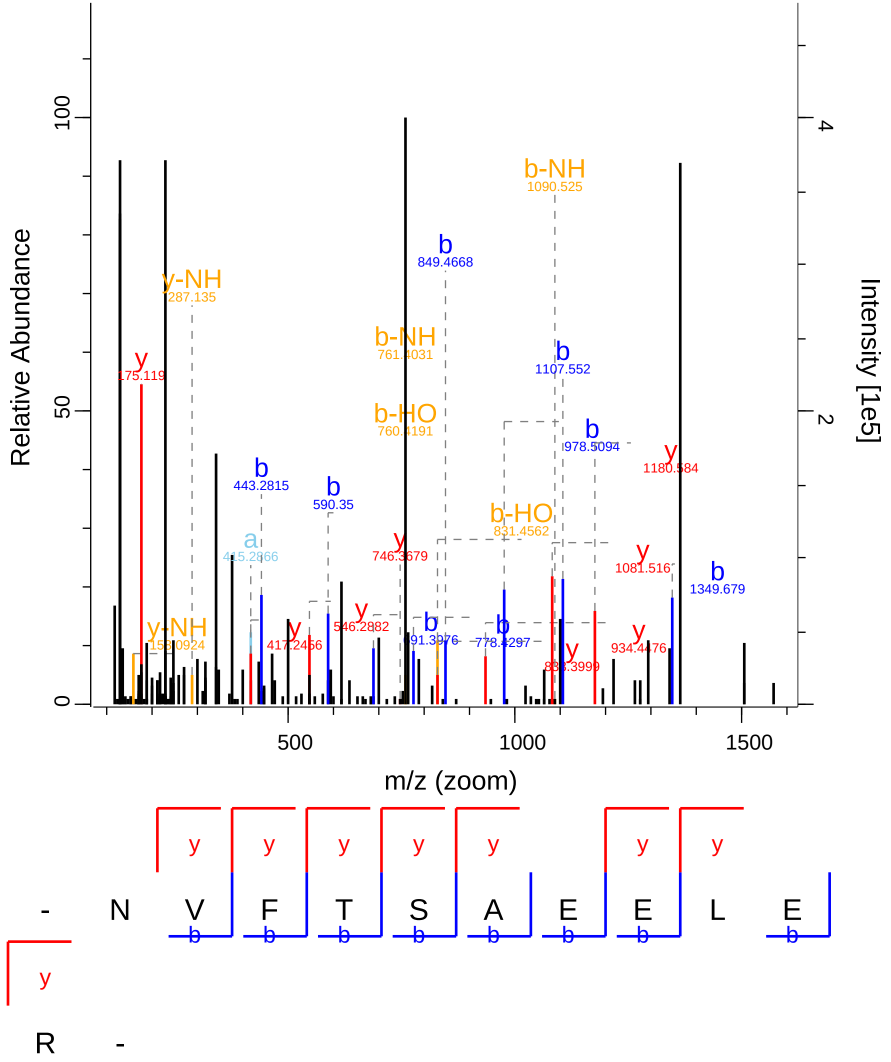

Raw File	Scan	Method	Score	m/z	Gene names
RO-QC-160121_ZackD_SS_Set_02_Fractio	26324	FTMS; HC	115.91	687.88	FIZ1

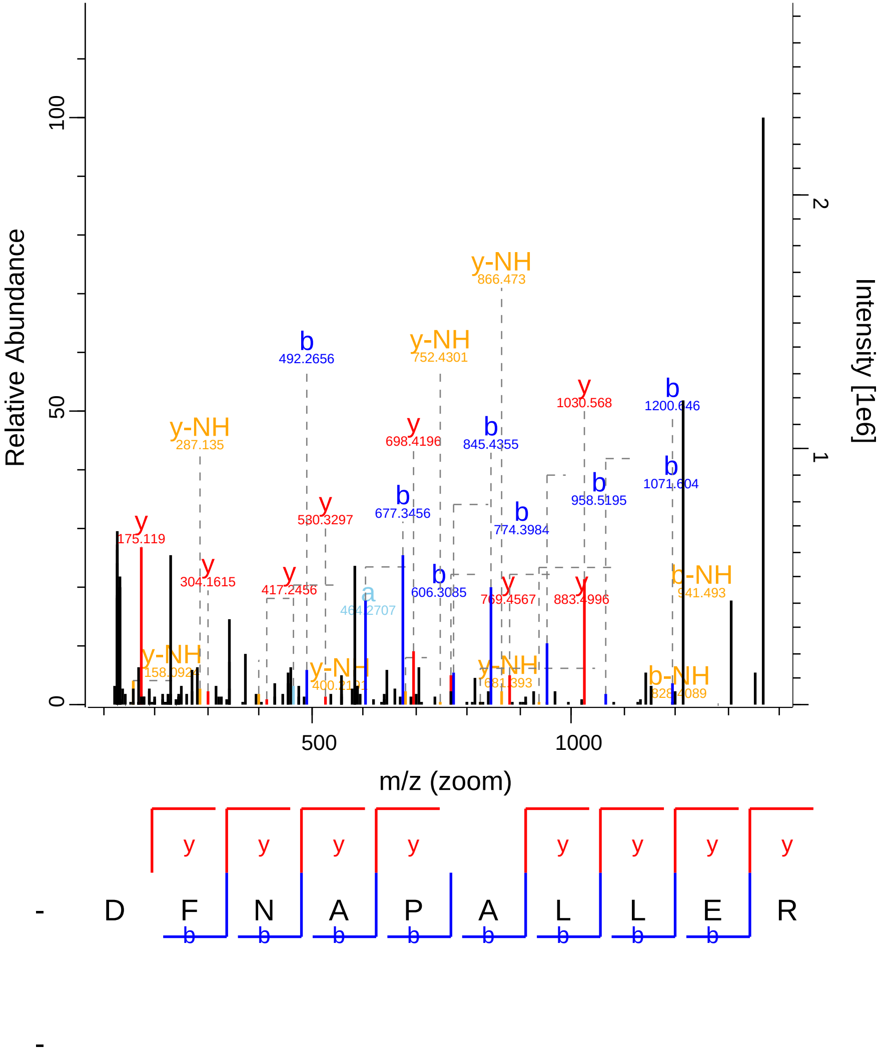

Raw File	Scan	Method	Score	m/z	Gene names
RO-QC-160121_ZackD_SS_Set_02_Fractio	26329	FTMS; HC	113.41	702.92	CHRDL1

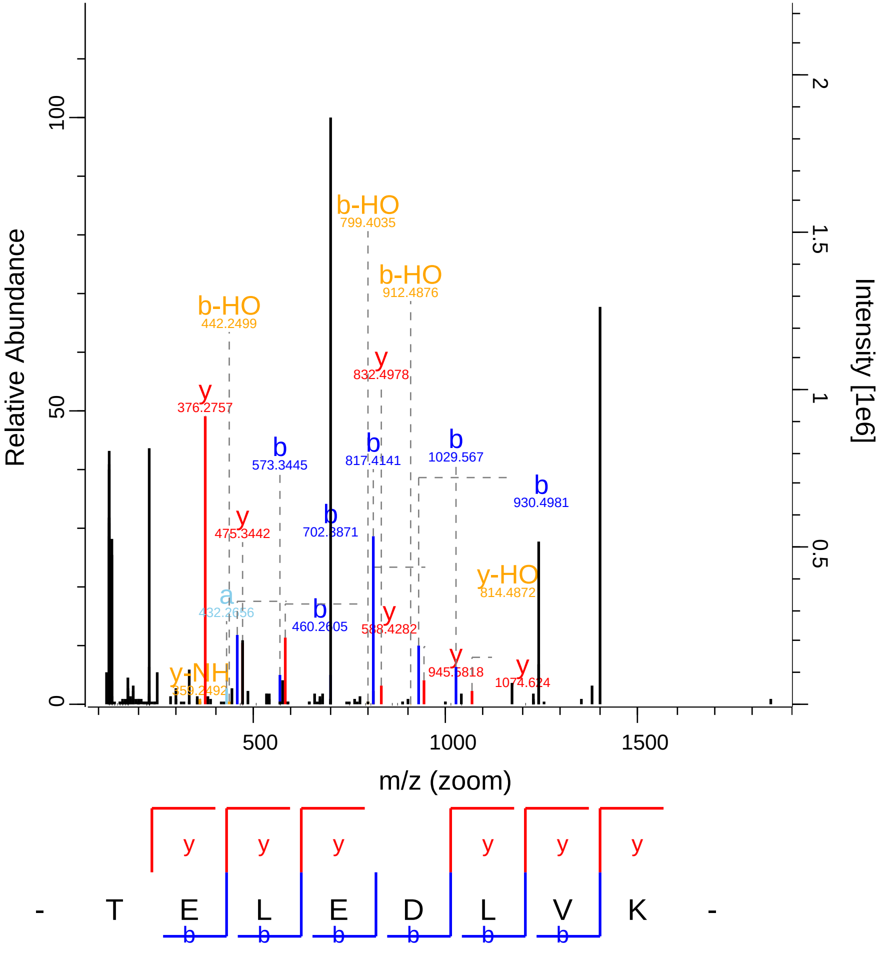

Raw File	Scan	Method	Score	m/z	Gene names
RO-QC-160121_ZackD_SS_Set_02_Fractior	26848	FTMS; HCI	120.32	909.8	DRAM2

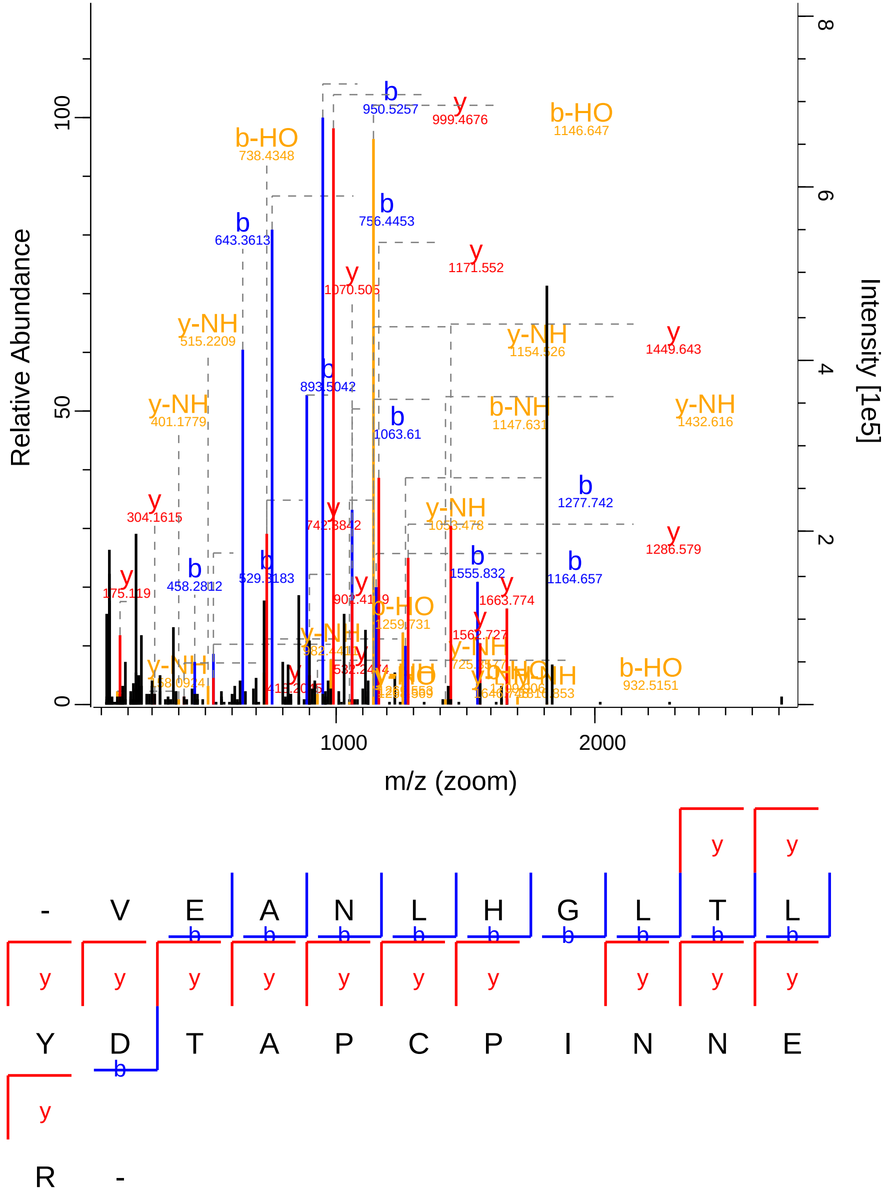

Raw File	Scan	Method	Score	m/z	Gene names
RO-QC-160121_ZackD_SS_Set_02_Fractior	27235	FTMS; HCI	98.94	926.7	FAM58A

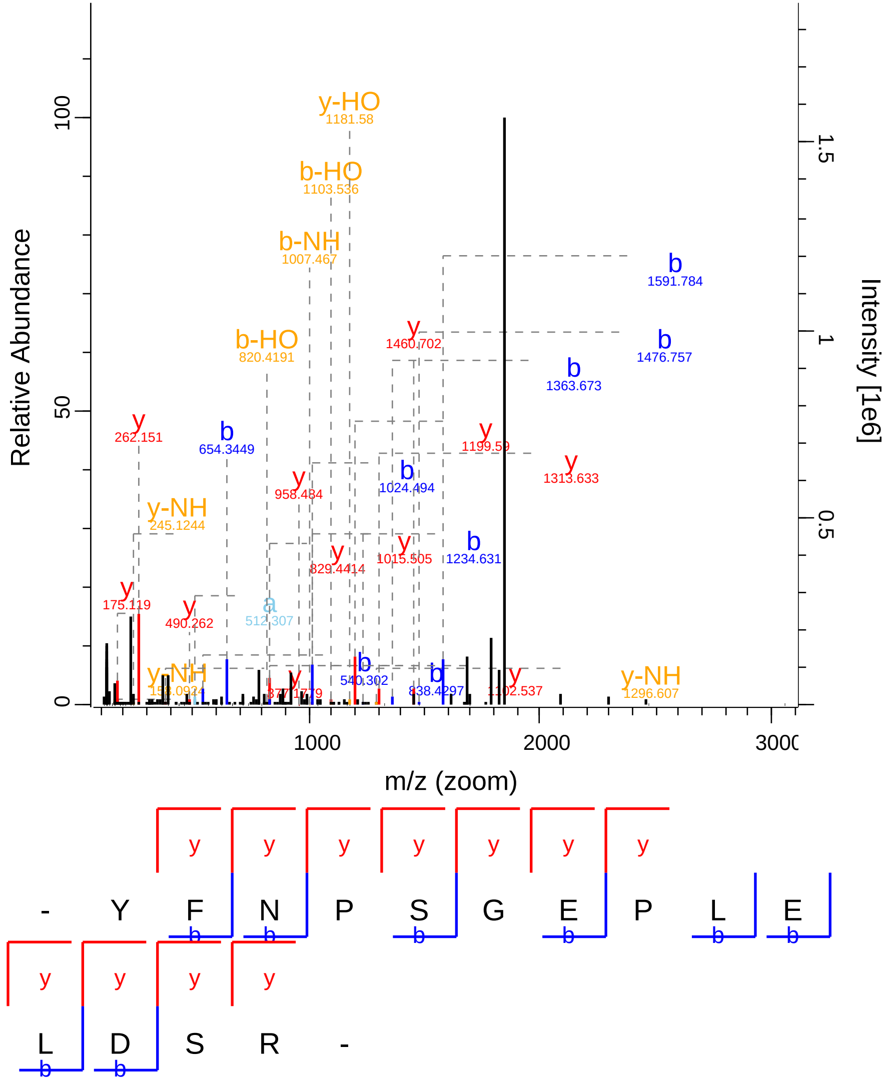

Raw File	Scan	Method	Score	m/z	Gene names
RO-QC-160121_ZackD_SS_Set_02_Fractio	27316	FTMS; HC	141.53	910.13	DRAM2

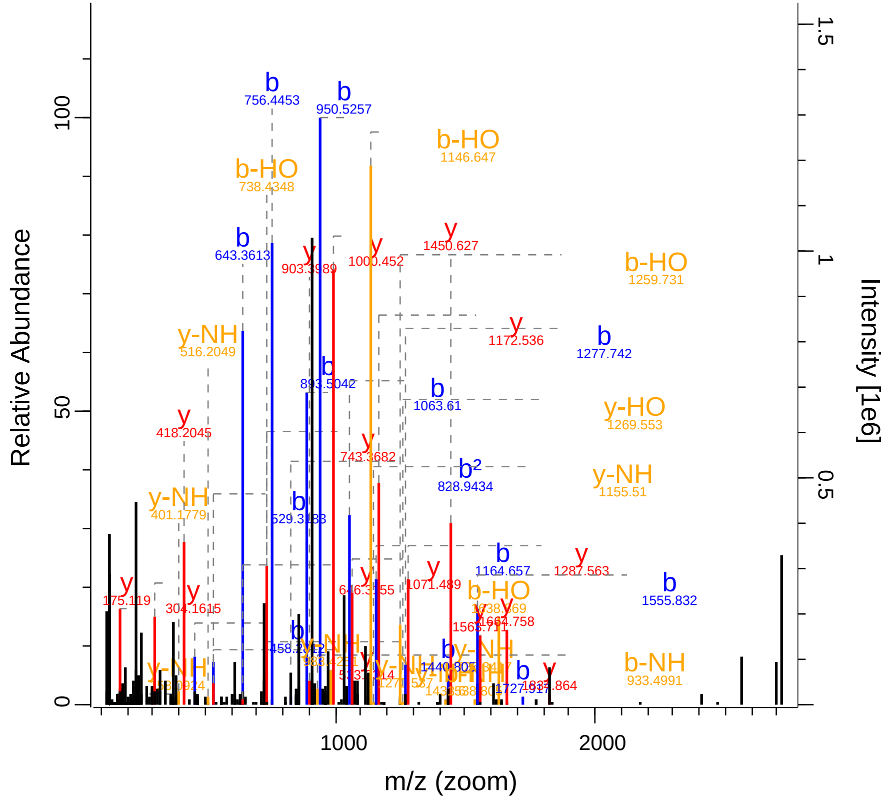

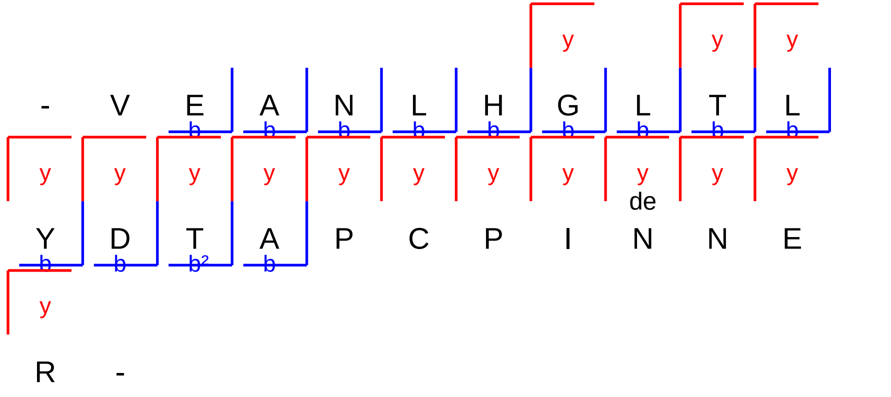

Raw File	Scan	Method	Score	m/z	Gene names
RO-QC-160121_ZackD_SS_Set_02_Fractio	27435	FTMS; HC	108.56	612.69	PLA2G16

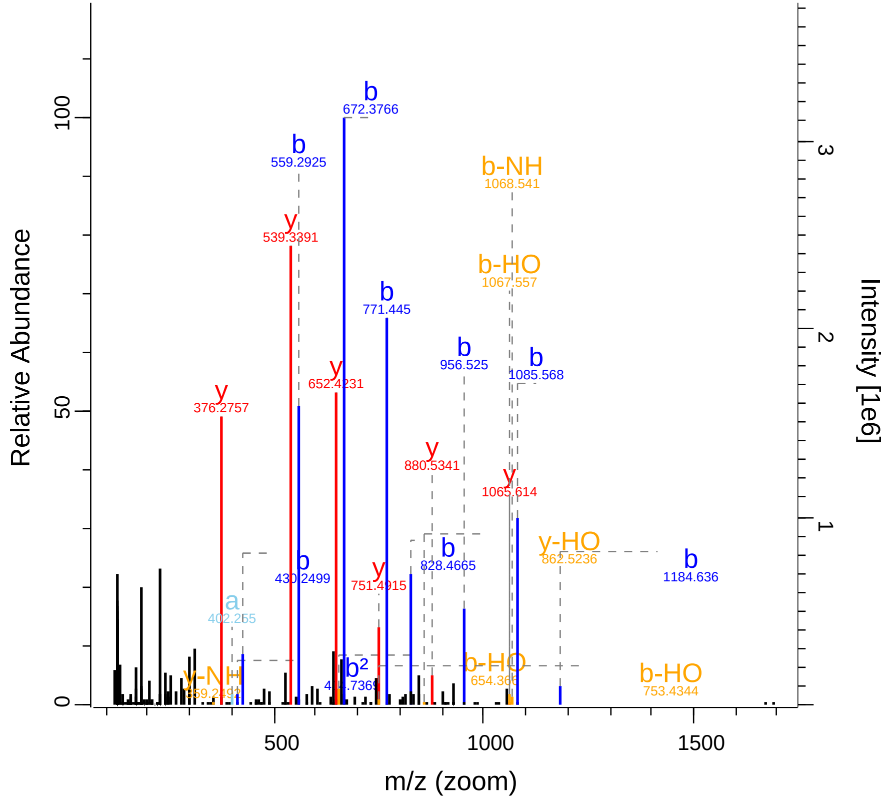

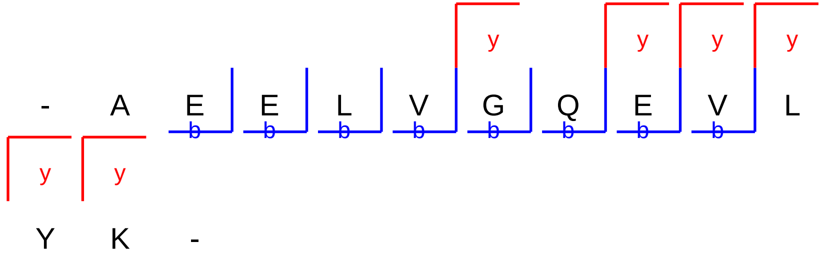

Raw File	Scan	Method	Score	m/z	Gene names
RO-QC-160121_ZackD_SS_Set_02_Fractio	27440	FTMS; HC	59.86	849.77	SLC31A1

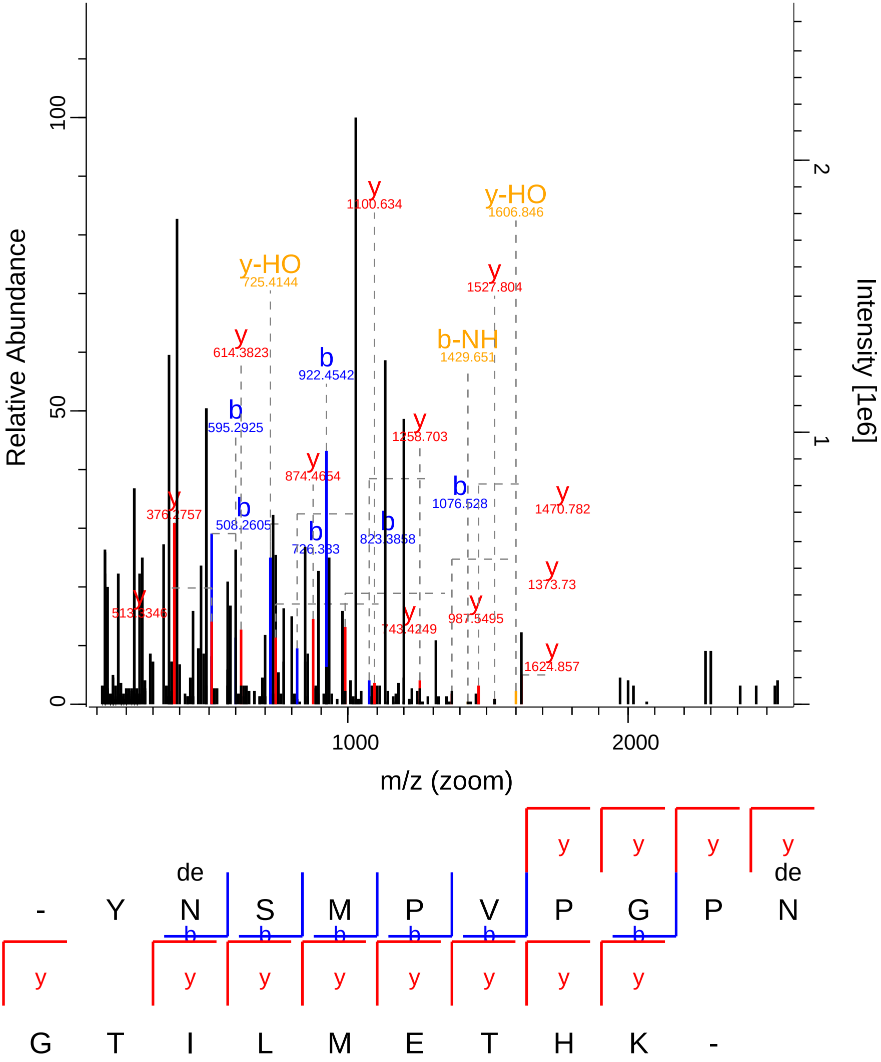

Raw File	Scan	Method	Score	m/z	Gene names
RO-QC-160121_ZackD_SS_Set_02_Fractio	27936	FTMS; HC	96.34	531.64	MRS2

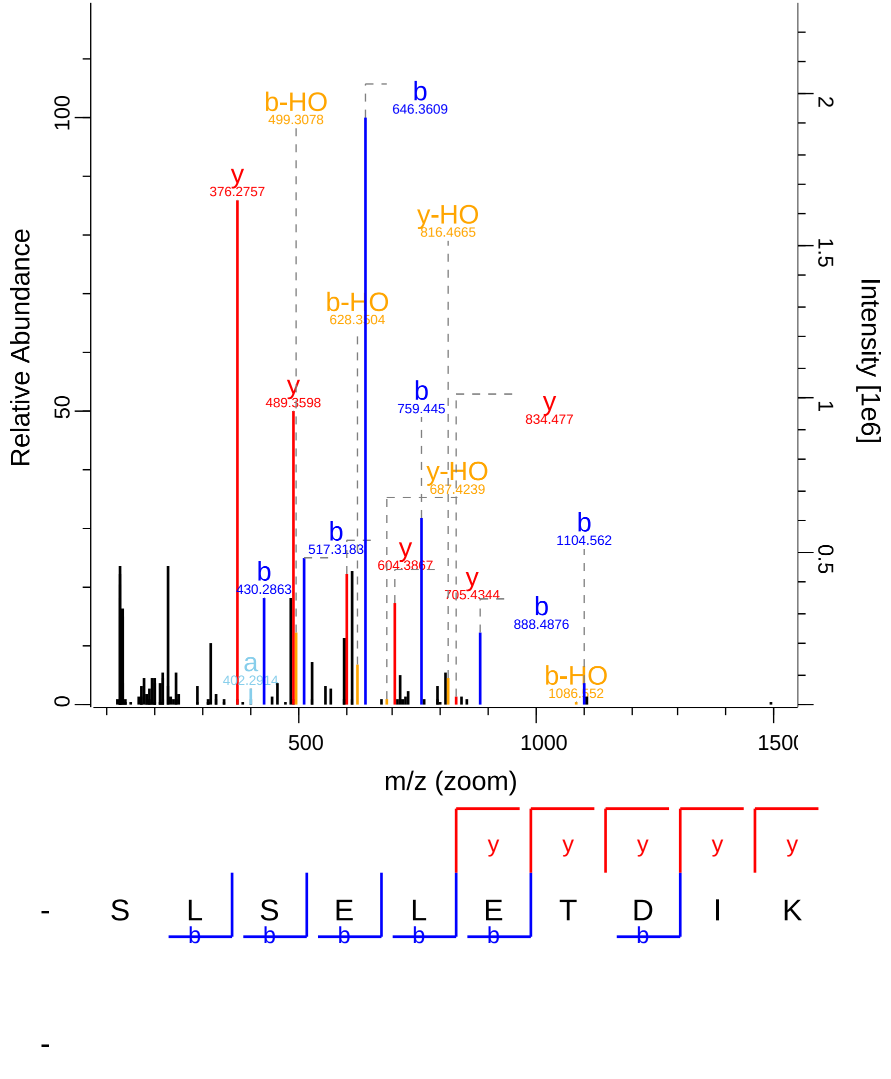

Raw File	Scan	Method	Score	m/z
RO-QC-160121_ZackD_SS_Set_02_Fraction_1	28137	FTMS; HCD	71.18	1262.15

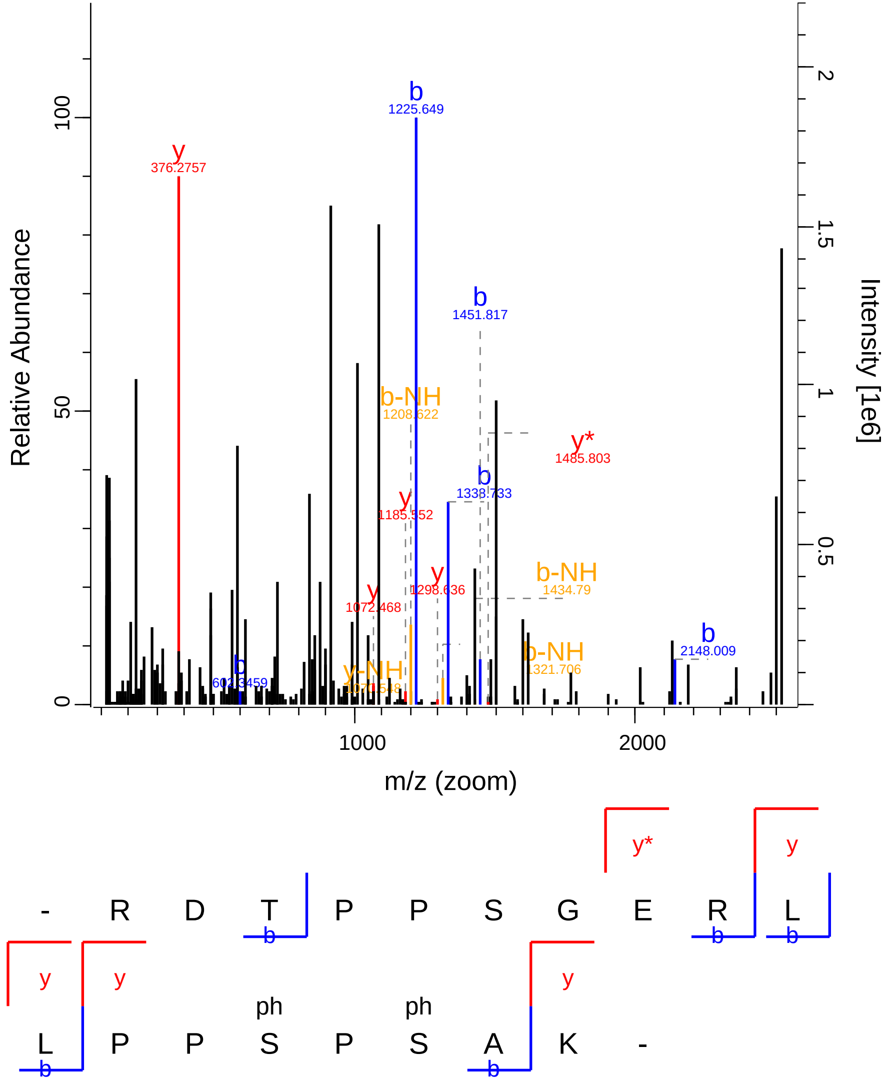

Raw File	Scan	Method	Score	m/z	Gene names
RO-QC-160121_ZackD_SS_Set_02_Fractic	28884	FTMS; HC	83.09	1077.54	PDSS1

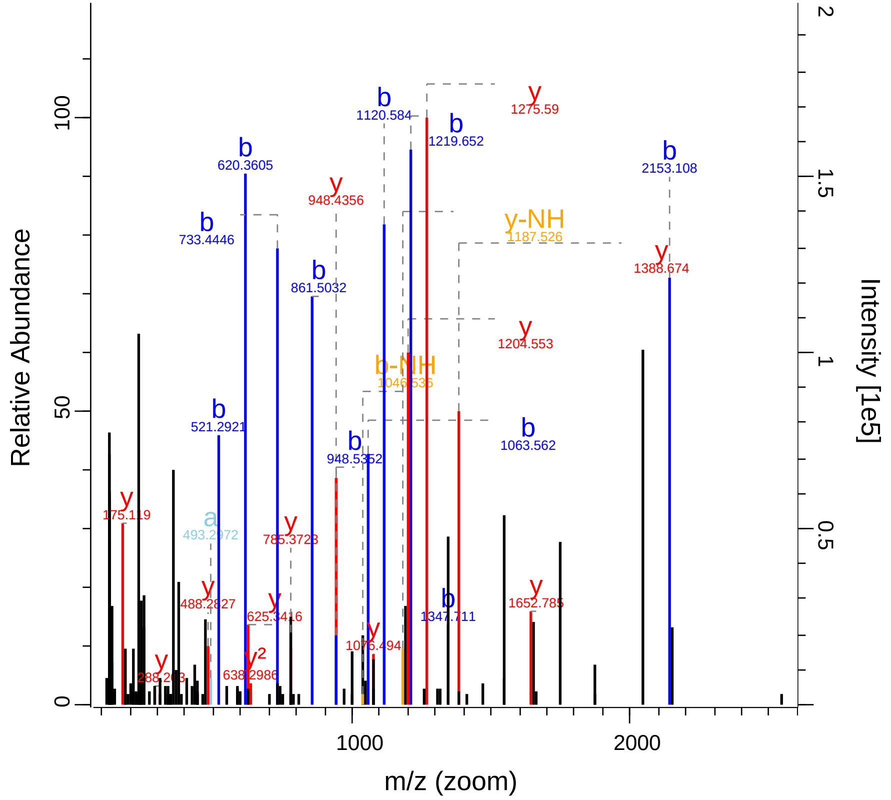

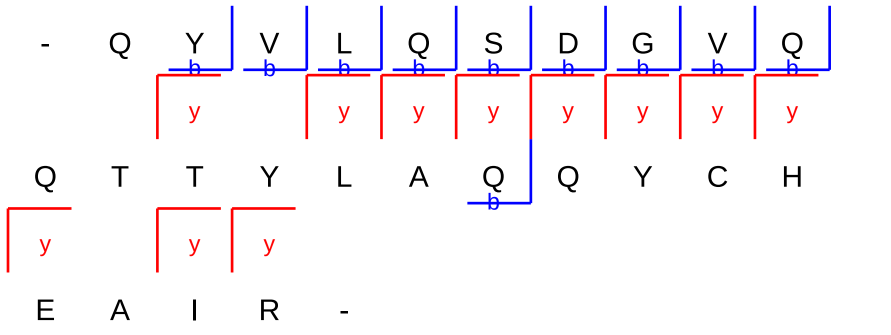

Raw File	Scan	Method	Score	m/z	Gene names
RO-QC-160121_ZackD_SS_Set_02_Fractic	29764	FTMS; HC	91.73	1029.86	MLX

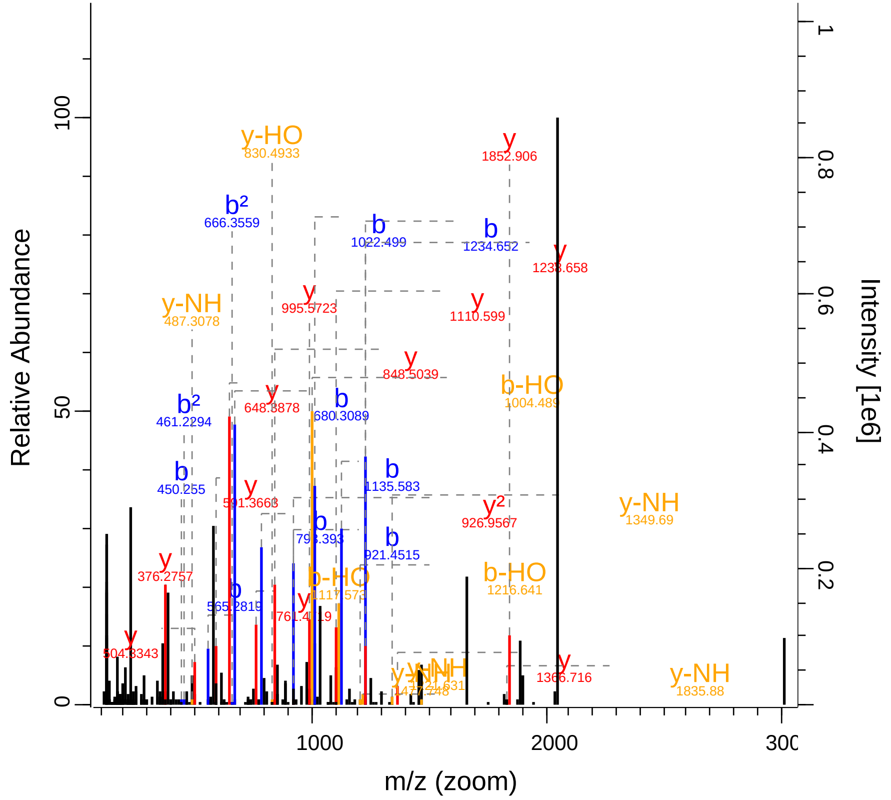

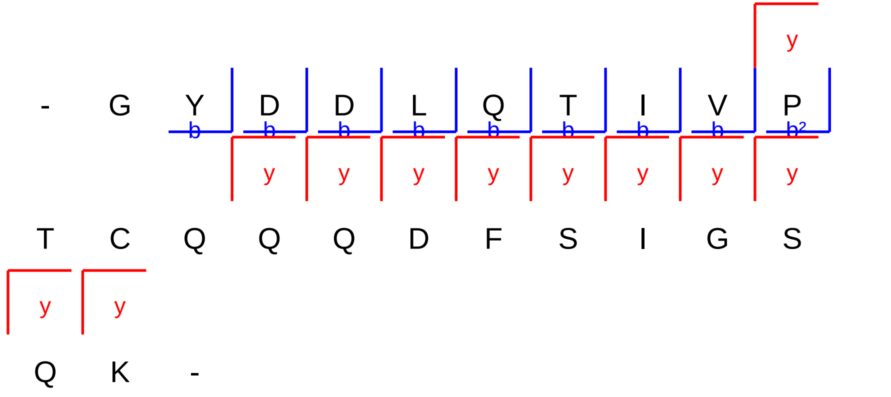

Raw File	Scan	Method	Score	m/z	Gene names
RO-QC-160121_ZackD_SS_Set_02_Fractio	30853	FTMS; HC	61.42	627.02	TMEM184C

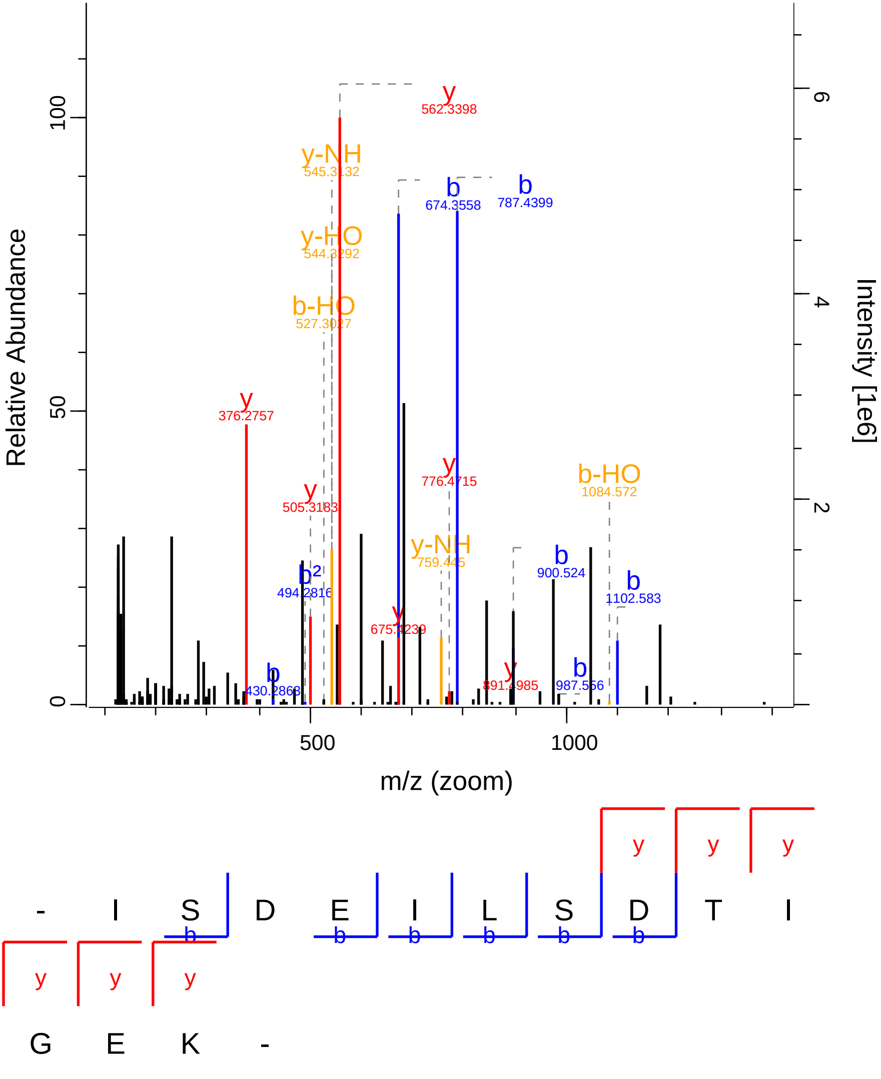

Raw File	Scan	Method	Score	m/z	Gene names
RO-QC-160121_ZackD_SS_Set_02_Fractio	30855	FTMS; HC	197.07	939.53	TMEM184C

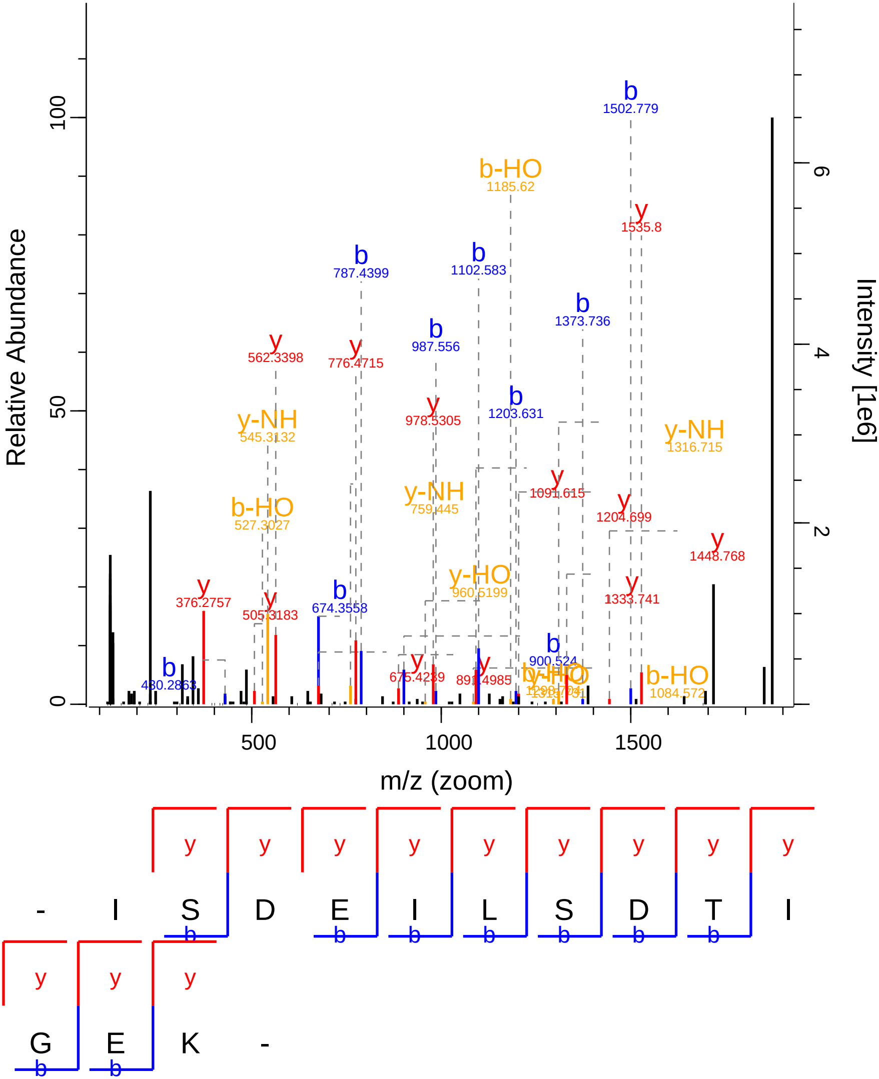

Raw File	Scan	Method	Score	m/z	Gene names
RO-QC-160121_ZackD_SS_Set_02_Fractio	31030	FTMS; HC	105.66	626.69	TMEM184C

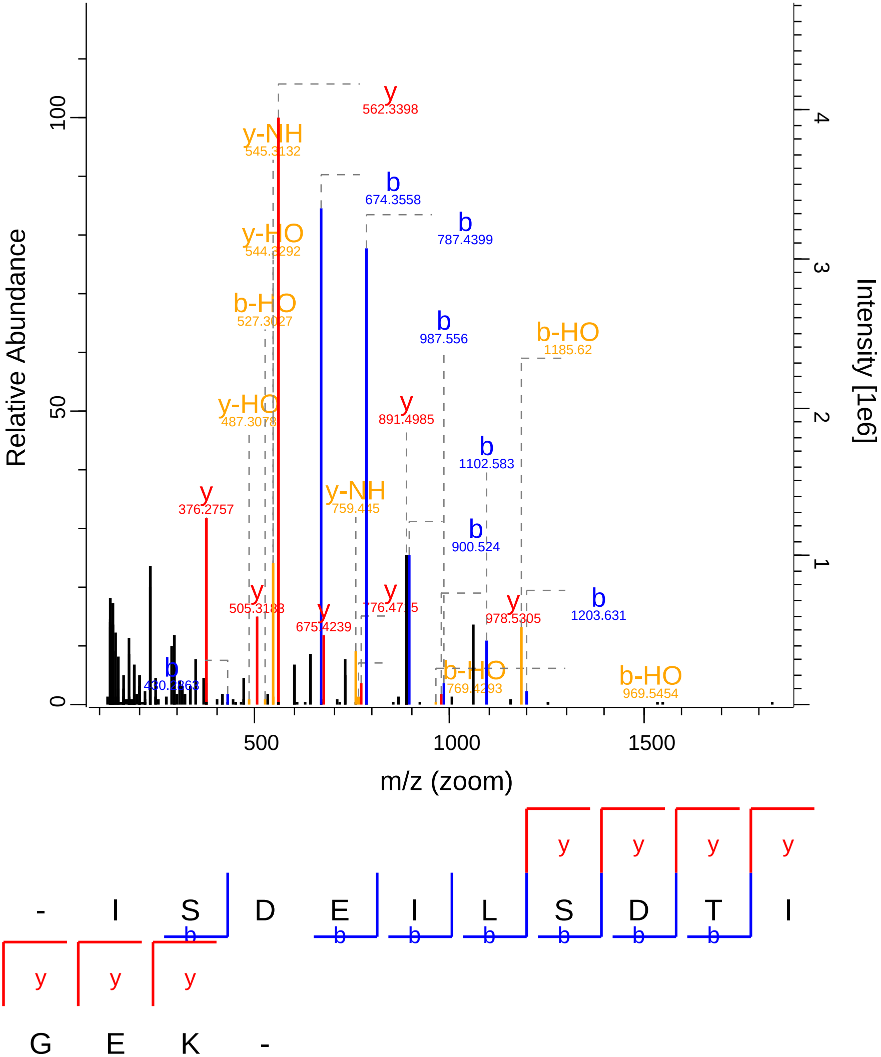

Raw File	Scan	Method	Score	m/z	Gene names
RO-QC-160121_ZackD_SS_Set_02_Fractio	31033	FTMS; HC	123.01	940.03	TMEM184C

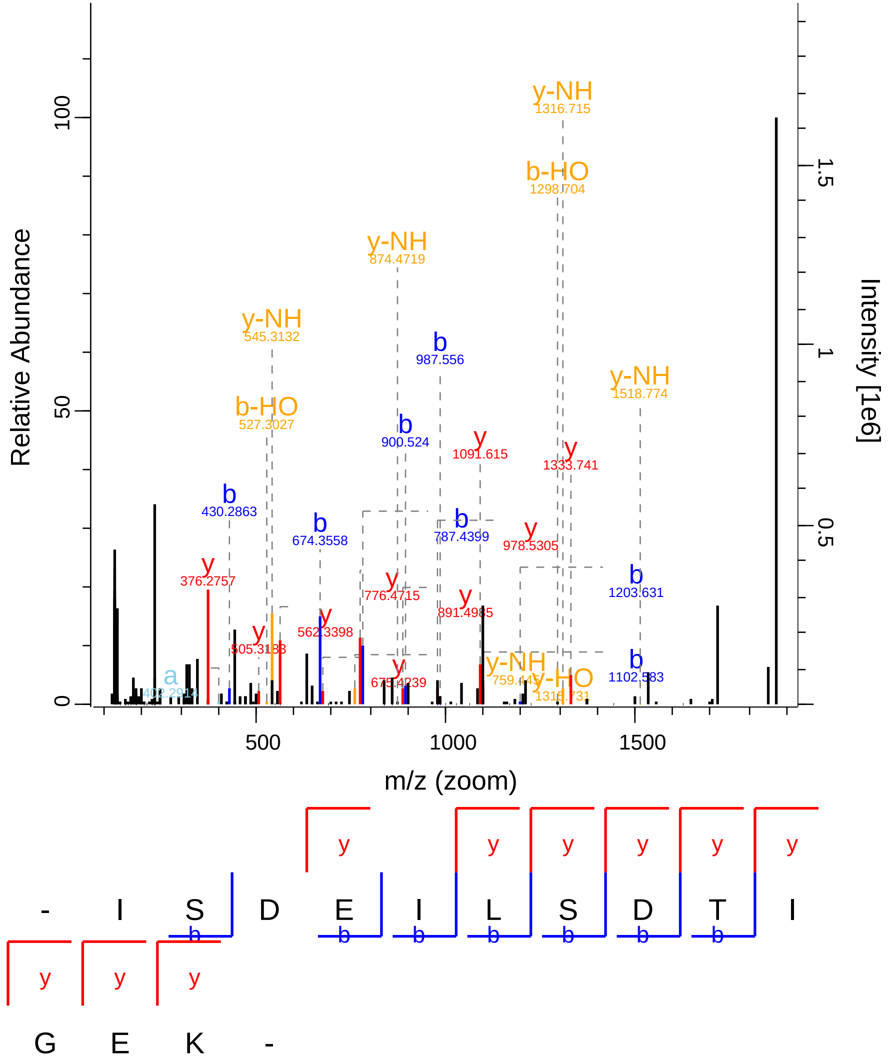

Raw File	Scan	Method	Score	m/z	Gene names
RO-QC-160121_ZackD_SS_Set_02_Fractio	31463	FTMS; HC	133.48	715.41	BCAS4

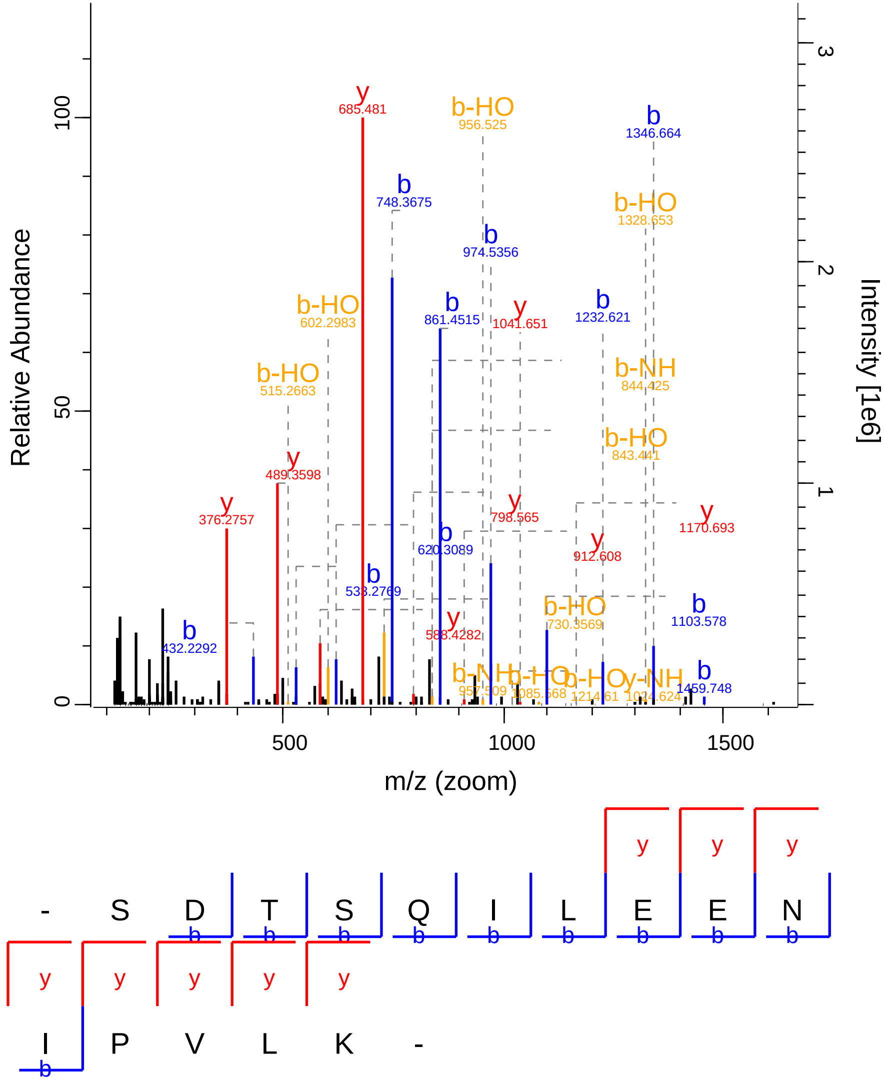

Raw File	Scan	Method	Score	m/z	Gene names
RO-QC-160121_ZackD_SS_Set_02_Fractio	31704	FTMS; HC	98.44	741.07	SMYD2

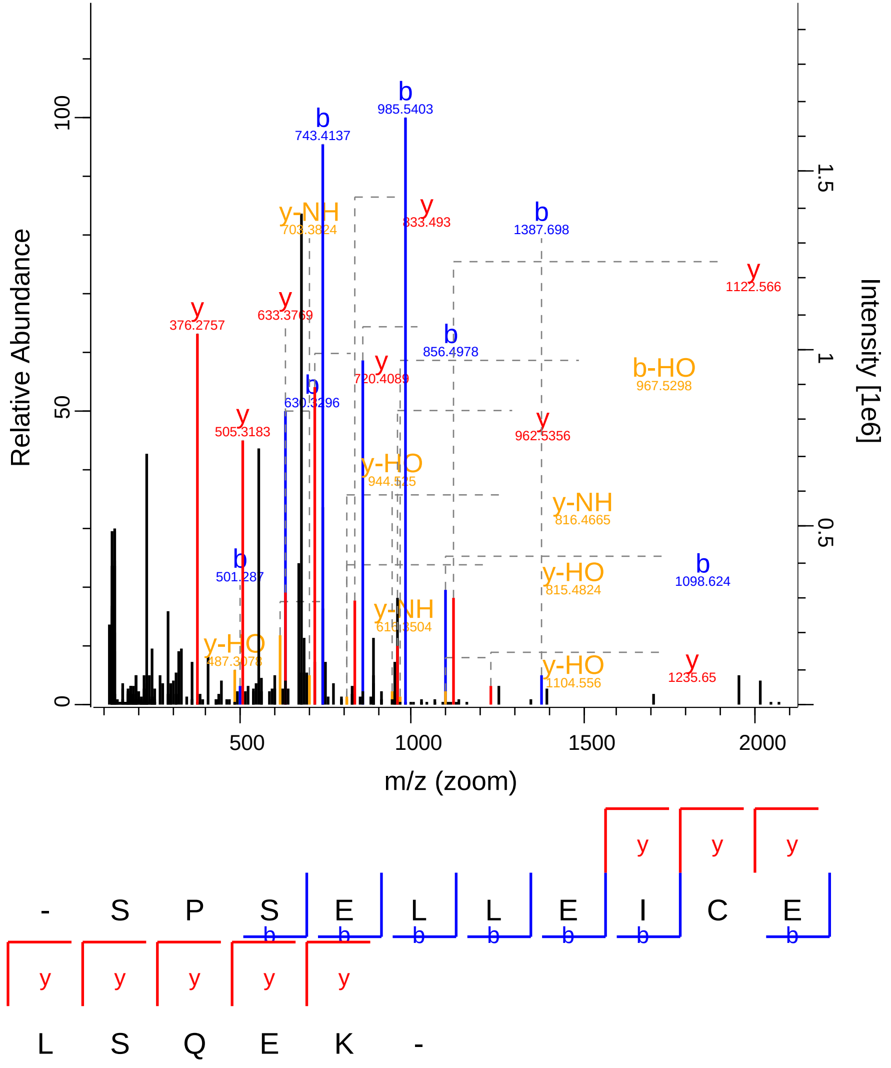

Raw File	Scan	Method	Score	m/z	Gene names
RO-QC-160121_ZackD_SS_Set_02_Fractio	32296	FTMS; HC	91.7	881.95	R3HDM4

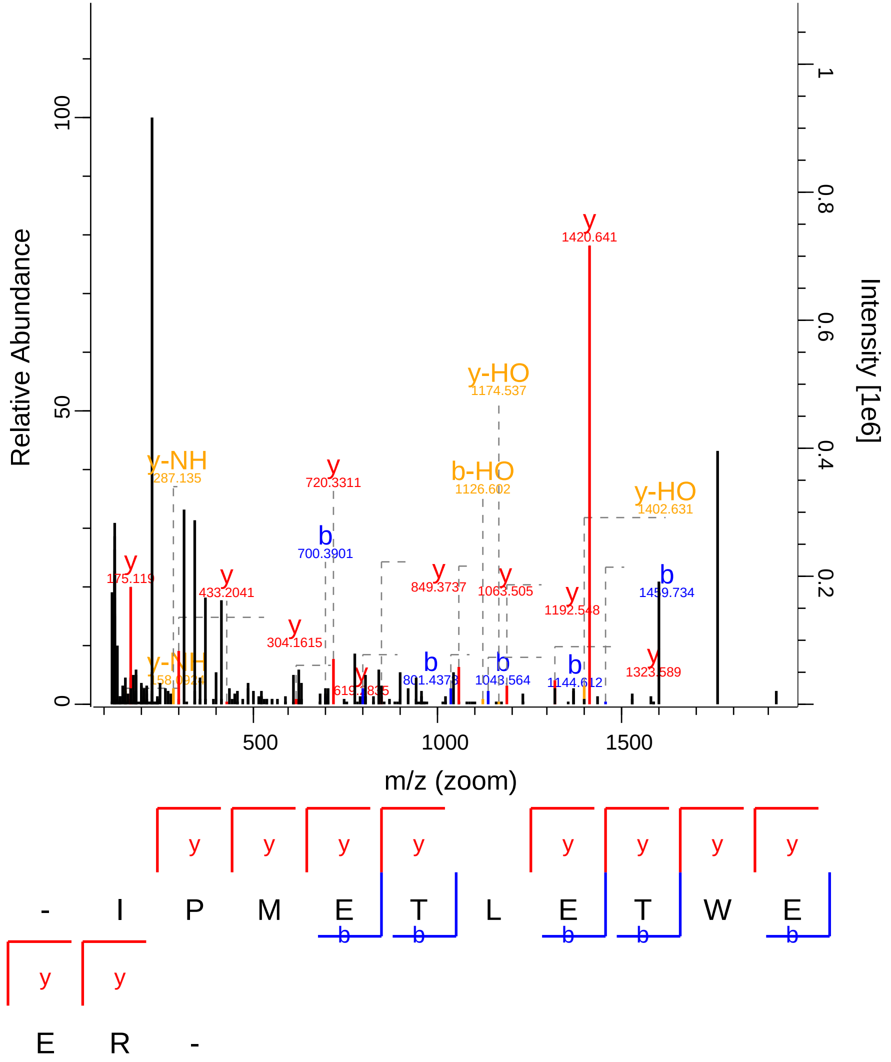

Raw File	Scan	Method	Score	m/z	Gene names
RO-QC-160121_ZackD_SS_Set_02_Fractio	32836	FTMS; HC	73.3	761.02	VCPKMT

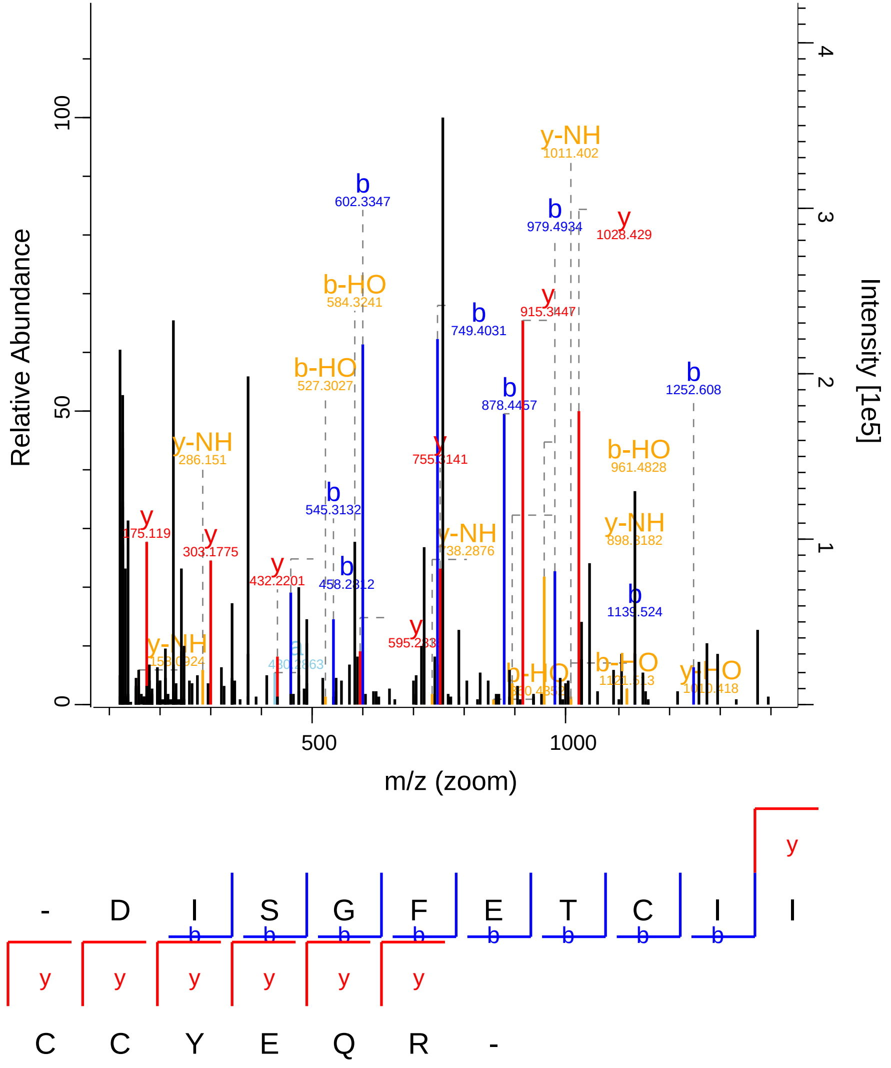

Raw File	Scan	Method	Score	m/z	Gene names
RO-QC-160121_ZackD_SS_Set_02_Fractio	32999	FTMS; HC	106.58	680.03	CCDC61

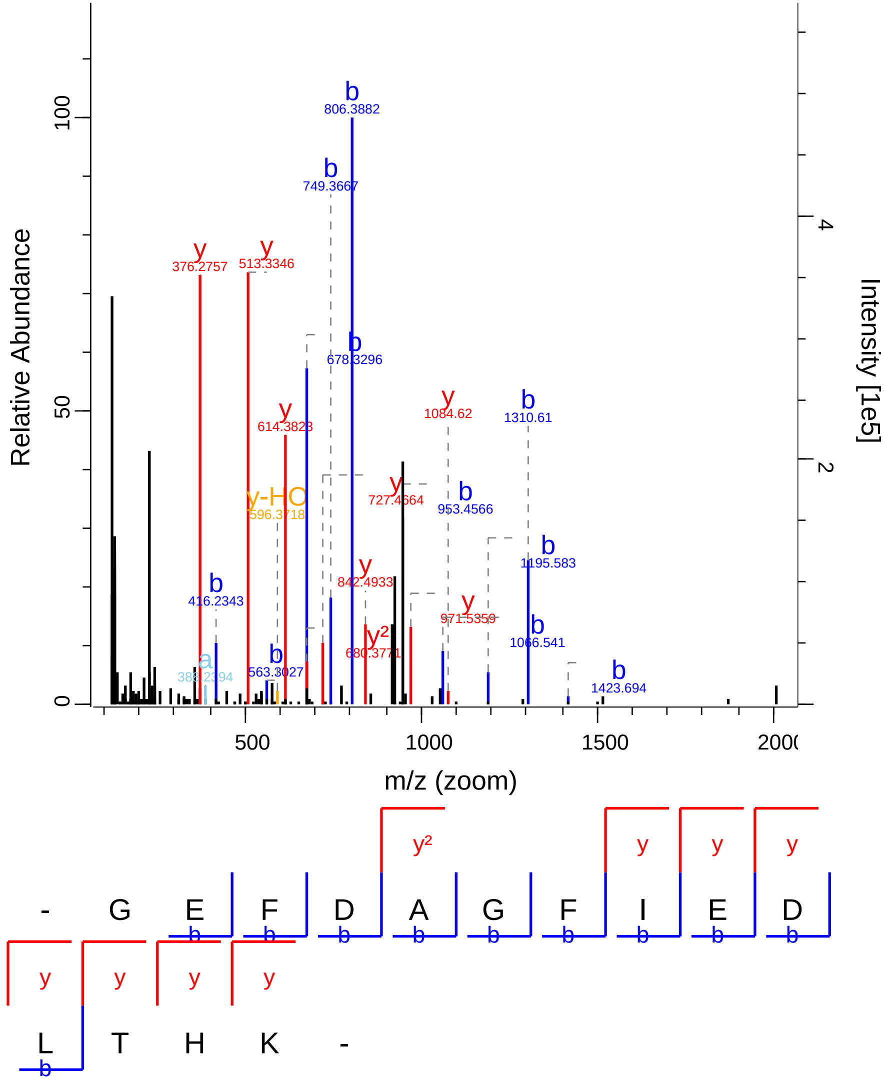

Raw File	Scan	Method	Score	m/z	Gene names
RO-QC-160121_ZackD_SS_Set_02_Fractio	33163	FTMS; HC	97.55	680.03	CCDC61

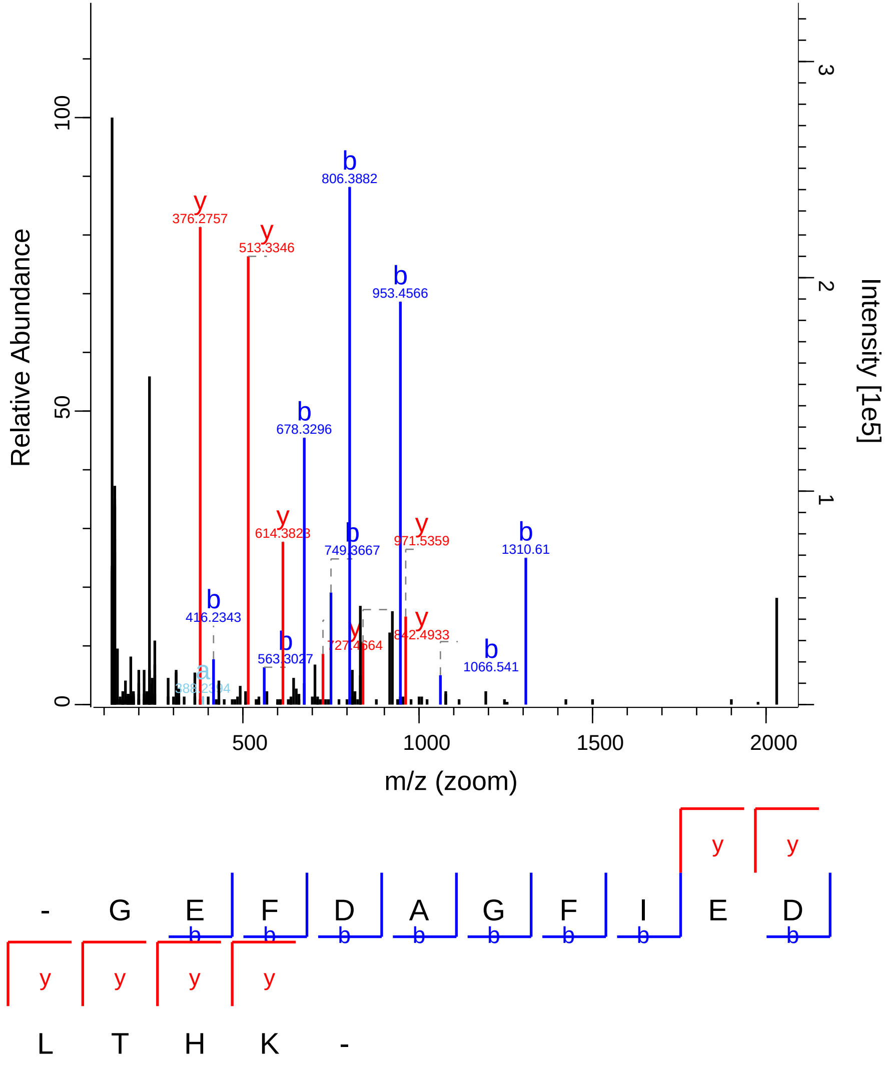

Raw File	Scan	Method	Score	m/z
RO-QC-160121_ZackD_SS_Set_02_Fraction_1	33277	FTMS; HCD	73.91	800.79

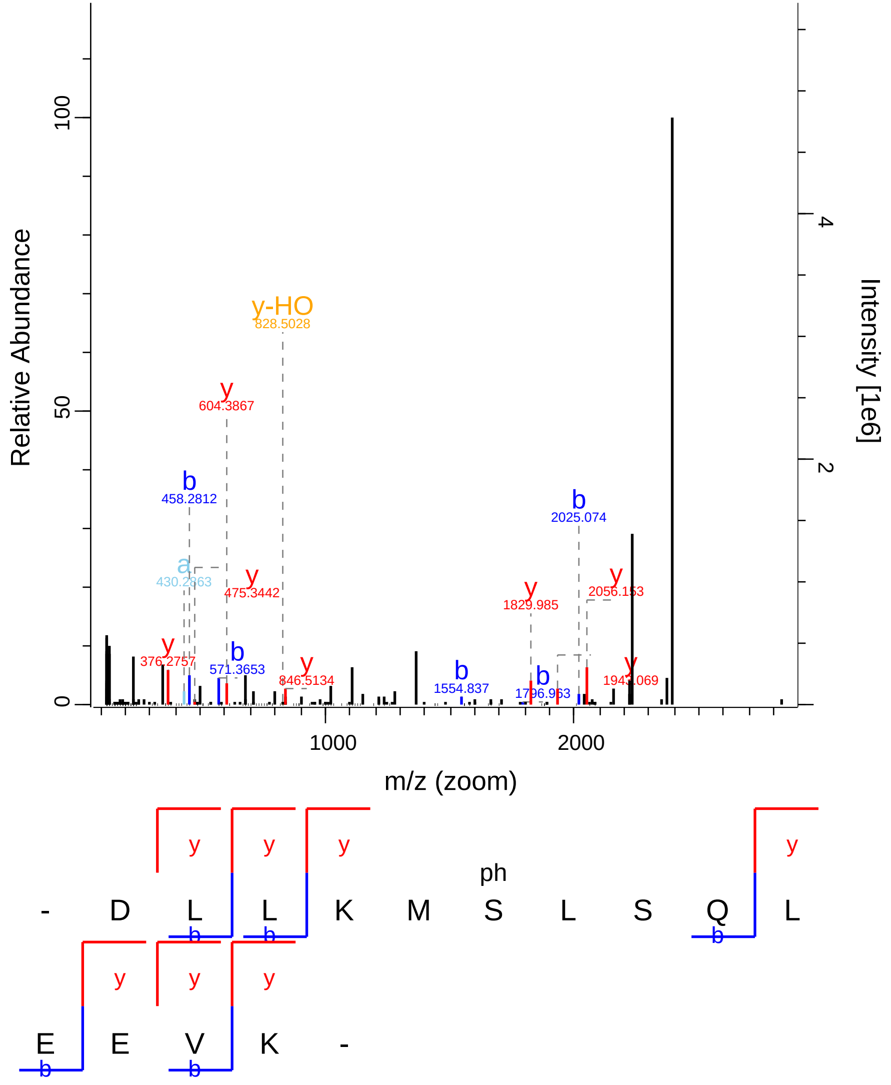

Raw File	Scan	Method	Score	m/z	Gene names
RO-QC-160121_ZackD_SS_Set_02_Fractio	34961	FTMS; HC	56.6	728.73	MILR1

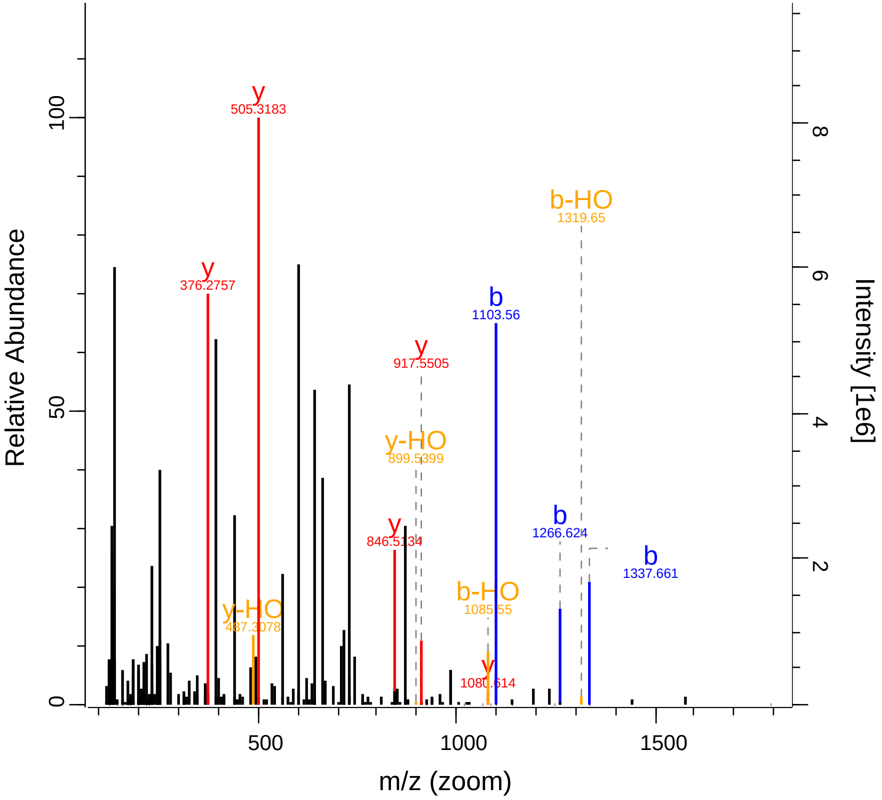

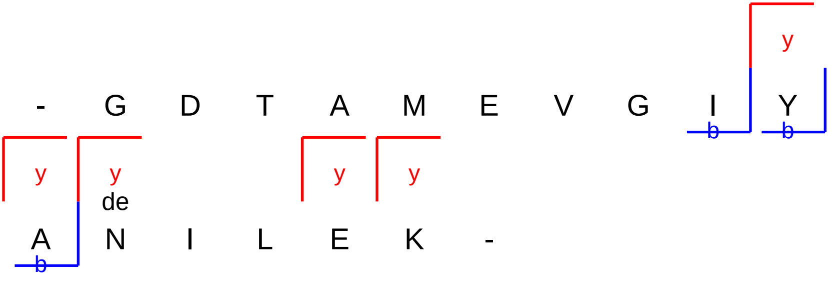

Raw File	Scan	Method	Score	m/z	Gene names
RO-QC-160121_ZackD_SS_Set_02_Fractior	3392	FTMS; HCI	40.59	376.45	ZCCHC7

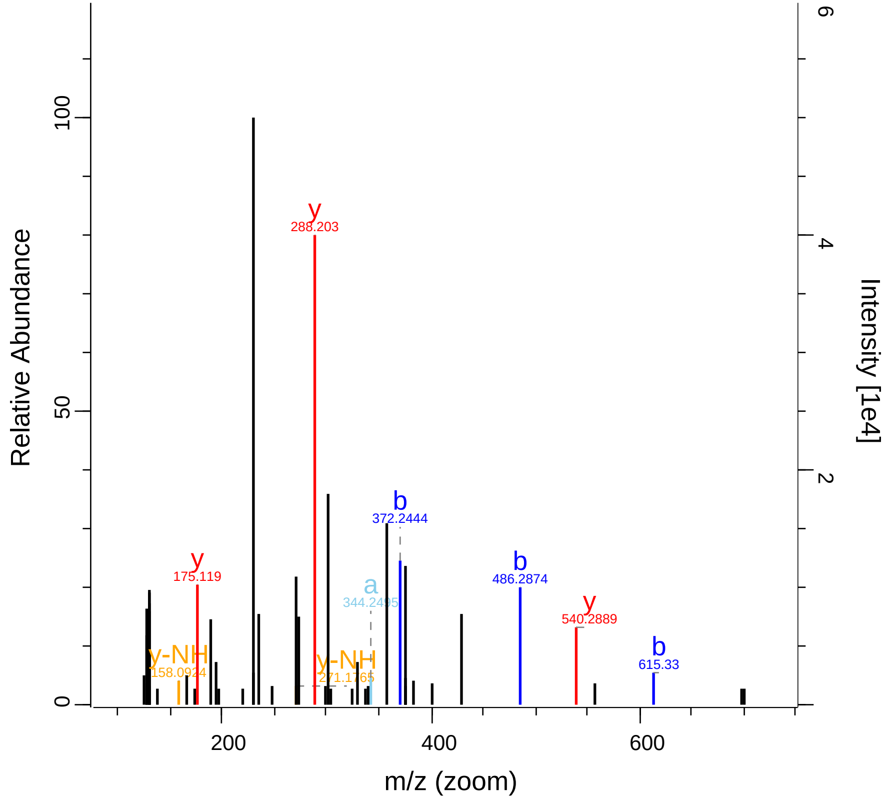

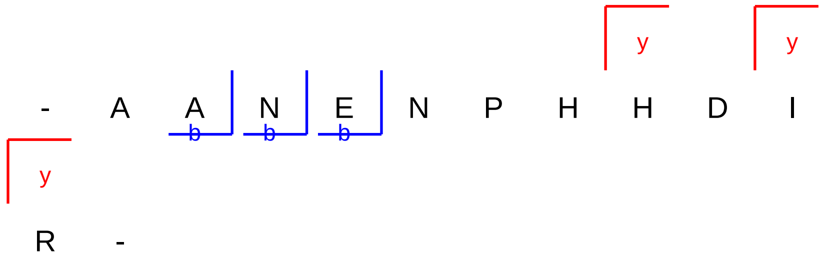

Raw File	Scan	Method	Score	m/z	Gene names
RO-QC-160121_ZackD_SS_Set_02_Fractior	3437	FTMS; HCI	61.44	501.59	ZCCHC7

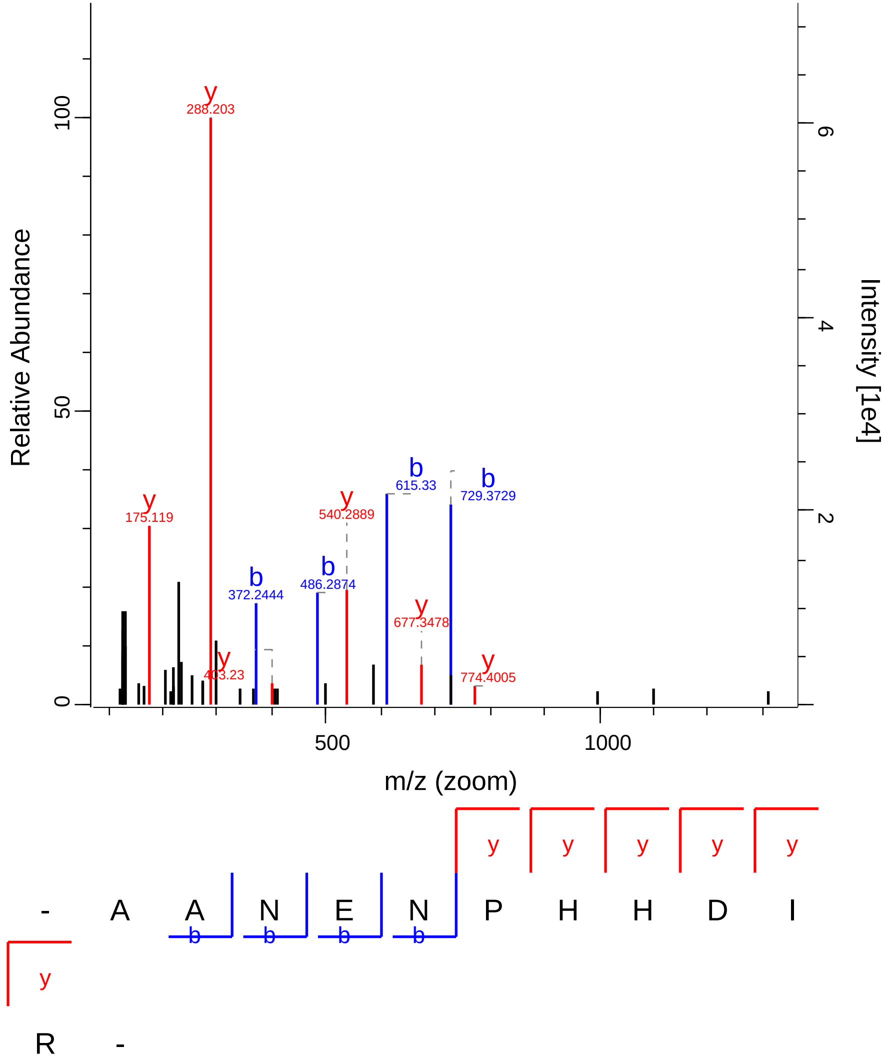

Raw File	Scan	Method	Score	m/z	Gene names
RO-QC-160121_ZackD_SS_Set_02_Fractior	4365	FTMS; HCI	72.55	444.59	TEX30

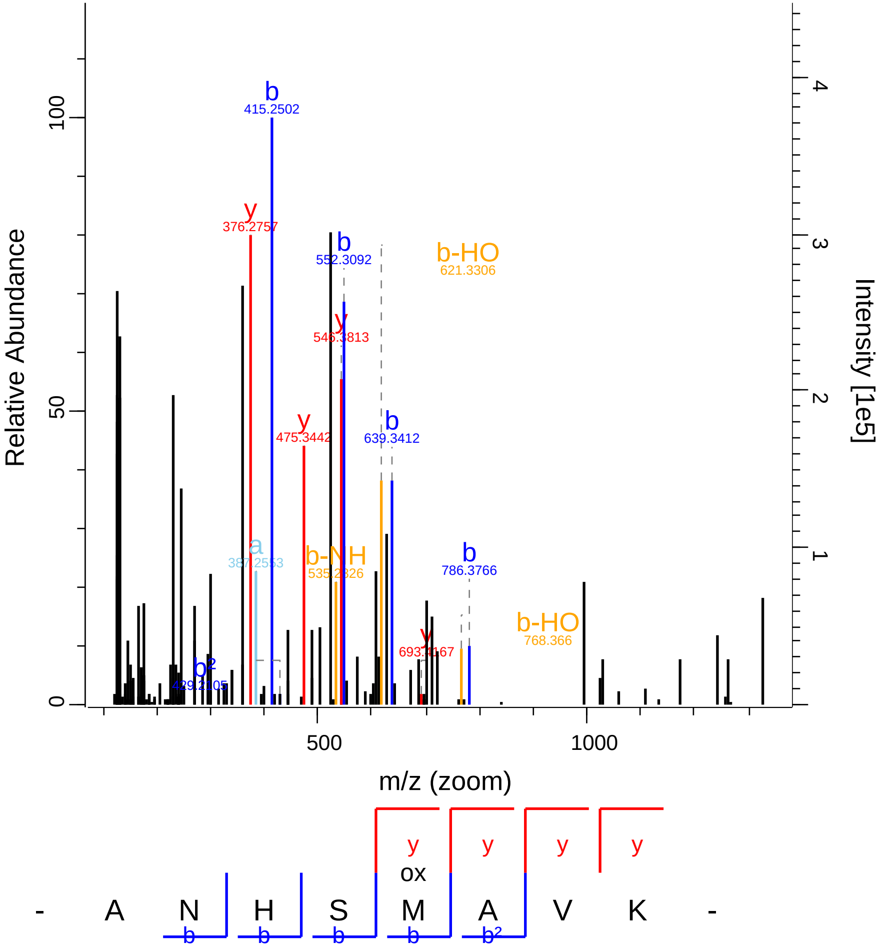

Raw File	Scan	Method	Score	m/z	Gene names
RO-QC-160121_ZackD_SS_Set_02_Fractior	4547	FTMS; HCI	83.23	520.91	GREM1

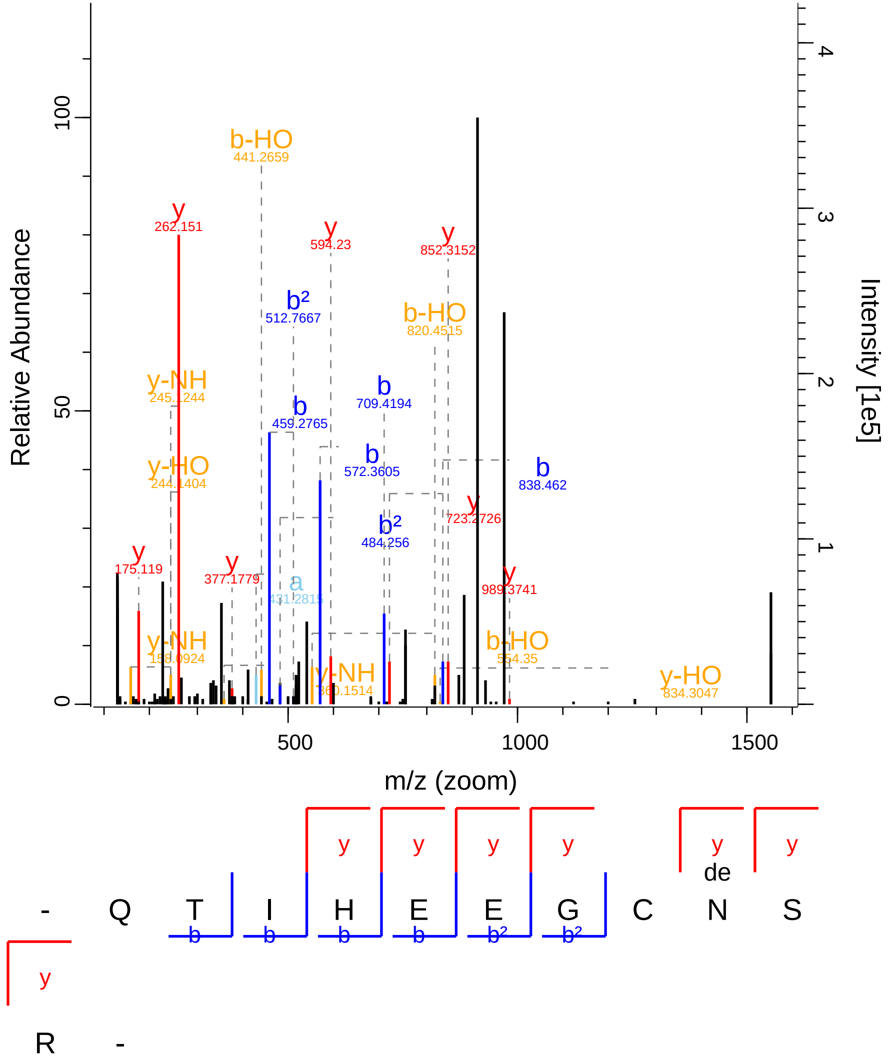

Raw File	Scan	Method	Score	m/z	Gene names
RO-QC-160121_ZackD_SS_Set_02_Fractio	4559	FTMS; HC	118.28	646.34	DENND1B

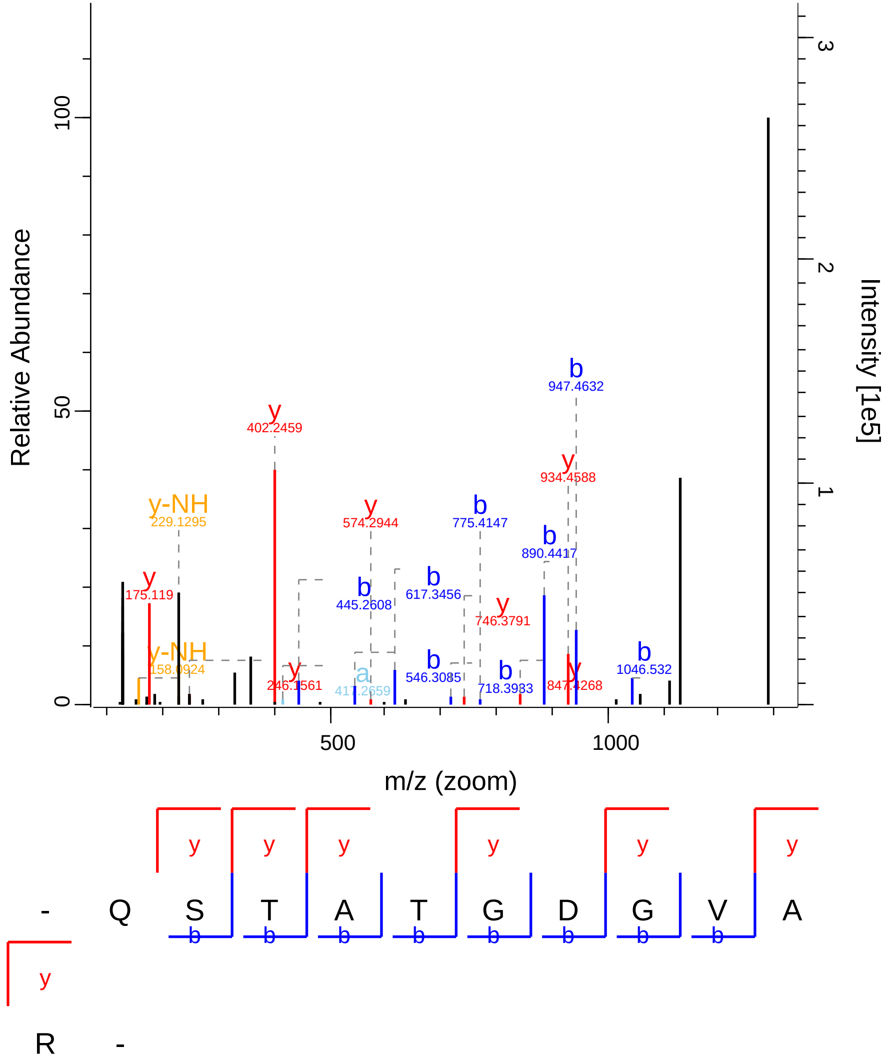

Raw File	Scan	Method	Score	m/z	Gene names
RO-QC-160121_ZackD_SS_Set_02_Fract	48482	FTMS; HC	55.75	643.68	ARHGEF35;ARHGE

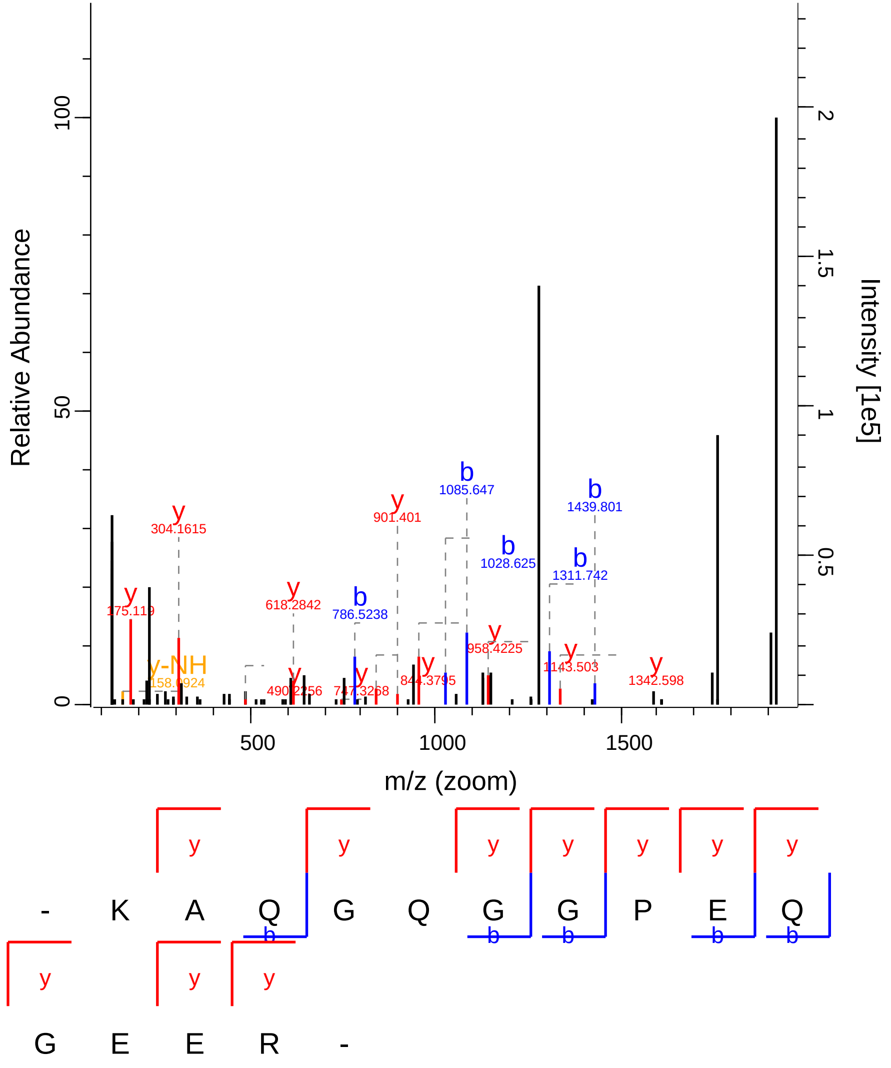

Raw File	Scan	Method	Score	m/z	Gene names
RO-QC-160121_ZackD_SS_Set_02_Fractio	4851	FTMS; HC	120.14	640.99	RUNDC1

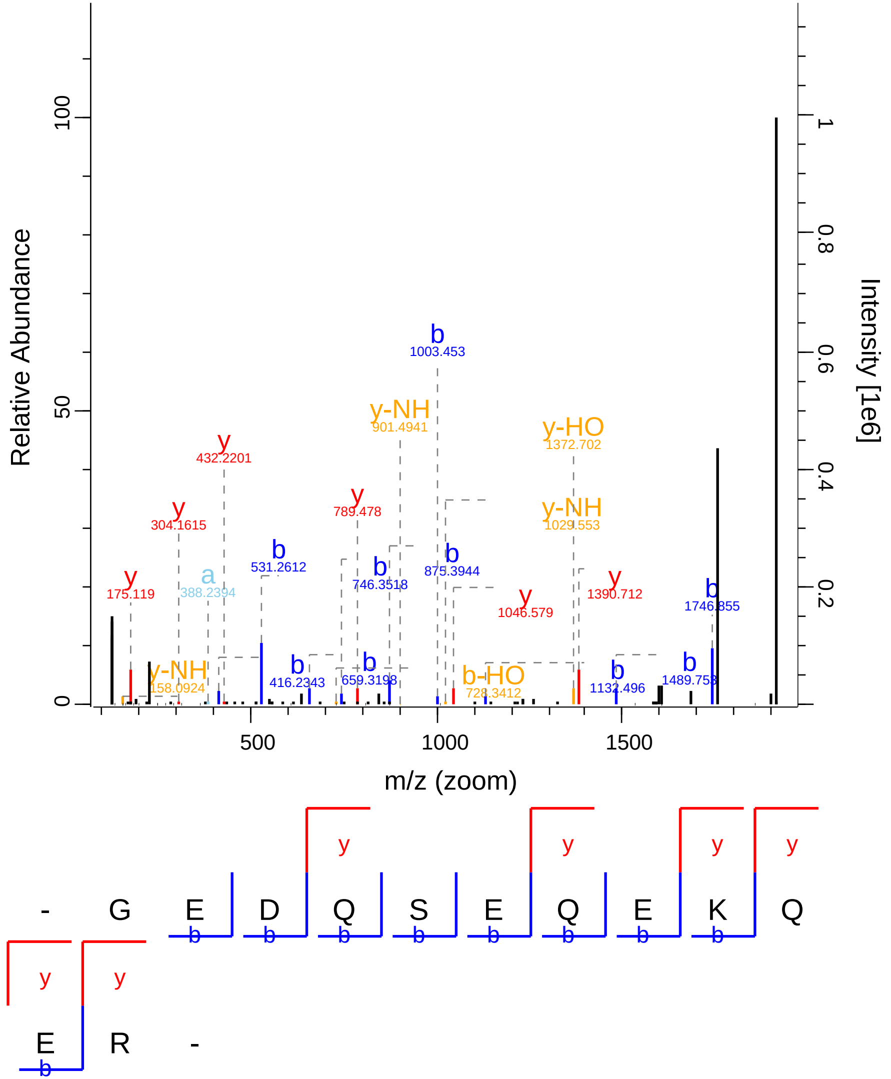

Raw File	Scan	Method	Score	m/z	Gene names
RO-QC-160121_ZackD_SS_Set_02_Fractior	4924	FTMS; HC	115.34	573.65	FAM110C

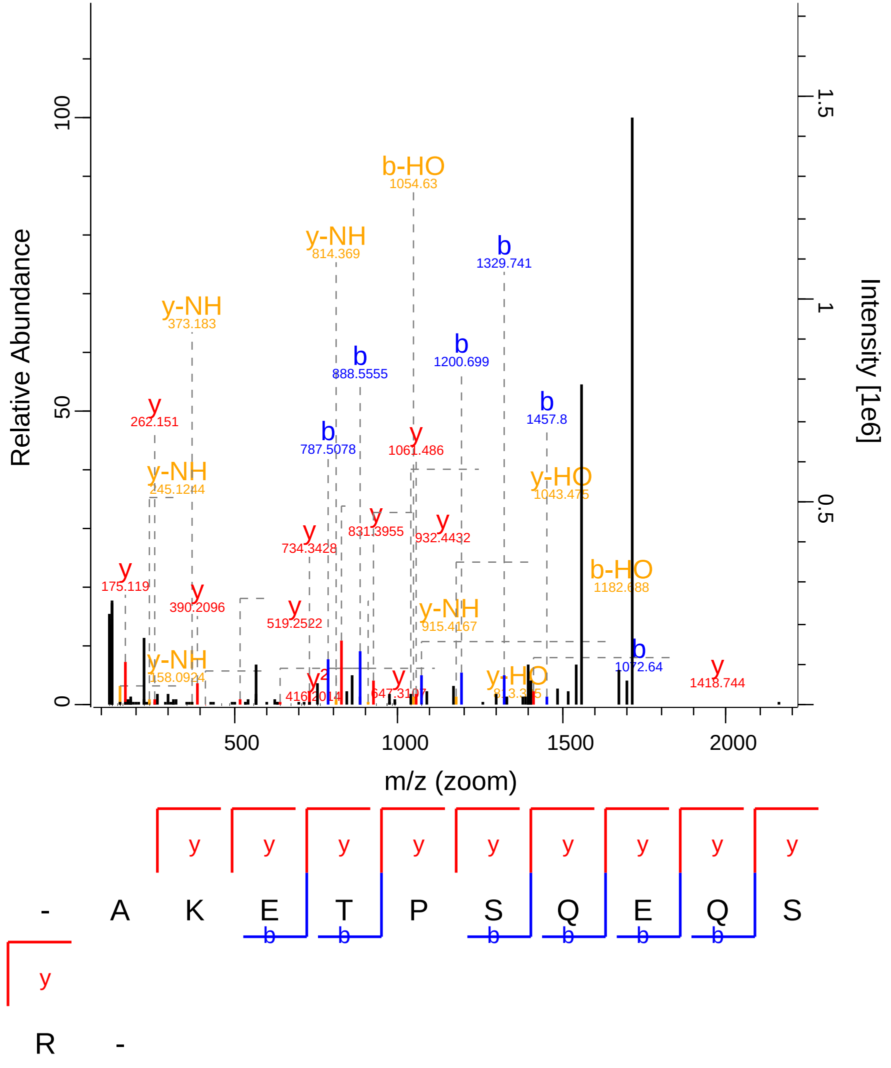

Raw File	Scan	Method	Score	m/z	Gene names
RO-QC-160121_ZackD_SS_Set_02_Fractior	5094	FTMS; HCI	57.11	435.55	C1orf109

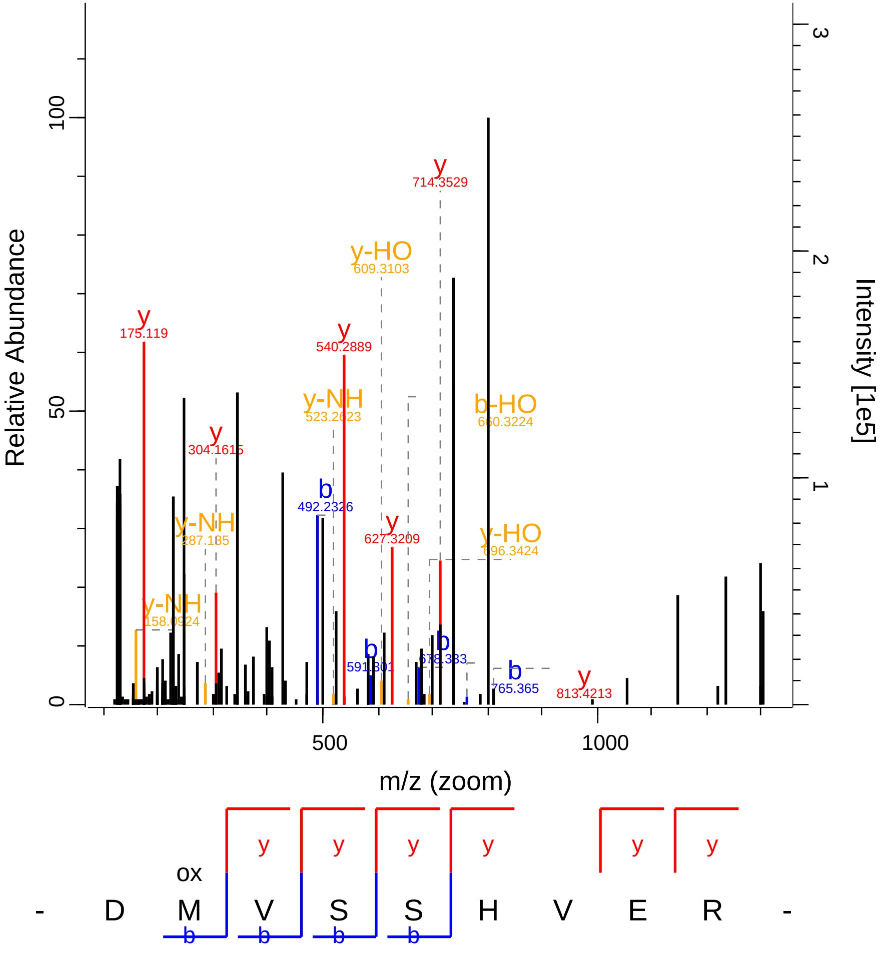

Raw File	Scan	Method	Score	m/z	Gene names
RO-QC-160121_ZackD_SS_Set_02_Fraction	5098	FTMS; HCI	65.25	548.8	WIPF1

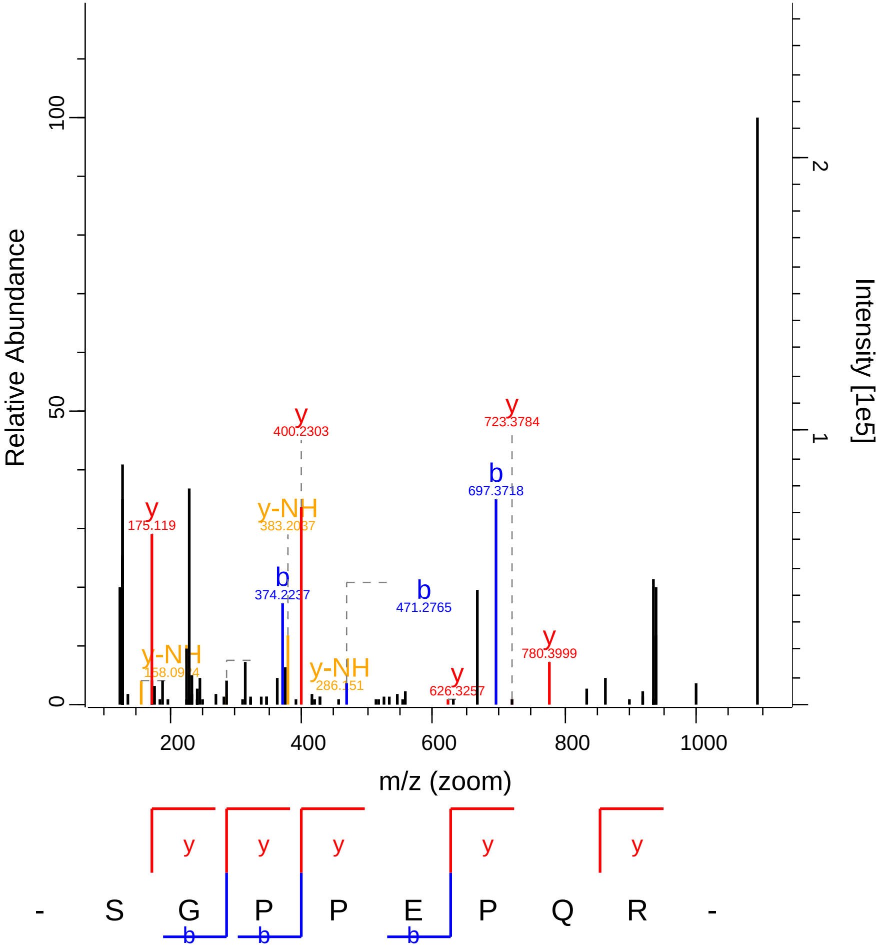

Raw File	Scan	Method	Score	m/z	Gene names
RO-QC-160121_ZackD_SS_Set_02_Fractior	5122	FTMS; HCl	90.91	669.83	TMEM47

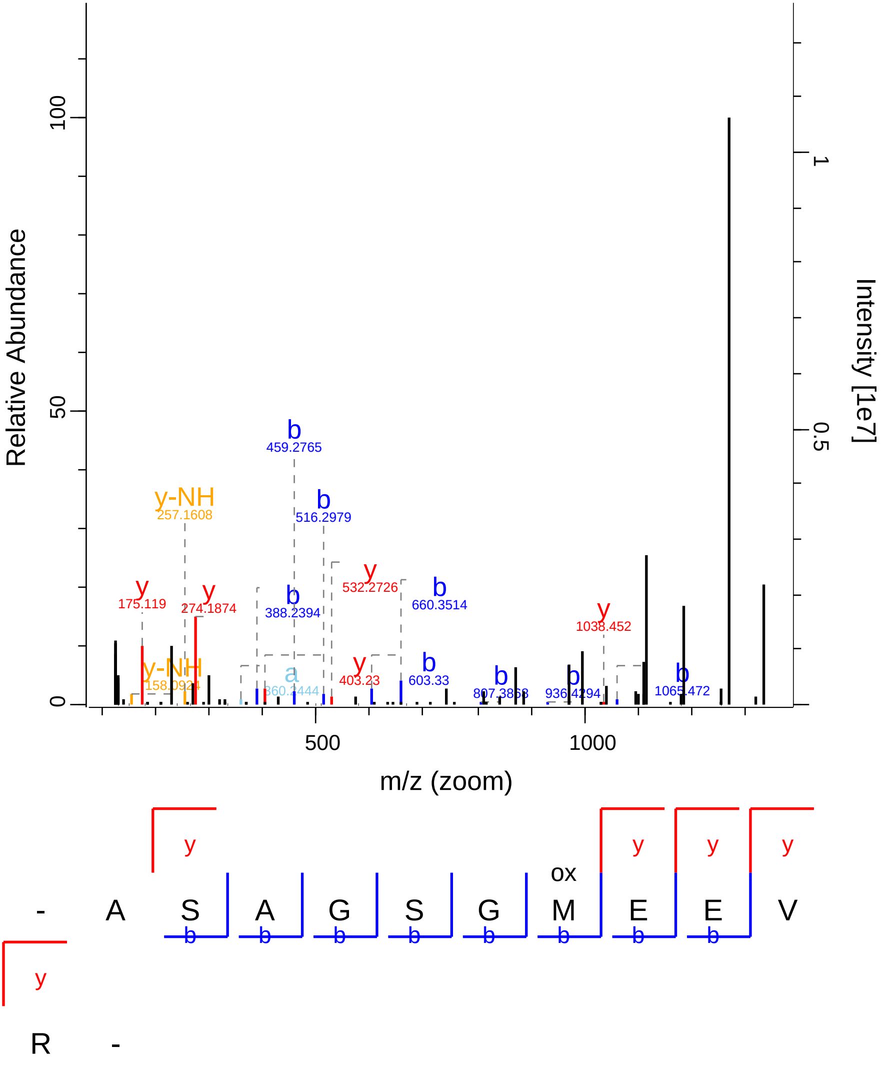

Raw File	Scan	Method	Score	m/z	Gene names
RO-QC-160121_ZackD_SS_Set_02_Fractio	5177	FTMS; HC	111.06	690.35	SLC2A12

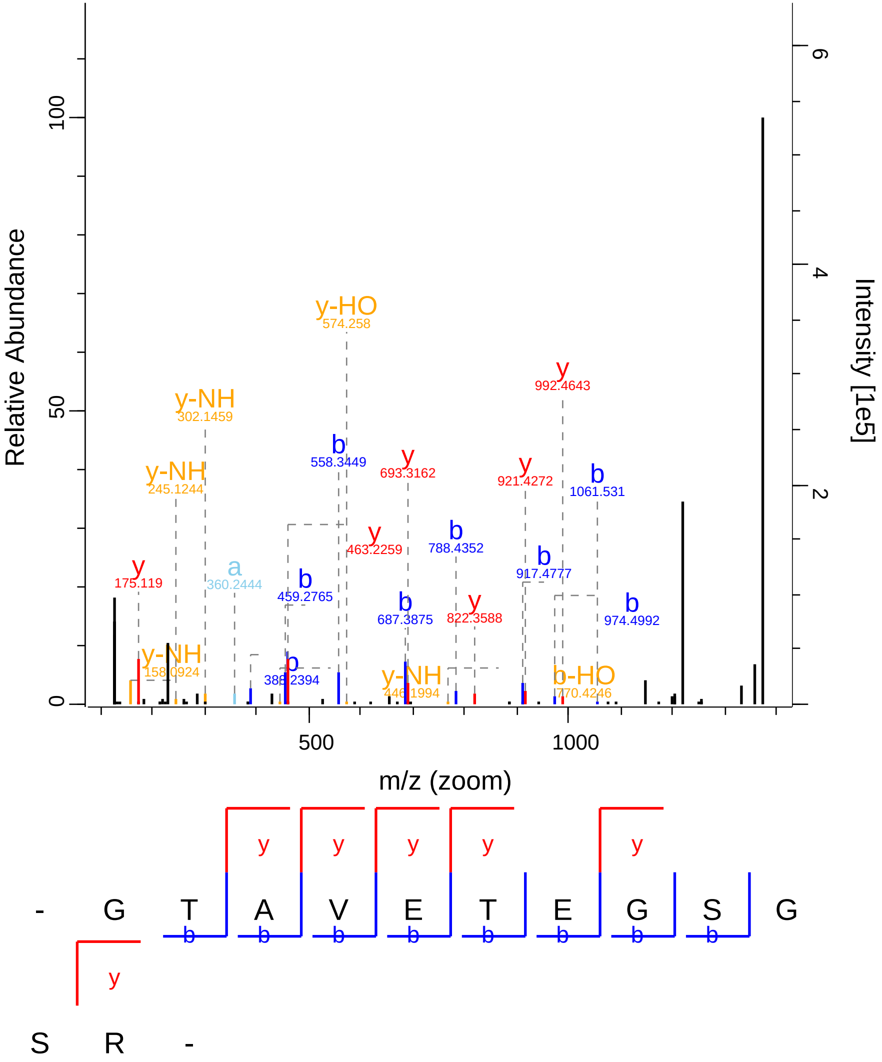

Raw File	Scan	Method	Score	m/z	Gene names
RO-QC-160121_ZackD_SS_Set_02_Fractior	5190	FTMS; HC	122.33	656.82	KIF24

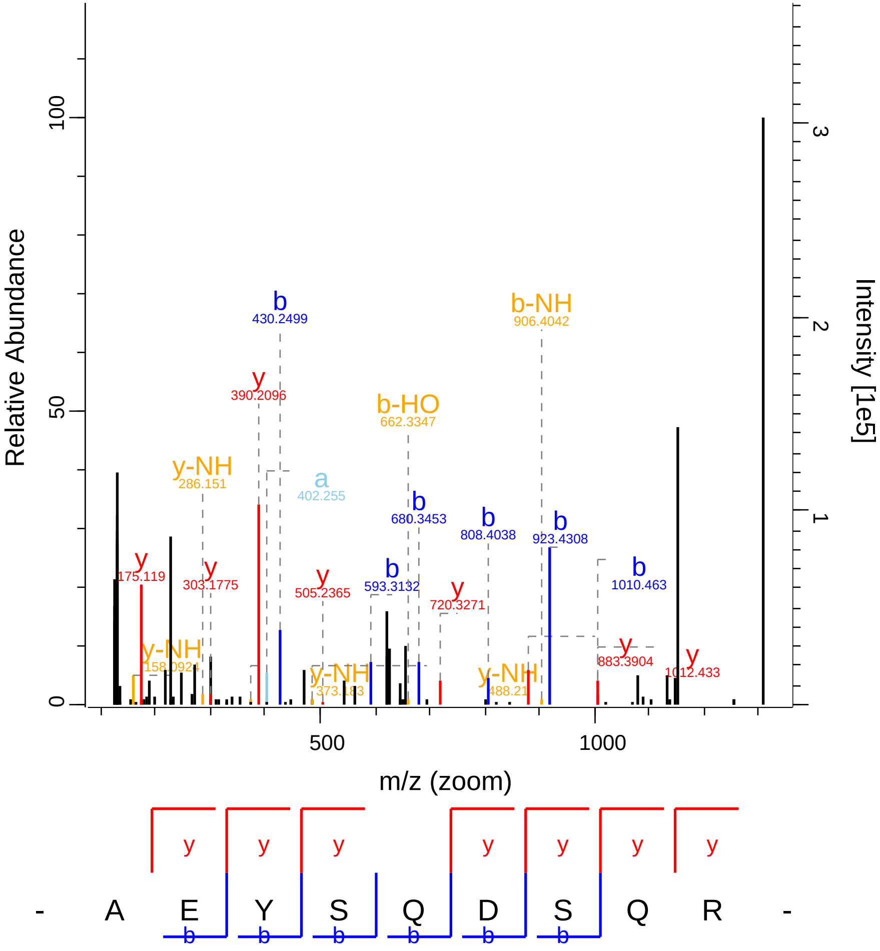

Raw File	Scan	Method	Score	m/z
RO-QC-160121_ZackD_SS_Set_02_Fraction_2	5263	FTMS; HCD	33.89	507.79

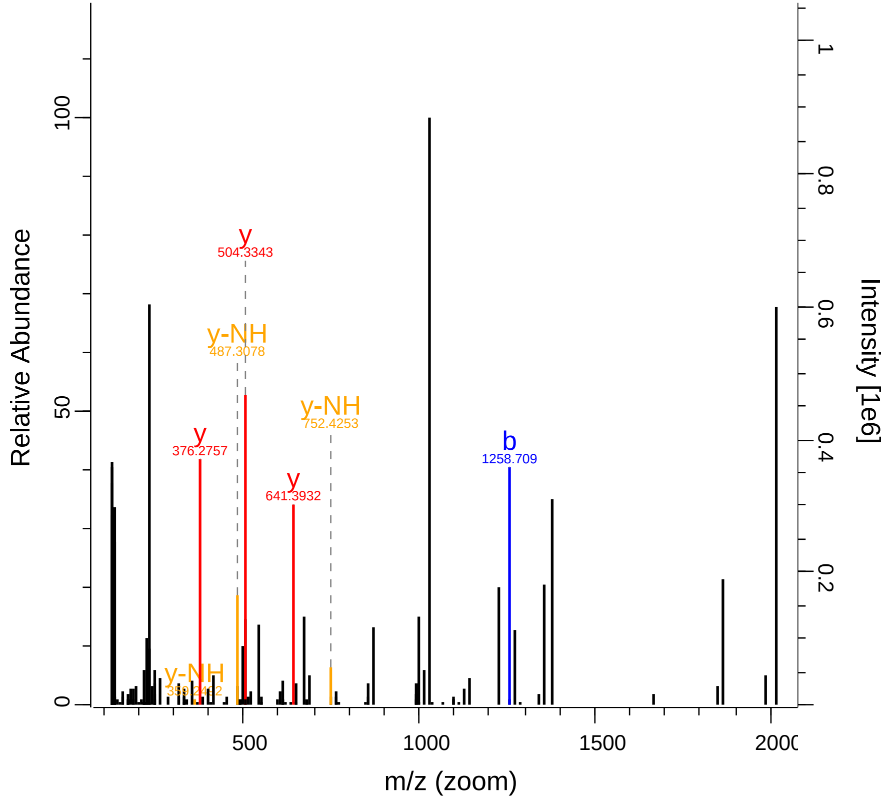

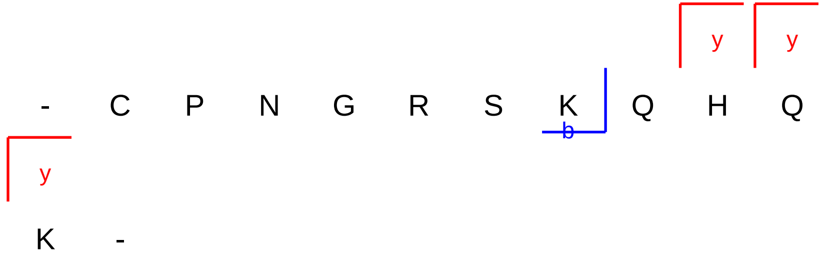

Raw File	Scan	Method	Score	m/z	Gene names
RO-QC-160121_ZackD_SS_Set_02_Fractio	5279	FTMS; HC	125.83	598.82	MTF2

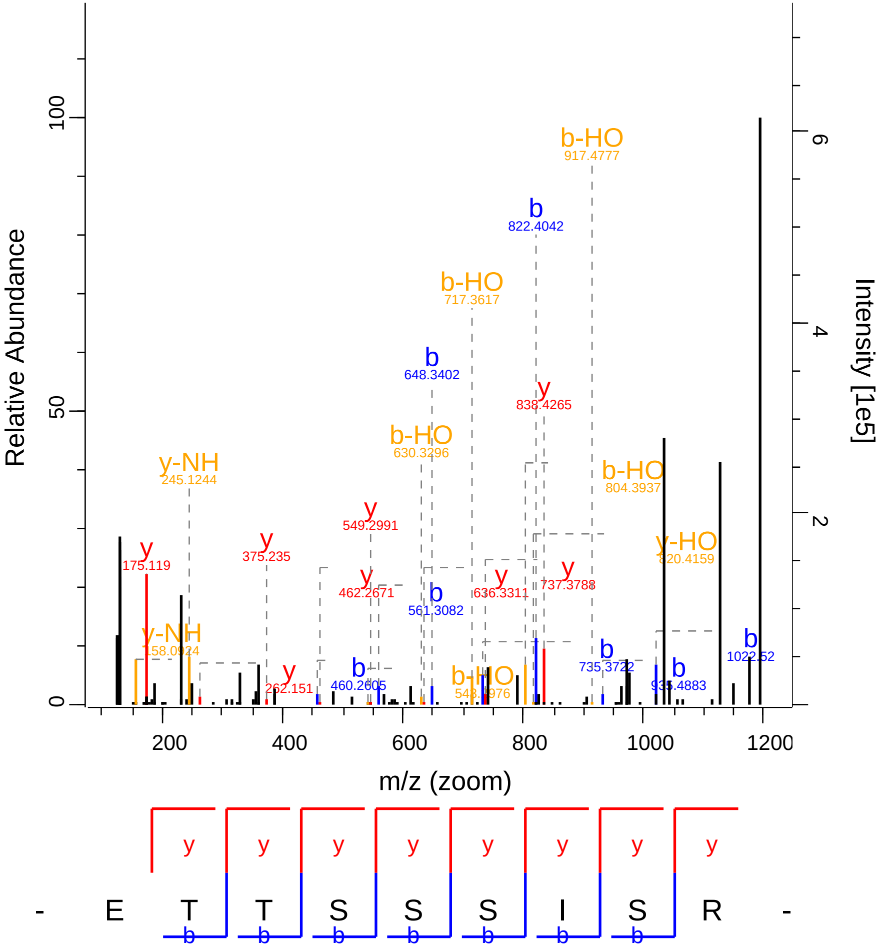

Raw File	Scan	Method	Score	m/z
RO-QC-160121_ZackD_SS_Set_02_Fraction_2	5286	FTMS; HCD	69.01	676.39

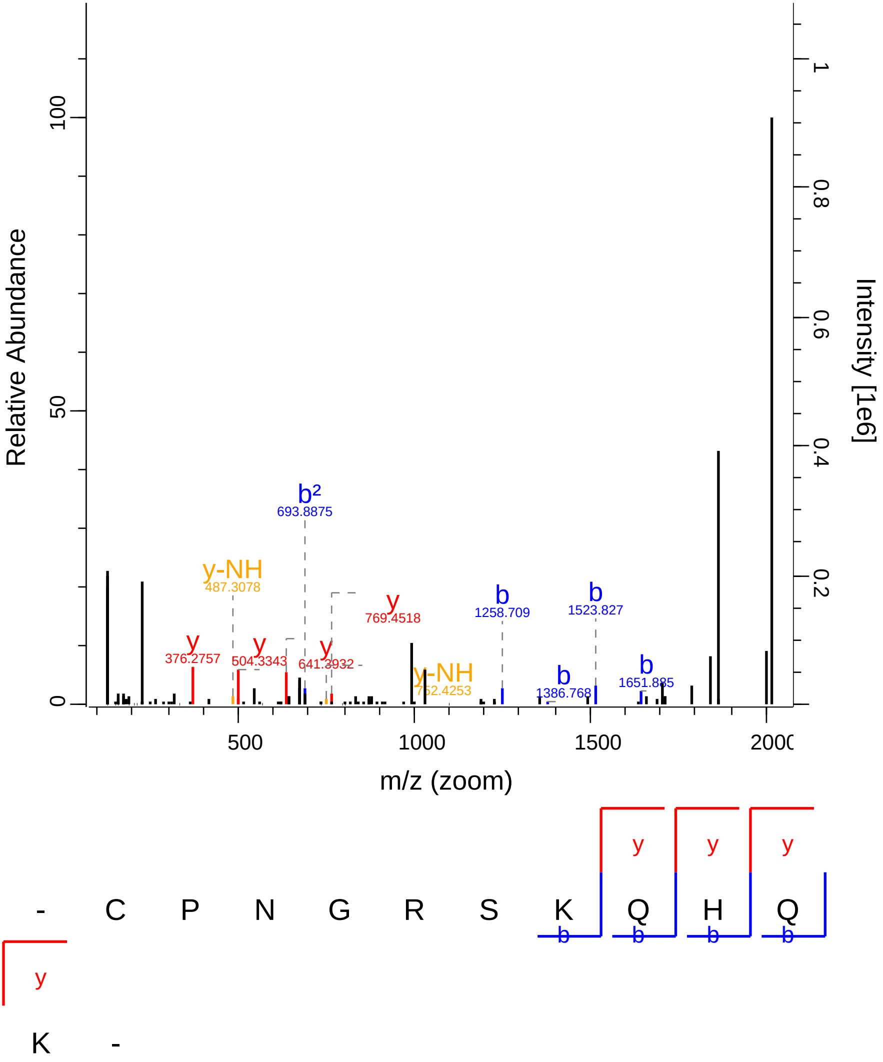

Raw File	Scan	Method	Score	m/z	Gene names
RO-QC-160121_ZackD_SS_Set_02_Fractior	5363	FTMS; HCI	86.34	755.85	RCN3

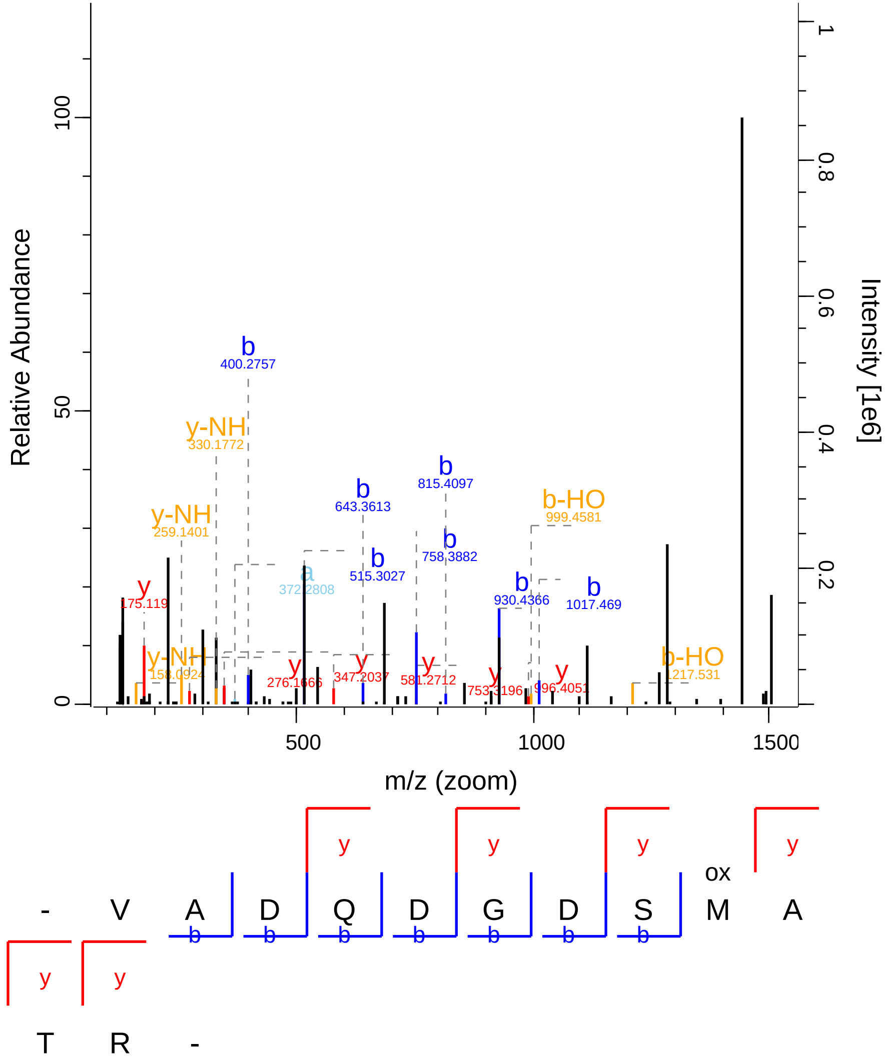

Raw File	Scan	Method	Score	m/z
RO-QC-160121_ZackD_SS_Set_02_Fraction_2	5438	FTMS; HCD	56.36	507.54

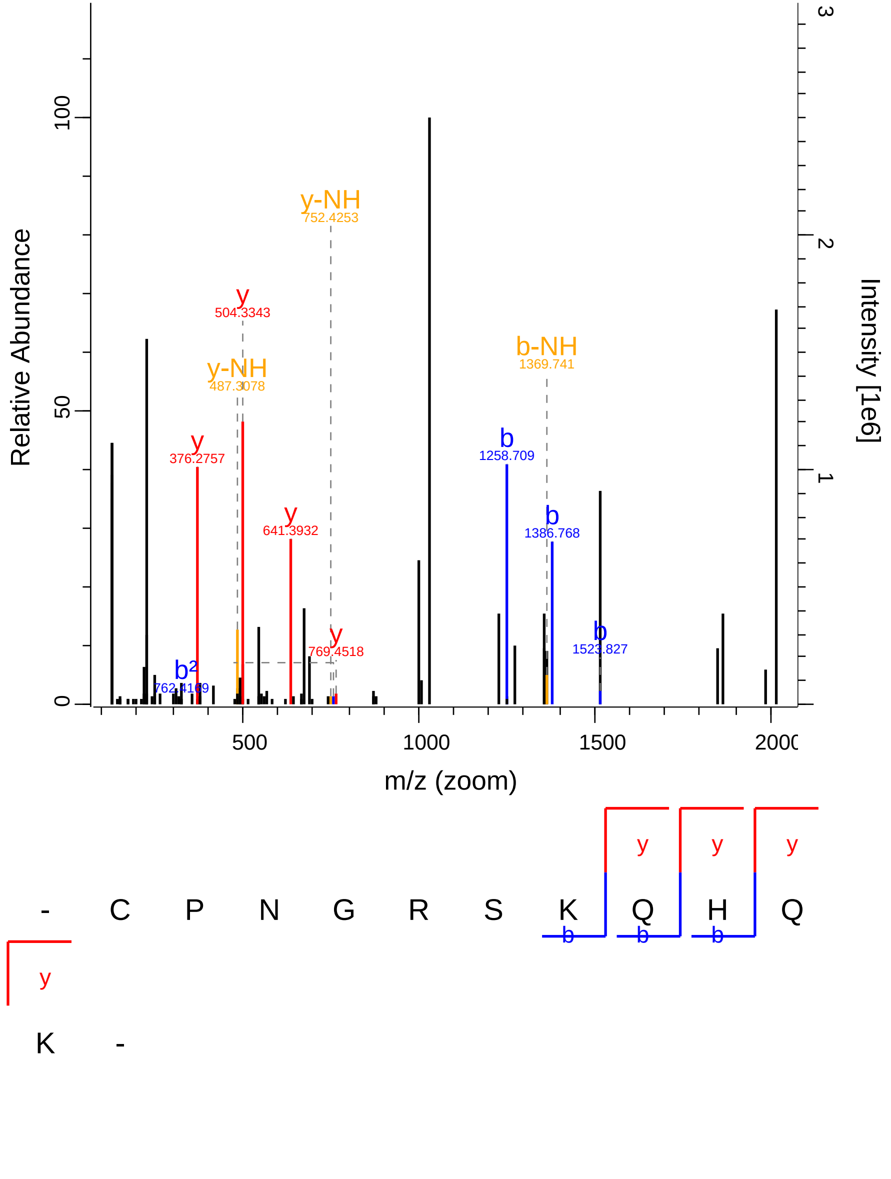

Raw File	Scan	Method	Score	m/z	Gene names
RO-QC-160121_ZackD_SS_Set_02_Fractio	5592	FTMS; HC	204.79	649.86	CD99

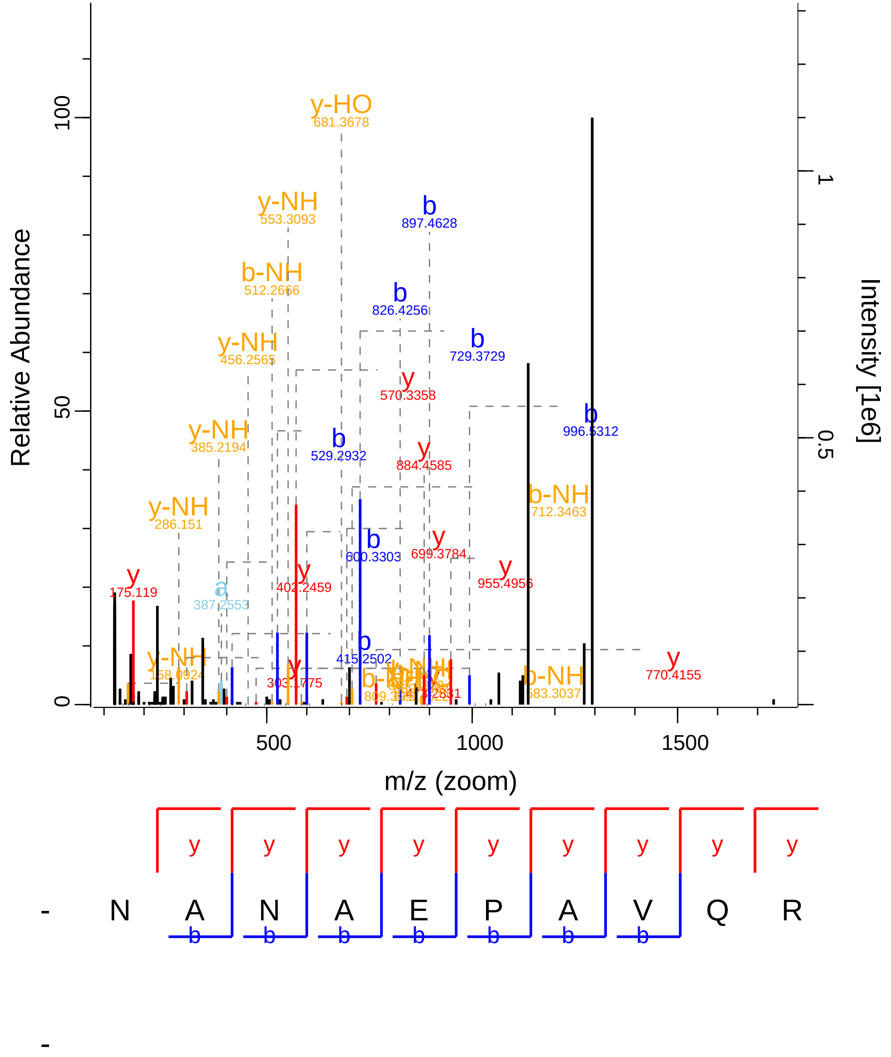

Raw File	Scan	Method	Score	m/z	Gene names
RO-QC-160121_ZackD_SS_Set_02_Fractior	5621	FTMS; HCI	76.94	433.57	CD99

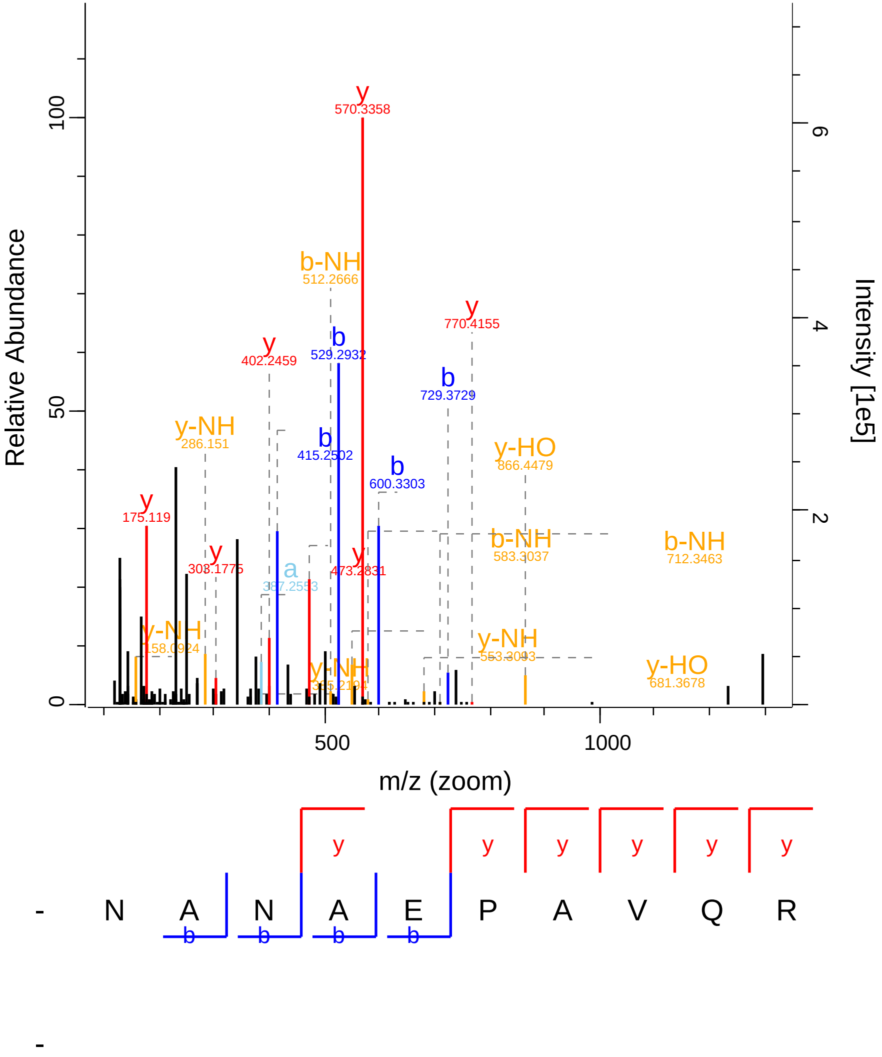

Raw File	Scan	Method	Score	m/z	Gene names
RO-QC-160121_ZackD_SS_Set_02_Fractior	5713	FTMS; HCI	56.92	713.39	LARP6

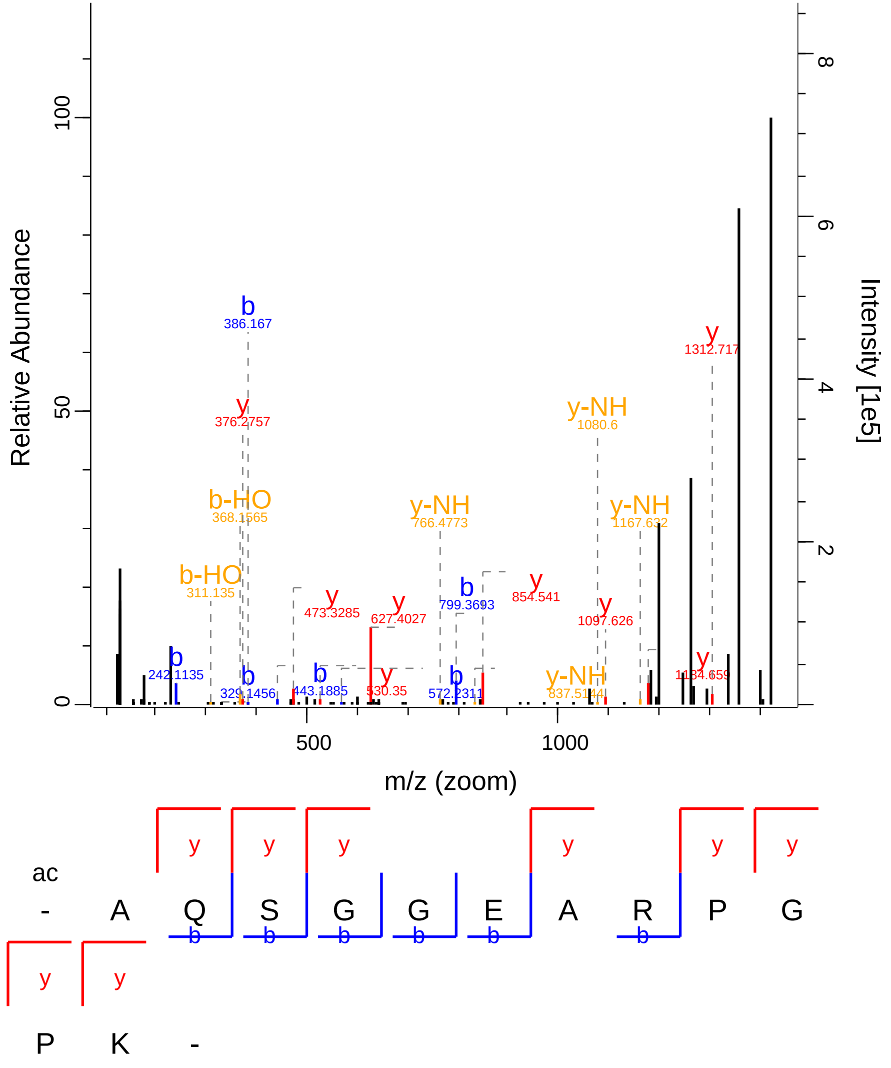

Raw File	Scan	Method	Score	m/z	Gene names
RO-QC-160121_ZackD_SS_Set_02_Fractior	5819	FTMS; HC	129.42	681.78	VOPP1

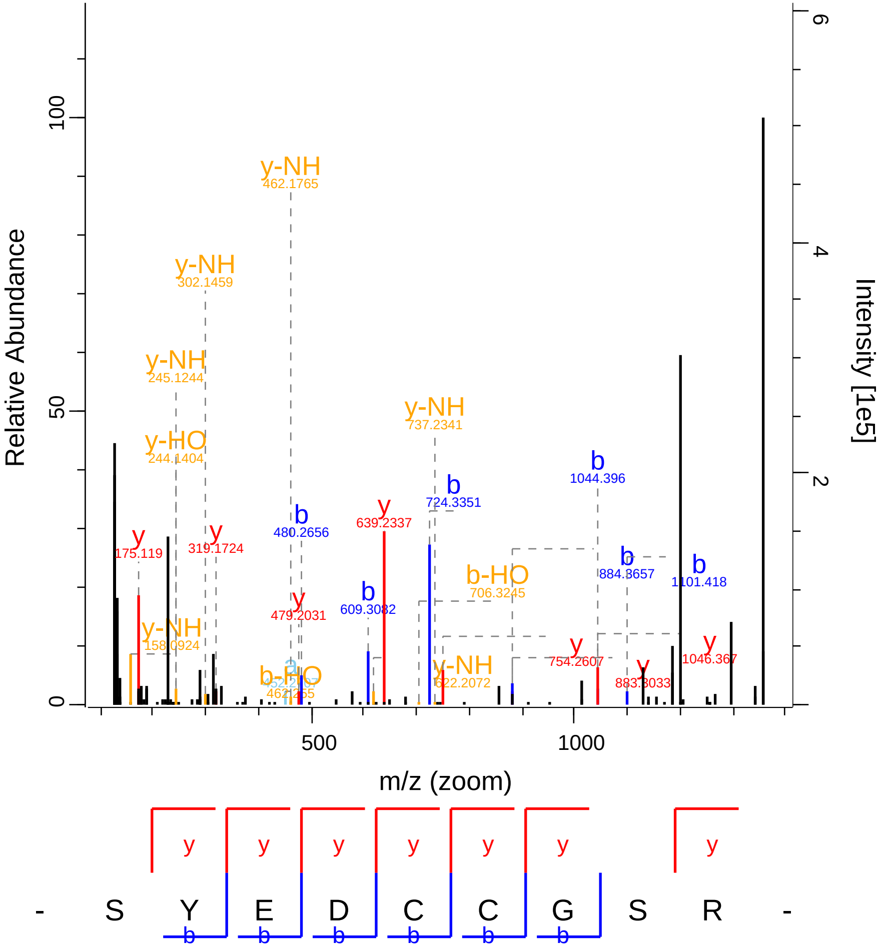

Raw File	Scan	Method	Score	m/z	Gene names
RO-QC-160121_ZackD_SS_Set_02_Fractior	5869	FTMS; HC	130.28	547.29	PI4K2B

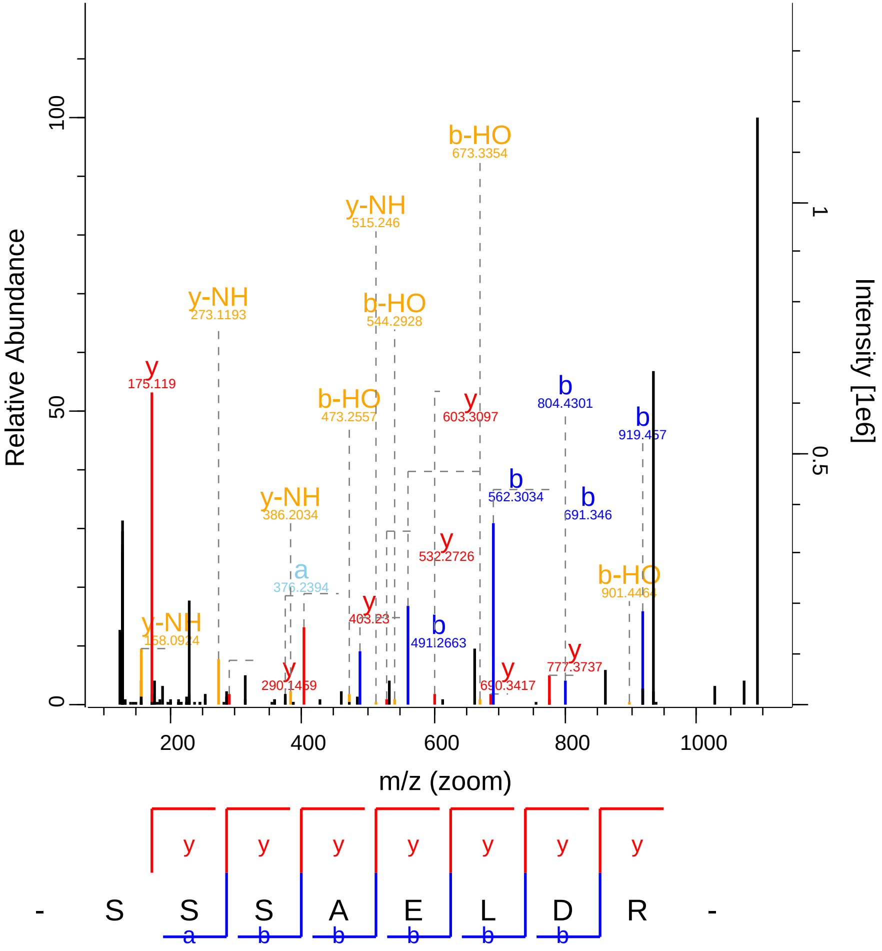

Raw File	Scan	Method	Score	m/z	Gene names
RO-QC-160121_ZackD_SS_Set_02_Fractioı	5977	FTMS; HC	125.47	681.79	VOPP1

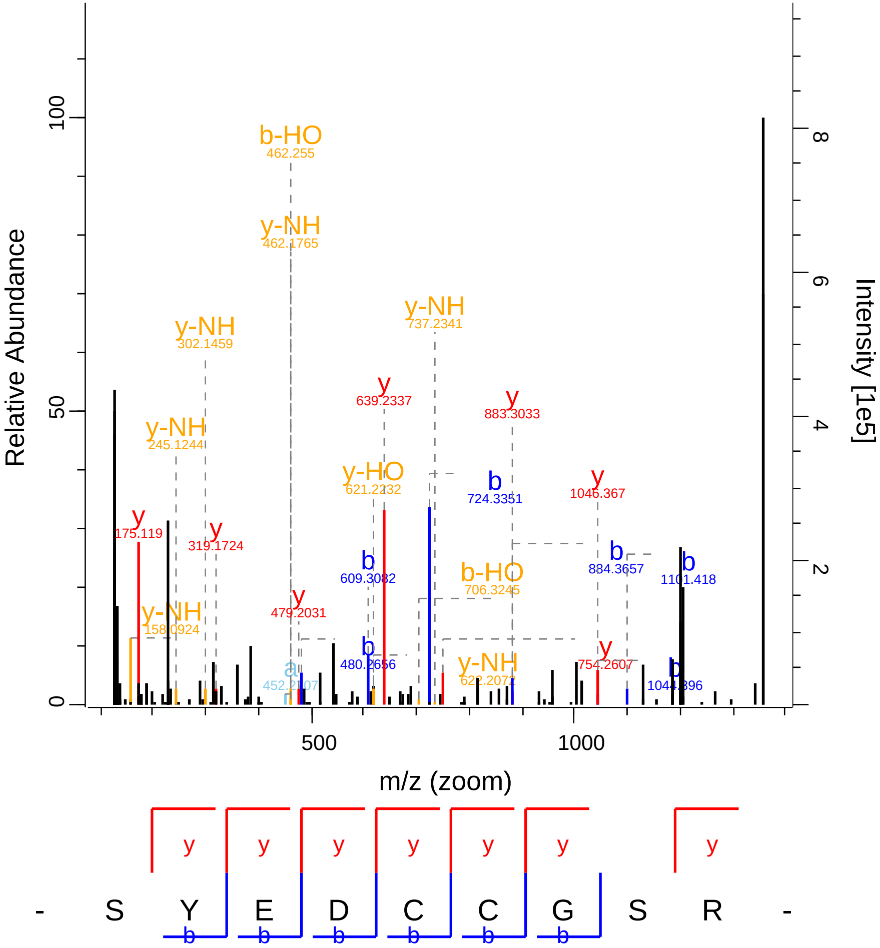

Raw File	Scan	Method	Score	m/z	Gene names
RO-QC-160121_ZackD_SS_Set_02_Fractio	6351	FTMS; HC	150.46	780.38	ZNF511

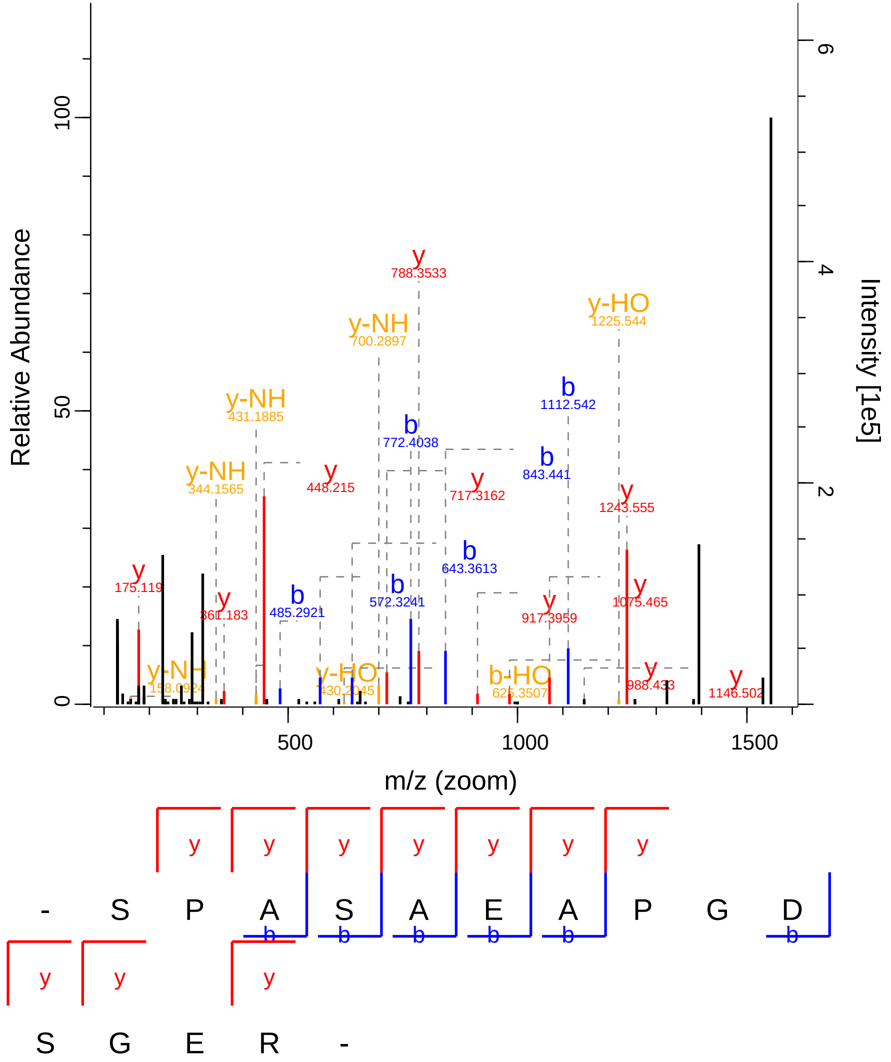

Raw File	Scan	Method	Score	m/z	Gene names
RO-QC-160121_ZackD_SS_Set_02_Fractior	6388	FTMS; HCI	86.94	520.59	ZNF511

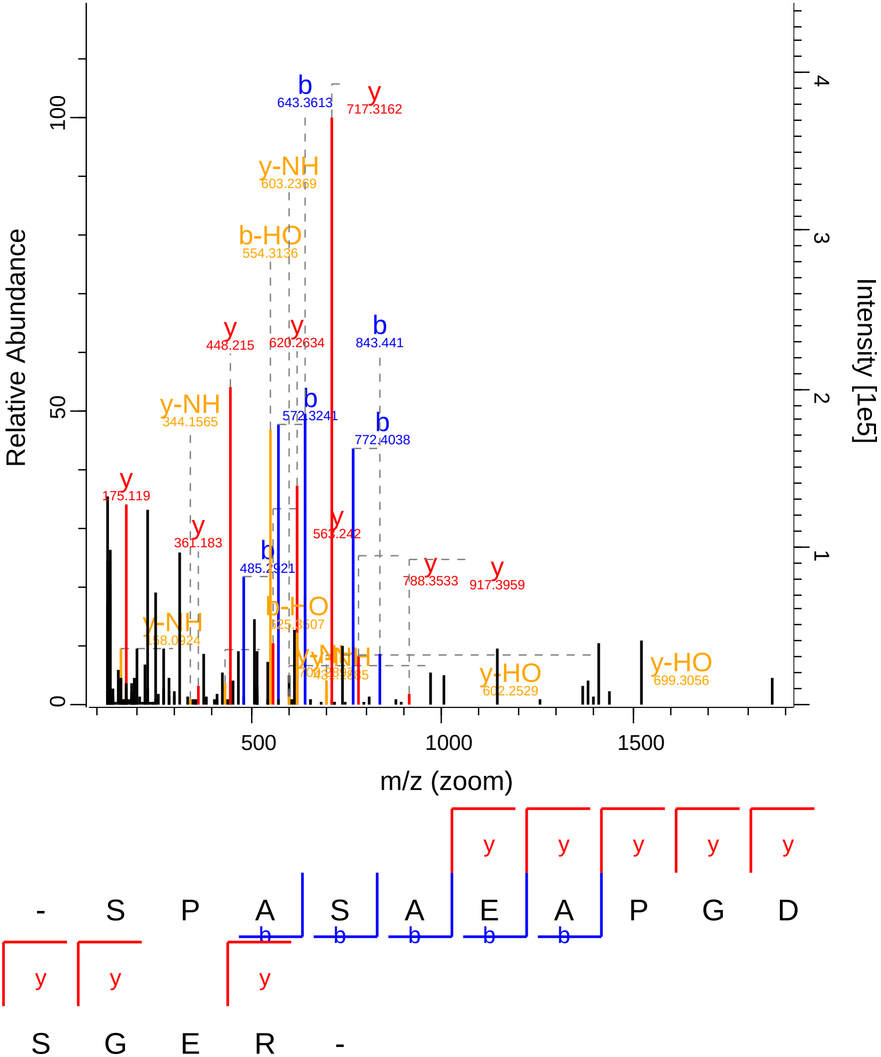

Raw File	Scan	Method	Score	m/z	Gene names
RO-QC-160121_ZackD_SS_Set_02_Fractior	6568	FTMS; HC	113.93	798.88	PROSER1

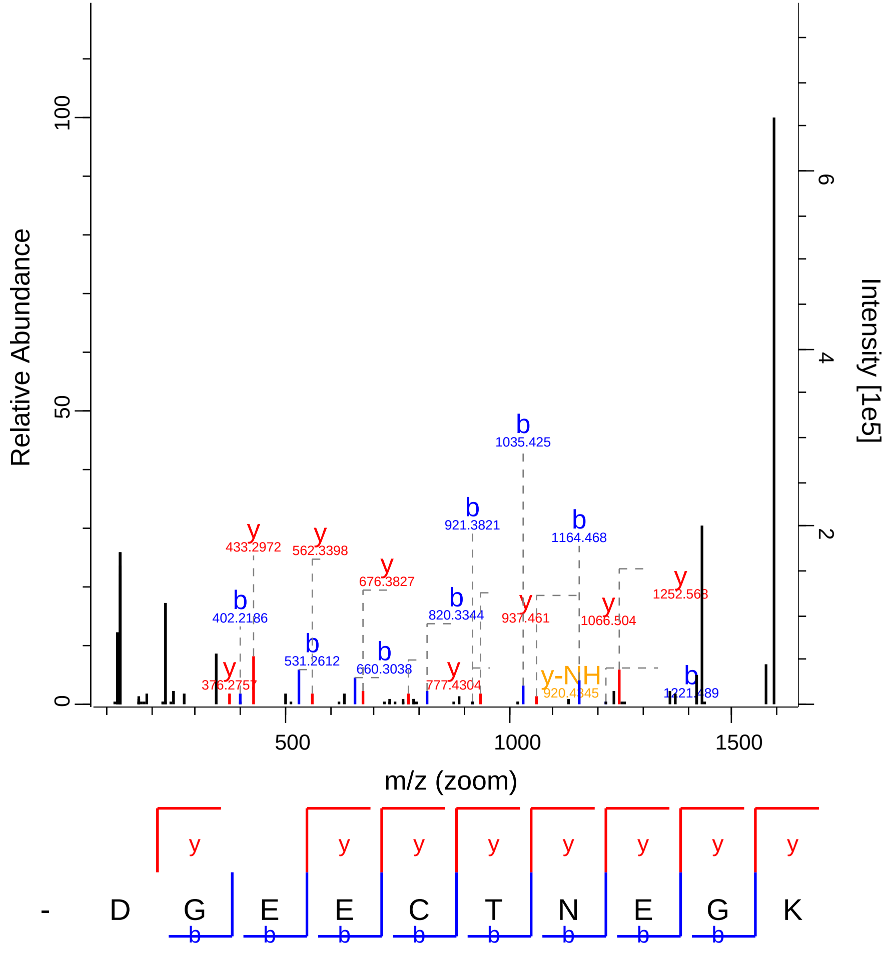

-

Raw File	Scan	Method	Score	m/z	Gene names
RO-QC-160121_ZackD_SS_Set_02_Fractio[illegible]	7238	FTMS; HC	124.23	743.86	CCBE1

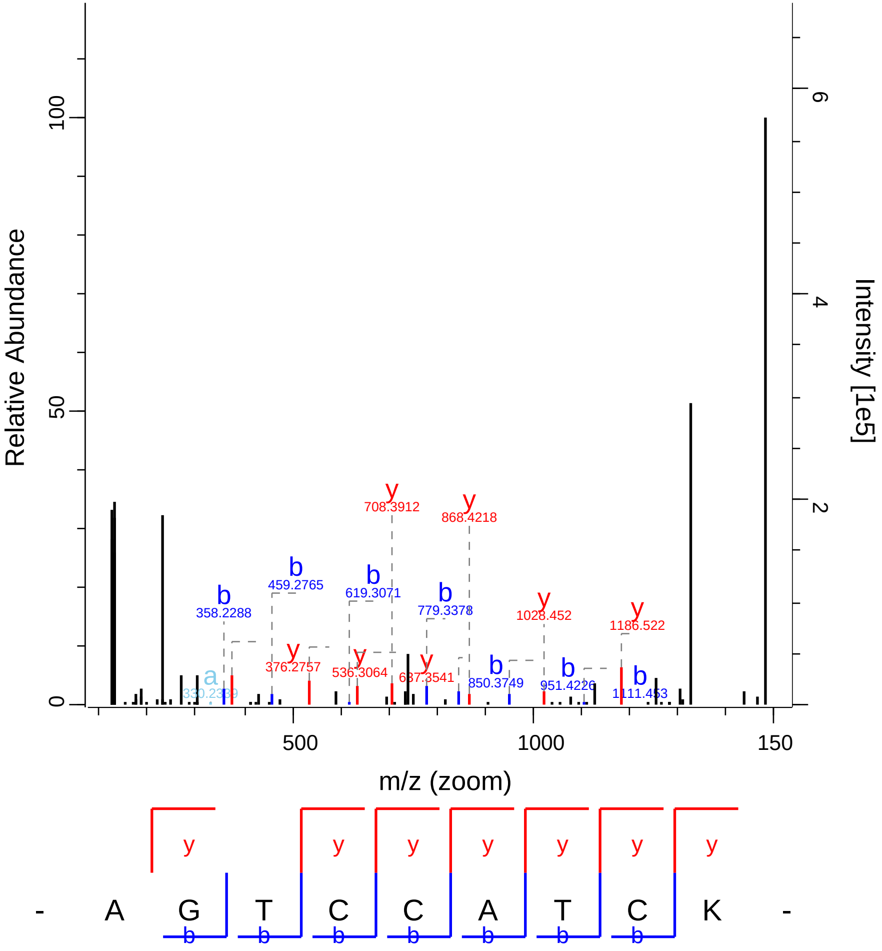

Raw File	Scan	Method	Score	m/z	Gene names
RO-QC-160121_ZackD_SS_Set_02_Fractior	7321	FTMS; HC	113.63	395.23	SHKBP1

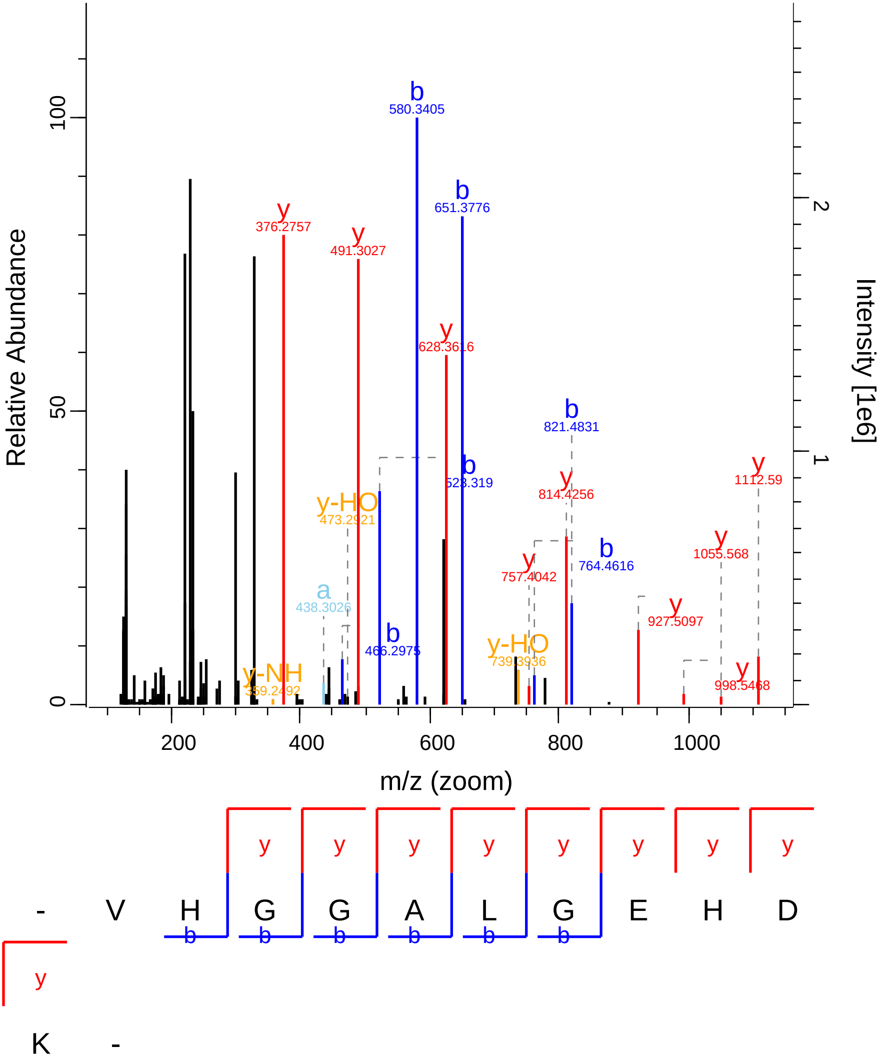

Raw File	Scan	Method	Score	m/z	Gene names
RO-QC-160121_ZackD_SS_Set_02_Fractioi	7820	FTMS; HC	162.36	772.92	FAM102B

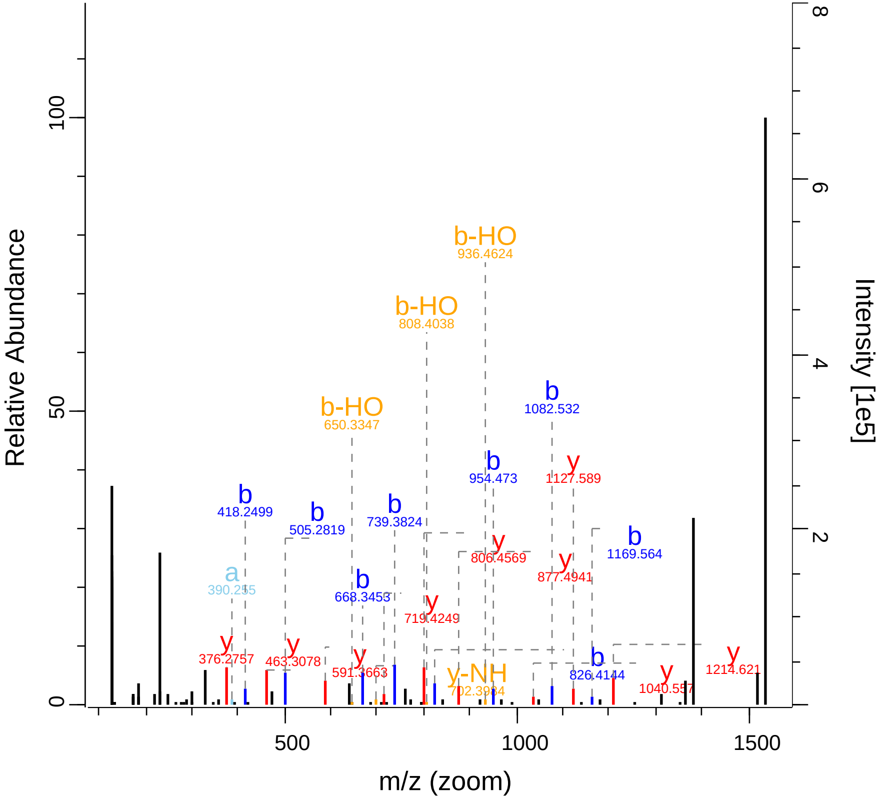

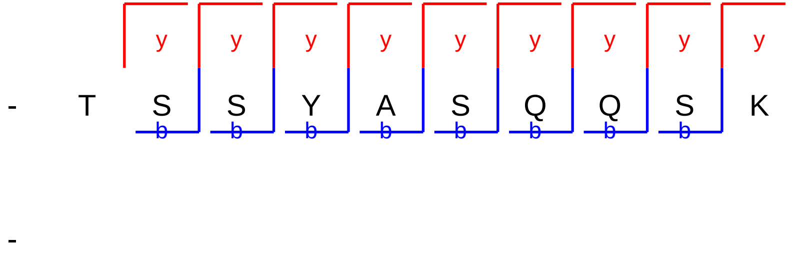

Raw File	Scan	Method	Score	m/z	Gene names
RO-QC-160121_ZackD_SS_Set_02_Fractior	7968	FTMS; HC	108.47	691.37	SH2B2

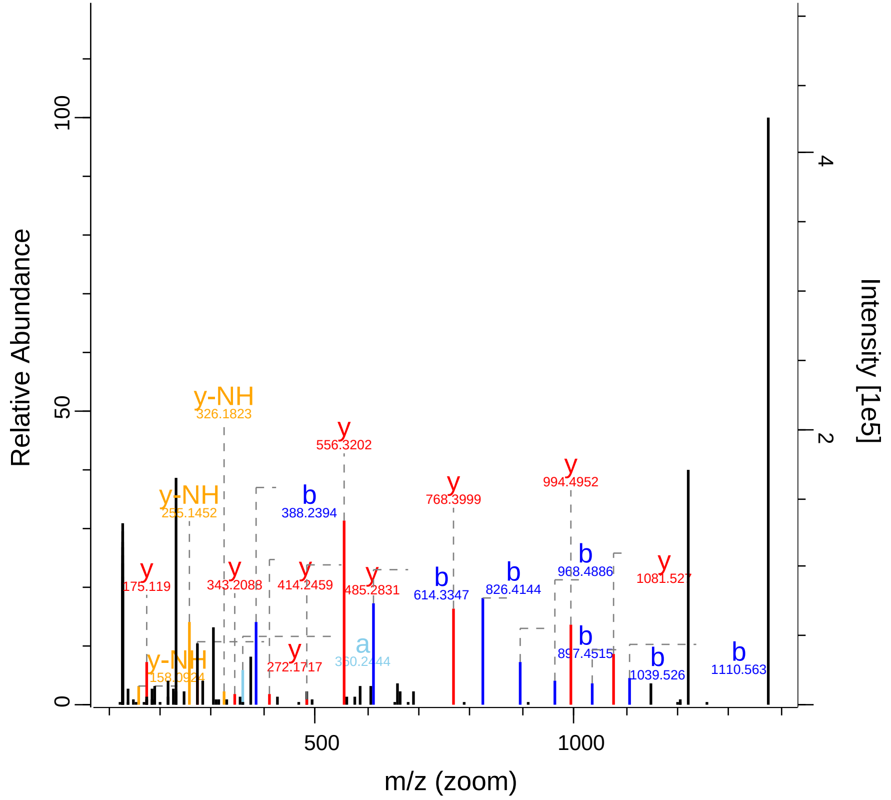

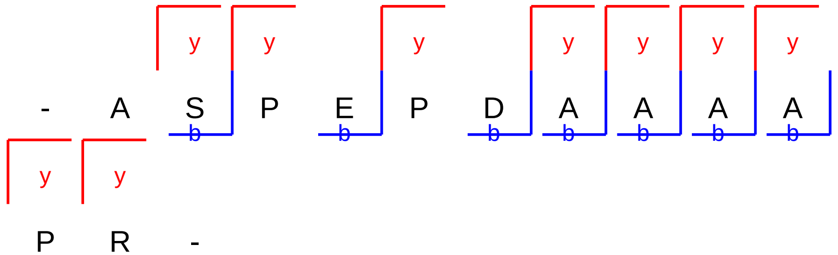

Raw File	Scan	Method	Score	m/z
RO-QC-160121_ZackD_SS_Set_02_Fraction_2	8094	FTMS; HCD	58.6	611.85

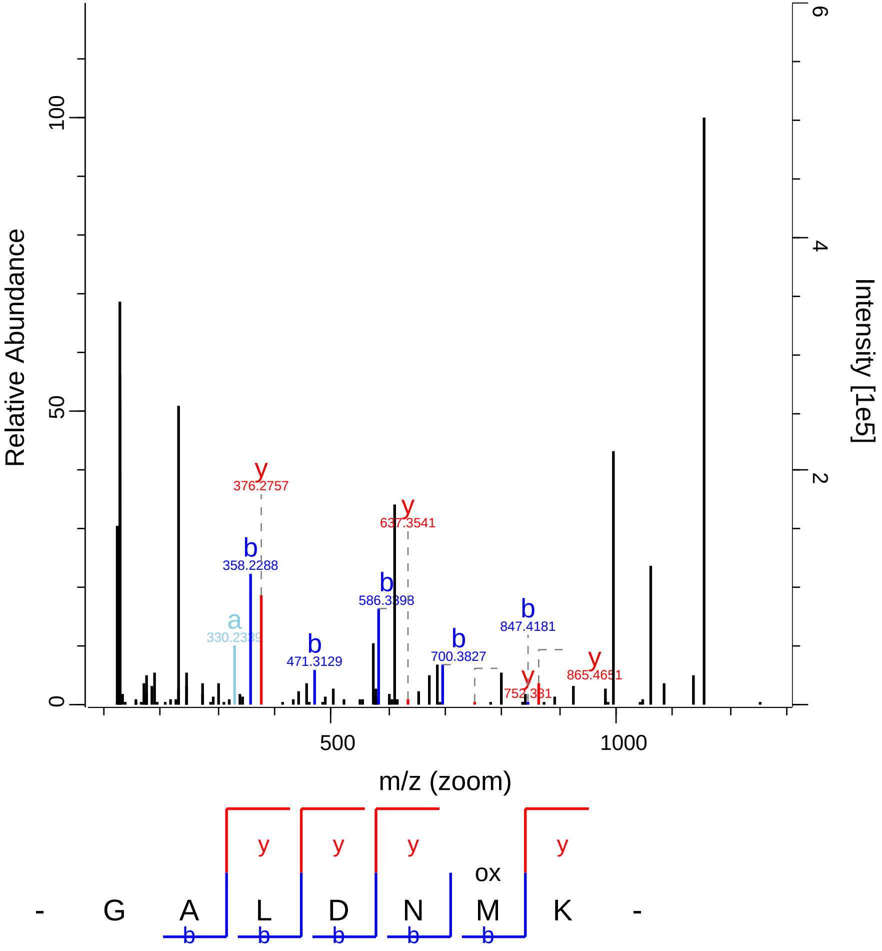

Raw File	Scan	Method	Score	m/z	Gene names
RO-QC-160121_ZackD_SS_Set_02_Fraction	8150	FTMS; HCI	133.98	781.9	SPICE1

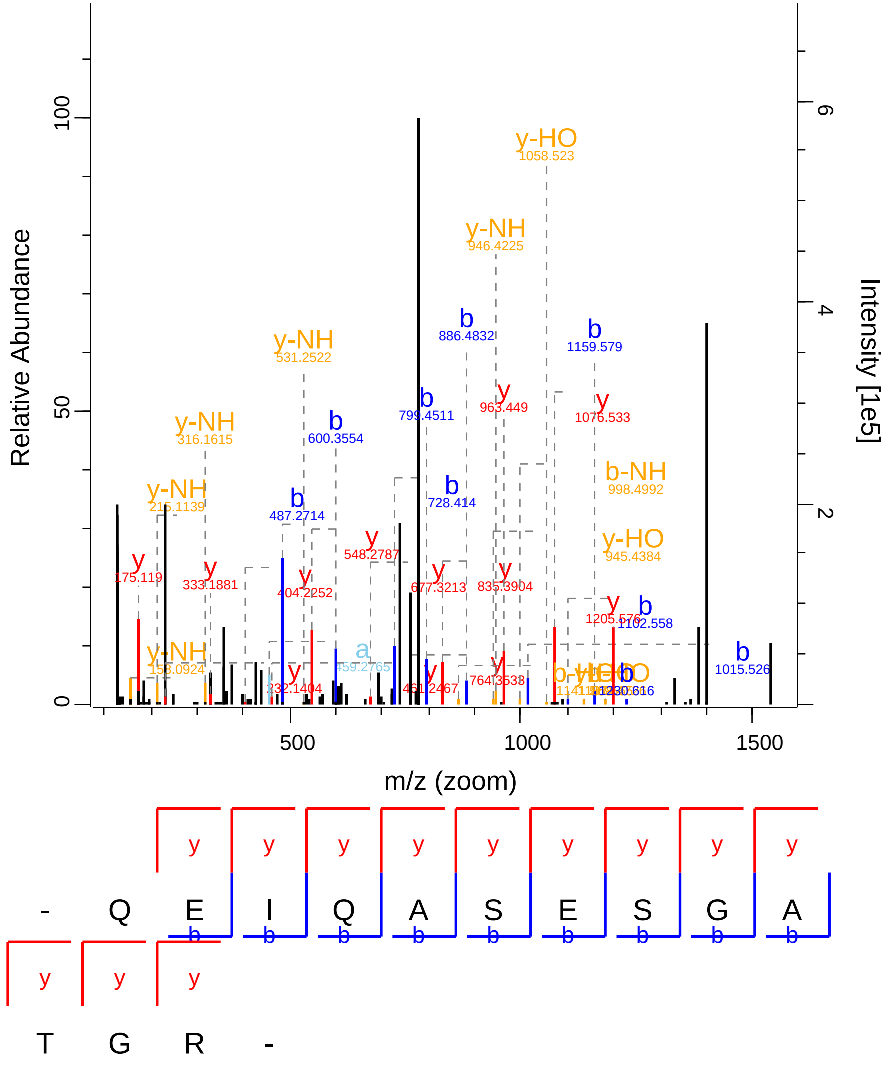

Raw File	Scan	Method	Score	m/z
RO-QC-160121_ZackD_SS_Set_02_Fraction_2	8282	FTMS; HCD	52.68	611.85

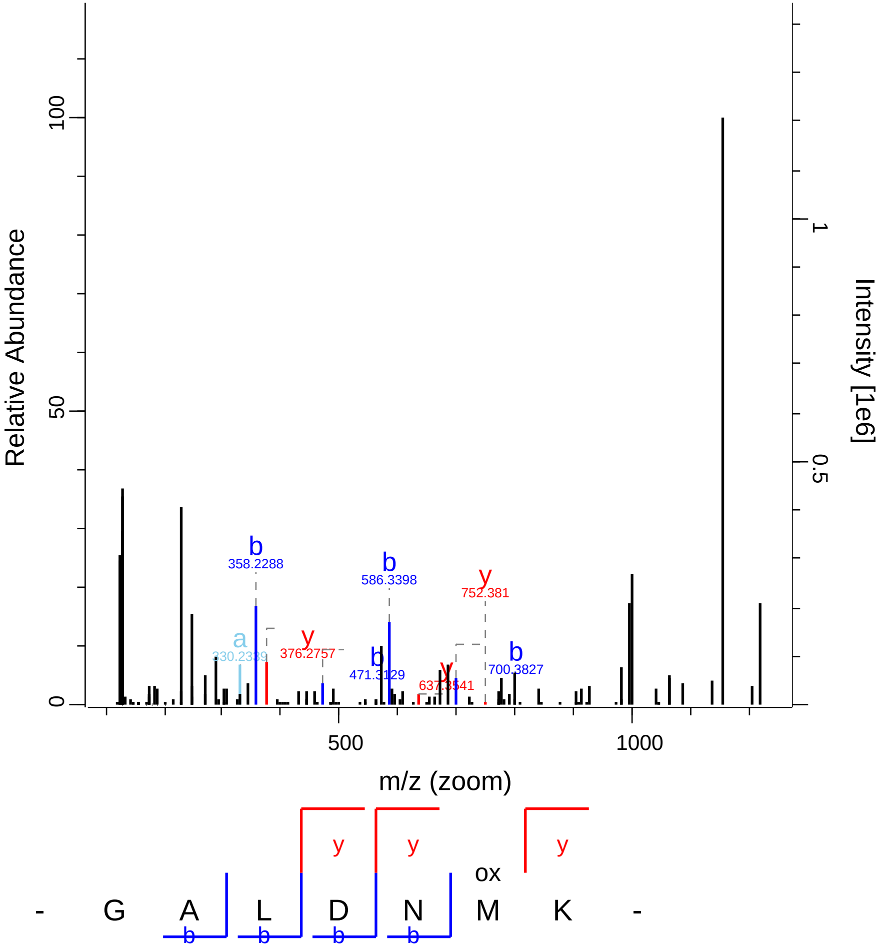

Raw File	Scan	Method	Score	m/z	Gene names
RO-QC-160121_ZackD_SS_Set_02_Fractio	8331	FTMS; HC	102.51	610.87	ICE2

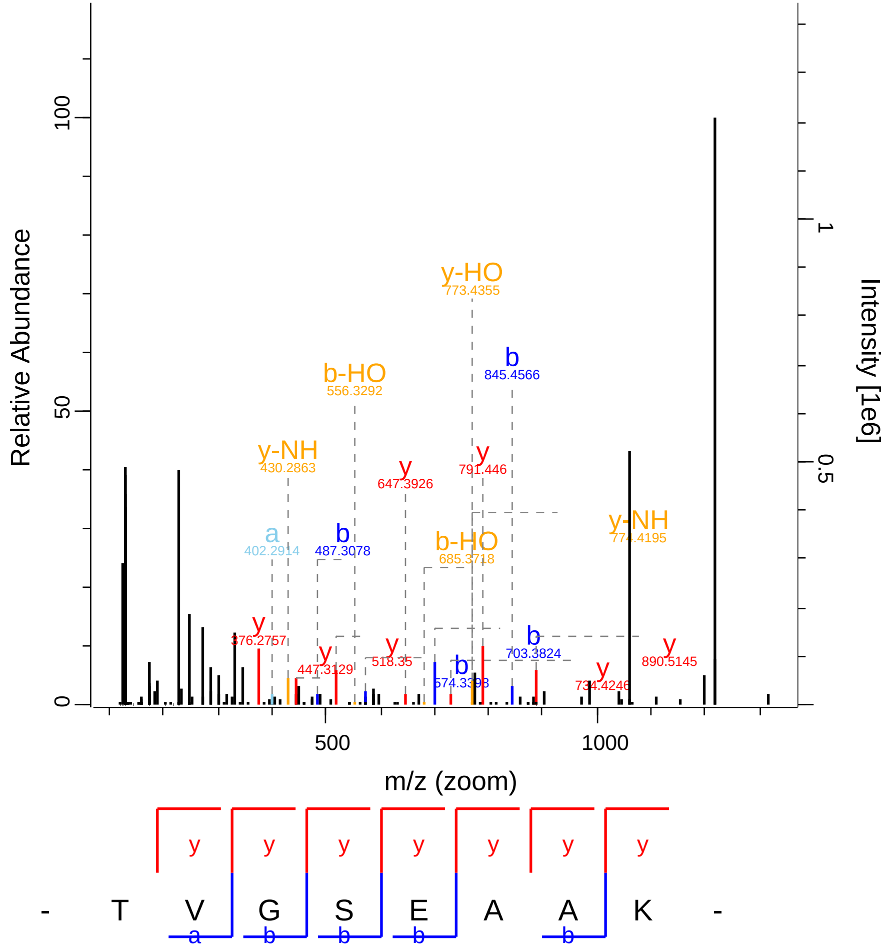

Raw File	Scan	Method	Score	m/z	Gene names
RO-QC-160121_ZackD_SS_Set_02_Fractior	8707	FTMS; HCI	95.5	482.29	C2orf44

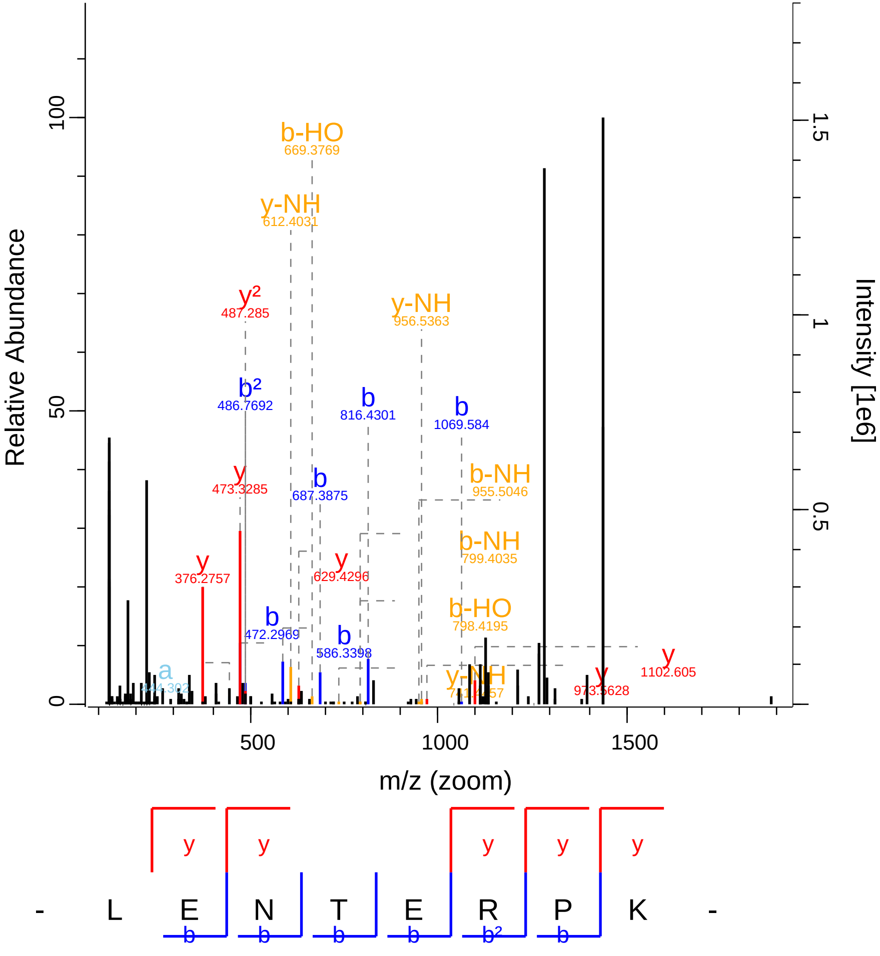

Raw File	Scan	Method	Score	m/z	Gene names
RO-QC-160121_ZackD_SS_Set_02_Fractio[illegible]	8792	FTMS; HC	120.86	730.37	WTIP

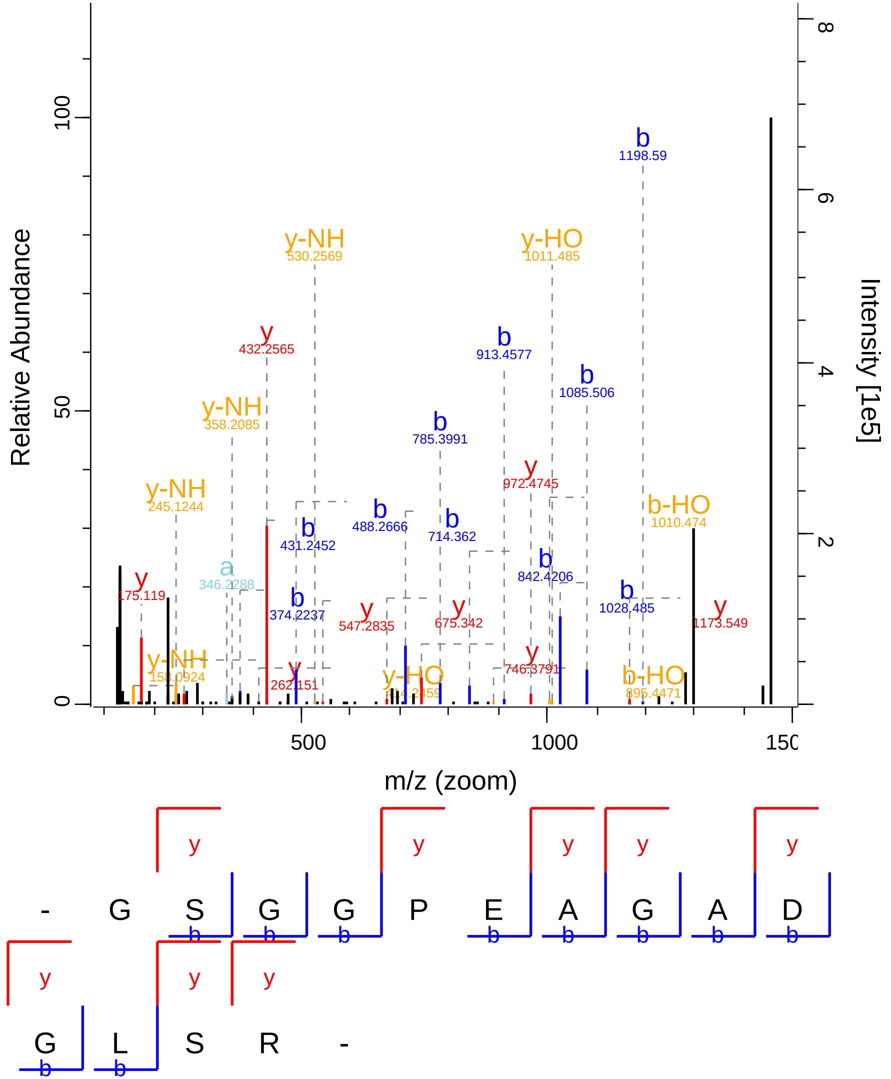

Raw File	Scan	Method	Score	m/z	Gene names
RO-QC-160121_ZackD_SS_Set_02_Fractior	9119	FTMS; HC	130.75	623.33	TFPT

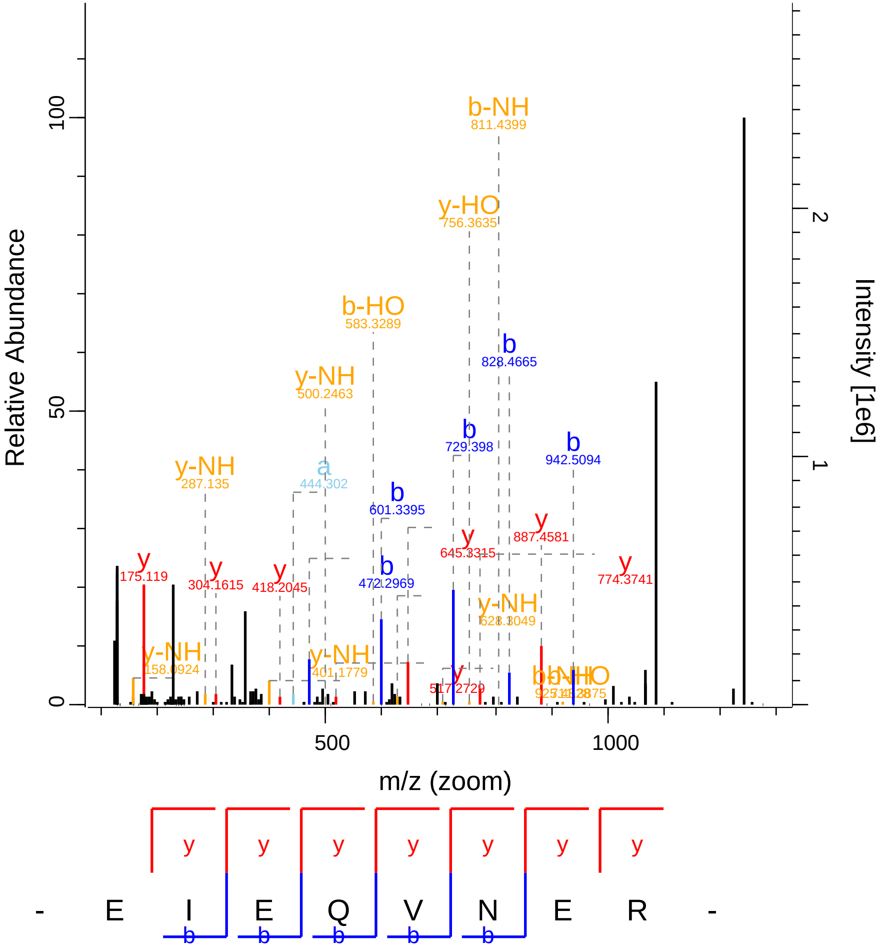

Raw File	Scan	Method	Score	m/z
RO-QC-160121_ZackD_SS_Set_02_Fraction_2	9363	FTMS; HCD	56.03	1289.69

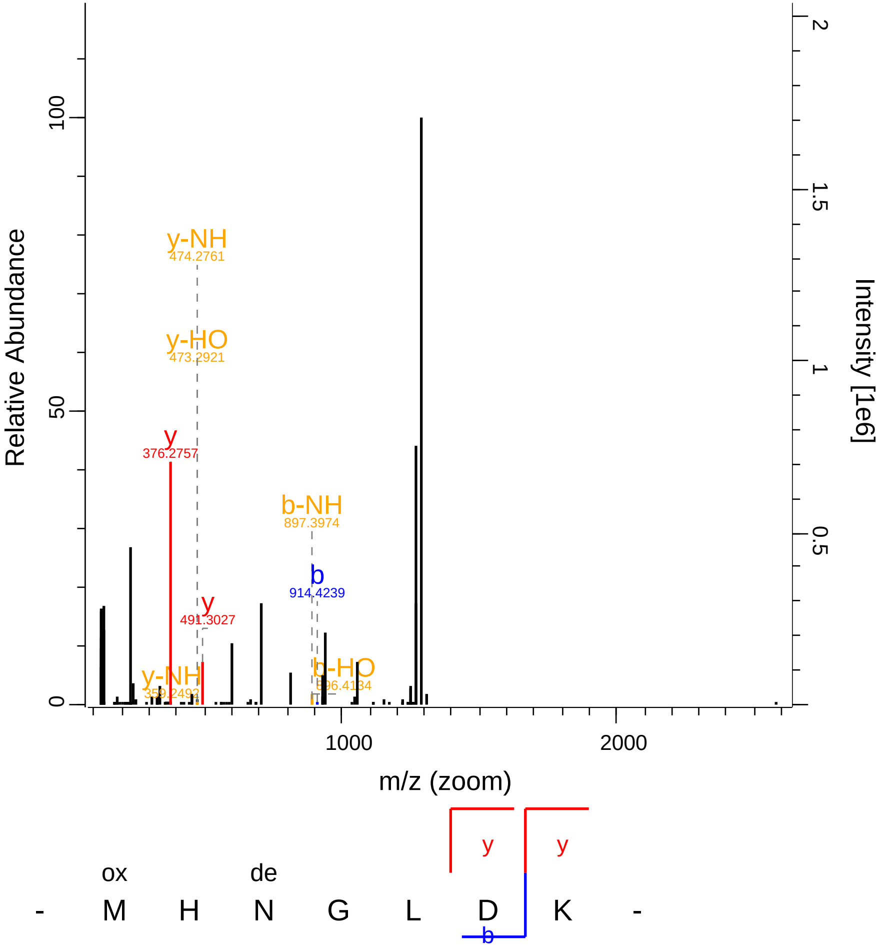

Raw File	Scan	Method	Score	m/z
RO-QC-160121_ZackD_SS_Set_02_Fraction_2	9519	FTMS; HCD	68.91	539.26

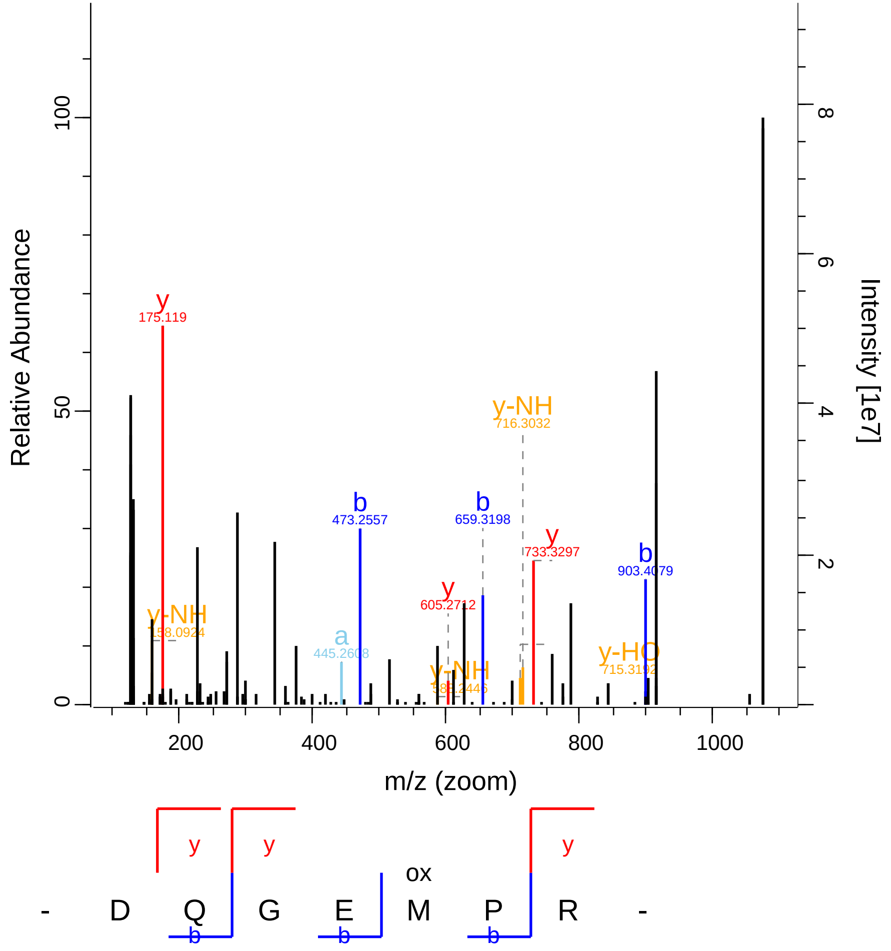

Raw File	Scan	Method	Score	m/z	Gene names
RO-QC-160121_ZackD_SS_Set_02_Fraction	9524	FTMS; HCI	43.08	841.9	GLRX2

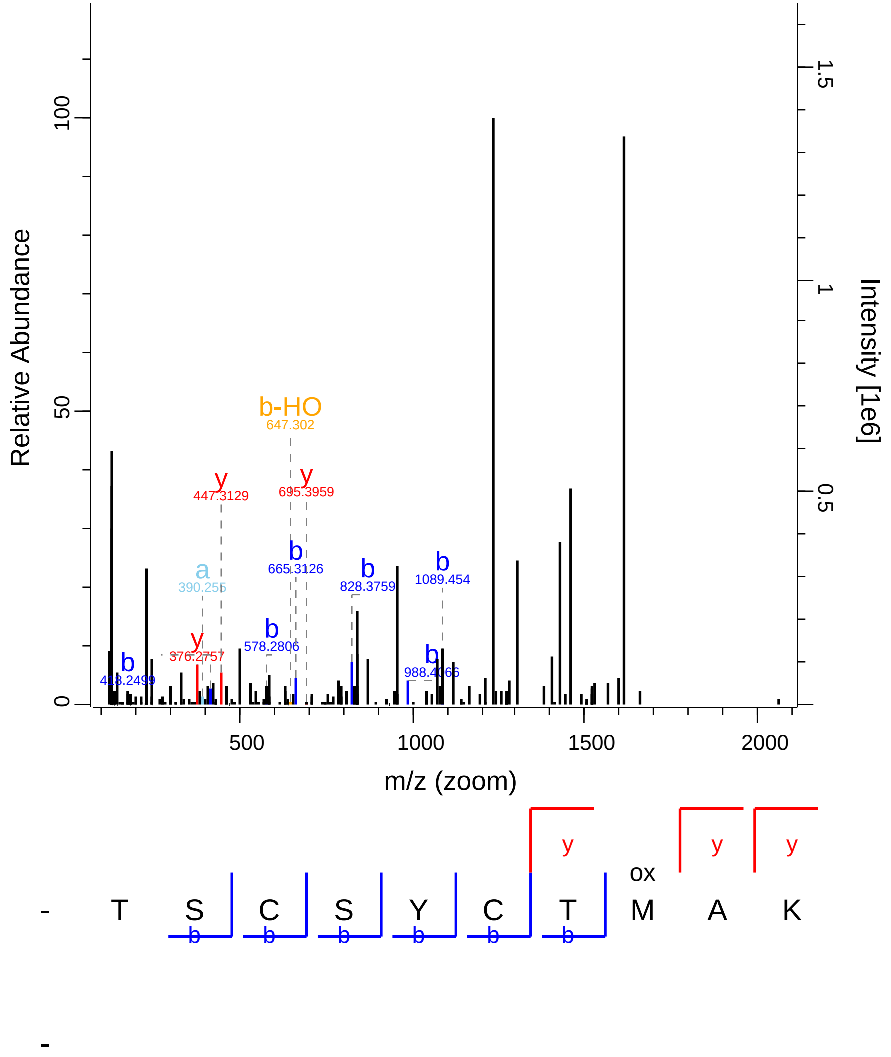

Raw File	Scan	Method	Score	m/z
RO-QC-160121_ZackD_SS_Set_02_Fraction_2	9572	FTMS; HCD	62.71	373.9

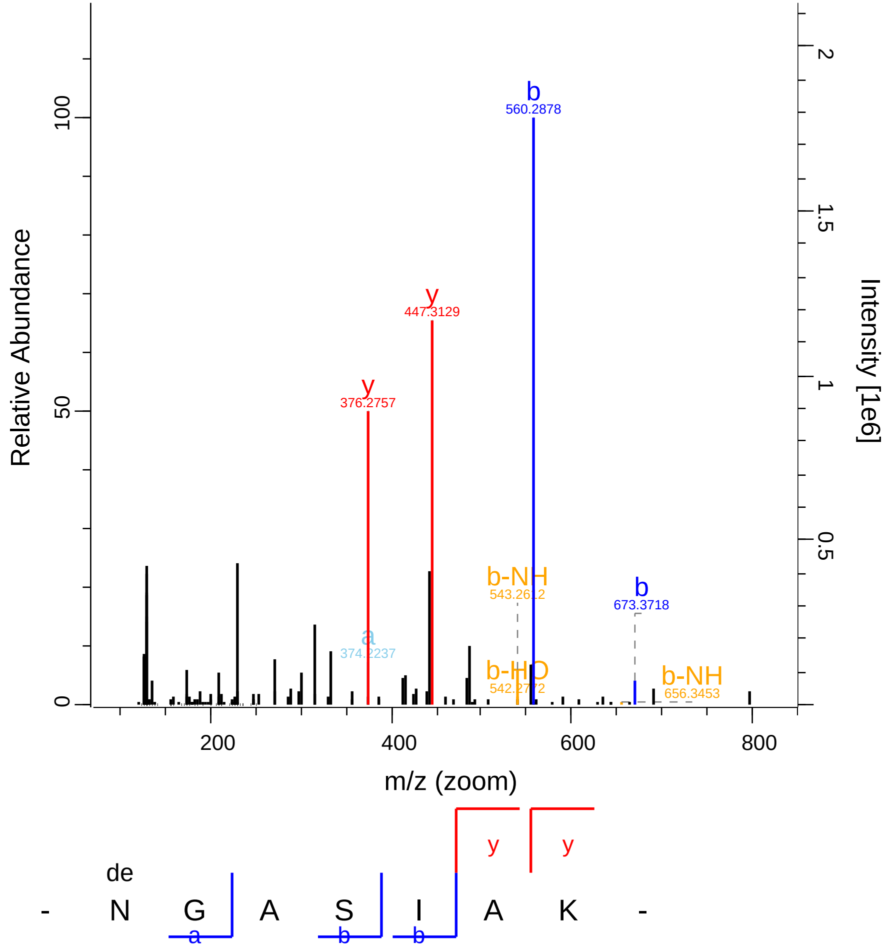

Raw File	Scan	Method	Score	m/z	Gene names
RO-QC-160121_ZackD_SS_Set_02_Fractior	9635	FTMS; HCI	87.08	571.31	SMIM7

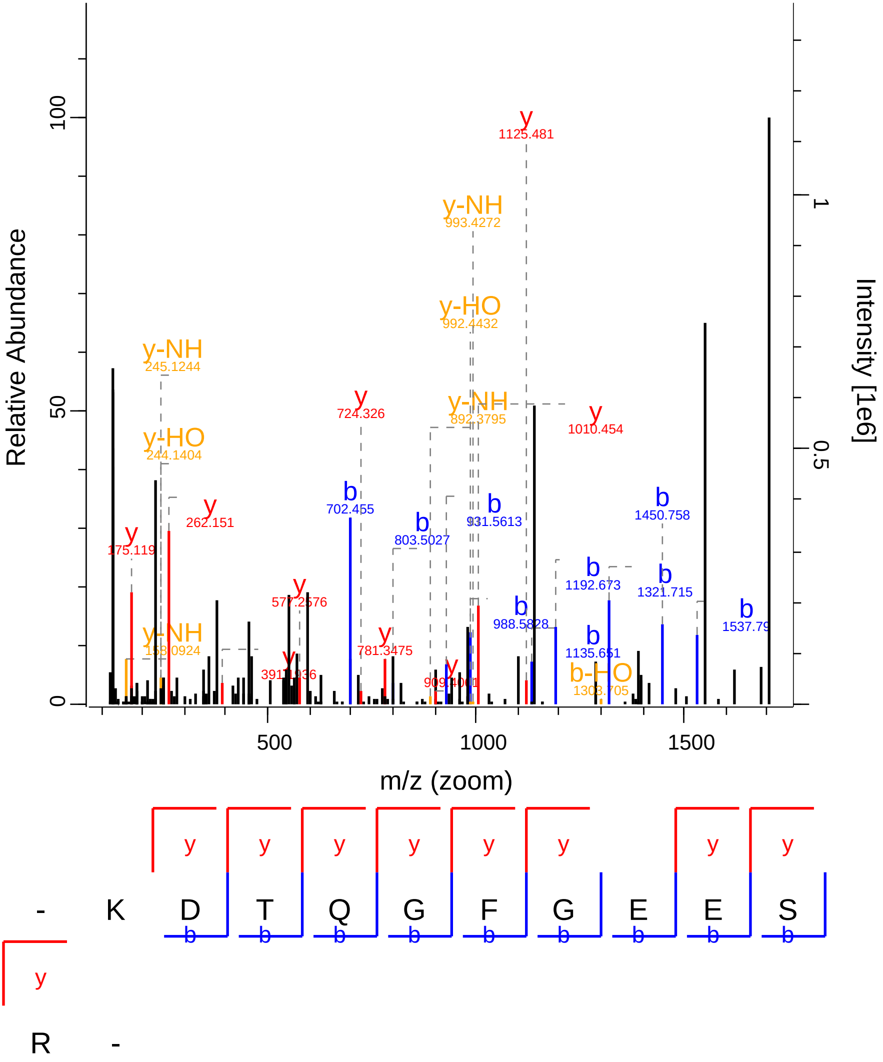

Raw File	Scan	Method	Score	m/z	Gene names
RO-QC-160121_ZackD_SS_Set_02_Fractio	10284	FTMS; HC	77.64	823.47	TMEM88

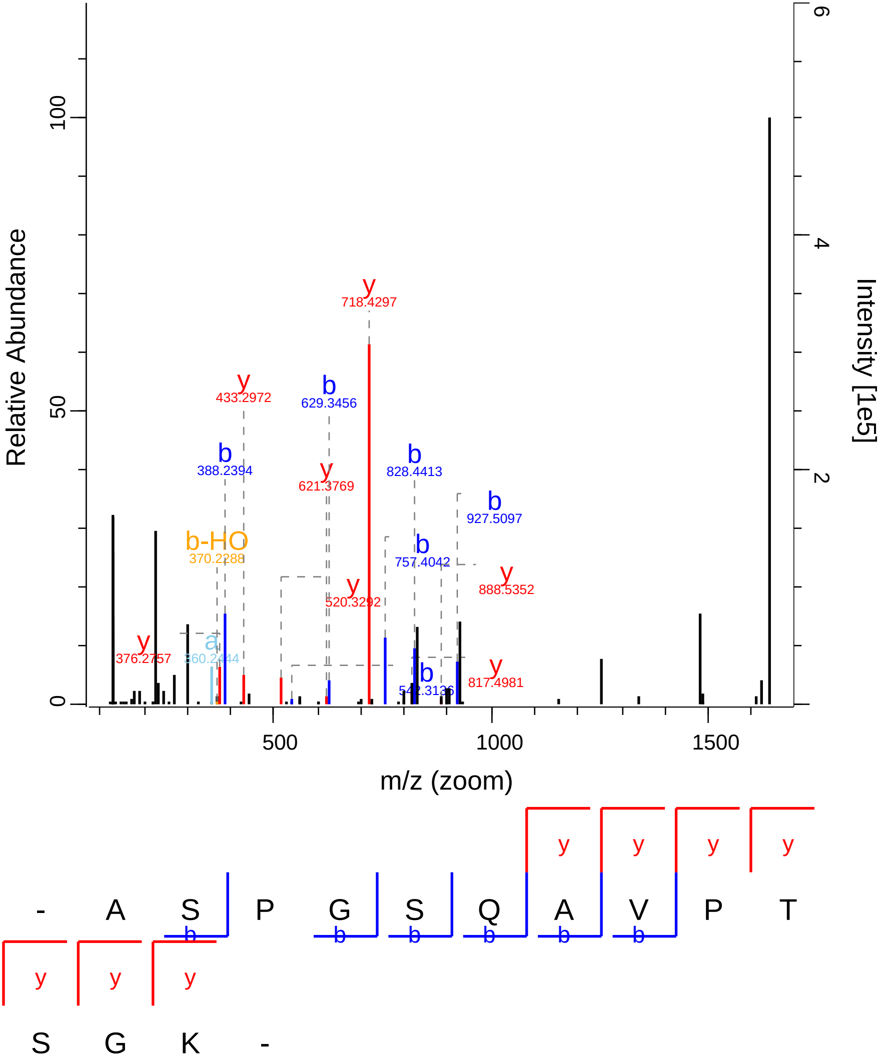

Raw File	Scan	Method	Score	m/z	Gene names
RO-QC-160121_ZackD_SS_Set_02_Fractio	10295	FTMS; HC	91.2	548.98	TMEM88

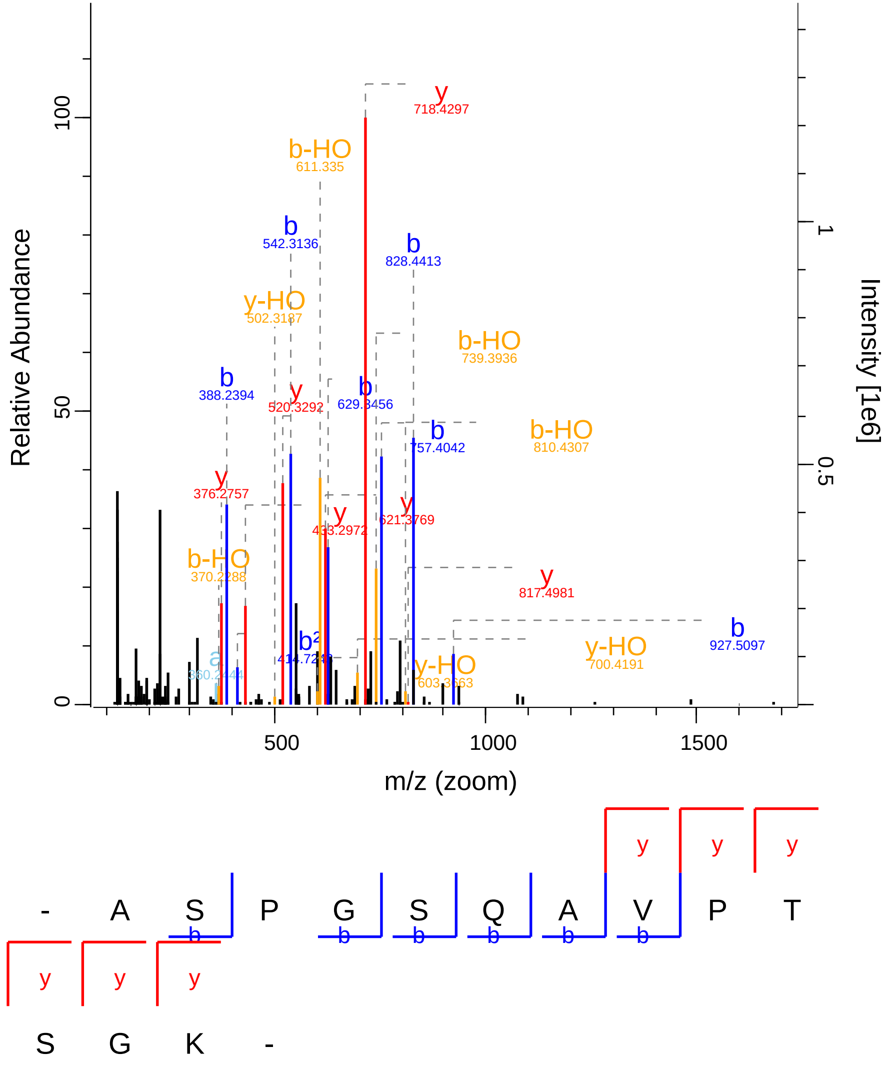

Raw File	Scan	Method	Score	m/z	Gene names
RO-QC-160121_ZackD_SS_Set_02_Fractio	10440	FTMS; HC	50.28	906.92	CC2D2B

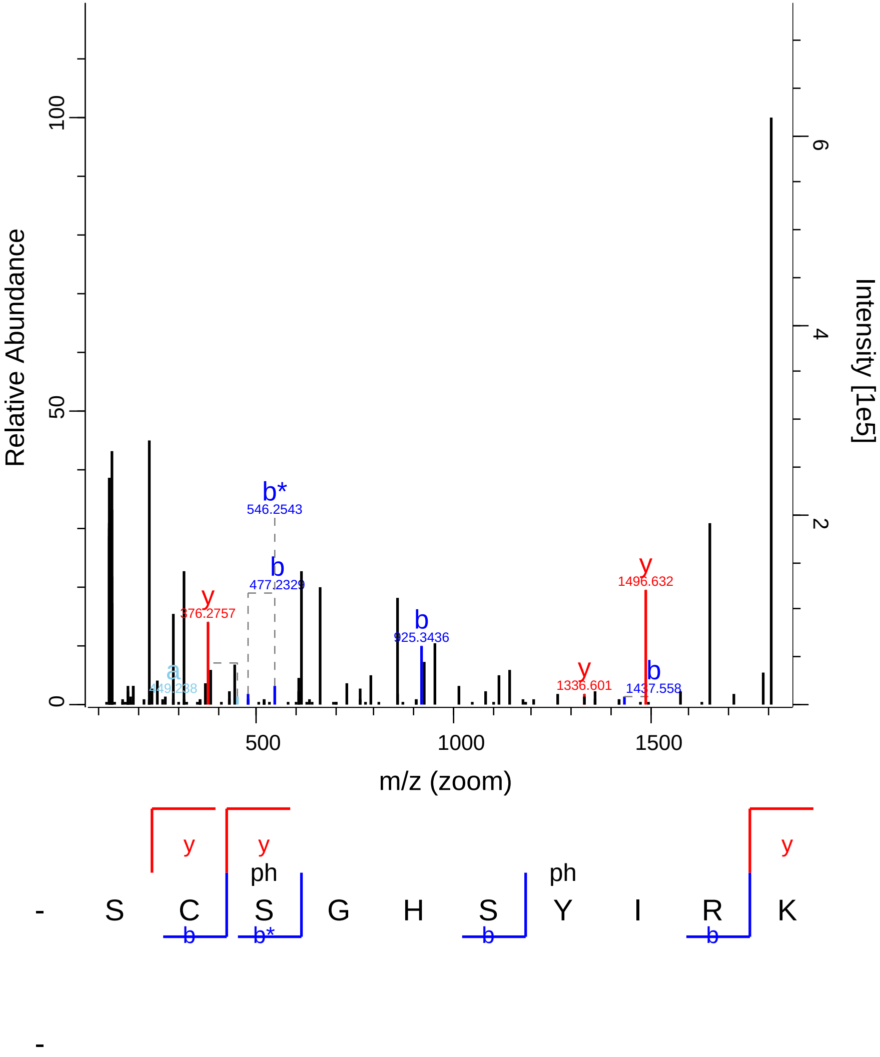

Raw File	Scan	Method	Score	m/z
RO-QC-160121_ZackD_SS_Set_02_Fraction_2	10445	FTMS; HCD	119.57	673.4

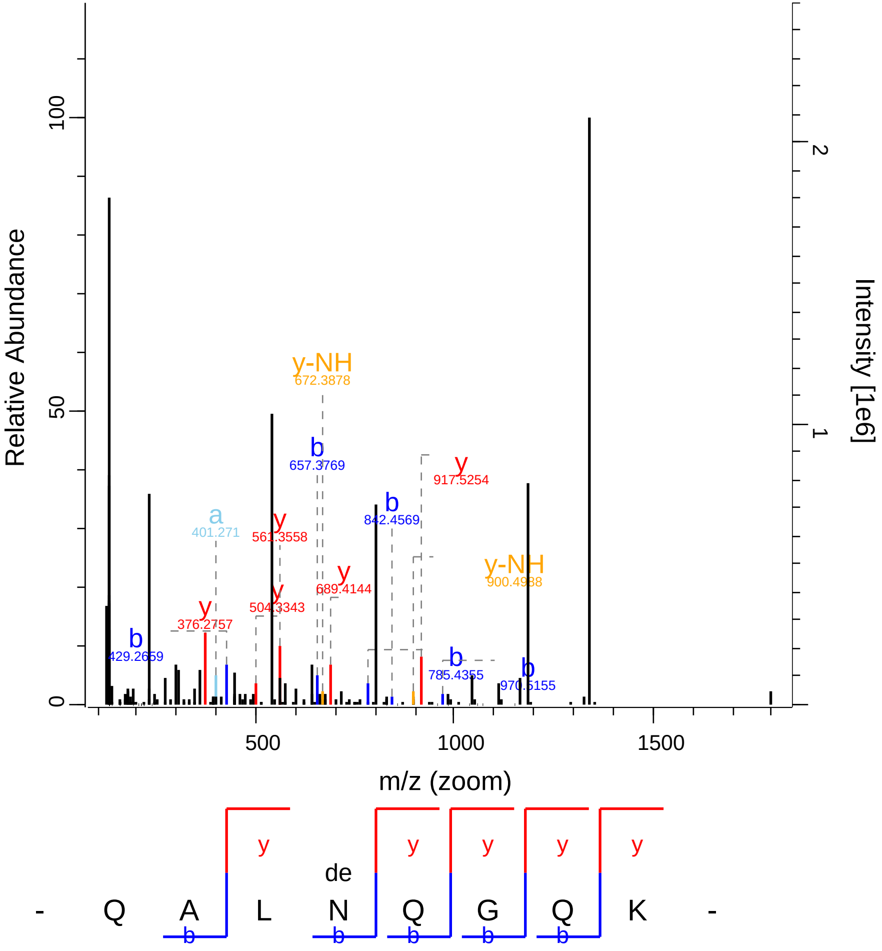

Raw File	Scan	Method	Score	m/z	Gene names
RO-QC-160121_ZackD_SS_Set_02_Fractio	10627	FTMS; HC	40.59	646.31	ATF7IP2

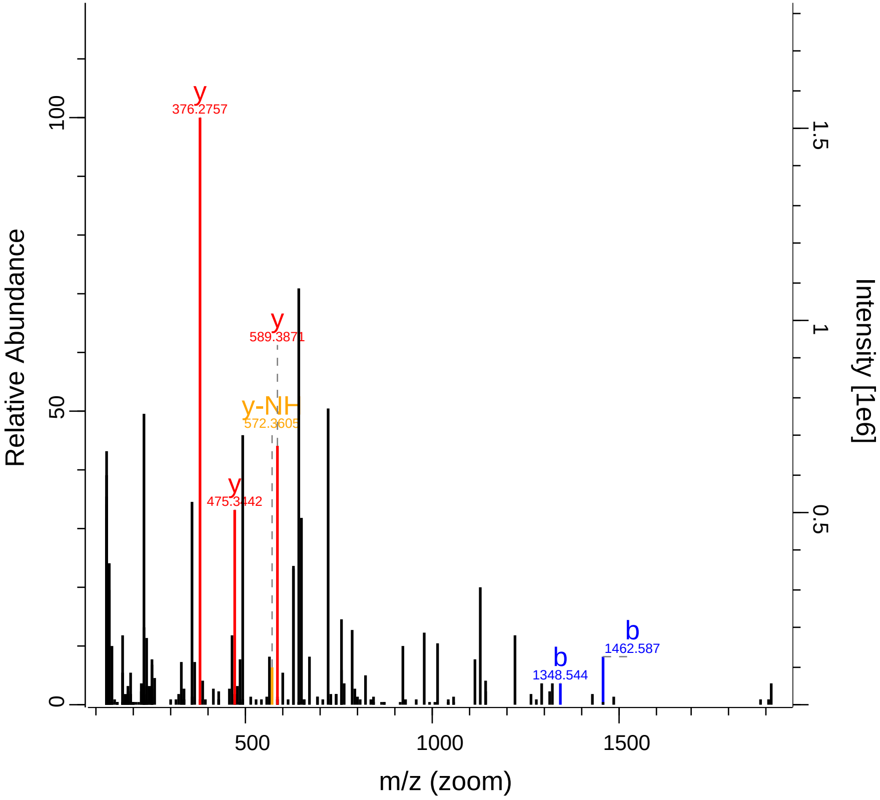

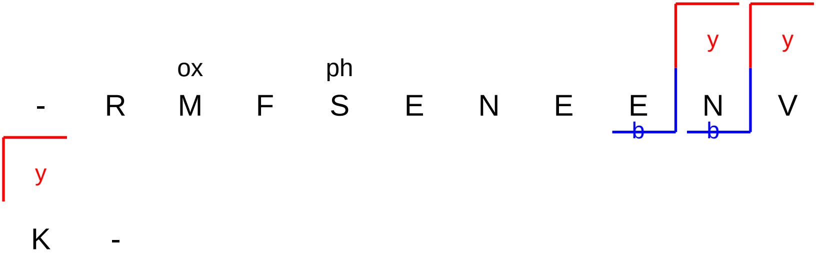

Raw File	Scan	Method	Score	m/z	Gene names
RO-QC-160121_ZackD_SS_Set_02_Fractio	10827	FTMS; HC	66.43	464.61	KCNV2

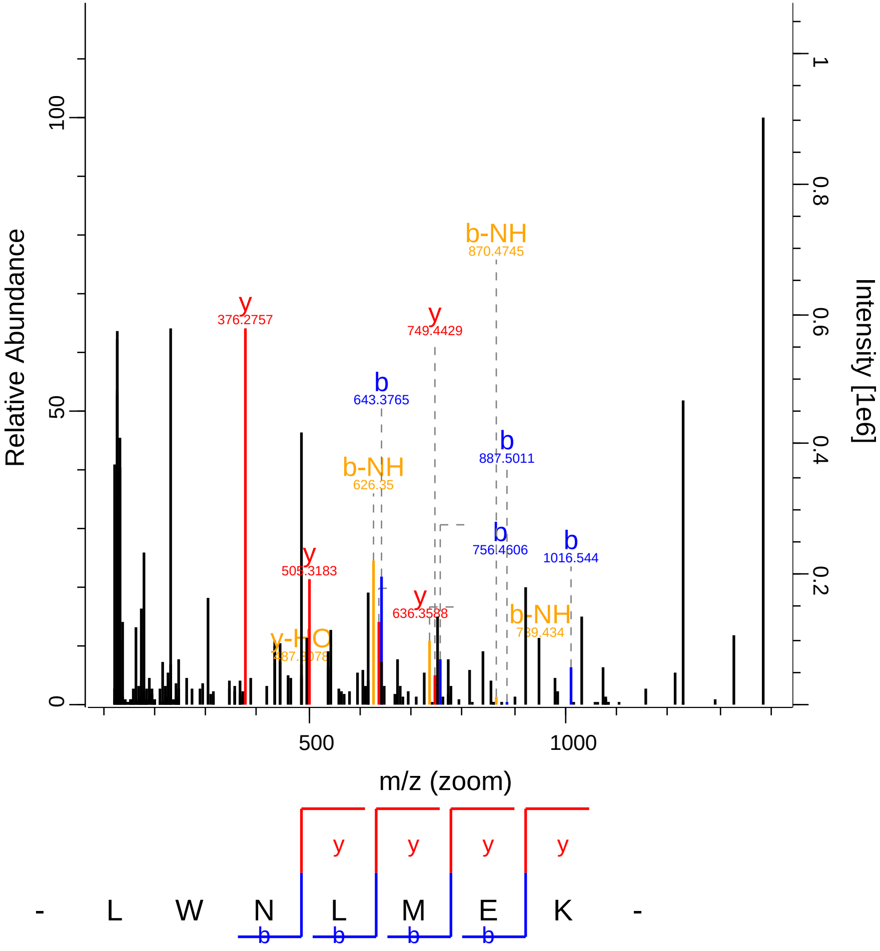

Raw File	Scan	Method	Score	m/z	Gene names
RO-QC-160121_ZackD_SS_Set_02_Fractio	11029	FTMS; HC	89.14	464.61	KCNV2

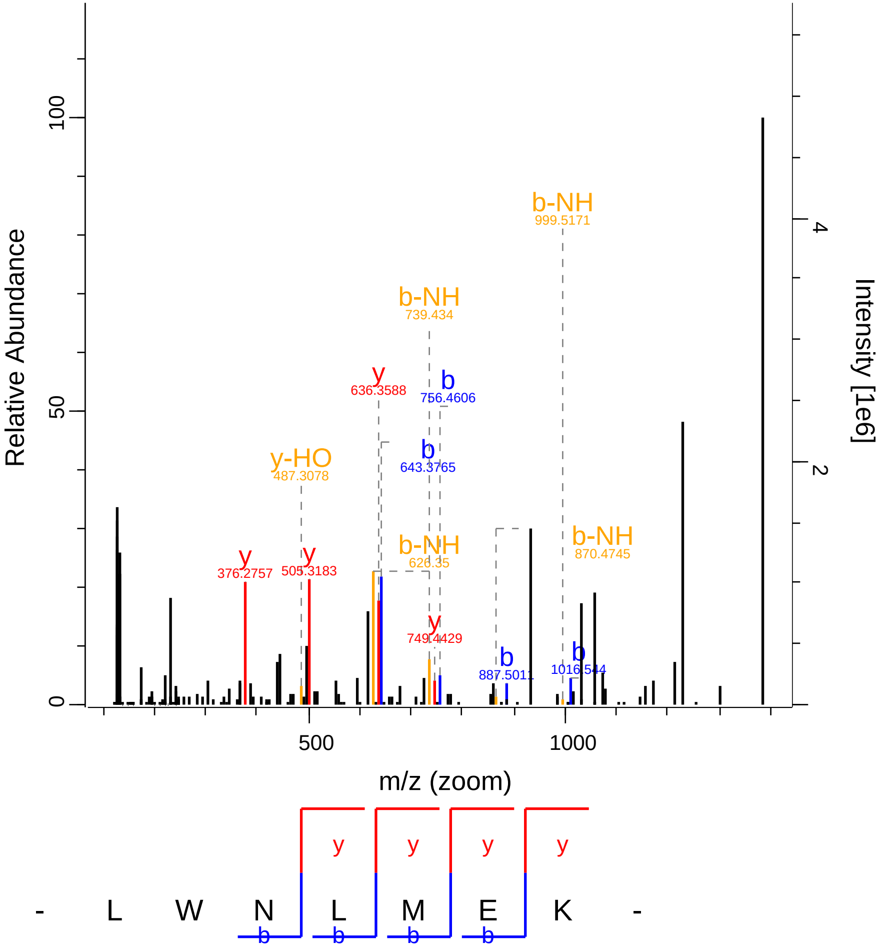

Raw File	Scan	Method	Score	m/z	Gene names
RO-QC-160121_ZackD_SS_Set_02_Fractic	11169	FTMS; HC	86.9	1035.99	MRC1

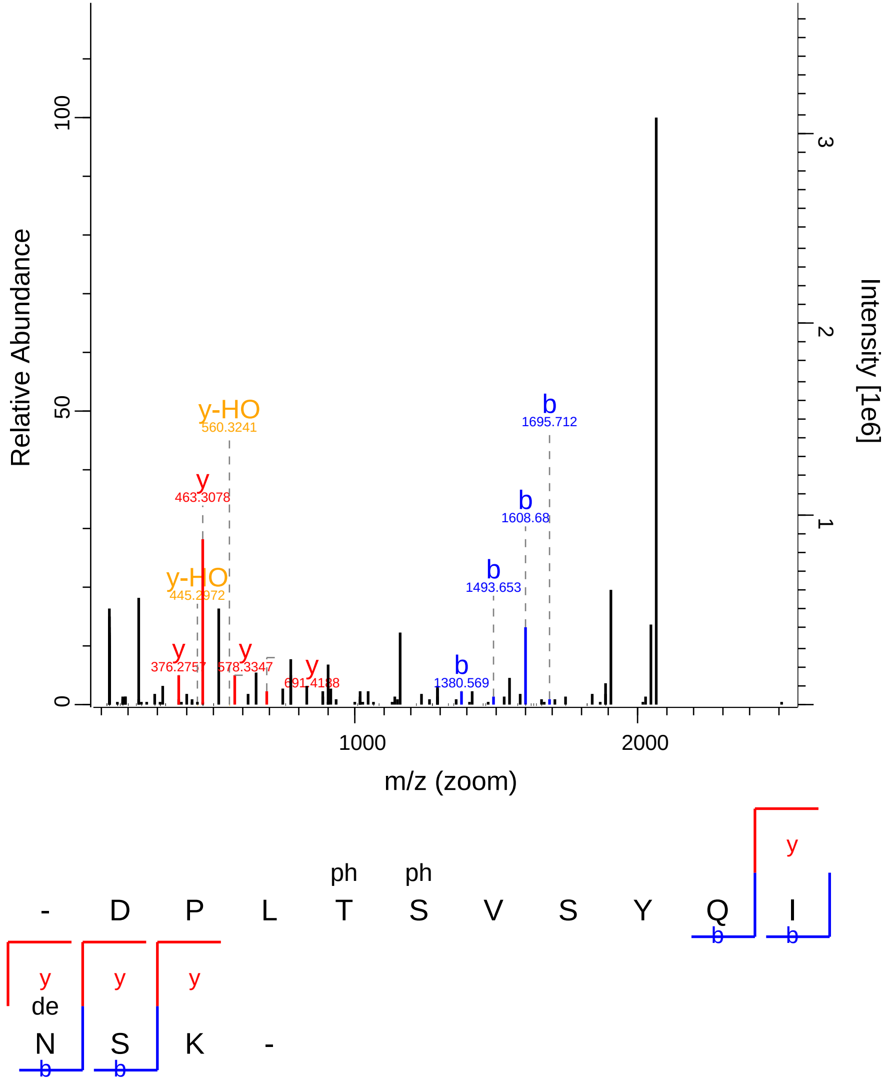

Raw File	Scan	Method	Score	m/z	Gene names
RO-QC-160121_ZackD_SS_Set_02_Fractio	11320	FTMS; HC	77.74	374.56	TXNDC16

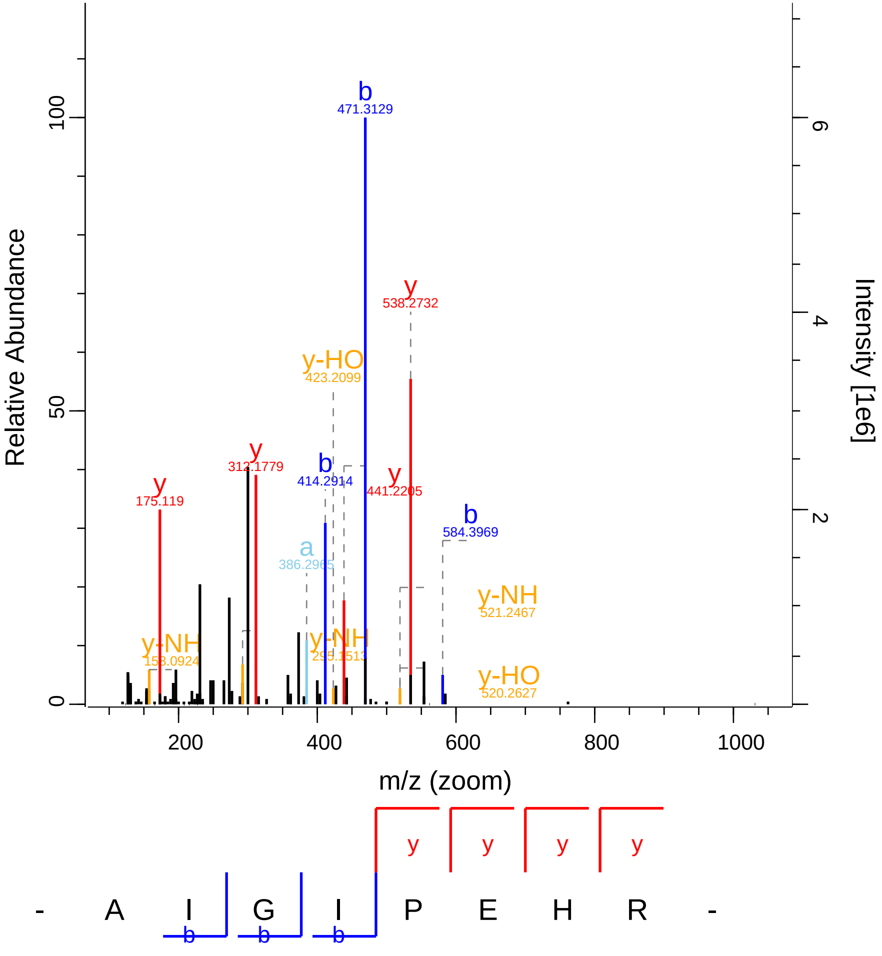

Raw File	Scan	Method	Score	m/z	Gene names
RO-QC-160121_ZackD_SS_Set_02_Fractic	11382	FTMS; HC	67.73	1035.99	MRC1

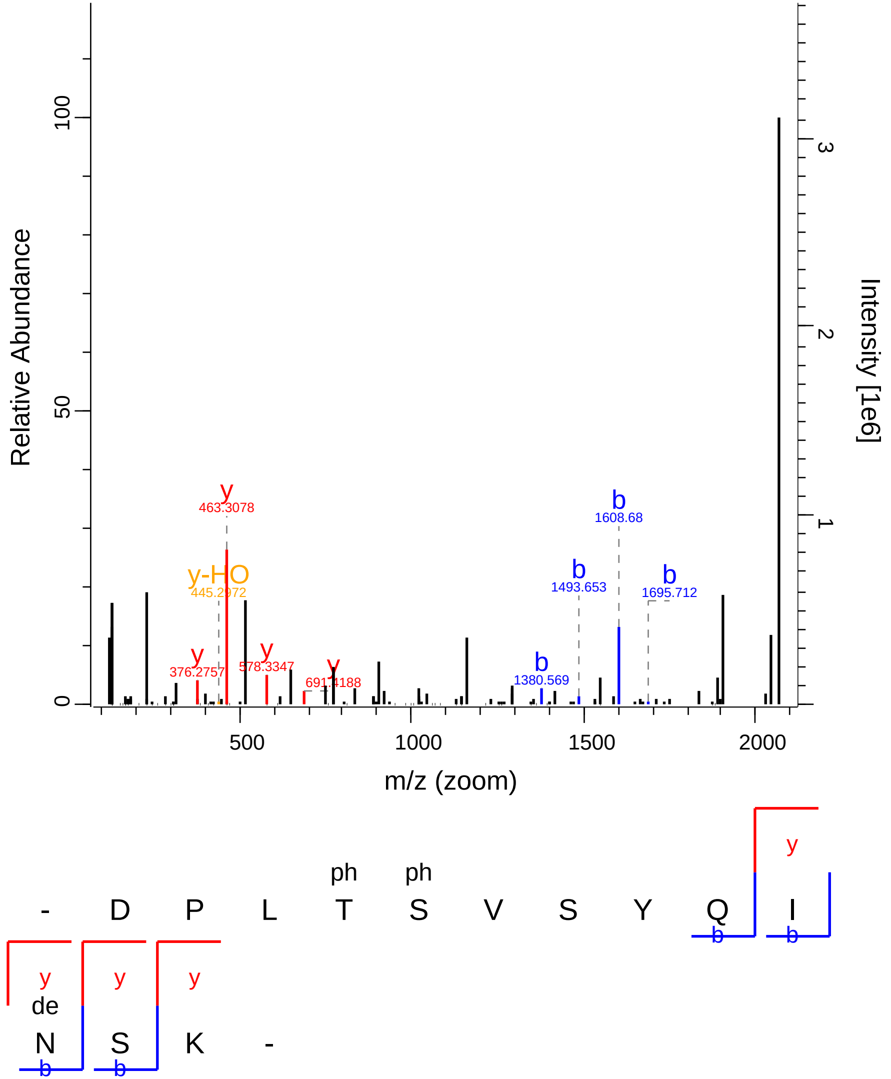

Raw File	Scan	Method	Score	m/z	Gene names
RO-QC-160121_ZackD_SS_Set_02_Fractio	12663	FTMS; HC	156.48	779.95	WRAP73

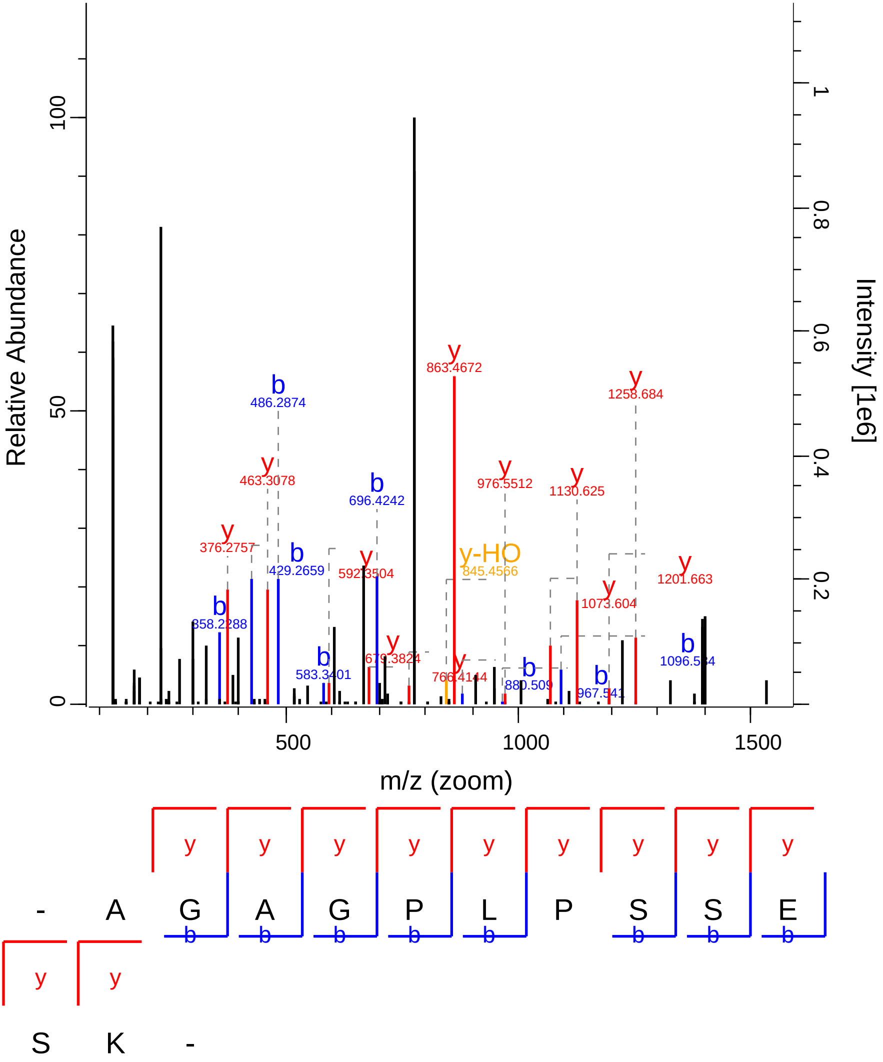

Raw File	Scan	Method	Score	m/z	Gene names
RO-QC-160121_ZackD_SS_Set_02_Fractio	13089	FTMS; HC	139.96	753.92	MED28

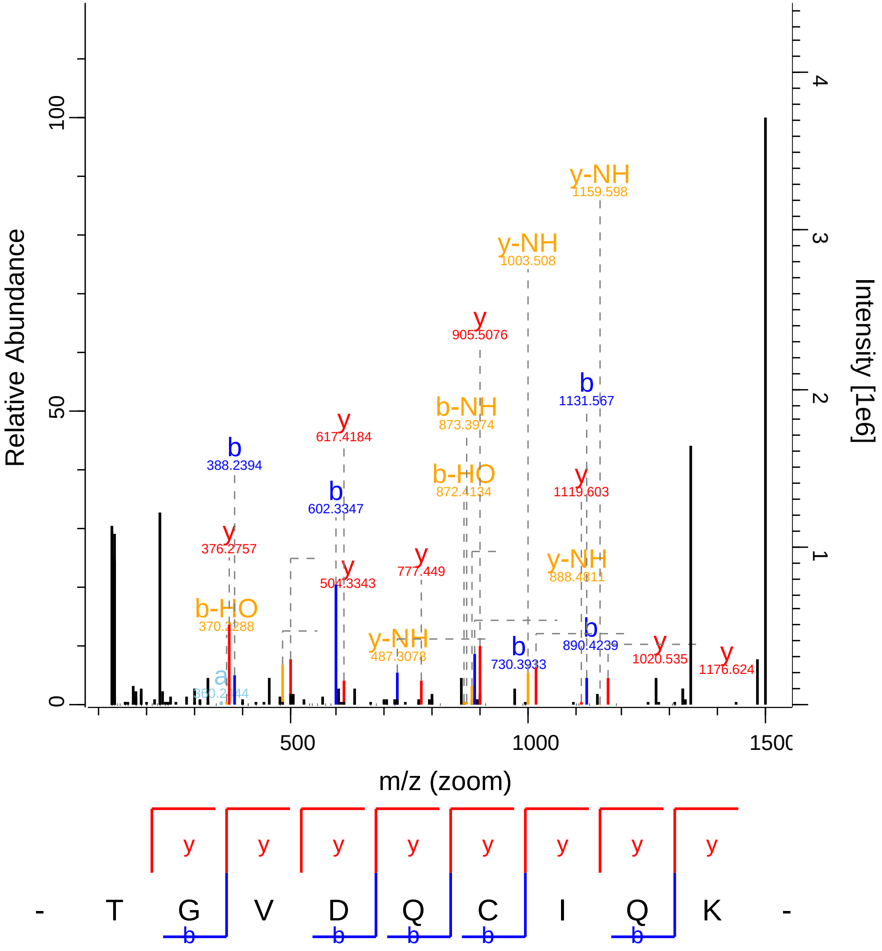

Raw File	Scan	Method	Score	m/z	Gene names
RO-QC-160121_ZackD_SS_Set_02_Fractio	13102	FTMS; HC	72.64	502.95	MED28

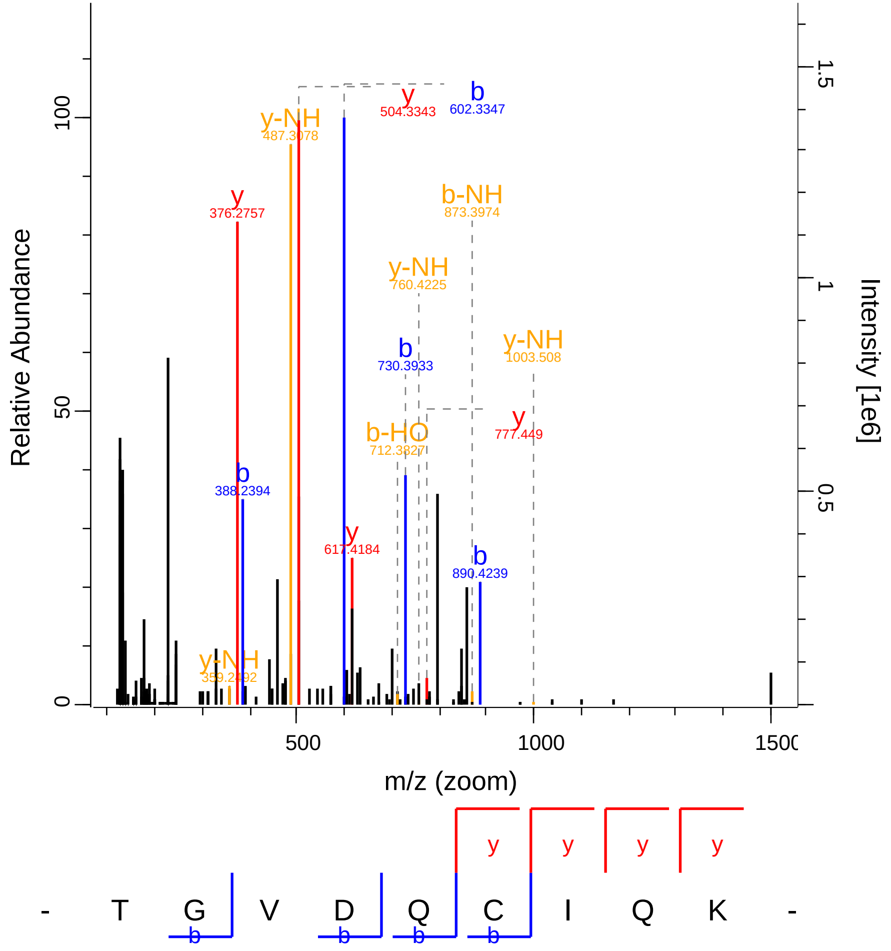

Raw File	Scan	Method	Score	m/z	Gene names
RO-QC-160121_ZackD_SS_Set_02_Fractio	13248	FTMS; HC	99.79	740.35	ANAPC16

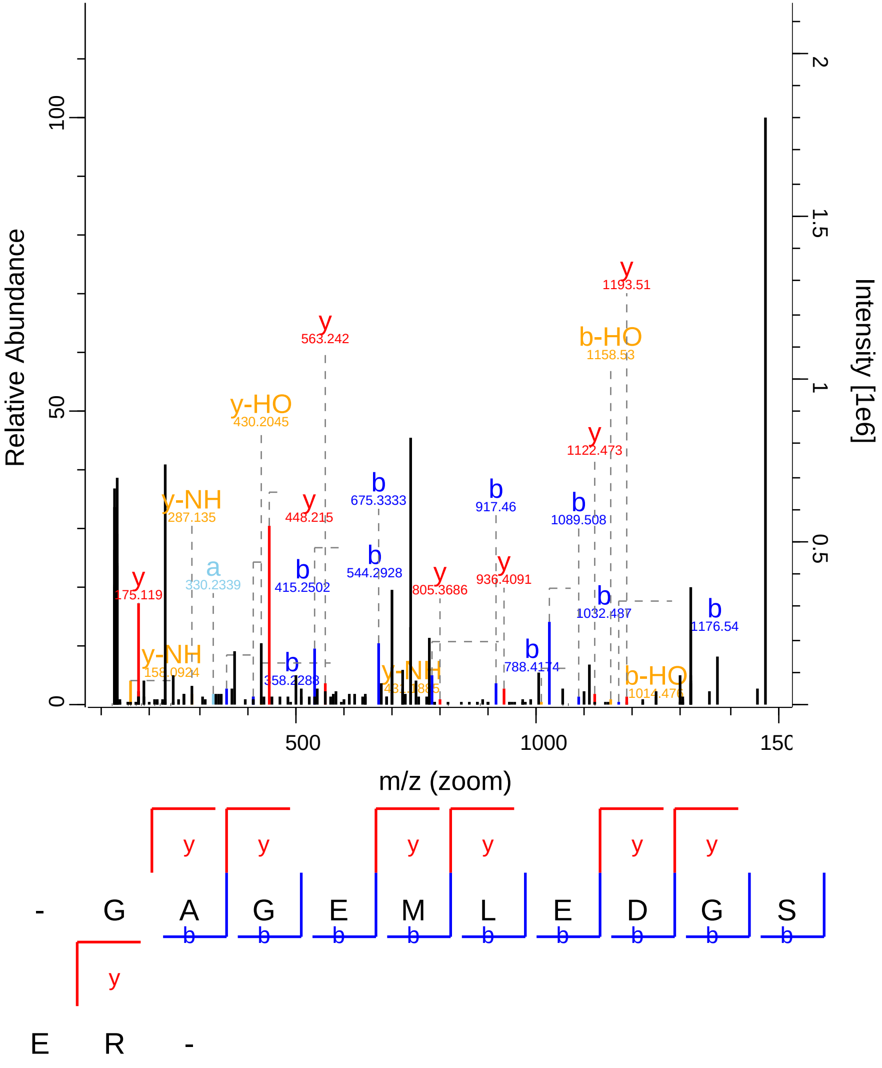

Raw File	Scan	Method	Score	m/z	Gene names
RO-QC-160121_ZackD_SS_Set_02_Fractio	13371	FTMS; HC	51.69	547.78	DEPDC7

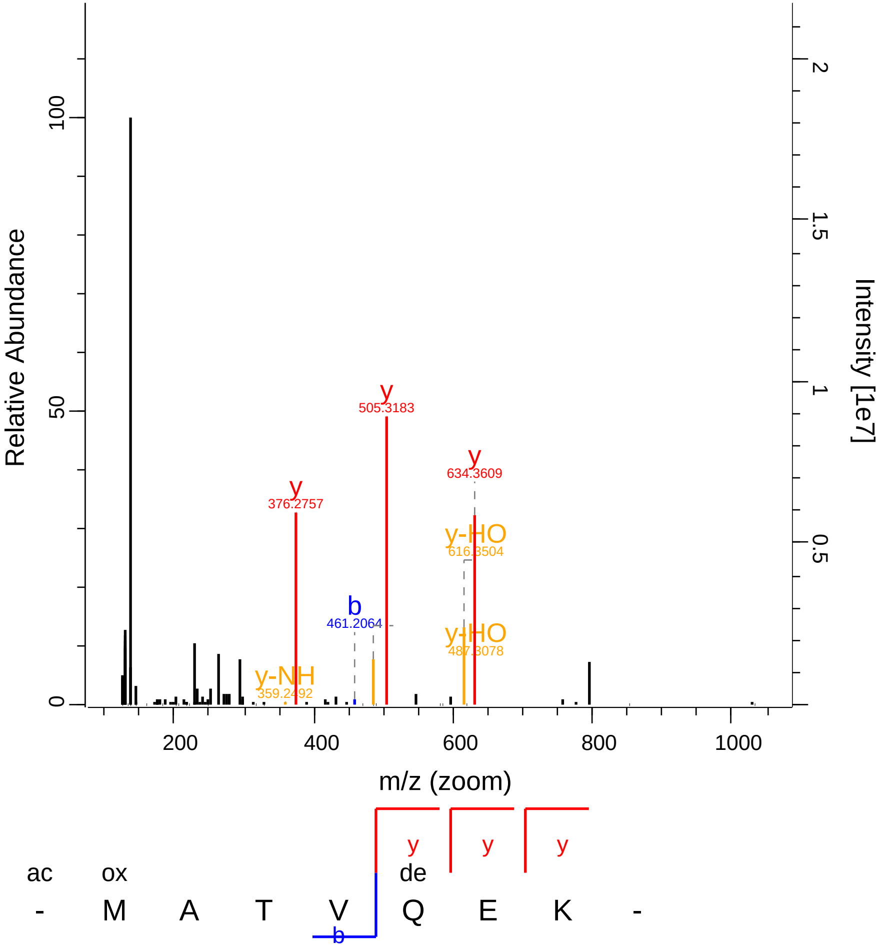

Raw File	Scan	Method	Score	m/z
RO-QC-160121_ZackD_SS_Set_02_Fraction_2	13394	FTMS; HCD	48.66	605.99

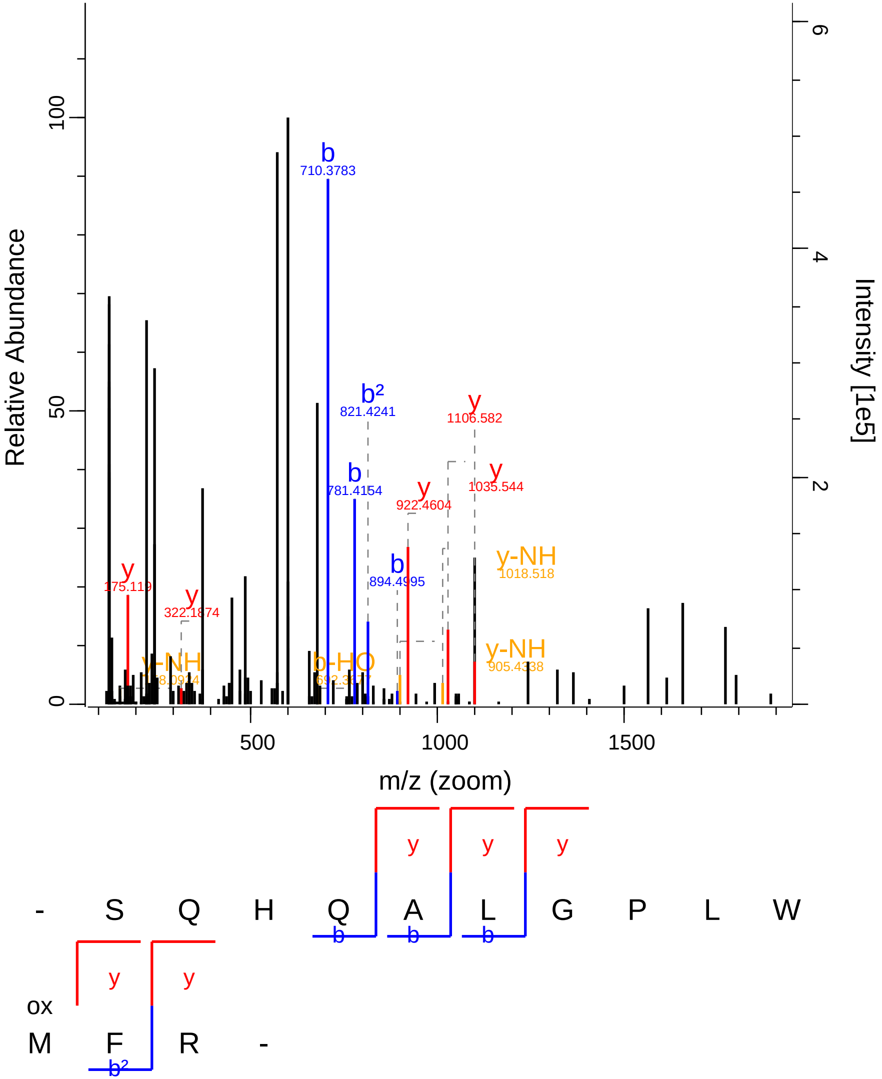

Raw File	Scan	Method	Score	m/z	Gene names
RO-QC-160121_ZackD_SS_Set_02_Fractio	14892	FTMS; HC	152.27	745.39	HS6ST1

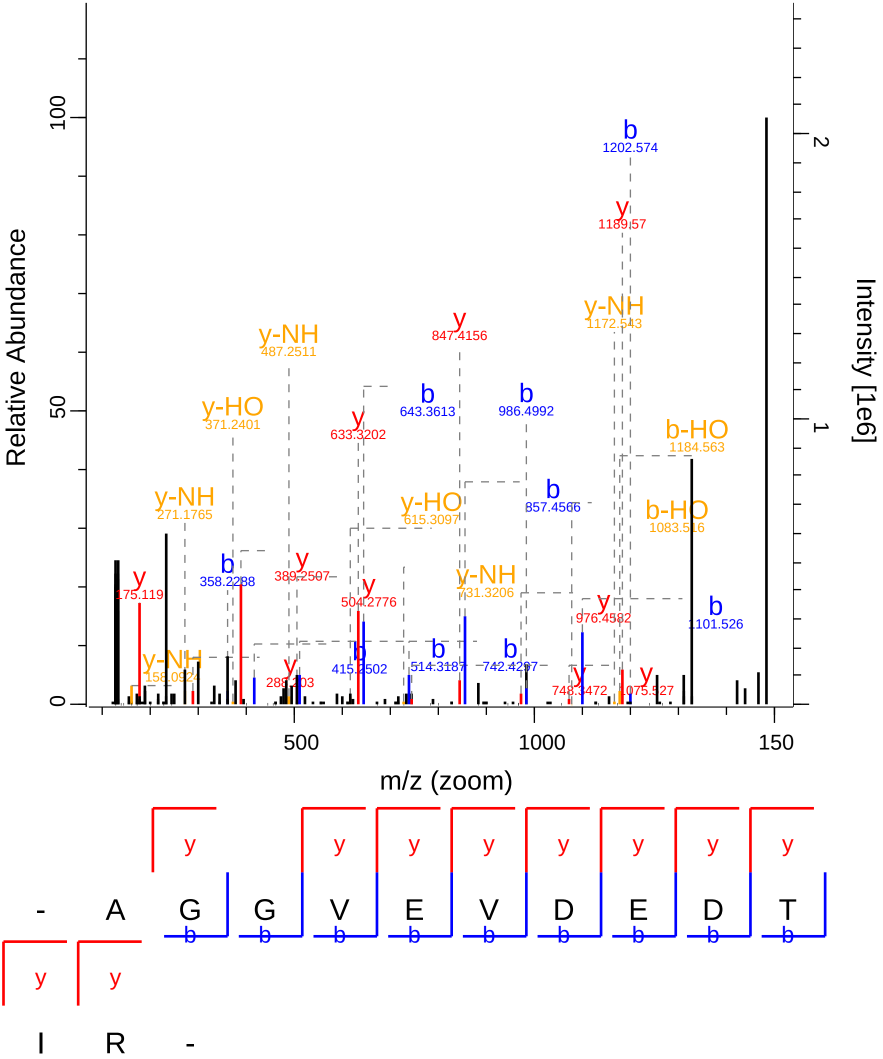

Raw File	Scan	Method	Score	m/z
RO-QC-160121_ZackD_SS_Set_02_Fraction_2	14926	FTMS; HCD	83.01	495.96

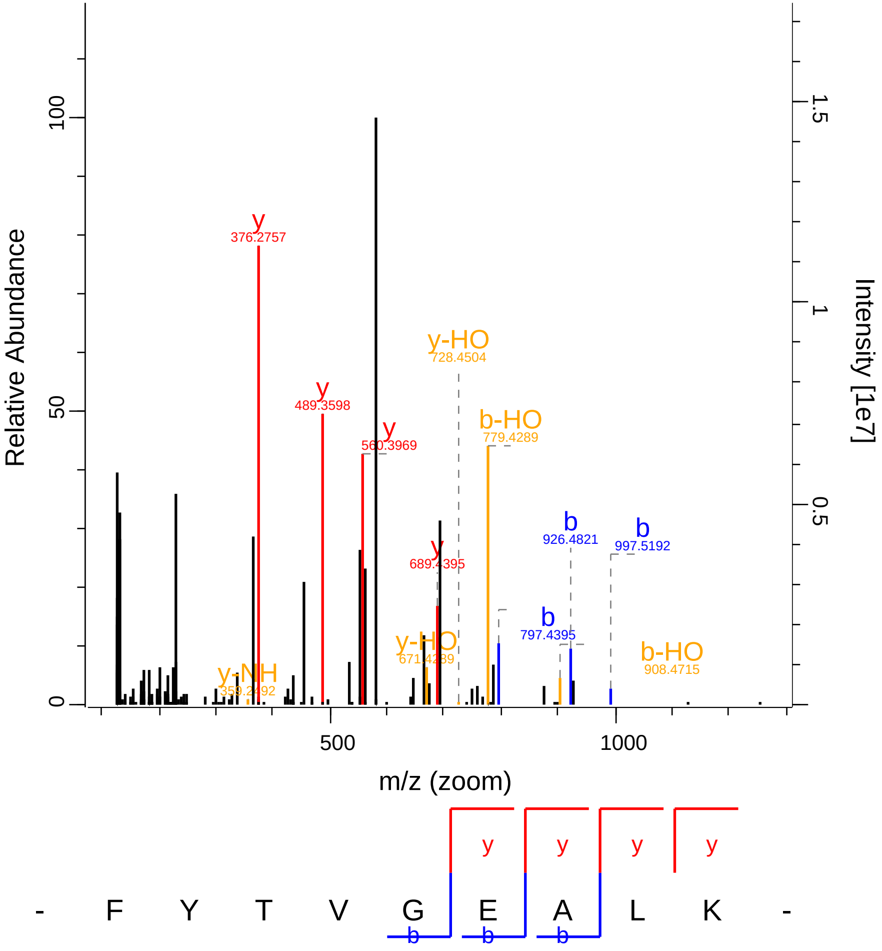

Raw File	Scan	Method	Score	m/z
RO-QC-160121_ZackD_SS_Set_02_Fraction_2	15142	FTMS; HCD	118.01	430.93

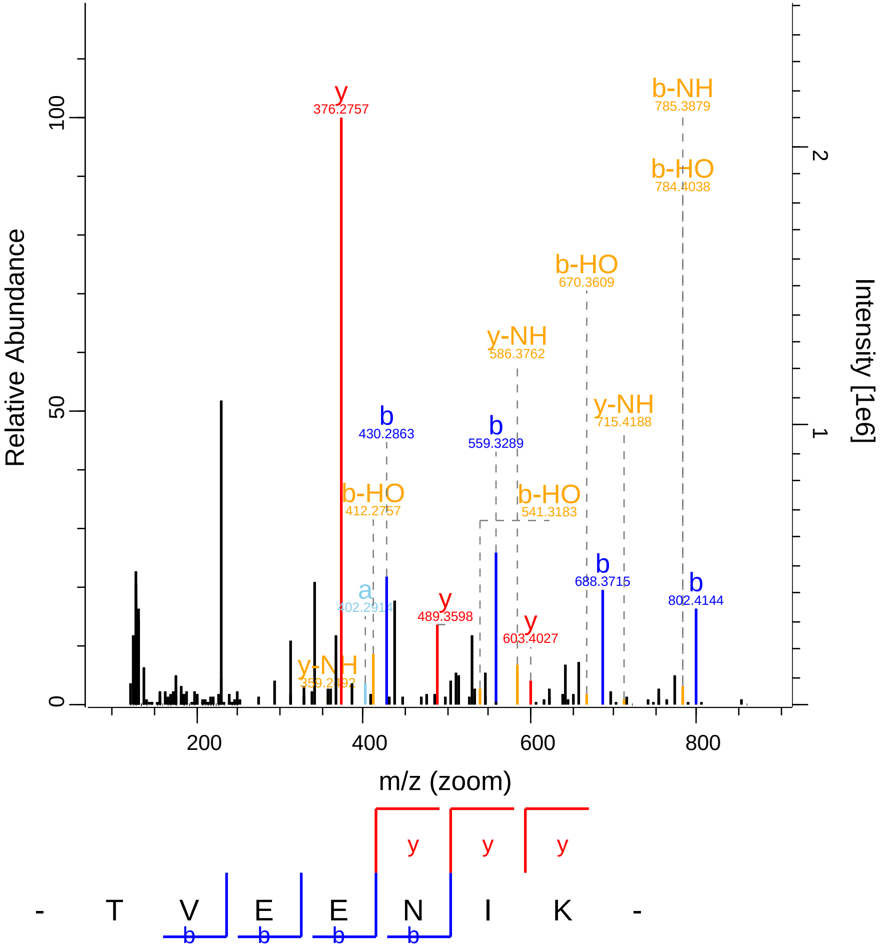

Raw File	Scan	Method	Score	m/z	Gene names
RO-QC-160121_ZackD_SS_Set_02_Fractio	15283	FTMS; HC	67.99	932.81	JAG1

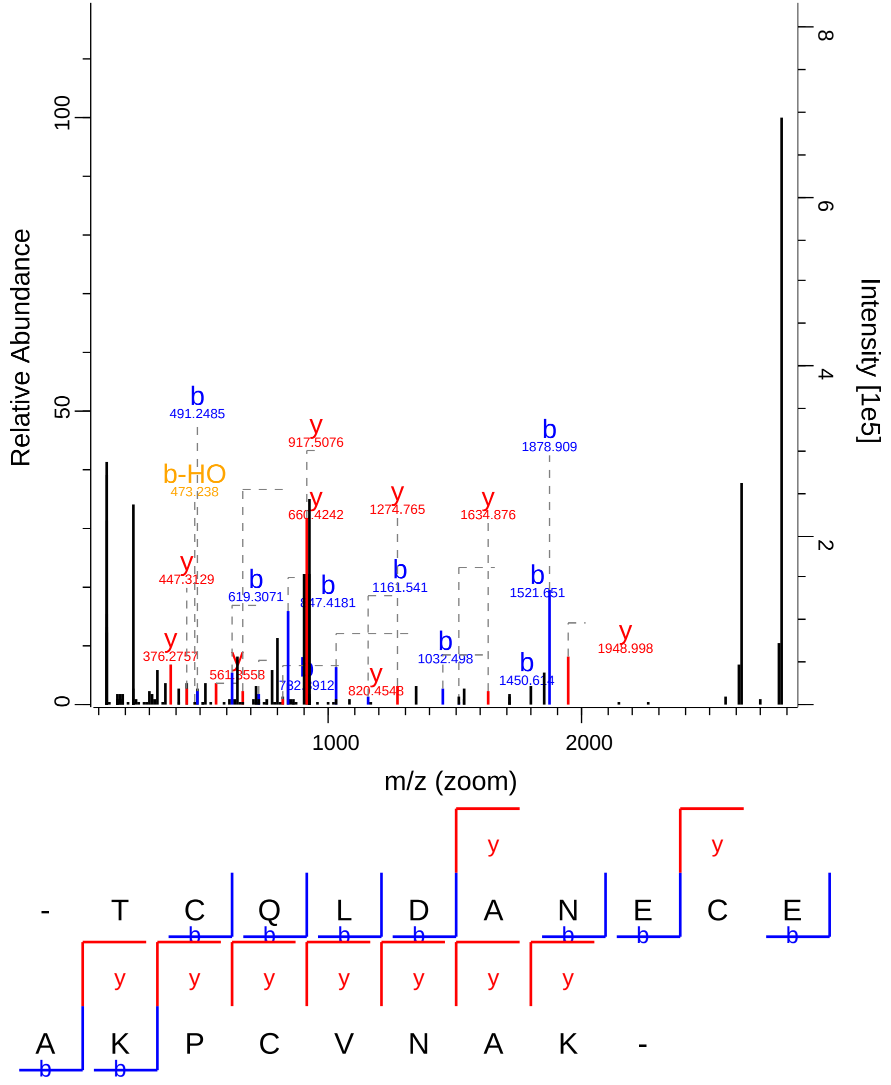

Raw File	Scan	Method	Score	m/z	Gene names
RO-QC-160121_ZackD_SS_Set_02_Fractio	15314	FTMS; HC	78.34	440.75	CCDC77

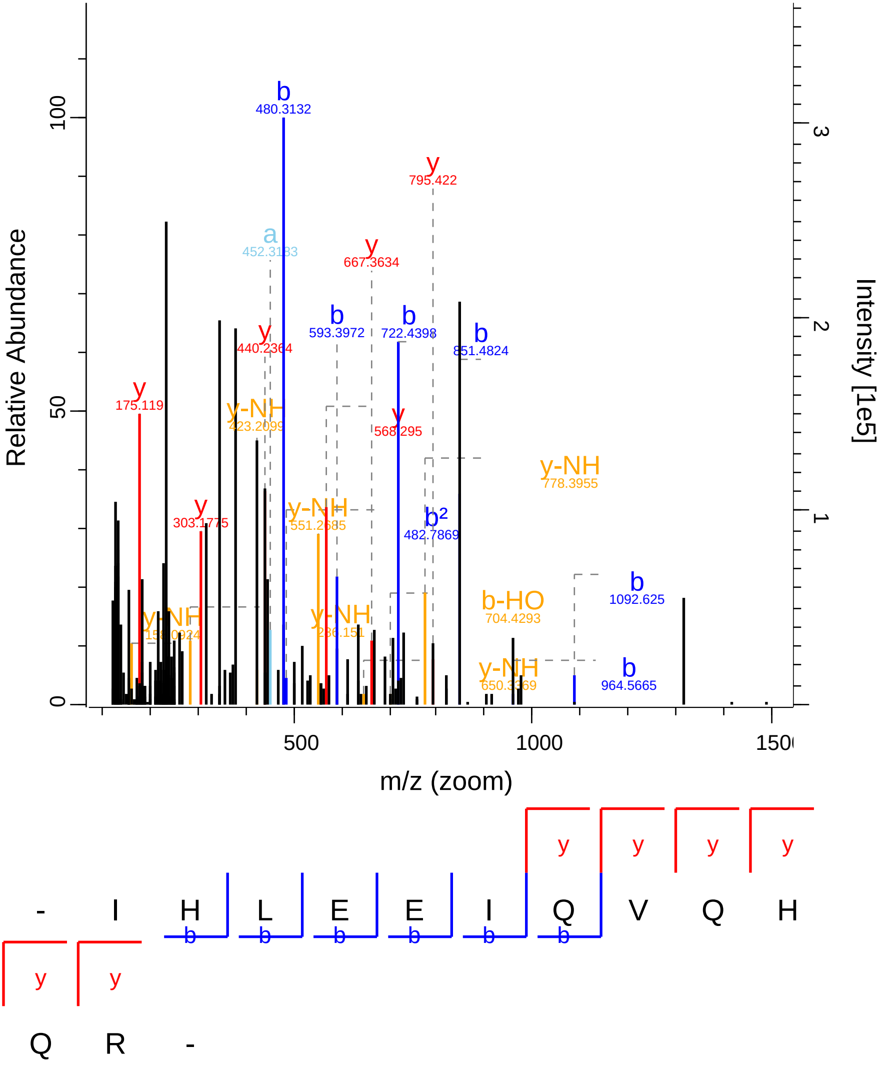

Raw File	Scan	Method	Score	m/z
RO-QC-160121_ZackD_SS_Set_02_Fraction_2	15333	FTMS; HCD	77.98	431.26

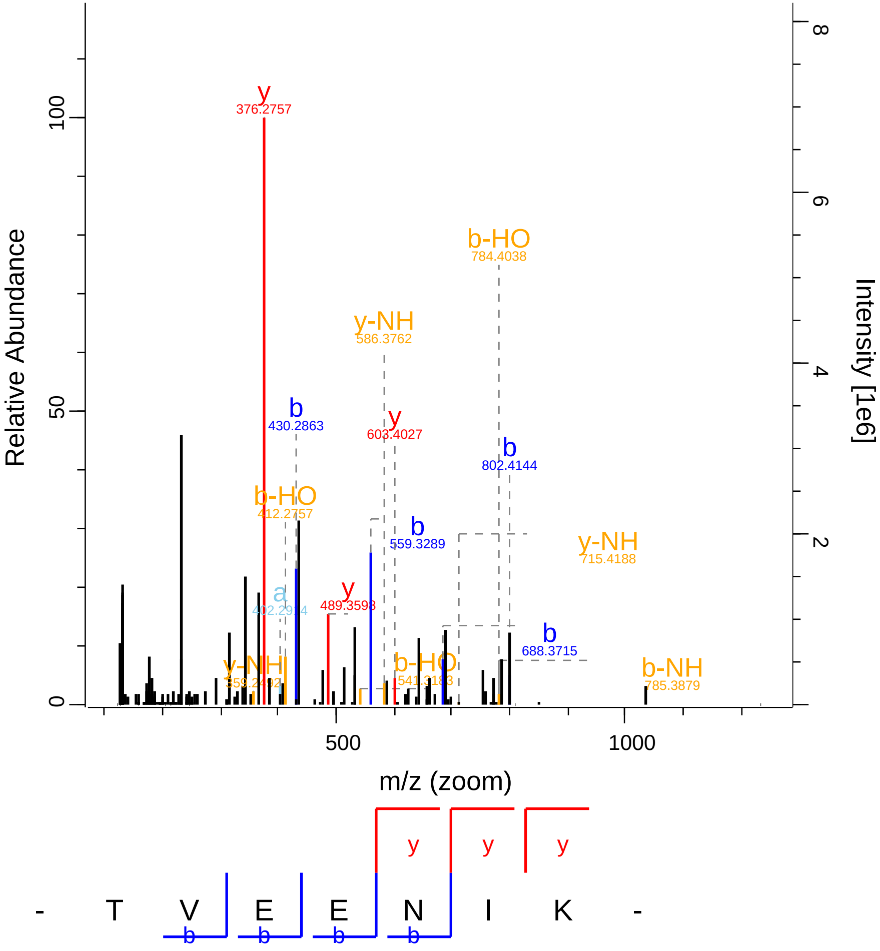

Raw File	Scan	Method	Score	m/z
RO-QC-160121_ZackD_SS_Set_02_Fraction_2	16099	FTMS; HCD	54.61	533.94

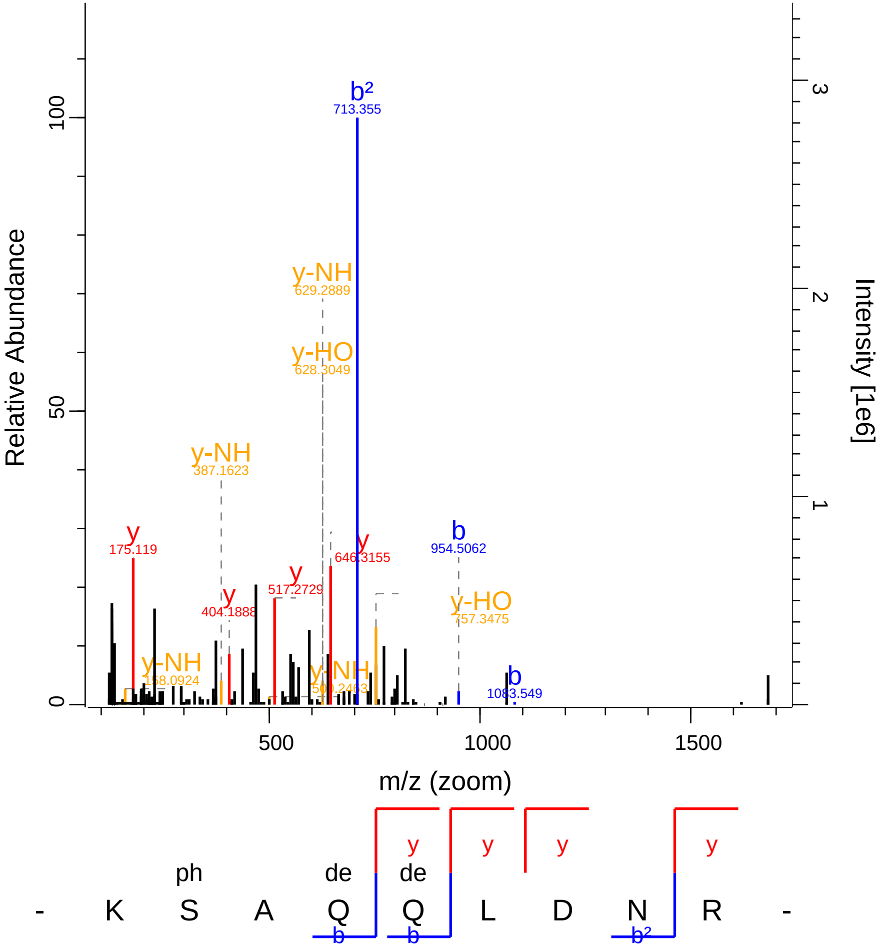

Raw File	Scan	Method	Score	m/z	Gene names
RO-QC-160121_ZackD_SS_Set_02_Fractio	16264	FTMS; HC	140.78	810.42	NR1H2

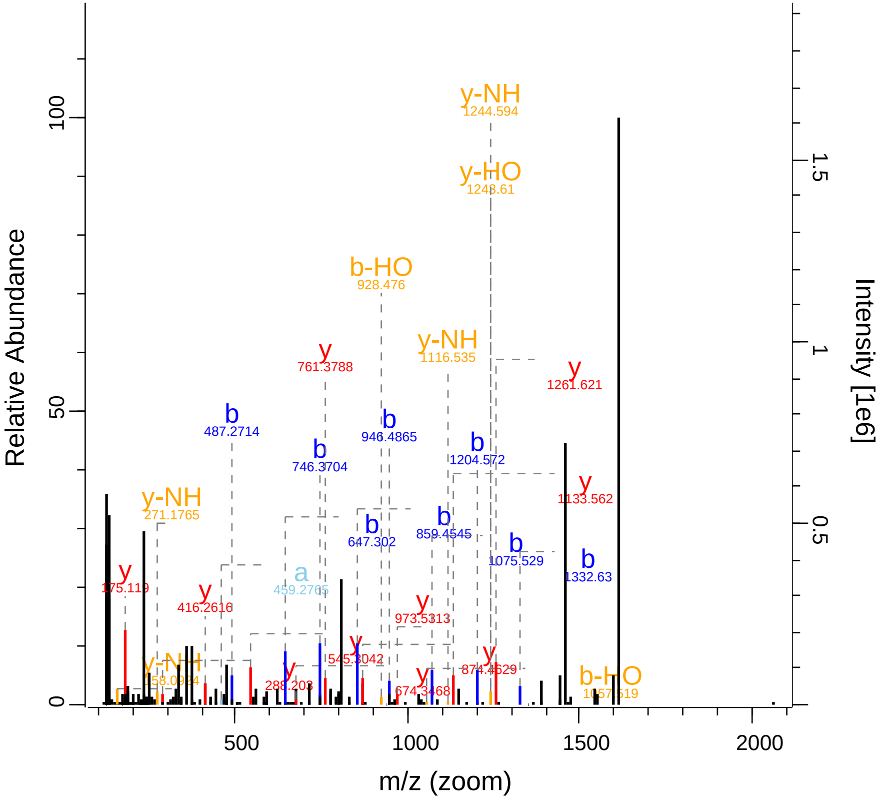

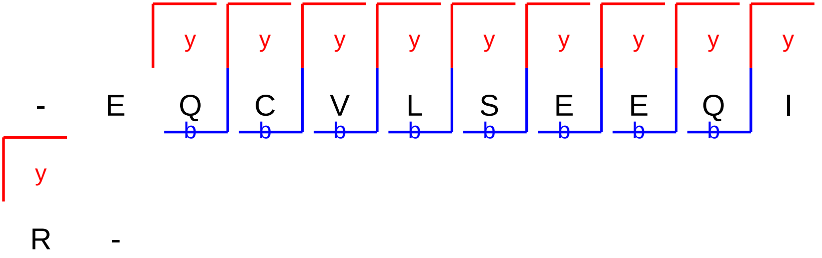

Raw File	Scan	Method	Score	m/z	Gene names
RO-QC-160121_ZackD_SS_Set_02_Fractio	16355	FTMS; HC	110.67	550.84	ZNF579

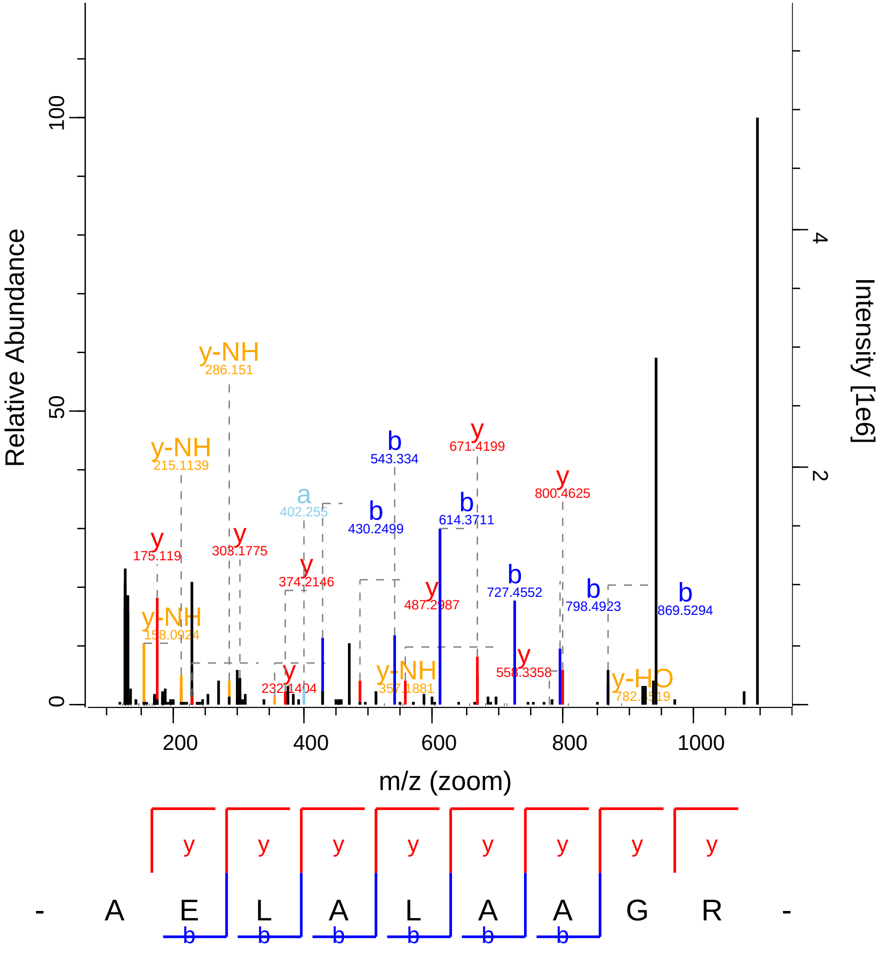

Raw File	Scan	Method	Score	m/z	Gene names
RO-QC-160121_ZackD_SS_Set_02_Fractio	16452	FTMS; HC	126.57	555.31	GINS2

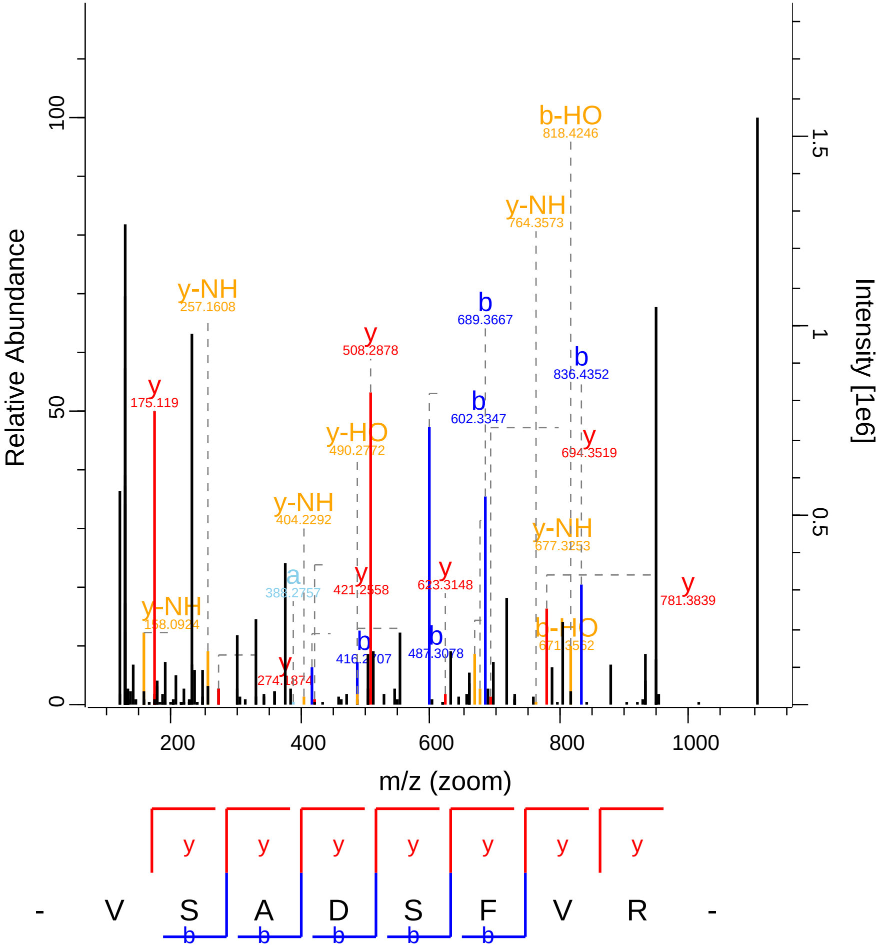

Raw File	Scan	Method	Score	m/z	Gene names
RO-QC-160121_ZackD_SS_Set_02_Fractio	16546	FTMS; HC	55.75	827.92	IQCB1

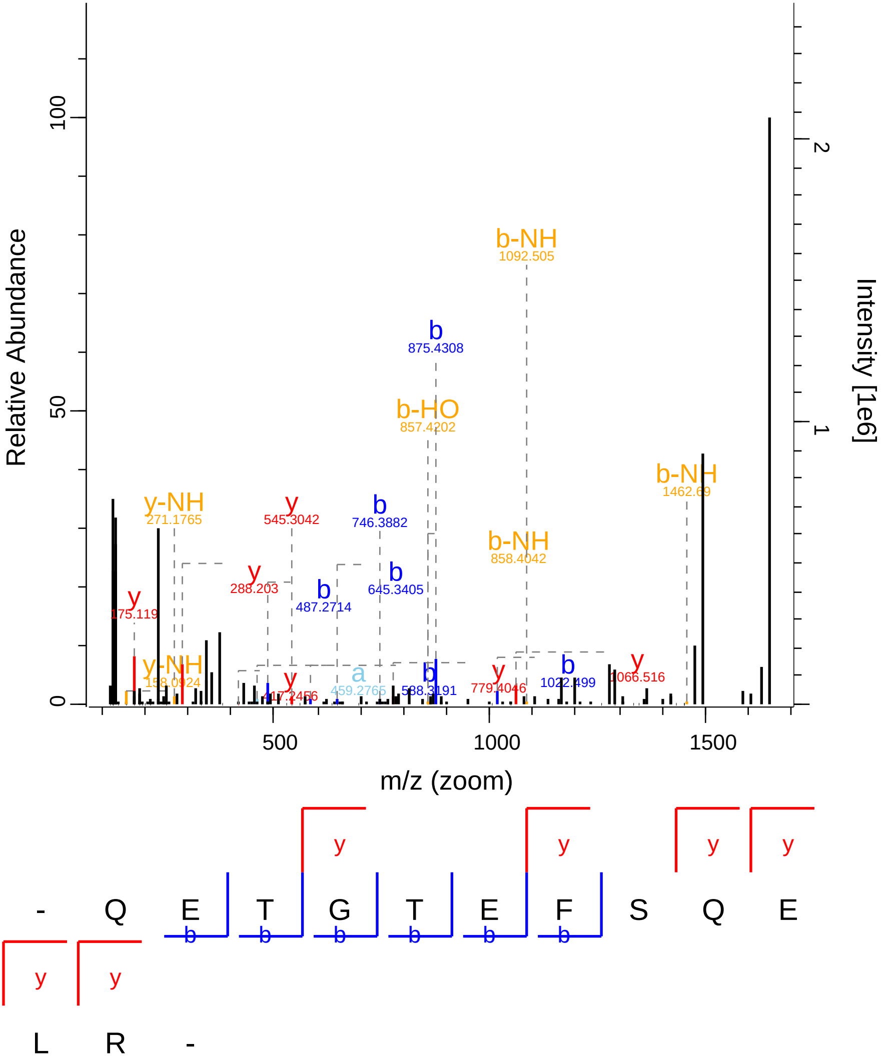

Raw File	Scan	Method	Score	m/z	Gene names
RO-QC-160121_ZackD_SS_Set_02_Fractio	16624	FTMS; HC	92.44	659.86	VWA5B1

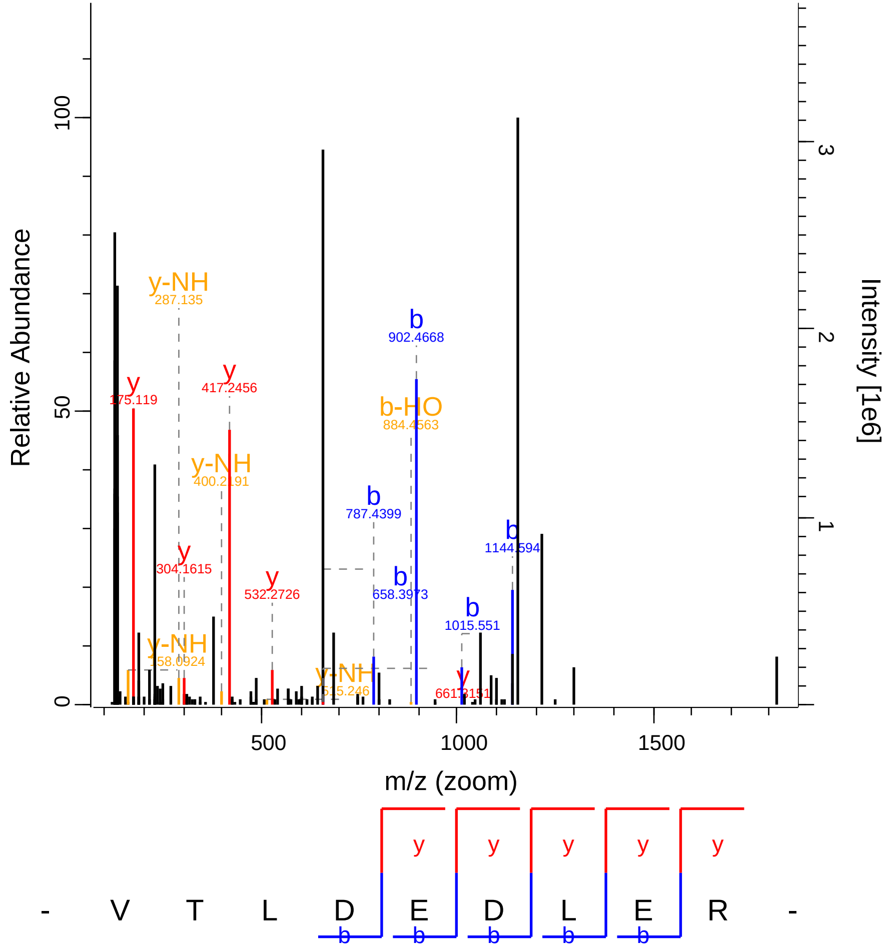

Raw File	Scan	Method	Score	m/z	Gene names
RO-QC-160121_ZackD_SS_Set_02_Fractio	17590	FTMS; HC	101.65	691.39	MTERF3

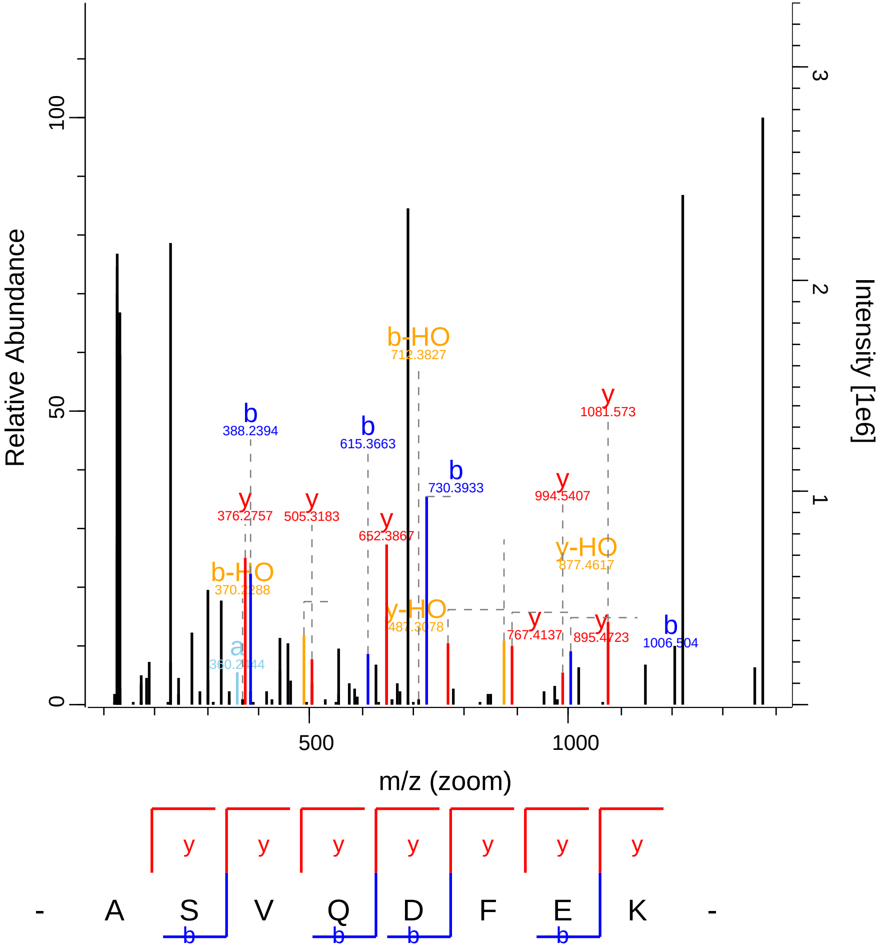

Raw File	Scan	Method	Score	m/z	Gene names
RO-QC-160121_ZackD_SS_Set_02_Fractio	17617	FTMS; HC	86.67	461.26	MTERF3

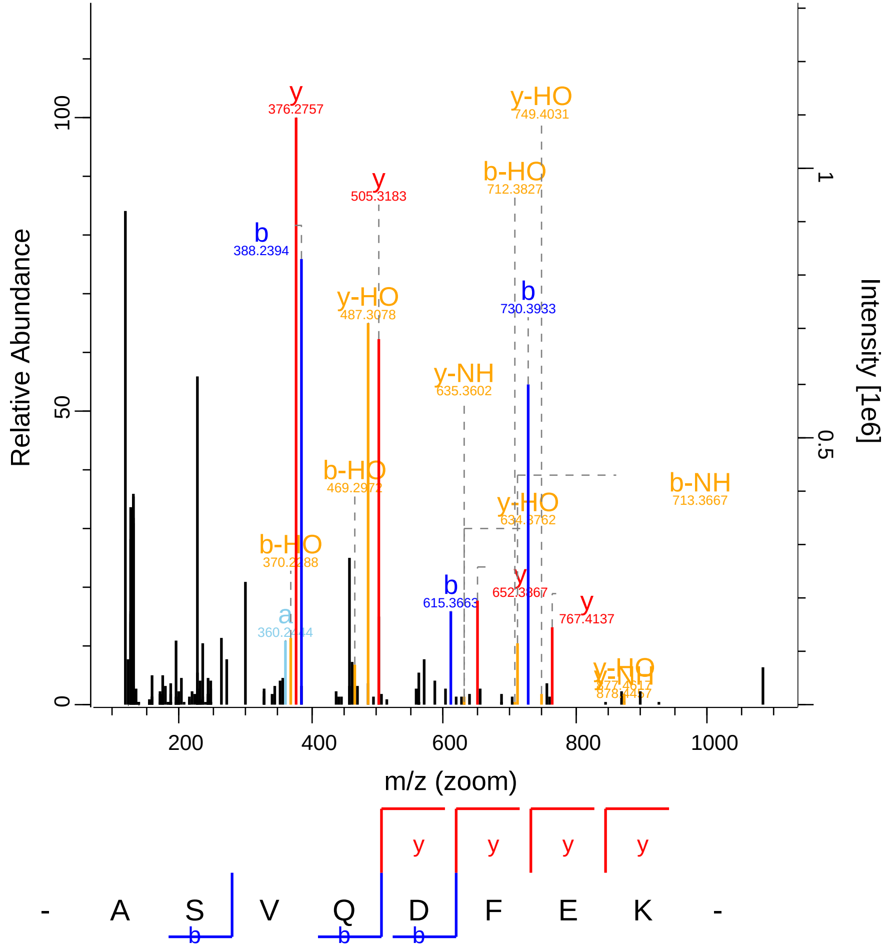

Raw File	Scan	Method	Score	m/z
RO-QC-160121_ZackD_SS_Set_02_Fraction_2	17714	FTMS; HCD	110.34	430.27

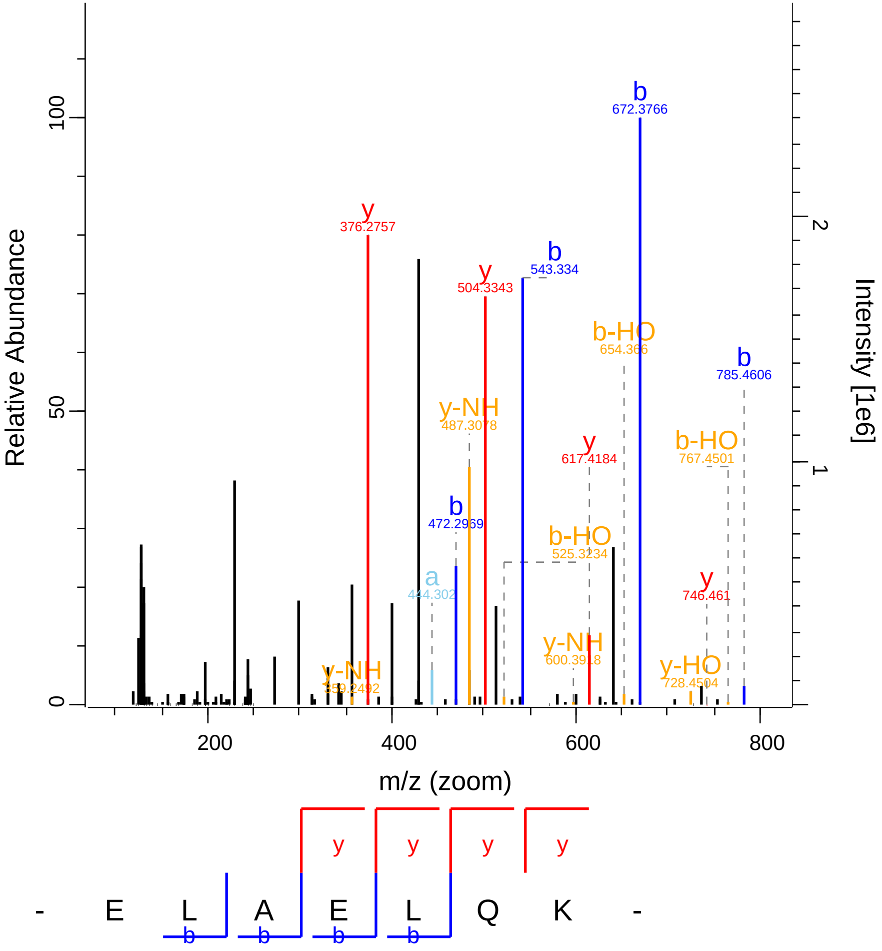

Raw File	Scan	Method	Score	m/z
RO-QC-160121_ZackD_SS_Set_02_Fraction_2	17973	FTMS; HCD	58.43	804.94

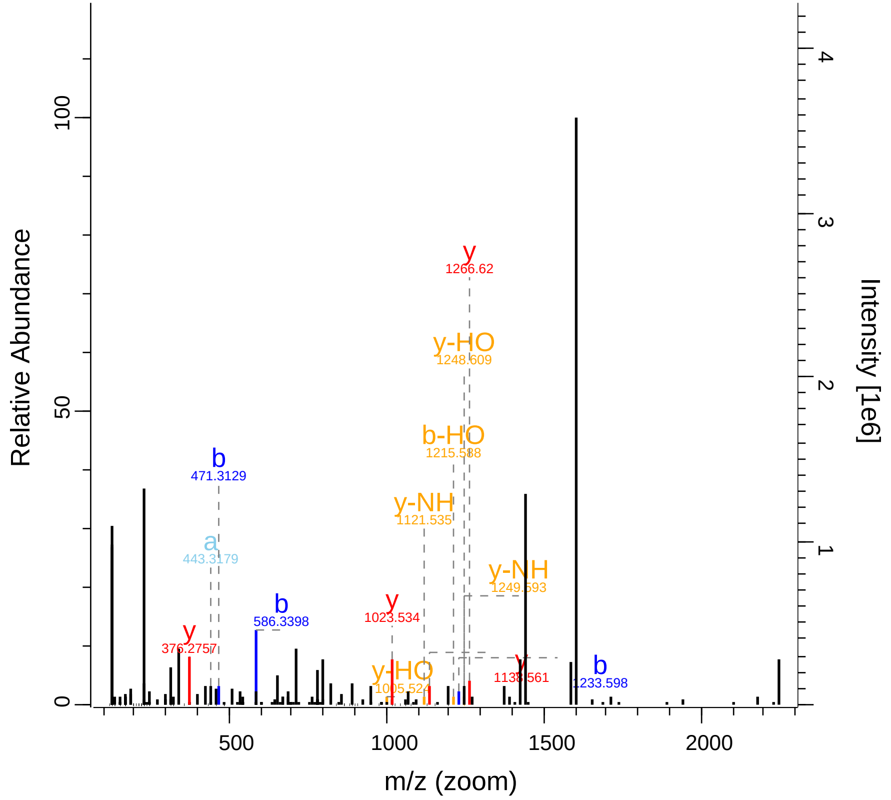

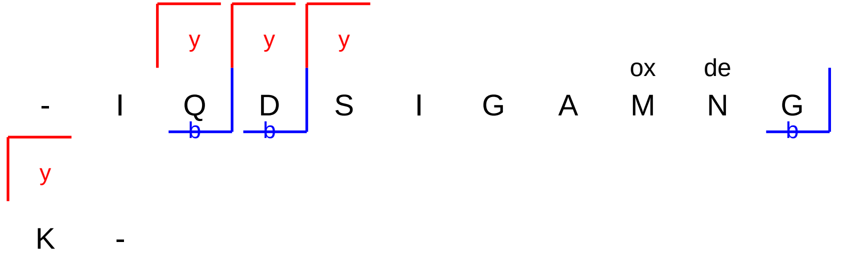

Raw File	Scan	Method	Score	m/z
RO-QC-160121_ZackD_SS_Set_02_Fraction_2	19165	FTMS; HCD	77.68	880.49

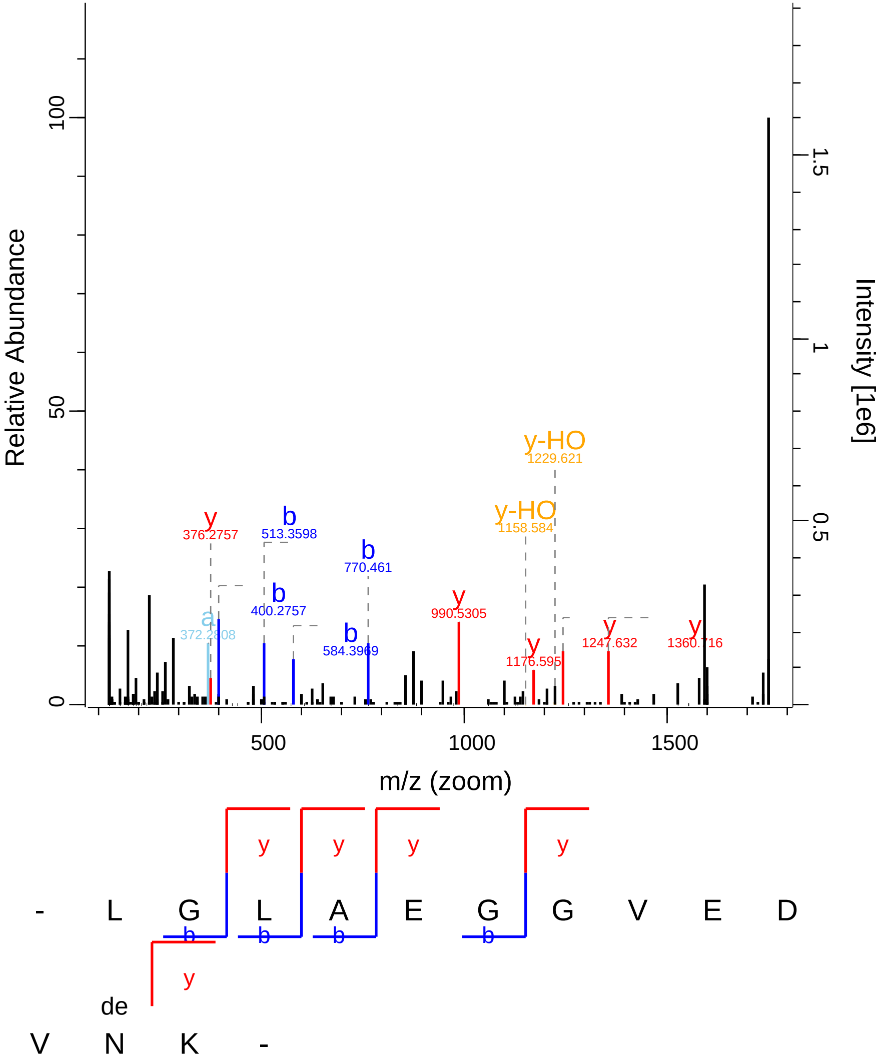

Raw File	Scan	Method	Score	m/z	Gene names
RO-QC-160121_ZackD_SS_Set_02_Fractio	19215	FTMS; HC	40.55	616.07	GRM4

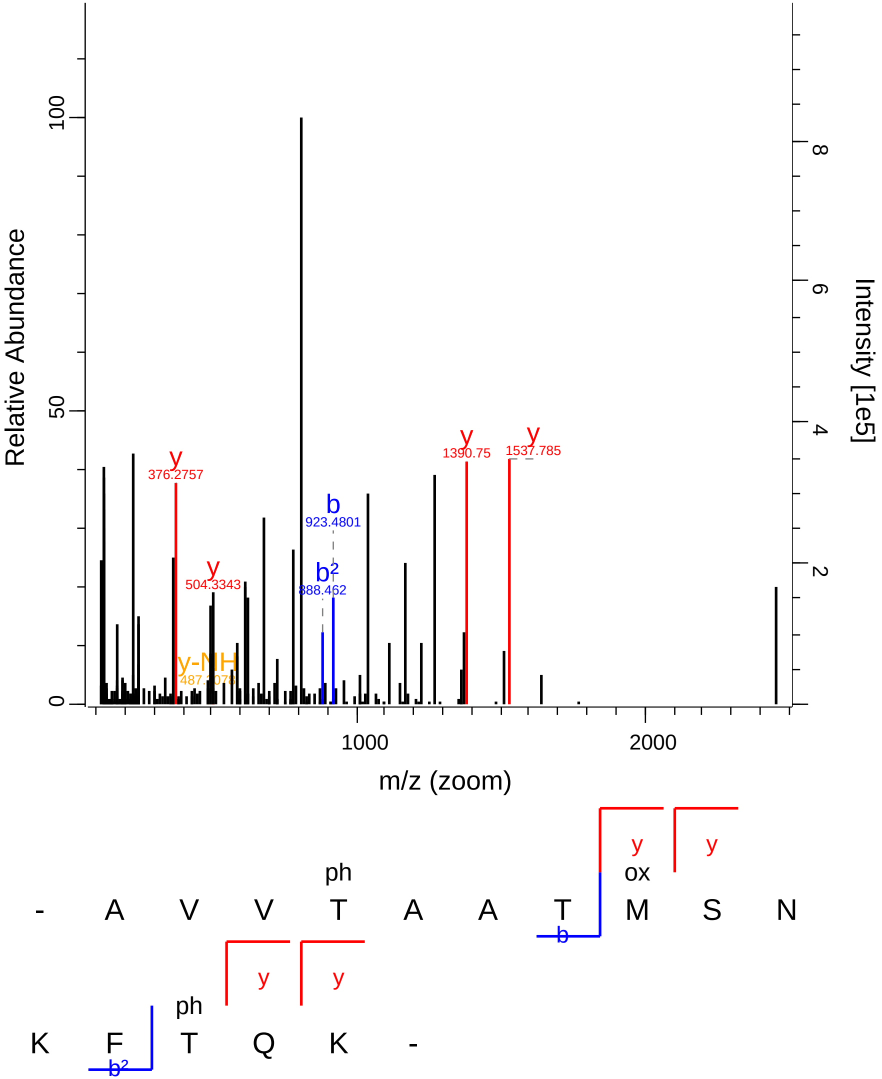

Raw File	Scan	Method	Score	m/z	Gene names
RO-QC-160121_ZackD_SS_Set_02_Fractio	19475	FTMS; HC	89.11	818.11	FIGNL1

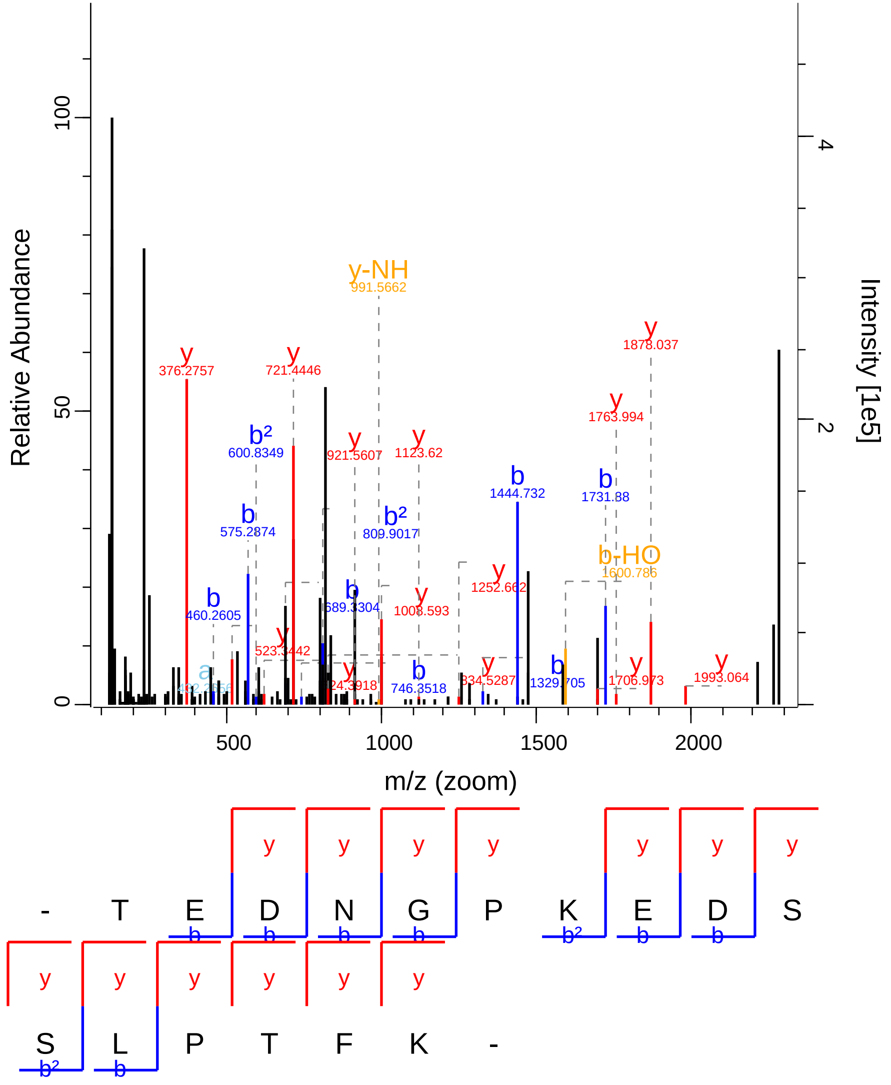

Raw File	Scan	Method	Score	m/z	Gene names
RO-QC-160121_ZackD_SS_Set_02_Fraction	19504	FTMS; HCI	73.93	546	ABHD6

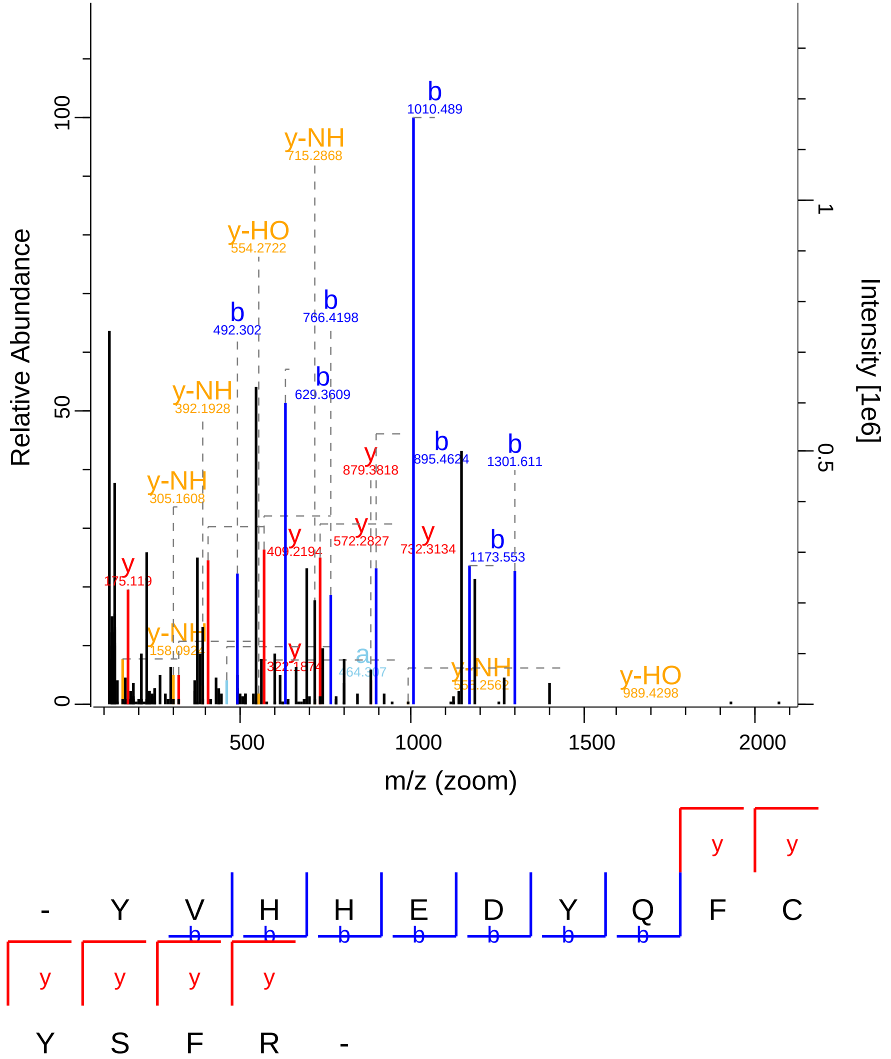

Raw File	Scan	Method	Score	m/z
RO-QC-160121_ZackD_SS_Set_02_Fraction_2	19613	FTMS; HCD	78.66	895.01

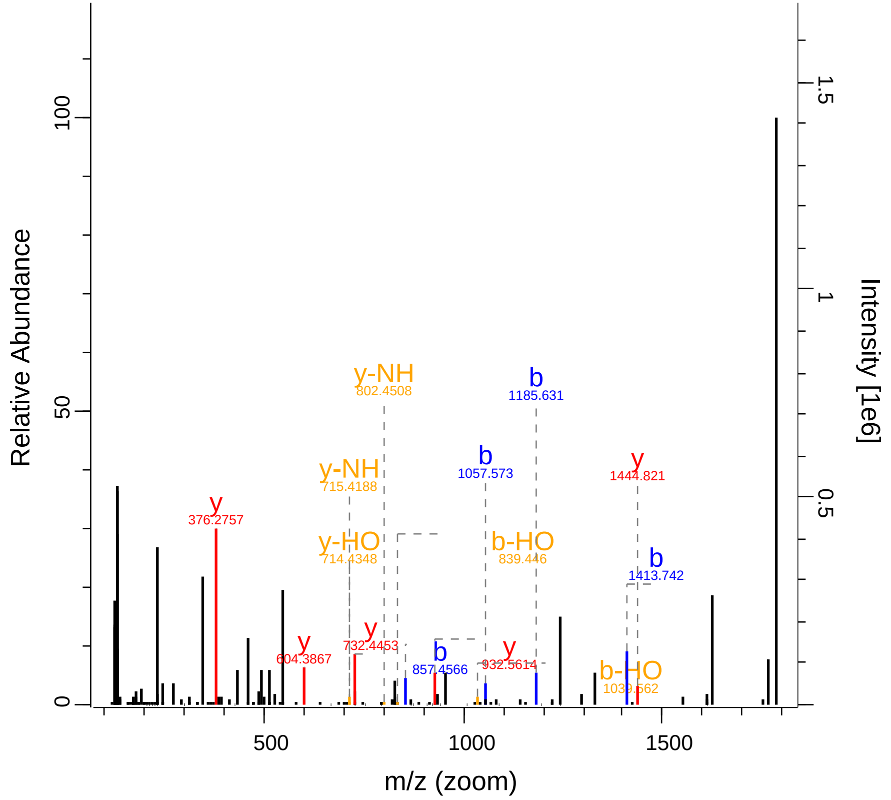

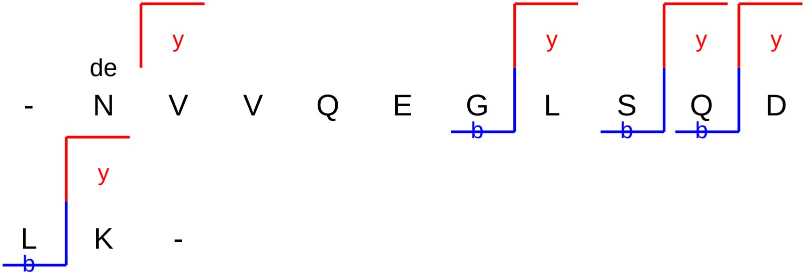

Raw File	Scan	Method	Score	m/z	Gene names
RO-QC-160121_ZackD_SS_Set_02_Fractio	19795	FTMS; HC	99.82	742.42	DGKE

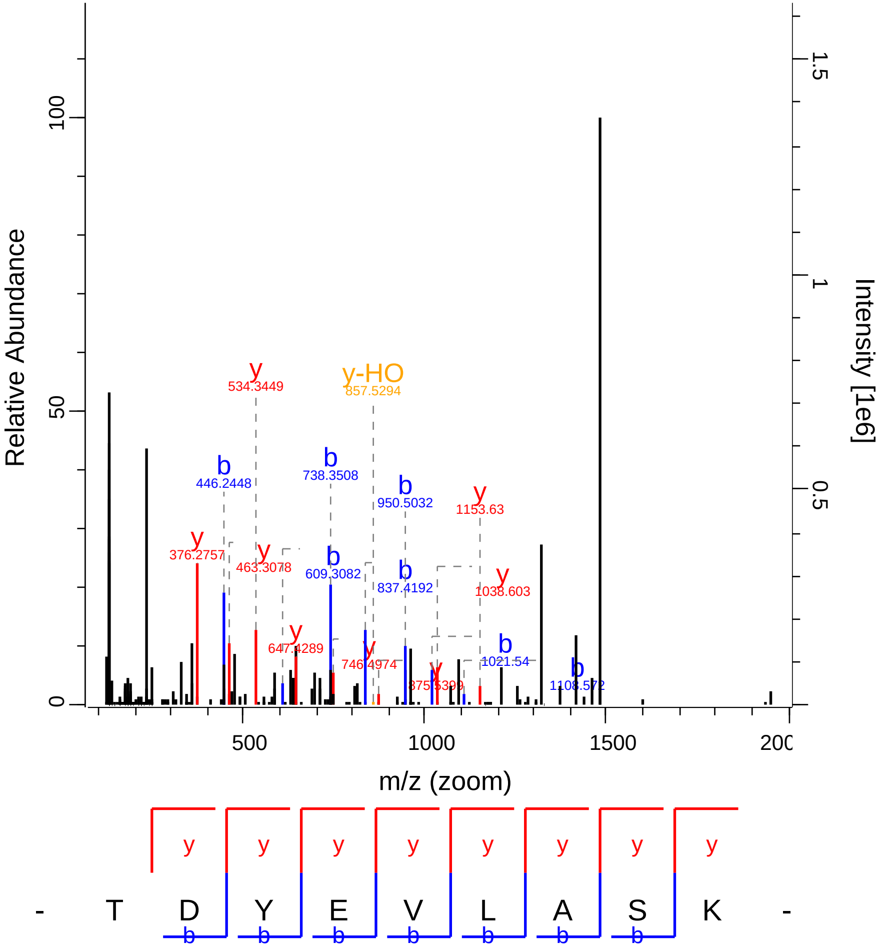

Raw File	Scan	Method	Score	m/z	Gene names
RO-QC-160121_ZackD_SS_Set_02_Fractio	19931	FTMS; HC	43.28	495.28	DGKE

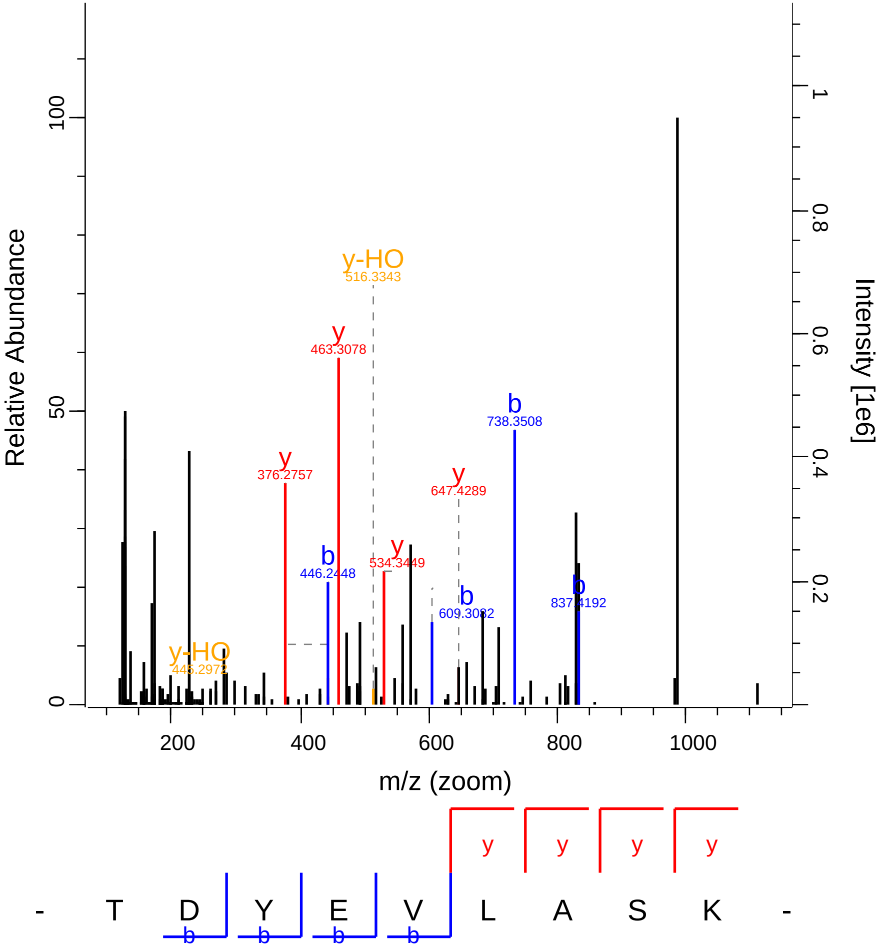

Raw File	Scan	Method	Score	m/z
RO-QC-160121_ZackD_SS_Set_02_Fraction_2	20098	FTMS; HCD	50.97	783.9

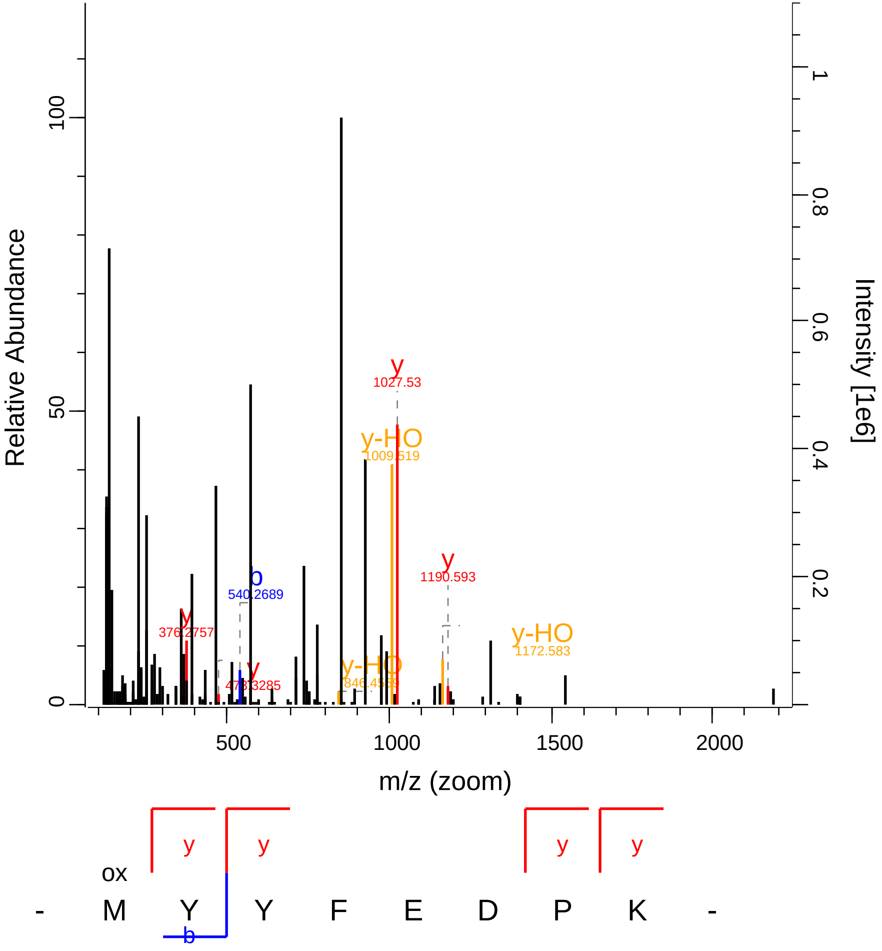

Raw File	Scan	Method	Score	m/z	Gene names
RO-QC-160121_ZackD_SS_Set_02_Fractio	20115	FTMS; HC	40.99	488.94	PROX1

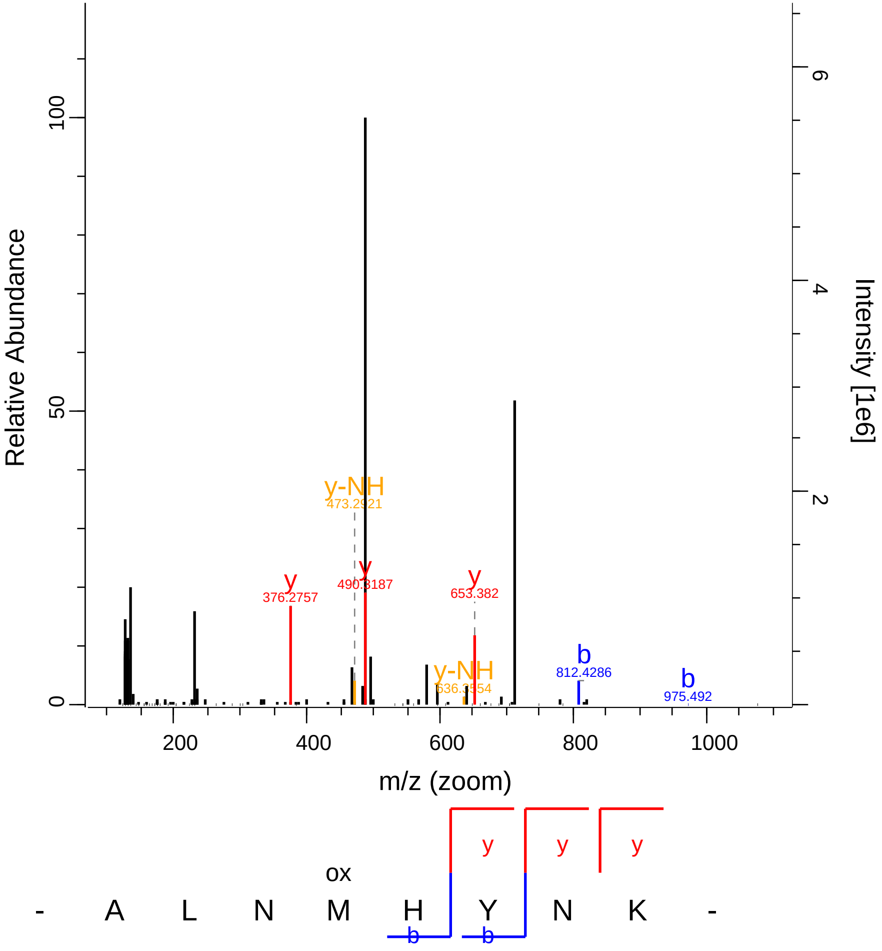

Raw File	Scan	Method	Score	m/z	Gene names
RO-QC-160121_ZackD_SS_Set_02_Fractio	20140	FTMS; HC	47.07	732.91	PROX1

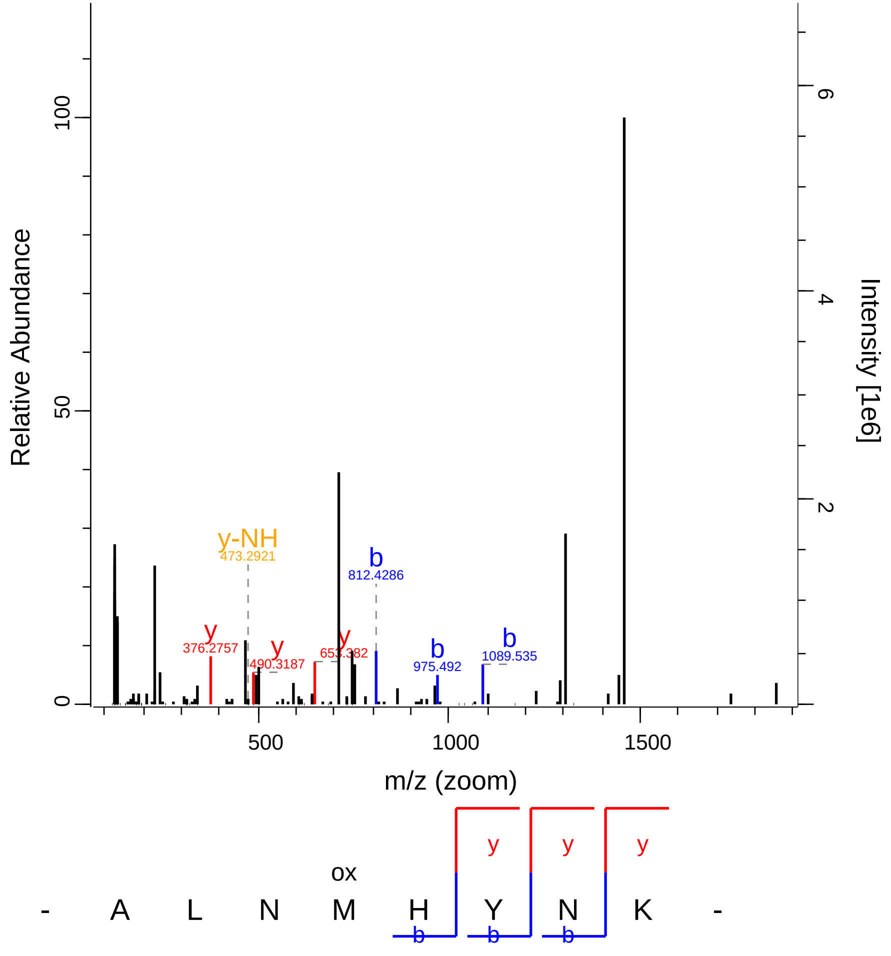

Raw File	Scan	Method	Score	m/z	Gene names
RO-QC-160121_ZackD_SS_Set_02_Fractio	20250	FTMS; HC	126.71	731.86	GSX2

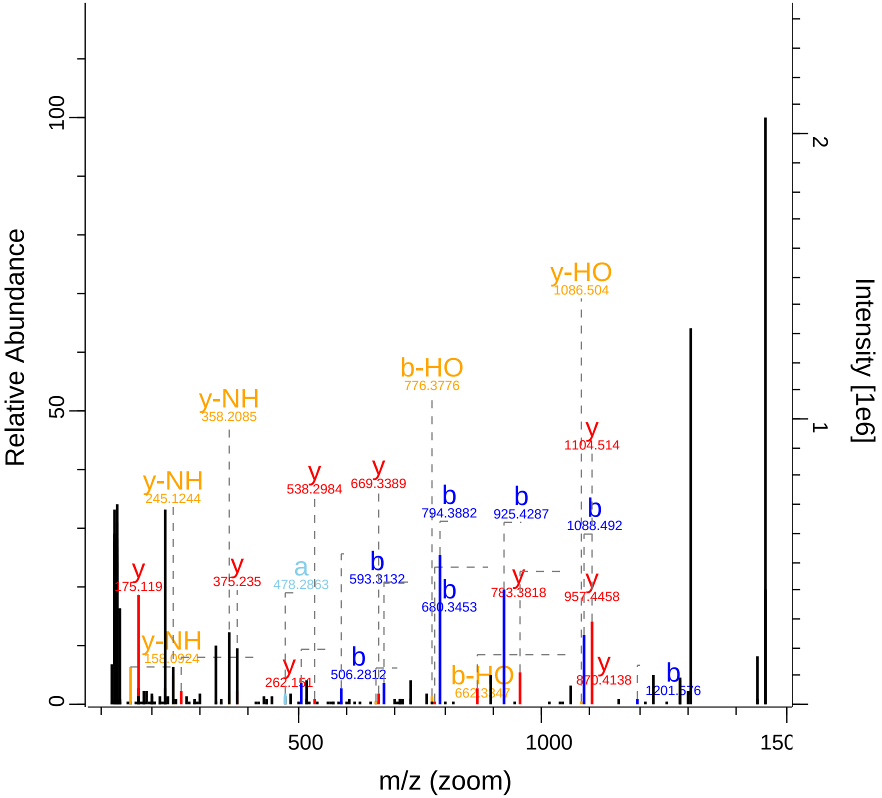

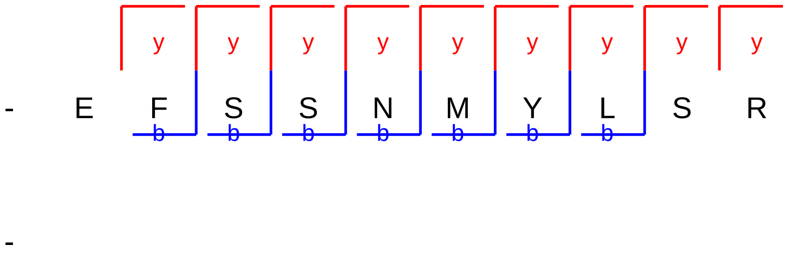

Raw File	Scan	Method	Score	m/z	Gene names
RO-QC-160121_ZackD_SS_Set_02_Fractic	20369	FTMS; HC	113.24	1086.01	HIGD1A

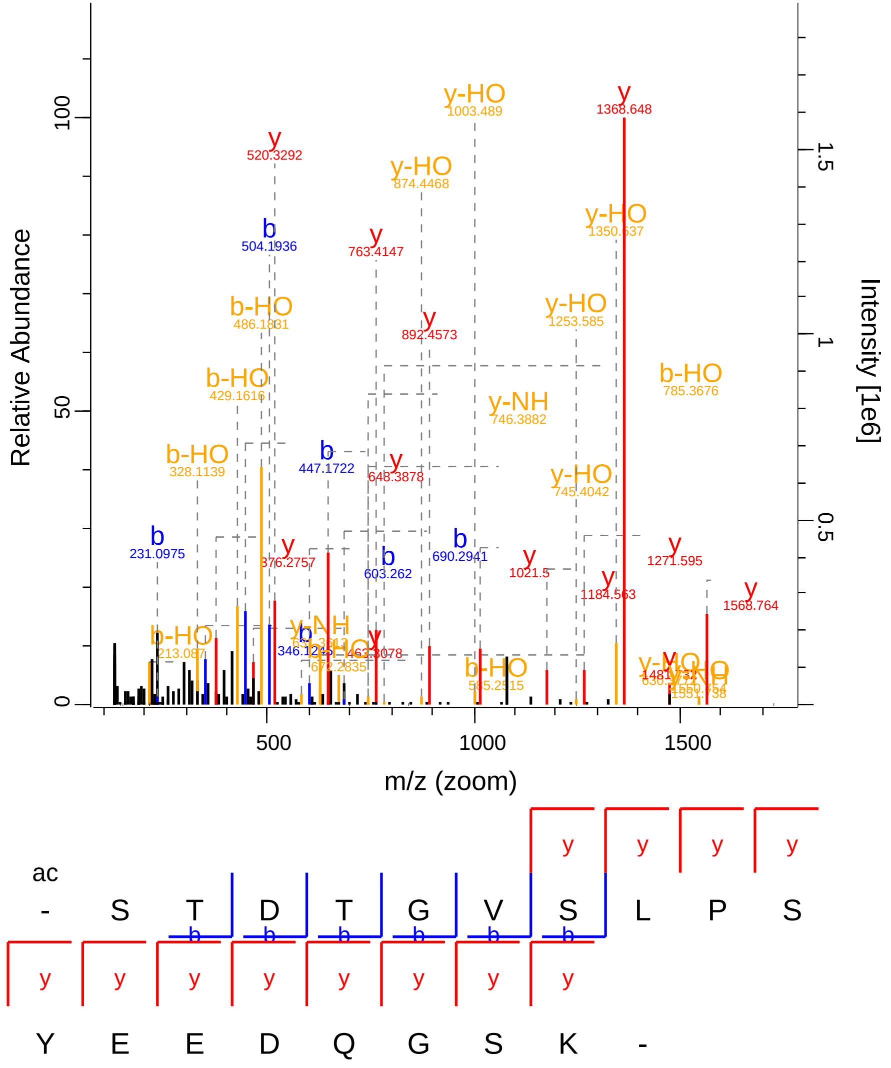

Raw File	Scan	Method	Score	m/z	Gene names
RO-QC-160121_ZackD_SS_Set_02_Fractio	20422	FTMS; HC	93.75	724.35	HIGD1A

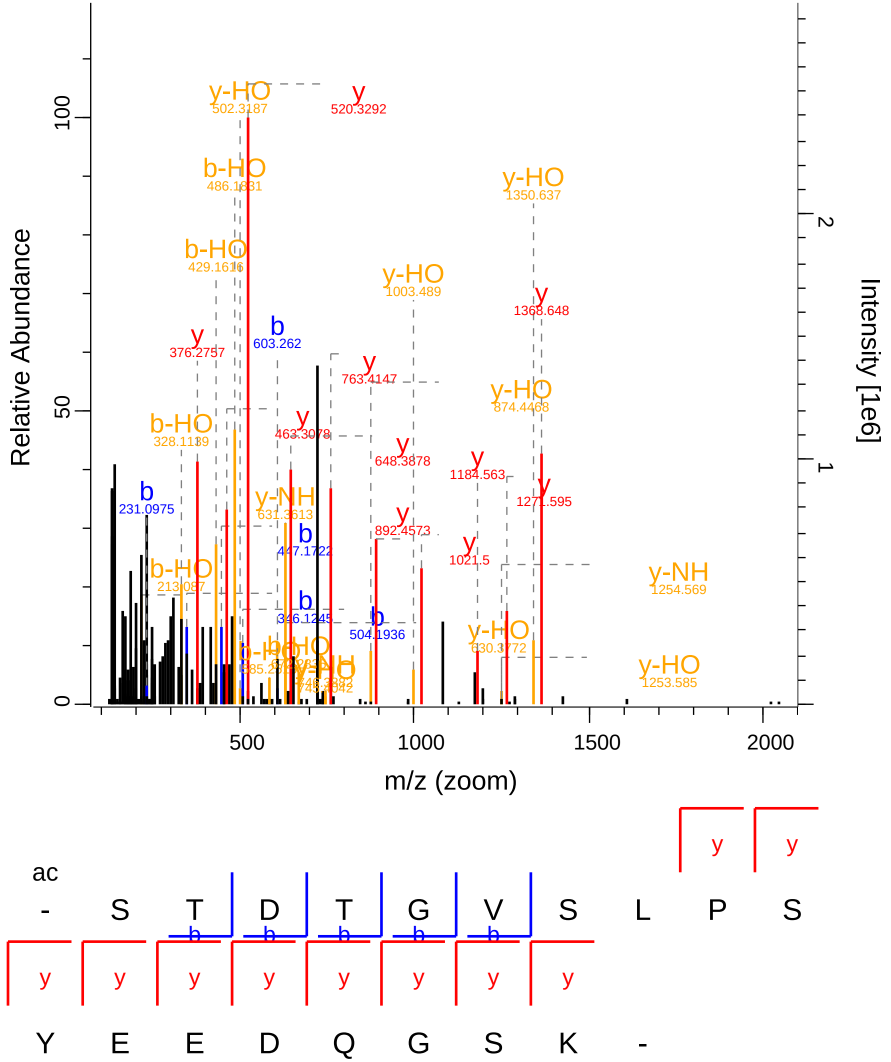

Raw File	Scan	Method	Score	m/z	Gene names
RO-QC-160121_ZackD_SS_Set_02_Fractic	20570	FTMS; HC	146.07	1086.52	HIGD1A

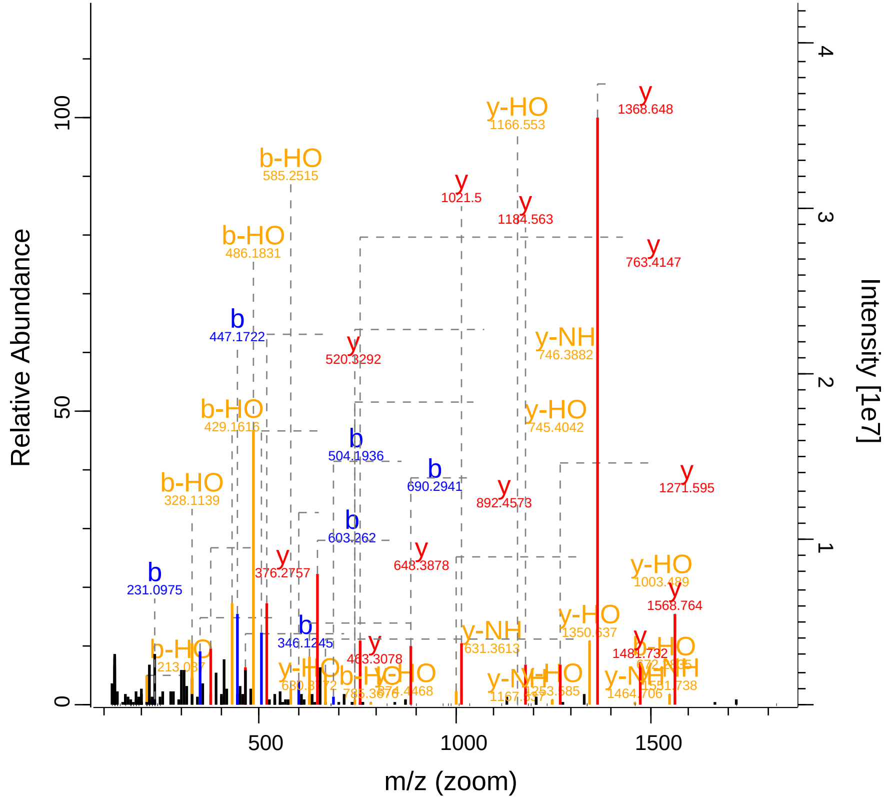

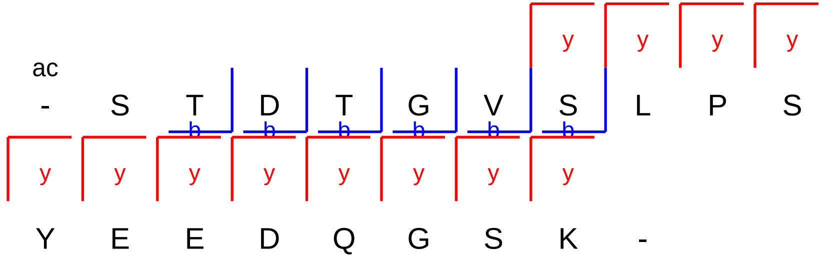

Raw File	Scan	Method	Score	m/z	Gene names
RO-QC-160121_ZackD_SS_Set_02_Fractio	20609	FTMS; HC	99.45	724.35	HIGD1A

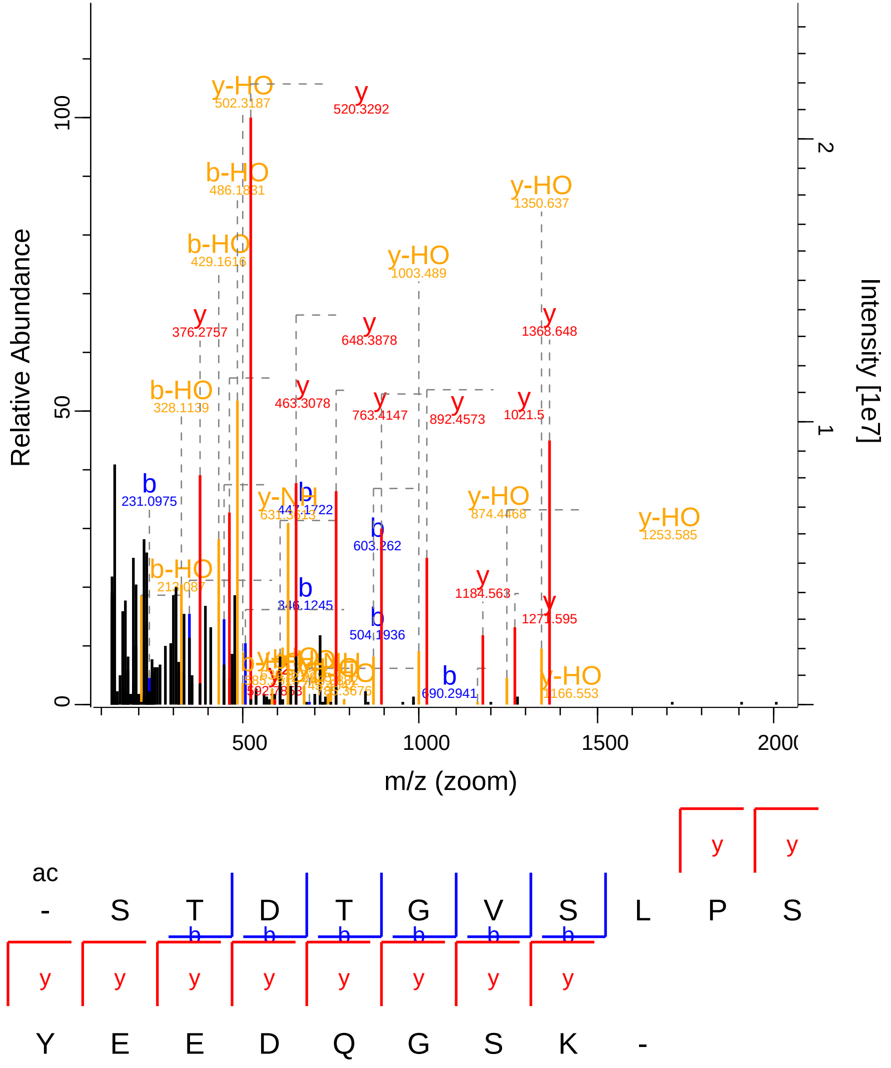

Raw File	Scan	Method	Score	m/z	Gene names
RO-QC-160121_ZackD_SS_Set_02_Fractio	20653	FTMS; HC	117.16	551.33	YBEY

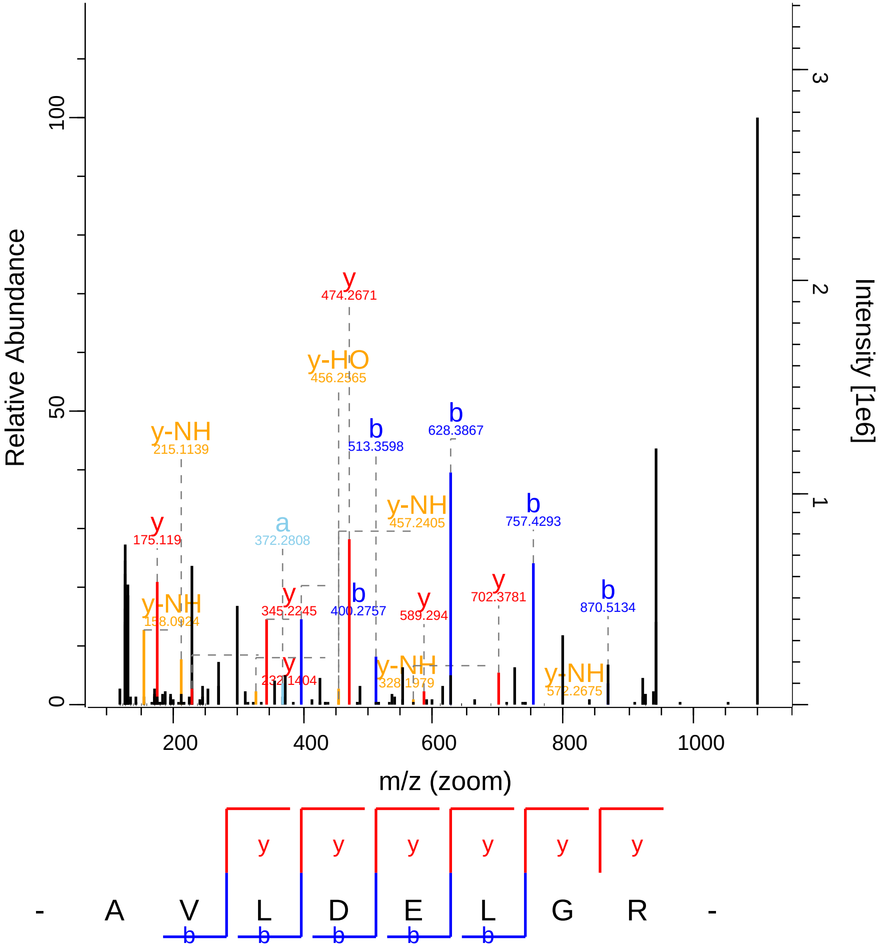

Raw File	Scan	Method	Score	m/z	Gene names
RO-QC-160121_ZackD_SS_Set_02_Fractio	20841	FTMS; HC	83.31	551.33	YBEY

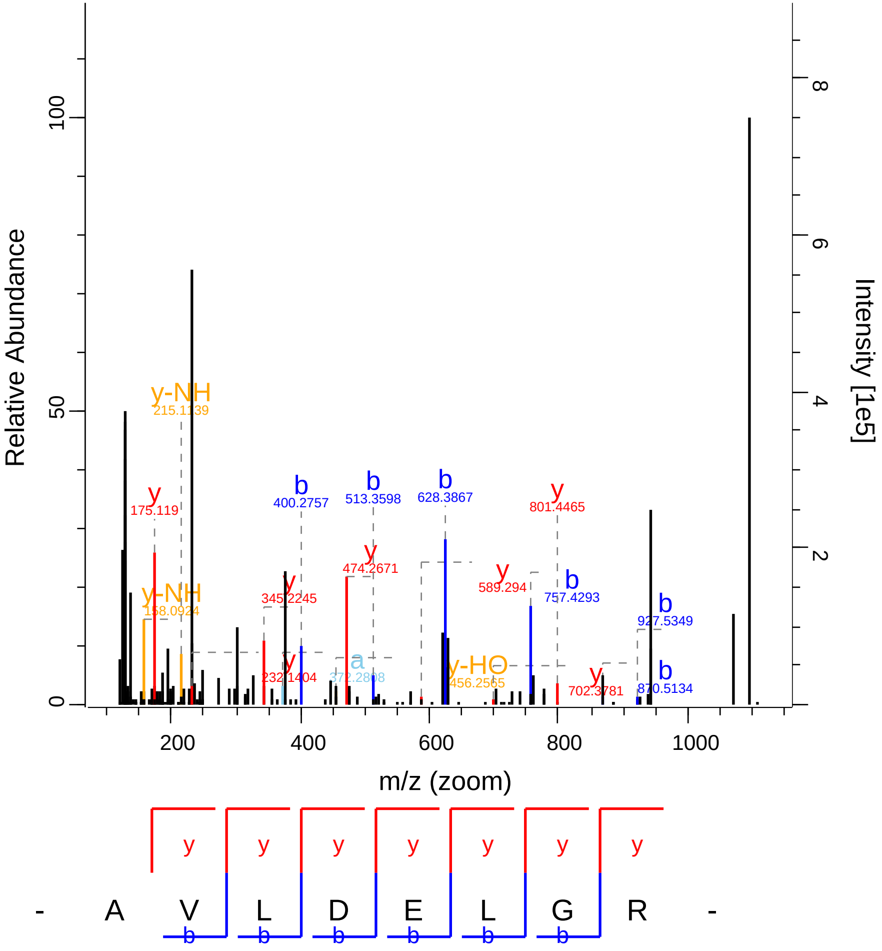

Raw File	Scan	Method	Score	m/z	Gene names
RO-QC-160121_ZackD_SS_Set_02_Fractic	21116	FTMS; HC	44.26	1086.19	SLX4

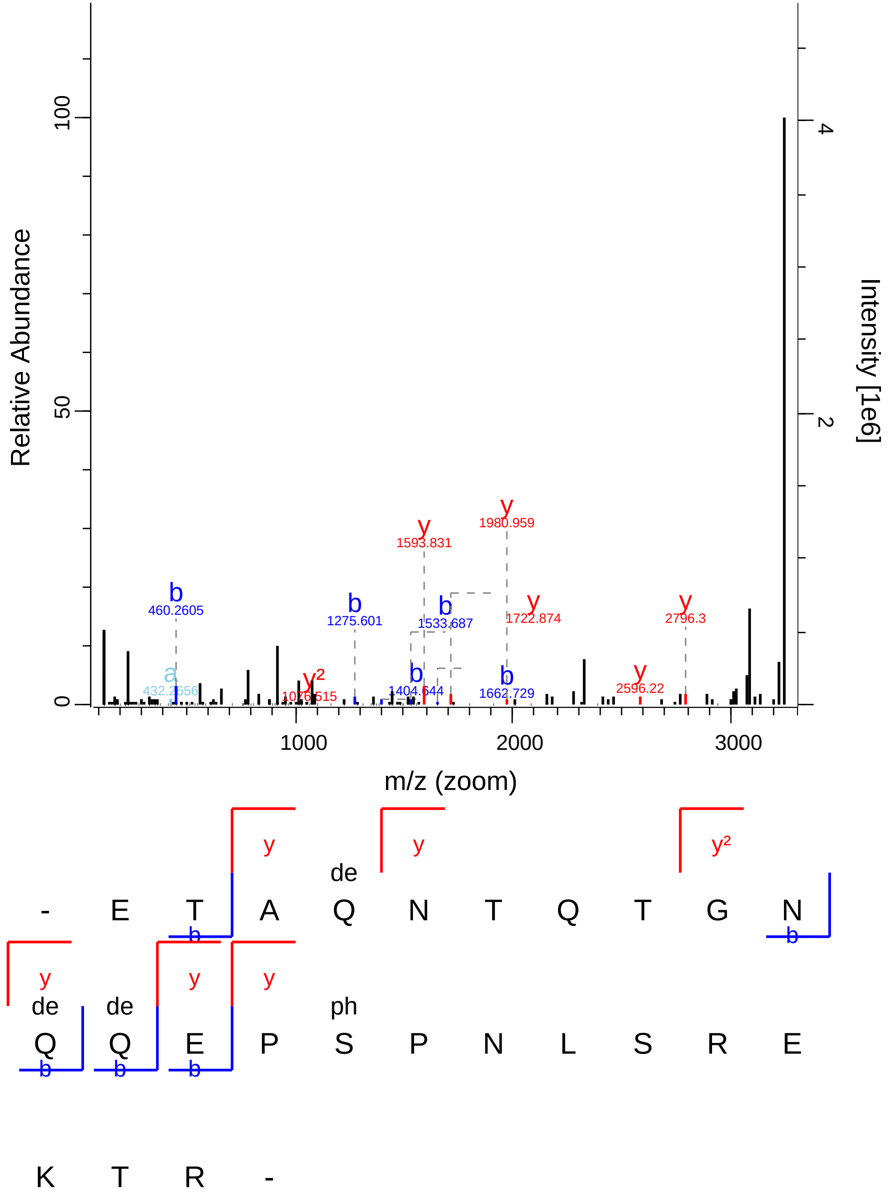

Raw File	Scan	Method	Score	m/z	Gene names
RO-QC-160121_ZackD_SS_Set_02_Fractio	21200	FTMS; HC	40.04	857.87	ERVK-16

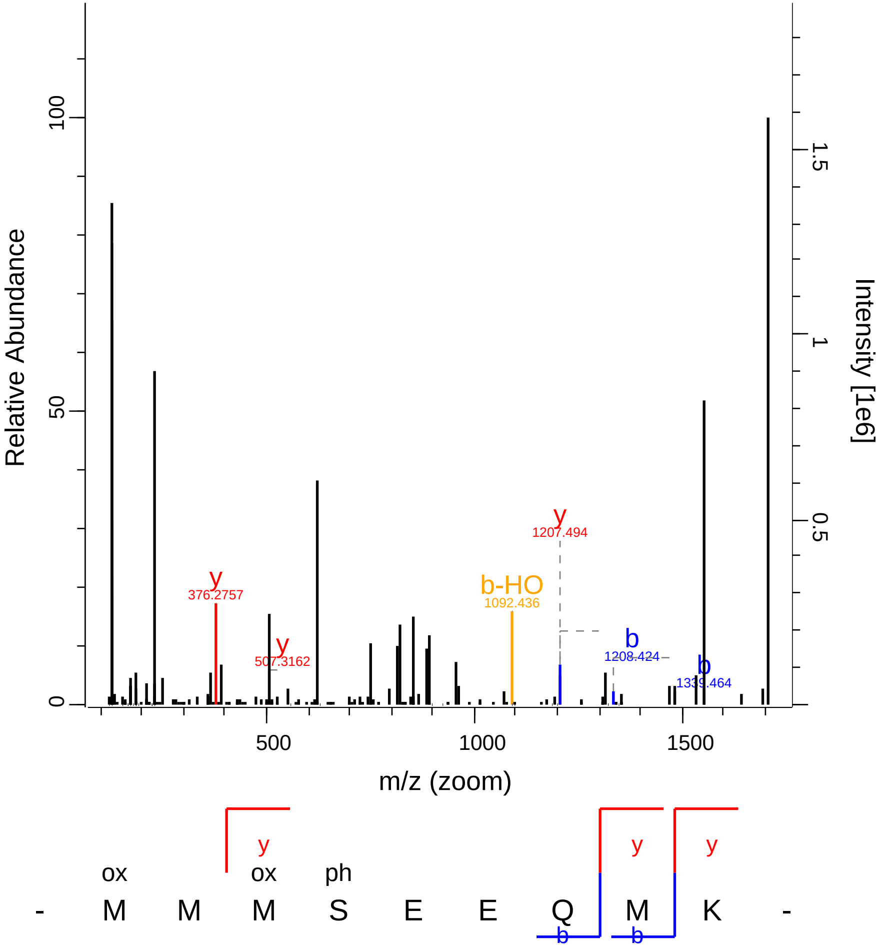

Raw File	Scan	Method	Score	m/z	Gene names
RO-QC-160121_ZackD_SS_Set_02_Fractio	21255	FTMS; HC	127.36	629.01	NATD1

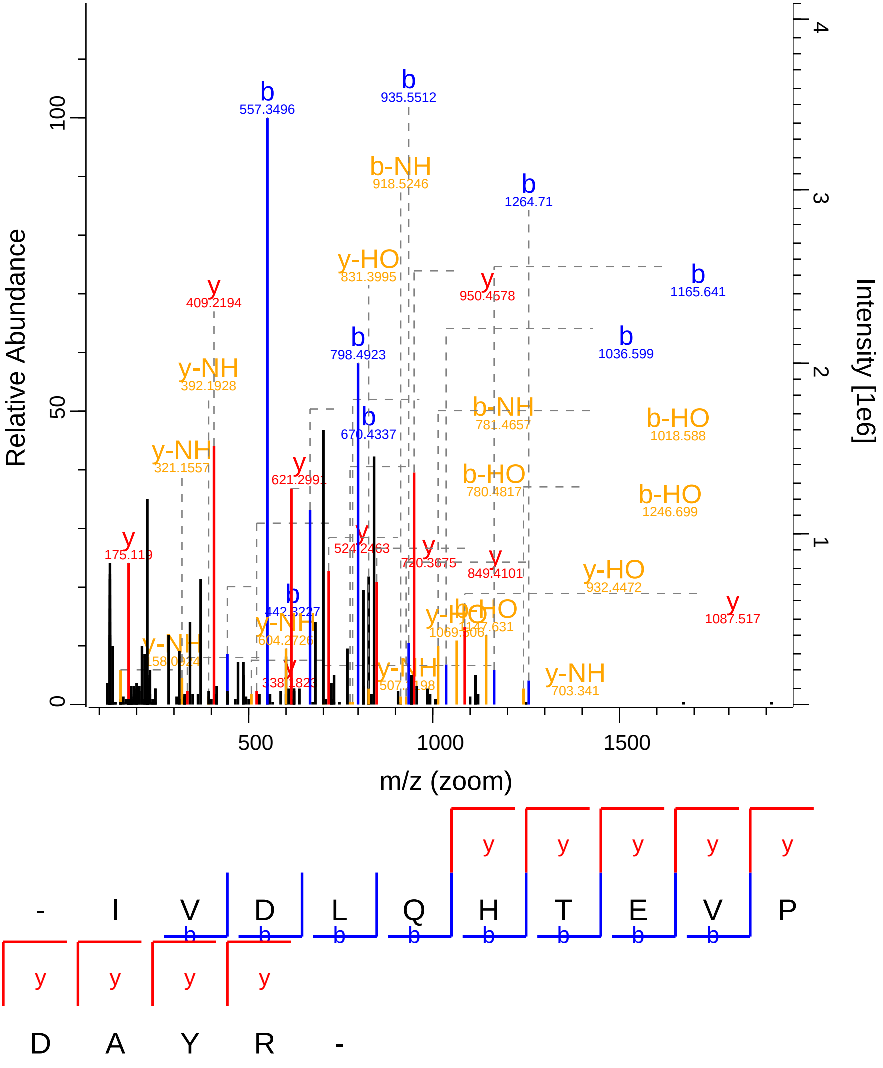

Raw File	Scan	Method	Score	m/z	Gene names
RO-QC-160121_ZackD_SS_Set_02_Fractio	21282	FTMS; HC	88.02	989.51	BRI3

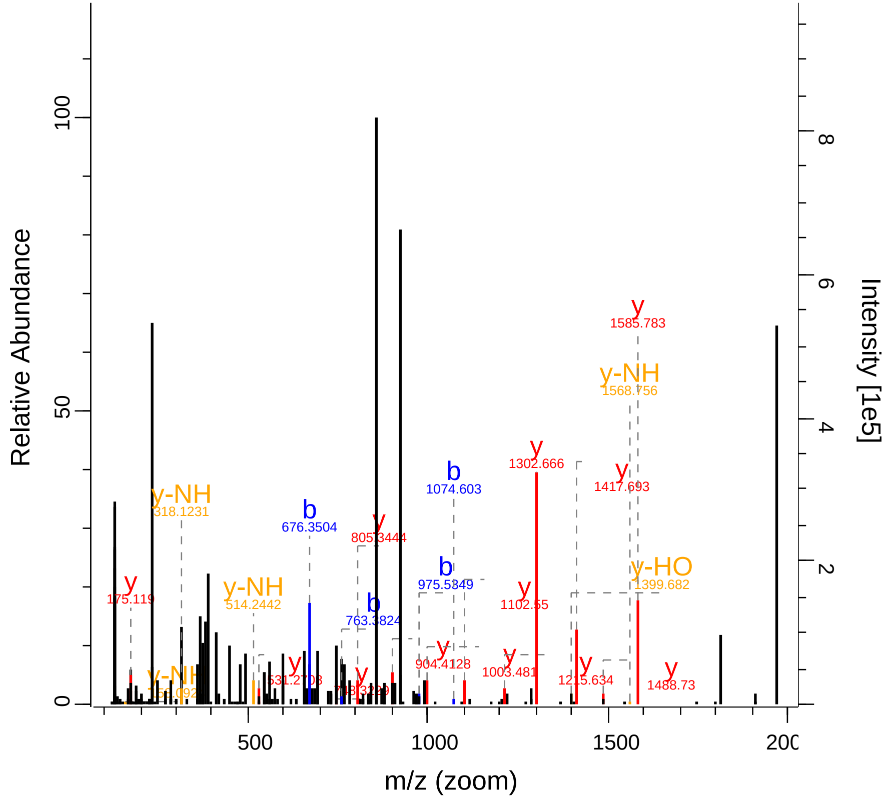

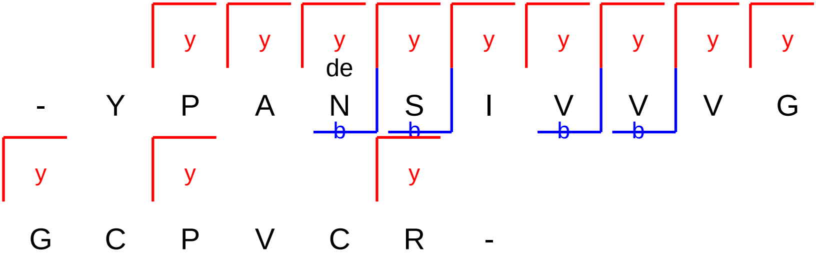

Raw File	Scan	Method	Score	m/z	Gene names
RO-QC-160121_ZackD_SS_Set_02_Fractio	21523	FTMS; HC	140.14	989.51	BRI3

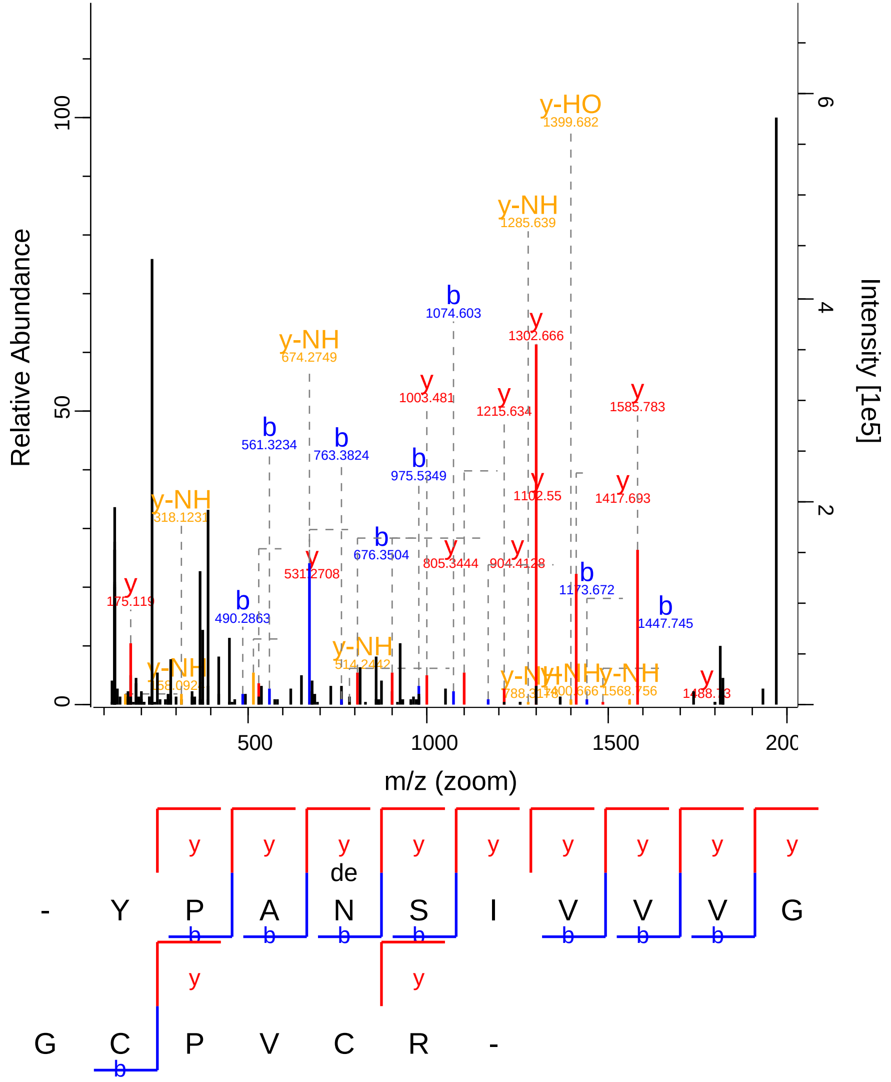

Raw File	Scan	Method	Score	m/z	Gene names
RO-QC-160121_ZackD_SS_Set_02_Fractio	21615	FTMS; HC	93.56	803.45	ATXN7L3B

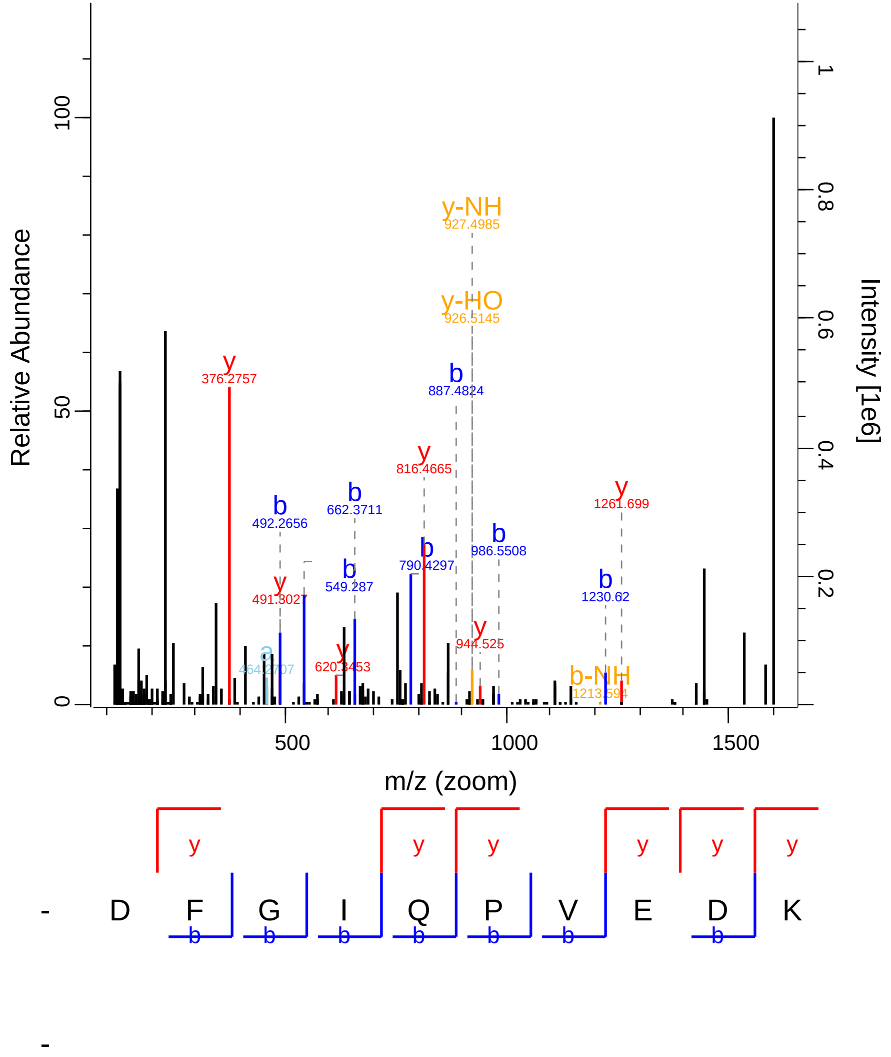

Raw File	Scan	Method	Score	m/z	Gene names
RO-QC-160121_ZackD_SS_Set_02_Fractio	21641	FTMS; HC	143.39	641.34	SLC31A1

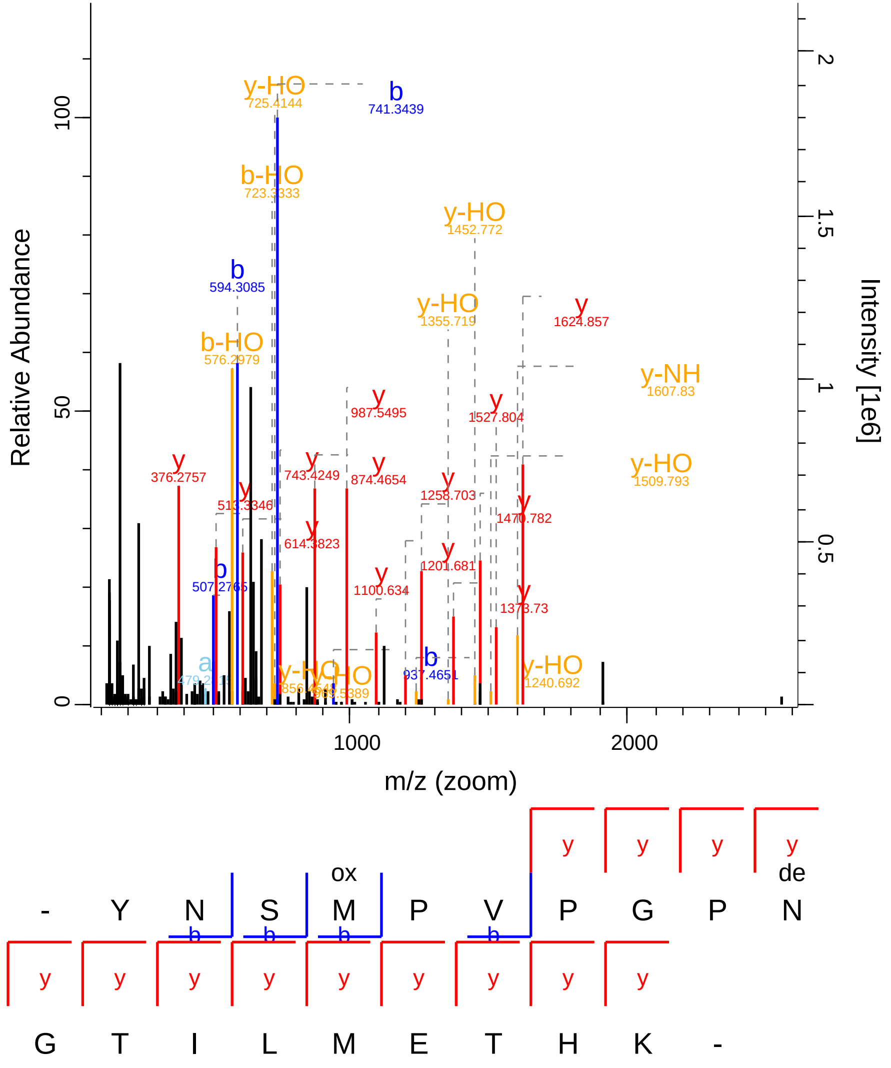

Raw File	Scan	Method	Score	m/z	Gene names
RO-QC-160121_ZackD_SS_Set_02_Fractio	22457	FTMS; HC	47.6	721.69	MOGAT2

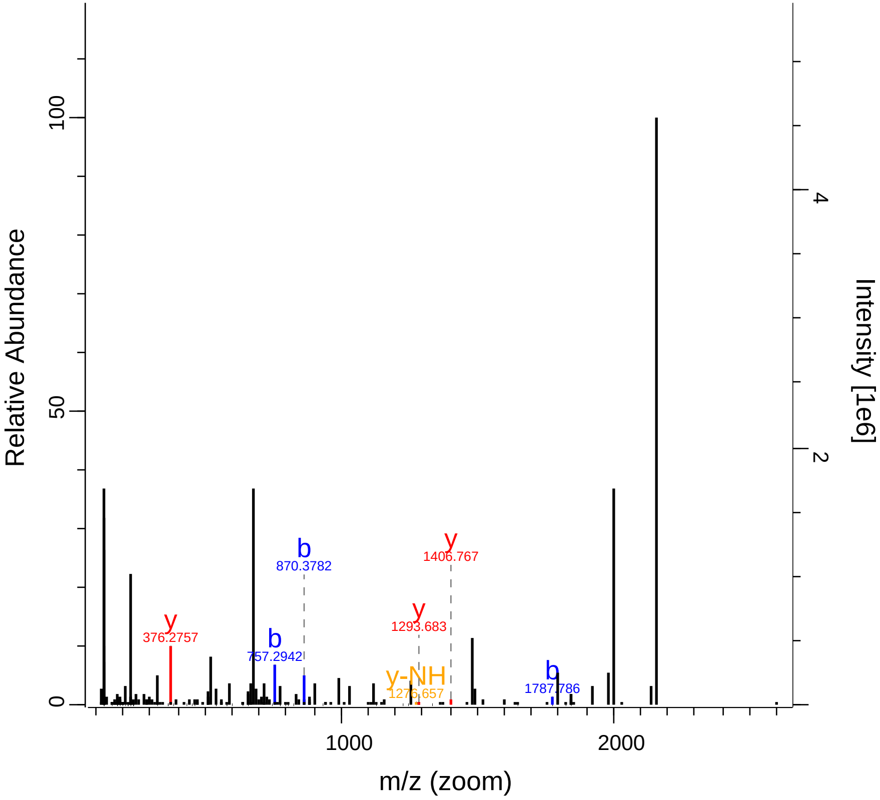

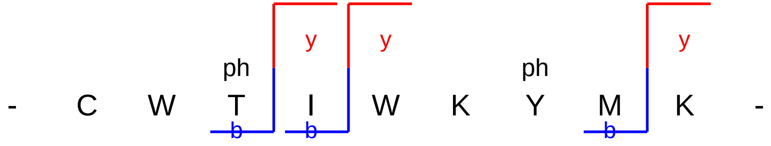

Raw File	Scan	Method	Score	m/z
RO-QC-160121_ZackD_SS_Set_02_Fraction_2	22491	FTMS; HCD	78.94	874.99

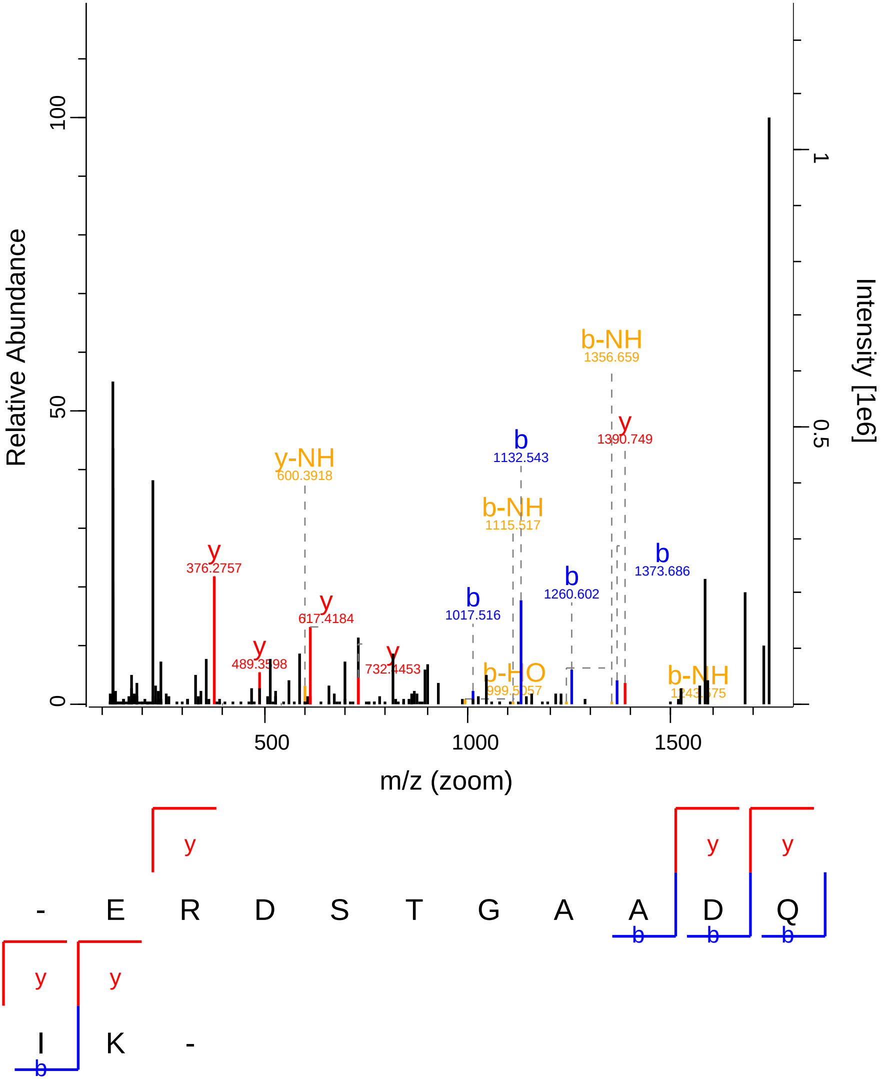

Raw File	Scan	Method	Score	m/z	Gene names
RO-QC-160121_ZackD_SS_Set_02_Fractio	22571	FTMS; HC	86.46	799.75	CDC42SE2

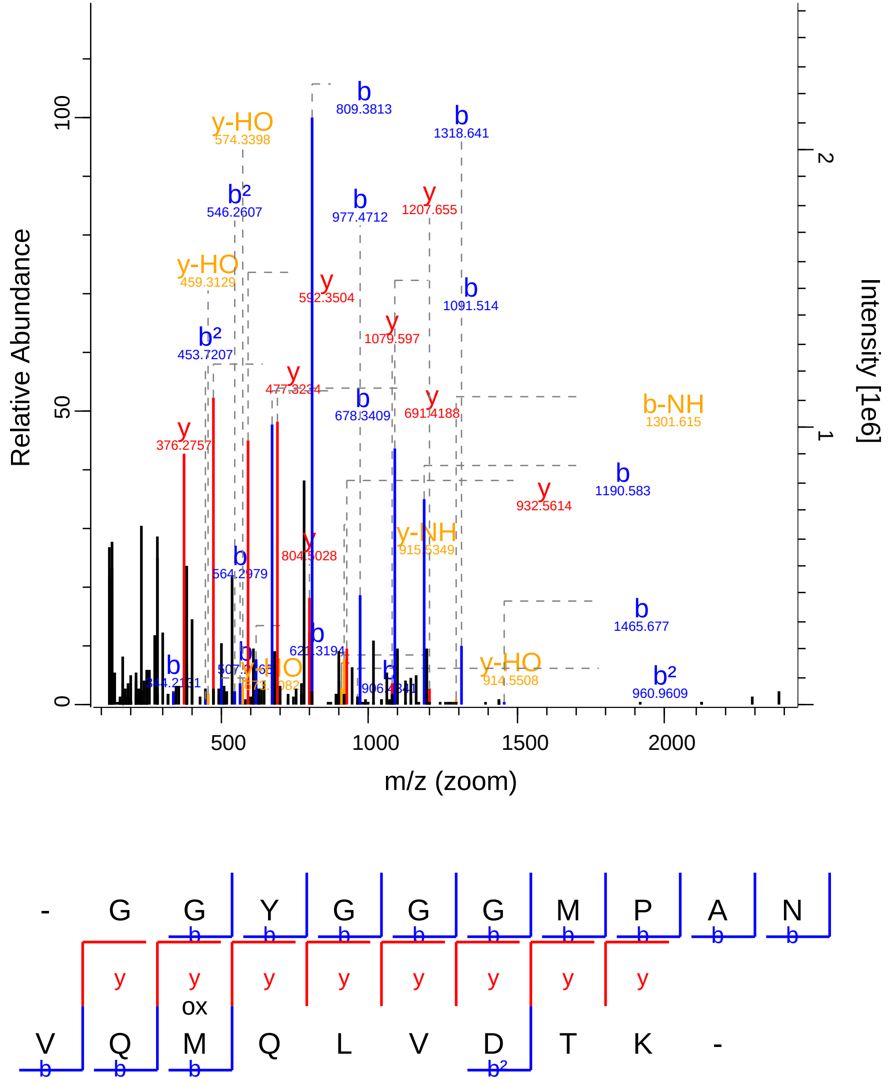

Raw File	Scan	Method	Score	m/z	Gene names
RO-QC-160121_ZackD_SS_Set_02_Fractio	23257	FTMS; HC	54.6	511.28	LAMB4

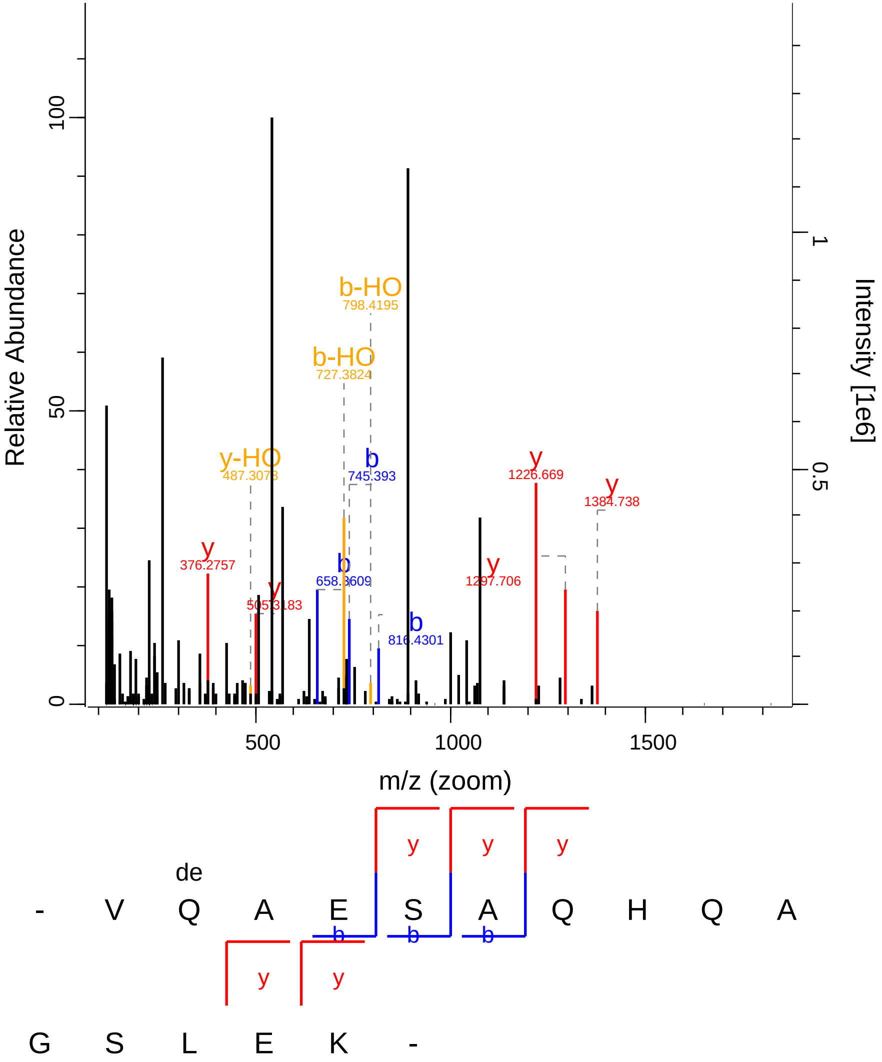

Raw File	Scan	Method	Score	m/z	Gene names
RO-QC-160121_ZackD_SS_Set_02_Fractio	23320	FTMS; HC	147.35	756.06	DCAF16

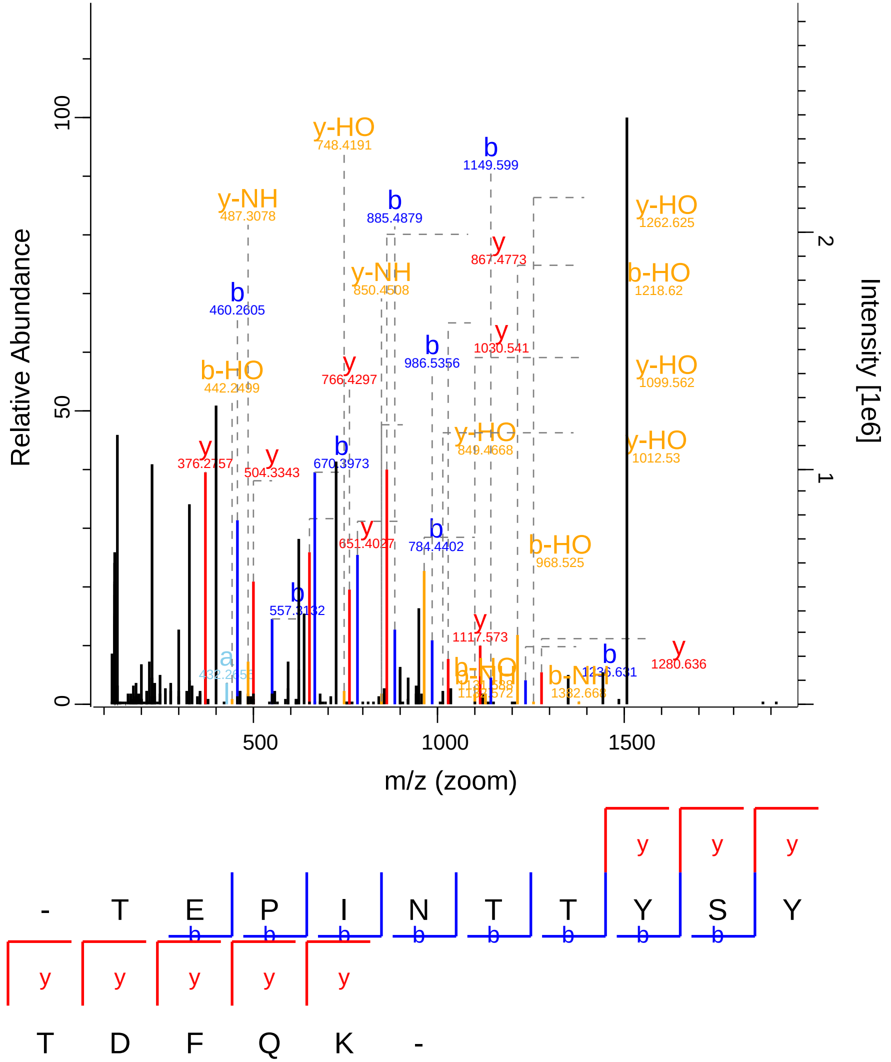

Raw File	Scan	Method	Score	m/z
RO-QC-160121_ZackD_SS_Set_02_Fraction_2	23611	FTMS; HCD	69.6	488.31

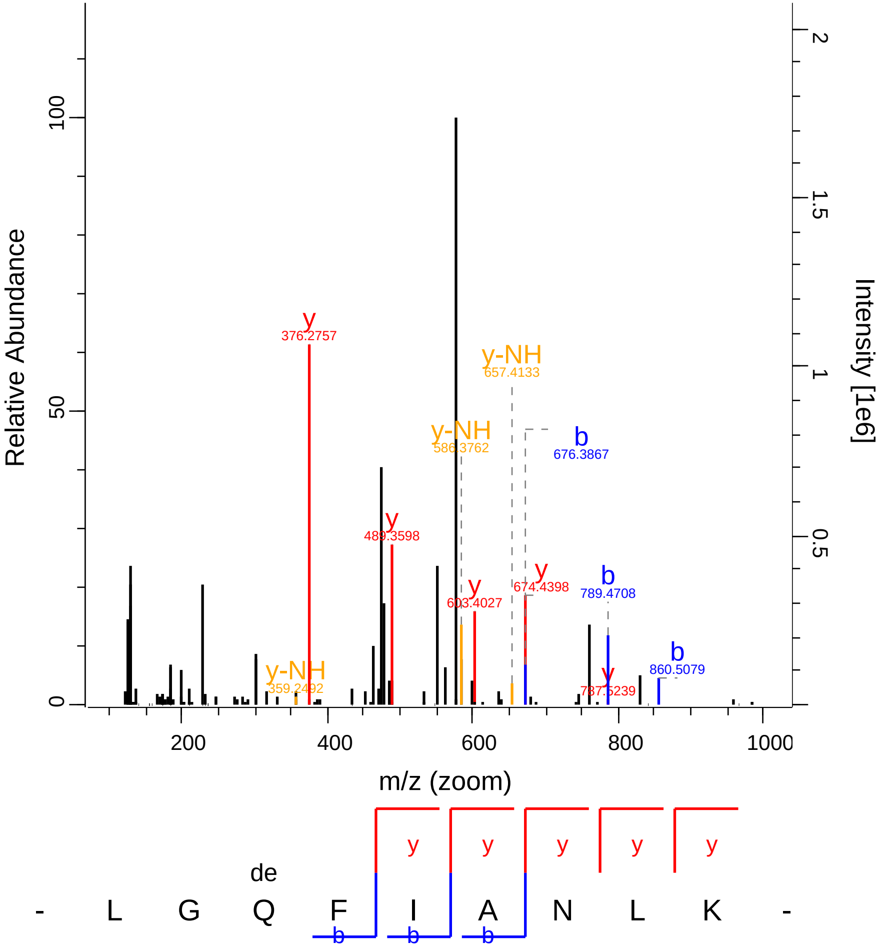

Raw File	Scan	Method	Score	m/z	Gene names
RO-QC-160121_ZackD_SS_Set_02_Fractio	24335	FTMS; HC	42.56	554.81	ADAMDEC1

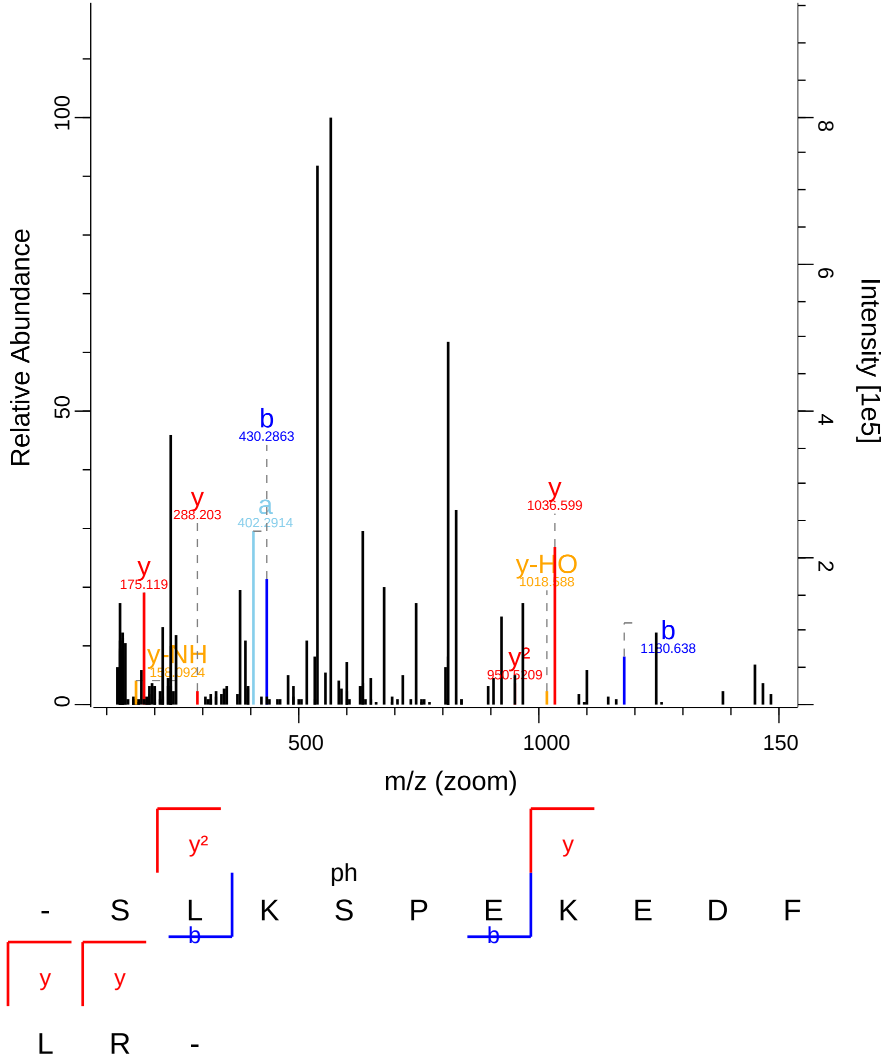

Raw File	Scan	Method	Score	m/z	Gene names
RO-QC-160121_ZackD_SS_Set_02_Fractio	24424	FTMS; HC	50.28	940.99	BAI2

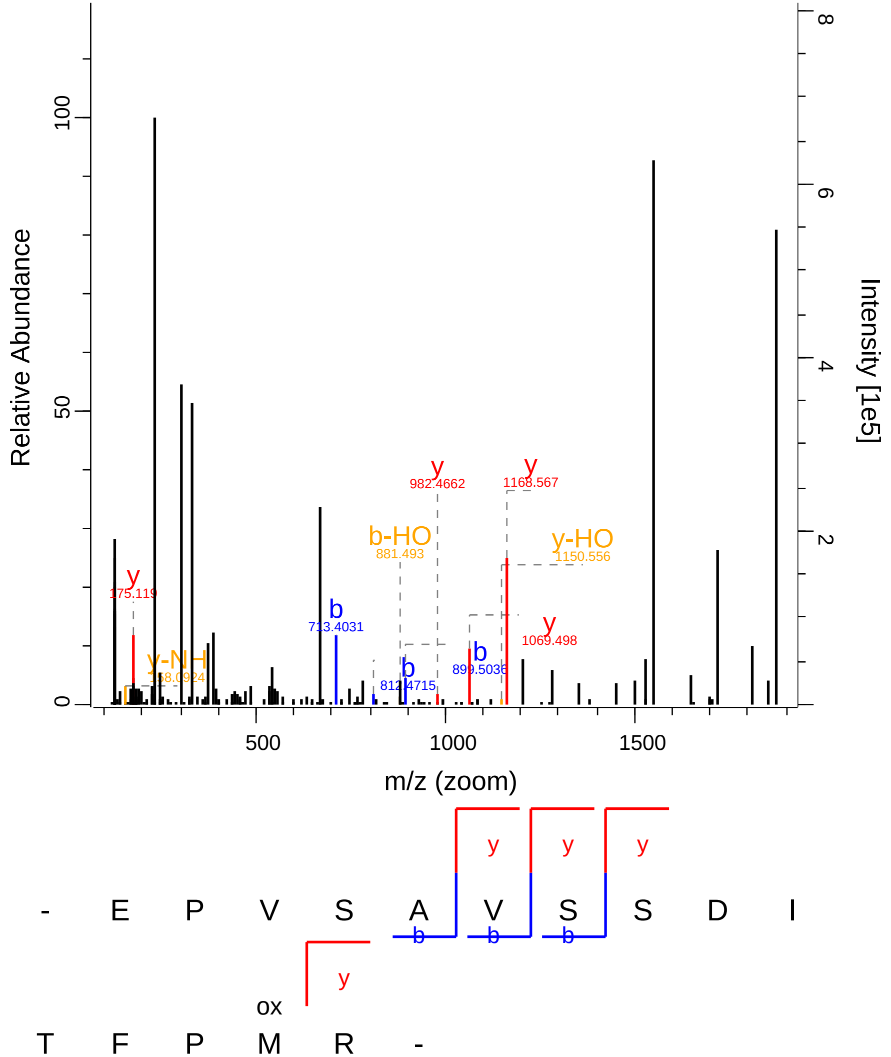

Raw File	Scan	Method	Score	m/z	Gene names
RO-QC-160121_ZackD_SS_Set_02_Fractio	24521	FTMS; HC	48.64	910.13	DRAM2

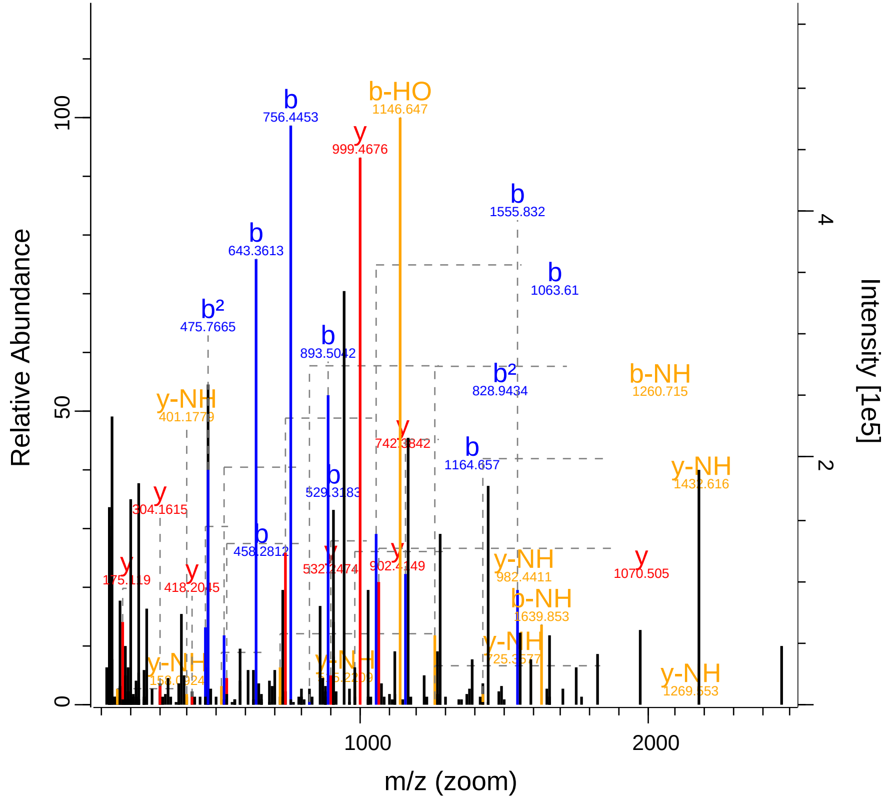

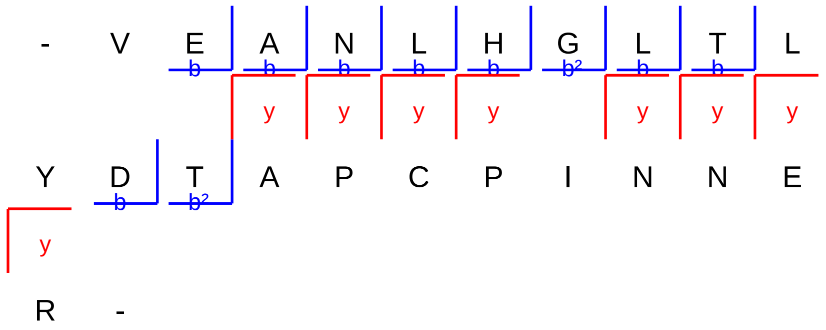

Raw File	Scan	Method	Score	m/z	Gene names
RO-QC-160121_ZackD_SS_Set_02_Fractio	25208	FTMS; HC	129.15	849.77	SLC31A1

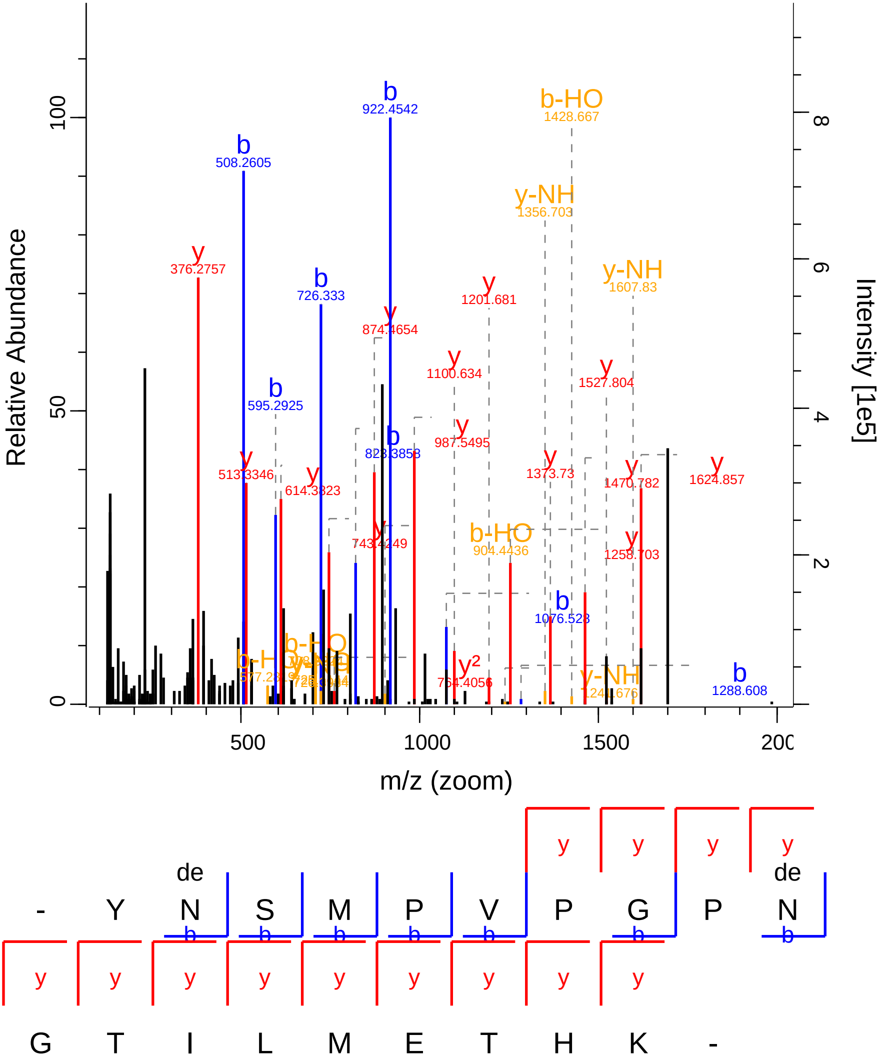

Raw File	Scan	Method	Score	m/z	Gene names
RO-QC-160121_ZackD_SS_Set_02_Fractio	25436	FTMS; HC	103.88	683.04	OSBPL7

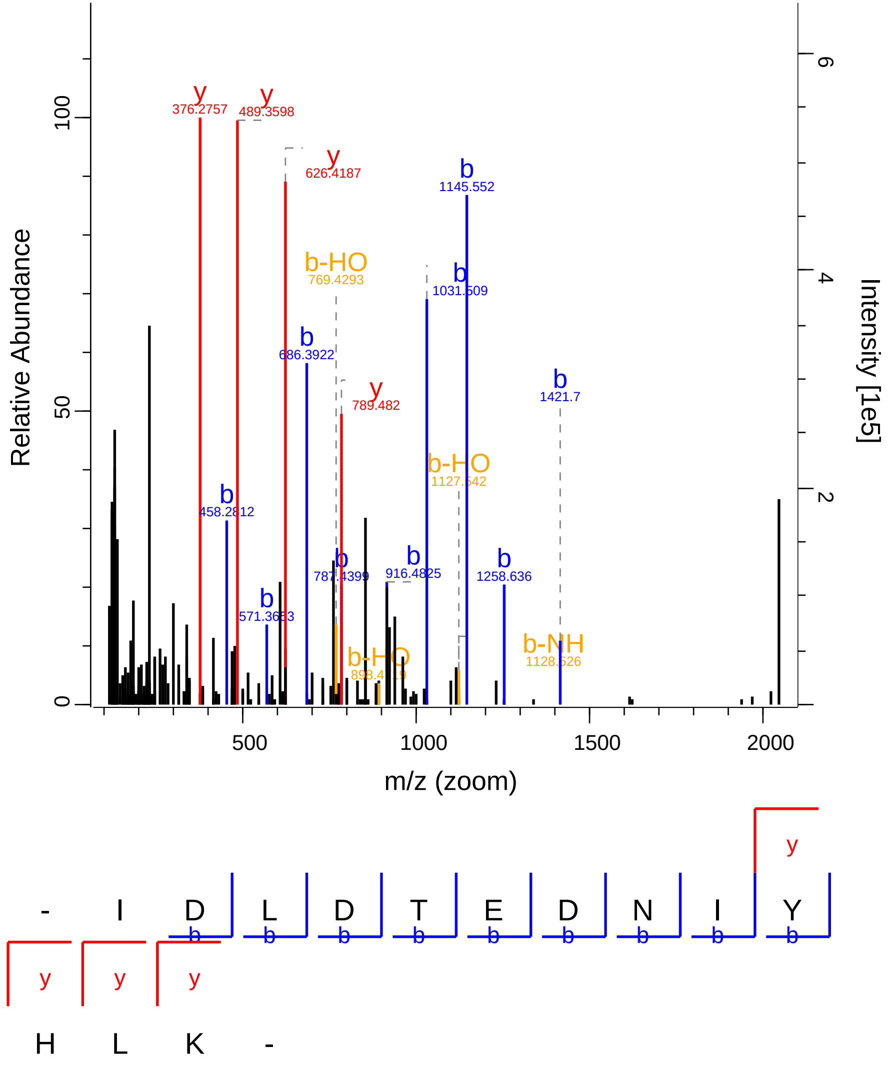

Raw File	Scan	Method	Score	m/z	Gene names
RO-QC-160121_ZackD_SS_Set_02_Fractio	25440	FTMS; HC	127.17	849.78	SLC31A1

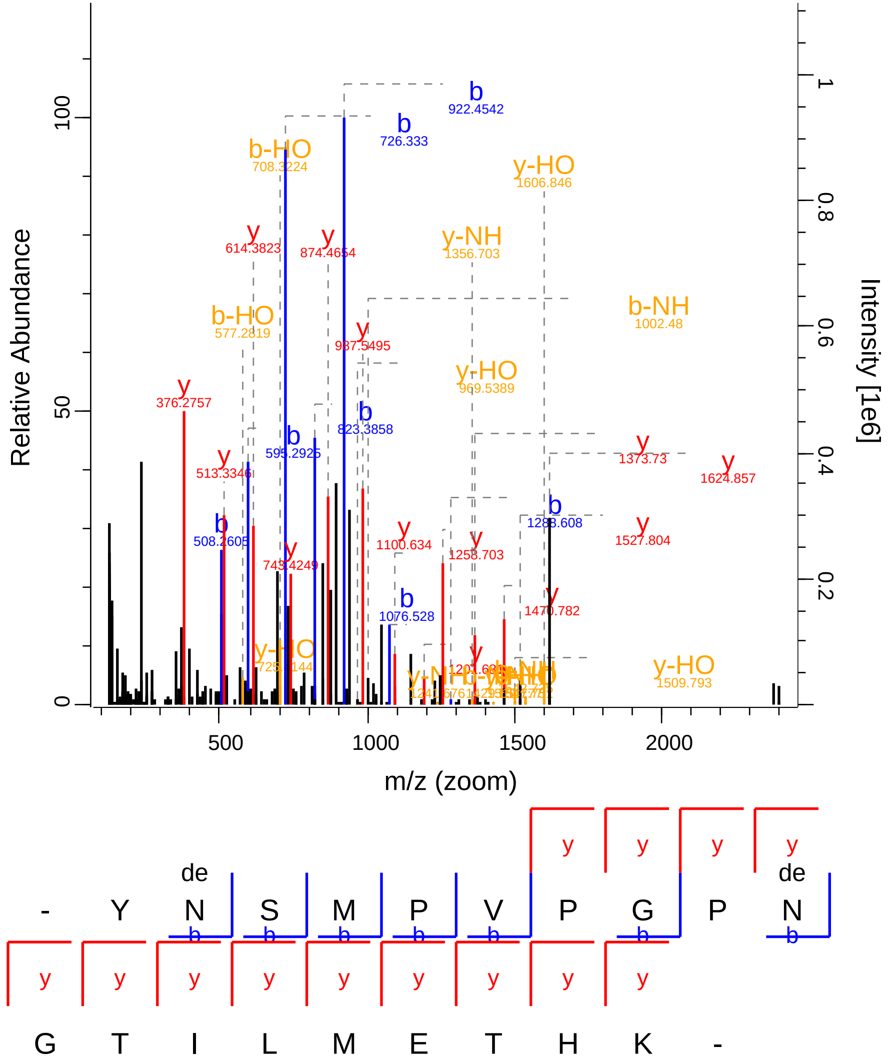

Raw File	Scan	Method	Score	m/z	Gene names
RO-QC-160121_ZackD_SS_Set_02_Fractio	25977	FTMS; HC	141.86	849.77	SLC31A1

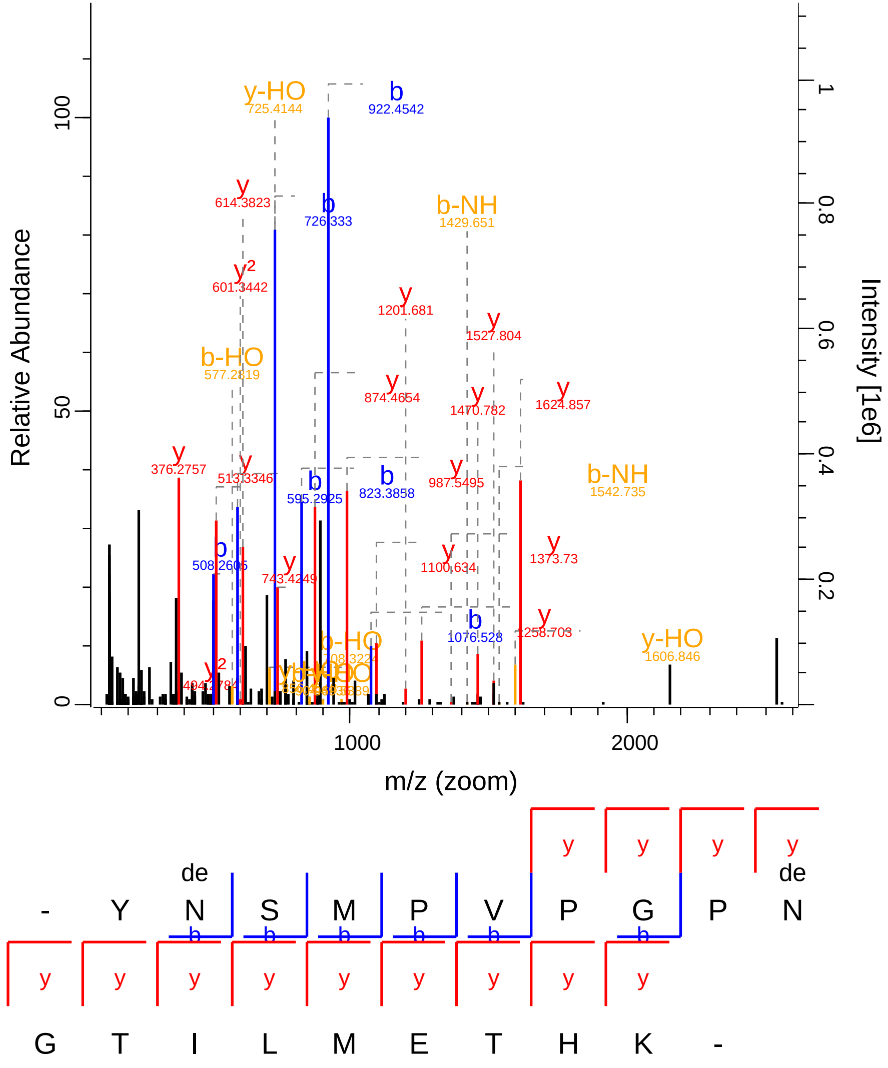

Raw File	Scan	Method	Score	m/z	Gene names
RO-QC-160121_ZackD_SS_Set_02_Fractio	26128	FTMS; HC	84.5	849.77	SLC31A1

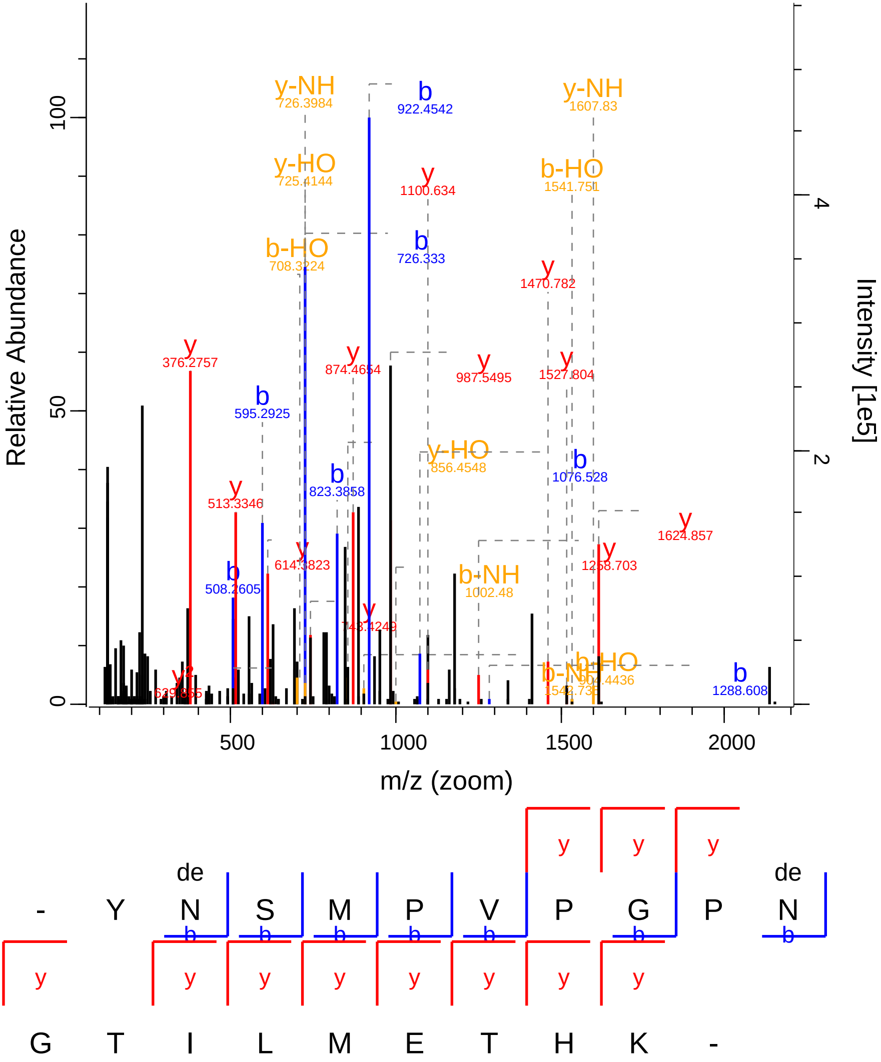

Raw File	Scan	Method	Score	m/z	Gene names
RO-QC-160121_ZackD_SS_Set_02_Fractio	26776	FTMS; HC	113.29	778.11	CHRAC1

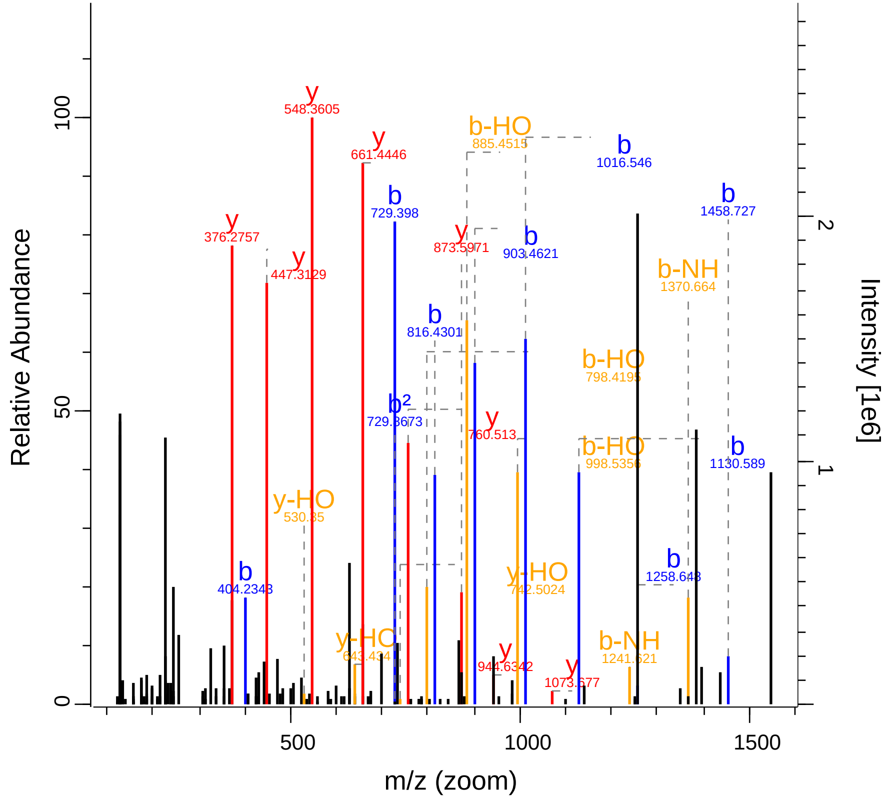

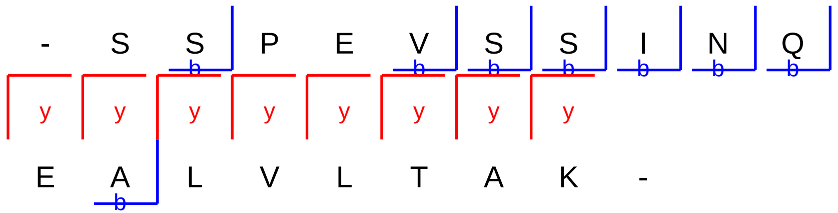

Raw File	Scan	Method	Score	m/z
RO-QC-160121_ZackD_SS_Set_02_Fraction_2	27459	FTMS; HCD	45.33	797.11

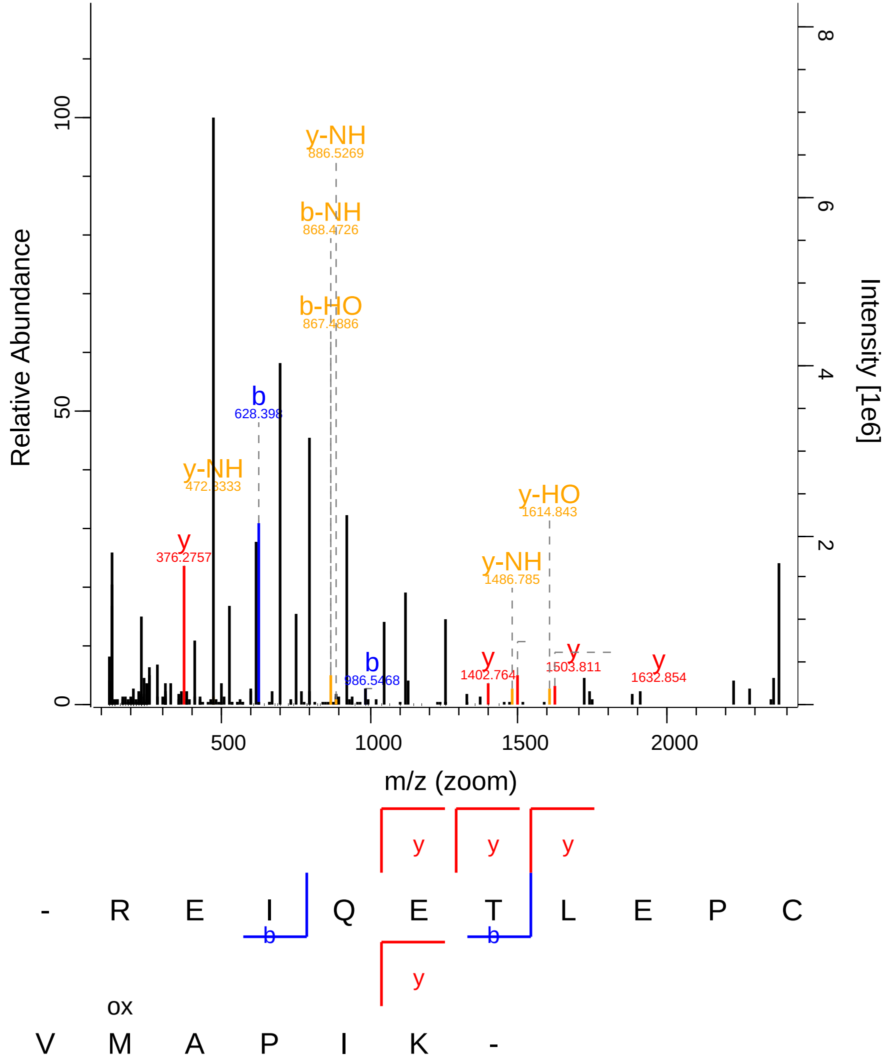

Raw File	Scan	Method	Score	m/z	Gene names
RO-QC-160121_ZackD_SS_Set_02_Fractio	27465	FTMS; HC	76.15	924.83	CLMP

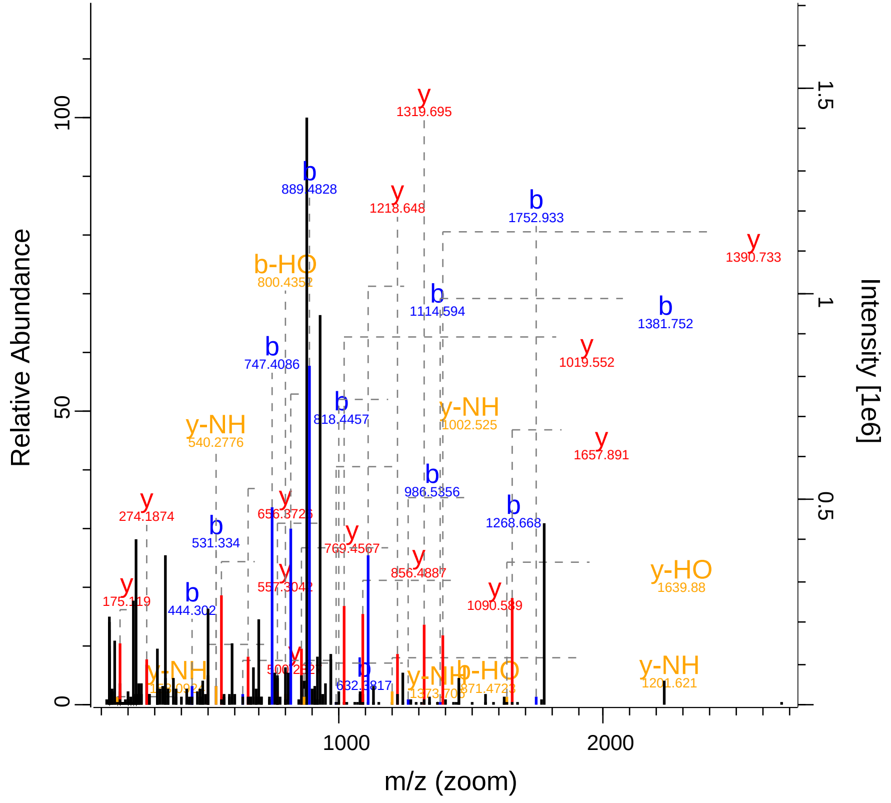

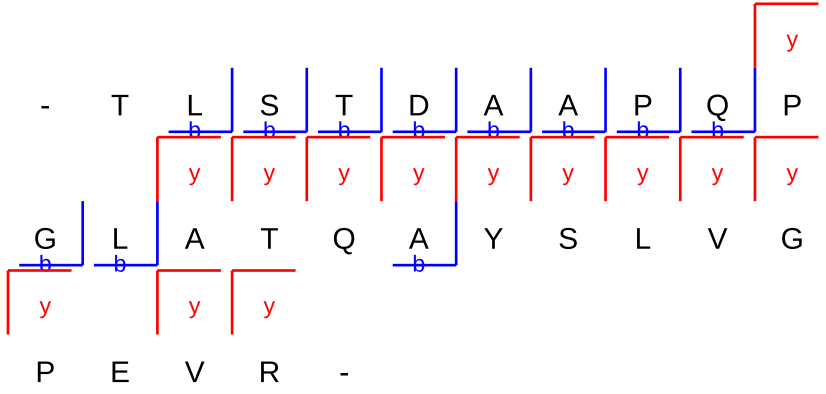

Raw File	Scan	Method	Score	m/z	Gene names
RO-QC-160121_ZackD_SS_Set_02_Fractio	27516	FTMS; HC	206.97	826.94	FBXL4

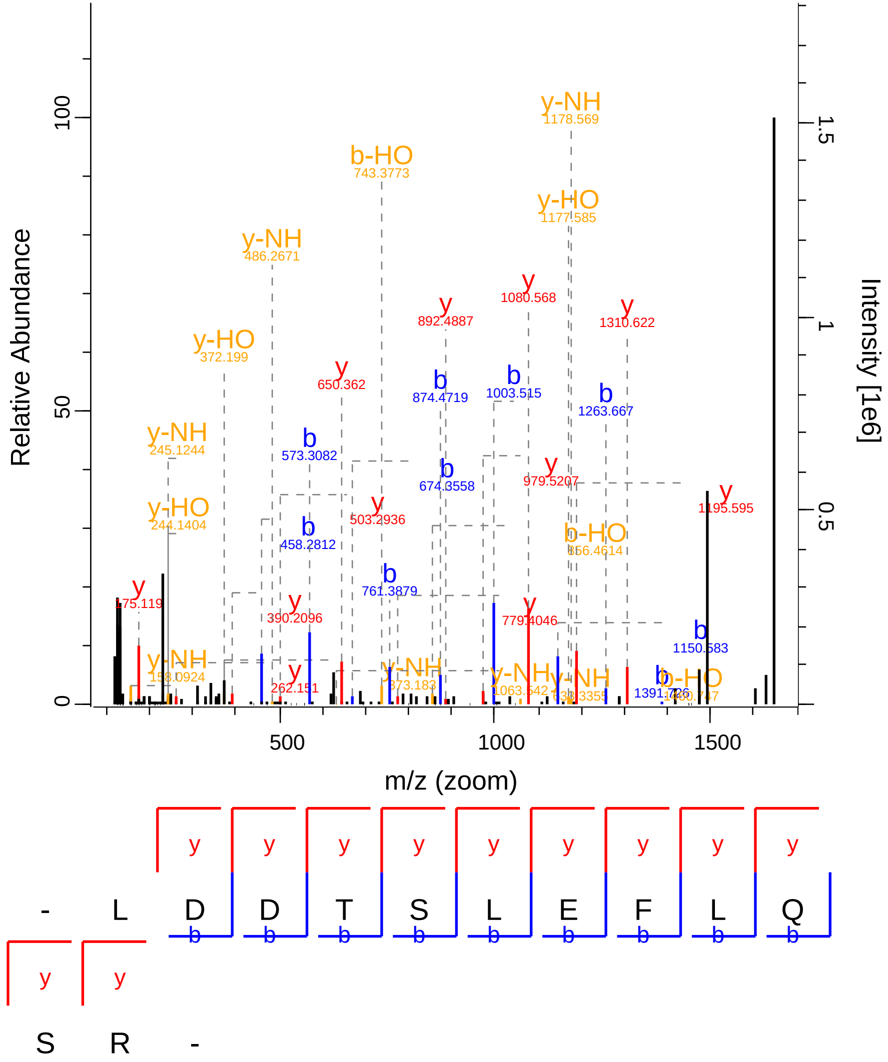

Raw File	Scan	Method	Score	m/z	Gene names
RO-QC-160121_ZackD_SS_Set_02_Fractio	27540	FTMS; HC	97.78	551.63	FBXL4

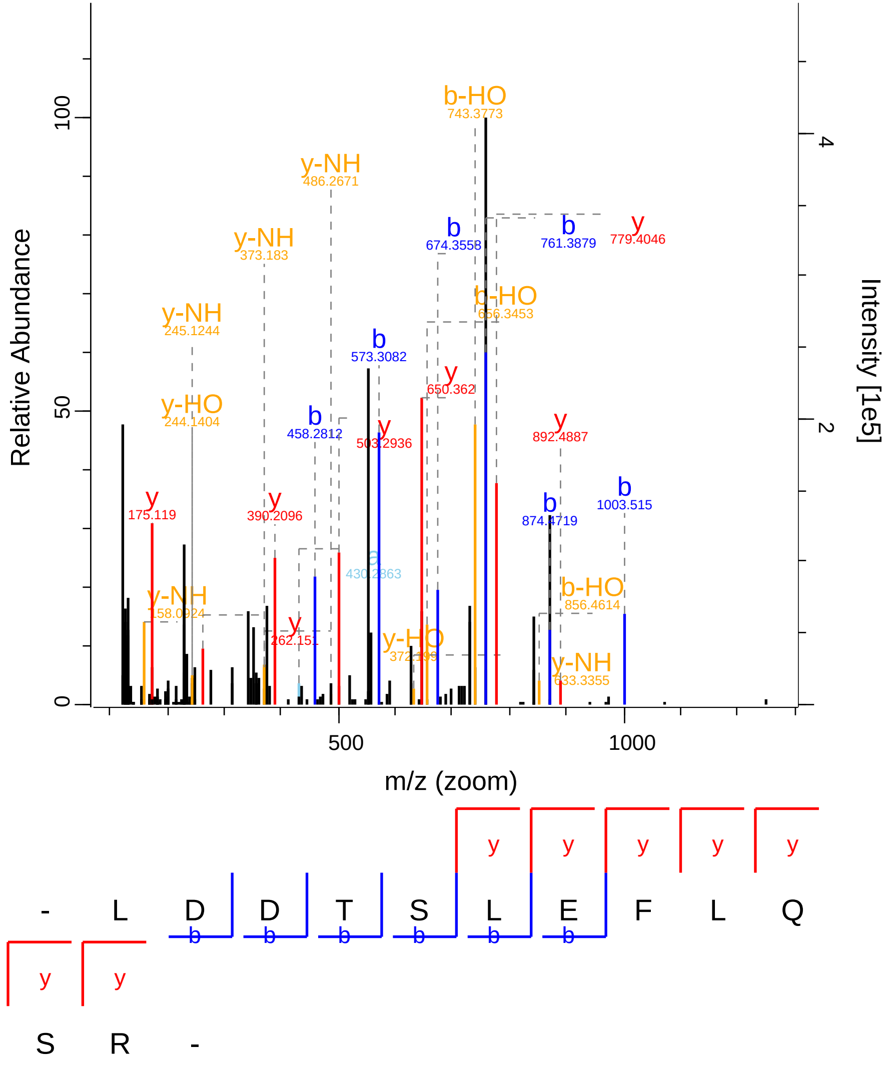

Raw File	Scan	Method	Score	m/z	Gene names
RO-QC-160121_ZackD_SS_Set_02_Fractio	27772	FTMS; HC	128.27	554.33	CYB5R4

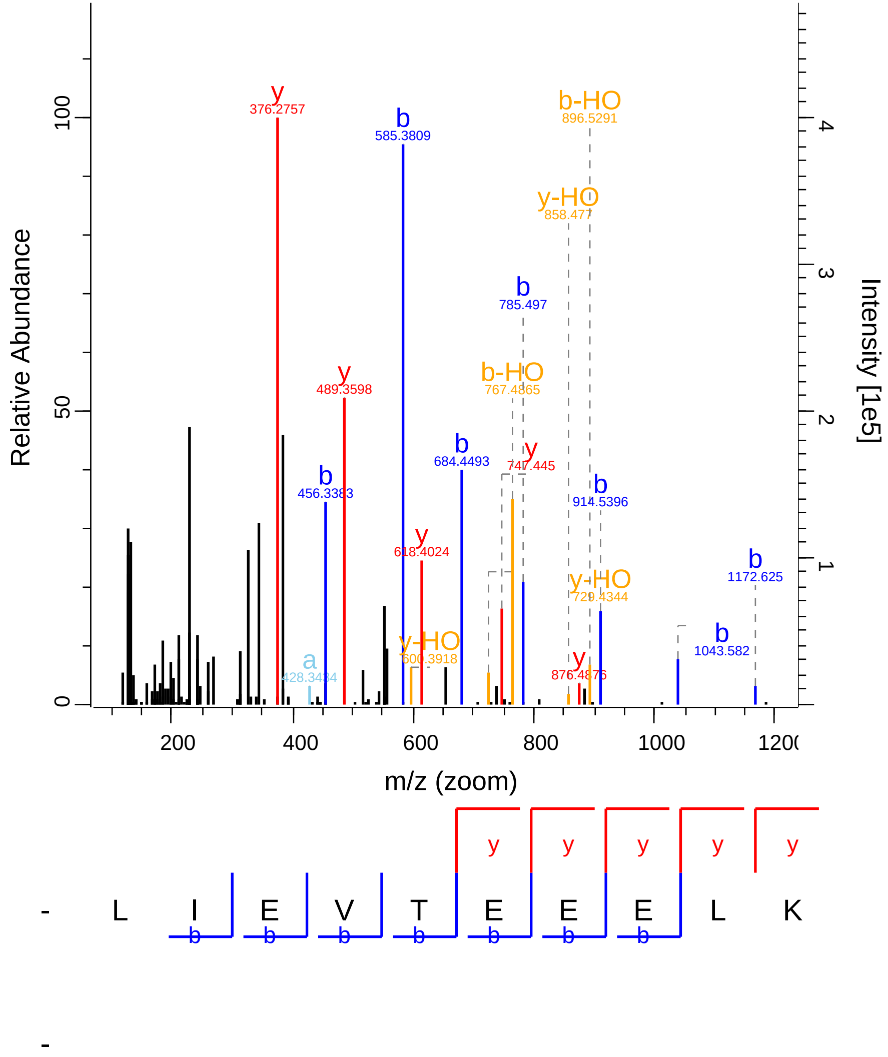

Raw File	Scan	Method	Score	m/z	Gene names
RO-QC-160121_ZackD_SS_Set_02_Fractio	27787	FTMS; HC	146.06	897.09	CRTC3

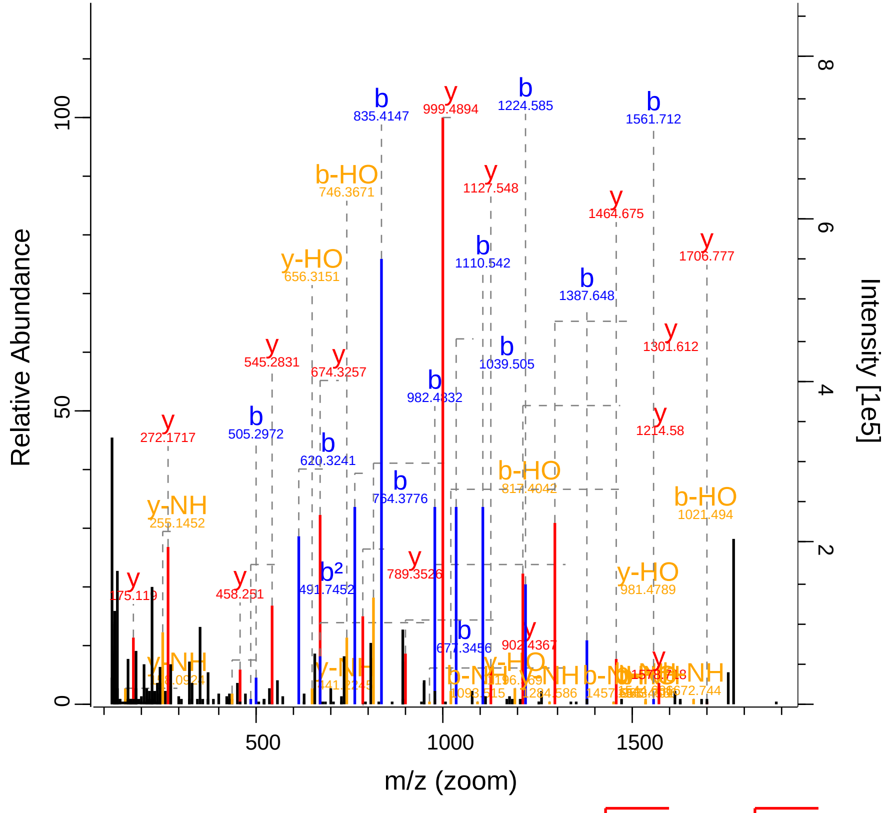

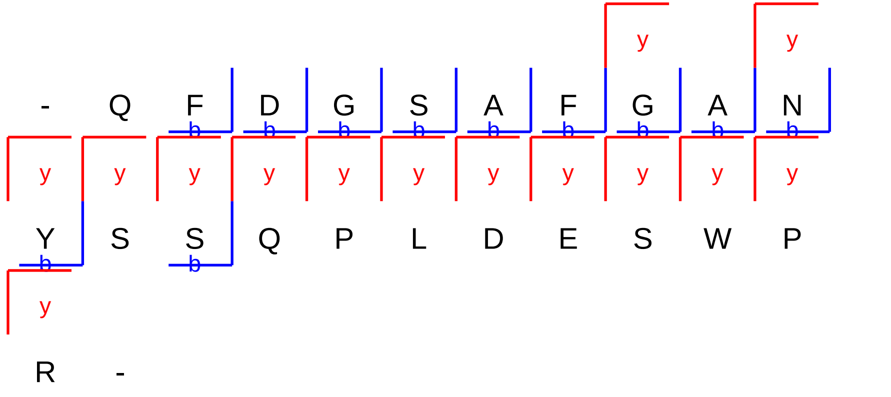

Raw File	Scan	Method	Score	m/z	Gene names
RO-QC-160121_ZackD_SS_Set_02_Fractio	29331	FTMS; HC	111.4	955.19	MAPKBP1

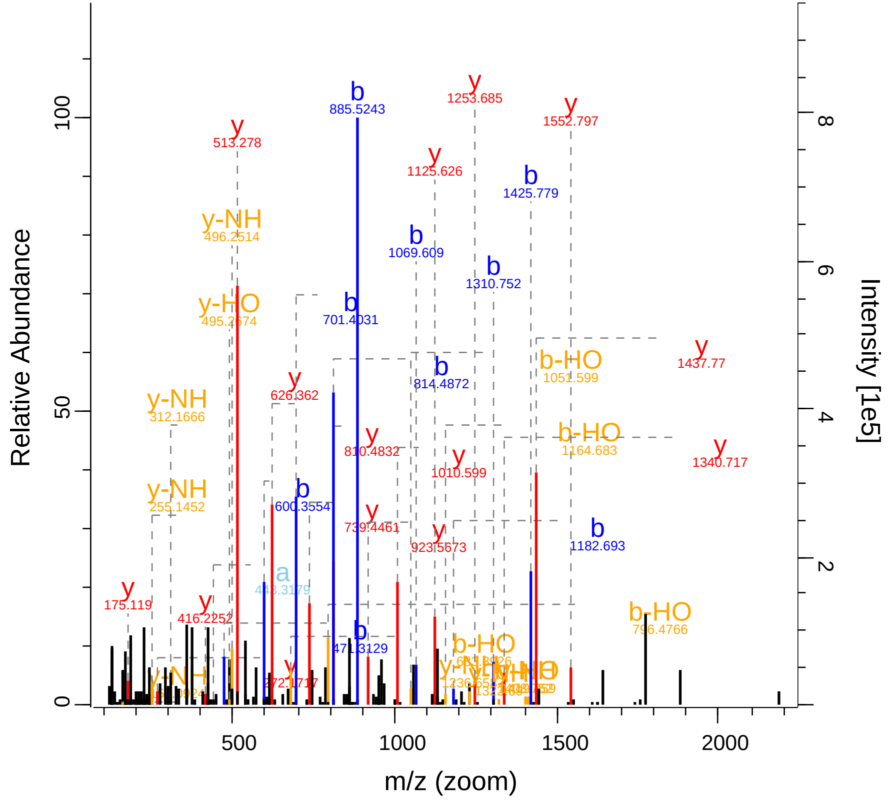

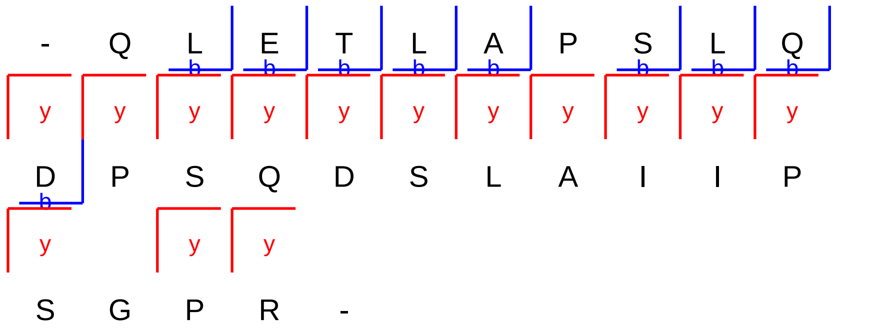

Raw File	Scan	Method	Score	m/z	Gene names
RO-QC-160121_ZackD_SS_Set_02_Fractio	29487	FTMS; HC	86.62	852.49	ESRRA

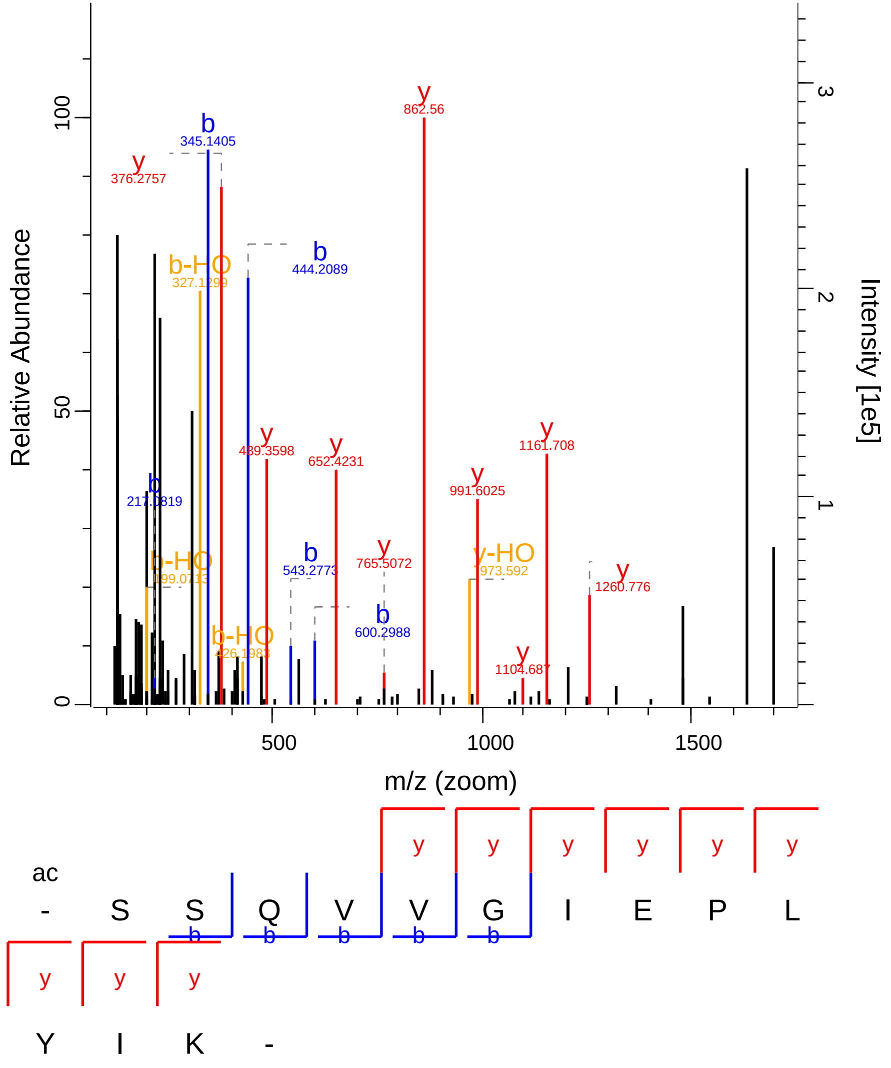

Raw File	Scan	Method	Score	m/z	Gene names
RO-QC-160121_ZackD_SS_Set_02_Fractio	30132	FTMS; HC	170.06	811.12	ANGEL1

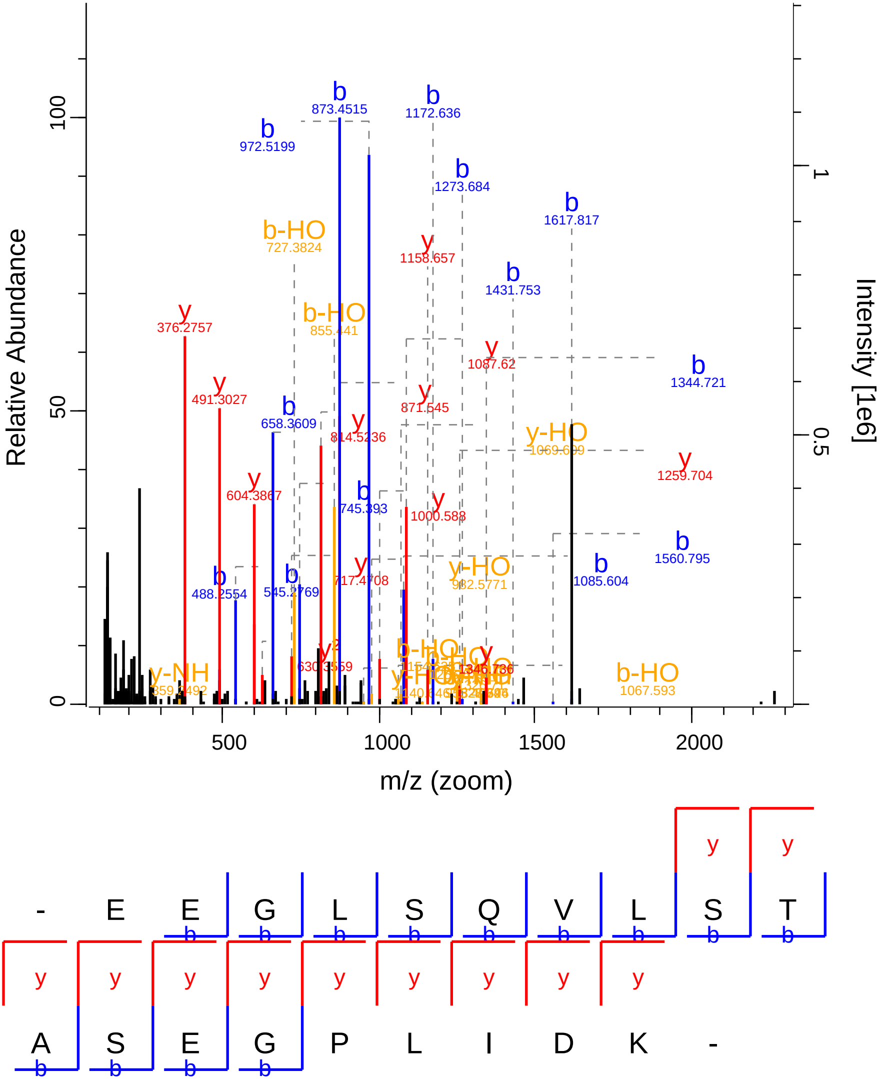

Raw File	Scan	Method	Score	m/z	Gene names
RO-QC-160121_ZackD_SS_Set_02_Fractio	30571	FTMS; HC	122.93	761.06	E4F1

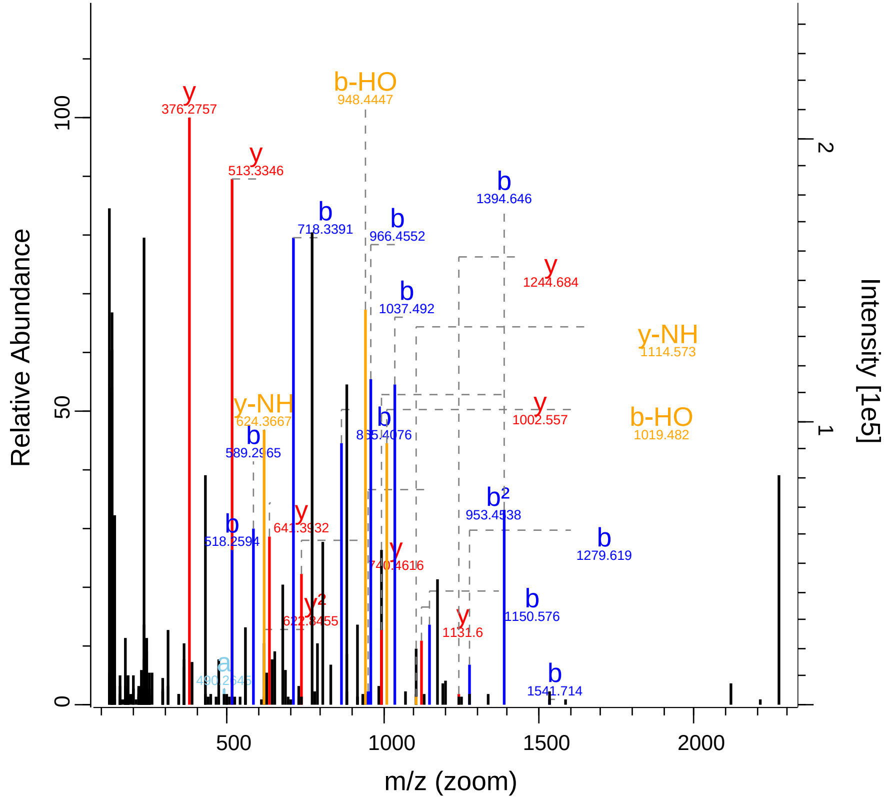

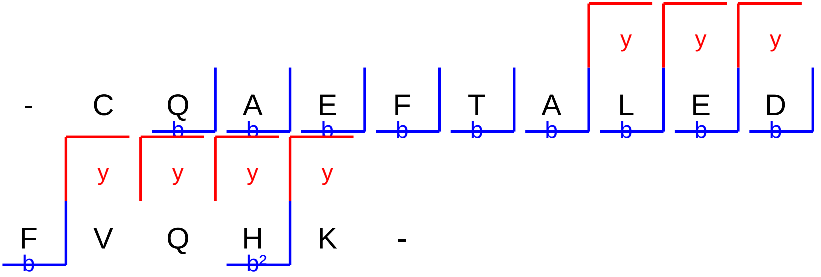

Raw File	Scan	Method	Score	m/z	Gene names
RO-QC-160121_ZackD_SS_Set_02_Fractio	31317	FTMS; HCI	170.53	999.2	LUM

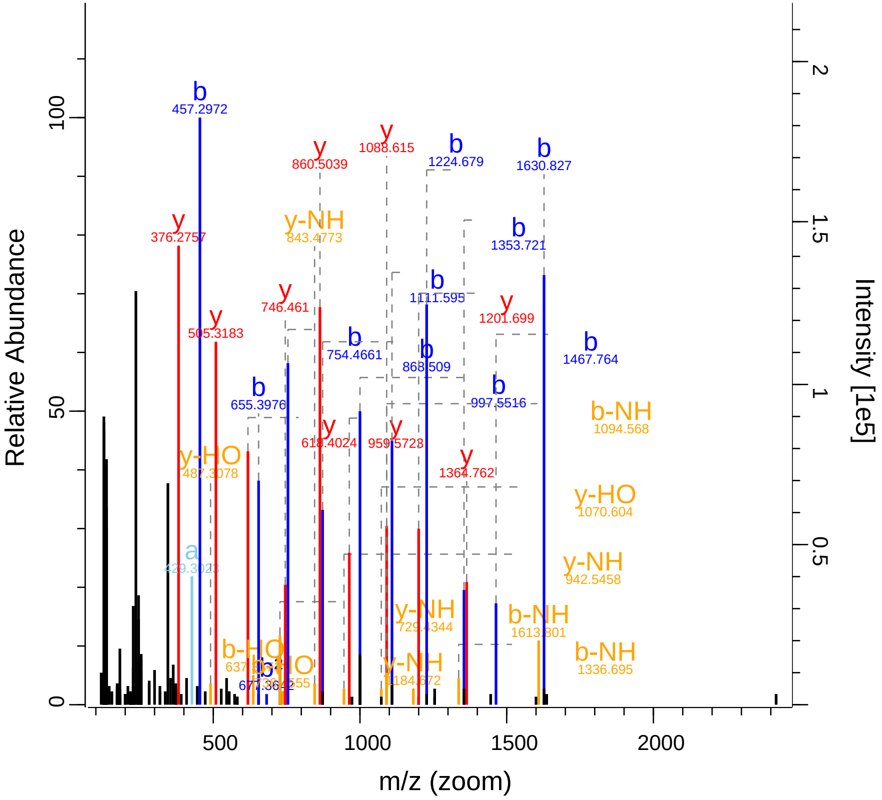

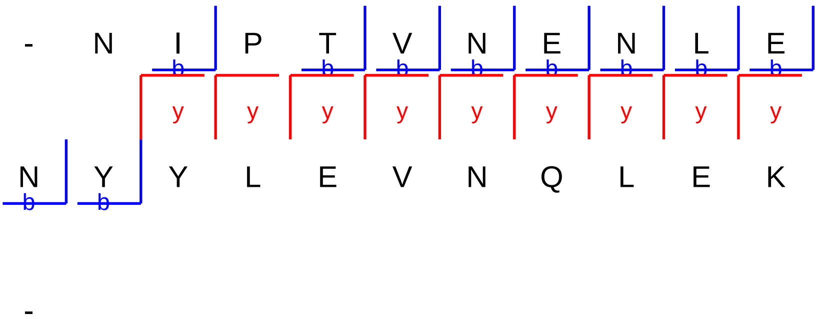

Raw File	Scan	Method	Score	m/z	Gene names
RO-QC-160121_ZackD_SS_Set_02_Fractio	31340	FTMS; HC	107.35	741.35	BEX4

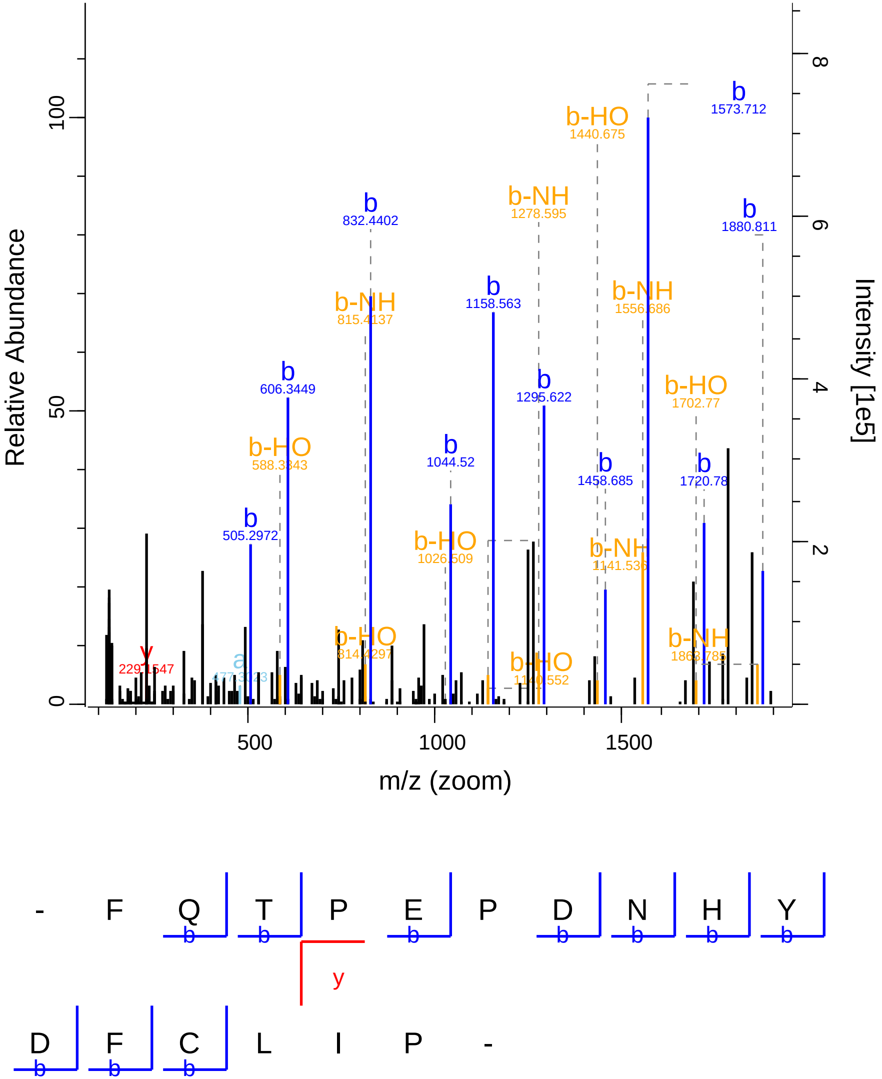

Raw File	Scan	Method	Score	m/z	Gene names
RO-QC-160121_ZackD_SS_Set_02_Fractio	31618	FTMS; HC	133.89	568.01	GRPEL2

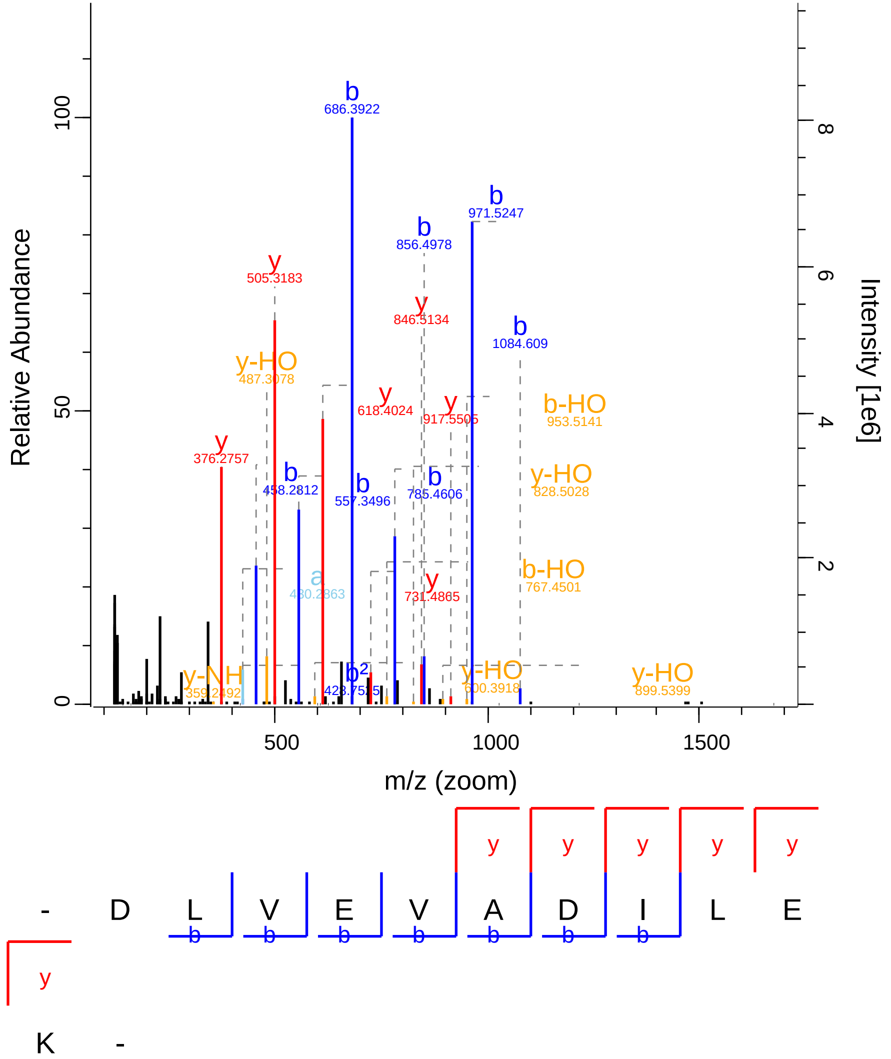

Raw File	Scan	Method	Score	m/z	Gene names
RO-QC-160121_ZackD_SS_Set_02_Fractio	31619	FTMS; HC	194.6	851.51	GRPEL2

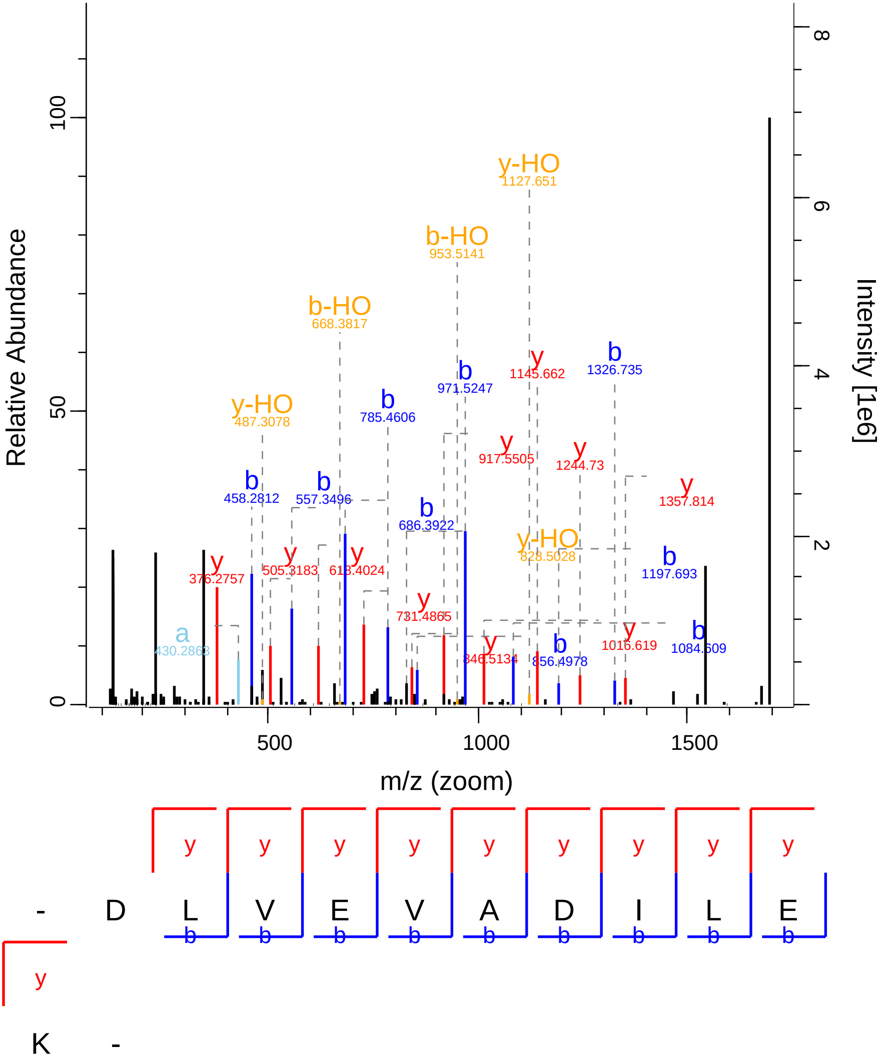

Raw File	Scan	Method	Score	m/z	Gene names
RO-QC-160121_ZackD_SS_Set_02_Fractior	31740	FTMS; HCl	36.02	728.4	MILR1

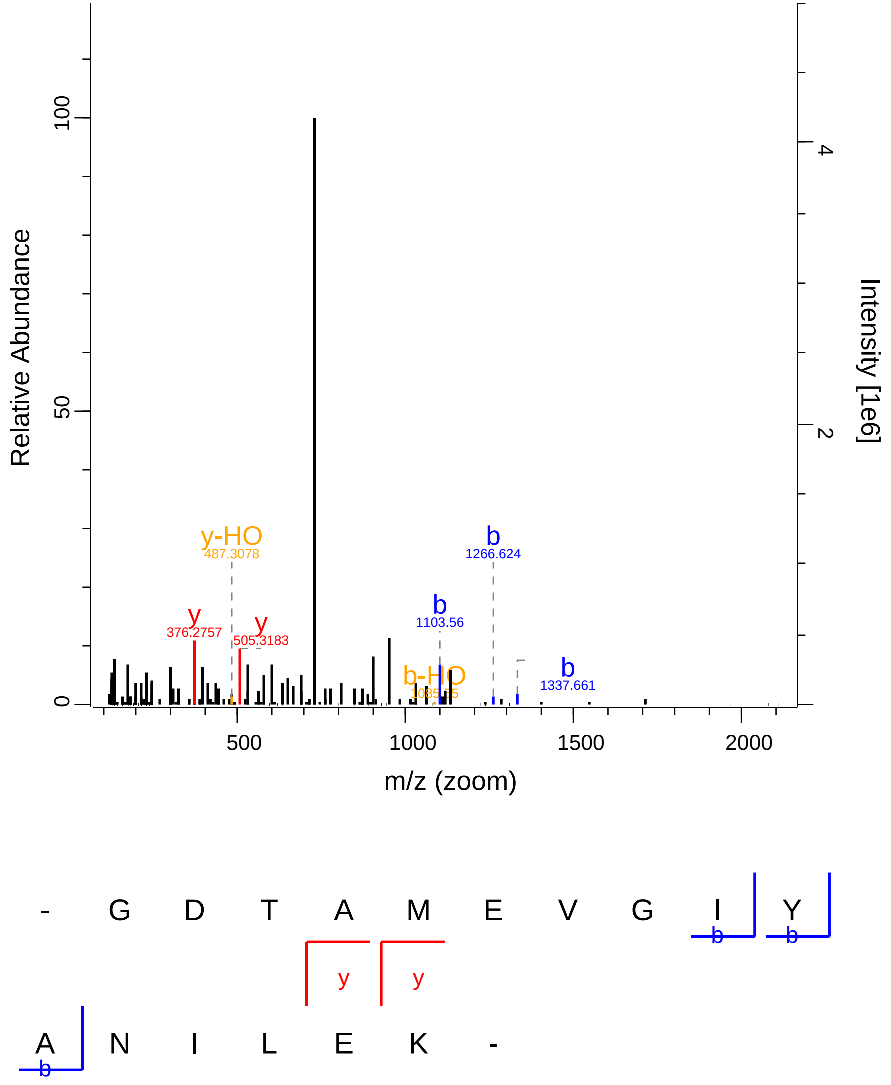

Raw File	Scan	Method	Score	m/z
RO-QC-160121_ZackD_SS_Set_02_Fraction_2	32016	FTMS; HCD	52.68	678.9

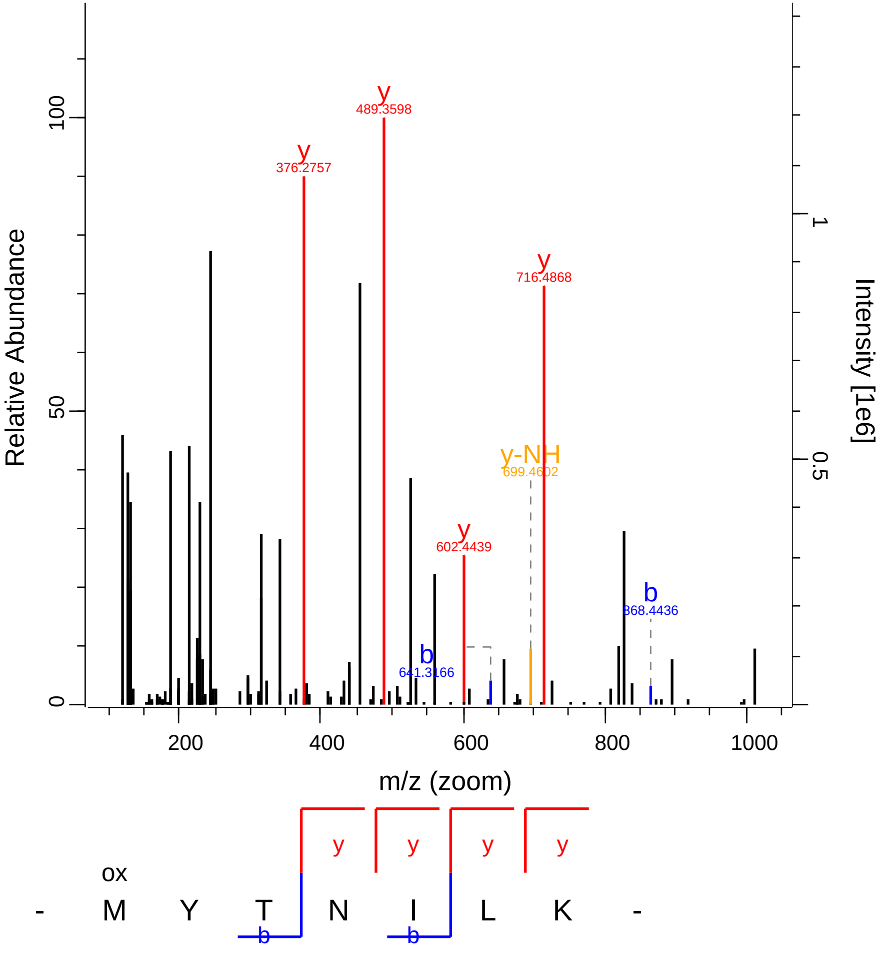

Raw File	Scan	Method	Score	m/z	Gene names
RO-QC-160121_ZackD_SS_Set_02_Fractior	3732	FTMS; HCI	68.07	412.22	ZSCAN26

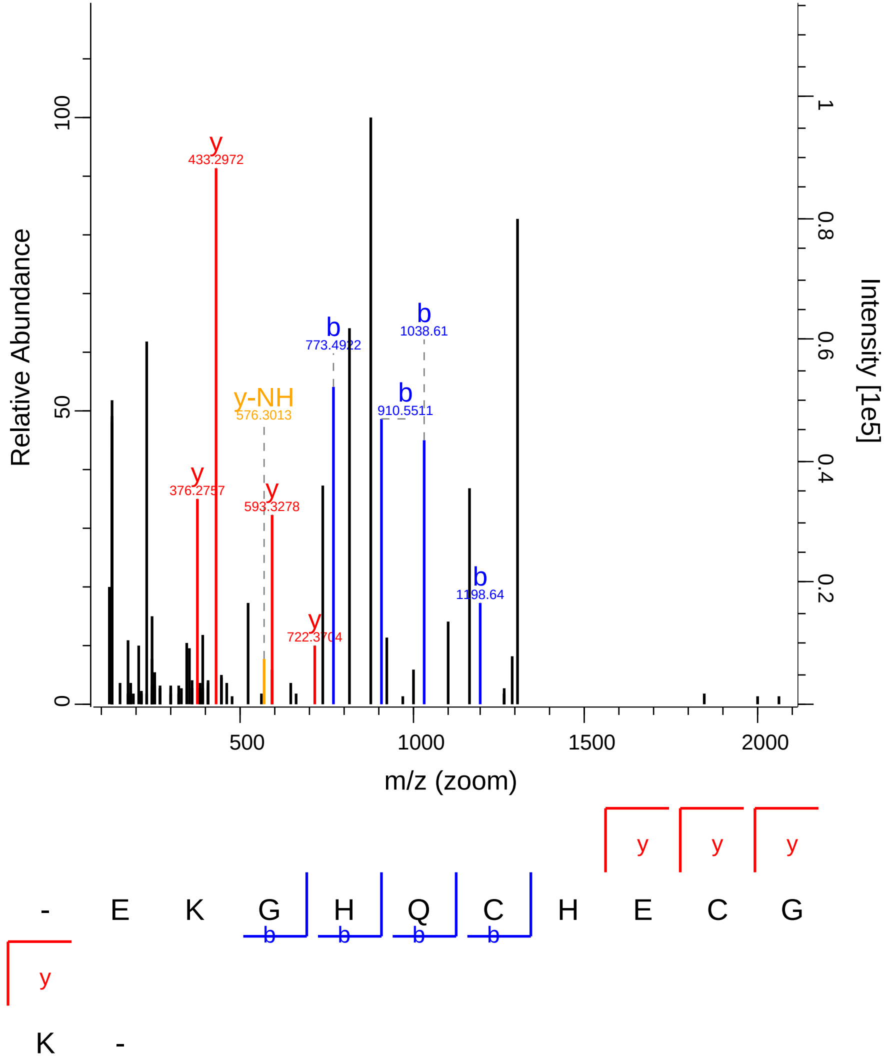

Raw File	Scan	Method	Score	m/z	Gene names
RO-QC-160121_ZackD_SS_Set_02_Fractio[illegible]	4585	FTMS; HC	155.48	522.95	TRAF3IP1

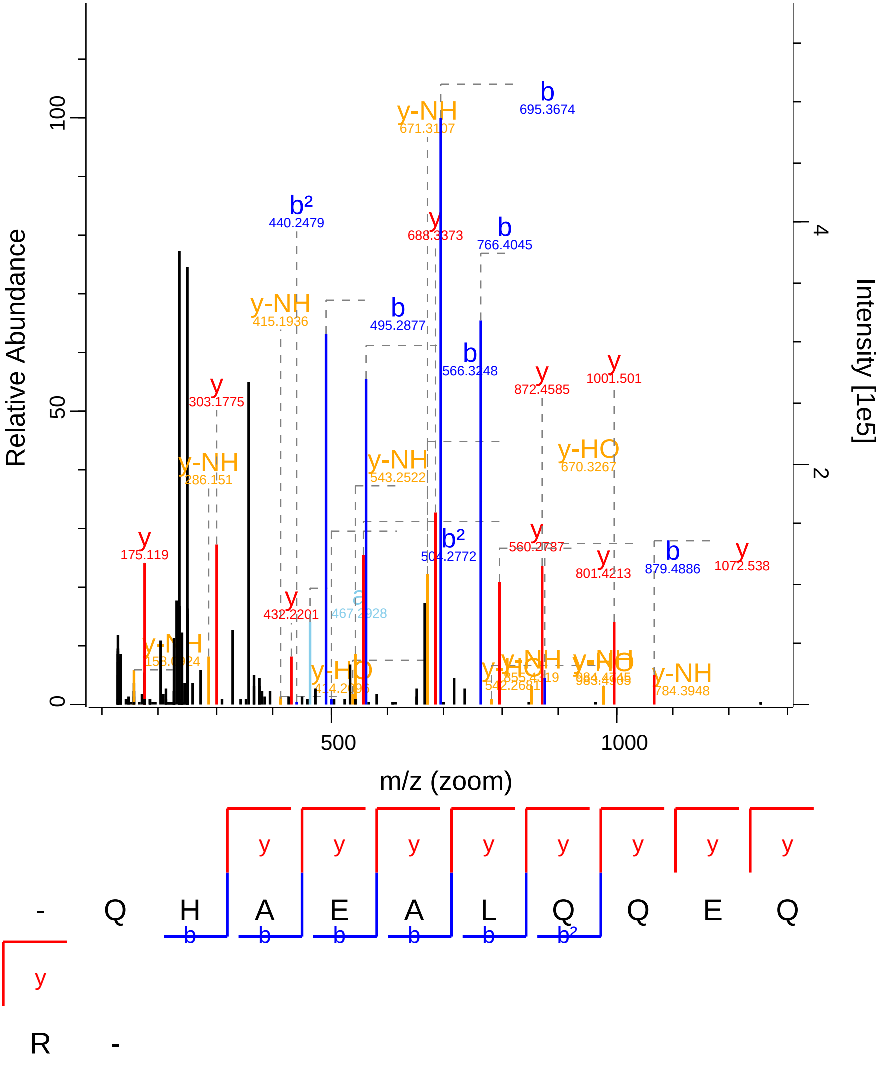

Raw File	Scan	Method	Score	m/z	Gene names
RO-QC-160121_ZackD_SS_Set_02_Fractior	4645	FTMS; HCl	75.82	496.28	MYC

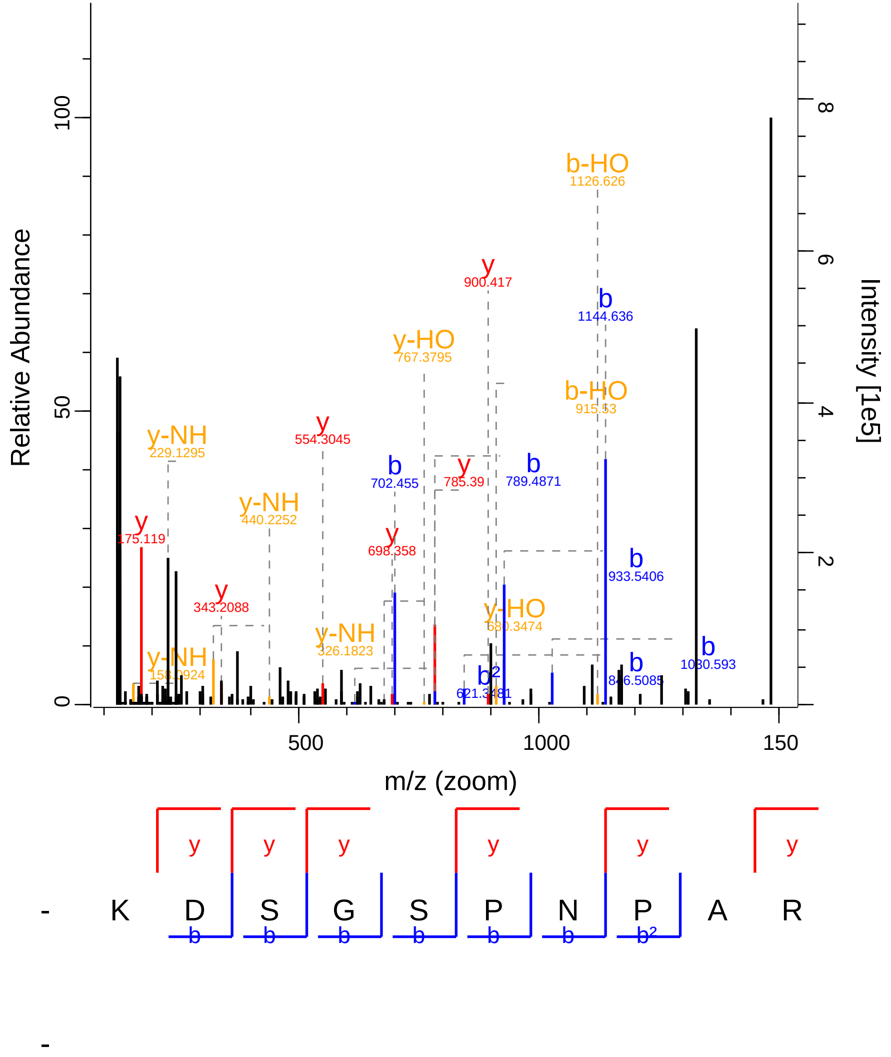

Raw File	Scan	Method	Score	m/z	Gene names
RO-QC-160121_ZackD_SS_Set_02_Fraction	4866	FTMS; HCI	76.77	548.8	WIPF1

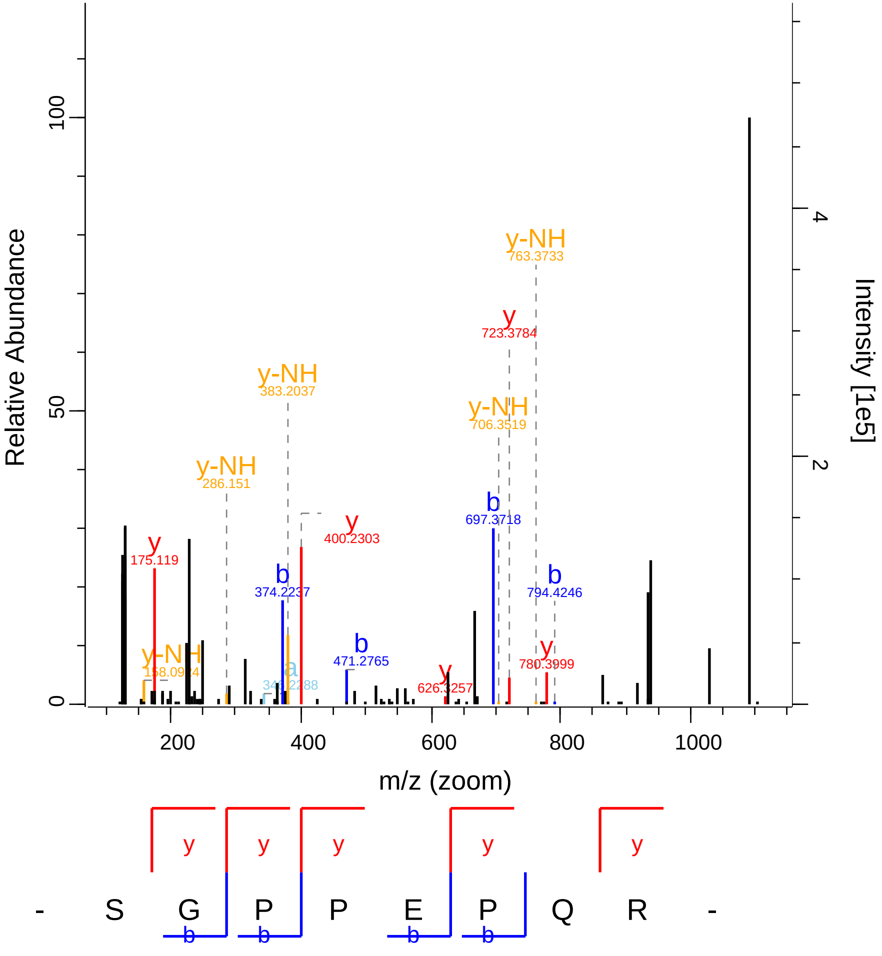

Raw File	Scan	Method	Score	m/z	Gene names
RO-QC-160121_ZackD_SS_Set_02_Fractior	5235	FTMS; HCI	66.6	495.03	MYSM1

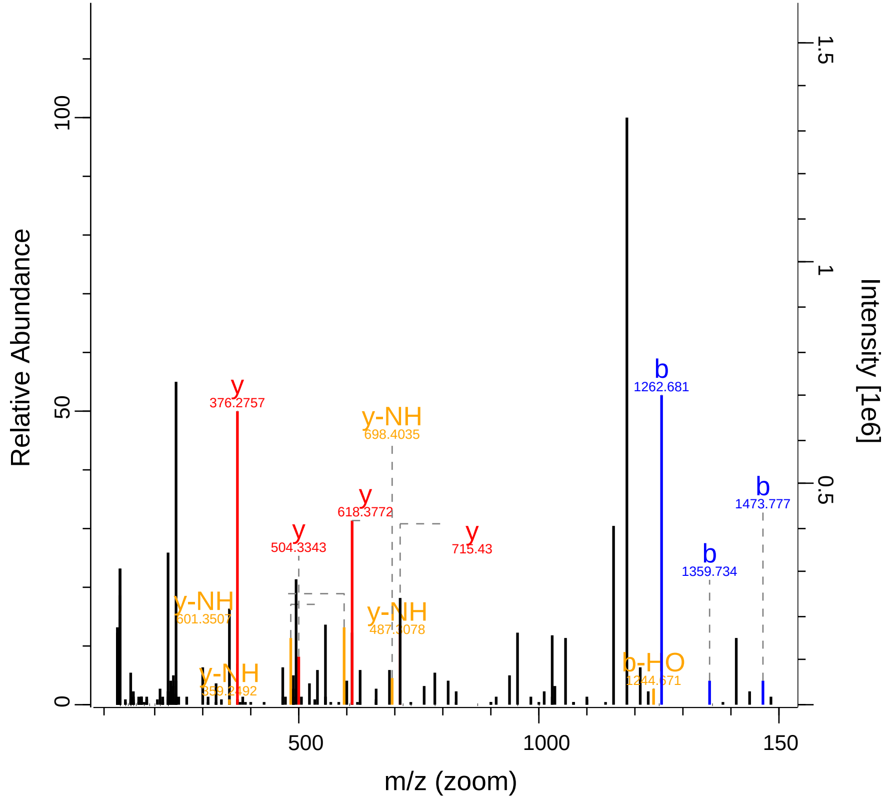

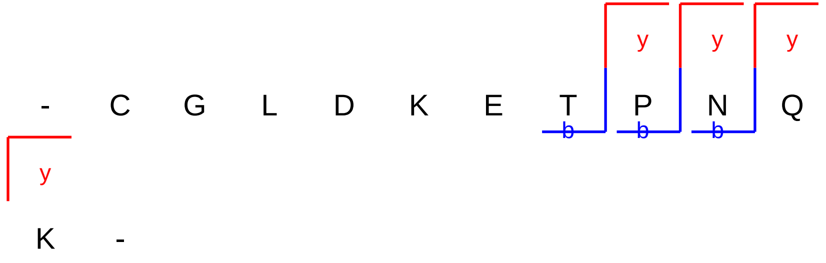

Raw File	Scan	Method	Score	m/z	Gene names
RO-QC-160121_ZackD_SS_Set_02_Fractior	5520	FTMS; HCI	55.26	566.62	SUN5

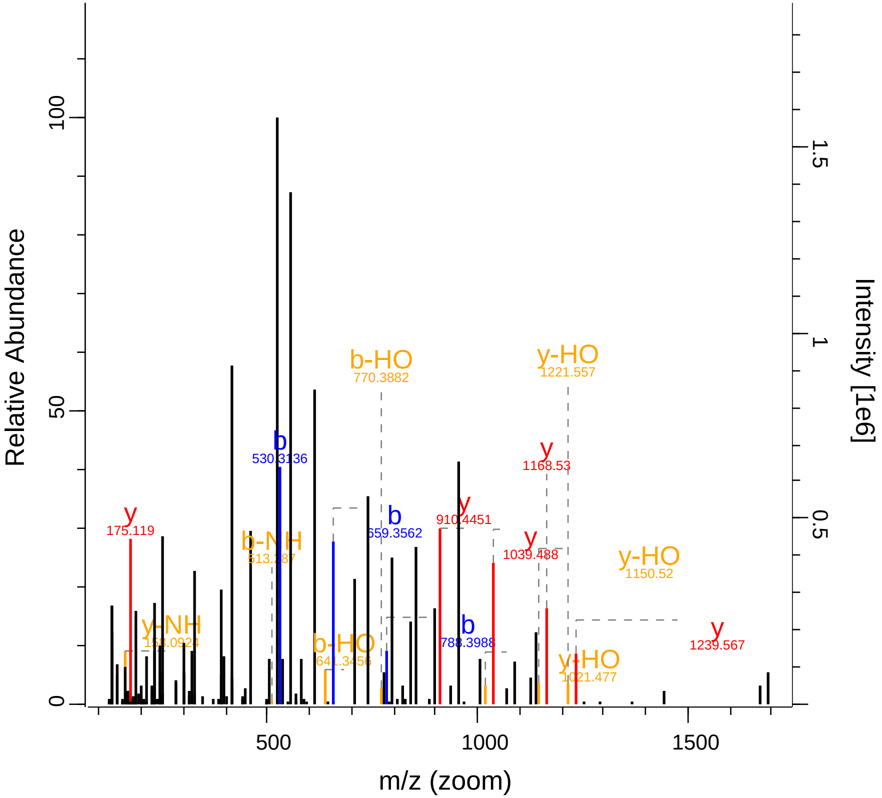

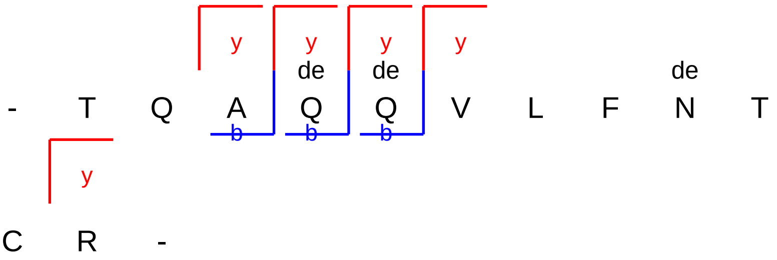

Raw File	Scan	Method	Score	m/z
RO-QC-160121_ZackD_SS_Set_02_Fraction_3	5521	FTMS; HCD	46.57	357.2

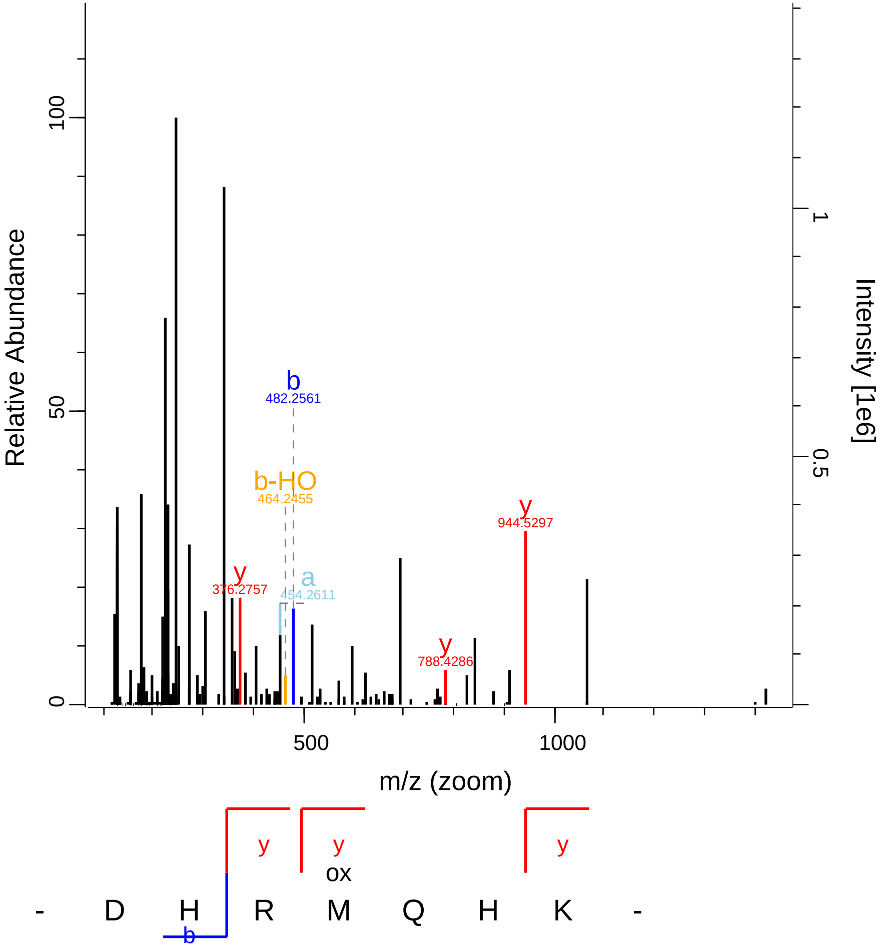

Raw File	Scan	Method	Score	m/z	Gene names
RO-QC-160121_ZackD_SS_Set_02_Fractior	5917	FTMS; HCI	99.82	686.31	C5orf45

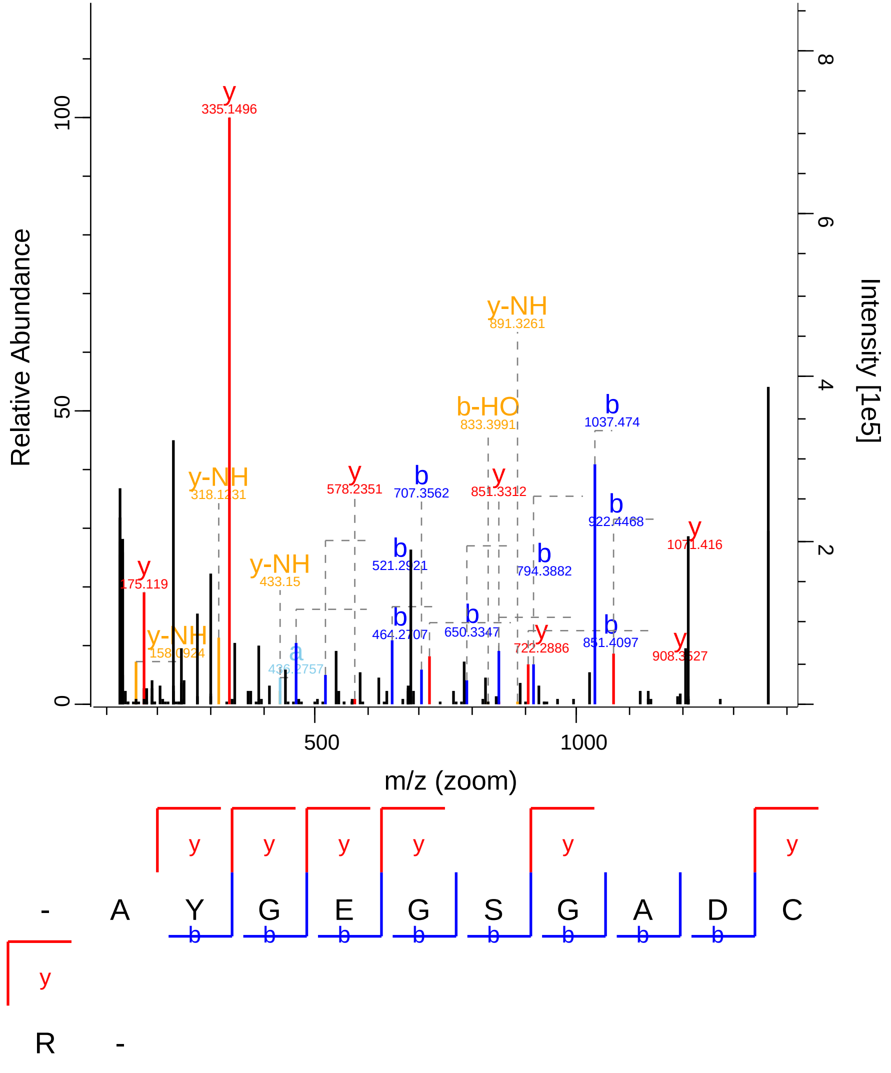

Raw File	Scan	Method	Score	m/z
RO-QC-160121_ZackD_SS_Set_02_Fraction_3	6181	FTMS; HCD	120.46	580.32

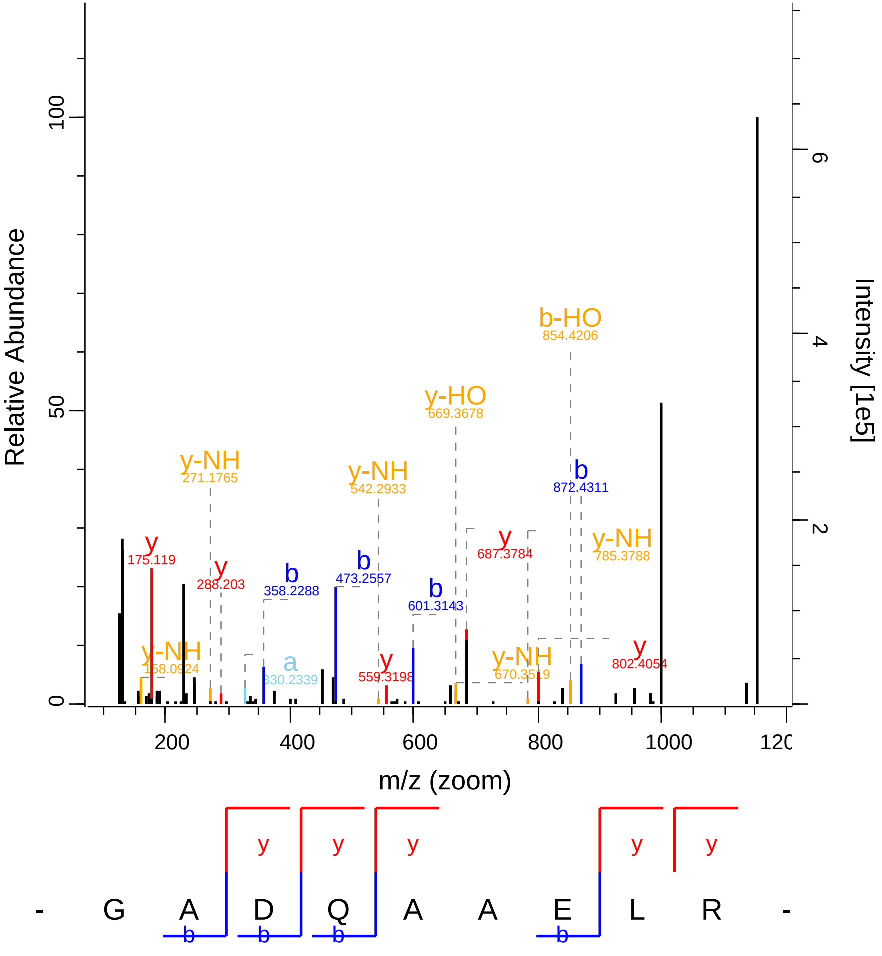

Raw File	Scan	Method	Score	m/z	Gene names
RO-QC-160121_ZackD_SS_Set_02_Fraction_	6697	FTMS; HCI	96.41	660	CASP2

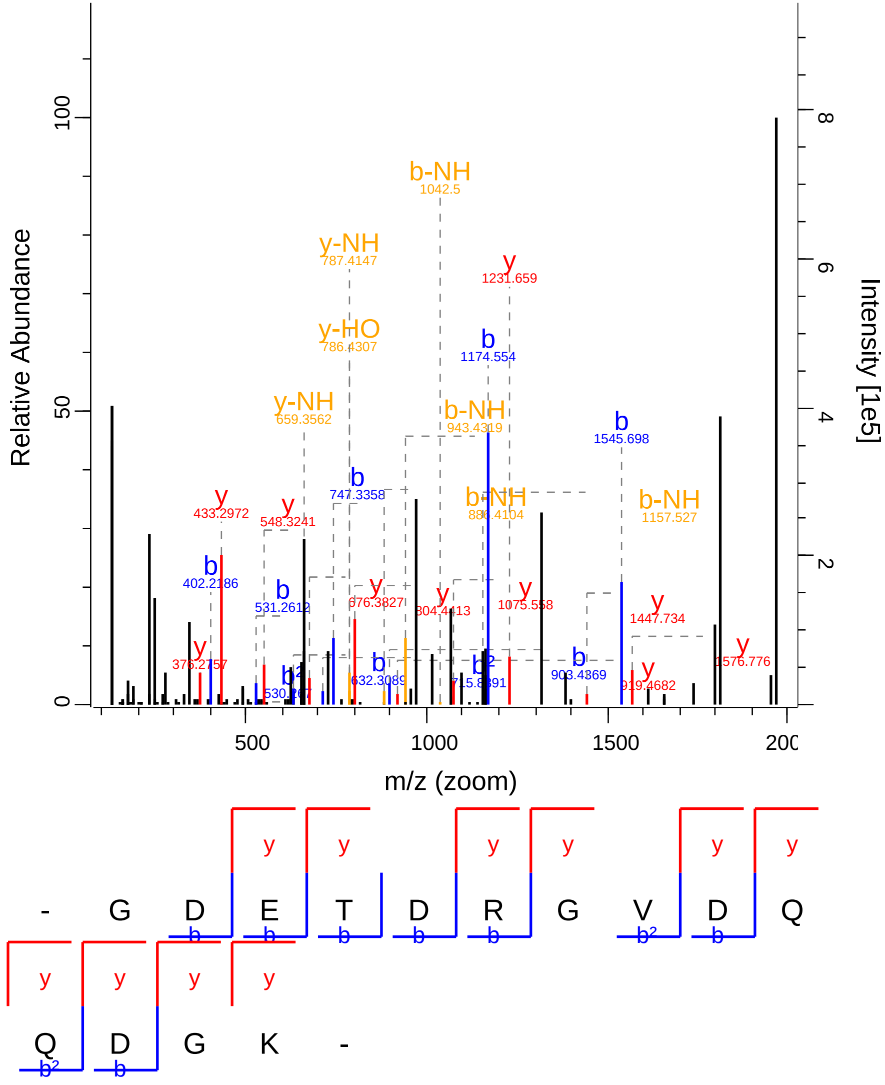

Raw File	Scan	Method	Score	m/z	Gene names
RO-QC-160121_ZackD_SS_Set_02_Fractior	7143	FTMS; HCI	70.44	618.02	SLC16A7

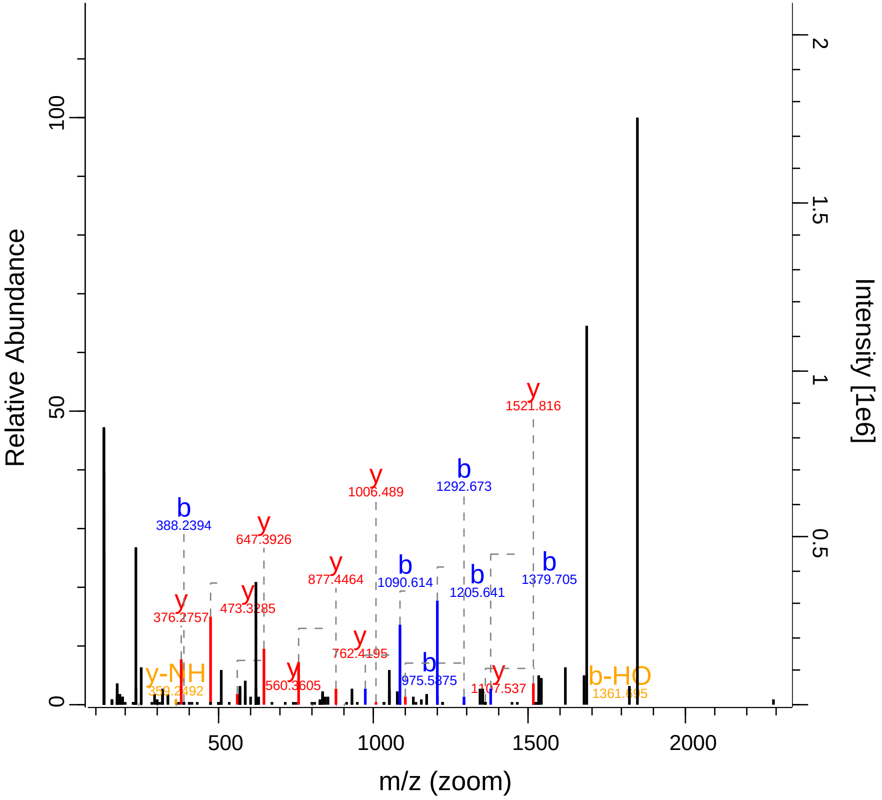

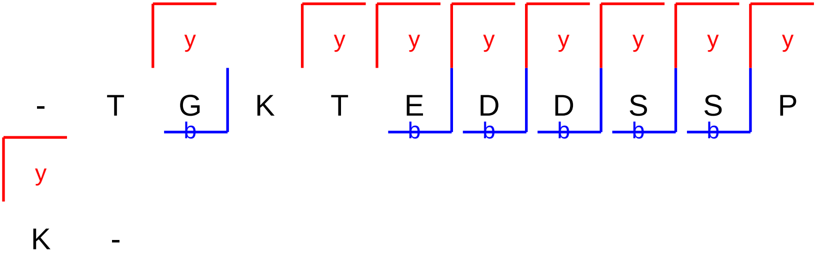

Raw File	Scan	Method	Score	m/z
RO-QC-160121_ZackD_SS_Set_02_Fraction_3	7512	FTMS; HCD	62.72	451.25

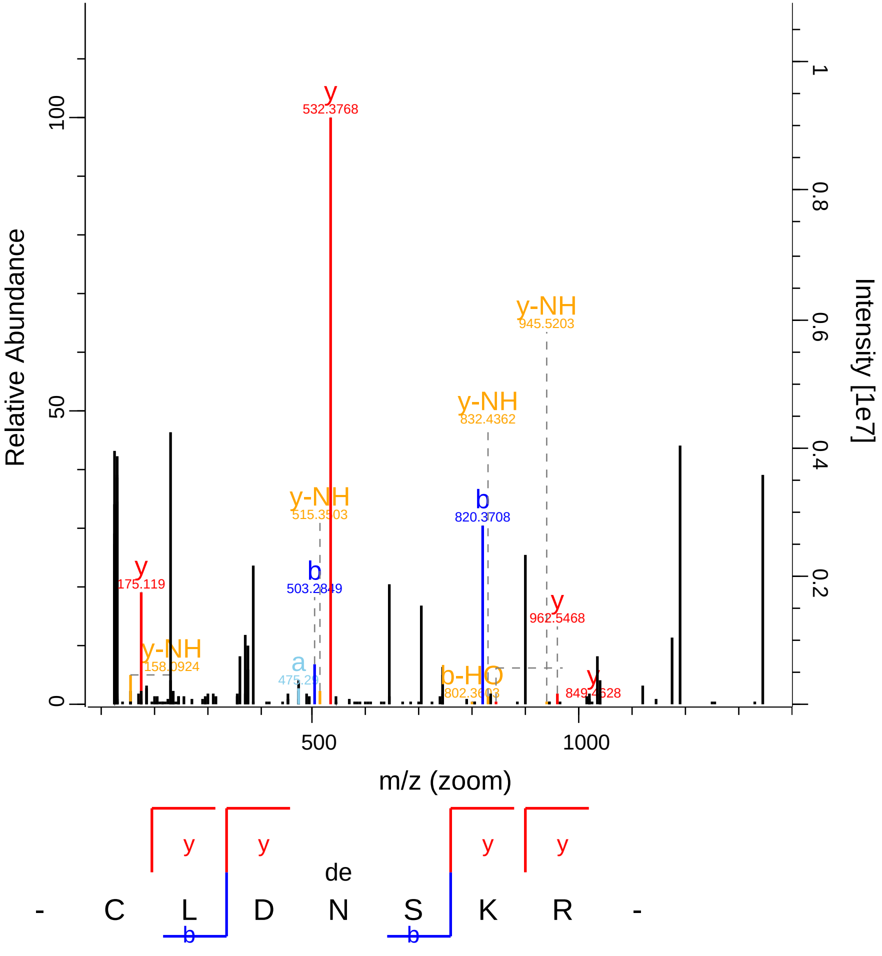

Raw File	Scan	Method	Score	m/z	Gene names
RO-QC-160121_ZackD_SS_Set_02_Fractior	7781	FTMS; HCI	70.86	370.56	NHLRC1

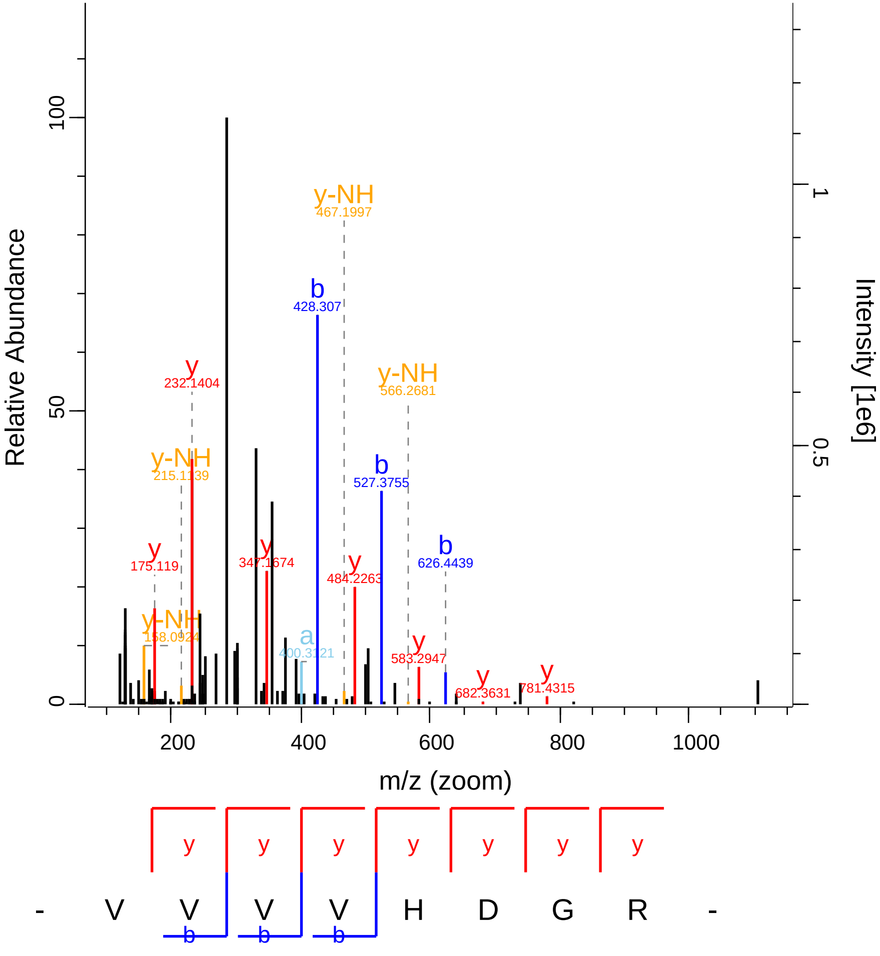

Raw File	Scan	Method	Score	m/z
RO-QC-160121_ZackD_SS_Set_02_Fraction_3	8509	FTMS; HCD	55.39	502.81

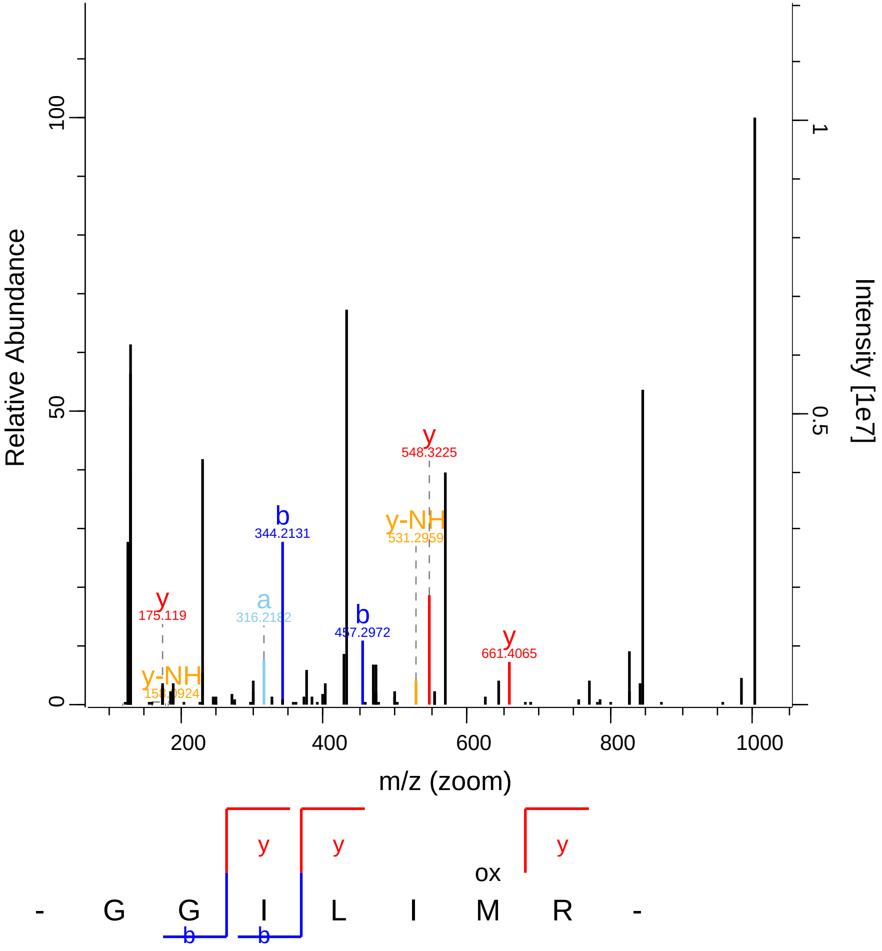

Raw File	Scan	Method	Score	m/z	Gene names
RO-QC-160121_ZackD_SS_Set_02_Fractior	8784	FTMS; HCI	76.34	638.33	ERICH1

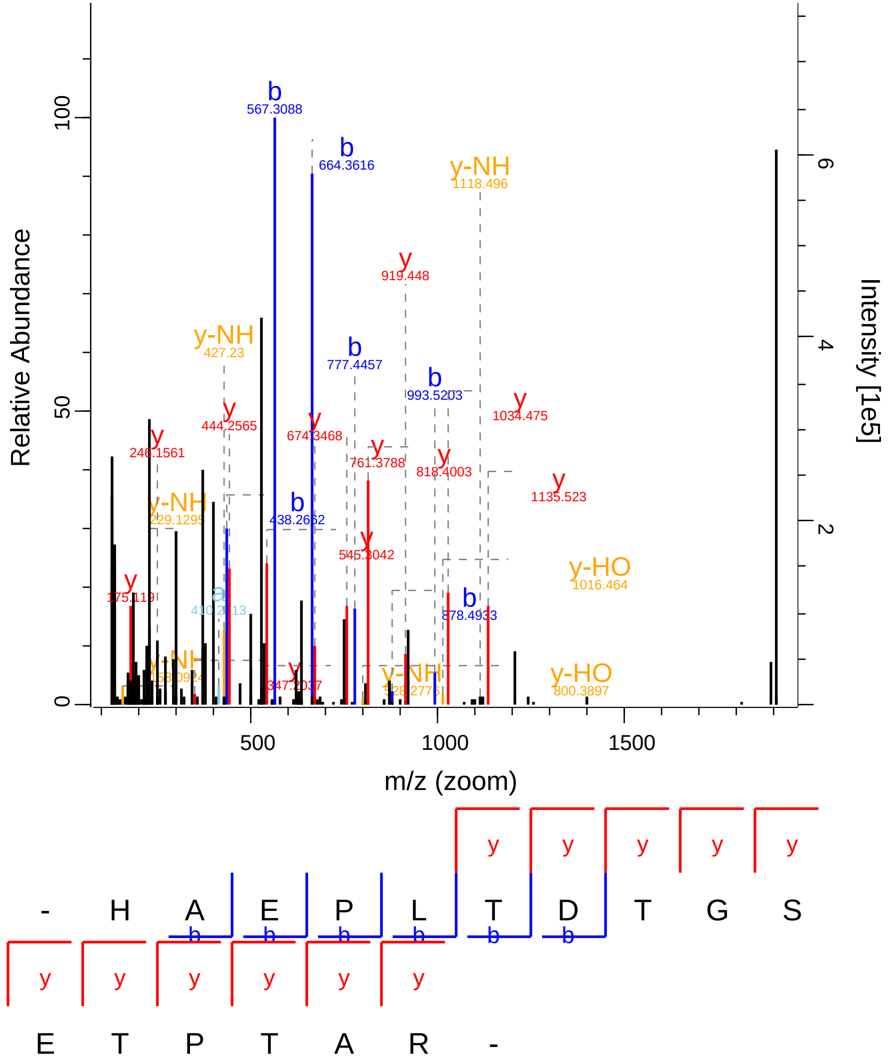

Raw File	Scan	Method	Score	m/z	Gene names
RO-QC-160121_ZackD_SS_Set_02_Fractior	9316	FTMS; HCI	78.82	623.83	TXNDC11

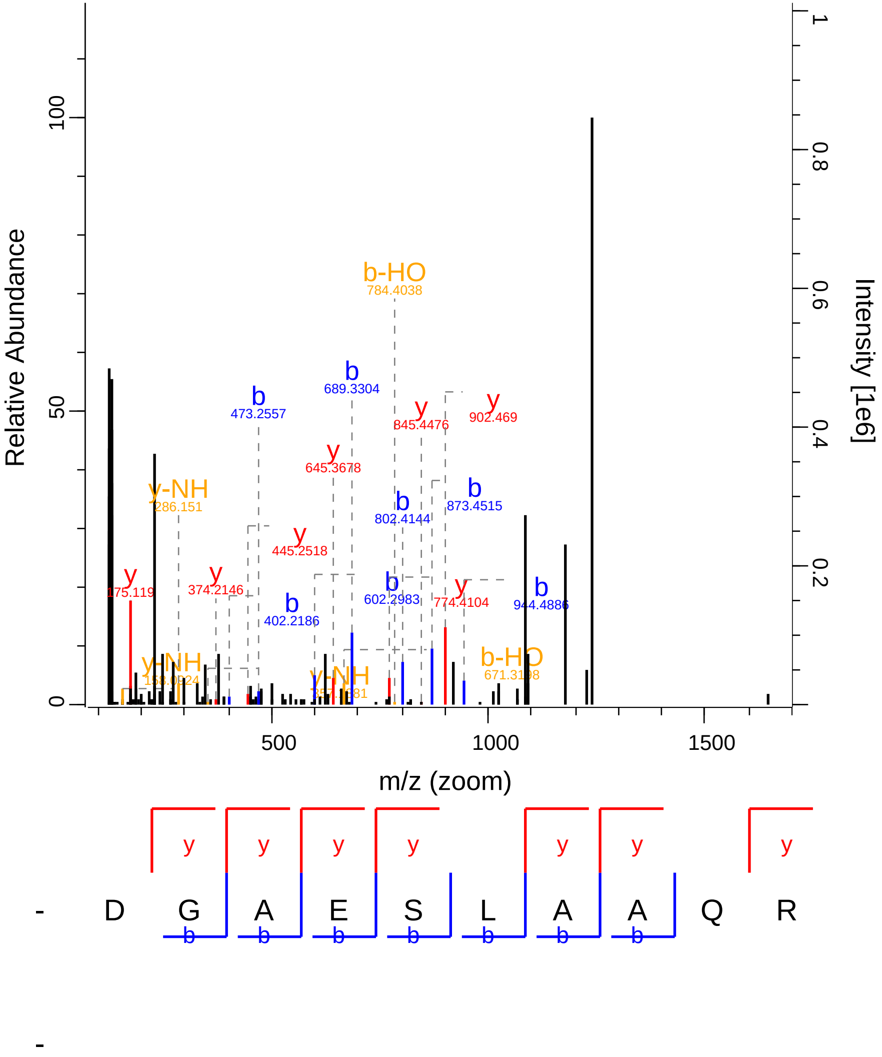

Raw File	Scan	Method	Score	m/z
RO-QC-160121_ZackD_SS_Set_02_Fraction_3	9659	FTMS; HCD	105.78	704.37

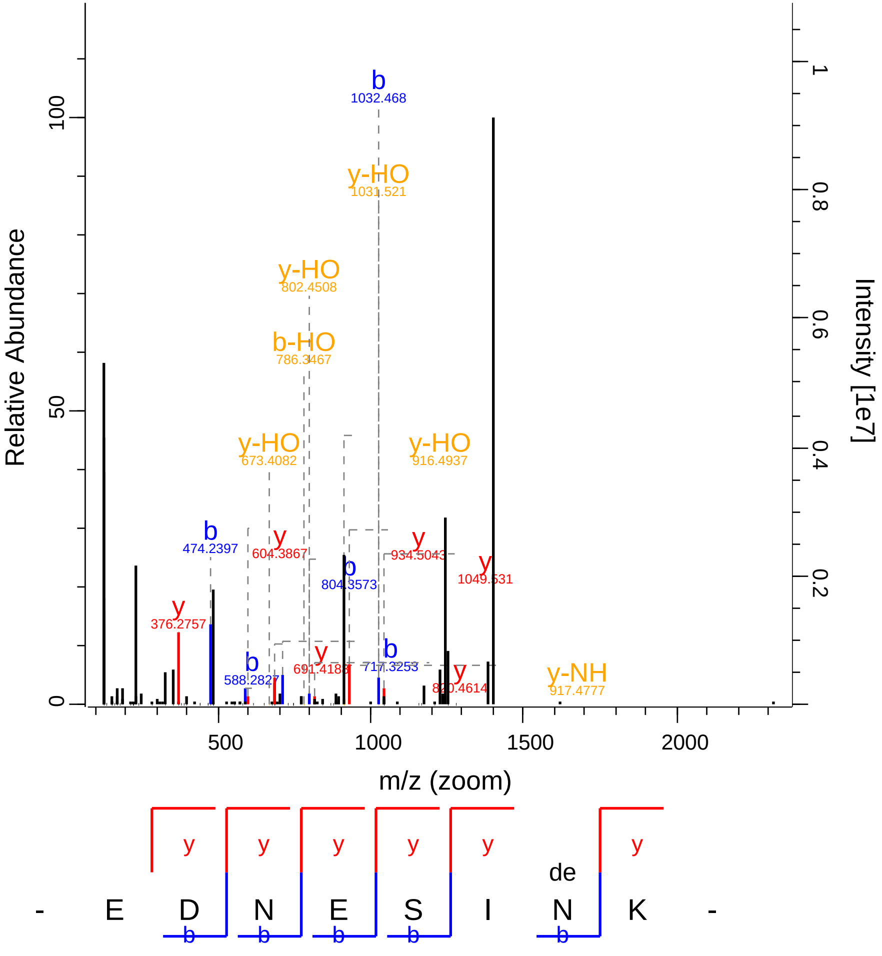

Raw File	Scan	Method	Score	m/z	Gene names
RO-QC-160121_ZackD_SS_Set_02_Fractio[illegible]	9783	FTMS; HC	139.28	637.88	G6PC3

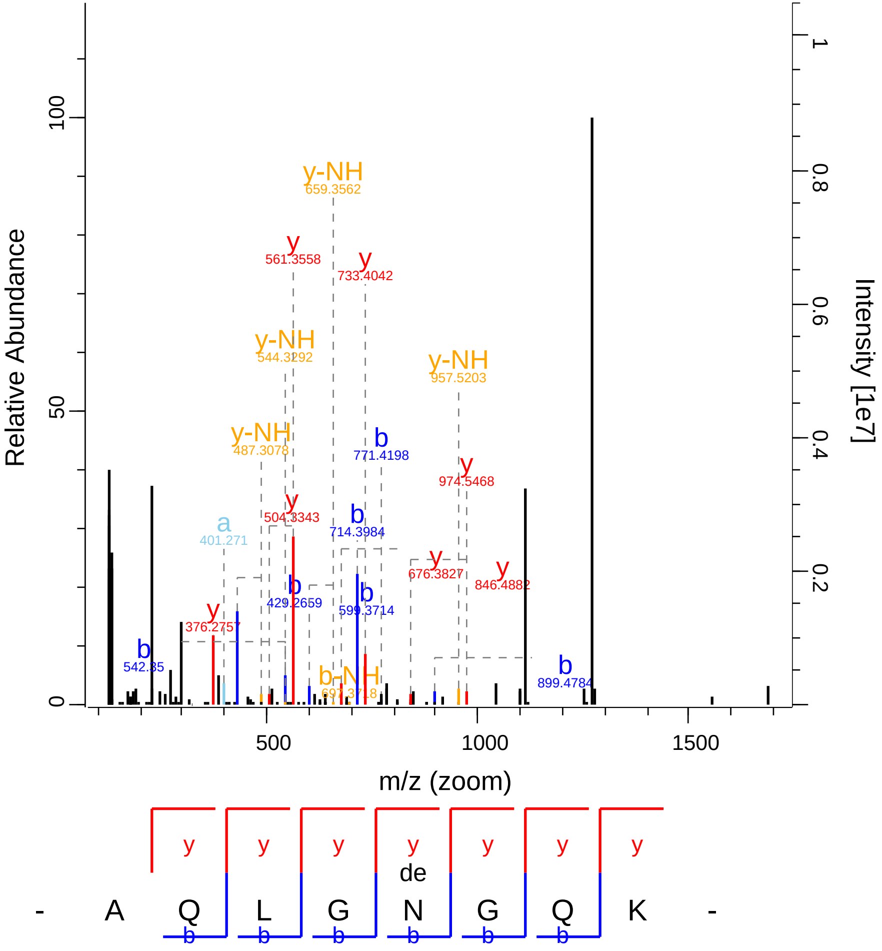

Raw File	Scan	Method	Score	m/z	Gene names
RO-QC-160121_ZackD_SS_Set_02_Fractior	9912	FTMS; HCI	42.82	584.67	OR2AE1

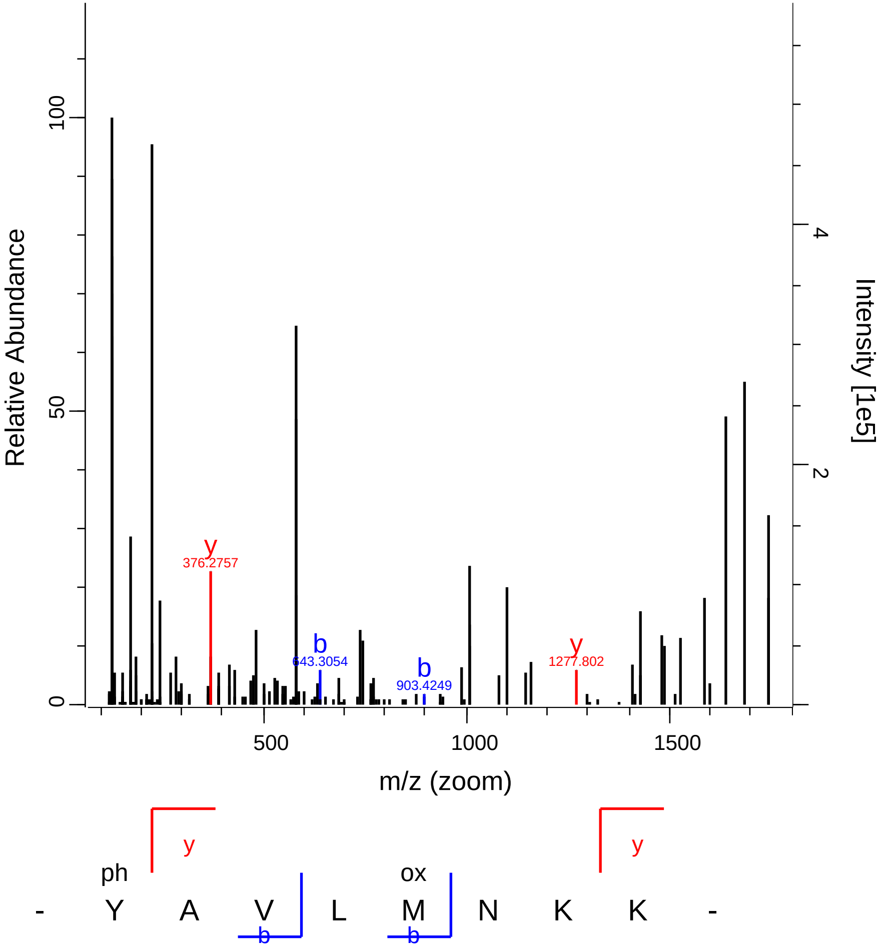

Raw File	Scan	Method	Score	m/z	Gene names
RO-QC-160121_ZackD_SS_Set_02_Fractio	10477	FTMS; HC	137.85	792.91	UBL3

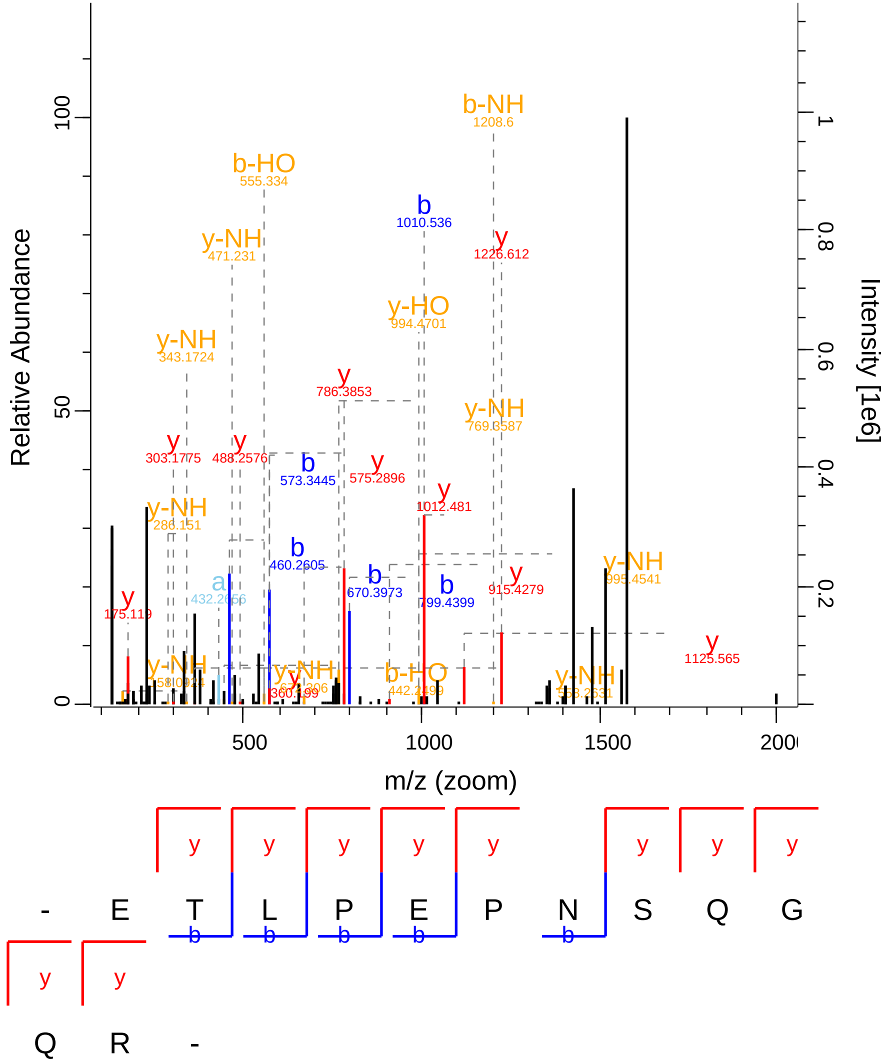

Raw File	Scan	Method	Score	m/z	Gene names
RO-QC-160121_ZackD_SS_Set_02_Fractio	10494	FTMS; HC	166.16	739.38	PLP2

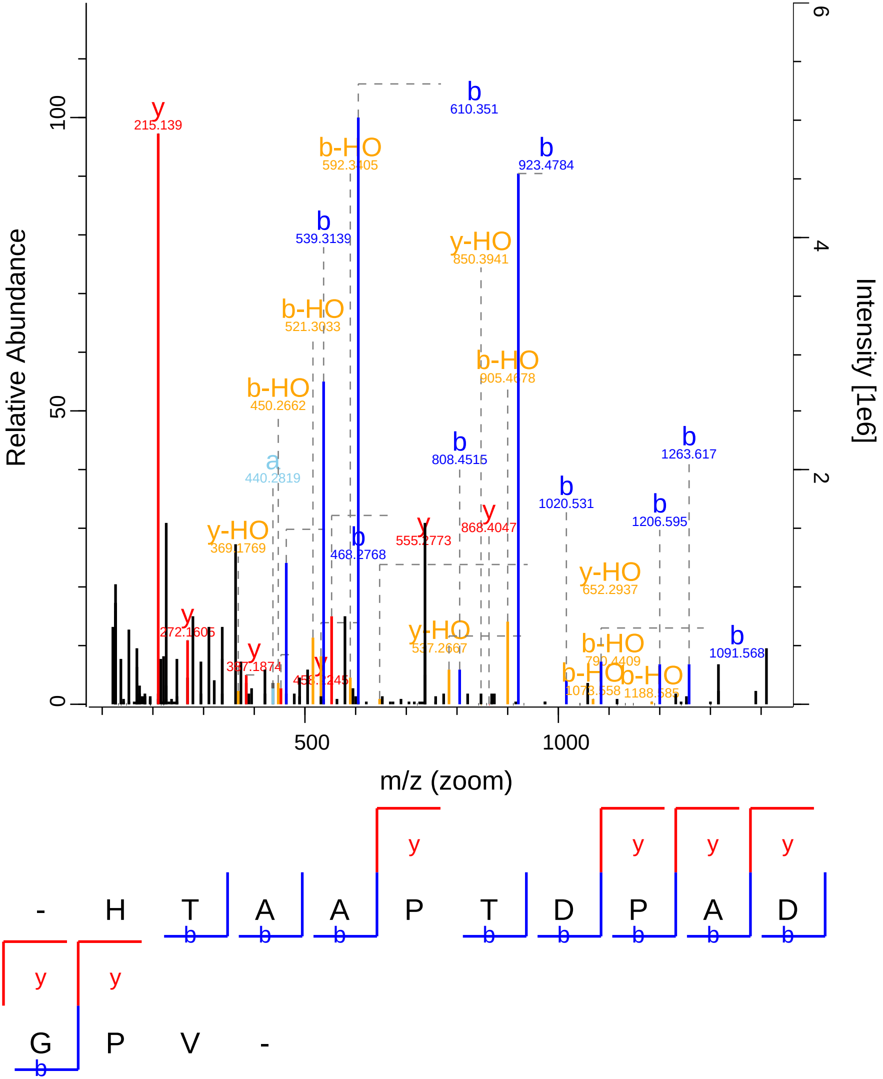

Raw File	Scan	Method	Score	m/z	Gene names
RO-QC-160121_ZackD_SS_Set_02_Fractio	10716	FTMS; HC	146.81	739.38	PLP2

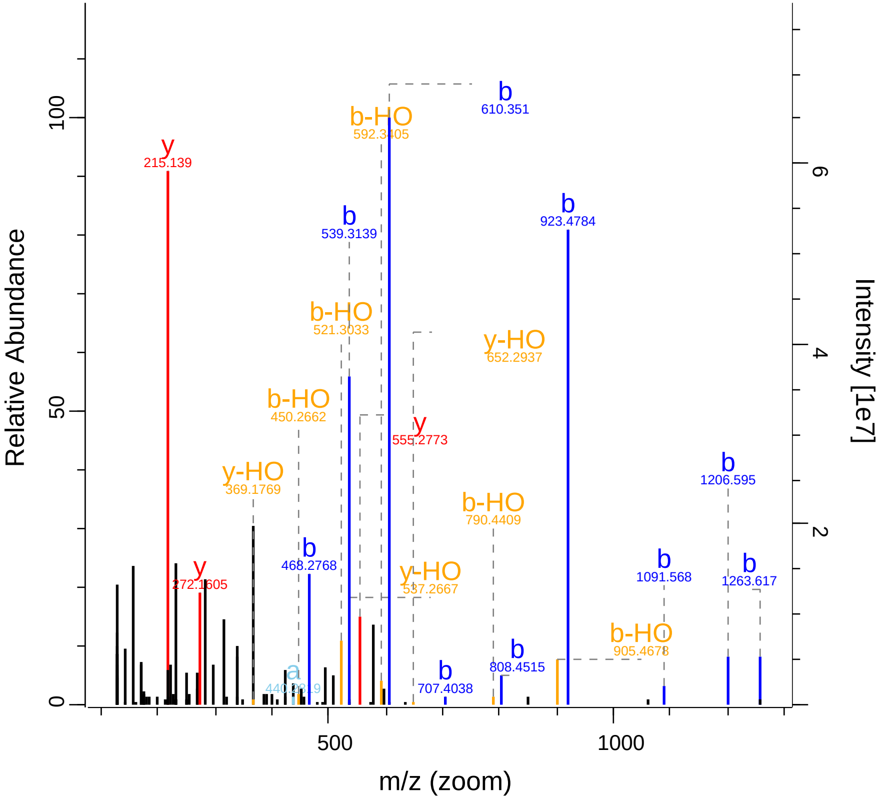

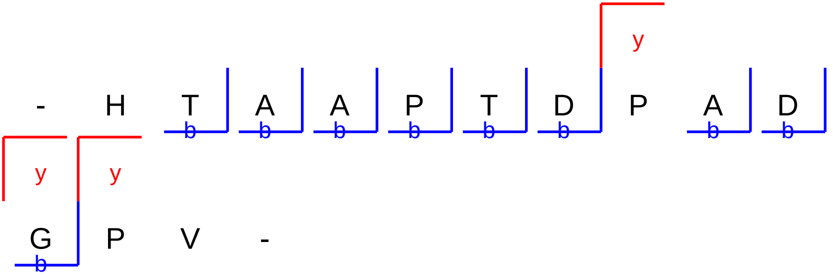

Raw File	Scan	Method	Score	m/z	Gene names
RO-QC-160121_ZackD_SS_Set_02_Fractio	10954	FTMS; HC	90.13	739.38	PLP2

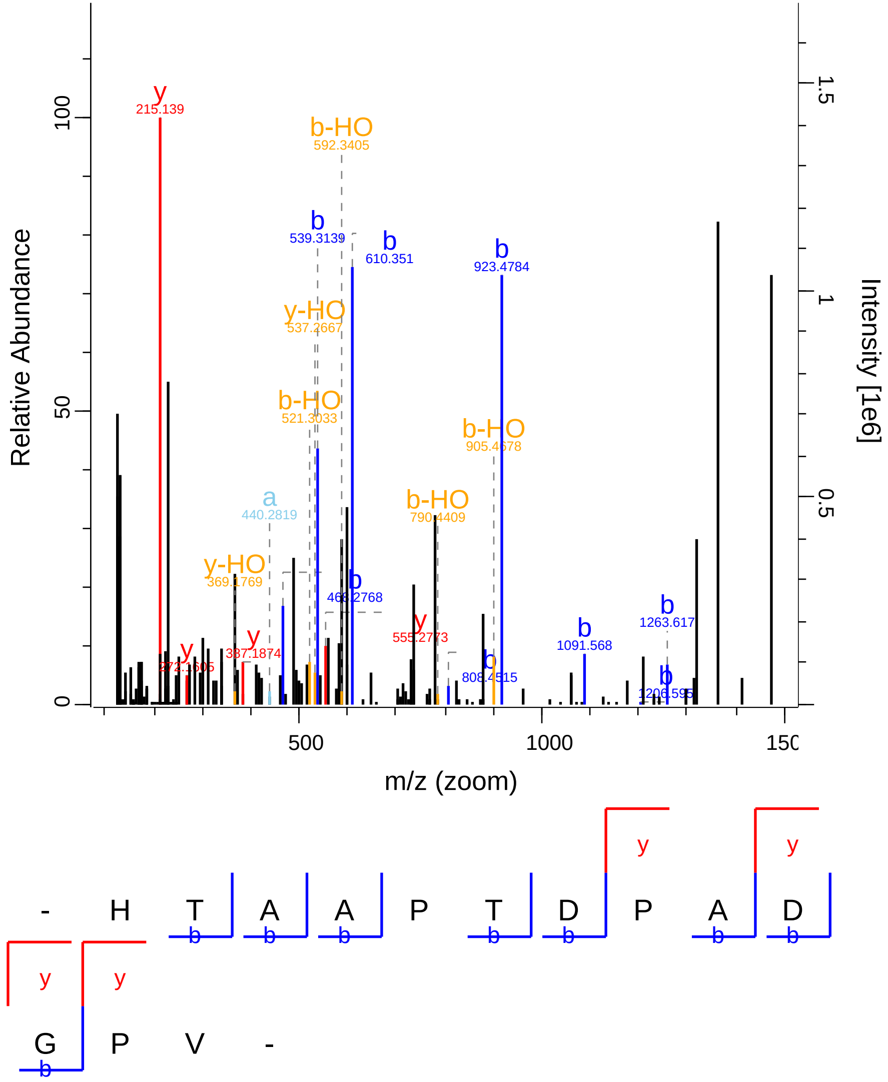

Raw File	Scan	Method	Score	m/z	Gene names
RO-QC-160121_ZackD_SS_Set_02_Fractio	12196	FTMS; HC	180.63	888.17	ZNF316

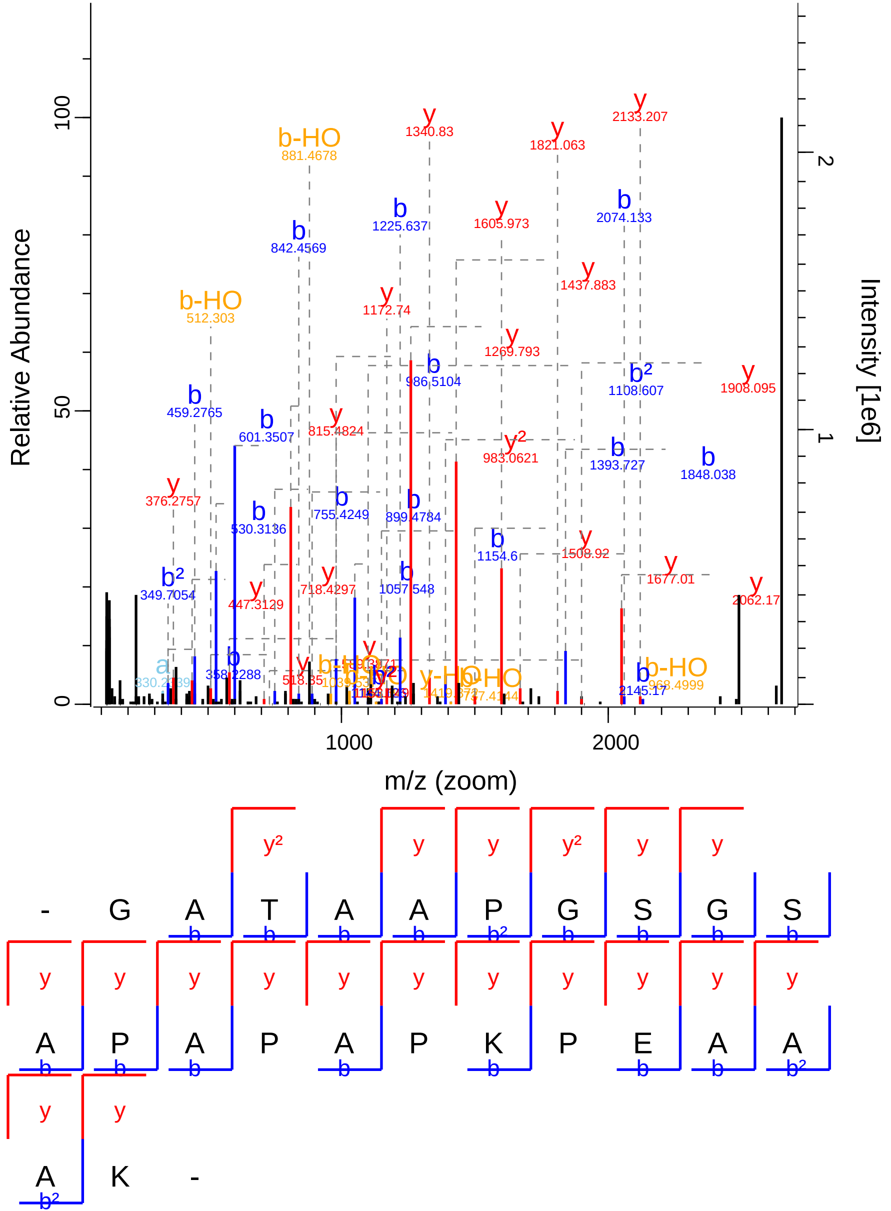

Raw File	Scan	Method	Score	m/z	Gene names
RO-QC-160121_ZackD_SS_Set_02_Fractio	12903	FTMS; HC	50.91	629.35	SLC7A14

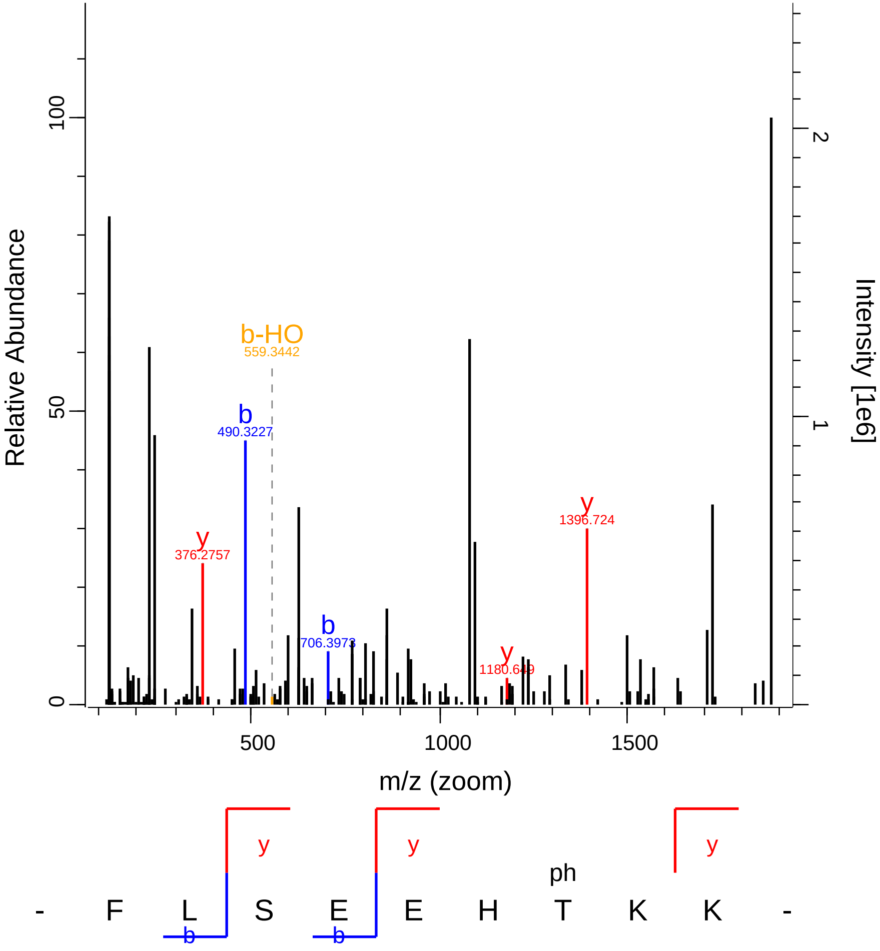

Raw File	Scan	Method	Score	m/z	Gene names
RO-QC-160121_ZackD_SS_Set_02_Fractio	13369	FTMS; HC	132.01	714.93	DSE

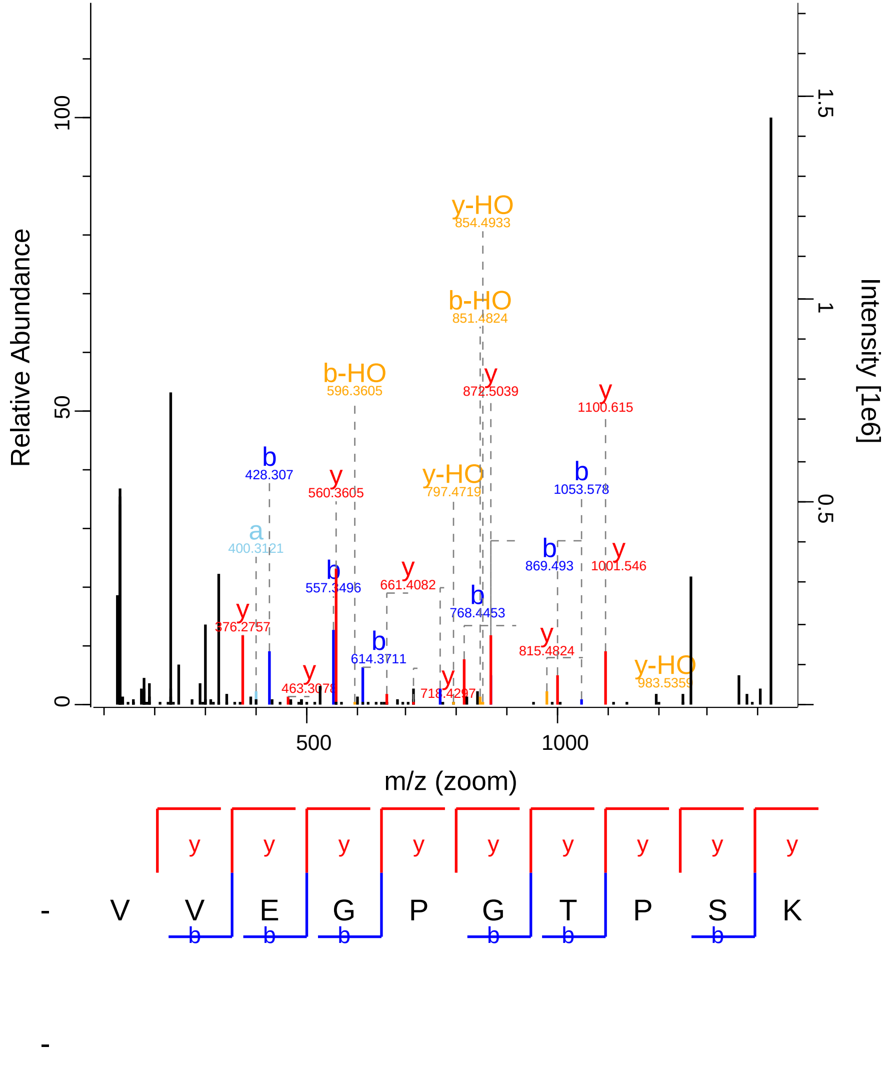

Raw File	Scan	Method	Score	m/z
RO-QC-160121_ZackD_SS_Set_02_Fraction_3	13973	FTMS; HCD	51.69	641.85

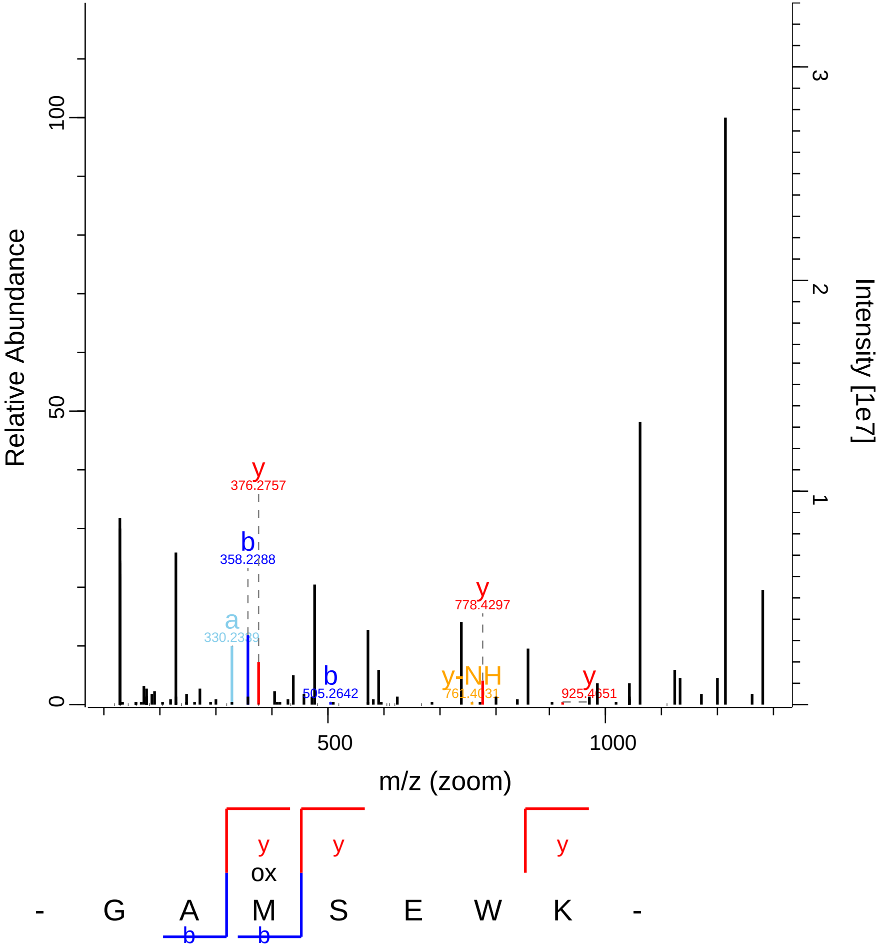

Raw File	Scan	Method	Score	m/z
RO-QC-160121_ZackD_SS_Set_02_Fraction_3	14193	FTMS; HCD	62	641.85

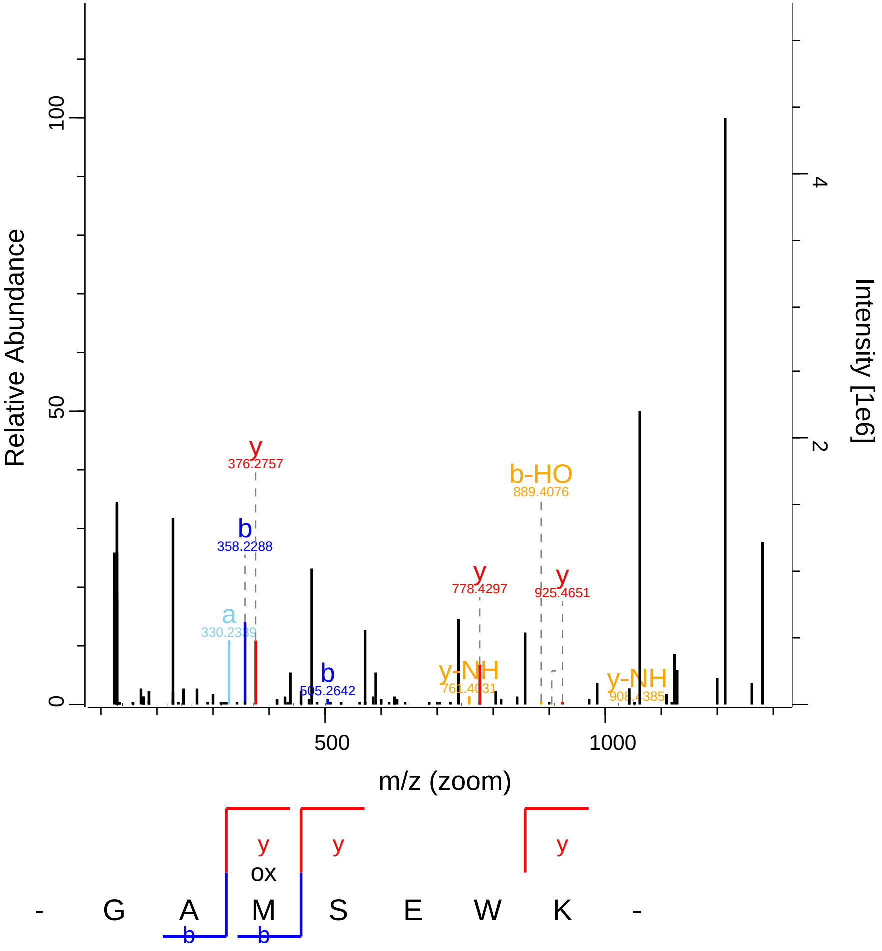

Raw File	Scan	Method	Score	m/z	Gene names
RO-QC-160121_ZackD_SS_Set_02_Fractio	14531	FTMS; HC	113.69	729.38	JOSD2

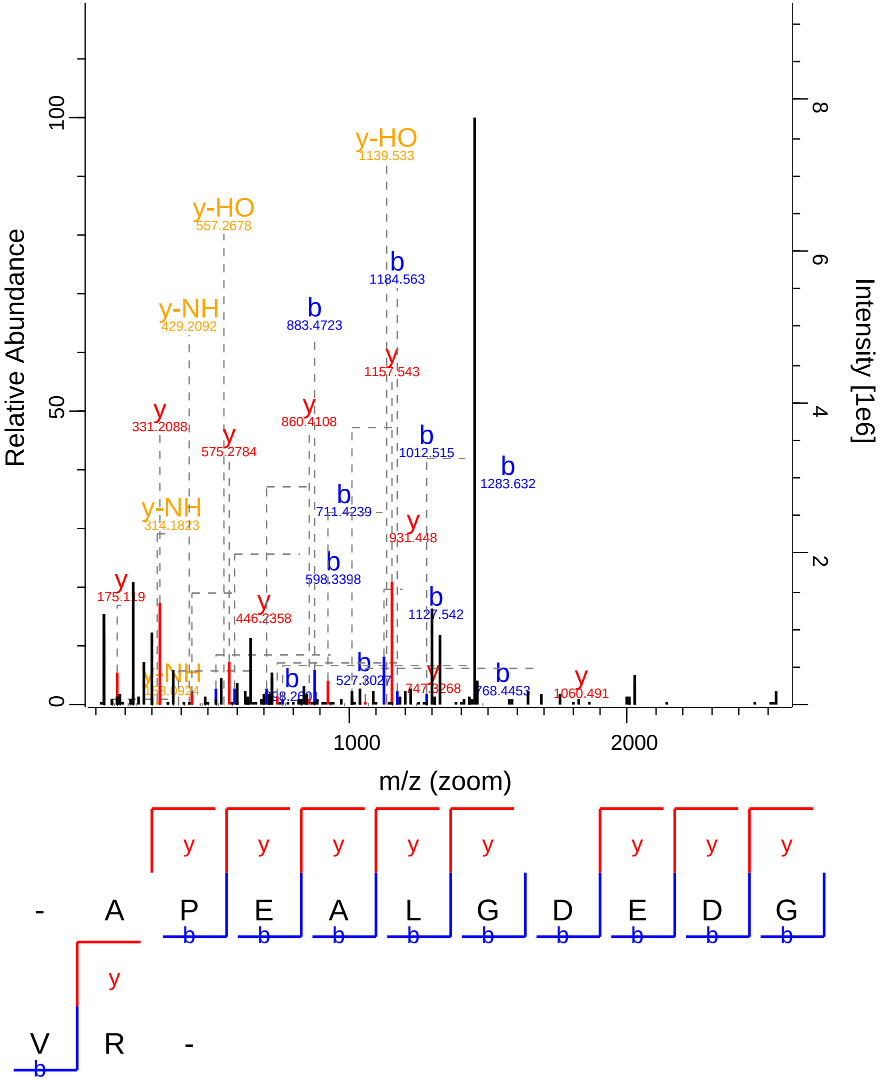

Raw File	Scan	Method	Score	m/z	Gene names
RO-QC-160121_ZackD_SS_Set_02_Fractio	15185	FTMS; HC	66.27	403.98	BPIFB4

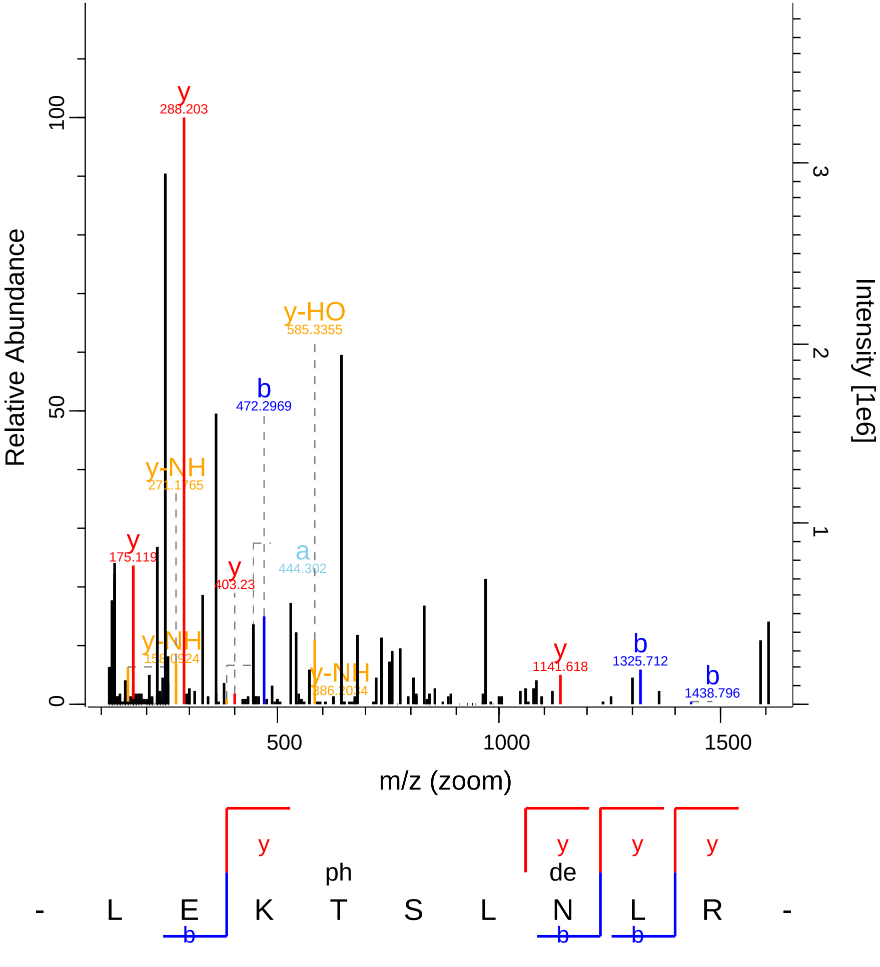

Raw File	Scan	Method	Score	m/z	Gene names
RO-QC-160121_ZackD_SS_Set_02_Fractio	15203	FTMS; HC	81.3	703.41	ATG12

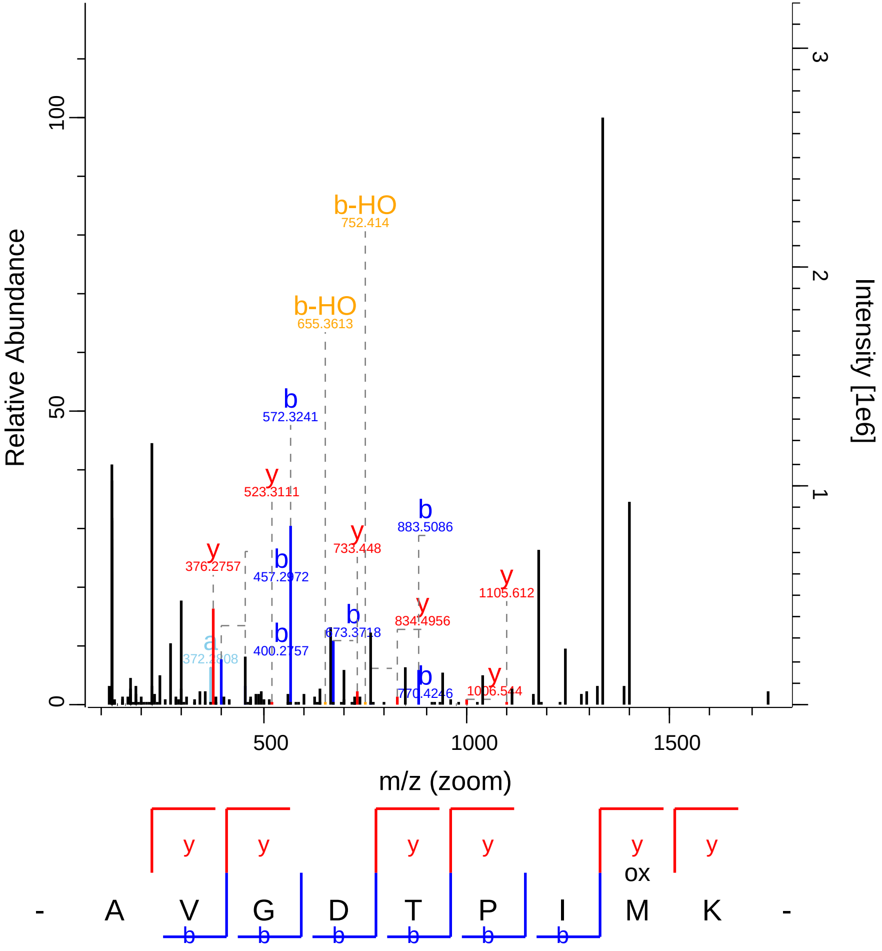

Raw File	Scan	Method	Score	m/z	Gene names
RO-QC-160121_ZackD_SS_Set_02_Fractio	15346	FTMS; HC	87.64	691.39	SLC7A6

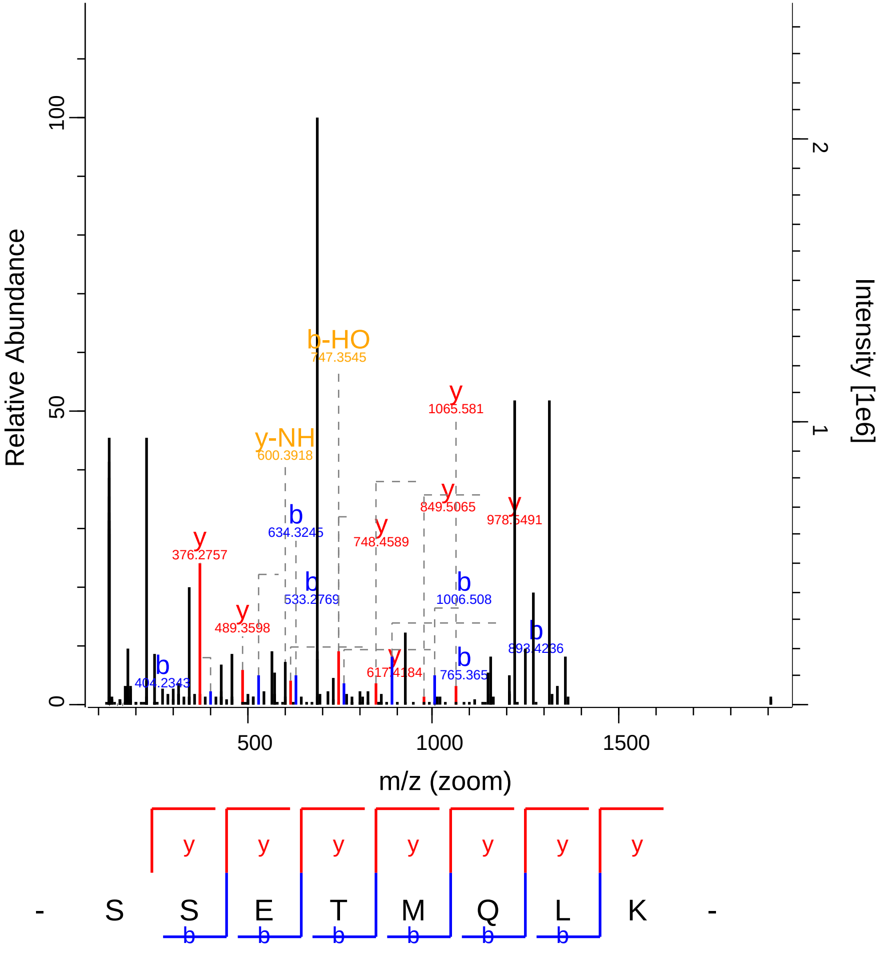

Raw File	Scan	Method	Score	m/z	Gene names
RO-QC-160121_ZackD_SS_Set_02_Fractio	15359	FTMS; HC	40.07	704.33	TBX19

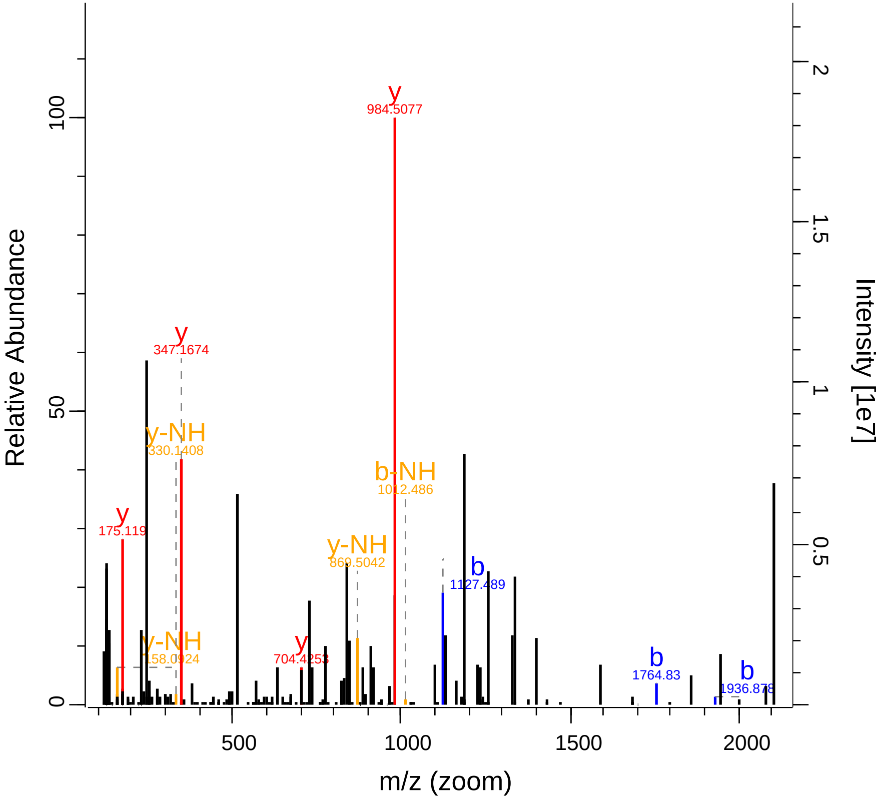

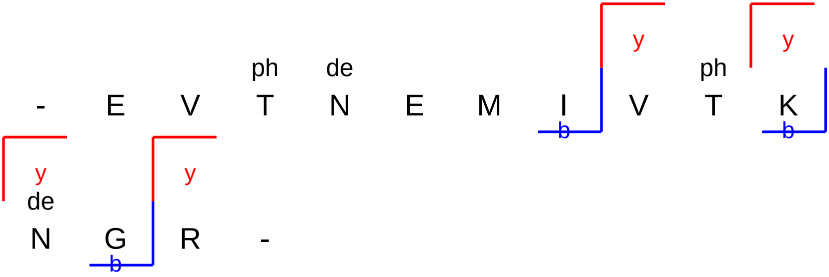

Raw File	Scan	Method	Score	m/z	Gene names
RO-QC-160121_ZackD_SS_Set_02_Fractio	16076	FTMS; HC	116.55	848.45	DTL

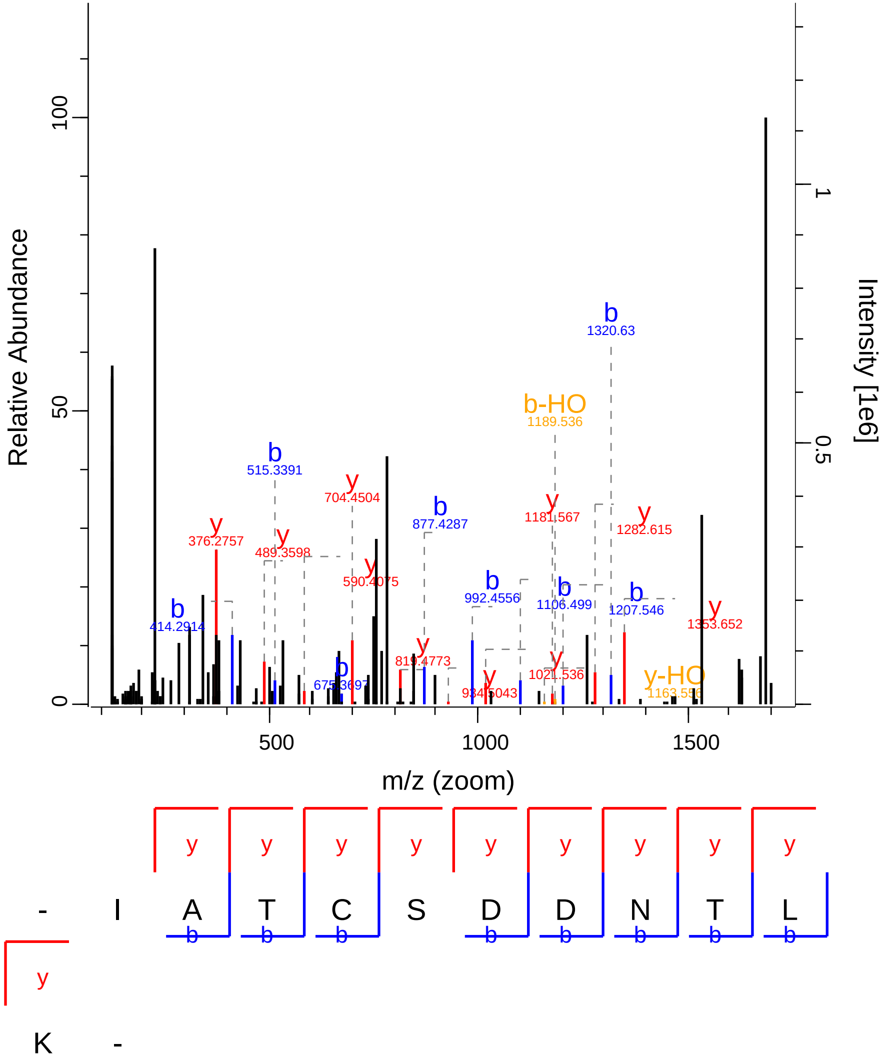

Raw File	Scan	Method	Score	m/z	Gene names
RO-QC-160121_ZackD_SS_Set_02_Fractio	16169	FTMS; HC	71.88	705.73	PQLC1

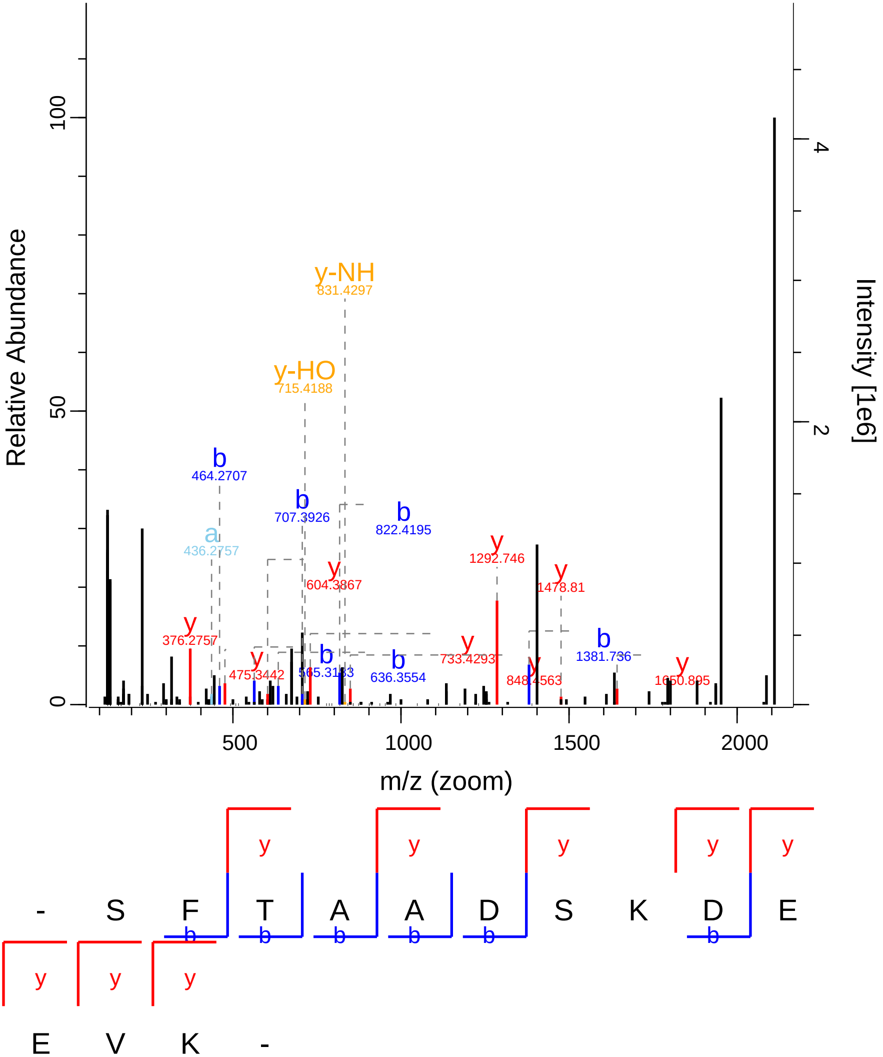

Raw File	Scan	Method	Score	m/z	Gene names
RO-QC-160121_ZackD_SS_Set_02_Fractic	16218	FTMS; HC	114.86	1036.45	TOR1AIP2

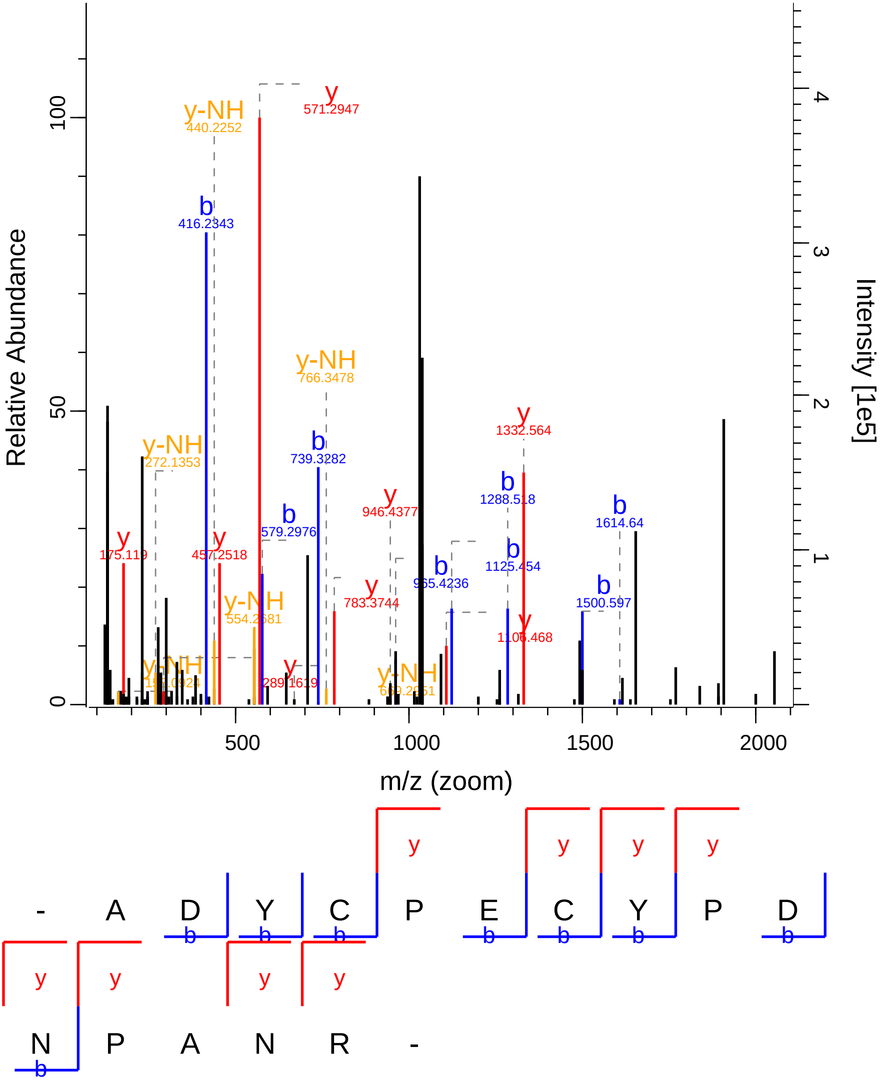

Raw File	Scan	Method	Score	m/z	Gene names
RO-QC-160121_ZackD_SS_Set_02_Fractio	16787	FTMS; HC	83.44	707.38	MFSD1

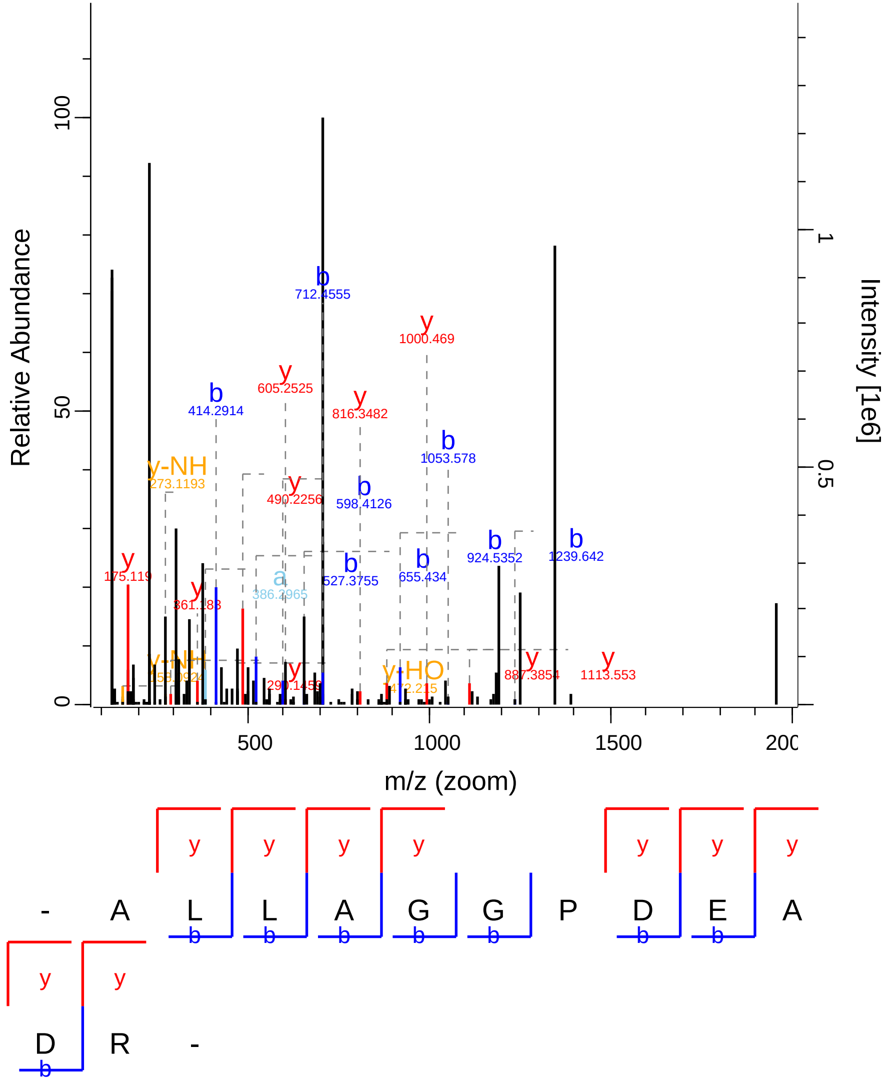

Raw File	Scan	Method	Score	m/z	Gene names
RO-QC-160121_ZackD_SS_Set_02_Fractio	16996	FTMS; HC	130.98	707.38	MFSD1

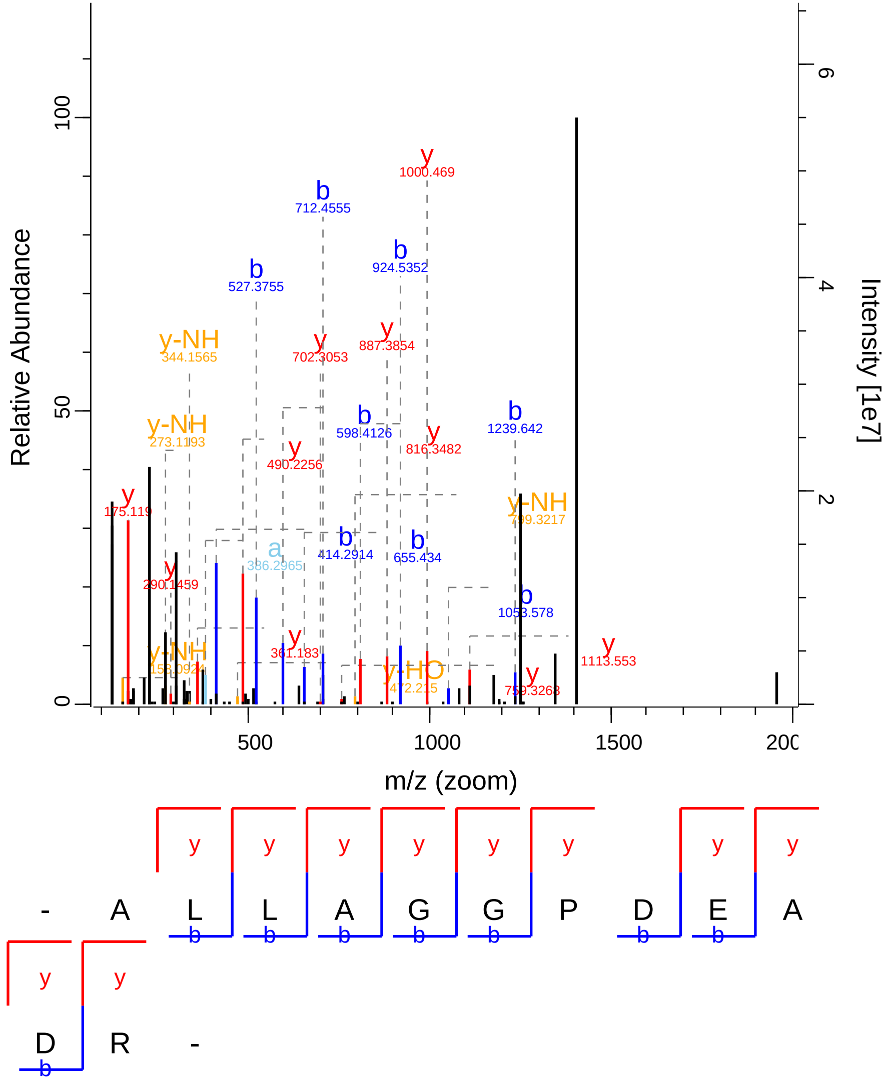

Raw File	Scan	Method	Score	m/z
RO-QC-160121_ZackD_SS_Set_02_Fraction_3	17029	FTMS; HCD	87.31	718.92

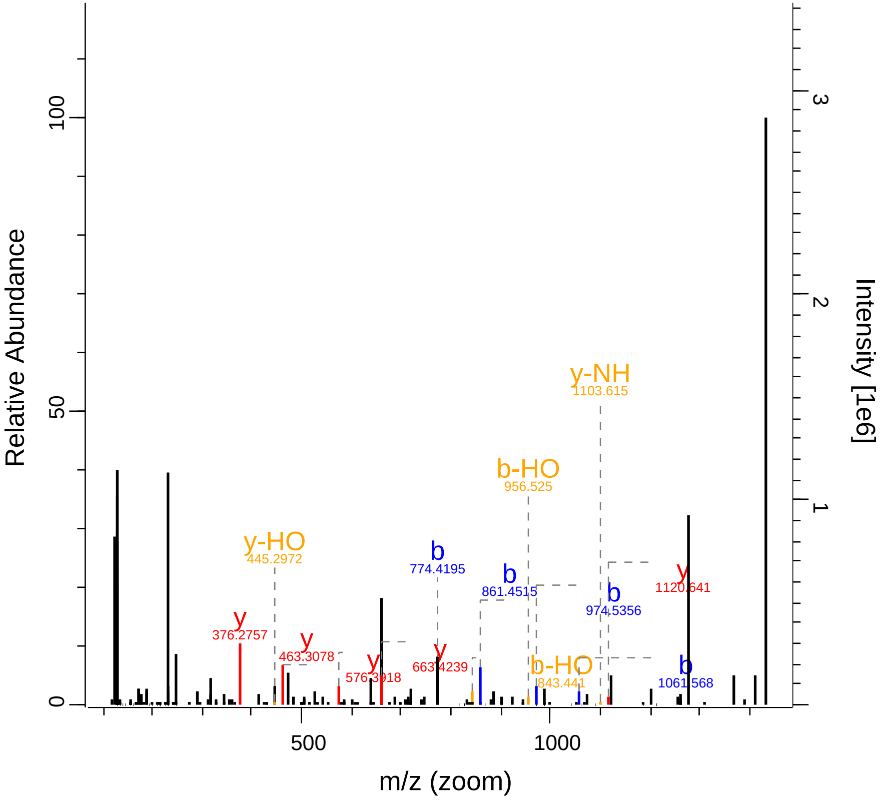

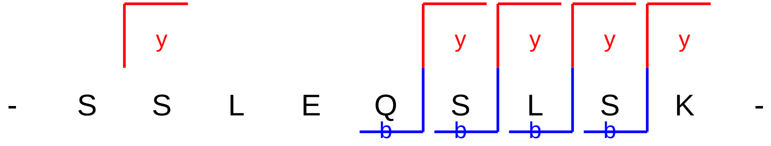

Raw File	Scan	Method	Score	m/z
RO-QC-160121_ZackD_SS_Set_02_Fraction_3	17192	FTMS; HCD	66.62	440.6

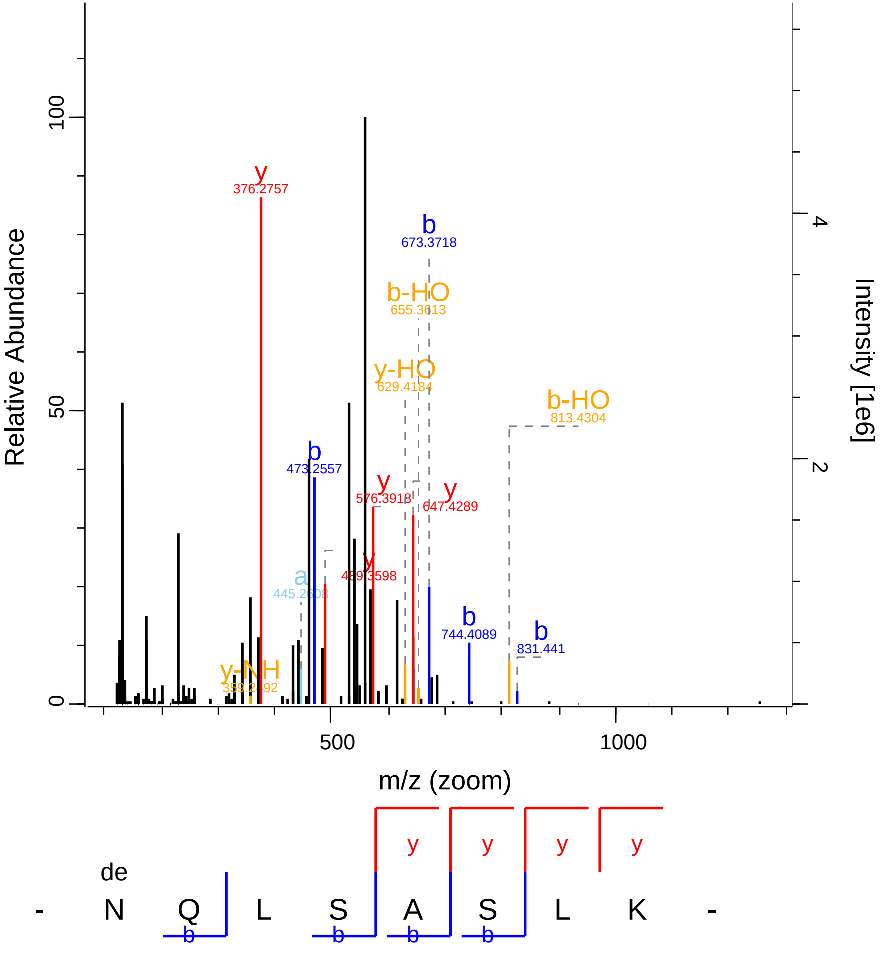

Raw File	Scan	Method	Score	m/z
RO-QC-160121_ZackD_SS_Set_02_Fraction_3	17203	FTMS; HCD	101.54	660.4

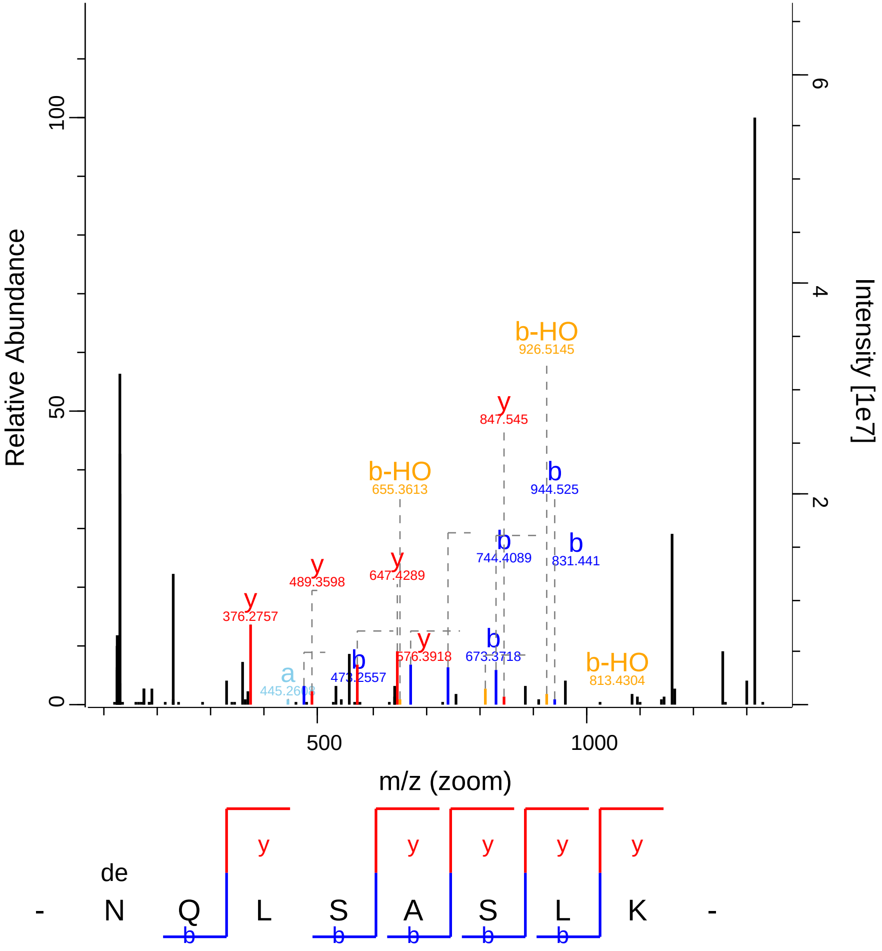

Raw File	Scan	Method	Score	m/z	Gene names
RO-QC-160121_ZackD_SS_Set_02_Fractio	17559	FTMS; HC	109.48	752.09	CERS1

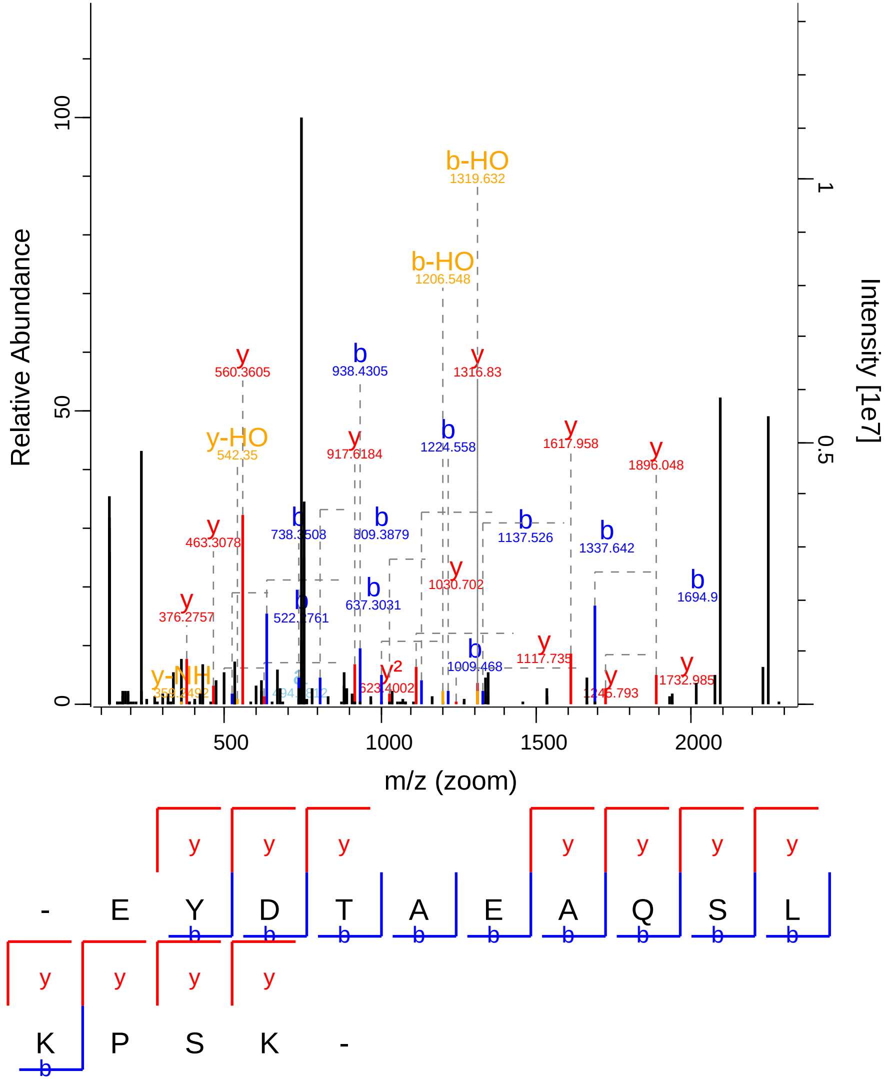

Raw File	Scan	Method	Score	m/z
RO-QC-160121_ZackD_SS_Set_02_Fraction_3	18240	FTMS; HCD	67.56	745.38

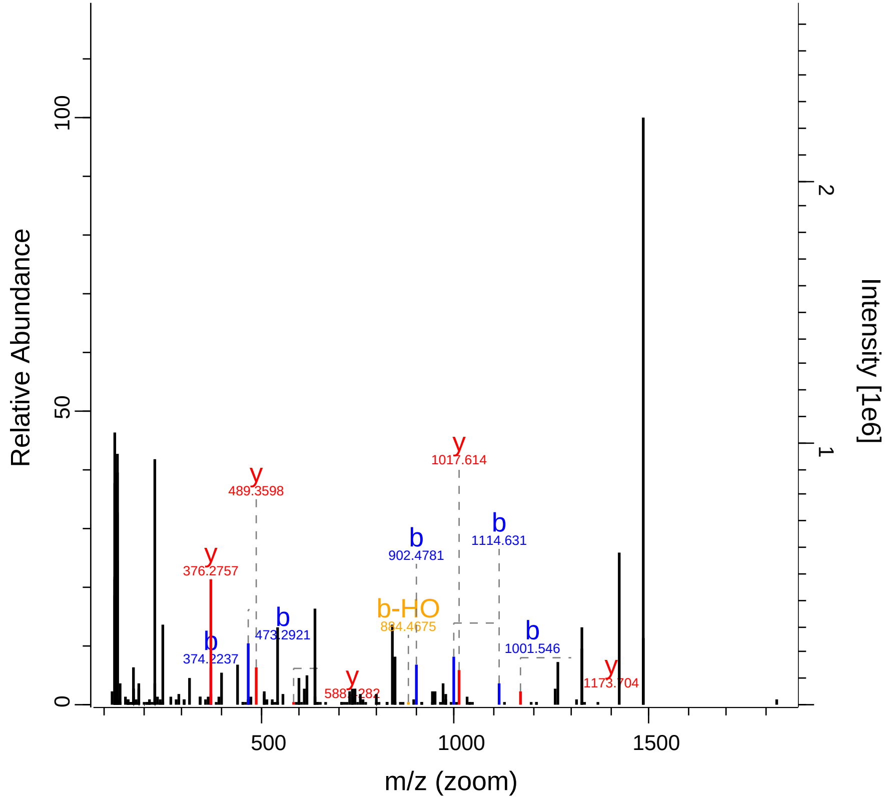

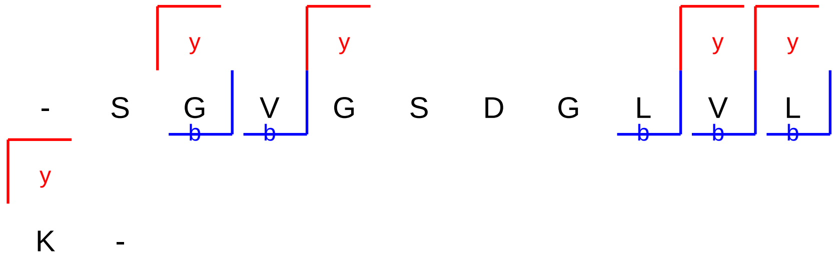

Raw File	Scan	Method	Score	m/z
RO-QC-160121_ZackD_SS_Set_02_Fraction_3	18283	FTMS; HCD	80.24	745.45

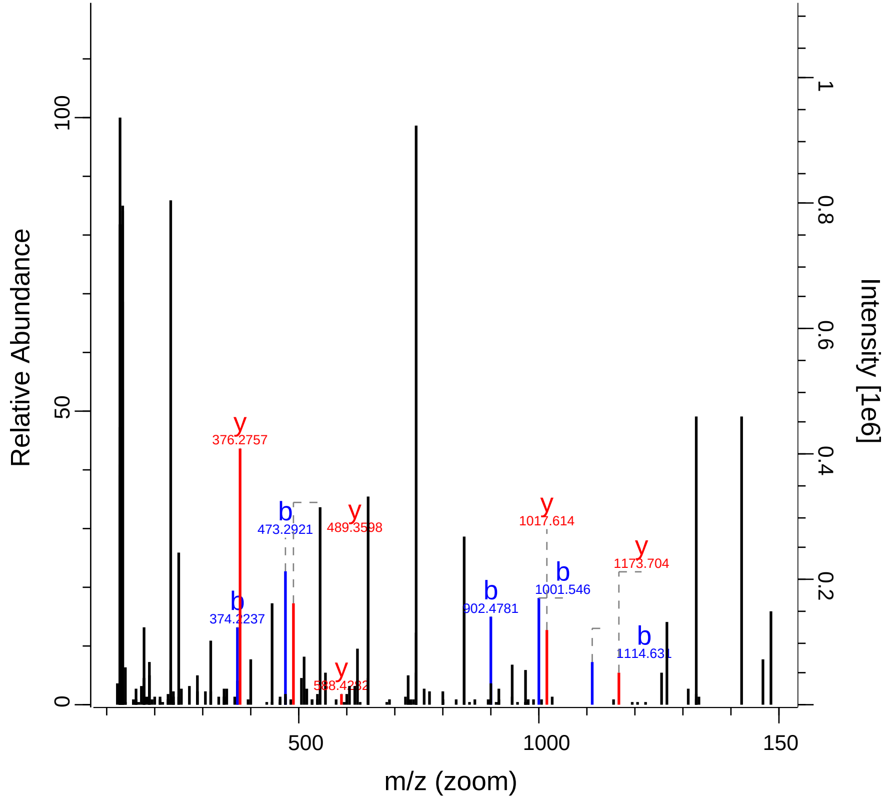

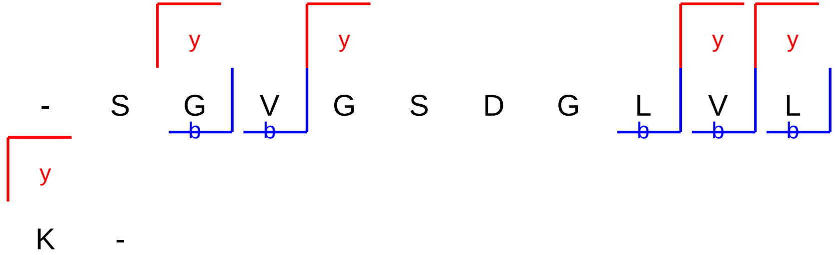

Raw File	Scan	Method	Score	m/z	Gene names
RO-QC-160121_ZackD_SS_Set_02_Fractio	18337	FTMS; HC	118.5	452.58	HBB;HBD

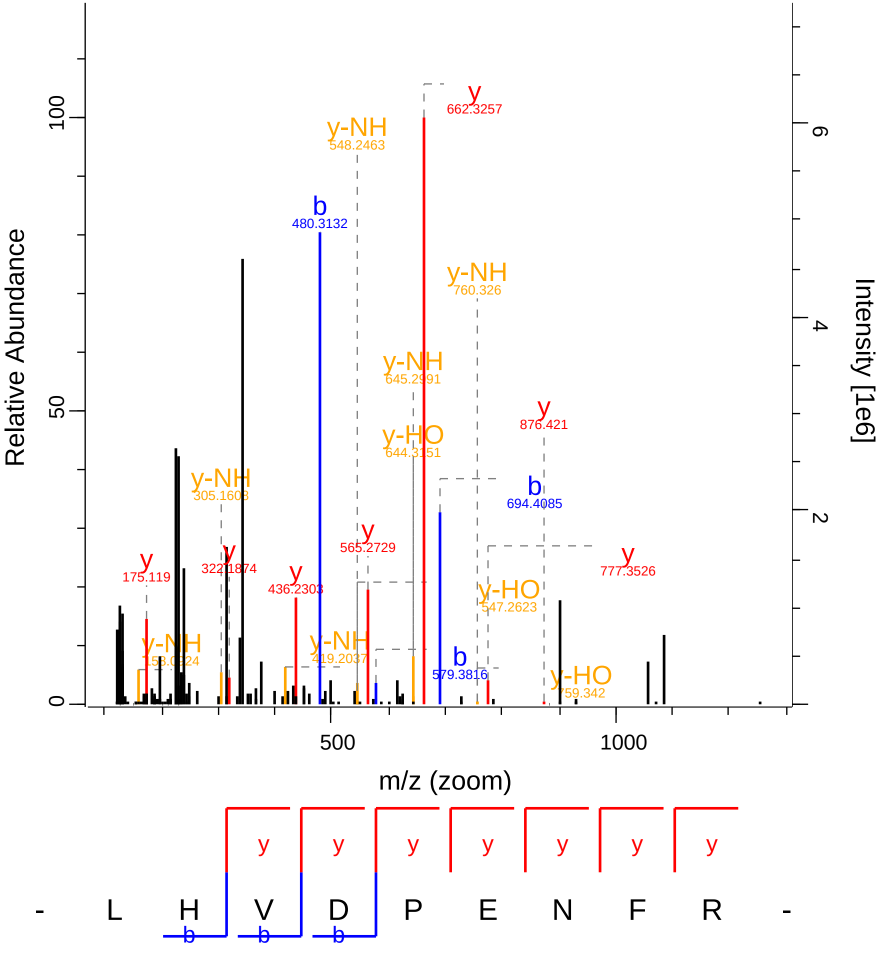

Raw File	Scan	Method	Score	m/z	Gene names
RO-QC-160121_ZackD_SS_Set_02_Fractior	18506	FTMS; HCI	41.32	800.1	ATP6V0C

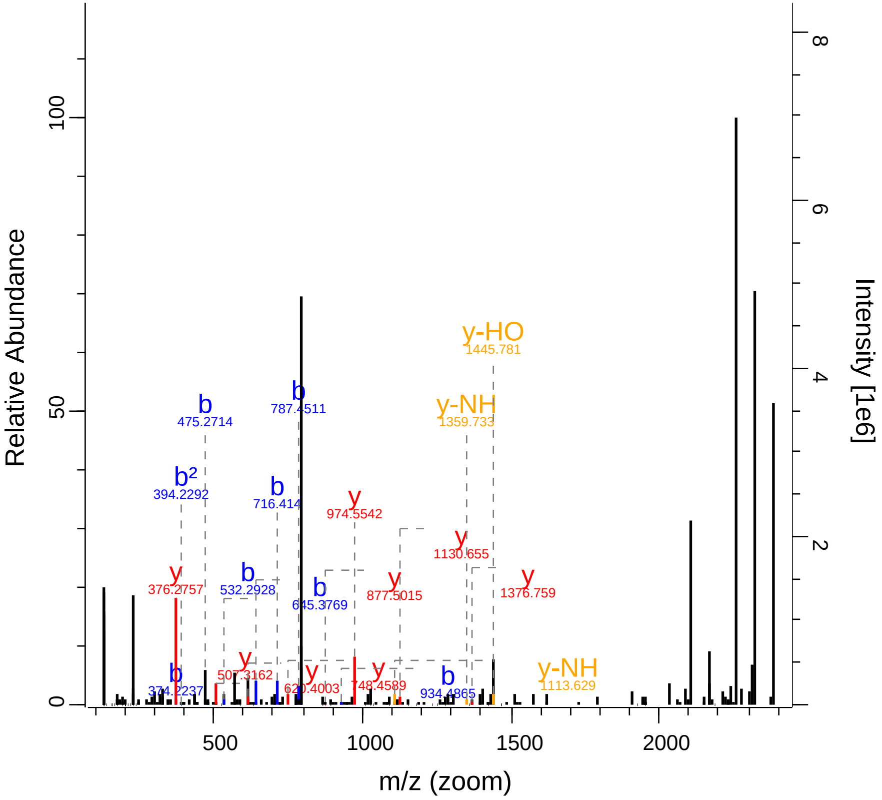

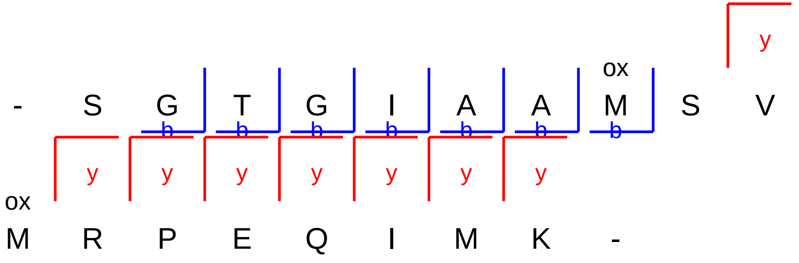

Raw File	Scan	Method	Score	m/z	Gene names
RO-QC-160121_ZackD_SS_Set_02_Fractior	18679	FTMS; HCI	49.56	610.8	PI15

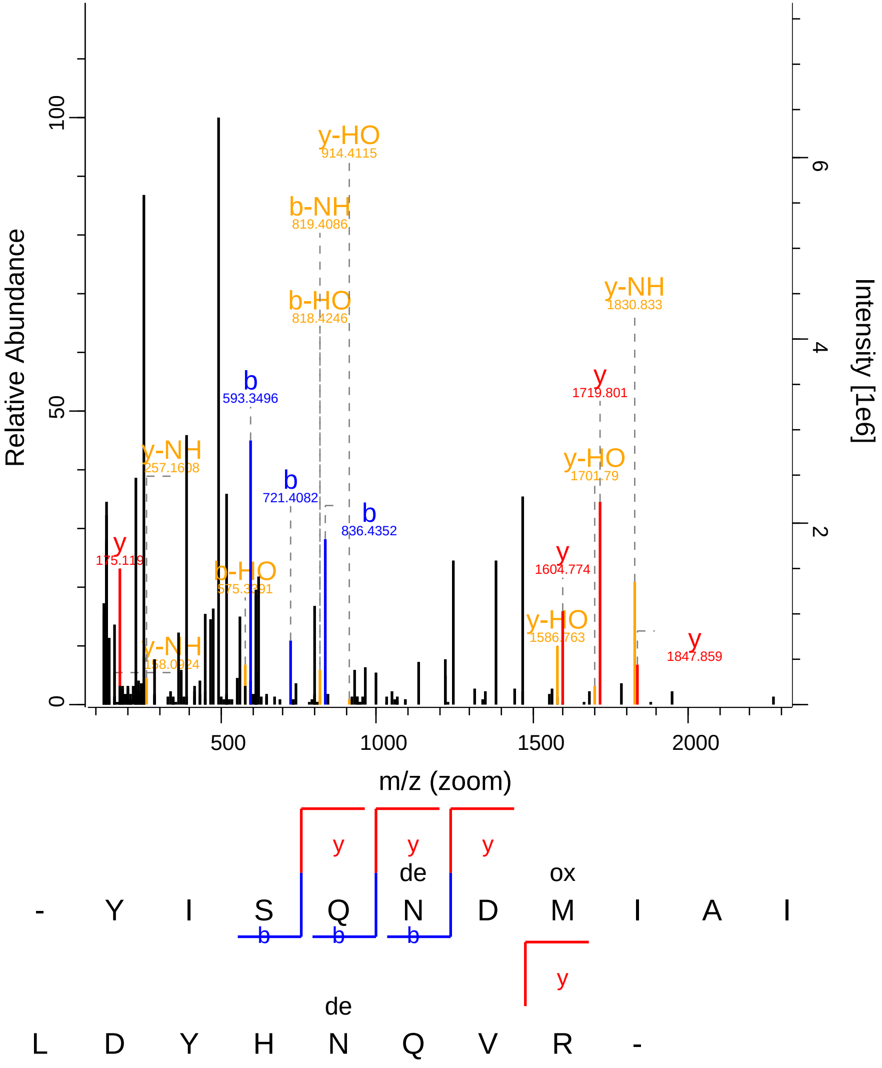

Raw File	Scan	Method	Score	m/z	Gene names
RO-QC-160121_ZackD_SS_Set_02_Fractior	18734	FTMS; HCI	50.05	800.1	ATP6V0C

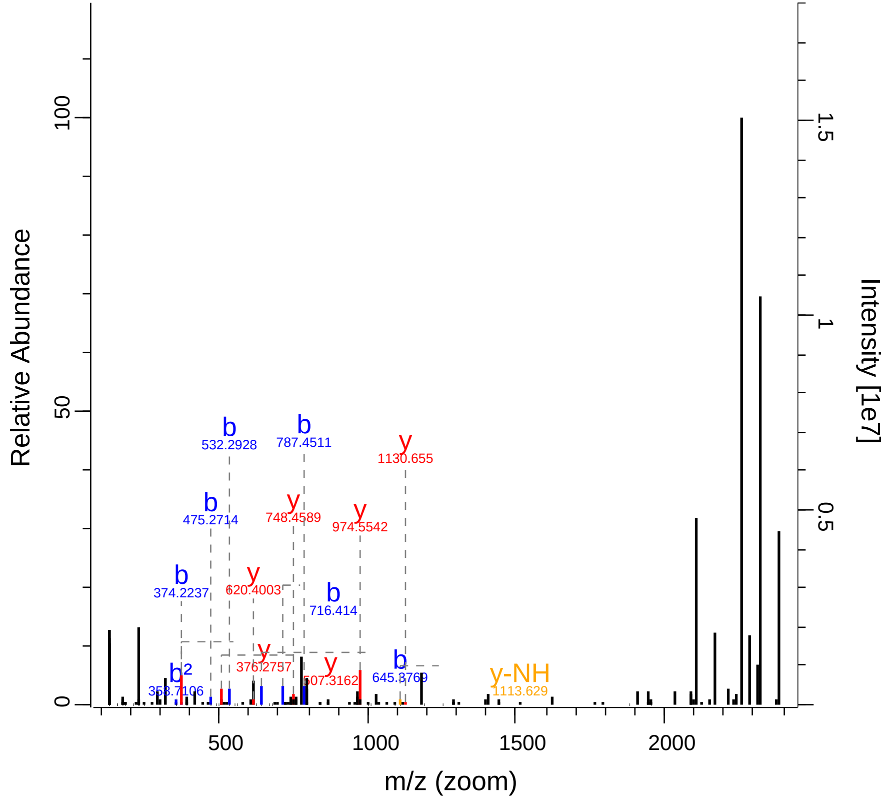

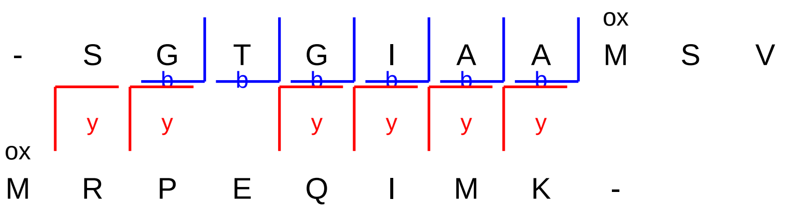

Raw File	Scan	Method	Score	m/z	Gene names
RO-QC-160121_ZackD_SS_Set_02_Fractio	18907	FTMS; HC	113.99	657.02	FGF1

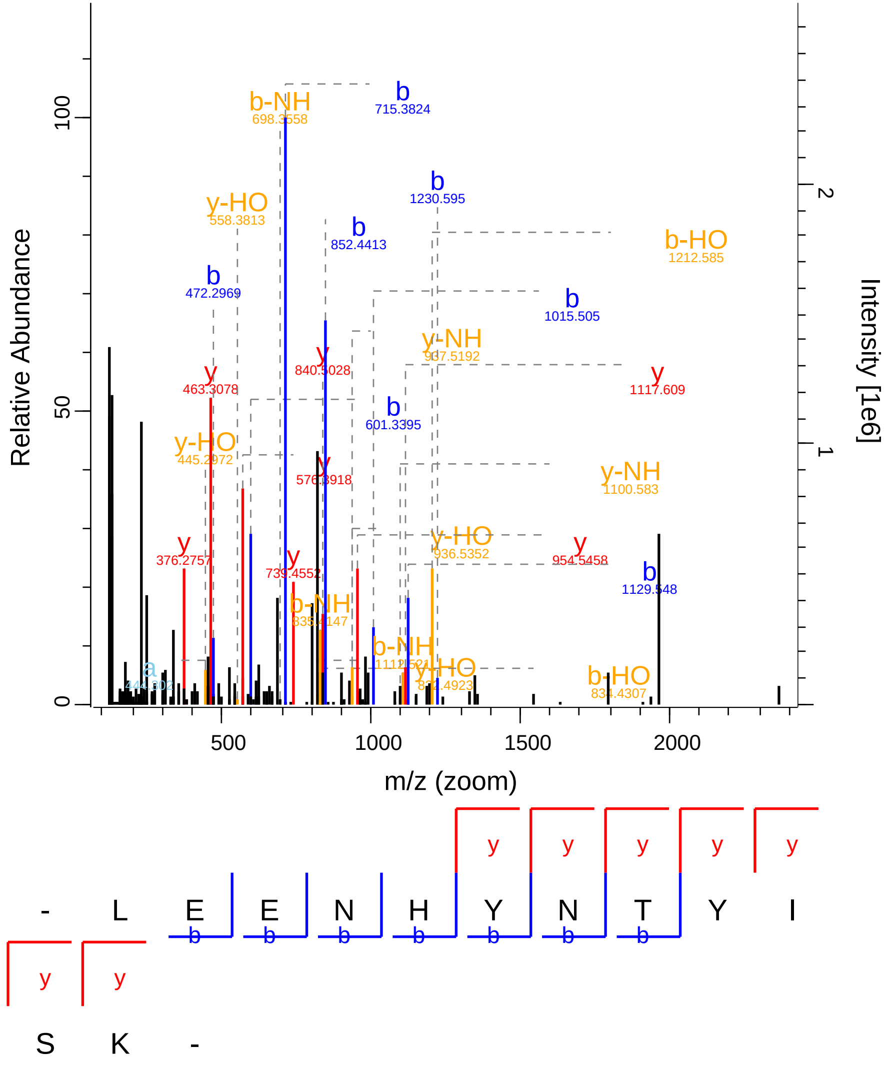

Raw File	Scan	Method	Score	m/z	Gene names
RO-QC-160121_ZackD_SS_Set_02_Fractio	19209	FTMS; HC	159.73	912.78	SOD3

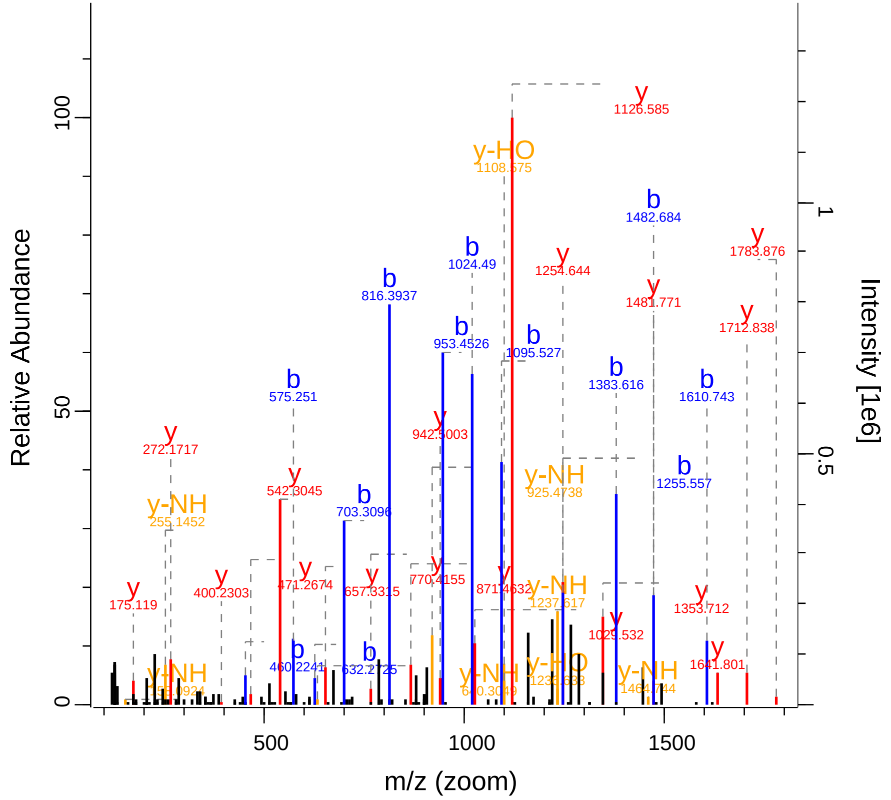

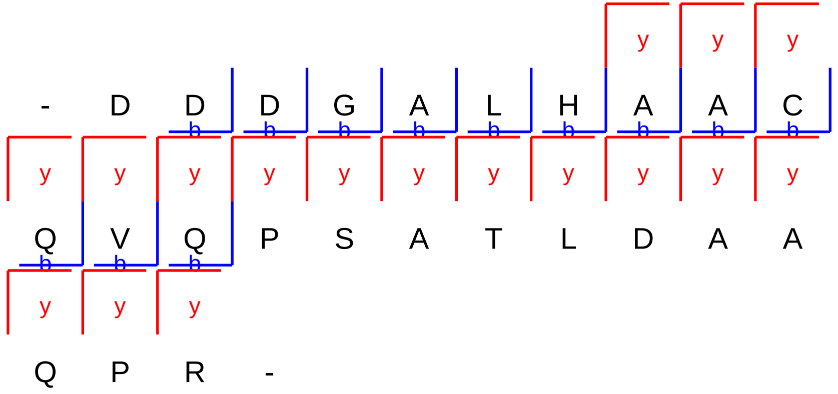

Raw File	Scan	Method	Score	m/z	Gene names
RO-QC-160121_ZackD_SS_Set_02_Fractio	19217	FTMS; HC	62.06	913.45	SOD3

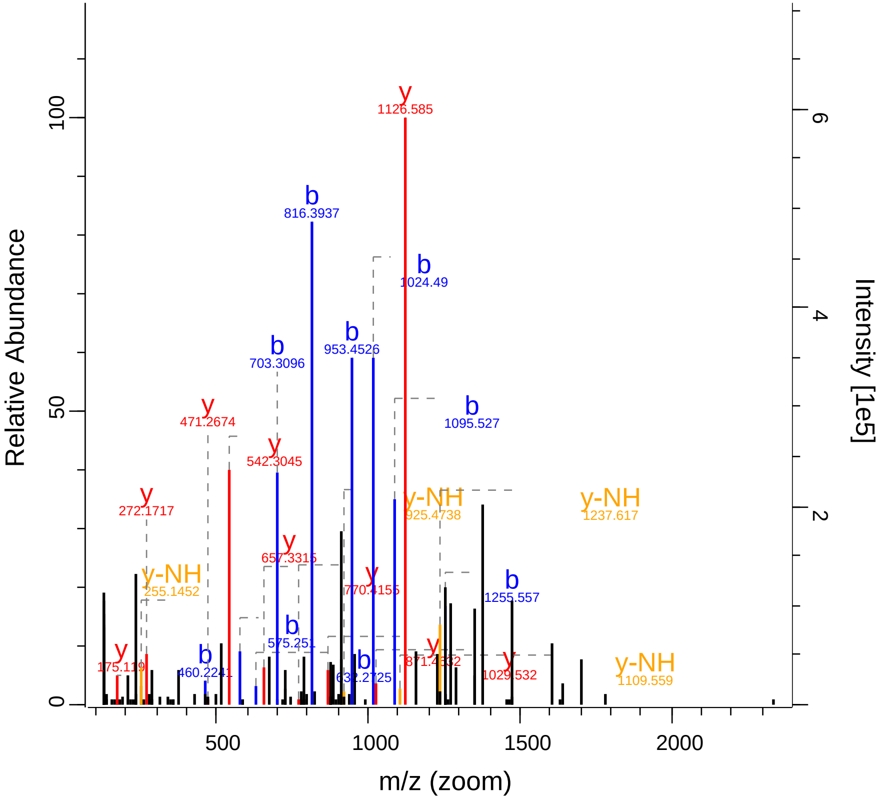

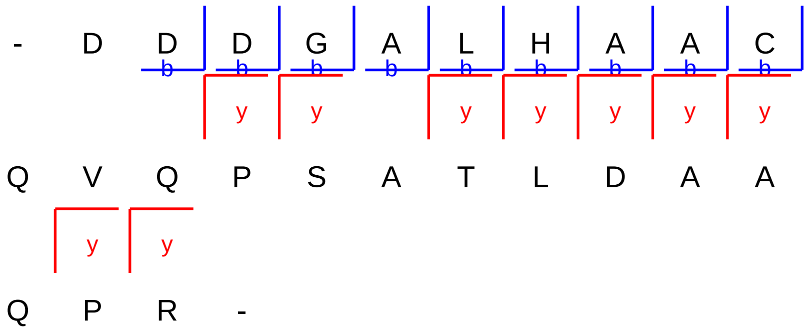

Raw File	Scan	Method	Score	m/z	Gene names
RO-QC-160121_ZackD_SS_Set_02_Fractio	19333	FTMS; HC	70.24	600.07	ATP6V0C

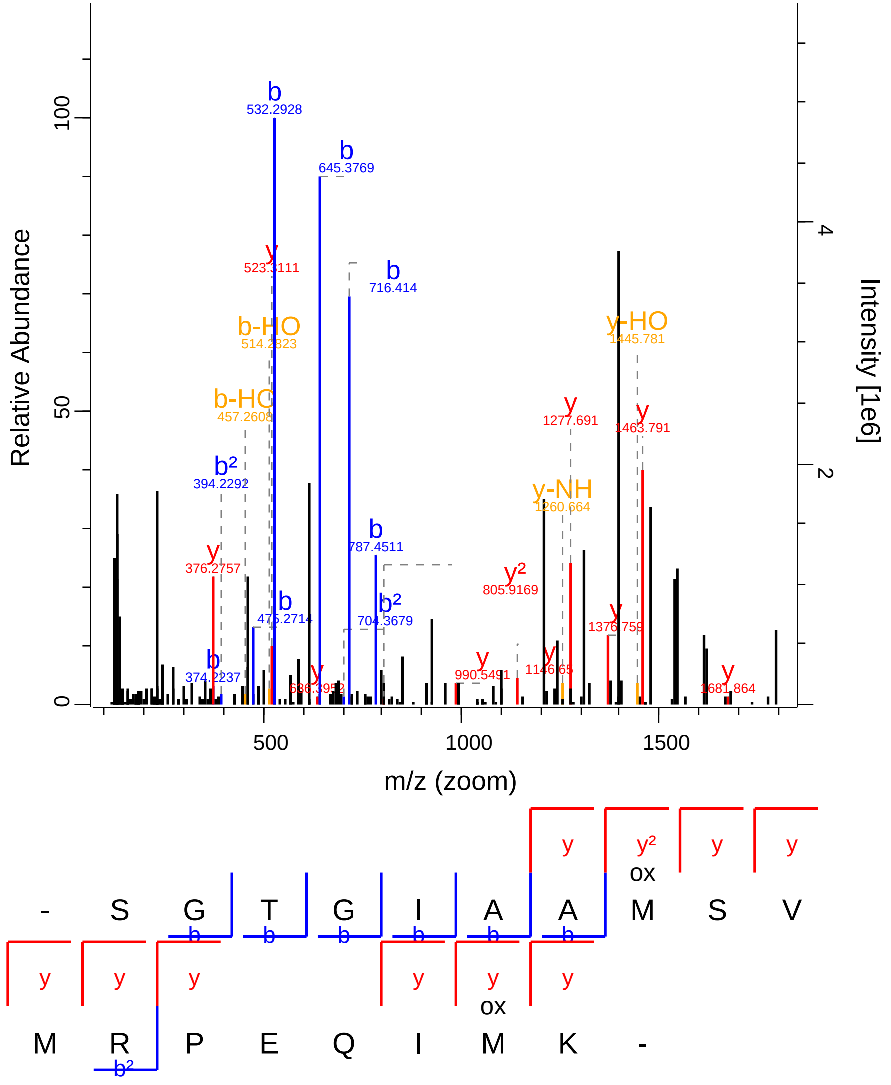

Raw File	Scan	Method	Score	m/z	Gene names
RO-QC-160121_ZackD_SS_Set_02_Fractio	19473	FTMS; HC	50.95	508.95	PTX3

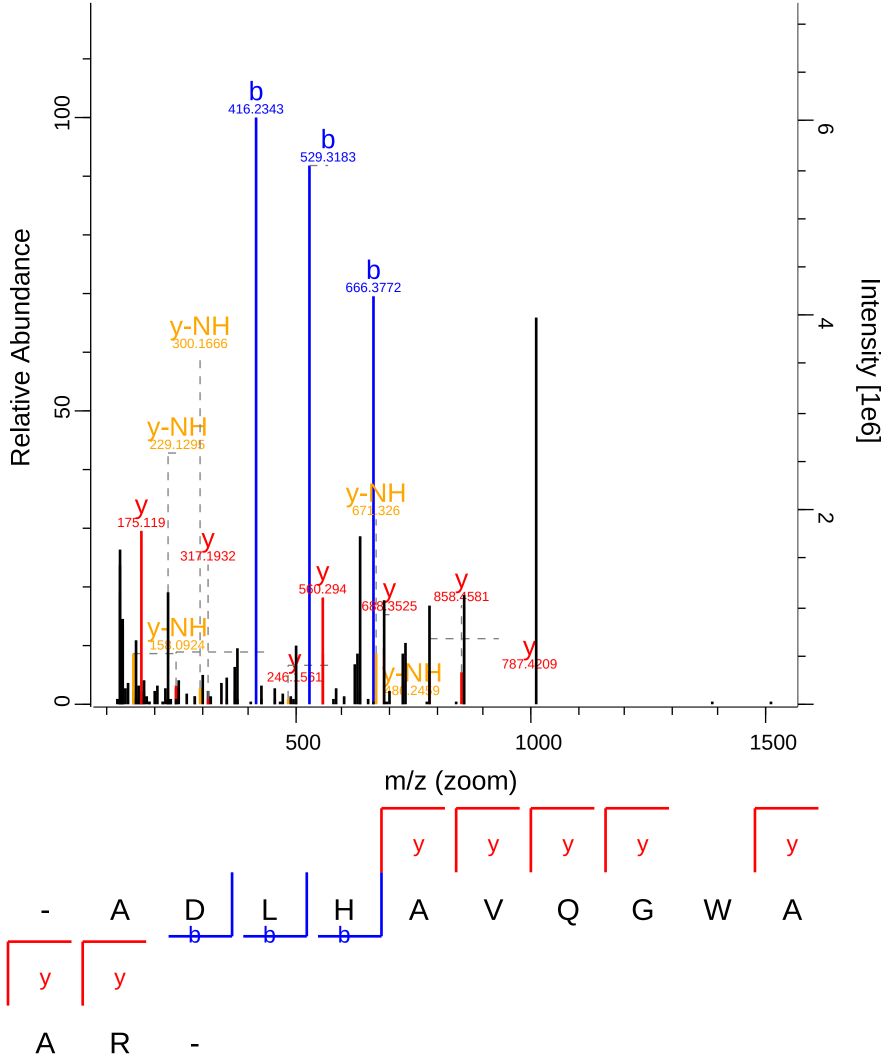

Raw File	Scan	Method	Score	m/z	Gene names
RO-QC-160121_ZackD_SS_Set_02_Fractio	19597	FTMS; HC	73.02	794.45	TRPV5

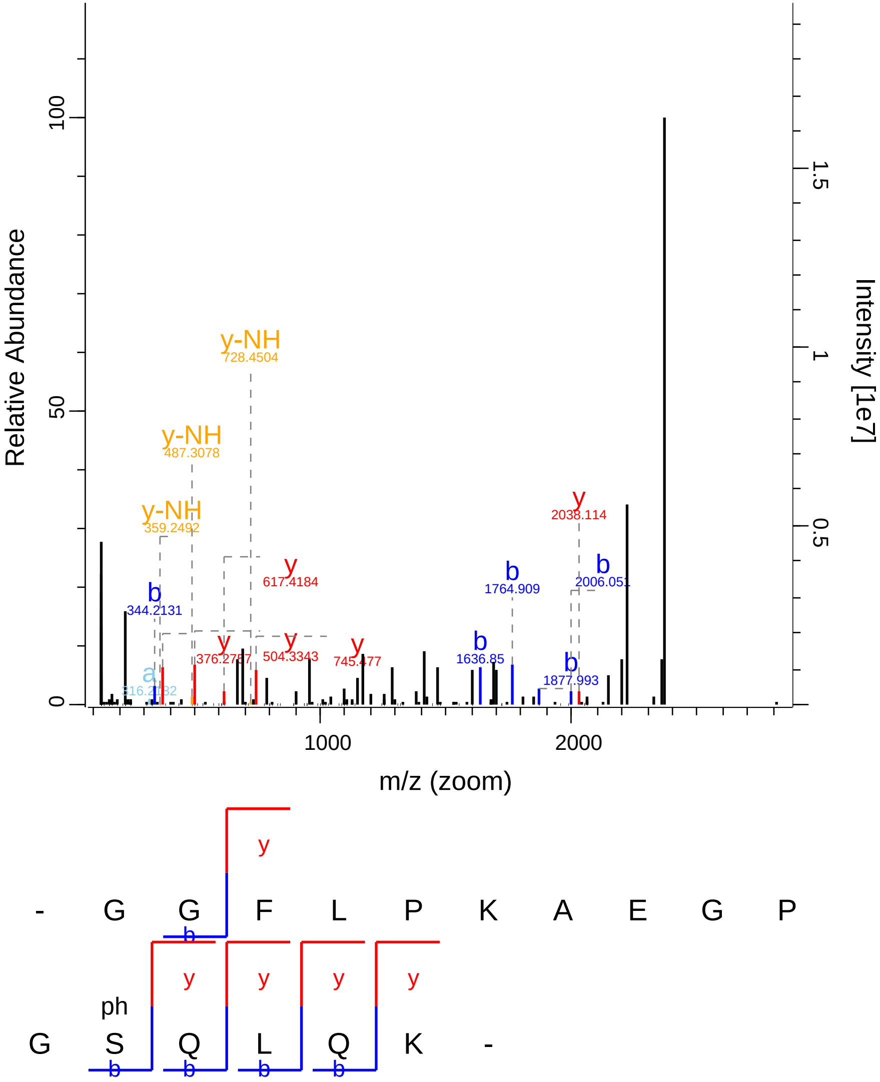

Raw File	Scan	Method	Score	m/z	Gene names
RO-QC-160121_ZackD_SS_Set_02_Fractio	19706	FTMS; HC	141.95	781.46	PASK

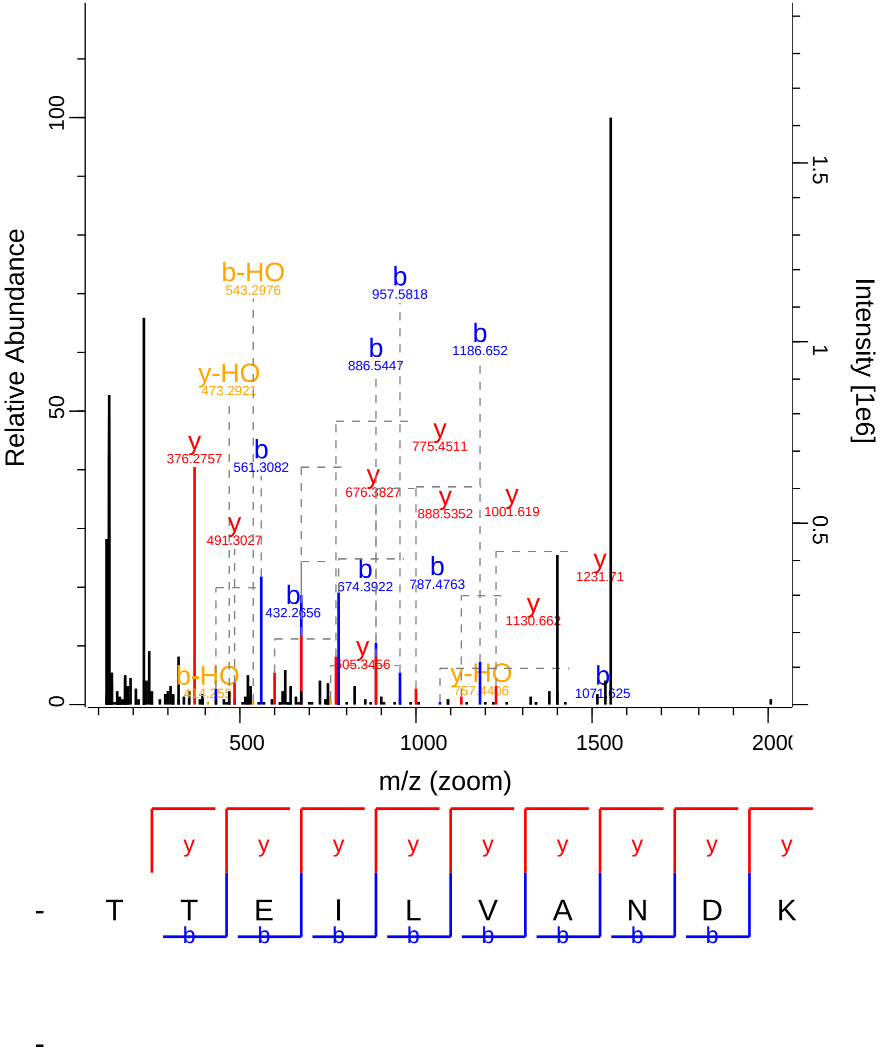

Raw File	Scan	Method	Score	m/z	Gene names
RO-QC-160121_ZackD_SS_Set_02_Fractio	19931	FTMS; HC	51.88	498.63	SIPA1L2

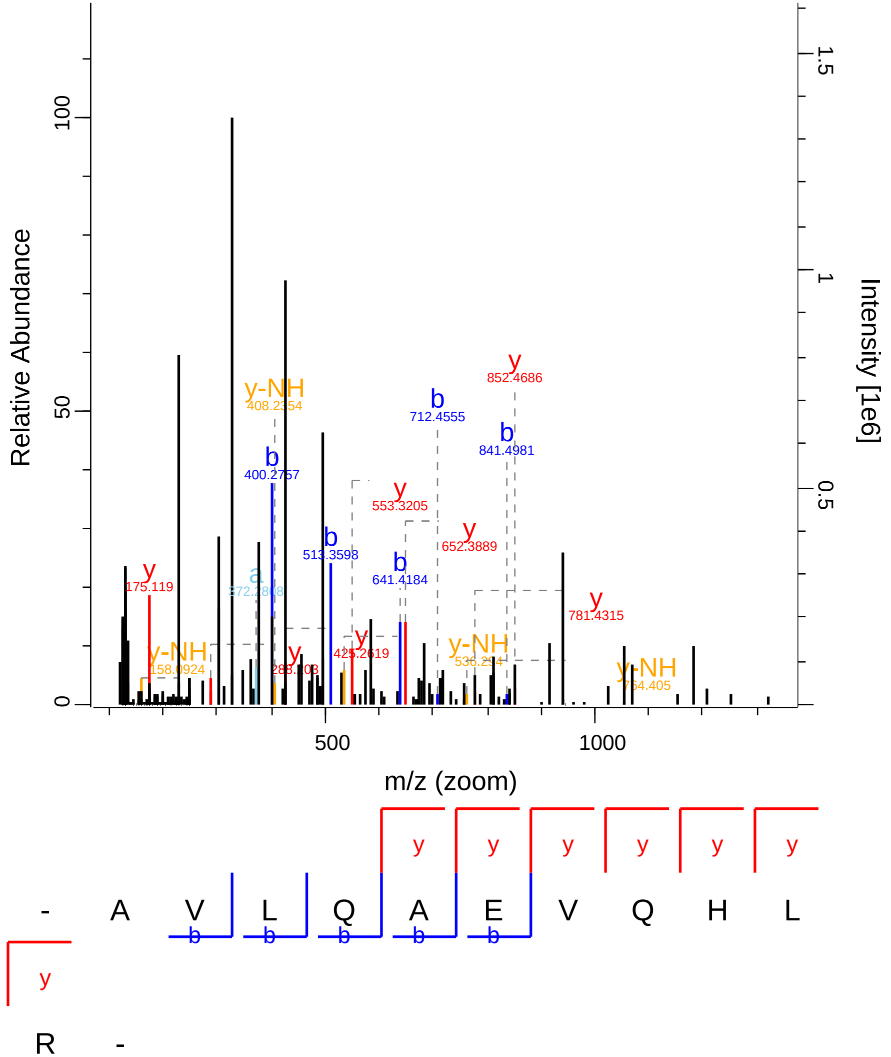

Raw File	Scan	Method	Score	m/z	Gene names
RO-QC-160121_ZackD_SS_Set_02_Fractio	20246	FTMS; HC	47.75	796.66	OR51F2

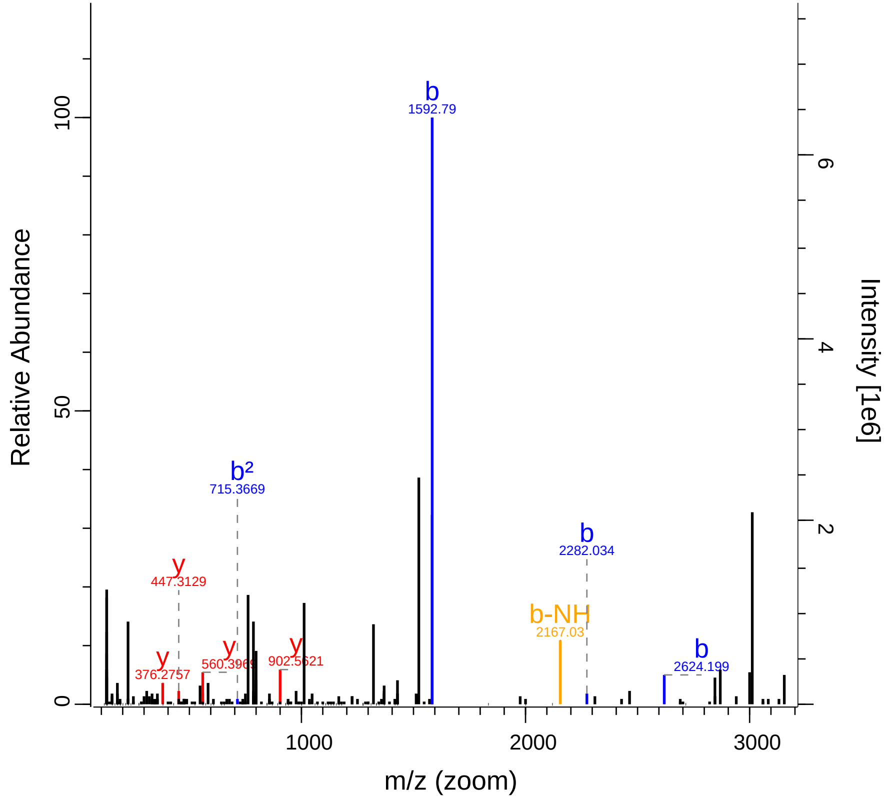

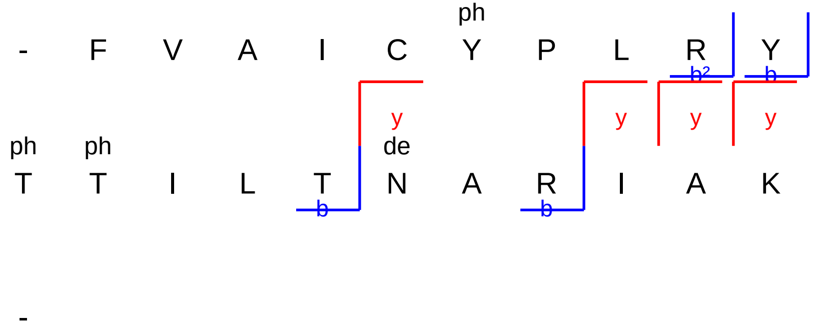

Raw File	Scan	Method	Score	m/z	Gene names
RO-QC-160121_ZackD_SS_Set_02_Fractior	21104	FTMS; HCI	82.42	486.6	LATS2

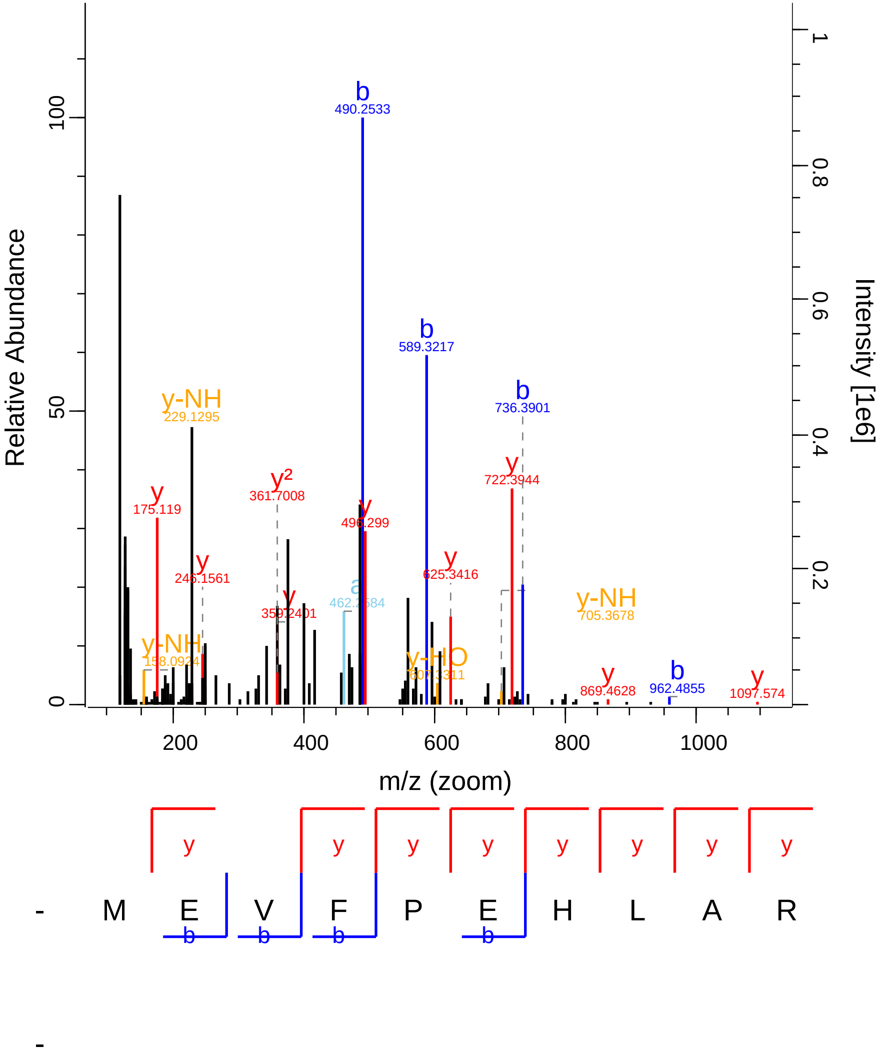

Raw File	Scan	Method	Score	m/z	Gene names
RO-QC-160121_ZackD_SS_Set_02_Fractio	21254	FTMS; HC	101.35	714.38	ZCCHC4

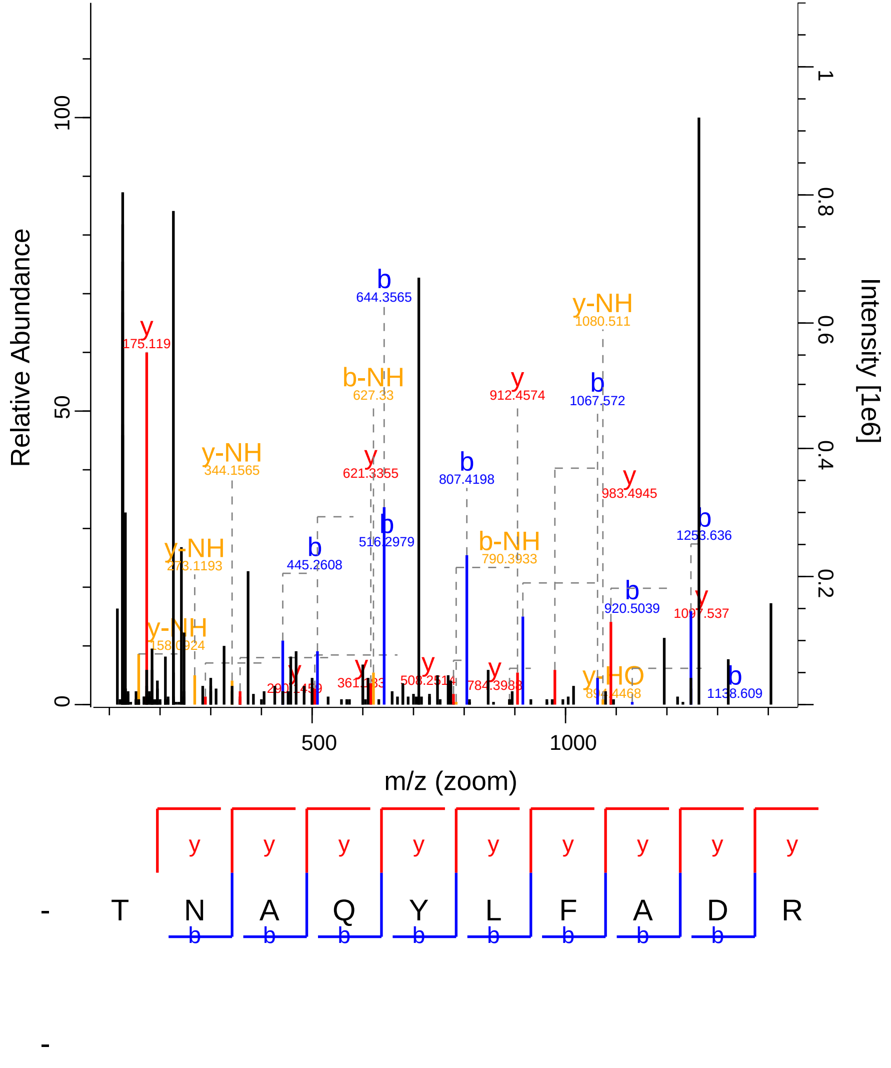

Raw File	Scan	Method	Score	m/z	Gene names
RO-QC-160121_ZackD_SS_Set_02_Fractio	21681	FTMS; HC	148.78	926.52	EPN2

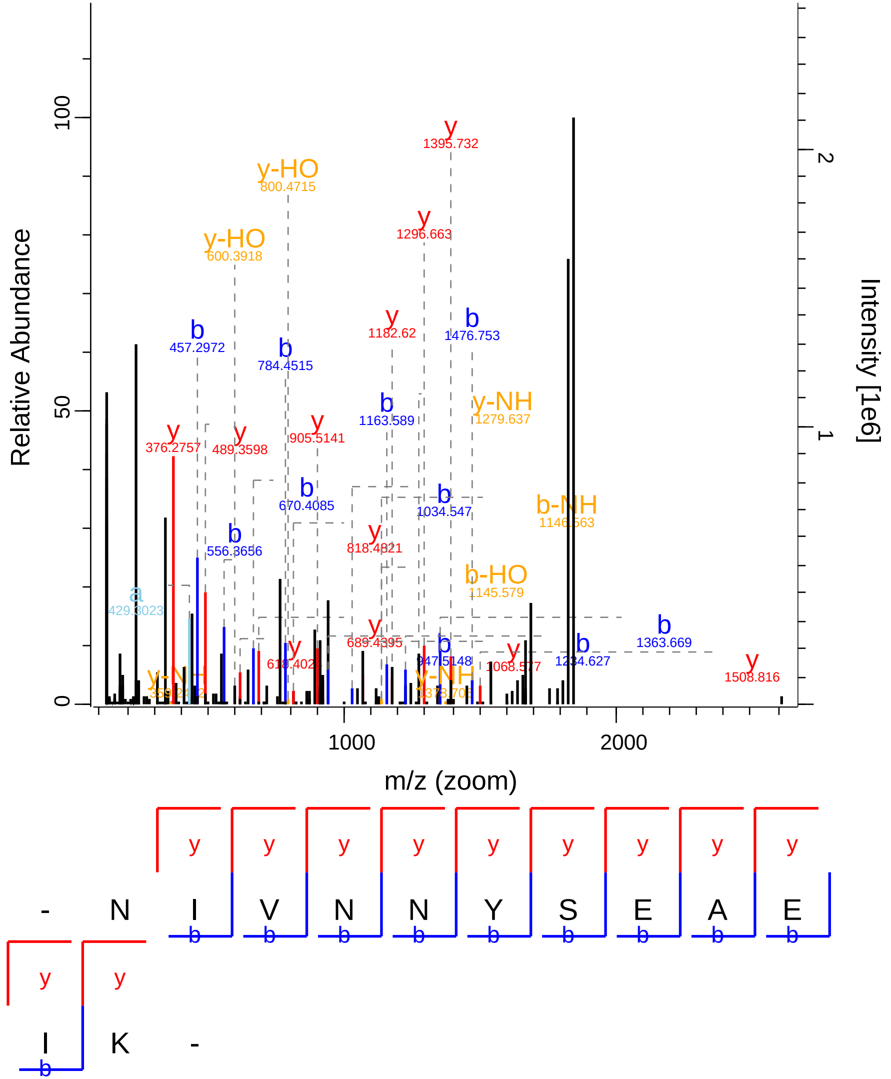

Raw File	Scan	Method	Score	m/z	Gene names
RO-QC-160121_ZackD_SS_Set_02_Fractior	22106	FTMS; HCI	108.98	689.9	CDC45

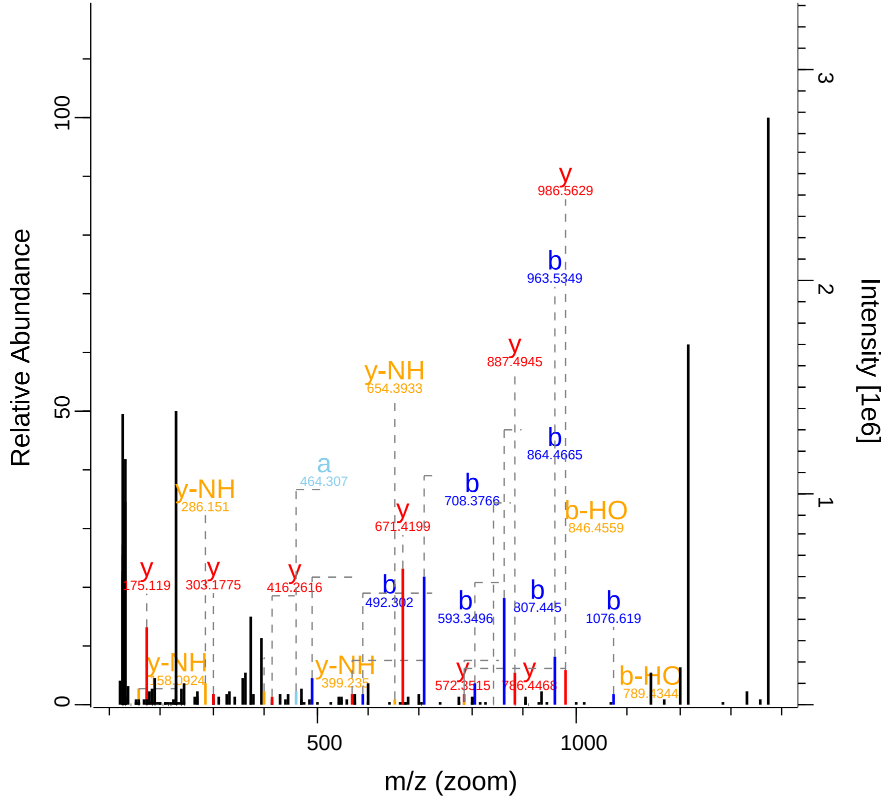

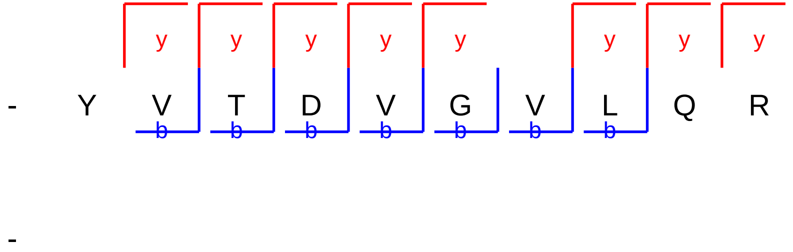

Raw File	Scan	Method	Score	m/z	Gene names
RO-QC-160121_ZackD_SS_Set_02_Fractio	22485	FTMS; HC	86.6	918.45	MRFAP1

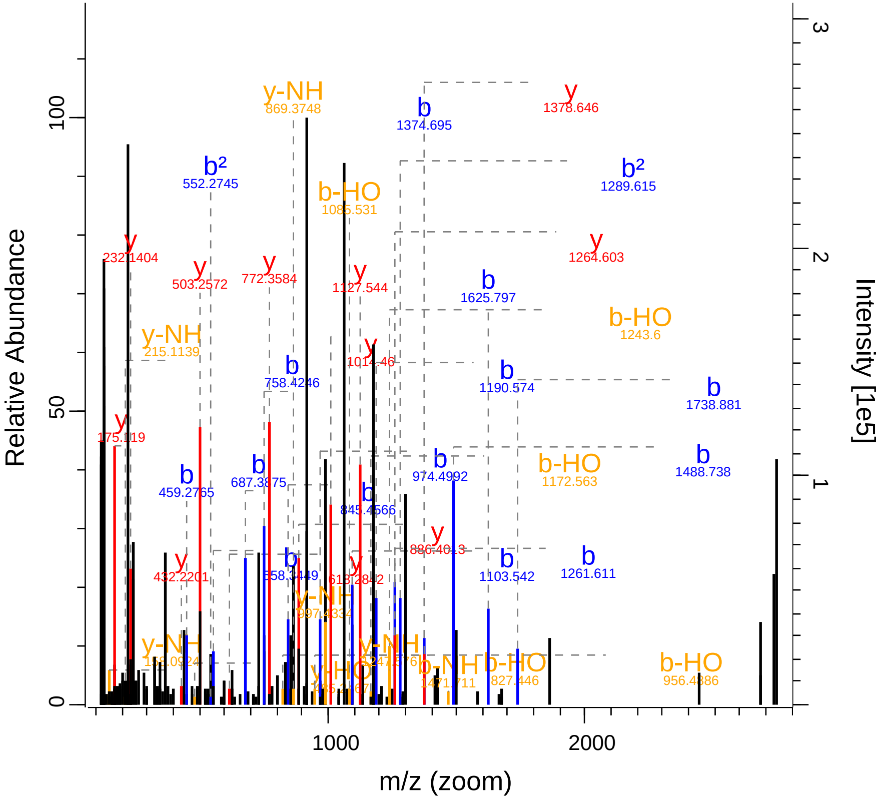

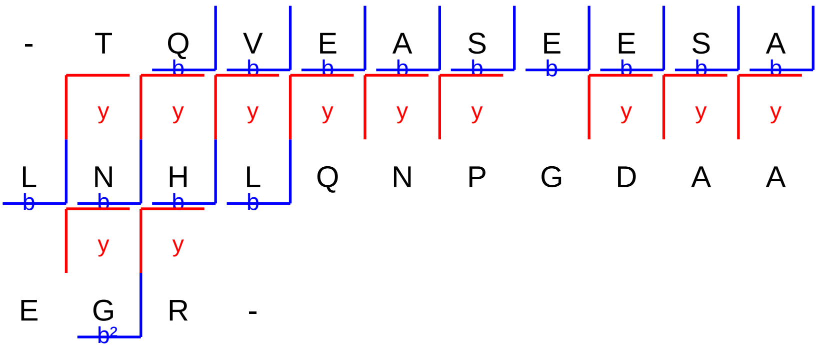

Raw File	Scan	Method	Score	m/z	Gene names
RO-QC-160121_ZackD_SS_Set_02_Fractio	22497	FTMS; HC	110.36	918.12	MRFAP1

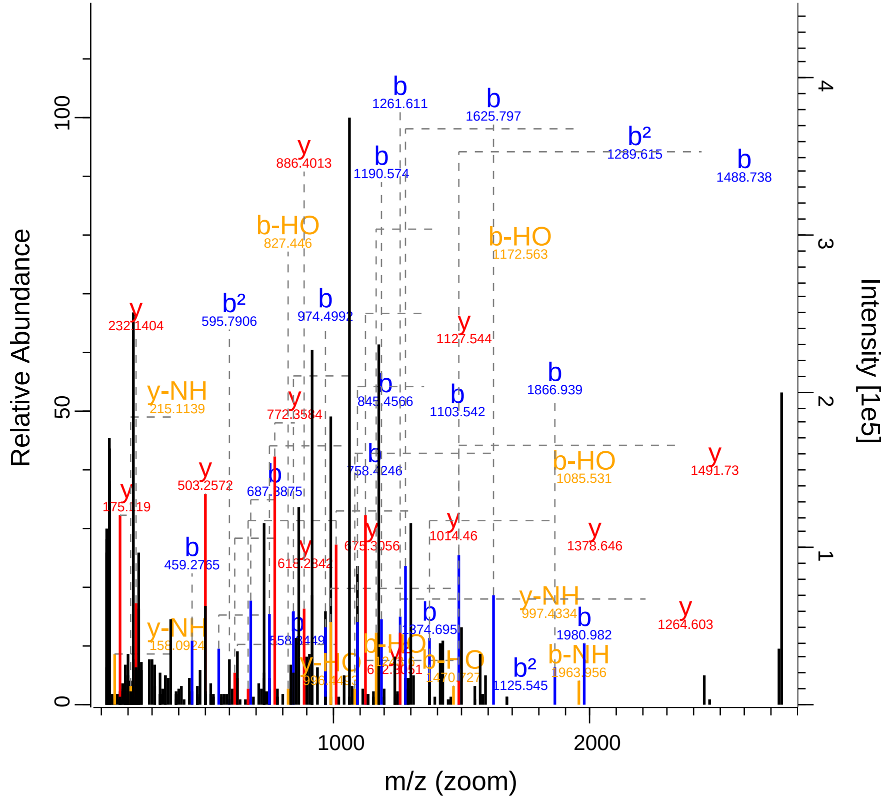

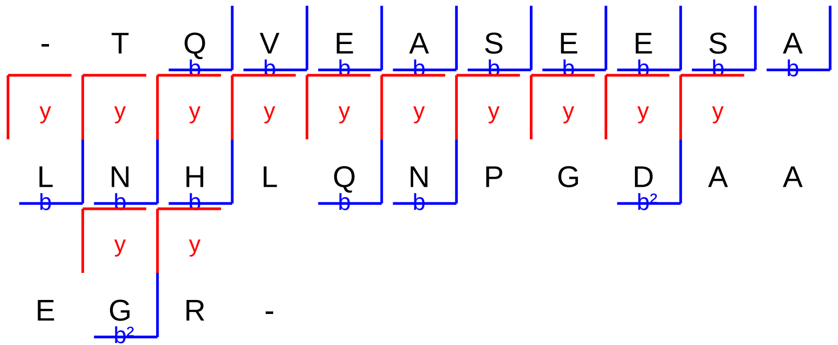

Raw File	Scan	Method	Score	m/z	Gene names
RO-QC-160121_ZackD_SS_Set_02_Fraction	22965	FTMS; HCI	101.64	829	TRDC

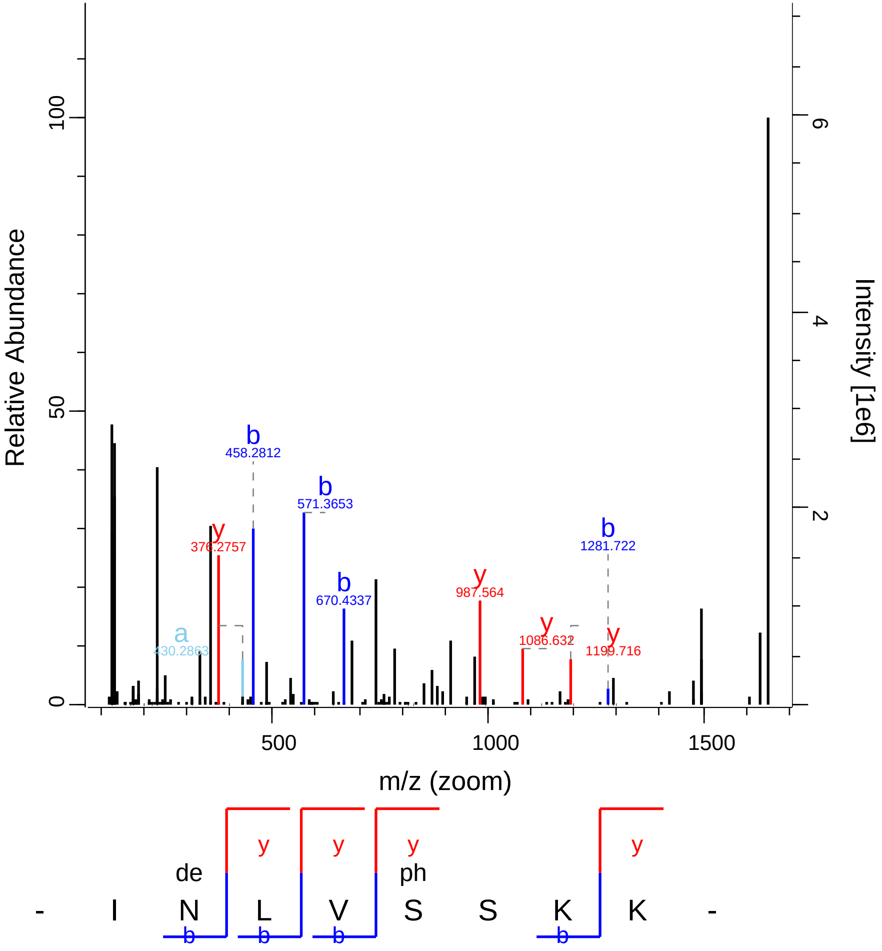

Raw File	Scan	Method	Score	m/z	Gene names
RO-QC-160121_ZackD_SS_Set_02_Fractio	23001	FTMS; HC	219.42	975.05	TRDMT1

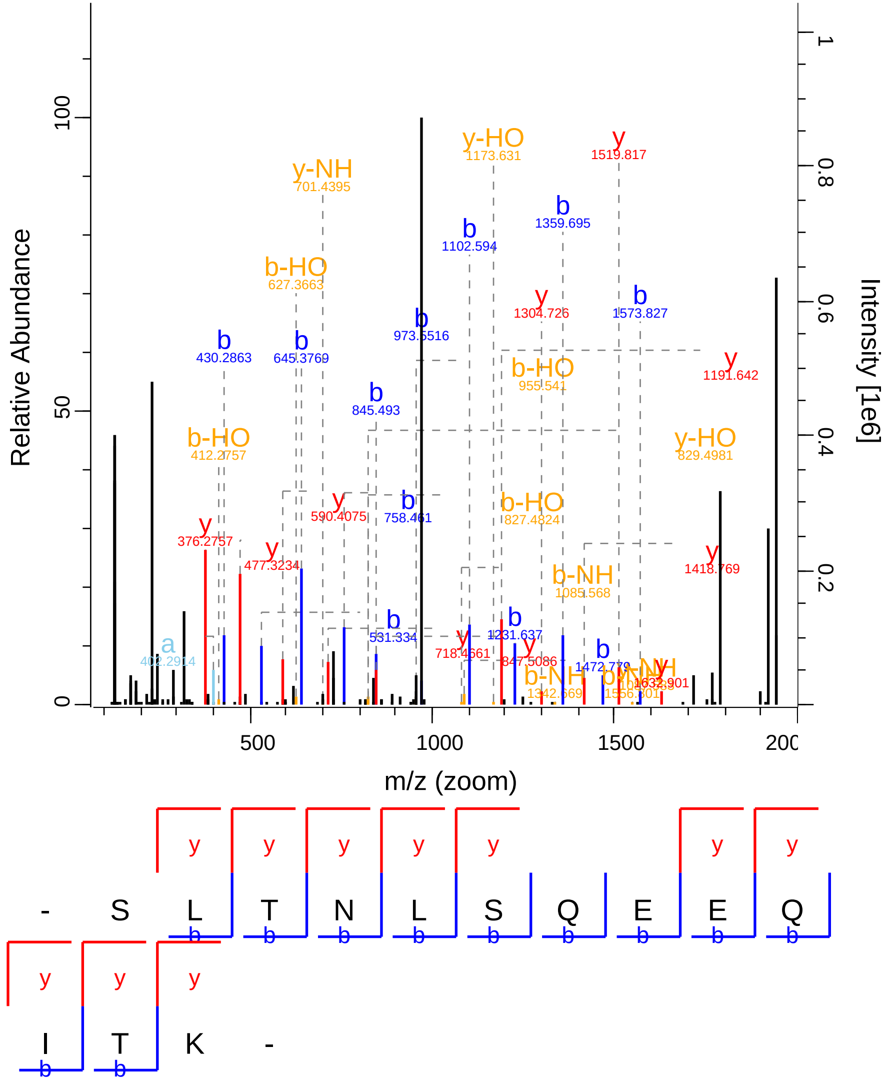

Raw File	Scan	Method	Score	m/z	Gene names
RO-QC-160121_ZackD_SS_Set_02_Fractio	23086	FTMS; HC	135.56	873.01	PGAP2

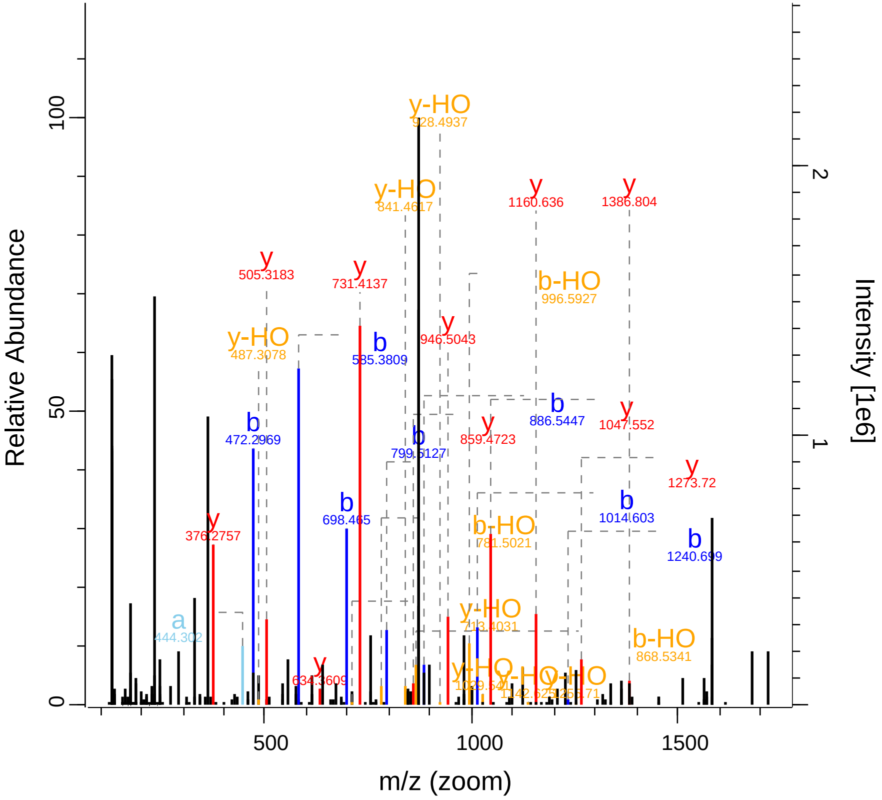

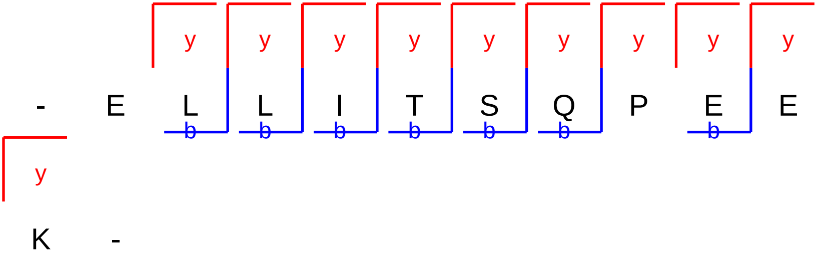

Raw File	Scan	Method	Score	m/z	Gene names
RO-QC-160121_ZackD_SS_Set_02_Fractio	23788	FTMS; HC	61.96	473.62	CD14

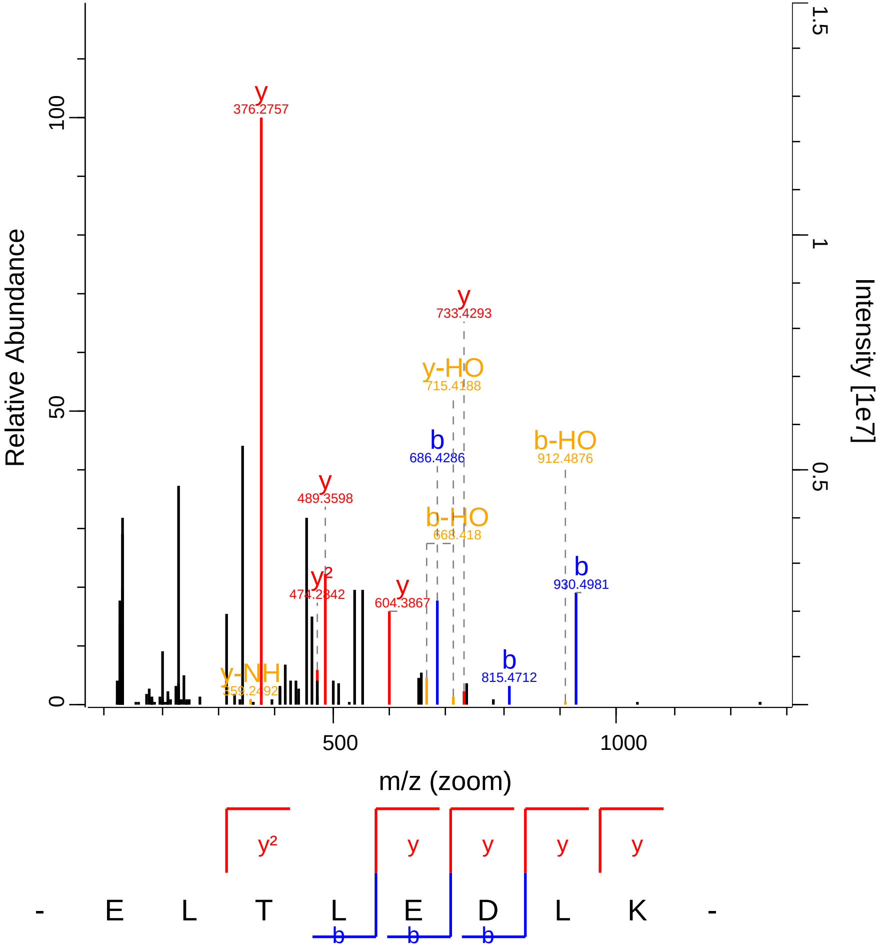

Raw File	Scan	Method	Score	m/z	Gene names
RO-QC-160121_ZackD_SS_Set_02_Fractio	24023	FTMS; HC	81.19	473.62	CD14

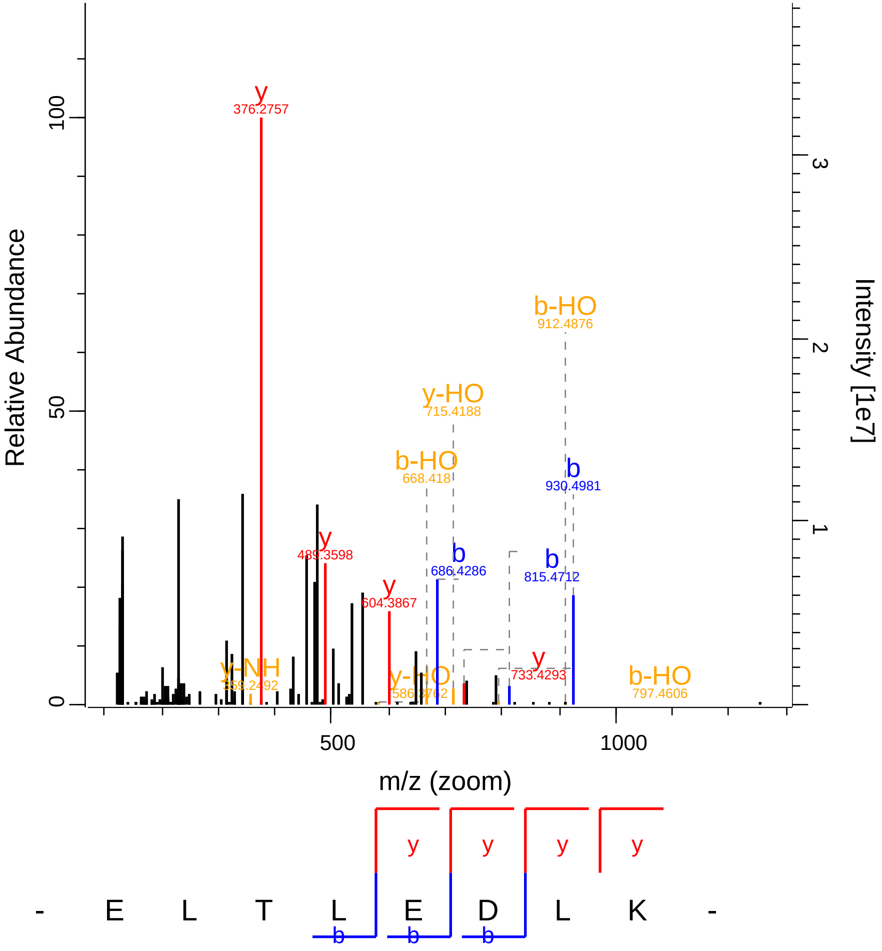

Raw File	Scan	Method	Score	m/z	Gene names
RO-QC-160121_ZackD_SS_Set_02_Fractio	24124	FTMS; HC	42.56	739.04	TRPM6

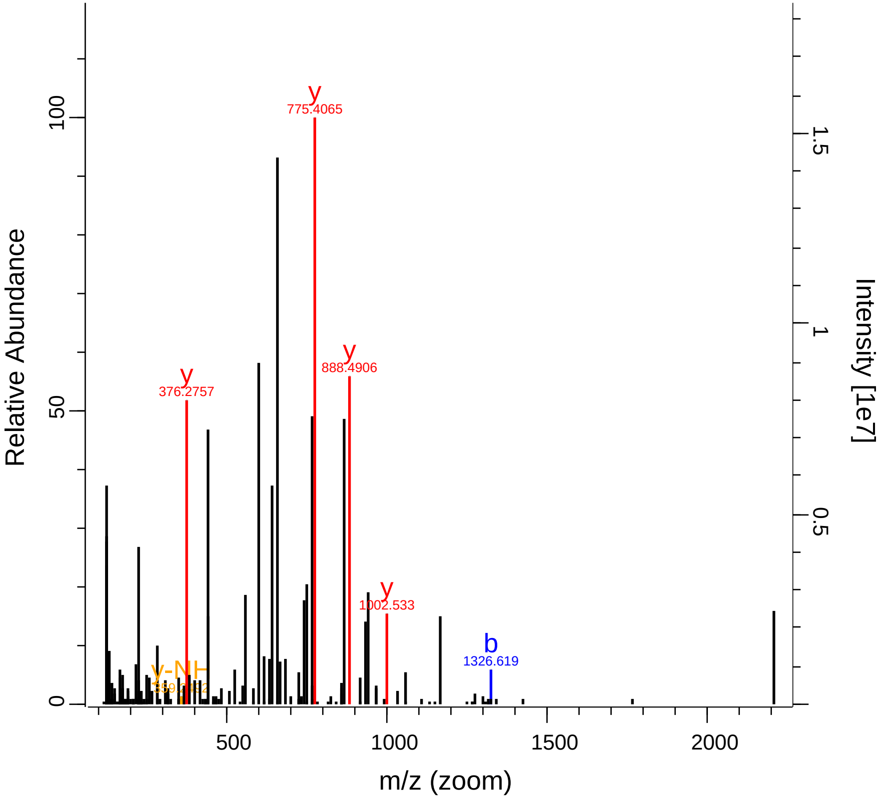

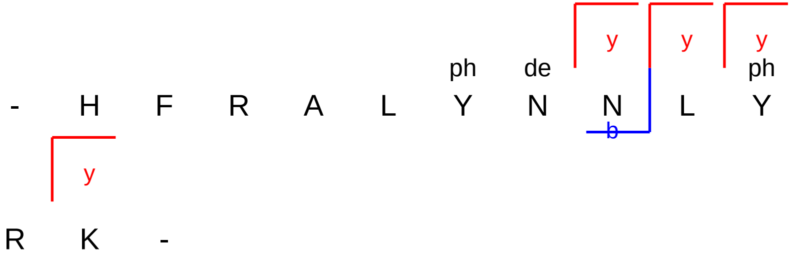

Raw File	Scan	Method	Score	m/z	Gene names
RO-QC-160121_ZackD_SS_Set_02_Fractio	24129	FTMS; HC	105.52	514.26	APOBEC3B

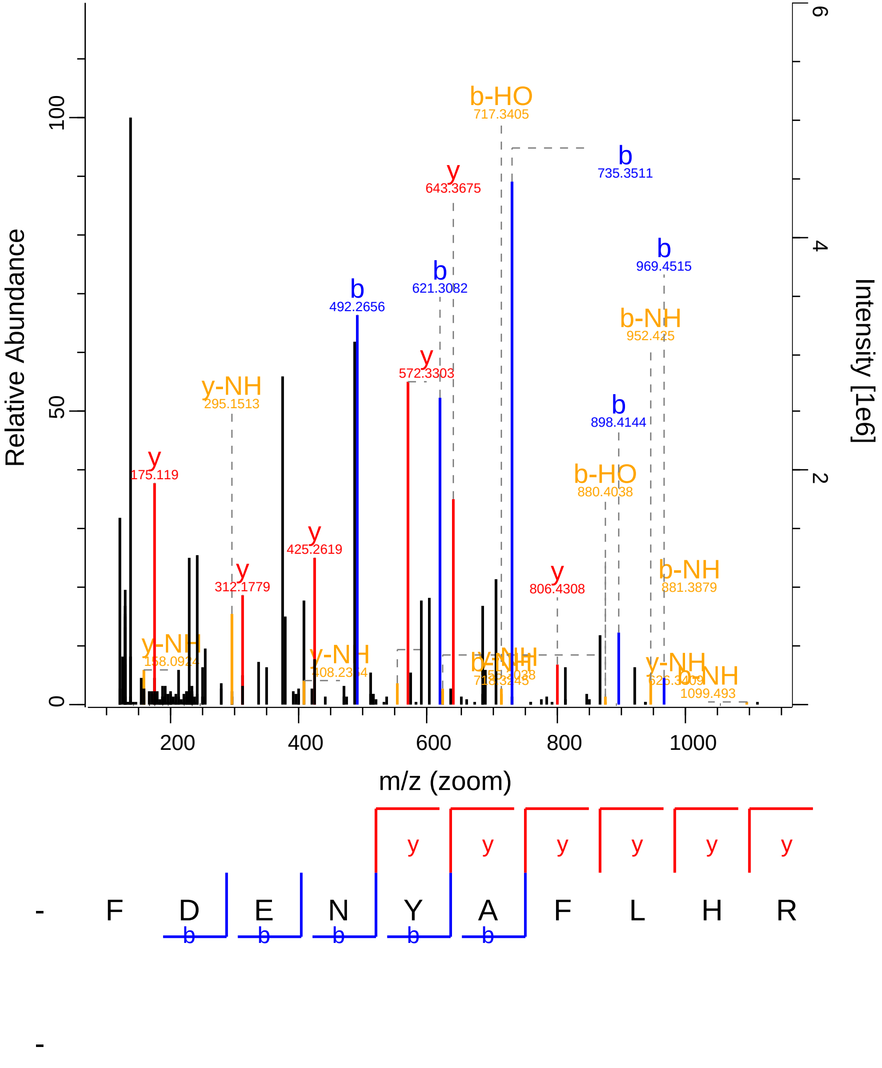

Raw File	Scan	Method	Score	m/z	Gene names
RO-QC-160121_ZackD_SS_Set_02_Fractio	24195	FTMS; HC	76.33	916.03	PRRX1

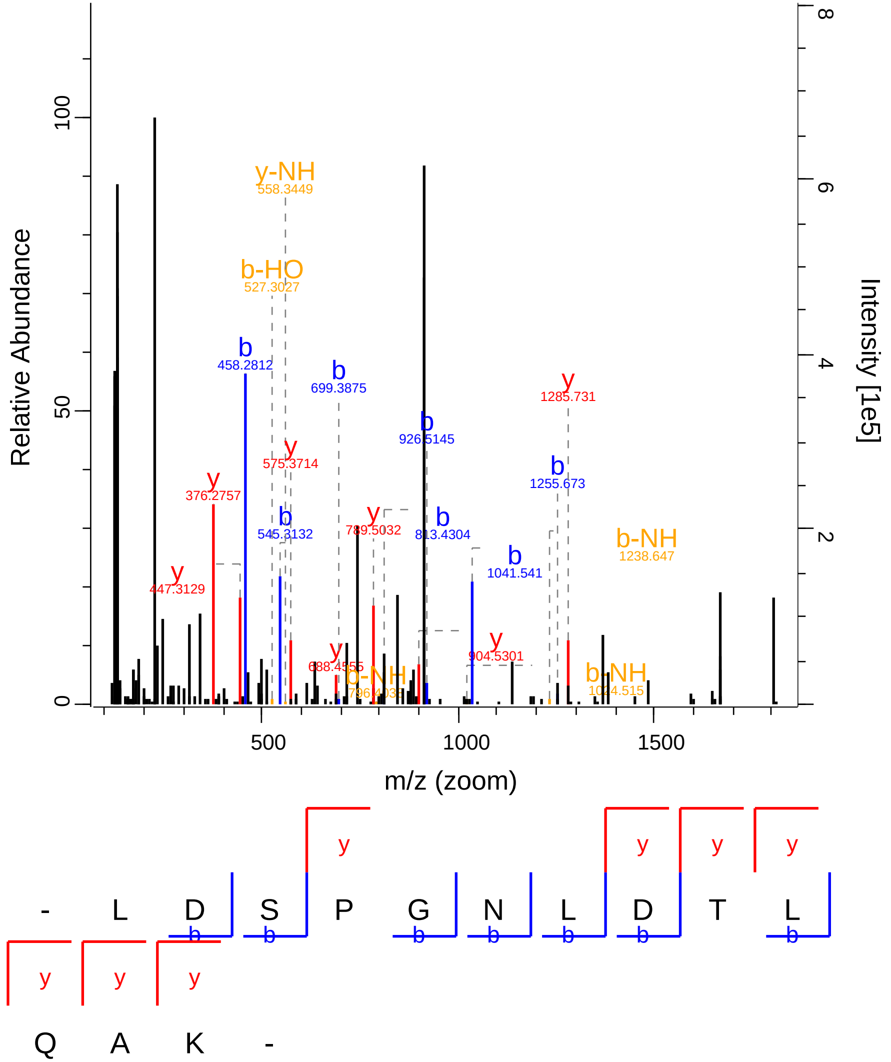

Raw File	Scan	Method	Score	m/z
RO-QC-160121_ZackD_SS_Set_02_Fraction_3	24295	FTMS; HCD	105.08	911.02

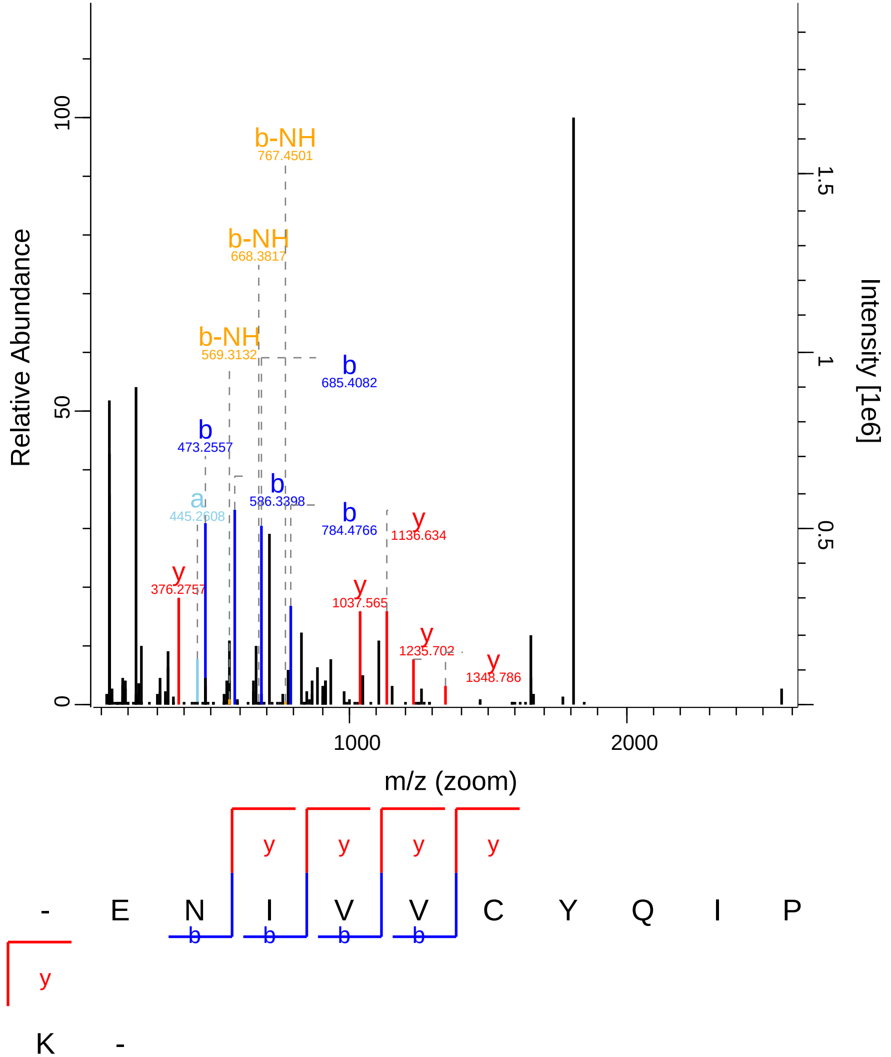

Raw File	Scan	Method	Score	m/z
RO-QC-160121_ZackD_SS_Set_02_Fraction_3	24327	FTMS; HCD	42.56	607.68

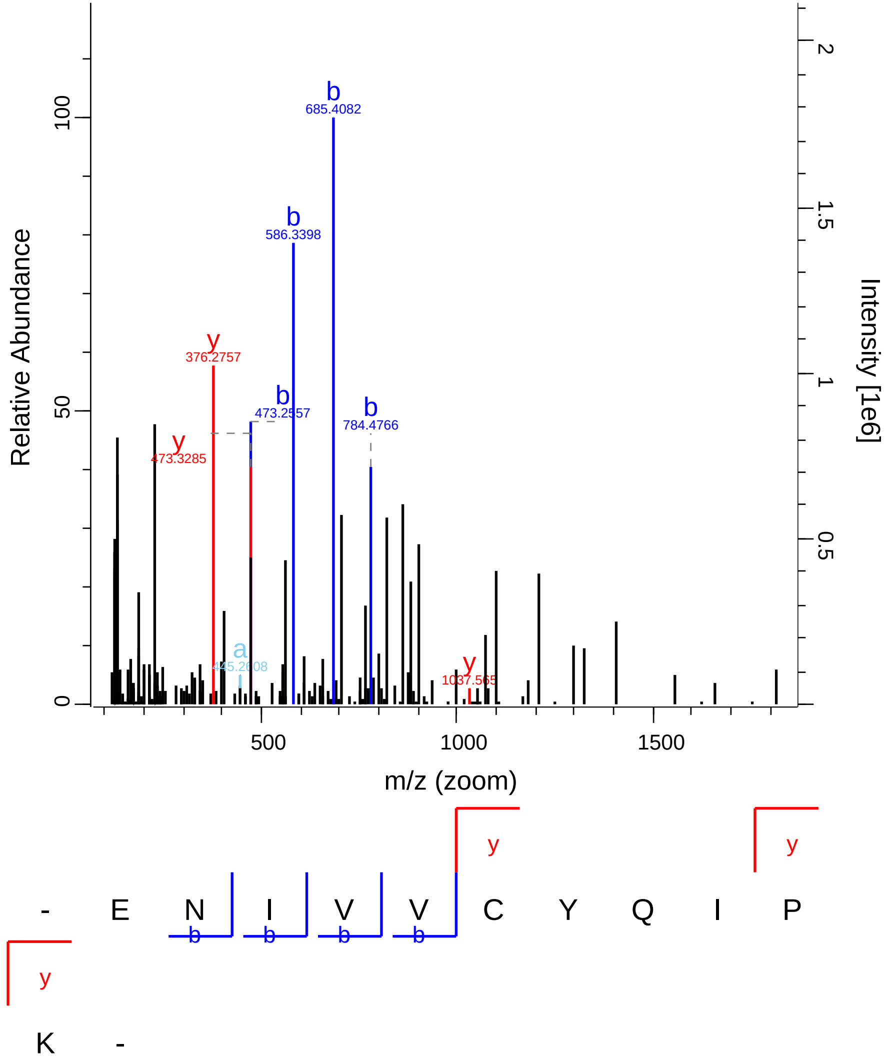

Raw File	Scan	Method	Score	m/z	Gene names
RO-QC-160121_ZackD_SS_Set_02_Fractio	25088	FTMS; HC	77.09	854.44	SLC31A1

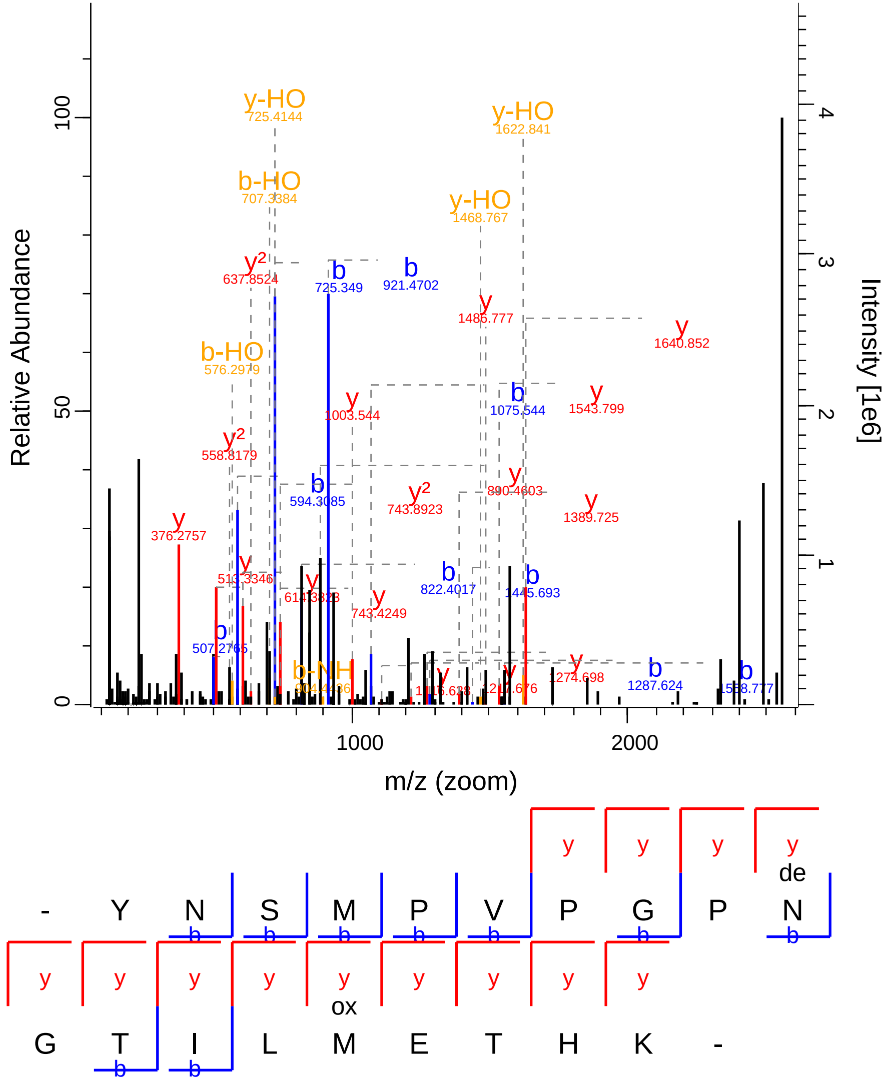

Raw File	Scan	Method	Score	m/z	Gene names
RO-QC-160121_ZackD_SS_Set_02_Fractio	25206	FTMS; HC	145.55	831.93	GPRC5B

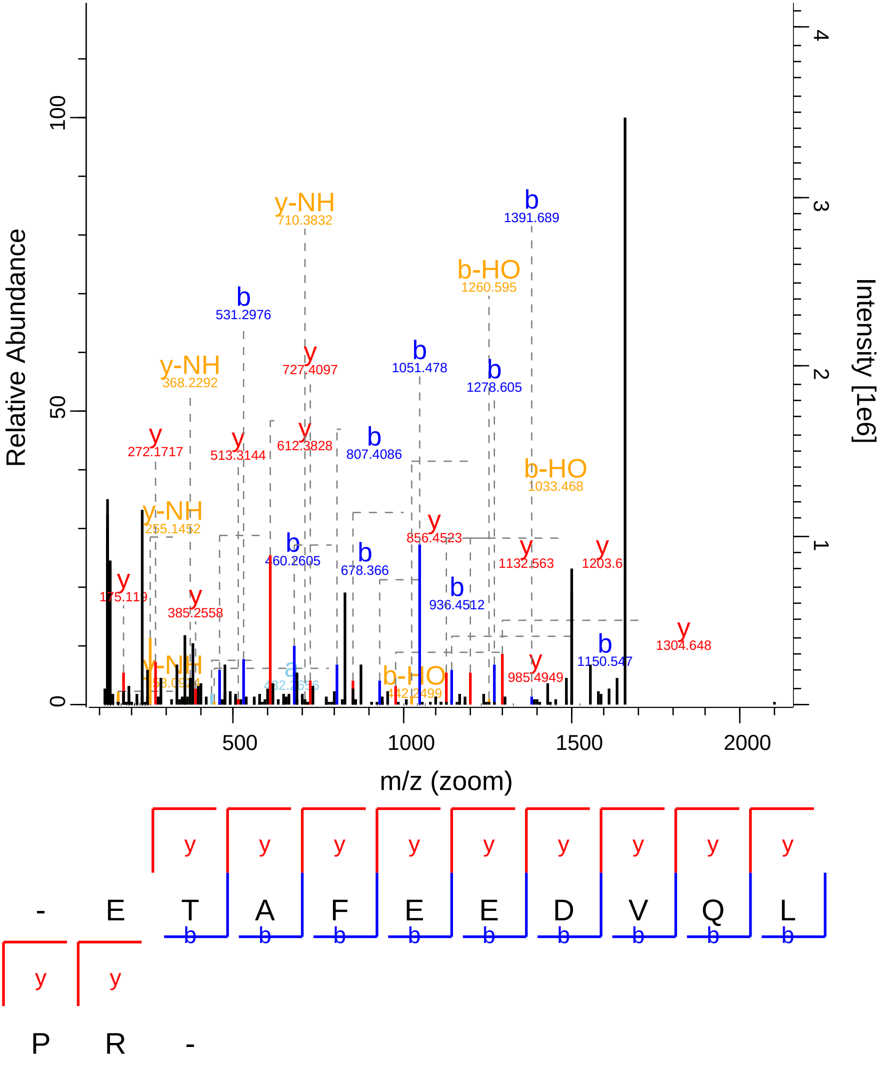

Raw File	Scan	Method	Score	m/z	Gene names
RO-QC-160121_ZackD_SS_Set_02_Fractio	25816	FTMS; HC	132.22	465.94	PTRH1

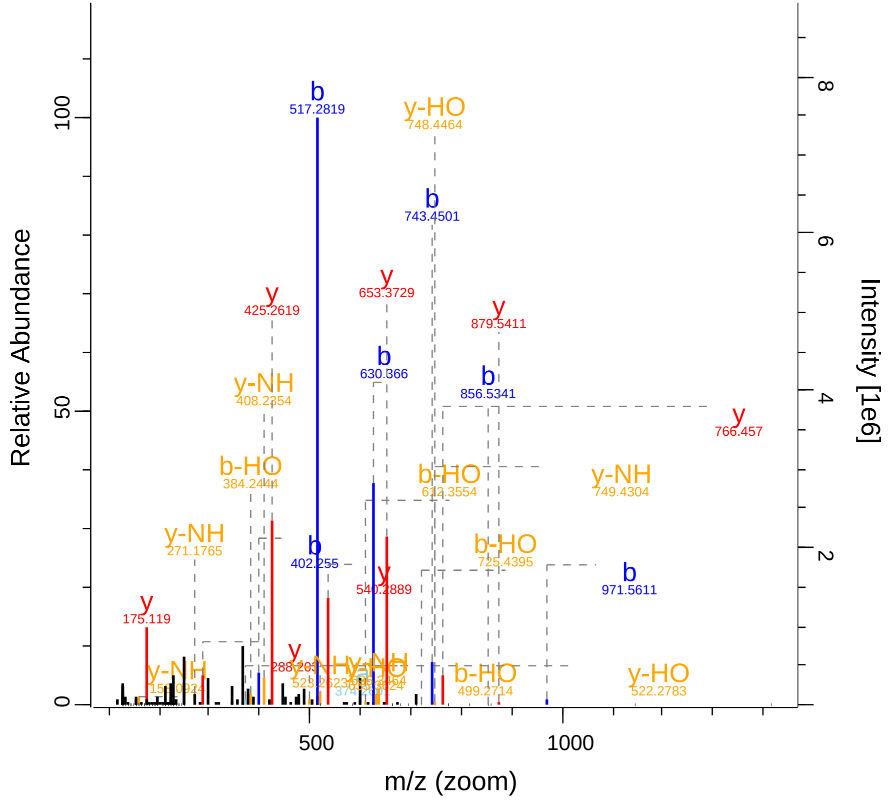

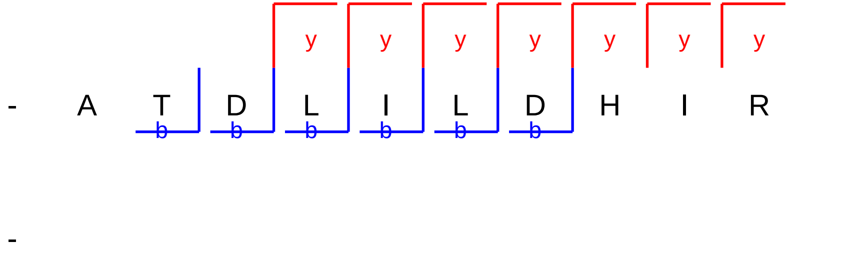

Raw File	Scan	Method	Score	m/z	Gene names
RO-QC-160121_ZackD_SS_Set_02_Fractio	26020	FTMS; HC	86.88	466.28	PTRH1

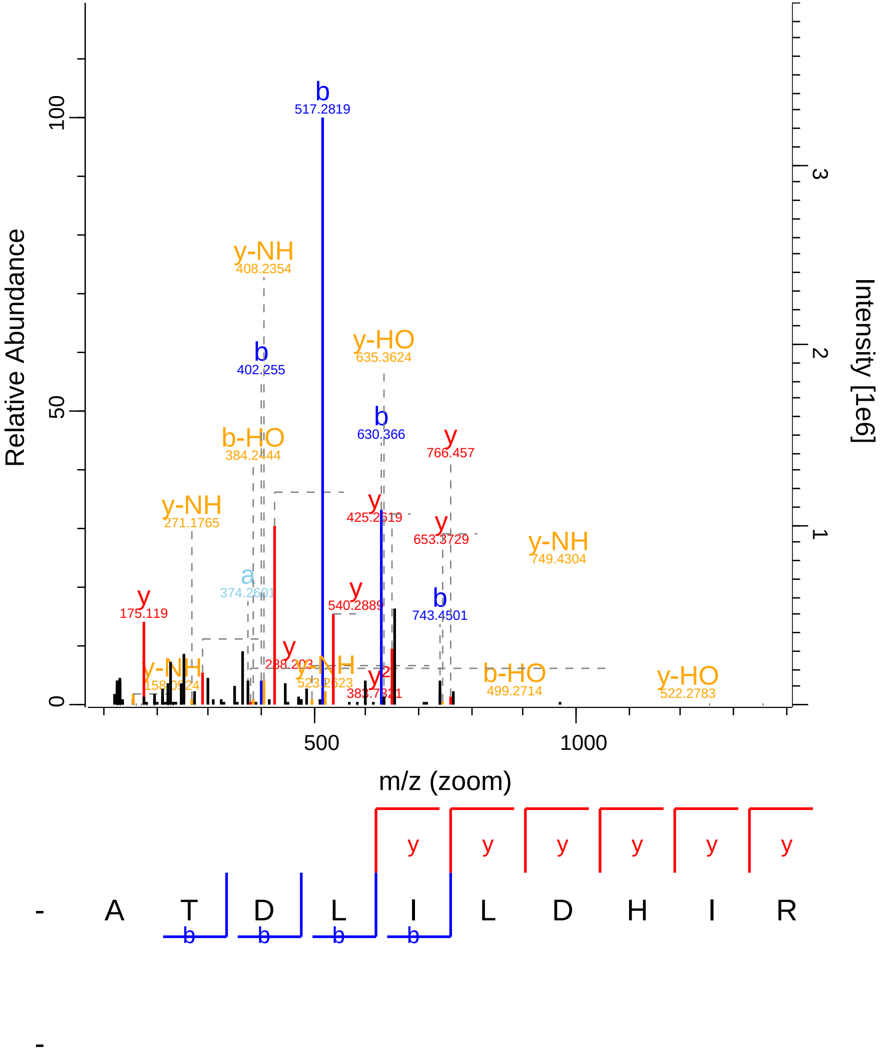

Raw File	Scan	Method	Score	m/z	Gene names
RO-QC-160121_ZackD_SS_Set_02_Fractio	26105	FTMS; HC	106.13	740.08	TMEM62

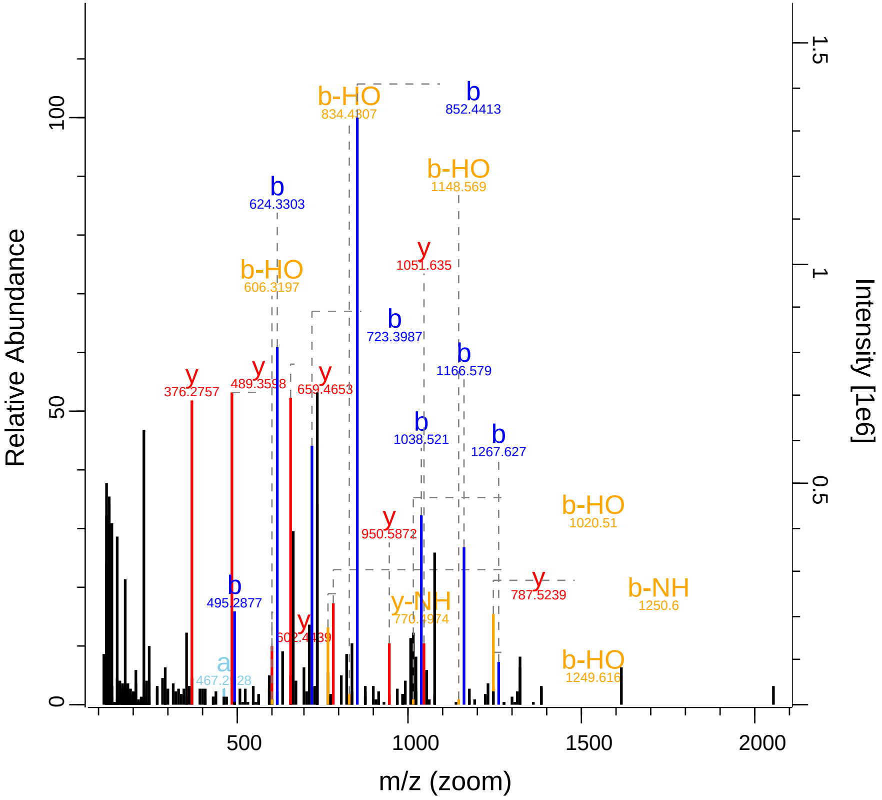

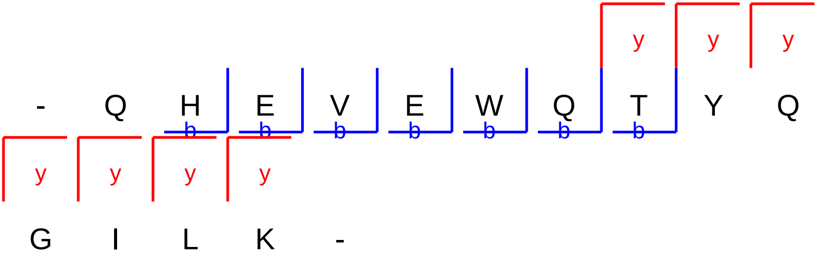

Raw File	Scan	Method	Score	m/z	Gene names
RO-QC-160121_ZackD_SS_Set_02_Fraction	26247	FTMS; HCI	75.29	1010	FTMT

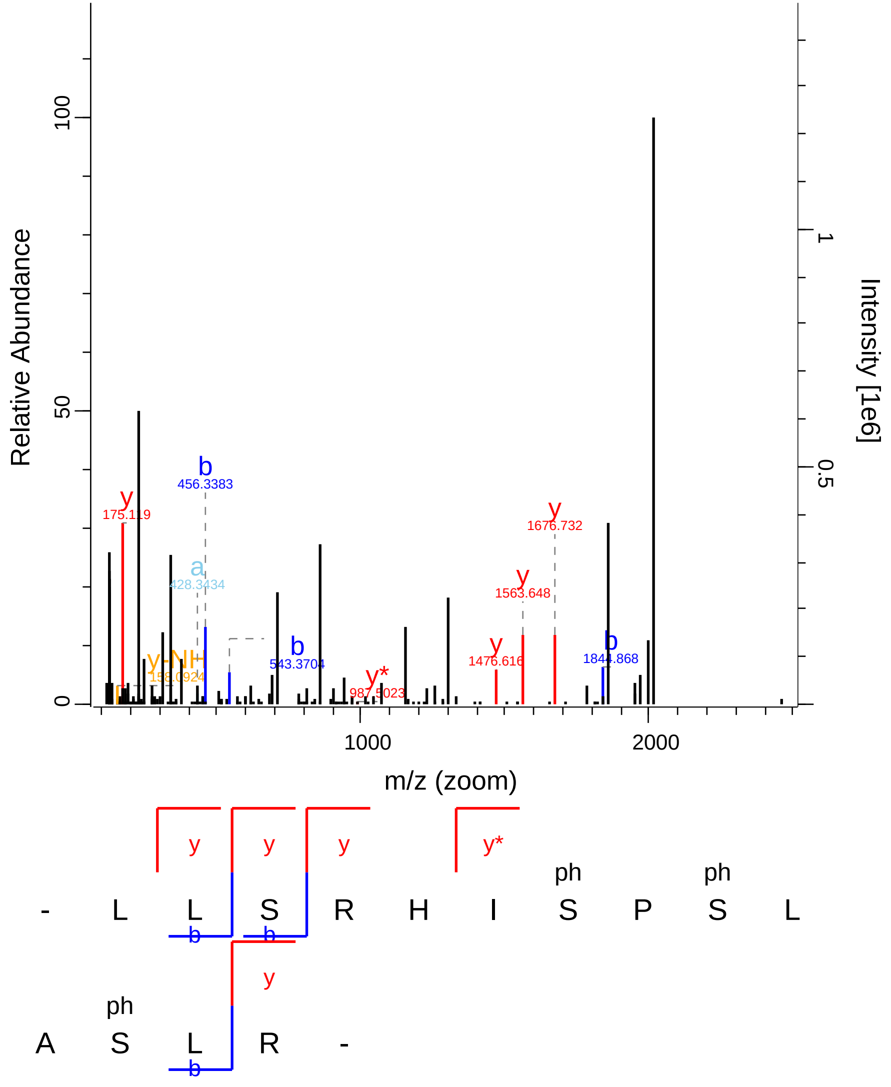

Raw File	Scan	Method	Score	m/z	Gene names
RO-QC-160121_ZackD_SS_Set_02_Fractio	26666	FTMS; HC	113.47	830.07	BTC

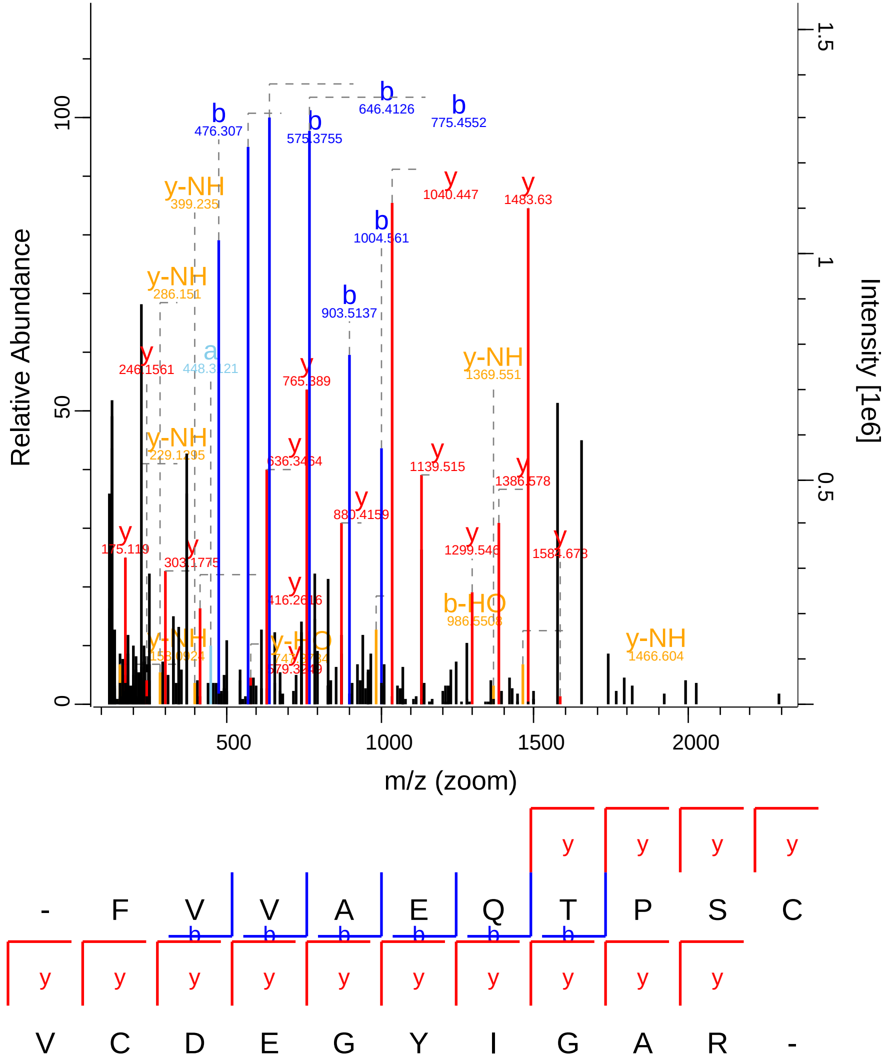

Raw File	Scan	Method	Score	m/z	Gene names
RO-QC-160121_ZackD_SS_Set_02_Fractic	27640	FTMS; HC	50.11	1226.12	LRRK1

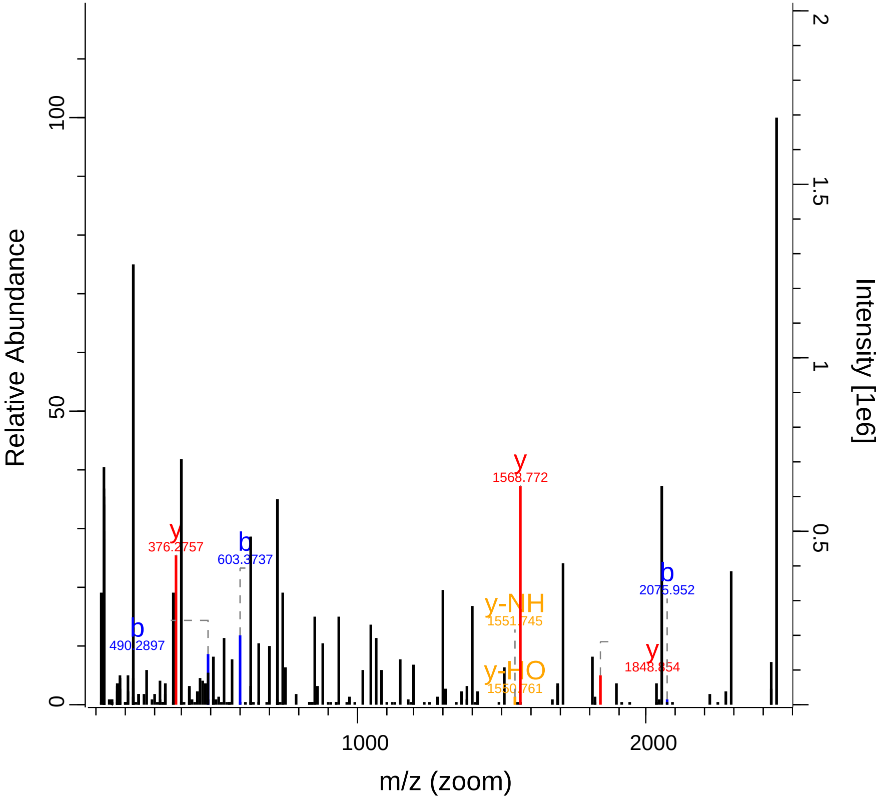

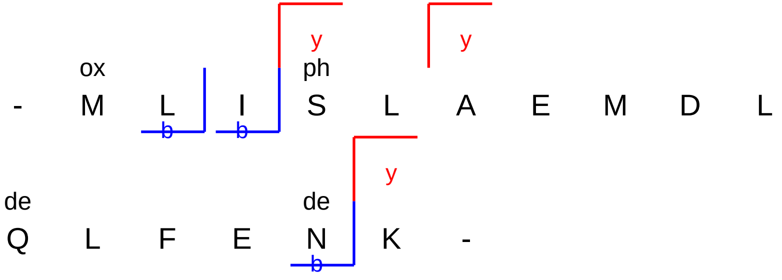

Raw File	Scan	Method	Score	m/z	Gene names
RO-QC-160121_ZackD_SS_Set_02_Fractio	27712	FTMS; HC	165.66	845.02	IFNGR1

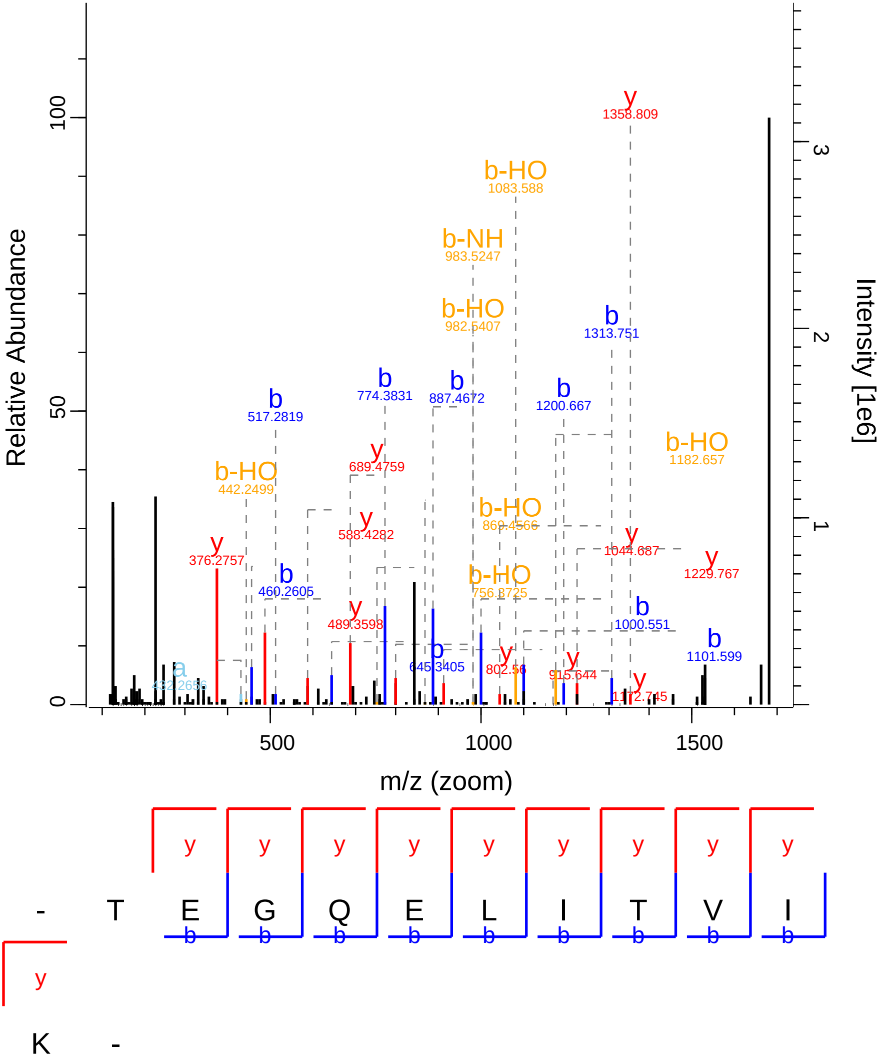

Raw File	Scan	Method	Score	m/z	Gene names
RO-QC-160121_ZackD_SS_Set_02_Fractio	27730	FTMS; HC	47.99	564.01	IFNGR1

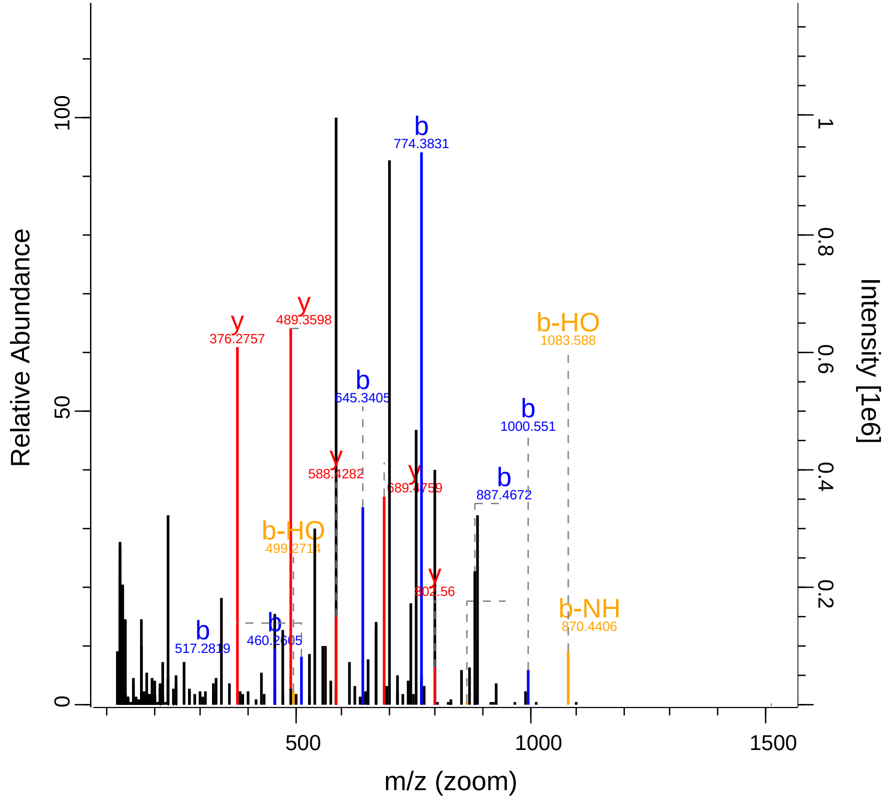

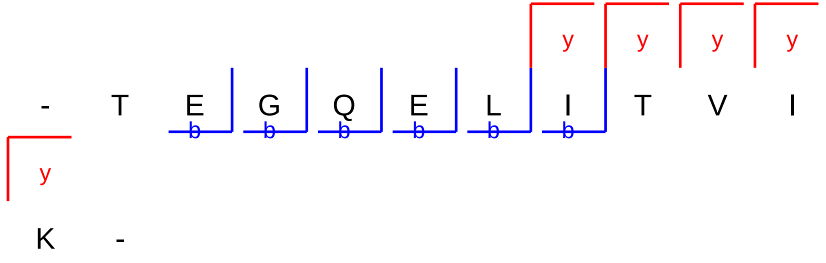

Raw File	Scan	Method	Score	m/z	Gene names
RO-QC-160121_ZackD_SS_Set_02_Fractio	28782	FTMS; HC	41.65	908.96	CCDC180

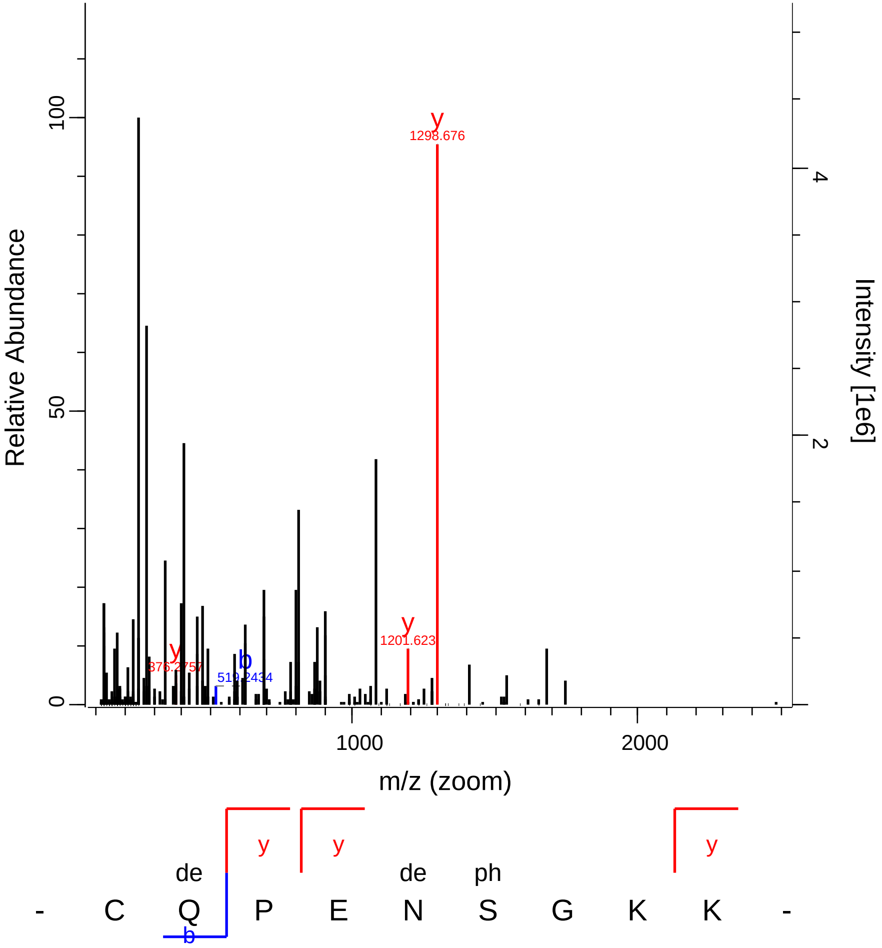

Raw File	Scan	Method	Score	m/z
RO-QC-160121_ZackD_SS_Set_02_Fraction_3	28953	FTMS; HCD	72.29	1251.19

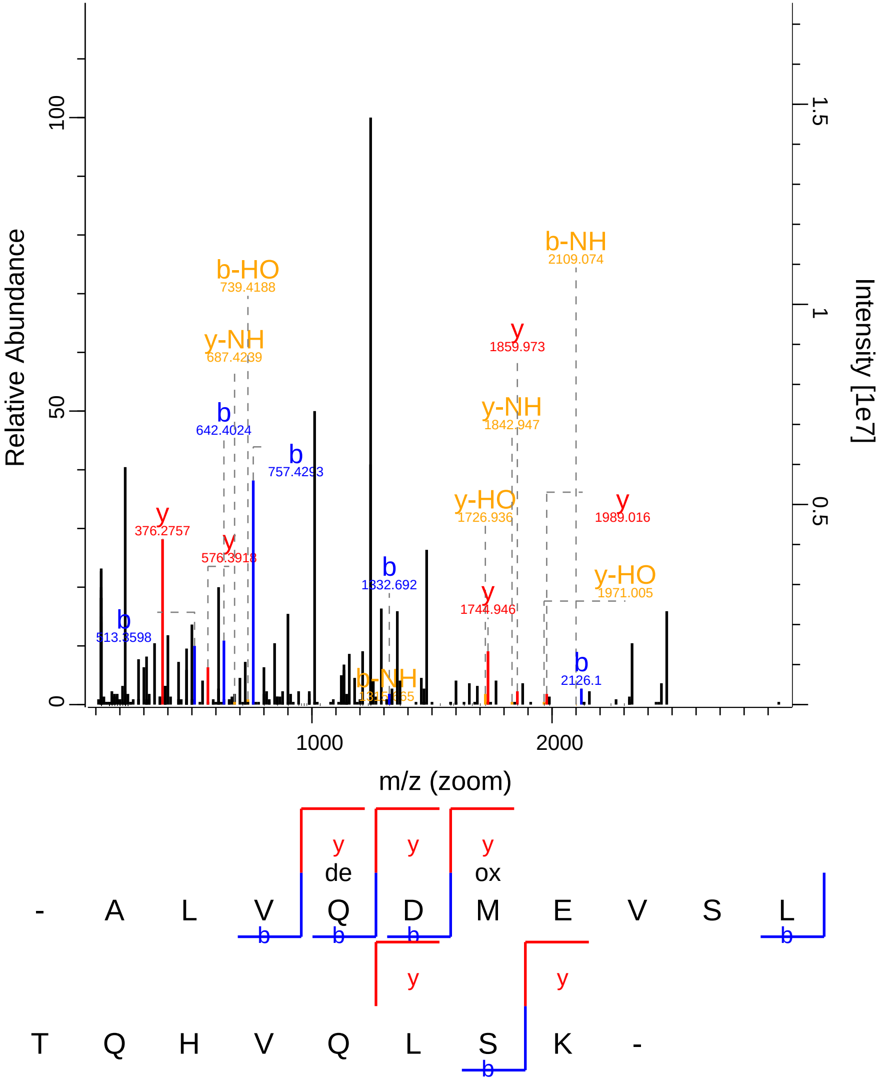

Raw File	Scan	Method	Score	m/z	Gene names
RO-QC-160121_ZackD_SS_Set_02_Fractio	29006	FTMS; HC	47.85	908.96	CCDC180

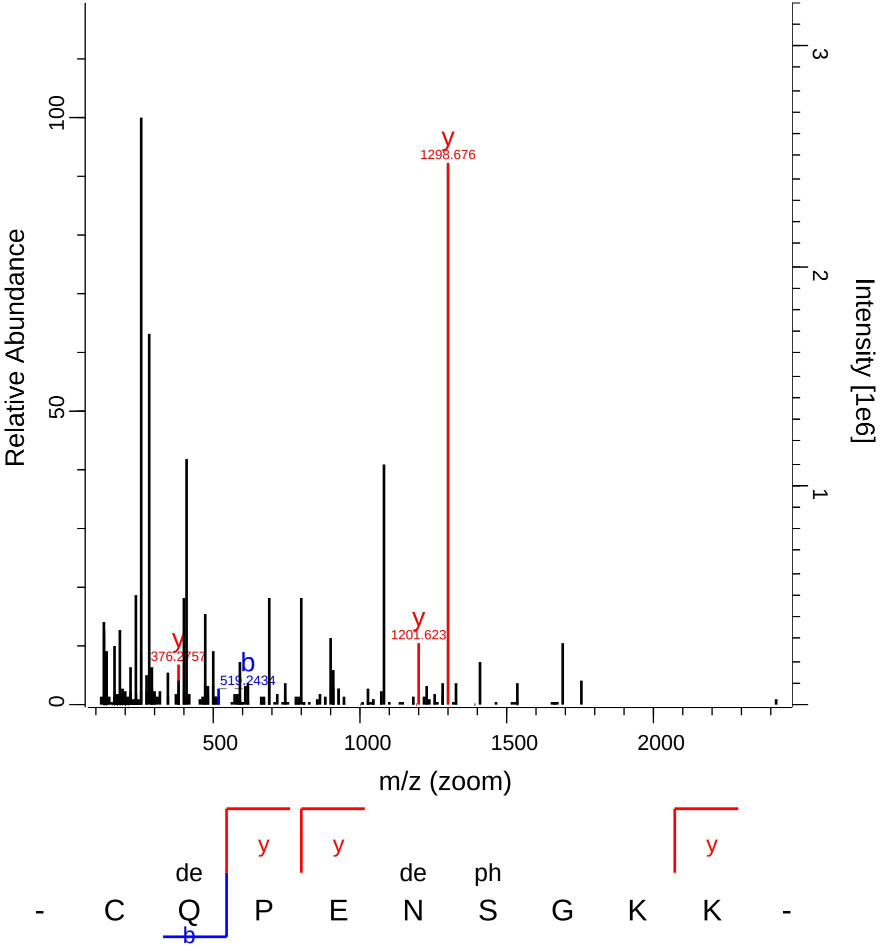

Raw File	Scan	Method	Score	m/z	Gene names
RO-QC-160121_ZackD_SS_Set_02_Fractio	30673	FTMS; HC	72.36	757.41	LBH

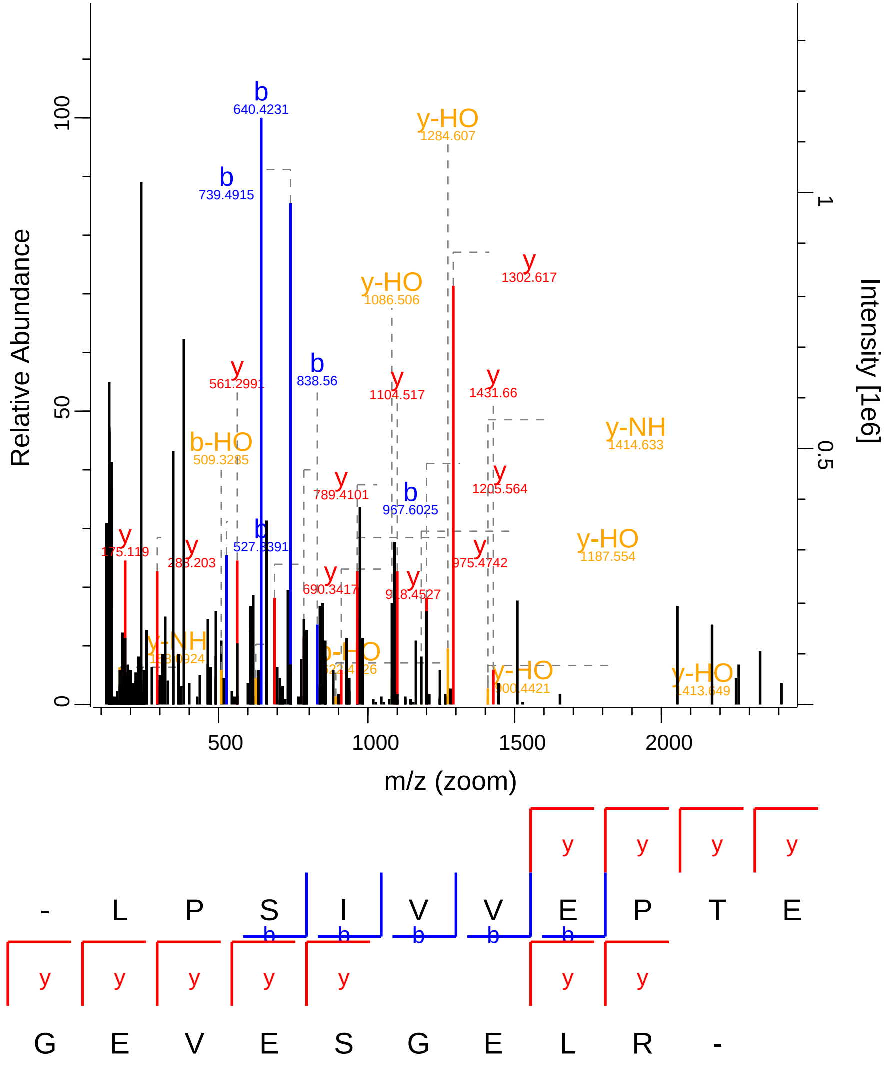

Raw File	Scan	Method	Score	m/z	Gene names
RO-QC-160121_ZackD_SS_Set_02_Fractic	30811	FTMS; HC	137.03	1027.51	SPC24

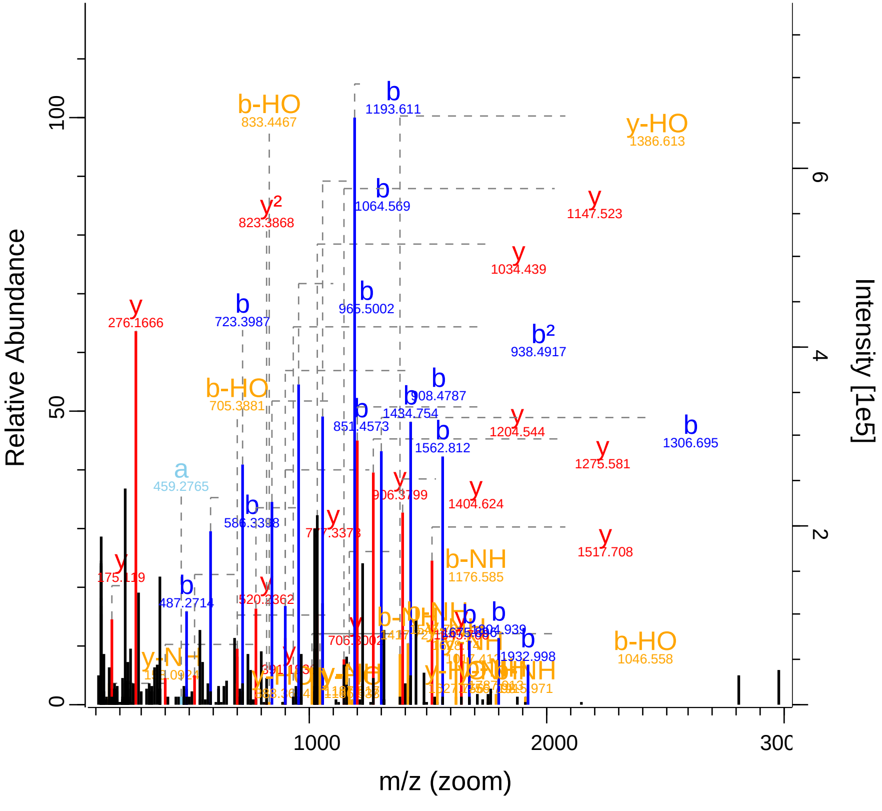

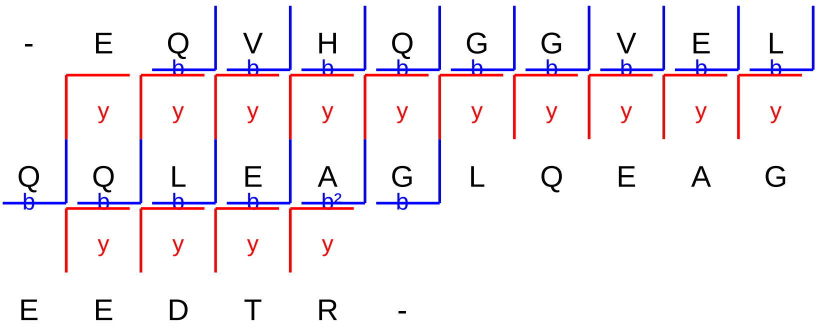

Raw File	Scan	Method	Score	m/z	Gene names
RO-QC-160121_ZackD_SS_Set_02_Fractic	31007	FTMS; HC	41.76	1046.18	ALPK3

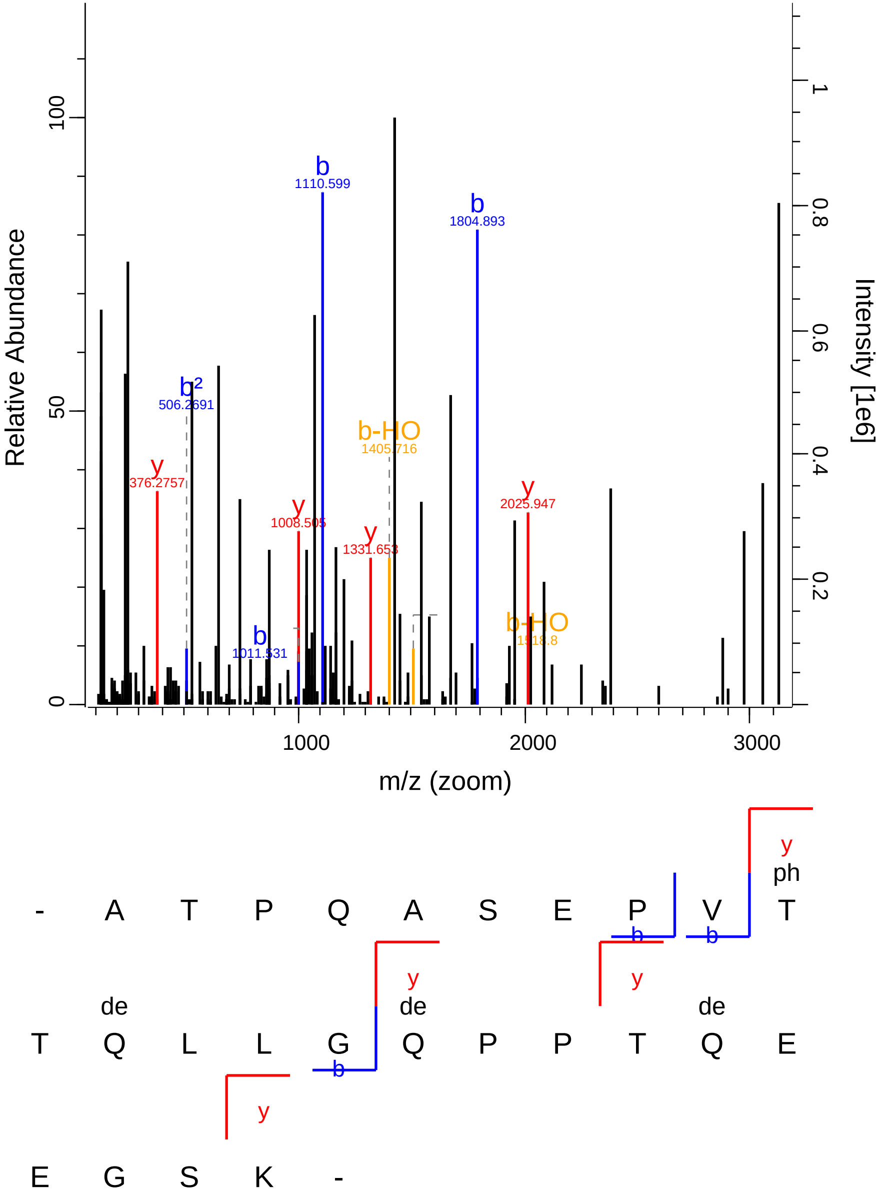

Raw File	Scan	Method	Score	m/z
RO-QC-160121_ZackD_SS_Set_02_Fraction_3	31896	FTMS; HCD	56.92	491.97

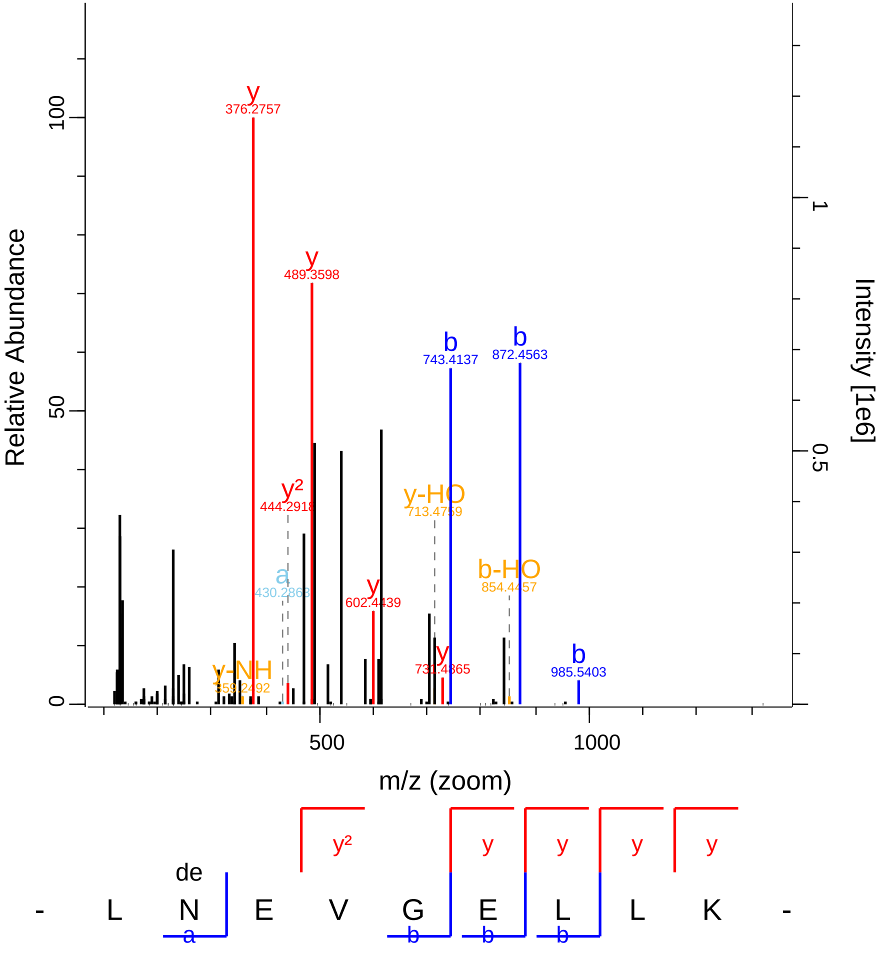

Raw File	Scan	Method	Score	m/z	Gene names
RO-QC-160121_ZackD_SS_Set_02_Fractio	32332	FTMS; HC	165.19	994.85	NDUFAF6

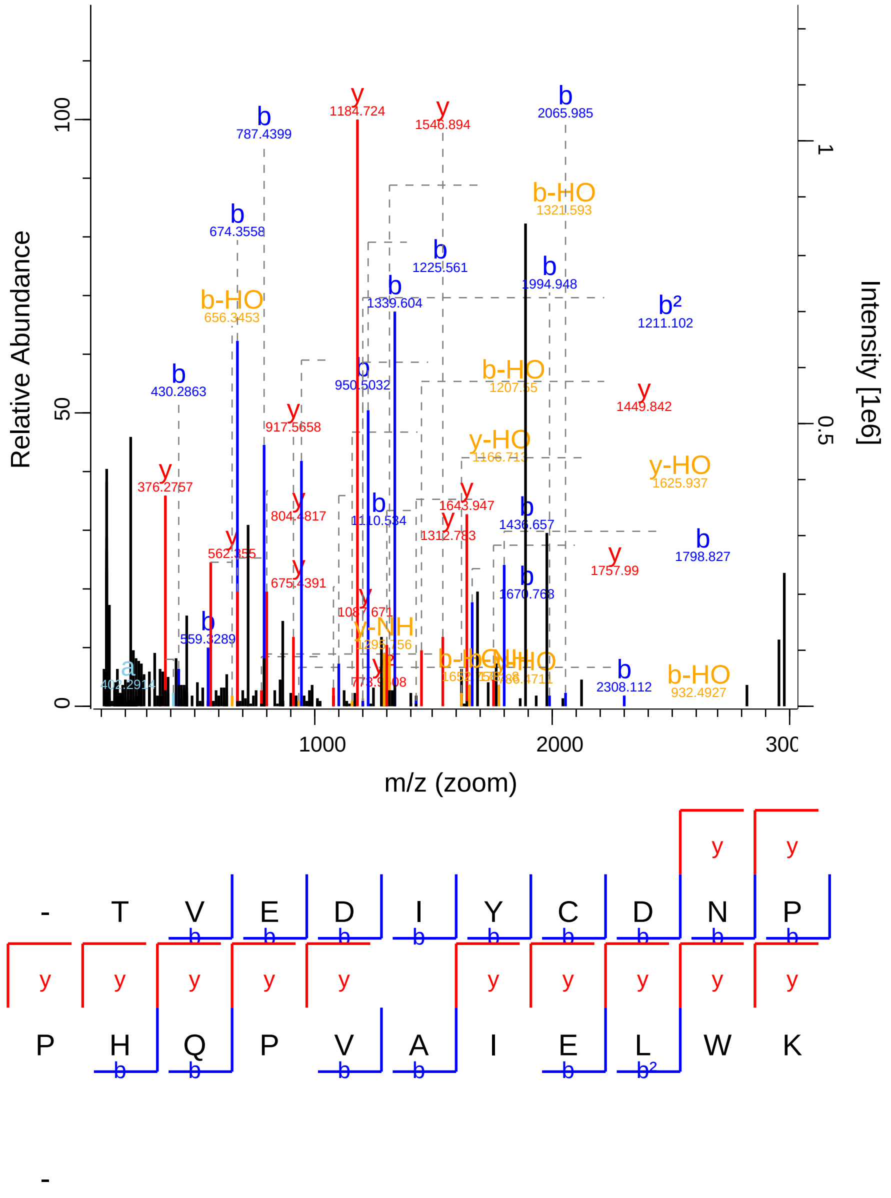

Raw File	Scan	Method	Score	m/z	Gene names
RO-QC-160121_ZackD_SS_Set_02_Fractio	32742	FTMS; HC	41.54	680.34	NPHS2

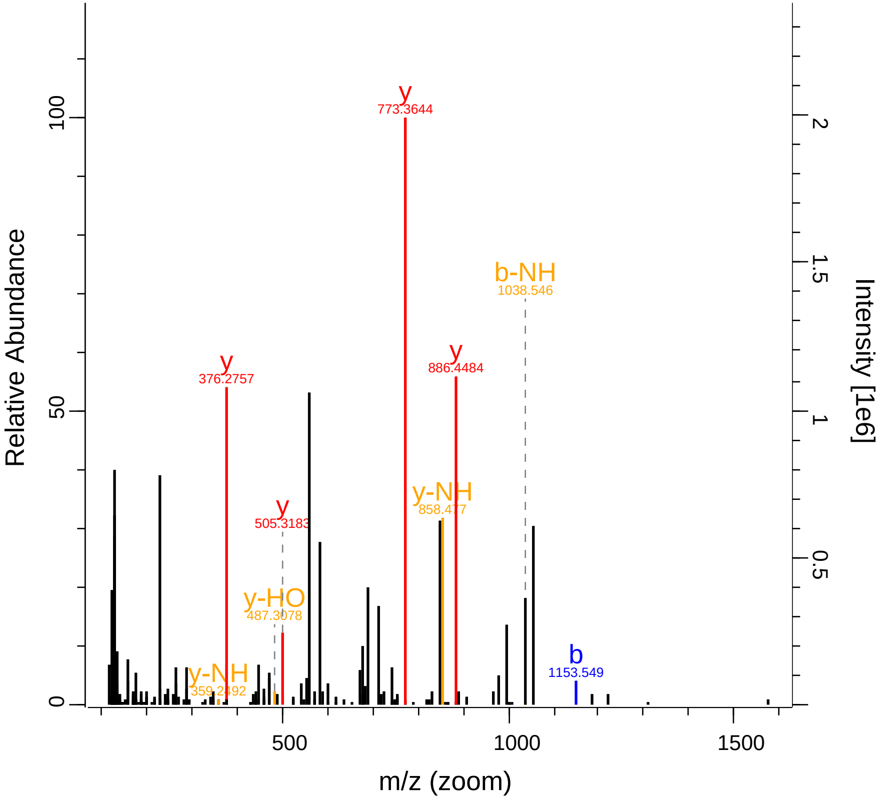

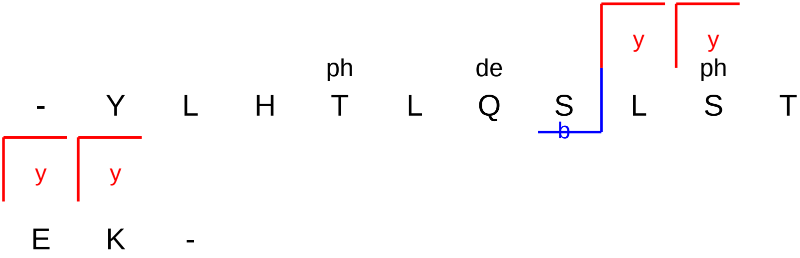

Raw File	Scan	Method	Score	m/z	Gene names
RO-QC-160121_ZackD_SS_Set_02_Fractio	34803	FTMS; HC	40.04	822.47	OR10G3

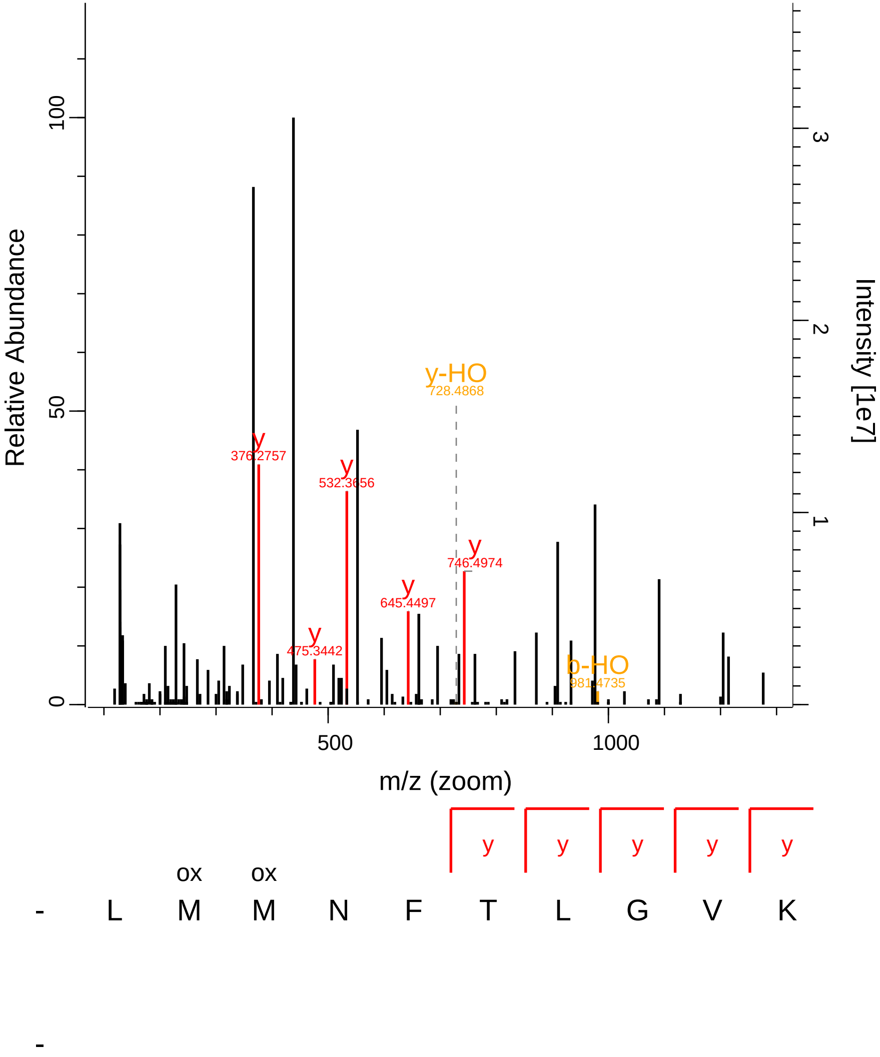

Raw File	Scan	Method	Score	m/z	Gene names
RO-QC-160121_ZackD_SS_Set_02_Fractic	35661	FTMS; HC	225.23	1032.51	CHCHD10

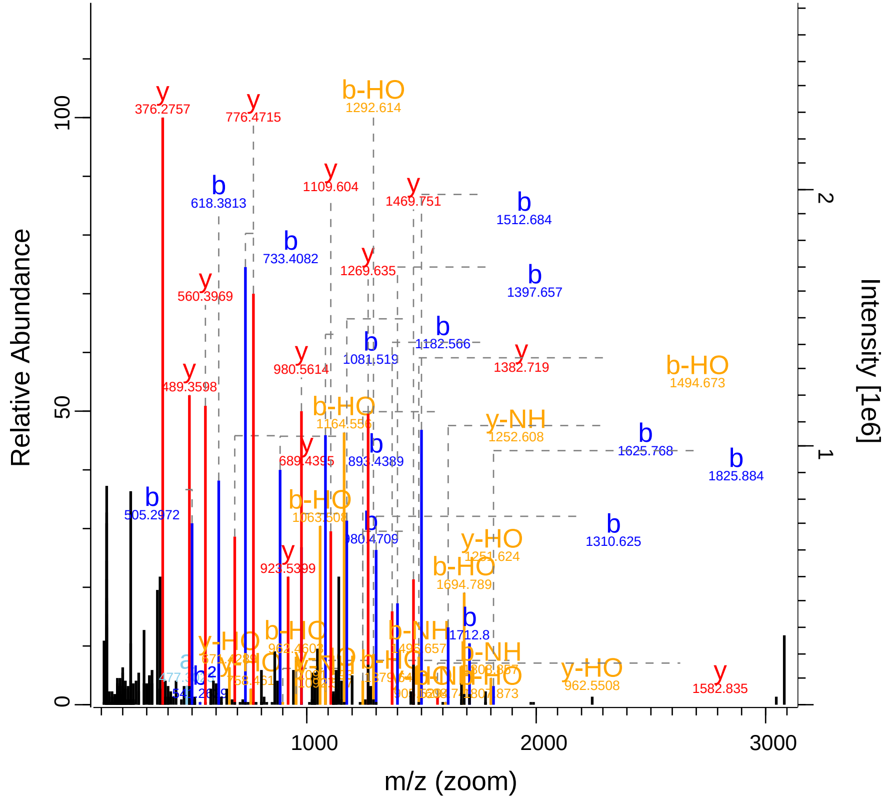

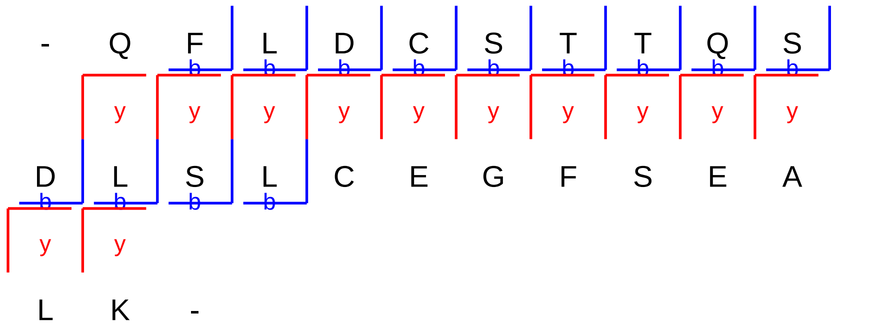

Raw File	Scan	Method	Score	m/z	Gene names
RO-QC-160121_ZackD_SS_Set_02_Fractio	35687	FTMS; HC	94.38	774.89	CHCHD10

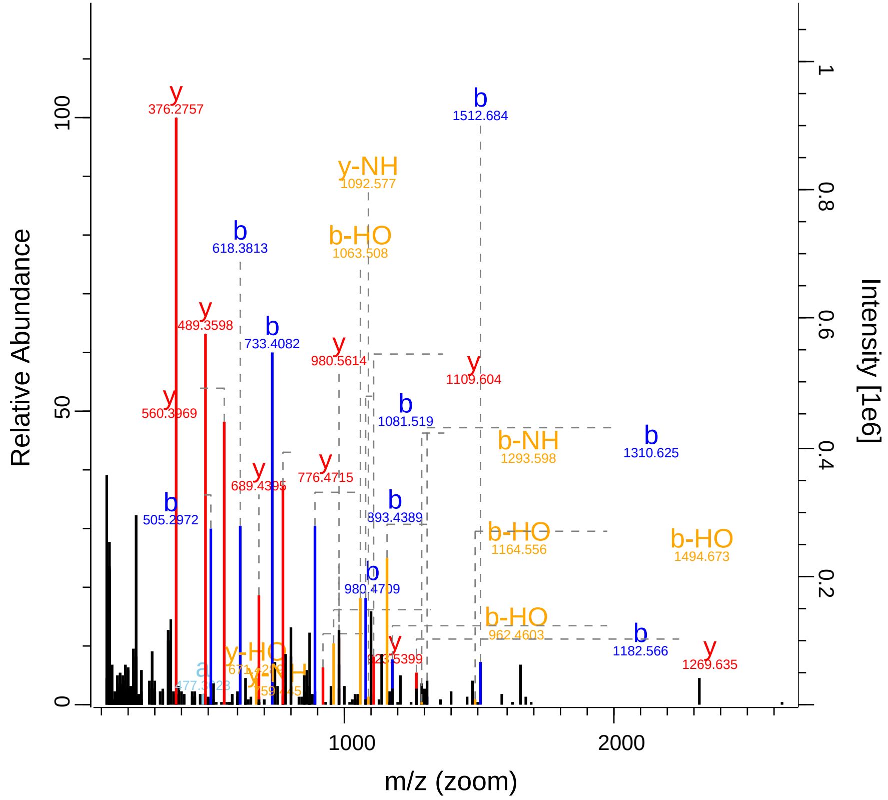

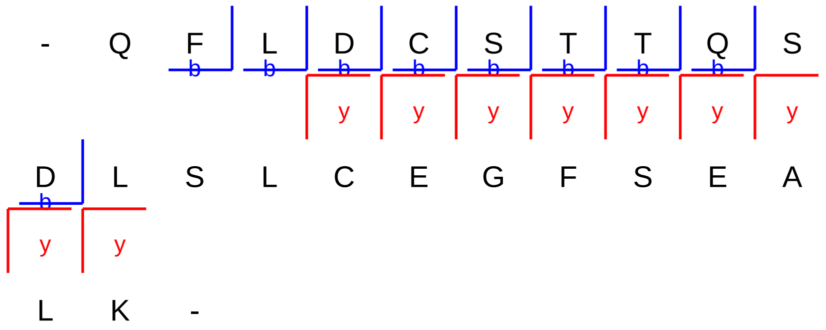

Raw File	Scan	Method	Score	m/z	Gene names
RO-QC-160121_ZackD_SS_Set_02_Fractio	35845	FTMS; HC	172.24	1032.51	CHCHD10

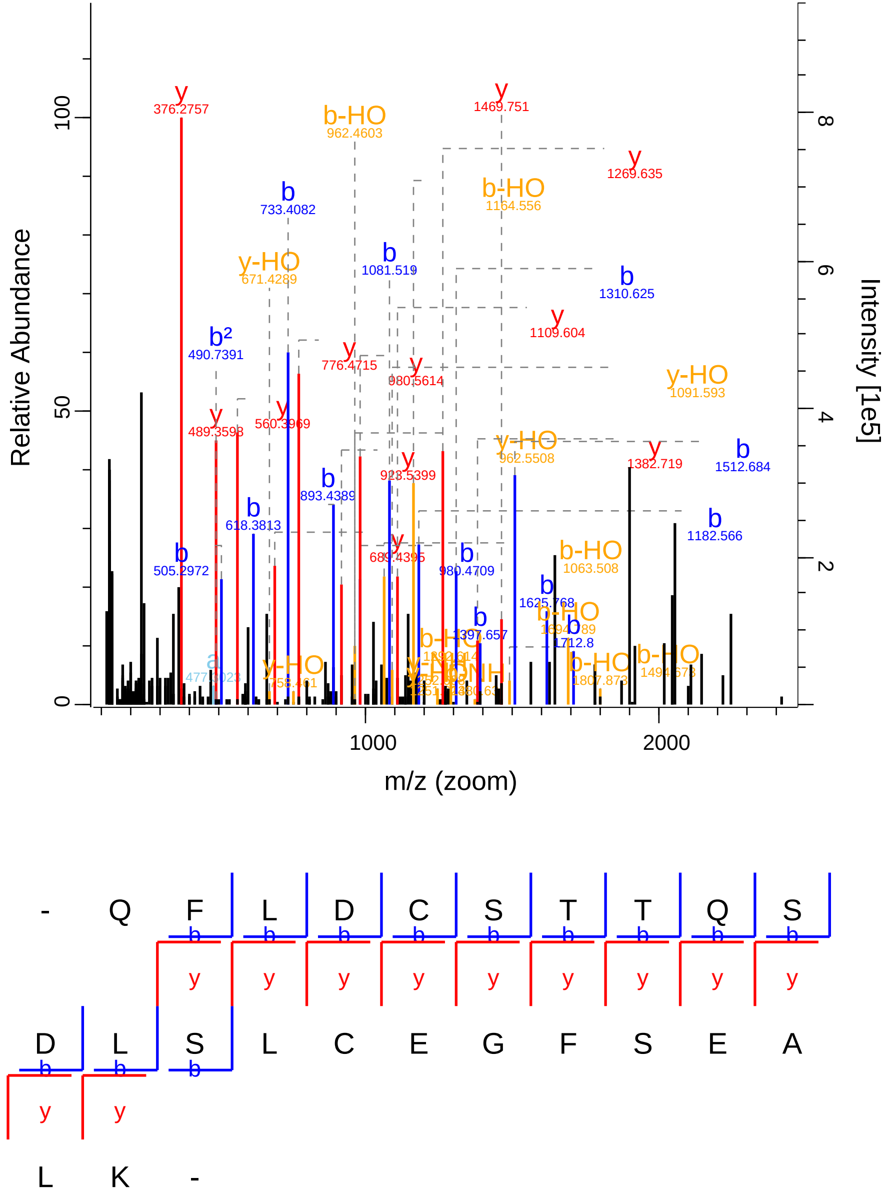

Raw File	Scan	Method	Score	m/z	Gene names
RO-QC-160121_ZackD_SS_Set_02_Fractic	35998	FTMS; HC	97.69	1065.02	EMB

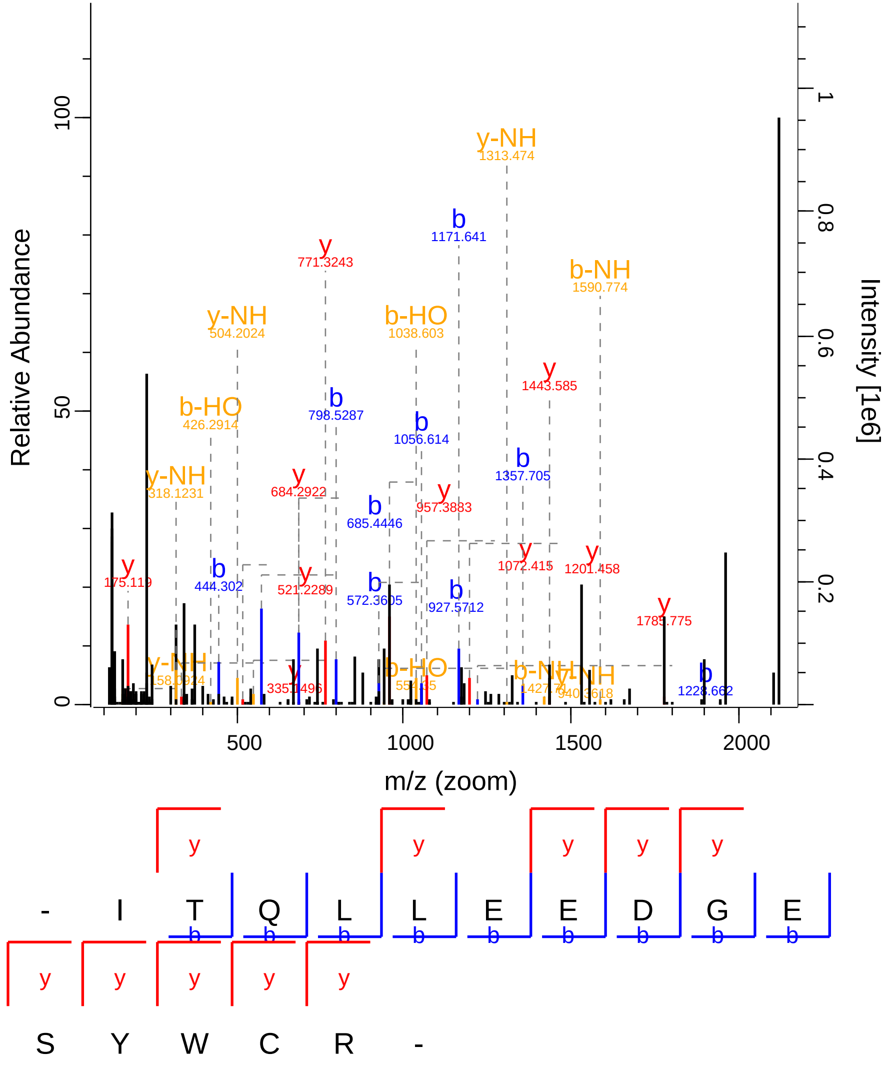

Raw File	Scan	Method	Score	m/z
RO-QC-160121_ZackD_SS_Set_02_Fraction_3	36341	FTMS; HCD	49.3	1054.61

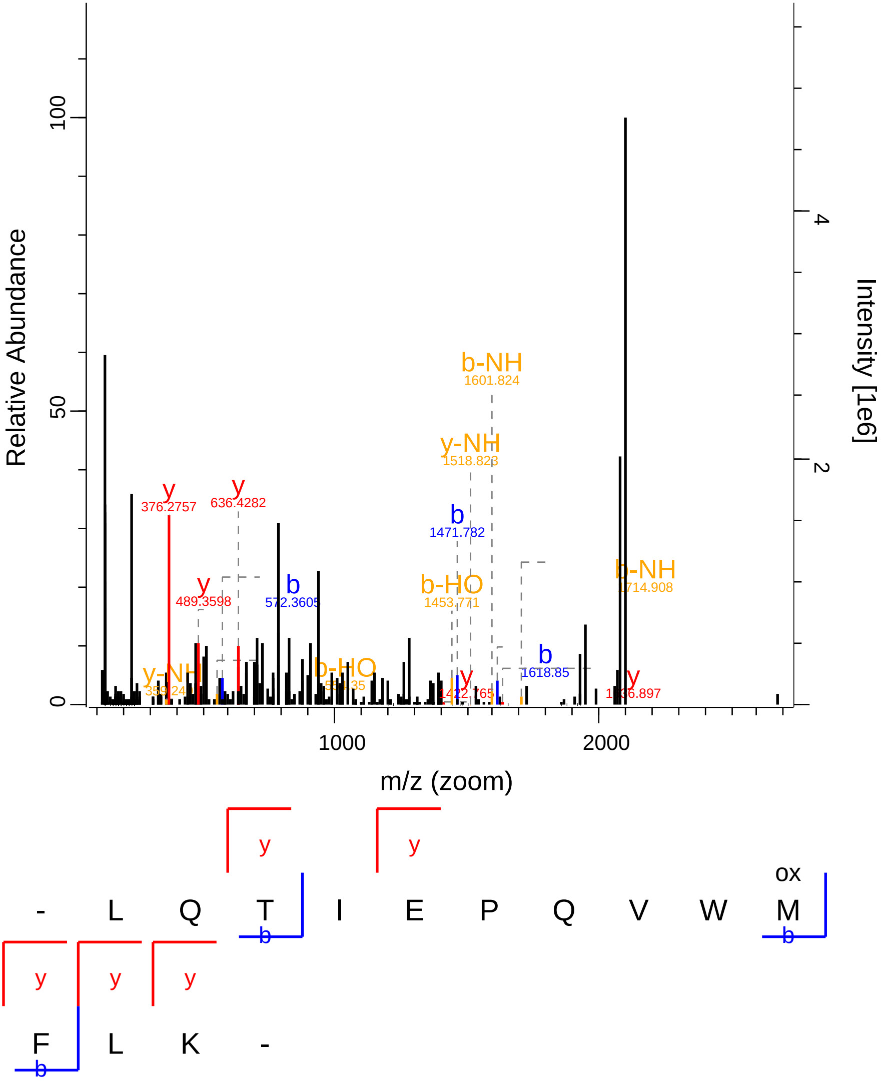

Raw File	Scan	Method	Score	m/z	Gene names
RO-QC-160121_ZackD_SS_Set_02_Fractic	37252	FTMS; HC	84.27	1001.96	C5orf45

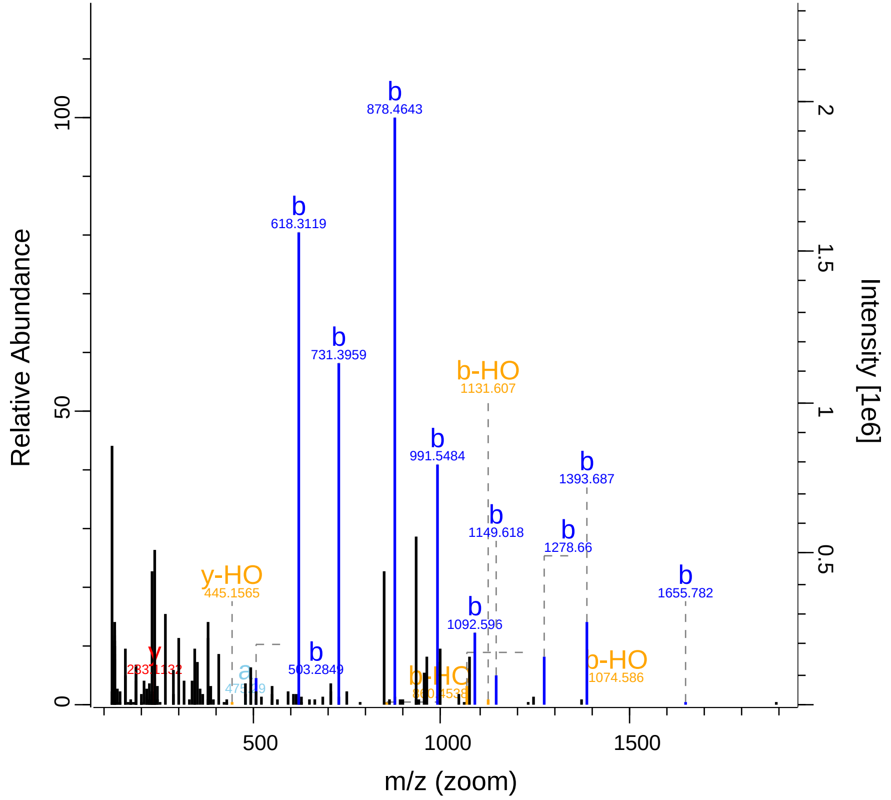

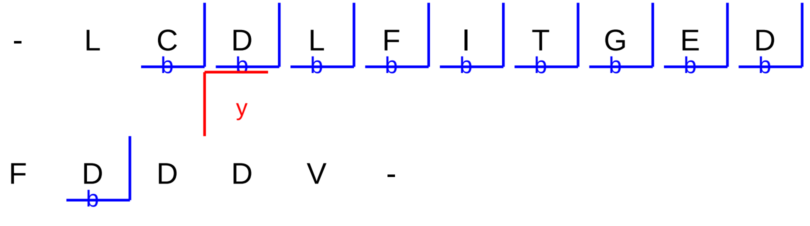

Raw File	Scan	Method	Score	m/z	Gene names
RO-QC-160121_ZackD_SS_Set_02_Fractio	37349	FTMS; HC	159.34	787.78	FUNDC1

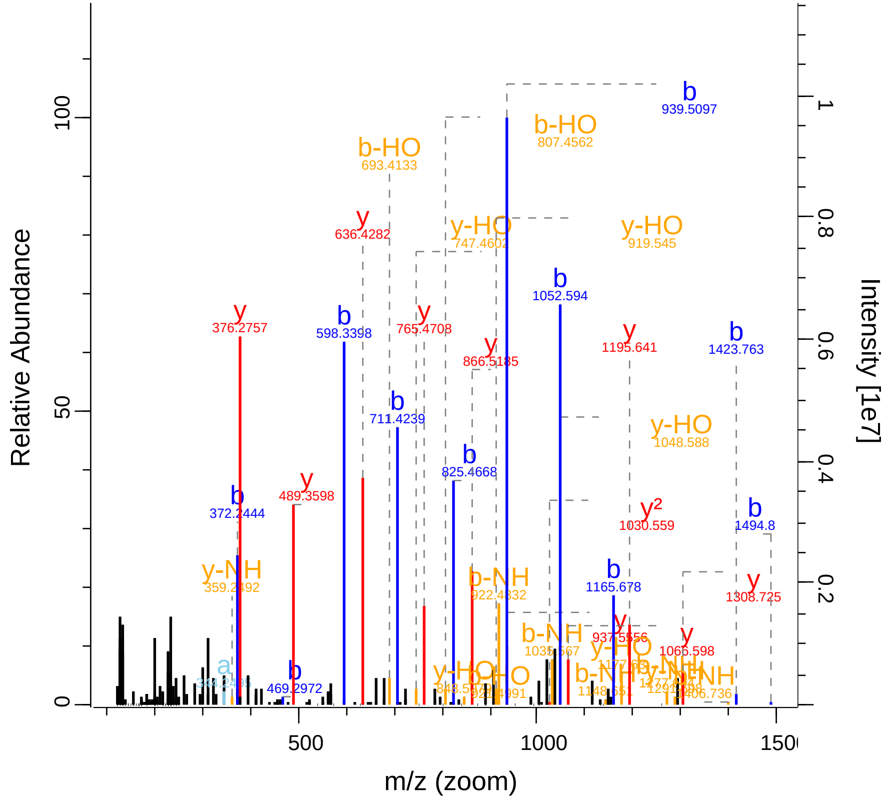

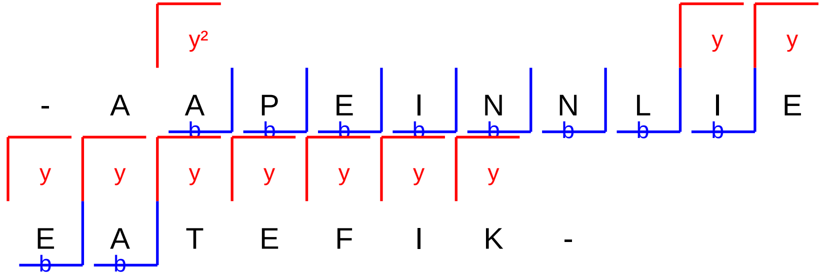

Raw File	Scan	Method	Score	m/z	Gene names
RO-QC-160121_ZackD_SS_Set_02_Fractior	5452	FTMS; HCI	45.16	580.98	PATZ1

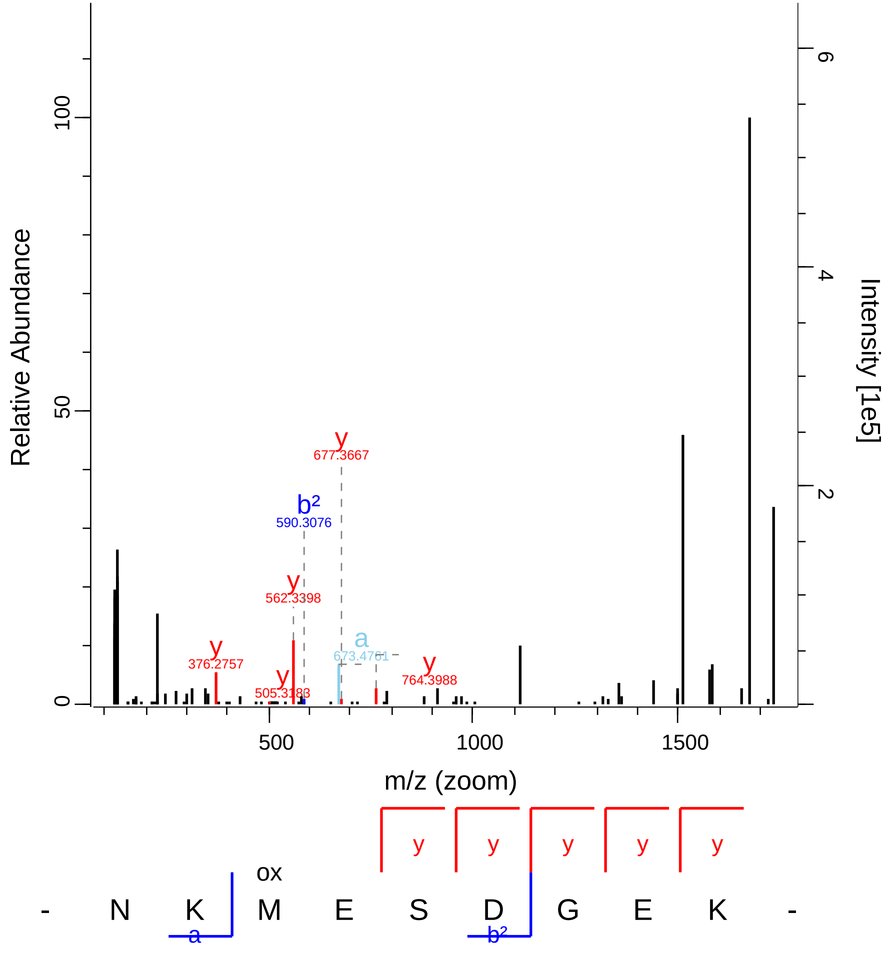

Raw File	Scan	Method	Score	m/z	Gene names
RO-QC-160121_ZackD_SS_Set_02_Fraction	5638	FTMS; HCI	52.79	440.5	FAM111B

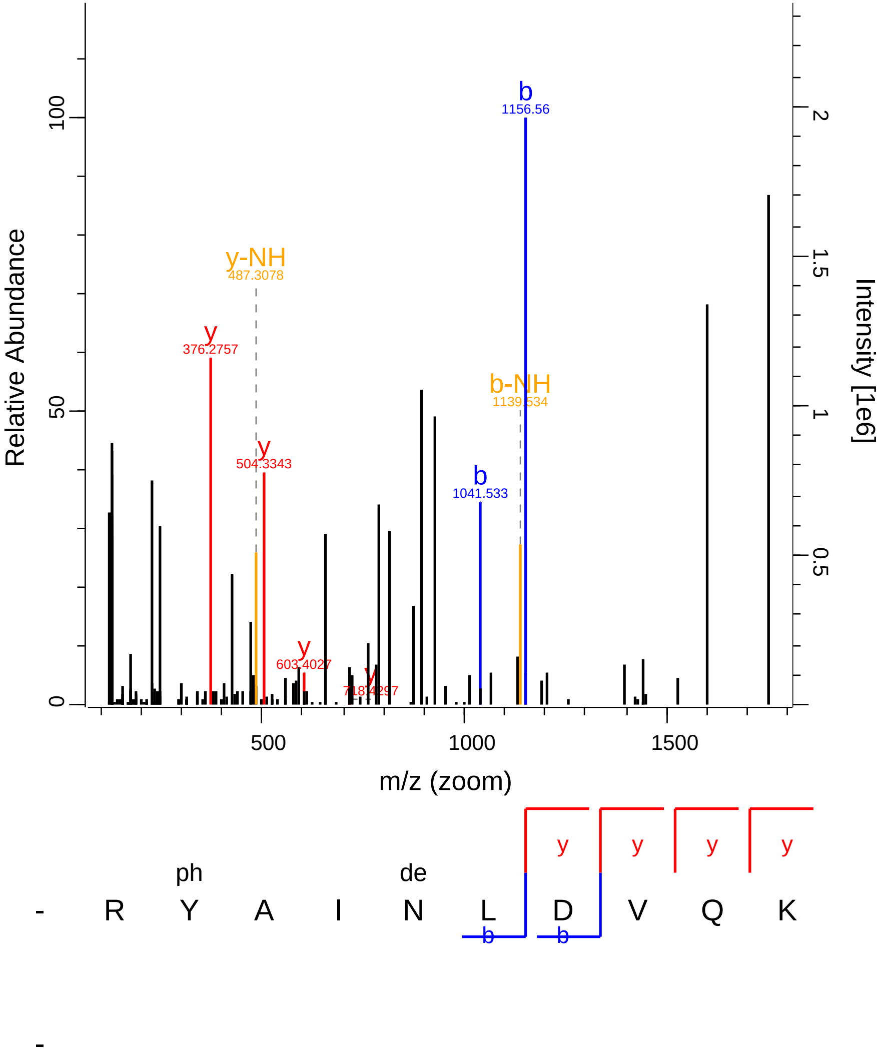

Raw File	Scan	Method	Score	m/z	Gene names
RO-QC-160121_ZackD_SS_Set_02_Fractior	6157	FTMS; HCI	64.57	467.94	SLCO4A1

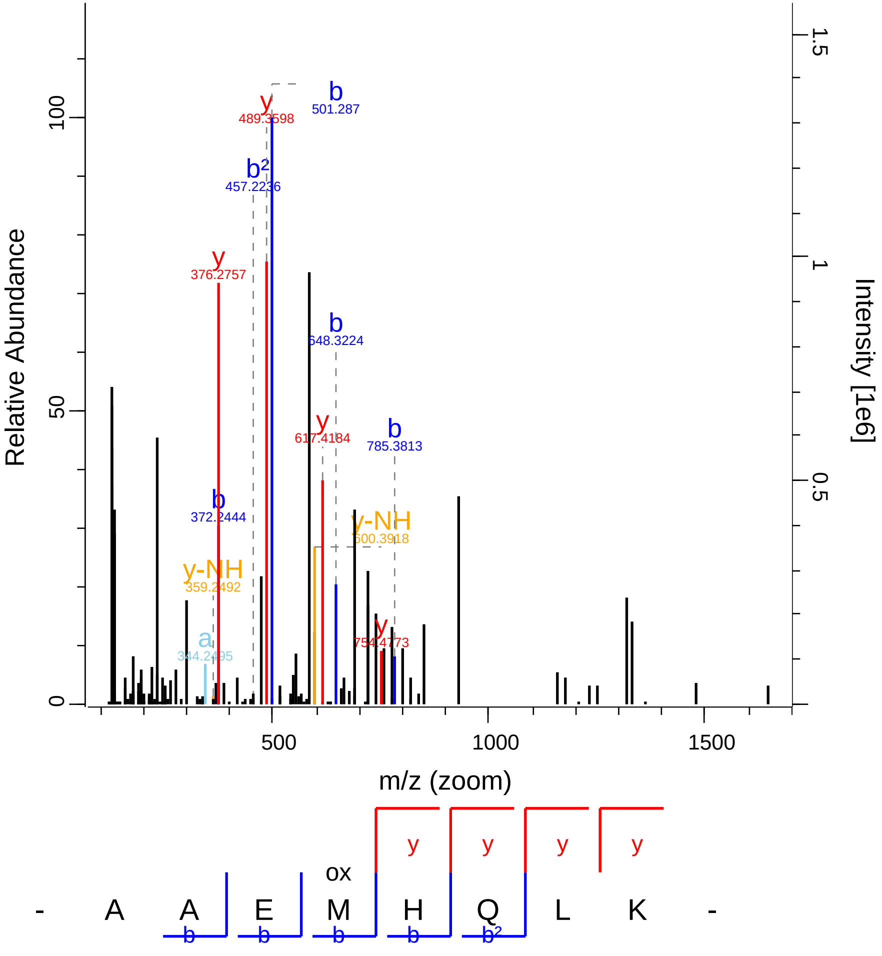

Raw File	Scan	Method	Score	m/z	Gene names
RO-QC-160121_ZackD_SS_Set_02_Fractio[illegible]	6420	FTMS; HC[illegible]	128.36	660.85	PEX26

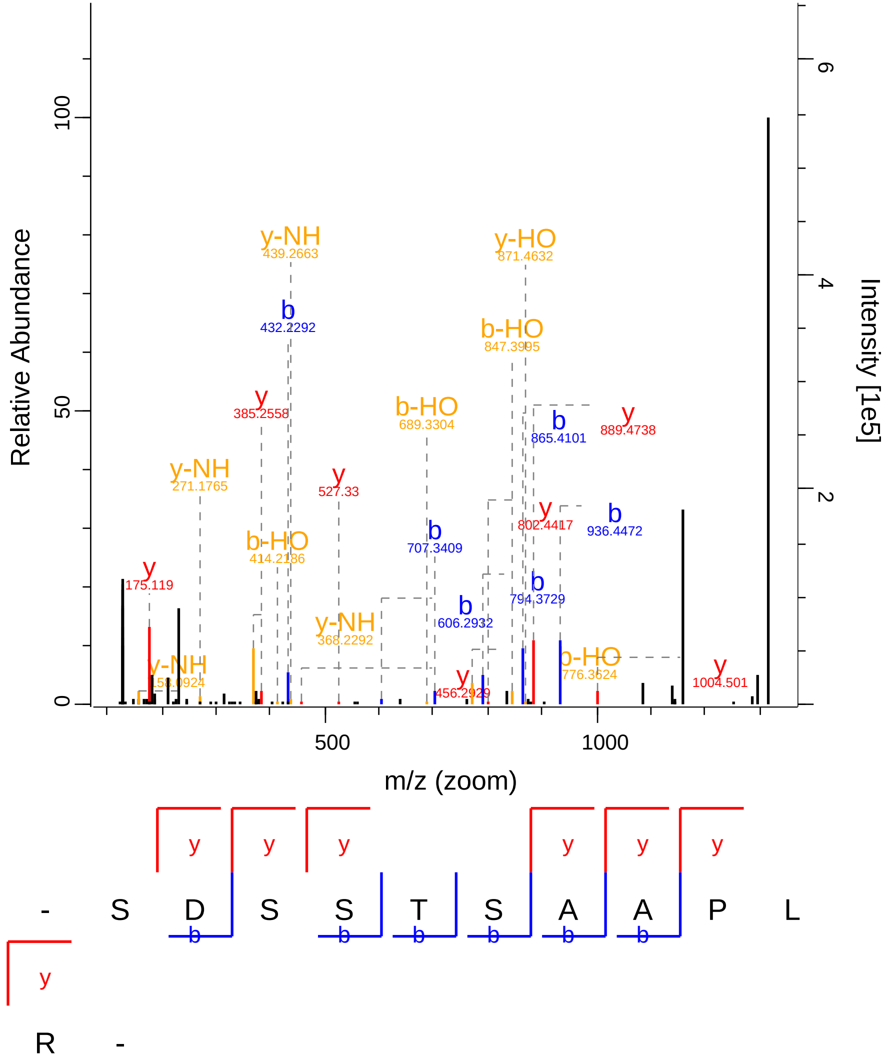

Raw File	Scan	Method	Score	m/z
RO-QC-160121_ZackD_SS_Set_02_Fraction_4	6502	FTMS; HCD	53.71	399.23

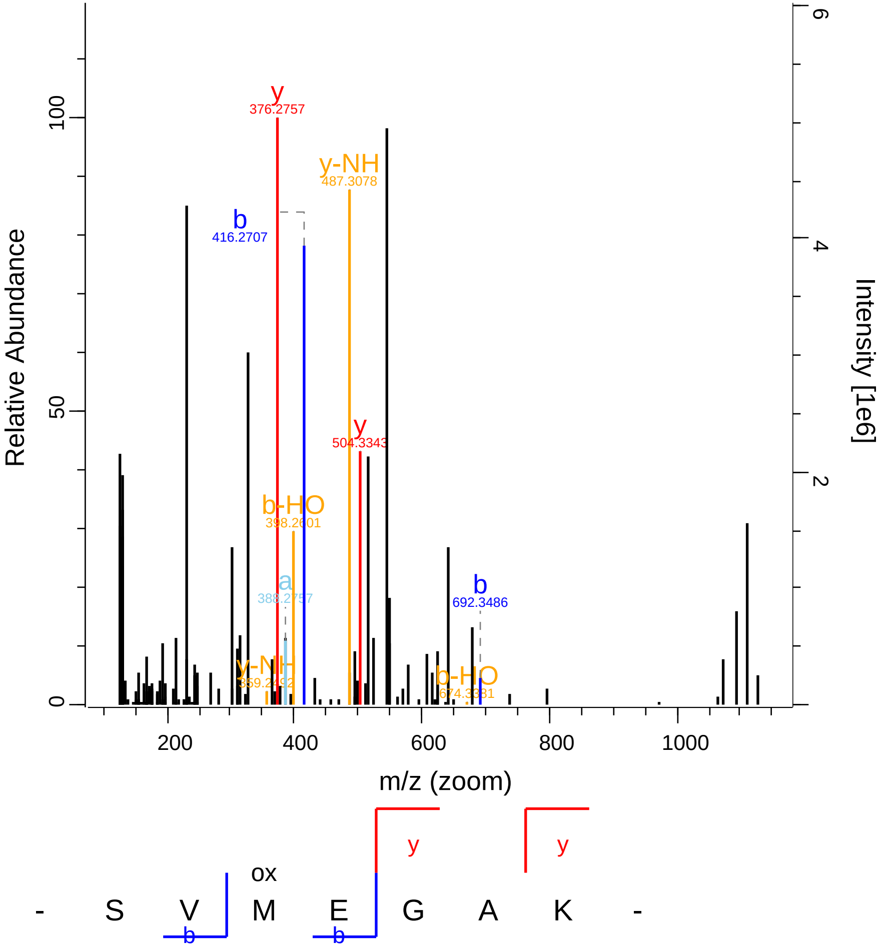

Raw File	Scan	Method	Score	m/z	Gene names
RO-QC-160121_ZackD_SS_Set_02_Fractior	6580	FTMS; HCI	109	429.24	ZNF668

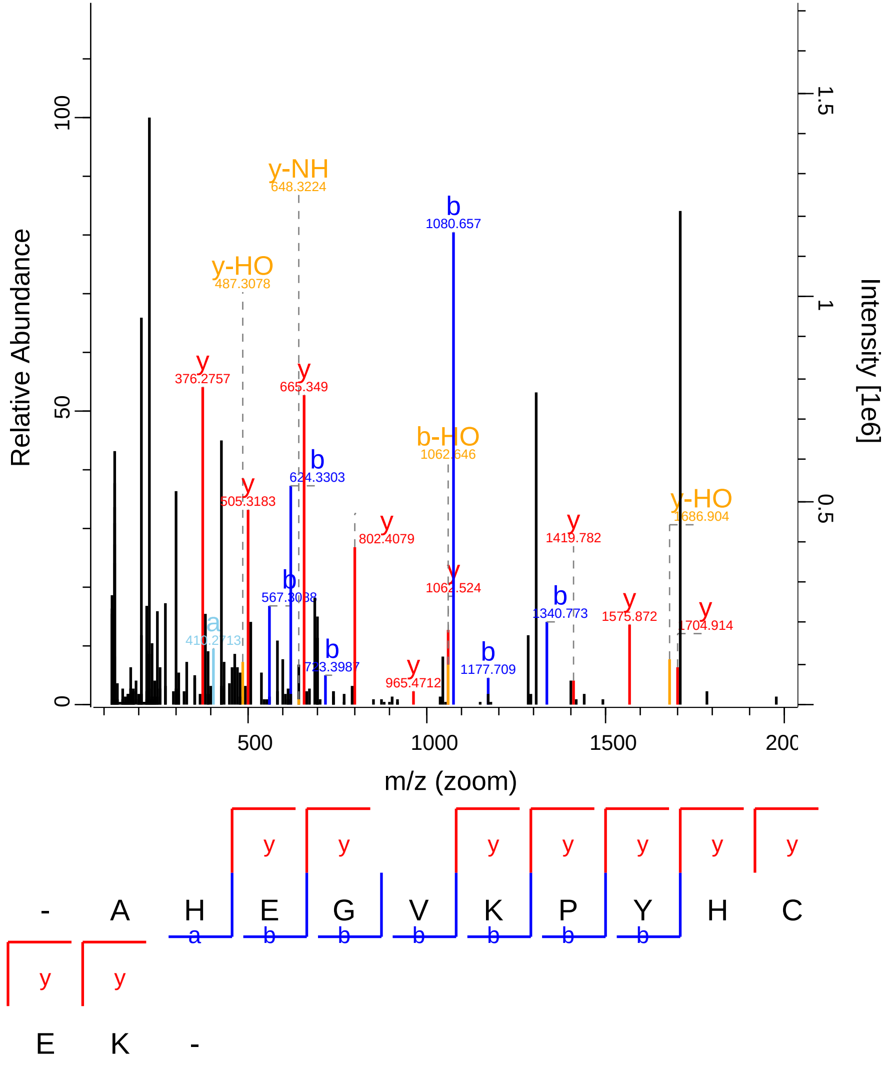

Raw File	Scan	Method	Score	m/z	Gene names
RO-QC-160121_ZackD_SS_Set_02_Fractioı	6820	FTMS; HC	213.98	699.34	TK2

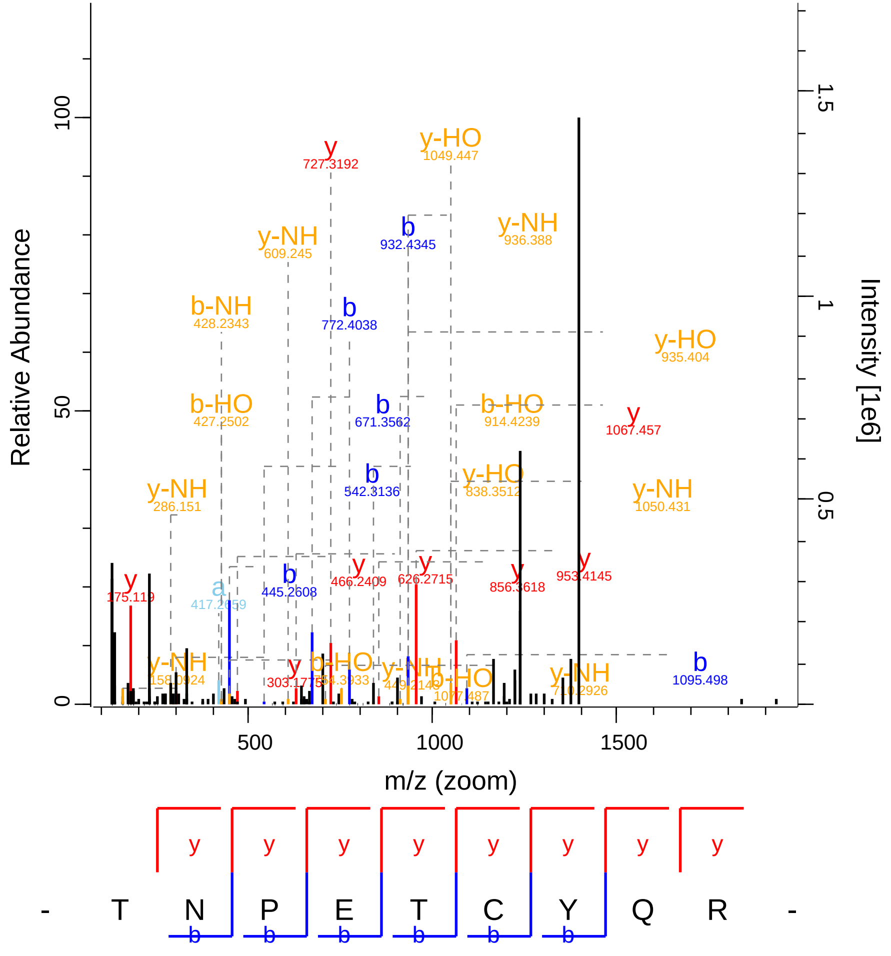

Raw File	Scan	Method	Score	m/z	Gene names
RO-QC-160121_ZackD_SS_Set_02_Fraction	7138	FTMS; HCI	170.47	682.4	PWWP2A

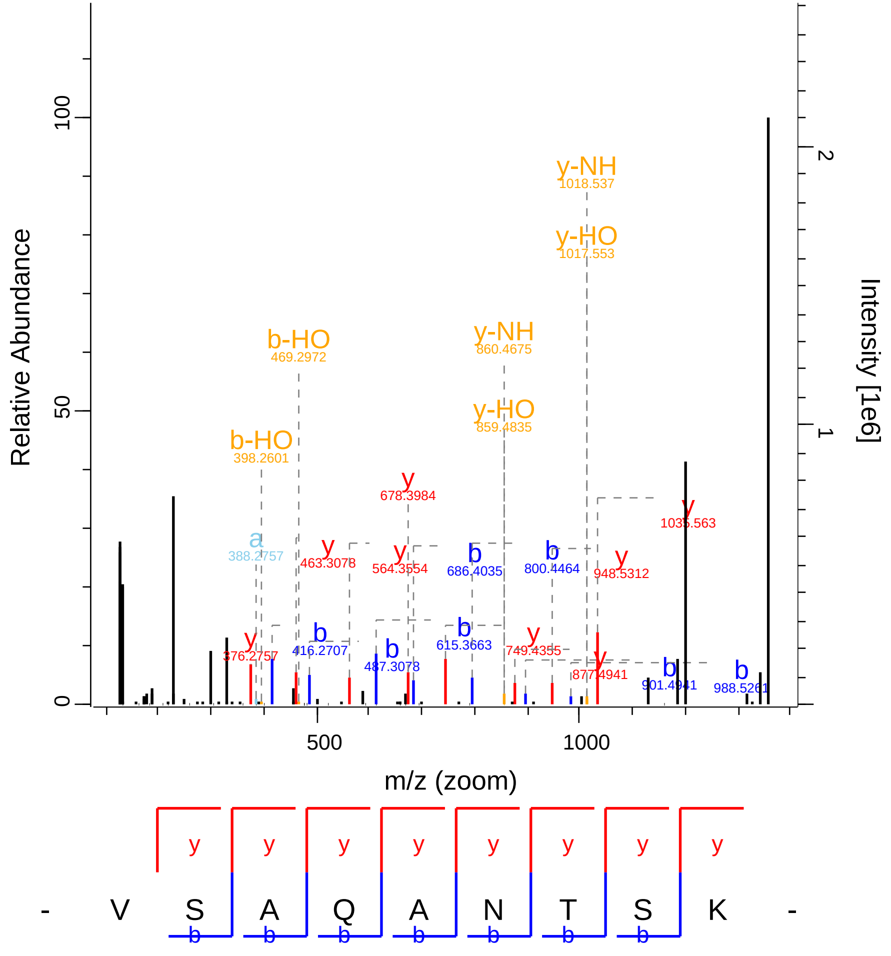

Raw File	Scan	Method	Score	m/z	Gene names
RO-QC-160121_ZackD_SS_Set_02_Fraction	7156	FTMS; HCI	105.98	786.9	SLC46A1

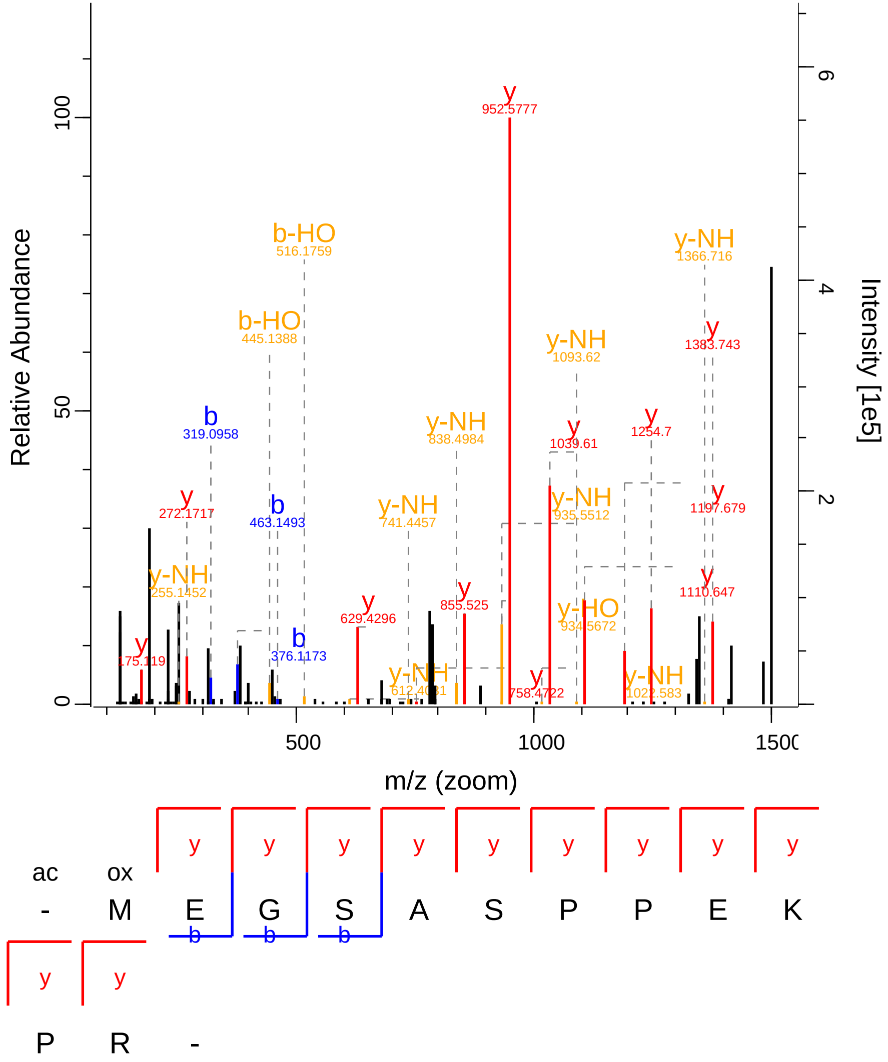

Raw File	Scan	Method	Score	m/z	Gene names
RO-QC-160121_ZackD_SS_Set_02_Fraction	7536	FTMS; HCI	85.81	547.3	DOLPP1

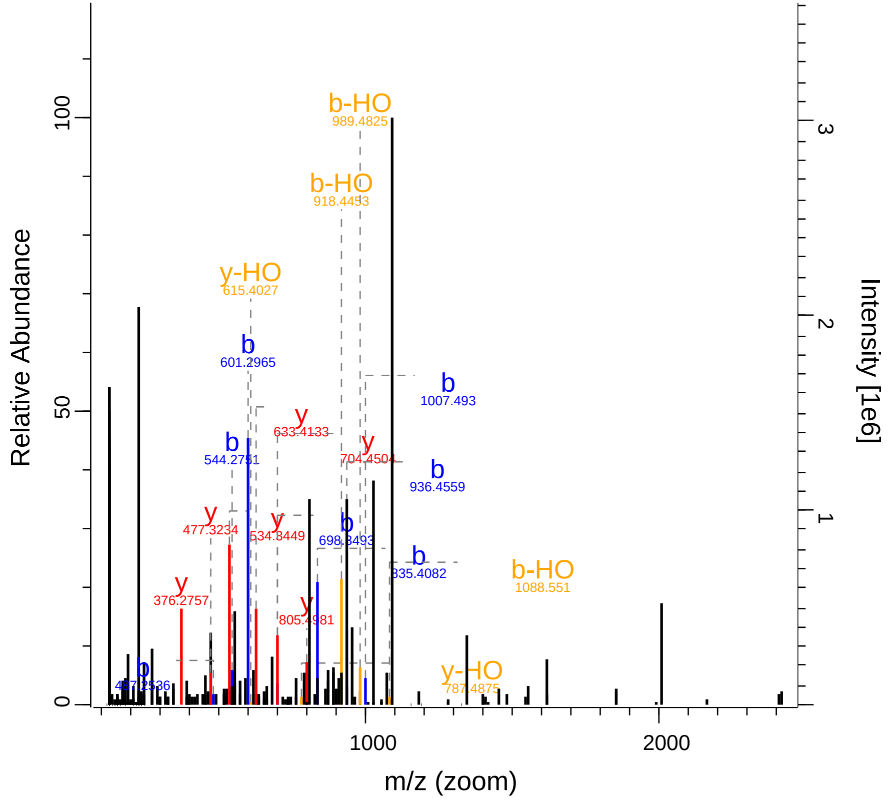

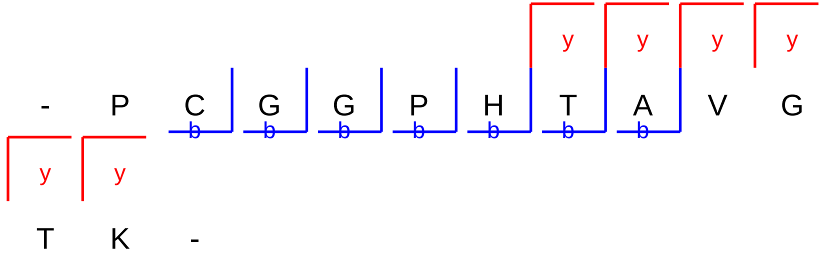

Raw File	Scan	Method	Score	m/z
RO-QC-160121_ZackD_SS_Set_02_Fraction_4	8605	FTMS; HCD	52.55	447

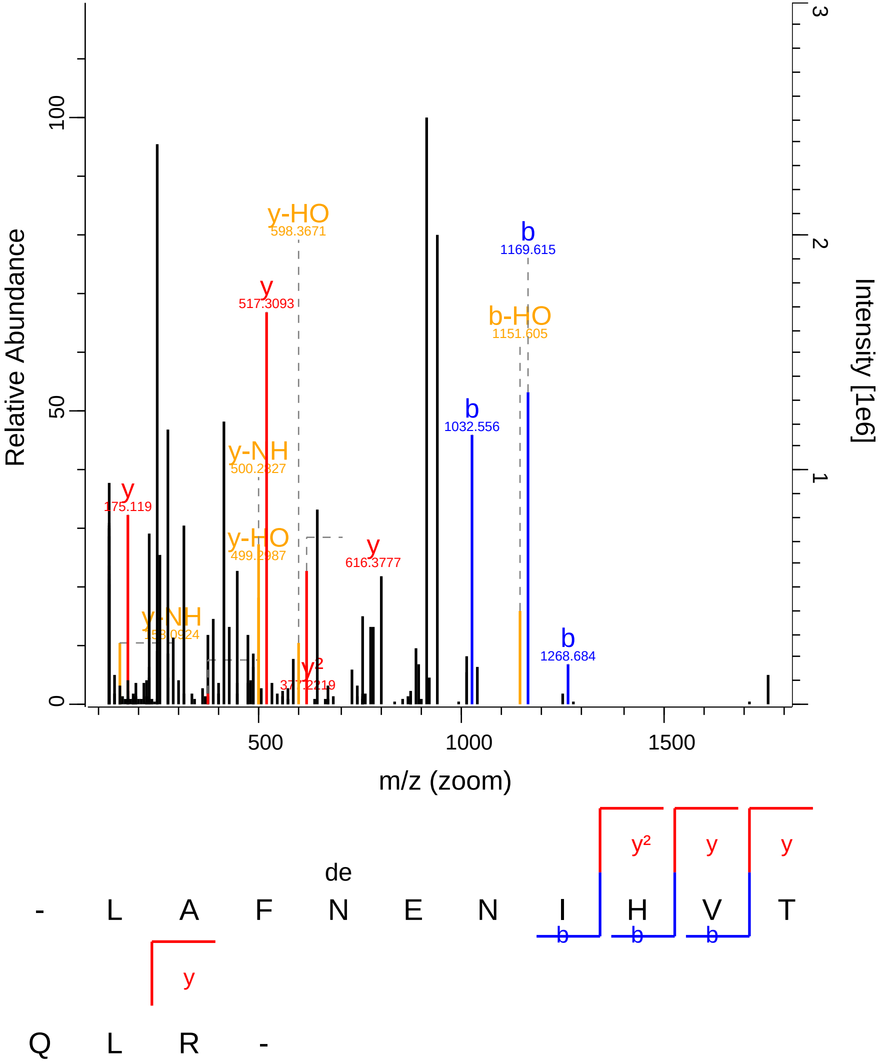

Raw File	Scan	Method	Score	m/z	Gene names
RO-QC-160121_ZackD_SS_Set_02_Fraction_	8653	FTMS; HCI	62.57	841.9	GLRX2

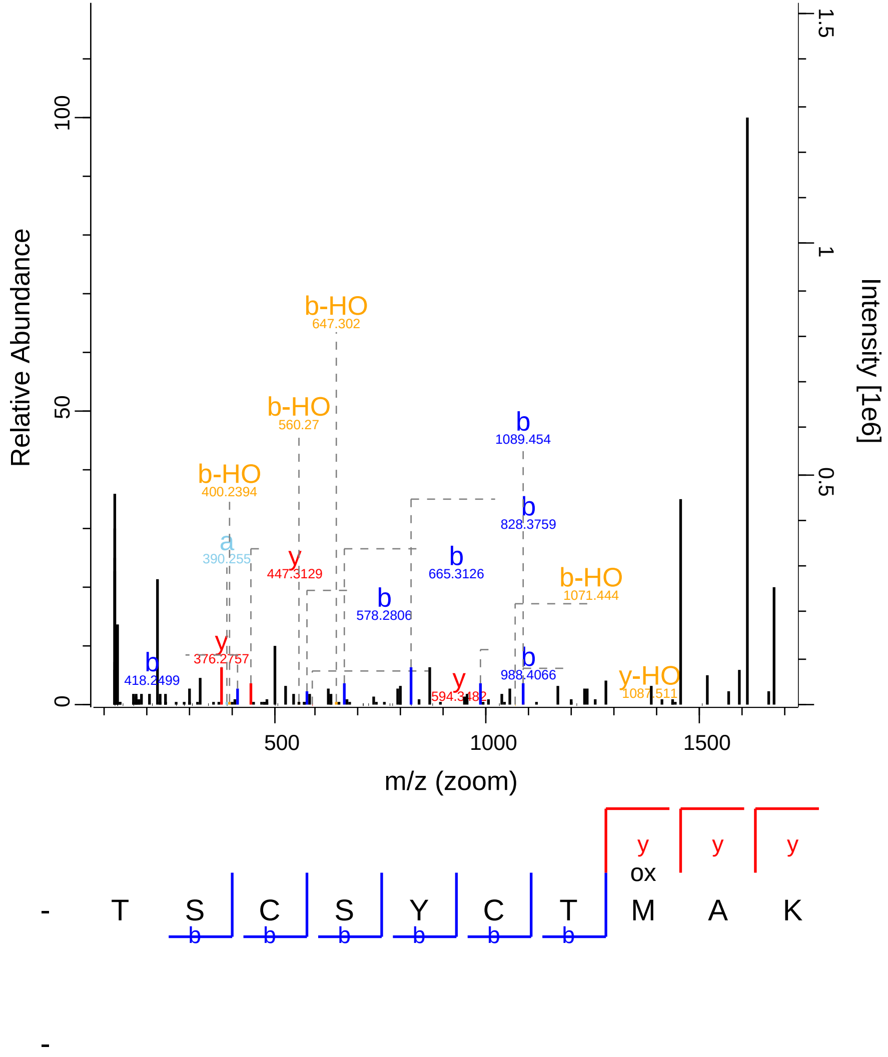

Raw File	Scan	Method	Score	m/z	Gene names
RO-QC-160121_ZackD_SS_Set_02_Fractior	8886	FTMS; HCI	62.72	699.39	SLC7A6

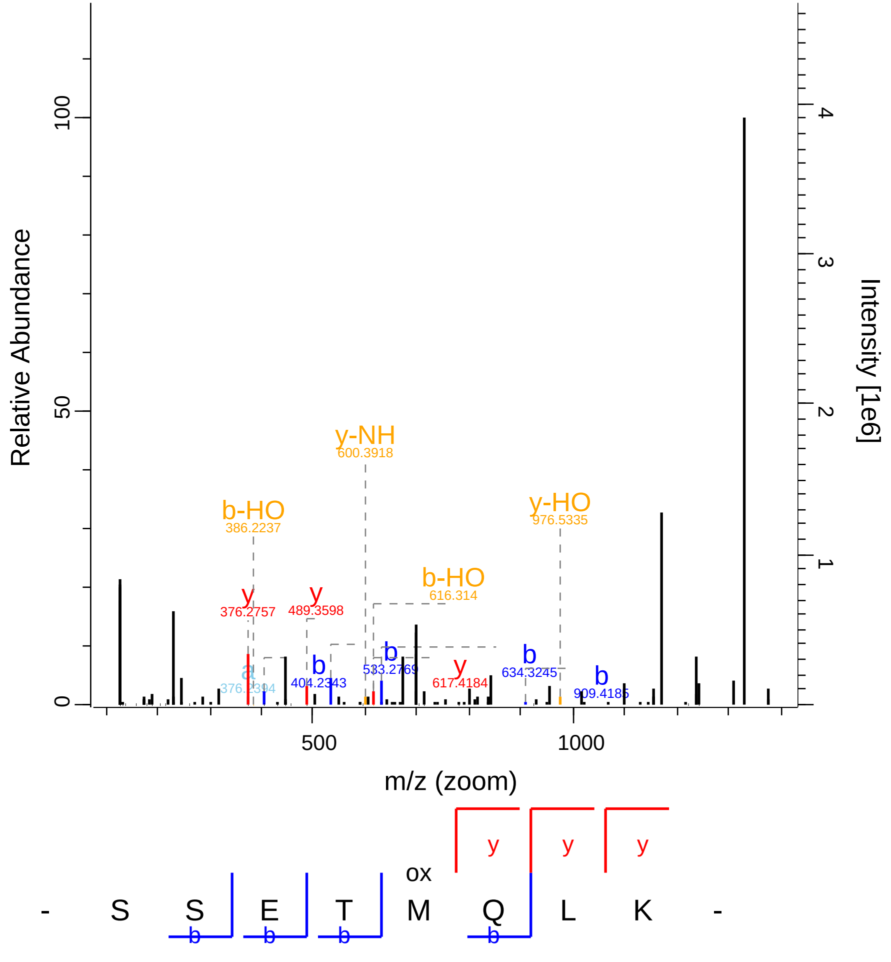

Raw File	Scan	Method	Score	m/z
RO-QC-160121_ZackD_SS_Set_02_Fraction_4	8956	FTMS; HCD	74.92	704.37

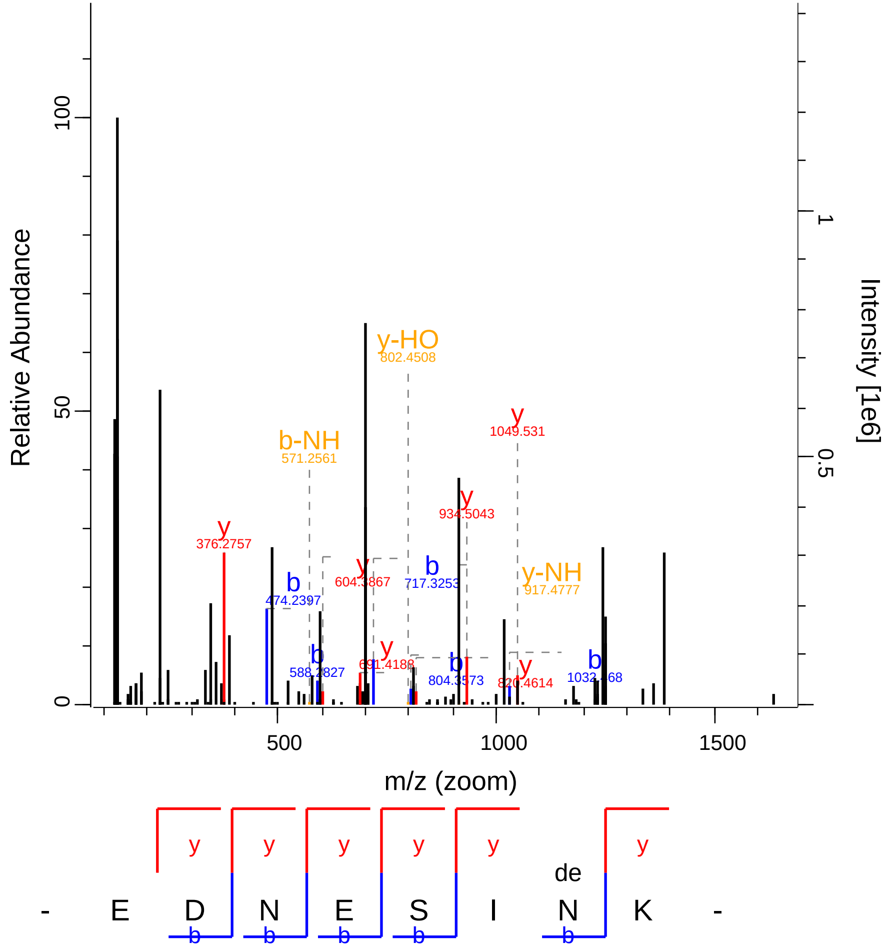

Raw File	Scan	Method	Score	m/z	Gene names
RO-QC-160121_ZackD_SS_Set_02_Fractior	9781	FTMS; HCI	46.96	604.29	CHSY1;CHSY3

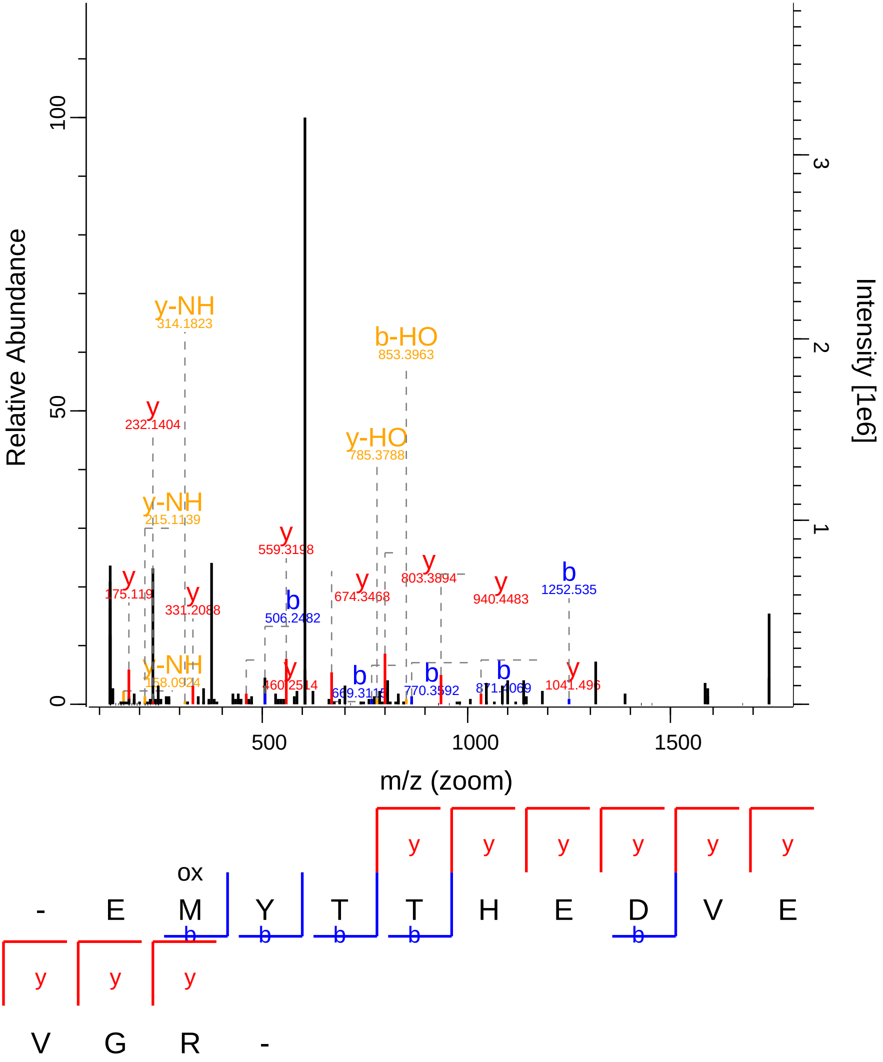

Raw File	Scan	Method	Score	m/z
RO-QC-160121_ZackD_SS_Set_02_Fraction_4	10085	FTMS; HCD	91.91	397.58

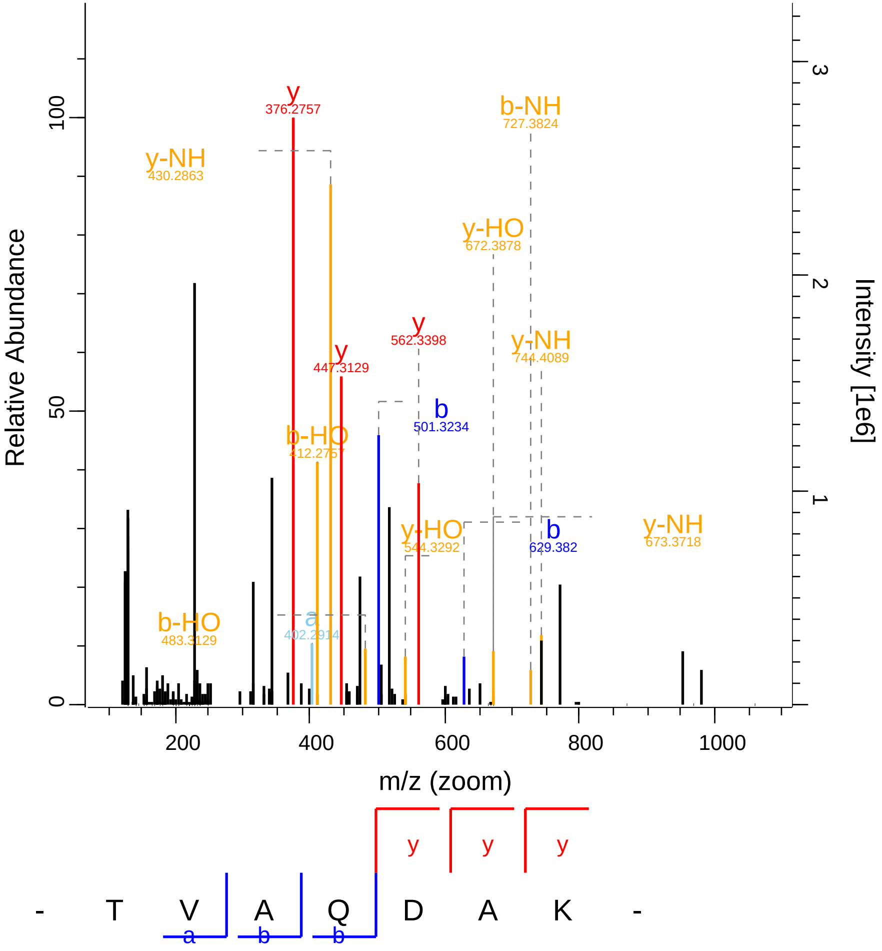

Raw File	Scan	Method	Score	m/z
RO-QC-160121_ZackD_SS_Set_02_Fraction_4	10294	FTMS; HCD	94.01	397.58

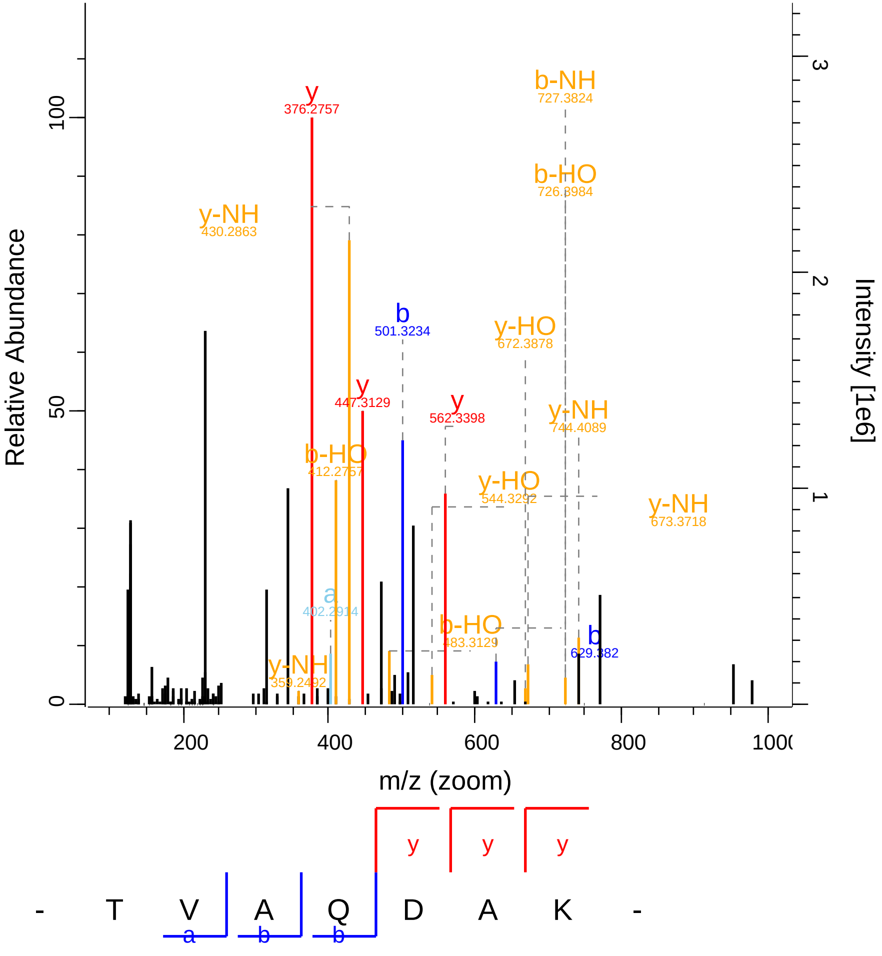

Raw File	Scan	Method	Score	m/z	Gene names
RO-QC-160121_ZackD_SS_Set_02_Fractio	10411	FTMS; HC	90.63	373.48	DENND4B

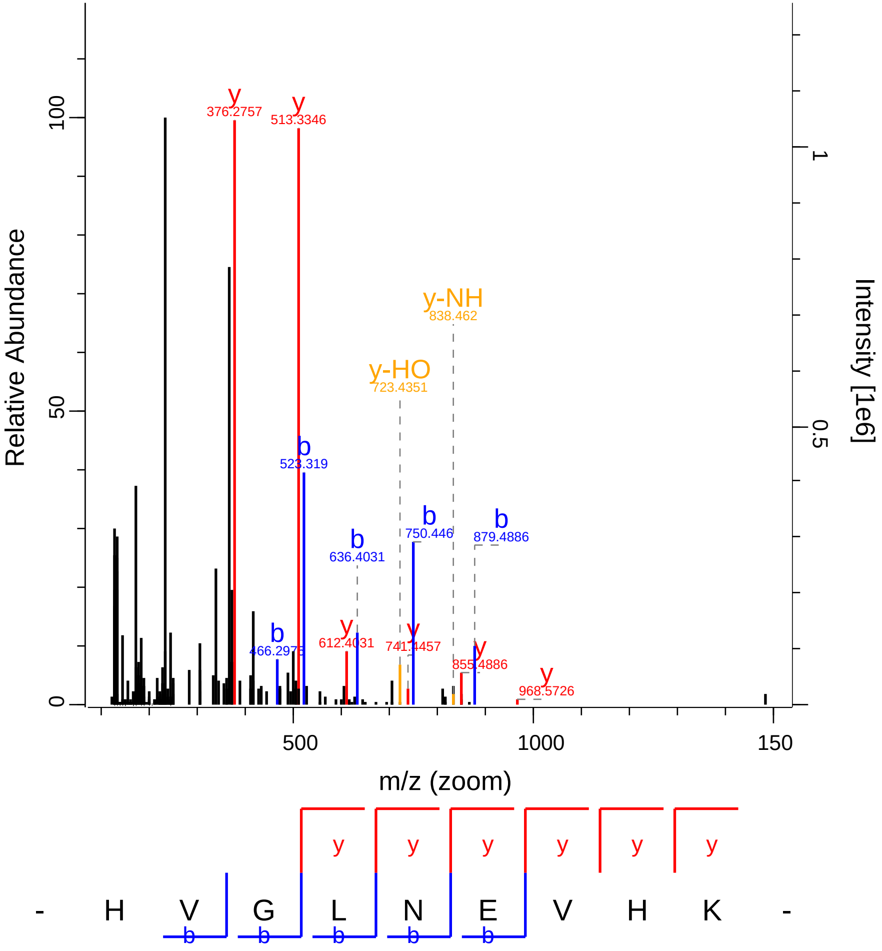

Raw File	Scan	Method	Score	m/z	Gene names
RO-QC-160121_ZackD_SS_Set_02_Fractio	10590	FTMS; HC	117.02	695.41	HES1

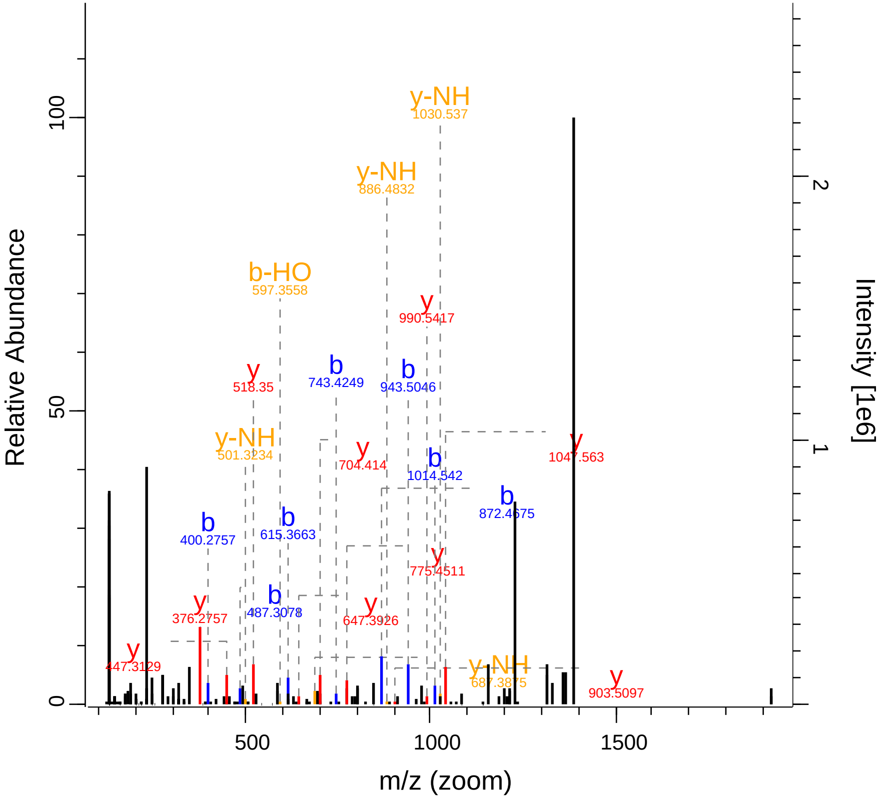

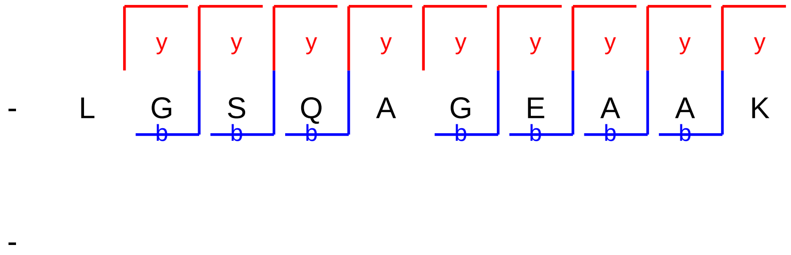

Raw File	Scan	Method	Score	m/z
RO-QC-160121_ZackD_SS_Set_02_Fraction_4	10676	FTMS; HCD	57.28	387.22

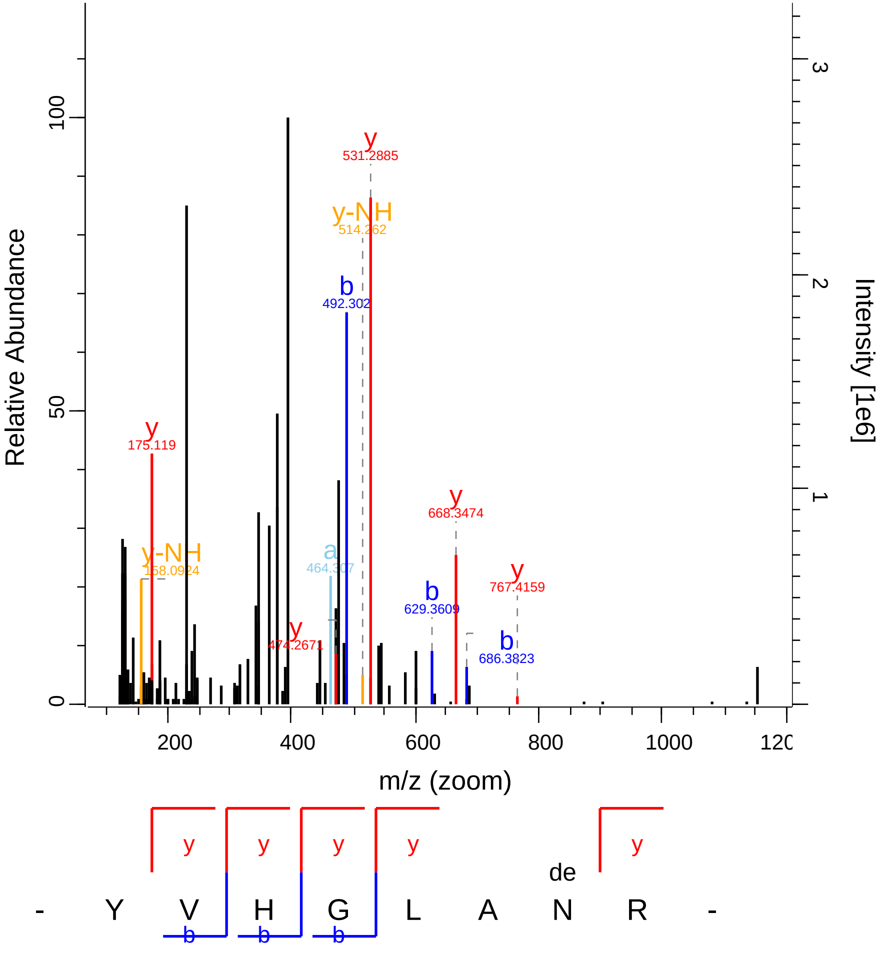

Raw File	Scan	Method	Score	m/z	Gene names
RO-QC-160121_ZackD_SS_Set_02_Fractio	11151	FTMS; HC	44.35	943.51	CDCA3

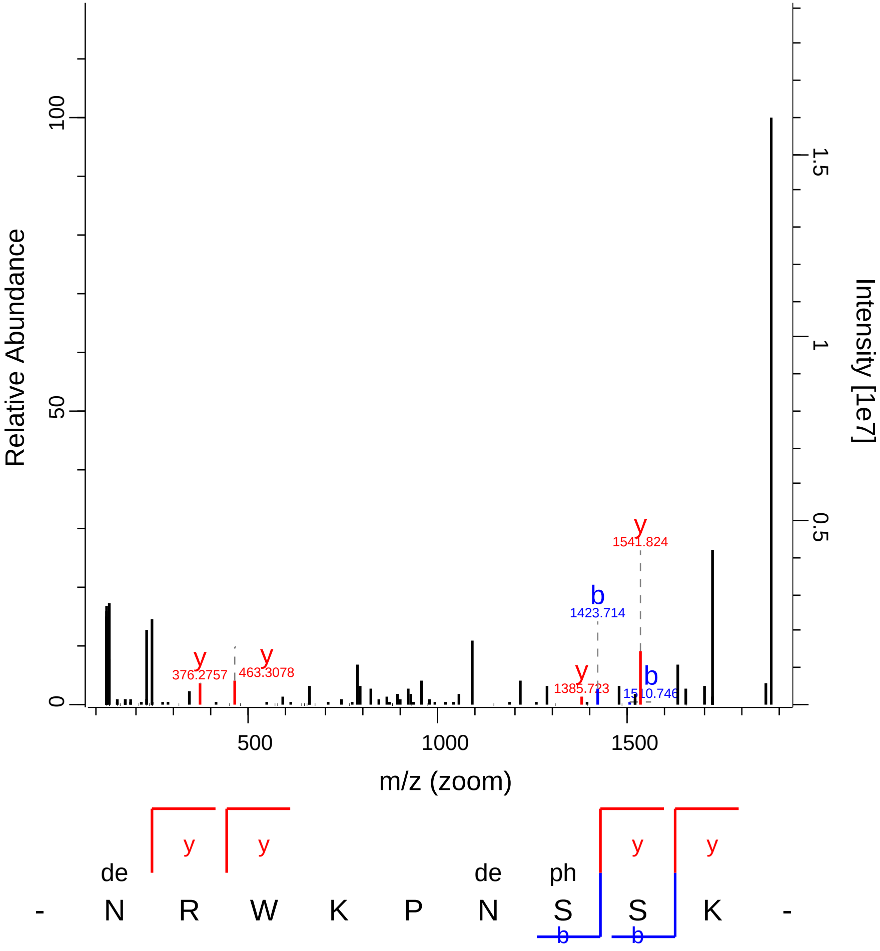

Raw File	Scan	Method	Score	m/z	Gene names
RO-QC-160121_ZackD_SS_Set_02_Fractio	11170	FTMS; HC	101.65	644.39	C6orf47

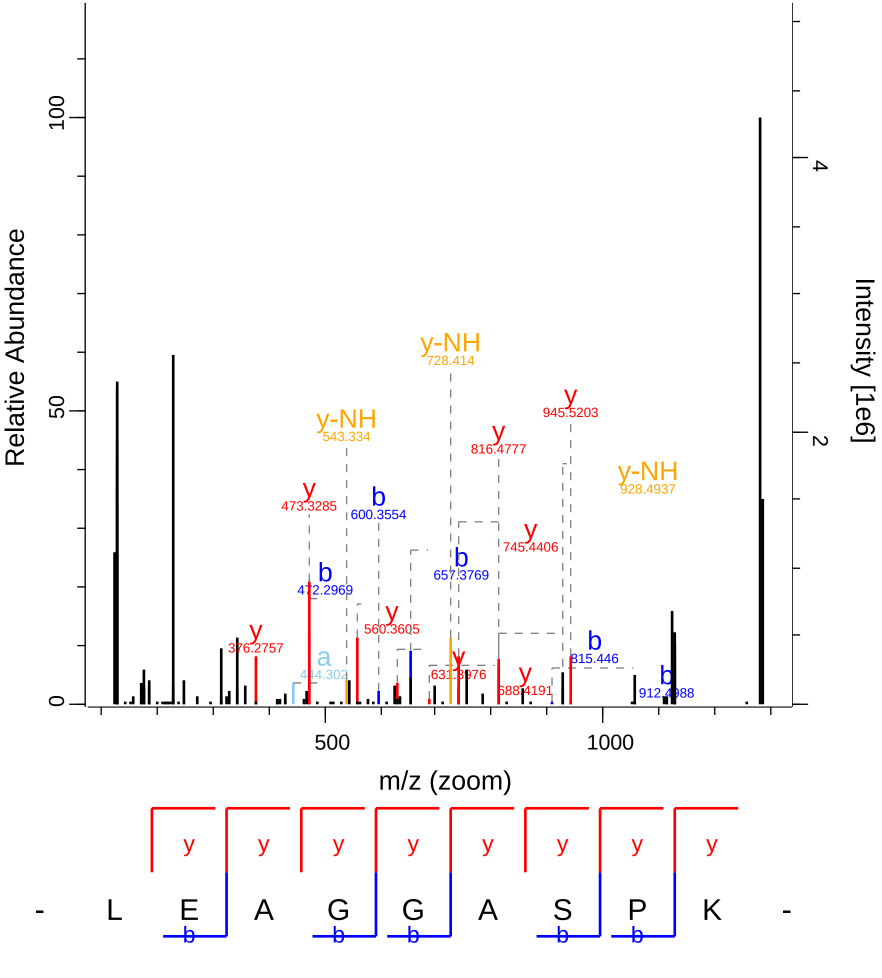

Raw File	Scan	Method	Score	m/z	Gene names
RO-QC-160121_ZackD_SS_Set_02_Fractio	11895	FTMS; HC	56.48	671.01	ZER1

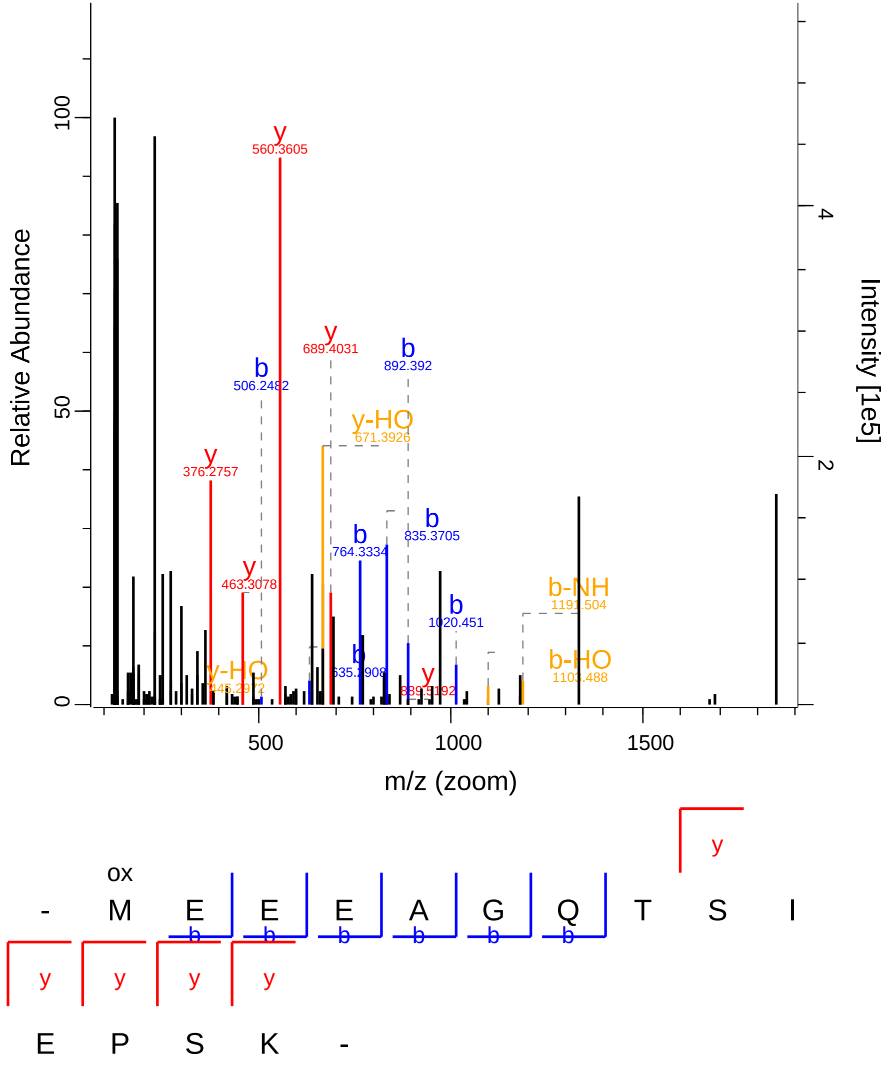

Raw File	Scan	Method	Score	m/z
RO-QC-160121_ZackD_SS_Set_02_Fraction_4	12054	FTMS; HCD	57.46	519.77

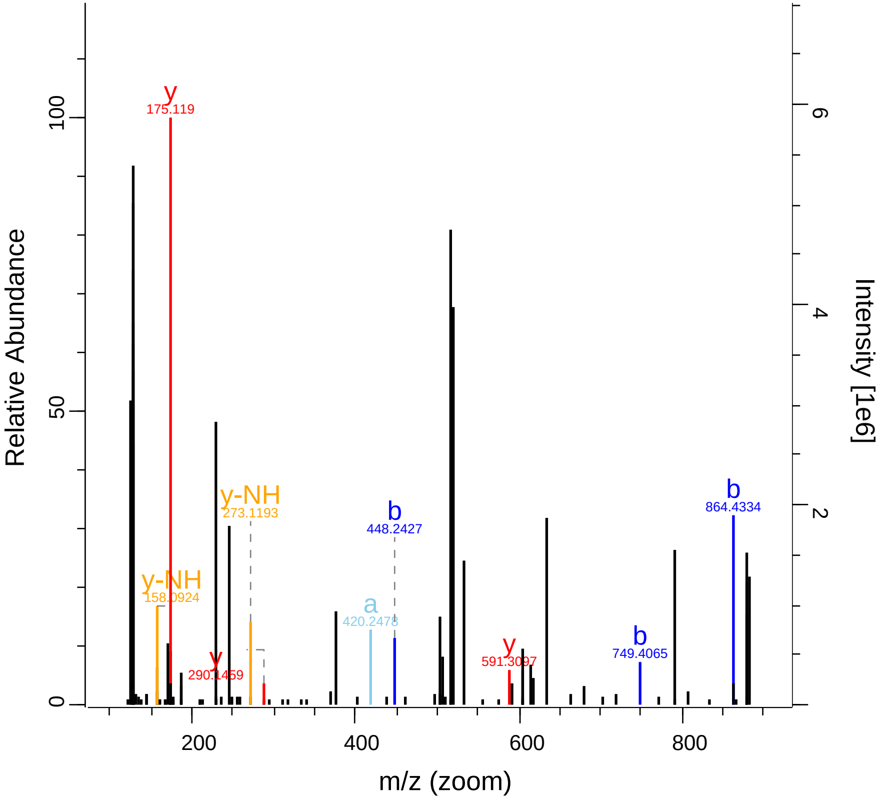

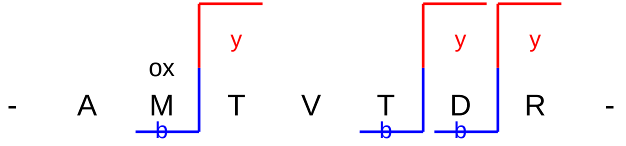

Raw File	Scan	Method	Score	m/z
RO-QC-160121_ZackD_SS_Set_02_Fraction_4	12061	FTMS; HCD	99.38	364.86

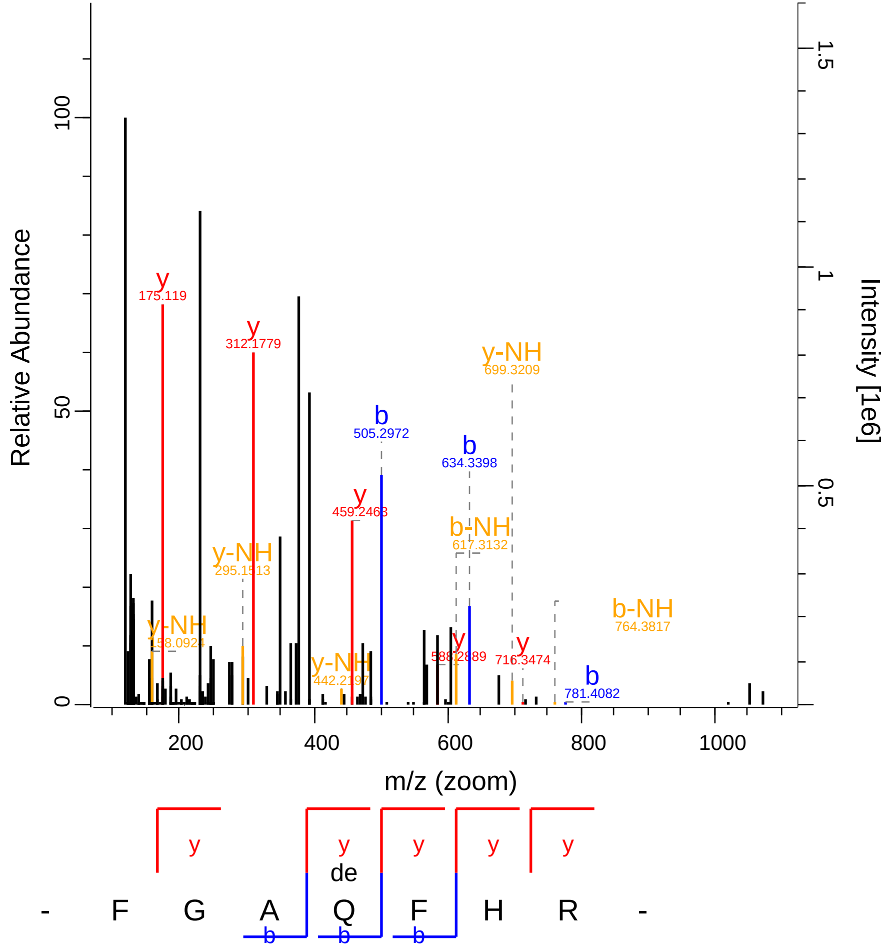

Raw File	Scan	Method	Score	m/z	Gene names
RO-QC-160121_ZackD_SS_Set_02_Fractio	14229	FTMS; HC	84.5	824.47	AIPL1

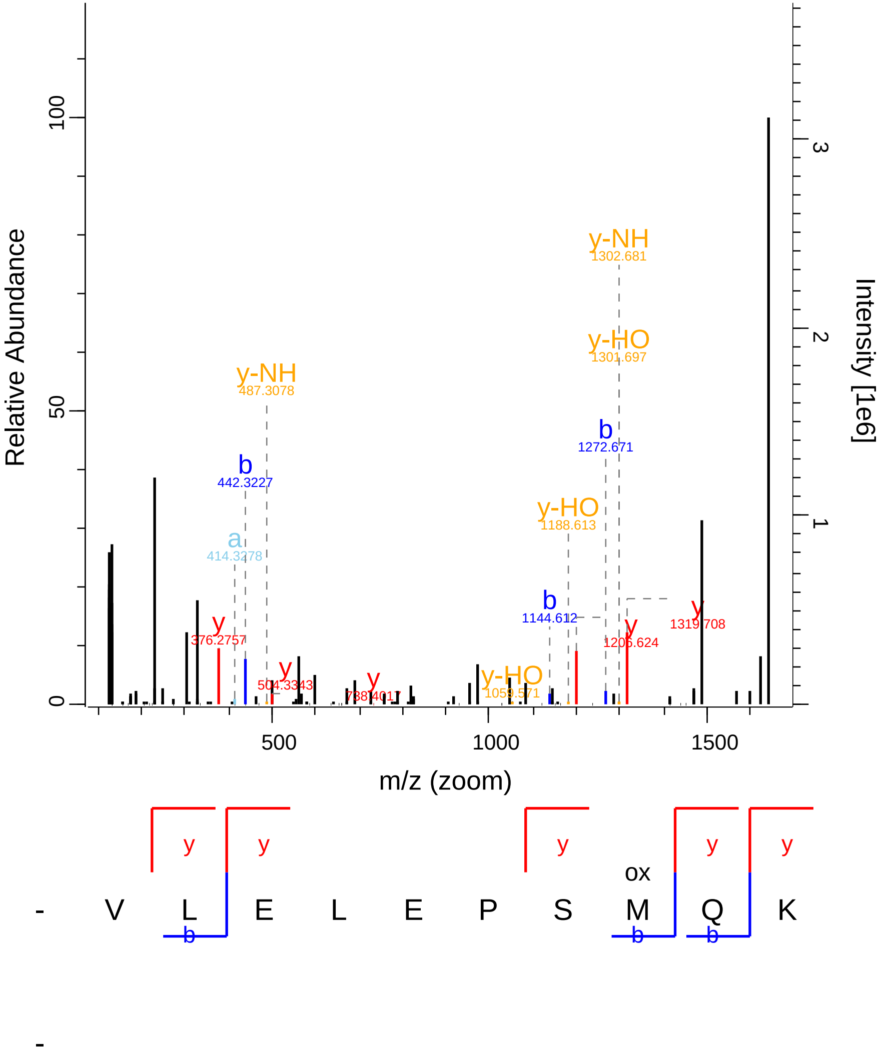

Raw File	Scan	Method	Score	m/z
RO-QC-160121_ZackD_SS_Set_02_Fraction_4	14359	FTMS; HCD	68.91	742.88

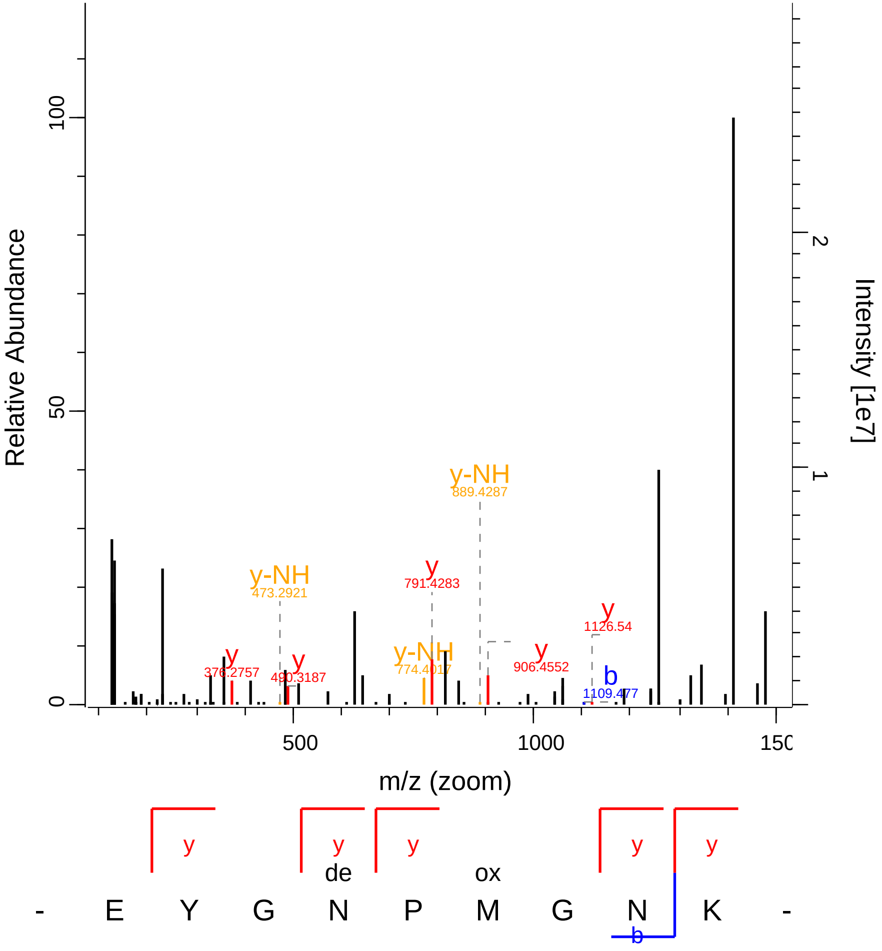

Raw File	Scan	Method	Score	m/z	Gene names
RO-QC-160121_ZackD_SS_Set_02_Fractio	14453	FTMS; HC	85.21	824.47	AIPL1

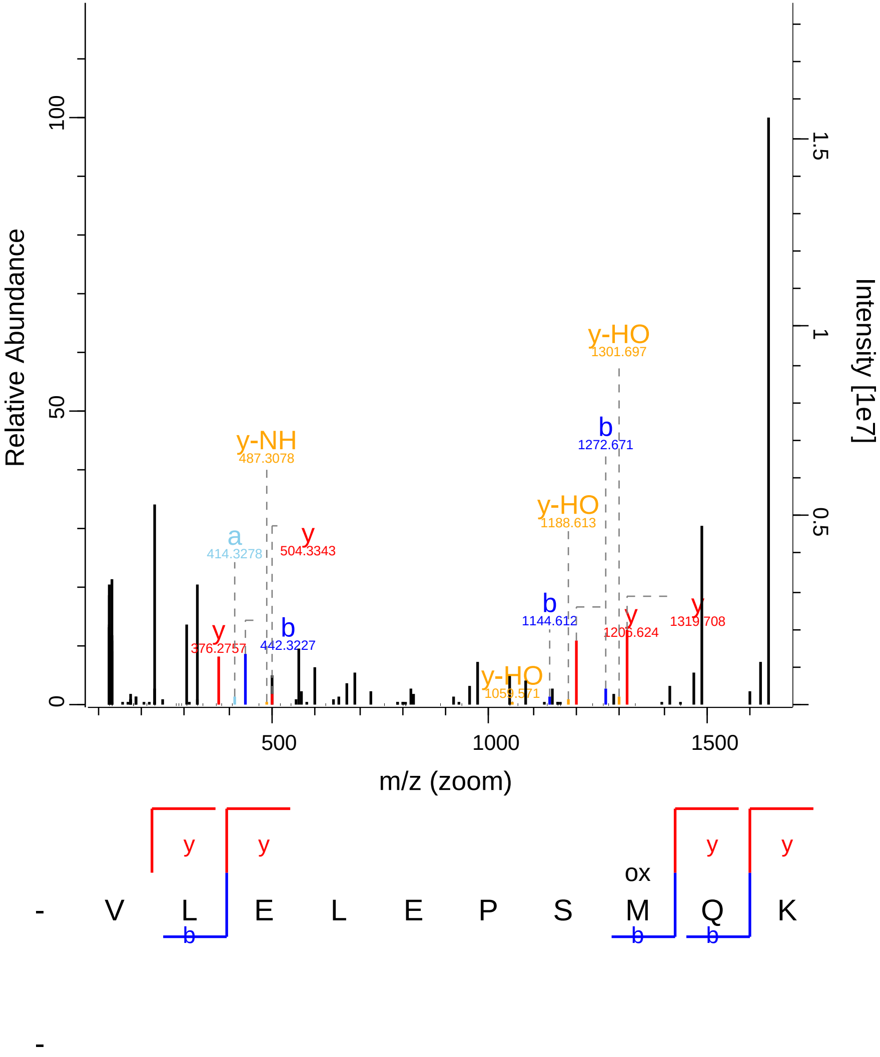

Raw File	Scan	Method	Score	m/z	Gene names
RO-QC-160121_ZackD_SS_Set_02_Fractio	15033	FTMS; HC	70.91	714.85	EID2

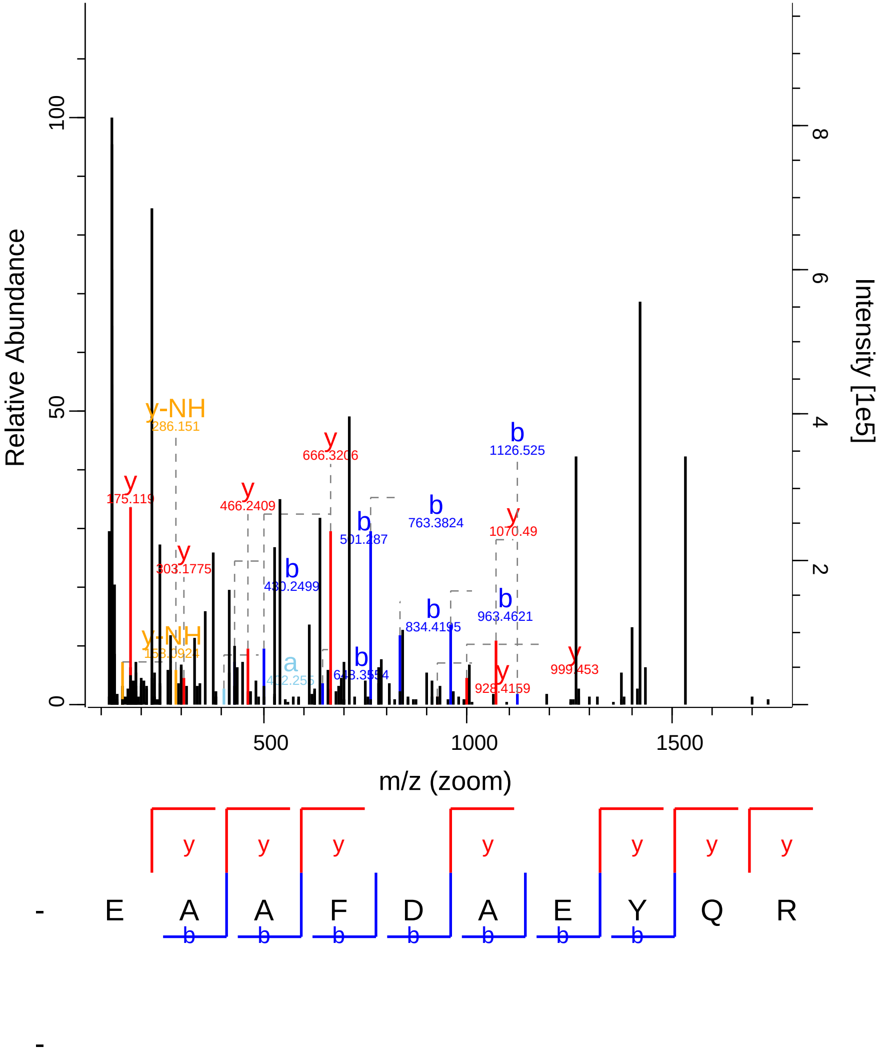

Raw File	Scan	Method	Score	m/z
RO-QC-160121_ZackD_SS_Set_02_Fraction_4	16231	FTMS; HCD	71.38	767.43

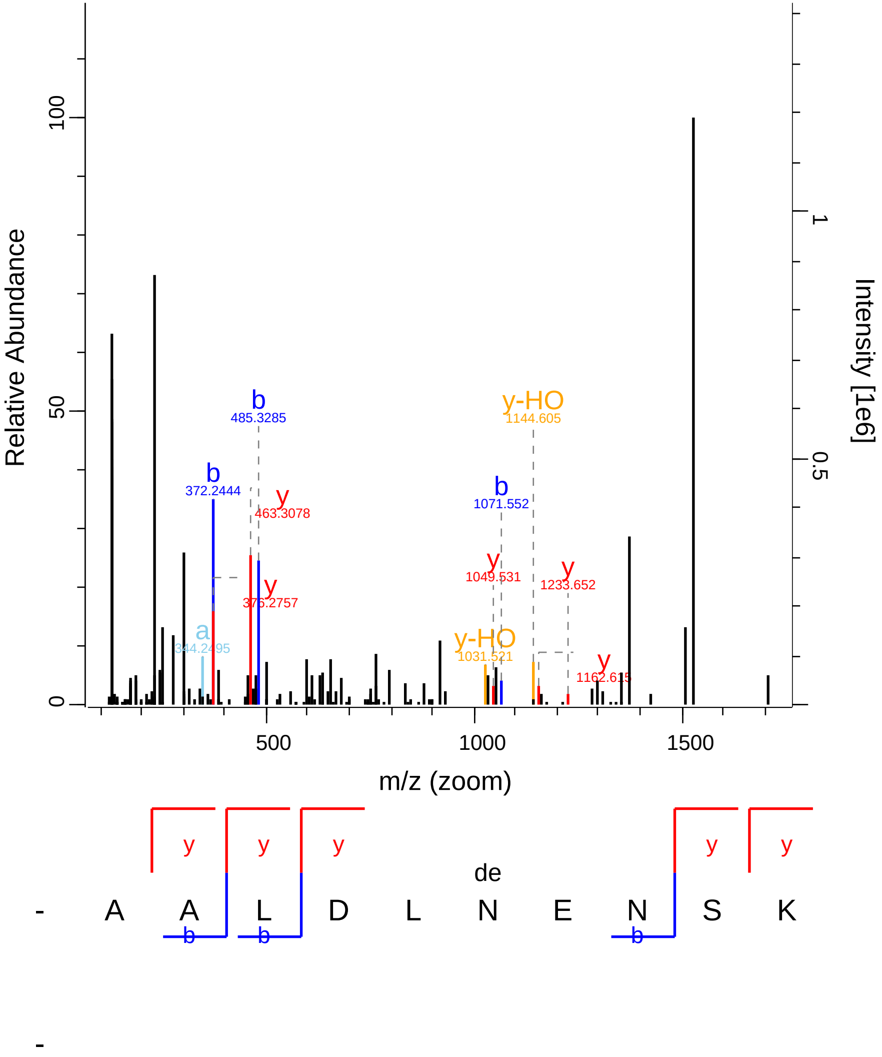

Raw File	Scan	Method	Score	m/z
RO-QC-160121_ZackD_SS_Set_02_Fraction_4	16294	FTMS; HCD	51.99	488.79

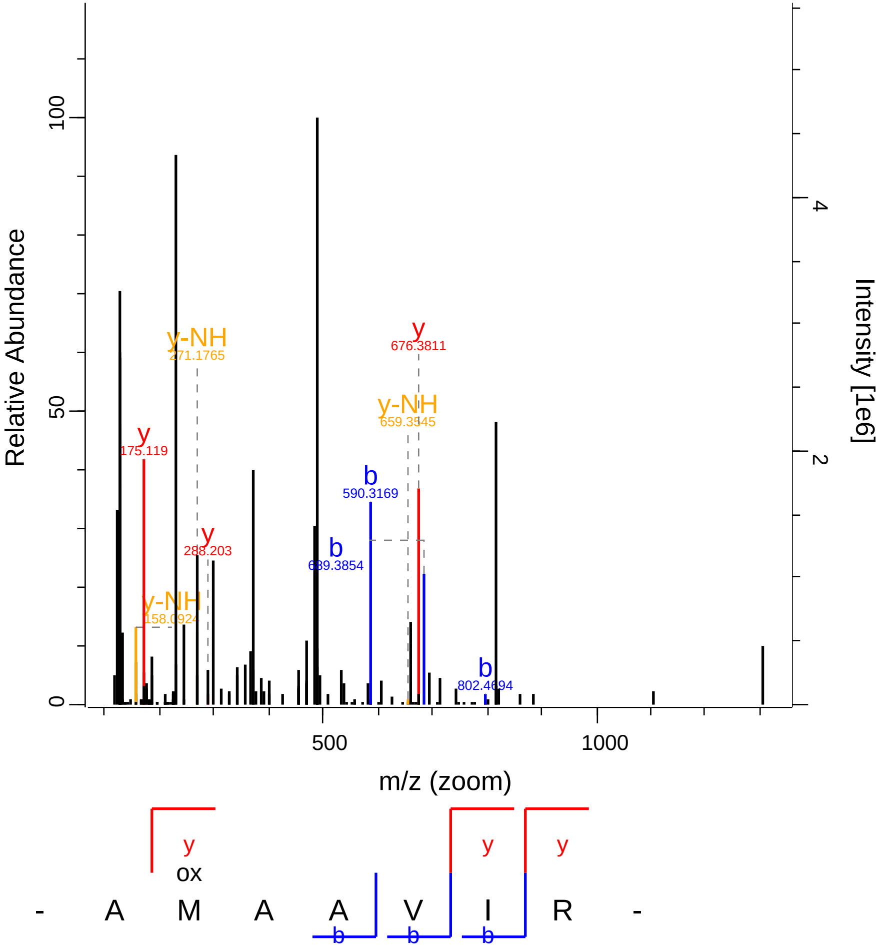

Raw File	Scan	Method	Score	m/z
RO-QC-160121_ZackD_SS_Set_02_Fraction_4	17079	FTMS; HCD	161.9	746.93

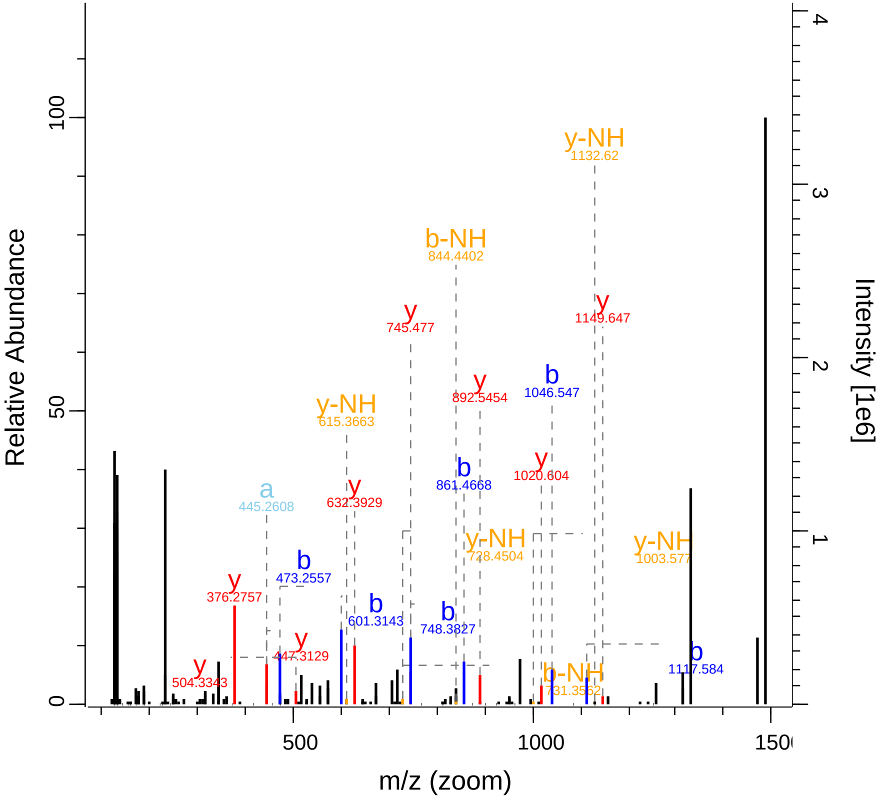

- N Q Q F I Q G A K -

de

Raw File	Scan	Method	Score	m/z	Gene names
RO-QC-160121_ZackD_SS_Set_02_Fraction	17109	FTMS; HCI	254.53	934	TBPL1

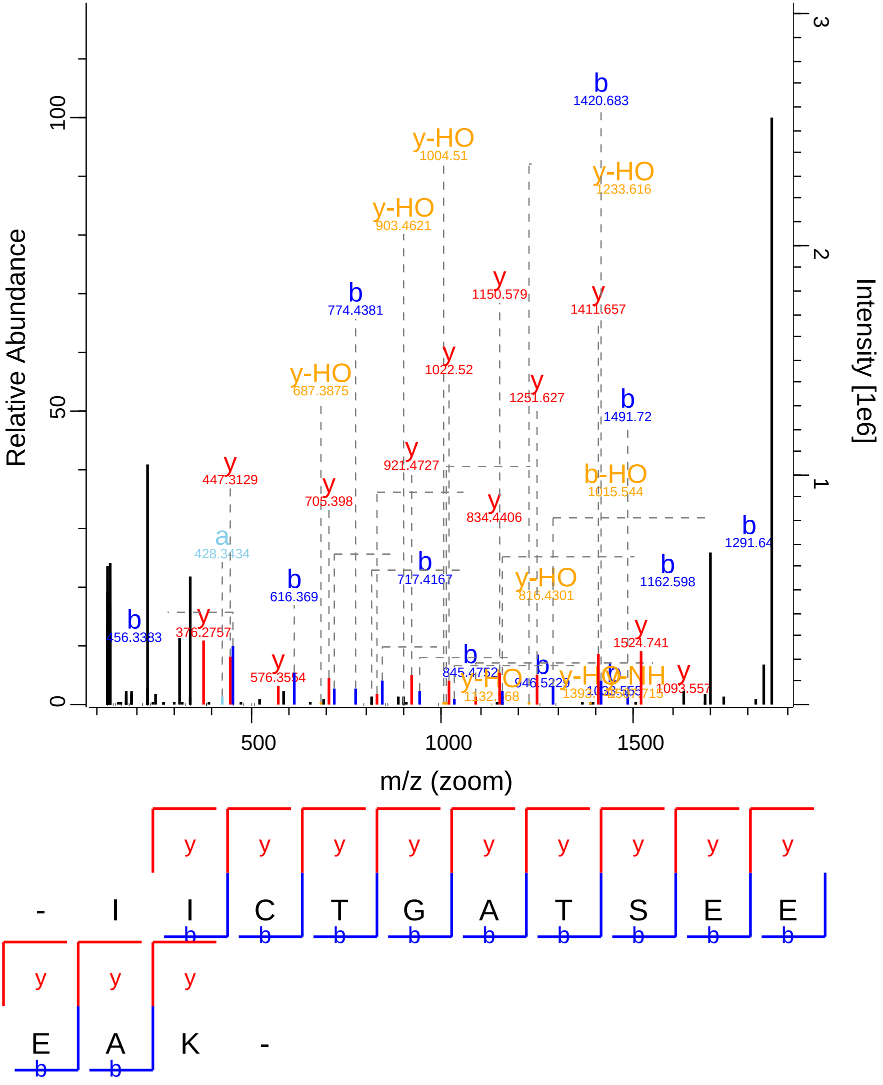

Raw File	Scan	Method	Score	m/z	Gene names
RO-QC-160121_ZackD_SS_Set_02_Fractio	17161	FTMS; HC	50.22	623.34	TBPL1

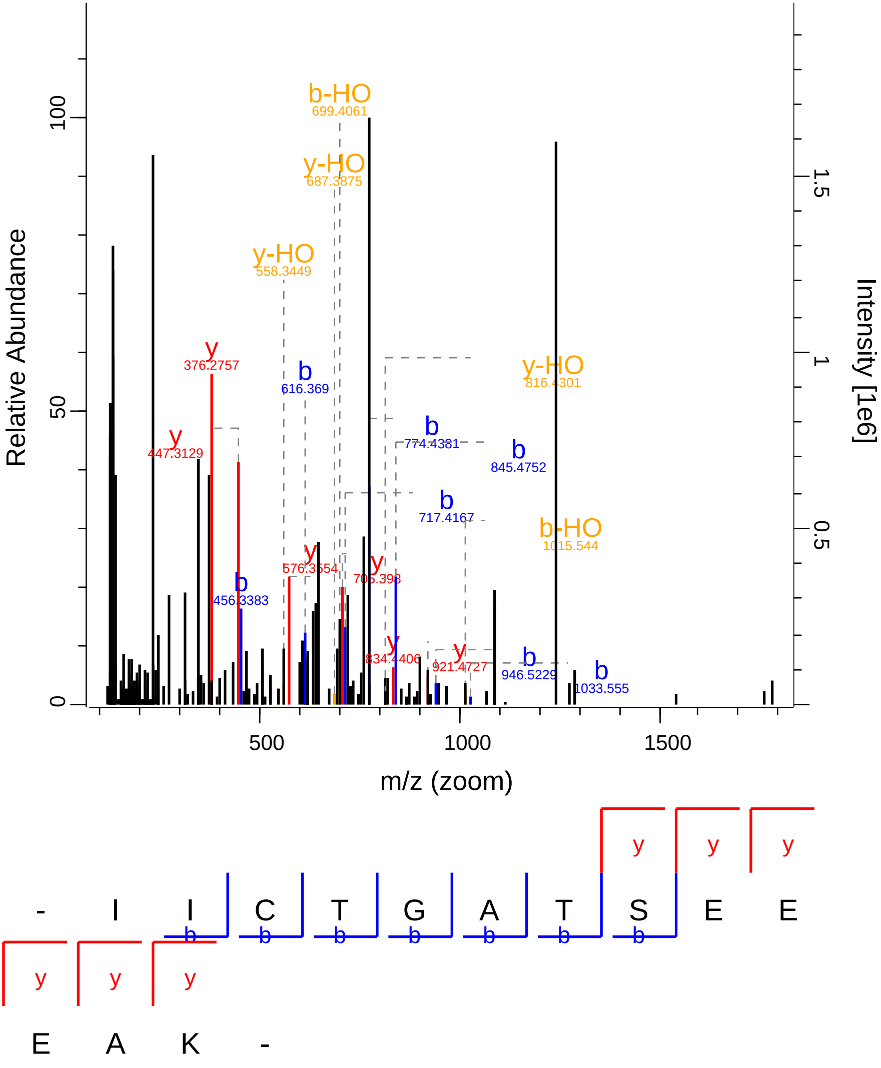

Raw File	Scan	Method	Score	m/z	Gene names
RO-QC-160121_ZackD_SS_Set_02_Fractio	17294	FTMS; HC	111.61	713.94	SYS1

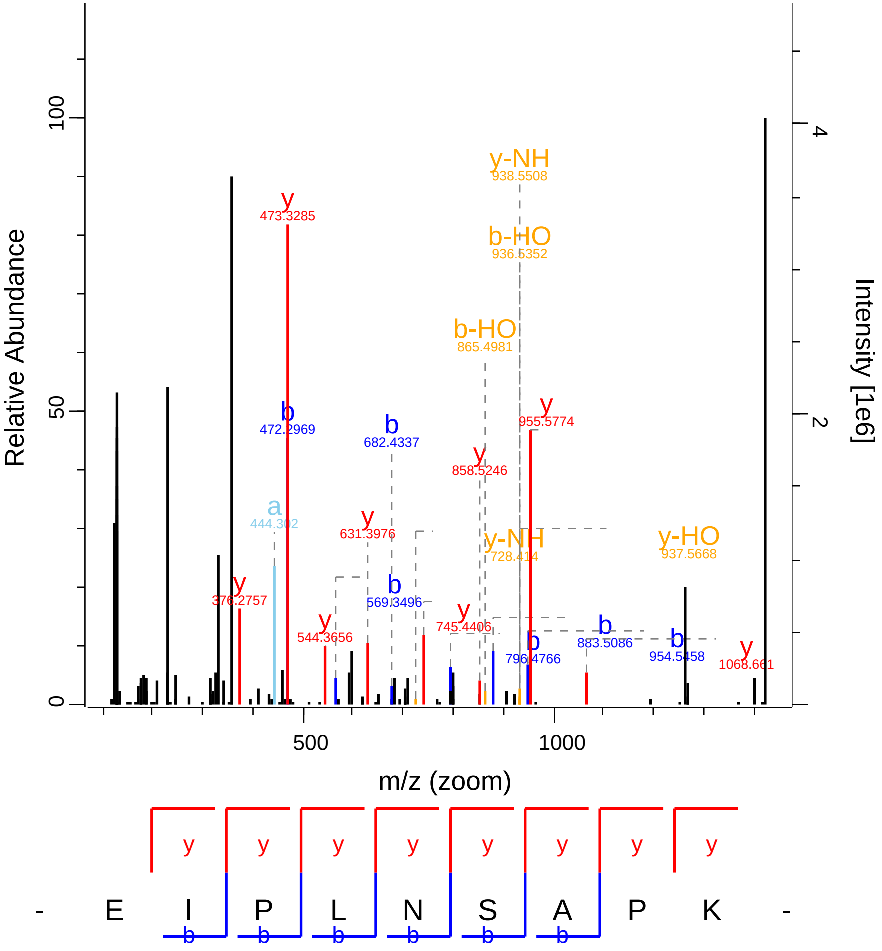

Raw File	Scan	Method	Score	m/z	Gene names
RO-QC-160121_ZackD_SS_Set_02_Fractio	17343	FTMS; HC	80.69	476.29	SYS1

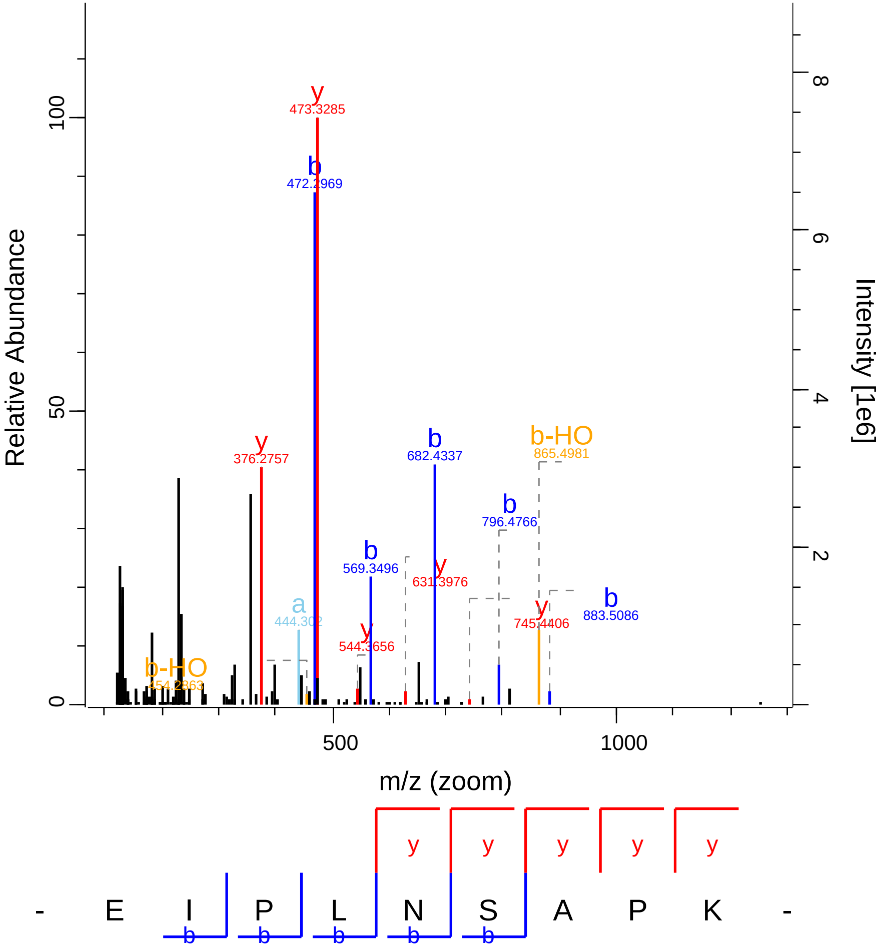

Raw File	Scan	Method	Score	m/z	Gene names
RO-QC-160121_ZackD_SS_Set_02_Fractio	17492	FTMS; HC	67.87	804.72	TTF1

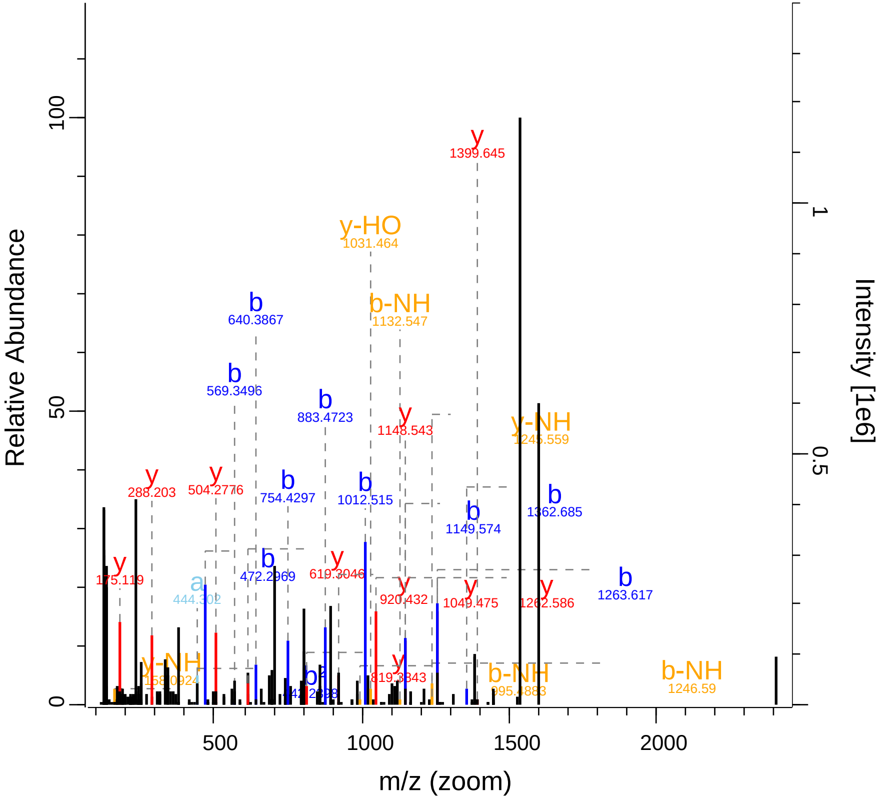

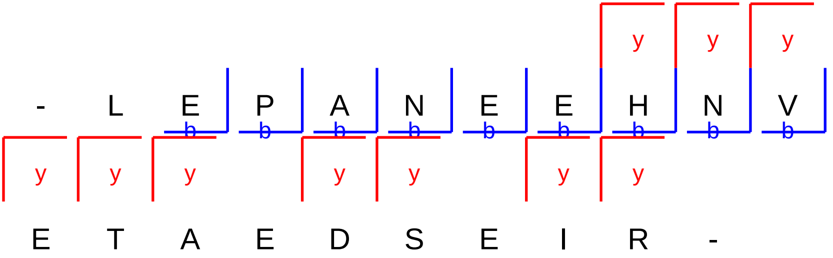

Raw File	Scan	Method	Score	m/z	Gene names
RO-QC-160121_ZackD_SS_Set_02_Fractio	17531	FTMS; HC	98.04	713.94	SYS1

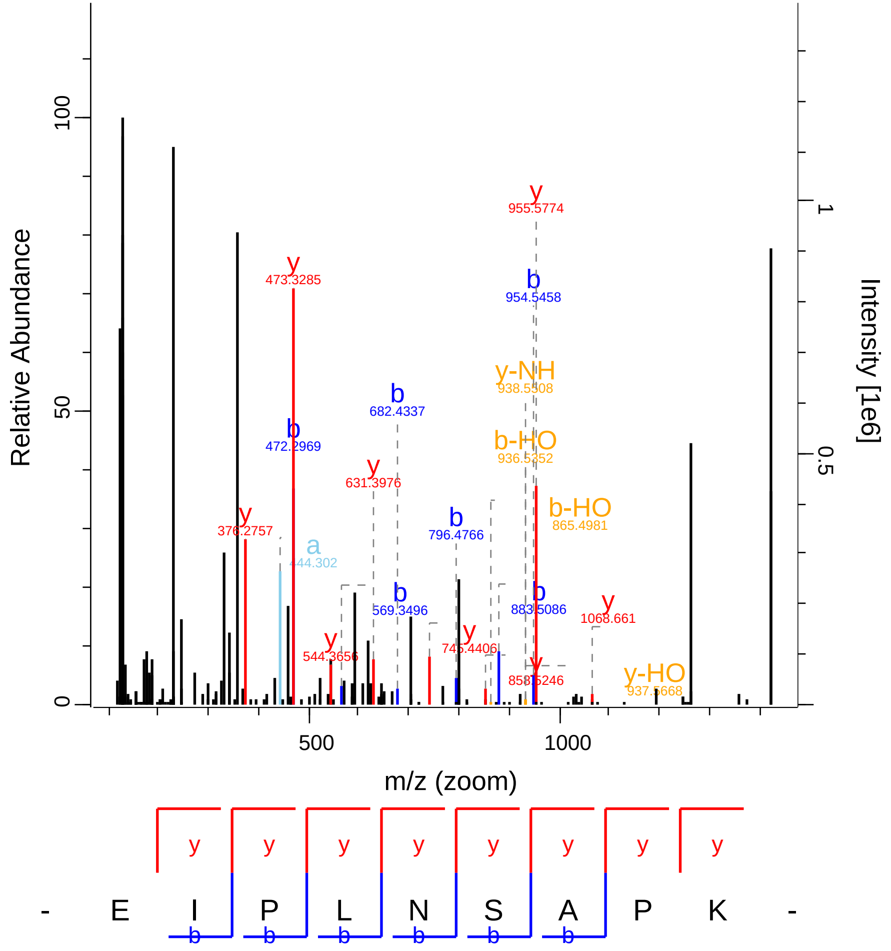

Raw File	Scan	Method	Score	m/z	Gene names
RO-QC-160121_ZackD_SS_Set_02_Fractio	17844	FTMS; HC	123.36	782.09	MED31

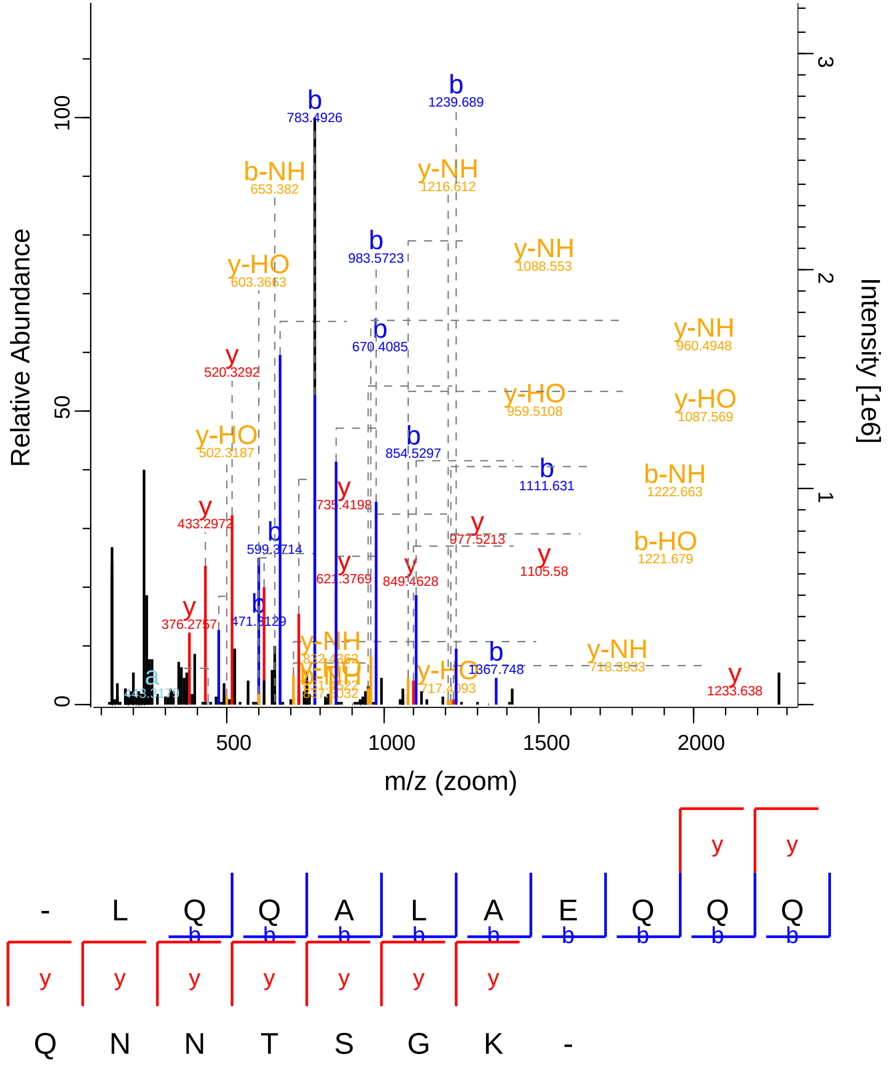

Raw File	Scan	Method	Score	m/z	Gene names
RO-QC-160121_ZackD_SS_Set_02_Fractio	18463	FTMS; HC	65.18	622.36	TMEM208

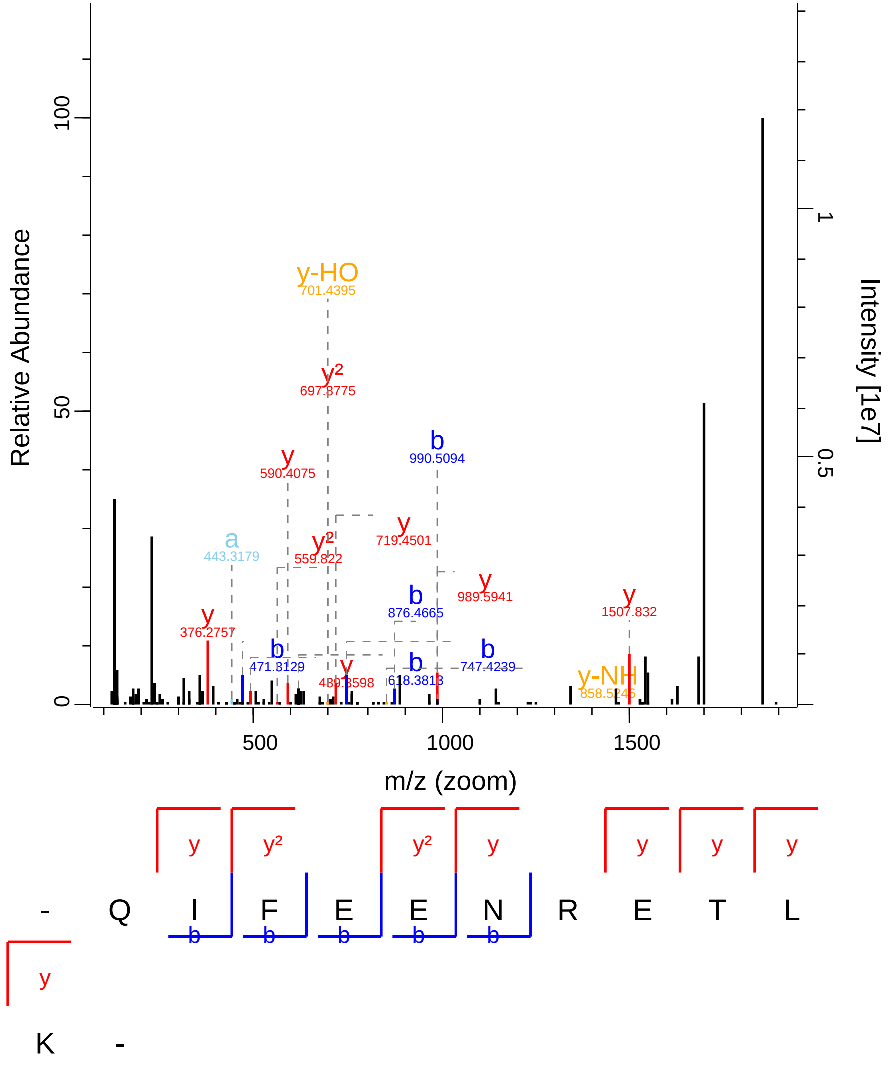

Raw File	Scan	Method	Score	m/z	Gene names
RO-QC-160121_ZackD_SS_Set_02_Fractio	18605	FTMS; HC	80.78	865.46	CBY1

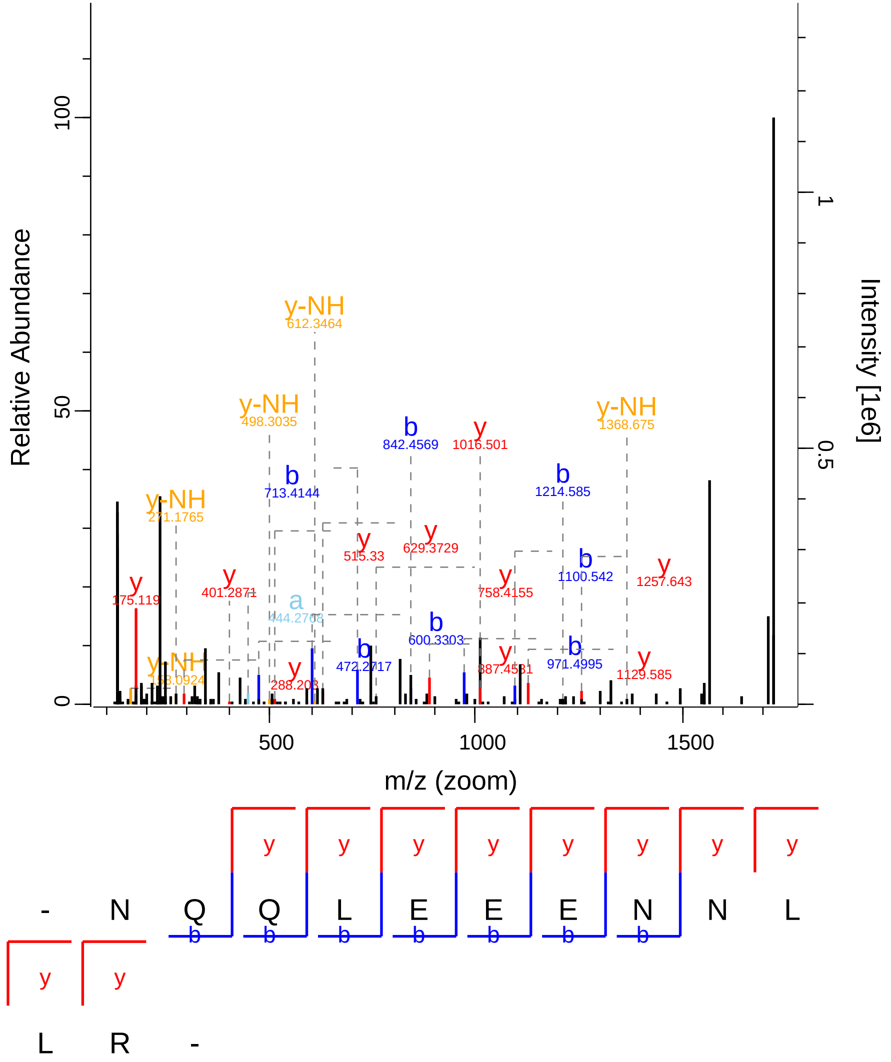

Raw File	Scan	Method	Score	m/z	Gene names
RO-QC-160121_ZackD_SS_Set_02_Fractic	18623	FTMS; HC	66.69	1029.98	ZNF215

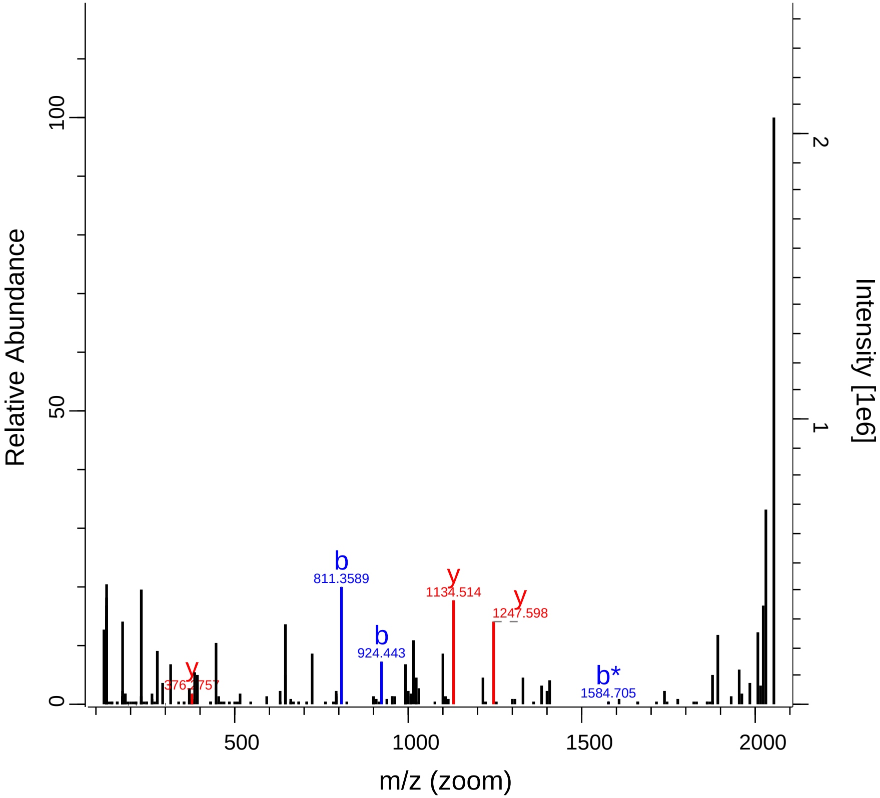

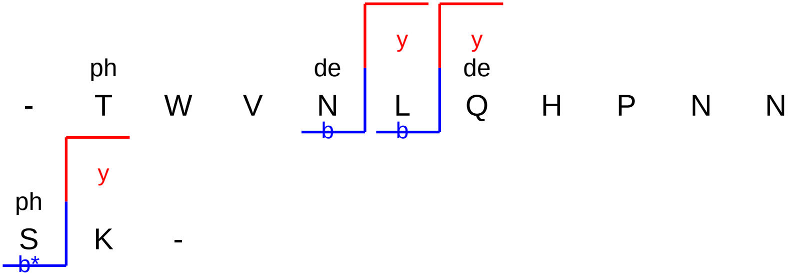

Raw File	Scan	Method	Score	m/z	Gene names
RO-QC-160121_ZackD_SS_Set_02_Fractio	18845	FTMS; HC	48.44	594.62	EXOC3L1

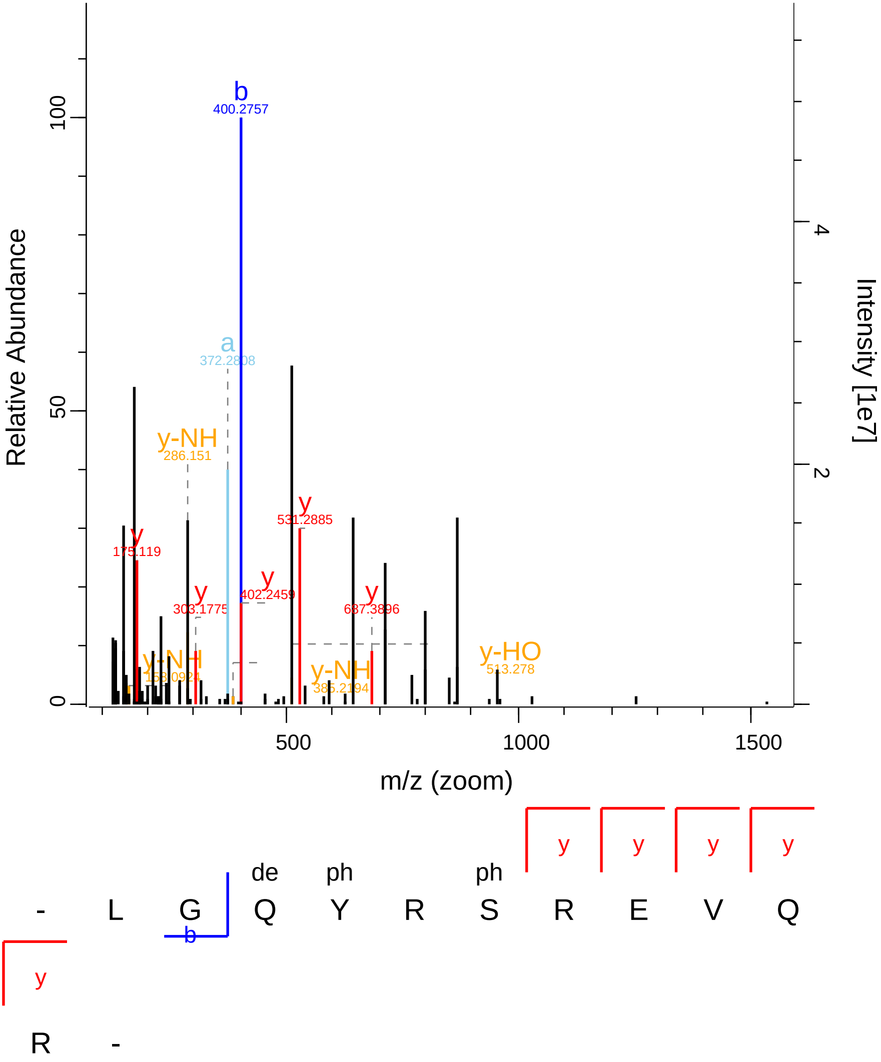

Raw File	Scan	Method	Score	m/z	Gene names
RO-QC-160121_ZackD_SS_Set_02_Fractio	19464	FTMS; HC	70.96	805.42	CDC42SE2

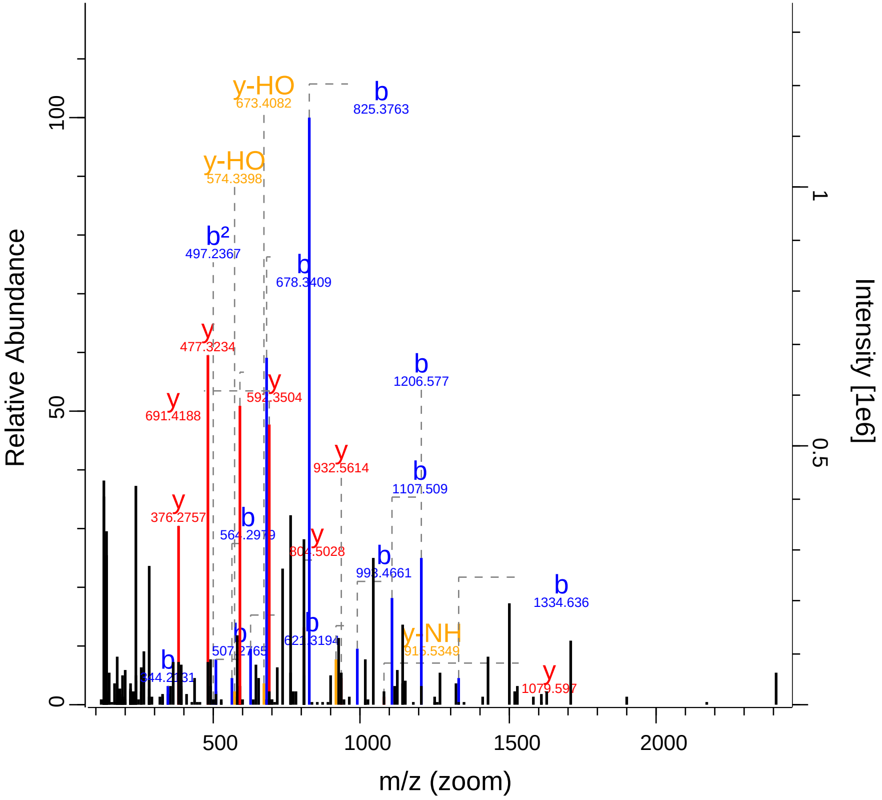

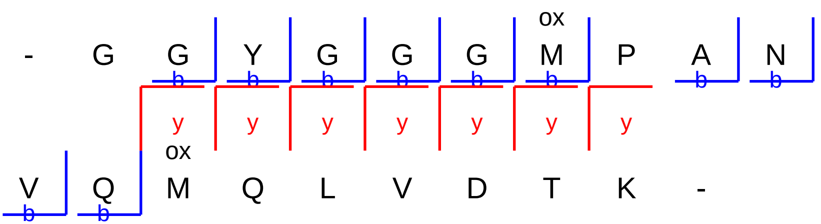

Raw File	Scan	Method	Score	m/z	Gene names
RO-QC-160121_ZackD_SS_Set_02_Fractio	19563	FTMS; HC	112.34	805.08	CDC42SE2

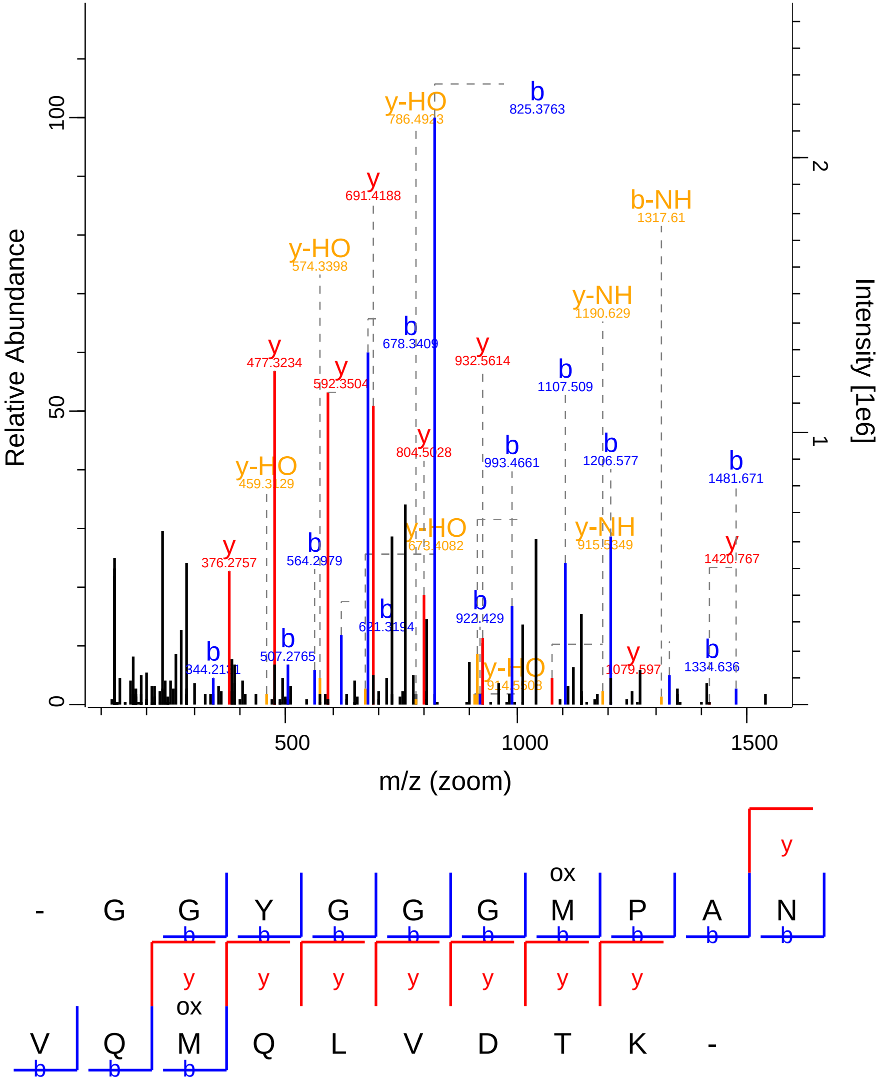

Raw File	Scan	Method	Score	m/z	Gene names
RO-QC-160121_ZackD_SS_Set_02_Fractic	19731	FTMS; HC	111.94	1207.12	CDC42SE2

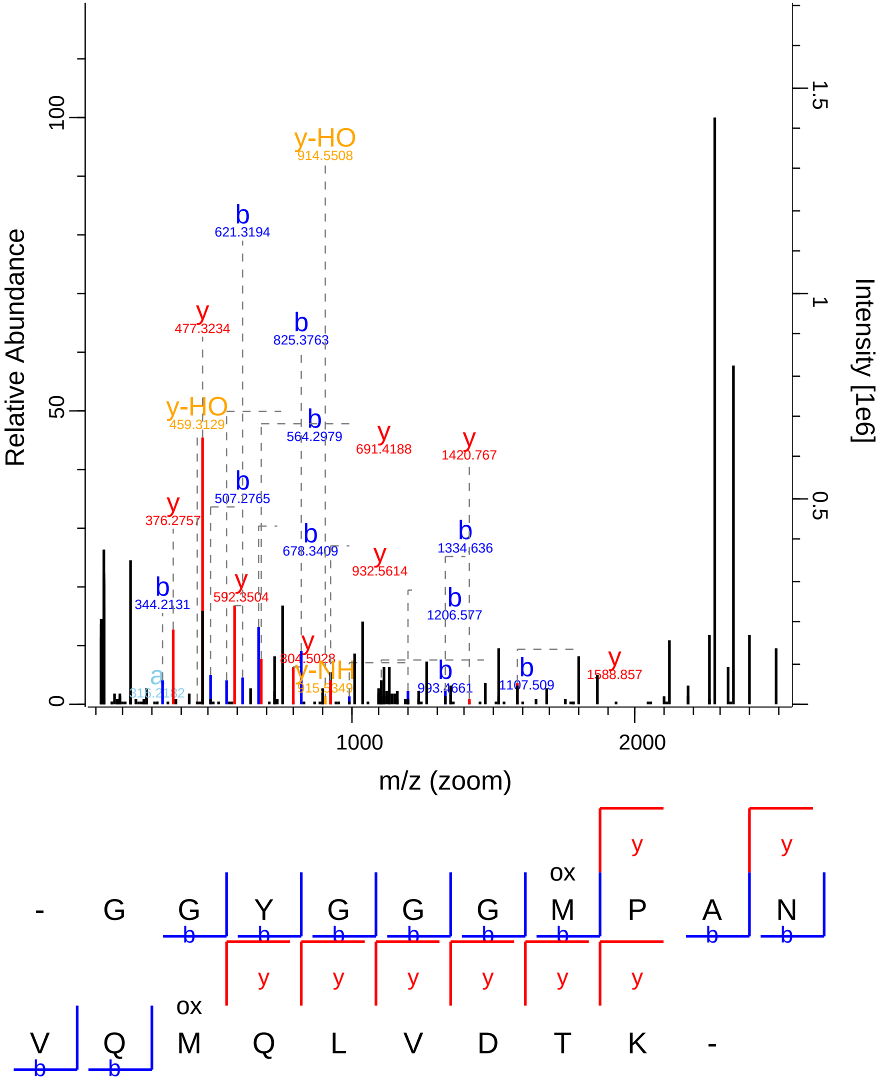

Raw File	Scan	Method	Score	m/z	Gene names
RO-QC-160121_ZackD_SS_Set_02_Fractio	19785	FTMS; HC	111.78	805.42	CDC42SE2

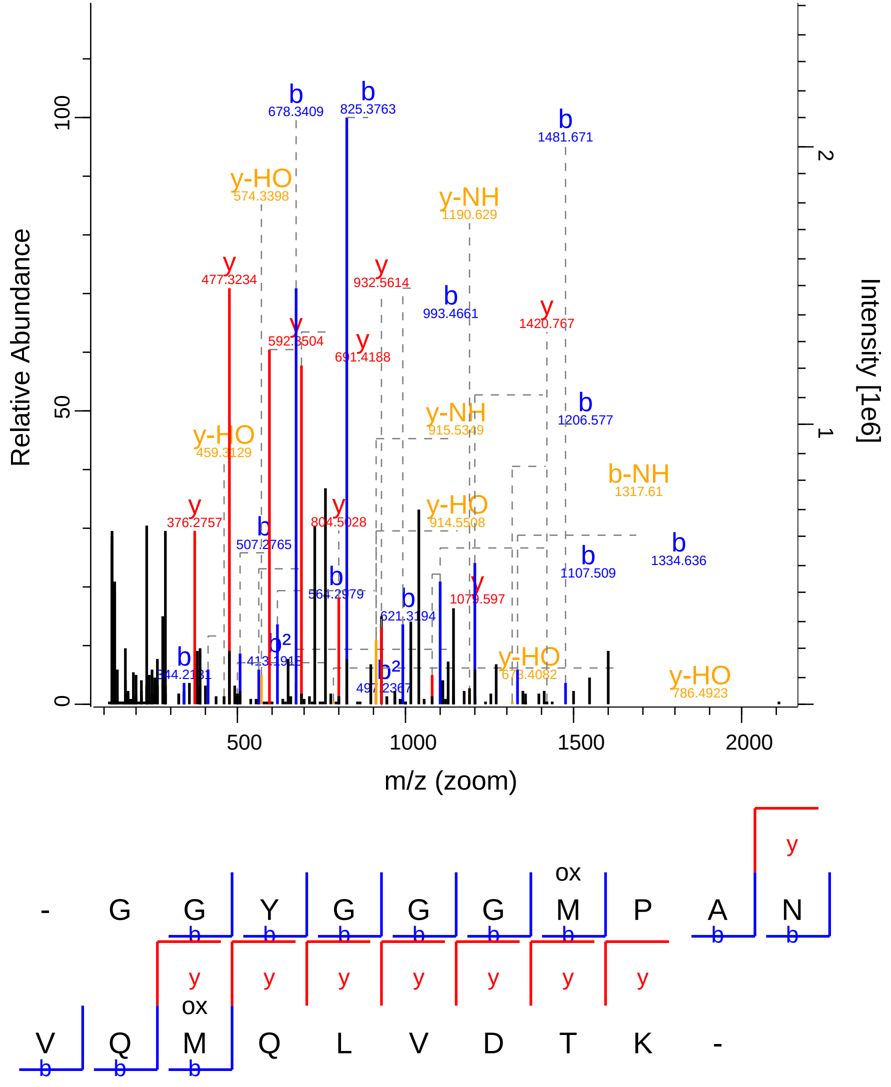

Raw File	Scan	Method	Score	m/z	Gene names
RO-QC-160121_ZackD_SS_Set_02_Fractio	20223	FTMS; HC	59.51	860.11	SLC31A1

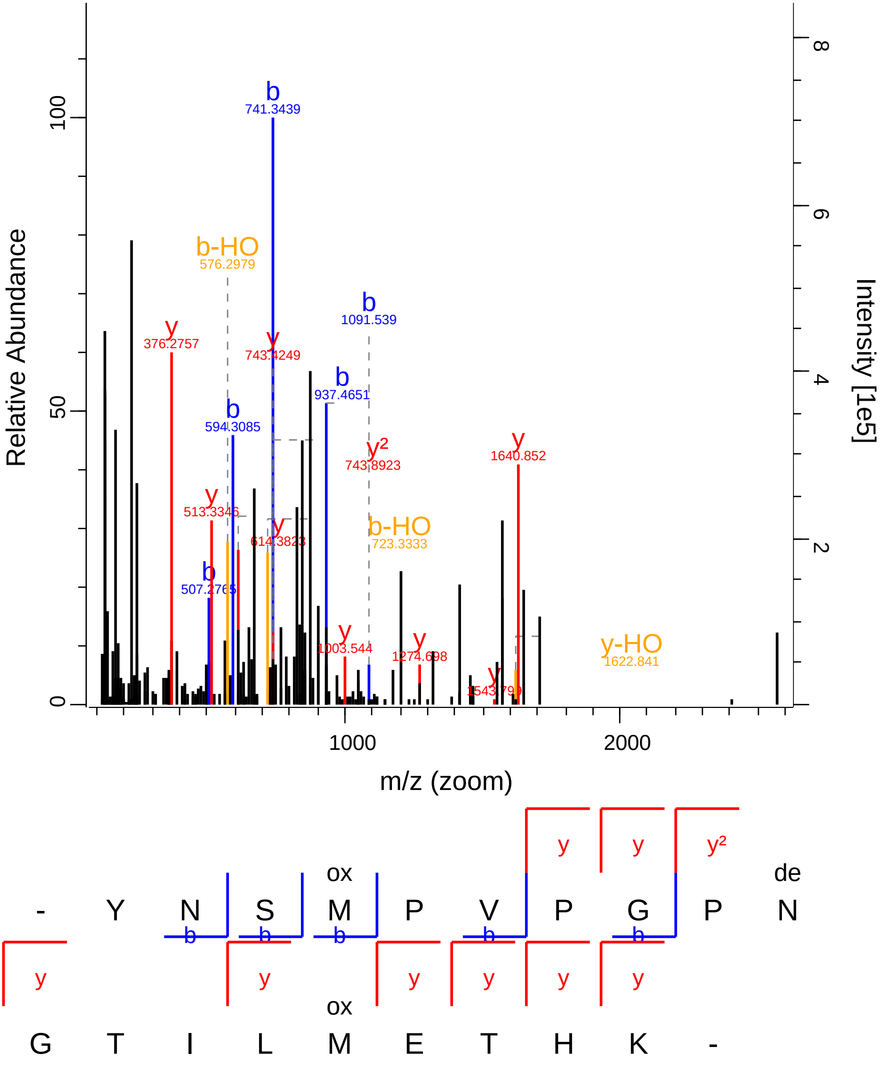

Raw File	Scan	Method	Score	m/z	Gene names
RO-QC-160121_ZackD_SS_Set_02_Fractio	20303	FTMS; HC	99.22	807.95	DMWD

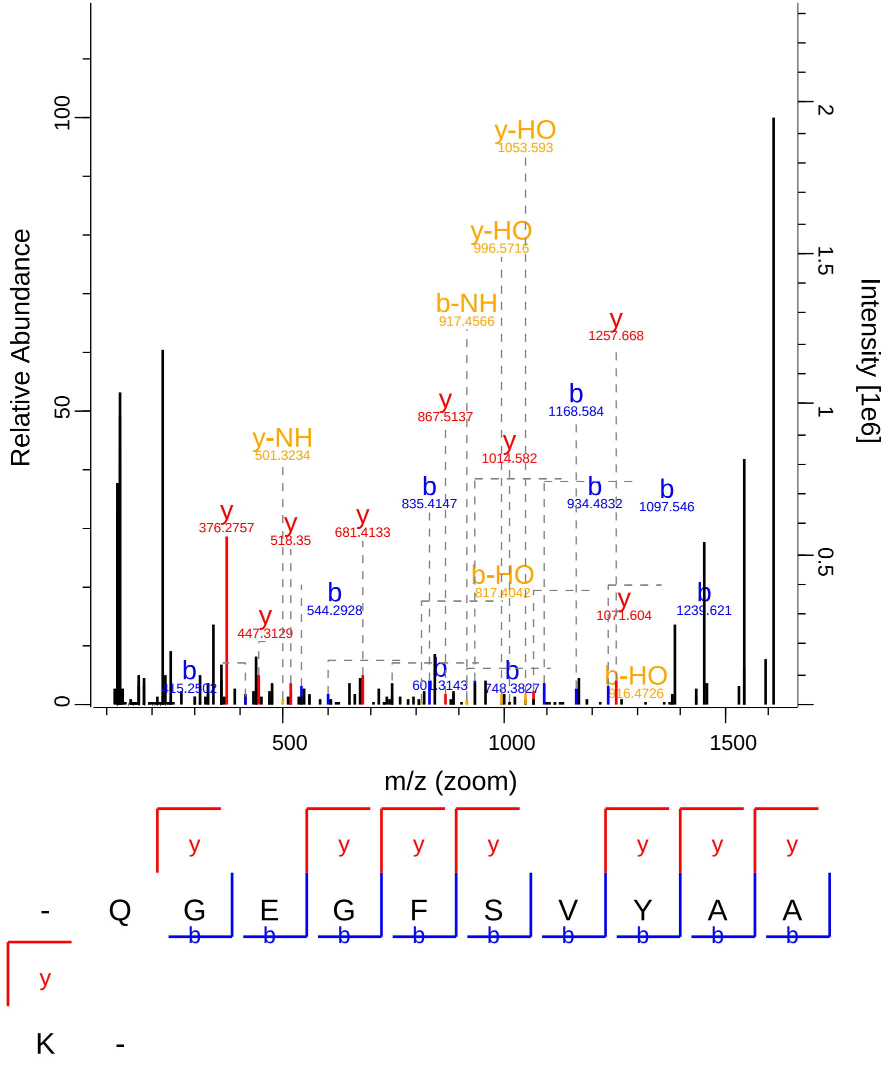

Raw File	Scan	Method	Score	m/z	Gene names
RO-QC-160121_ZackD_SS_Set_02_Fractior	20318	FTMS; HCl	54.76	530.8	ZIK1

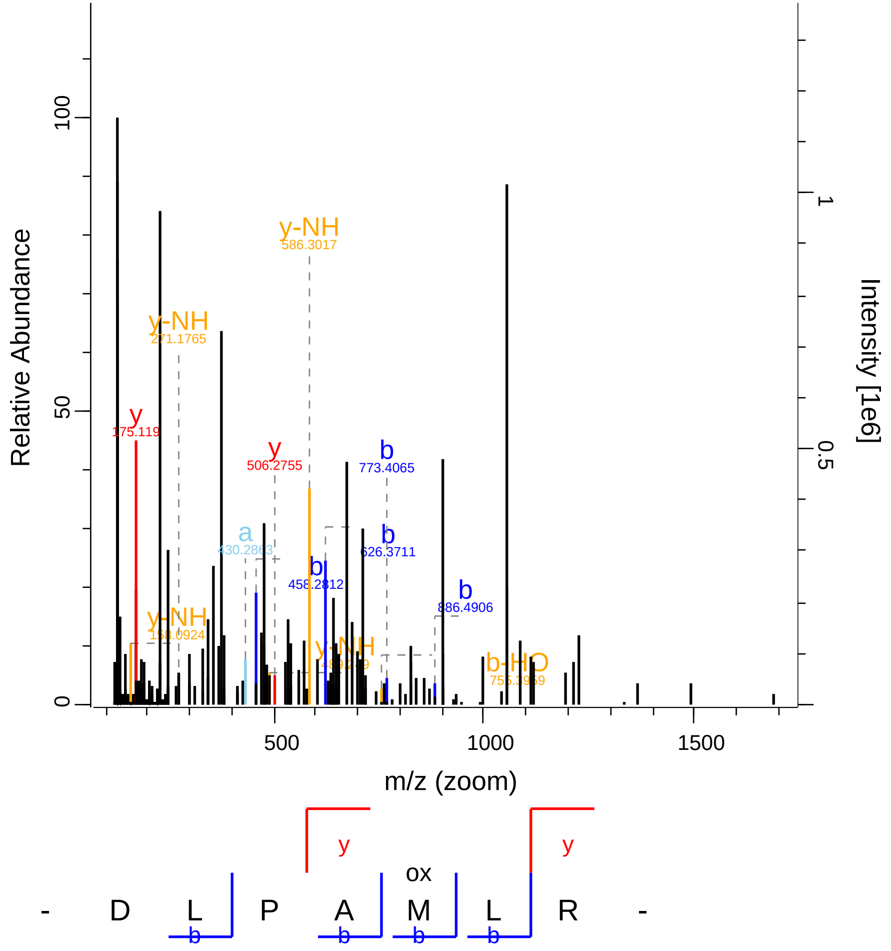

Raw File	Scan	Method	Score	m/z	Gene names
RO-QC-160121_ZackD_SS_Set_02_Fractio	20339	FTMS; HC	47.29	942.49	DYRK4

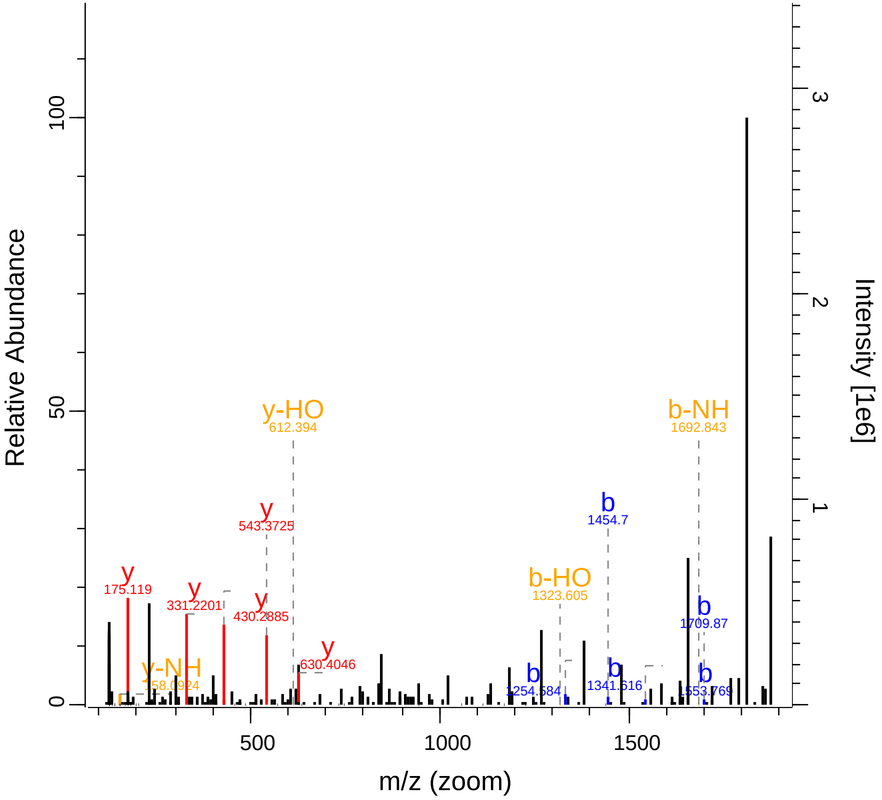

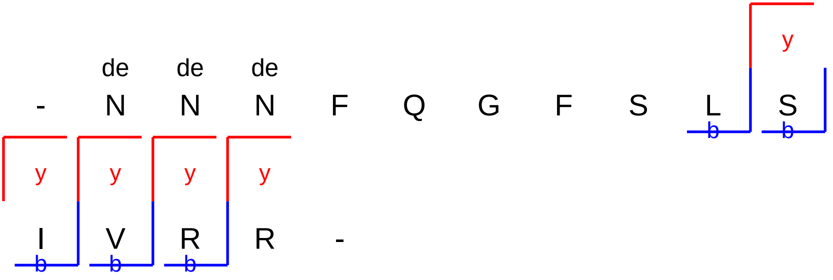

Raw File	Scan	Method	Score	m/z
RO-QC-160121_ZackD_SS_Set_02_Fraction_4	20378	FTMS; HCD	79.89	710.4

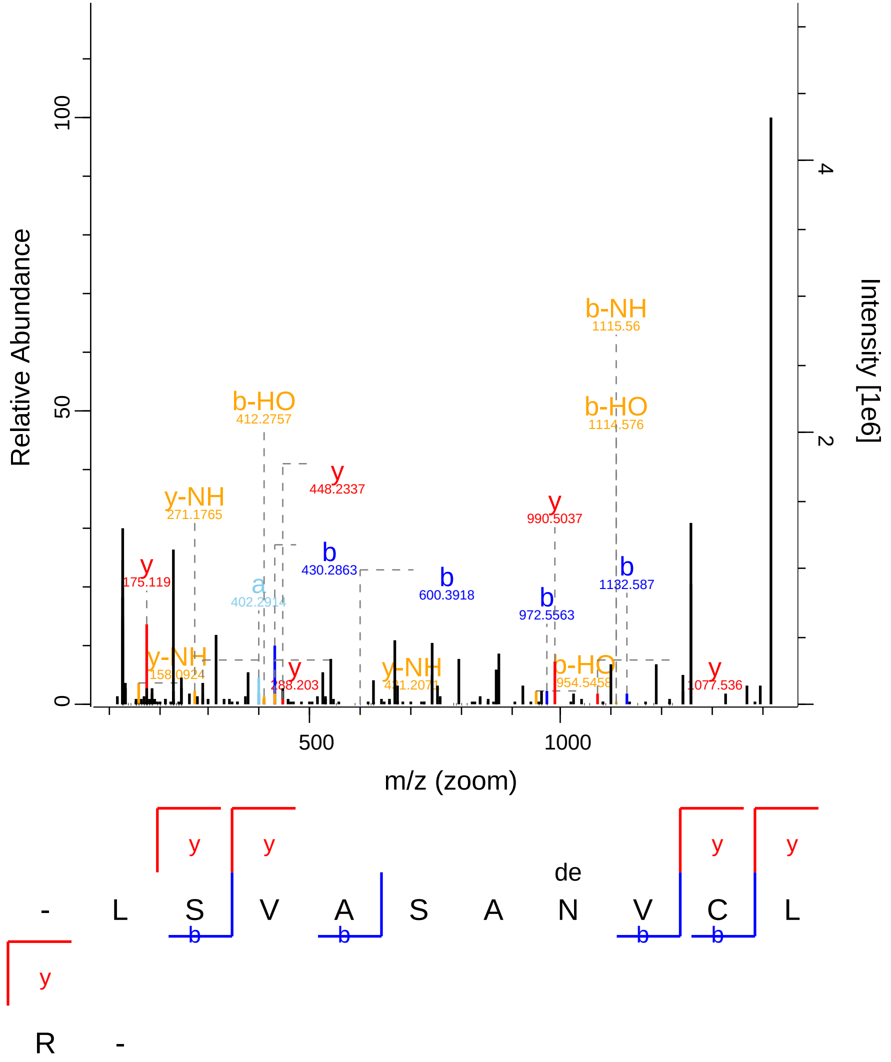

Raw File	Scan	Method	Score	m/z	Gene names
RO-QC-160121_ZackD_SS_Set_02_Fractio	20439	FTMS; HC	69.36	860.11	SLC31A1

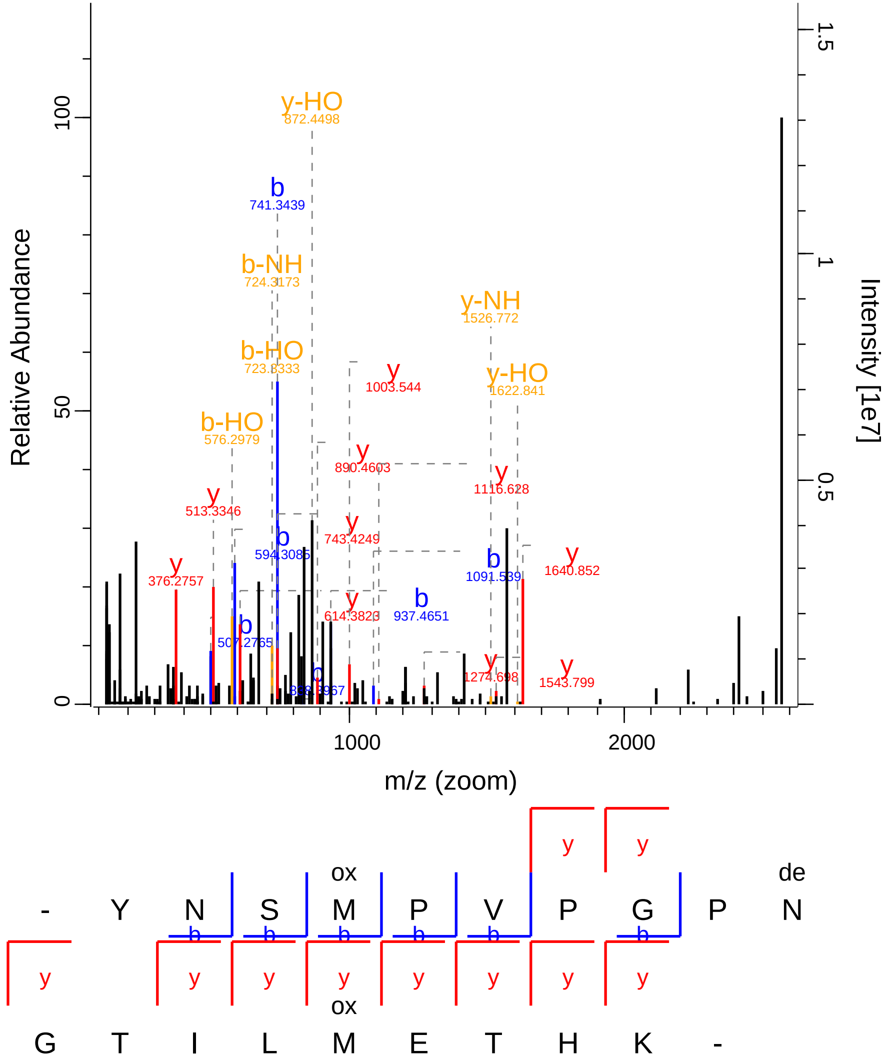

Raw File	Scan	Method	Score	m/z	Gene names
RO-QC-160121_ZackD_SS_Set_02_Fractic	20635	FTMS; HC	64.04	1029.98	ZNF215

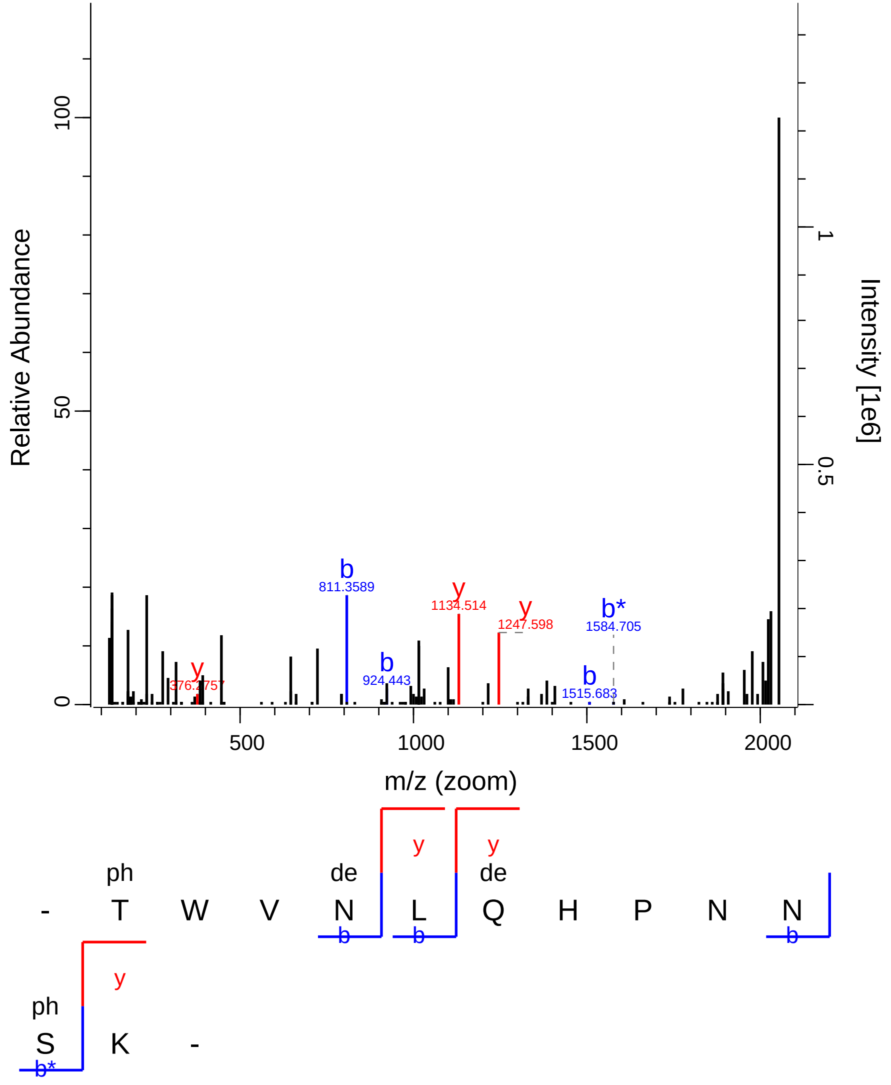

Raw File	Scan	Method	Score	m/z	Gene names
RO-QC-160121_ZackD_SS_Set_02_Fractio	20667	FTMS; HC	75.33	860.11	SLC31A1

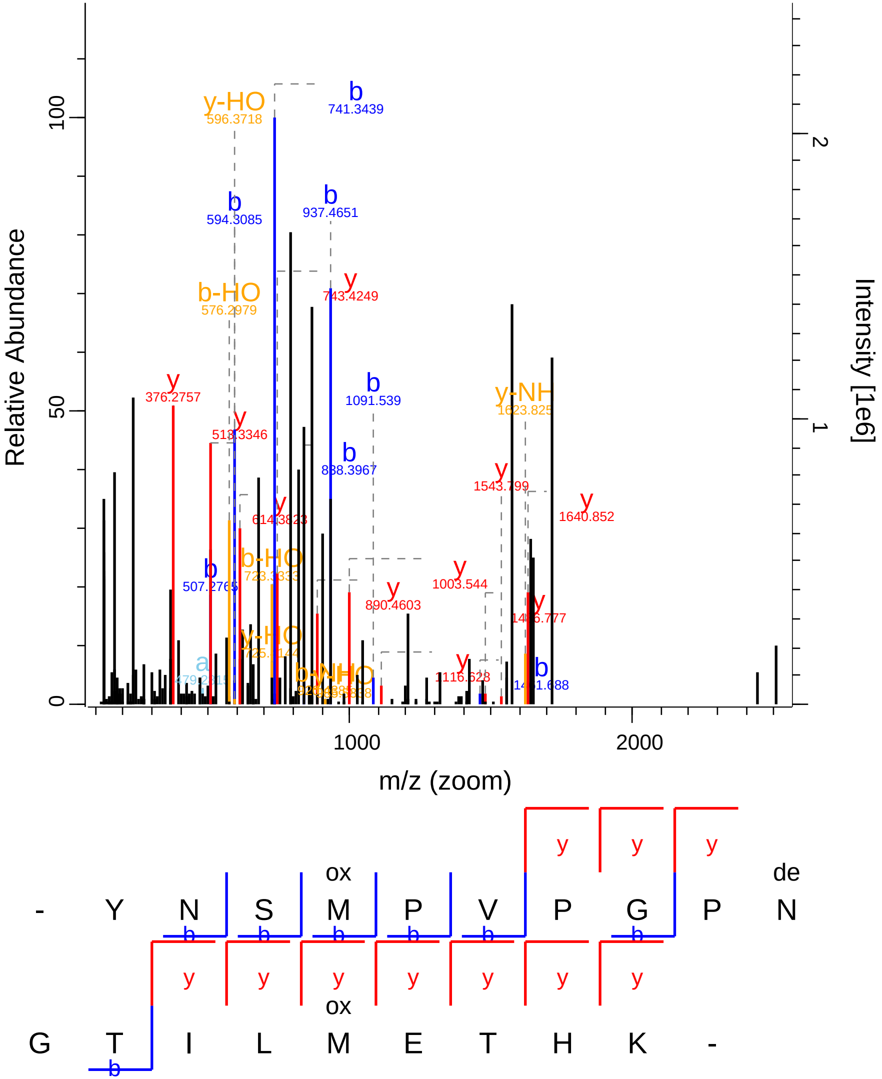

Raw File	Scan	Method	Score	m/z
RO-QC-160121_ZackD_SS_Set_02_Fraction_4	21055	FTMS; HCD	42.8	549.99

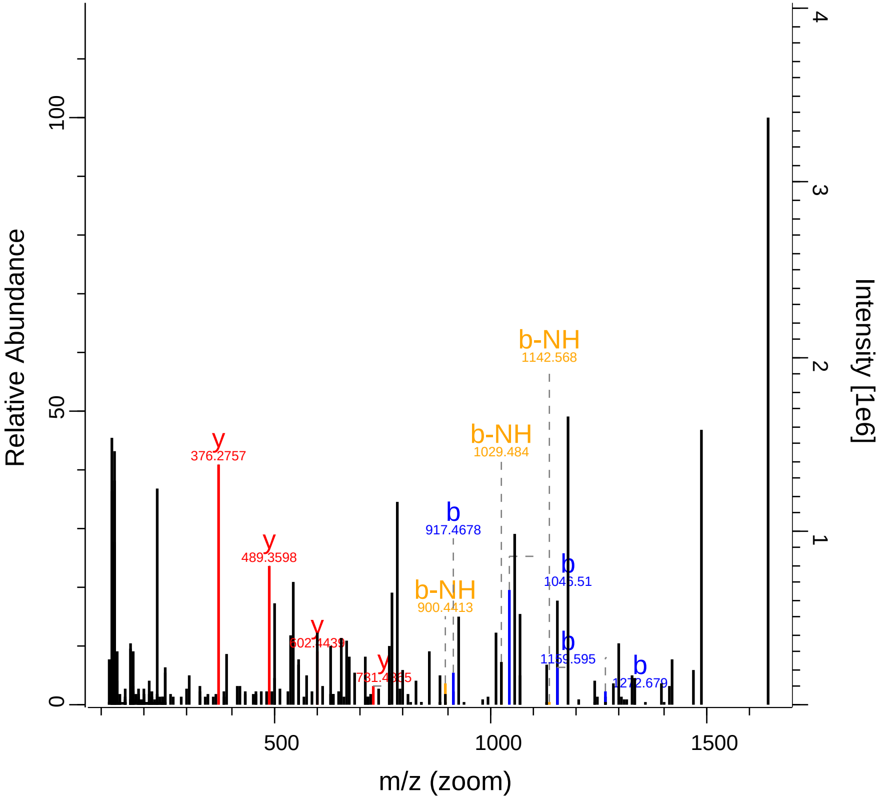

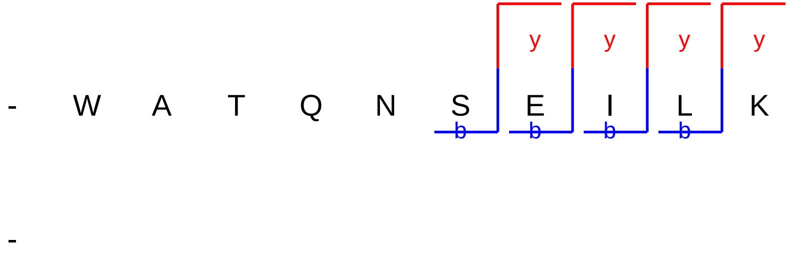

Raw File	Scan	Method	Score	m/z	Gene names
RO-QC-160121_ZackD_SS_Set_02_Fractior	21162	FTMS; HCI	53.97	712.4	STAP1

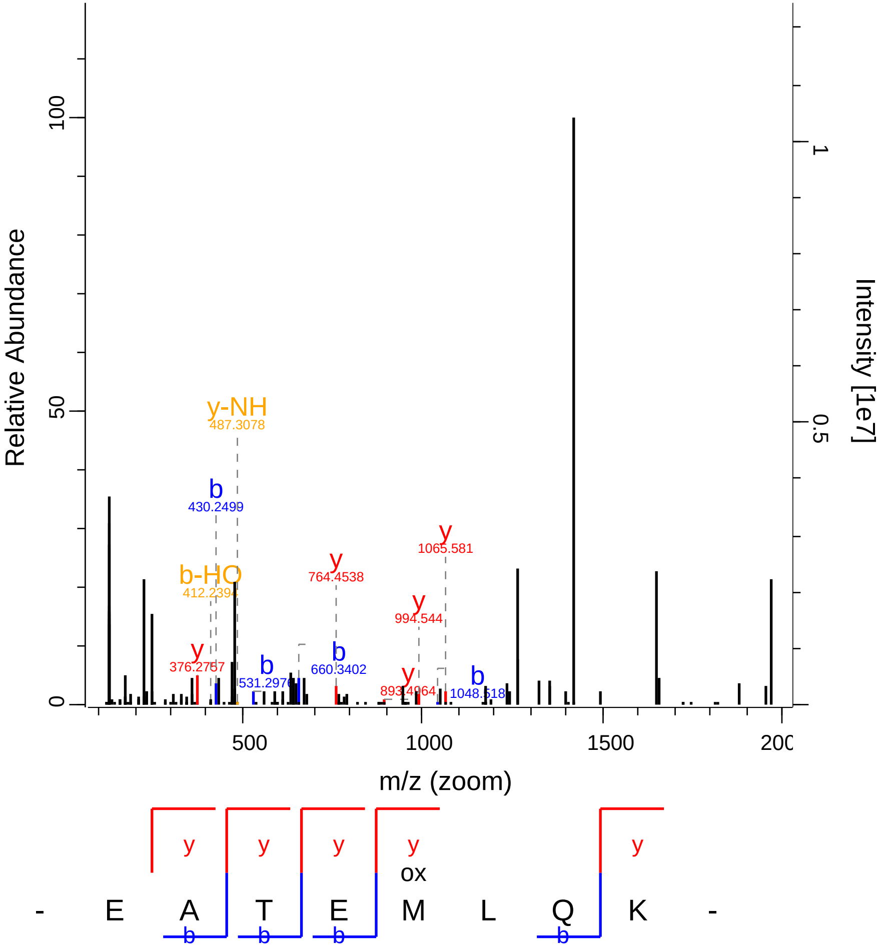

Raw File	Scan	Method	Score	m/z
RO-QC-160121_ZackD_SS_Set_02_Fraction_4	21188	FTMS; HCD	66.69	824.48

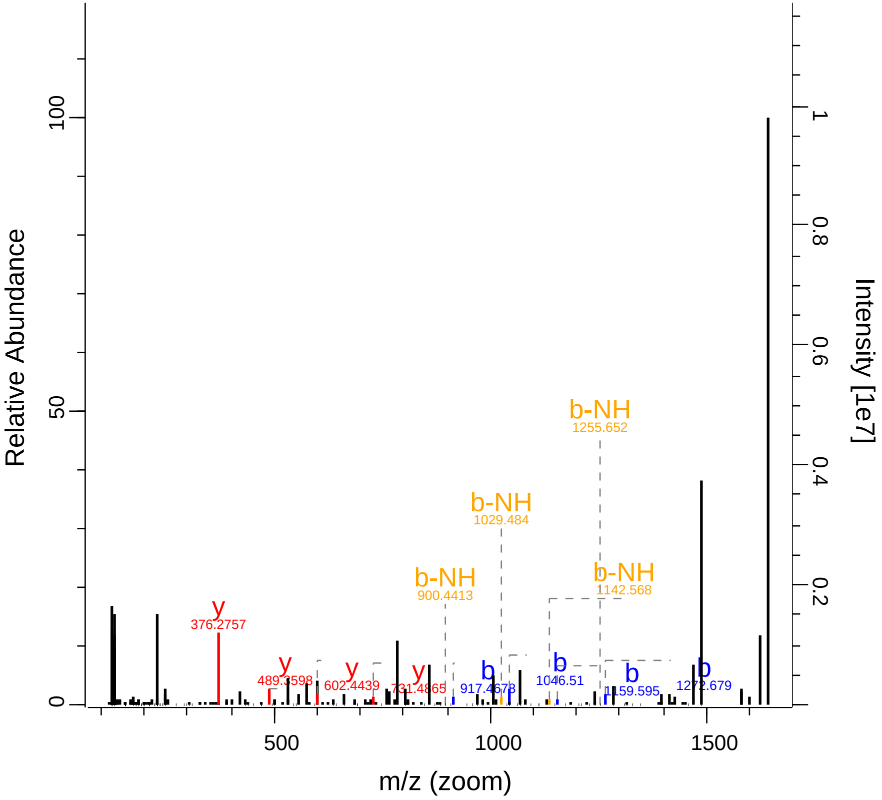

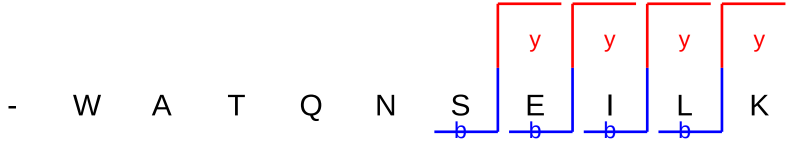

Raw File	Scan	Method	Score	m/z
RO-QC-160121_ZackD_SS_Set_02_Fraction_4	21280	FTMS; HCD	76.23	549.99

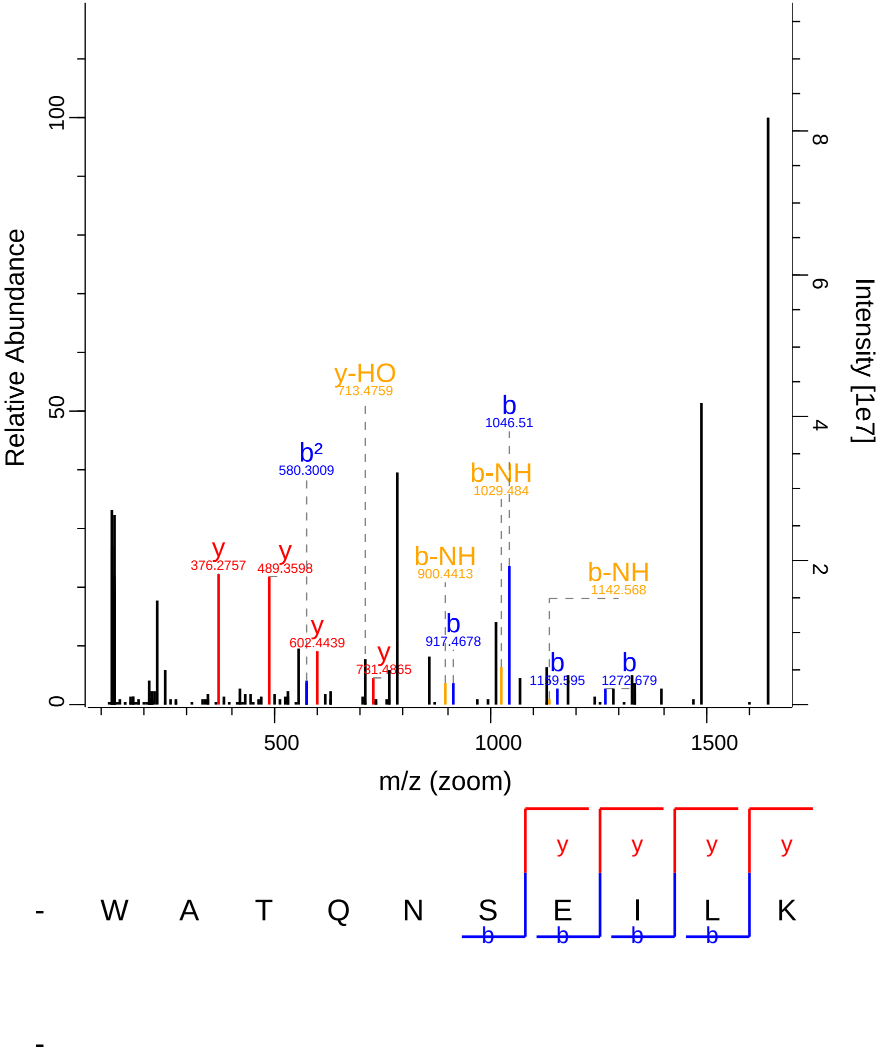

Raw File	Scan	Method	Score	m/z	Gene names
RO-QC-160121_ZackD_SS_Set_02_Fractio	21589	FTMS; HC	41.05	562.56	CYP17A1

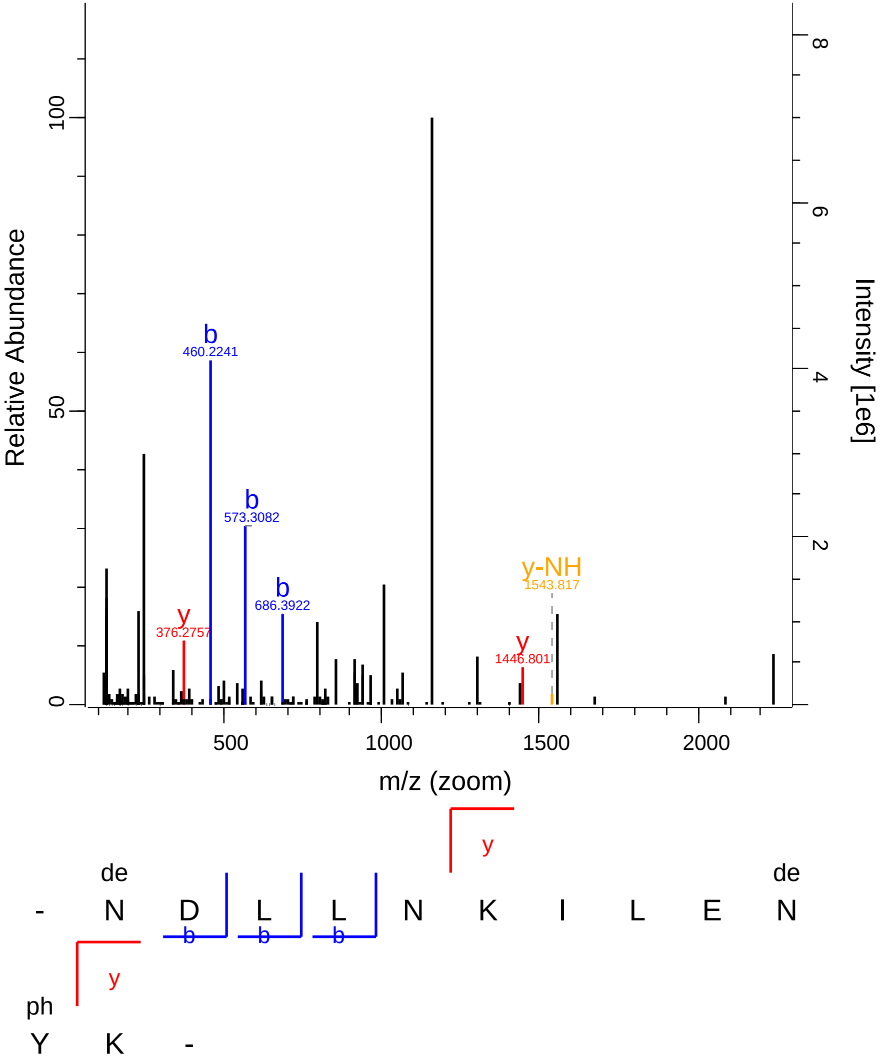

Raw File	Scan	Method	Score	m/z
RO-QC-160121_ZackD_SS_Set_02_Fraction_4	22042	FTMS; HCD	84.48	470.95

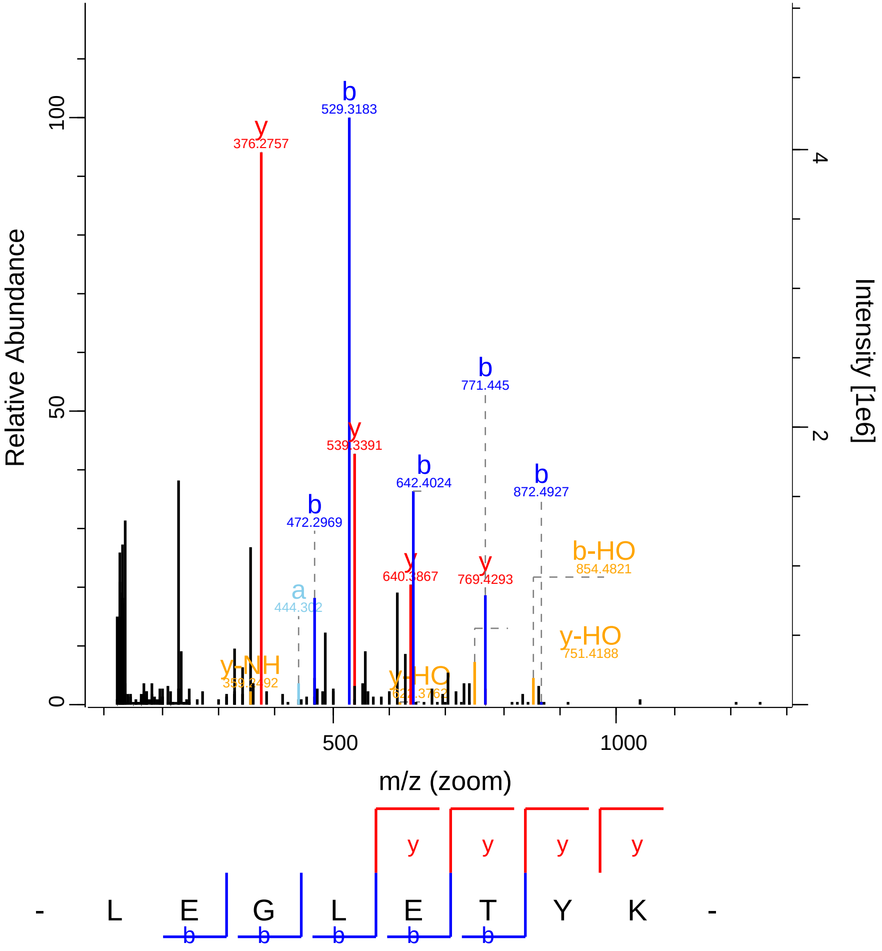

Raw File	Scan	Method	Score	m/z	Gene names
RO-QC-160121_ZackD_SS_Set_02_Fractio	22683	FTMS; HC	60.48	473.62	CD14

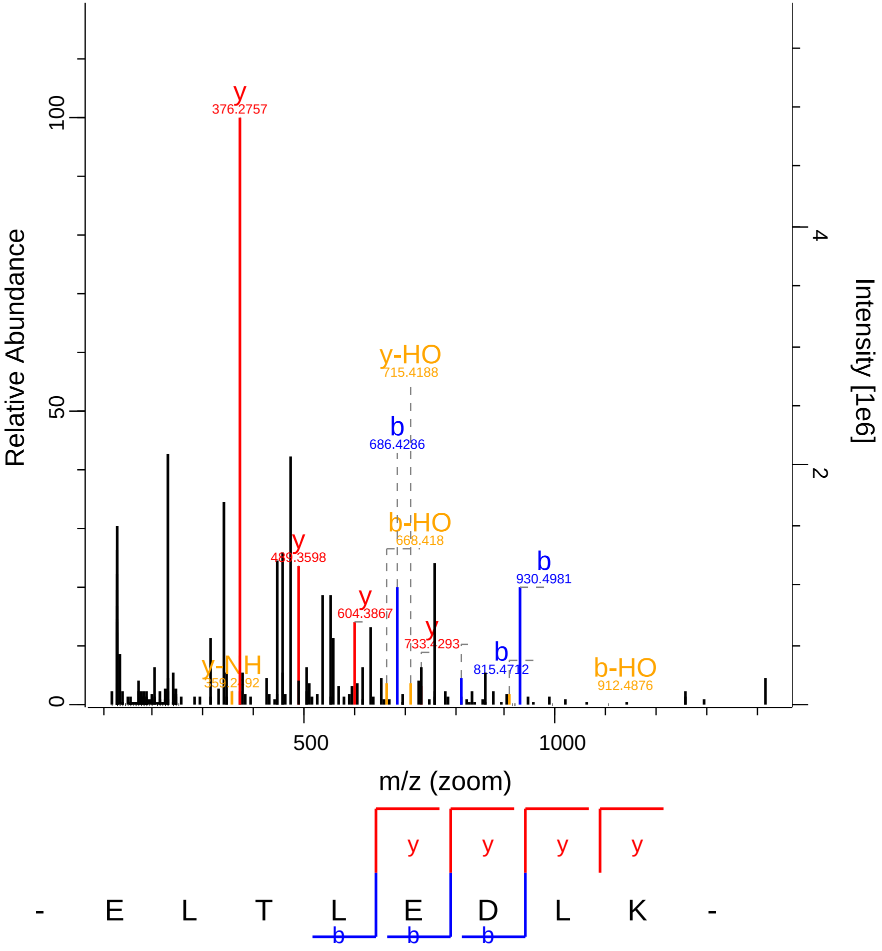

Raw File	Scan	Method	Score	m/z
RO-QC-160121_ZackD_SS_Set_02_Fraction_4	23341	FTMS; HCD	107.79	914.46

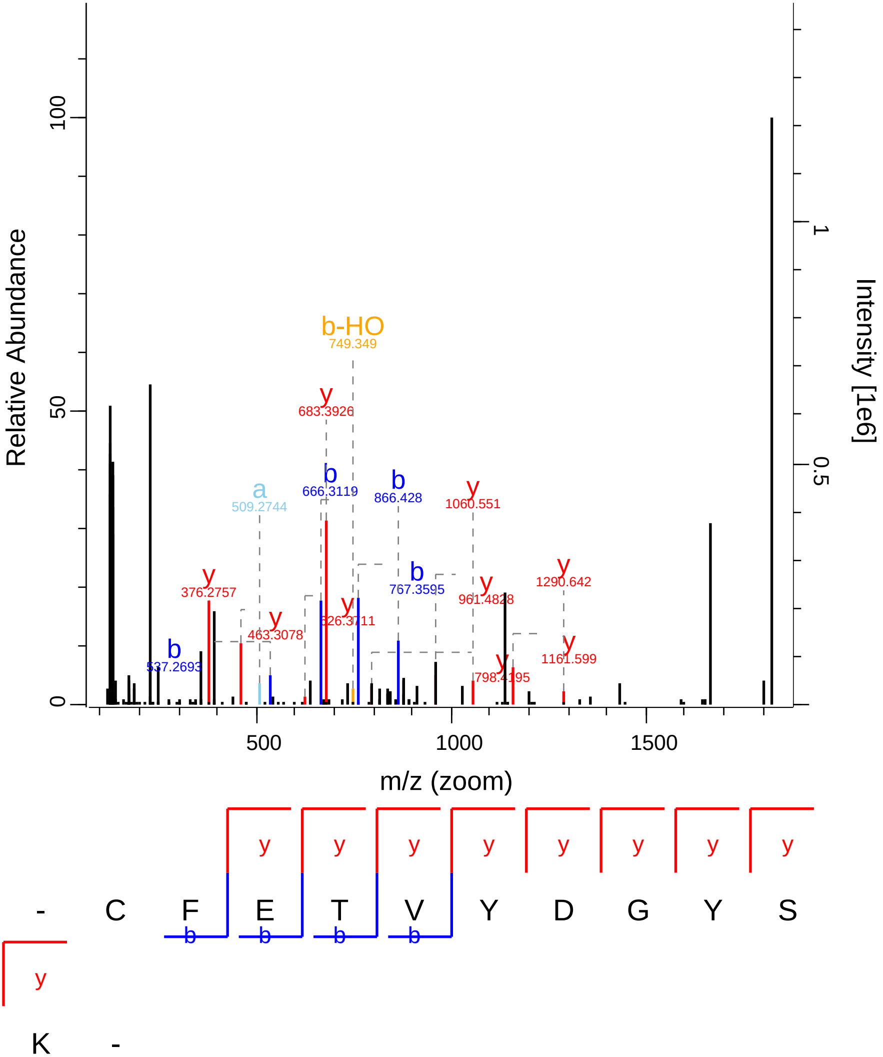

Raw File	Scan	Method	Score	m/z	Gene names
RO-QC-160121_ZackD_SS_Set_02_Fractior	23886	FTMS; HCI	117.07	799.1	FAM103A1

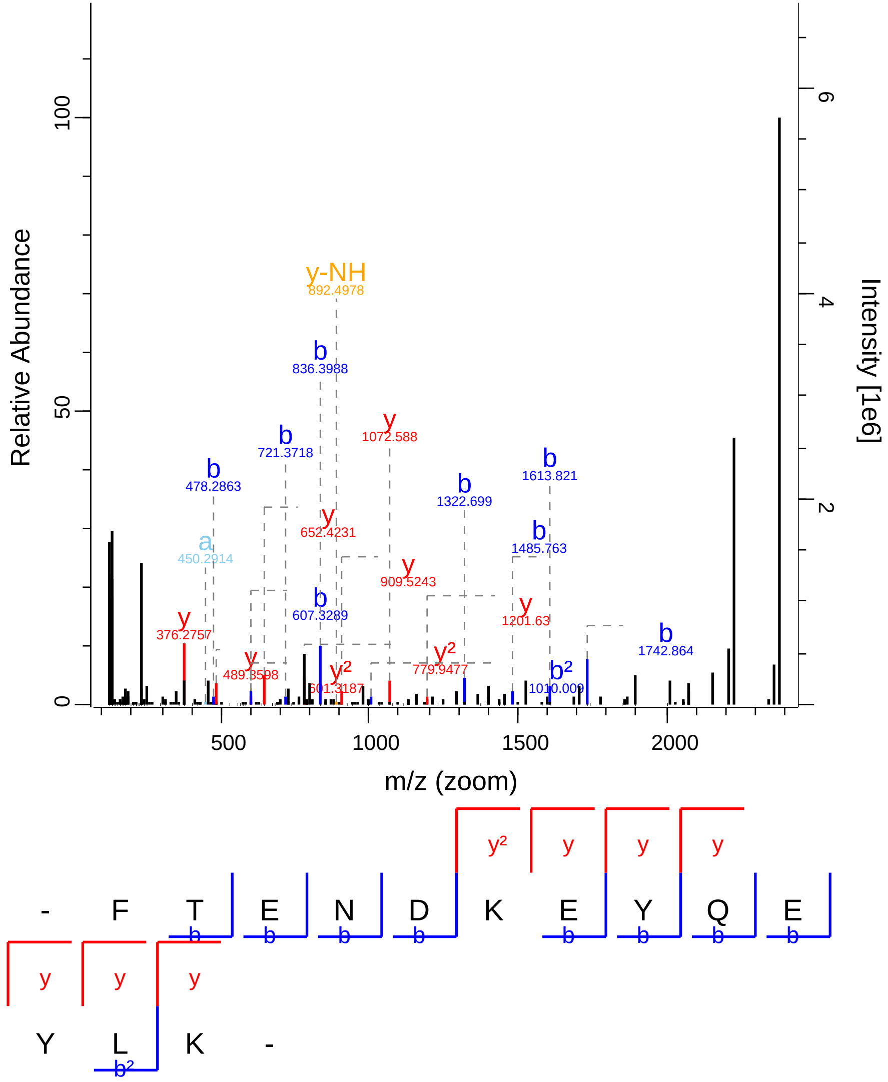

Raw File	Scan	Method	Score	m/z	Gene names
RO-QC-160121_ZackD_SS_Set_02_Fractio	23966	FTMS; HC	85.54	599.58	FAM103A1

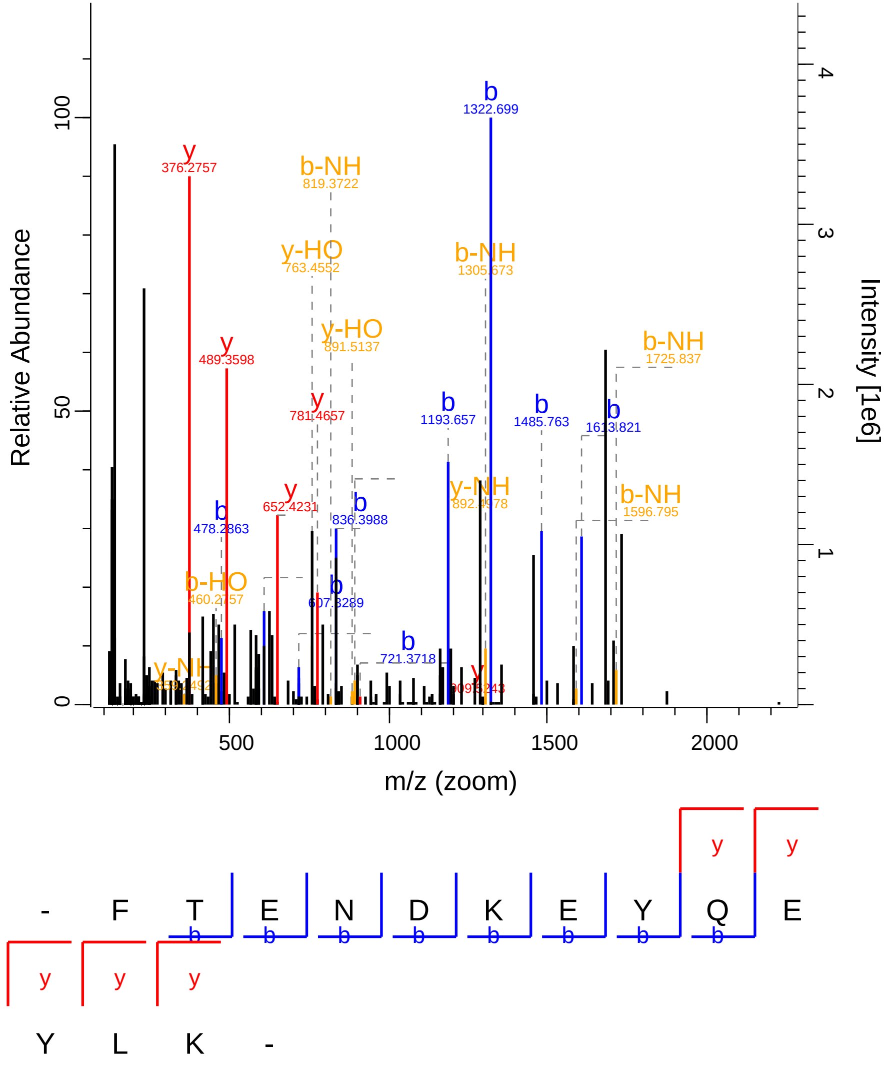

Raw File	Scan	Method	Score	m/z	Gene names
RO-QC-160121_ZackD_SS_Set_02_Fractic	23969	FTMS; HC	161.1	1198.15	FAM103A1

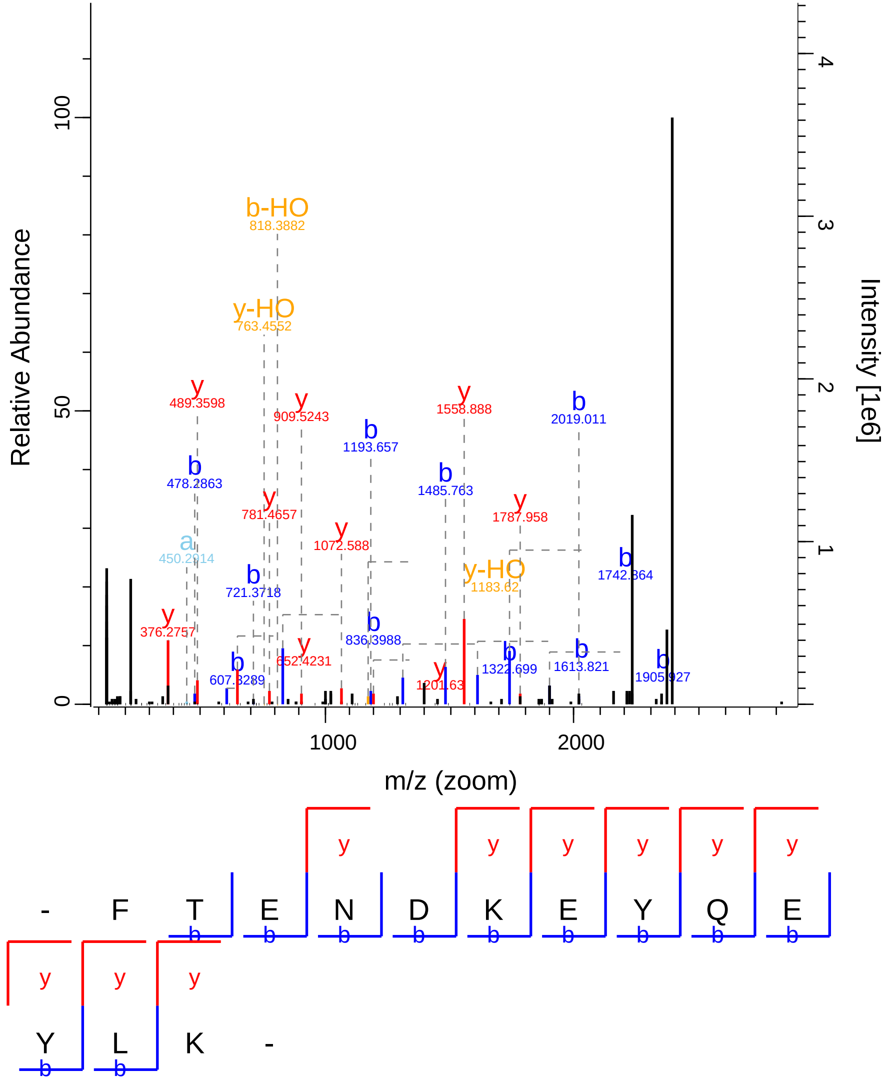

Raw File	Scan	Method	Score	m/z	Gene names
RO-QC-160121_ZackD_SS_Set_02_Fractio	24490	FTMS; HC	119.27	710.93	PUS7L

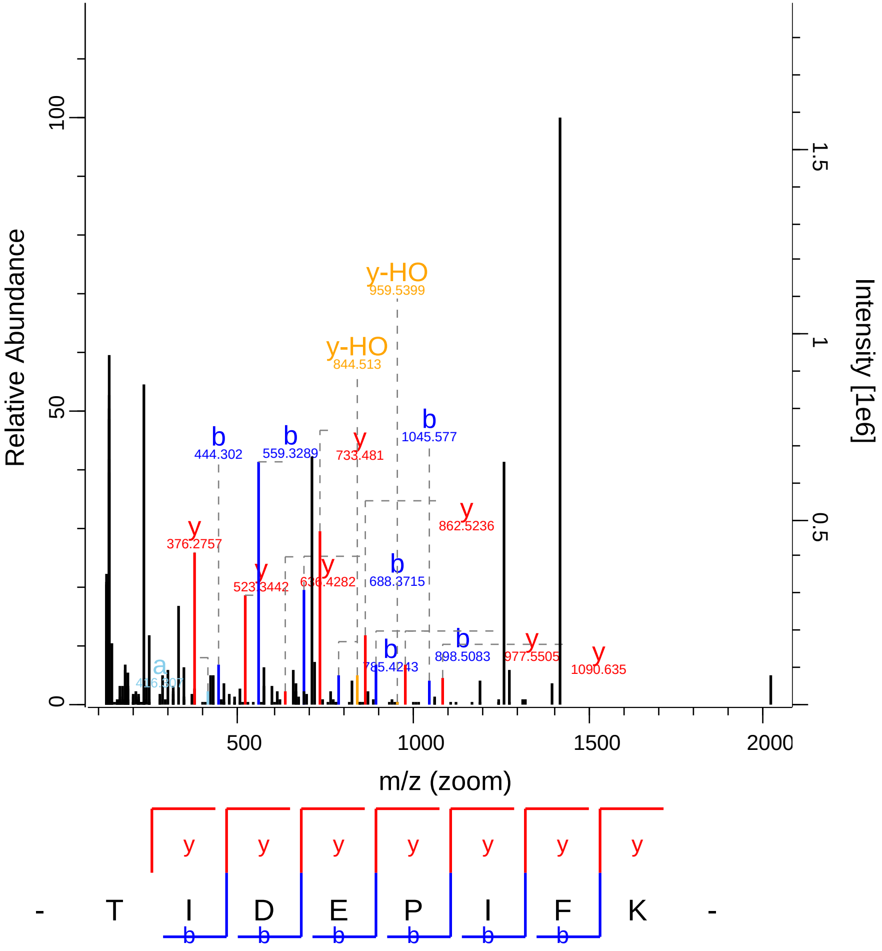

Raw File	Scan	Method	Score	m/z	Gene names
RO-QC-160121_ZackD_SS_Set_02_Fractio	25287	FTMS; HC	88.28	847.08	CNIH4

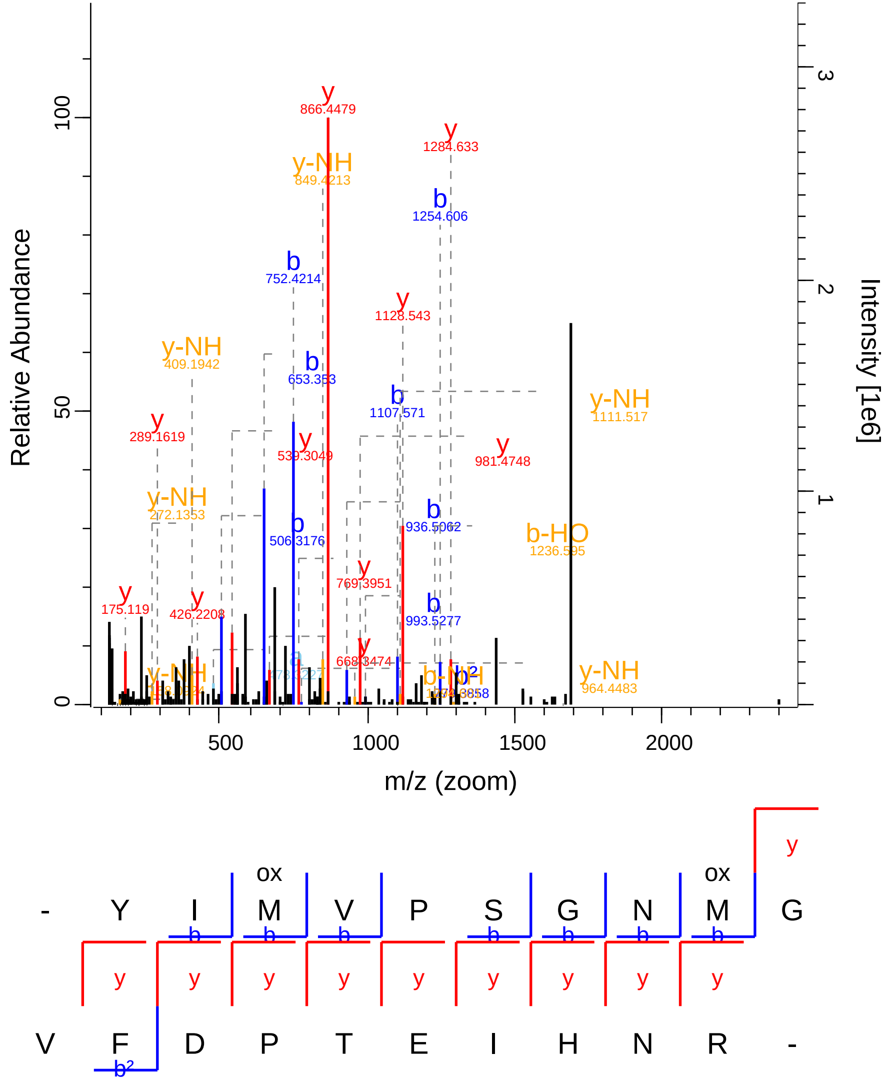

Raw File	Scan	Method	Score	m/z	Gene names
RO-QC-160121_ZackD_SS_Set_02_Fraction	25290	FTMS; HCI	71.22	542	CDC42EP2

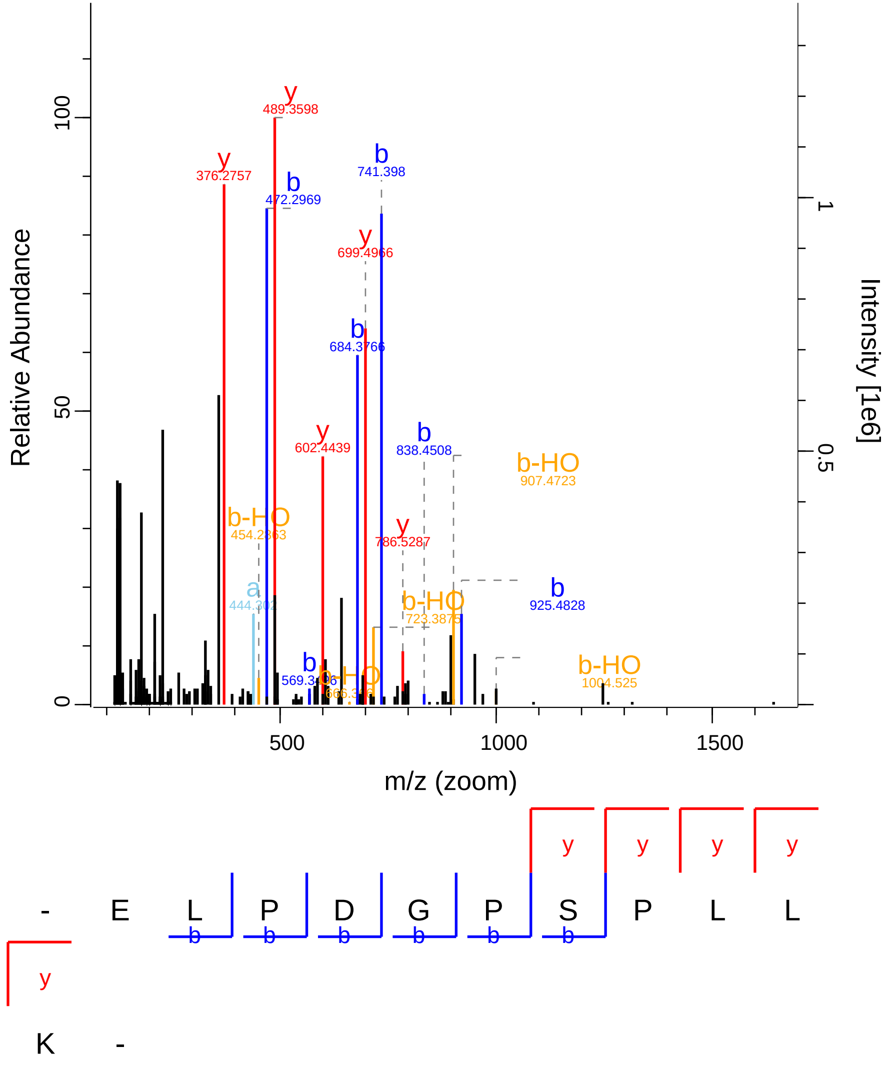

Raw File	Scan	Method	Score	m/z
RO-QC-160121_ZackD_SS_Set_02_Fraction_4	25638	FTMS; HCD	50.11	546.98

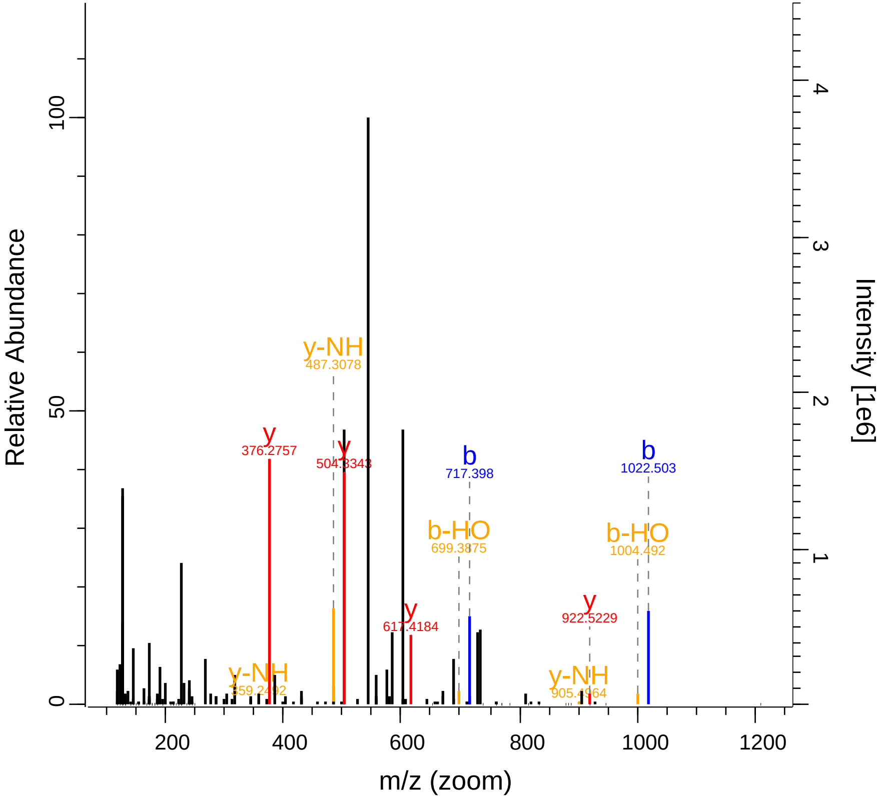

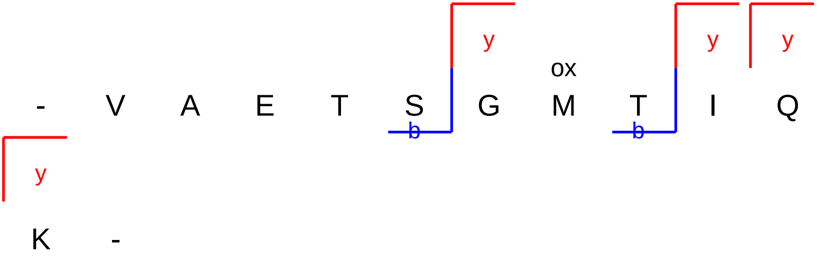

Raw File	Scan	Method	Score	m/z	Gene names
RO-QC-160121_ZackD_SS_Set_02_Fractio	26428	FTMS; HC	60.02	666.39	CDNF

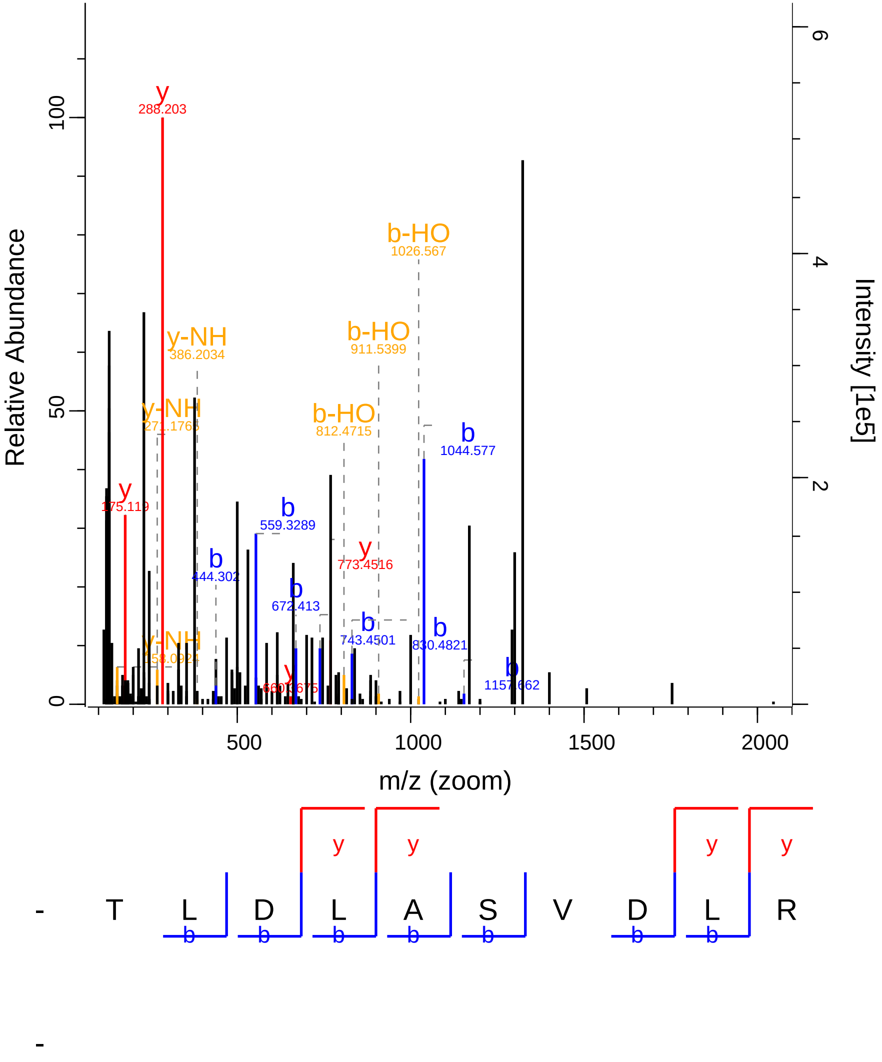

Raw File	Scan	Method	Score	m/z	Gene names
RO-QC-160121_ZackD_SS_Set_02_Fractio	27254	FTMS; HC	117.71	888.45	ANKRD39

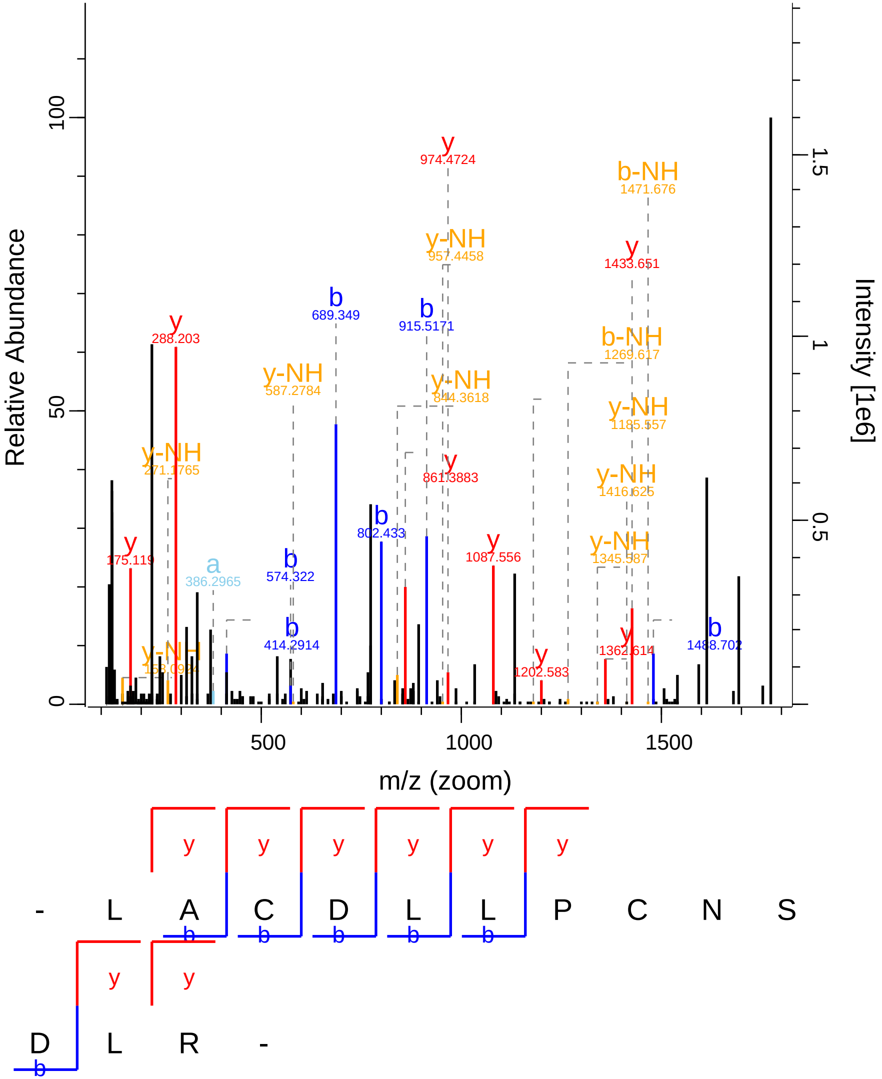

Raw File	Scan	Method	Score	m/z	Gene names
RO-QC-160121_ZackD_SS_Set_02_Fractio	28062	FTMS; HC	106.18	770.42	PAXIP1

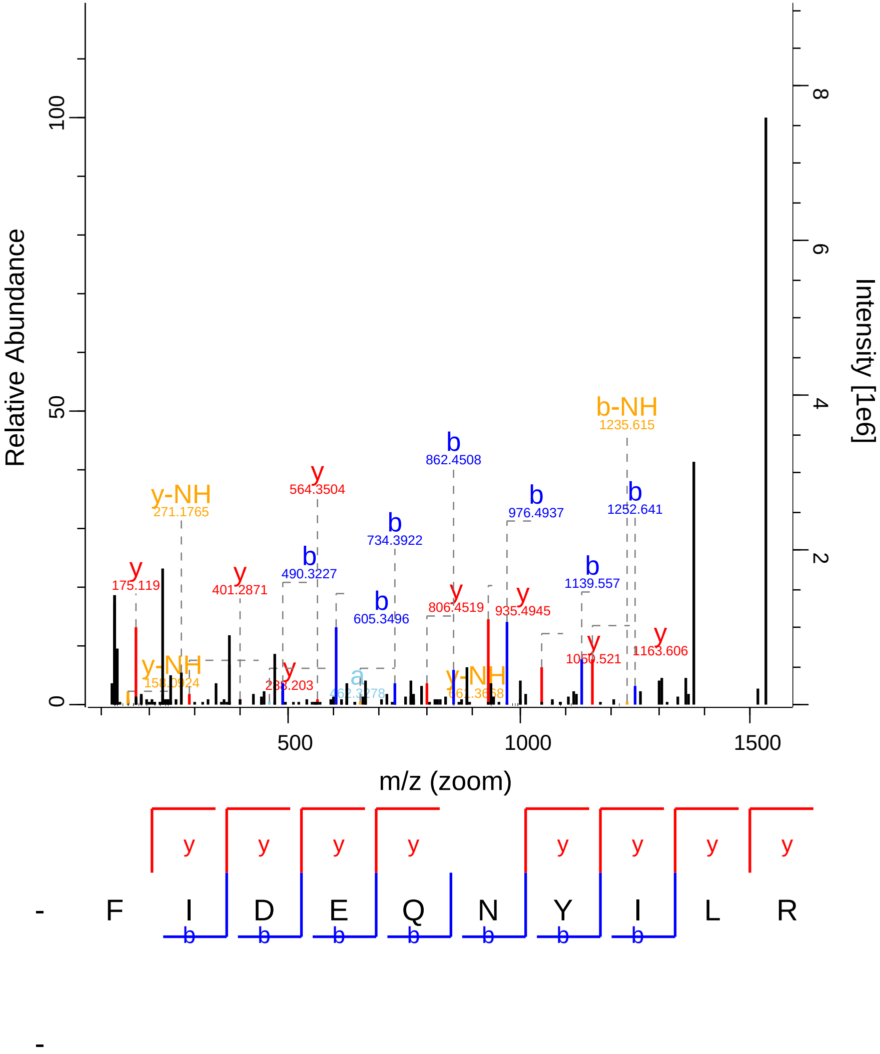

Raw File	Scan	Method	Score	m/z	Gene names
RO-QC-160121_ZackD_SS_Set_02_Fractio	28121	FTMS; HC	153.34	841.43	IL17D

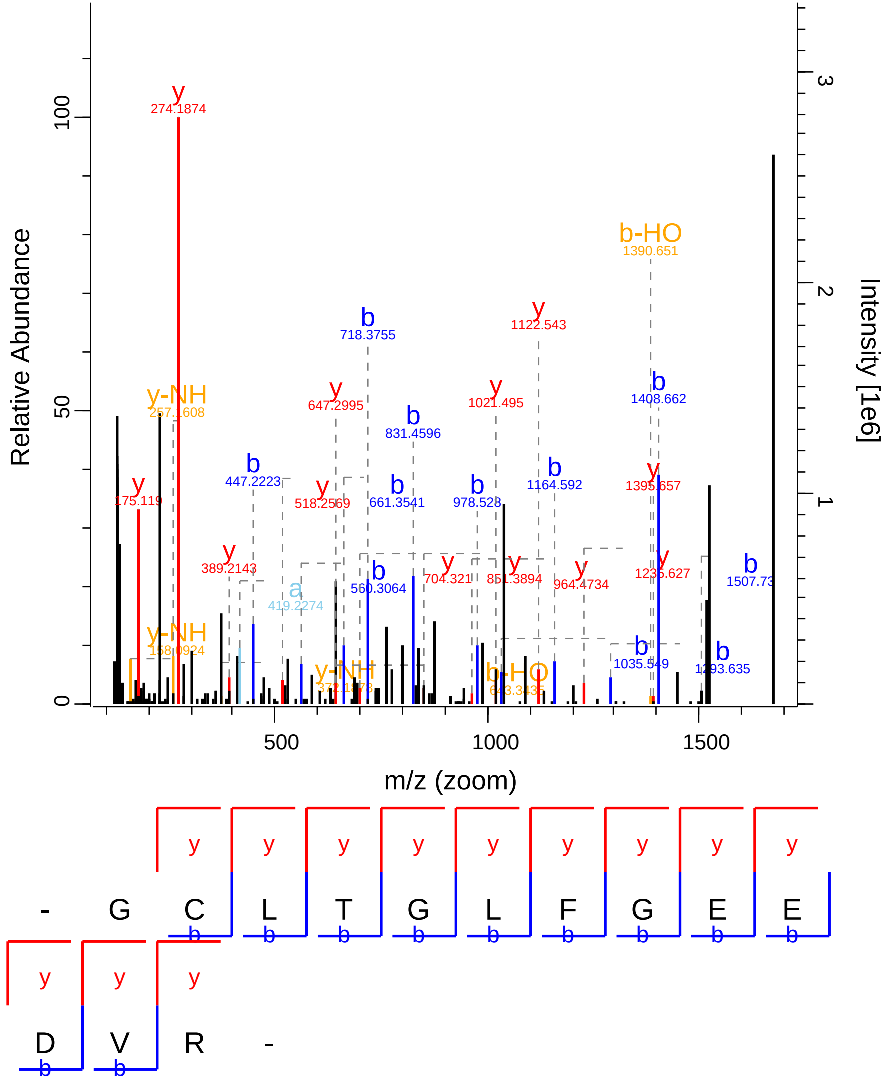

Raw File	Scan	Method	Score	m/z	Gene names
RO-QC-160121_ZackD_SS_Set_02_Fractio	28172	FTMS; HC	83.2	561.29	IL17D

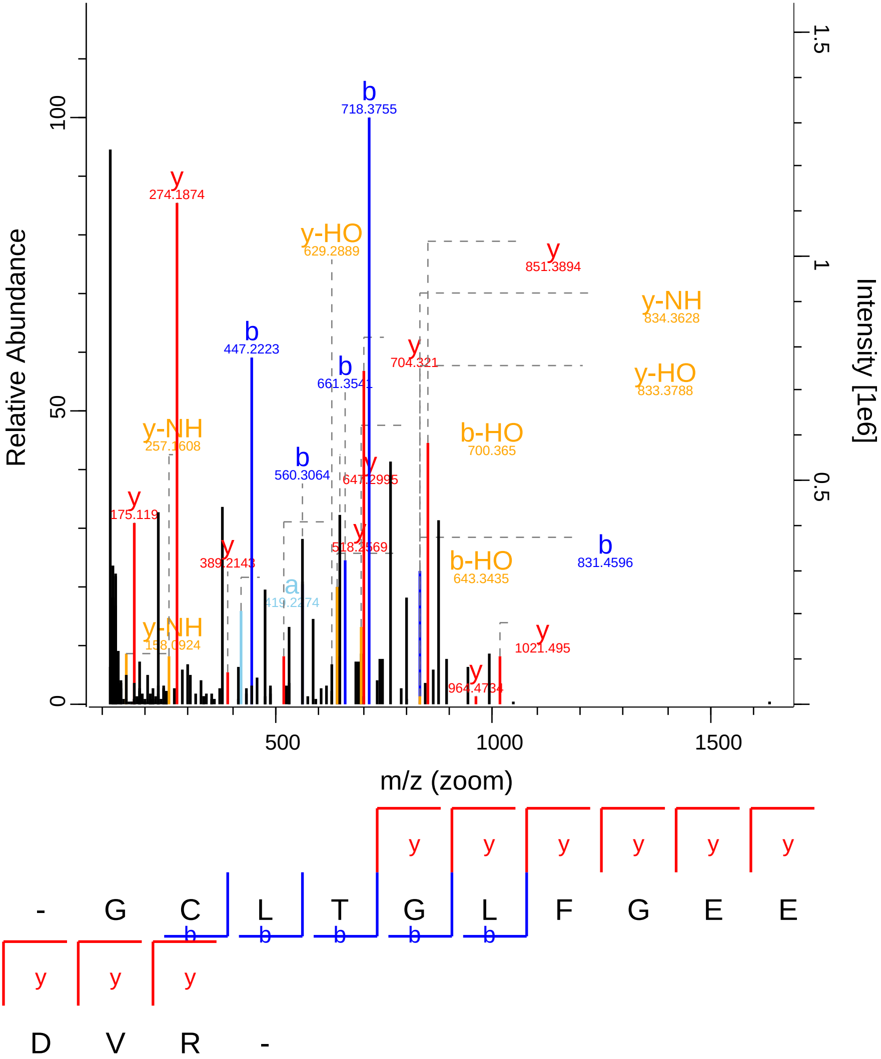

Raw File	Scan	Method	Score	m/z	Gene names
RO-QC-160121_ZackD_SS_Set_02_Fractio	28213	FTMS; HC	46.13	737.86	TRIL

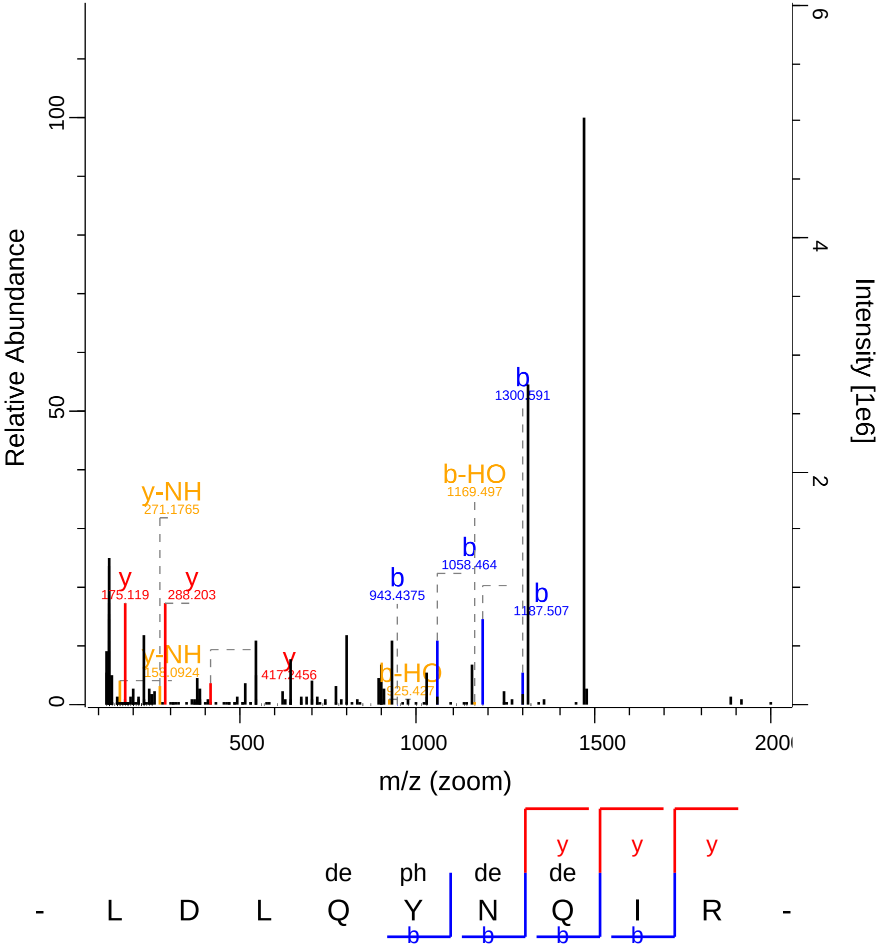

Raw File	Scan	Method	Score	m/z	Gene names
RO-QC-160121_ZackD_SS_Set_02_Fractio	28751	FTMS; HC	75.89	800.47	PRTFDC1

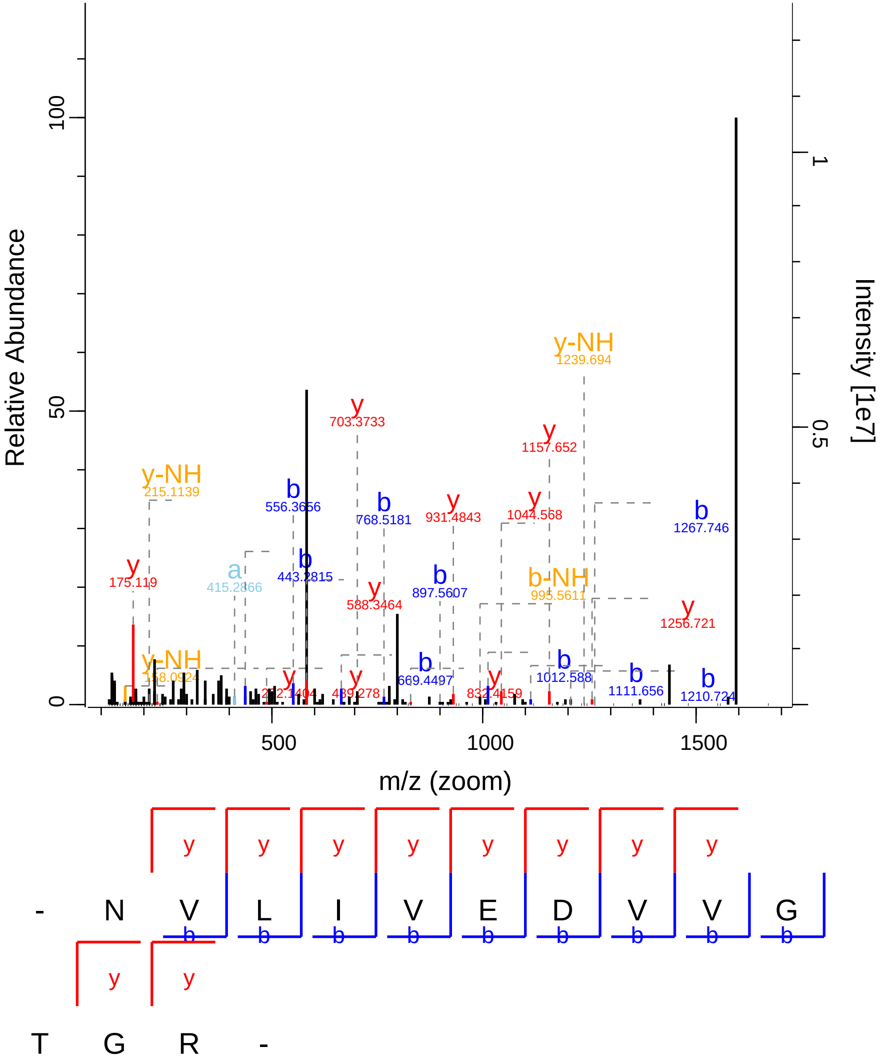

Raw File	Scan	Method	Score	m/z	Gene names
RO-QC-160121_ZackD_SS_Set_02_Fractio	28848	FTMS; HC	66.14	845.14	CABLES2

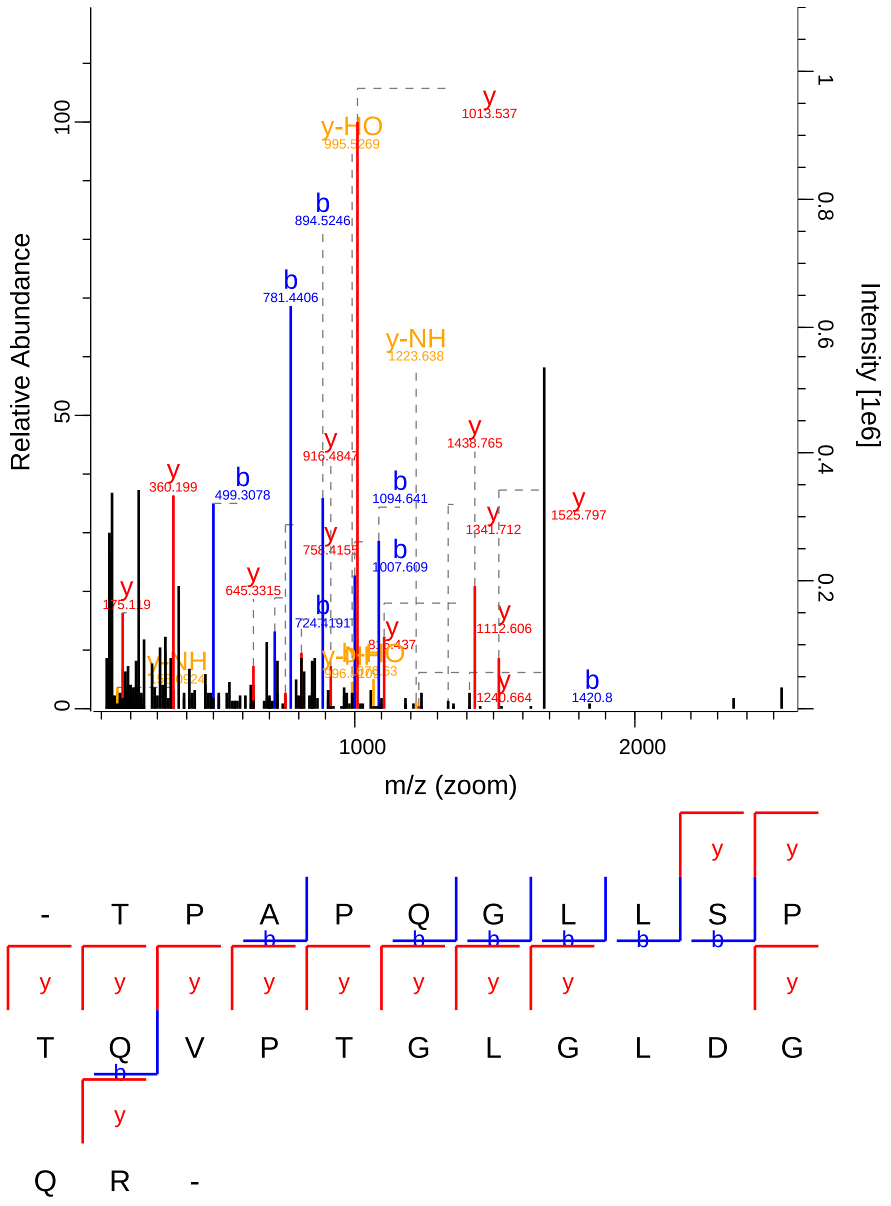

Raw File	Scan	Method	Score	m/z	Gene names
RO-QC-160121_ZackD_SS_Set_02_Fractio	28877	FTMS; HC	44.16	815.69	SLITRK4

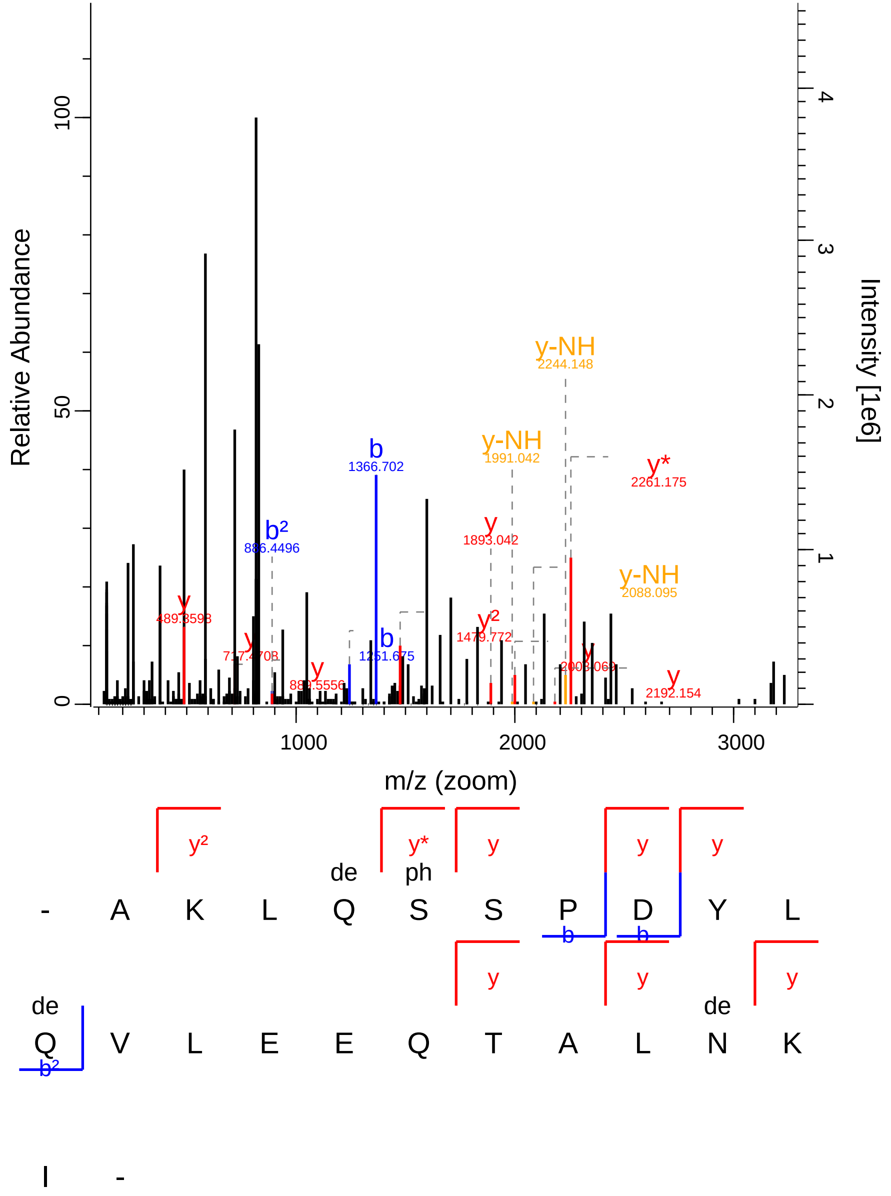

Raw File	Scan	Method	Score	m/z	Gene names
RO-QC-160121_ZackD_SS_Set_02_Fractio	30126	FTMS; HC	40.94	750.38	CLEC12A

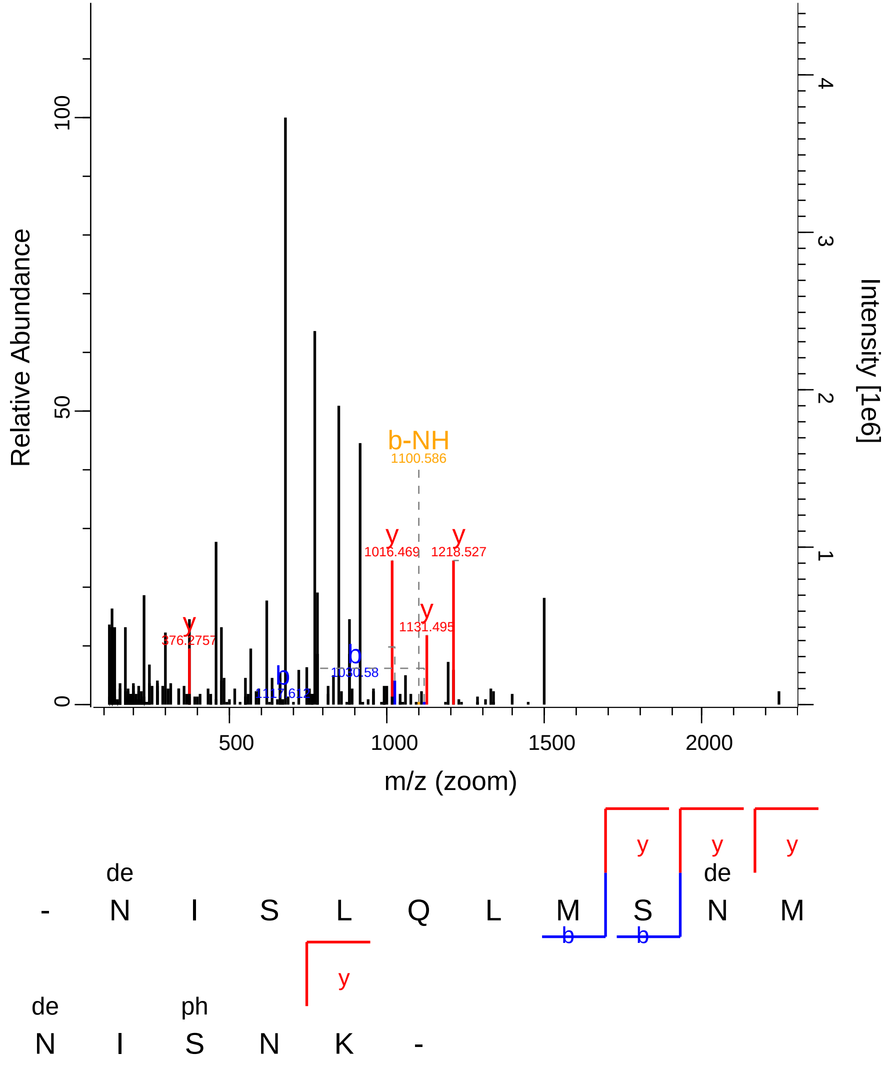

Raw File	Scan	Method	Score	m/z	Gene names
RO-QC-160121_ZackD_SS_Set_02_Fractic	30151	FTMS; HC	53.24	1125.06	CLEC12A

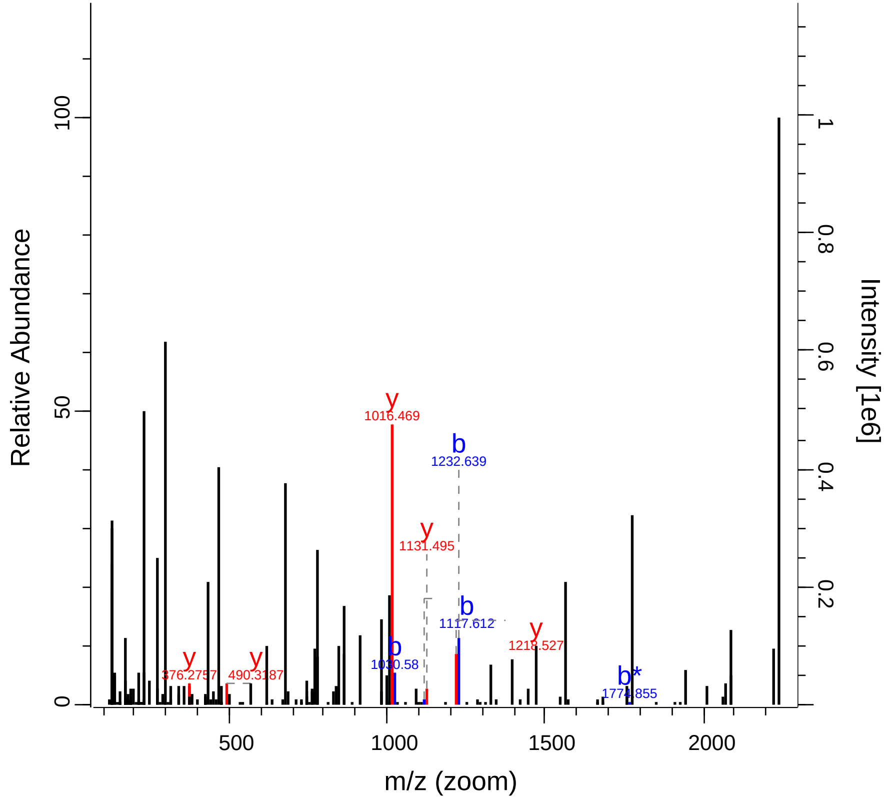

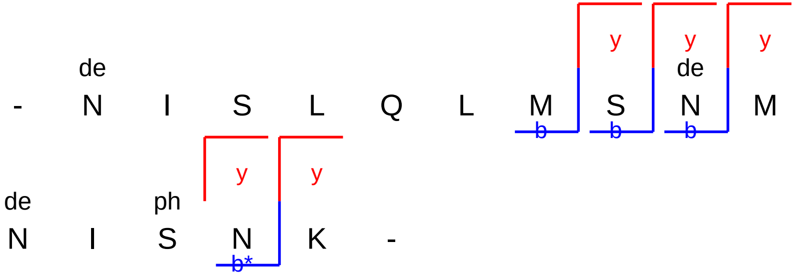

Raw File	Scan	Method	Score	m/z	Gene names
RO-QC-160121_ZackD_SS_Set_02_Fractio	30468	FTMS; HC	71.93	565.33	IGFALS

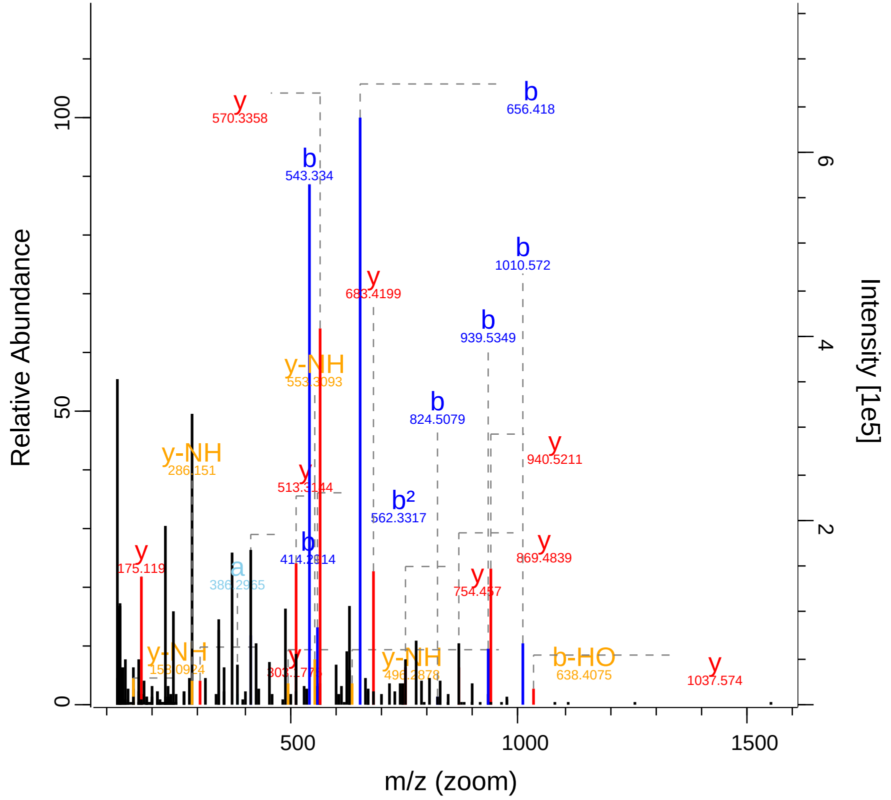

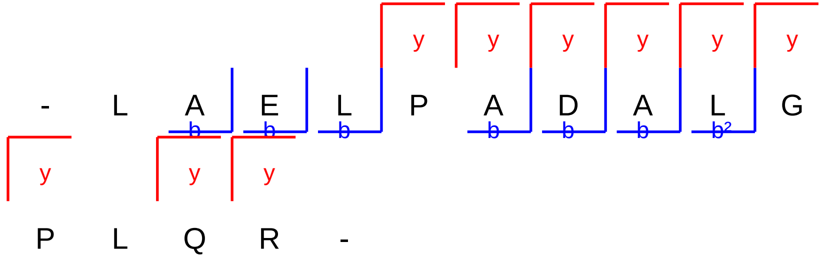

Raw File	Scan	Method	Score	m/z	Gene names
RO-QC-160121_ZackD_SS_Set_02_Fractio	30646	FTMS; HC	59.57	601.36	FAM184B

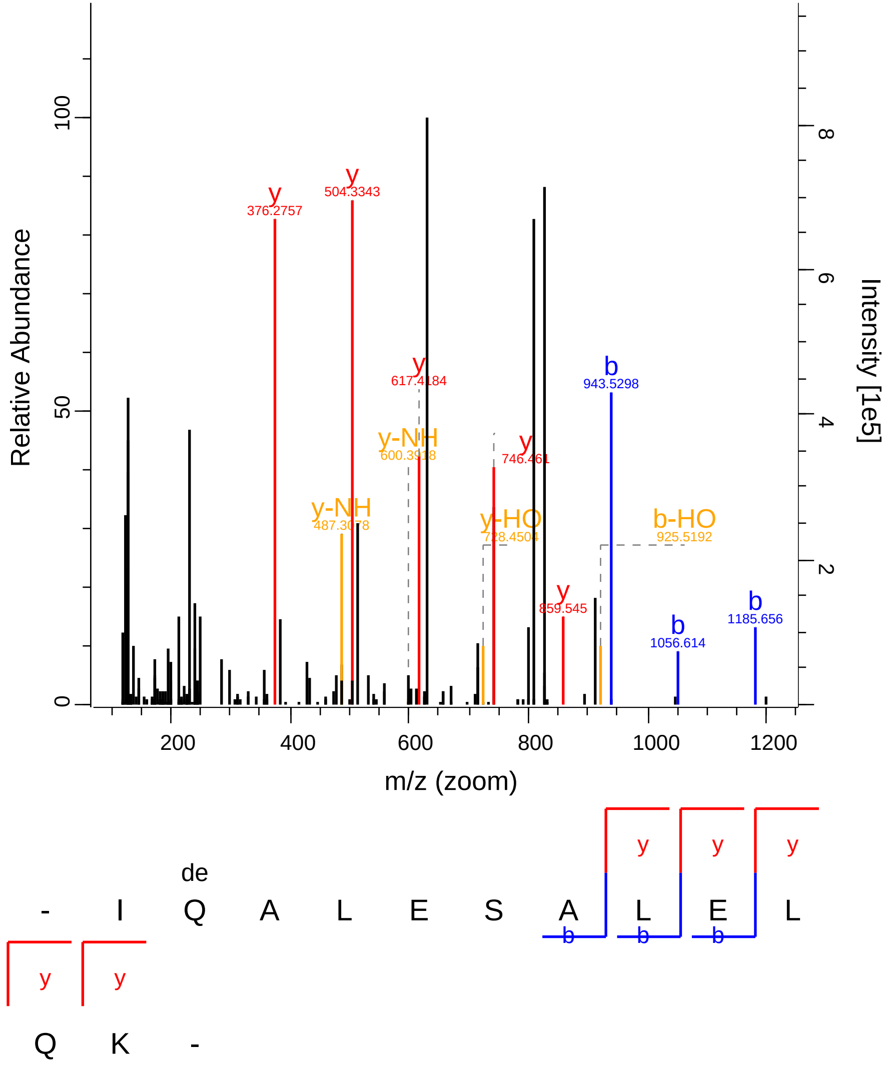

Raw File	Scan	Method	Score	m/z	Gene names
RO-QC-160121_ZackD_SS_Set_02_Fractio	30726	FTMS; HC	203.64	687.38	TMUB2

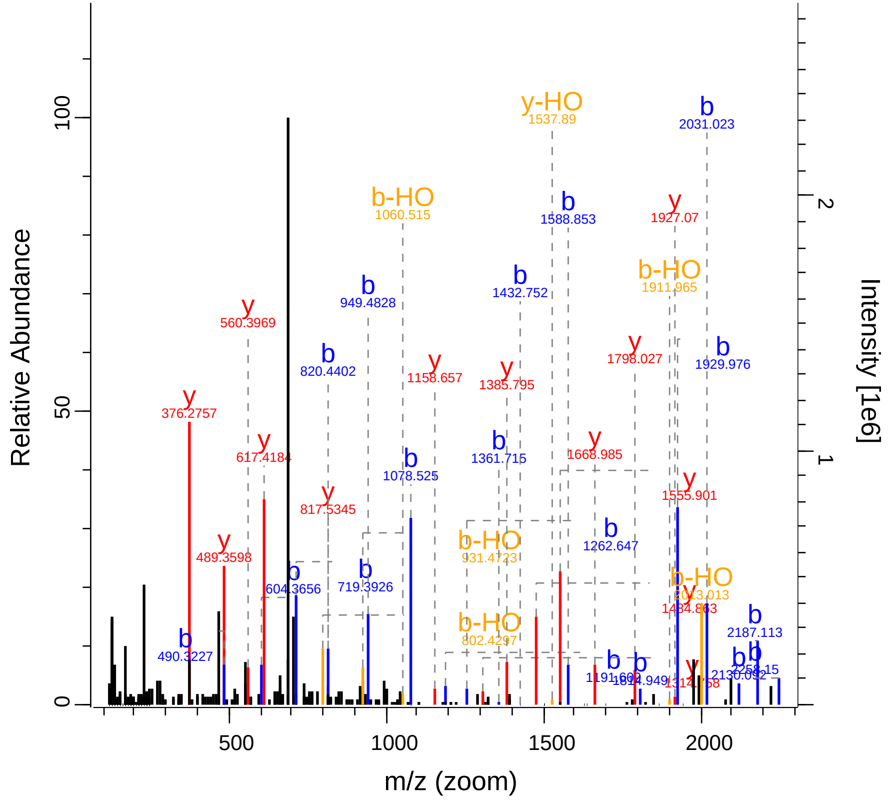

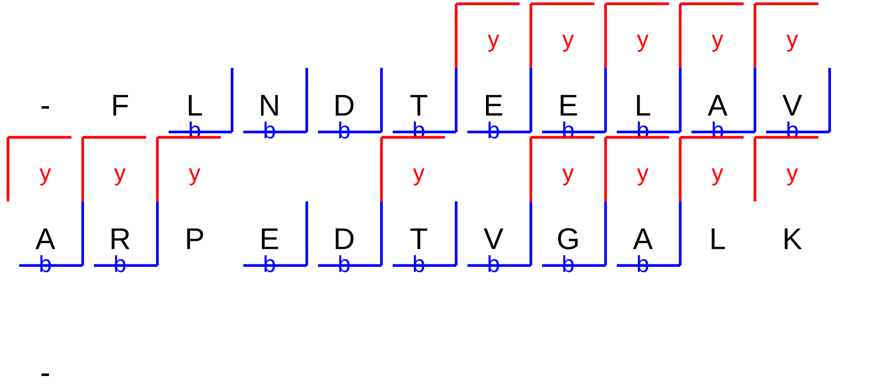

Raw File	Scan	Method	Score	m/z	Gene names
RO-QC-160121_ZackD_SS_Set_02_Fractio	31004	FTMS; HC	54.81	669.33	ABRACL

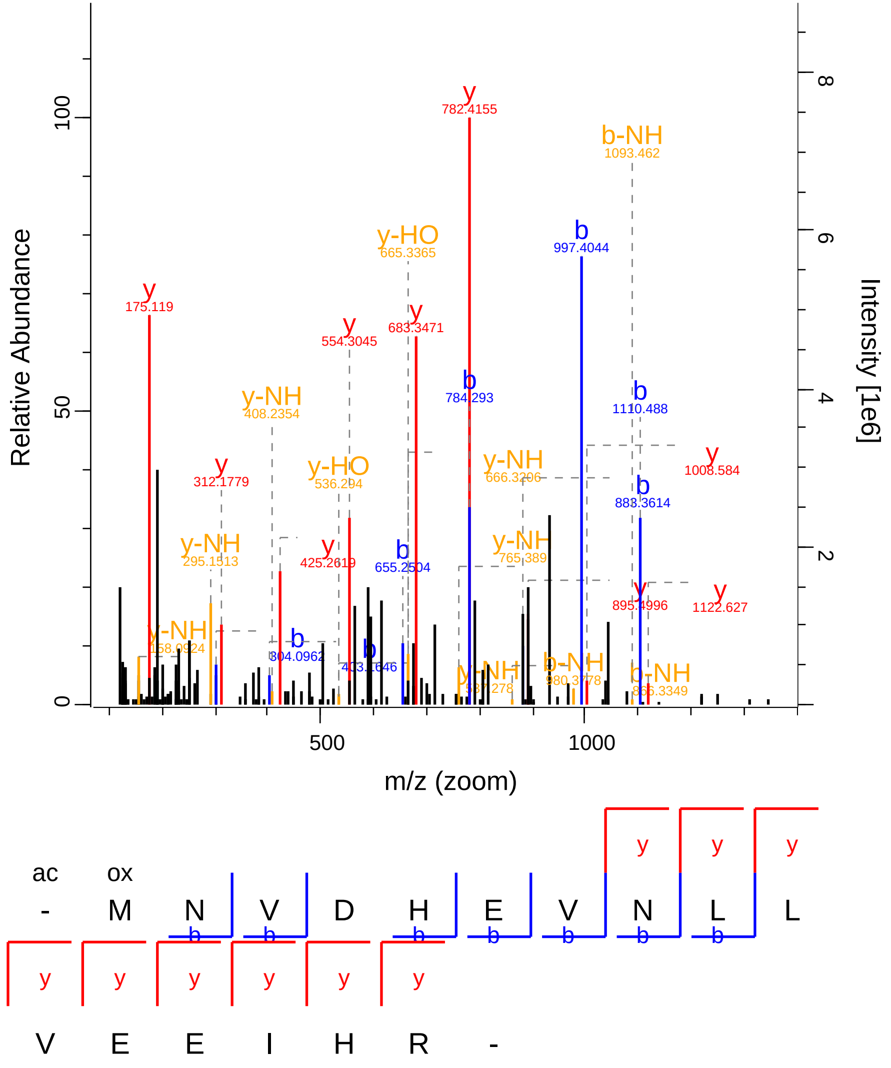

Raw File	Scan	Method	Score	m/z	Gene names
RO-QC-160121_ZackD_SS_Set_02_Fractio	31199	FTMS; HC	46.32	669.33	ABRACL

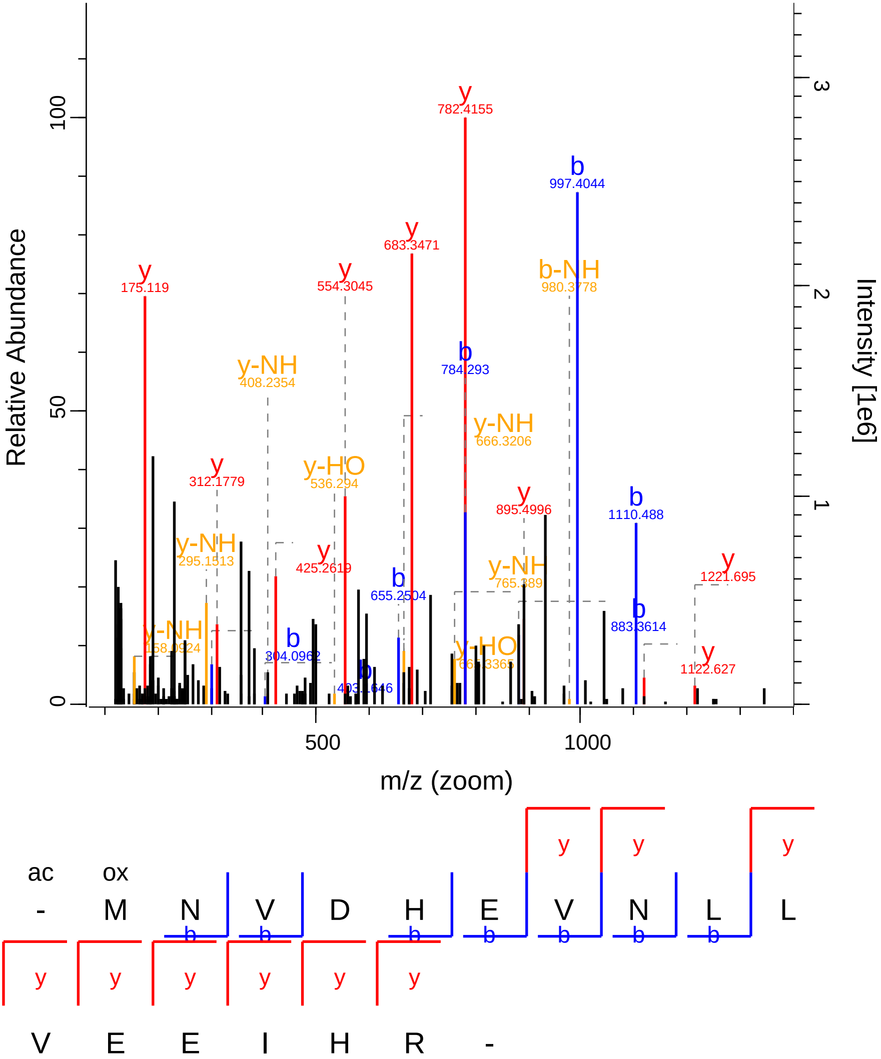

Raw File	Scan	Method	Score	m/z	Gene names
RO-QC-160121_ZackD_SS_Set_02_Fractio	31519	FTMS; HC	45.37	919.13	FOXC2

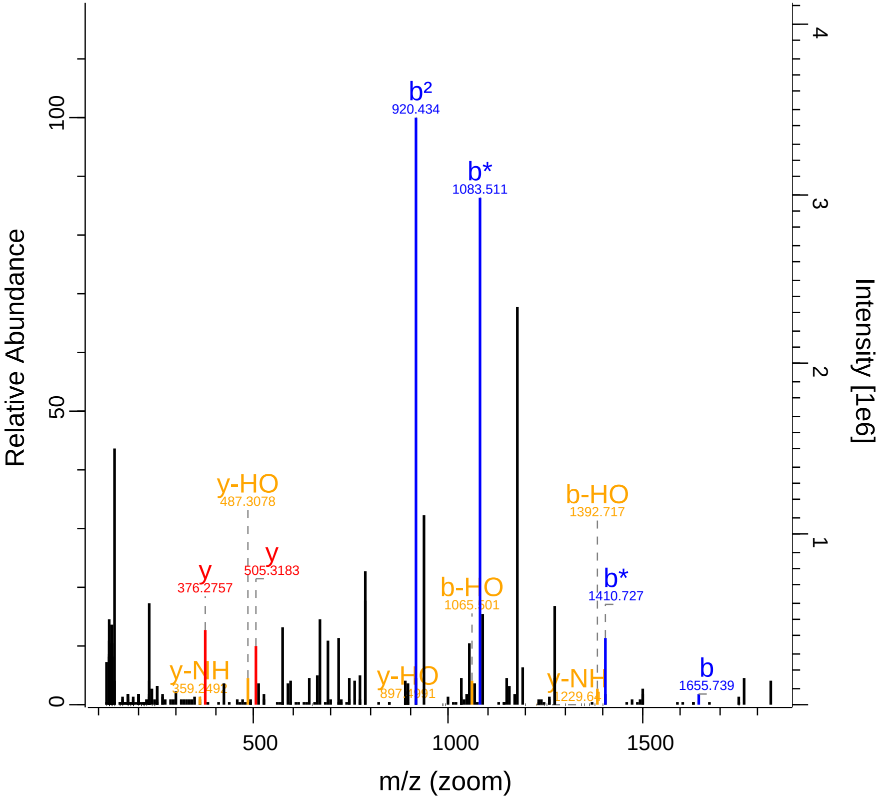

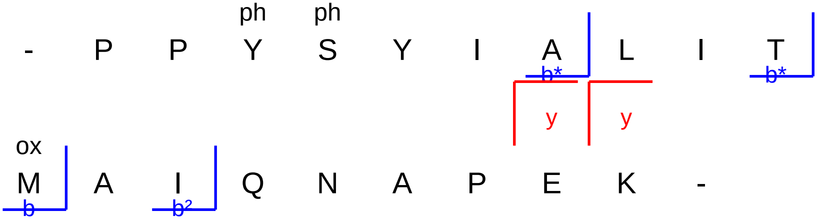

Raw File	Scan	Method	Score	m/z	Gene names
RO-QC-160121_ZackD_SS_Set_02_Fractio	31938	FTMS; HC	80.1	541.31	SYNGAP1

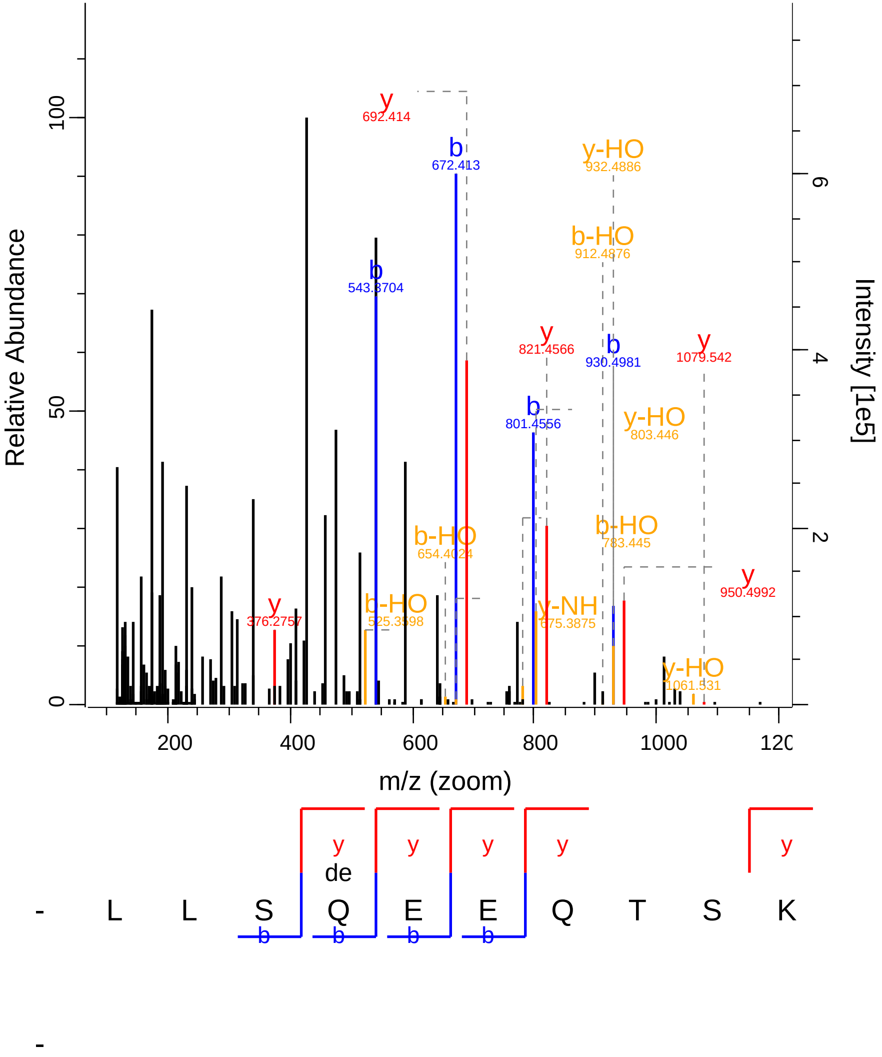

Raw File	Scan	Method	Score	m/z	Gene names
RO-QC-160121_ZackD_SS_Set_02_Fractior	33631	FTMS; HCI	84.25	737.4	MON1A

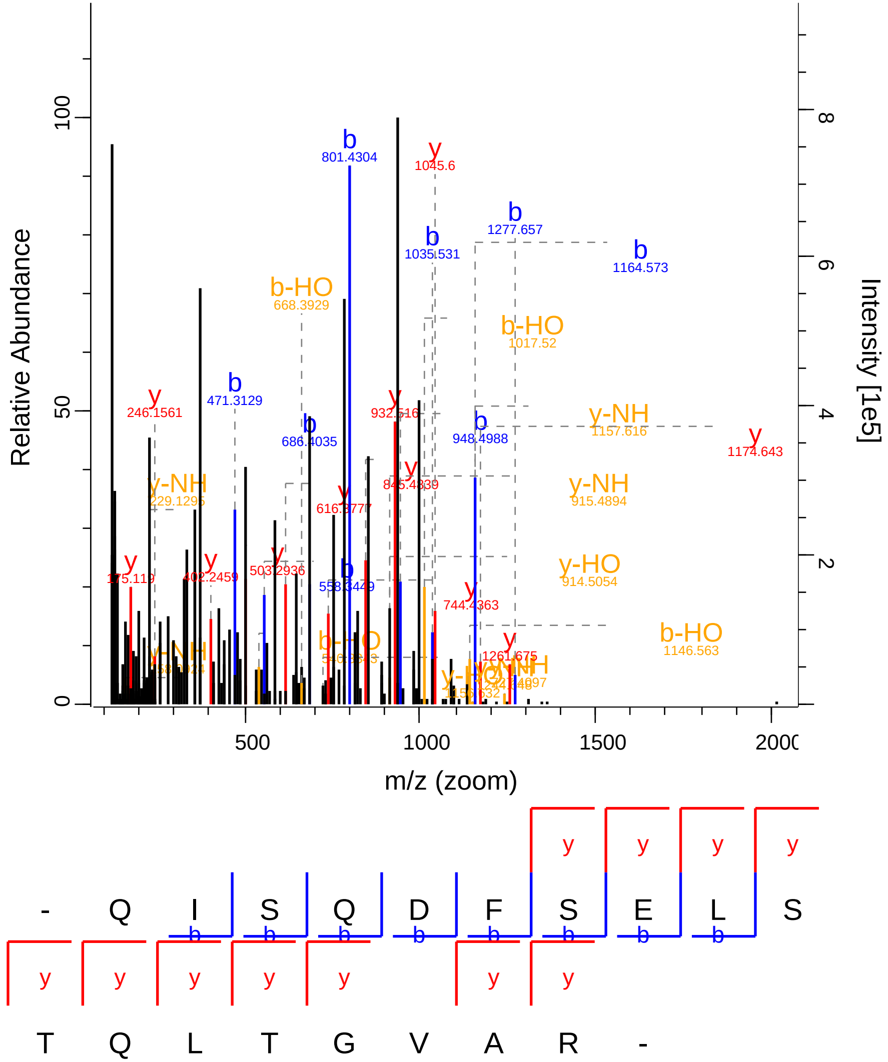

Raw File	Scan	Method	Score	m/z	Gene names
RO-QC-160121_ZackD_SS_Set_02_Fractio	33743	FTMS; HC	80.13	801.68	FAM122A

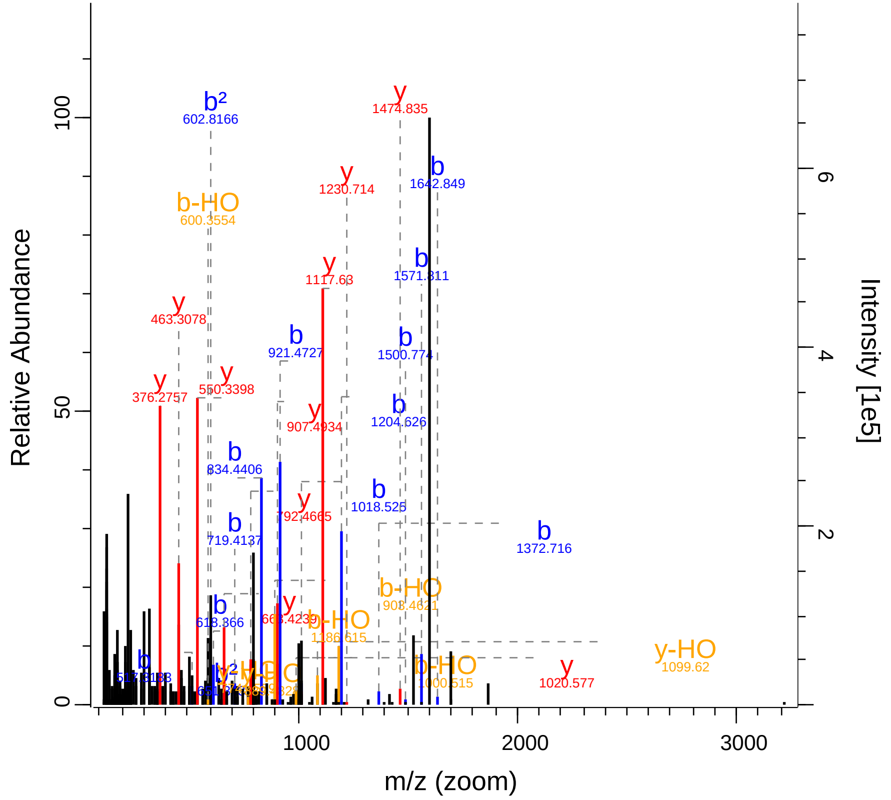

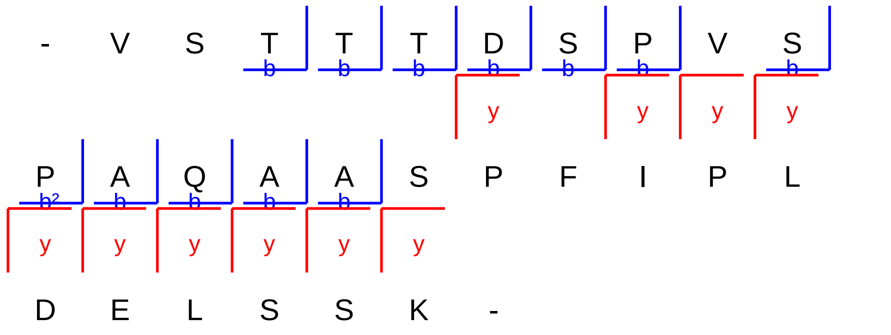

Raw File	Scan	Method	Score	m/z	Gene names
RO-QC-160121_ZackD_SS_Set_02_Fractio	33791	FTMS; HC	74.68	620.36	WNT11

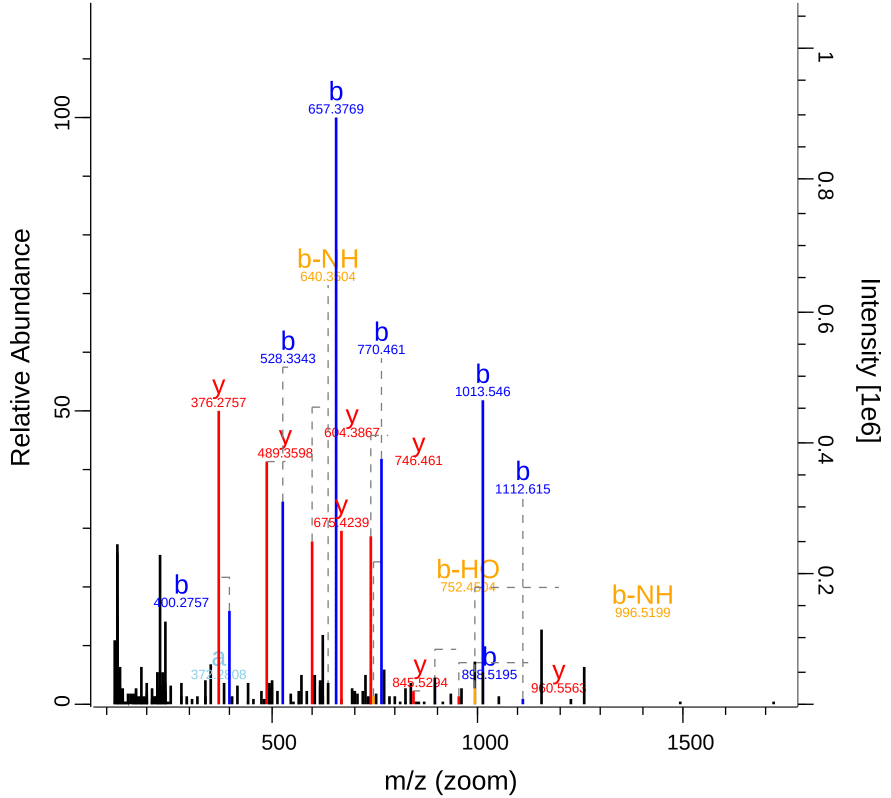

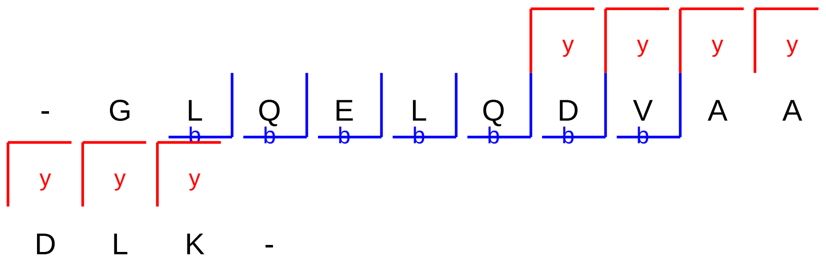

Raw File	Scan	Method	Score	m/z	Gene names
RO-QC-160121_ZackD_SS_Set_02_Fractic	33864	FTMS; HC	148.87	1068.57	FAM122A

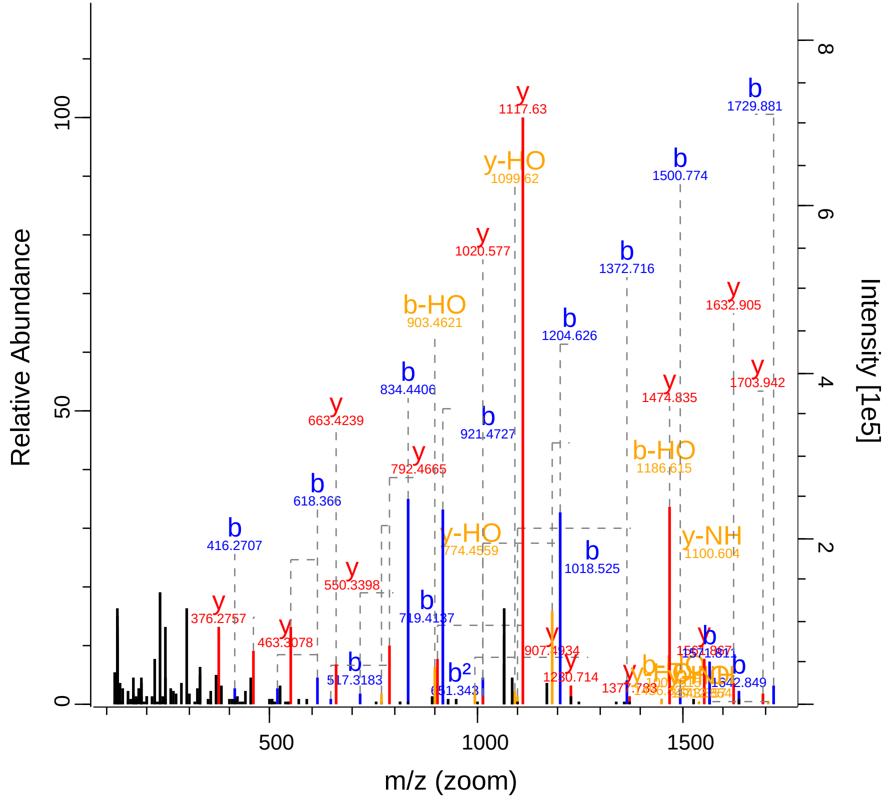

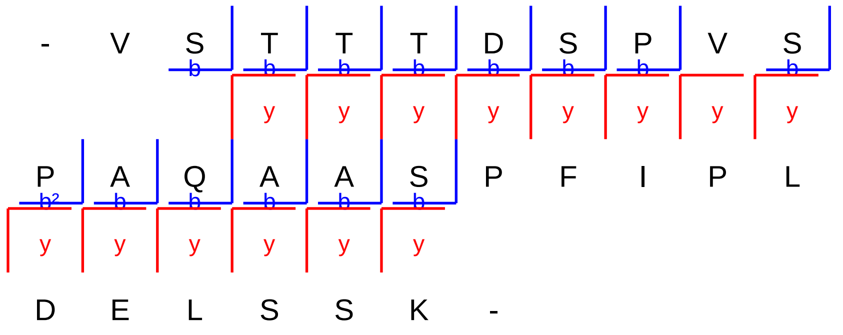

Raw File	Scan	Method	Score	m/z
RO-QC-160121_ZackD_SS_Set_02_Fraction_4	34751	FTMS; HCD	51.46	1280.6

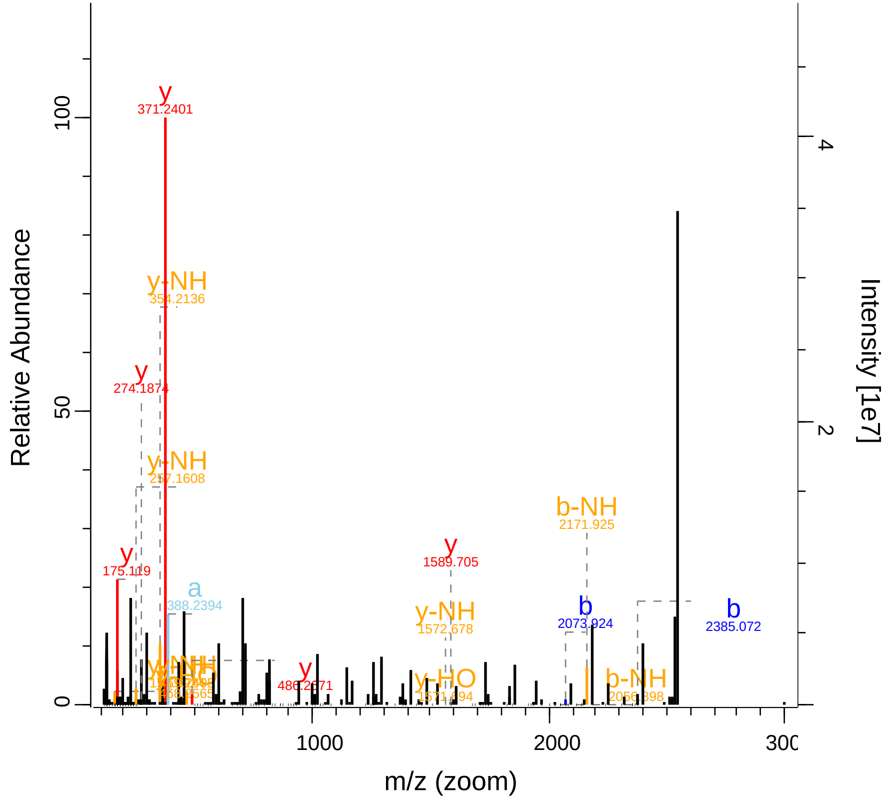

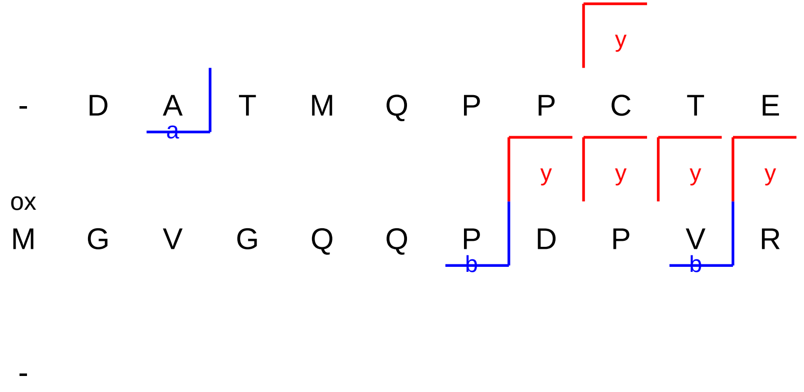

Raw File	Scan	Method	Score	m/z	Gene names
RO-QC-160121_ZackD_SS_Set_02_Fractio	34822	FTMS; HC	134.18	996.05	LRIG1

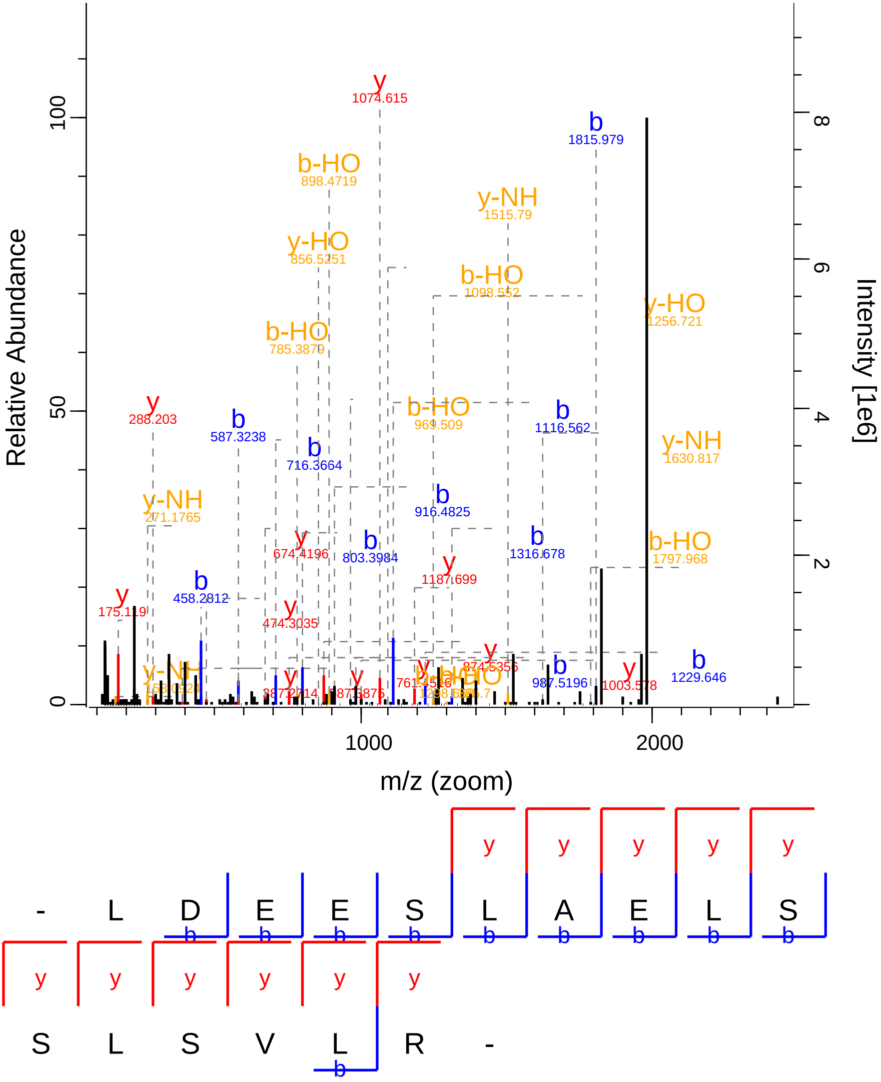

Raw File	Scan	Method	Score	m/z
RO-QC-160121_ZackD_SS_Set_02_Fraction_4	35233	FTMS; HCD	62.2	548.68

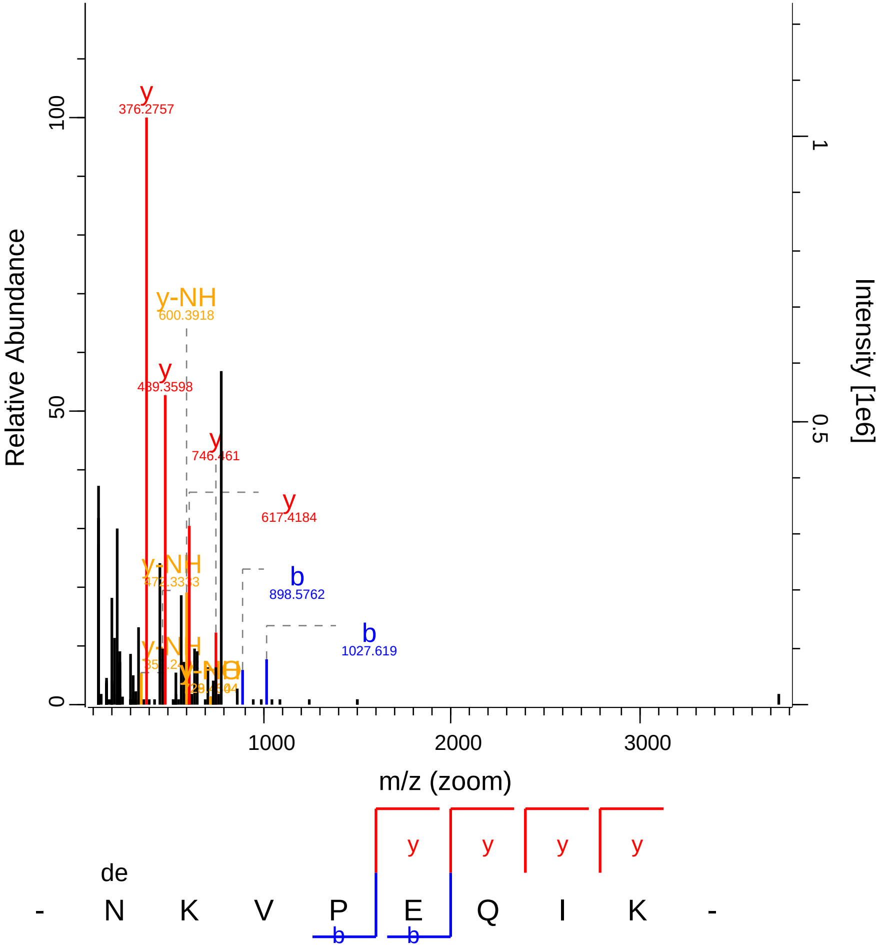

Raw File	Scan	Method	Score	m/z
RO-QC-160121_ZackD_SS_Set_02_Fraction_4	35524	FTMS; HCD	60.96	625.37

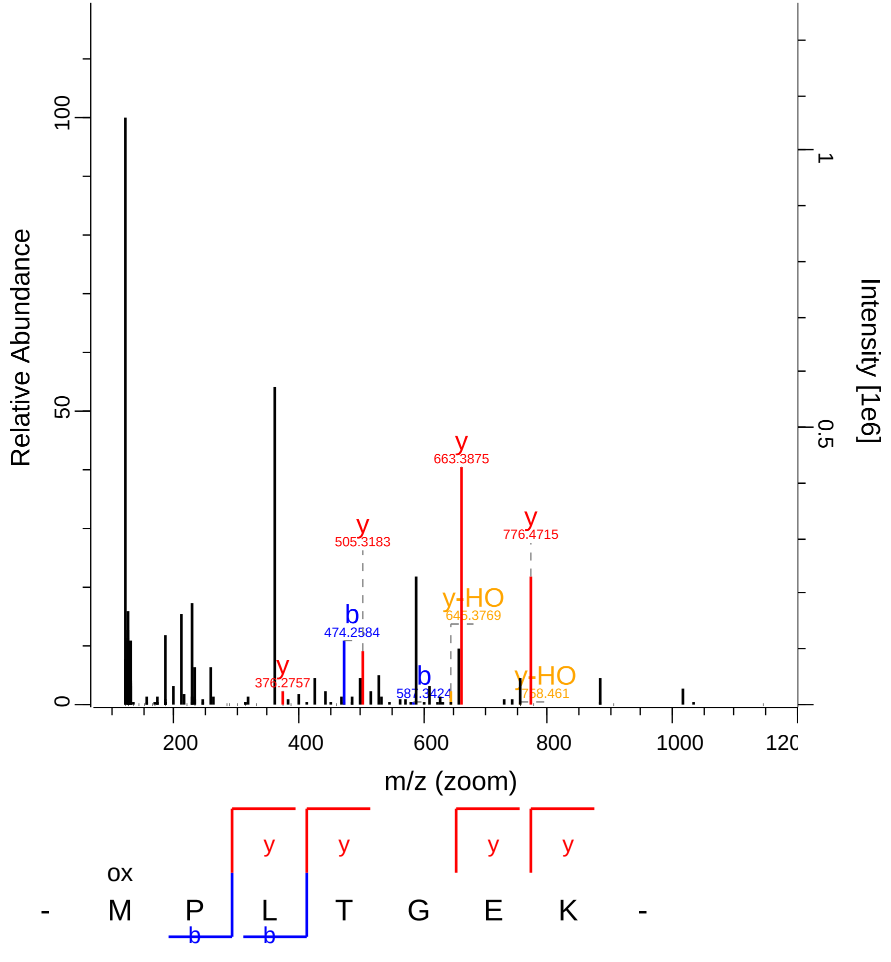

Raw File	Scan	Method	Score	m/z	Gene names
RO-QC-160121_ZackD_SS_Set_02_Fractior	5023	FTMS; HCI	92.92	723.39	SLC30A8

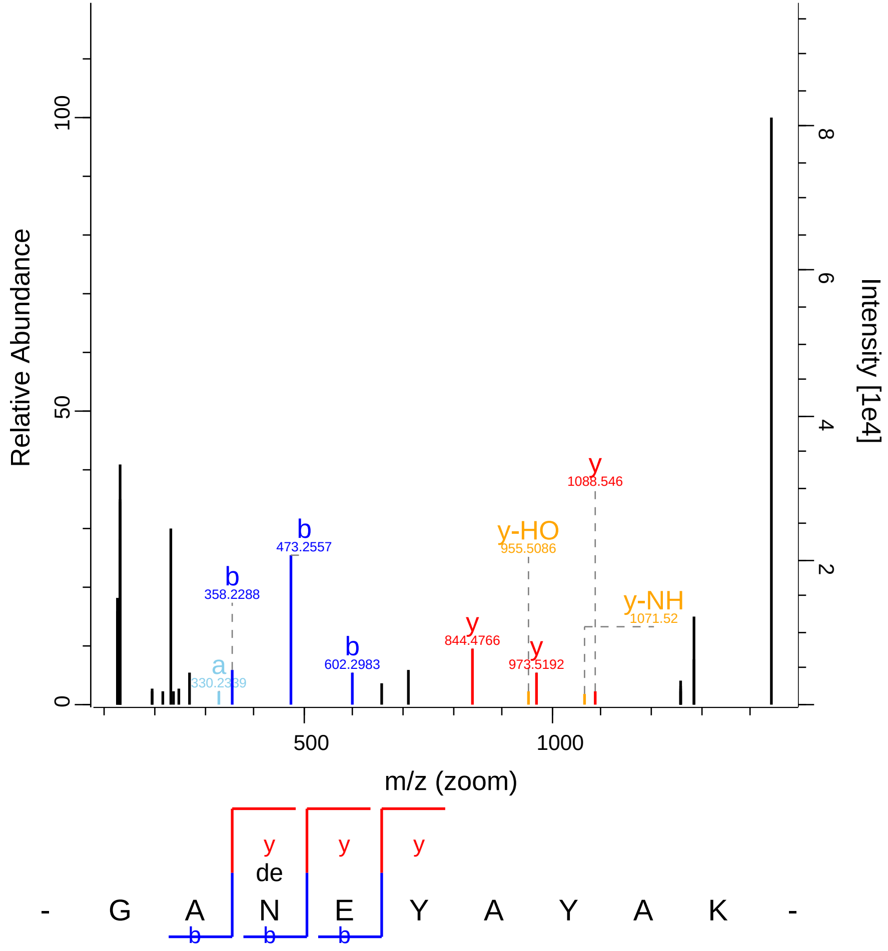

Raw File	Scan	Method	Score	m/z	Gene names
RO-QC-160121_ZackD_SS_Set_02_Fractior	5073	FTMS; HCI	99.75	577.29	SPAG5

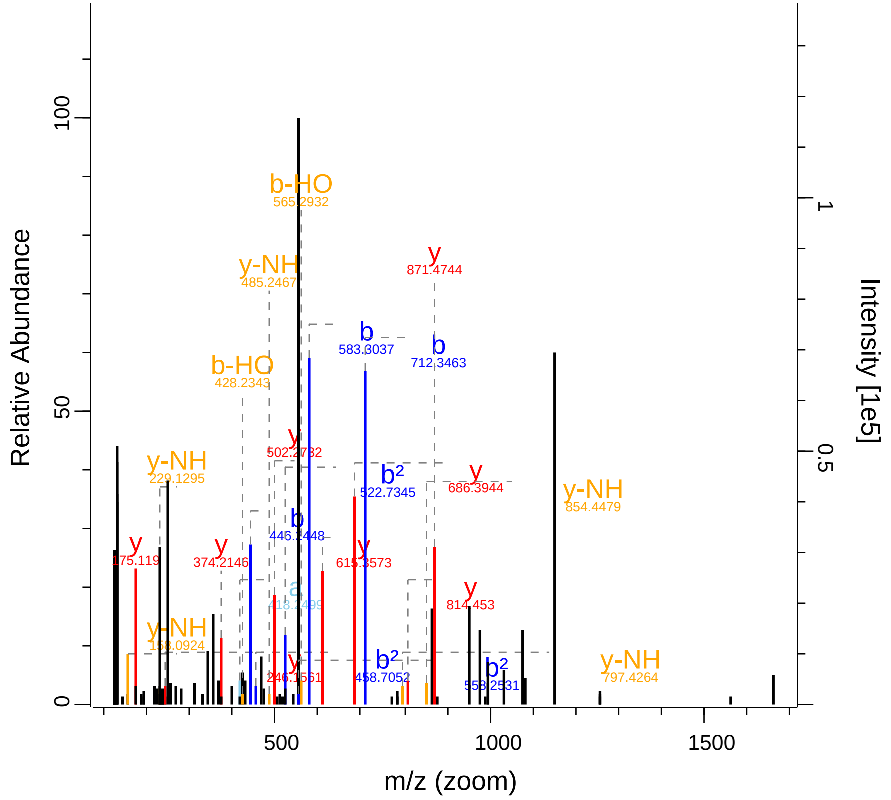

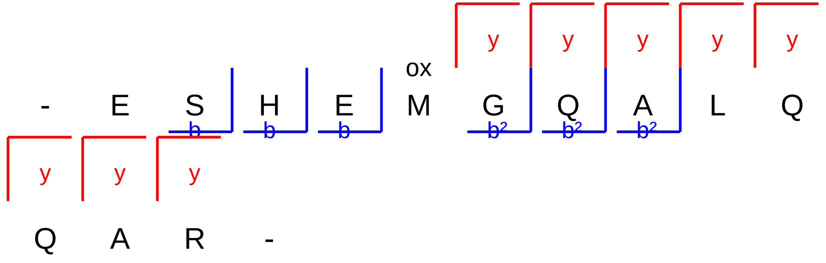

Raw File	Scan	Method	Score	m/z	Gene names
RO-QC-160121_ZackD_SS_Set_02_Fractioı	5238	FTMS; HC	105.25	577.29	SPAG5

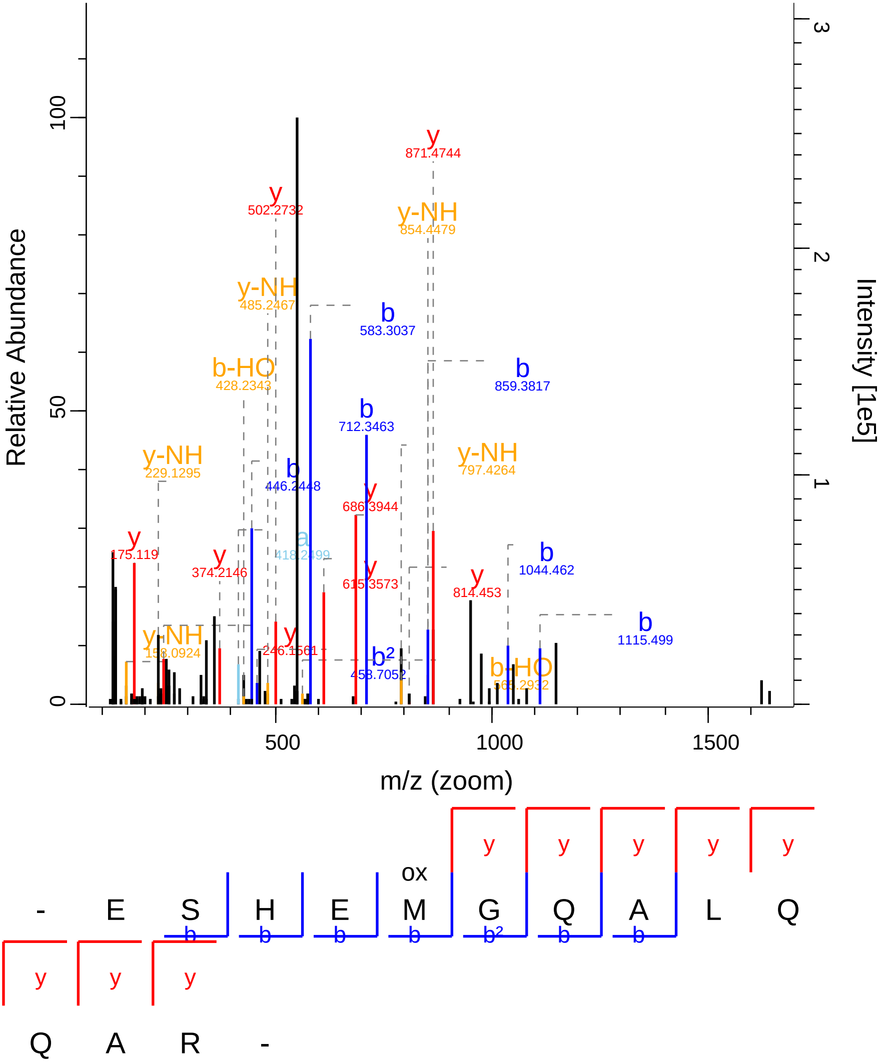

Raw File	Scan	Method	Score	m/z	Gene names
RO-QC-160121_ZackD_SS_Set_02_Fractio	5628	FTMS; HC	175.44	621.34	FAM89A

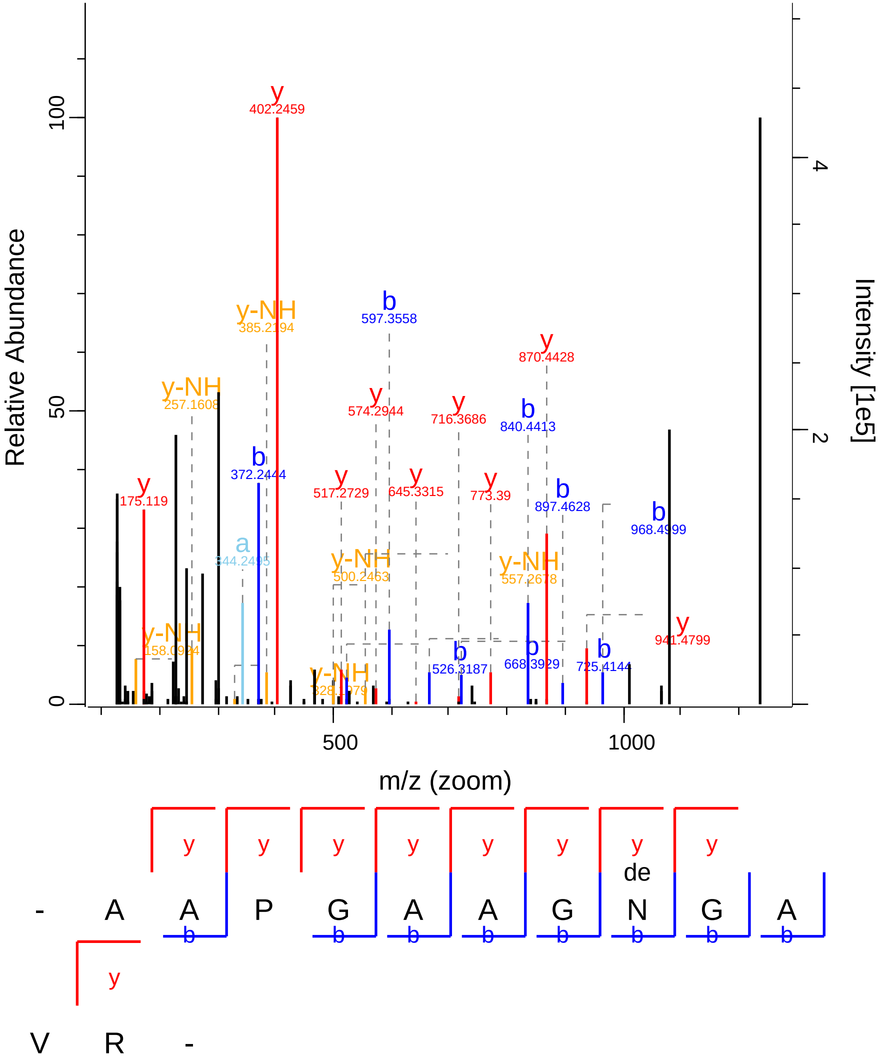

Raw File	Scan	Method	Score	m/z	Gene names
RO-QC-160121_ZackD_SS_Set_02_Fractior	5757	FTMS; HCI	93.11	367.42	GTF3A

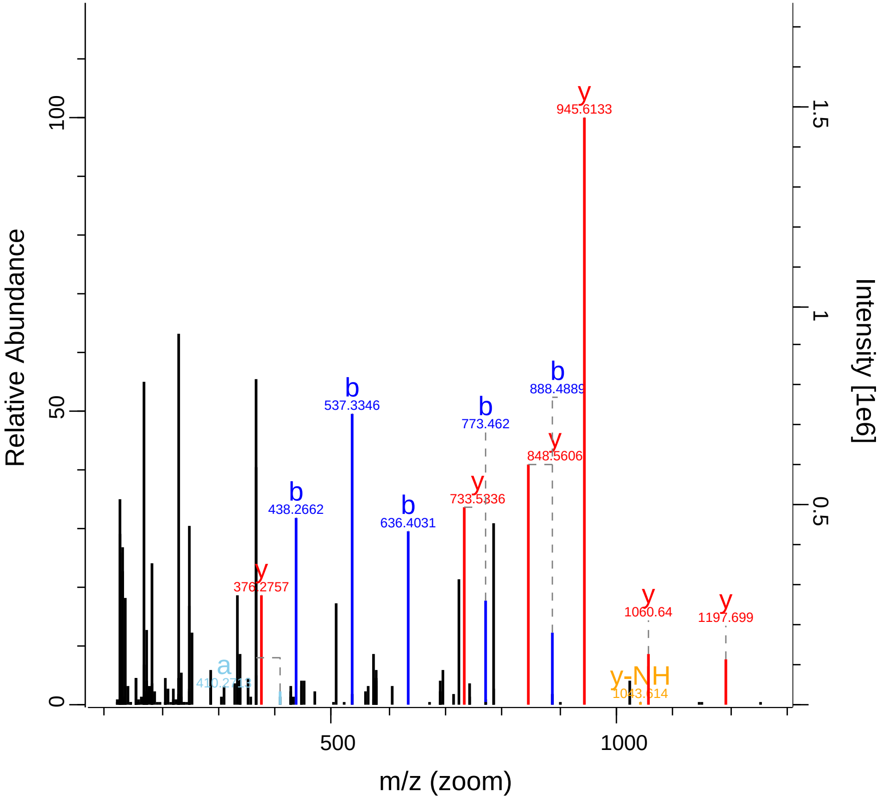

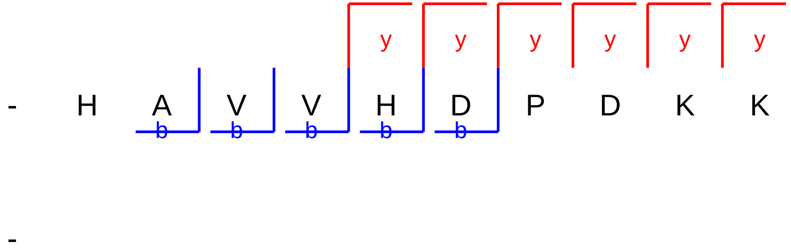

Raw File	Scan	Method	Score	m/z	Gene names
RO-QC-160121_ZackD_SS_Set_02_Fractior	6144	FTMS; HCI	67.56	407.97	CBX2

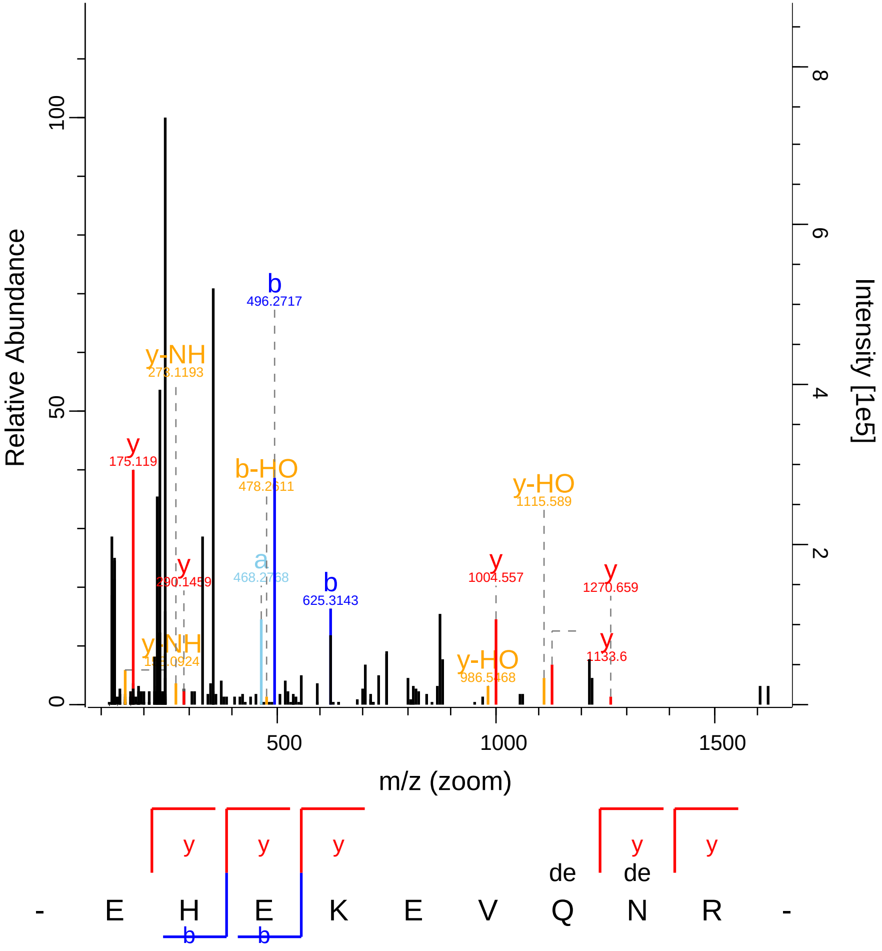

Raw File	Scan	Method	Score	m/z	Gene names
RO-QC-160121_ZackD_SS_Set_02_Fractior	6381	FTMS; HCI	81.31	658.01	KIF26B

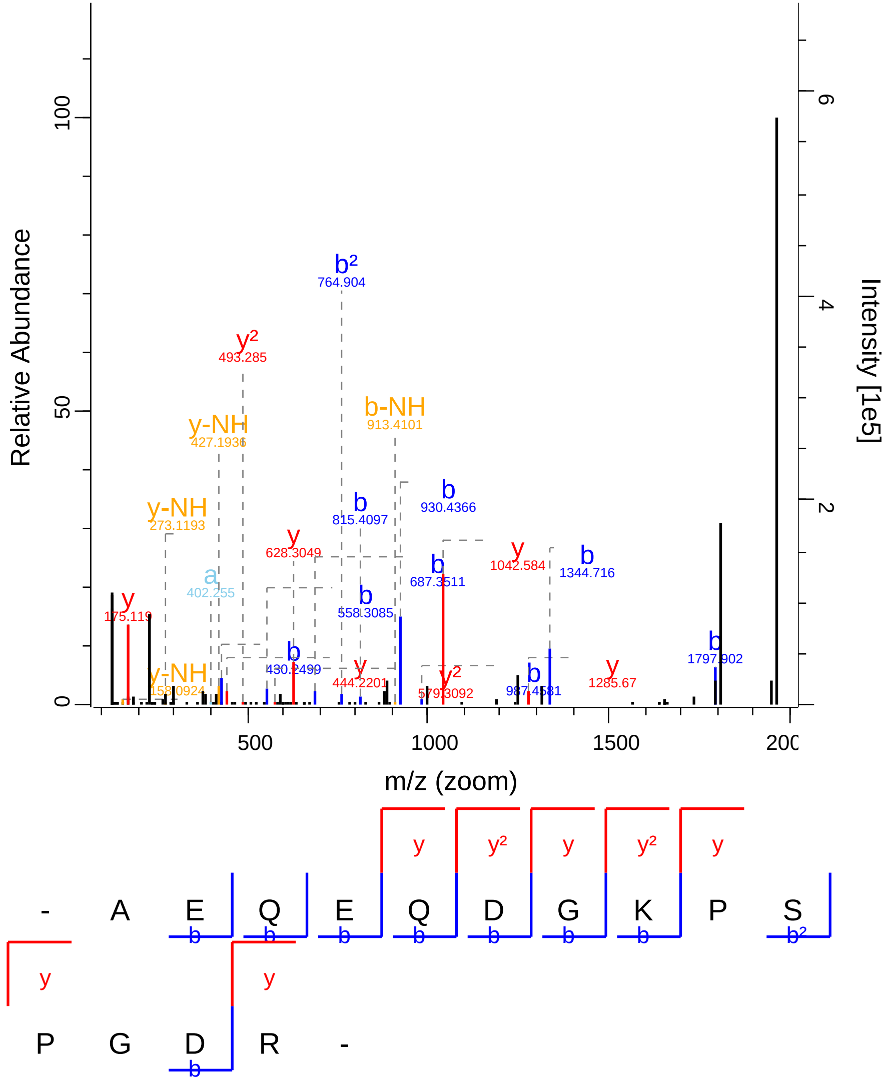

Raw File	Scan	Method	Score	m/z	Gene names
RO-QC-160121_ZackD_SS_Set_02_Fractior	6661	FTMS; HCI	99.52	539.61	SEMA5A

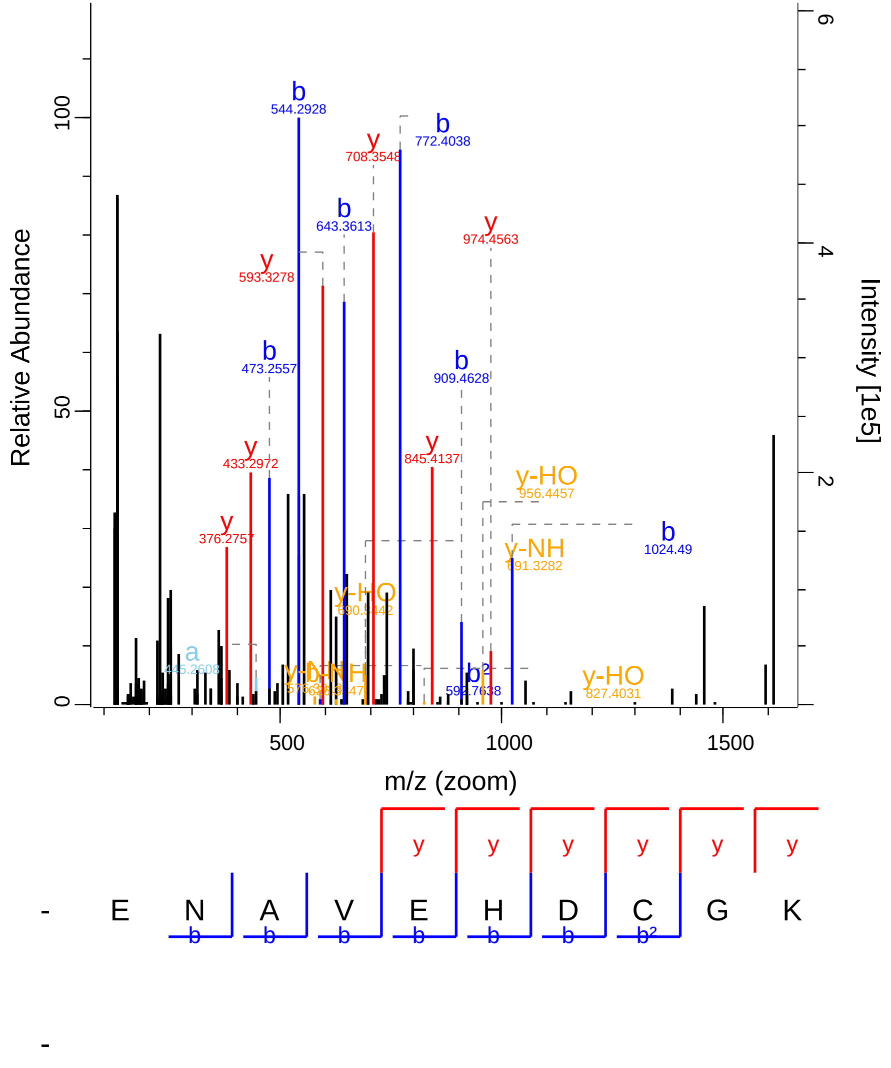

Raw File	Scan	Method	Score	m/z	Gene names
RO-QC-160121_ZackD_SS_Set_02_Fractioı	6823	FTMS; HC	161.21	646.04	UTP23

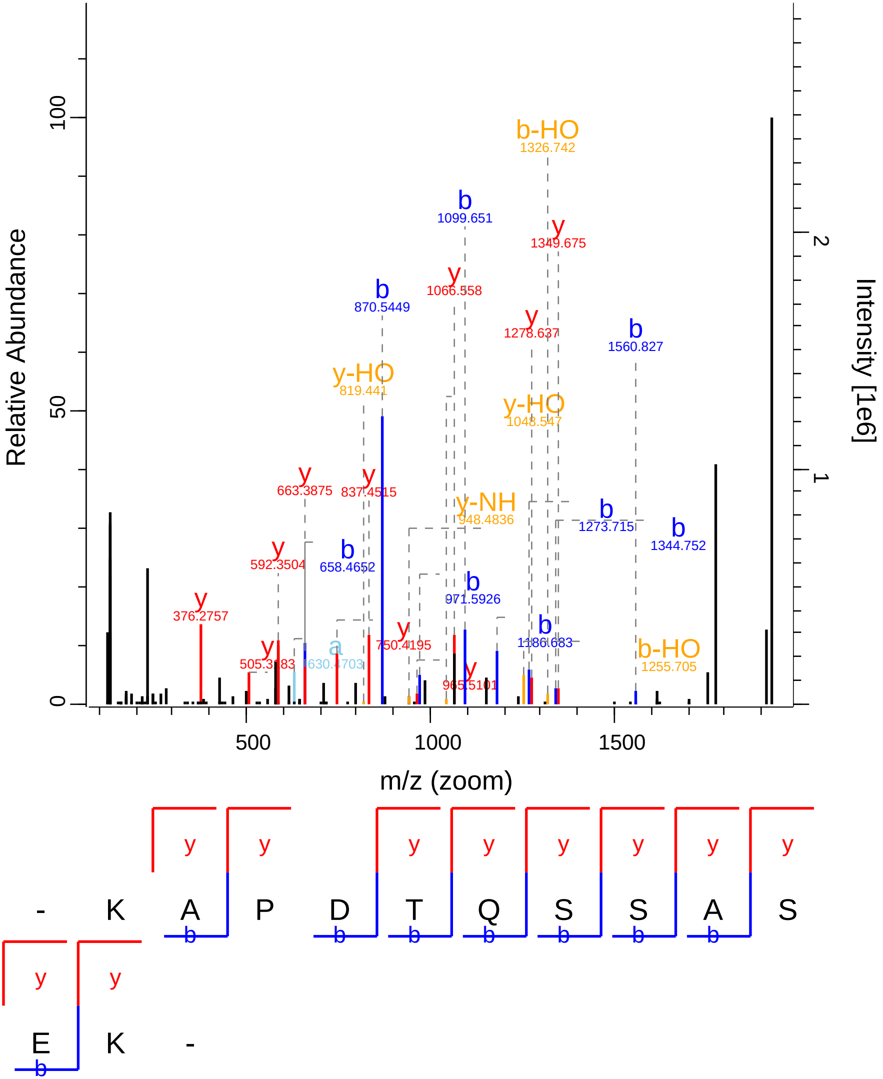

Raw File	Scan	Method	Score	m/z	Gene names
RO-QC-160121_ZackD_SS_Set_02_Fractior	7114	FTMS; HCI	69.95	652.33	NIPAL3

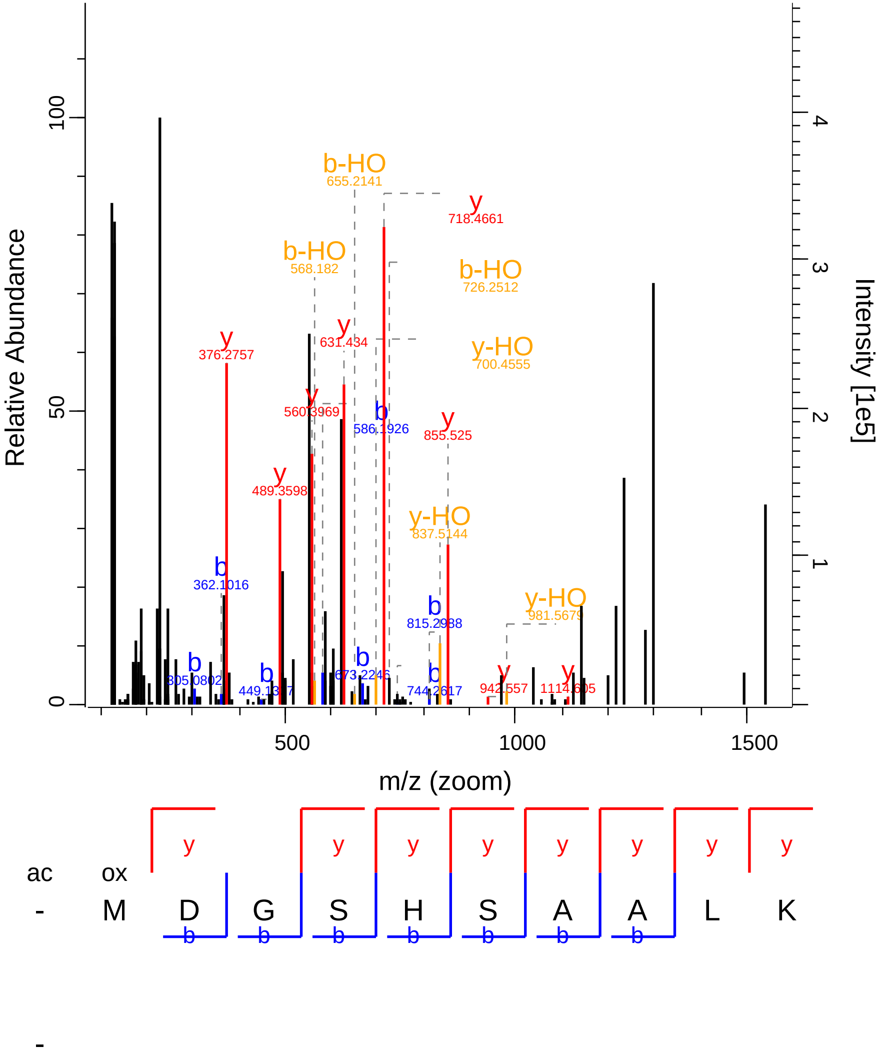

Raw File	Scan	Method	Score	m/z	Gene names
RO-QC-160121_ZackD_SS_Set_02_Fractio	10291	FTMS; HC	69.44	633.84	CFHR2

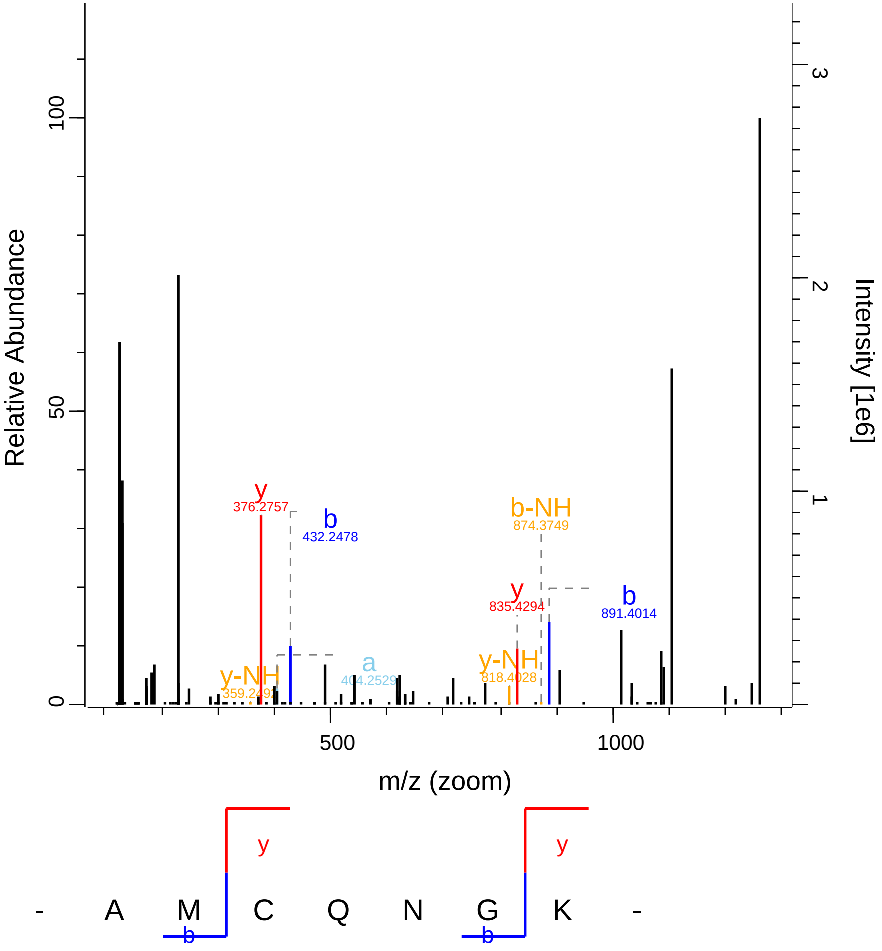

Raw File	Scan	Method	Score	m/z	Gene names
RO-QC-160121_ZackD_SS_Set_02_Fractio	10685	FTMS; HC	145.89	397.48	ZFP64

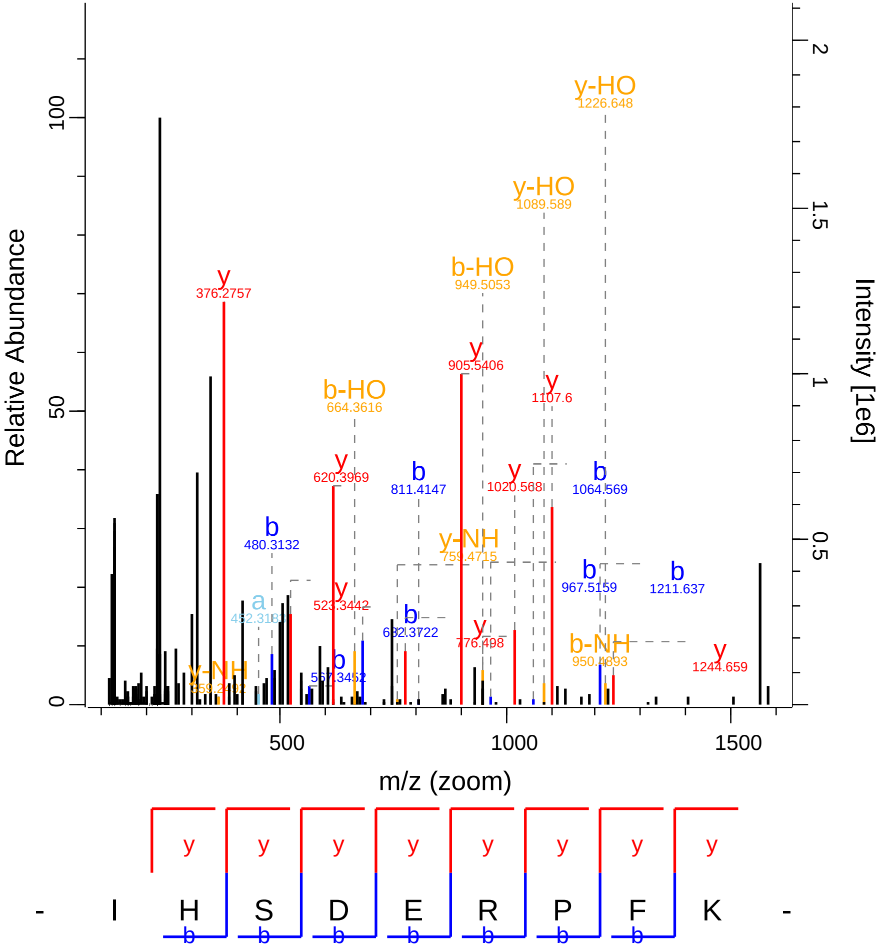

Raw File	Scan	Method	Score	m/z	Gene names
RO-QC-160121_ZackD_SS_Set_02_Fractior	10793	FTMS; HCI	82.48	733.7	SSPN

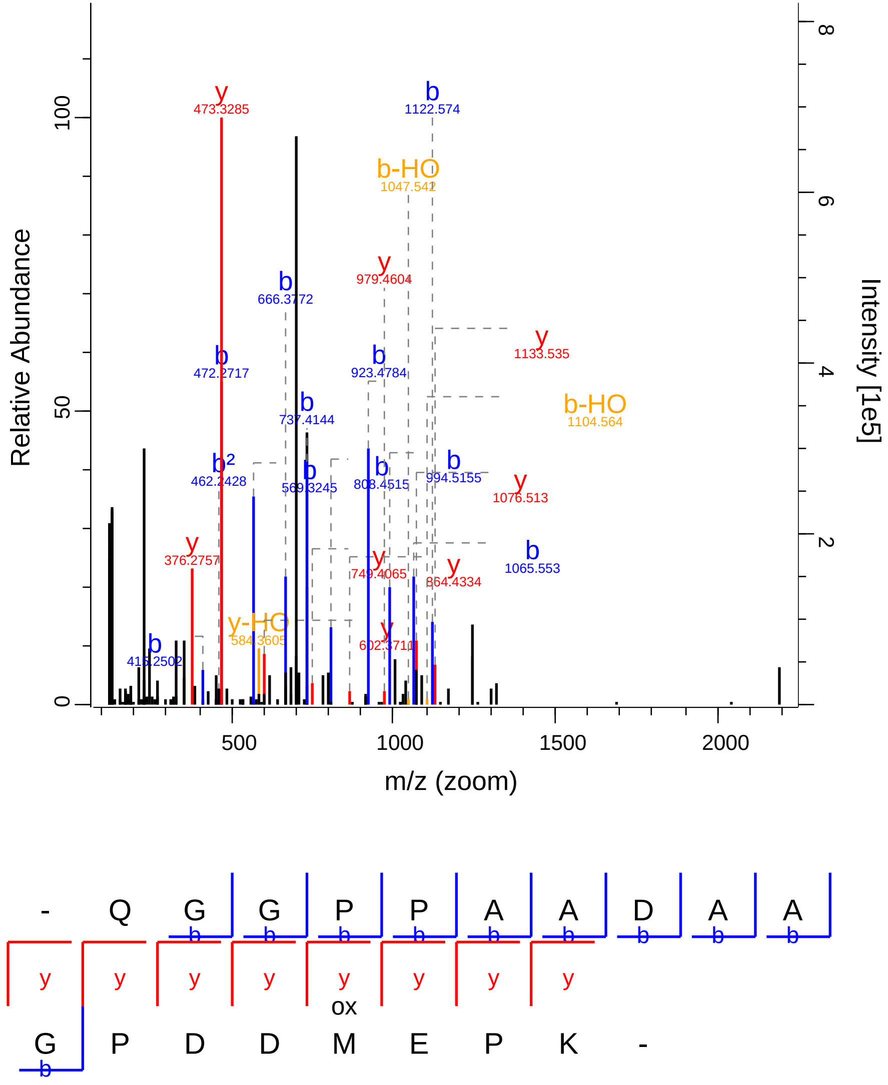

Raw File	Scan	Method	Score	m/z	Gene names
RO-QC-160121_ZackD_SS_Set_02_Fractio	11138	FTMS; HC	52.23	641.84	CFHR2

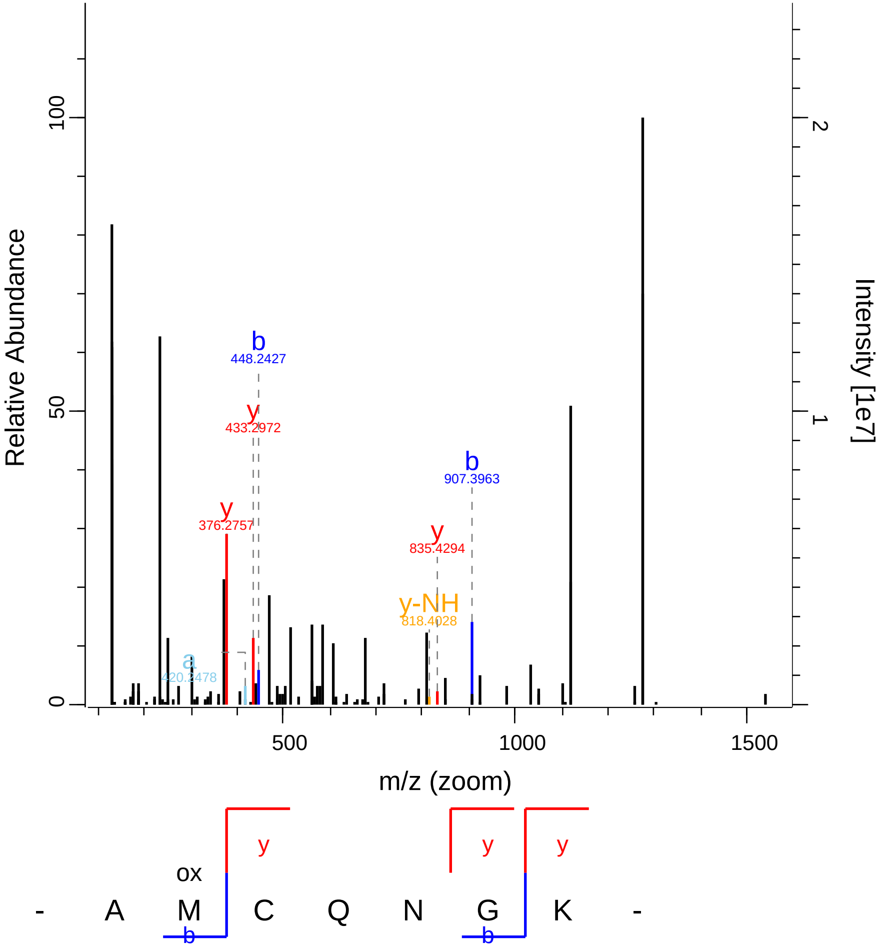

Raw File	Scan	Method	Score	m/z	Gene names
RO-QC-160121_ZackD_SS_Set_02_Fractio	11243	FTMS; HC	42.81	717.36	TRAF1

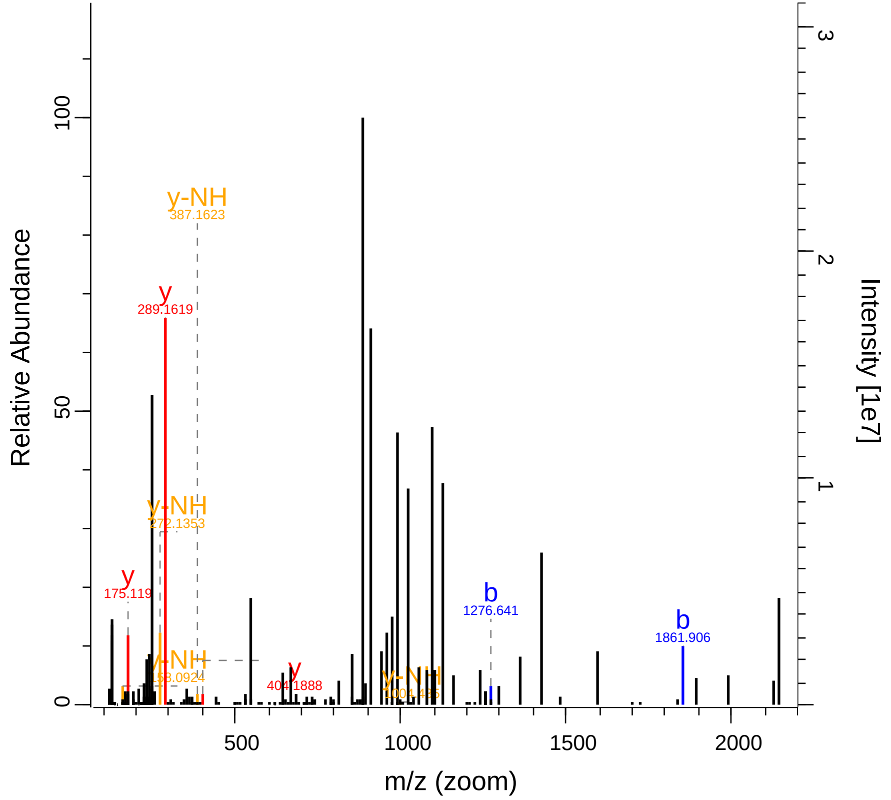

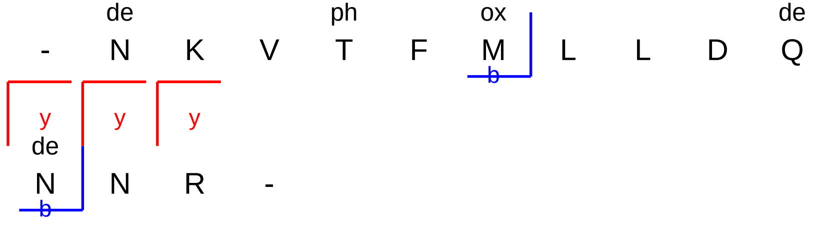

Raw File	Scan	Method	Score	m/z	Gene names
RO-QC-160121_ZackD_SS_Set_02_Fractio	11573	FTMS; HC	85.21	654.39	DNAJC15

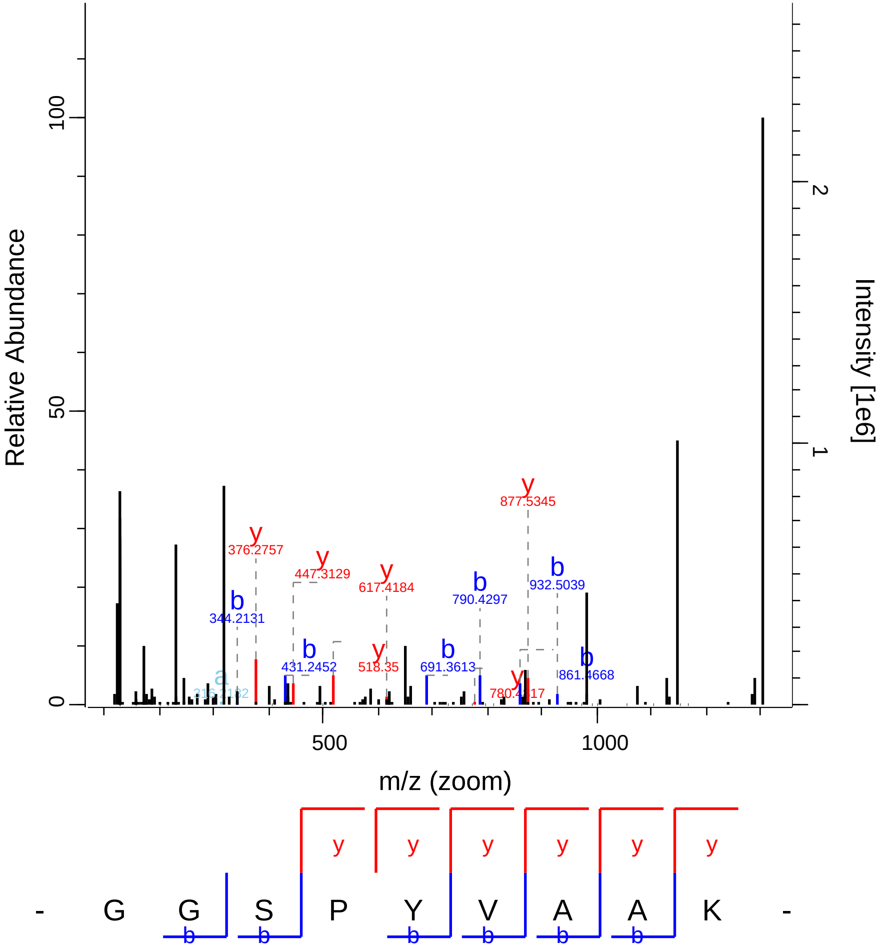

Raw File	Scan	Method	Score	m/z	Gene names
RO-QC-160121_ZackD_SS_Set_02_Fractio	12308	FTMS; HC	56.26	403.74	CCDC146

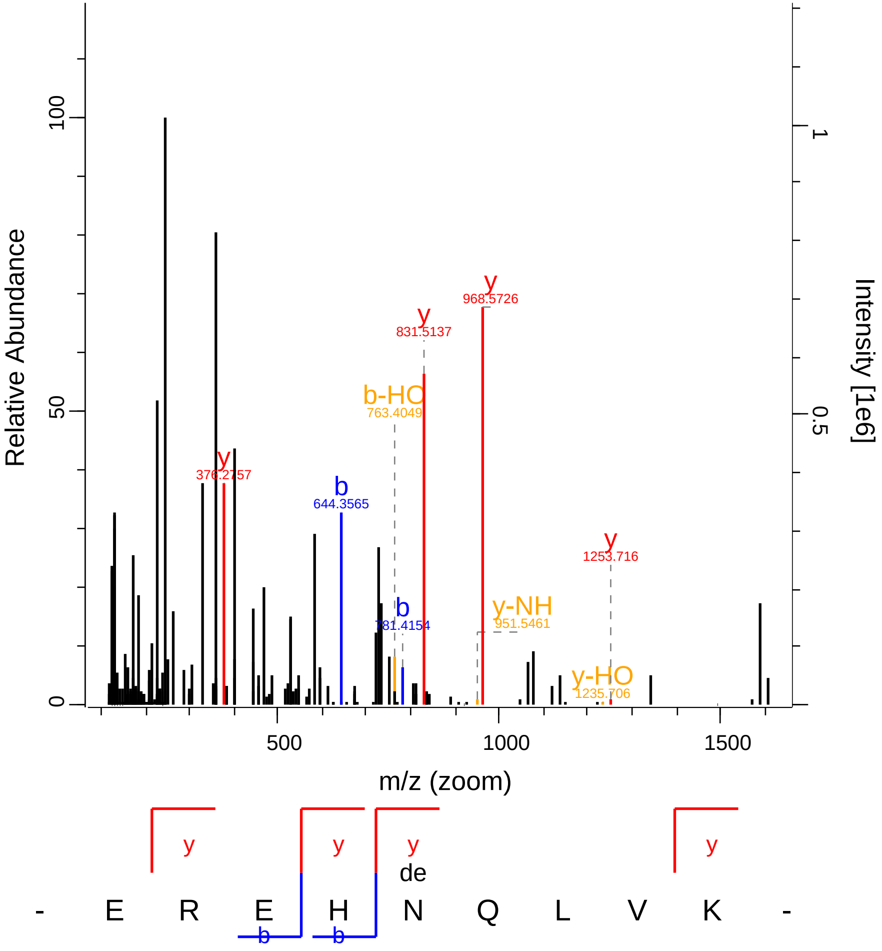

Raw File	Scan	Method	Score	m/z
RO-QC-160121_ZackD_SS_Set_02_Fraction_5	14214	FTMS; HCD	59.64	398.57

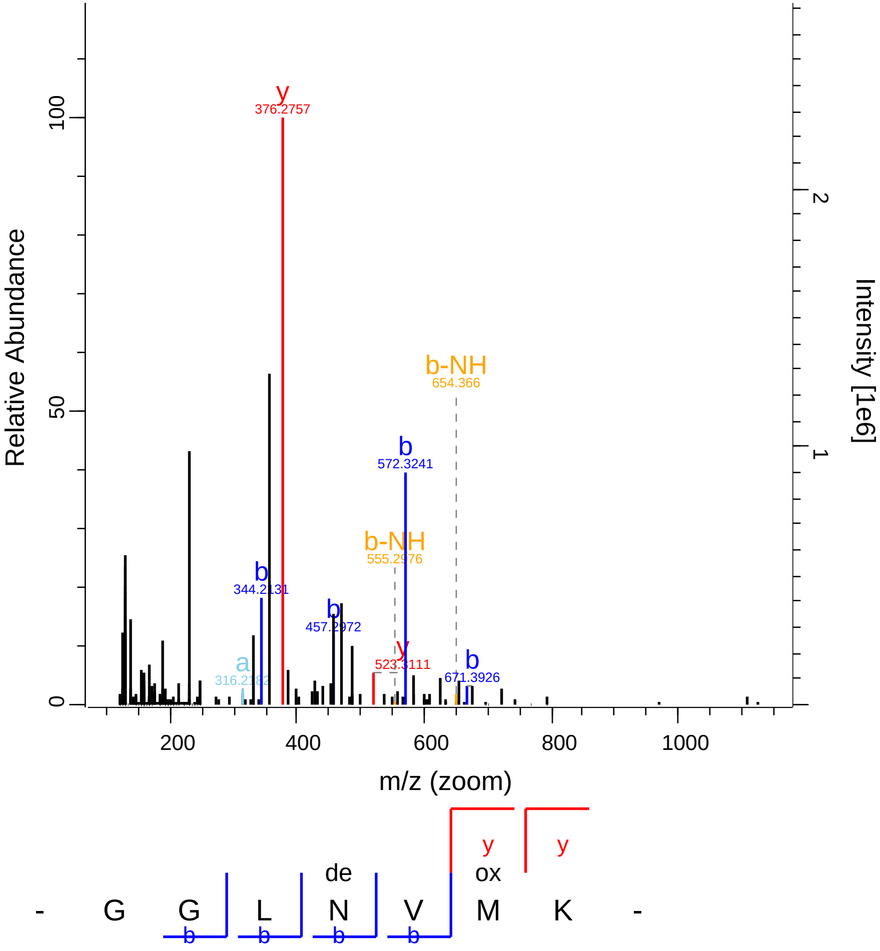

Raw File	Scan	Method	Score	m/z	Gene names
RO-QC-160121_ZackD_SS_Set_02_Fractio	14553	FTMS; HC	85.21	824.47	AIPL1

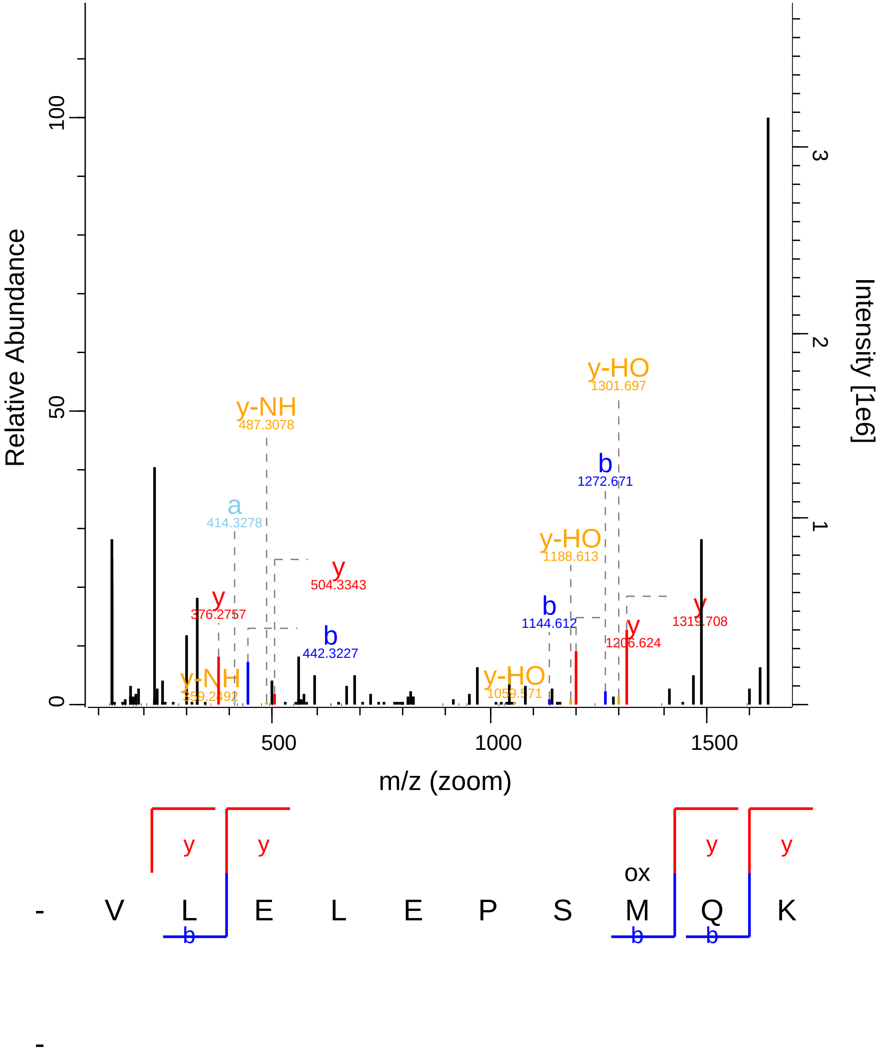

Raw File	Scan	Method	Score	m/z	Gene names
RO-QC-160121_ZackD_SS_Set_02_Fractic	14622	FTMS; HC	49.63	1207.64	ATP6V0C

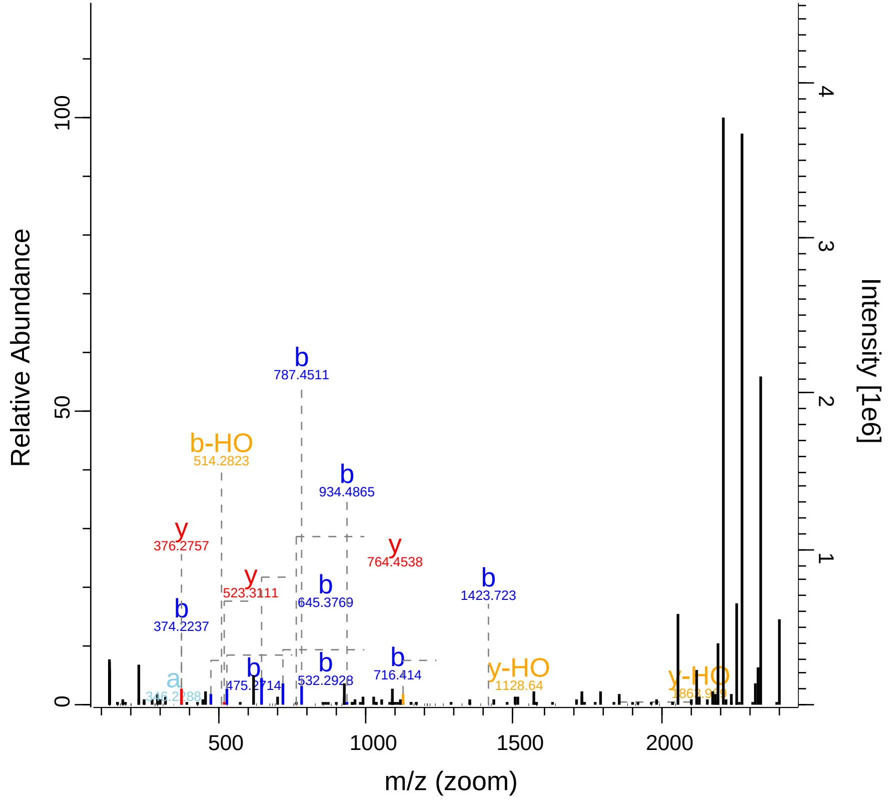

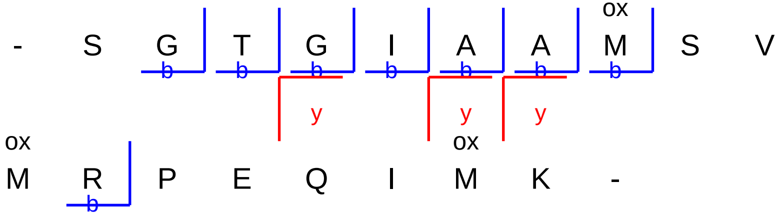

Raw File	Scan	Method	Score	m/z	Gene names
RO-QC-160121_ZackD_SS_Set_02_Fractio	14643	FTMS; HC	44.3	604.32	ATP6V0C

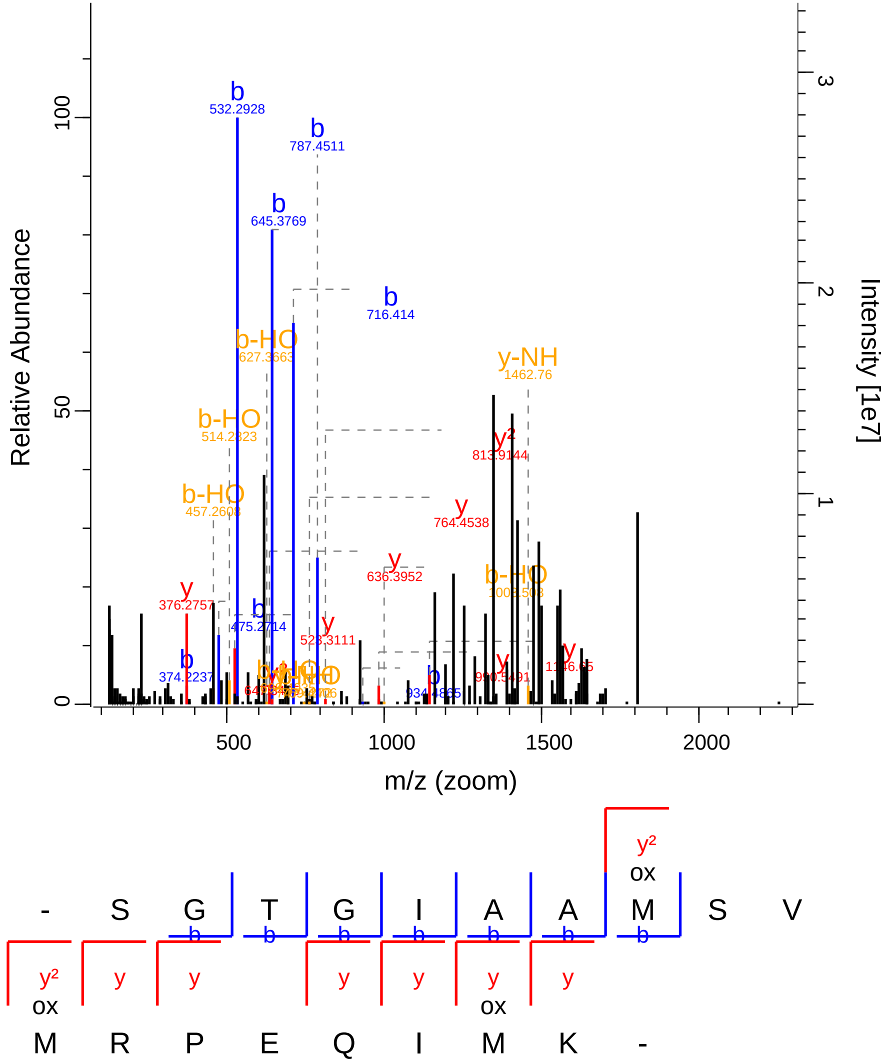

Raw File	Scan	Method	Score	m/z	Gene names
RO-QC-160121_ZackD_SS_Set_02_Fractio	14801	FTMS; HC	73.26	824.47	AIPL1

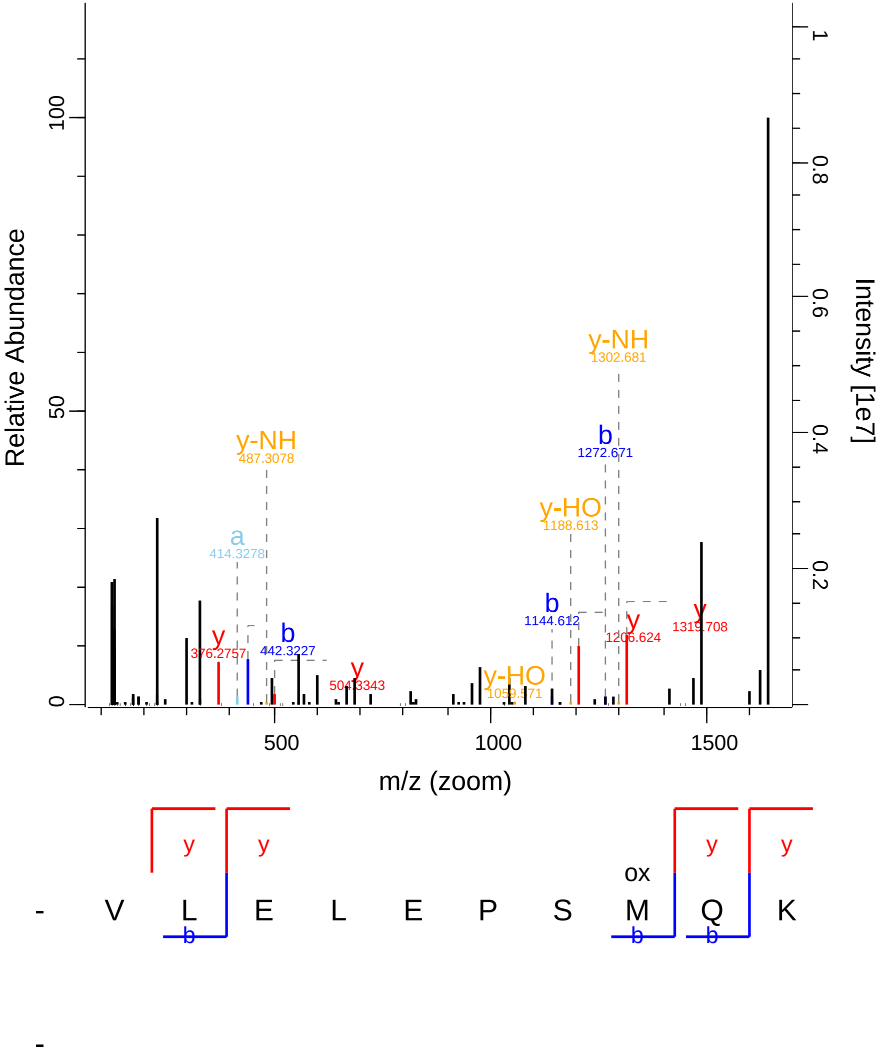

Raw File	Scan	Method	Score	m/z	Gene names
RO-QC-160121_ZackD_SS_Set_02_Fractio	15584	FTMS; HC	116.73	602.82	NPC1L1

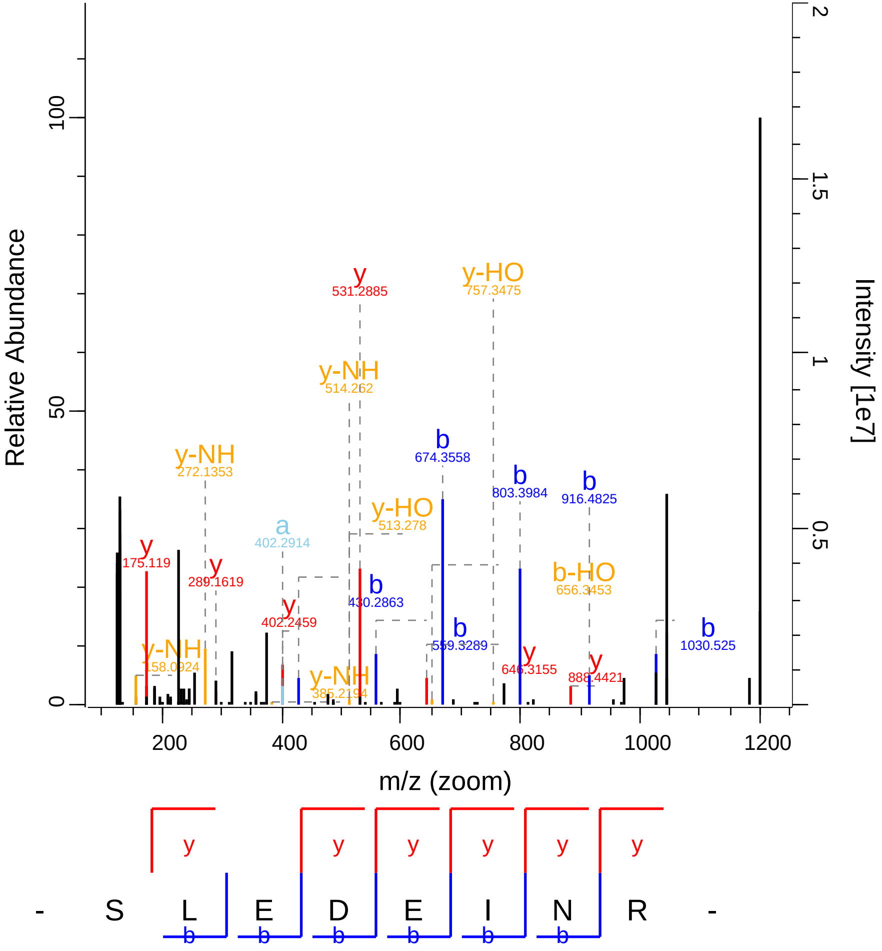

Raw File	Scan	Method	Score	m/z	Gene names
RO-QC-160121_ZackD_SS_Set_02_Fractio	15624	FTMS; HC	58.98	432.91	TEKT5

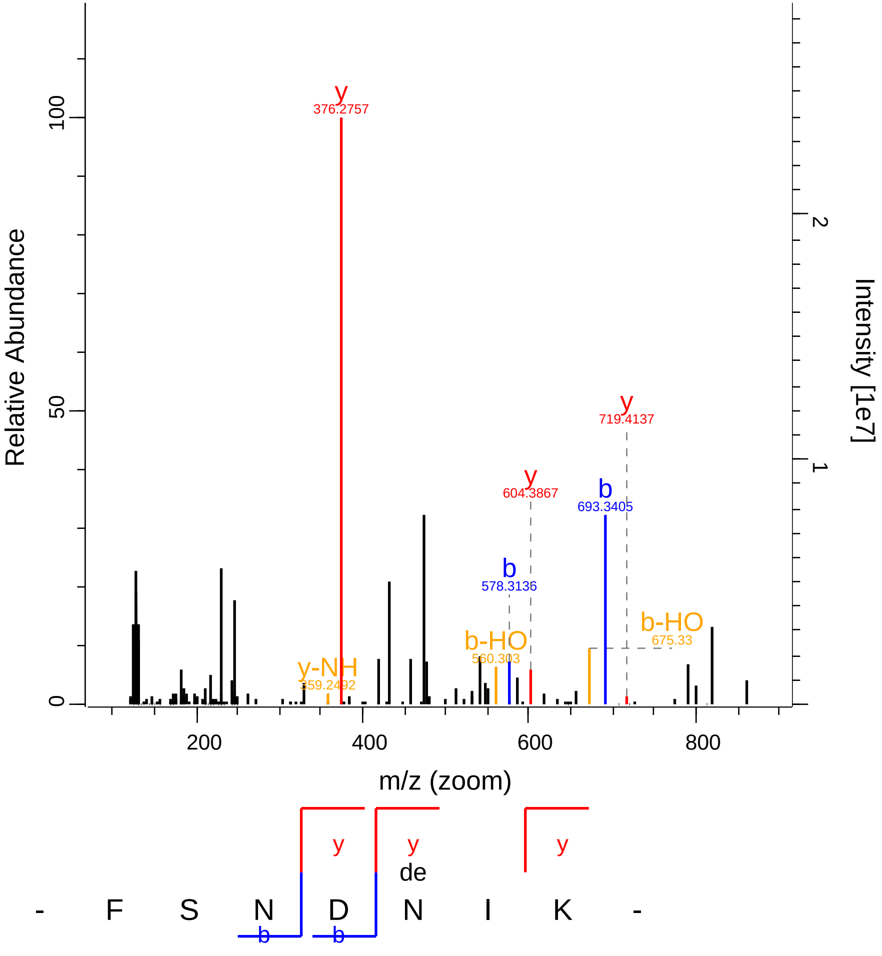

Raw File	Scan	Method	Score	m/z	Gene names
RO-QC-160121_ZackD_SS_Set_02_Fractio	15799	FTMS; HC	94.87	602.82	NPC1L1

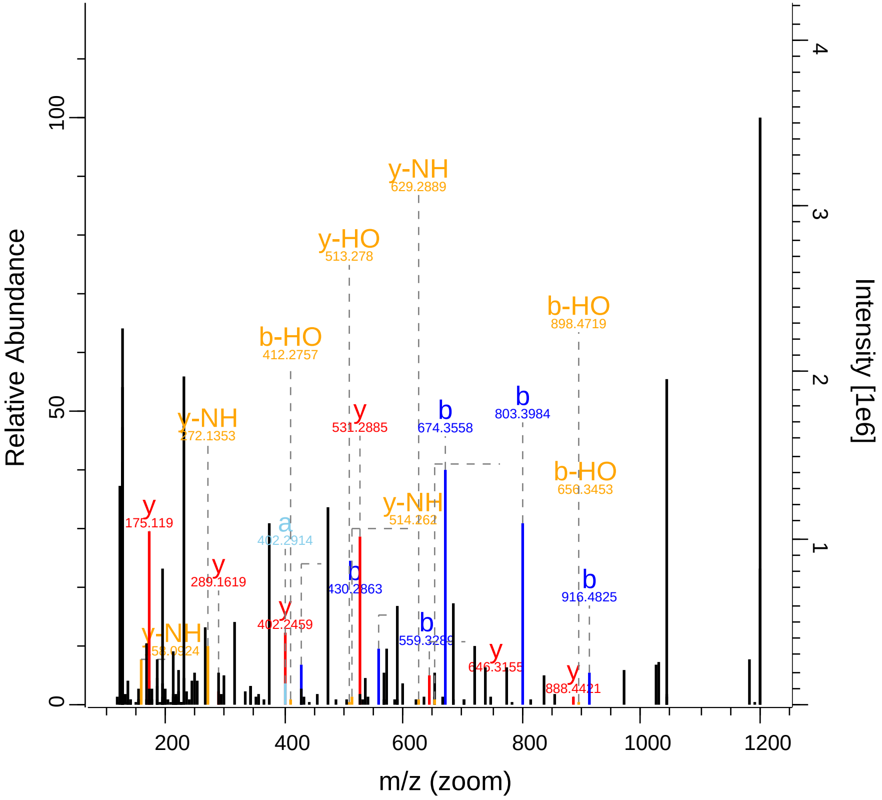

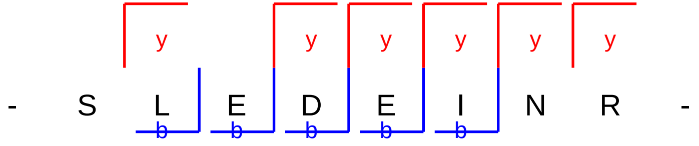

Raw File	Scan	Method	Score	m/z
RO-QC-160121_ZackD_SS_Set_02_Fraction_5	17593	FTMS; HCD	47.07	604.83

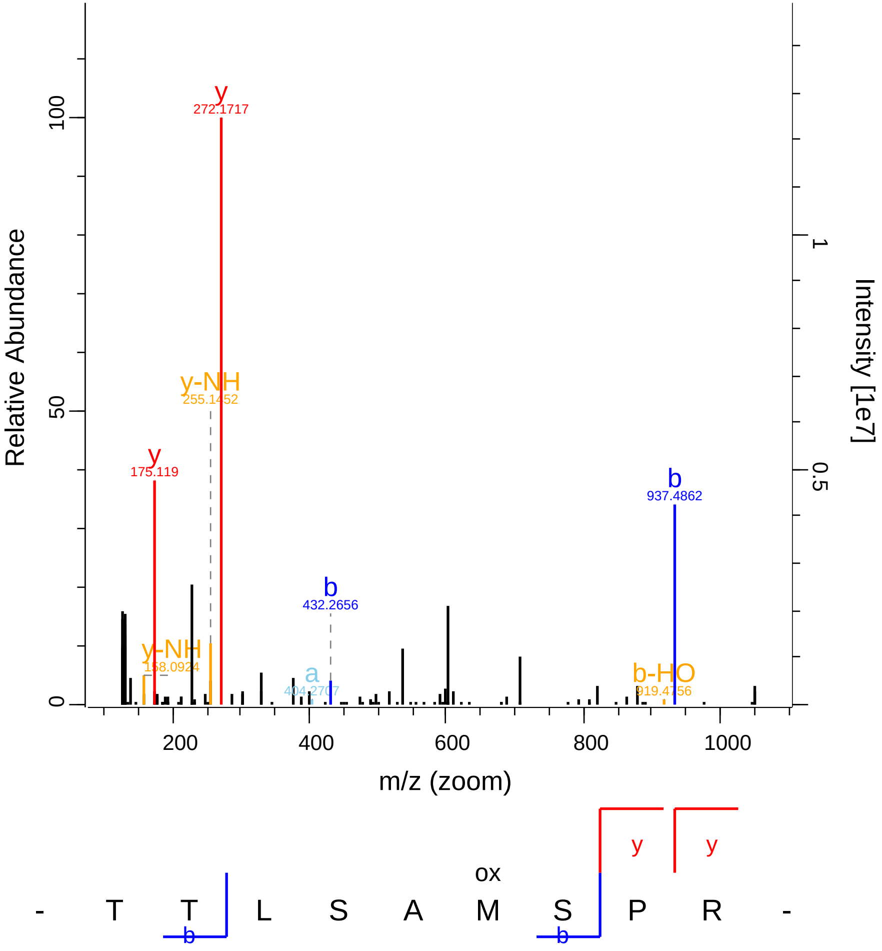

Raw File	Scan	Method	Score	m/z
RO-QC-160121_ZackD_SS_Set_02_Fraction_5	17606	FTMS; HCD	41.03	403.55

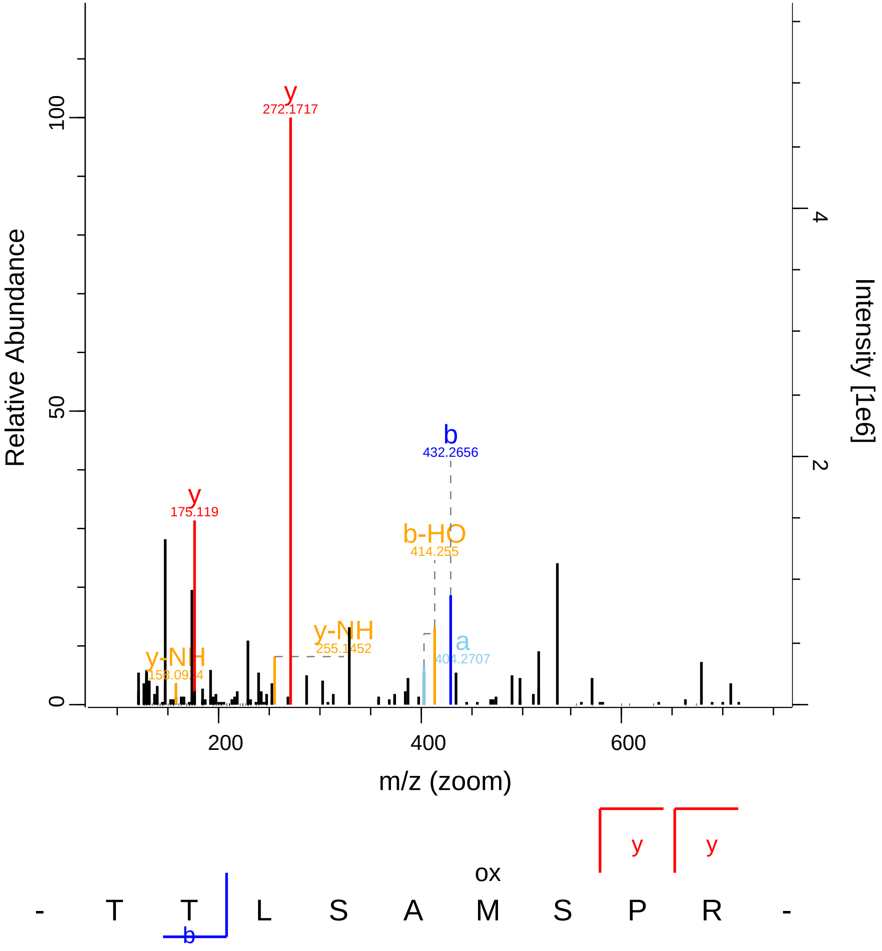

Raw File	Scan	Method	Score	m/z	Gene names
RO-QC-160121_ZackD_SS_Set_02_Fractio	19477	FTMS; HC	43.38	755.08	GIMAP7

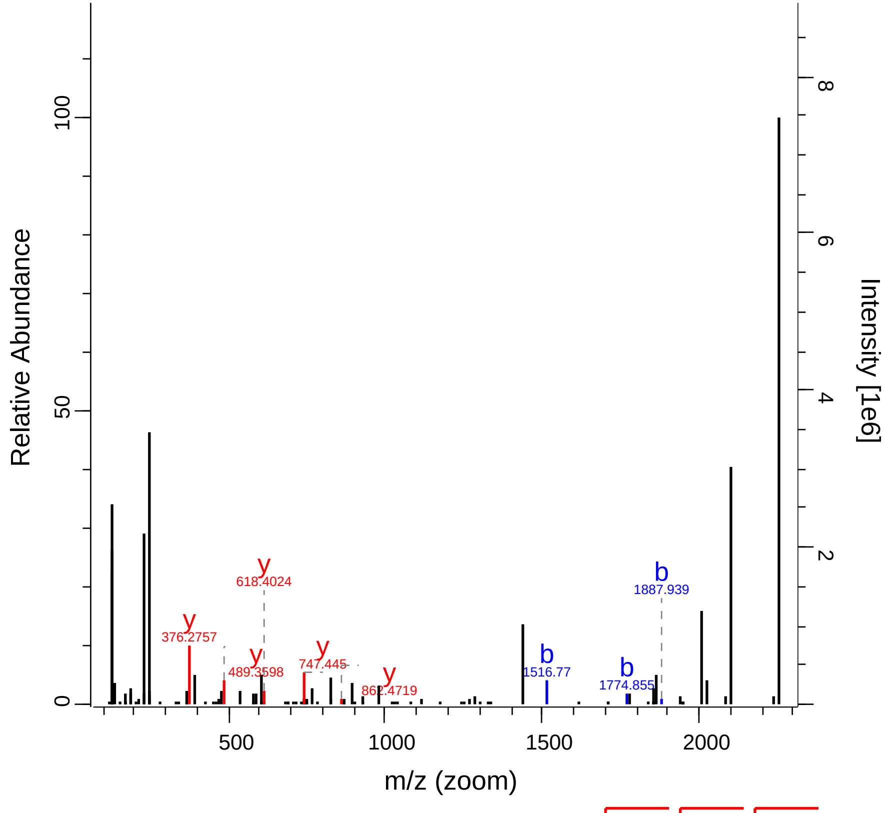

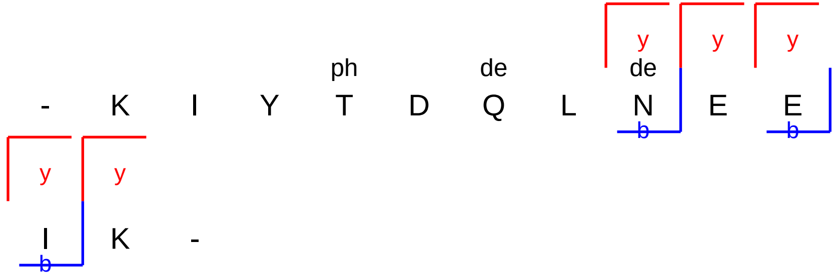

Raw File	Scan	Method	Score	m/z	Gene names
RO-QC-160121_ZackD_SS_Set_02_Fractio	19780	FTMS; HC	43	471.91	NKPD1

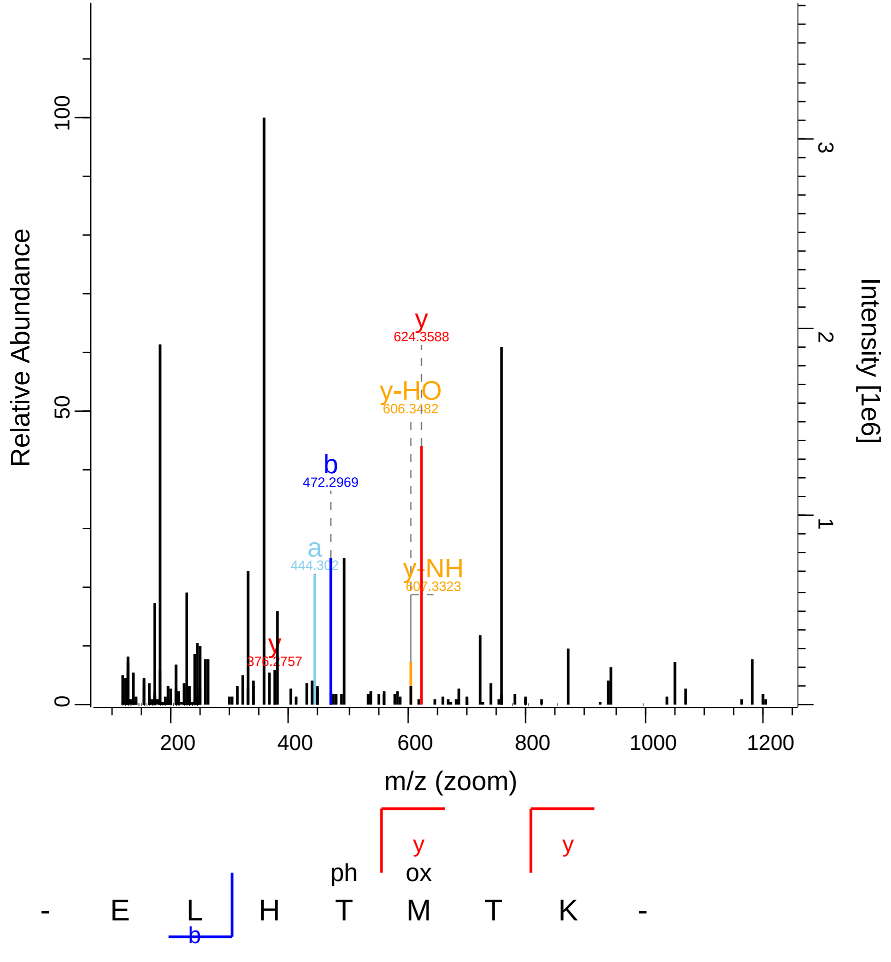

Raw File	Scan	Method	Score	m/z
RO-QC-160121_ZackD_SS_Set_02_Fraction_5	19843	FTMS; HCD	141.78	693.94

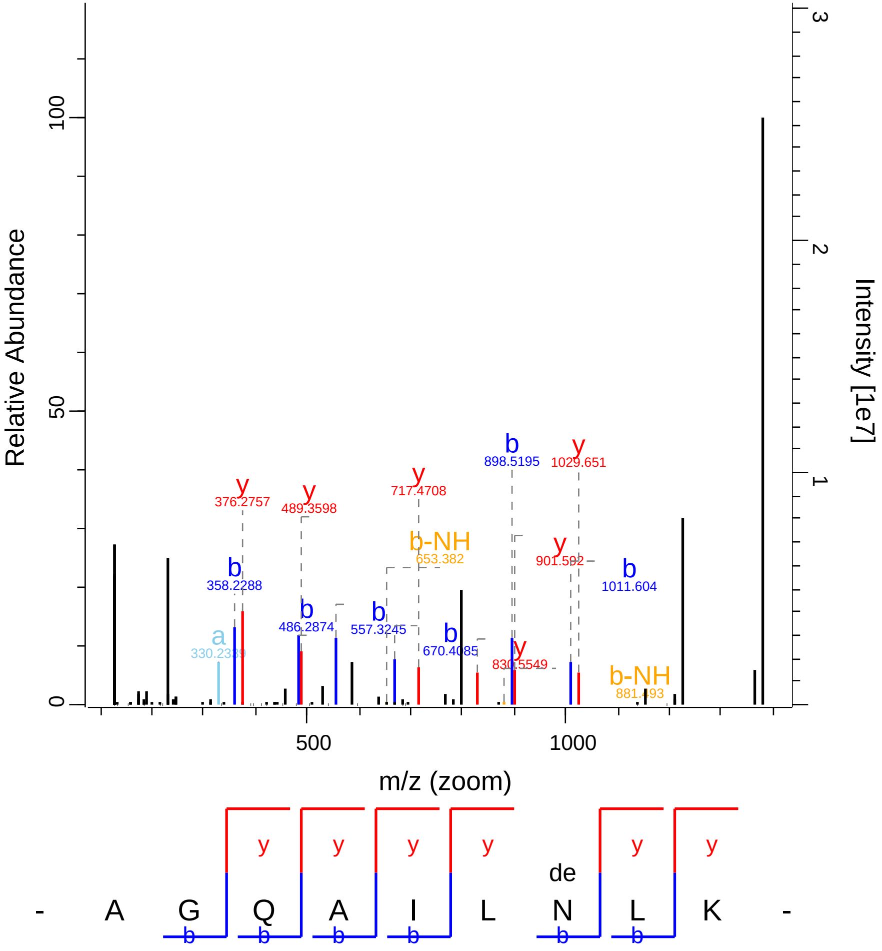

Raw File	Scan	Method	Score	m/z	Gene names
RO-QC-160121_ZackD_SS_Set_02_Fractio	20119	FTMS; HC	63.82	819.47	MIS18A

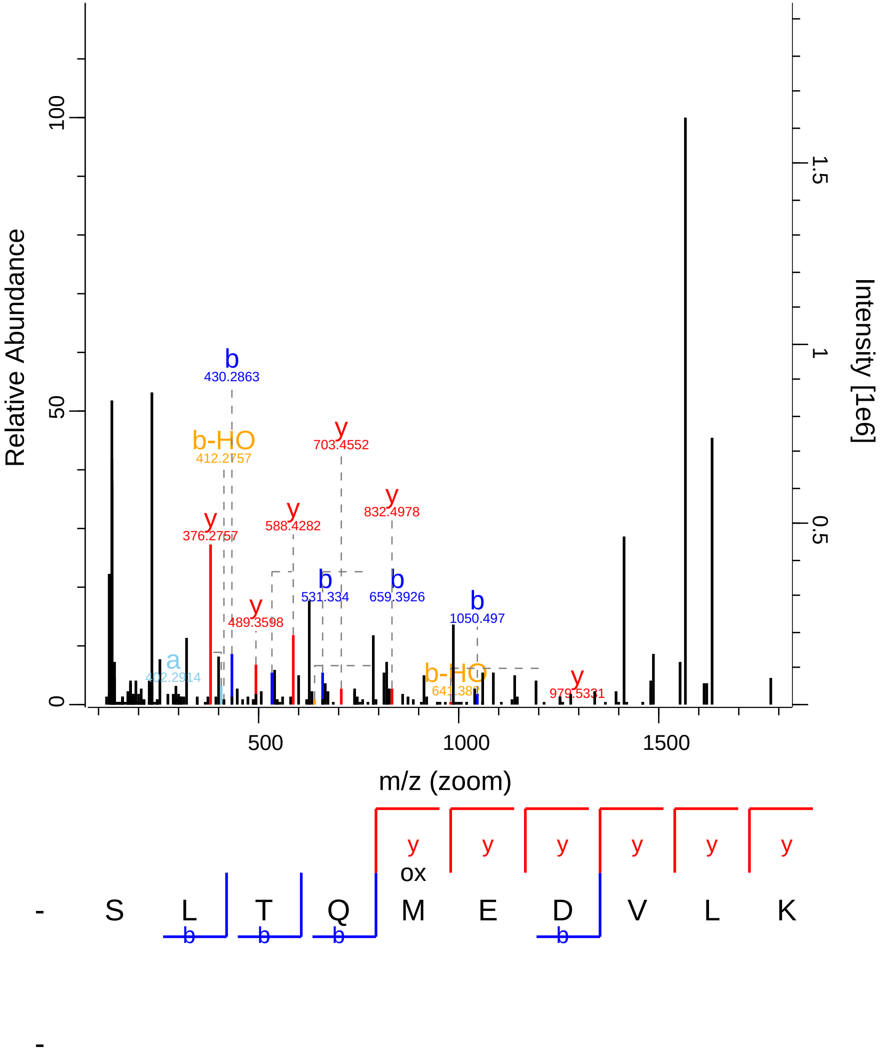

Raw File	Scan	Method	Score	m/z	Gene names
RO-QC-160121_ZackD_SS_Set_02_Fractio	20743	FTMS; HC	150.85	818.44	GPATCH2

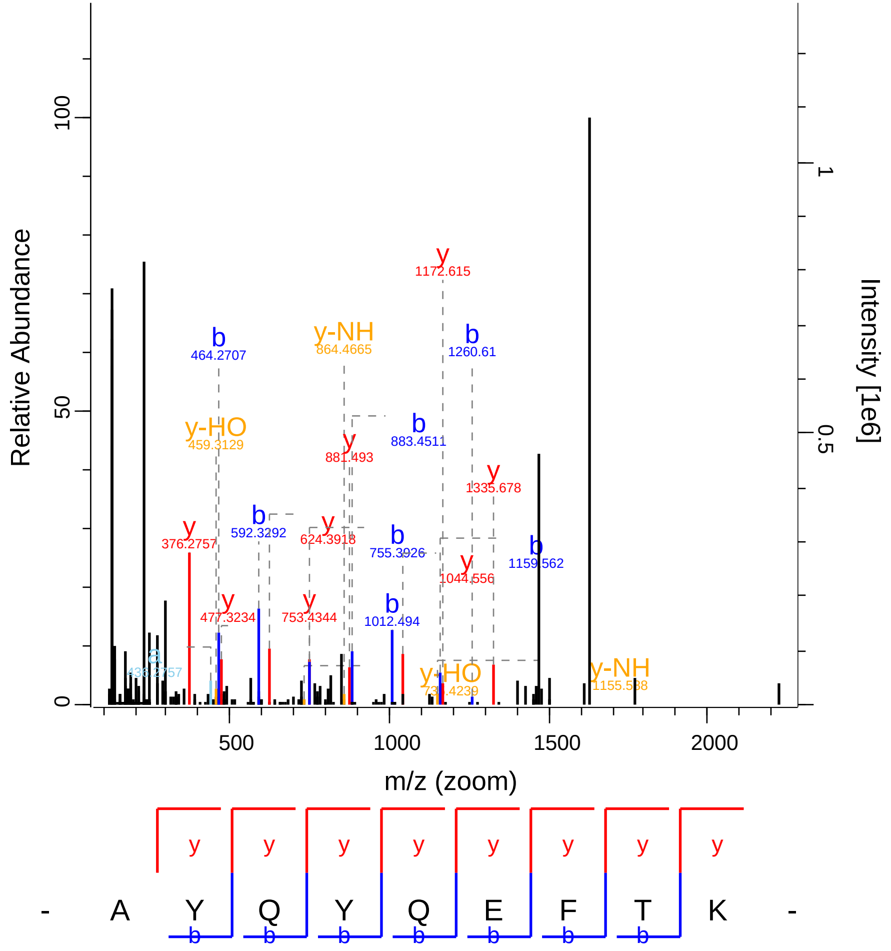

Raw File	Scan	Method	Score	m/z	Gene names
RO-QC-160121_ZackD_SS_Set_02_Fractio	20924	FTMS; HC	70.82	741.42	SMPDL3A

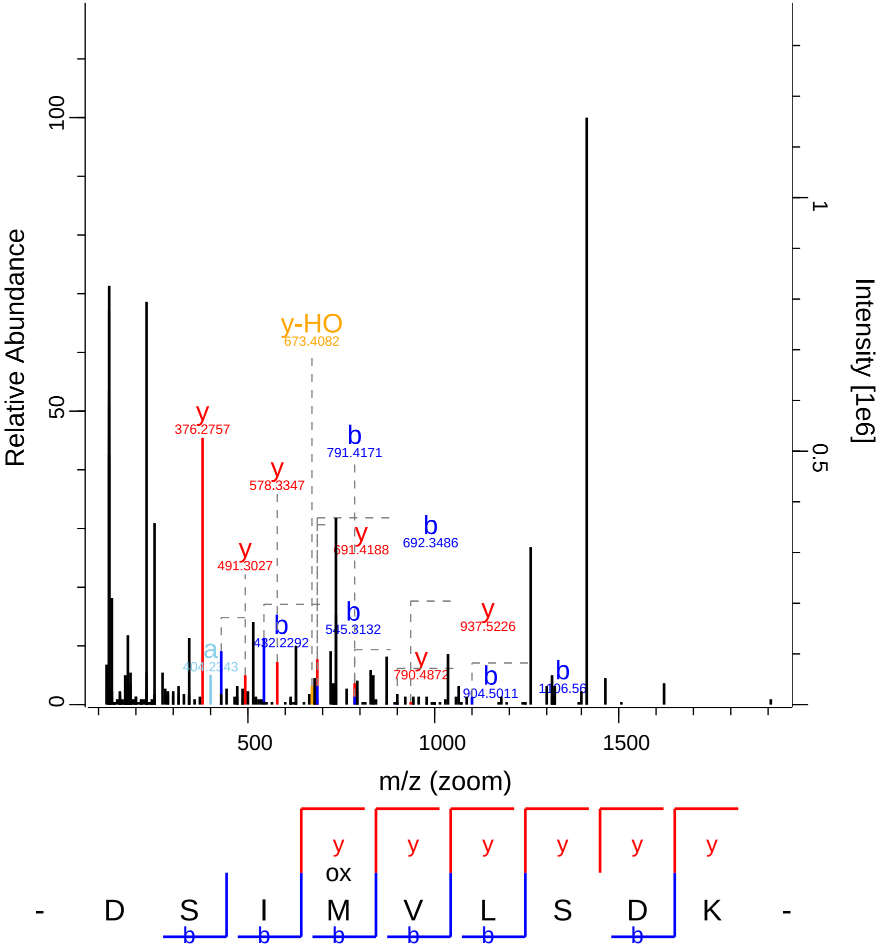

Raw File	Scan	Method	Score	m/z	Gene names
RO-QC-160121_ZackD_SS_Set_02_Fractio	21197	FTMS; HC	43.54	589.33	SEC22A

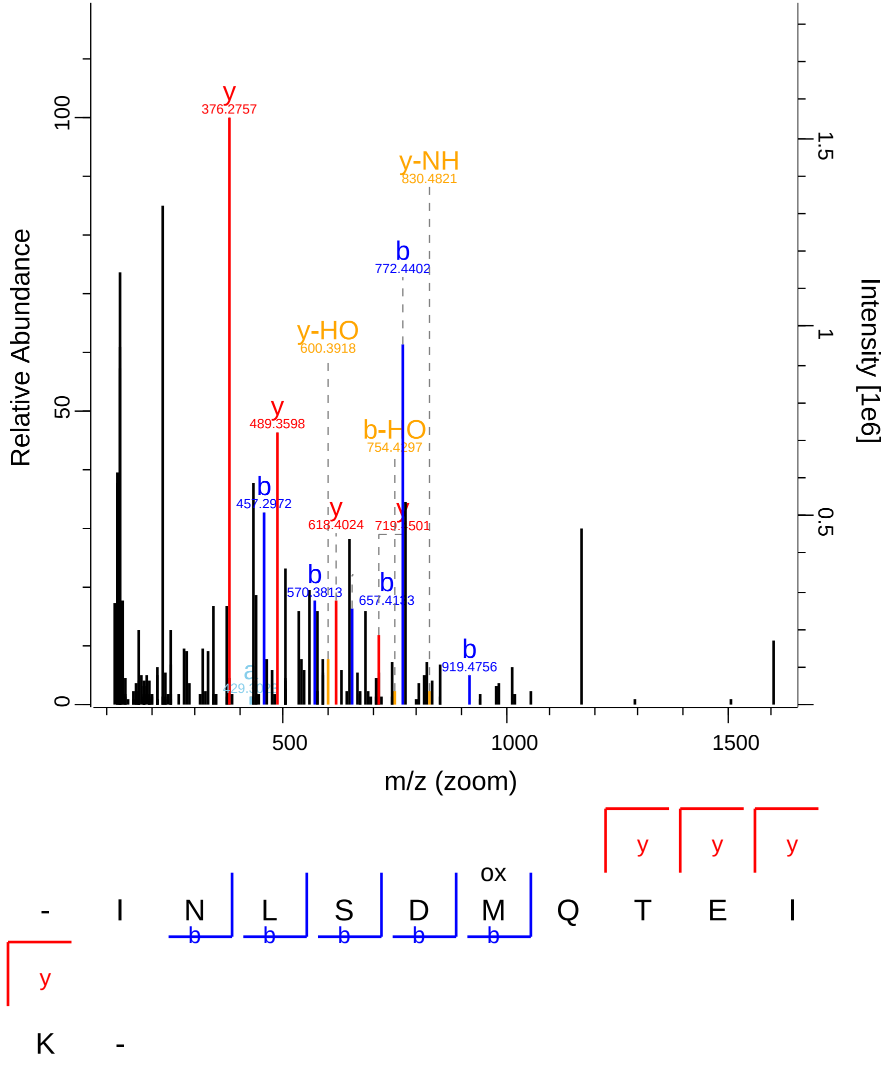

Raw File	Scan	Method	Score	m/z
RO-QC-160121_ZackD_SS_Set_02_Fraction_5	21646	FTMS; HCD	51.85	701.37

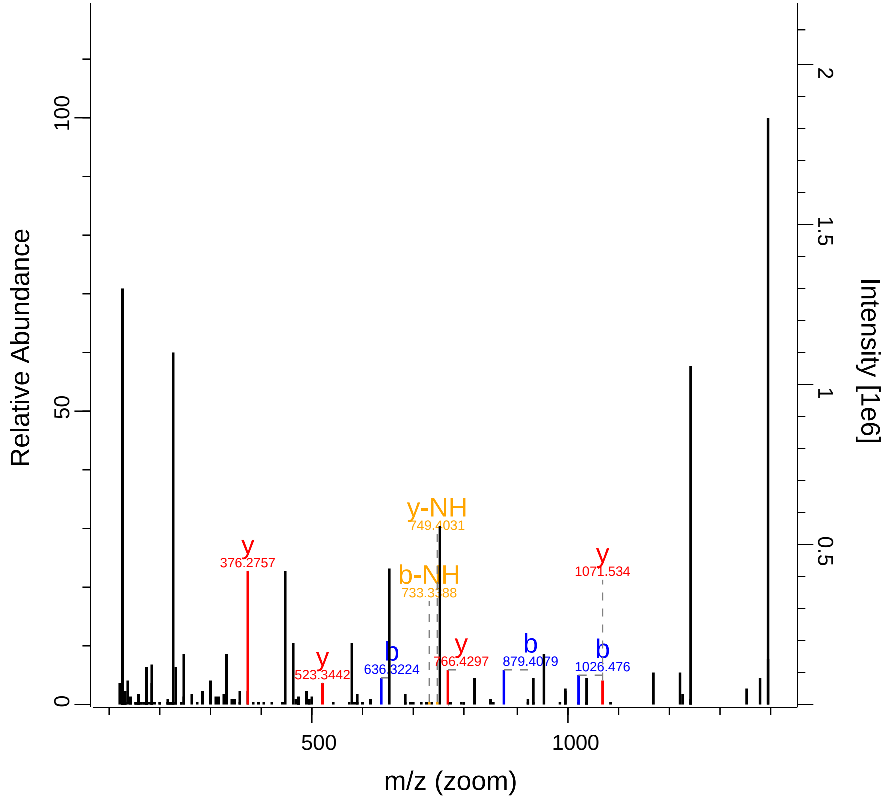

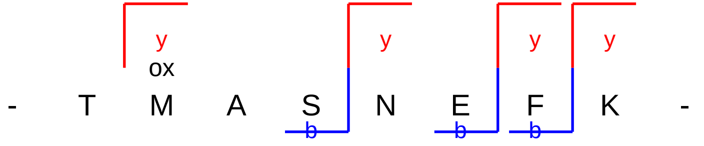

Raw File	Scan	Method	Score	m/z	Gene names
RO-QC-160121_ZackD_SS_Set_02_Fractio	22090	FTMS; HC	92.49	600.86	TMEM167A

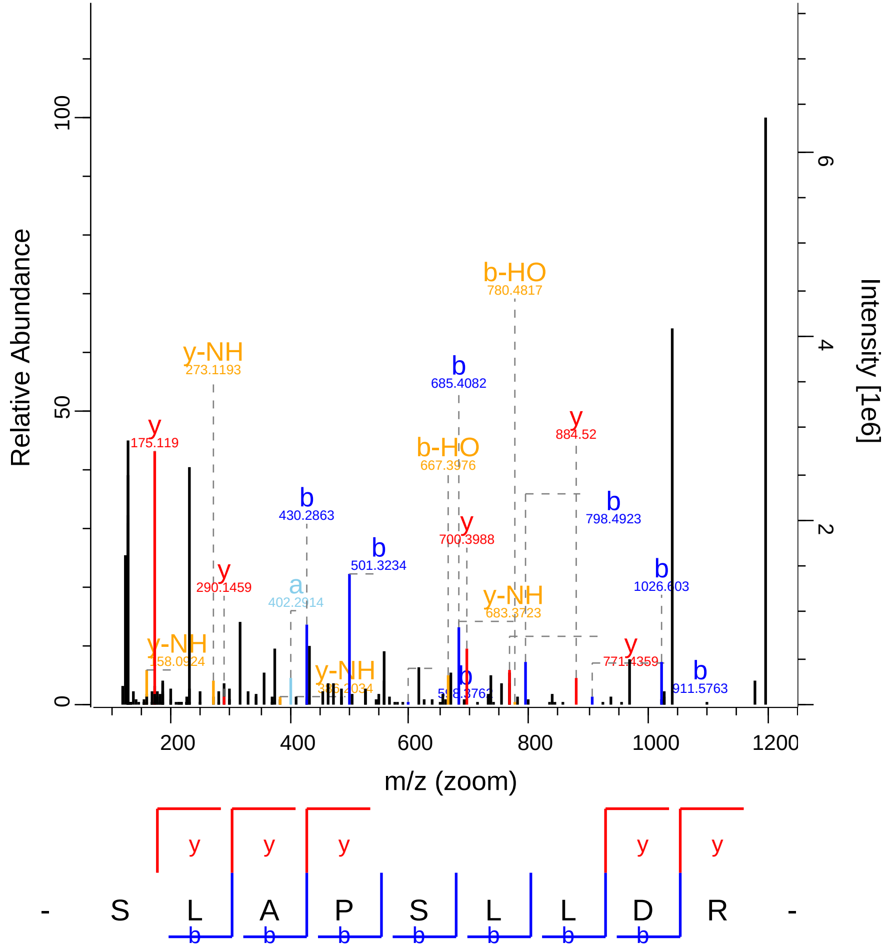

Raw File	Scan	Method	Score	m/z	Gene names
RO-QC-160121_ZackD_SS_Set_02_Fractior	22255	FTMS; HCI	47.07	750.9	TIAF1

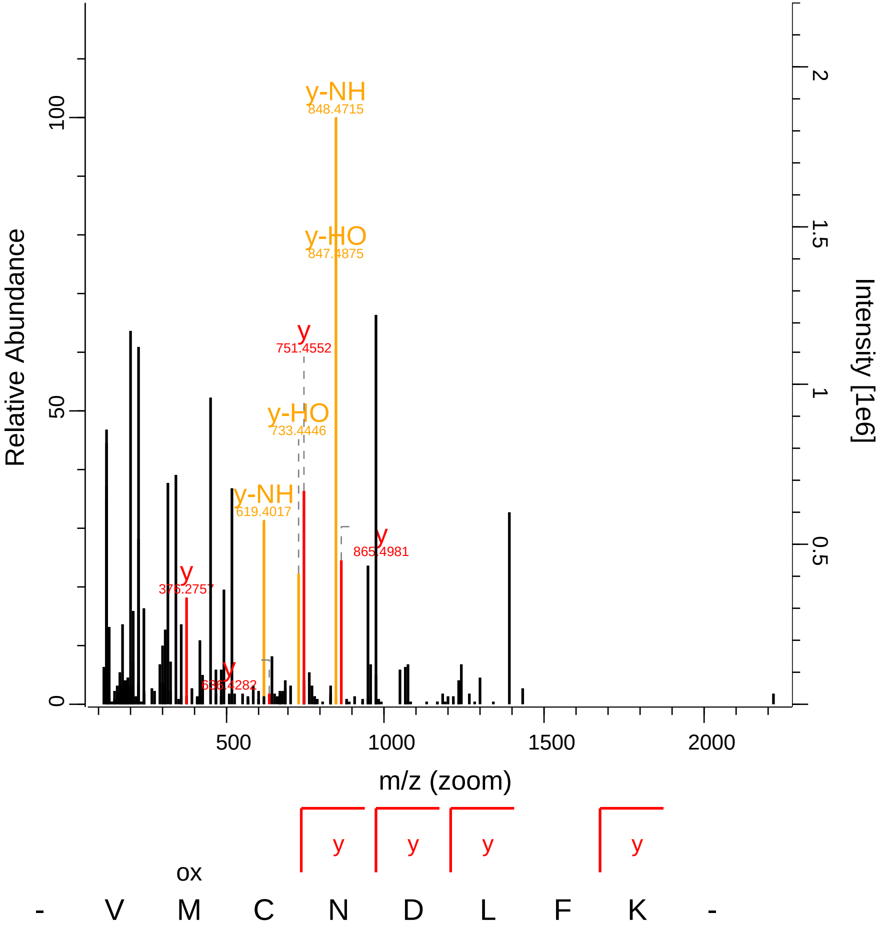

Raw File	Scan	Method	Score	m/z	Gene names
RO-QC-160121_ZackD_SS_Set_02_Fractio	22312	FTMS; HC	125.04	600.86	TMEM167A

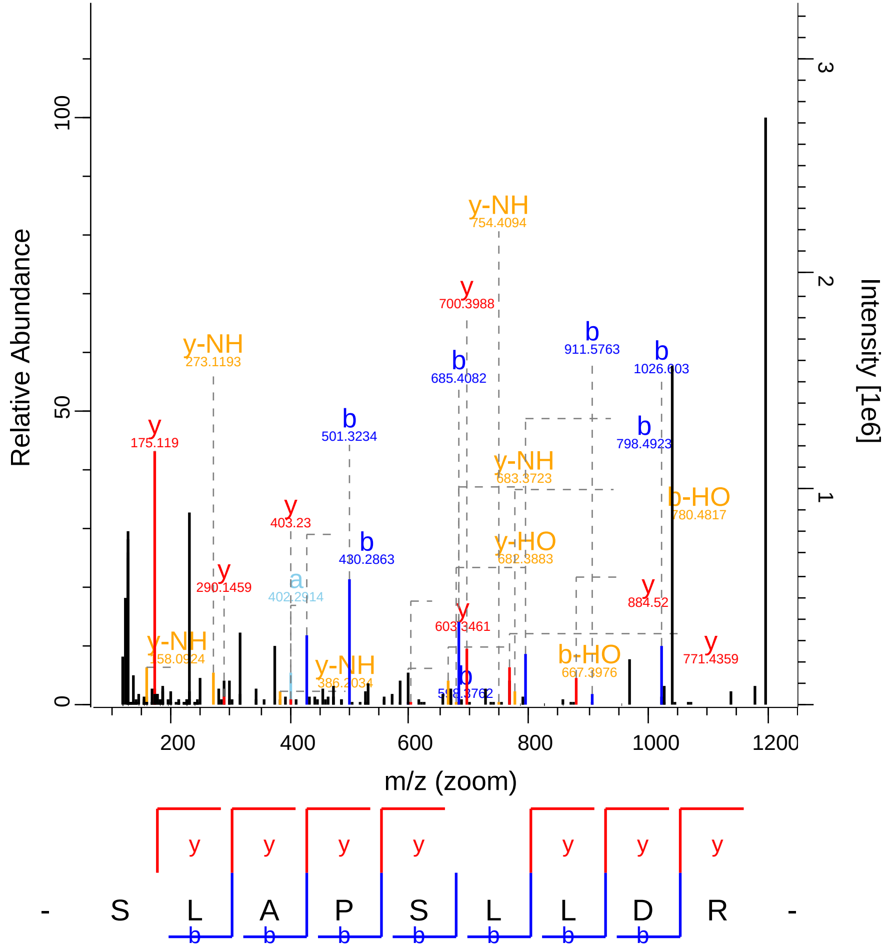

Raw File	Scan	Method	Score	m/z
RO-QC-160121_ZackD_SS_Set_02_Fraction_5	22768	FTMS; HCD	59.15	860.46

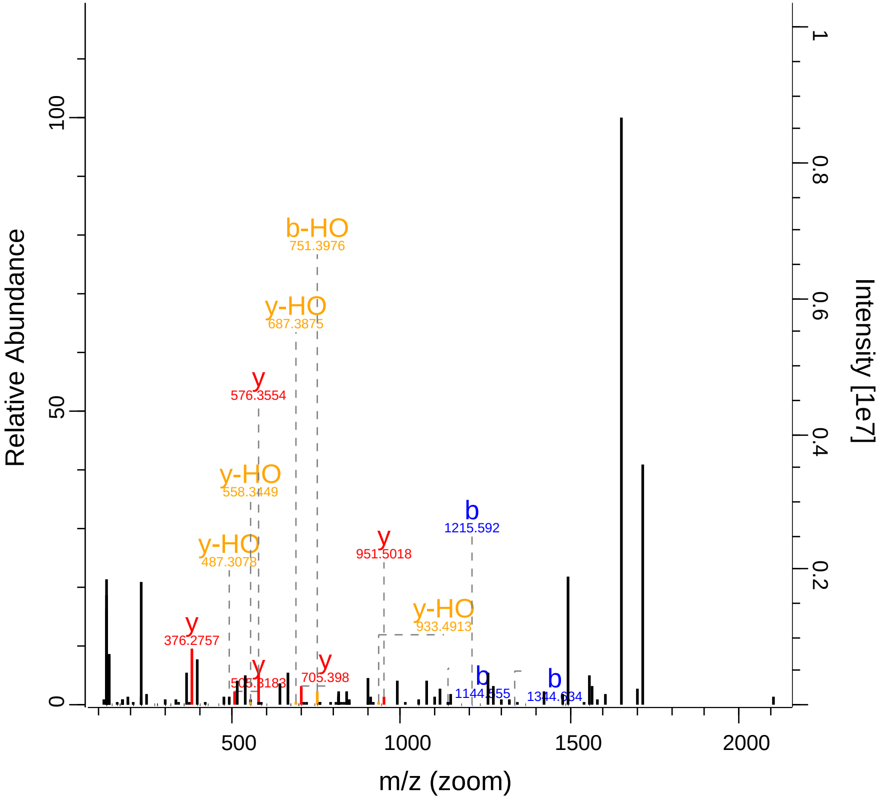

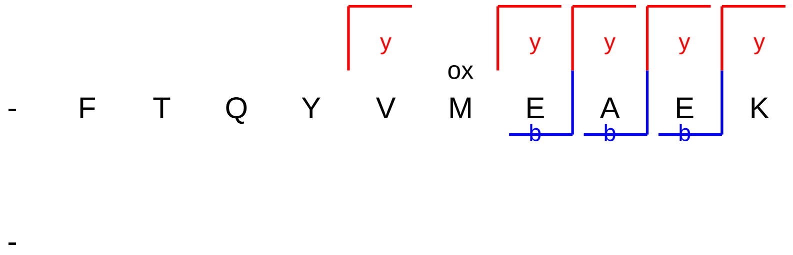

Raw File	Scan	Method	Score	m/z	Gene names
RO-QC-160121_ZackD_SS_Set_02_Fractio	22880	FTMS; HC	149.72	722.45	ARL4C

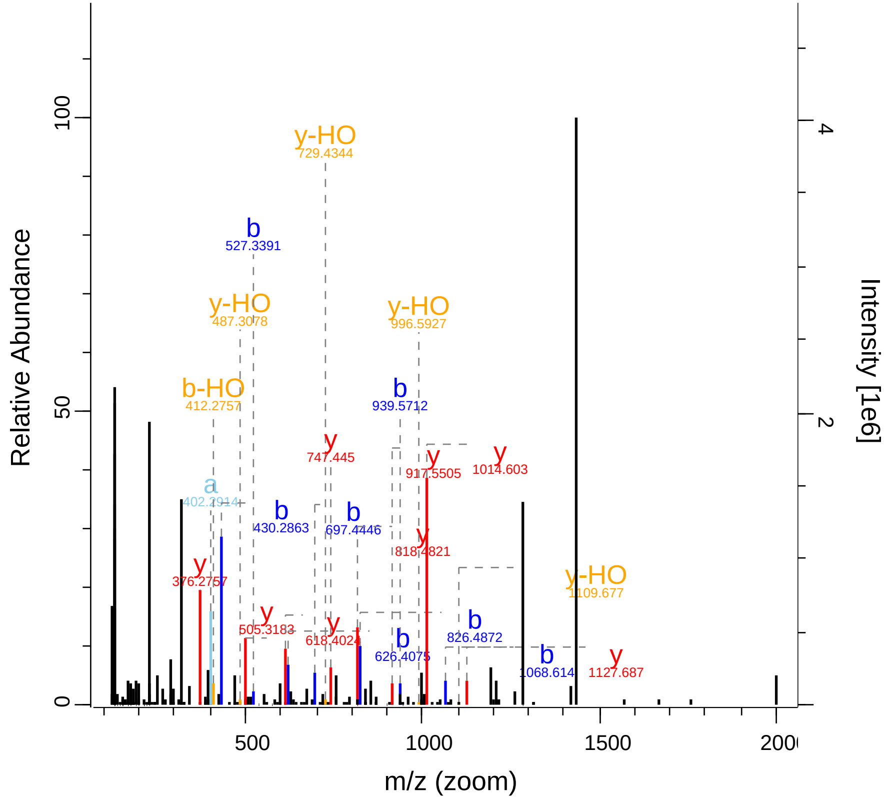

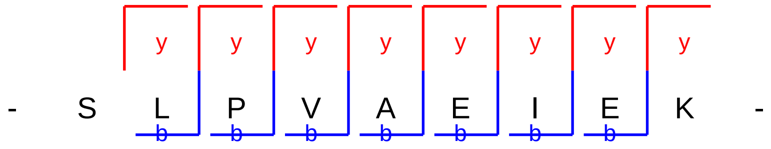

Raw File	Scan	Method	Score	m/z	Gene names
RO-QC-160121_ZackD_SS_Set_02_Fractio	23637	FTMS; HC	94.97	750.03	RHBDD1

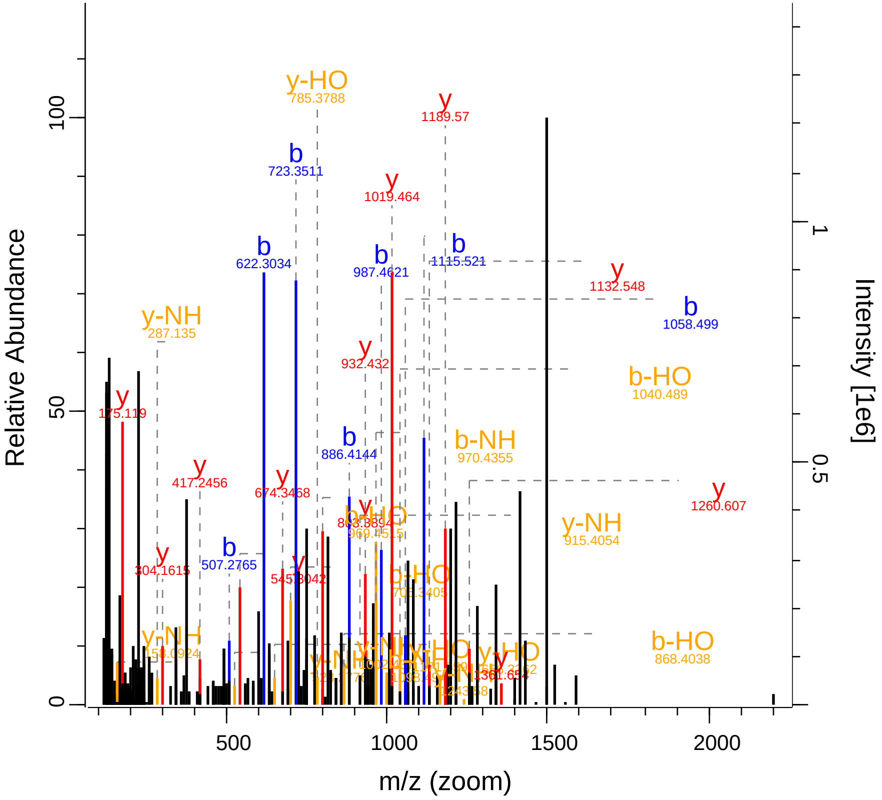

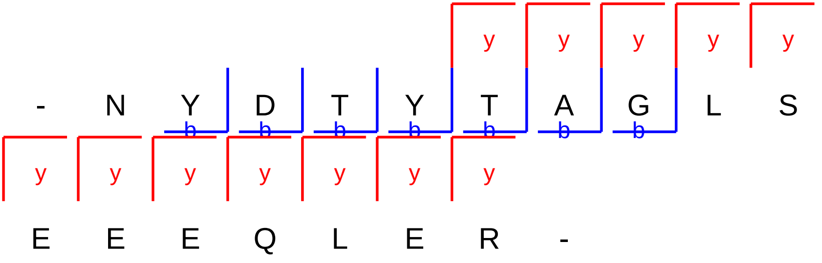

Raw File	Scan	Method	Score	m/z	Gene names
RO-QC-160121_ZackD_SS_Set_02_Fractio	23640	FTMS; HC	189.09	1124.54	RHBDD1

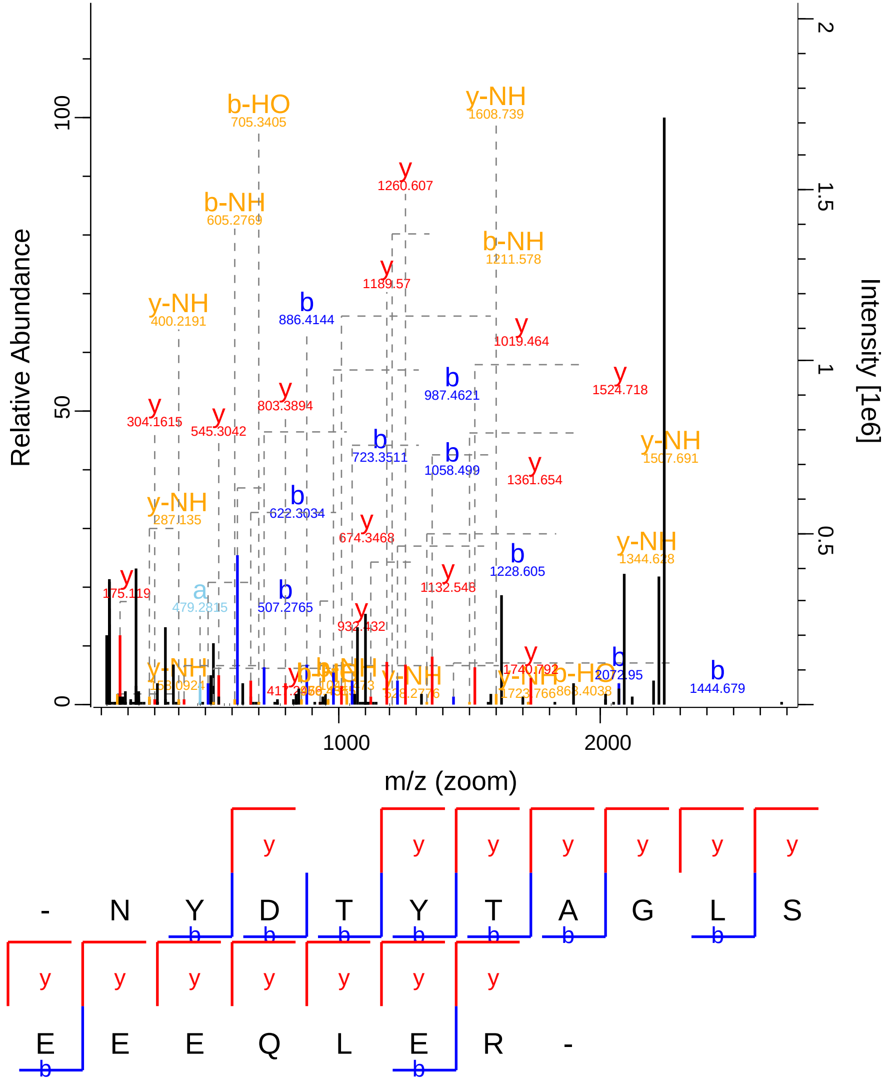

Raw File	Scan	Method	Score	m/z	Gene names
RO-QC-160121_ZackD_SS_Set_02_Fractior	24147	FTMS; HCI	63.13	799.1	FAM103A1

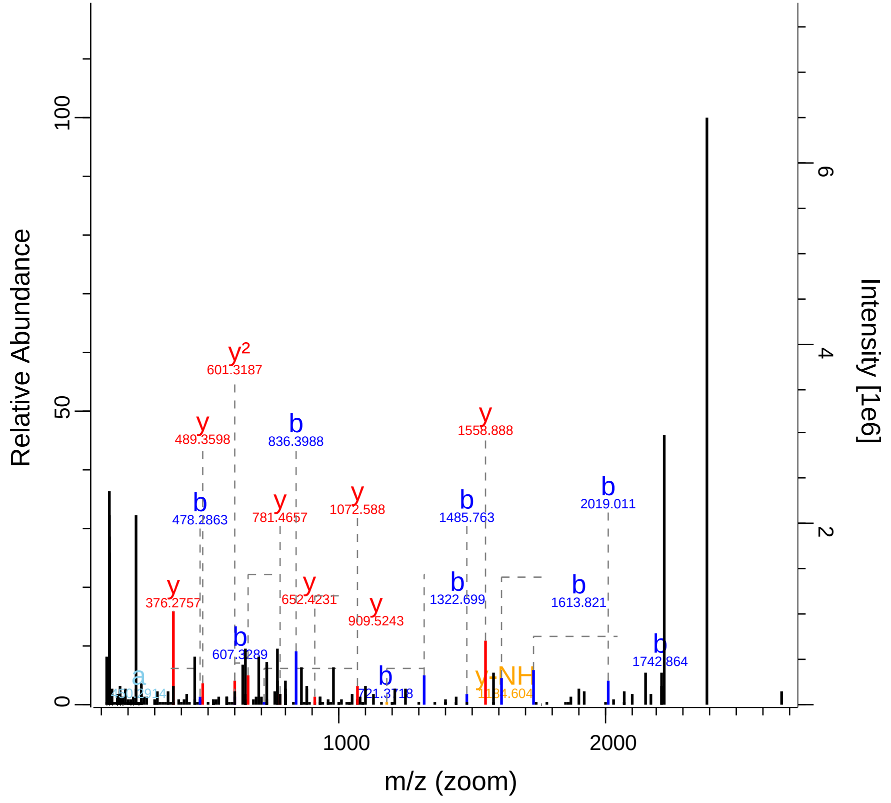

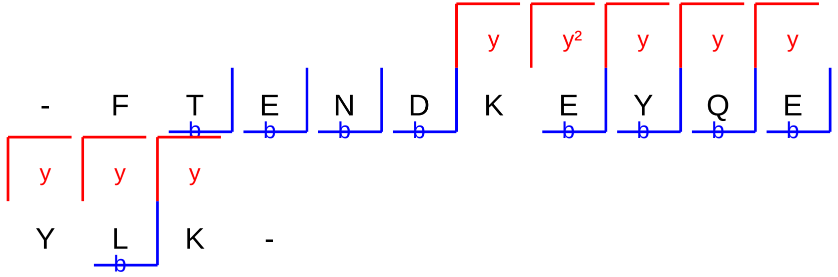

Raw File	Scan	Method	Score	m/z	Gene names
RO-QC-160121_ZackD_SS_Set_02_Fractio	25039	FTMS; HC	61.84	717.43	AIM2

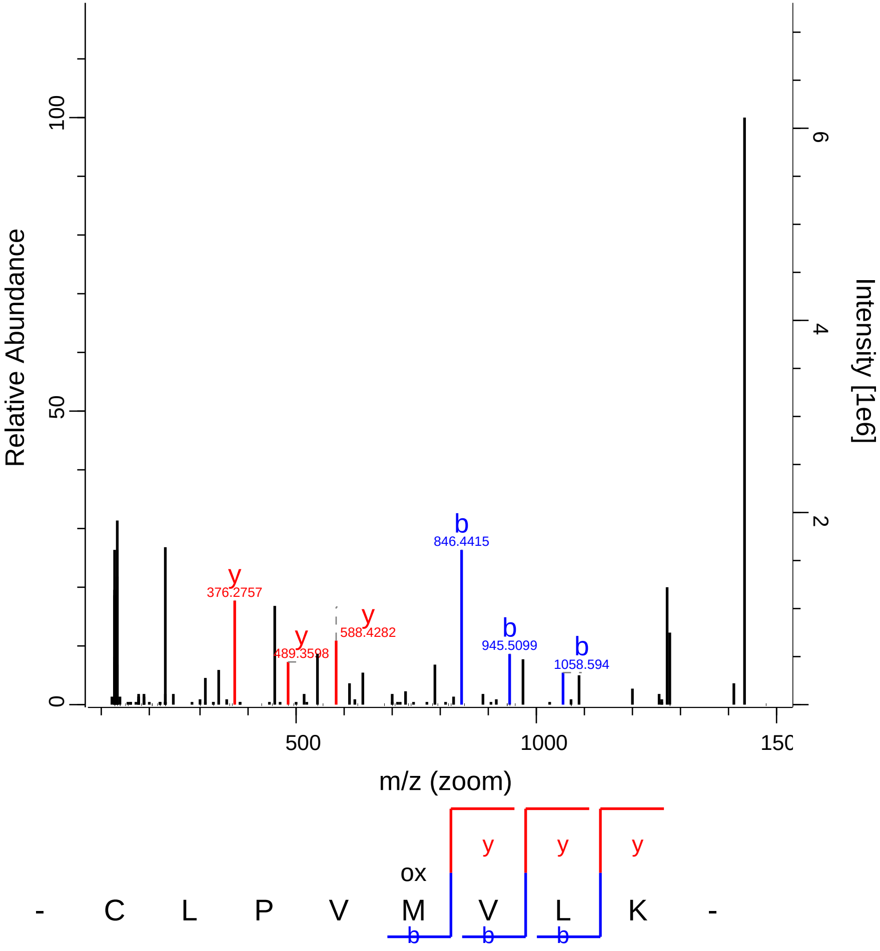

Raw File	Scan	Method	Score	m/z	Gene names
RO-QC-160121_ZackD_SS_Set_02_Fractio	25898	FTMS; HC	178.07	801.47	RASSF3

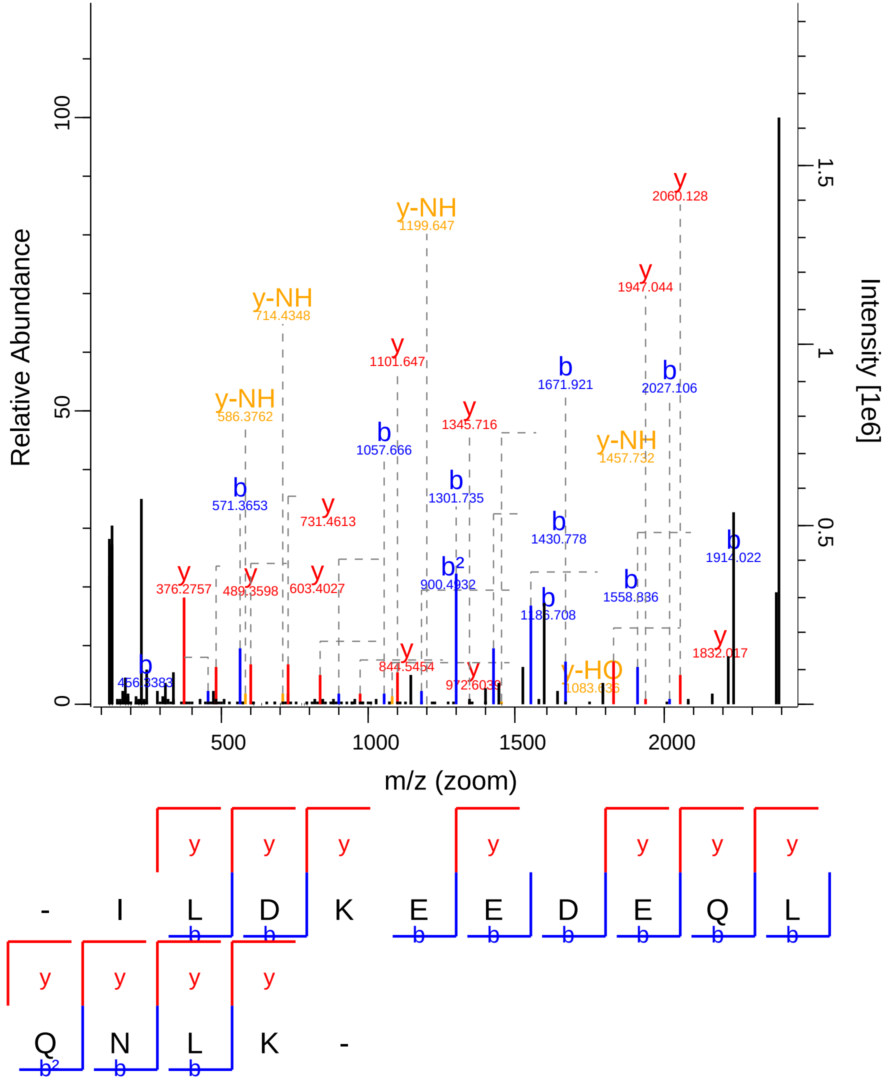

Raw File	Scan	Method	Score	m/z	Gene names
RO-QC-160121_ZackD_SS_Set_02_Fractio	26455	FTMS; HC	48.82	847.41	CNIH4

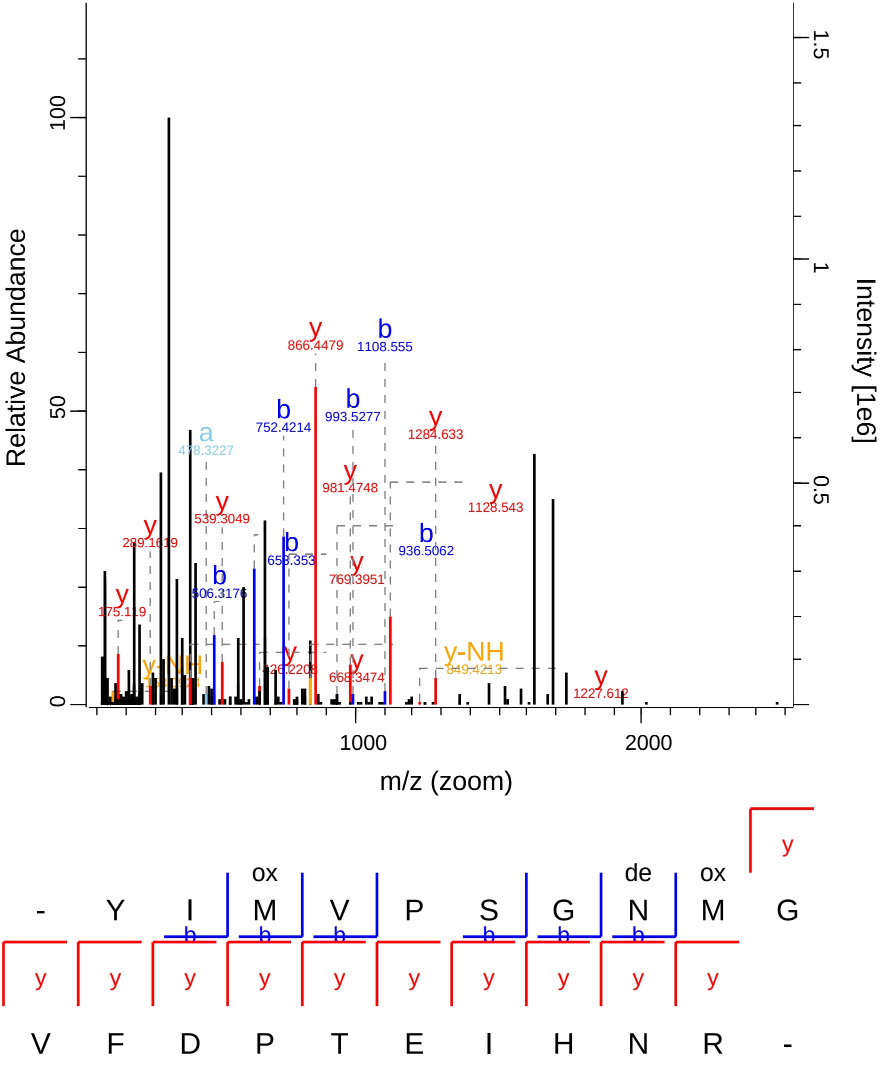

Raw File	Scan	Method	Score	m/z	Gene names
RO-QC-160121_ZackD_SS_Set_02_Fractio	26560	FTMS; HC	113.77	494.29	GATC

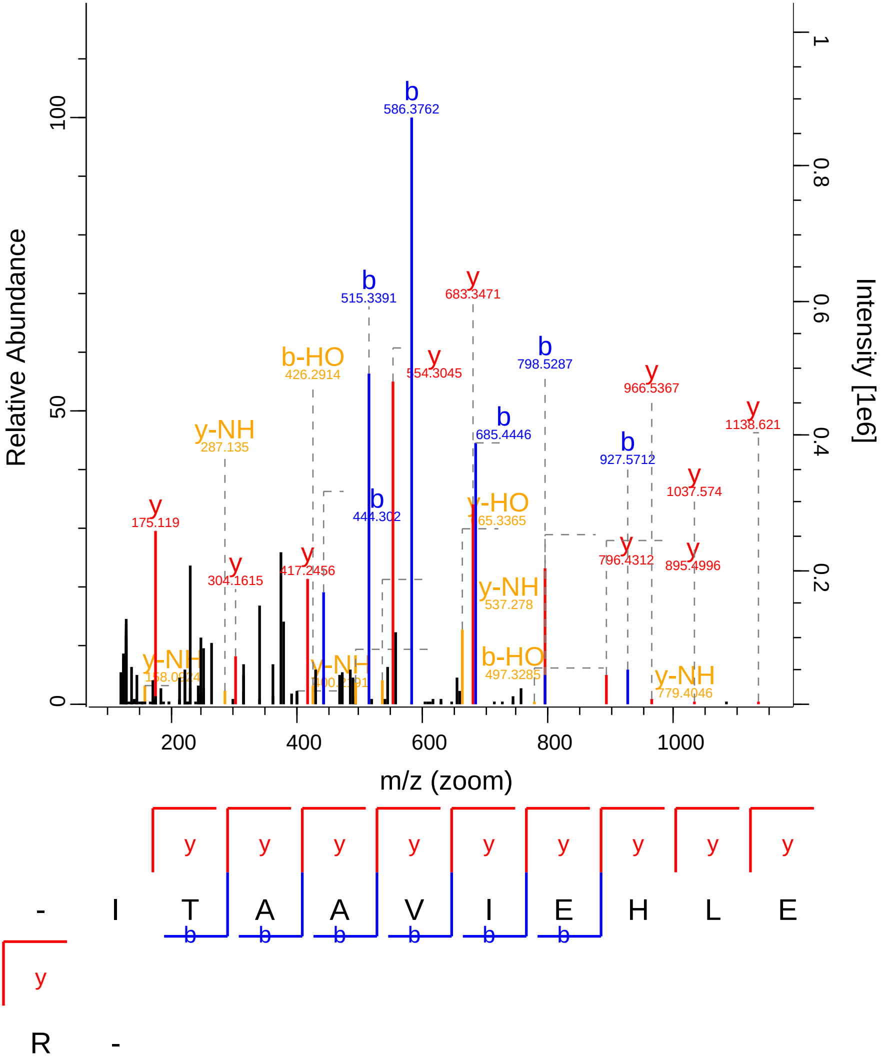

Raw File	Scan	Method	Score	m/z	Gene names
RO-QC-160121_ZackD_SS_Set_02_Fractio	26816	FTMS; HC	51.01	594.99	IFI44

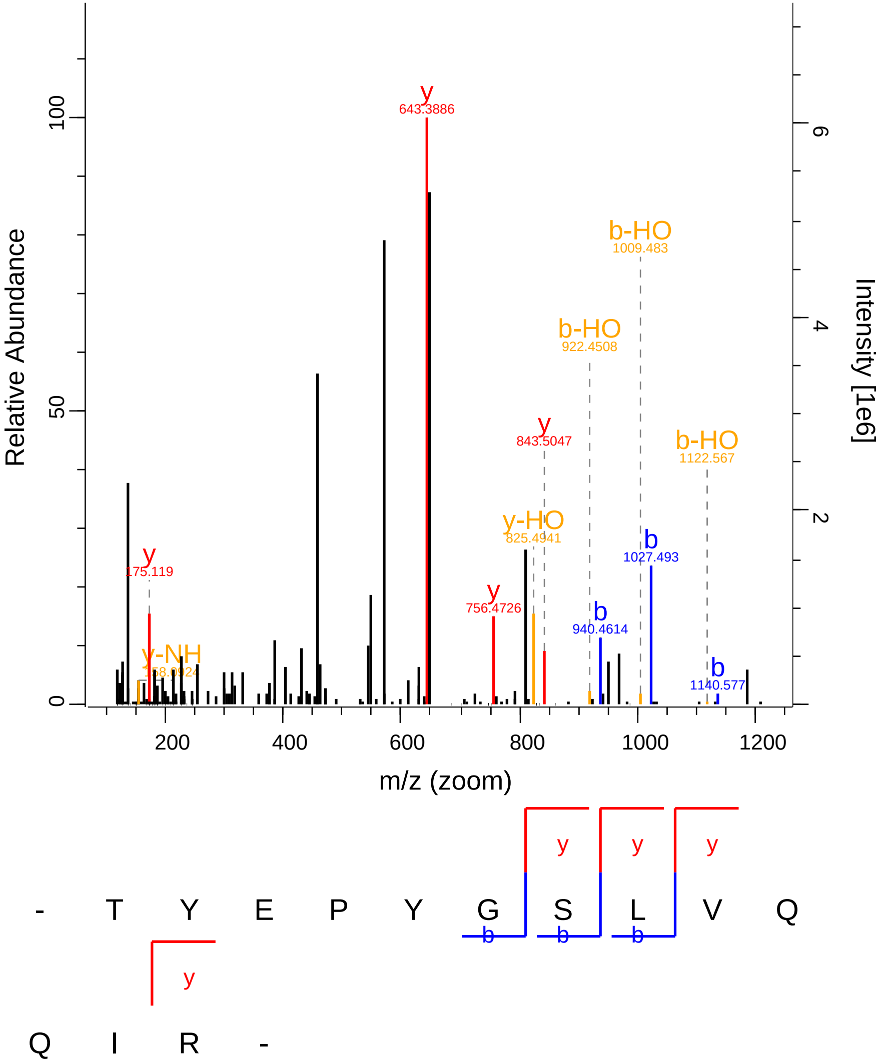

Raw File	Scan	Method	Score	m/z	Gene names
RO-QC-160121_ZackD_SS_Set_02_Fractio	27021	FTMS; HC	44.62	594.99	IFI44

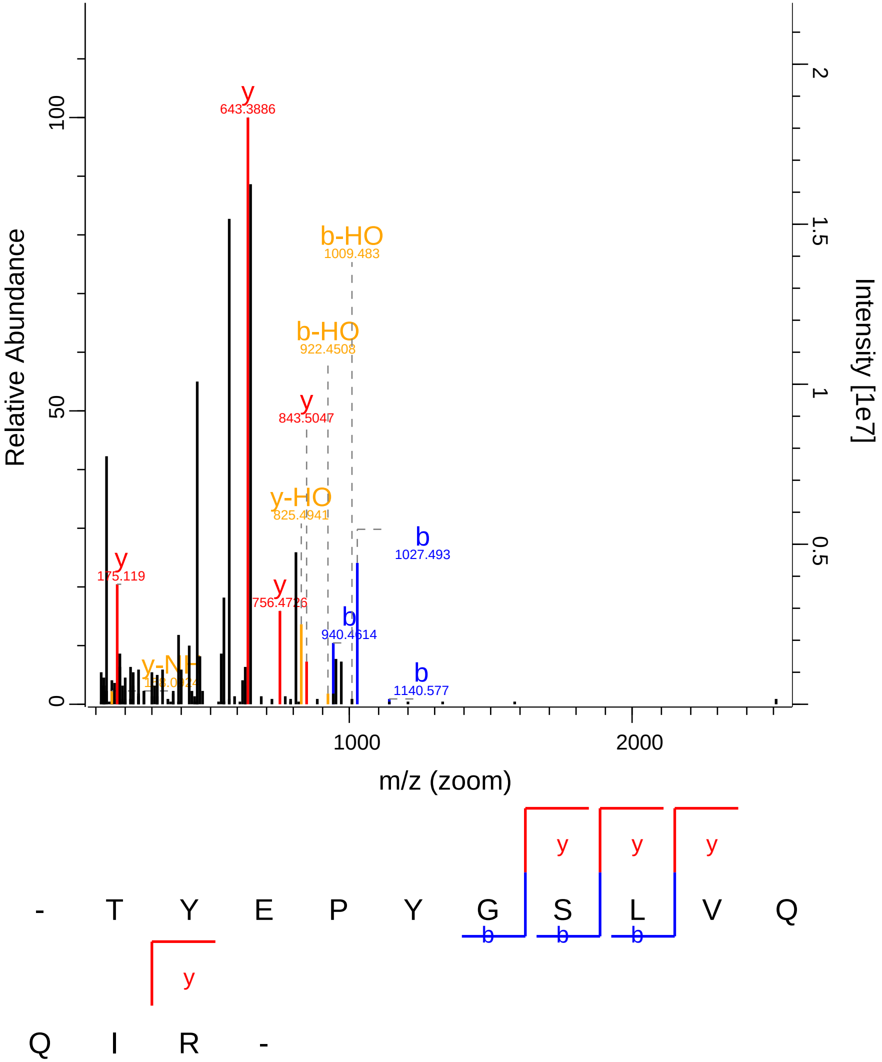

Raw File	Scan	Method	Score	m/z	Gene names
RO-QC-160121_ZackD_SS_Set_02_Fractio	27706	FTMS; HC	107.21	644.38	DNLZ

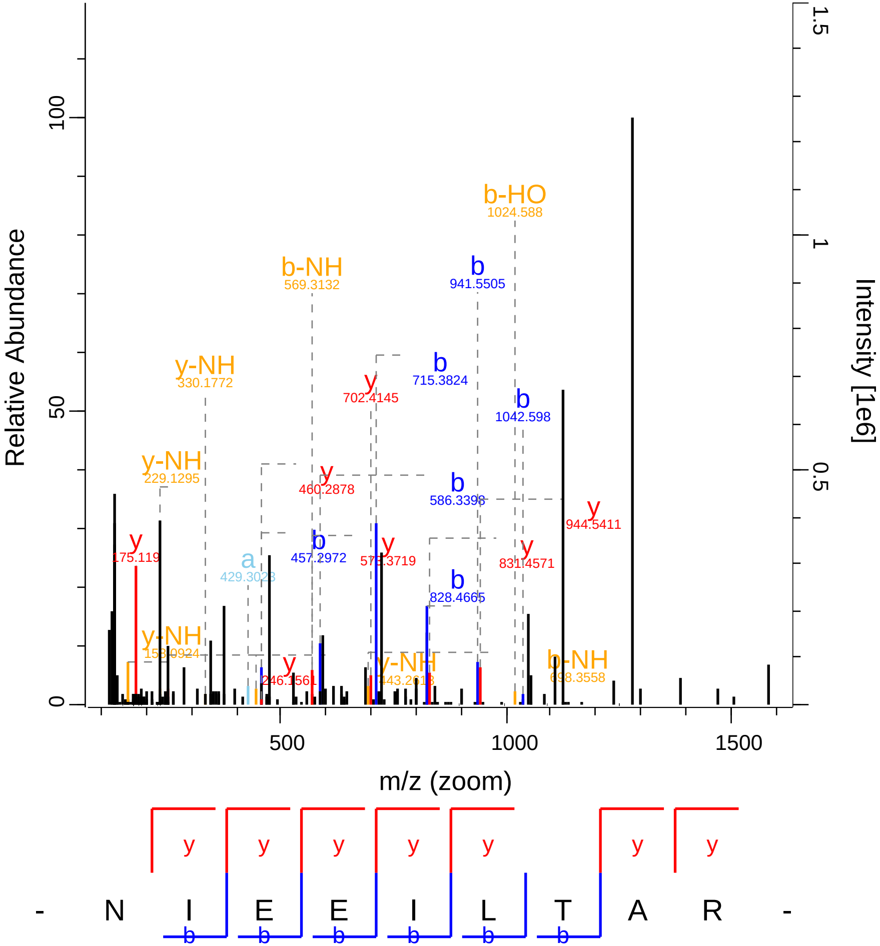

Raw File	Scan	Method	Score	m/z	Gene names
RO-QC-160121_ZackD_SS_Set_02_Fractio	27738	FTMS; HC	159.74	983.52	C1GALT1C1

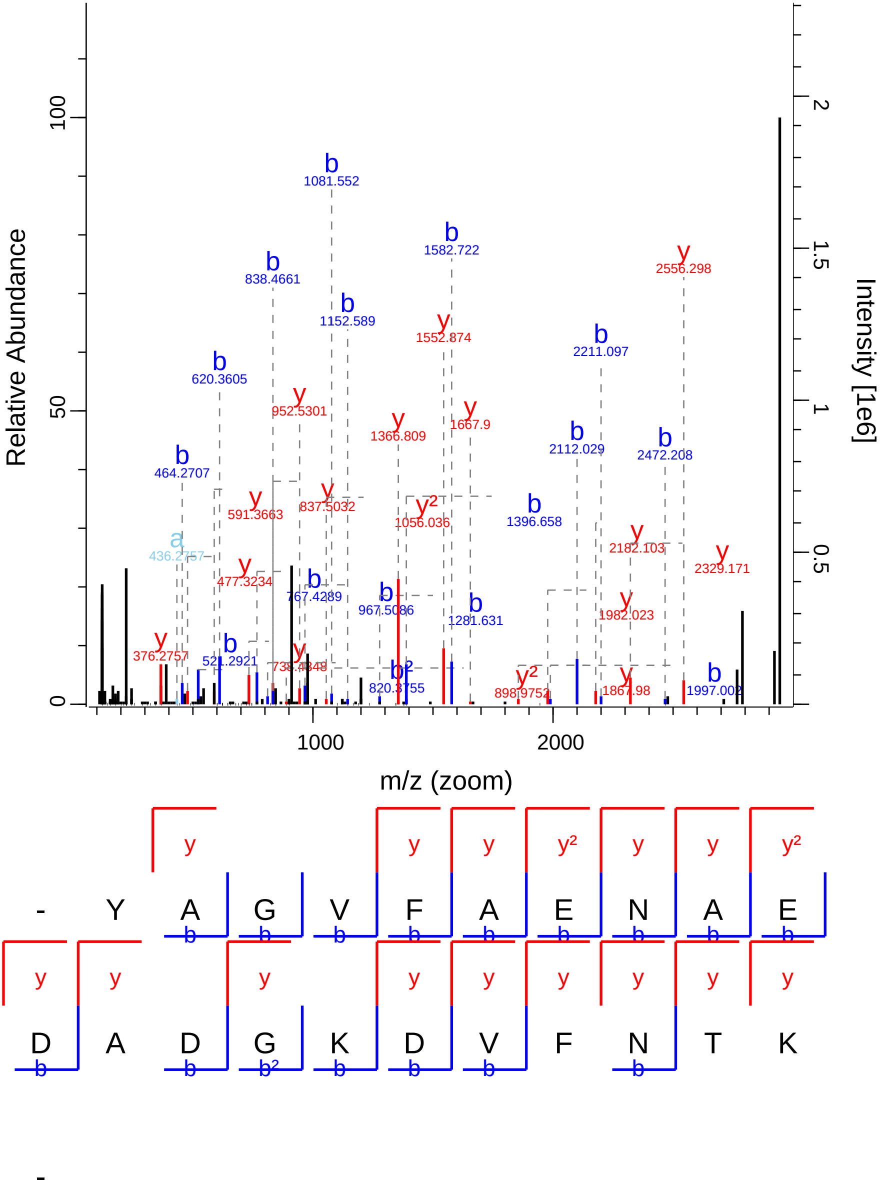

Raw File	Scan	Method	Score	m/z	Gene names
RO-QC-160121_ZackD_SS_Set_02_Fractio	27753	FTMS; HC	135.73	738.14	C1GALT1C1

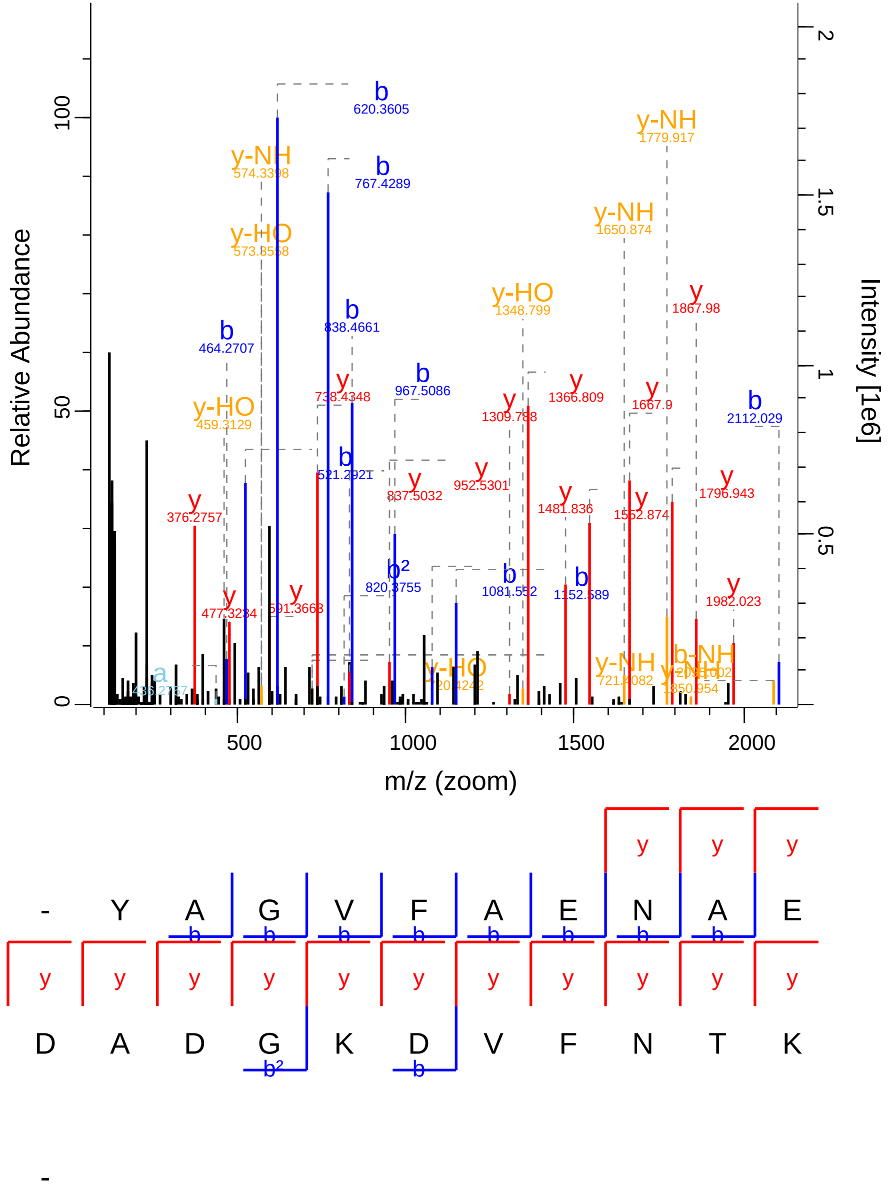

Raw File	Scan	Method	Score	m/z	Gene names
RO-QC-160121_ZackD_SS_Set_02_Fractio	28259	FTMS; HC	26.15	666.05	B3GALNT1

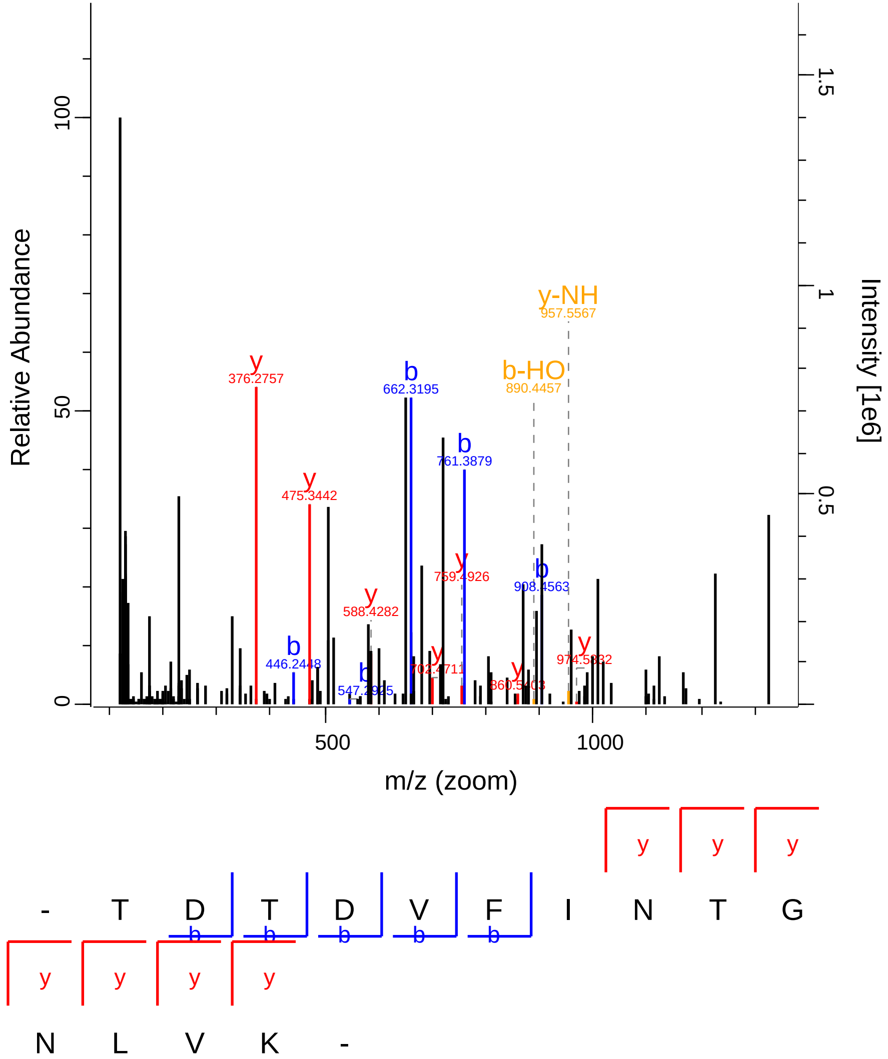

Raw File	Scan	Method	Score	m/z	Gene names
RO-QC-160121_ZackD_SS_Set_02_Fractio	28419	FTMS; HC	85.29	884.47	USP21

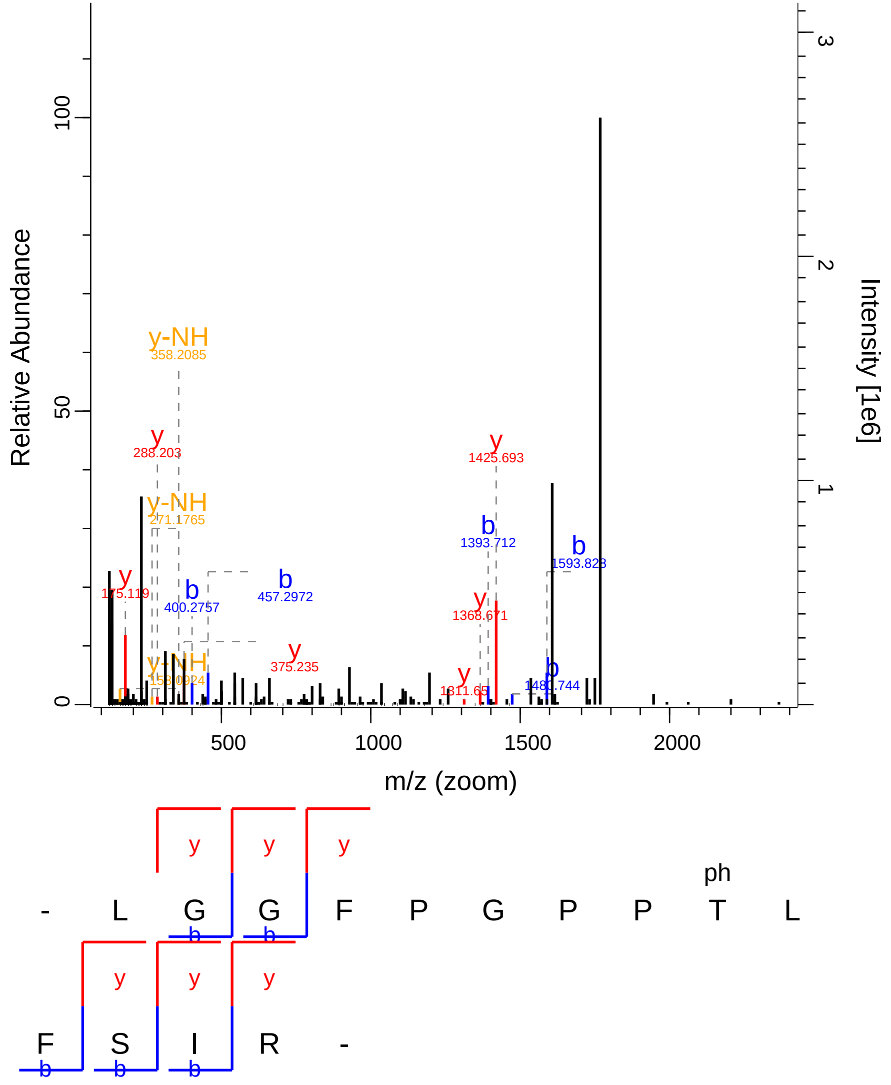

Raw File	Scan	Method	Score	m/z
RO-QC-160121_ZackD_SS_Set_02_Fraction_5	28424	FTMS; HCD	61.68	459.23

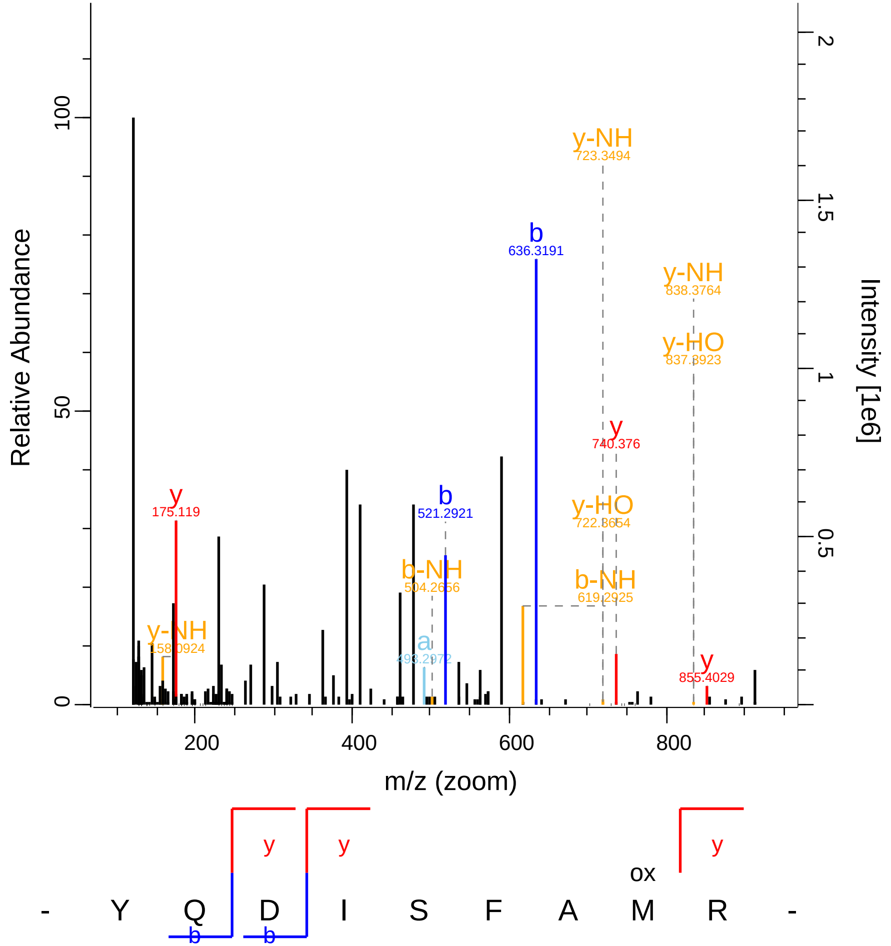

Raw File	Scan	Method	Score	m/z
RO-QC-160121_ZackD_SS_Set_02_Fraction_5	28465	FTMS; HCD	62.46	688.35

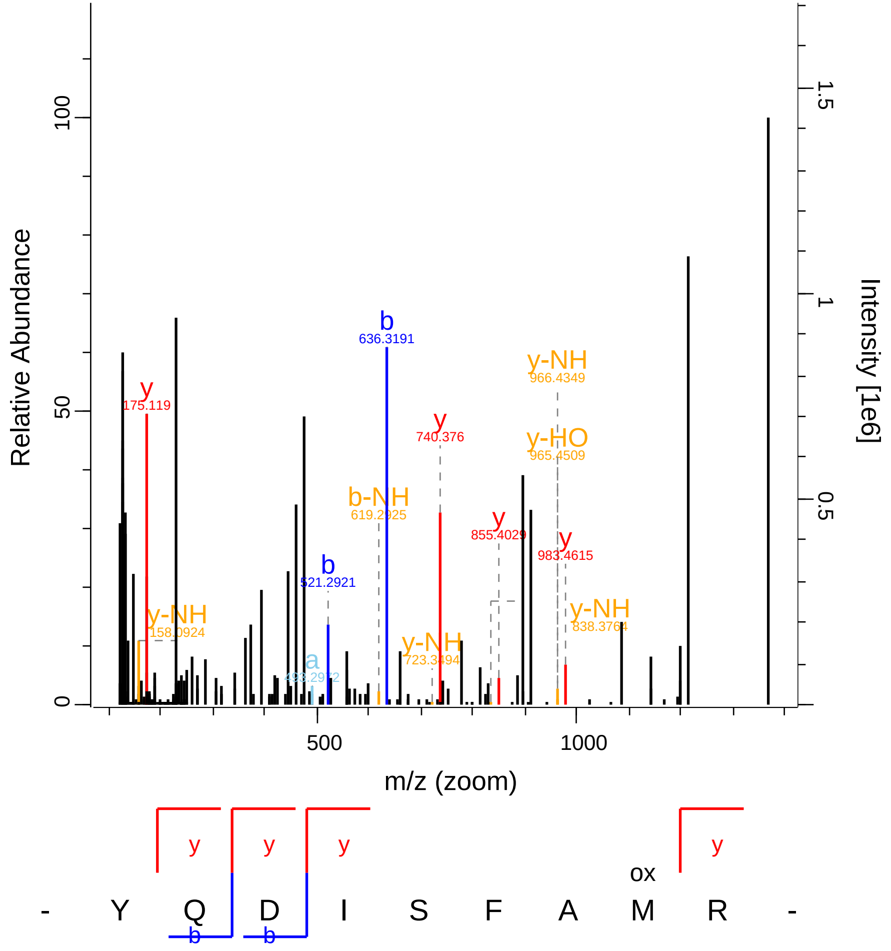

Raw File	Scan	Method	Score	m/z	Gene names
RO-QC-160121_ZackD_SS_Set_02_Fractior	29061	FTMS; HCI	84.72	612.7	RAB20

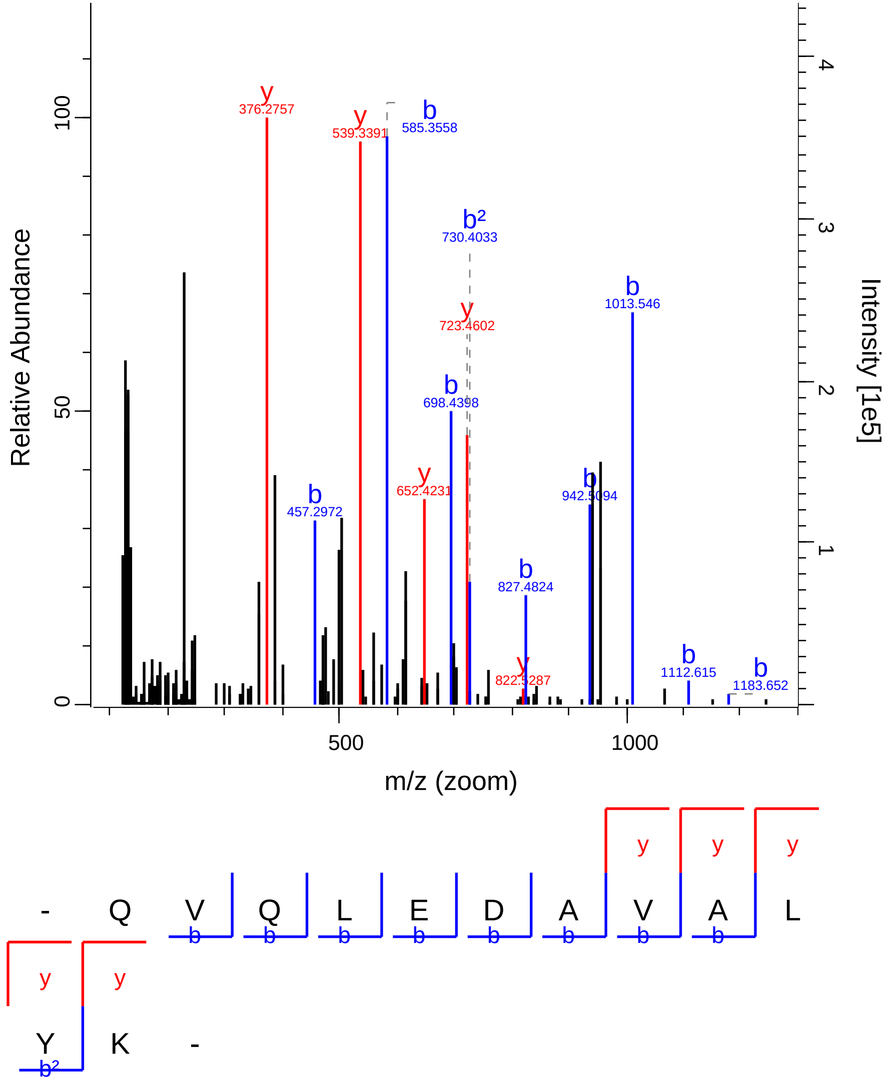

Raw File	Scan	Method	Score	m/z	Gene names
RO-QC-160121_ZackD_SS_Set_02_Fractio	29800	FTMS; HC	84.17	901.54	FAM184B

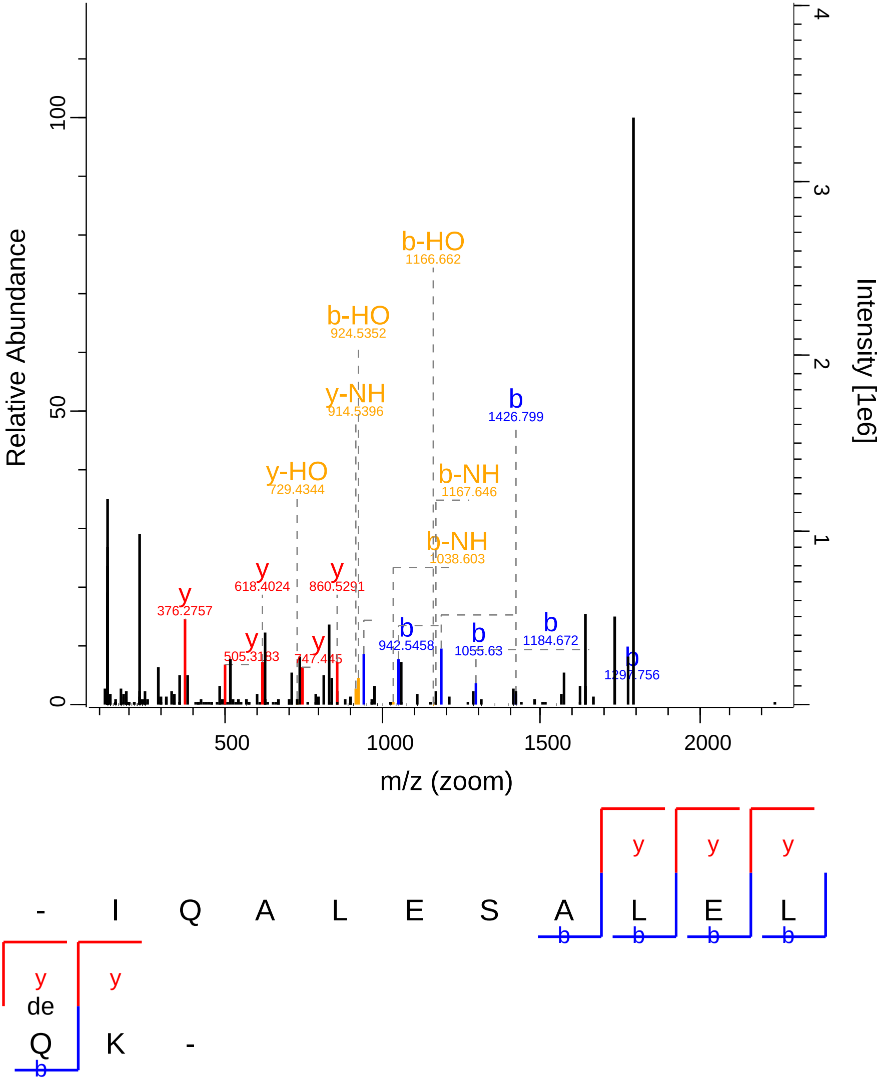

Raw File	Scan	Method	Score	m/z	Gene names
RO-QC-160121_ZackD_SS_Set_02_Fractio	30642	FTMS; HC	60.44	963.54	OR7A10

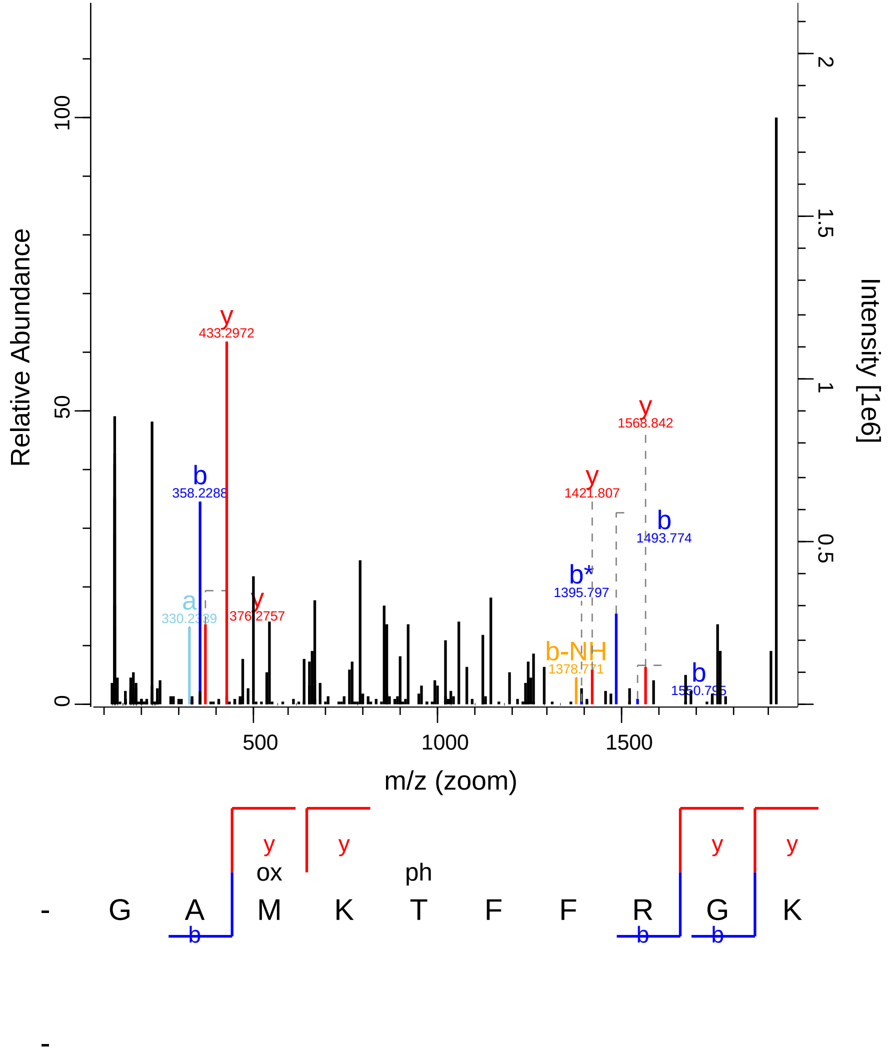

Raw File	Scan	Method	Score	m/z	Gene names
RO-QC-160121_ZackD_SS_Set_02_Fractic	31004	FTMS; HC	61.65	1160.56	SGOL2

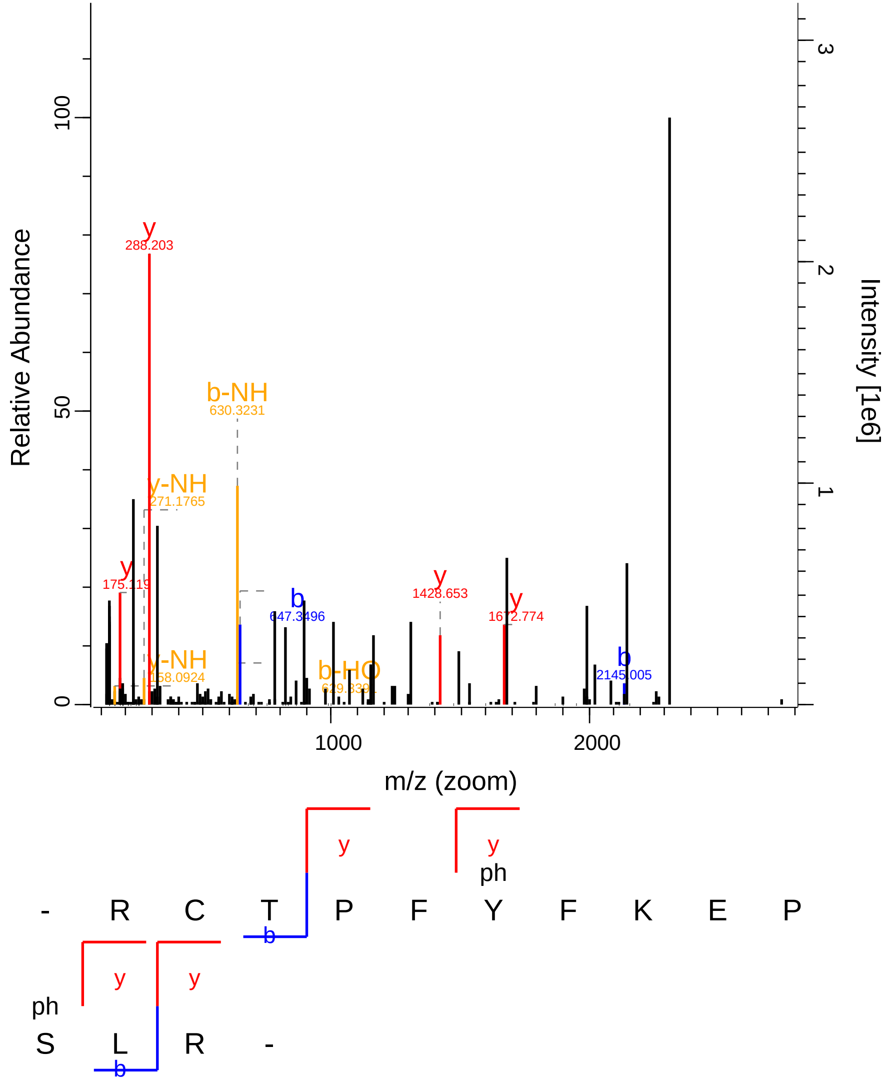

Raw File	Scan	Method	Score	m/z	Gene names
RO-QC-160121_ZackD_SS_Set_02_Fractio	31125	FTMS; HC	138.66	782.95	PUS10

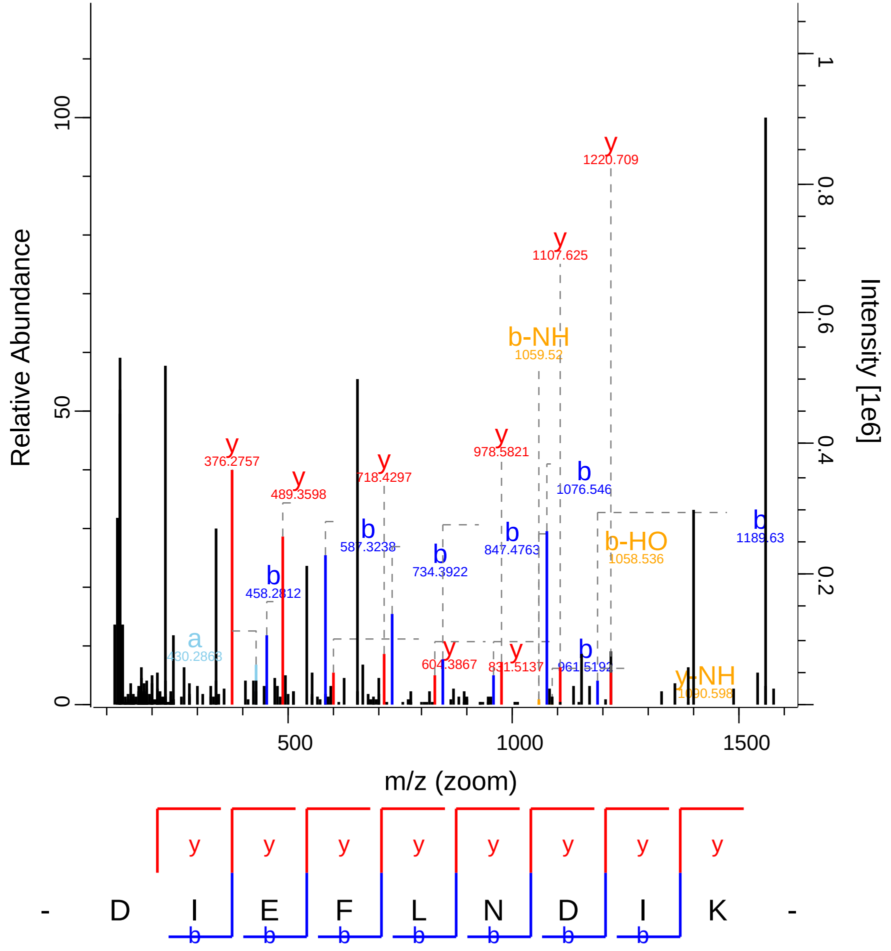

Raw File	Scan	Method	Score	m/z	Gene names
RO-QC-160121_ZackD_SS_Set_02_Fractio	31187	FTMS; HC	87.09	717.05	GPT2;GPT

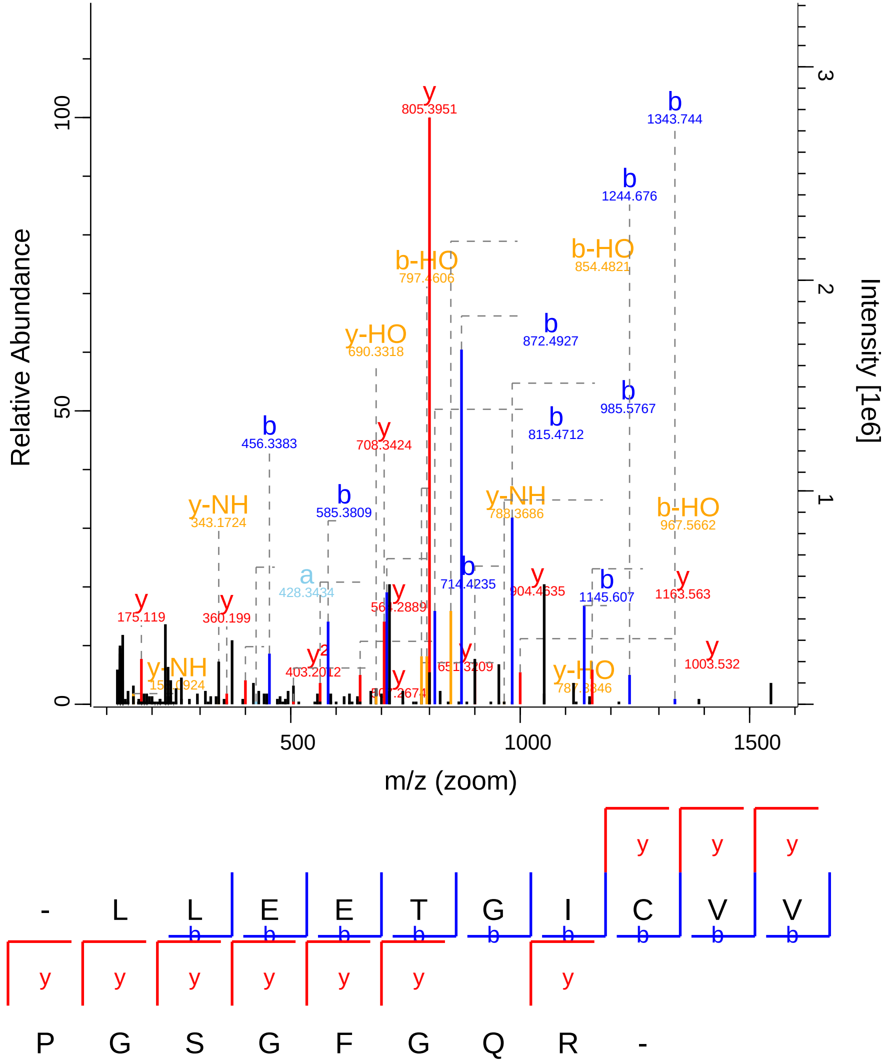

Raw File	Scan	Method	Score	m/z	Gene names
RO-QC-160121_ZackD_SS_Set_02_Fractio	31218	FTMS; HC	131.22	660.89	FAM96A

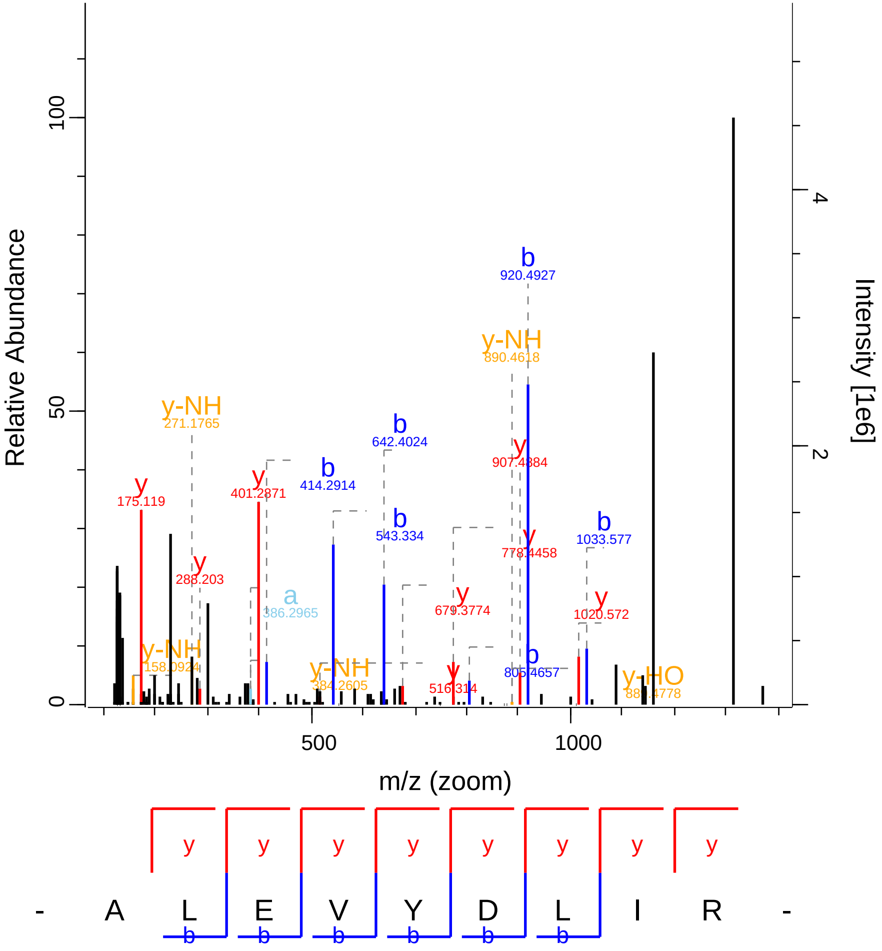

Raw File	Scan	Method	Score	m/z	Gene names
RO-QC-160121_ZackD_SS_Set_02_Fractio	31239	FTMS; HC	176.91	1075.07	GPT2;GPT

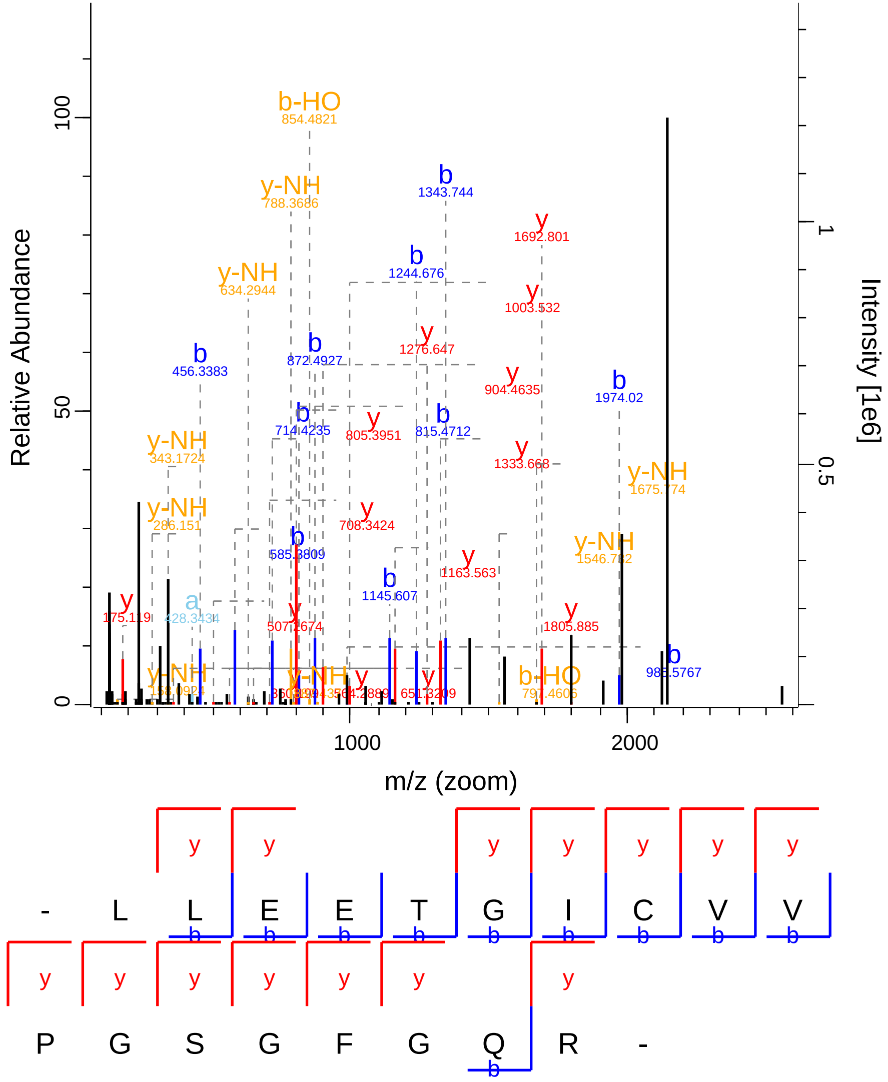

Raw File	Scan	Method	Score	m/z	Gene names
RO-QC-160121_ZackD_SS_Set_02_Fractio	32105	FTMS; HC	72.94	657.37	CD96

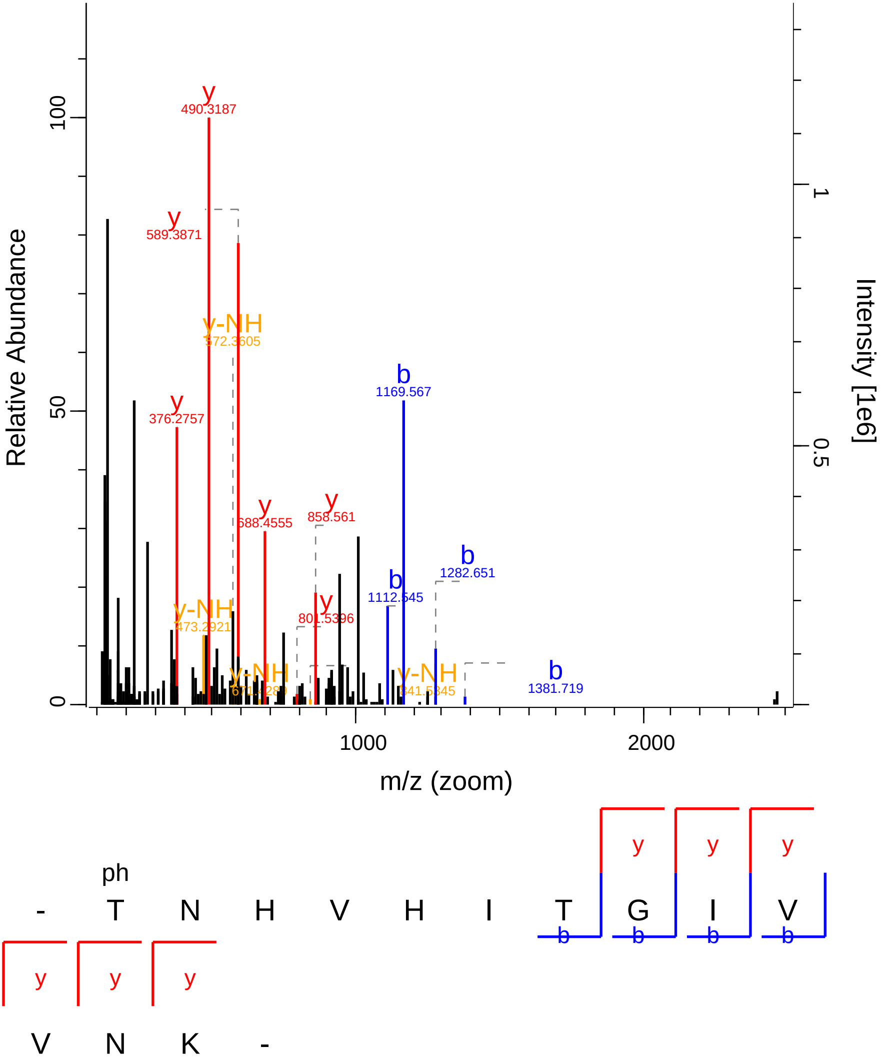

Raw File	Scan	Method	Score	m/z	Gene names
RO-QC-160121_ZackD_SS_Set_02_Fractior	32129	FTMS; HCI	117.02	731.9	PEX7

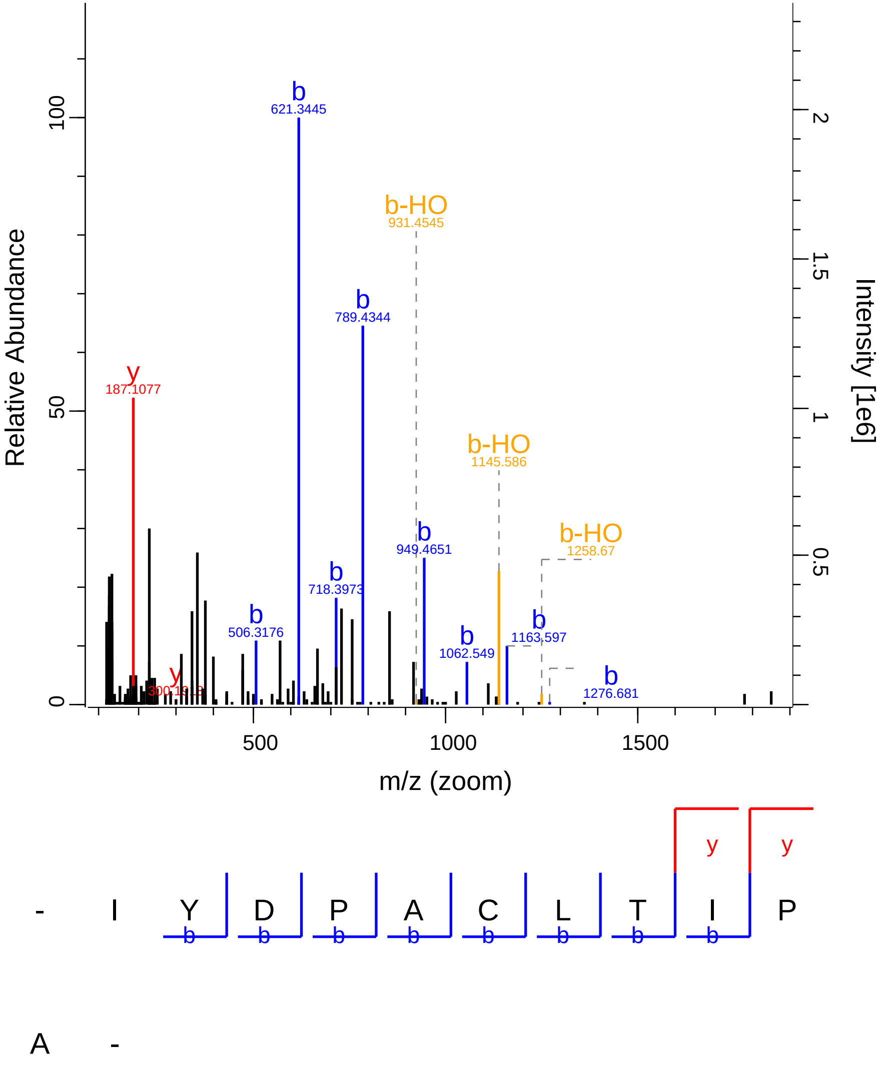

Raw File	Scan	Method	Score	m/z	Gene names
RO-QC-160121_ZackD_SS_Set_02_Fractio	33559	FTMS; HC	120.29	770.75	DNASE1L1

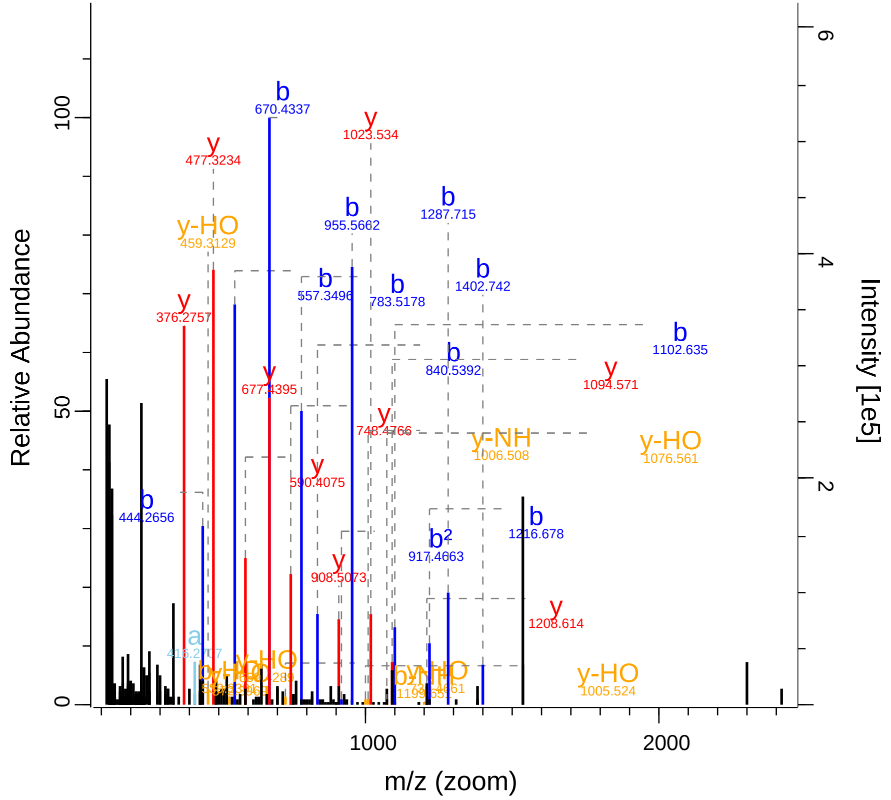

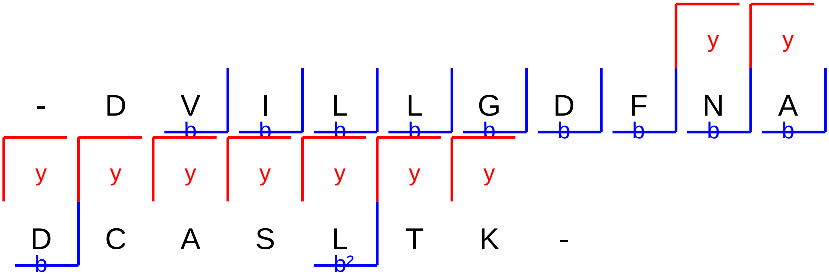

Raw File	Scan	Method	Score	m/z	Gene names
RO-QC-160121_ZackD_SS_Set_02_Fractio	34258	FTMS; HC	73.21	895.03	PPEF1

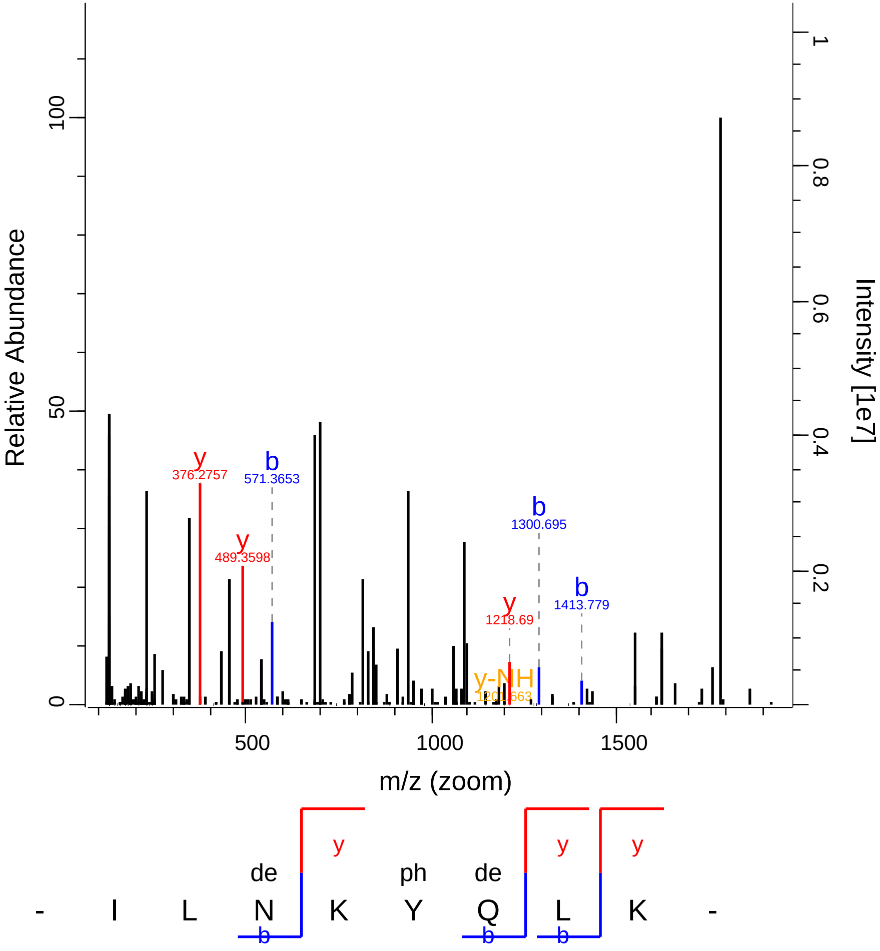

Raw File	Scan	Method	Score	m/z	Gene names
RO-QC-160121_ZackD_SS_Set_02_Fractio	35203	FTMS; HC	101.65	796.93	KLHDC9

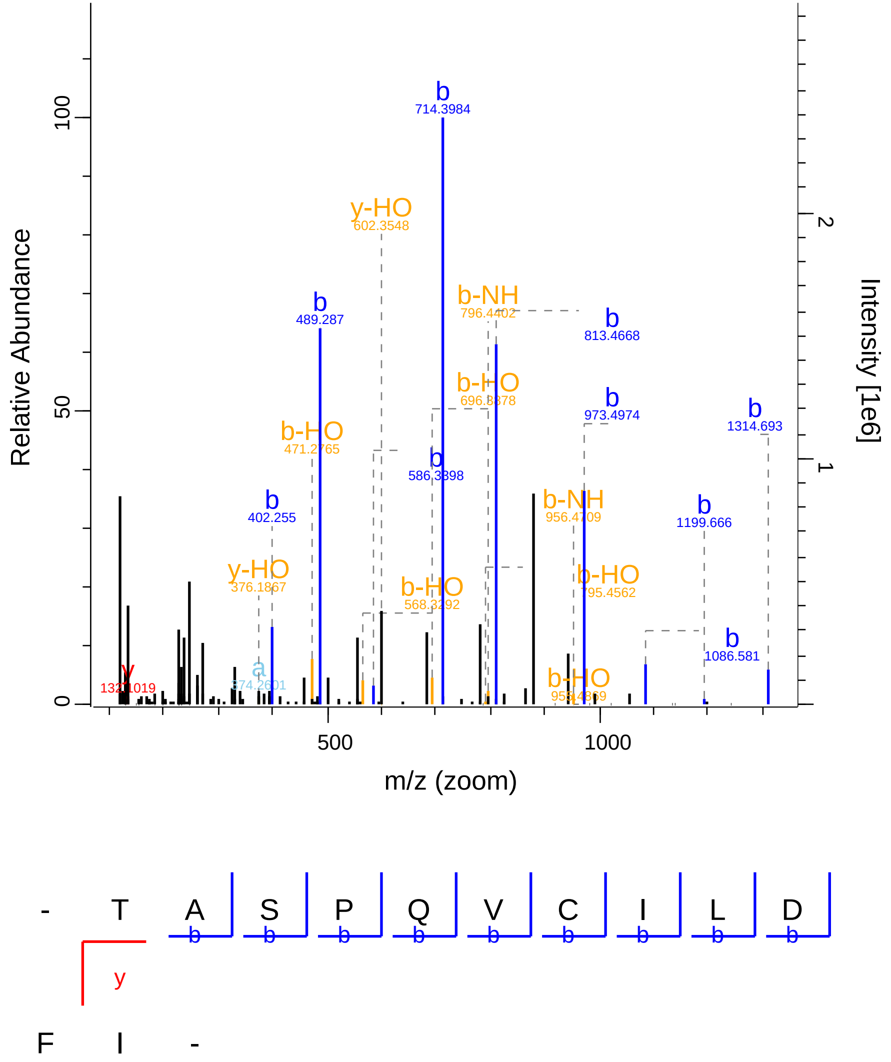

Raw File	Scan	Method	Score	m/z
RO-QC-160121_ZackD_SS_Set_02_Fraction_6	4165	FTMS; HCD	40.99	466.25

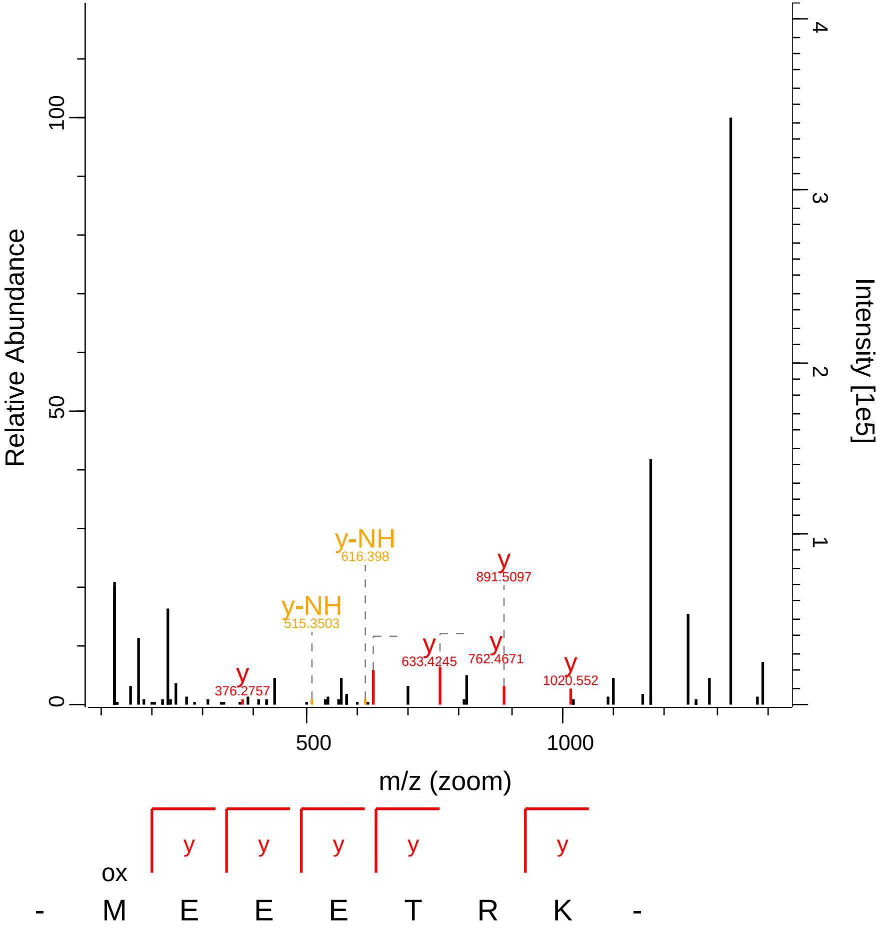

Raw File	Scan	Method	Score	m/z	Gene names
RO-QC-160121_ZackD_SS_Set_02_Fractior	4392	FTMS; HCI	88.58	507.95	METTL25

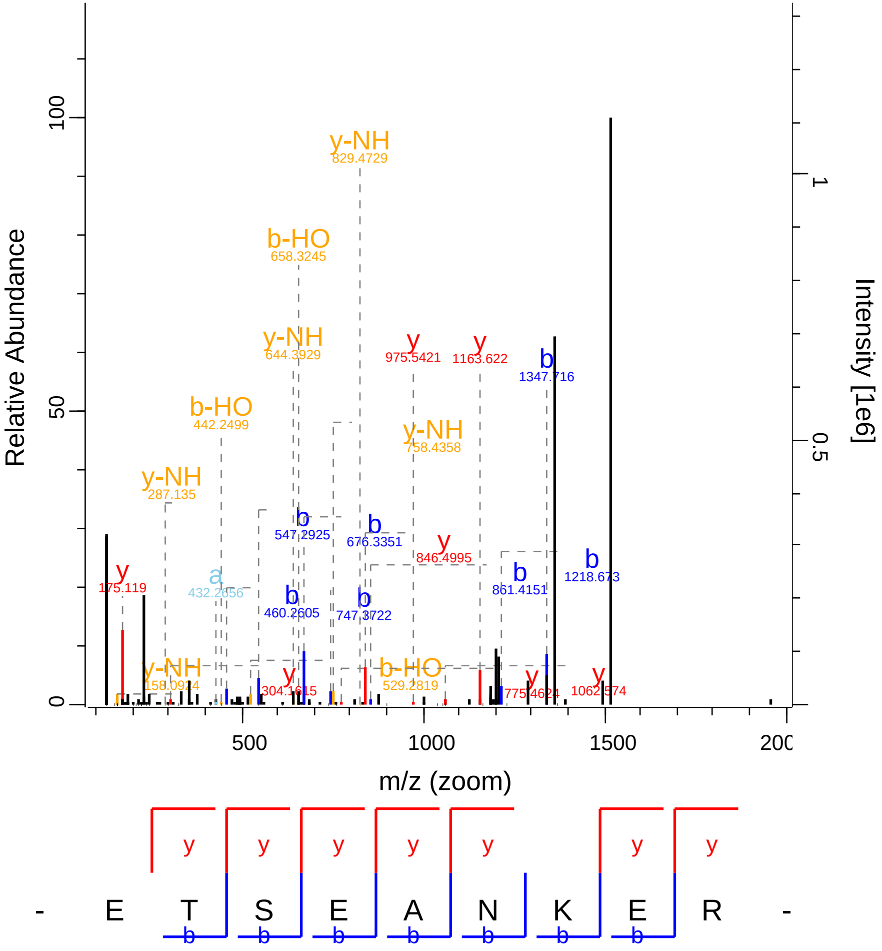

Raw File	Scan	Method	Score	m/z	Gene names
RO-QC-160121_ZackD_SS_Set_02_Fractior	4460	FTMS; HCI	90.71	675.66	TRPM1

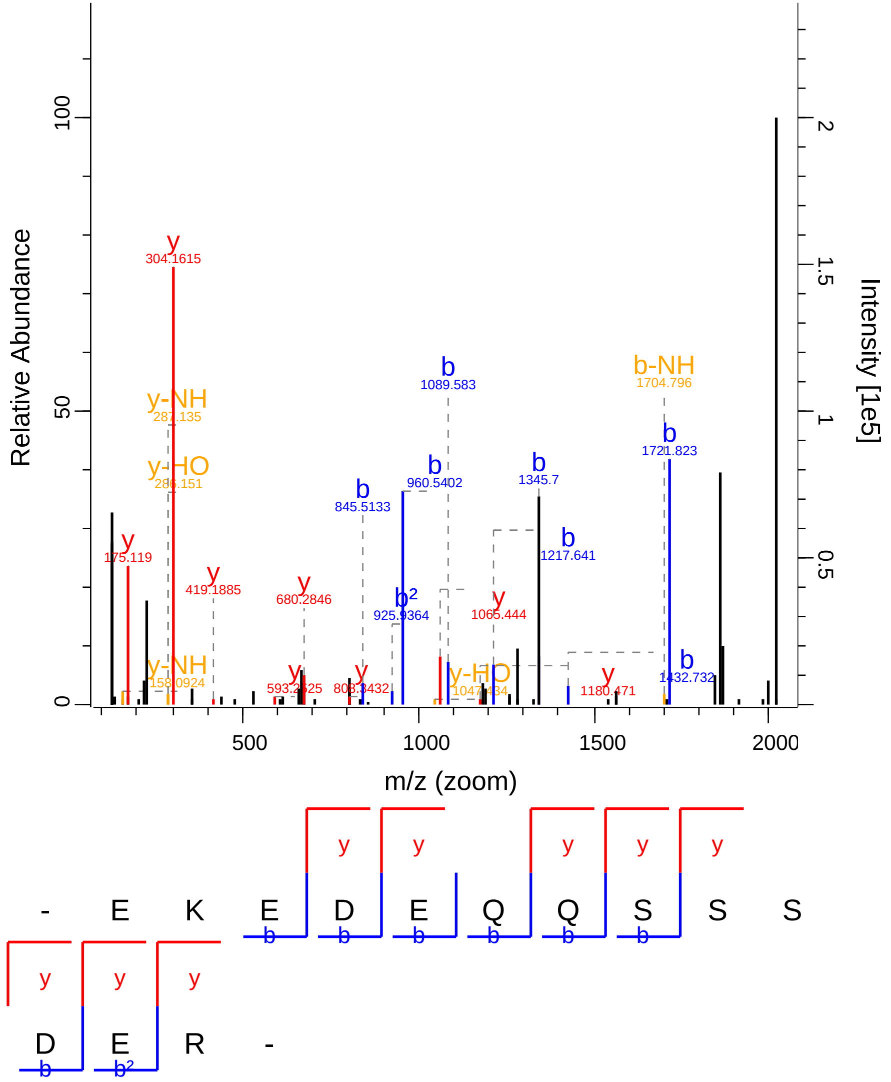

Raw File	Scan	Method	Score	m/z	Gene names
RO-QC-160121_ZackD_SS_Set_02_Fractior	4866	FTMS; HCI	54.28	638.97	SUV420H1

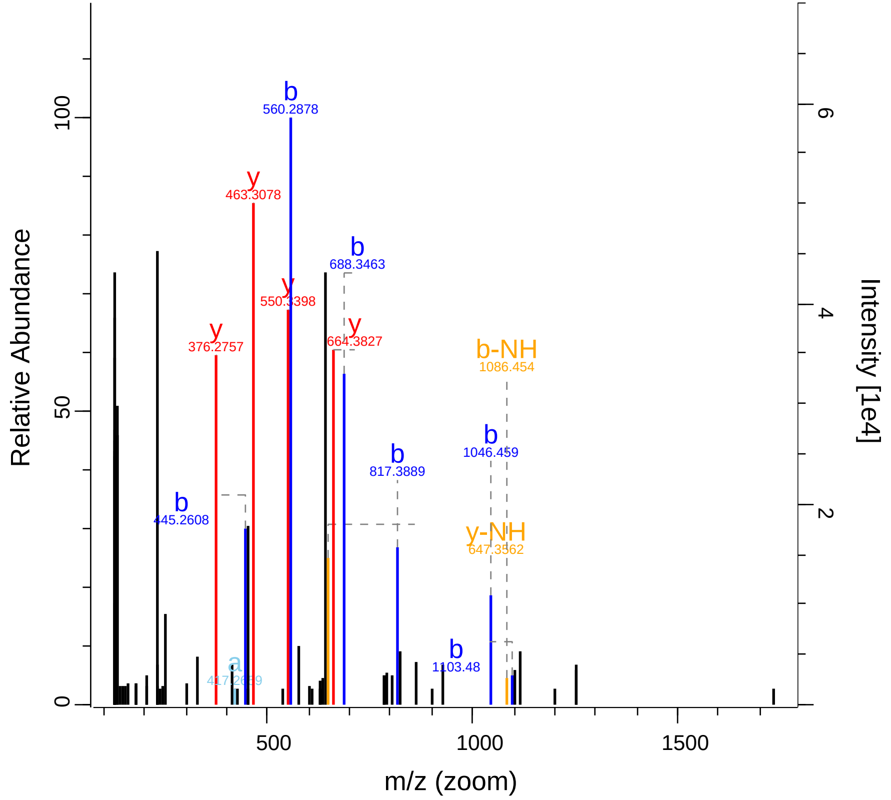

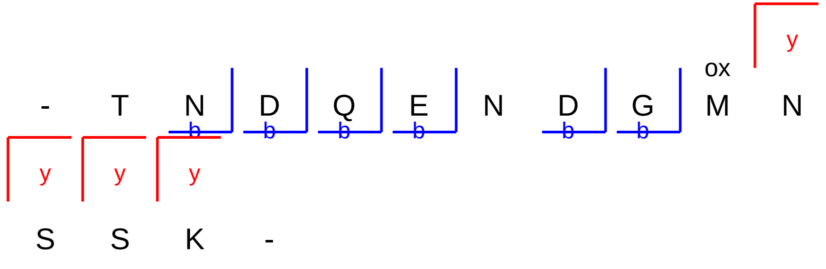

Raw File	Scan	Method	Score	m/z	Gene names
RO-QC-160121_ZackD_SS_Set_02_Fractior	4879	FTMS; HCI	47.44	611.31	INPP5B

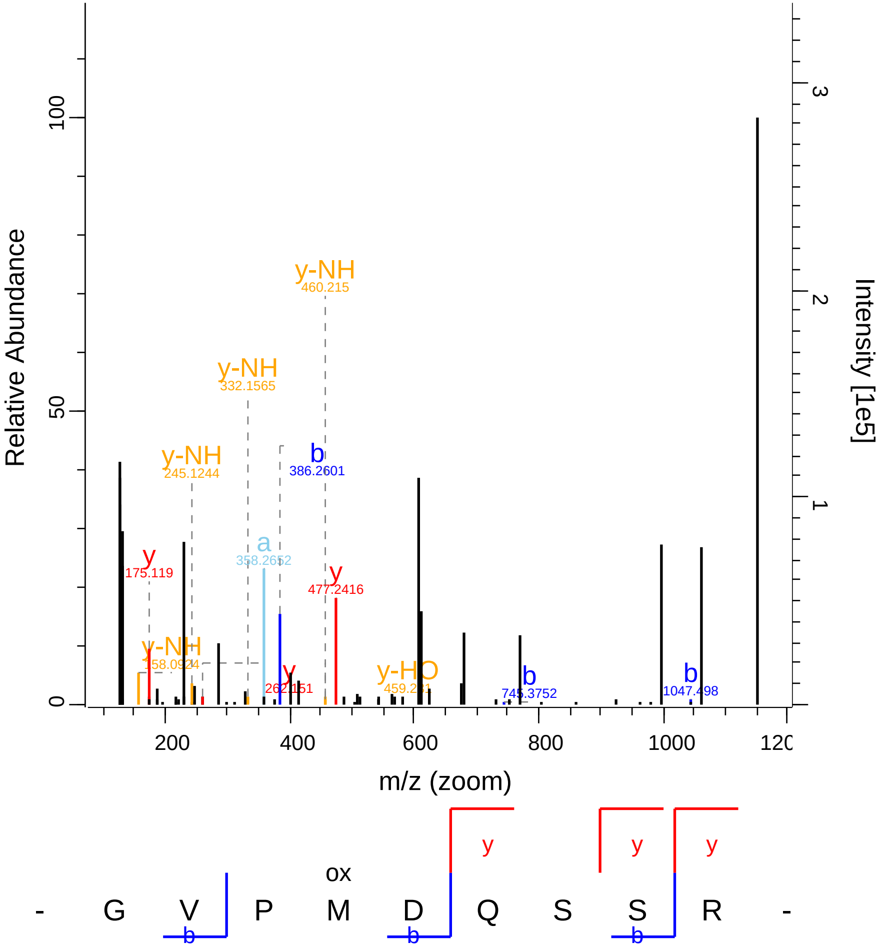

Raw File	Scan	Method	Score	m/z	Gene names
RO-QC-160121_ZackD_SS_Set_02_Fractior	5209	FTMS; HCI	96.71	636.28	OLFML2A

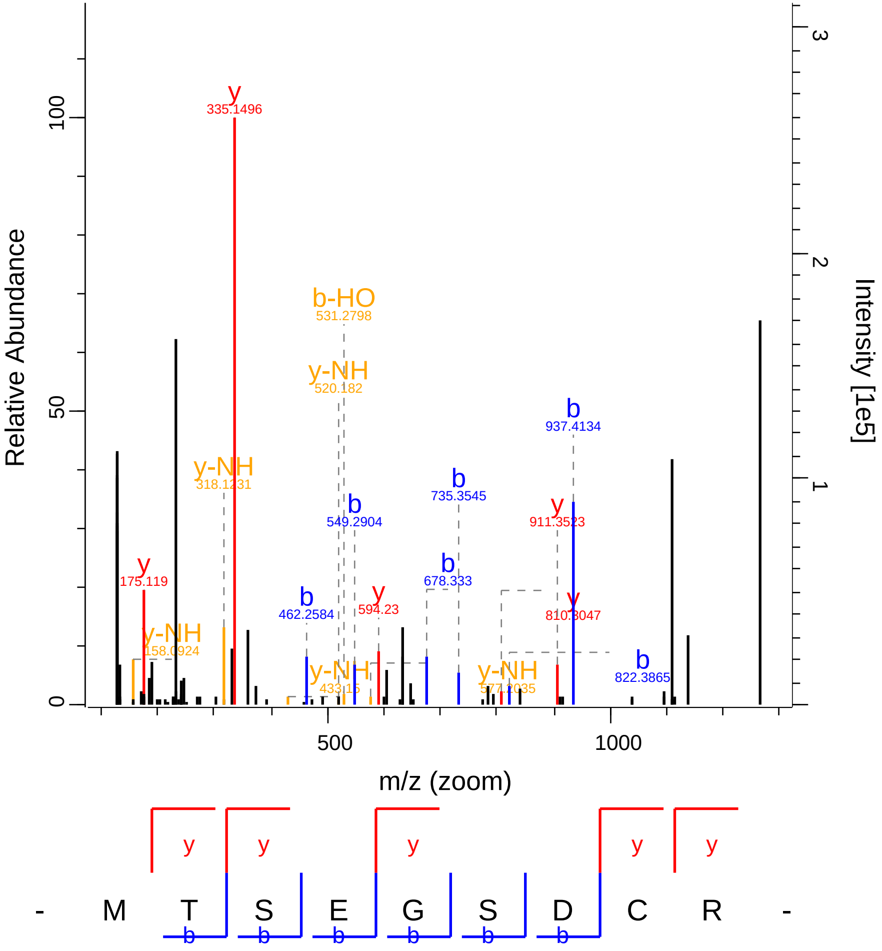

Raw File	Scan	Method	Score	m/z	Gene names
RO-QC-160121_ZackD_SS_Set_02_Fractio	5415	FTMS; HC	113.41	566.79	NXNL1

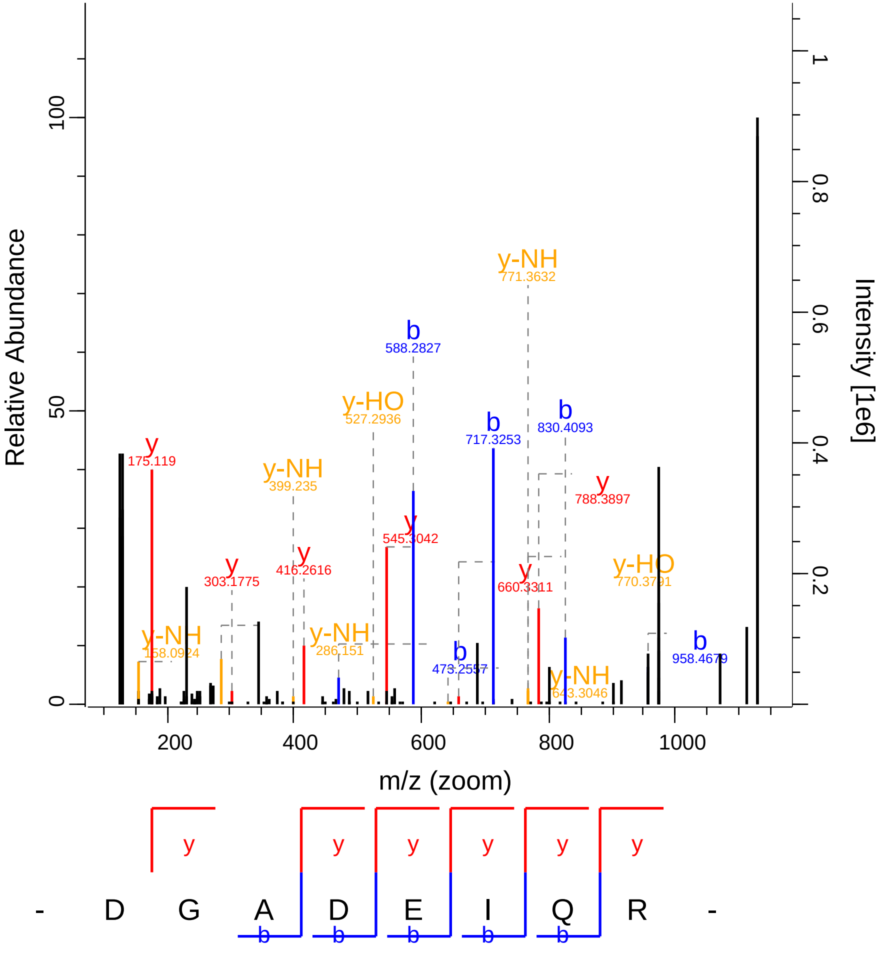

Raw File	Scan	Method	Score	m/z	Gene names
RO-QC-160121_ZackD_SS_Set_02_Fractio	5838	FTMS; HC	110.66	787.88	PCSK1N

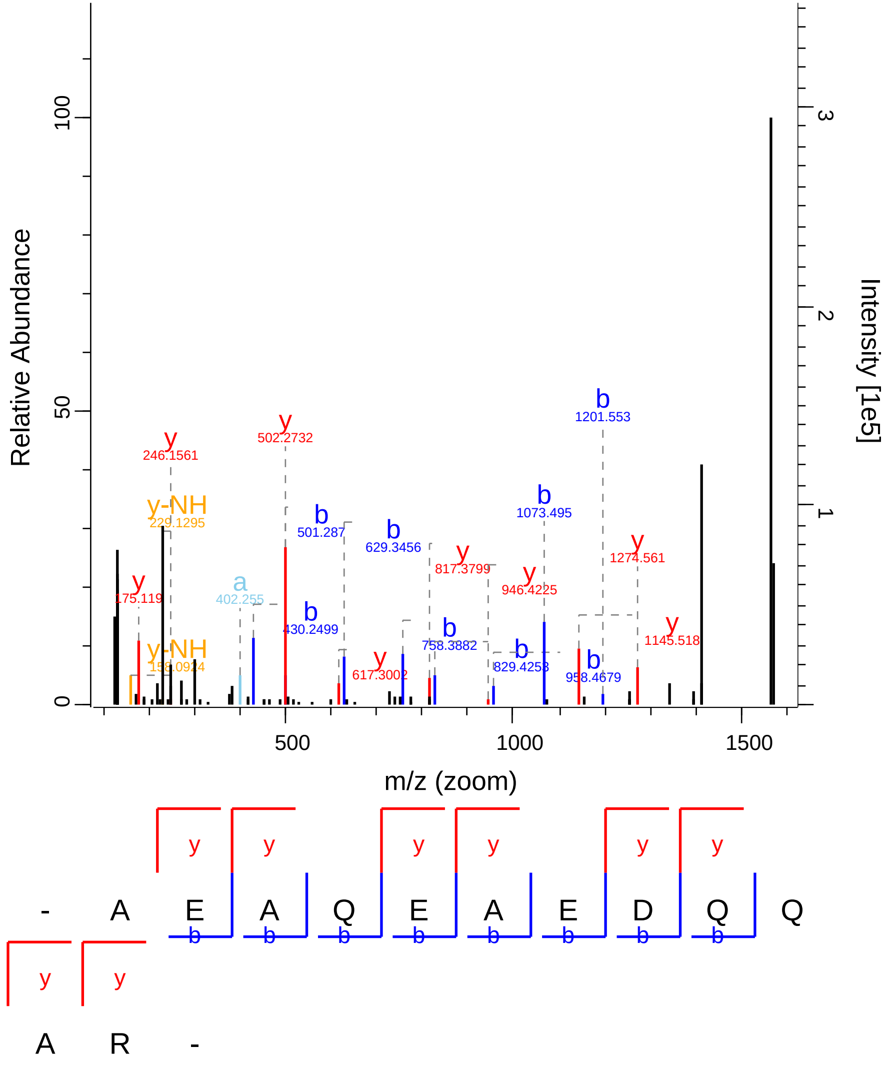

Raw File	Scan	Method	Score	m/z	Gene names
RO-QC-160121_ZackD_SS_Set_02_Fractior	6052	FTMS; HCl	81.97	819.42	TMEM132B

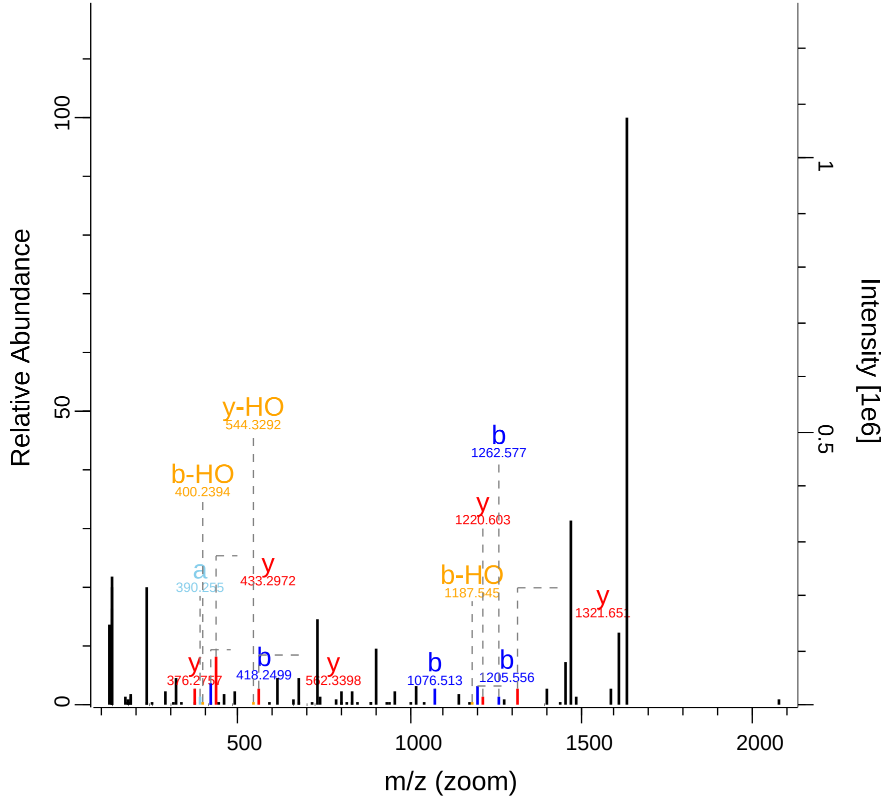

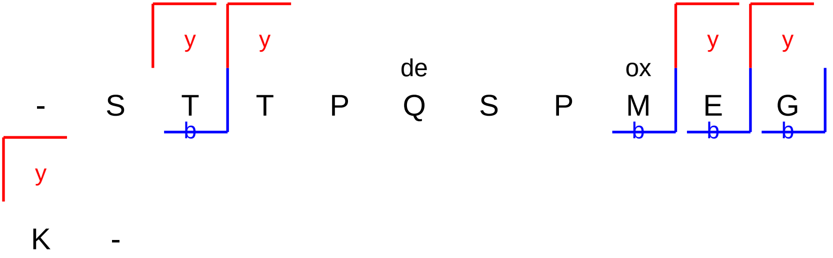

Raw File	Scan	Method	Score	m/z
RO-QC-160121_ZackD_SS_Set_02_Fraction_6	6070	FTMS; HCD	40.72	606.66

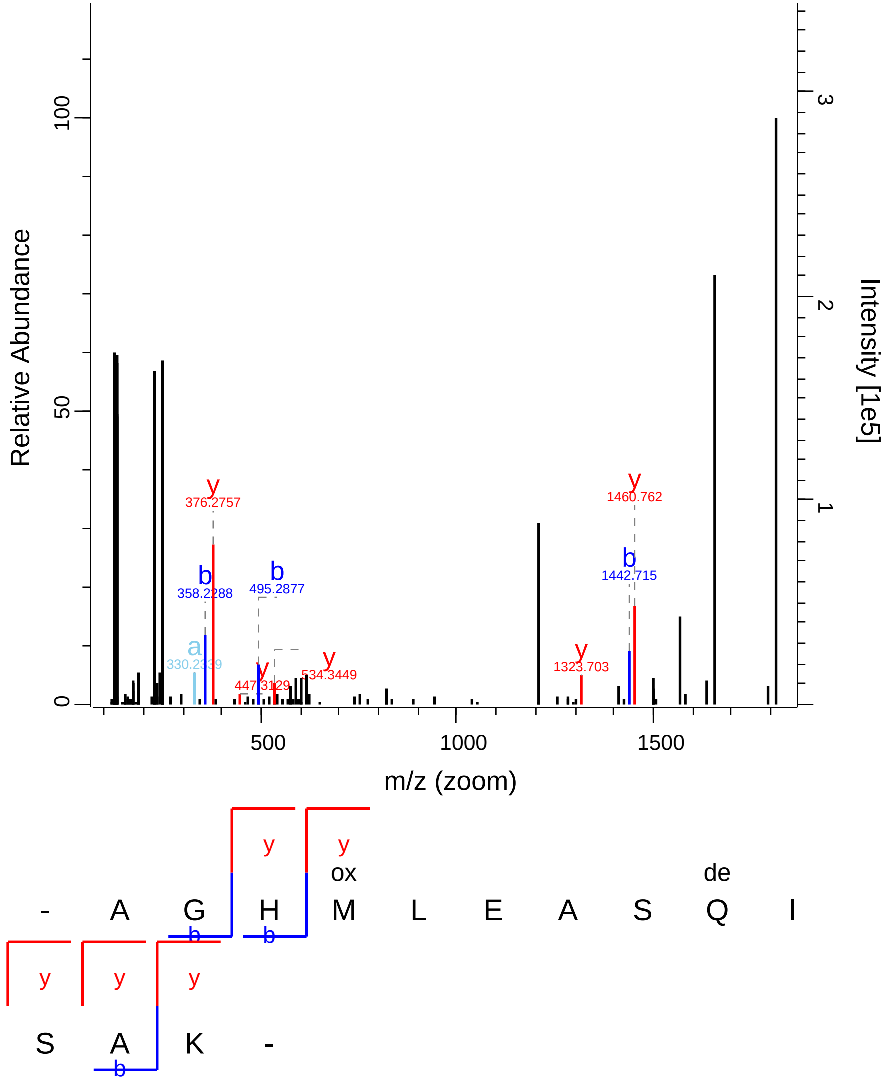

Raw File	Scan	Method	Score	m/z	Gene names
RO-QC-160121_ZackD_SS_Set_02_Fractior	6083	FTMS; HCI	69.31	672.34	ADAMTS3

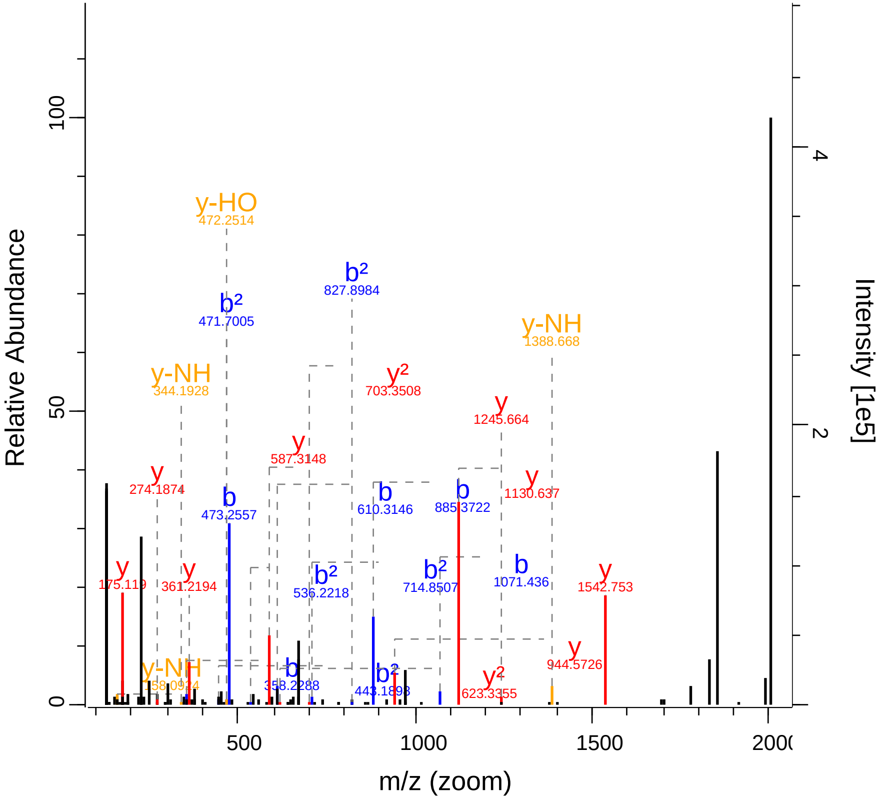

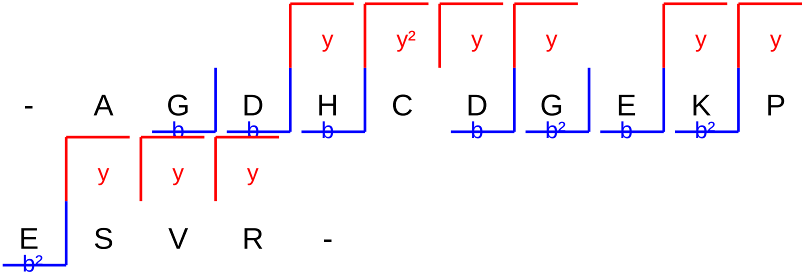

Raw File	Scan	Method	Score	m/z	Gene names
RO-QC-160121_ZackD_SS_Set_02_Fractio	6289	FTMS; HC	102.52	385.24	MGP

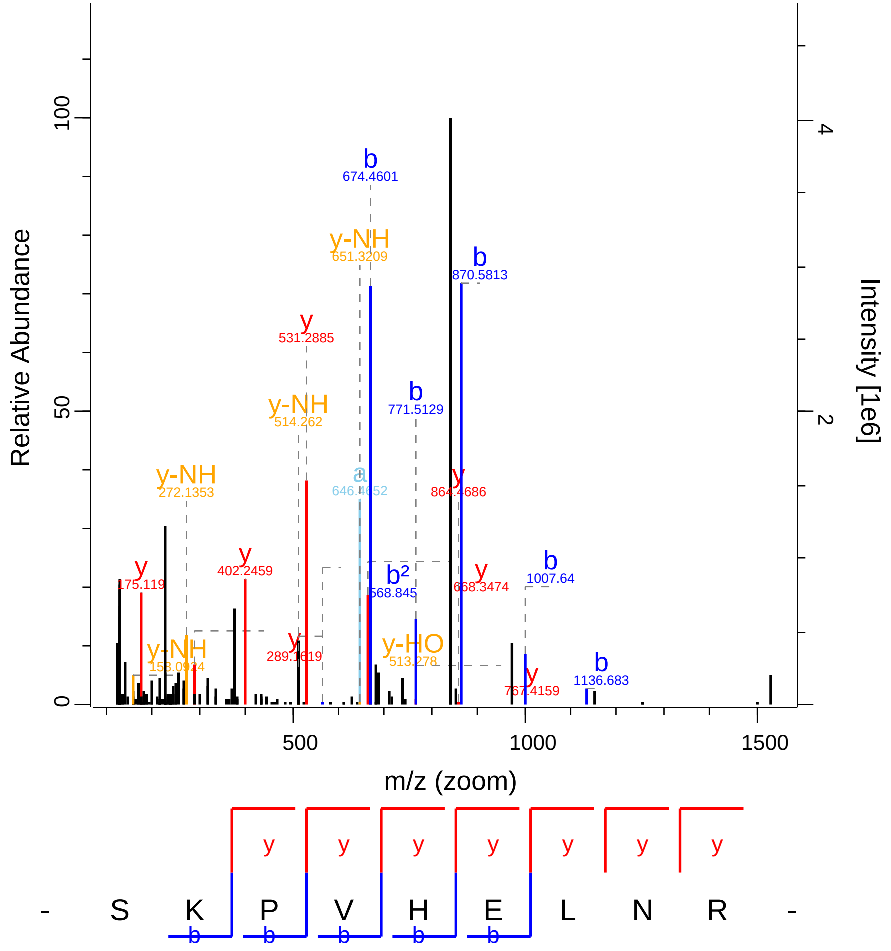

Raw File	Scan	Method	Score	m/z	Gene names
RO-QC-160121_ZackD_SS_Set_02_Fractior	6618	FTMS; HCI	83.87	551.66	HAND2

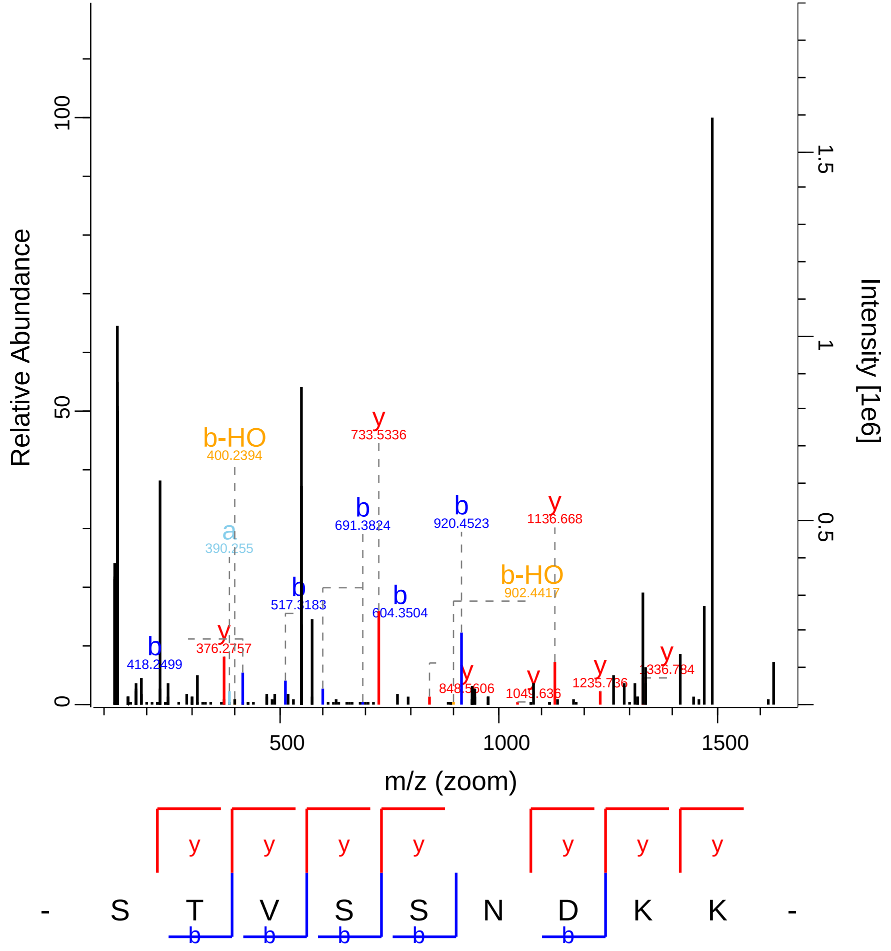

Raw File	Scan	Method	Score	m/z	Gene names
RO-QC-160121_ZackD_SS_Set_02_Fractio	6674	FTMS; HC	103.82	674.36	TIPIN

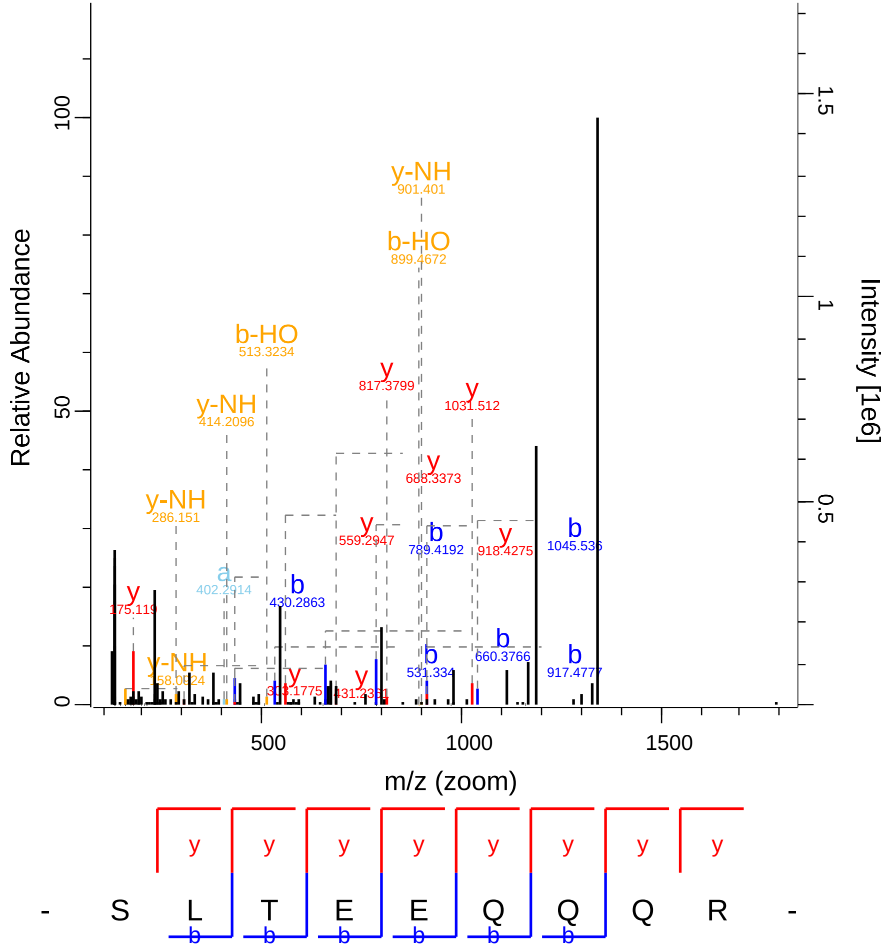

Raw File	Scan	Method	Score	m/z	Gene names
RO-QC-160121_ZackD_SS_Set_02_Fractior	6829	FTMS; HCI	87.18	581.82	AIFM3

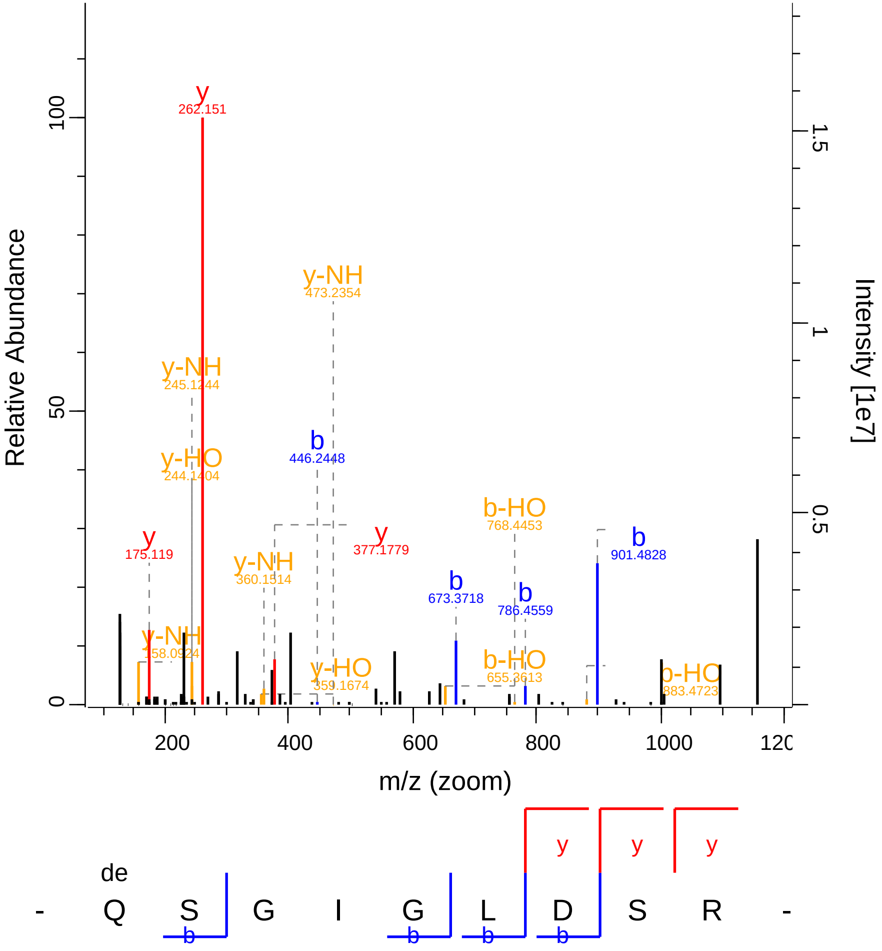

Raw File	Scan	Method	Score	m/z	Gene names
RO-QC-160121_ZackD_SS_Set_02_Fractioı	6945	FTMS; HC	131.06	682.87	ING5

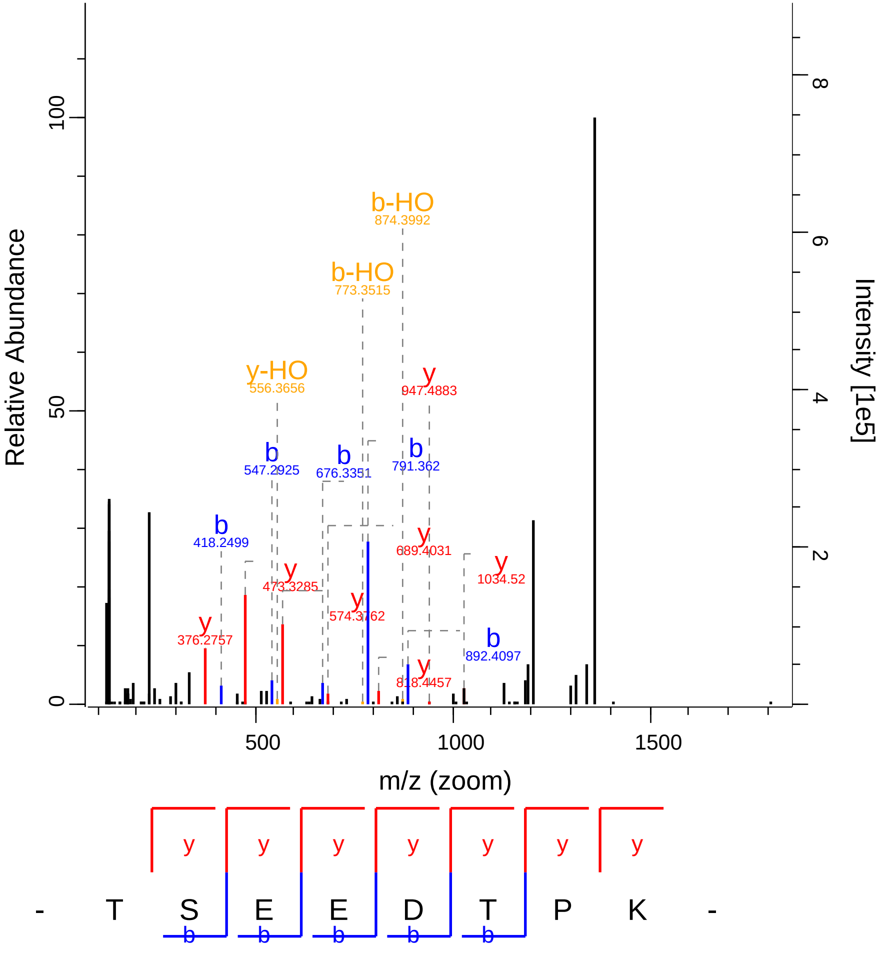

Raw File	Scan	Method	Score	m/z	Gene names
RO-QC-160121_ZackD_SS_Set_02_Fractior	7072	FTMS; HCI	47.68	761.38	ADRA2B

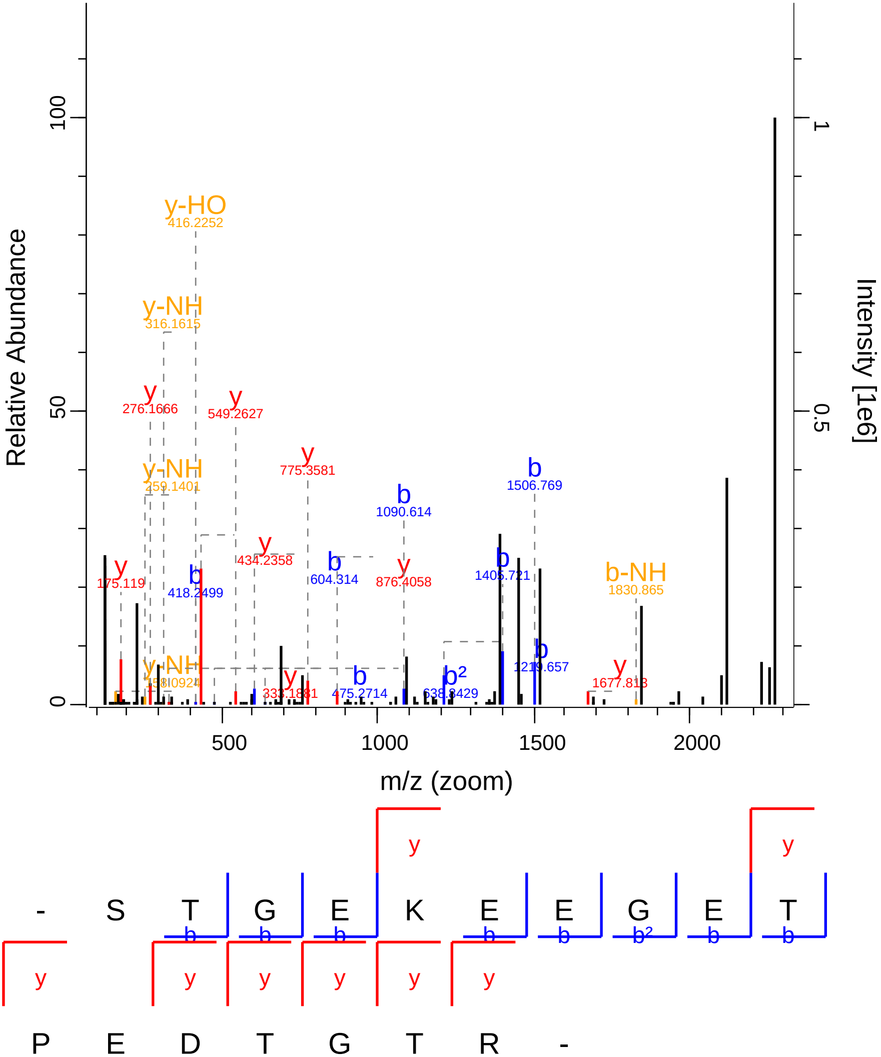

Raw File	Scan	Method	Score	m/z	Gene names
RO-QC-160121_ZackD_SS_Set_02_Fractior	7470	FTMS; HCl	95.48	596.63	EXT1

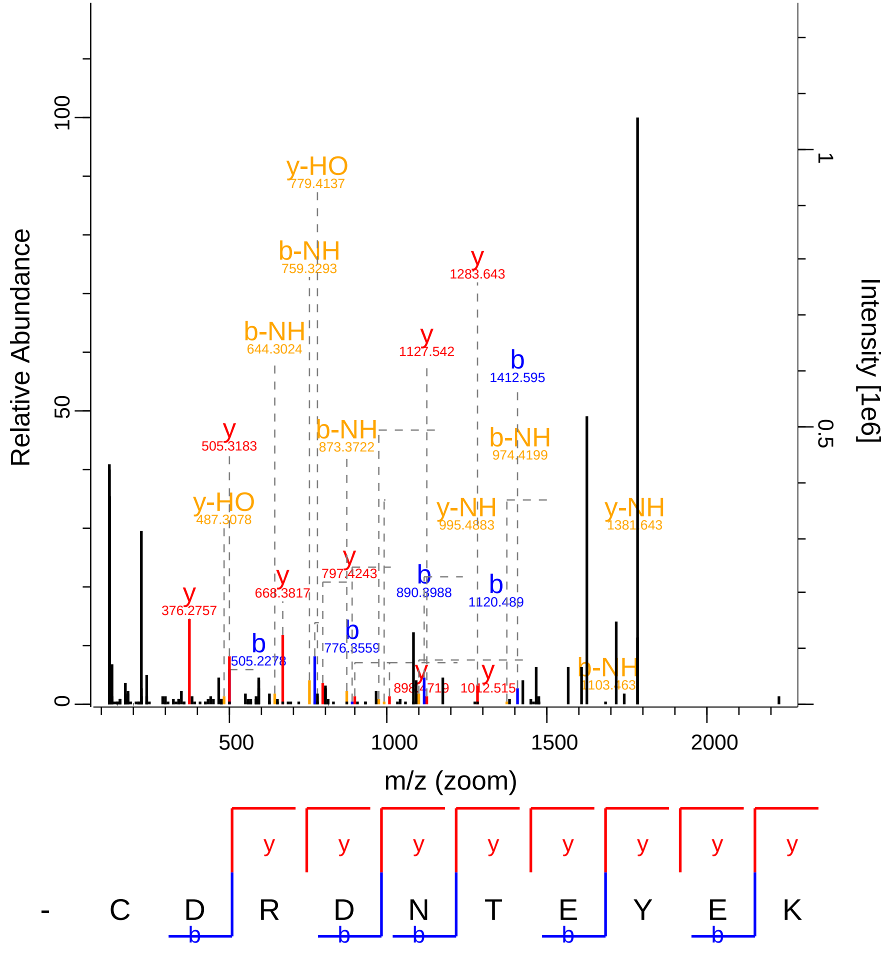

Raw File	Scan	Method	Score	m/z	Gene names
RO-QC-160121_ZackD_SS_Set_02_Fraction	7823	FTMS; HCI	160.15	768.4	PLEKHH2

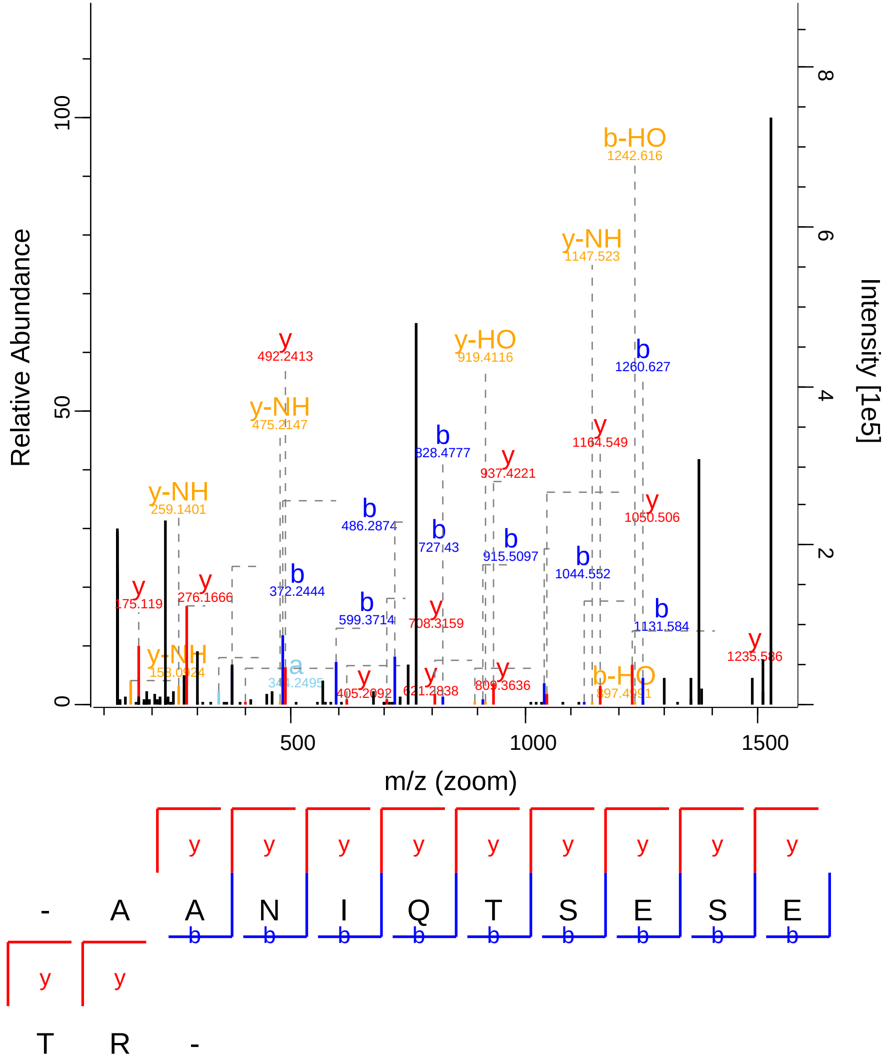

Raw File	Scan	Method	Score	m/z	Gene names
RO-QC-160121_ZackD_SS_Set_02_Fractio	8013	FTMS; HC	155.06	780.42	INSIG2

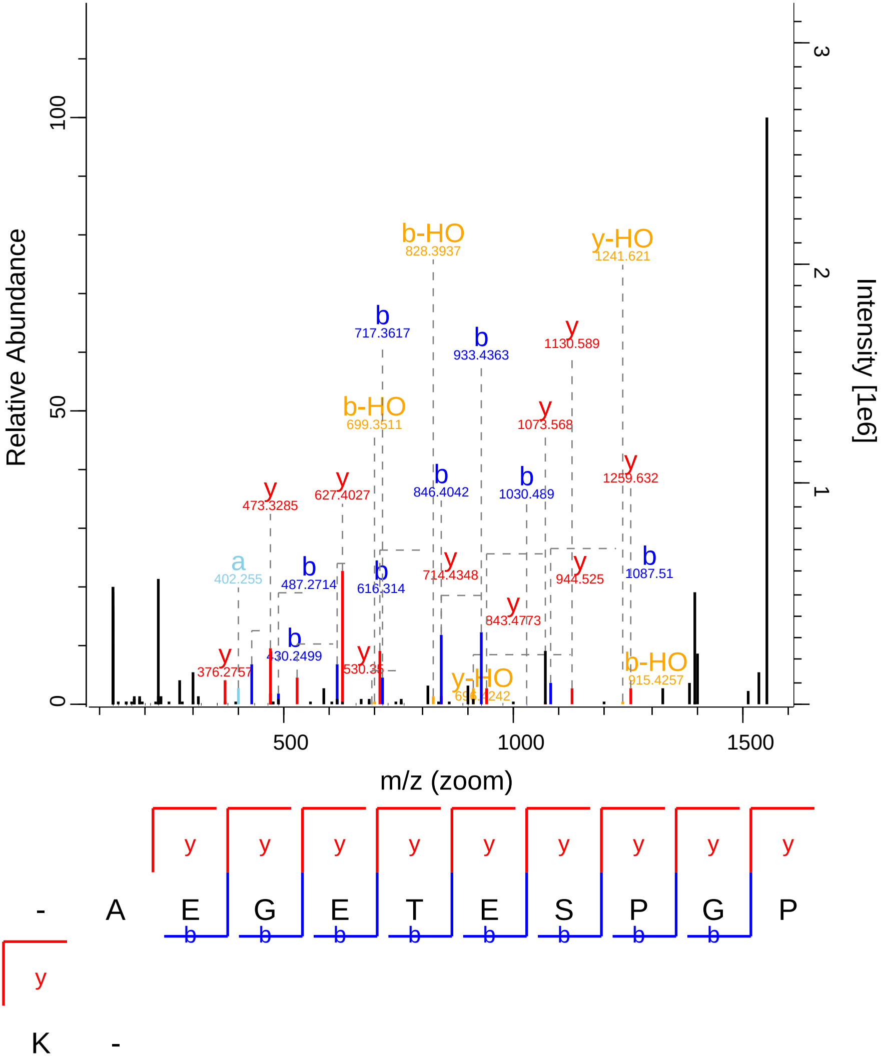

Raw File	Scan	Method	Score	m/z	Gene names
RO-QC-160121_ZackD_SS_Set_02_Fractio	8698	FTMS; HC	155.51	637.39	G6PC3

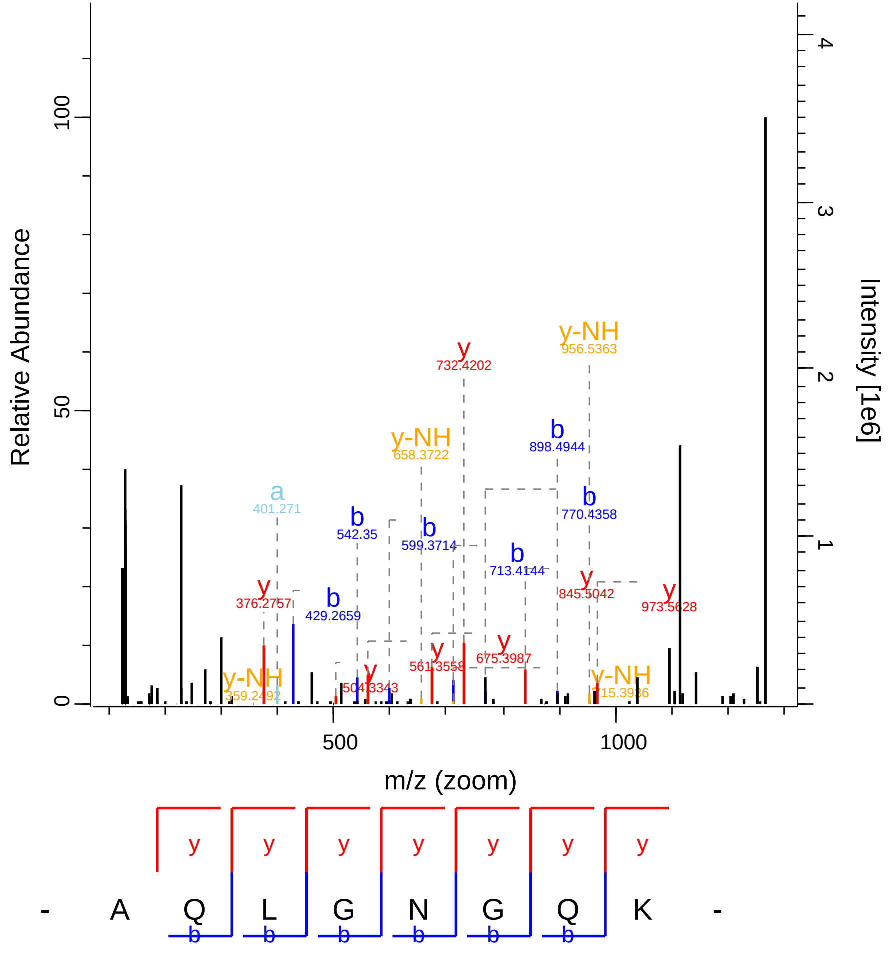

Raw File	Scan	Method	Score	m/z	Gene names
RO-QC-160121_ZackD_SS_Set_02_Fractior	8842	FTMS; HCI	90.05	567.34	SLC35C1

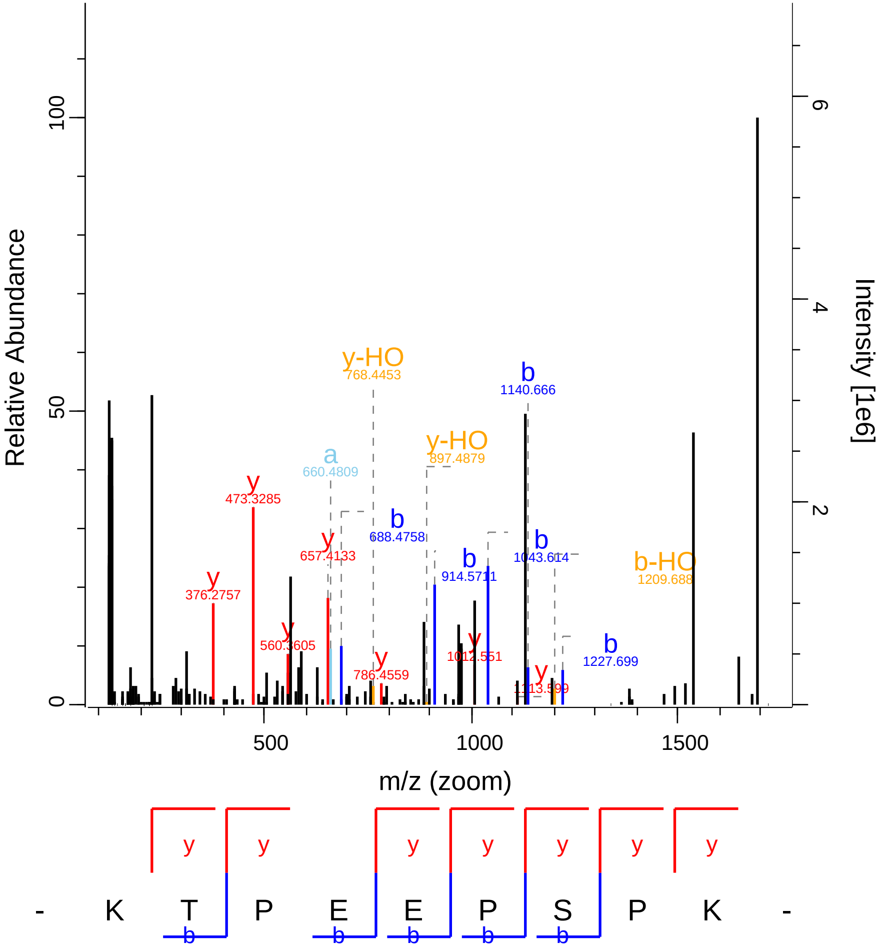

Raw File	Scan	Method	Score	m/z	Gene names
RO-QC-160121_ZackD_SS_Set_02_Fractior	9067	FTMS; HCI	94.26	428.27	AZIN2

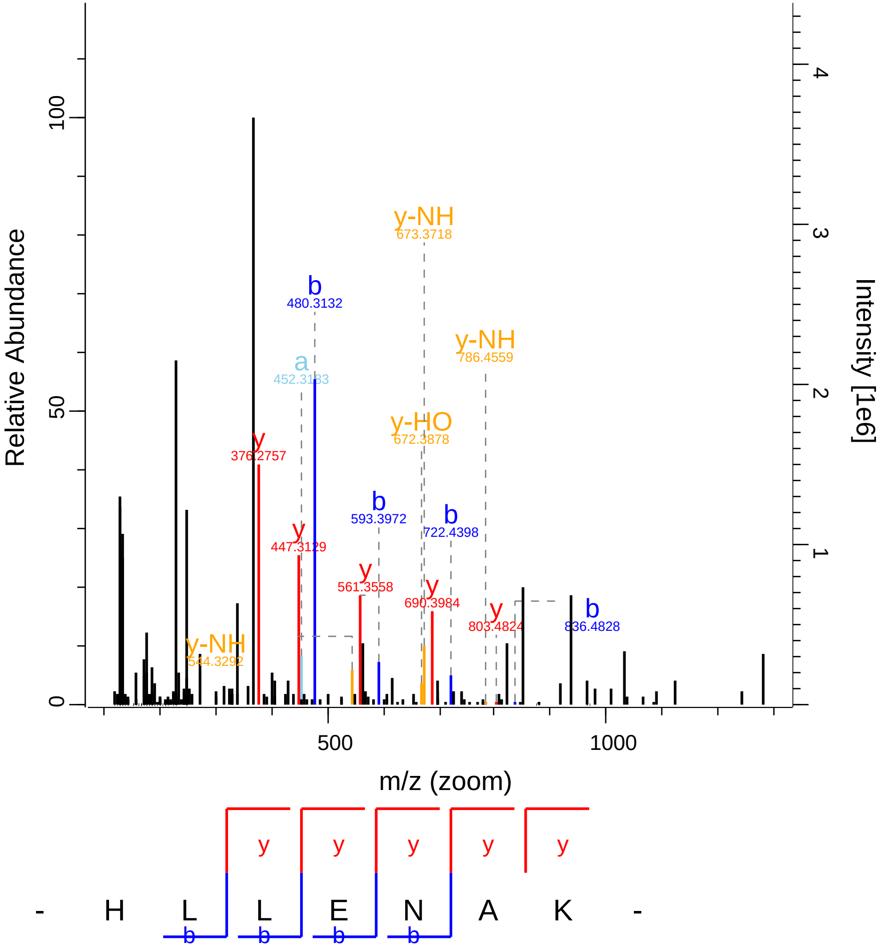

Raw File	Scan	Method	Score	m/z	Gene names
RO-QC-160121_ZackD_SS_Set_02_Fractior	9905	FTMS; HCI	56.51	685.85	CRIPT

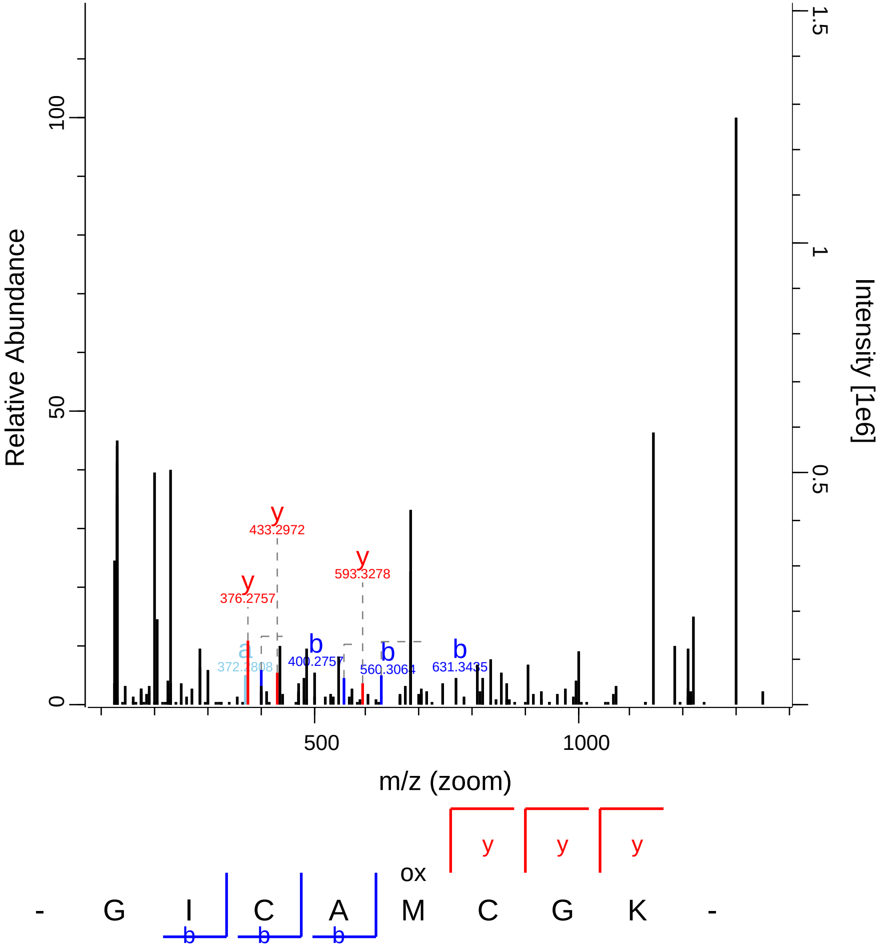

Raw File	Scan	Method	Score	m/z	Gene names
RO-QC-160121_ZackD_SS_Set_02_Fractio	9919	FTMS; HC	185.33	680.38	ANKRD16

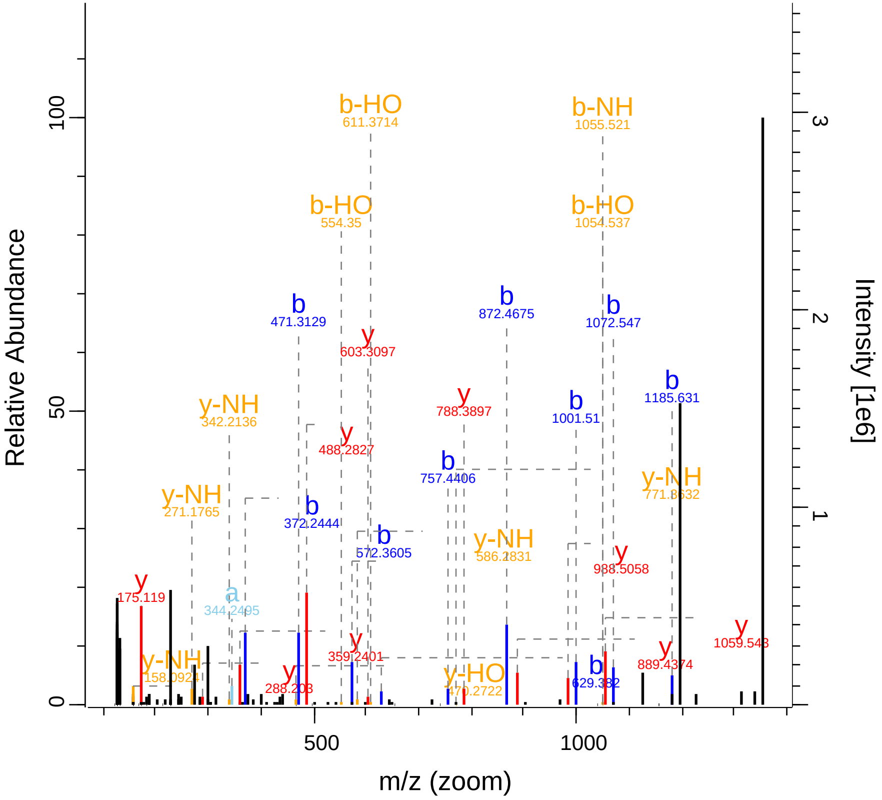

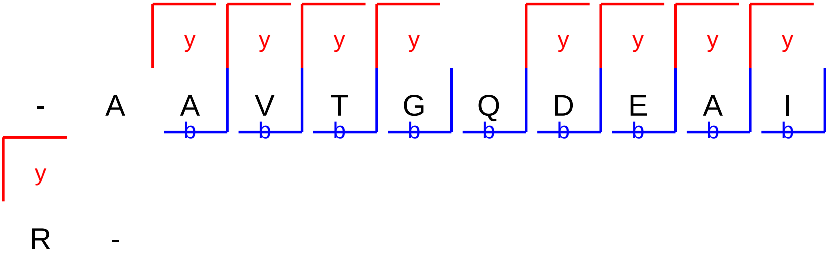

Raw File	Scan	Method	Score	m/z	Gene names
RO-QC-160121_ZackD_SS_Set_02_Fractio	10667	FTMS; HC	73.21	722.02	C16orf70

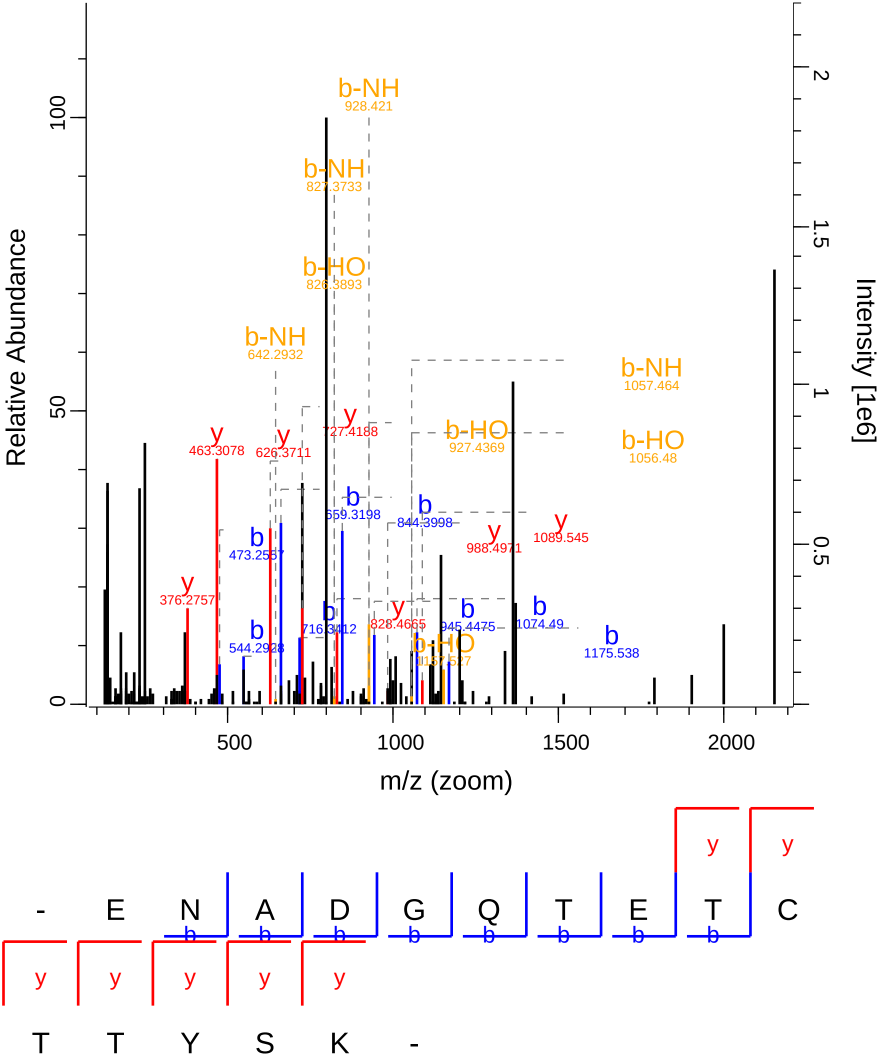

Raw File	Scan	Method	Score	m/z	Gene names
RO-QC-160121_ZackD_SS_Set_02_Fractior	10965	FTMS; HCI	111.73	356.9	CENPA

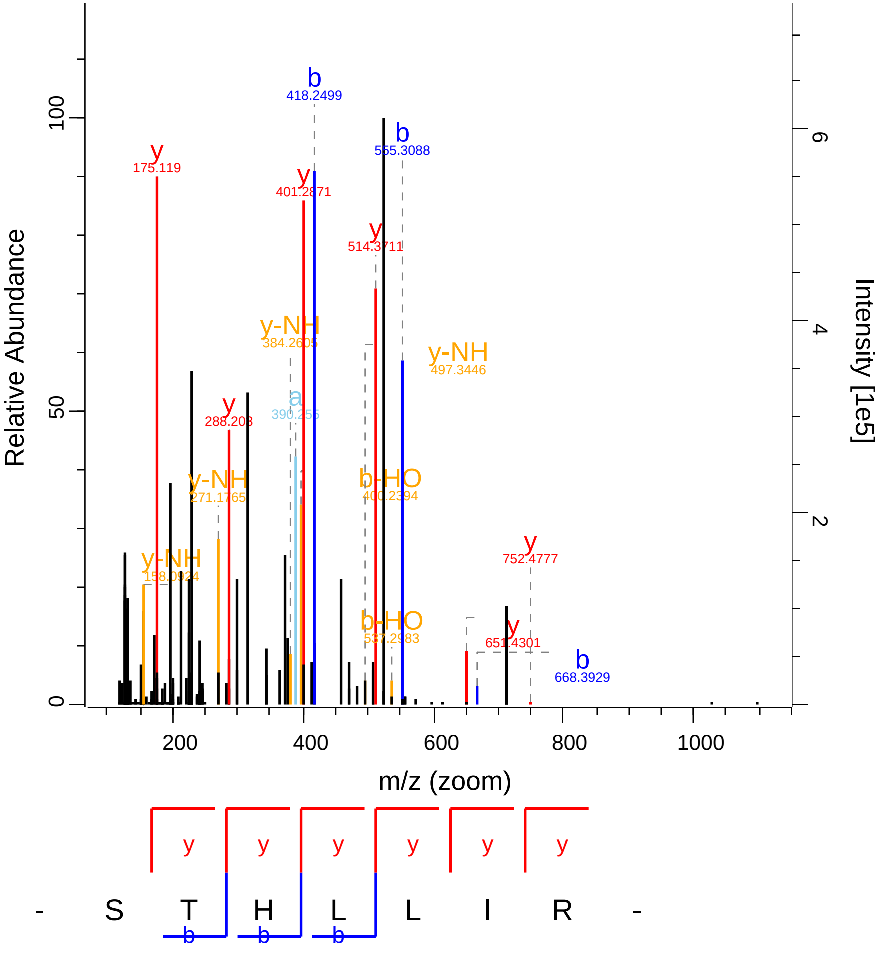

Raw File	Scan	Method	Score	m/z	Gene names
RO-QC-160121_ZackD_SS_Set_02_Fra	11395	FTMS; HC	76.77	596.35	ATP5G1;ATP5G2;ATP

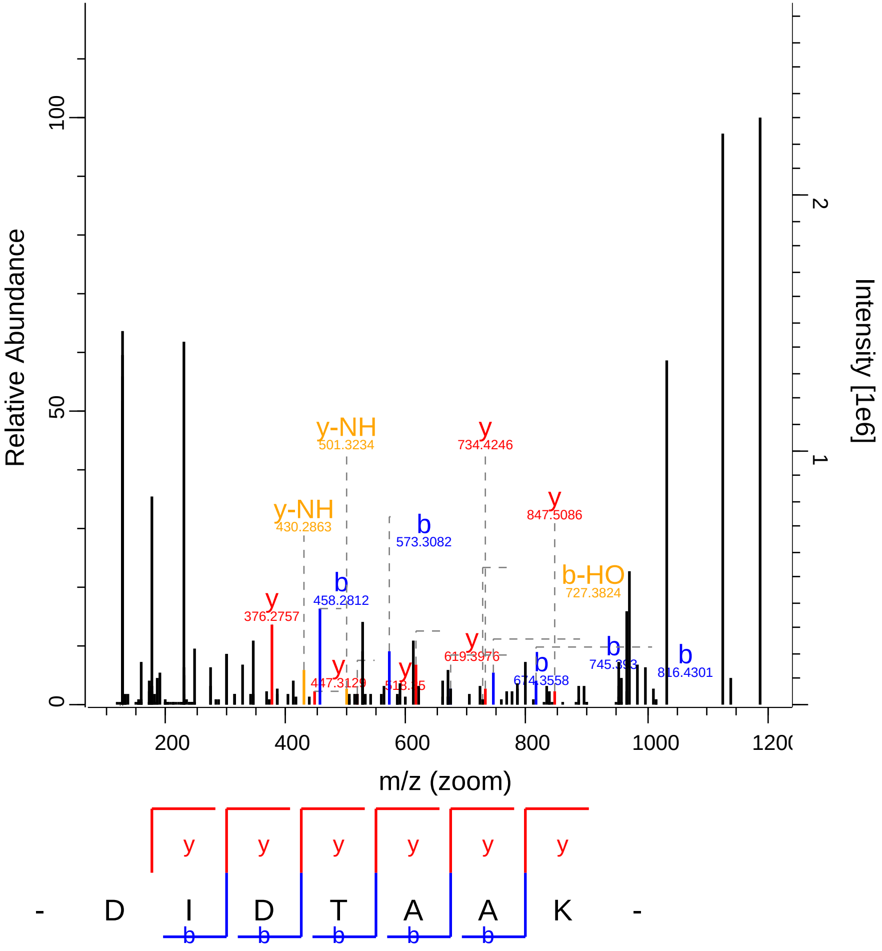

Raw File	Scan	Method	Score	m/z	Gene names
RO-QC-160121_ZackD_SS_Set_02_Fractio	12432	FTMS; HCI	108.46	379.9	FRMD4B

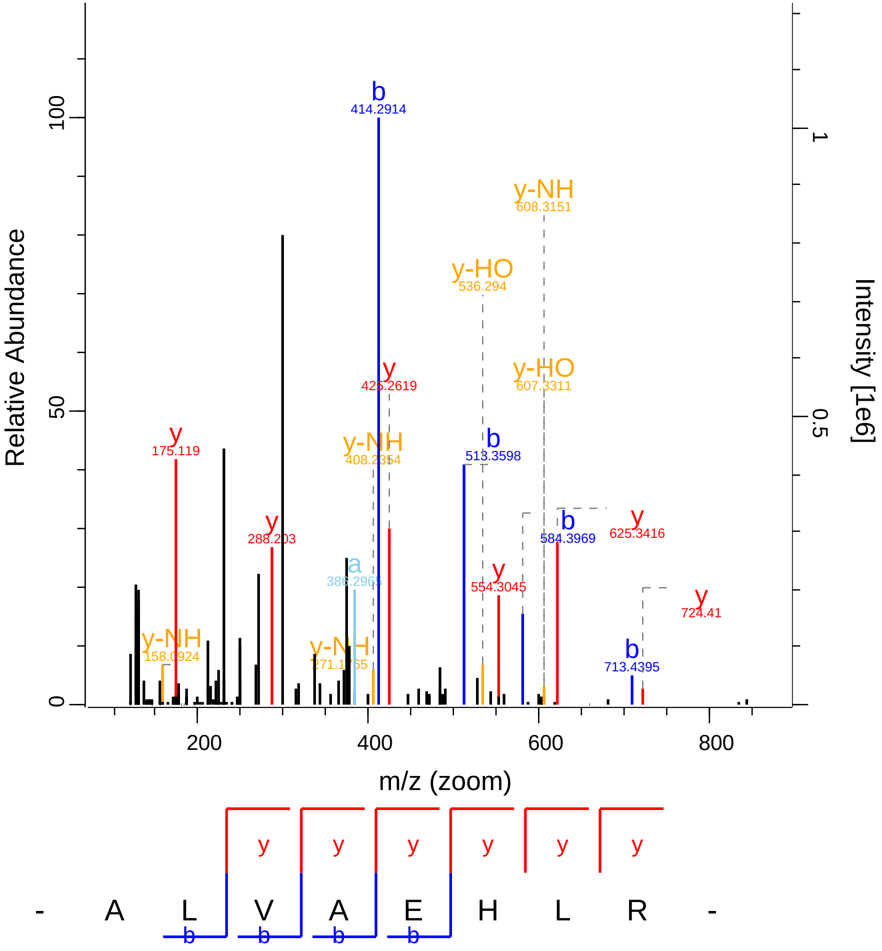

Raw File	Scan	Method	Score	m/z	Gene names
RO-QC-160121_ZackD_SS_Set_02_Fractio	13056	FTMS; HC	110.31	714.86	FRAS1

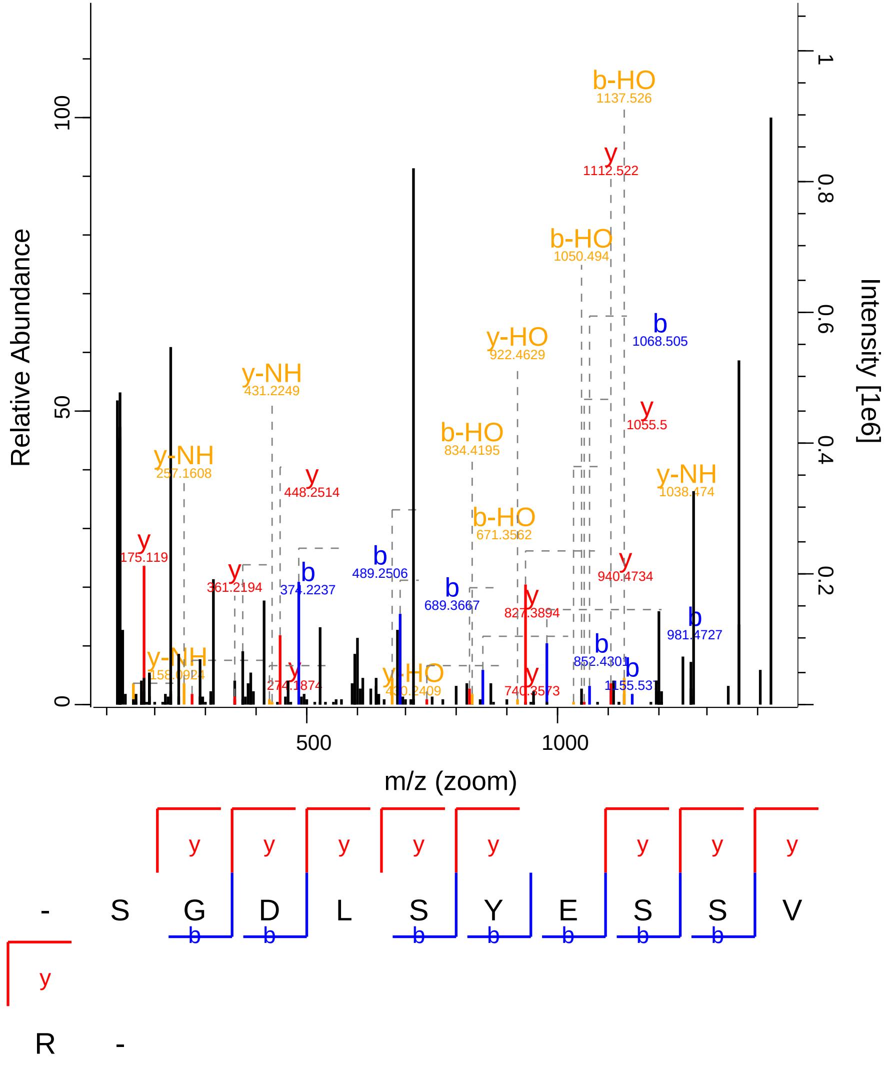

Raw File	Scan	Method	Score	m/z	Gene names
RO-QC-160121_ZackD_SS_Set_02_Fractio	13616	FTMS; HC	86.01	569.36	RIC8B

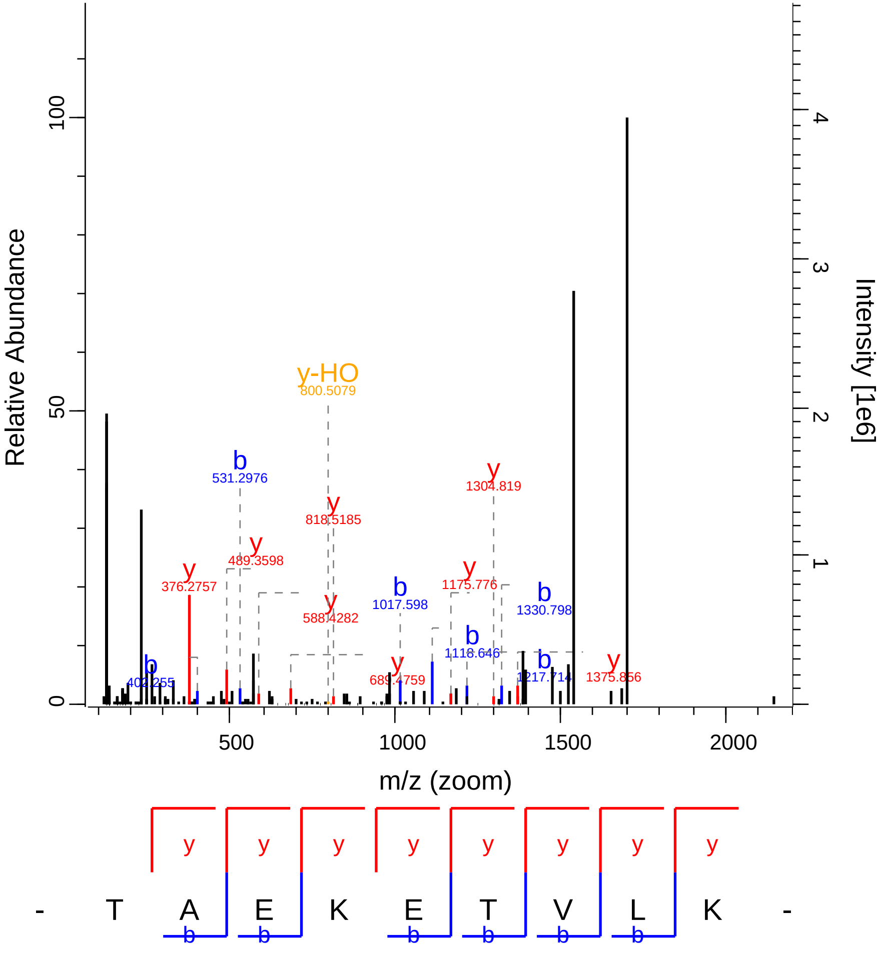

Raw File	Scan	Method	Score	m/z
RO-QC-160121_ZackD_SS_Set_02_Fraction_6	13636	FTMS; HCD	73.26	434.27

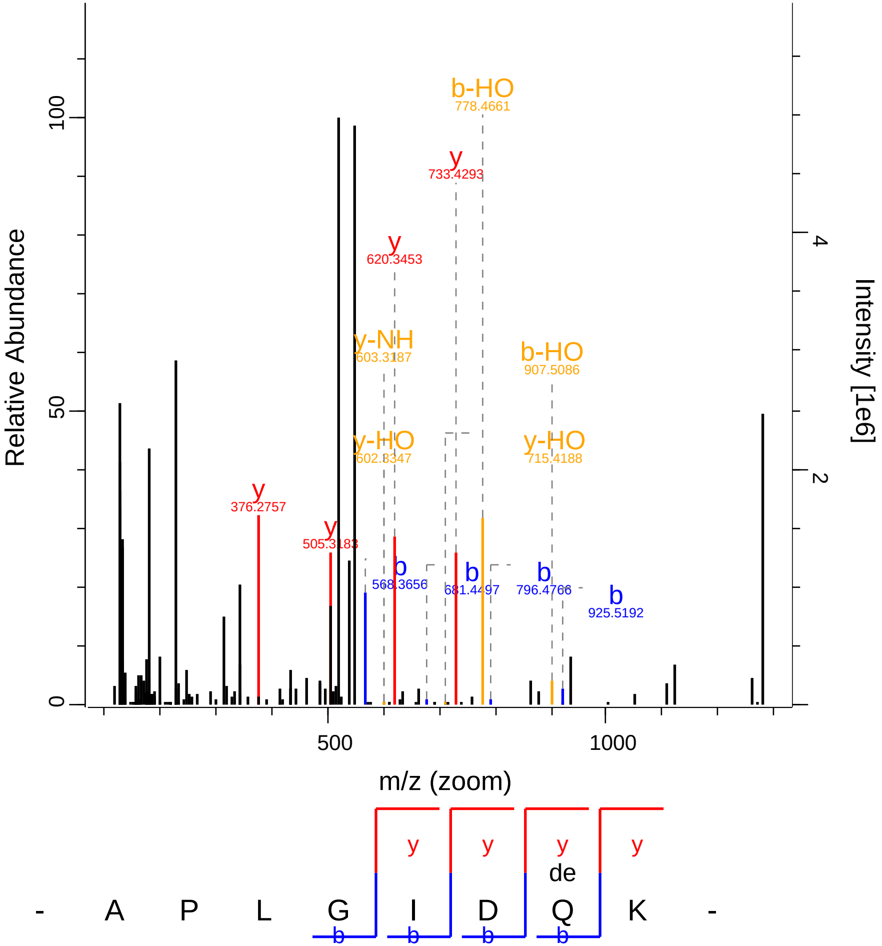

Raw File	Scan	Method	Score	m/z	Gene names
RO-QC-160121_ZackD_SS_Set_02_Fractio	13817	FTMS; HC	110.53	701.38	ANKH

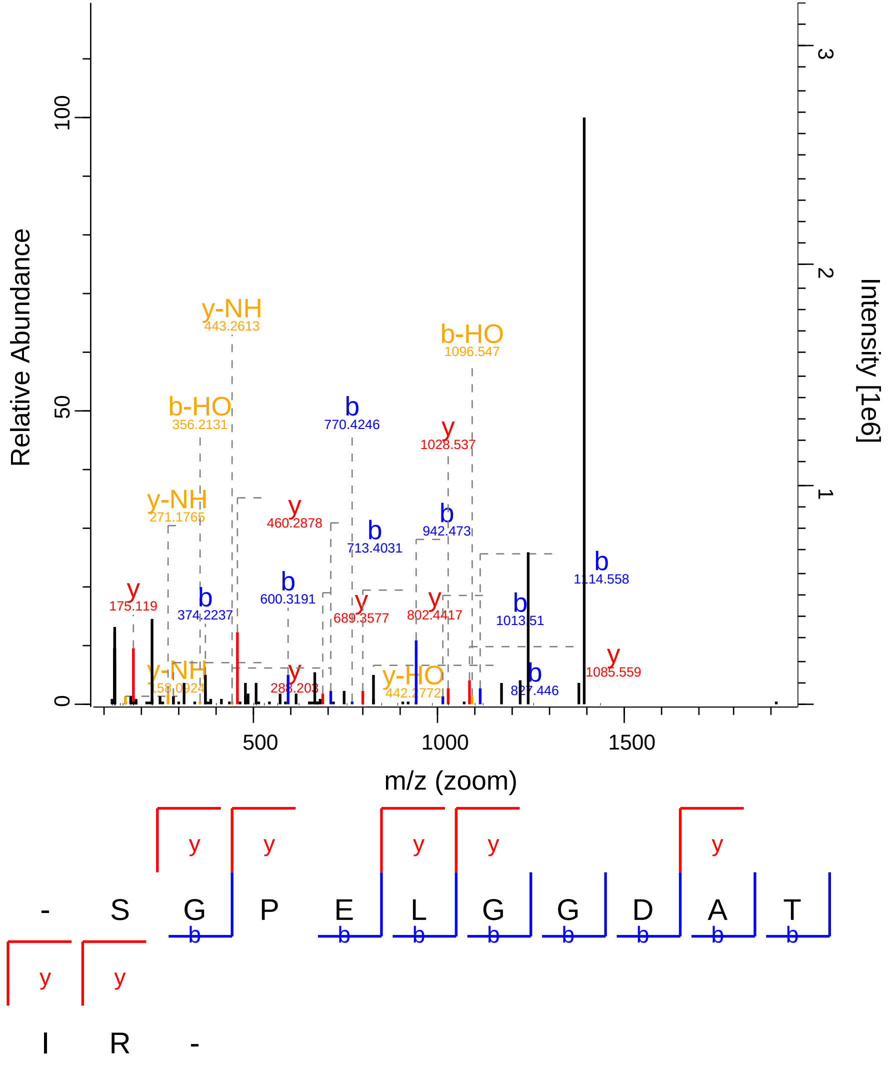

Raw File	Scan	Method	Score	m/z	Gene names
RO-QC-160121_ZackD_SS_Set_02_Fractio	14688	FTMS; HC	146.48	763.88	FCF1

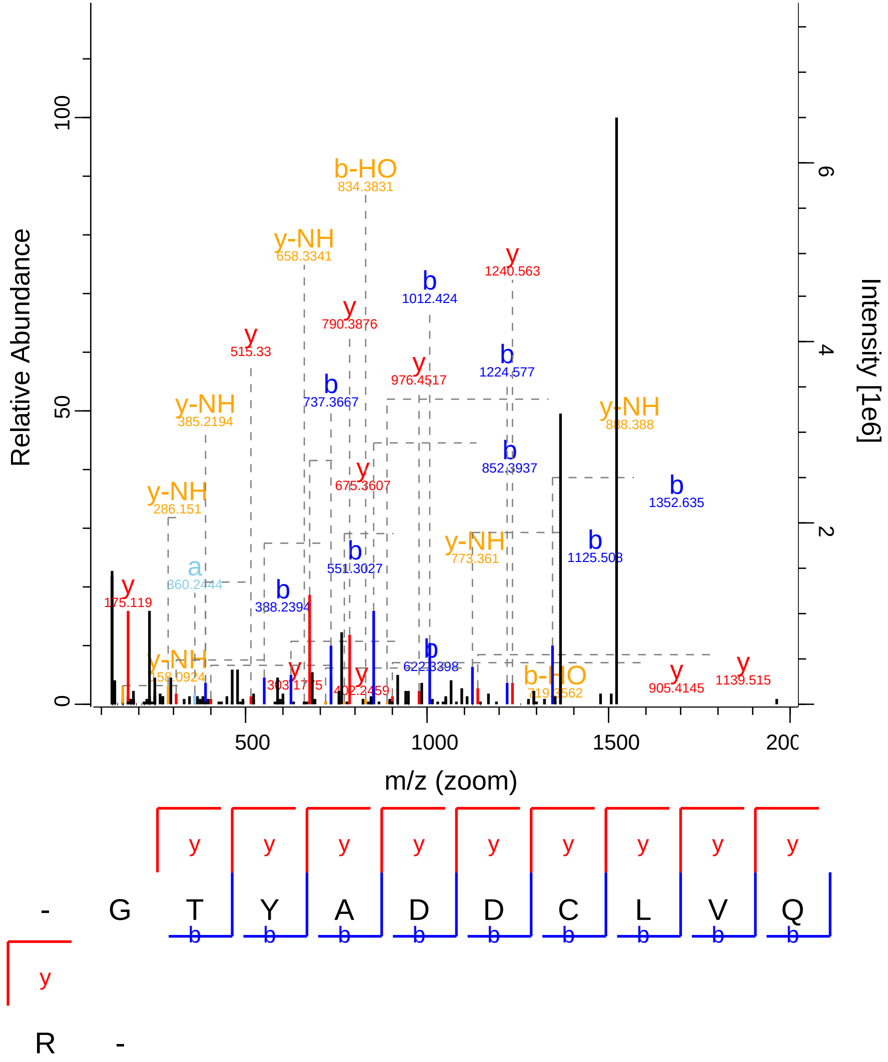

Raw File	Scan	Method	Score	m/z	Gene names
RO-QC-160121_ZackD_SS_Set_02_Fractio	15242	FTMS; HC	112.83	441.94	DALRD3

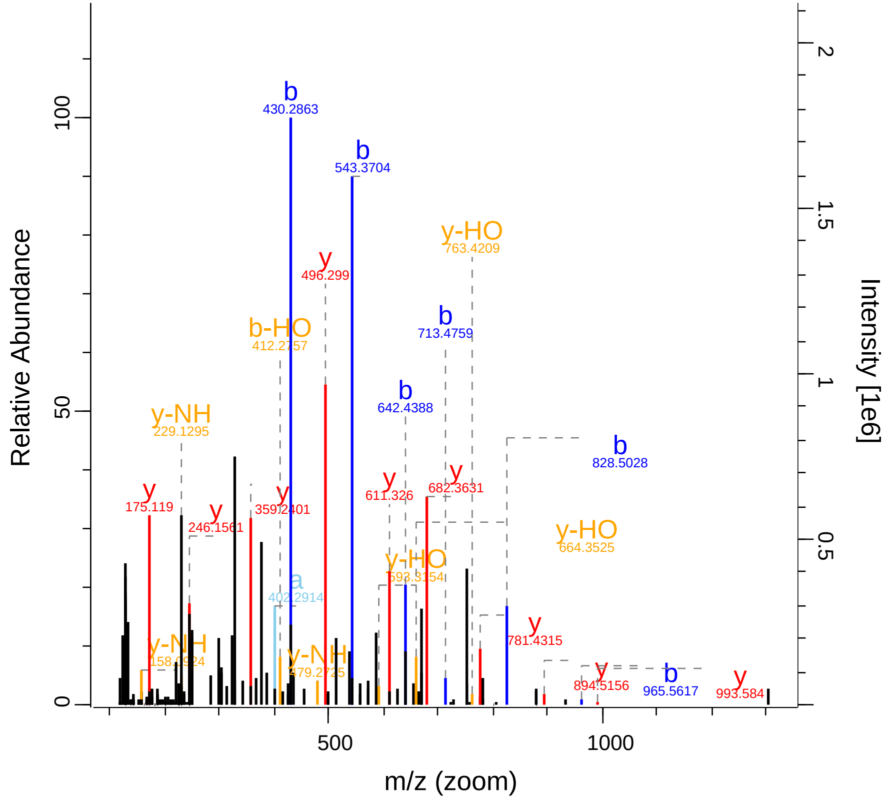

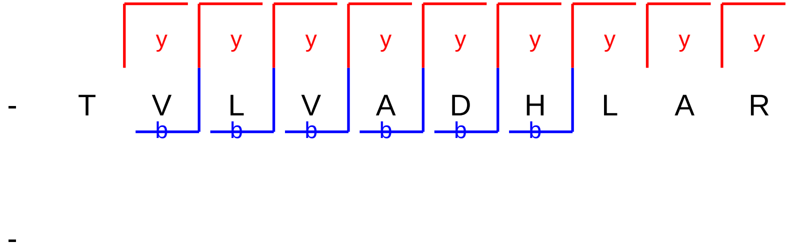

Raw File	Scan	Method	Score	m/z
RO-QC-160121_ZackD_SS_Set_02_Fraction_6	15329	FTMS; HCD	53.97	680.34

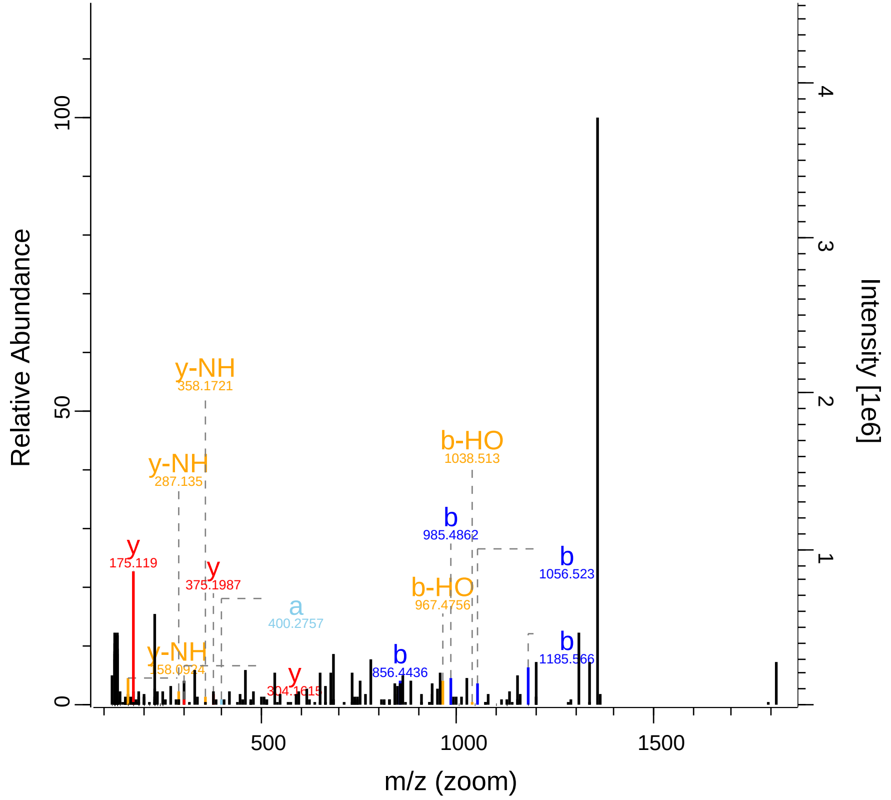

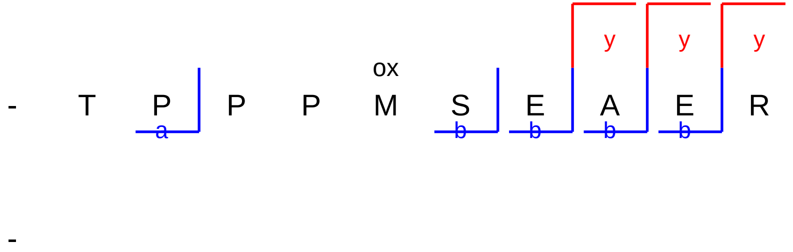

Raw File	Scan	Method	Score	m/z	Gene names
RO-QC-160121_ZackD_SS_Set_02_Fractio	15348	FTMS; HC	55.81	538.65	ATP5SL

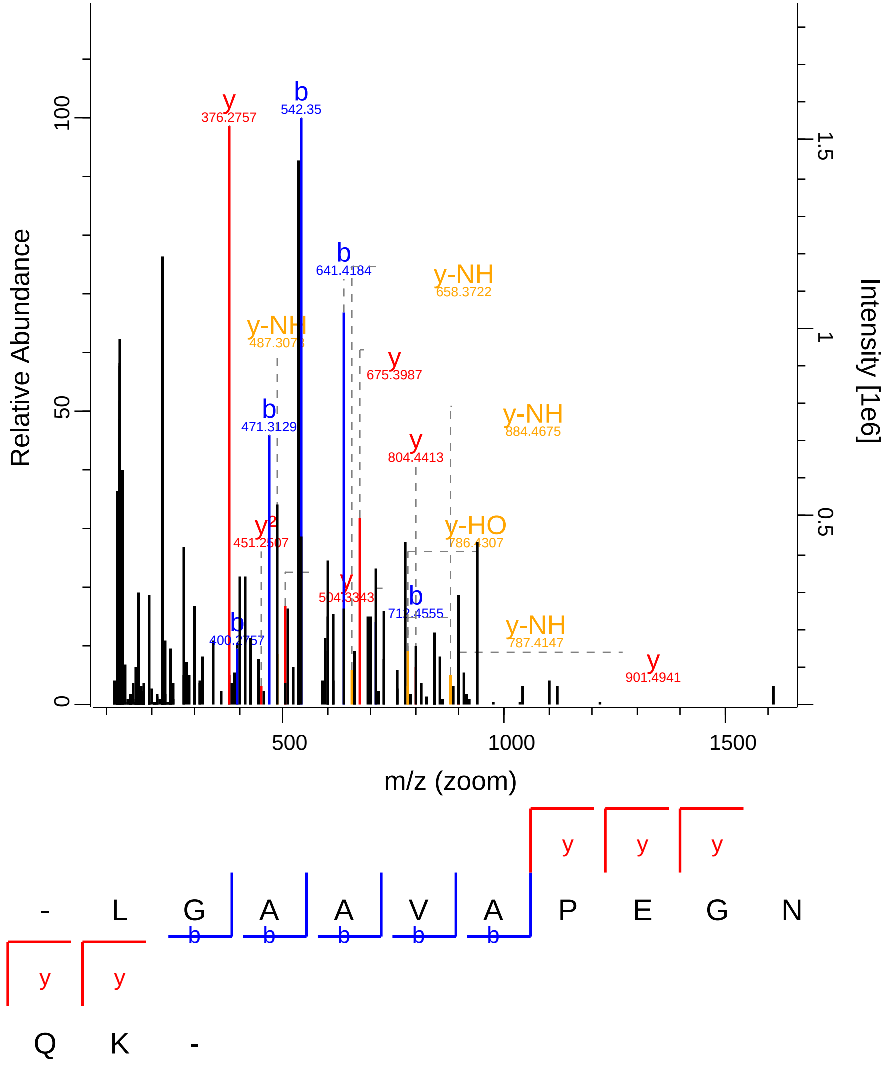

Raw File	Scan	Method	Score	m/z
RO-QC-160121_ZackD_SS_Set_02_Fraction_6	16093	FTMS; HCD	98.52	681.91

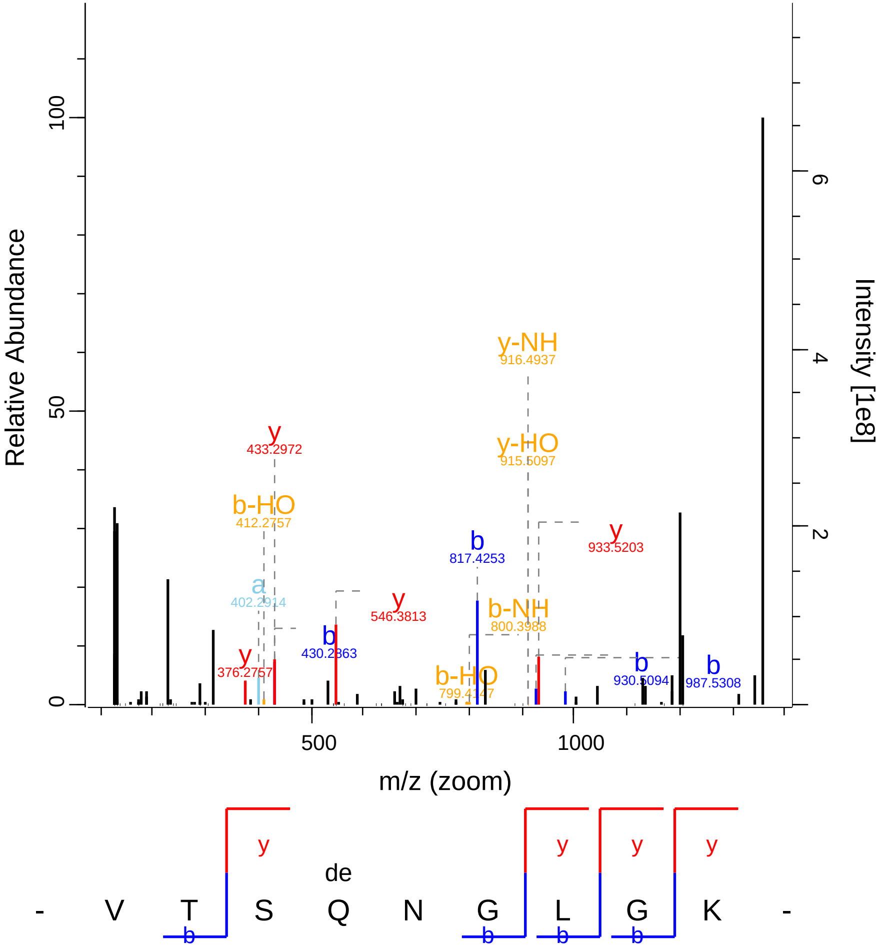

Raw File	Scan	Method	Score	m/z	Gene names
RO-QC-160121_ZackD_SS_Set_02_Fractio	16254	FTMS; HC	67.73	572.82	CCDC173

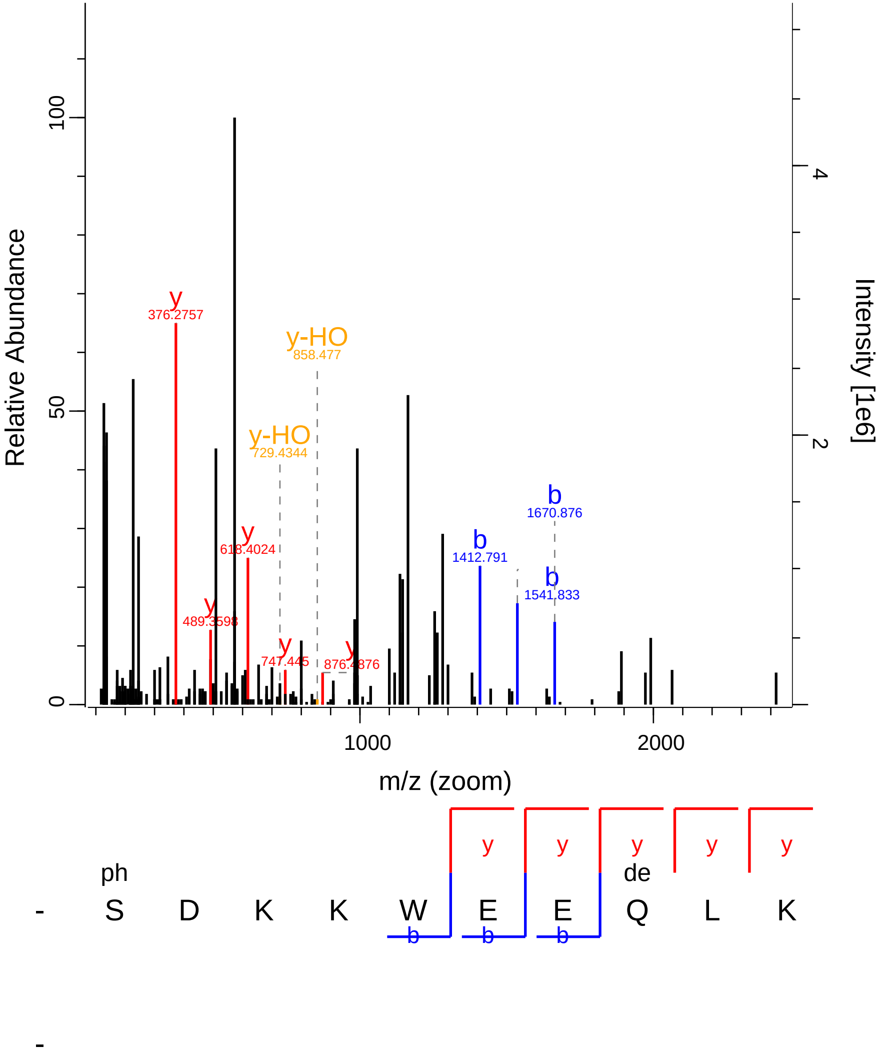

Raw File	Scan	Method	Score	m/z
RO-QC-160121_ZackD_SS_Set_02_Fraction_6	16312	FTMS; HCD	122.74	667.41

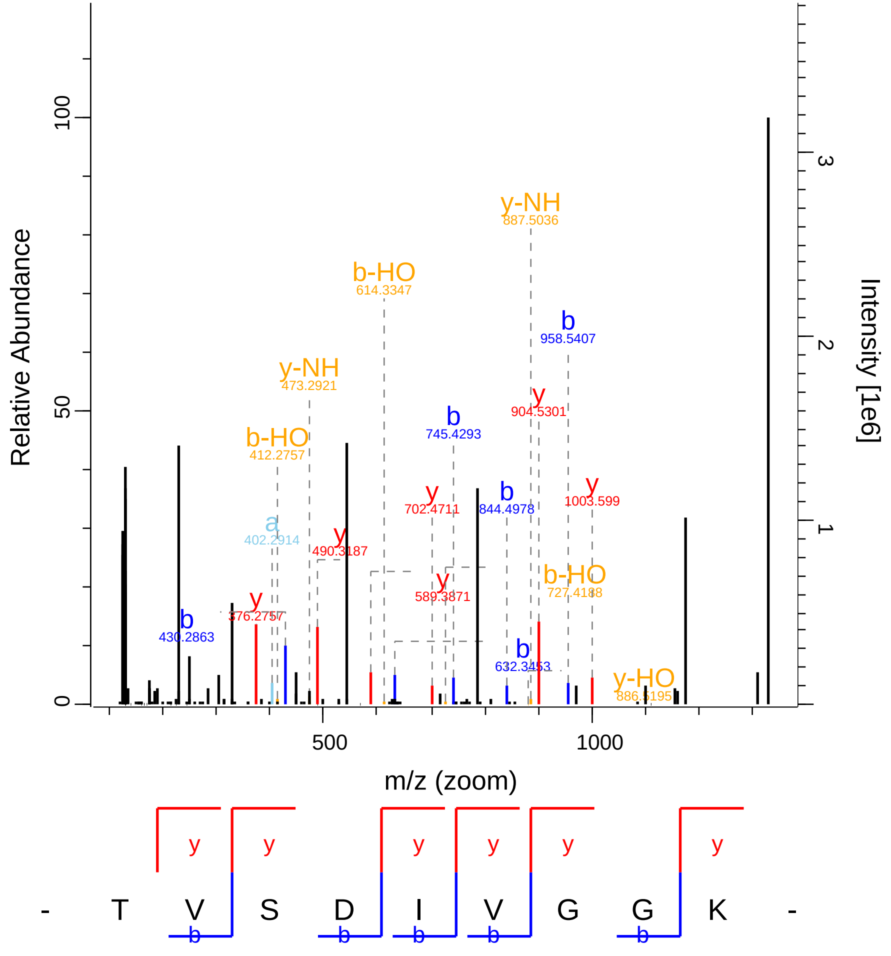

Raw File	Scan	Method	Score	m/z	Gene names
RO-QC-160121_ZackD_SS_Set_02_Fractio	16440	FTMS; HC	112.3	760.73	ZFYVE27

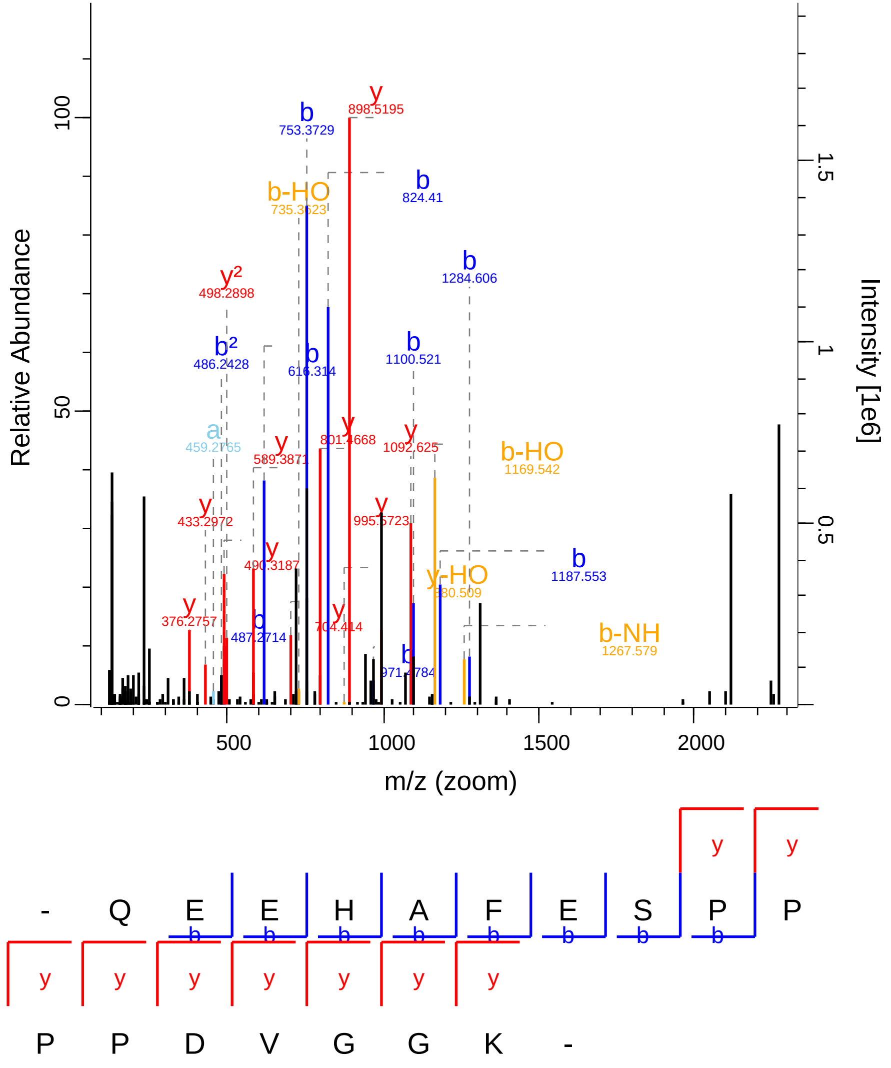

Raw File	Scan	Method	Score	m/z	Gene names
RO-QC-160121_ZackD_SS_Set_02_Fractio	16637	FTMS; HC	253.79	955.01	NIPA1

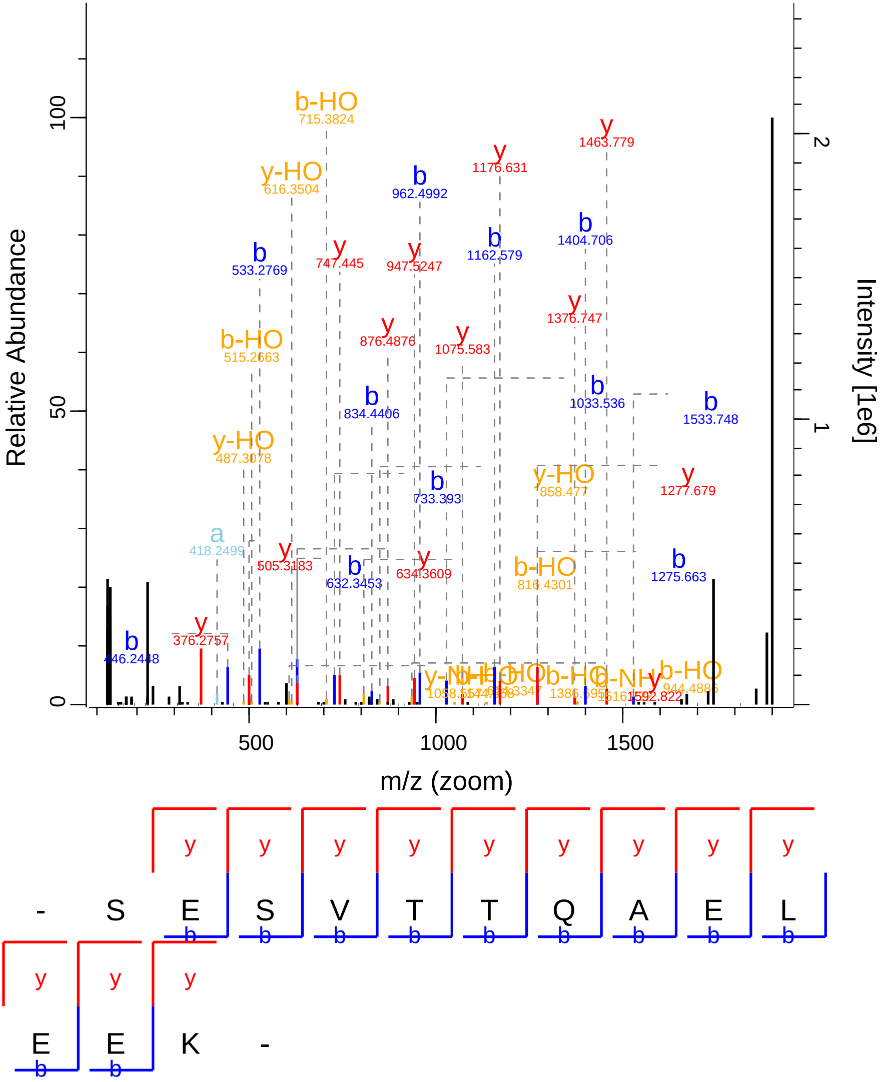

Raw File	Scan	Method	Score	m/z	Gene names
RO-QC-160121_ZackD_SS_Set_02_Fractio	16742	FTMS; HC	141.55	484.83	CLIP4

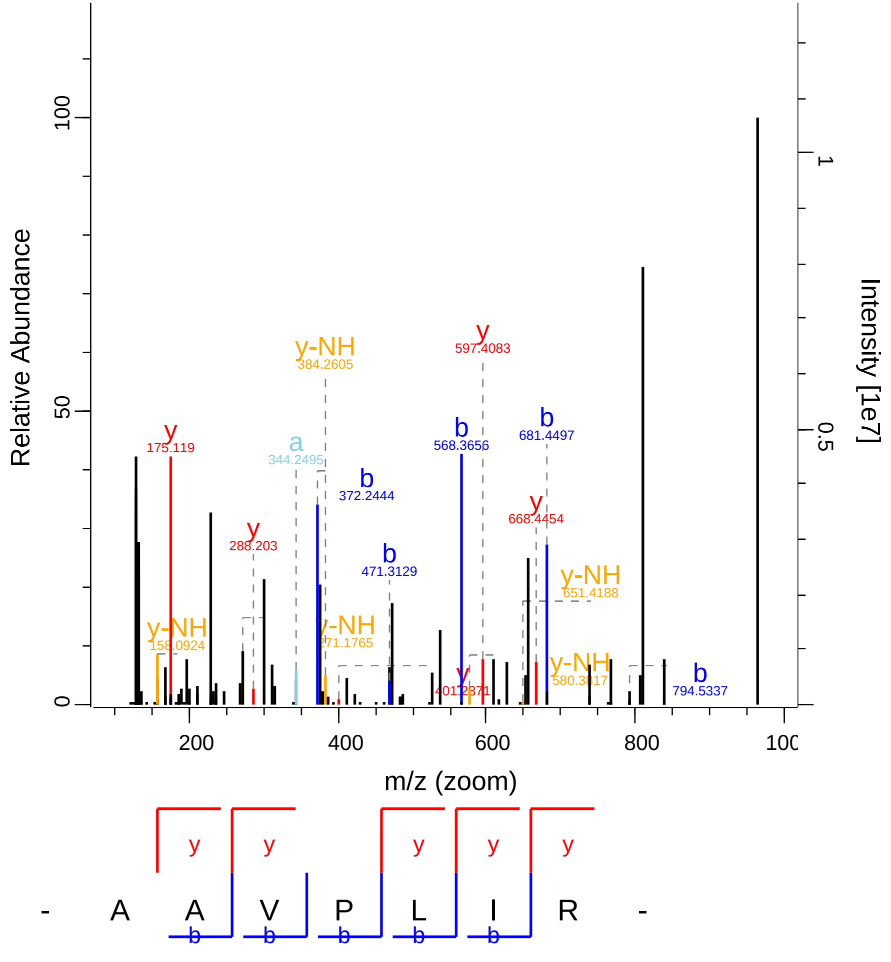

Raw File	Scan	Method	Score	m/z	Gene names
RO-QC-160121_ZackD_SS_Set_02_Fractio	17377	FTMS; HC	78.33	461.29	IL33

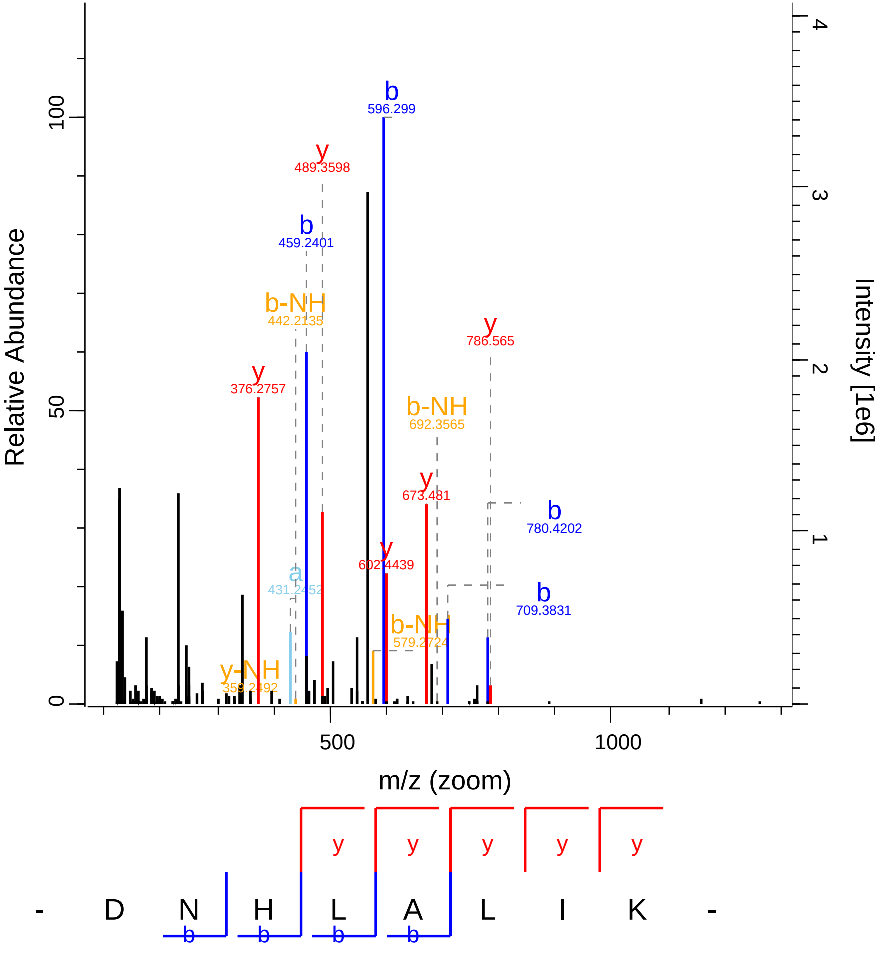

Raw File Sc Metl Sc m/ Gene names

RO-QC-160121_Z 1€ FT 2 8 4 6 USP17L1;USP17L24;USP17L20;USP17L19;USP17L17;USP1

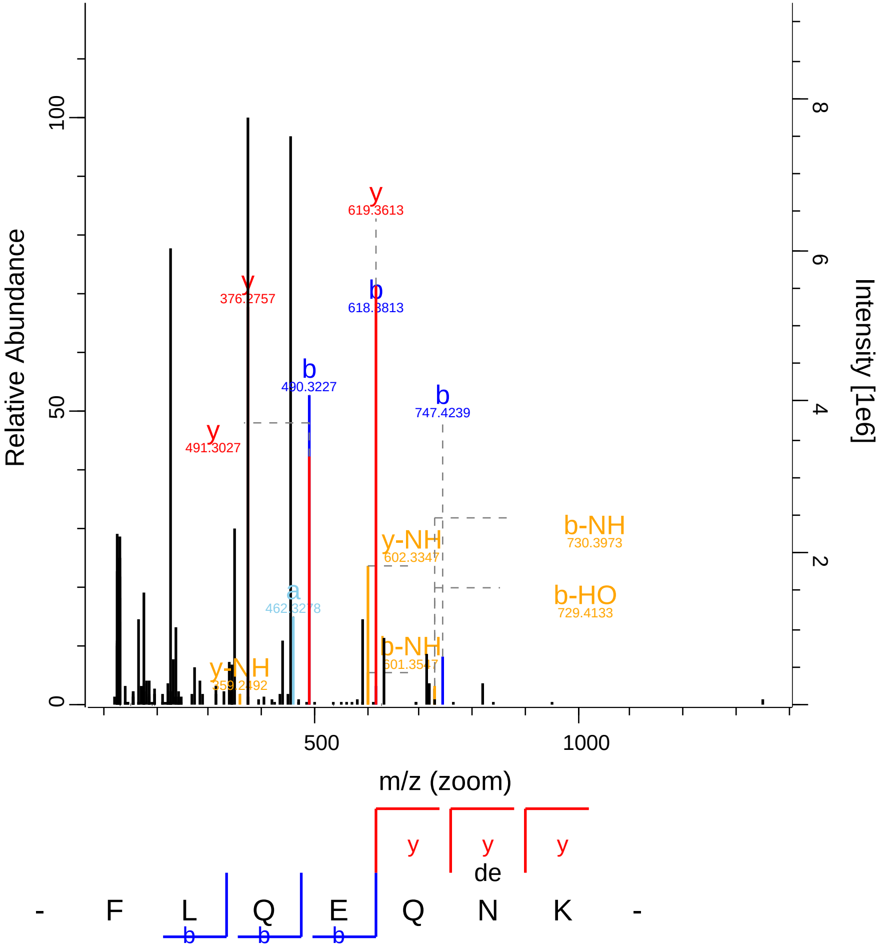

Raw File	Scan	Method	Score	m/z	Gene names
RO-QC-160121_ZackD_SS_Set_02_Fractio	18789	FTMS; HC	227.94	806.91	YIPF6

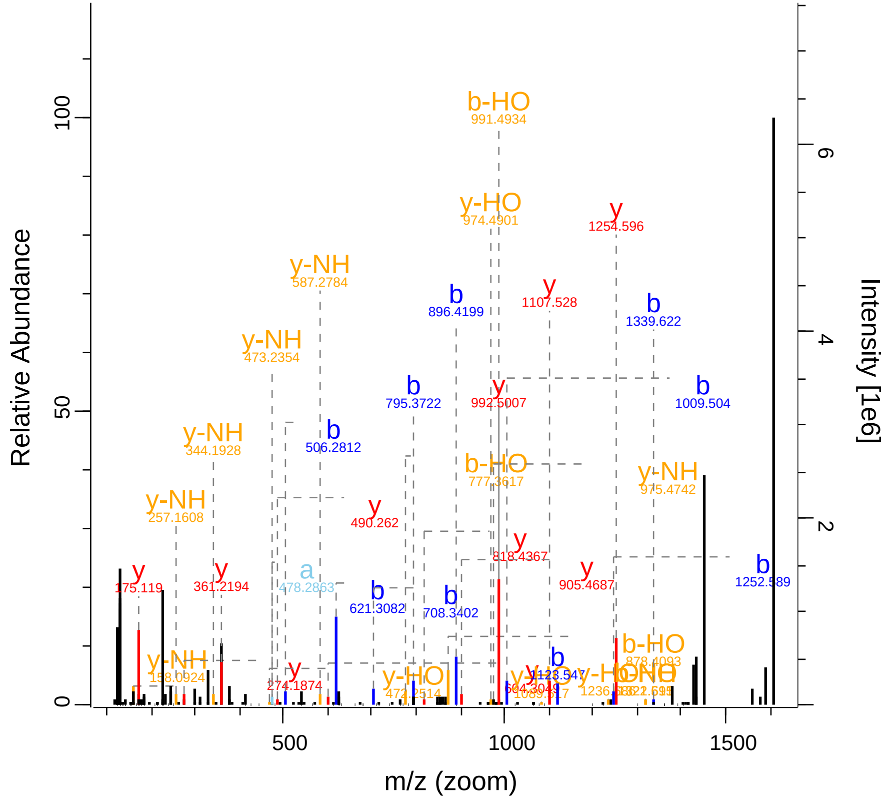

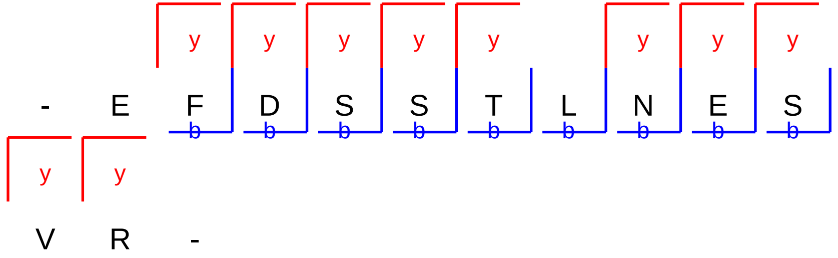

Raw File	Scan	Method	Score	m/z	Gene names
RO-QC-160121_ZackD_SS_Set_02_Fractio	18878	FTMS; HC	90.7	538.27	YIPF6

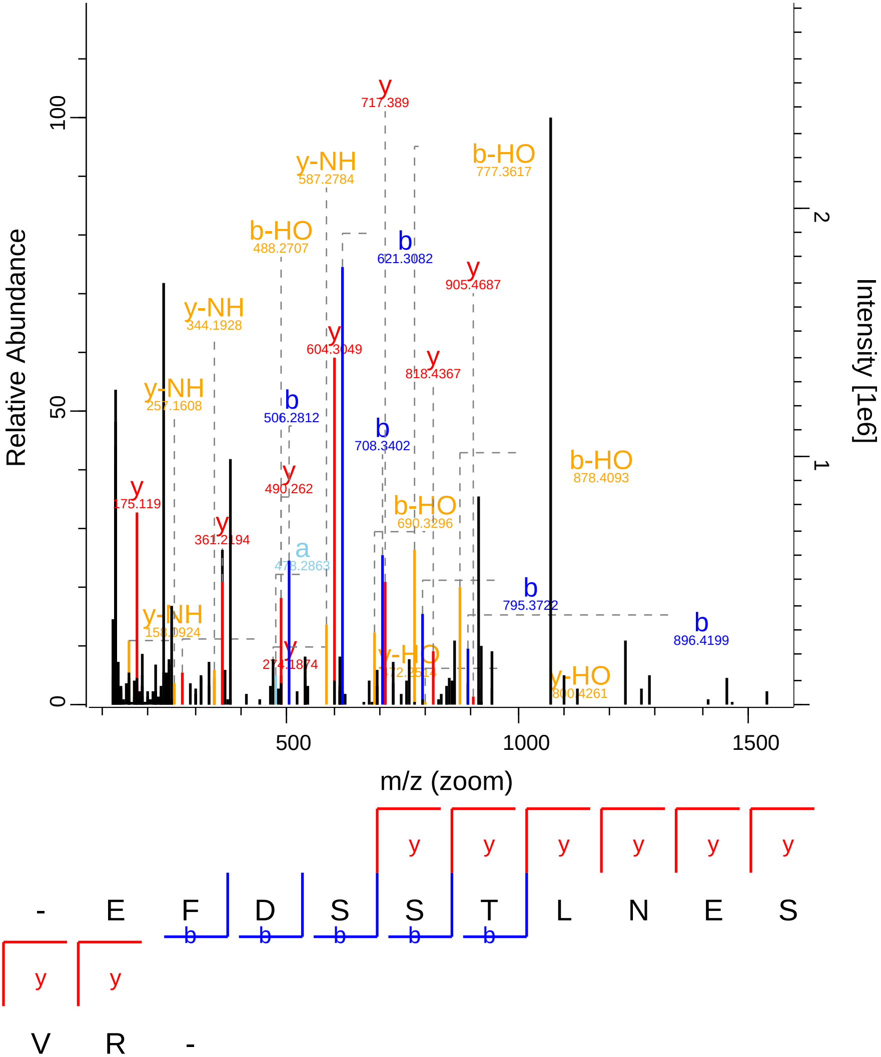

Raw File	Scan	Method	Score	m/z	Gene names
RO-QC-160121_ZackD_SS_Set_02_Fractio	19058	FTMS; HC	105.35	748.88	CES2

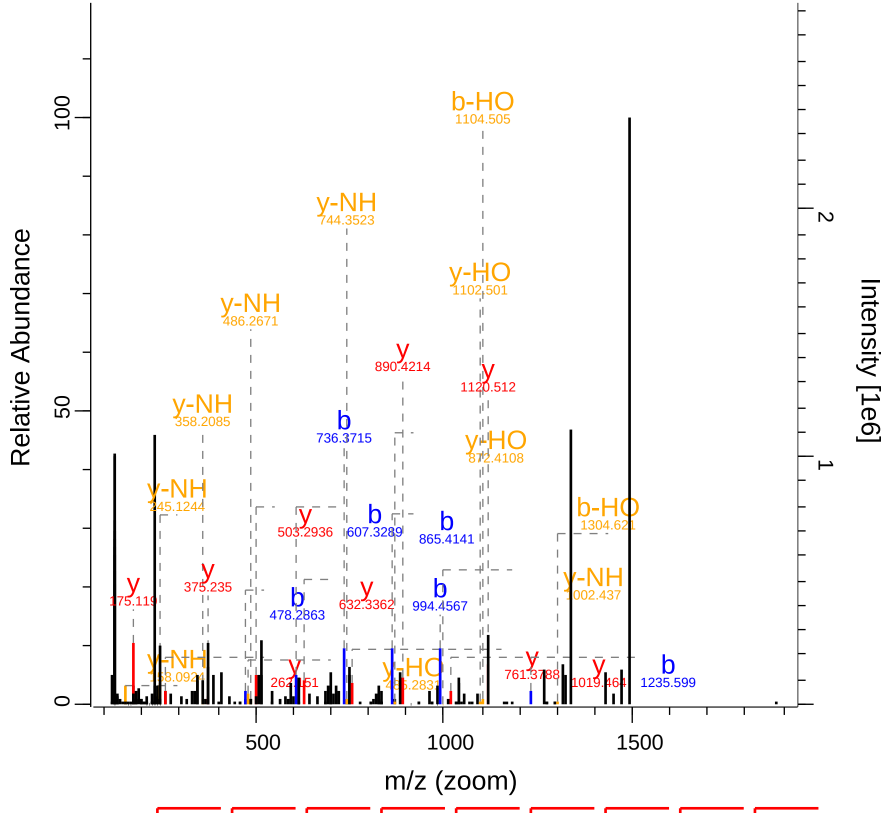

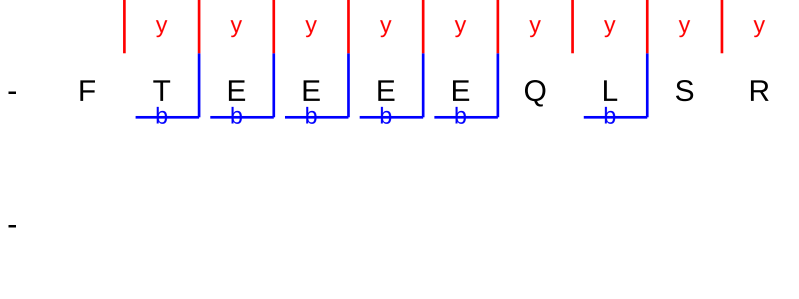

Raw File	Scan	Method	Score	m/z	Gene names
RO-QC-160121_ZackD_SS_Set_02_Fractio	19320	FTMS; HC	46.43	817.77	RTTN

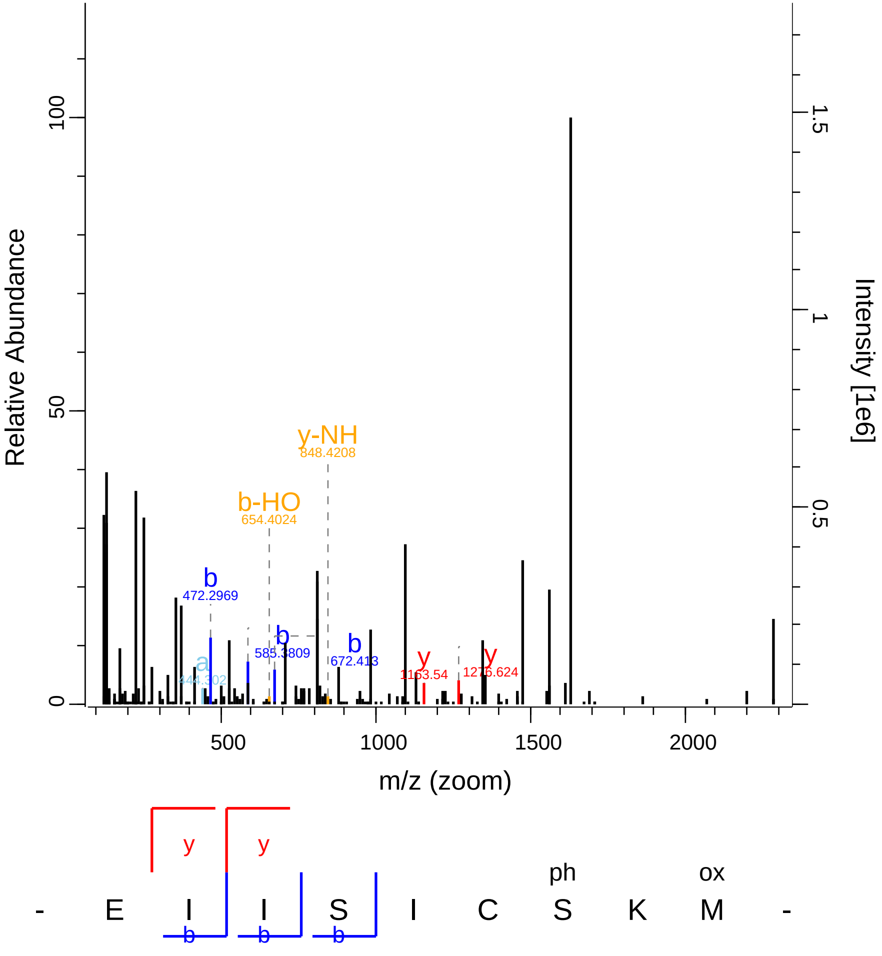

Raw File	Scan	Method	Score	m/z
RO-QC-160121_ZackD_SS_Set_02_Fraction_6	19398	FTMS; HCD	73.04	429.94

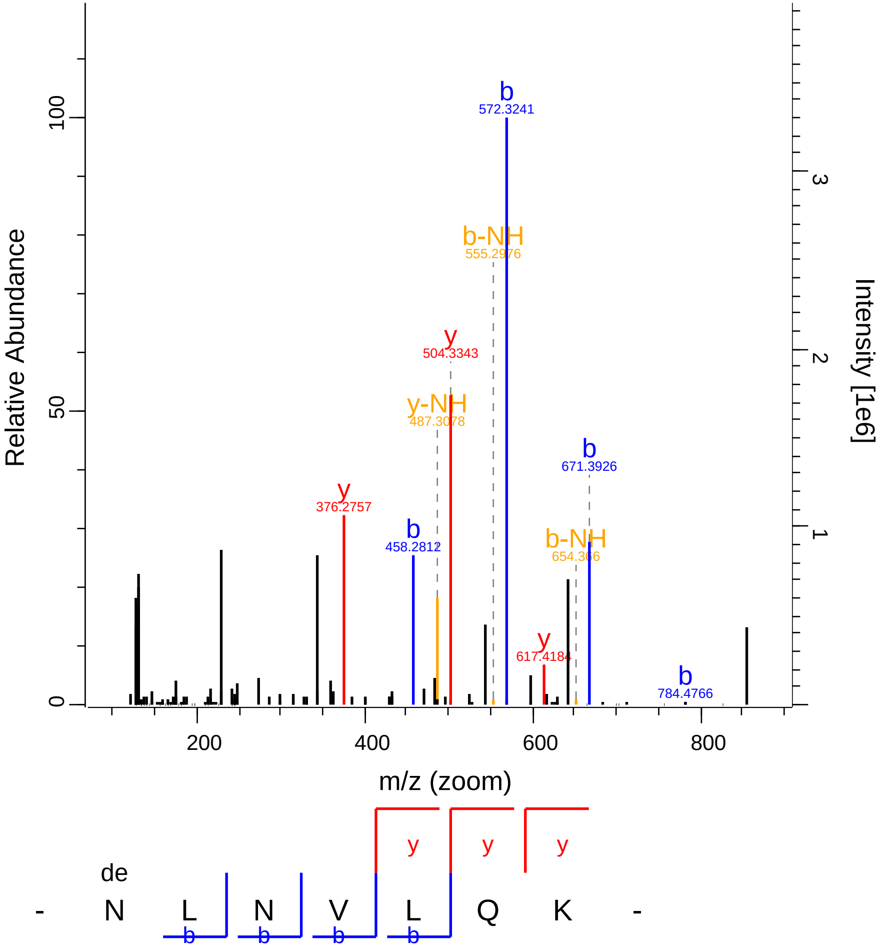

Raw File	Scan	Method	Score	m/z	Gene names
RO-QC-160121_ZackD_SS_Set_02_Fractio	19425	FTMS; HC	103.56	743.89	KLHL7

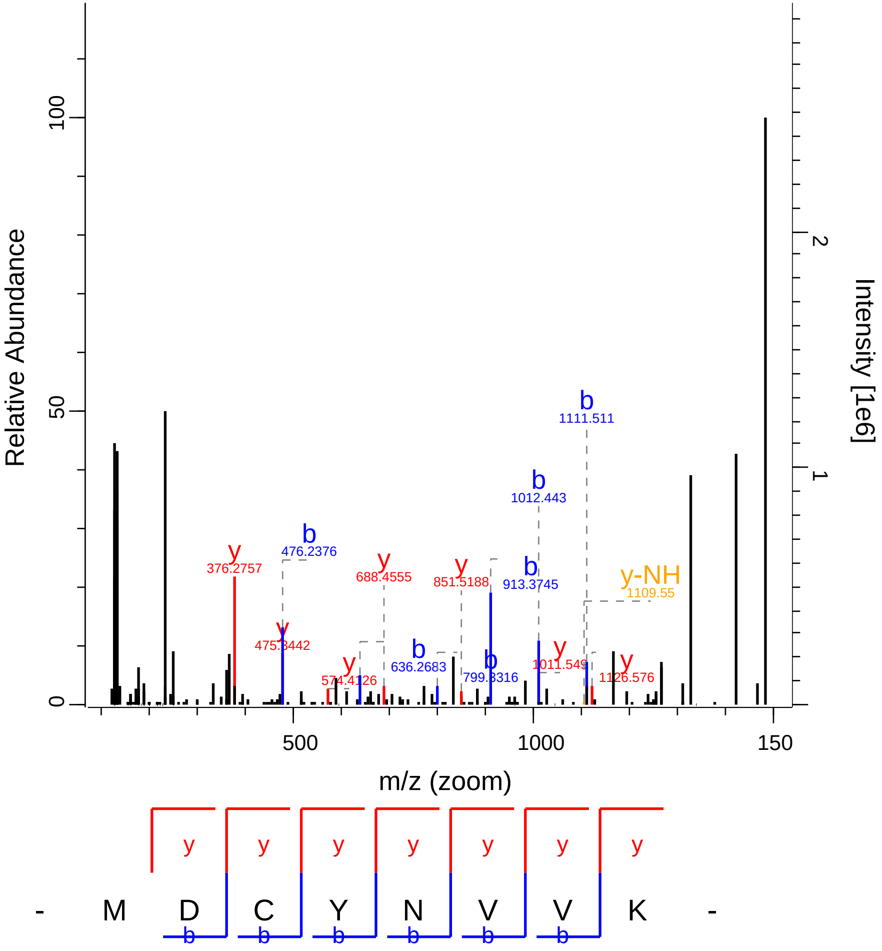

Raw File	Scan	Method	Score	m/z	Gene names
RO-QC-160121_ZackD_SS_Set_02_Fractio	19460	FTMS; HC	50.56	755.07	GIMAP7

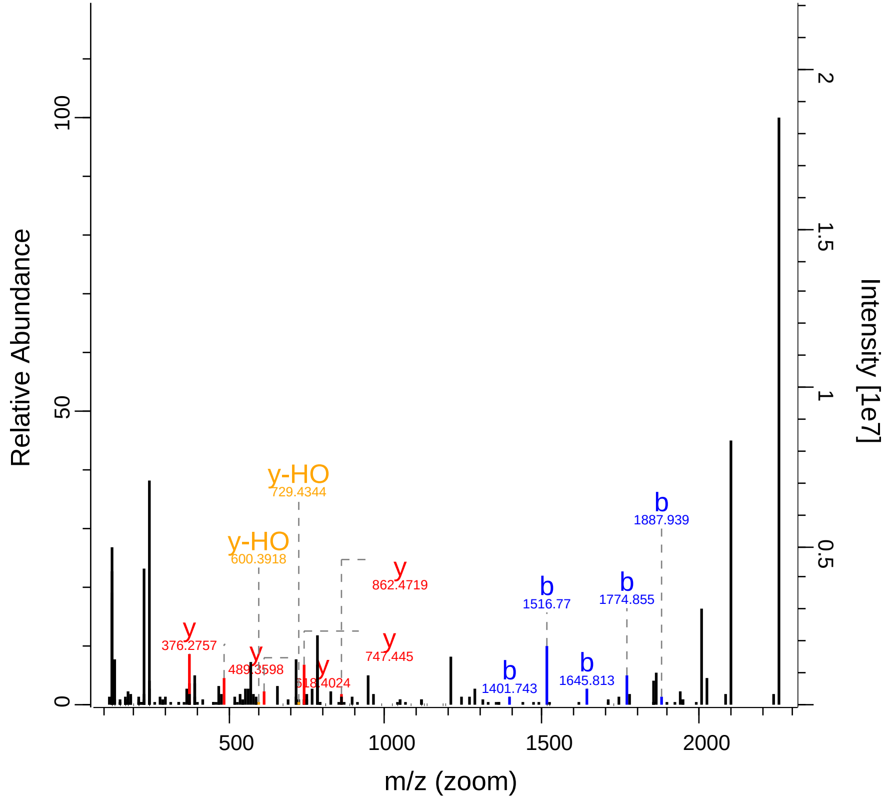

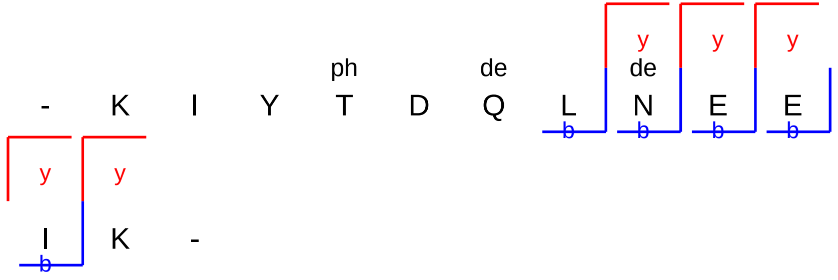

Raw File	Scan	Method	Score	m/z	Gene names
RO-QC-160121_ZackD_SS_Set_02_Fractio	19945	FTMS; HC	77.79	813.45	DDIT3

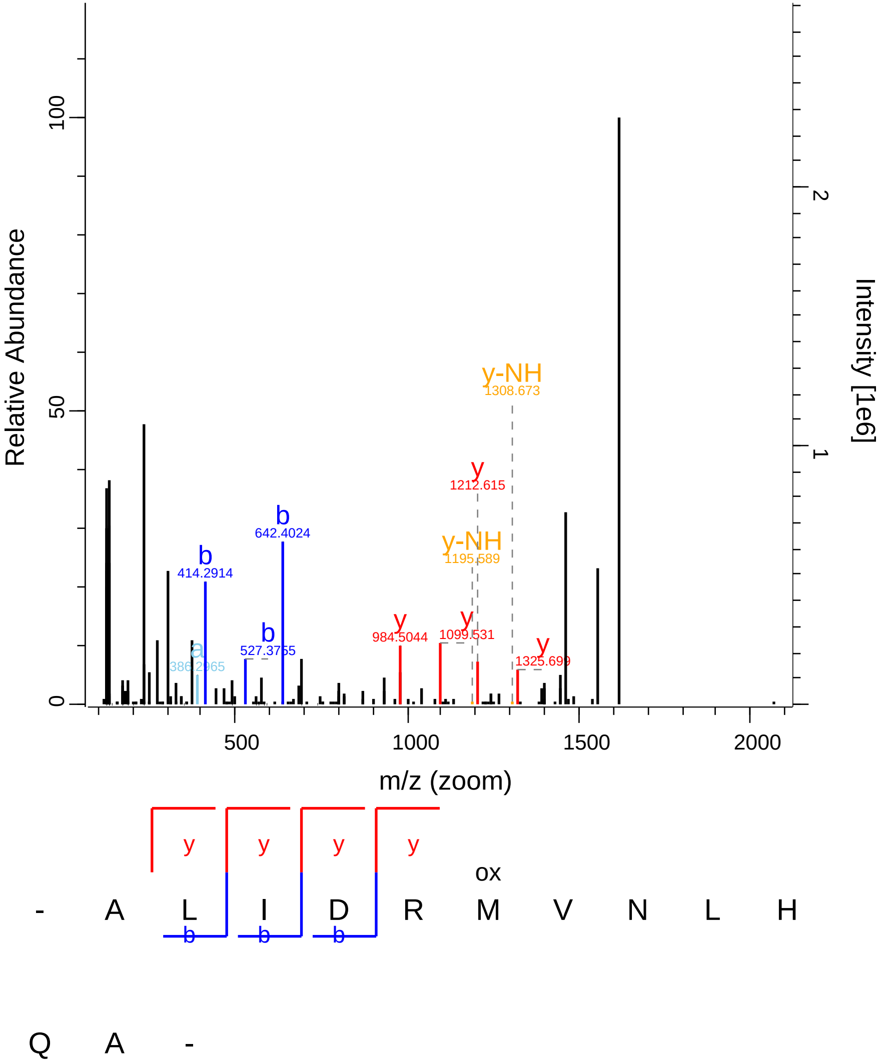

Raw File	Scan	Method	Score	m/z	Gene names
RO-QC-160121_ZackD_SS_Set_02_Fractic	20674	FTMS; HC	42.79	1264.15	FSCN3

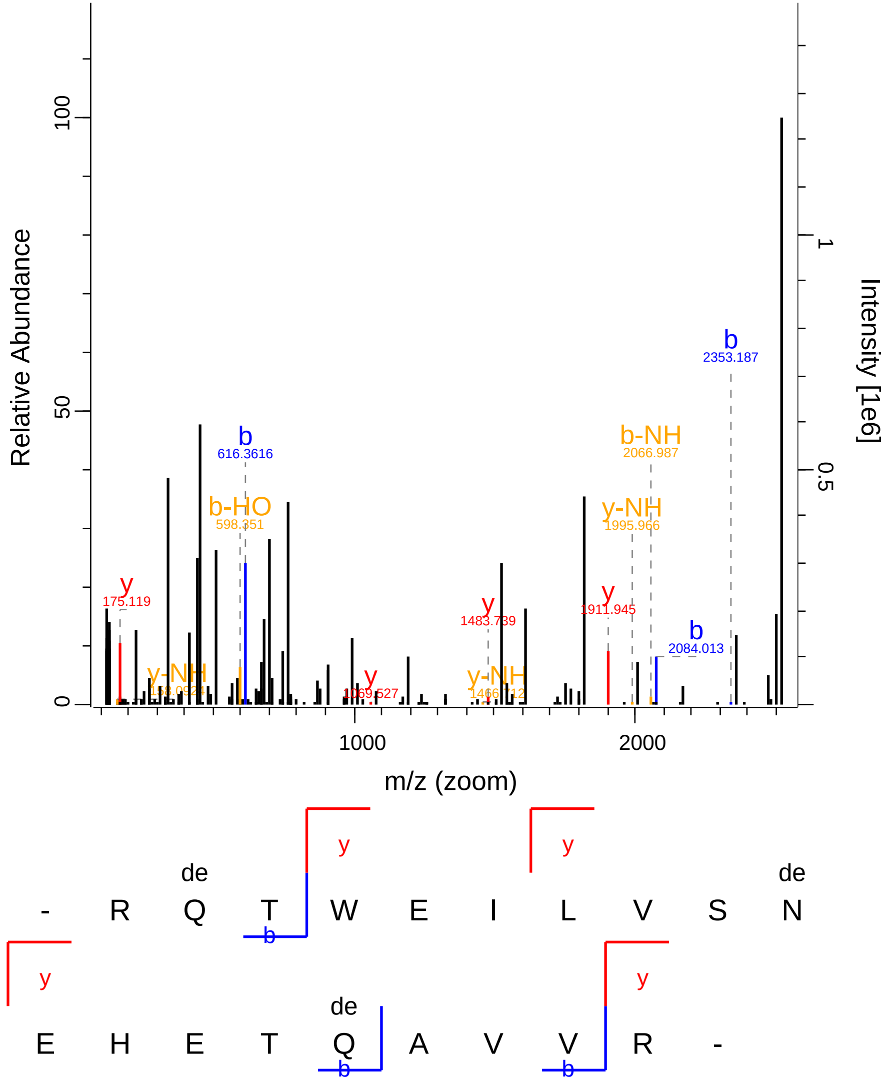

Raw File	Scan	Method	Score	m/z	Gene names
RO-QC-160121_ZackD_SS_Set_02_Fractio	20802	FTMS; HC	83.36	652.03	SLC25A17

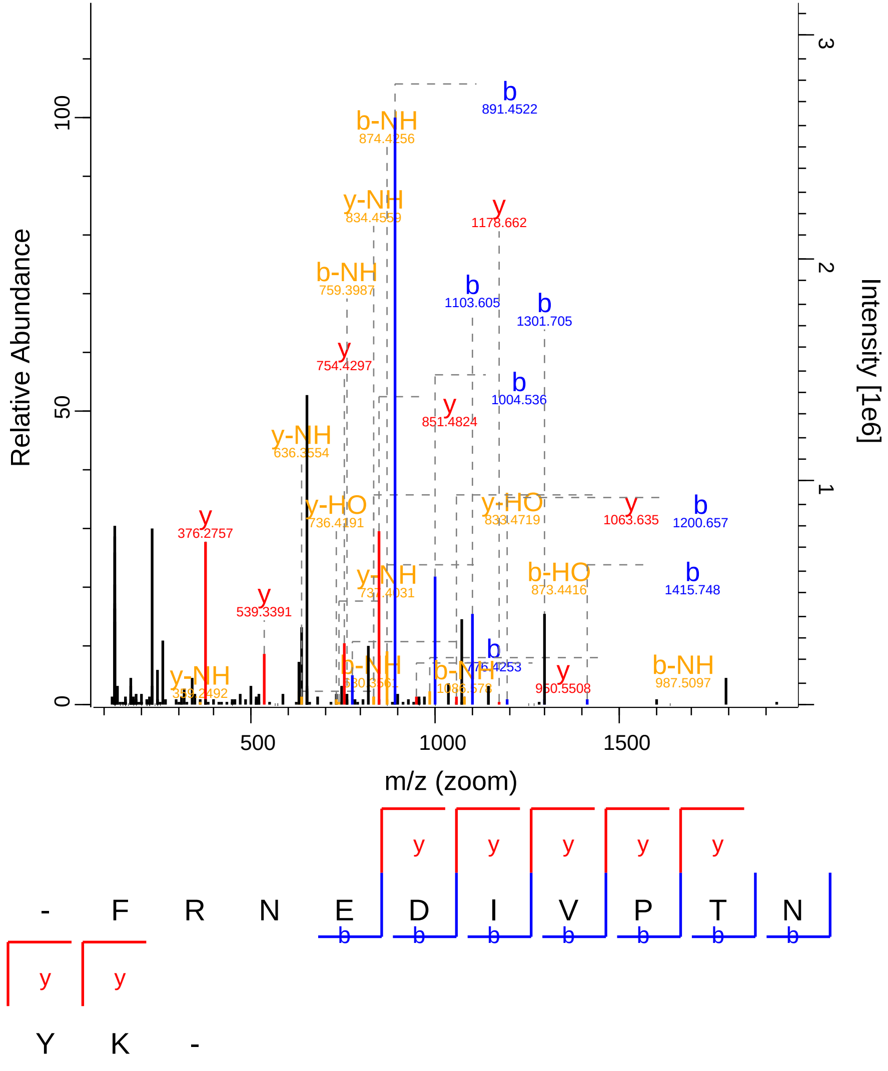

Raw File	Scan	Method	Score	m/z	Gene names
RO-QC-160121_ZackD_SS_Set_02_Fractio	21093	FTMS; HC	159.58	935.47	NUDT6

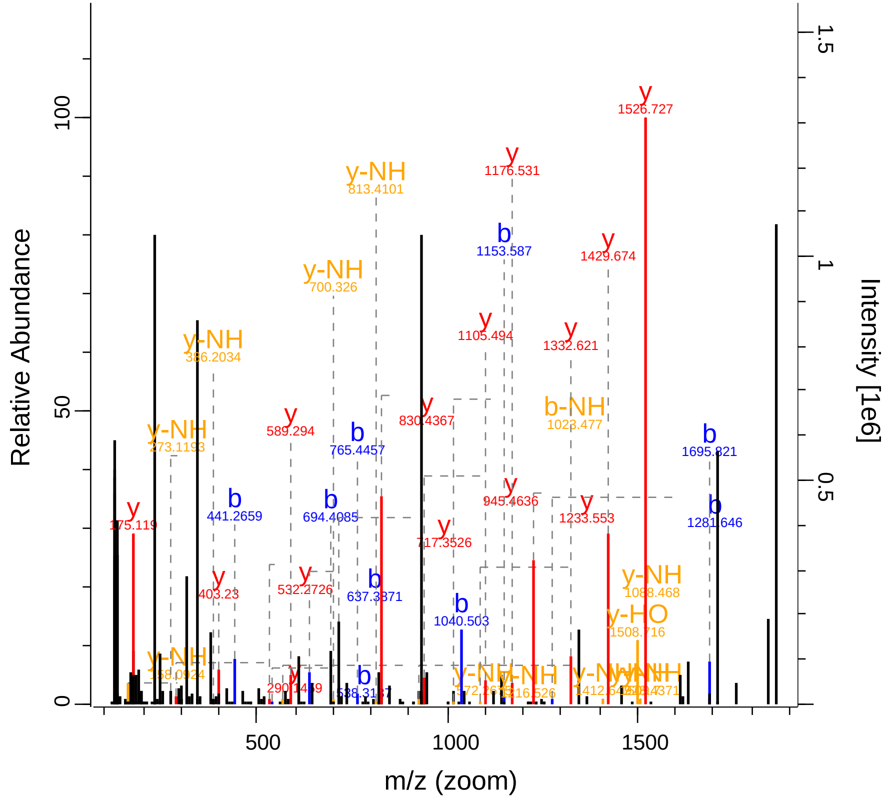

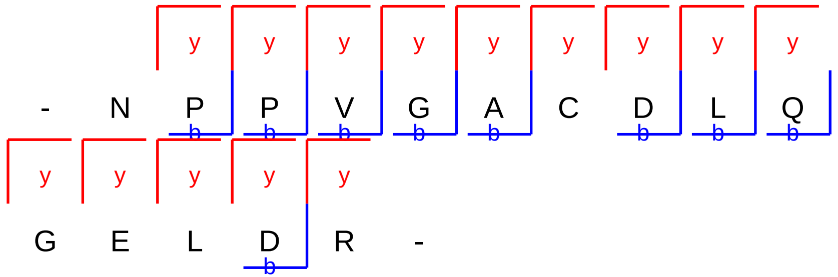

Raw File	Scan	Method	Score	m/z	Gene names
RO-QC-160121_ZackD_SS_Set_02_Fractic	22375	FTMS; HC	116.24	1191.14	ATP6V0C

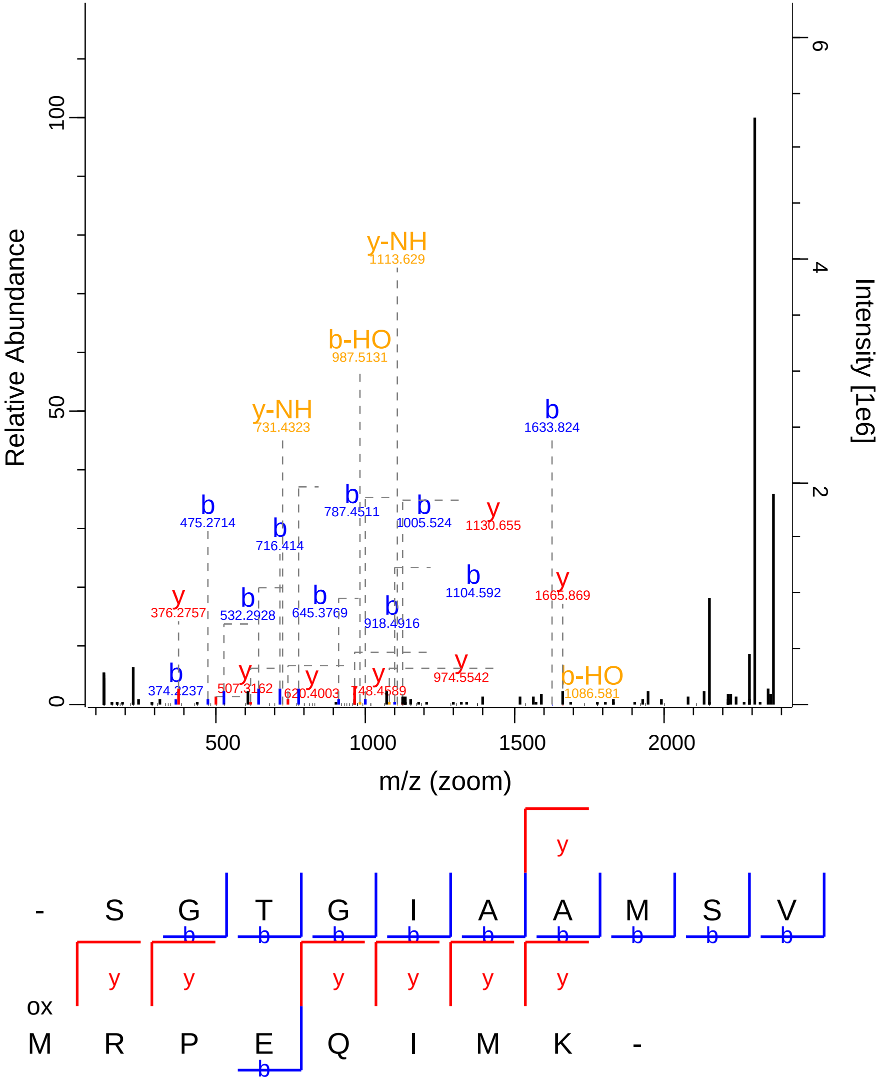

Raw File	Scan	Method	Score	m/z	Gene names
RO-QC-160121_ZackD_SS_Set_02_Fractio	22394	FTMS; HC	128	600.86	TMEM167A

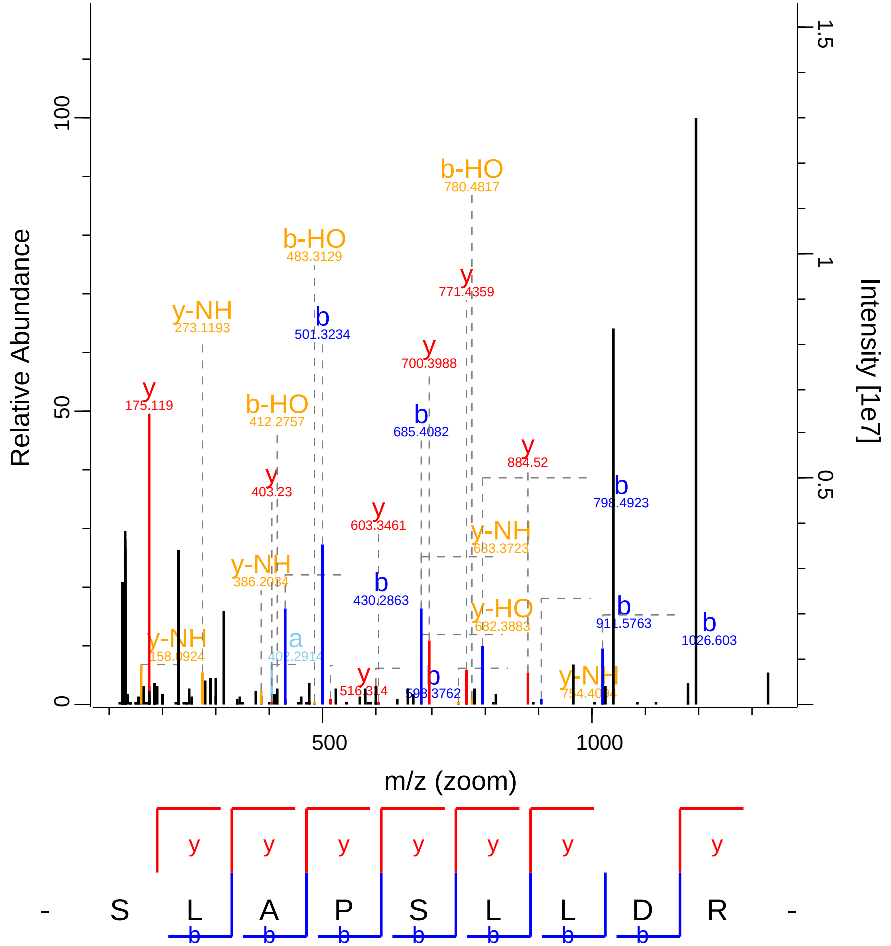

Raw File	Scan	Method	Score	m/z	Gene names
RO-QC-160121_ZackD_SS_Set_02_Fractic	22441	FTMS; HC	85.84	1192.15	ATP6V0C

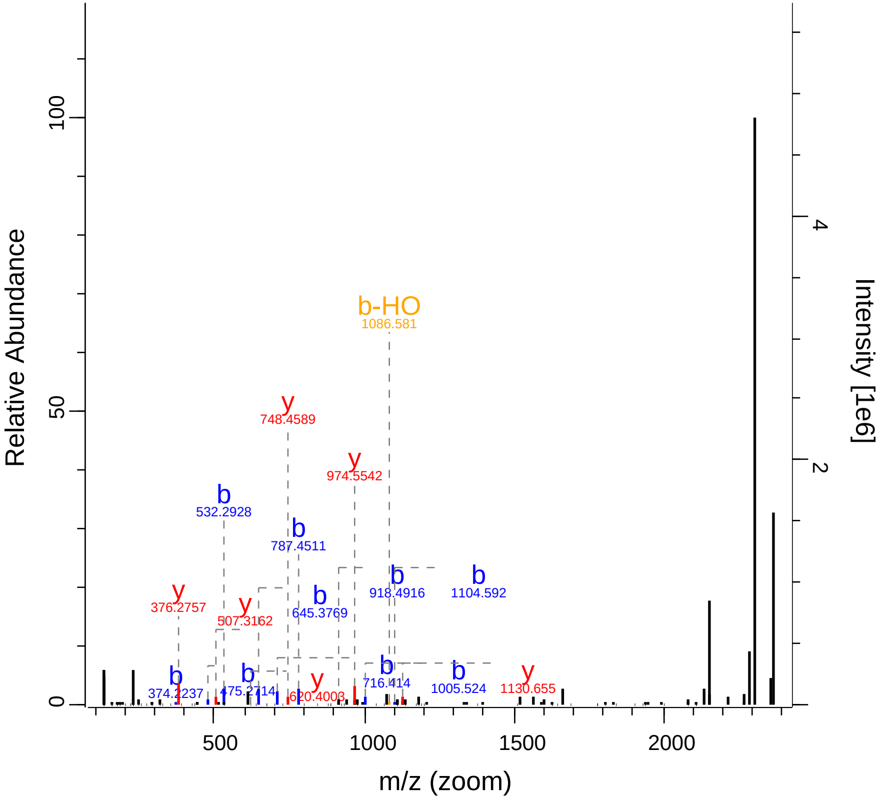

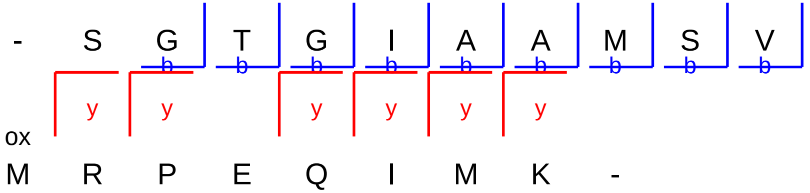

Raw File	Scan	Method	Score	m/z	Gene names
RO-QC-160121_ZackD_SS_Set_02_Fractio	22486	FTMS; HC	72.18	794.76	ATP6V0C

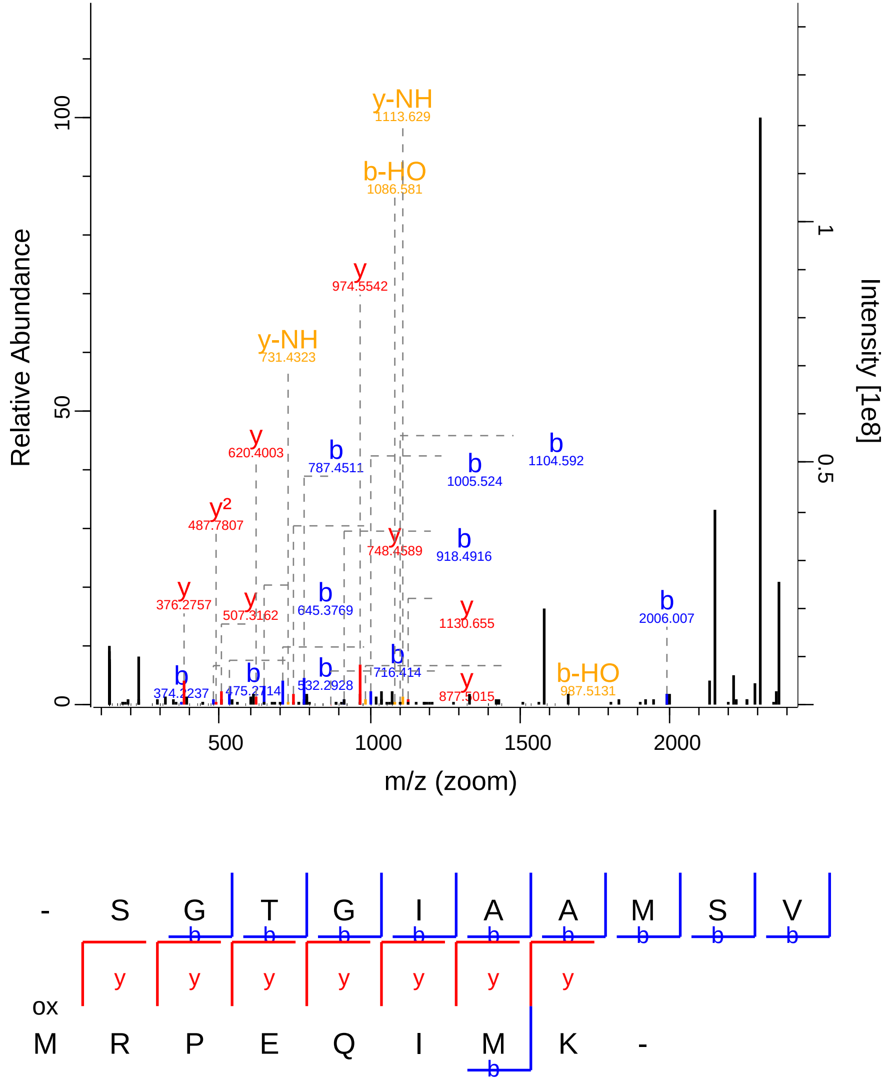

Raw File	Scan	Method	Score	m/z	Gene names
RO-QC-160121_ZackD_SS_Set_02_Fractior	22996	FTMS; HCI	143.85	638.4	CABP7

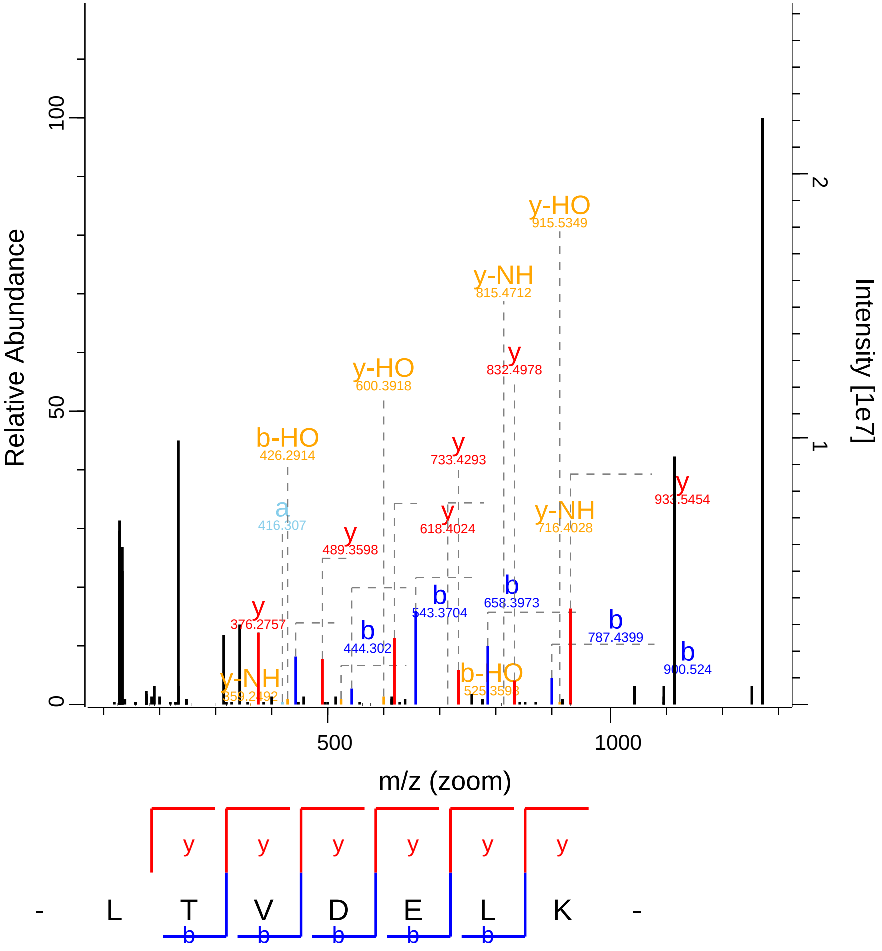

Raw File	Scan	Method	Score	m/z	Gene names
RO-QC-160121_ZackD_SS_Set_02_Fractio	23140	FTMS; HC	38.96	459.61	PTPN21

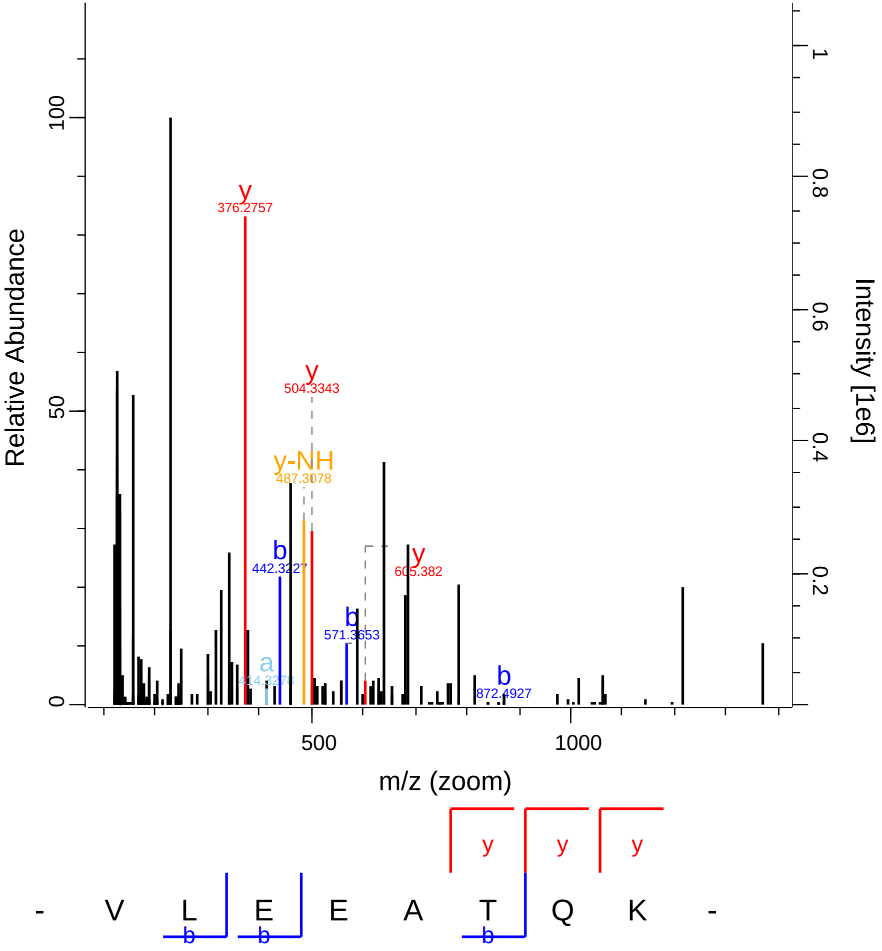

Raw File	Scan	Method	Score	m/z	Gene names
RO-QC-160121_ZackD_SS_Set_02_Fractio	23171	FTMS; HC	87.28	425.94	CABP7

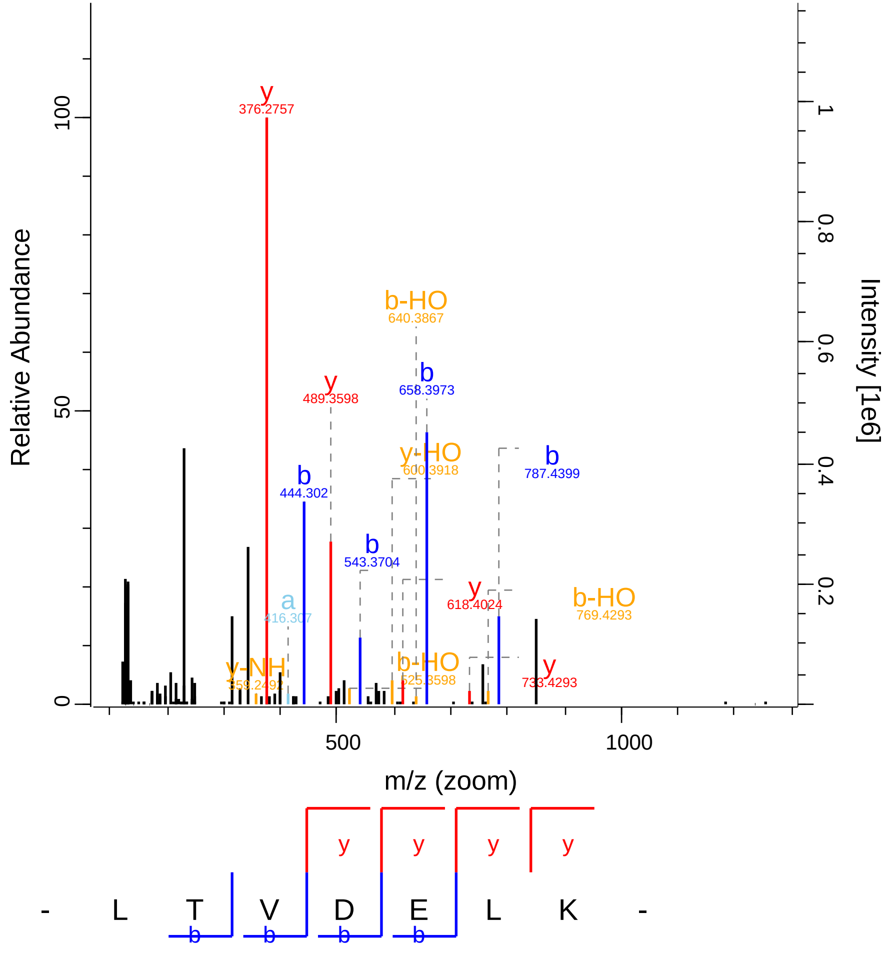

Raw File	Scan	Method	Score	m/z	Gene names
RO-QC-160121_ZackD_SS_Set_02_Fractioı	23202	FTMS; HCI	104.37	638.4	CABP7

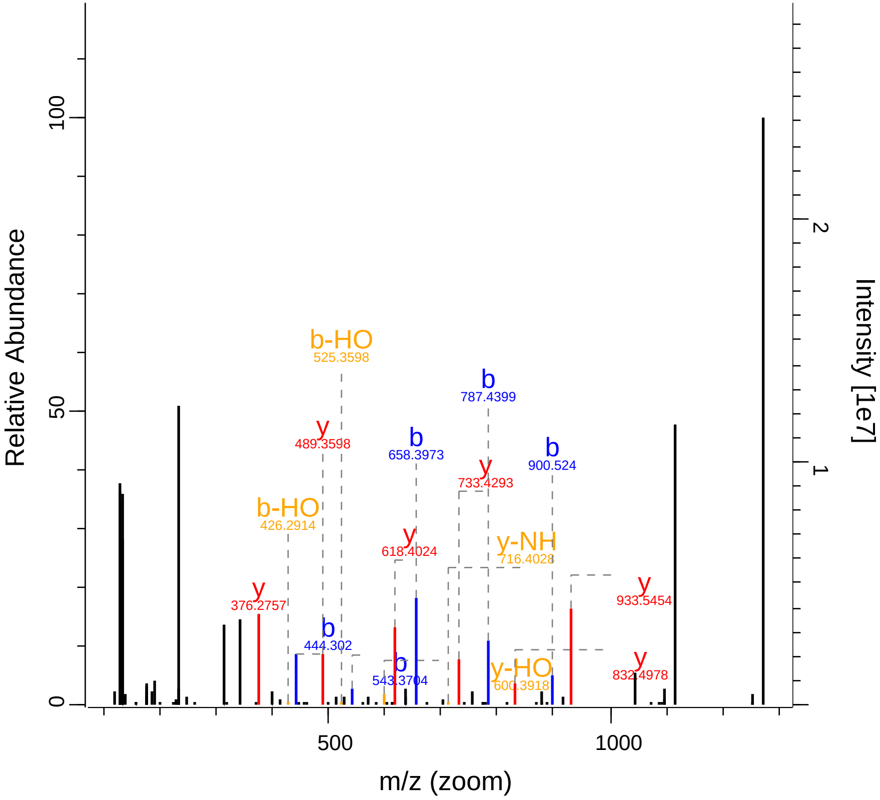

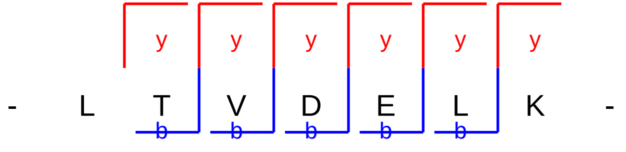

Raw File	Scan	Method	Score	m/z	Gene names
RO-QC-160121_ZackD_SS_Set_02_Fractio	24677	FTMS; HC	104.79	905.42	ACTR6

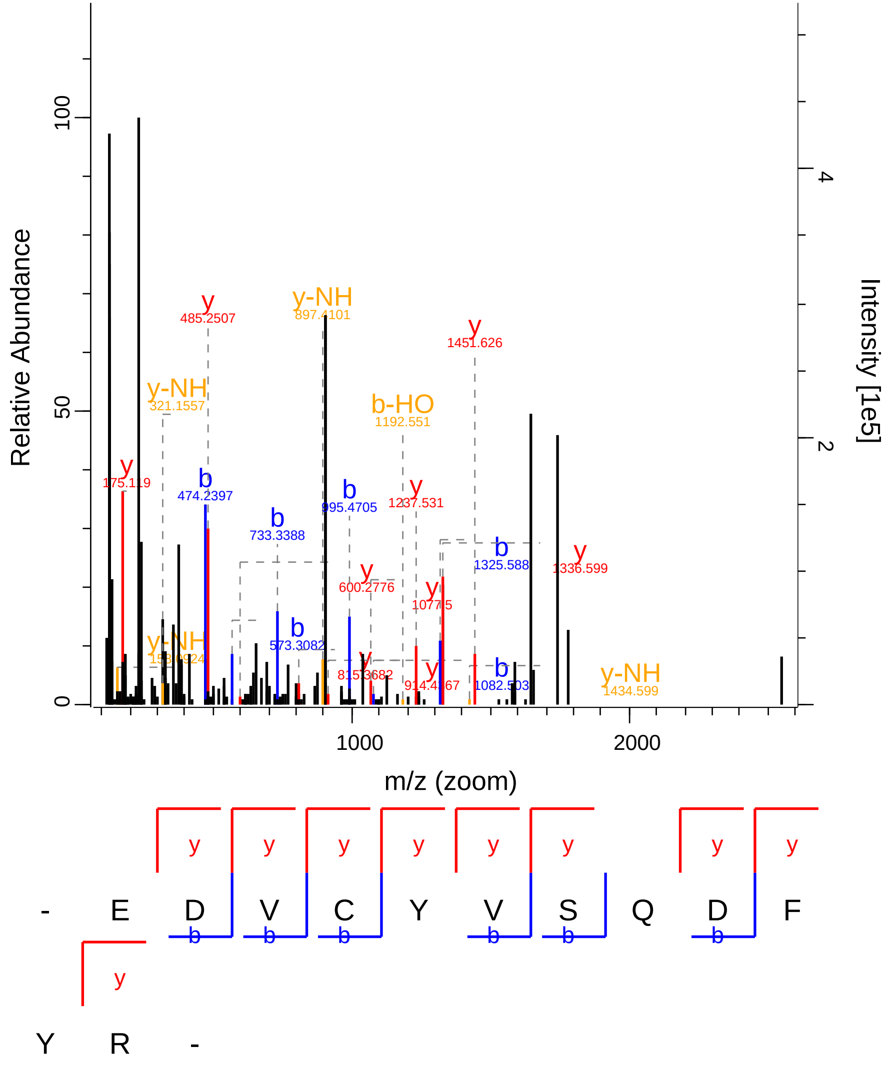

Raw File	Scan	Method	Score	m/z	Gene names
RO-QC-160121_ZackD_SS_Set_02_Fractic	24933	FTMS; HC	43.68	1209.06	LUZP4

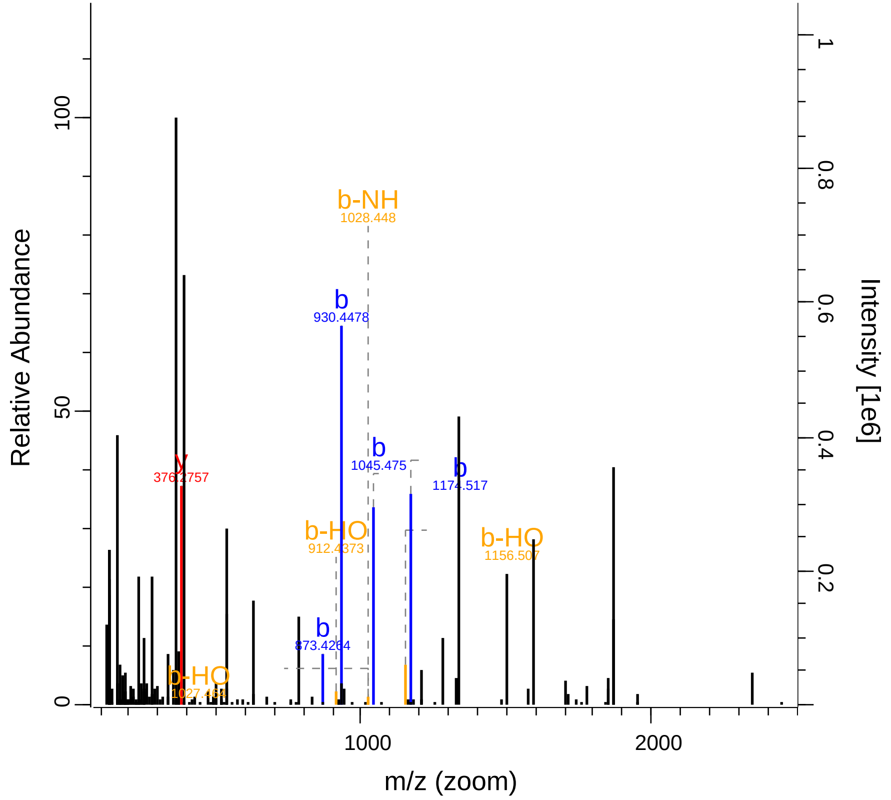

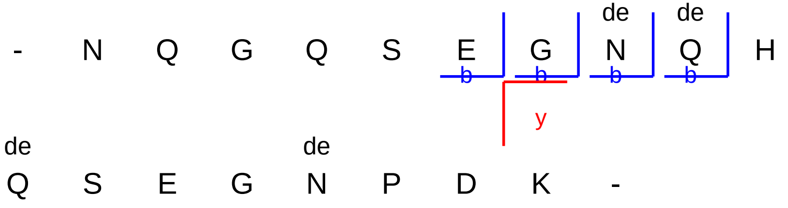

Raw File	Scan	Method	Score	m/z	Gene names
RO-QC-160121_ZackD_SS_Set_02_Fractio	25587	FTMS; HC	168.74	893.47	MRPL35

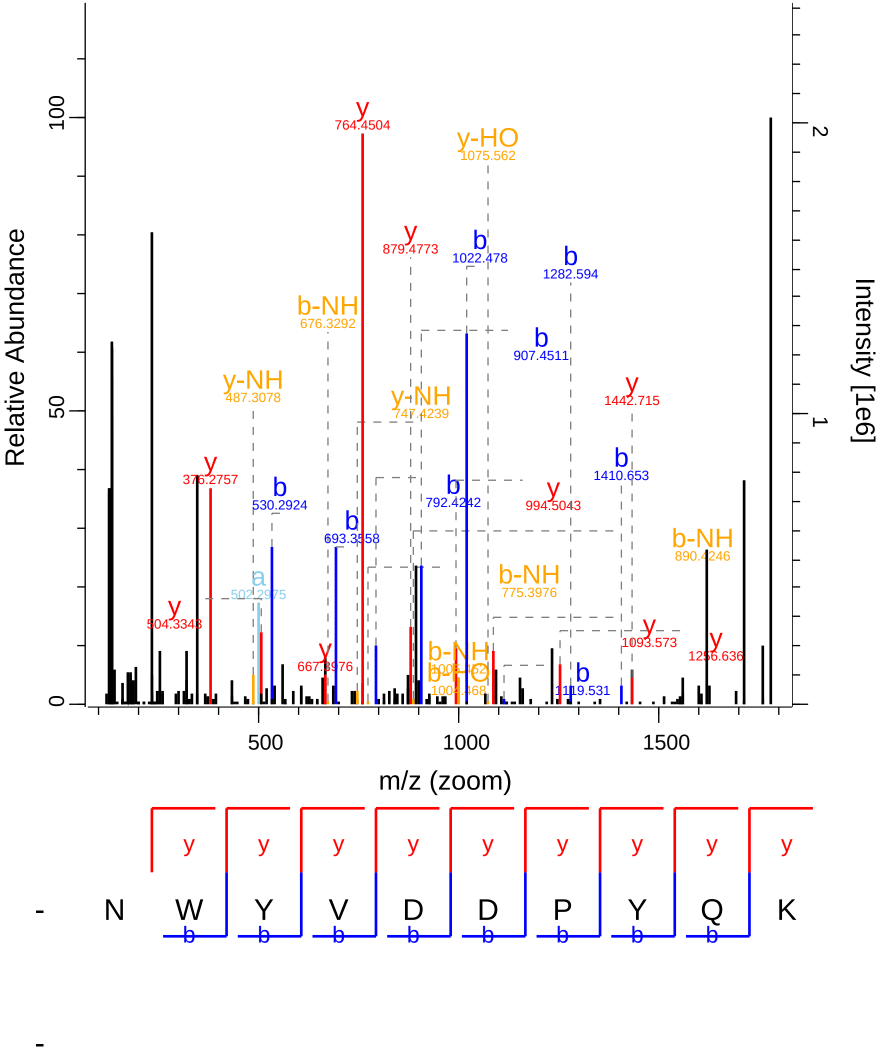

Raw File	Scan	Method	Score	m/z	Gene names
RO-QC-160121_ZackD_SS_Set_02_Fractio	25605	FTMS; HC	51.09	596.31	MRPL35

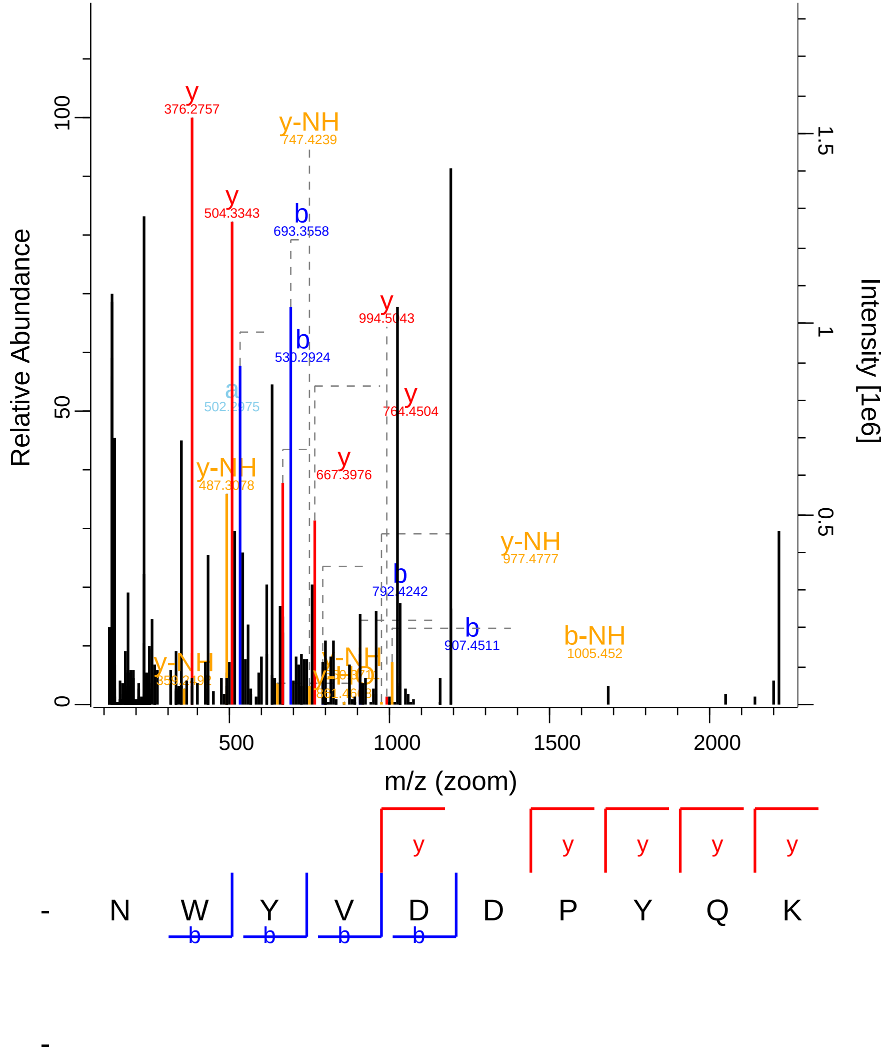

Raw File	Scan	Method	Score	m/z
RO-QC-160121_ZackD_SS_Set_02_Fraction_6	25893	FTMS; HCD	136.8	681.92

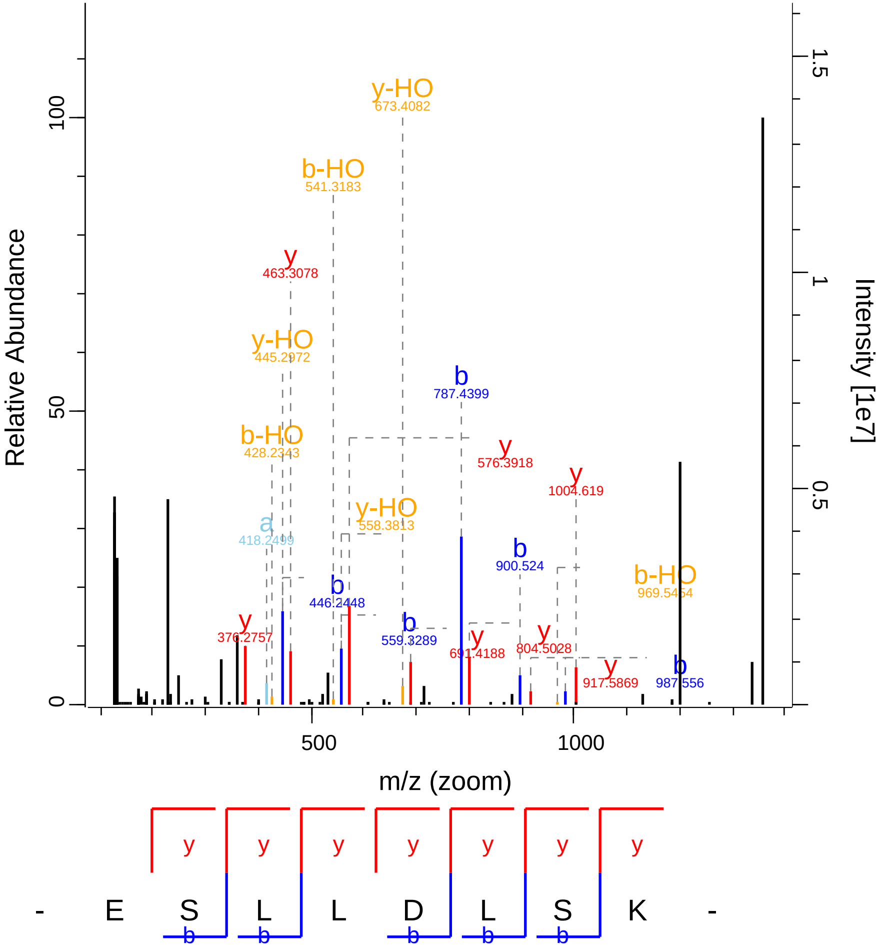

Raw File	Scan	Method	Score	m/z
RO-QC-160121_ZackD_SS_Set_02_Fraction_6	25911	FTMS; HCD	111.17	454.95

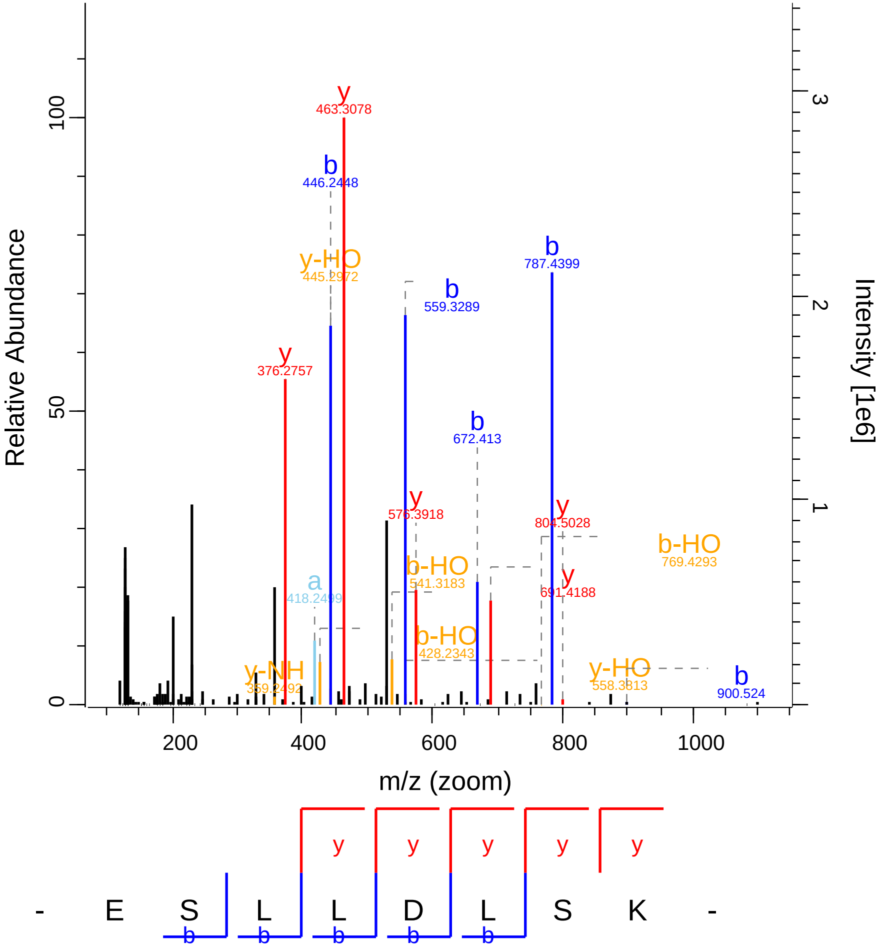

Raw File	Scan	Method	Score	m/z
RO-QC-160121_ZackD_SS_Set_02_Fraction_6	26093	FTMS; HCD	123.72	681.92

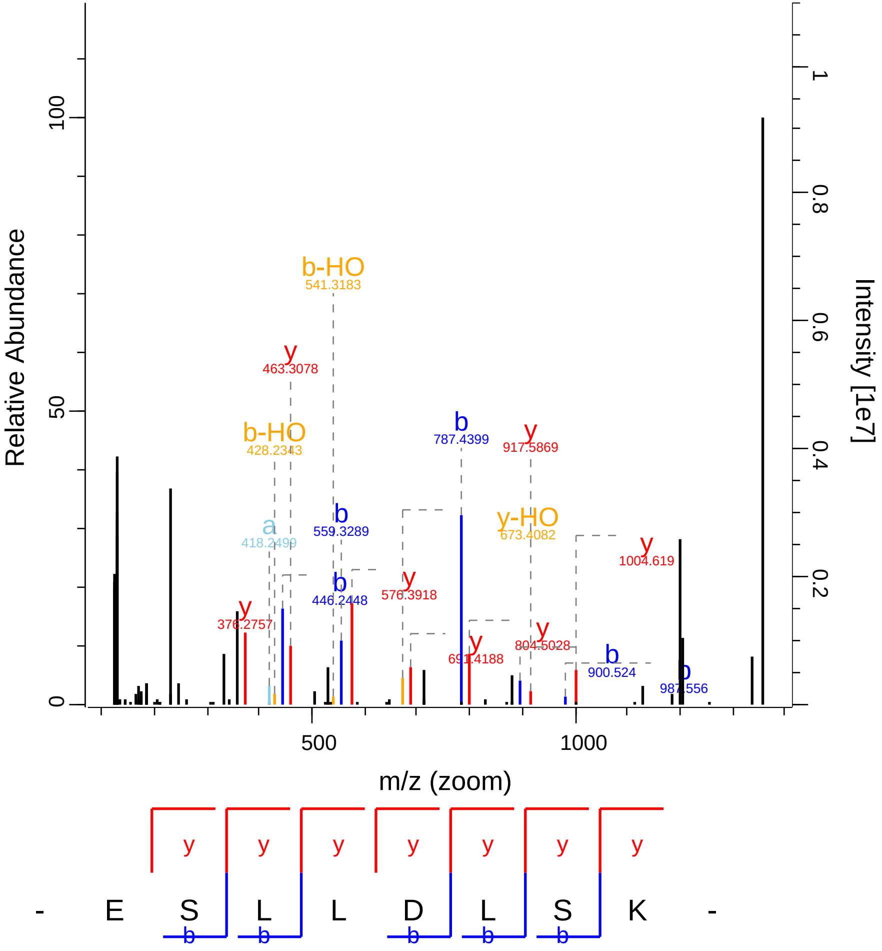

Raw File	Scan	Method	Score	m/z	Gene names
RO-QC-160121_ZackD_SS_Set_02_Fractio	26569	FTMS; HC	86.09	405.21	SCTR

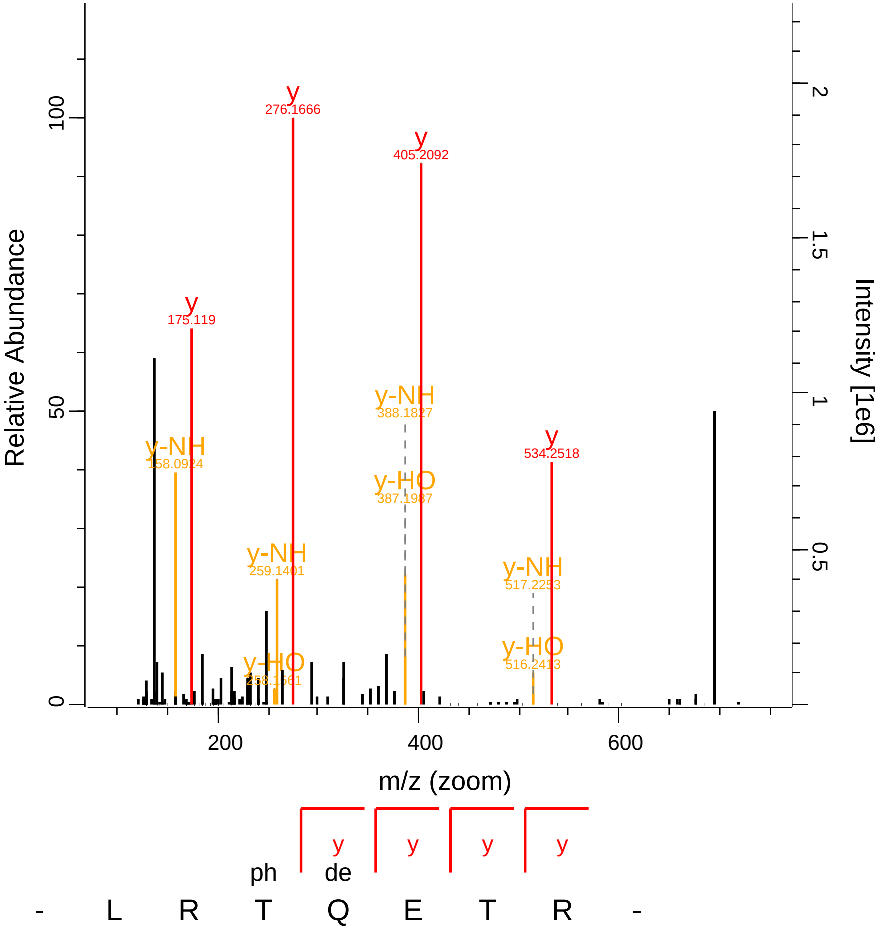

Raw File	Scan	Method	Score	m/z	Gene names
RO-QC-160121_ZackD_SS_Set_02_Fractio	28116	FTMS; HC	100.42	612.85	MELK

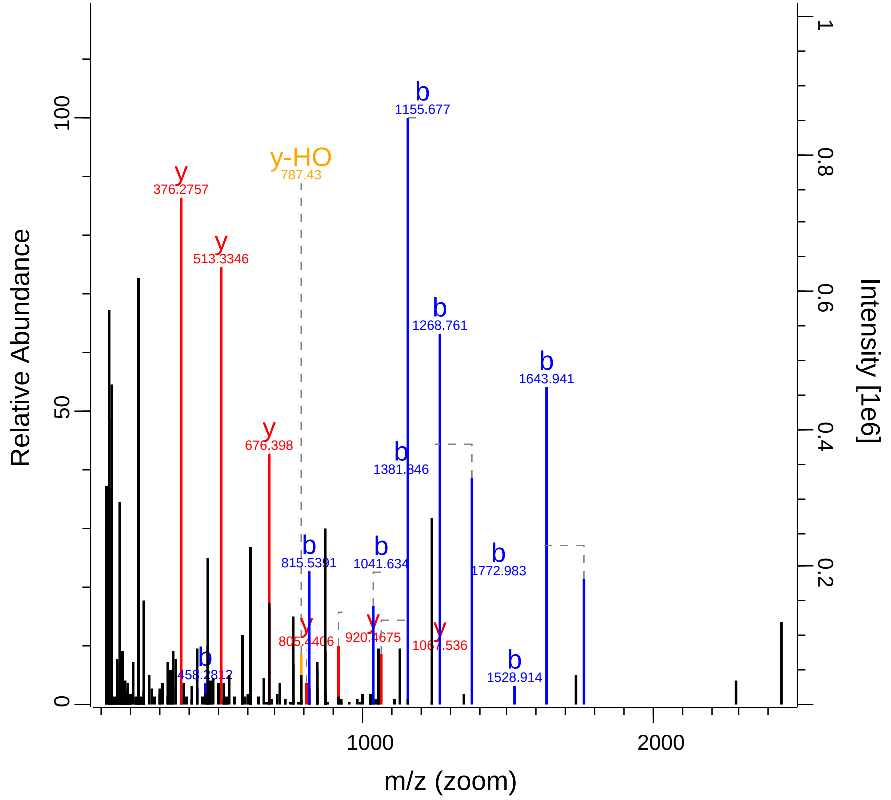

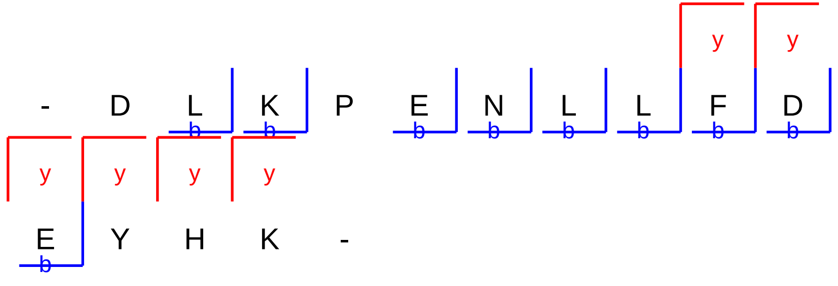

Raw File	Scan	Method	Score	m/z	Gene names
RO-QC-160121_ZackD_SS_Set_02_Fractio	29156	FTMS; HC	102.52	585.66	GSKIP

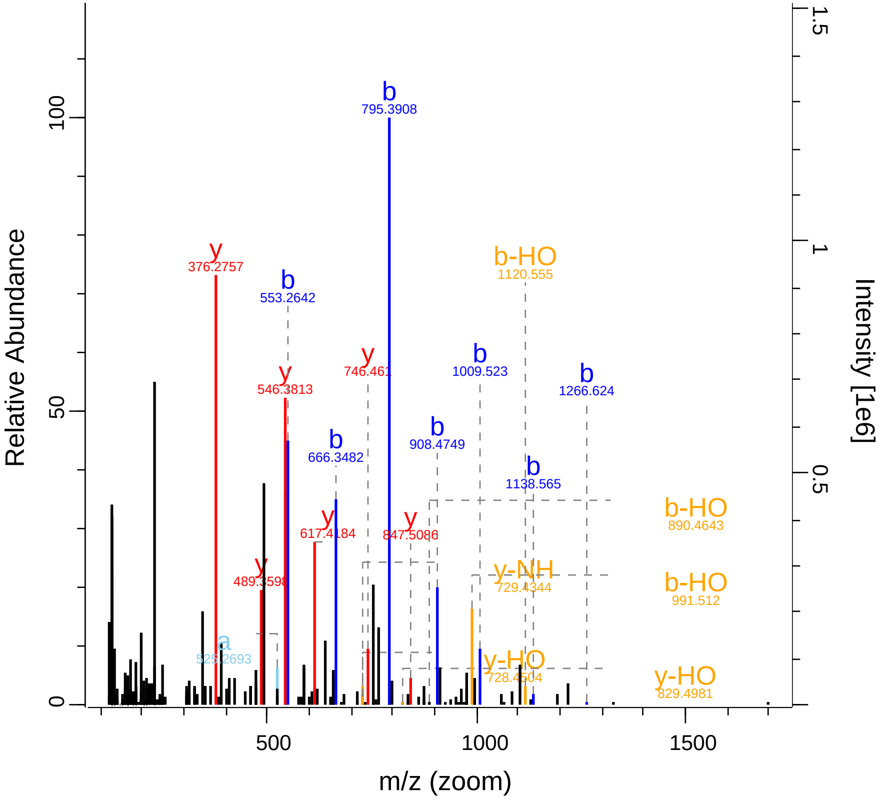

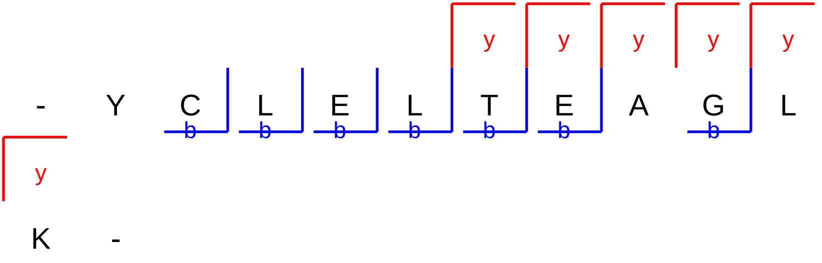

Raw File	Scan	Method	Score	m/z	Gene names
RO-QC-160121_ZackD_SS_Set_02_Fractio	29166	FTMS; HC	179.1	877.99	GSKIP

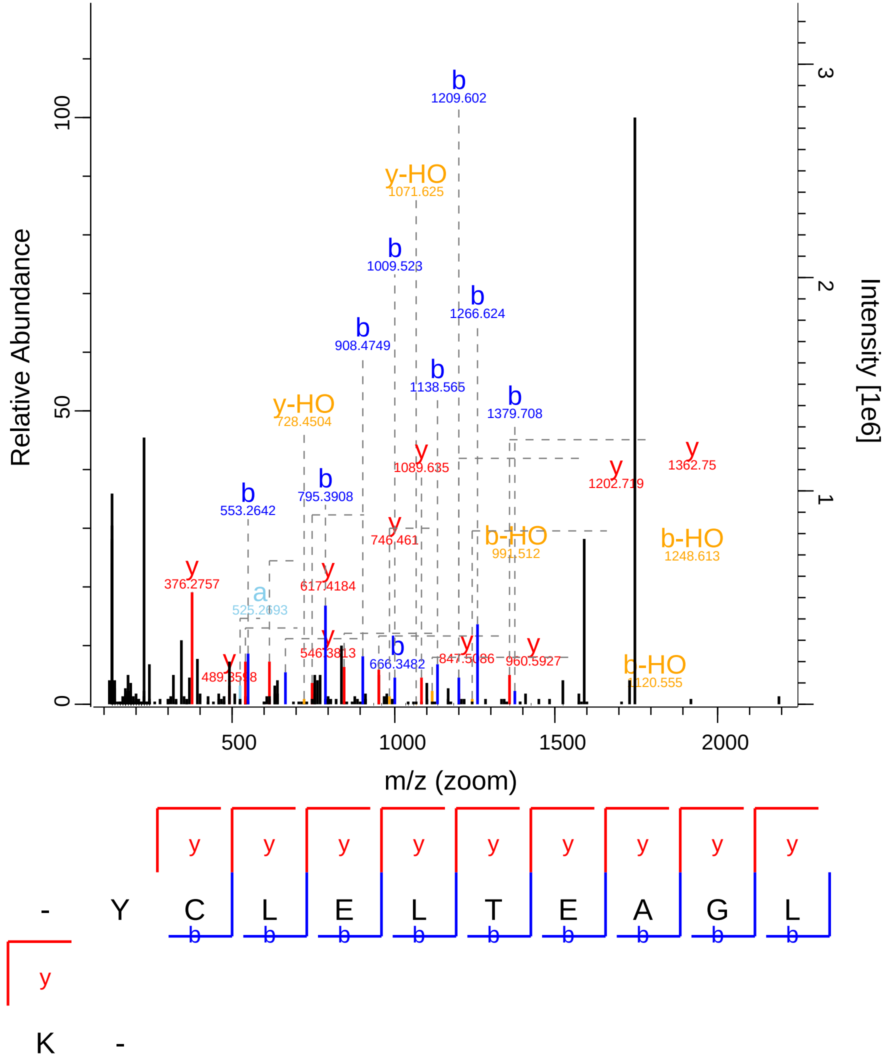

Raw File	Scan	Method	Score	m/z	Gene names
RO-QC-160121_ZackD_SS_Set_02_Fractio	29354	FTMS; HC	111.46	682.88	MT-CYB

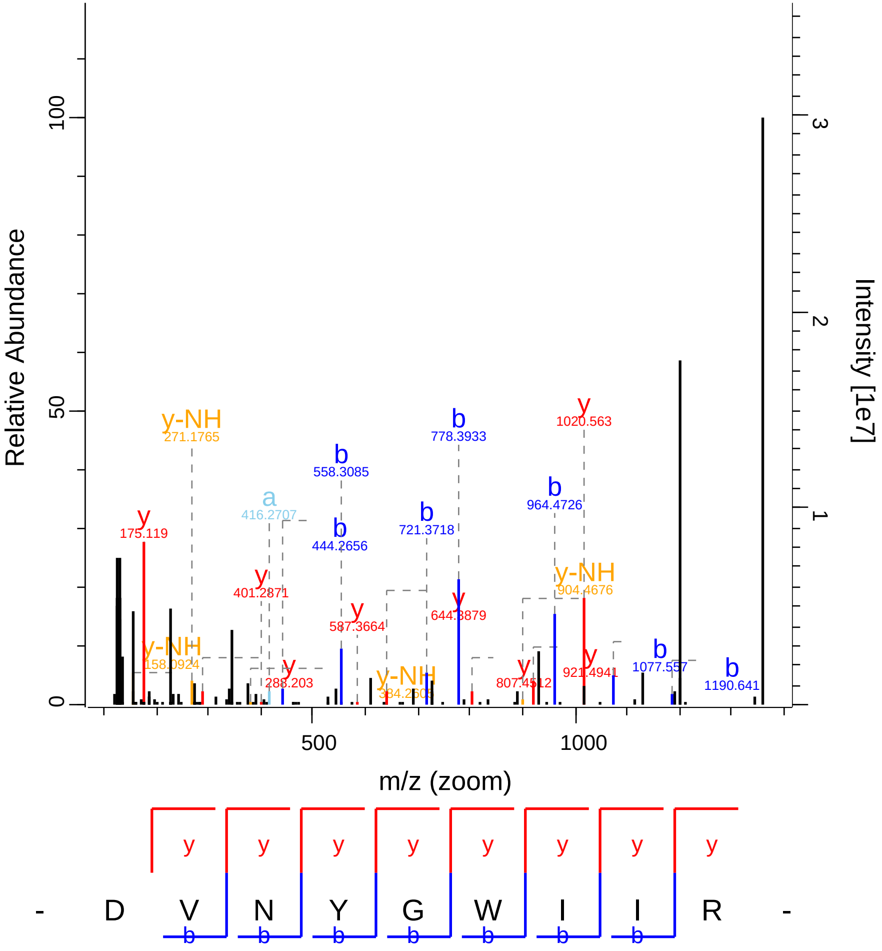

Raw File	Scan	Method	Score	m/z	Gene names
RO-QC-160121_ZackD_SS_Set_02_Fractio	29615	FTMS; HC	136.04	841.42	CNIH4

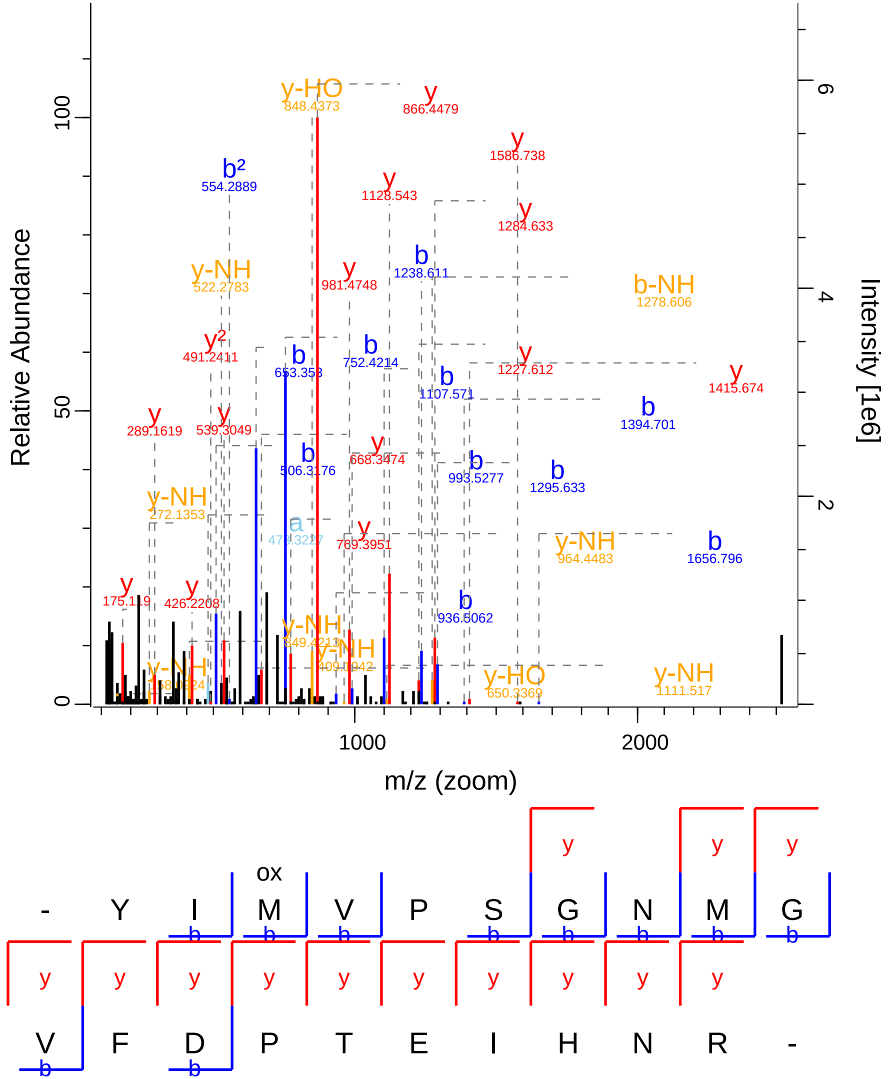

Raw File	Scan	Method	Score	m/z	Gene names
RO-QC-160121_ZackD_SS_Set_02_Fractio	29815	FTMS; HC	137.69	841.75	CNIH4

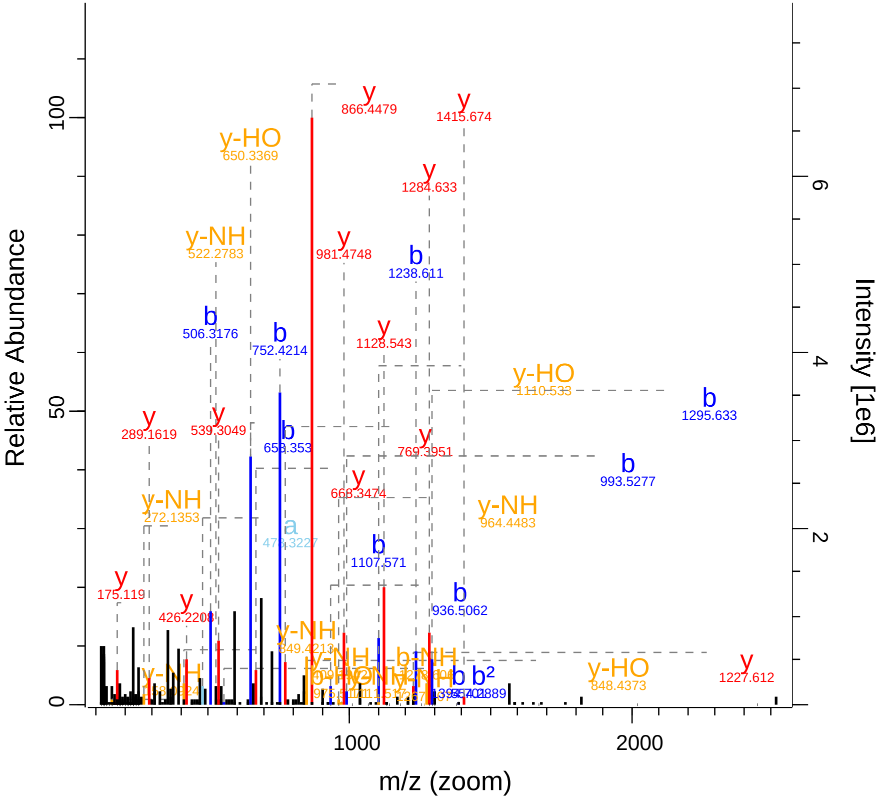

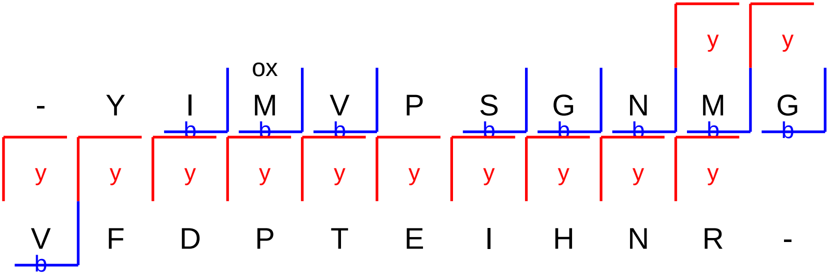

Raw File	Scan	Method	Score	m/z	Gene names
RO-QC-160121_ZackD_SS_Set_02_Fractio	29892	FTMS; HC	140.82	655.38	ALKBH8

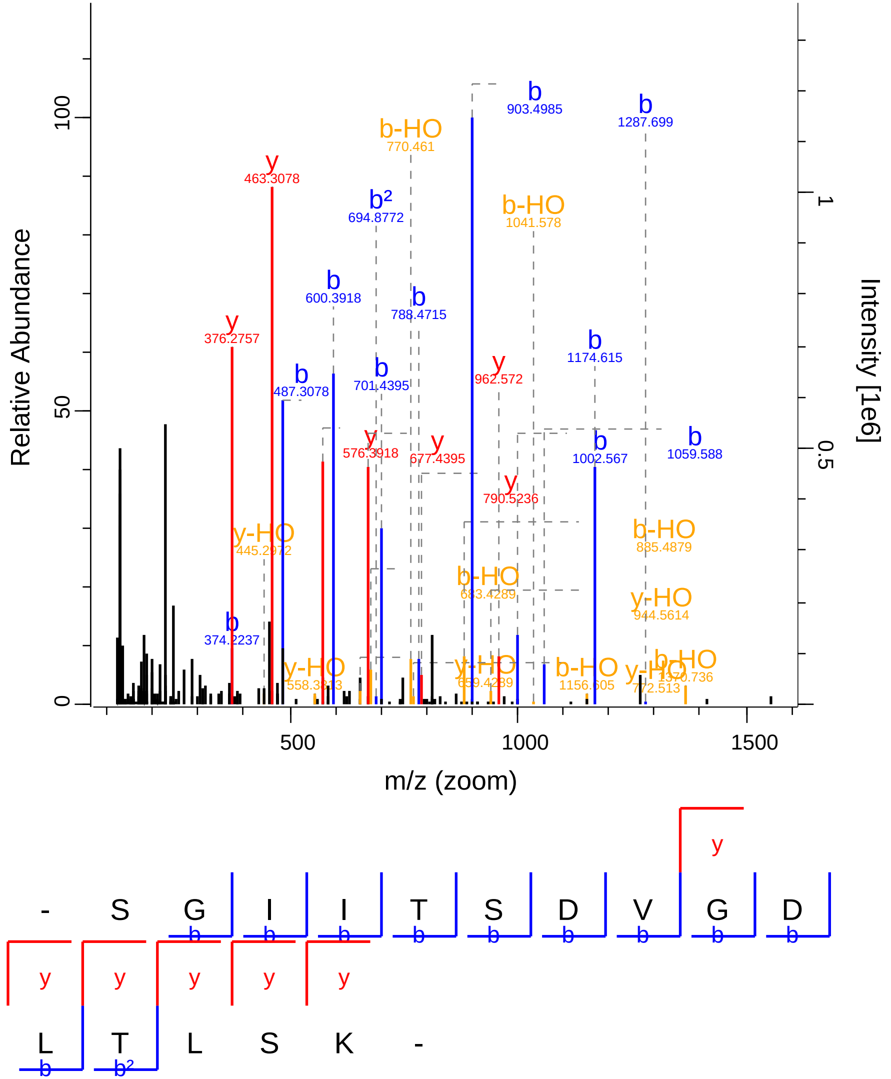

Raw File	Scan	Method	Score	m/z	Gene names
RO-QC-160121_ZackD_SS_Set_02_Fractio	29969	FTMS; HC	117.86	946.51	POP7

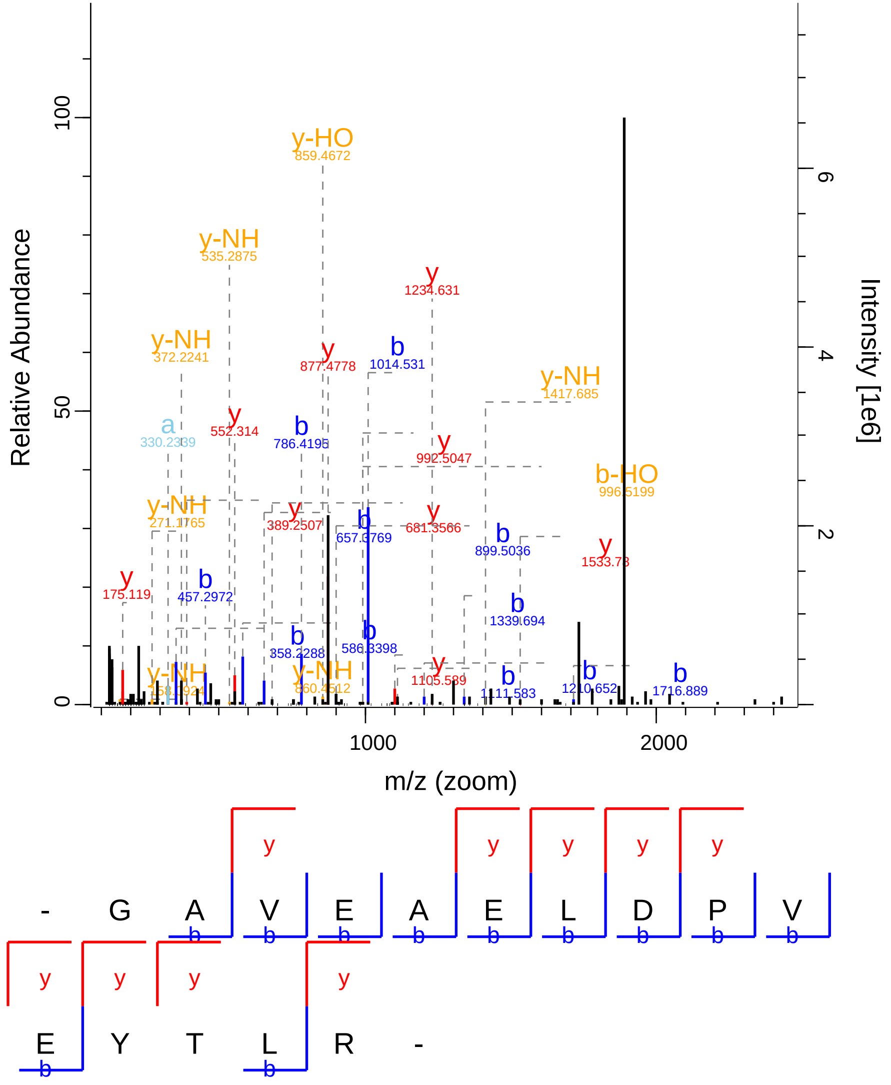

Raw File	Scan	Method	Score	m/z	Gene names
RO-QC-160121_ZackD_SS_Set_02_Fractio	29979	FTMS; HC	244.18	946.01	POP7

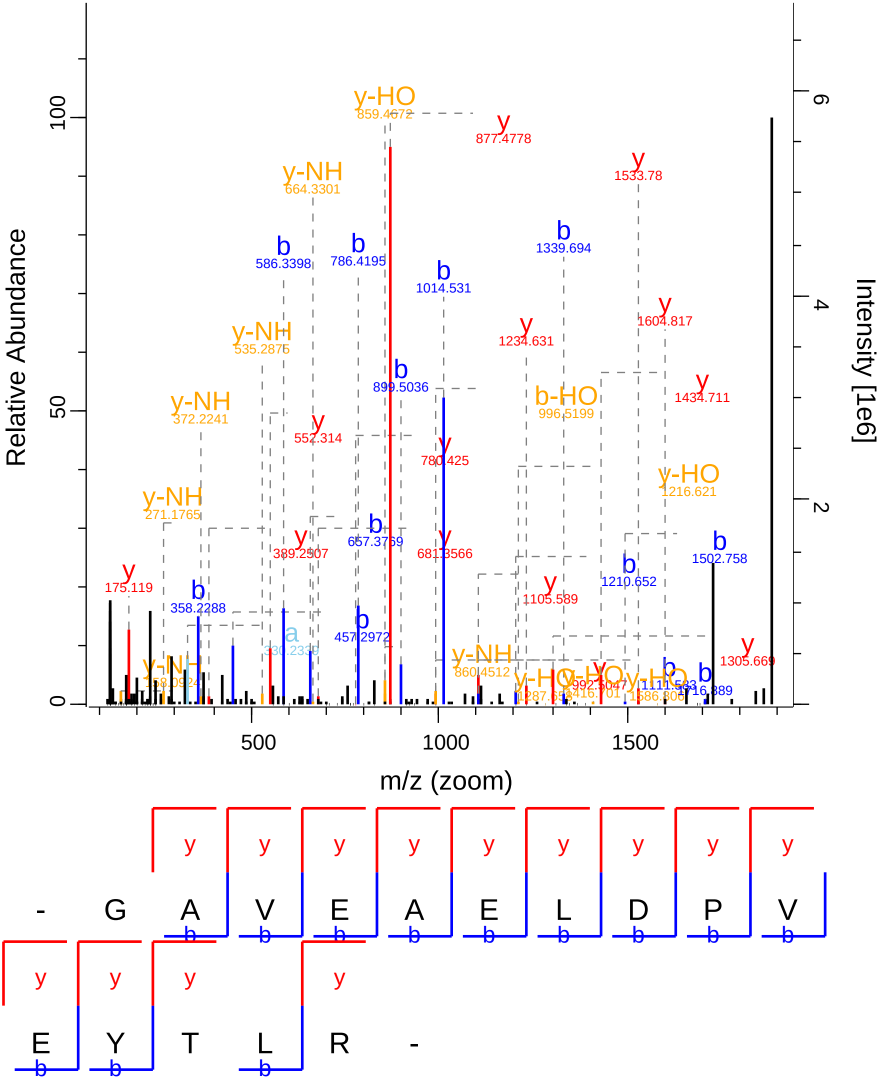

Raw File	Scan	Method	Score	m/z	Gene names
RO-QC-160121_ZackD_SS_Set_02_Fractio	30004	FTMS; HC	133.86	839.48	GTF3C6

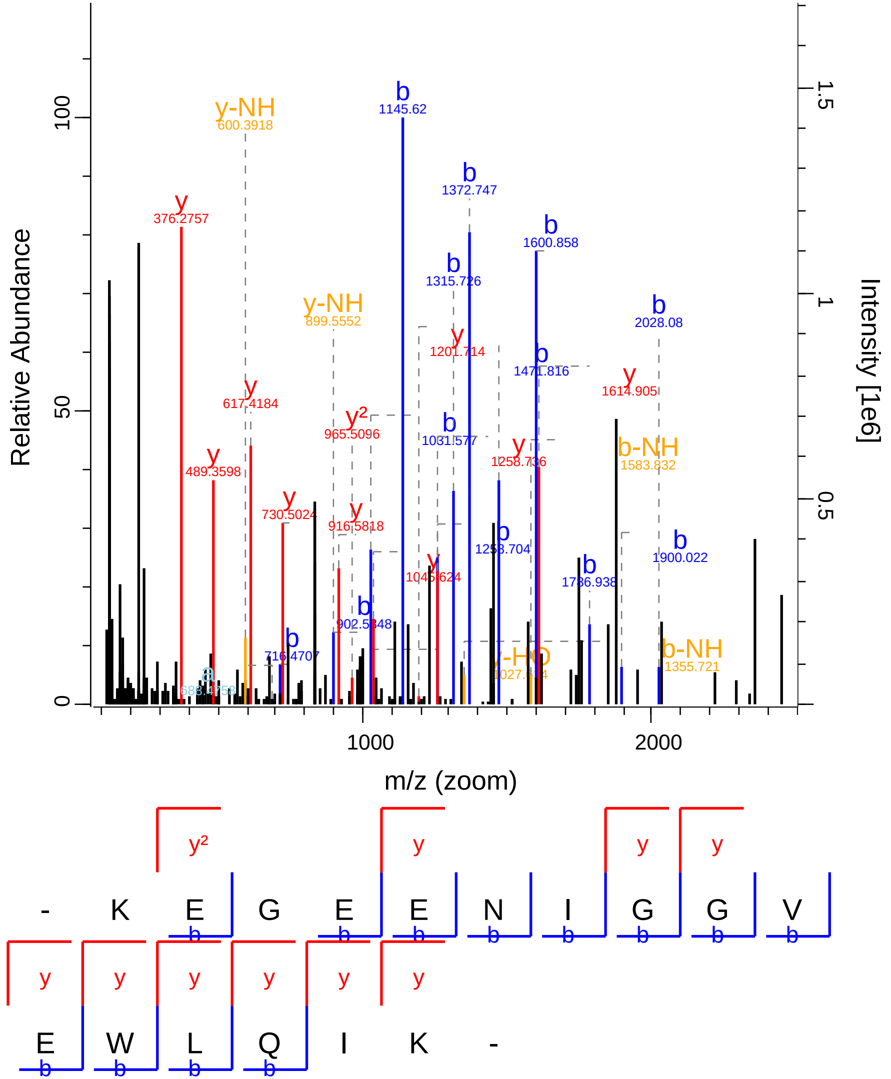

Raw File	Scan	Method	Score	m/z	Gene names
RO-QC-160121_ZackD_SS_Set_02_Fractio	30127	FTMS; HC	188.23	828.84	C4orf33

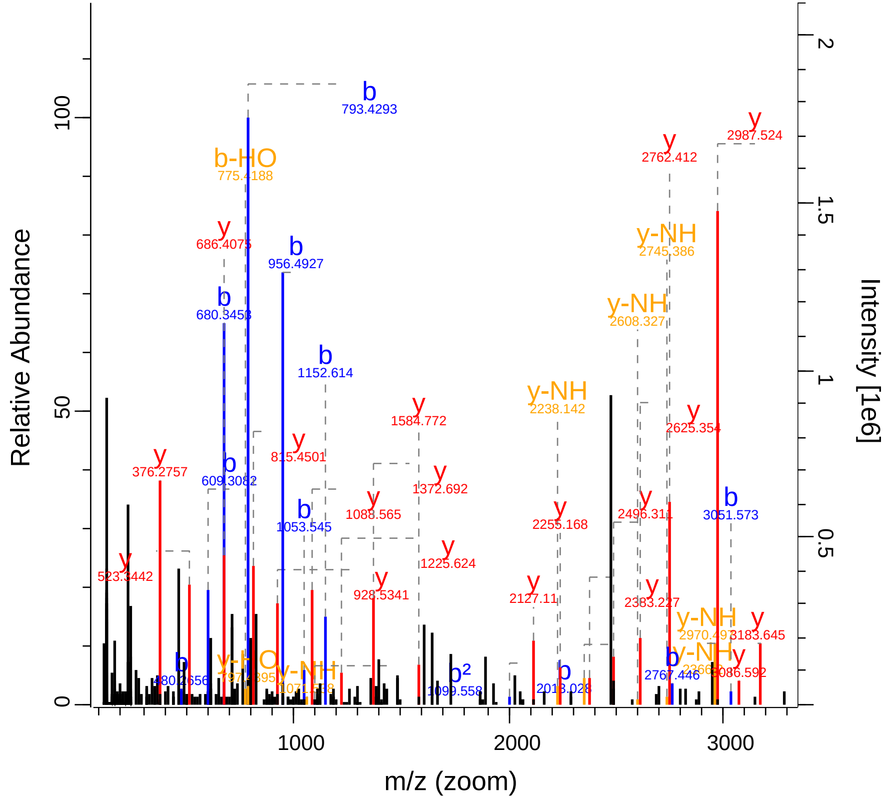

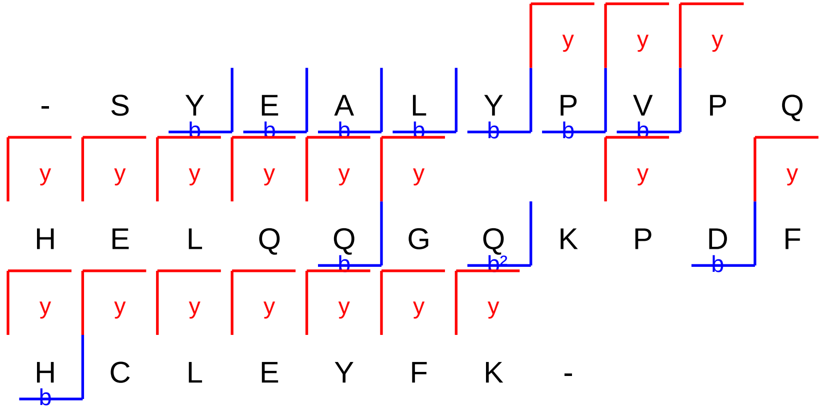

Raw File	Scan	Method	Score	m/z	Gene names
RO-QC-160121_ZackD_SS_Set_02_Fractior	30526	FTMS; HCI	79.51	872.5	KBTBD2

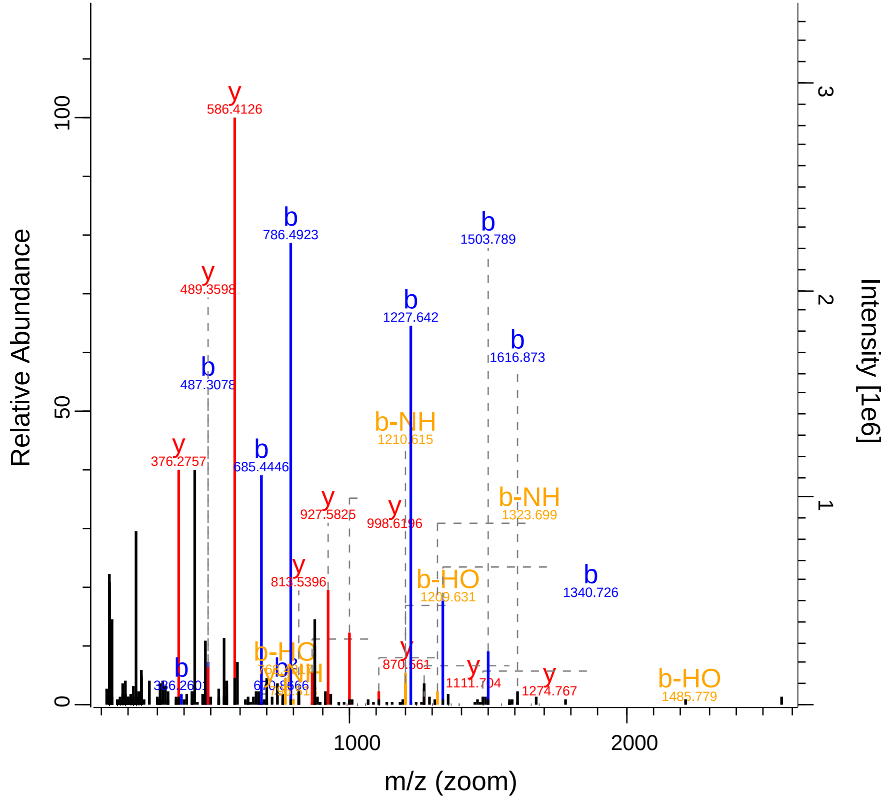

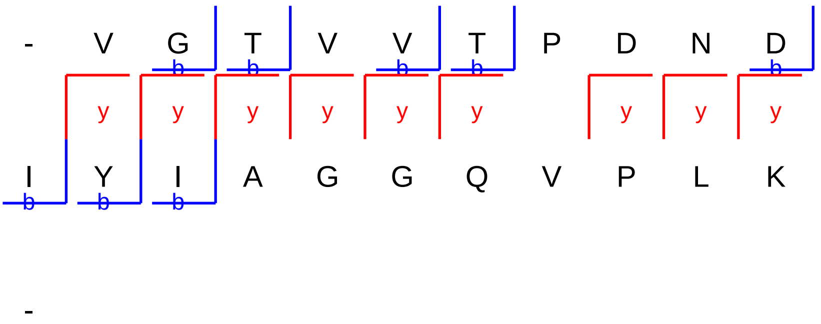

Raw File	Scan	Method	Score	m/z
RO-QC-160121_ZackD_SS_Set_02_Fraction_6	30855	FTMS; HCD	81.59	736.96

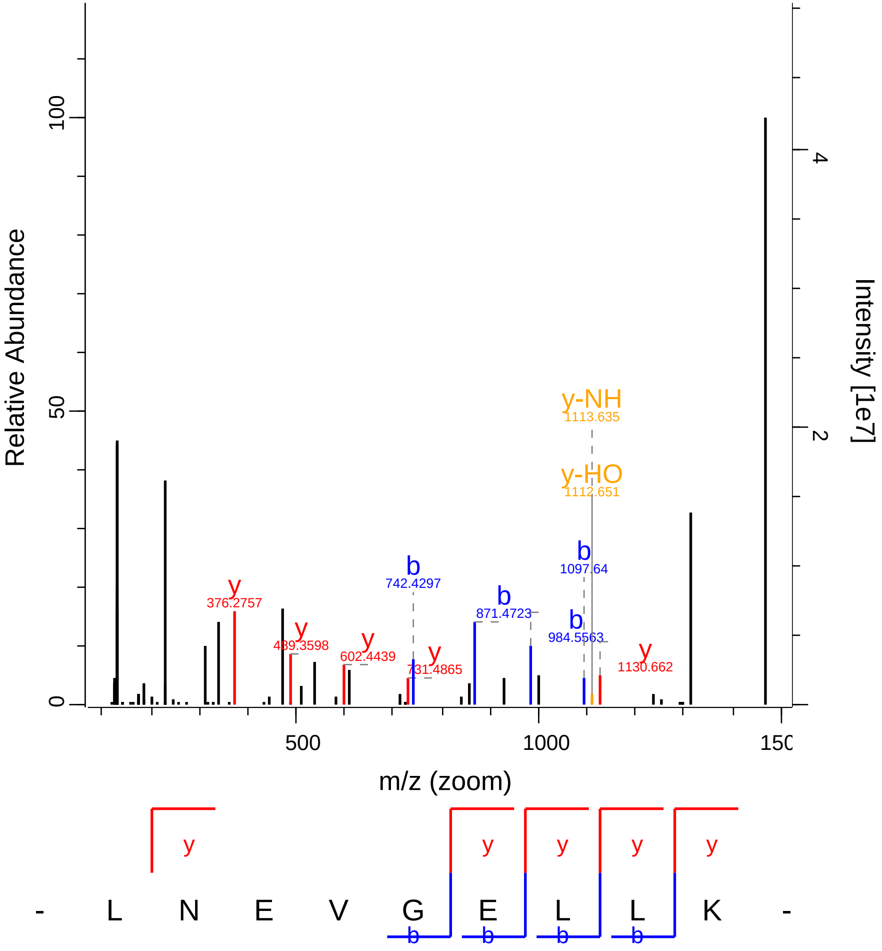

Raw File	Scan	Method	Score	m/z
RO-QC-160121_ZackD_SS_Set_02_Fraction_6	30872	FTMS; HCD	53.76	491.64

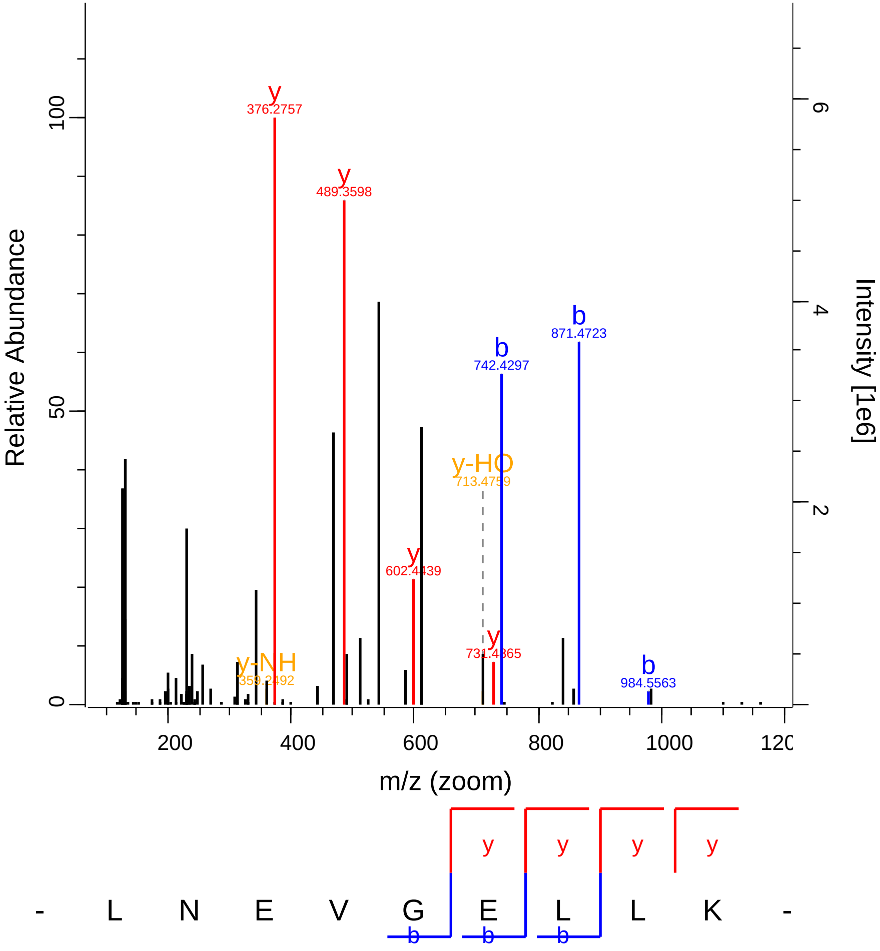

Raw File	Scan	Method	Score	m/z	Gene names
RO-QC-160121_ZackD_SS_Set_02_Fractio	31147	FTMS; HC	230.95	856.96	UBE2S

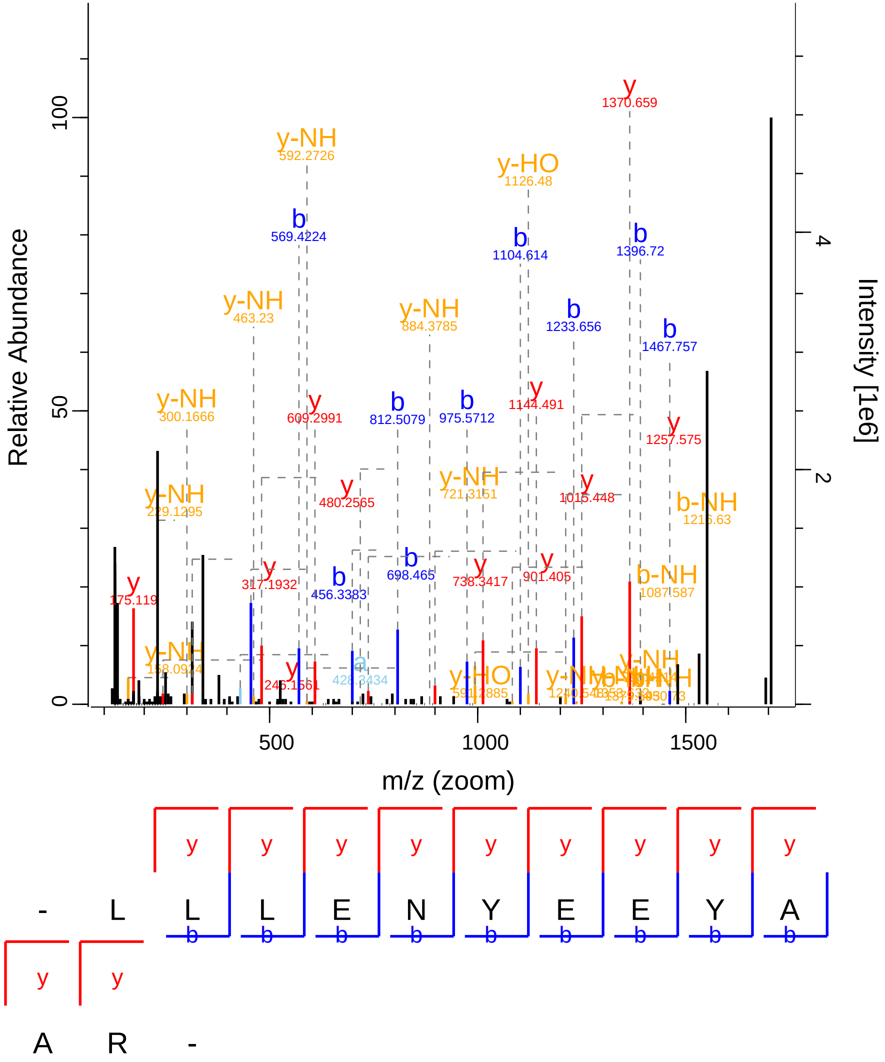

Raw File	Scan	Method	Score	m/z
RO-QC-160121_ZackD_SS_Set_02_Fraction_6	31596	FTMS; HCD	47.82	529.64

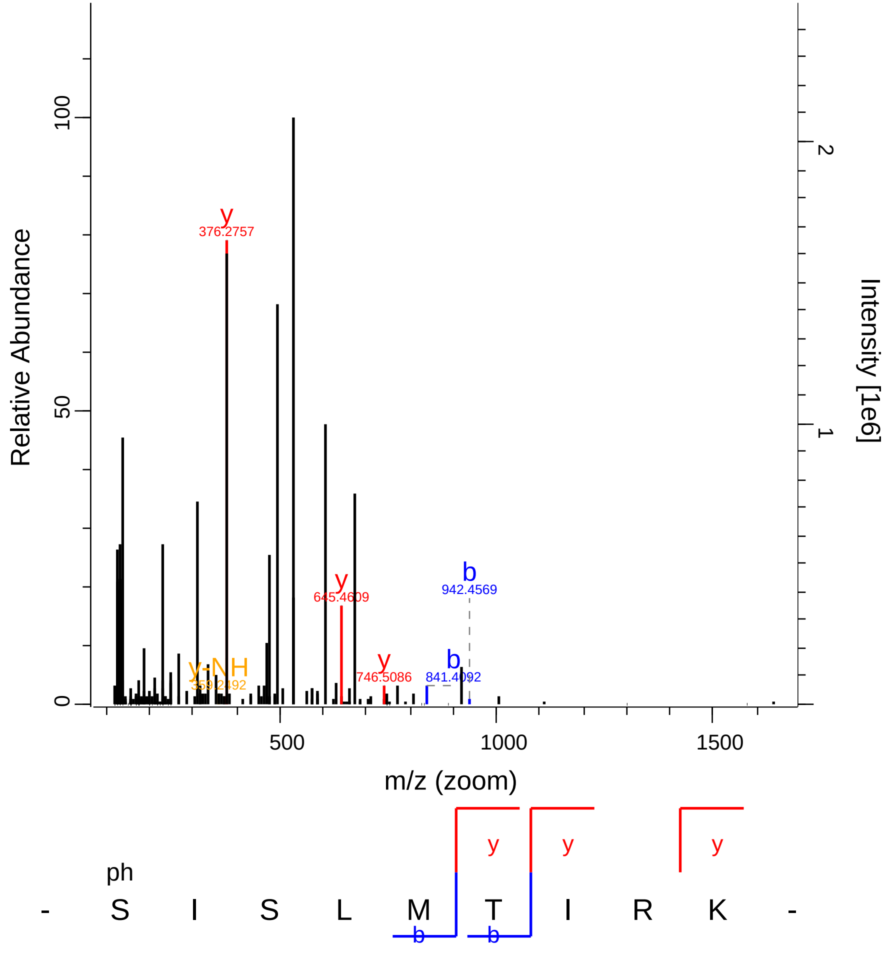

Raw File	Scan	Method	Score	m/z	Gene names
RO-QC-160121_ZackD_SS_Set_02_Fractio	31637	FTMS; HC	74.15	729.43	BOLA1

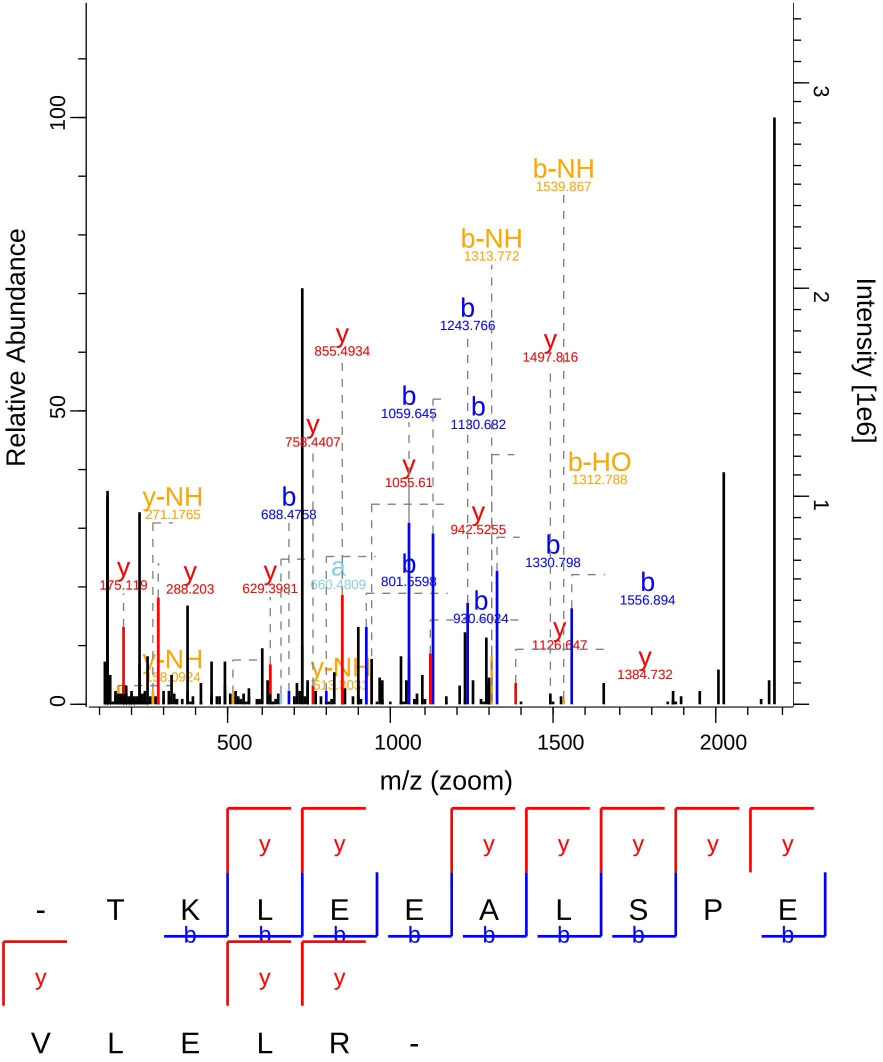

Raw File	Scan	Method	Score	m/z	Gene names
RO-QC-160121_ZackD_SS_Set_02_Fractio	32112	FTMS; HC	105.16	911.73	C12orf29

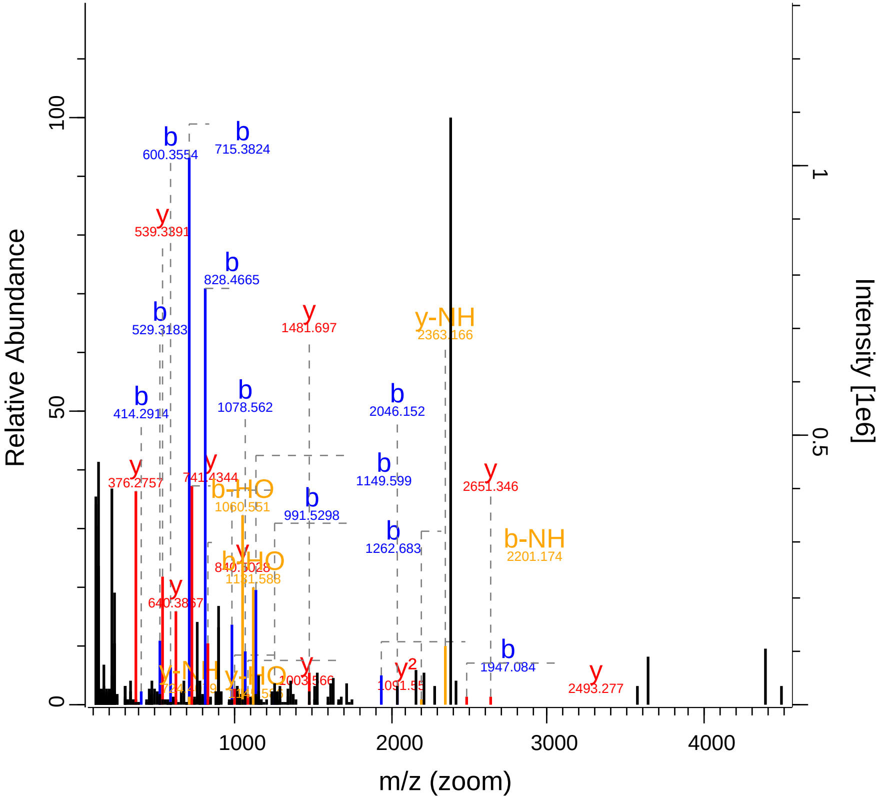

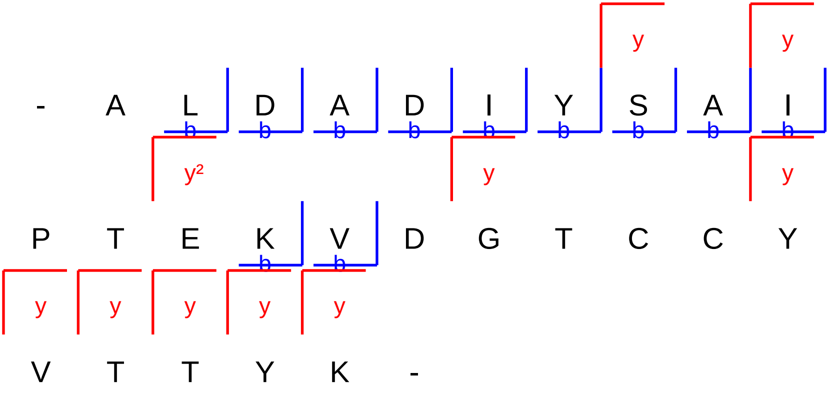

Raw File	Scan	Method	Score	m/z
RO-QC-160121_ZackD_SS_Set_02_Fraction_6	32283	FTMS; HCD	101.64	733.44

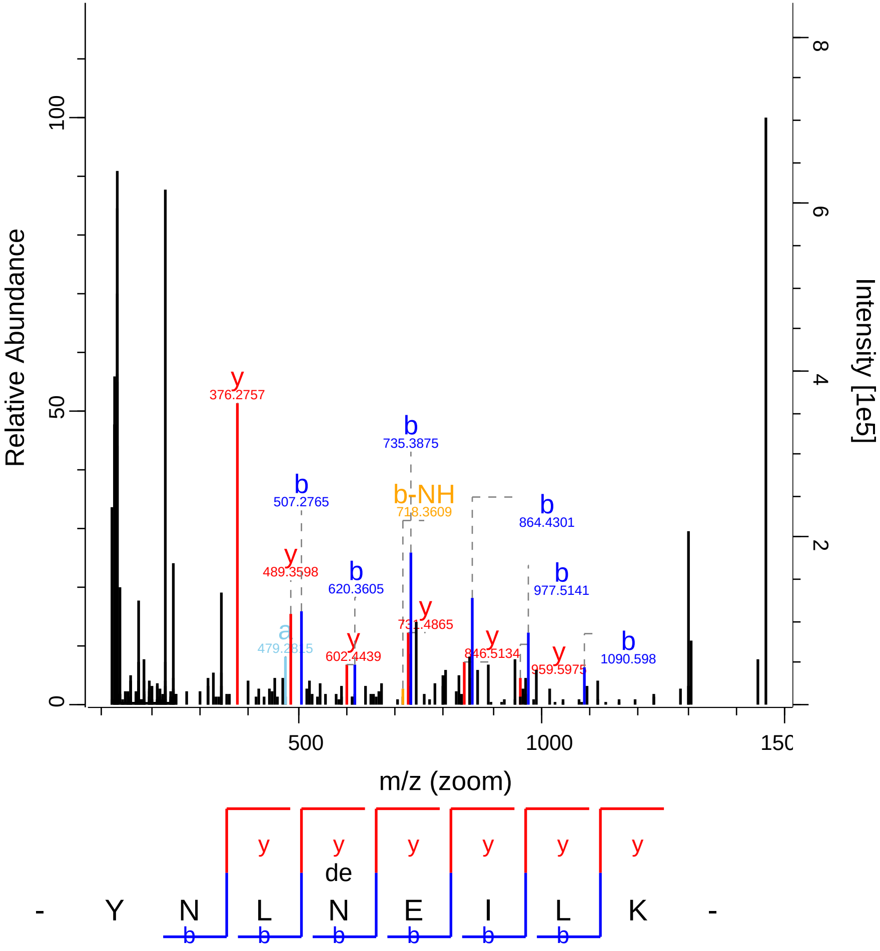

Raw File	Scan	Method	Score	m/z	Gene names
RO-QC-160121_ZackD_SS_Set_02_Fractio	32314	FTMS; HC	98.51	739.42	MZT1

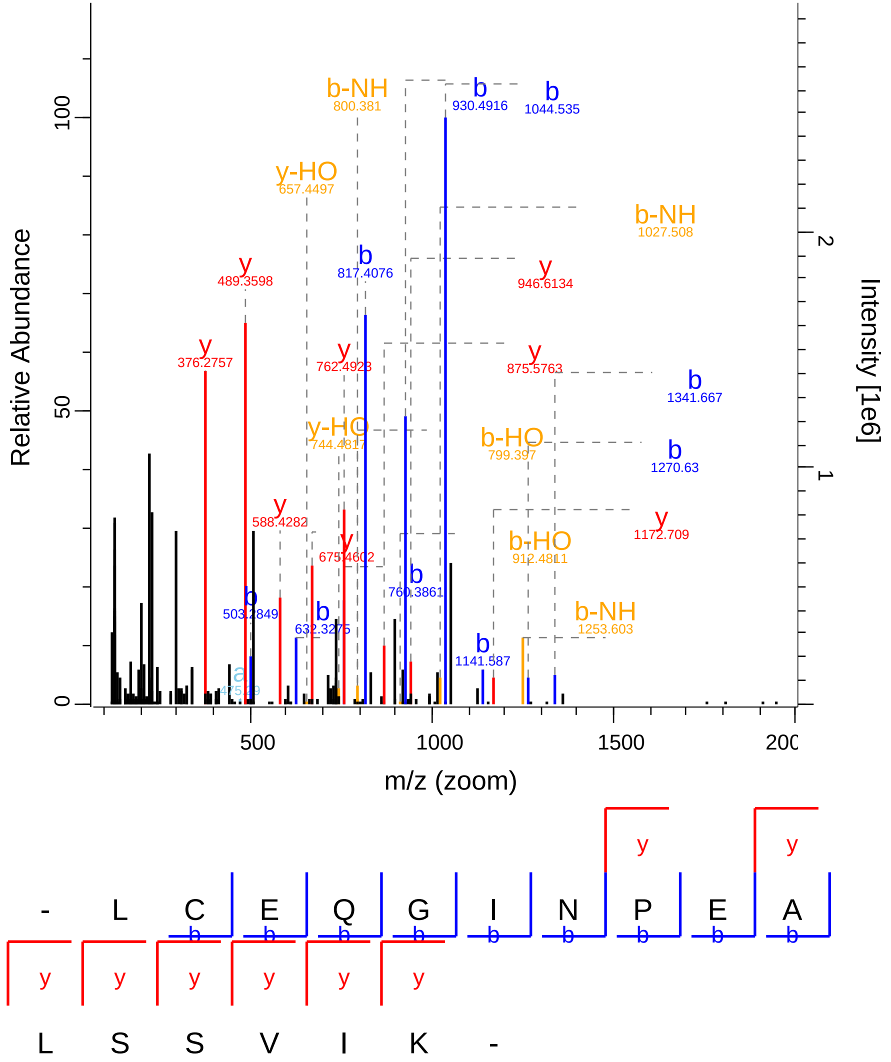

Raw File	Scan	Method	Score	m/z	Gene names
RO-QC-160121_ZackD_SS_Set_02_Fractior	32736	FTMS; HCI	45.26	754.9	CFTR

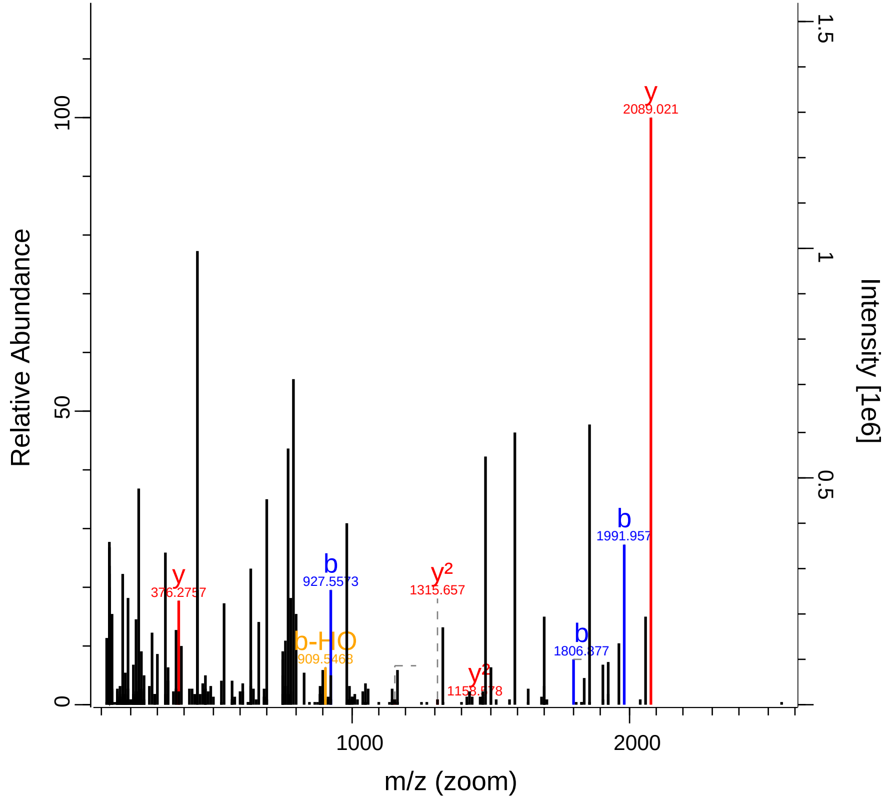

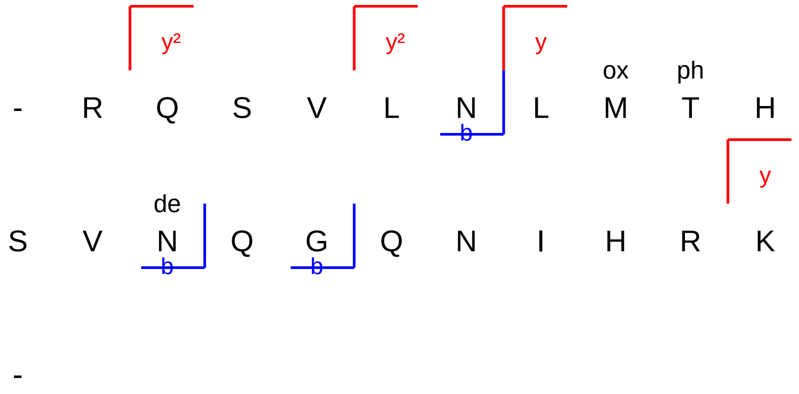

Raw File	Scan	Method	Score	m/z	Gene names
RO-QC-160121_ZackD_SS_Set_02_Fractic	34655	FTMS; HC	143.31	1030.47	DENND6A

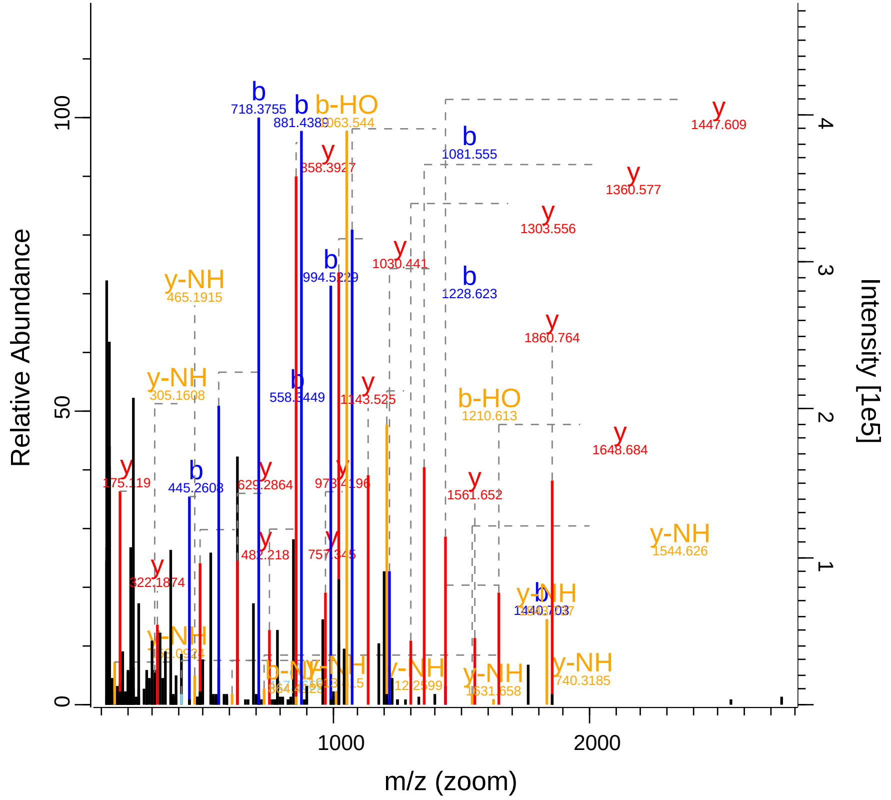

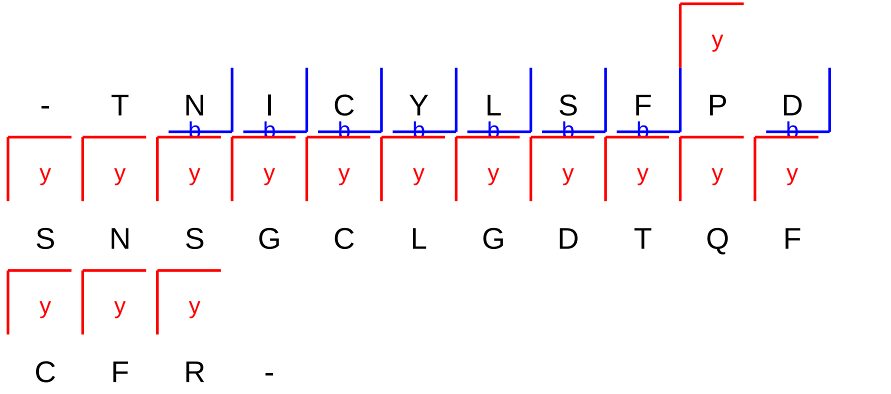

Raw File	Scan	Method	Score	m/z	Gene names
RO-QC-160121_ZackD_SS_Set_02_Fractic	35005	FTMS; HC	45.55	1243.66	LPPR5

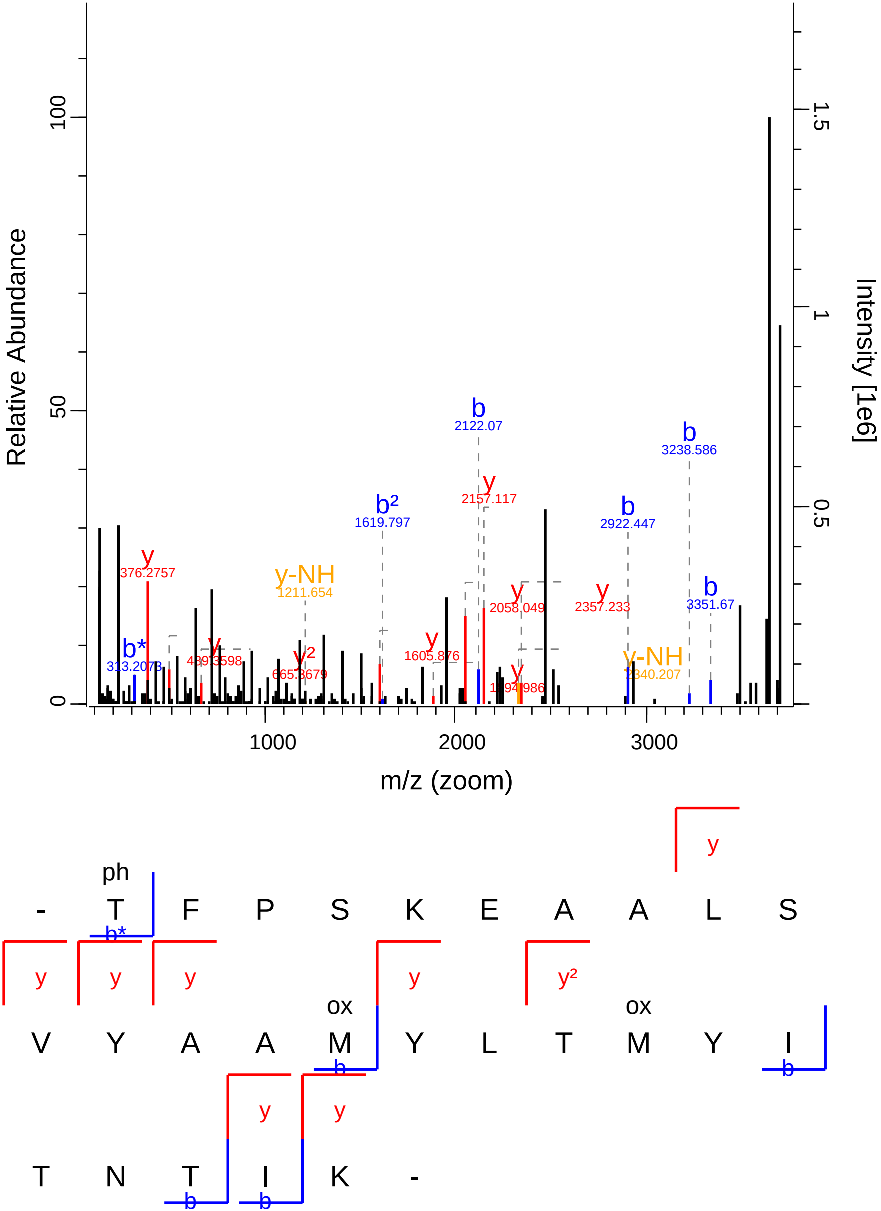

Raw File	Scan	Method	Score	m/z	Gene names
RO-QC-160121_ZackD_SS_Set_02_Fractic	36159	FTMS; HC	193.14	1066.27	MT-CO1

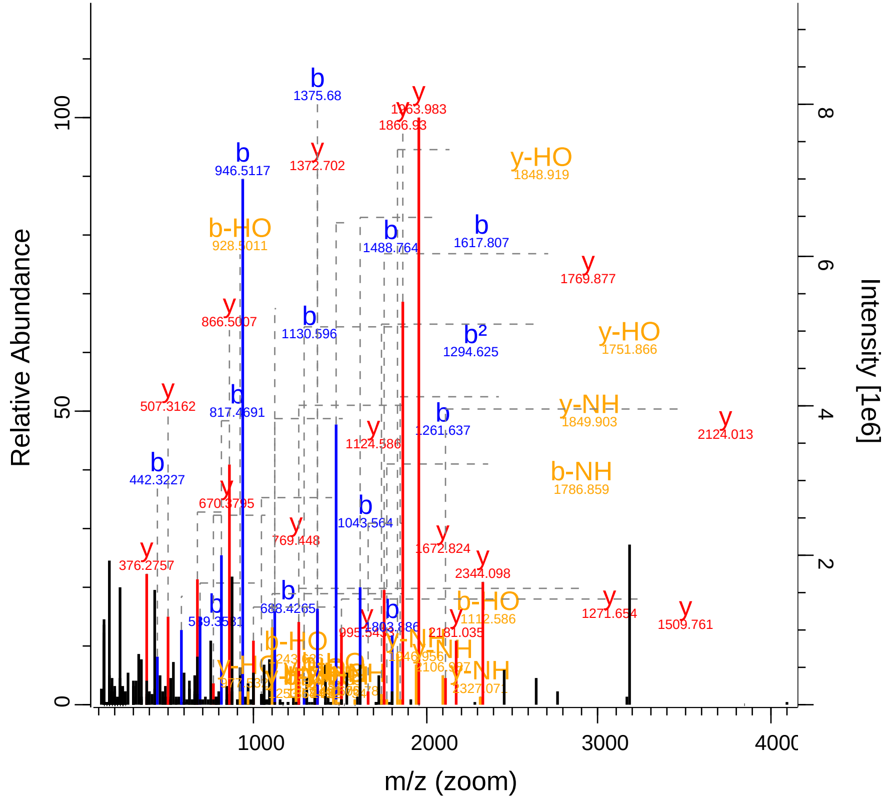

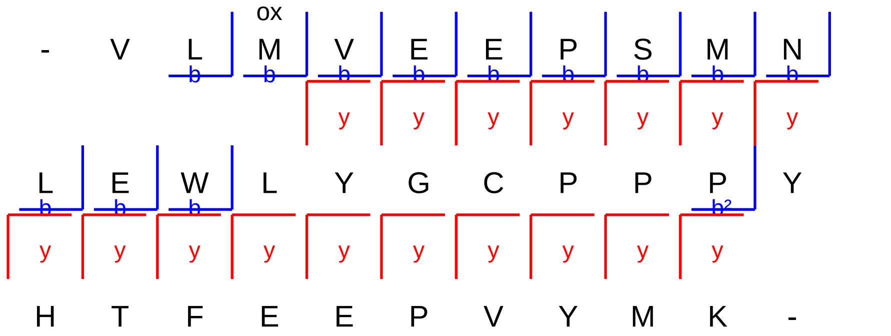

Raw File	Scan	Method	Score	m/z	Gene names
RO-QC-160121_ZackD_SS_Set_02_Fractio	36532	FTMS; HC	79.51	912.16	GID4

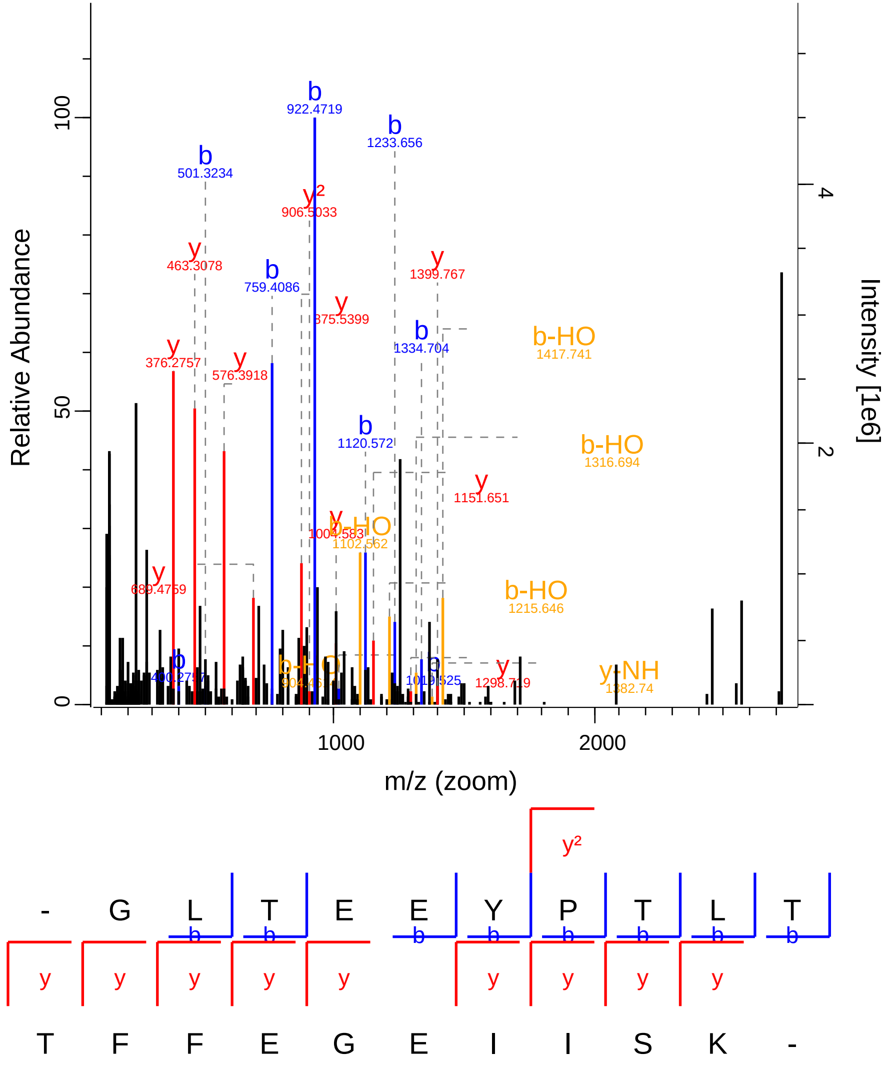

Raw File	Scan	Method	Score	m/z	Gene names
RO-QC-160121_ZackD_SS_Set_02_Fractio	36599	FTMS; HC	116.73	860.44	PRSS58

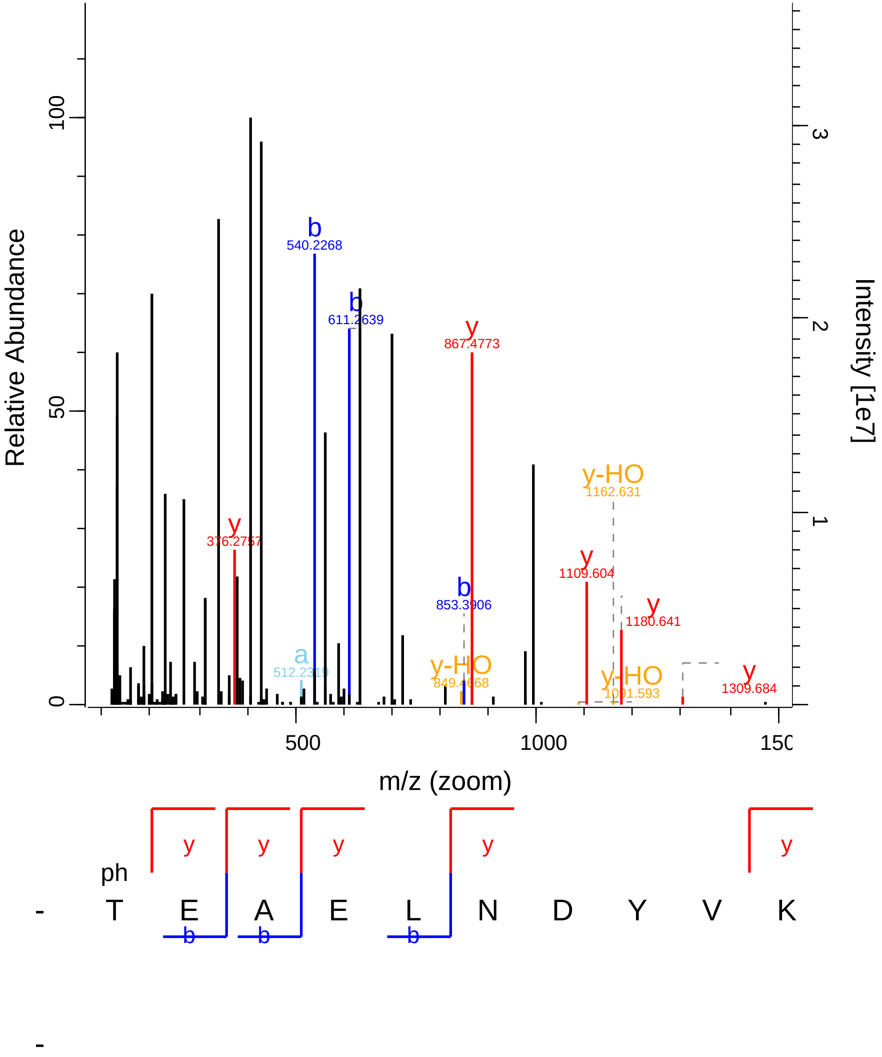

Raw File	Scan	Method	Score	m/z
RO-QC-160121_ZackD_SS_Set_02_Fraction_6	36906	FTMS; HCD	49.36	682.35

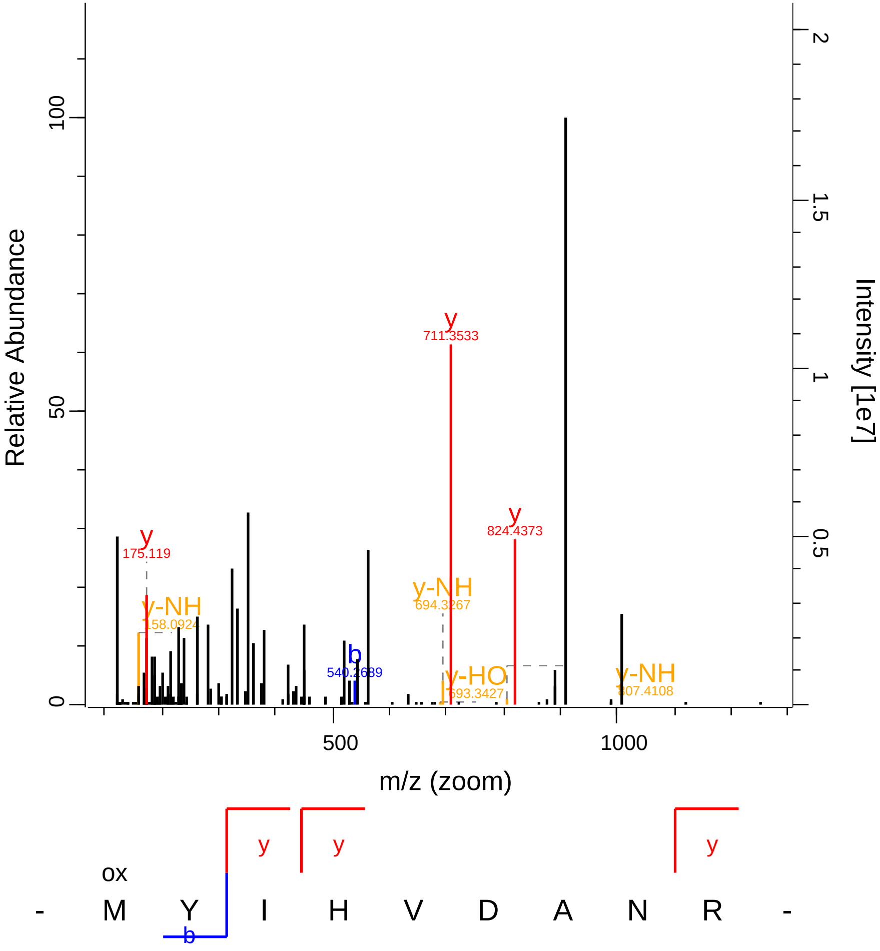

Raw File	Scan	Method	Score	m/z	Gene names
RO-QC-160121_ZackD_SS_Set_02_Fractio	37294	FTMS; HC	161.17	929.53	GRK6;GRK5

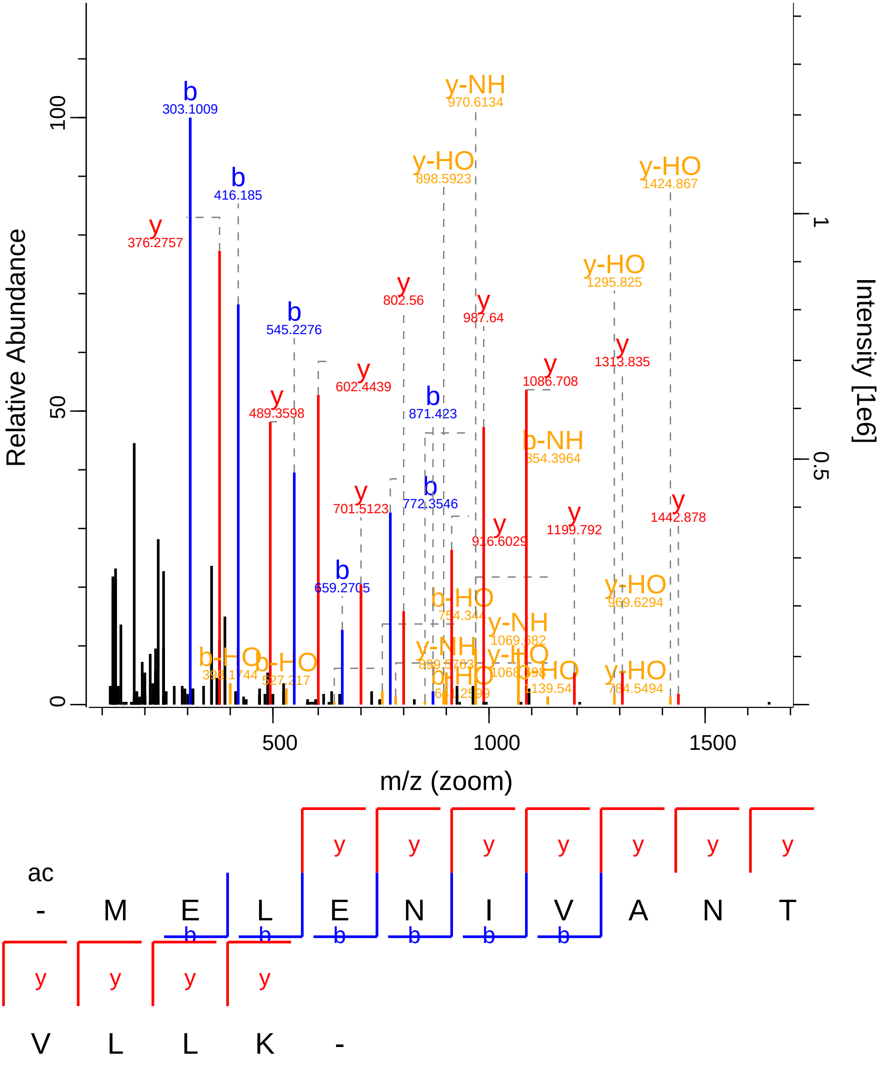

Raw File	Scan	Method	Score	m/z
RO-QC-160121_ZackD_SS_Set_02_Fraction_7	5253	FTMS; HCD	71.35	543.94

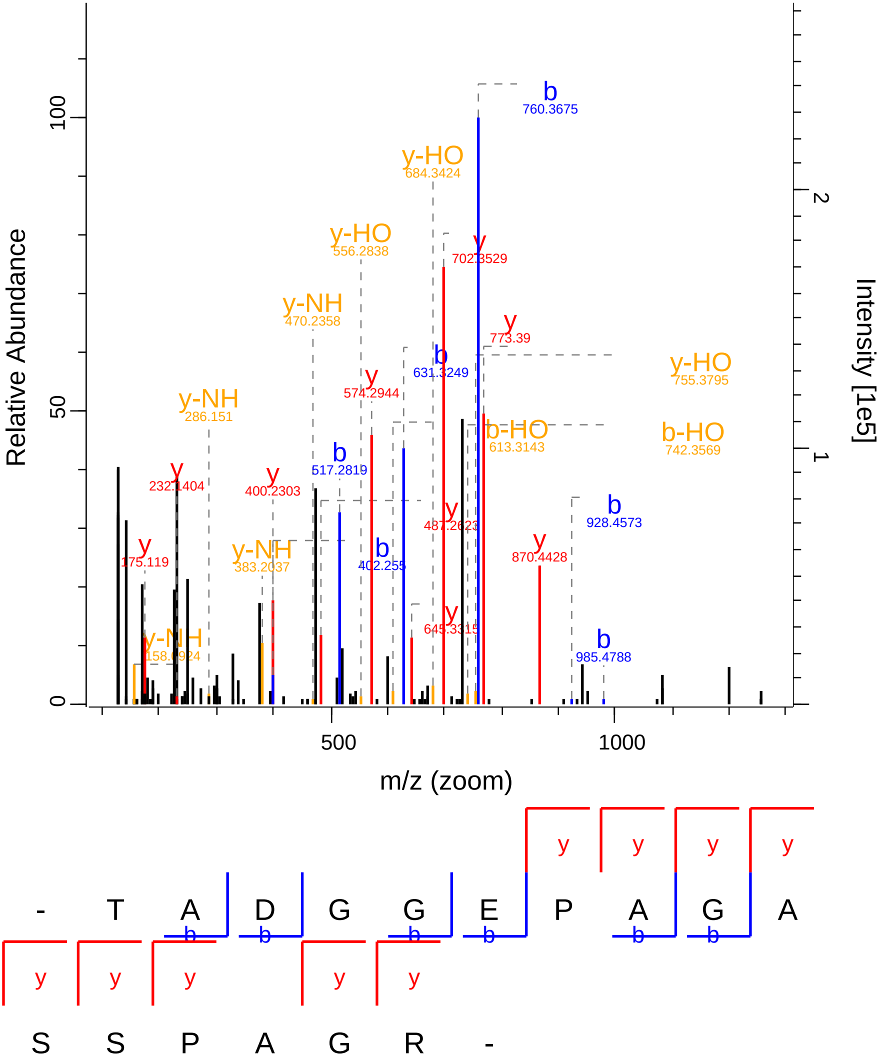

Raw File	Scan	Method	Score	m/z
RO-QC-160121_ZackD_SS_Set_02_Fraction_7	5272	FTMS; HCD	142.91	815.41

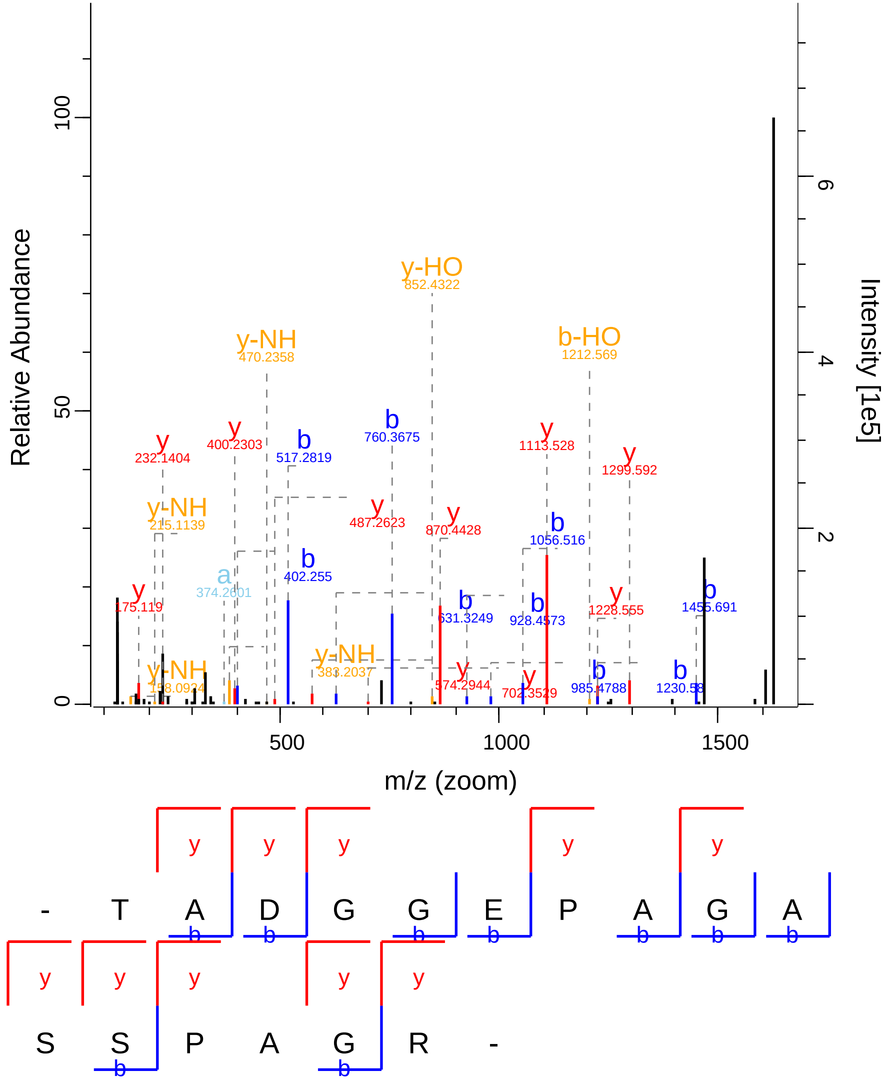

Raw File	Scan	Method	Score	m/z	Gene names
RO-QC-160121_ZackD_SS_Set_02_Fractior	5391	FTMS; HCI	68.07	490.79	TEX9

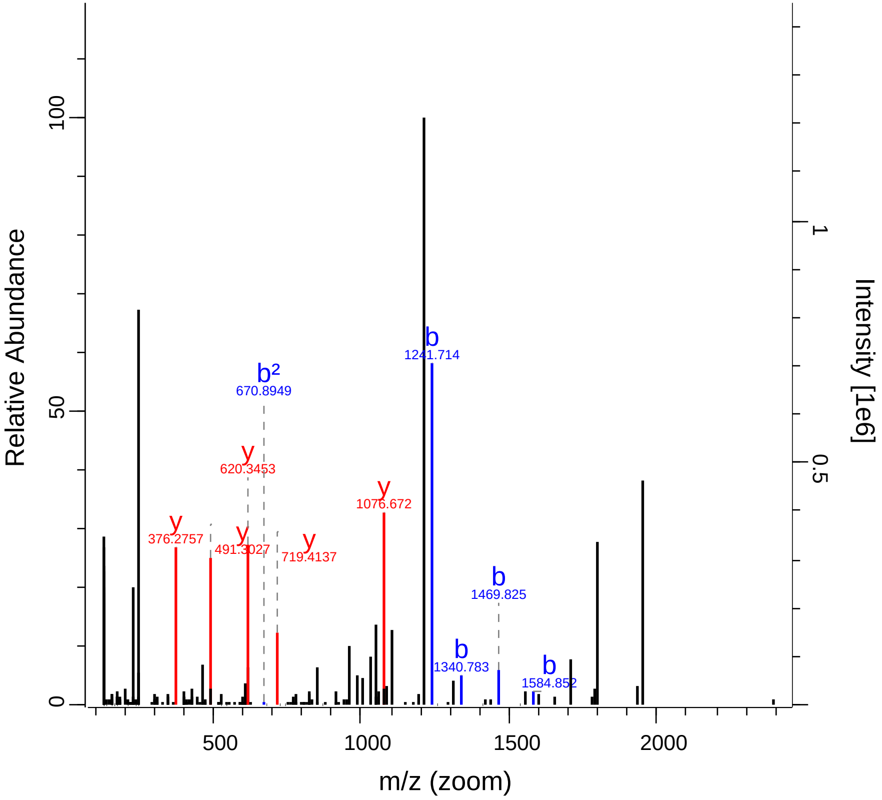

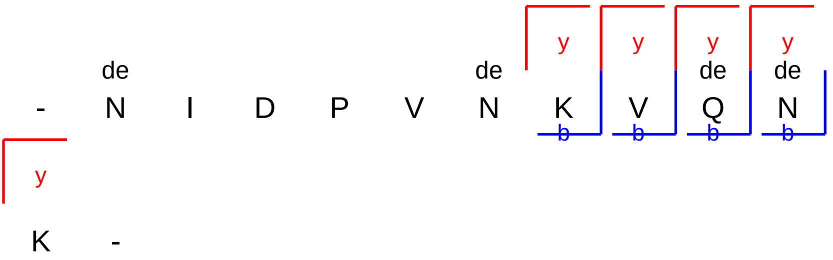

Raw File	Scan	Method	Score	m/z	Gene names
RO-QC-160121_ZackD_SS_Set_02_Fractior	6023	FTMS; HCI	164.1	709.86	SLCO3A1

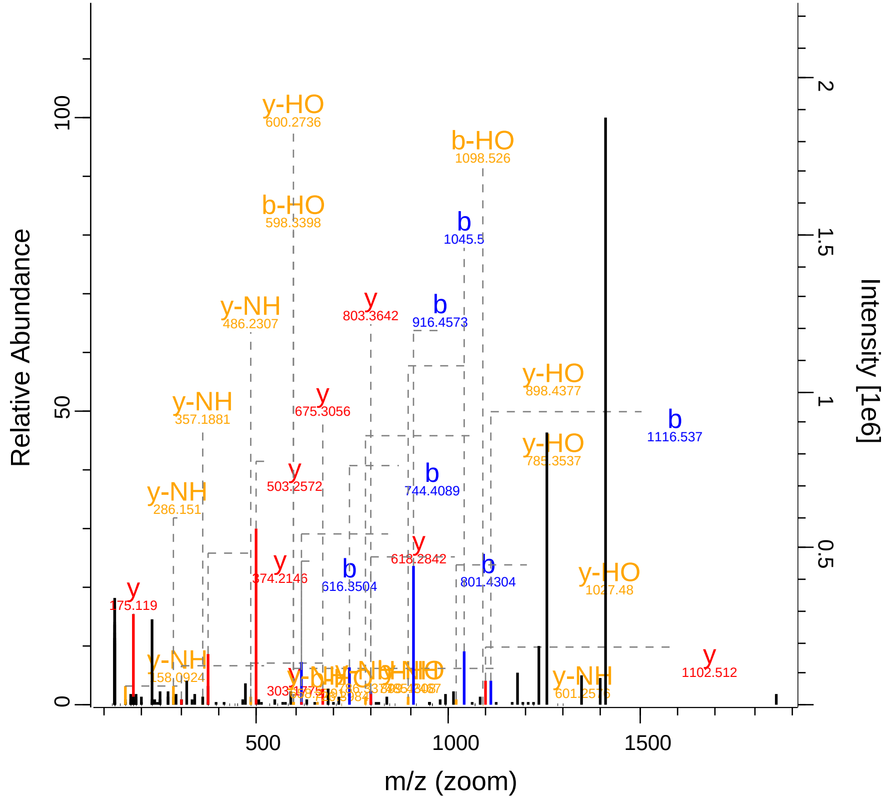

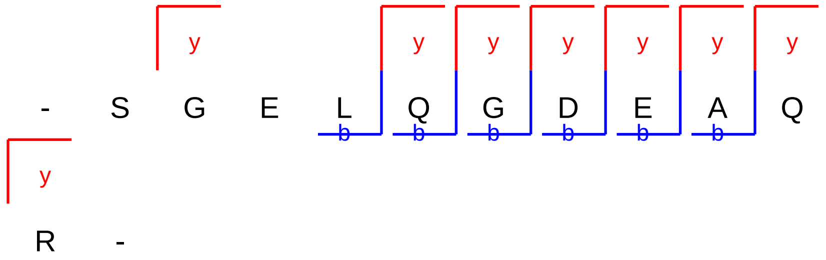

Raw File	Scan	Method	Score	m/z	Gene names
RO-QC-160121_ZackD_SS_Set_02_Fractior	6228	FTMS; HCI	51.01	861.42	SECISBP2

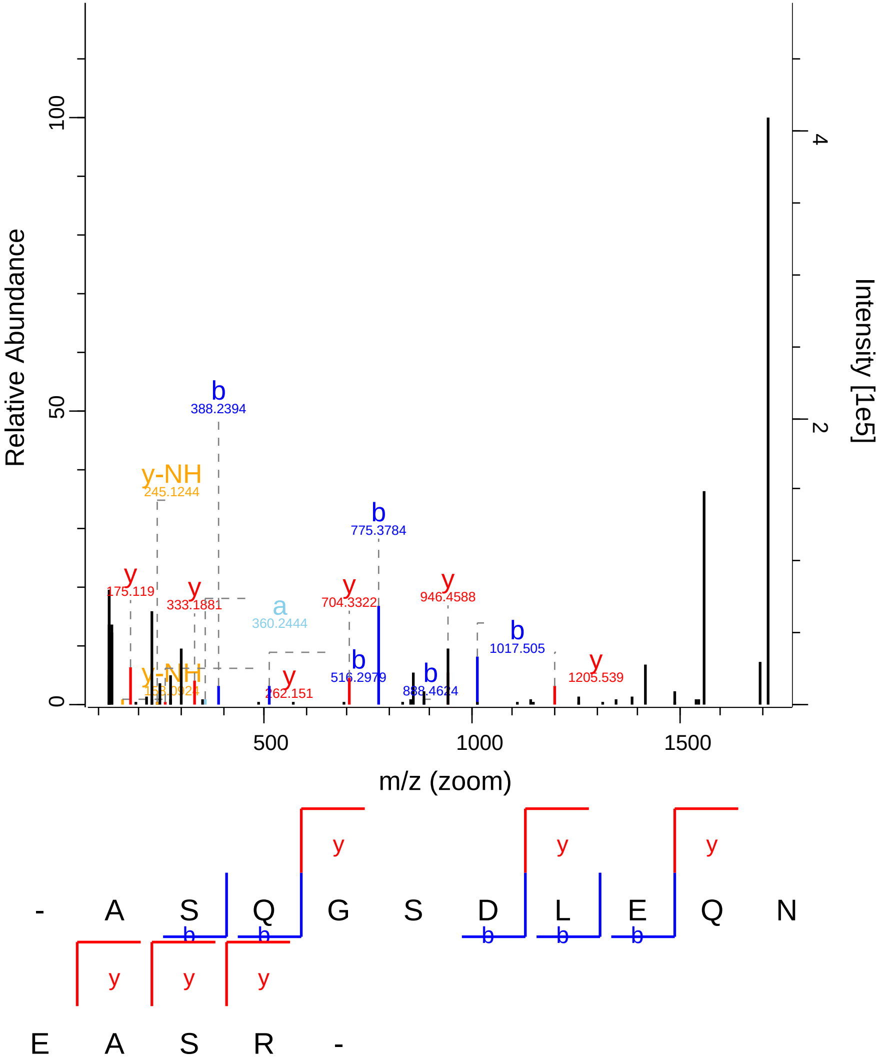

Raw File	Scan	Method	Score	m/z
RO-QC-160121_ZackD_SS_Set_02_Fraction_7	6387	FTMS; HCD	81.55	459.27

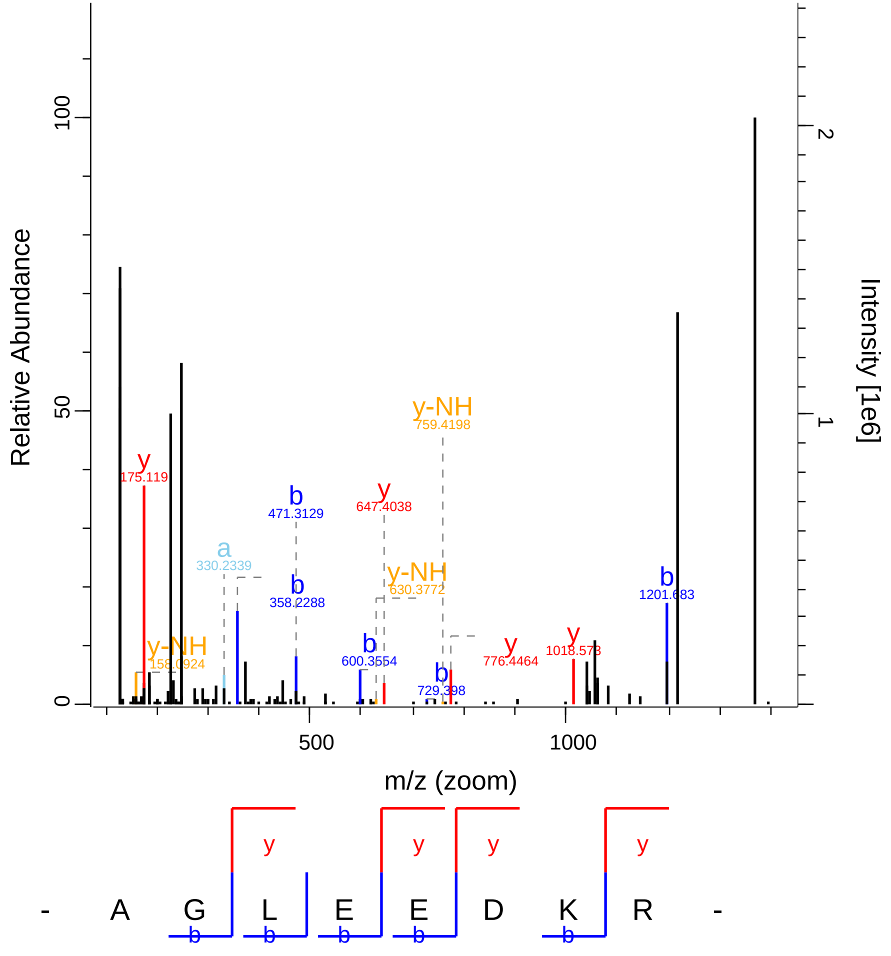

Raw File	Scan	Method	Score	m/z
RO-QC-160121_ZackD_SS_Set_02_Fraction_7	6976	FTMS; HCD	104.17	691.72

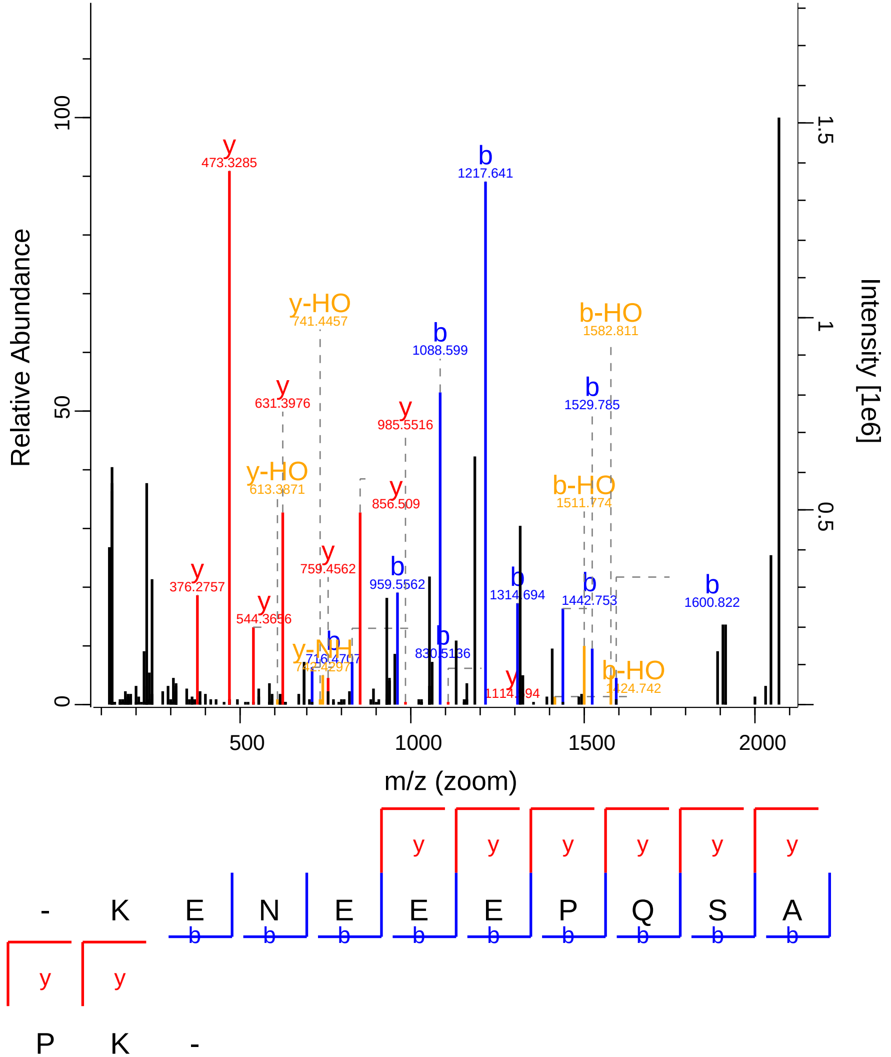

Raw File	Scan	Method	Score	m/z	Gene names
RO-QC-160121_ZackD_SS_Set_02_Fractior	7090	FTMS; HC	120.06	739.42	BBX

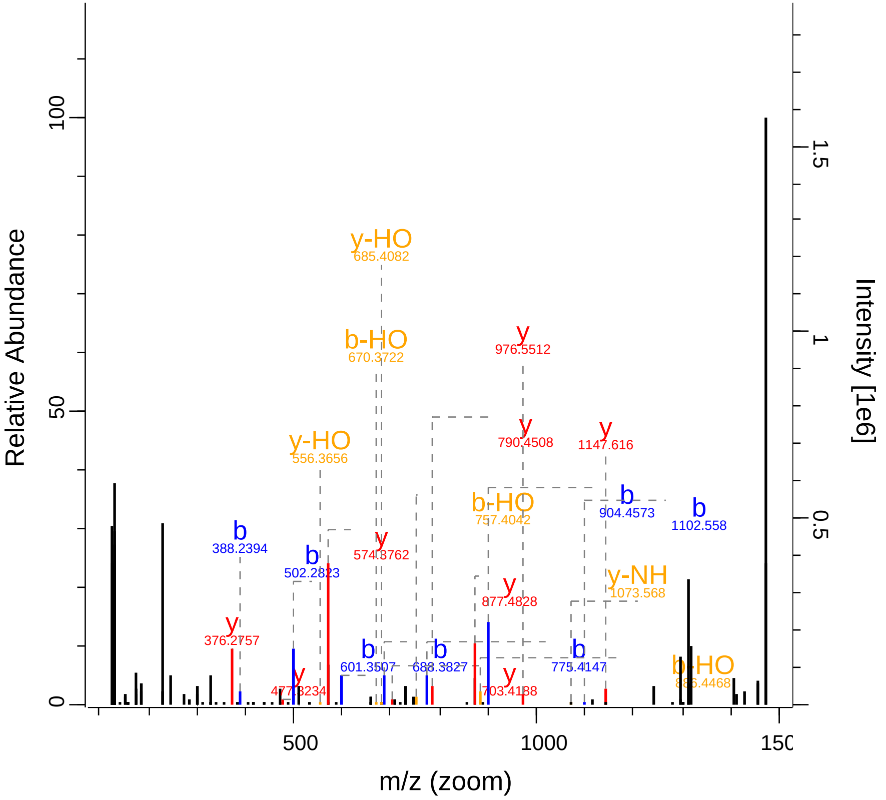

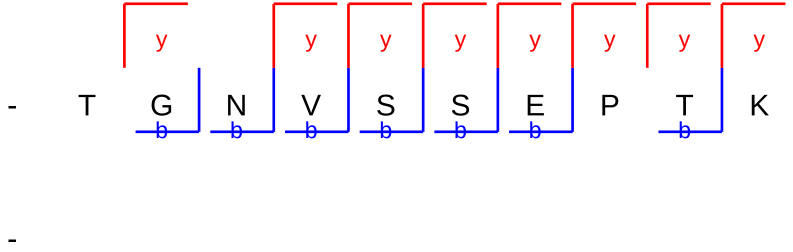

Raw File	Scan	Method	Score	m/z	Gene names
RO-QC-160121_ZackD_SS_Set_02_Fractior	8087	FTMS; HCI	51.79	699.39	SLC7A6

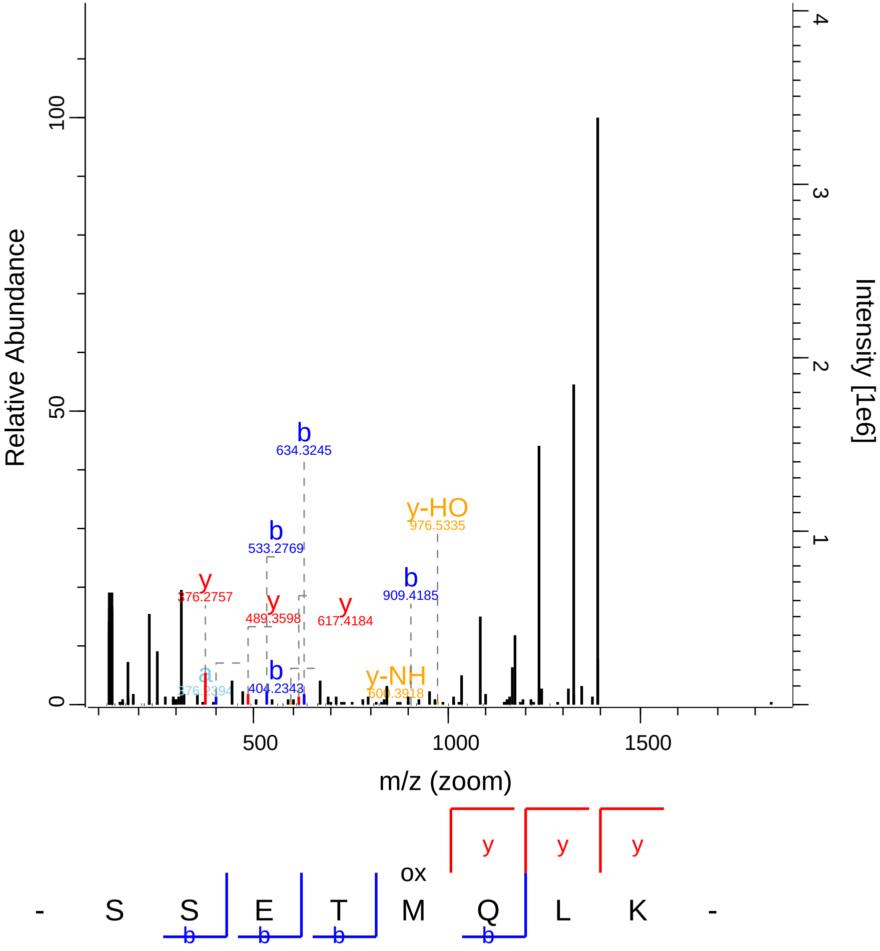

Raw File	Scan	Method	Score	m/z	Gene names
RO-QC-160121_ZackD_SS_Set_02_Fraction	9442	FTMS; HCI	169.33	779.9	SLC39A1

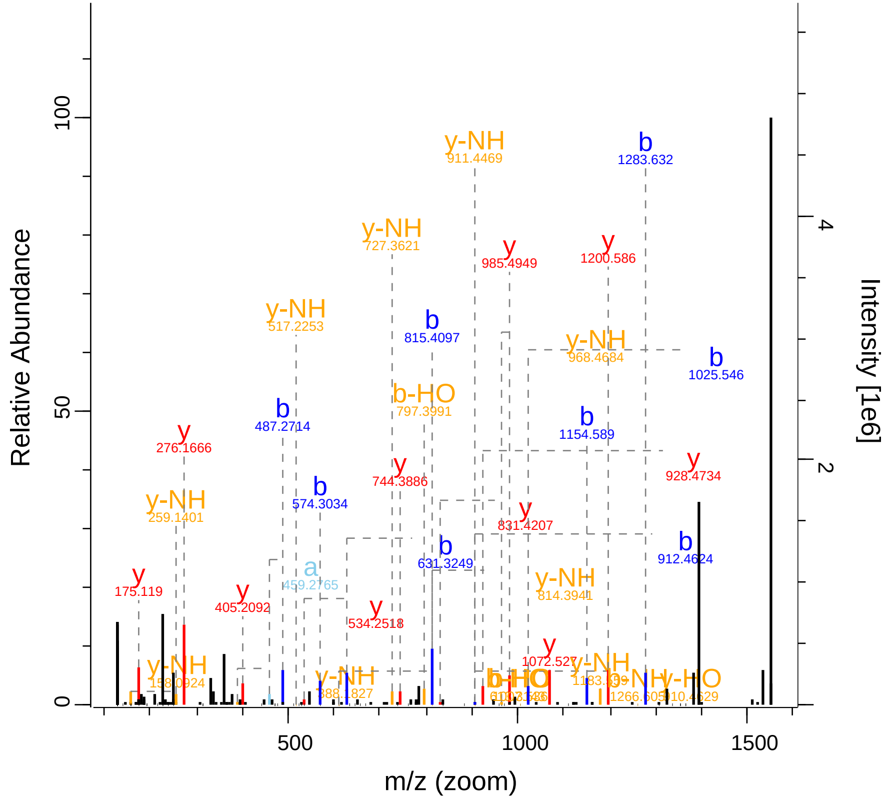

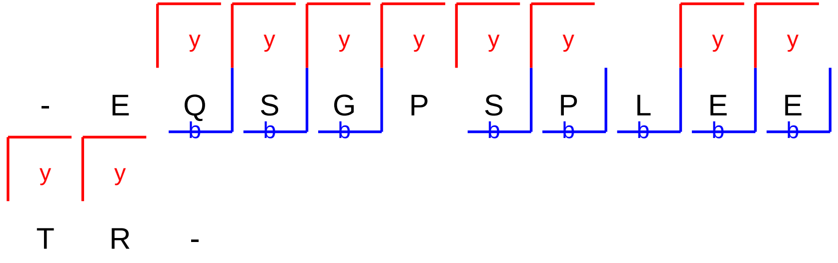

Raw File	Scan Method	Score	m/z	Gene names
RO-QC-160121_ZackD_SS_Set_02_Fra	9783 FTMS; HC	96.28	596.35	ATP5G1;ATP5G2;ATP5

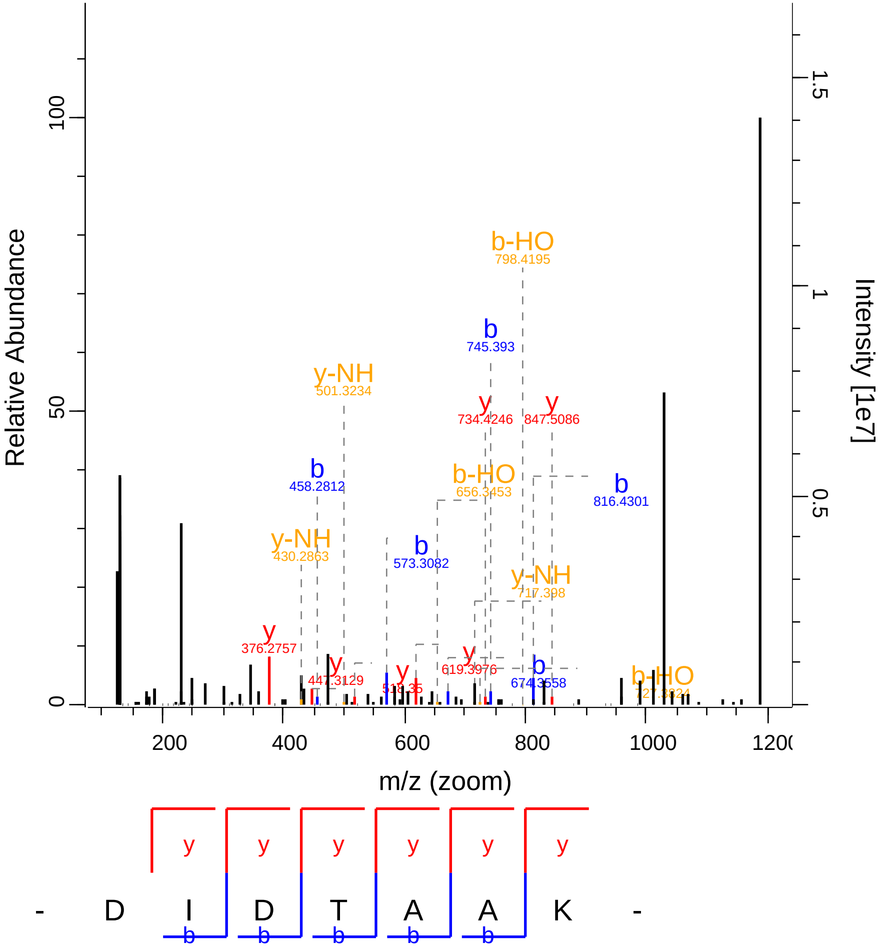

Raw File	Scan	Method	Score	m/z
RO-QC-160121_ZackD_SS_Set_02_Fraction_7	10570	FTMS; HCD	51.86	724.91

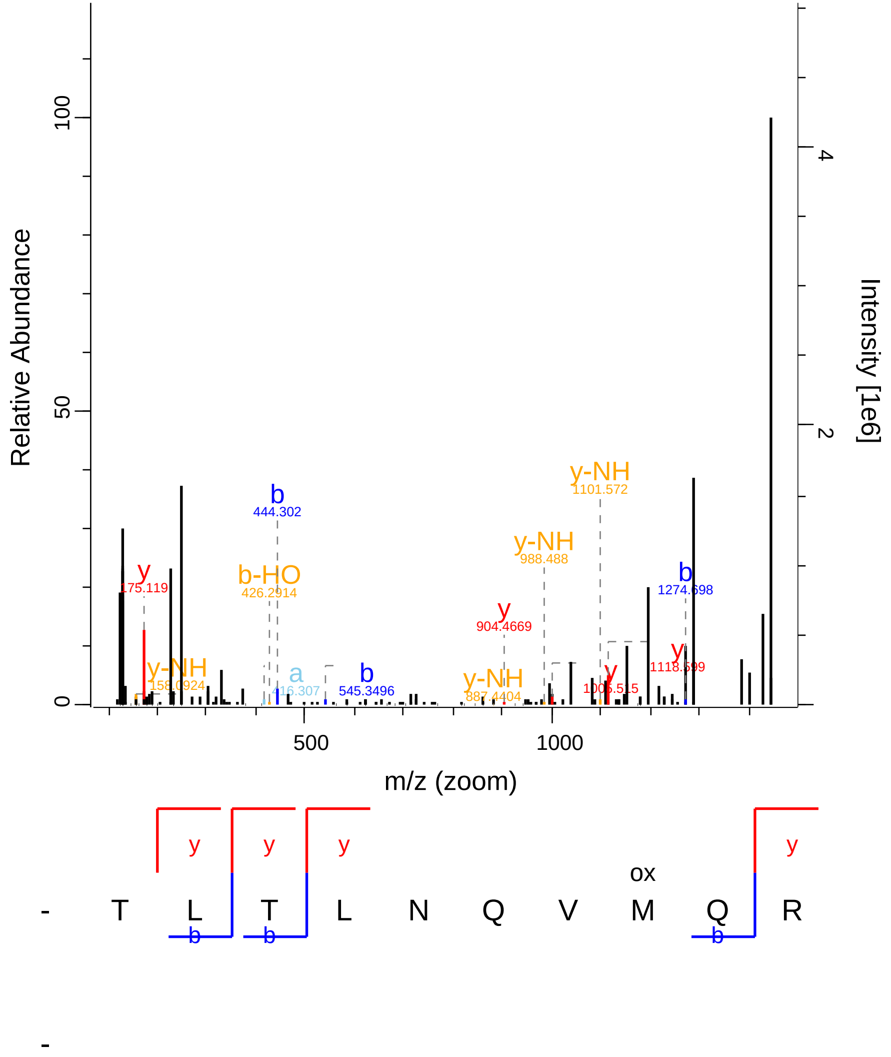

Raw File	Scan	Method	Score	m/z	Gene names
RO-QC-160121_ZackD_SS_Set_02_Fr	10678	FTMS; HC	117.16	596.35	ATP5G1;ATP5G2;ATP

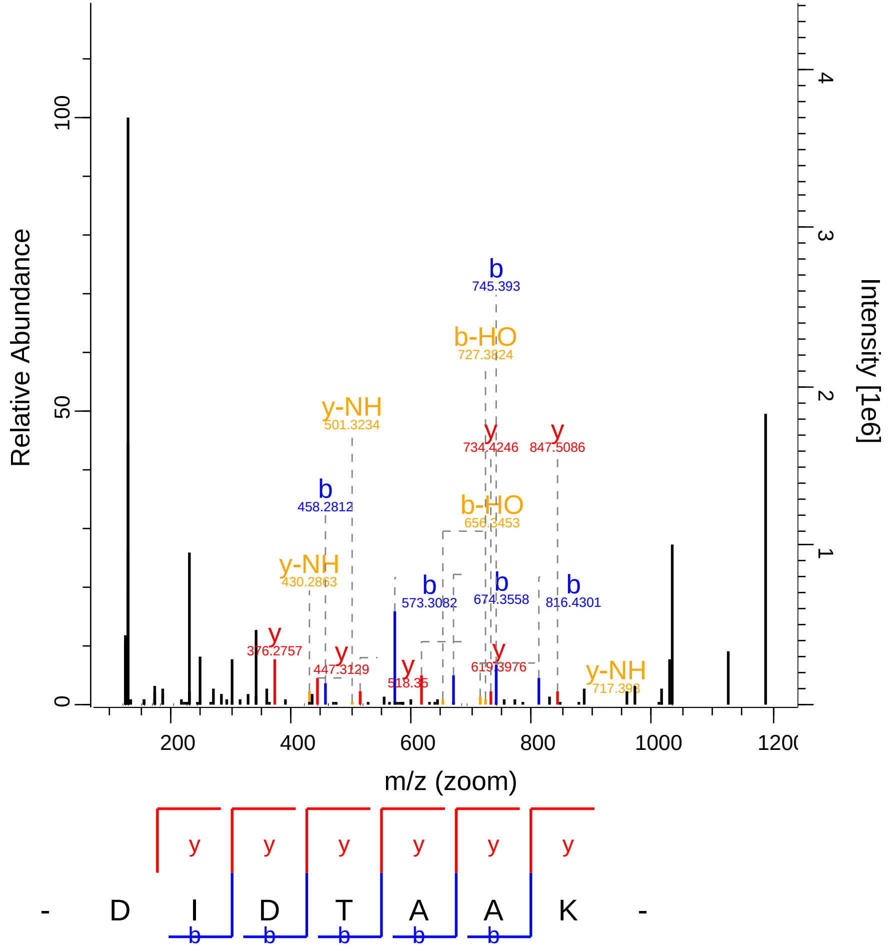

Raw File	Scan	Method	Score	m/z	Gene names
RO-QC-160121_ZackD_SS_Set_02_	1104	dt FTMS; H	42.6	378.48	GOLGA6L1;GOLGA6L6;GOL

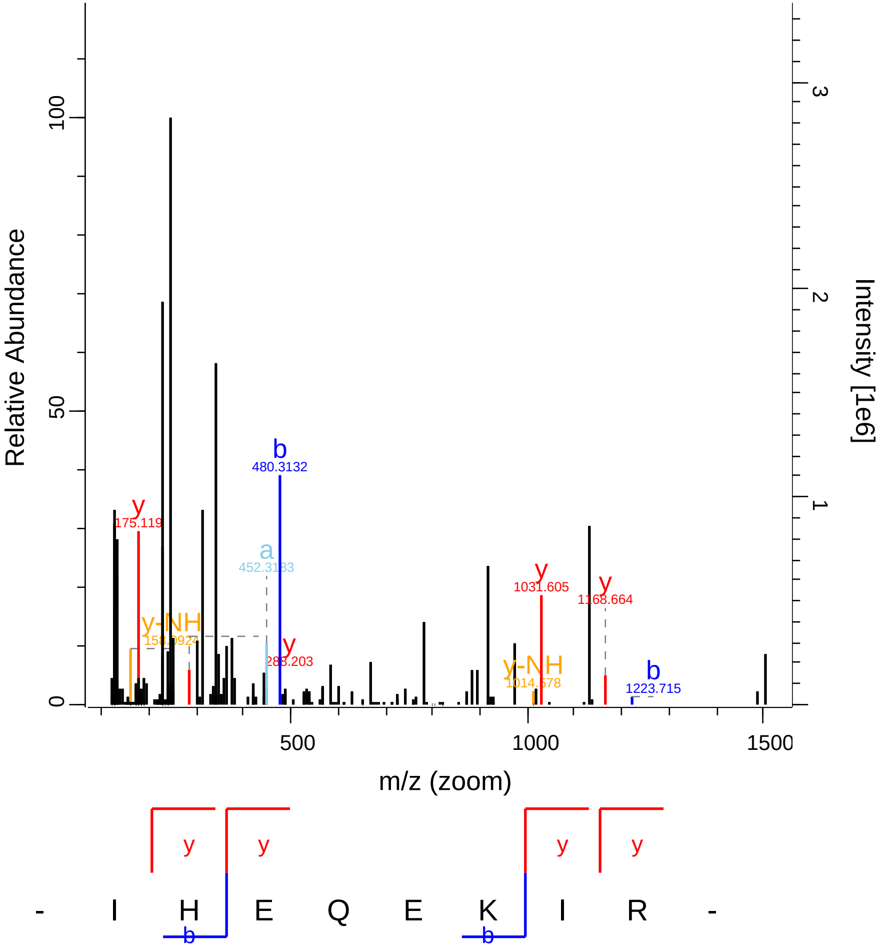

Raw File	Scan	Method	Score	m/z	Gene names
RO-QC-160121_ZackD_SS_Set_02_Fractio	11949	FTMS; HC	82.45	623.87	TMEM141

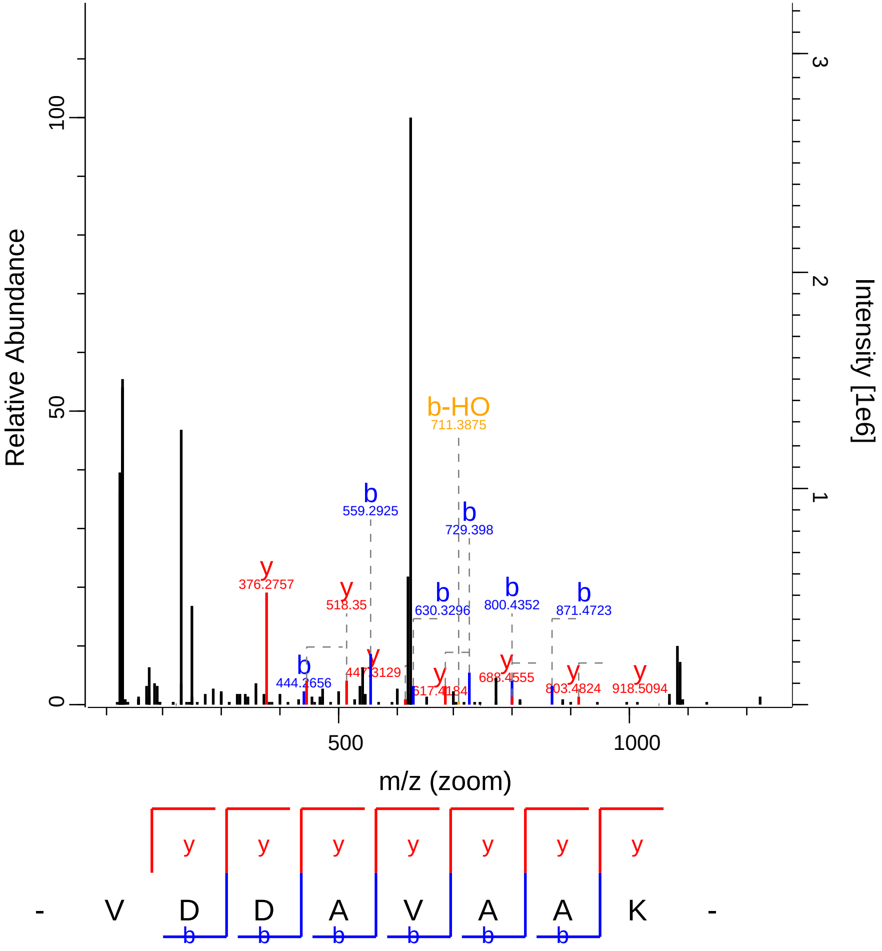

Raw File	Scan	Method	Score	m/z	Gene names
RO-QC-160121_ZackD_SS_Set_02_Fractio	12003	FTMS; HC	157.24	804.44	MRPL34

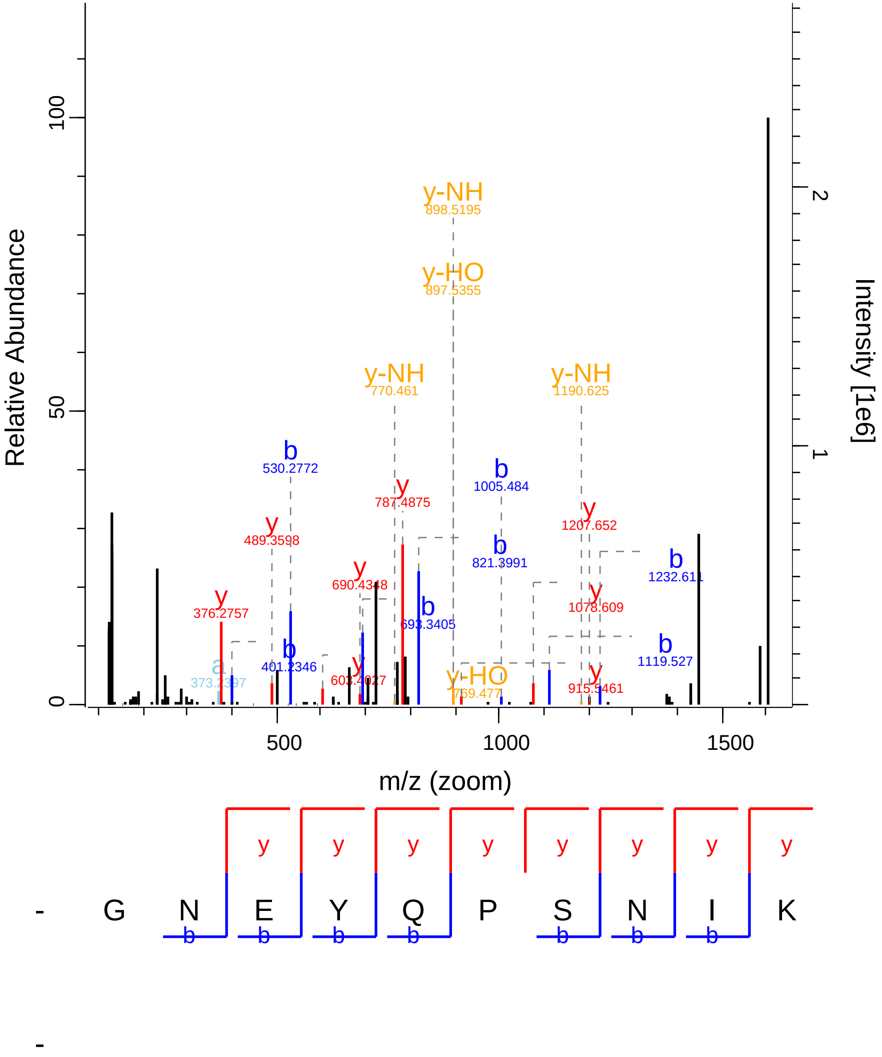

Raw File	Scan	Method	Score	m/z
RO-QC-160121_ZackD_SS_Set_02_Fraction_7	14164	FTMS; HCD	82.17	1057.58

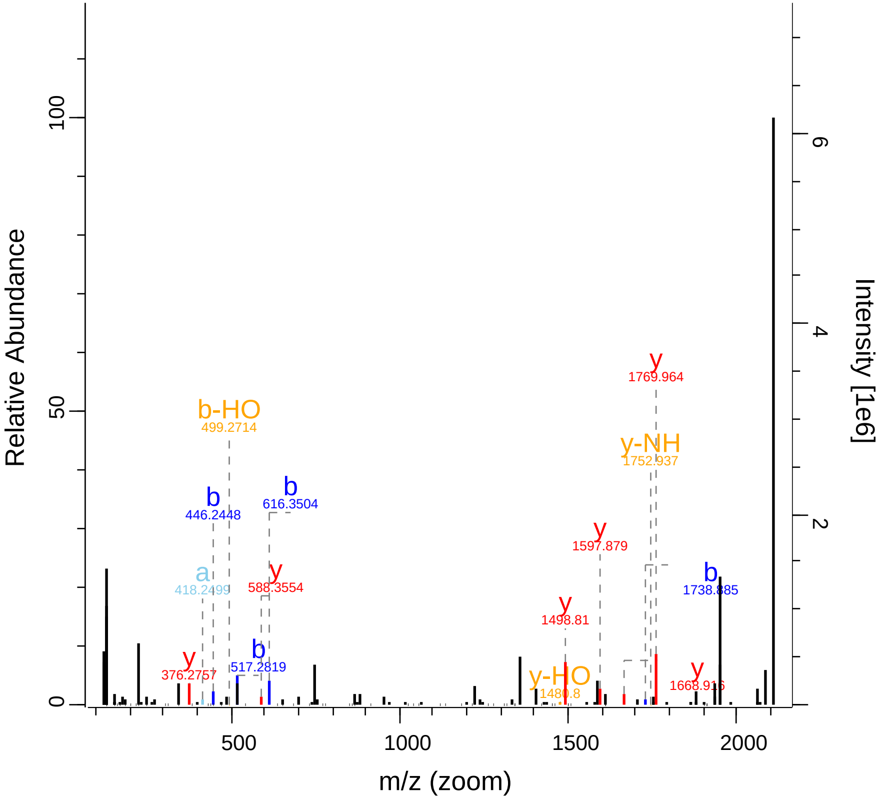

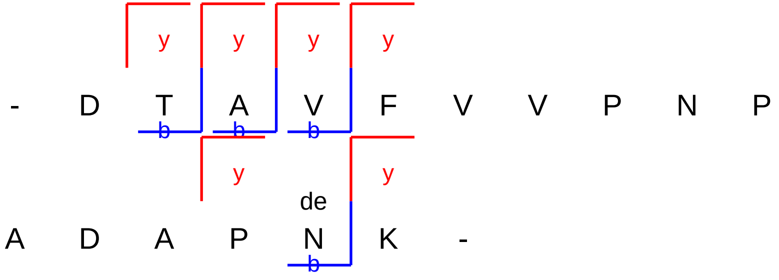

Raw File	Scan	Method	Score	m/z	Gene names
RO-QC-160121_ZackD_SS_Set_02_Fractio	17392	FTMS; HC	61.96	1269.7	FAM81A

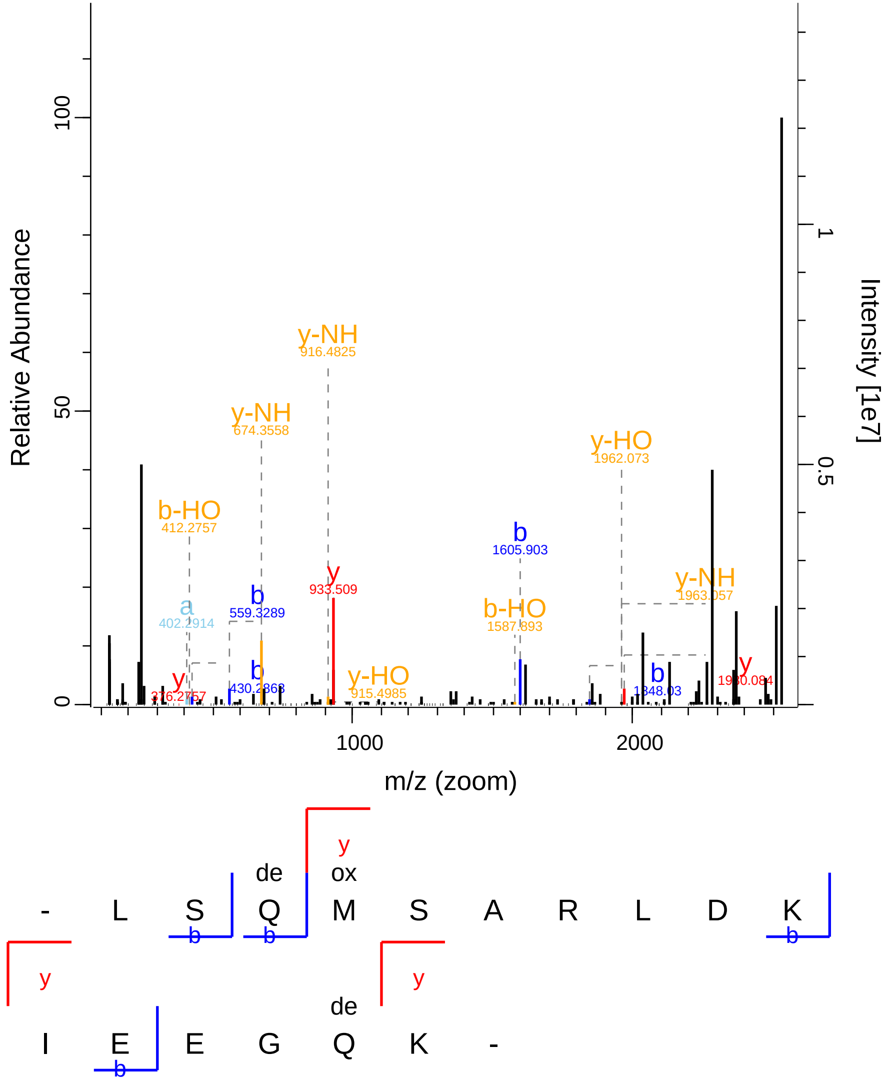

Raw File	Scan	Method	Score	m/z	Gene names
RO-QC-160121_ZackD_SS_Set_02_Fractior	18800	FTMS; HCI	74.34	799.5	GVINP1

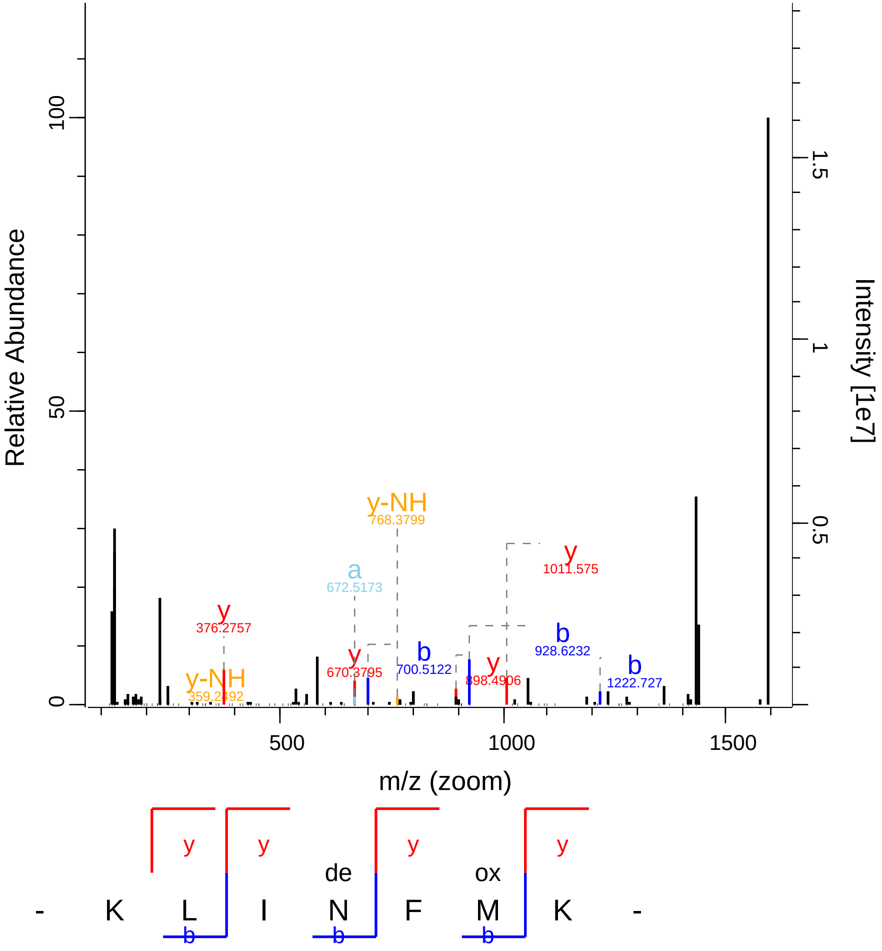

Raw File	Scan	Method	Score	m/z	Gene names
RO-QC-160121_ZackD_SS_Set_02_Fractio	18819	FTMS; HC	60.79	533.34	GVINP1

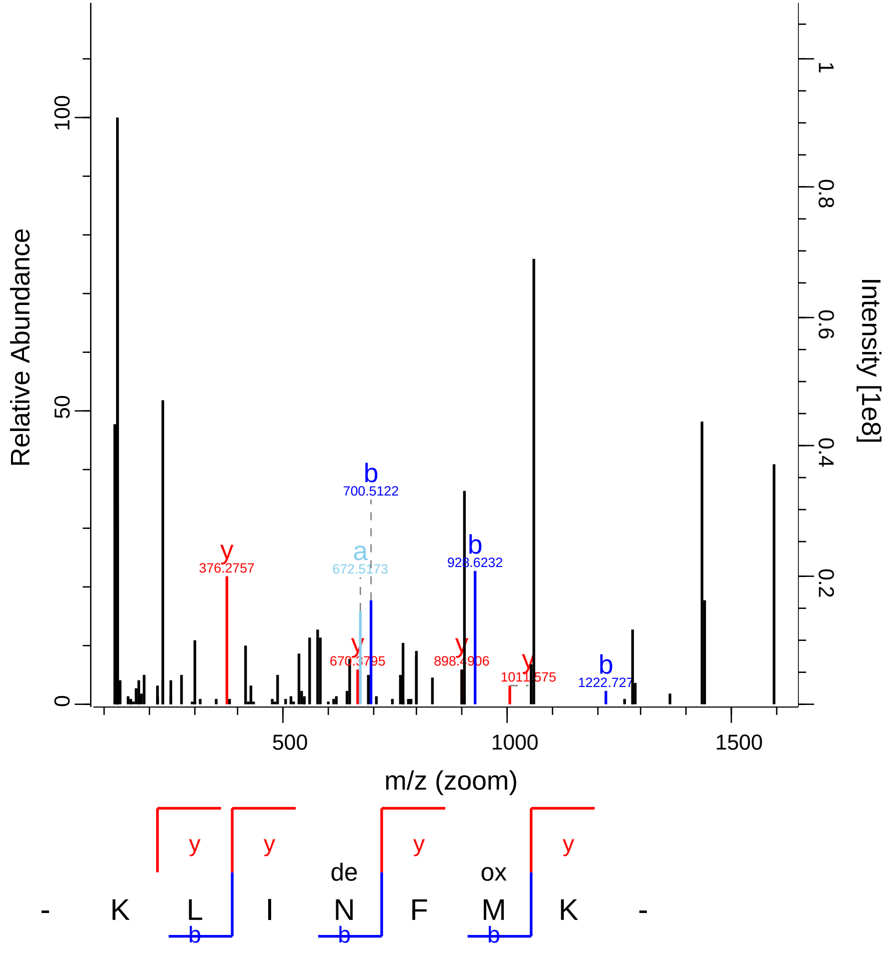

Raw File	Scan	Method	Score	m/z	Gene names
RO-QC-160121_ZackD_SS_Set_02_Fra	19421	FTMS; HC	40.5	989.46	SPATA31C2;SPATA31

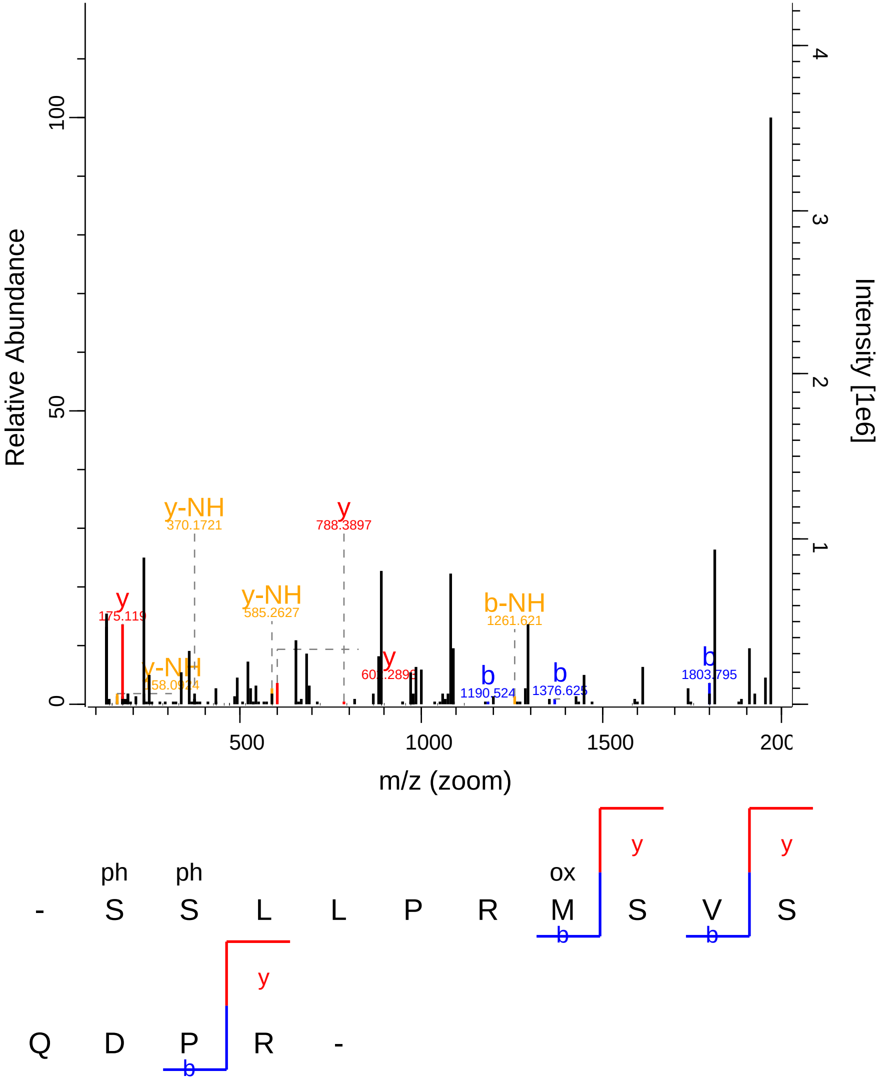

Raw File	Scan	Method	Score	m/z	Gene names
RO-QC-160121_ZackD_SS_Set_02_Fractio	19903	FTMS; HC	162.49	858.46	MGST2

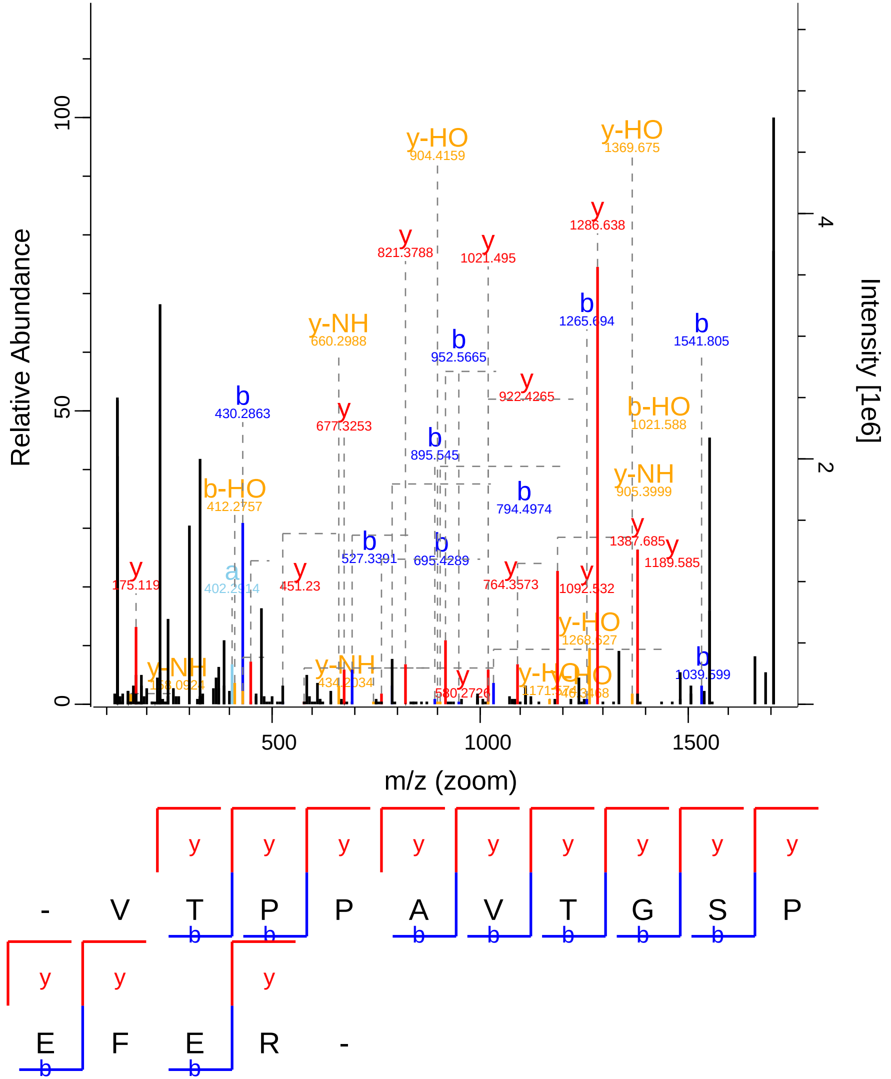

Raw File	Scan	Method	Score	m/z	Gene names
RO-QC-160121_ZackD_SS_Set_02_Fractio	20208	FTMS; HC	45.61	723.35	SVEP1

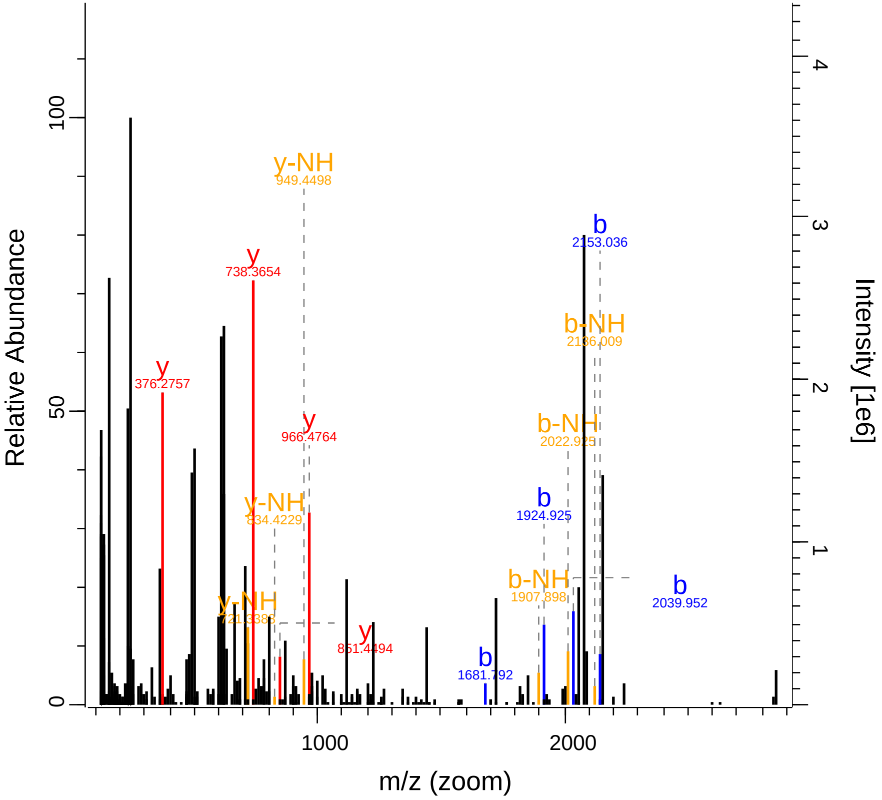

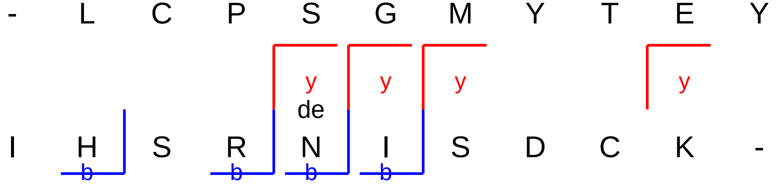

Raw File	Scan	Method	Score	m/z	Gene names
RO-QC-160121_ZackD_SS_Set_02_Fractio	20854	FTMS; HC	51.87	702.07	ZNF518A

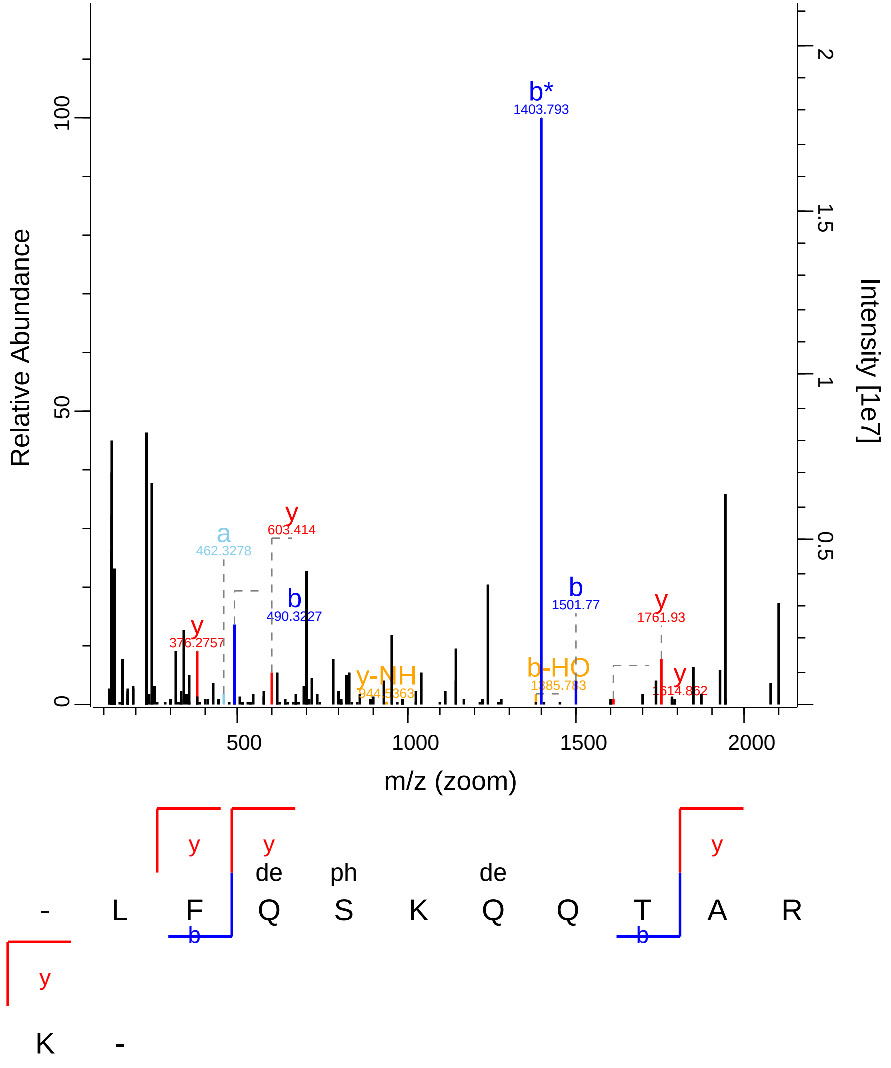

Raw File	Scan	Method	Score	m/z	Gene names
RO-QC-160121_ZackD_SS_Set_02_Fractic	21645	FTMS; HC	62.47	1078.05	NACA2

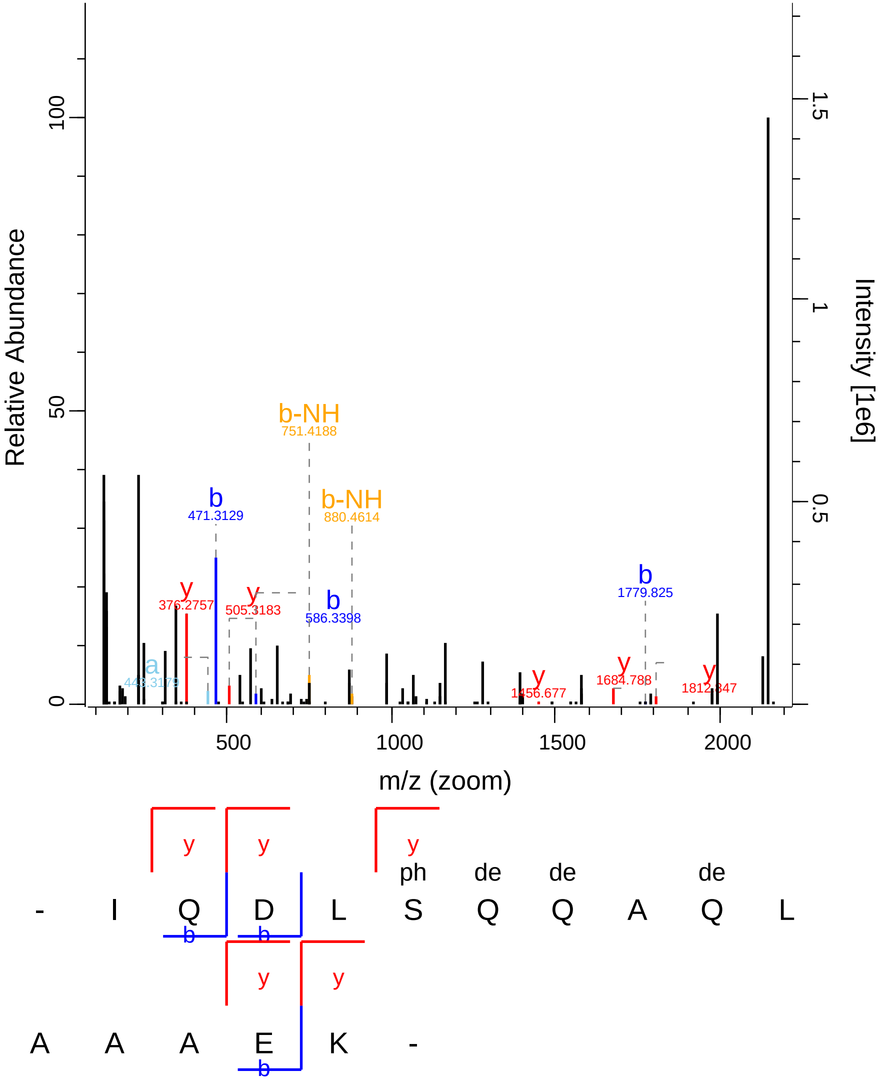

Raw File	Scan	Method	Score	m/z
RO-QC-160121_ZackD_SS_Set_02_Fraction_7	21875	FTMS; HCD	113.22	732.42

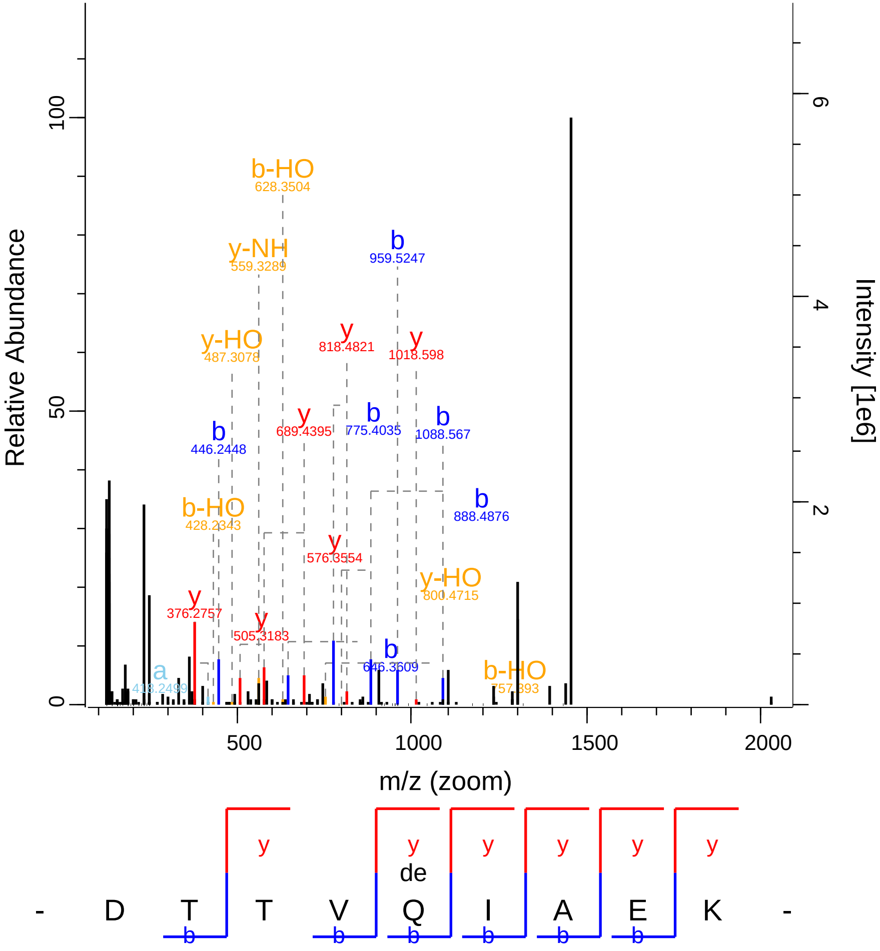

Raw File	Scan	Method	Score	m/z	Gene names
RO-QC-160121_ZackD_SS_Set_02_Fractio	22454	FTMS; HC	71.52	453.26	YPEL5

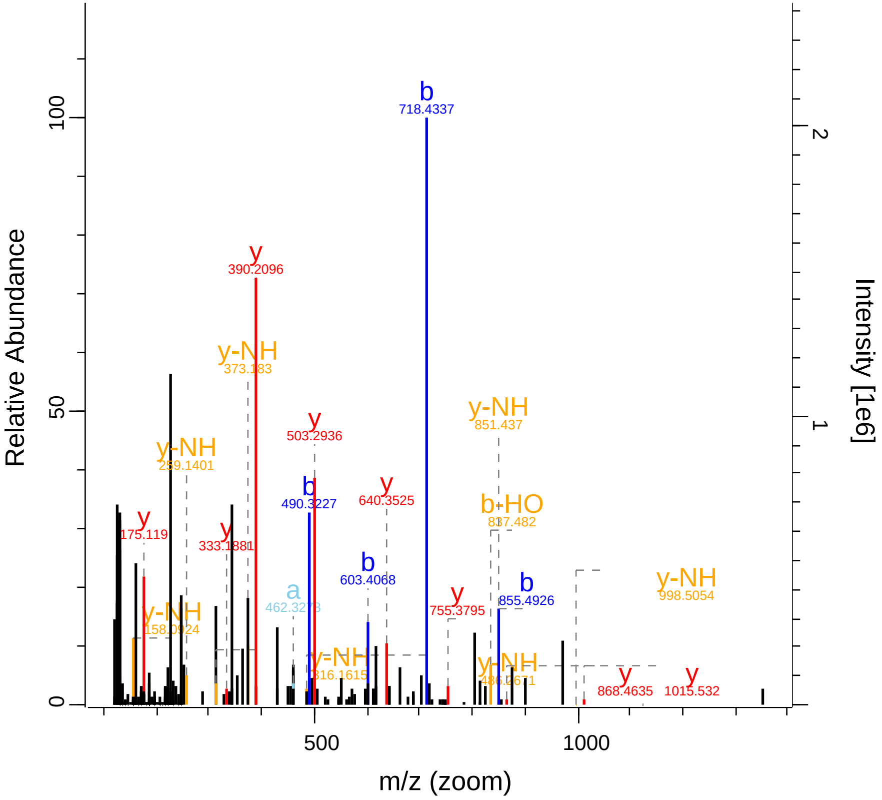

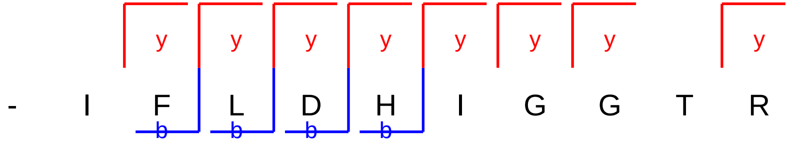

-

Raw File	Scan	Method	Score	m/z	Gene names
RO-QC-160121_ZackD_SS_Set_02_Fra	23320	FTMS; HC	81.97	960.06	ANKRD18A;ANKRD1

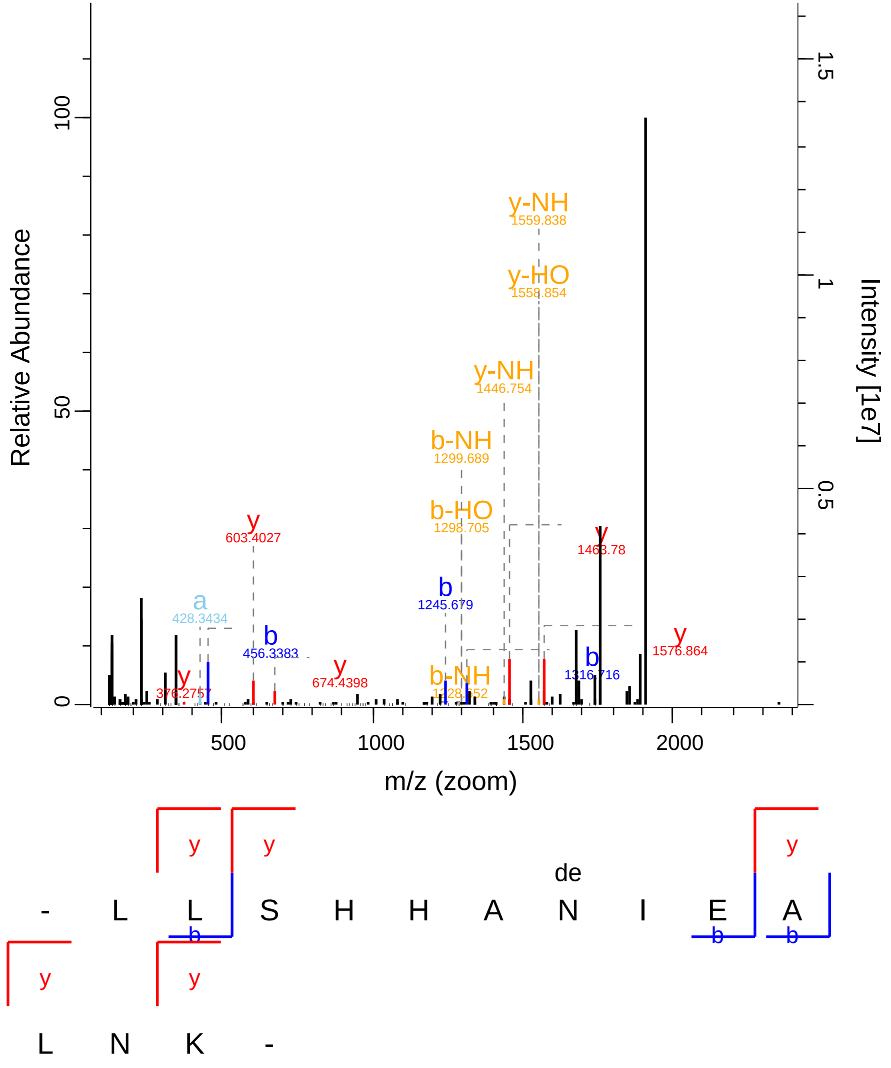

Raw File	Scan	Method	Score	m/z	Gene names
RO-QC-160121_ZackD_SS_Set_02_Fractio	24463	FTMS; HC	53.52	803.91	SMIM4

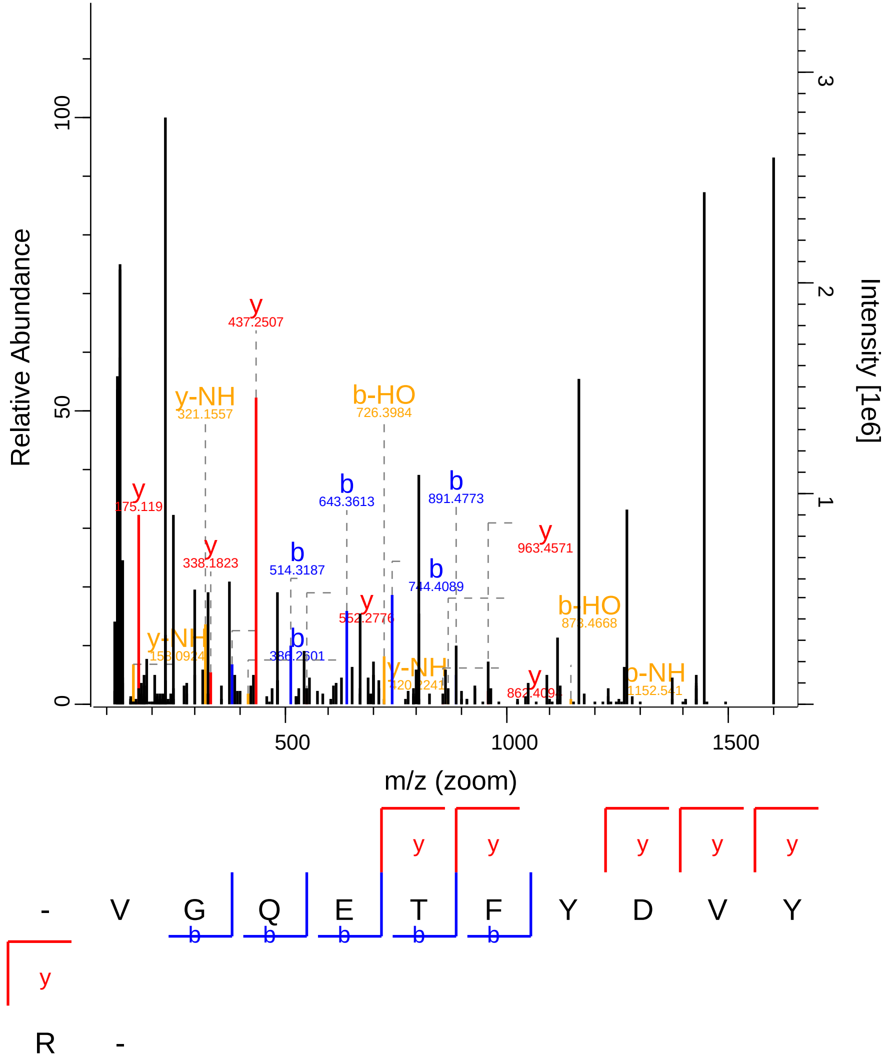

Raw File	Scan	Method	Score	m/z	Gene names
RO-QC-160121_ZackD_SS_Set_02_Fractic	25858	FTMS; HC	52.61	1065.22	CLC

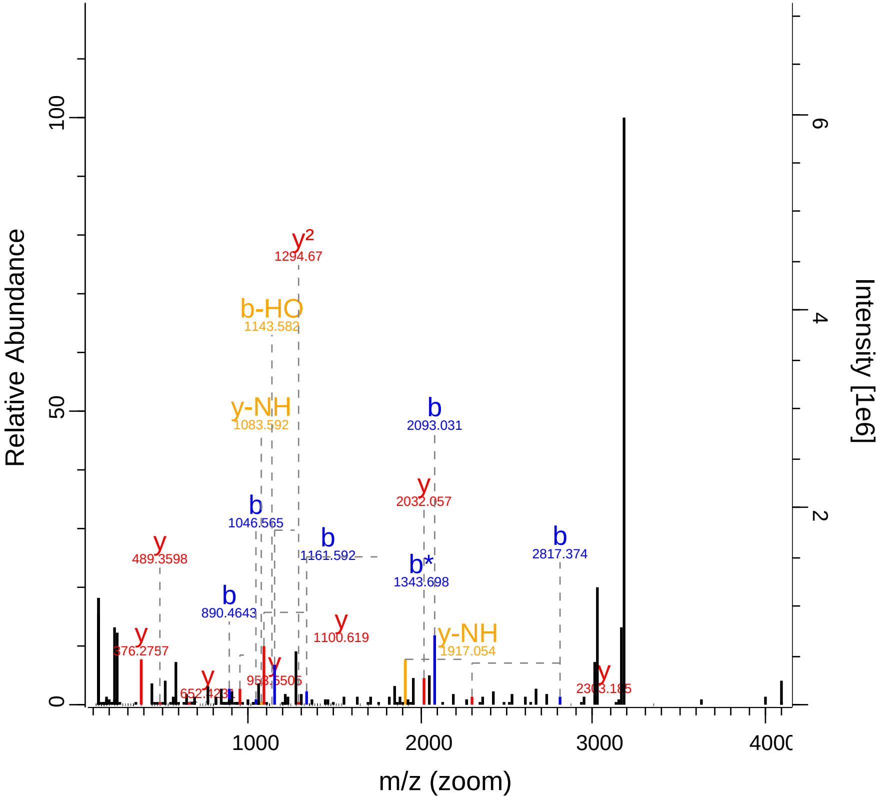

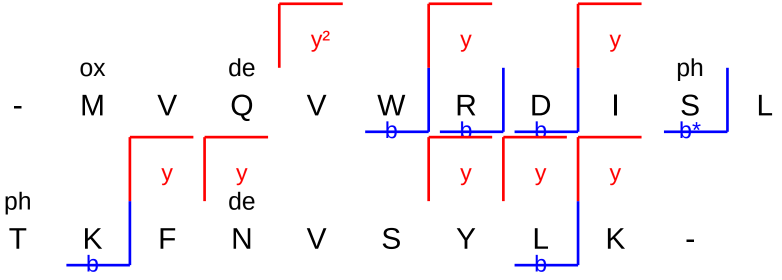

Raw File	Scan	Method	Score	m/z
RO-QC-160121_ZackD_SS_Set_02_Fraction_7	26013	FTMS; HCD	42.57	742.08

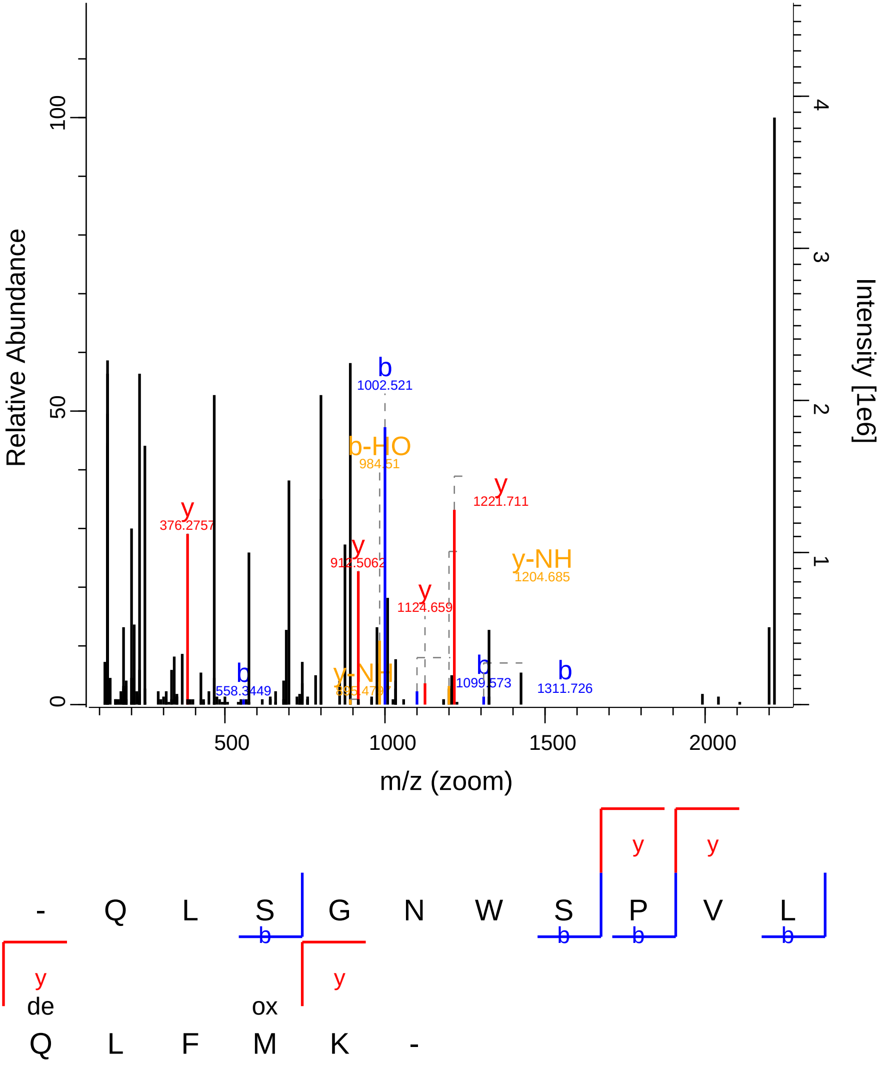

Raw File	Scan	Method	Score	m/z	Gene names
RO-QC-160121_ZackD_SS_Set_02_Fractio	26481	FTMS; HC	56.48	823.44	CHDC2

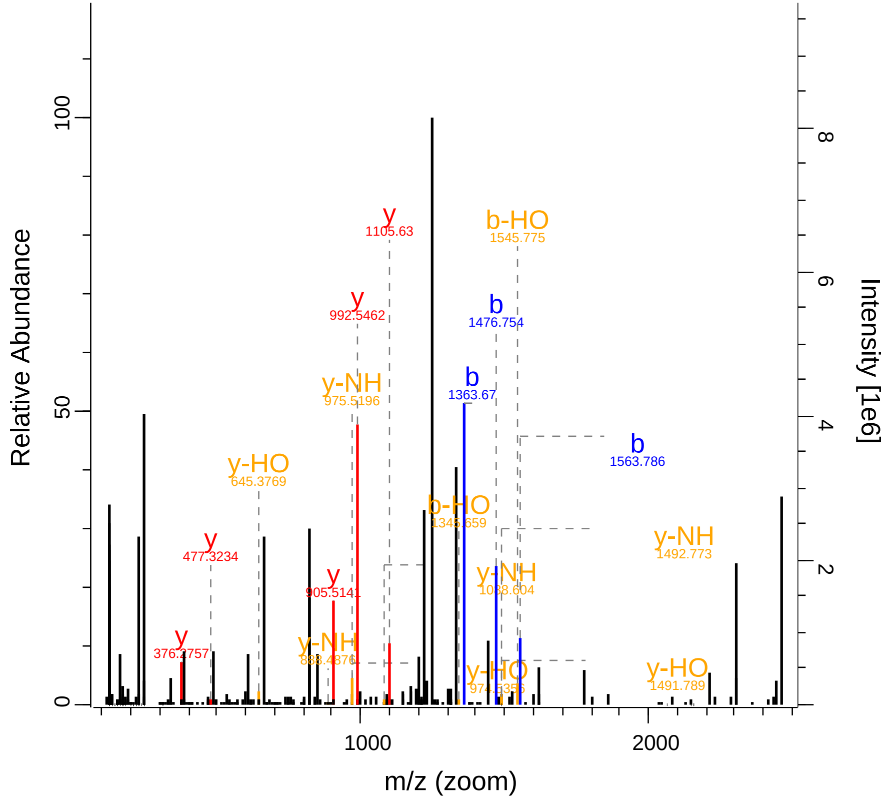

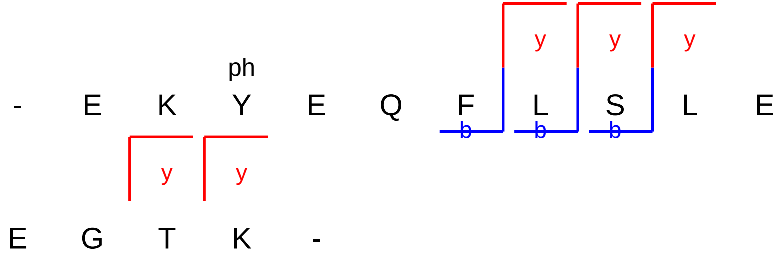

Raw File	Scan	Method	Score	m/z
RO-QC-160121_ZackD_SS_Set_02_Fraction_7	26567	FTMS; HCD	42.56	489.55

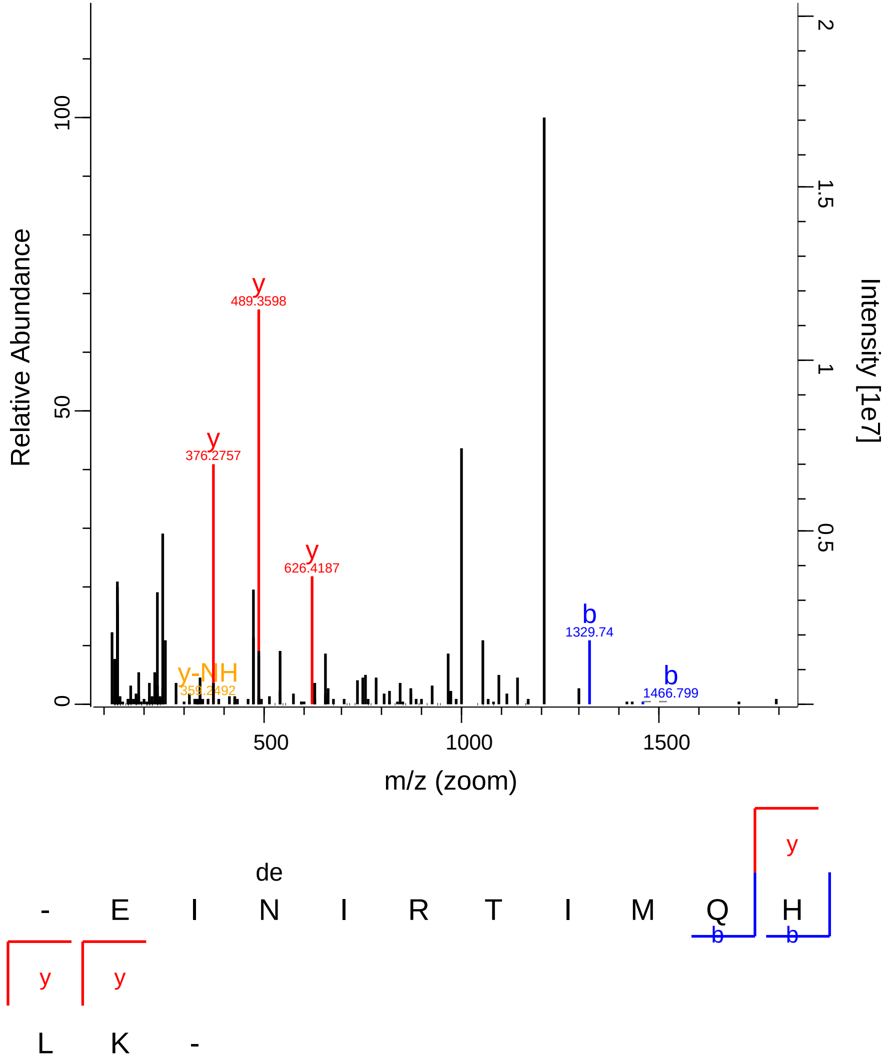

Raw File	Scan	Method	Score	m/z	Gene names
RO-QC-160121_ZackD_SS_Set_02_Fractio	27209	FTMS; HC	40.02	660.12	ABCA13

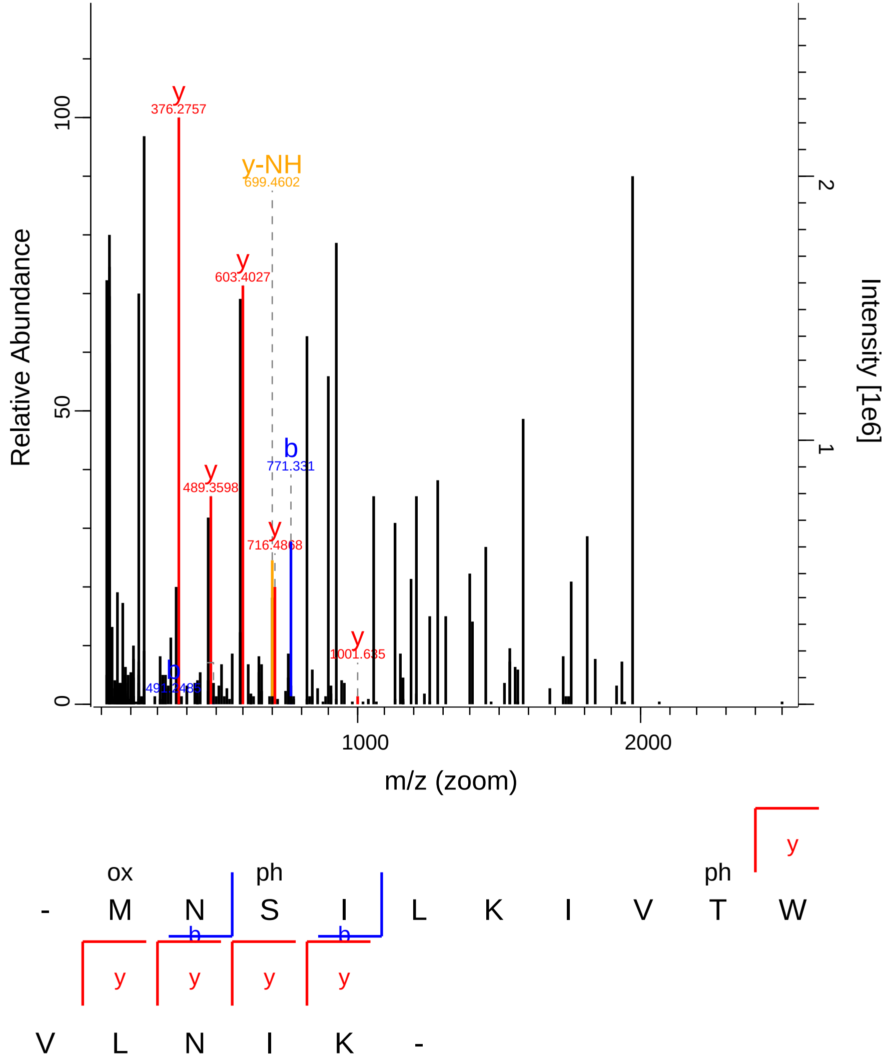

Raw File	Scan	Method	Score	m/z	Gene names
RO-QC-160121_ZackD_SS_Set_02_Fractio	27242	FTMS; HC	47.94	562.66	PDE6A

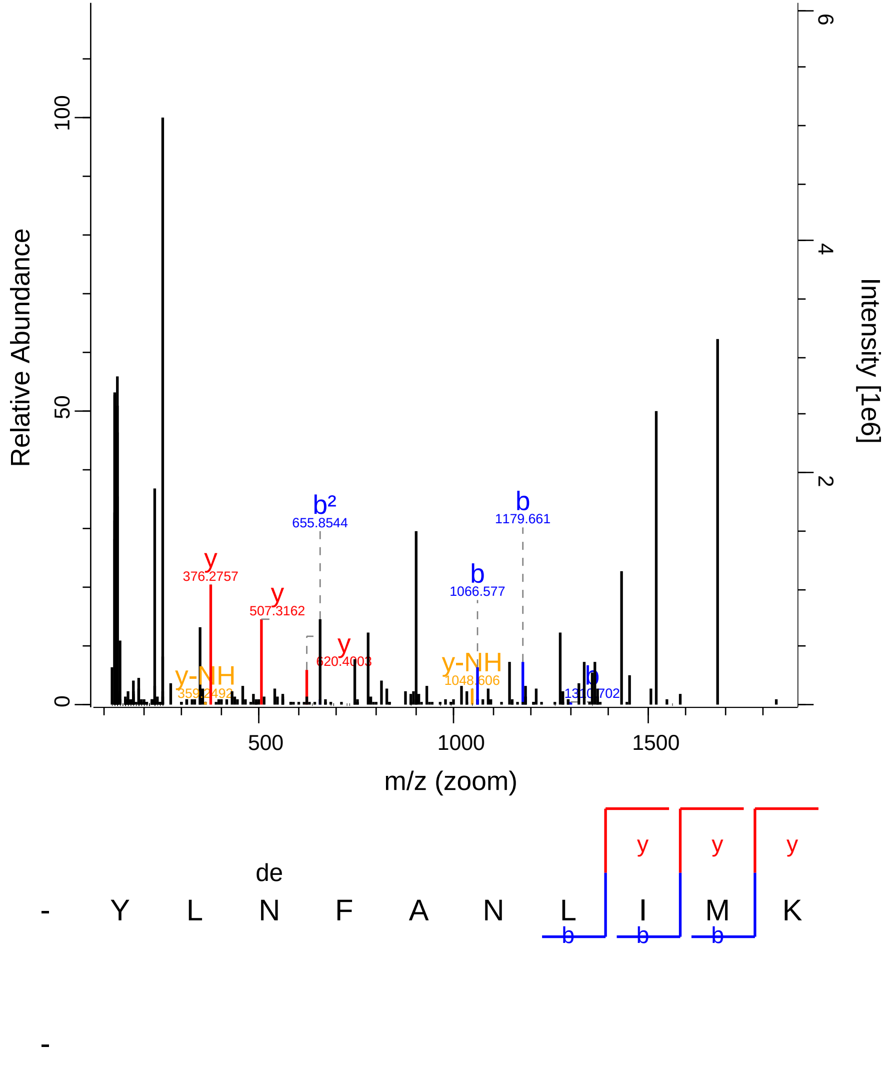

Raw File	Scan	Method	Score	m/z	Gene names
RO-QC-160121_ZackD_SS_Set_02_Fractio	27366	FTMS; HC	153.05	837.97	RGR

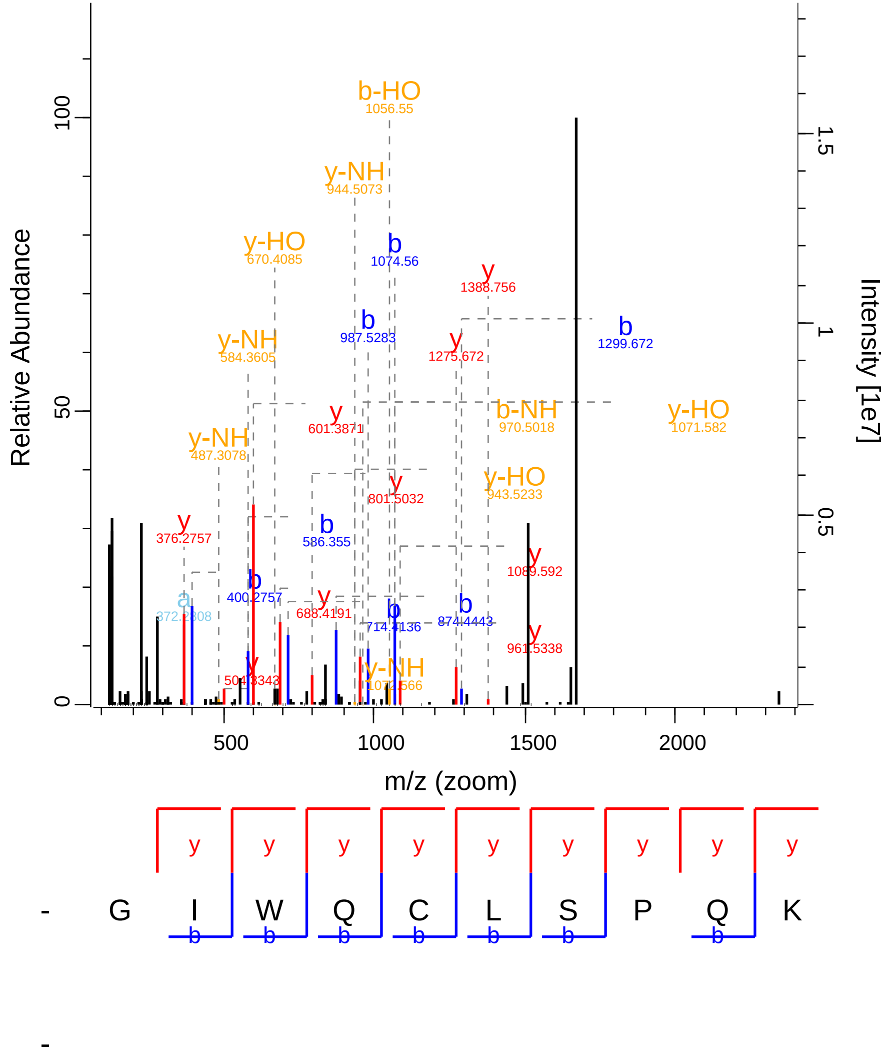

Raw File	Scan	Method	Score	m/z	Gene names
RO-QC-160121_ZackD_SS_Set_02_Fractio	27394	FTMS; HC	99.82	558.99	RGR

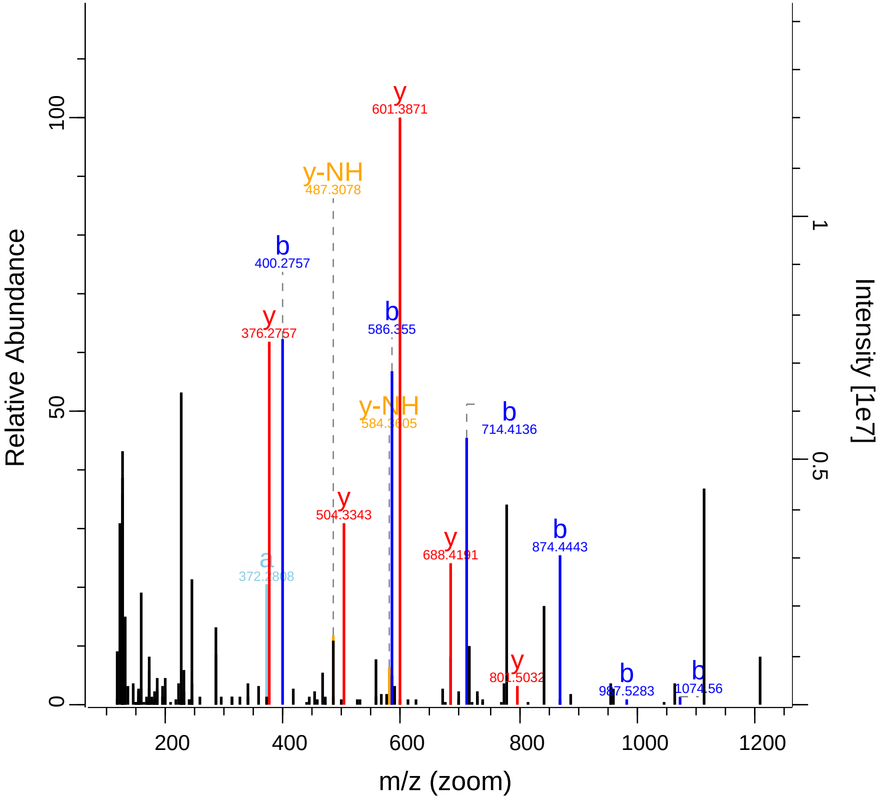

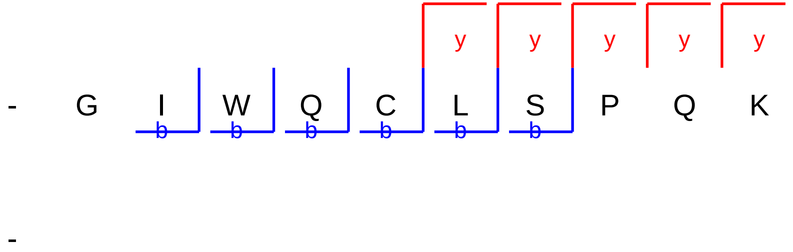

Raw File	Scan	Method	Score	m/z	Gene names
RO-QC-160121_ZackD_SS_Set_02_Fractio	27993	FTMS; HC	89.47	692.39	NACAP1

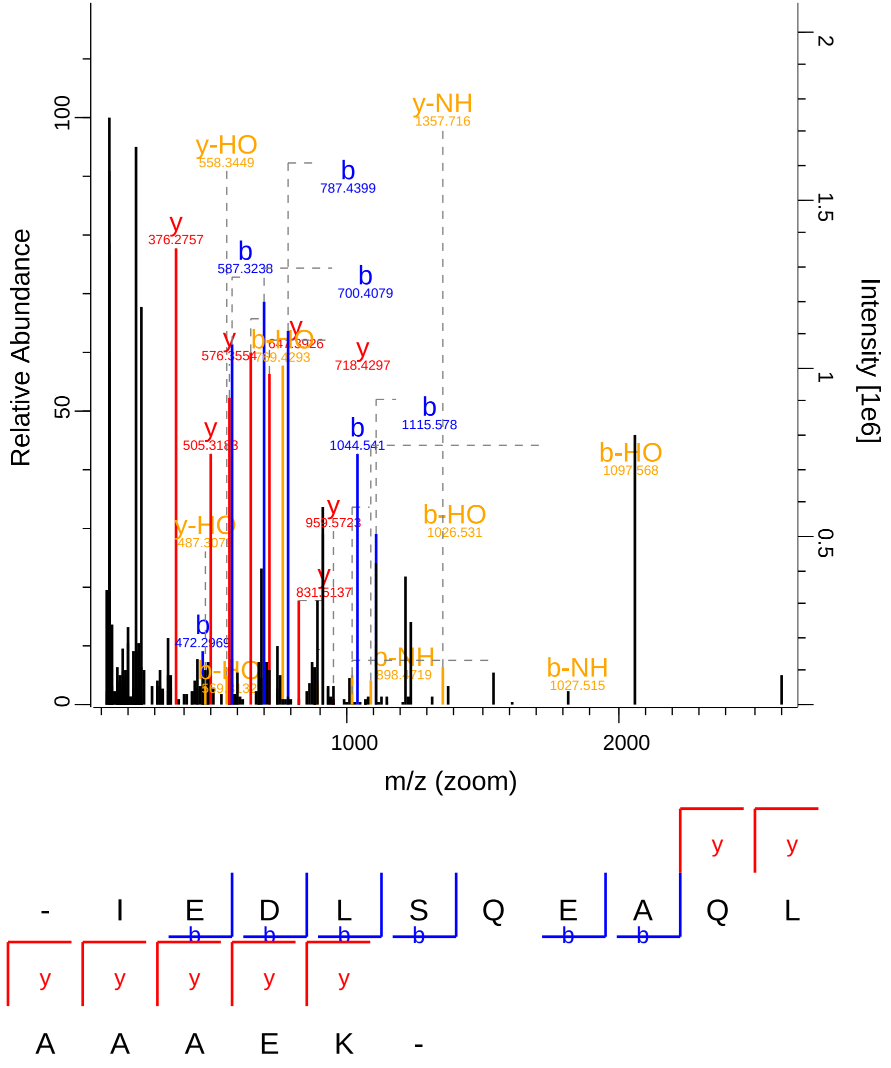

Raw File	Scan	Method	Score	m/z	Gene names
RO-QC-160121_ZackD_SS_Set_02_Fractic	28661	FTMS; HC	63.22	1124.62	FABP2

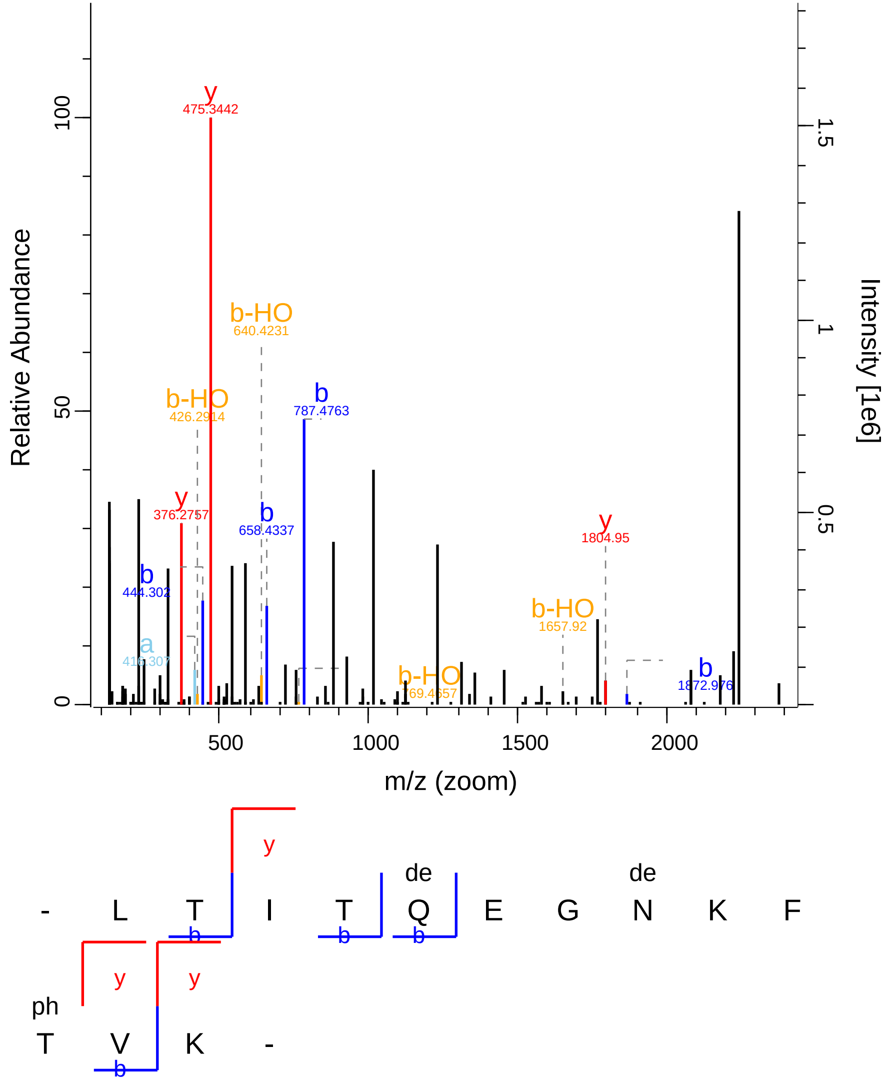

Raw File	Scan	Method	Score	m/z	Gene names
RO-QC-160121_ZackD_SS_Set_02_Fractio	28703	FTMS; HC	88.73	429.26	KRT82

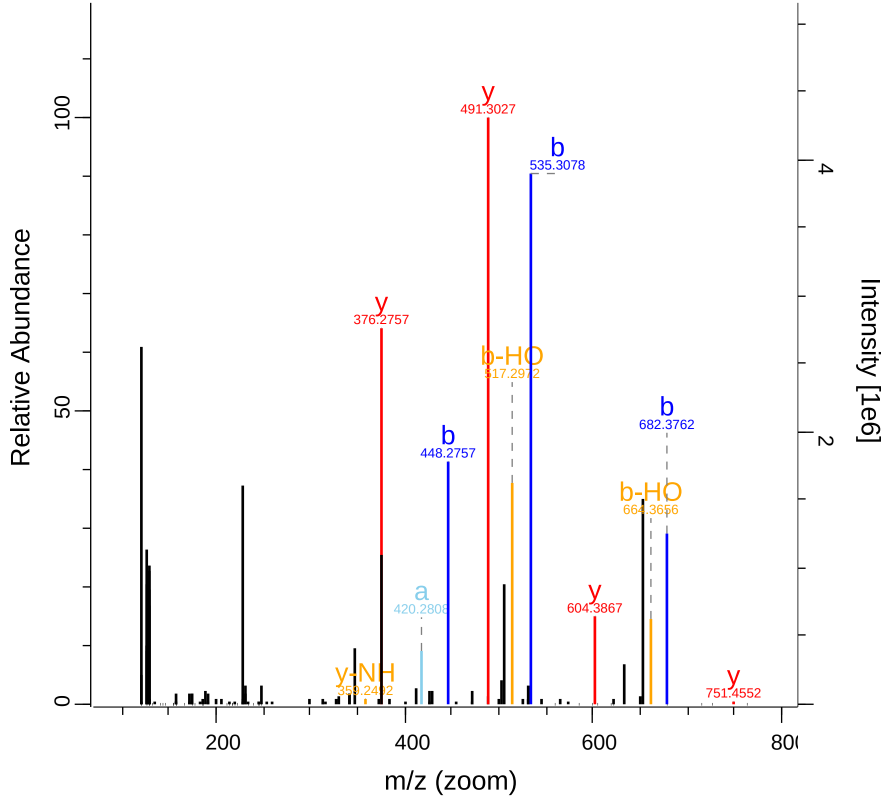

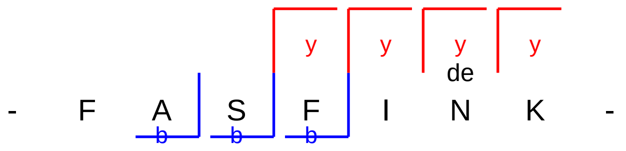

Raw File	Scan	Method	Score	m/z	Gene names
RO-QC-160121_ZackD_SS_Set_02_Fractio	28821	FTMS; HC	117.93	643.38	KRT82

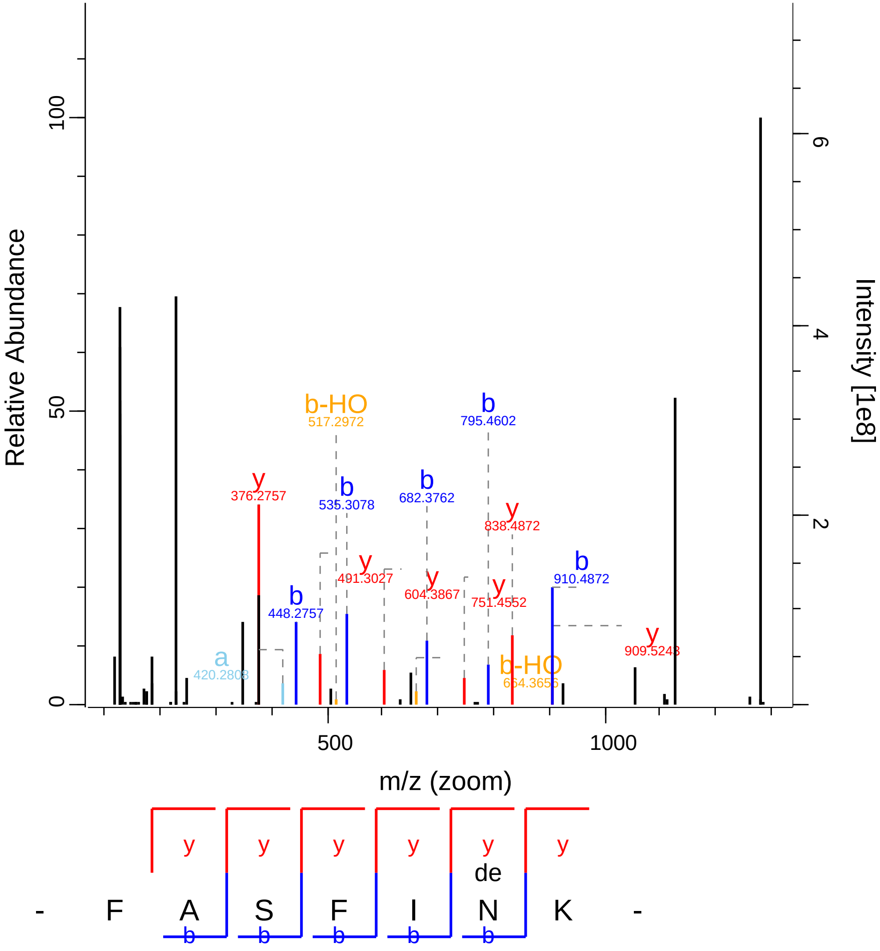

Raw File	Scan	Method	Score	m/z	Gene names
RO-QC-160121_ZackD_SS_Set_02_Fractio	29045	FTMS; HC	124.34	643.38	KRT82

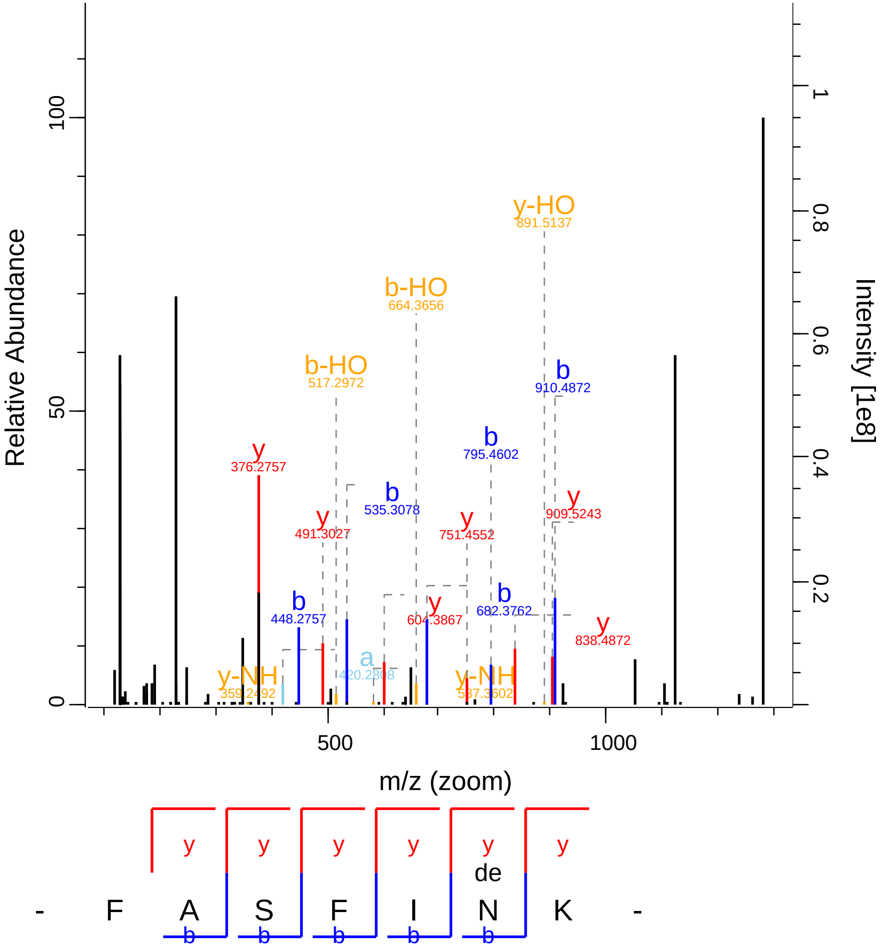

Raw File	Scan	Method	Score	m/z	Gene names
RO-QC-160121_ZackD_SS_Set_02_Fractio	30976	FTMS; HC	119.27	701.46	UNC79

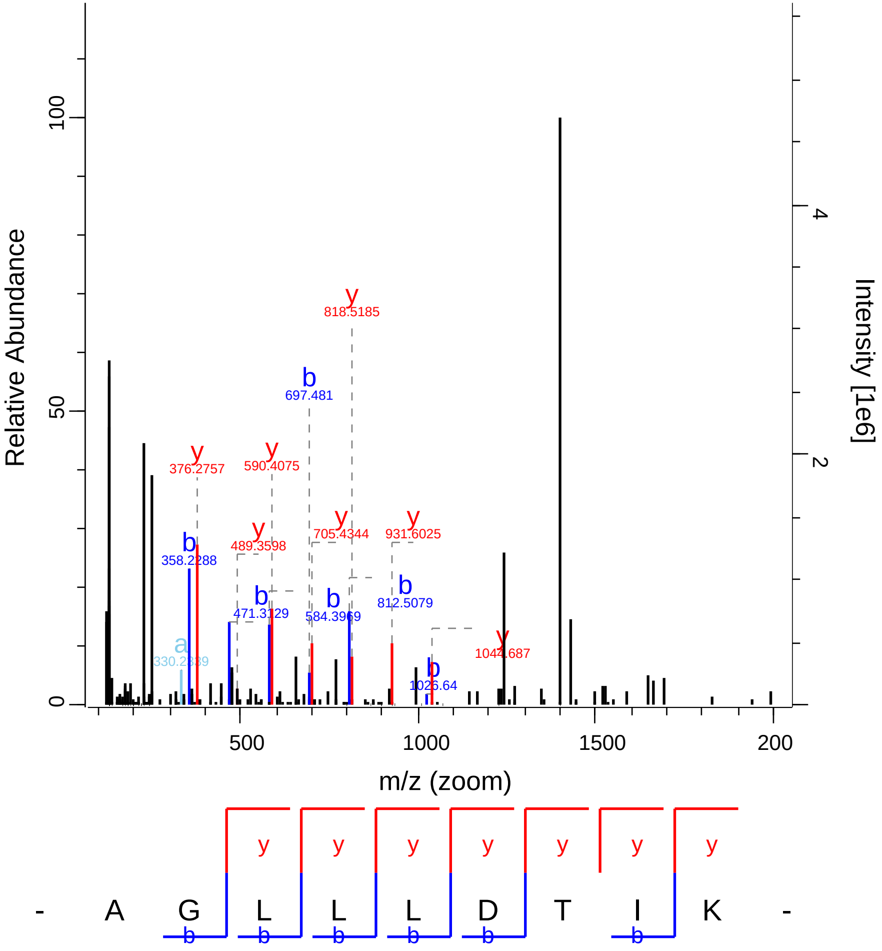

Raw File	Scan	Method	Score	m/z	Gene names
RO-QC-160121_ZackD_SS_Set_02_Fractio	32381	FTMS; HC	206.45	1095.65	KRTCAP2

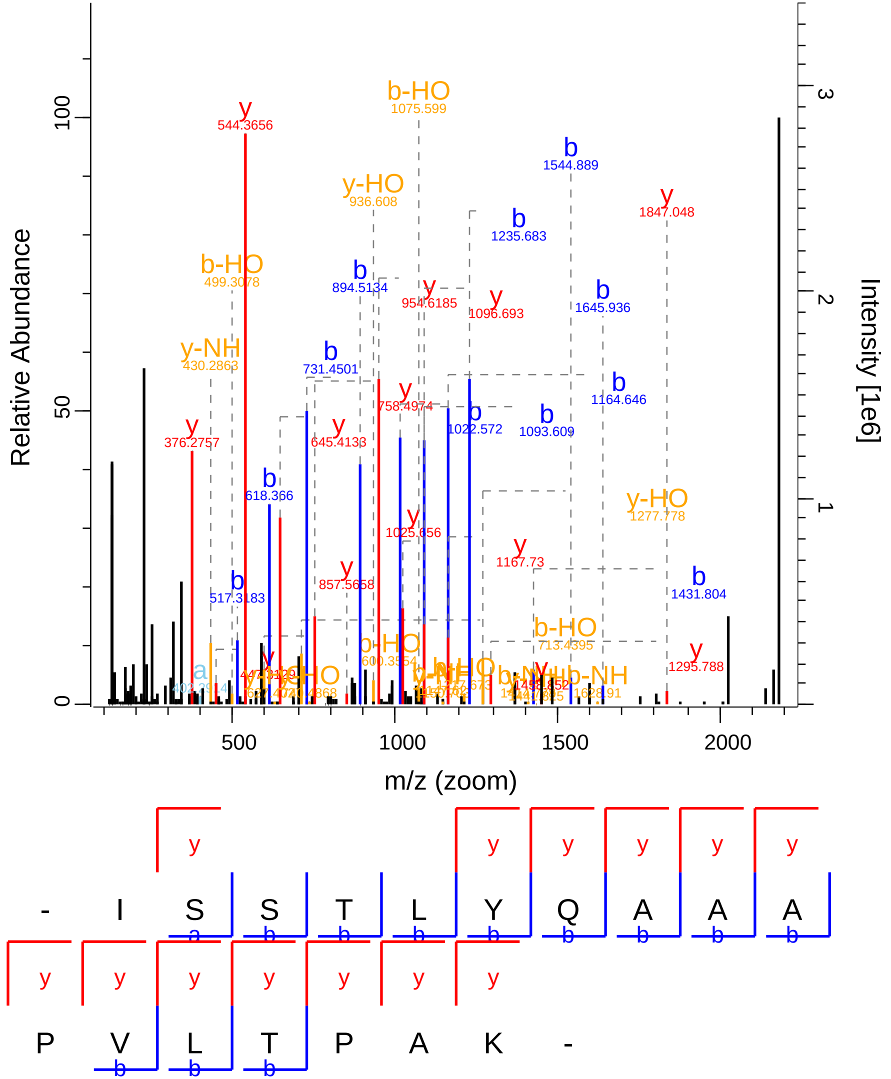

Raw File	Scan	Method	Score	m/z	Gene names
RO-QC-160121_ZackD_SS_Set_02_Fractio	32444	FTMS; HC	65.72	548.33	KRTCAP2

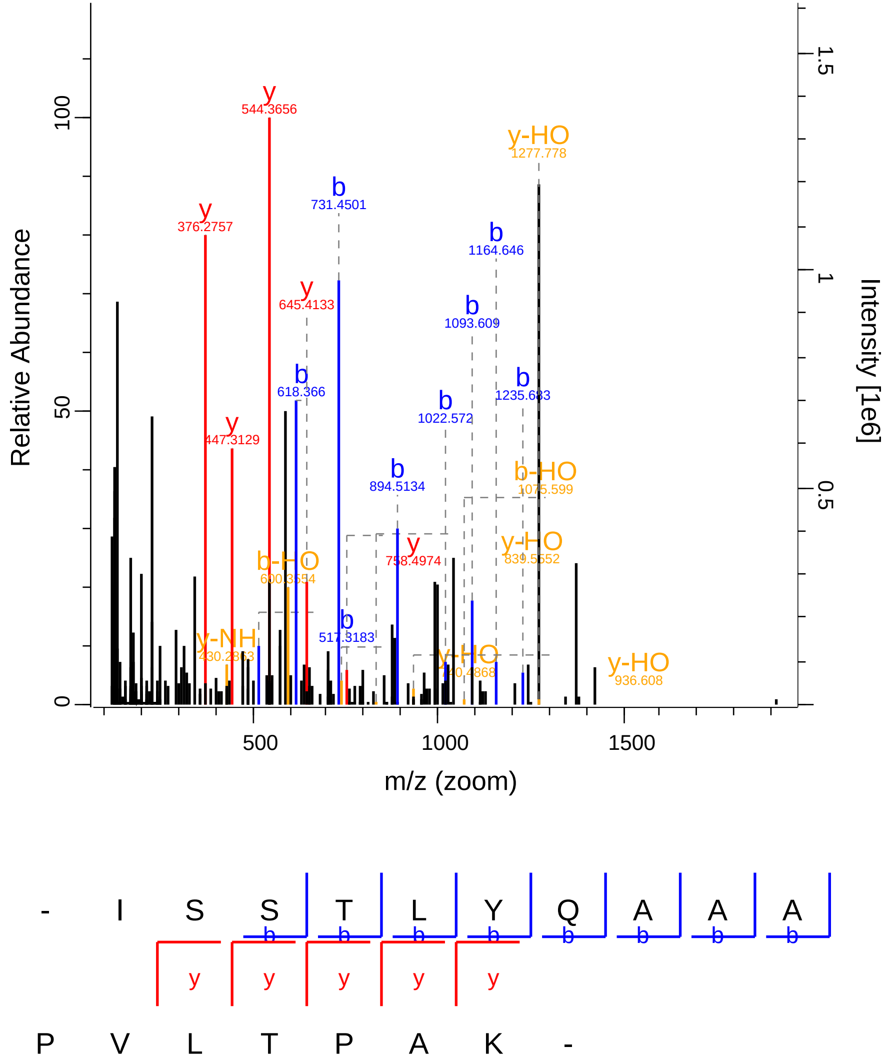

Raw File	Scan	Method	Score	m/z	Gene names
RO-QC-160121_ZackD_SS_Set_02_Fractio	32567	FTMS; HC	55.33	730.77	KRTCAP2

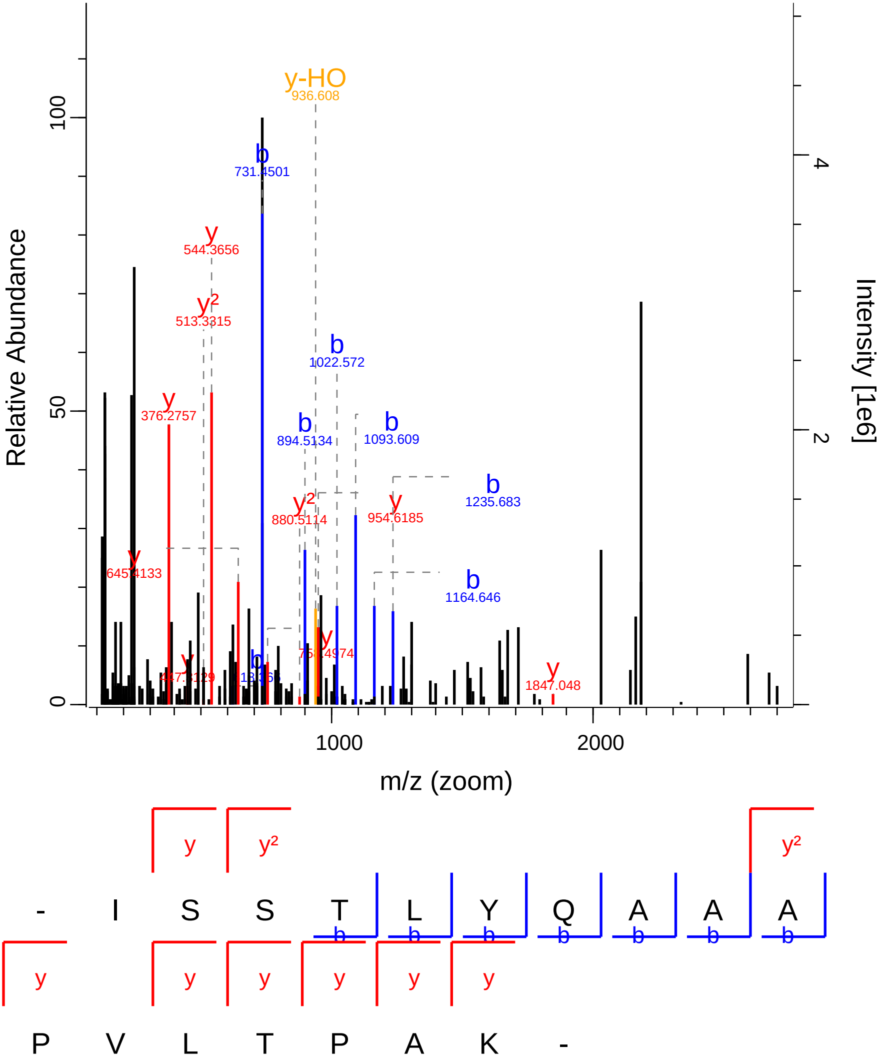

Raw File	Scan	Method	Score	m/z
RO-QC-160121_ZackD_SS_Set_02_Fraction_7	32906	FTMS; HCD	43.96	752.17

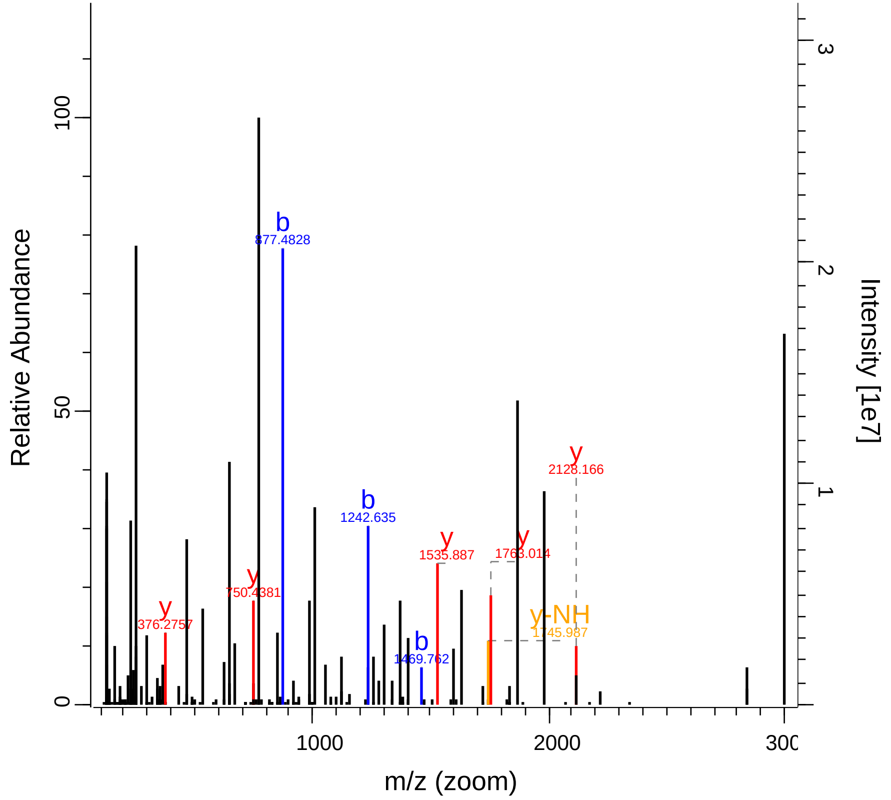

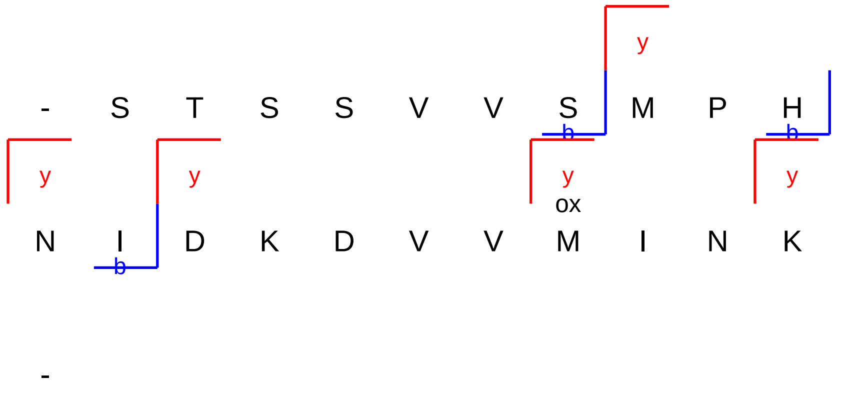

Raw File	Scan	Method	Score	m/z
RO-QC-160121_ZackD_SS_Set_02_Fraction_7	33004	FTMS; HCD	59	1002.56

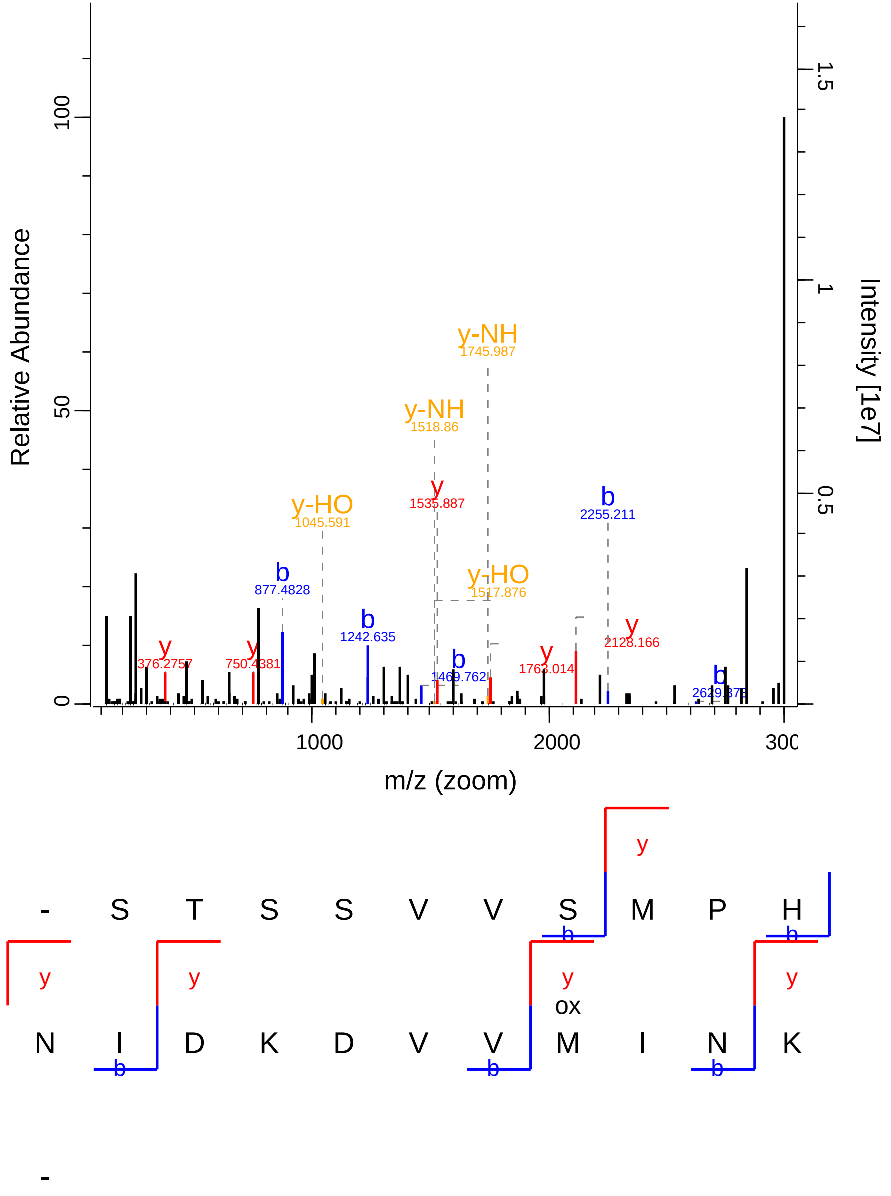

Raw File	Scan	Method	Score	m/z
RO-QC-160121_ZackD_SS_Set_02_Fraction_7	33308	FTMS; HCD	73.04	464.95

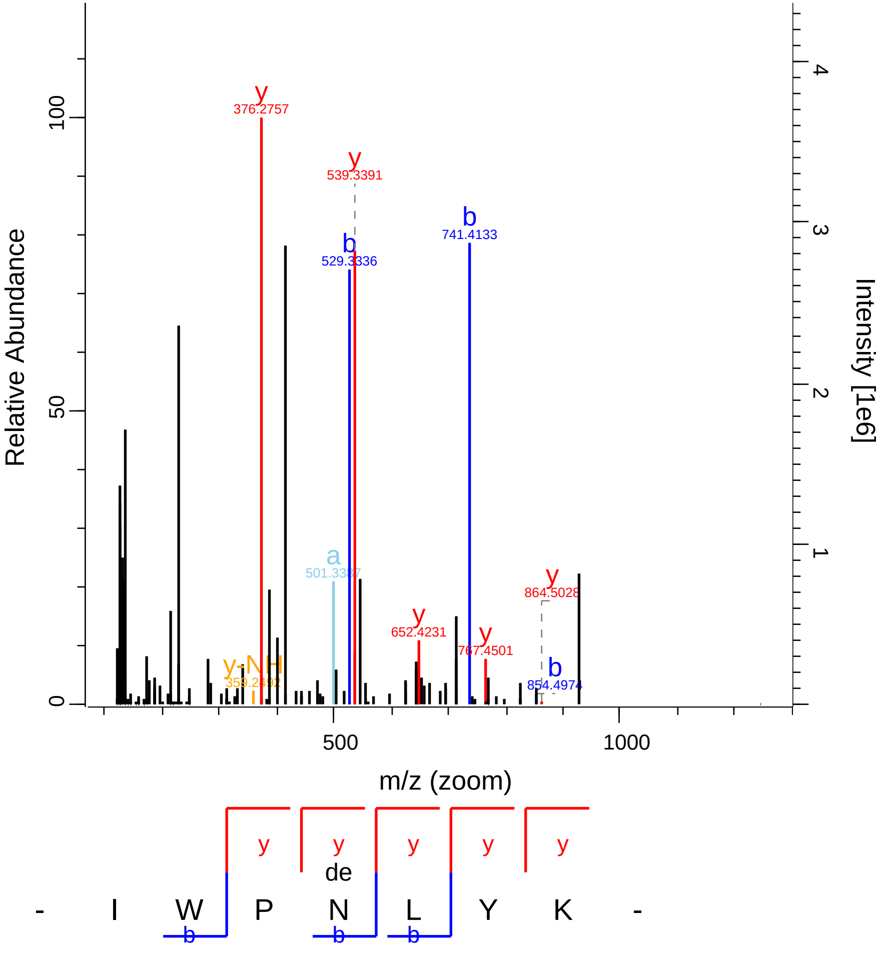

Raw File	Scan	Method	Score	m/z	Gene names
RO-QC-160121_ZackD_SS_Set_02_Fractio	33514	FTMS; HC	77.74	552.01	KLHDC4

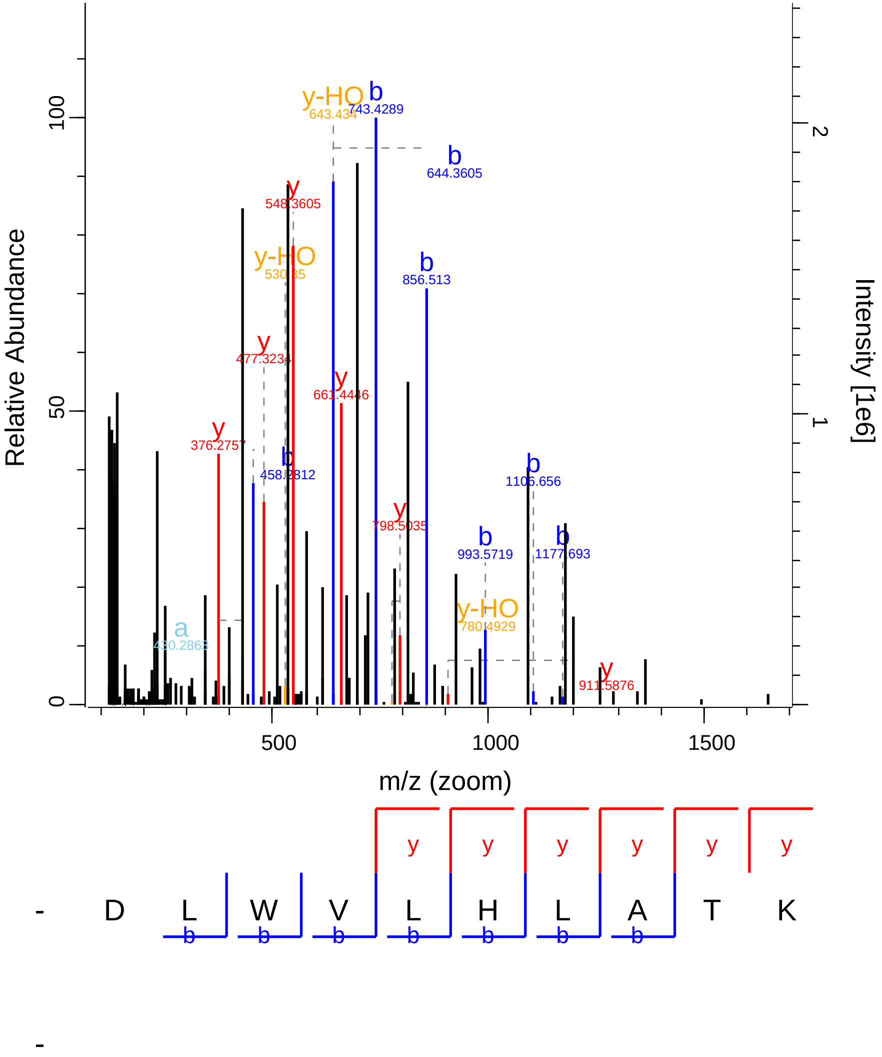

Raw File	Scan	Method	Score	m/z	Gene names
RO-QC-160121_ZackD_SS_Set_02_Fractio	33613	FTMS; HC	48.29	975.53	NCOA1

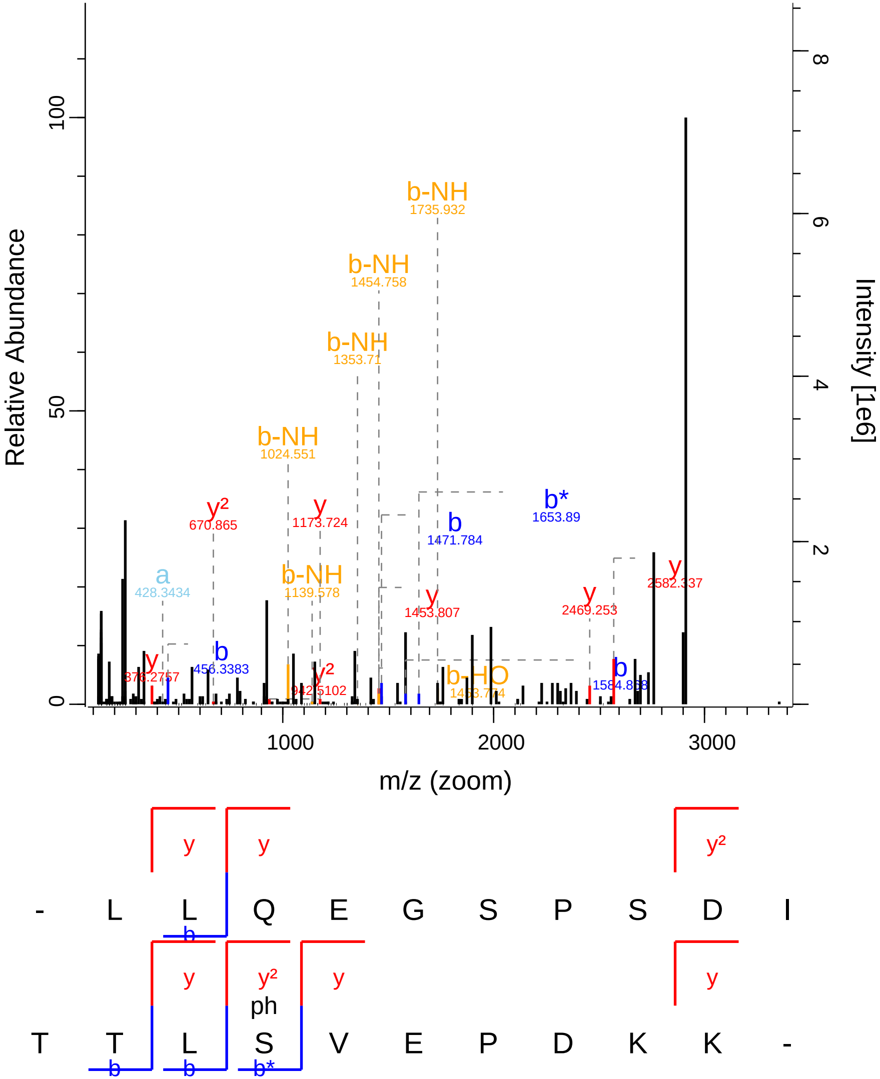

Raw File	Scan	Method	Score	m/z	Gene names
RO-QC-160121_ZackD_SS_Set_02_Fractic	34186	FTMS; HC	50.21	1155.63	EBF2

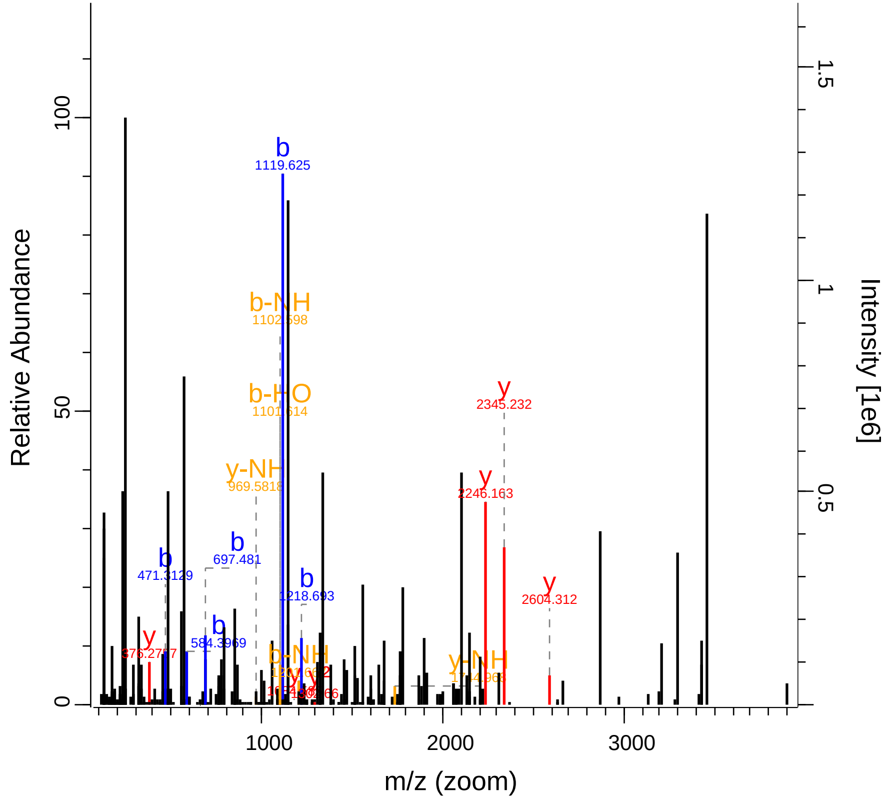

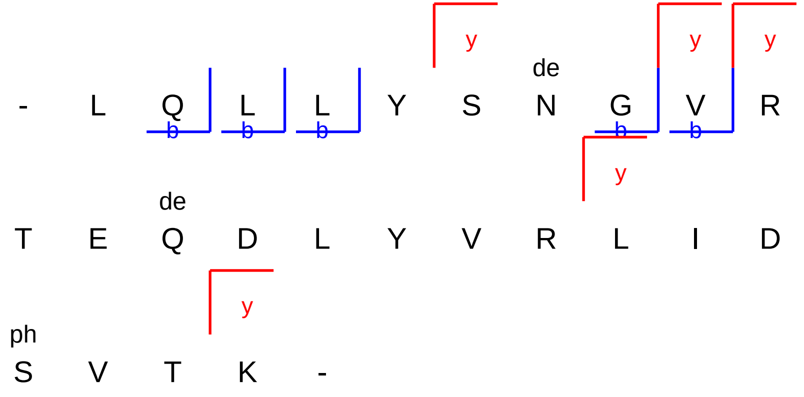

Raw File	Scan	Method	Score	m/z
RO-QC-160121_ZackD_SS_Set_02_Fraction_7	34240	FTMS; HCD	41.26	808.94

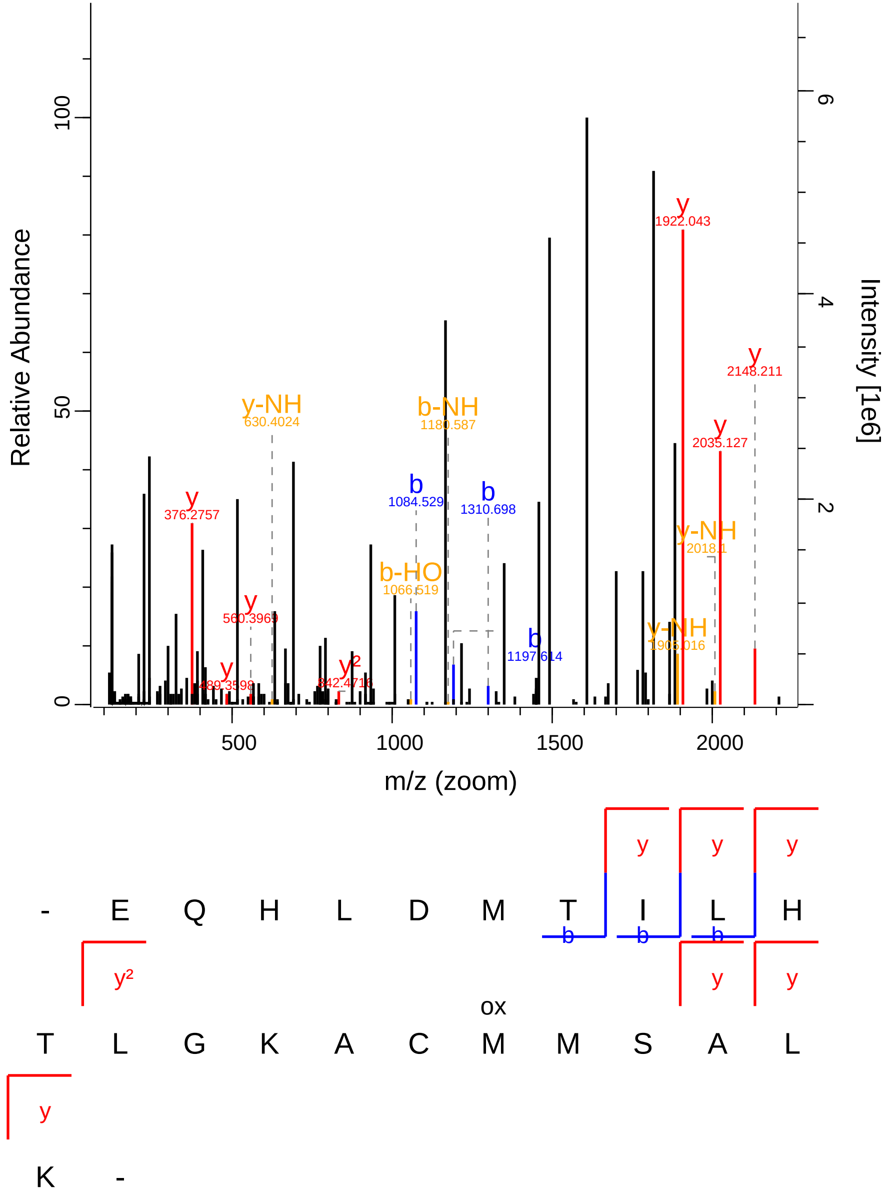

Raw File	Scan	Method	Score	m/z	Gene names
RO-QC-160121_ZackD_SS_Set_02_Fractio	35625	FTMS; HC	55.98	496.95	GTF2A1

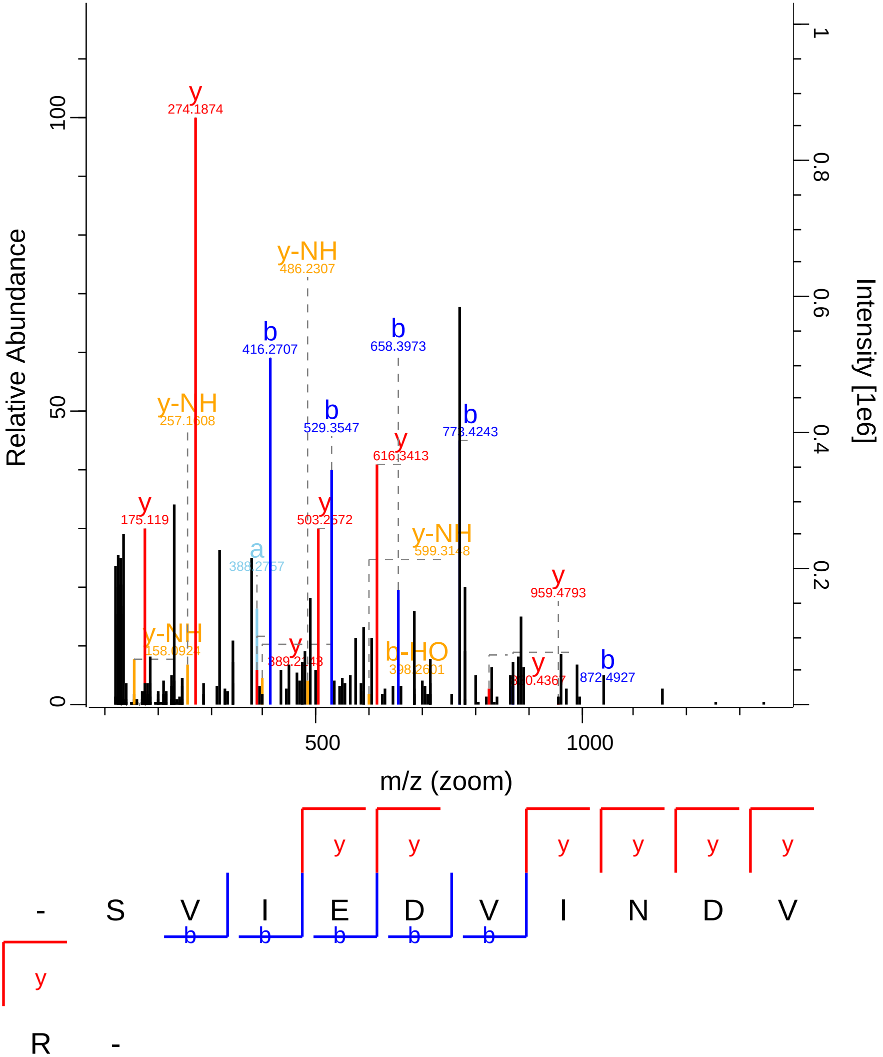

Raw File	Scan	Method	Score	m/z	Gene names
RO-QC-160121_ZackD_SS_Set_02_Fractio	35754	FTMS; HC	62.23	636.32	NOP10

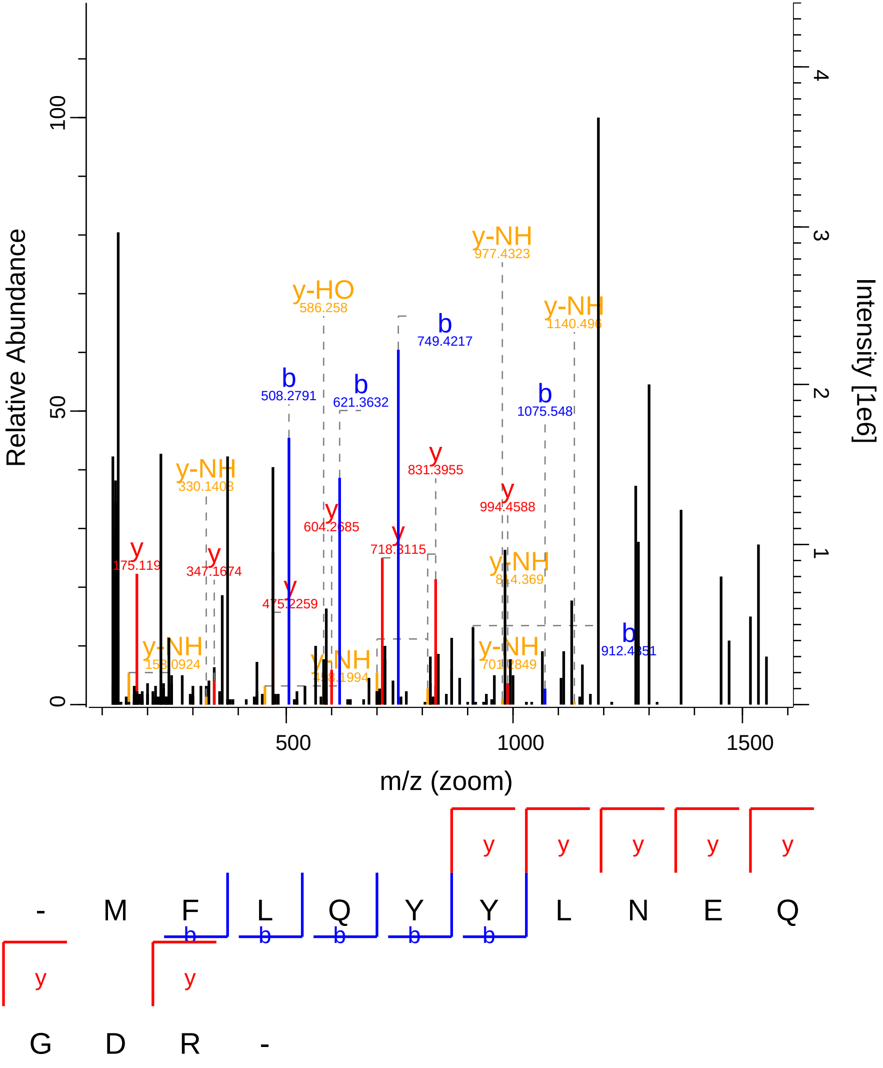

Raw File	Scan	Method	Score	m/z	Gene names
RO-QC-160121_ZackD_SS_Set_02_Fractior	35997	FTMS; HCI	38.89	875.1	KCNK1

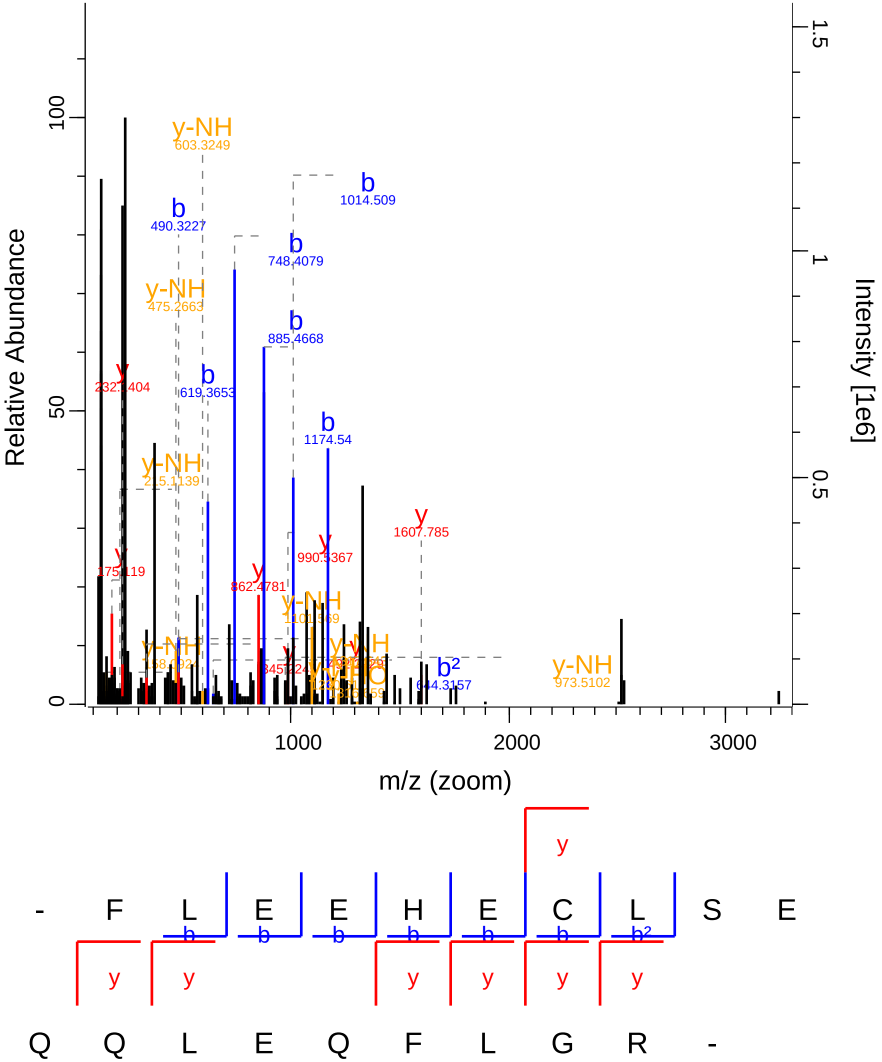

Raw File	Scan	Method	Score	m/z	Gene names
RO-QC-160121_ZackD_SS_Set_02_Fractio	36079	FTMS; HC	71.43	696.44	TMBIM6

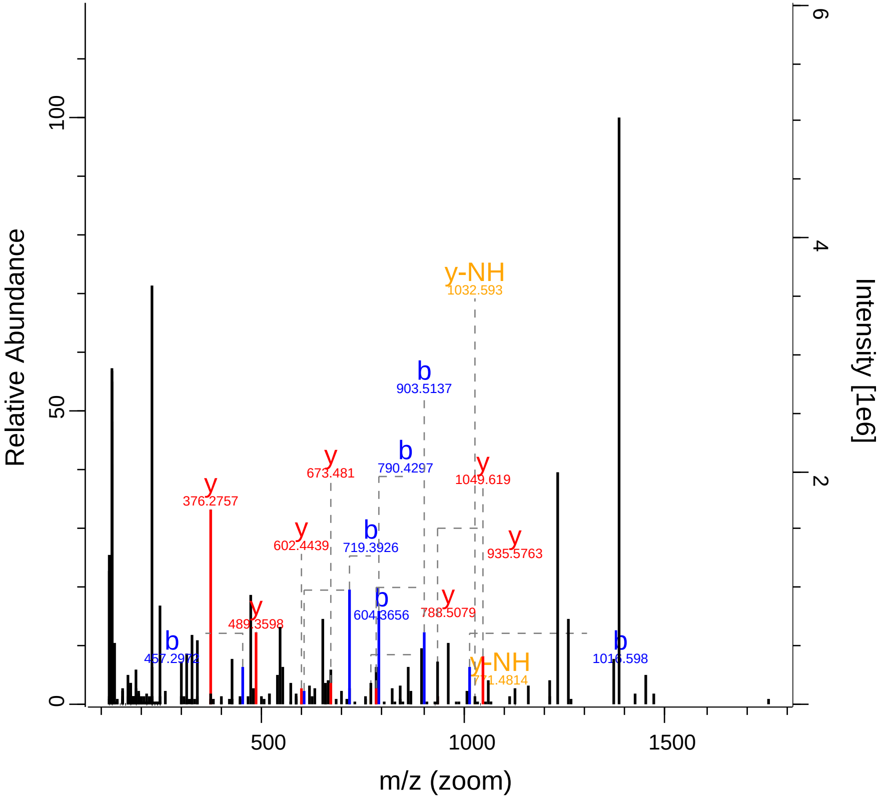

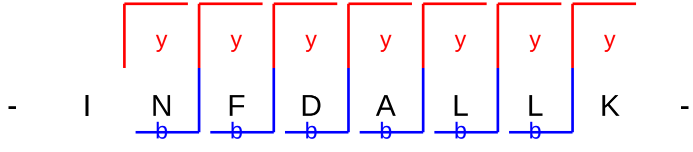

Raw File	Scan	Method	Score	m/z	Gene names
RO-QC-160121_ZackD_SS_Set_02_Fractio	36206	FTMS; HC	84.74	464.63	TMBIM6

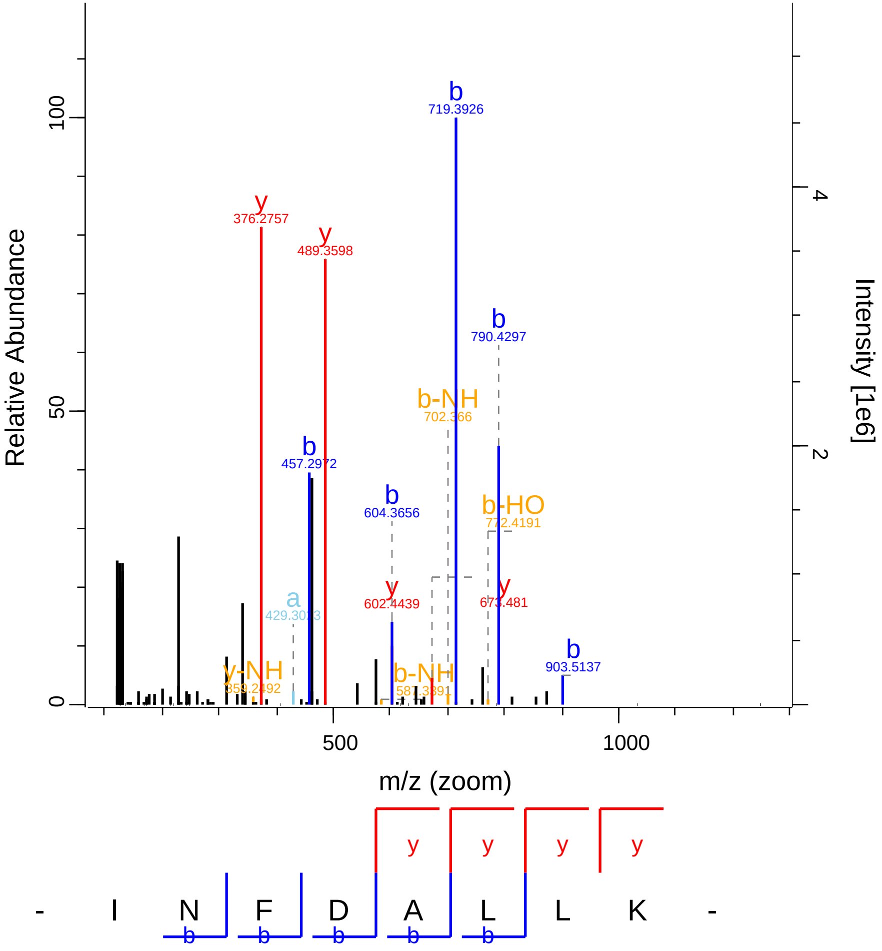

Raw File	Scan	Method	Score	m/z	Gene names
RO-QC-160121_ZackD_SS_Set_02_Fractio	36378	FTMS; HC	116.73	603.38	SLC37A3

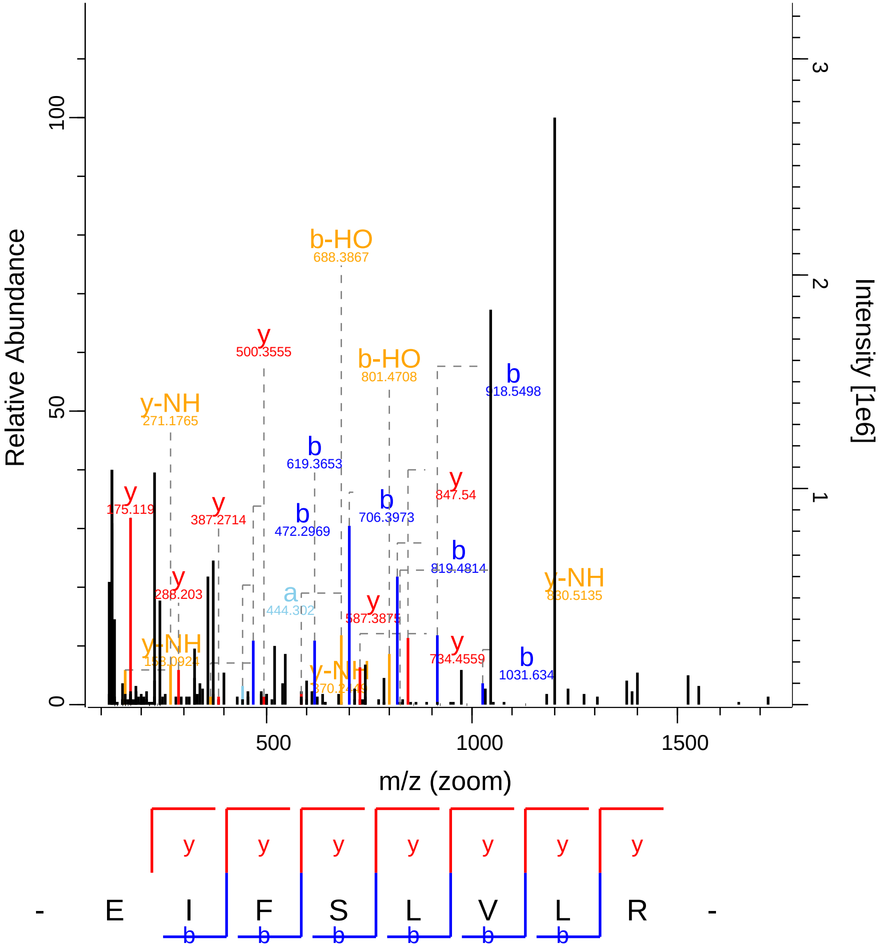

Raw File	Scan	Method	Score	m/z	Gene names
RO-QC-160121_ZackD_SS_Set_02_Fractio	36947	FTMS; HC	40.4	1091.9	MPHOSPH9

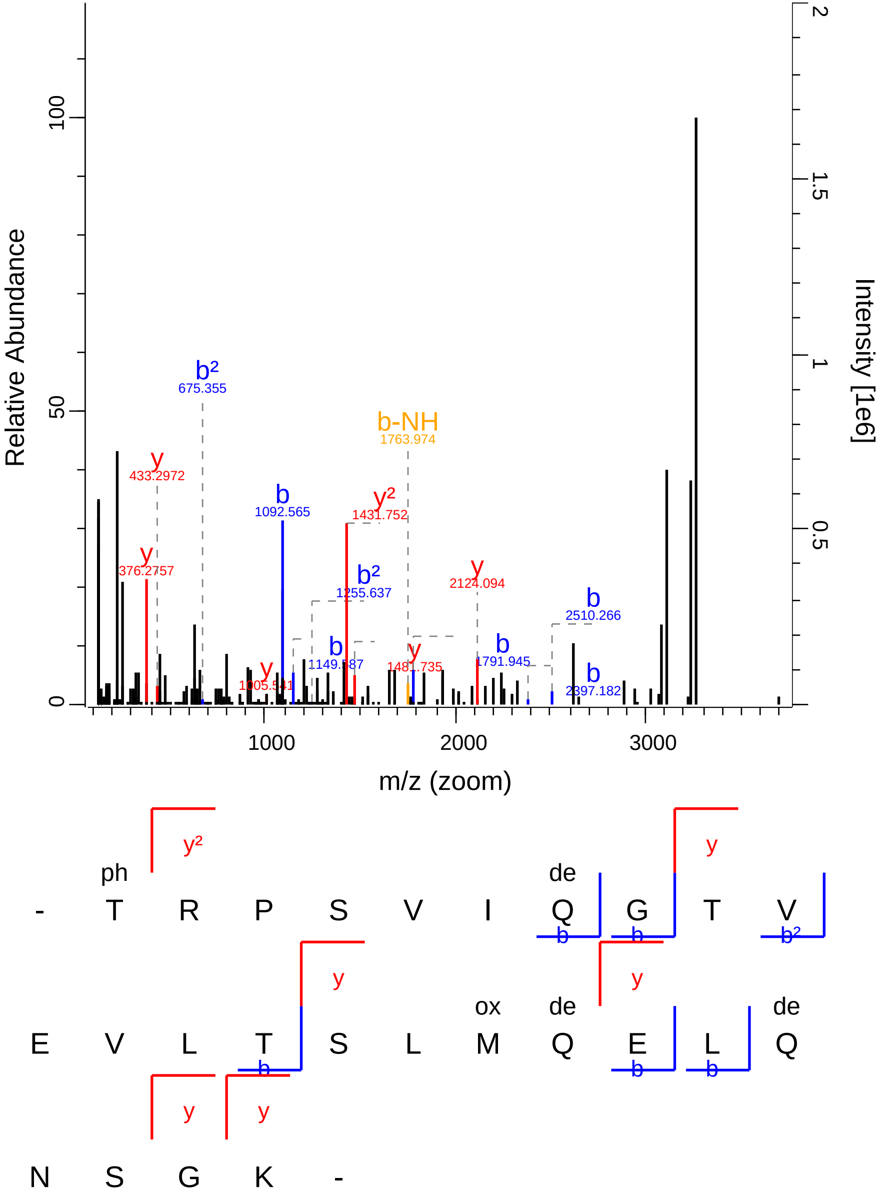

Raw File	Scan	Method	Score	m/z	Gene names
RO-QC-160121_ZackD_SS_Set_02_Fractic	37552	FTMS; HC	177.11	1037.53	SFT2D1

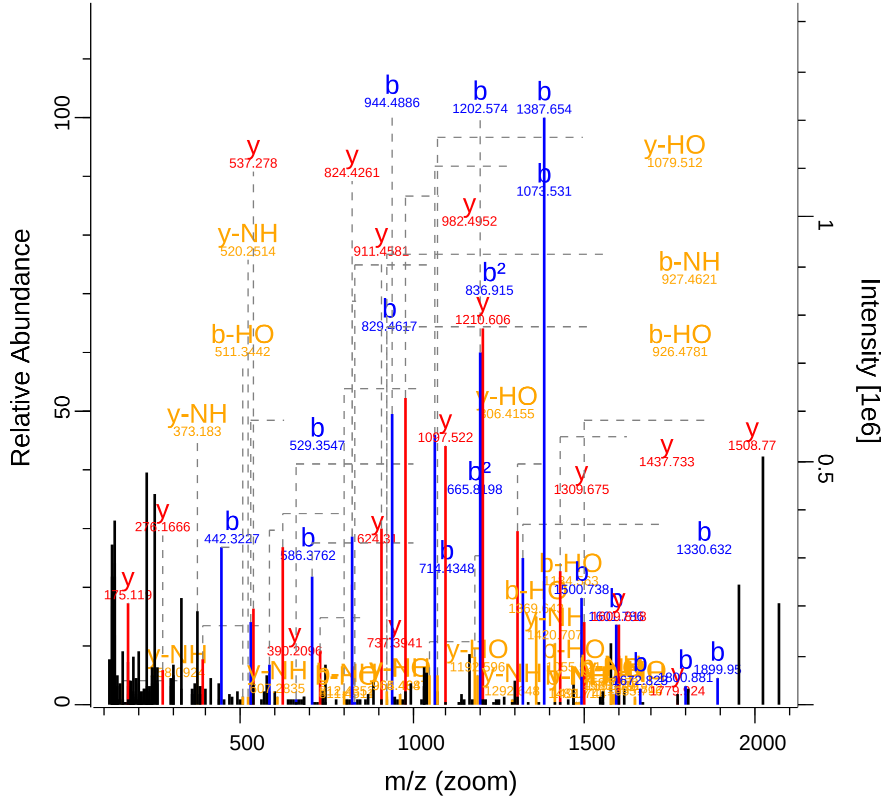

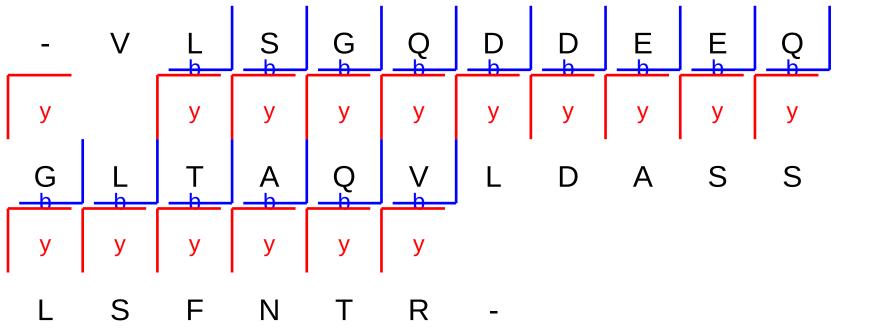

Raw File	Scan	Method	Score	m/z	Gene names
RO-QC-160121_ZackD_SS_Set_02_Fracti(	37883	FTMS; HC	156.51	1098.06	DAZAP2

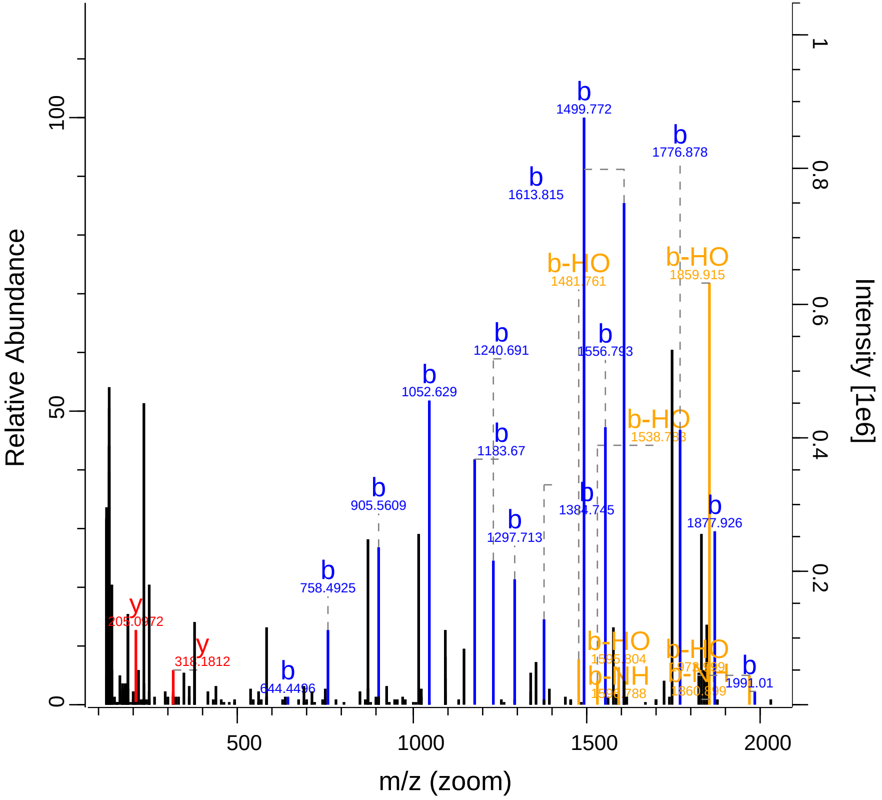

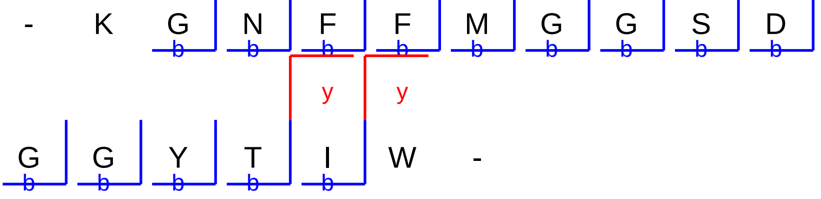

Raw File	Scan	Method	Score	m/z	Gene names
RO-QC-160121_ZackD_SS_Set_02_Fractio	37899	FTMS; HC	71.34	732.37	DAZAP2

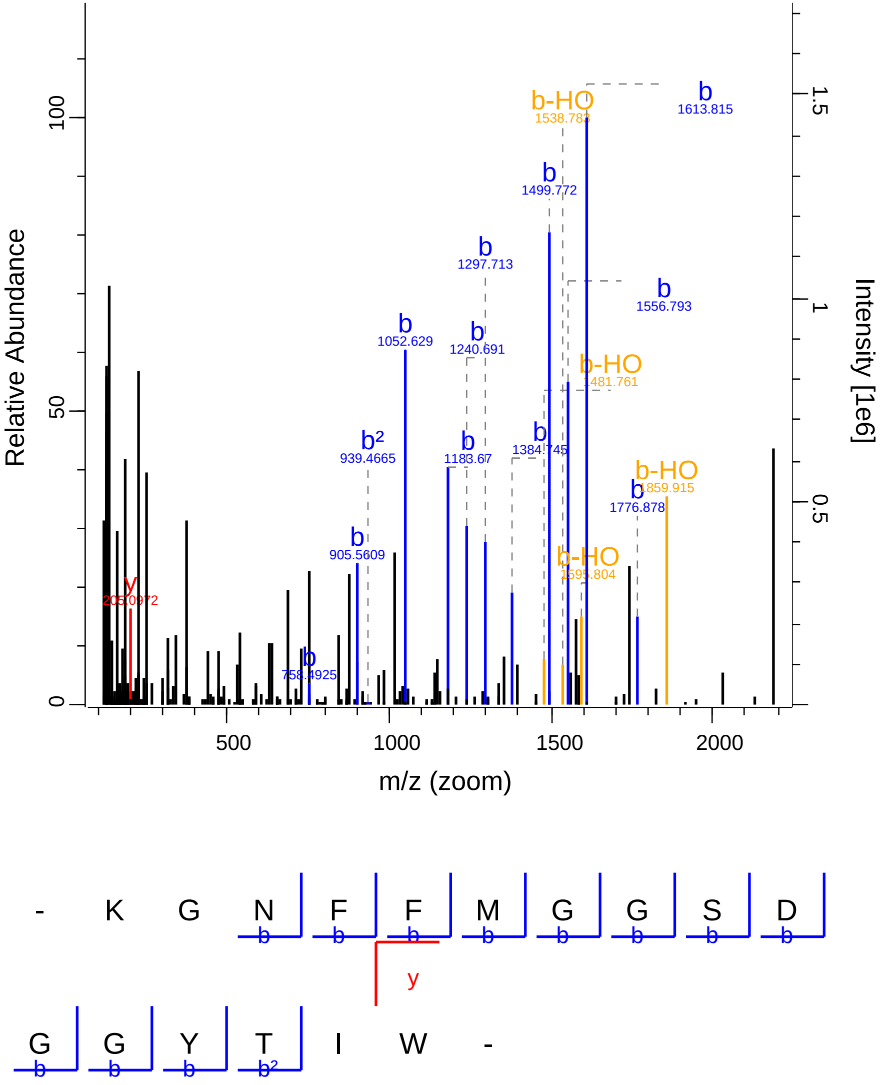

Raw File	Scan	Method	Score	m/z	Gene names
RO-QC-160121_ZackD_SS_Set_02_Fractio	39812	FTMS; HC	116.77	630.71	UQCR11

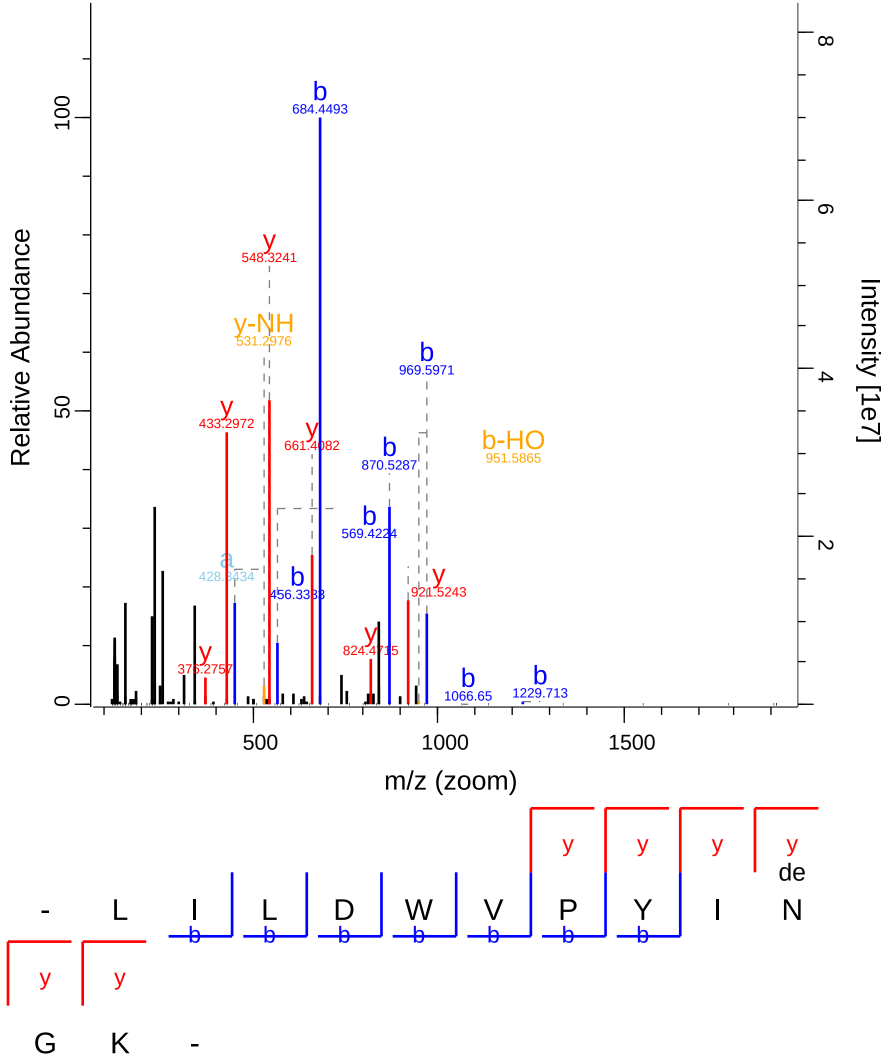

Raw File	Scan	Method	Score	m/z	Gene names
RO-QC-160121_ZackD_SS_Set_02_Fractio	39818	FTMS; HC	116.05	946.06	UQCR11

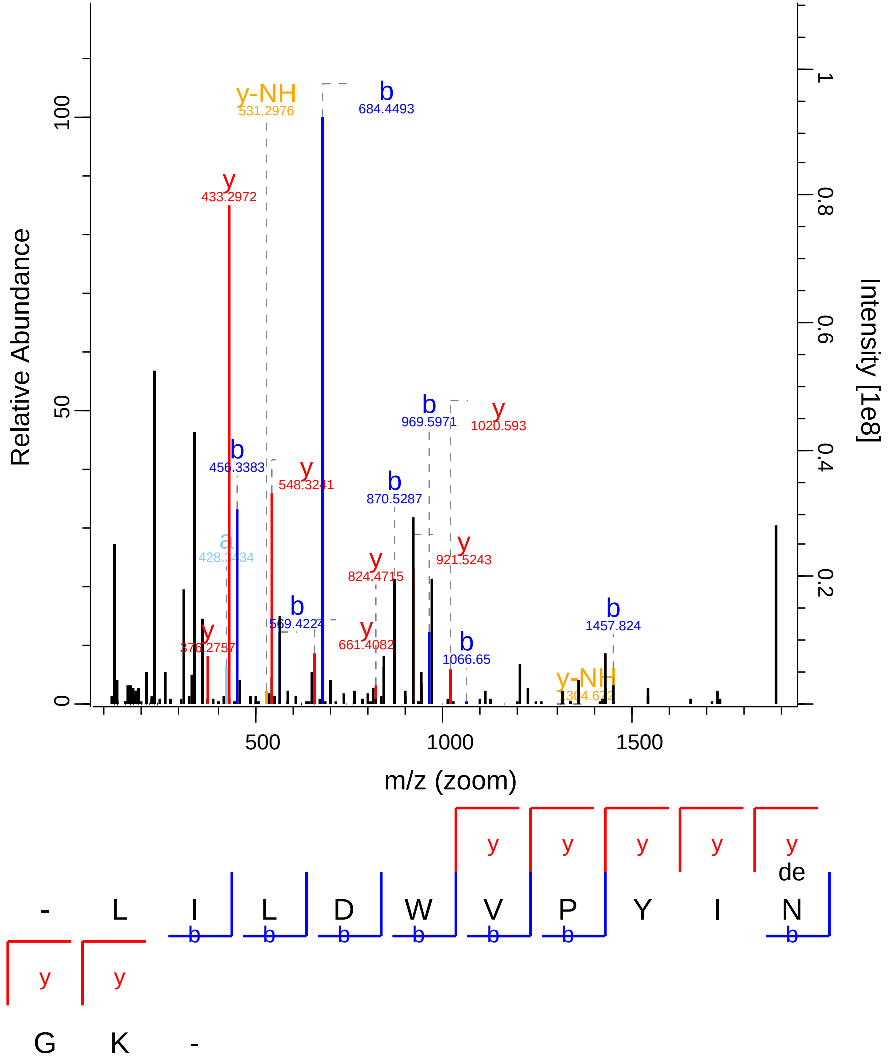

Raw File	Scan	Method	Score	m/z	Gene names
RO-QC-160121_ZackD_SS_Set_02_Fractio	40383	FTMS; HC	99.09	846.13	NINJ1

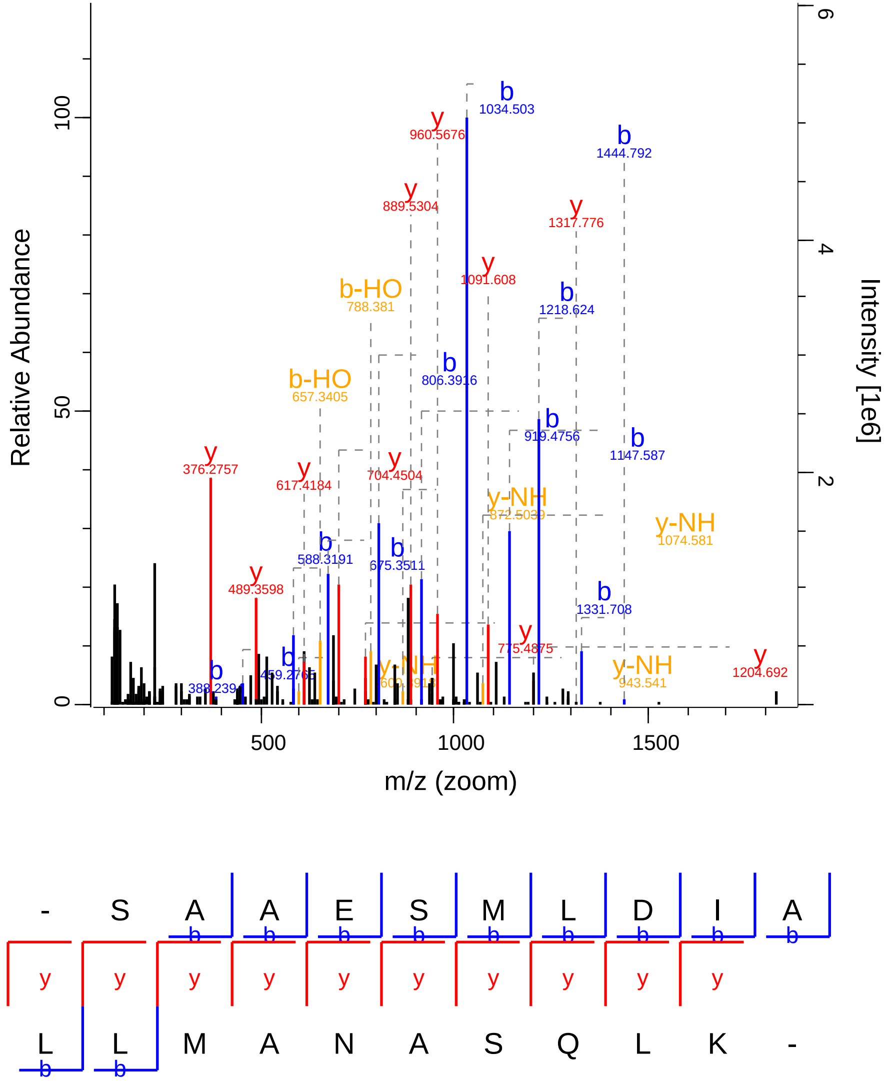

Raw File	Scan	Method	Score	m/z	Gene names
RO-QC-160121_ZackD_SS_Set_02_Fractio	40596	FTMS; HC	95.65	679.68	PSENEN

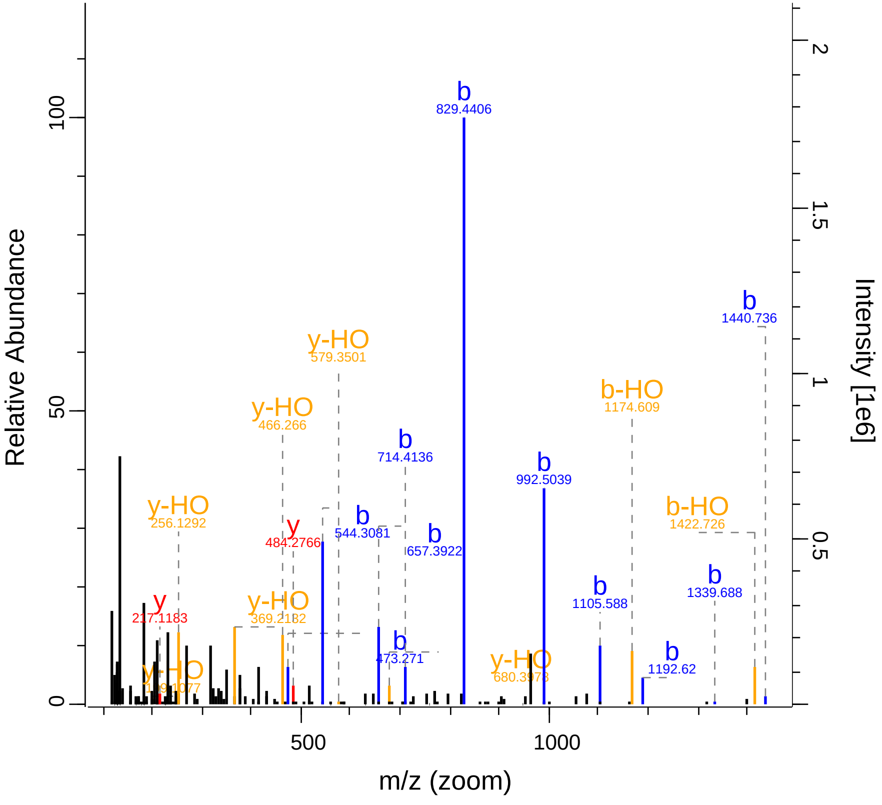

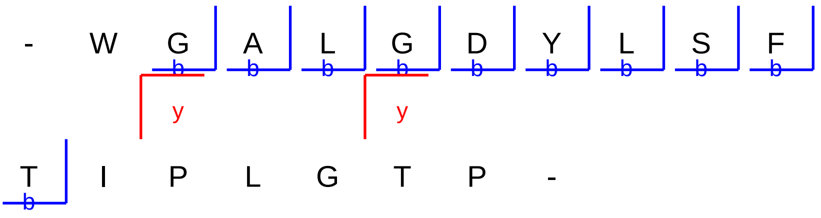

Raw File	Scan	Method	Score	m/z	Gene names
RO-QC-160121_ZackD_SS_Set_02_Fractior	40740	FTMS; HCl	108.24	643.9	STRA13

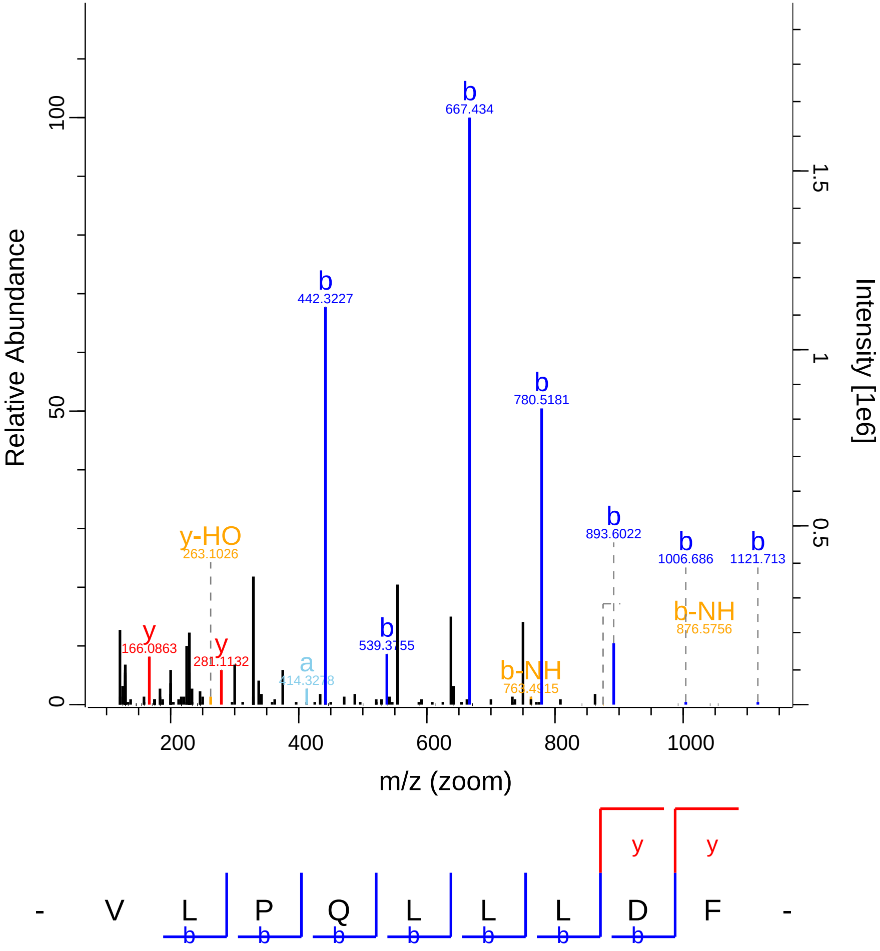

Raw File	Scan	Method	Score	m/z	Gene names
RO-QC-160121_ZackD_SS_Set_02_Fractio	5901	FTMS; HC	154.24	752.92	CDK10

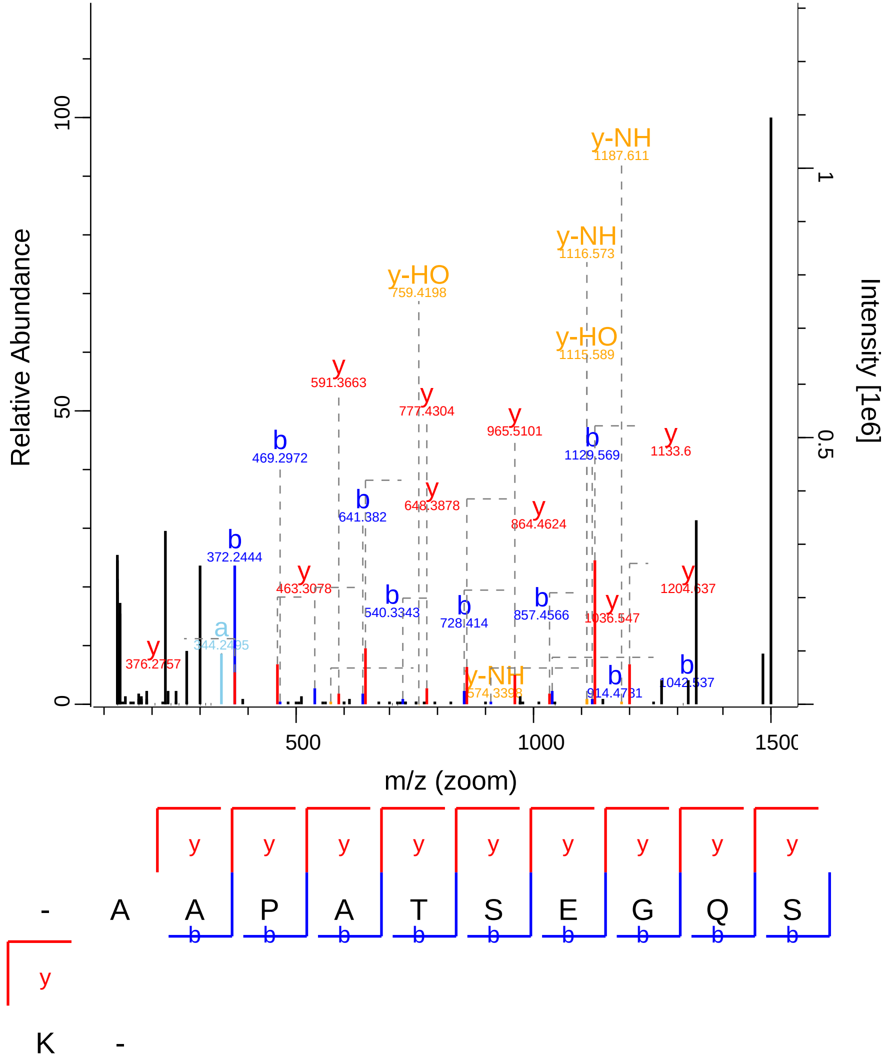

Raw File	Scan	Method	Score	m/z	Gene names
RO-QC-160121_ZackD_SS_Set_02_Fractior	5968	FTMS; HCI	35.33	502.28	CDK10

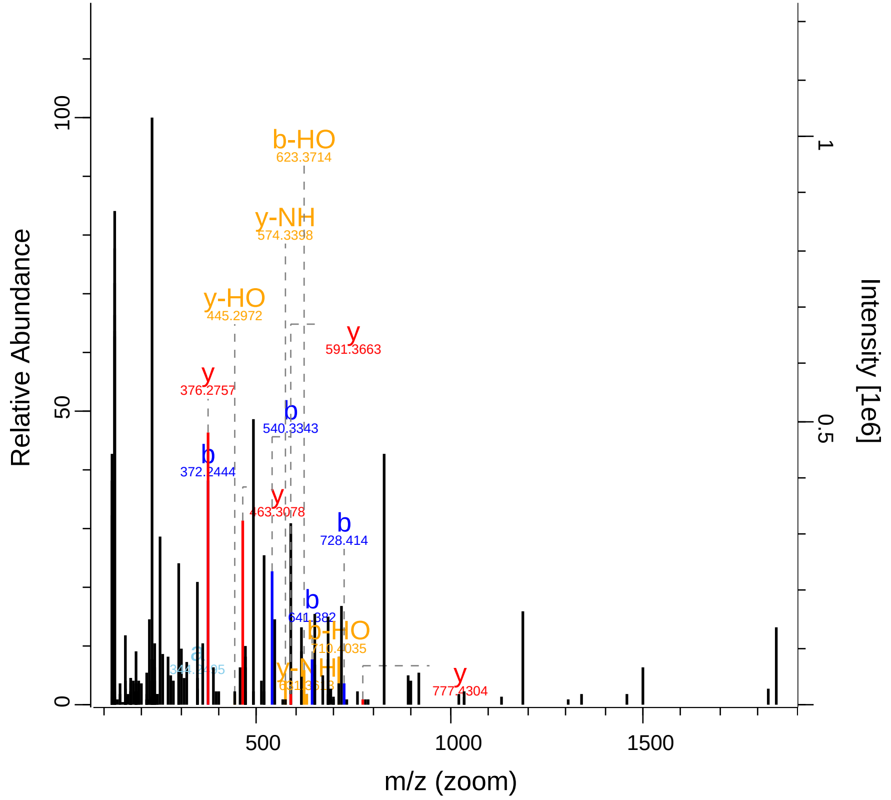

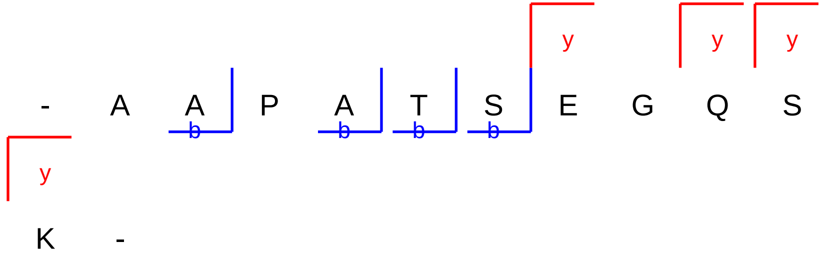

Raw File	Scan	Method	Score	m/z	Gene names
RO-QC-160121_ZackD_SS_Set_02_Fractio	6015	FTMS; HC	108.32	579.64	TATDN2

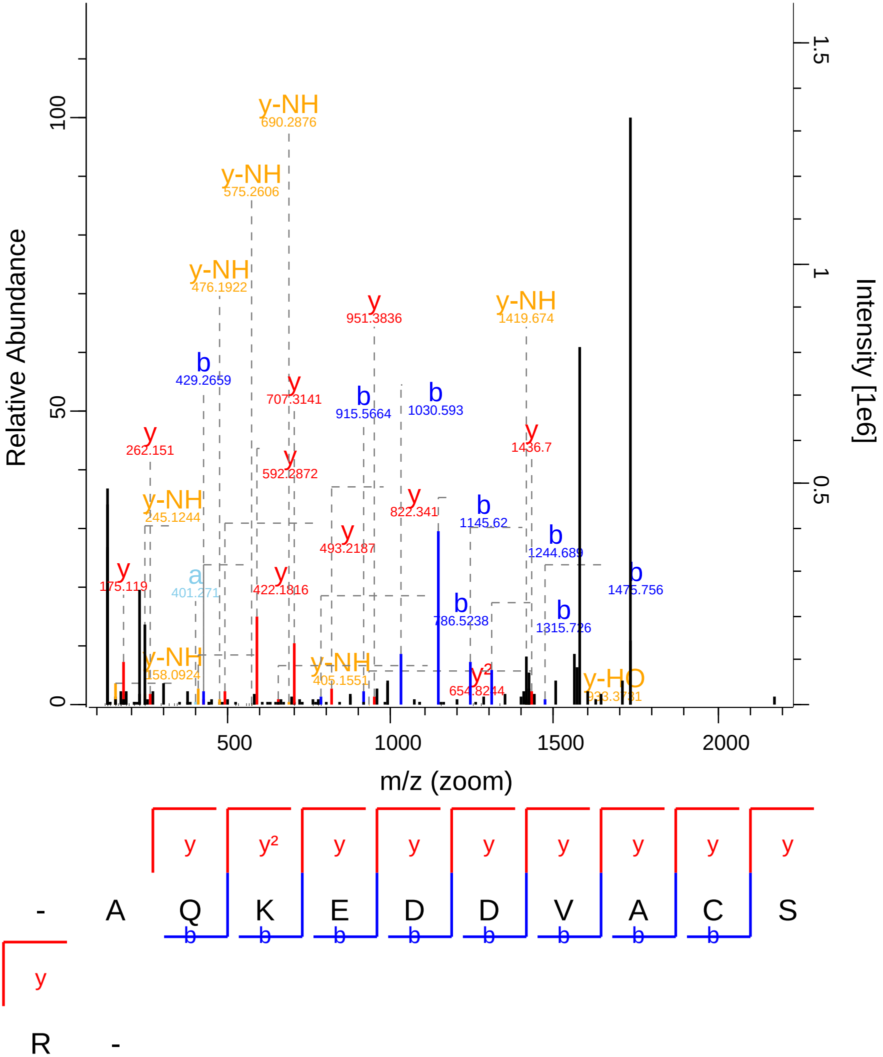

Raw File	Scan	Method	Score	m/z	Gene names
RO-QC-160121_ZackD_SS_Set_02_Fractior	6055	FTMS; HCI	53.17	645.86	ZNF706

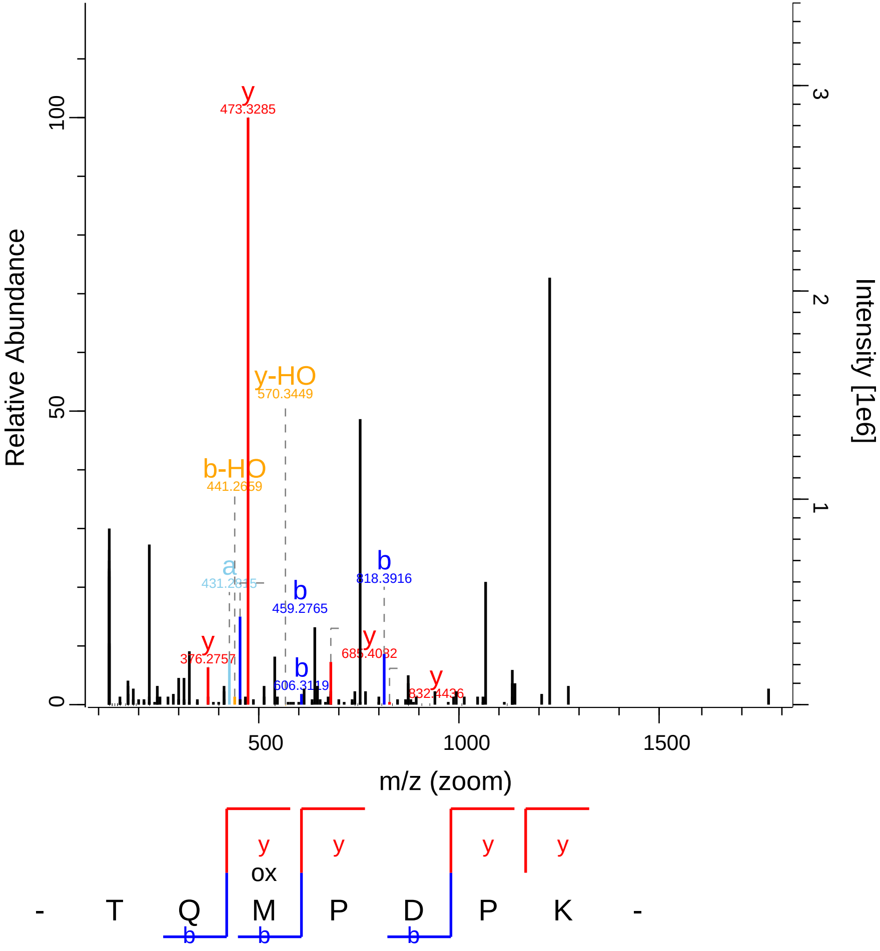

Raw File	Scan	Method	Score	m/z	Gene names
RO-QC-160121_ZackD_SS_Set_02_Fractior	6182	FTMS; HCI	88.95	664.88	IMUP

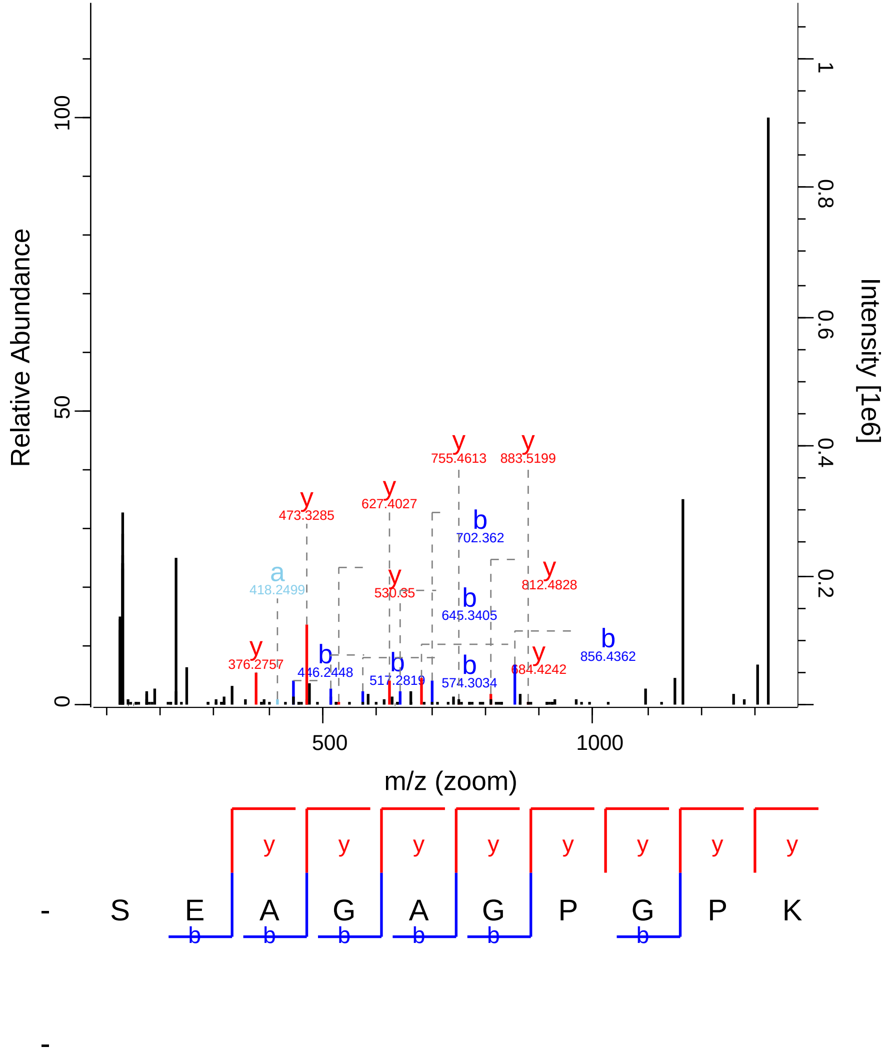

Raw File	Scan	Method	Score	m/z	Gene names
RO-QC-160121_ZackD_SS_Set_02_Fractior	6256	FTMS; HCI	78.69	861.42	SECISBP2

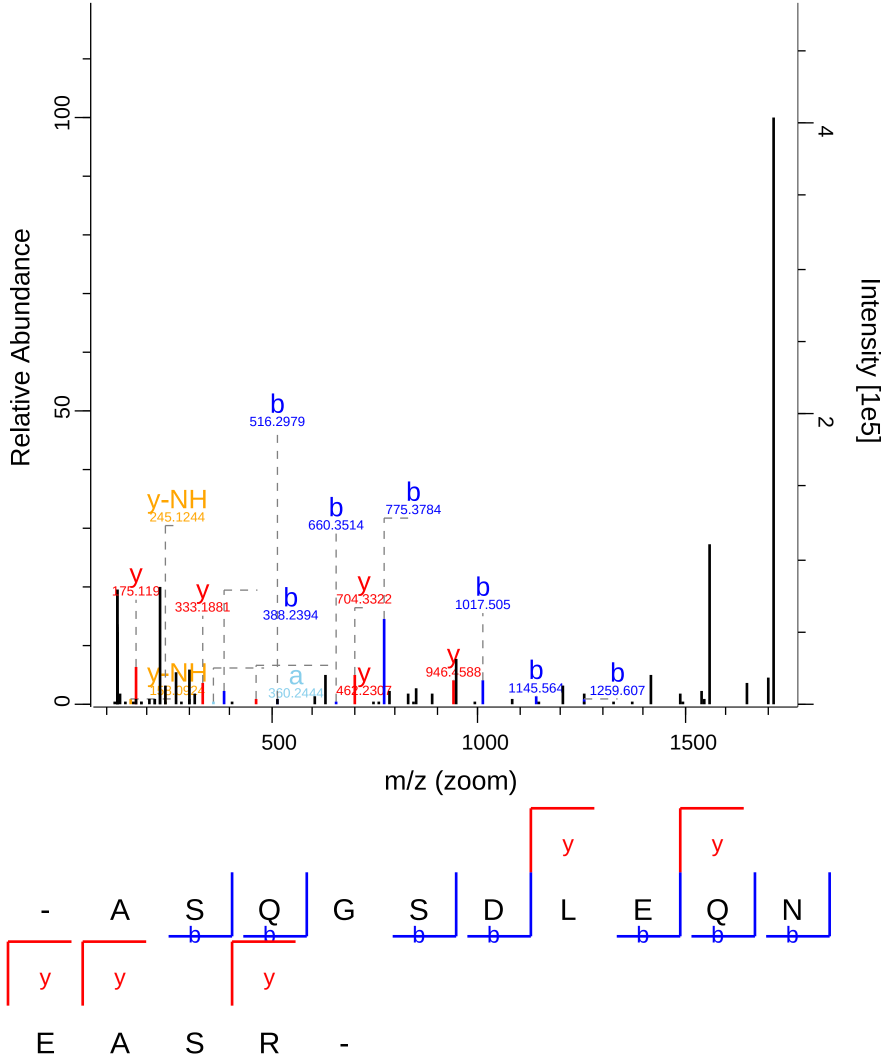

Raw File	Scan	Method	Score	m/z	Gene names
RO-QC-160121_ZackD_SS_Set_02_Fractior	6307	FTMS; HCI	45.26	622.32	PECAM1

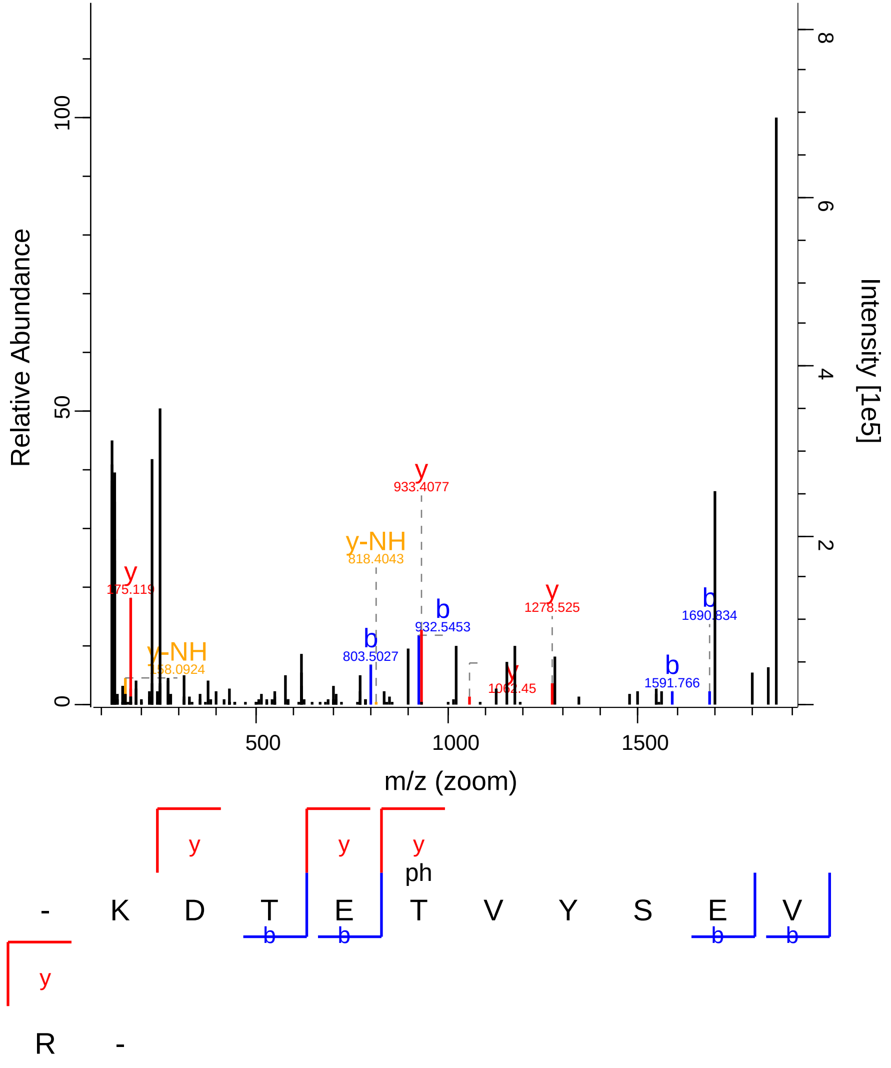

Raw File	Scan	Method	Score	m/z	Gene names
RO-QC-160121_ZackD_SS_Set_02_Fractior	6407	FTMS; HCl	52.34	598.34	USP31

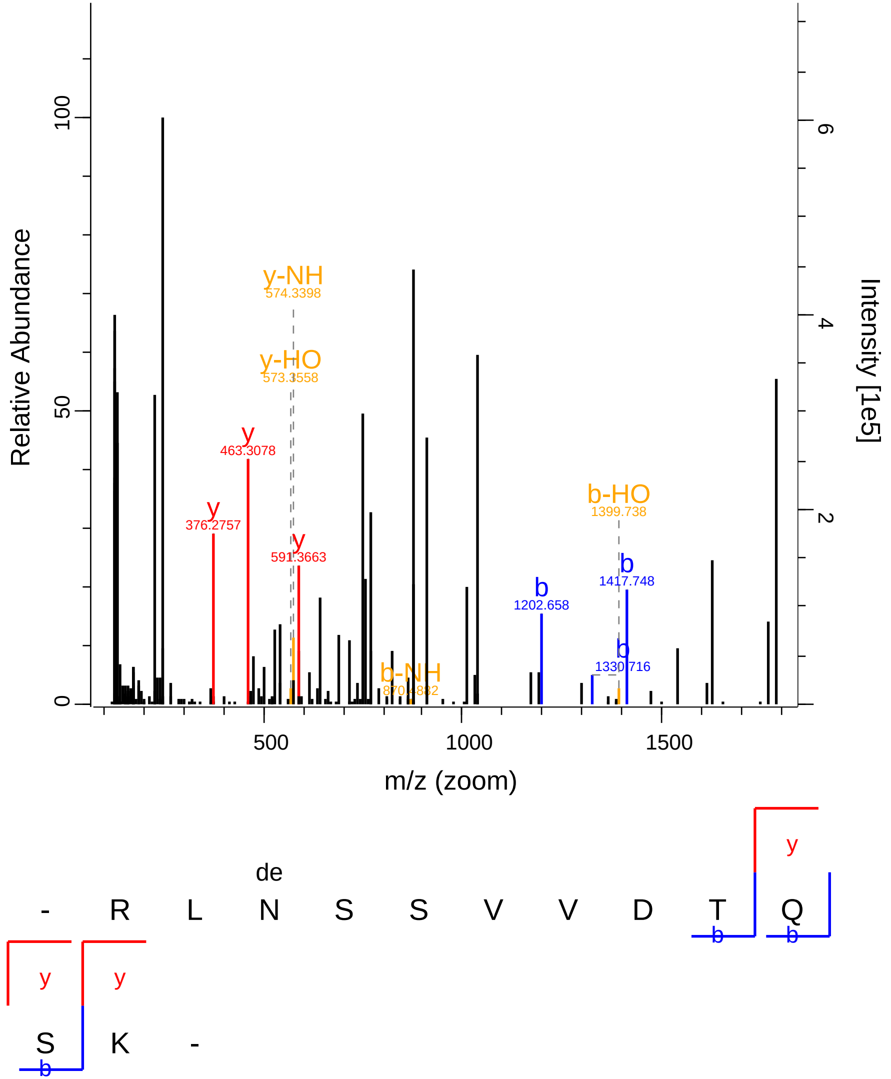

Raw File	Scan	Method	Score	m/z	Gene names
RO-QC-160121_ZackD_SS_Set_02_Fractio[illegible]	6684	FTMS; HC	117.98	549.28	MACROD2

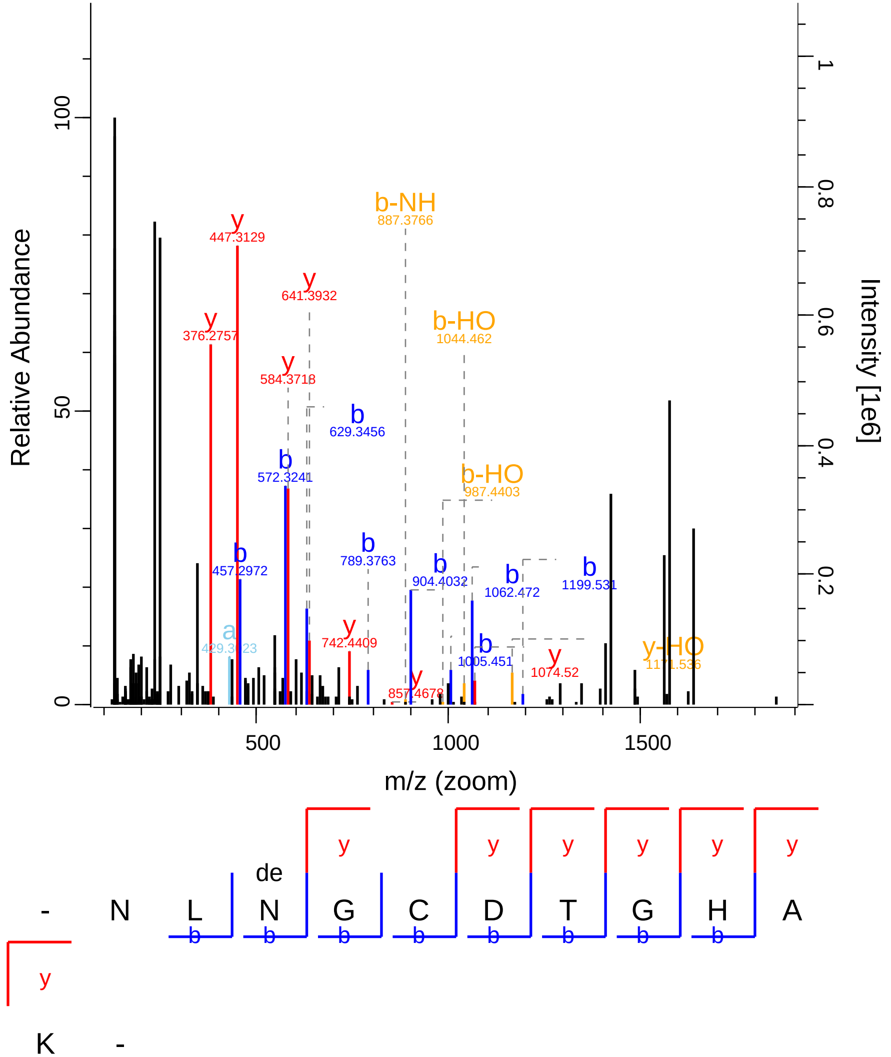

Raw File	Scan	Method	Score	m/z	Gene names
RO-QC-160121_ZackD_SS_Set_02_Fractior	7021	FTMS; HC	128.61	721.86	TMEFF1

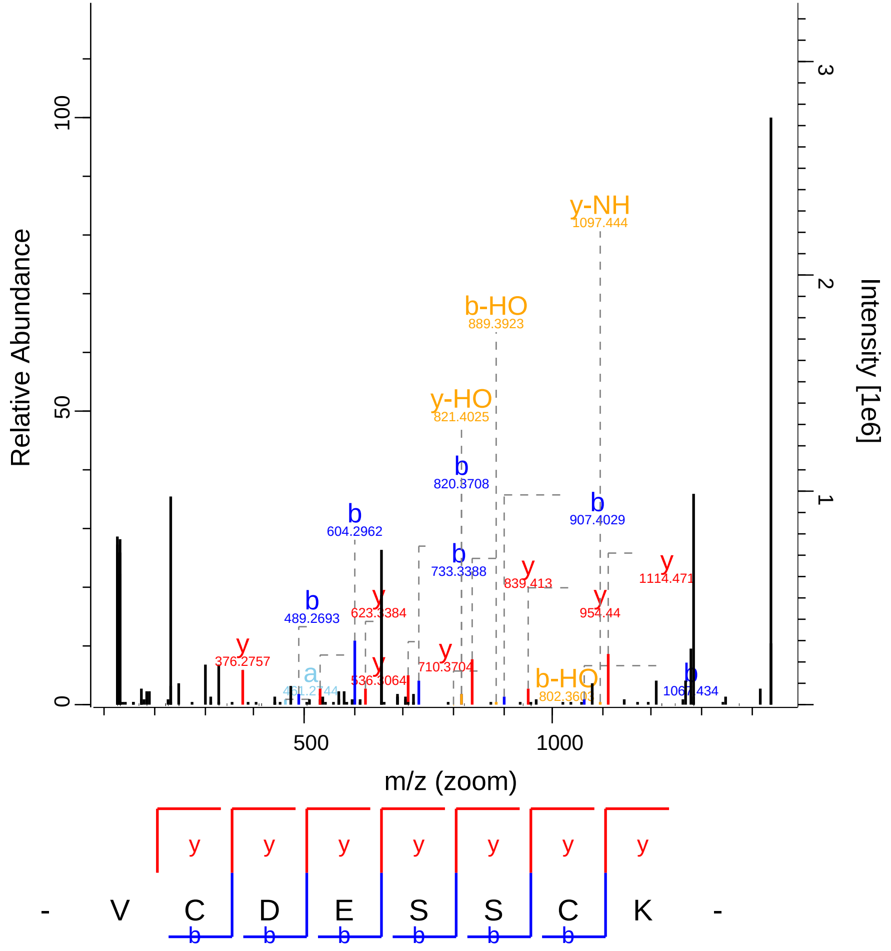

Raw File	Scan	Method	Score	m/z
RO-QC-160121_ZackD_SS_Set_02_Fraction_8	7221	FTMS; HCD	58.51	400.73

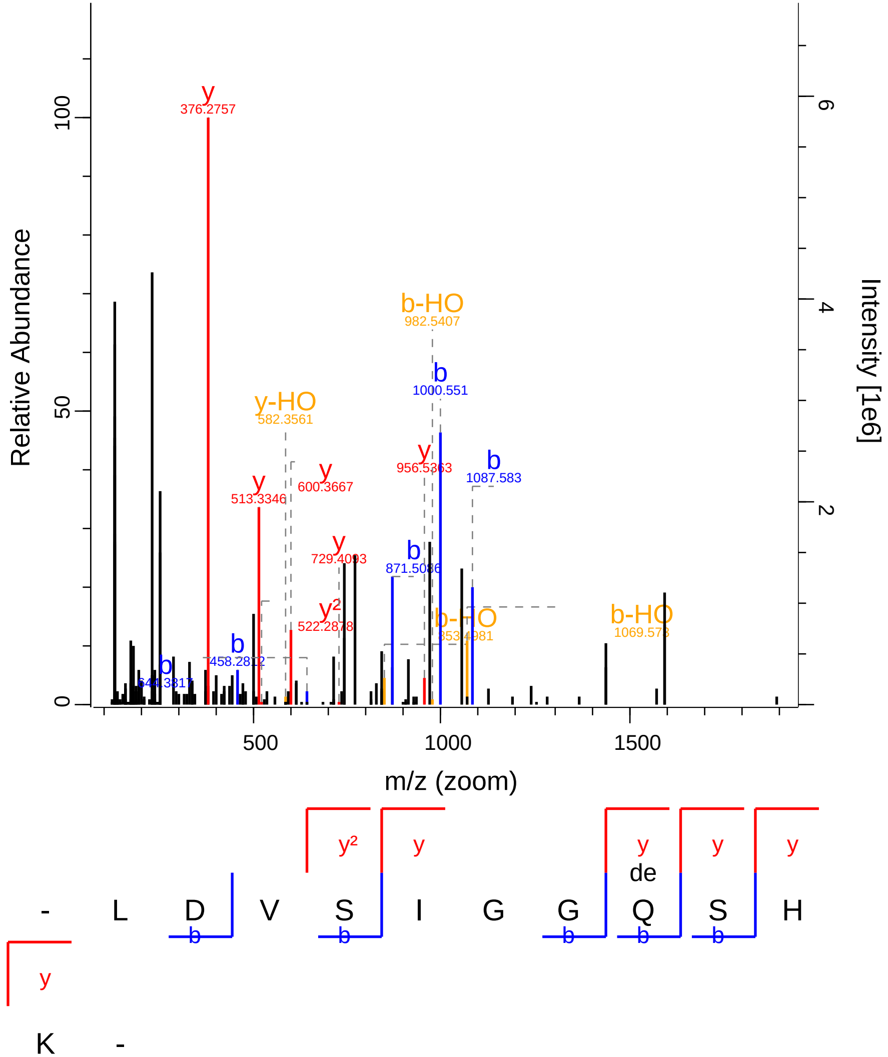

Raw File	Scan	Method	Score	m/z	Gene names
RO-QC-160121_ZackD_SS_Set_02_Fractior	7517	FTMS; HCI	42.74	562.68	GLOD5

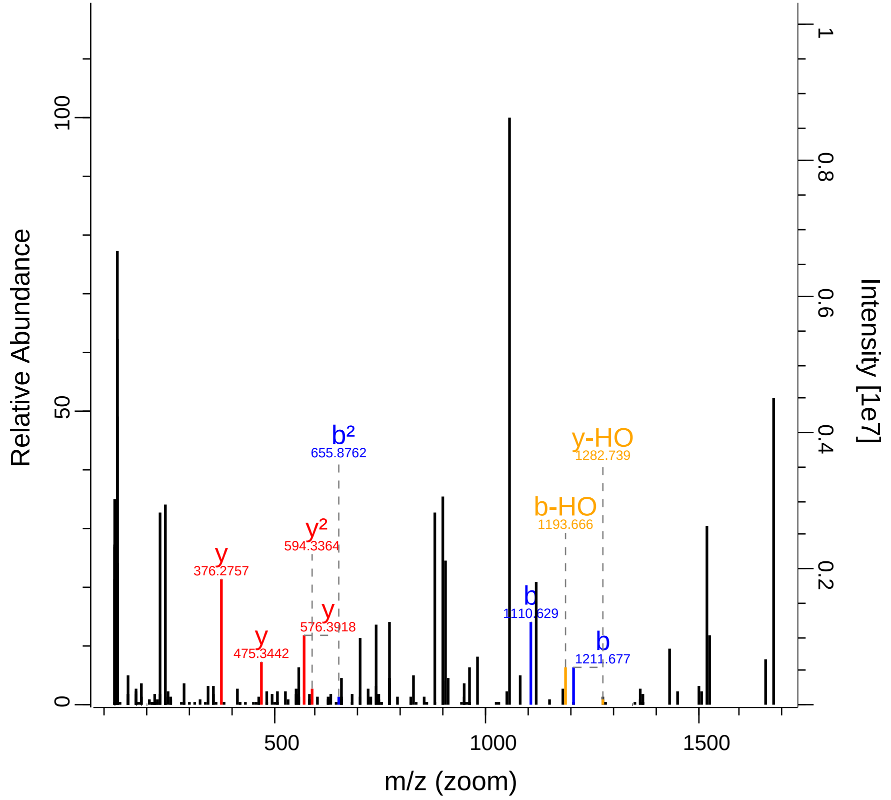

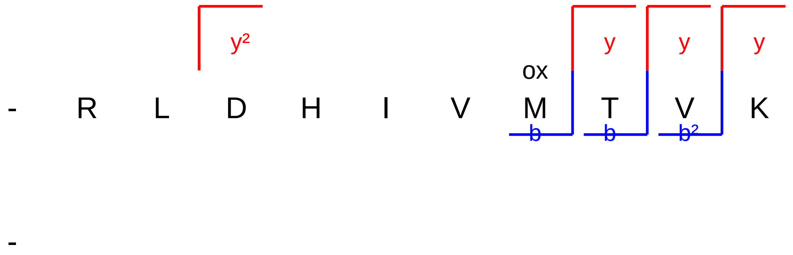

Raw File	Scan	Method	Score	m/z	Gene names
RO-QC-160121_ZackD_SS_Set_02_Fractior	7552	FTMS; HCI	65.06	843.51	GLOD5

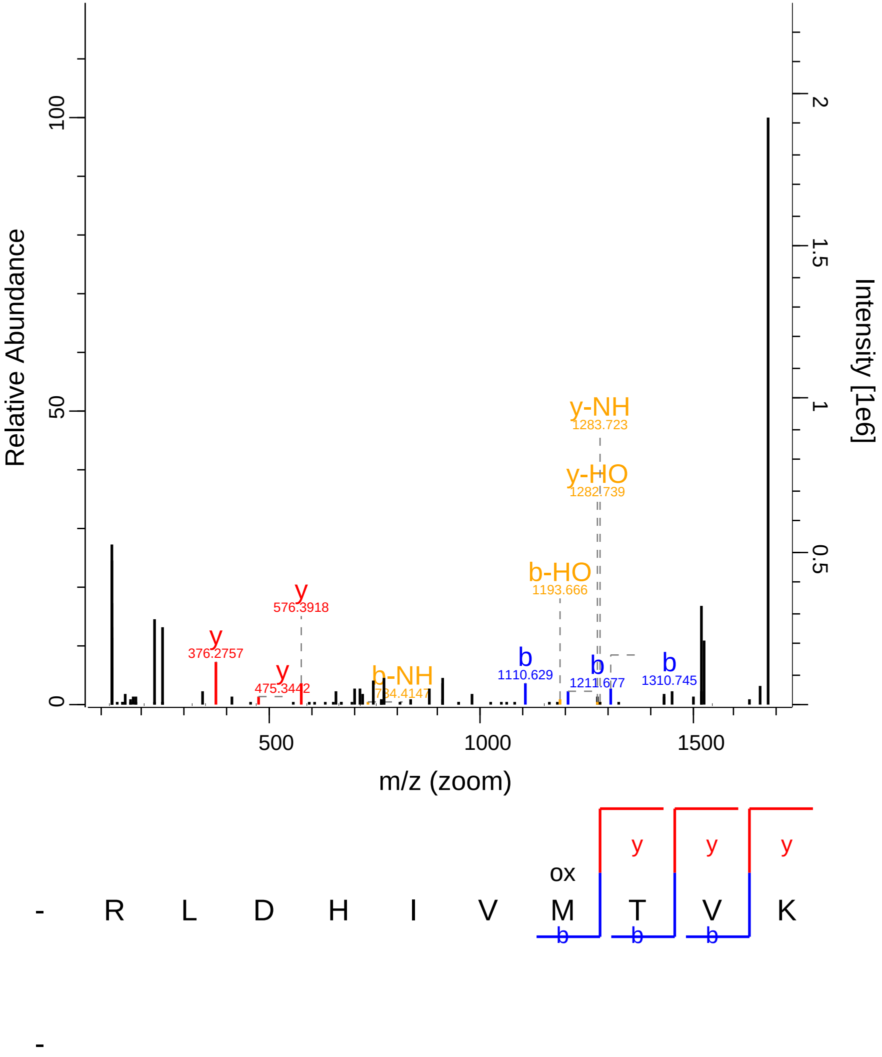

Raw File	Scan	Method	Score	m/z	Gene names
RO-QC-160121_ZackD_SS_Set_02_Fractior	9088	FTMS; HCI	93.55	440.77	ZNF66

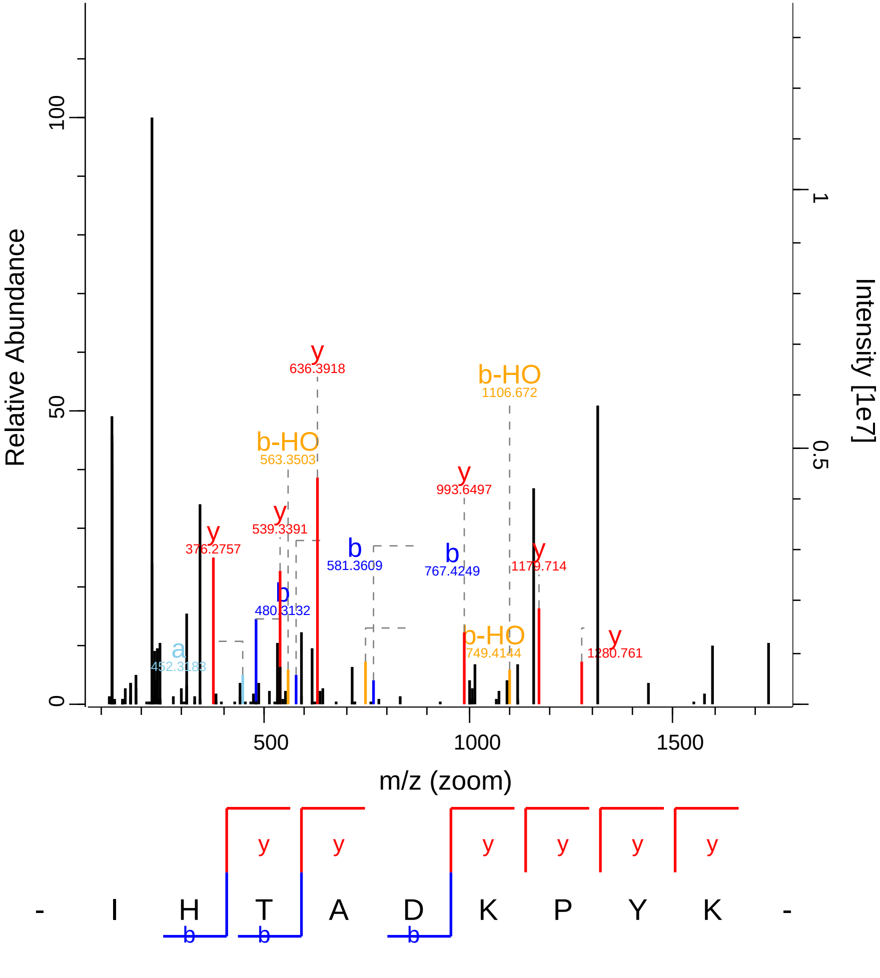

Raw File	Scan Method	Score	m/z	Gene names
RO-QC-160121_ZackD_SS_Set_02_Fra 9205	b FTMS; HC	124.59	596.35	ATP5G1;ATP5G2;ATP

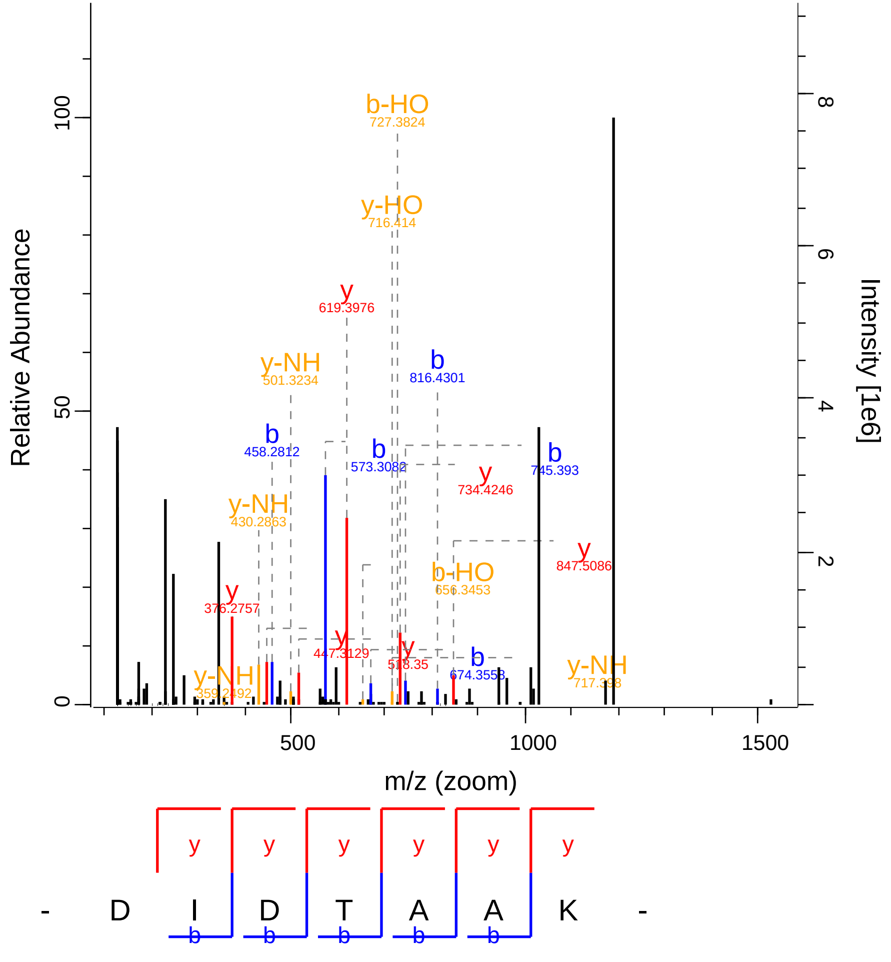

Raw File	Scan	Method	Score	m/z	Gene names
RO-QC-160121_ZackD_SS_Set_02_Fraction	9469	FTMS; HCI	158.04	779.9	SLC39A1

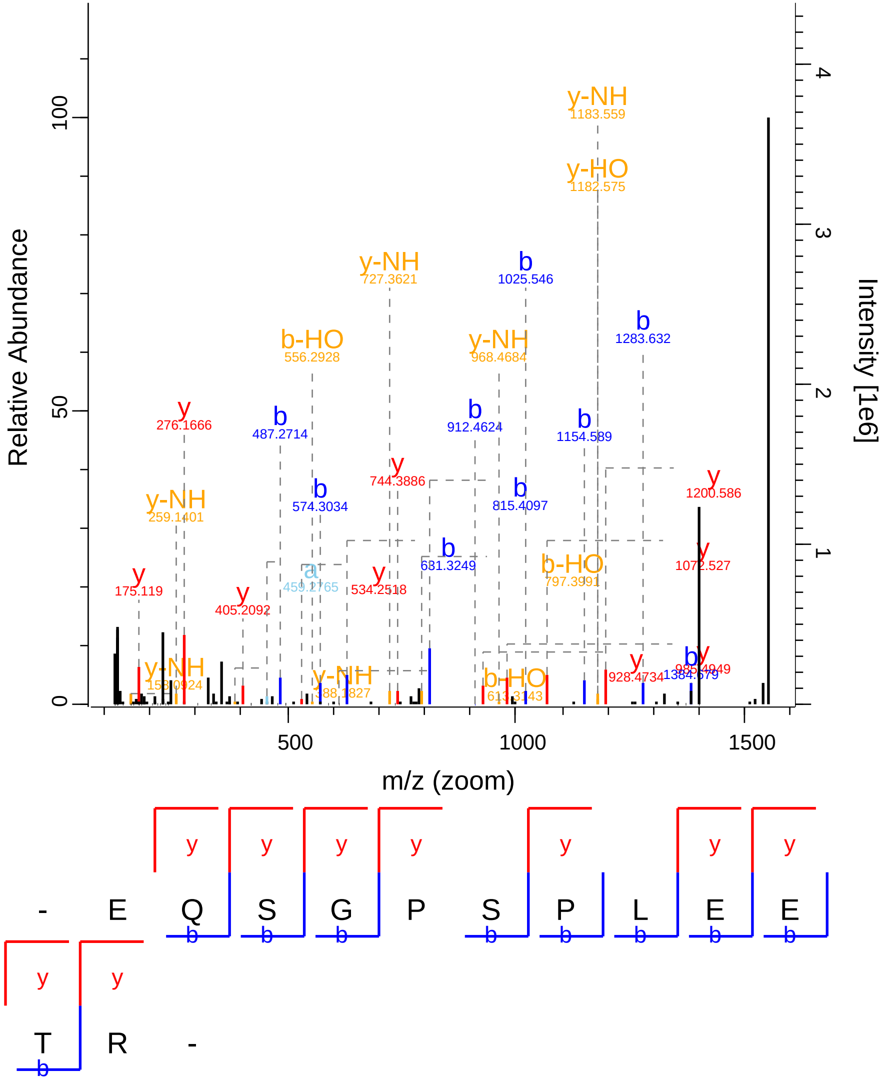

Raw File	Scan Method	Score	m/z	Gene names
RO-QC-160121_ZackD_SS_Set_02_Fra	9713 FTMS; HC	143	596.35	ATP5G1;ATP5G2;ATP

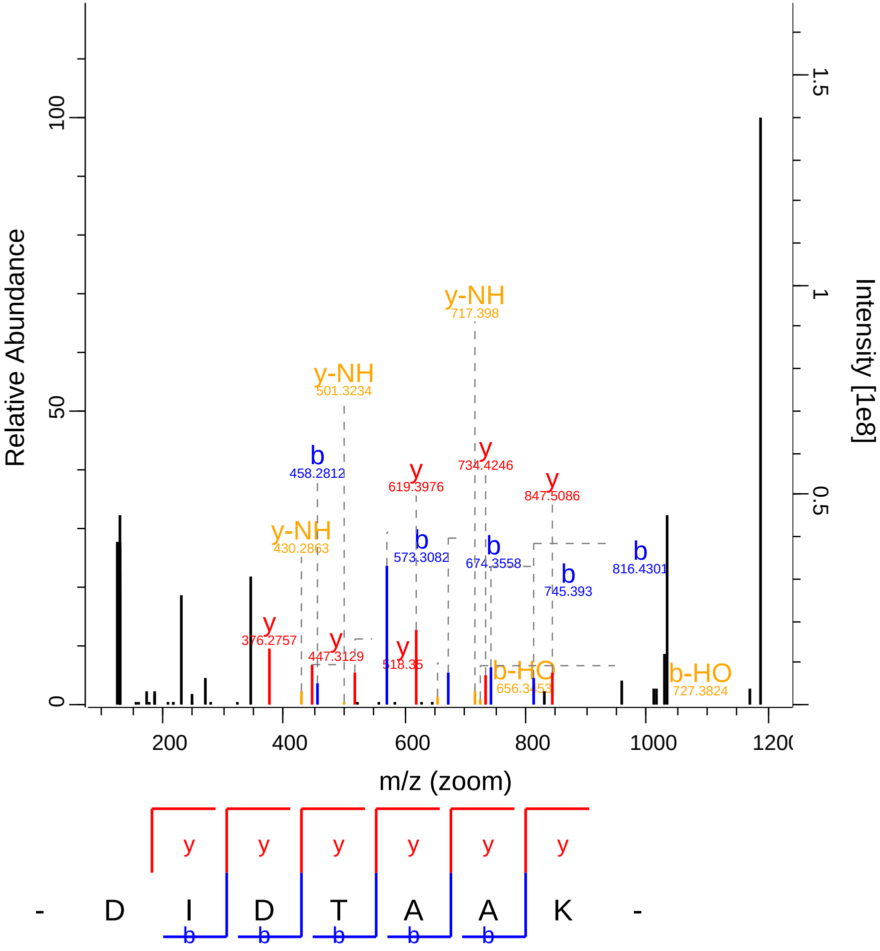

Raw File	Scan	Method	Score	m/z	Gene names
RO-QC-160121_ZackD_SS_Set_02_Fractior	9734	FTMS; HCI	67.99	663.38	RBM20

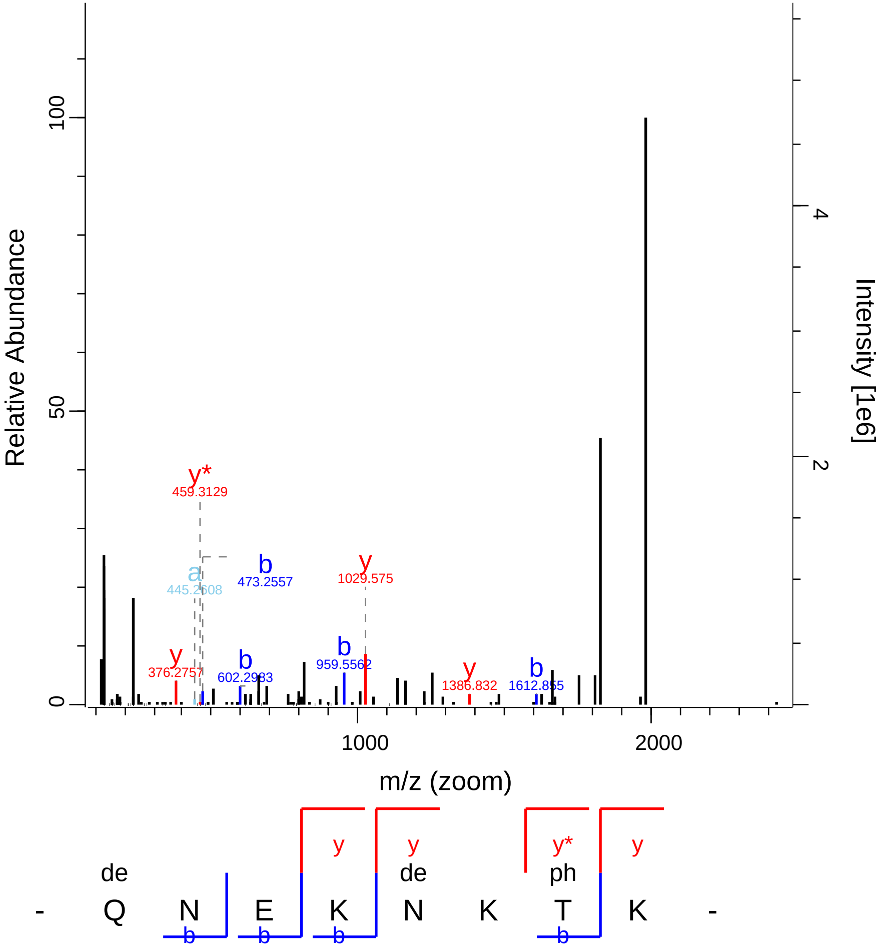

Raw File	Scan Method	Score m/z	Gene names
RO-QC-160121_ZackD_SS_Set_02_Fra 9943	FTMS; HC 143	596.35	ATP5G1;ATP5G2;ATP5

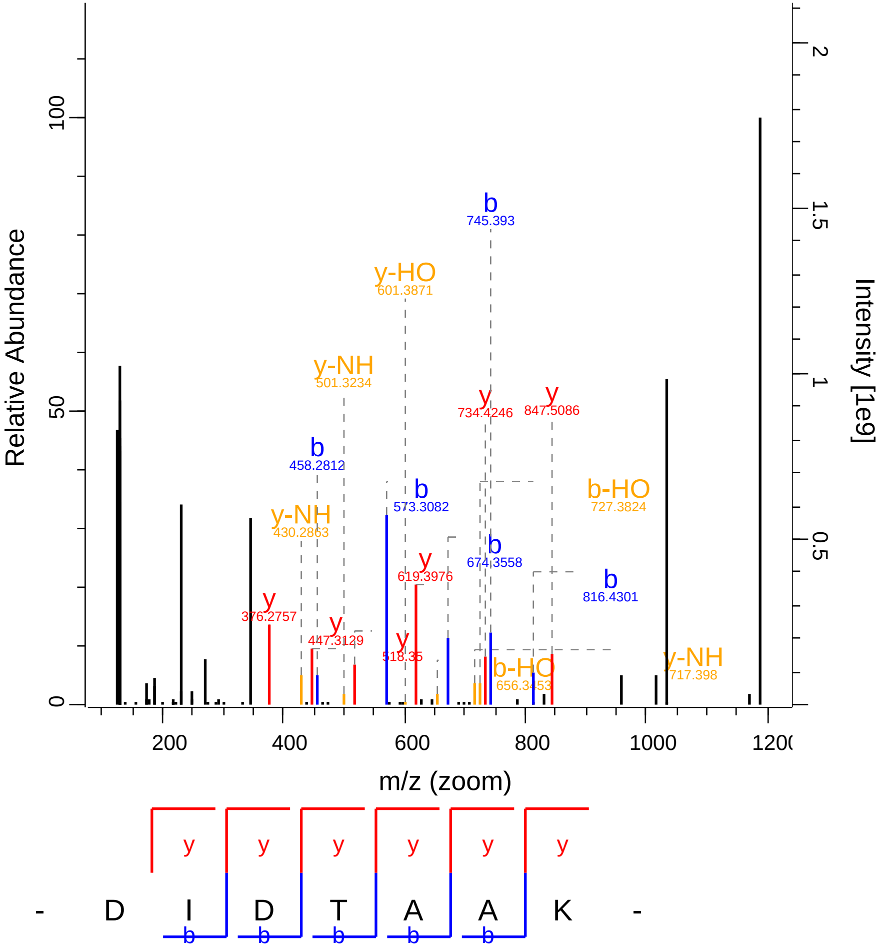

Raw File	Scan	Method	Score	m/z	Gene names
RO-QC-160121_ZackD_SS_Set_02_Fra	10041	FTMS; HC	97.47	397.9	ATP5G1;ATP5G2;ATP5

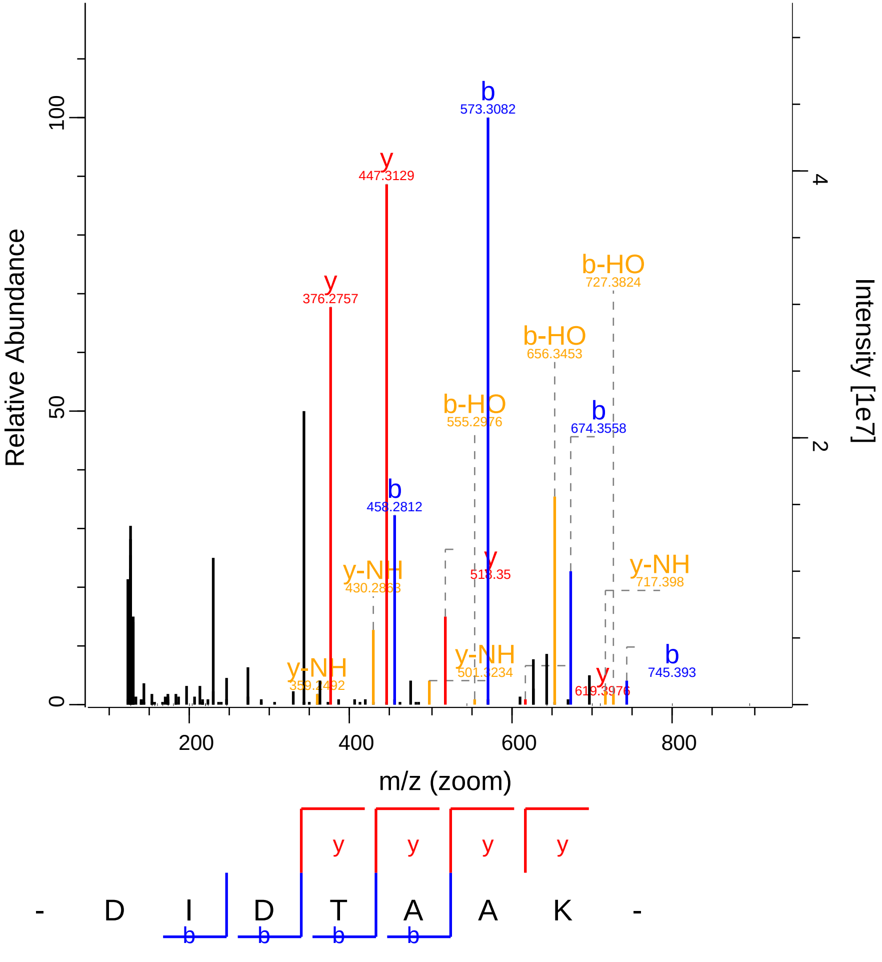

Raw File	Scan	Method	Score	m/z	Gene names
RO-QC-160121_ZackD_SS_Set_02_Fr	10187	FTMS; H(	104.21	596.35	ATP5G1;ATP5G2;ATP

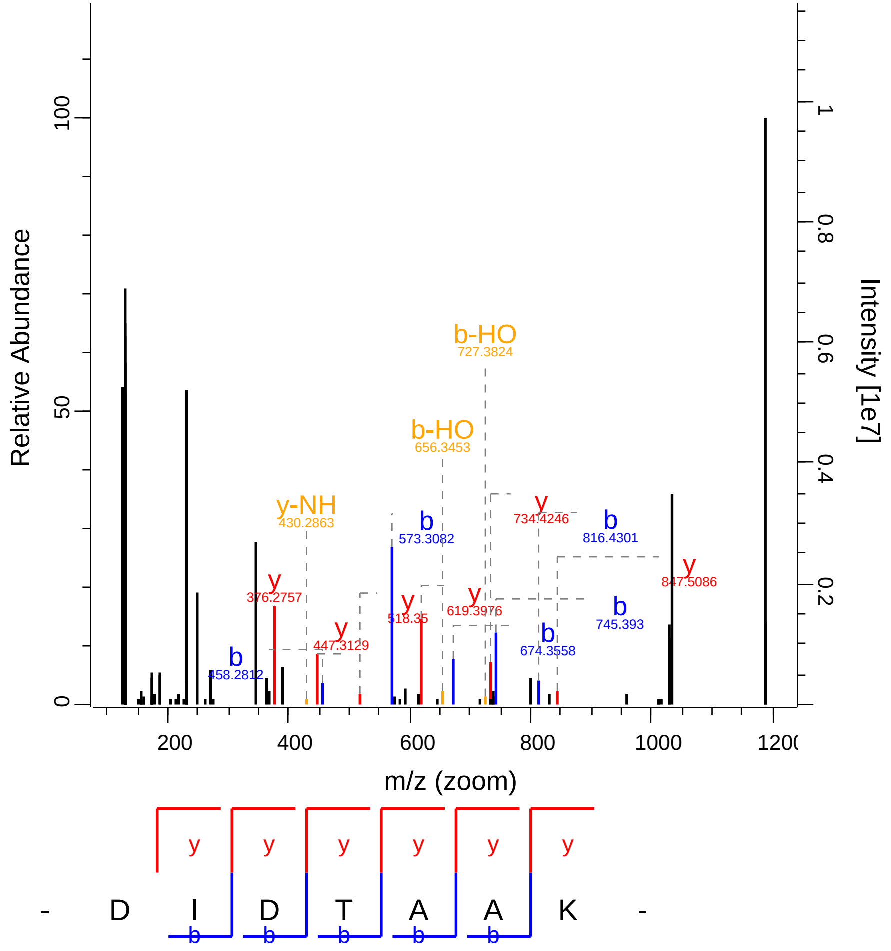

Raw File	Scan	Method	Score	m/z	Gene names
RO-QC-160121_ZackD_SS_Set_02_Fr	10572	FTMS; HC	114.78	596.35	ATP5G1;ATP5G2;ATP

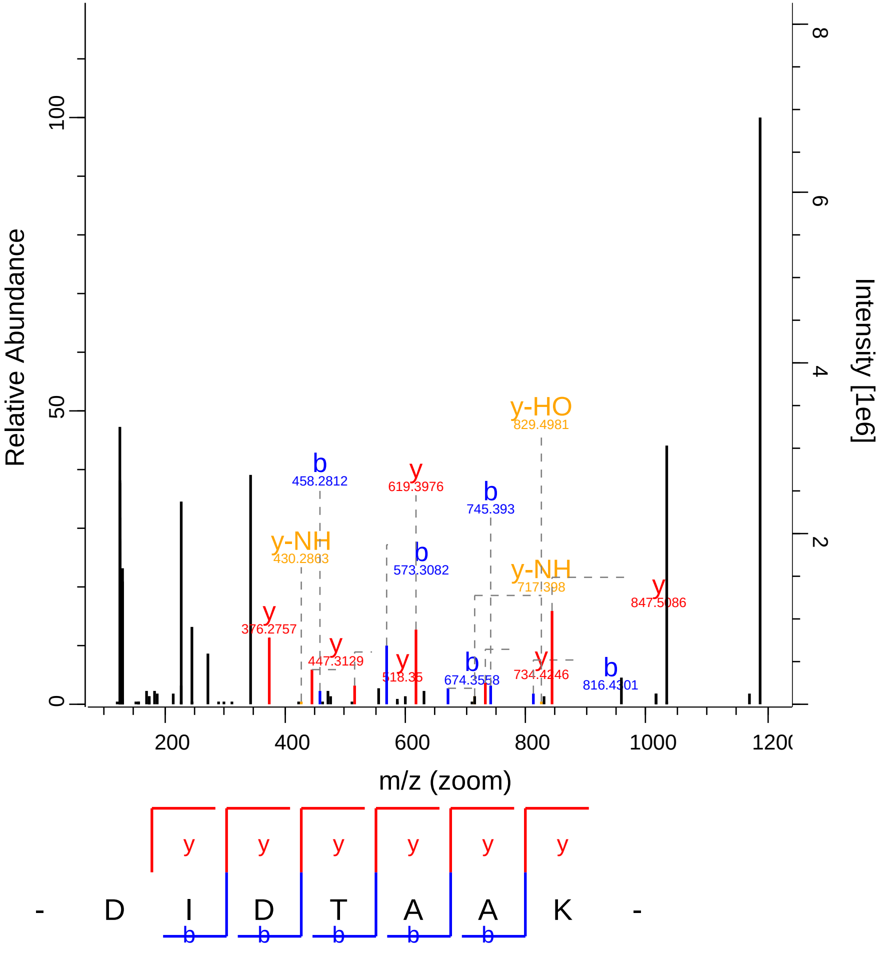

Raw File	Scan	Method	Score	m/z
RO-QC-160121_ZackD_SS_Set_02_Fraction_8	11518	FTMS; HCD	114.89	688.4

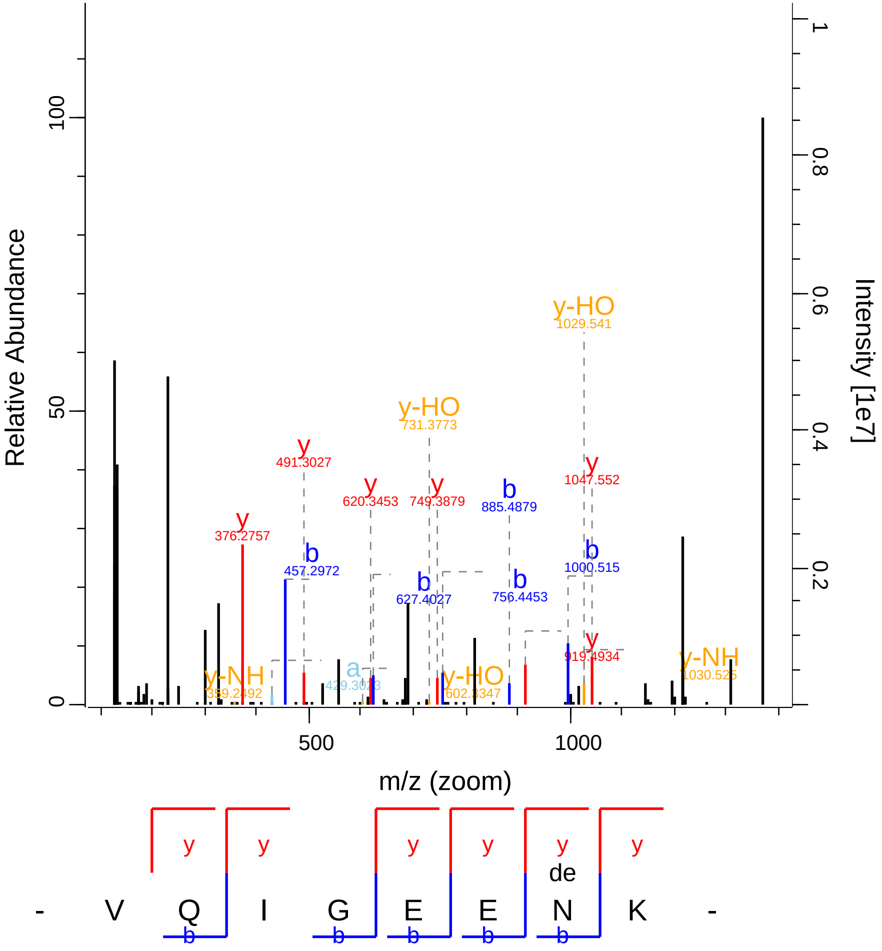

Raw File	Scan	Method	Score	m/z	Gene names
RO-QC-160121_ZackD_SS_Set_02_Fractio	12724	FTMS; HC	127.02	718.89	SERF2

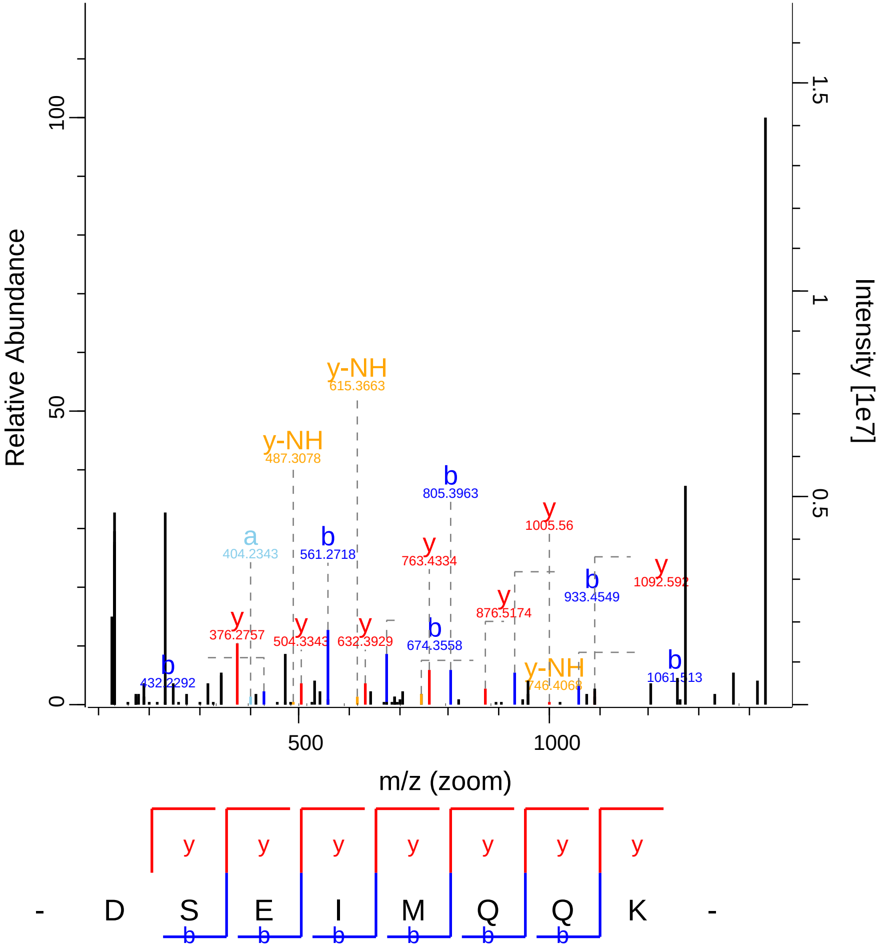

Raw File	Scan	Method	Score	m/z	Gene names
RO-QC-160121_ZackD_SS_Set_02_Fractio	13265	FTMS; HC	51.77	493.51	GABRE

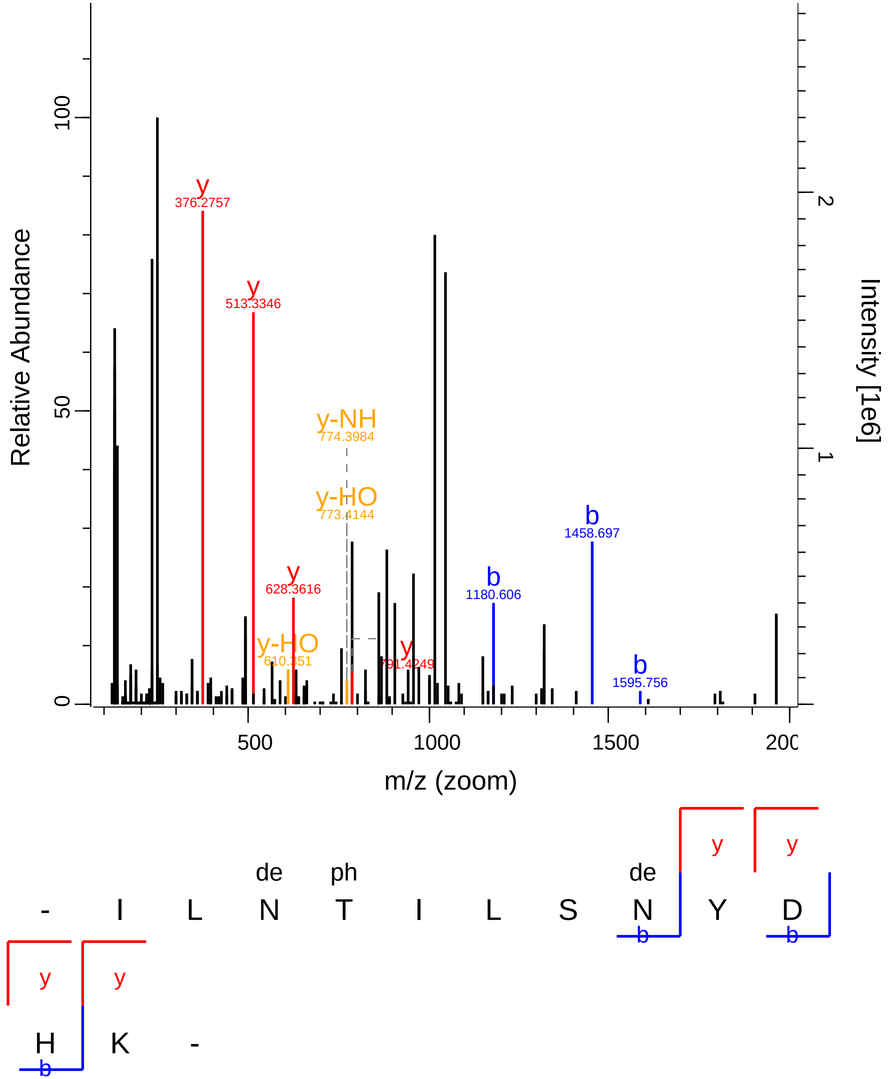

Raw File	Scan	Method	Score	m/z	Gene names
RO-QC-160121_ZackD_SS_Set_02_Fractio	13373	FTMS; HC	130.6	801.43	ALKBH2

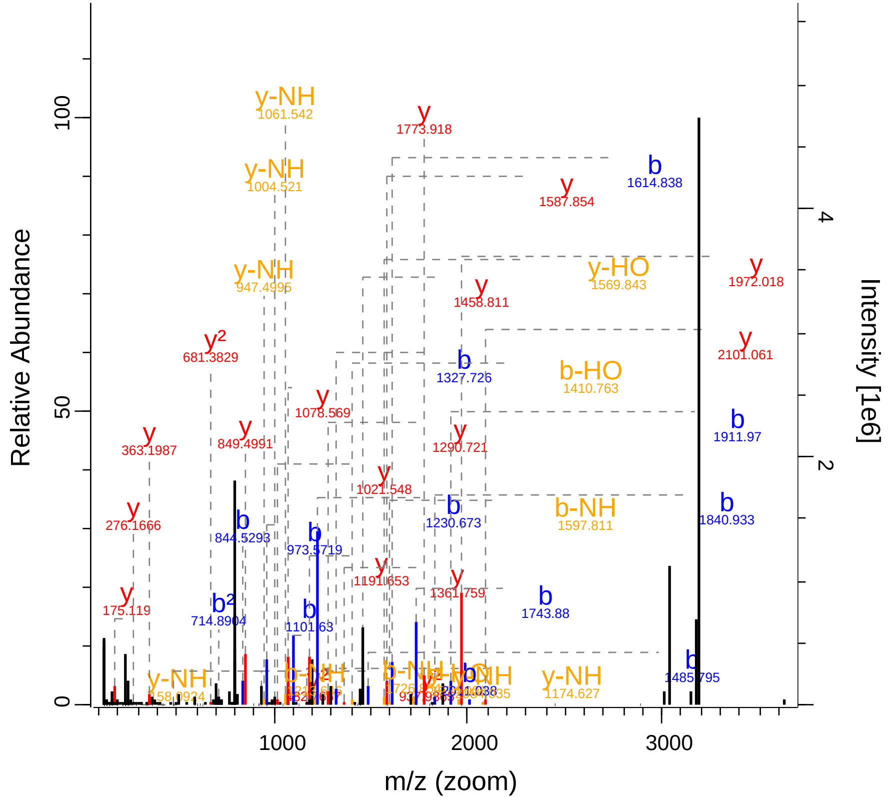

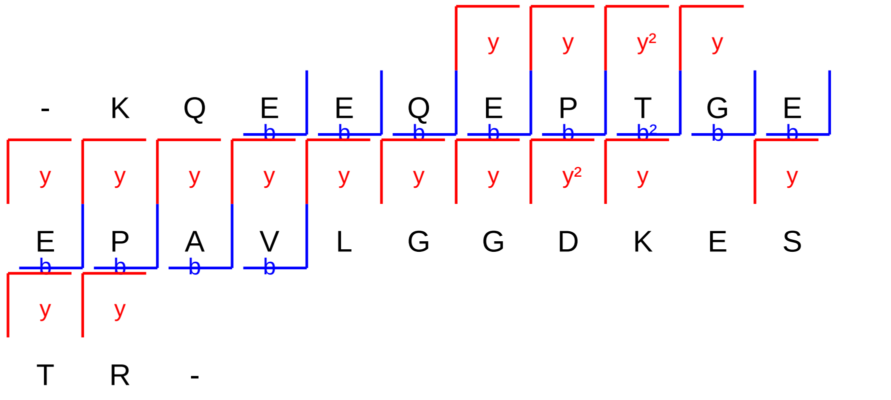

Raw File	Scan	Method	Score	m/z	Gene names
RO-QC-160121_ZackD_SS_Set_02_Fractio	13391	FTMS; HC	83.78	767.93	ZNF251

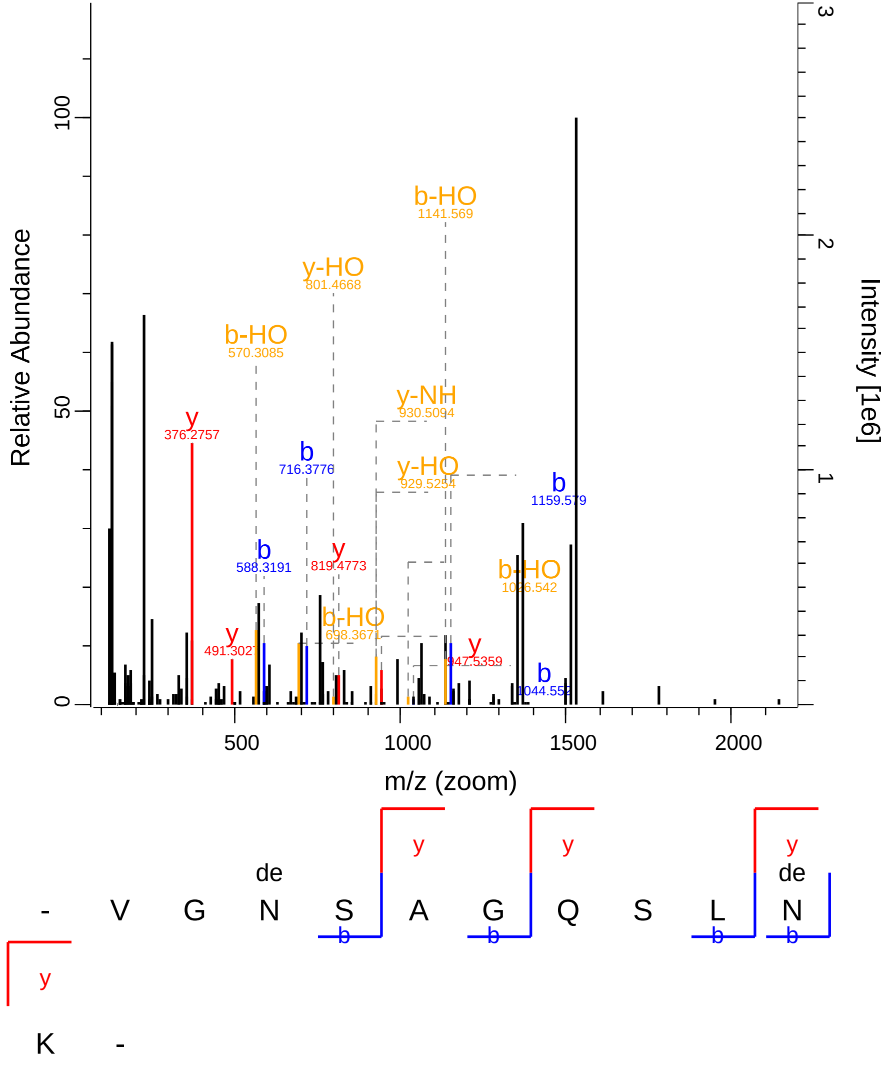

Raw File	Scan	Method	Score	m/z	Gene names
RO-QC-160121_ZackD_SS_Set_02_Fractio	14136	FTMS; HC	47.26	976.58	NEGR1

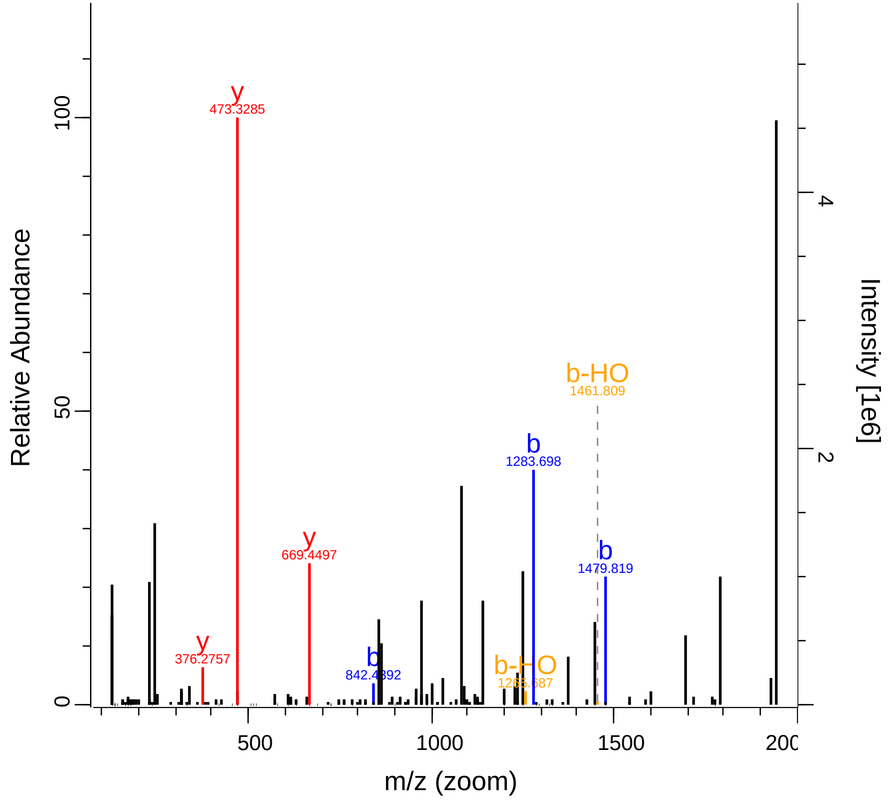

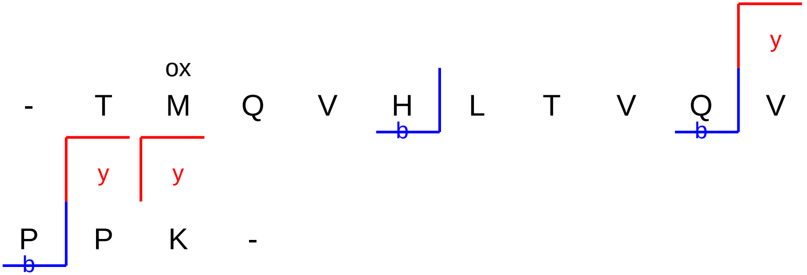

Raw File	Scan	Method	Score	m/z
RO-QC-160121_ZackD_SS_Set_02_Fraction_8	14989	FTMS; HCD	48.24	582.36

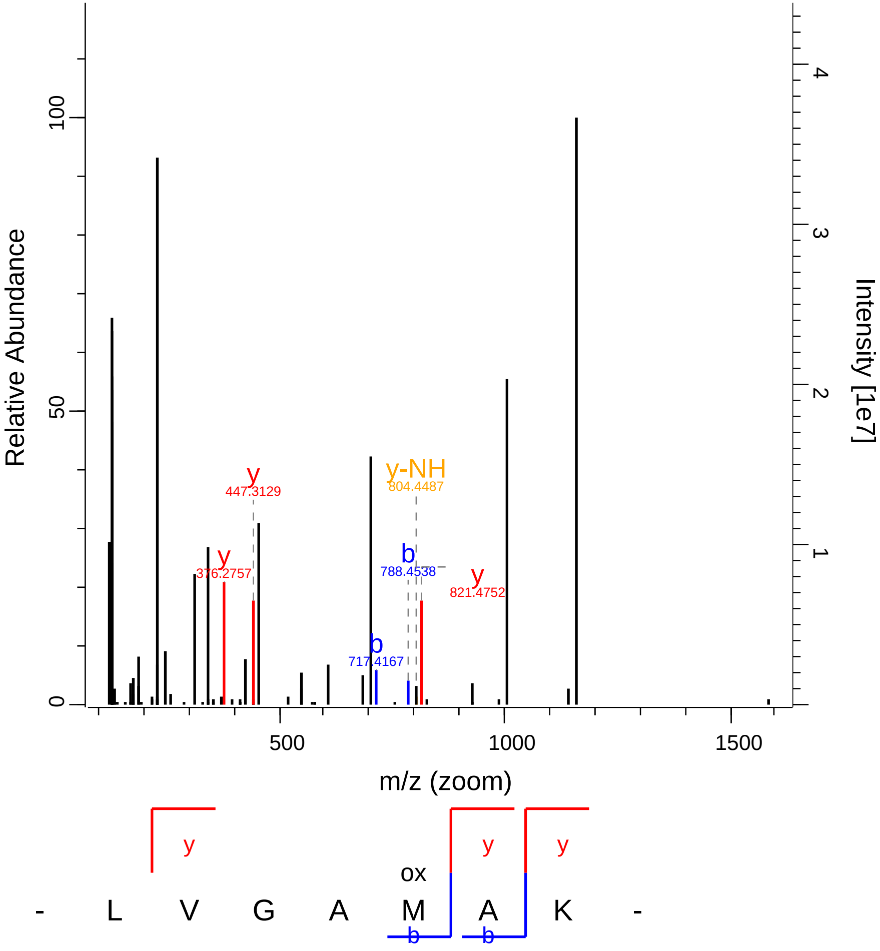

Raw File	Scan	Method	Score	m/z	Gene names
RO-QC-160121_ZackD_SS_Set_02_Fractio	15058	FTMS; HC	47.26	977.08	NEGR1

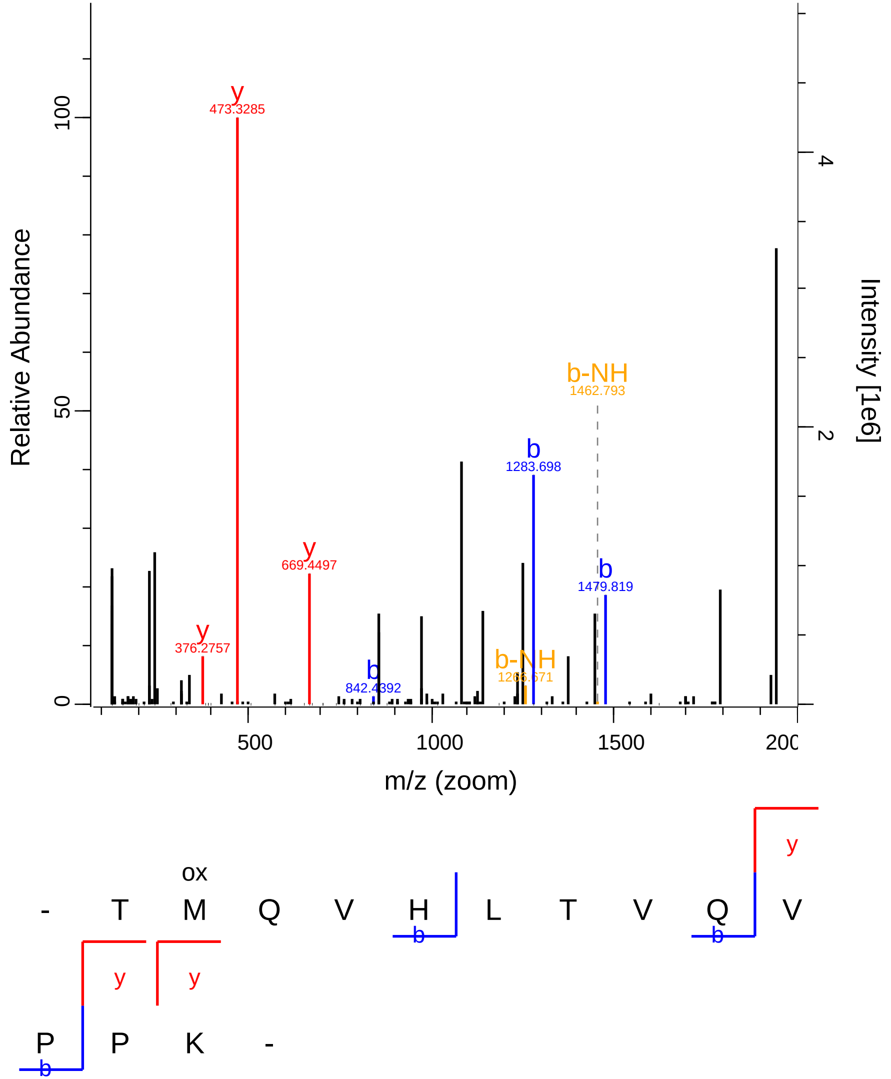

Raw File	Scan	Method	Score	m/z	Gene names
RO-QC-160121_ZackD_SS_Set_02_Fractio	15119	FTMS; HC	53.57	676.07	ZNF425

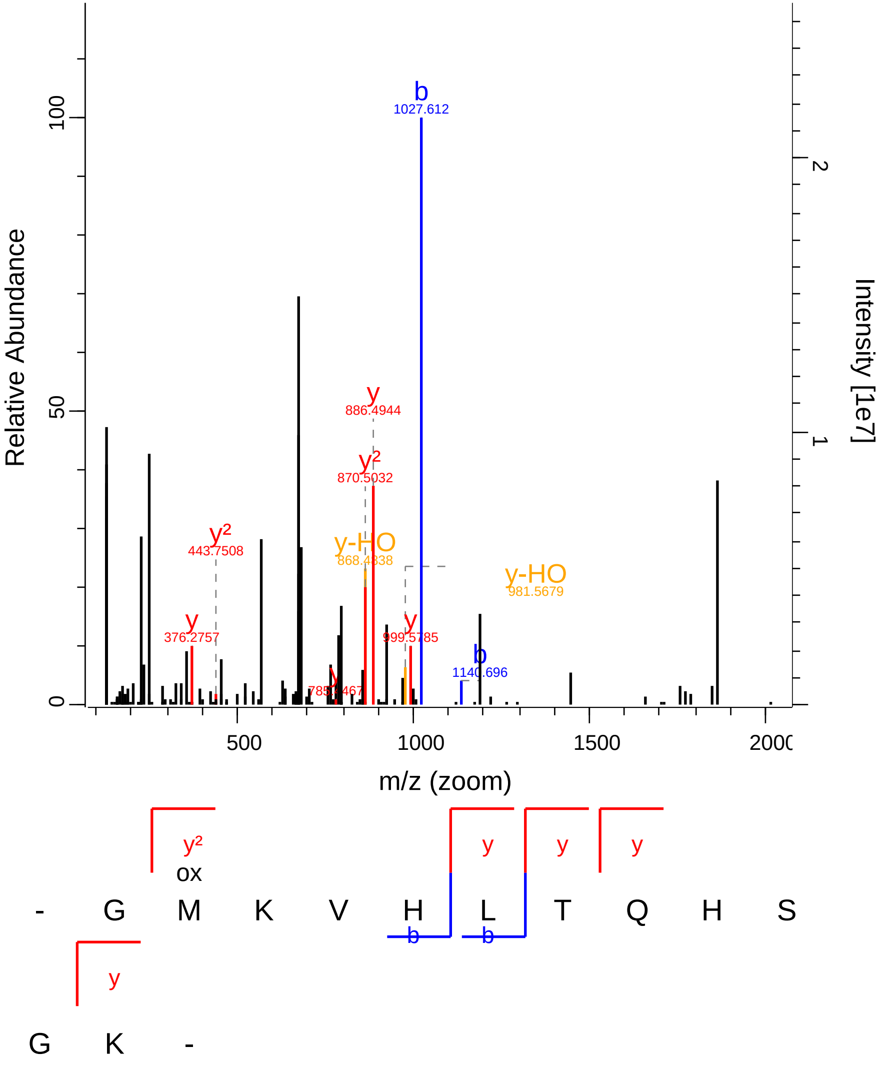

Raw File	Scan	Method	Score	m/z	Gene names
RO-QC-160121_ZackD_SS_Set_02_Fractio	17161	FTMS; HC	122.79	714.38	BIRC2;BIRC3

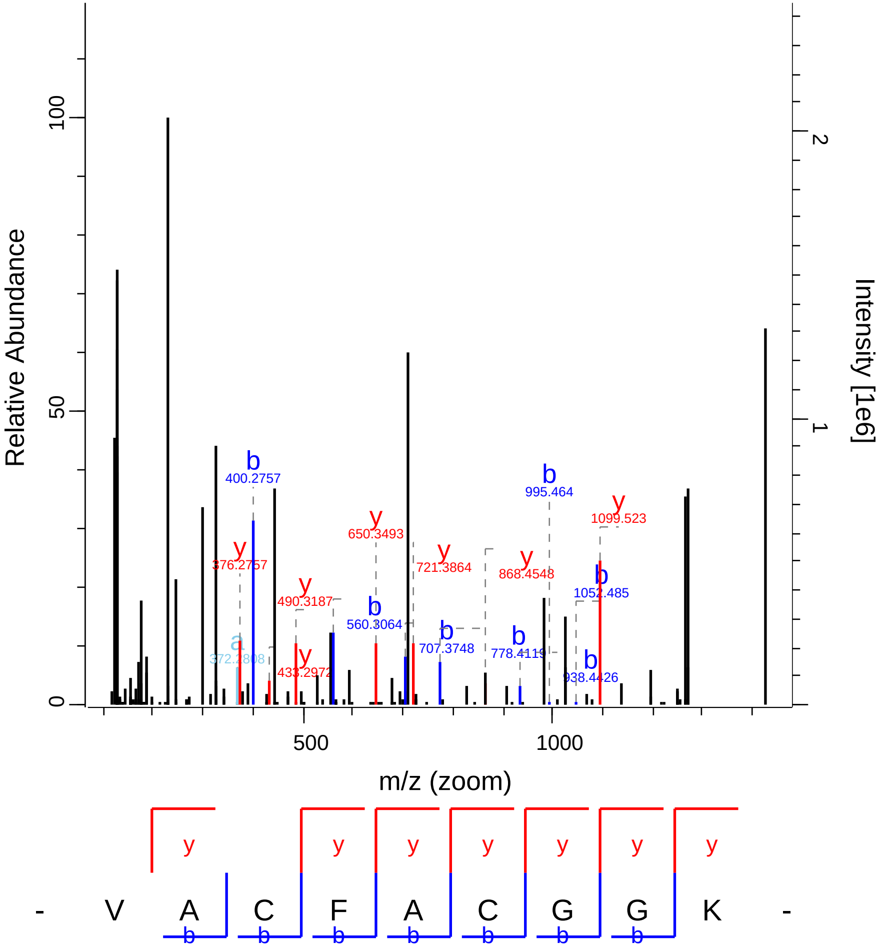

Raw File	Scan	Method	Score	m/z	Gene names
RO-QC-160121_ZackD_SS_Set_02_Fractior	19096	FTMS; HCI	53.28	723.6	SVEP1

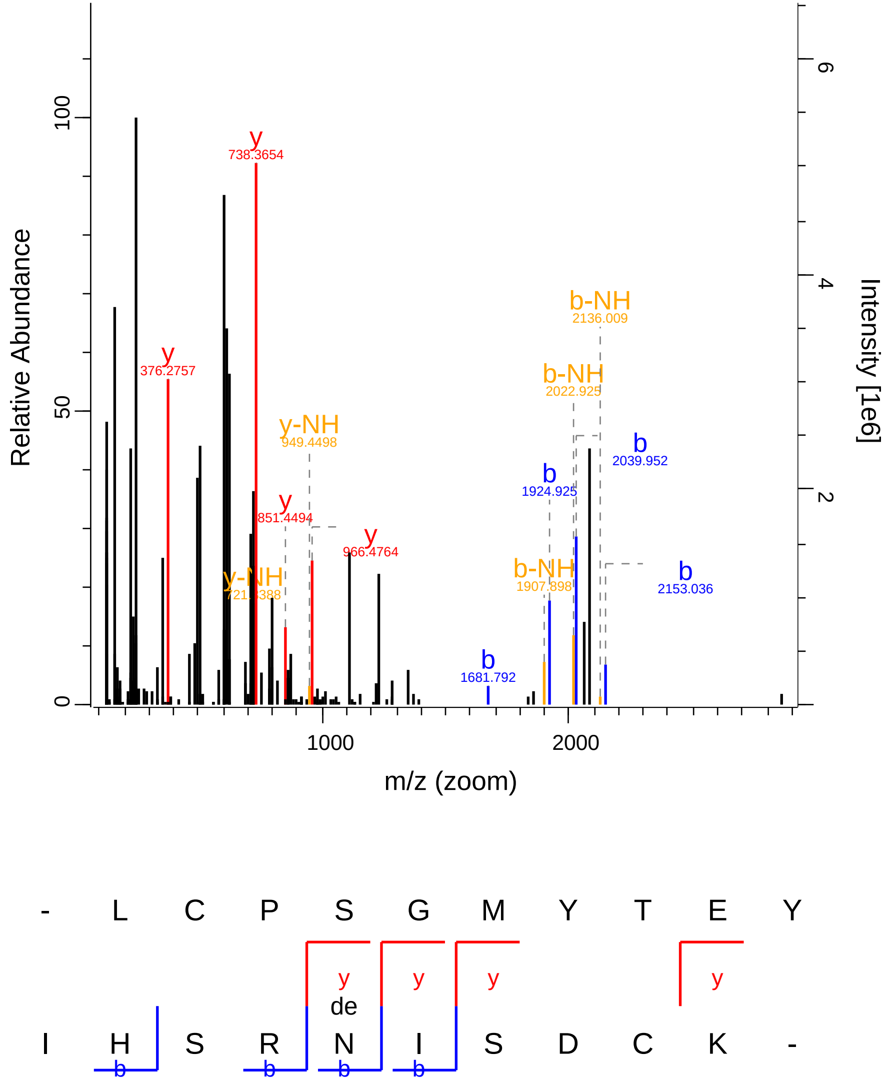

Raw File	Scan	Method	Score	m/z	Gene names
RO-QC-160121_ZackD_SS_Set_02_Fractio	19365	FTMS; HC	52.3	964.13	SVEP1

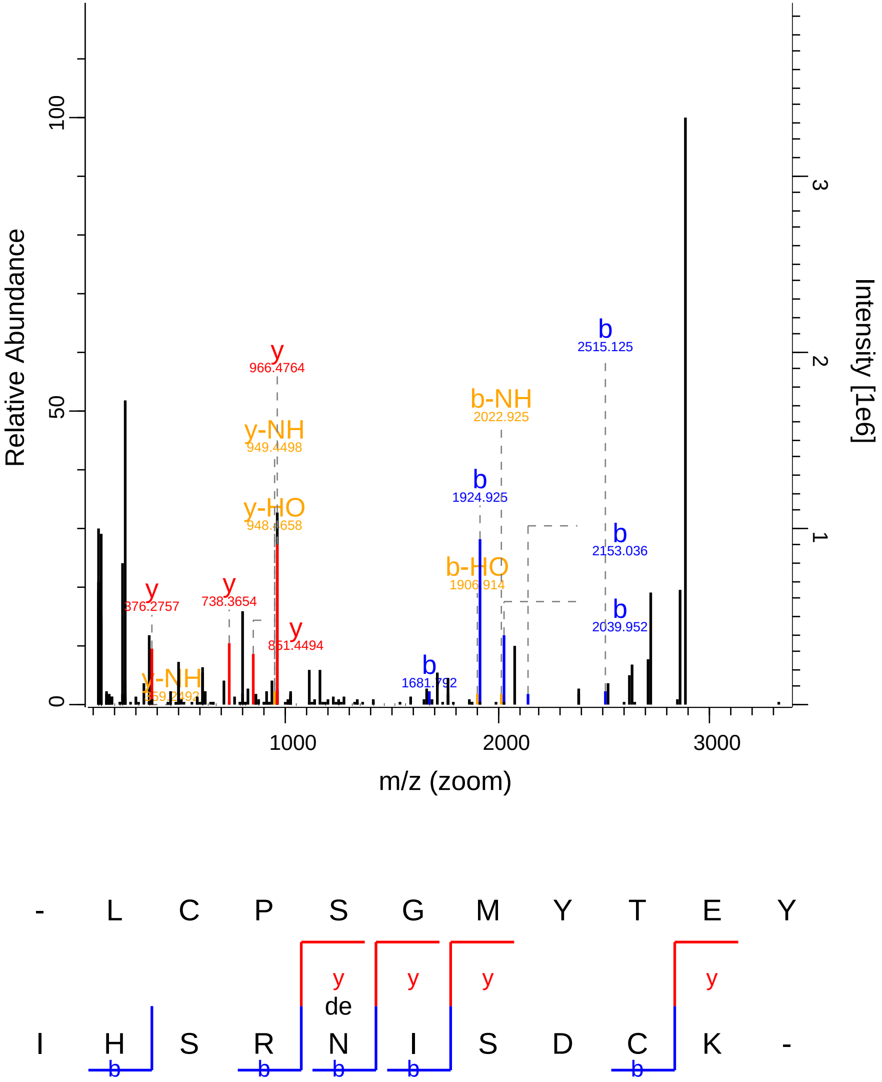

Raw File	Scan	Method	Score	m/z	Gene names
RO-QC-160121_ZackD_SS_Set_02_Fractior	20864	FTMS; HCI	94.69	831.5	C11orf71

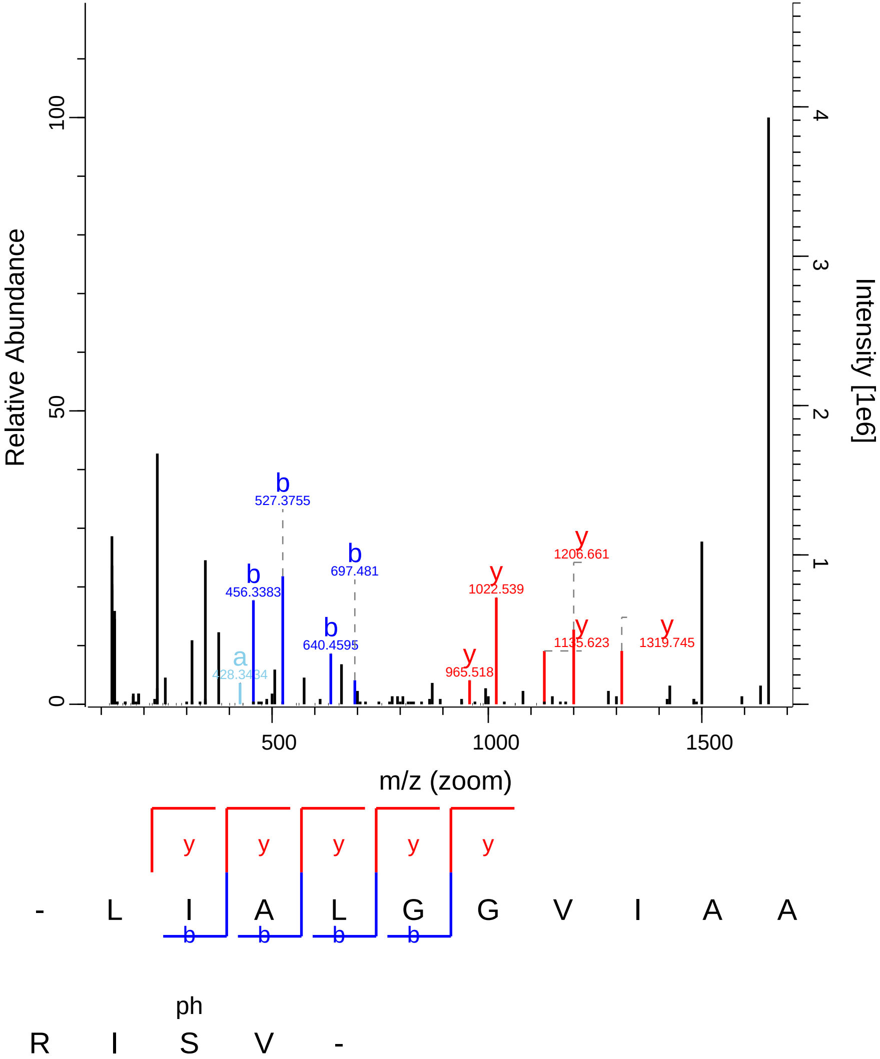

Raw File	Scan	Method	Score	m/z	Gene names
RO-QC-160121_ZackD_SS_Set_02_Fractio	22312	FTMS; HC	49.12	739.08	RIMS1

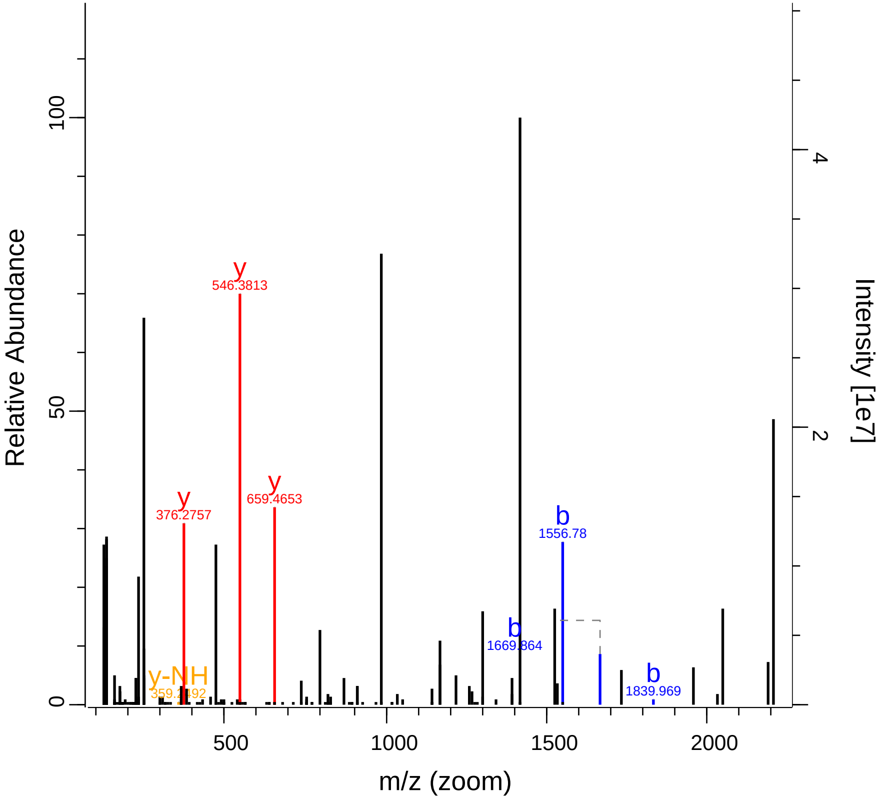

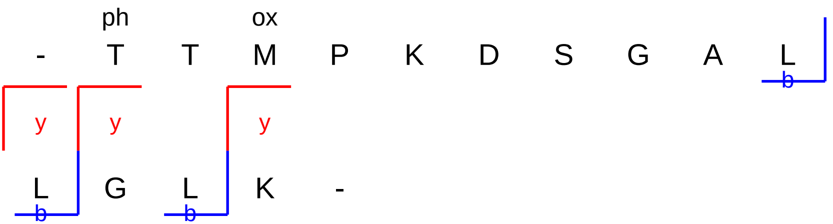

Raw File	Scan	Method	Score	m/z	Gene names
RO-QC-160121_ZackD_SS_Set_02_Fractio	22737	FTMS; HC	203.35	780.46	CAGE1

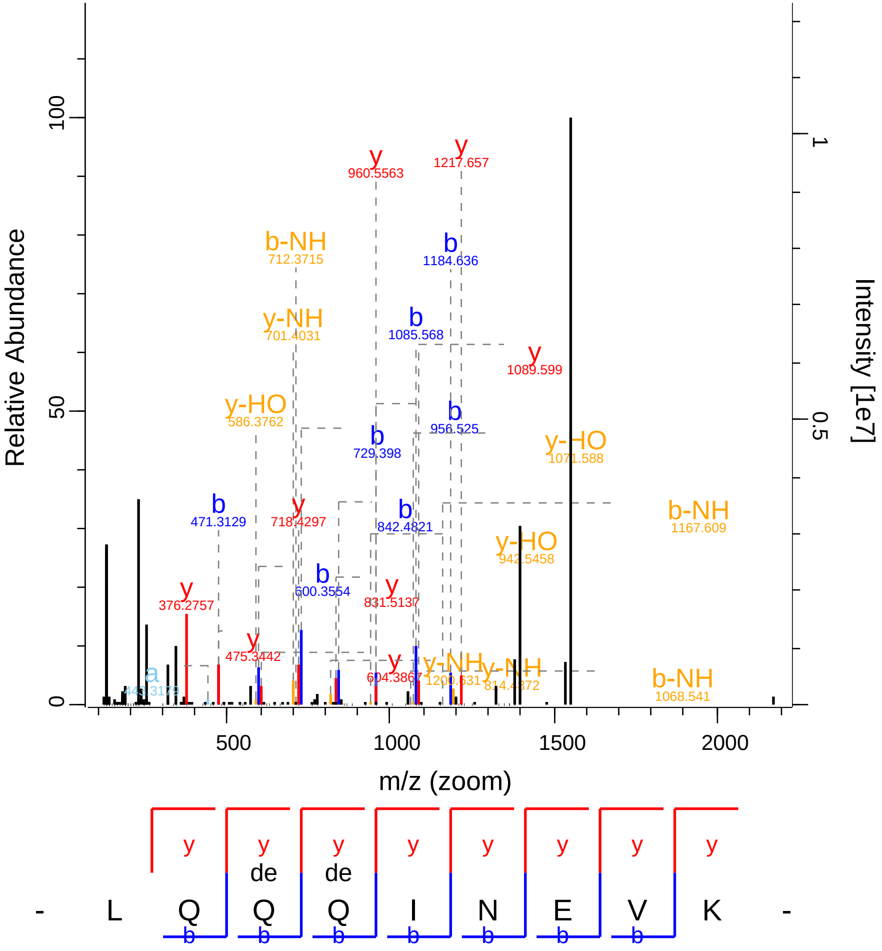

Raw File	Scan	Method	Score	m/z
RO-QC-160121_ZackD_SS_Set_02_Fraction_8	22891	FTMS; HCD	49.42	883.01

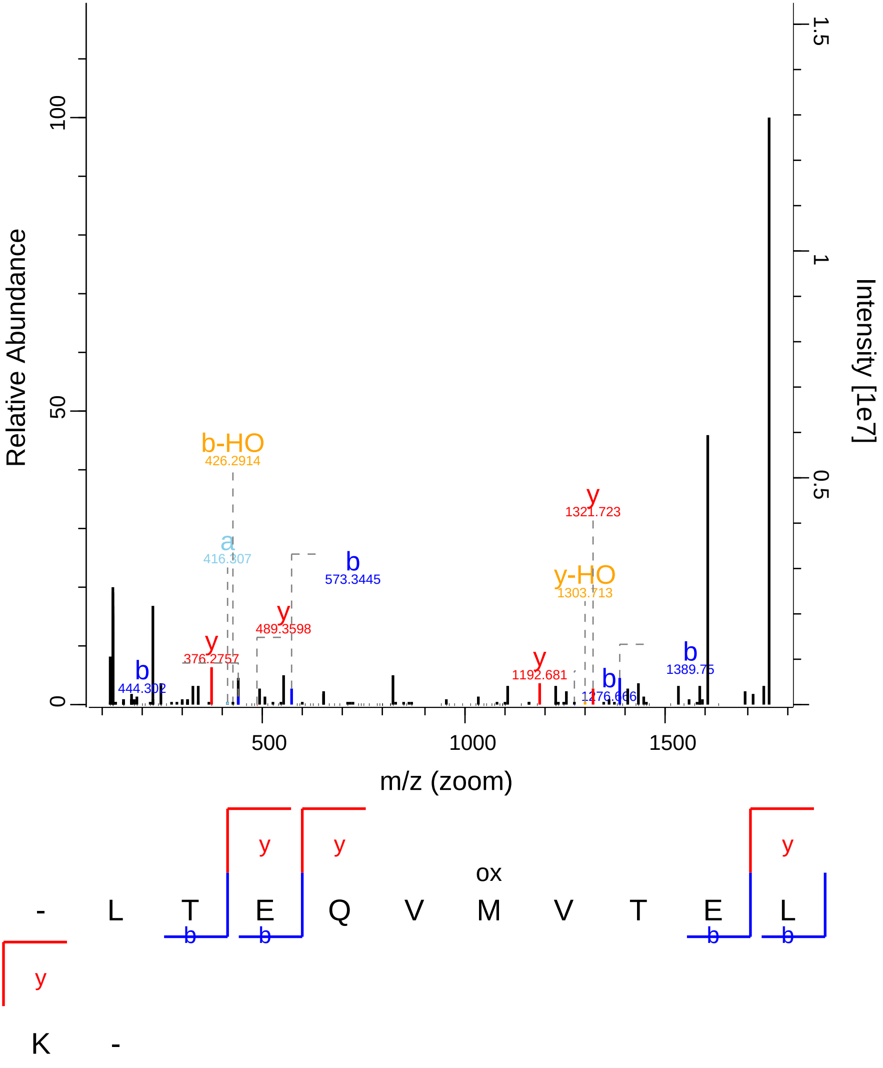

Raw File	Scan	Method	Score	m/z
RO-QC-160121_ZackD_SS_Set_02_Fraction_8	23013	FTMS; HCD	82.26	396.6

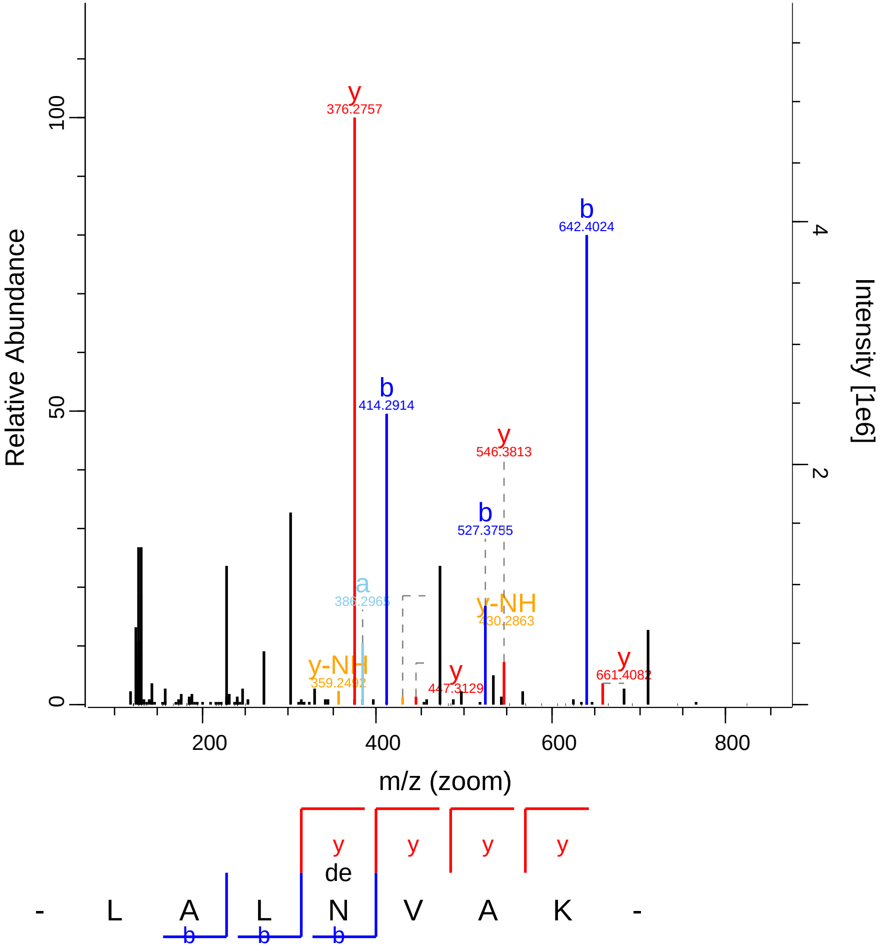

Raw File	Scan	Method	Score	m/z
RO-QC-160121_ZackD_SS_Set_02_Fraction_8	23094	FTMS; HCD	85.53	895.98

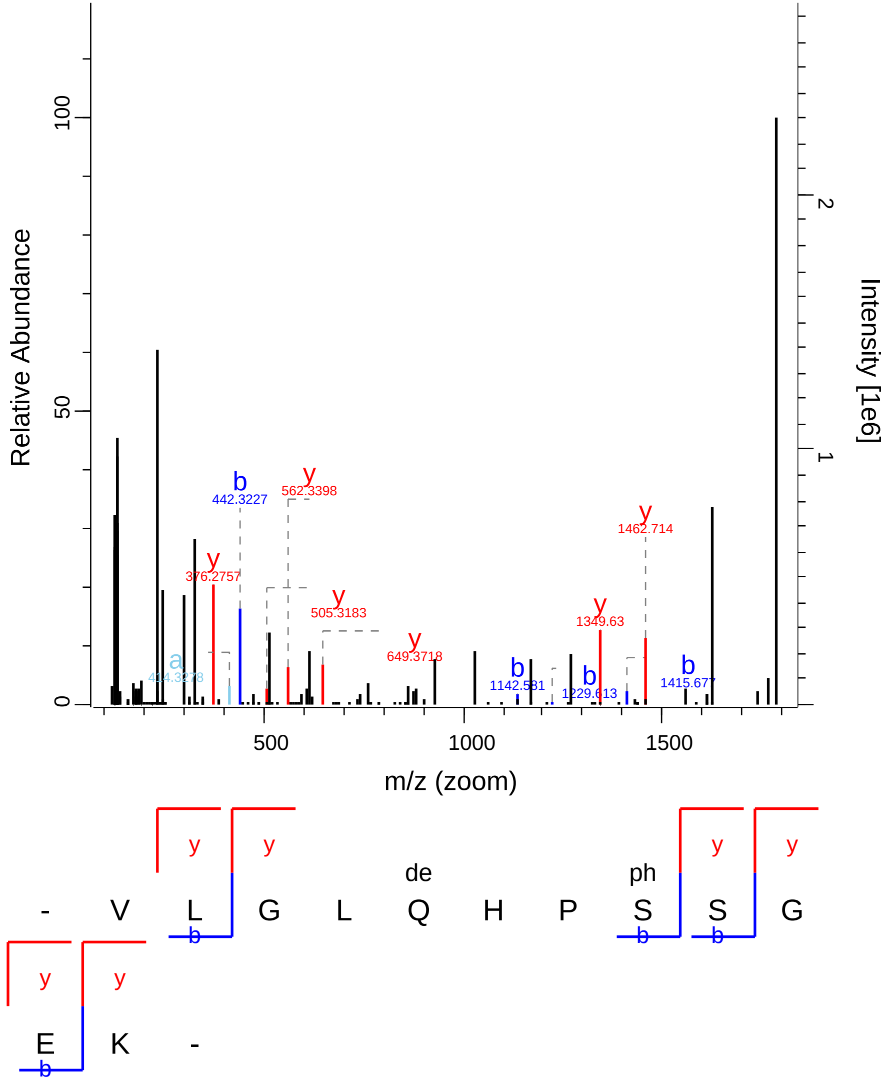

Raw File	Scan	Method	Score	m/z
RO-QC-160121_ZackD_SS_Set_02_Fraction_8	23634	FTMS; HCD	64.8	406.6

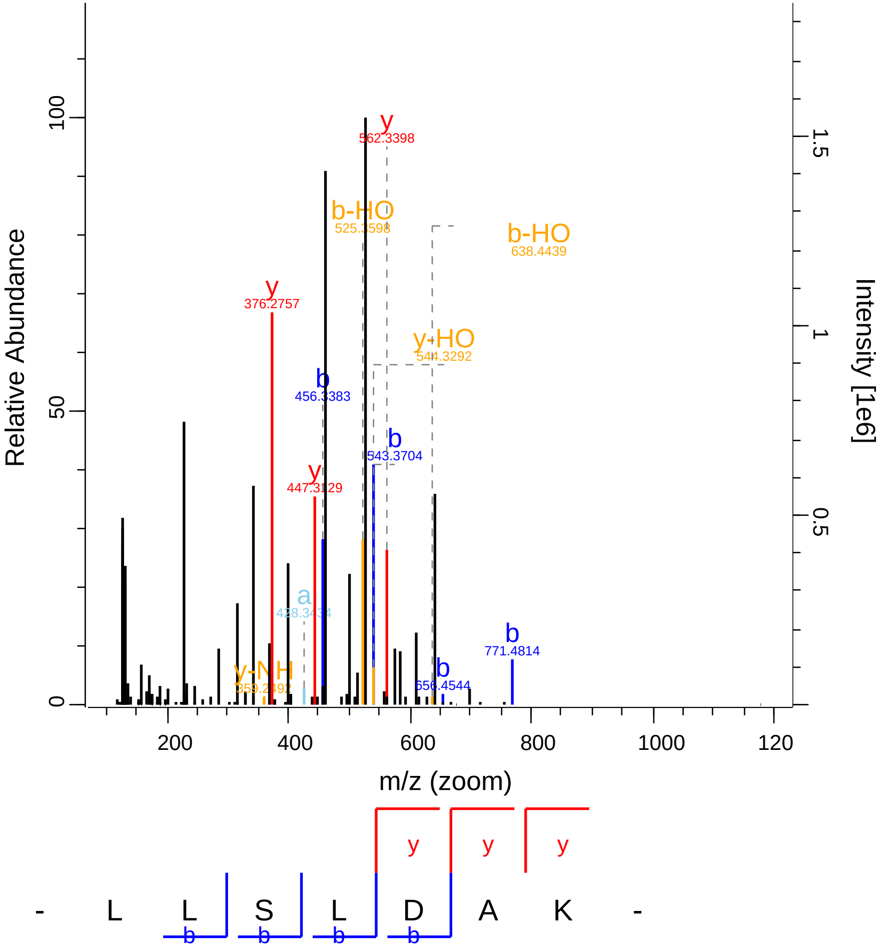

Raw File	Scan	Method	Score	m/z	Gene names
RO-QC-160121_ZackD_SS_Set_02_Fractic	24088	FTMS; HC	183.91	1105.87	C6orf120

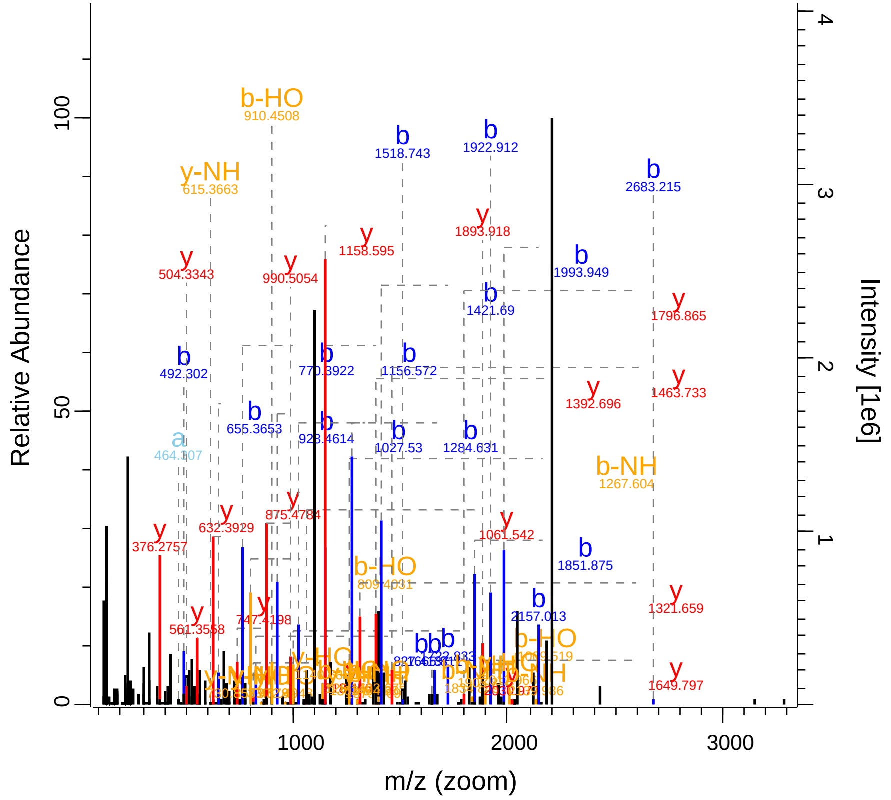

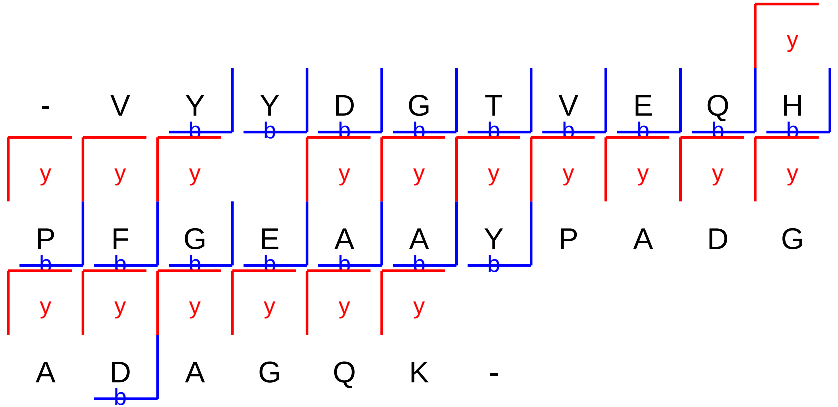

Raw File	Scan	Method	Score	m/z
RO-QC-160121_ZackD_SS_Set_02_Fraction_8	25049	FTMS; HCD	132.09	430.28

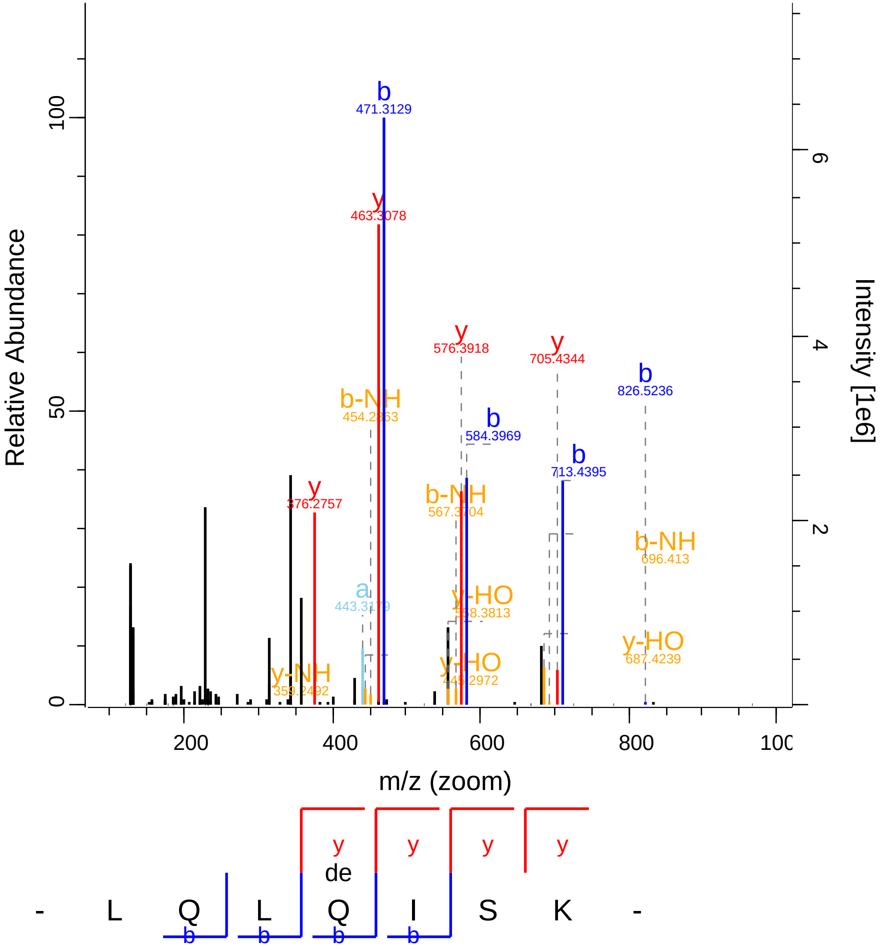

Raw File	Scan	Method	Score	m/z
RO-QC-160121_ZackD_SS_Set_02_Fraction_8	25599	FTMS; HCD	41.21	1000.88

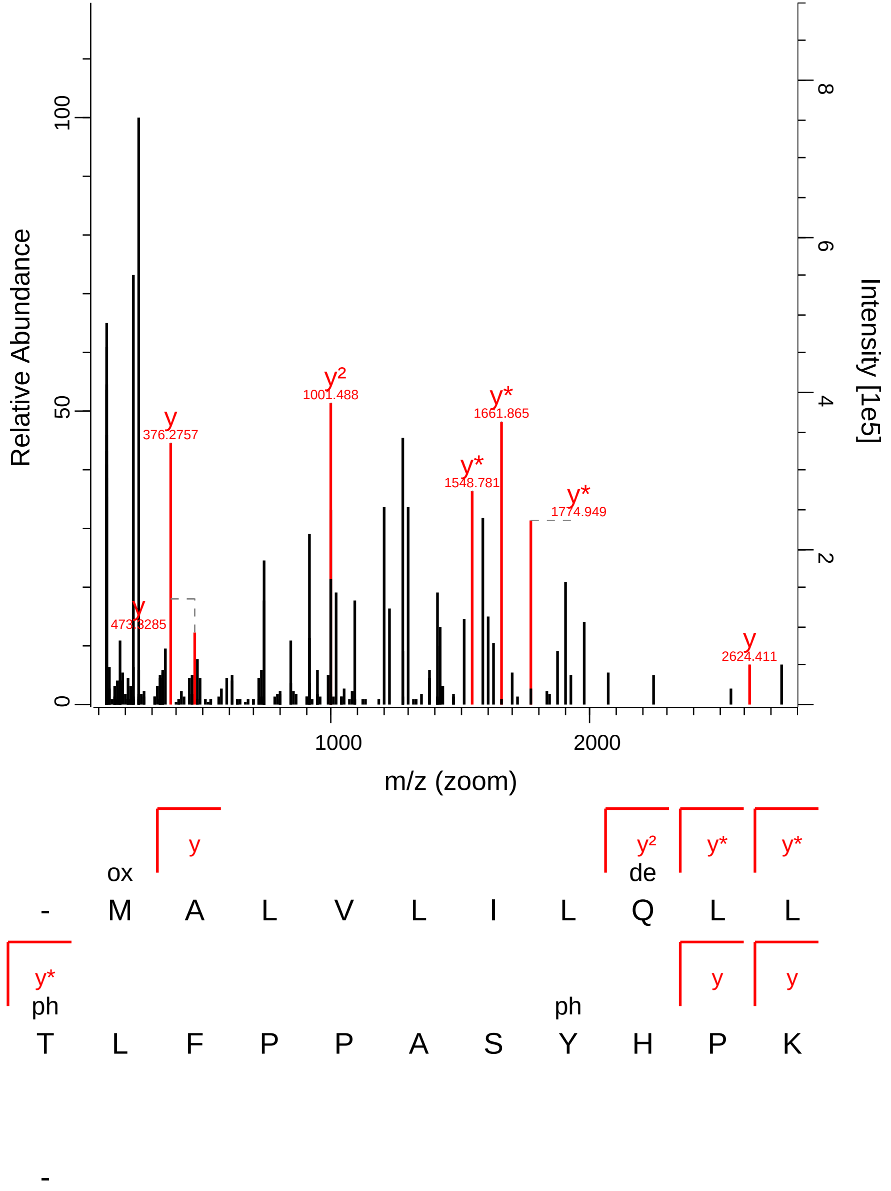

Raw File	Scan	Method	Score	m/z	Gene names
RO-QC-160121_ZackD_SS_Set_02_Fractior	28125	FTMS; HCI	46.75	870.8	CCDC168

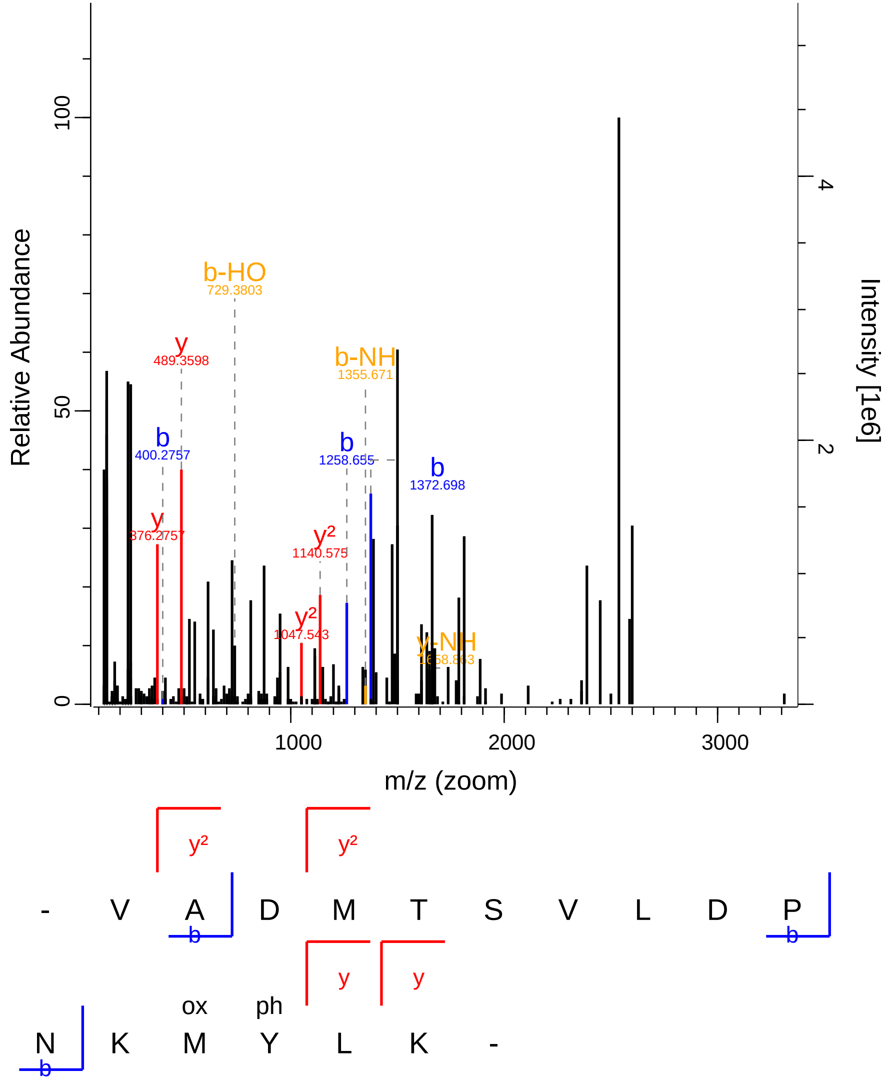

Raw File	Scan	Method	Score	m/z	Gene names
RO-QC-160121_ZackD_SS_Set_02_Fractio	28227	FTMS; HC	145.28	904.01	UGCG

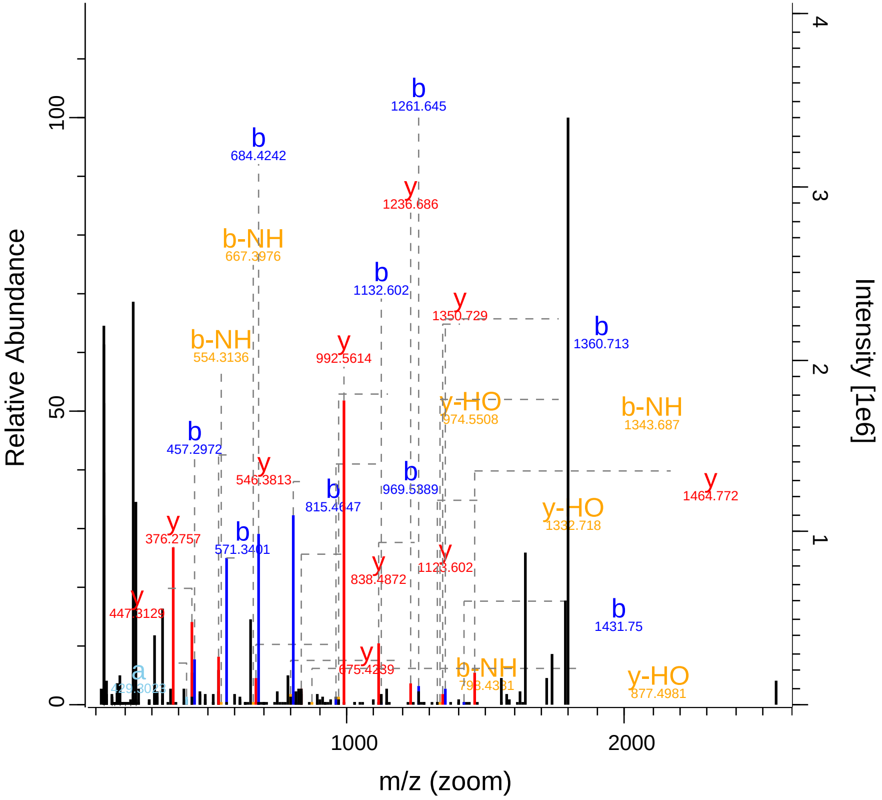

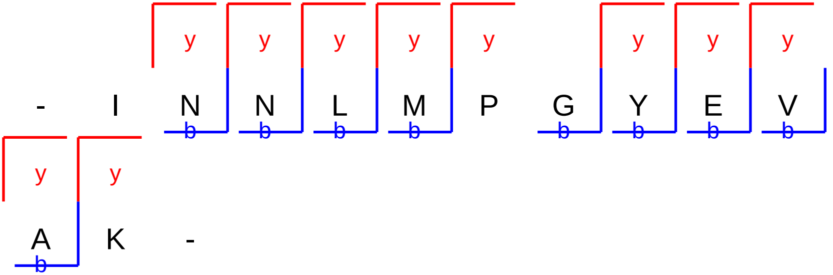

Raw File	Scan	Method	Score	m/z
RO-QC-160121_ZackD_SS_Set_02_Fraction_8	28989	FTMS; HCD	40.72	675.36

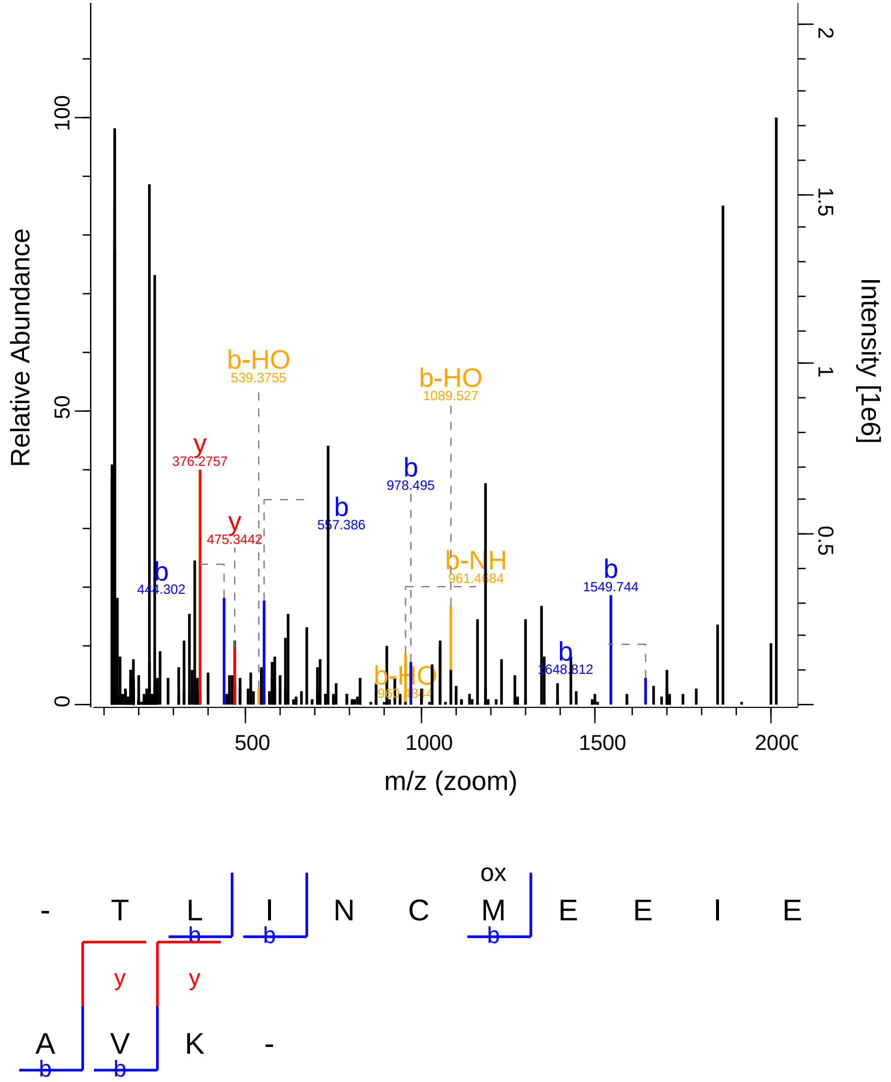

Raw File	Scan	Method	Score	m/z	Gene names
RO-QC-160121_ZackD_SS_Set_02_Fractio	30995	FTMS; HC	83.42	843.46	ABHD5

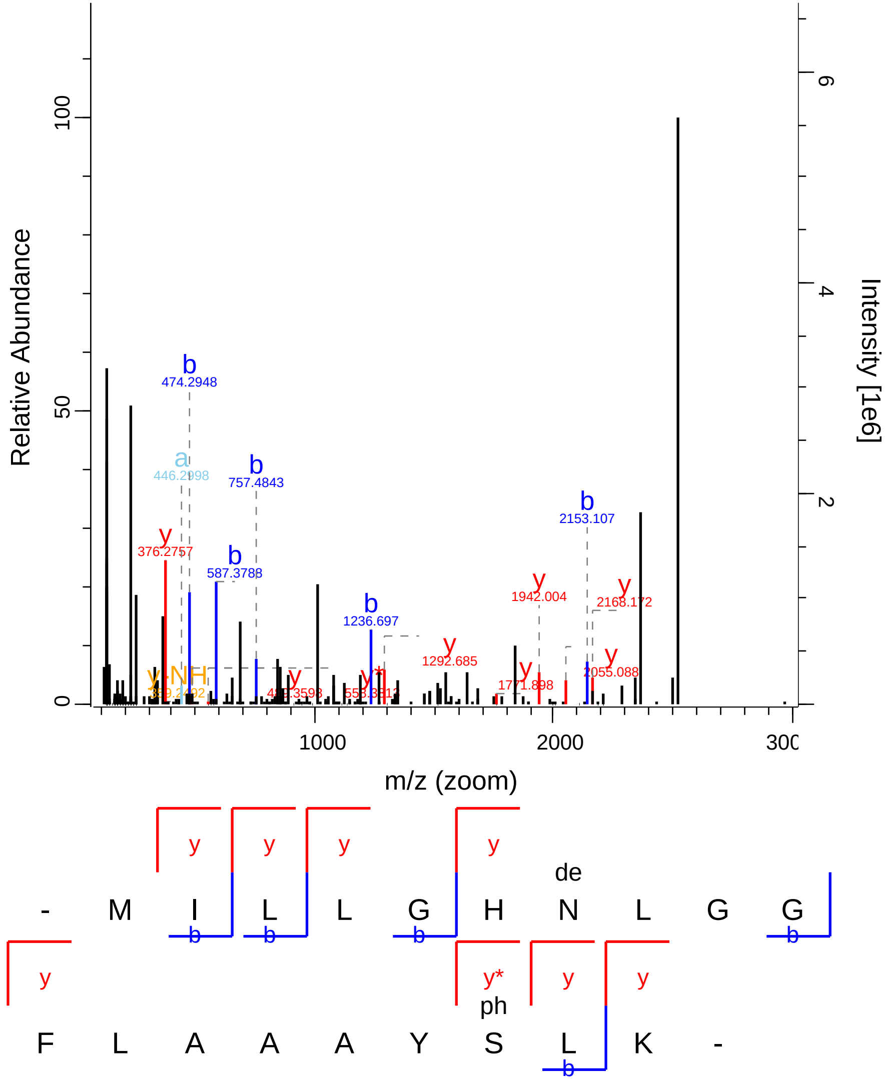

Raw File	Scan	Method	Score	m/z
RO-QC-160121_ZackD_SS_Set_02_Fraction_8	31128	FTMS; HCD	53.97	457.97

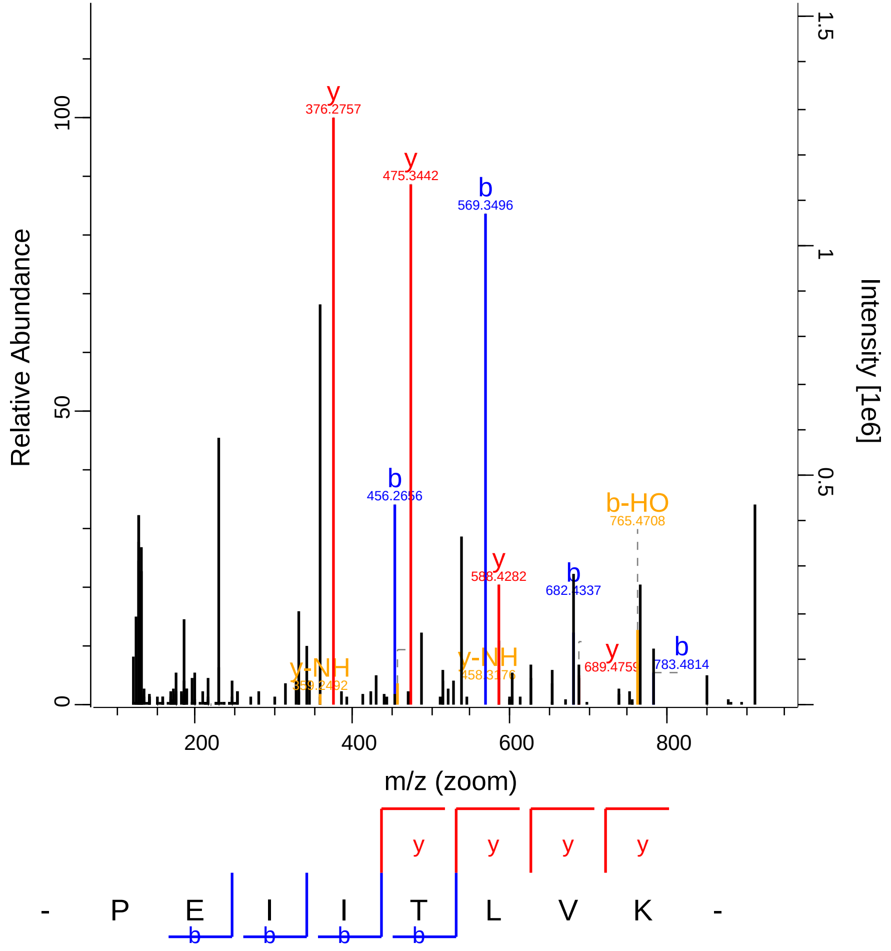

Raw File	Scan	Method	Score	m/z	Gene names
RO-QC-160121_ZackD_SS_Set_02_Fractio	32650	FTMS; HC	61.96	963.03	CFAP99

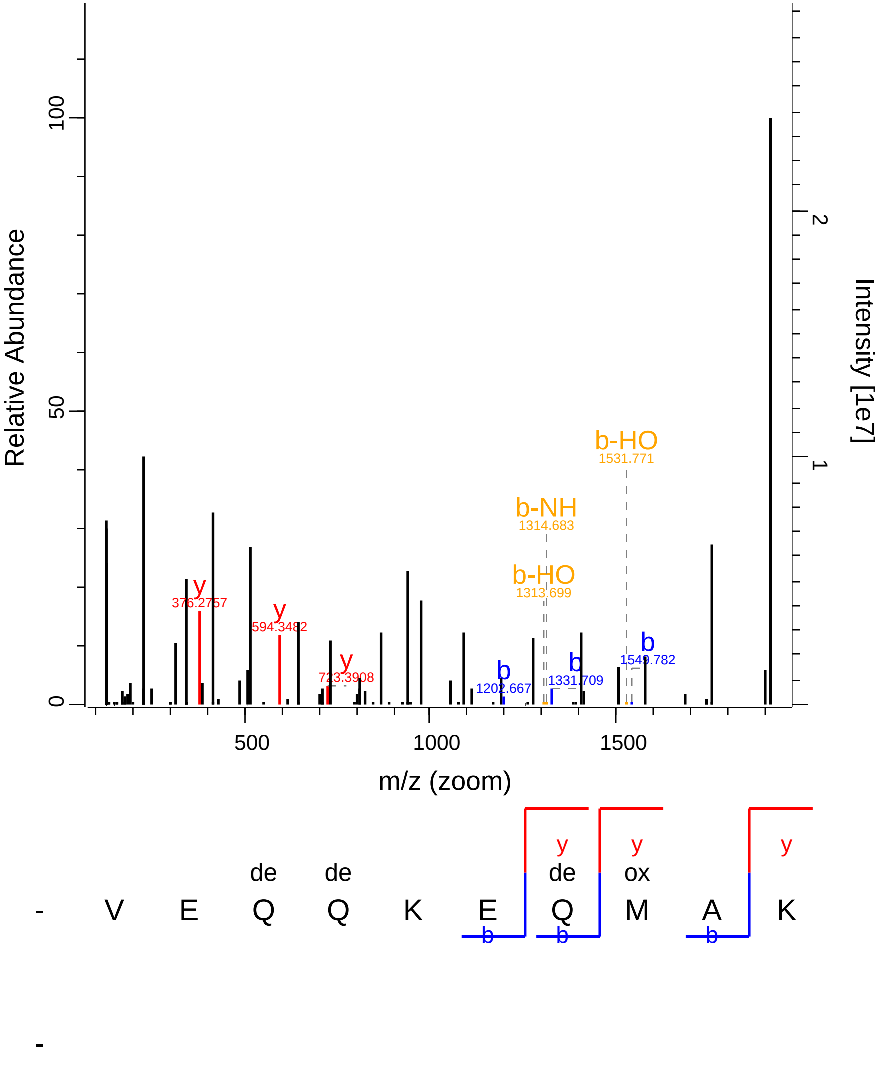

Raw File	Scan	Method	Score	m/z
RO-QC-160121_ZackD_SS_Set_02_Fraction_8	34468	FTMS; HCD	51.85	789.96

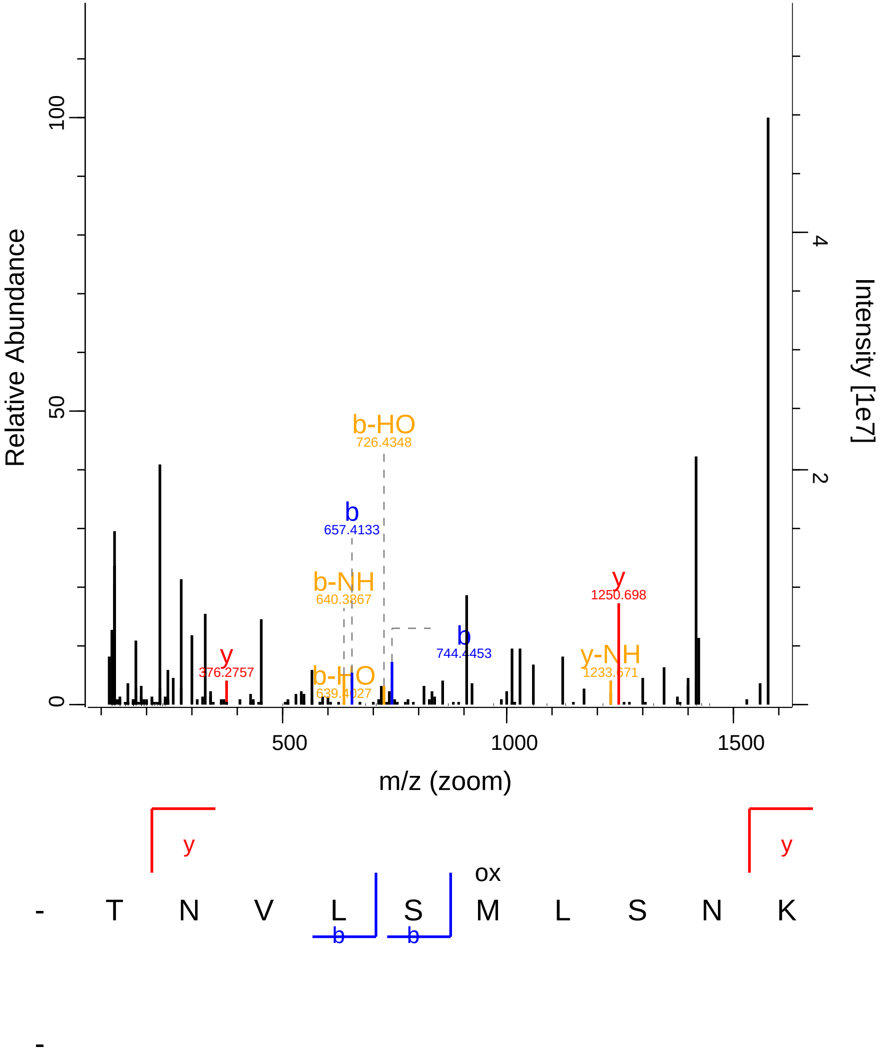

Raw File	Scan	Method	Score	m/z	Gene names
RO-QC-160121_ZackD_SS_Set_02_Fractio	34973	FTMS; HC	92.84	890.03	ASH1L

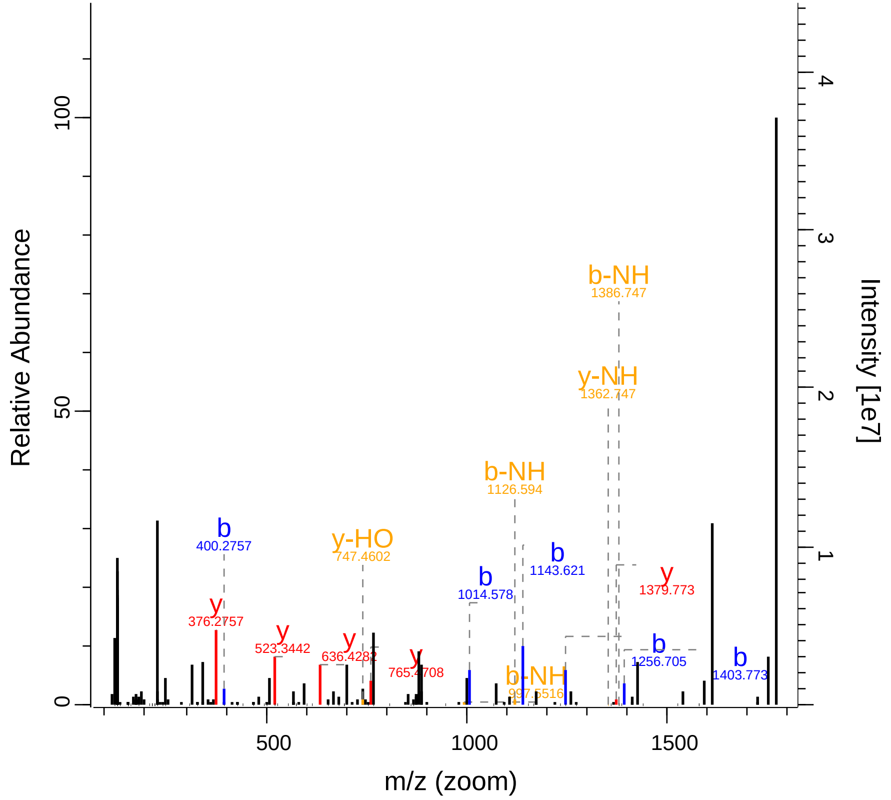

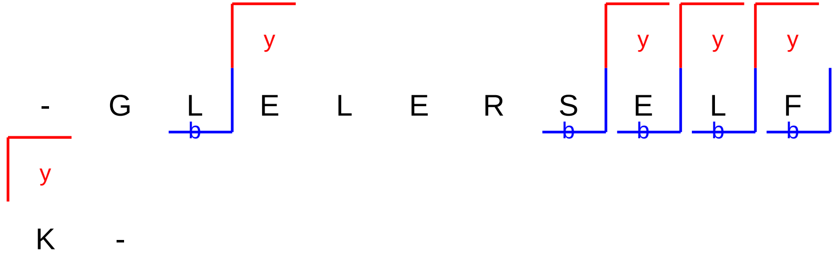

Raw File	Scan	Method	Score	m/z	Gene names
RO-QC-160121_ZackD_SS_Set_02_Fractio	36925	FTMS; HC	114.19	439.29	VCAM1

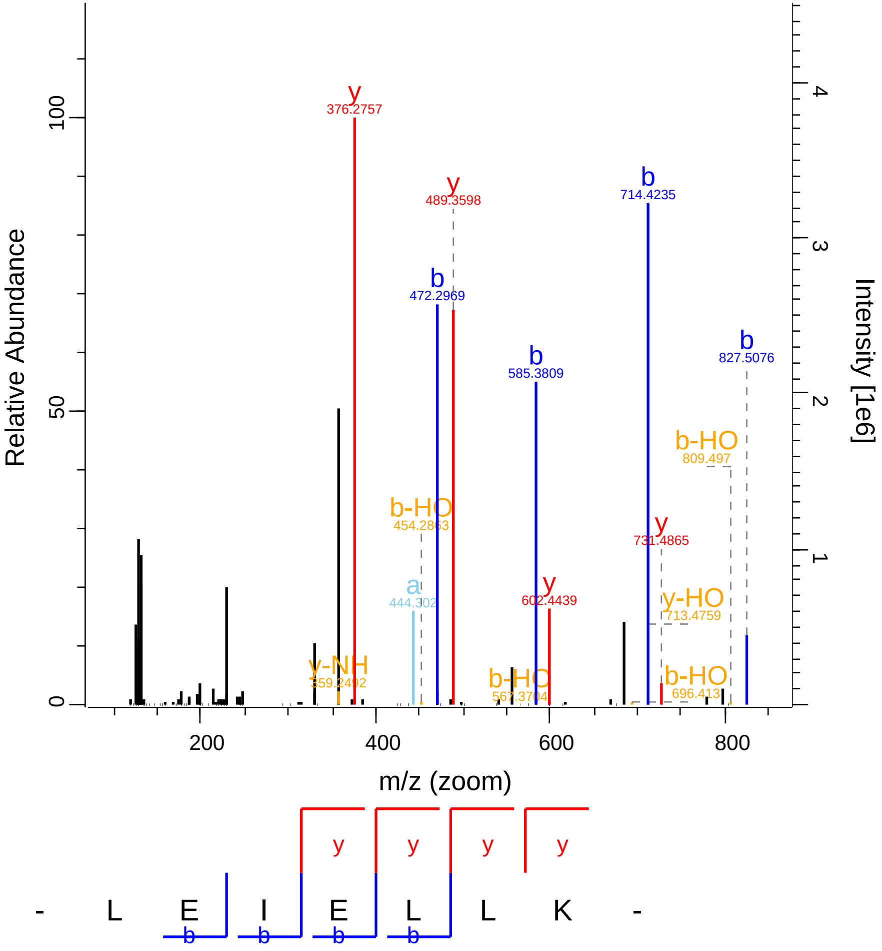

Raw File	Scan	Method	Score	m/z	Gene names
RO-QC-160121_ZackD_SS_Set_02_Fractio	38037	FTMS; HC	94.45	627.05	ALKBH7

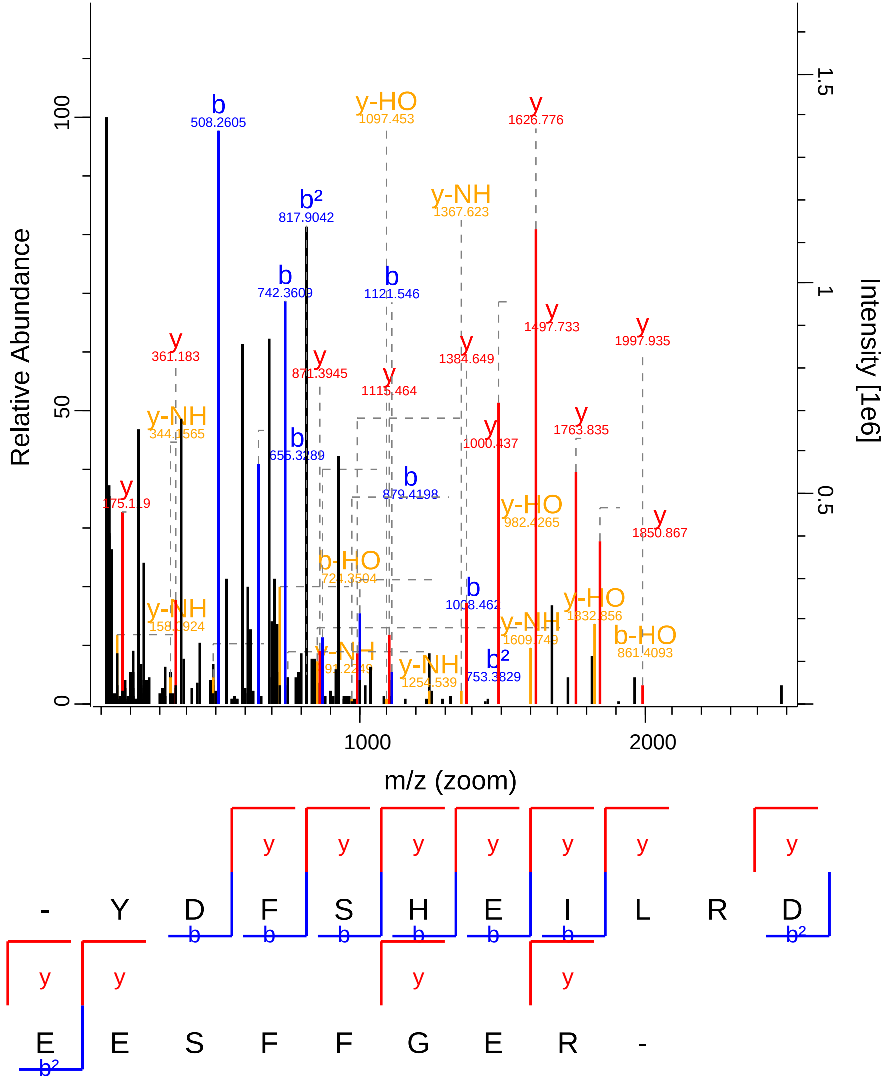

Raw File	Scan	Method	Score	m/z	Gene names
RO-QC-160121_ZackD_SS_Set_02_Fractio	40964	FTMS; HC	110.77	744.08	C11orf84

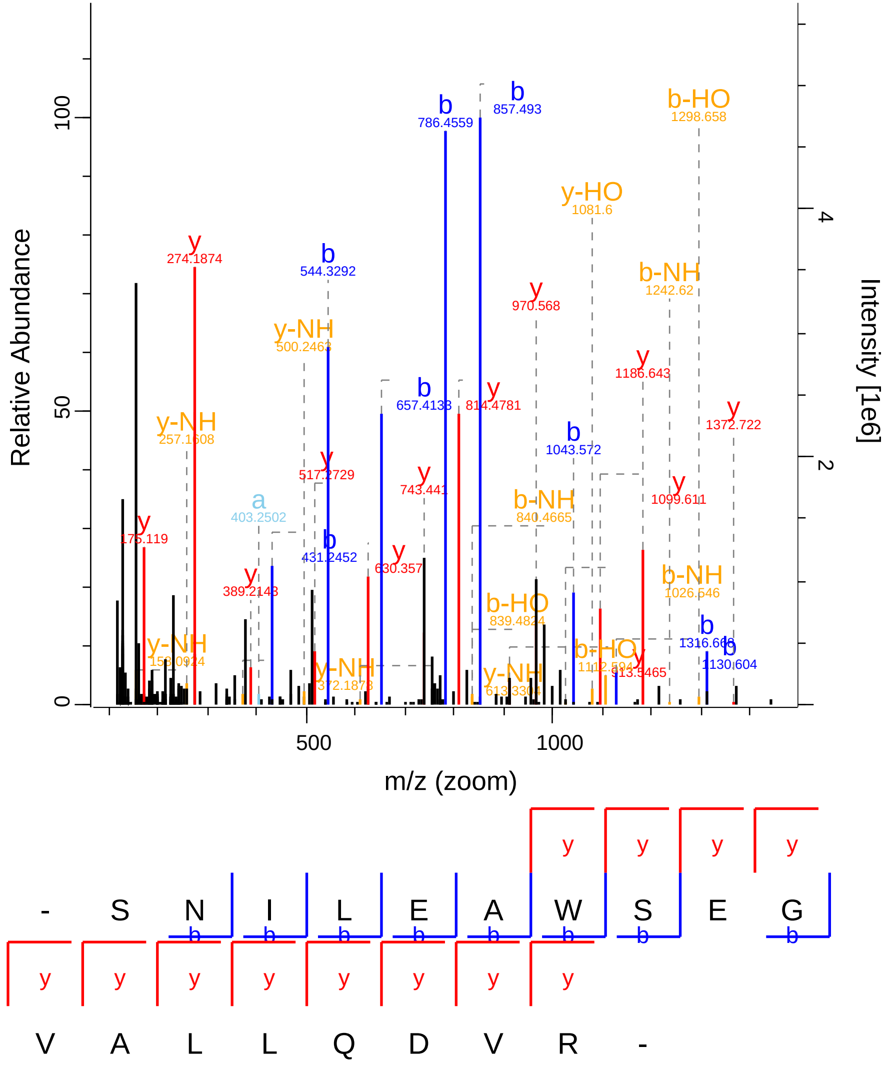

Raw File	Scan	Method	Score	m/z	Gene names
RO-QC-160121_ZackD_SS_Set_02_Fractic	40984	FTMS; HC	69.38	1115.61	C11orf84

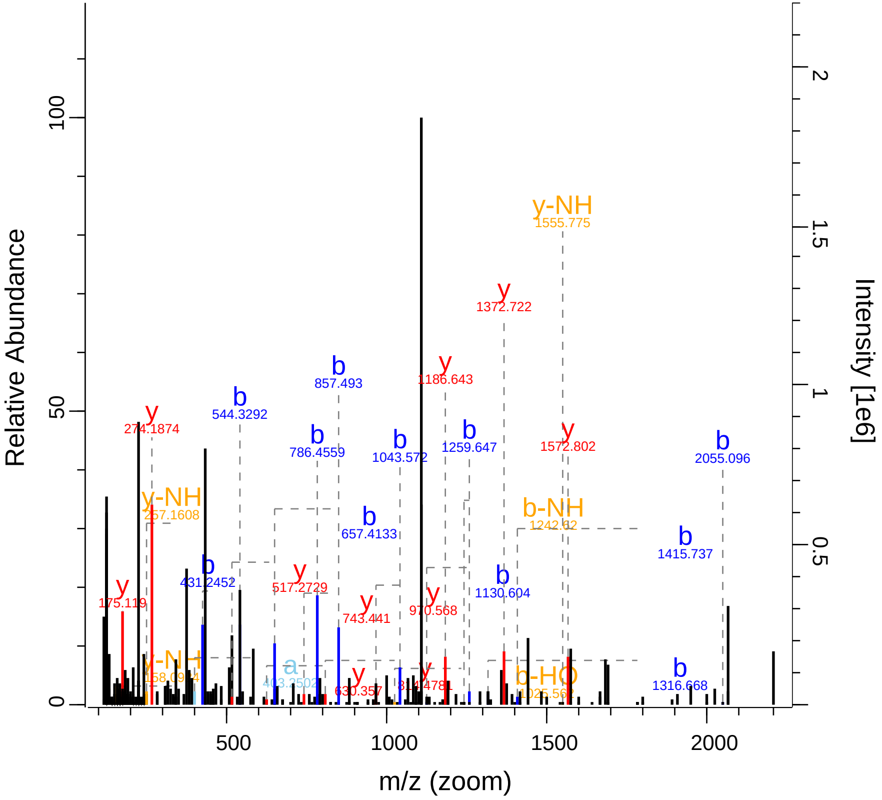

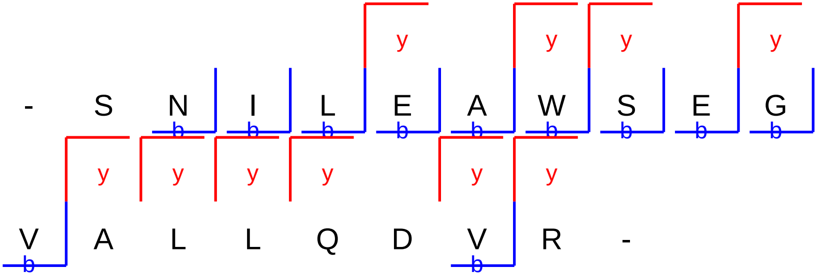

Raw File	Scan	Method	Score	m/z	Gene names
RO-QC-160121_ZackD_SS_Set_02_Fractior	5472	FTMS; HCI	71.35	448.52	S100PBP

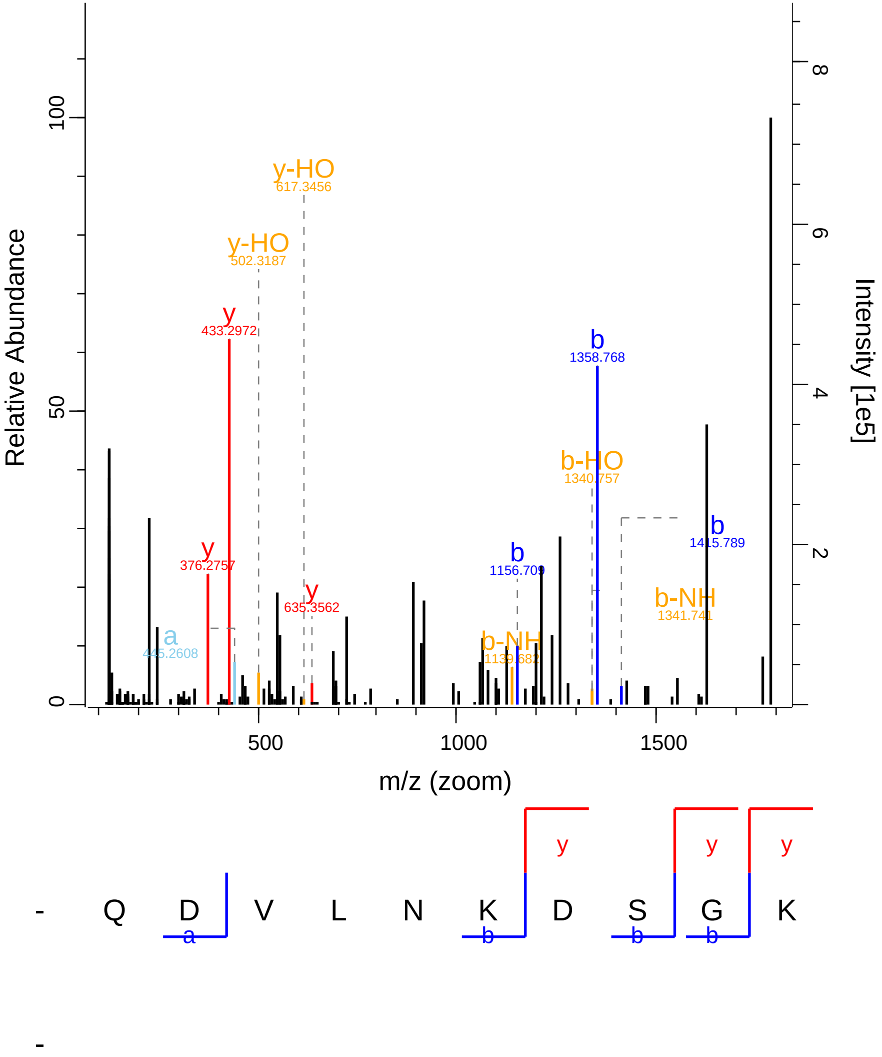

Raw File	Scan	Method	Score	m/z	Gene names
RO-QC-160121_ZackD_SS_Set_02_Fractio[illegible]	5839	FTMS; HC	172.17	876.92	GLIPR1

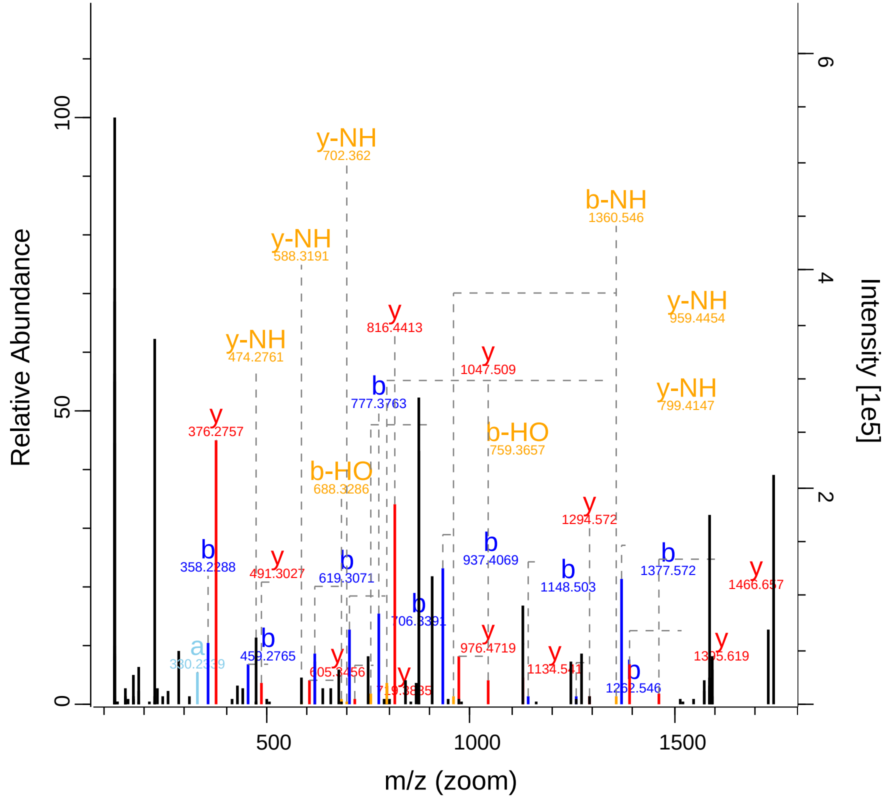

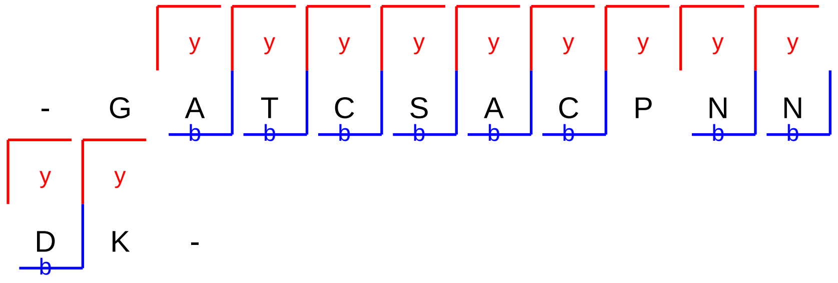

Raw File	Scan	Method	Score	m/z	Gene names
RO-QC-160121_ZackD_SS_Set_02_Fraction	6115	FTMS; HCI	119.17	685.4	ARHGAP31

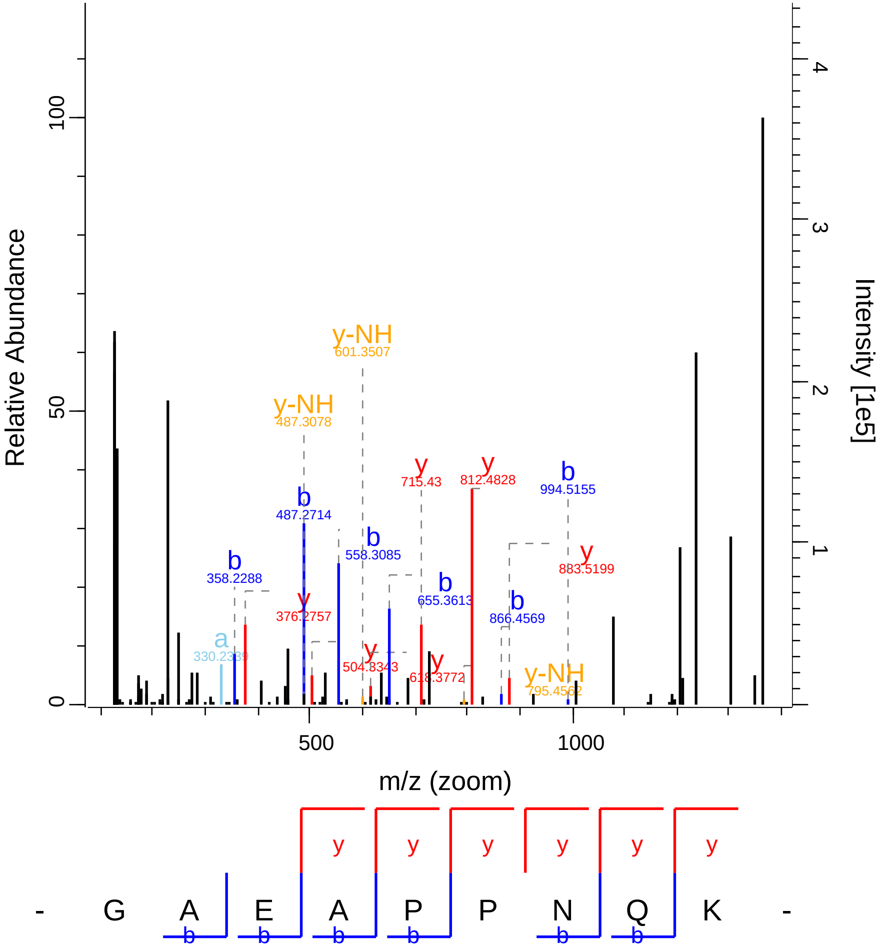

Raw File	Scan	Method	Score	m/z	Gene names
RO-QC-160121_ZackD_SS_Set_02_Fractior	6238	FTMS; HCI	138.1	664.88	IMUP

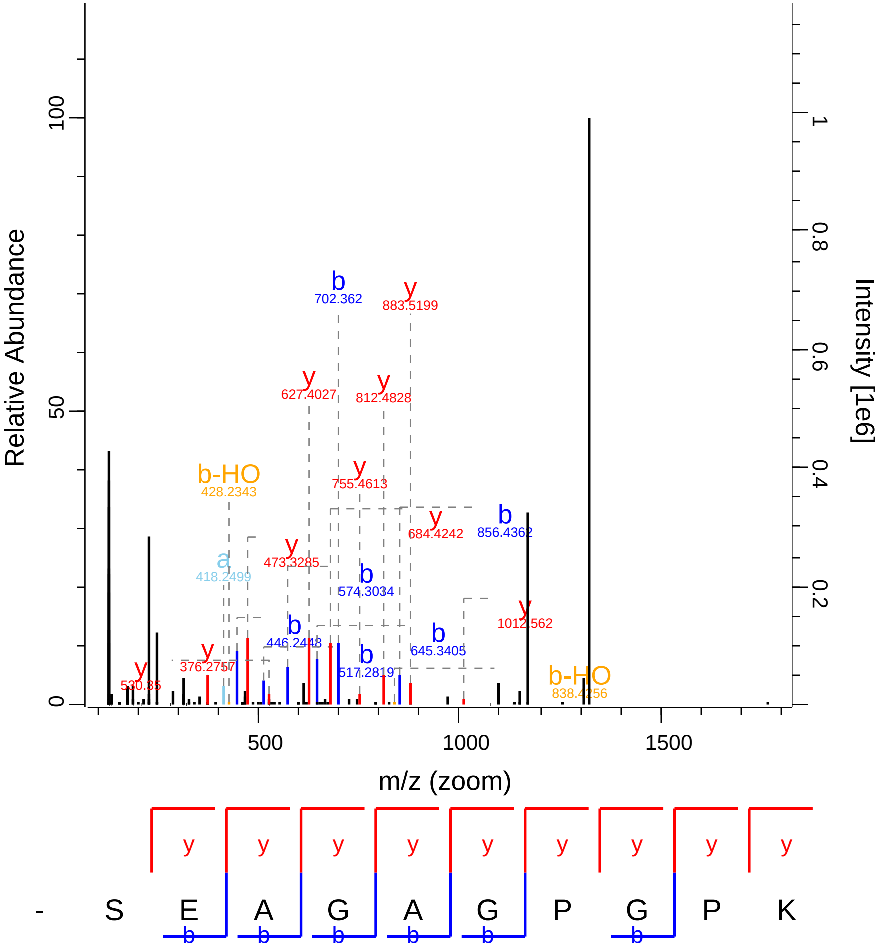

Raw File	Scan	Method	Score	m/z
RO-QC-160121_ZackD_SS_Set_02_Fraction_9	7106	FTMS; HCD	33.59	352.79

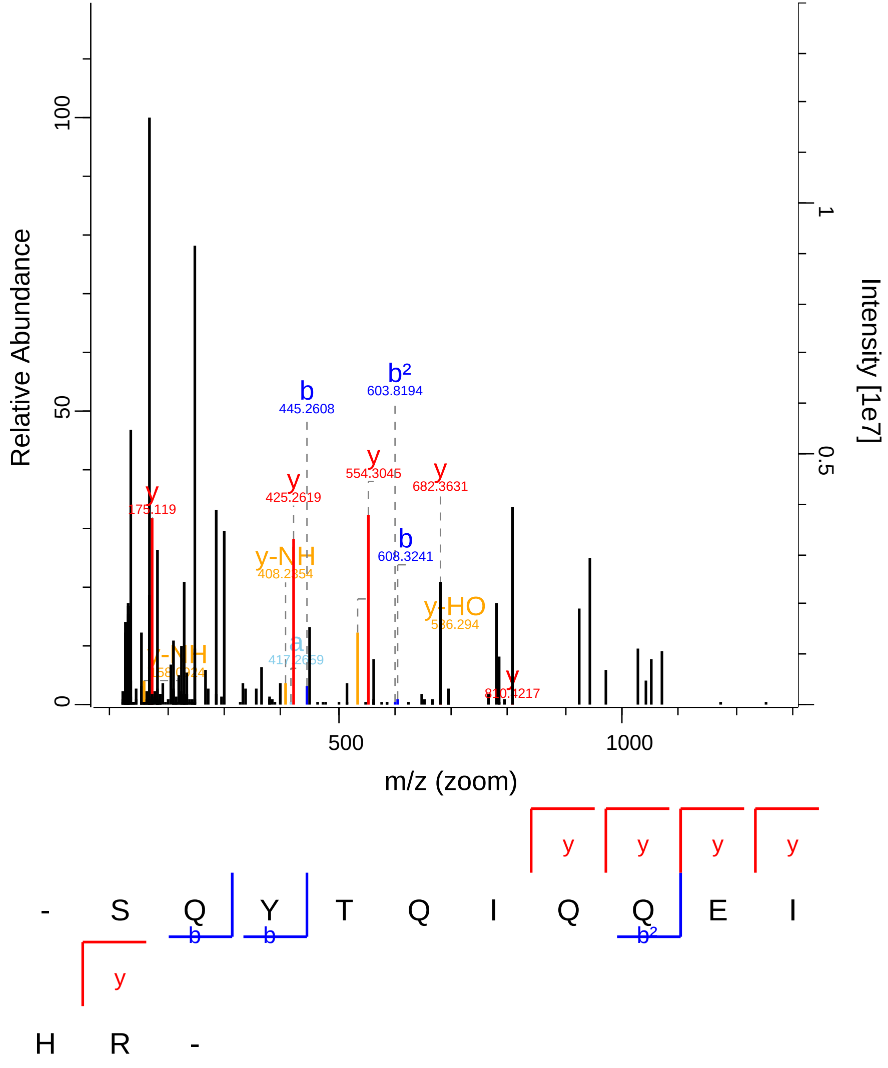

Raw File	Scan	Method	Score	m/z	Gene names
RO-QC-160121_ZackD_SS_Set_02_Fractior	7607	FTMS; HCI	80.24	496.32	SLC35D1

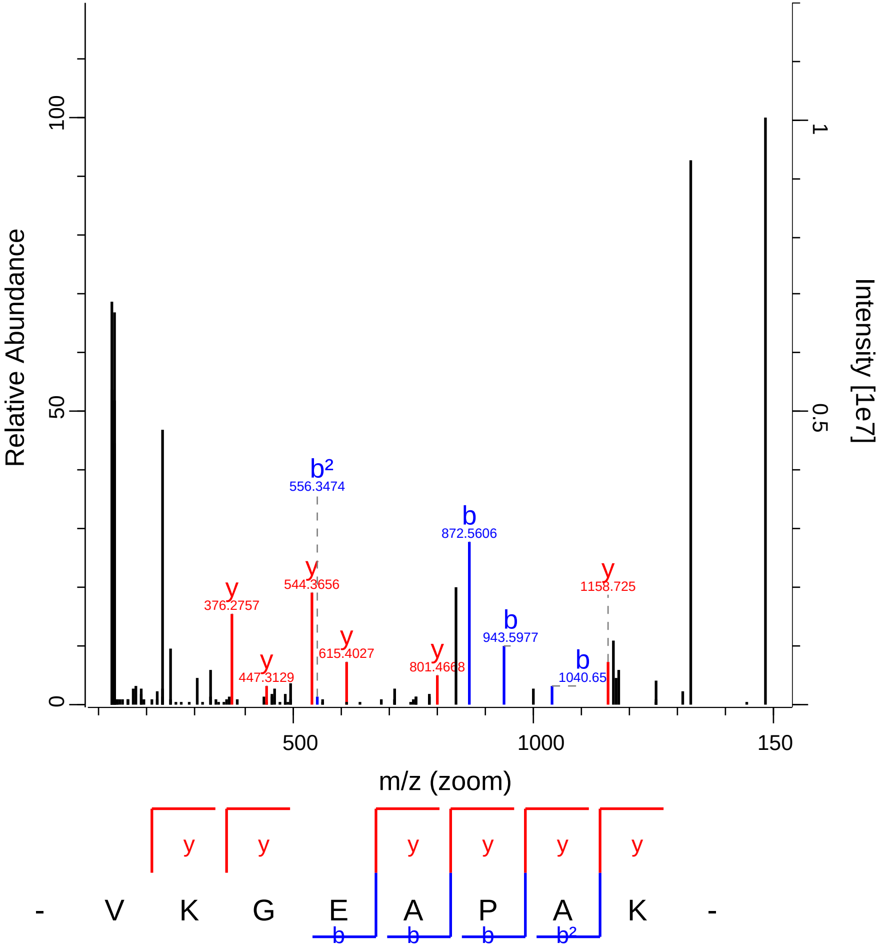

Raw File	Scan	Method	Score	m/z	Gene names
RO-QC-160121_ZackD_SS_Set_02_Fractio	7633	FTMS; HC	180.97	796.94	ZMYM1

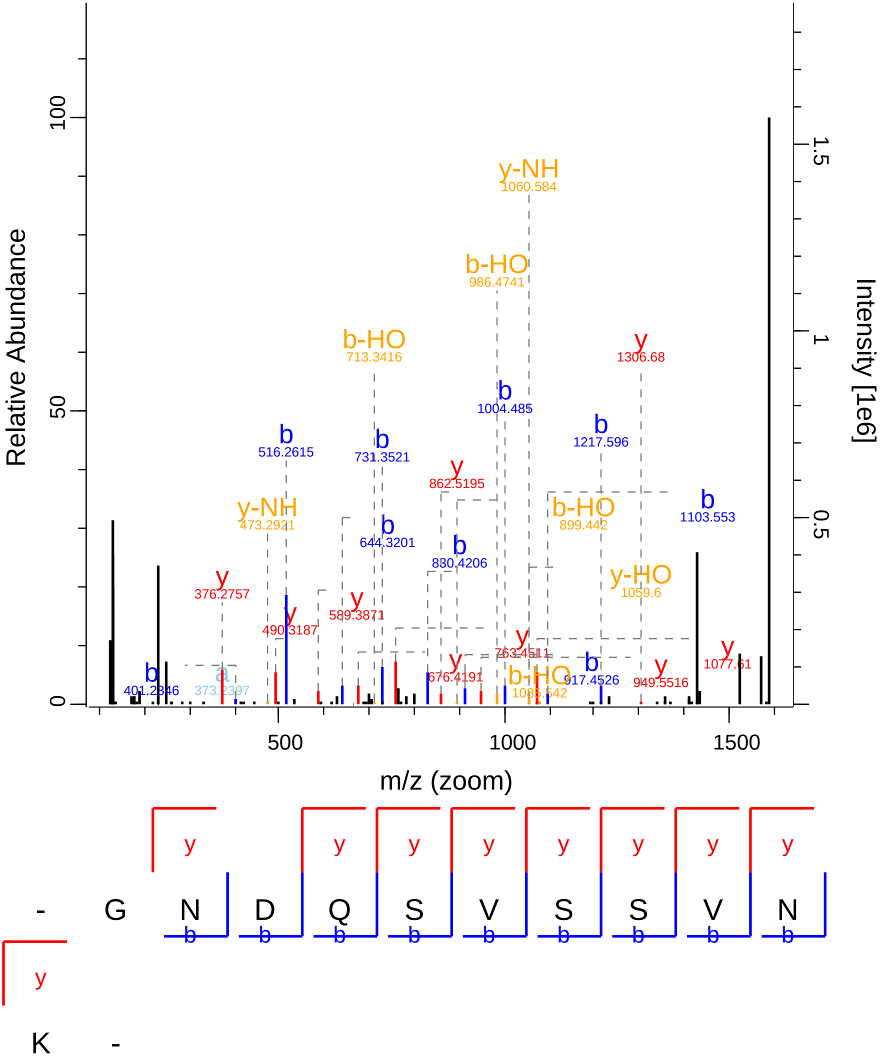

Raw File	Scan	Method	Score	m/z	Gene names
RO-QC-160121_ZackD_SS_Set_02_Fractior	8557	FTMS; HCI	53.17	861.04	GLYCTK

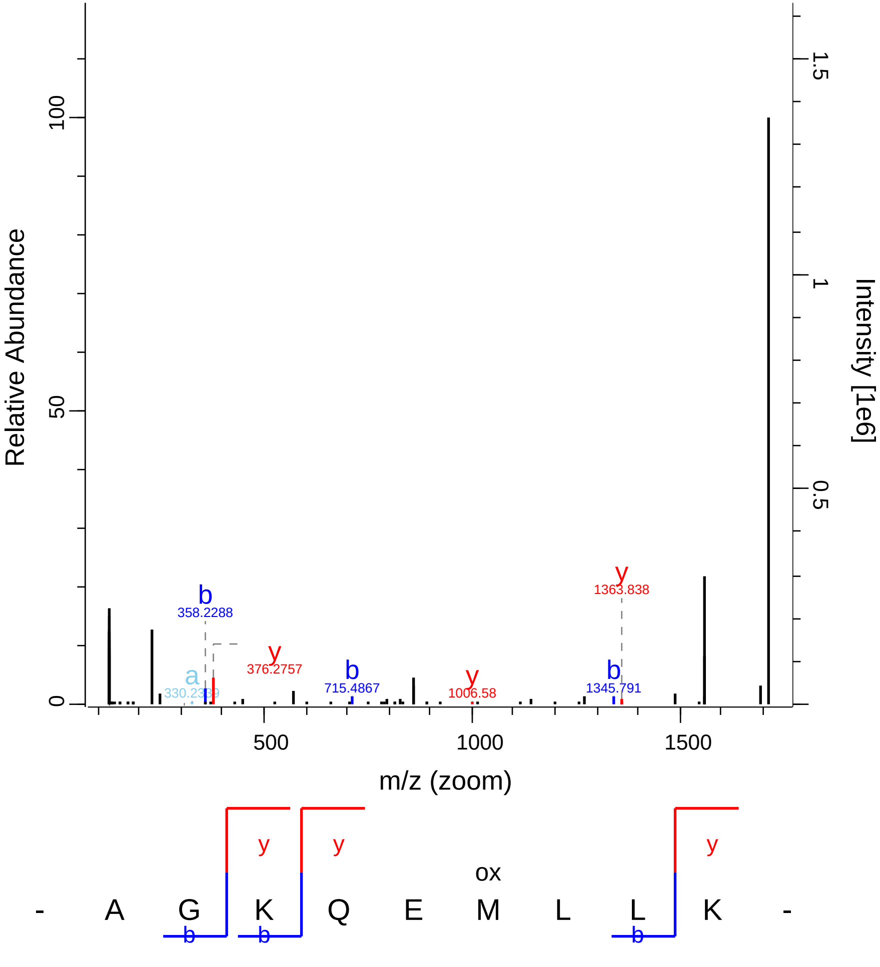

Raw File	Scan	Method	Score	m/z
RO-QC-160121_ZackD_SS_Set_02_Fraction_9	8725	FTMS; HCD	59.21	634.35

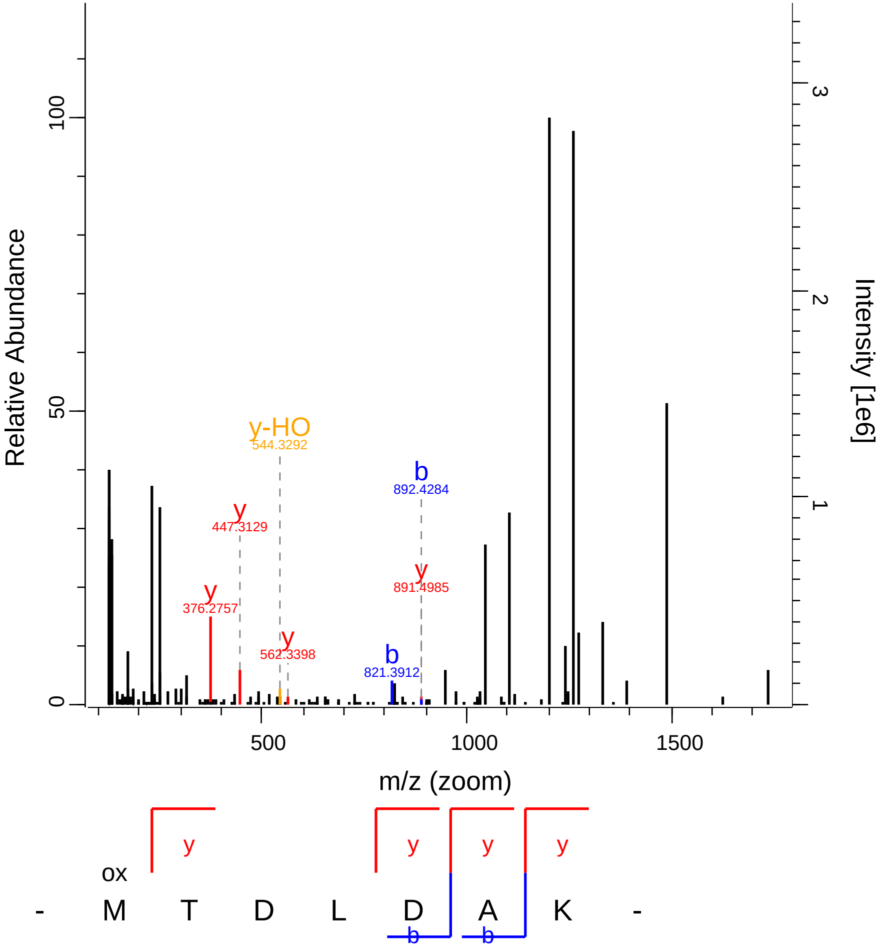

Raw File	Scan	Method	Score	m/z	Gene names
RO-QC-160121_ZackD_SS_Set_02_Fractior	8962	FTMS; HCI	58.68	390.48	RUNDC3B

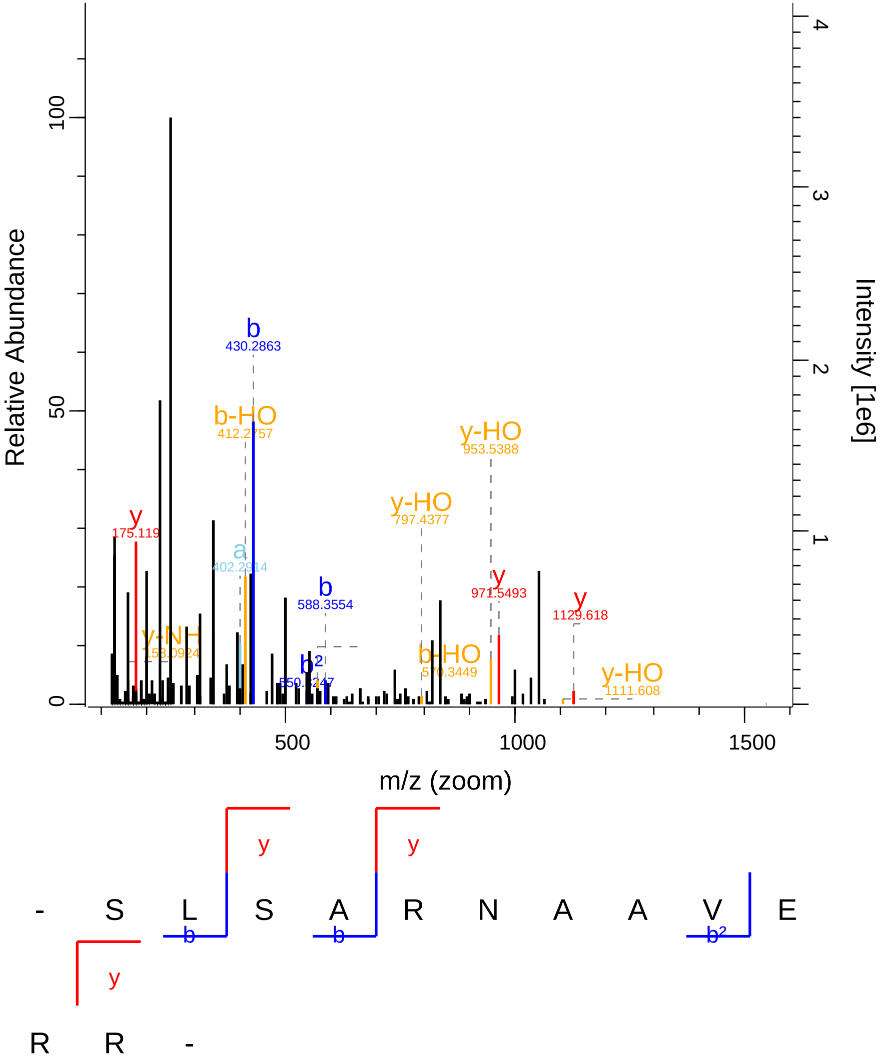

Raw File	Scan	Method	Score	m/z	Gene names
RO-QC-160121_ZackD_SS_Set_02_Fractio	9116	FTMS; HC	107.66	652.39	PAX9

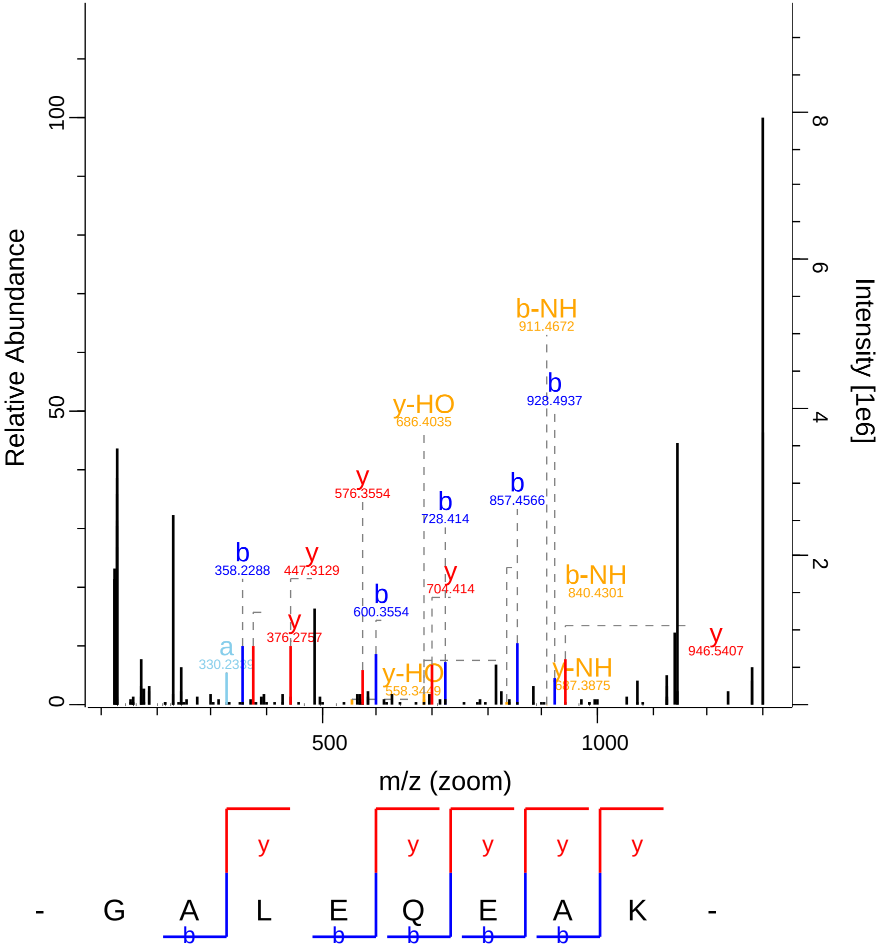

Raw File	Scan	Method	Score	m/z	Gene names
RO-QC-160121_ZackD_SS_Set_02_Fractior	9521	FTMS; HCI	71.98	531.97	PEX16

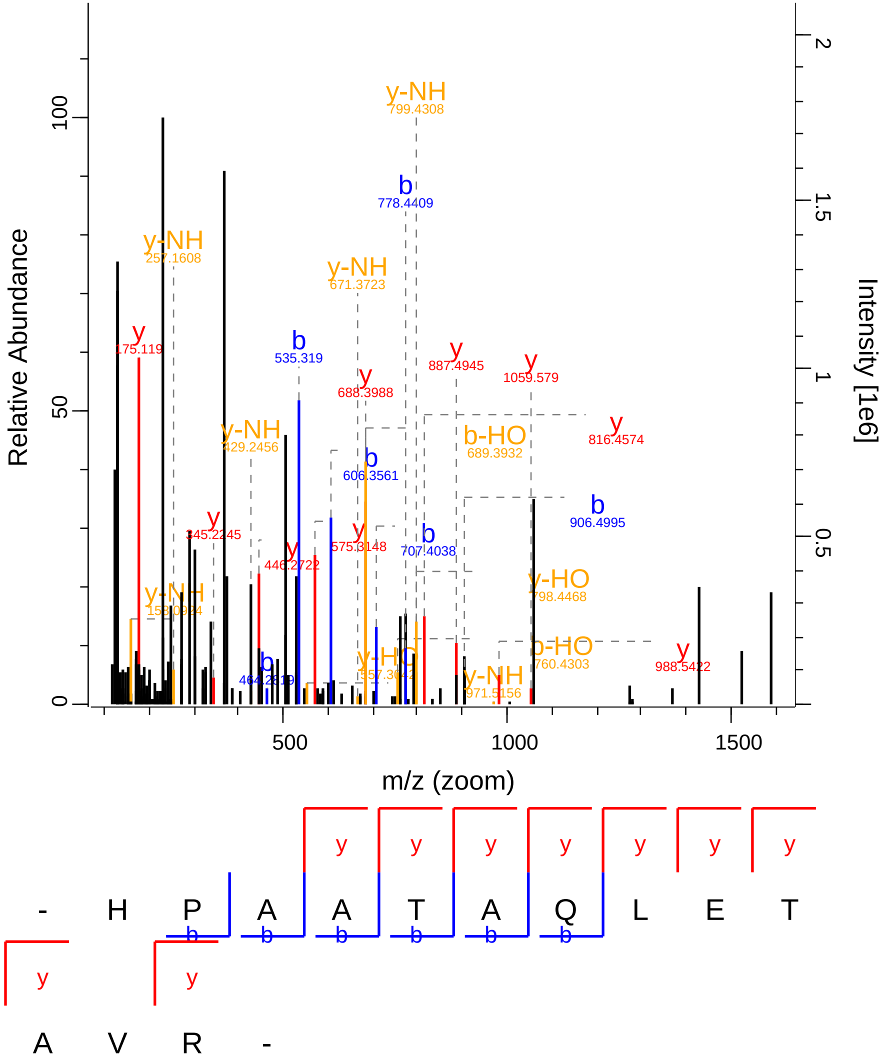

Raw File	Scan	Method	Score	m/z	Gene names
RO-QC-160121_ZackD_SS_Set_02_Fr	10083	FTMS; H(	128.57	596.35	ATP5G1;ATP5G2;ATP

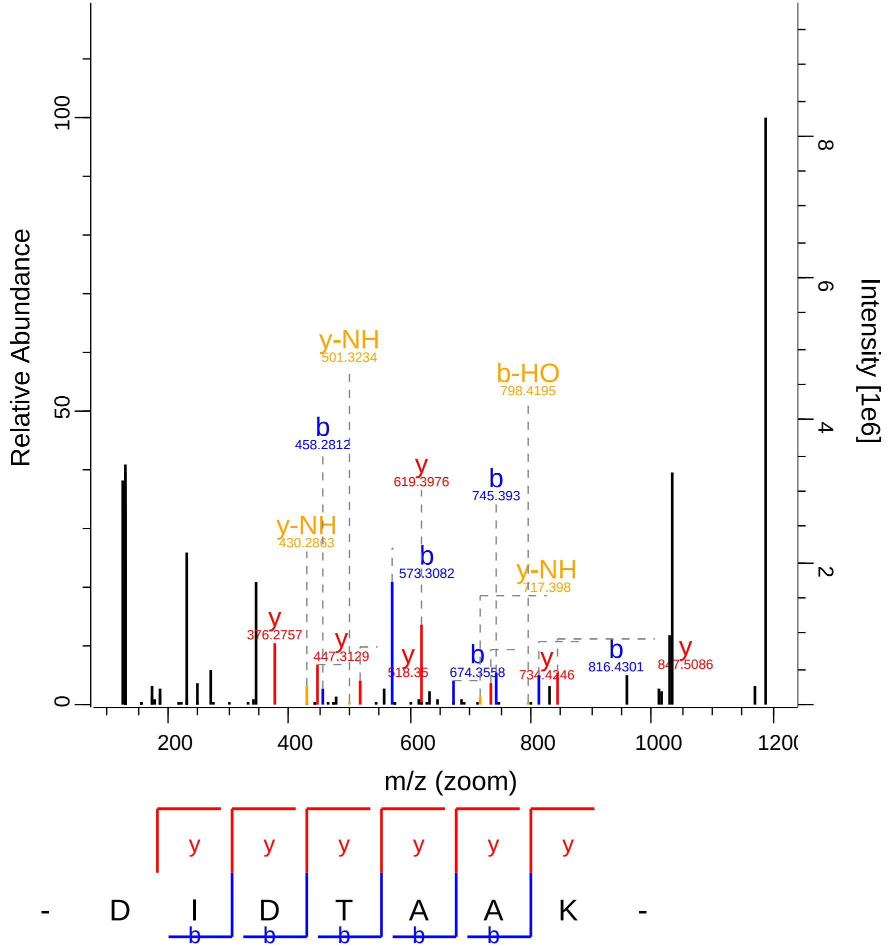

Raw File	Scan	Method	Score	m/z	Gene names
RO-QC-160121_ZackD_SS_Set_02_Fr	10304	FTMS; HC	130.77	596.35	ATP5G1;ATP5G2;ATP

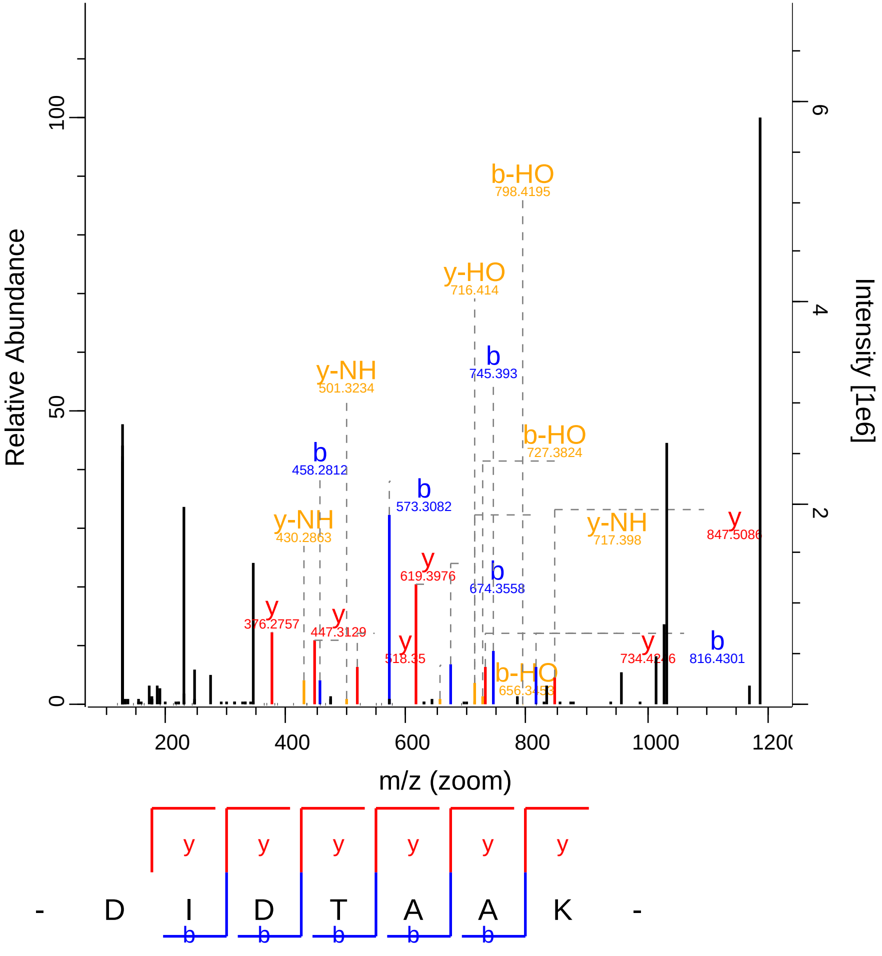

Raw File	Scan	Method	Score	m/z	Gene names
RO-QC-160121_ZackD_SS_Set_02_Fractic	10800	FTMS; HC	51.19	1038.53	USP43

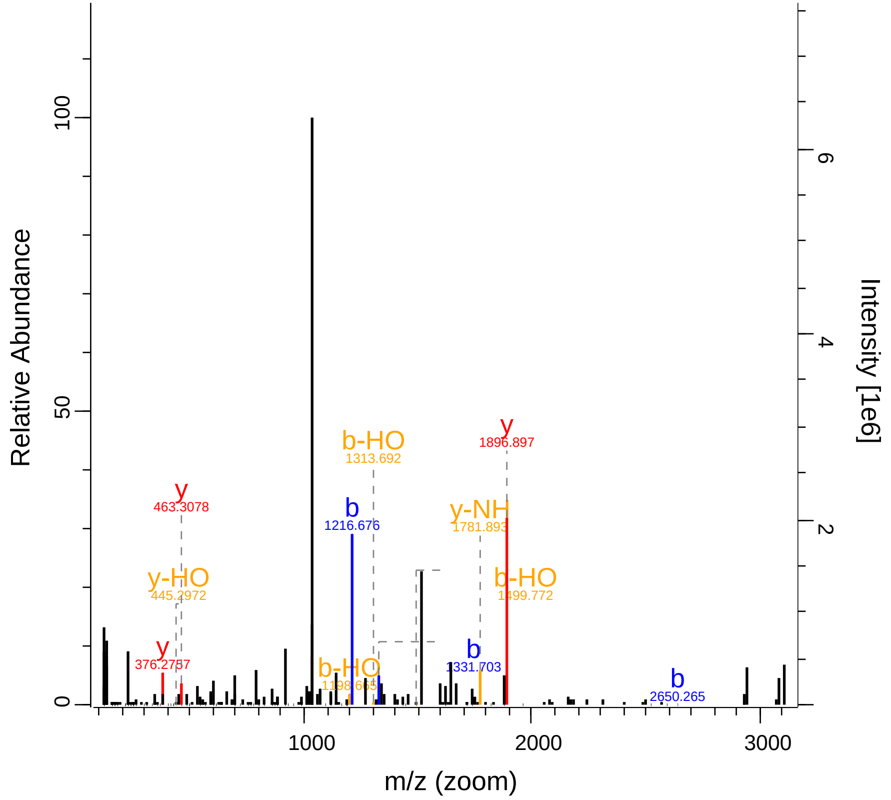

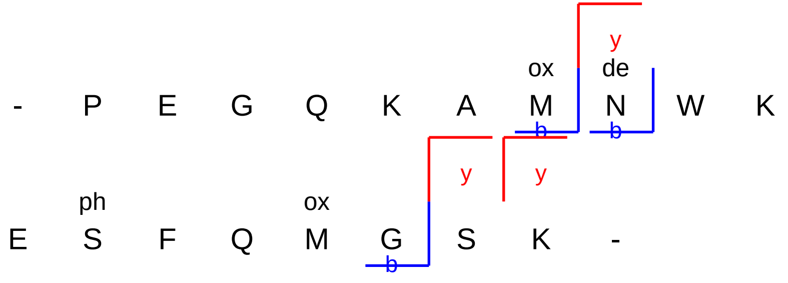

Raw File	Scan	Method	Score	m/z	Gene names
RO-QC-160121_ZackD_SS_Set_02_Fractio	13040	FTMS; HC	137.16	744.89	APH1B

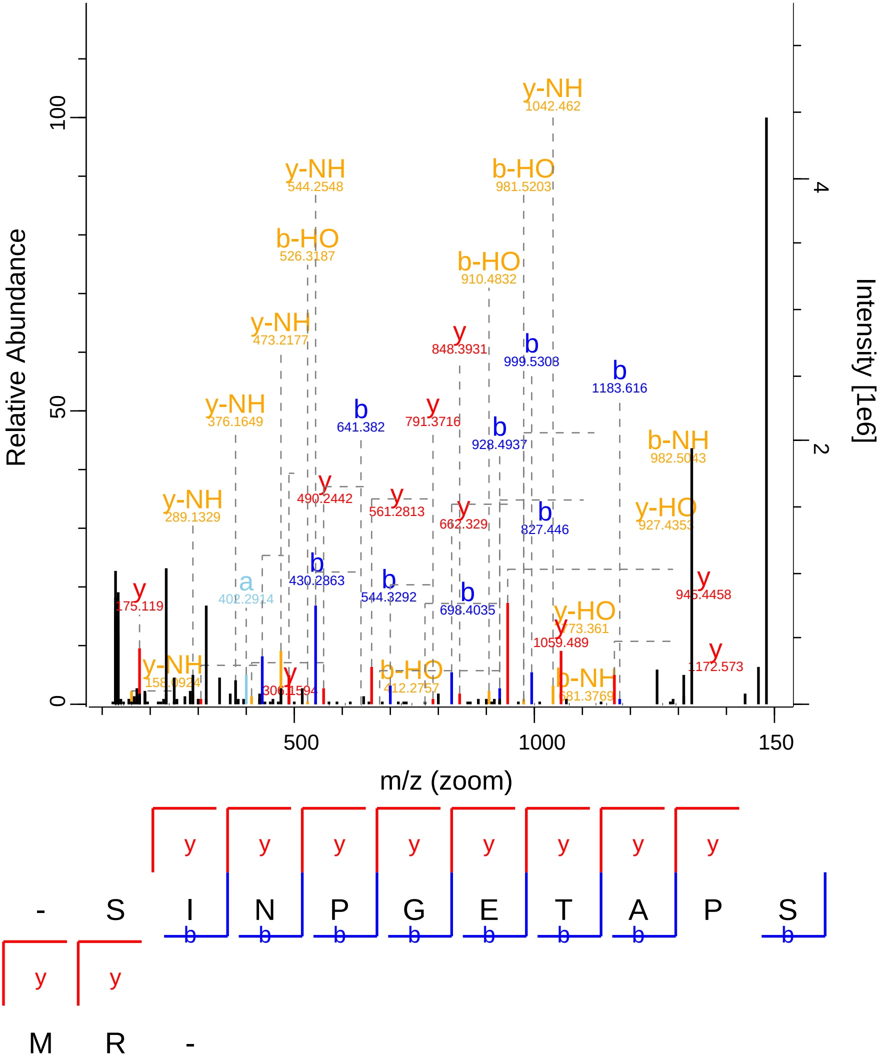

Raw File	Scan	Method	Score	m/z	Gene names
RO-QC-160121_ZackD_SS_Set_02_Fractio	13948	FTMS; HC	145.9	719.89	CYSTM1

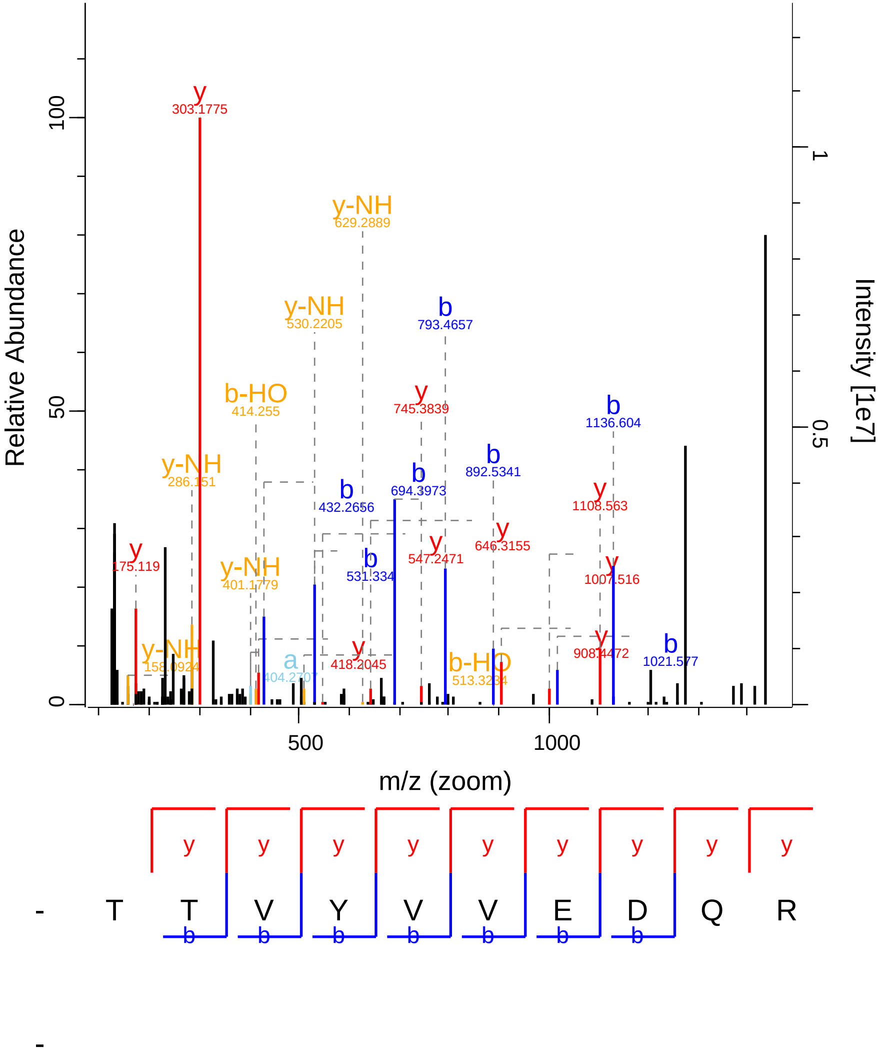

Raw File	Scan	Method	Score	m/z	Gene names
RO-QC-160121_ZackD_SS_Set_02_Fractio	14209	FTMS; HC	74.2	720.39	CYSTM1

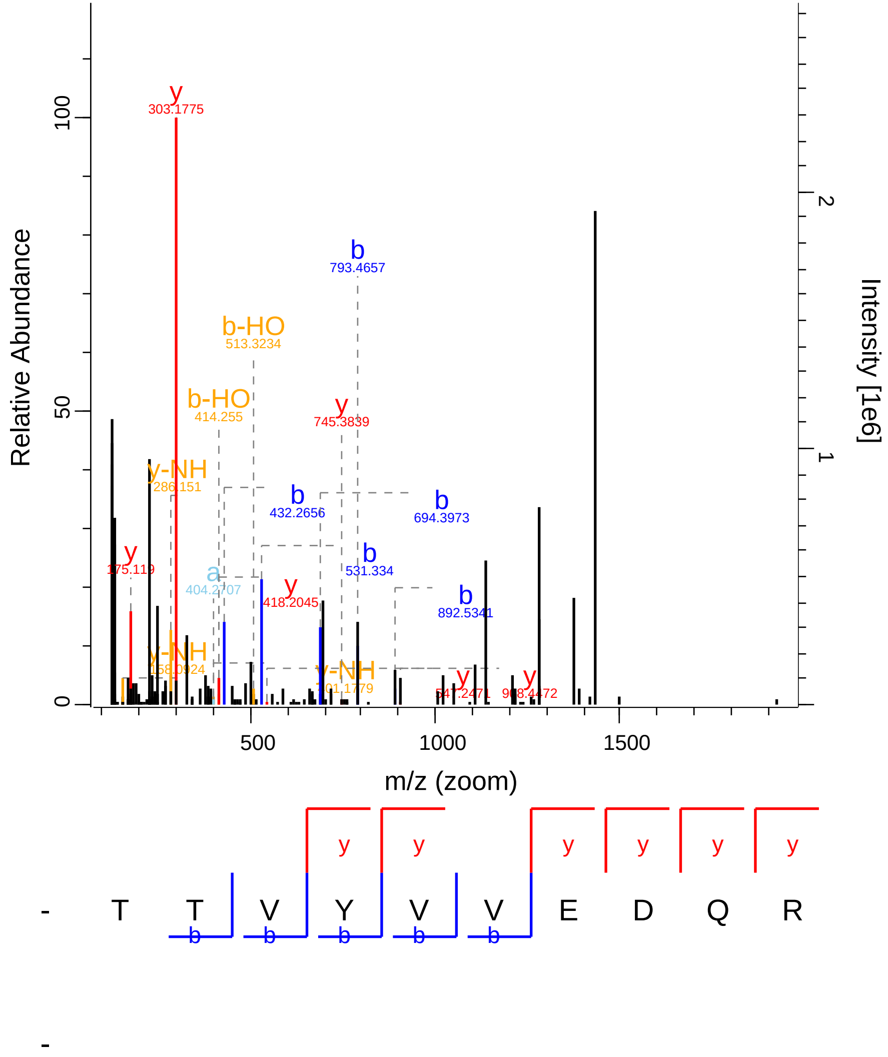

Raw File	Scan	Method	Score	m/z	Gene names
RO-QC-160121_ZackD_SS_Set_02_Fractio	14306	FTMS; HC	68.38	709.89	FBXL19

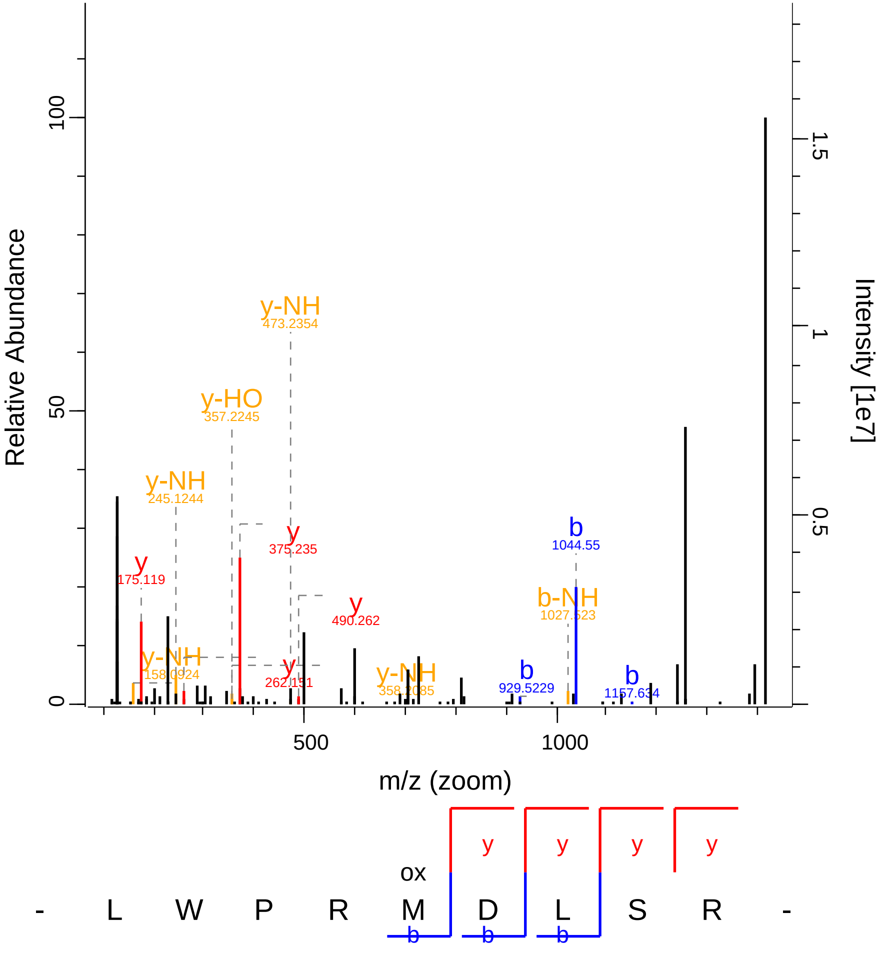

Raw File	Scan	Method	Score	m/z	Gene names
RO-QC-160121_ZackD_SS_Set_02_Fractio	14360	FTMS; HC	82.28	403.73	MED7

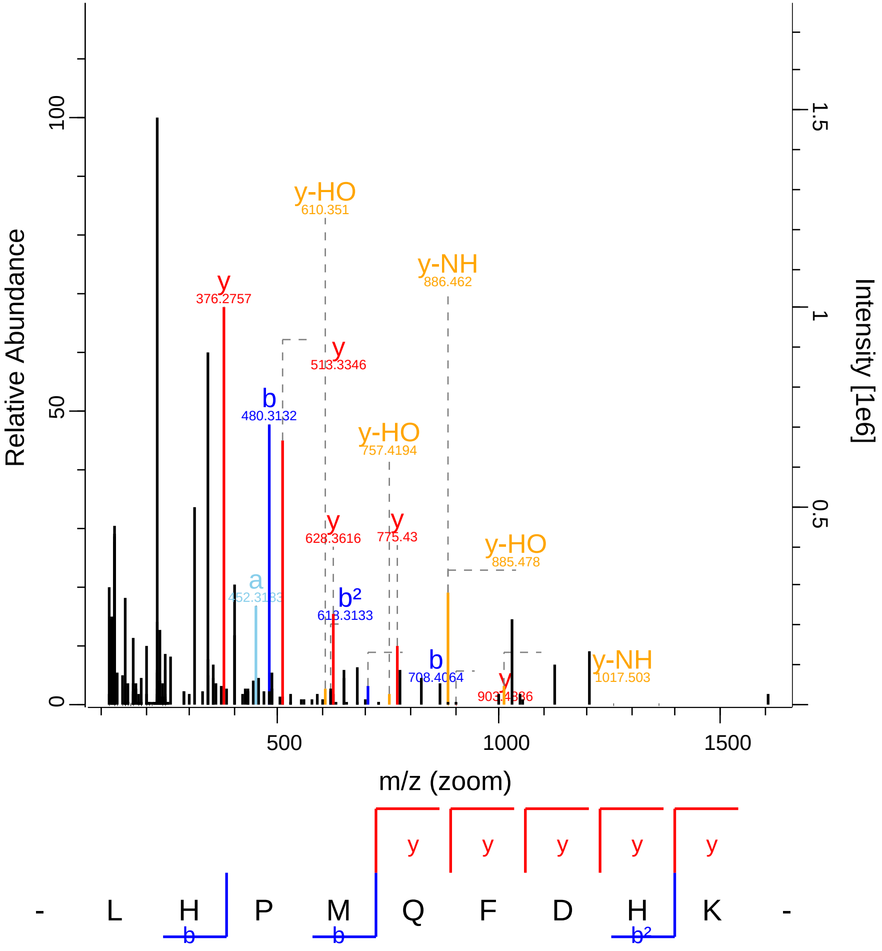

Raw File	Scan	Method	Score	m/z	Gene names
RO-QC-160121_ZackD_SS_Set_02_Fractio	15997	FTMS; HC	136.57	463.31	TMC1

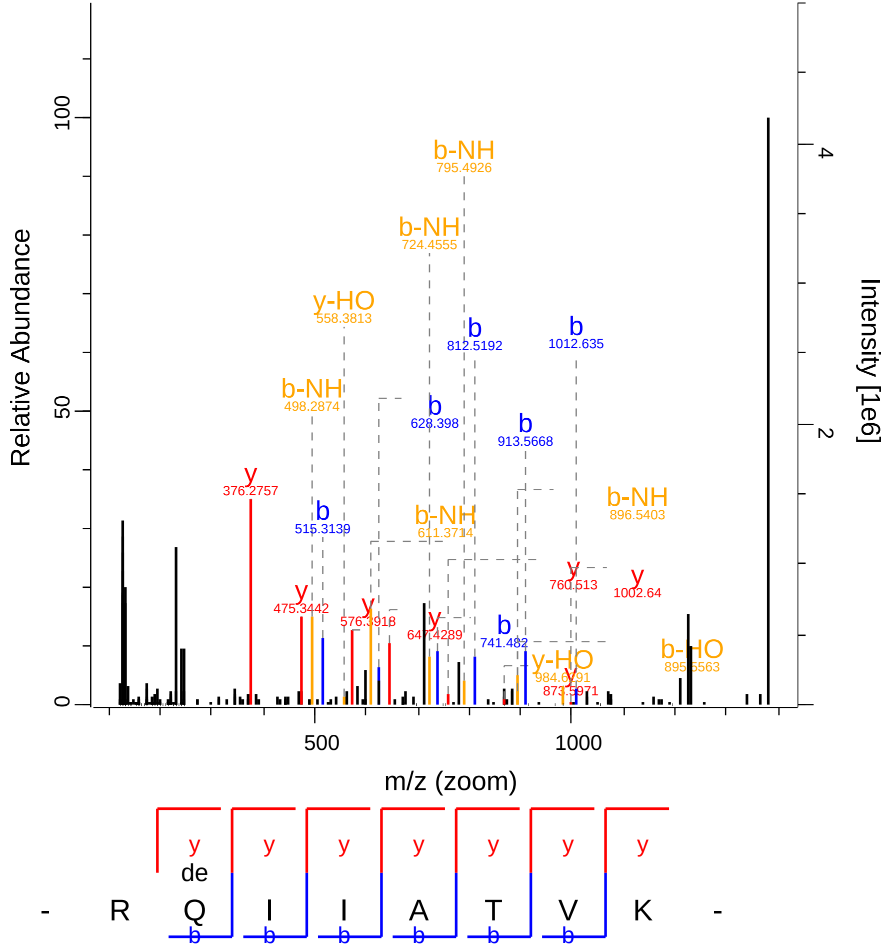

Raw File	Scan	Method	Score	m/z
RO-QC-160121_ZackD_SS_Set_02_Fraction_9	16130	FTMS; HCD	116.35	580.38

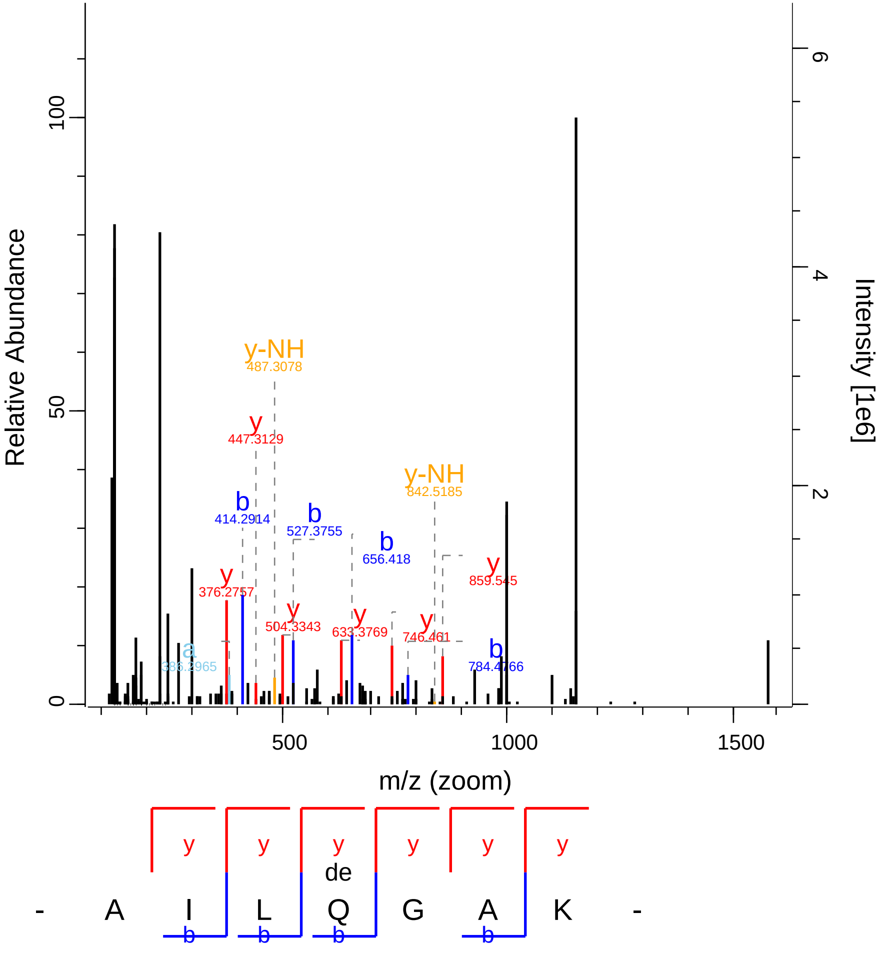

Raw File	Scan	Method	Score	m/z	Gene names
RO-QC-160121_ZackD_SS_Set_02_Fractio	17178	FTMS; HC	113.81	778.38	F2R

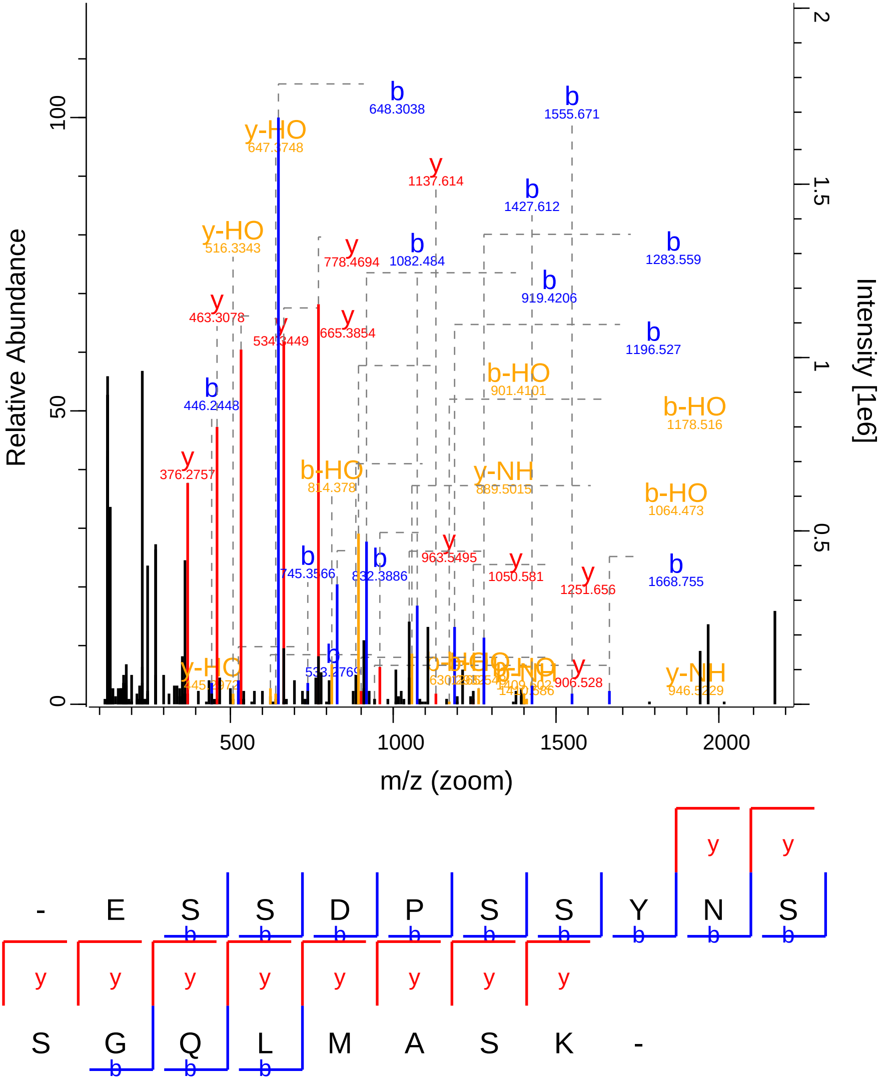

Raw File	Scan	Method	Score	m/z	Gene names
RO-QC-160121_ZackD_SS_Set_02_Fractio	18414	FTMS; HC	50.04	640.86	PROK2

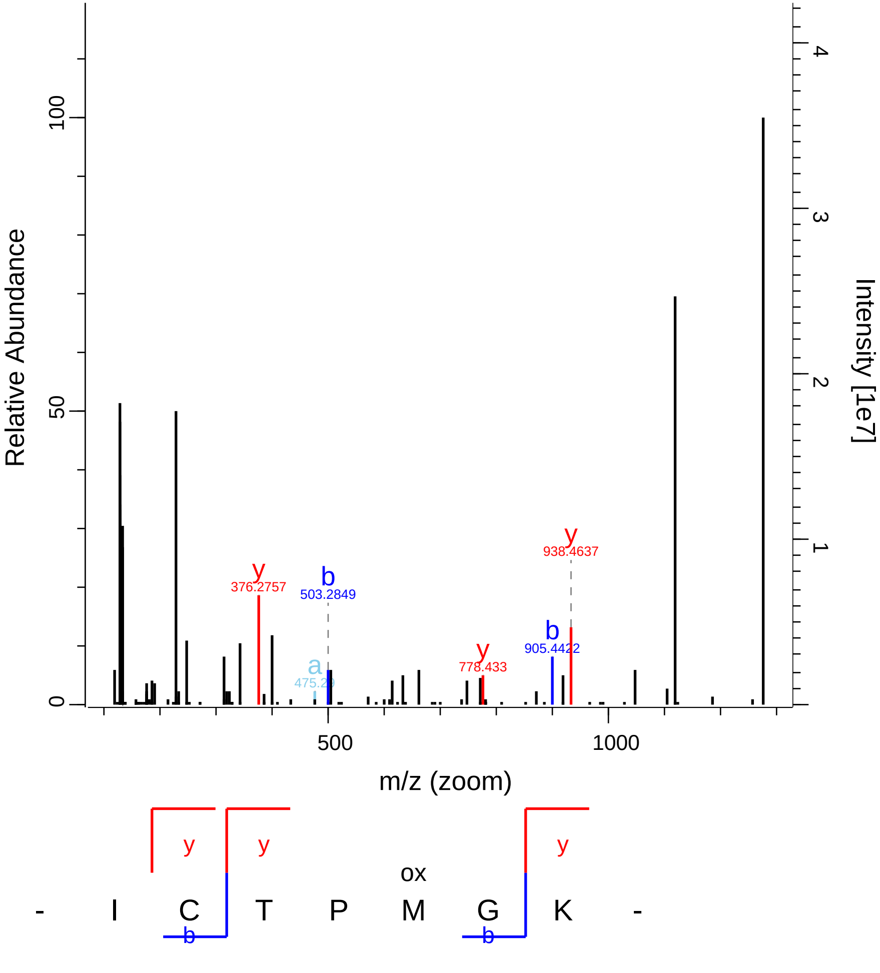

Raw File	Scan	Method	Score	m/z	Gene names
RO-QC-160121_ZackD_SS_Set_02_Fractio	18660	FTMS; HC	50.04	640.86	PROK2

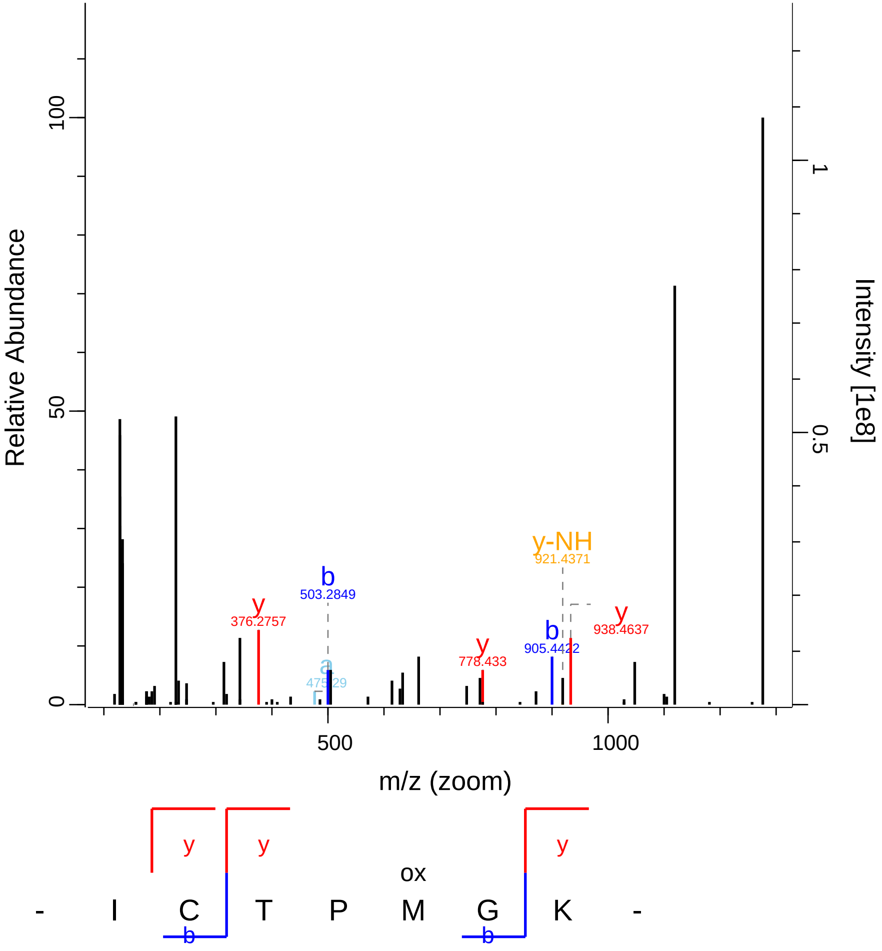

Raw File	Scan	Method	Score	m/z	Gene names
RO-QC-160121_ZackD_SS_Set_02_Fractio	18710	FTMS; HC	43.51	537.56	CCDC60

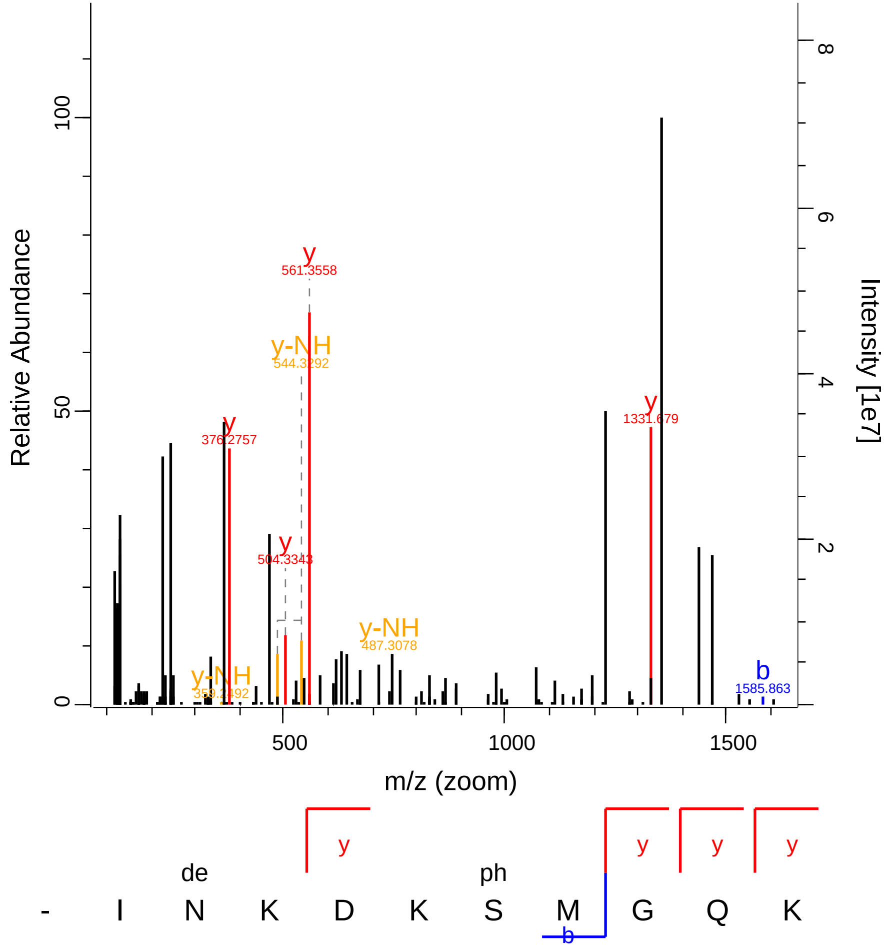

-

Raw File	Scan	Method	Score	m/z	Gene names
RO-QC-160121_ZackD_SS_Set_02_Fractio	18824	FTMS; HC	130.94	737.93	SLC29A3

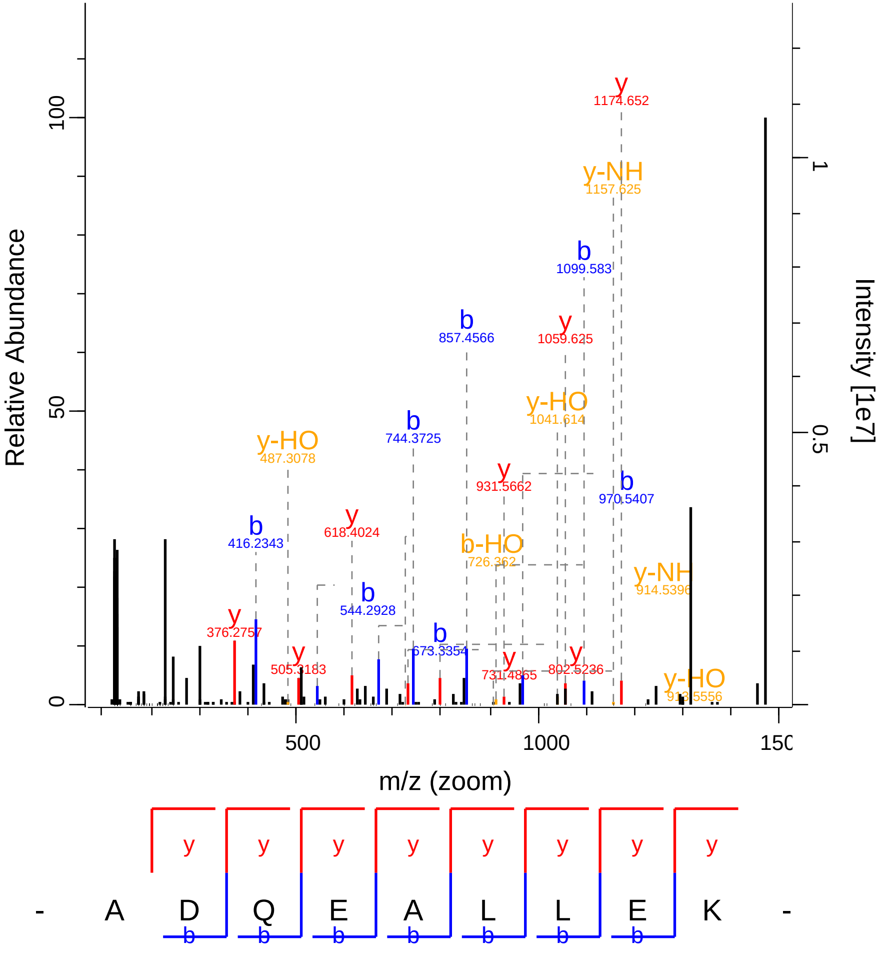

Raw File	Scan	Method	Score	m/z	Gene names
RO-QC-160121_ZackD_SS_Set_02_Fractio	19561	FTMS; HC	45.43	651.11	DNAH9

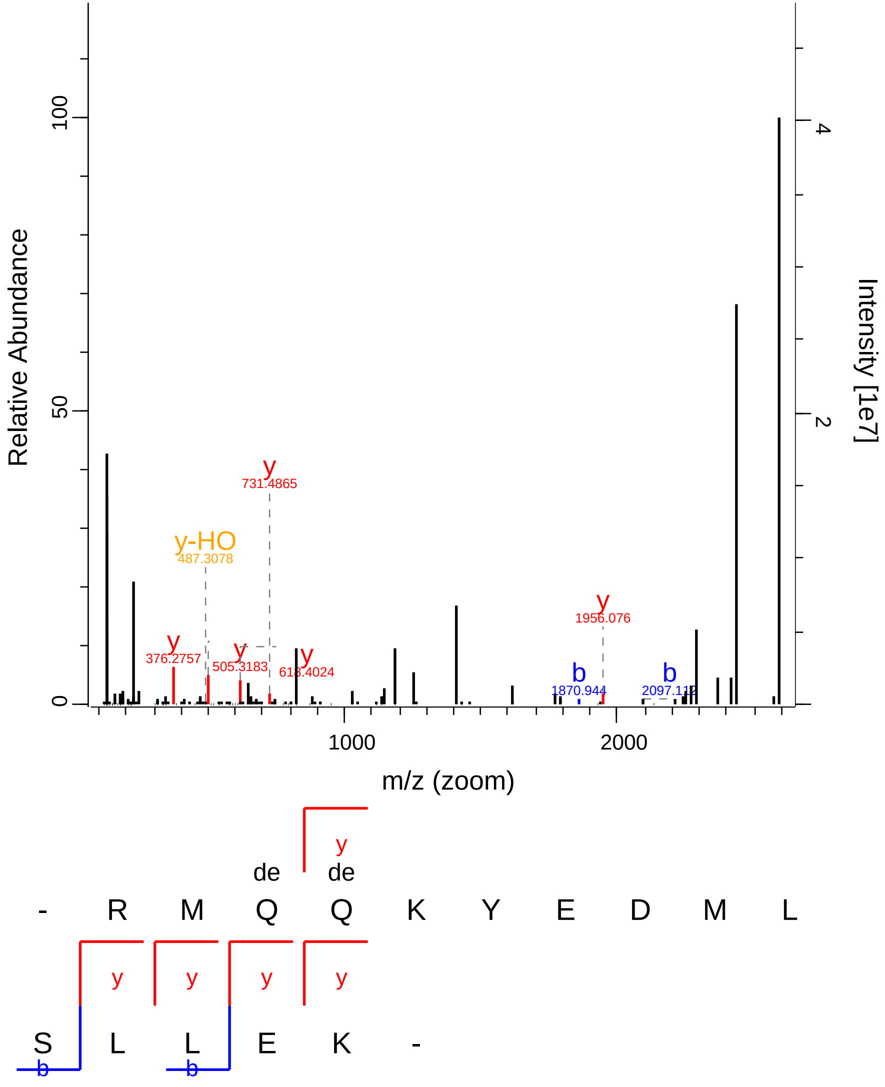

Raw File	Scan	Method	Score	m/z	Gene names
RO-QC-160121_ZackD_SS_Set_02_Fractio	20228	FTMS; HC	41.54	451.51	TMCC2

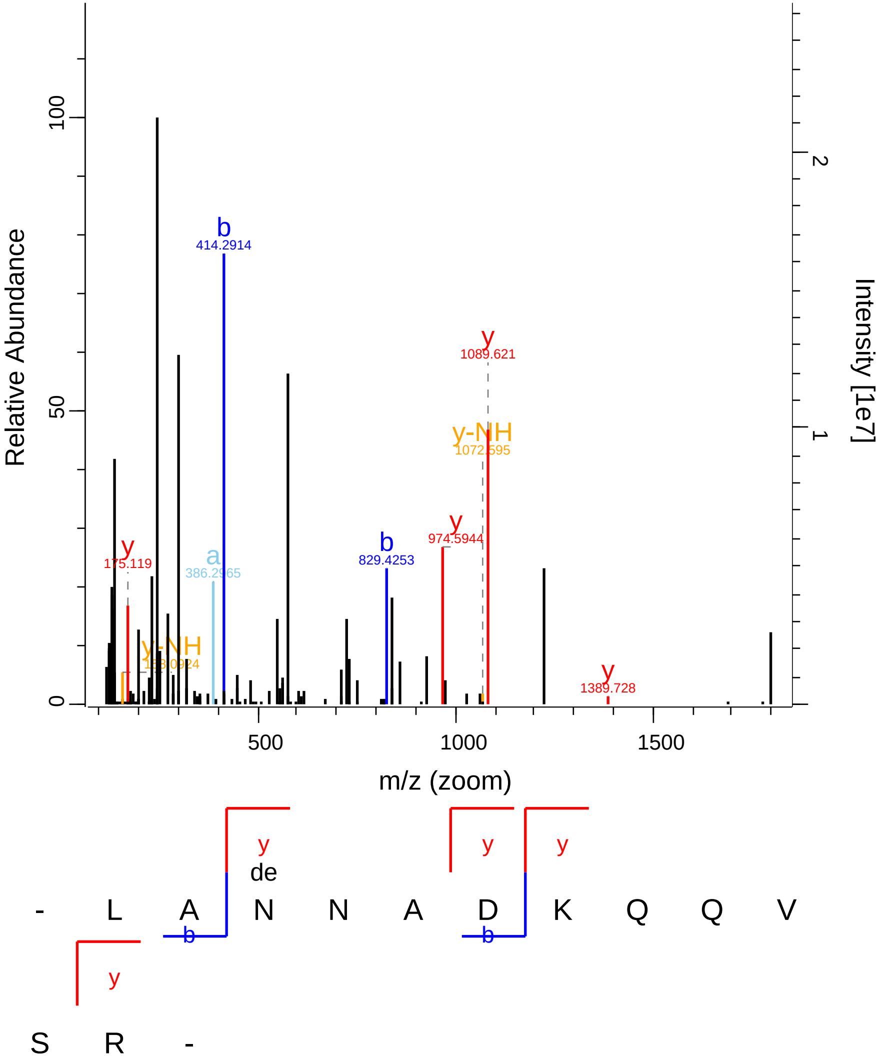

Raw File	Scan	Method	Score	m/z	Gene names
RO-QC-160121_ZackD_SS_Set_02_Fractio	21442	FTMS; HC	62.3	516.98	FAM53C

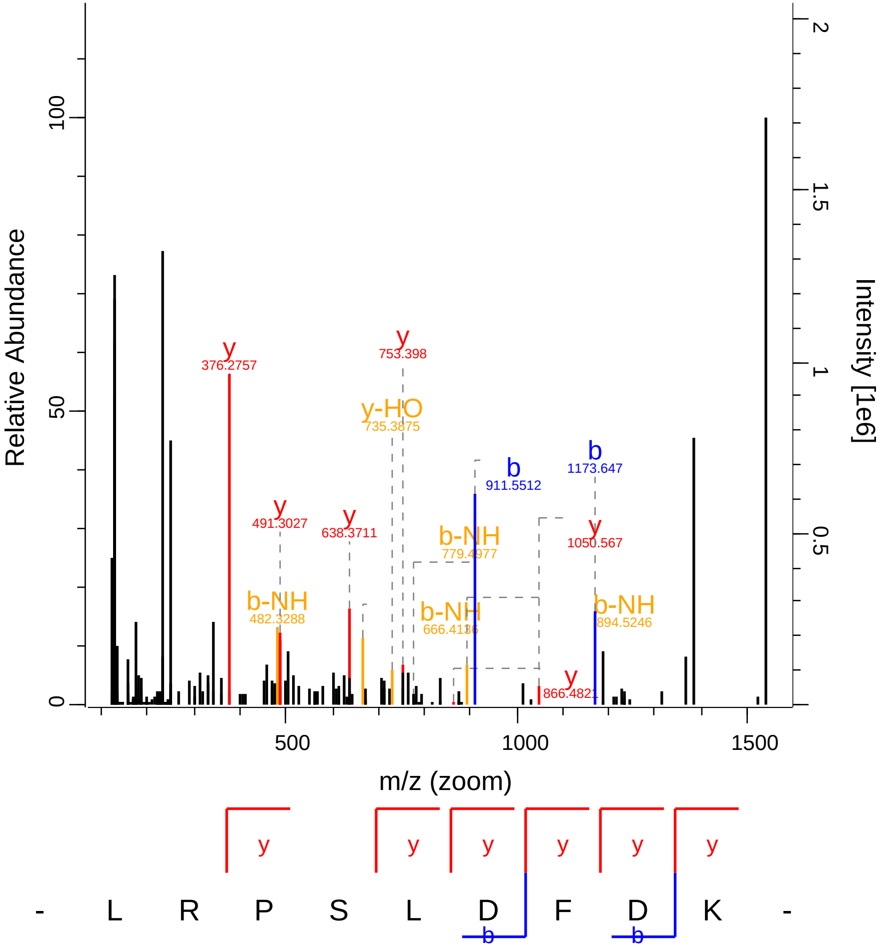

Raw File	Scan	Method	Score	m/z	Gene names
RO-QC-160121_ZackD_SS_Set_02_Fractio	22462	FTMS; HC	43.08	498.79	FLG2

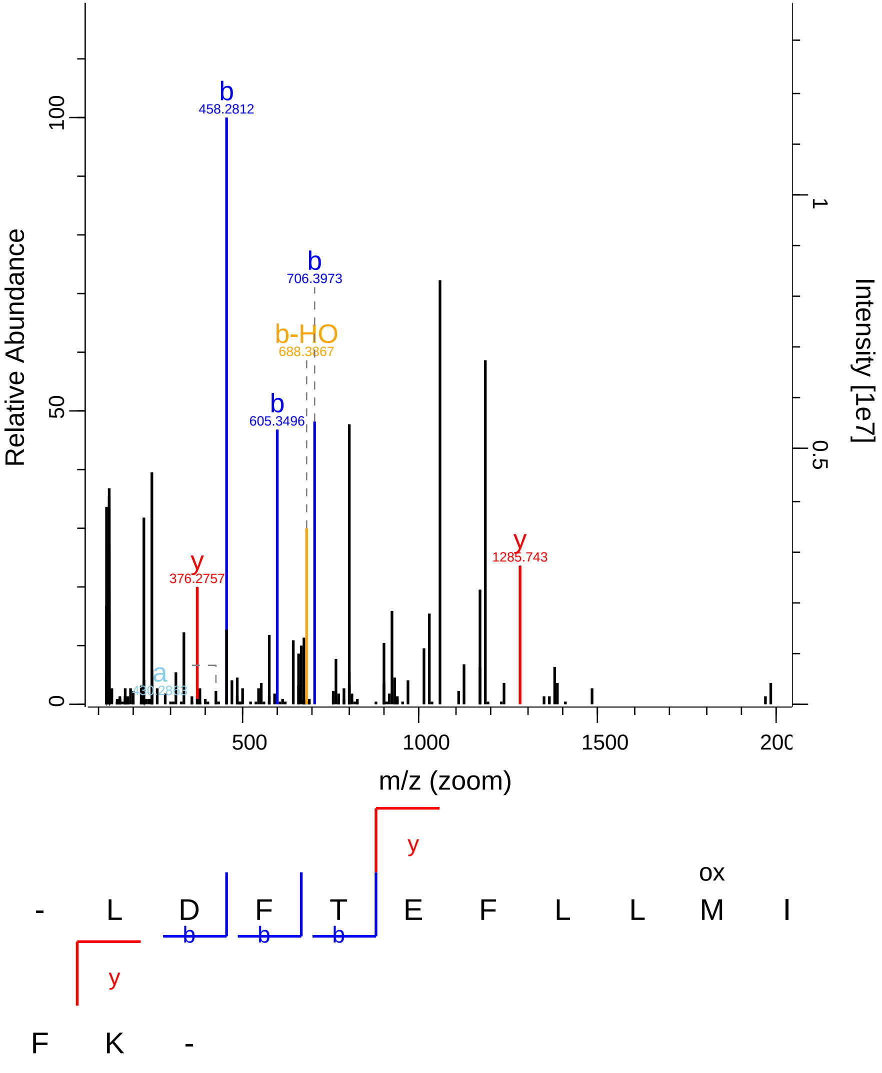

Raw File	Scan	Method	Score	m/z	Gene names
RO-QC-160121_ZackD_SS_Set_02_Fractio	22598	FTMS; HC	102.53	751.41	EMC6

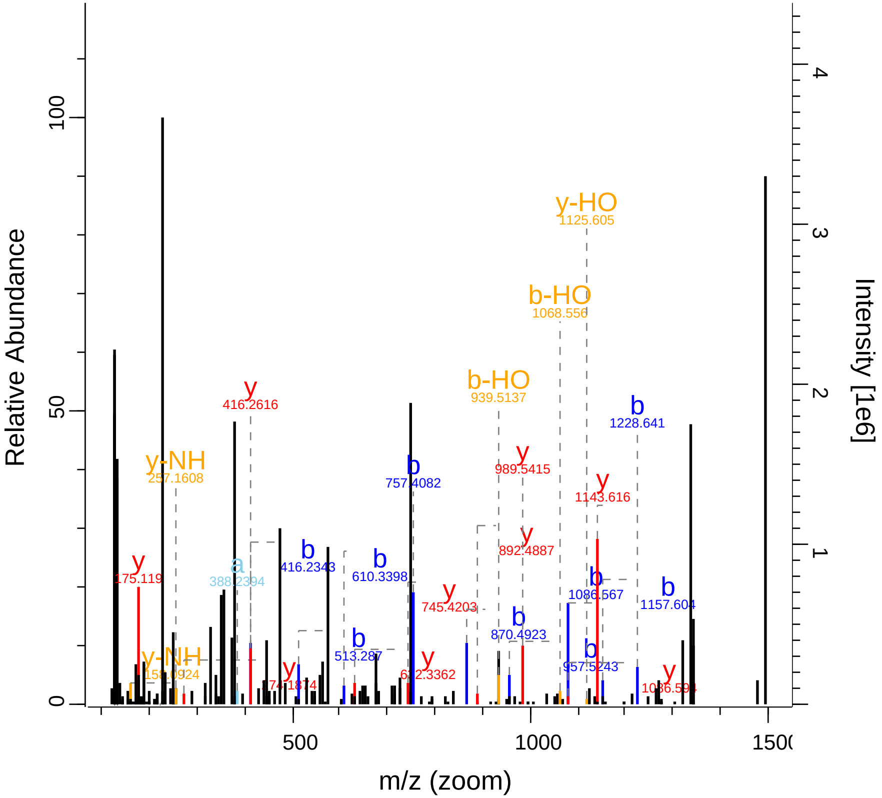

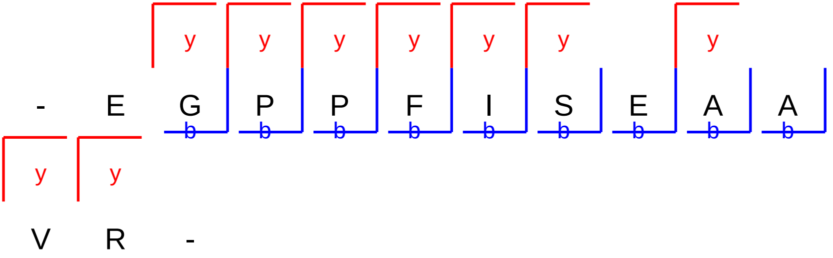

Raw File	Scan	Method	Score	m/z
RO-QC-160121_ZackD_SS_Set_02_Fraction_9	22707	FTMS; HCD	50.97	410.6

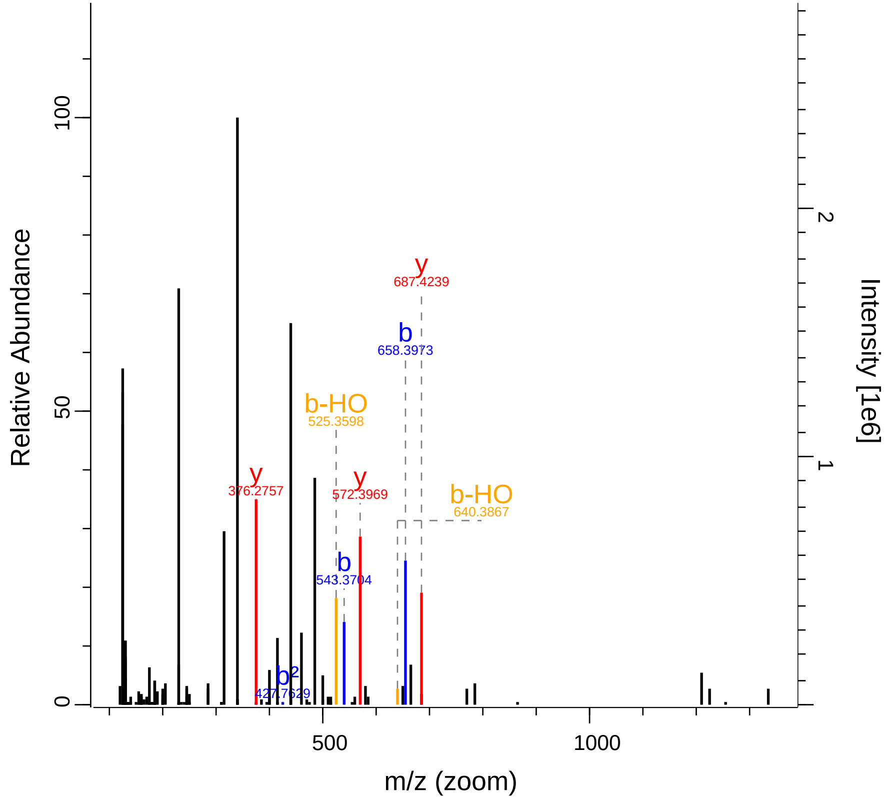

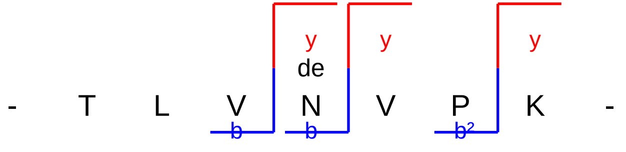

Raw File	Scan	Method	Score	m/z
RO-QC-160121_ZackD_SS_Set_02_Fraction_9	22972	FTMS; HCD	68.42	410.6

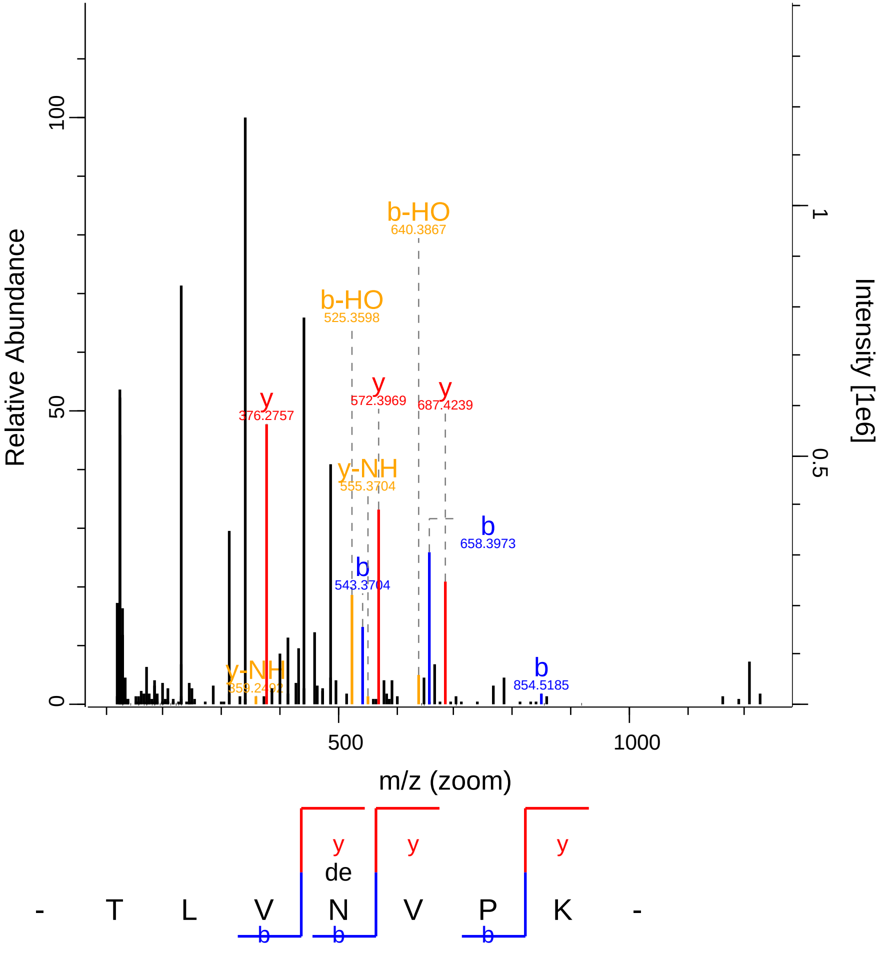

Raw File	Scan	Method	Score	m/z	Gene names
RO-QC-160121_ZackD_SS_Set_02_Fractio	25282	FTMS; HC	141.39	649.89	EBP

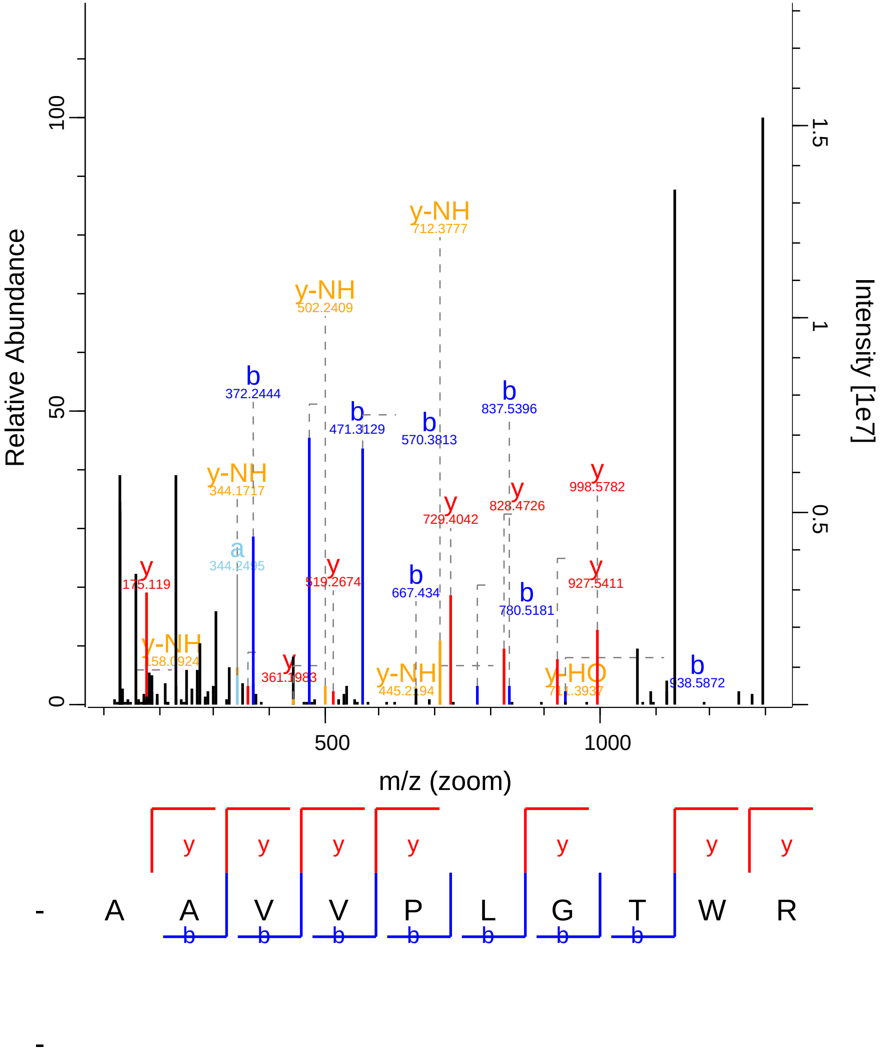

Raw File	Scan	Method	Score	m/z	Gene names
RO-QC-160121_ZackD_SS_Set_02_Fractio	25470	FTMS; HC	103.55	984.04	NALCN

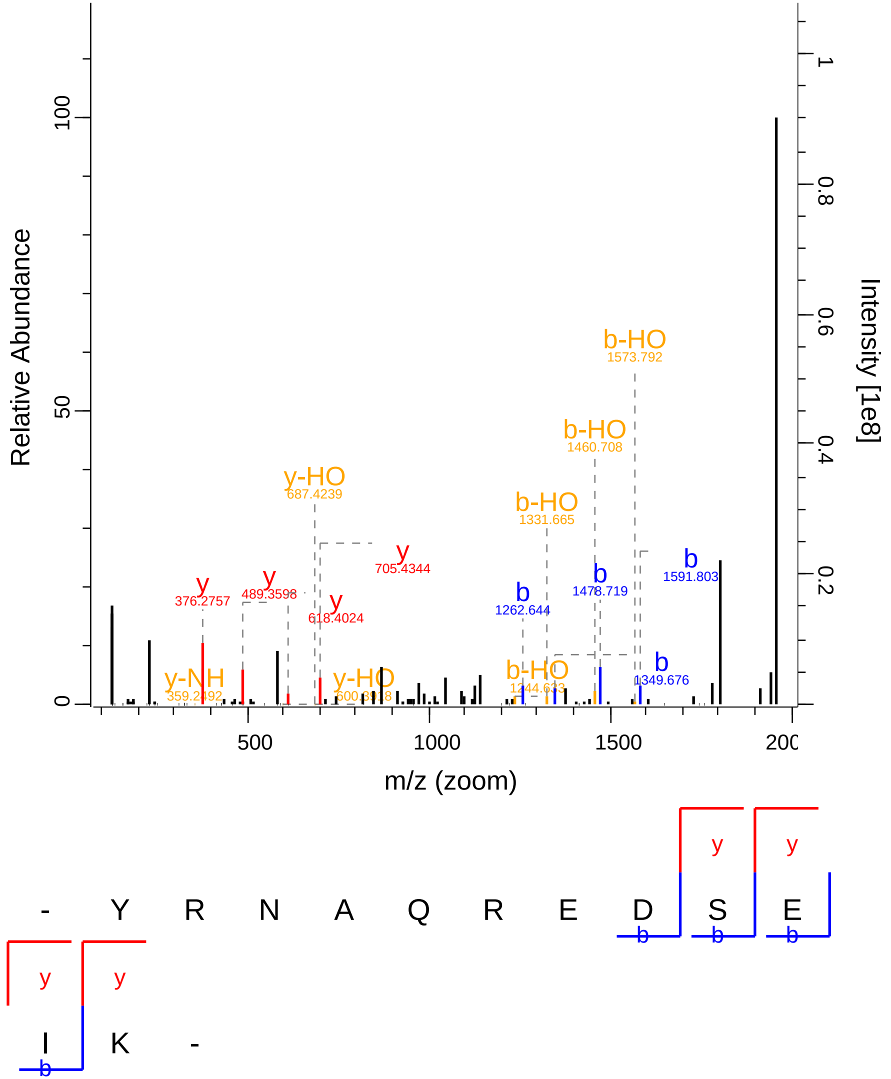

Raw File	Scan	Method	Score	m/z	Gene names
RO-QC-160121_ZackD_SS_Set_02_Fractic	27556	FTMS; HC	96.82	1184.15	ATP6V0C

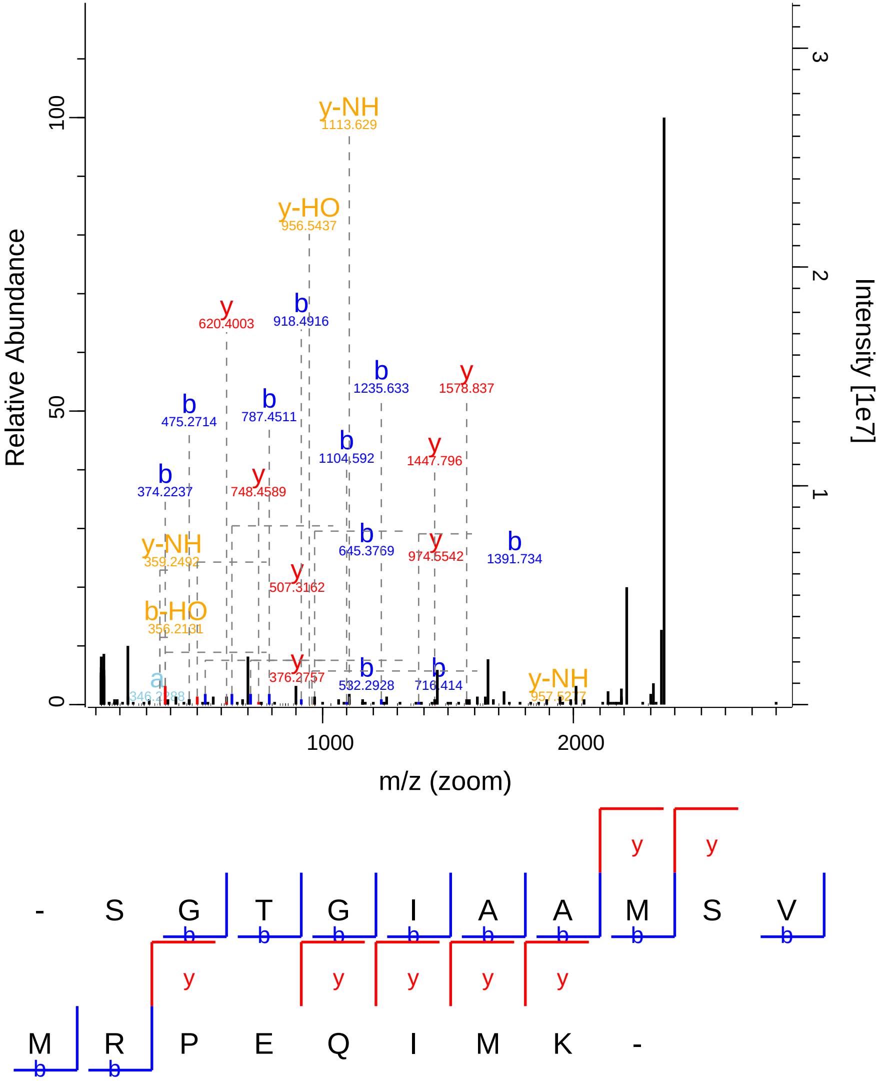

Raw File	Scan	Method	Score	m/z	Gene names
RO-QC-160121_ZackD_SS_Set_02_Fractio	27561	FTMS; HC	49.99	592.33	ATP6V0C

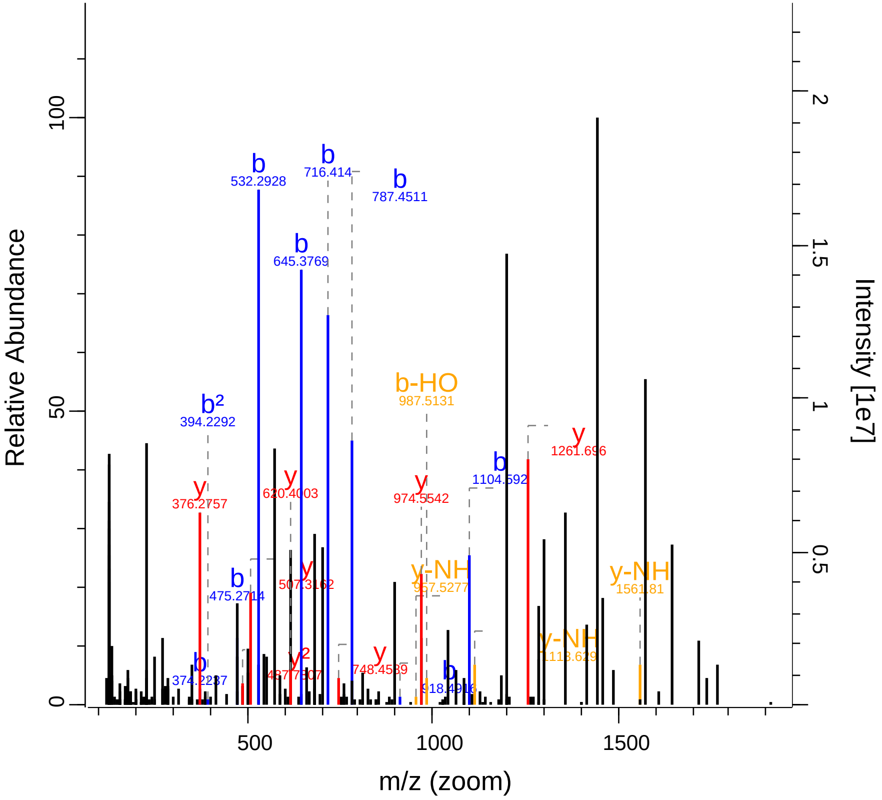

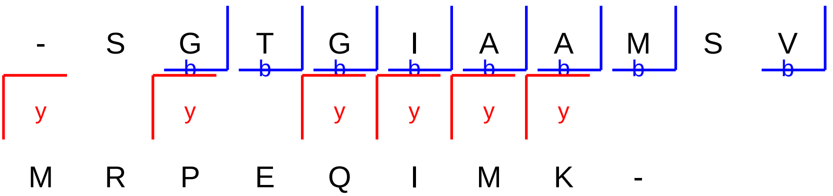

Raw File	Scan	Method	Score	m/z
RO-QC-160121_ZackD_SS_Set_02_Fraction_9	27613	FTMS; HCD	44.14	515.99

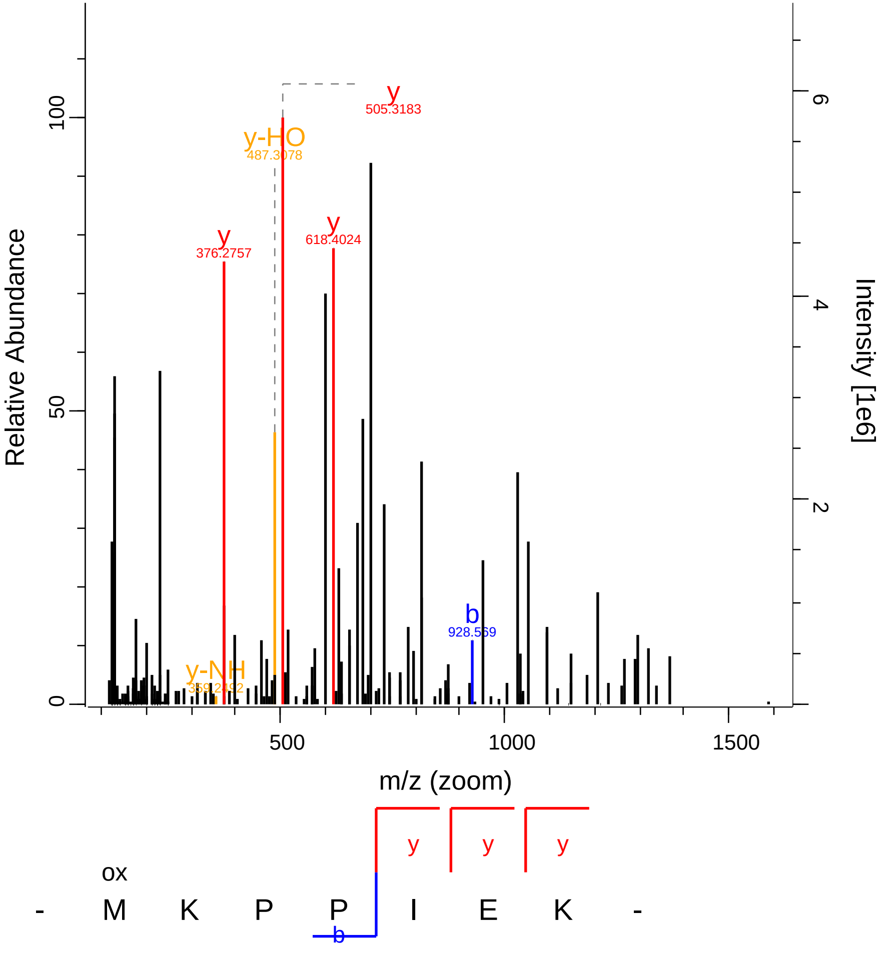

Raw File	Scan	Method	Score	m/z	Gene names
RO-QC-160121_ZackD_SS_Set_02_Fractio	27752	FTMS; HC	100.69	789.44	ATP6V0C

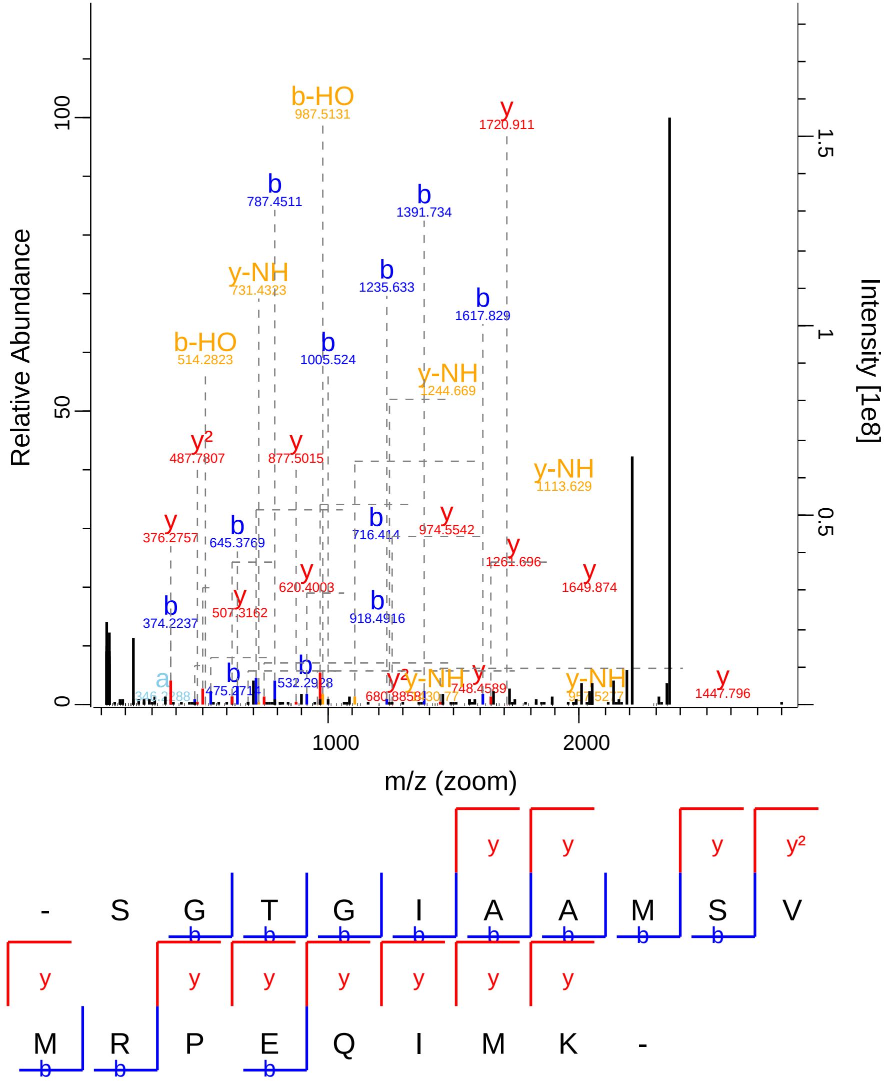

Raw File	Scan	Method	Score	m/z	Gene names
RO-QC-160121_ZackD_SS_Set_02_Fractio	27815	FTMS; HC	32.19	592.58	ATP6V0C

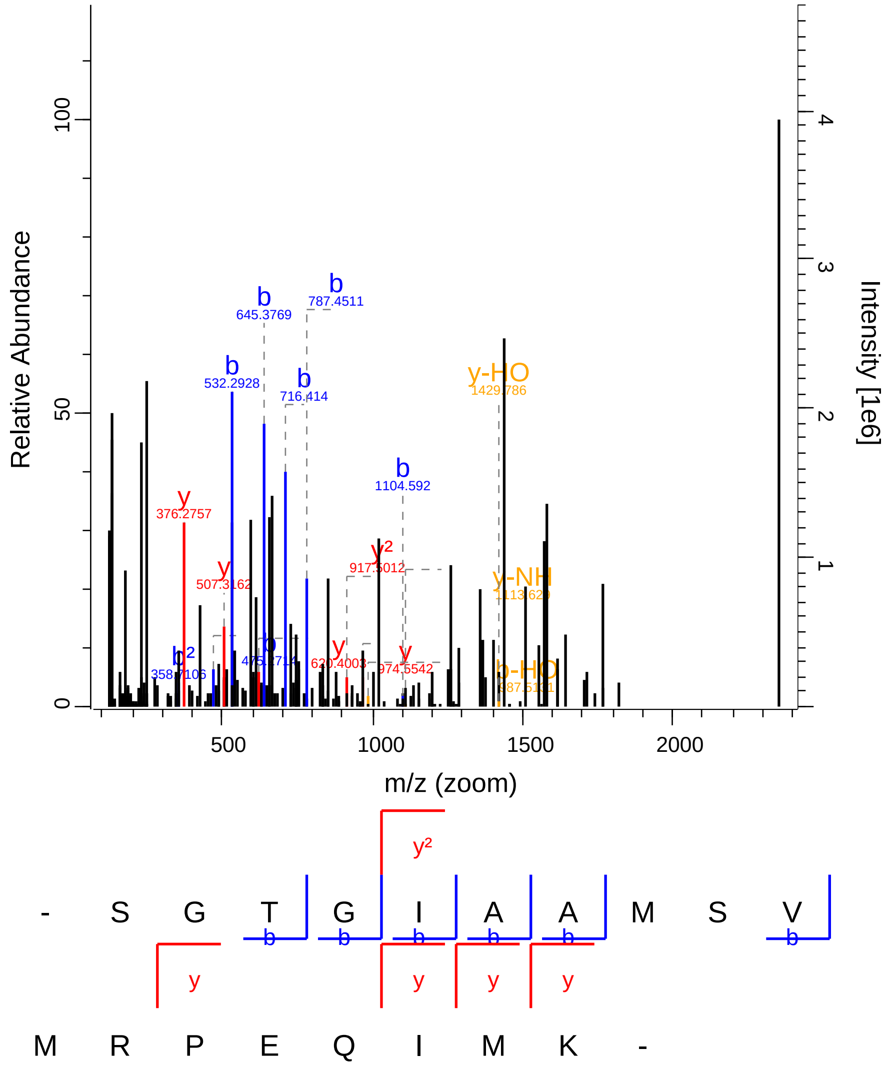

Raw File	Scan	Method	Score	m/z	Gene names
RO-QC-160121_ZackD_SS_Set_02_Fractio	28133	FTMS; HC	200.14	1259.3	COX6A1

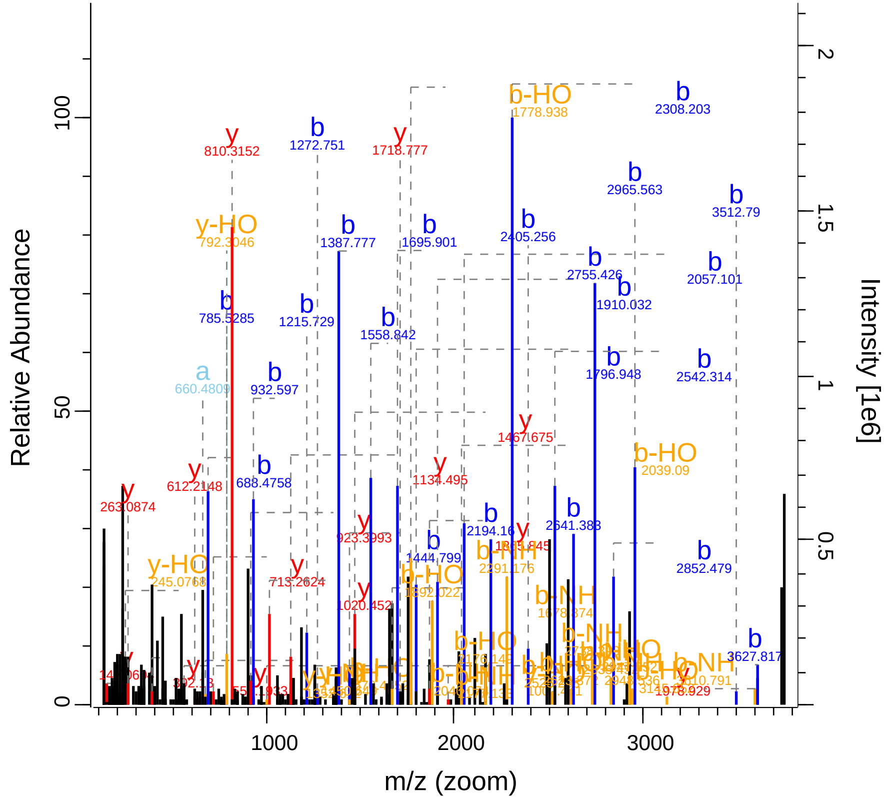

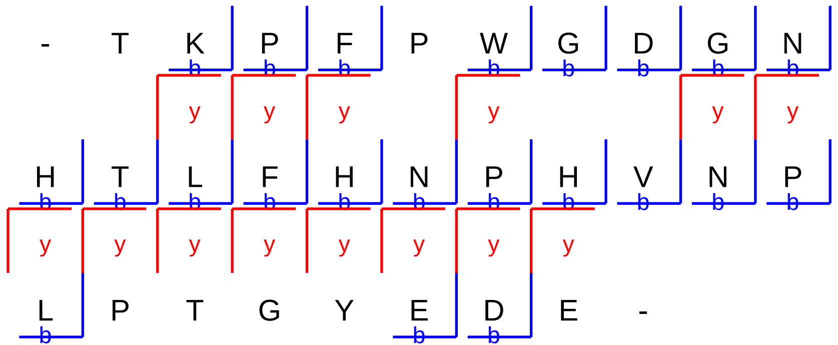

Raw File	Scan	Method	Score	m/z	Gene names
RO-QC-160121_ZackD_SS_Set_02_Fractio	28176	FTMS; HC	93.88	756.18	COX6A1

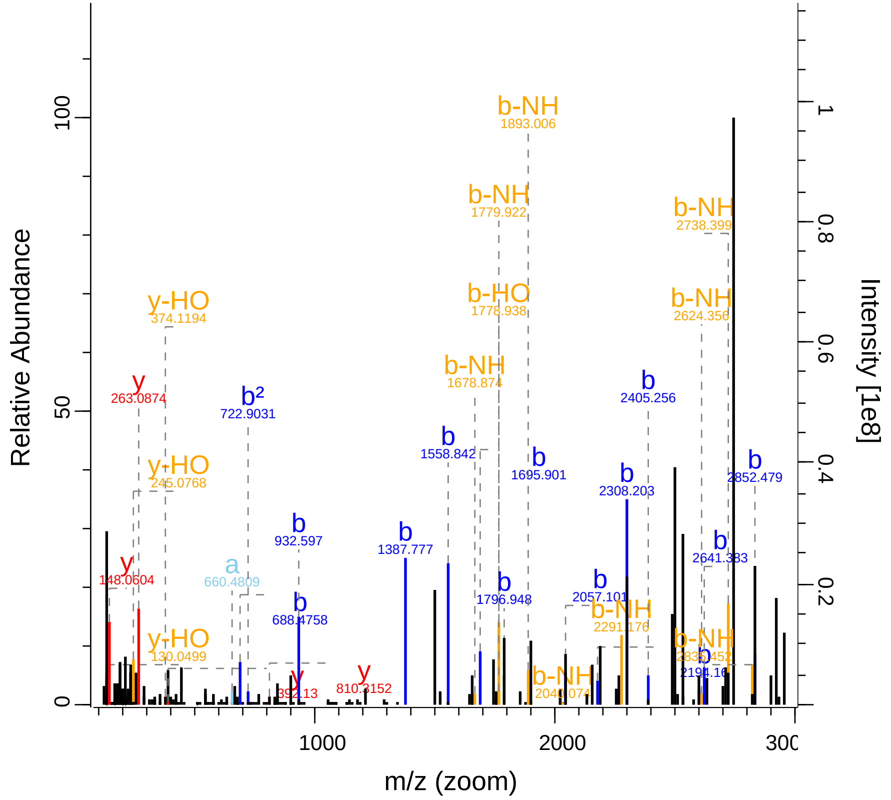

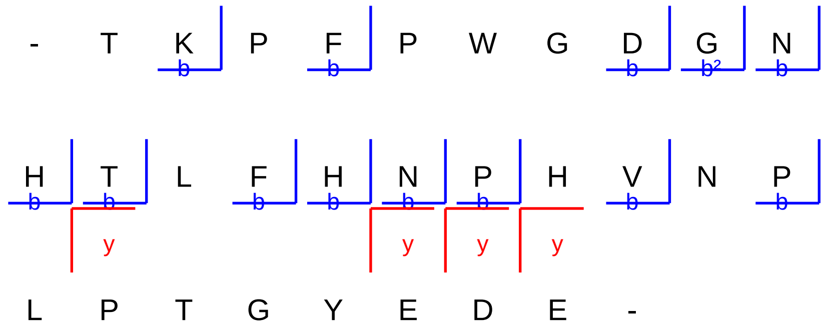

Raw File	Scan	Method	Score	m/z	Gene names
RO-QC-160121_ZackD_SS_Set_02_Fractic	28332	FTMS; HC	77.12	1001.53	DUSP6

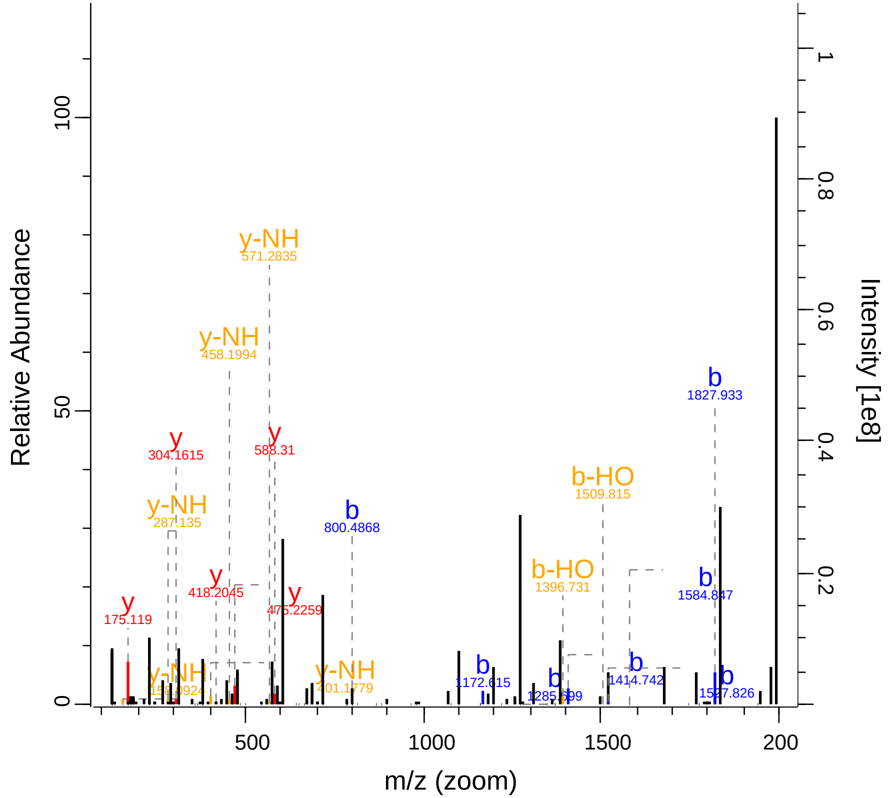

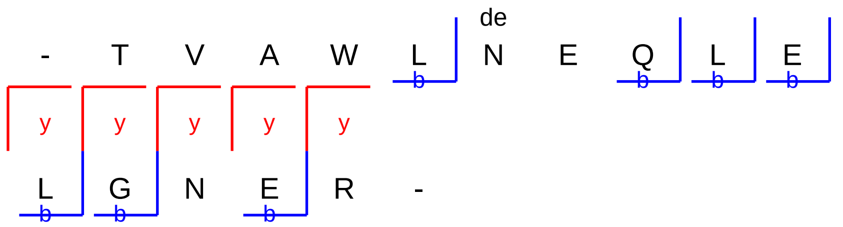

Raw File	Scan	Method	Score	m/z	Gene names
RO-QC-160121_ZackD_SS_Set_02_Fractio	30203	FTMS; HC	45.61	899.13	CEMIP

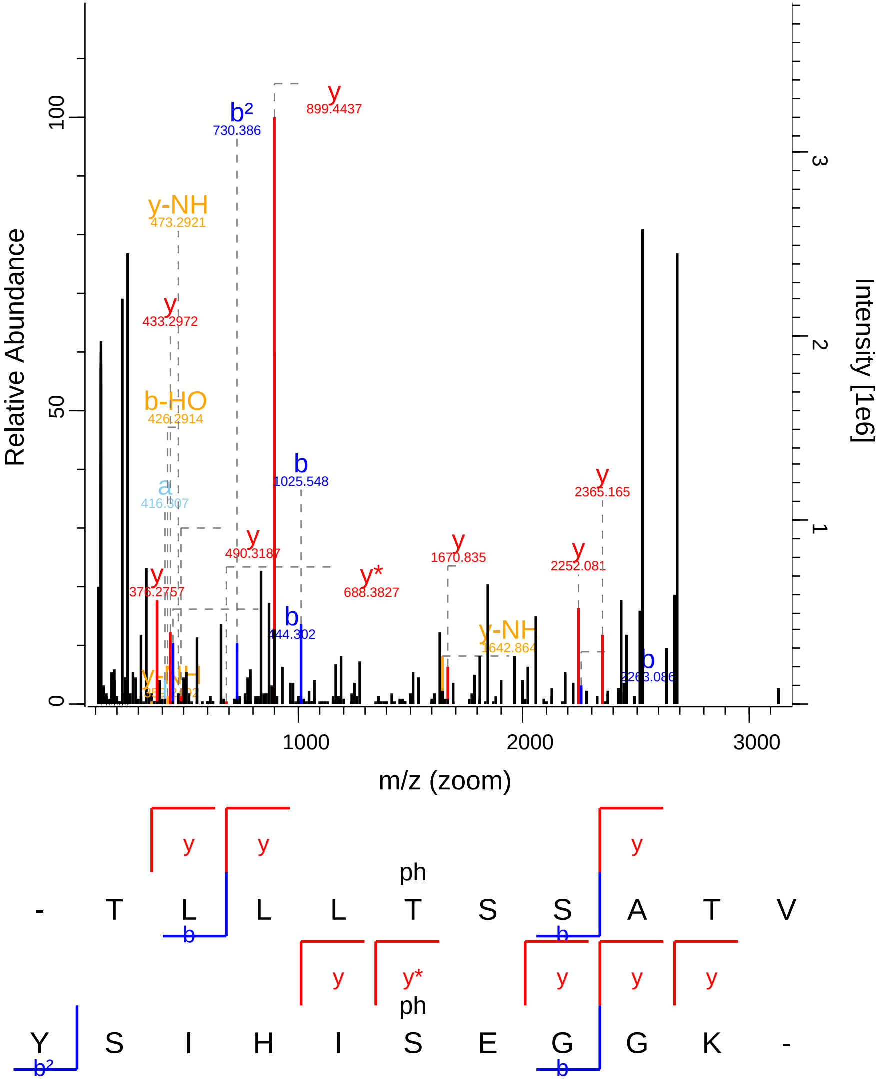

Raw File	Scan	Method	Score	m/z
RO-QC-160121_ZackD_SS_Set_02_Fraction_9	31311	FTMS; HCD	105.95	719.91

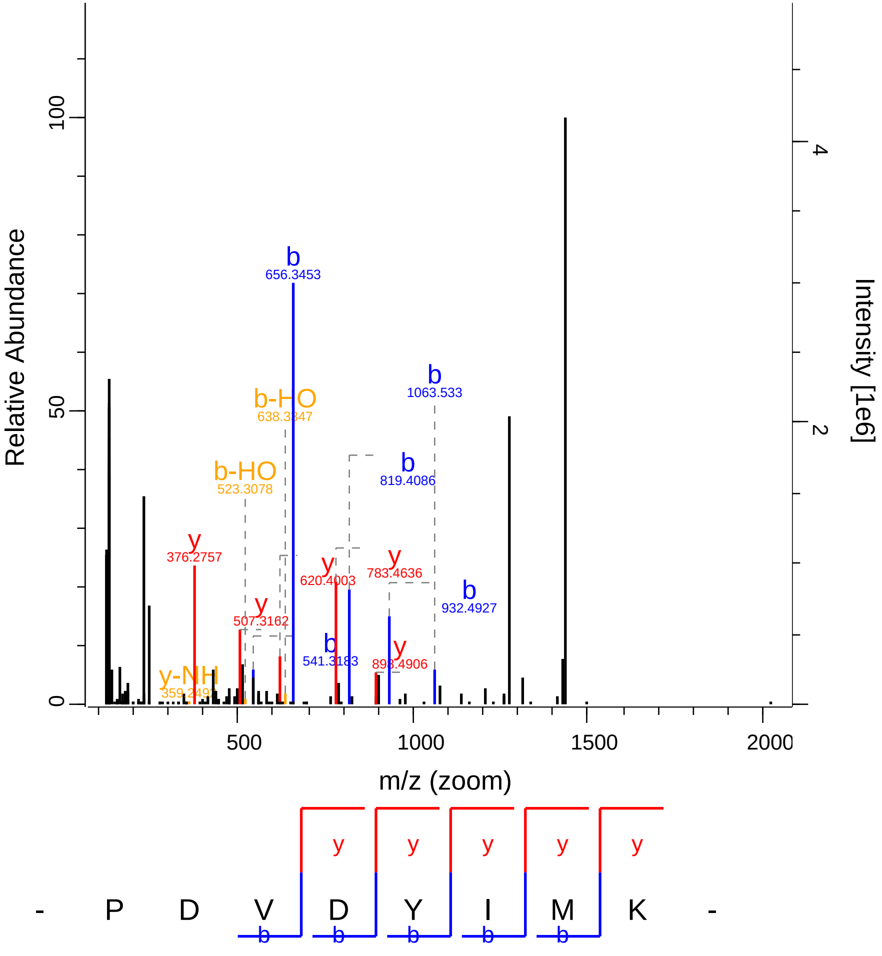

Raw File	Scan	Method	Score	m/z
RO-QC-160121_ZackD_SS_Set_02_Fraction_9	31487	FTMS; HCD	49.5	544

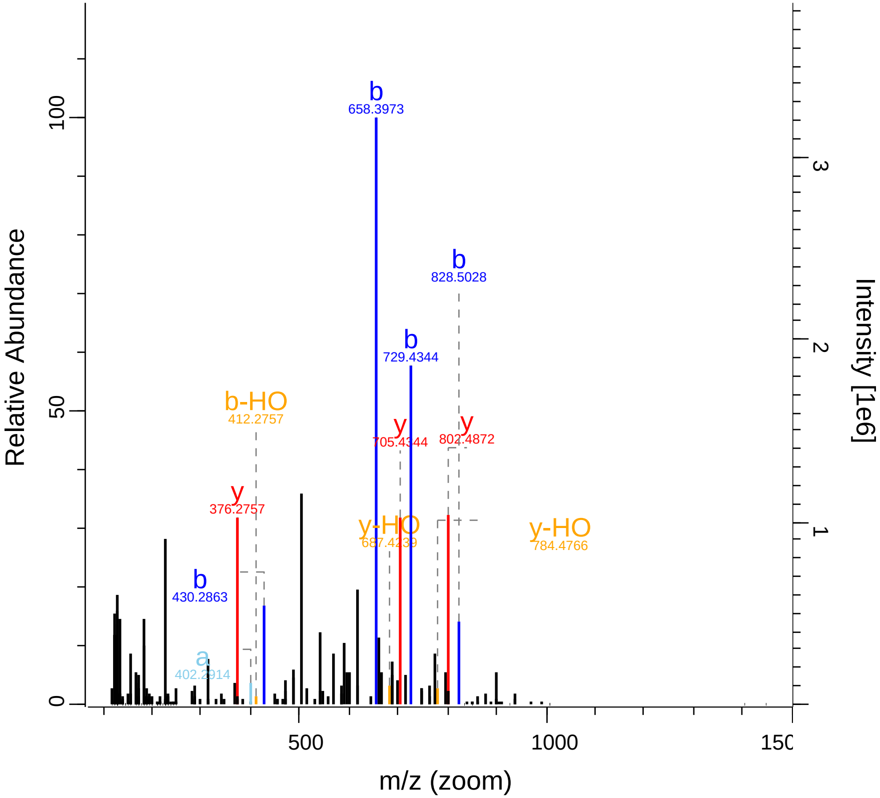

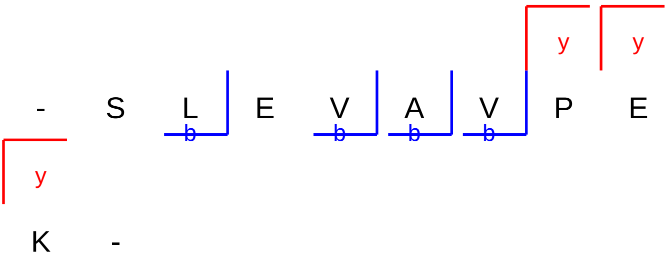

Raw File	Scan	Method	Score	m/z
RO-QC-160121_ZackD_SS_Set_02_Fraction_9	31508	FTMS; HCD	89.55	719.91

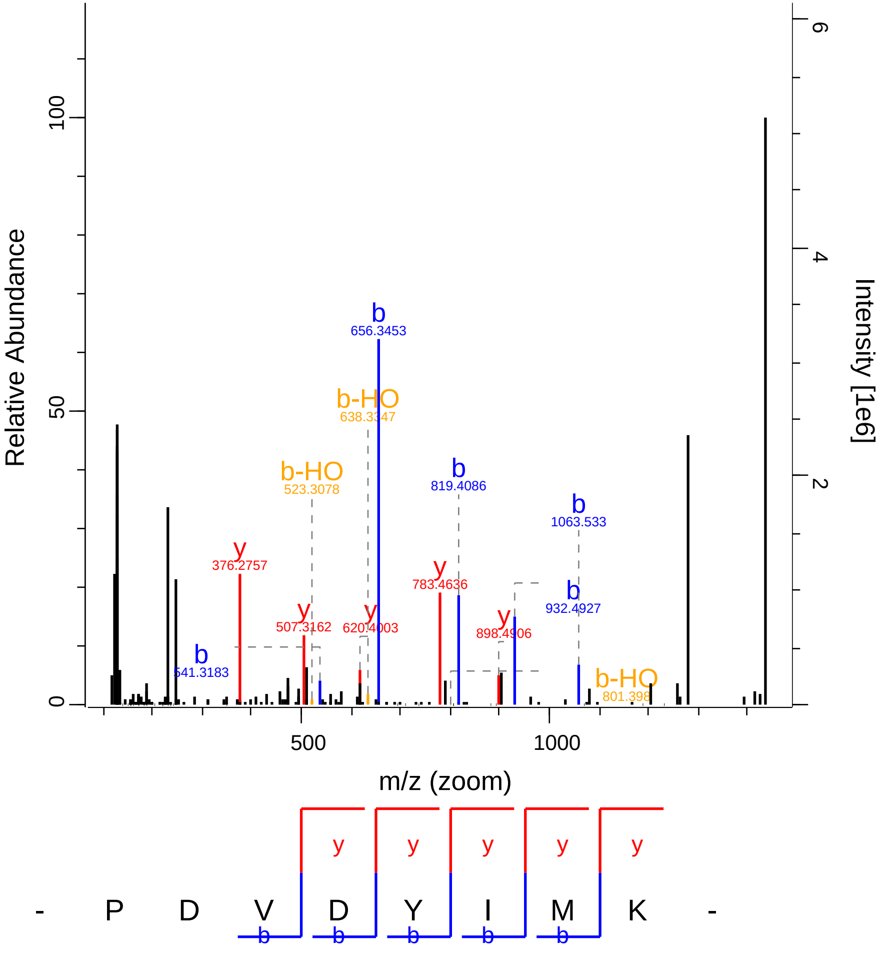

Raw File	Scan	Method	Score	m/z
RO-QC-160121_ZackD_SS_Set_02_Fraction_9	31532	FTMS; HCD	99.65	815.5

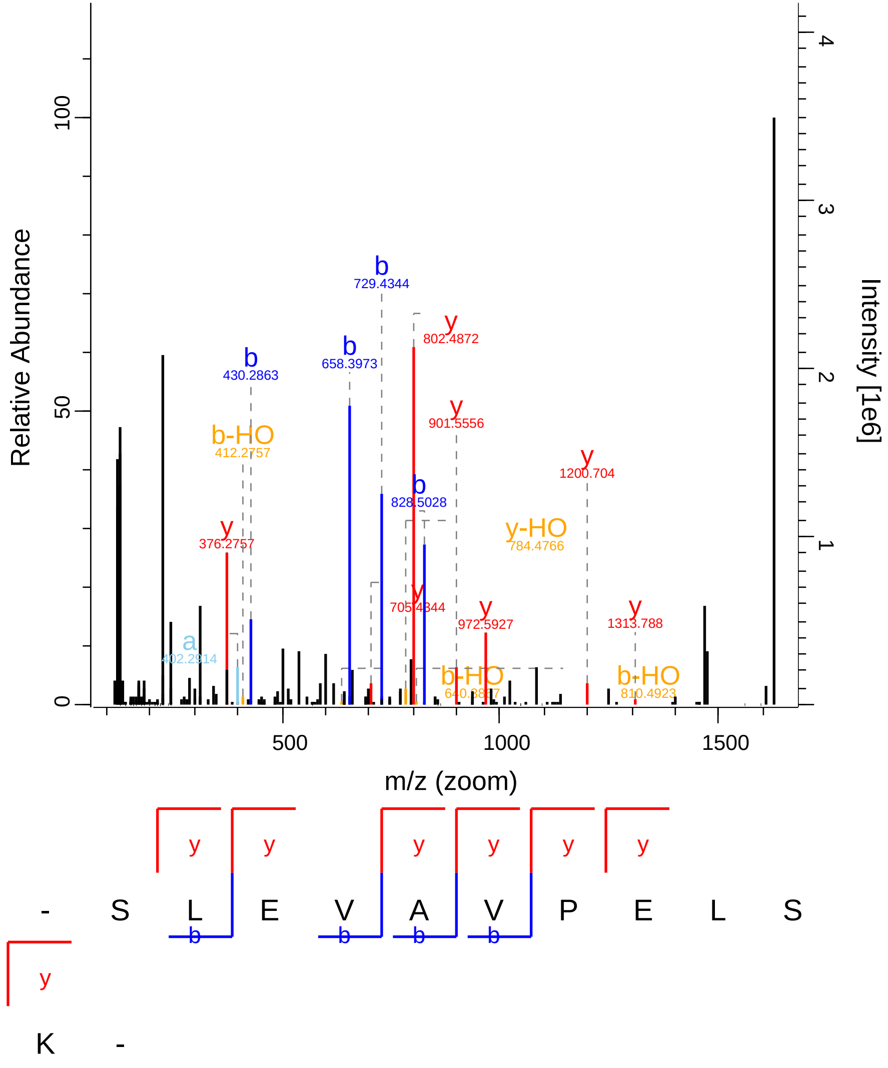

Raw File	Scan	Method	Score	m/z
RO-QC-160121_ZackD_SS_Set_02_Fraction_9	31575	FTMS; HCD	48	544.33

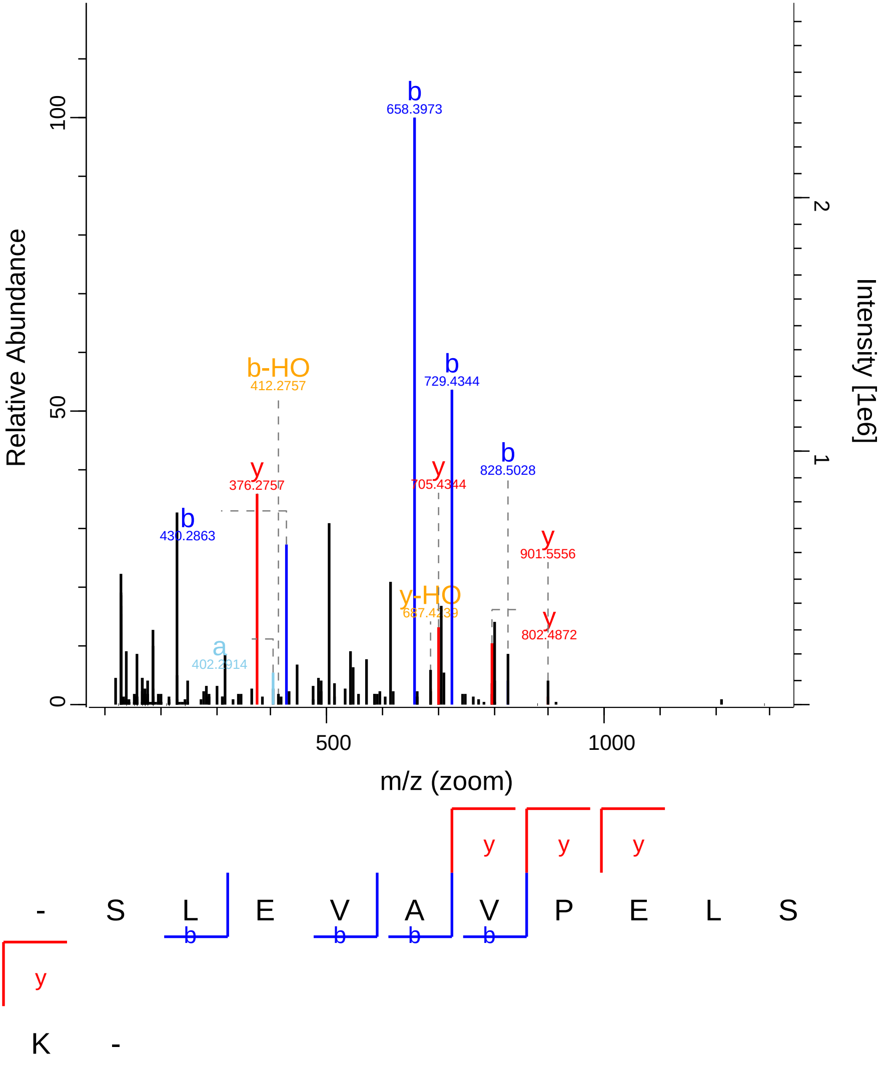

Raw File	Scan	Method	Score	m/z
RO-QC-160121_ZackD_SS_Set_02_Fraction_9	31813	FTMS; HCD	61.34	544

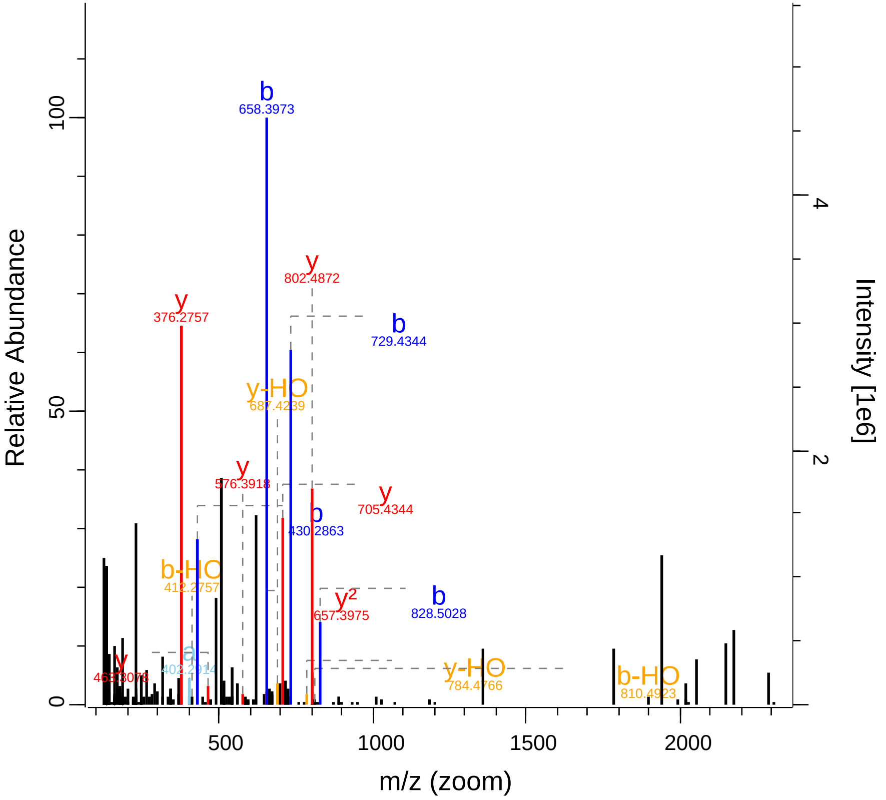

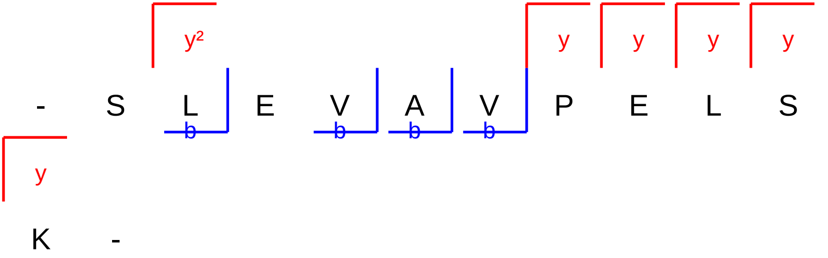

Raw File	Scan	Method	Score	m/z	Gene names
RO-QC-160121_ZackD_SS_Set_02_Fraction	32475	FTMS; HCD	45.7	821.2	MMP1

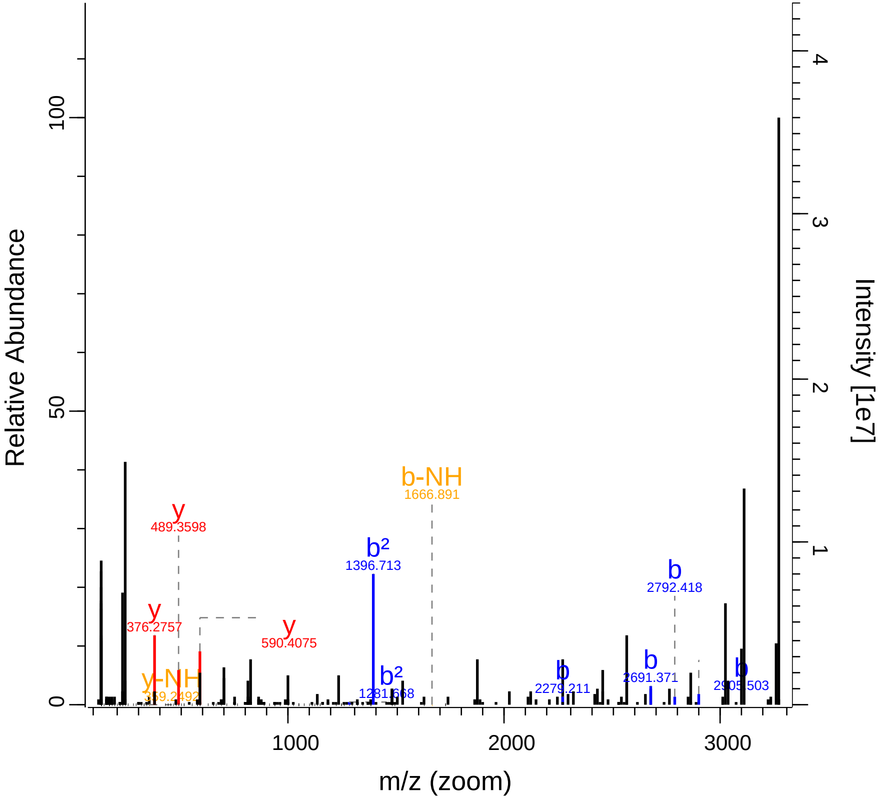

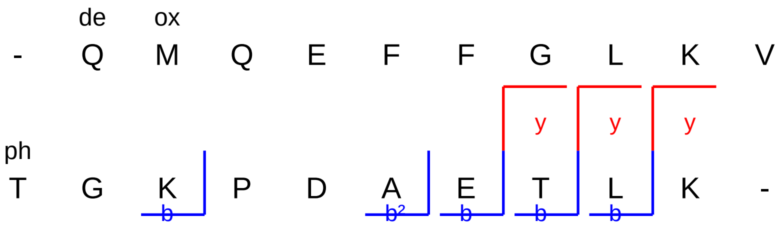

Raw File	Scan	Method	Score	m/z	Gene names
RO-QC-160121_ZackD_SS_Set_02_Fractio	32482	FTMS; HC	102.89	836.42	CNIH4

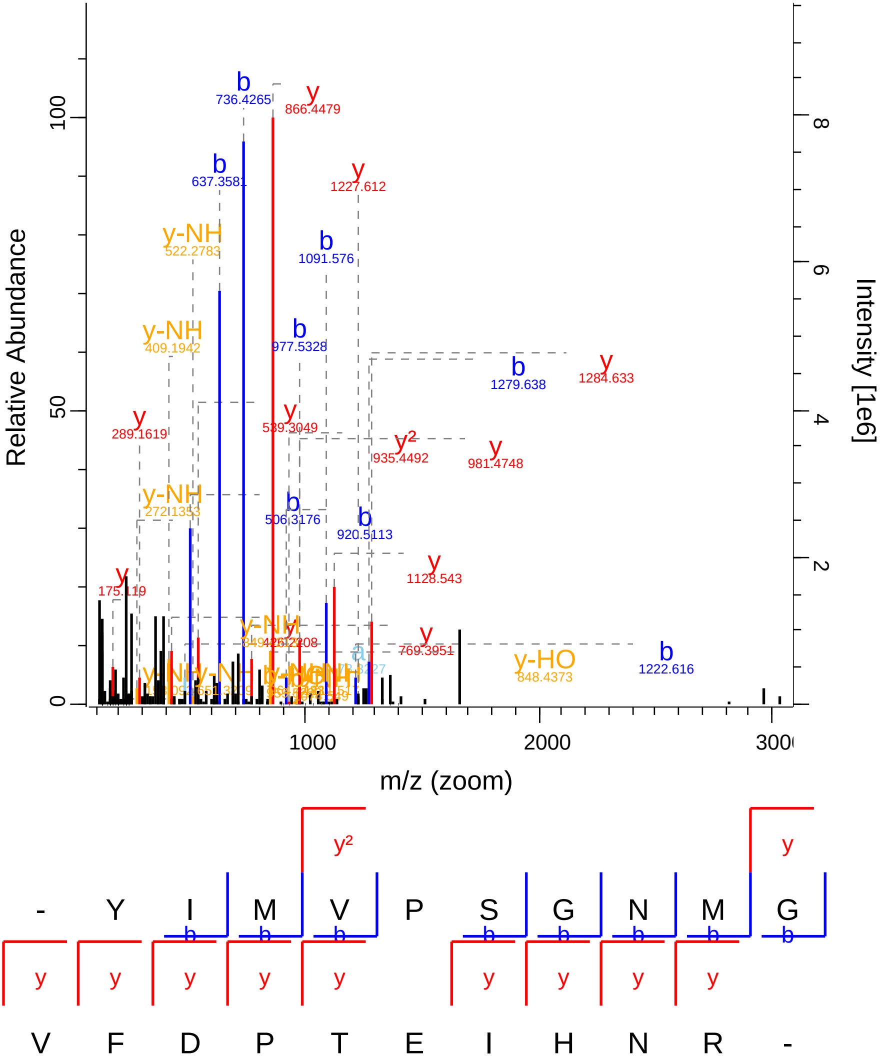

Raw File	Scan	Method	Score	m/z	Gene names
RO-QC-160121_ZackD_SS_Set_02_Fractio	32699	FTMS; HC	140.28	836.42	CNIH4

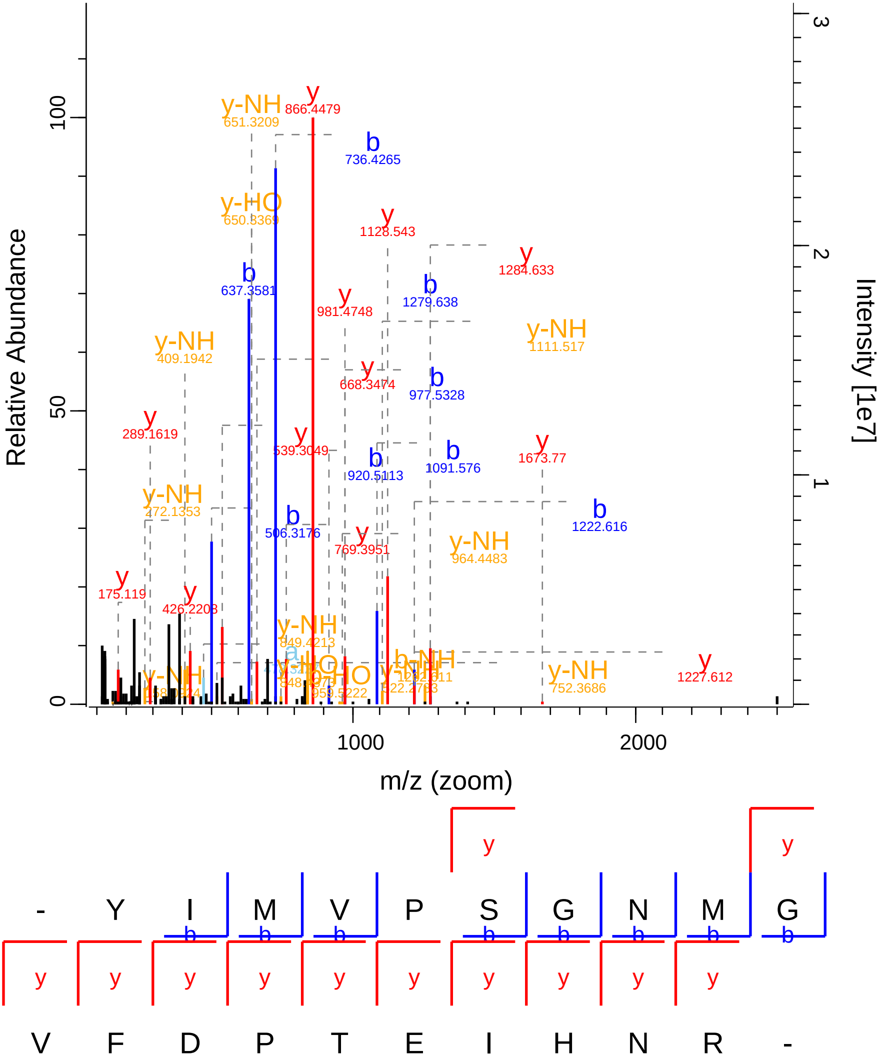

Raw File	Scan	Method	Score	m/z	Gene names
RO-QC-160121_ZackD_SS_Set_02_Fractio	33488	FTMS; HCI	114.39	673.9	C19orf12

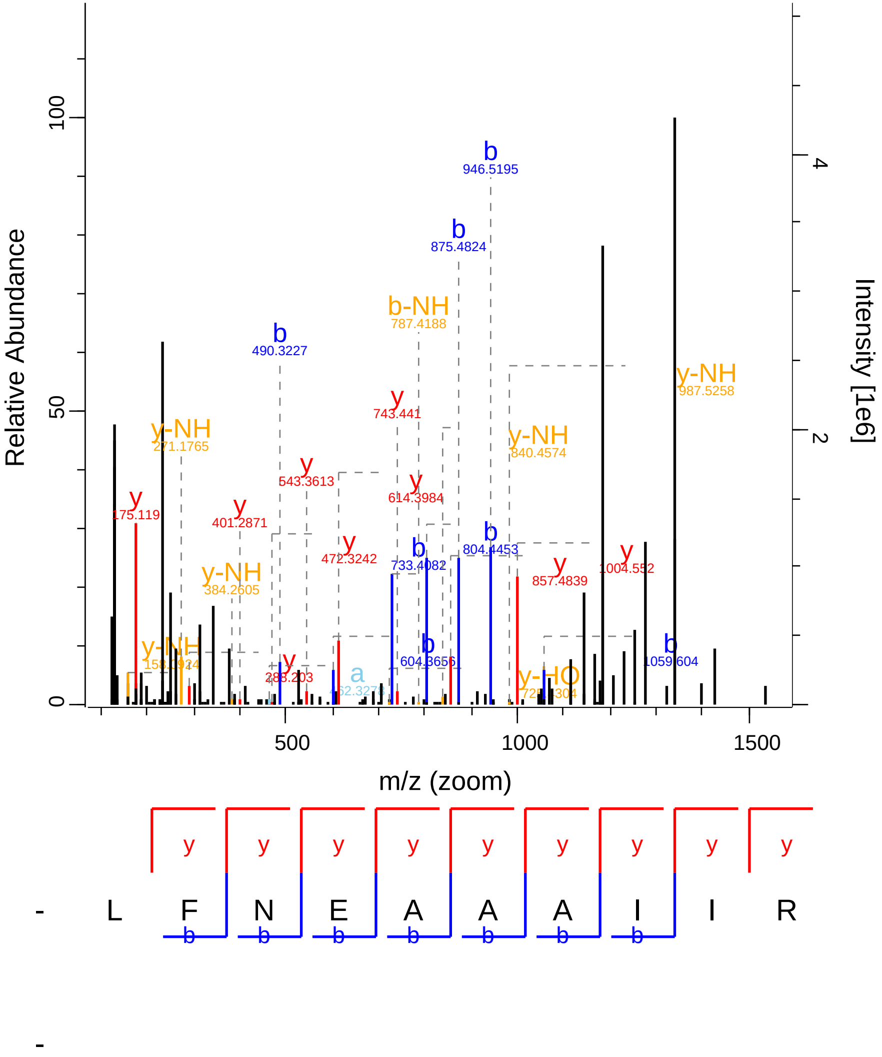

Raw File	Scan	Method	Score	m/z
RO-QC-160121_ZackD_SS_Set_02_Fraction_9	35414	FTMS; HCD	44.2	676.62

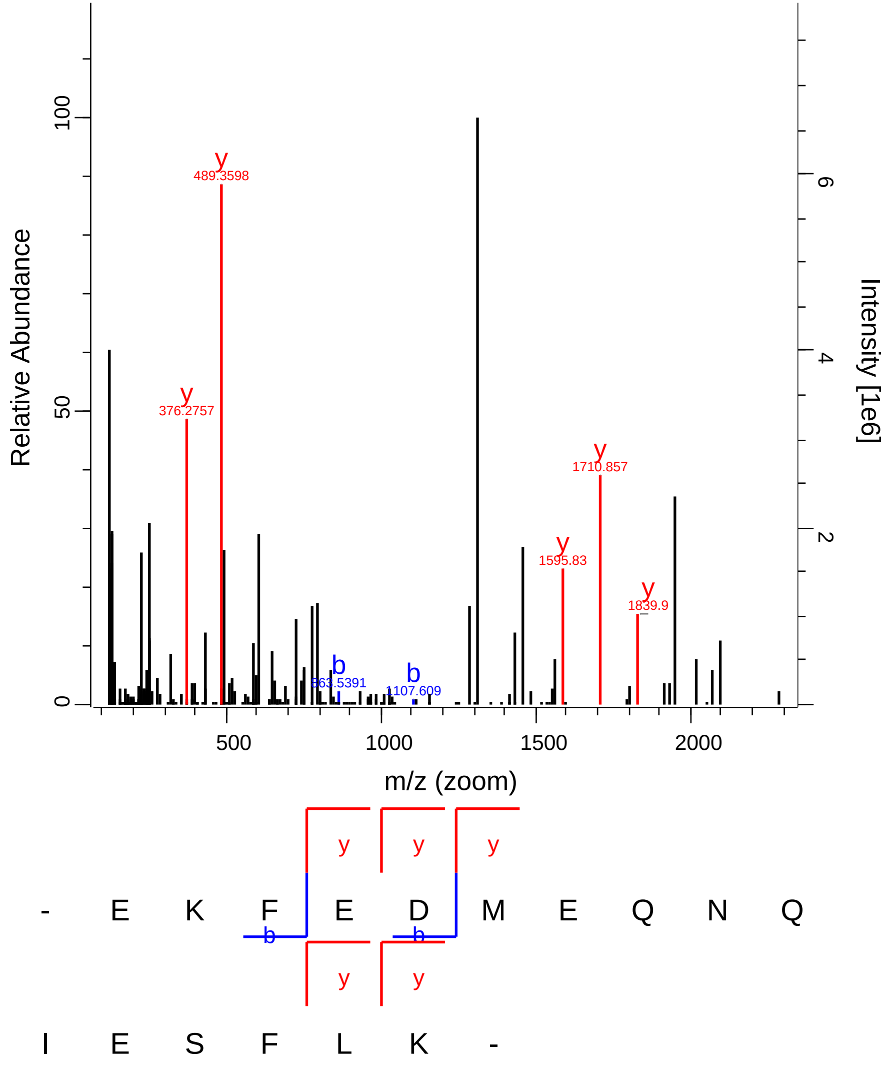

Raw File	Scan	Method	Score	m/z	Gene names
RO-QC-160121_ZackD_SS_Set_02_Fractic	36911	FTMS; HC	80.23	1081.62	C6

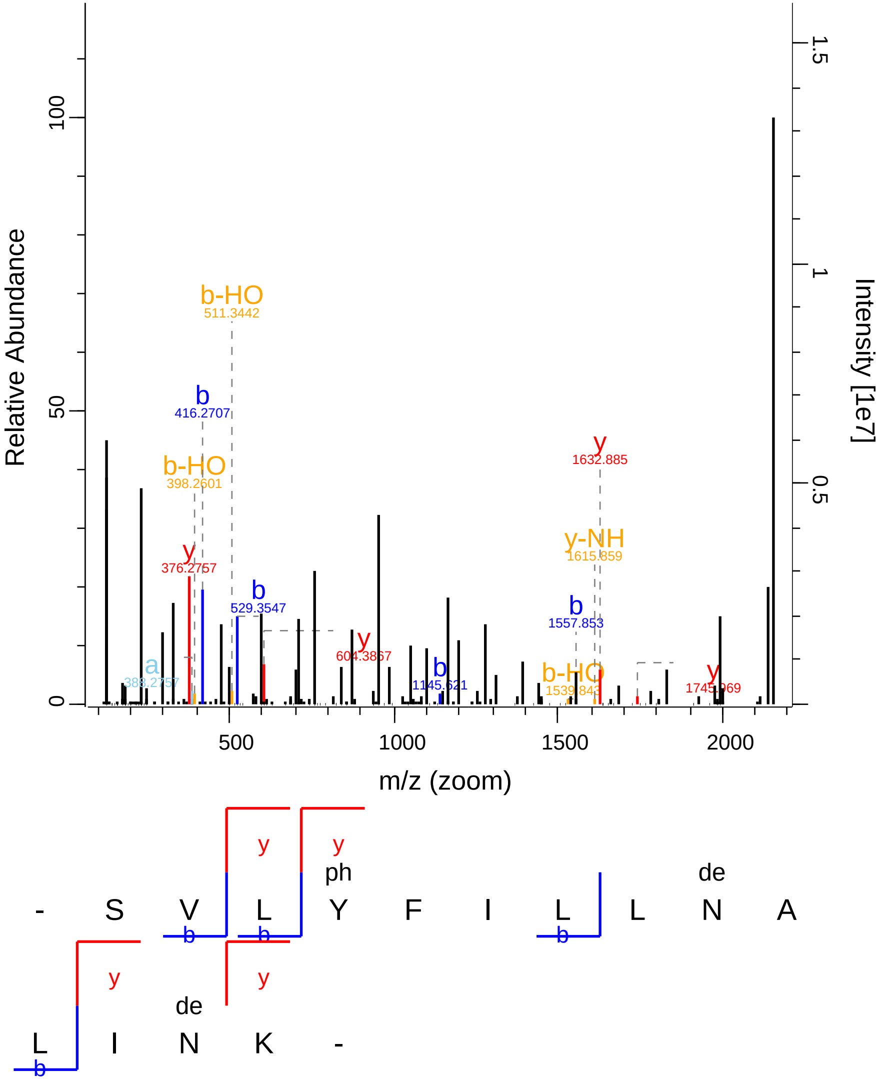

Raw File	Scan	Method	Score	m/z	Gene names
RO-QC-160121_ZackD_SS_Set_02_Fractic	37127	FTMS; HC	67.11	1081.62	C6

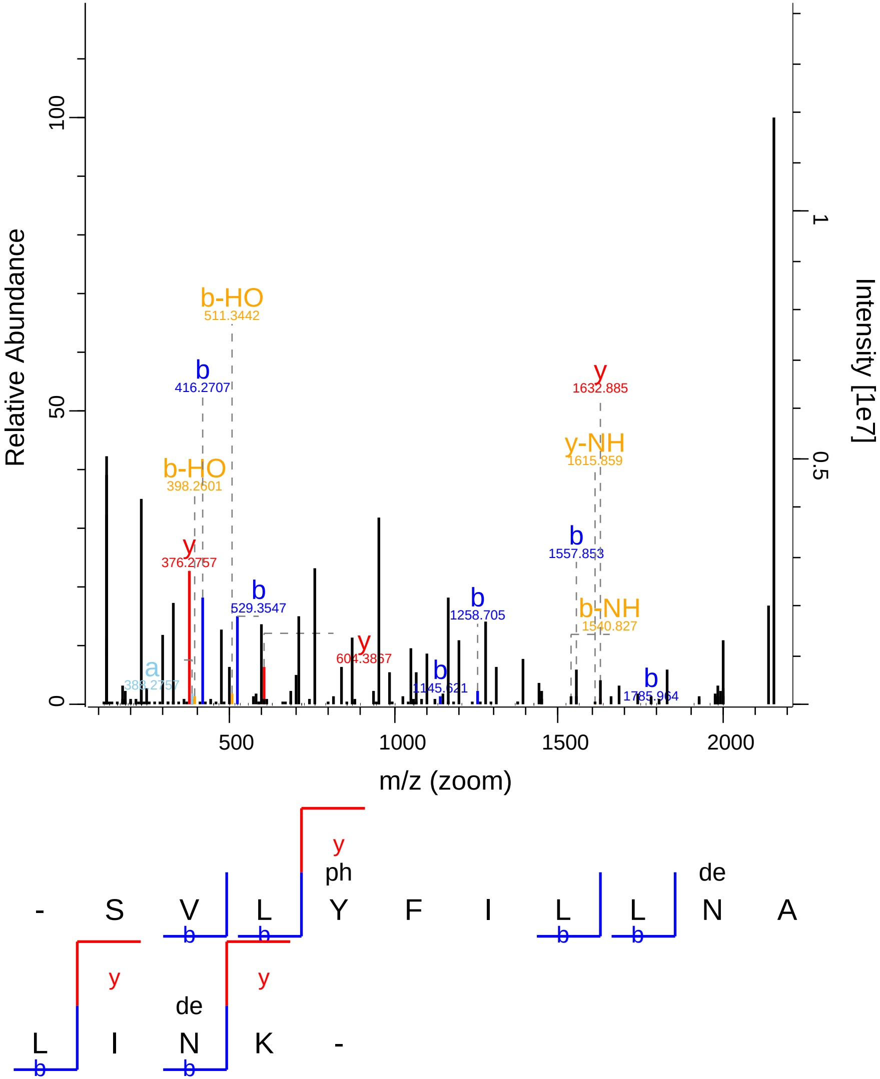

Raw File	Scan	Method	Score	m/z	Gene names
RO-QC-160121_ZackD_SS_Set_02_Fracti	37470	FTMS; HC	111.82	1028.59	ZKSCAN2

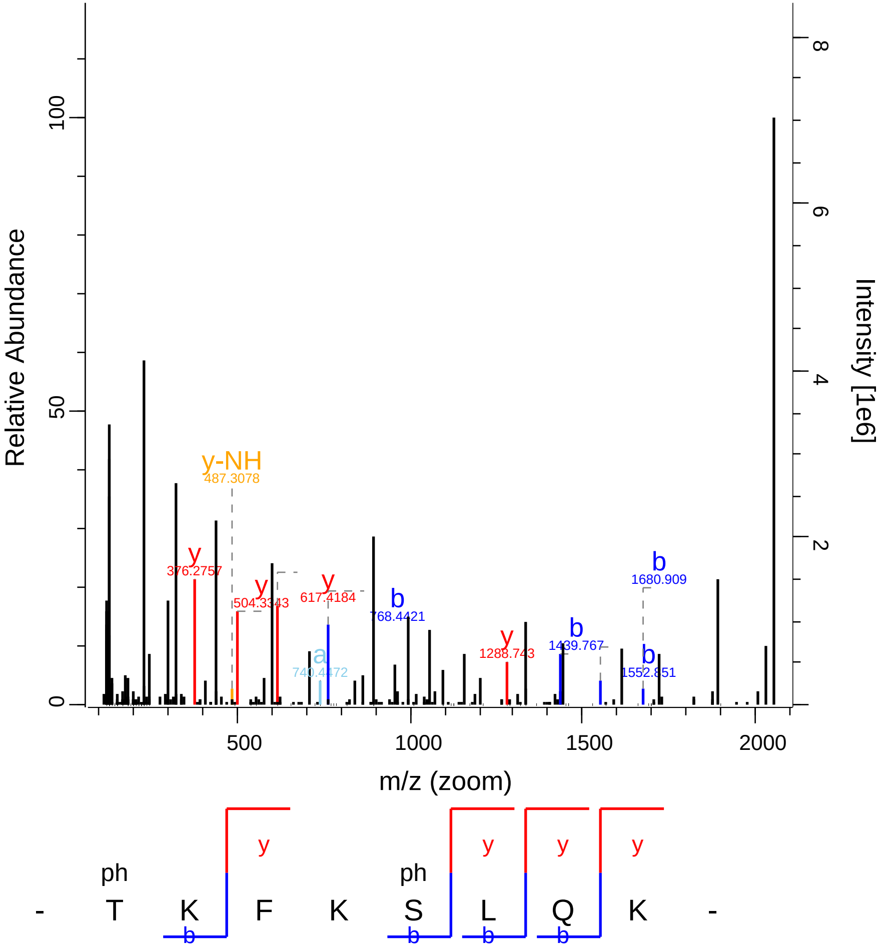

Raw File	Scan	Method	Score	m/z	Gene names
RO-QC-160121_ZackD_SS_Set_02_Fractio	40517	FTMS; HC	53.06	848.43	S100A1

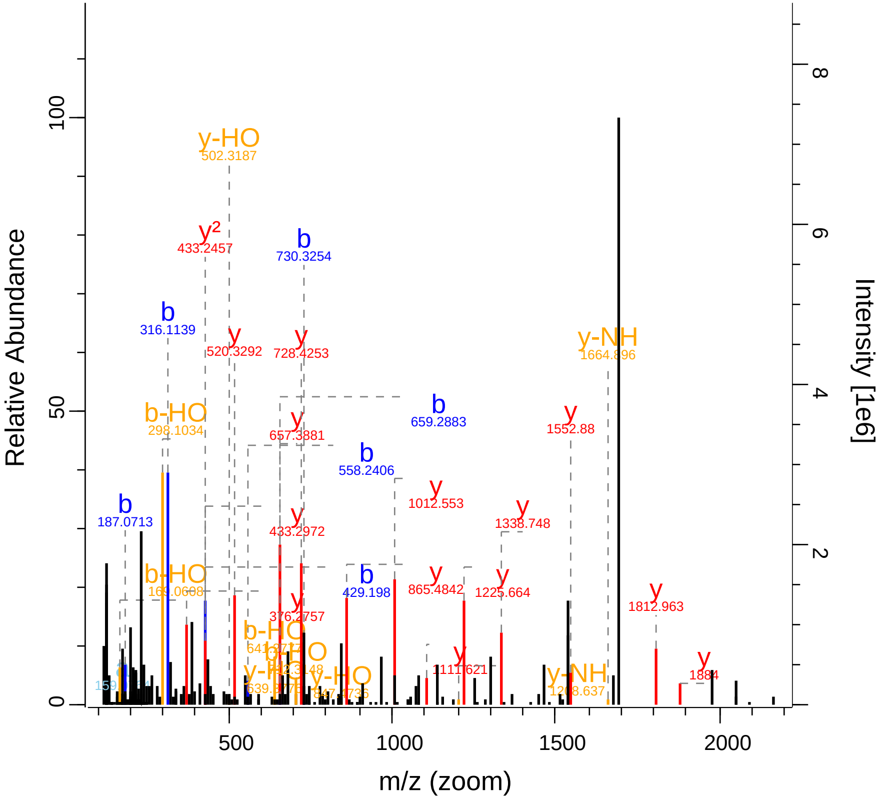

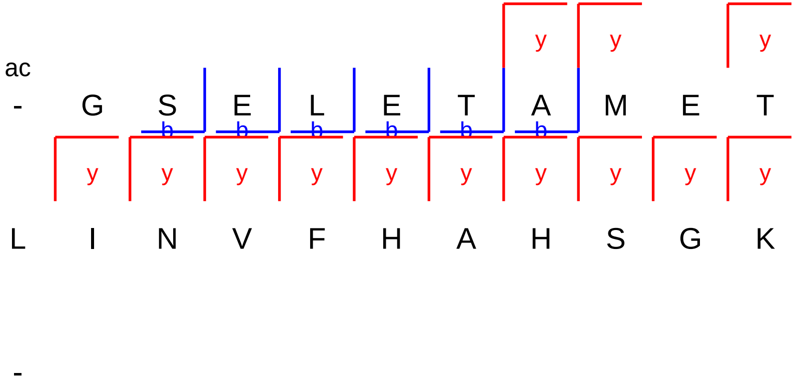

Raw File	Scan	Method	Score	m/z	Gene names
RO-QC-160121_ZackD_SS_Set_02_Fractio	40545	FTMS; HC	141.55	766.09	NPW

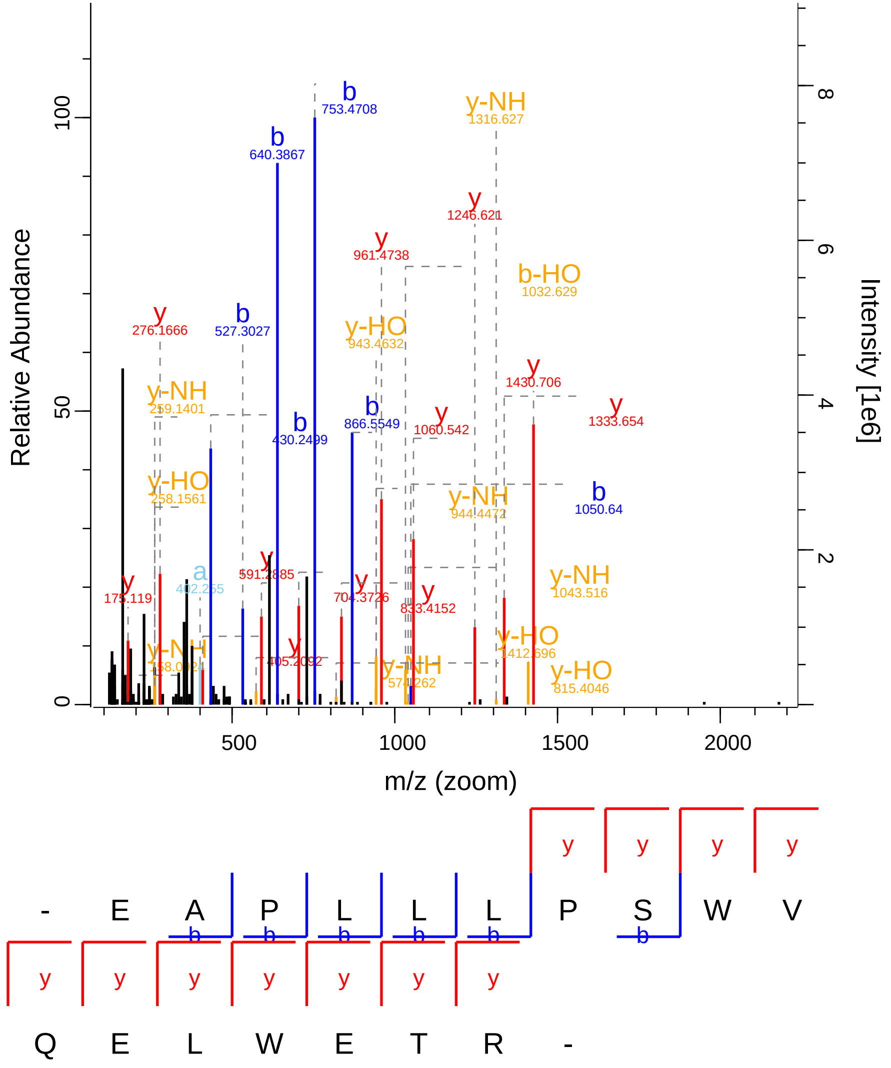

Raw File	Scan	Method	Score	m/z	Gene names
RO-QC-160121_ZackD_SS_Set_02_Fractic	40547	FTMS; HC	139.78	1149.13	NPW

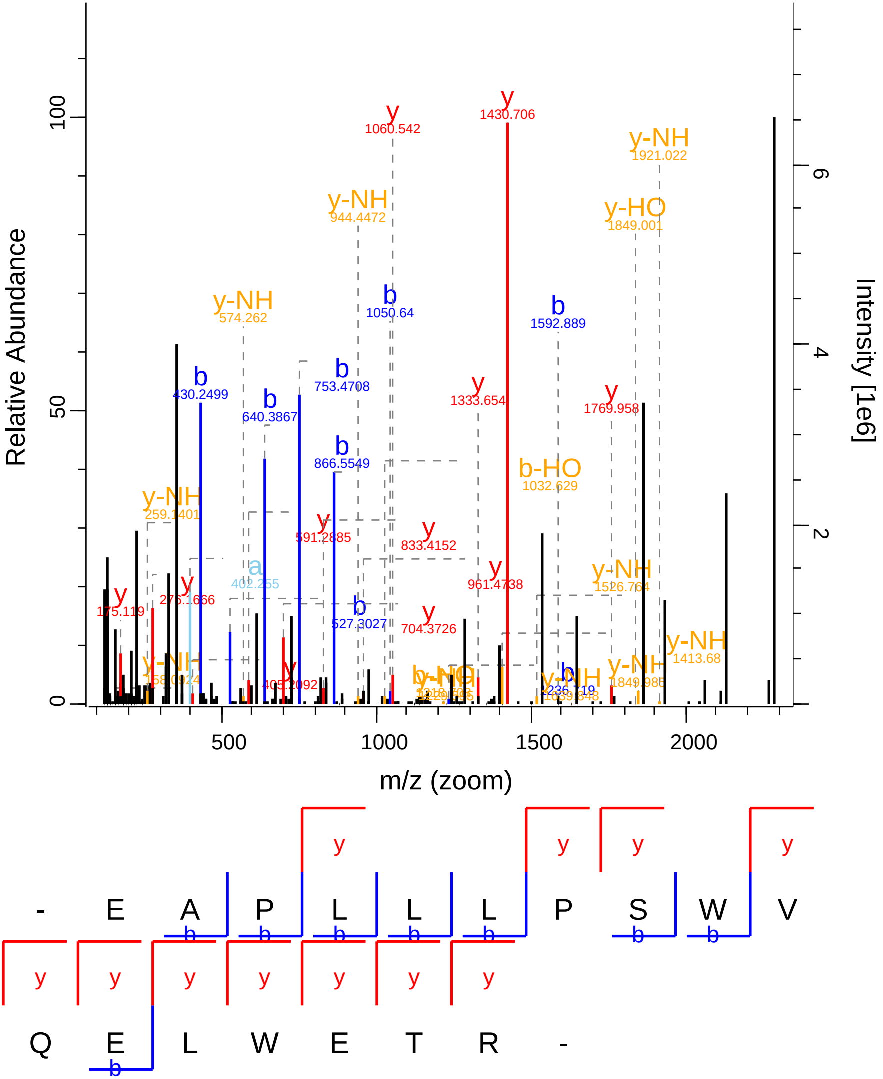

Raw File	Scan	Method	Score	m/z	Gene names
RO-QC-160121_ZackD_SS_Set_02_Fraction	4678	FTMS; HC	53.68	776.86	USP42

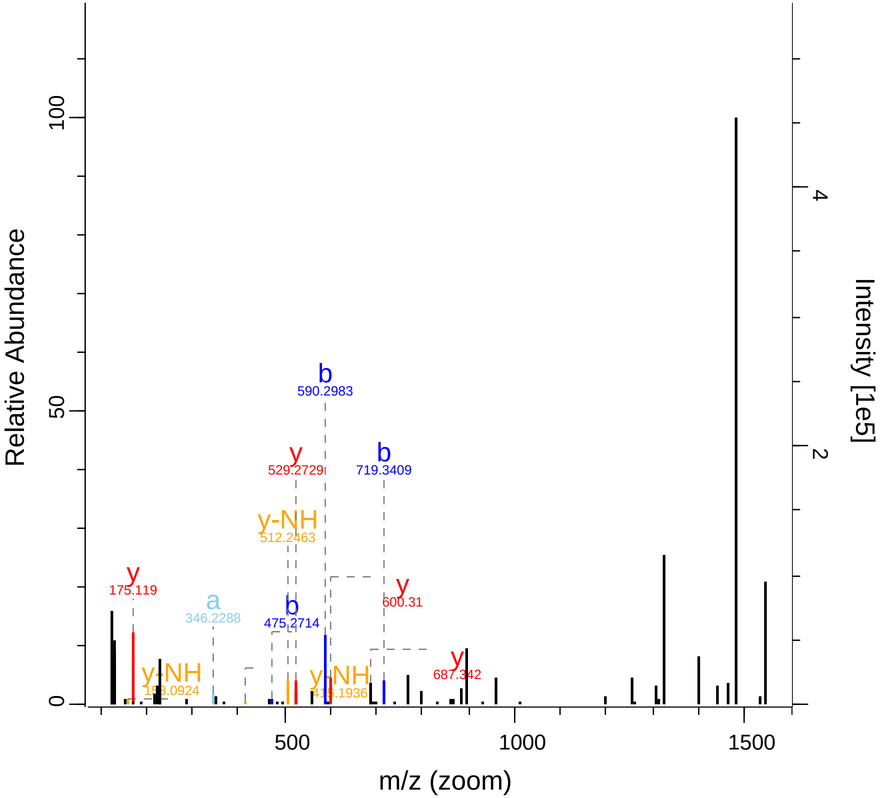

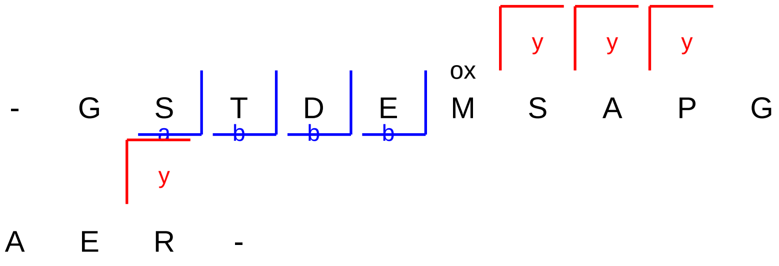

Raw File	Scan	Method	Score	m/z	Gene names
RO-QC-160121_ZackD_SS_Set_02_Fraction	4834	FTMS; HC	71.22	480.27	C10orf12

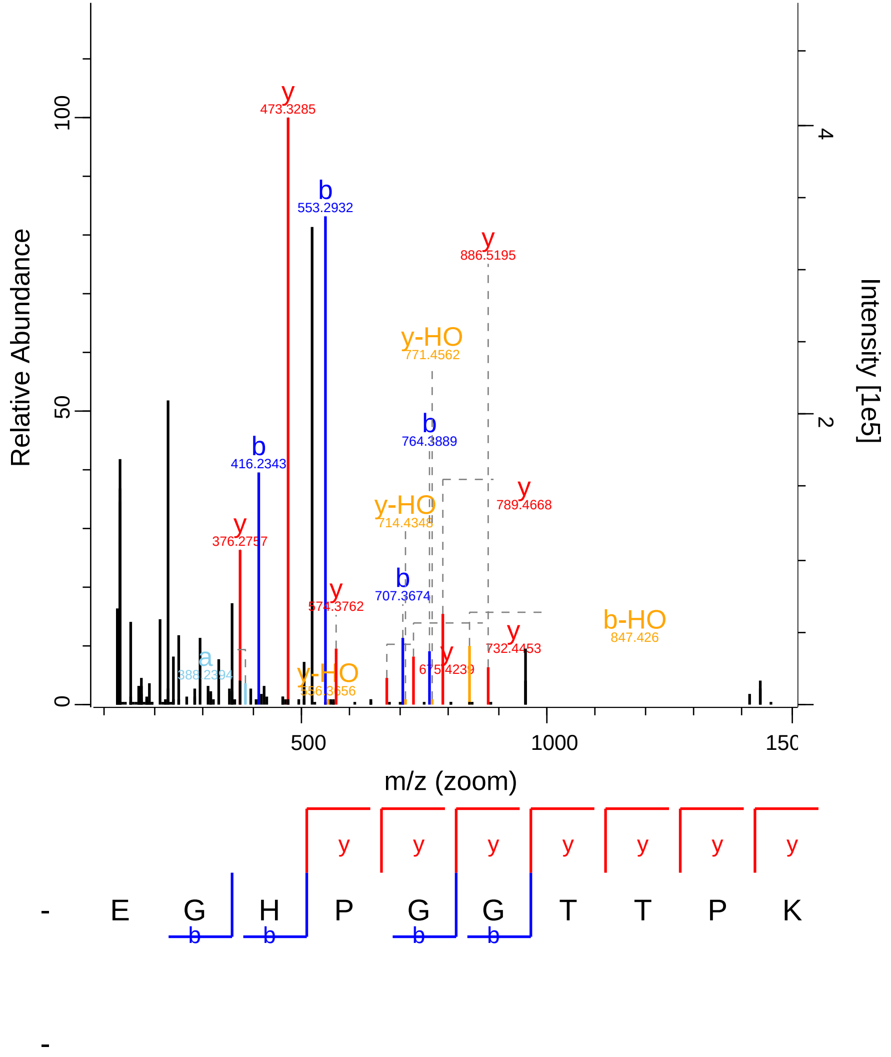

Raw File	Scan	Method	Score	m/z	Gene names
RO-QC-160121_ZackD_SS_Set_02_Fraction_	5056	FTMS; HC	91.86	578.28	EPC2

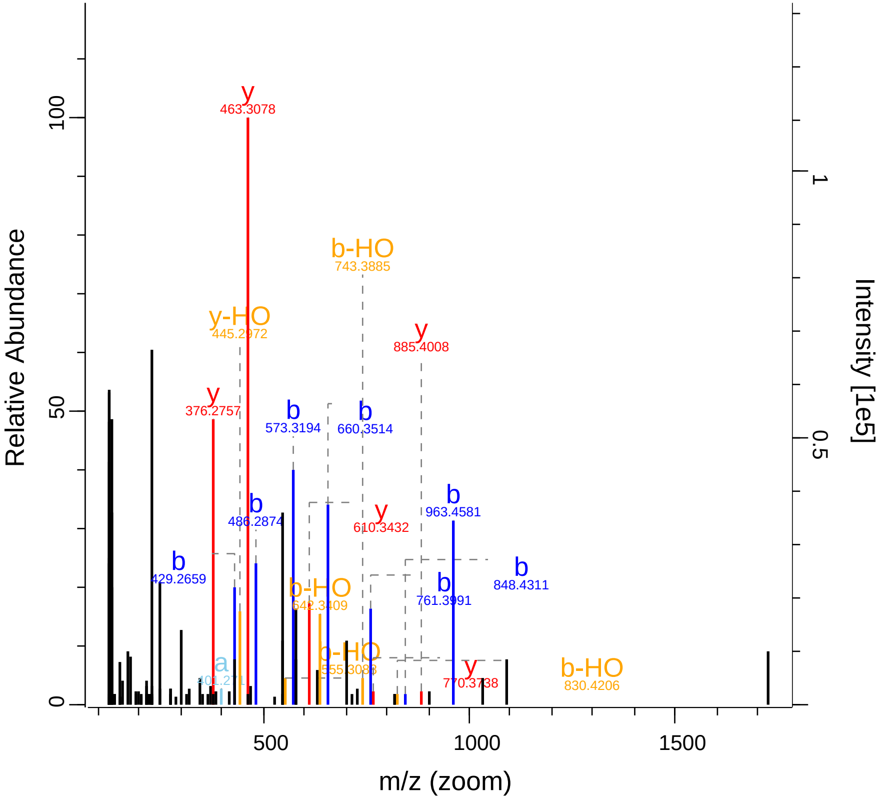

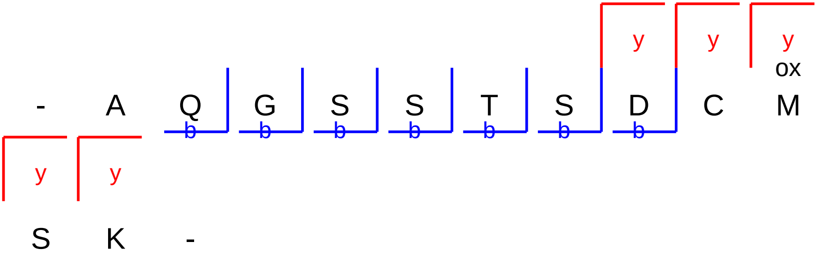

Raw File	Scan	Method	Score	m/z	Gene names
RO-QC-160121_ZackD_SS_Set_02_Fraction	5104	FTMS; HC	87.18	486.58	CEP68

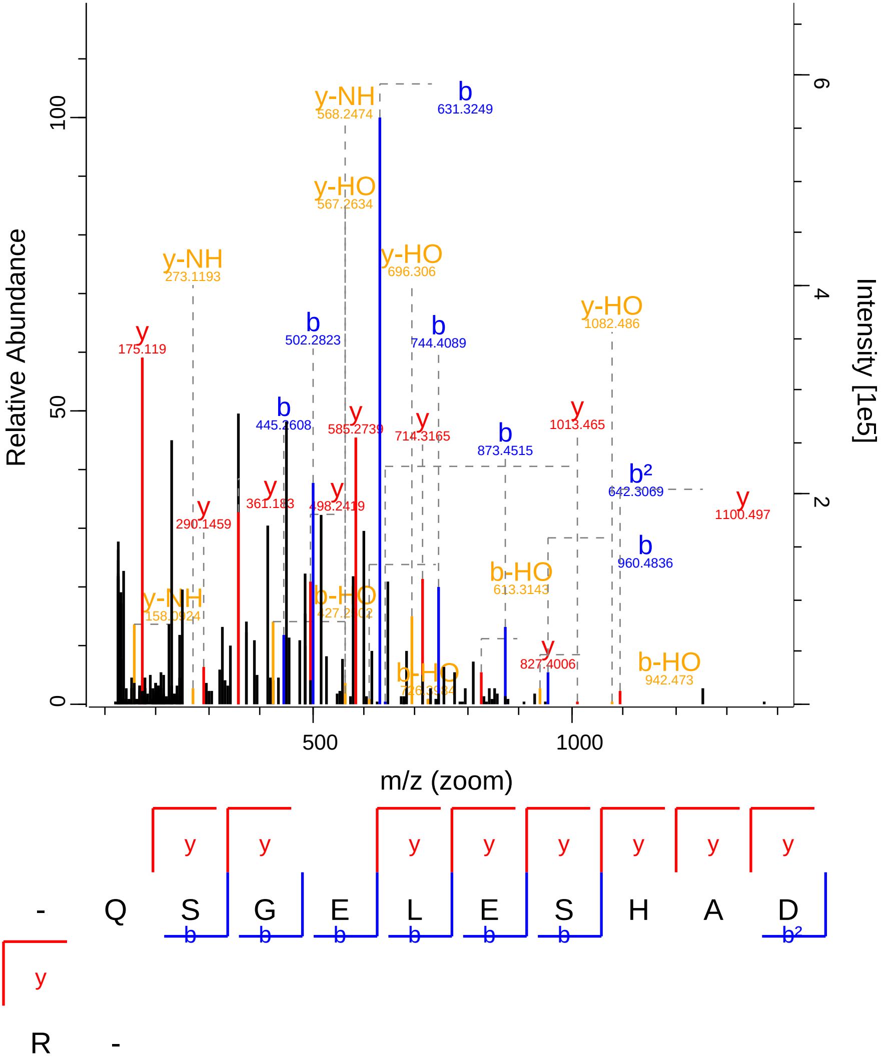

Raw File	Scan	Method	Score	m/z	Gene names
RO-QC-160121_ZackD_SS_Set_02_Fraction	5287	FTMS; HC	131.42	494.92	TMCO6

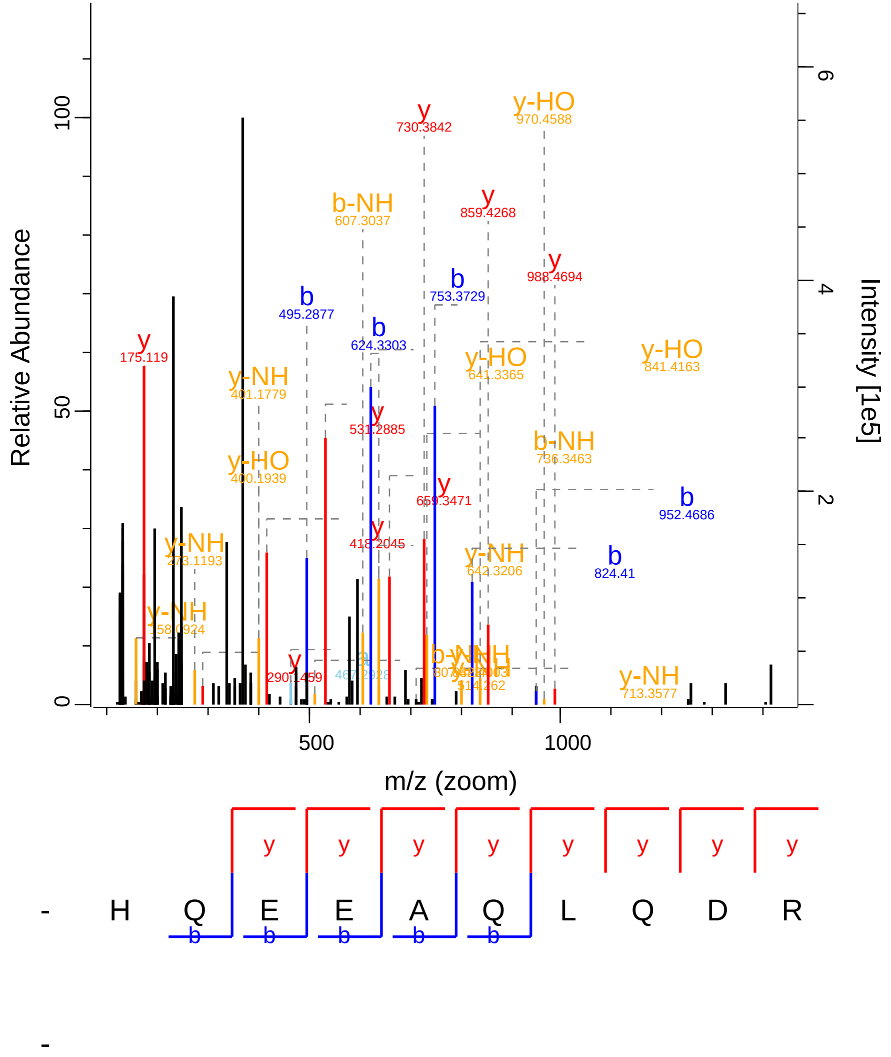

Raw File	Scan	Method	Score	m/z	Gene names
RO-QC-160121_ZackD_SS_Set_02_Fraction	5360	FTMS; HC	77.66	741.88	TMCO6

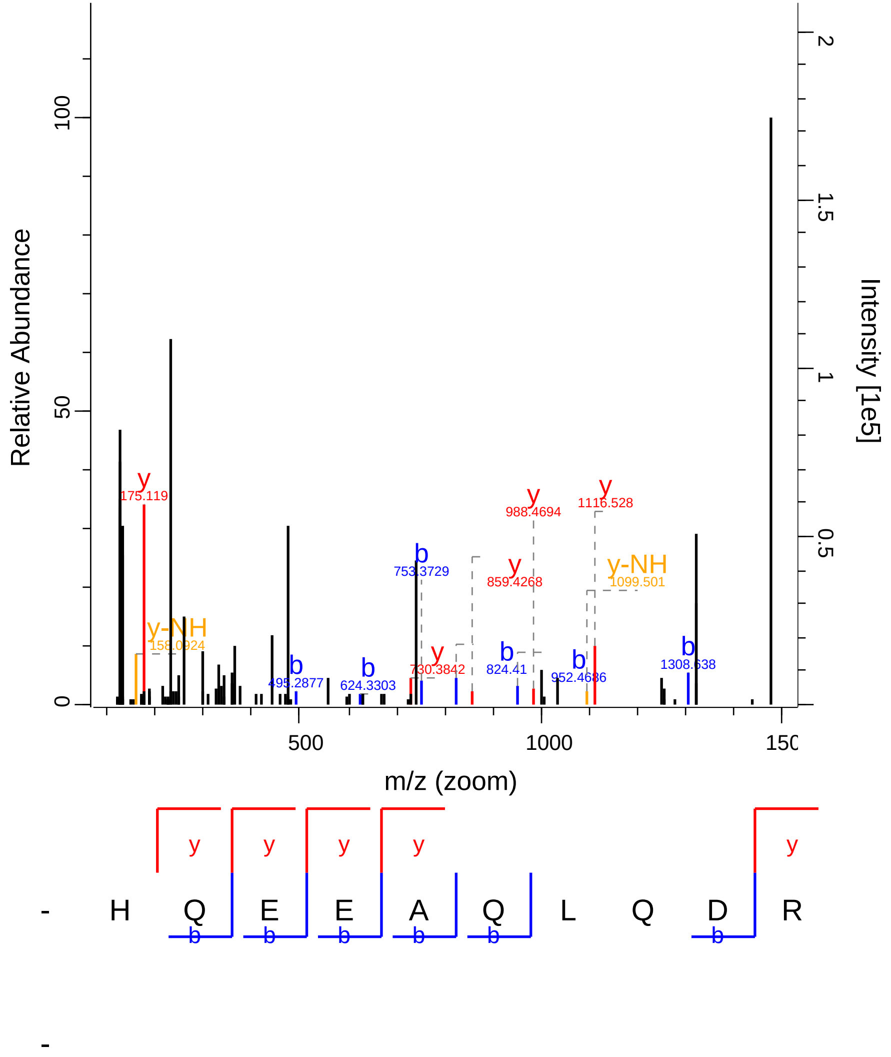

Raw File	Scan	Method	Score	m/z	Gene names
RO-QC-160121_ZackD_SS_Set_02_Fraction	5569	FTMS; HC	130.98	629.81	RUSC2

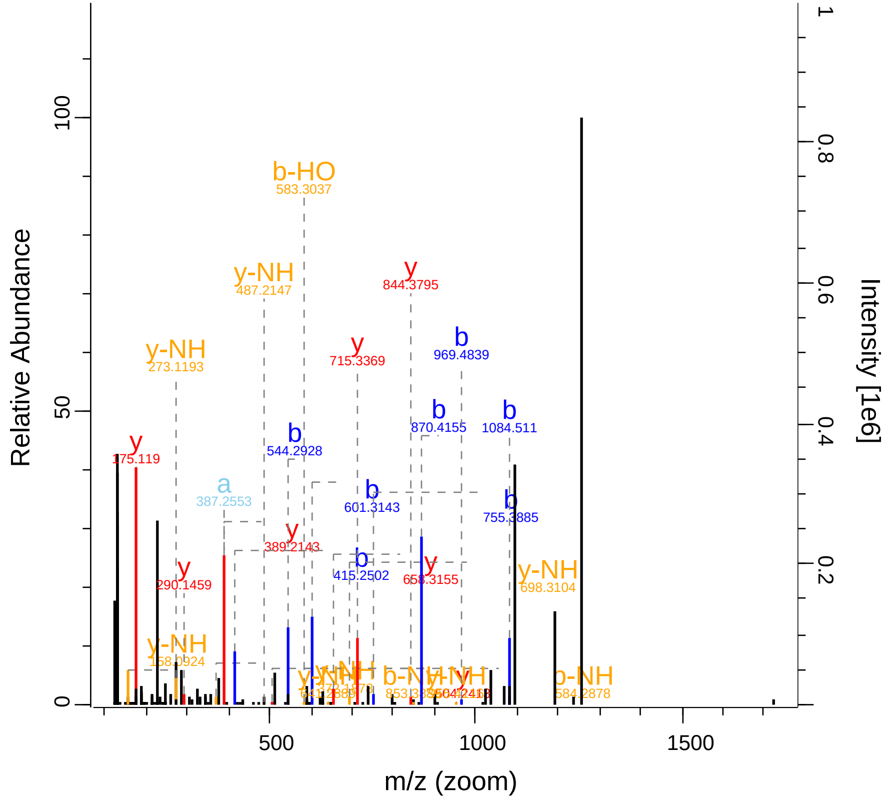

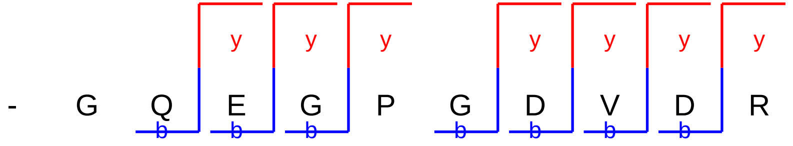

-

Raw File	Scan	Method	Score	m/z
RO-QC-160121_ZackD_SS_Set_02_Fraction_10	5637	FTMS; HCD	82.45	447.59

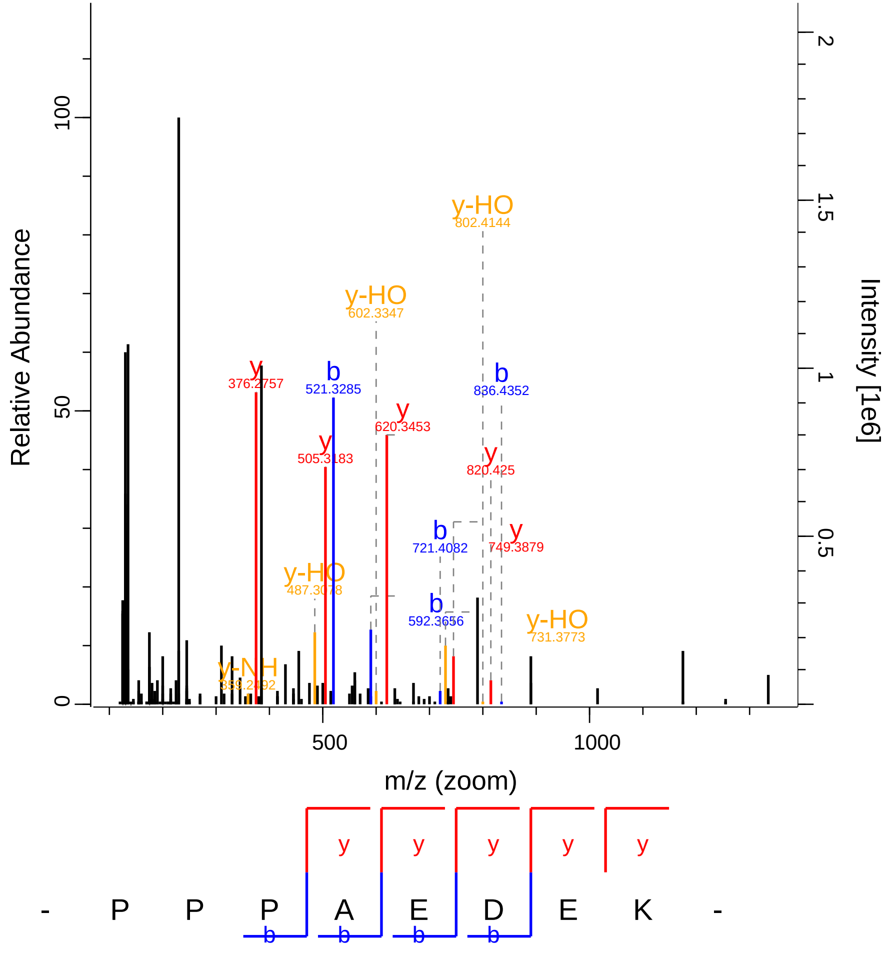

Raw File	Scan	Method	Score	m/z	Gene names
RO-QC-160121_ZackD_SS_Set_02_Fraction	5693	FTMS; HC	129.63	650.35	CD99

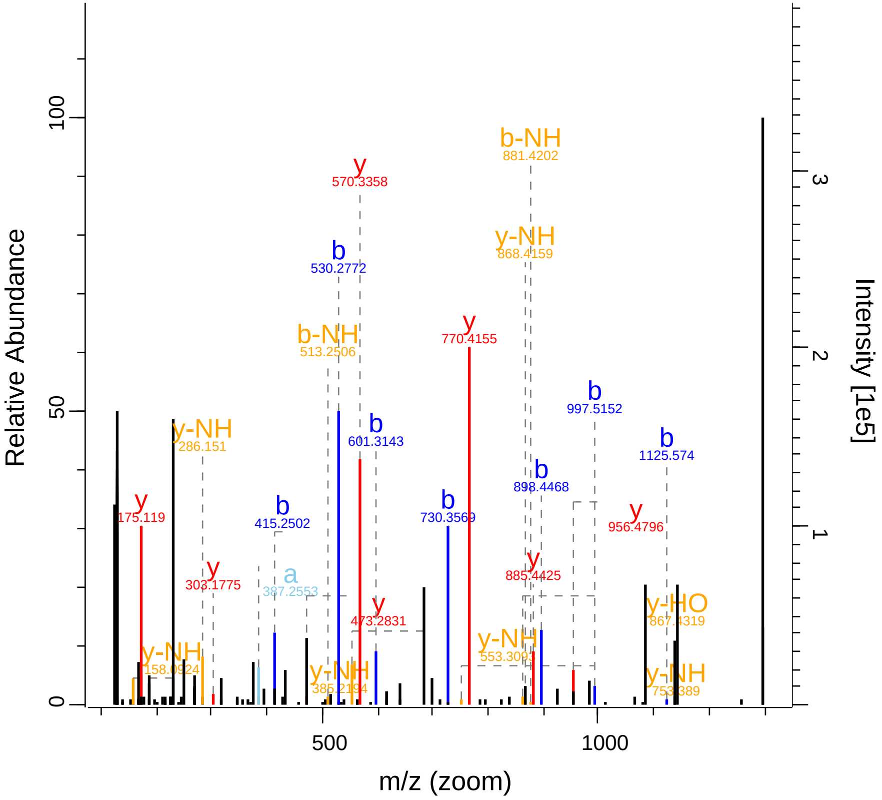

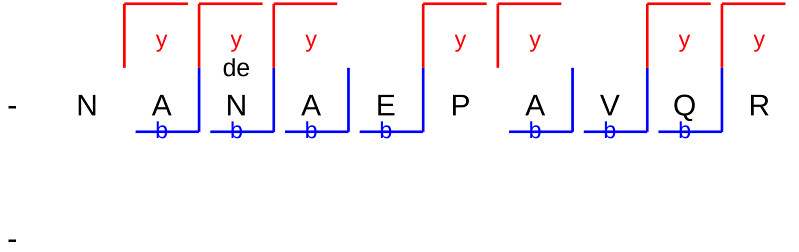

Raw File	Scan	Method	Score	m/z	Gene names
RO-QC-160121_ZackD_SS_Set_02_Fraction_	5840	FTMS; HC	113.62	674.3	FZD7

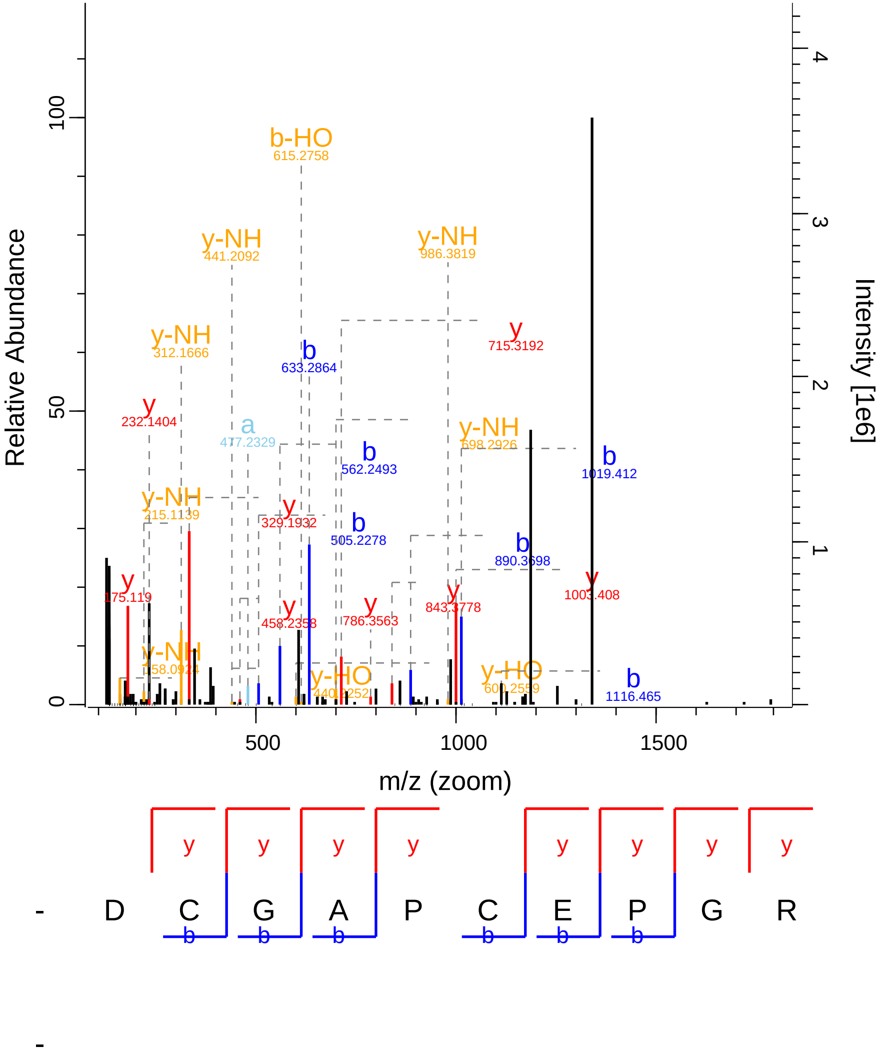

Raw File	Scan	Method	Score	m/z	Gene names
RO-QC-160121_ZackD_SS_Set_02_Fraction	5951	FTMS; HC	82.64	630.32	SPRED3

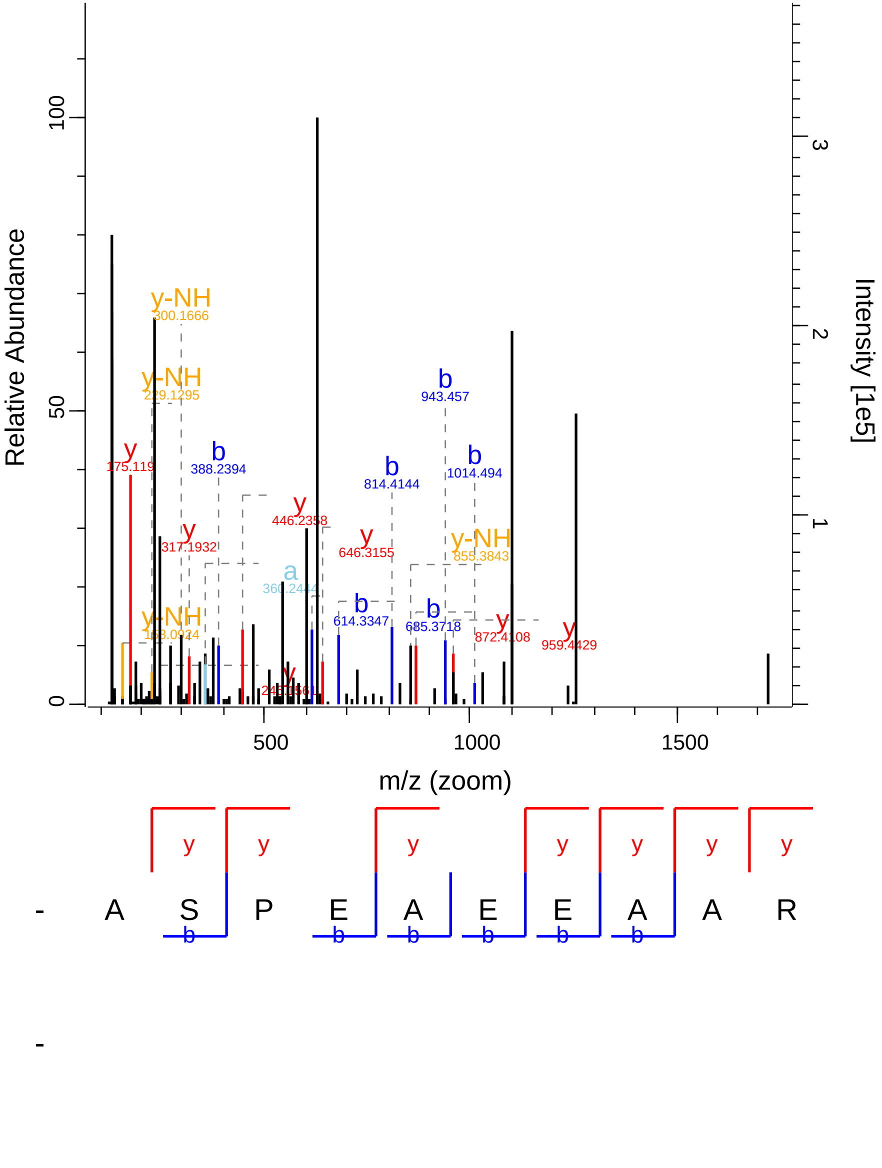

Raw File	Scan	Method	Score	m/z	Gene names
RO-QC-160121_ZackD_SS_Set_02_Fraction	5967	FTMS; HC	81.26	650.35	CD99

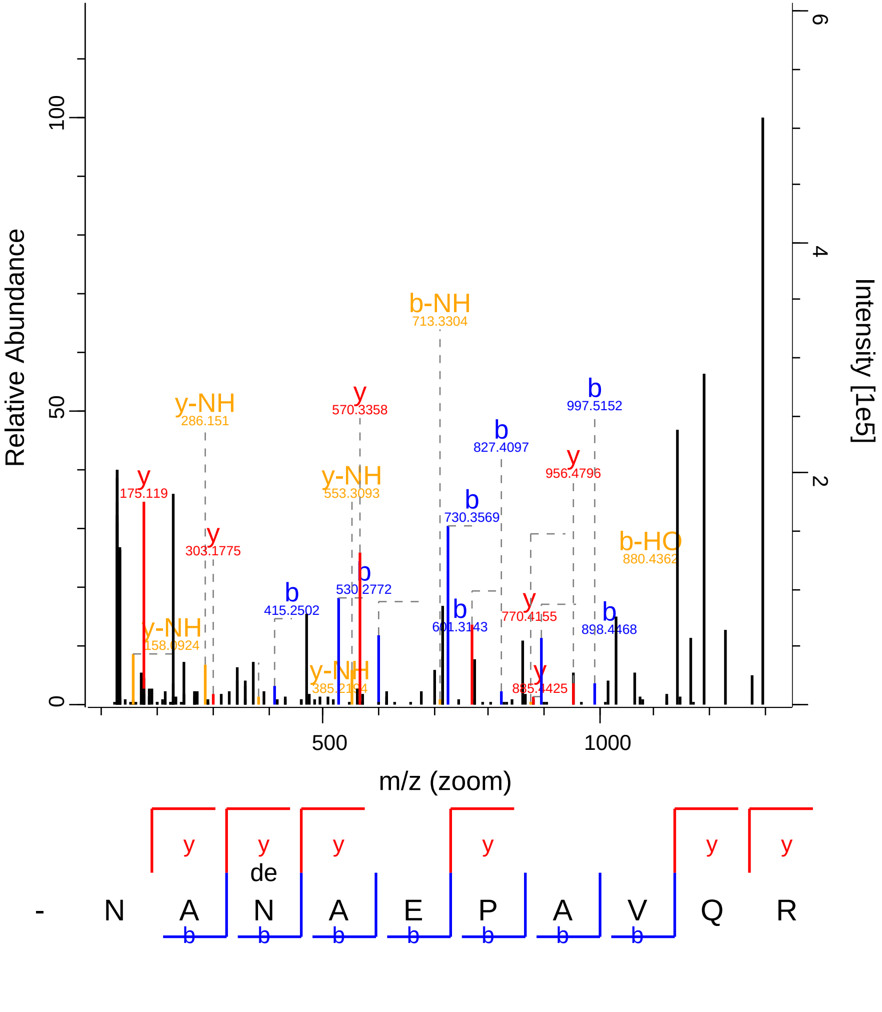

Raw File	Scan	Method	Score	m/z	Gene names
RO-QC-160121_ZackD_SS_Set_02_Fraction	6180	FTMS; HC	124.98	678.39	ADAT2

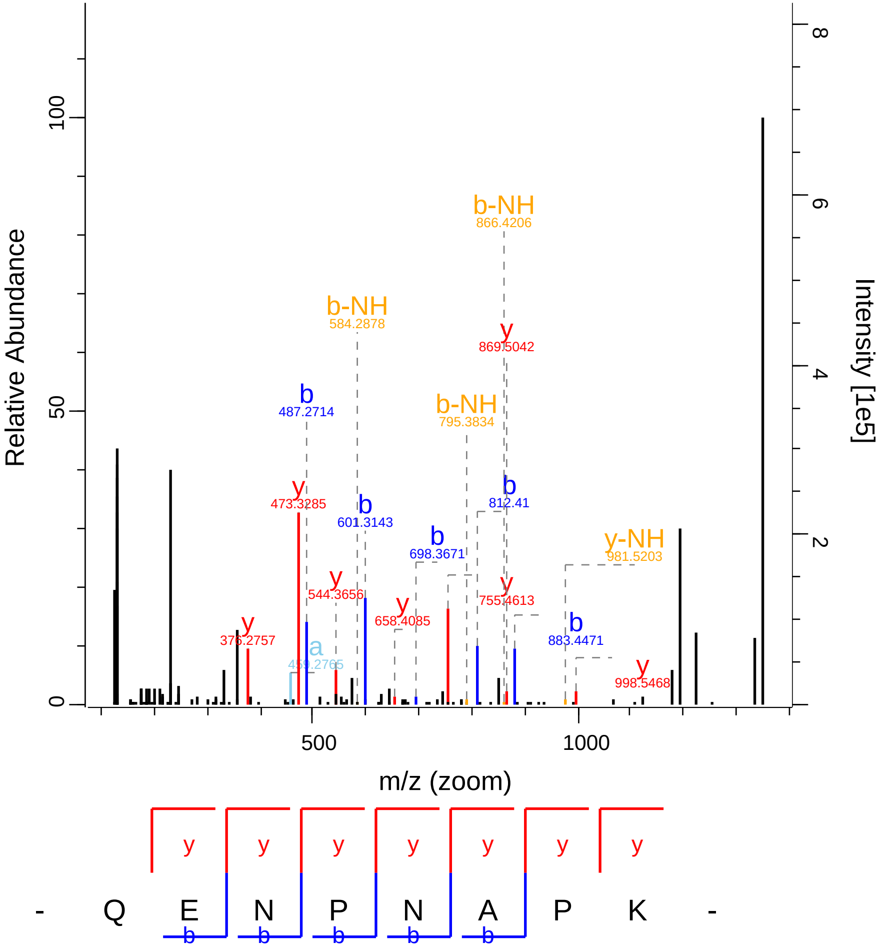

Raw File	Scan	Method	Score	m/z	Gene names
RO-QC-160121_ZackD_SS_Set_02_Fraction	6193	FTMS; HC	76.35	668.82	DSG1

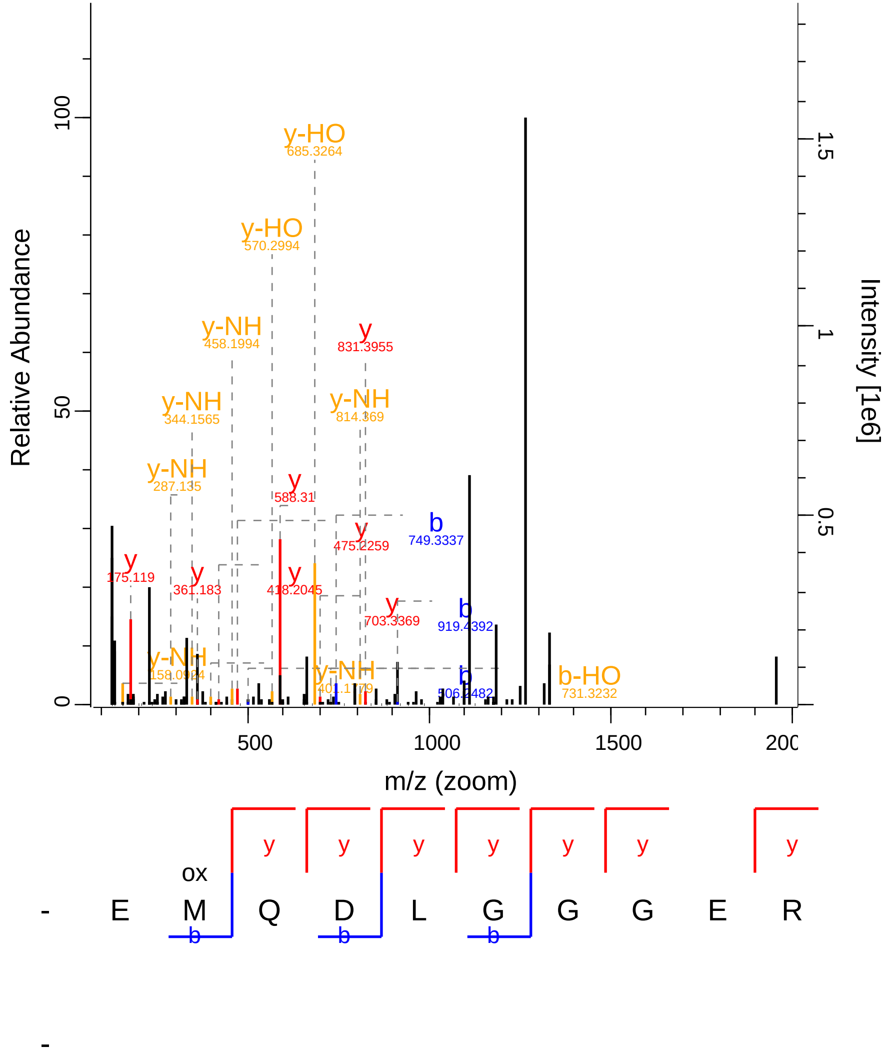

Raw File	Scan	Method	Score	m/z	Gene names
RO-QC-160121_ZackD_SS_Set_02_Fraction	6407	FTMS; HC	137.85	650.35	CD99

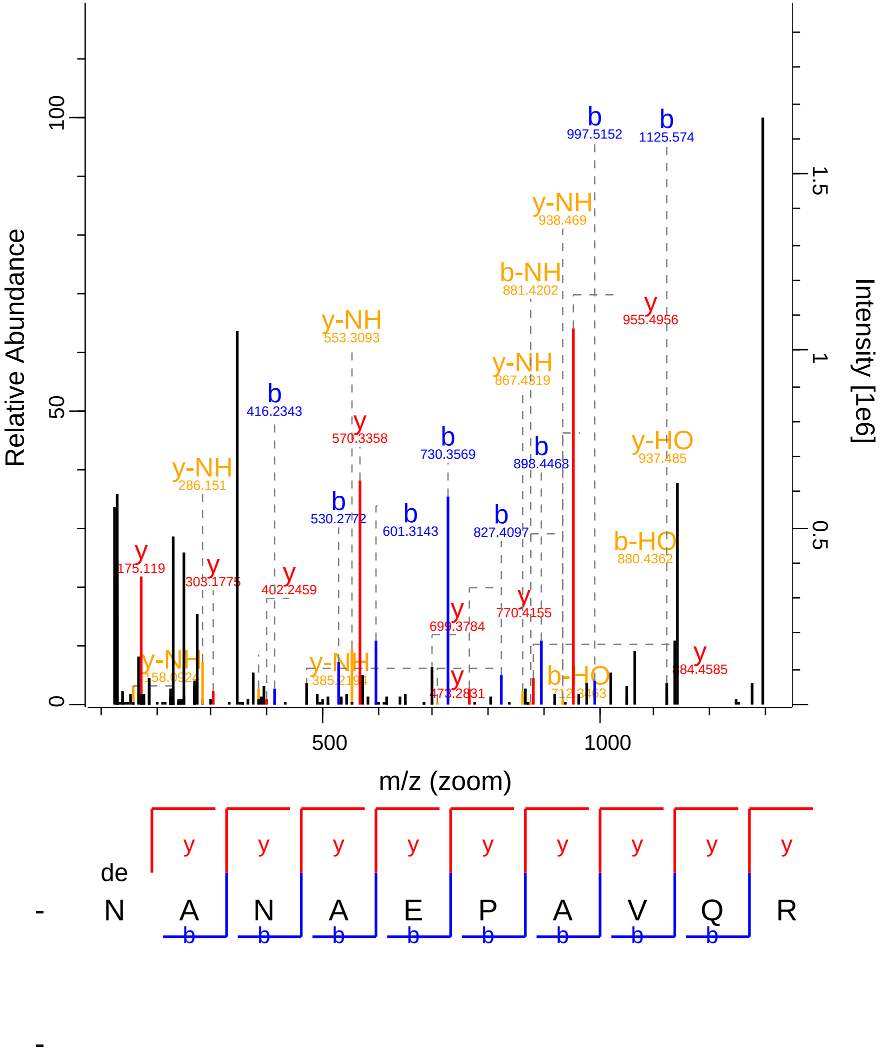

Raw File	Scan	Method	Score	m/z
RO-QC-160121_ZackD_SS_Set_02_Fraction_10	6687	FTMS; HCD	89.75	460.92

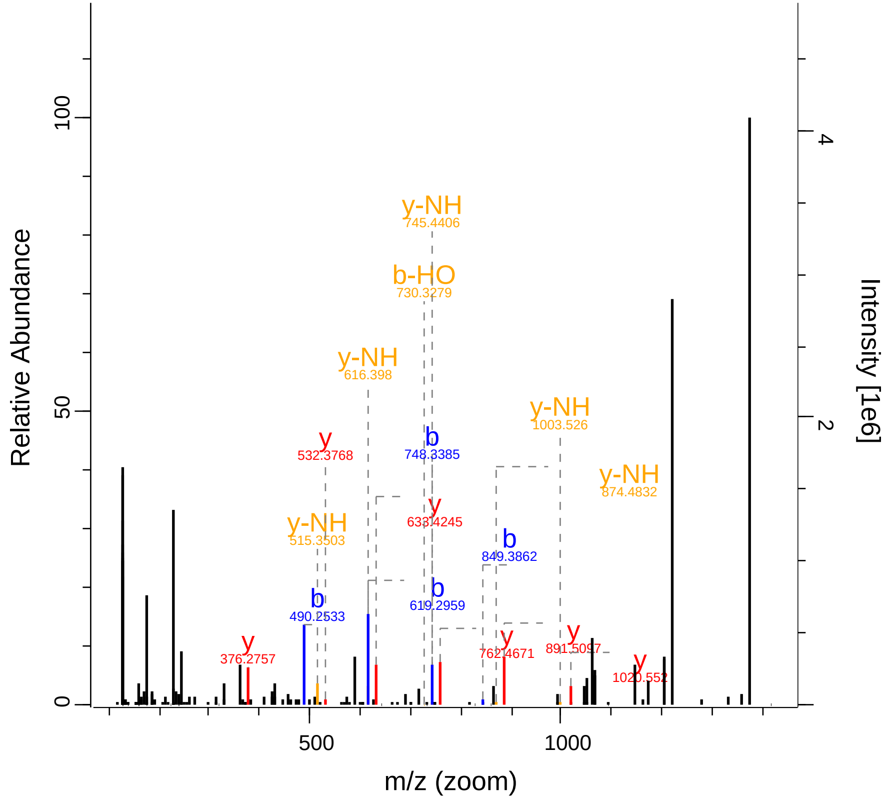

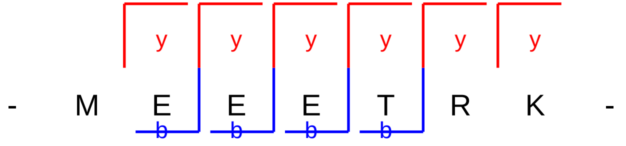

Raw File	Scan	Method	Score	m/z
RO-QC-160121_ZackD_SS_Set_02_Fraction_10	6724	FTMS; HCD	134.06	690.88

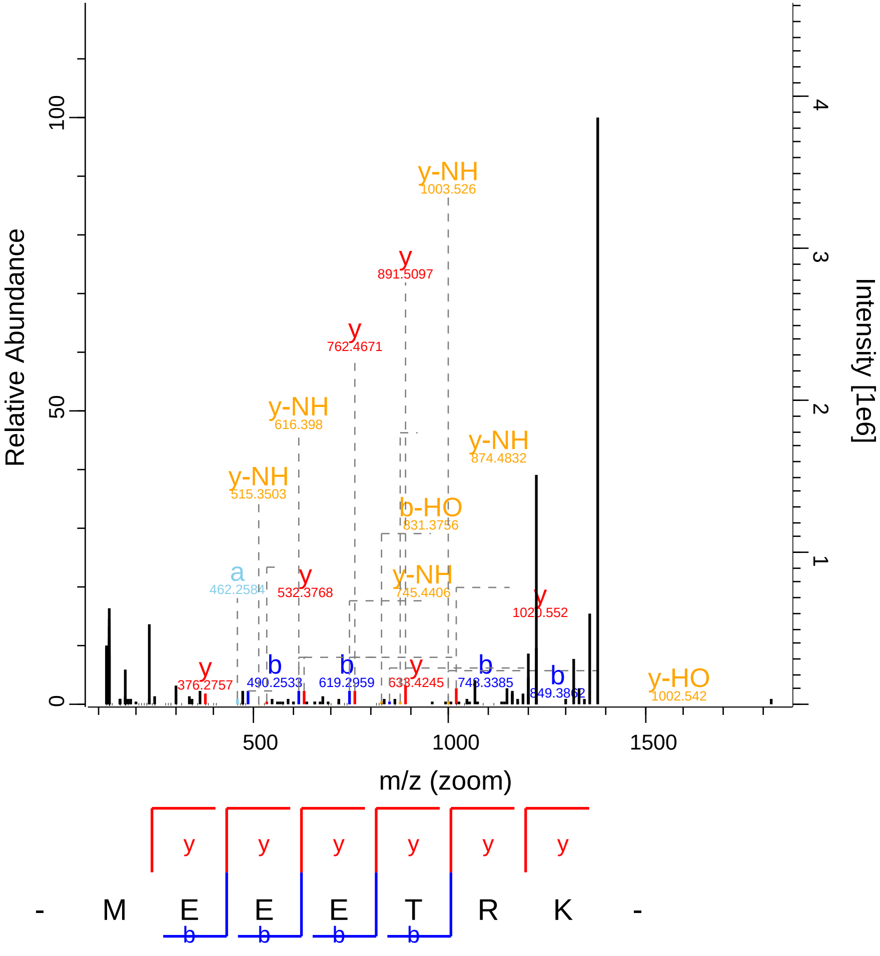

Raw File	Scan	Method	Score	m/z
RO-QC-160121_ZackD_SS_Set_02_Fraction_10	7146	FTMS; HCD	39.7	352.79

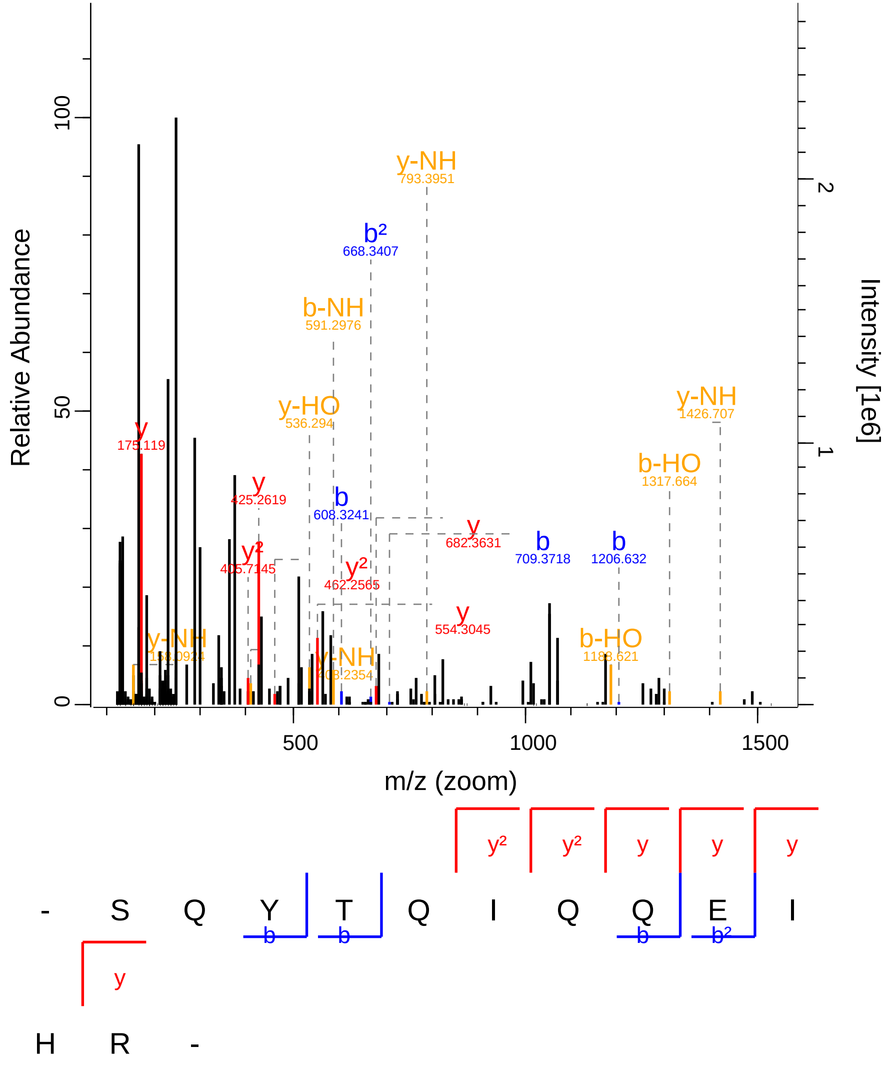

Raw File	Scan	Method	Score	m/z	Gene names
RO-QC-160121_ZackD_SS_Set_02_Fraction	7397	FTMS; HC	61.65	498.95	PARP16

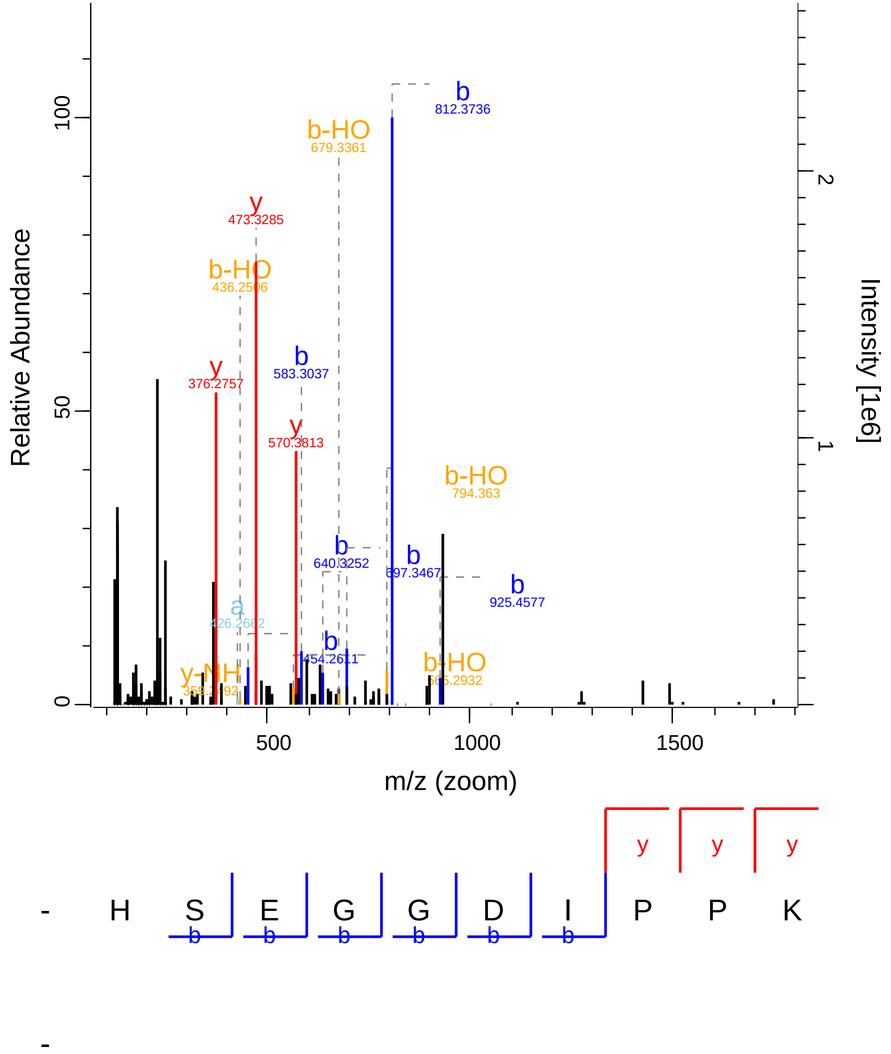

Raw File	Scan	Method	Score	m/z	Gene names
RO-QC-160121_ZackD_SS_Set_02_Fraction	7672	FTMS; HC	76.81	748.35	ANAPC16

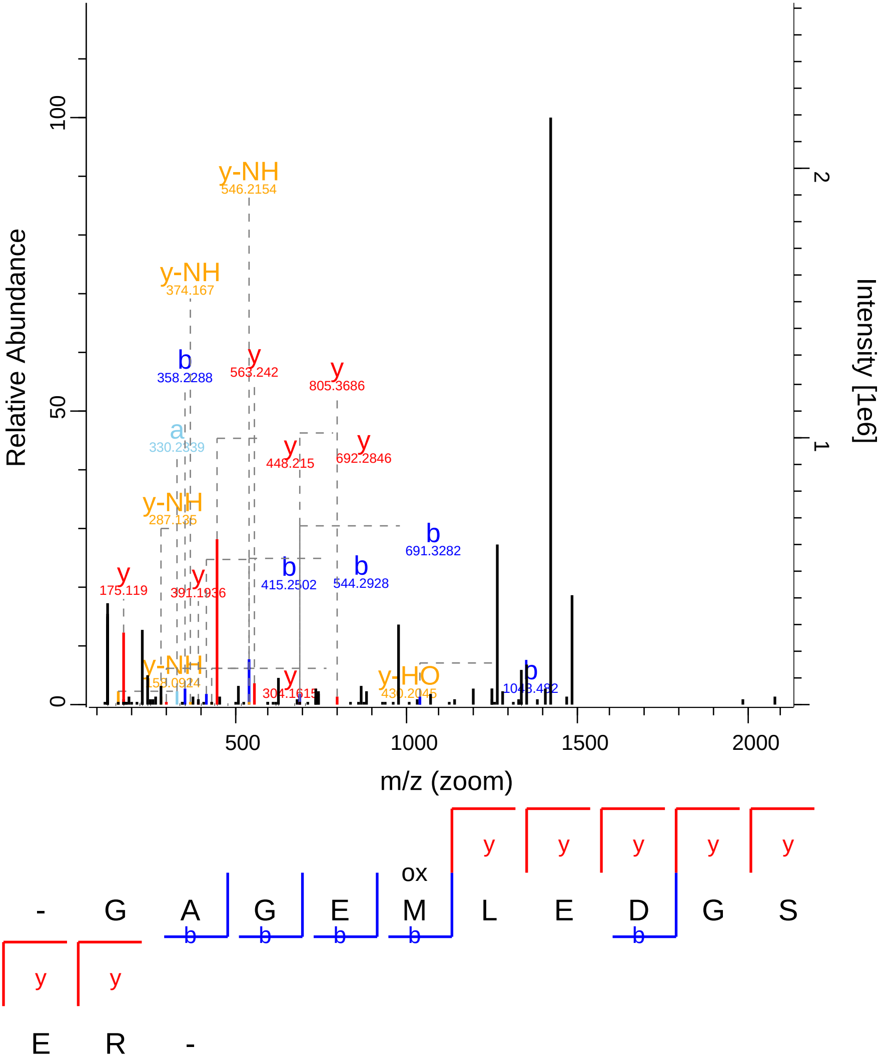

Raw File	Scan	Method	Score	m/z	Gene names
RO-QC-160121_ZackD_SS_Set_02_Fraction	8927	FTMS; HC	95.77	699.82	C11orf57

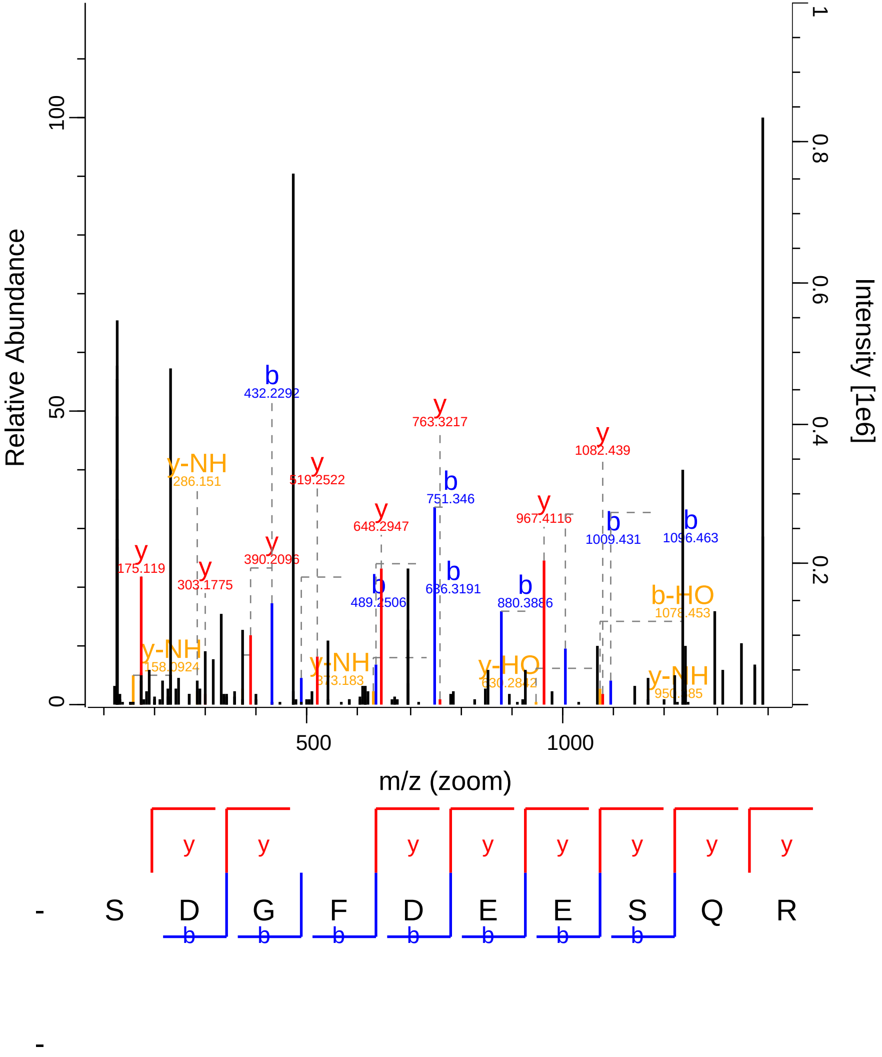

Raw File	Scan	Method	Score	m/z	Gene names
RO-QC-160121_ZackD_SS_Set_02_Fractior	11981	FTMS; HC	109.11	730.38	SLC16A10

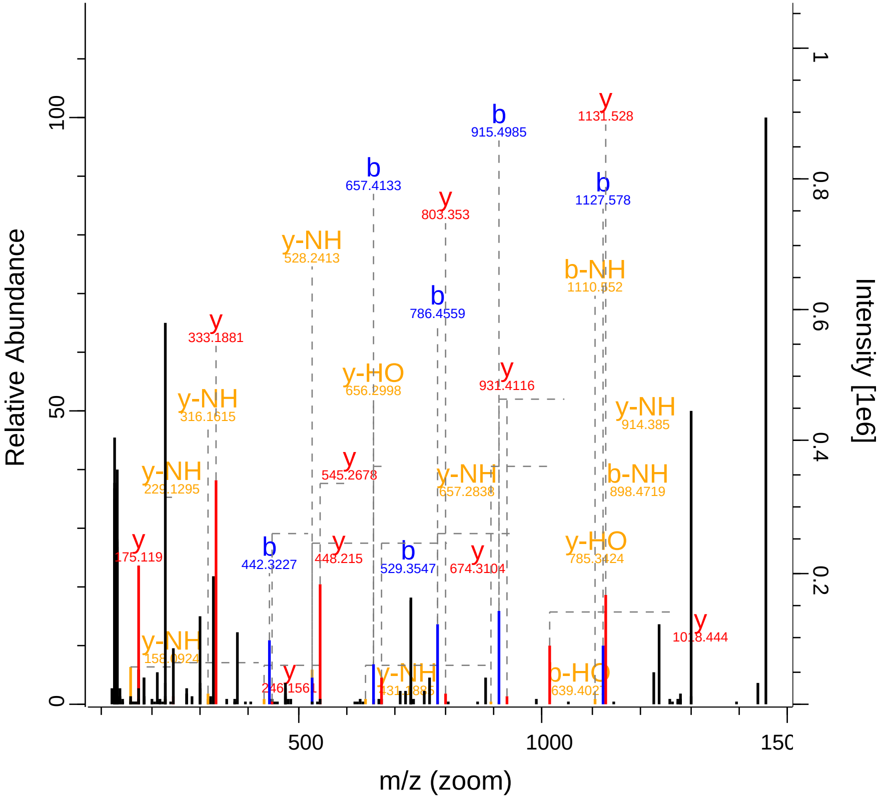

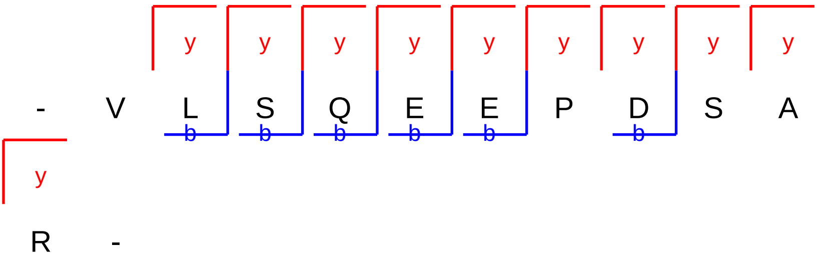

Raw File	Scan	Method	Score	m/z	Gene names
RO-QC-160121_ZackD_SS_Set_02_Fractior	12040	FTMS; HC	191.55	798.37	FXYD1

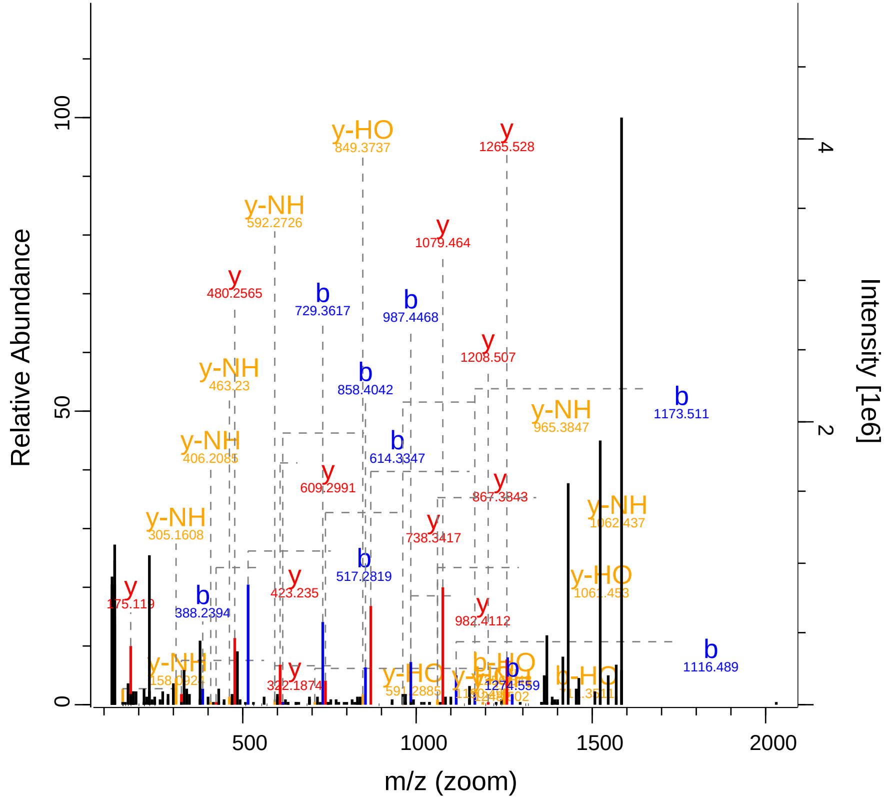

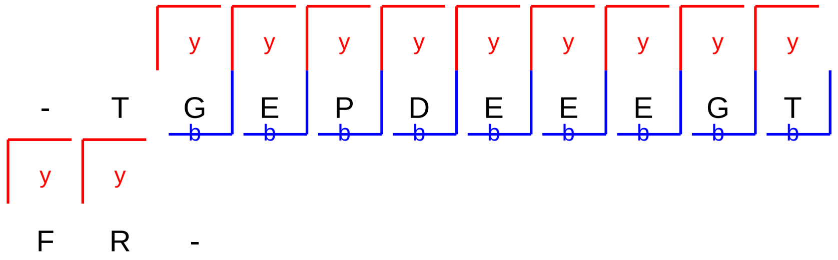

Raw File	Scan	Method	Score	m/z	Gene names
RO-QC-160121_ZackD_SS_Set_02_Fractior	12170	FTMS; HC	68.91	729.45	SFMBT2

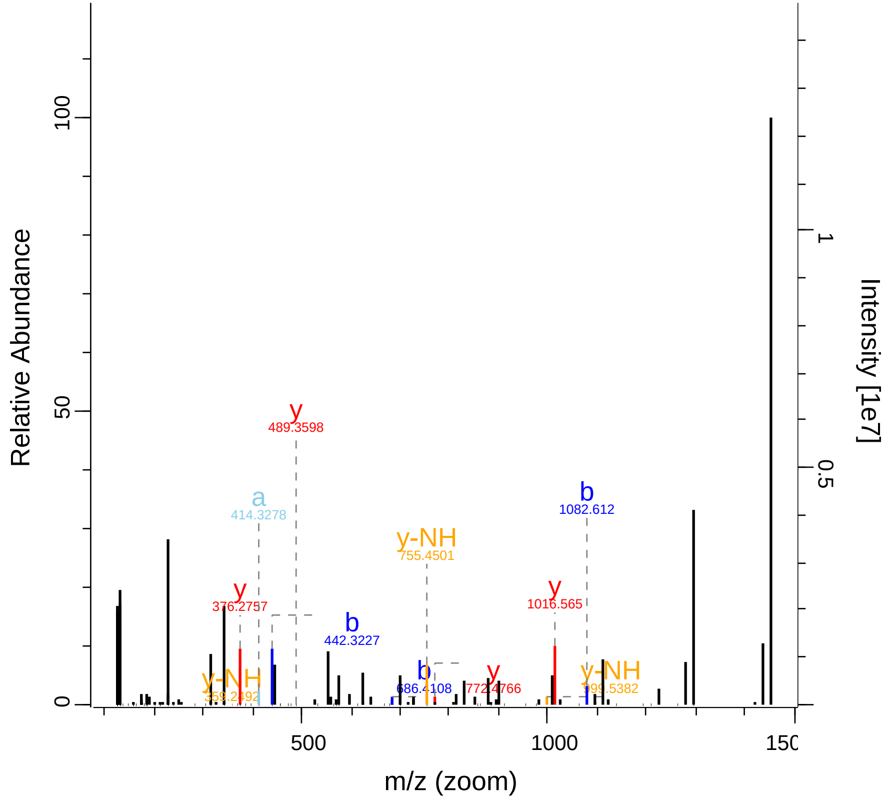

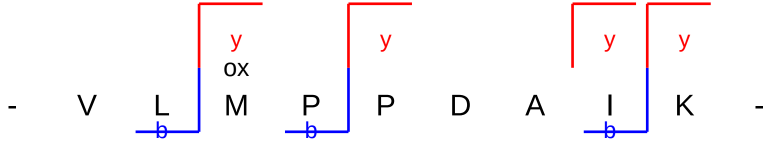

Raw File	Scan	Method	Score	m/z	Gene names
RO-QC-160121_ZackD_SS_Set_02_Fractior	12294	FTMS; HC	252.16	798.37	FXYD1

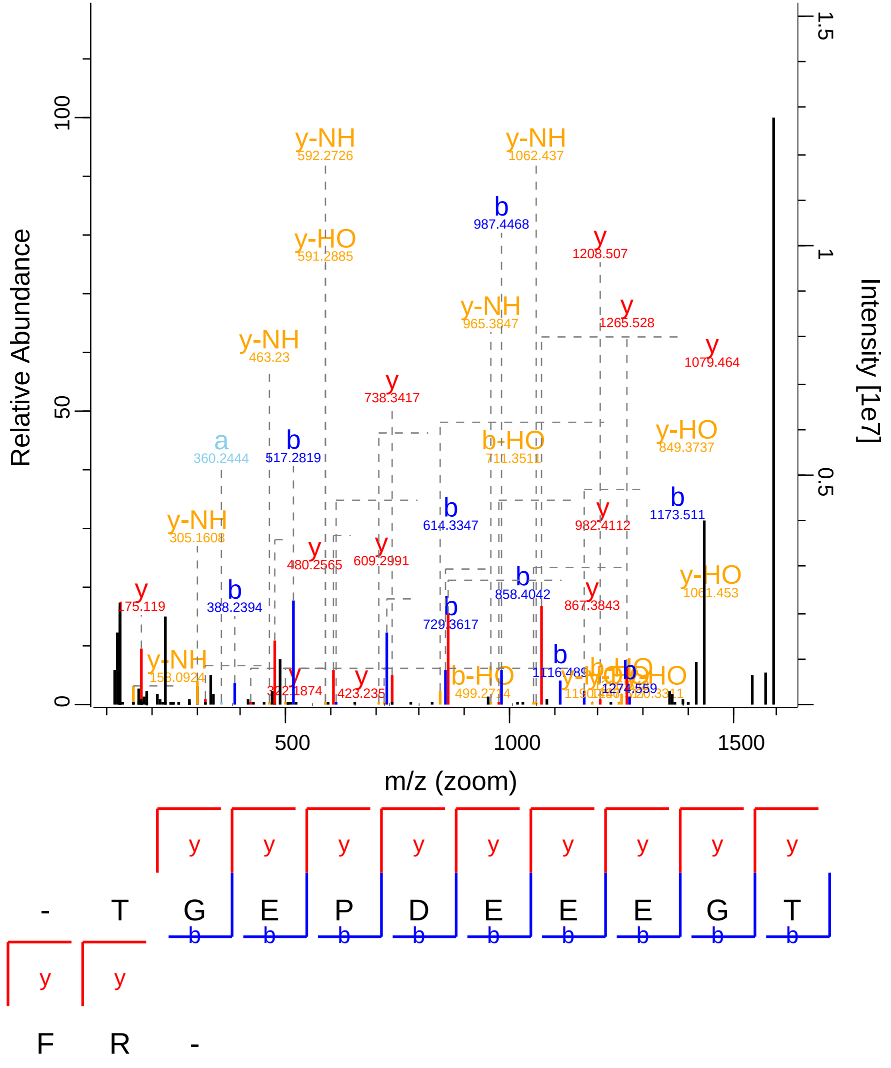

Raw File	Scan	Method	Score	m/z	Gene names
RO-QC-160121_ZackD_SS_Set_02_Fractior	12441	FTMS; HC	99.28	826.45	PDPN

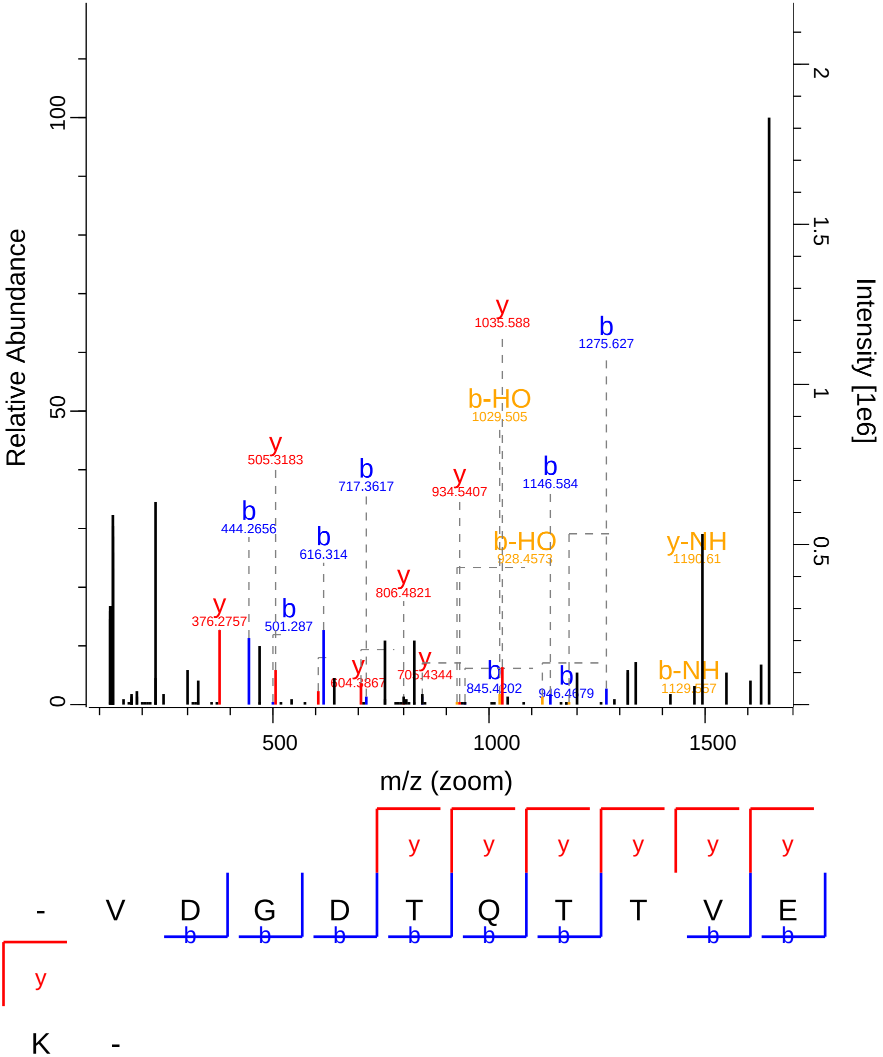

Raw File	Scan	Method	Score	m/z
RO-QC-160121_ZackD_SS_Set_02_Fraction_10	13050	FTMS; HCD	41.09	490.88

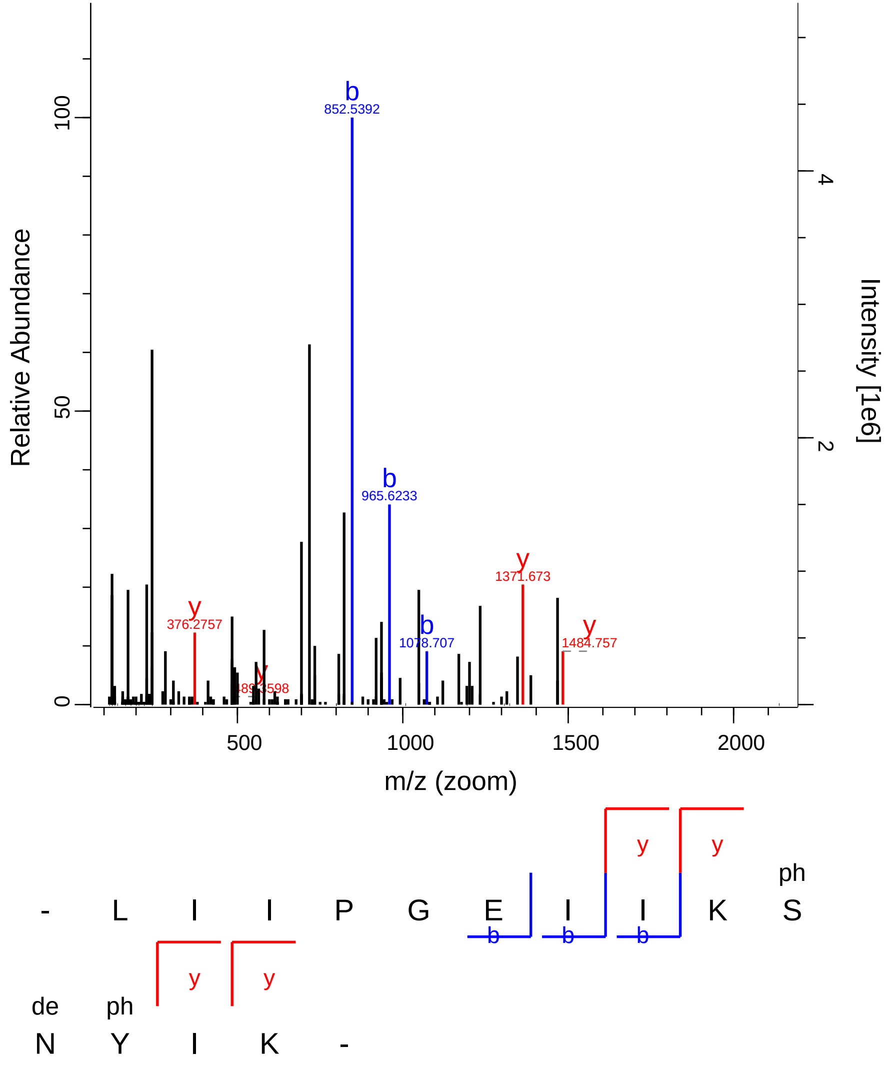

Raw File	Scan	Method	Score	m/z	Gene names
RO-QC-160121_ZackD_SS_Set_02_Fractior	13434	FTMS; HC	144.38	602.89	EXOC3L4

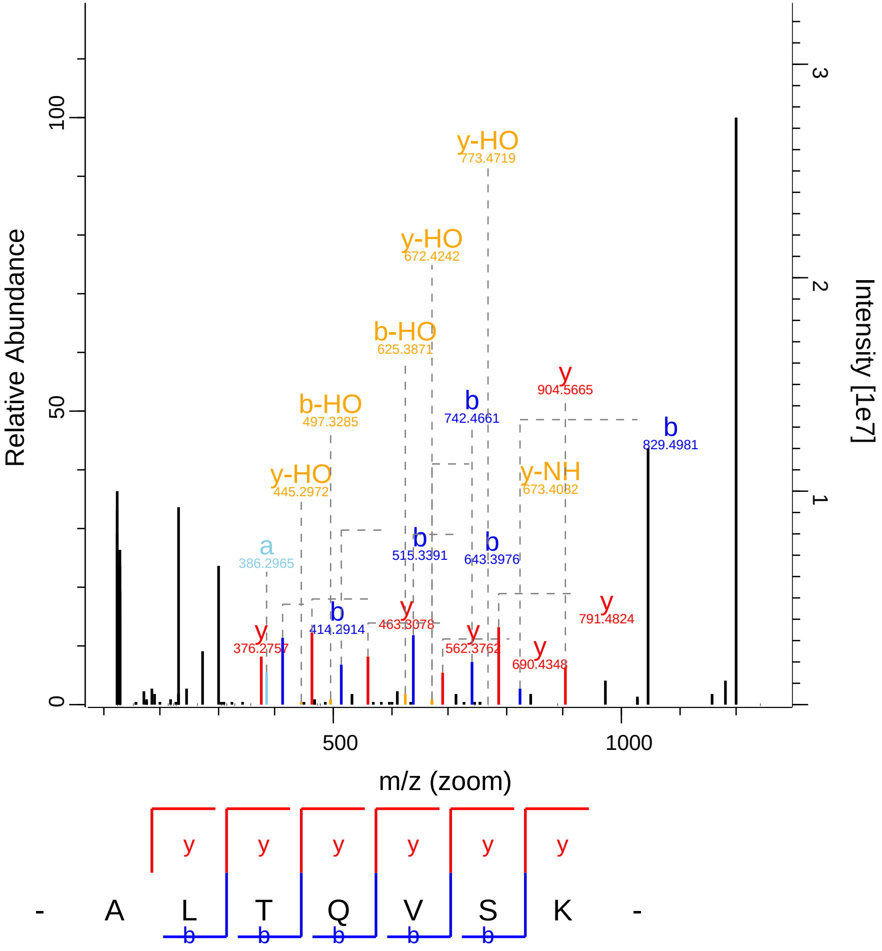

Raw File	Scan	Method	Score	m/z	Gene names
RO-QC-160121_ZackD_SS_Set_02_Fractior	13638	FTMS; HC	125.16	671.36	MKS1

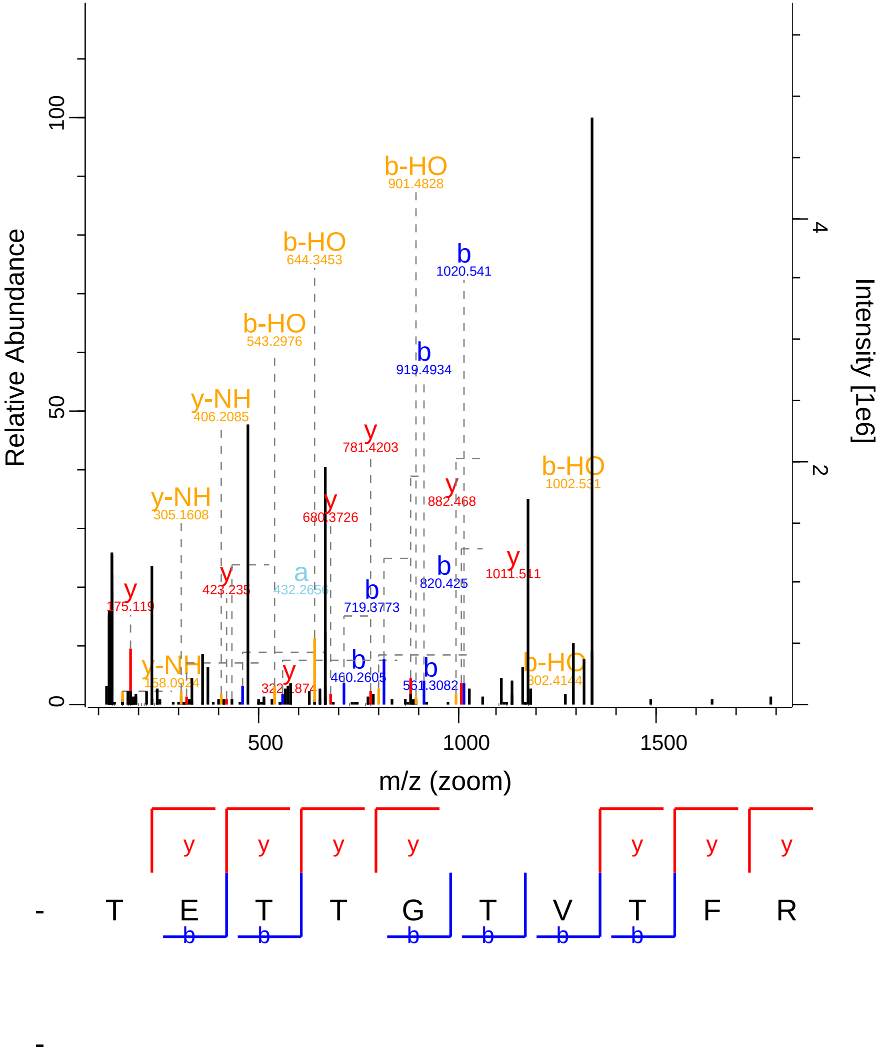

Raw File	Scan	Method	Score	m/z	Gene names
RO-QC-160121_ZackD_SS_Set_02_Fractior	13703	FTMS; HC	116.73	602.89	EXOC3L4

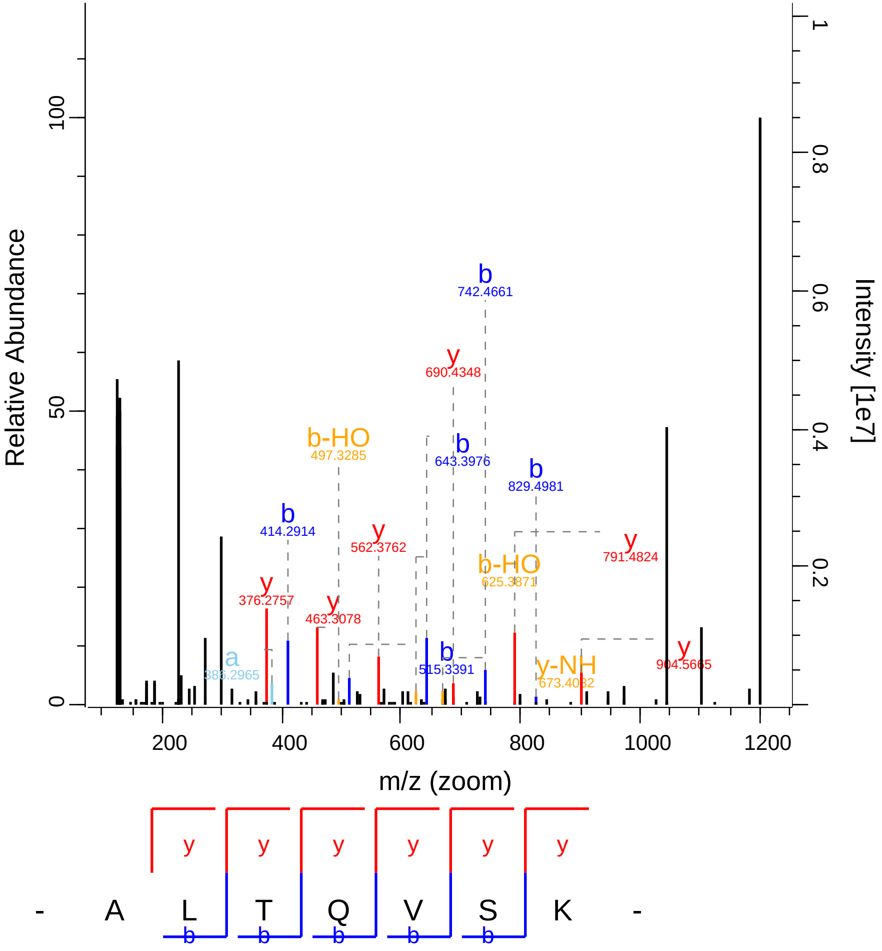

Raw File	Scan	Method	Score	m/z
RO-QC-160121_ZackD_SS_Set_02_Fraction_10	15013	FTMS; HCD	49.36	378.21

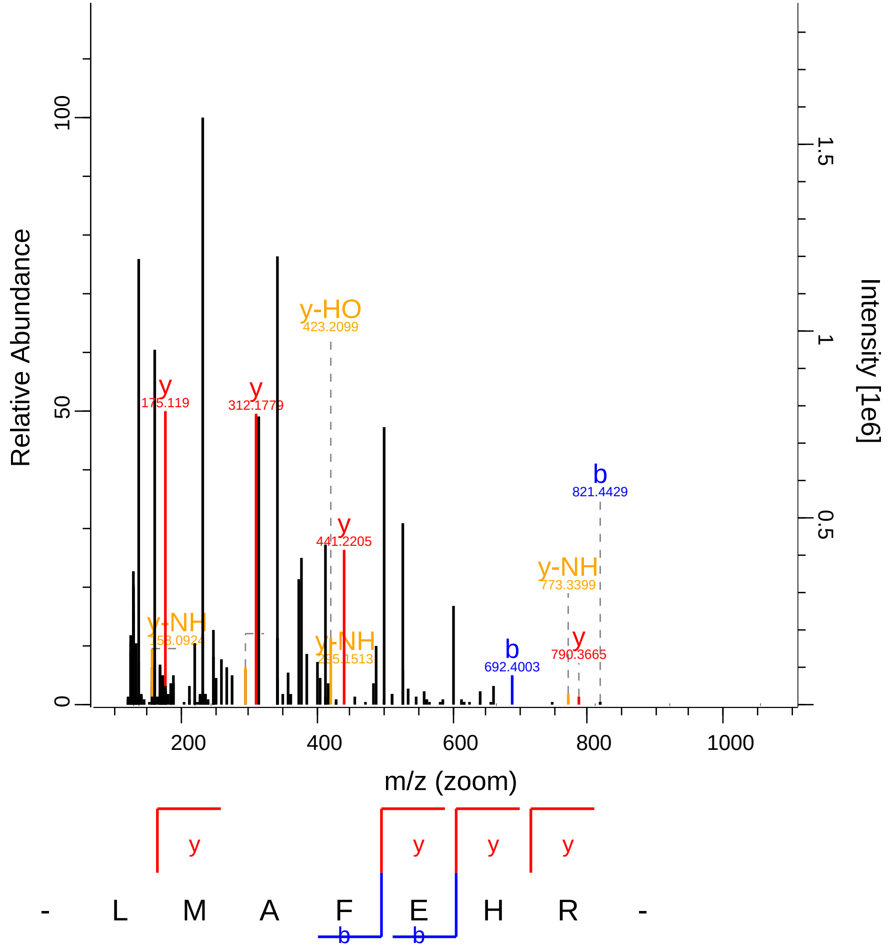

Raw File	Scan	Method	Score	m/z	Gene names
RO-QC-160121_ZackD_SS_Set_02_Fraction_	15168	FTMS; HC	93.45	719.9	CYSTM1

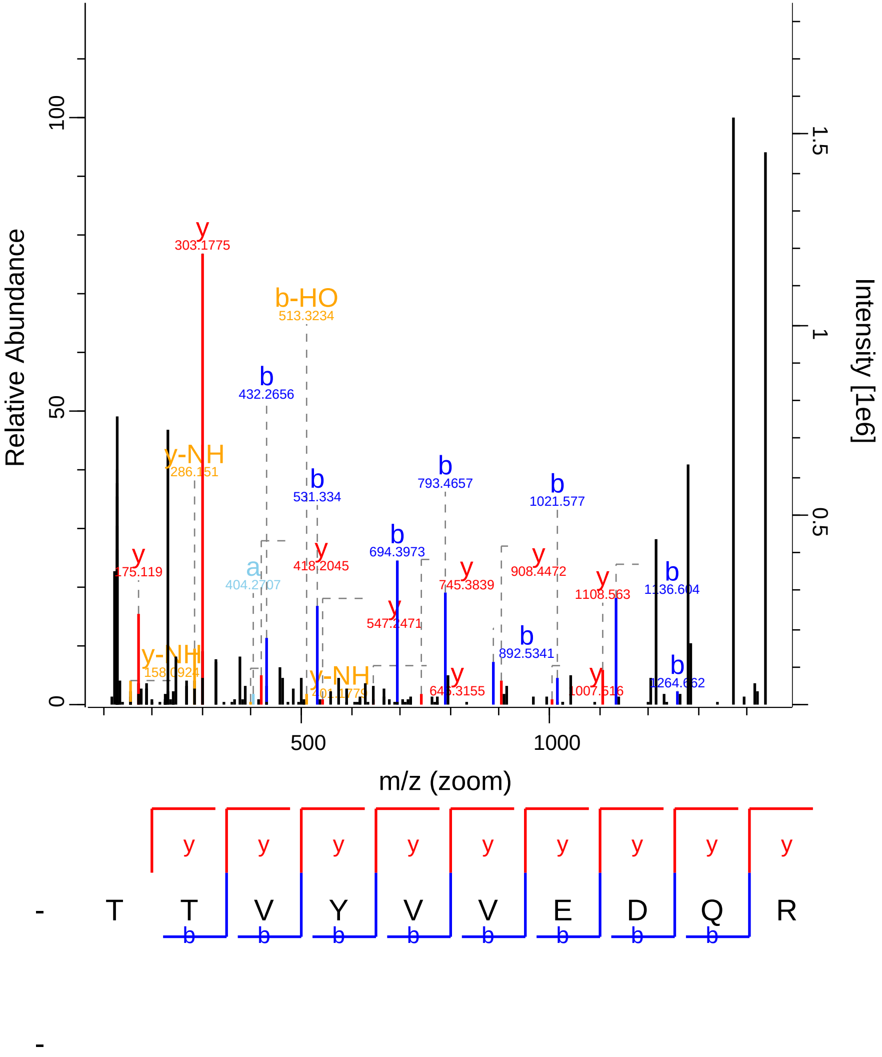

Raw File	Scan	Method	Score	m/z	Gene names
RO-QC-160121_ZackD_SS_Set_02_Fractior	15170	FTMS; HC	77.68	413.57	TAF12

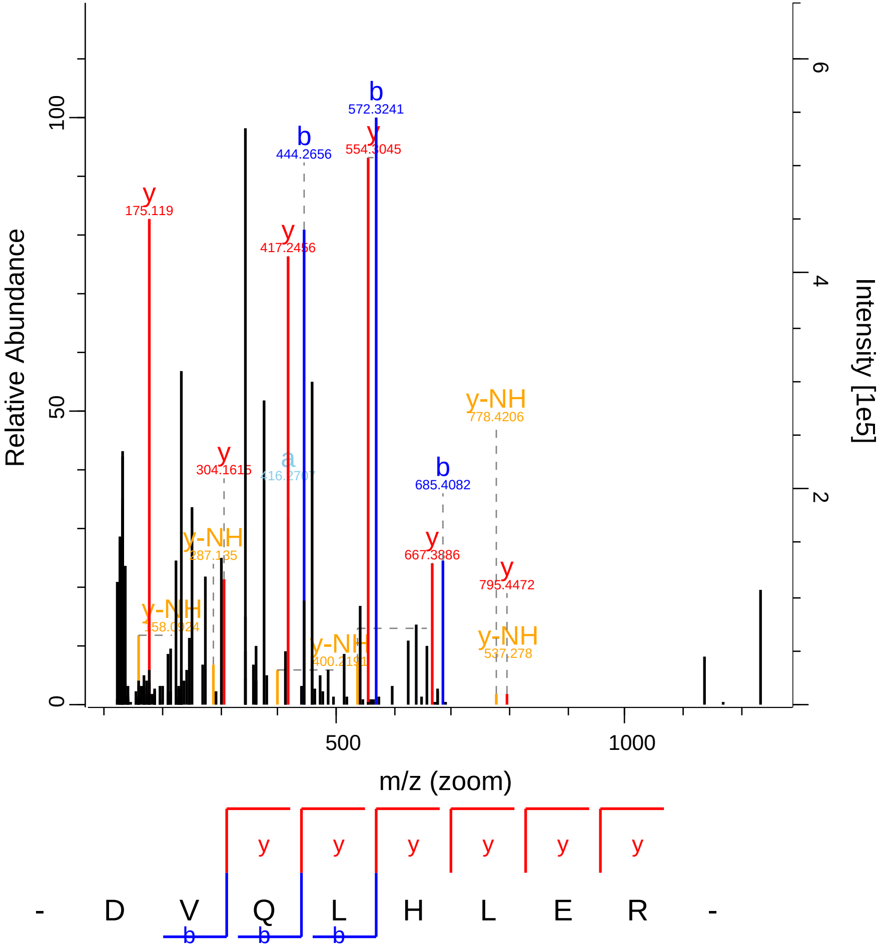

Raw File	Scan	Method	Score	m/z	Gene names
RO-QC-160121_ZackD_SS_Set_02_Fractior	15399	FTMS; HC	74.2	720.39	CYSTM1

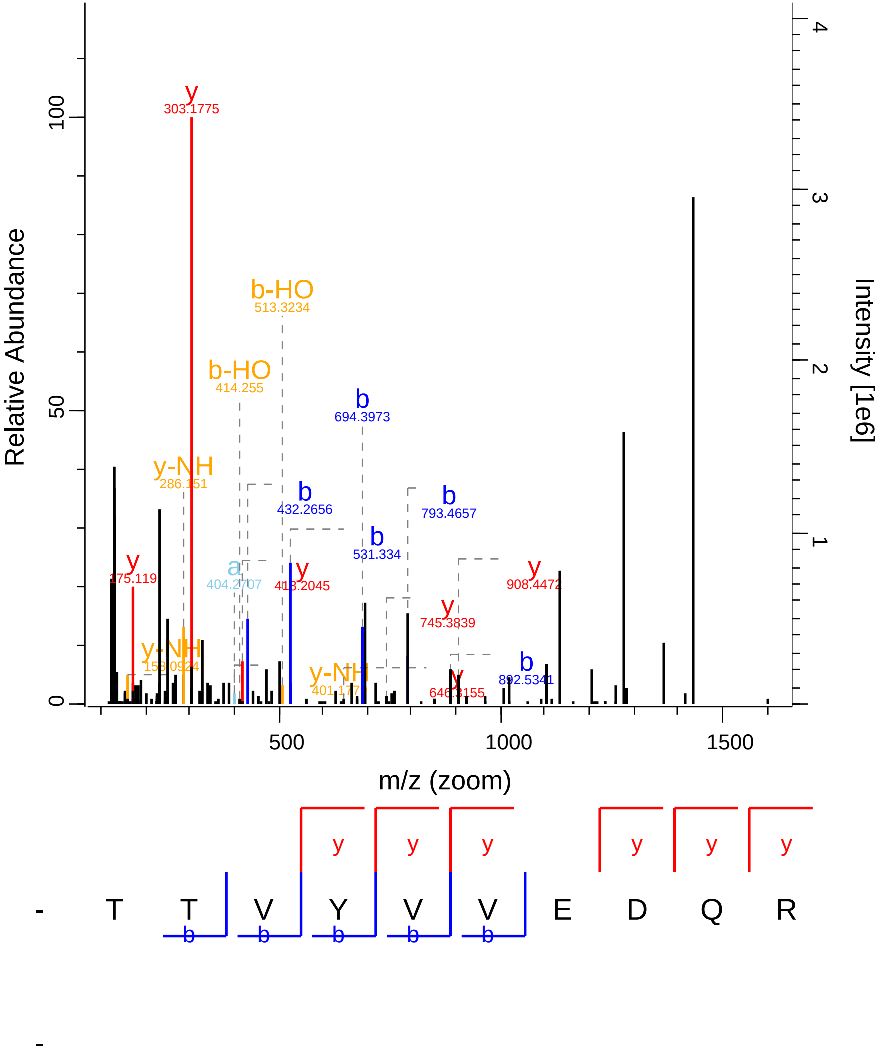

Raw File	Scan	Method	Score	m/z	Gene names
RO-QC-160121_ZackD_SS_Set_02_Fractio[illegible]	16627	FTMS; HC	134.32	865.44	IGKC

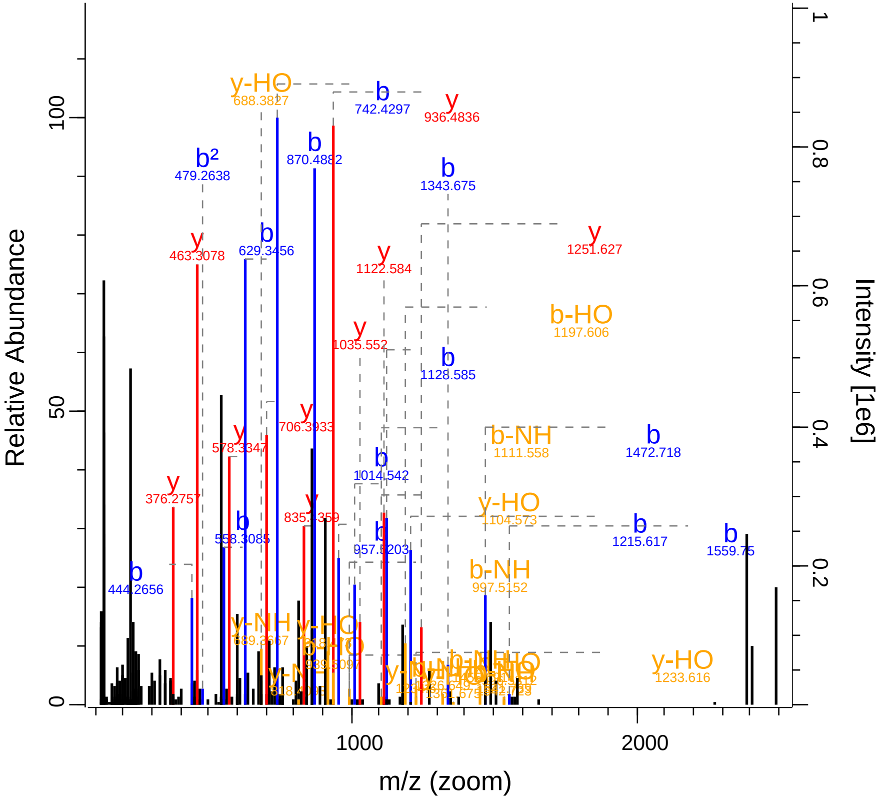

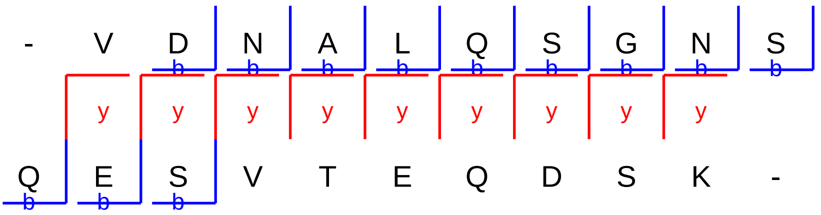

Raw File	Scan	Method	Score	m/z	Gene names
RO-QC-160121_ZackD_SS_Set_02_Fractior	16961	FTMS; HC	87.48	608.34	UTS2

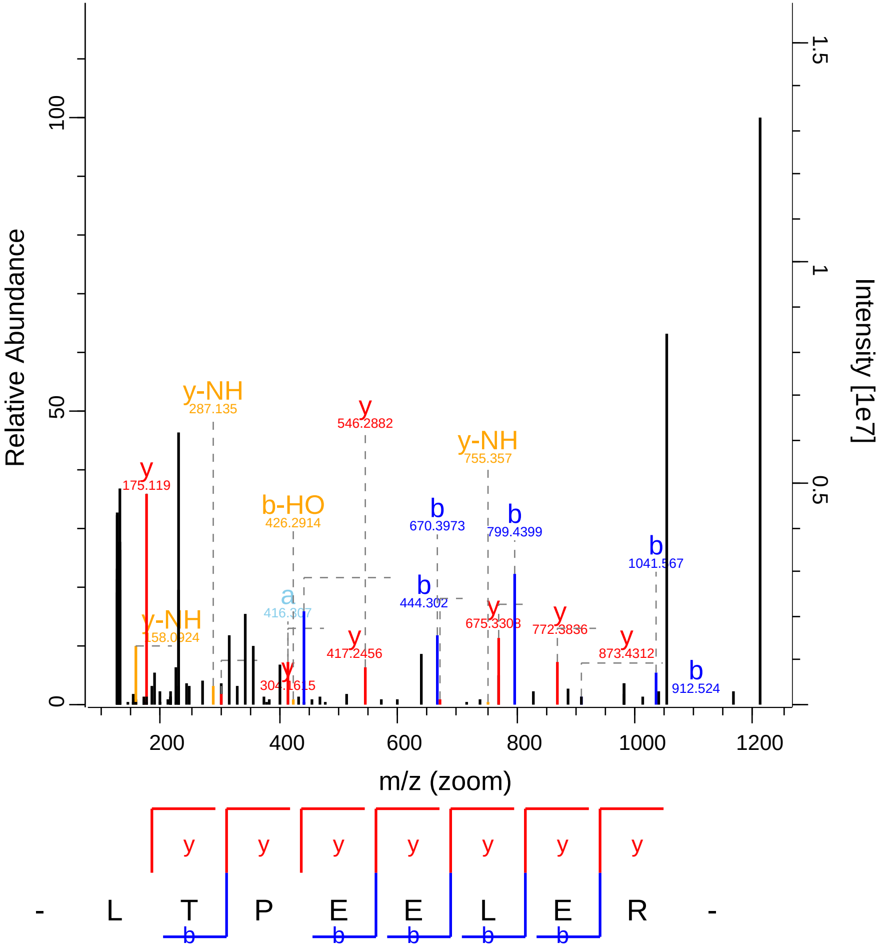

Raw File	Scan	Method	Score	m/z
RO-QC-160121_ZackD_SS_Set_02_Fraction_10	17975	FTMS; HCD	42.11	744.89

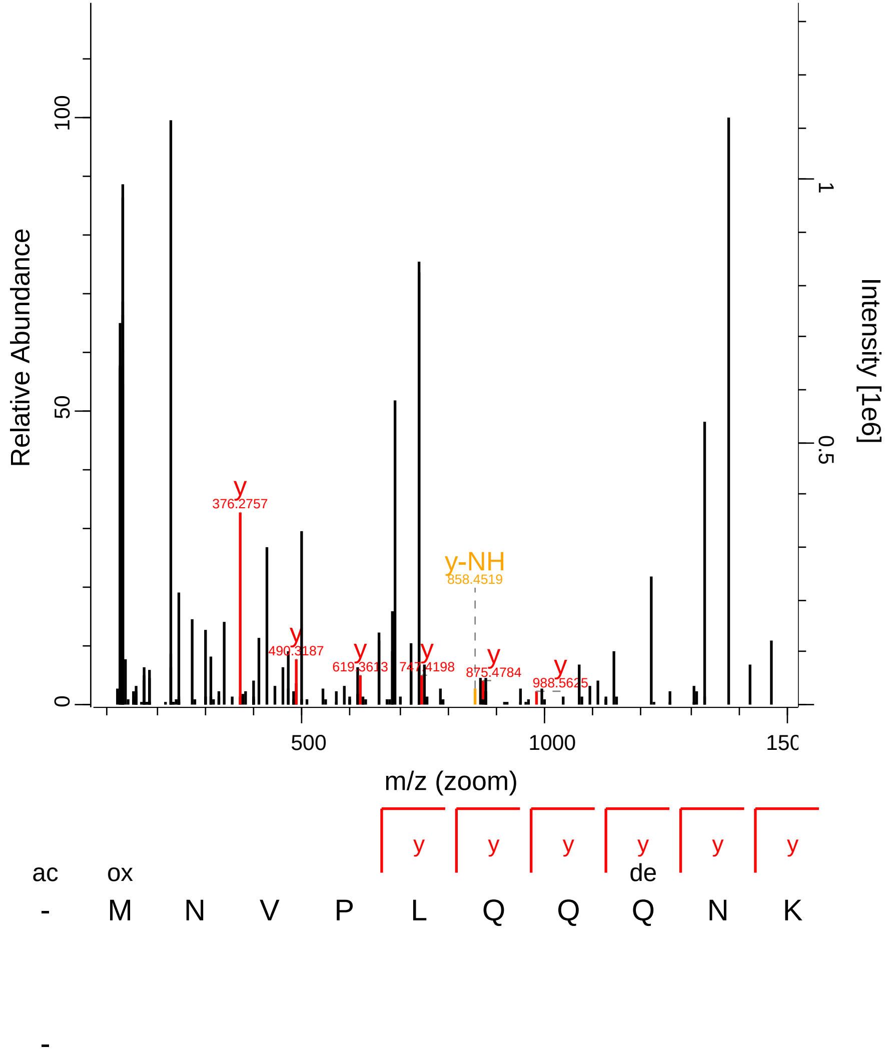

Raw File	Scan	Method	Score	m/z
RO-QC-160121_ZackD_SS_Set_02_Fraction_10	18262	FTMS; HCD	72.01	1055.04

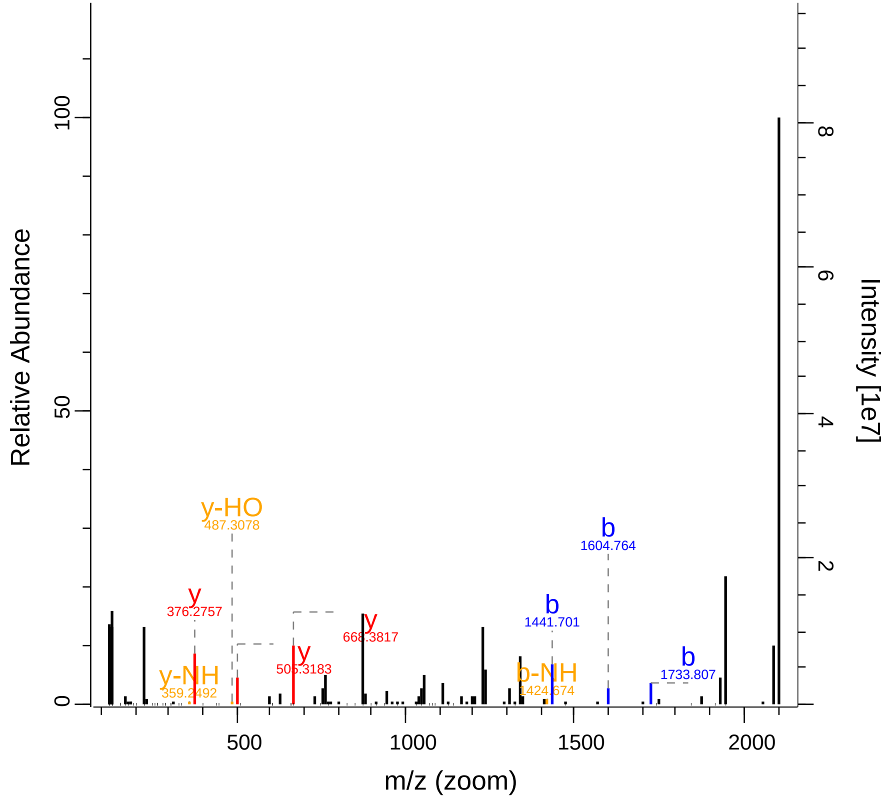

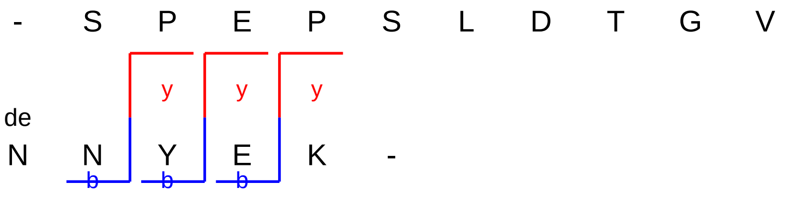

Raw File	Scan	Method	Score	m/z
RO-QC-160121_ZackD_SS_Set_02_Fraction_10	18580	FTMS; HCD	79.12	587.38

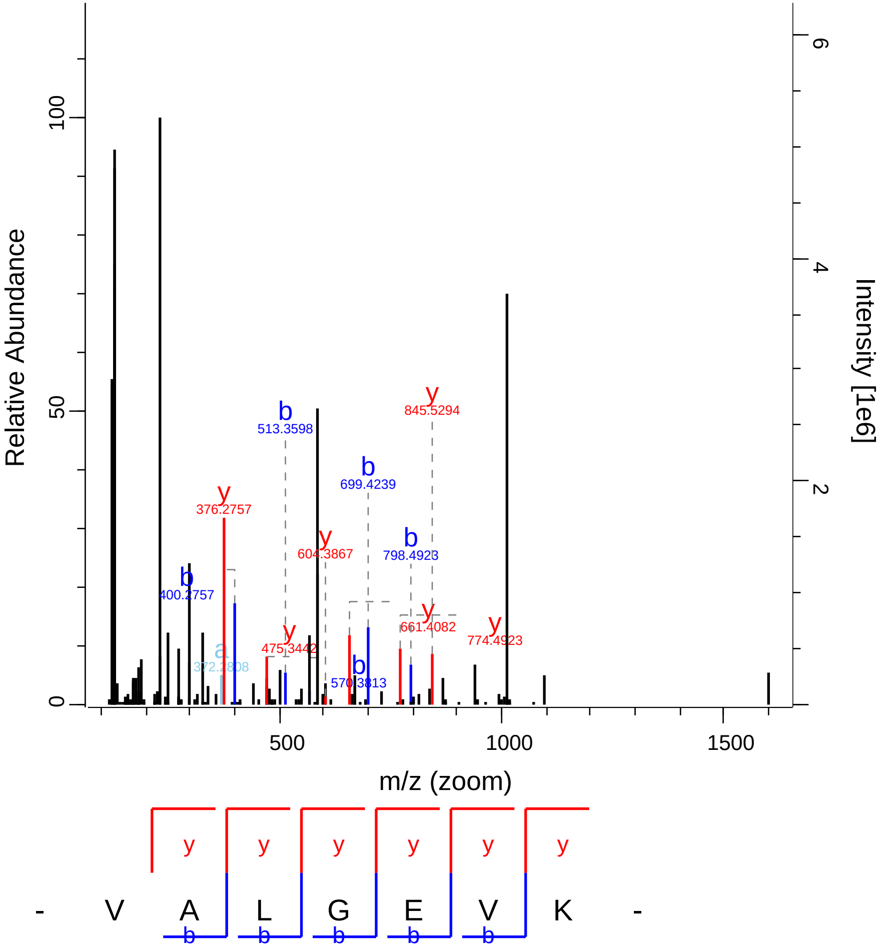

Raw File	Scan	Method	Score	m/z
RO-QC-160121_ZackD_SS_Set_02_Fraction_10	18659	FTMS; HCD	103.29	391.92

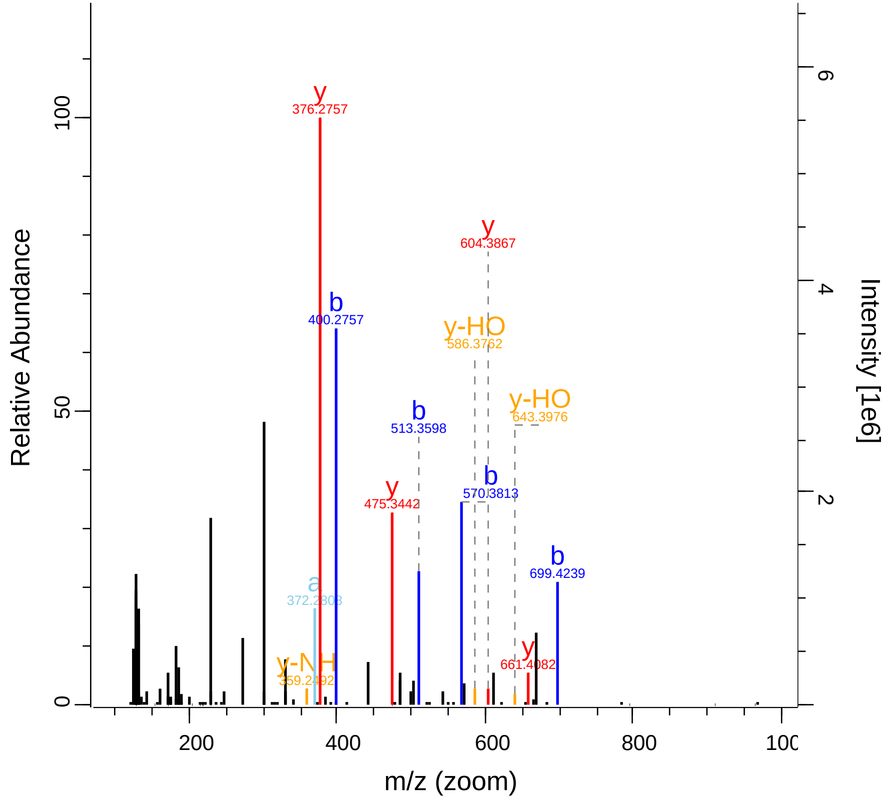

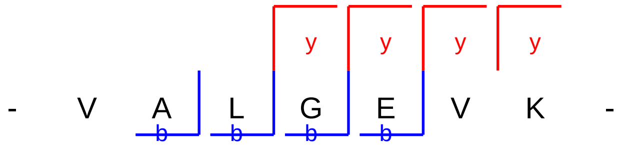

Raw File	Scan	Method	Score	m/z	Gene names
RO-QC-160121_ZackD_SS_Set_02_Fractior	18713	FTMS; HC	87.48	721.92	PPP1R2P1

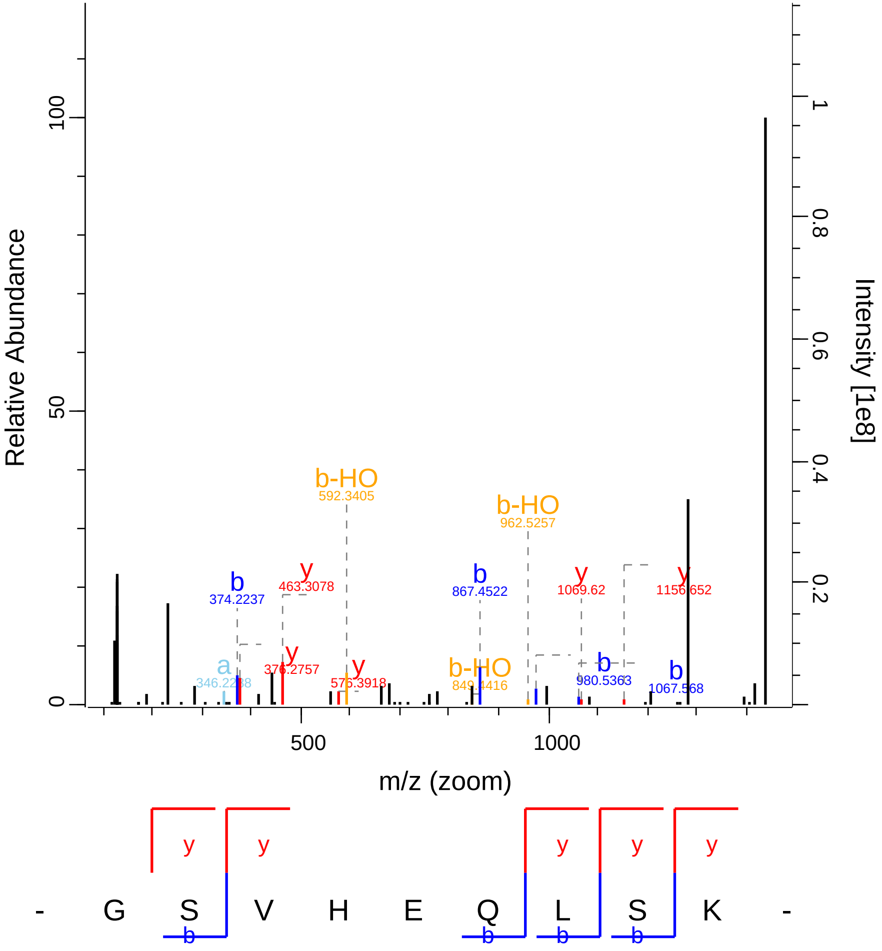

Raw File	Scan	Method	Score	m/z	Gene names
RO-QC-160121_ZackD_SS_Set_02_Fractior	18724	FTMS; HC	84.52	768.43	NAT1

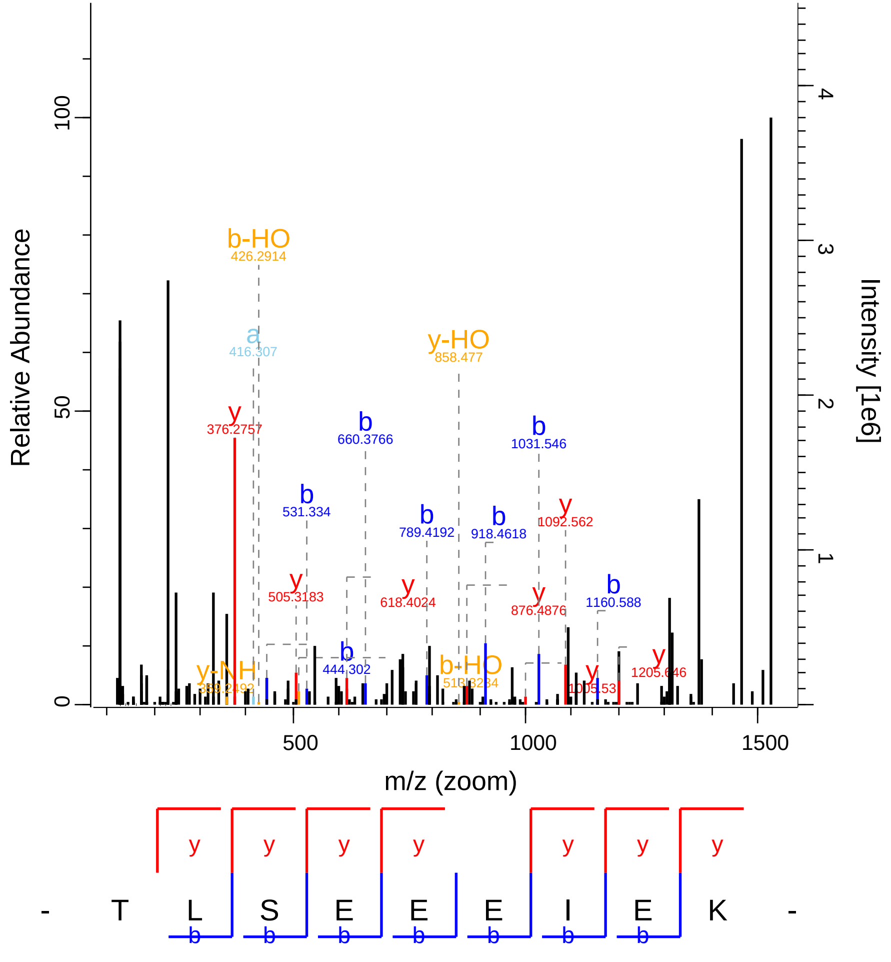

Raw File	Scan	Method	Score	m/z	Gene names
RO-QC-160121_ZackD_SS_Set_02_Fractior	18943	FTMS; HC	93.6	754.92	POC1B

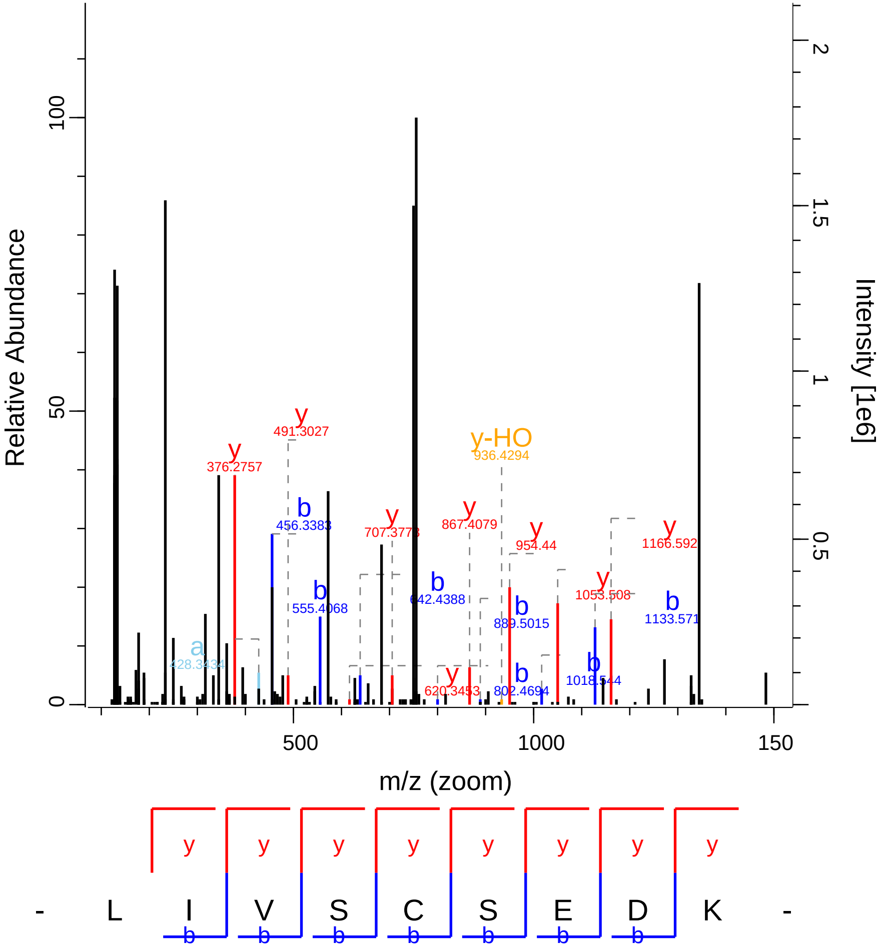

Raw File	Scan	Method	Score	m/z	Gene names
RO-QC-160121_ZackD_SS_Set_02_Fractior	21008	FTMS; HC	62	843.97	NRIP3

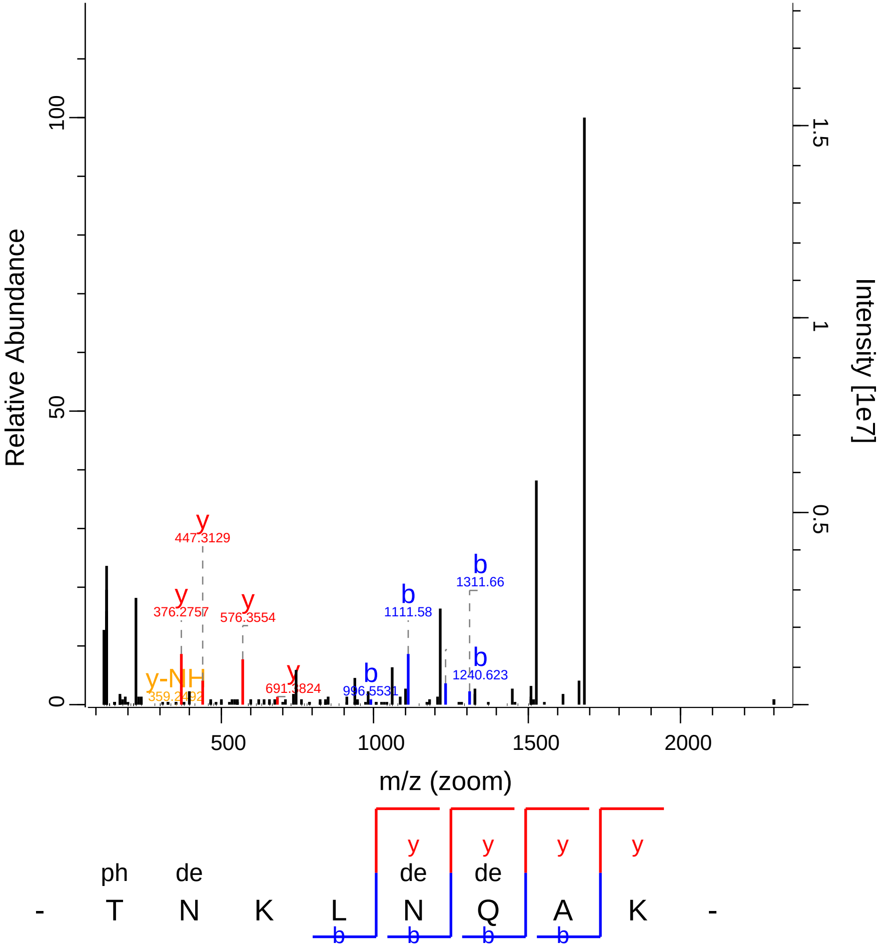

Raw File	Scan	Method	Score	m/z
RO-QC-160121_ZackD_SS_Set_02_Fraction_10	21610	FTMS; HCD	62.84	597.37

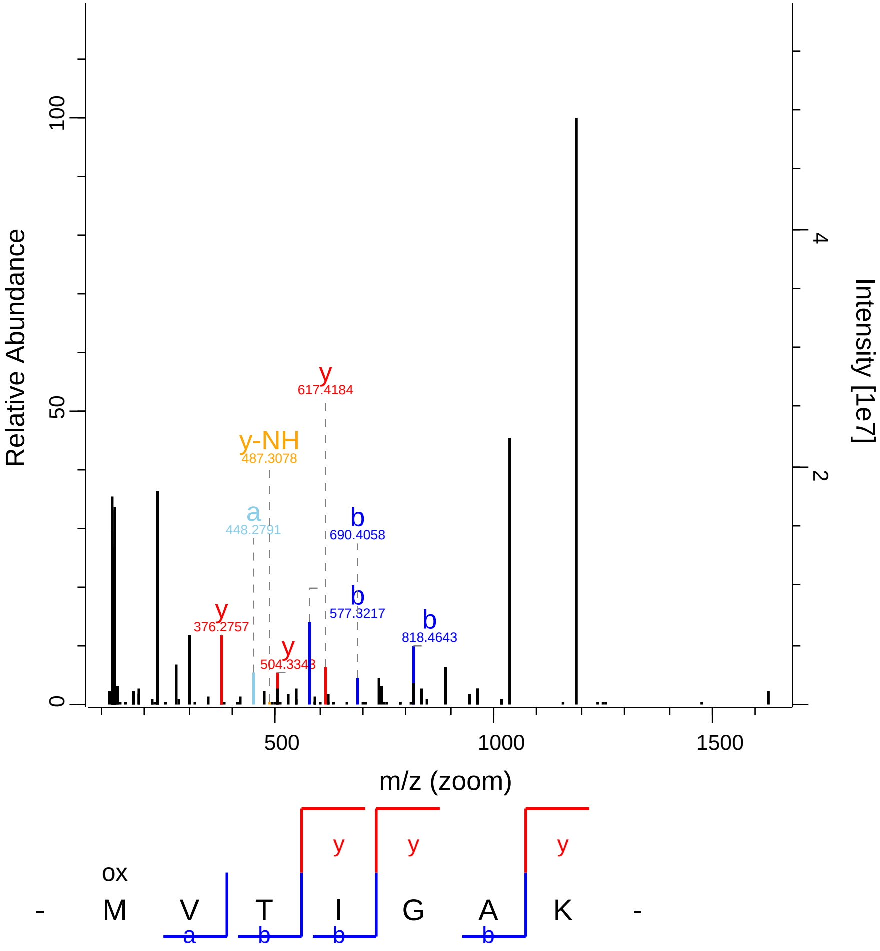

Raw File	Scan	Method	Score	m/z	Gene names
RO-QC-160121_ZackD_SS_Set_02_Fractior	21738	FTMS; HC	53.24	755.05	ADCY10

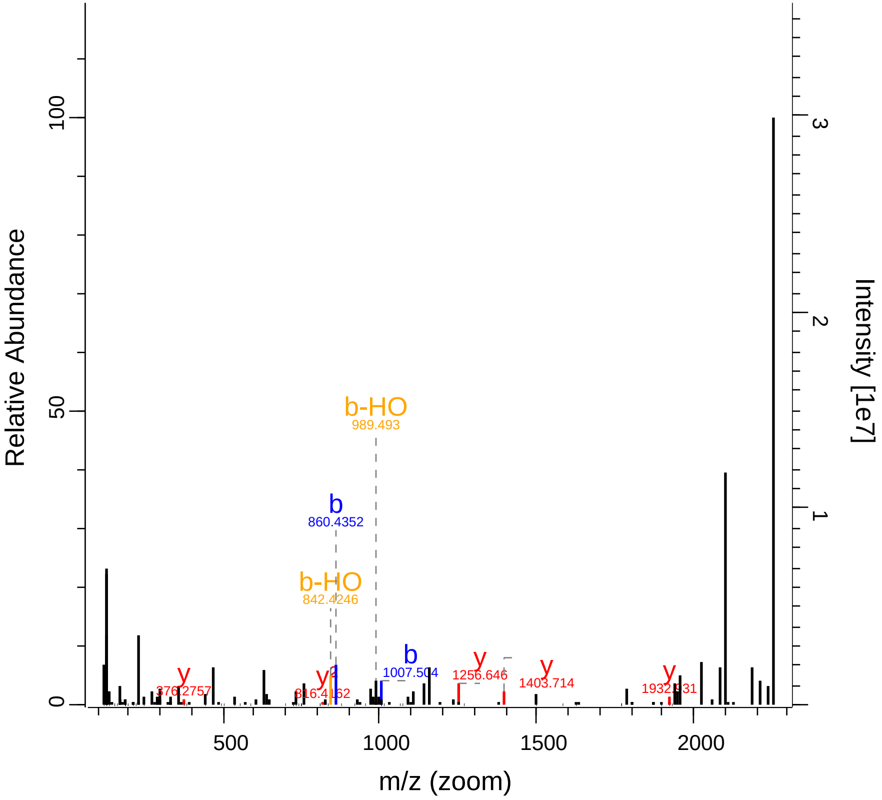

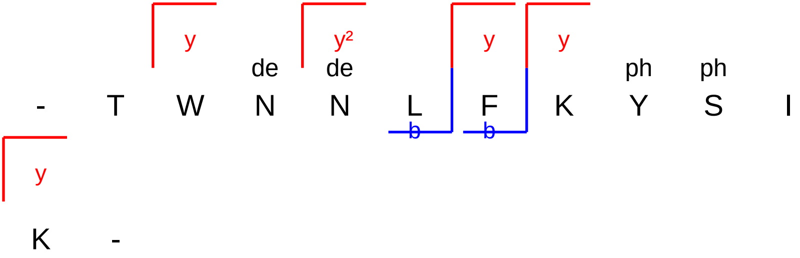

Raw File	Scan	Method	Score	m/z	Gene names
RO-QC-160121_ZackD_SS_Set_02_Fractior	22512	FTMS; HC	135.34	900.97	STK17A

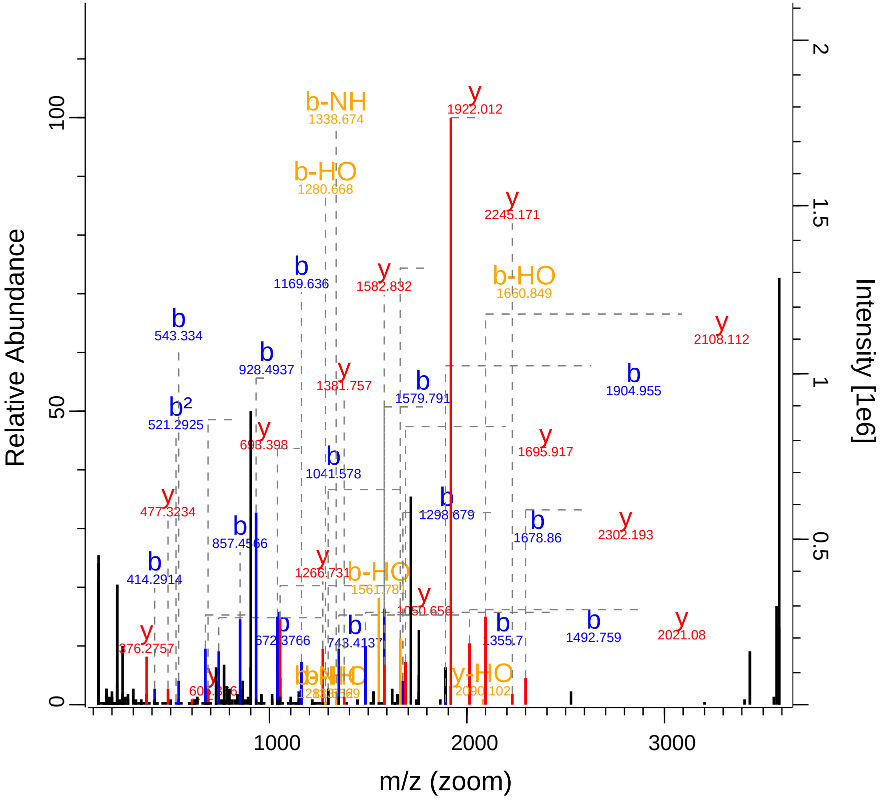

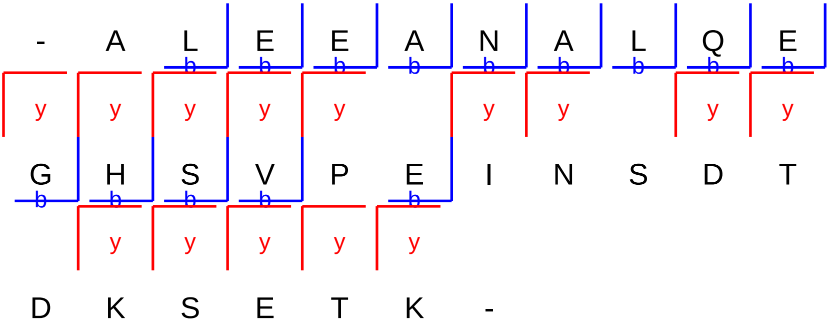

Raw File	Scan	Method	Score	m/z	Gene names
RO-QC-160121_ZackD_SS_Set_02_Fraction	23835	FTMS; HC	110.77	989.2	DPY19L3

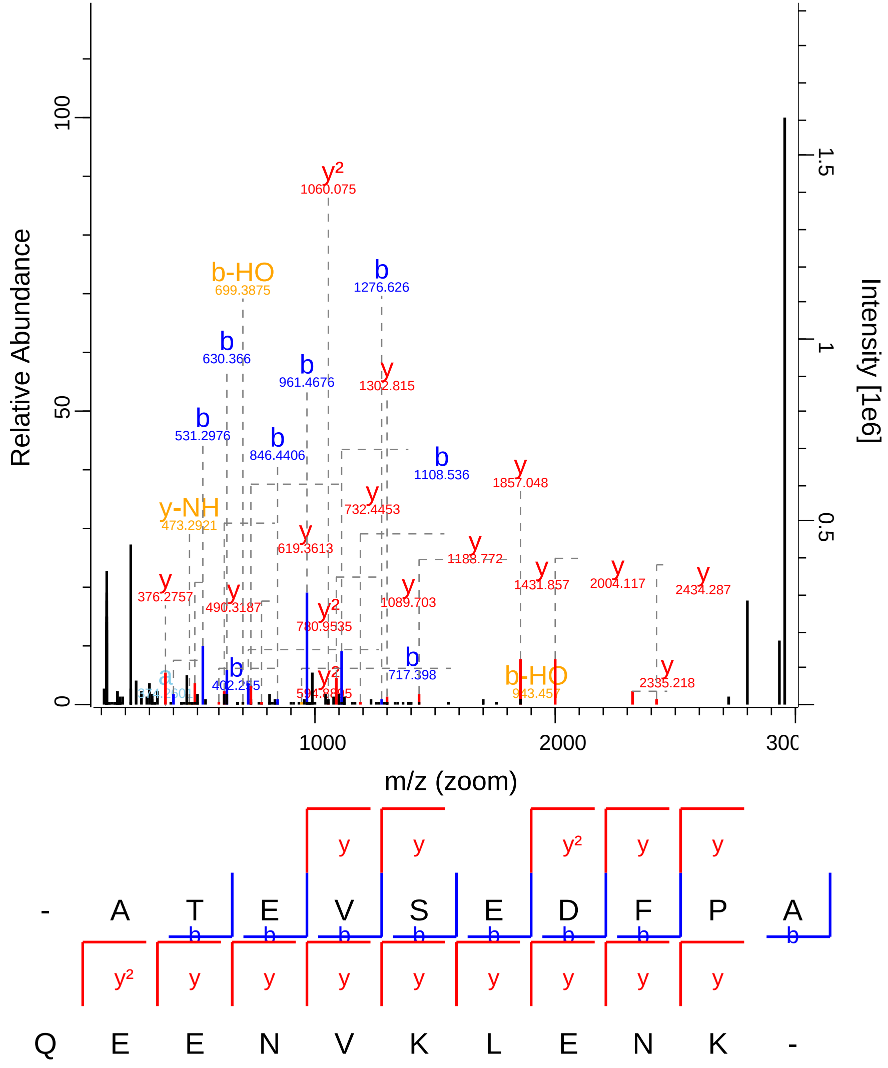

Raw File	Scan	Method	Score	m/z	Gene names
RO-QC-160121_ZackD_SS_Set_02_Fractior	23986	FTMS; HC	122.52	708.92	ALG12

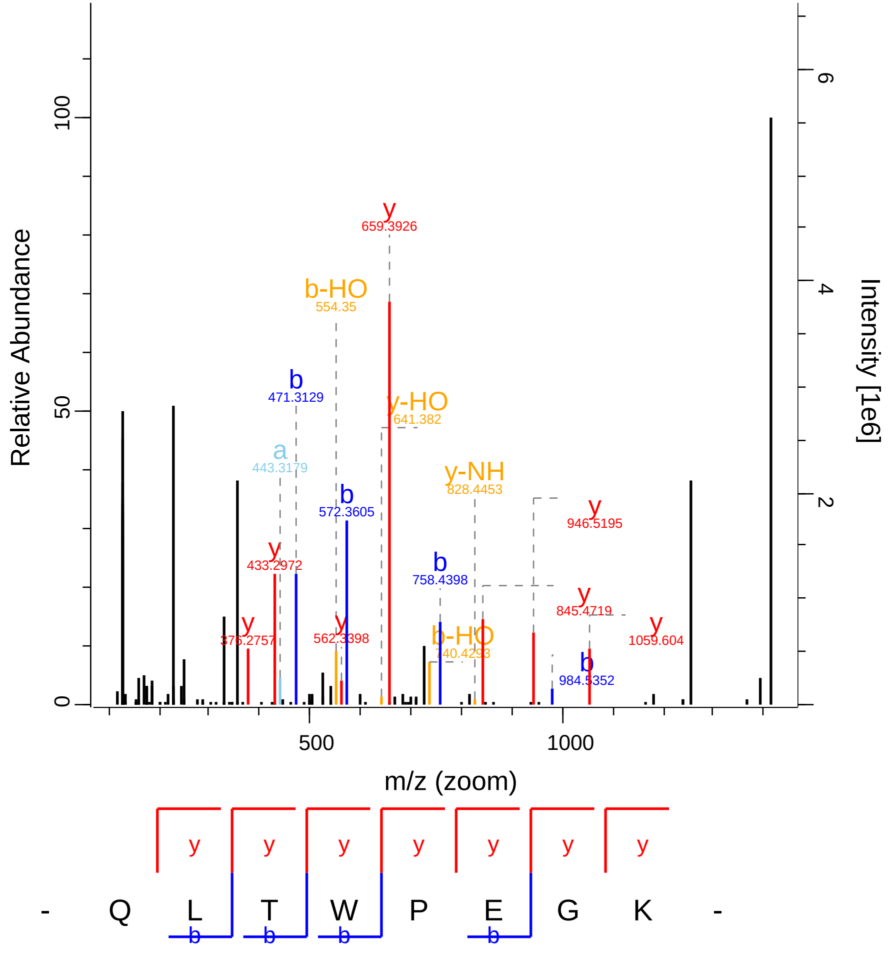

Raw File	Scan	Method	Score	m/z	Gene names
RO-QC-160121_ZackD_SS_Set_02_Fractior	24008	FTMS; HC	99.75	972.95	MT-ND3

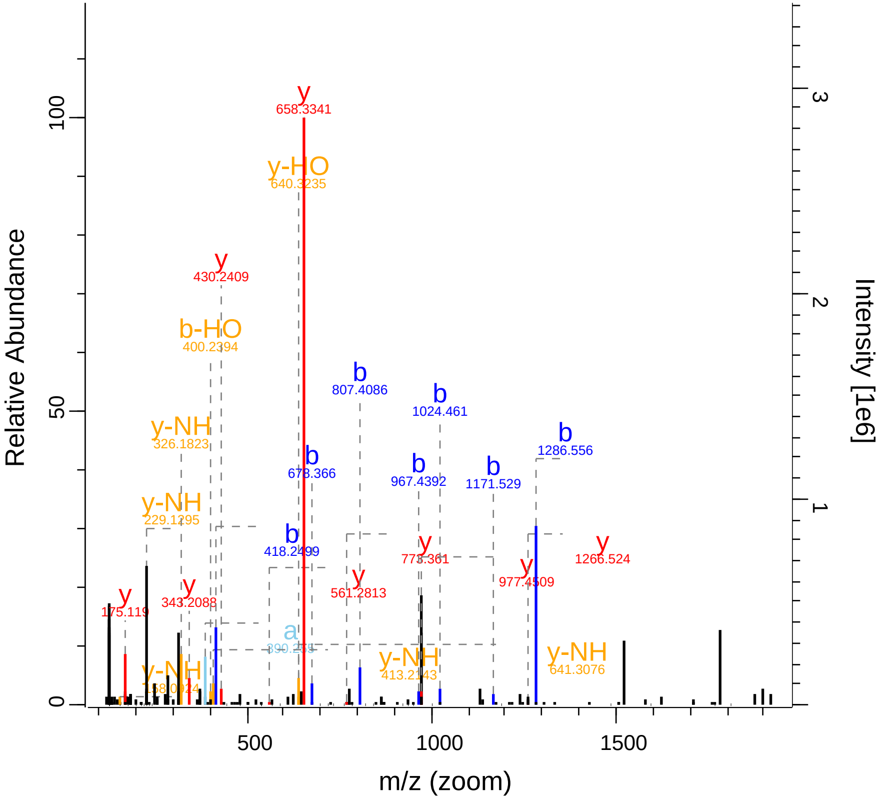

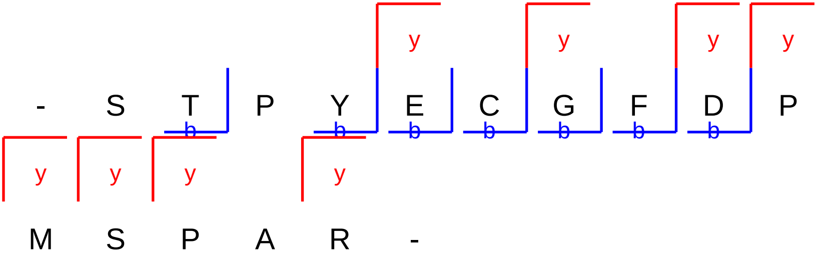

Raw File	Scan	Method	Score	m/z	Gene names
RO-QC-160121_ZackD_SS_Set_02_Fractior	24038	FTMS; HC	77.22	648.63	MT-ND3

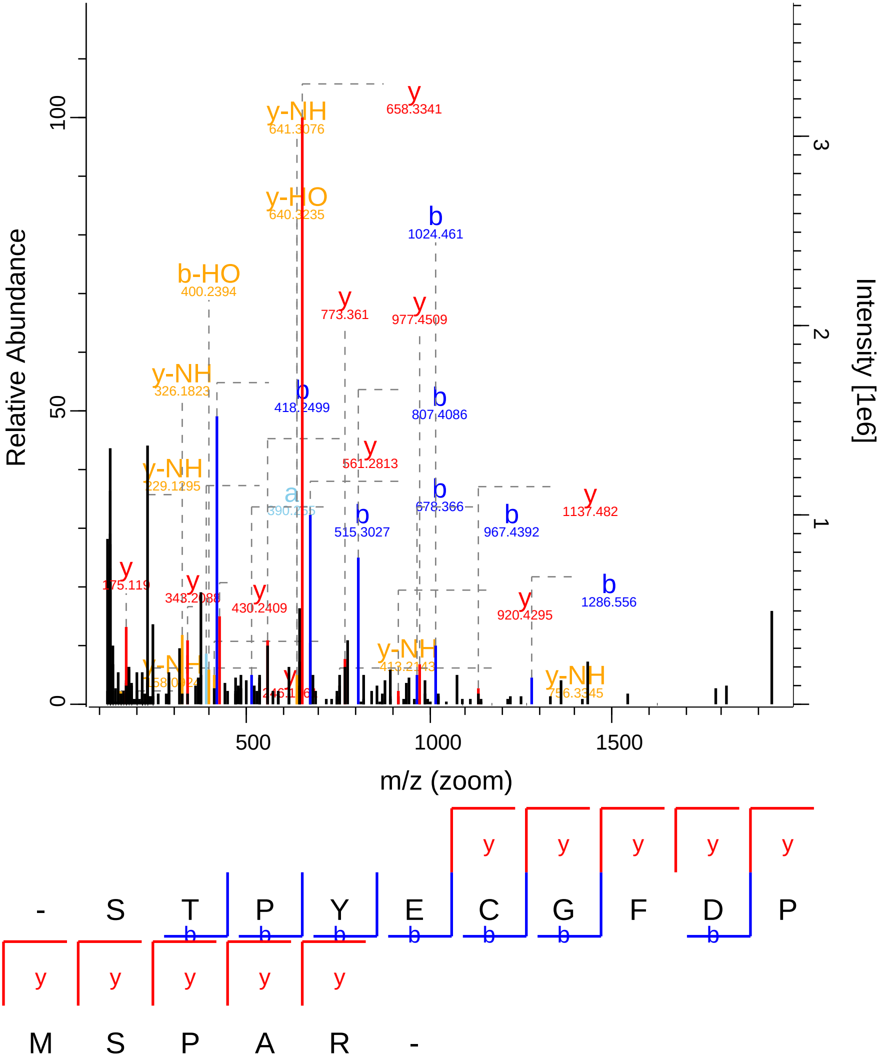

Raw File	Scan	Method	Score	m/z	Gene names
RO-QC-160121_ZackD_SS_Set_02_Fractior	24081	FTMS; HC	67.9	472.95	ALG12

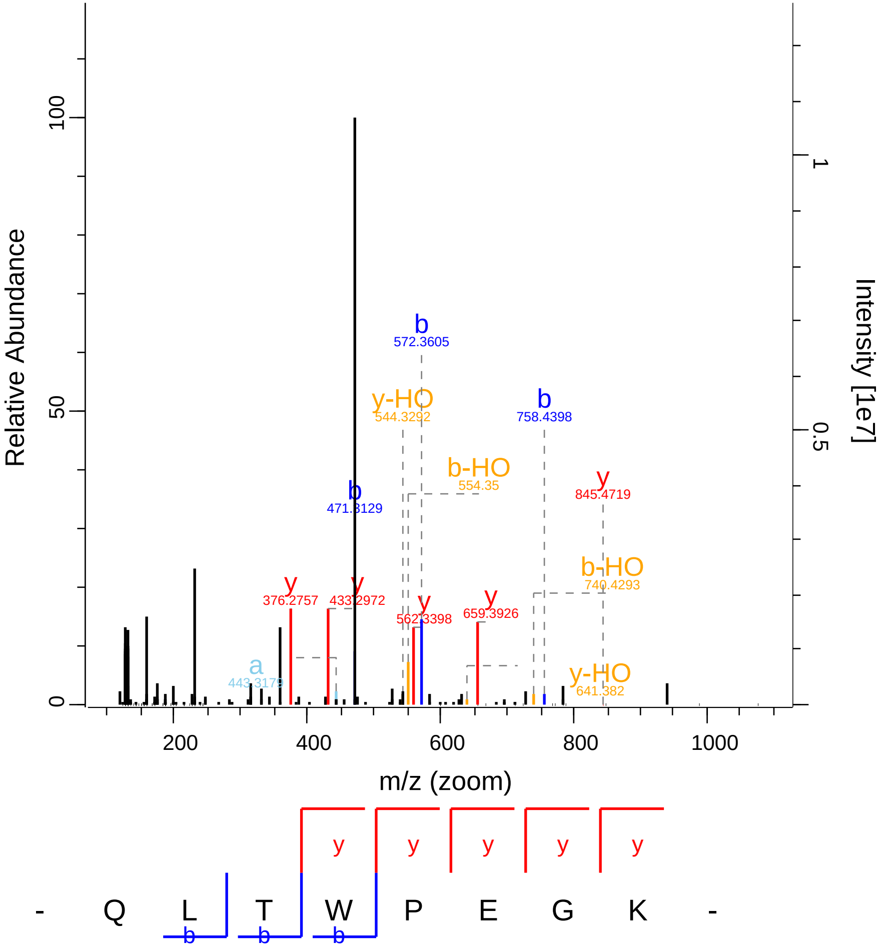

Raw File	Scan	Method	Score	m/z
RO-QC-160121_ZackD_SS_Set_02_Fraction_10	26308	FTMS; HCD	83.87	1010.02

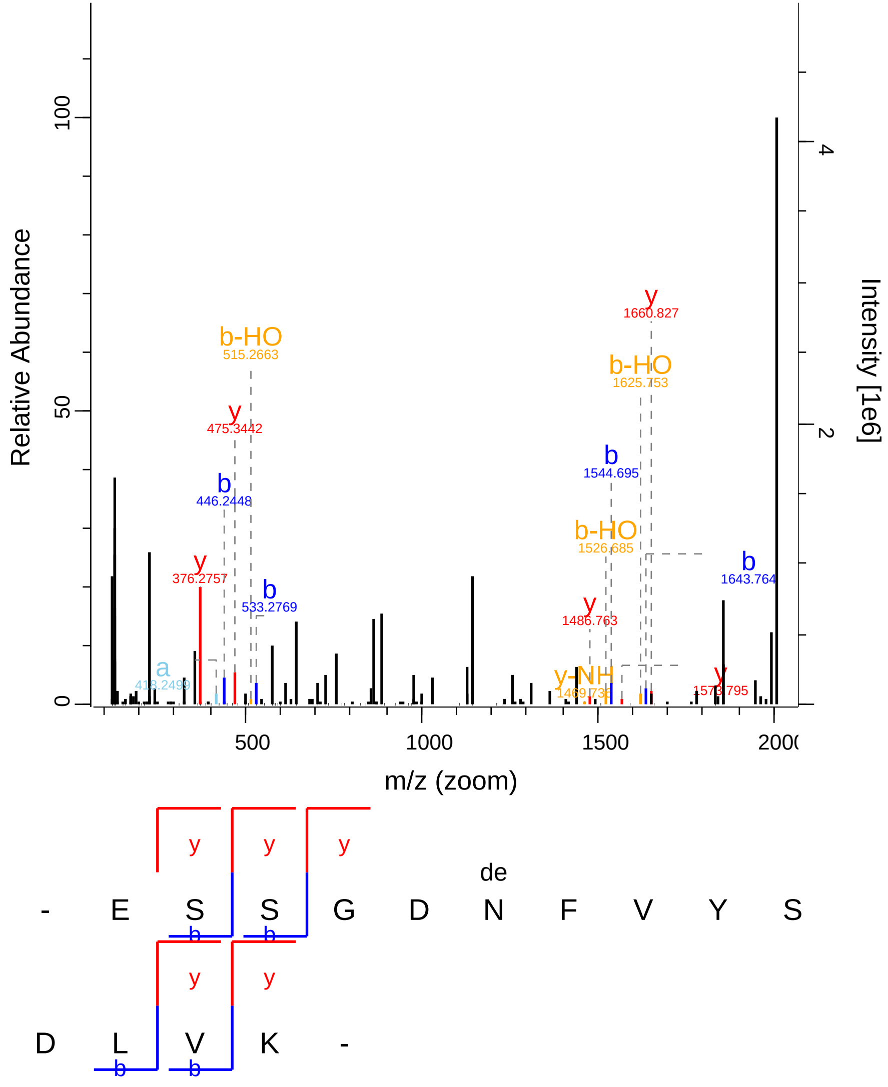

Raw File	Scan	Method	Score	m/z
RO-QC-160121_ZackD_SS_Set_02_Fraction_10	26320	FTMS; HCD	56.05	494.79

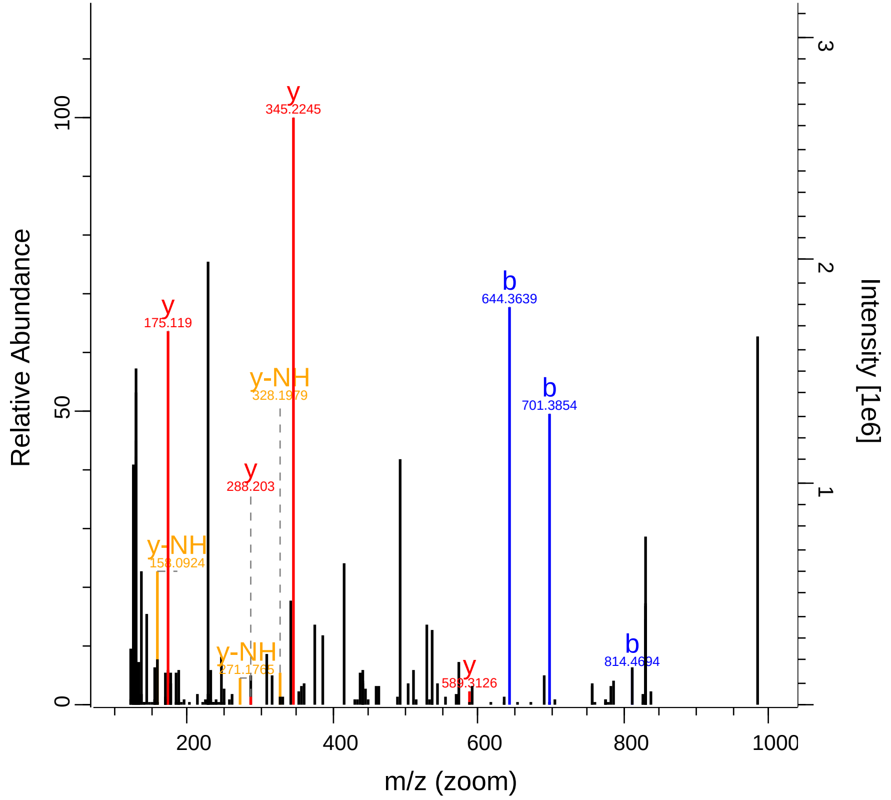

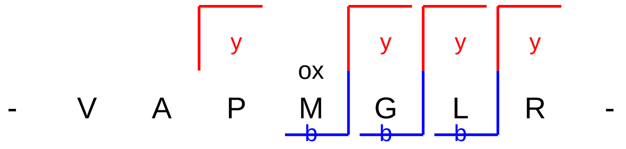

Raw File	Scan	Method	Score	m/z	Gene names
RO-QC-160121_ZackD_SS_Set_02_Fractior	26395	FTMS; HC	61.62	818.78	LMO4

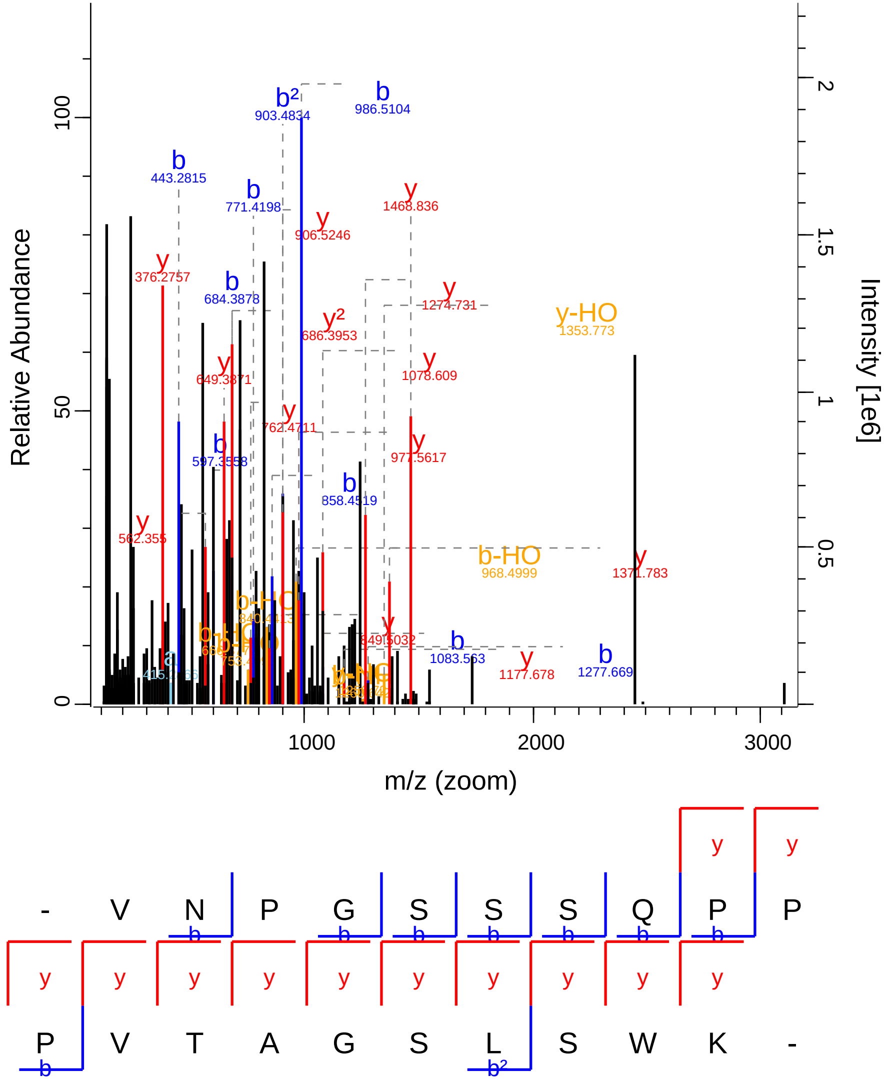

Raw File	Scan	Method	Score	m/z	Gene names
RO-QC-160121_ZackD_SS_Set_02_Fractior	26779	FTMS; HC	46.43	464.28	RASGRF1

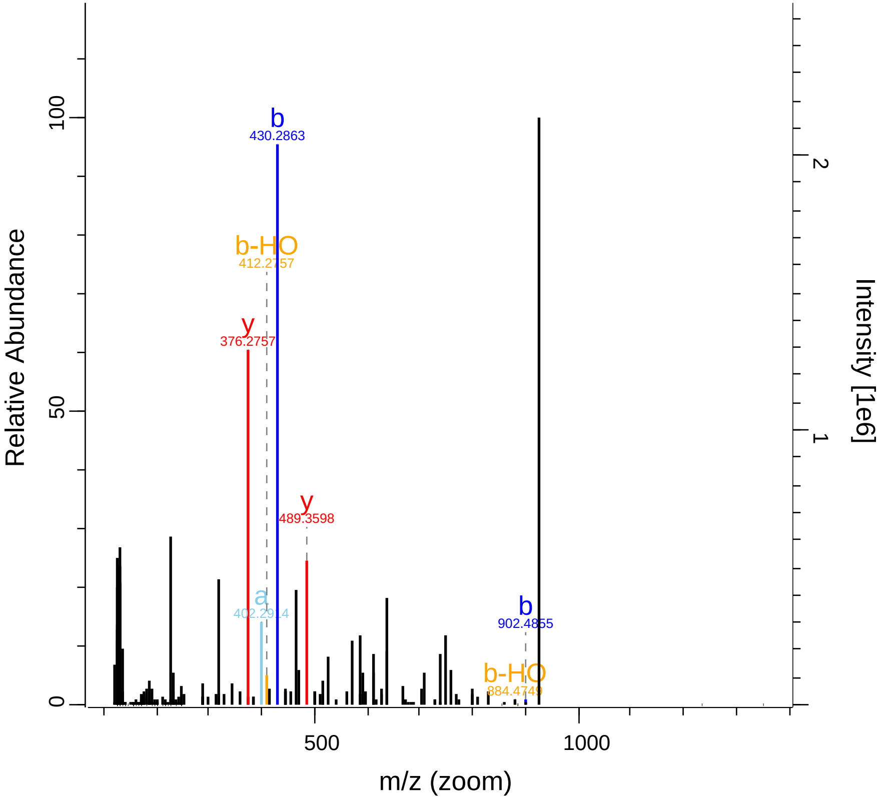

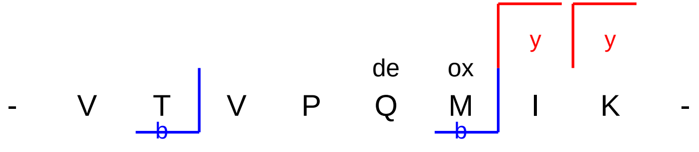

Raw File	Scan	Method	Score	m/z
RO-QC-160121_ZackD_SS_Set_02_Fraction_10	27767	FTMS; HCD	132.29	656.93

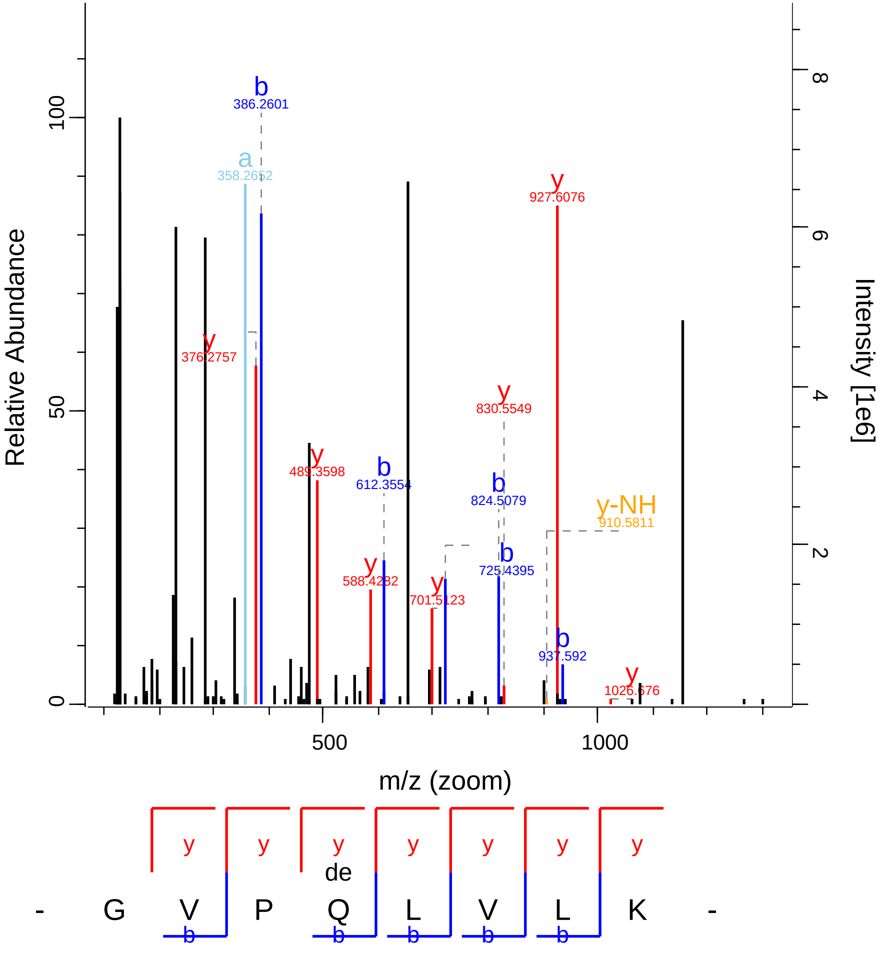

Raw File	Scan	Method	Score	m/z
RO-QC-160121_ZackD_SS_Set_02_Fraction_10	27822	FTMS; HCD	68.07	1060.09

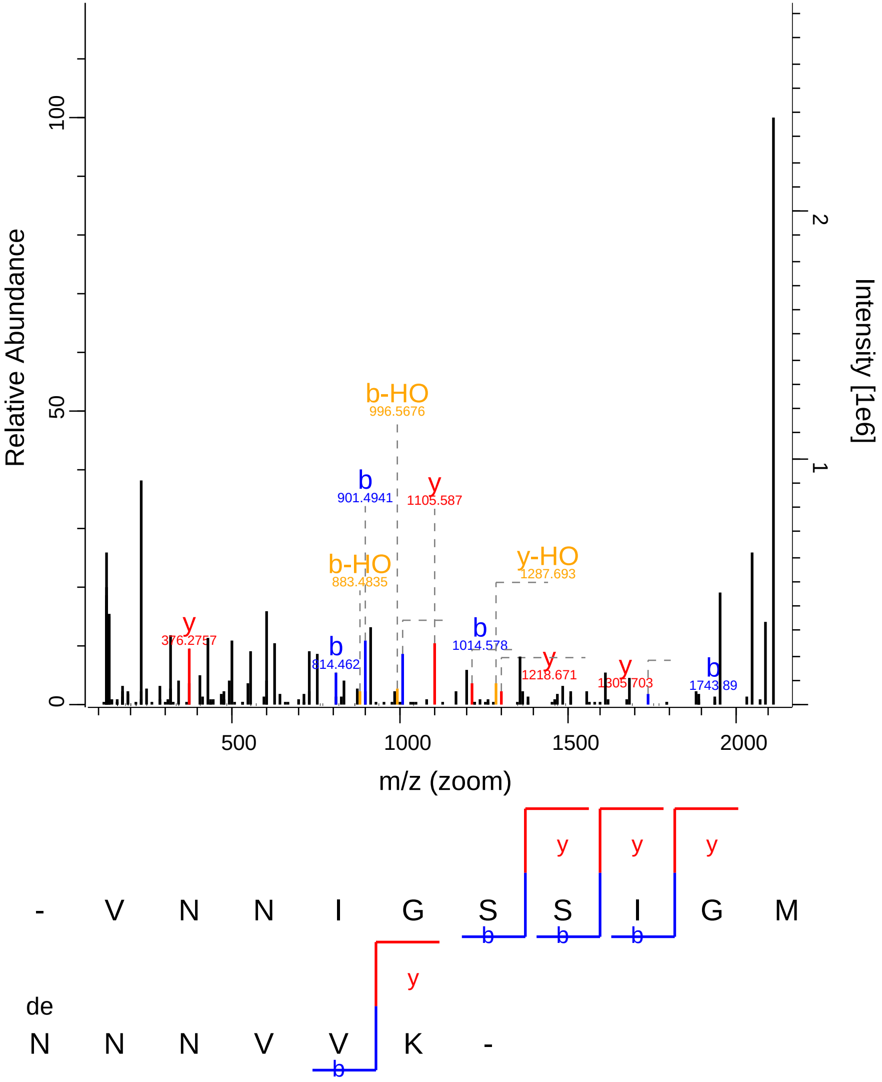

Raw File	Scan	Method	Score	m/z
RO-QC-160121_ZackD_SS_Set_02_Fraction_10	28642	FTMS; HCD	53.17	727.9

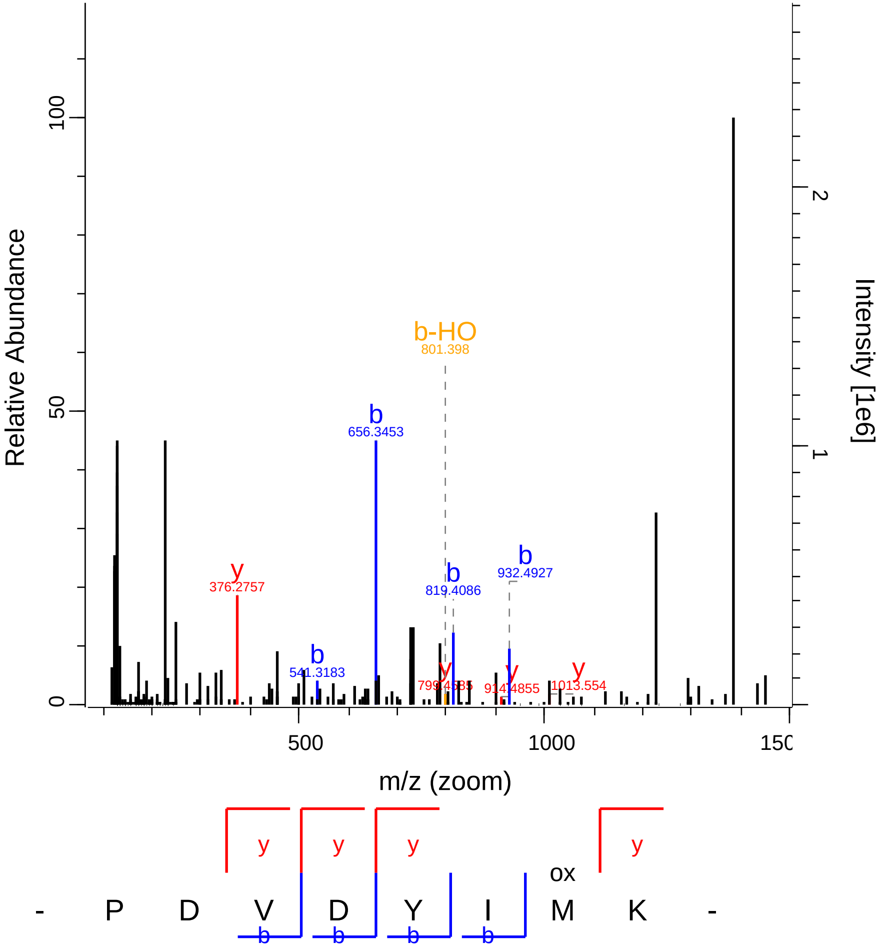

Raw File	Scan	Method	Score	m/z
RO-QC-160121_ZackD_SS_Set_02_Fraction_10	28835	FTMS; HCD	95.52	851.02

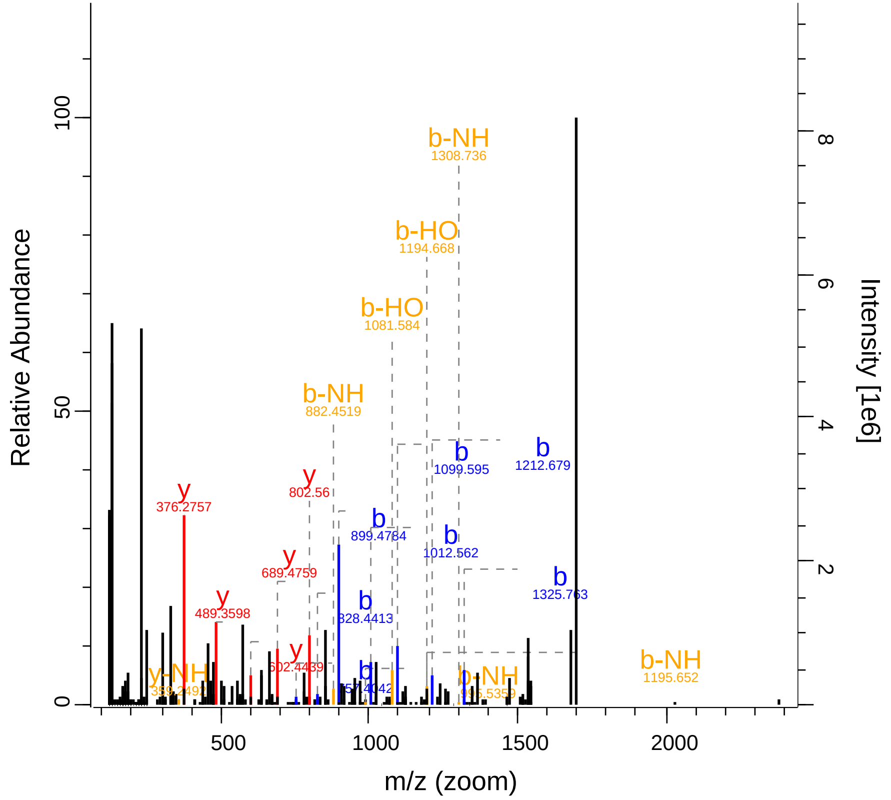

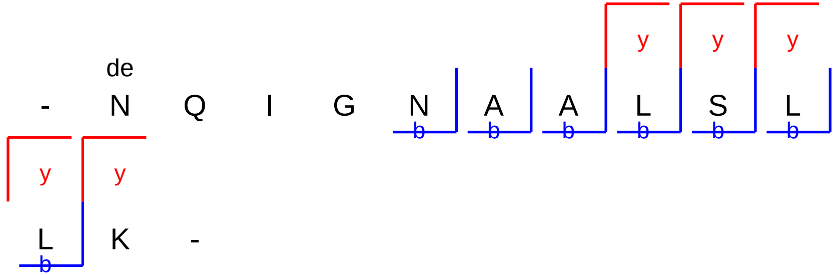

Raw File	Scan	Method	Score	m/z	Gene names
RO-QC-160121_ZackD_SS_Set_02_Fractior	29120	FTMS; HC	53.87	558.67	ZNRF1

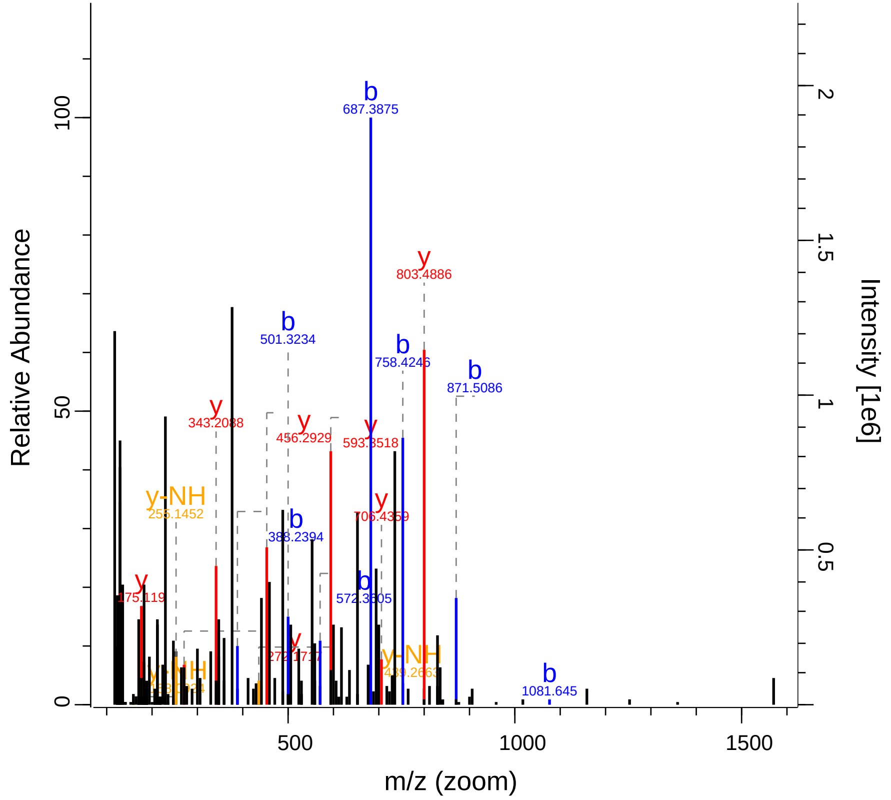

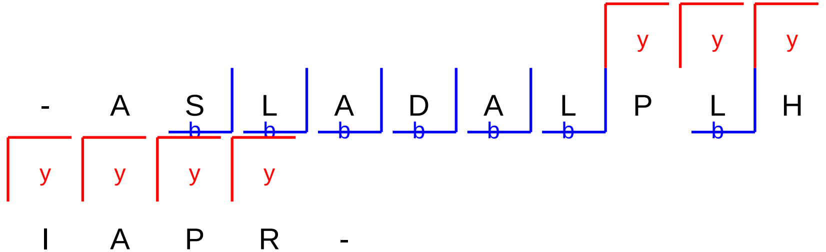

Raw File	Scan	Method	Score	m/z
RO-QC-160121_ZackD_SS_Set_02_Fraction_10	29475	FTMS; HCD	51.09	568.64

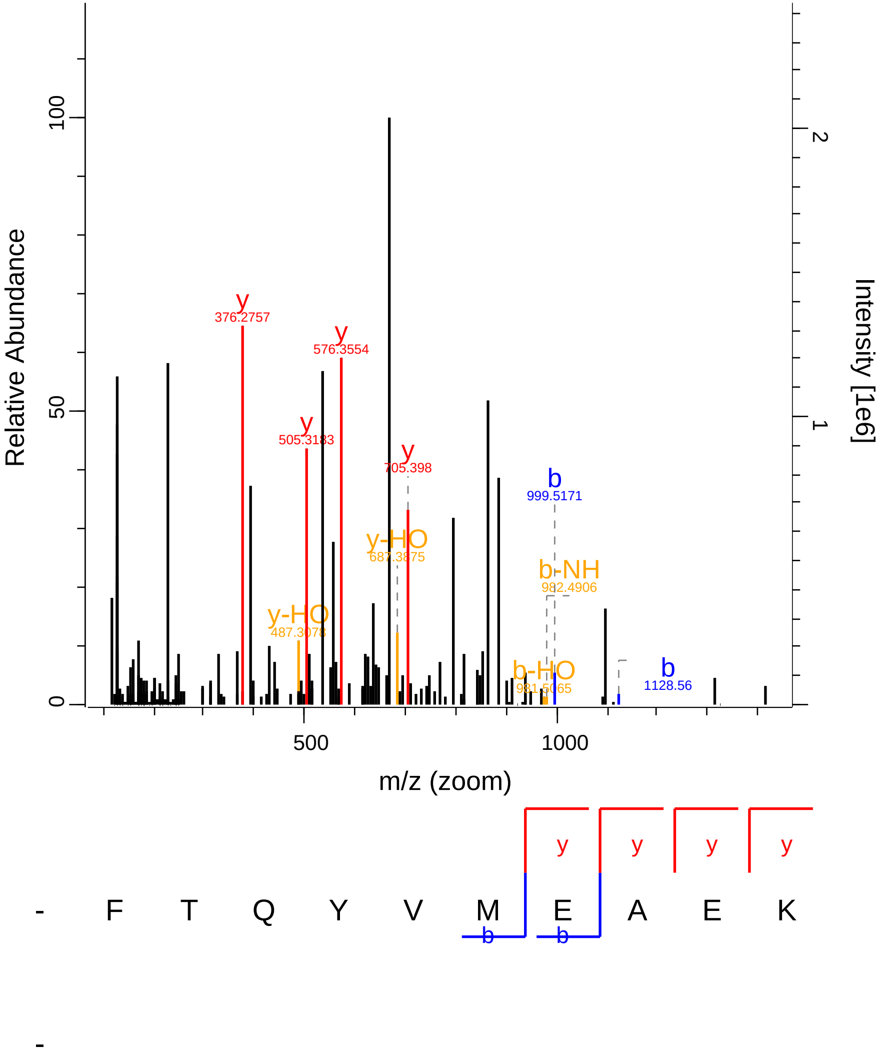

Raw File	Scan	Method	Score	m/z	Gene names
RO-QC-160121_ZackD_SS_Set_02_Fractio	30492	FTMS; HC	109.44	619.99	METAP1D

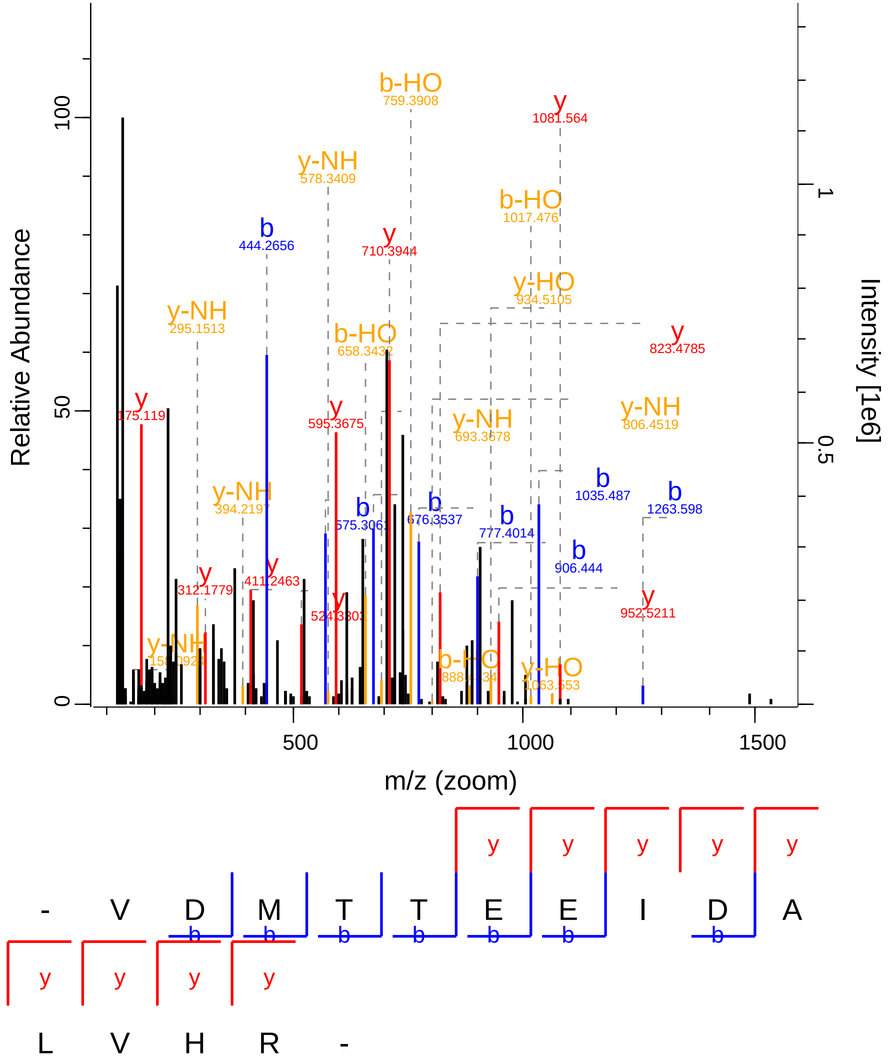

Raw File	Scan	Method	Score	m/z	Gene names
RO-QC-160121_ZackD_SS_Set_02_Fractior	31345	FTMS; HC	70.94	814.08	HN1

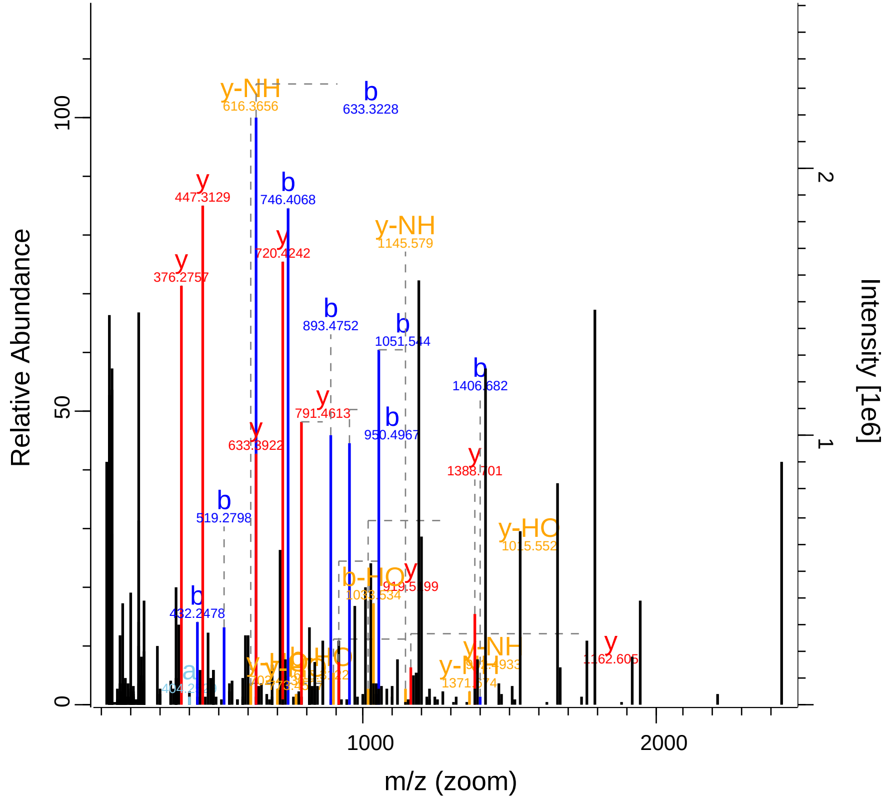

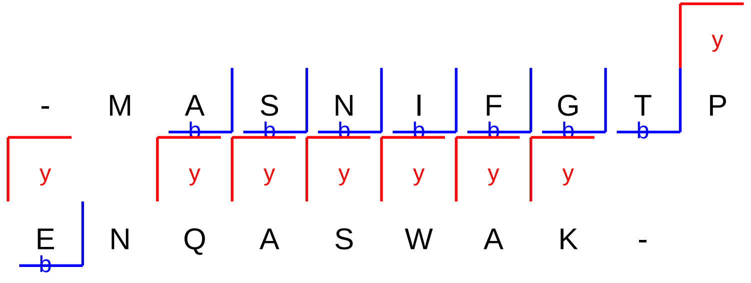

Raw File	Scan	Method	Score	m/z	Gene names
RO-QC-160121_ZackD_SS_Set_02_Fractio	31388	FTMS; HC	175.29	1220.63	HN1

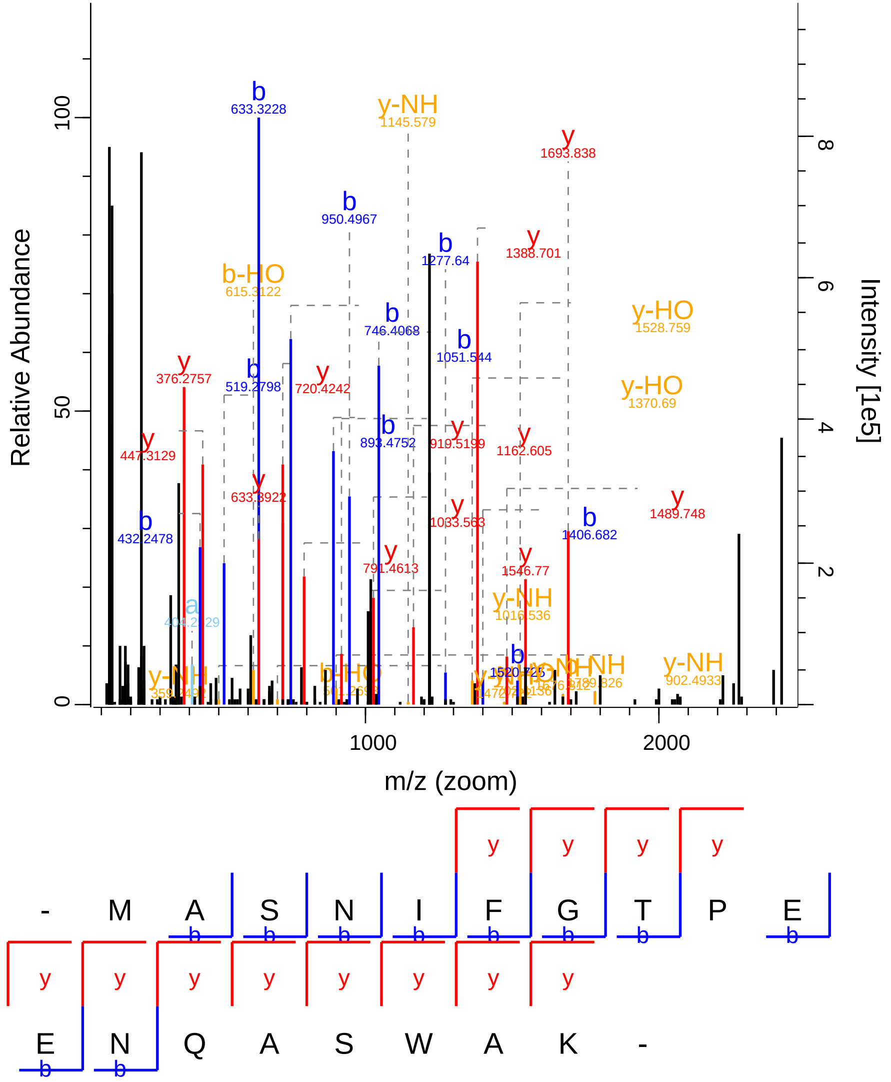

Raw File	Scan	Method	Score	m/z	Gene names
RO-QC-160121_ZackD_SS_Set_02_Fractior	32041	FTMS; HC	50.12	978.51	ARHGAP22

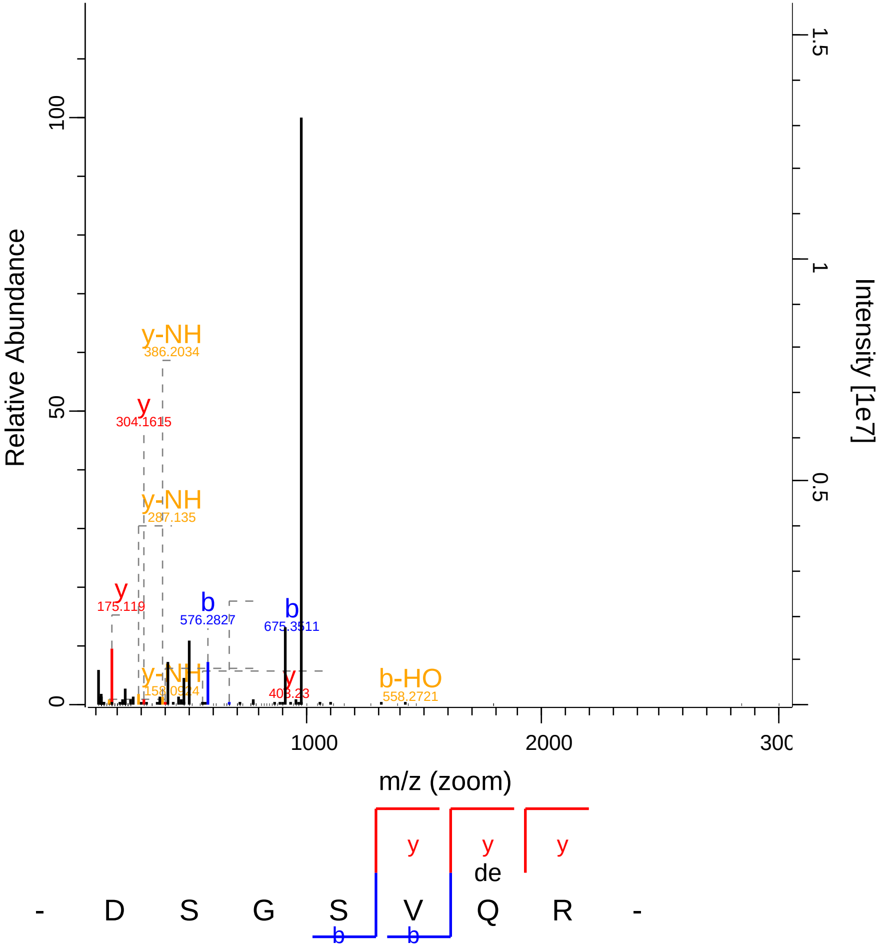

Raw File	Scan	Method	Score	m/z	Gene names
RO-QC-160121_ZackD_SS_Set_02_Fractior	32206	FTMS; HC	71.45	508.64	PNLDC1

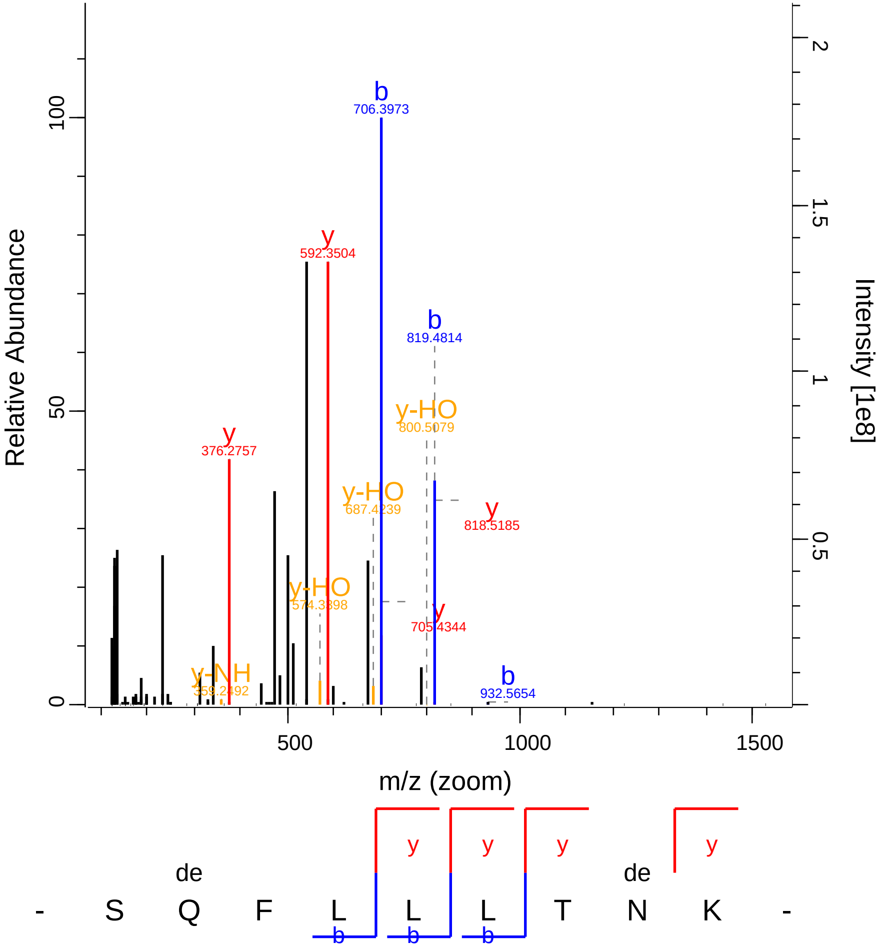

Raw File	Scan	Method	Score	m/z	Gene names
RO-QC-160121_ZackD_SS_Set_02_Fractior	32306	FTMS; HC	87.36	515.29	PQLC2

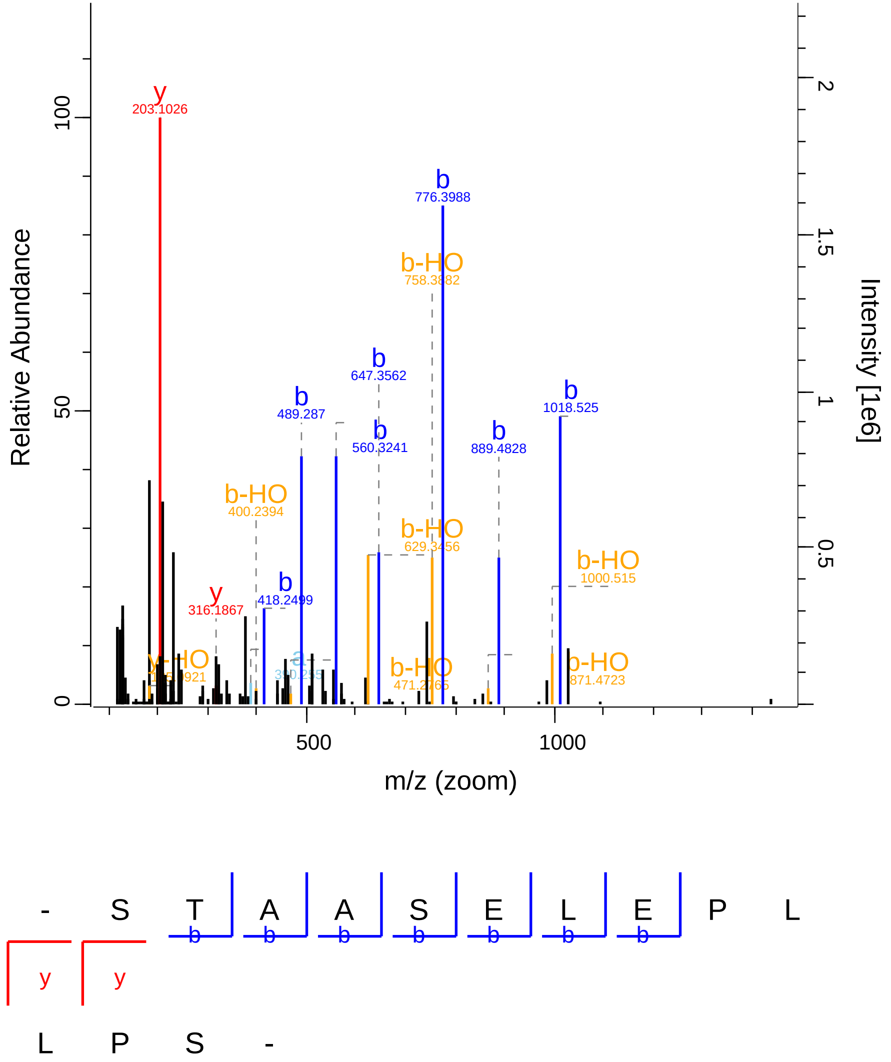

Raw File	Scan	Method	Score	m/z
RO-QC-160121_ZackD_SS_Set_02_Fraction_10	33733	FTMS; HCD	57.56	521.66

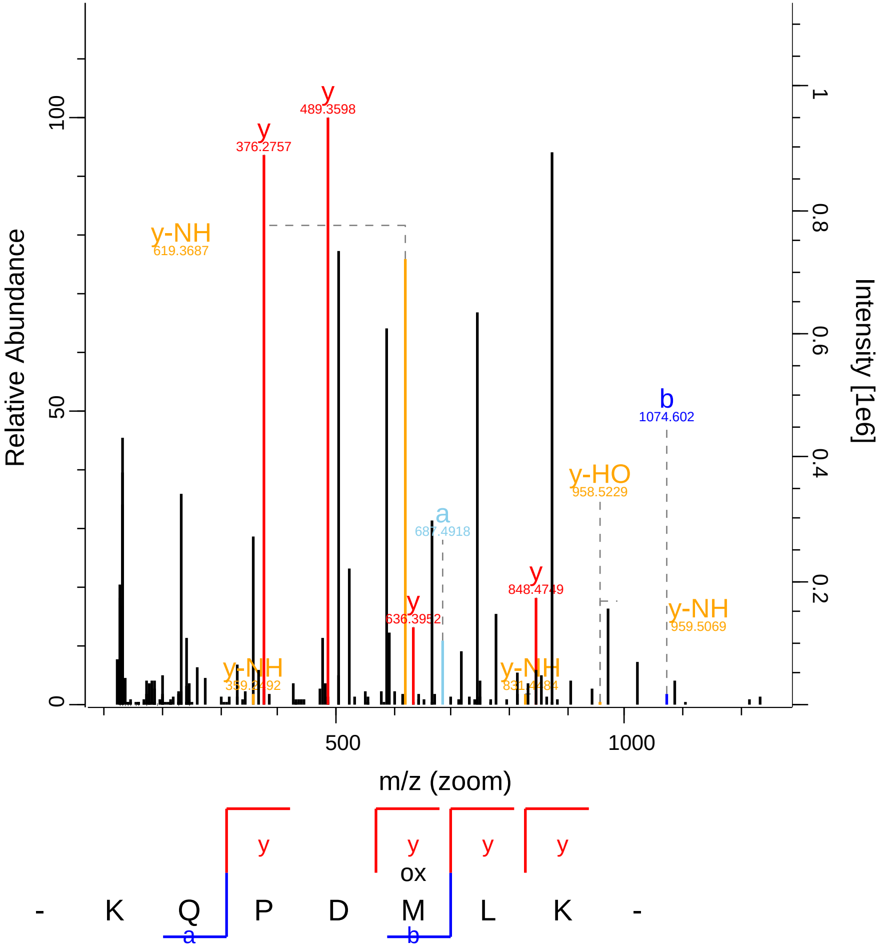

Raw File	Scan	Method	Score	m/z	Gene names
RO-QC-160121_ZackD_SS_Set_02_Fractior	35510	FTMS; HC	43.53	814.42	CCDC7

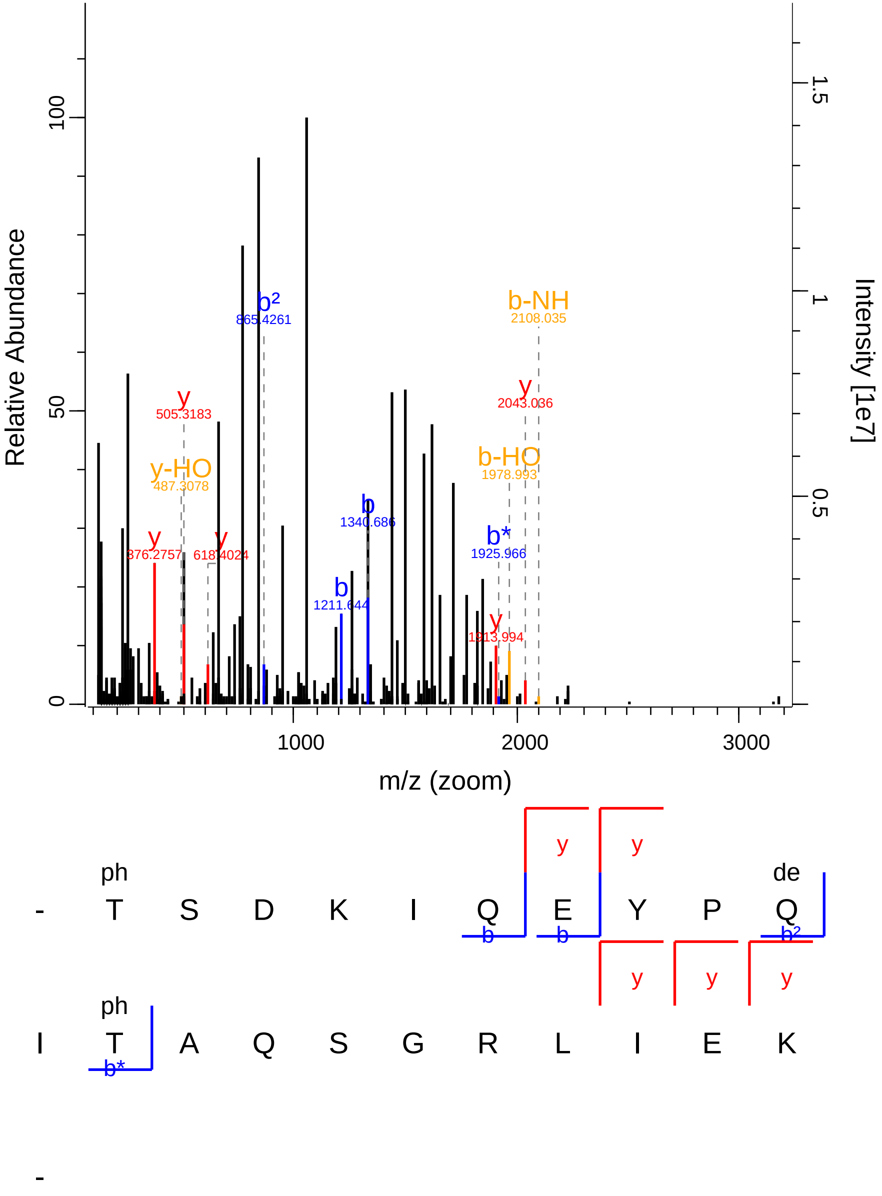

Raw File	Scan	Method	Score	m/z	Gene names
RO-QC-160121_ZackD_SS_Set_02_Fractioı	36902	FTMS; HC	124.08	955.14	RABIF

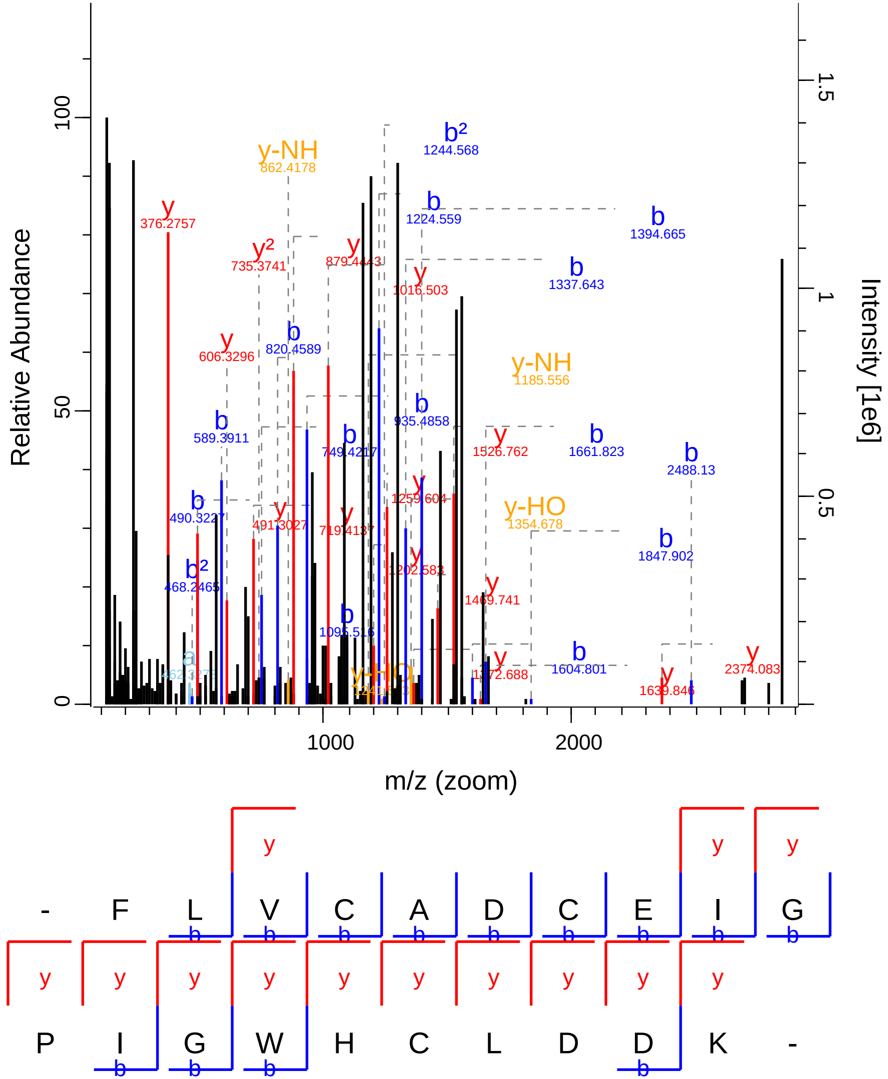

Raw File	Scan	Method	Score	m/z	Gene names
RO-QC-160121_ZackD_SS_Set_02_Fractio	37365	FTMS; HC	202.62	1045.73	FAM174A

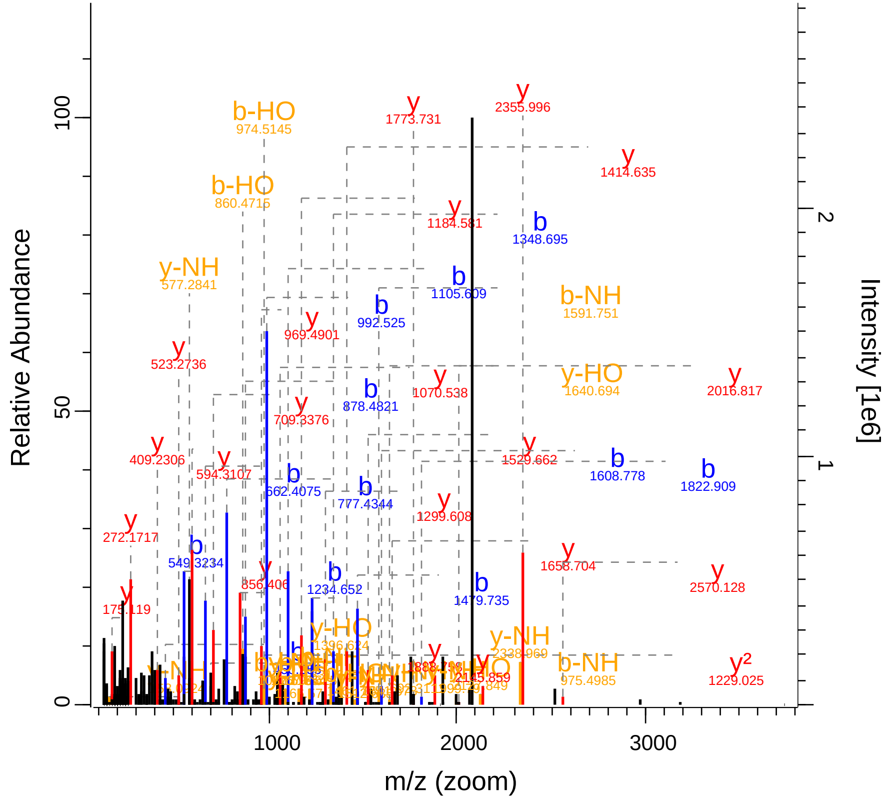

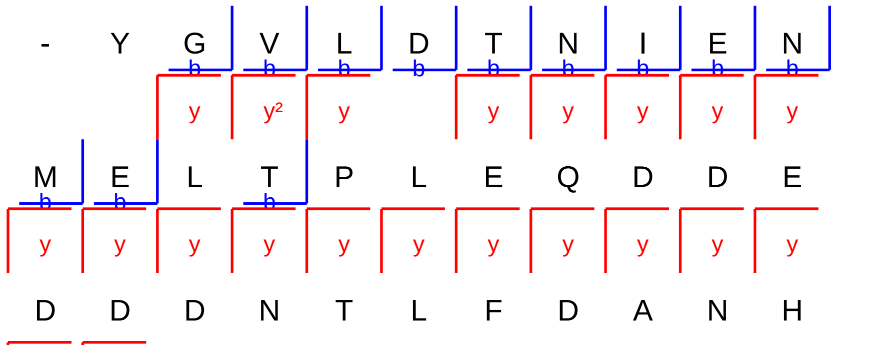

Raw File	Scan	Method	Score	m/z	Gene names
RO-QC-160121_ZackD_SS_Set_02_Fractior	37982	FTMS; HC	87.15	618.02	OR4E1

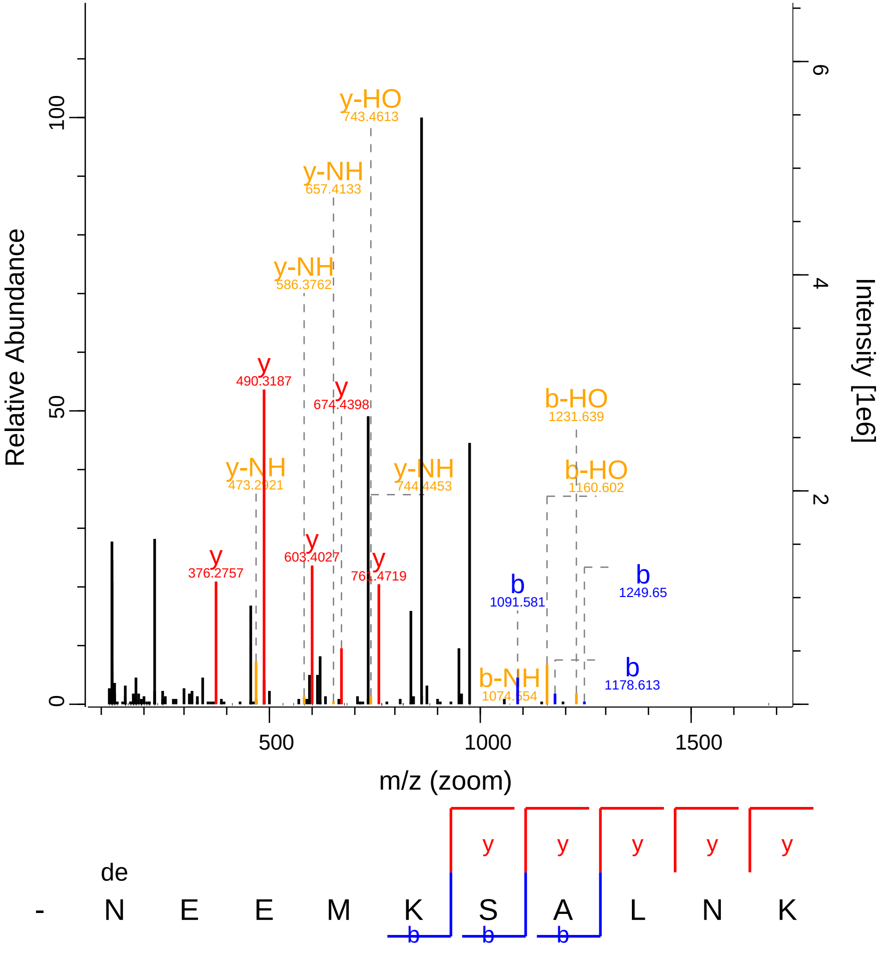

-

Raw File	Scan	Method	Score	m/z	Gene names
RO-QC-160121_ZackD_SS_Set_02_Fractio	38400	FTMS; HC	126.31	1094.22	B9D1

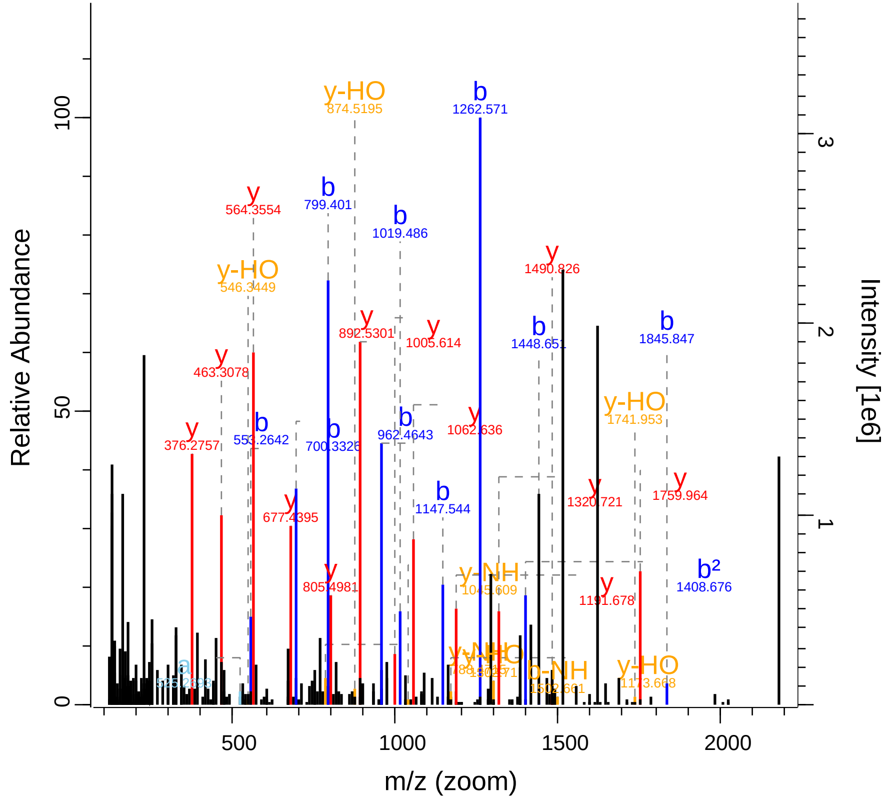

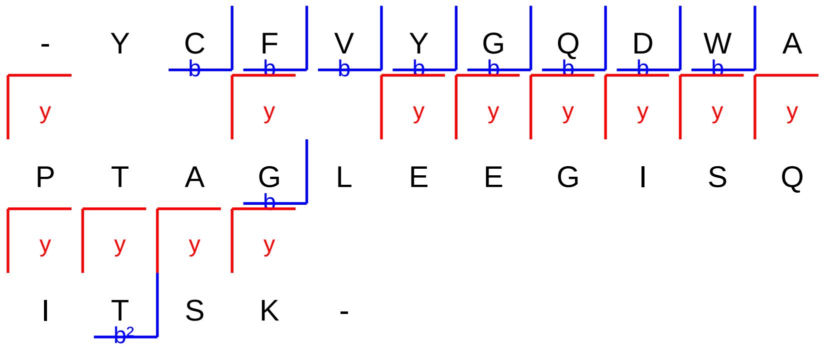

Raw File	Scan	Method	Score	m/z	Gene names
RO-QC-160121_ZackD_SS_Set_02_Fractio	39599	FTMS; HC	56.12	1440.69	TCF7L1

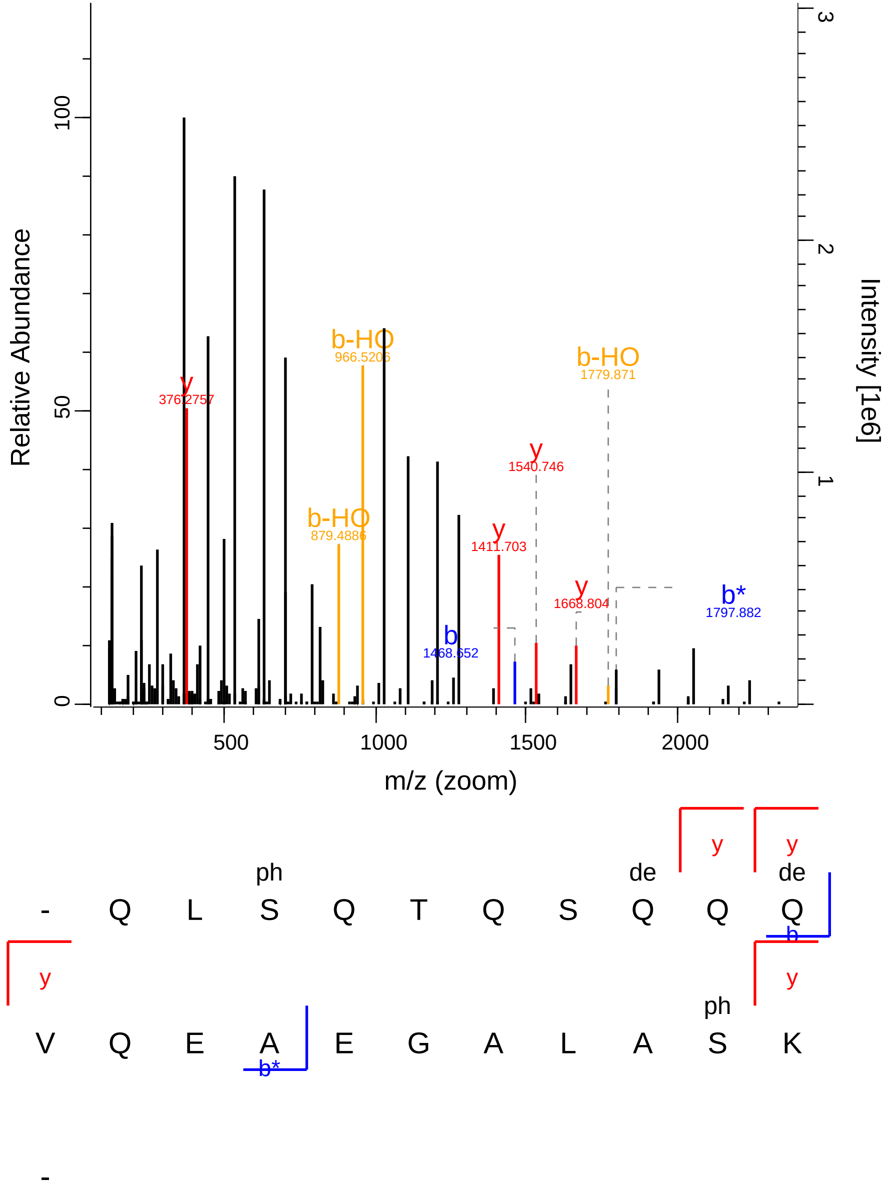

Raw File	Scan	Method	Score	m/z	Gene names
RO-QC-160121_ZackD_SS_Set_02_Fractior	39692	FTMS; HC	85.74	835.11	BSCL2

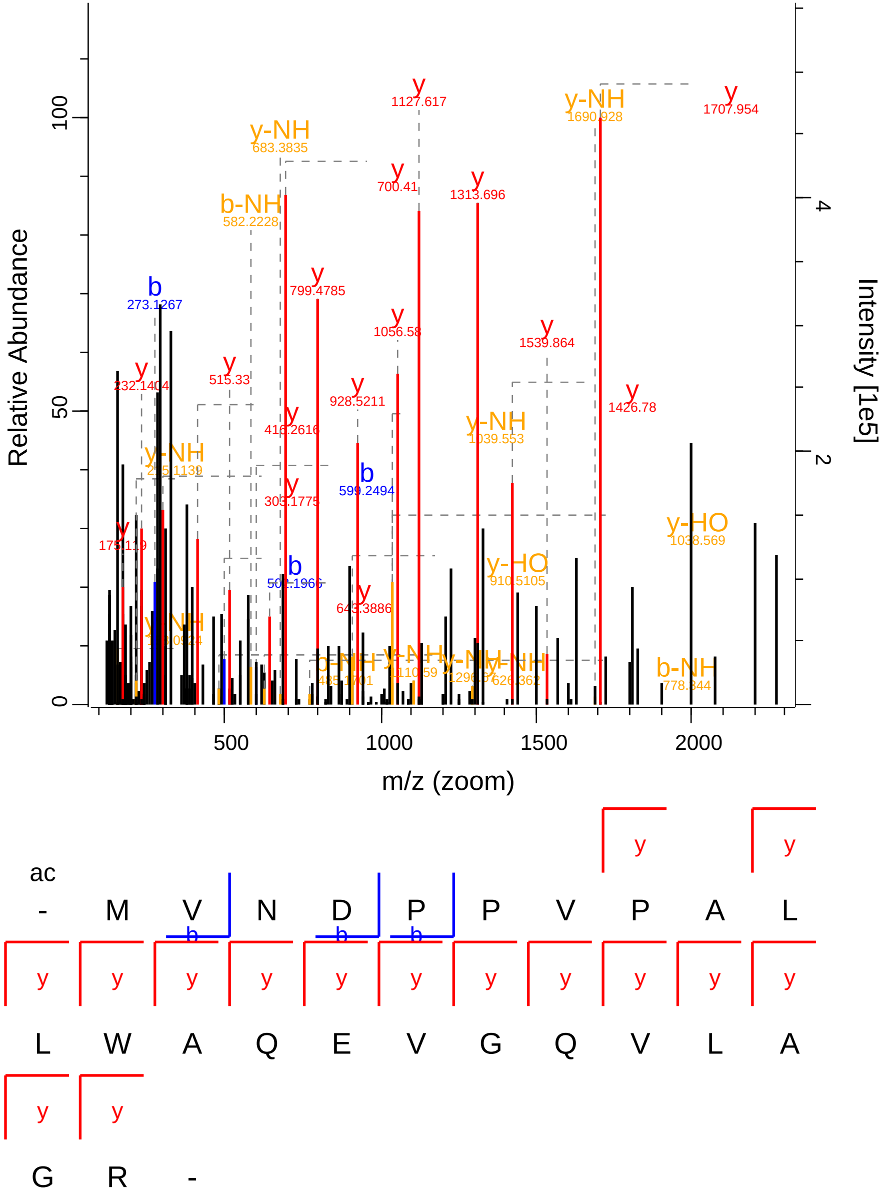

Raw File	Scan	Method	Score	m/z	Gene names
RO-QC-160121_ZackD_SS_Set_02_Fractio	39695	FTMS; HC	89.47	1252.17	BSCL2

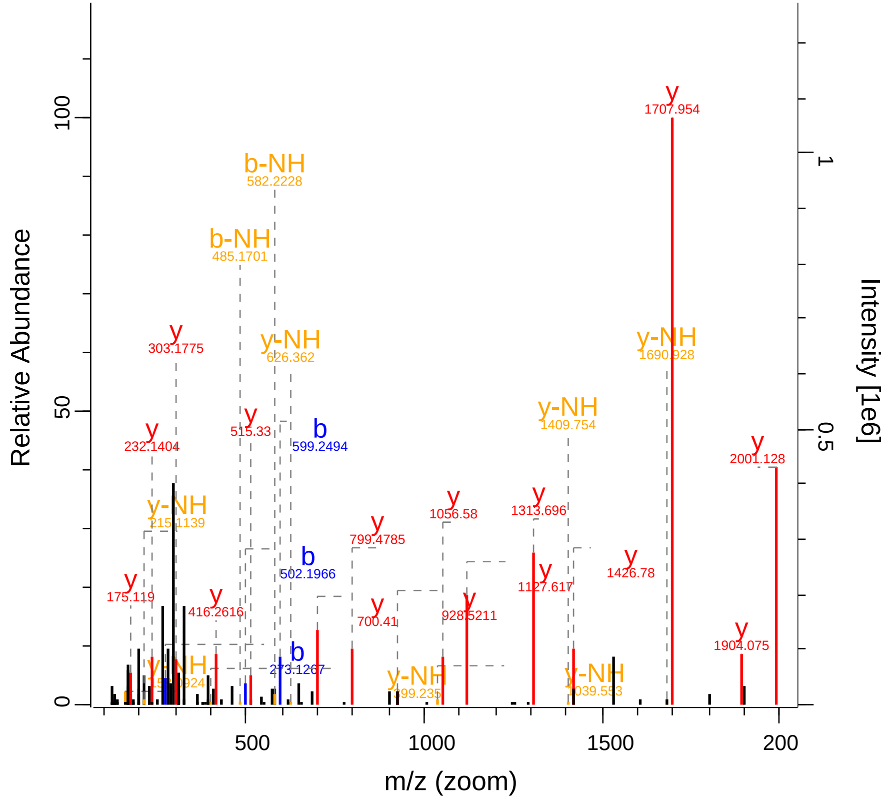

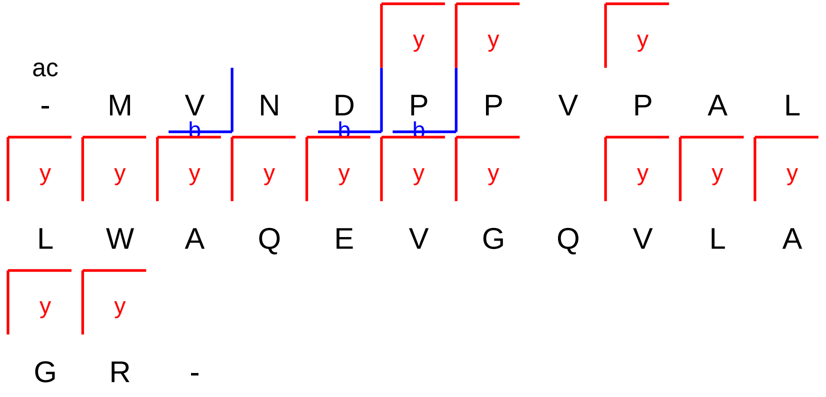

Raw File	Scan	Method	Score	m/z	Gene names
RO-QC-160121_ZackD_SS_Set_02_Fraction	4789	FTMS; HC	168.05	619.29	ZBTB1

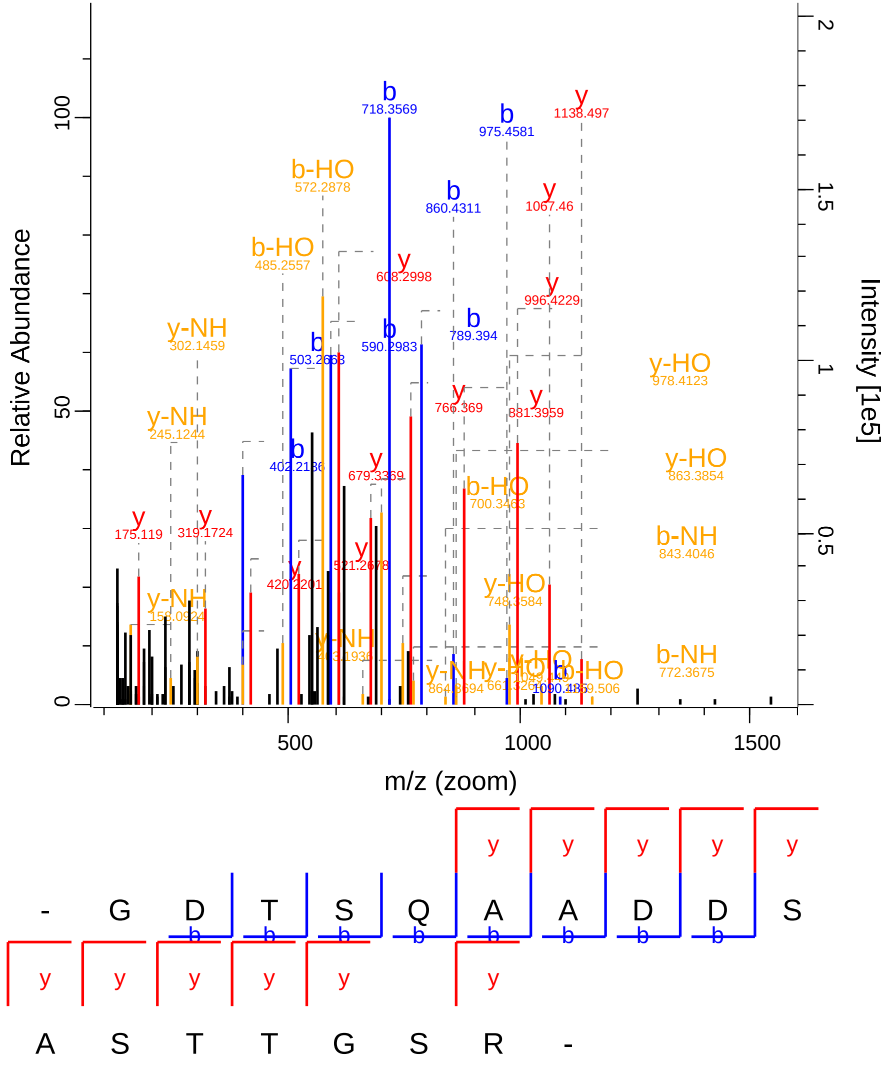

Raw File	Scan	Method	Score	m/z	Gene names
RO-QC-160121_ZackD_SS_Set_02_Fraction	4790	FTMS; HC	99.53	928.43	ZBTB1

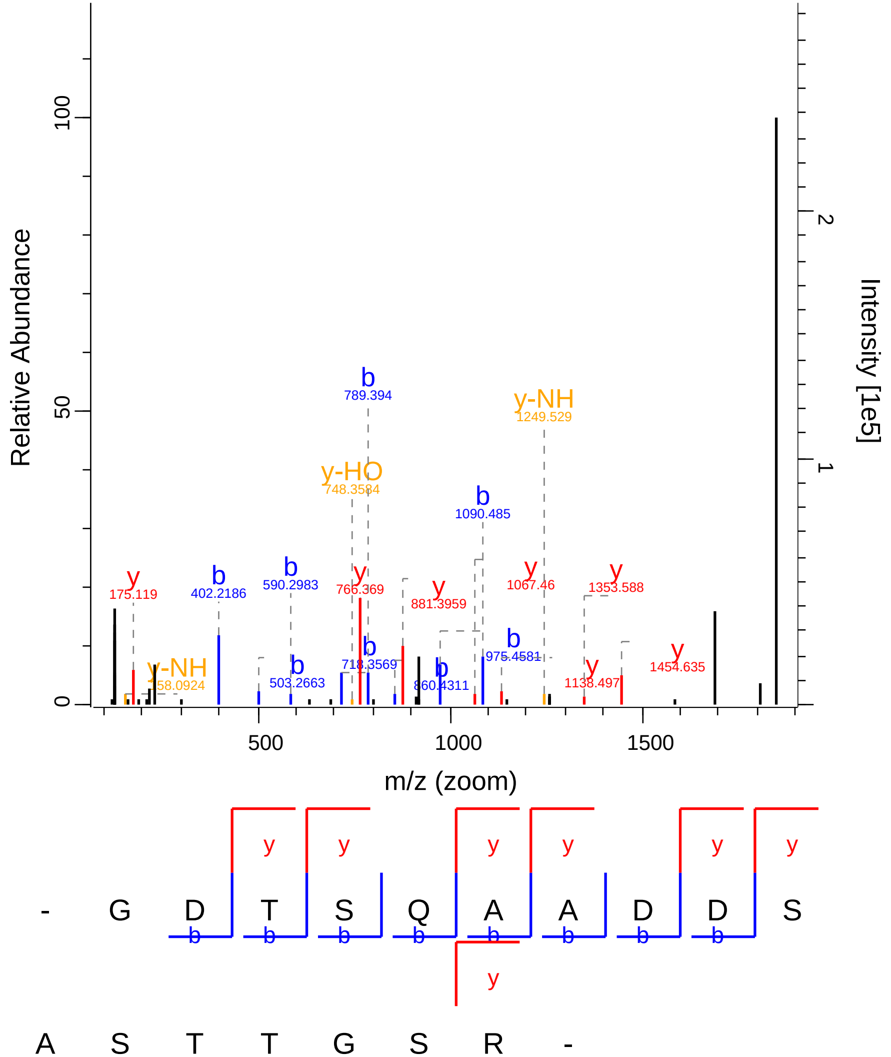

Raw File	Scan	Method	Score	m/z	Gene names
RO-QC-160121_ZackD_SS_Set_02_Fraction	5332	FTMS; HC	70.1	390.47	UNC45B

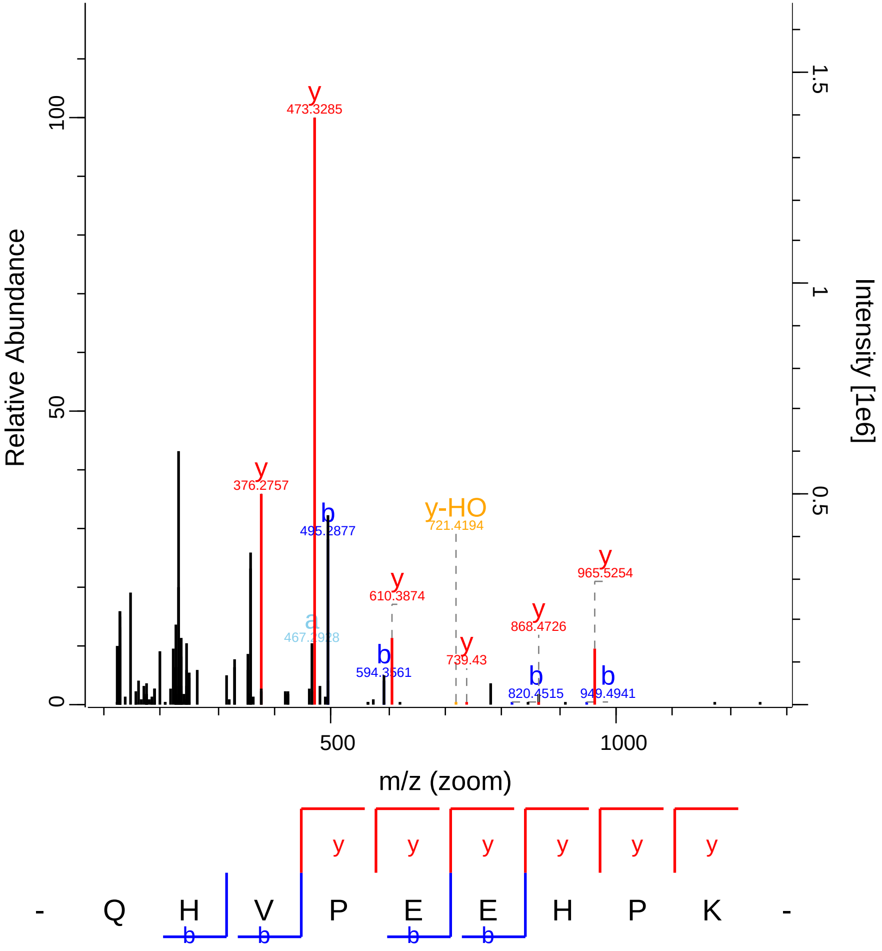

Raw File	Scan	Method	Score	m/z
RO-QC-160121_ZackD_SS_Set_02_Fraction_11	5611	FTMS; HCD	60.91	447.59

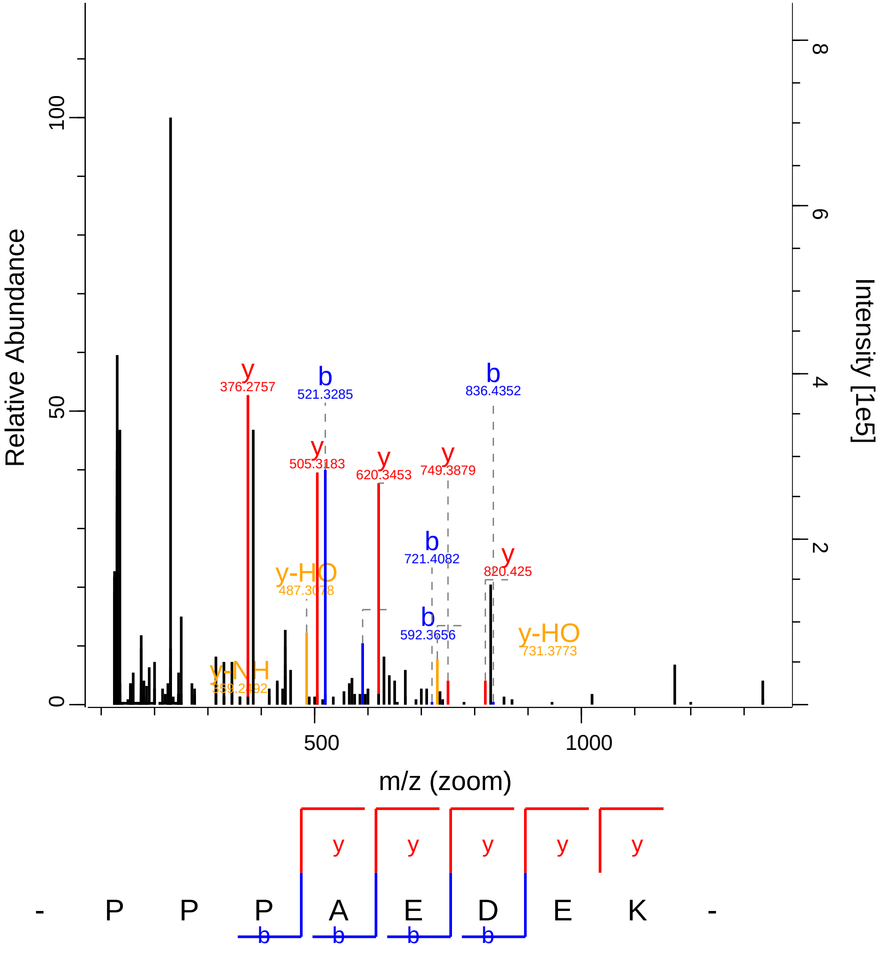

Raw File	Scan	Method	Score	m/z
RO-QC-160121_ZackD_SS_Set_02_Fraction_11	5612	FTMS; HCD	47.91	676.37

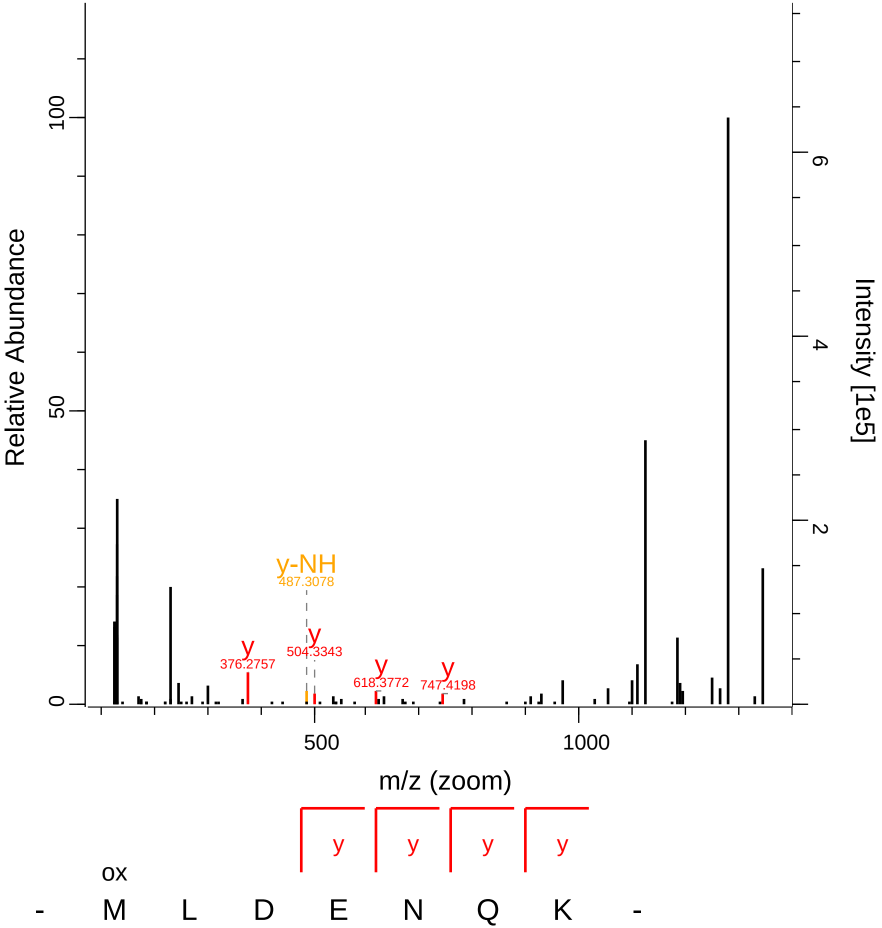

Raw File	Scan	Method	Score	m/z	Gene names
RO-QC-160121_ZackD_SS_Set_02_Fraction	5641	FTMS; HC	56.23	506.04	MOSPD1

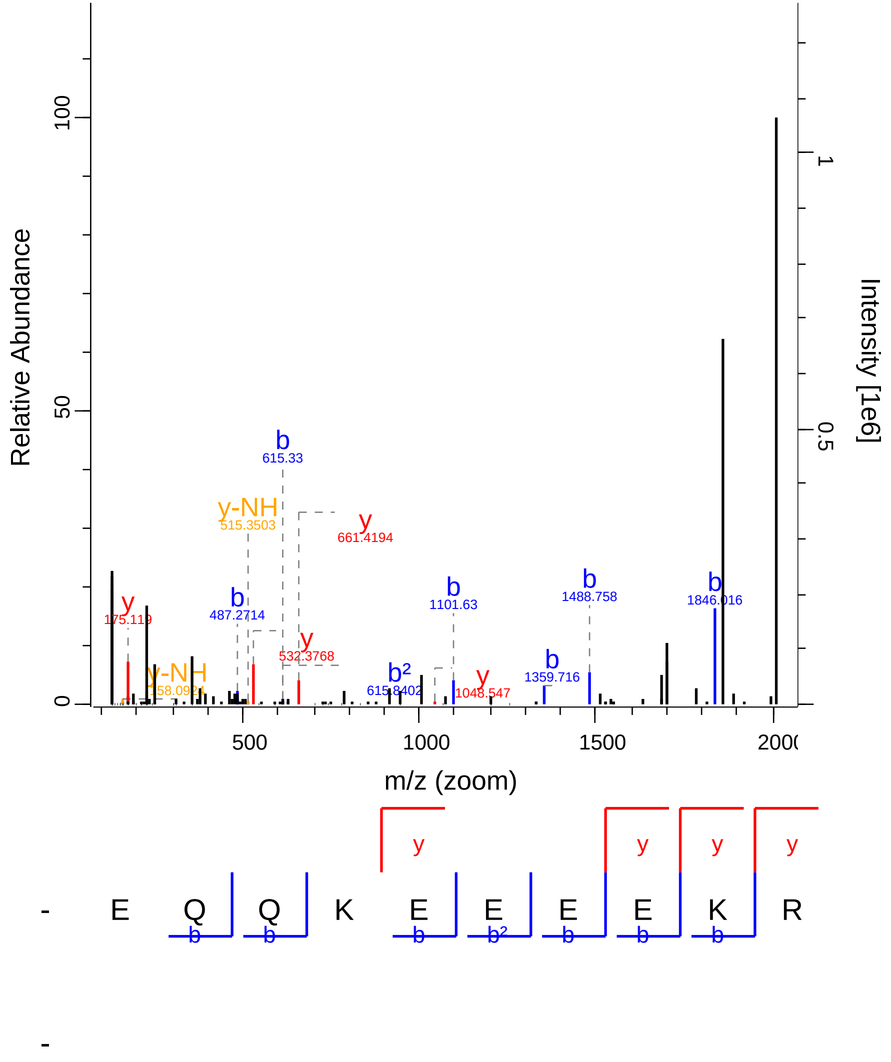

Raw File	Scan	Method	Score	m/z	Gene names
RO-QC-160121_ZackD_SS_Set_02_Fraction	6126	FTMS; HC	71.35	510.94	ARHGAP39

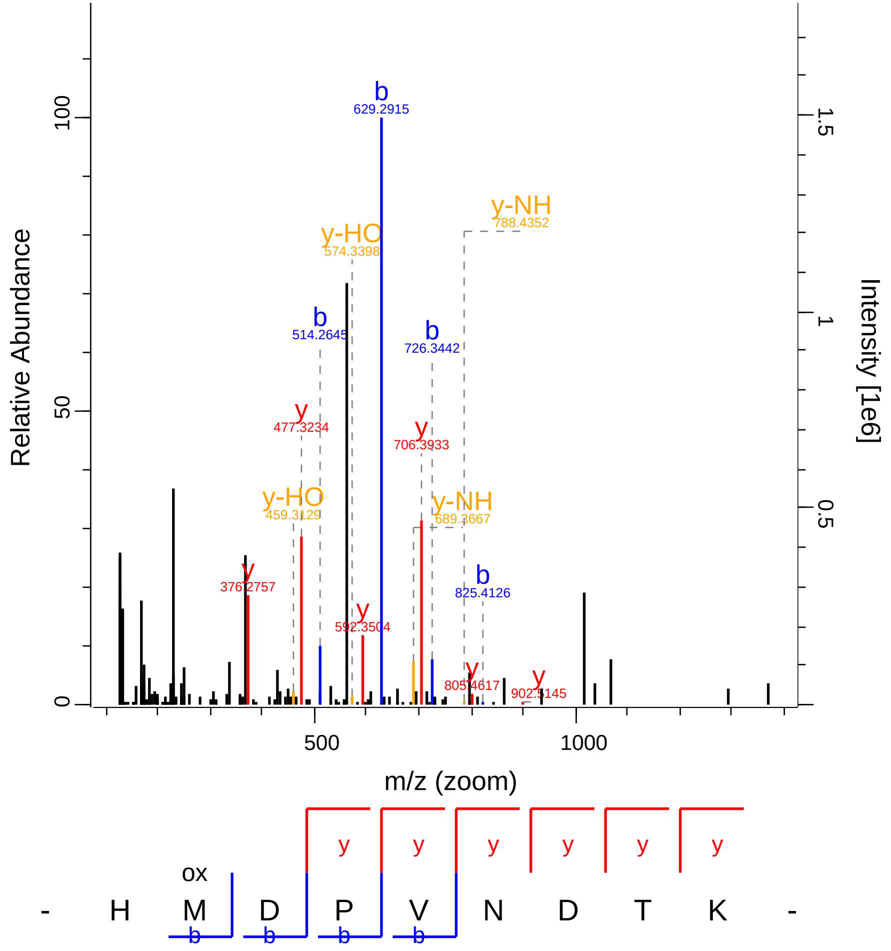

Raw File	Scan	Method	Score	m/z	Gene names
RO-QC-160121_ZackD_SS_Set_02_Fraction	6160	FTMS; HC	78.12	560.95	TASP1

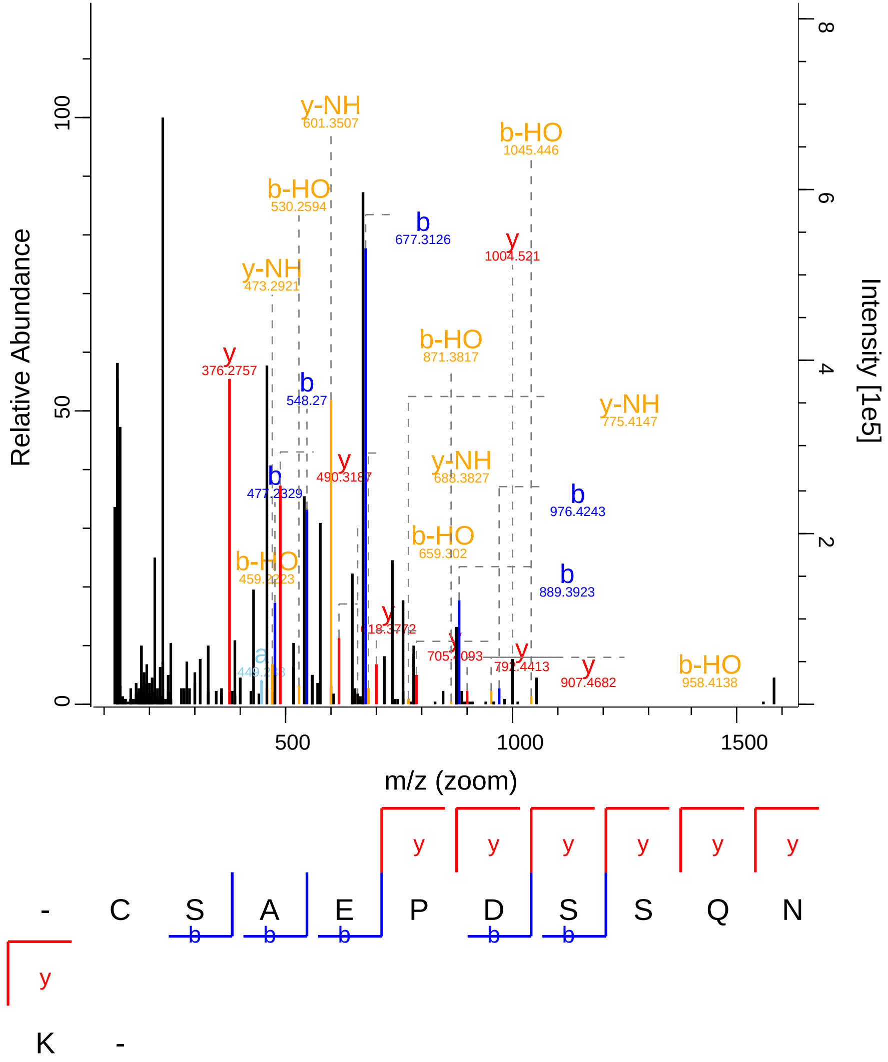

Raw File	Scan	Method	Score	m/z	Gene names
RO-QC-160121_ZackD_SS_Set_02_Fraction	6172	FTMS; HC	56.73	841.42	TASP1

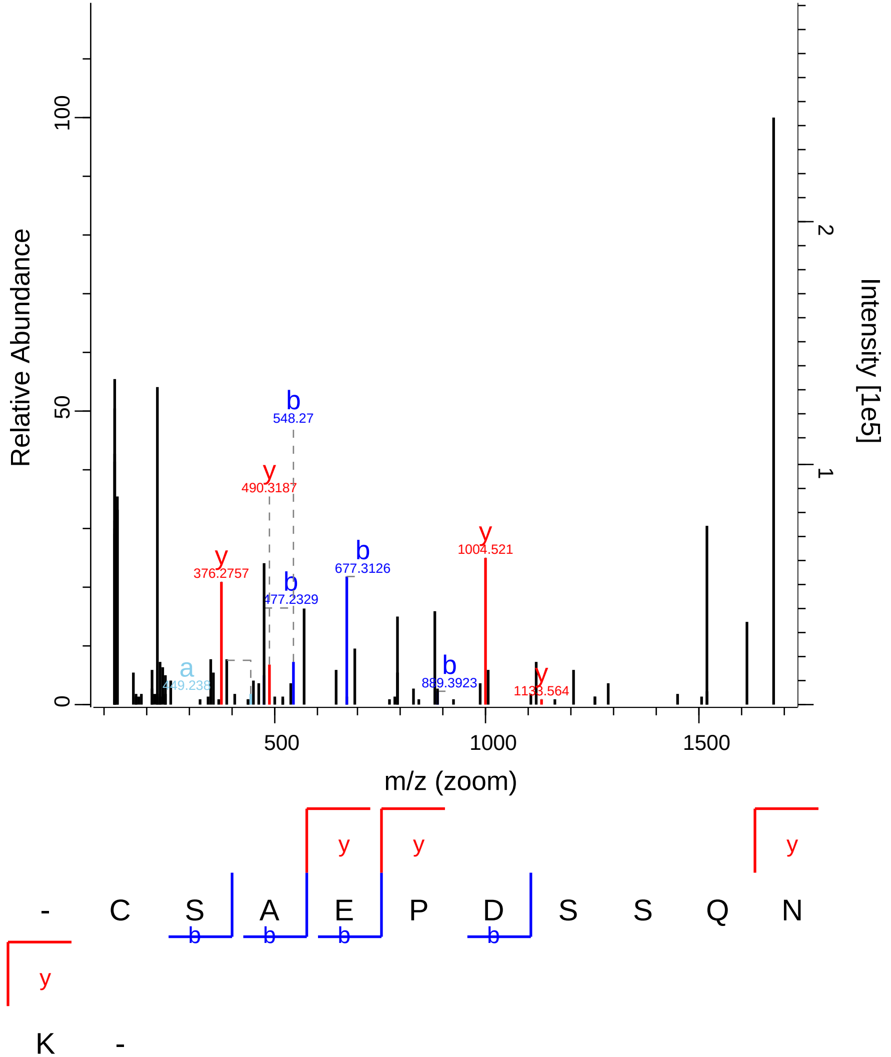

Raw File	Scan	Method	Score	m/z	Gene names
RO-QC-160121_ZackD_SS_Set_02_Fraction	6273	FTMS; HC	93.35	602.66	CCSAP

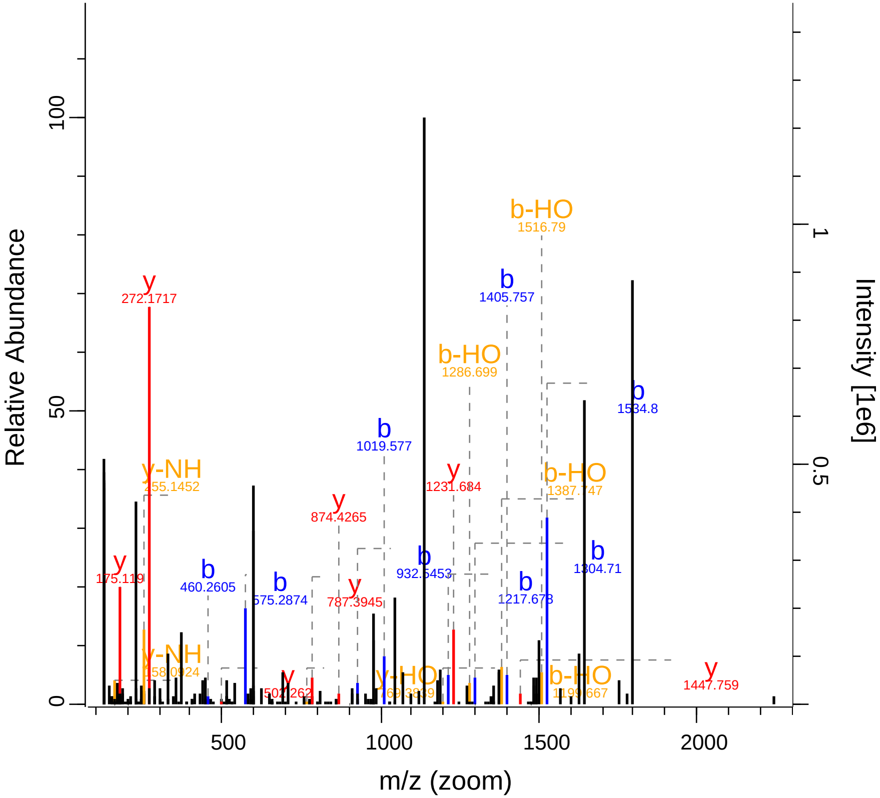

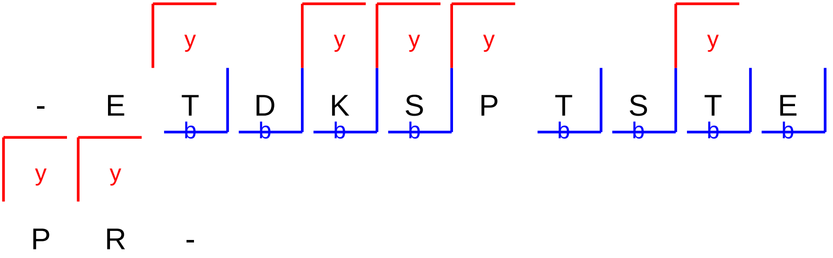

Raw File	Scan	Method	Score	m/z	Gene names
RO-QC-160121_ZackD_SS_Set_02_Fraction	7171	FTMS; HC	134.75	780.89	GKAP1

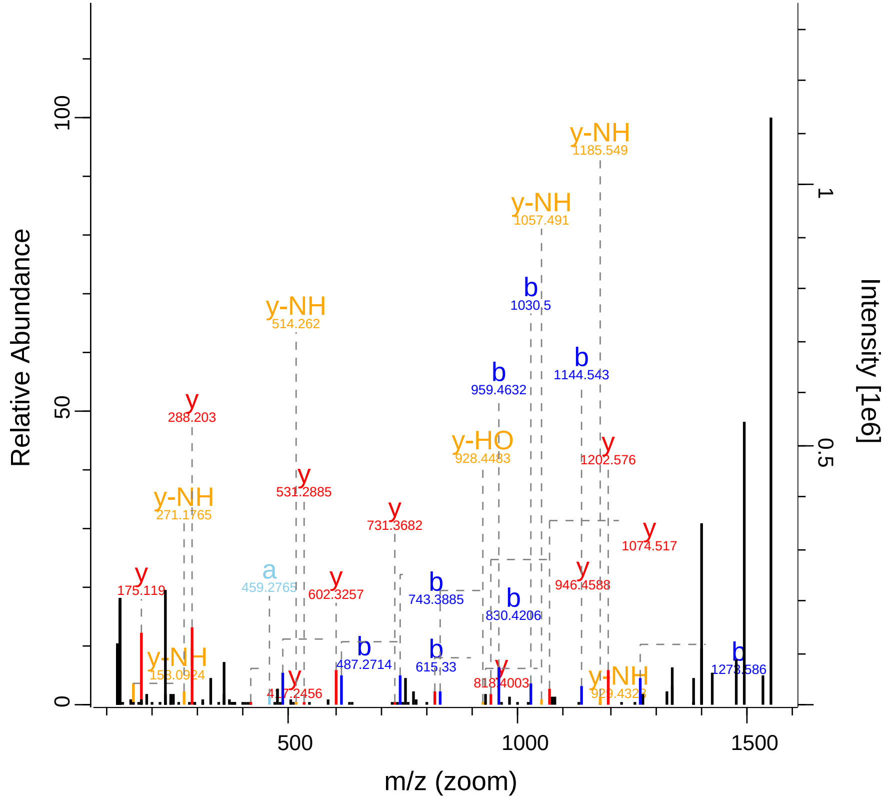

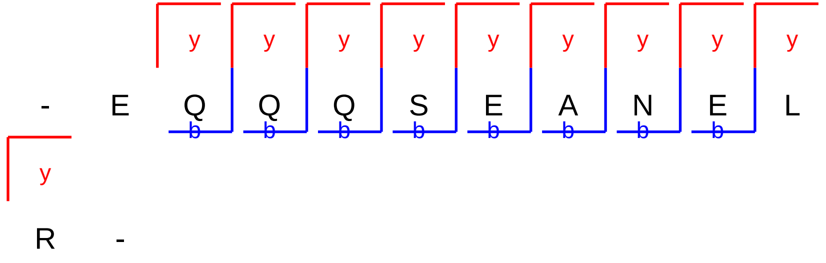

Raw File	Scan	Method	Score	m/z	Gene names
RO-QC-160121_ZackD_SS_Set_02_Fraction	7413	FTMS; HC	91.08	704.83	TAB3

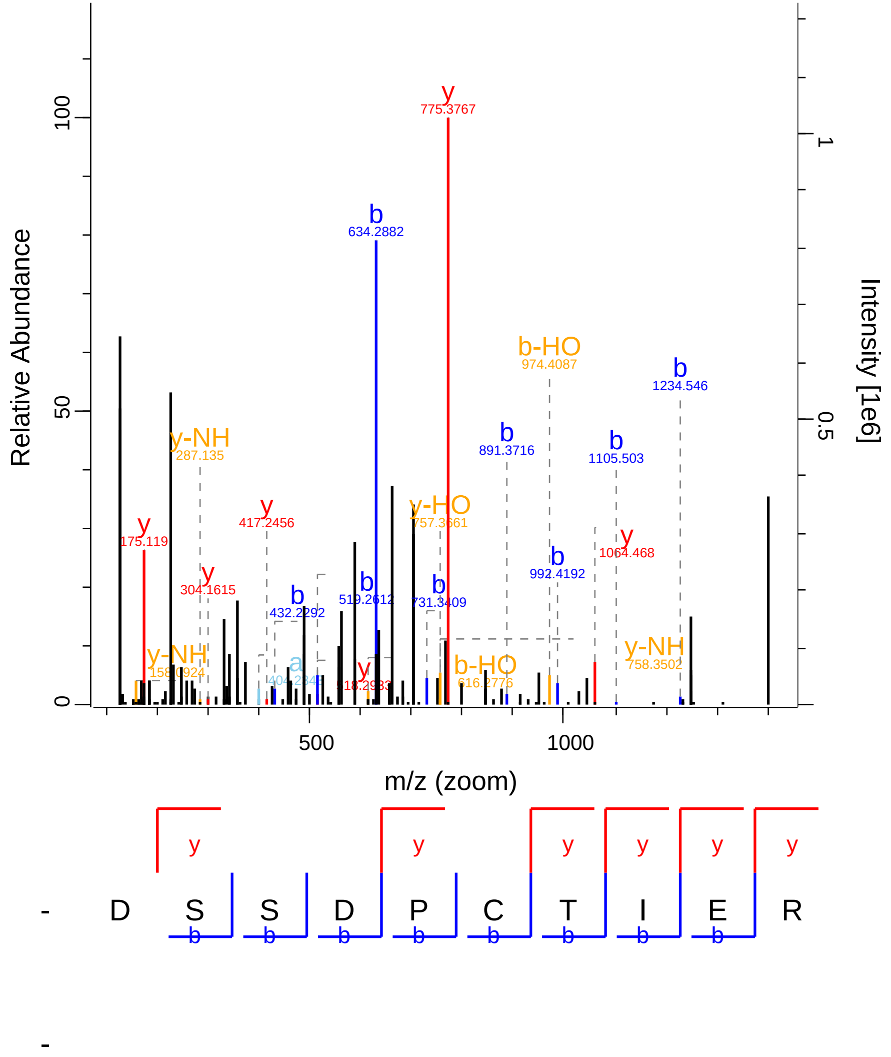

Raw File	Scan	Method	Score	m/z	Gene names
RO-QC-160121_ZackD_SS_Set_02_Fraction	7510	FTMS; HC	72.23	694.34	MLF2

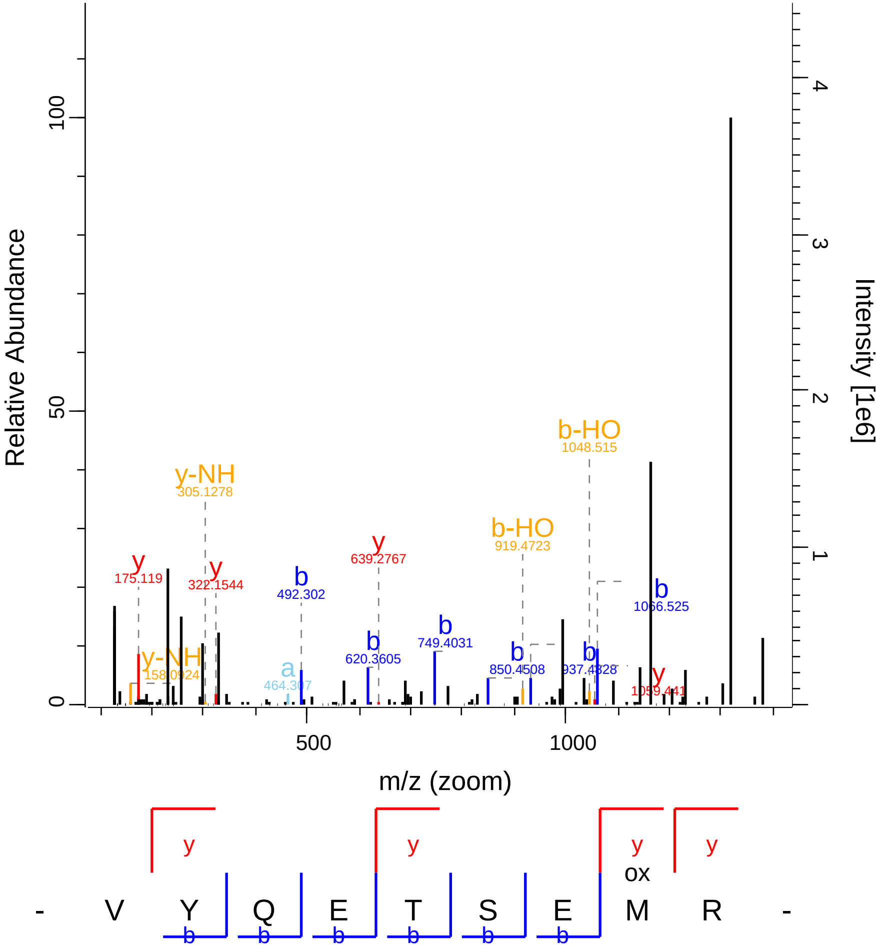

Raw File	Scan	Method	Score	m/z	Gene names
RO-QC-160121_ZackD_SS_Set_02_Fraction_	8194	FTMS; HC	45.16	664.66	KLF2;KLF4

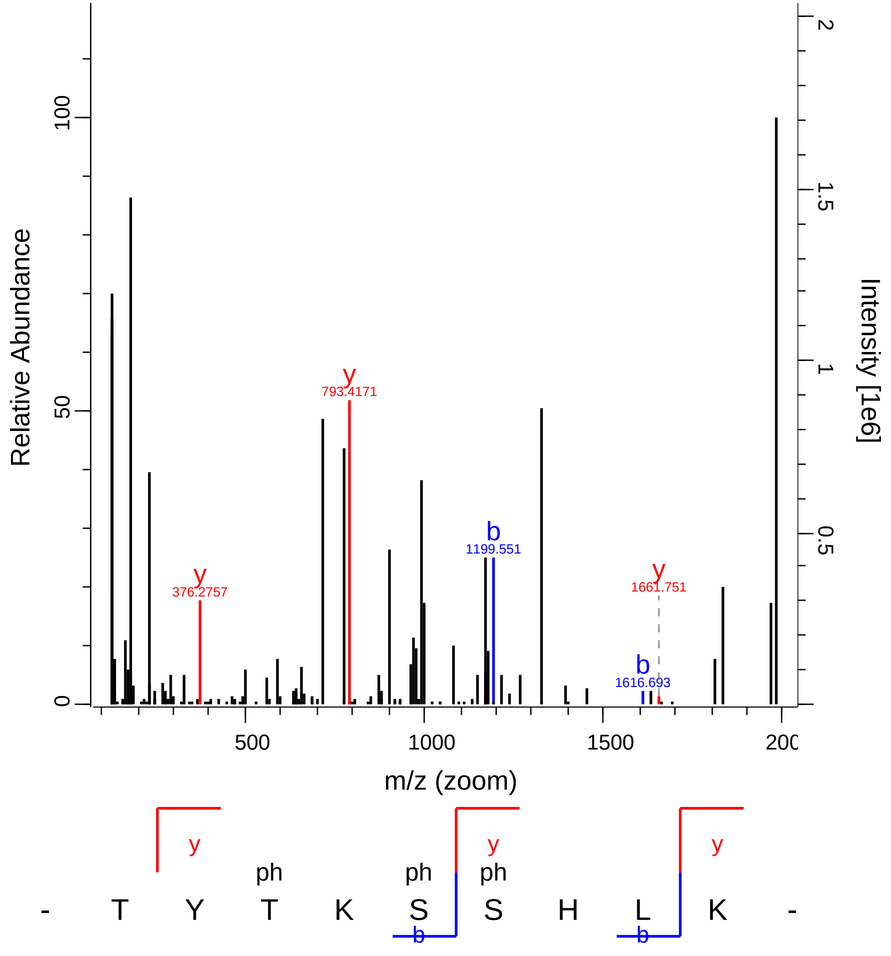

Raw File	Scan	Method	Score	m/z	Gene names
RO-QC-160121_ZackD_SS_Set_02_Fraction	8502	FTMS; HC	101.64	726.89	SERF2

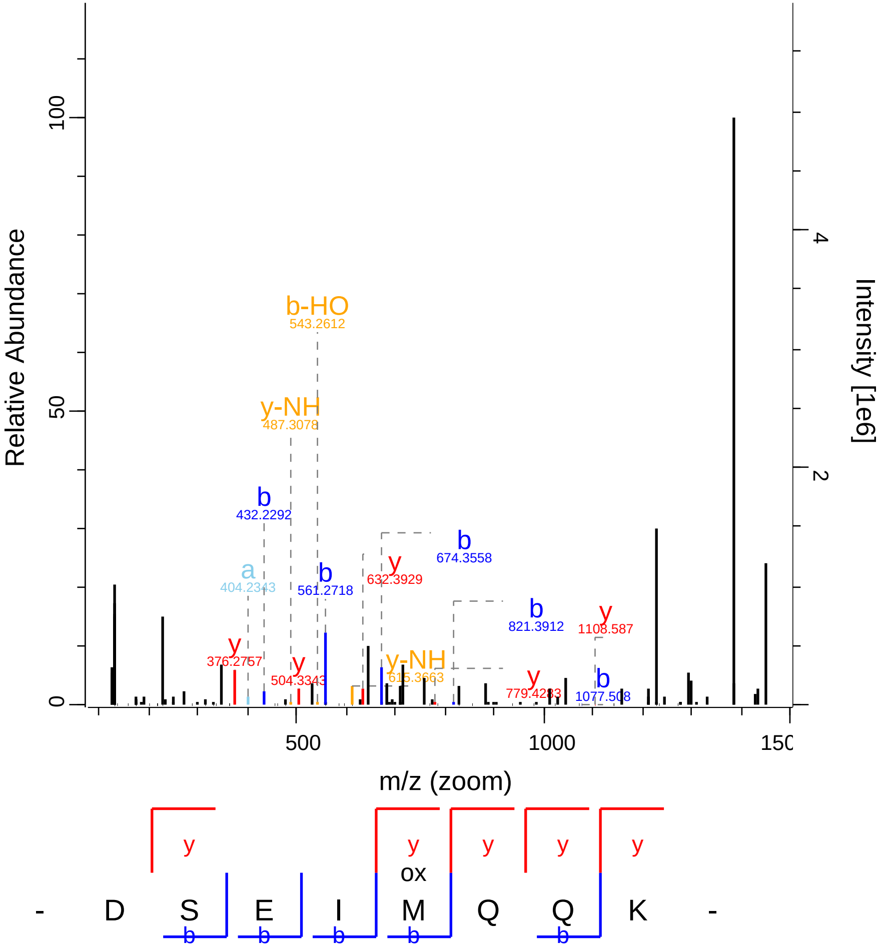

Raw File	Scan	Method	Score	m/z	Gene names
RO-QC-160121_ZackD_SS_Set_02_Fractior	10007	FTMS; HC	195.8	831.39	LEPROT

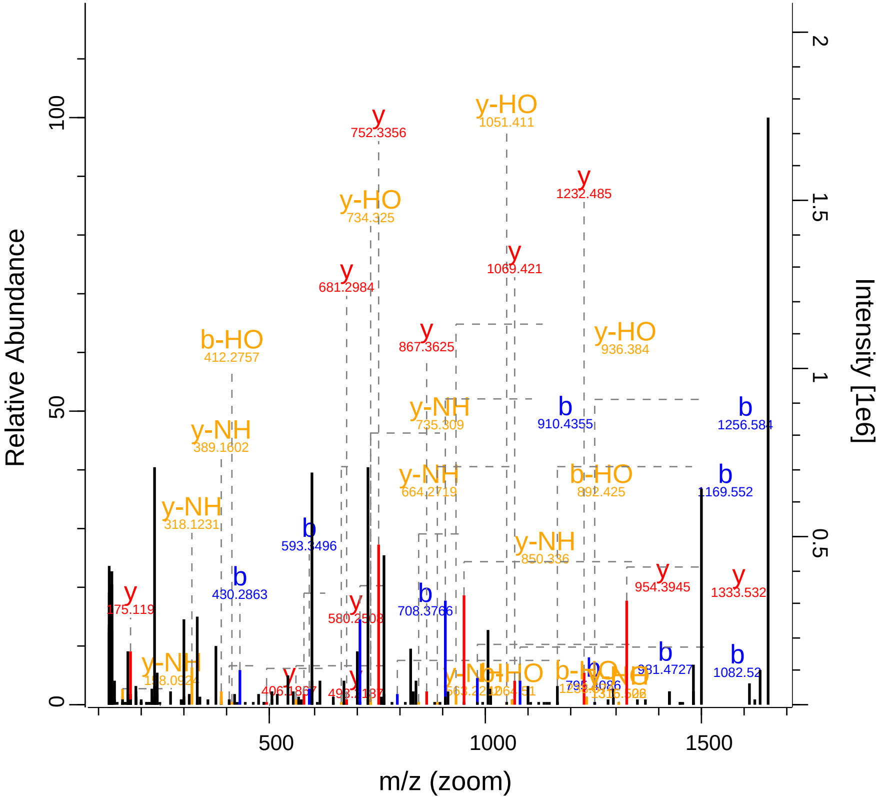

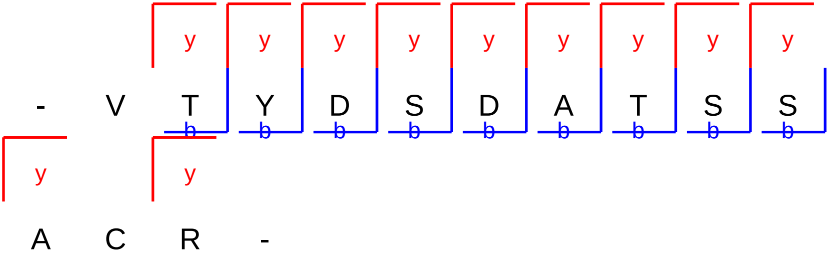

Raw File	Scan	Method	Score	m/z	Gene names
RO-QC-160121_ZackD_SS_Set_02_Fractior	10177	FTMS; HC	94.41	366.22	C2orf15

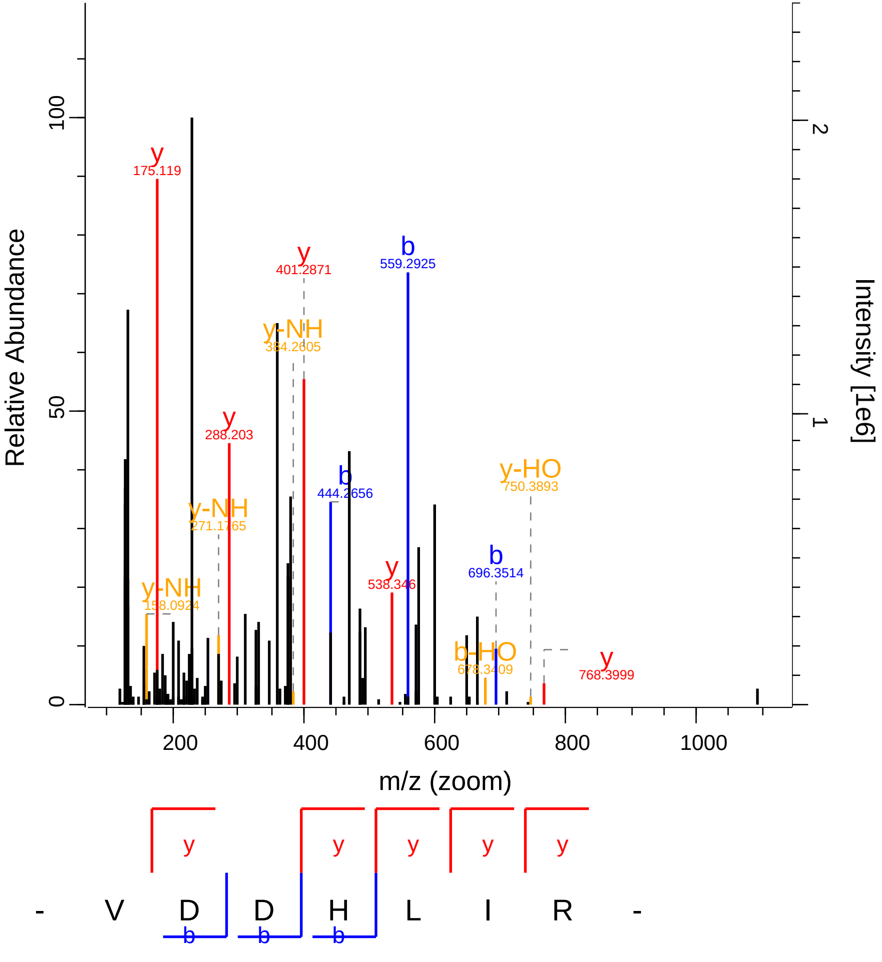

Raw File	Scan	Method	Score	m/z	Gene names
RO-QC-160121_ZackD_SS_Set_02_Fractior	10216	FTMS; HC	284.47	831.39	LEPROT

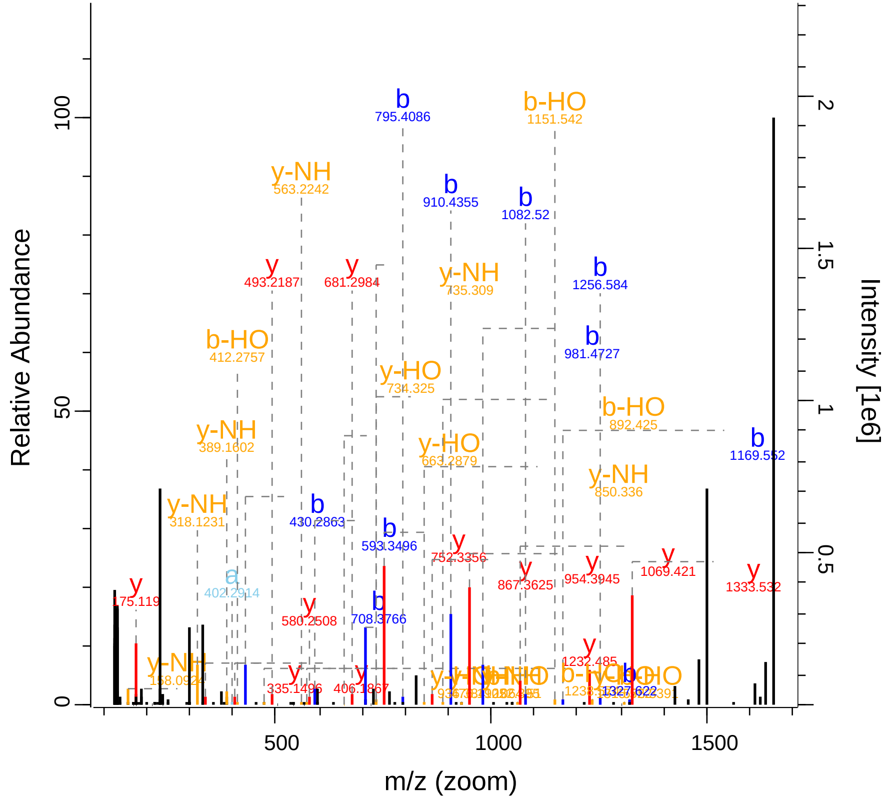

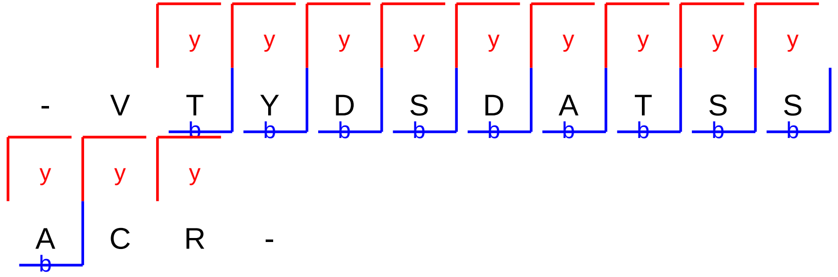

Raw File	Scan	Method	Score	m/z	Gene names
RO-QC-160121_ZackD_SS_Set_02_Fract	10301	FTMS; HC	47.82	427.74	GOLGA8B;GOLGA

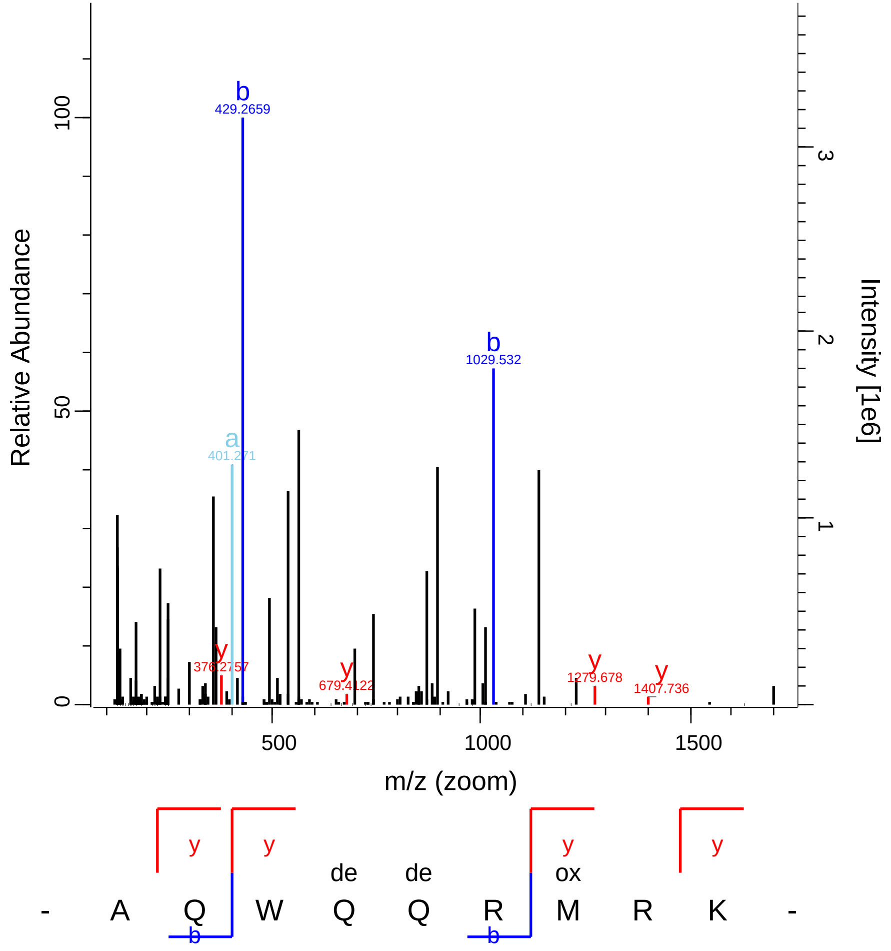

Raw File	Scan	Method	Score	m/z	Gene names
RO-QC-160121_ZackD_SS_Set_02_Fractior	10579	FTMS; HC	54.26	400.49	USH1C

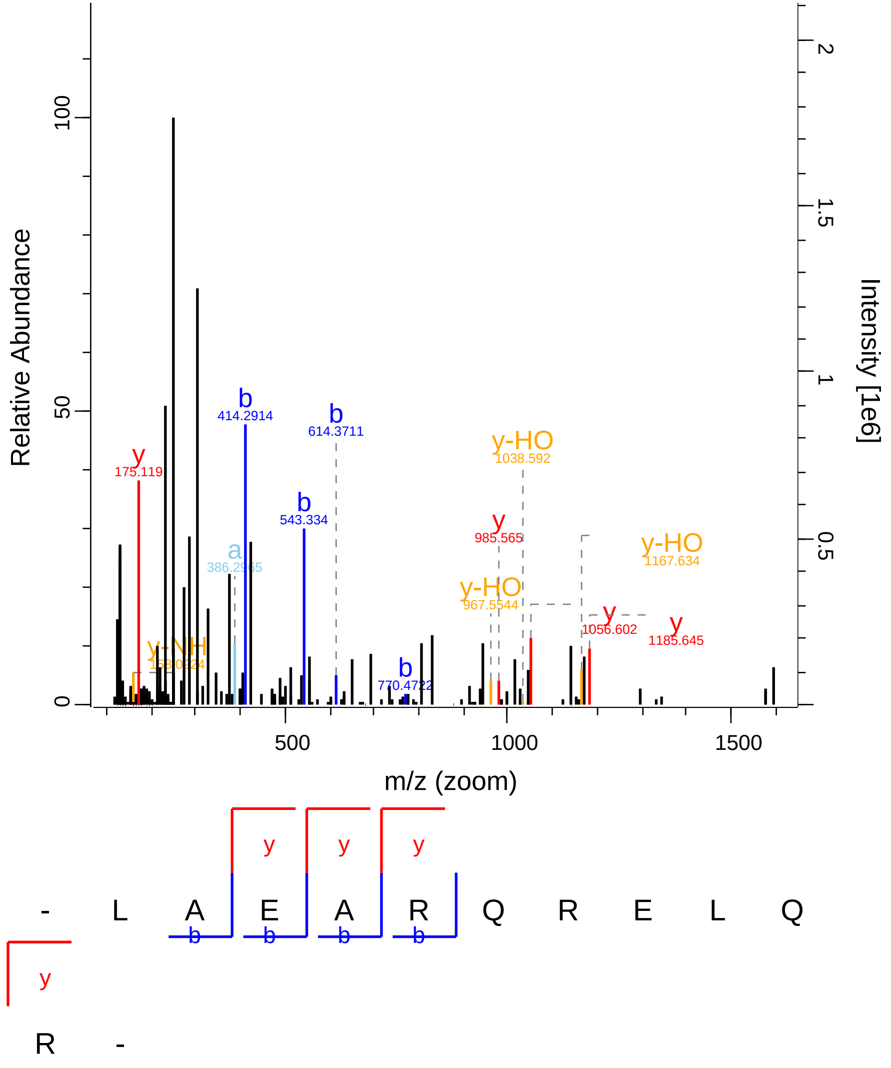

Raw File	Scan	Method	Score	m/z	Gene names
RO-QC-160121_ZackD_SS_Set_02_Fraction	12543	FTMS; HC	244.01	832.9	YIPF5

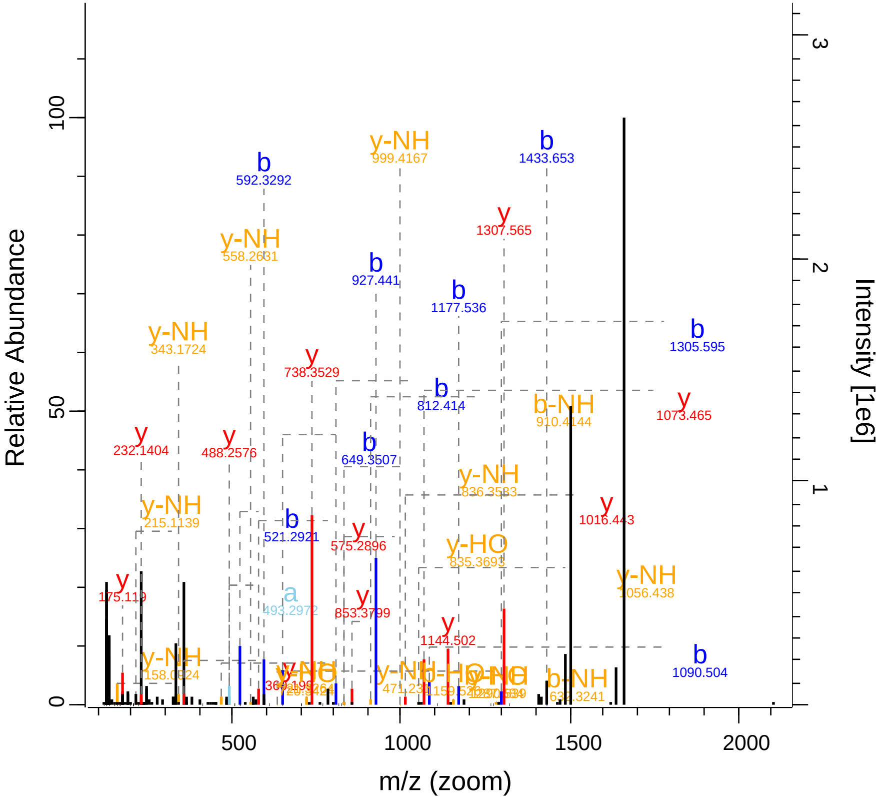

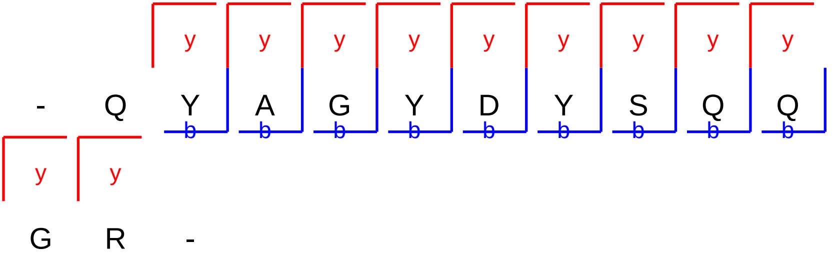

Raw File	Scan	Method	Score	m/z	Gene names
RO-QC-160121_ZackD_SS_Set_02_Fractior	12716	FTMS; HC	66.46	686.84	MLF2

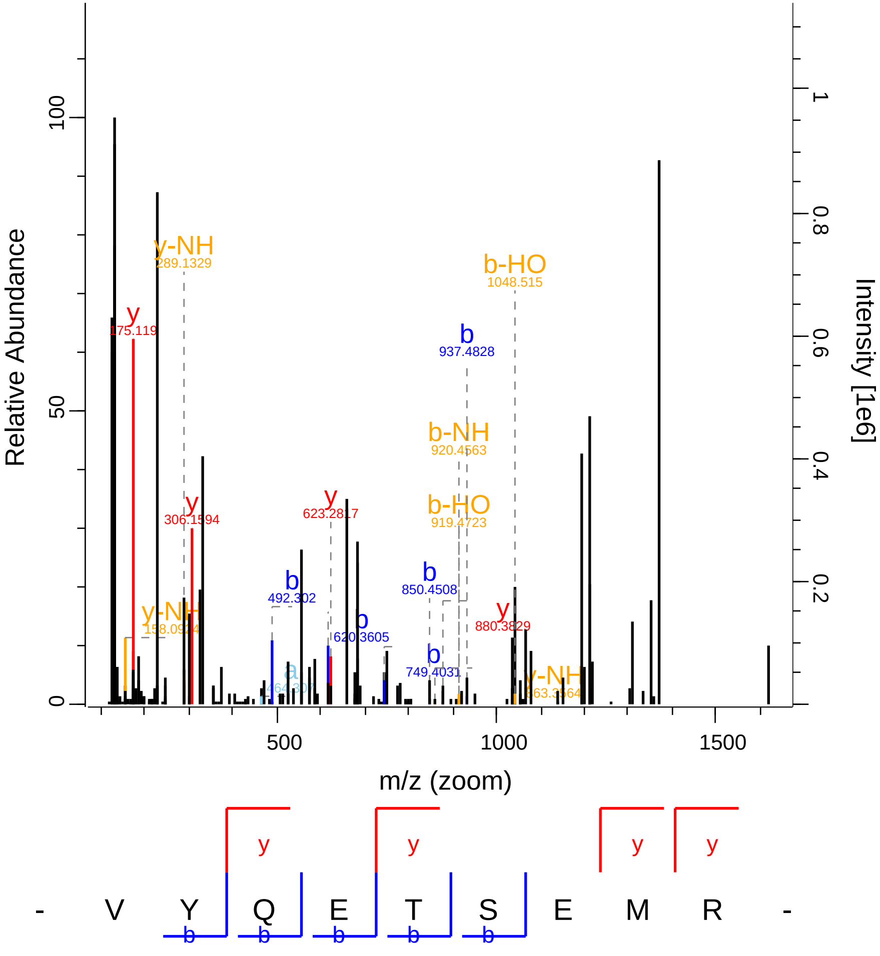

Raw File	Scan	Method	Score	m/z	Gene names
RO-QC-160121_ZackD_SS_Set_02_Fraction	12753	FTMS; HC	81.26	833.4	YIPF5

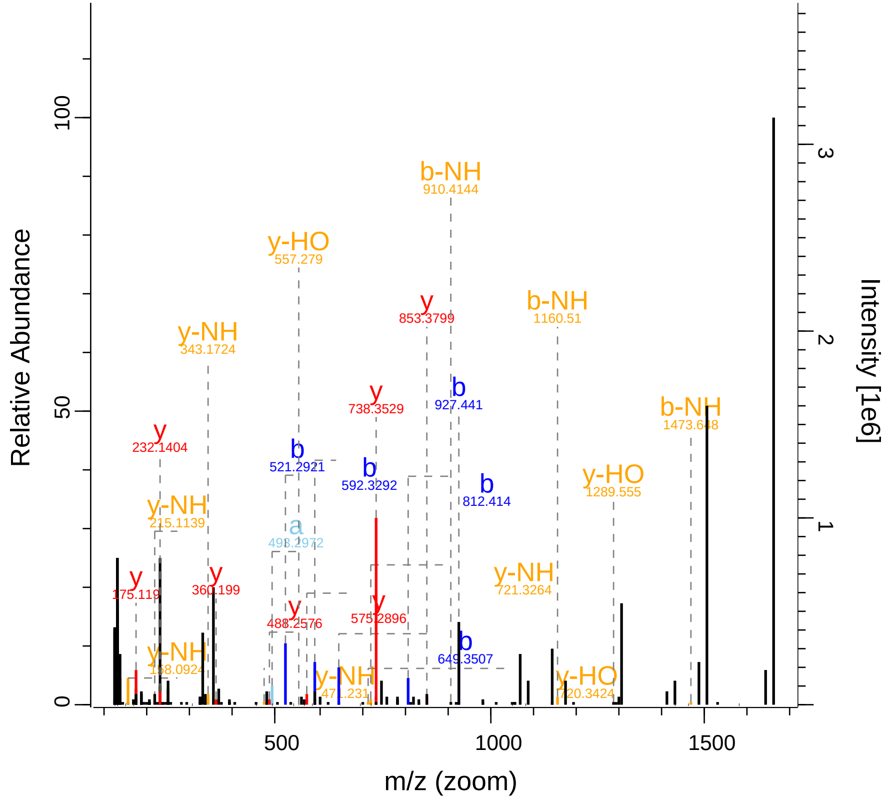

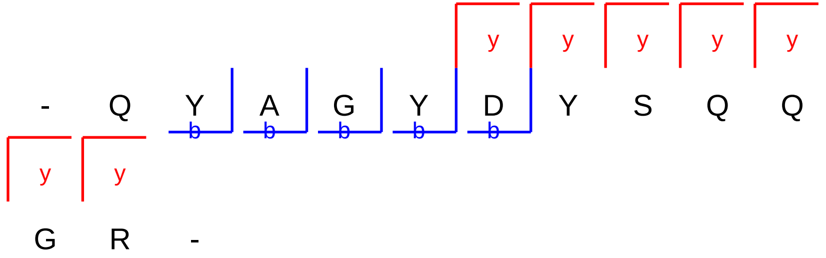

Raw File	Scan	Method	Score	m/z	Gene names
RO-QC-160121_ZackD_SS_Set_02_Fractior	12979	FTMS; HC	54.7	725.41	ZNF350

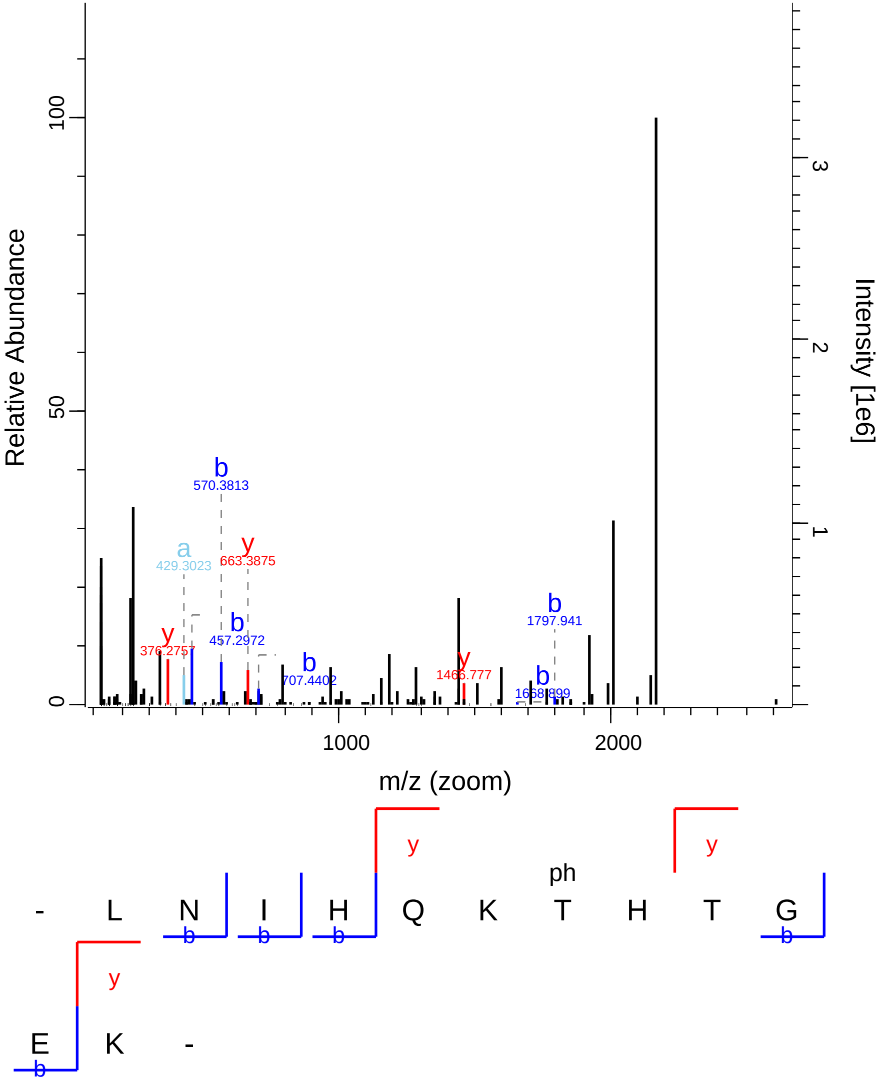

Raw File	Scan	Method	Score	m/z	Gene names
RO-QC-160121_ZackD_SS_Set_02_Fractior	13196	FTMS; HC	82.36	725.07	ZNF350

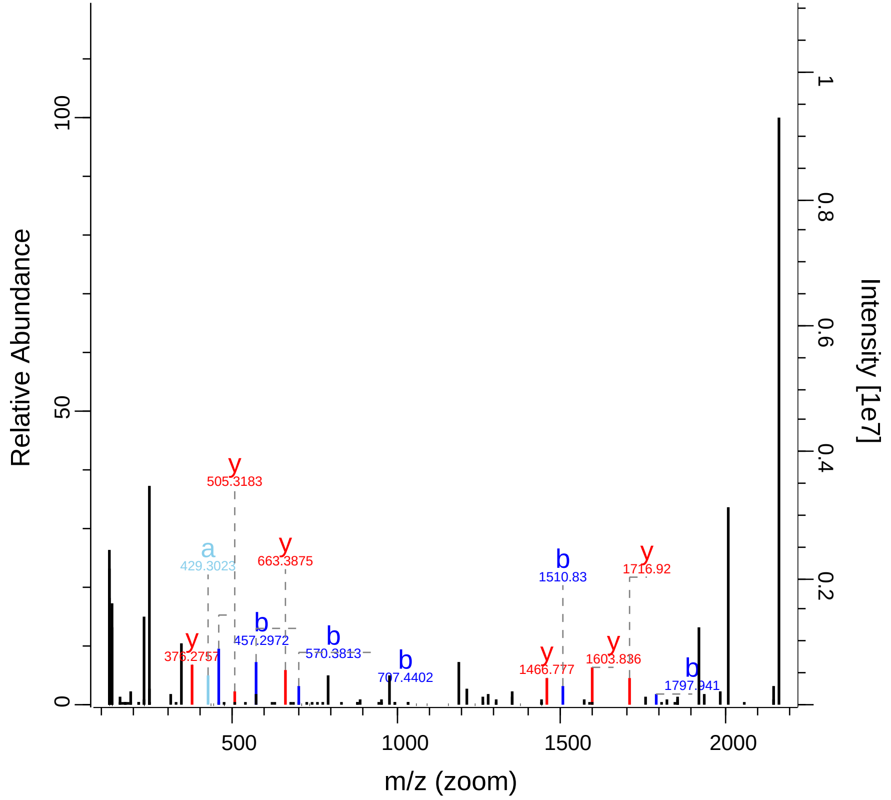

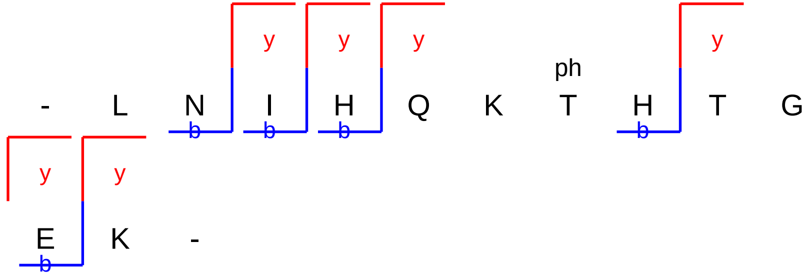

Raw File	Scan	Method	Score	m/z	Gene names
RO-QC-160121_ZackD_SS_Set_02_Fractior	13765	FTMS; HC	150.1	745.95	GPR155

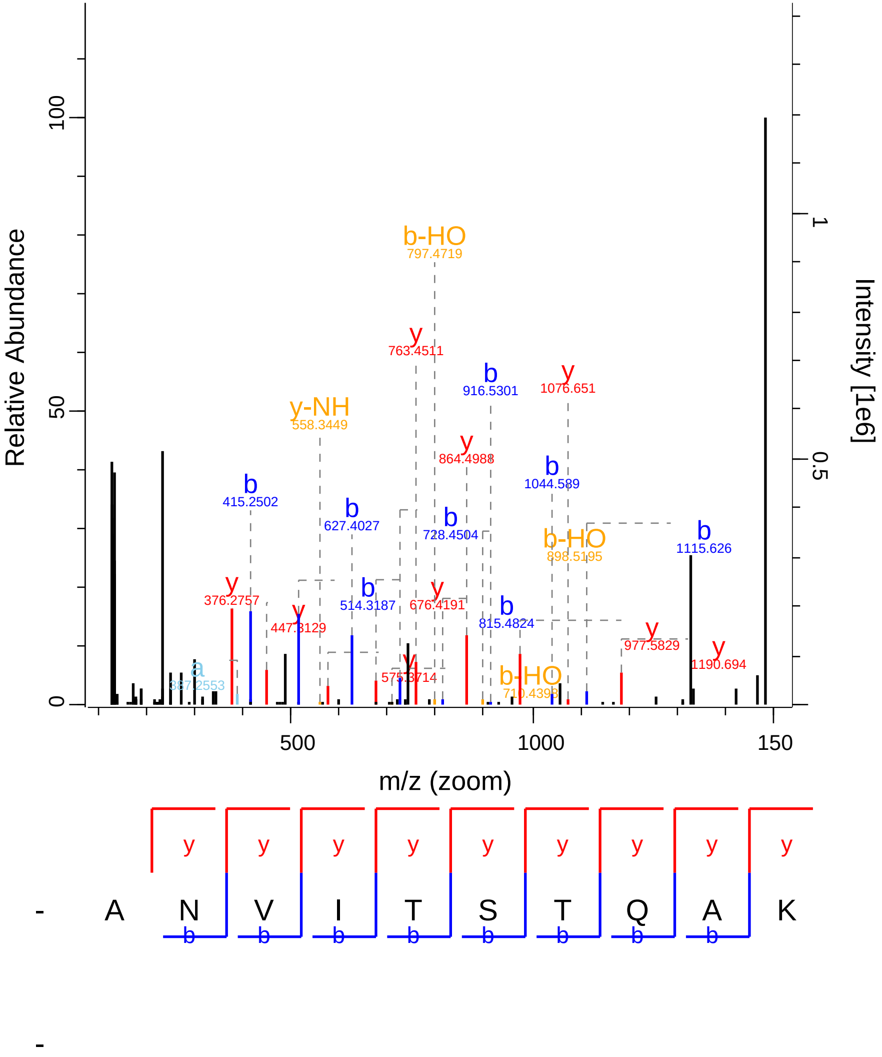

Raw File	Scan	Method	Score	m/z	Gene names
RO-QC-160121_ZackD_SS_Set_02_Fractior	13767	FTMS; HC	94.32	783.71	F2R

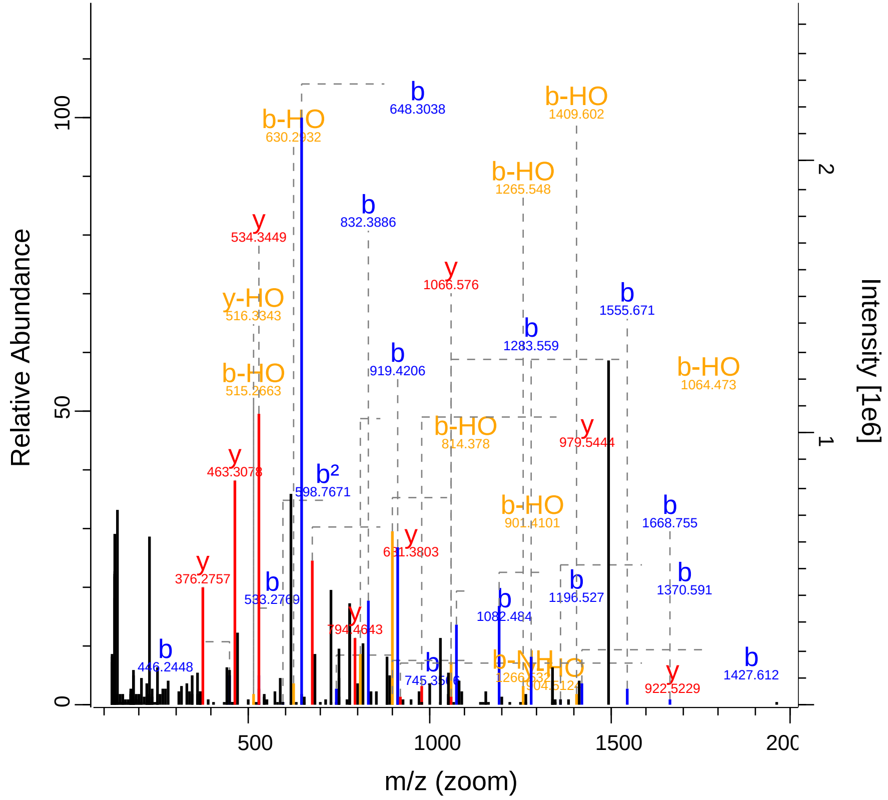

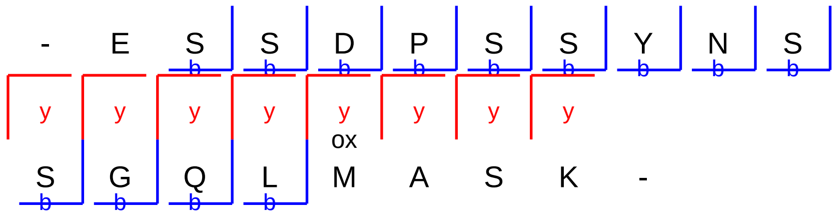

Raw File	Scan	Method	Score	m/z
RO-QC-160121_ZackD_SS_Set_02_Fraction_11	14160	FTMS; HCD	43.81	378.21

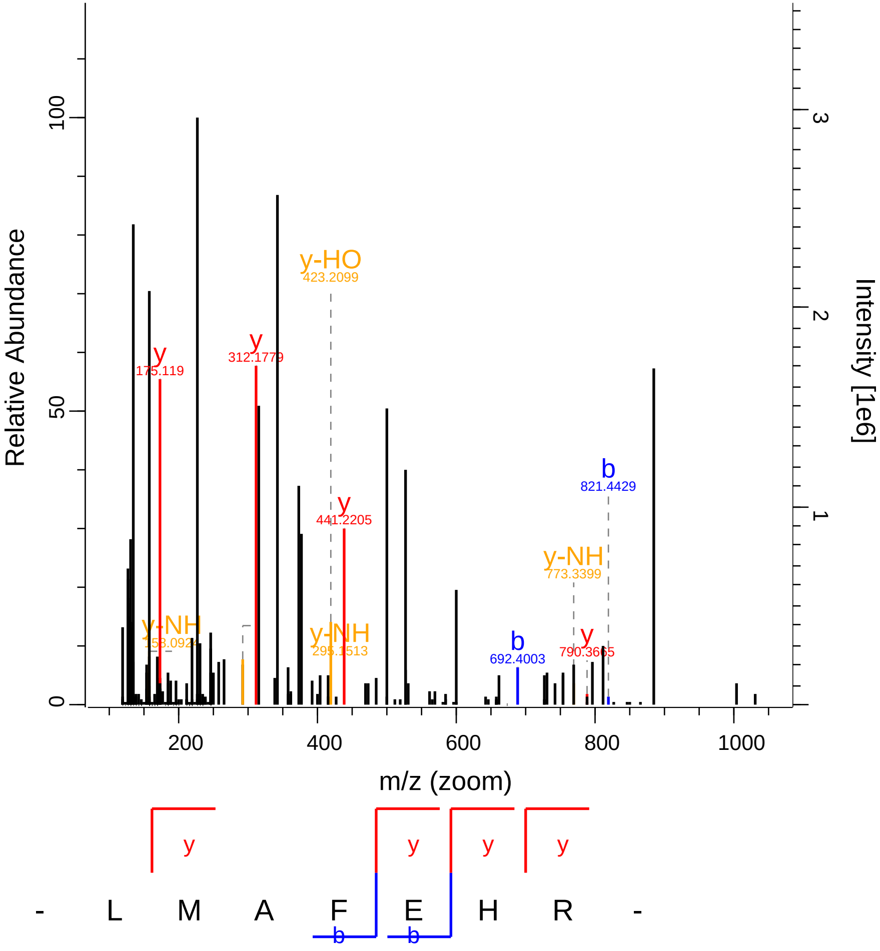

Raw File	Scan	Method	Score	m/z
RO-QC-160121_ZackD_SS_Set_02_Fraction_11	14395	FTMS; HCD	59.21	378.21

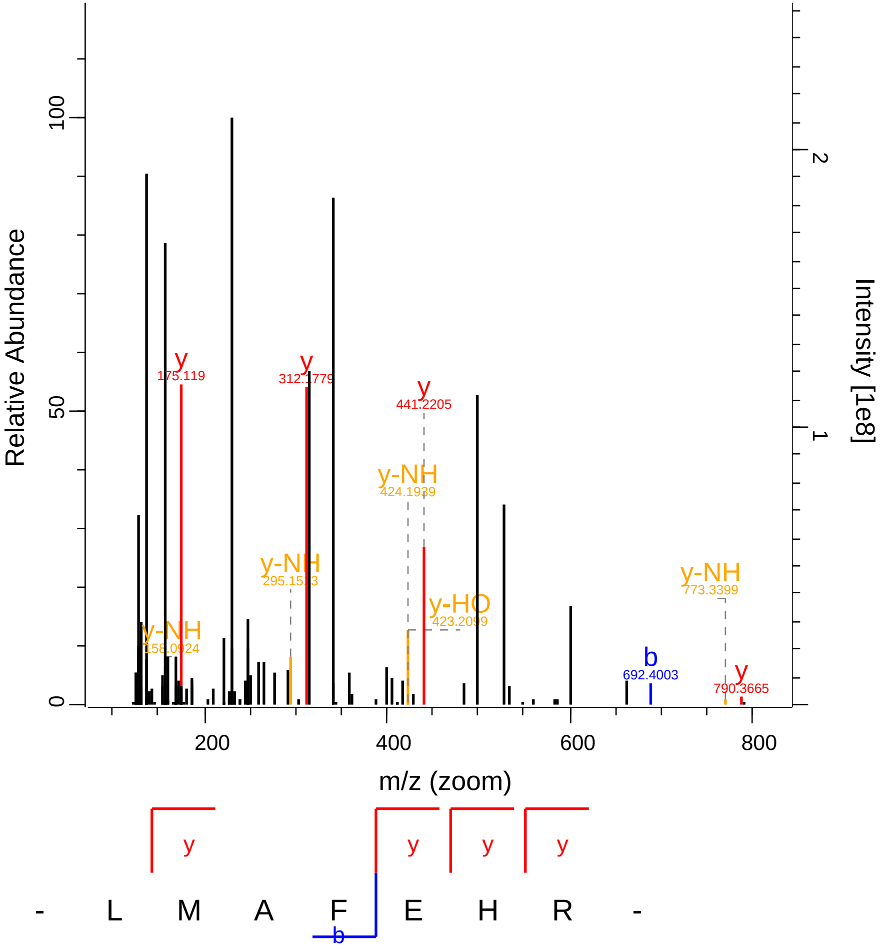

Raw File	Scan	Method	Score	m/z
RO-QC-160121_ZackD_SS_Set_02_Fraction_11	14628	FTMS; HCD	57.56	378.21

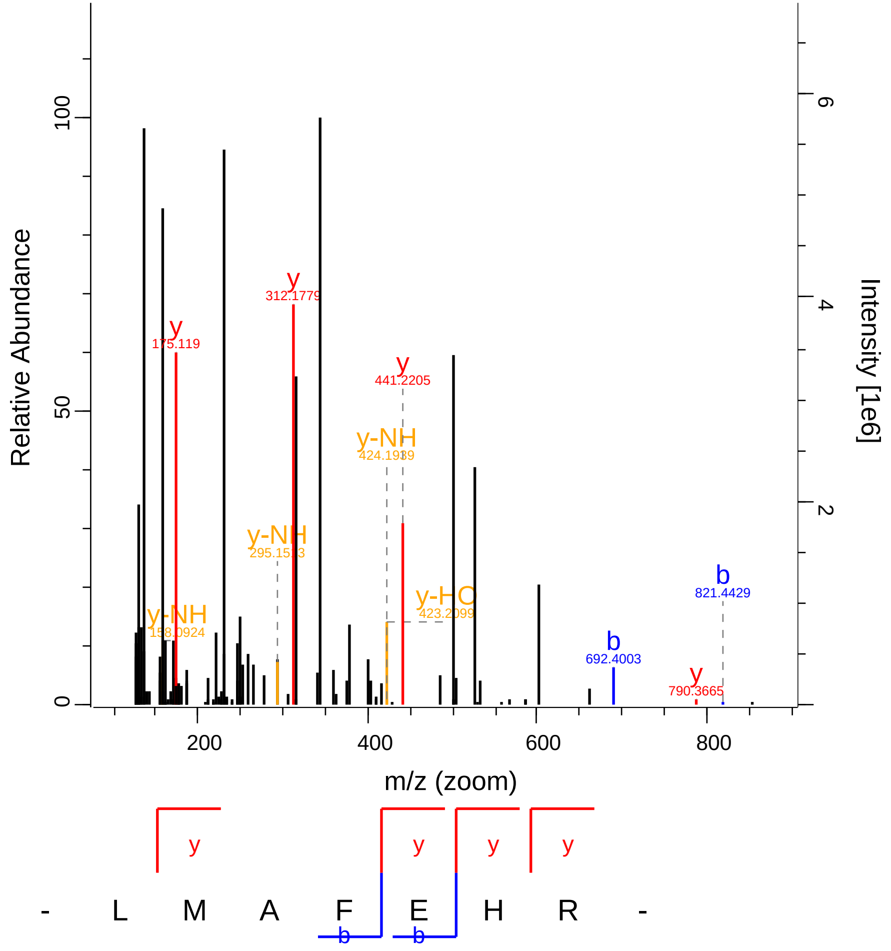

Raw File	Scan	Method	Score	m/z
RO-QC-160121_ZackD_SS_Set_02_Fraction_11	14907	FTMS; HCD	50.97	378.21

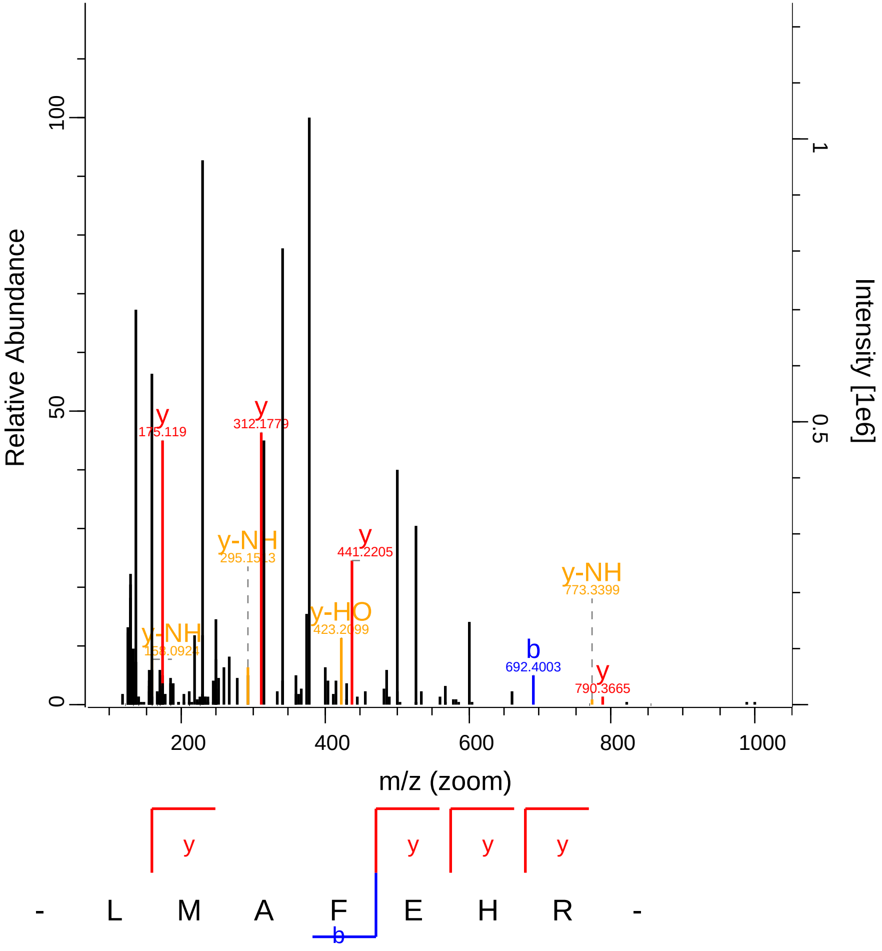

Raw File	Scan	Method	Score	m/z	Gene names
RO-QC-160121_ZackD_SS_Set_02_Fractio	15785	FTMS; HC	93.11	1063.04	LRRC8B

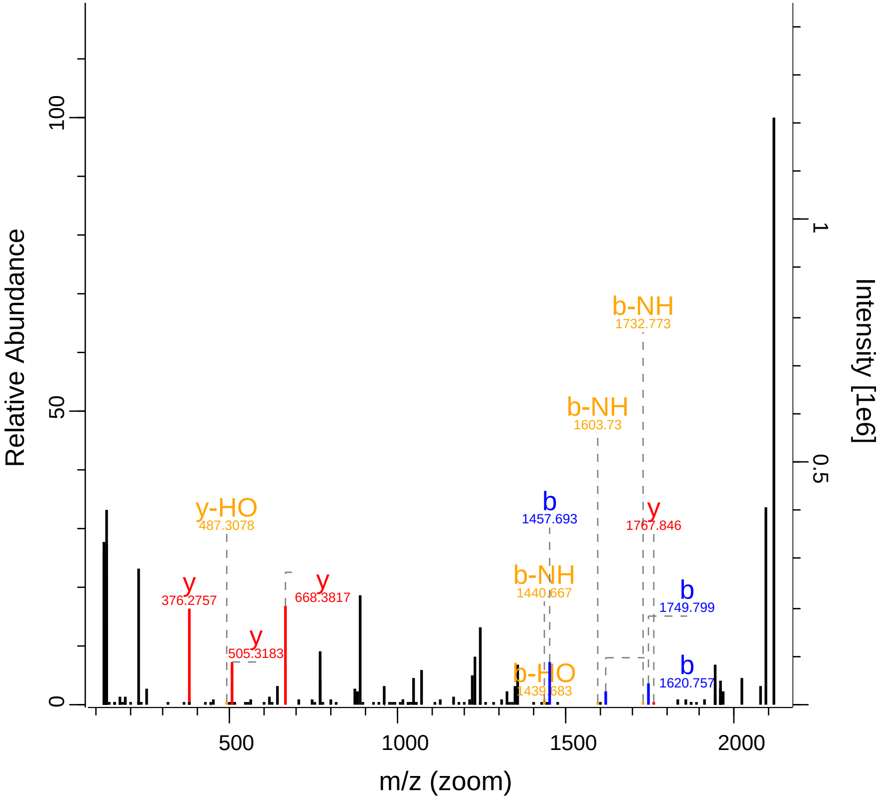

- Q Q Y S Y I D A V C

Y E K -

Raw File	Scan	Method	Score	m/z	Gene names
RO-QC-160121_ZackD_SS_Set_02_Fractior	16489	FTMS; HC	92.49	608.34	UTS2

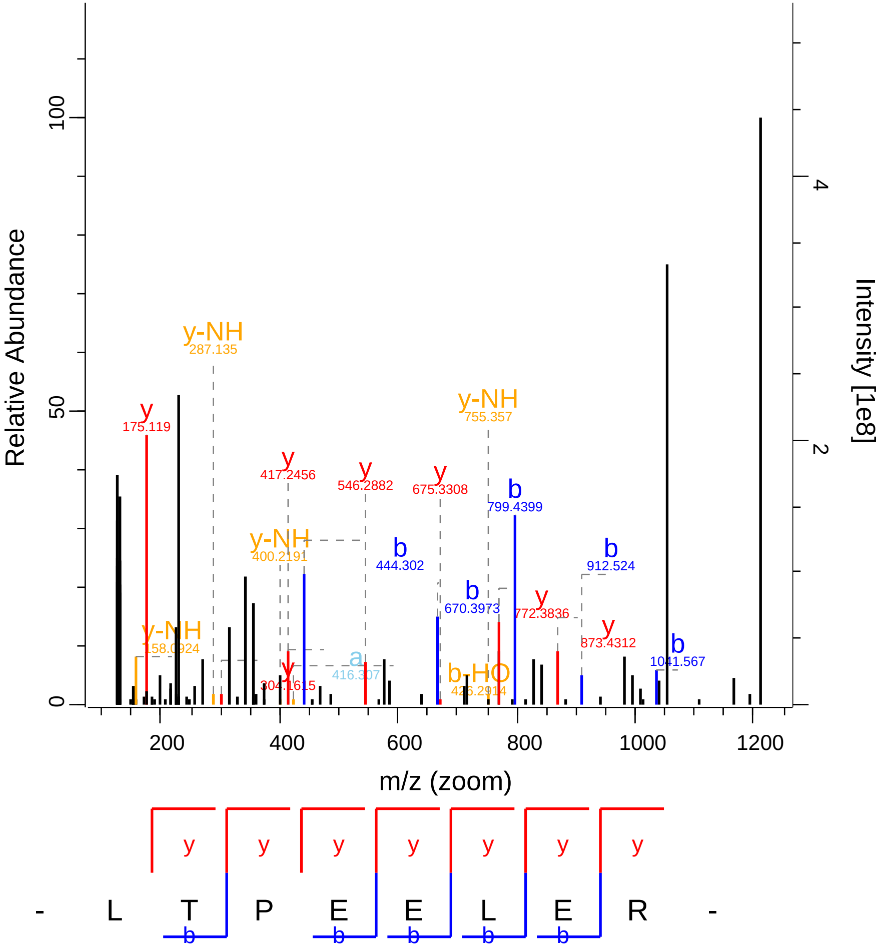

Raw File	Scan	Method	Score	m/z	Gene names
RO-QC-160121_ZackD_SS_Set_02_Fractior	16716	FTMS; HC	110.12	608.34	UTS2

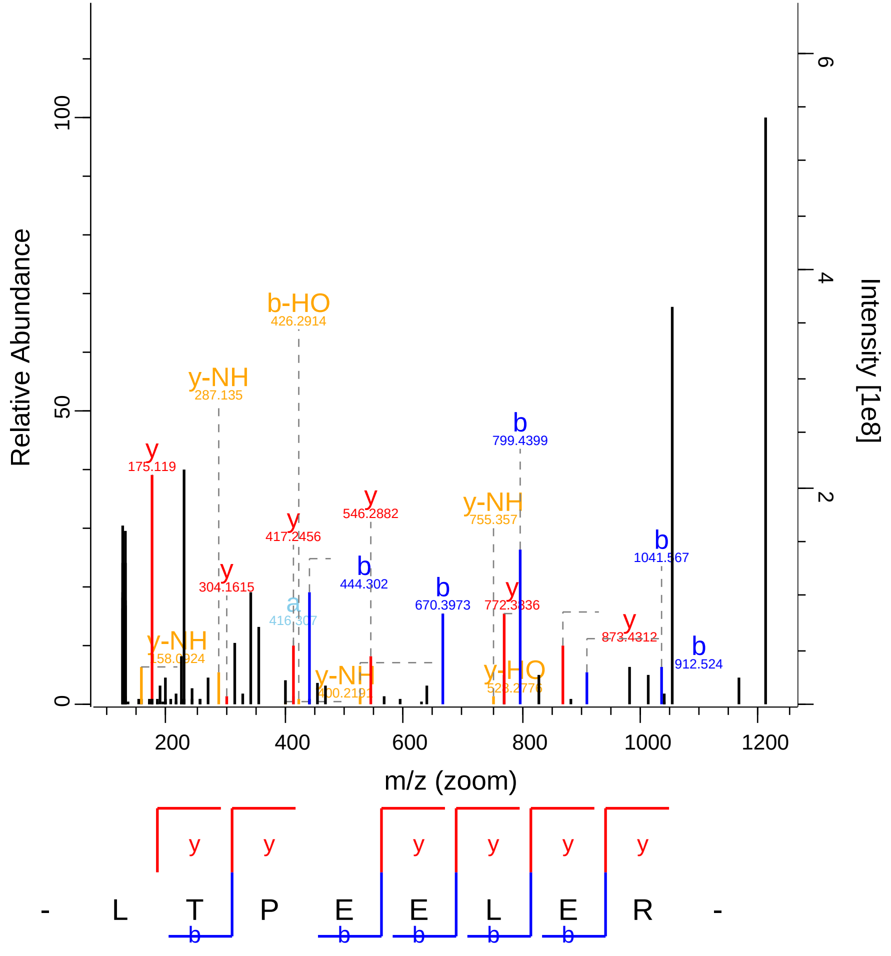

Raw File	Scan	Method	Score	m/z	Gene names
RO-QC-160121_ZackD_SS_Set_02_Fractior	16950	FTMS; HC	58.72	608.34	UTS2

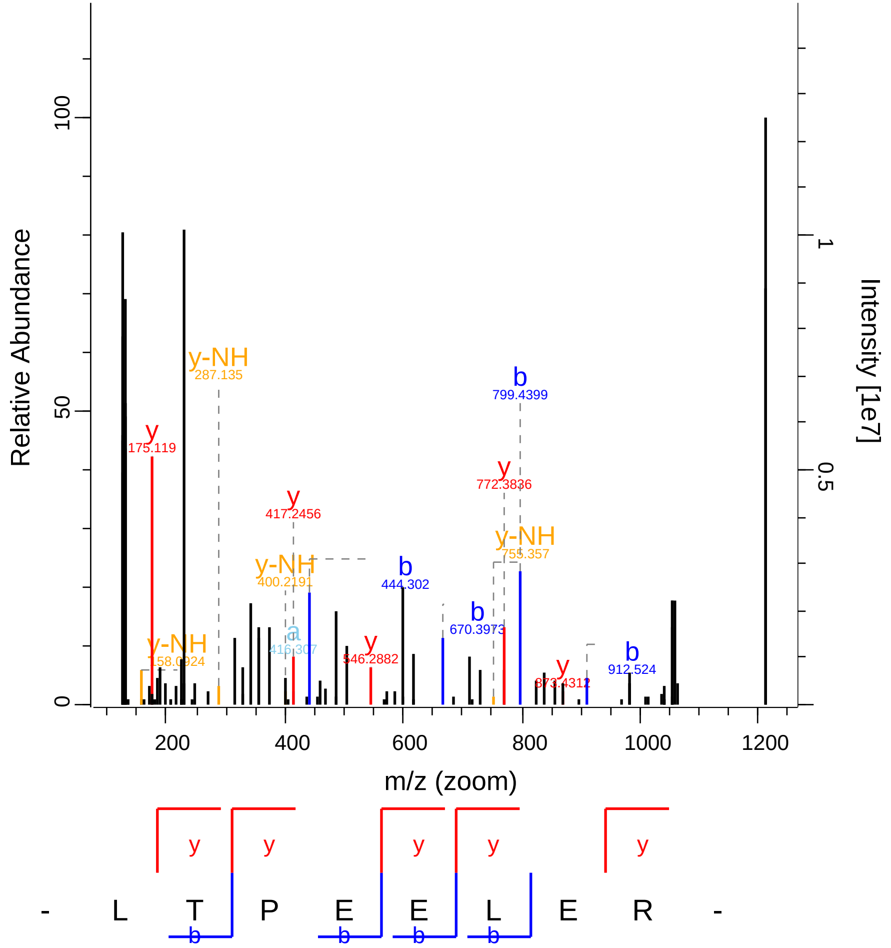

Raw File	Scan	Method	Score	m/z	Gene names
RO-QC-160121_ZackD_SS_Set_02_Fractior	16962	FTMS; HC	87.5	649.05	MAD2L1BP

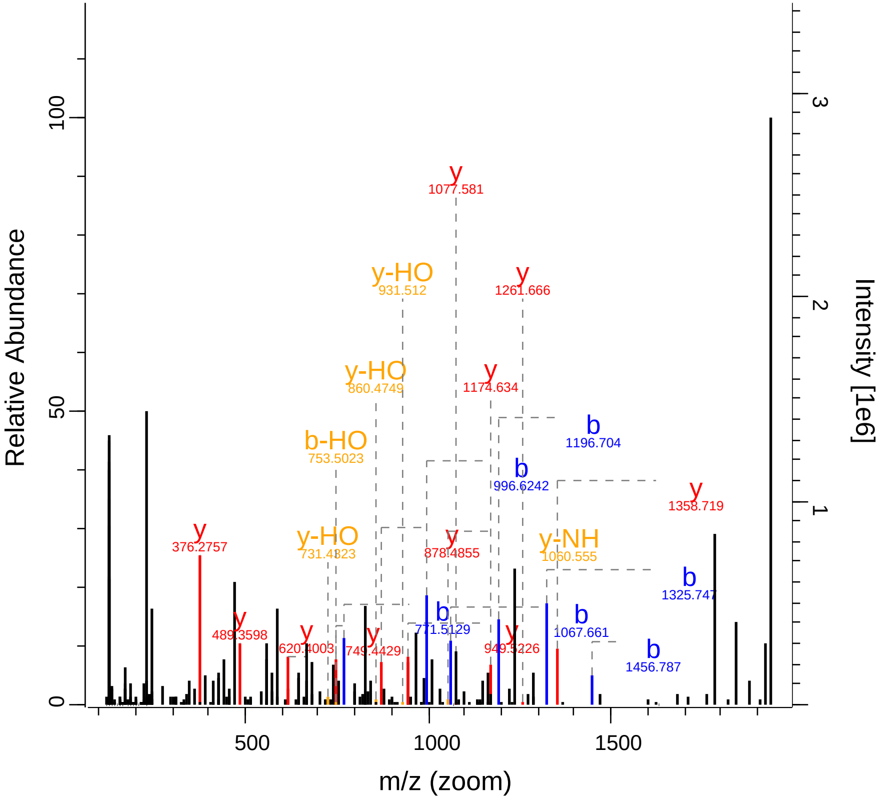

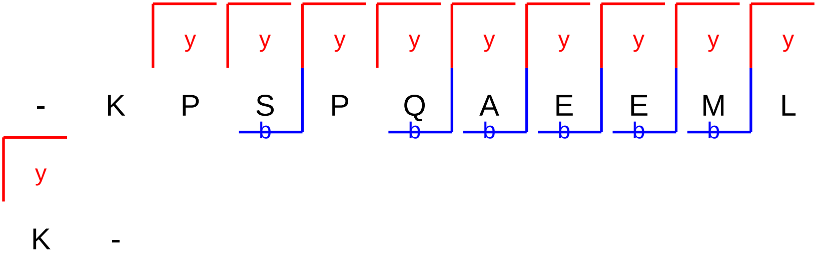

Raw File	Scan	Method	Score	m/z
RO-QC-160121_ZackD_SS_Set_02_Fraction_11	17252	FTMS; HCD	105.78	710.41

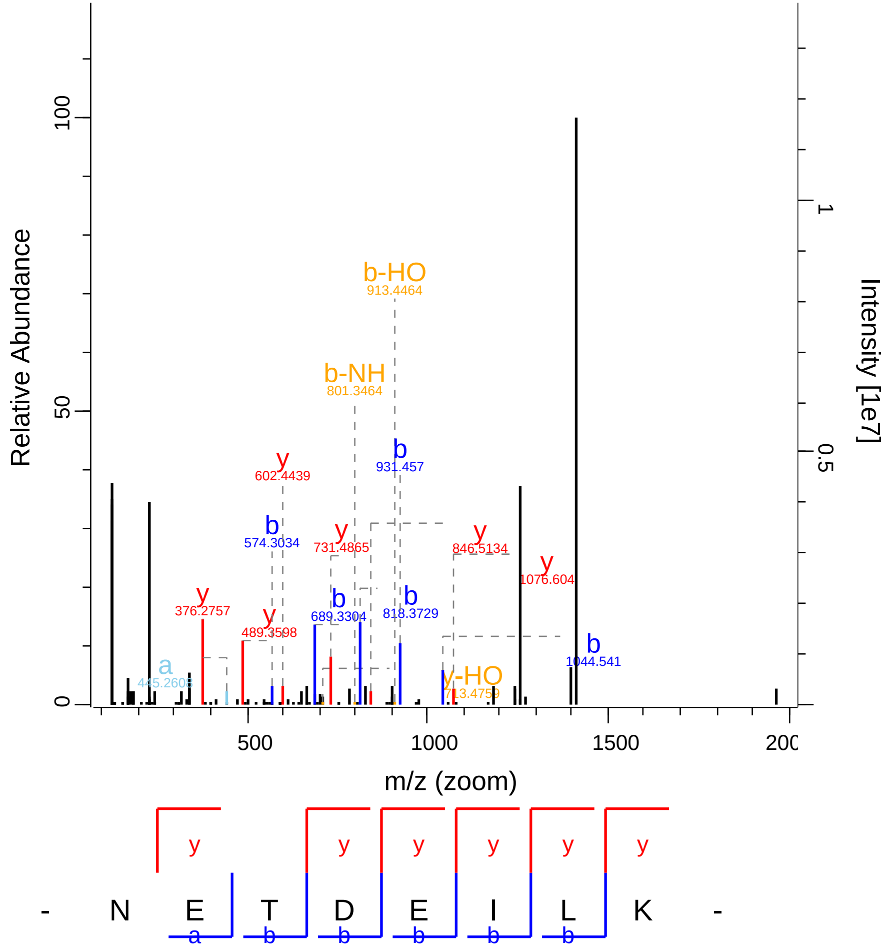

Raw File	Scan	Method	Score	m/z	Gene names
RO-QC-160121_ZackD_SS_Set_02_Fractior	17291	FTMS; HC	172.8	897.99	CCDC59

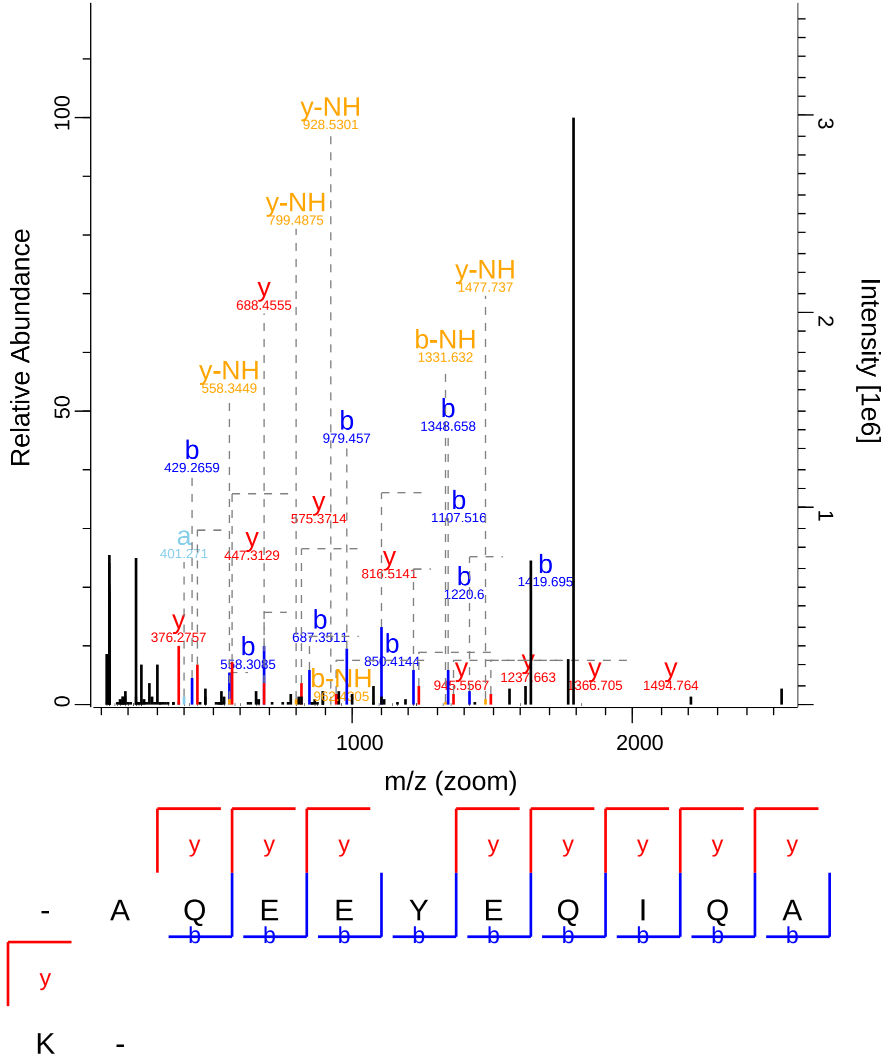

Raw File	Scan	Method	Score	m/z	Gene names
RO-QC-160121_ZackD_SS_Set_02_Fractior	18185	FTMS; HC	81.55	709.44	PCDH17

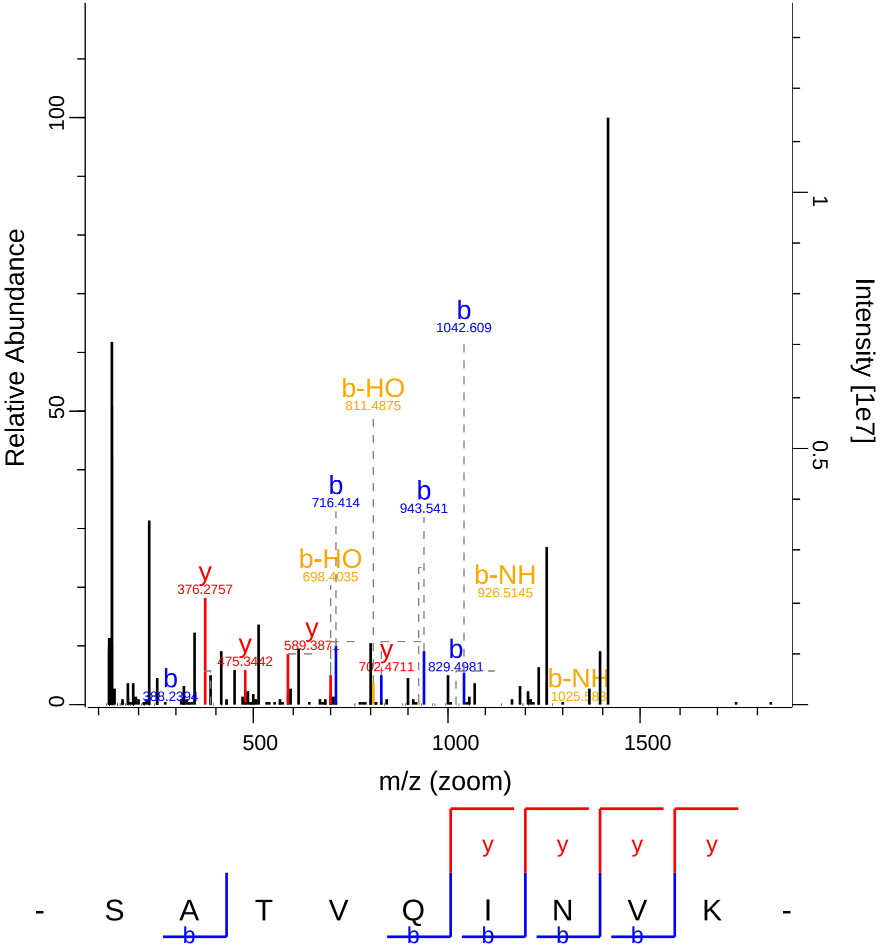

Raw File	Scan	Method	Score	m/z	Gene names
RO-QC-160121_ZackD_SS_Set_02_Fraction	18198	FTMS; HC	47.96	473.3	PCDH17

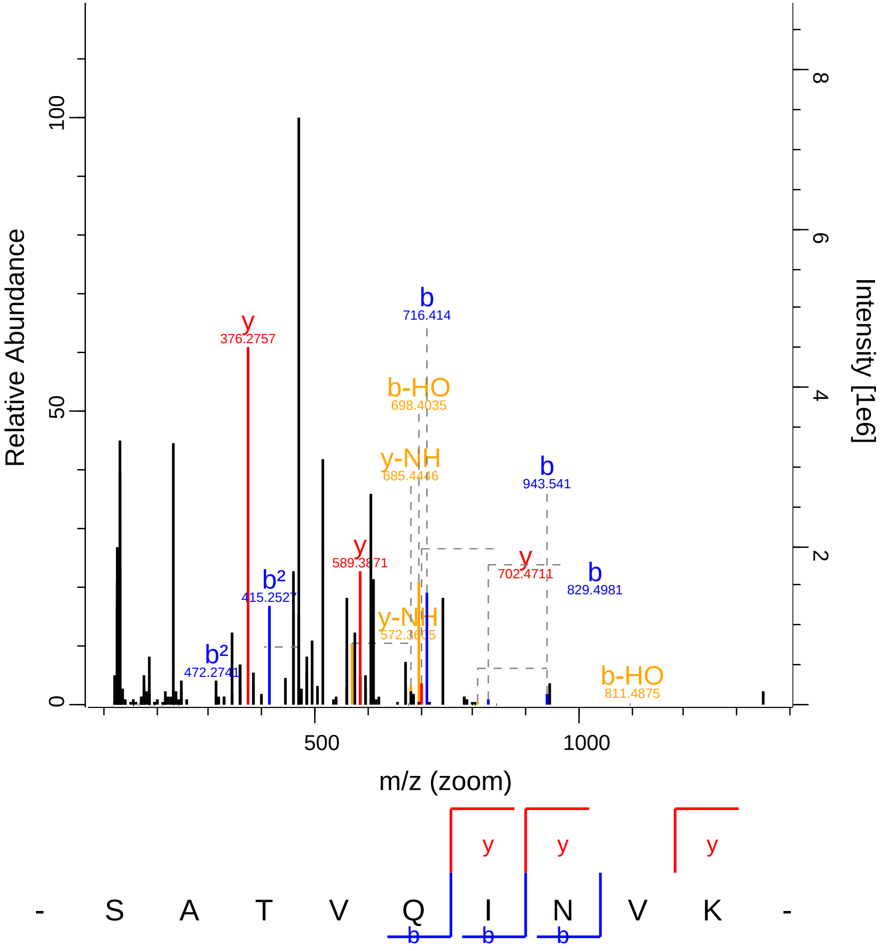

Raw File	Scan	Method	Score	m/z
RO-QC-160121_ZackD_SS_Set_02_Fraction_11	18511	FTMS; HCD	62.84	633.85

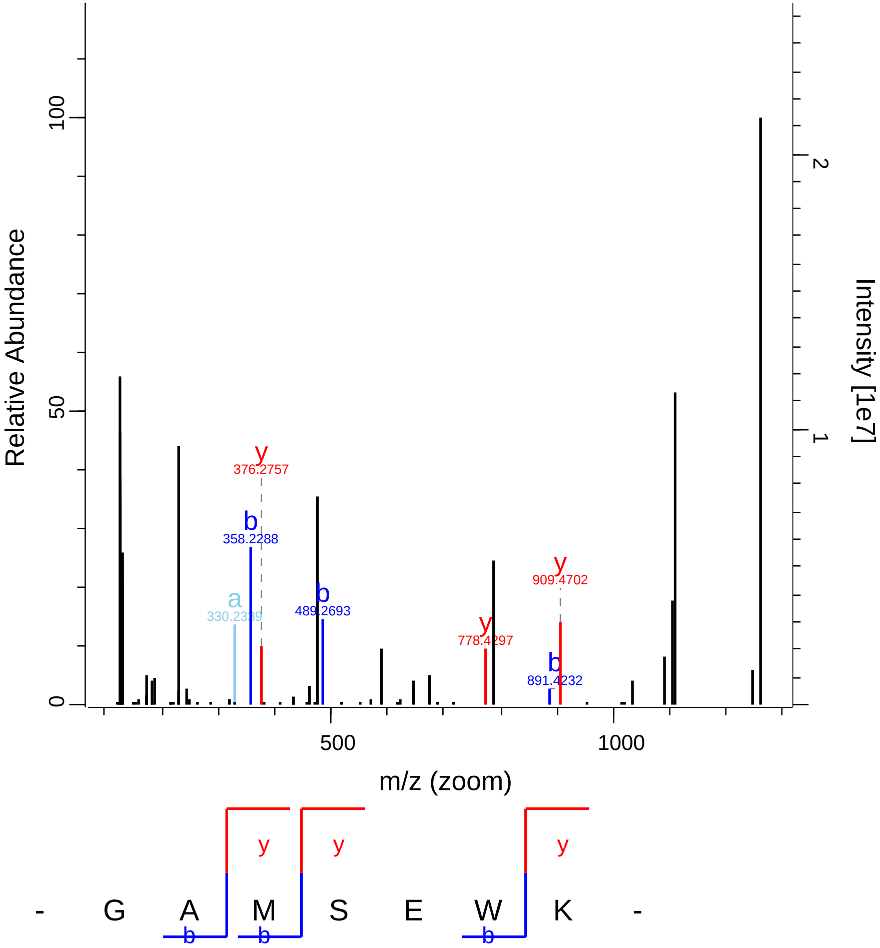

Raw File	Scan	Method	Score	m/z
RO-QC-160121_ZackD_SS_Set_02_Fraction_11	18729	FTMS; HCD	62.84	633.85

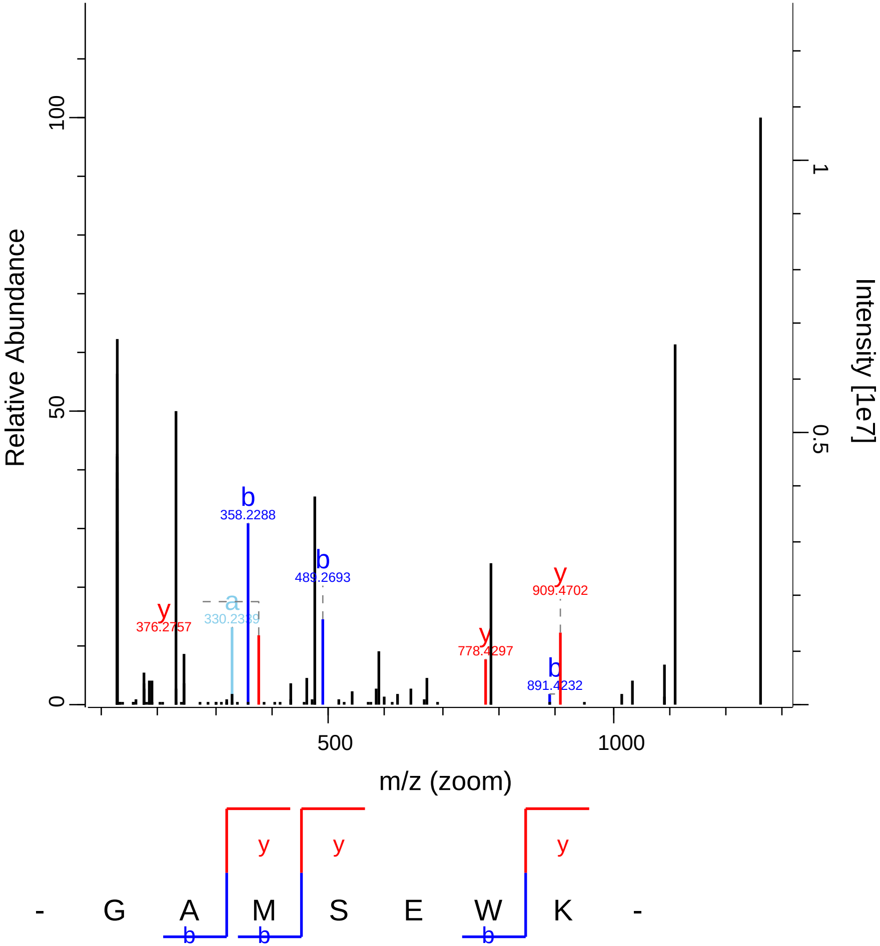

Raw File	Scan	Method	Score	m/z
RO-QC-160121_ZackD_SS_Set_02_Fraction_11	18914	FTMS; HCD	132.57	667.41

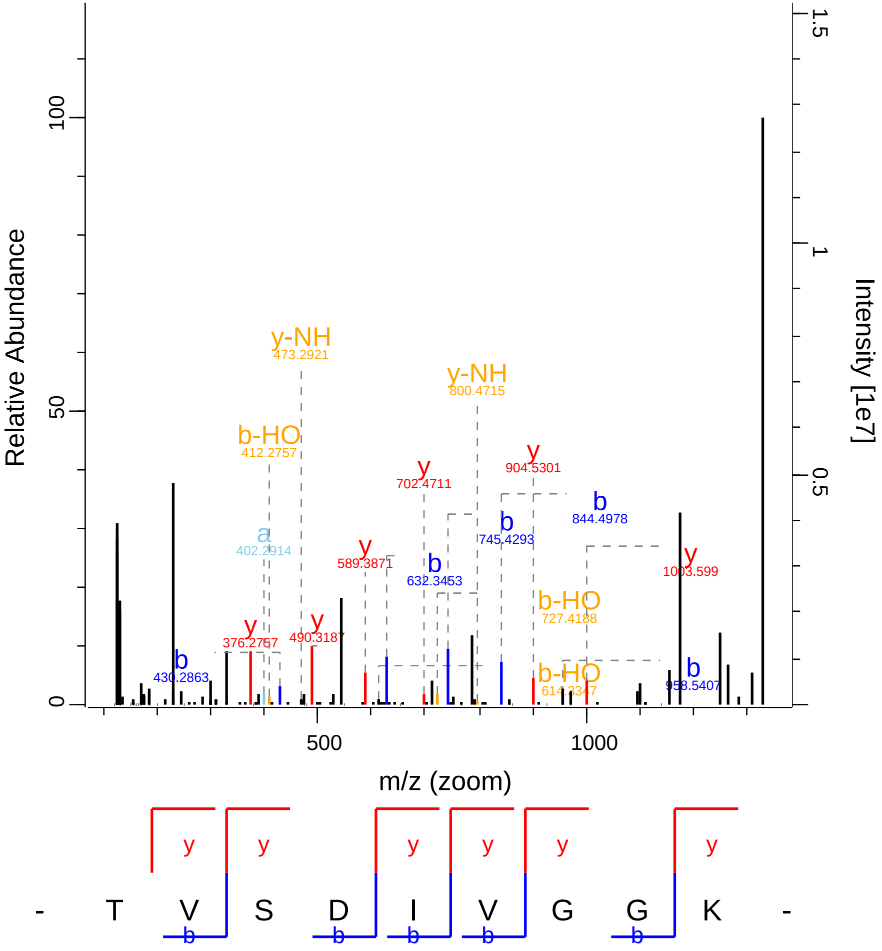

Raw File	Scan	Method	Score	m/z
RO-QC-160121_ZackD_SS_Set_02_Fraction_11	18934	FTMS; HCD	34.93	445.61

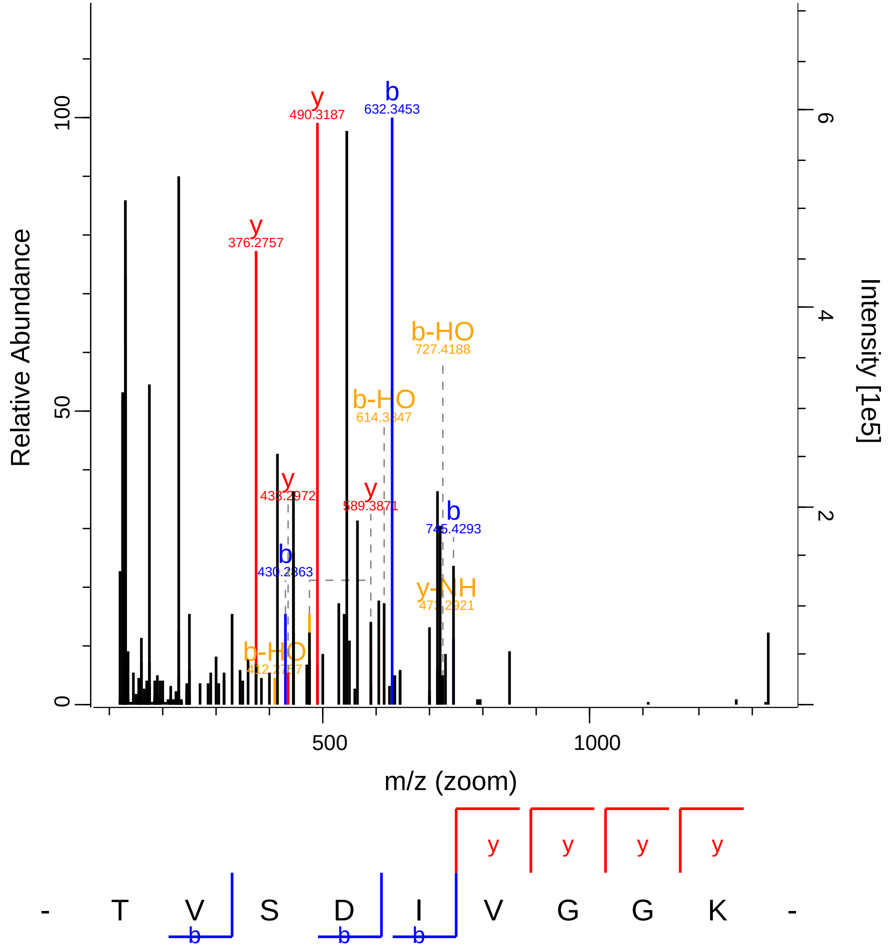

Raw File	Scan	Method	Score	m/z	Gene names
RO-QC-160121_ZackD_SS_Set_02_Fractio	19353	FTMS; HC	175.15	779.92	FKBP1B

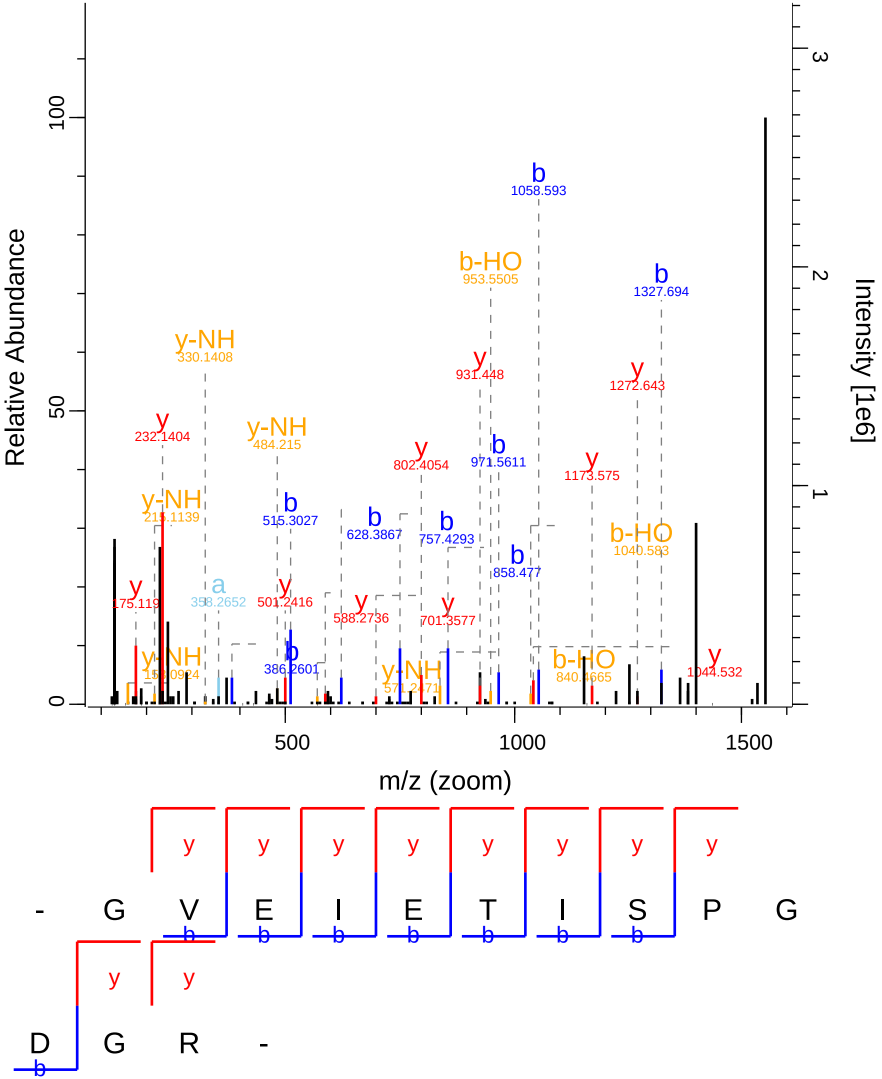

Raw File	Scan	Method	Score	m/z
RO-QC-160121_ZackD_SS_Set_02_Fraction_11	19576	FTMS; HCD	54.55	692.9

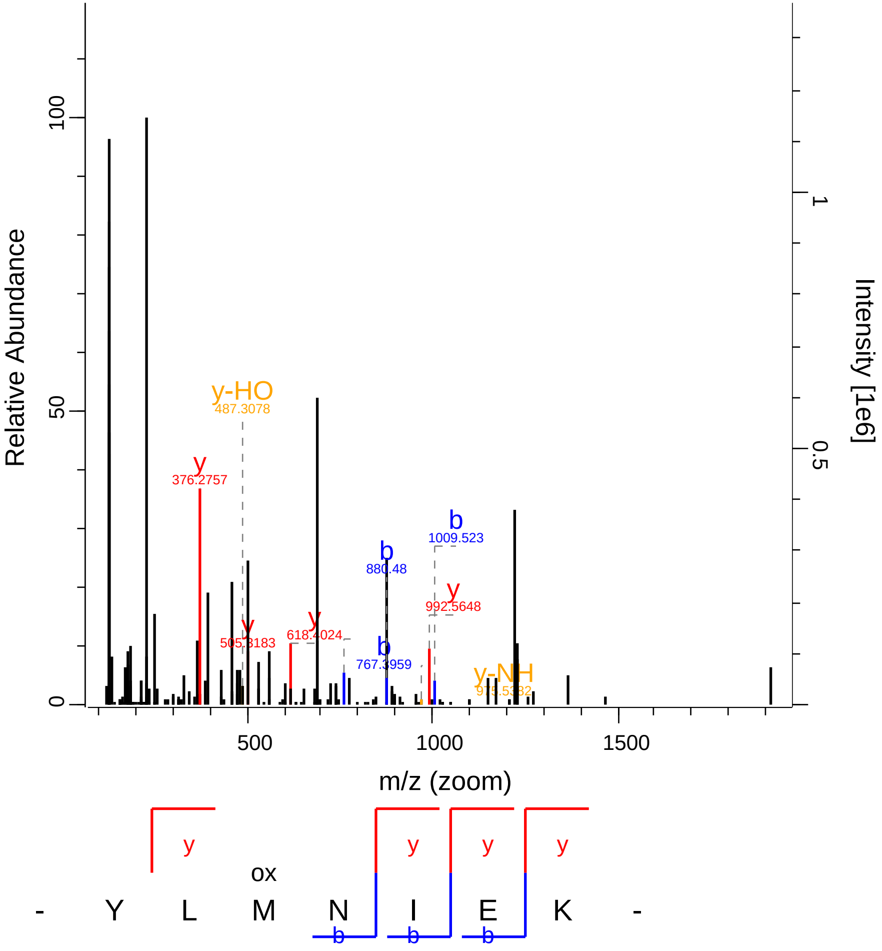

Raw File	Scan	Method	Score	m/z
RO-QC-160121_ZackD_SS_Set_02_Fraction_11	19817	FTMS; HCD	66.26	692.9

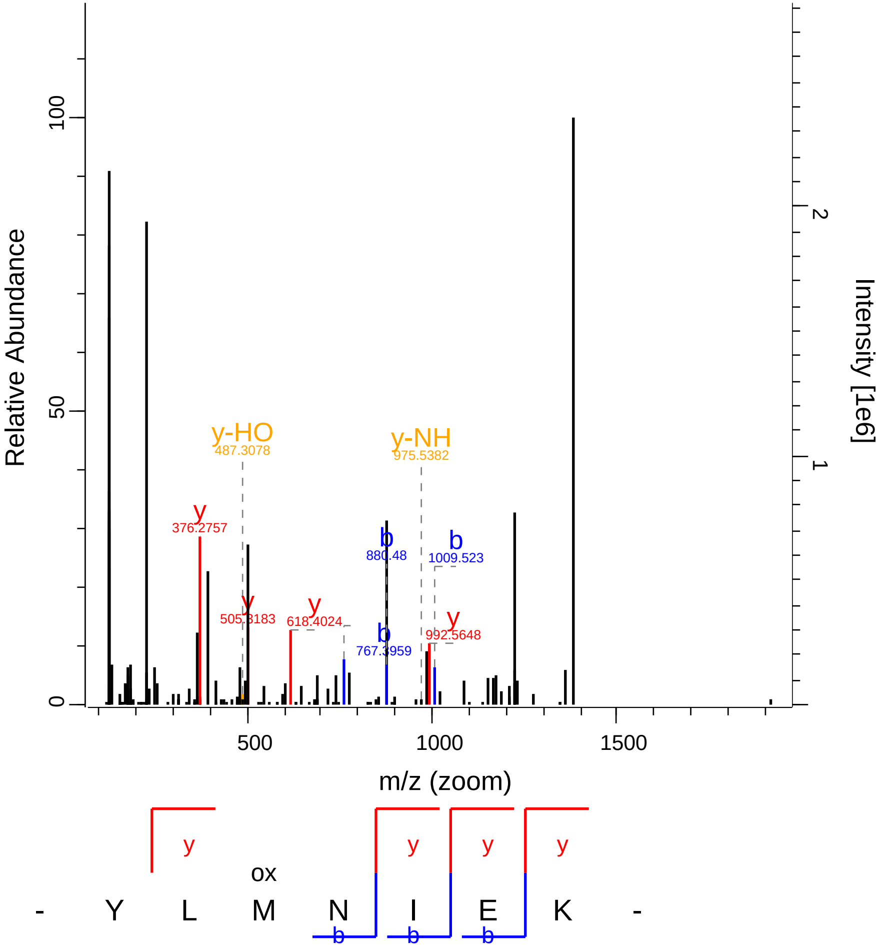

Raw File	Scan	Method	Score	m/z	Gene names
RO-QC-160121_ZackD_SS_Set_02_Fractior	20486	FTMS; HC	142.83	509.61	CPA6

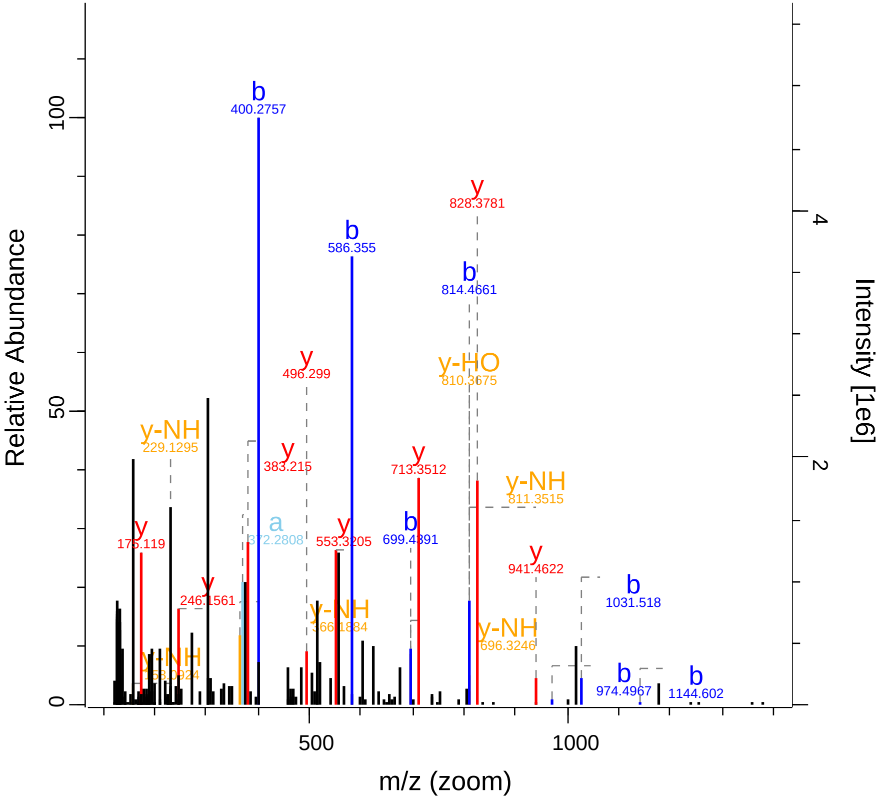

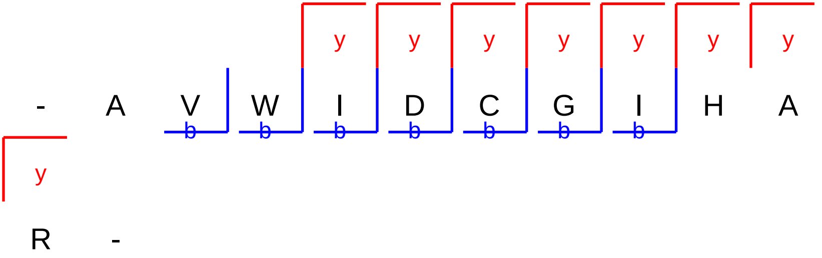

Raw File	Scan	Method	Score	m/z	Gene names
RO-QC-160121_ZackD_SS_Set_02_Fractio[illegible]	21908	FTMS; HC	158.79	855.45	RCBTB1

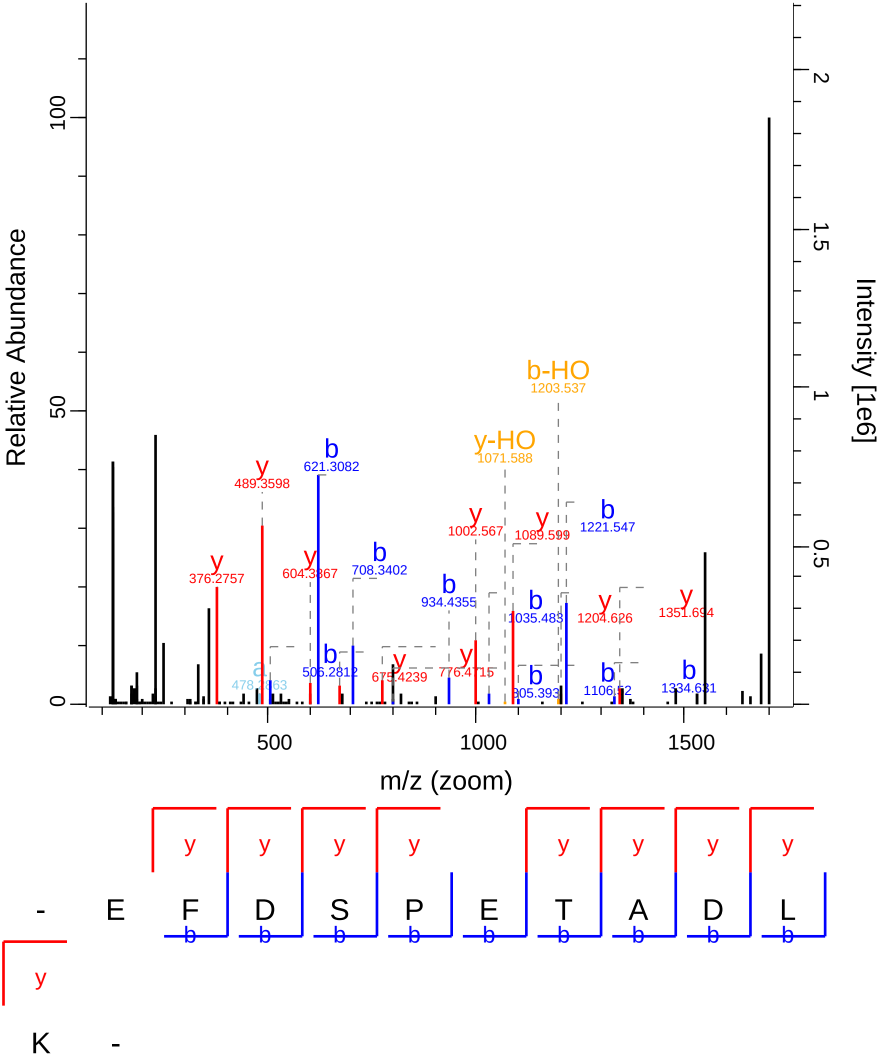

Raw File	Scan	Method	Score	m/z	Gene names
RO-QC-160121_ZackD_SS_Set_02_Fraction_	22245	FTMS; HC	40.06	522.3	SAMD8

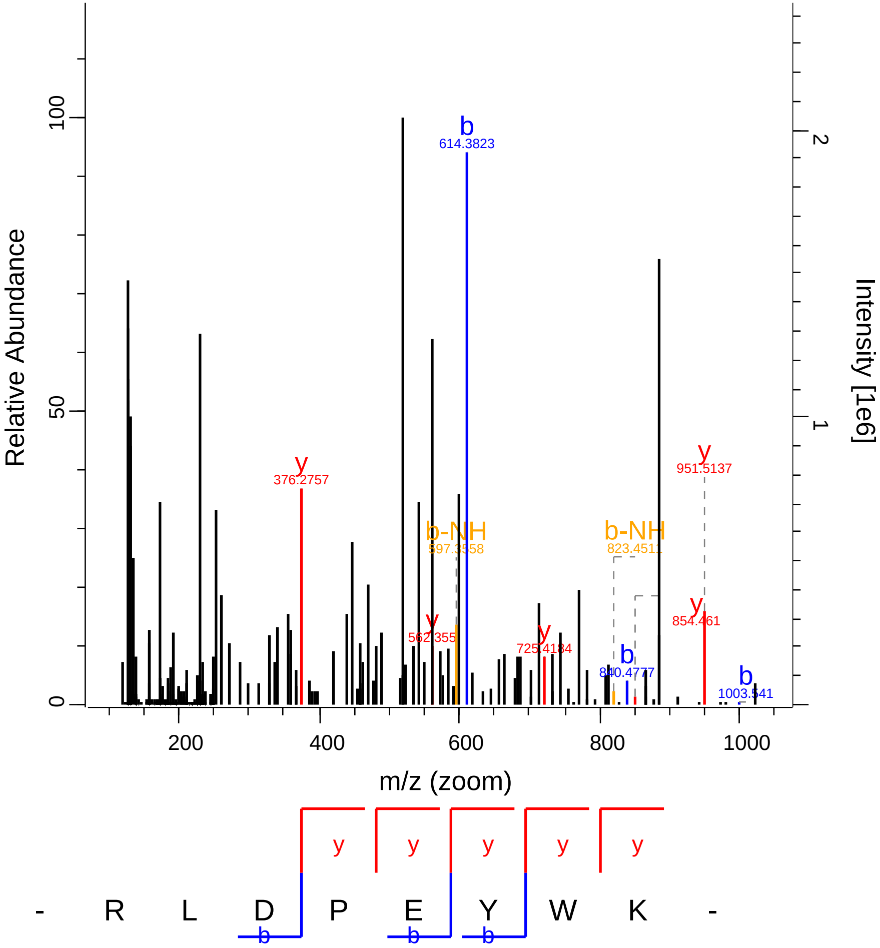

Raw File	Scan	Method	Score	m/z	Gene names
RO-QC-160121_ZackD_SS_Set_02_Fractior	22371	FTMS; HC	108.57	779.13	GPR176

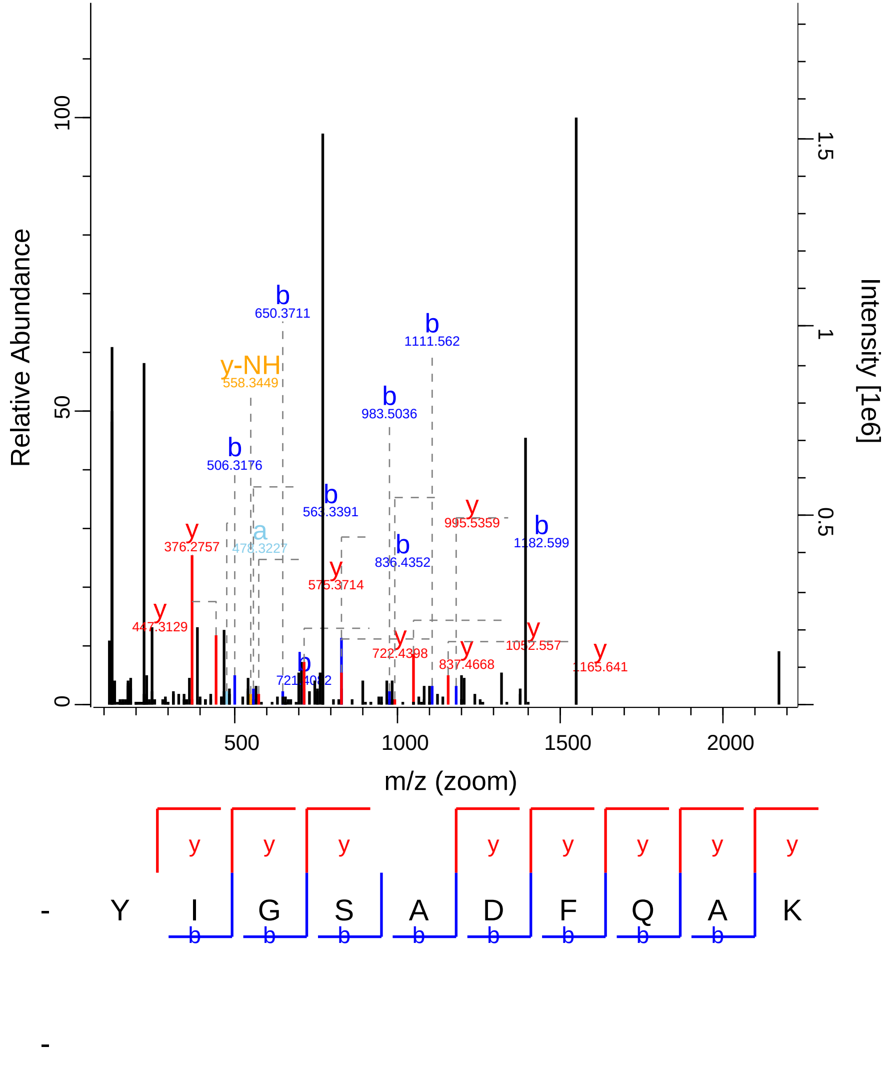

Raw File	Scan	Method	Score	m/z	Gene names
RO-QC-160121_ZackD_SS_Set_02_Fractior	22755	FTMS; HC	85.96	513.65	LNX2

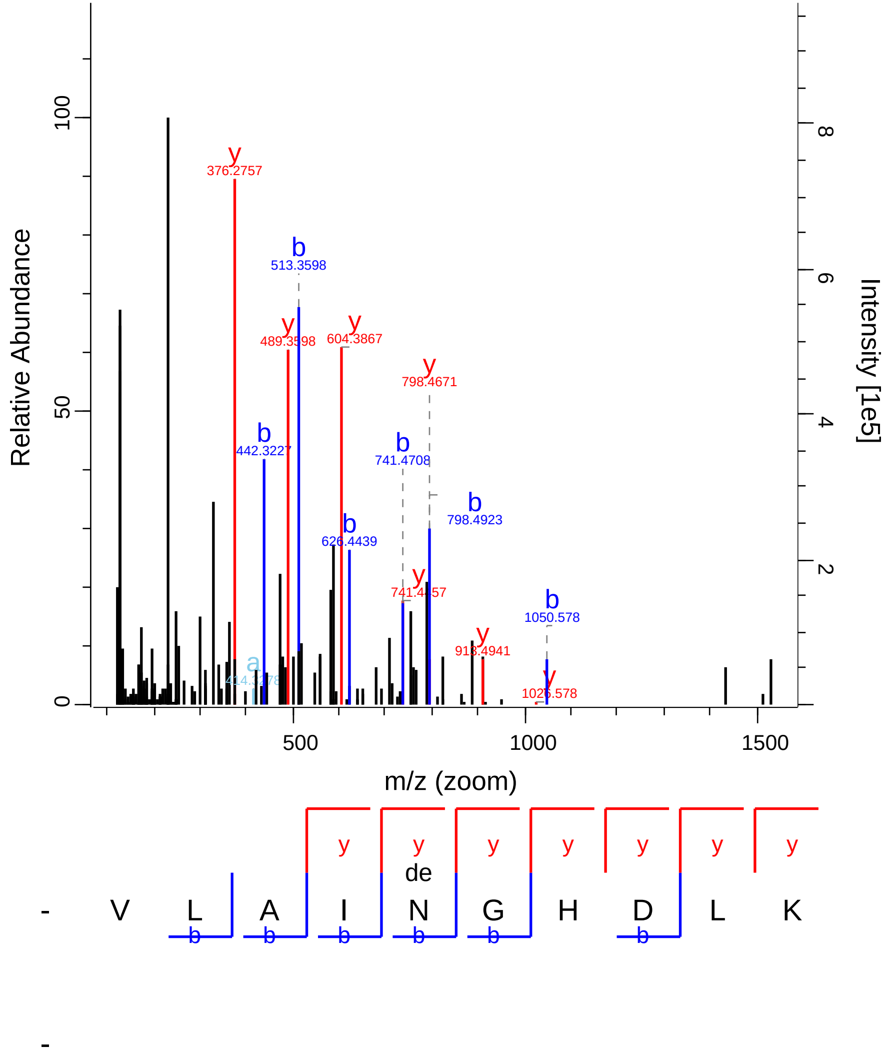

Raw File	Scan	Method	Score	m/z
RO-QC-160121_ZackD_SS_Set_02_Fraction_11	23128	FTMS; HCD	94.41	431.26

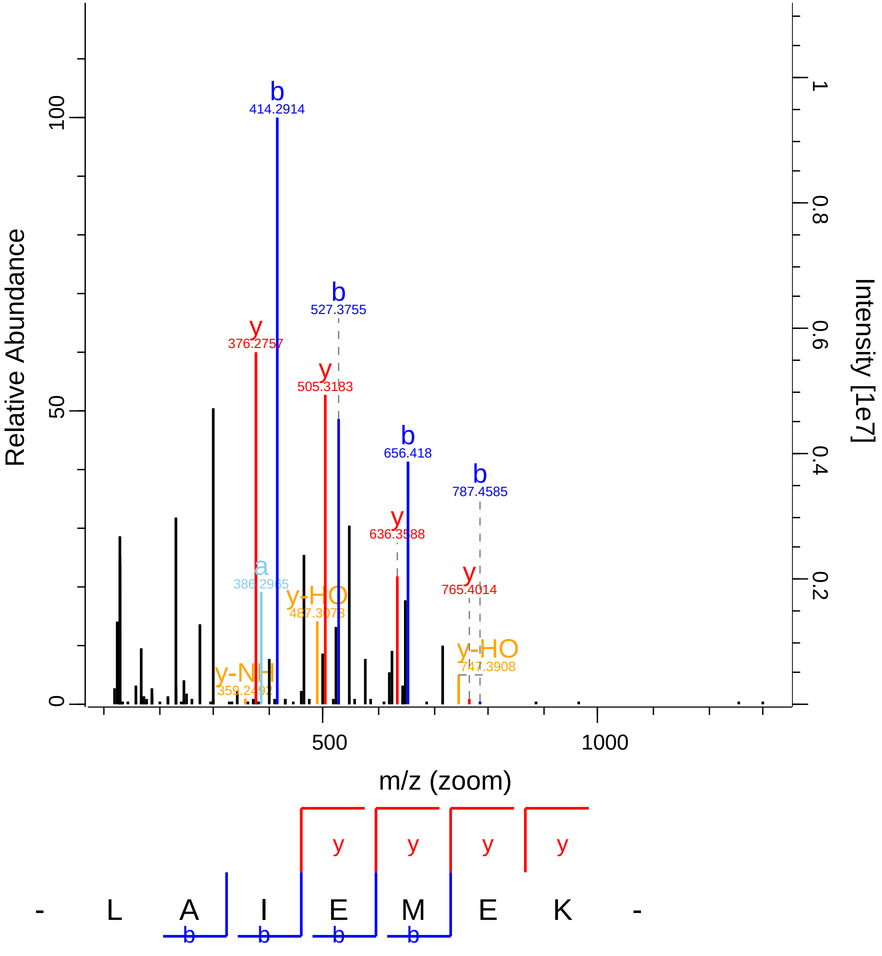

Raw File	Scan	Method	Score	m/z	Gene names
RO-QC-160121_ZackD_SS_Set_02_Fractior	23373	FTMS; HC	63.83	689.11	BIRC5

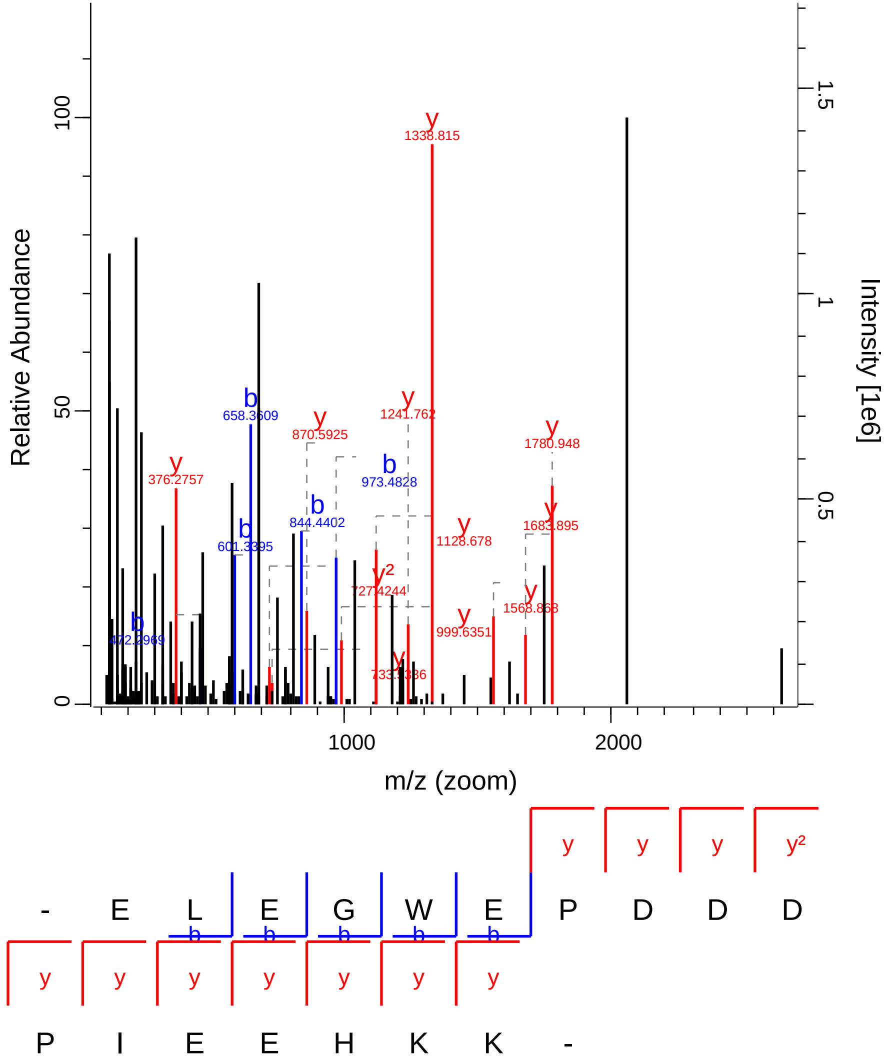

Raw File	Scan	Method	Score	m/z	Gene names
RO-QC-160121_ZackD_SS_Set_02_Fractior	24347	FTMS; HC	87.43	794.43	ATP6V0C

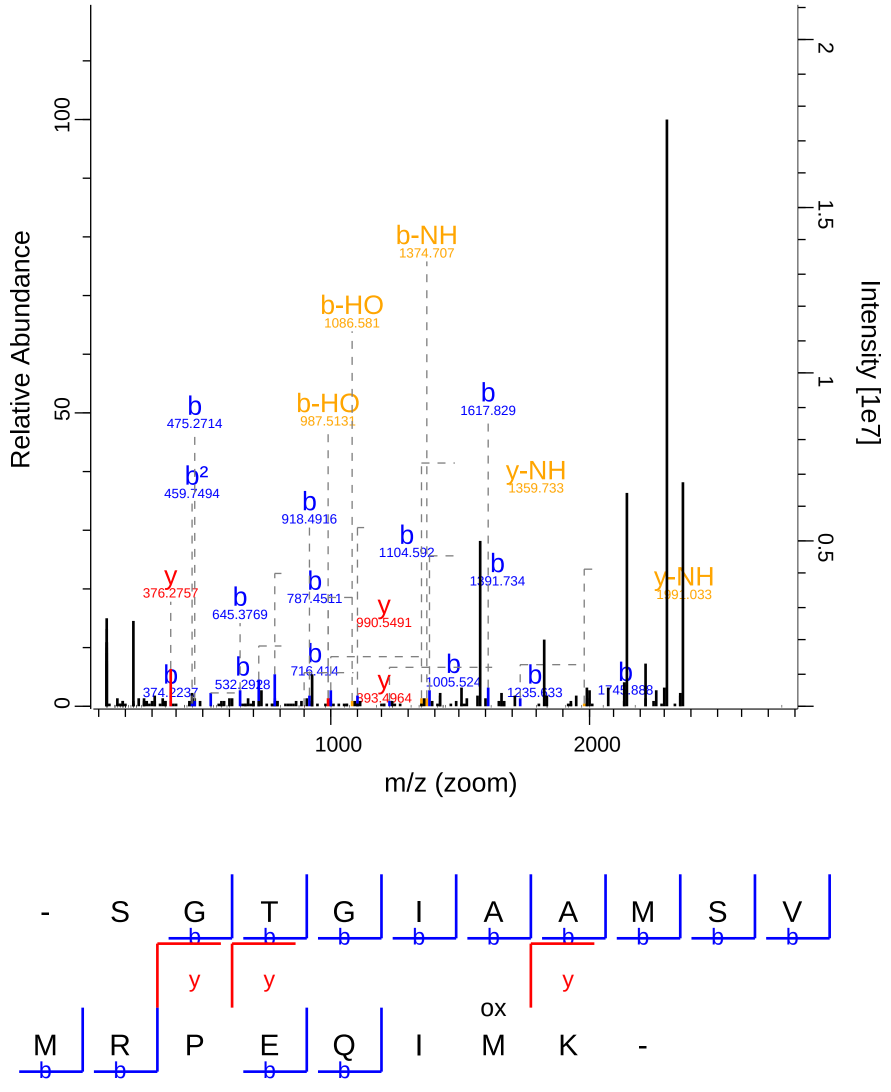

Raw File	Scan	Method	Score	m/z	Gene names
RO-QC-160121_ZackD_SS_Set_02_Fractio	24458	FTMS; HC	147.73	1191.14	ATP6V0C

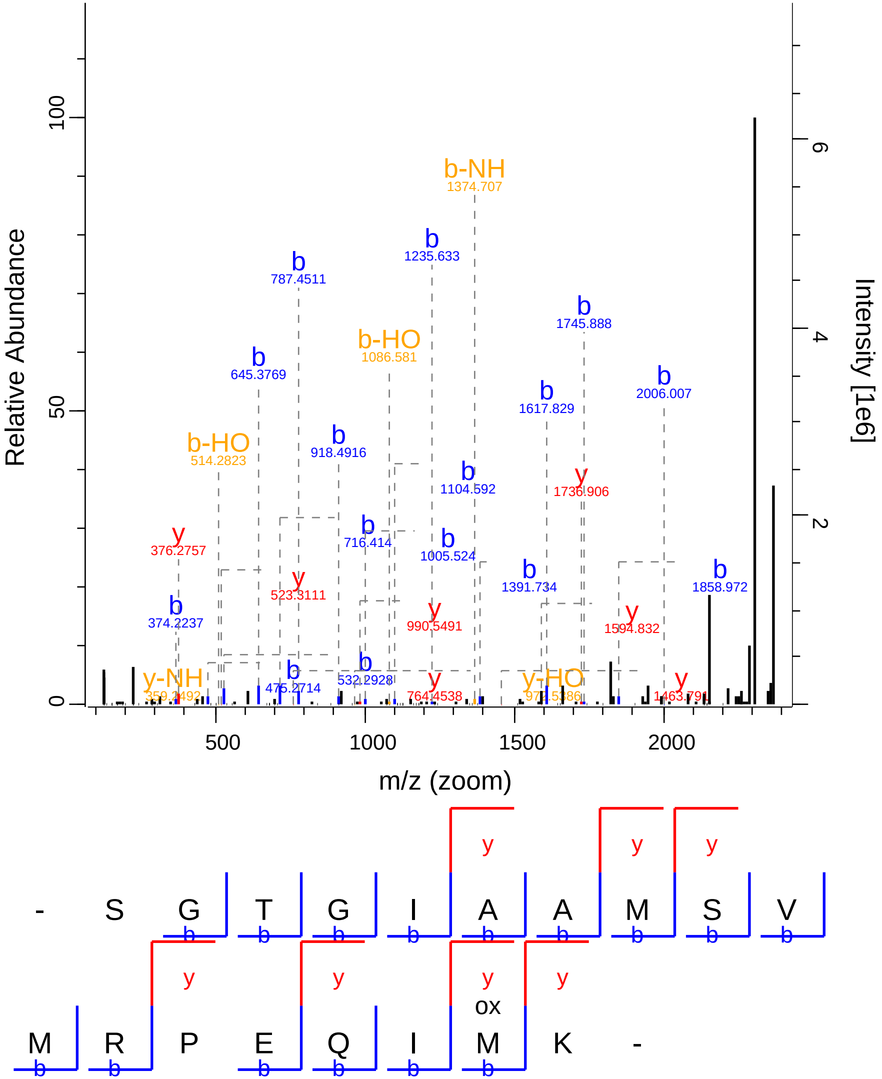

Raw File	Scan	Method	Score	m/z	Gene names
RO-QC-160121_ZackD_SS_Set_02_Fractior	24550	FTMS; HC	79.8	794.76	ATP6V0C

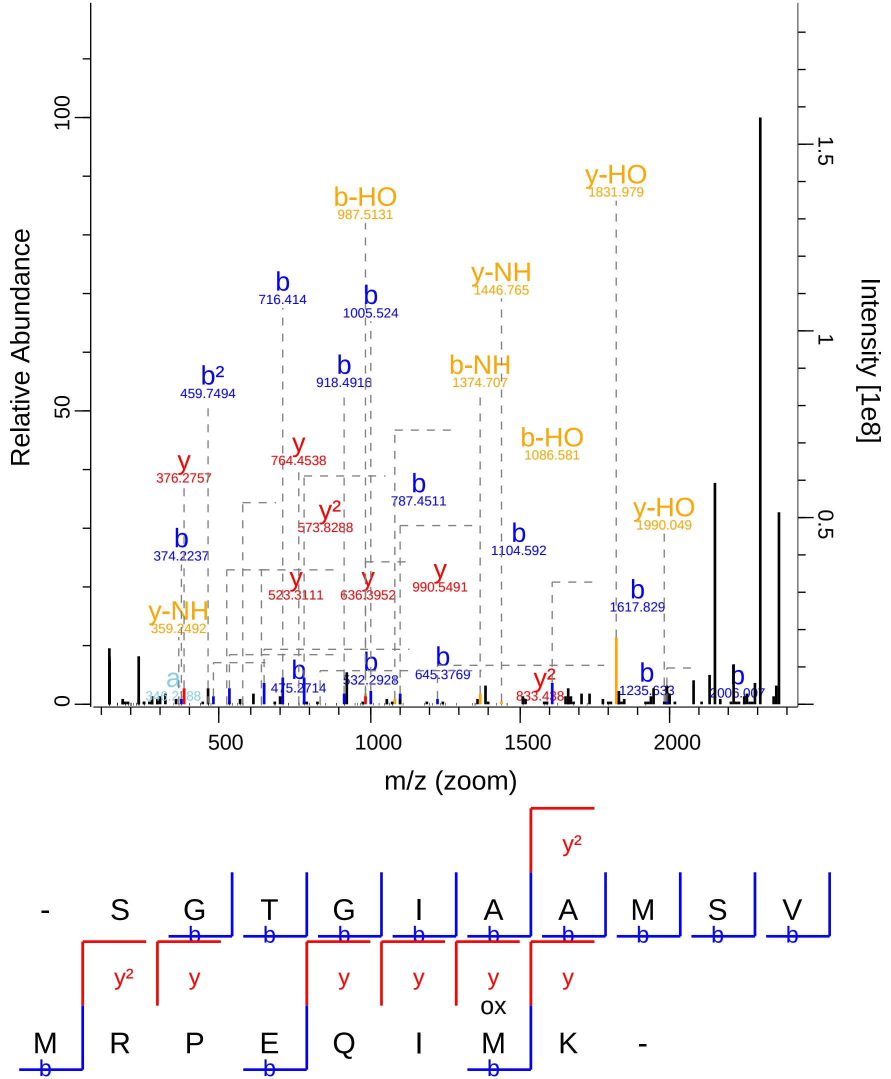

Raw File	Scan	Method	Score	m/z	Gene names
RO-QC-160121_ZackD_SS_Set_02_Fractior	24612	FTMS; HC	87.07	884.42	TMBIM1

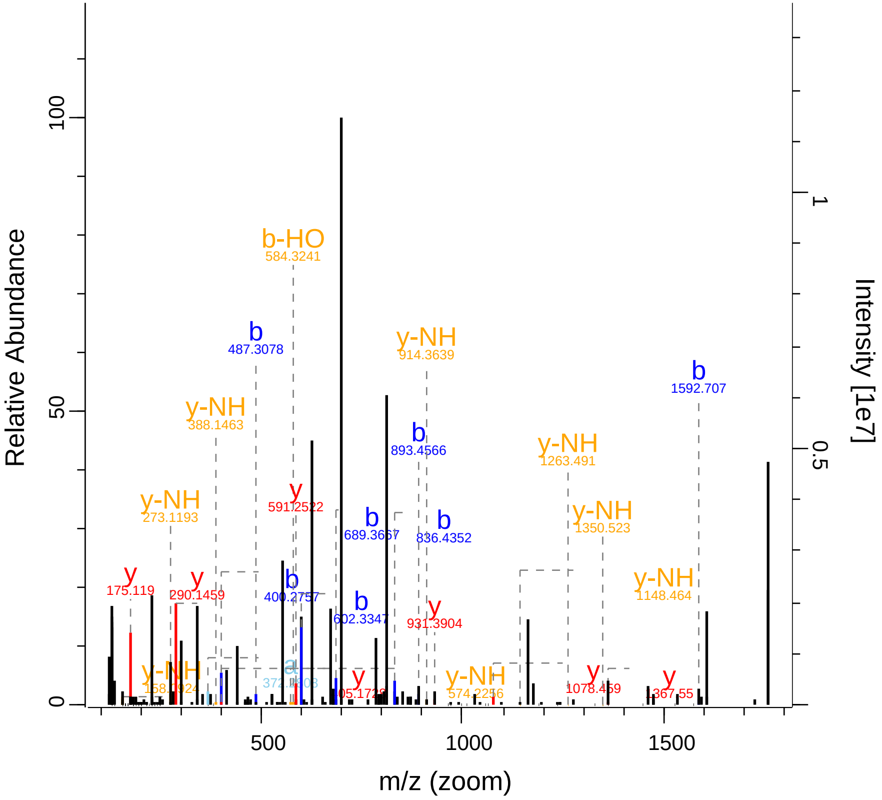

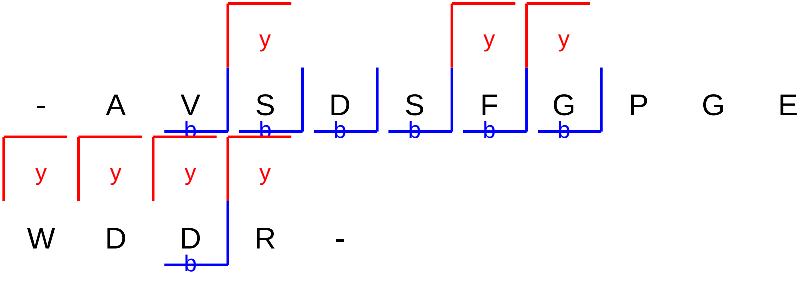

Raw File	Scan	Method	Score	m/z	Gene names
RO-QC-160121_ZackD_SS_Set_02_Fractior	25281	FTMS; HC	163.71	880.47	CACUL1

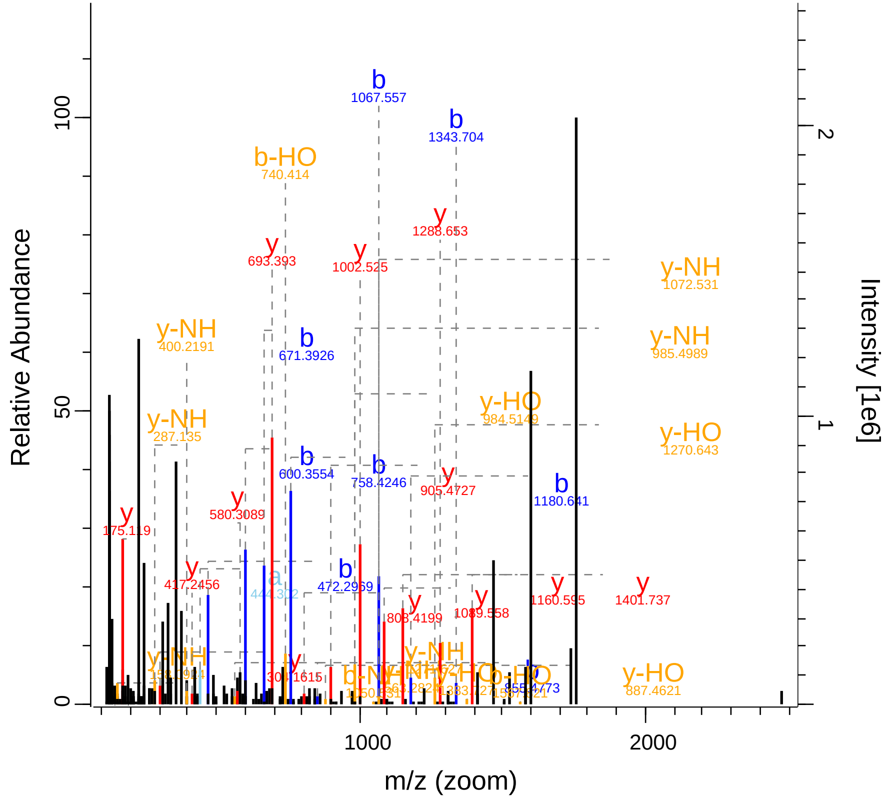

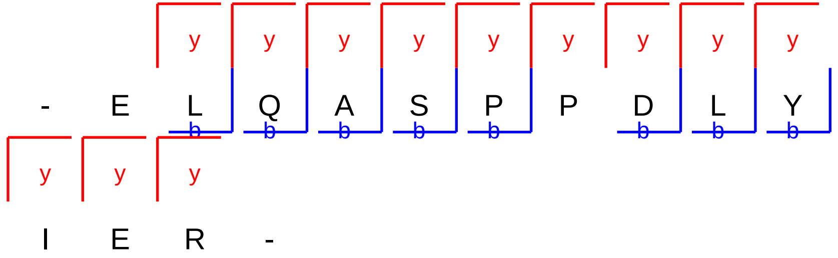

Raw File	Scan	Method	Score	m/z	Gene names
RO-QC-160121_ZackD_SS_Set_02_Fractior	25393	FTMS; HC	161.63	818.99	DGCR8

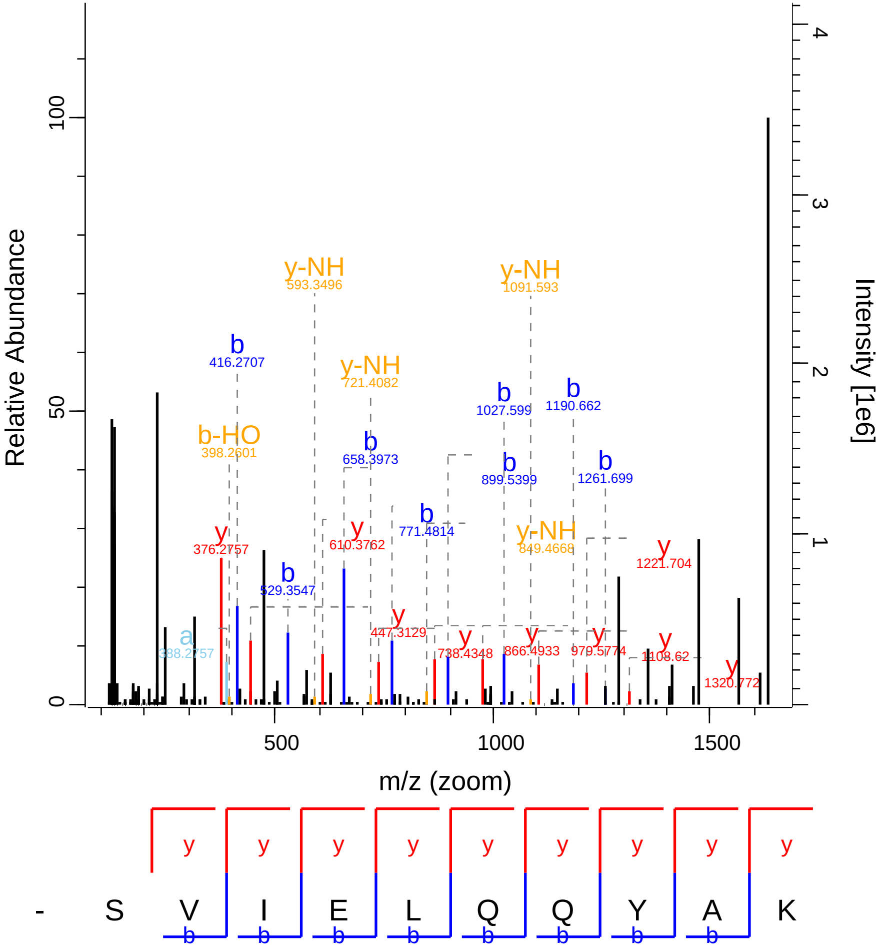

Raw File	Scan	Method	Score	m/z
RO-QC-160121_ZackD_SS_Set_02_Fraction_11	27246	FTMS; HCD	49.35	639.85

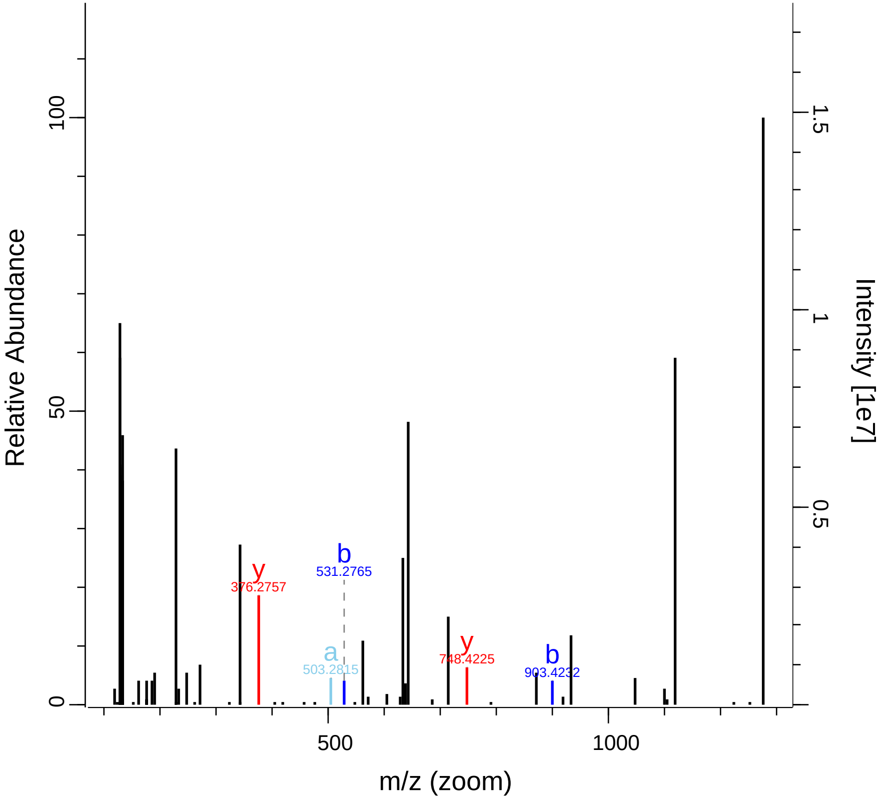

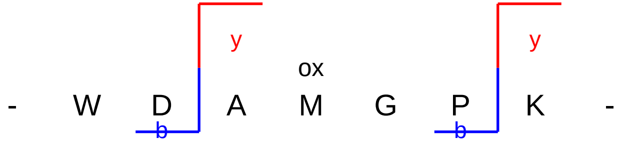

Raw File	Scan	Method	Score	m/z	Gene names
RO-QC-160121_ZackD_SS_Set_02_Fractior	28067	FTMS; HC	109.72	841.75	CNIH4

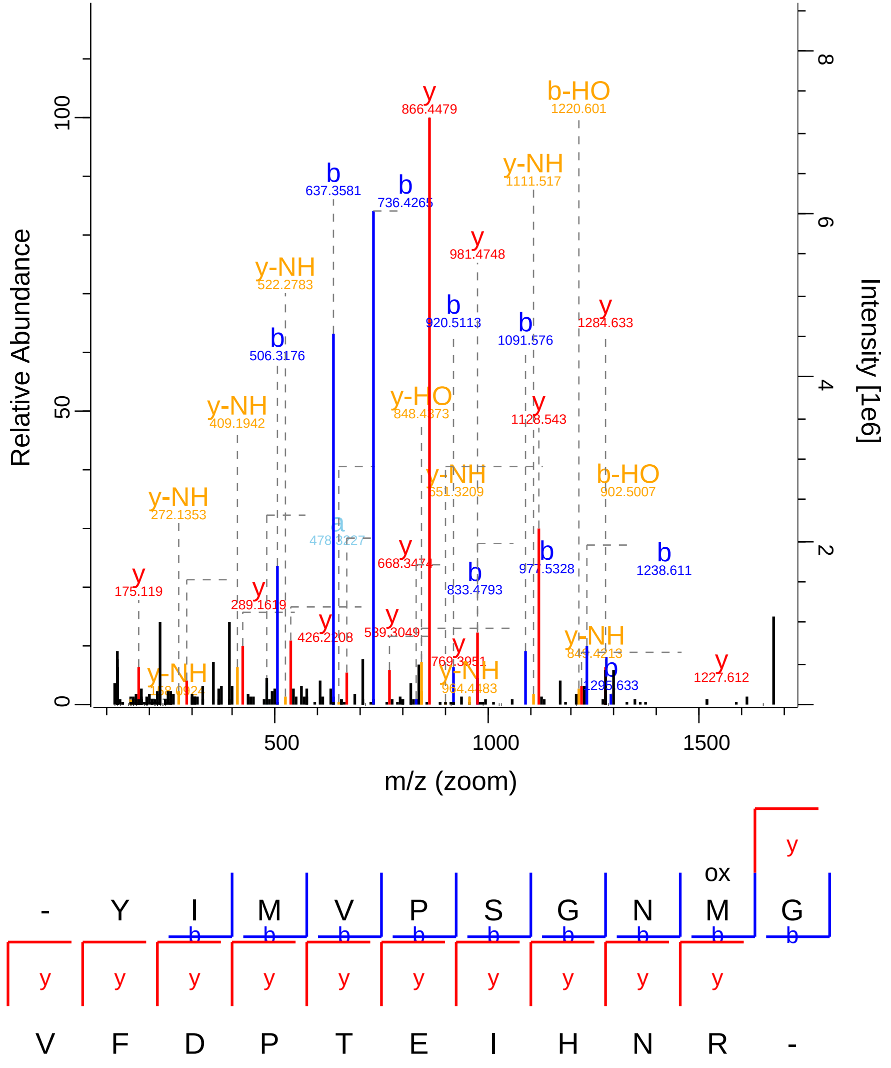

Raw File	Scan	Method	Score	m/z	Gene names
RO-QC-160121_ZackD_SS_Set_02_Fractior	28100	FTMS; HC	47.73	631.57	CNIH4

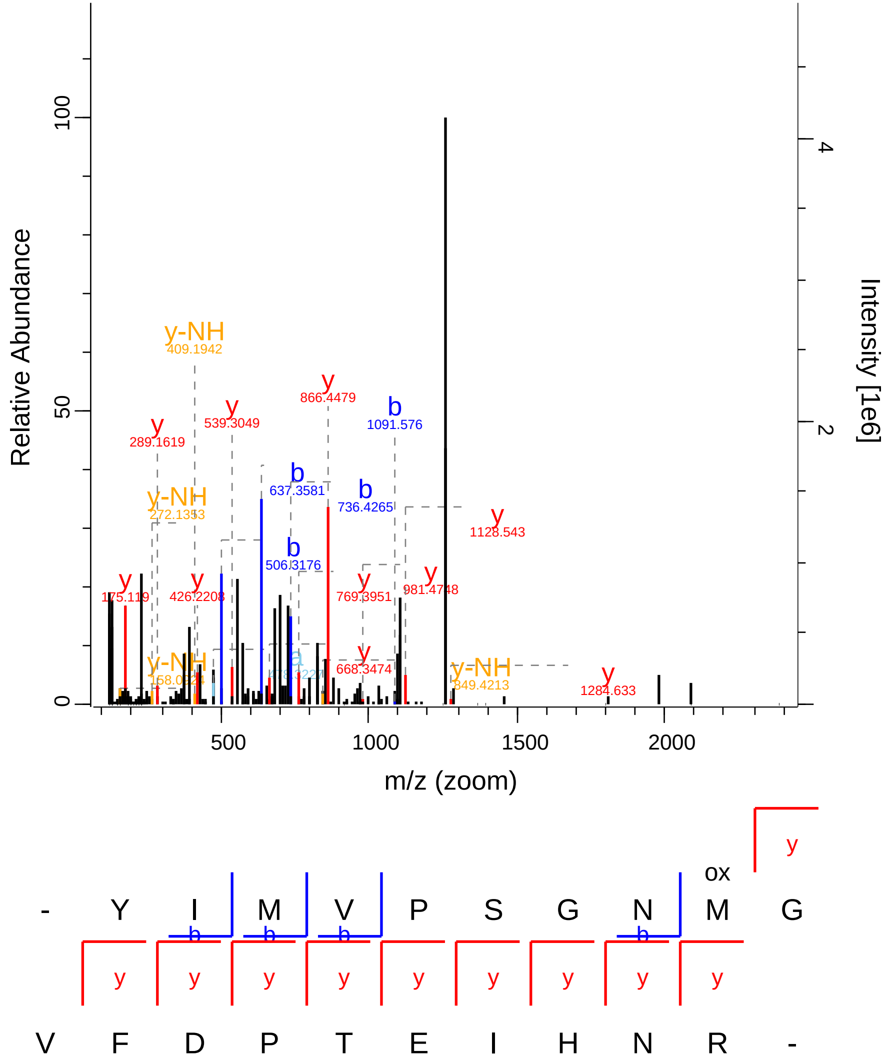

Raw File	Scan	Method	Score	m/z	Gene names
RO-QC-160121_ZackD_SS_Set_02_Fractior	28617	FTMS; HC	60.15	666.36	TRAF4

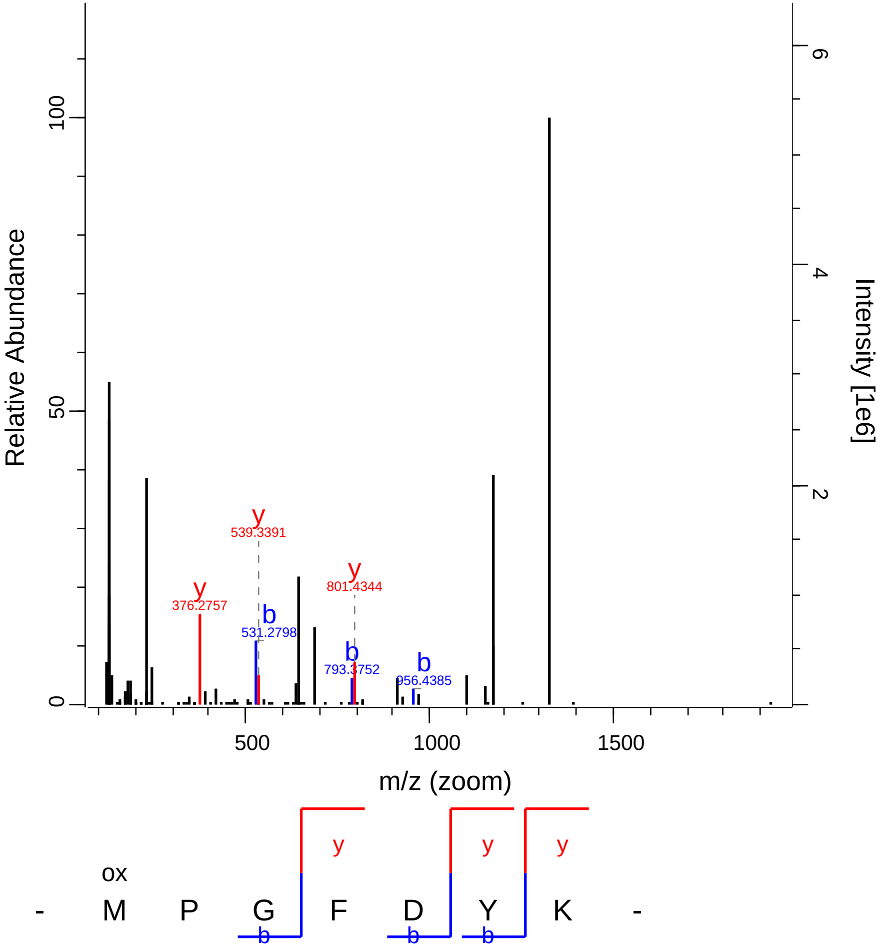

Raw File	Scan	Method	Score	m/z	Gene names
RO-QC-160121_ZackD_SS_Set_02_Fractior	29182	FTMS; HC	122.64	704.36	EMP3

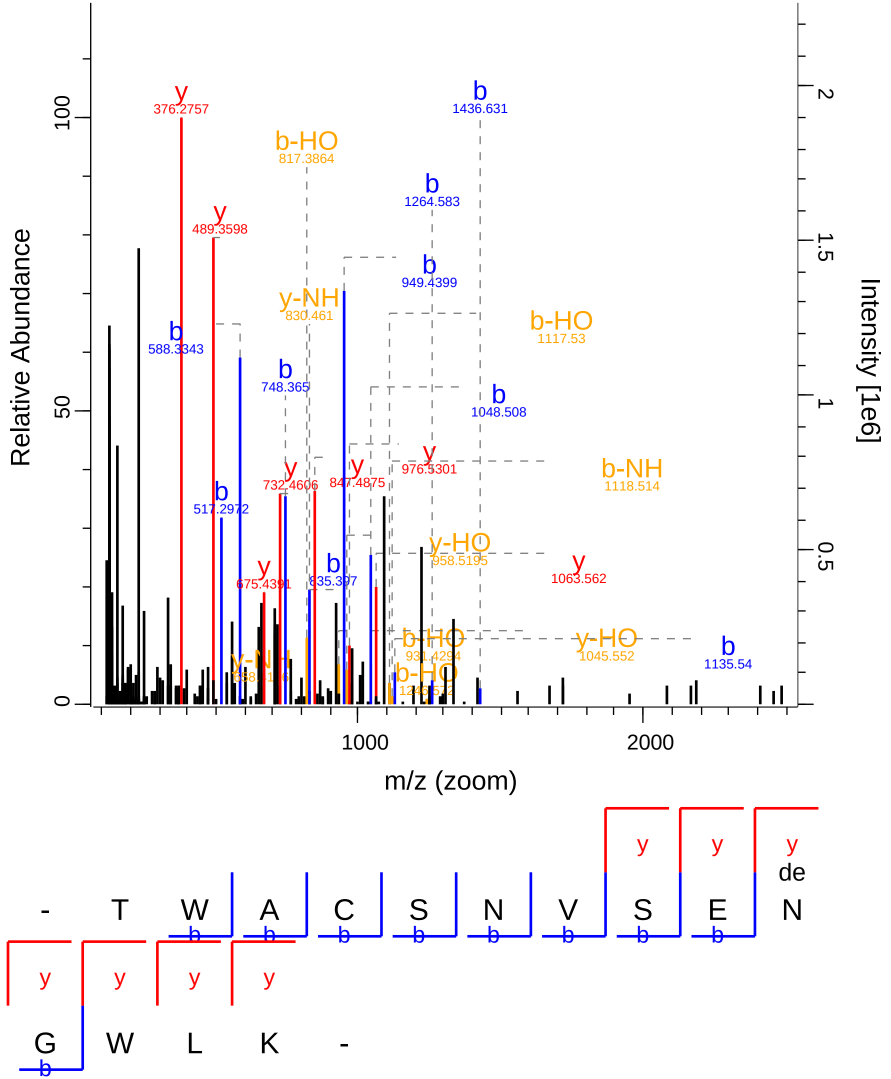

Raw File	Scan	Method	Score	m/z	Gene names
RO-QC-160121_ZackD_SS_Set_02_Fractio	29196	FTMS; HC	232.6	1056.04	EMP3

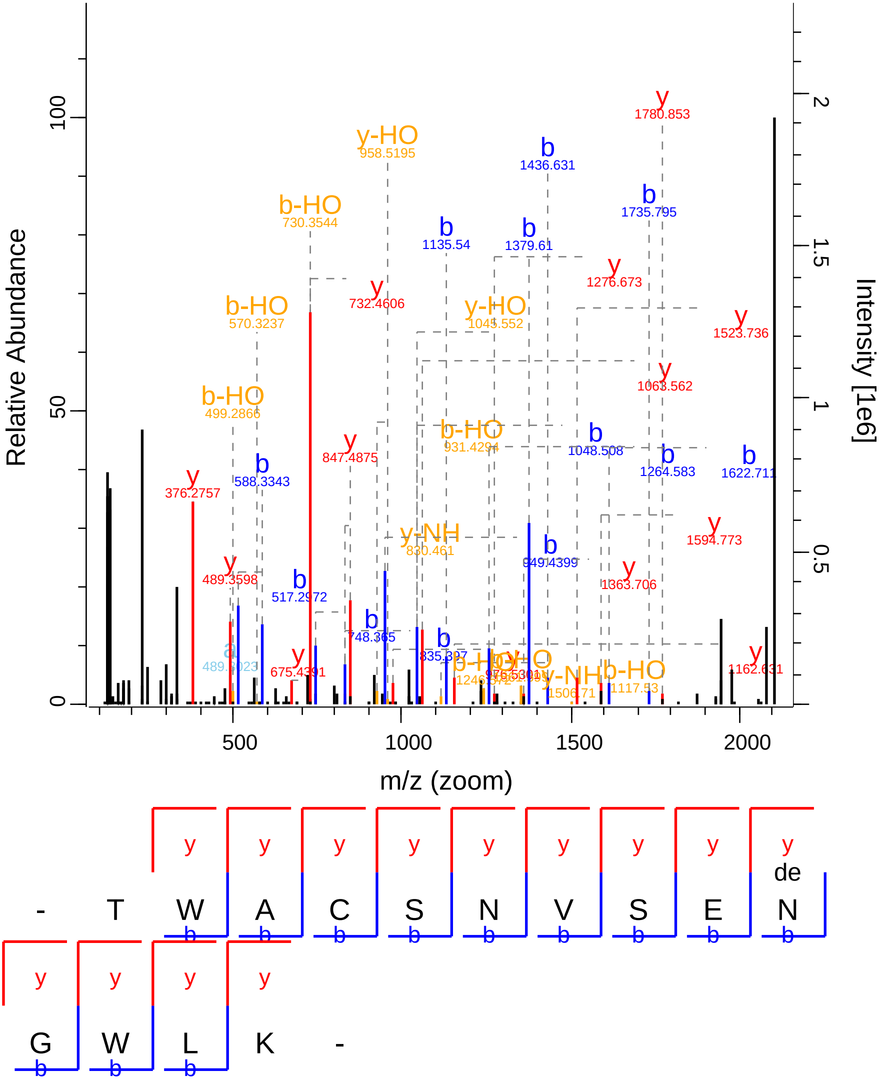

Raw File	Scan	Method	Score	m/z	Gene names
RO-QC-160121_ZackD_SS_Set_02_Fractior	29673	FTMS; HC	57.29	715.74	SEPHS2

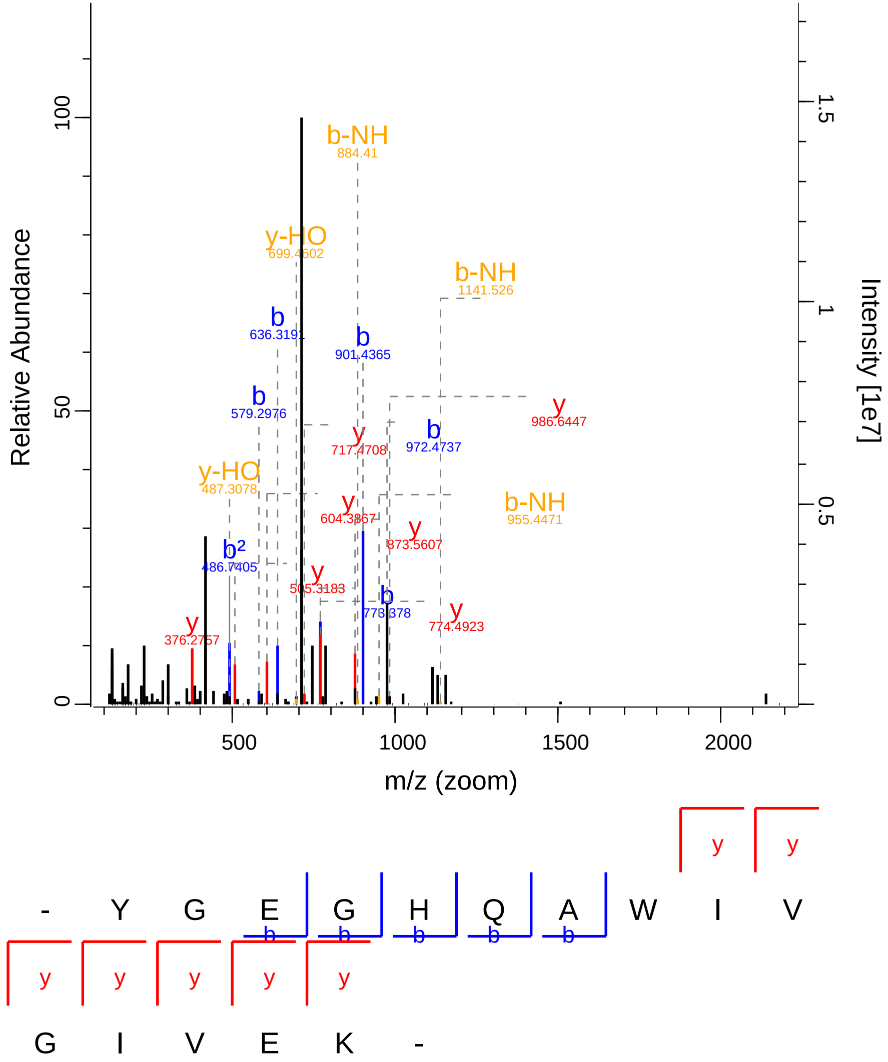

Raw File	Scan	Method	Score	m/z	Gene names
RO-QC-160121_ZackD_SS_Set_02_Fractior	29722	FTMS; HC	80.97	825.41	TFDP1

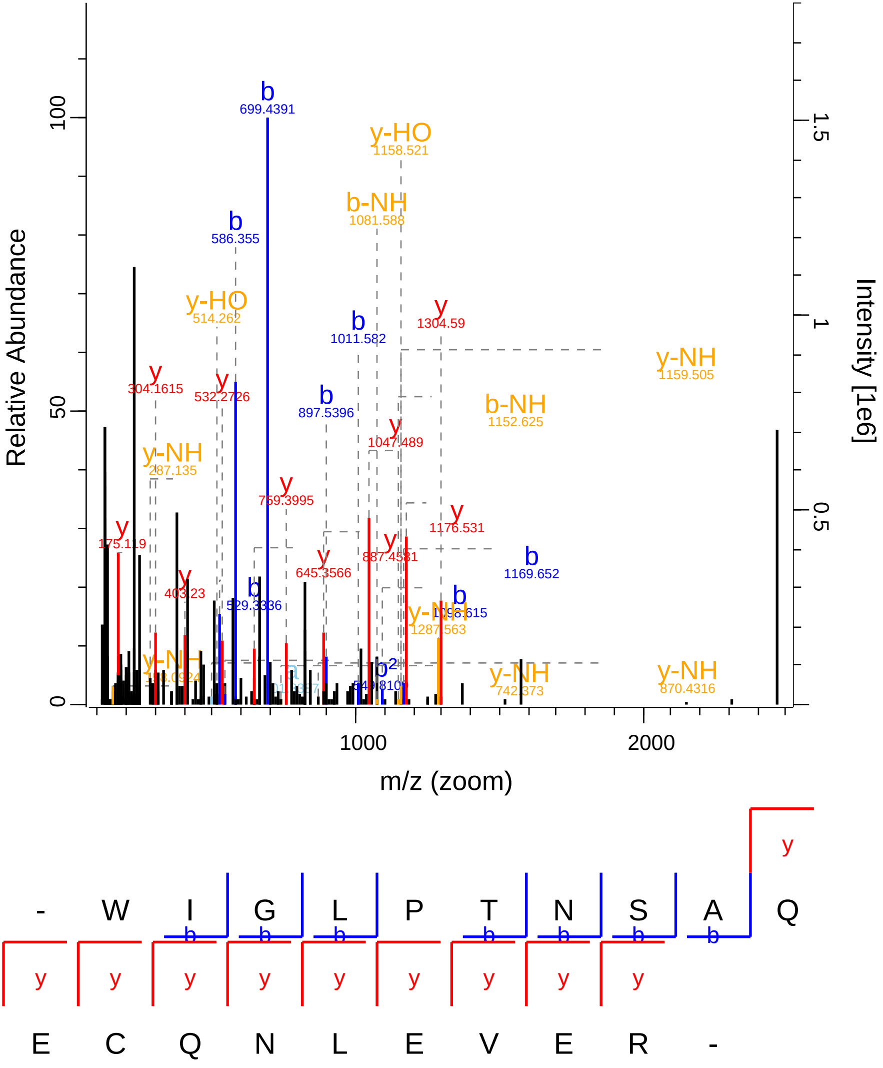

Raw File	Scan	Method	Score	m/z	Gene names
RO-QC-160121_ZackD_SS_Set_02_Fraction_	30660	FTMS; HC	149.96	815	ARL10

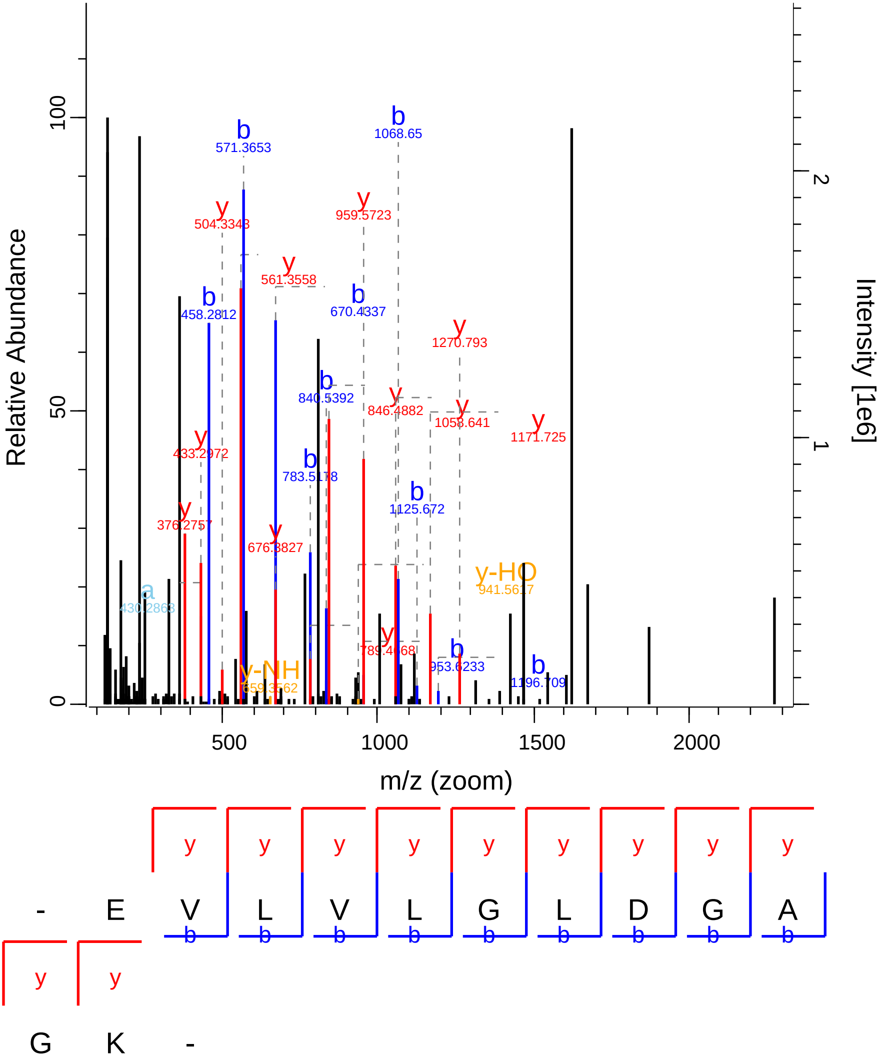

Raw File	Scan	Method	Score	m/z	Gene names
RO-QC-160121_ZackD_SS_Set_02_Fractior	30702	FTMS; HC	55.84	648.36	CLK3

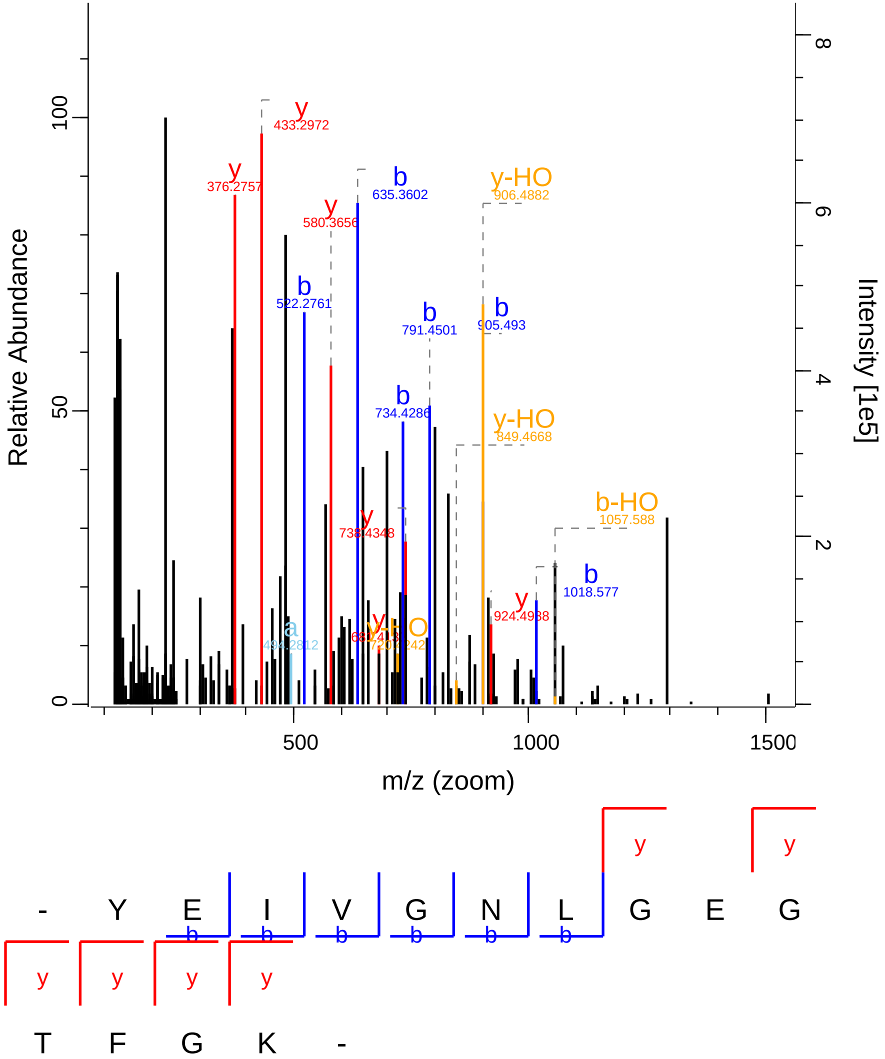

Raw File	Scan	Method	Score	m/z	Gene names
RO-QC-160121_ZackD_SS_Set_02_Fraction	31368	FTMS; HC	177.36	651.9	SHISA4

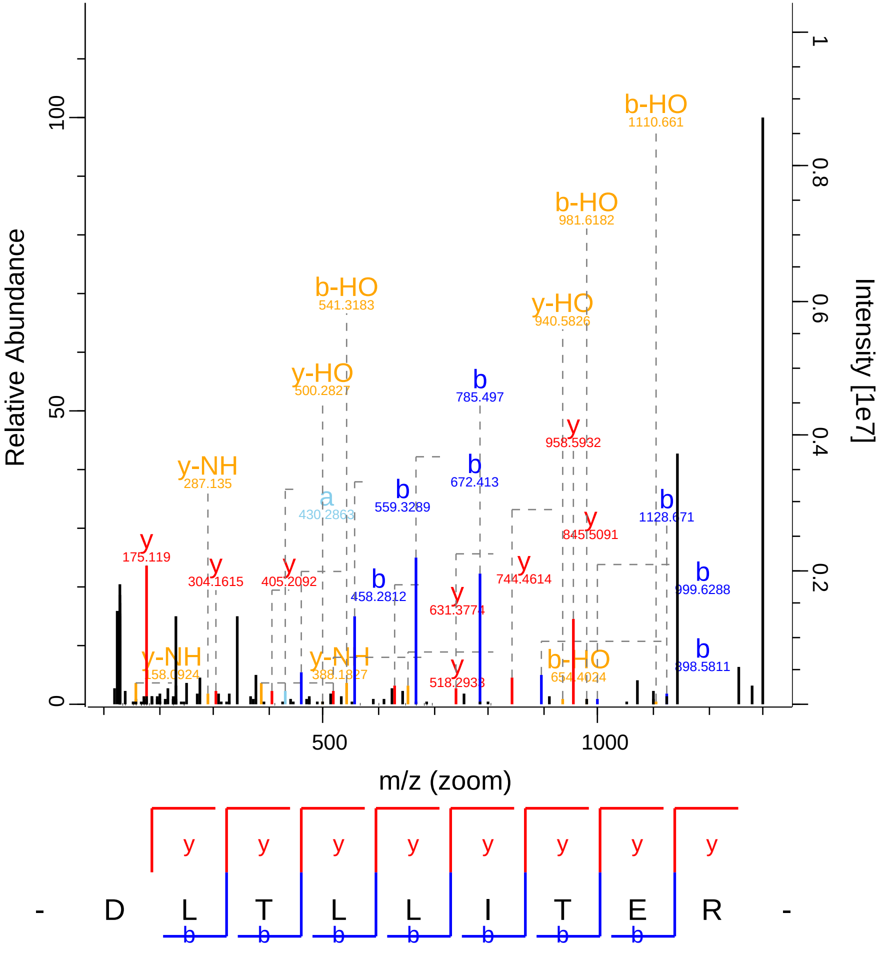

Raw File	Scan	Method	Score	m/z	Gene names
RO-QC-160121_ZackD_SS_Set_02_Fractior	32764	FTMS; HC	40.35	562.97	LMO2

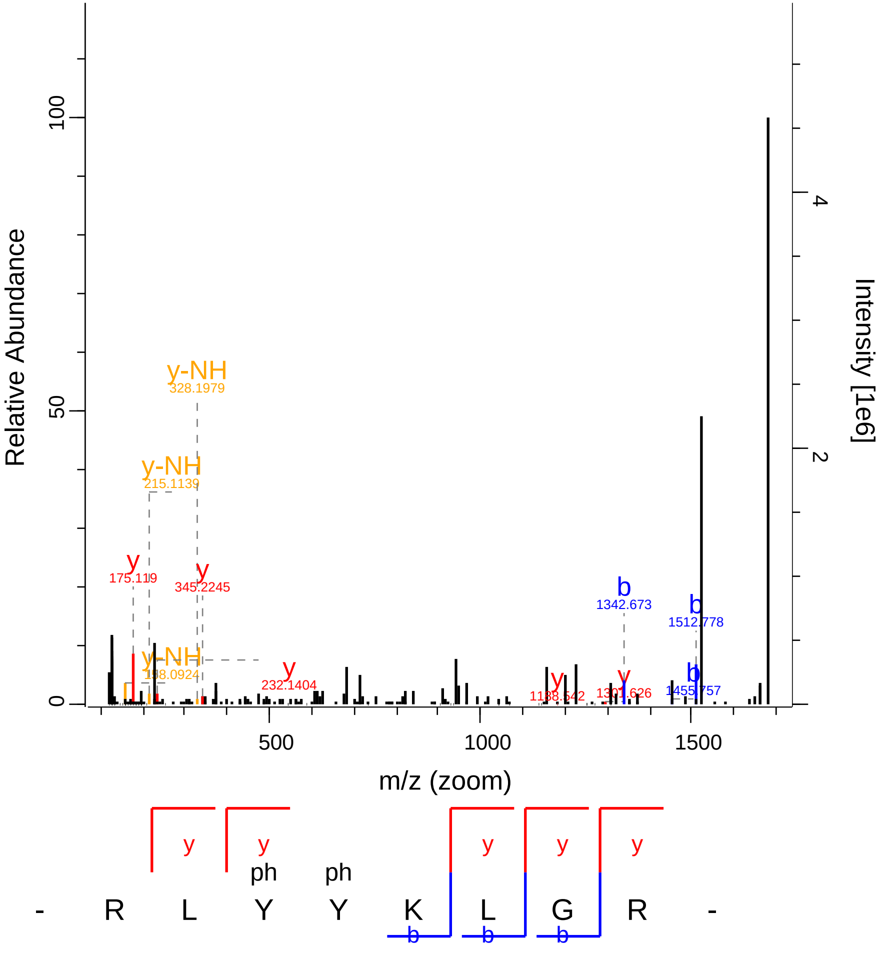

Raw File	Scan	Method	Score	m/z	Gene names
RO-QC-160121_ZackD_SS_Set_02_Fractior	34581	FTMS; HC	84.41	734.77	EFCAB14

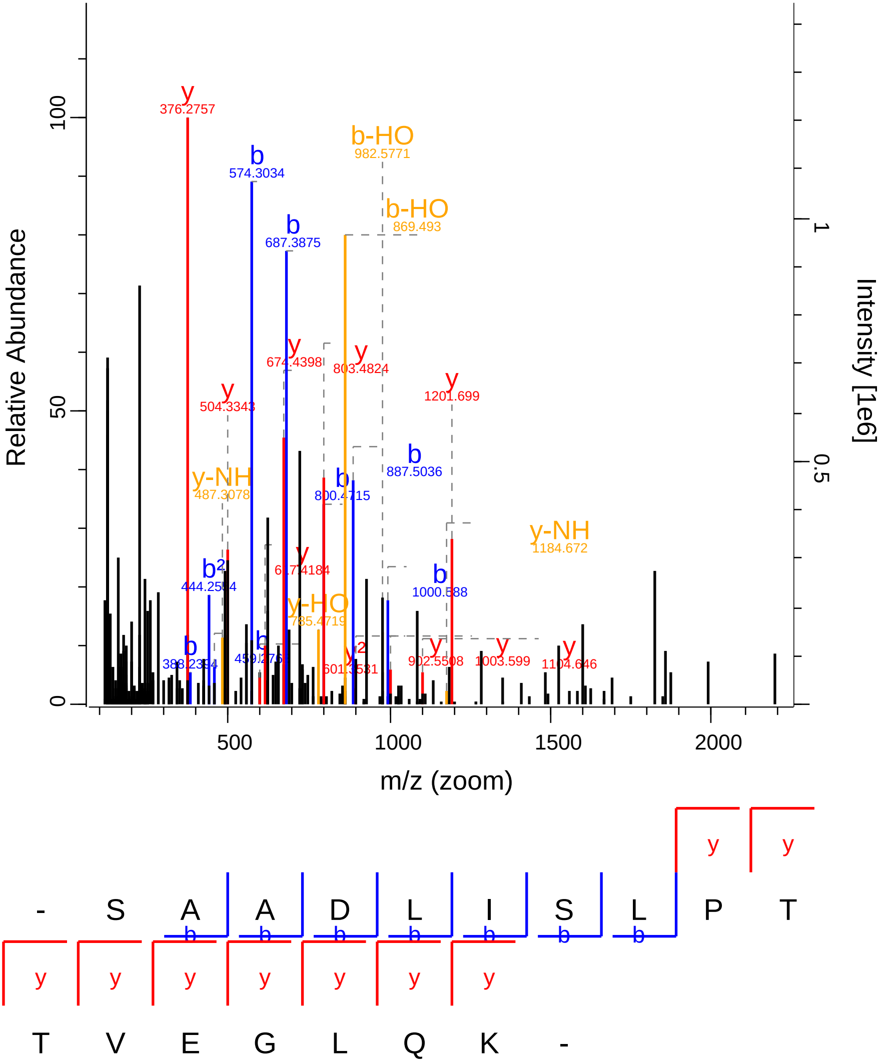

Raw File	Scan	Method	Score	m/z	Gene names
RO-QC-160121_ZackD_SS_Set_02_Fractior	35175	FTMS; HC	83.11	778.42	IGHG1

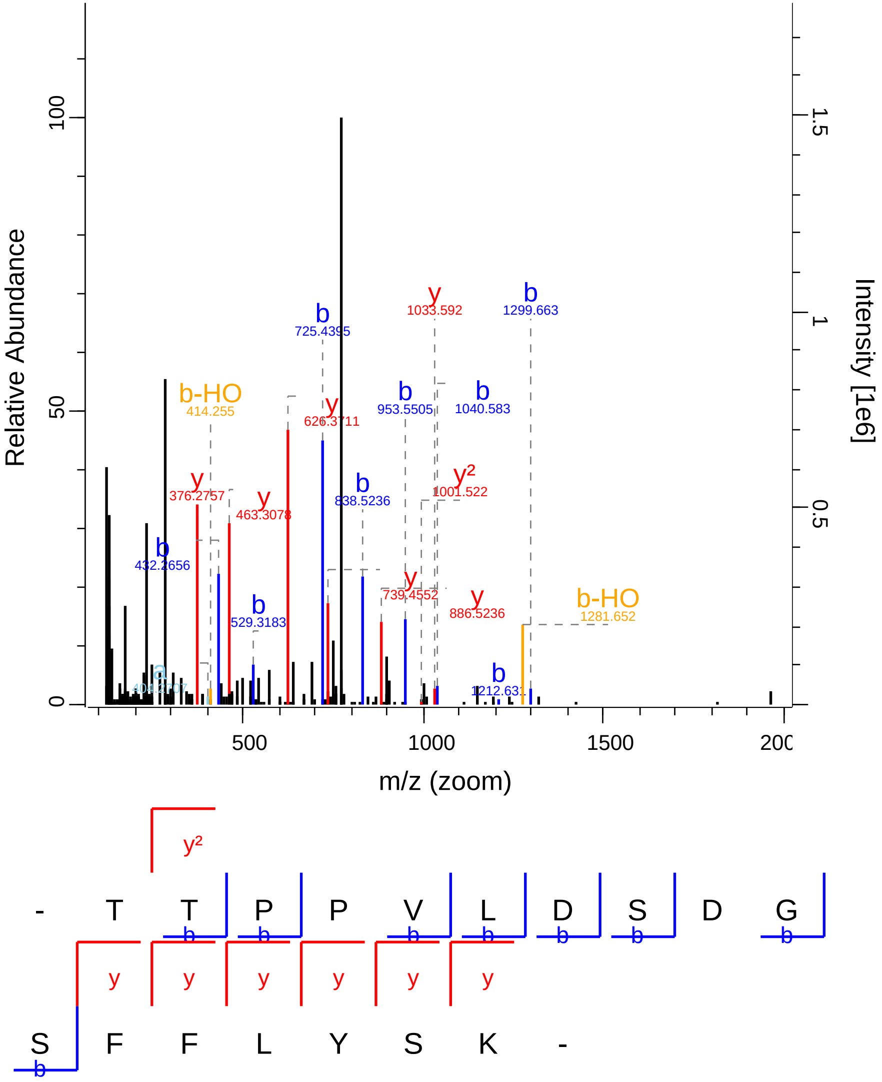

Raw File	Scan	Method	Score	m/z	Gene names
RO-QC-160121_ZackD_SS_Set_02_Fractior	35267	FTMS; HC	69.19	837.79	C7orf26

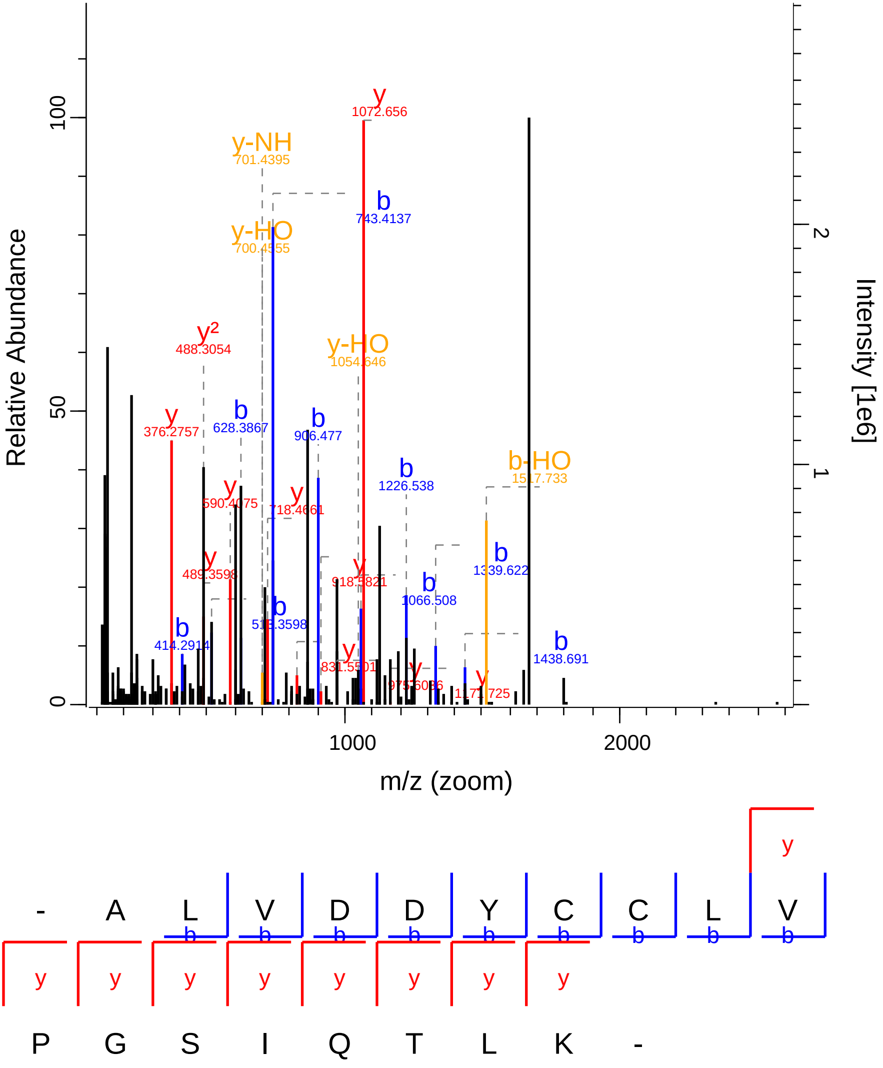

Raw File	Scan	Method	Score	m/z	Gene names
RO-QC-160121_ZackD_SS_Set_02_Fractio[illegible]	35737	FTMS; HC[illegible]	107.72	853.47	CLN8

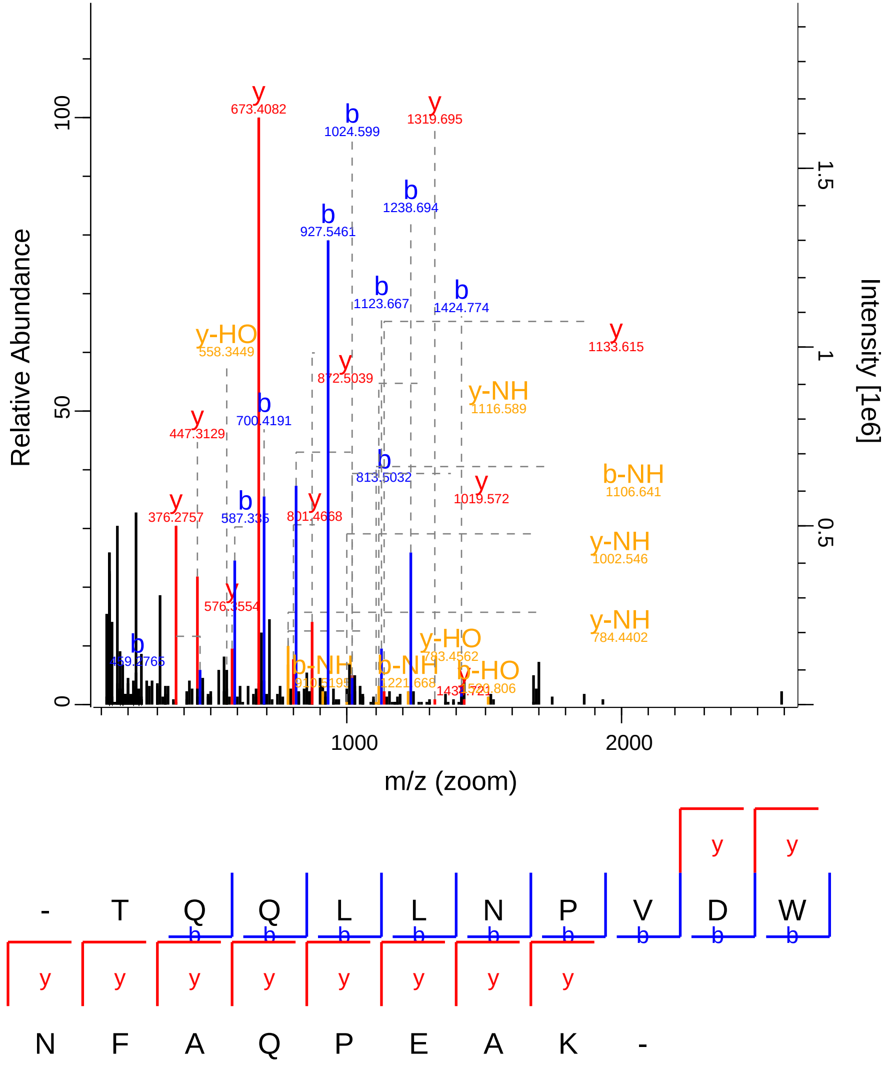

Raw File	Scan	Method	Score	m/z	Gene names
RO-QC-160121_ZackD_SS_Set_02_Fractioı	35761	FTMS; HC	149.47	853.13	CLN8

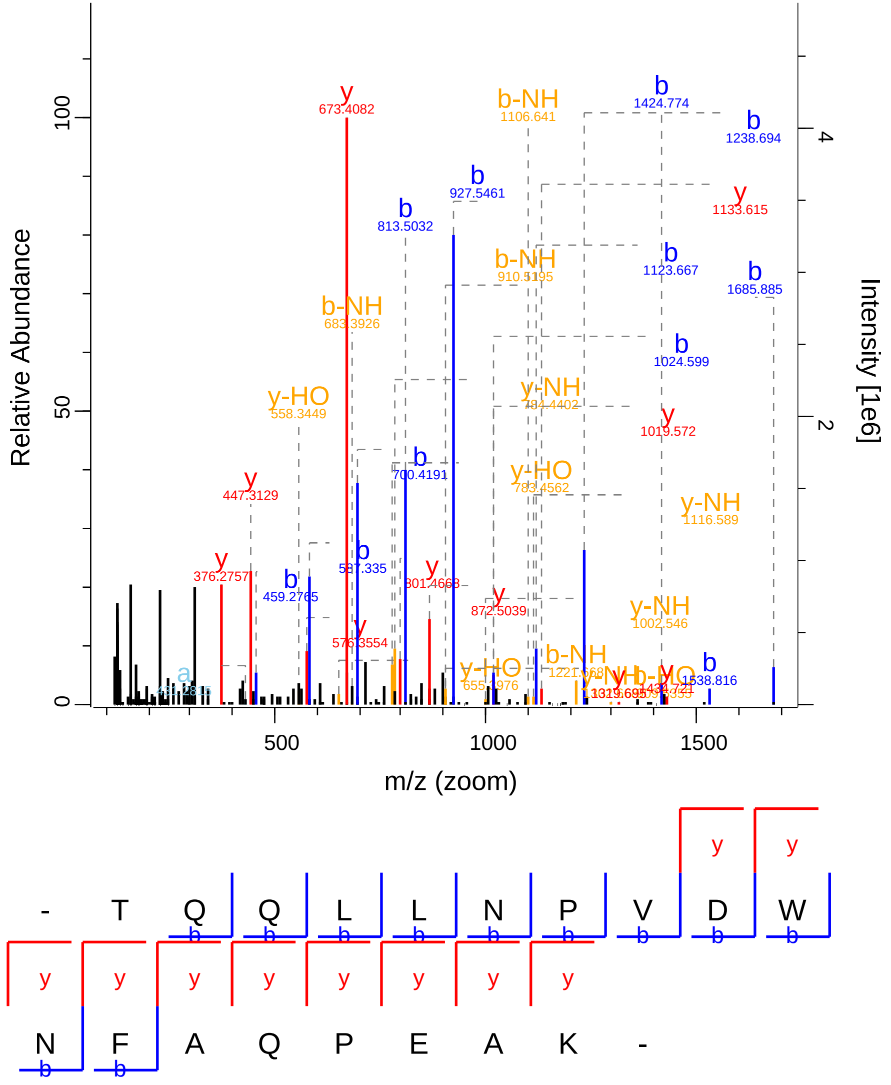

Raw File	Scan	Method	Score	m/z	Gene names
RO-QC-160121_ZackD_SS_Set_02_Fractio	38066	FTMS; HC	44.3	1359.21	RADIL

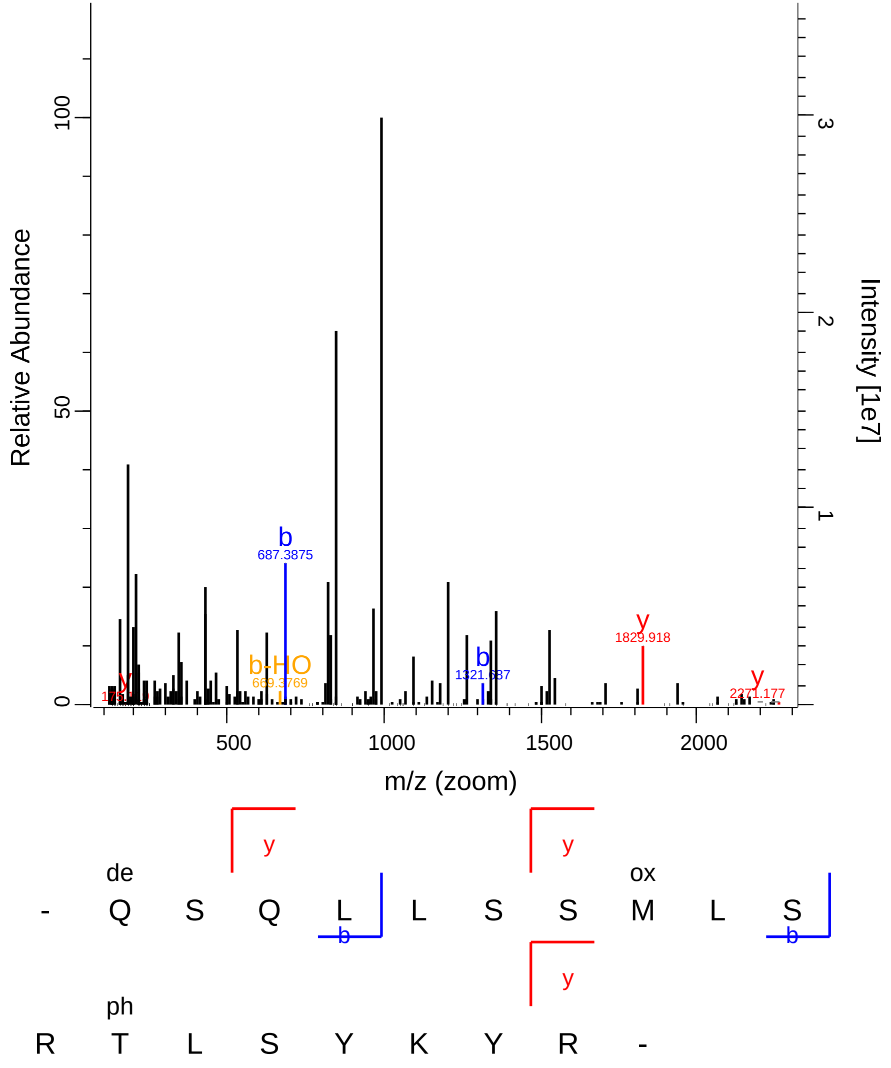

Raw File	Scan	Method	Score	m/z	Gene names
RO-QC-160121_ZackD_SS_Set_02_Fractior	38192	FTMS; HC	94.3	993.52	TOMM7

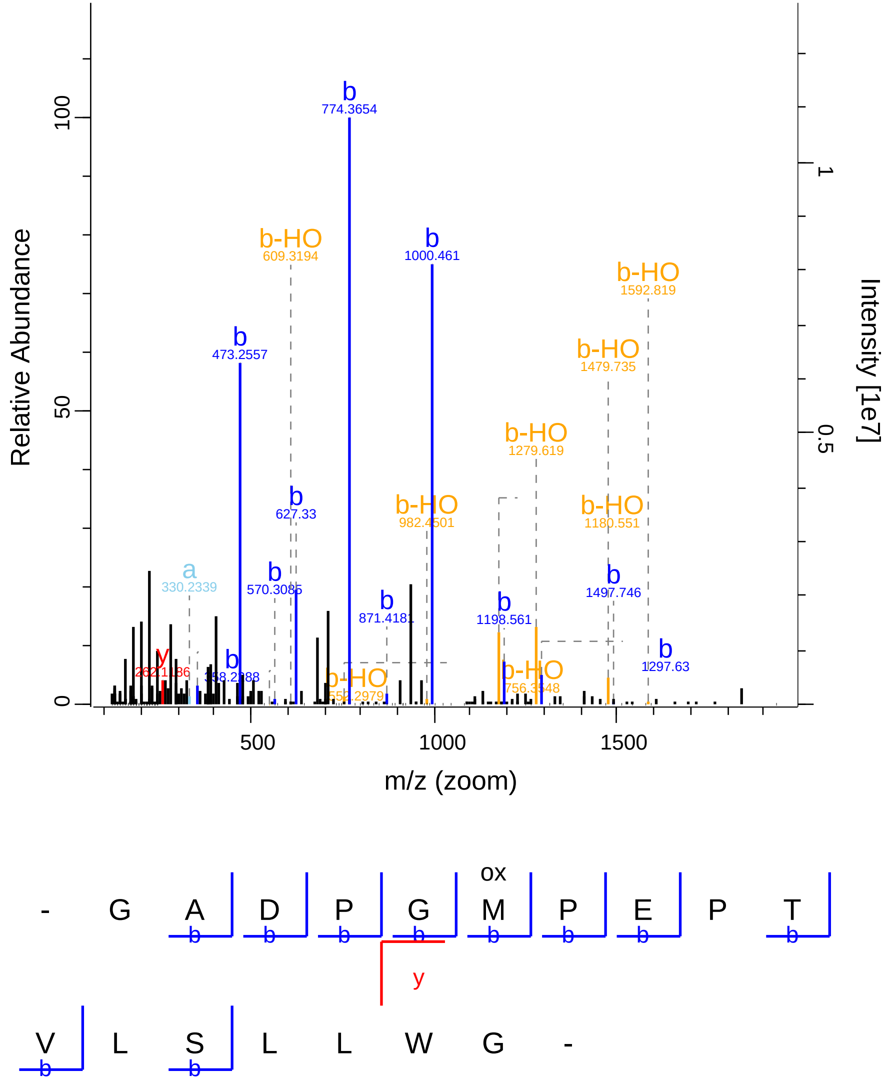

Raw File	Scan	Method	Score	m/z	Gene names
RO-QC-160121_ZackD_SS_Set_02_Fractior	38304	FTMS; HC	40.8	657.35	TOMM7

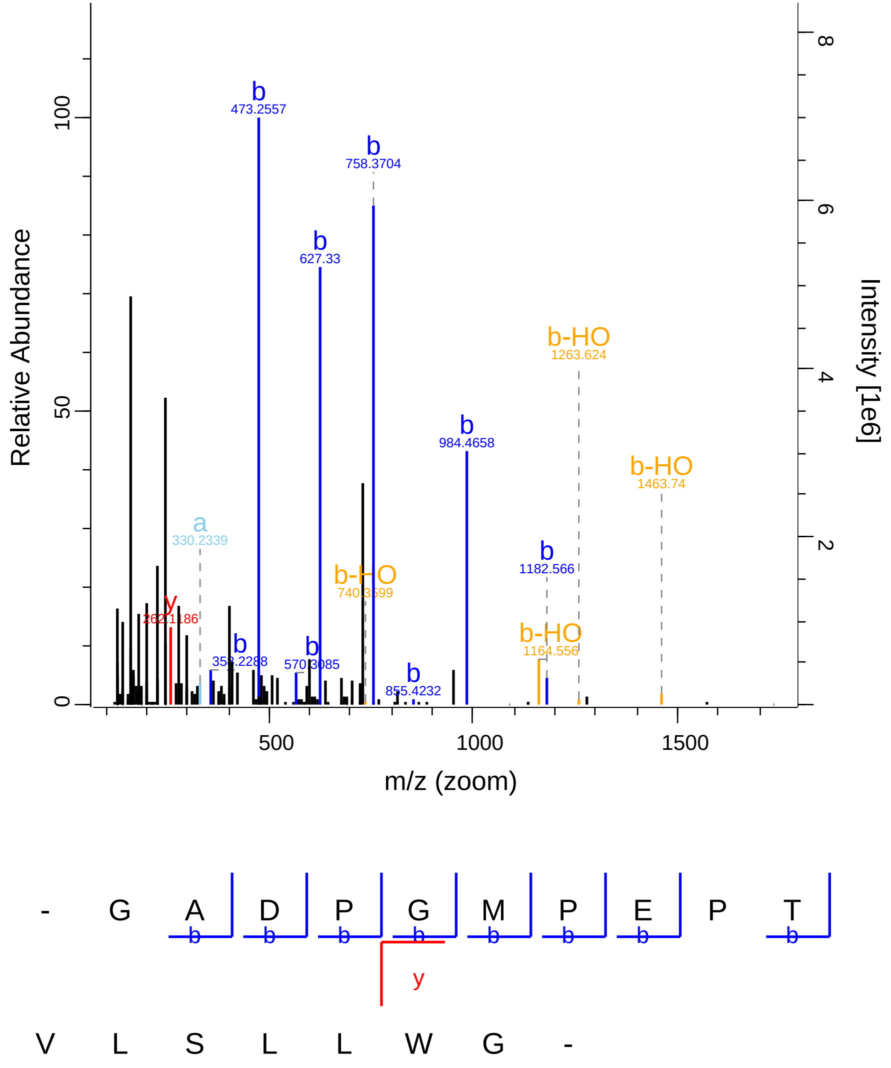

Raw File	Scan	Method	Score	m/z	Gene names
RO-QC-160121_ZackD_SS_Set_02_Fraction	4997	FTMS; HC	64.82	488.27	PITPNM2

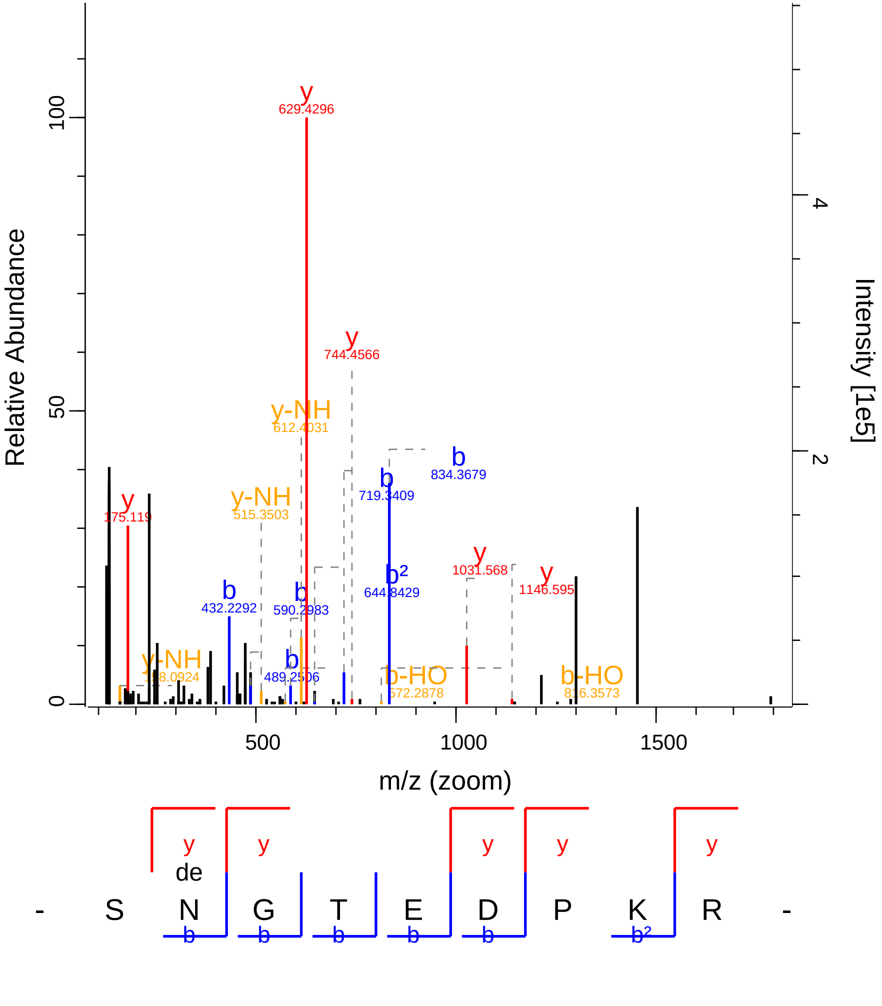

Raw File	Scan	Method	Score	m/z	Gene names
RO-QC-160121_ZackD_SS_Set_02_Fraction	5142	FTMS; HC	56.01	641.84	PDE5A

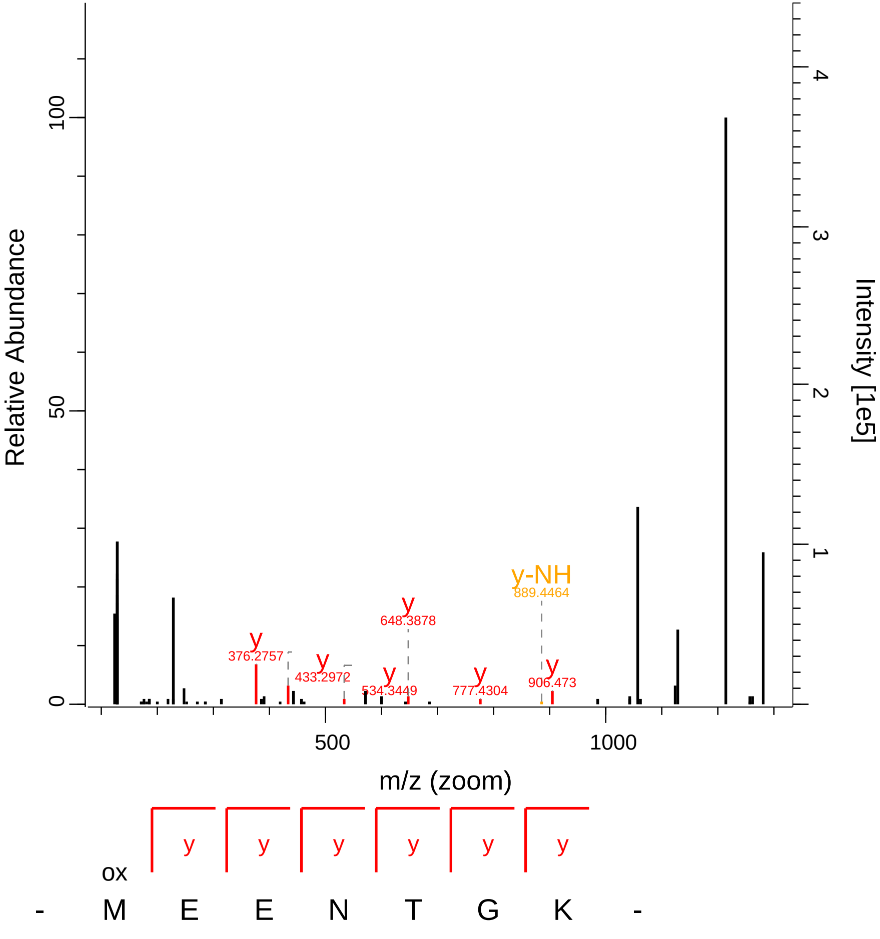

Raw File	Scan	Method	Score	m/z	Gene names
RO-QC-160121_ZackD_SS_Set_02_Fraction_	5235	FTMS; HCI	100.45	390	ODC1

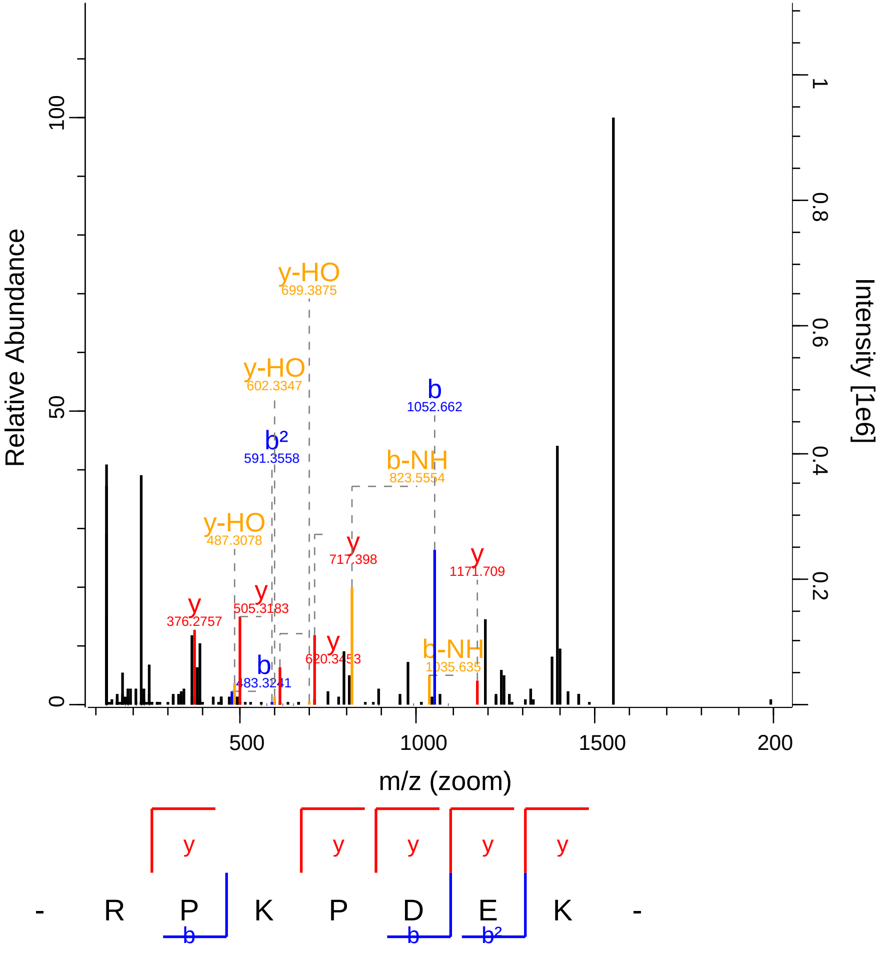

Raw File	Scan	Method	Score	m/z	Gene names
RO-QC-160121_ZackD_SS_Set_02_Fraction_	5304	FTMS; HCI	84.48	397	ADAM28

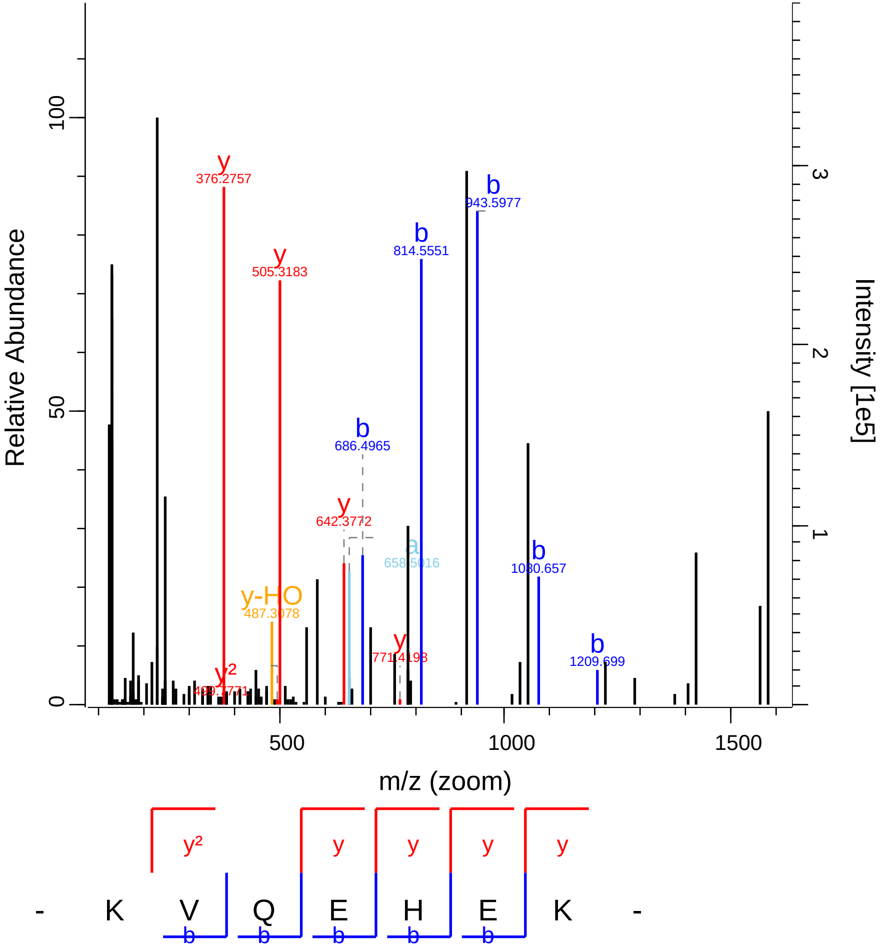

Raw File	Scan	Method	Score	m/z	Gene names
RO-QC-160121_ZackD_SS_Set_02_Fraction_	5366	FTMS; HC	58.92	722.37	TRPS1

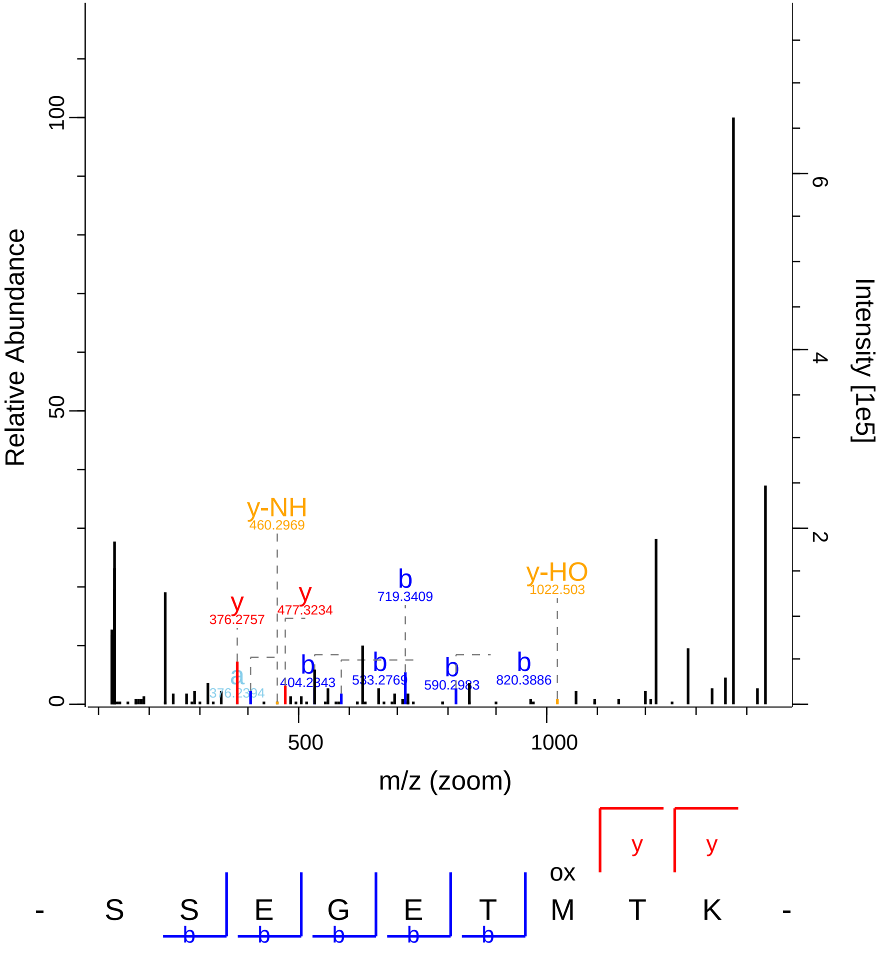

Raw File	Scan	Method	Score	m/z	Gene names
RO-QC-160121_ZackD_SS_Set_02_Fraction	5370	FTMS; HC	81.95	683.83	LMBR1

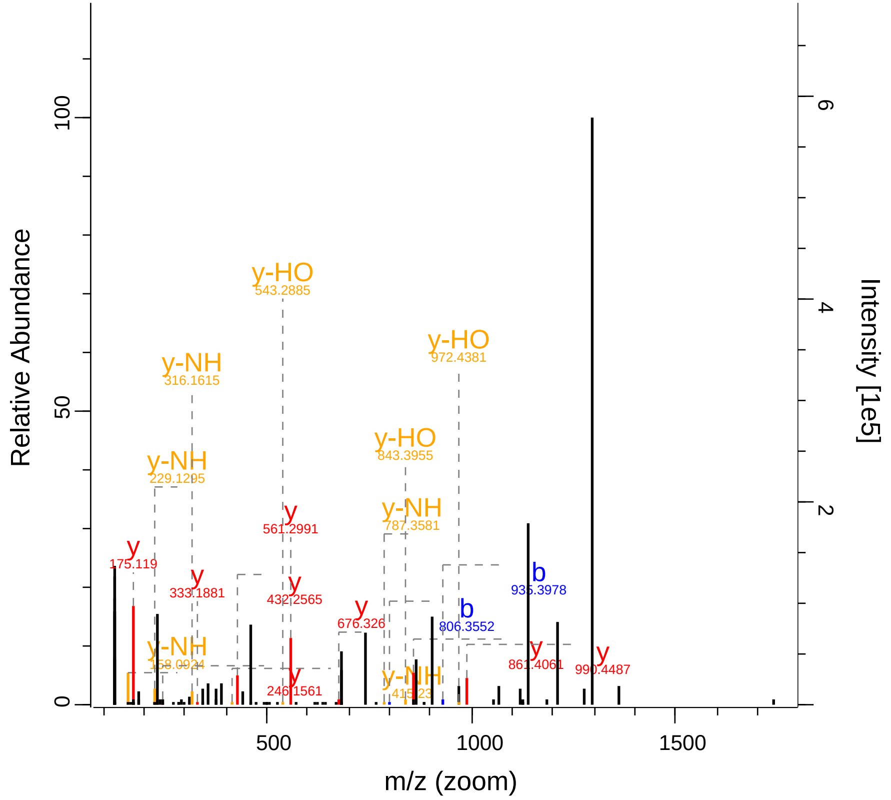

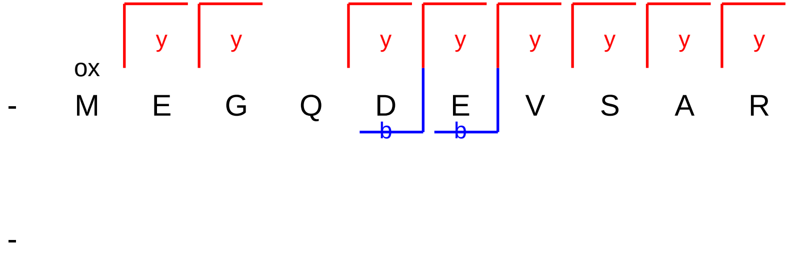

Raw File	Scan	Method	Score	m/z	Gene names
RO-QC-160121_ZackD_SS_Set_02_Fraction_	5509	FTMS; HCI	64.44	370.6	PRDM5

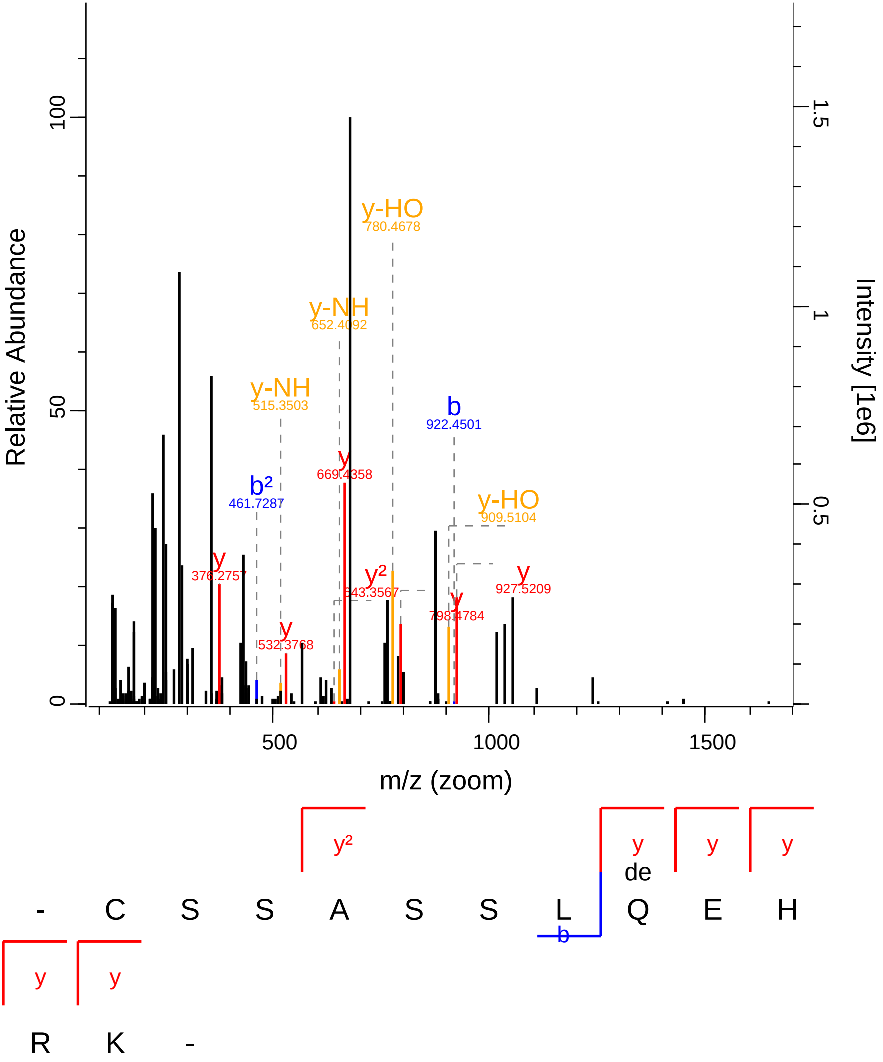

Raw File	Scan	Method	Score	m/z	Gene names
RO-QC-160121_ZackD_SS_Set_02_Fraction	5761	FTMS; HC	77.38	788.39	PCSK1N

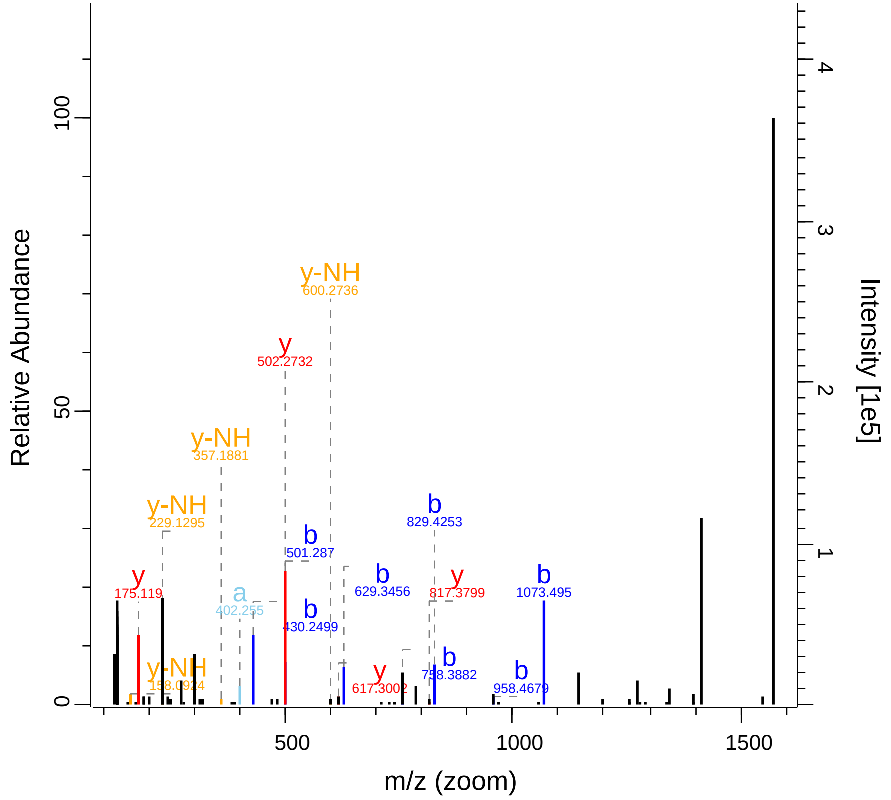

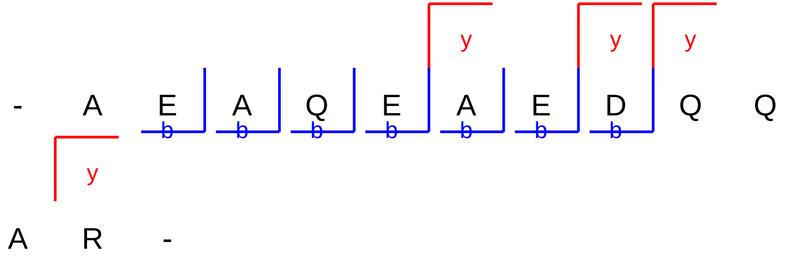

Raw File	Scan	Method	Score	m/z	Gene names
RO-QC-160121_ZackD_SS_Set_02_Fraction	5811	FTMS; HC	137.4	525.59	PCSK1N

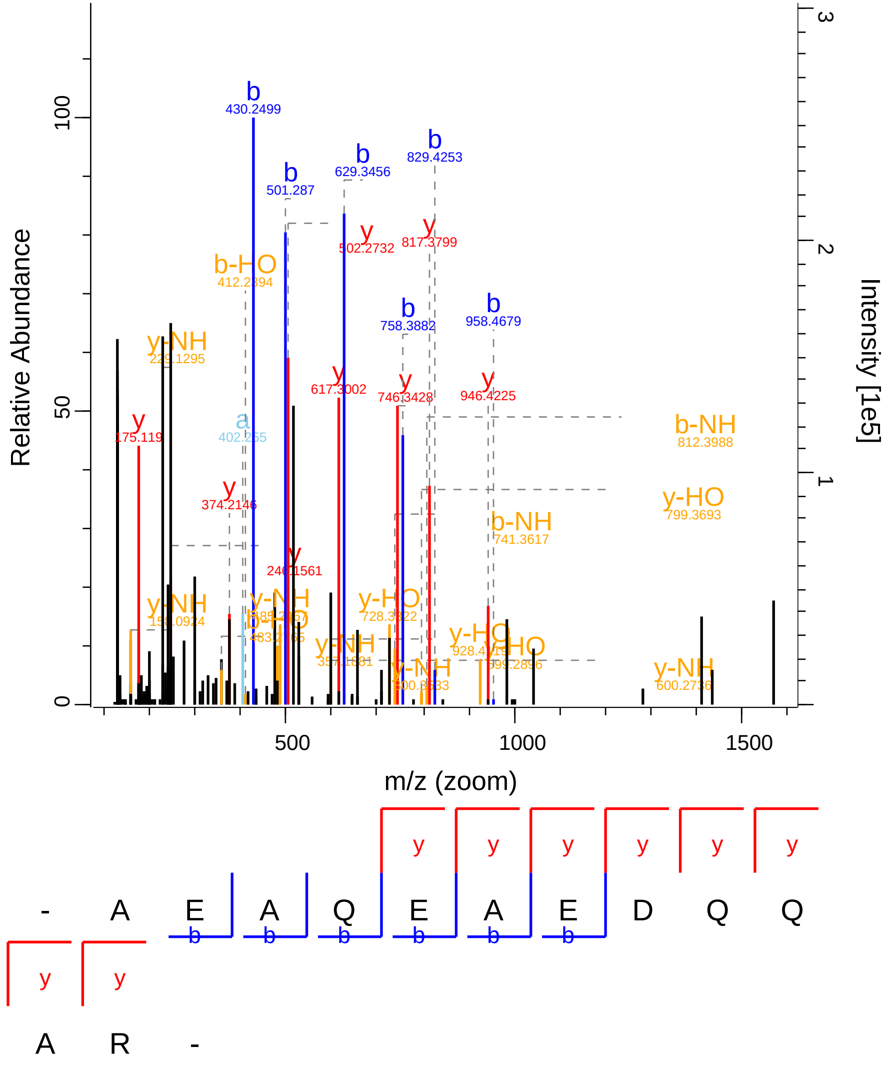

Raw File	Scan	Method	Score	m/z	Gene names
RO-QC-160121_ZackD_SS_Set_02_Fraction_	5910	FTMS; HC	98.05	771.35	ABCA7

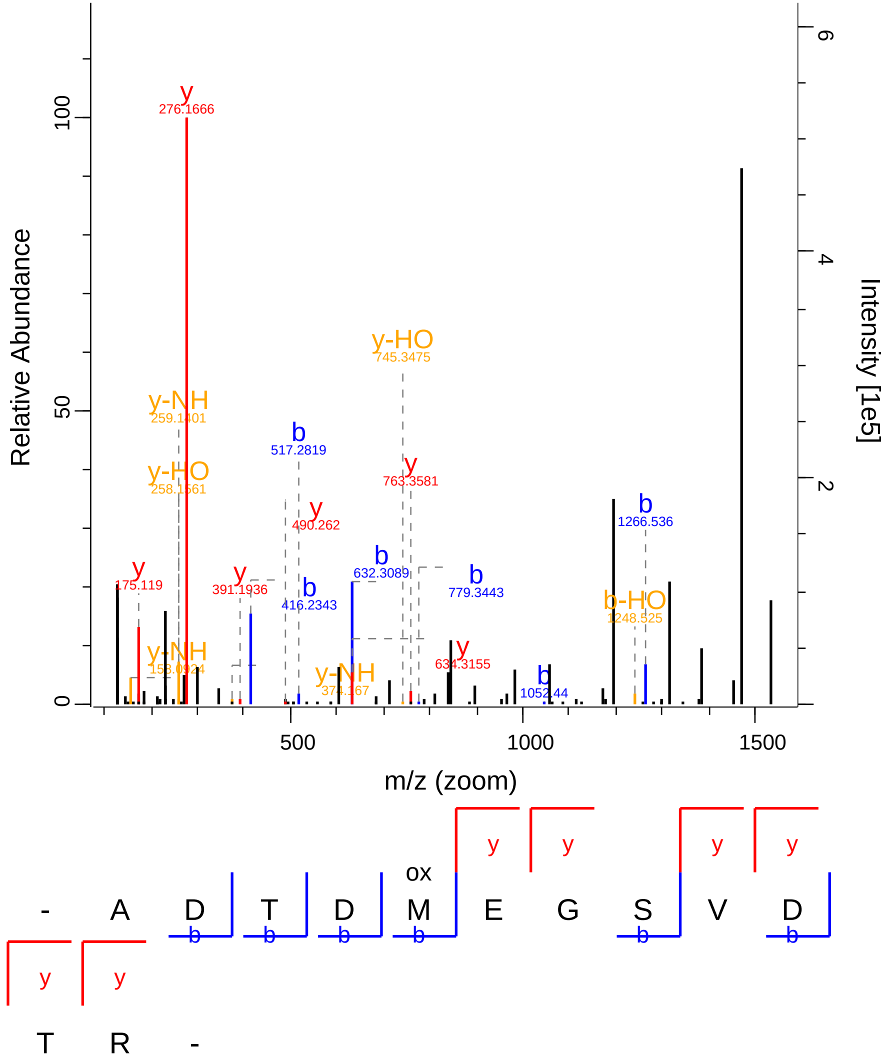

Raw File	Scan	Method	Score	m/z
RO-QC-160121_ZackD_SS_Set_02_Fraction_12	6060	FTMS; HCD	44.3	475.51

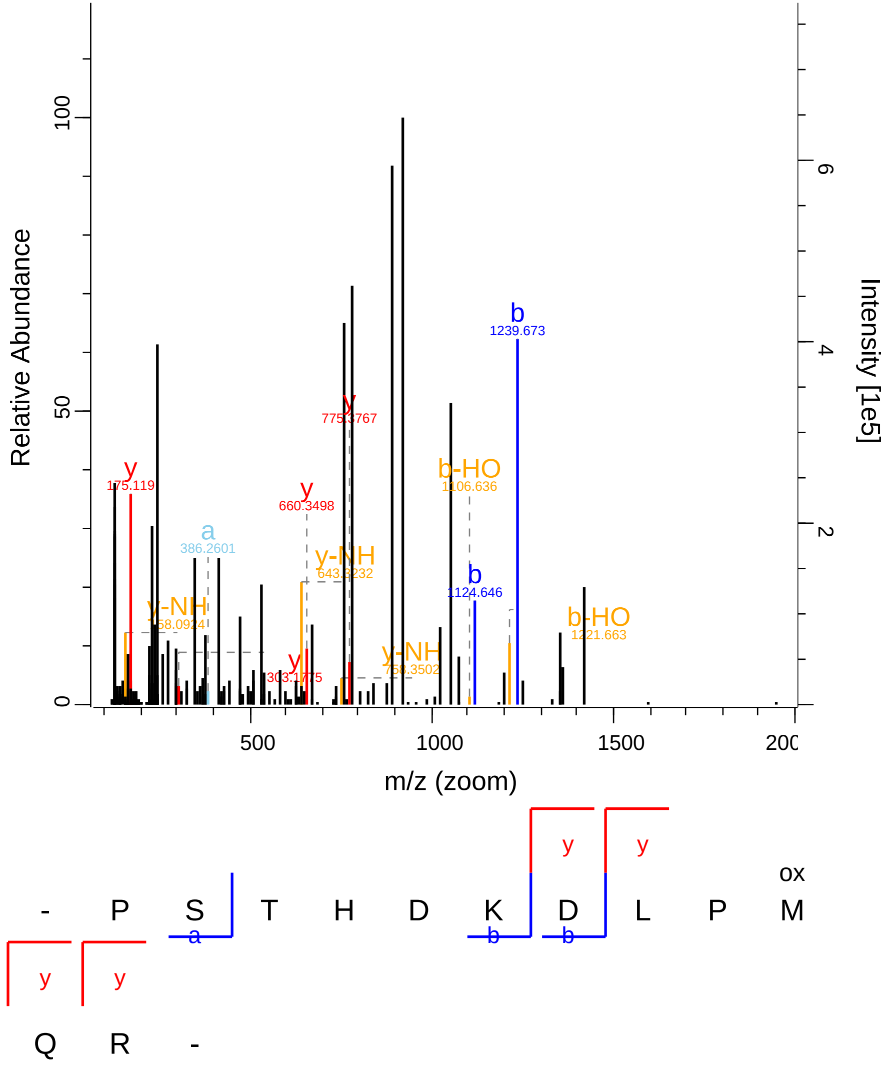

Raw File	Scan	Method	Score	m/z	Gene names
RO-QC-160121_ZackD_SS_Set_02_Fraction	6239	FTMS; HC	109.11	724.85	FAM189B

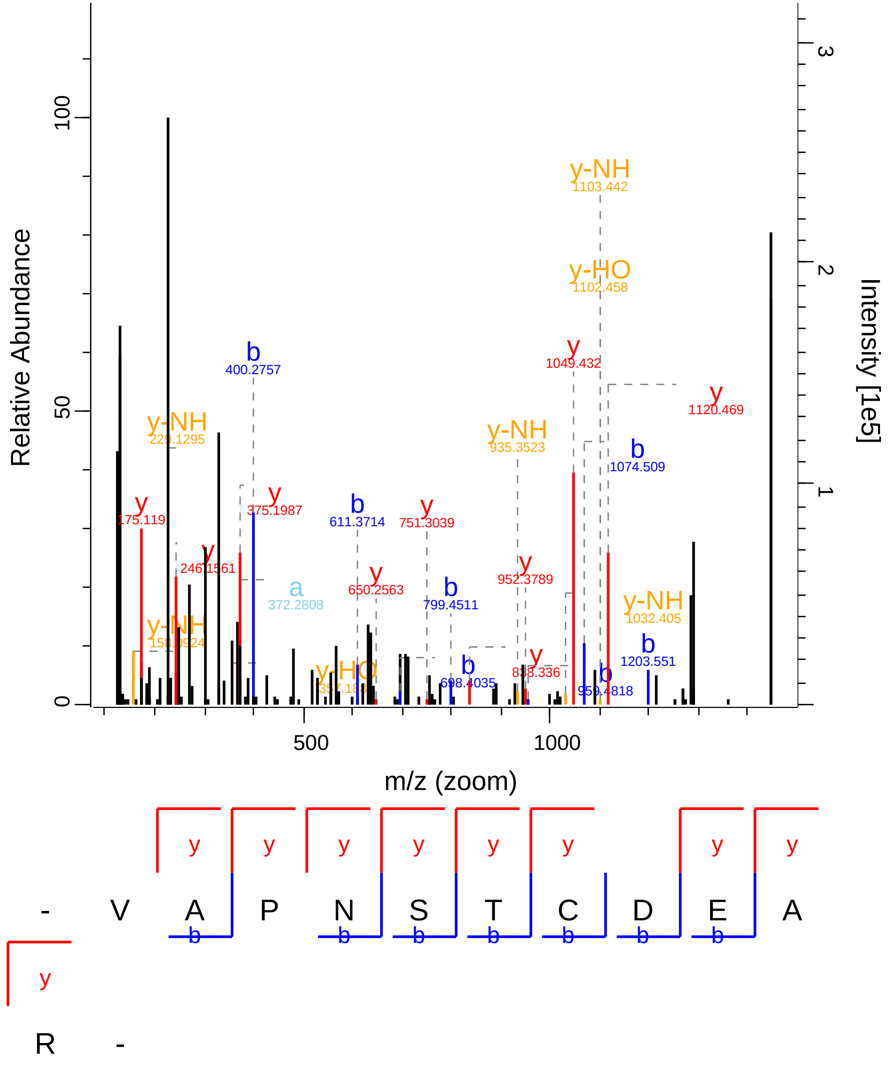

Raw File	Scan	Method	Score	m/z	Gene names
RO-QC-160121_ZackD_SS_Set_02_Fraction	6499	FTMS; HC	97.57	658.87	MAGI3

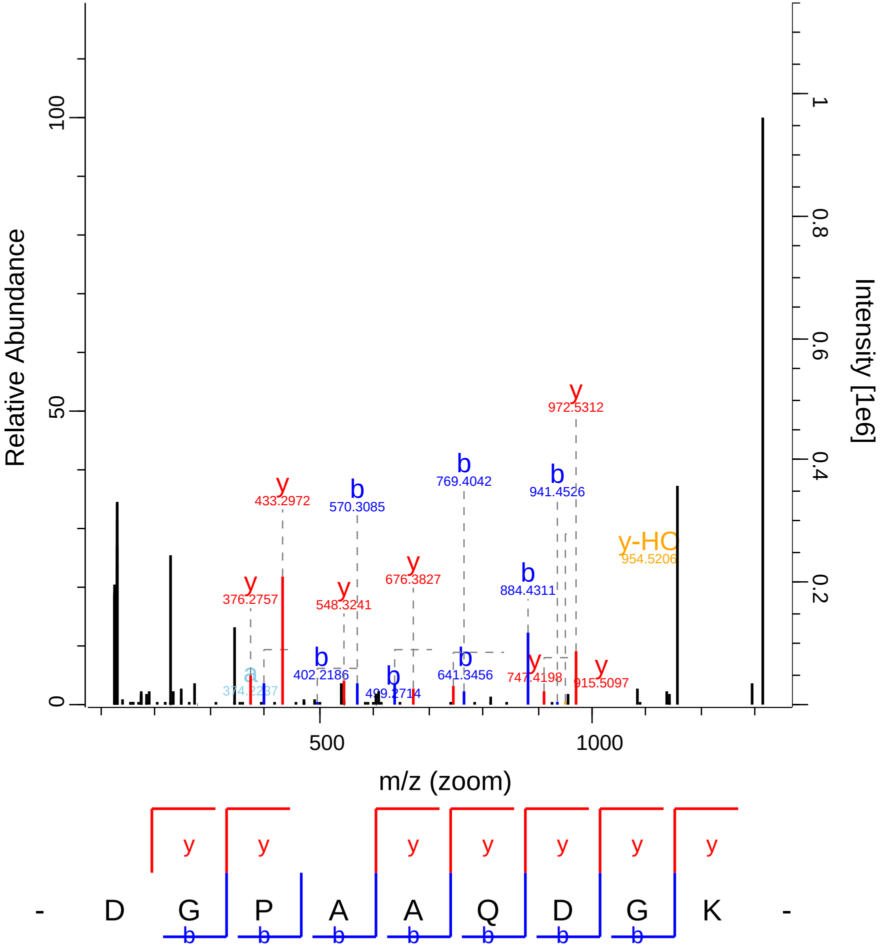

Raw File	Scan	Method	Score	m/z	Gene names
RO-QC-160121_ZackD_SS_Set_02_Fraction	6640	FTMS; HC	50.04	564.79	IGSF21

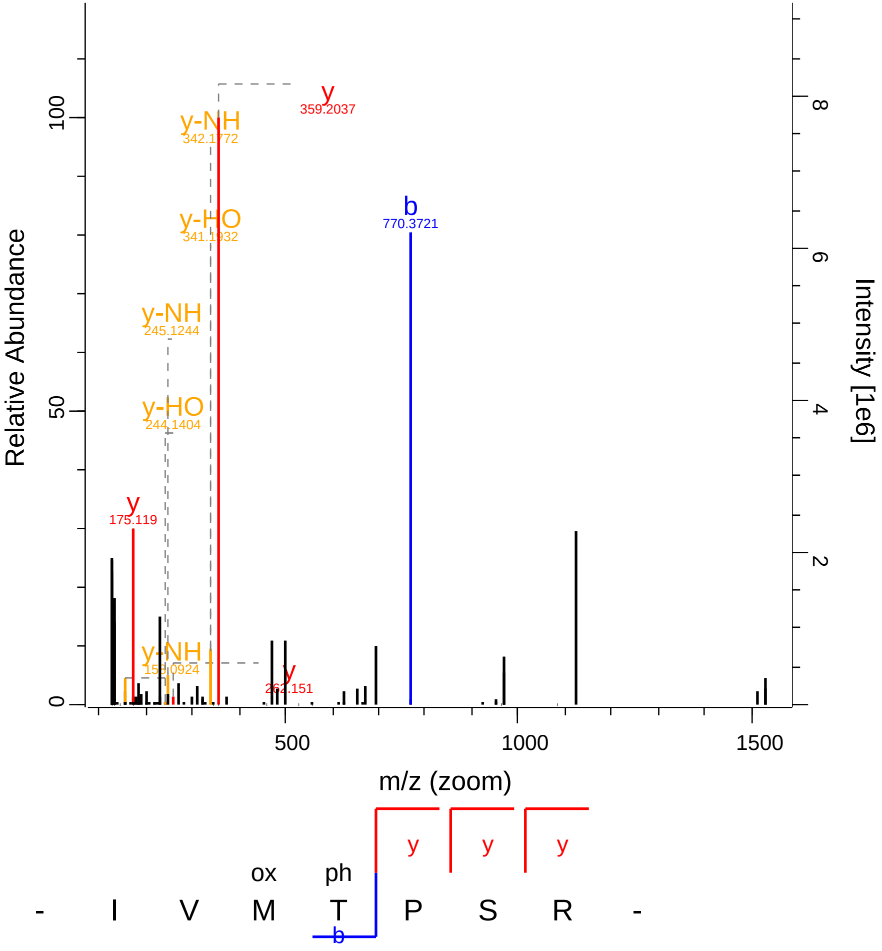

Raw File	Scan	Method	Score	m/z	Gene names
RO-QC-160121_ZackD_SS_Set_02_Fraction	6699	FTMS; HC	118.71	679.89	LENG9

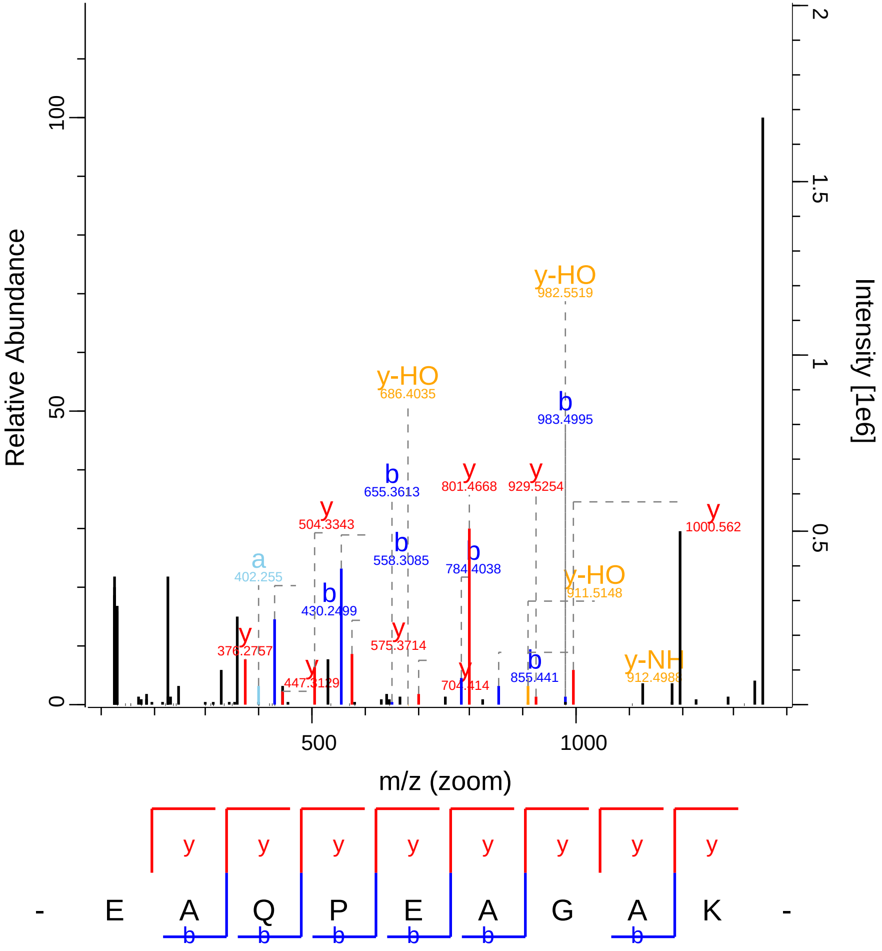

Raw File	Scan	Method	Score	m/z	Gene names
RO-QC-160121_ZackD_SS_Set_02_Fraction	6842	FTMS; HC	54.76	556.27	PLEKHF1

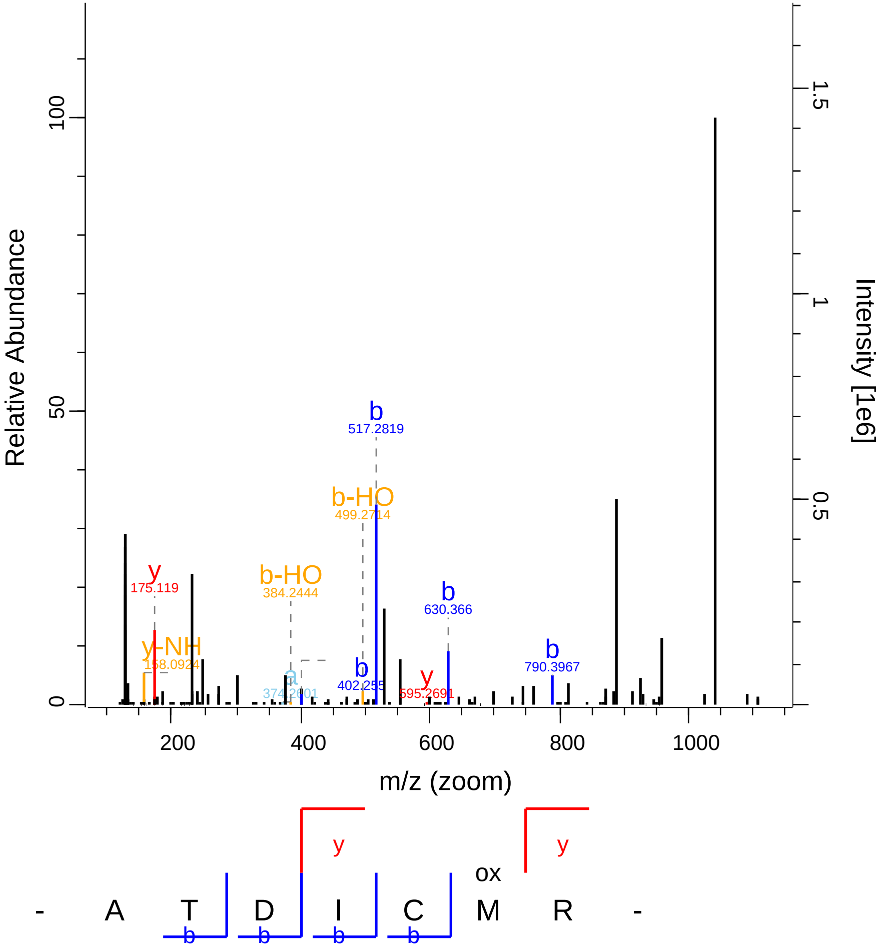

Raw File	Scan	Method	Score	m/z
RO-QC-160121_ZackD_SS_Set_02_Fraction_12	6999	FTMS; HCD	80.22	435.59

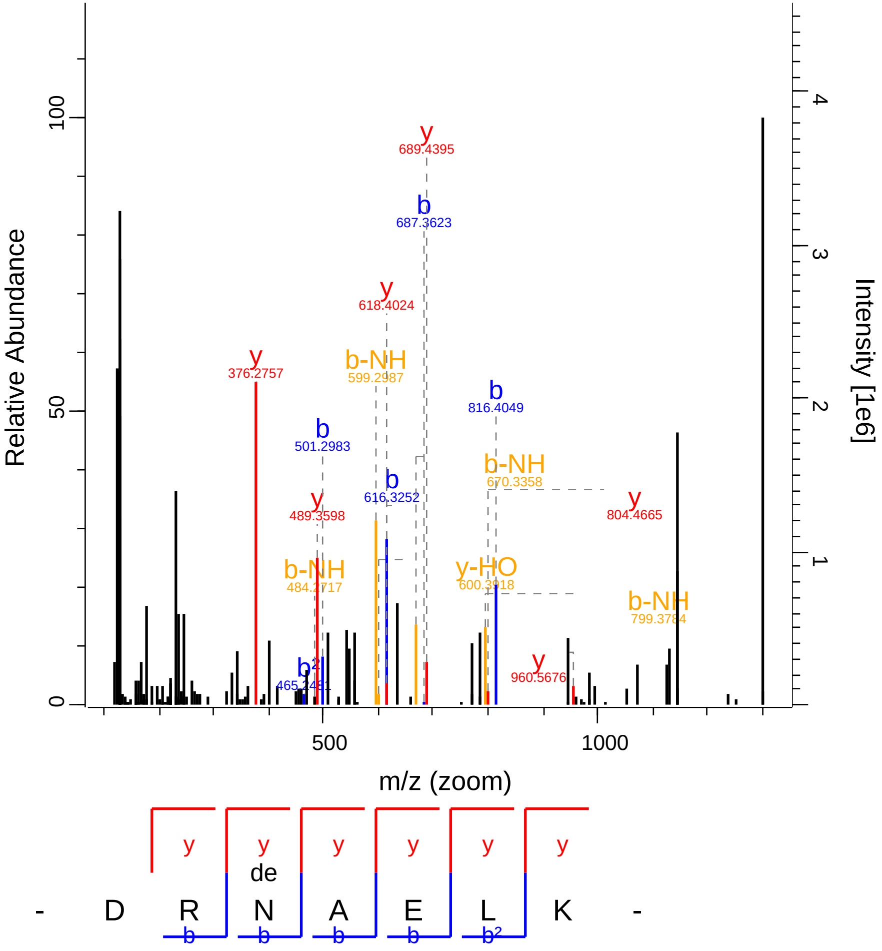

Raw File	Scan	Method	Score	m/z	Gene names
RO-QC-160121_ZackD_SS_Set_02_Fraction_	7413	FTMS; HCI	64.73	781.4	GKAP1

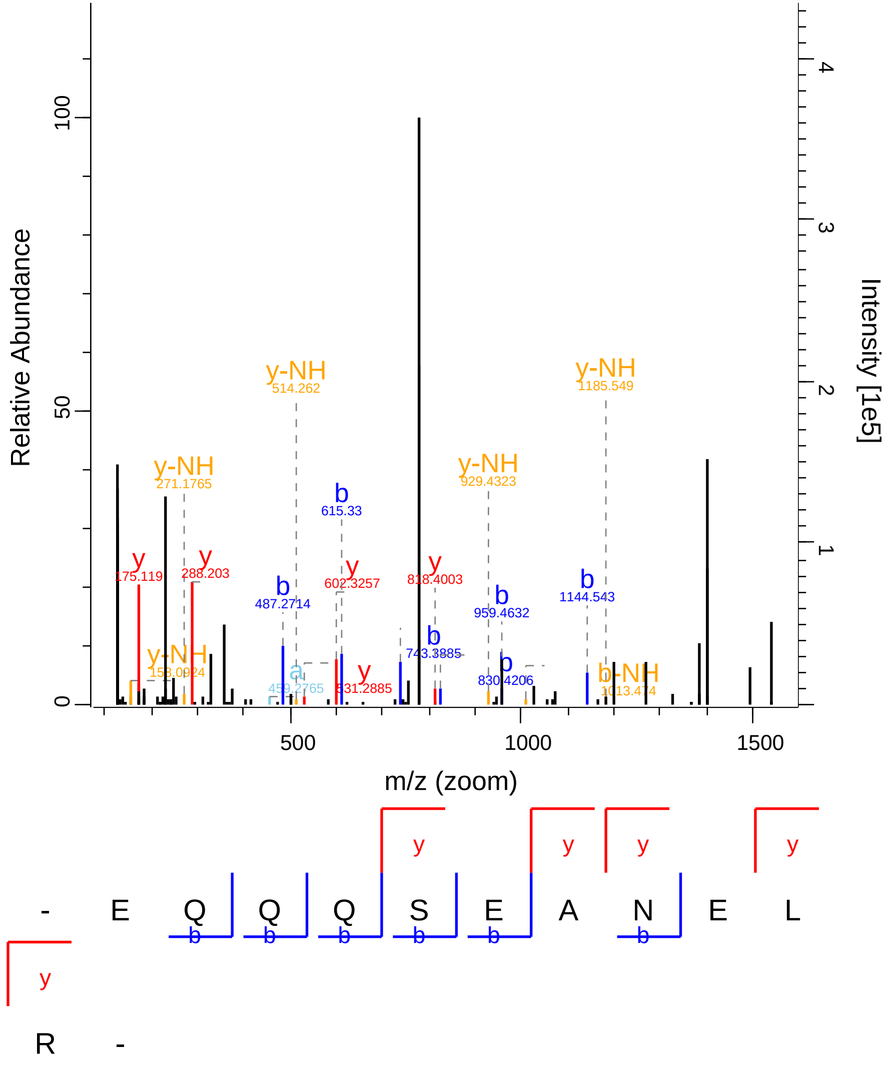

Raw File	Scan	Method	Score	m/z	Gene names
RO-QC-160121_ZackD_SS_Set_02_Fraction	7746	FTMS; HCD	48	433.75	KCNA10

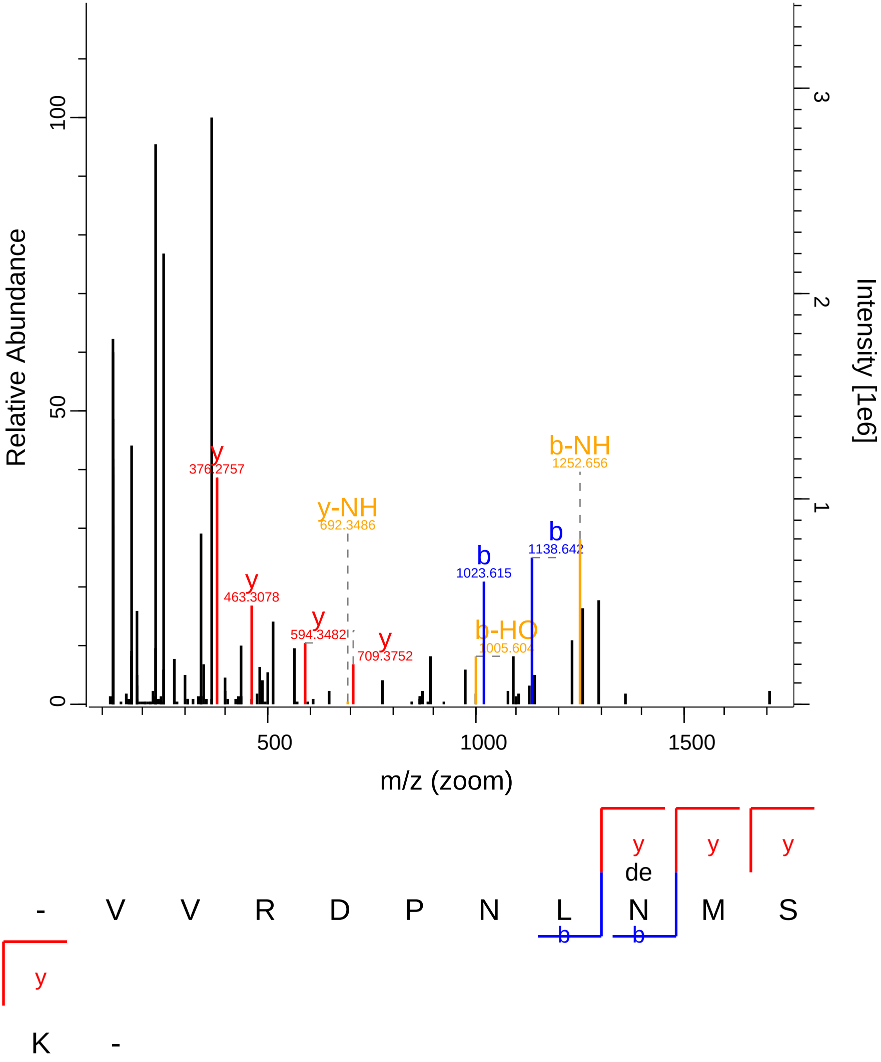

Raw File	Scan	Method	Score	m/z	Gene names
RO-QC-160121_ZackD_SS_Set_02_Fraction	8137	FTMS; HC	48	433.75	KCNA10

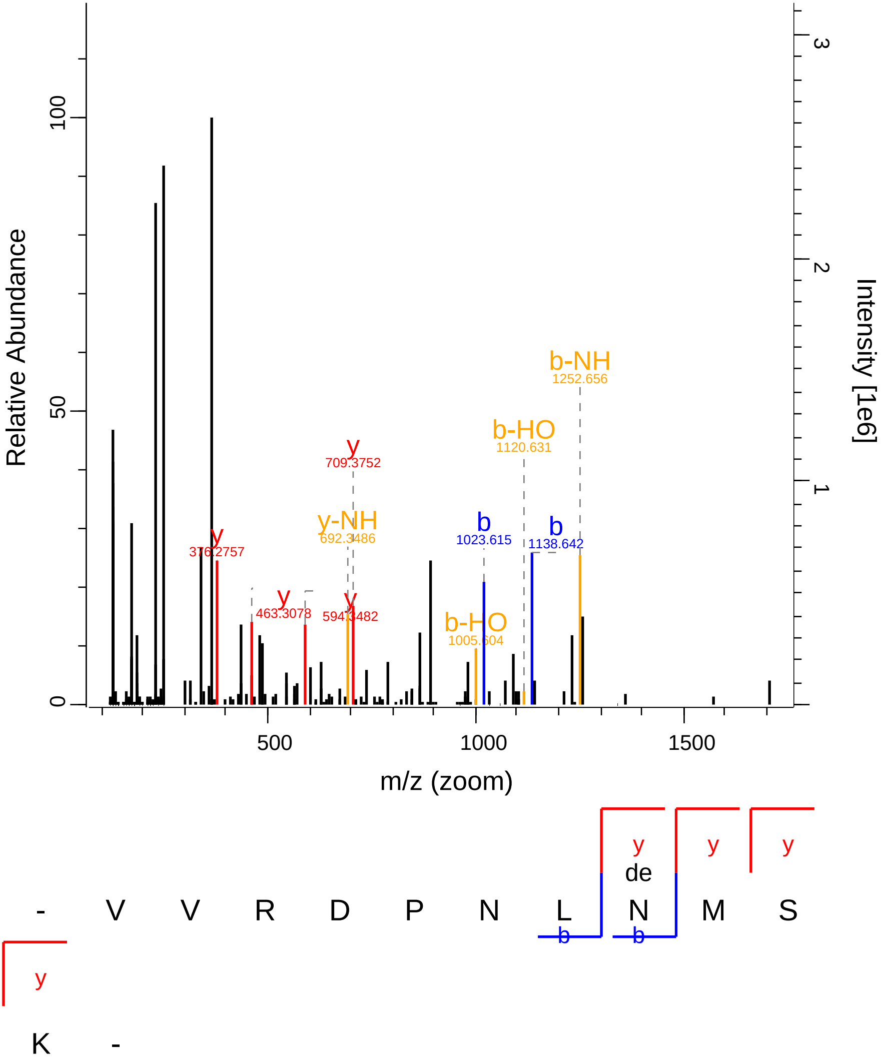

Raw File	Scan	Method	Score	m/z	Gene names
RO-QC-160121_ZackD_SS_Set_02_Fraction	8264	FTMS; HC	144.09	631.84	MBTPS2

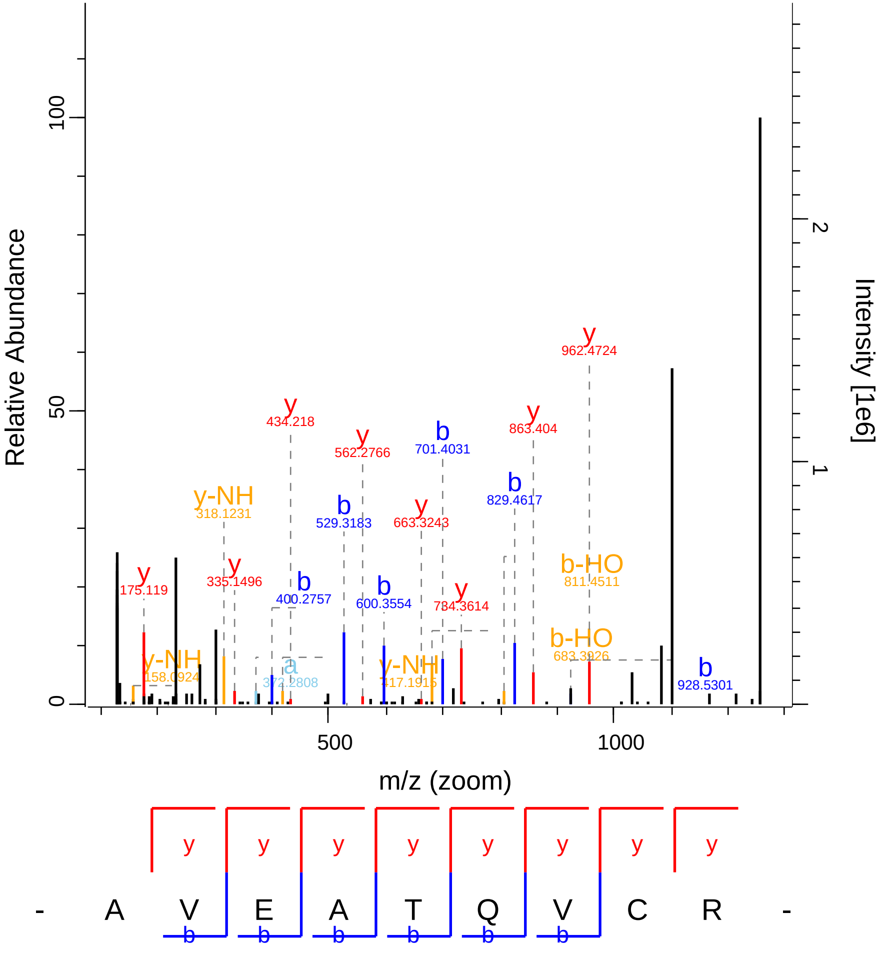

Raw File	Scan	Method	Score	m/z	Gene names
RO-QC-160121_ZackD_SS_Set_02_Fraction	8520	FTMS; HC	97.86	661.34	RIN2

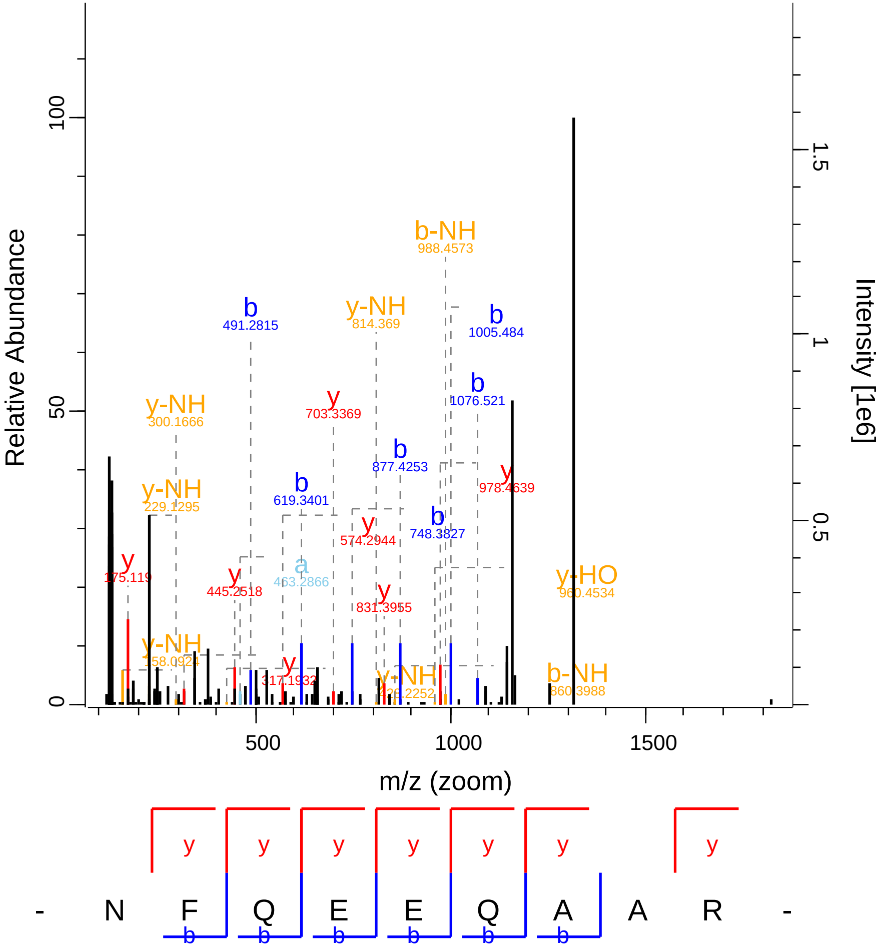

Raw File	Scan	Method	Score	m/z	Gene names
RO-QC-160121_ZackD_SS_Set_02_Fraction	8948	FTMS; HC	136.99	630.34	LIPT2

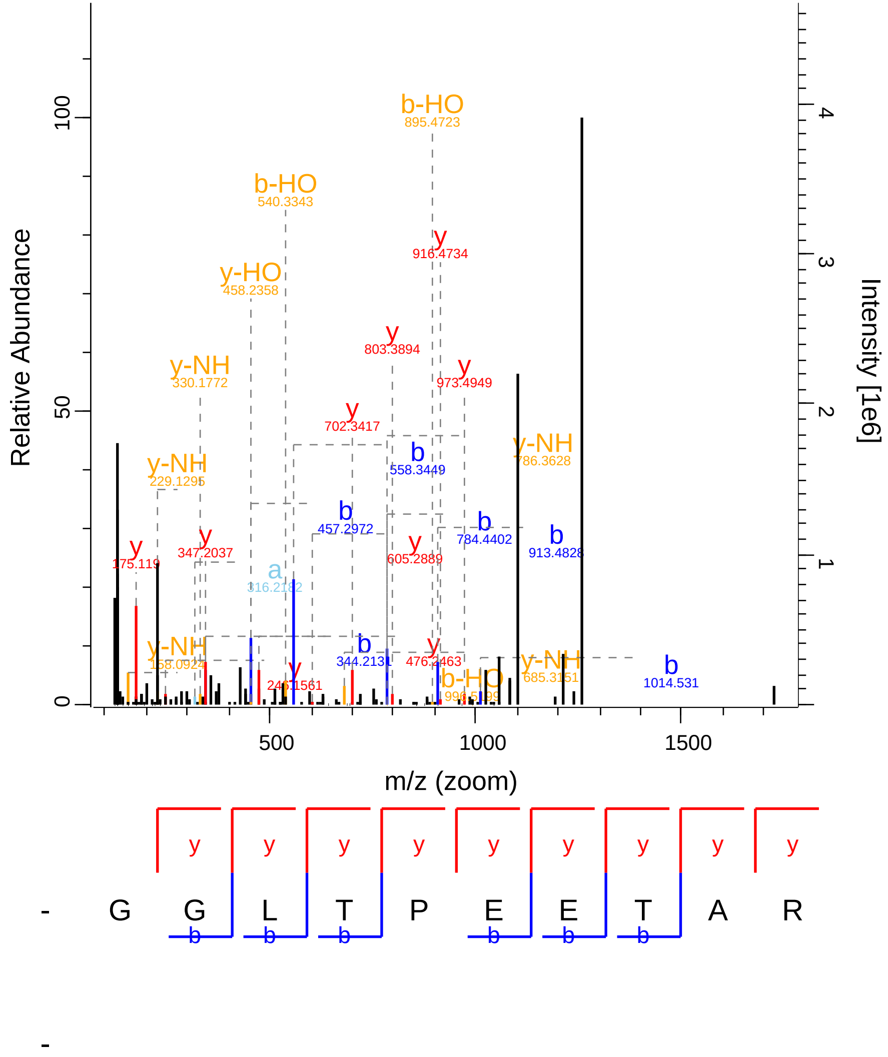

Raw File	Scan	Method	Score	m/z	Gene names
RO-QC-160121_ZackD_SS_Set_02_Fraction	9142	FTMS; HC	105.95	350.21	SAMD1

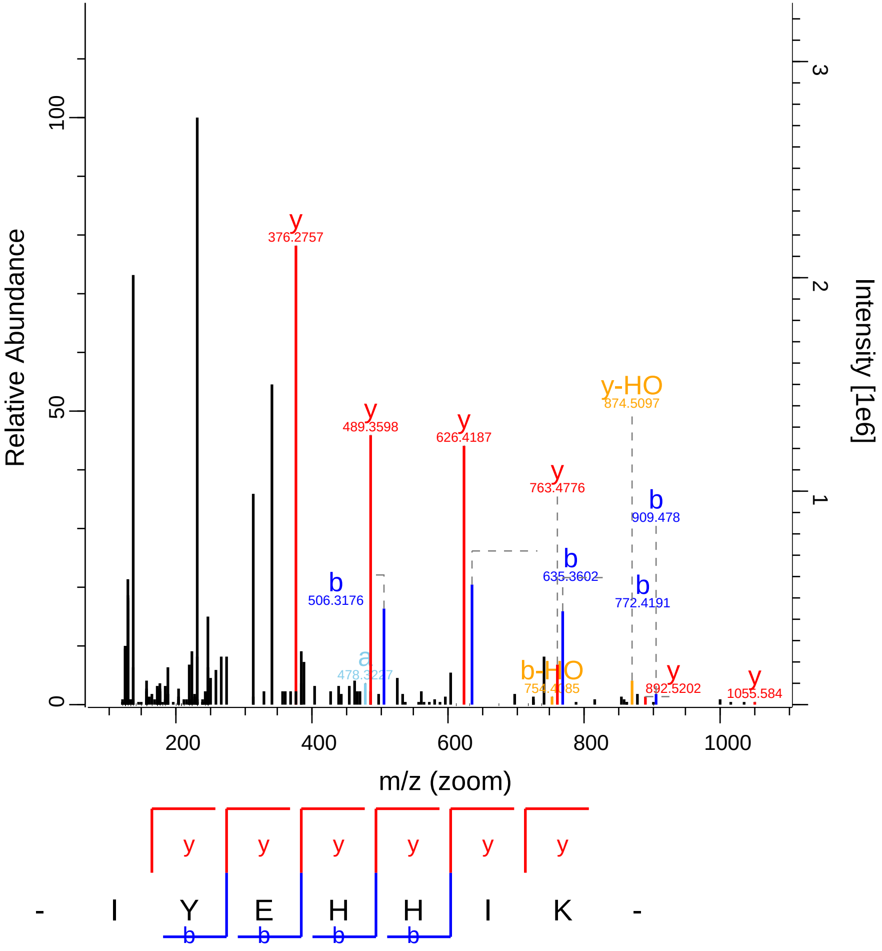

Raw File	Scan	Method	Score	m/z
RO-QC-160121_ZackD_SS_Set_02_Fraction_12	9215	FTMS; HCD	46.66	389.24

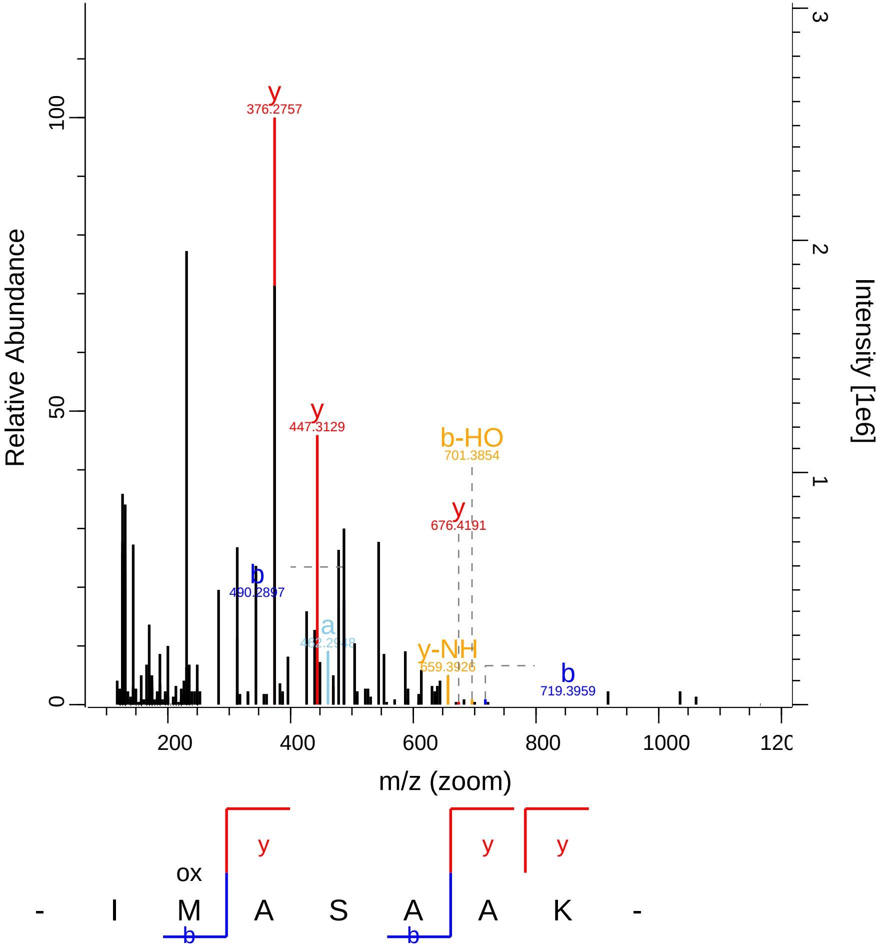

Raw File	Scan	Method	Score	m/z	Gene names
RO-QC-160121_ZackD_SS_Set_02_Fraction	9845	FTMS; HC	109.11	566.81	CCDC167

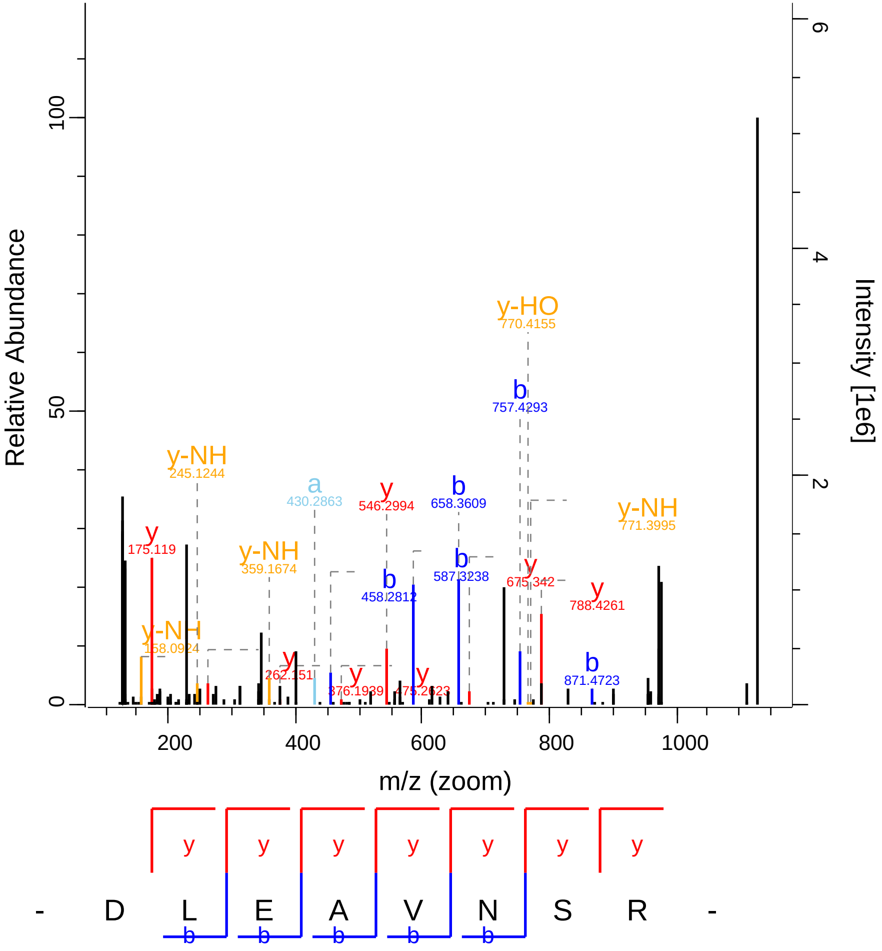

Raw File	Scan	Method	Score	m/z
RO-QC-160121_ZackD_SS_Set_02_Fraction_12	10283	FTMS; HCD	57.45	562.33

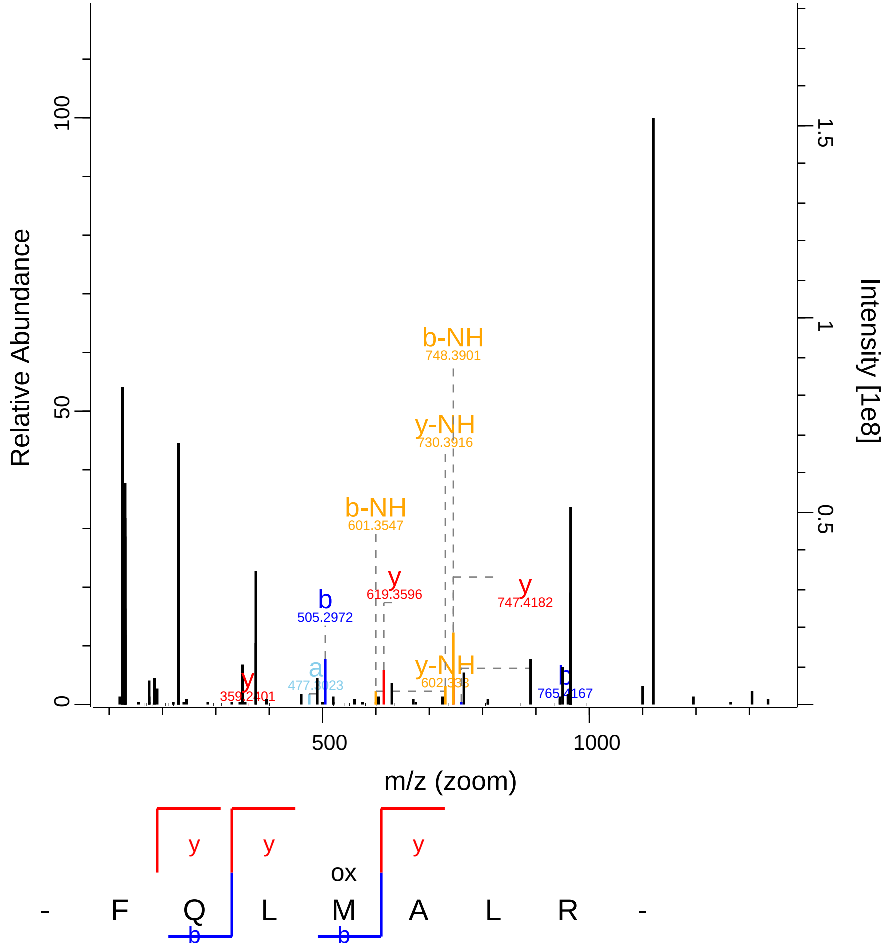

Raw File	Scan	Method	Score	m/z	Gene names
RO-QC-160121_ZackD_SS_Set_02_Fraction	10743	FTMS; HC	132.96	733.9	PIGB

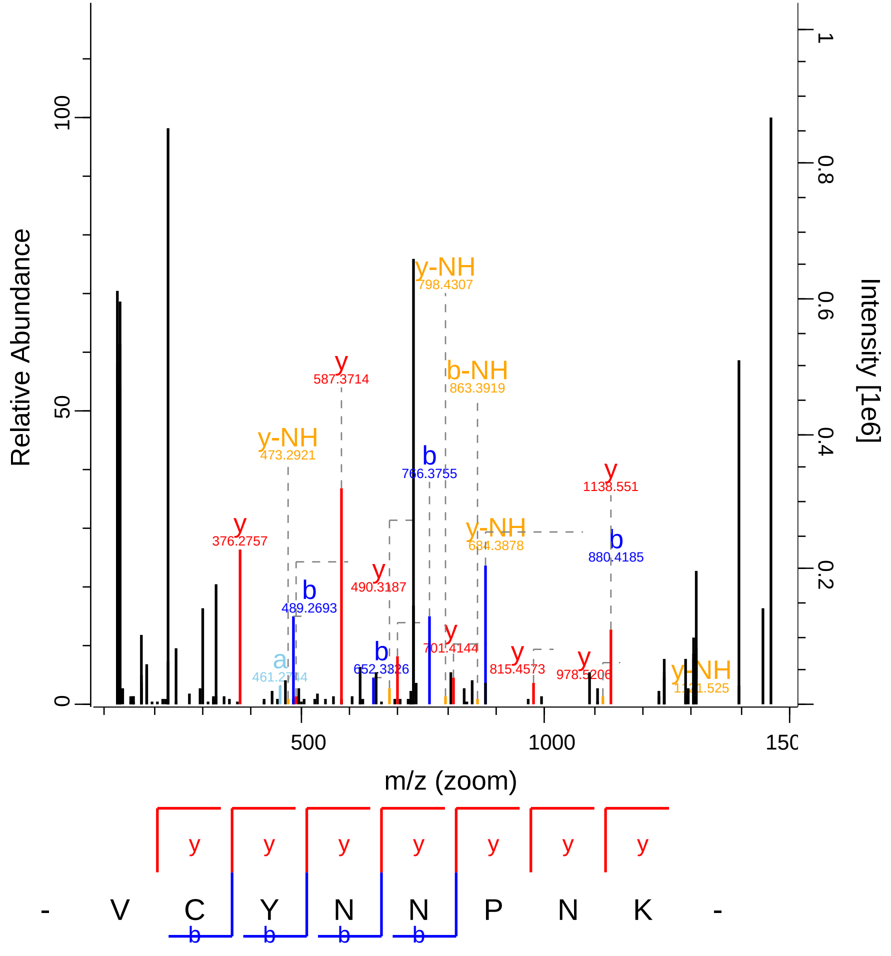

Raw File	Scan	Method	Score	m/z	Gene names
RO-QC-160121_ZackD_SS_Set_02_Fractior	11618	FTMS; HC	112.17	637.42	NOVA2

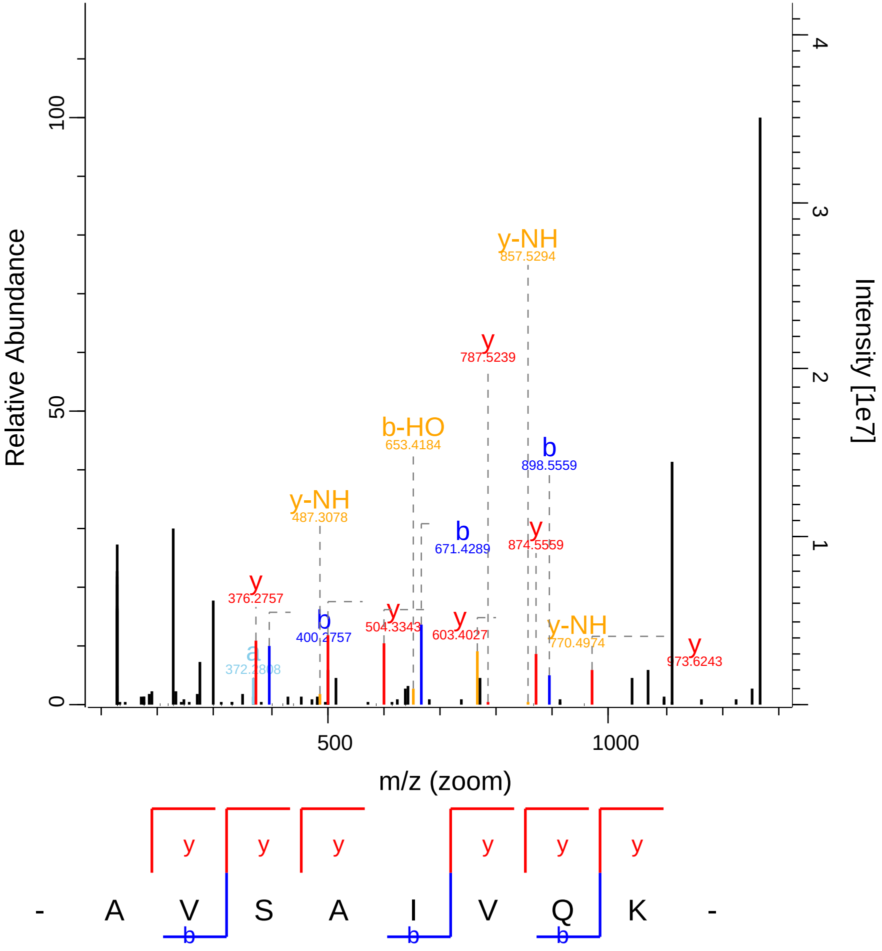

Raw File	Scan	Method	Score	m/z	Gene names
RO-QC-160121_ZackD_SS_Set_02_Fractior	11687	FTMS; HC	58.92	425.28	NOVA2

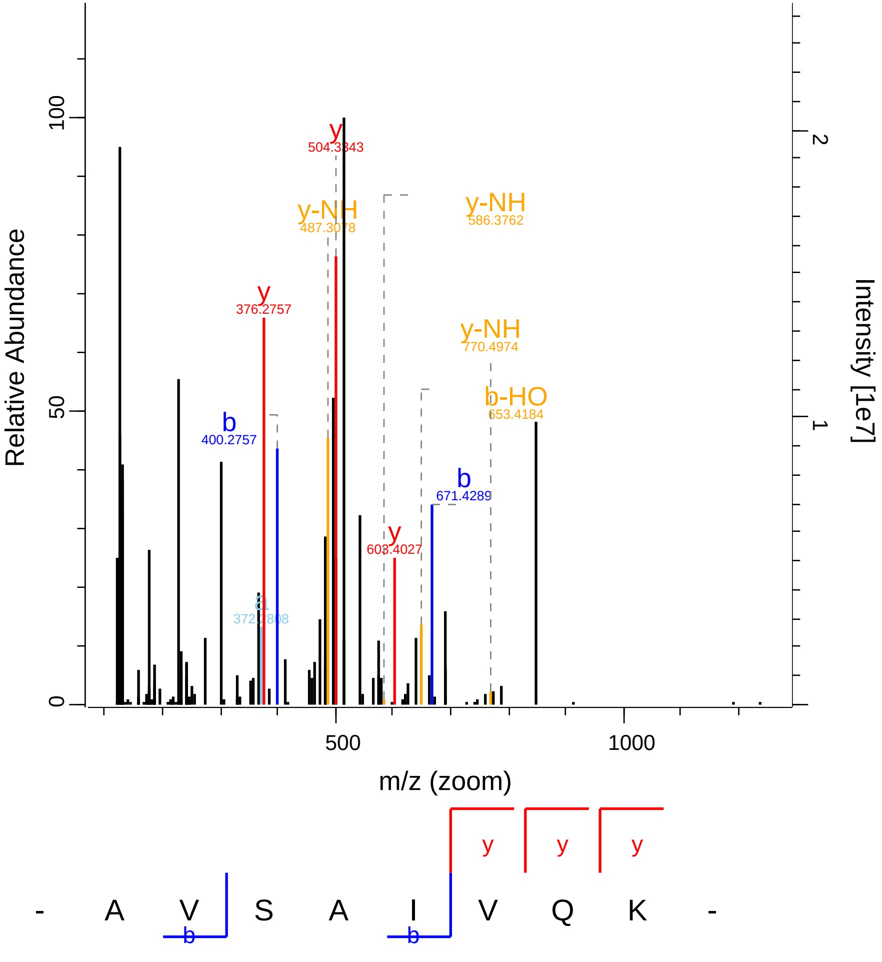

Raw File	Scan	Method	Score	m/z	Gene names
RO-QC-160121_ZackD_SS_Set_02_Fraction	12851	FTMS; HC	107.06	843.9	PLLP

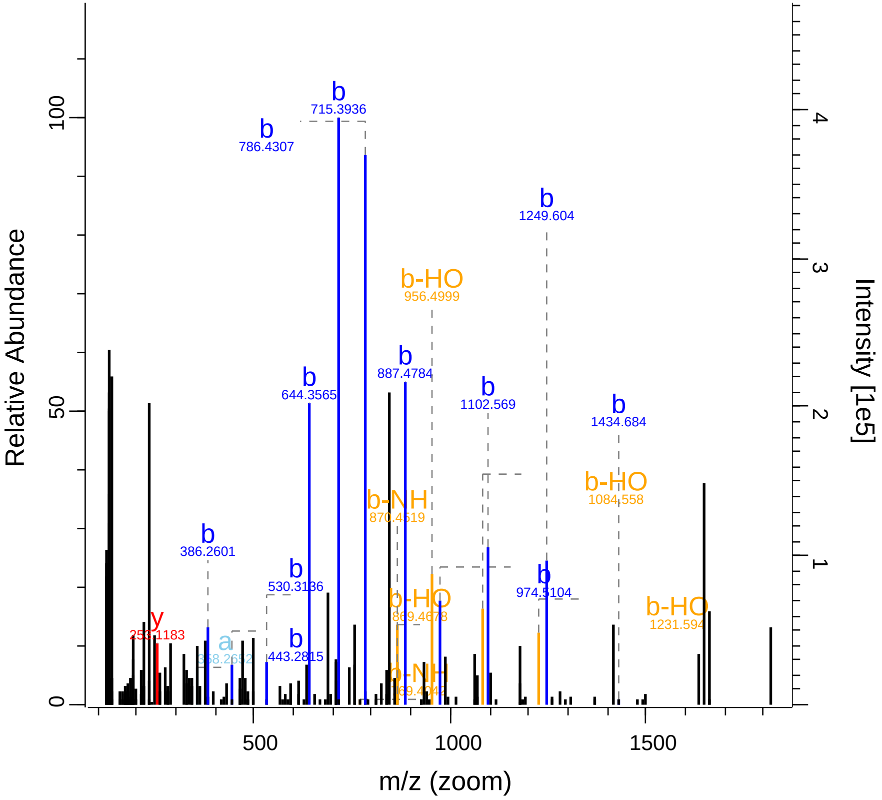

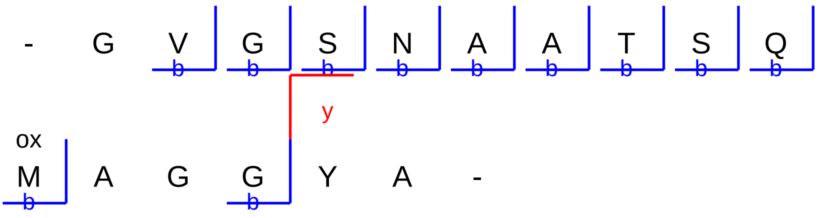

Raw File	Scan	Method	Score	m/z	Gene names
RO-QC-160121_ZackD_SS_Set_02_Fractior	12917	FTMS; HC	48.42	403.74	CCDC146

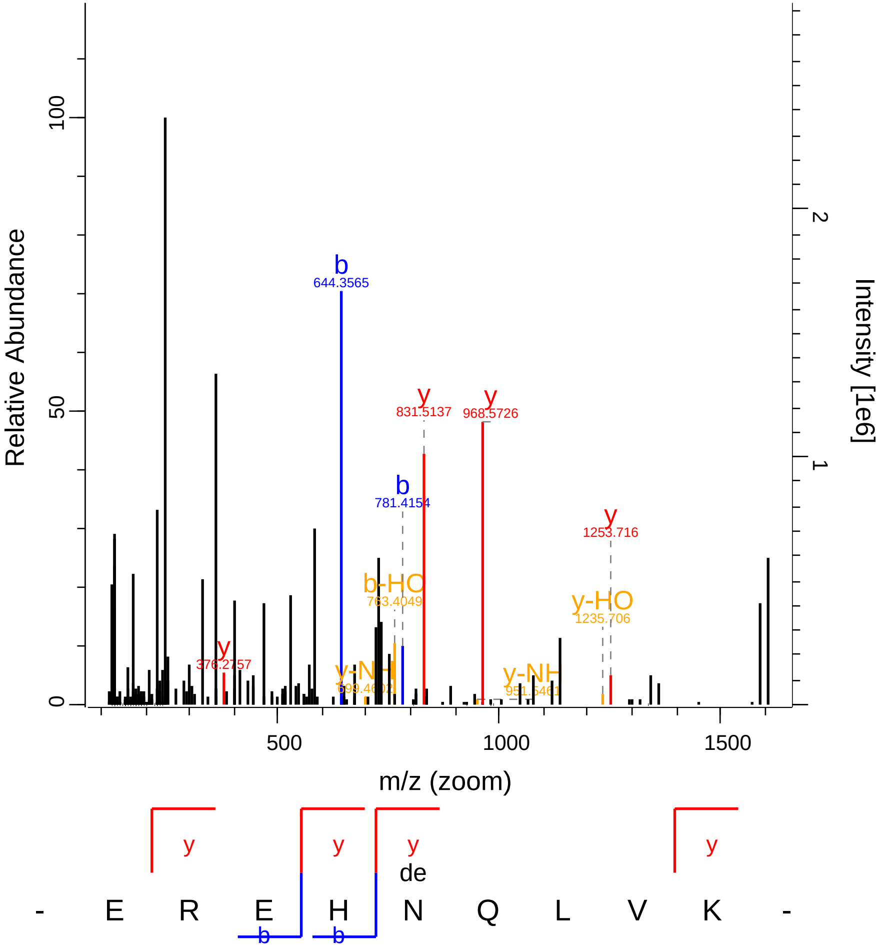

Raw File	Scan	Method	Score	m/z	Gene names
RO-QC-160121_ZackD_SS_Set_02_Fractior	13106	FTMS; HC	87.48	479.29	COX8A

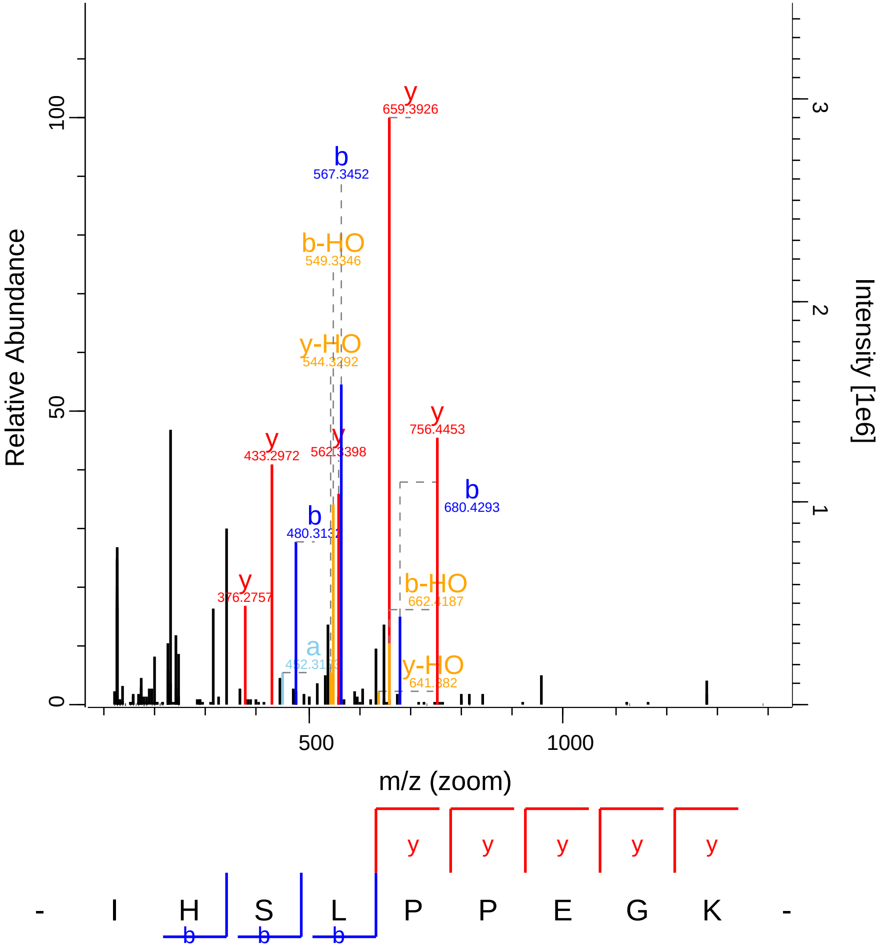

Raw File	Scan	Method	Score	m/z
RO-QC-160121_ZackD_SS_Set_02_Fraction_12	13994	FTMS; HCD	43.12	453.6

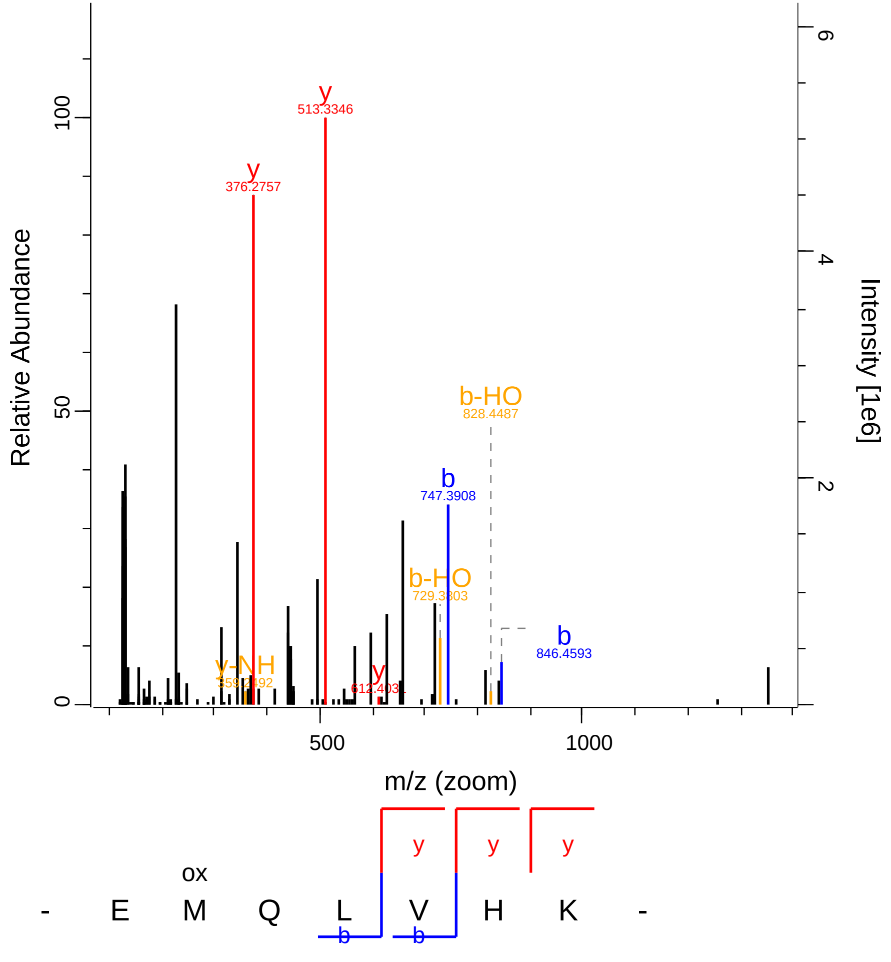

Raw File	Scan	Method	Score	m/z
RO-QC-160121_ZackD_SS_Set_02_Fraction_12	14010	FTMS; HCD	57.1	423.28

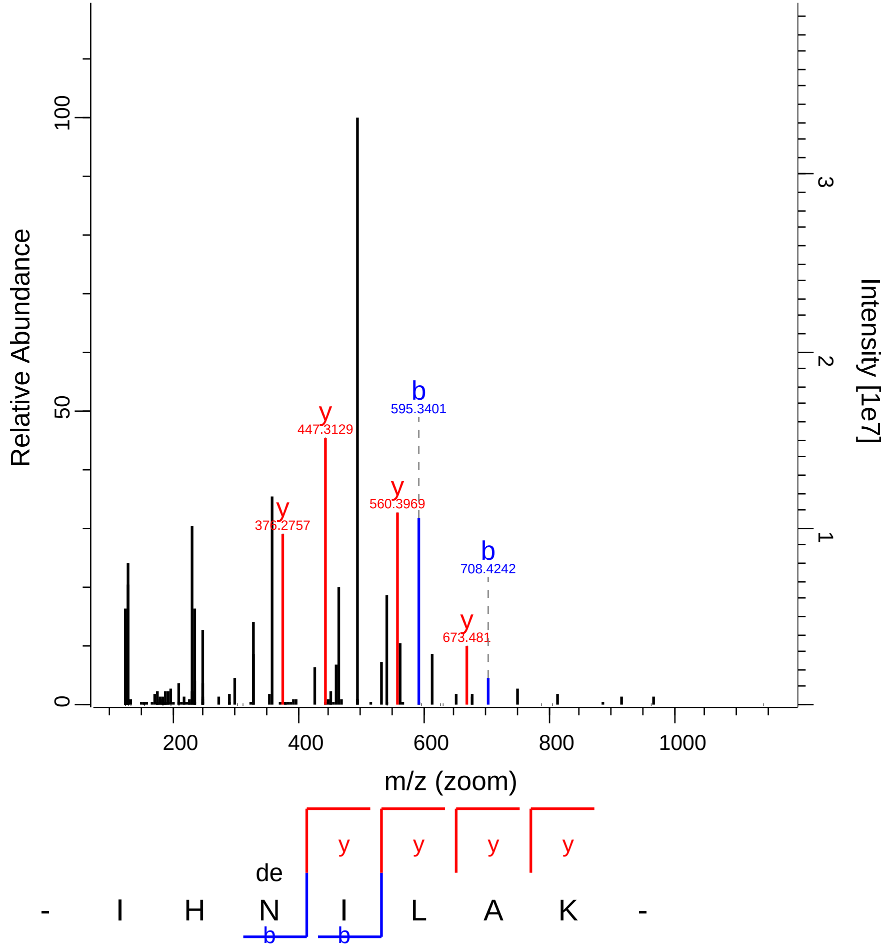

Raw File	Scan	Method	Score	m/z
RO-QC-160121_ZackD_SS_Set_02_Fraction_12	14236	FTMS; HCD	57.1	423.28

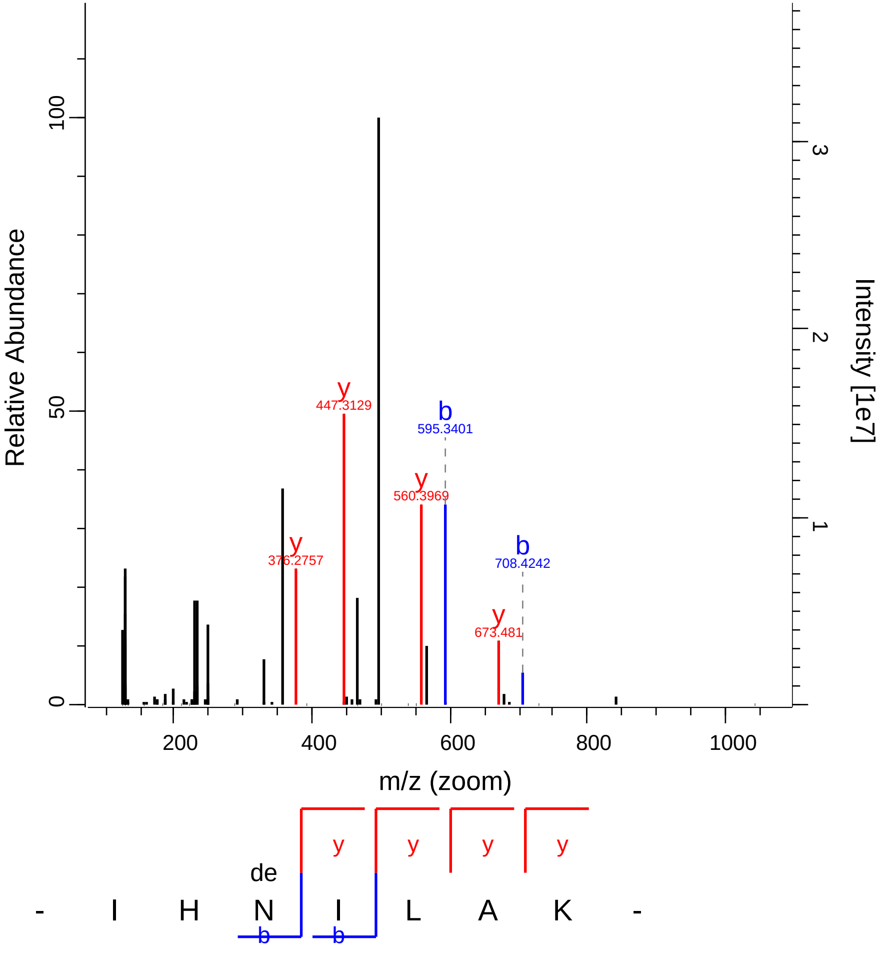

Raw File	Scan	Method	Score	m/z	Gene names
RO-QC-160121_ZackD_SS_Set_02_Fraction	15193	FTMS; HC	41.26	697.6	CCSMST1

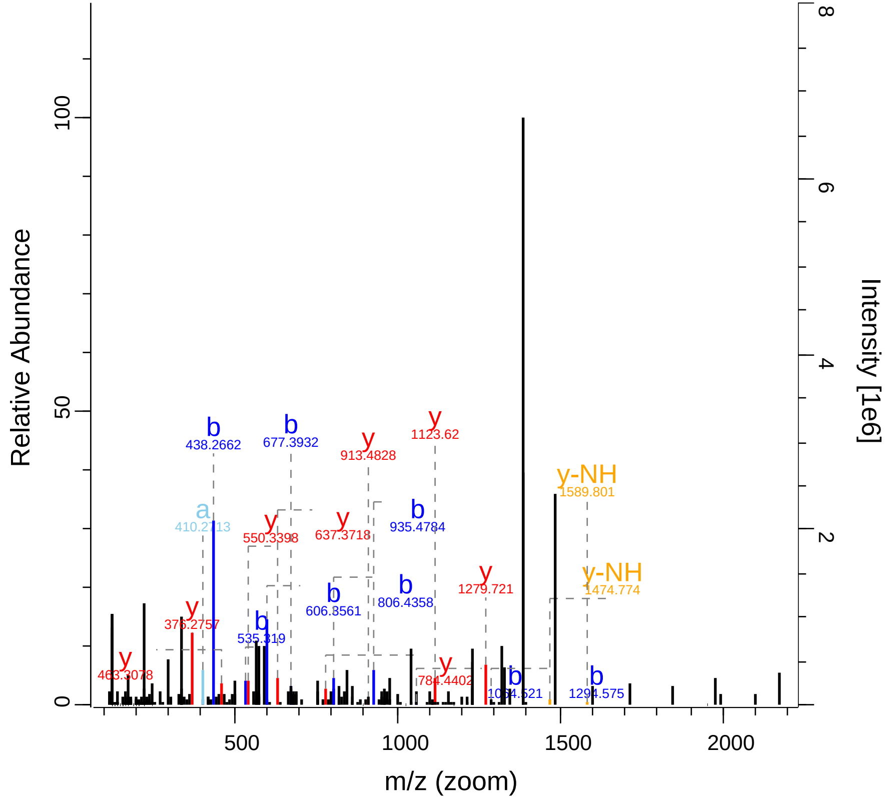

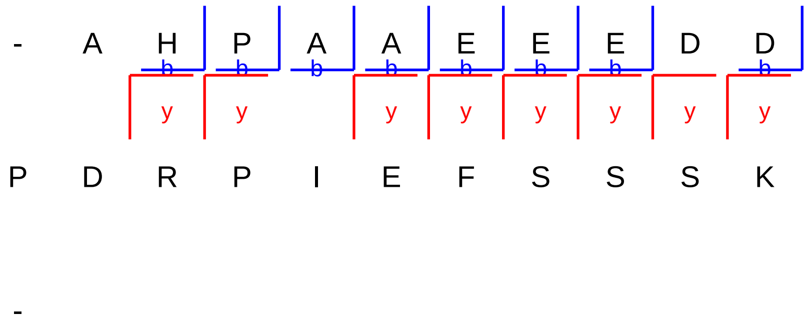

Raw File	Scan	Method	Score	m/z	Gene names
RO-QC-160121_ZackD_SS_Set_02_Fractior	15408	FTMS; HC	171.92	819.98	PLK2

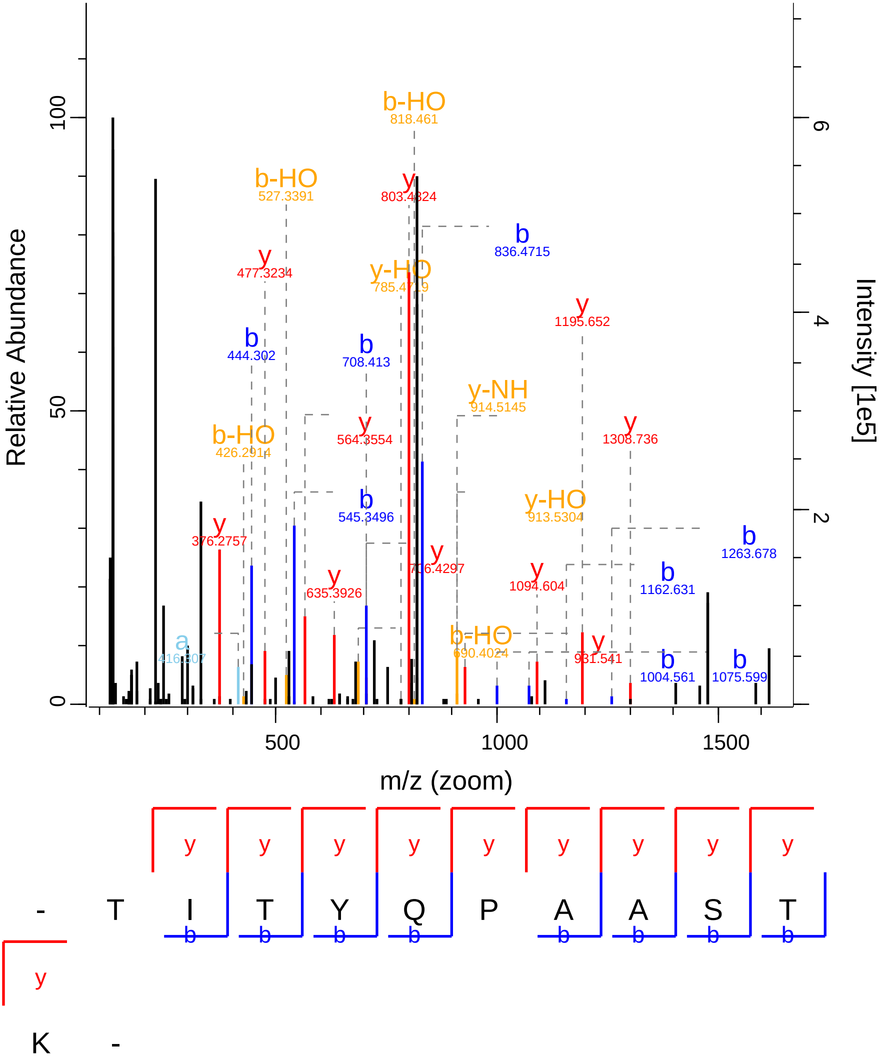

Raw File	Scan	Method	Score	m/z	Gene names
RO-QC-160121_ZackD_SS_Set_02_Fractio	15519	FTMS; HC	120.15	797.42	CLDN1

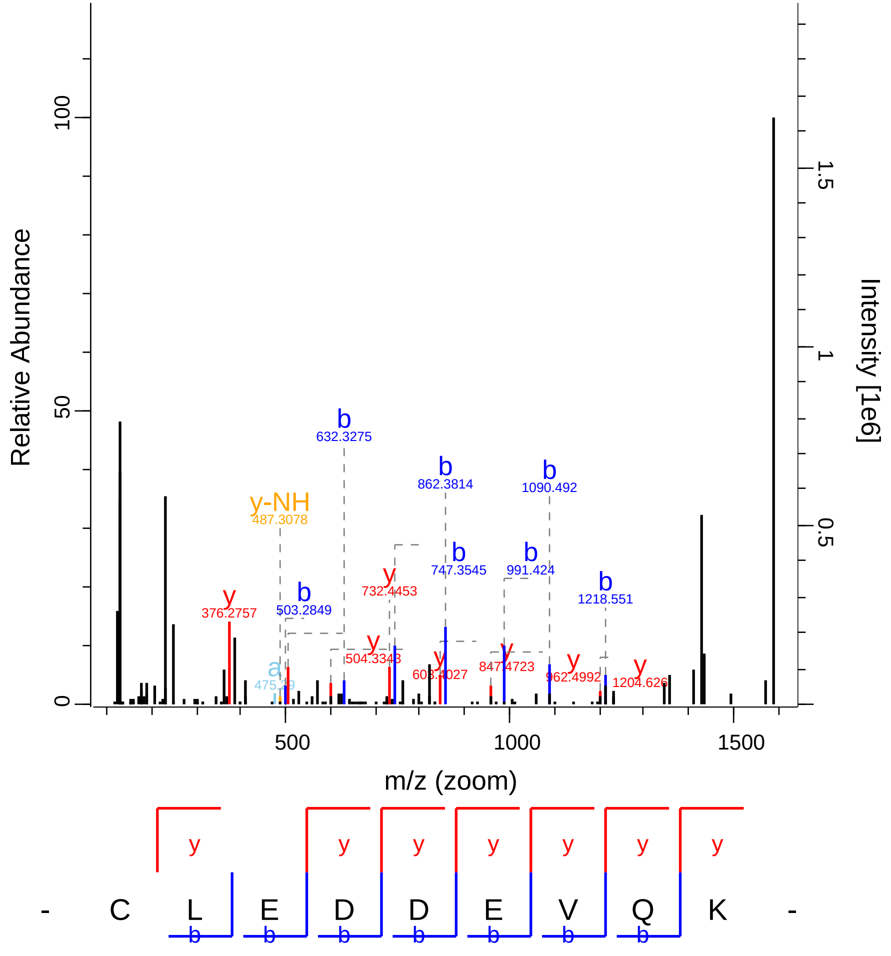

Raw File	Scan	Method	Score	m/z	Gene names
RO-QC-160121_ZackD_SS_Set_02_Fractior	16219	FTMS; HC	84.17	749.44	LRRC32

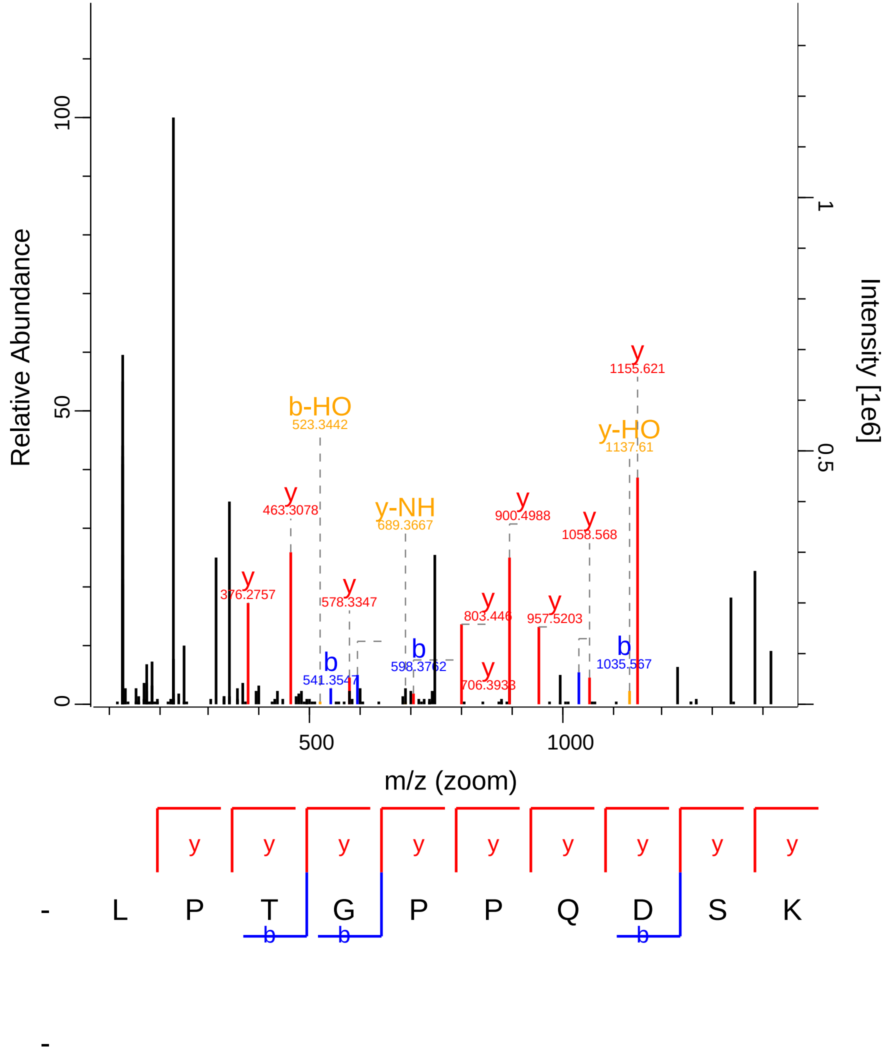

Raw File	Scan	Method	Score	m/z	Gene names
RO-QC-160121_ZackD_SS_Set_02_Fractio	16873	FTMS; HC	107.66	781.49	LRRIQ1

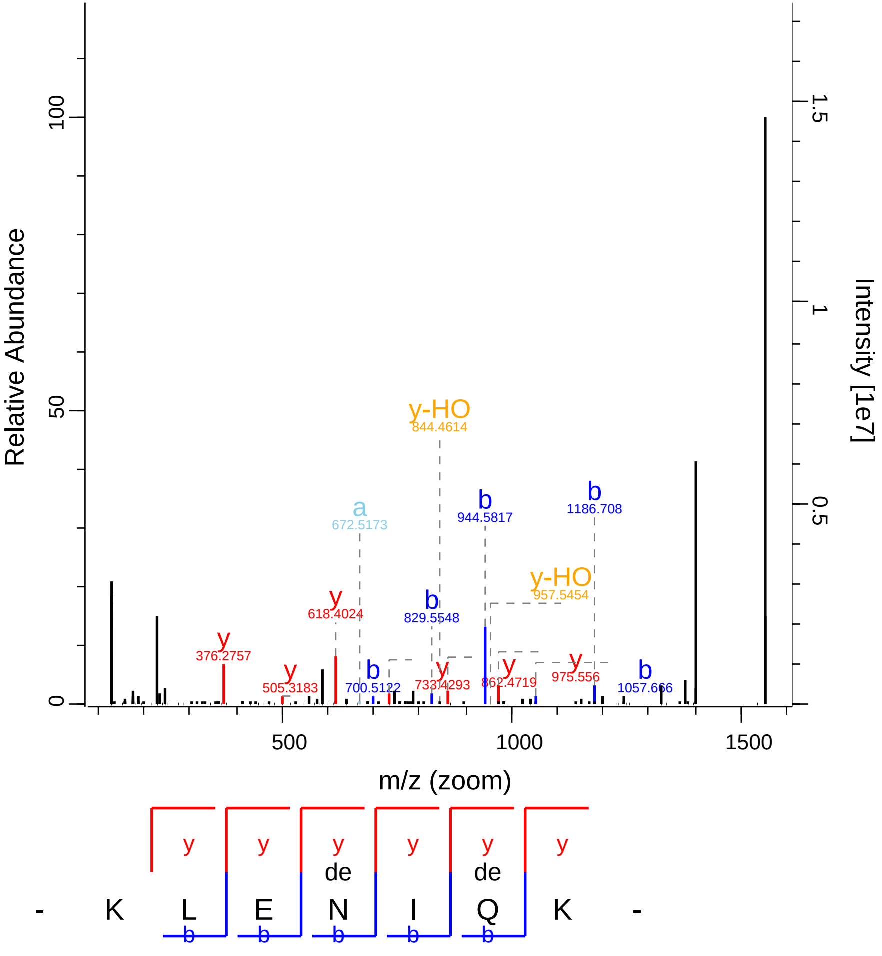

Raw File	Scan	Method	Score	m/z	Gene names
RO-QC-160121_ZackD_SS_Set_02_Fractior	17729	FTMS; HC	278.84	897.46	SMIM1

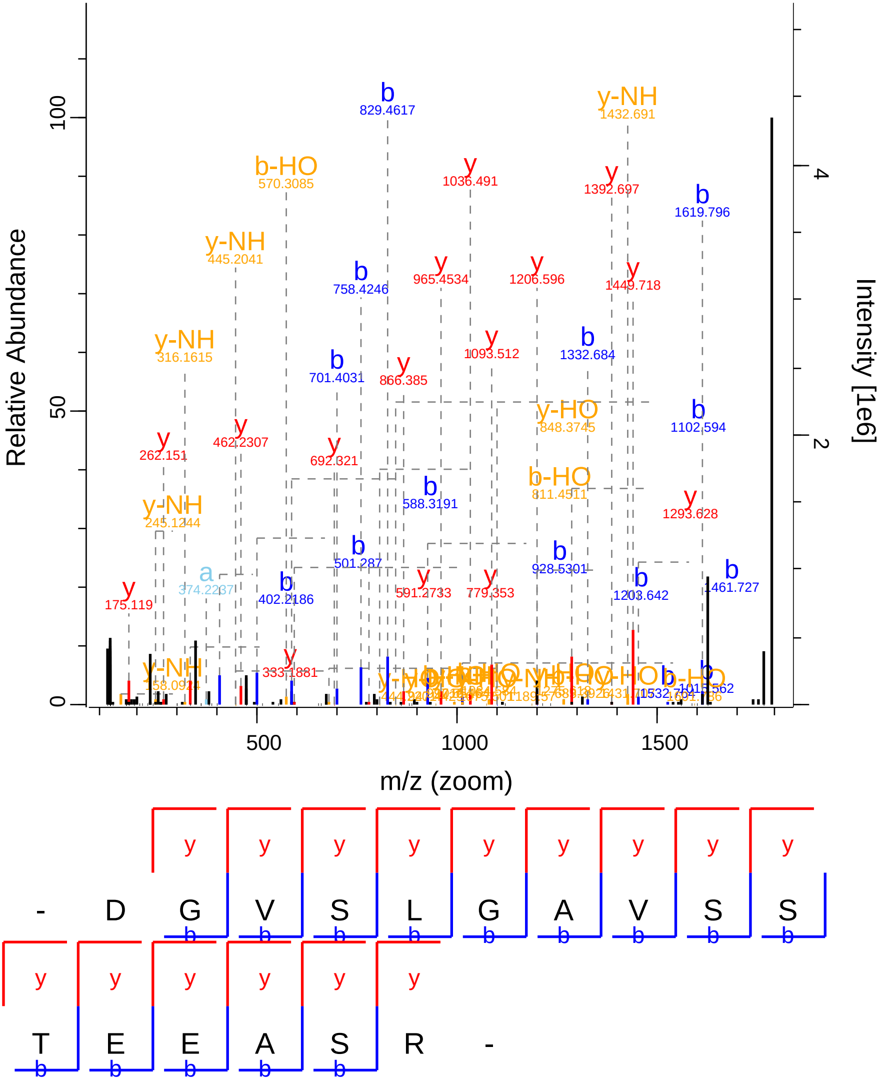

Raw File	Scan	Method	Score	m/z	Gene names
RO-QC-160121_ZackD_SS_Set_02_Fractior	17917	FTMS; HC	92.44	732.91	POMP

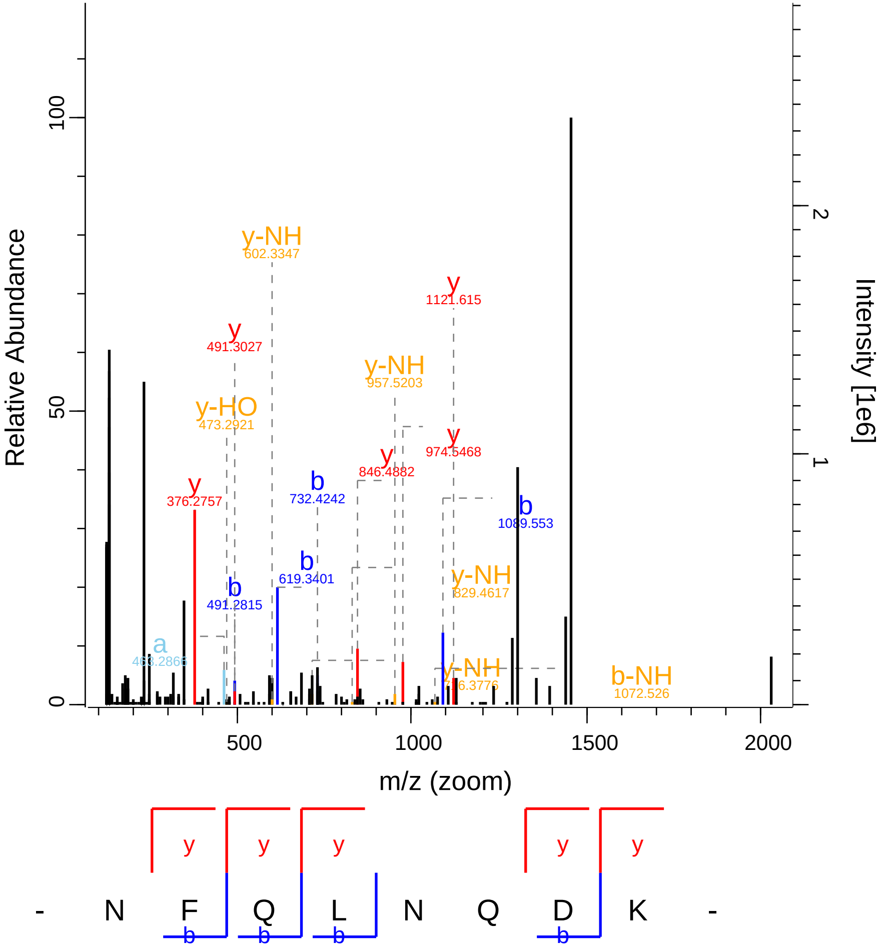

Raw File	Scan	Method	Score	m/z	Gene names
RO-QC-160121_ZackD_SS_Set_02_Fractior	17972	FTMS; HC	176.91	897.46	SMIM1

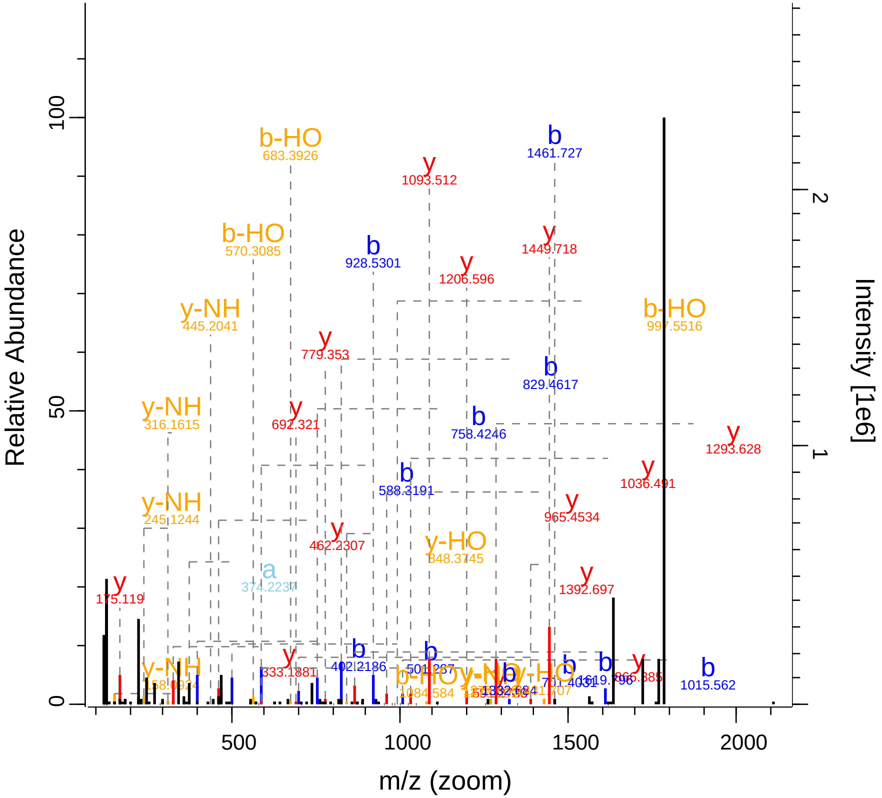

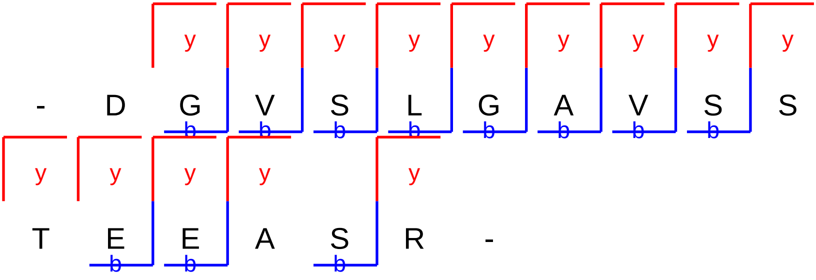

Raw File	Scan	Method	Score	m/z	Gene names
RO-QC-160121_ZackD_SS_Set_02_Fractior	18375	FTMS; HC	44.14	725.37	TTLL6

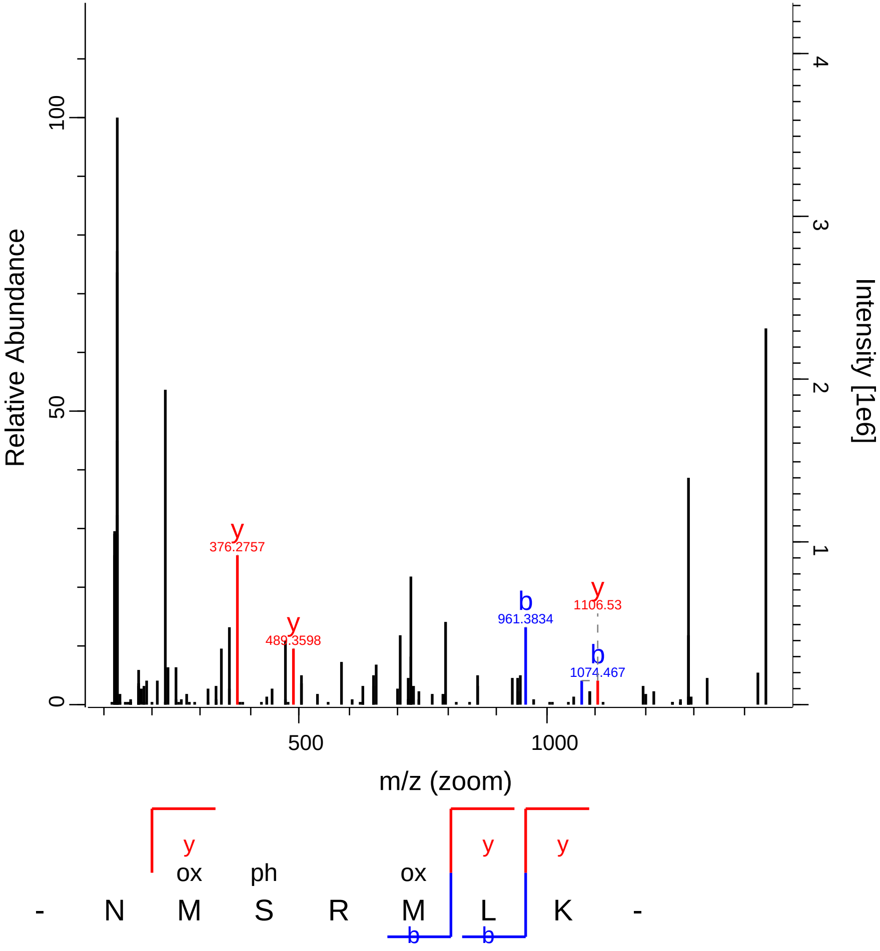

Raw File	Scan	Method	Score	m/z
RO-QC-160121_ZackD_SS_Set_02_Fraction_12	18684	FTMS; HCD	73.93	786.94

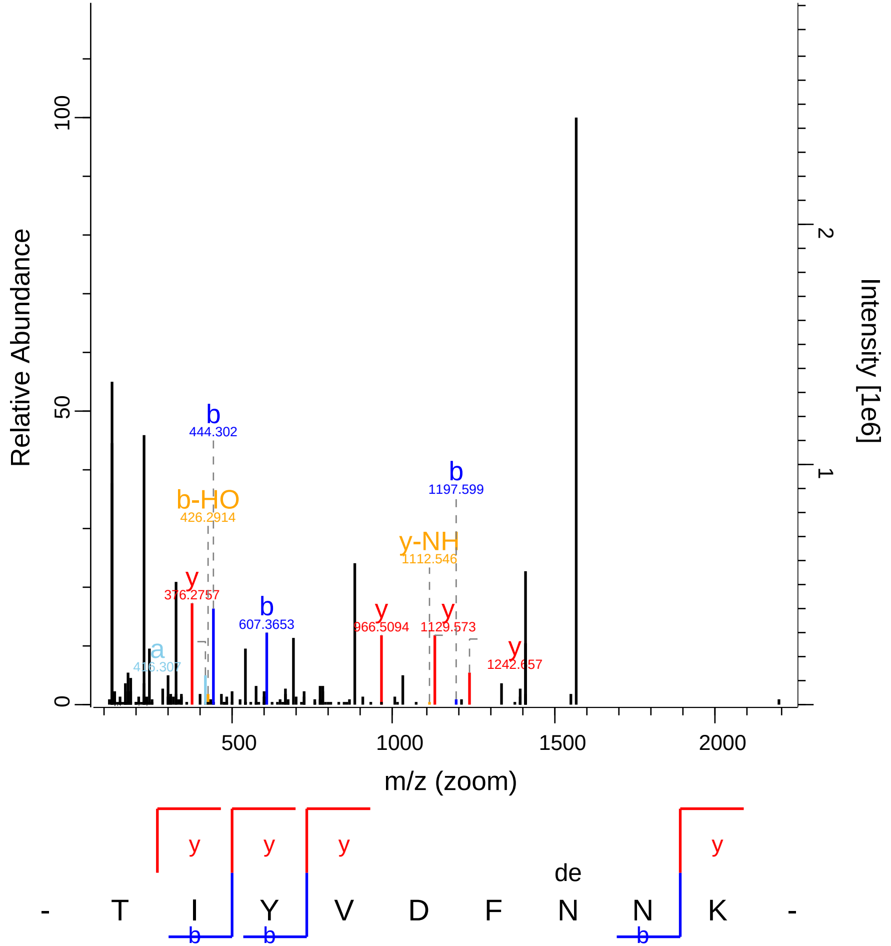

Raw File	Scan	Method	Score	m/z
RO-QC-160121_ZackD_SS_Set_02_Fraction_12	18957	FTMS; HCD	47.2	482.62

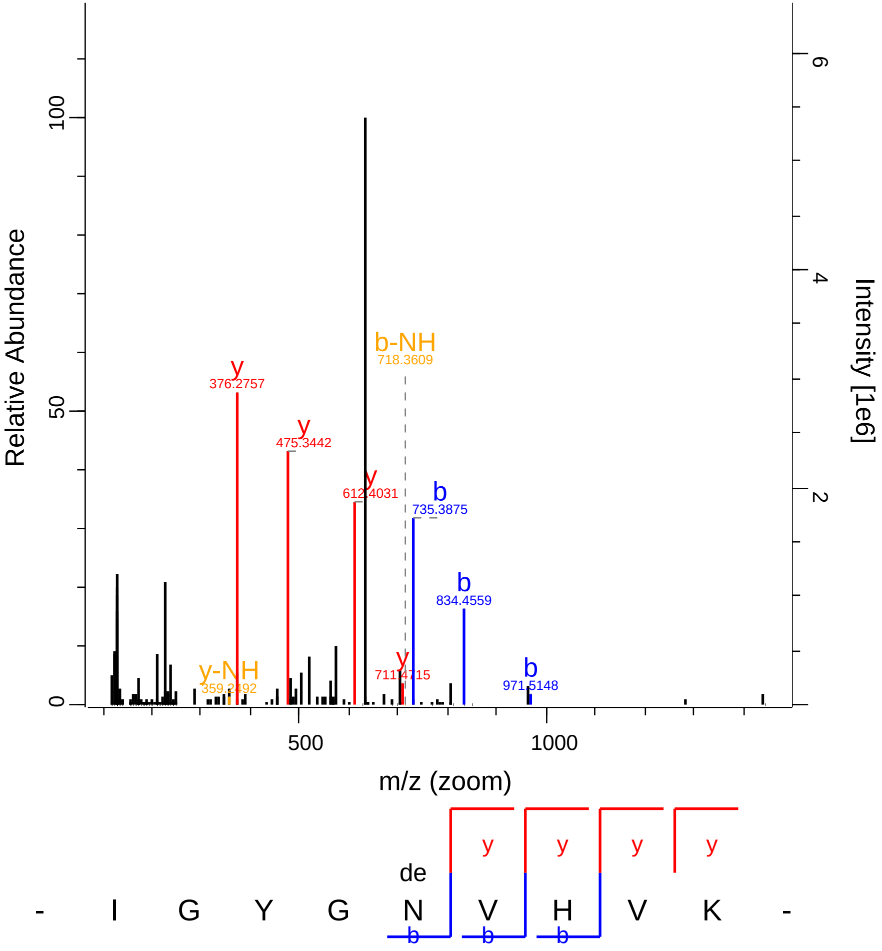

Raw File	Scan	Method	Score	m/z	Gene names
RO-QC-160121_ZackD_SS_Set_02_Fractio	19552	FTMS; HC	106.42	783.43	KIAA0226

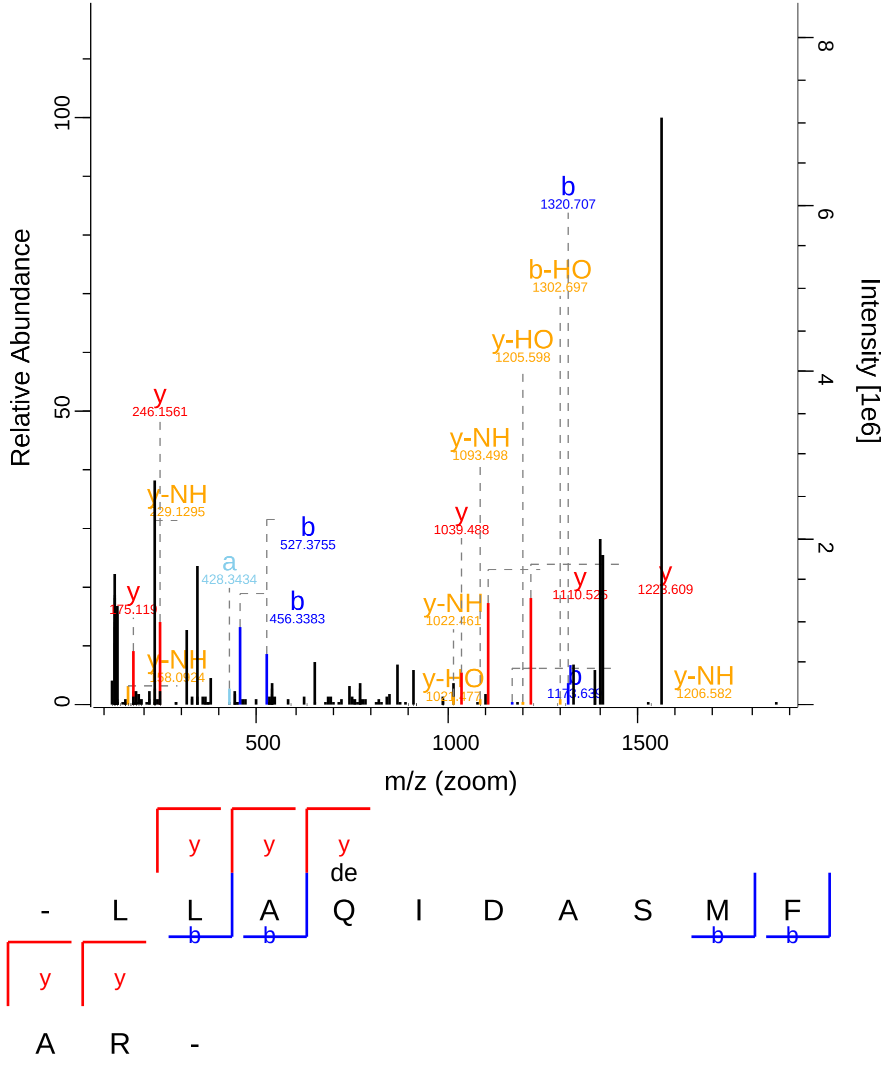

Raw File	Scan	Method	Score	m/z	Gene names
RO-QC-160121_ZackD_SS_Set_02_Fractior	19811	FTMS; HC	144.1	751.92	GFOD2

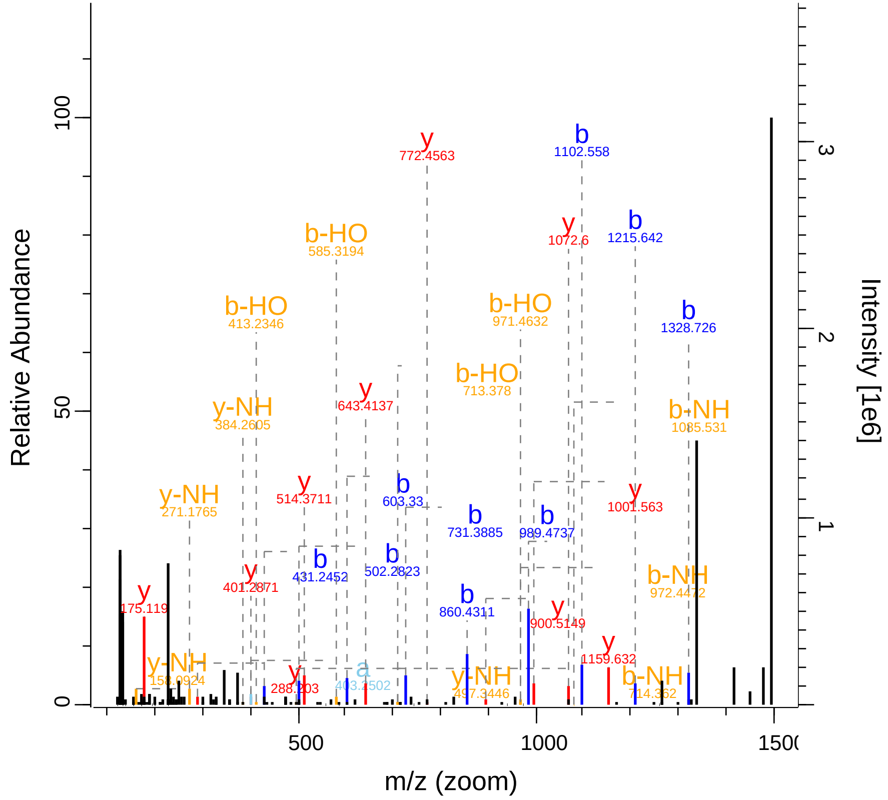

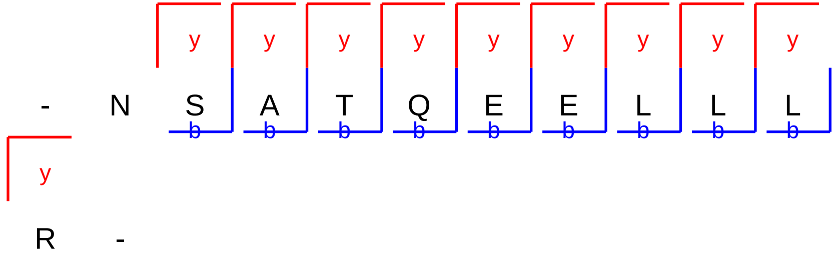

Raw File	Scan	Method	Score	m/z	Gene names
RO-QC-160121_ZackD_SS_Set_02_Fractior	20247	FTMS; HC	64.09	964.47	C3orf49

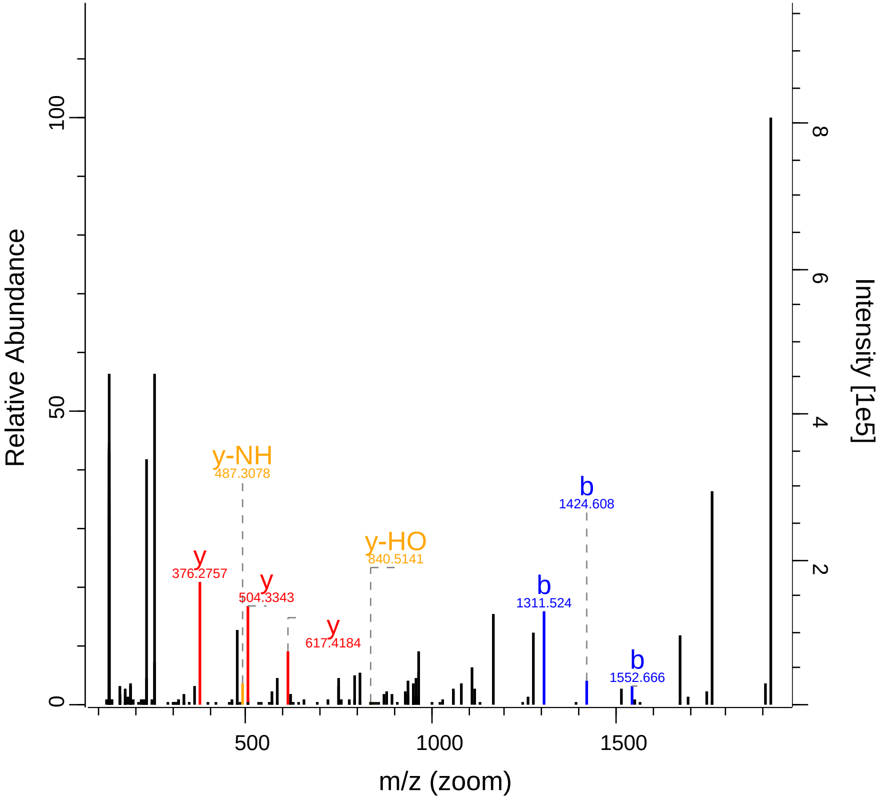

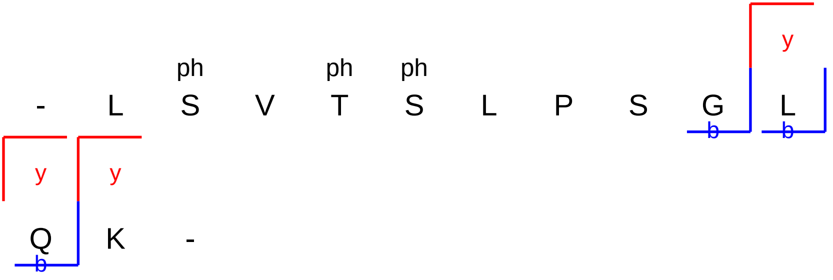

Raw File	Scan	Method	Score	m/z
RO-QC-160121_ZackD_SS_Set_02_Fraction_12	21092	FTMS; HCD	121.96	724.92

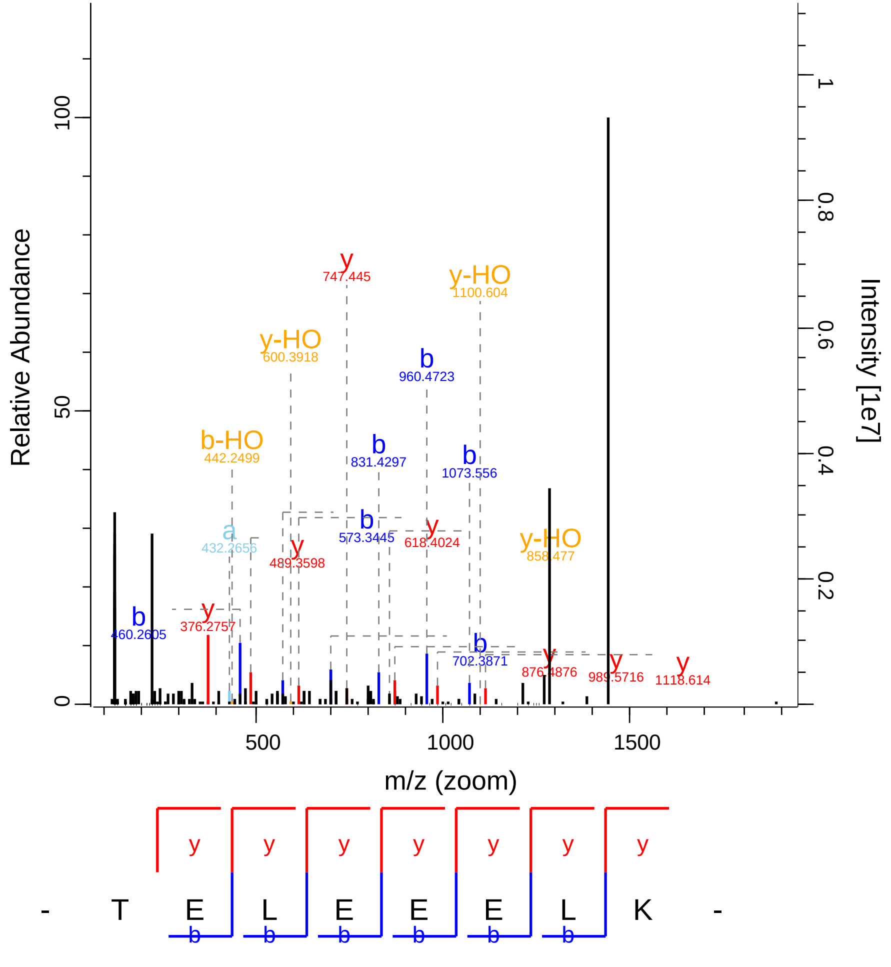

Raw File	Scan	Method	Score	m/z	Gene names
RO-QC-160121_ZackD_SS_Set_02_Fractior	21671	FTMS; HC	66.39	746.06	PRDM11

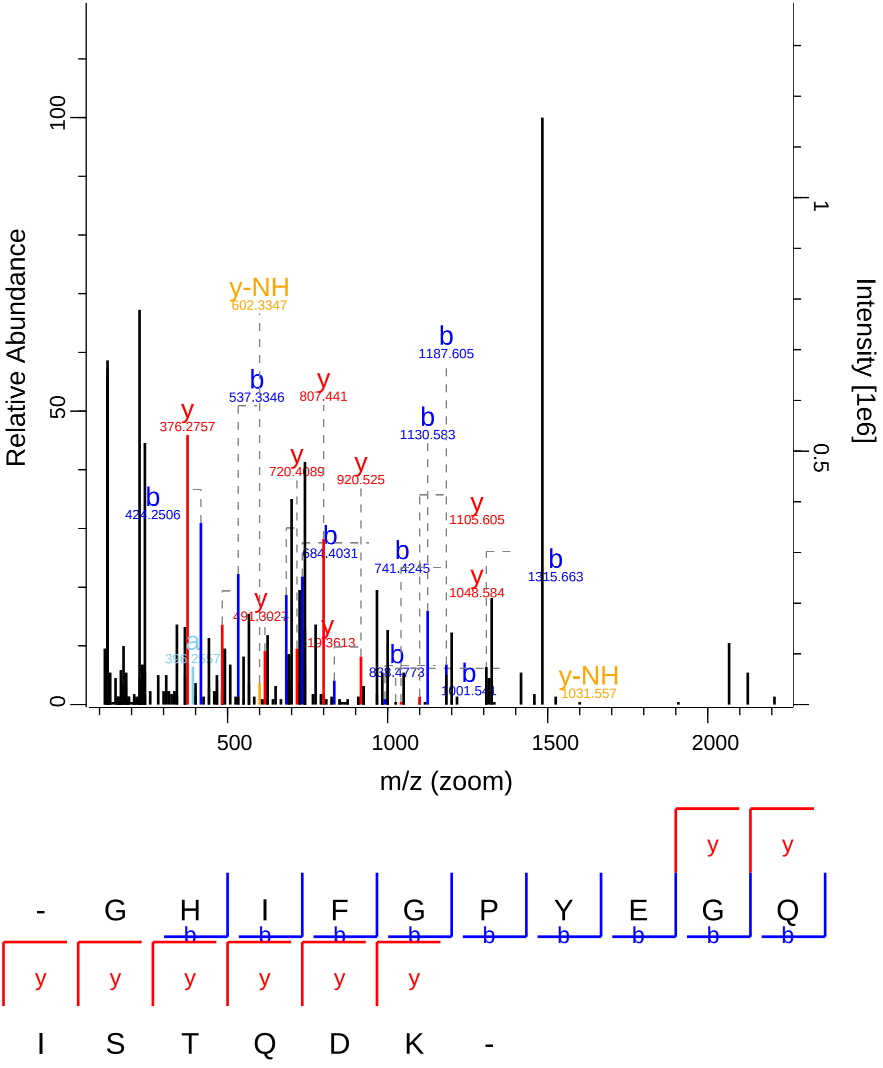

Raw File	Scan	Method	Score	m/z
RO-QC-160121_ZackD_SS_Set_02_Fraction_12	22645	FTMS; HCD	54.77	439.28

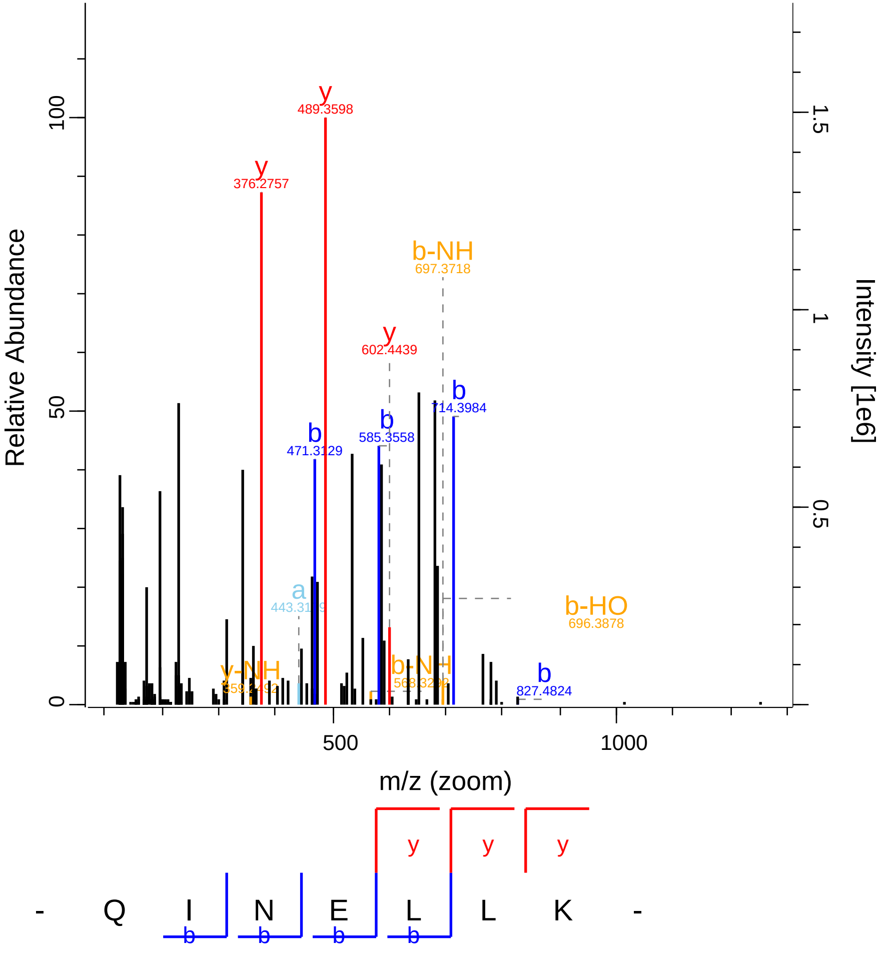

Raw File	Scan	Method	Score	m/z	Gene names
RO-QC-160121_ZackD_SS_Set_02_Fractio	22703	FTMS; HC	115.49	429.59	KANSL2

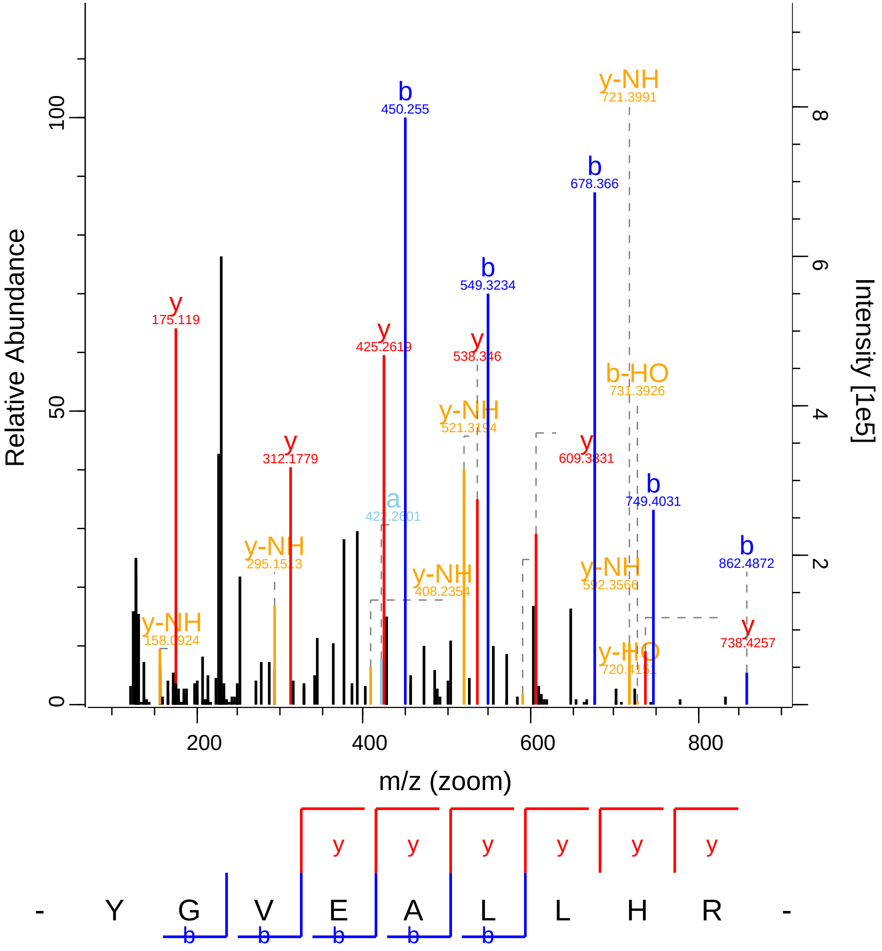

Raw File	Scan	Method	Score	m/z	Gene names
RO-QC-160121_ZackD_SS_Set_02_Fractioı	22950	FTMS; HC	140.75	538.97	SEC61B

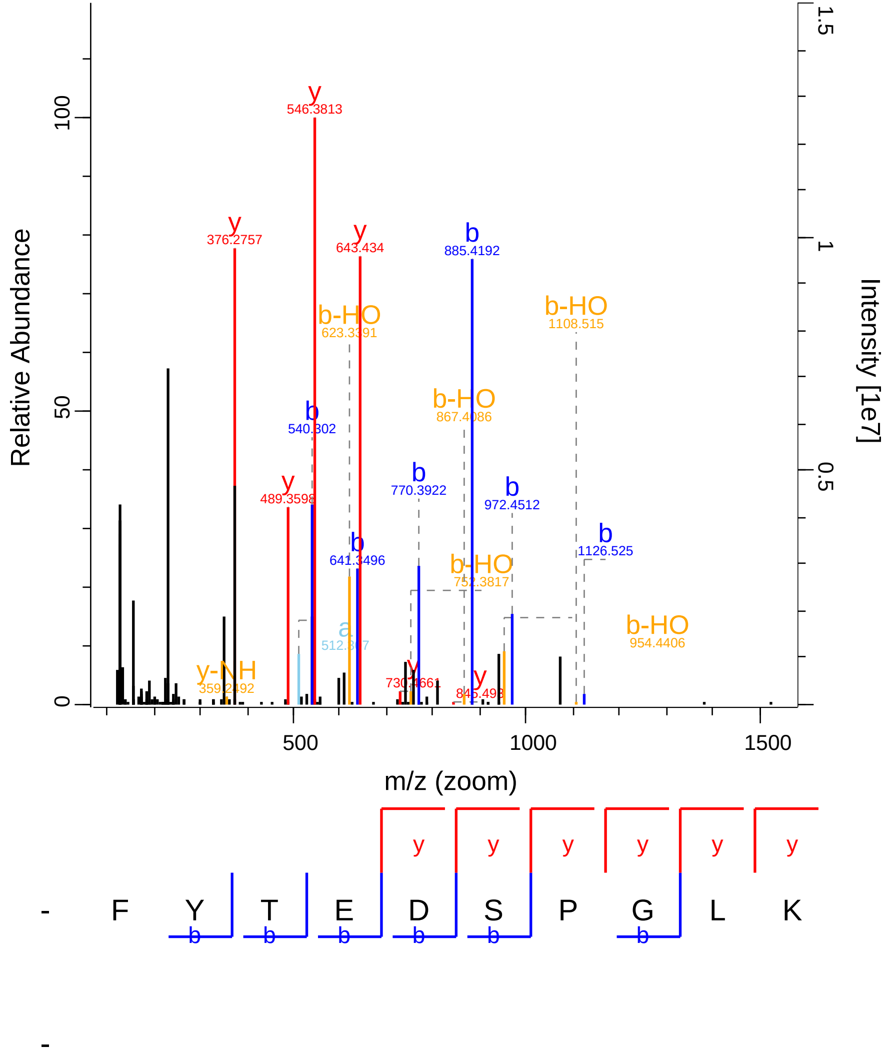

Raw File	Scan	Method	Score	m/z	Gene names
RO-QC-160121_ZackD_SS_Set_02_Fractior	22982	FTMS; HC	169.44	807.94	SEC61B

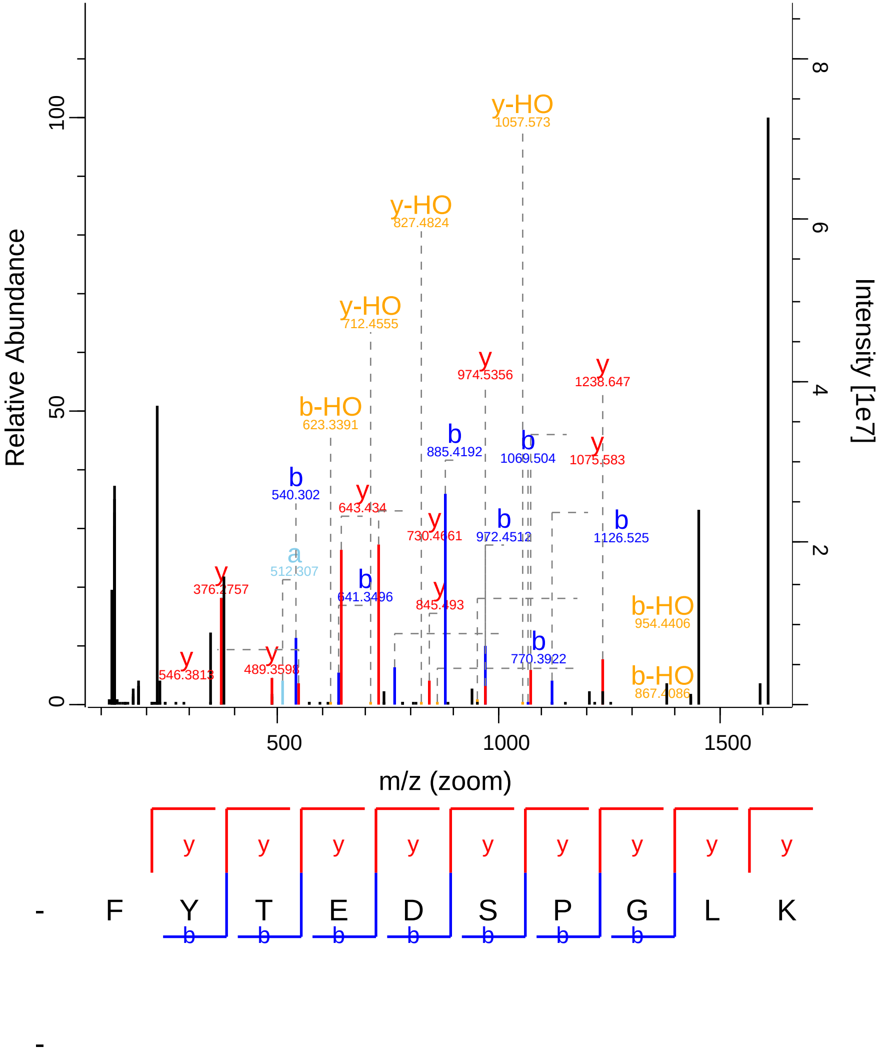

Raw File	Scan	Method	Score	m/z	Gene names
RO-QC-160121_ZackD_SS_Set_02_Fractio	23369	FTMS; HC	129.89	793.98	POLR3F

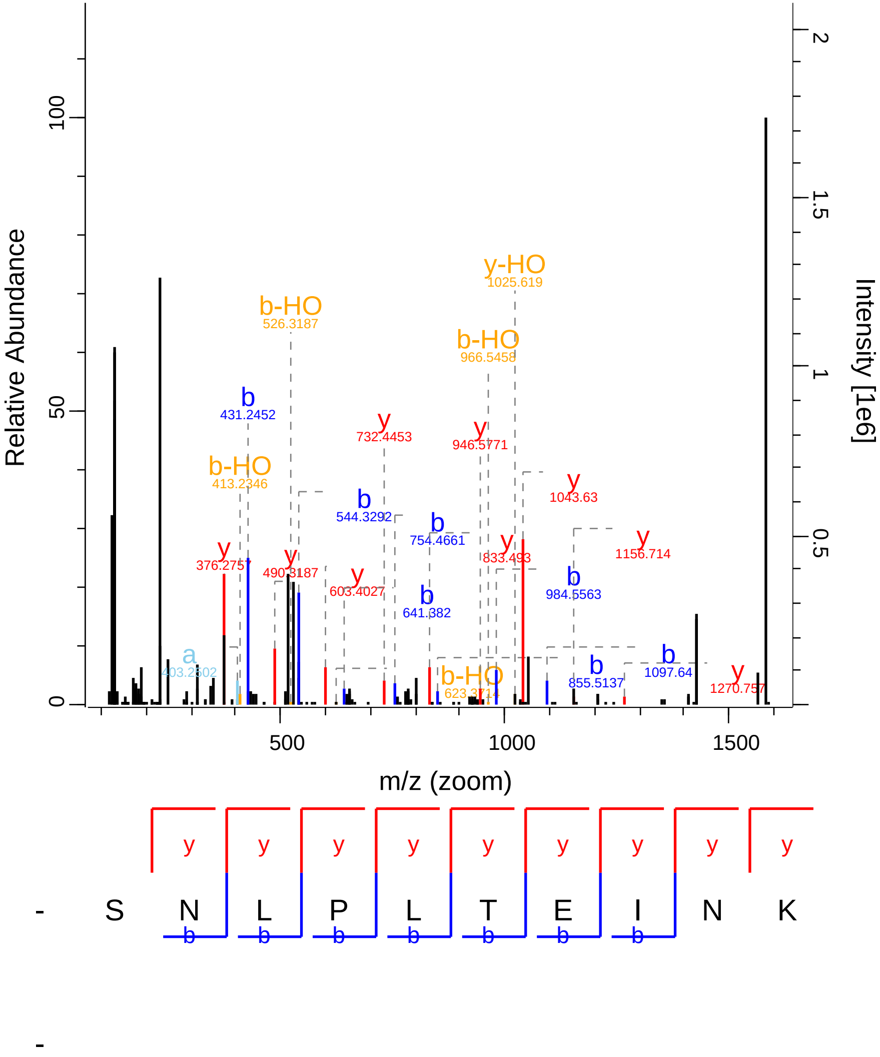

Raw File	Scan	Method	Score	m/z
RO-QC-160121_ZackD_SS_Set_02_Fraction_12	23640	FTMS; HCD	40.59	689.07

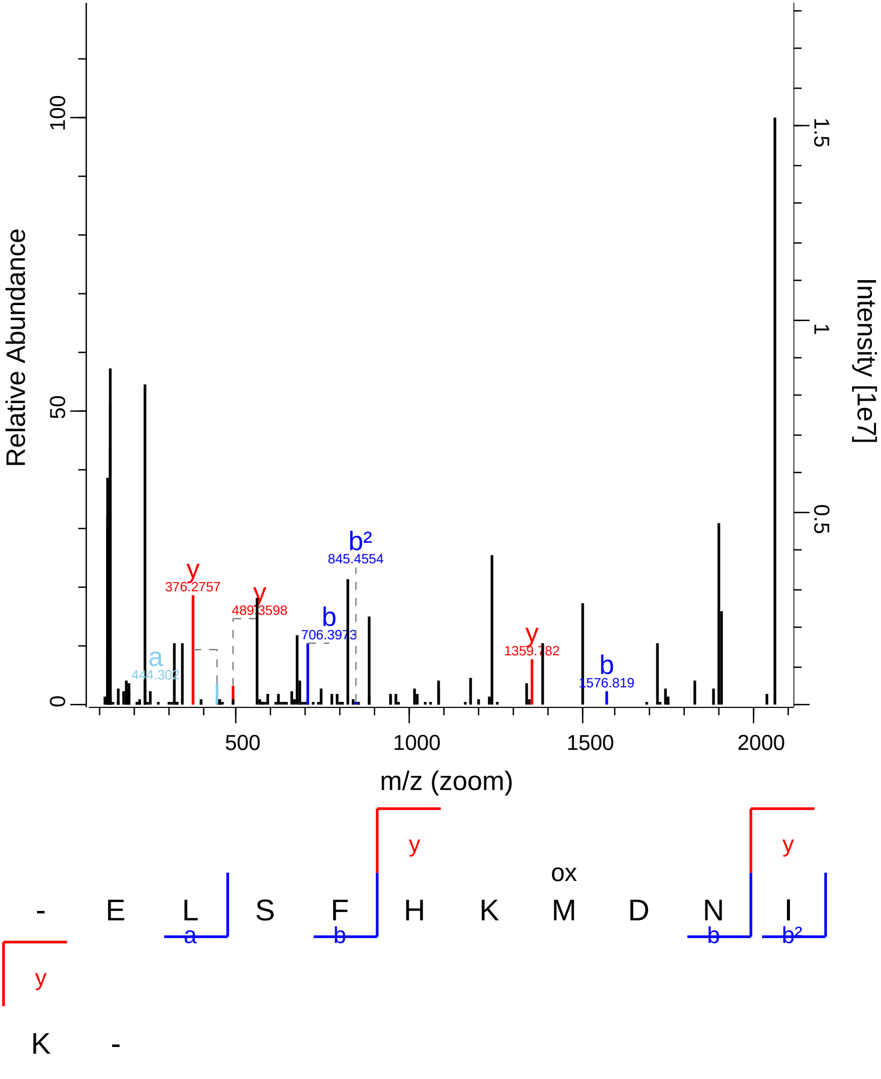

Raw File	Scan	Method	Score	m/z	Gene names
RO-QC-160121_ZackD_SS_Set_02_Fractio	25168	FTMS; HC	138.02	1080.58	CD99L2

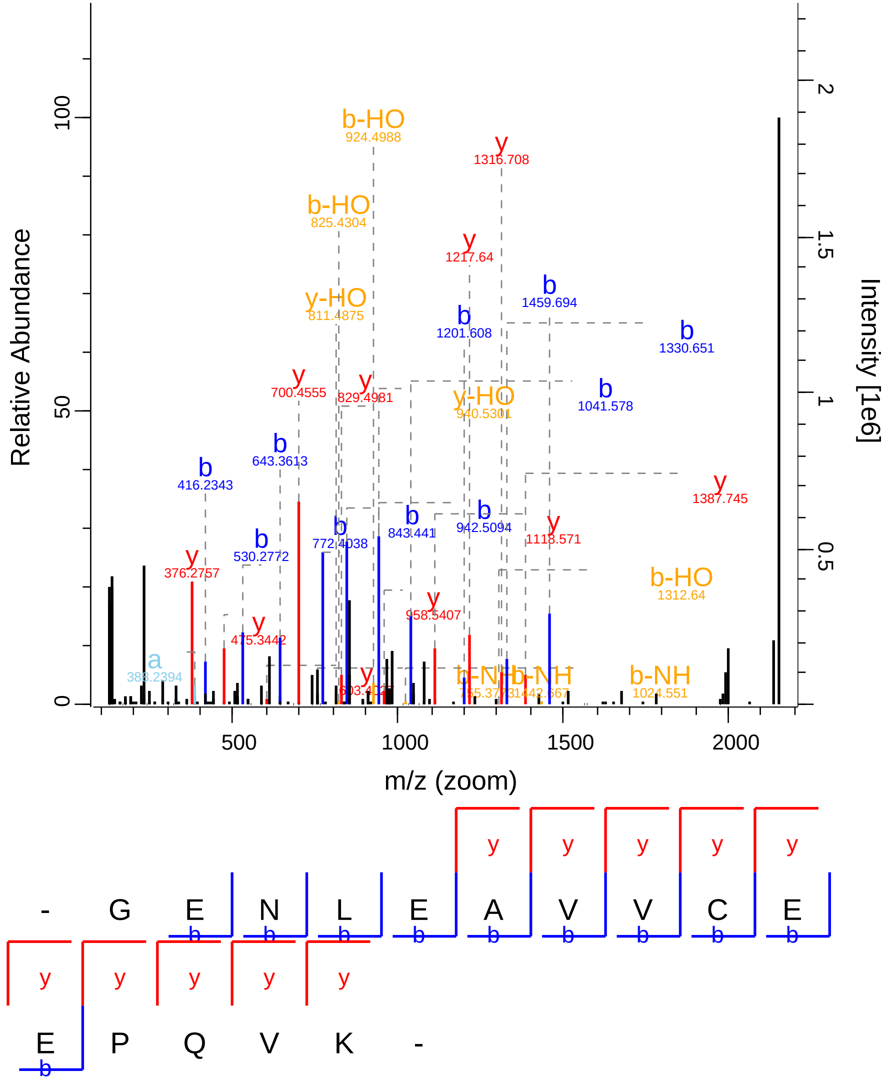

Raw File	Scan	Method	Score	m/z	Gene names
RO-QC-160121_ZackD_SS_Set_02_Fractior	25336	FTMS; HC	82.59	720.38	CD99L2

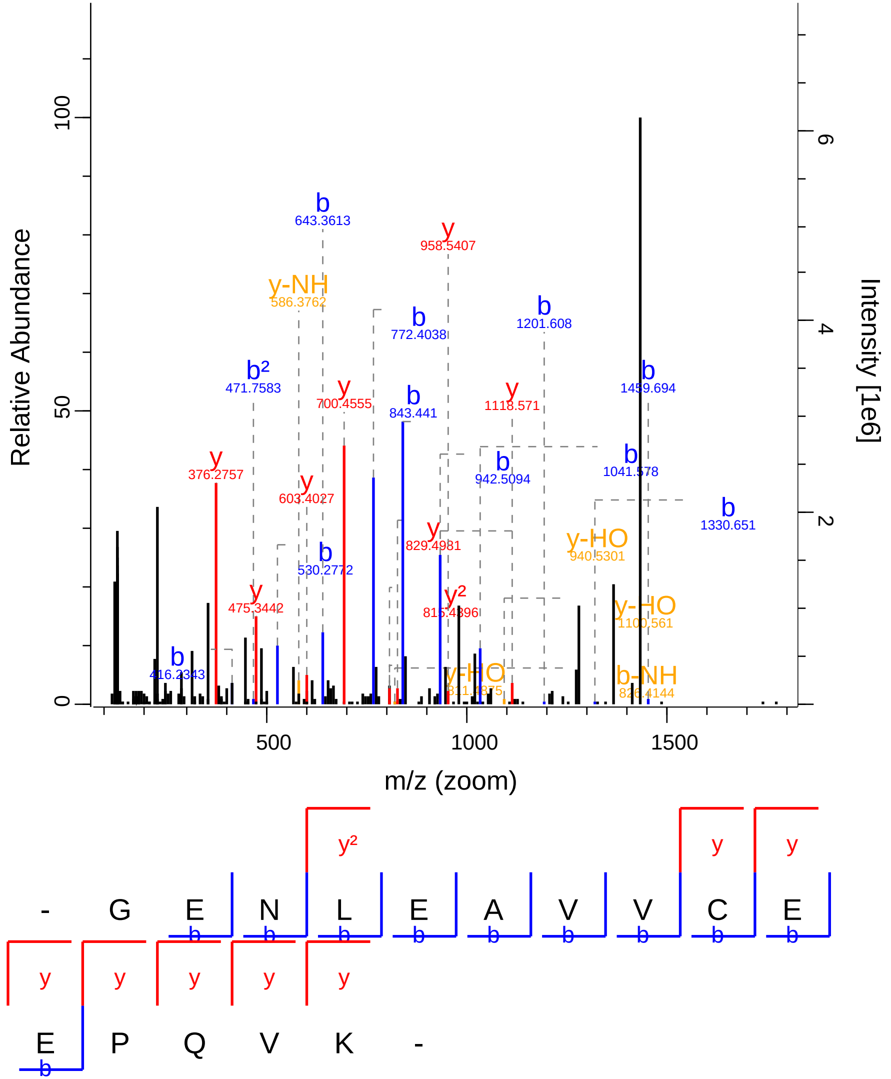

Raw File	Scan	Method	Score	m/z	Gene names
RO-QC-160121_ZackD_SS_Set_02_Fractio	25420	FTMS; HC	138.78	1009.17	MIB2

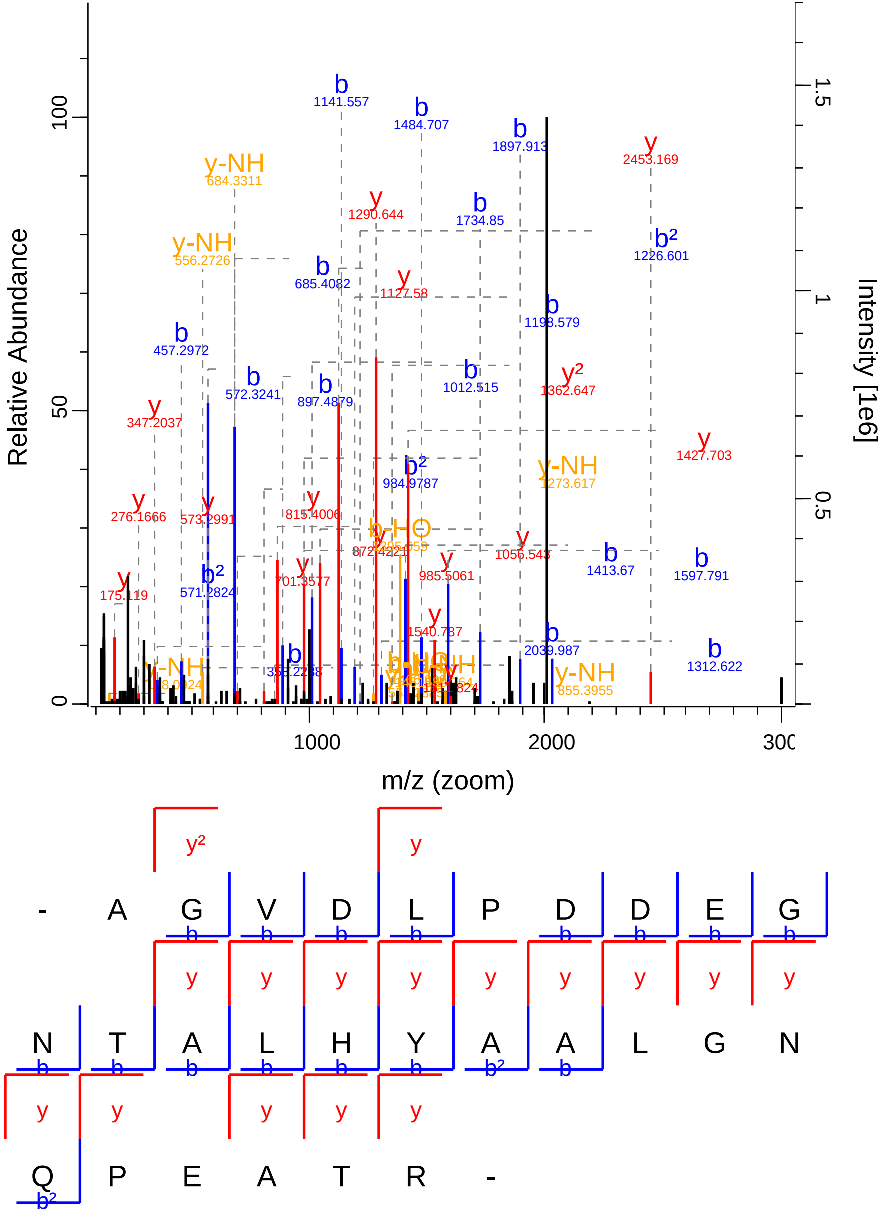

Raw File	Scan	Method	Score	m/z	Gene names
RO-QC-160121_ZackD_SS_Set_02_Fractio	25472	FTMS; HC	188.22	1008.83	MIB2

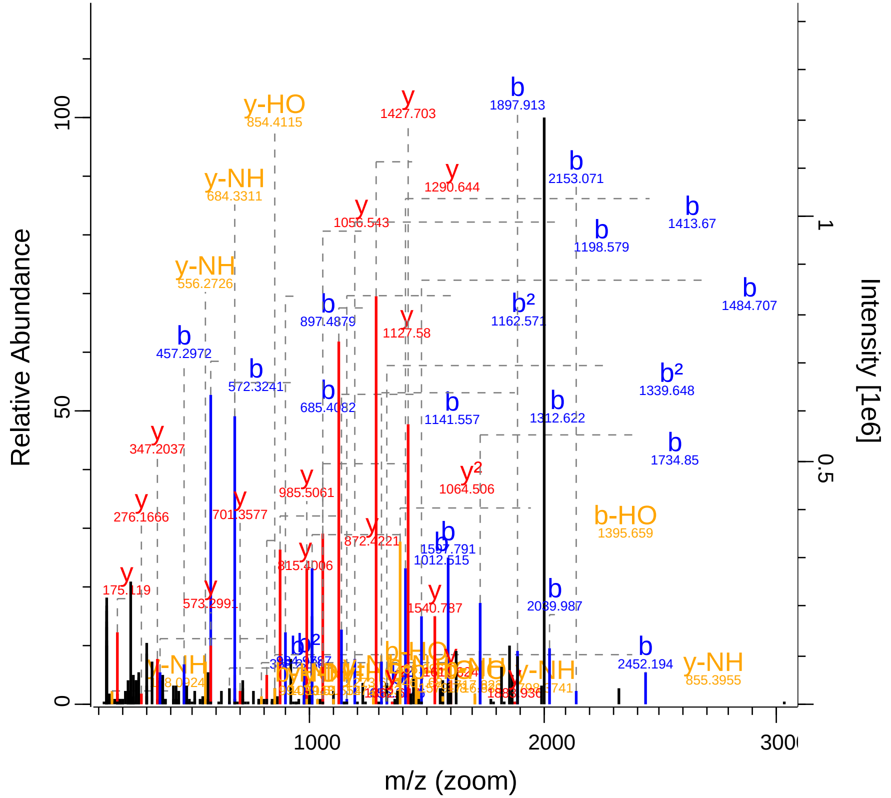

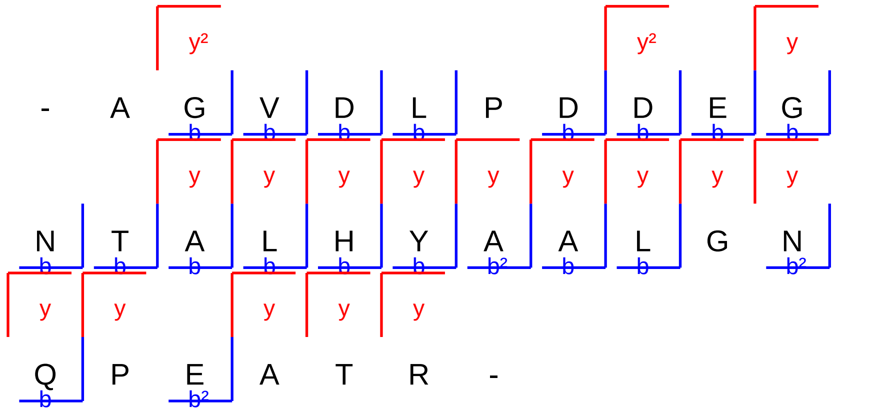

Raw File	Scan	Method	Score	m/z	Gene names
RO-QC-160121_ZackD_SS_Set_02_Fractior	25989	FTMS; HC	44.2	595.98	MRPL35

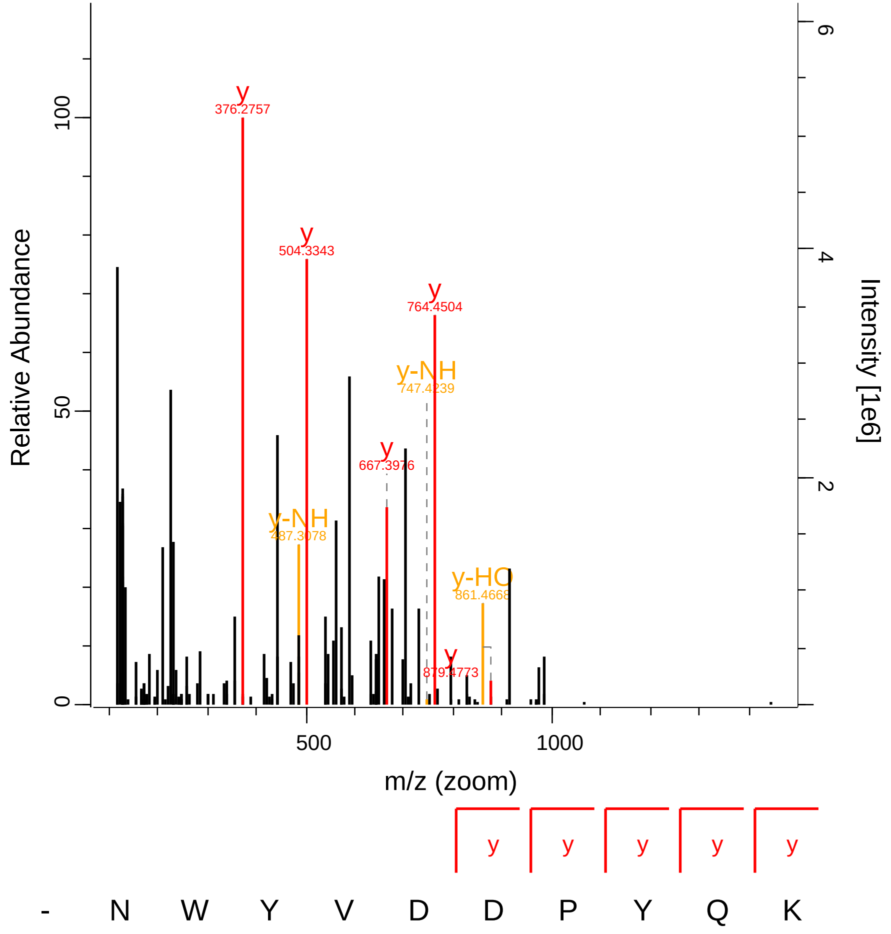

-

Raw File	Scan	Method	Score	m/z
RO-QC-160121_ZackD_SS_Set_02_Fraction_12	26028	FTMS; HCD	85.85	678.39

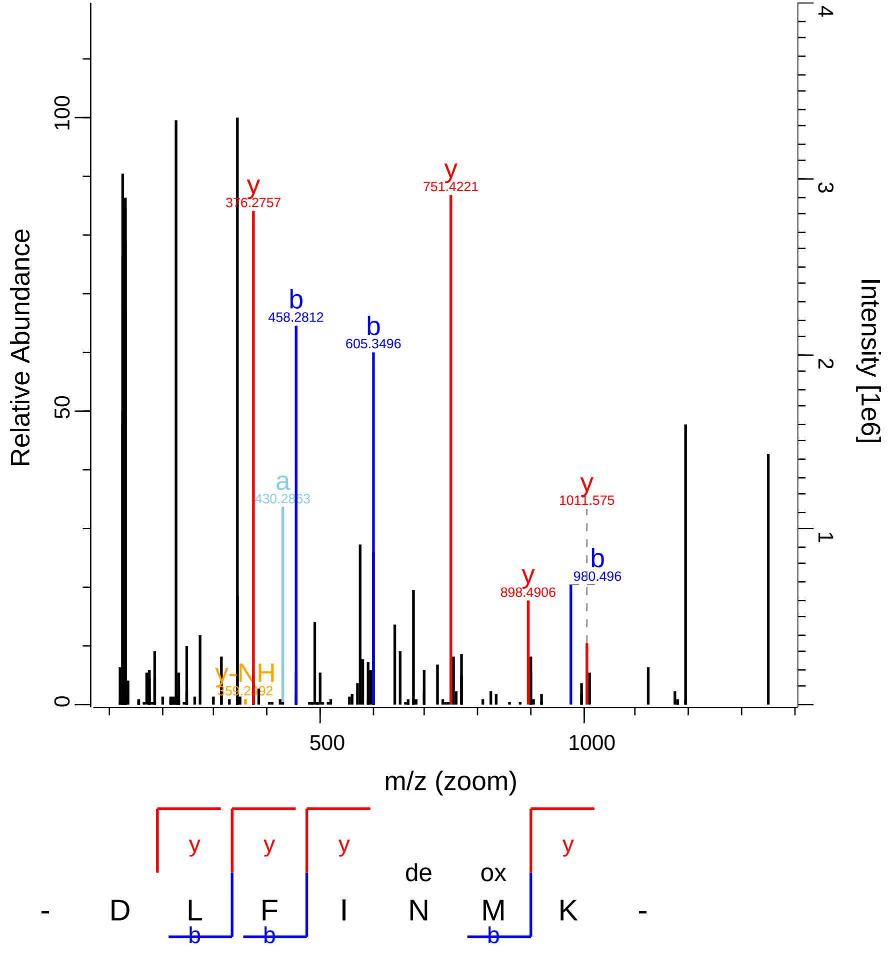

Raw File	Scan	Method	Score	m/z	Gene names
RO-QC-160121_ZackD_SS_Set_02_Fraction	27321	FTMS; HC	88.09	544.3	SLC2A8

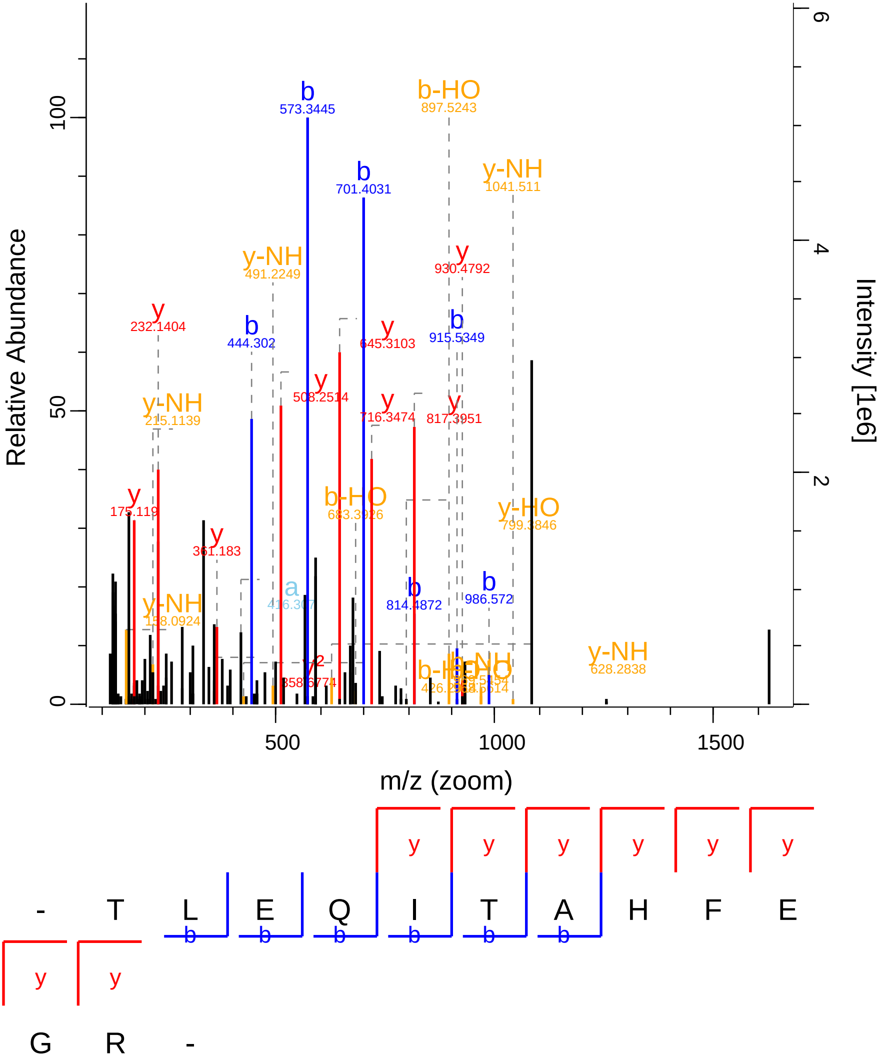

Raw File	Scan	Method	Score	m/z	Gene names
RO-QC-160121_ZackD_SS_Set_02_Fraction	28930	FTMS; HC	156.01	875.5	SEC22A

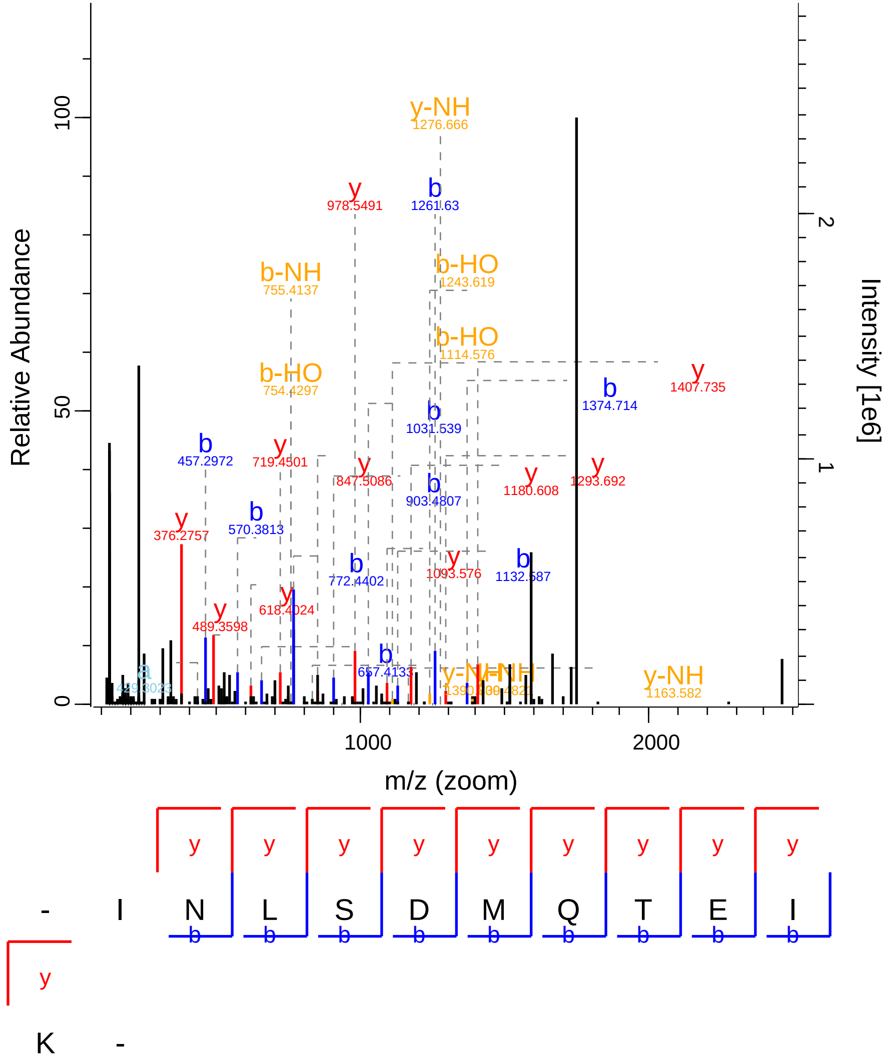

Raw File	Scan	Method	Score	m/z	Gene names
RO-QC-160121_ZackD_SS_Set_02_Fraction_	29117	FTMS; HC	53.55	550.3	FOXN3

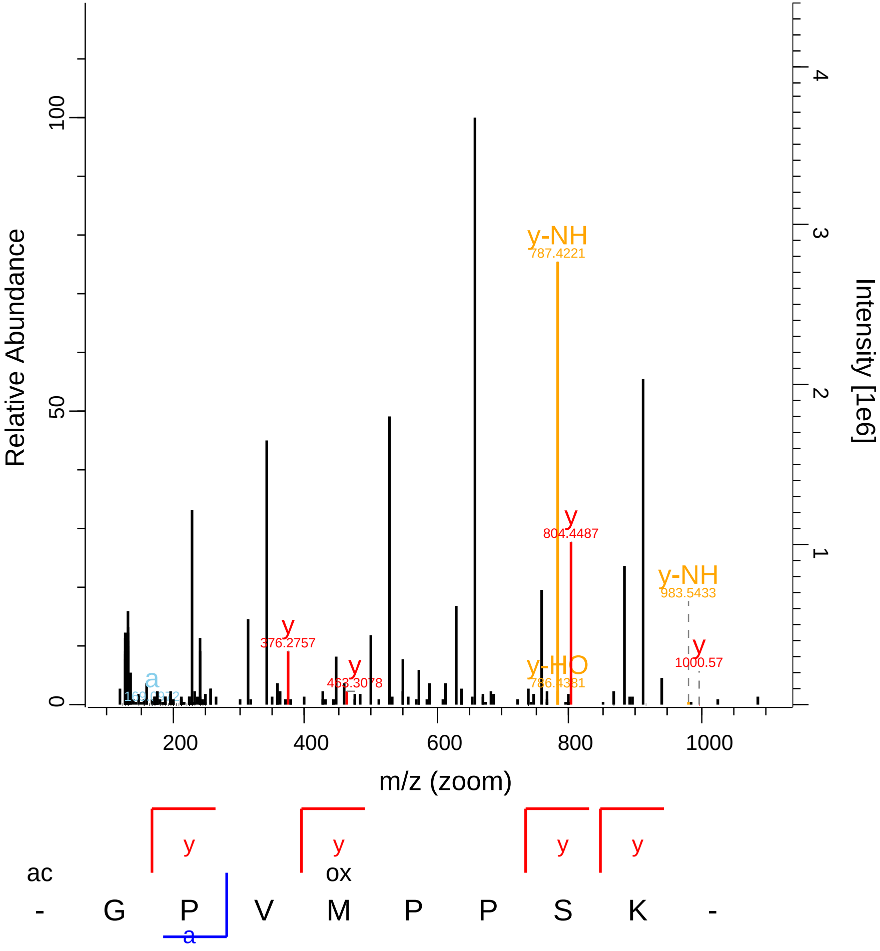

Raw File	Scan	Method	Score	m/z	Gene names
RO-QC-160121_ZackD_SS_Set_02_Fractior	30405	FTMS; HC	64.55	601.03	FAM184B

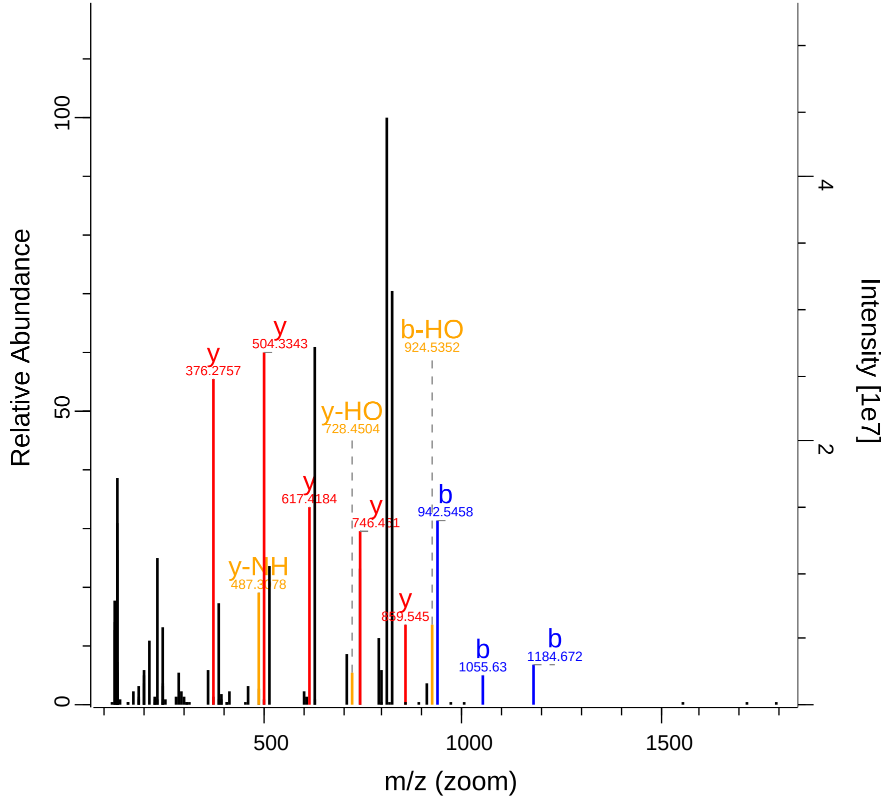

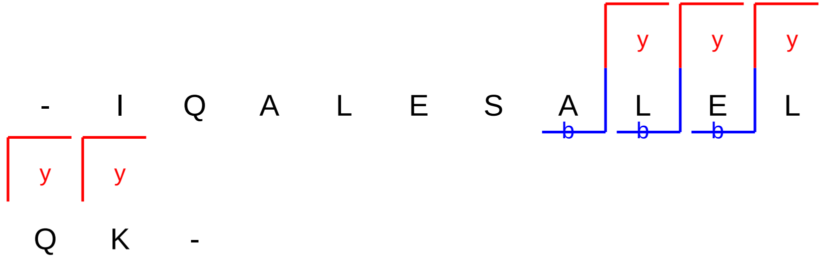

Raw File	Scan	Method	Score	m/z	Gene names
RO-QC-160121_ZackD_SS_Set_02_Fractior	30406	FTMS; HC	86.9	901.05	FAM184B

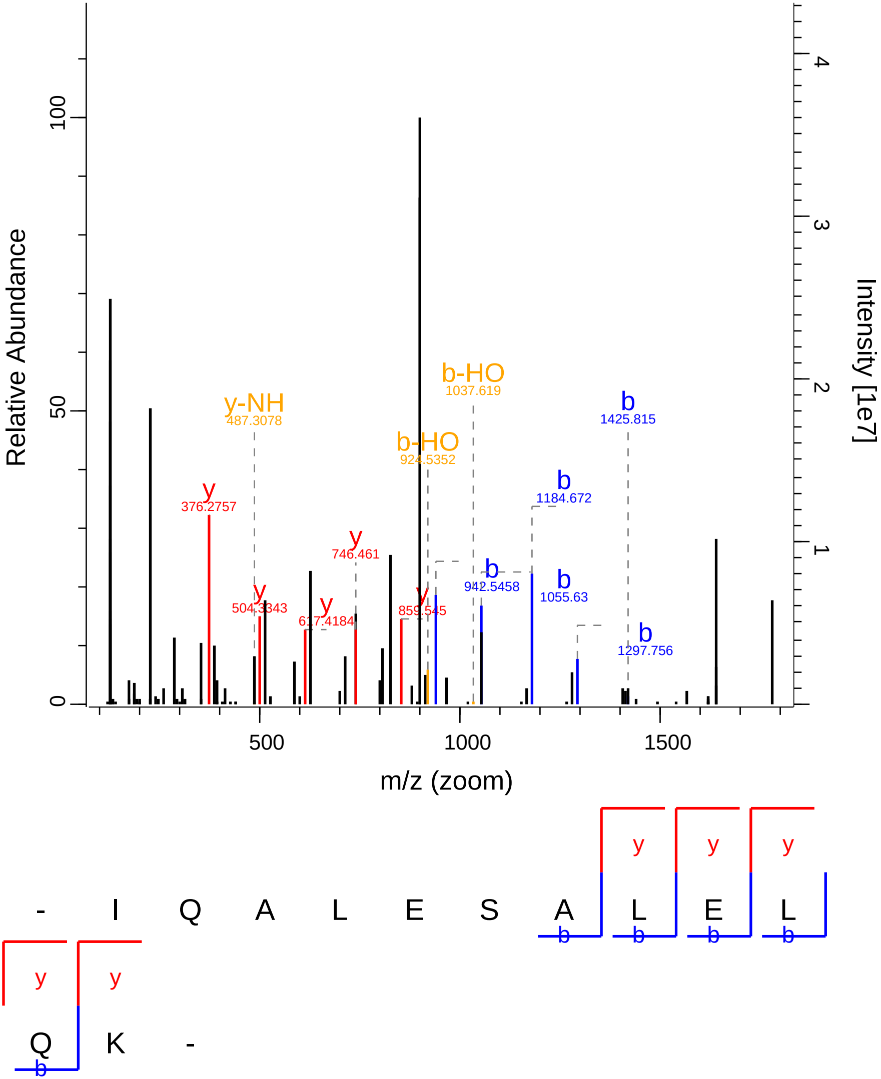

Raw File	Scan	Method	Score	m/z	Gene names
RO-QC-160121_ZackD_SS_Set_02_Fractio[illegible]	30543	FTMS; HC	137.16	820.94	LRP10

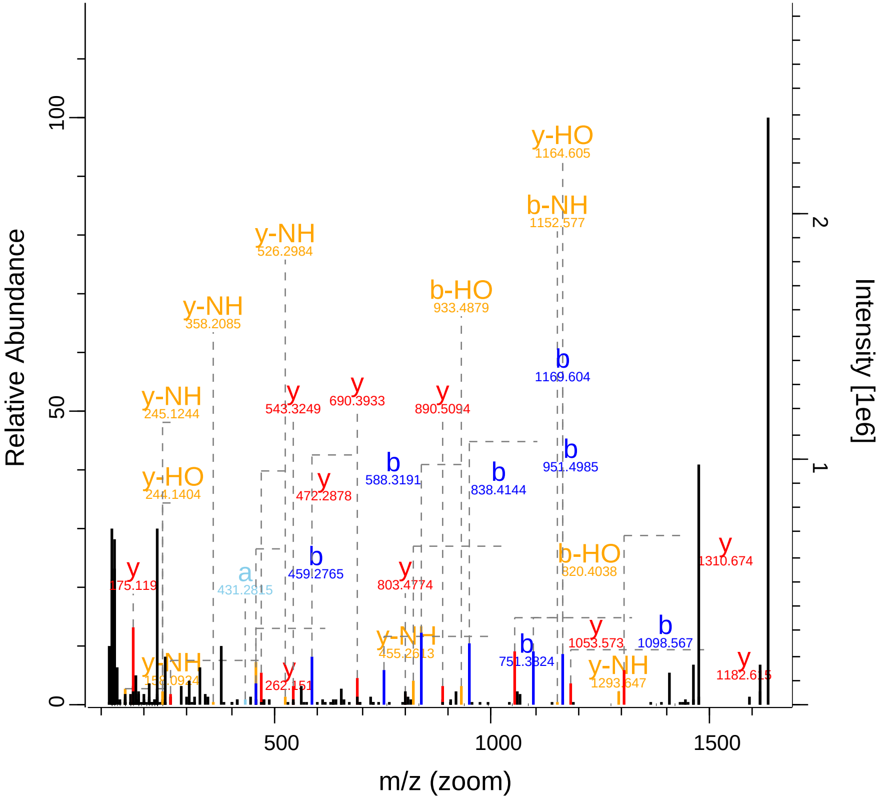

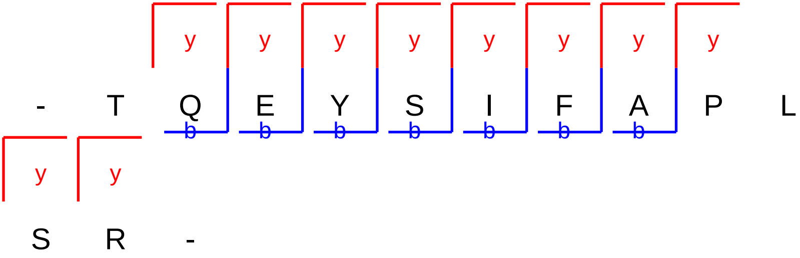

Raw File	Scan	Method	Score	m/z	Gene names
RO-QC-160121_ZackD_SS_Set_02_Fractior	30632	FTMS; HC	68.28	547.63	LRP10

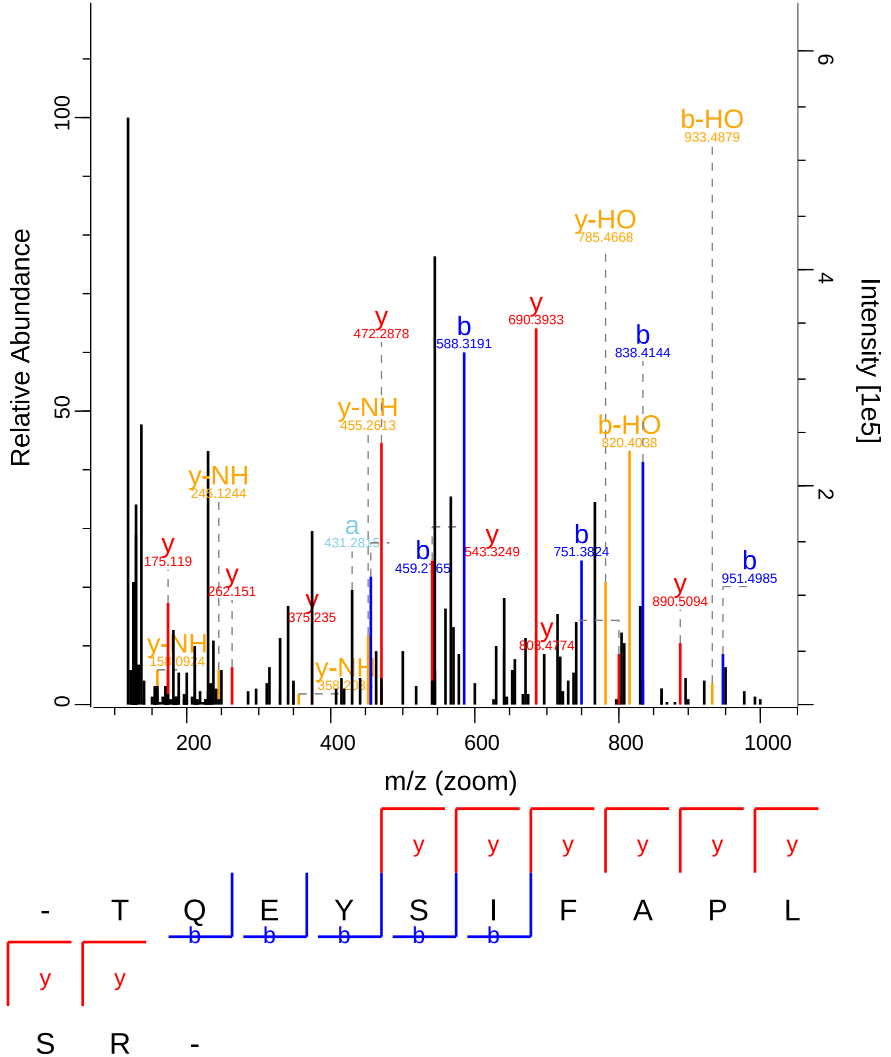

Raw File	Scan	Method	Score	m/z	Gene names
RO-QC-160121_ZackD_SS_Set_02_Fractior	30730	FTMS; HC	40.38	601.37	FAM184B

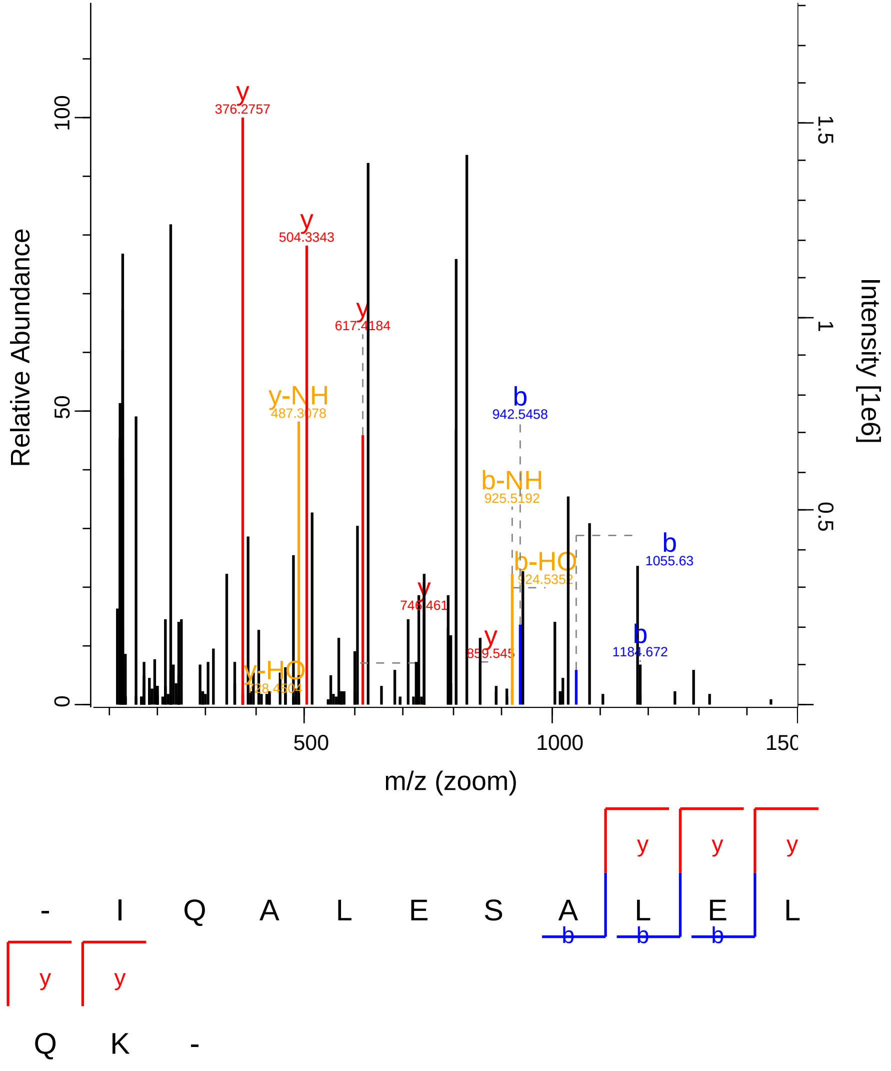

Raw File	Scan	Method	Score	m/z	Gene names
RO-QC-160121_ZackD_SS_Set_02_Fraction	30781	FTMS; HC	54.81	758.4	EFCAB10

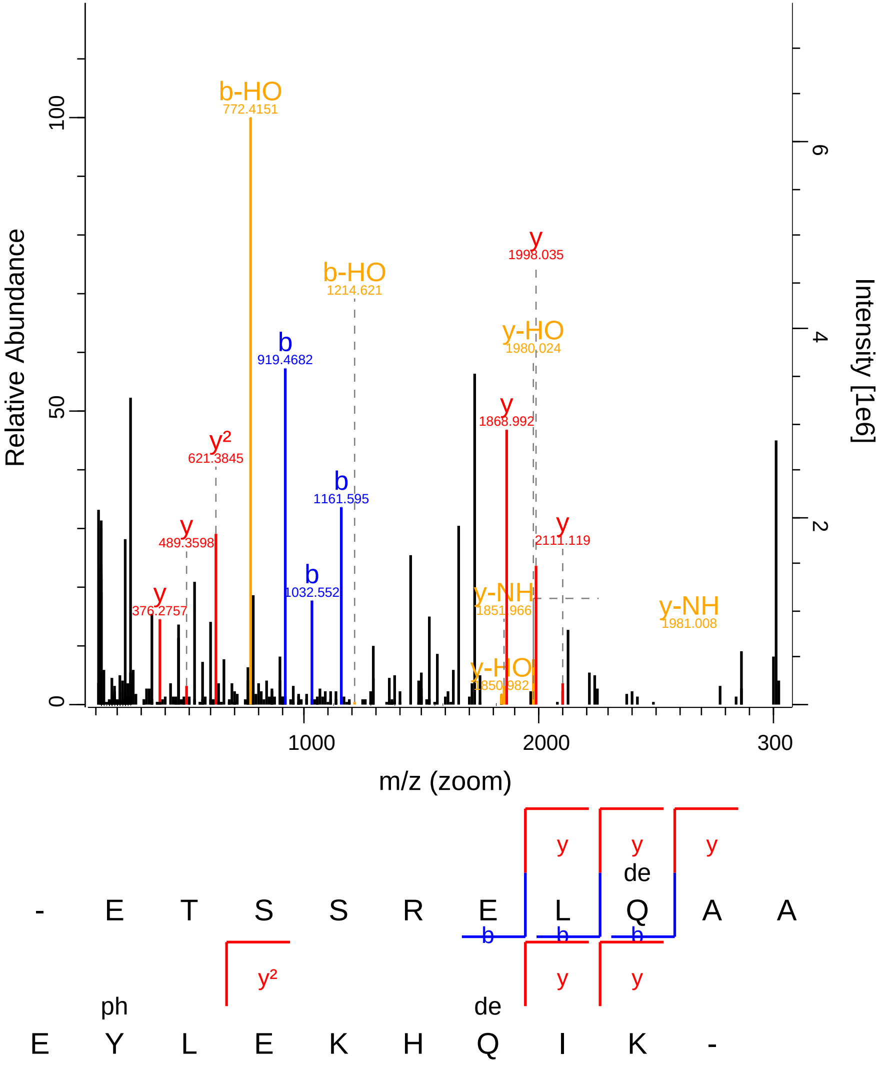

Raw File	Scan	Method	Score	m/z	Gene names
RO-QC-160121_ZackD_SS_Set_02_Fractior	30927	FTMS; HC	64.55	601.03	FAM184B

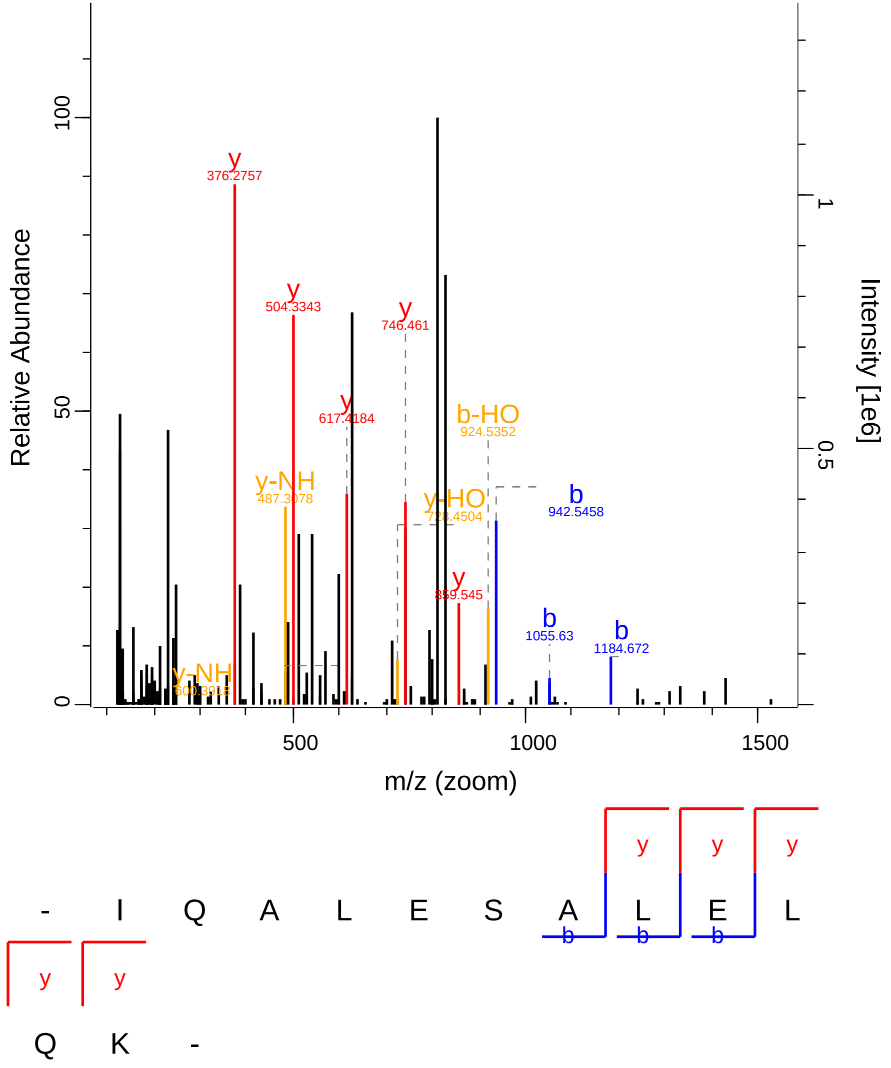

Raw File	Scan	Method	Score	m/z	Gene names
RO-QC-160121_ZackD_SS_Set_02_Fractior	31511	FTMS; HC	44.43	657.04	SLC46A3

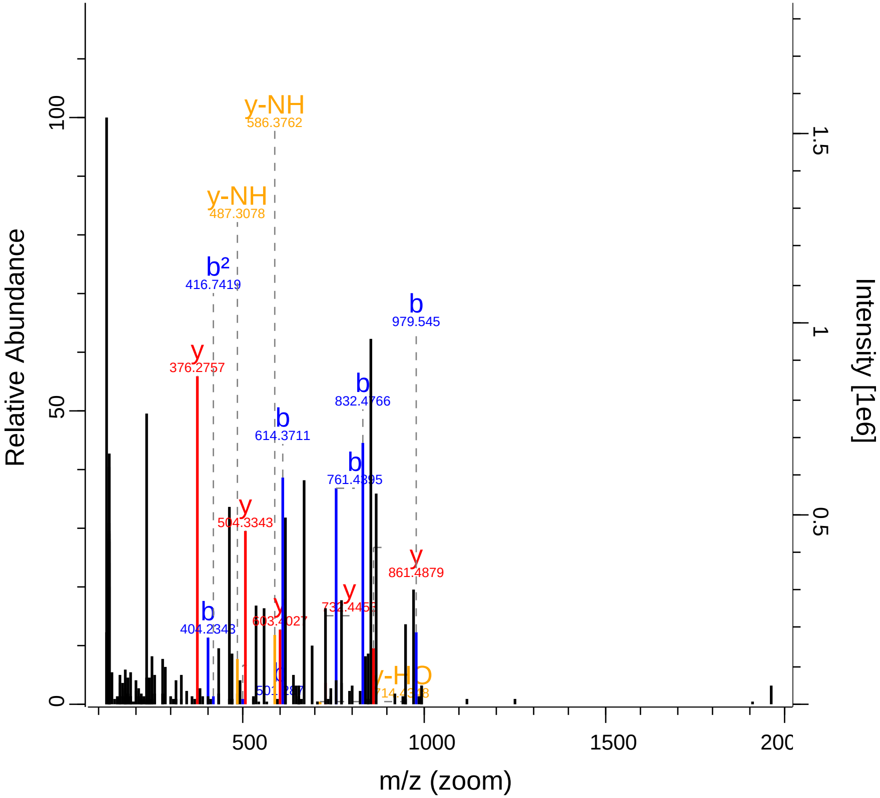

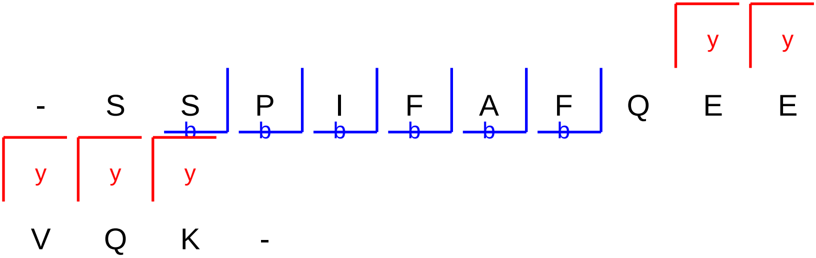

Raw File	Scan	Method	Score	m/z	Gene names
RO-QC-160121_ZackD_SS_Set_02_Fractior	31531	FTMS; HC	104.79	985.05	SLC46A3

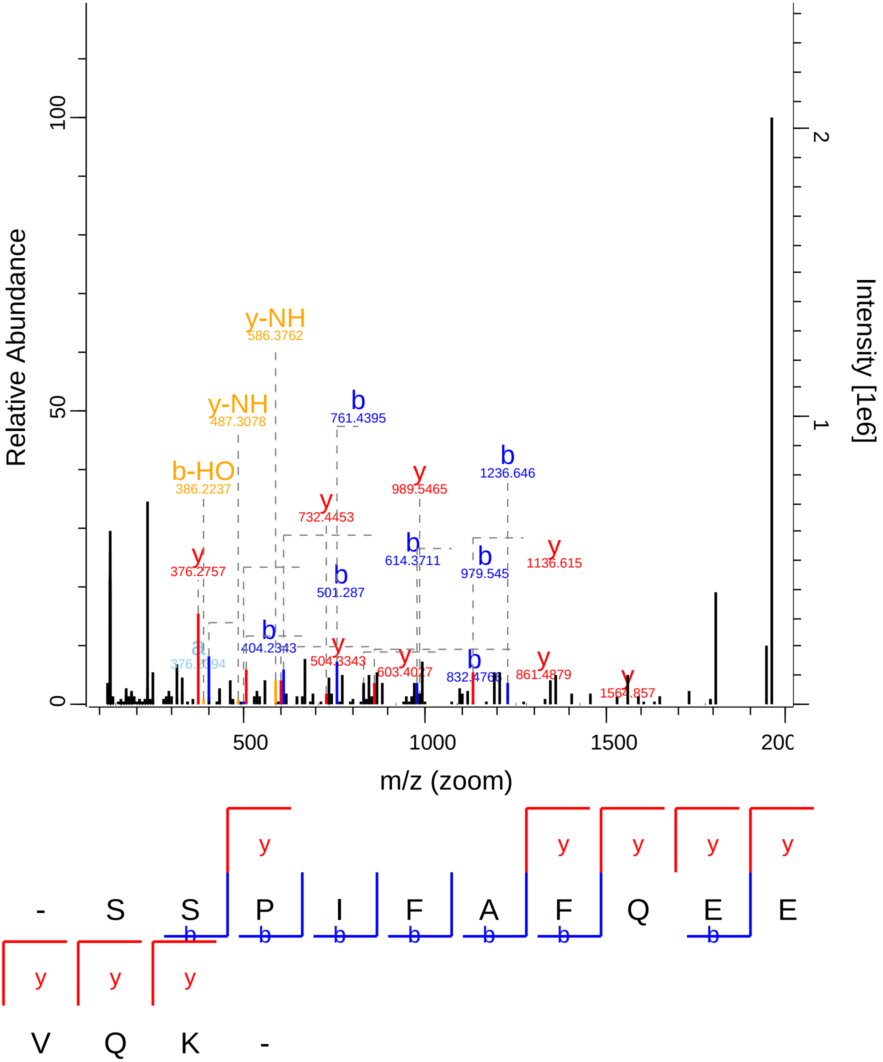

Raw File	Scan	Method	Score	m/z
RO-QC-160121_ZackD_SS_Set_02_Fraction_12	31652	FTMS; HCD	133.55	701.93

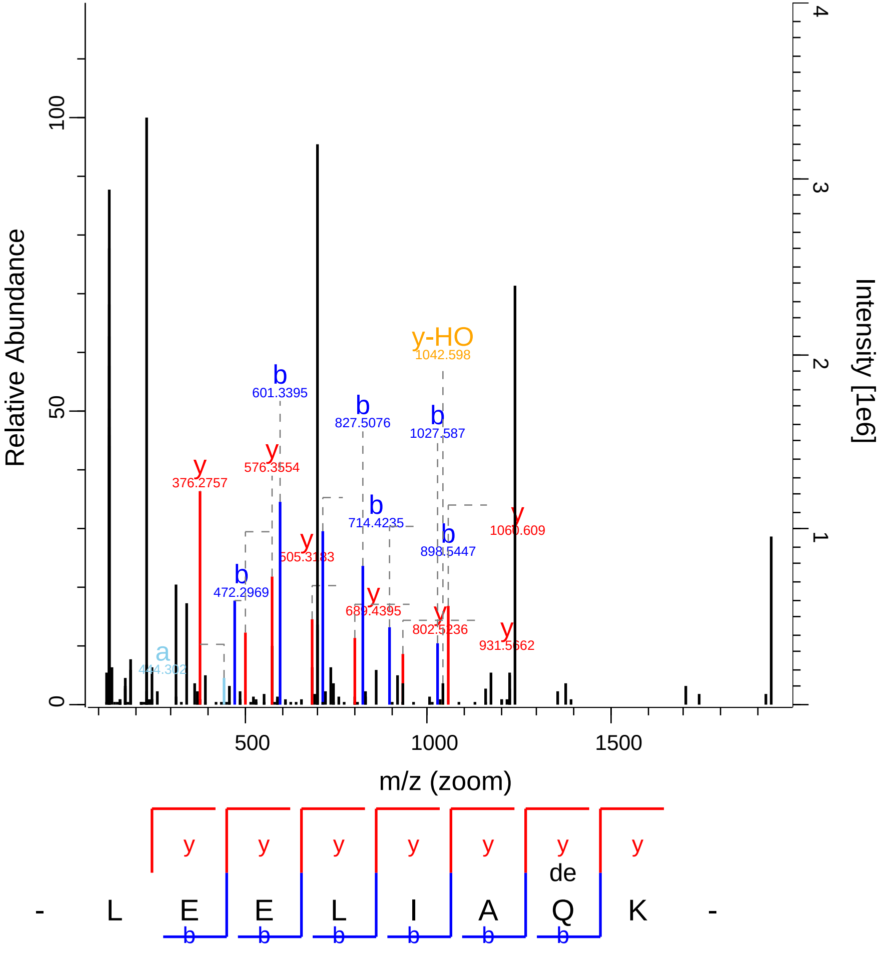

Raw File	Scan	Method	Score	m/z	Gene names
RO-QC-160121_ZackD_SS_Set_02_Fractior	31699	FTMS; HC	64.22	657.04	SLC46A3

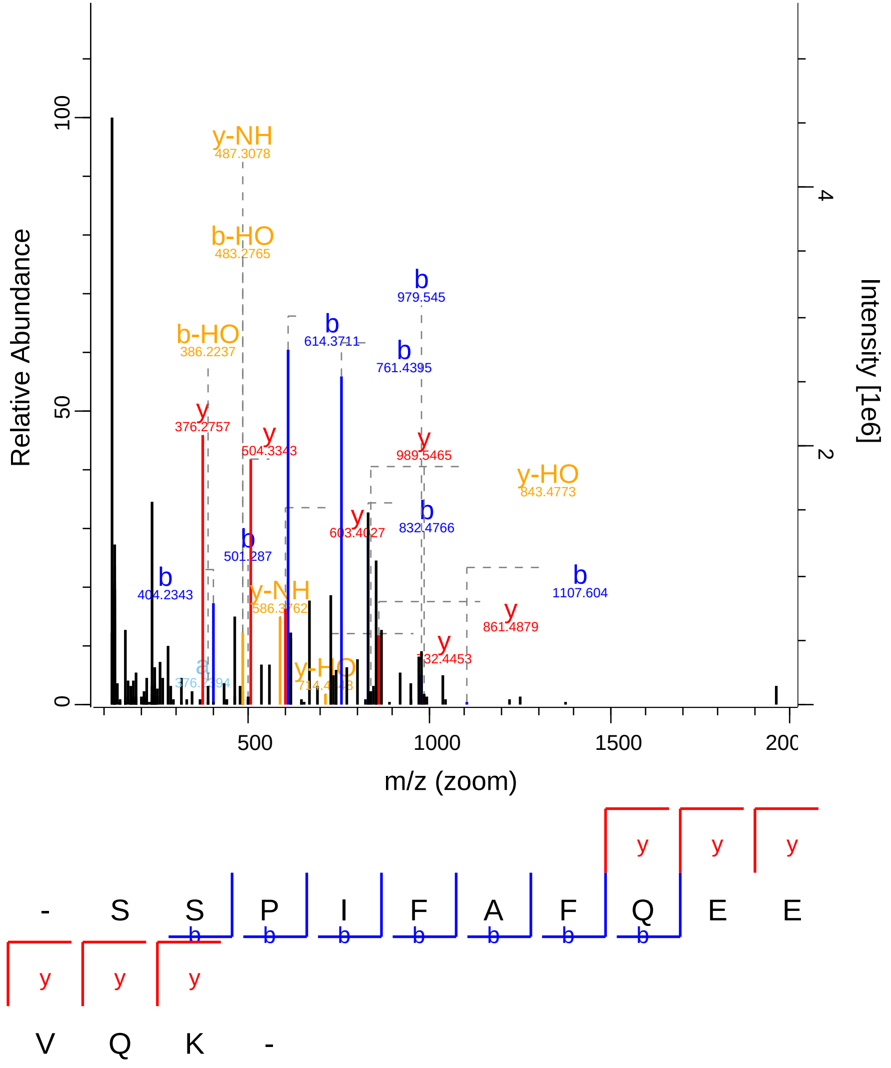

Raw File	Scan	Method	Score	m/z	Gene names
RO-QC-160121_ZackD_SS_Set_02_Fractior	31781	FTMS; HC	120.12	715.46	HAUS2

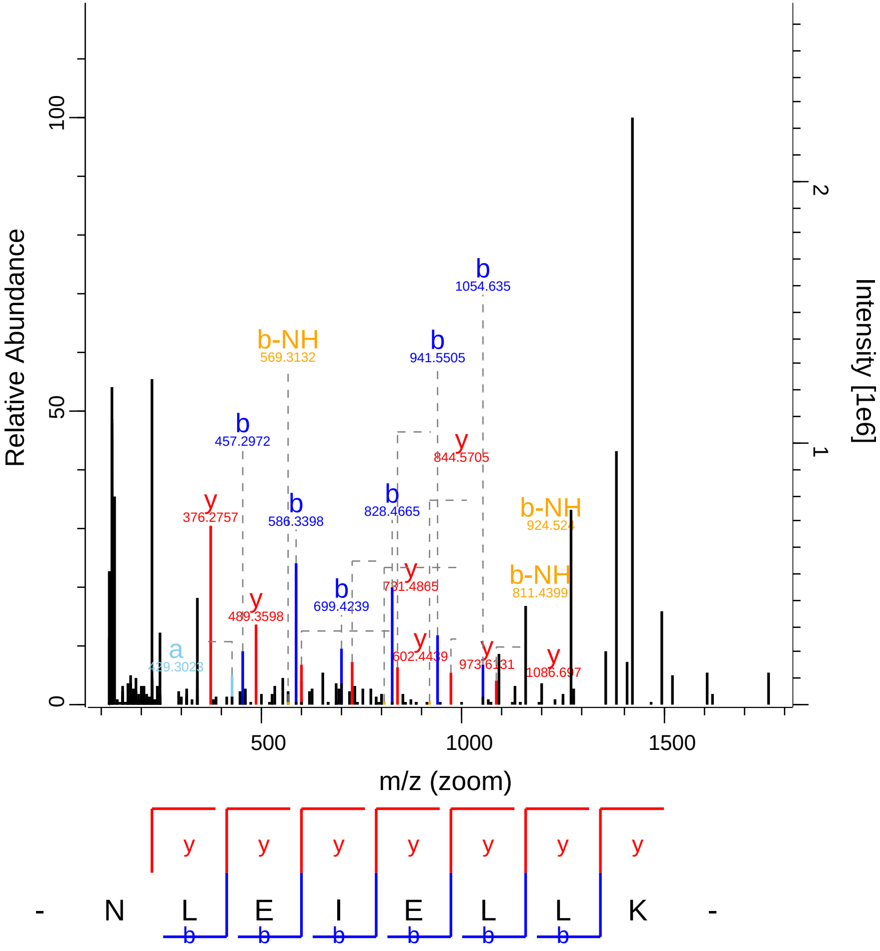

Raw File	Scan	Method	Score	m/z
RO-QC-160121_ZackD_SS_Set_02_Fraction_12	32057	FTMS; HCD	64.04	1035.58

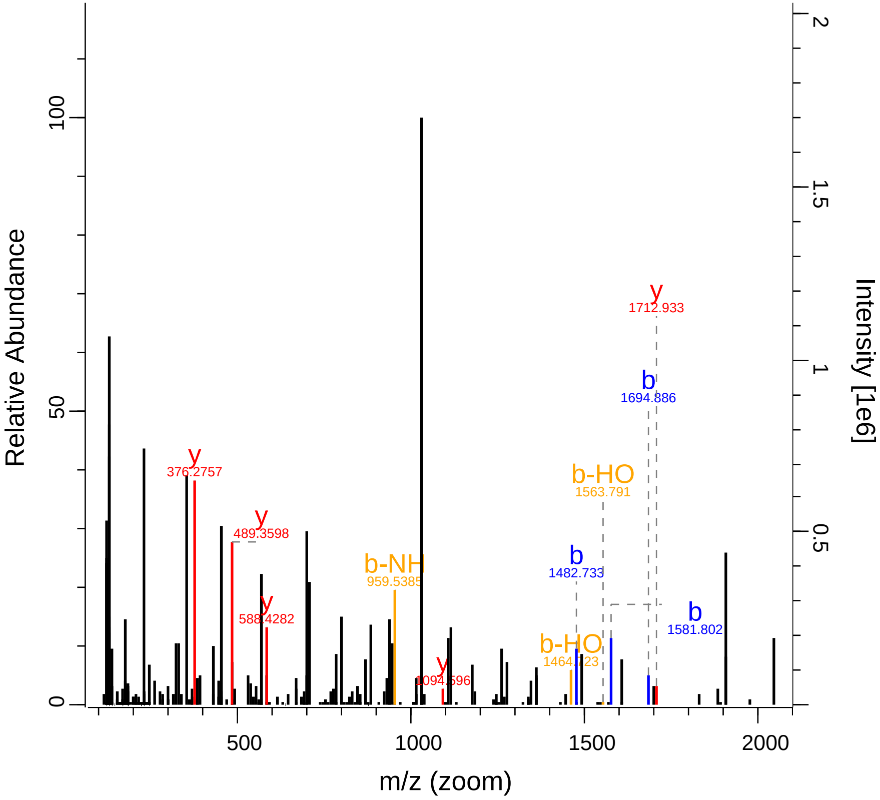

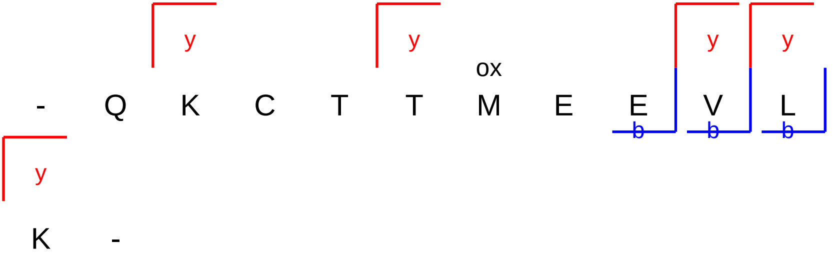

Raw File	Scan	Method	Score	m/z	Gene names
RO-QC-160121_ZackD_SS_Set_02_Fractior	32486	FTMS; HC	70.98	563.67	SLC35A2

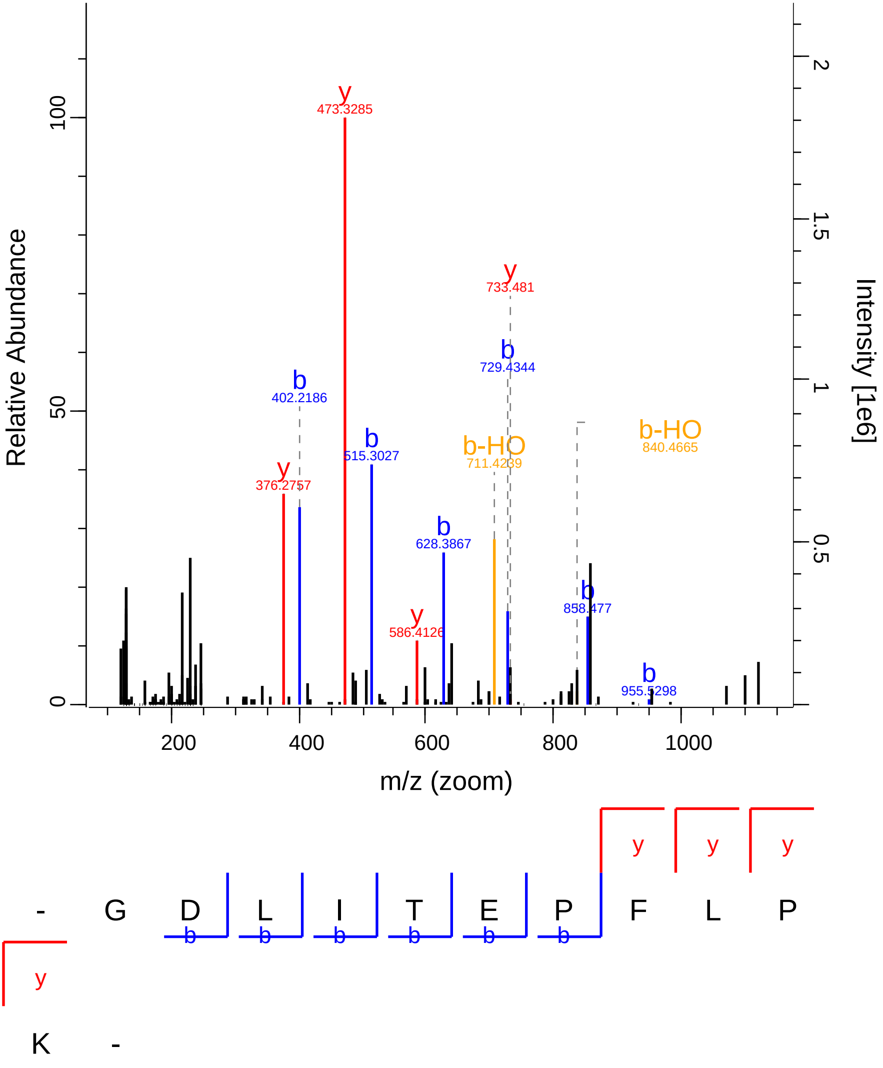

Raw File	Scan	Method	Score	m/z	Gene names
RO-QC-160121_ZackD_SS_Set_02_Fractio	34128	FTMS; HC	41.09	1351.63	ALPI

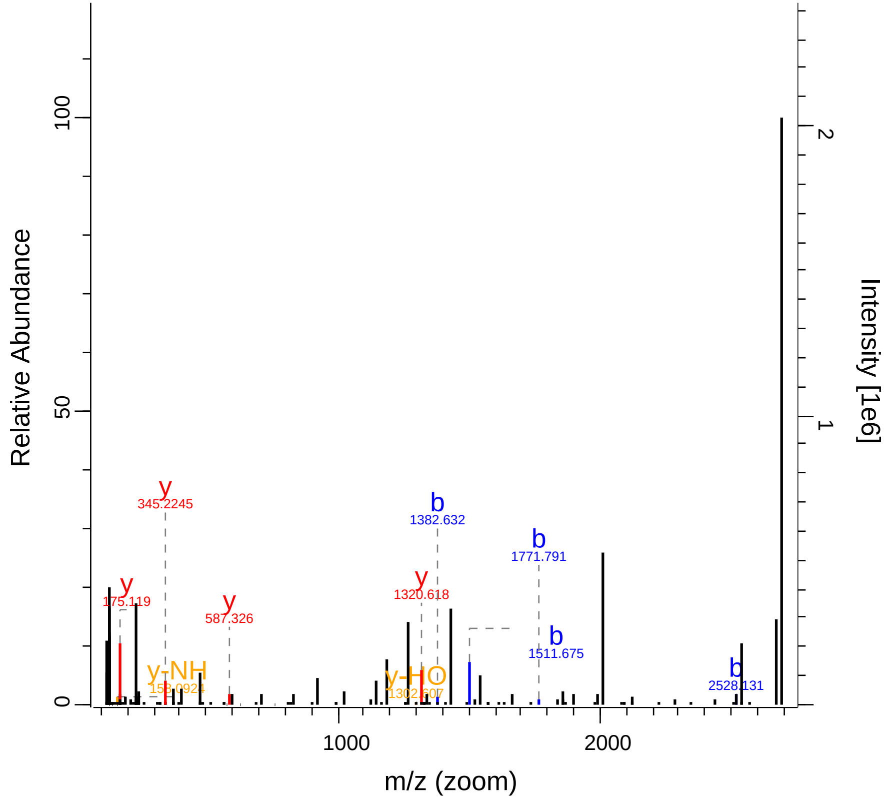

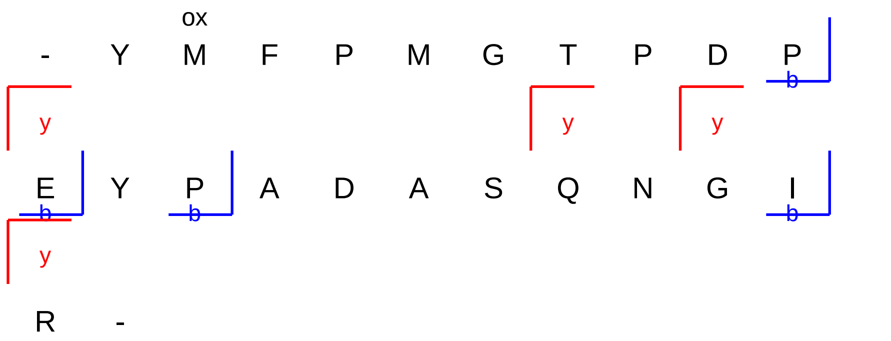

Raw File	Scan	Method	Score	m/z
RO-QC-160121_ZackD_SS_Set_02_Fraction_12	34315	FTMS; HCD	41.38	679.03

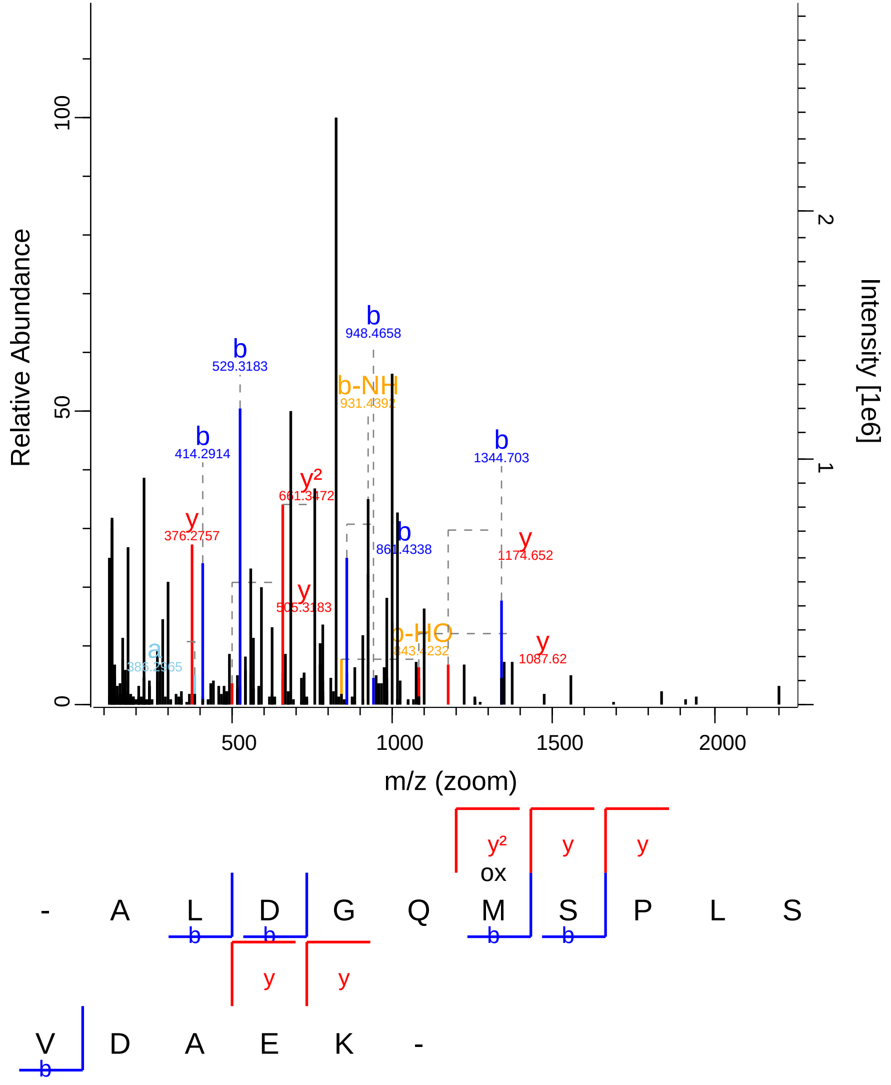

Raw File	Scan	Method	Score	m/z	Gene names
RO-QC-160121_ZackD_SS_Set_02_Fractior	34498	FTMS; HC	37.99	570.63	POP5

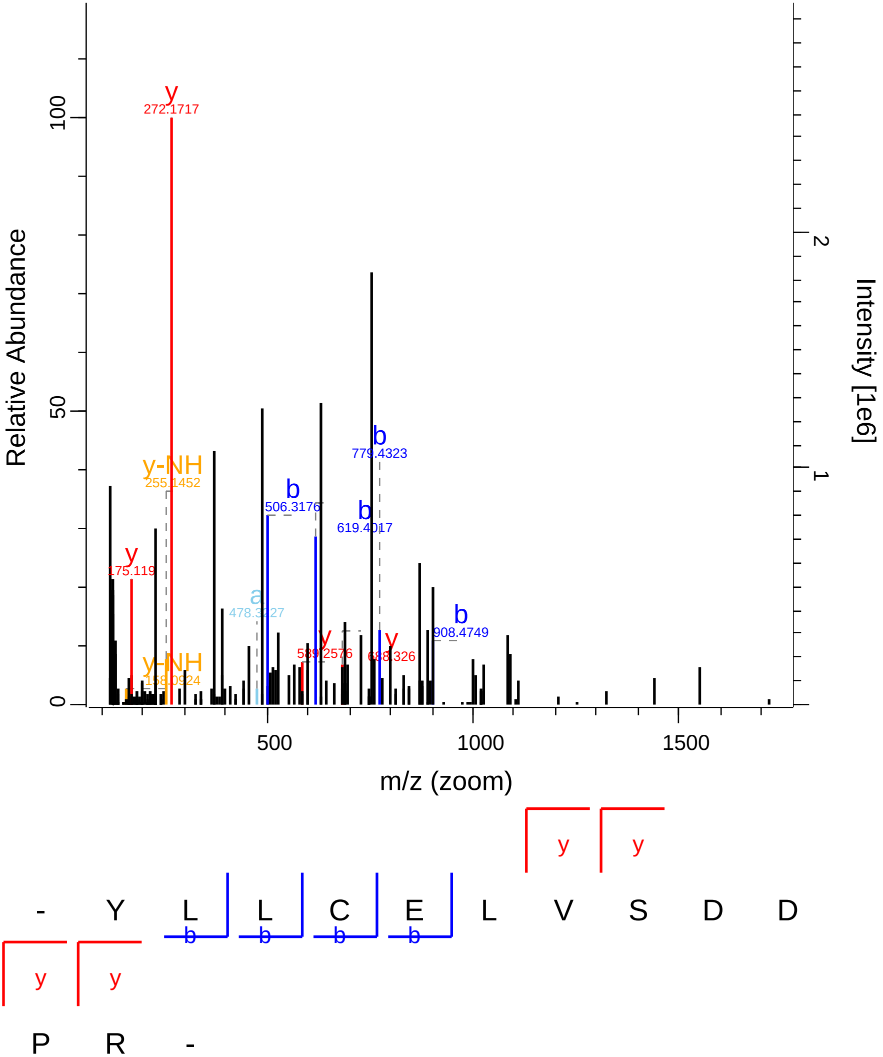

Raw File	Scan	Method	Score	m/z
RO-QC-160121_ZackD_SS_Set_02_Fraction_12	35011	FTMS; HCD	48.05	882.46

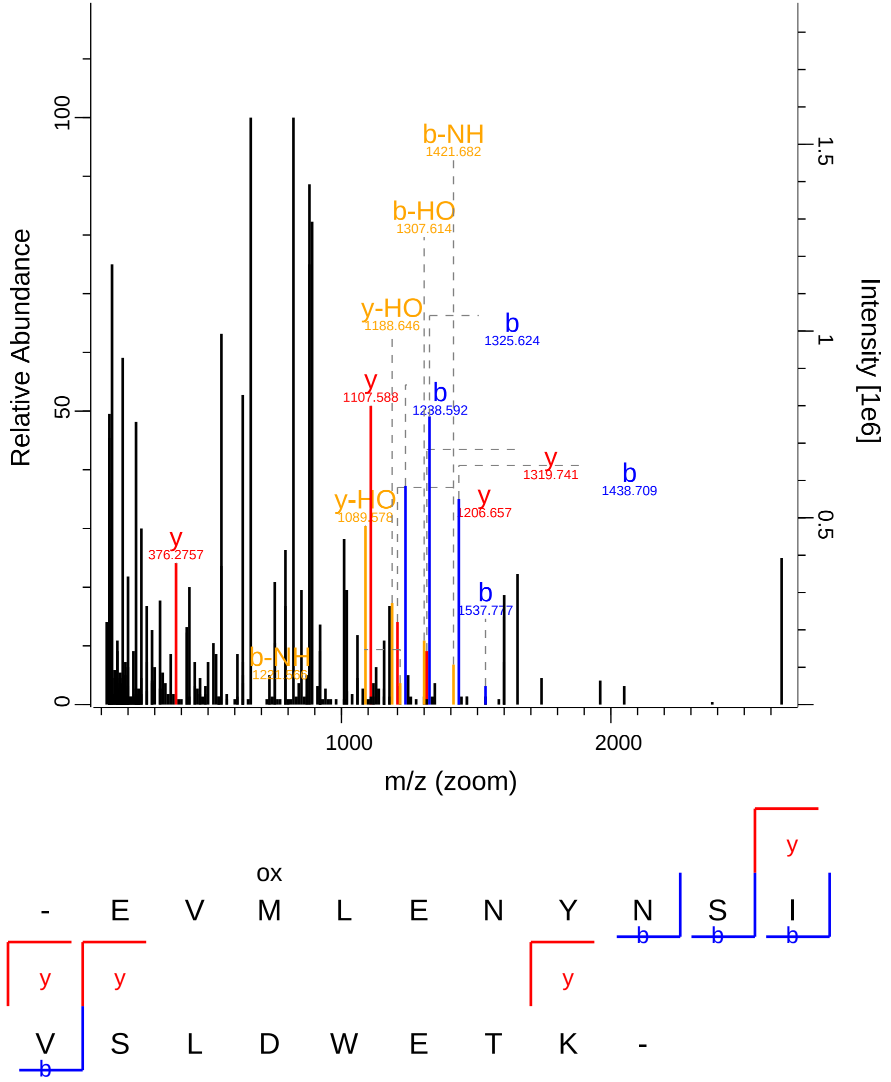

Raw File	Scan	Method	Score	m/z	Gene names
RO-QC-160121_ZackD_SS_Set_02_Fractior	35485	FTMS; HC	93.23	646.03	METRN

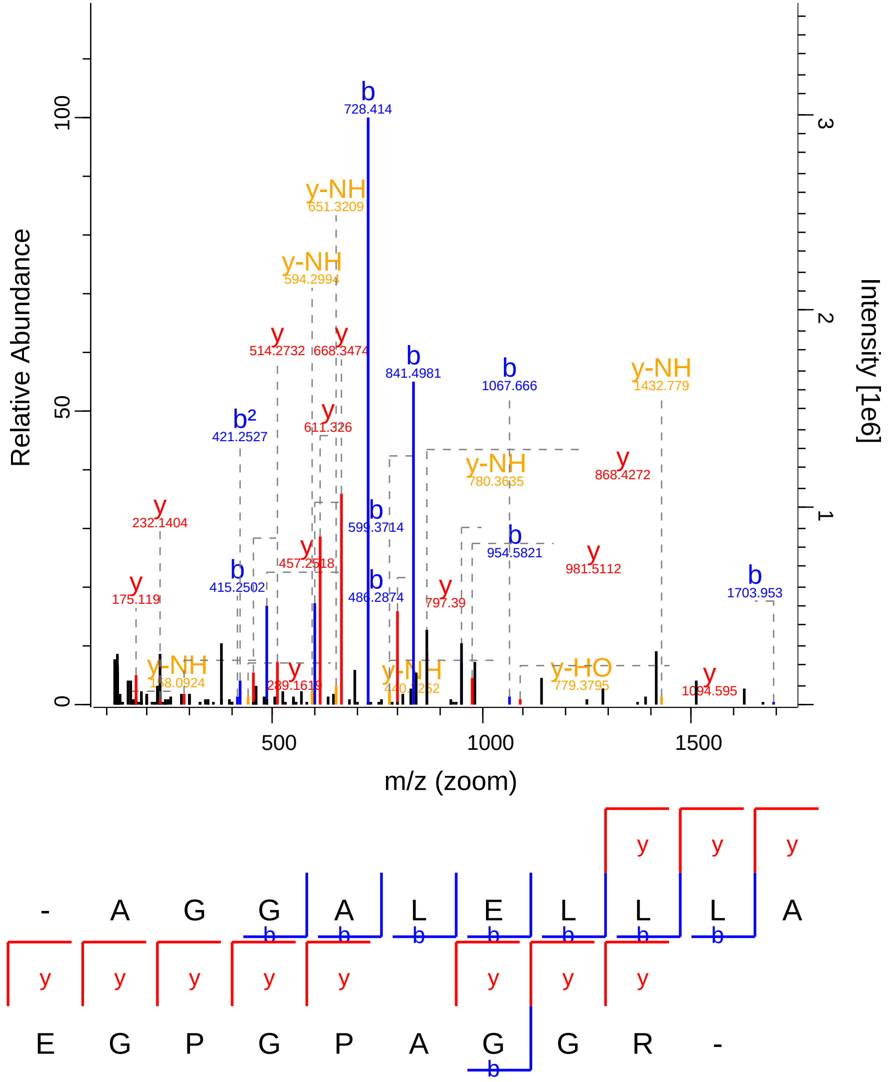

Raw File	Scan	Method	Score	m/z	Gene names
RO-QC-160121_ZackD_SS_Set_02_Fraction_	35892	FTMS; HC	45.33	600.1	HMGXB3

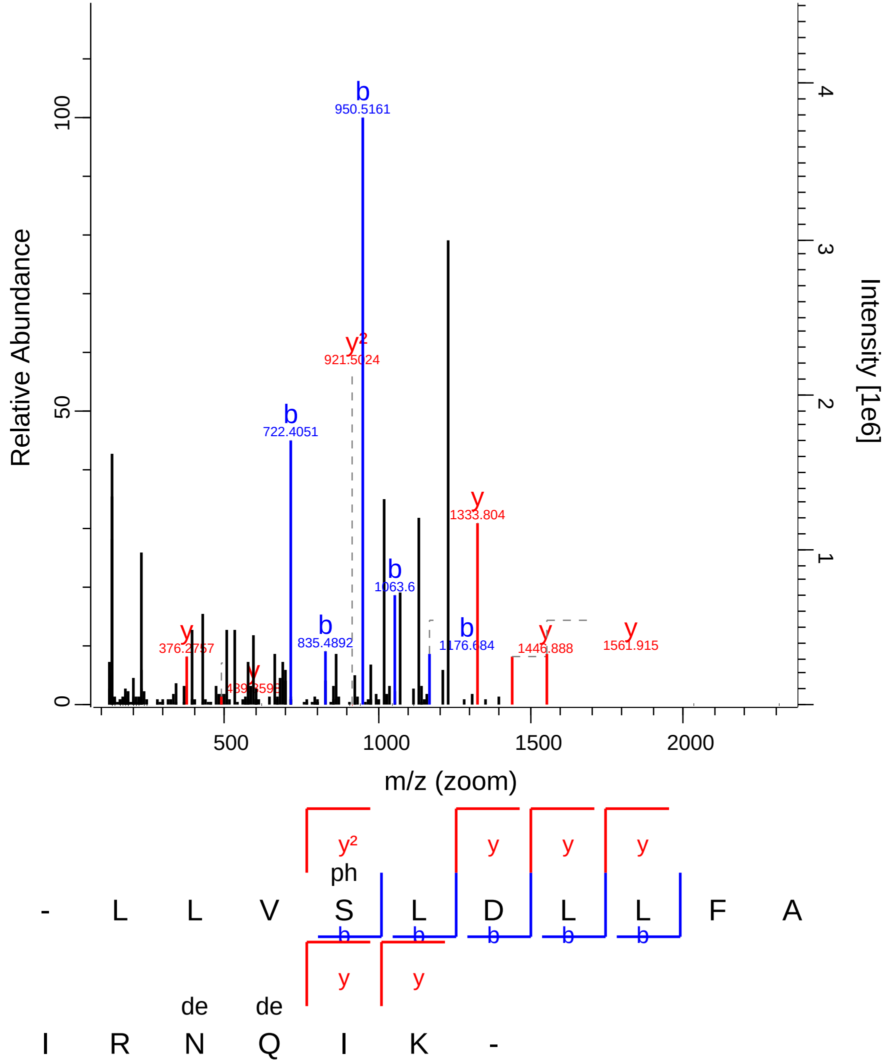

Raw File	Scan	Method	Score	m/z	Gene names
RO-QC-160121_ZackD_SS_Set_02_Fraction	37331	FTMS; HC	114.33	717.7	DEGS1

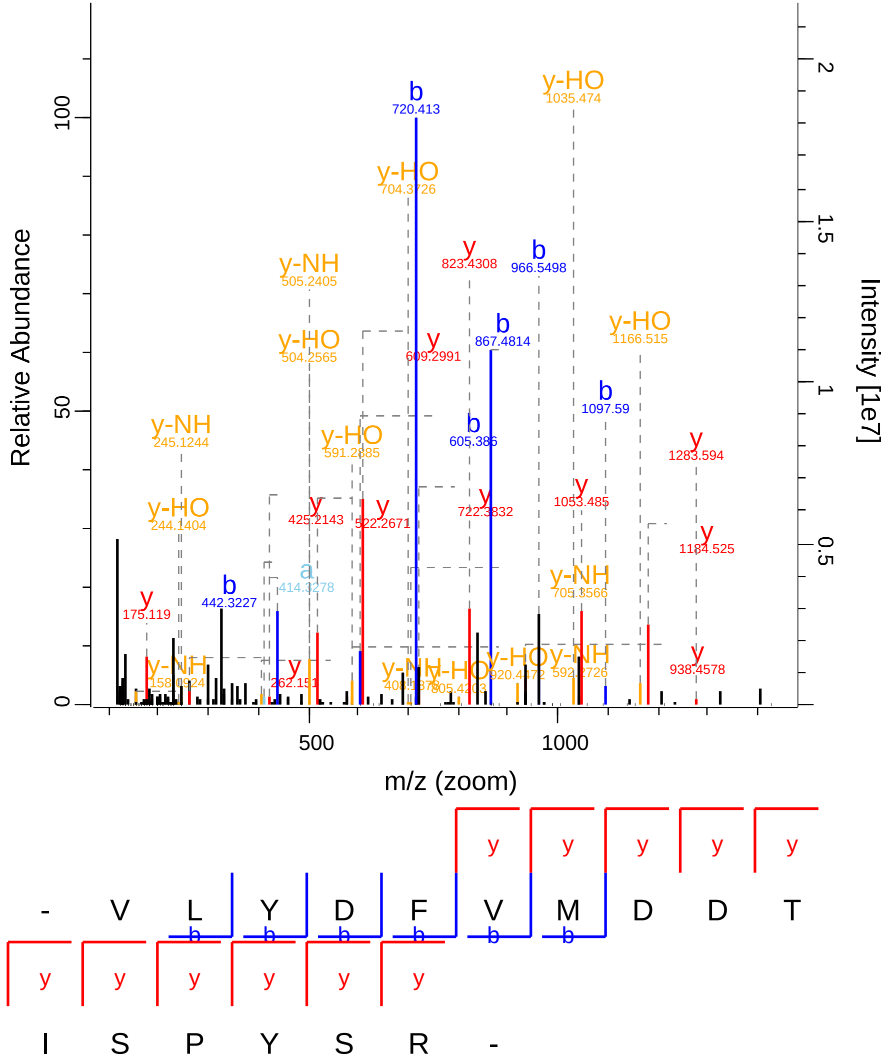

Raw File	Scan	Method	Score	m/z
RO-QC-160121_ZackD_SS_Set_02_Fraction_12	37333	FTMS; HCD	87.31	888.5

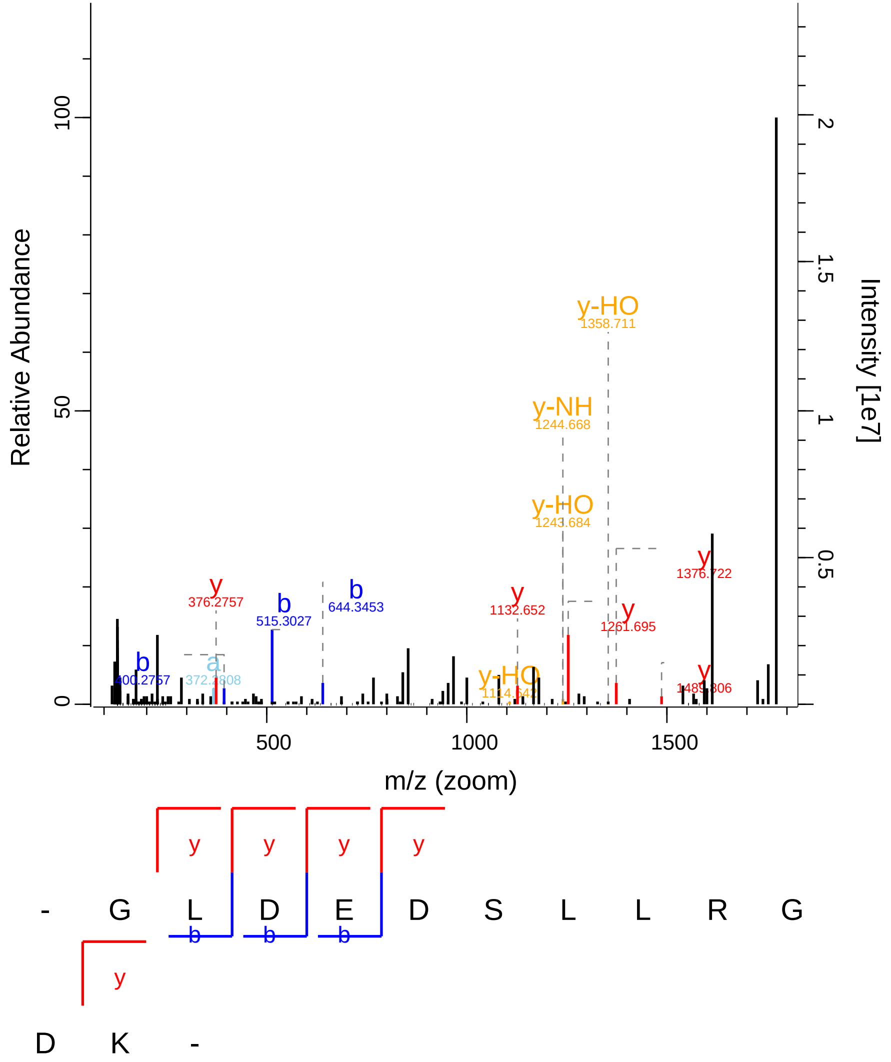

Raw File	Scan	Method	Score	m/z	Gene names
RO-QC-160121_ZackD_SS_Set_02_Fractior	37979	FTMS; HC	46.32	662.68	TOMM7

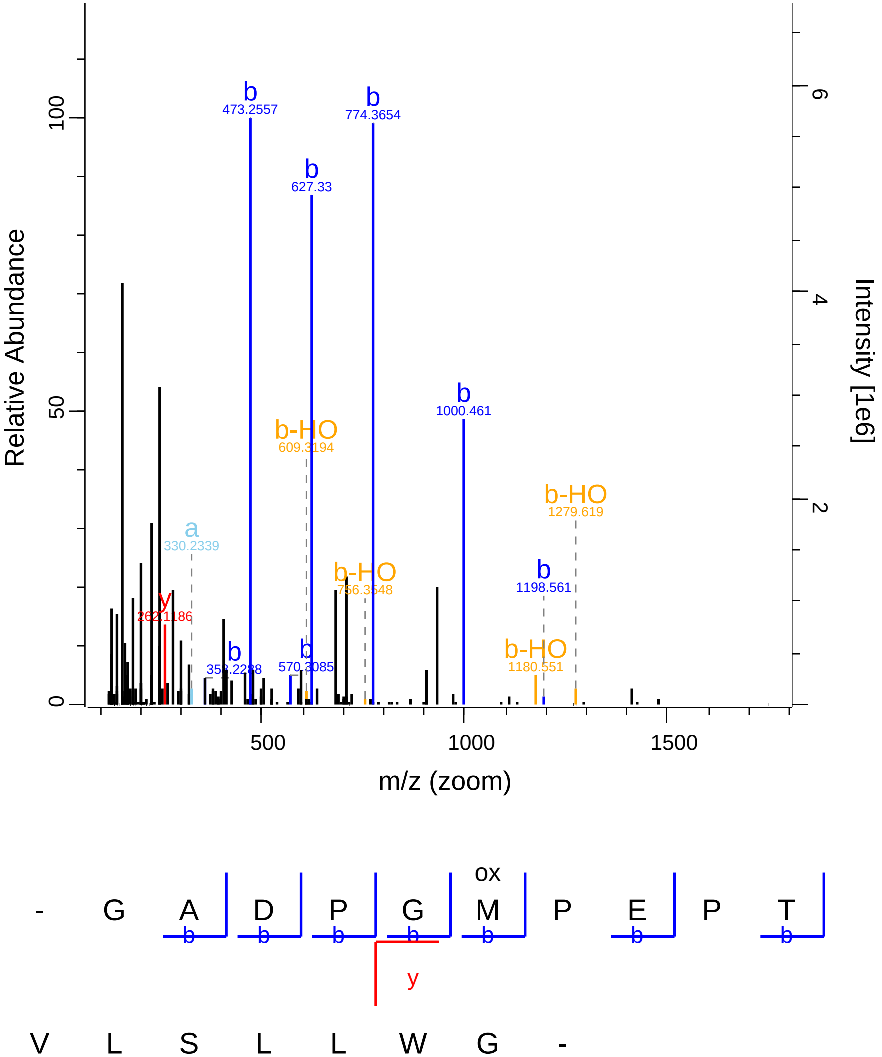

Raw File	Scan	Method	Score	m/z	Gene names
RO-QC-160121_ZackD_SS_Set_02_Fractior	37981	FTMS; HC	117.02	993.52	TOMM7

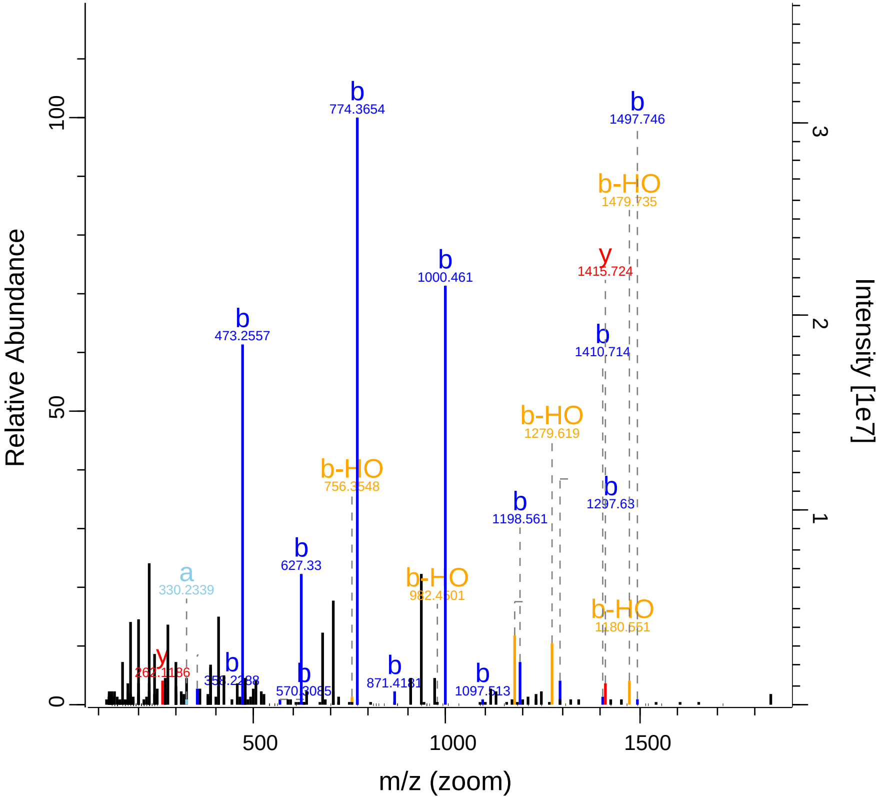

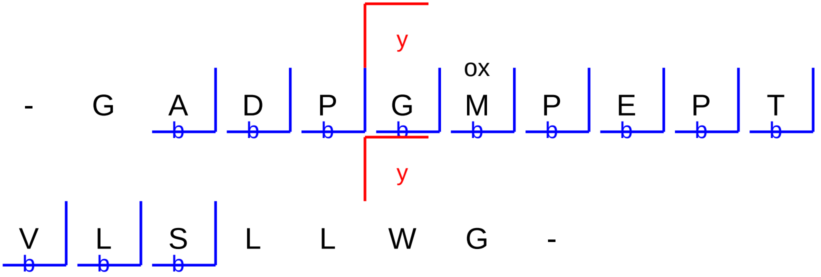

Raw File	Scan	Method	Score	m/z	Gene names
RO-QC-160121_ZackD_SS_Set_02_Fractio	38243	FTMS; HC	94.46	1260.16	BSCL2

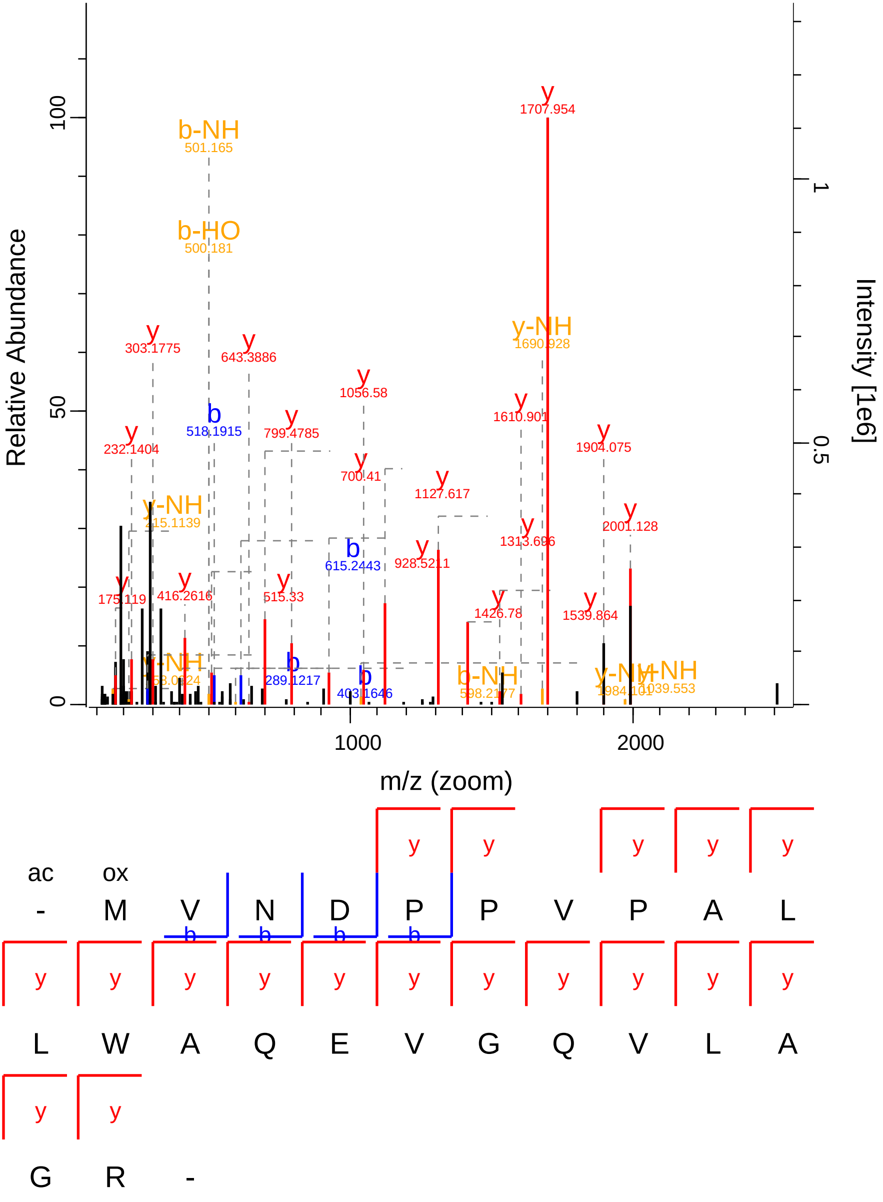

Supplement: Supplemental Figure S16 [file mmc16.pdf]
