# Supplementary material for: Proteome Landscape of Epithelial-to-Mesenchymal Transition (EMT) of Retinal Pigment Epithelium Shares Commonalities With Malignancy-Associated EMT
Source: Mol Cell Proteomics. 2021 Aug 27;20:100131. doi: 10.1016/j.mcpro.2021.100131 (PMC8482521; doi:10.1016/j.mcpro.2021.100131)

Supplemental Figure. S17: MS2 spectra for single peptide identifications and phospho STY

Raw File	Scan	Method	Score	m/z	Gene names
RO-QC-160121_ZackD_SS_Set_01_Fractior	7132	FTMS; HCI	80.52	485.27	SRSF4

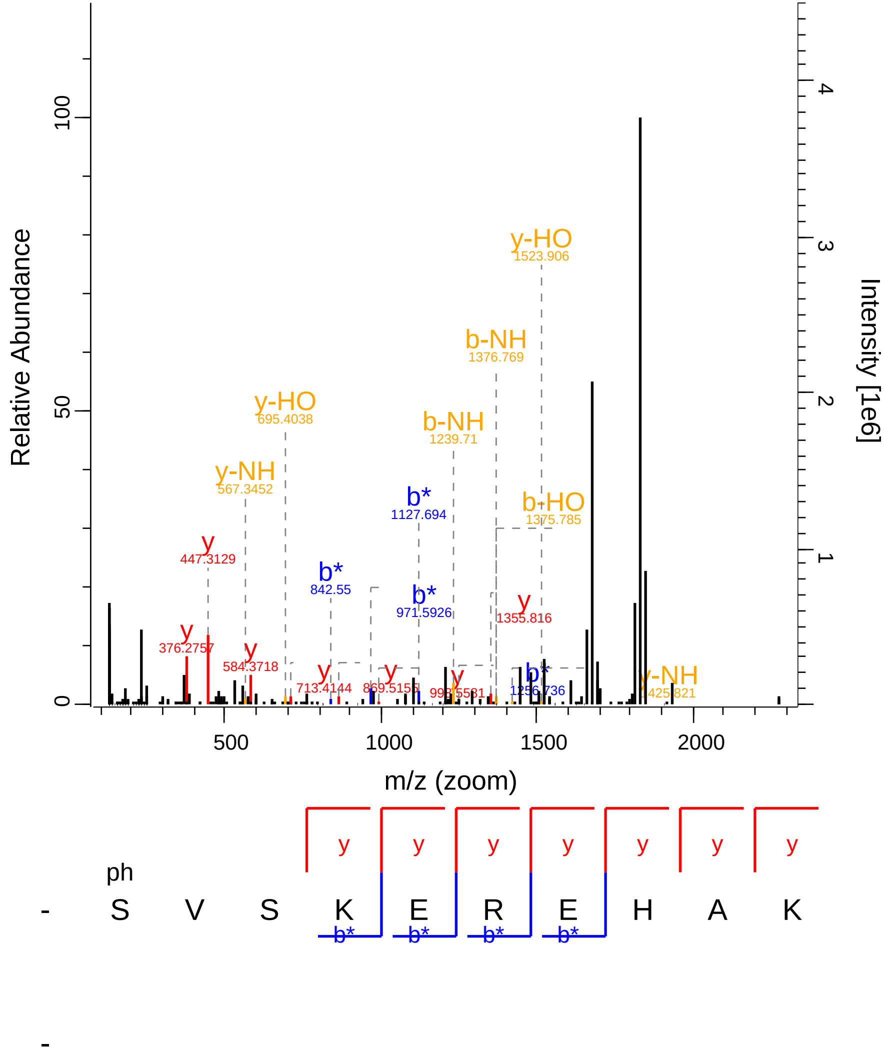

Raw File	Scan	Method	Score	m/z	Gene names
RO-QC-160121_ZackD_SS_Set_01_Fractior	7306	FTMS; HCI	92.06	696.36	TLK1

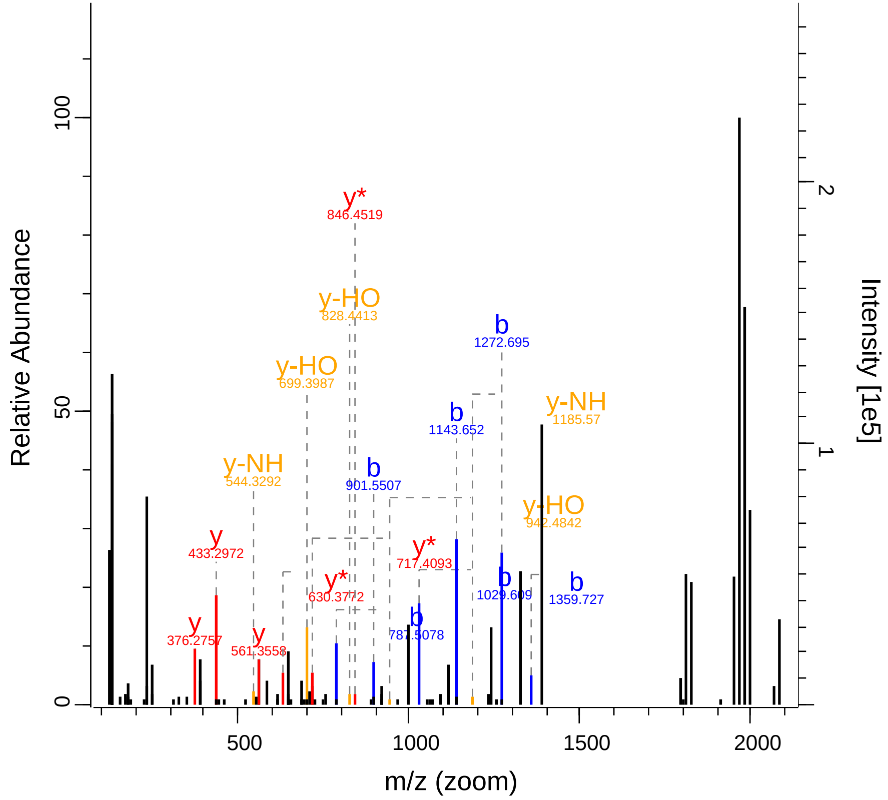

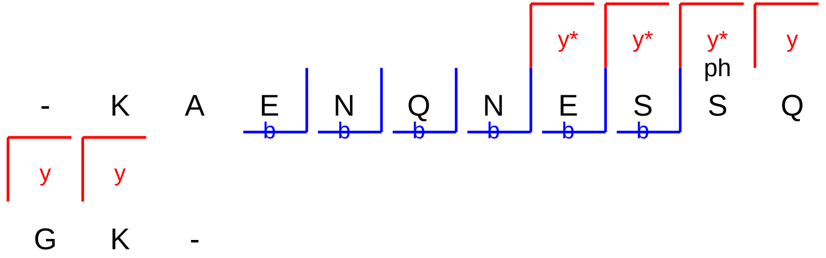

Raw File	Scan	Method	Score	m/z	Gene names
RO-QC-160121_ZackD_SS_Set_01_Fraction_	7333	FTMS; HCI	40.35	486.6	MEFV

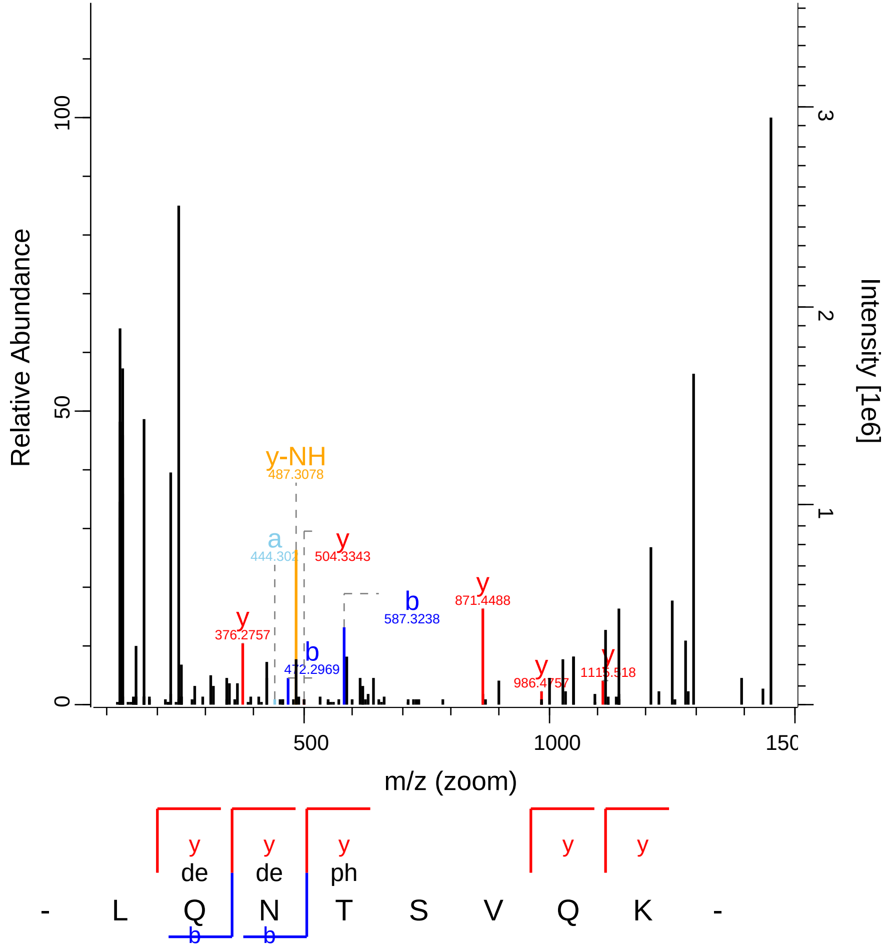

Raw File	Scan	Method	Score	m/z	Gene names
RO-QC-160121_ZackD_SS_Set_01_Fractior	8128	FTMS; HCI	59.73	723.73	PPIG

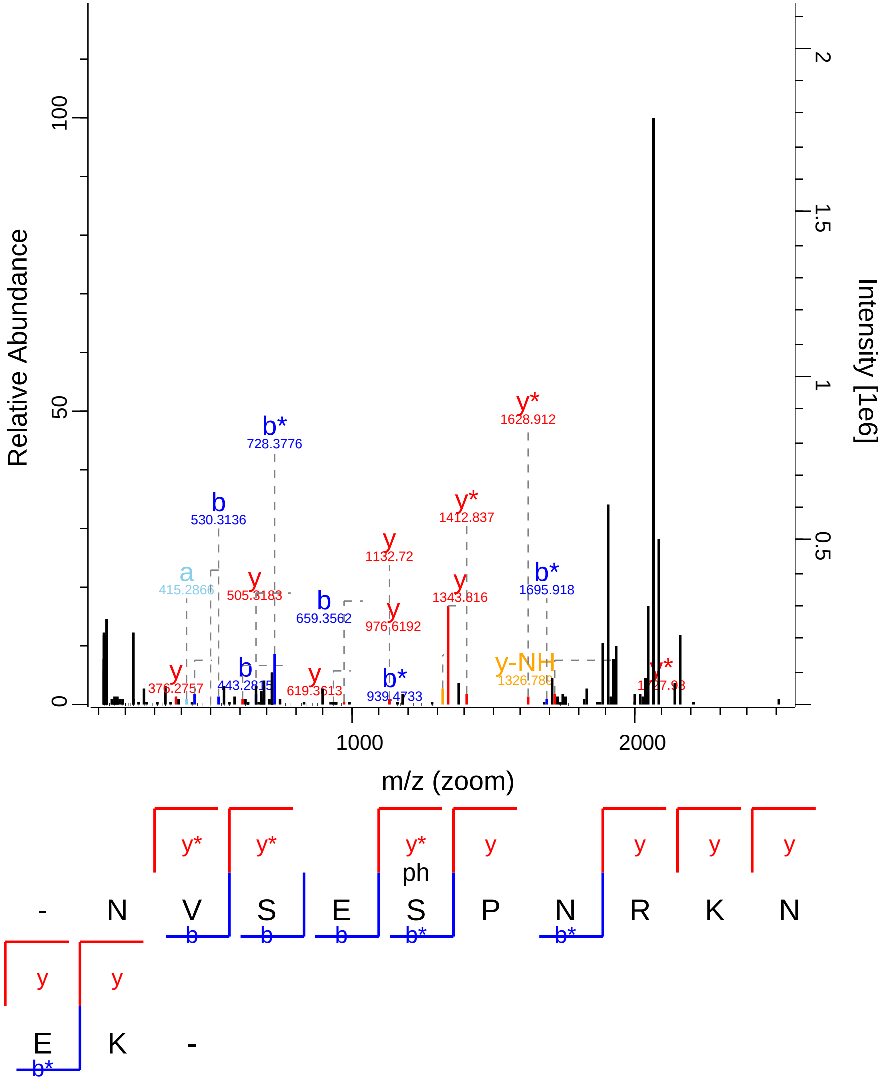

Raw File	Scan	Method	Score	m/z	Gene names
RO-QC-160121_ZackD_SS_Set_01_Fraction	8937	FTMS; HCI	95.36	496.6	LMNA

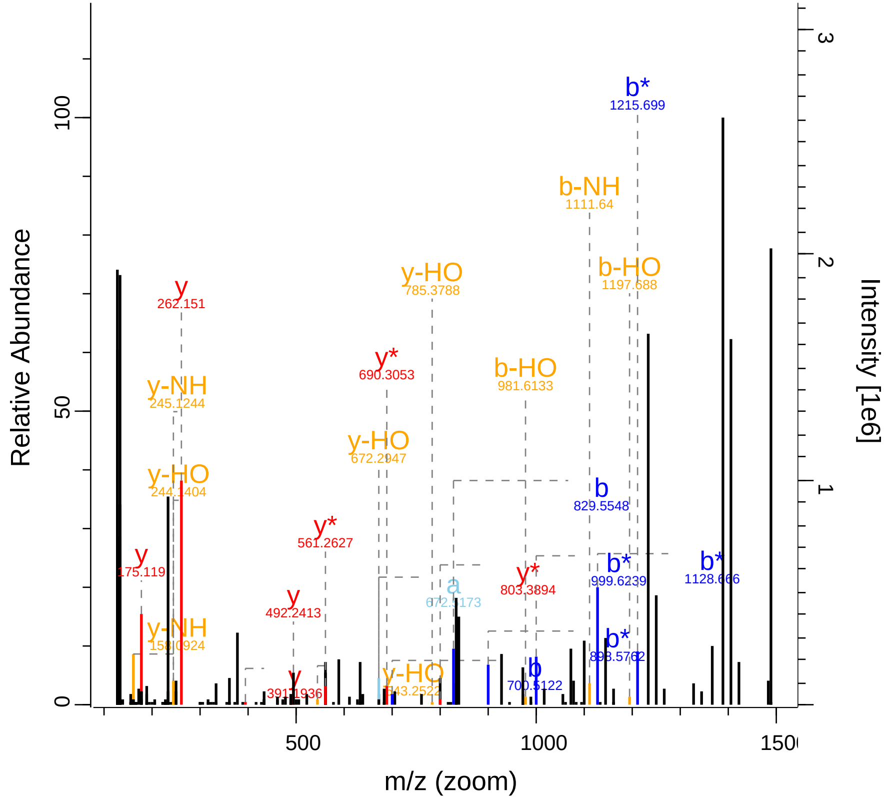

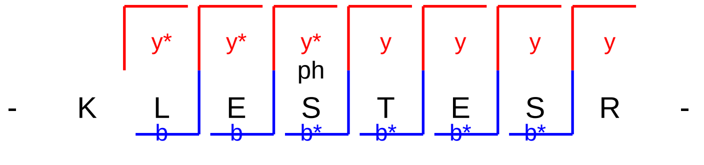

Raw File	Scan	Method	Score	m/z	Gene names
RO-QC-160121_ZackD_SS_Set_01_Fractior	9165	FTMS; HCI	47.07	776.86	CCNL1

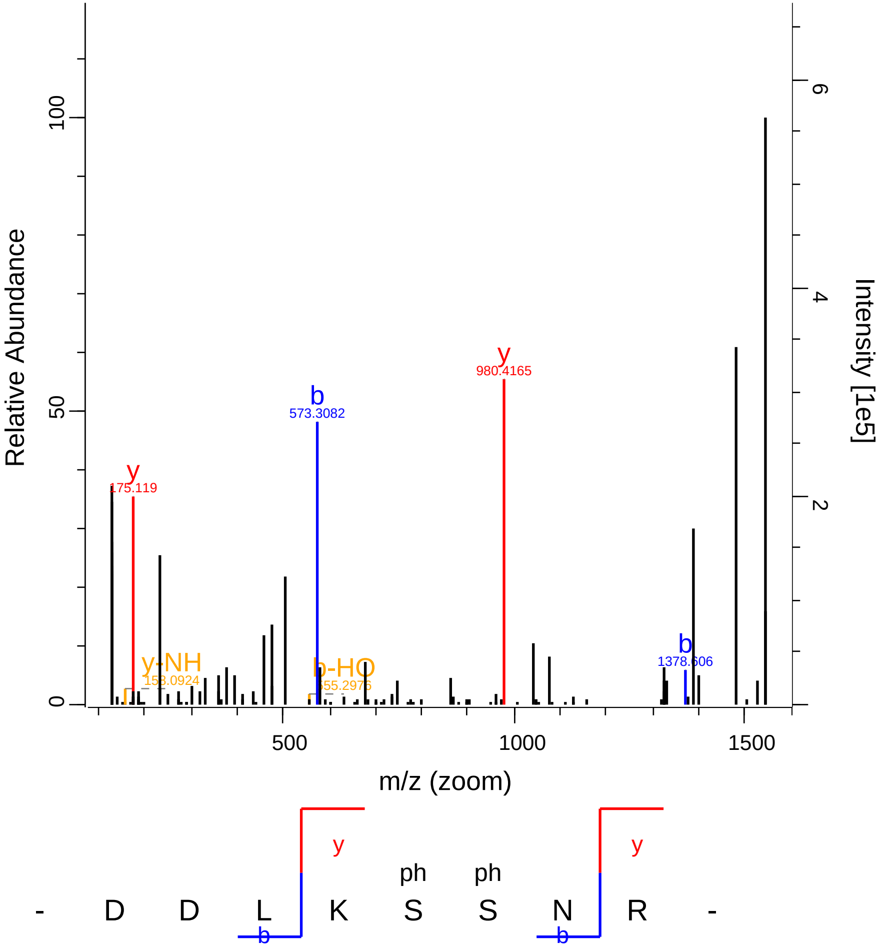

Raw File	Scan	Method	Score	m/z	Gene names
RO-QC-160121_ZackD_SS_Set_01_Fractior	9201	FTMS; HCI	82.07	471.77	PRPF4B

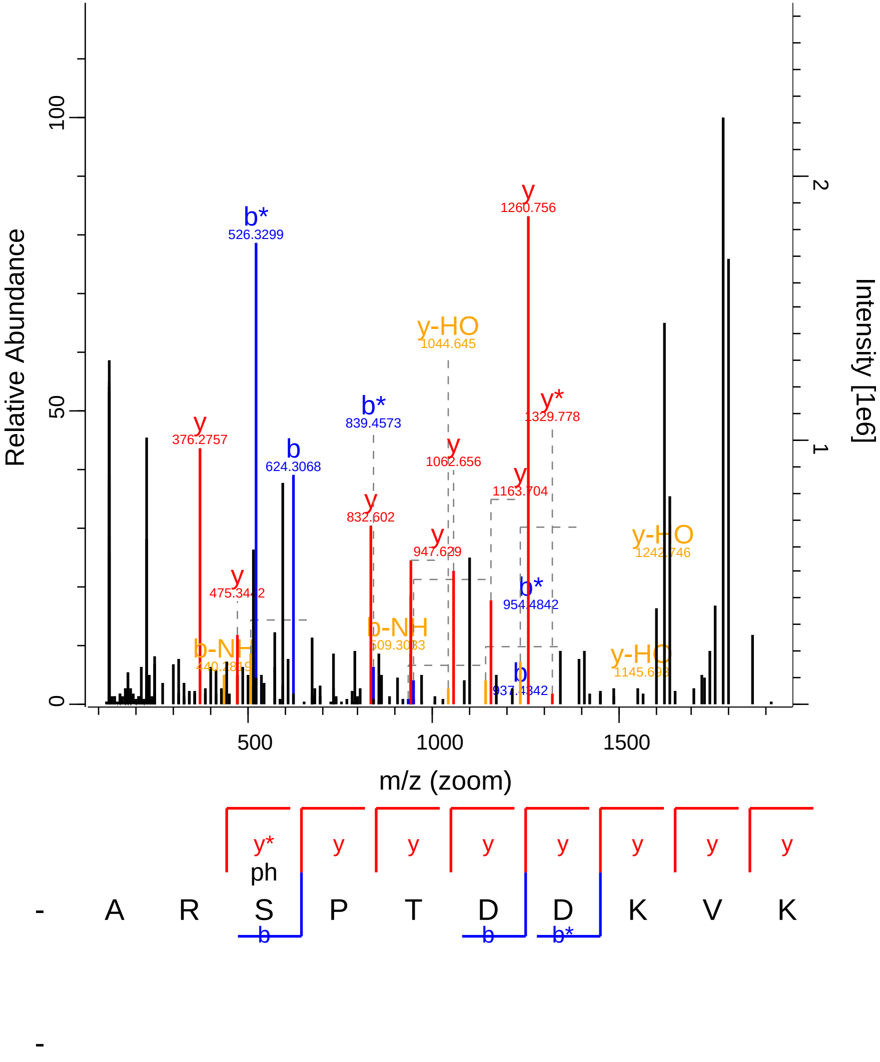

Raw File	Scan	Method	Score	m/z	Gene names
RO-QC-160121_ZackD_SS_Set_01_Fractior	9449	FTMS; HCI	72.43	671.35	AKAP8L

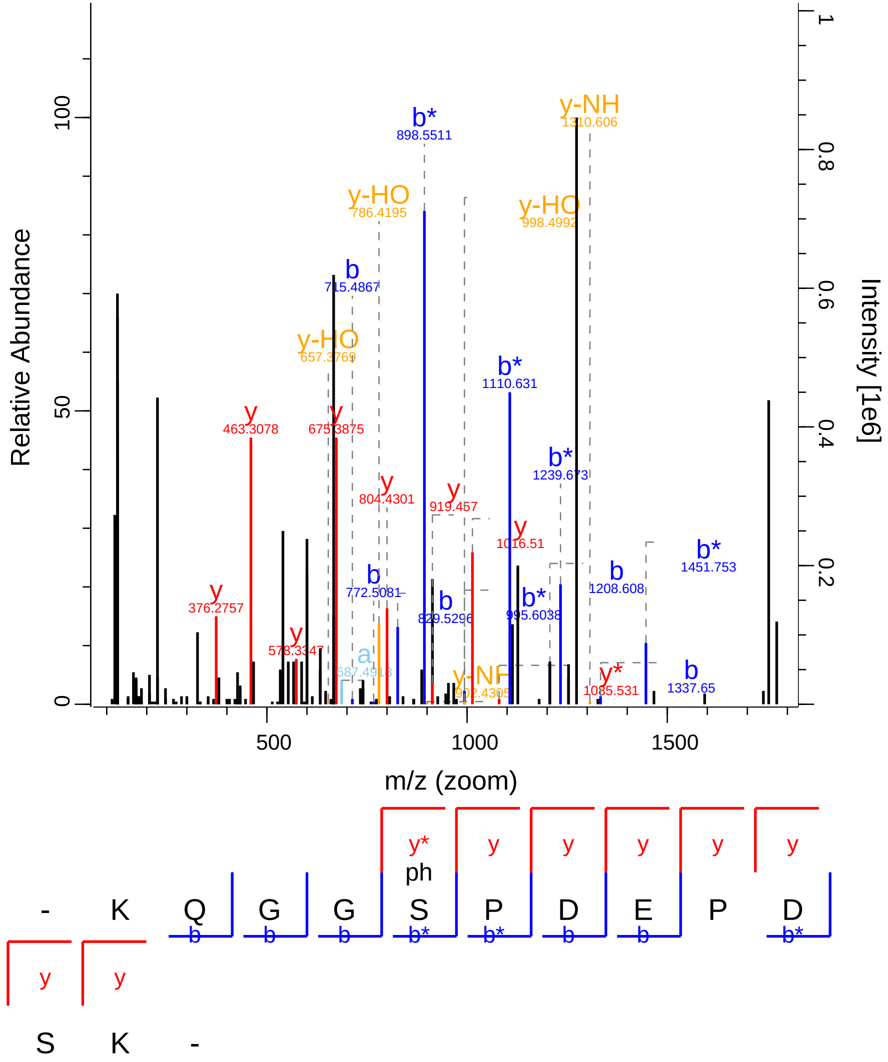

Raw File	Scan	Method	Score	m/z	Gene names
RO-QC-160121_ZackD_SS_Set_01_Fractior	9784	FTMS; HCI	52.58	527.62	TCOF1

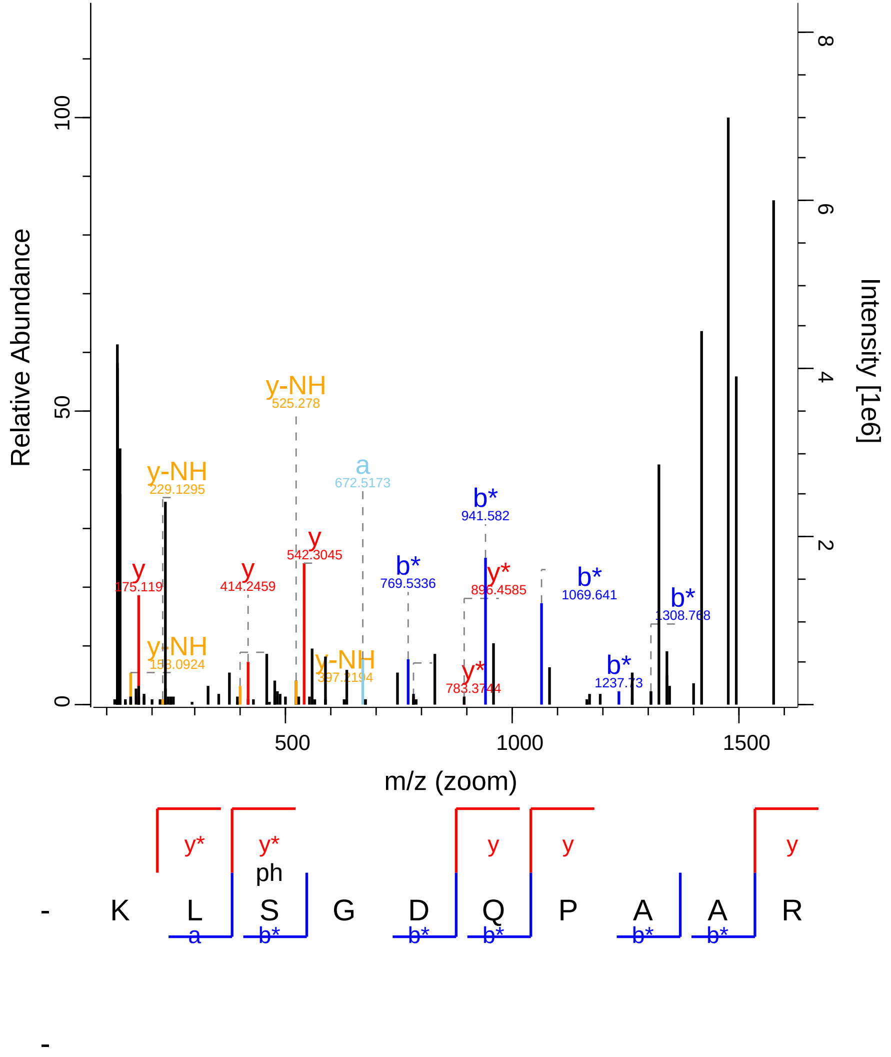

Raw File	Scan	Method	Score	m/z	Gene names
RO-QC-160121_ZackD_SS_Set_01_Fractior	11053	FTMS; HCI	101.64	551.7	MARCKS

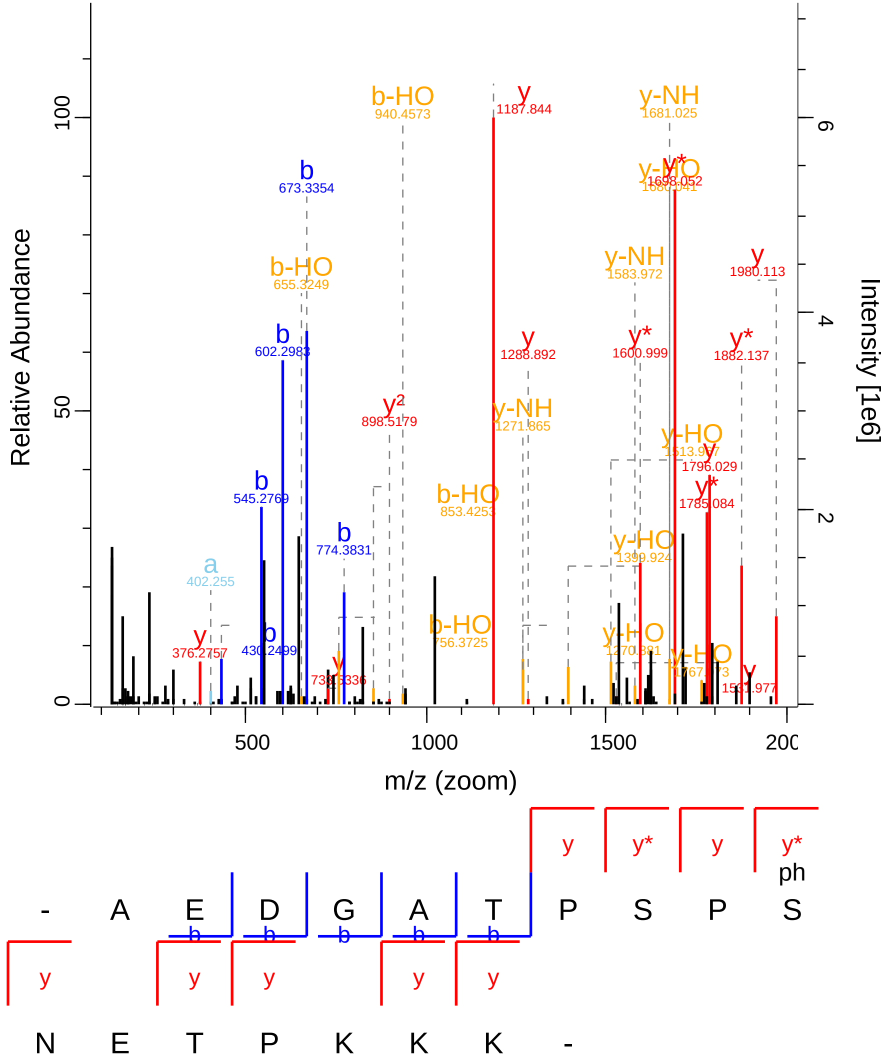

Raw File	Scan	Method	Score	m/z	Gene names
RO-QC-160121_ZackD_SS_Set_01_Fractio	12721	FTMS; HC	66.83	845.12	SRRM2

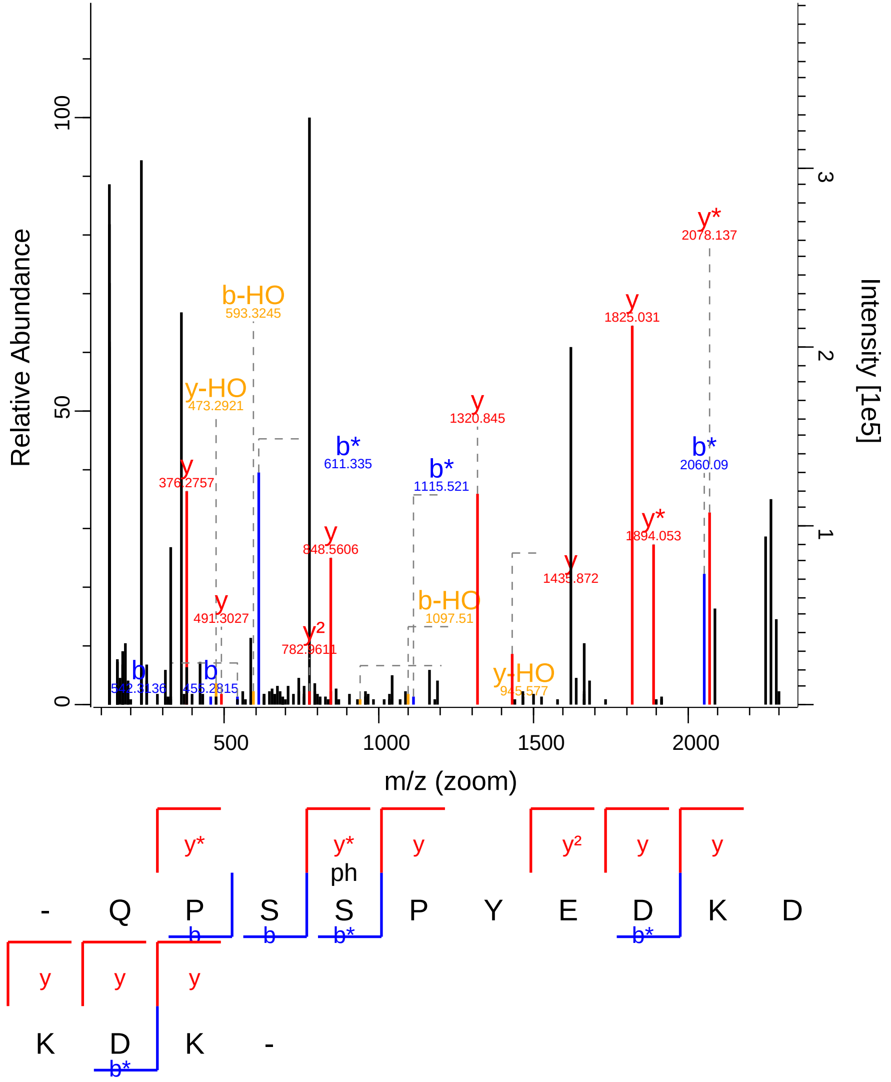

Raw File	Scan	Method	Score	m/z	Gene names
RO-QC-160121_ZackD_SS_Set_01_Fractior	13054	FTMS; HCI	91.86	878.9	FNBP4

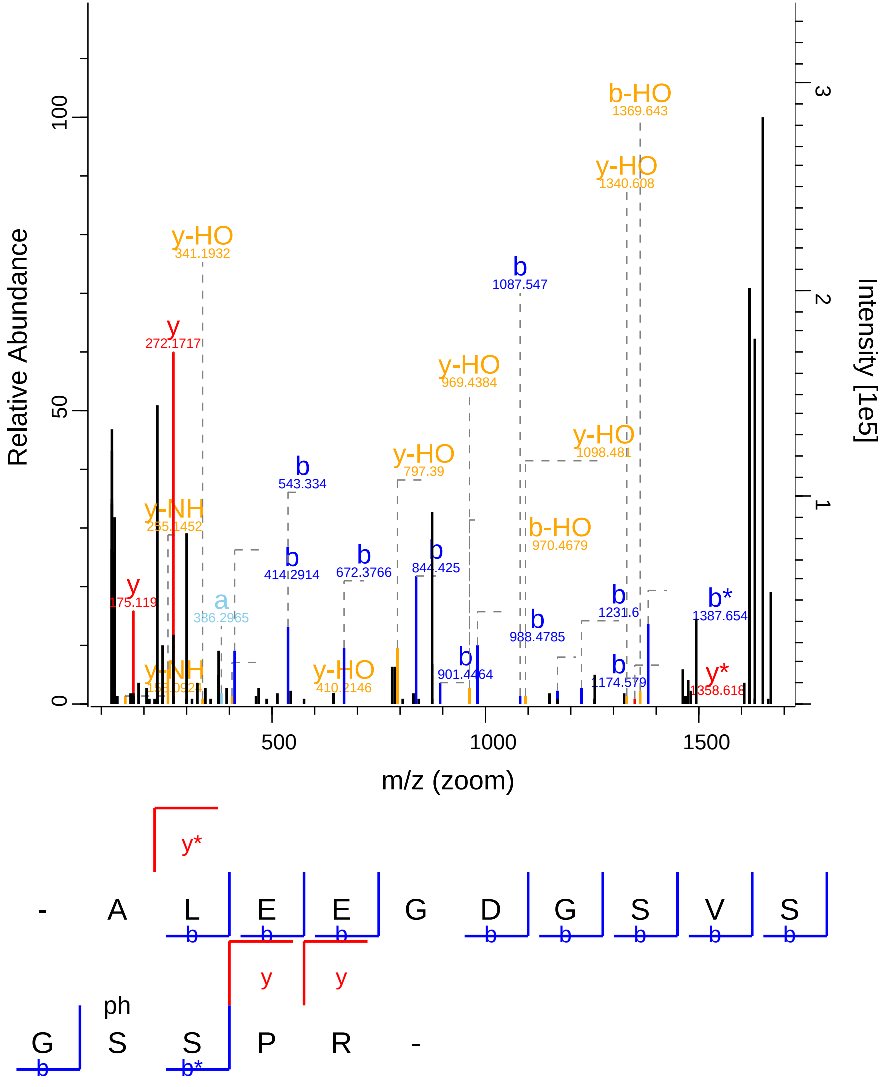

Raw File	Scan	Method	Score	m/z	Gene names
RO-QC-160121_ZackD_SS_Set_01_Fractio	14462	FTMS; HC	66.02	715.37	LEO1

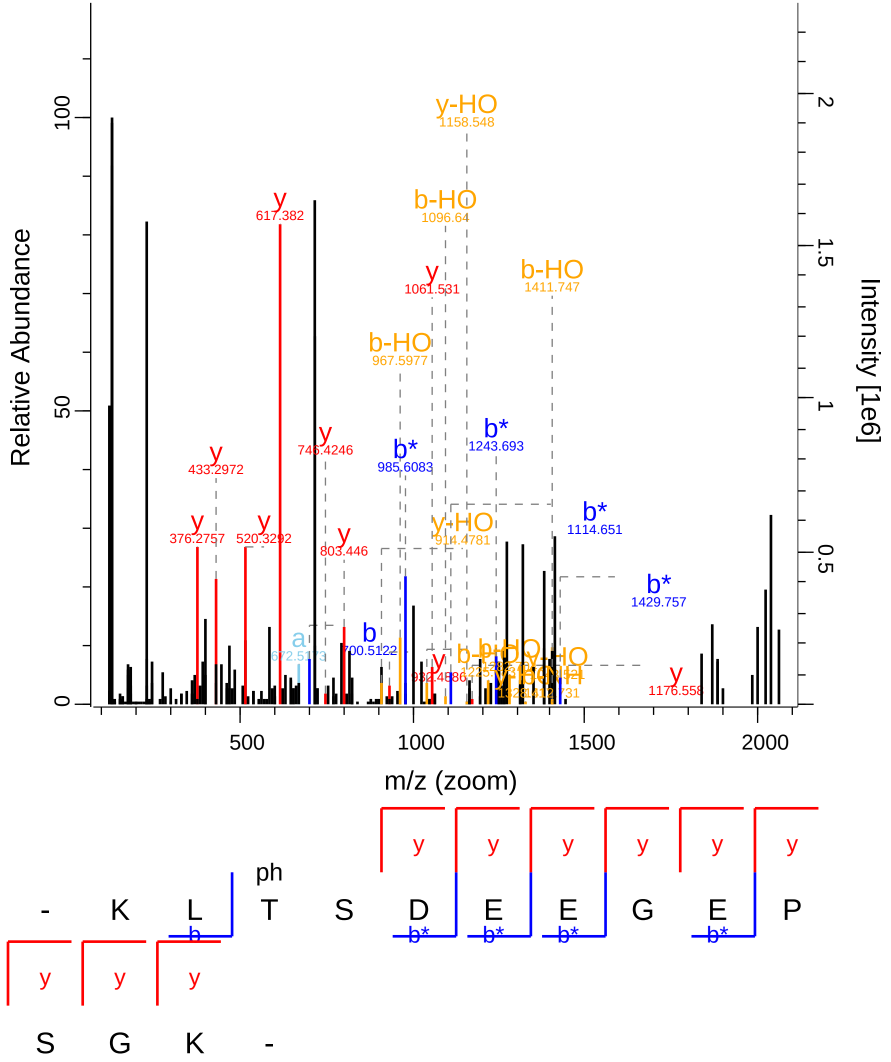

Raw File	Scan	Method	Score	m/z	Gene names
RO-QC-160121_ZackD_SS_Set_01_Fractio	14560	FTMS; HC	134.25	846.91	ERCC5

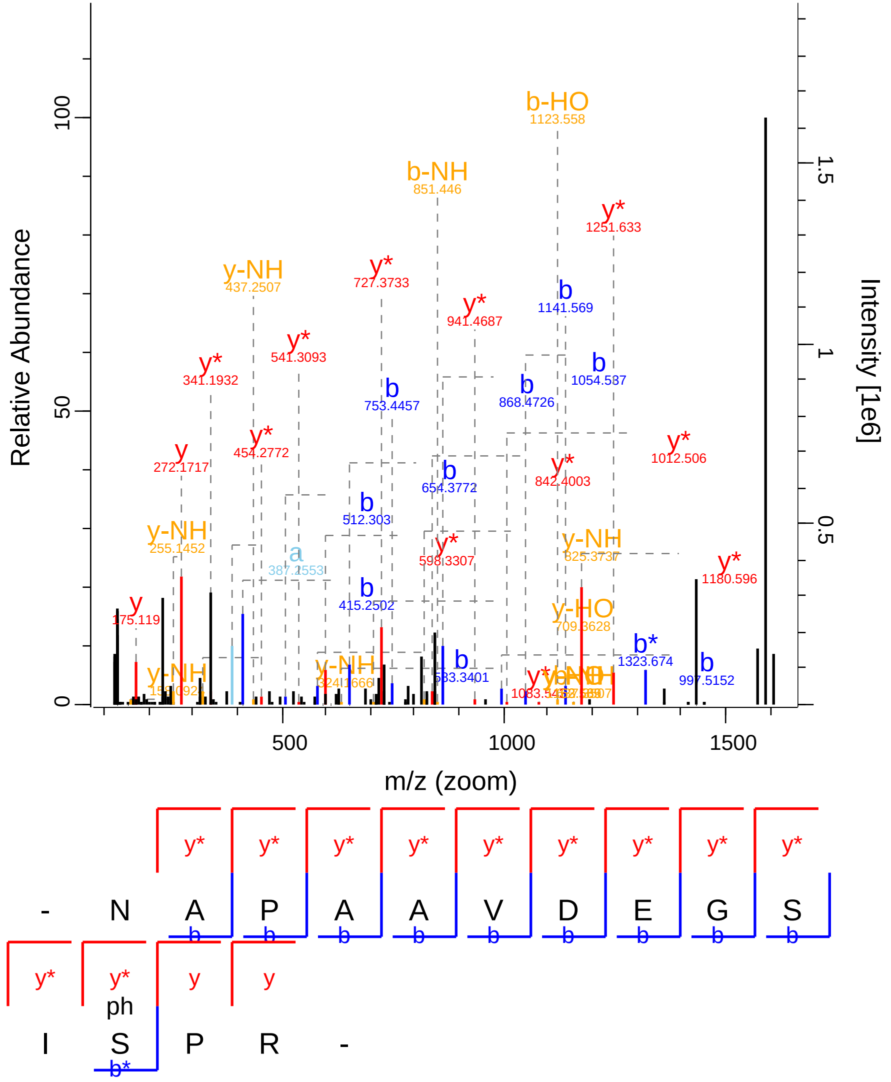

Raw File	Scan	Method	Score	m/z	Gene names
RO-QC-160121_ZackD_SS_Set_01_Fractio	14683	FTMS; HC	132.14	835.39	PPAN

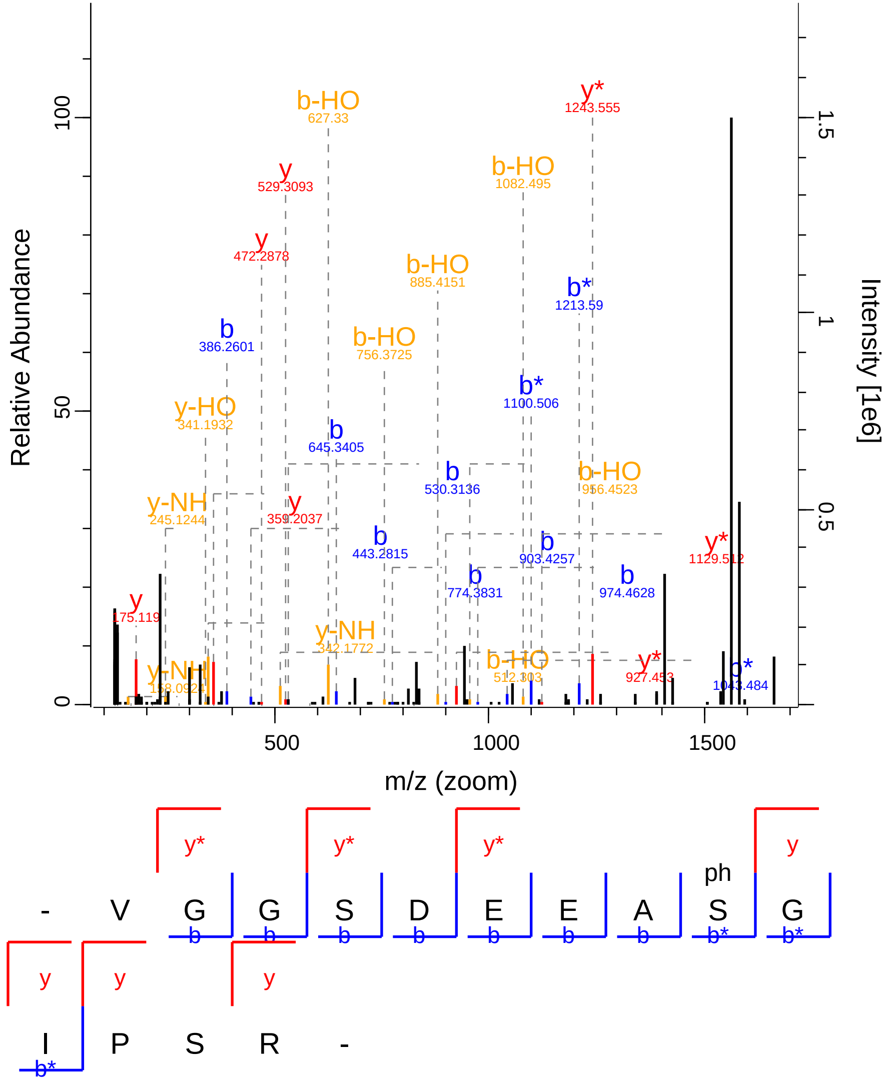

Raw File	Scan	Method	Score	m/z	Gene names
RO-QC-160121_ZackD_SS_Set_01_Fractio	14897	FTMS; HC	198.48	1032.87	AKAP2

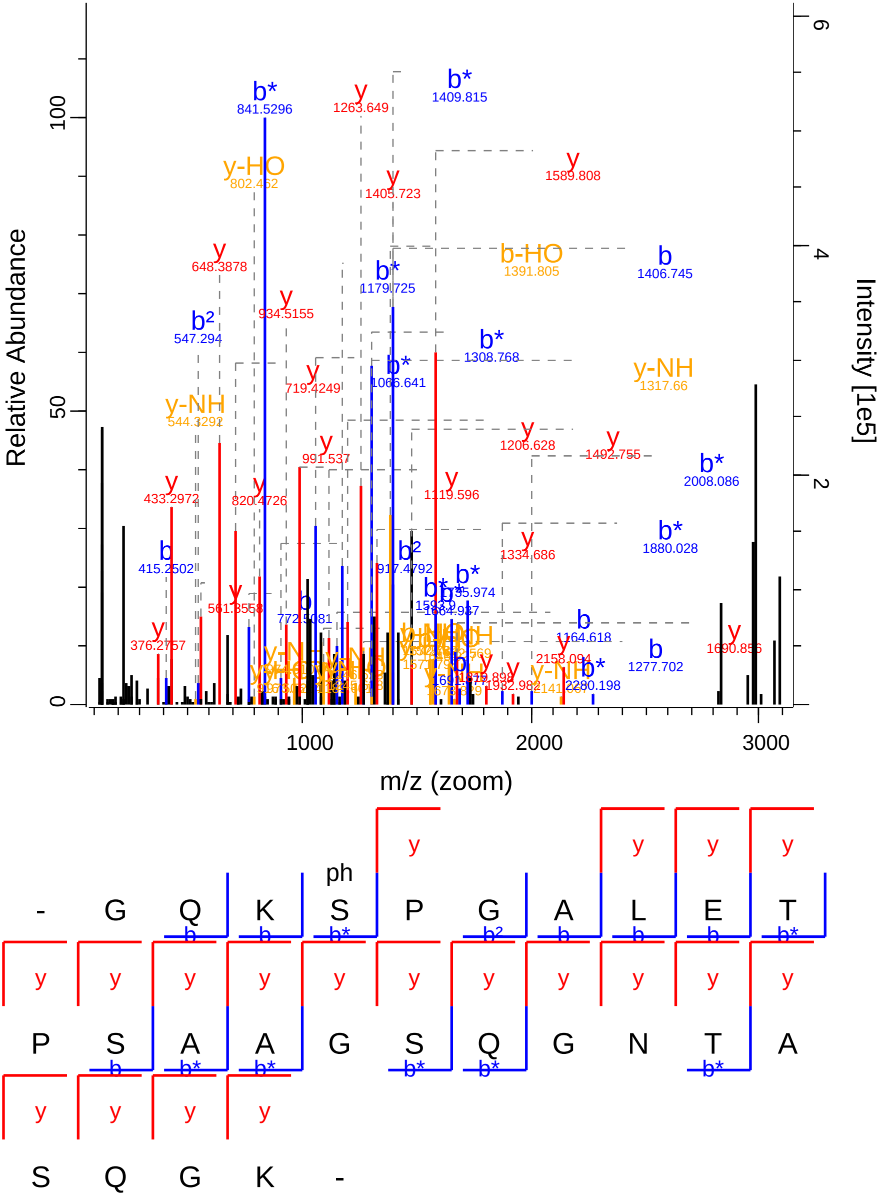

Raw File	Scan	Method	Score	m/z	Gene names
RO-QC-160121_ZackD_SS_Set_01_Fractic	15640	FTMS; HC	71.56	1231.05	TINAG

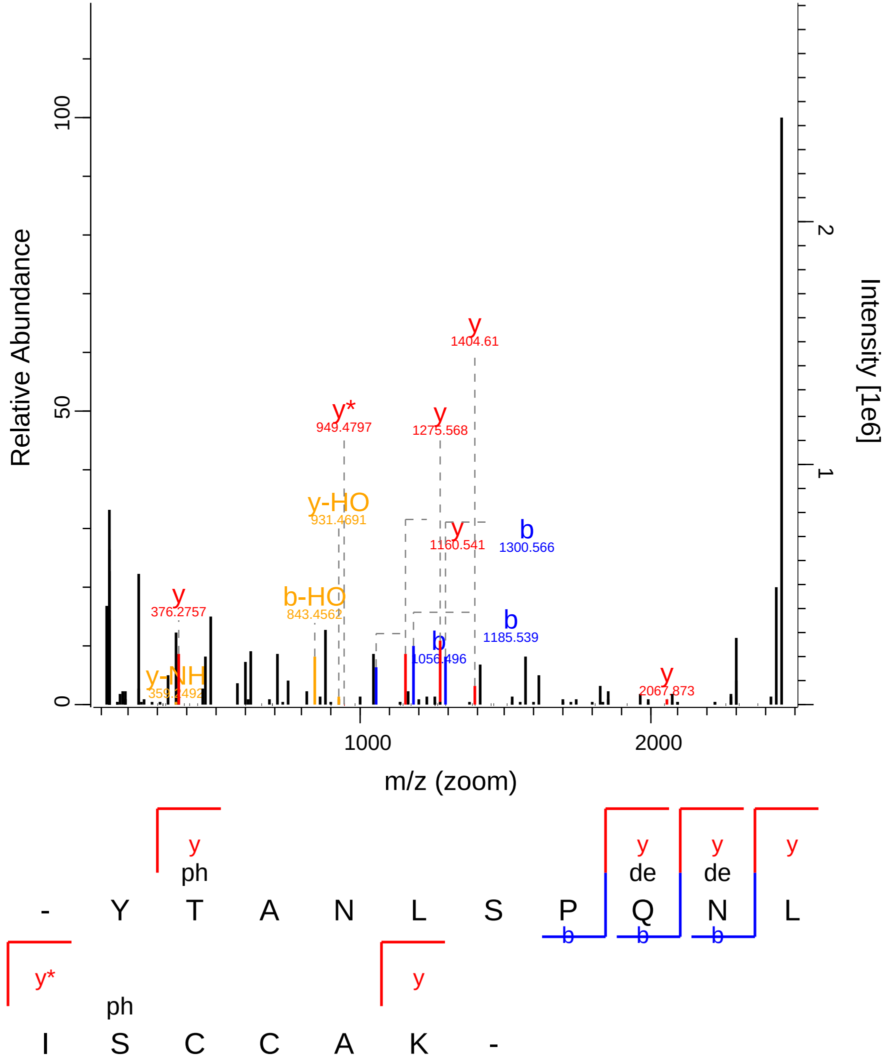

Raw File	Scan	Method	Score	m/z	Gene names
RO-QC-160121_ZackD_SS_Set_01_Fractic	15851	FTMS; HC	89.66	1118.04	NUMA1

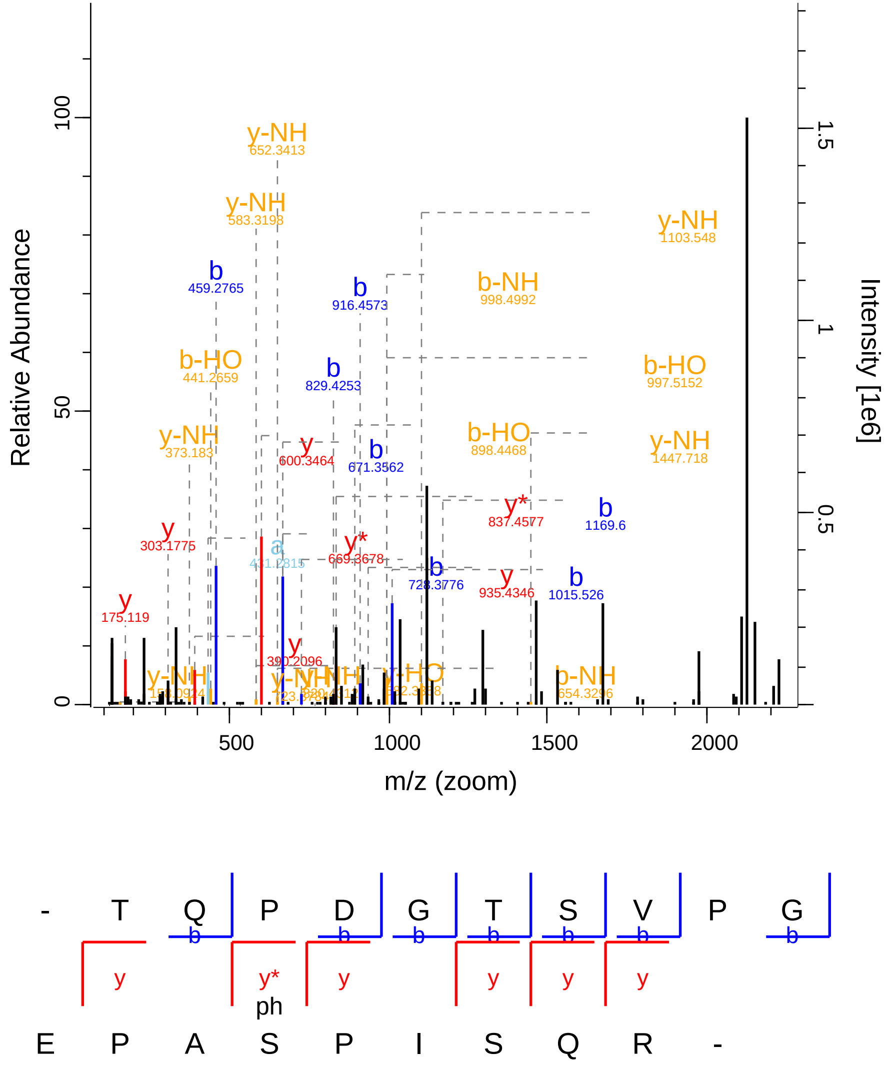

Raw File	Scan	Method	Score	m/z	Gene names
RO-QC-160121_ZackD_SS_Set_01_Fractio	18941	FTMS; HC	41.24	515.78	MMRN1

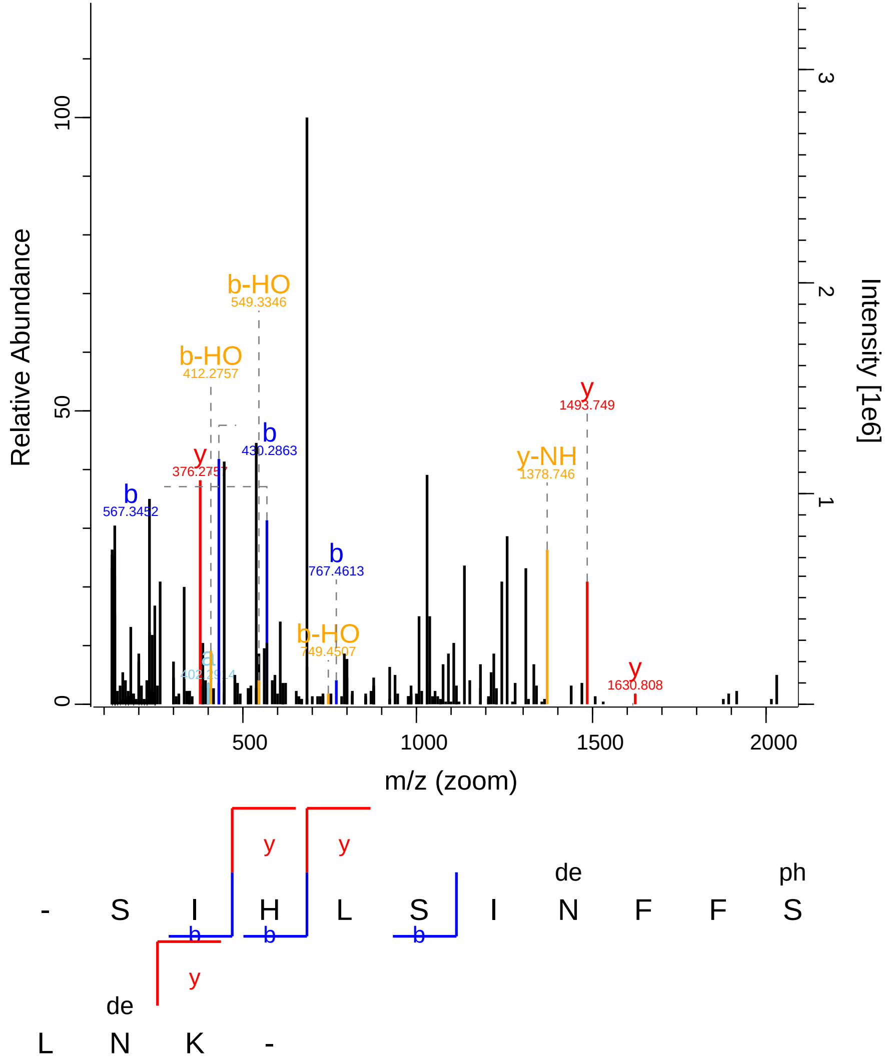

Raw File	Scan	Method	Score	m/z	Gene names
RO-QC-160121_ZackD_SS_Set_01_Fractio	18974	FTMS; HC	188.69	783.74	FLNA

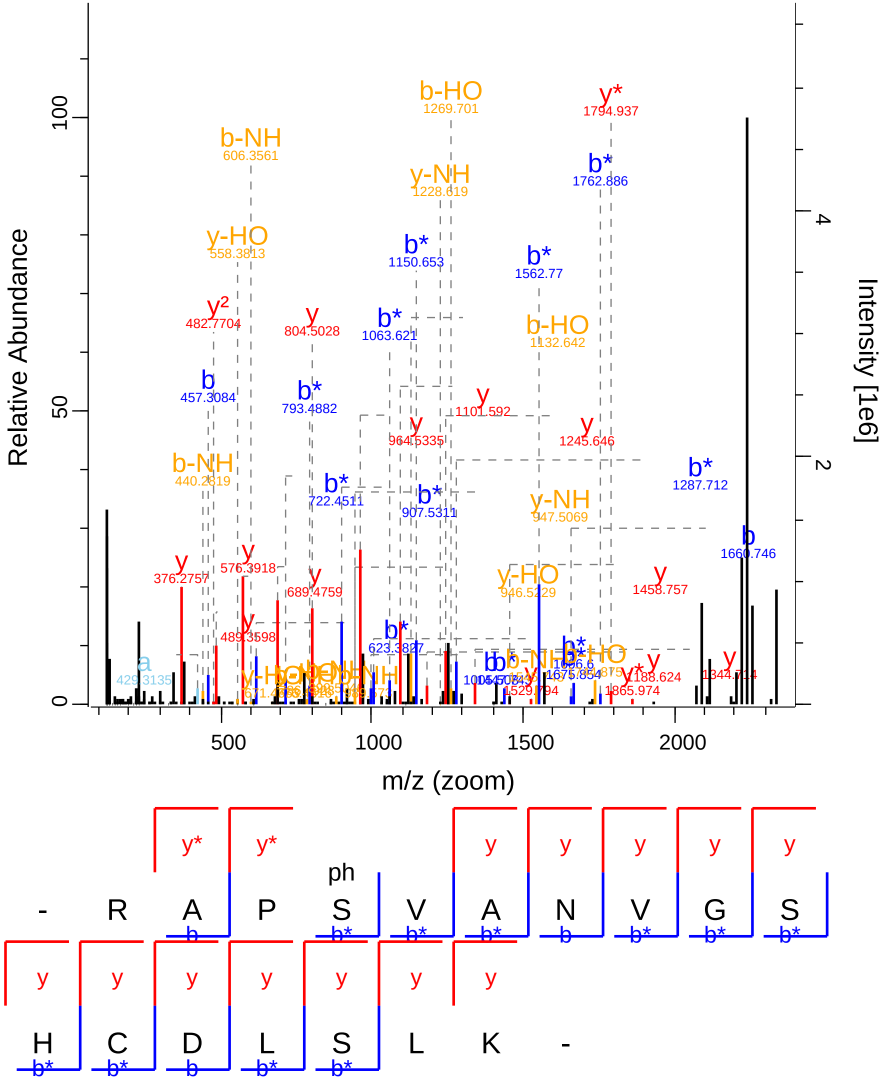

Raw File	Scan	Method	Score	m/z	Gene names
RO-QC-160121_ZackD_SS_Set_01_Fractio	19644	FTMS; HC	113.31	988.17	TANC1

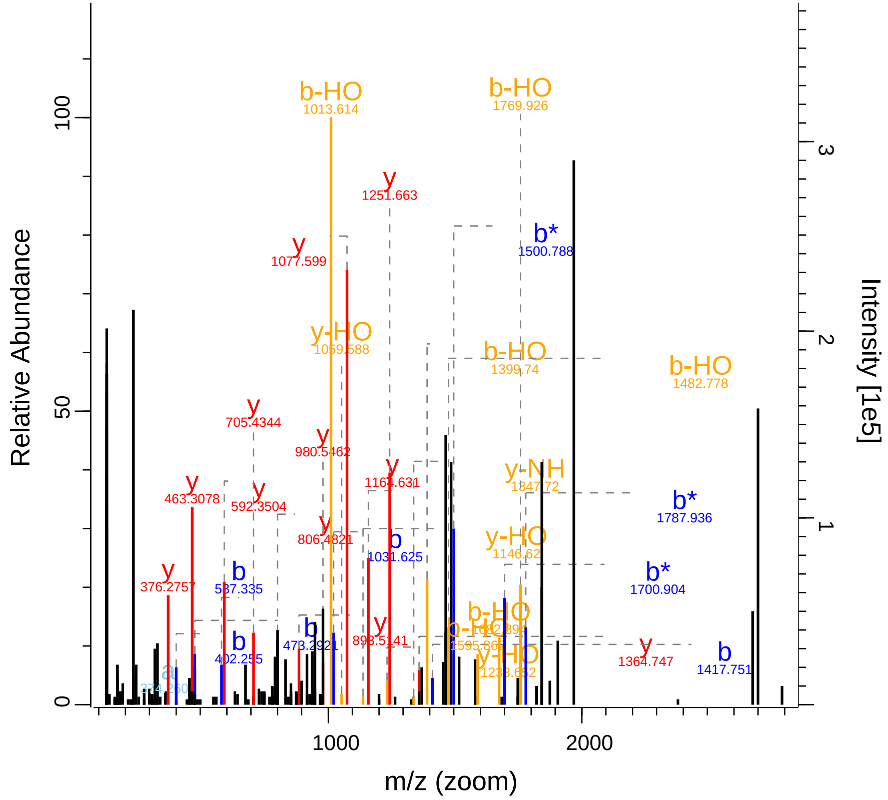

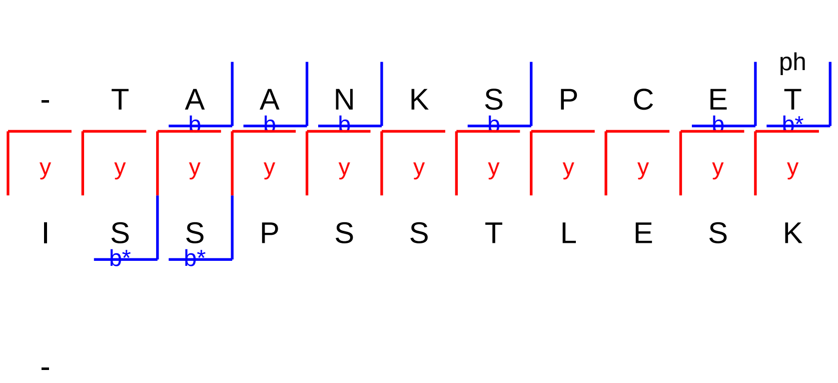

Raw File	Scan	Method	Score	m/z	Gene names
RO-QC-160121_ZackD_SS_Set_01_Fractio	19849	FTMS; HC	120.85	838.79	AHNAK

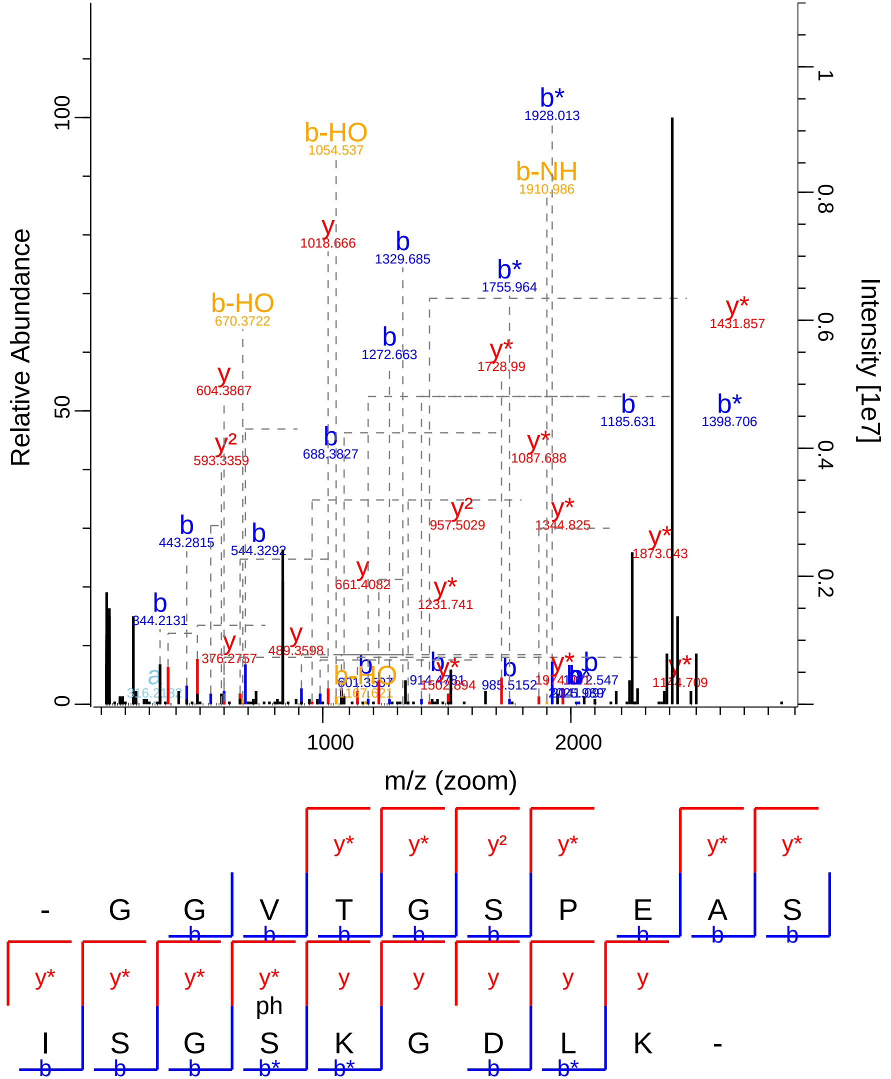

Raw File	Scan	Method	Score	m/z	Gene names
RO-QC-160121_ZackD_SS_Set_01_Fractio	20167	FTMS; HC	99.6	766.41	DIP2C

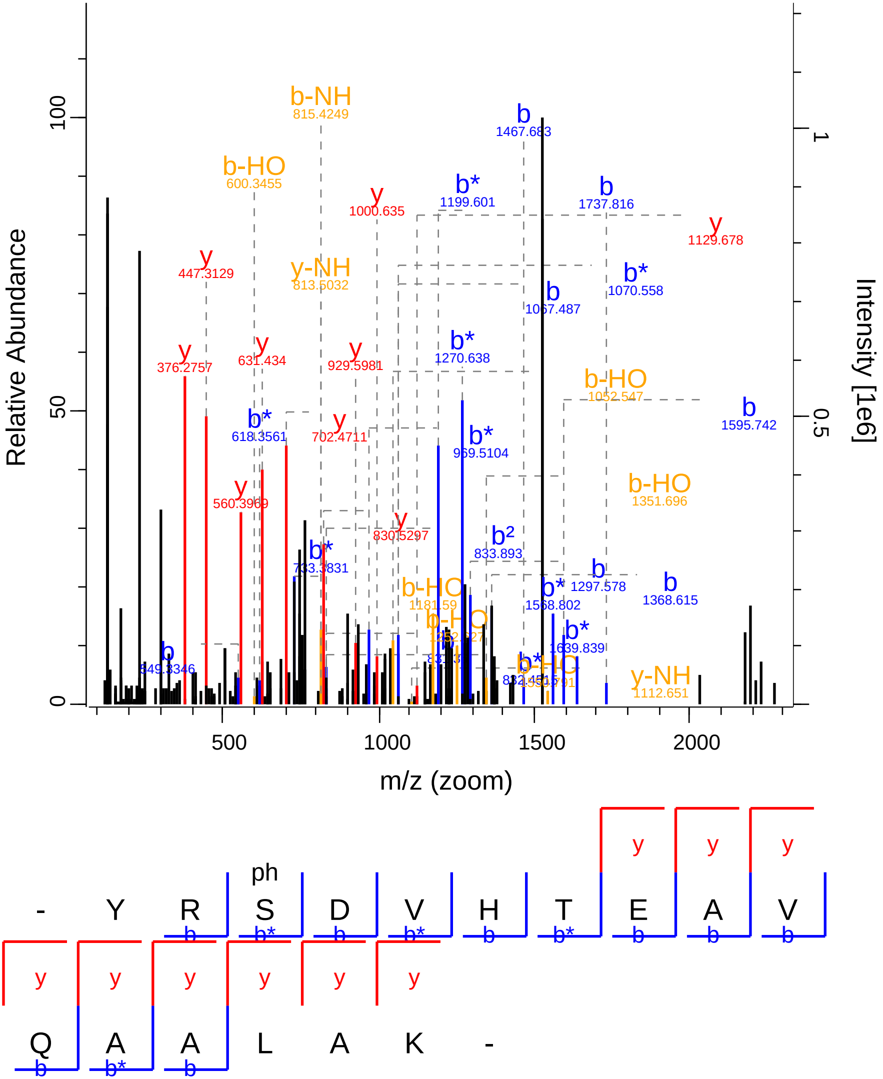

Raw File	Scan	Method	Score	m/z	Gene names
RO-QC-160121_ZackD_SS_Set_01_Fractio	20858	FTMS; HC	123.09	933.74	CDK13

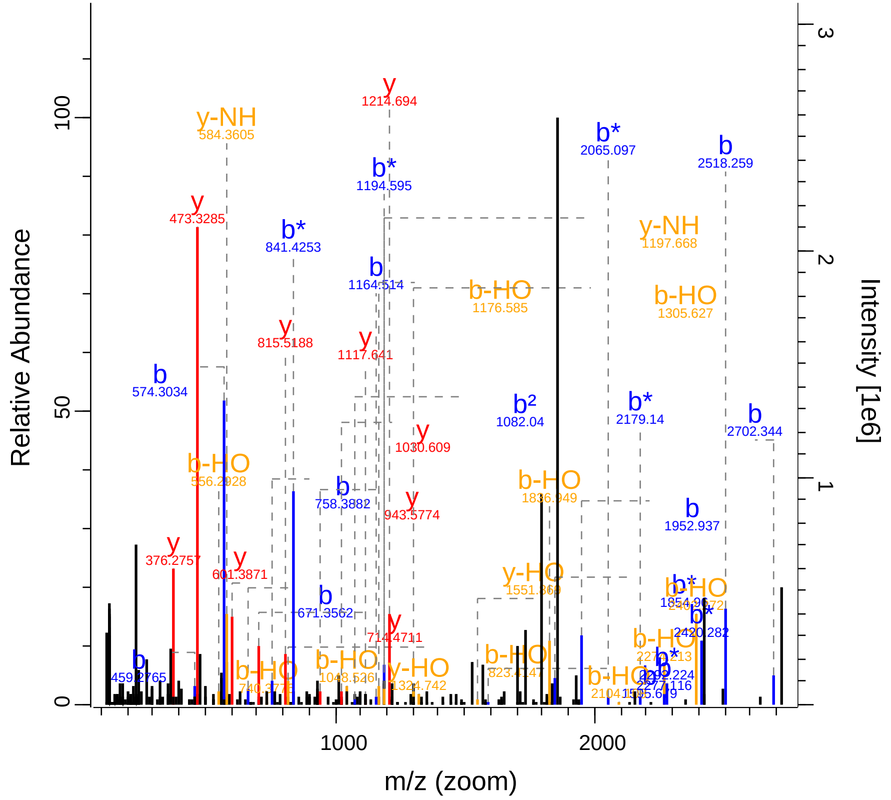

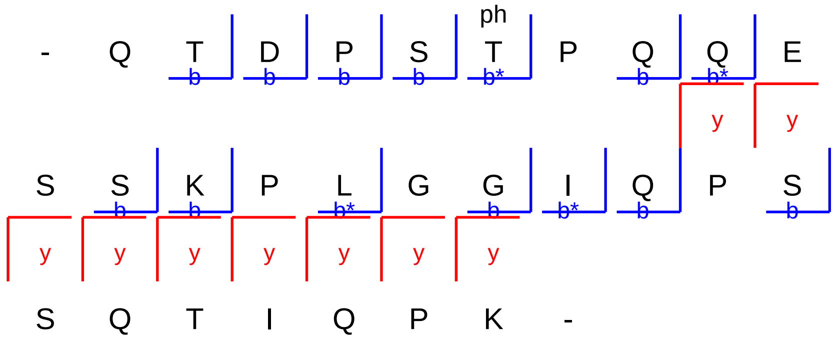

Raw File	Scan	Method	Score	m/z	Gene names
RO-QC-160121_ZackD_SS_Set_01_Fractio	21834	FTMS; HC	60.03	827.81	LARP1

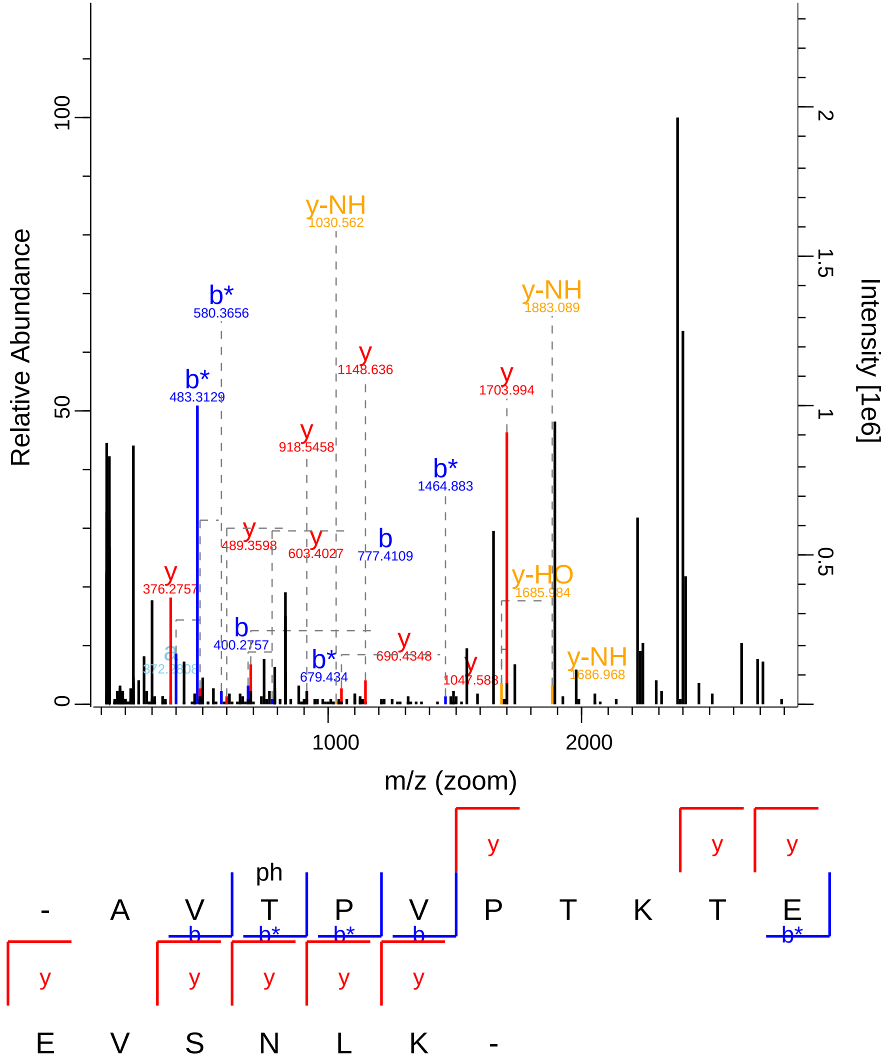

Raw File	Scan	Method	Score	m/z	Gene names
RO-QC-160121_ZackD_SS_Set_01_Fractio	21971	FTMS; HC	74.77	601.01	RAB3IL1

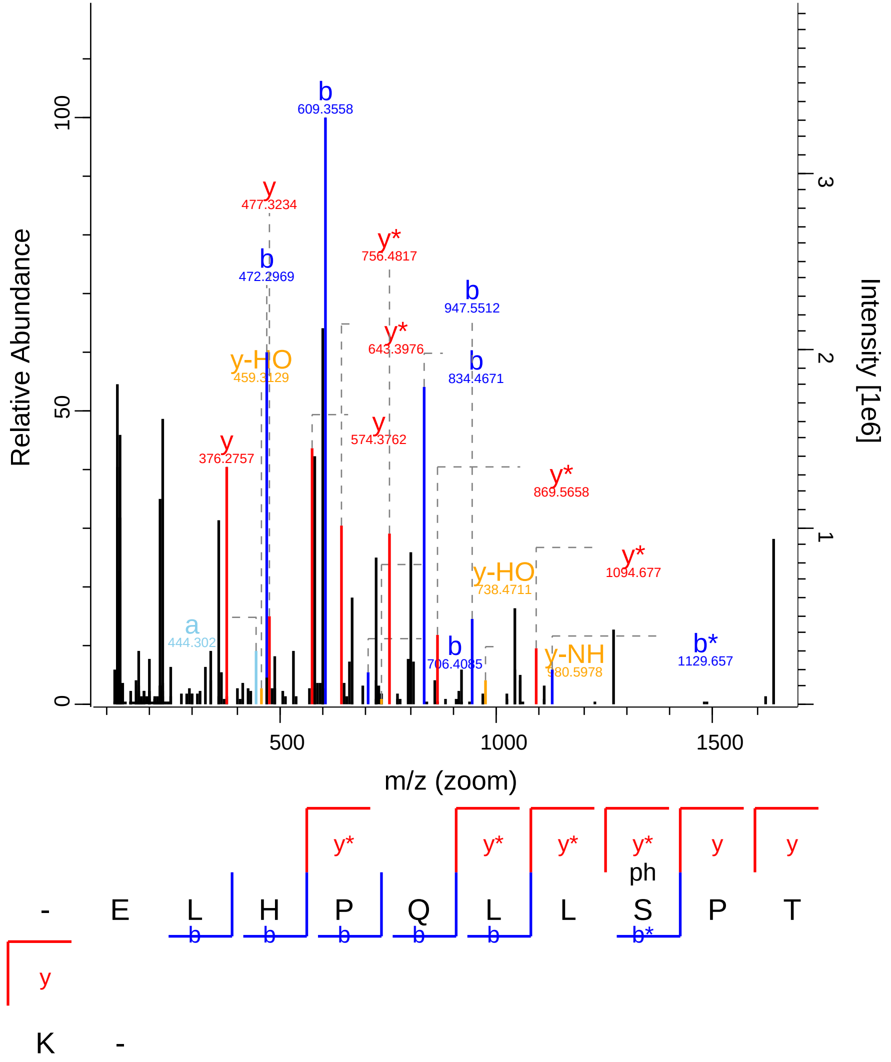

Raw File	Scan	Method	Score	m/z	Gene names
RO-QC-160121_ZackD_SS_Set_01_Fractio	22135	FTMS; HC	45.26	597.28	ERMP1

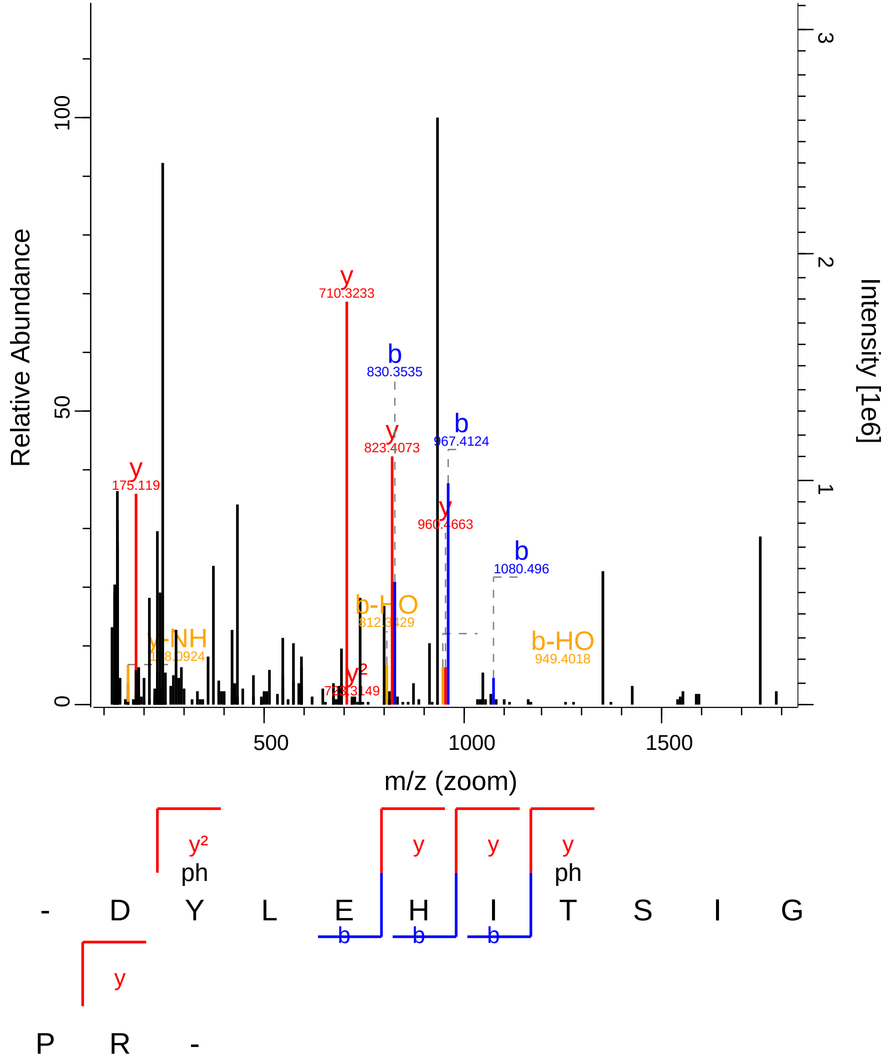

Raw File	Scan	Method	Score	m/z	Gene names
RO-QC-160121_ZackD_SS_Set_01_Fractio	22662	FTMS; HC	64.65	922.98	CLIP1

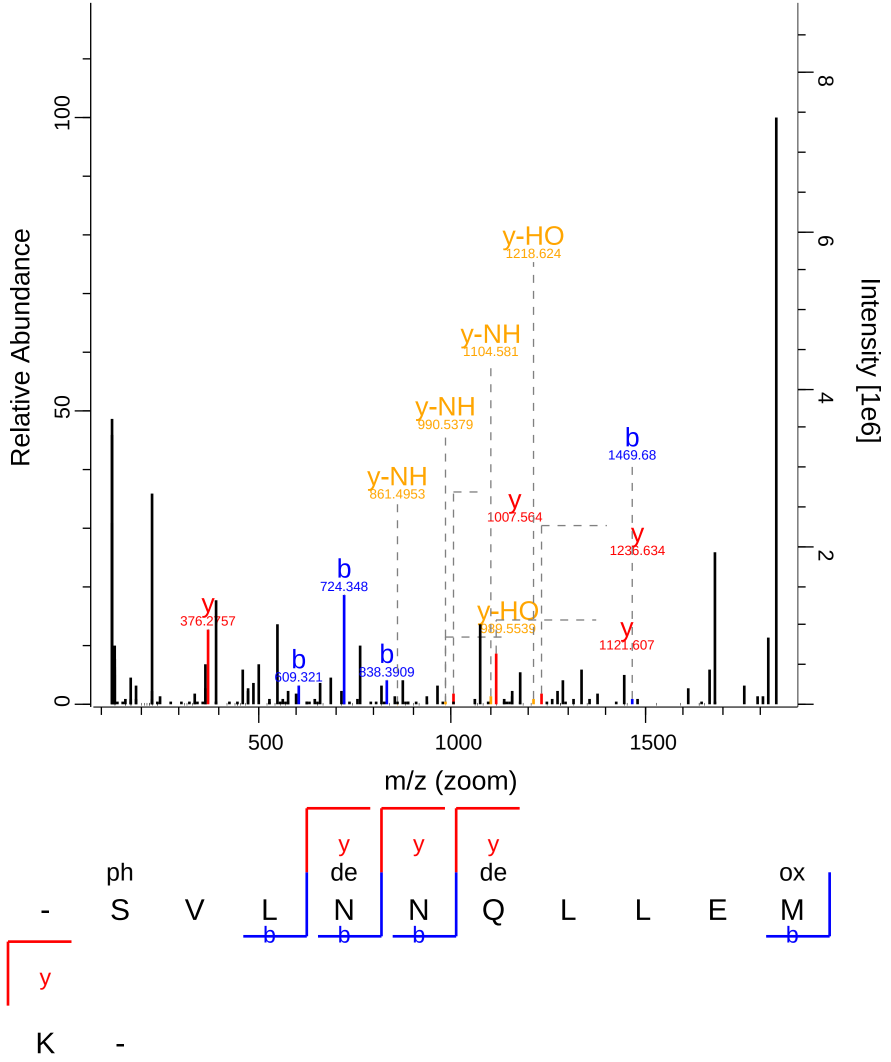

Raw File	Scan	Method	Score	m/z	Gene names
RO-QC-160121_ZackD_SS_Set_01_Fractic	23158	FTMS; HC	55.57	1193.16	SETD1A

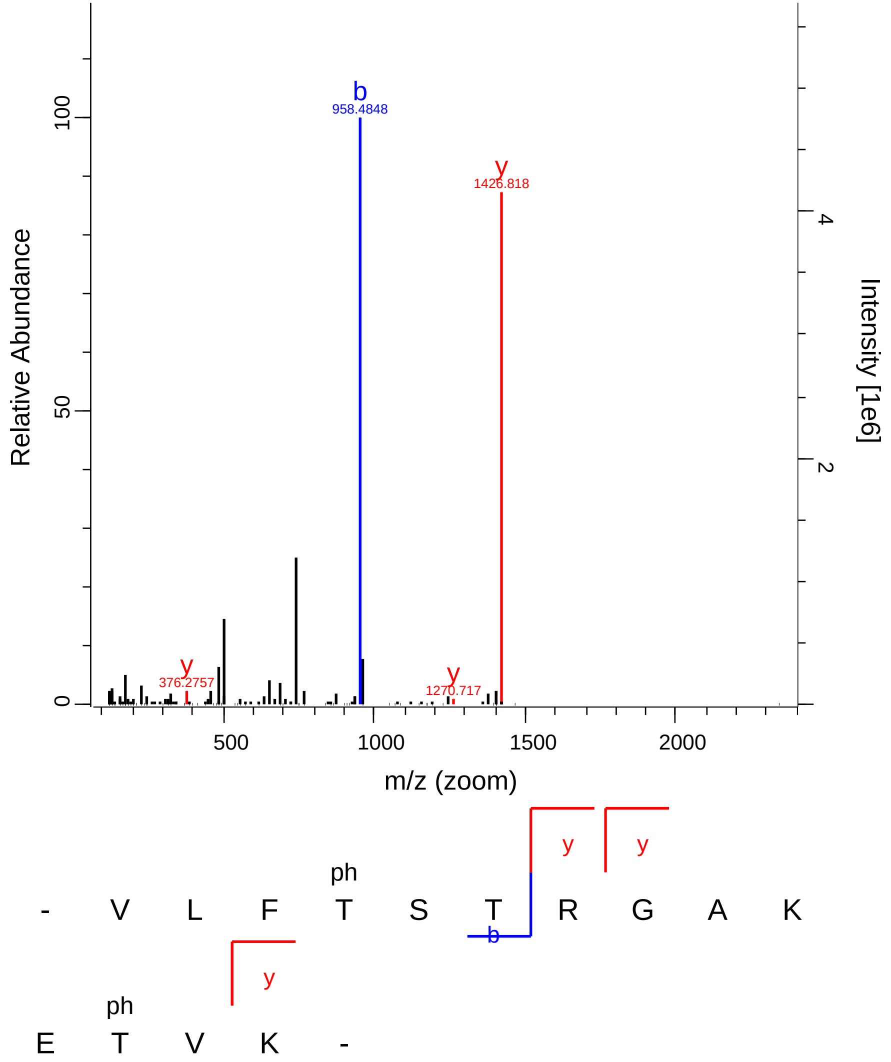

Raw File	Scan	Method	Score	m/z	Gene names
RO-QC-160121_ZackD_SS_Set_01_Fractio	23406	FTMS; HC	103.68	787.12	PCBP1

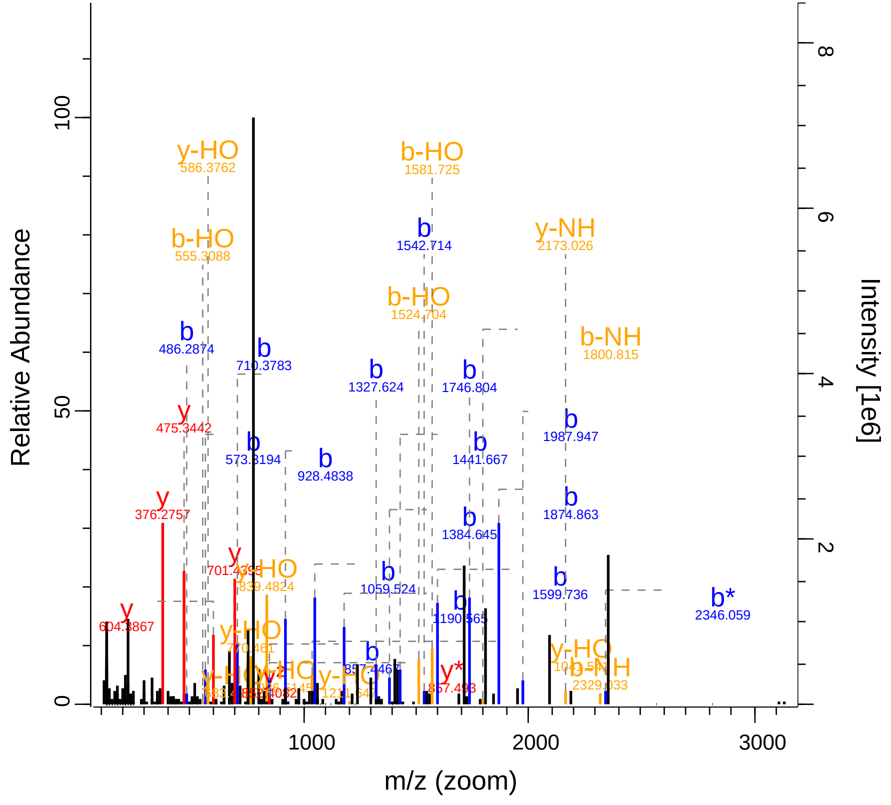

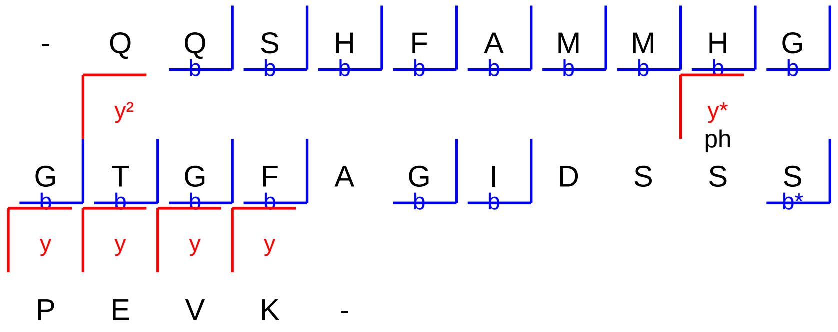

Raw File	Scan	Method	Score	m/z	Gene names
RO-QC-160121_ZackD_SS_Set_01_Fractio	23428	FTMS; HC	113.37	1049.16	PCBP1

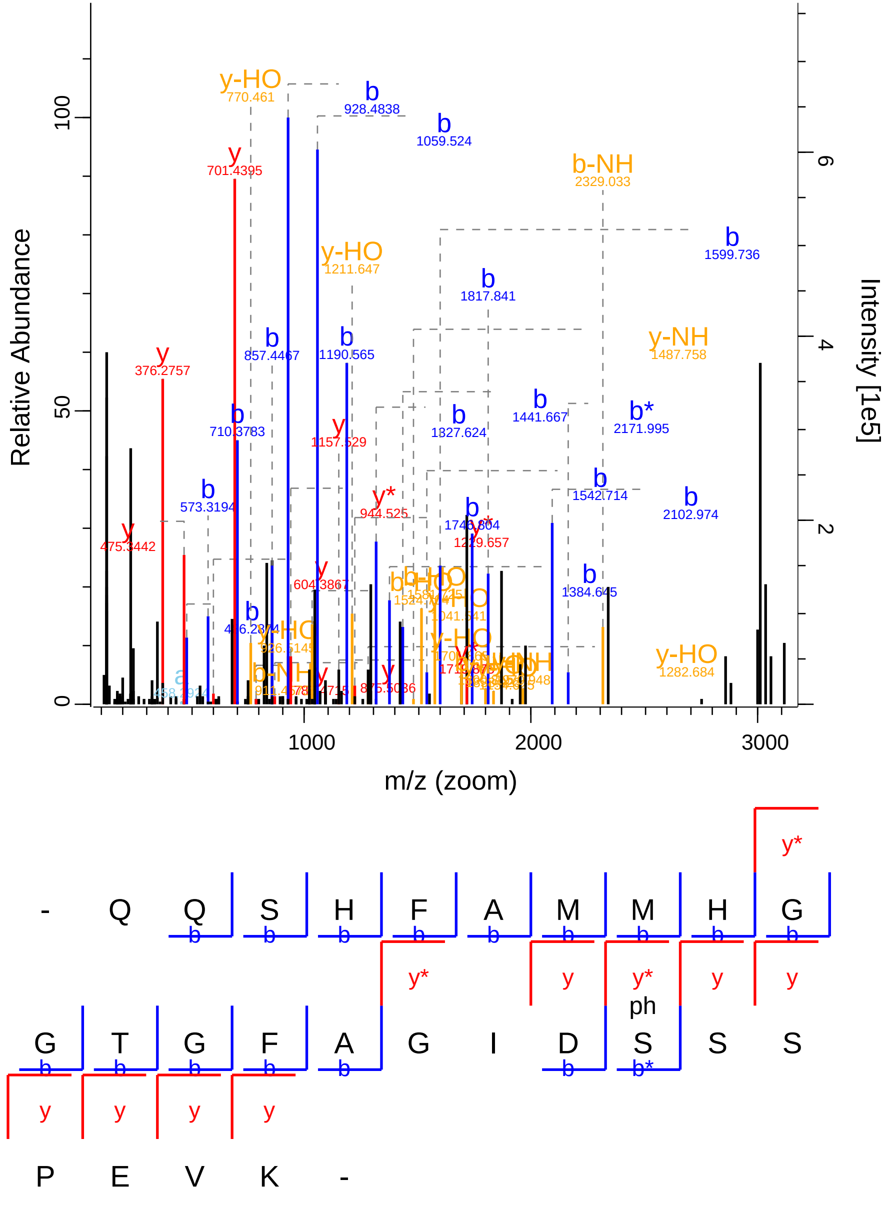

Raw File	Scan	Method	Score	m/z	Gene names
RO-QC-160121_ZackD_SS_Set_01_Fractio	24027	FTMS; HC	51.04	823.43	ZC3HC1

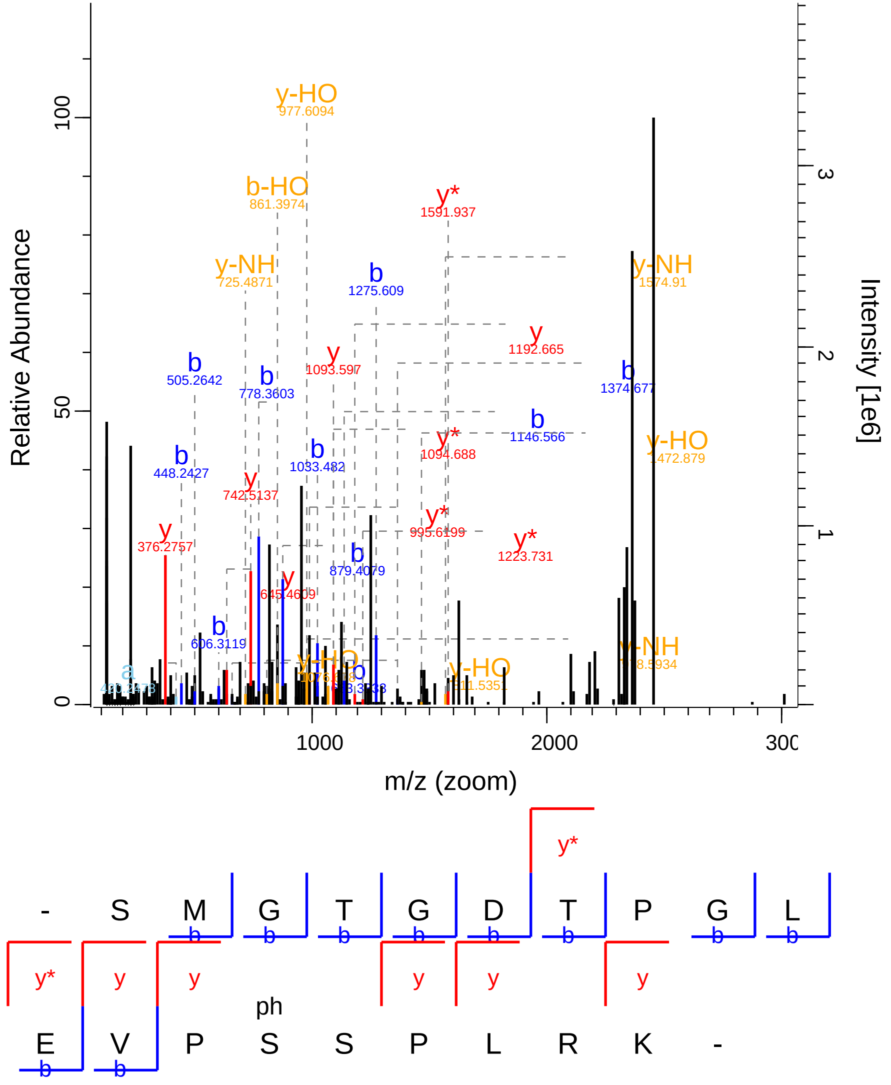

Raw File	Scan	Method	Score	m/z	Gene names
RO-QC-160121_ZackD_SS_Set_01_Fractio	27150	FTMS; HC	49.63	696.03	RASEF

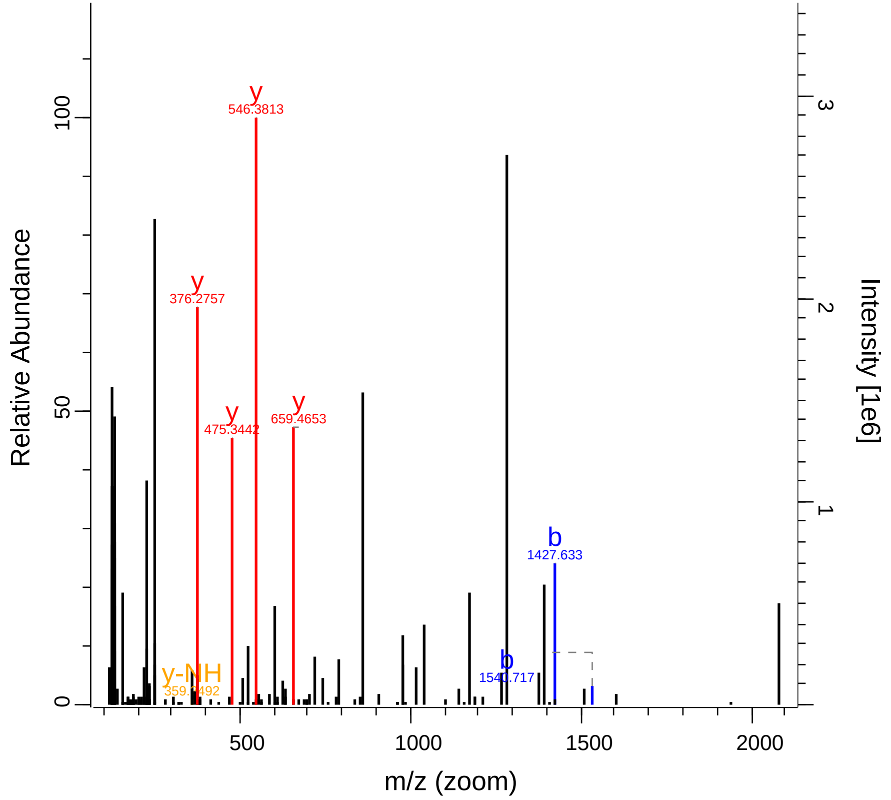

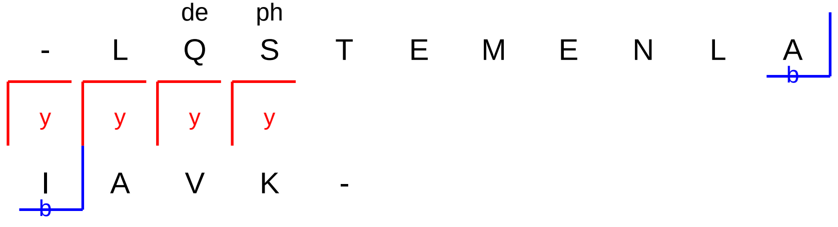

Raw File	Scan	Method	Score	m/z	Gene names
RO-QC-160121_ZackD_SS_Set_01_Fractio	27316	FTMS; HC	194.27	838.19	NEXN

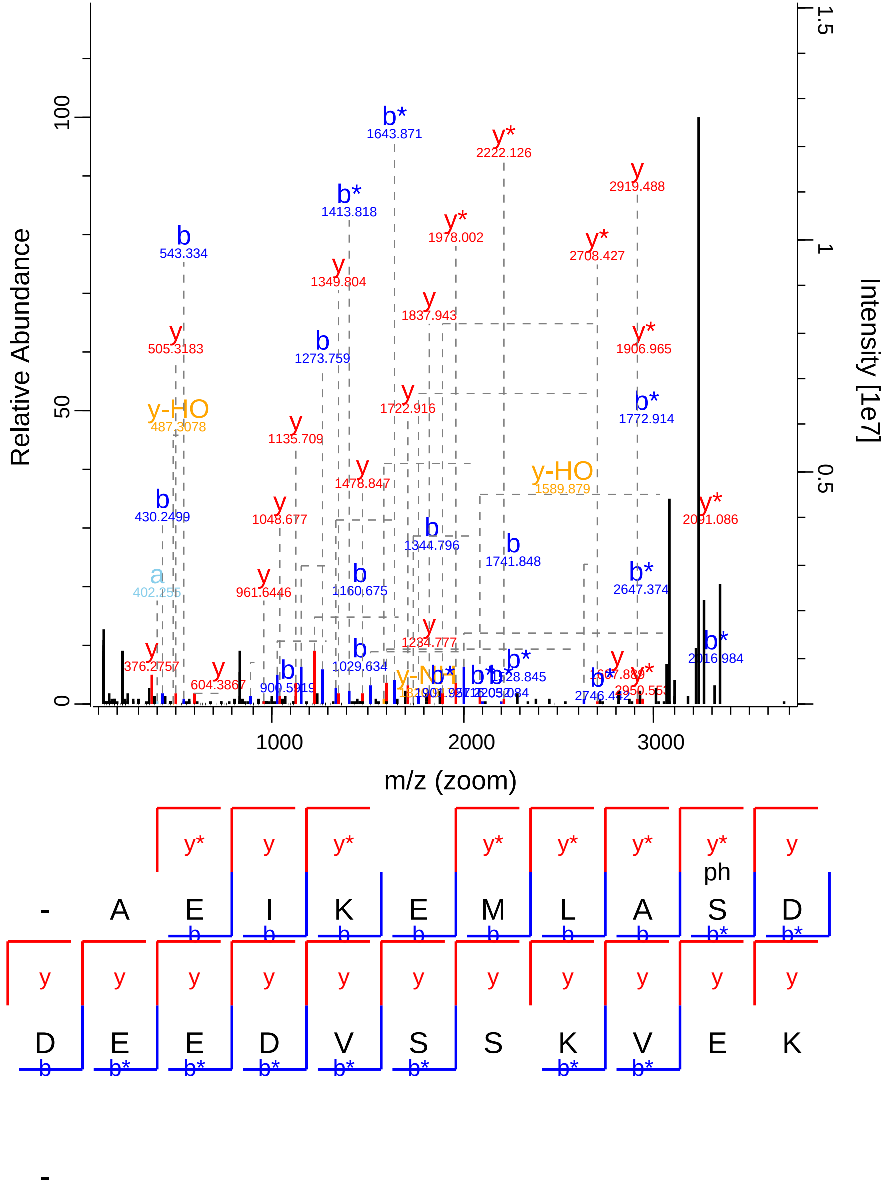

Raw File	Scan	Method	Score	m/z	Gene names
RO-QC-160121_ZackD_SS_Set_01_Fracti	29170	FTMS; HC	112.72	1075.54	RRAGC;RRAG

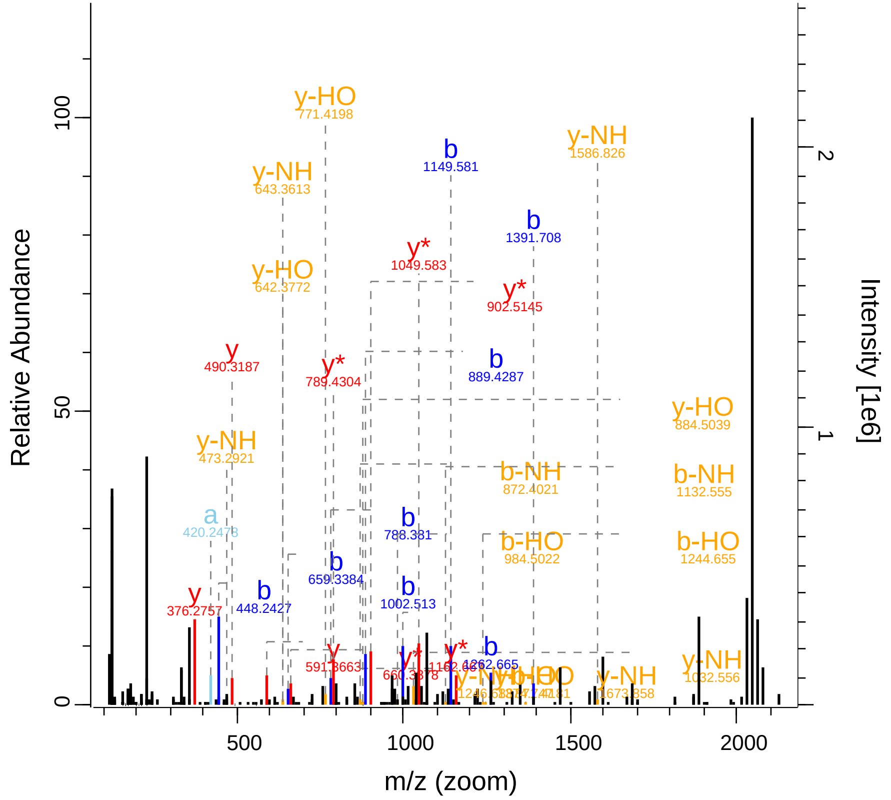

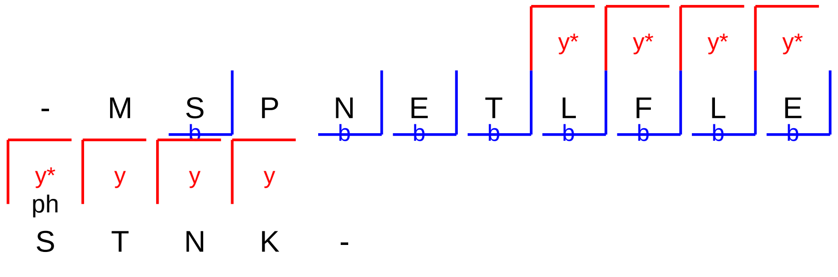

Raw File	Scan	Method	Score	m/z	Gene names
RO-QC-160121_ZackD_SS_Set_01_Fractic	31117	FTMS; HC	186.7	1098.04	G3BP1

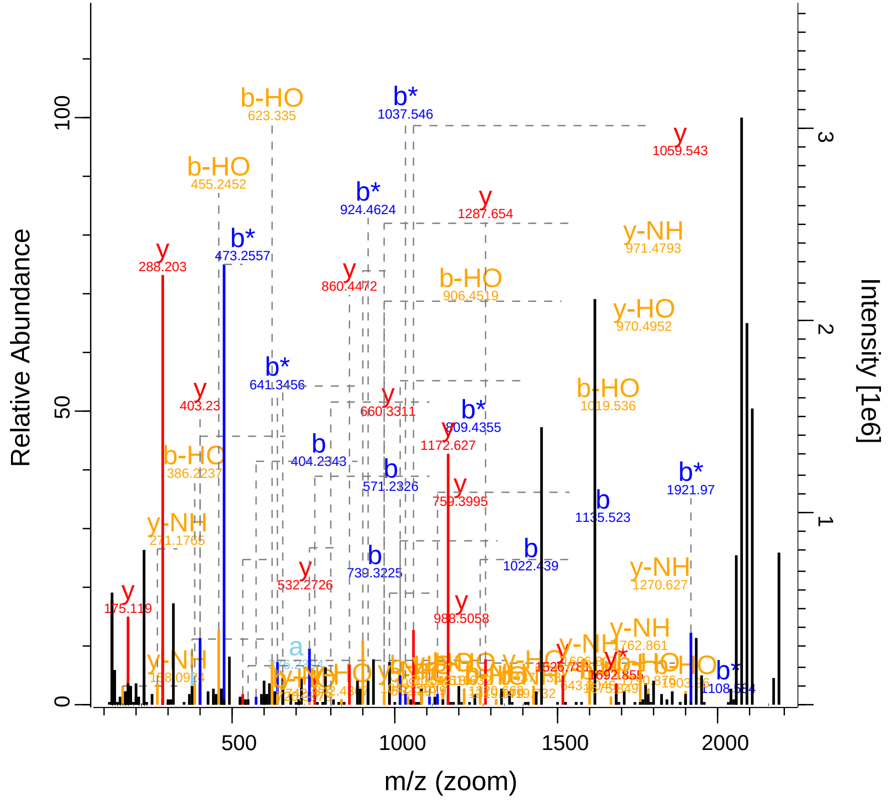

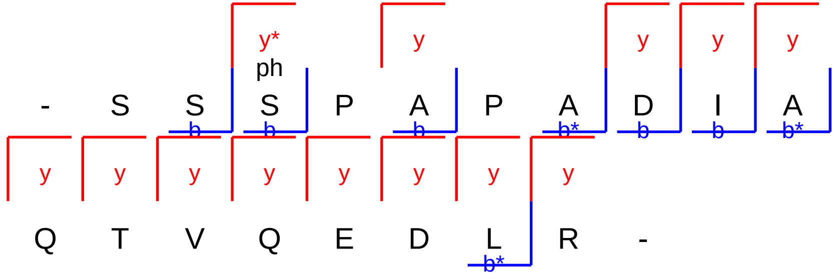

Raw File	Scan	Method	Score	m/z	Gene names
RO-QC-160121_ZackD_SS_Set_01_Fractio	31638	FTMS; HC	120.47	1020.53	MAP1A

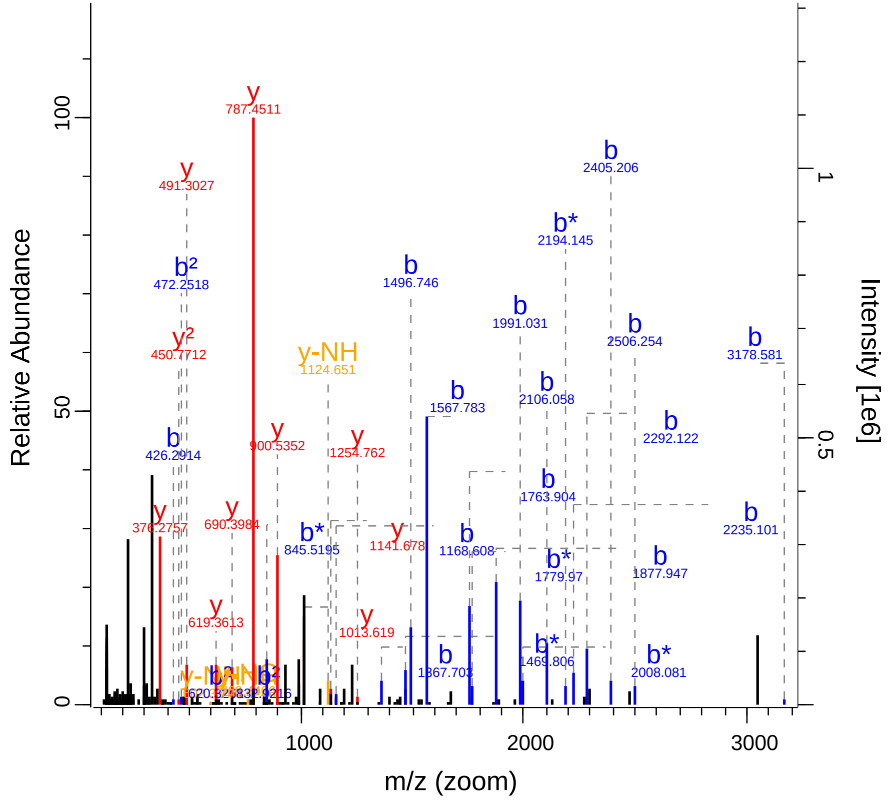

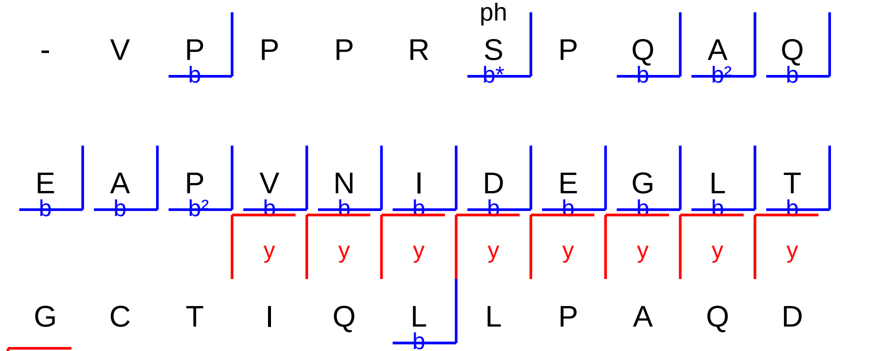

Raw File	Scan	Method	Score	m/z	Gene names
RO-QC-160121_ZackD_SS_Set_01_Fractio	32310	FTMS; HC	81.84	714.86	TMCC3

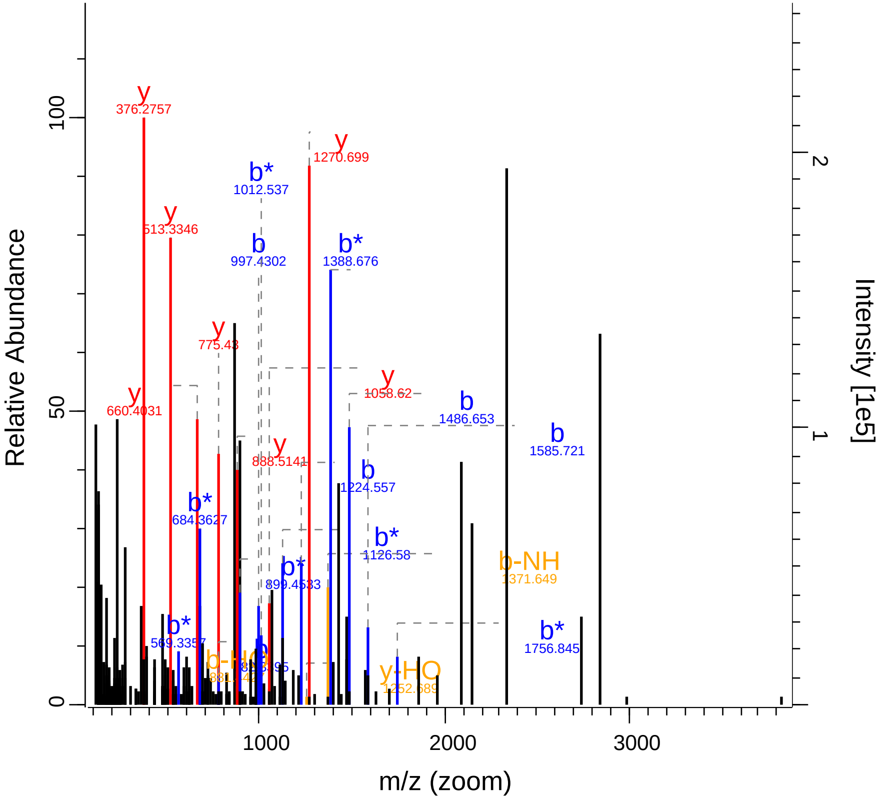

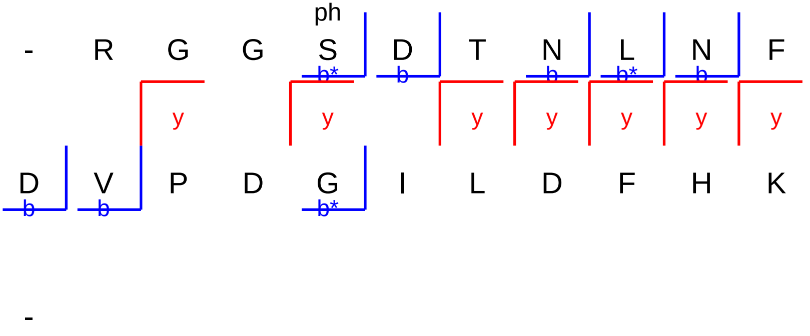

Raw File	Scan	Method	Score	m/z	Gene names
RO-QC-160121_ZackD_SS_Set_01_Fractic	32515	FTMS; HC	68.15	1021.54	HUWE1

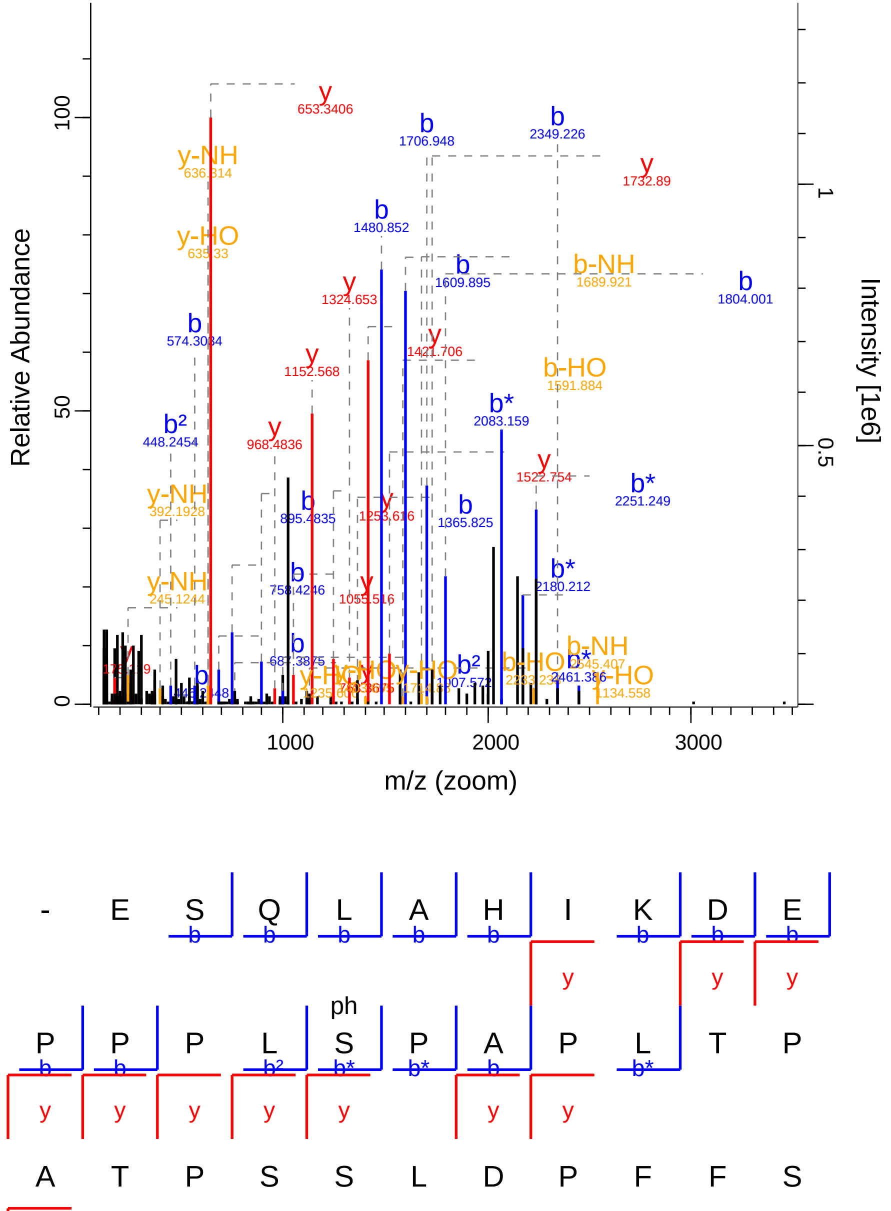

Raw File	Scan	Method	Score	m/z	Gene names
RO-QC-160121_ZackD_SS_Set_01_Fractic	32550	FTMS; HC	46.97	1034.51	KIAA1191

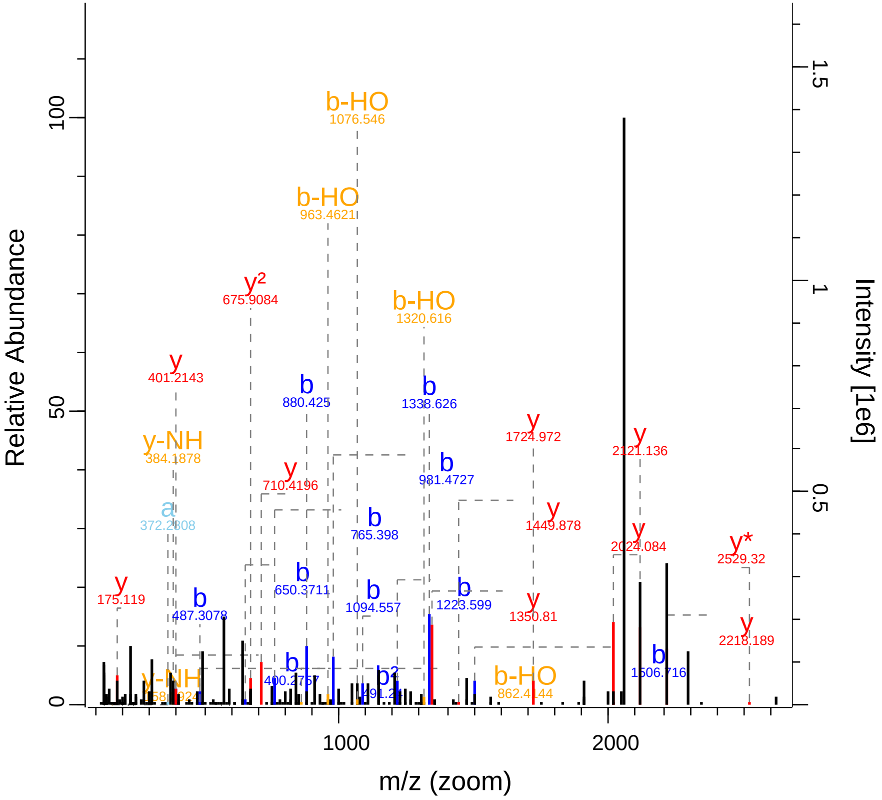

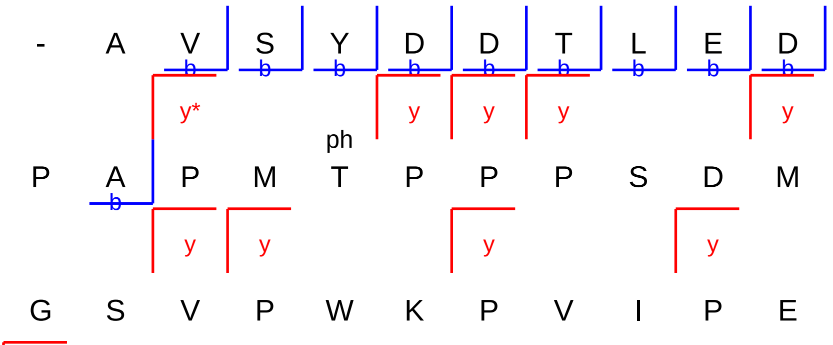

Raw File	Scan	Method	Score	m/z	Gene names
RO-QC-160121_ZackD_SS_Set_01_Fractio	32670	FTMS; HC	208.15	1070.53	TMEM230

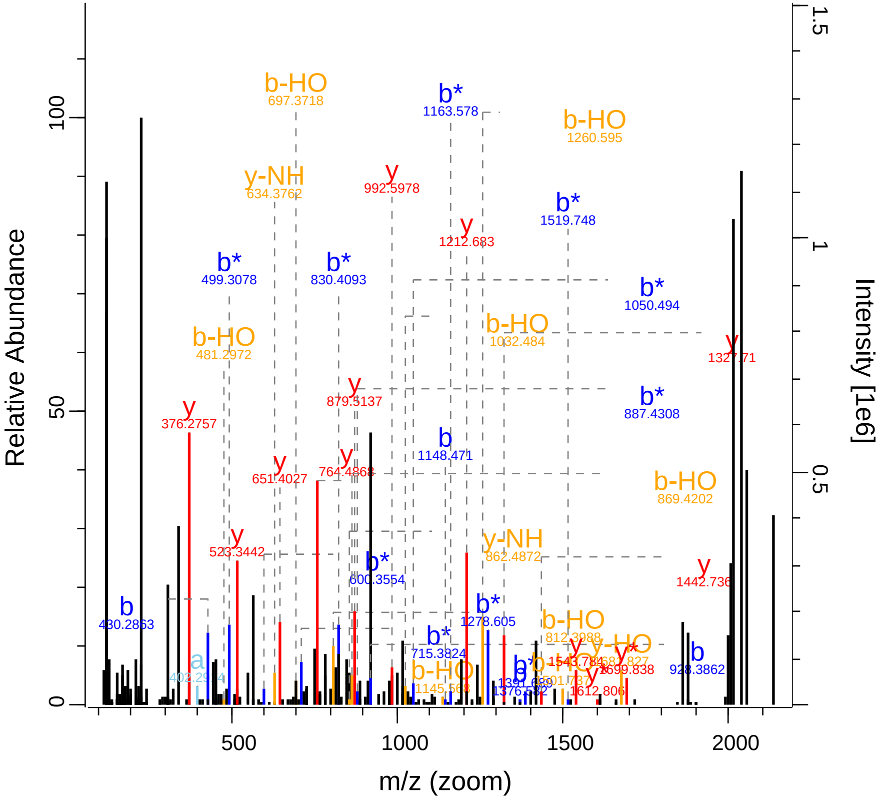

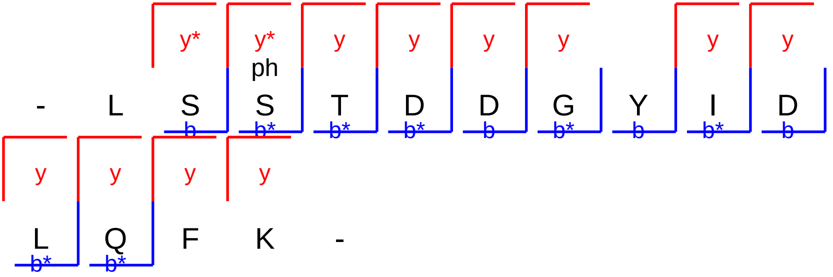

Raw File	Scan	Method	Score	m/z	Gene names
RO-QC-160121_ZackD_SS_Set_01_Fractio	33022	FTMS; HC	148.7	869.93	TOP2B

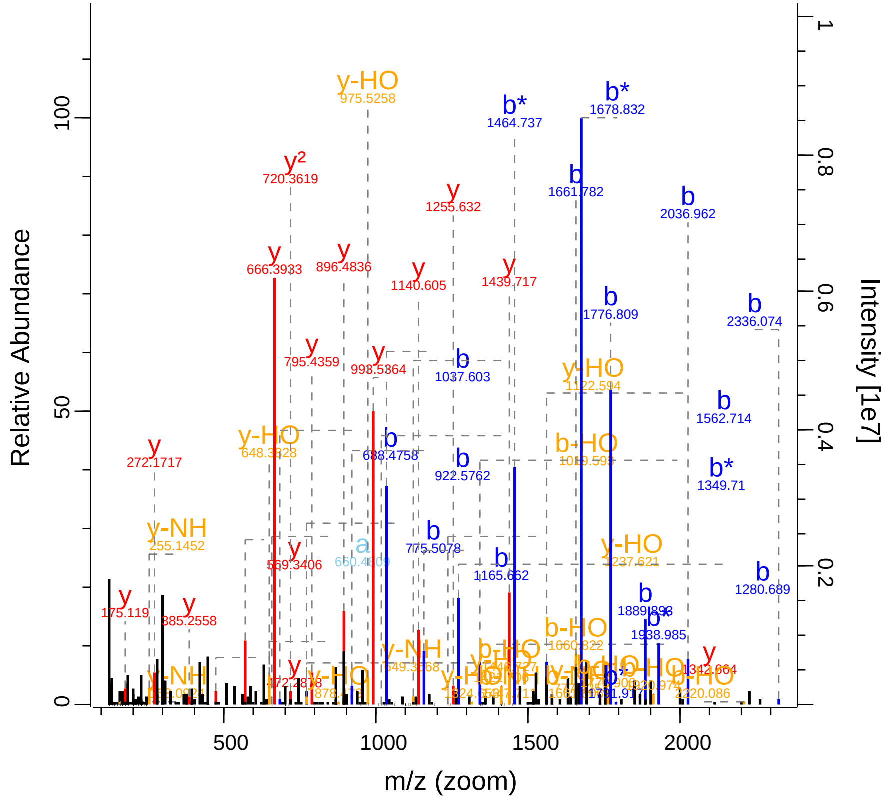

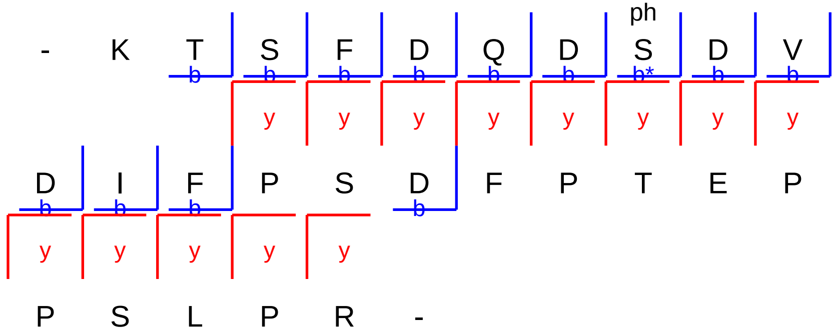

Raw File	Scan	Method	Score	m/z	Gene names
RO-QC-160121_ZackD_SS_Set_01_Fractio	33237	FTMS; HC	53.33	759.04	PLCXD3

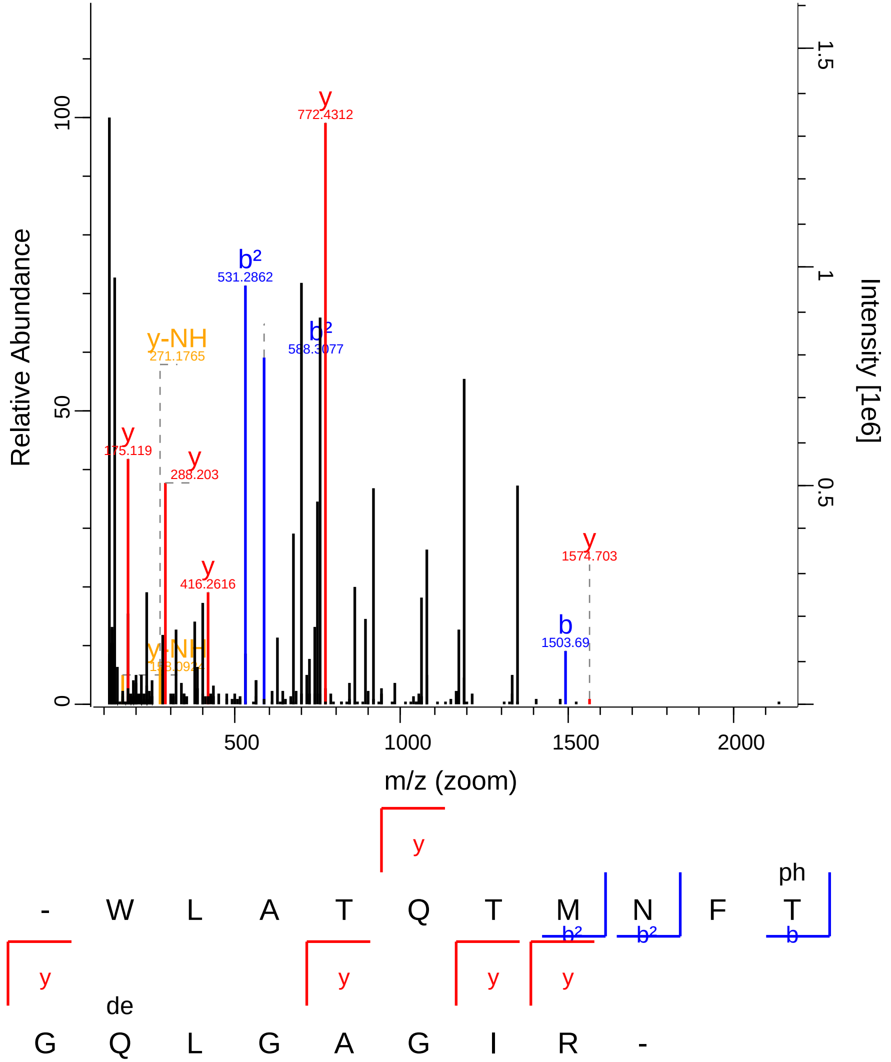

Raw File	Scan	Method	Score	m/z	Gene names
RO-QC-160121_ZackD_SS_Set_01_Fractic	33685	FTMS; HC	92.49	1000.85	SMAP2

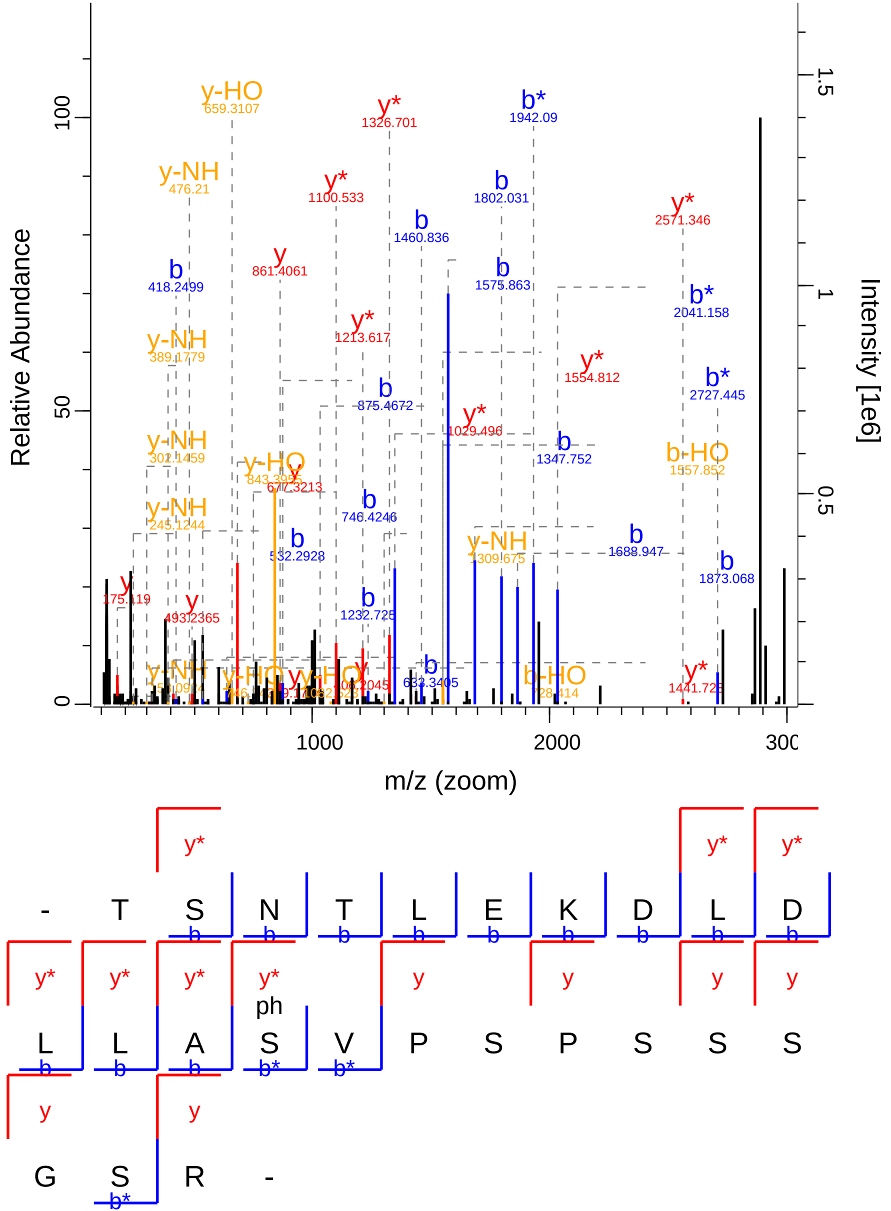

Raw File	Scan	Method	Score	m/z	Gene names
RO-QC-160121_ZackD_SS_Set_01_Fractio	33950	FTMS; HC	61.96	838.94	PARP4

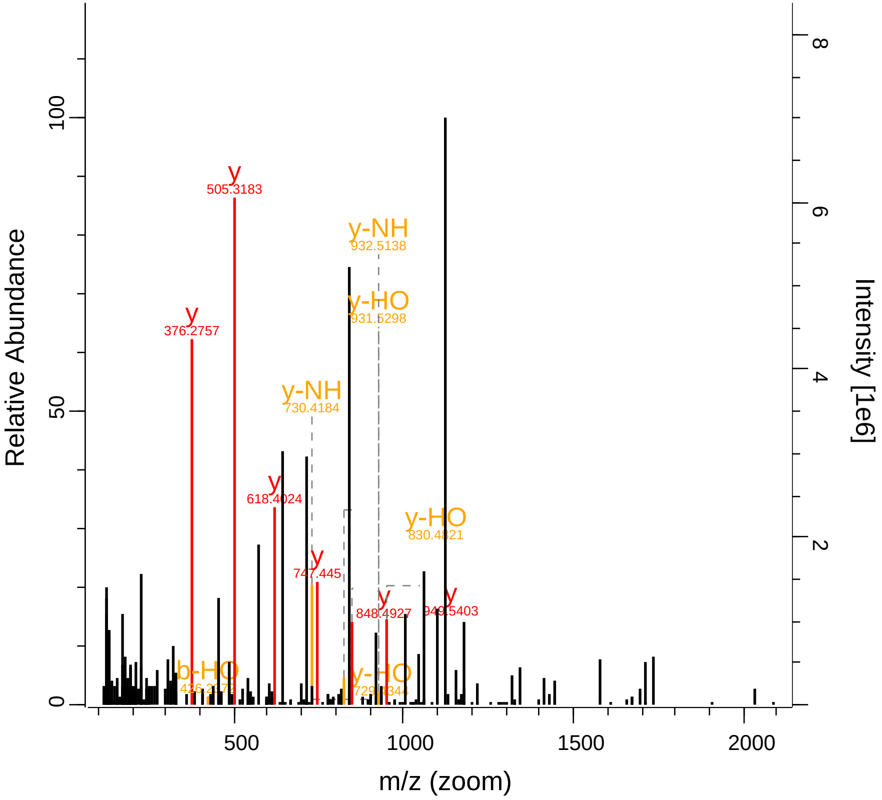

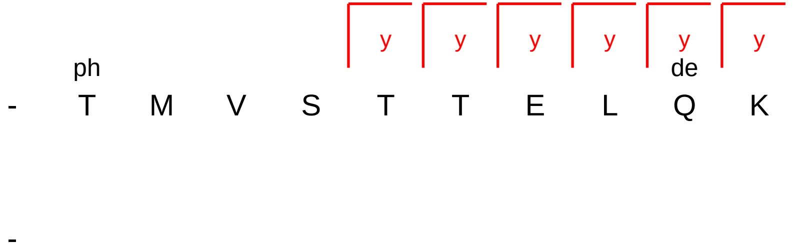

Raw File	Scan	Method	Score	m/z	Gene names
RO-QC-160121_ZackD_SS_Set_01_Fractic	34058	FTMS; HC	56.23	1339.18	C17orf104

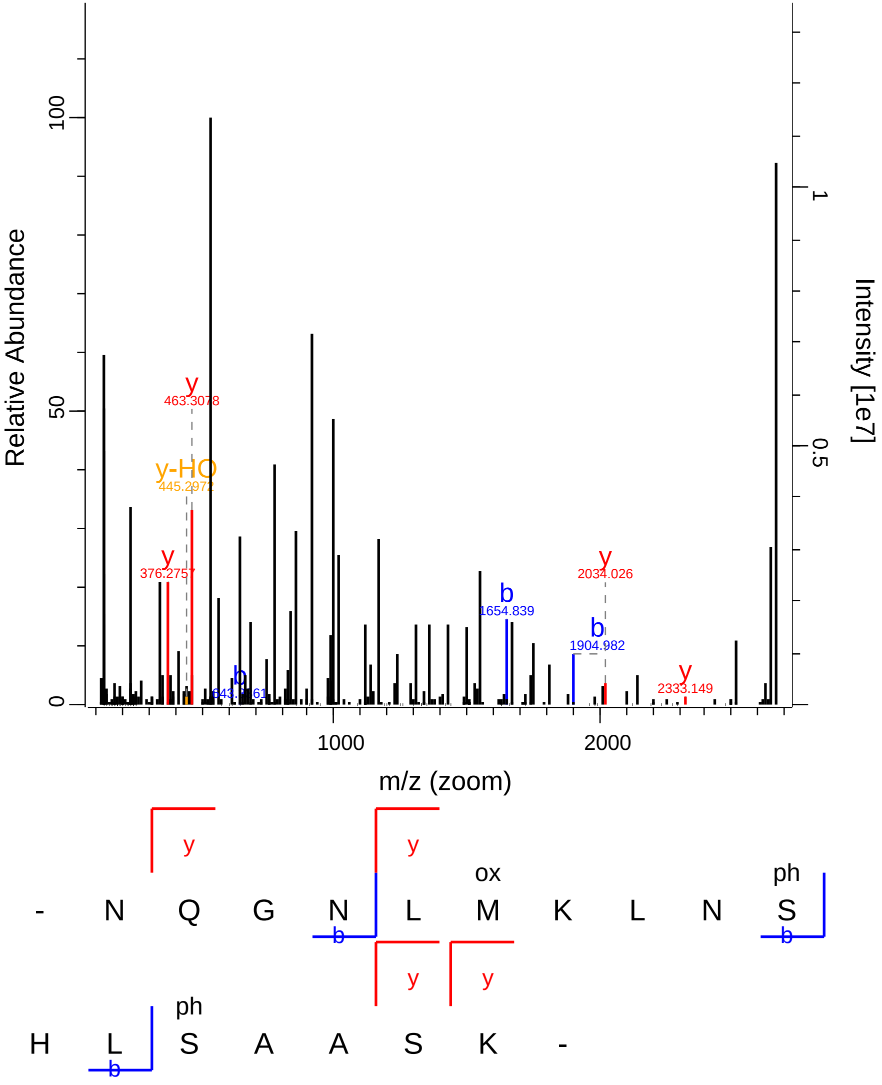

Raw File	Scan	Method	Score	m/z	Gene names
RO-QC-160121_ZackD_SS_Set_01_Fractio	34388	FTMS; HC	88.33	870.09	TOM1

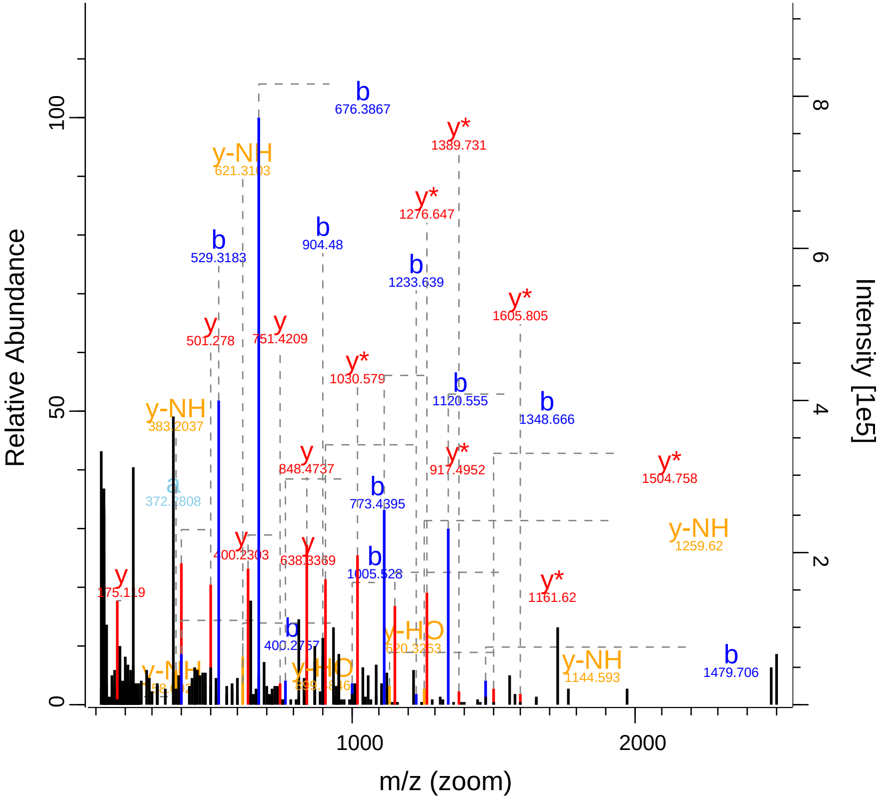

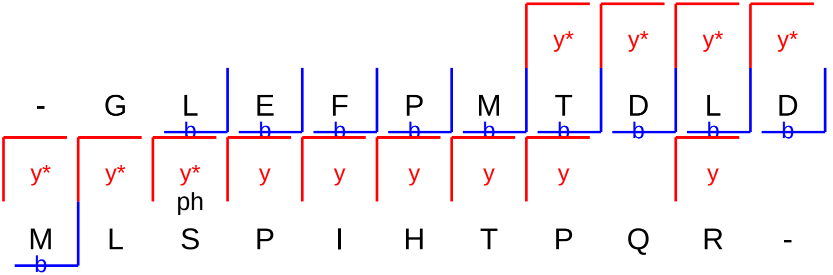

Raw File	Scan	Method	Score	m/z	Gene names
RO-QC-160121_ZackD_SS_Set_01_Fractio	34635	FTMS; HC	147.11	928.12	WRAP53

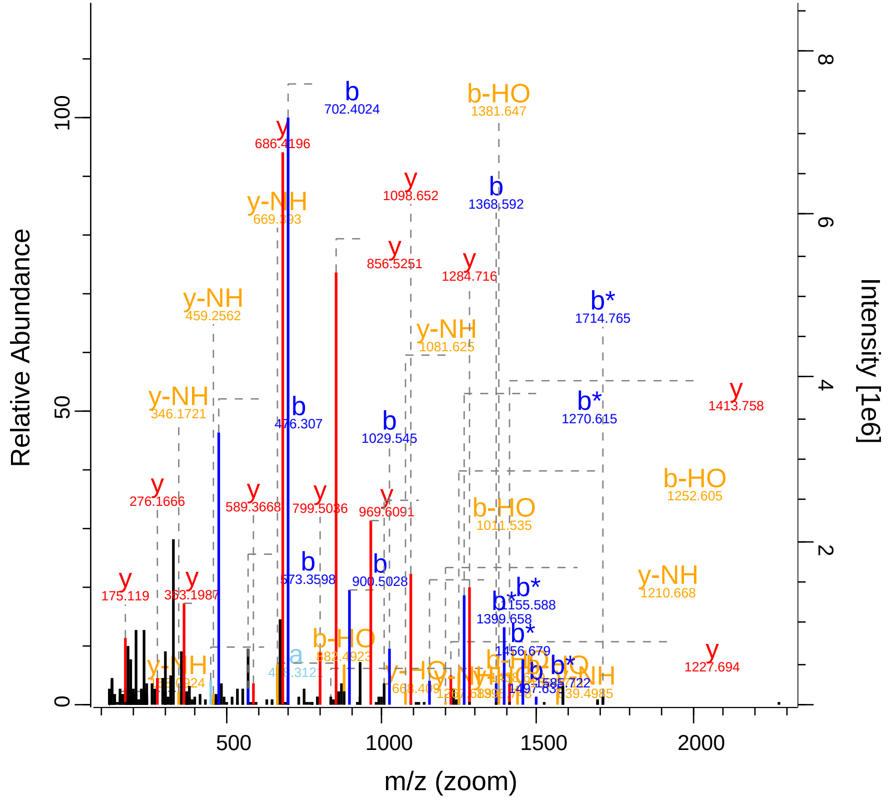

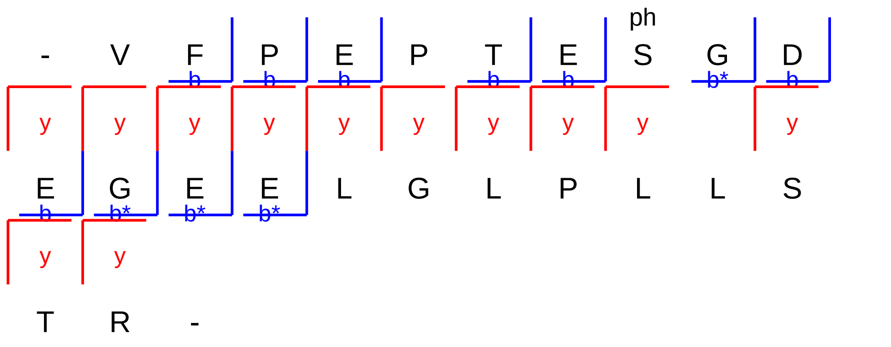

Raw File	Scan	Method	Score	m/z	Gene names
RO-QC-160121_ZackD_SS_Set_01_Fractior	5250	FTMS; HCI	54.28	498.78	PLEKHA7

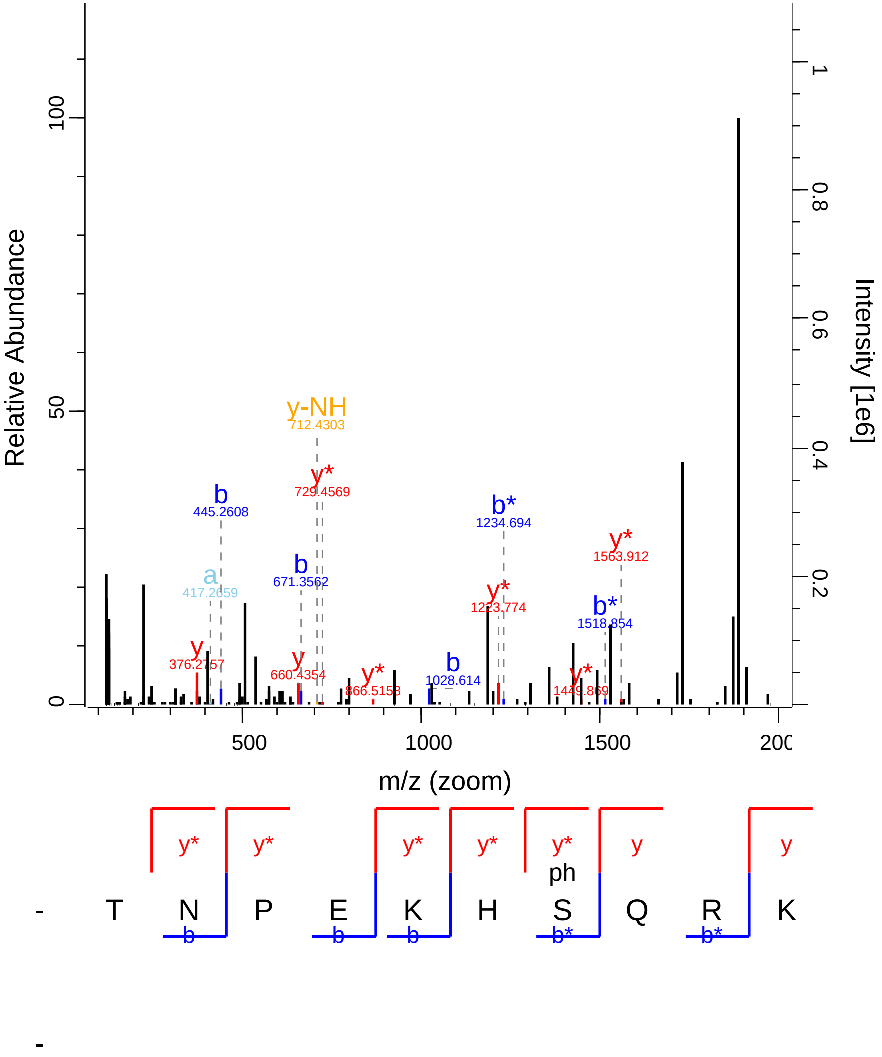

Raw File	Scan	Method	Score	m/z	Gene names
RO-QC-160121_ZackD_SS_Set_01_Fractio[illegible]	5640	FTMS; HC	105.46	685.83	AAK1

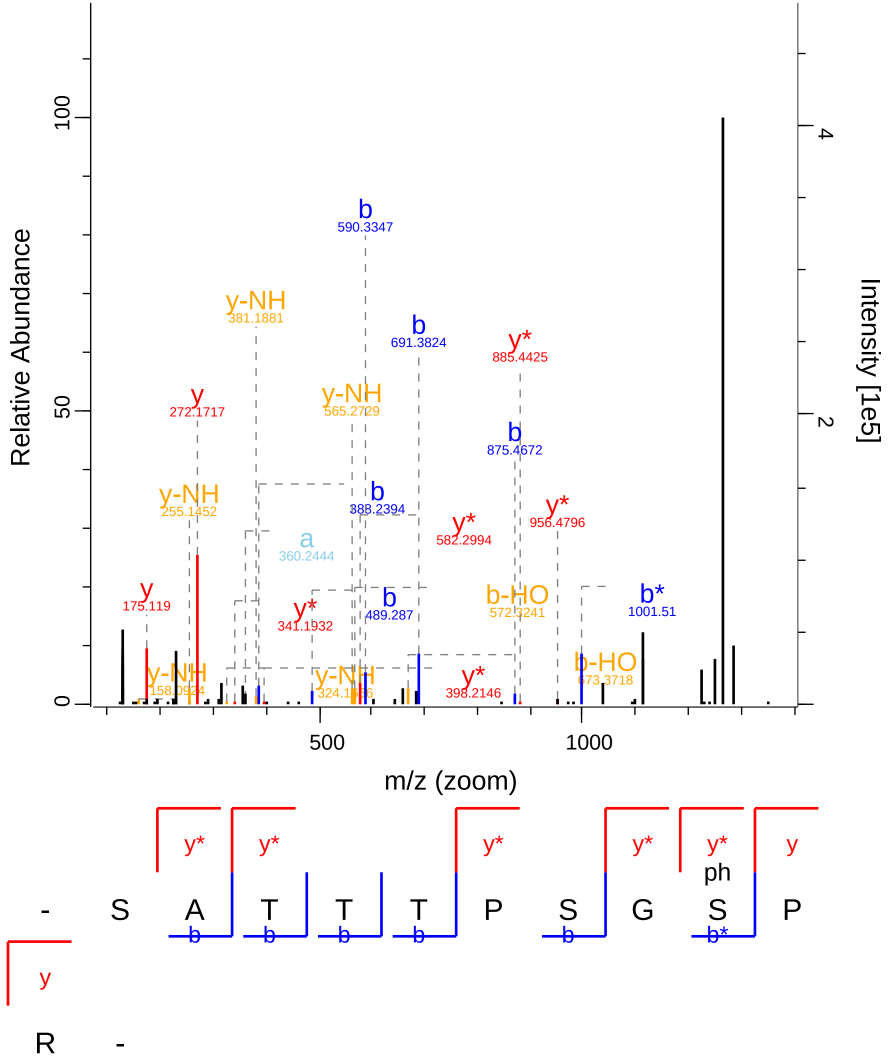

Raw File	Scan	Method	Score	m/z	Gene names
RO-QC-160121_ZackD_SS_Set_01_Fractio	6719	FTMS; HC	147.52	791.38	WWP2

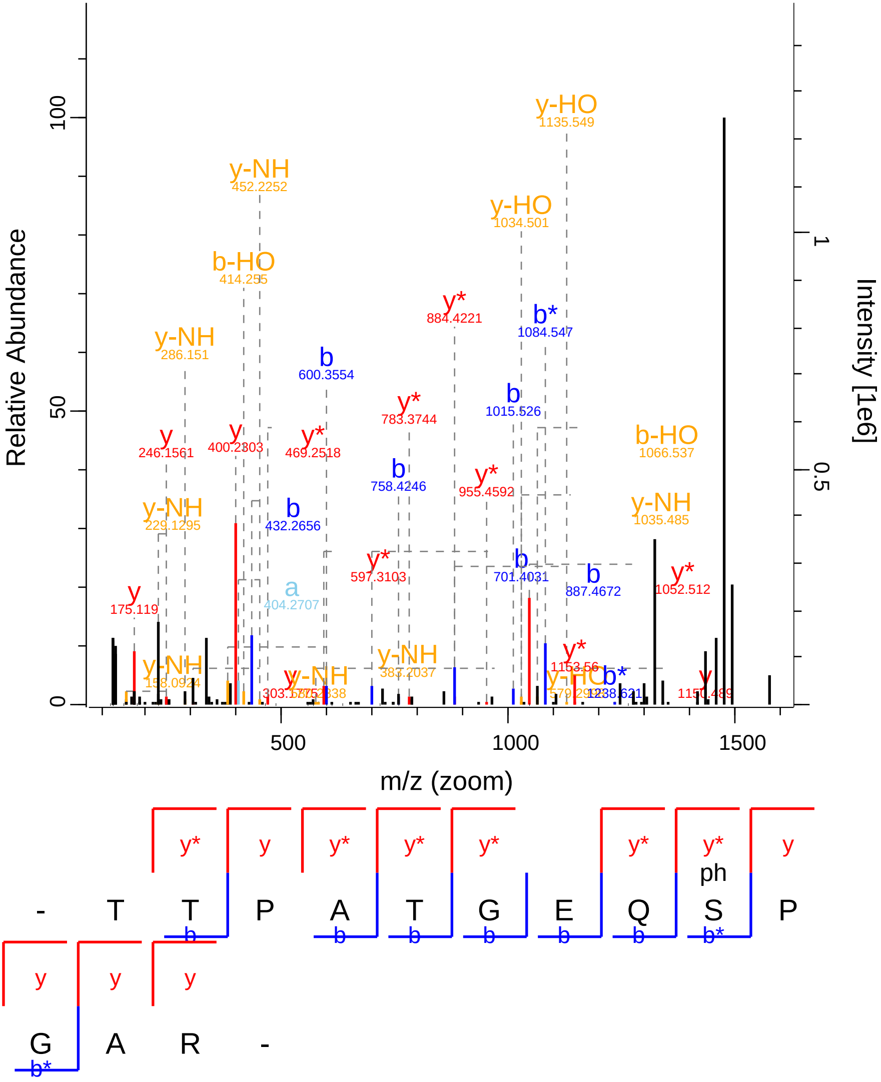

Raw File	Scan	Method	Score	m/z	Gene names
RO-QC-160121_ZackD_SS_Set_01_Fractior	6786	FTMS; HCI	94.19	638.35	HMGN3

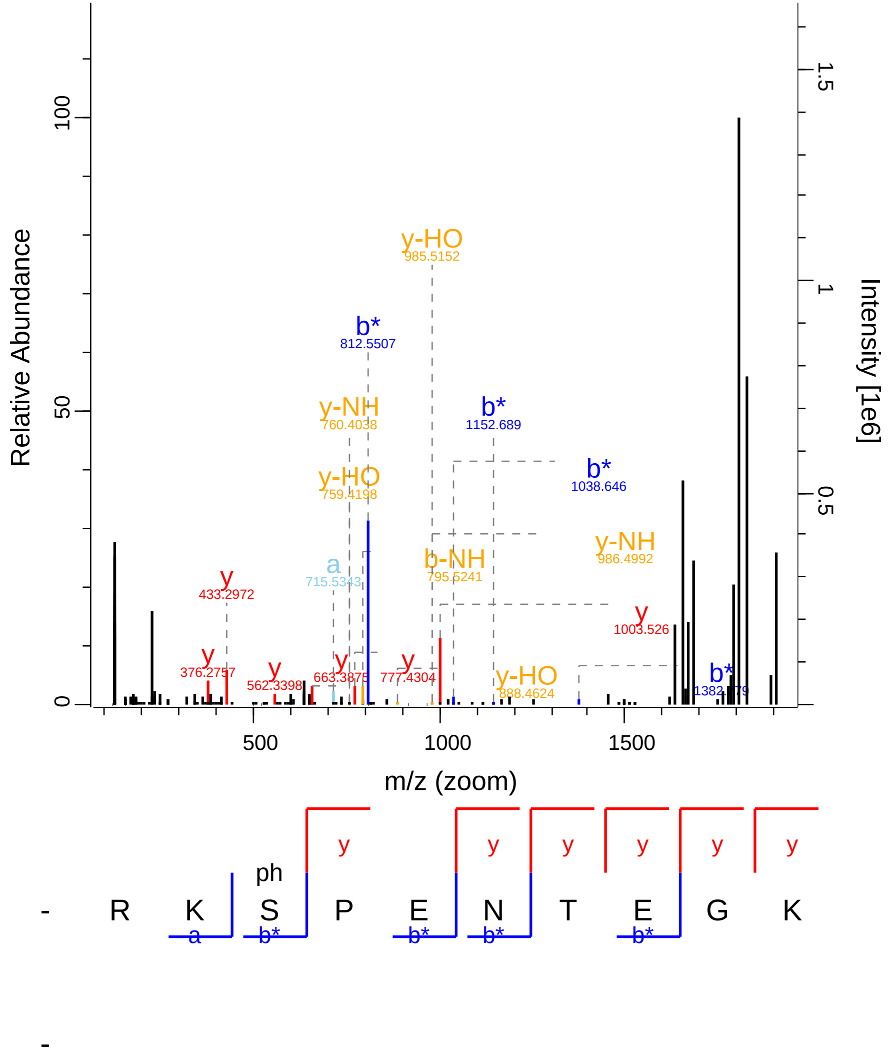

Raw File	Scan	Method	Score	m/z	Gene names
RO-QC-160121_ZackD_SS_Set_01_Fractio	10107	FTMS; HC	77.89	491.79	STMN1

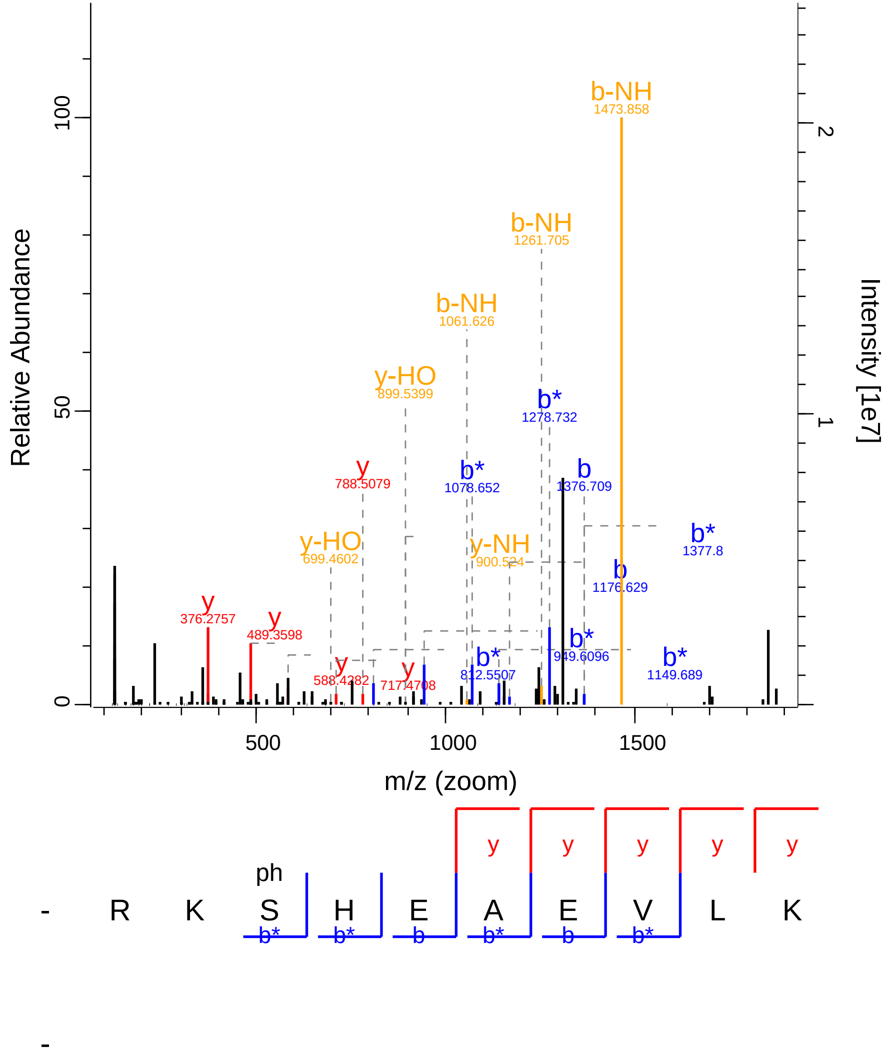

Raw File	Scan	Method	Score	m/z	Gene names
RO-QC-160121_ZackD_SS_Set_01_Fractio	10305	FTMS; HC	113.43	984.95	CD276

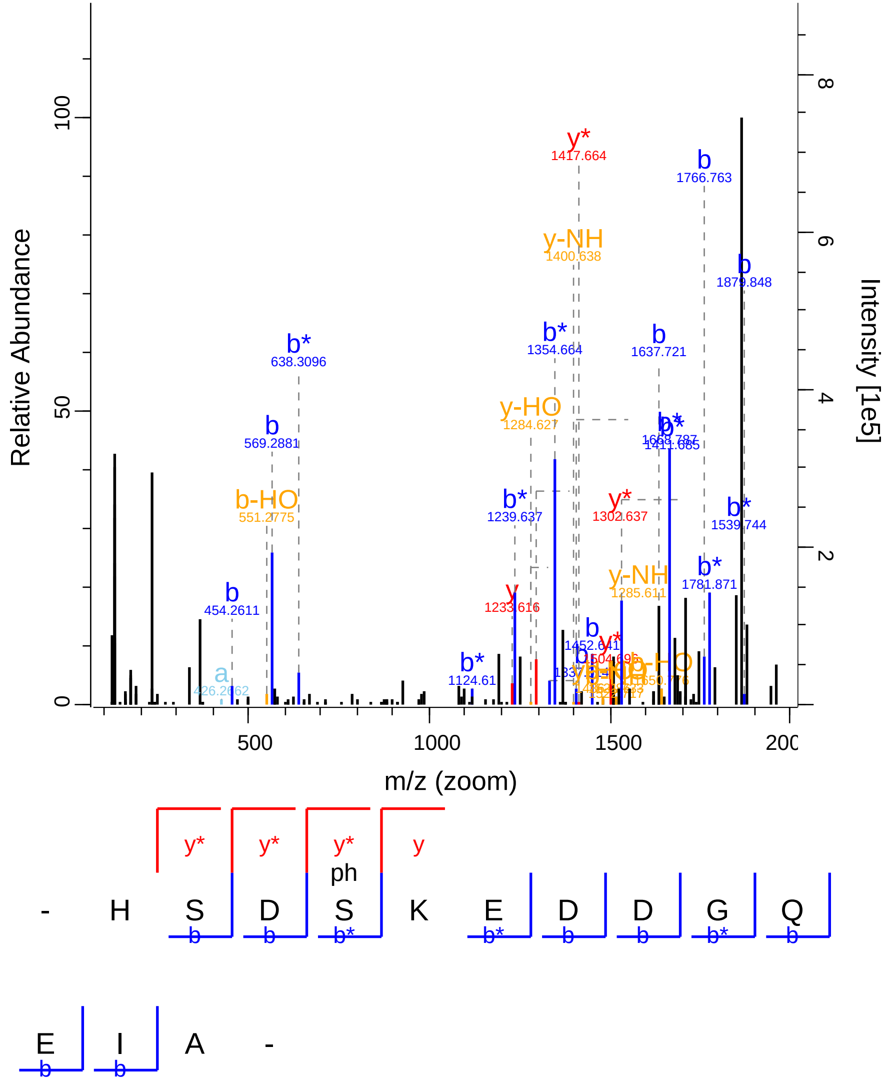

Raw File	Scan	Method	Score	m/z	Gene names
RO-QC-160121_ZackD_SS_Set_01_Fractio	11201	FTMS; HC	115.66	680.33	MARCKS

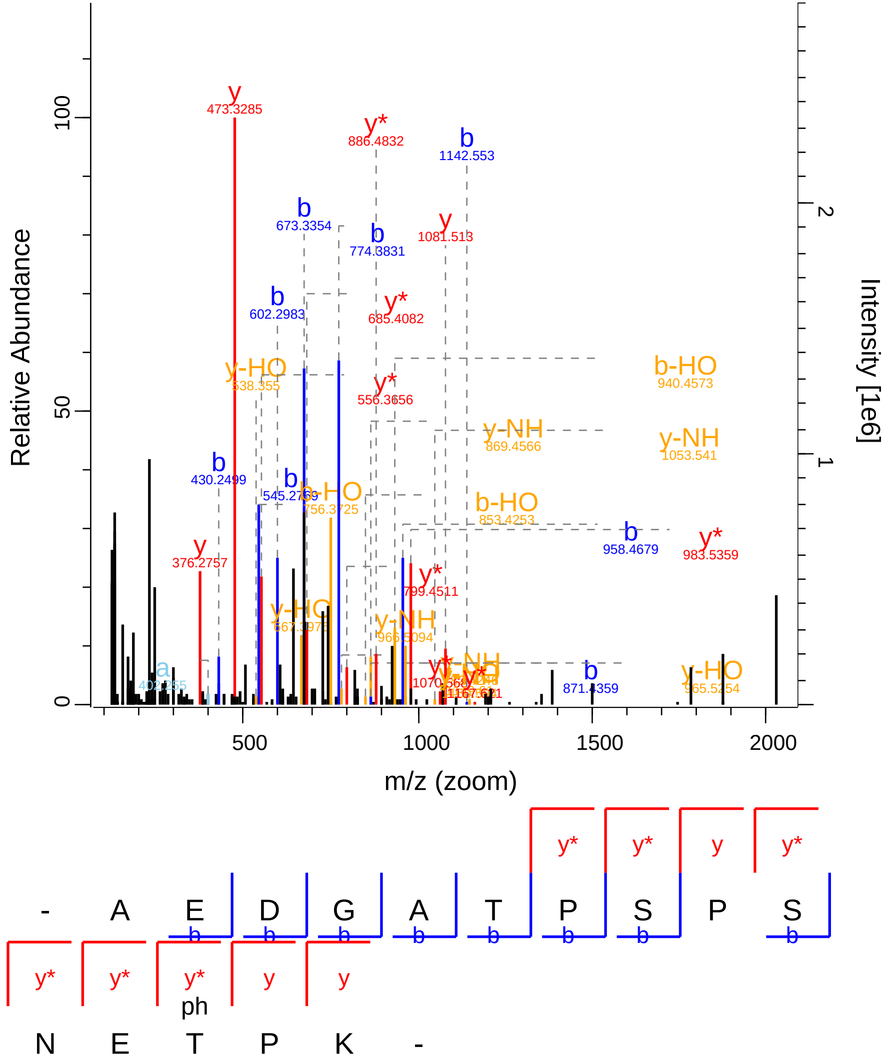

Raw File	Scan	Method	Score	m/z	Gene names
RO-QC-160121_ZackD_SS_Set_01_Fractic	12017	FTMS; HC	75.51	1035.99	MRC1

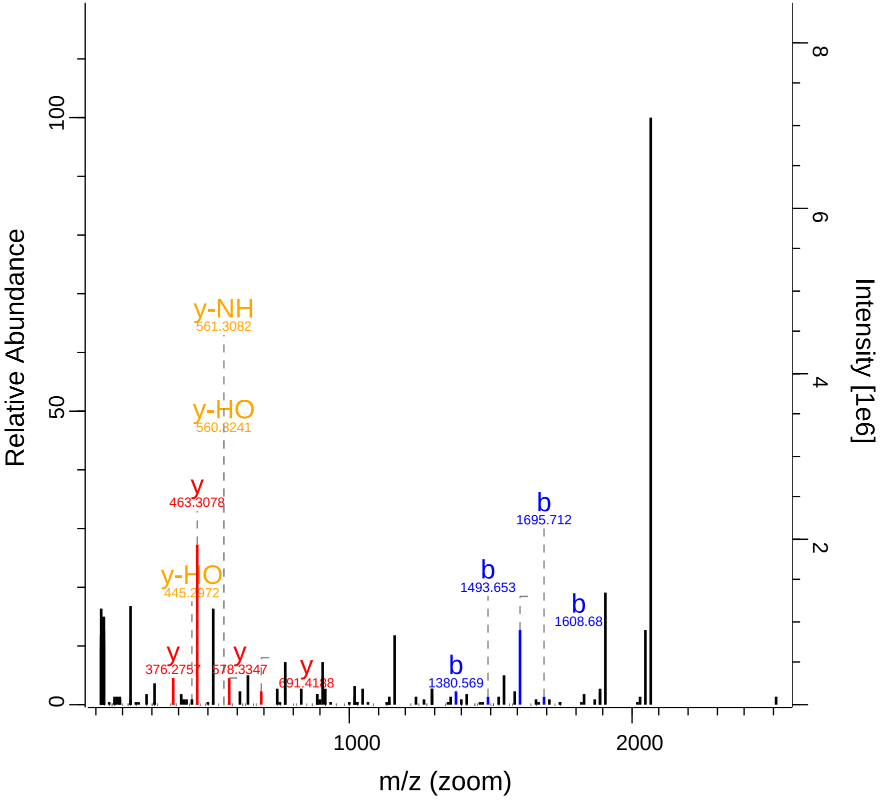

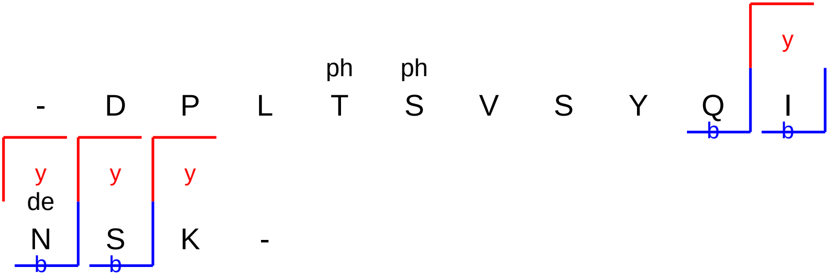

Raw File	Scan	Method	Score	m/z	Gene names
RO-QC-160121_ZackD_SS_Set_01_Fractio	12501	FTMS; HC	181.58	1019.99	MARCKS

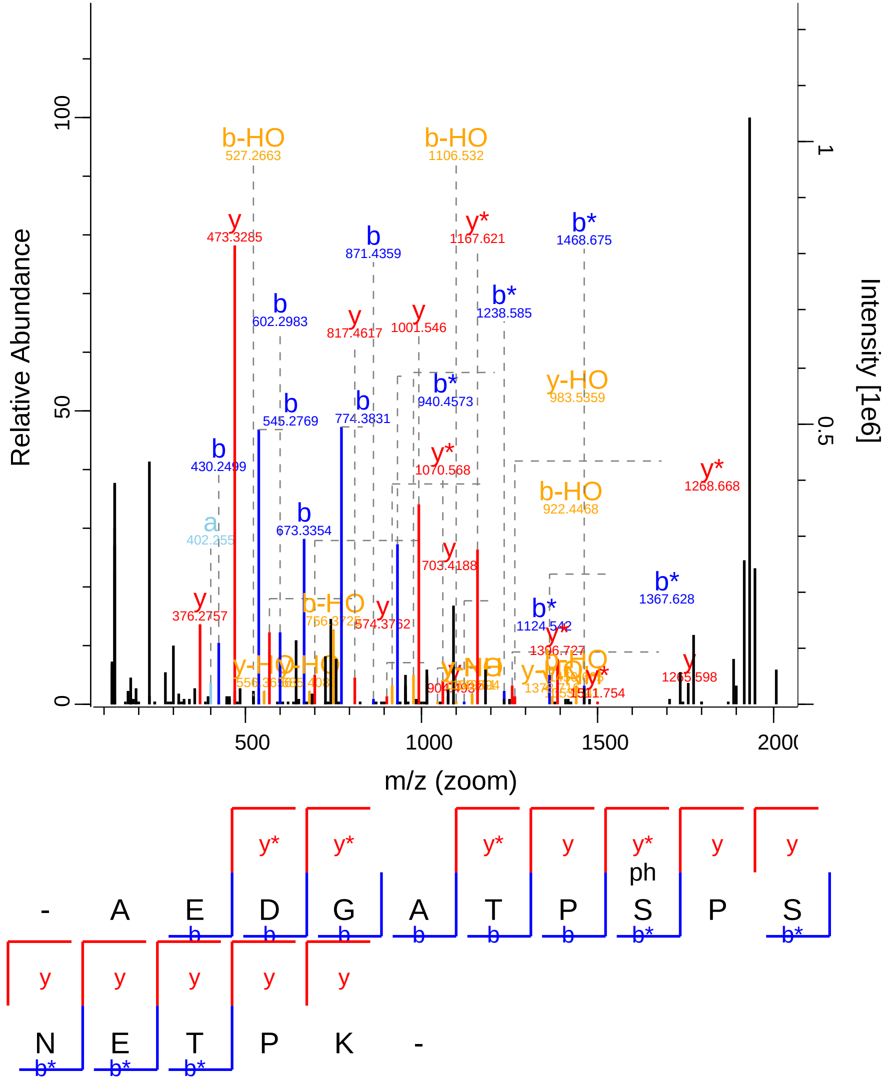

Raw File	Scan	Method	Score	m/z	Gene names
RO-QC-160121_ZackD_SS_Set_01_Fractio	12530	FTMS; HC	154.82	841.12	MATR3

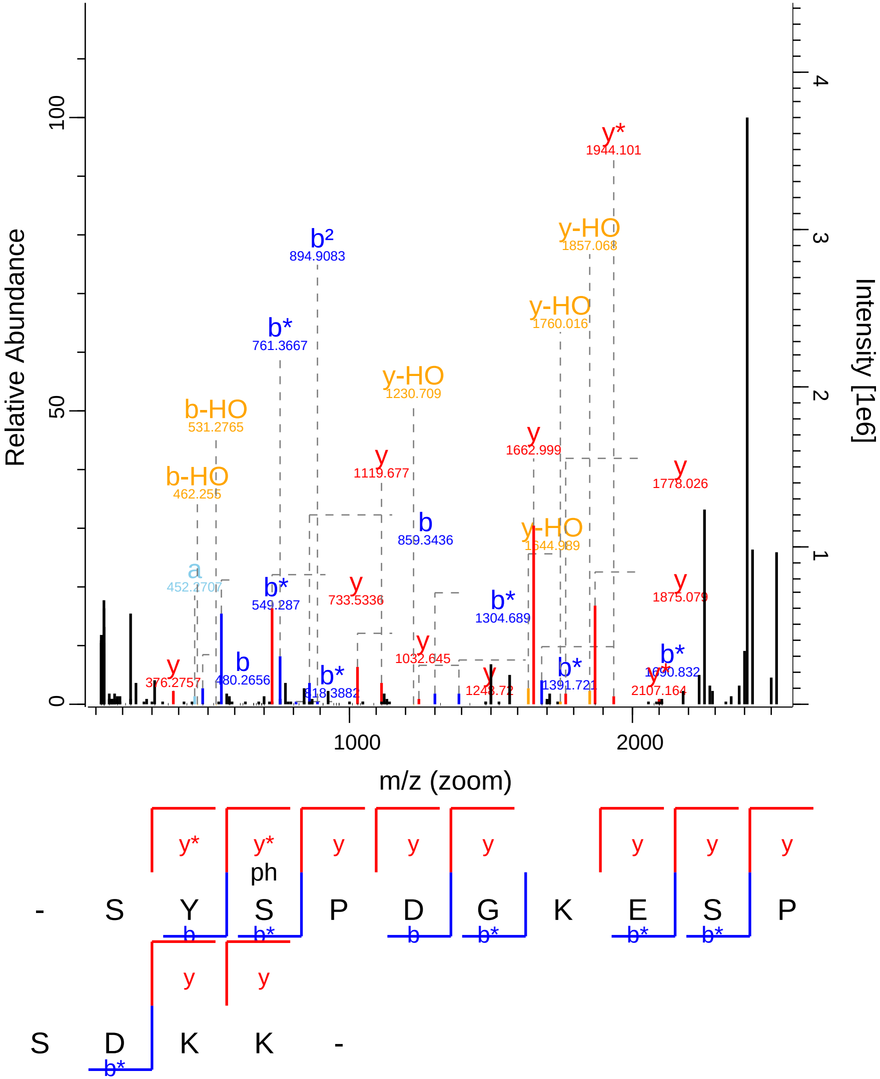

Raw File	Scan	Method	Score	m/z	Gene names
RO-QC-160121_ZackD_SS_Set_01_Fractio	12709	FTMS; HC	107.97	705.34	MCM2

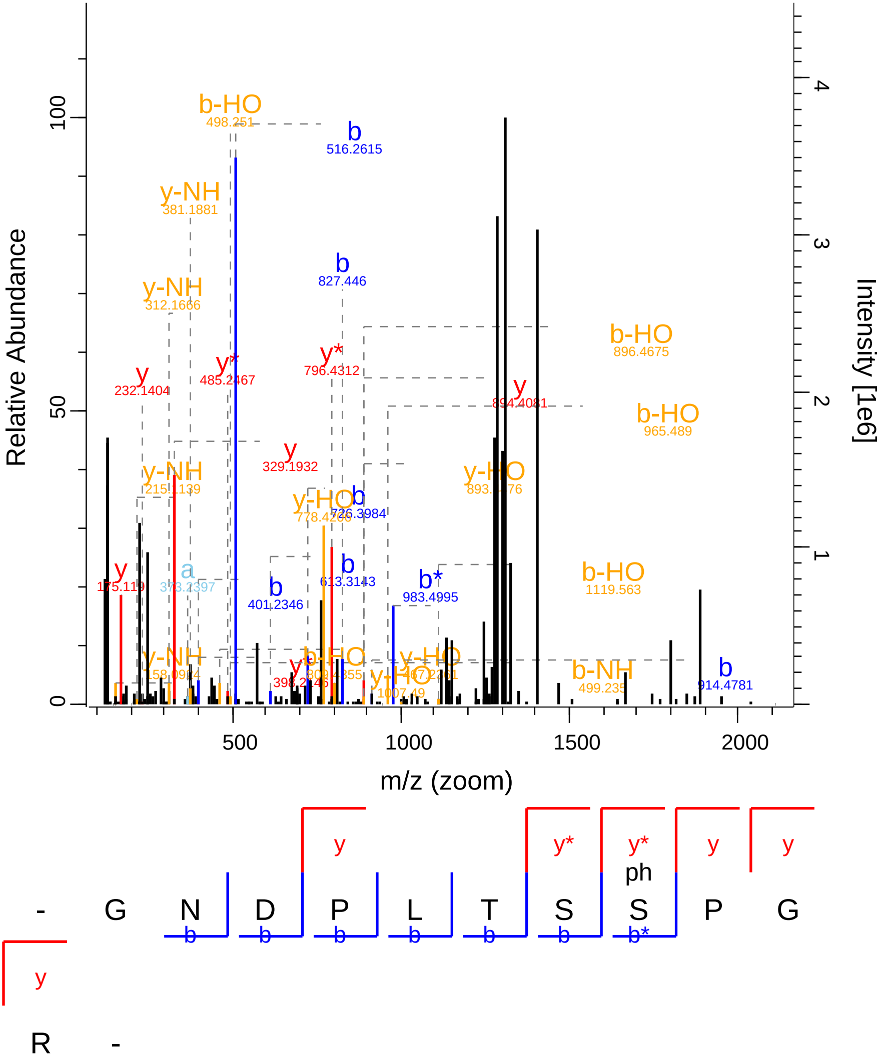

Raw File	Scan	Method	Score	m/z	Gene names
RO-QC-160121_ZackD_SS_Set_01_Fractio	13234	FTMS; HC	154.47	1022.06	PIKFYVE

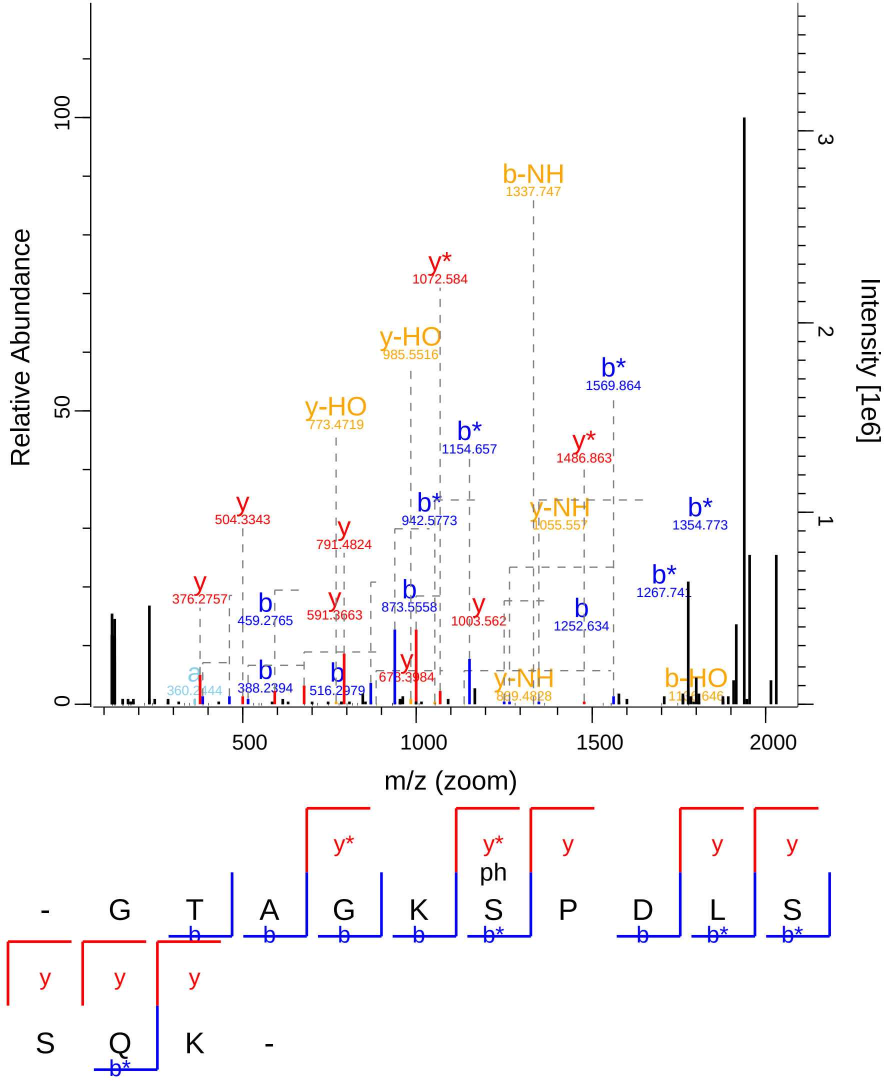

Raw File	Scan	Method	Score	m/z	Gene names
RO-QC-160121_ZackD_SS_Set_01_Fractio	13853	FTMS; HC	136.02	865.47	PSMD2

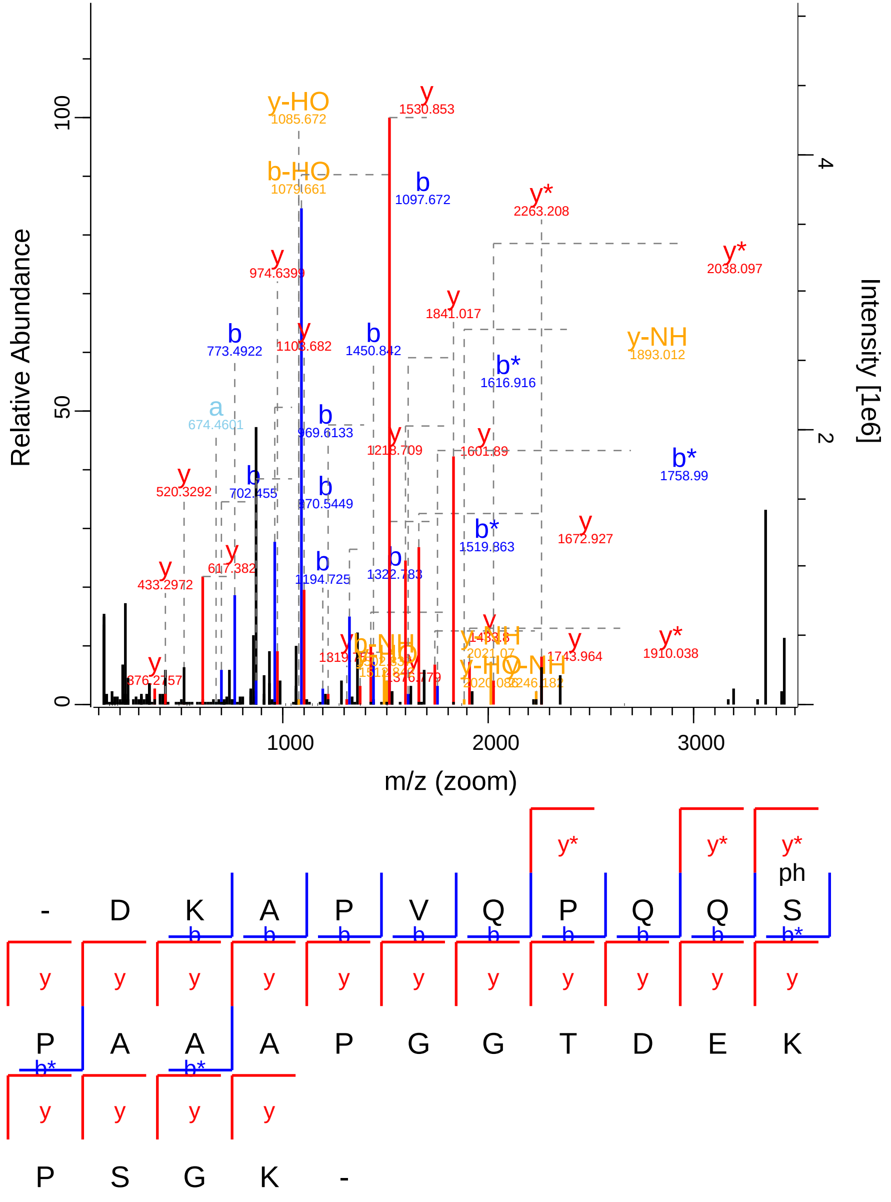

Raw File	Scan	Method	Score	m/z	Gene names
RO-QC-160121_ZackD_SS_Set_01_Fractio	14201	FTMS; HC	84.66	956.01	GPR110

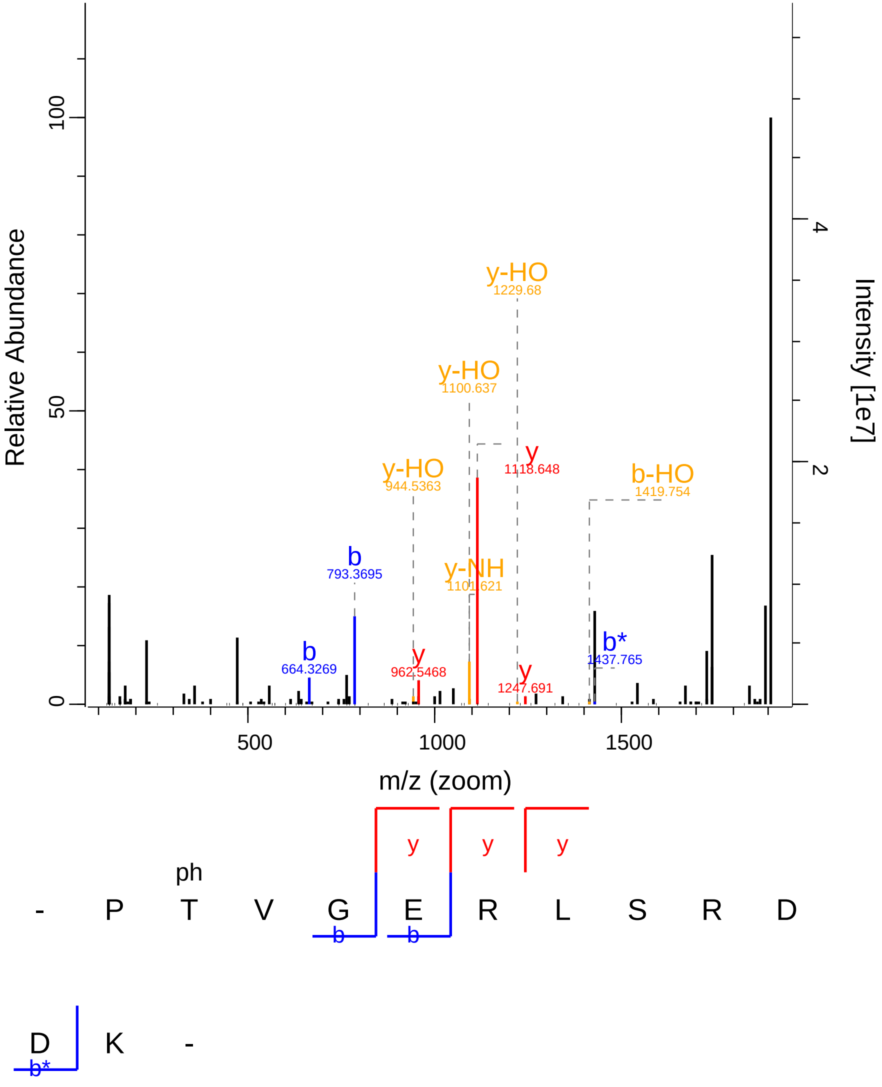

Raw File	Scan	Method	Score	m/z	Gene names
RO-QC-160121_ZackD_SS_Set_01_Fractio	14492	FTMS; HC	74.7	852.11	RPP30

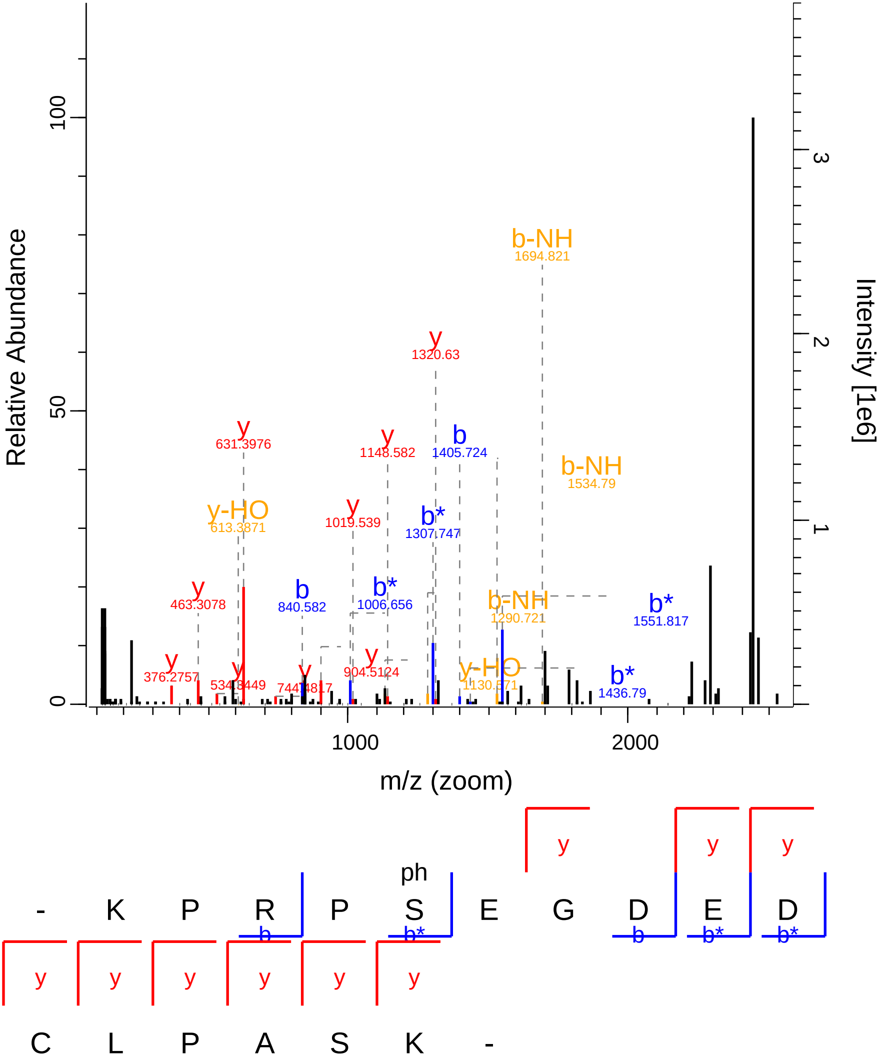

Raw File	Scan	Method	Score	m/z	Gene names
RO-QC-160121_ZackD_SS_Set_01_Fractio	15216	FTMS; HC	66.39	558.32	SORBS2

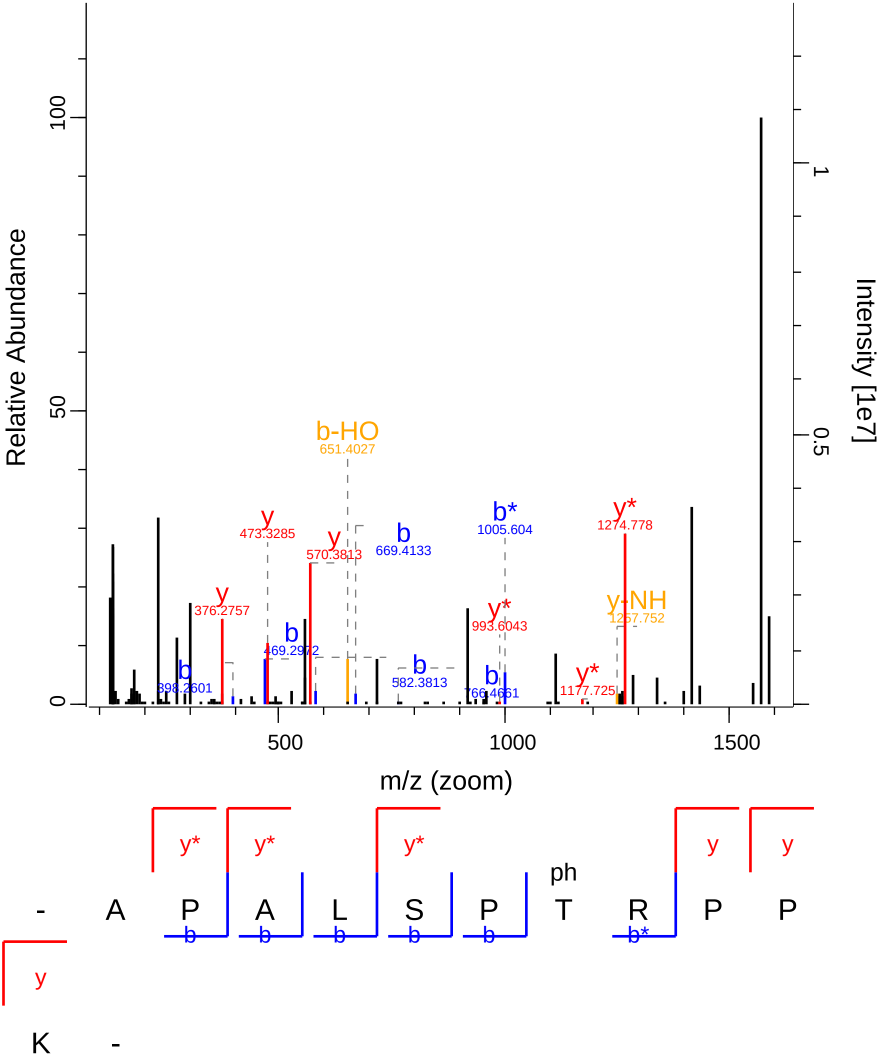

Raw File	Scan	Method	Score	m/z	Gene names
RO-QC-160121_ZackD_SS_Set_01_Fractio	15238	FTMS; HC	134.13	836.98	SORBS2

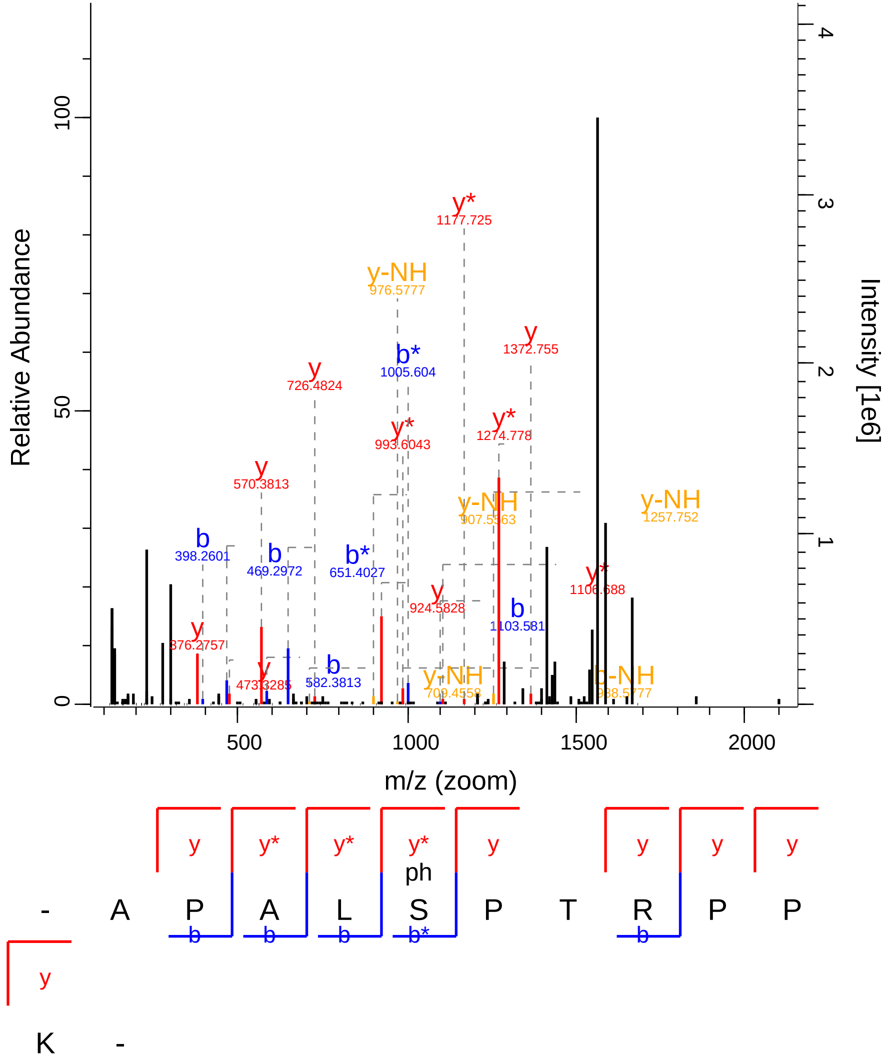

Raw File	Scan	Method	Score	m/z	Gene names
RO-QC-160121_ZackD_SS_Set_01_Fractio	15448	FTMS; HC	73.17	825.76	UBR4

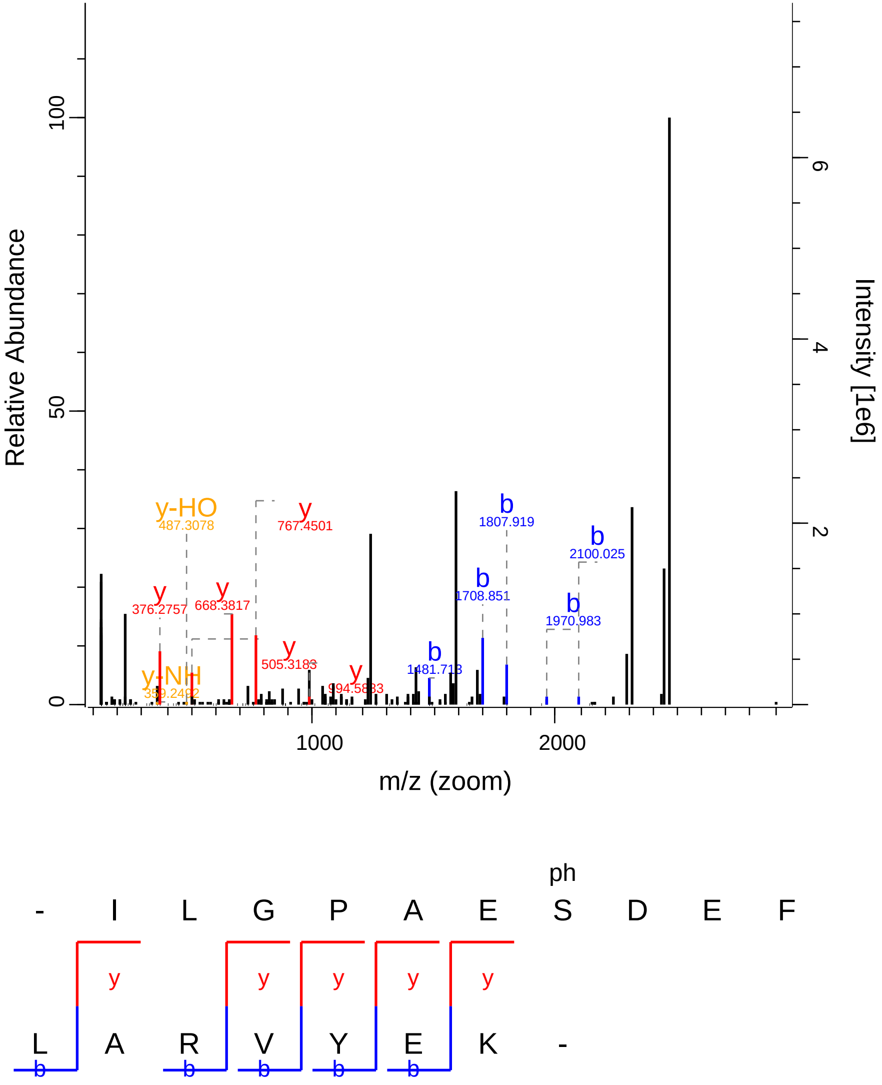

Raw File	Scan	Method	Score	m/z	Gene names
RO-QC-160121_ZackD_SS_Set_01_Fractio	15816	FTMS; HC	134.08	984.06	CCNK

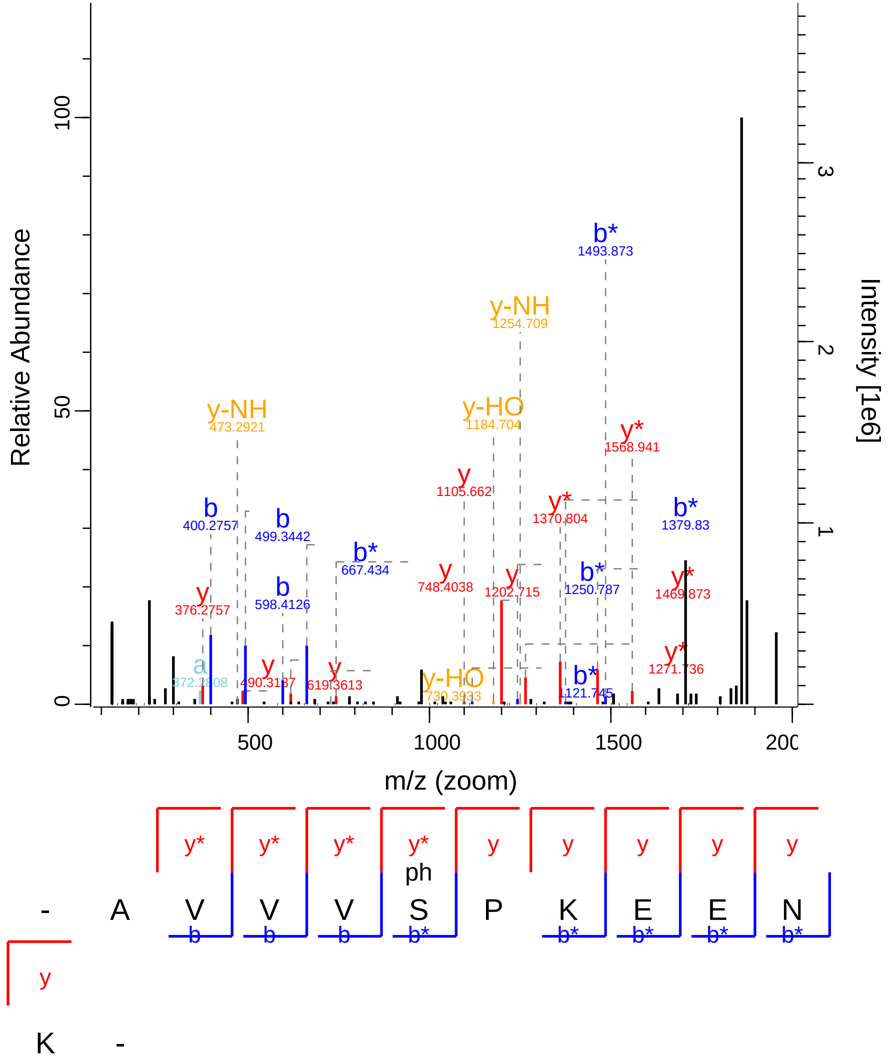

Raw File	Scan	Method	Score	m/z	Gene names
RO-QC-160121_ZackD_SS_Set_01_Fractic	15985	FTMS; HC	465.48	1209.06	SLC9A3R1

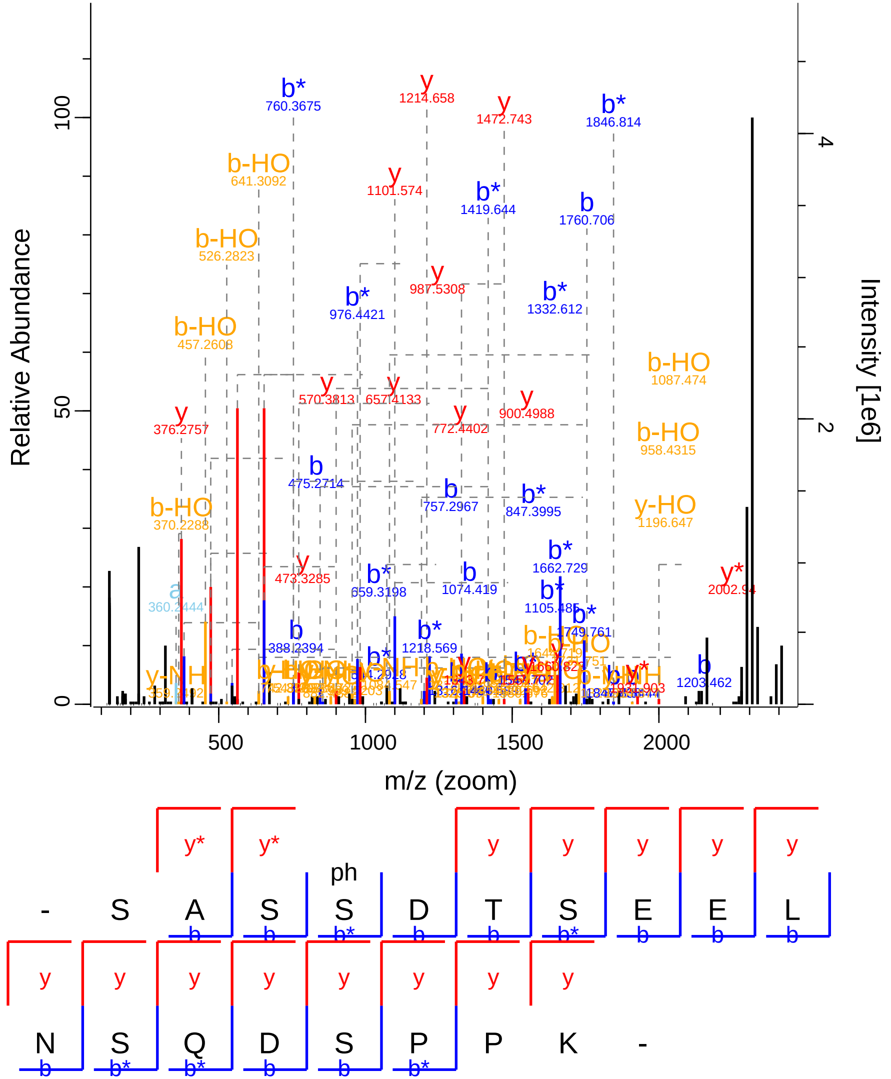

Raw File	Scan	Method	Score	m/z	Gene names
RO-QC-160121_ZackD_SS_Set_01_Fractio	16085	FTMS; HC	137.73	806.71	SLC9A3R1

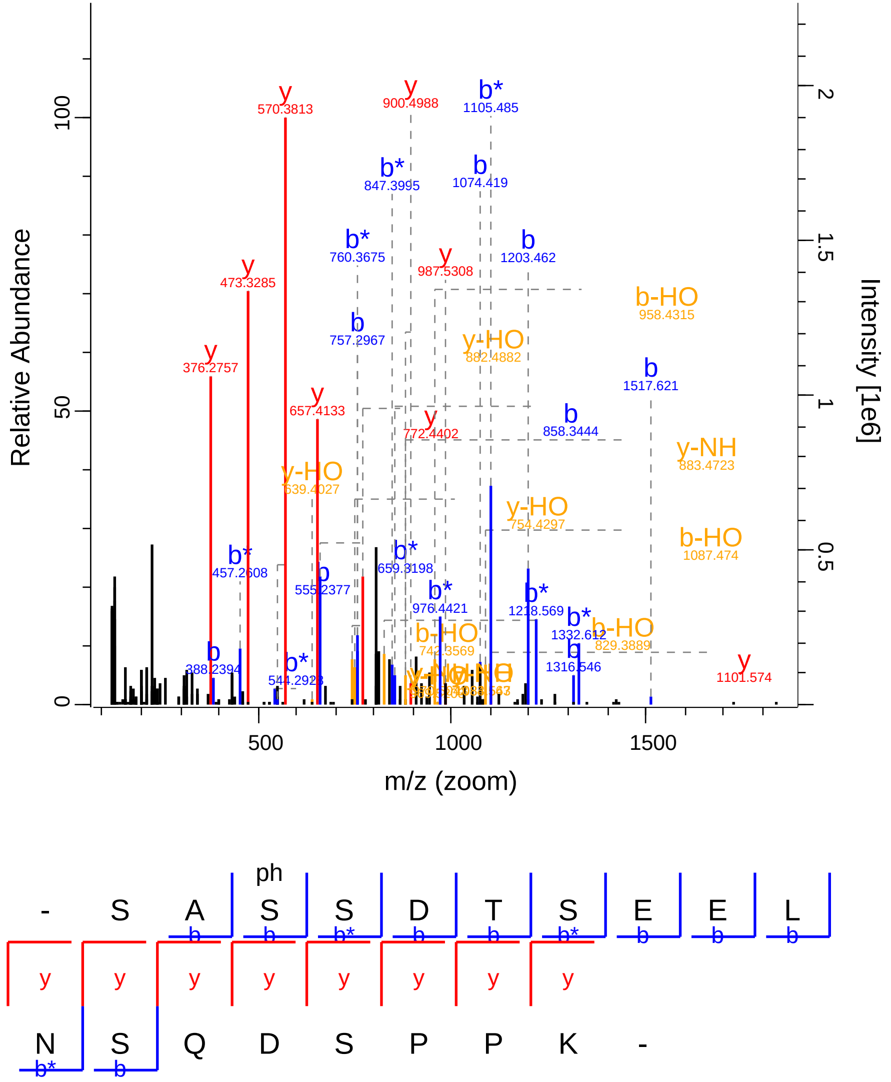

Raw File	Scan	Method	Score	m/z	Gene names
RO-QC-160121_ZackD_SS_Set_01_Fractior	16105	FTMS; HCI	213.73	947.5	RBM25

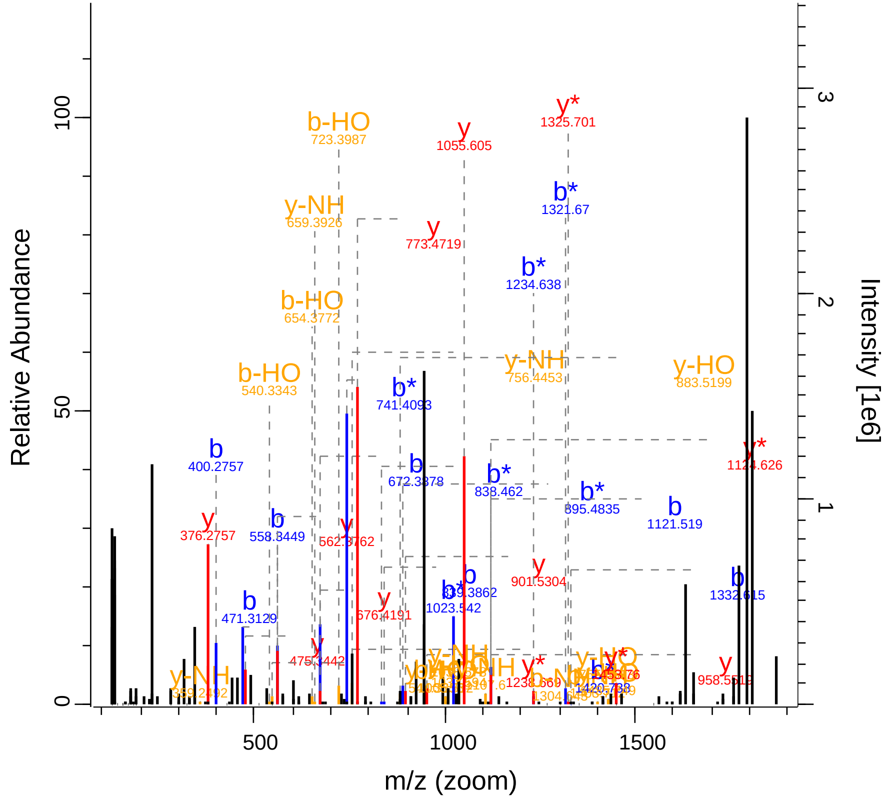

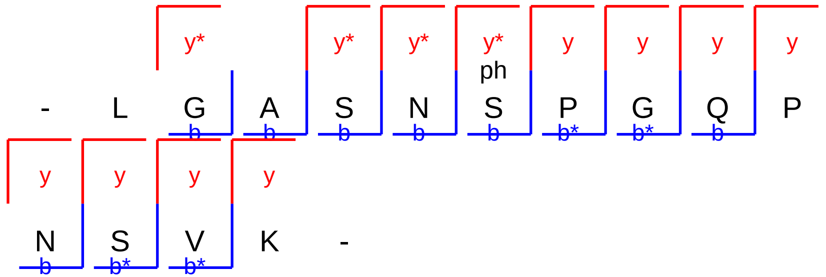

Raw File	Scan	Method	Score	m/z	Gene names
RO-QC-160121_ZackD_SS_Set_01_Fractio	16562	FTMS; HC	216.72	1141.98	CTNND1

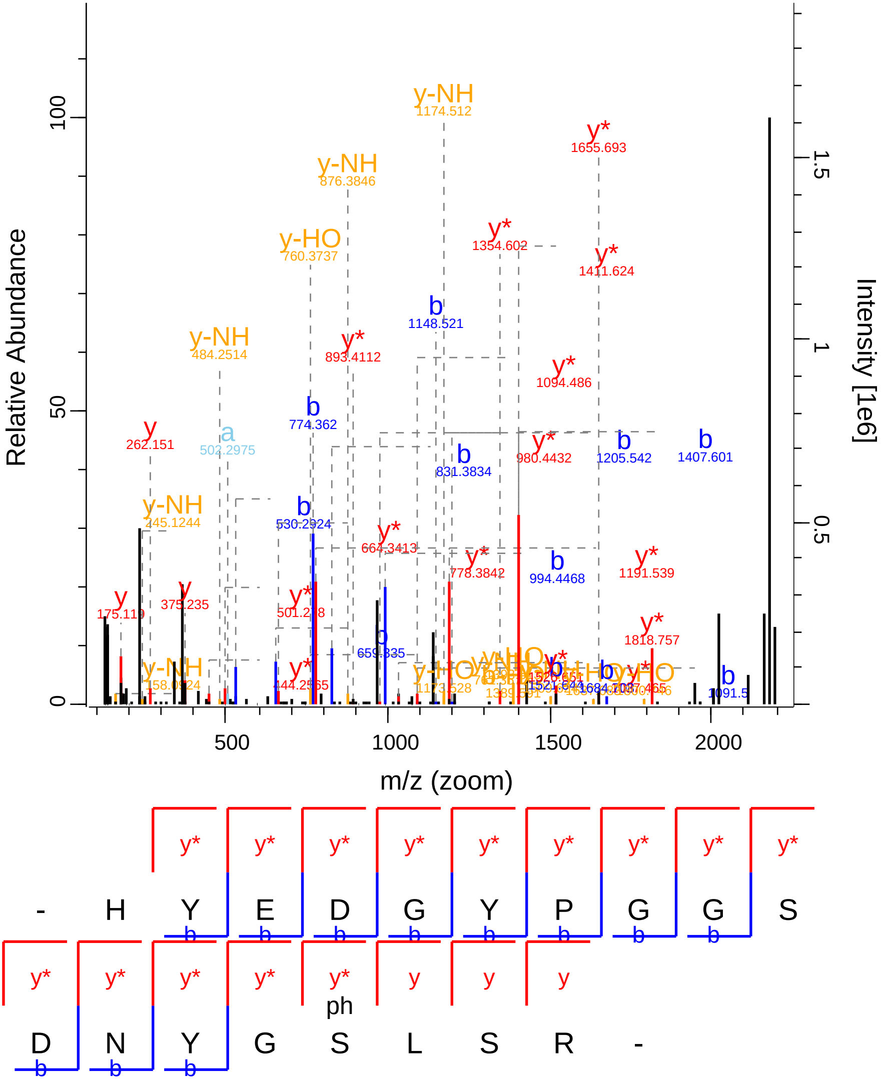

Raw File	Scan	Method	Score	m/z	Gene names
RO-QC-160121_ZackD_SS_Set_01_Fractic	16767	FTMS; HC	88.55	1029.09	CALD1

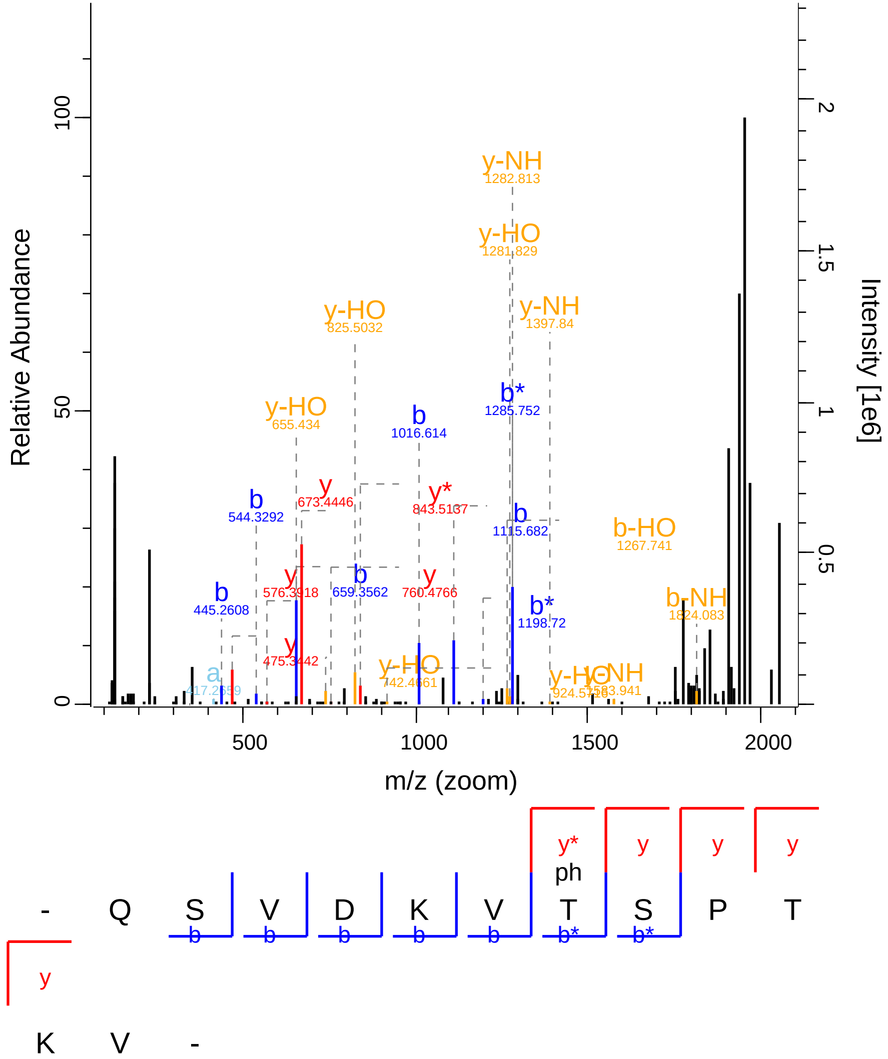

Raw File	Scan	Method	Score	m/z	Gene names
RO-QC-160121_ZackD_SS_Set_01_Fractic	17061	FTMS; HC	43.63	1007.49	KIF23

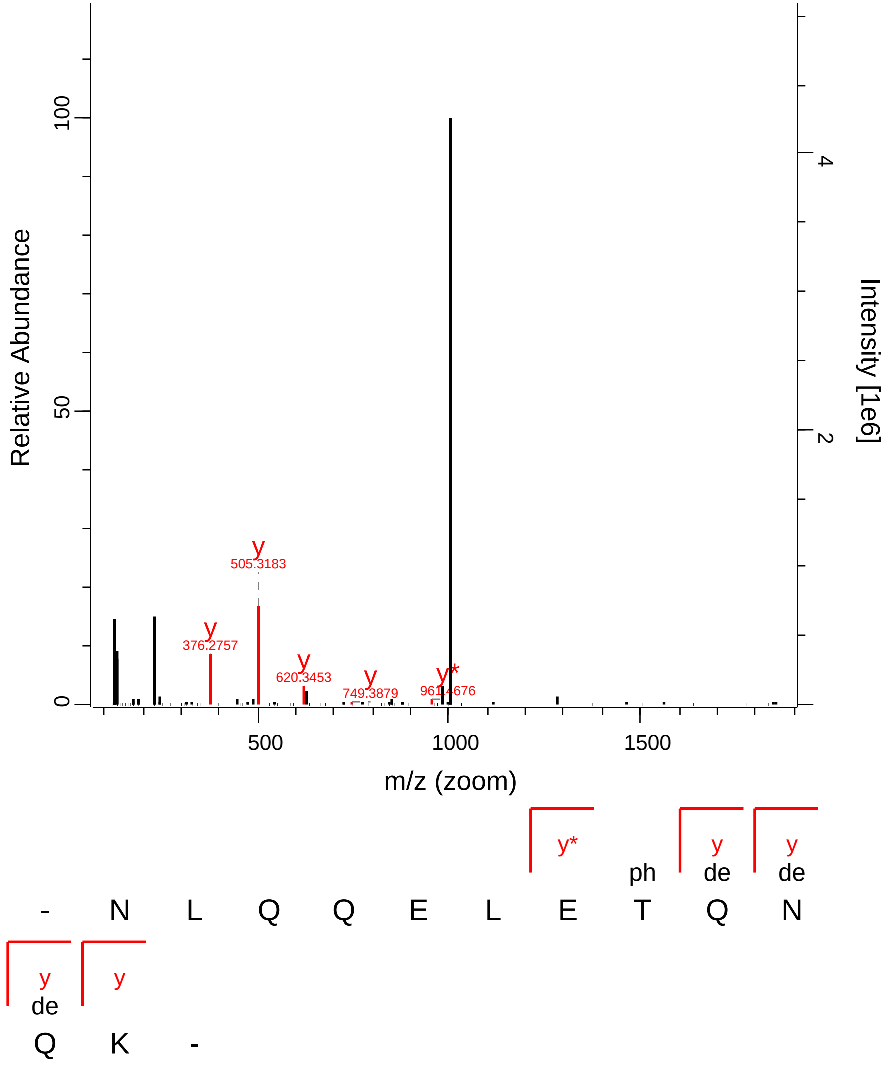

Raw File	Scan	Method	Score	m/z	Gene names
RO-QC-160121_ZackD_SS_Set_01_Fractio	18475	FTMS; HC	137.11	892.43	MYEF2

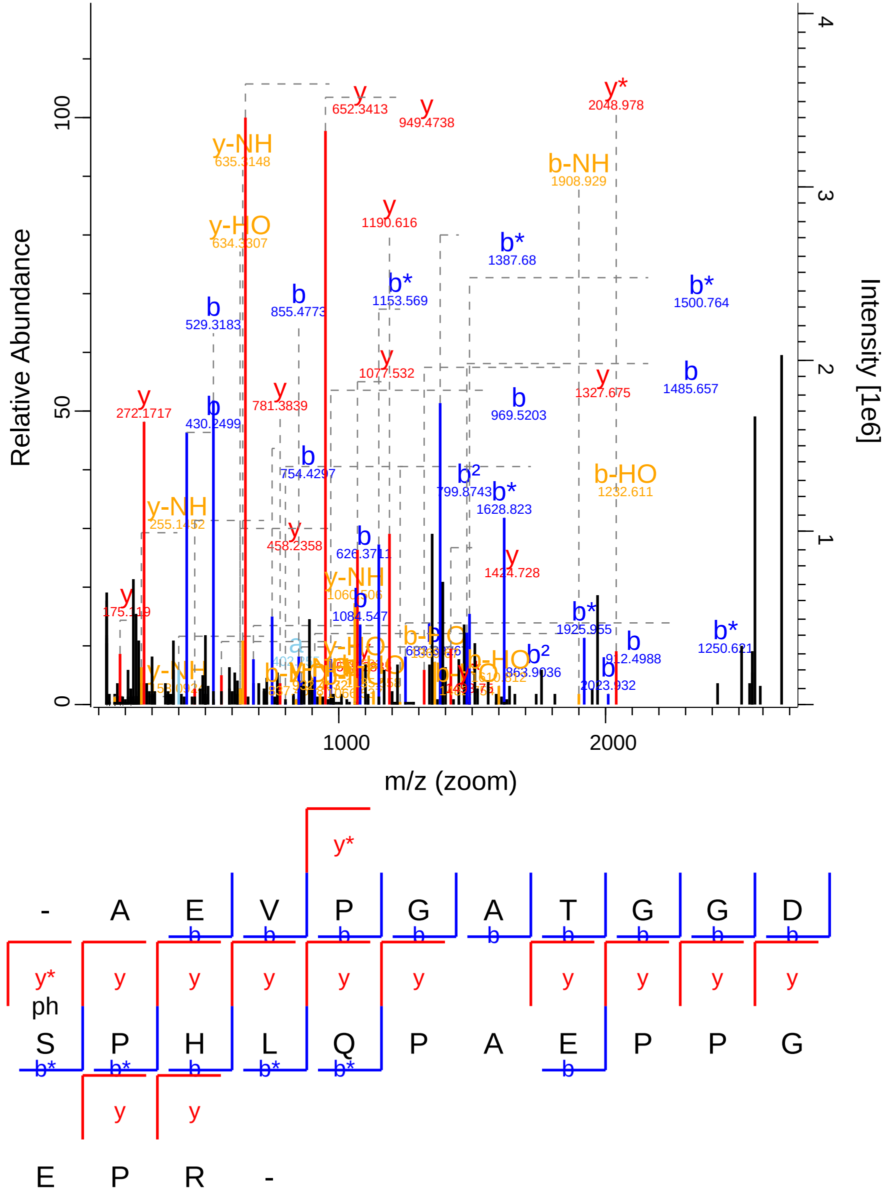

Raw File	Scan	Method	Score	m/z	Gene names
RO-QC-160121_ZackD_SS_Set_01_Fractio	18818	FTMS; HC	60.4	929.98	MPP5

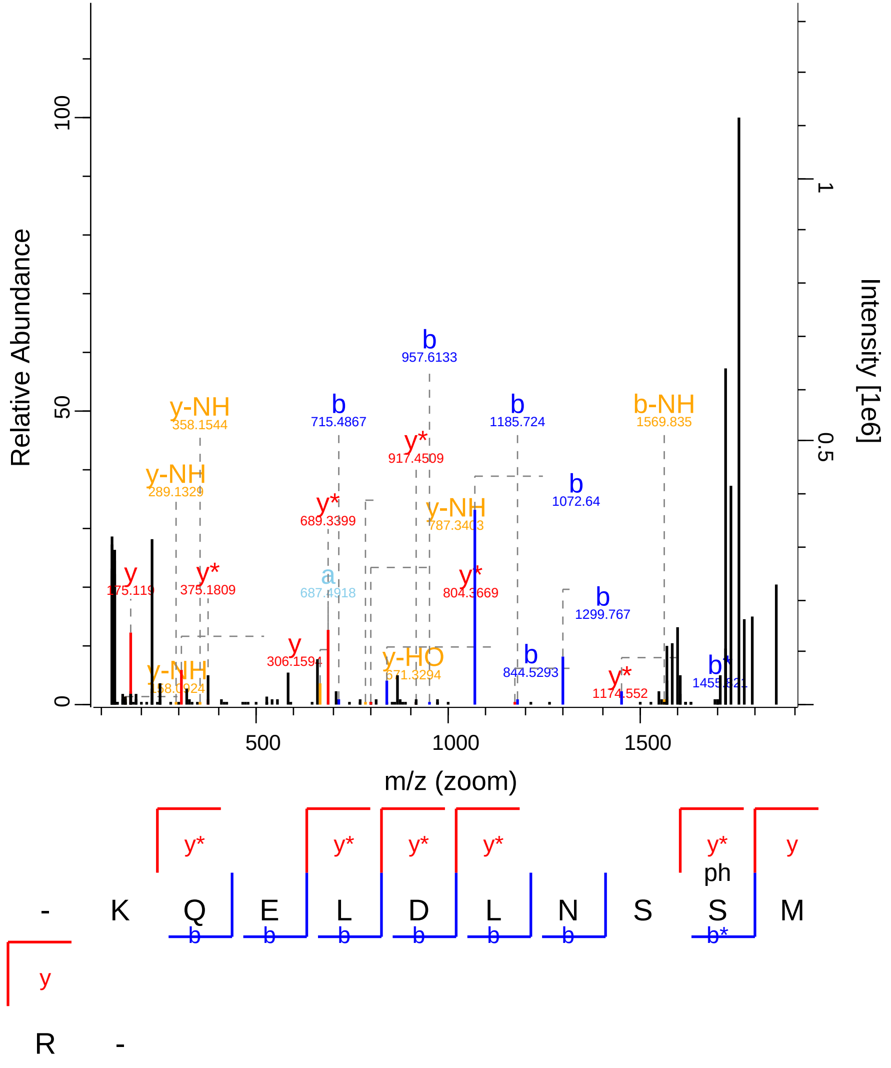

Raw File	Scan	Method	Score	m/z	Gene names
RO-QC-160121_ZackD_SS_Set_01_Fractio	19394	FTMS; HC	109.16	764.92	RAI14

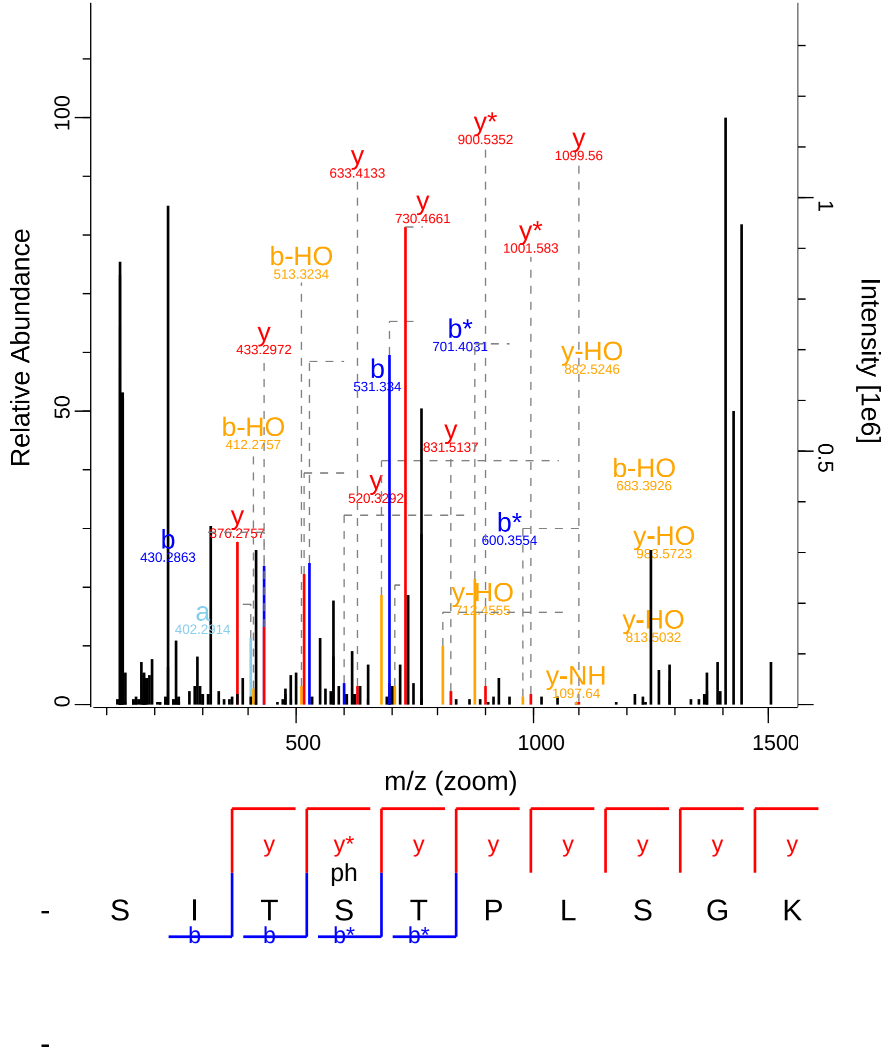

Raw File	Scan	Method	Score	m/z	Gene names
RO-QC-160121_ZackD_SS_Set_01_Fractio	19580	FTMS; HC	63.28	742.59	NOP58

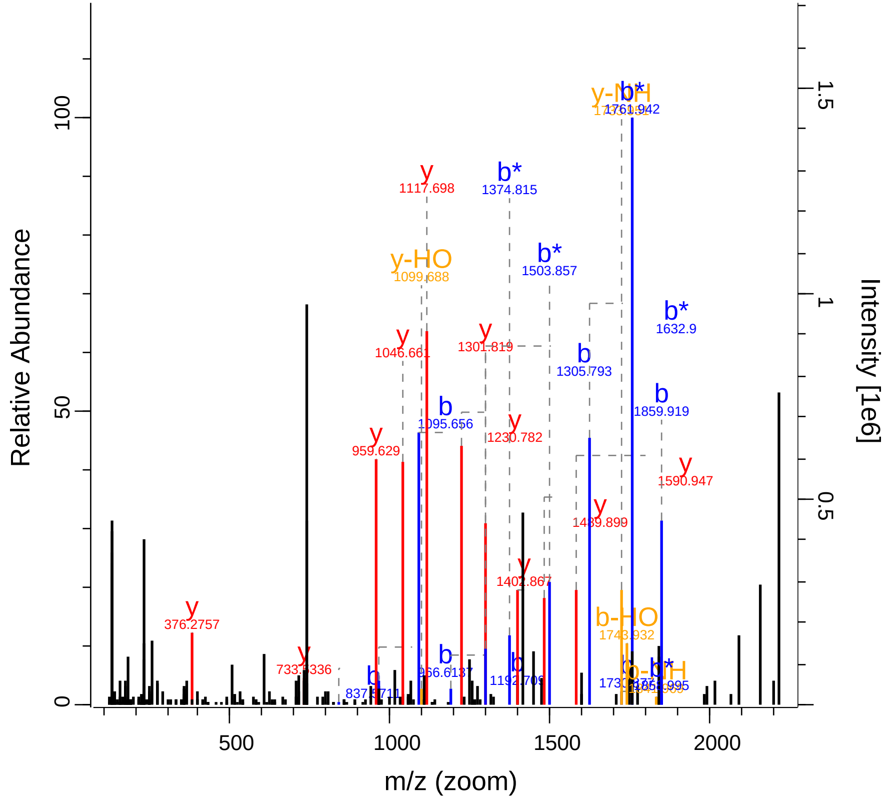

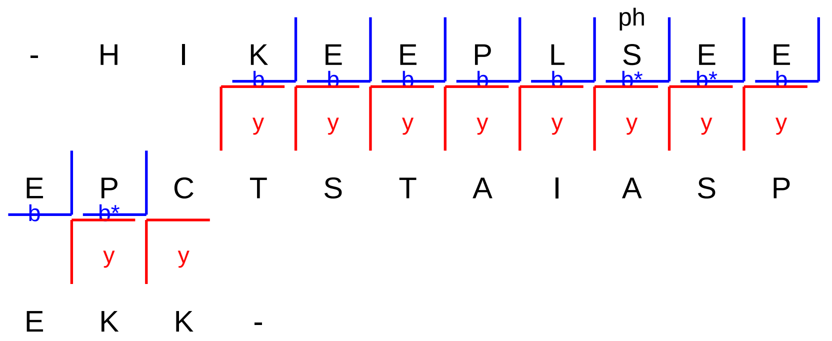

Raw File	Scan	Method	Score	m/z	Gene names
RO-QC-160121_ZackD_SS_Set_01_Fractio	19802	FTMS; HC	226.25	1240.67	STUB1

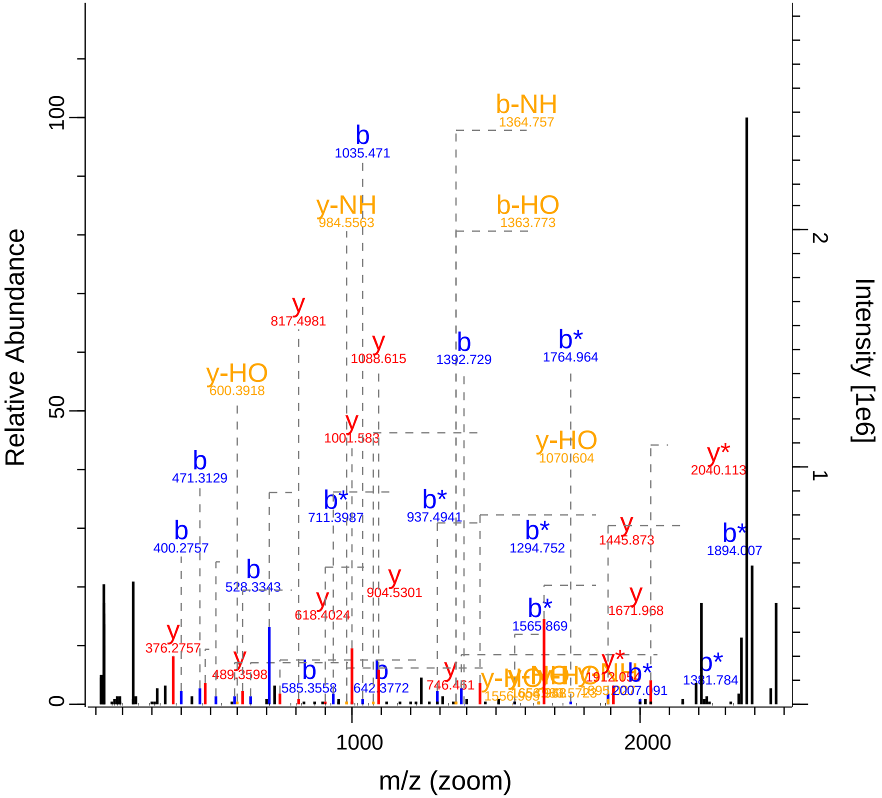

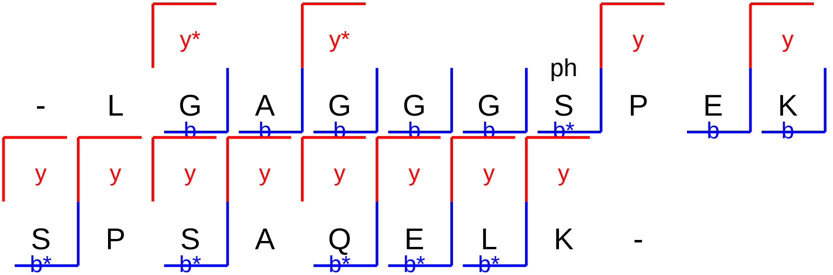

Raw File	Scan	Method	Score	m/z	Gene names
RO-QC-160121_ZackD_SS_Set_01_Fractic	20785	FTMS; HC	40.49	1015.84	DCST1

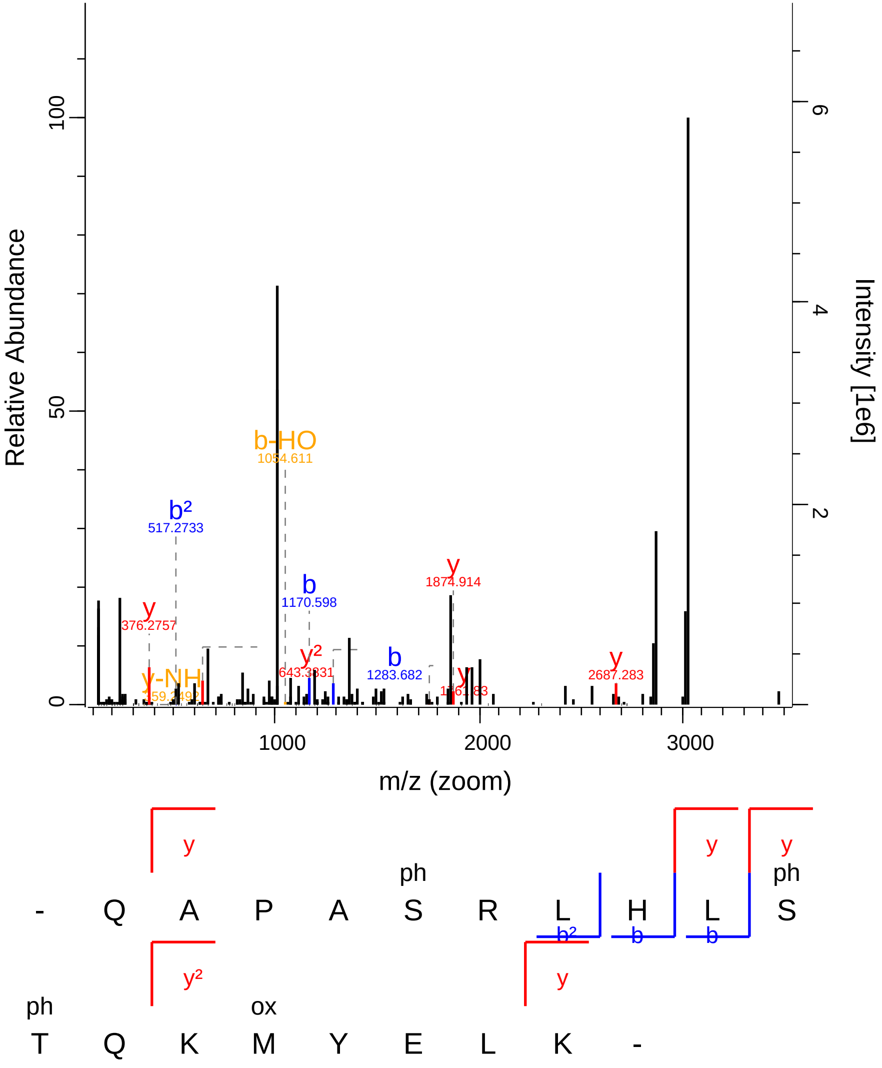

Raw File	Scan	Method	Score	m/z	Gene names
RO-QC-160121_ZackD_SS_Set_01_Fractic	21200	FTMS; HC	46.73	1108.05	EEF1G

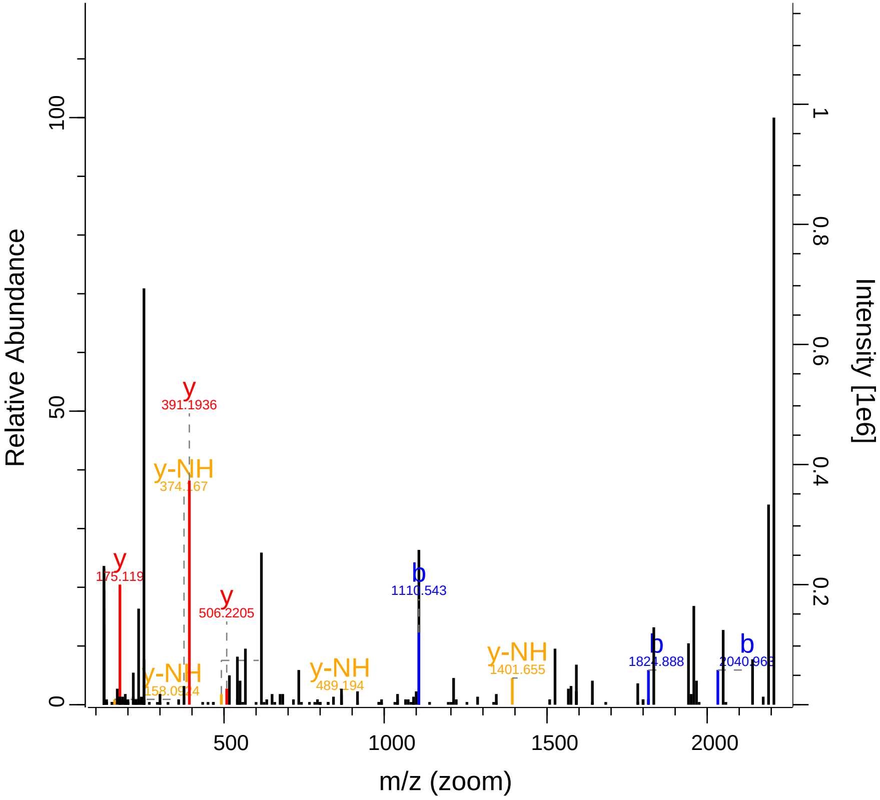

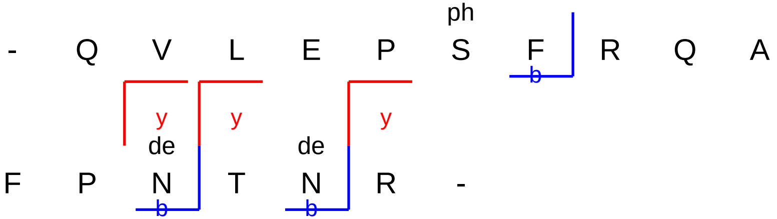

Raw File	Scan	Method	Score	m/z	Gene names
RO-QC-160121_ZackD_SS_Set_01_Fractio	21256	FTMS; HC	174.59	922.83	HABP4

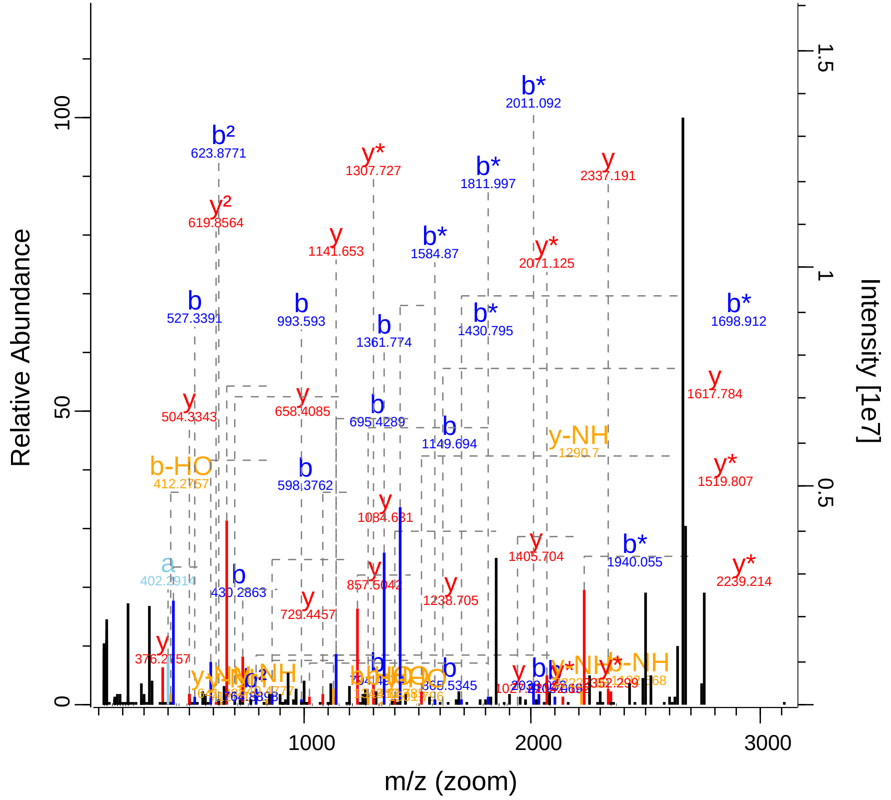

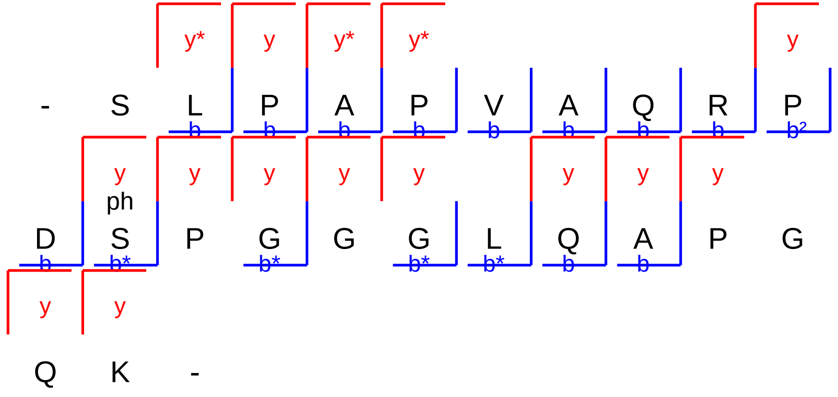

Raw File	Scan	Method	Score	m/z	Gene names
RO-QC-160121_ZackD_SS_Set_01_Fractio	22603	FTMS; HC	74.16	998.56	MTUS2

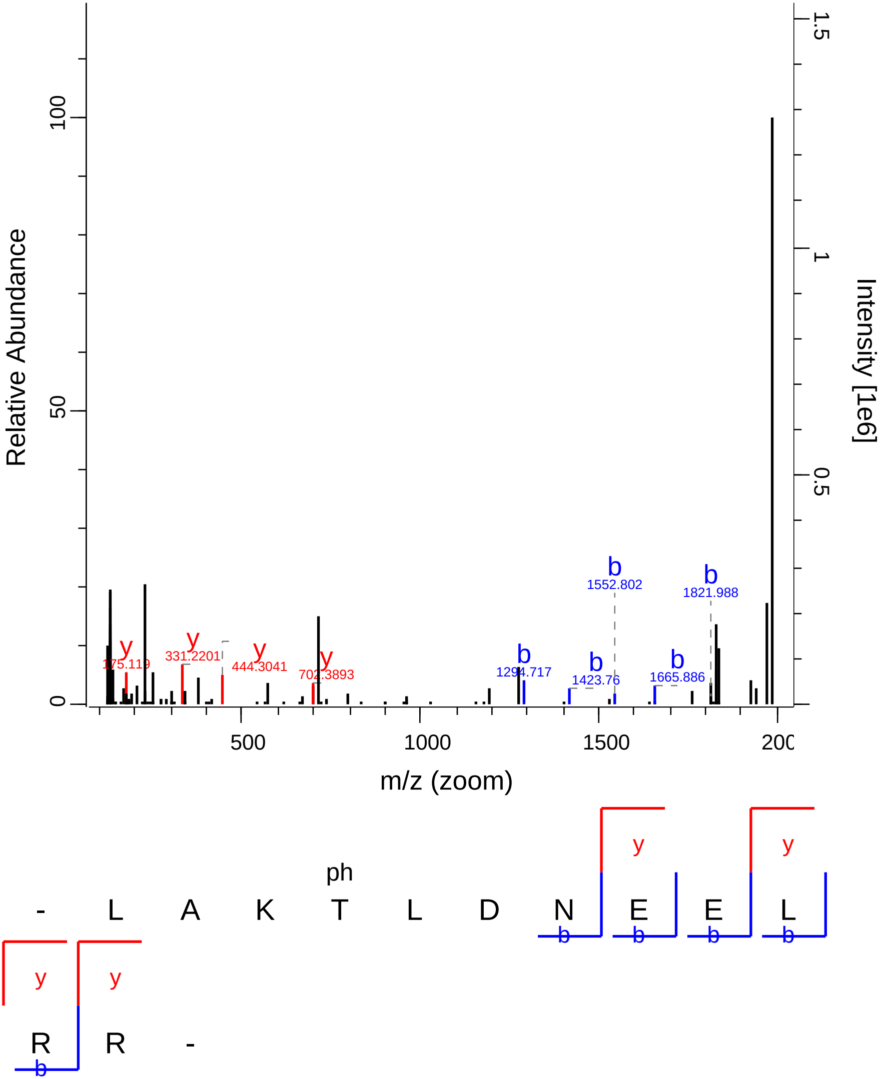

Raw File	Scan	Method	Score	m/z	Gene names
RO-QC-160121_ZackD_SS_Set_01_Fractic	23487	FTMS; HC	55.23	1086.19	SLX4

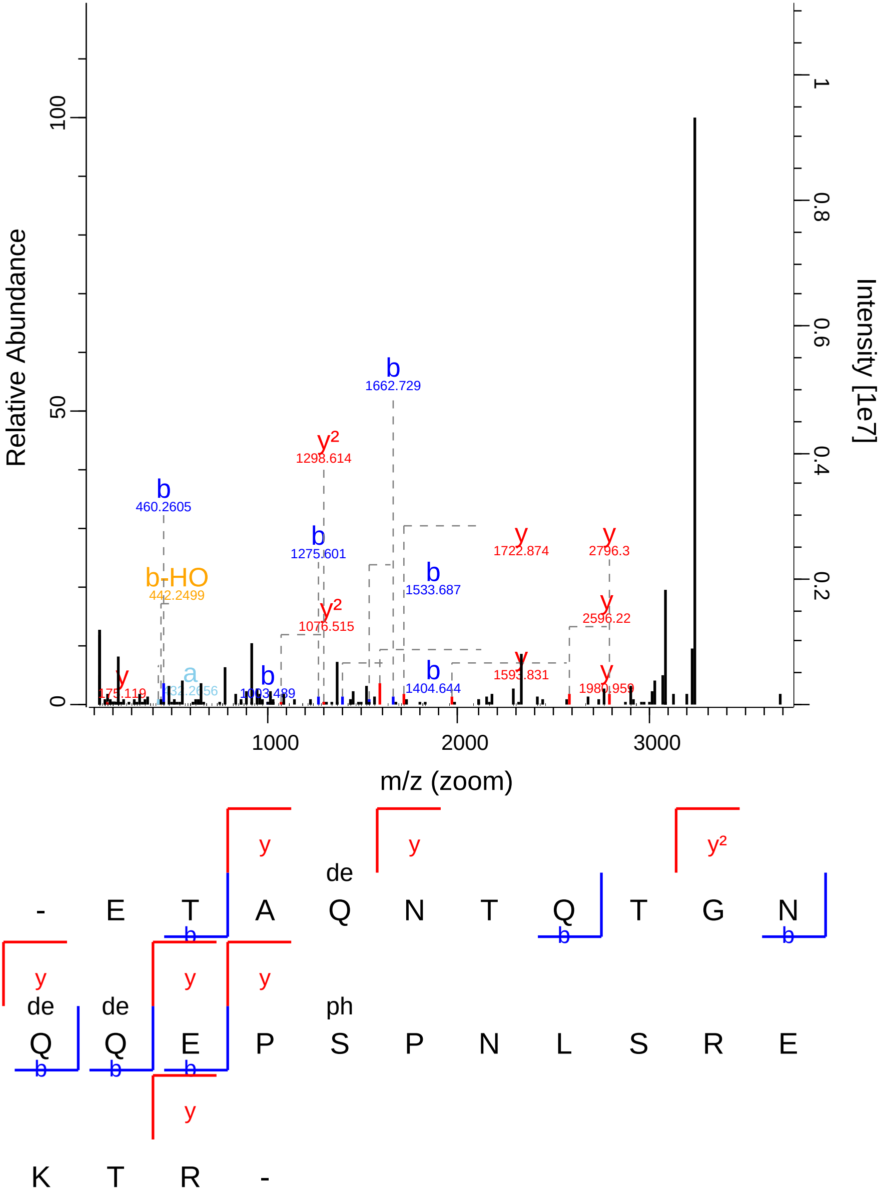

Raw File	Scan	Method	Score	m/z	Gene names
RO-QC-160121_ZackD_SS_Set_01_Fractic	24320	FTMS; HC	72.53	1220.25	THUMPD1

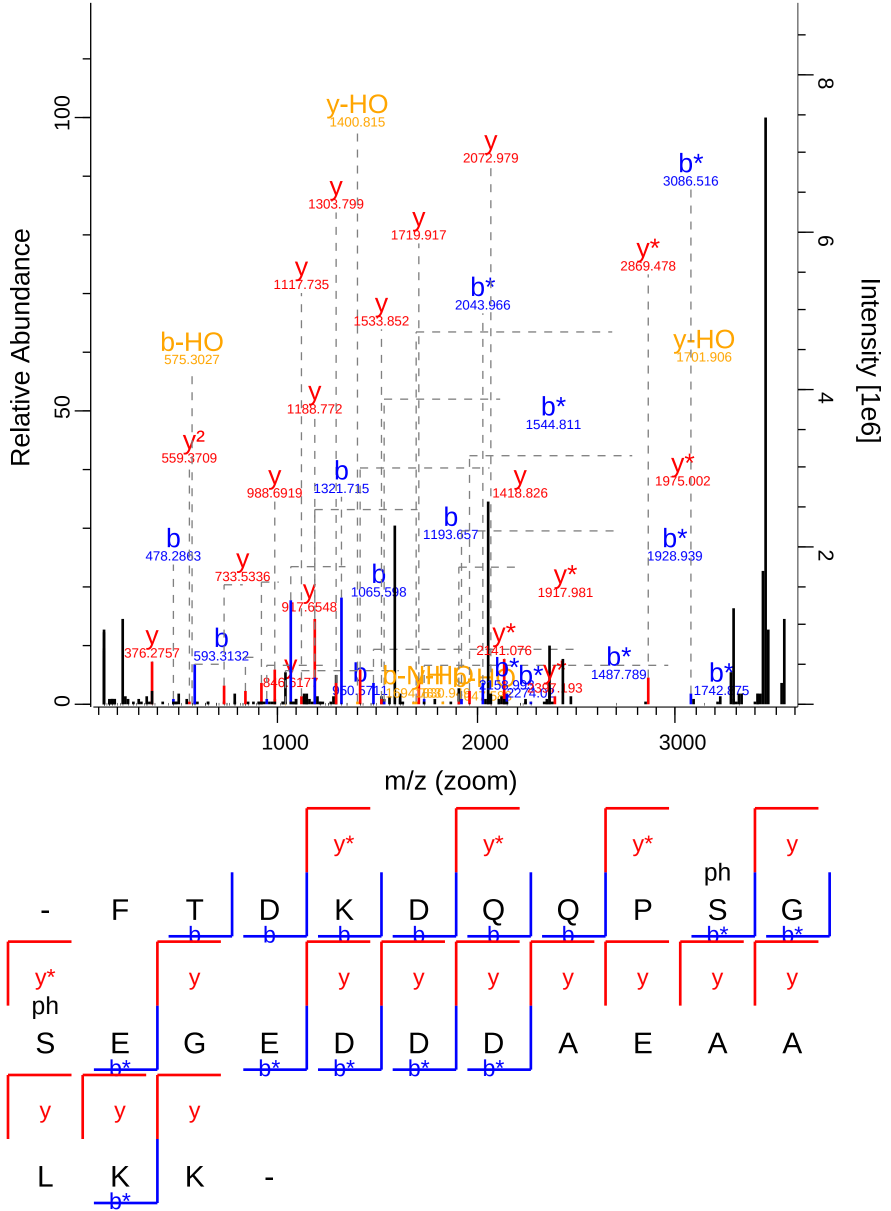

Raw File	Scan	Method	Score	m/z	Gene names
RO-QC-160121_ZackD_SS_Set_01_Fraction	24927	FTMS; HCl	224.05	936	ALDOA

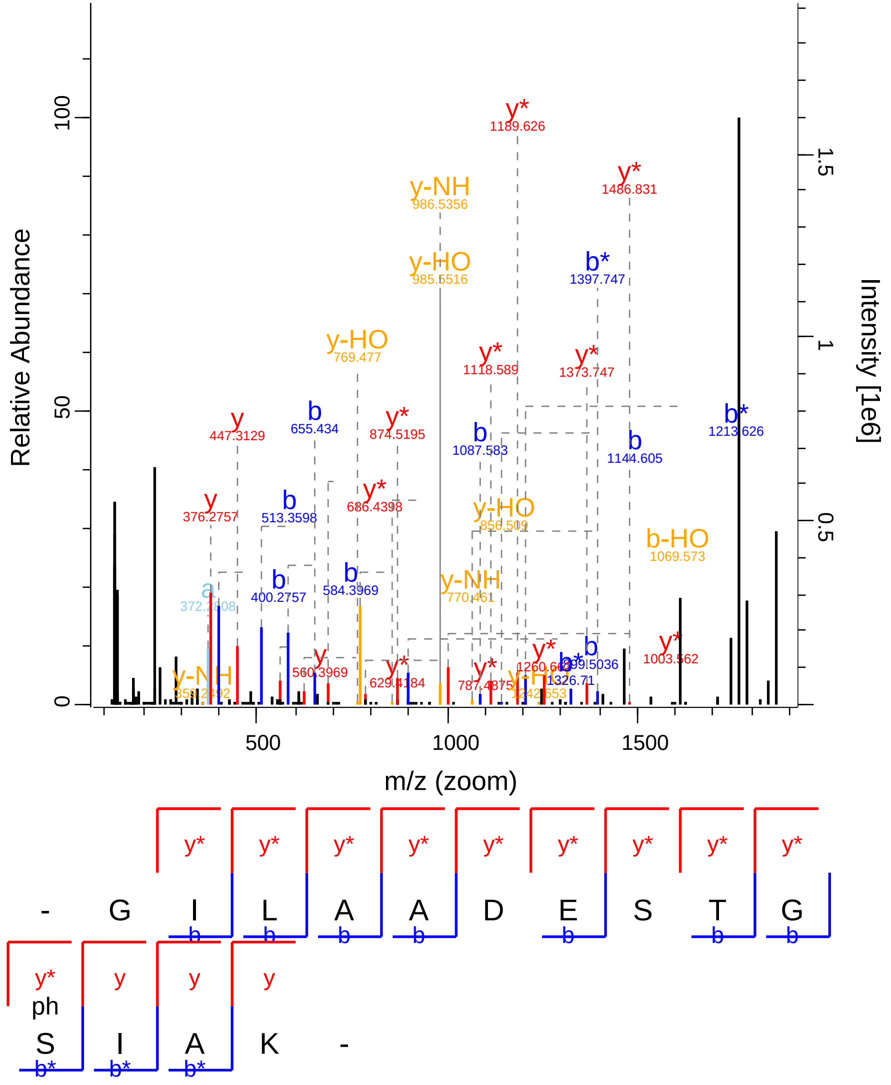

Raw File	Scan	Method	Score	m/z	Gene names
RO-QC-160121_ZackD_SS_Set_01_Fractio	25671	FTMS; HC	56.91	717.13	LARP1

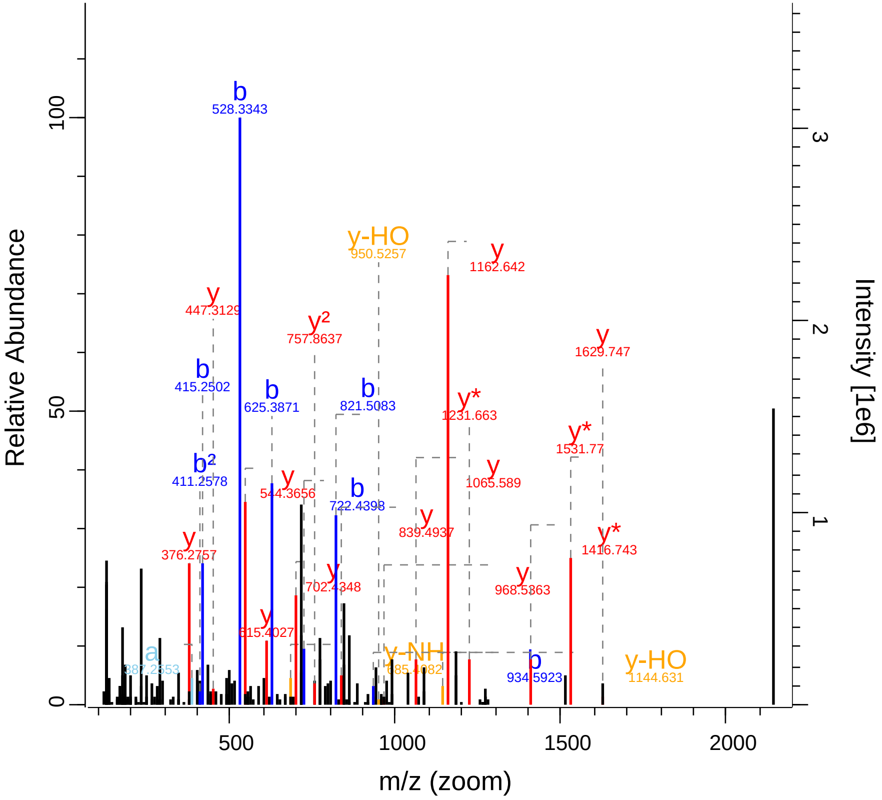

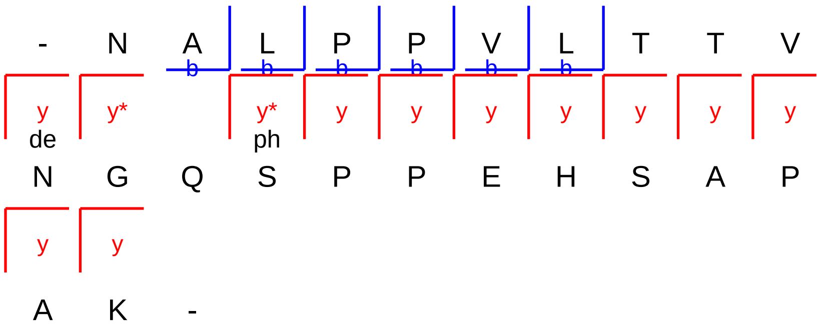

Raw File	Scan	Method	Score	m/z	Gene names
RO-QC-160121_ZackD_SS_Set_01_Fractio	27159	FTMS; HC	49.5	920.88	EIF2S2

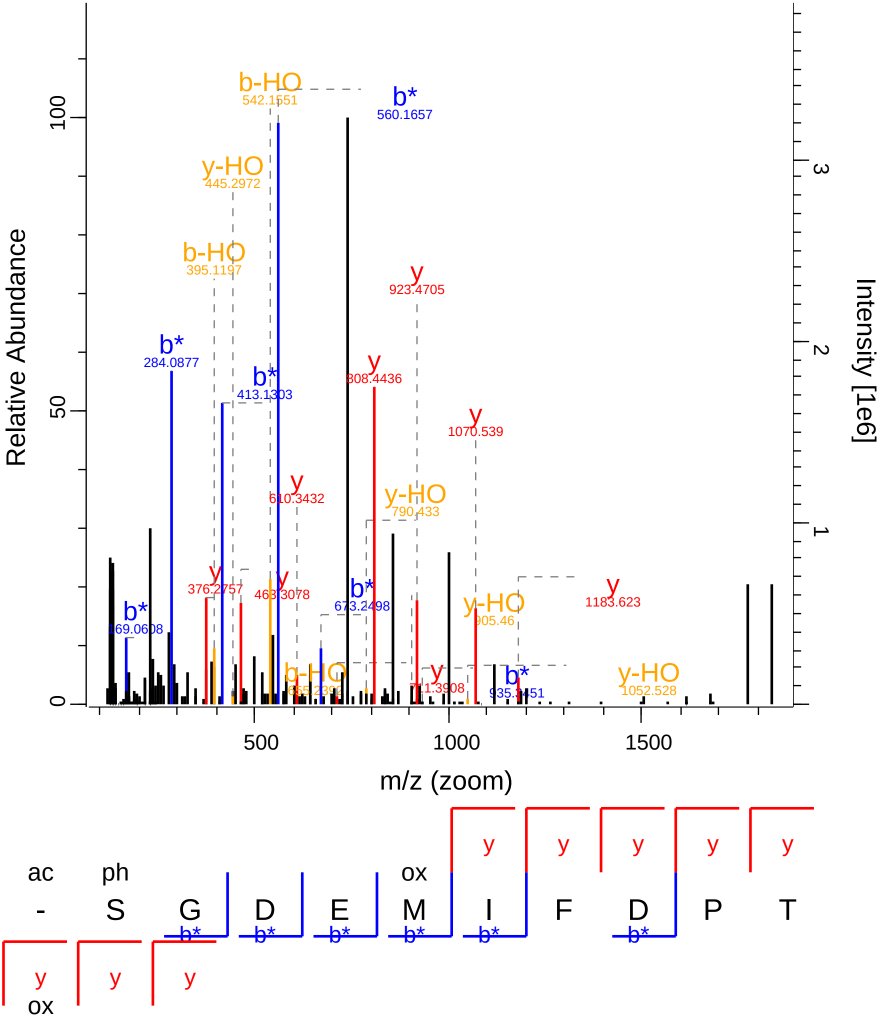

Raw File	Scan	Method	Score	m/z	Gene names
RO-QC-160121_ZackD_SS_Set_01_Fractio	27207	FTMS; HC	40.49	992.85	PLCH1

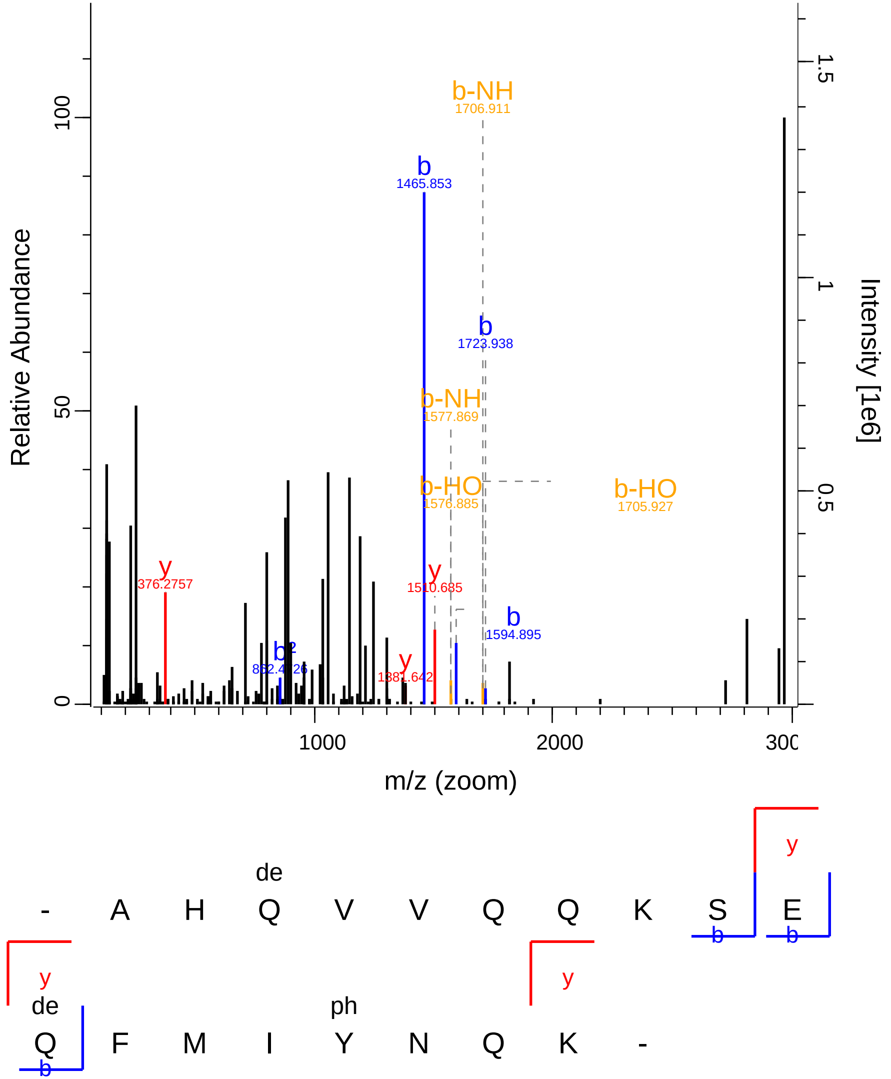

Raw File	Scan	Method	Score	m/z	Gene names
RO-QC-160121_ZackD_SS_Set_01_Fractio	27497	FTMS; HC	54.78	554.81	ADAMDEC1

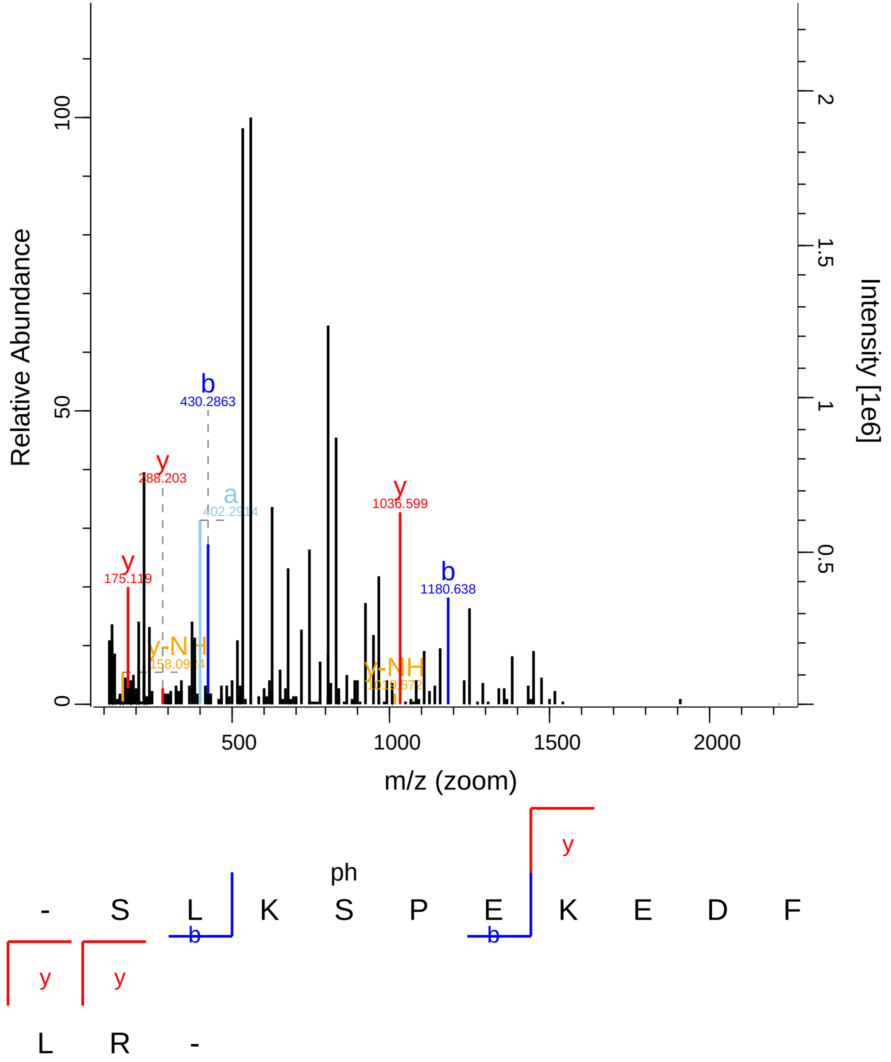

Raw File	Scan	Method	Score	m/z	Gene names
RO-QC-160121_ZackD_SS_Set_01_Fractic	27835	FTMS; HC	69.62	1060.86	MAP1B

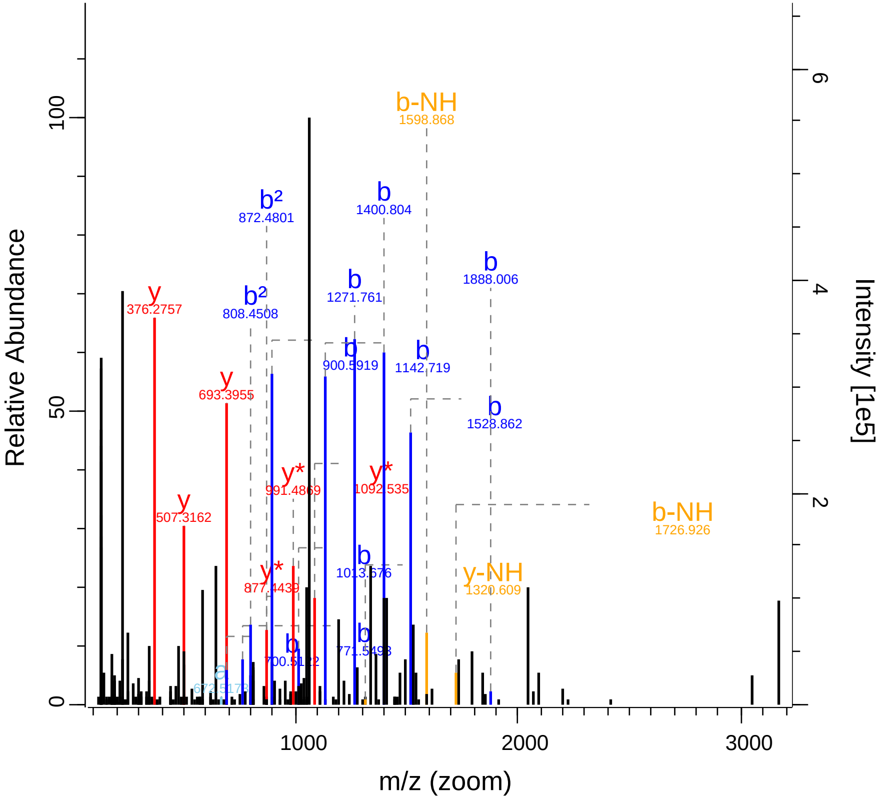

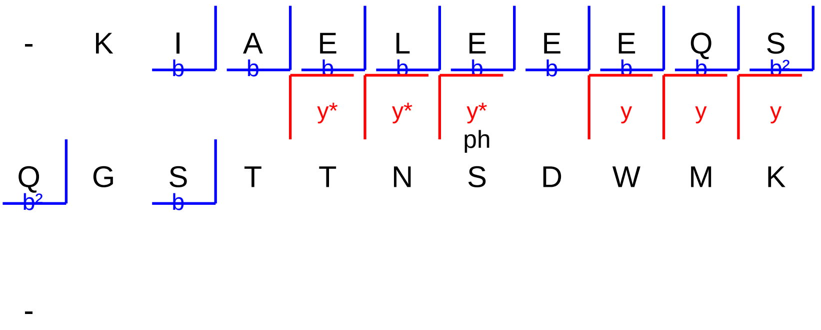

Raw File	Scan	Method	Score	m/z	Gene names
RO-QC-160121_ZackD_SS_Set_01_Fractic	27940	FTMS; HC	205.33	1158.13	MAP4

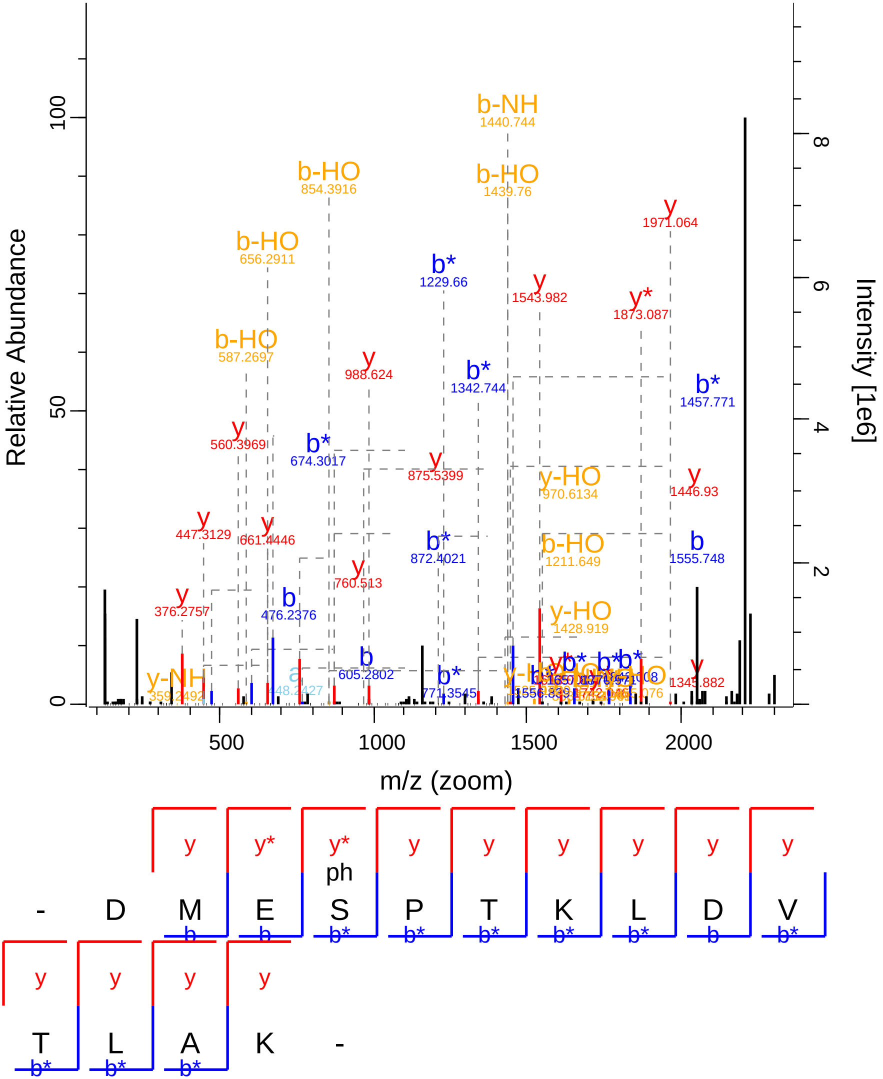

Raw File	Scan	Method	Score	m/z	Gene names
RO-QC-160121_ZackD_SS_Set_01_Fractic	28043	FTMS; HC	100.17	1216.63	TCOF1

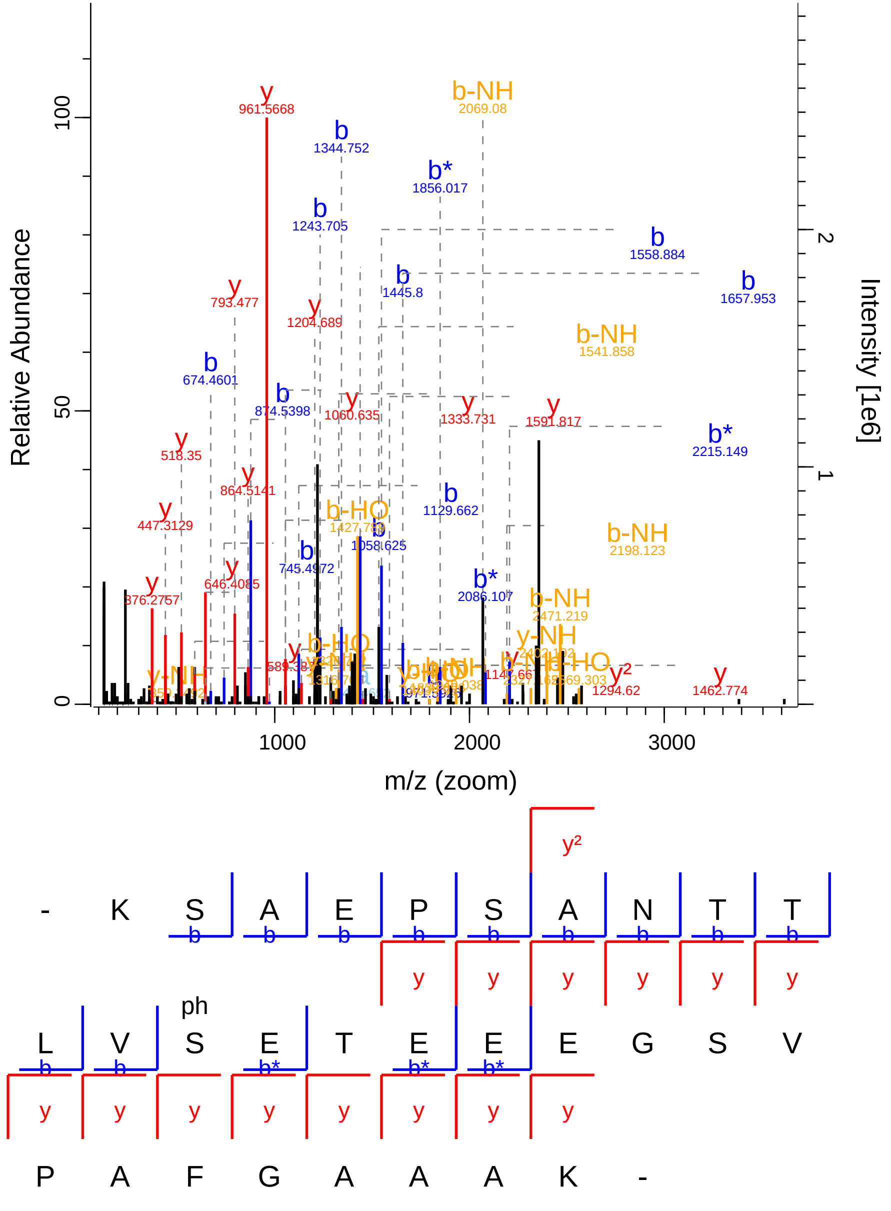

Raw File	Scan	Method	Score	m/z	Gene names
RO-QC-160121_ZackD_SS_Set_01_Fractio	28330	FTMS; HC	186.02	941.45	TOM1L1

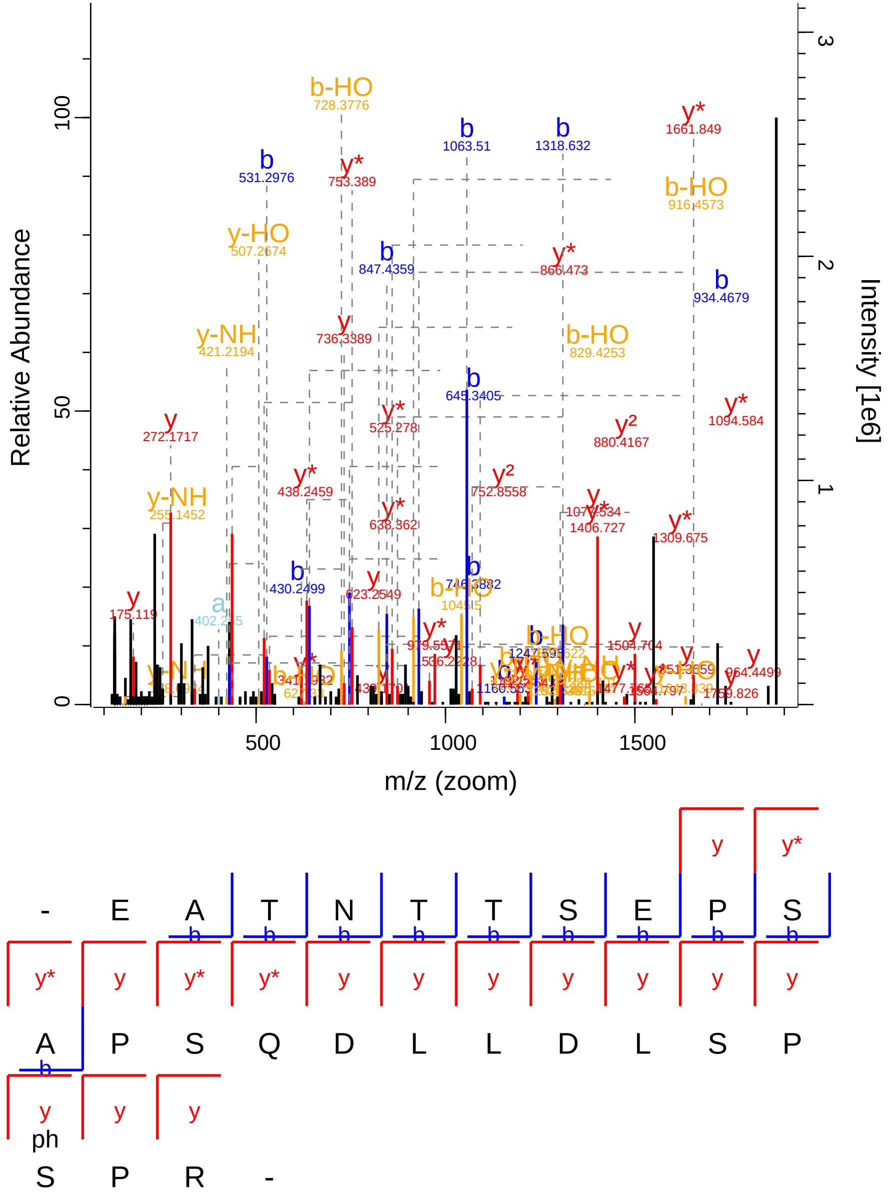

Raw File	Scan	Method	Score	m/z	Gene names
RO-QC-160121_ZackD_SS_Set_01_Fractio	30061	FTMS; HC	222.45	942.23	FAM13A

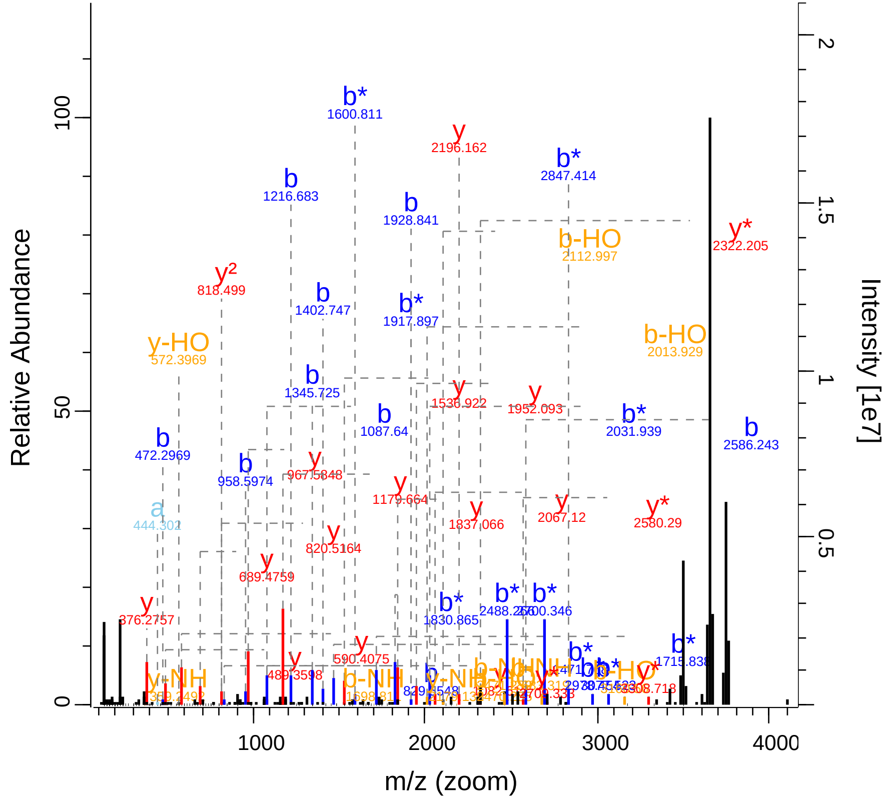

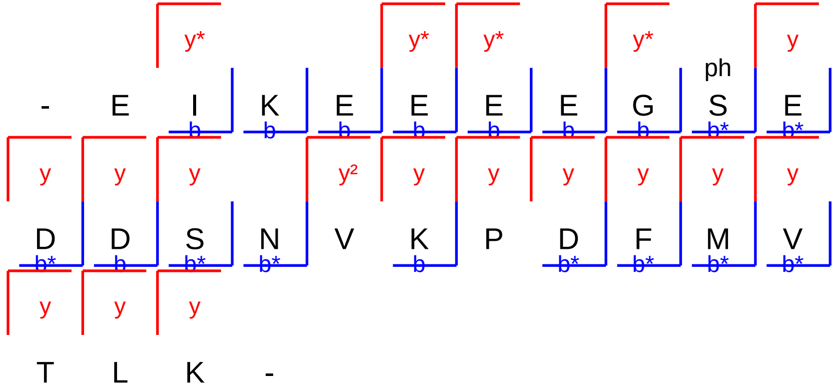

Raw File	Scan	Method	Score	m/z	Gene names
RO-QC-160121_ZackD_SS_Set_01_Fractic	30399	FTMS; HC	46.98	1090.05	TOP2B

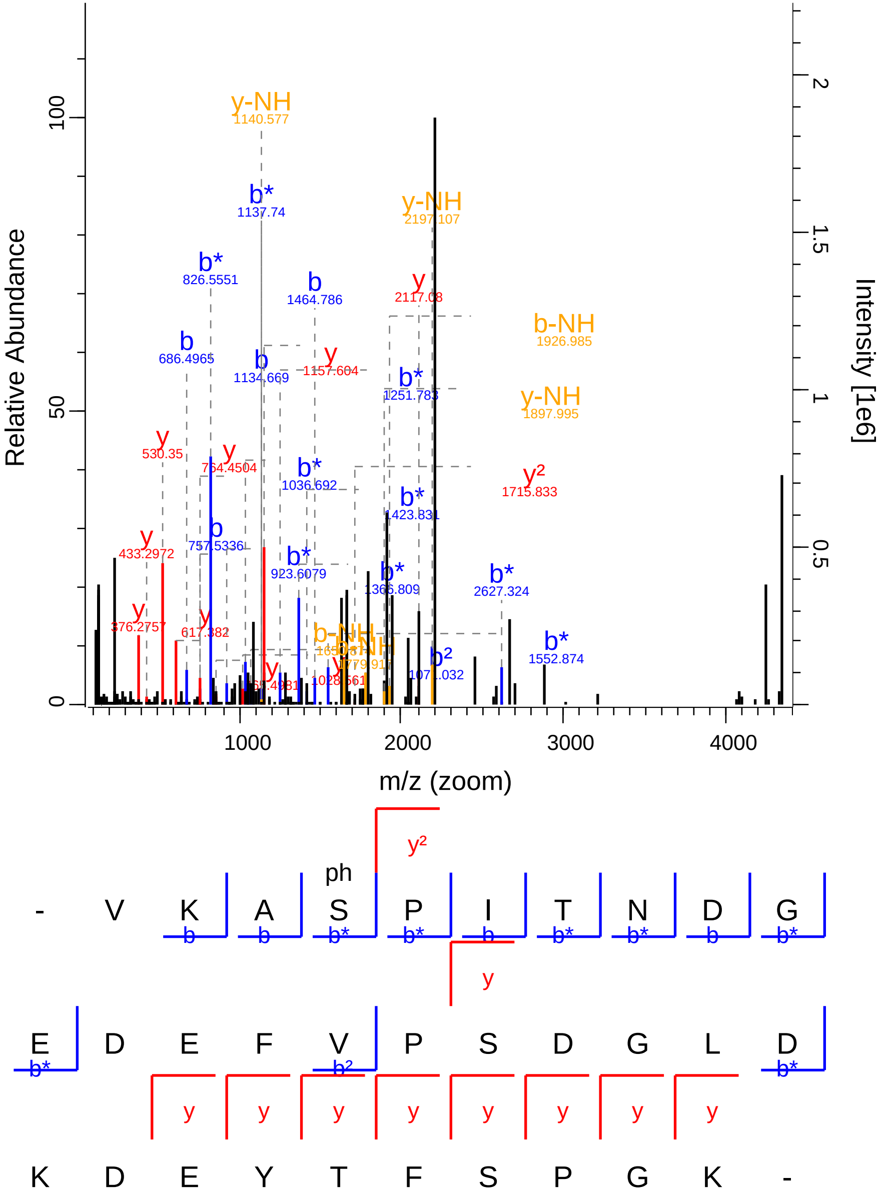

Raw File	Scan	Method	Score	m/z	Gene names
RO-QC-160121_ZackD_SS_Set_01_Fractio	30597	FTMS; HC	154.74	732.73	NEDD4L

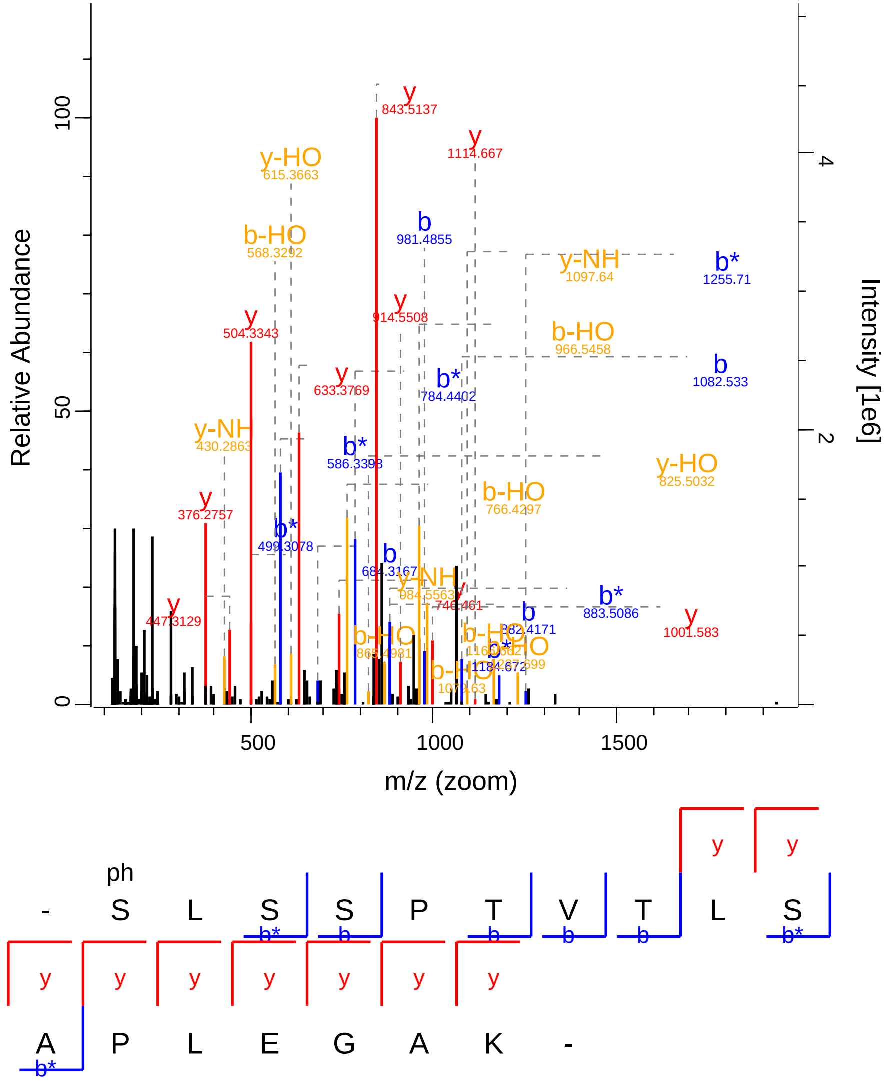

Raw File	Scan	Method	Score	m/z	Gene names
RO-QC-160121_ZackD_SS_Set_01_Fractio	30665	FTMS; HC	319.74	1098.6	NEDD4L

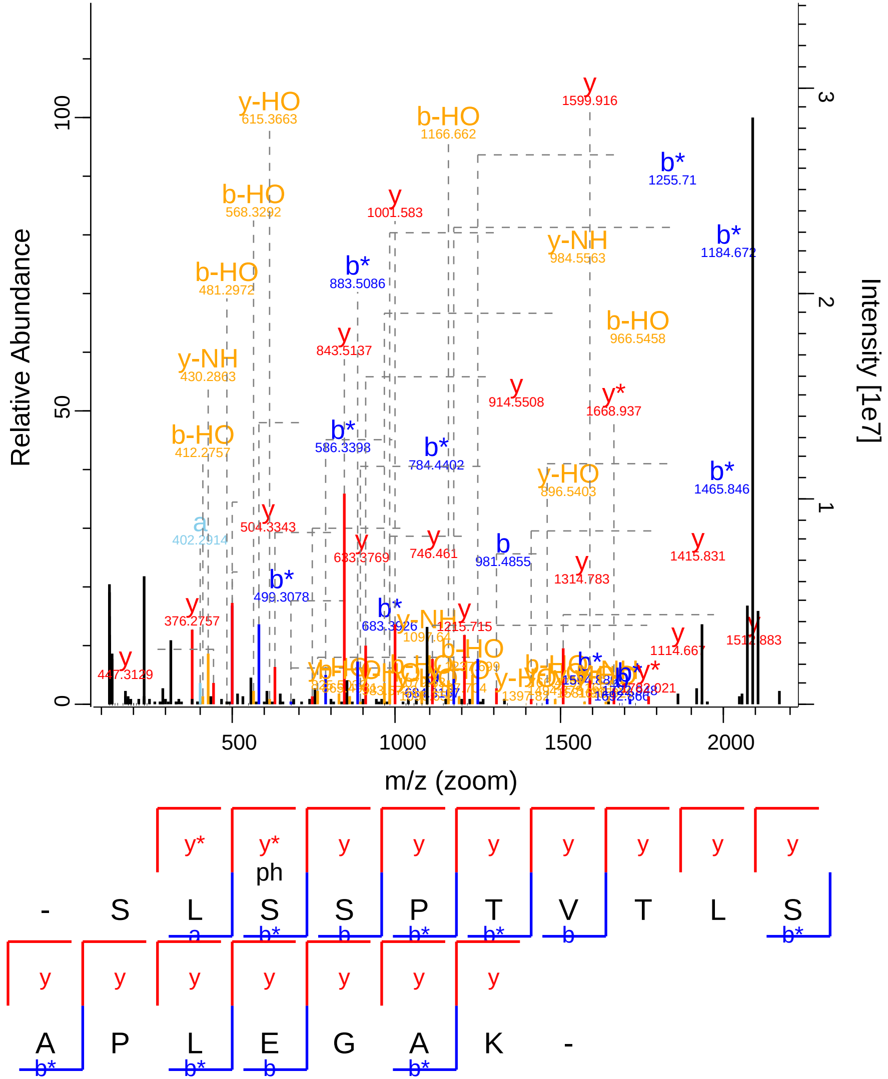

Raw File	Scan	Method	Score	m/z	Gene names
RO-QC-160121_ZackD_SS_Set_01_Fractic	31738	FTMS; HC	51.77	1465.21	RABAC1

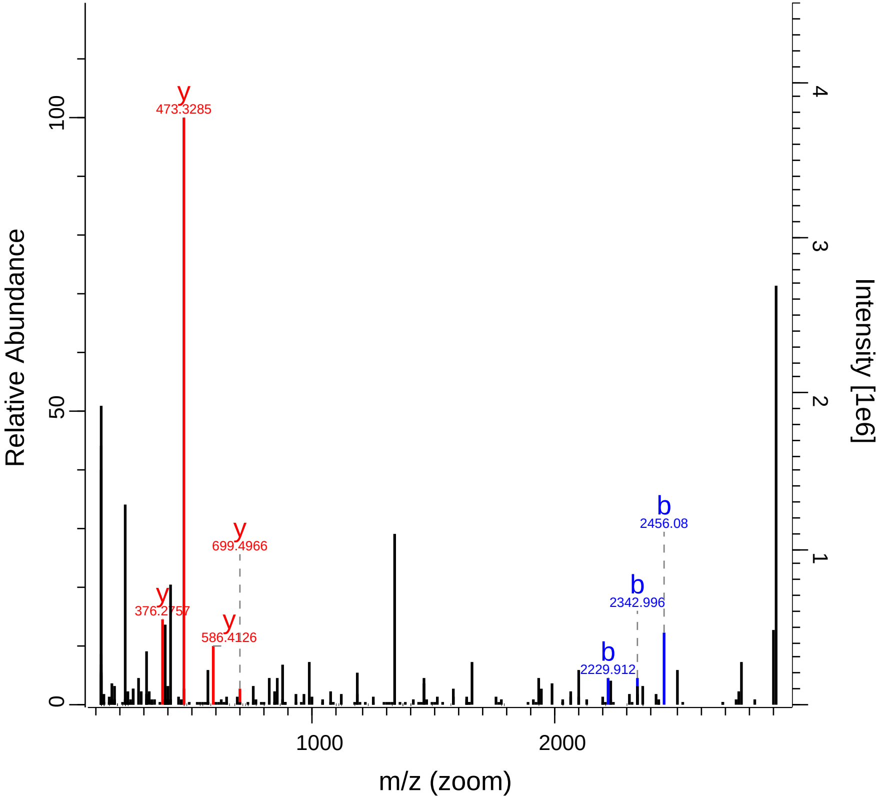

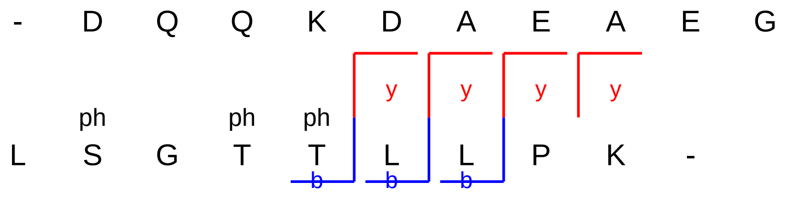

Raw File	Scan	Method	Score	m/z	Gene names
RO-QC-160121_ZackD_SS_Set_01_Fractio	32393	FTMS; HC	52.79	1307.7	EFCAB3

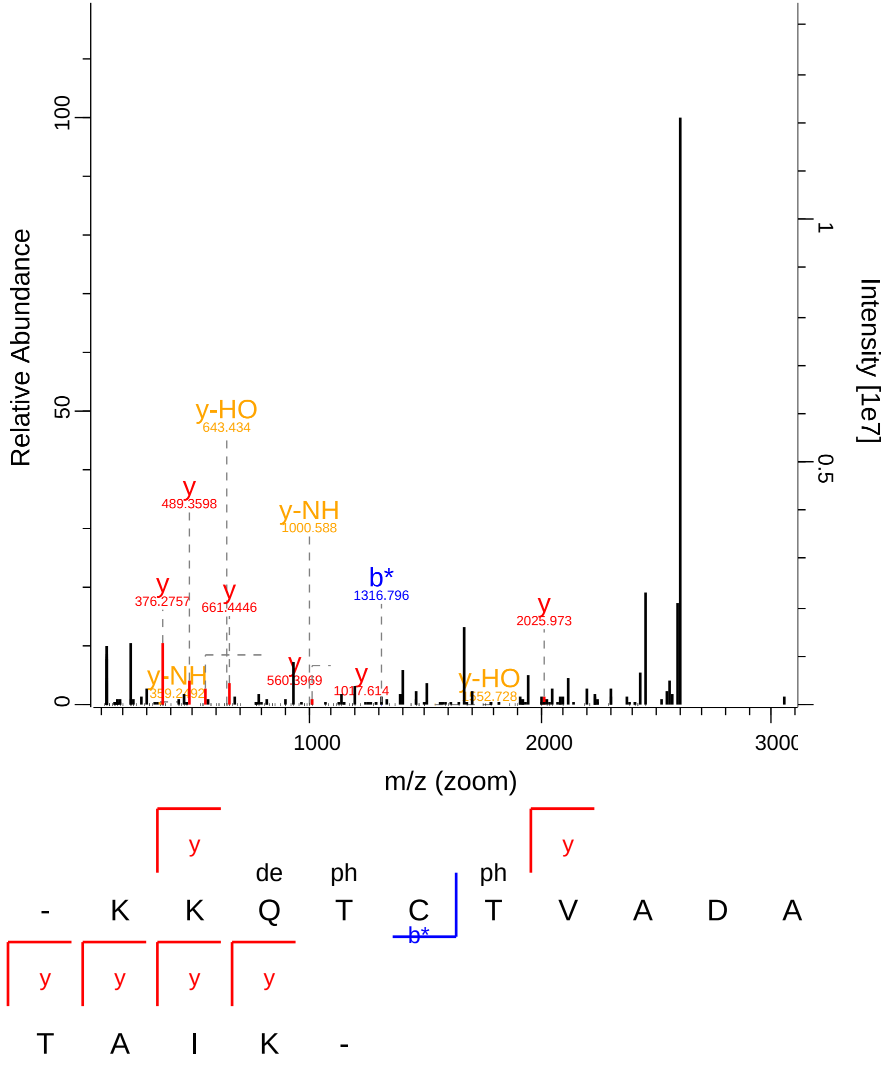

Raw File	Scan	Method	Score	m/z	Gene names
RO-QC-160121_ZackD_SS_Set_01_Fractio	33020	FTMS; HC	128.25	923.22	AKAP13

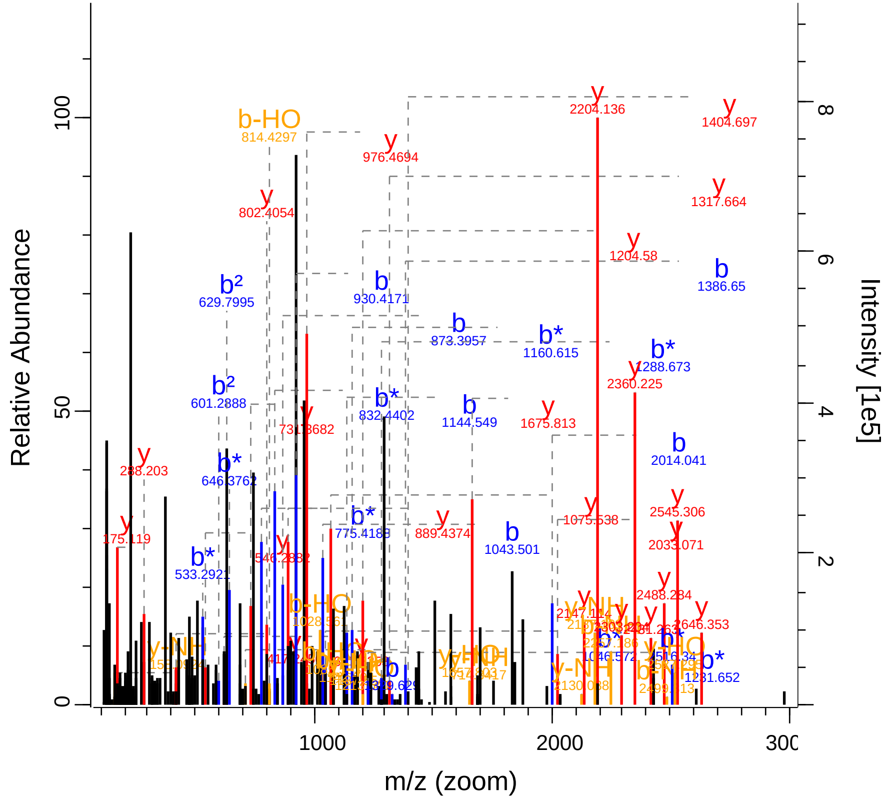

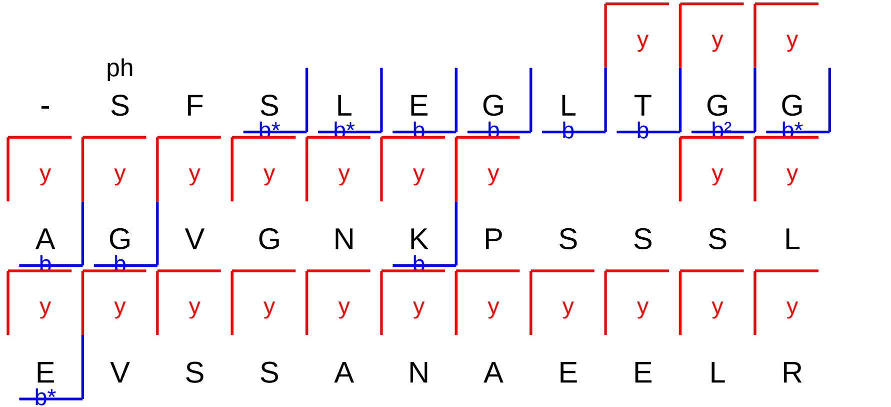

Raw File	Scan	Method	Score	m/z	Gene names
RO-QC-160121_ZackD_SS_Set_01_Fractio	34197	FTMS; HC	65.42	952.83	EHD1

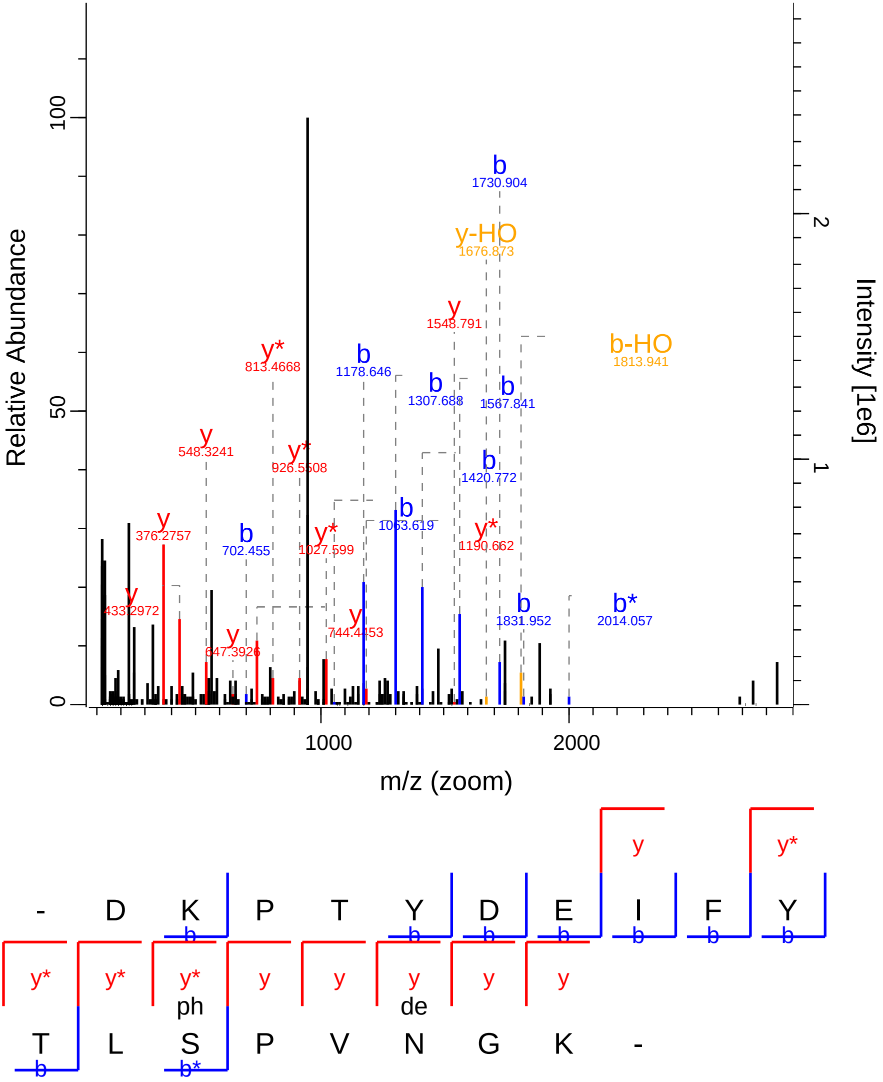

Raw File	Scan	Method	Score	m/z	Gene names
RO-QC-160121_ZackD_SS_Set_01_Fractio	34430	FTMS; HC	132.47	1236.08	PA2G4

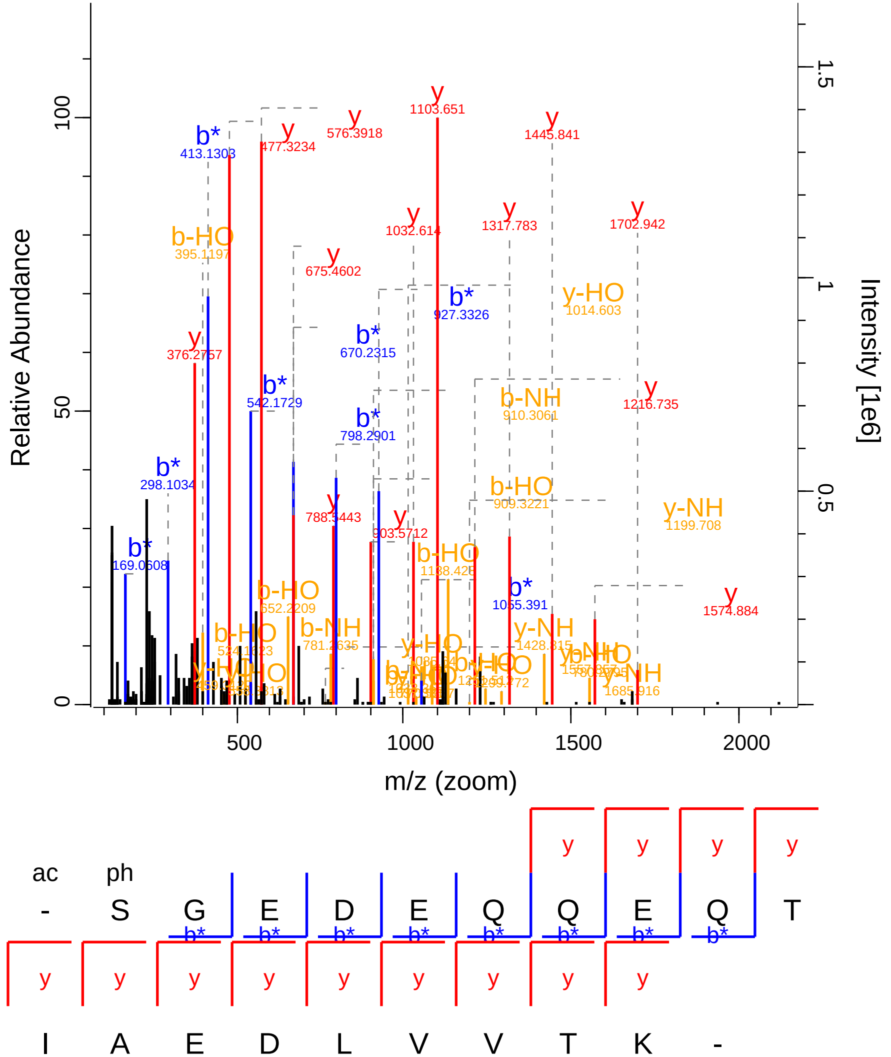

Raw File	Scan	Method	Score	m/z	Gene names
RO-QC-160121_ZackD_SS_Set_01_Frac	34652	FTMS; HC	49.31	994.02	FAM120A;C9orf129

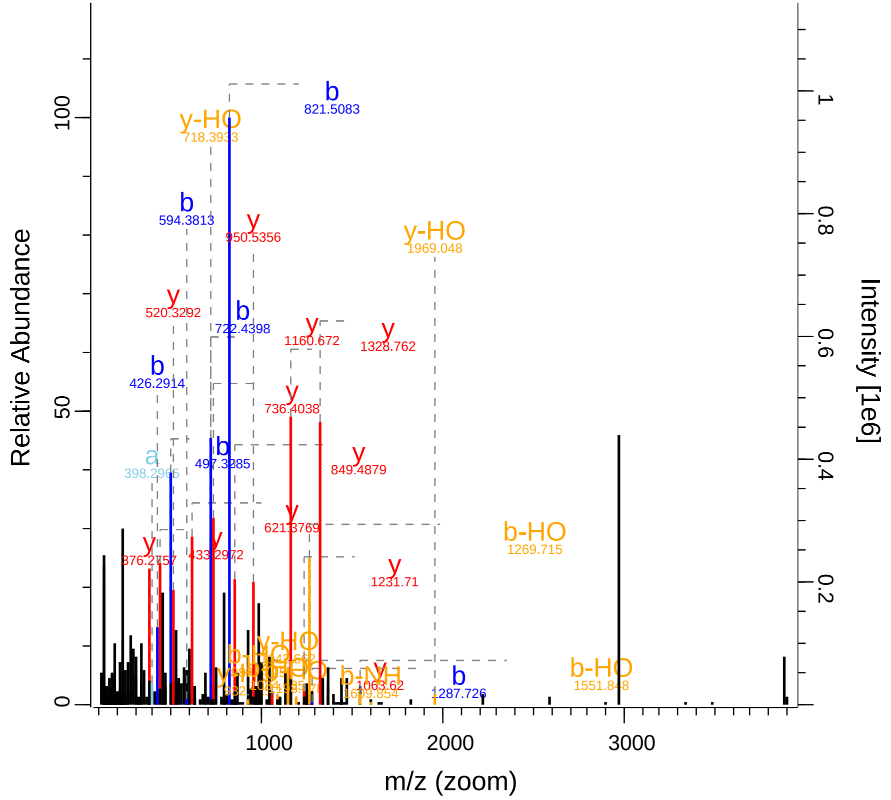

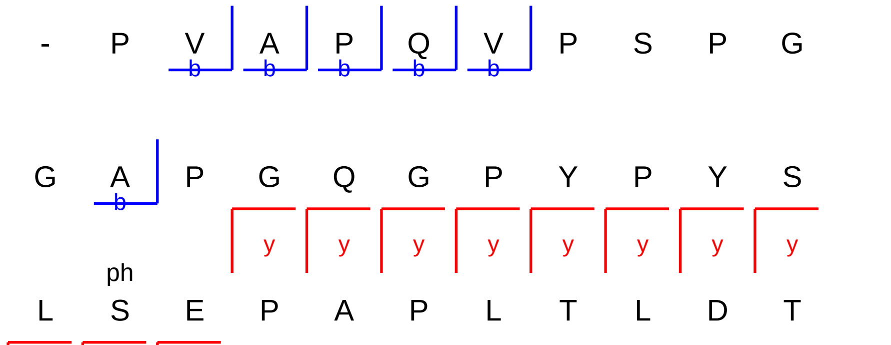

Raw File	Scan	Method	Score	m/z	Gene names
RO-QC-160121_ZackD_SS_Set_01_Fractio	35303	FTMS; HC	47.55	779.08	VAV1

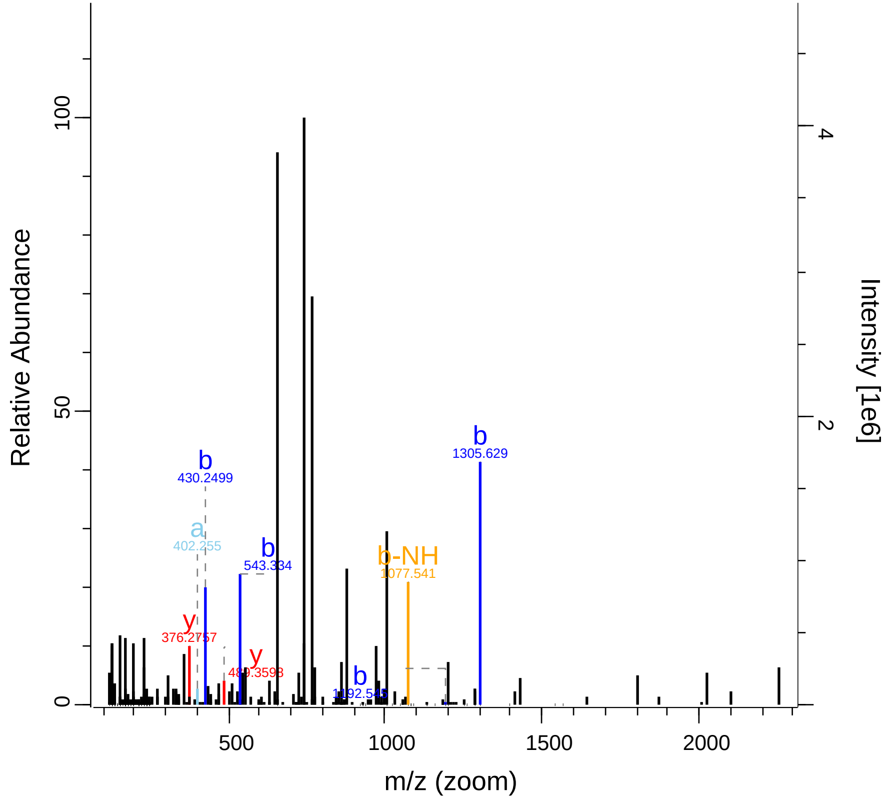

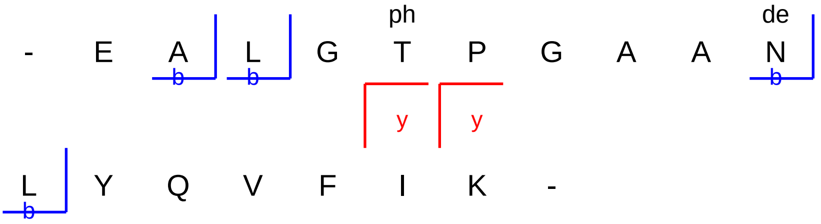

Raw File	Scan	Method	Score	m/z	Gene names
RO-QC-160121_ZackD_SS_Set_01_Fractic	36034	FTMS; HC	82.85	1001.88	EIF3G

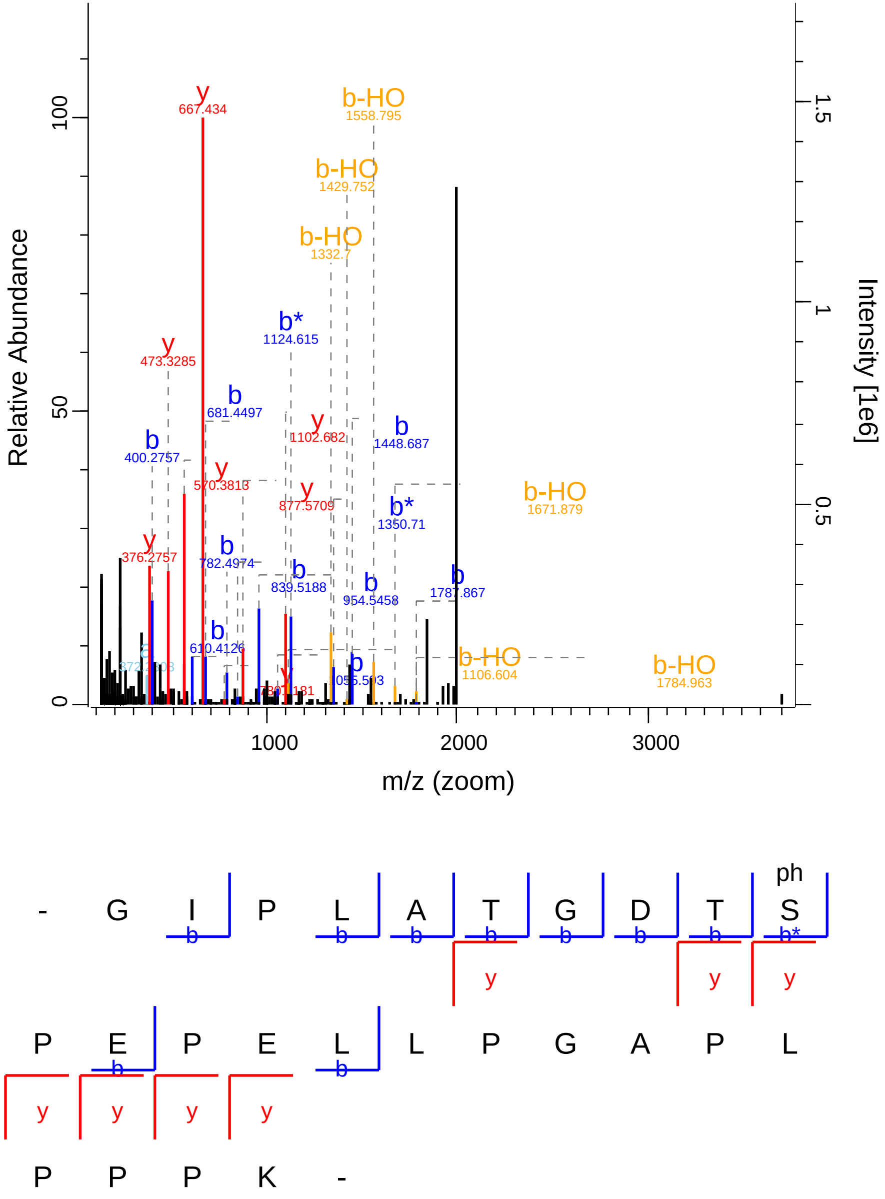

Raw File	Scan	Method	Score	m/z	Gene names
RO-QC-160121_ZackD_SS_Set_01_Fractio	36767	FTMS; HC	262.77	1048.5	SLK

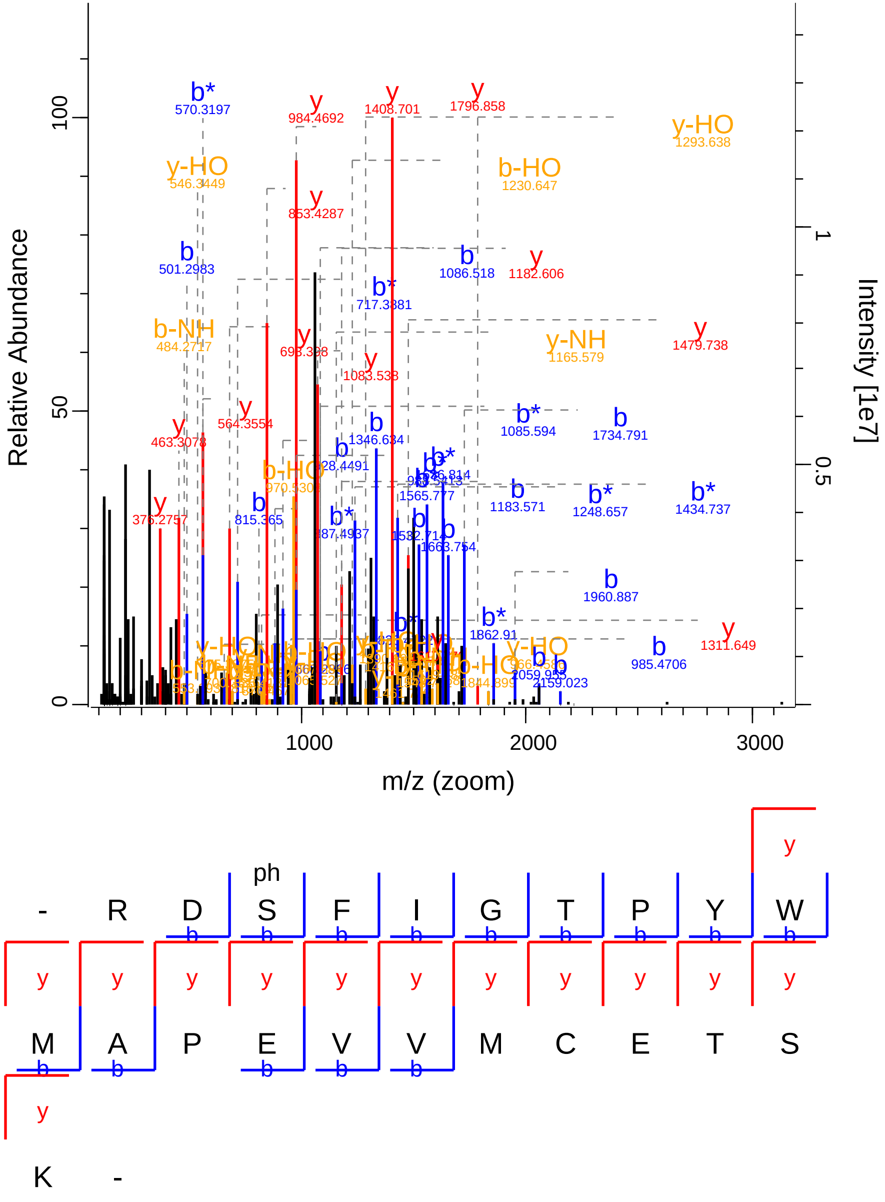

Raw File	Scan	Method	Score	m/z	Gene names
RO-QC-160121_ZackD_SS_Set_01_Fractioı	36827	FTMS; HCI	148.04	865.7	PRKCB

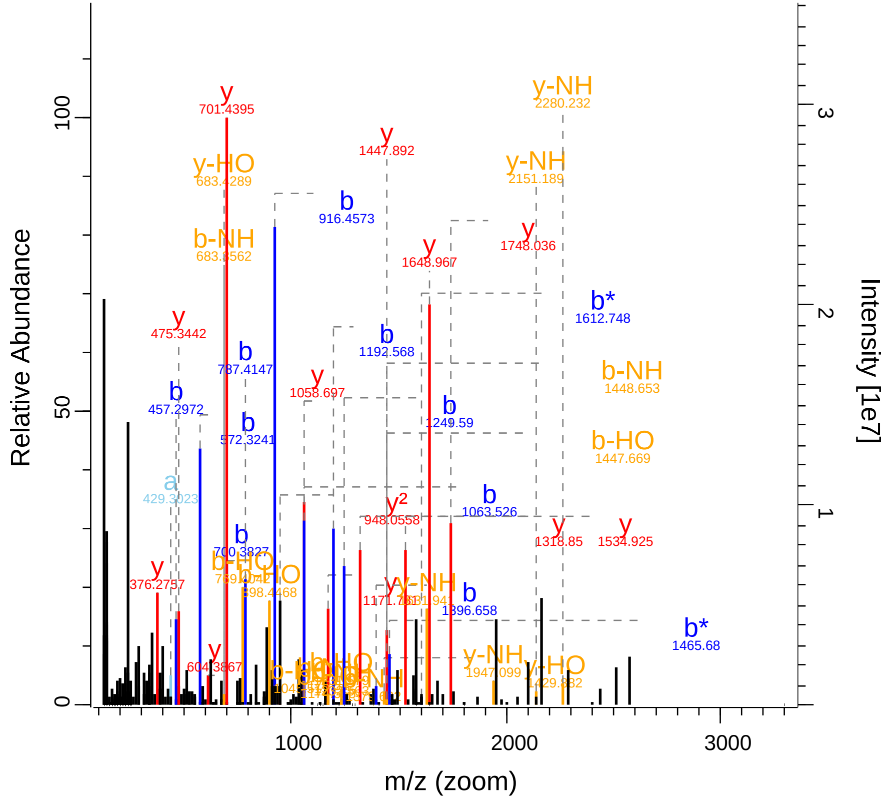

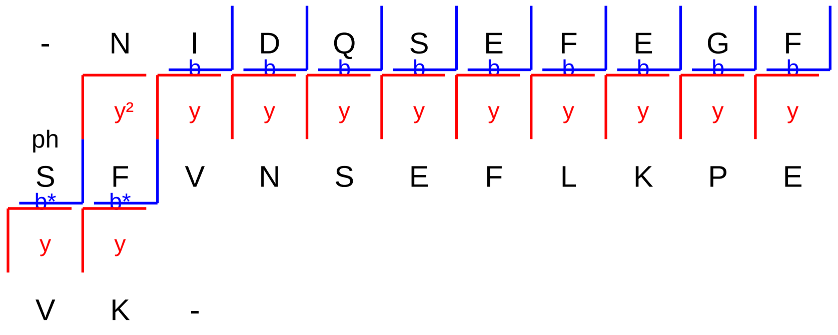

Raw File	Scan	Method	Score	m/z	Gene names
RO-QC-160121_ZackD_SS_Set_01_Fractic	36842	FTMS; HC	53.4	1323.69	SNCAIP

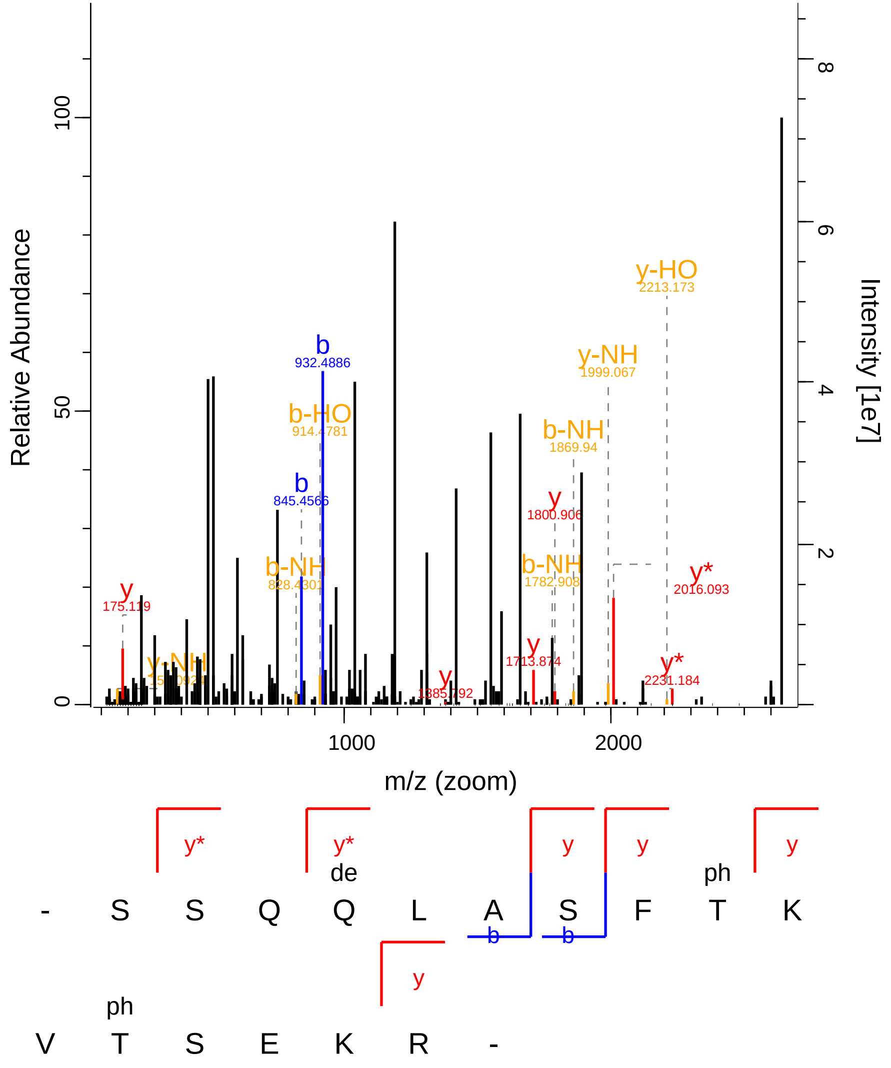

Raw File	Scan	Method	Score	m/z	Gene names
RO-QC-160121_ZackD_SS_Set_01_Fractio	37034	FTMS; HC	220.28	907.47	PRKCD

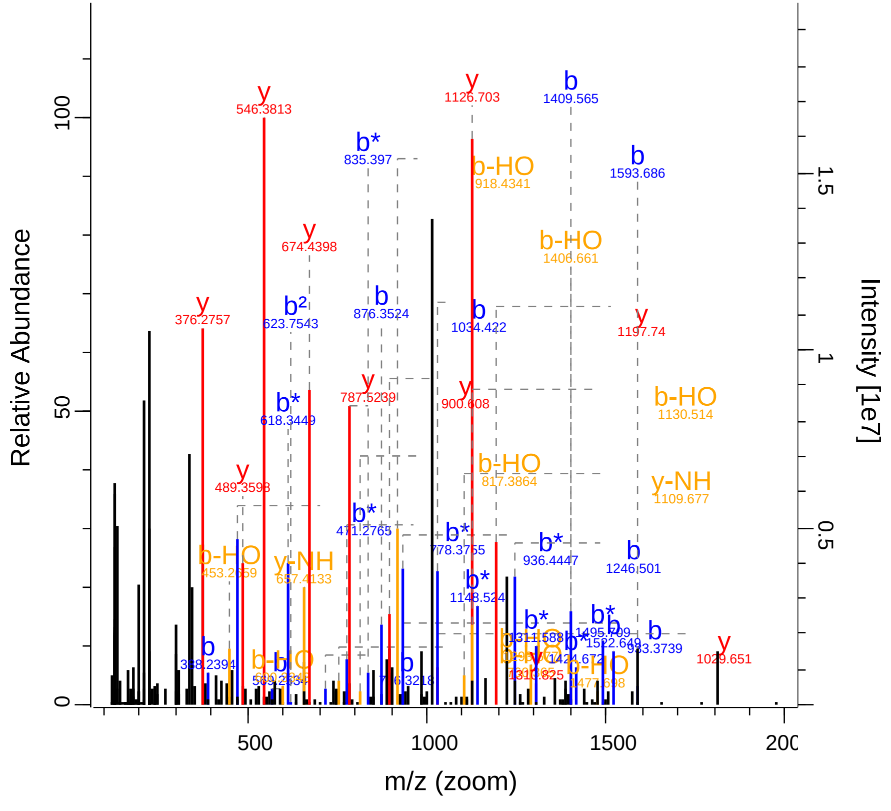

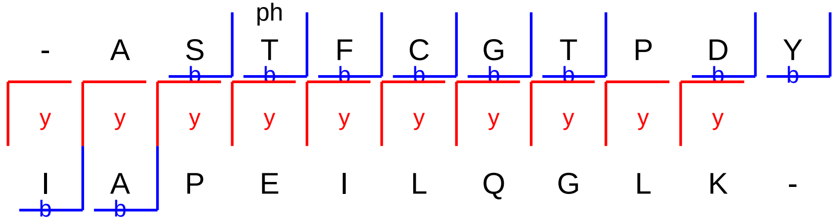

Raw File	Scan	Method	Score	m/z	Gene names
RO-QC-160121_ZackD_SS_Set_01_Fractio	37088	FTMS; HC	66.53	545.97	SMAD2

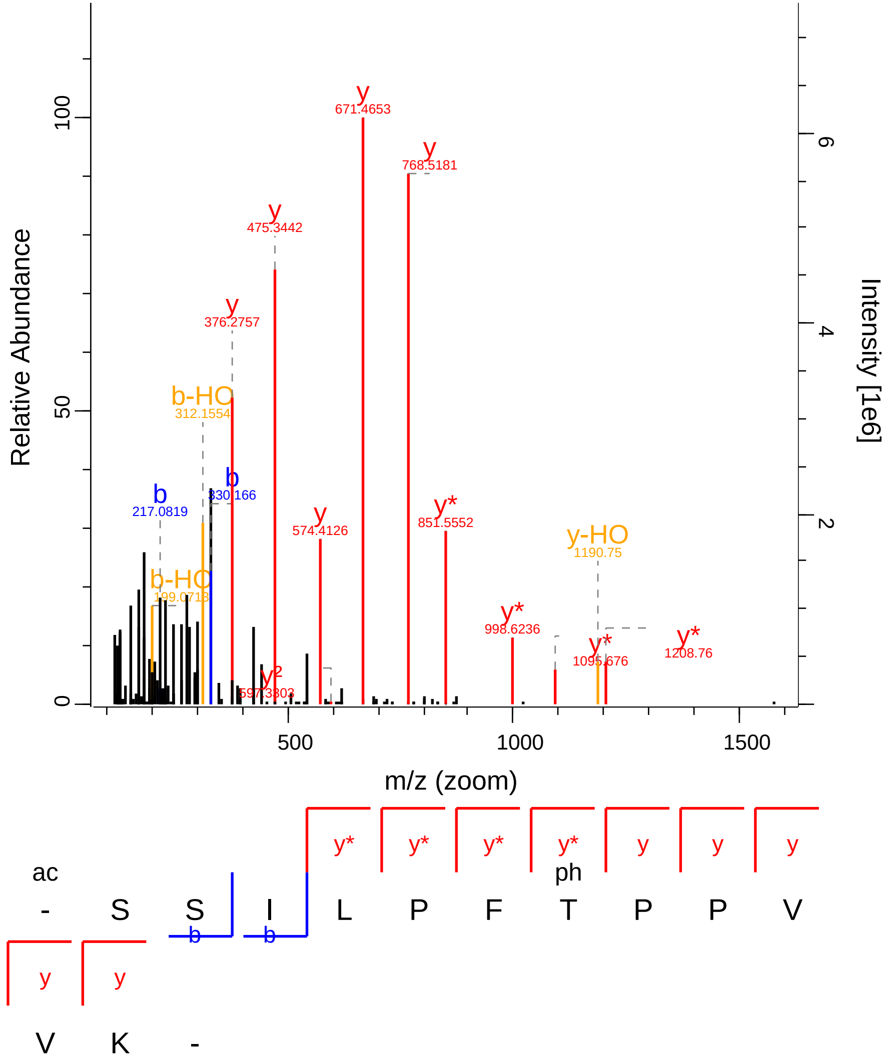

Raw File	Scan	Method	Score	m/z	Gene names
RO-QC-160121_ZackD_SS_Set_01_Fractic	37102	FTMS; HC	46.16	1097.57	HHIP

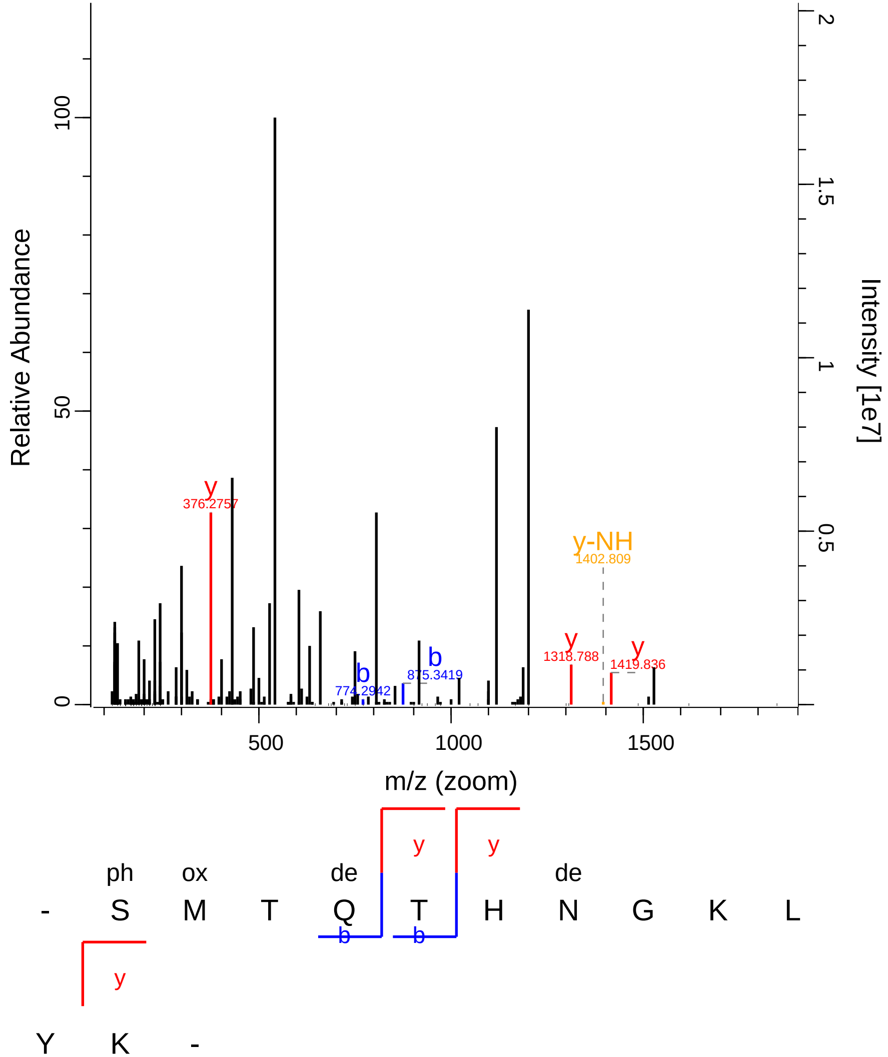

Raw File	Scan	Method	Score	m/z	Gene names
RO-QC-160121_ZackD_SS_Set_01_Fractic	37449	FTMS; HC	62.67	1140.86	PSMF1

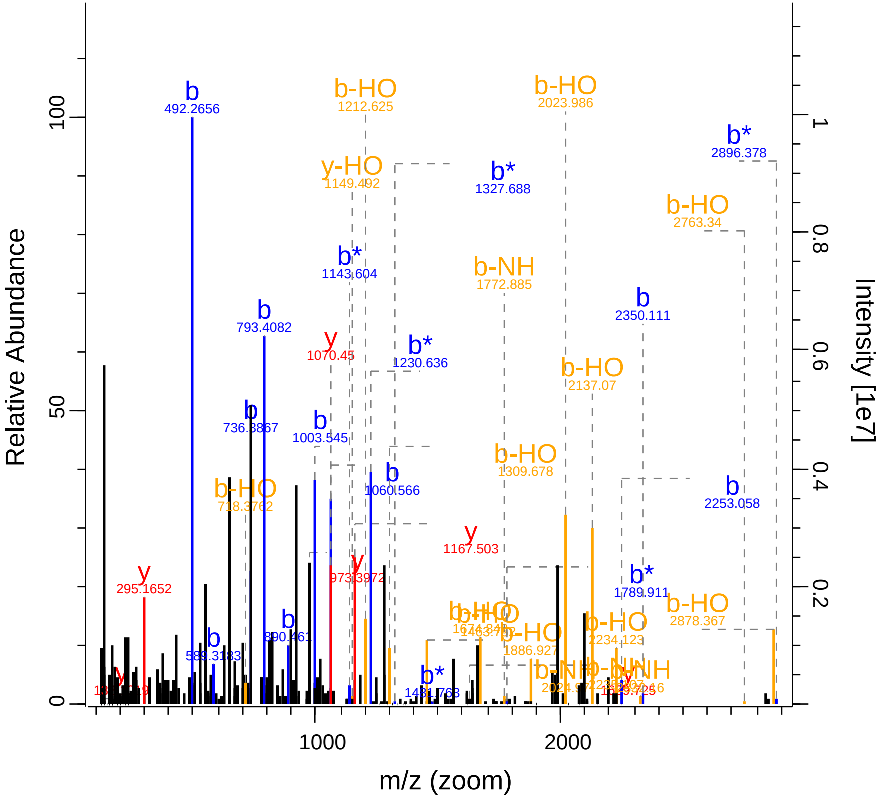

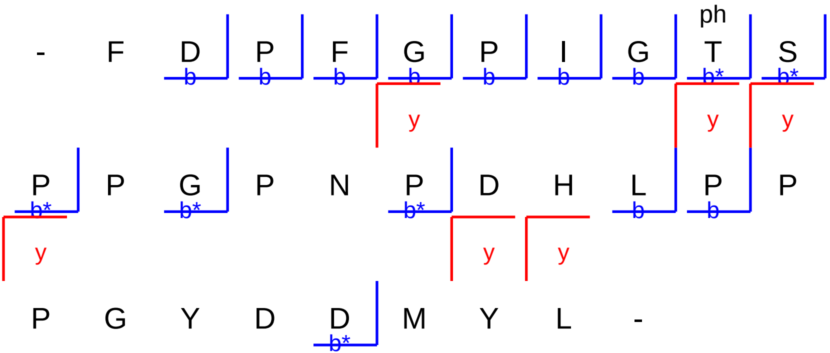

Raw File	Scan	Method	Score	m/z	Gene names
RO-QC-160121_ZackD_SS_Set_01_Fractio	37585	FTMS; HC	68.89	853.95	SHROOM3

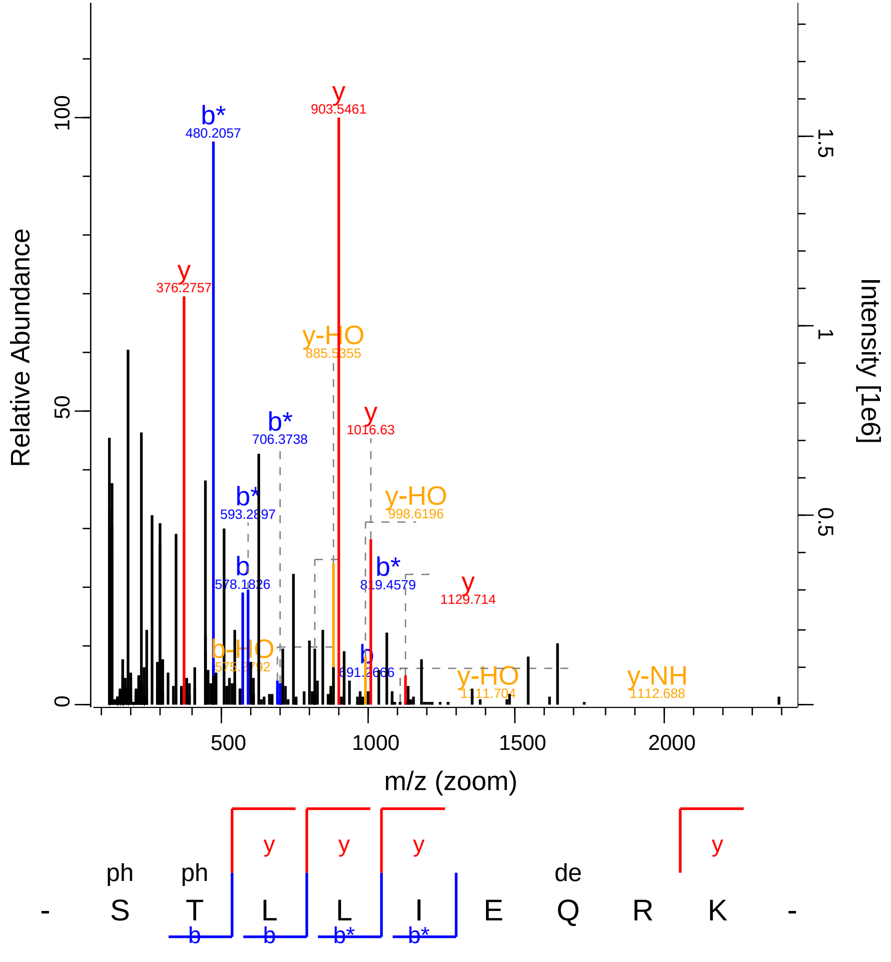

Raw File	Scan	Method	Score	m/z	Gene names
RO-QC-160121_ZackD_SS_Set_01_Fractio	38148	FTMS; HC	41.87	872.14	LRRK2

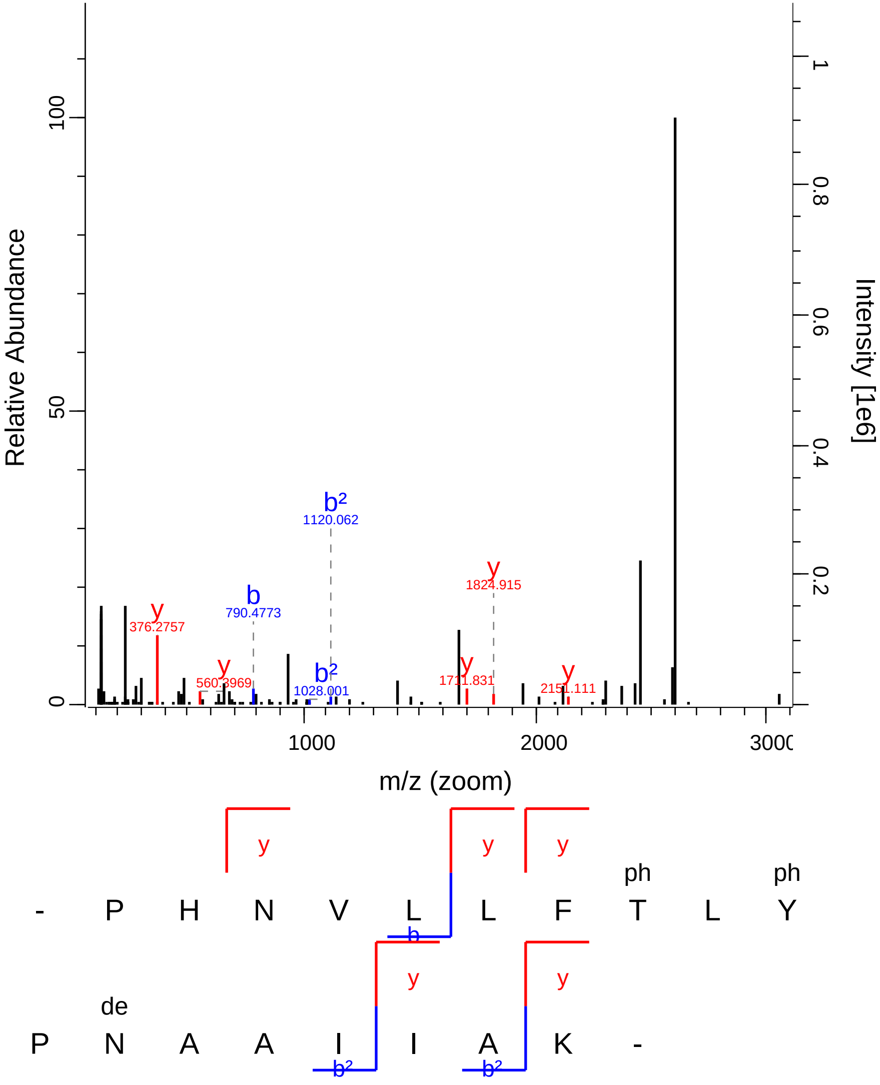

Raw File	Scan	Method	Score	m/z	Gene names
RO-QC-160121_ZackD_SS_Set_01_Fractior	4897	FTMS; HCI	74.77	428.24	SCLY

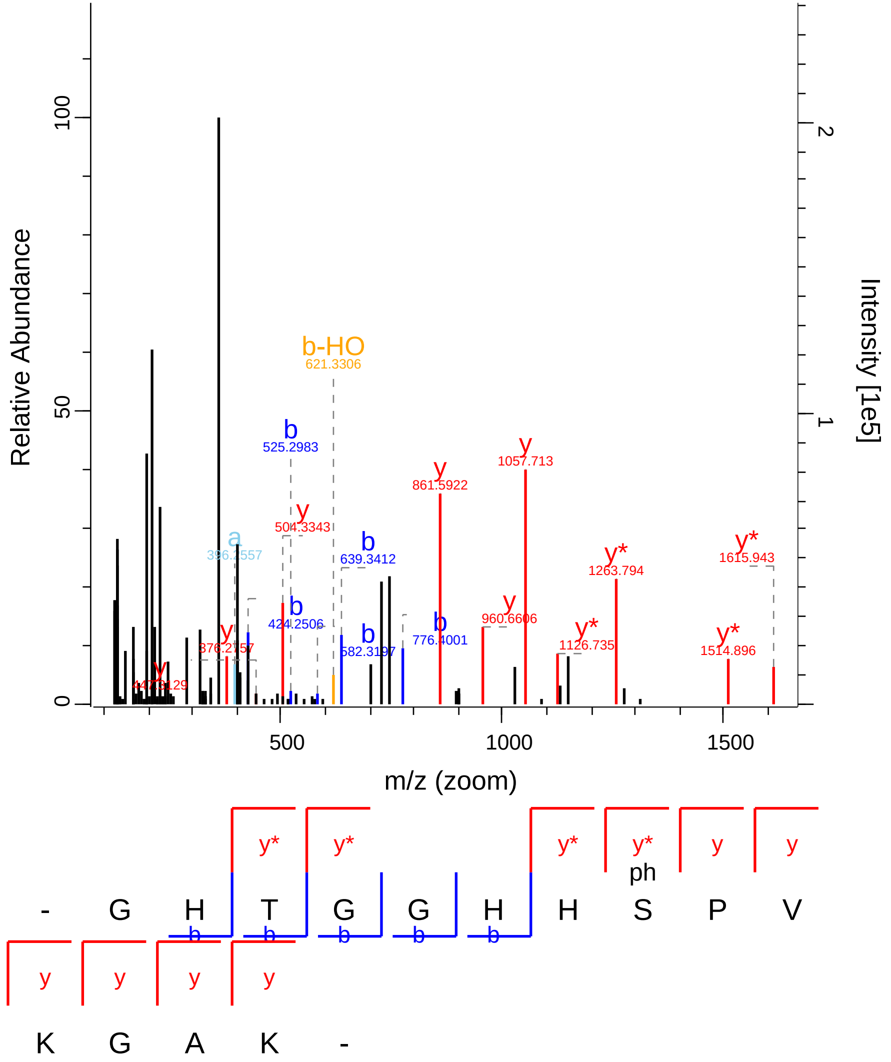

Raw File	Scan	Method	Score	m/z	Gene names
RO-QC-160121_ZackD_SS_Set_01_Fractio[illegible]	7694	FTMS; HC	150.09	736.86	SORBS2

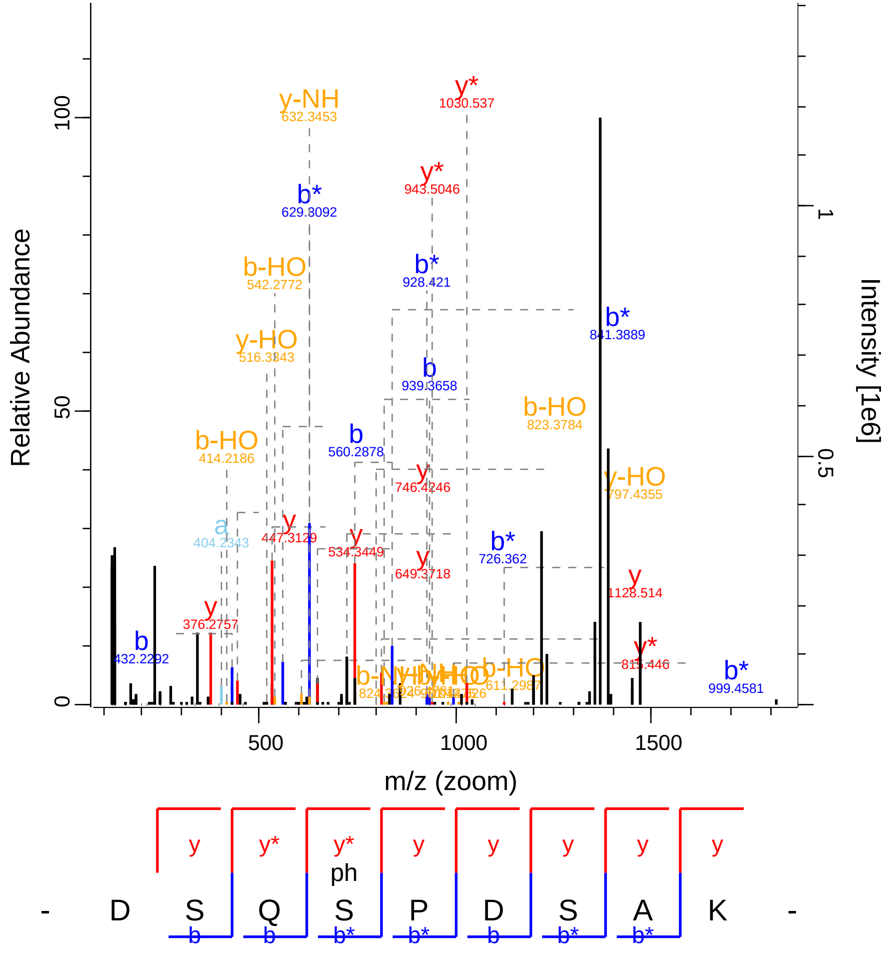

Raw File	Scan	Method	Score	m/z	Gene names
RO-QC-160121_ZackD_SS_Set_01_Fractior	8336	FTMS; HCI	86.62	910.97	KAT6A

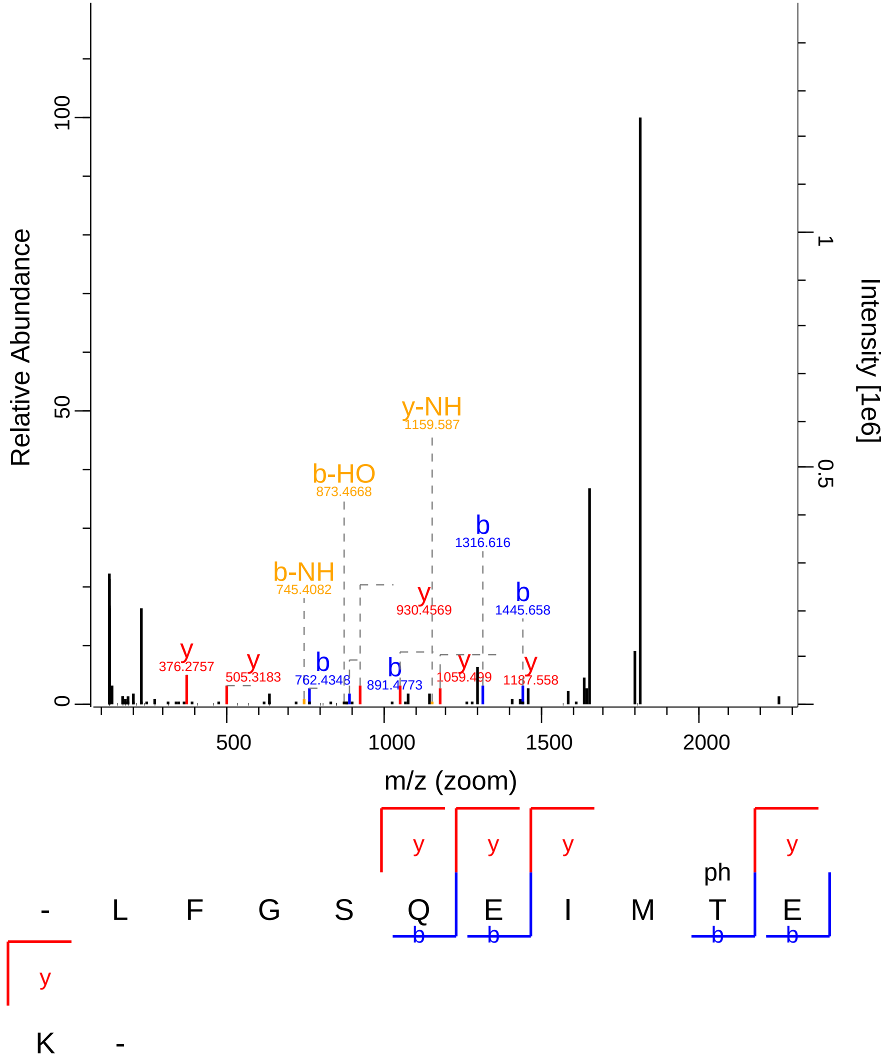

Raw File	Scan	Method	Score	m/z	Gene names
RO-QC-160121_ZackD_SS_Set_01_Fractior	8590	FTMS; HCI	80.16	929.44	LDB1

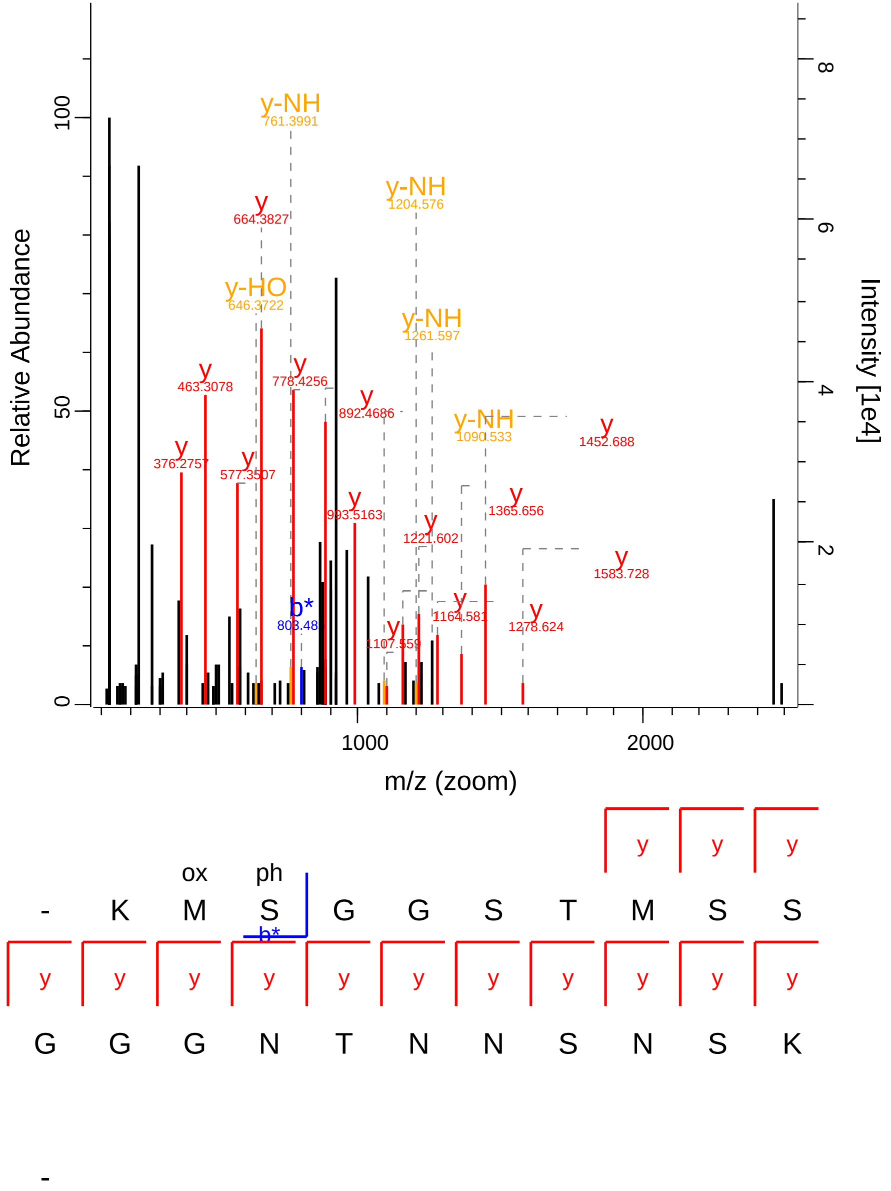

Raw File	Scan	Method	Score	m/z	Gene names
RO-QC-160121_ZackD_SS_Set_01_Fractio	10268	FTMS; HC	55.68	352.46	SDF2L1

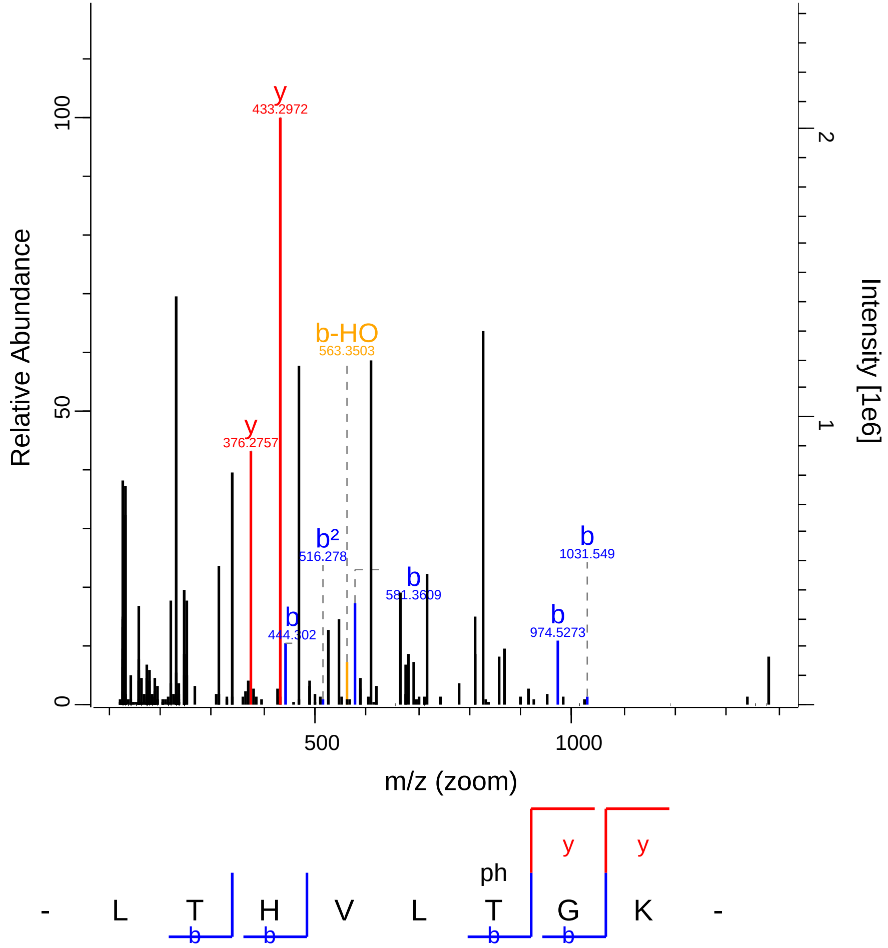

Raw File	Scan	Method	Score	m/z
RO-QC-160121_ZackD_SS_Set_01_Fraction_3	10509	FTMS; HCD	43.5	540.29

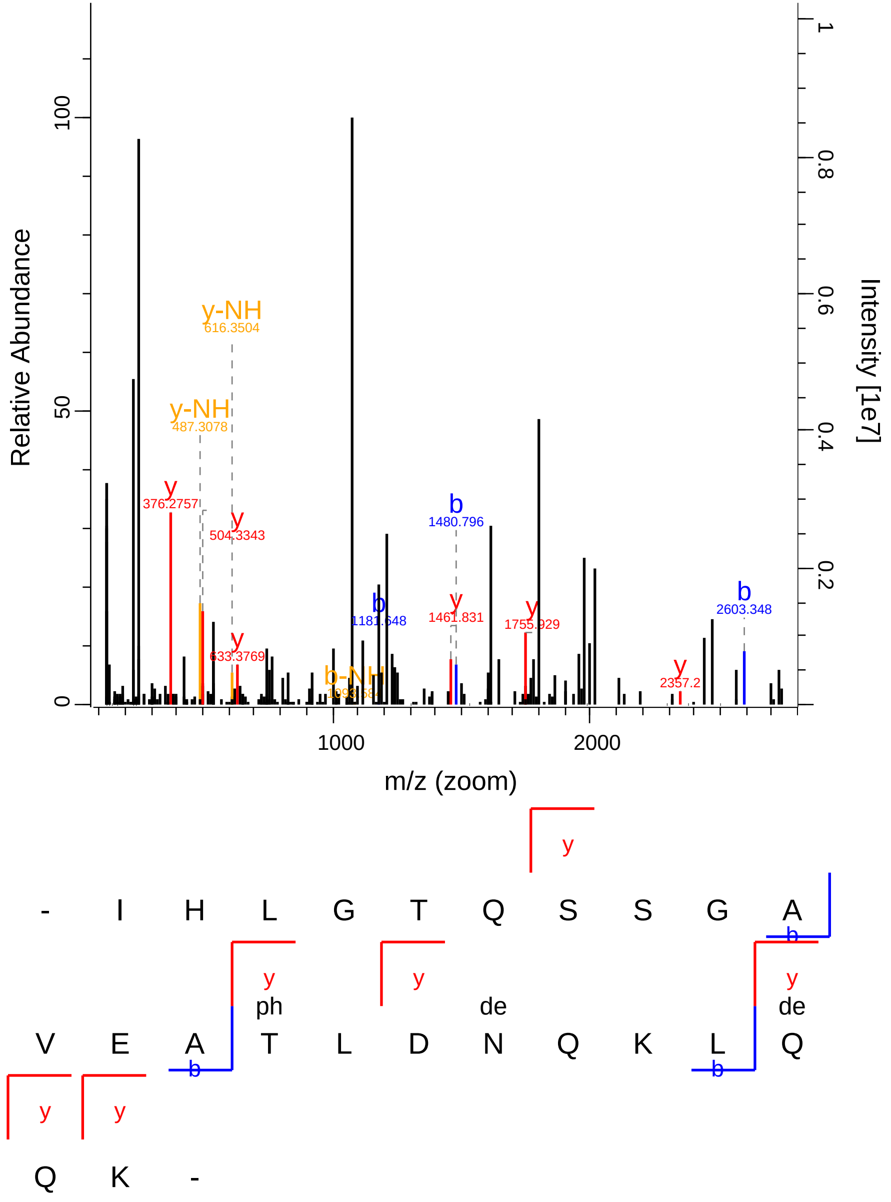

Raw File	Scan	Method	Score	m/z	Gene names
RO-QC-160121_ZackD_SS_Set_01_Fractio	10679	FTMS; HC	93.26	469.92	EFHC2

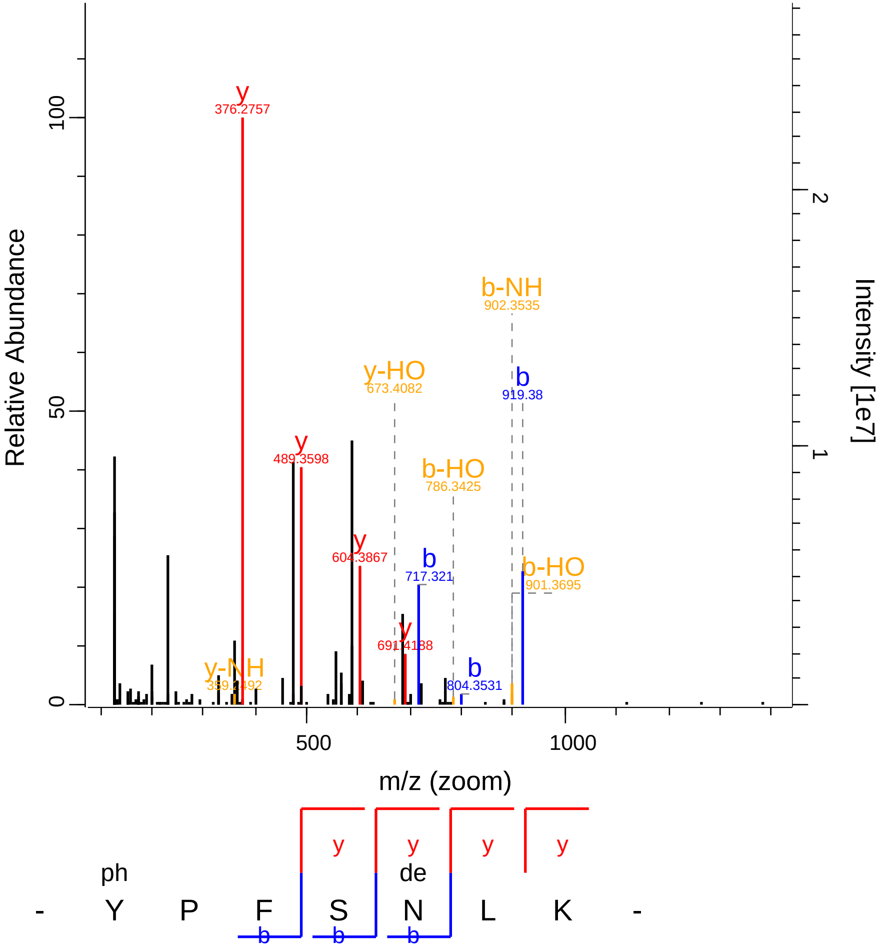

Raw File	Scan	Method	Score	m/z	Gene names
RO-QC-160121_ZackD_SS_Set_01_Fractio	11022	FTMS; HC	188	701.99	DSG2

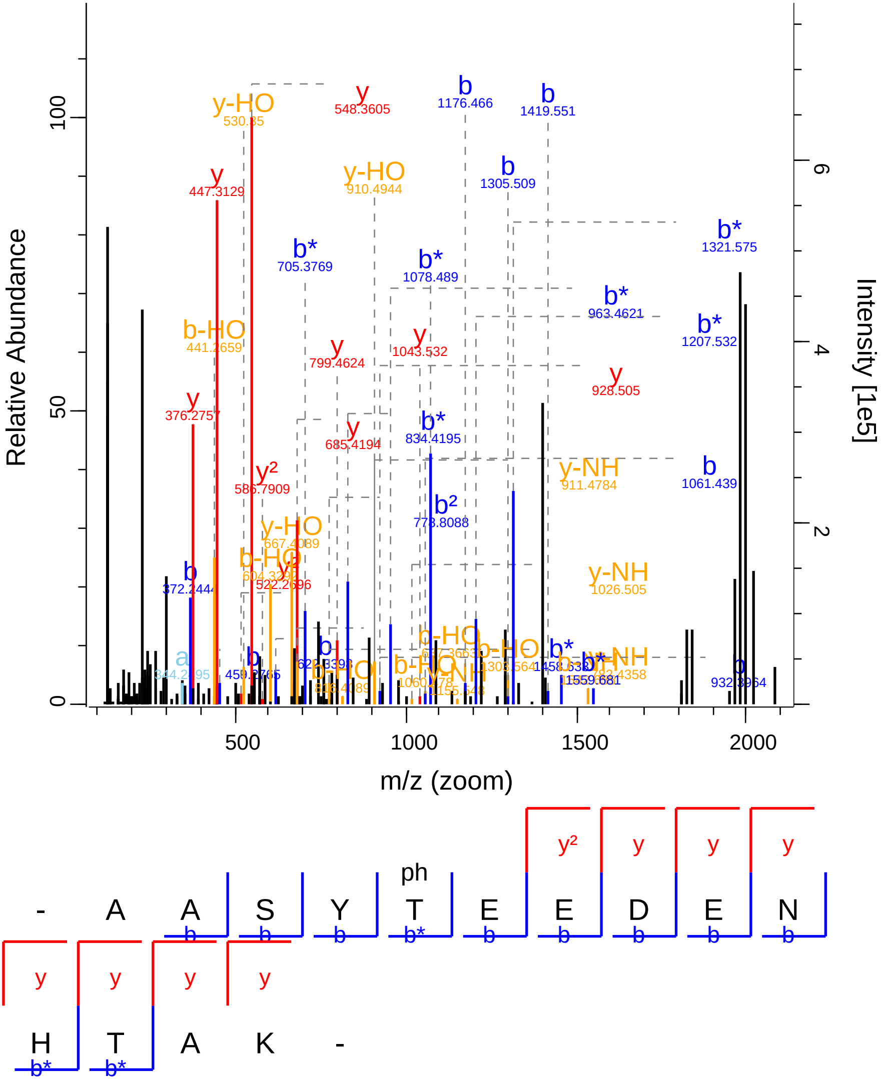

Raw File	Scan	Method	Score	m/z	Gene names
RO-QC-160121_ZackD_SS_Set_01_Fractio	11090	FTMS; HC	104.2	707.38	PRRC2A

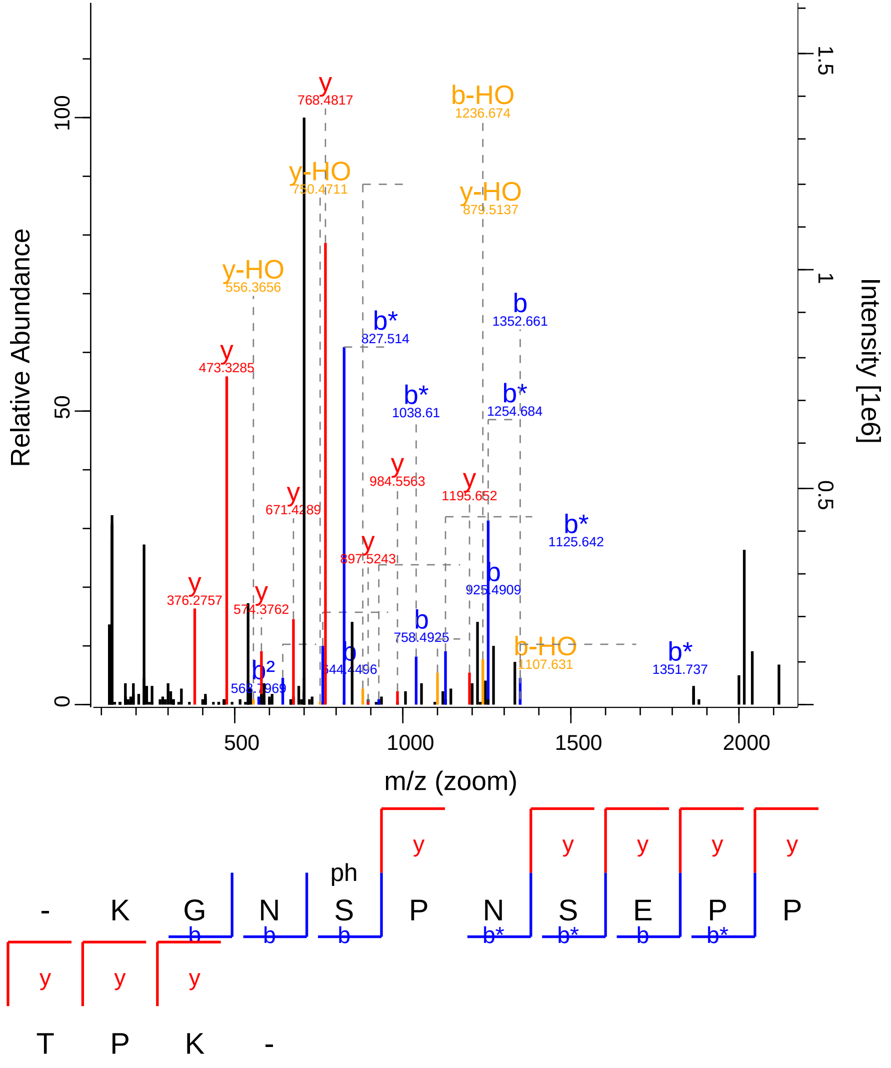

Raw File	Scan	Method	Score	m/z	Gene names
RO-QC-160121_ZackD_SS_Set_01_Fractio	12028	FTMS; HC	171.26	919.95	MAP7D1

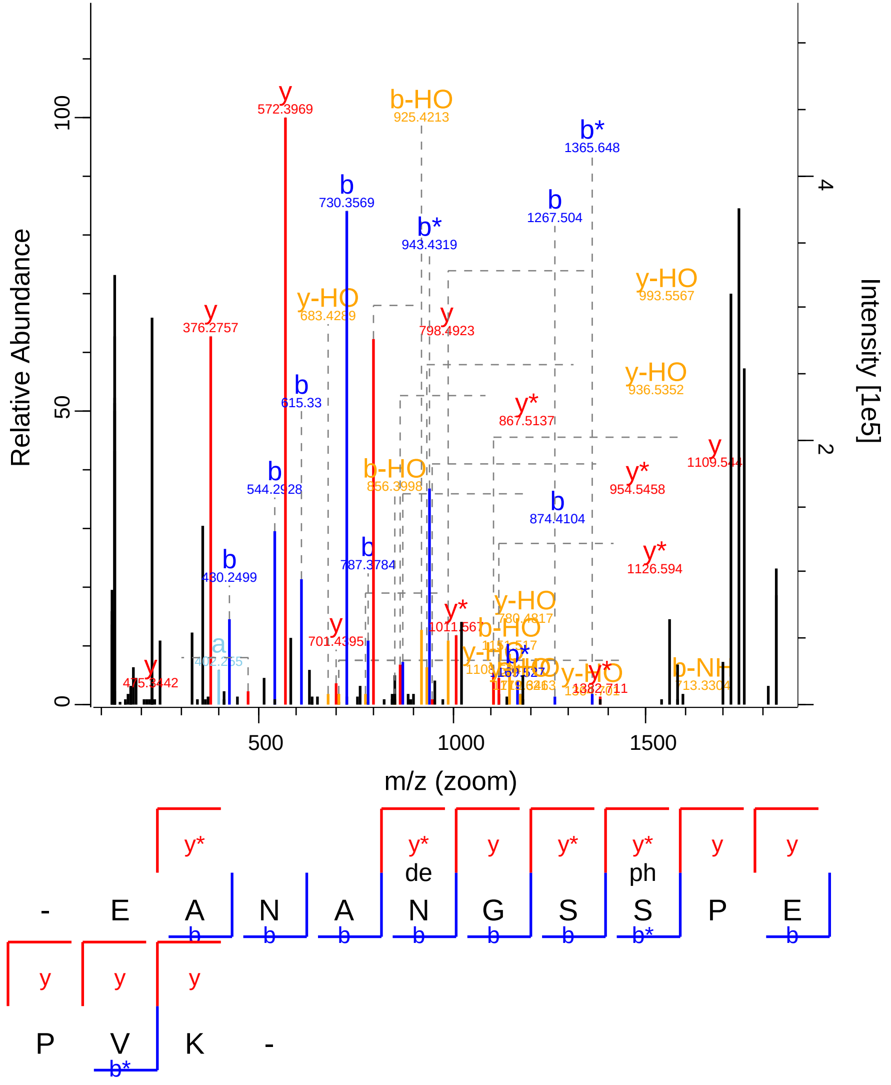

Raw File	Scan	Method	Score	m/z	Gene names
RO-QC-160121_ZackD_SS_Set_01_Fractio	12178	FTMS; HC	95.95	749.09	APC

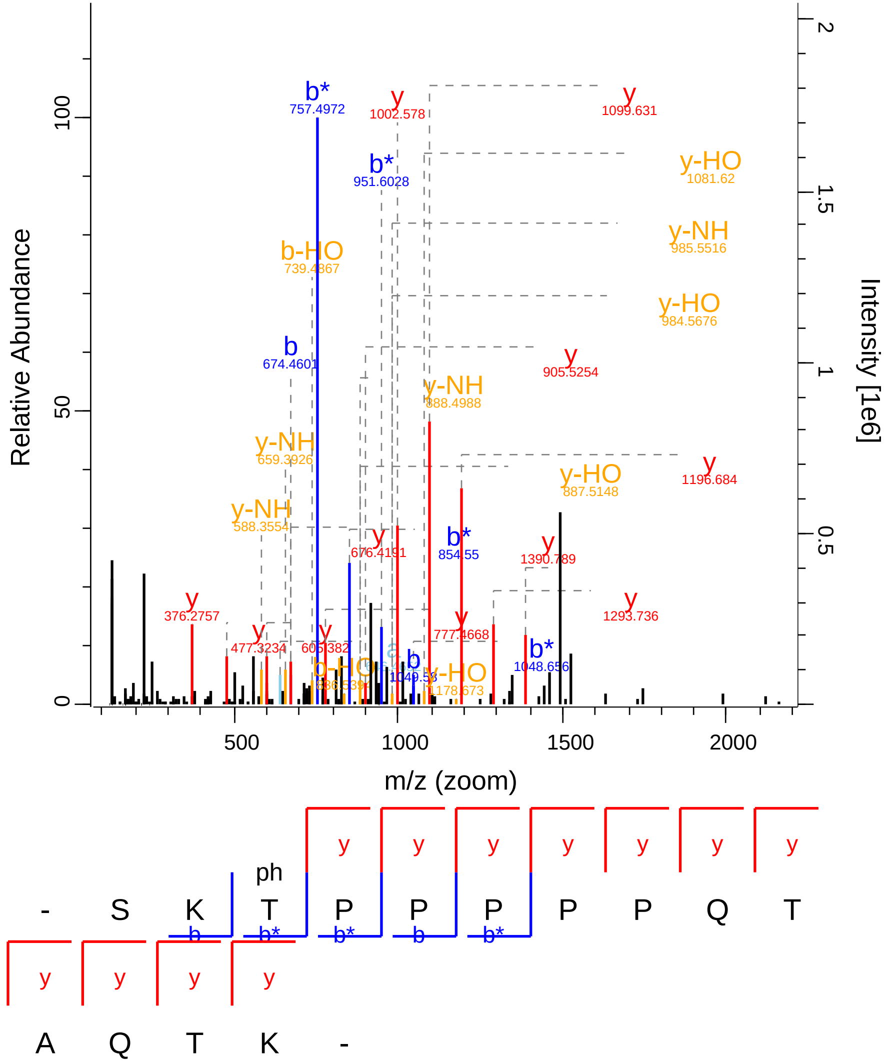

Raw File	Scan	Method	Score	m/z	Gene names
RO-QC-160121_ZackD_SS_Set_01_Fractio	13061	FTMS; HC	83.4	661.26	PDHA1;PDHA

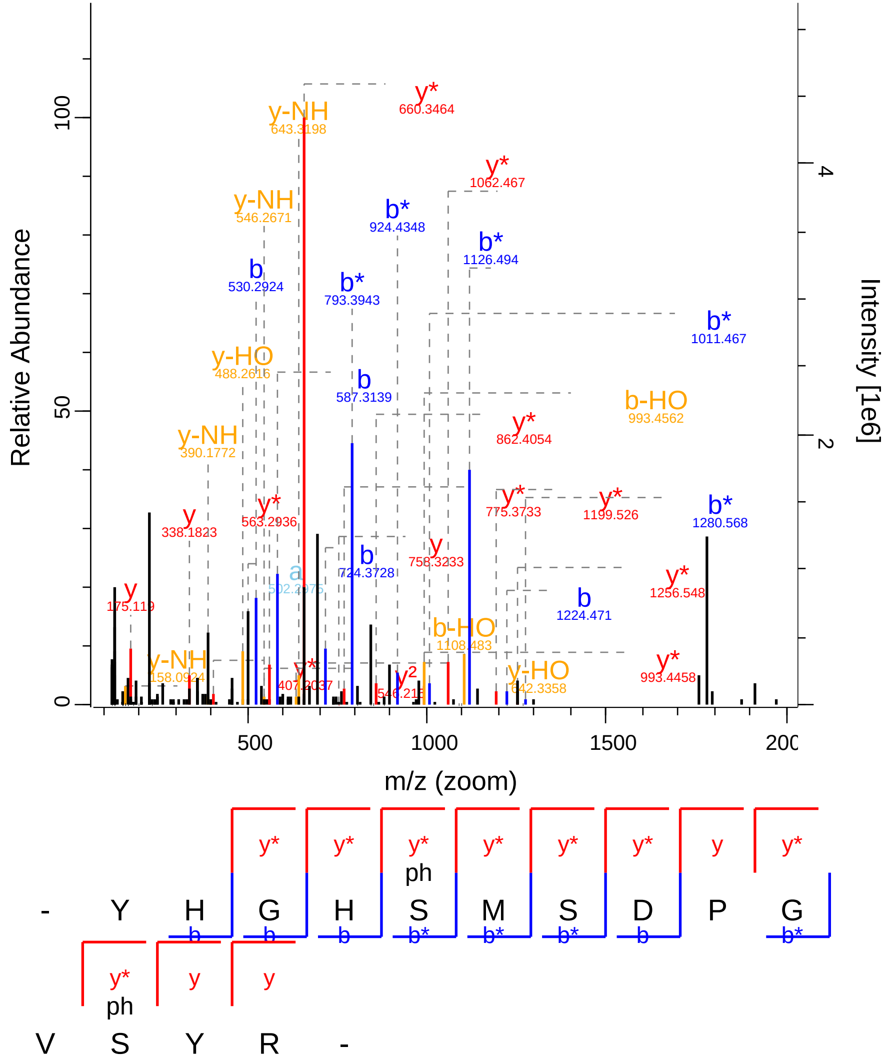

Raw File	Scan	Method	Score	m/z	Gene names
RO-QC-160121_ZackD_SS_Set_01_Fractio	13309	FTMS; HC	61.38	600.62	CCHCR1

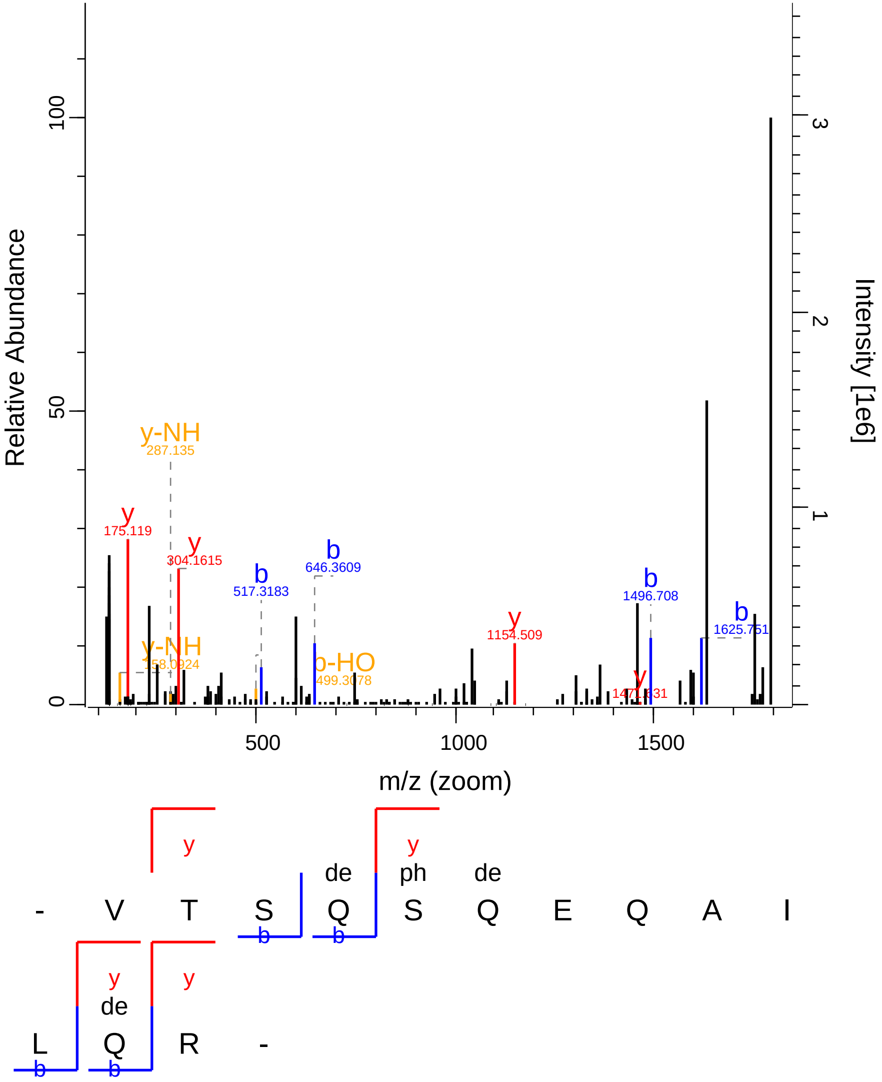

Raw File	Scan	Method	Score	m/z	Gene names
RO-QC-160121_ZackD_SS_Set_01_Fractio	13336	FTMS; HC	124.42	915.95	PRPF38B

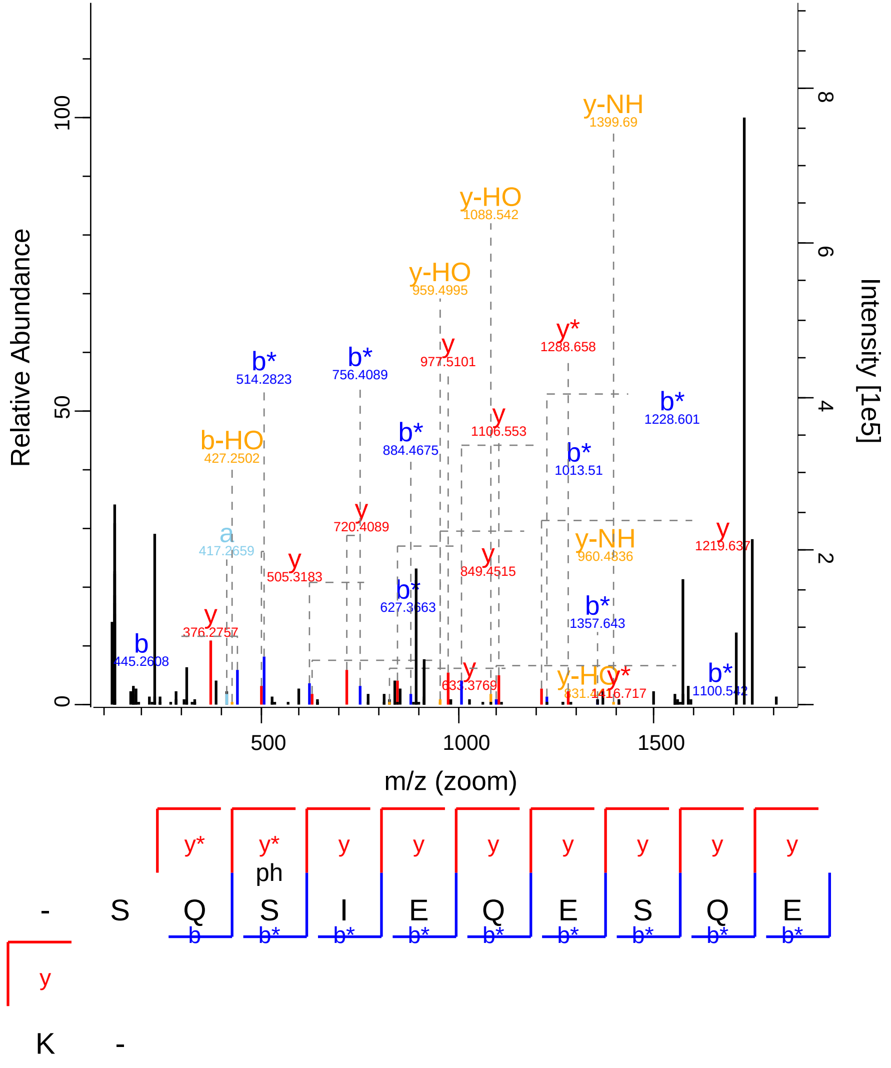

Raw File	Scan	Method	Score	m/z	Gene names
RO-QC-160121_ZackD_SS_Set_01_Fractio	13597	FTMS; HC	129.03	898.49	CDK12

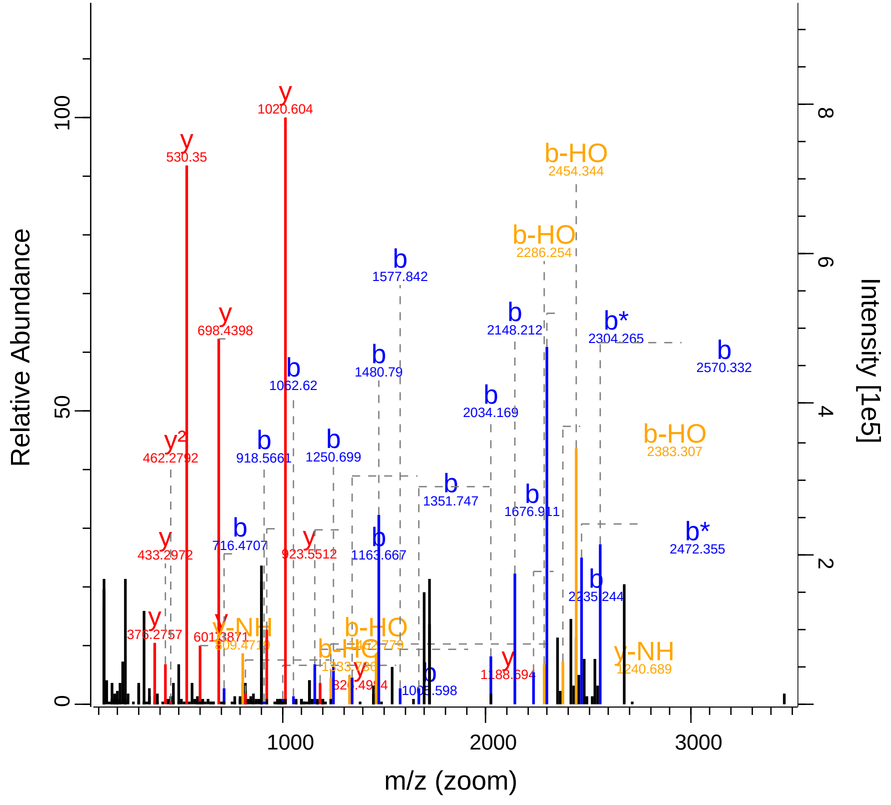

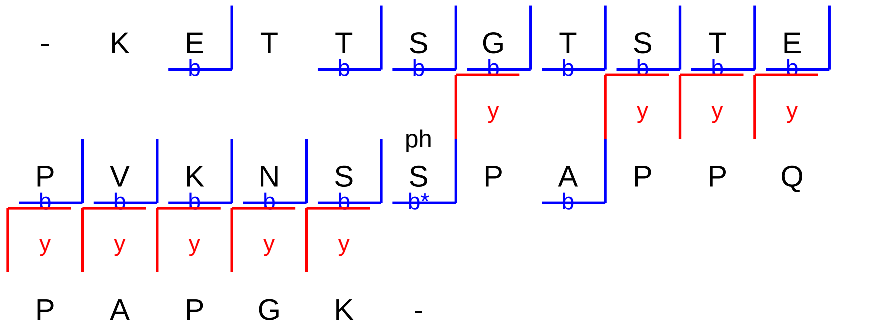

Raw File	Scan	Method	Score	m/z	Gene names
RO-QC-160121_ZackD_SS_Set_01_Fractio	13642	FTMS; HC	51.64	601.34	MAP1B

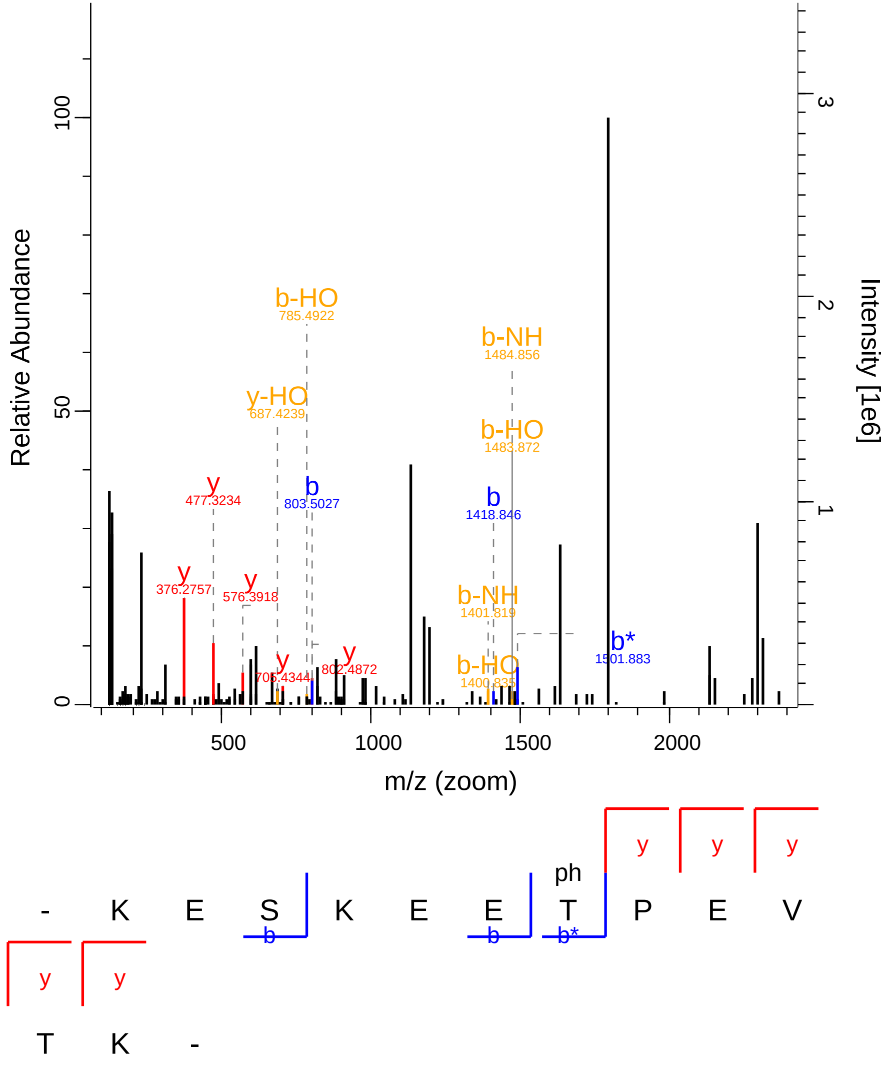

Raw File	Scan	Method	Score	m/z	Gene names
RO-QC-160121_ZackD_SS_Set_01_Fractio	13830	FTMS; HC	53.17	587.99	EPB41L1

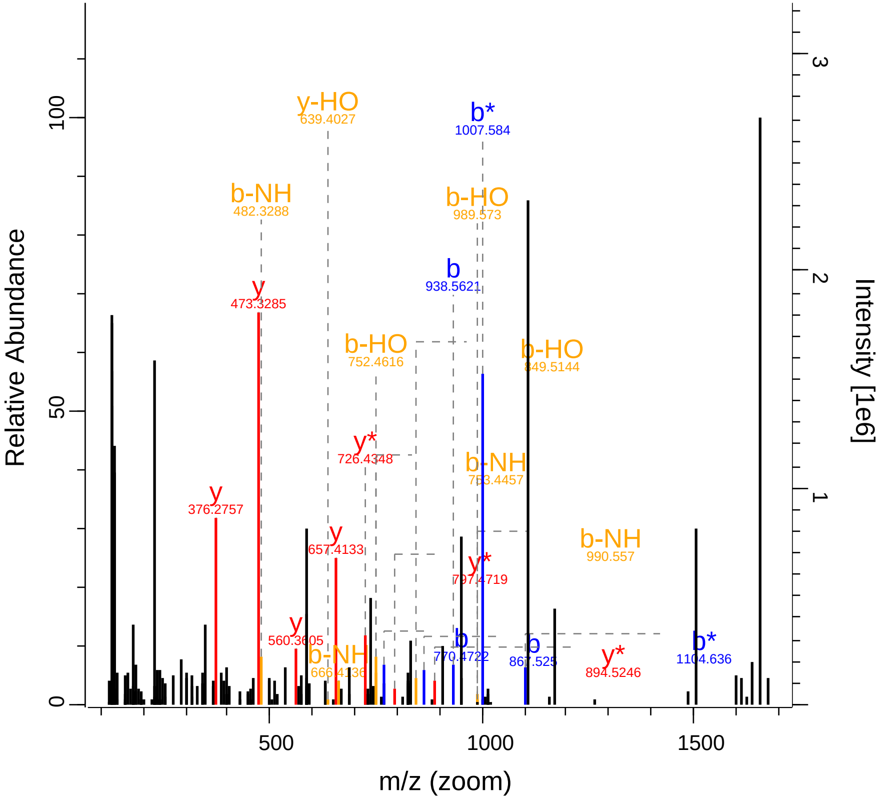

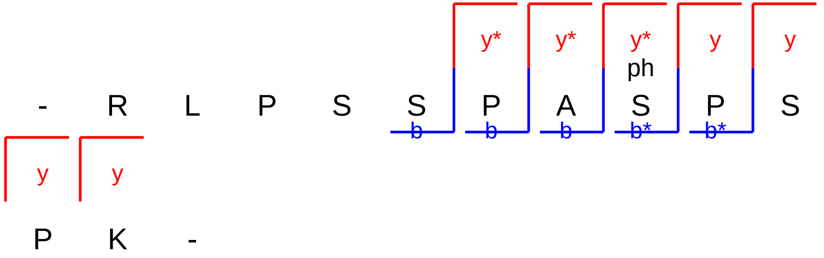

Raw File	Scan	Method	Score	m/z	Gene names
RO-QC-160121_ZackD_SS_Set_01_Fractio	15046	FTMS; HC	40.35	849.88	SLC4A7

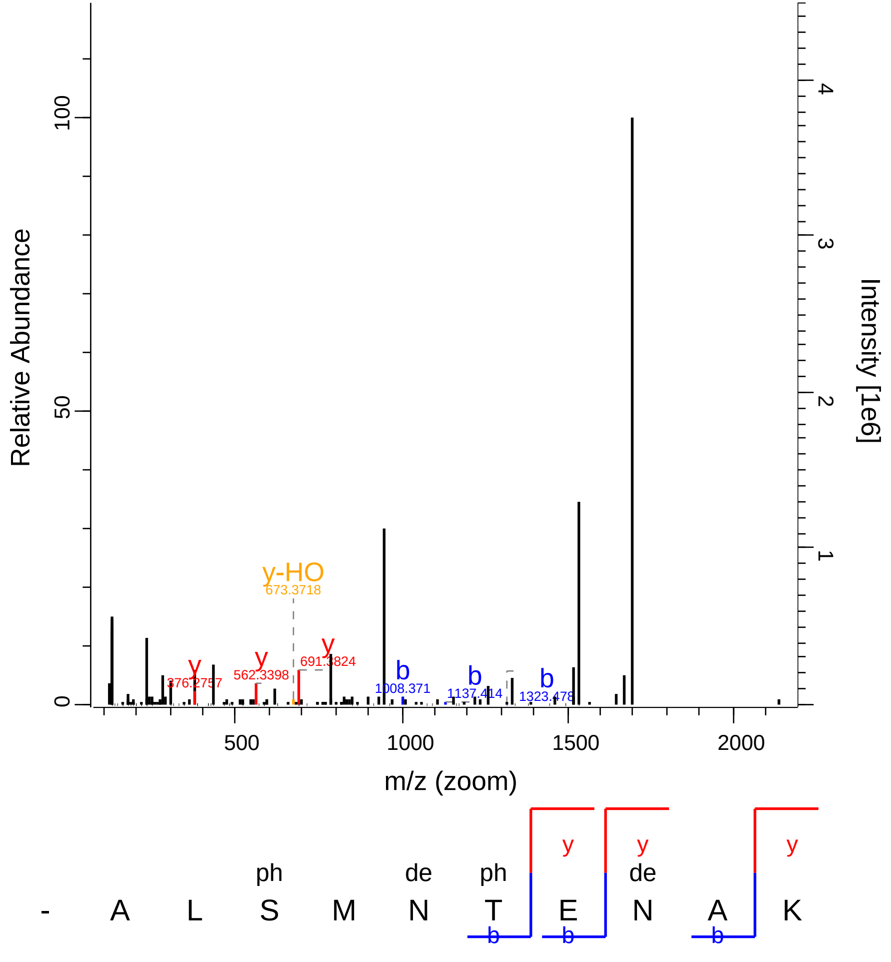

-

Raw File	Scan	Method	Score	m/z	Gene names
RO-QC-160121_ZackD_SS_Set_01_Fractior	15912	FTMS; HCI	145.83	617.8	MECP2

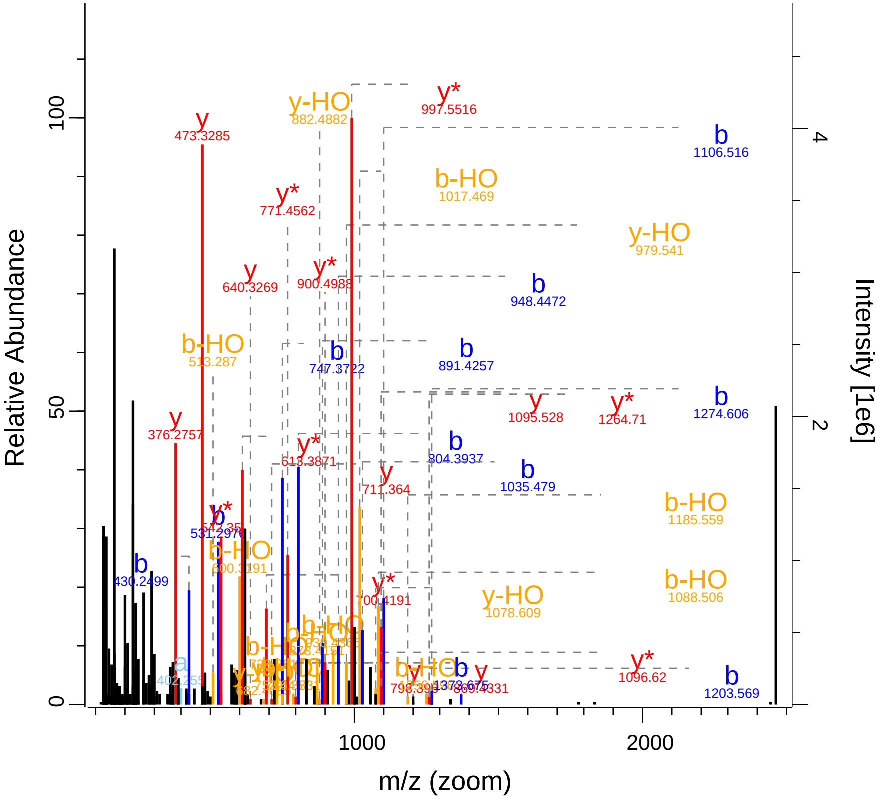

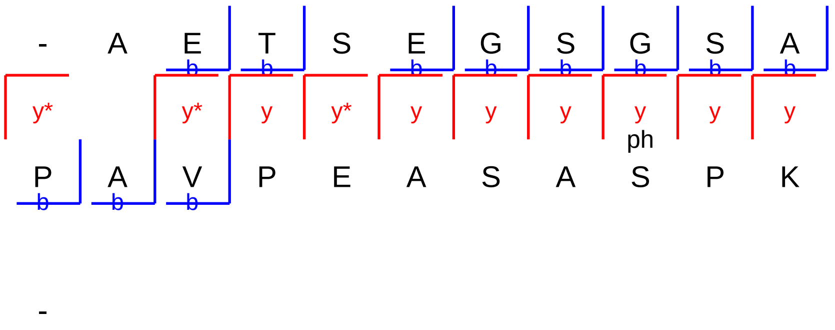

Raw File	Scan	Method	Score	m/z	Gene names
RO-QC-160121_ZackD_SS_Set_01_Fractior	16014	FTMS; HCI	100.97	799.7	PDS5B

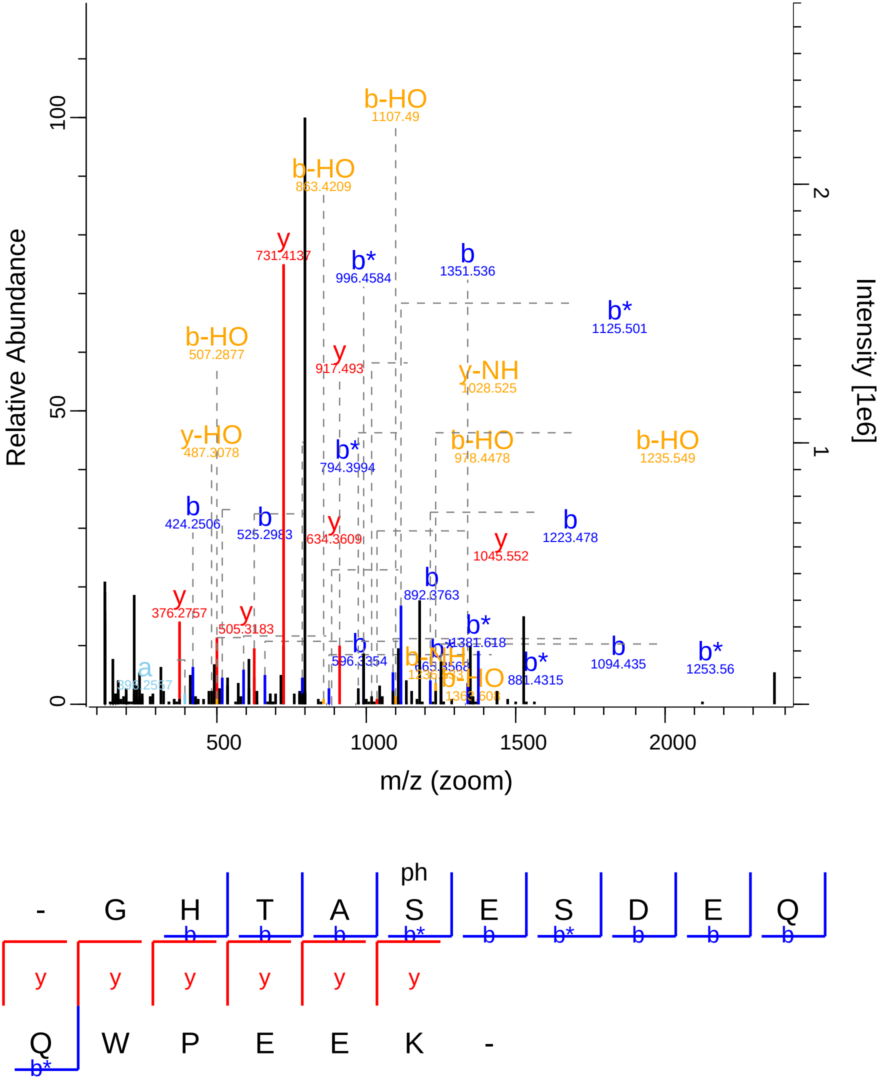

Raw File	Scan	Method	Score	m/z	Gene names
RO-QC-160121_ZackD_SS_Set_01_Fractio	16252	FTMS; HC	141.08	871.43	IRS2

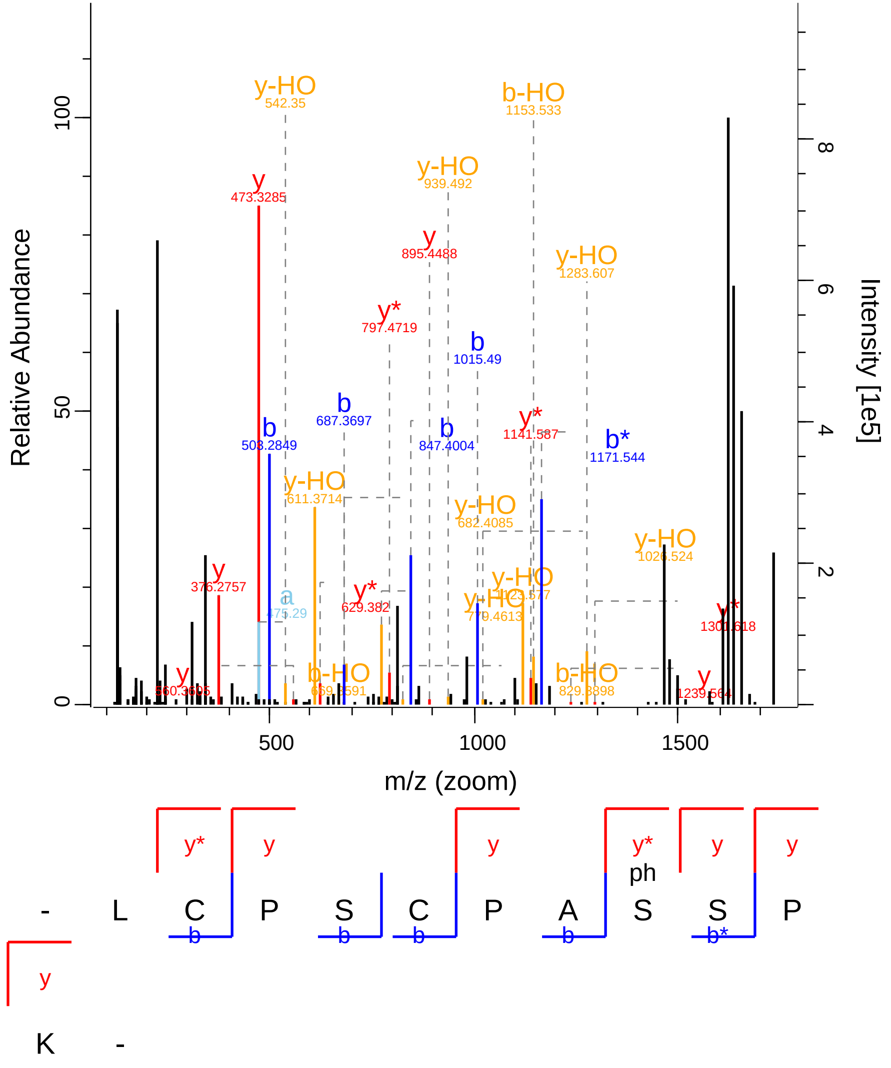

Raw File	Scan	Method	Score	m/z	Gene names
RO-QC-160121_ZackD_SS_Set_01_Fraction	16379	FTMS; HCI	64.24	604	AKTIP

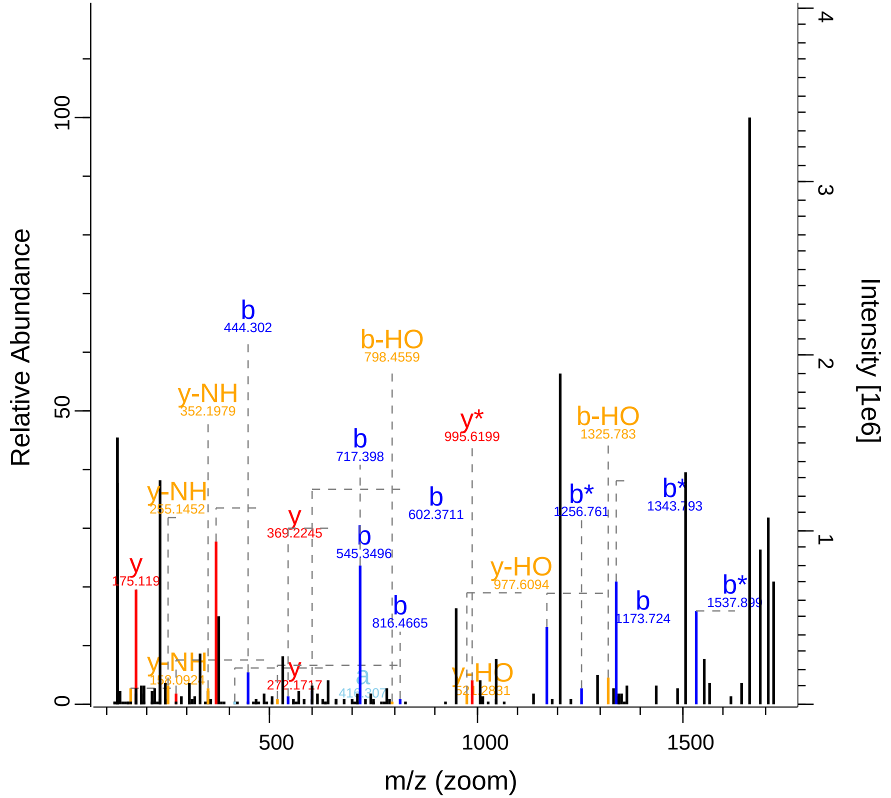

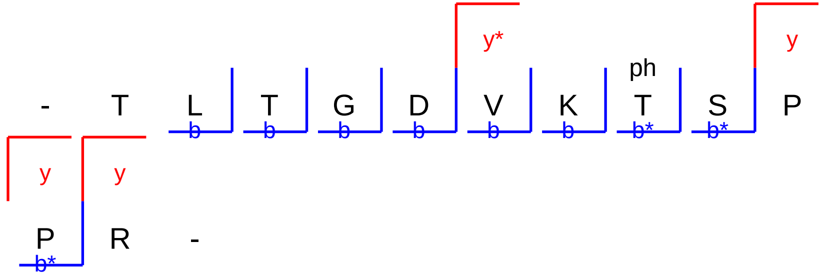

Raw File	Scan	Method	Score	m/z	Gene names
RO-QC-160121_ZackD_SS_Set_01_Fractio	17308	FTMS; HC	117.64	1002.15	HMGA1

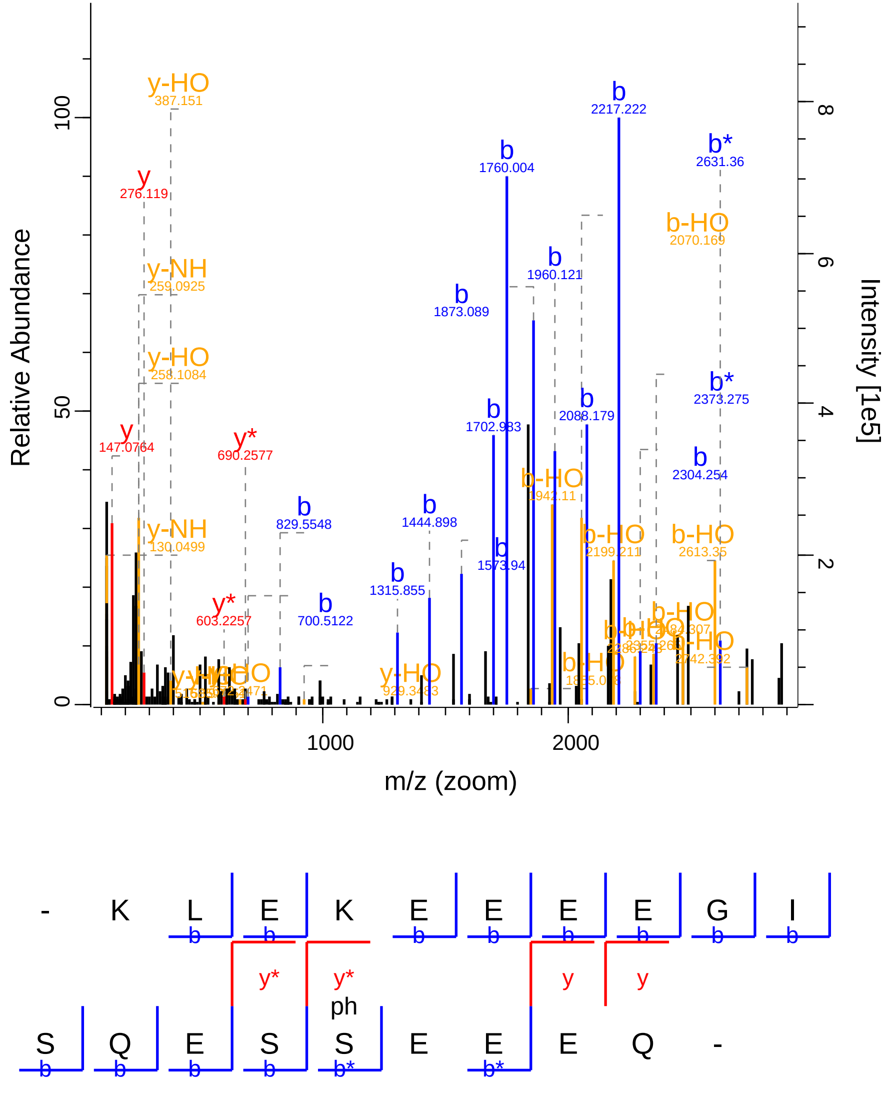

Raw File	Scan	Method	Score	m/z	Gene names
RO-QC-160121_ZackD_SS_Set_01_Fractio	17418	FTMS; HC	73.21	853.44	TERF1

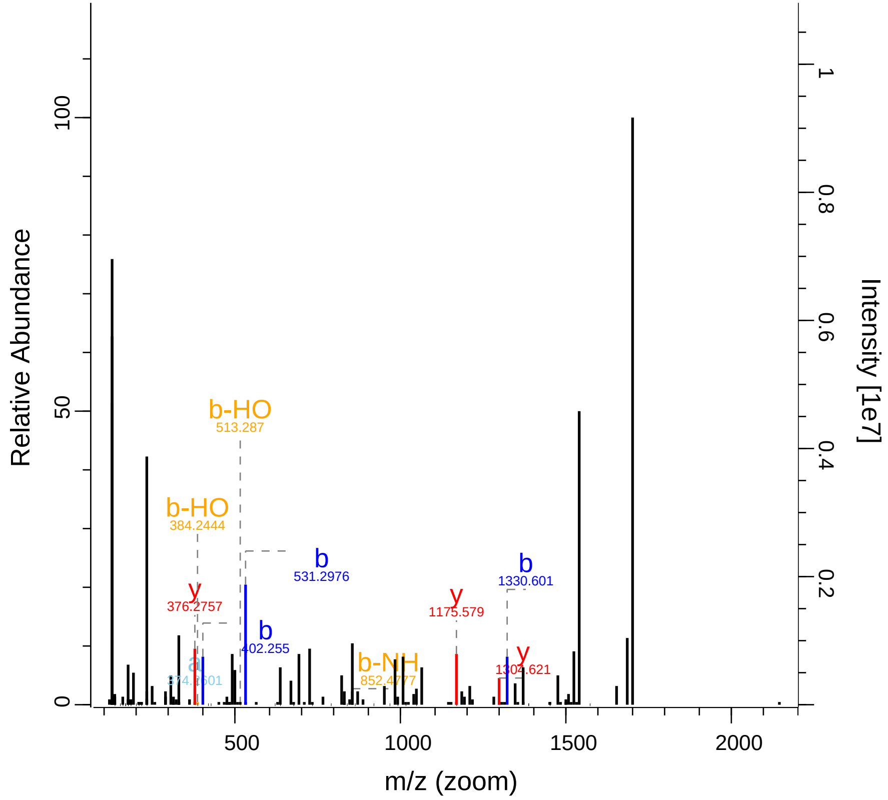

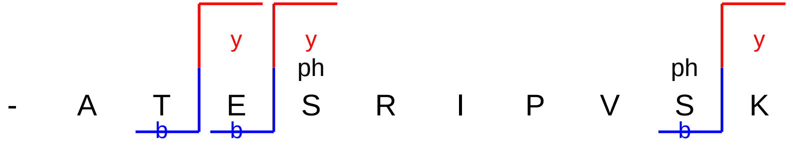

-

Raw File	Scan	Method	Score	m/z	Gene names
RO-QC-160121_ZackD_SS_Set_01_Fractio	17793	FTMS; HC	209.7	1230.6	ATP8A1

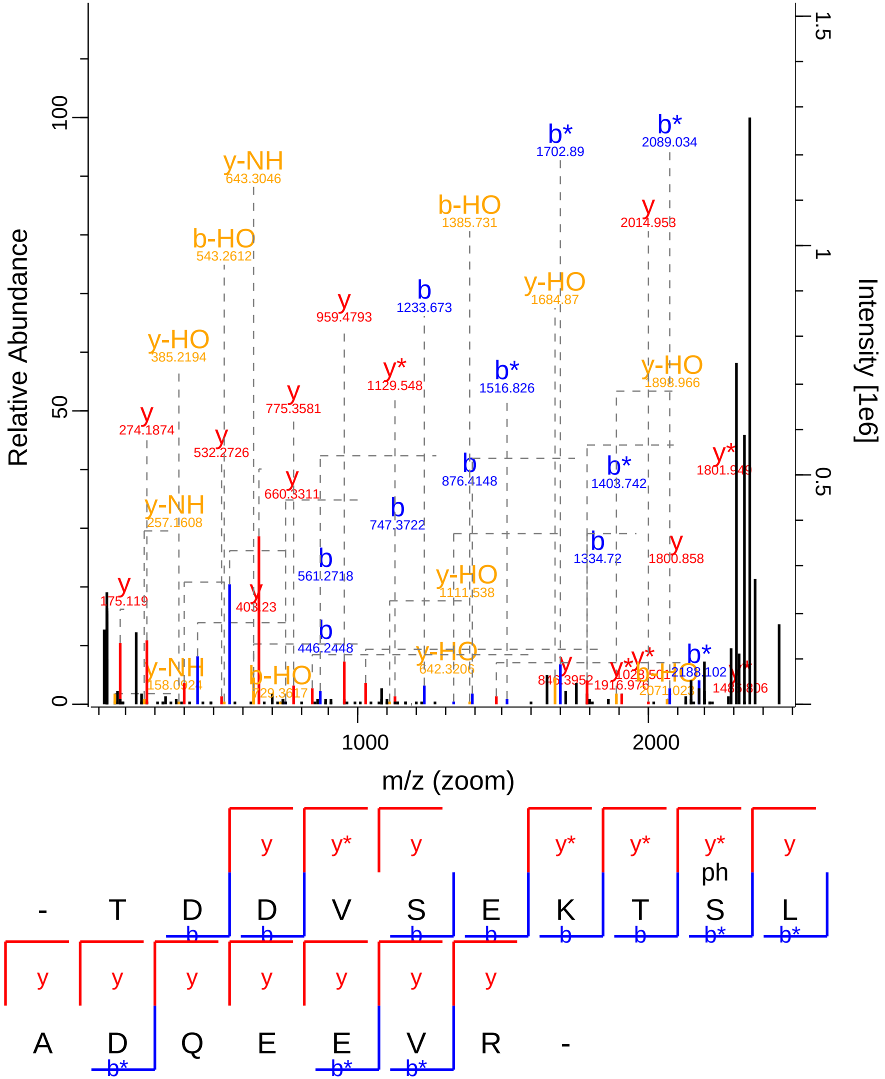

Raw File	Scan	Method	Score	m/z	Gene names
RO-QC-160121_ZackD_SS_Set_01_Fractio	18038	FTMS; HC	79.69	898.13	PALM

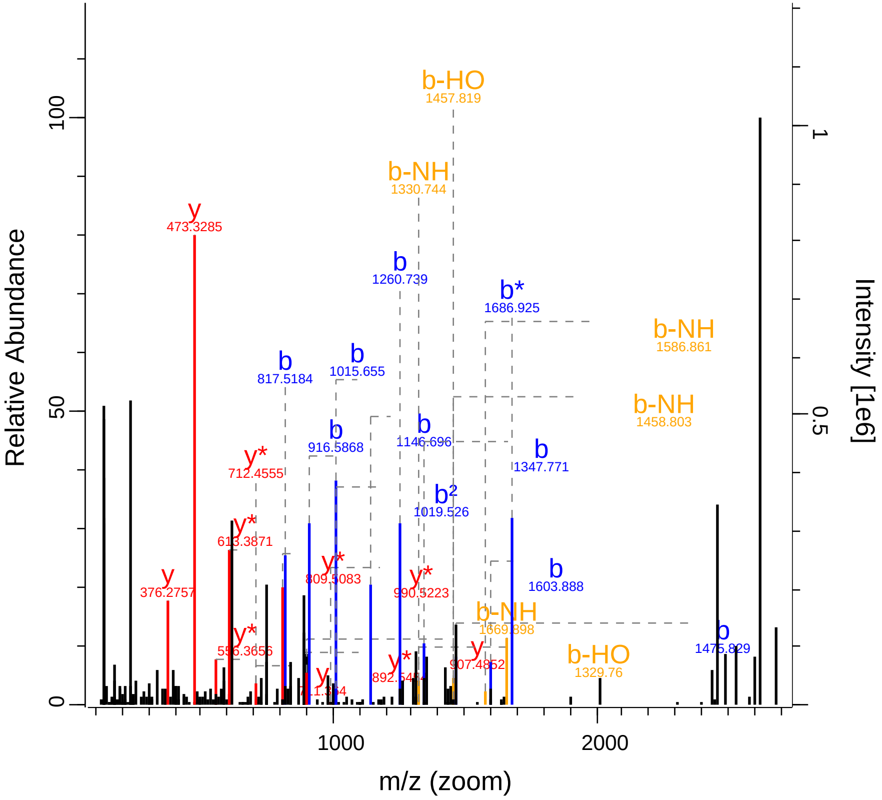

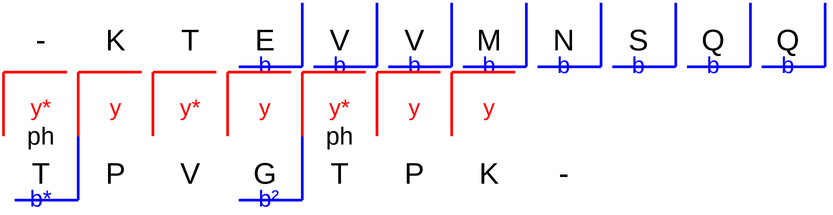

Raw File	Scan	Method	Score	m/z	Gene names
RO-QC-160121_ZackD_SS_Set_01_Fractio	18977	FTMS; HC	311.97	1249.57	DOCK7

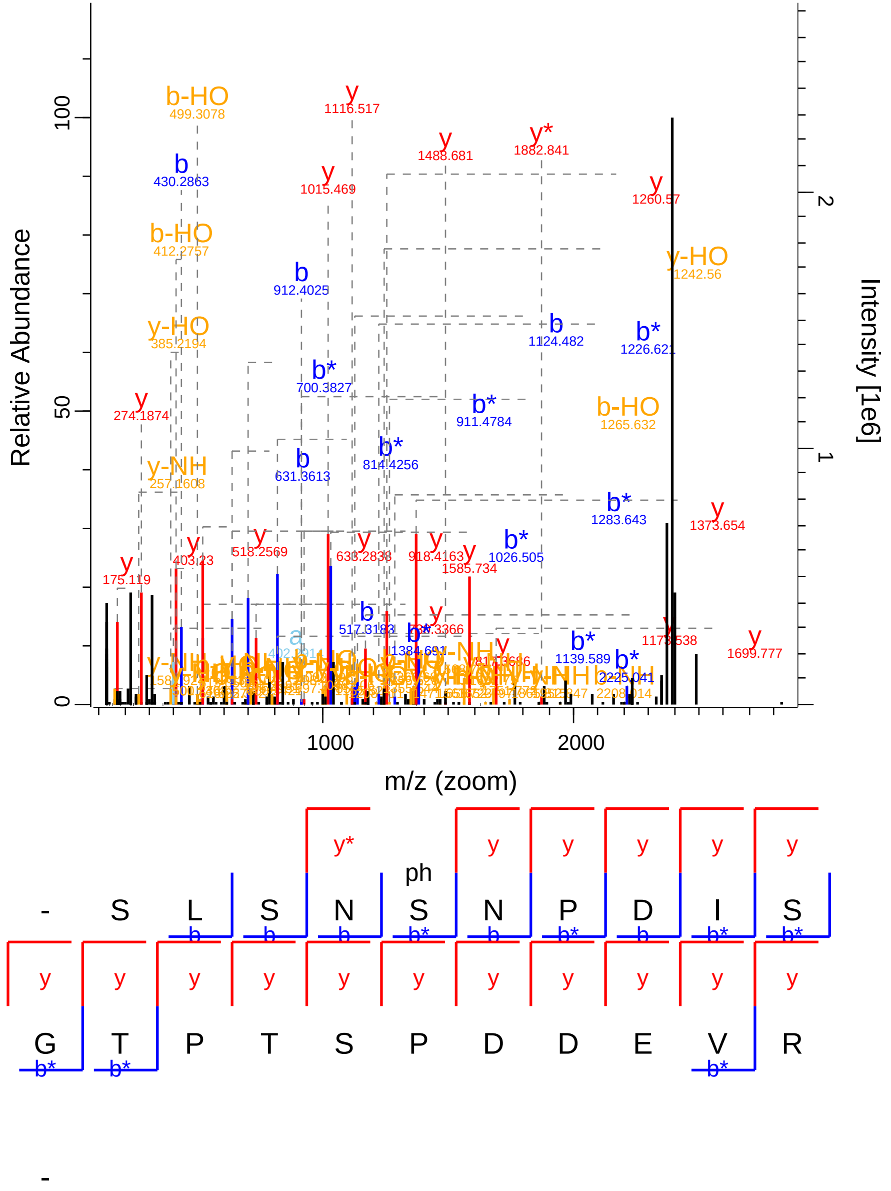

Raw File	Scan	Method	Score	m/z	Gene names
RO-QC-160121_ZackD_SS_Set_01_Fractio	19475	FTMS; HC	118.91	802.03	TBC1D2B

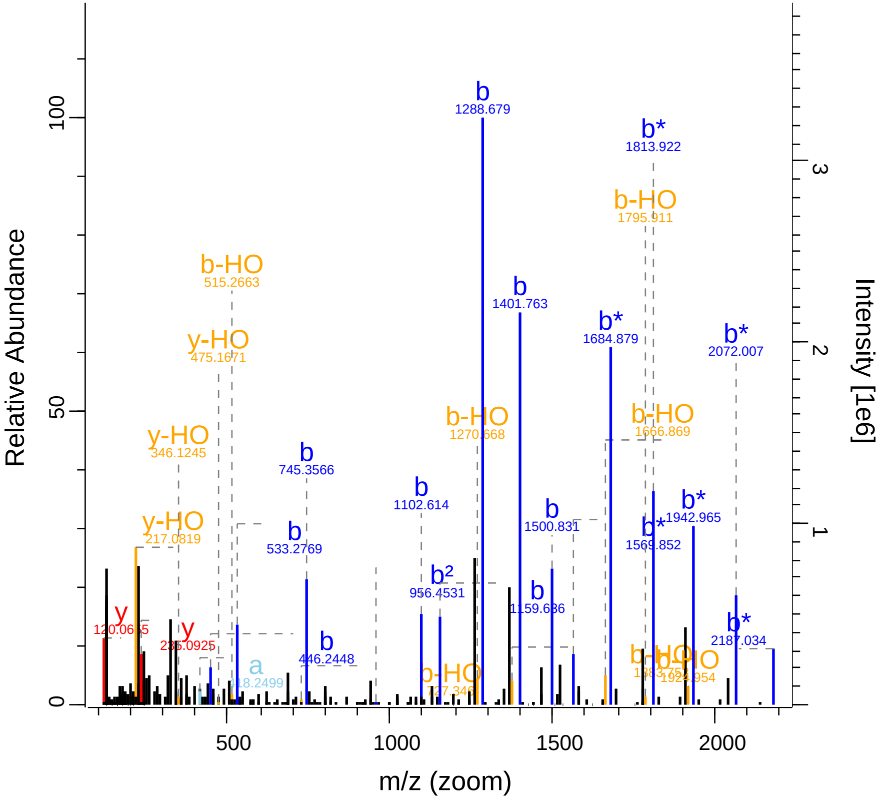

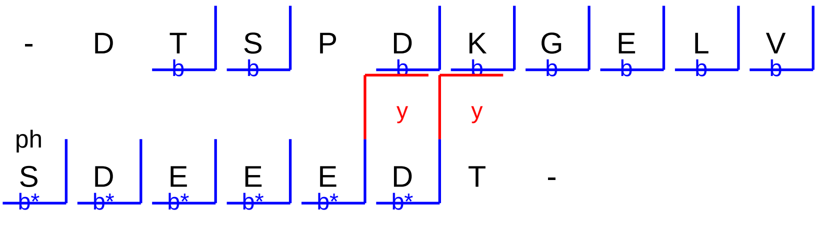

Raw File	Scan	Method	Score	m/z	Gene names
RO-QC-160121_ZackD_SS_Set_01_Fractio	19965	FTMS; HC	96.17	762.93	PDCD4

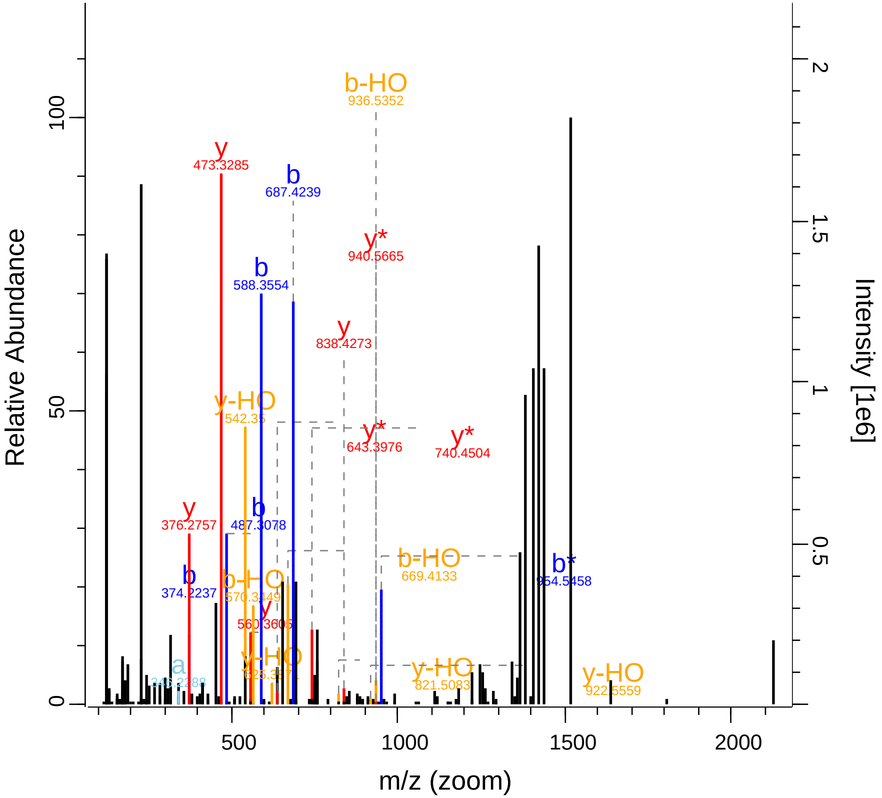

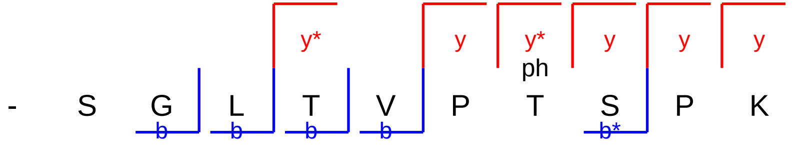

-

Raw File	Scan	Method	Score	m/z	Gene names
RO-QC-160121_ZackD_SS_Set_01_Fractio	20399	FTMS; HC	67.25	863.78	MAP1B

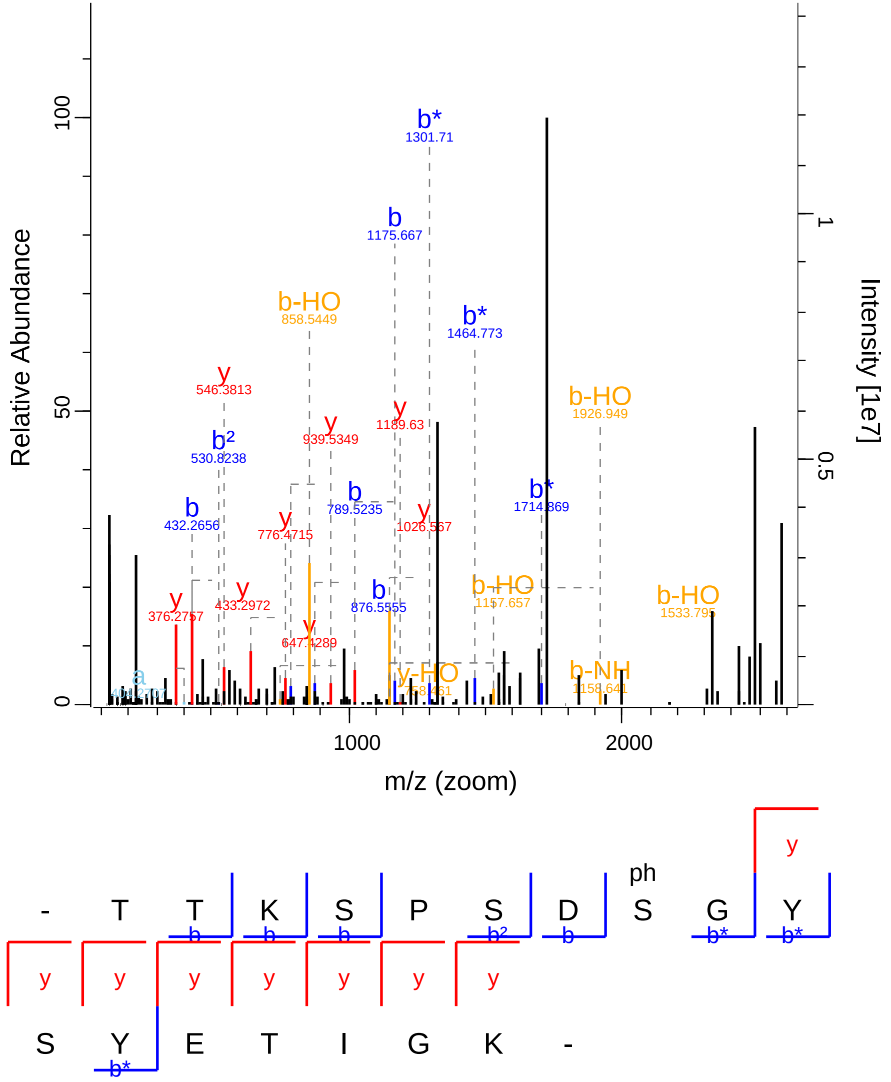

Raw File	Scan	Method	Score	m/z	Gene names
RO-QC-160121_ZackD_SS_Set_01_Fractic	21597	FTMS; HC	81.62	1260.64	KCNH5

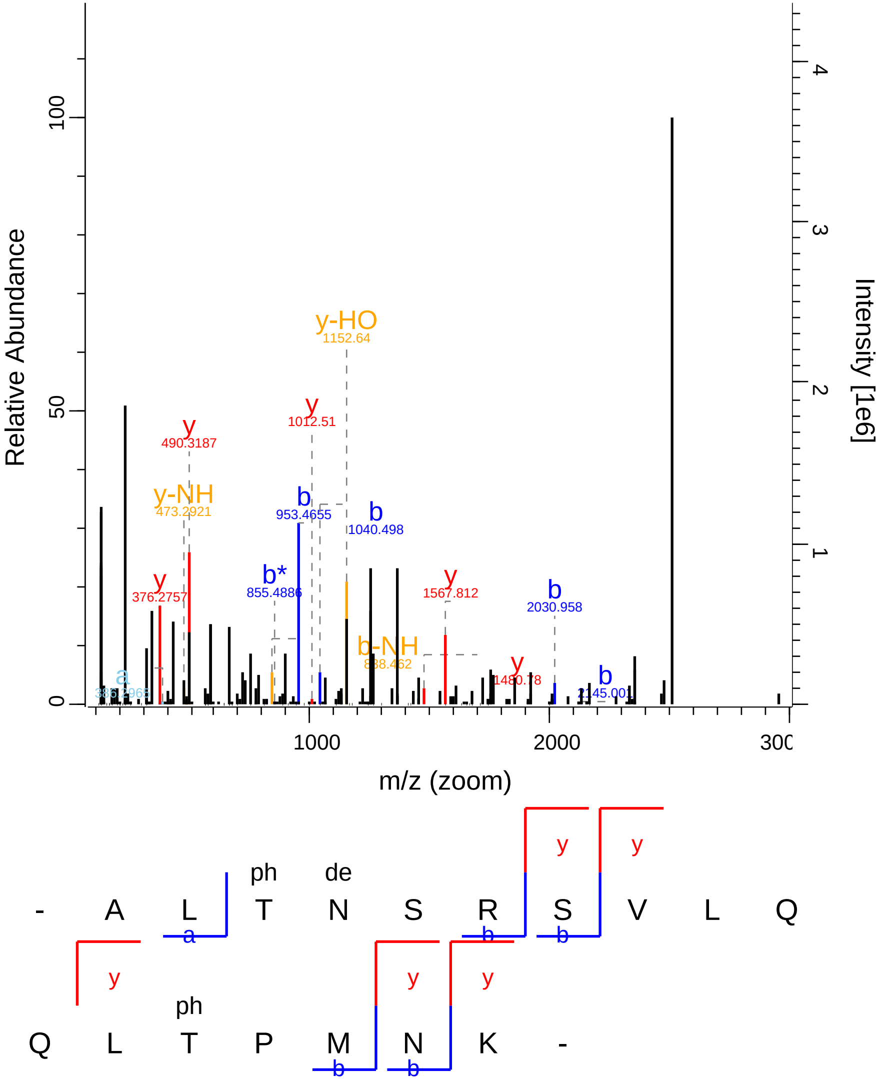

Raw File	Scan	Method	Score	m/z	Gene names
RO-QC-160121_ZackD_SS_Set_01_Fractic	21877	FTMS; HC	241.46	1140.24	PGRMC1

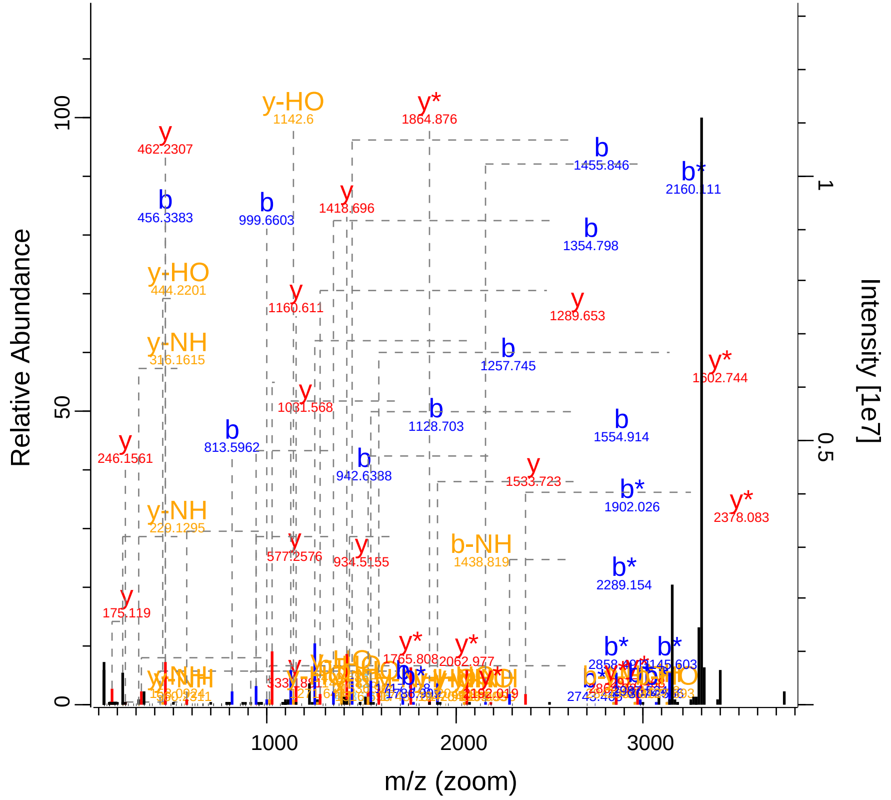

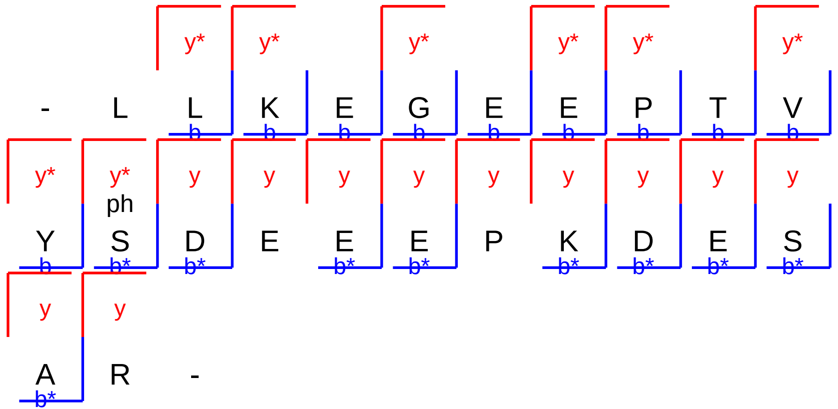

Raw File	Scan	Method	Score	m/z	Gene names
RO-QC-160121_ZackD_SS_Set_01_Fractio	22348	FTMS; HC	149.33	1112.81	FBN1

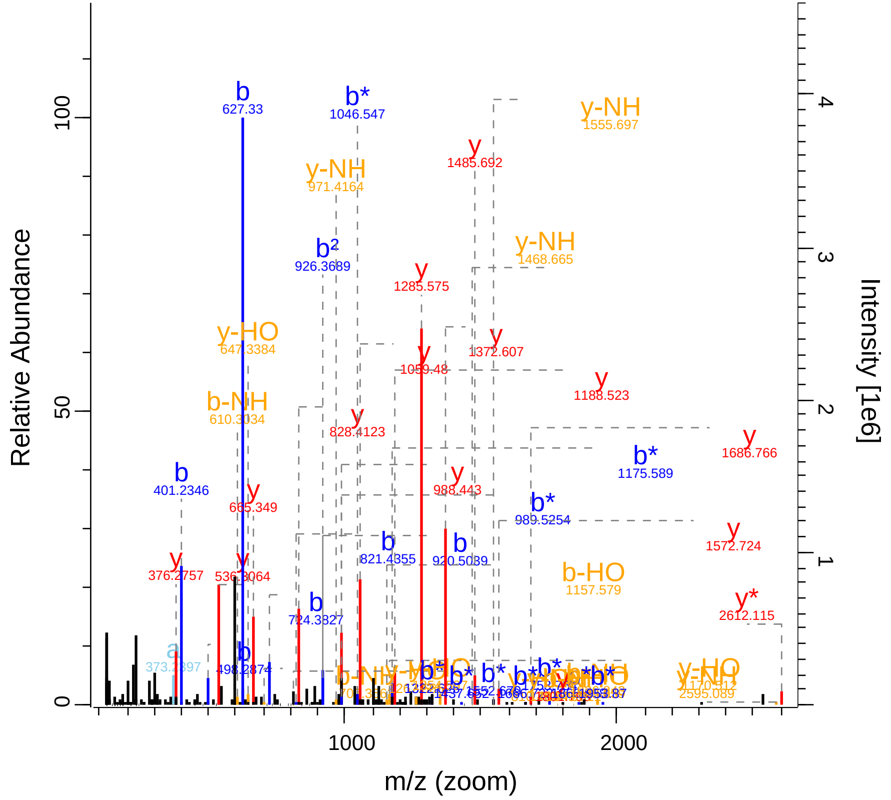

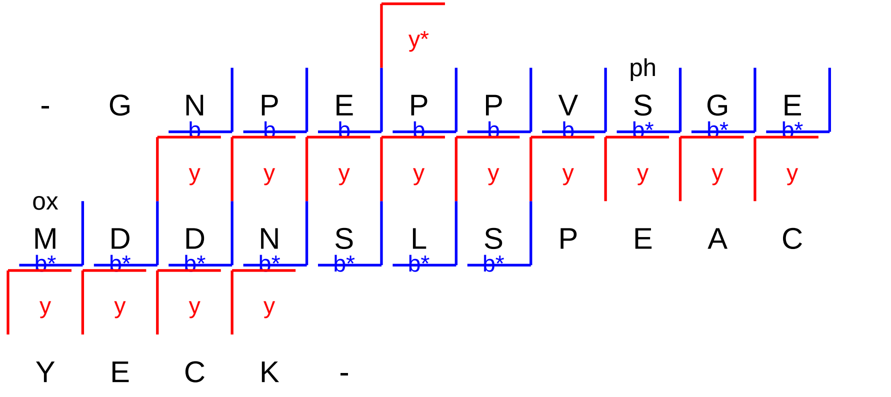

Raw File	Scan	Method	Score	m/z	Gene names
RO-QC-160121_ZackD_SS_Set_01_Fractio	22833	FTMS; HC	71.43	845.11	PRRC2A

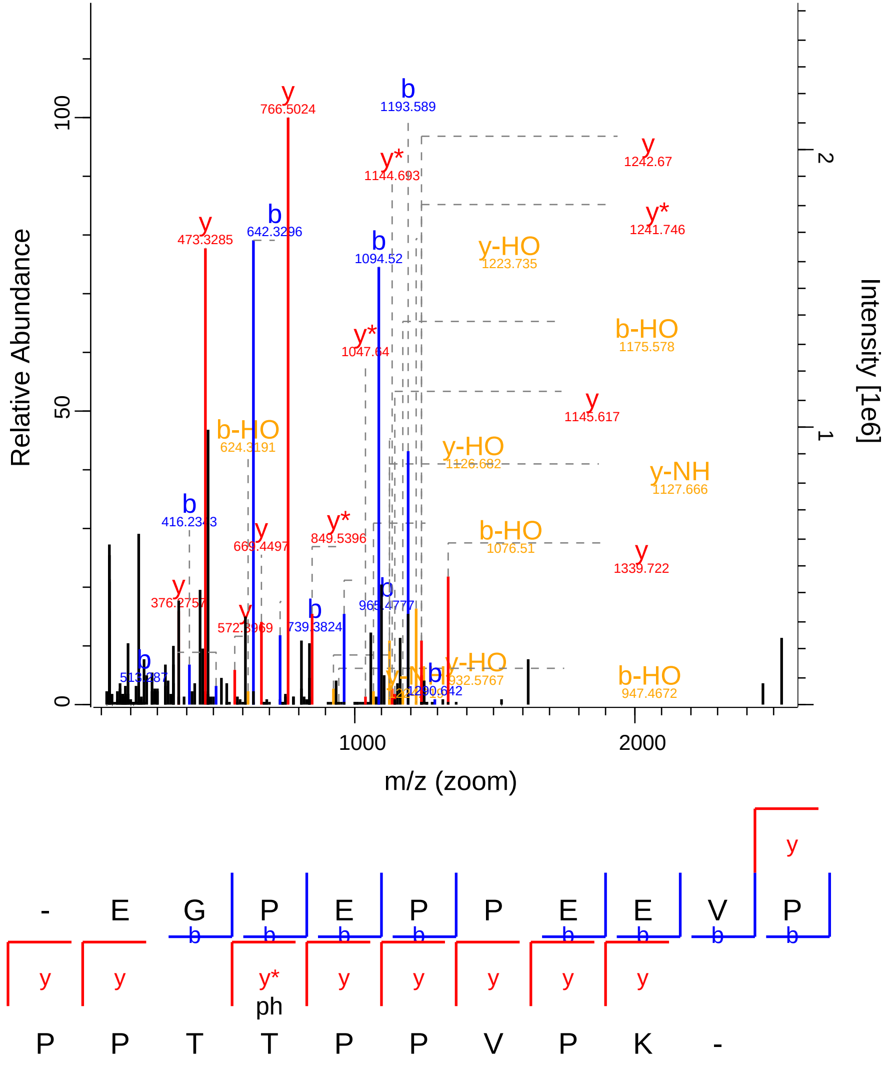

Raw File	Scan	Method	Score	m/z	Gene names
RO-QC-160121_ZackD_SS_Set_01_Fractio	22870	FTMS; HC	70.09	906.76	ANTXR1

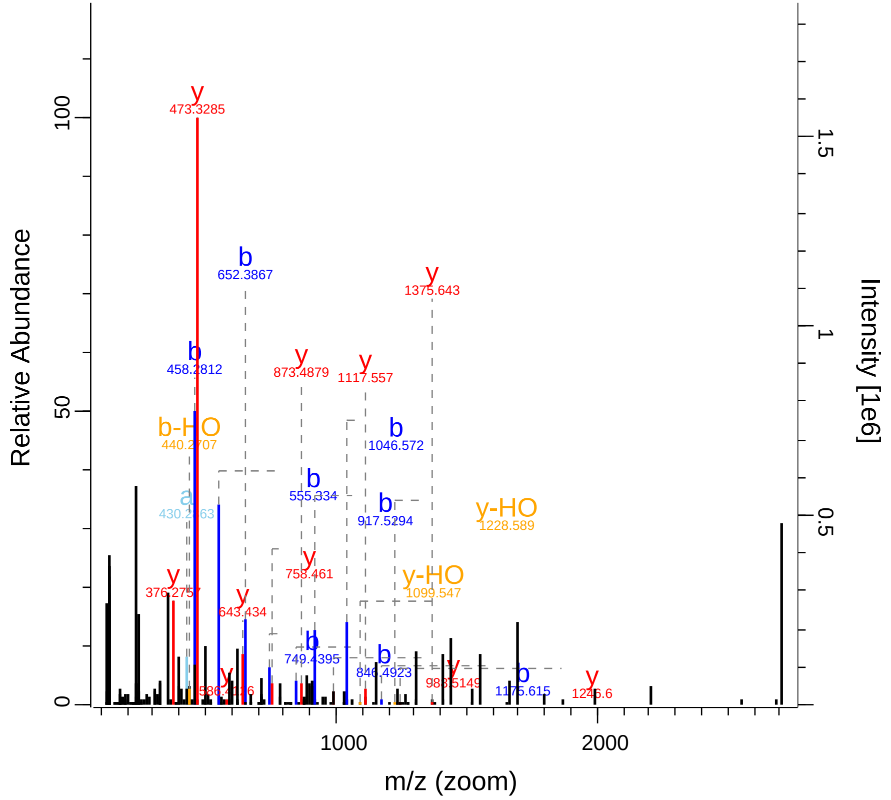

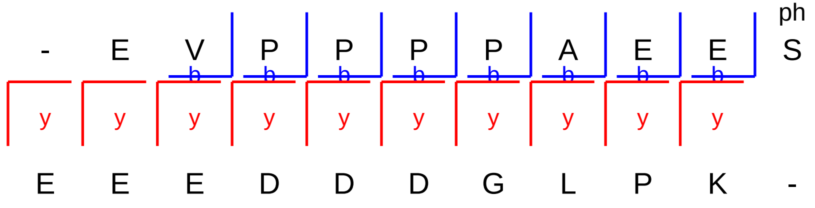

Raw File	Scan	Method	Score	m/z	Gene names
RO-QC-160121_ZackD_SS_Set_01_Fractic	23320	FTMS; HC	74.4	1022.03	CLIP1

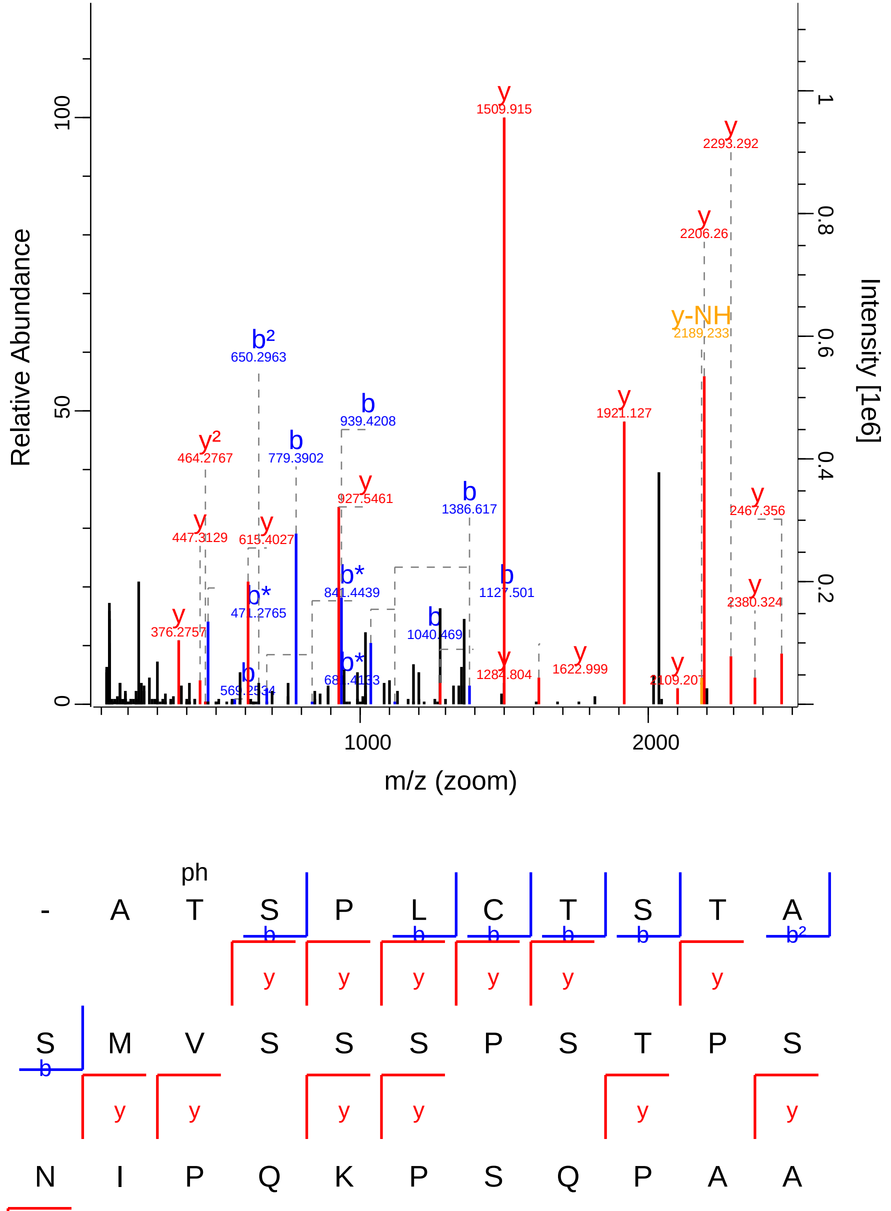

Raw File	Scan	Method	Score	m/z	Gene names
RO-QC-160121_ZackD_SS_Set_01_Fractio	25658	FTMS; HC	69.6	713.34	CNTRL

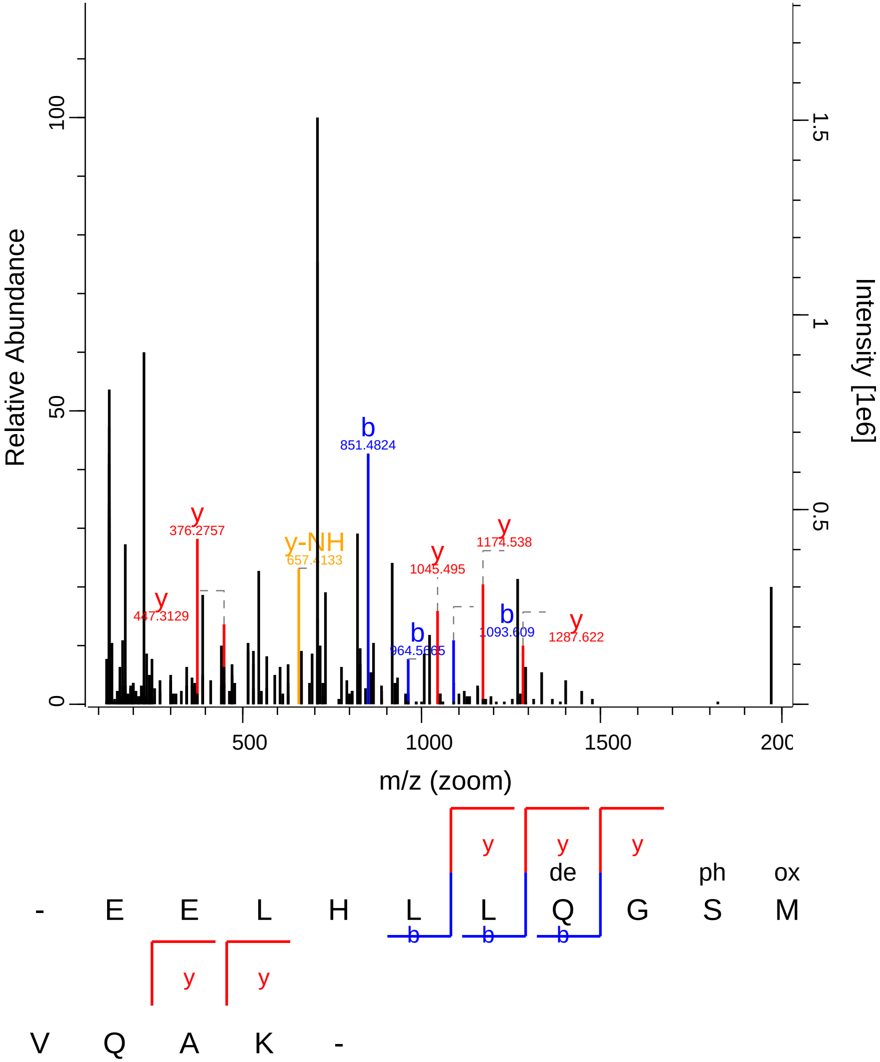

Raw File	Scan	Method	Score	m/z	Gene names
RO-QC-160121_ZackD_SS_Set_01_Fractio	26204	FTMS; HC	150.79	1221.25	MAP1B

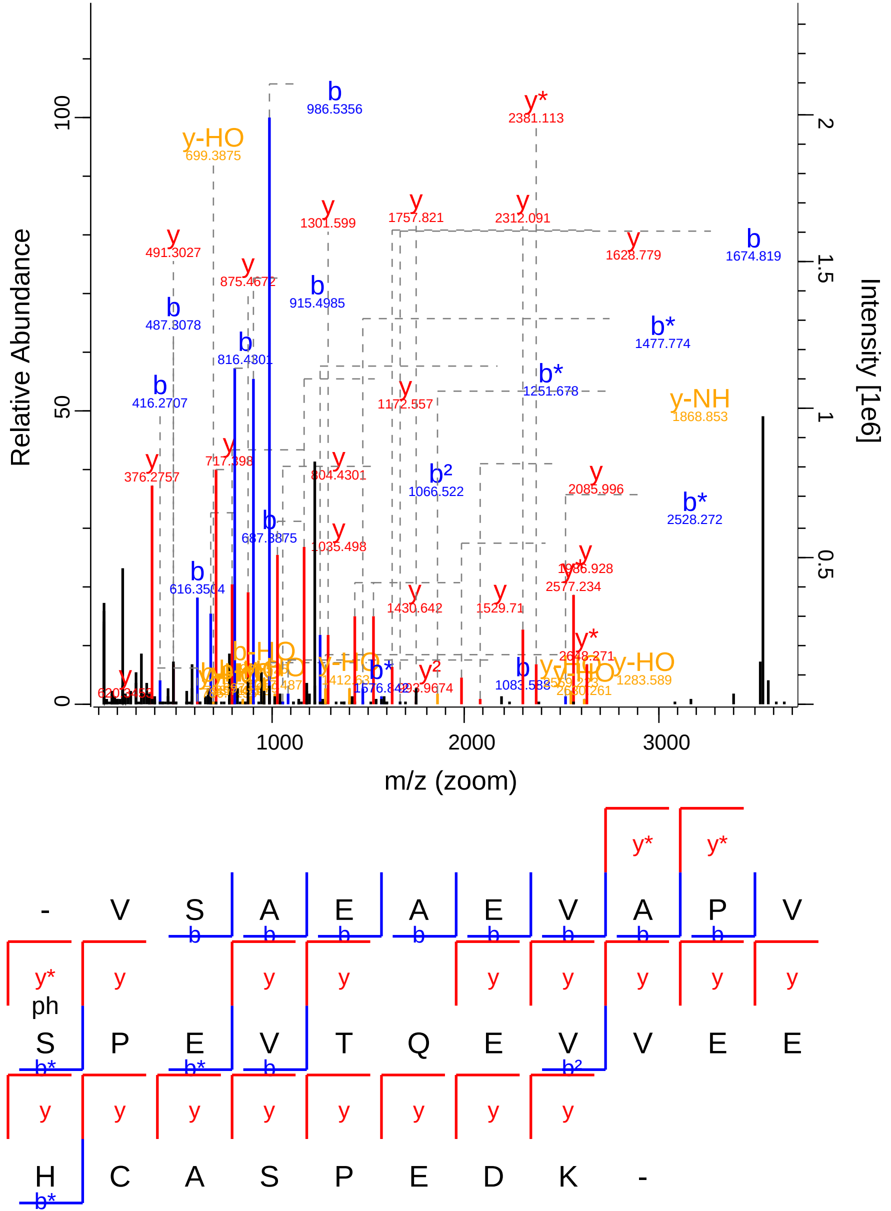

Raw File	Scan	Method	Score	m/z	Gene names
RO-QC-160121_ZackD_SS_Set_01_Fractio	27476	FTMS; HC	125.52	815.16	NOP56

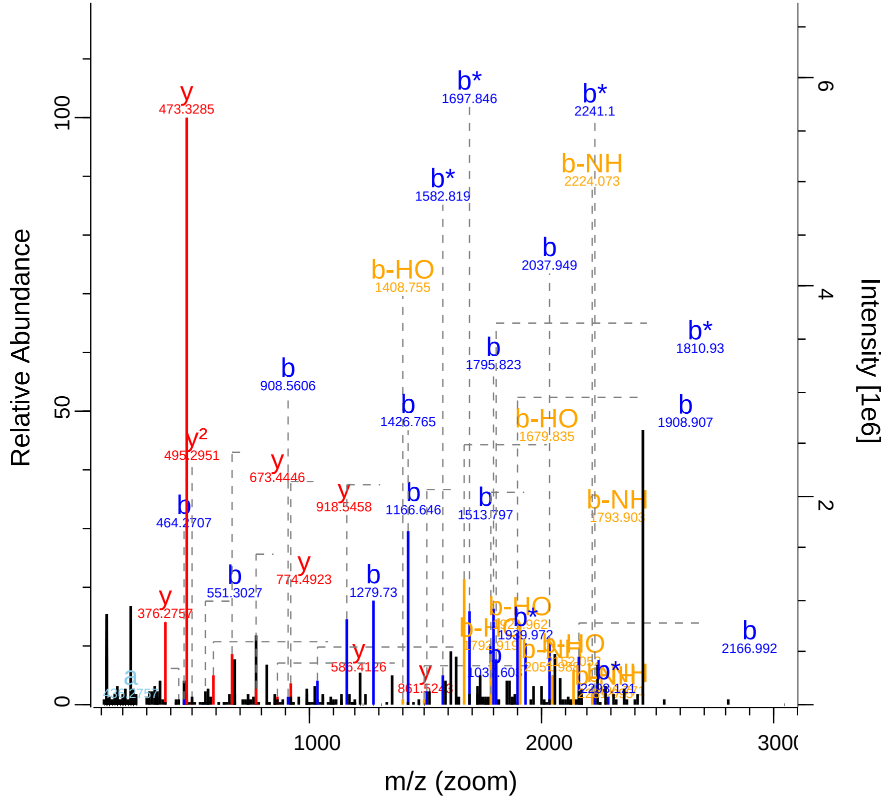

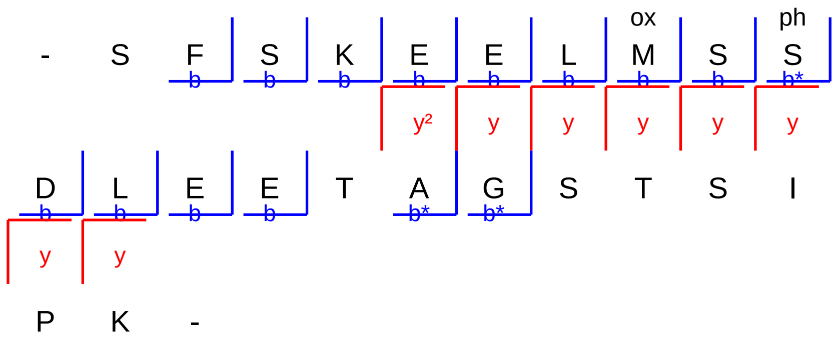

Raw File	Scan	Method	Score	m/z	Gene names
RO-QC-160121_ZackD_SS_Set_01_Fractio	27800	FTMS; HC	96.02	986.49	CCNYL1

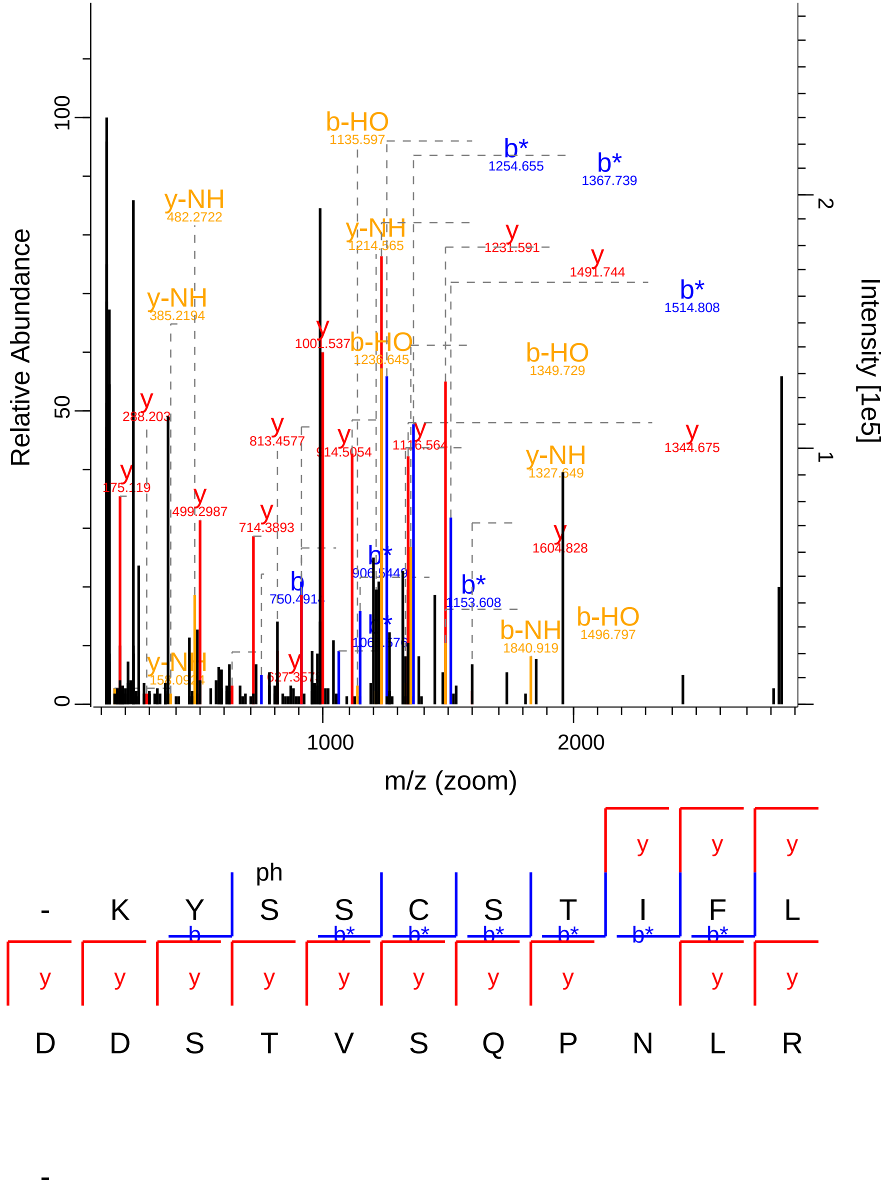

Raw File	Scan	Method	Score	m/z	Gene names
RO-QC-160121_ZackD_SS_Set_01_Fractio	28159	FTMS; HC	93.16	882.41	SRSF9

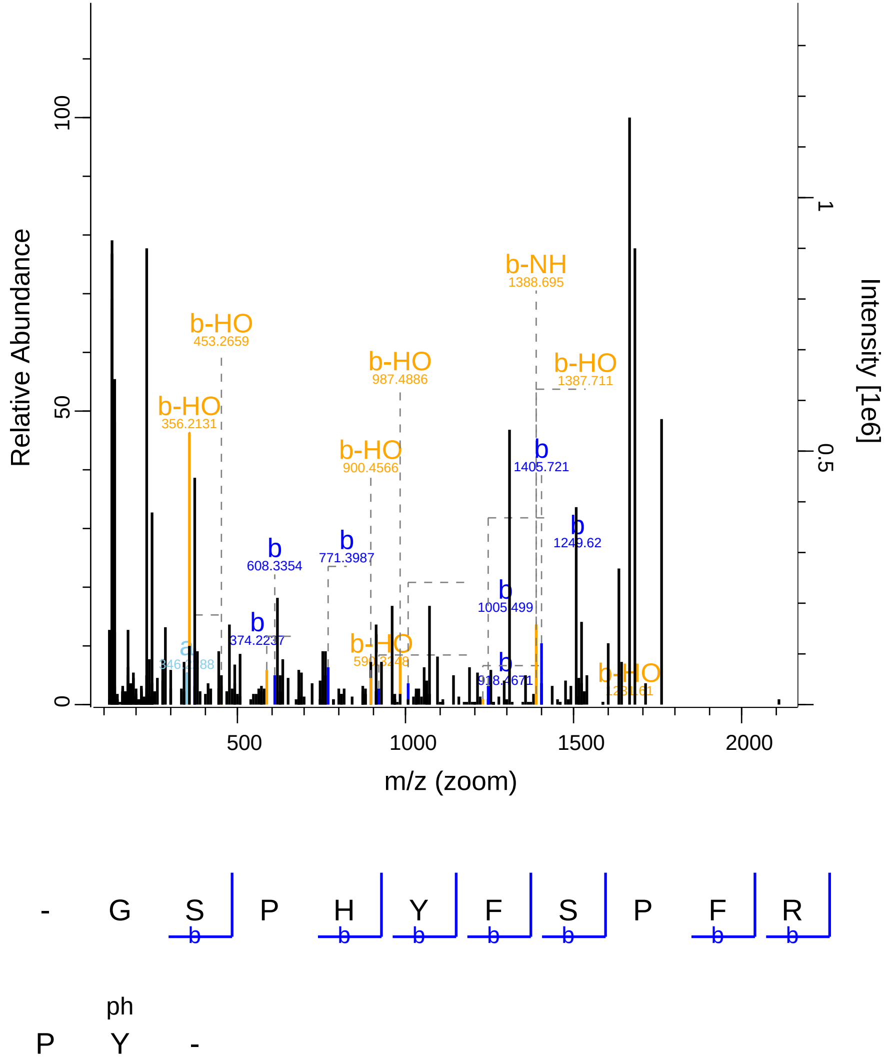

Raw File	Scan	Method	Score	m/z	Gene names
RO-QC-160121_ZackD_SS_Set_01_Fractio	28375	FTMS; HC	90.32	836.41	RAB11FIP1

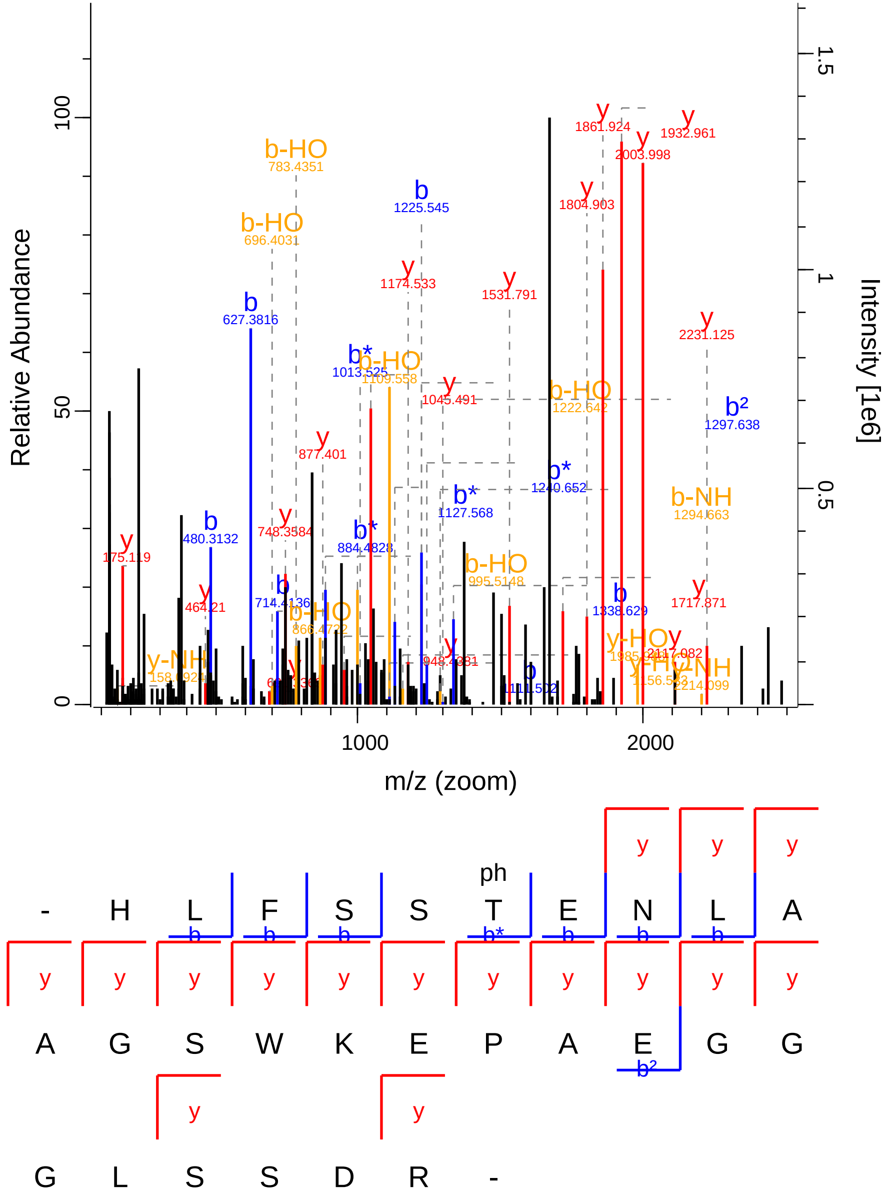

Raw File	Scan	Method	Score	m/z	Gene names
RO-QC-160121_ZackD_SS_Set_01_Fraction	29143	FTMS; HCI	61.96	907	GOLGA4

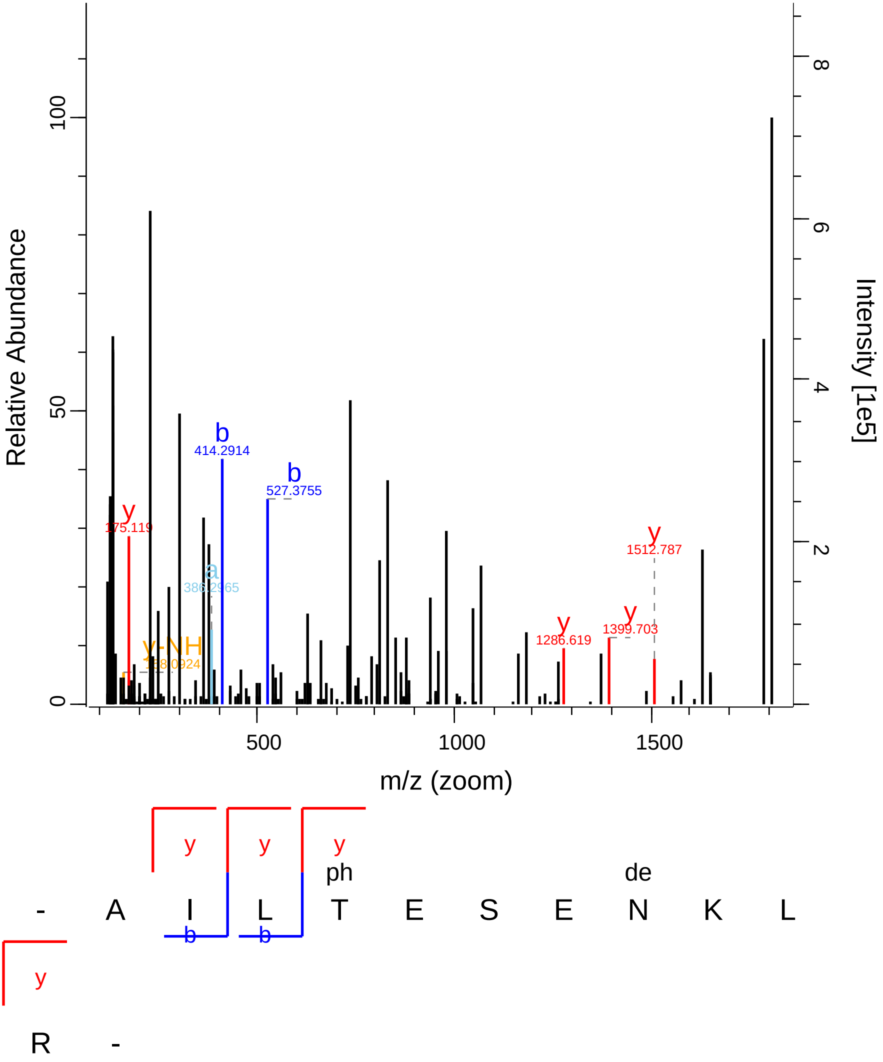

Raw File	Scan	Method	Score	m/z	Gene names
RO-QC-160121_ZackD_SS_Set_01_Fractio	29747	FTMS; HC	350.23	948.98	AQP1

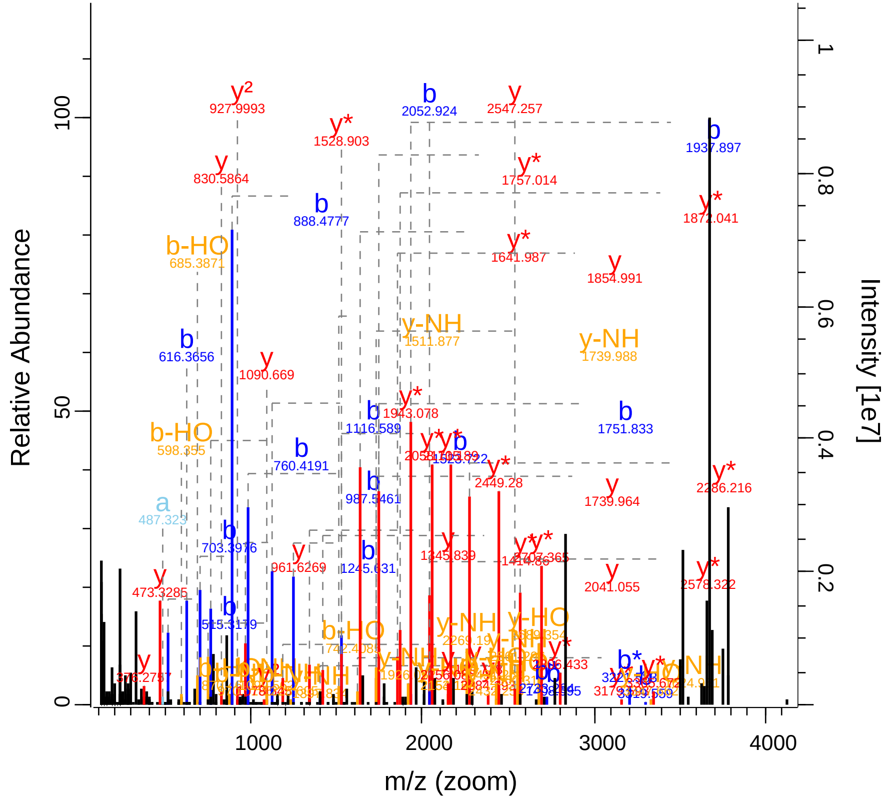

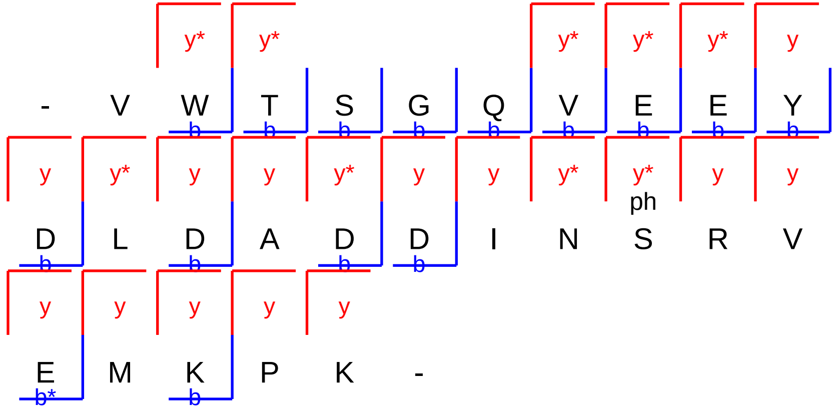

Raw File	Scan	Method	Score	m/z	Gene names
RO-QC-160121_ZackD_SS_Set_01_Fractic	29958	FTMS; HC	71.68	1017.14	GJA1

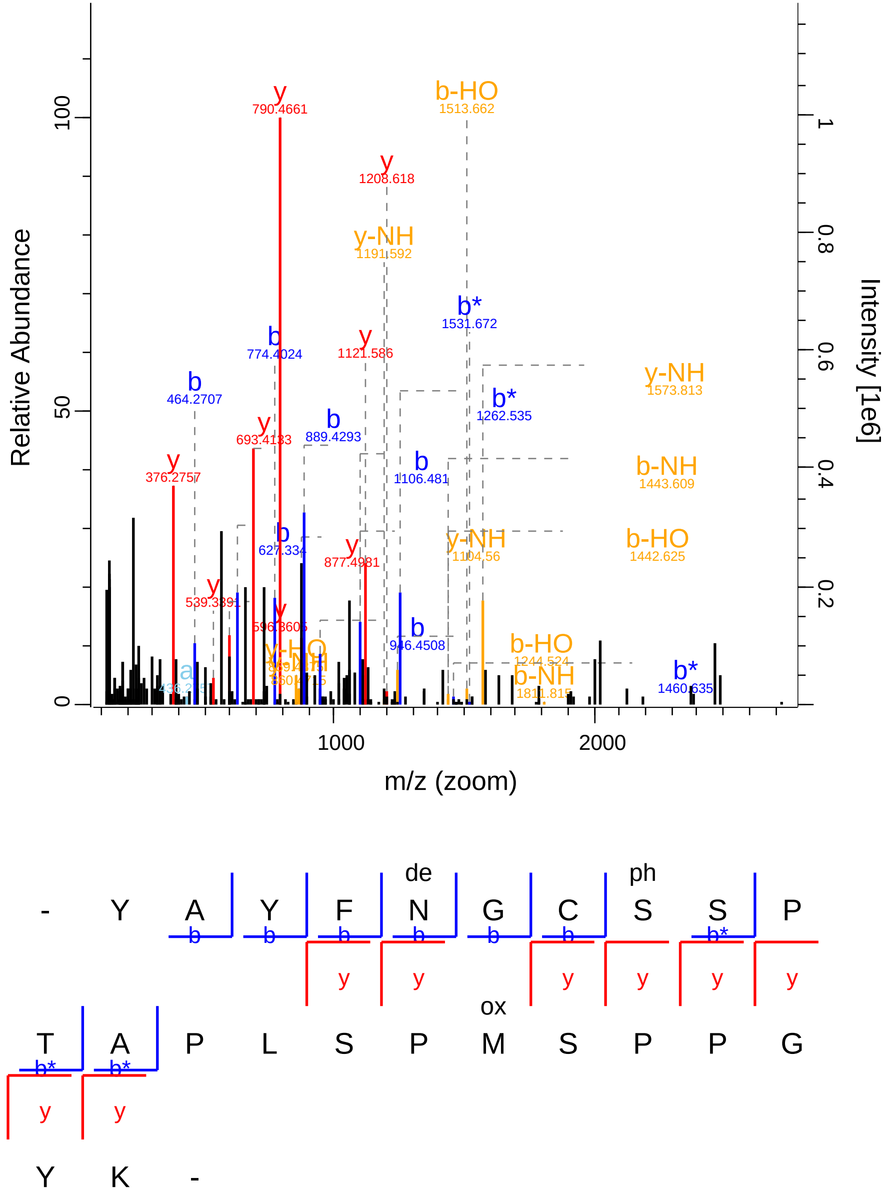

Raw File	Scan	Method	Score	m/z	Gene names
RO-QC-160121_ZackD_SS_Set_01_Fractio	30836	FTMS; HC	140.89	1349.6	MPHOSPH8

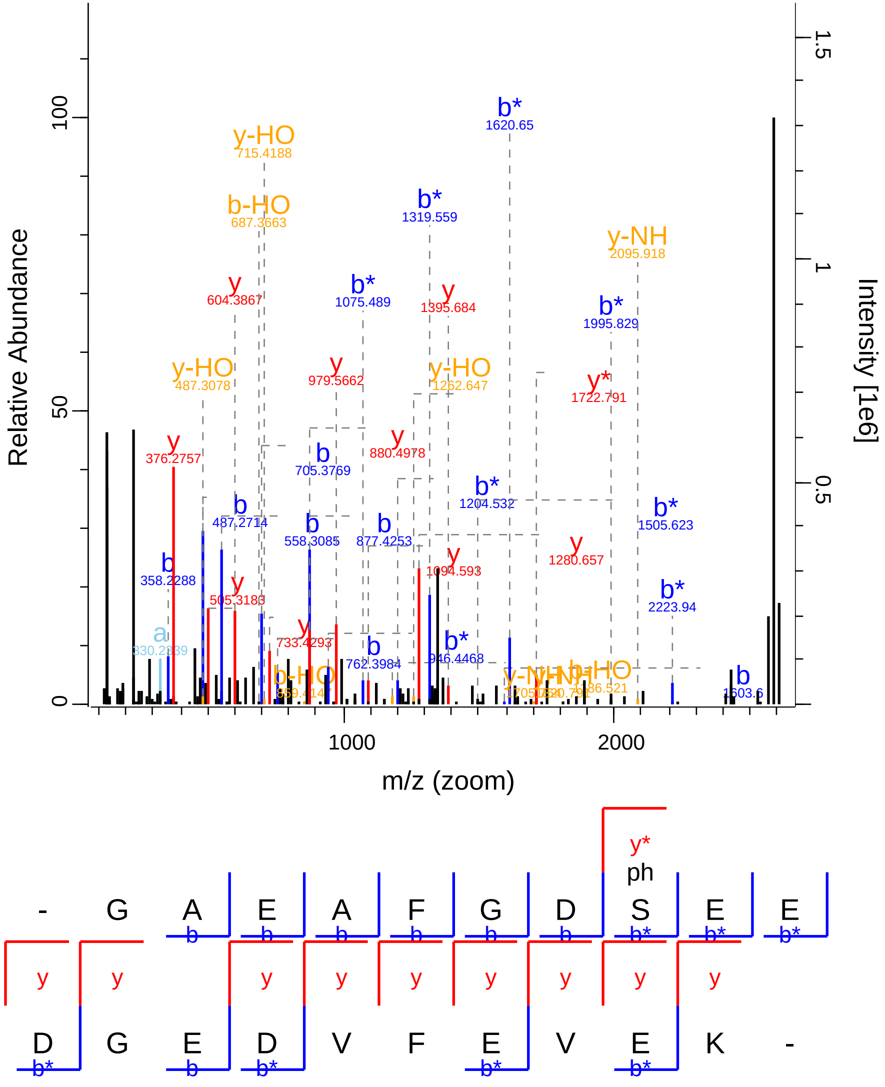

Raw File	Scan	Method	Score	m/z	Gene names
RO-QC-160121_ZackD_SS_Set_01_Fractic	31848	FTMS; HC	40.35	1196.56	SPNS1

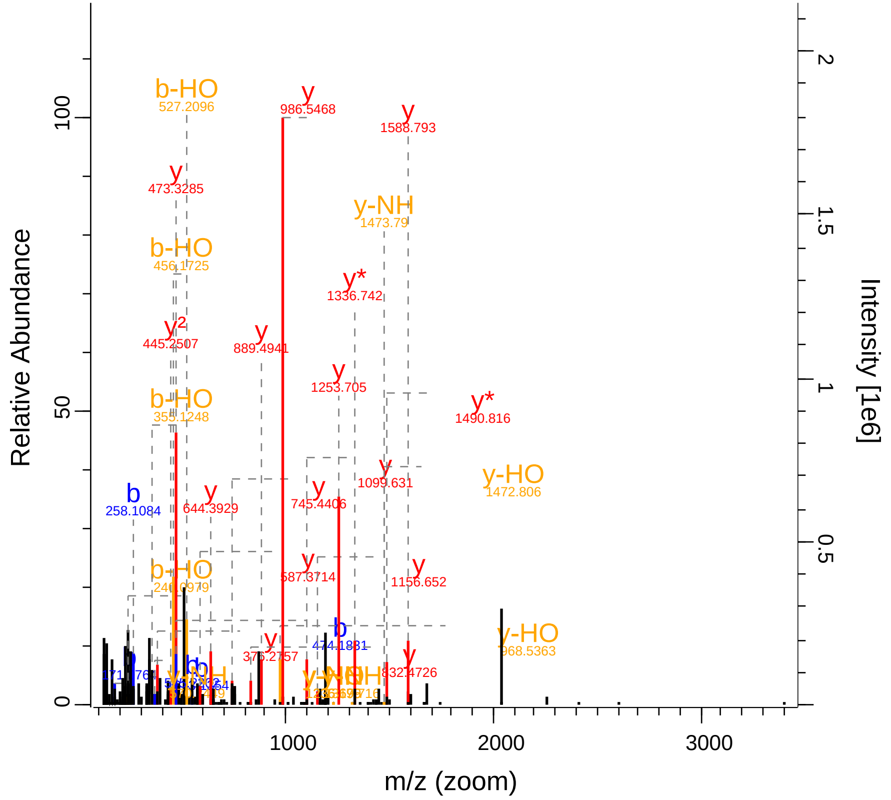

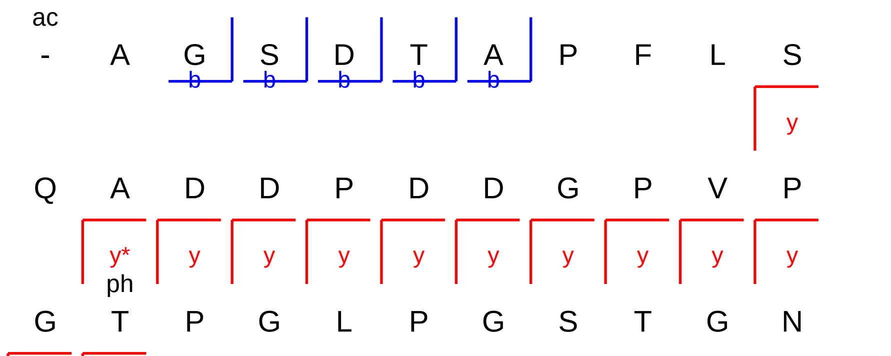

Raw File	Scan	Method	Score	m/z	Gene names
RO-QC-160121_ZackD_SS_Set_01_Fractio	31877	FTMS; HC	42.45	772.39	TOMM22

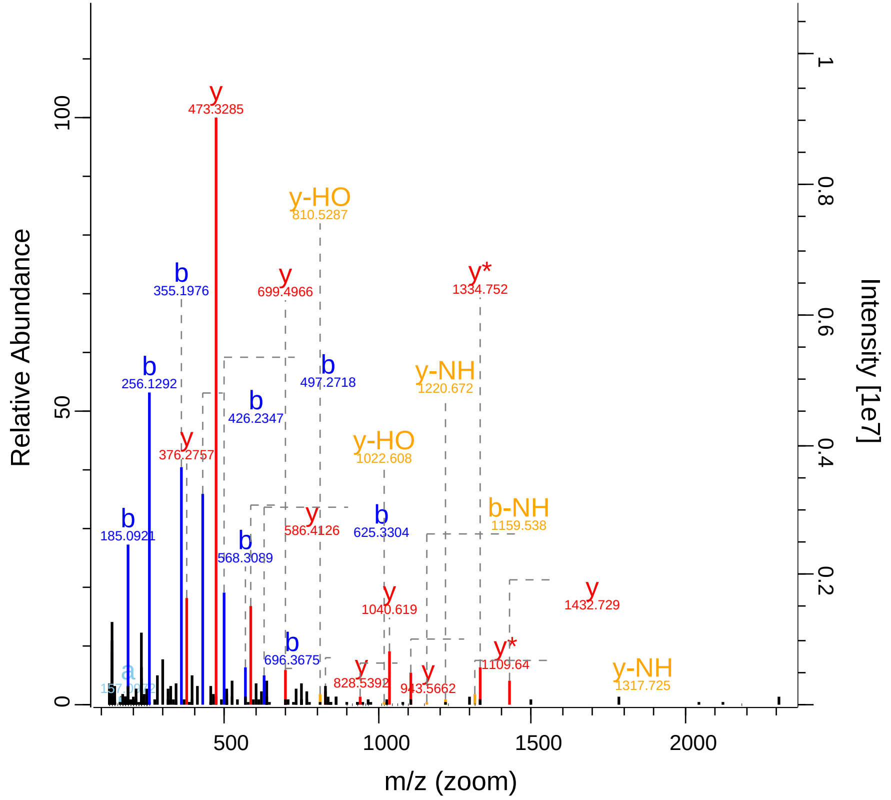

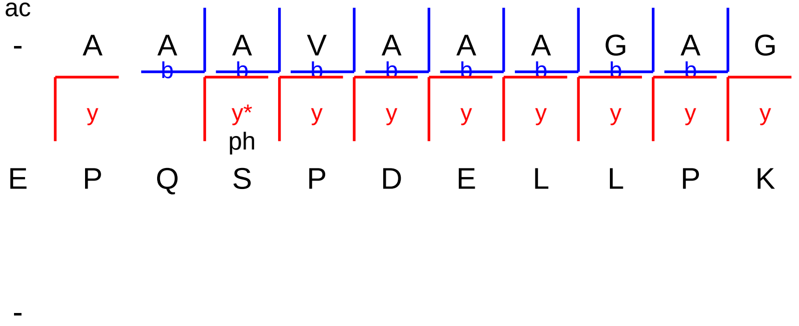

Raw File	Scan	Method	Score	m/z	Gene names
RO-QC-160121_ZackD_SS_Set_01_Fractio	32904	FTMS; HC	54.05	946.73	LIMD1

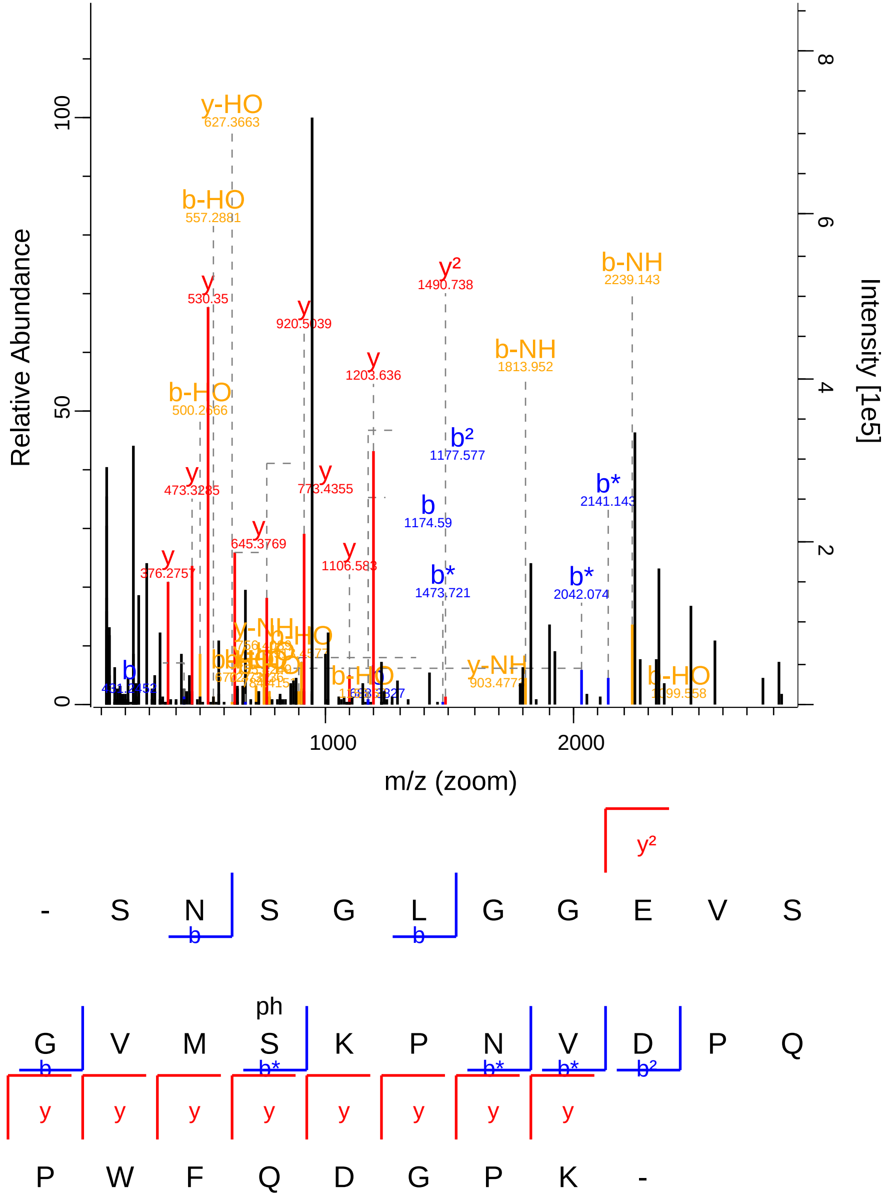

Raw File	Scan	Method	Score	m/z	Gene names
RO-QC-160121_ZackD_SS_Set_01_Fractic	34307	FTMS; HC	66.43	1016.59	RPSA

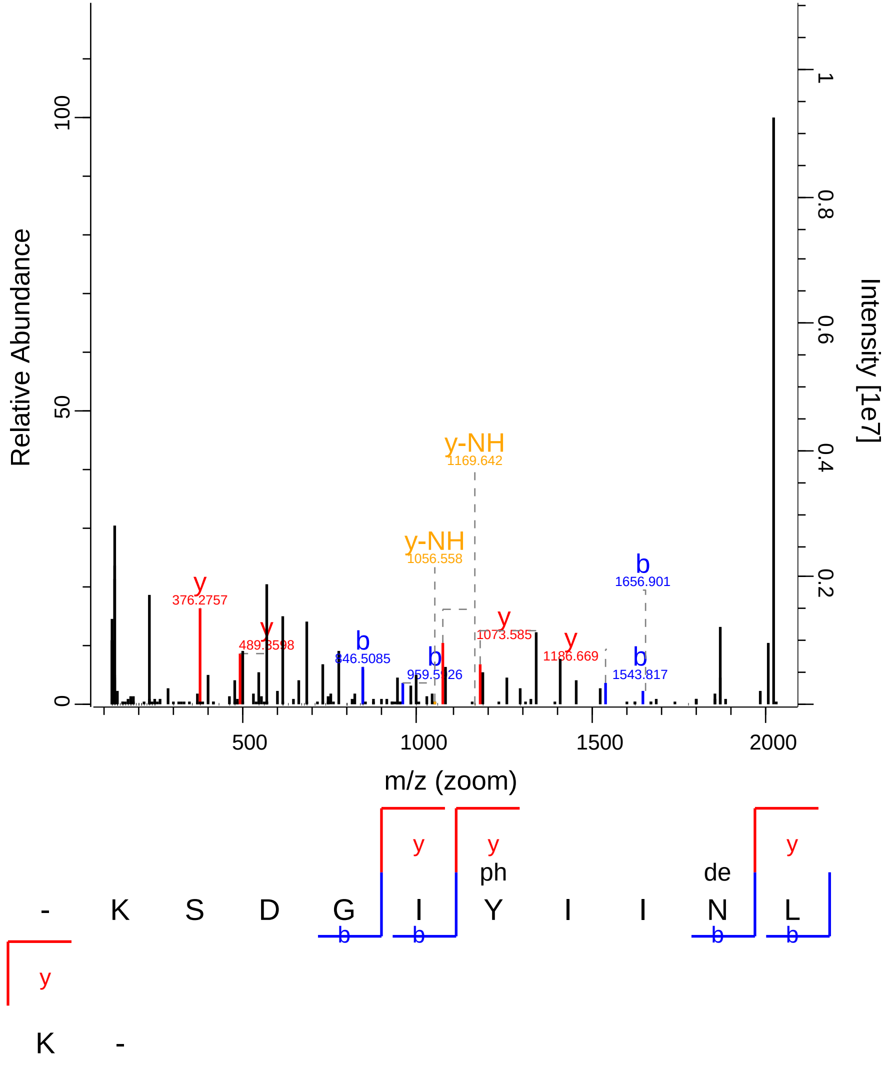

Raw File	Scan	Method	Score	m/z	Gene names
RO-QC-160121_ZackD_SS_Set_01_Fractic	34374	FTMS; HC	57.84	1290.13	ZZEF1

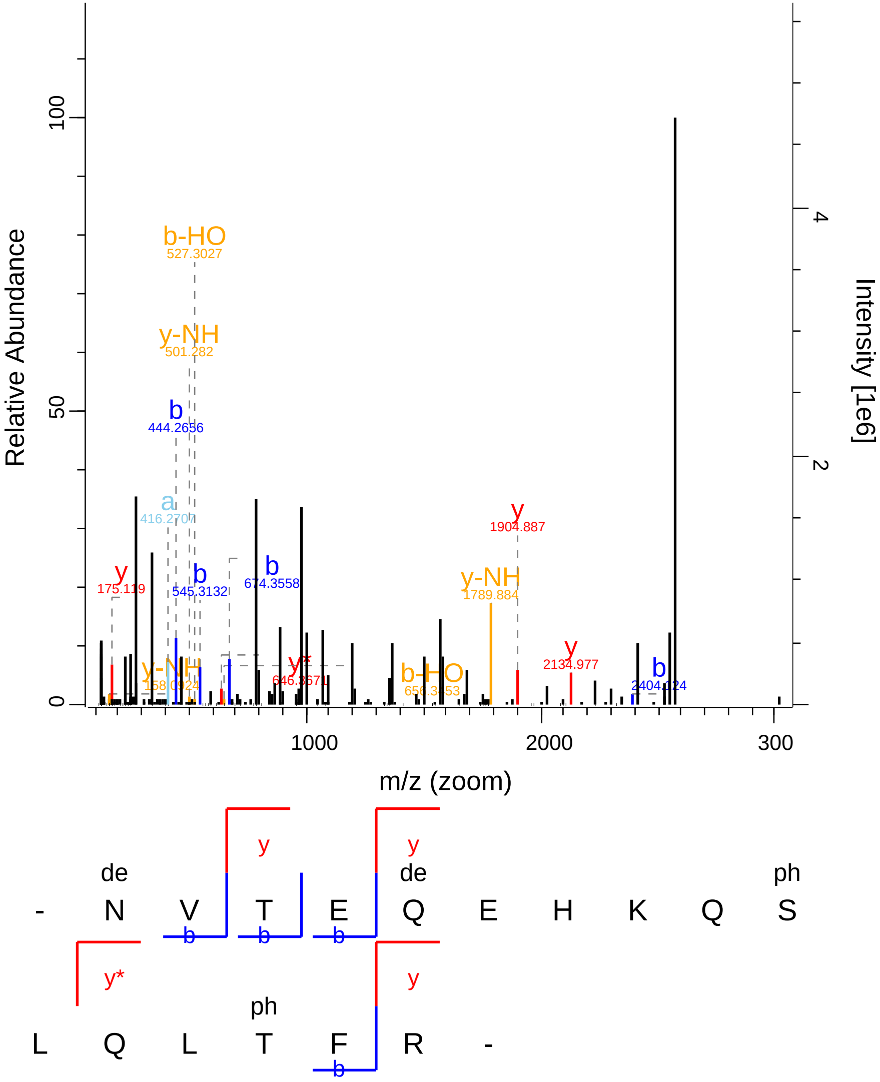

Raw File	Scan	Method	Score	m/z	Gene names
RO-QC-160121_ZackD_SS_Set_01_Fractic	34467	FTMS; HC	75.23	1002.22	EIF3G

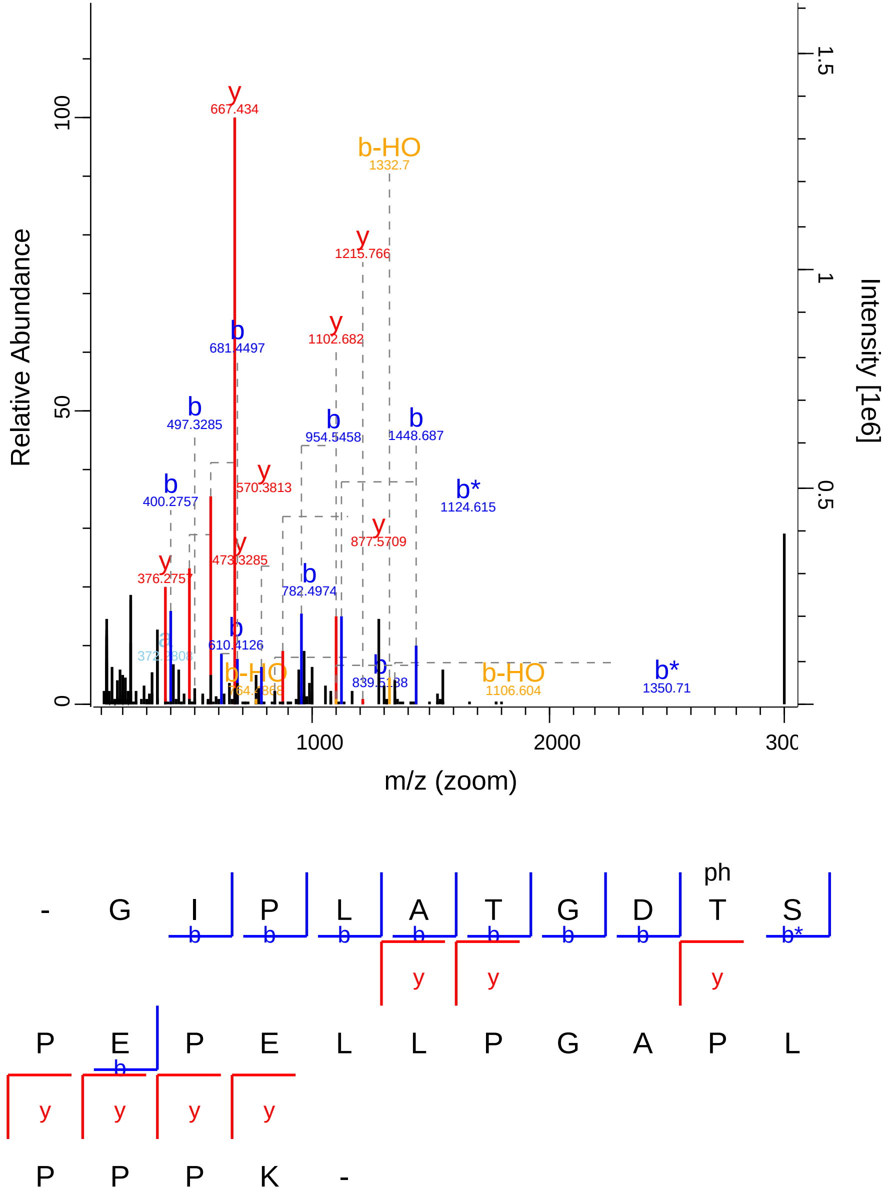

Raw File	Scan	Method	Score	m/z	Gene names
RO-QC-160121_ZackD_SS_Set_01_Fractio	35069	FTMS; HC	45.51	806.38	FTH1

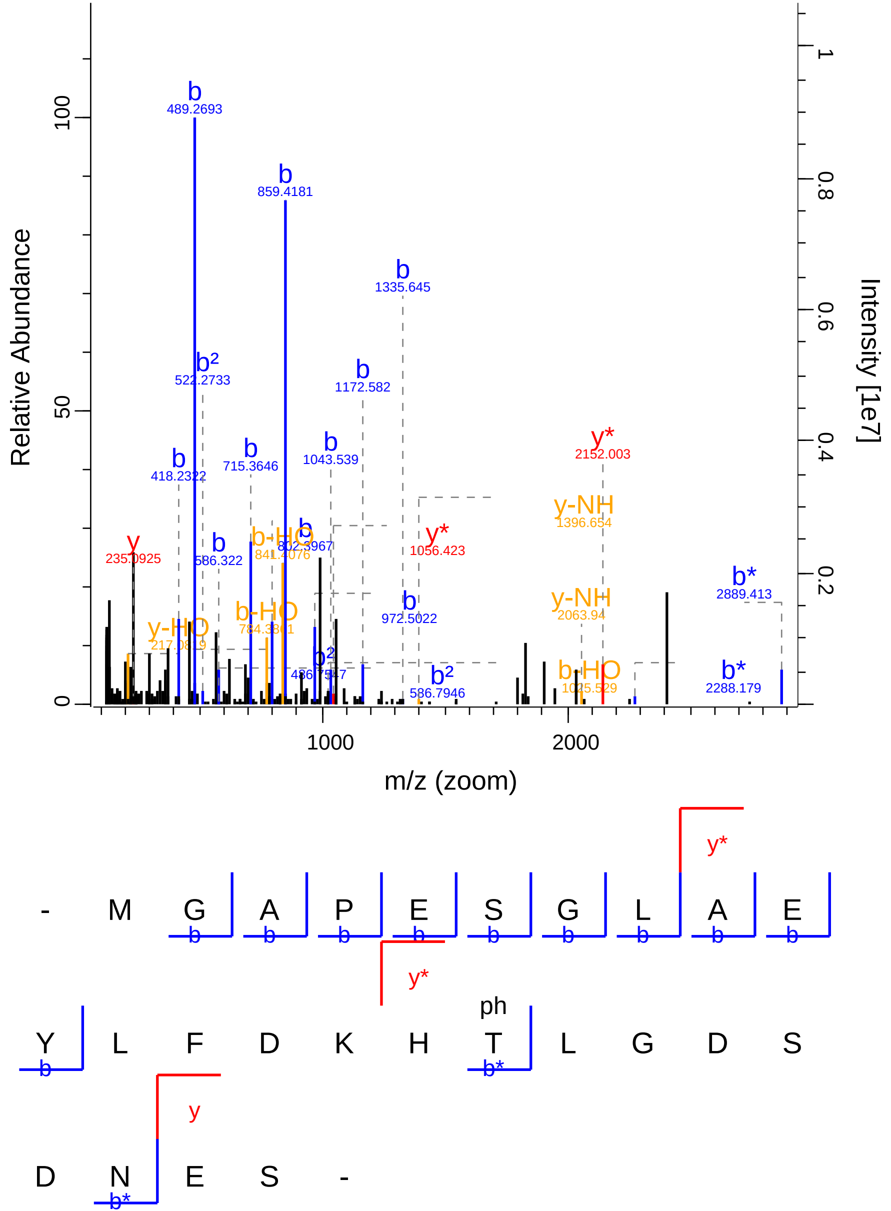

Raw File	Scan	Method	Score	m/z	Gene names
RO-QC-160121_ZackD_SS_Set_01_Fractic	35559	FTMS; HC	104.44	1126.91	NOTCH2

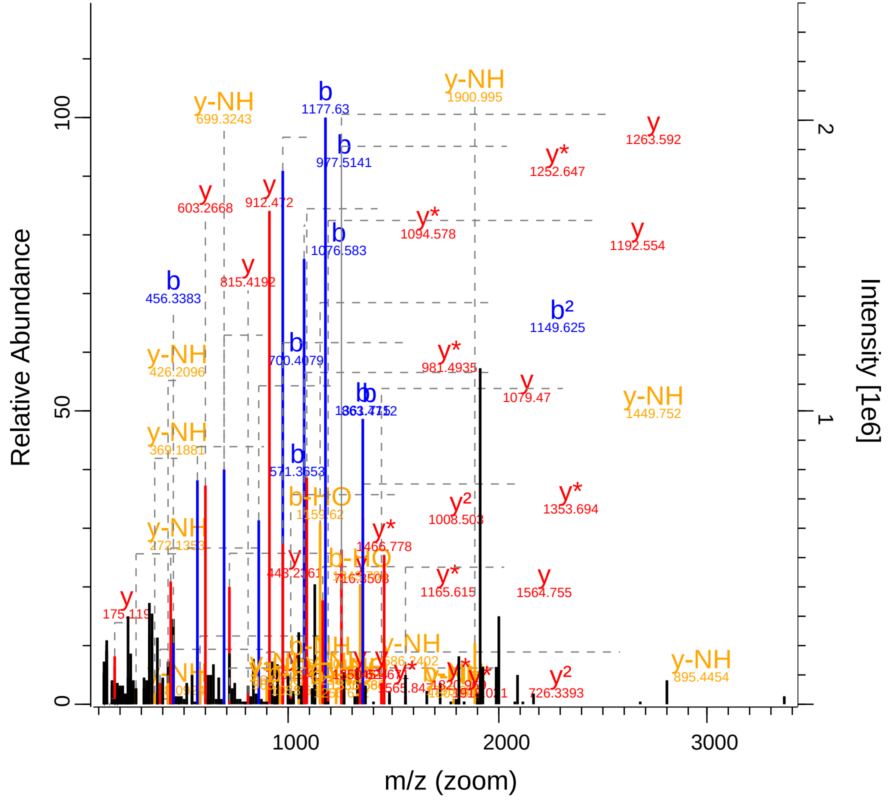

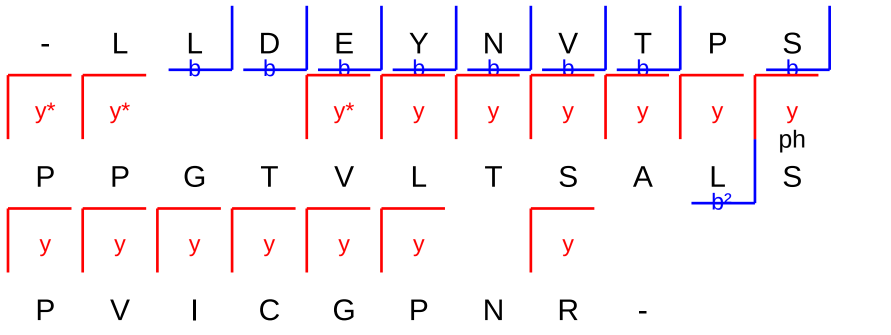

Raw File	Scan	Method	Score	m/z	Gene names
RO-QC-160121_ZackD_SS_Set_01_Fra	35783	FTMS; H(	256.79	1315.35	MYL12B;MYL12A;MY

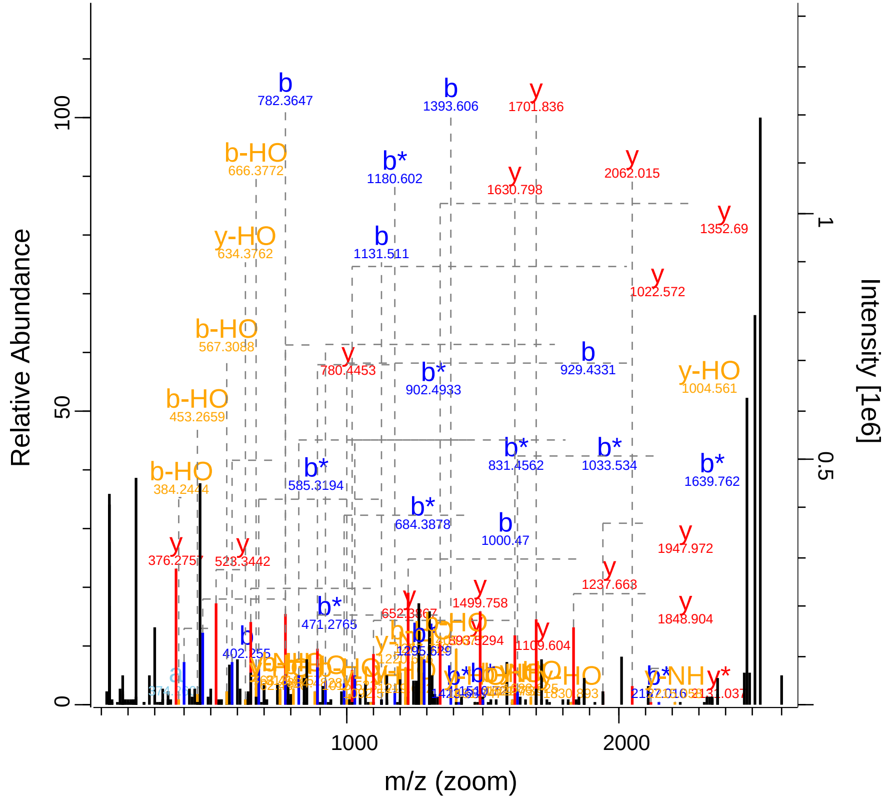

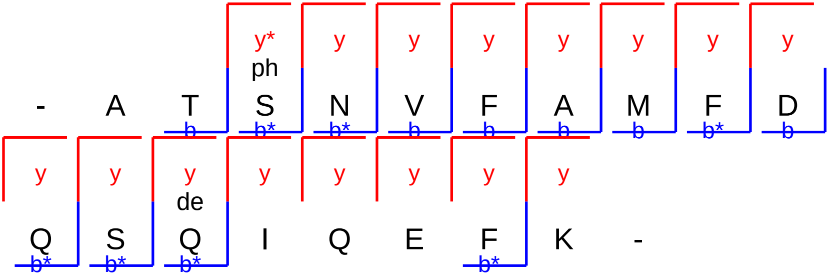

Raw File	Scan	Method	Score	m/z	Gene names
RO-QC-160121_ZackD_SS_Set_01_Fractior	4858	FTMS; HCI	42.56	444.06	RBM17

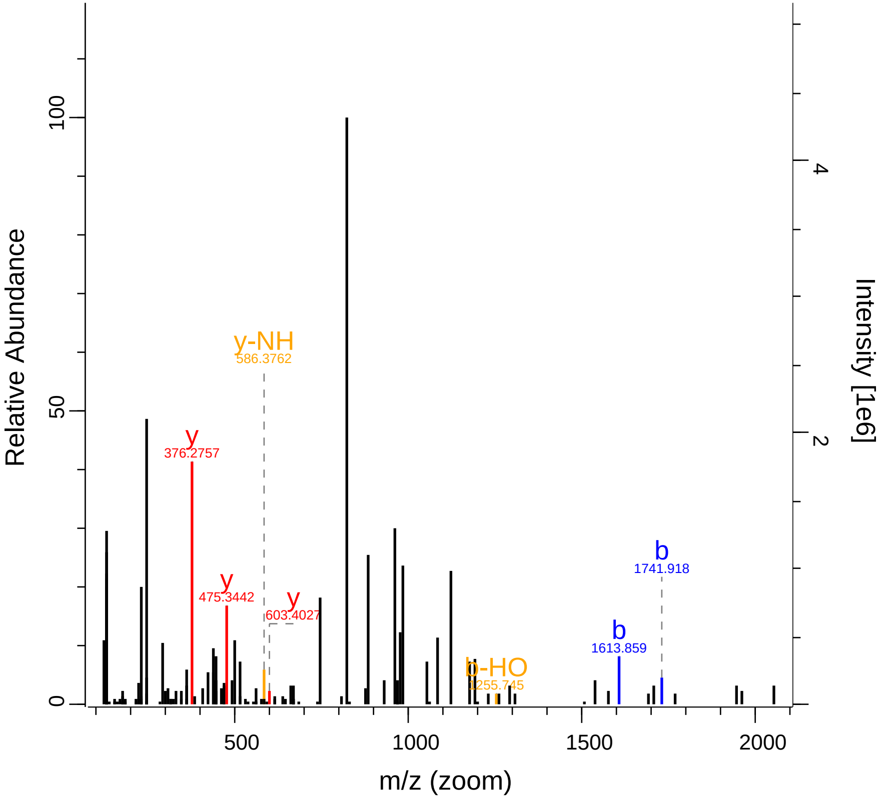

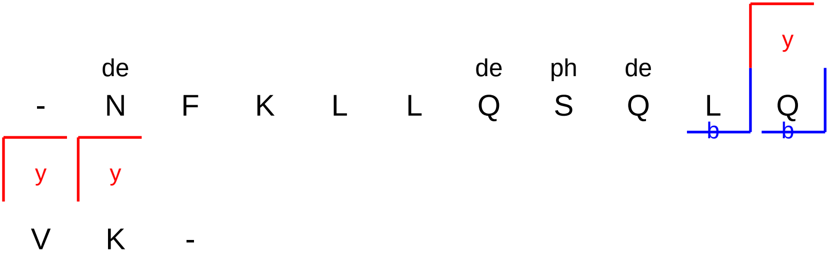

Raw File	Scan	Method	Score	m/z	Gene names
RO-QC-160121_ZackD_SS_Set_01_Fraction_	6347	FTMS; HCI	108.96	668	SHROOM2

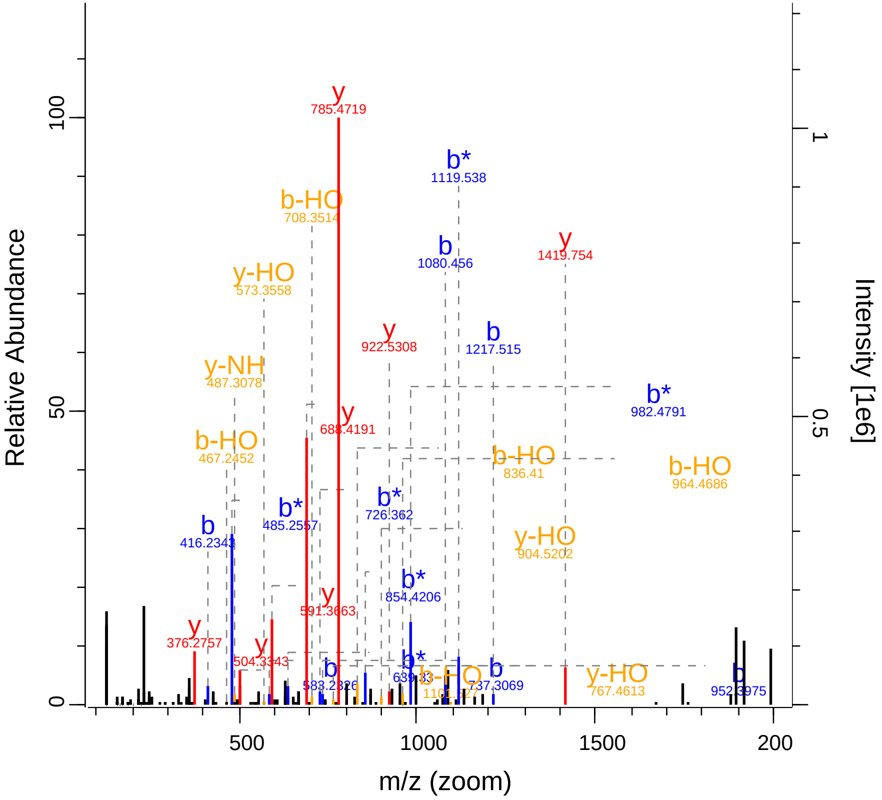

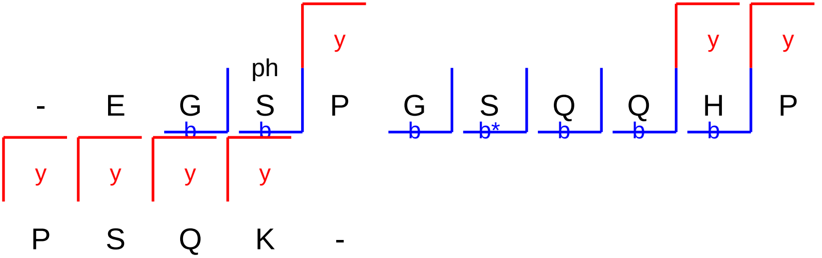

Raw File	Scan	Method	Score	m/z	Gene names
RO-QC-160121_ZackD_SS_Set_01_Fractio	7560	FTMS; HC	159.81	1110.02	EIF4B

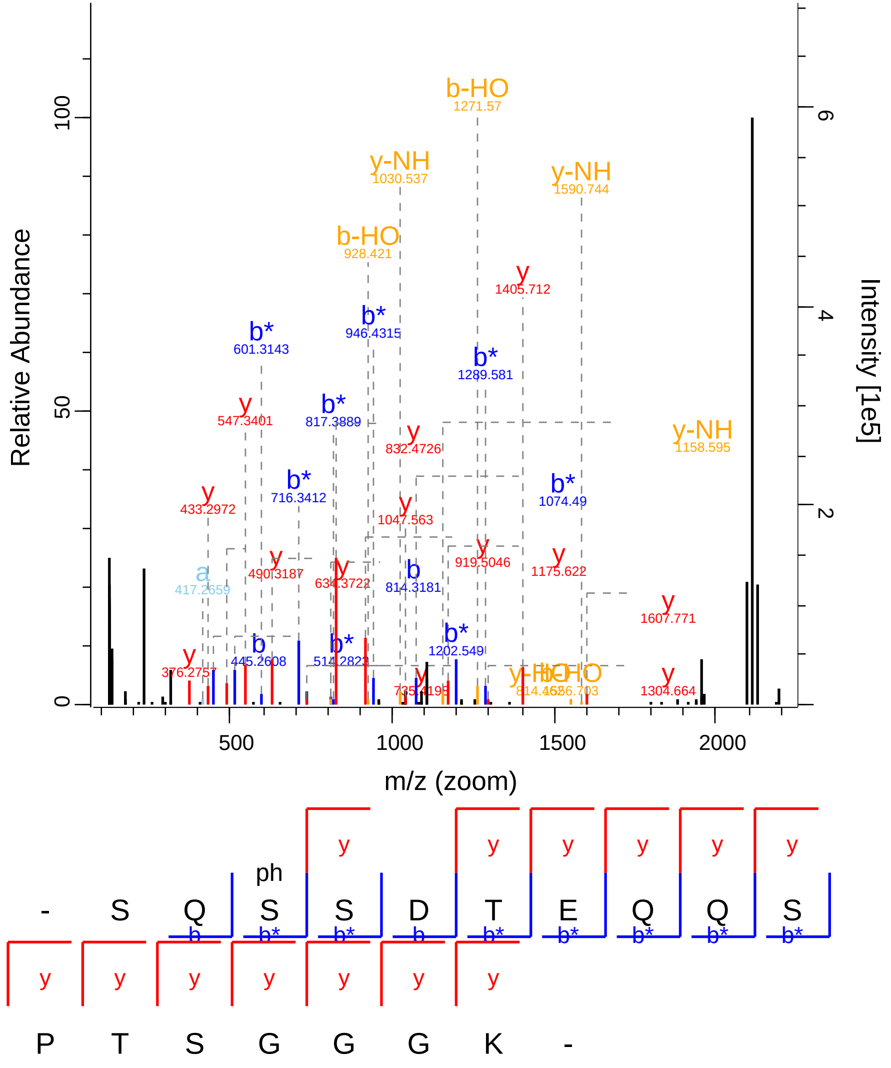

Raw File	Scan	Method	Score	m/z	Gene names
RO-QC-160121_ZackD_SS_Set_01_Fractior	8215	FTMS; HC	160.38	942.03	ARHGAP1

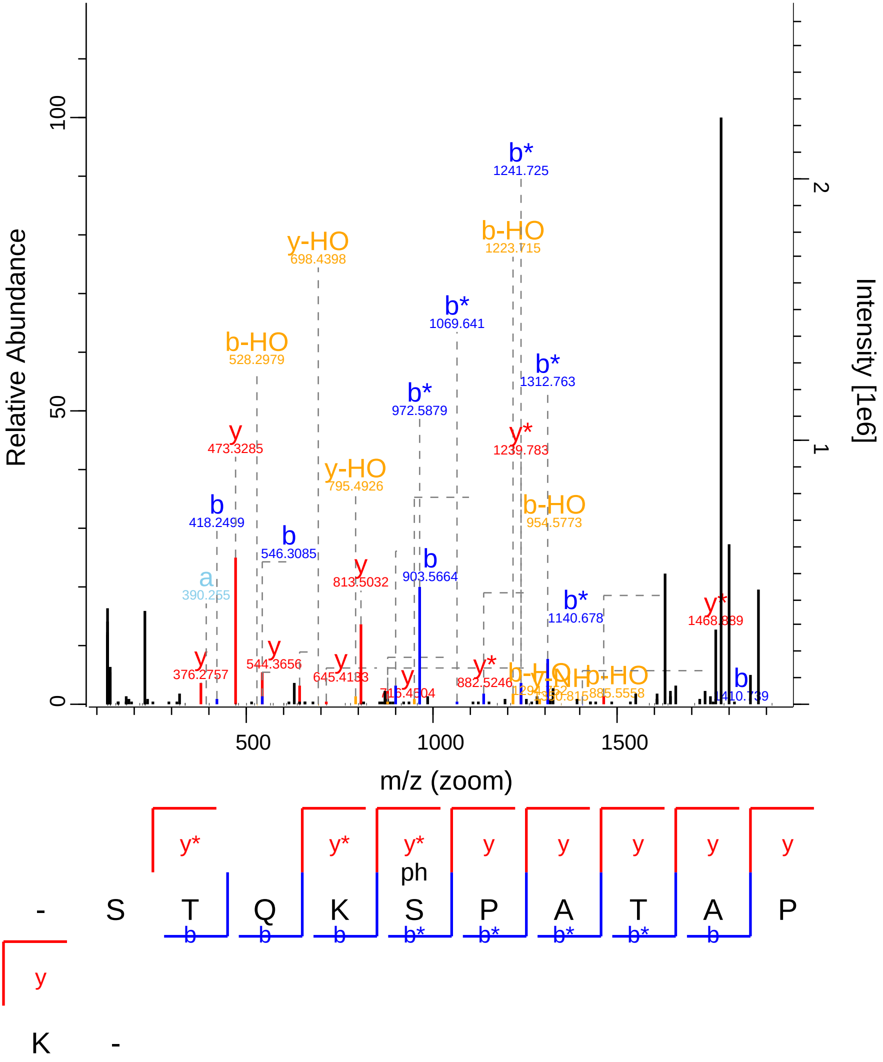

Raw File	Scan	Method	Score	m/z	Gene names
RO-QC-160121_ZackD_SS_Set_01_Fractior	8801	FTMS; HC	122.46	735.35	PLEC

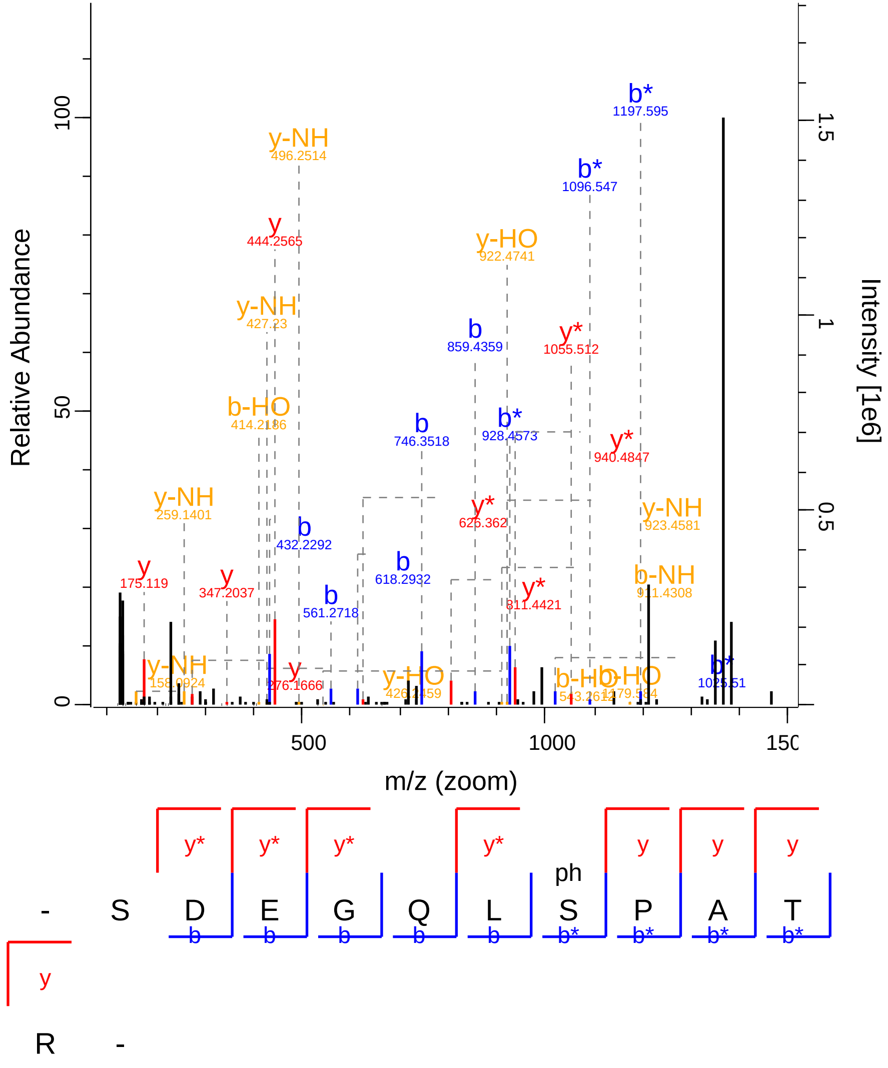

Raw File	Scan	Method	Score	m/z	Gene names
RO-QC-160121_ZackD_SS_Set_01_Fractior	9140	FTMS; HCI	98.3	720.08	VPS4A

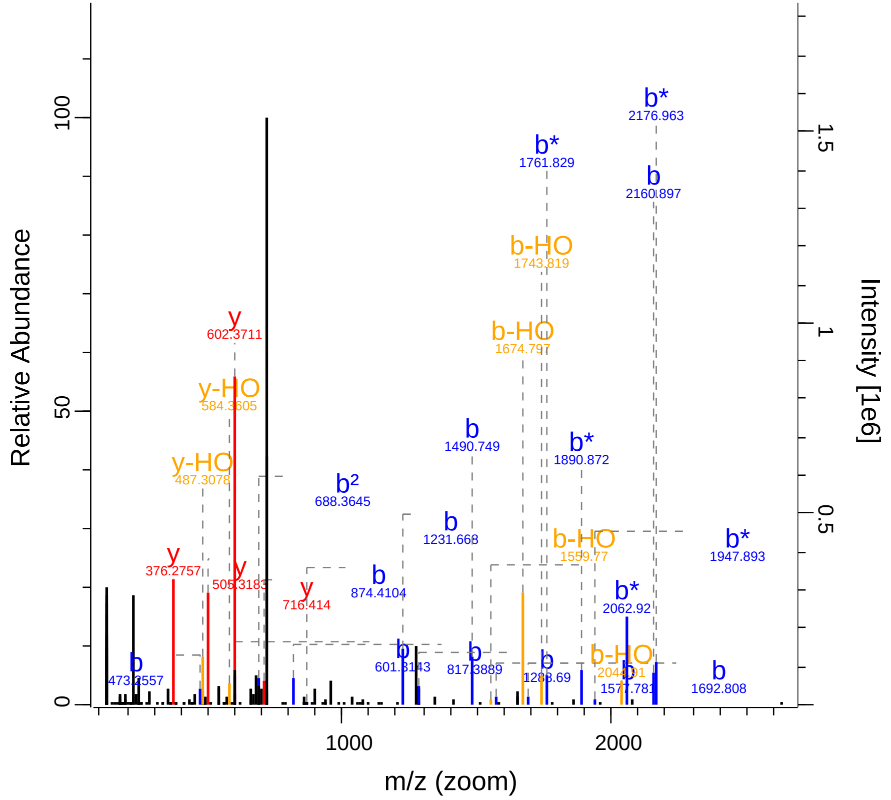

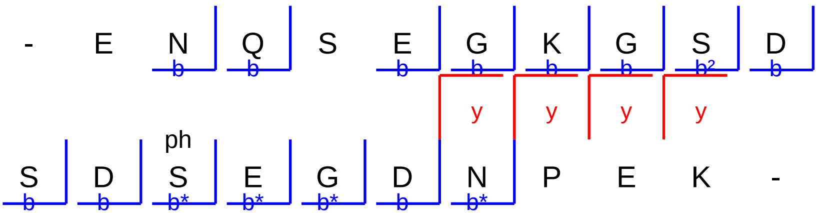

Raw File	Scan	Method	Score	m/z	Gene names
RO-QC-160121_ZackD_SS_Set_01_Fractio	10514	FTMS; HC	184.44	792.41	BCLAF1

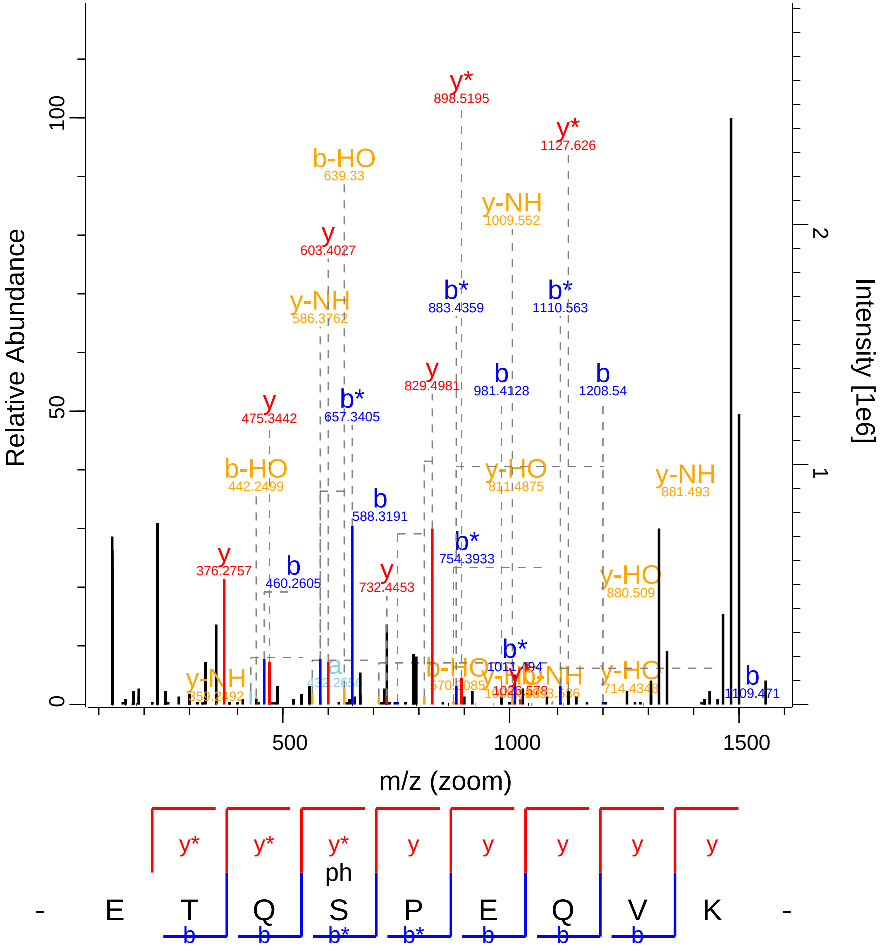

Raw File	Scan	Method	Score	m/z	Gene names
RO-QC-160121_ZackD_SS_Set_01_Fractio	11456	FTMS; HC	52.55	460.02	PFKFB3

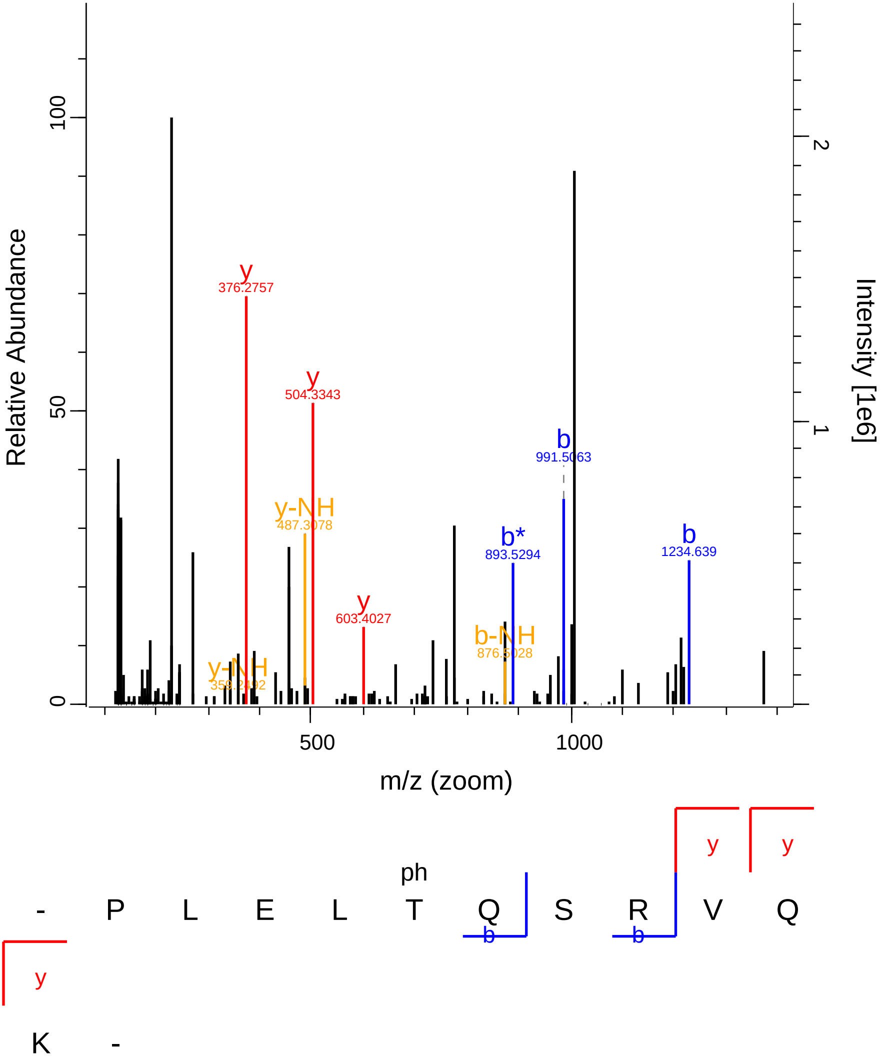

Raw File	Scan	Method	Score	m/z	Gene names
RO-QC-160121_ZackD_SS_Set_01_Fractio	11487	FTMS; HC	88.99	642.33	BASP1

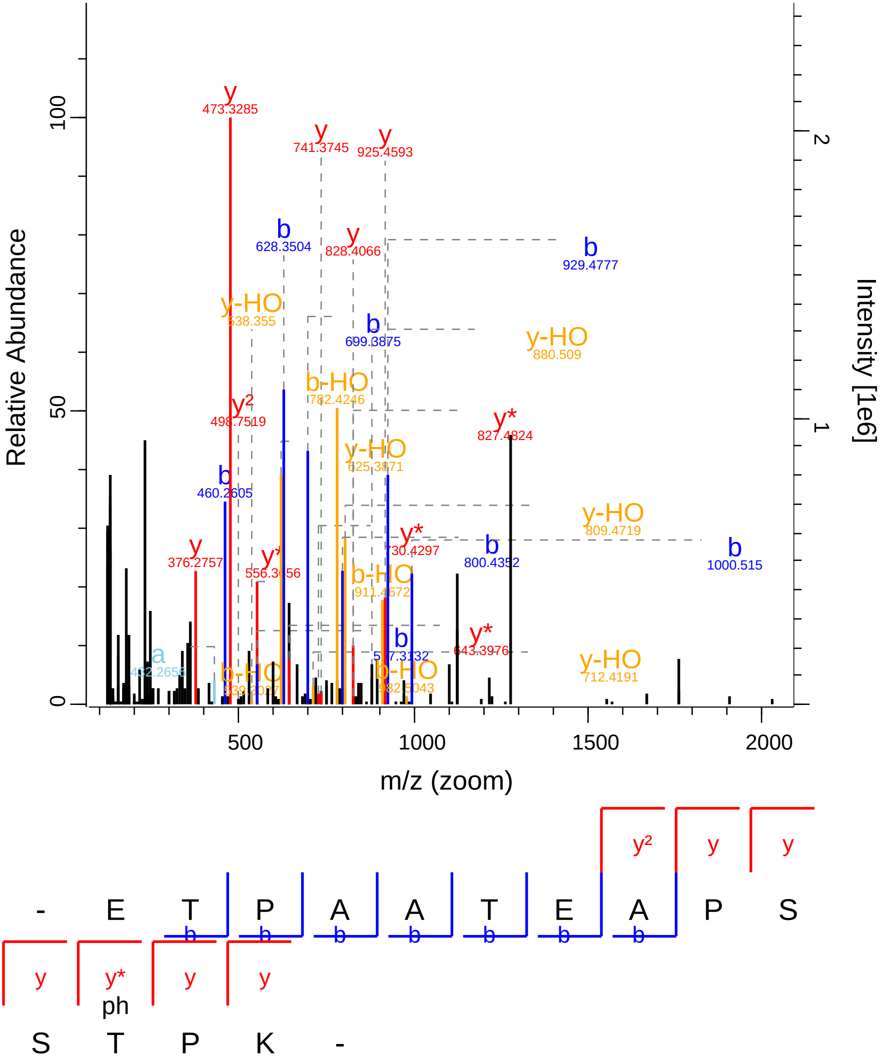

Raw File	Scan	Method	Score	m/z	Gene names
RO-QC-160121_ZackD_SS_Set_01_Fractio	12071	FTMS; HC	47.08	943.51	CDCA3

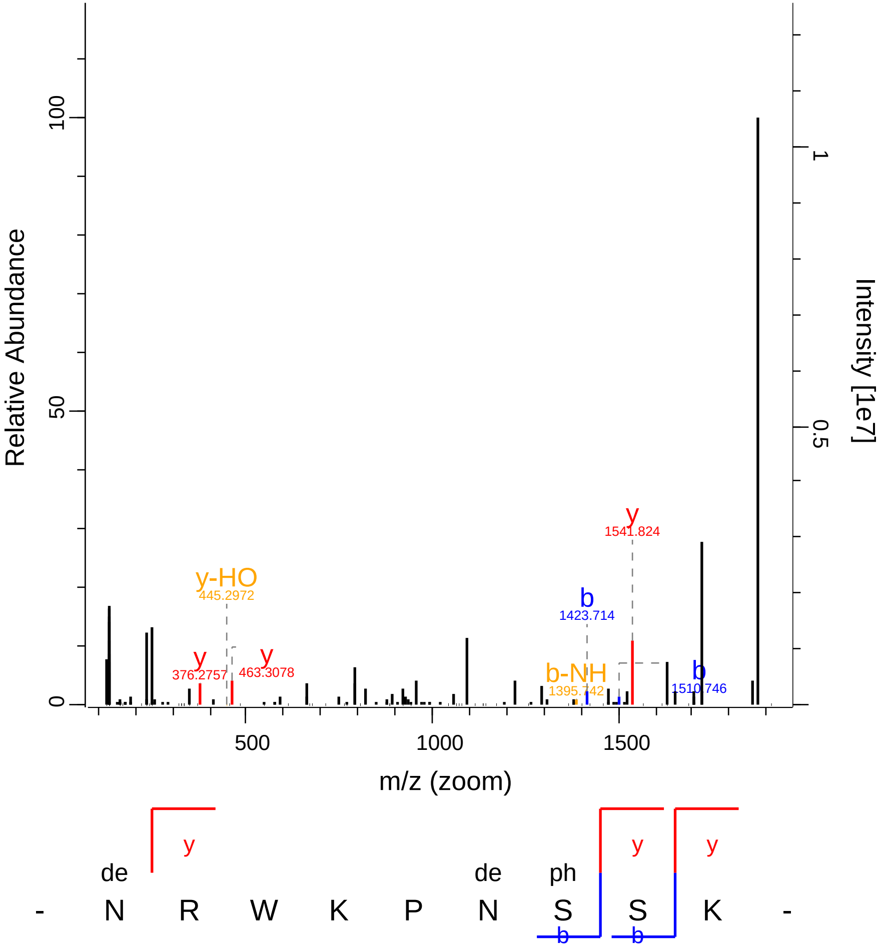

Raw File	Scan	Method	Score	m/z	Gene names
RO-QC-160121_ZackD_SS_Set_01_Fractio	13018	FTMS; HC	217.22	854.39	CBX3

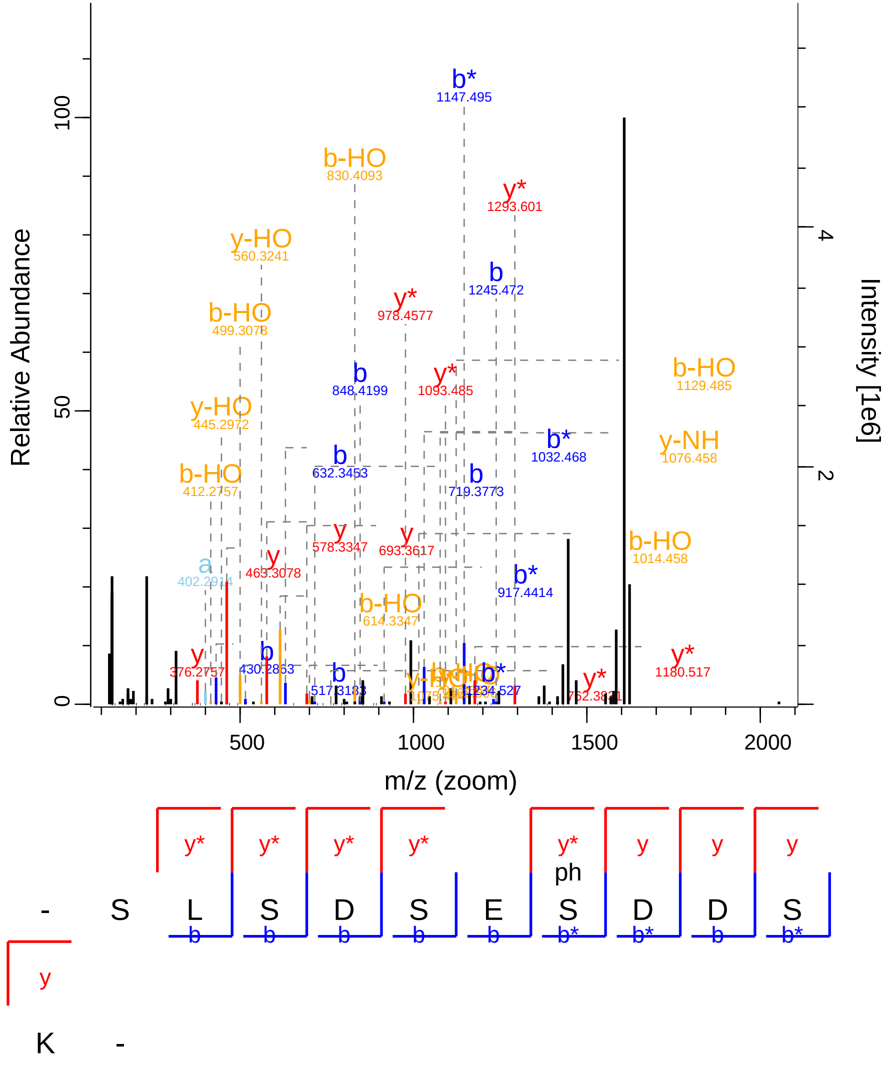

Raw File	Scan	Method	Score	m/z	Gene names
RO-QC-160121_ZackD_SS_Set_01_Fractior	13177	FTMS; HCl	122.3	882.1	HMGN3

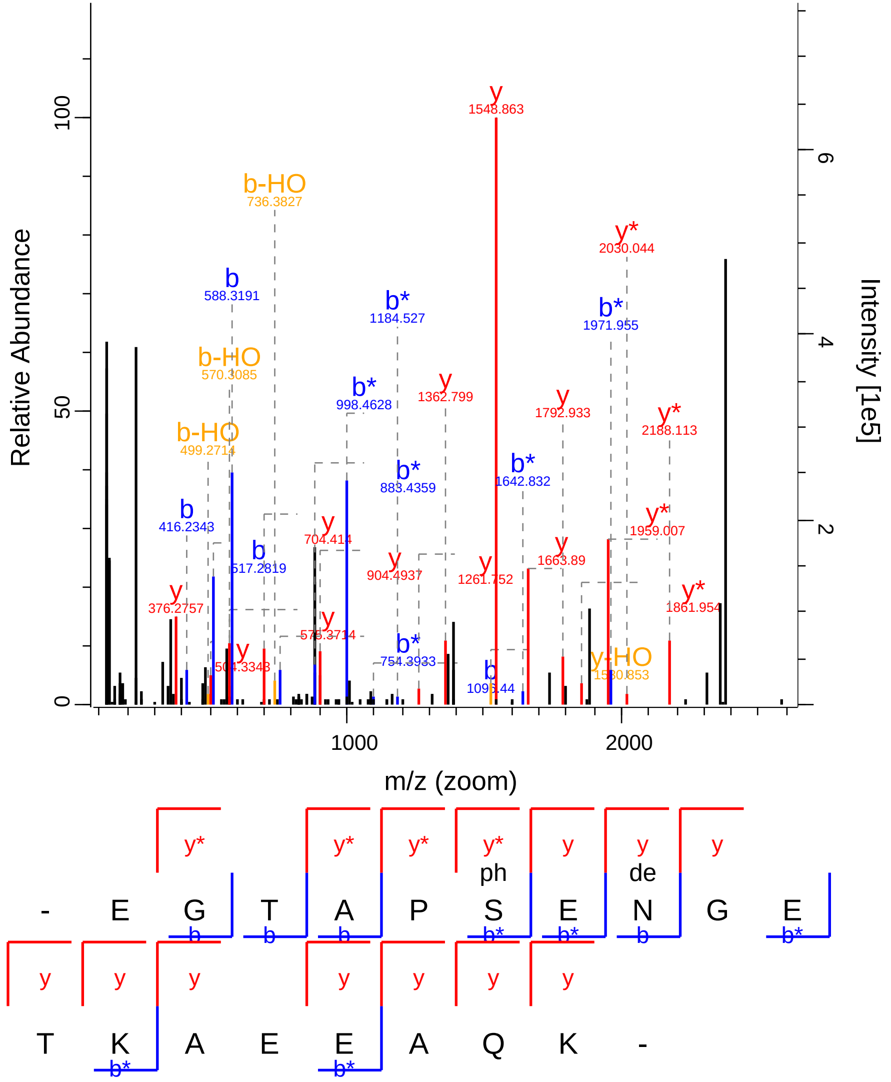

Raw File	Scan	Method	Score	m/z	Gene names
RO-QC-160121_ZackD_SS_Set_01_Fractio	14341	FTMS; HC	96.1	964.05	GAS2

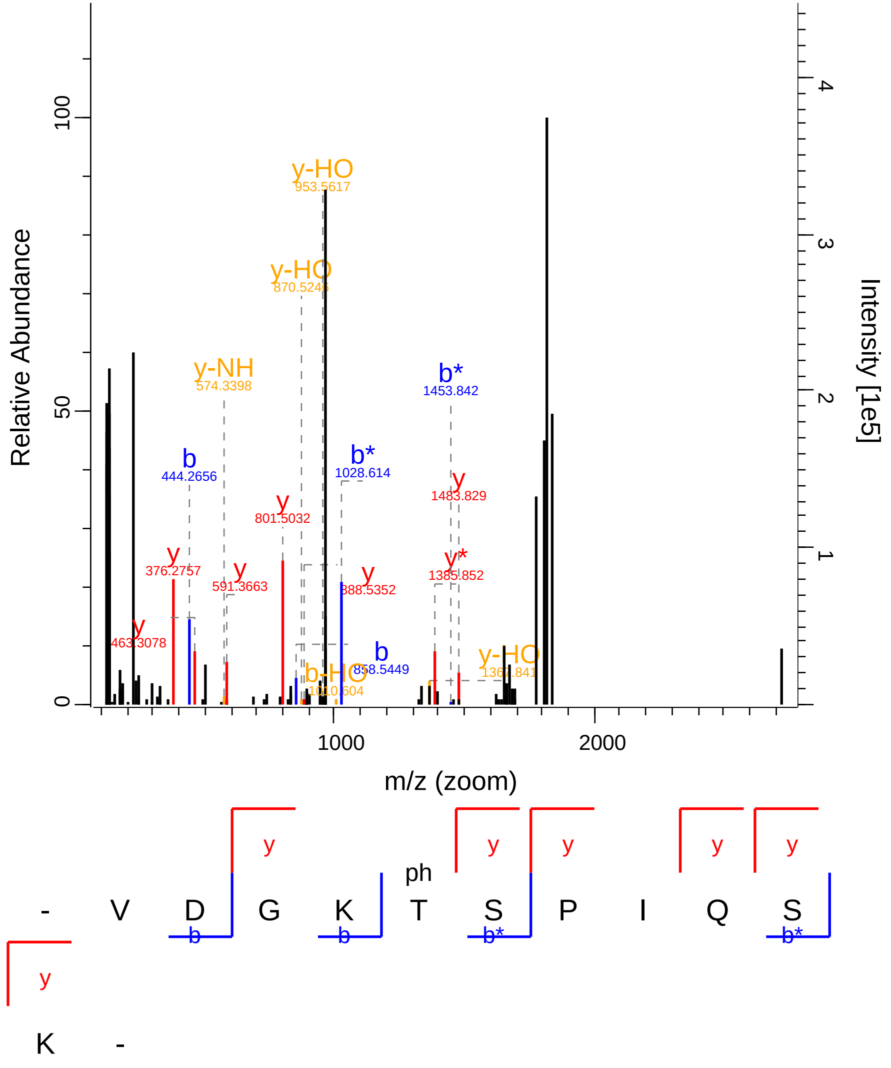

Raw File	Scan	Method	Score	m/z	Gene names
RO-QC-160121_ZackD_SS_Set_01_Fractio	14583	FTMS; HC	210.79	905.43	HTATSF1

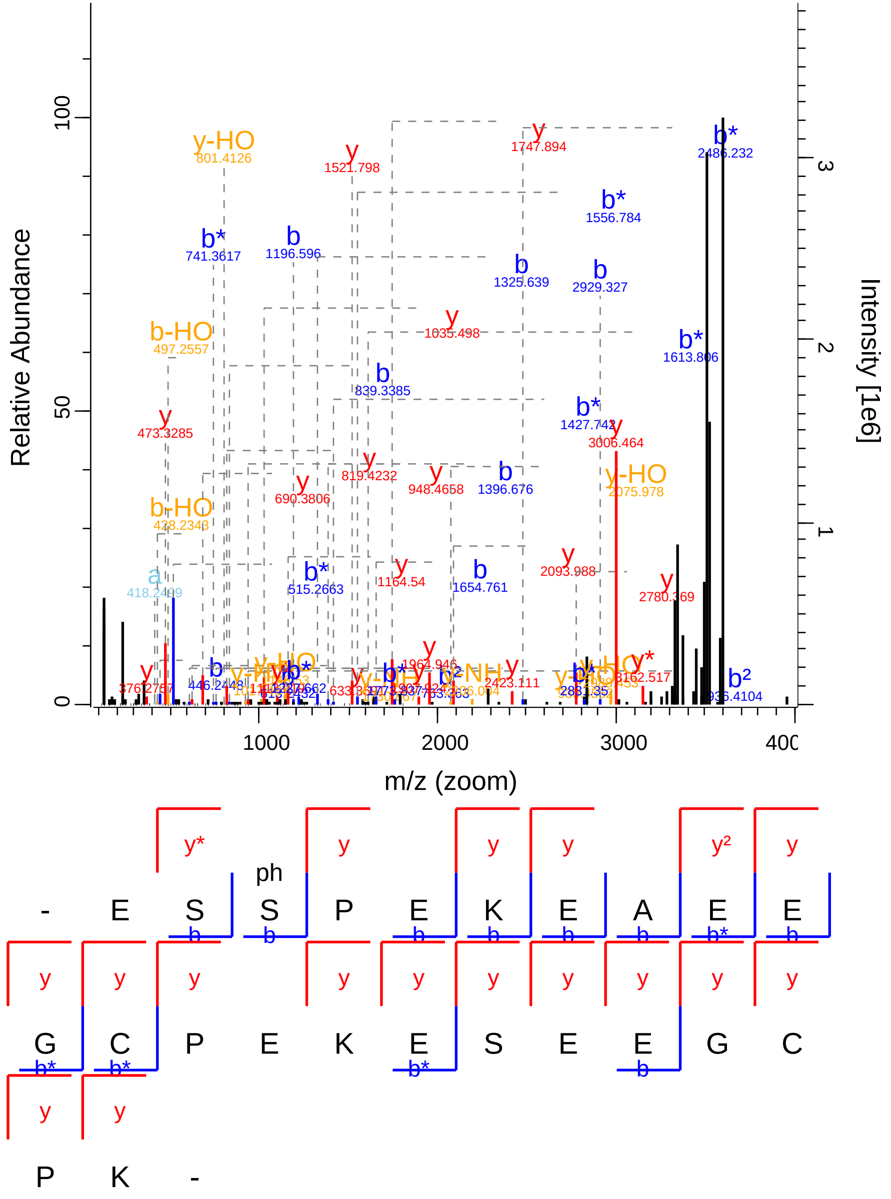

Raw File	Scan	Method	Score	m/z	Gene names
RO-QC-160121_ZackD_SS_Set_01_Fractio	14755	FTMS; HC	175.66	824.39	NDRG1

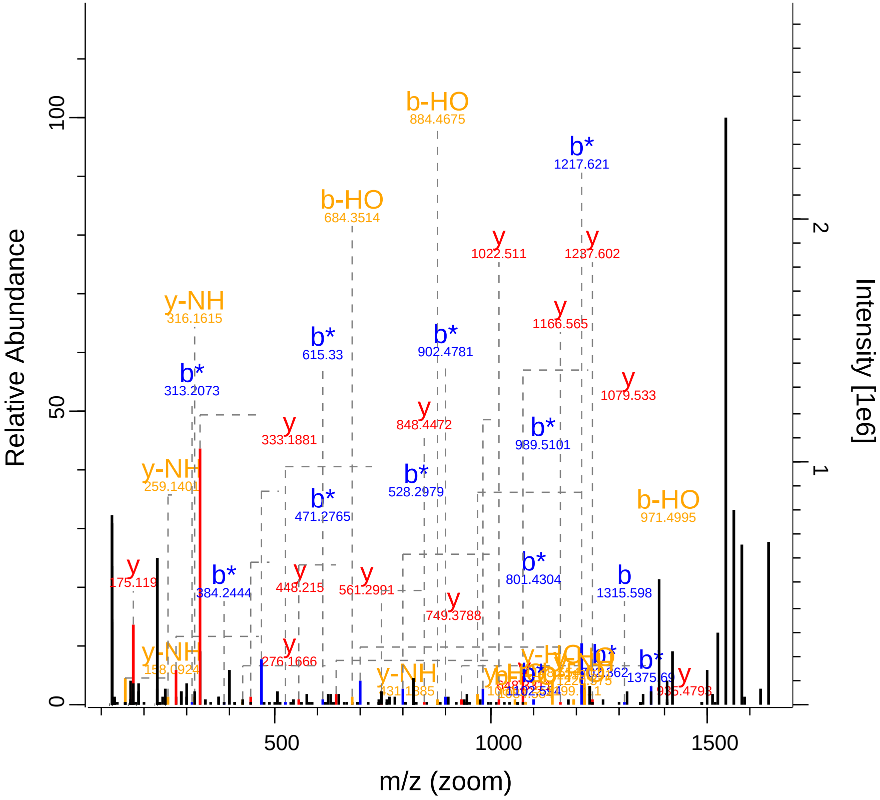

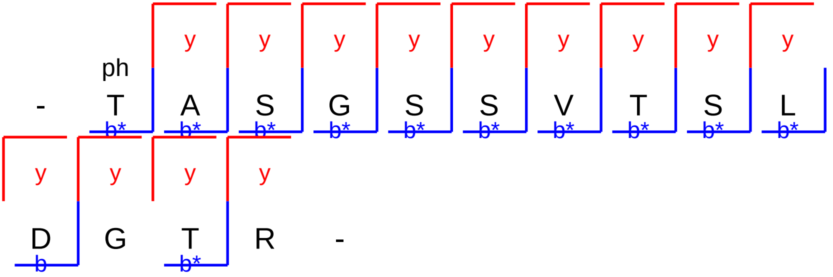

Raw File	Scan	Method	Score	m/z	Gene names
RO-QC-160121_ZackD_SS_Set_01_Fractio	14952	FTMS; HC	138.76	663.34	THRAP3

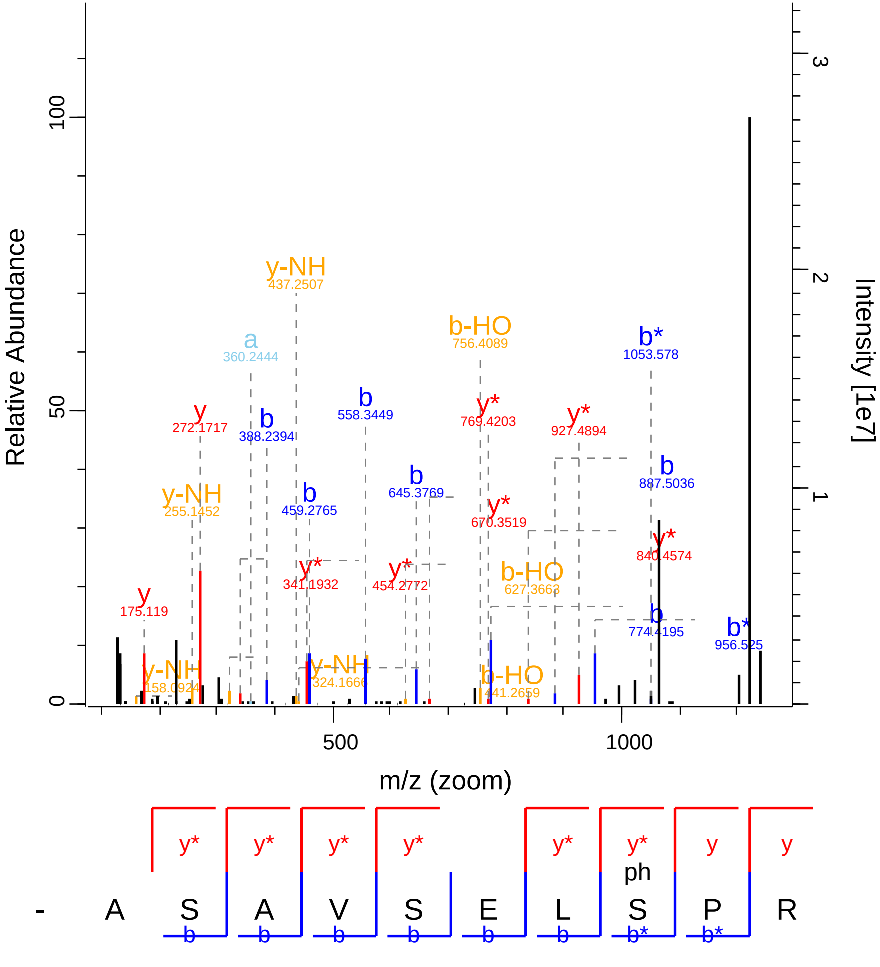

Raw File	Scan	Method	Score	m/z	Gene names
RO-QC-160121_ZackD_SS_Set_01_Fractio	15476	FTMS; HC	62.92	831.47	KIF21A

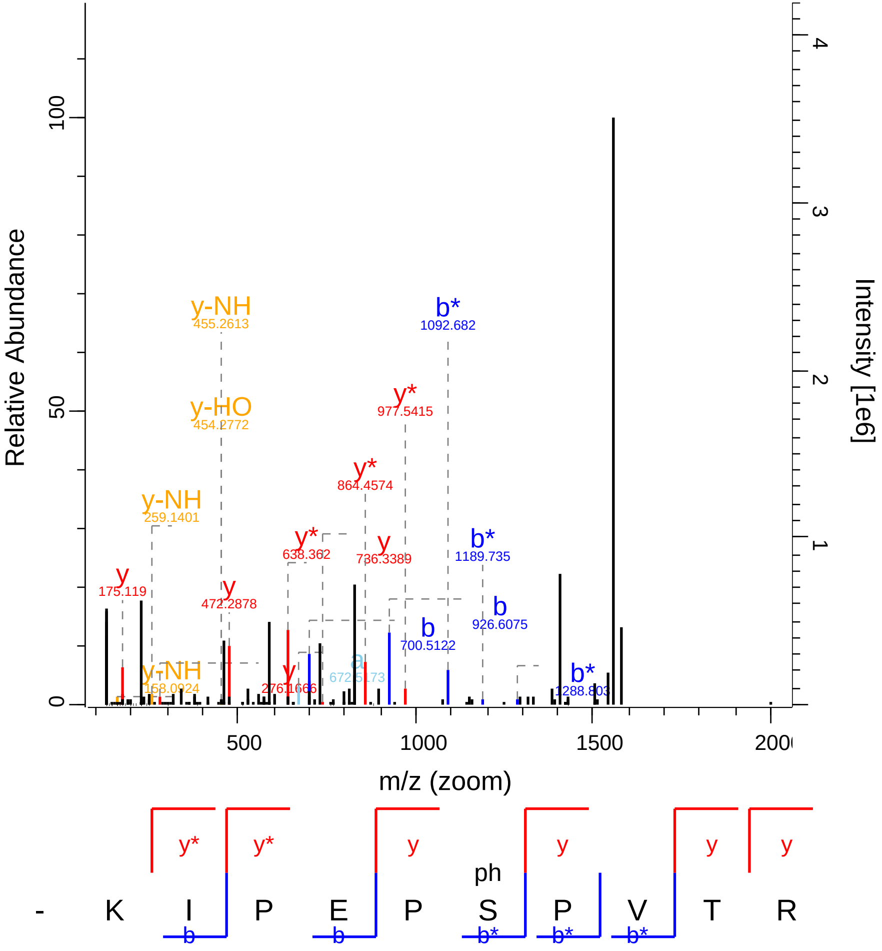

Raw File	Scan	Method	Score	m/z	Gene names
RO-QC-160121_ZackD_SS_Set_01_Fractio	15773	FTMS; HC	48.14	677.36	ANKRD26

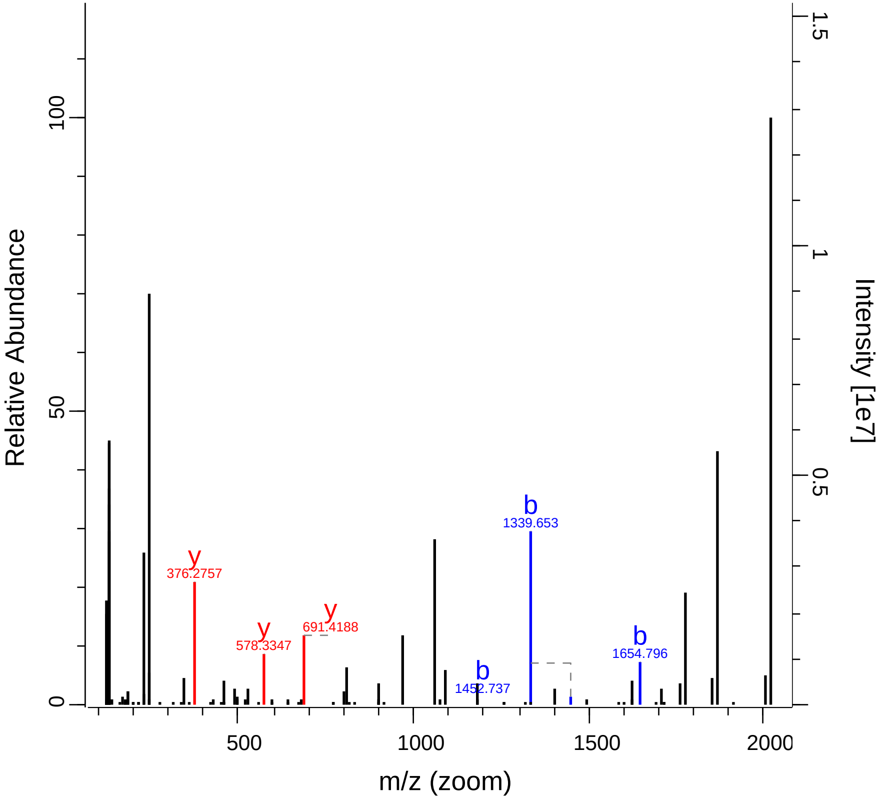

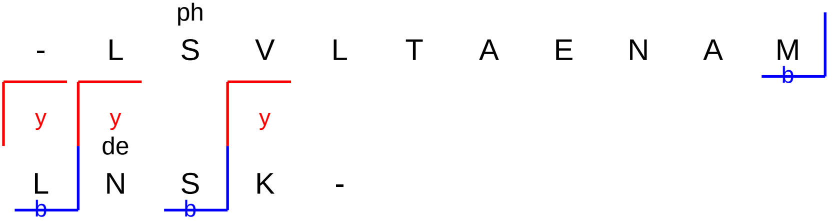

Raw File	Scan	Method	Score	m/z	Gene names
RO-QC-160121_ZackD_SS_Set_01_Fractic	15799	FTMS; HC	46.78	1118.19	LUC7L3

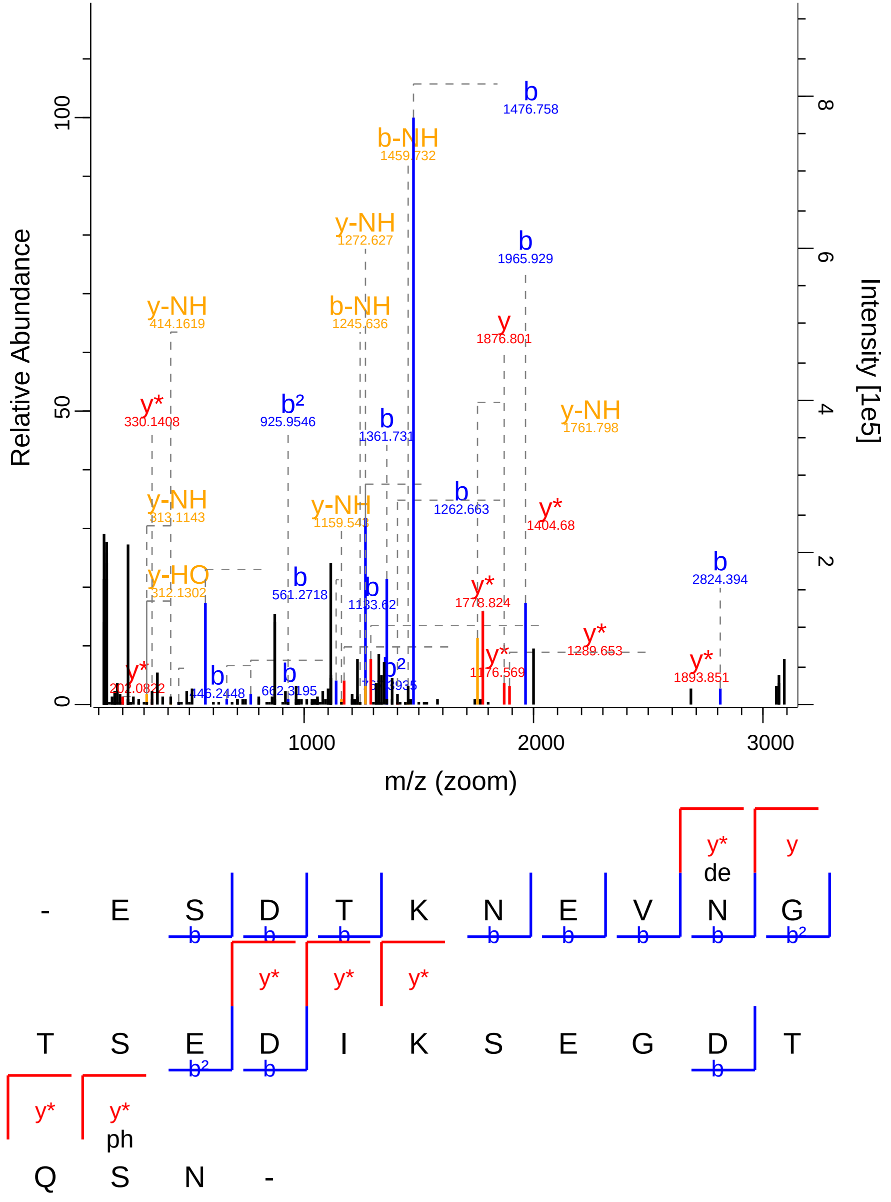

Raw File	Scan	Method	Score	m/z	Gene names
RO-QC-160121_ZackD_SS_Set_01_Fractio	15847	FTMS; HC	78.2	739.36	SLC16A1

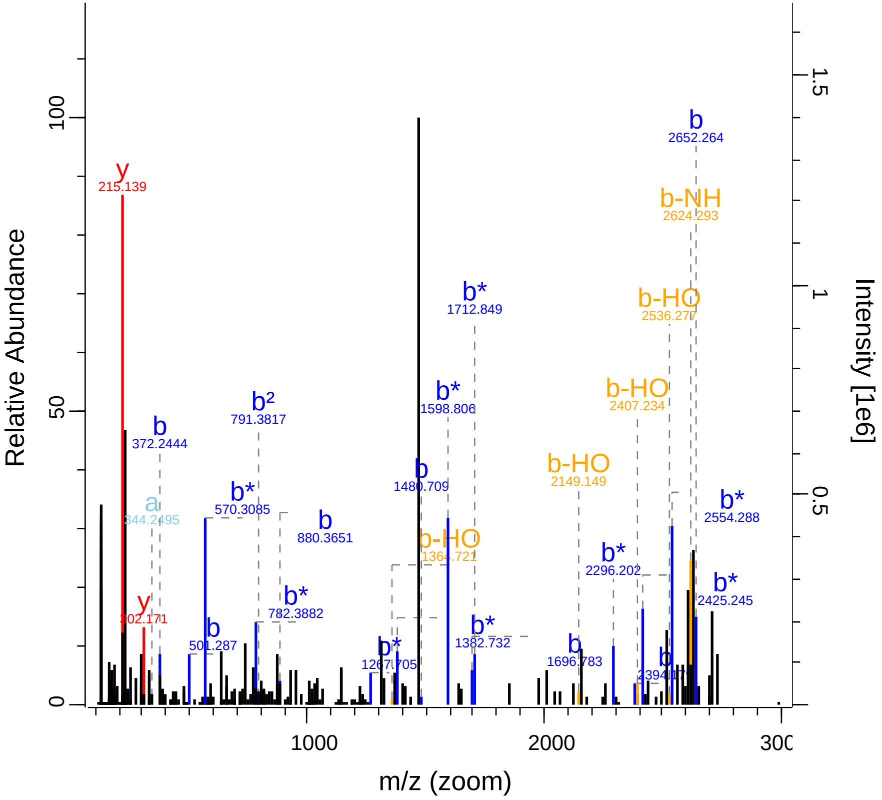

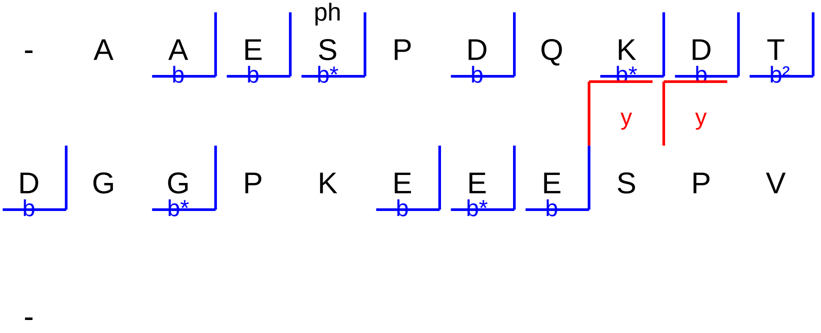

Raw File	Scan	Method	Score	m/z	Gene names
RO-QC-160121_ZackD_SS_Set_01_Fractio	15924	FTMS; HC	113.65	796.04	TOE1

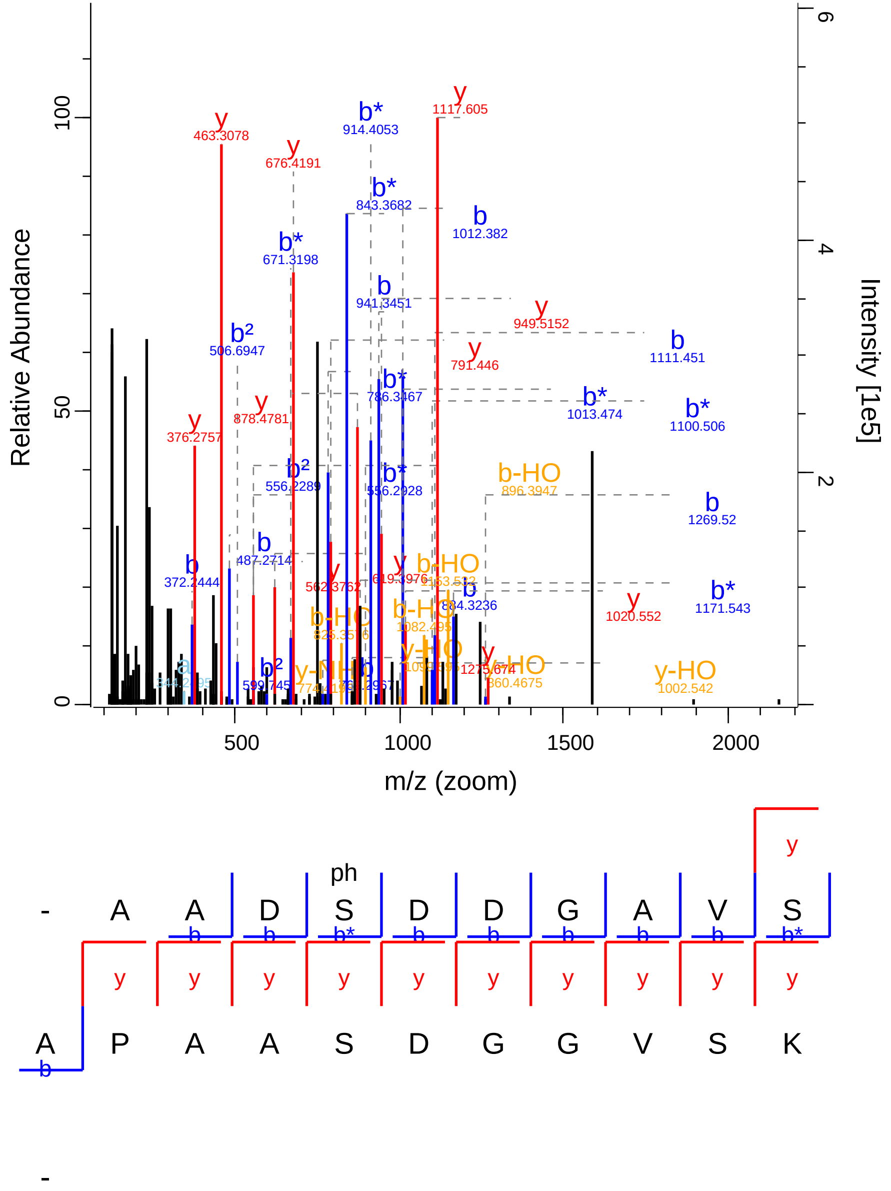

Raw File	Scan	Method	Score	m/z	Gene names
RO-QC-160121_ZackD_SS_Set_01_Fractio	16636	FTMS; HC	187.16	990.49	TNS3

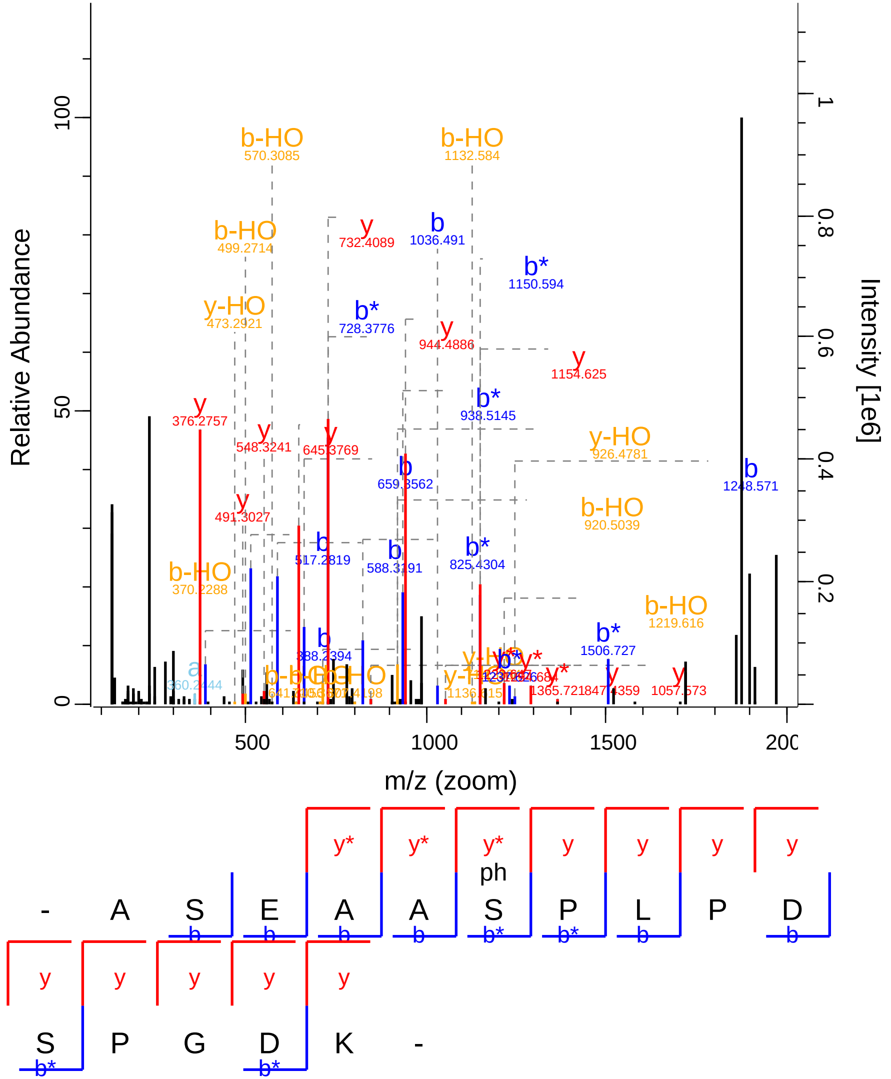

Raw File	Scan	Method	Score	m/z	Gene names
RO-QC-160121_ZackD_SS_Set_01_Fractio	16984	FTMS; HC	75.36	788.06	SLC16A3

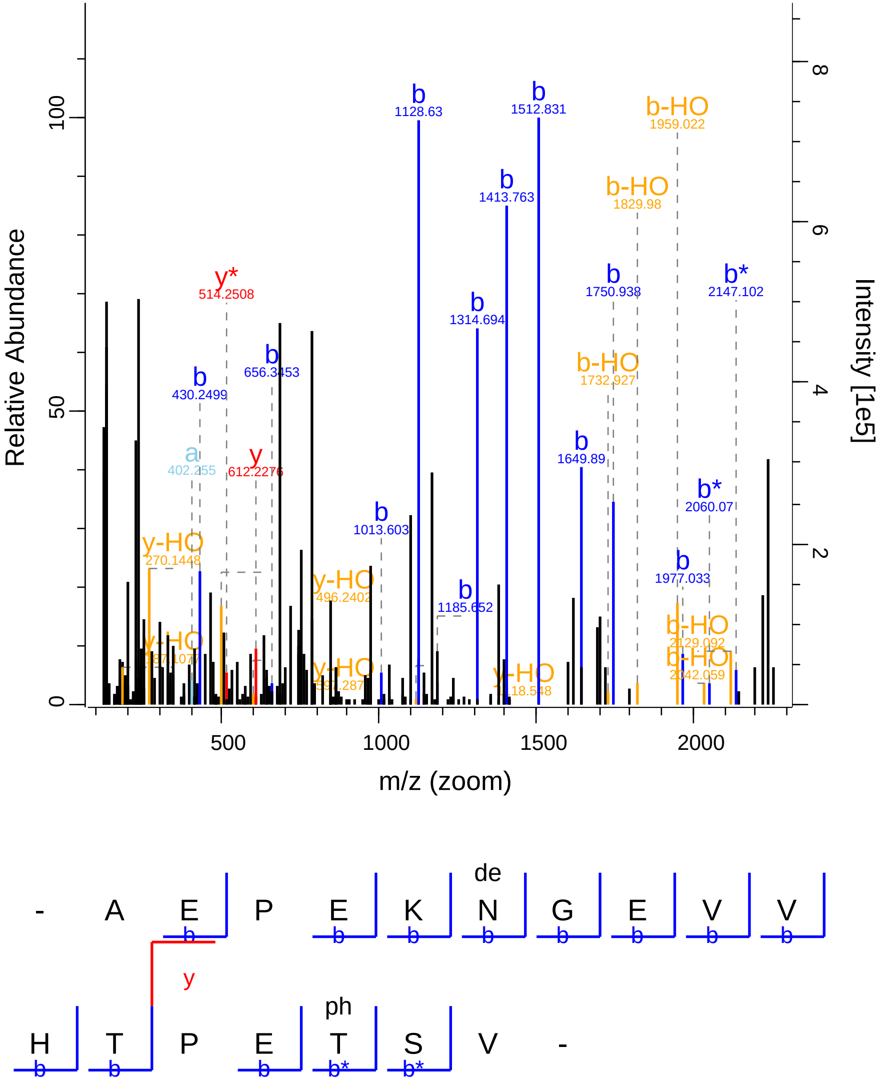

Raw File	Scan	Method	Score	m/z	Gene names
RO-QC-160121_ZackD_SS_Set_01_Fractio	17301	FTMS; HC	96.06	811.37	PGAM1

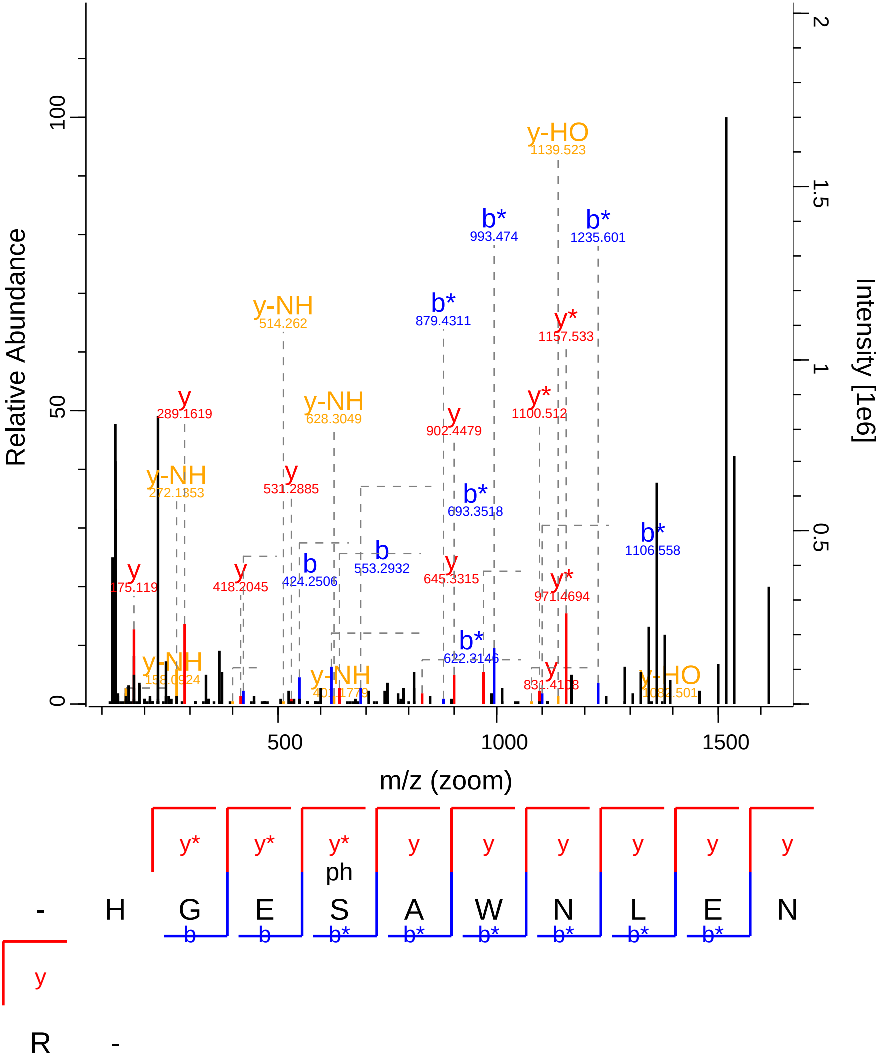

Raw File	Scan	Method	Score	m/z	Gene names
RO-QC-160121_ZackD_SS_Set_01_Fractic	17390	FTMS; HC	109.02	1017.06	PLEKHA7

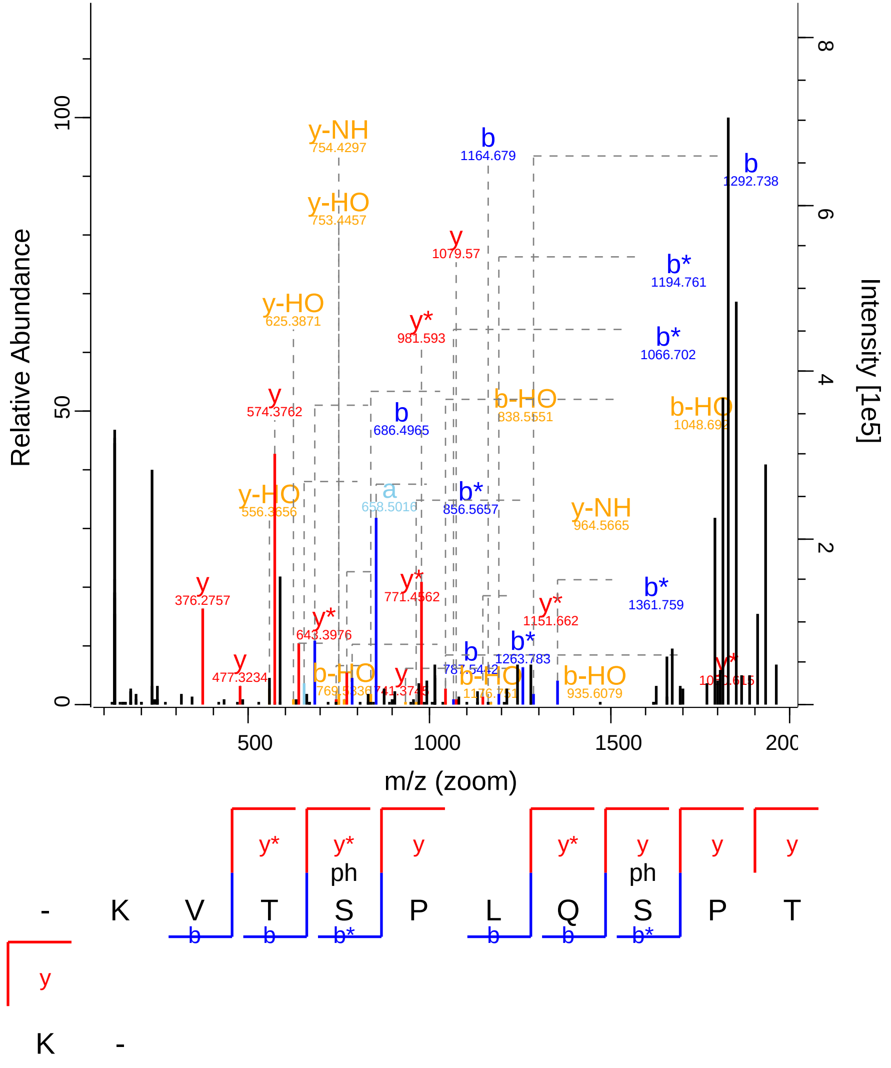

Raw File	Scan	Method	Score	m/z	Gene names
RO-QC-160121_ZackD_SS_Set_01_Fractio	17469	FTMS; HC	73.8	852.72	AKT2

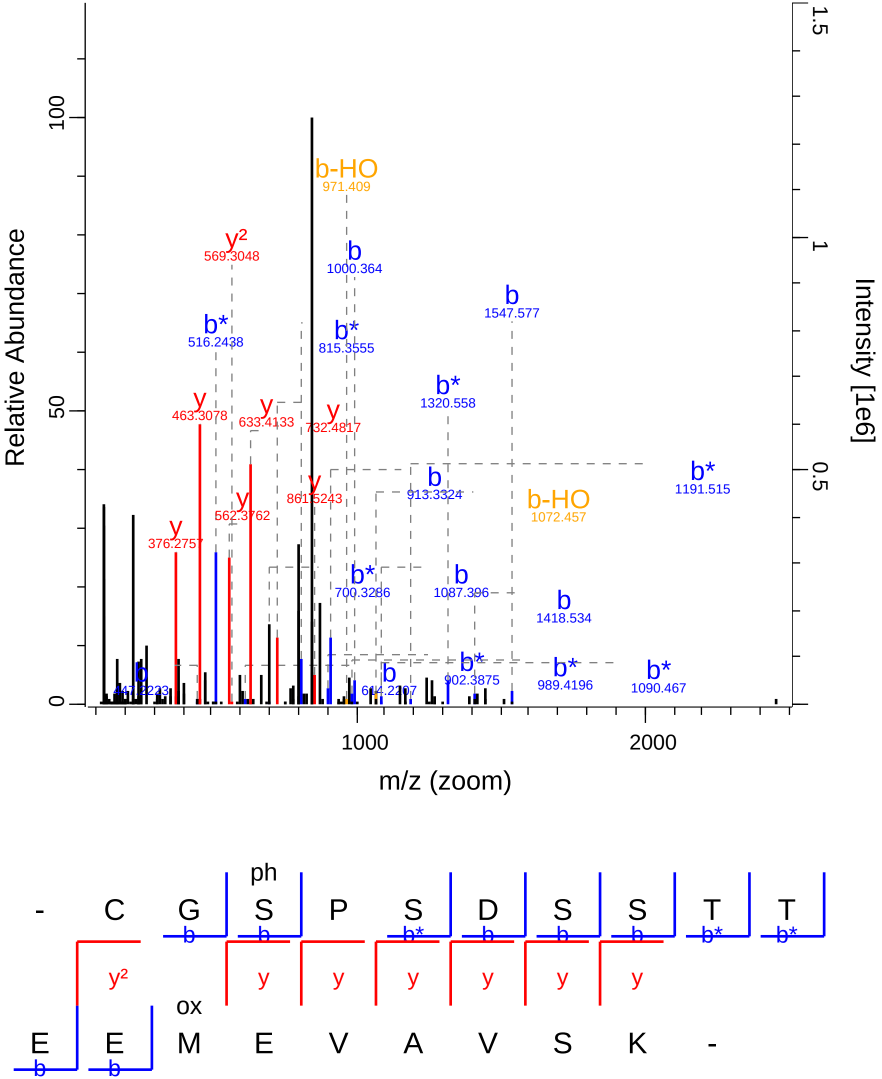

Raw File	Scan	Method	Score	m/z	Gene names
RO-QC-160121_ZackD_SS_Set_01_Fractio	18149	FTMS; HC	107.83	766.38	MAP1B

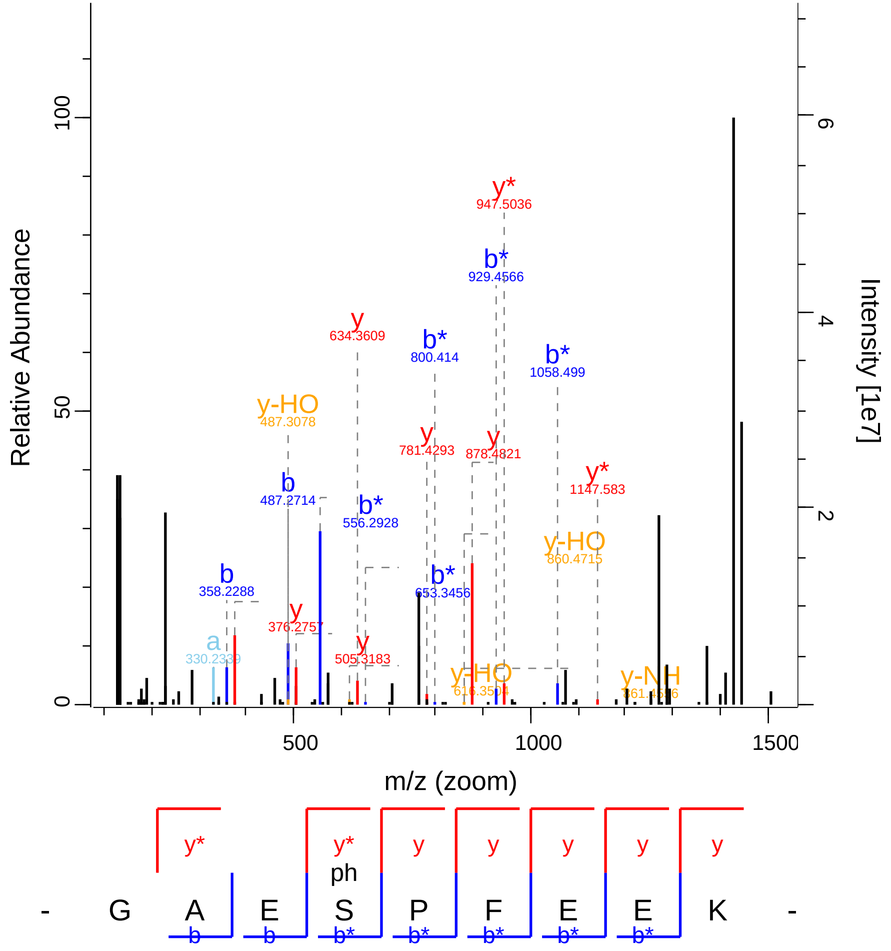

Raw File	Scan	Method	Score	m/z	Gene names
RO-QC-160121_ZackD_SS_Set_01_Fractio	18408	FTMS; HC	79.15	643.82	TSPYL4

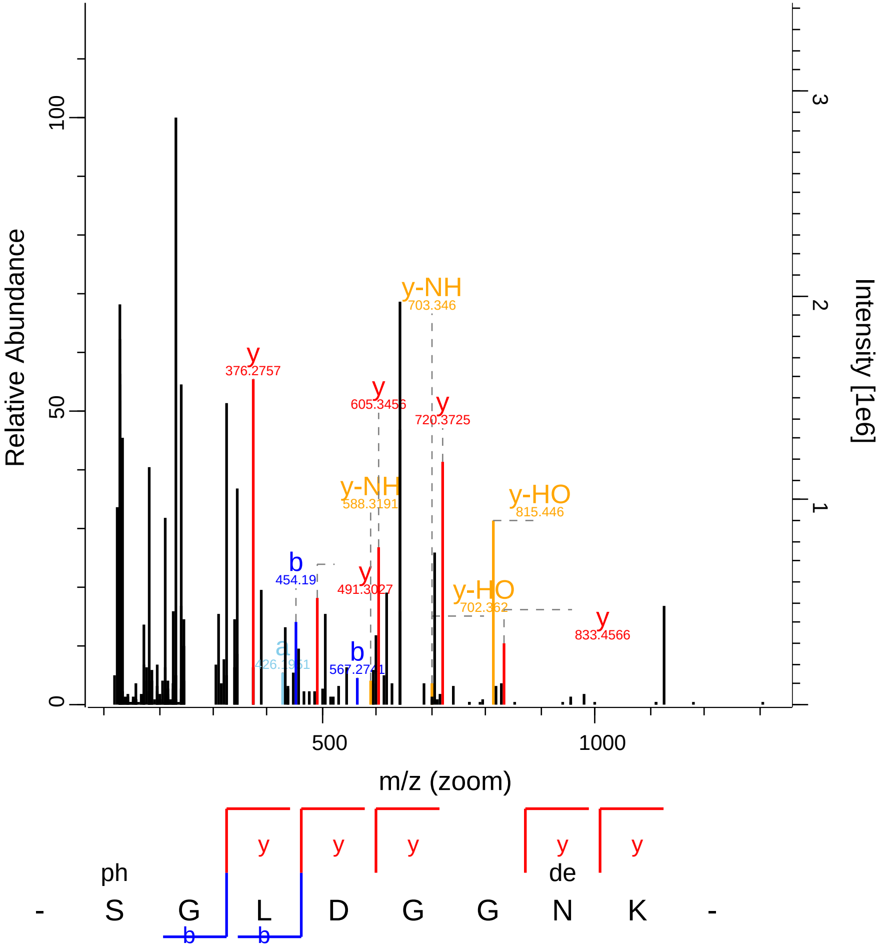

Raw File	Scan	Method	Score	m/z	Gene names
RO-QC-160121_ZackD_SS_Set_01_Fractior	18628	FTMS; HCI	51.61	638.6	MCTP2

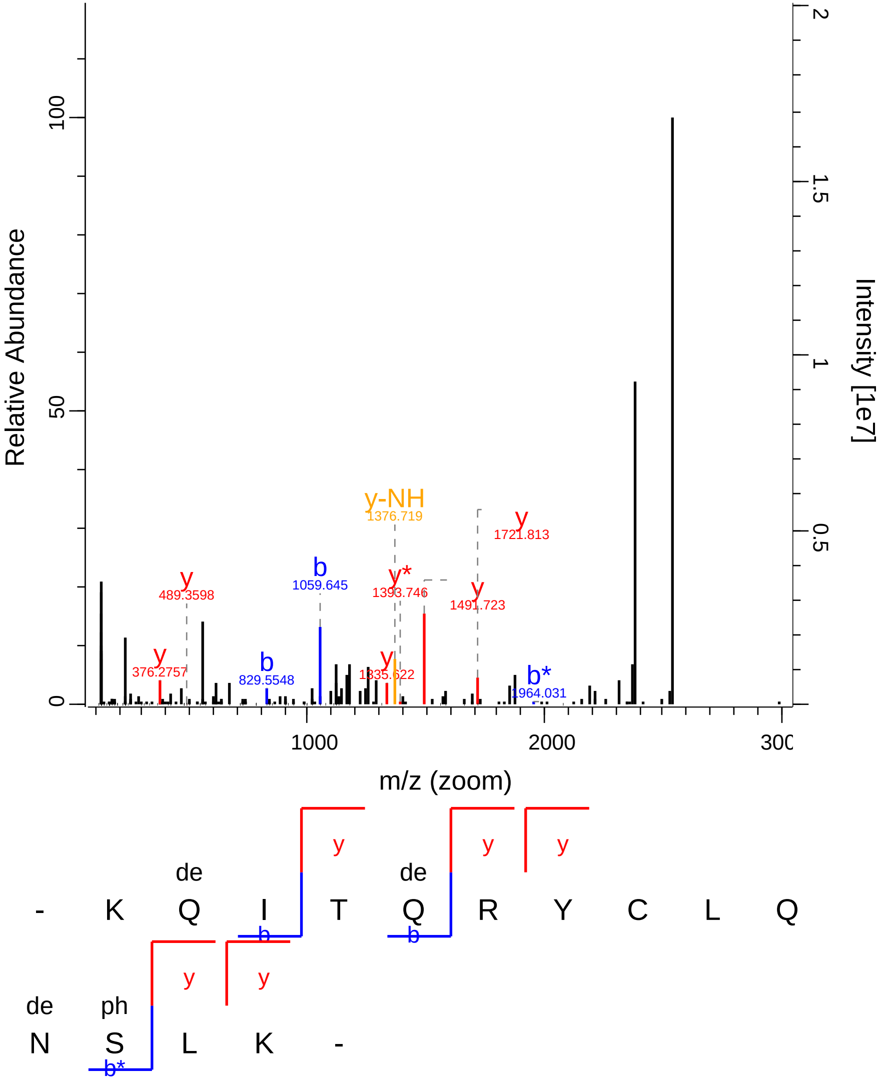

Raw File	Scan	Method	Score	m/z	Gene names
RO-QC-160121_ZackD_SS_Set_01_Fraction	19182	FTMS; HCI	58.98	891	ELP4

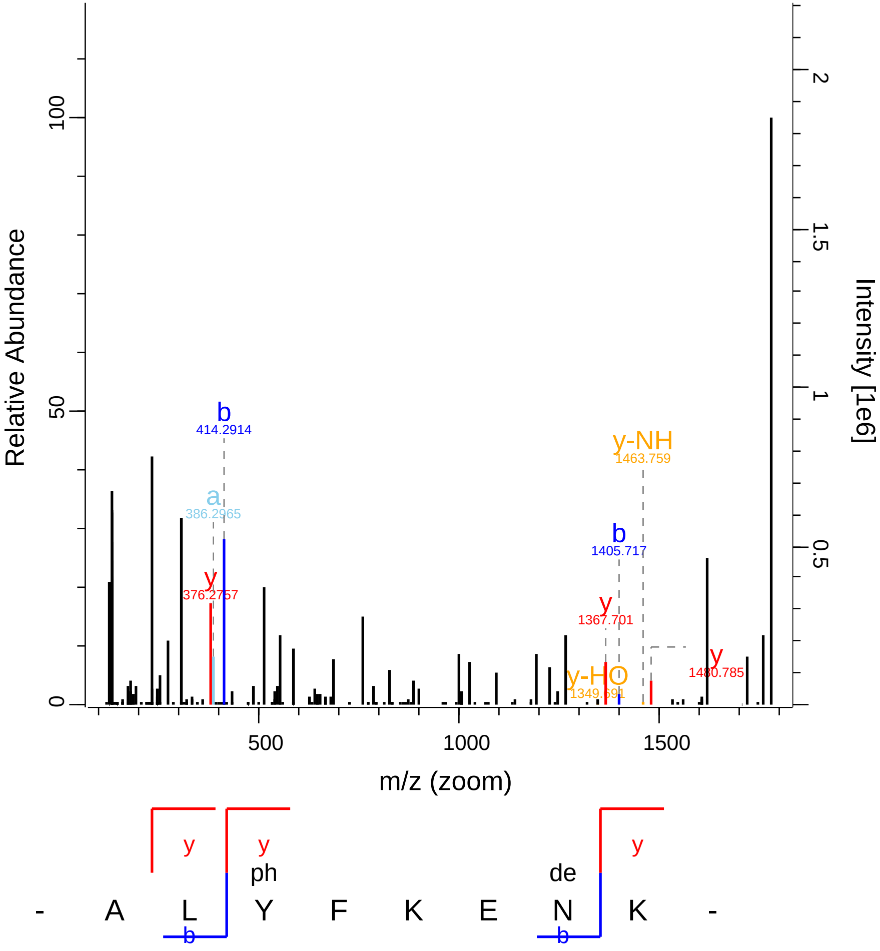

Raw File	Scan	Method	Score	m/z	Gene names
RO-QC-160121_ZackD_SS_Set_01_Fractio	20445	FTMS; HC	73.98	622.66	PFKP

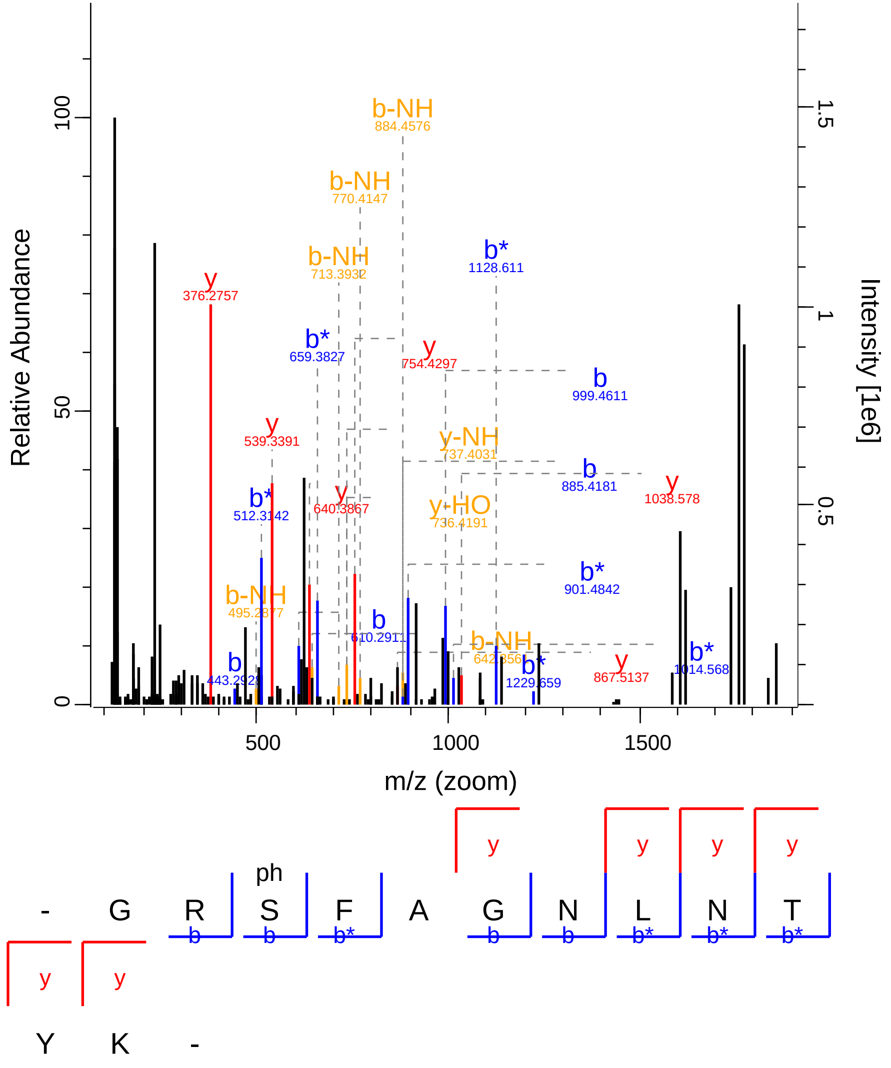

Raw File	Scan	Method	Score	m/z	Gene names
RO-QC-160121_ZackD_SS_Set_01_Fractio	20651	FTMS; HC	142.89	735.38	PRPH

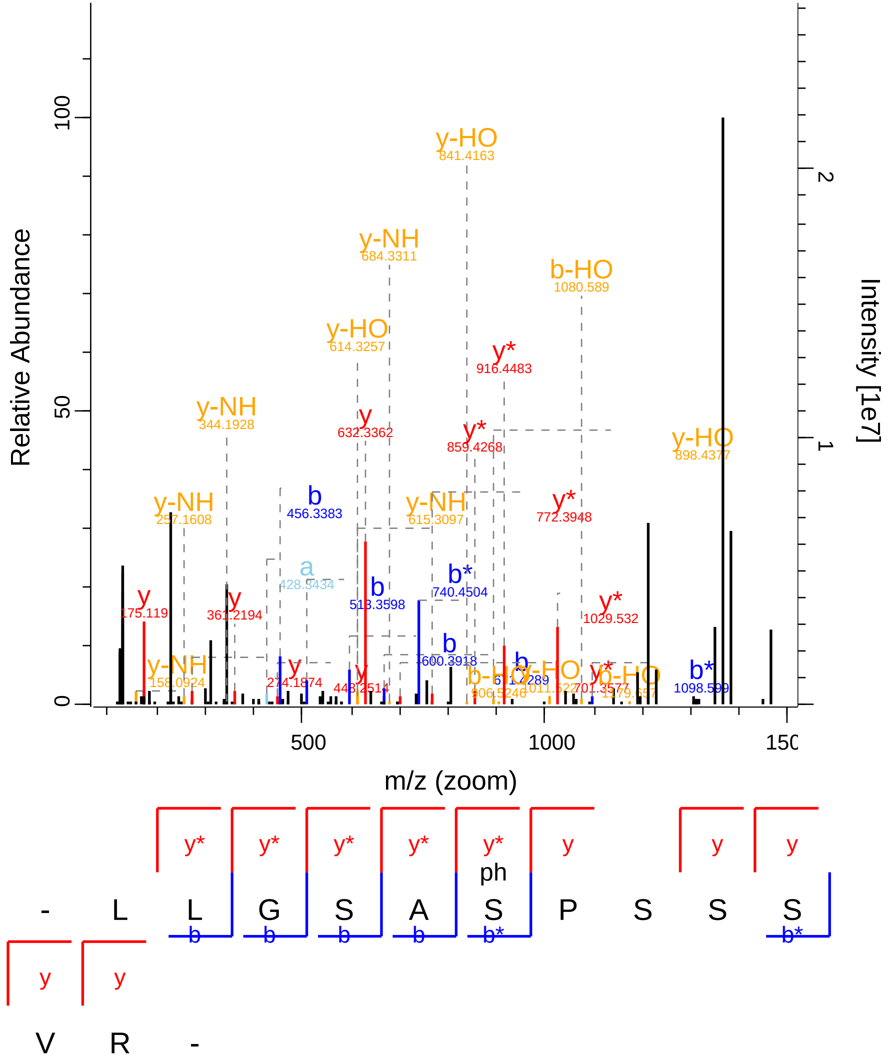

Raw File	Scan	Method	Score	m/z	Gene names
RO-QC-160121_ZackD_SS_Set_01_Fractio	21245	FTMS; HC	45.83	623.68	SOWAHC

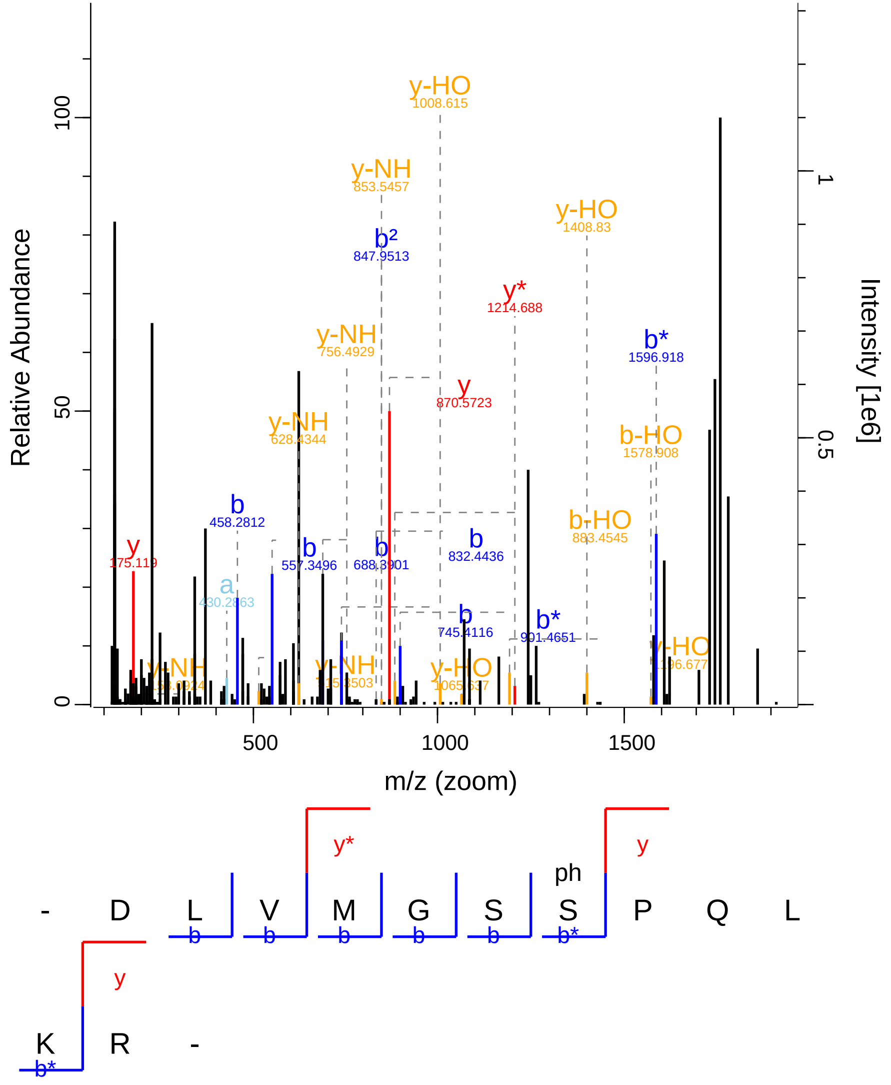

Raw File	Scan	Method	Score	m/z	Gene names
RO-QC-160121_ZackD_SS_Set_01_Fractic	21387	FTMS; HC	108.49	1138.53	RMDN3

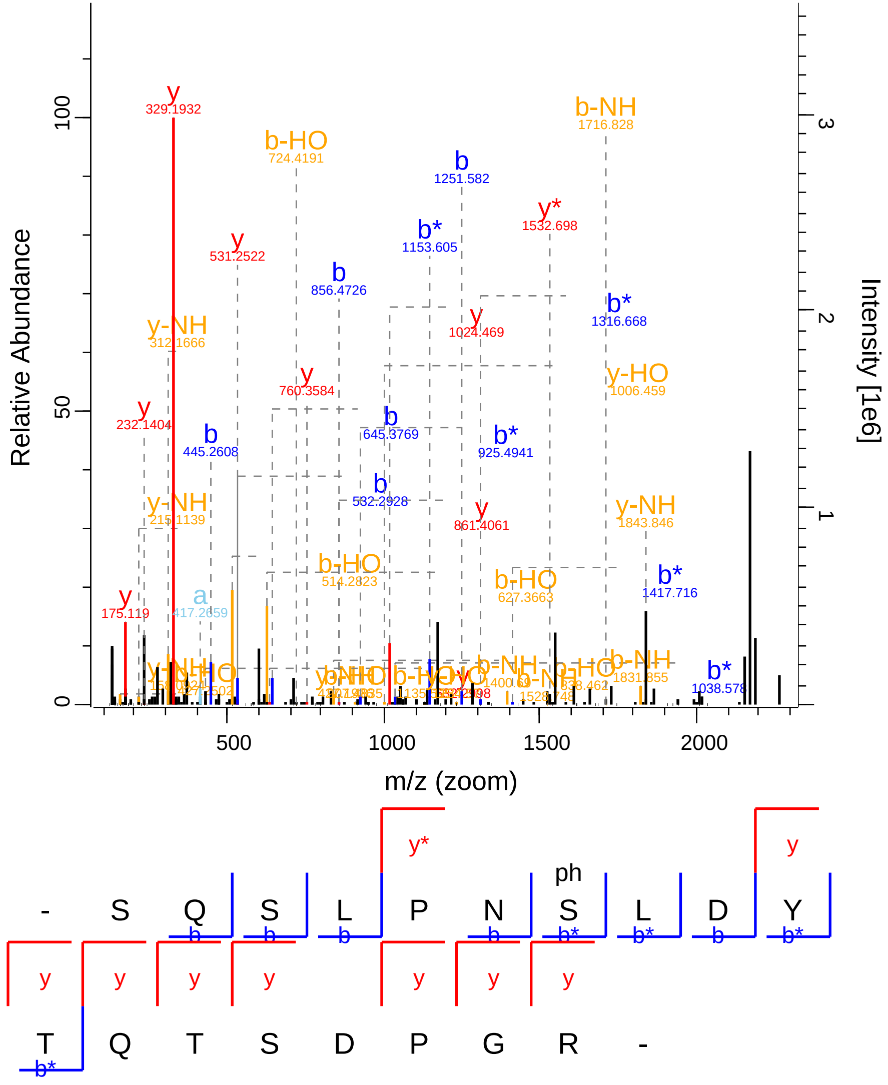

Raw File	Scan	Method	Score	m/z	Gene names
RO-QC-160121_ZackD_SS_Set_01_Fractio	21474	FTMS; HC	136.77	923.11	HUWE1

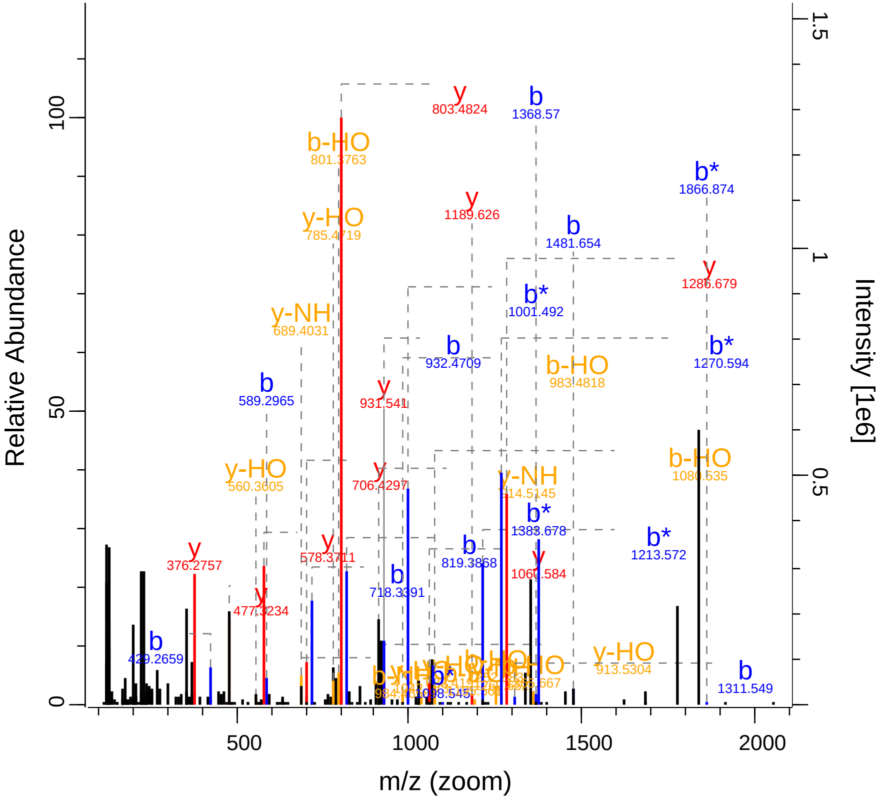

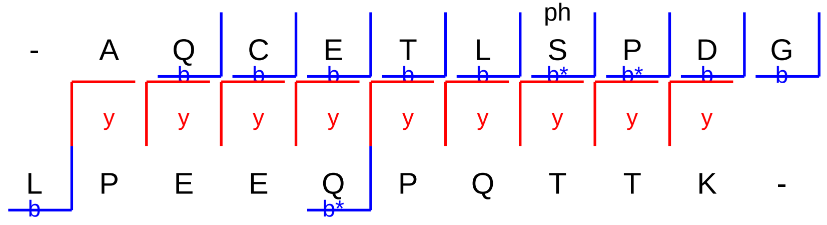

Raw File	Scan	Method	Score	m/z	Gene names
RO-QC-160121_ZackD_SS_Set_01_Fractio	21609	FTMS; HC	78.49	927.47	MIOS

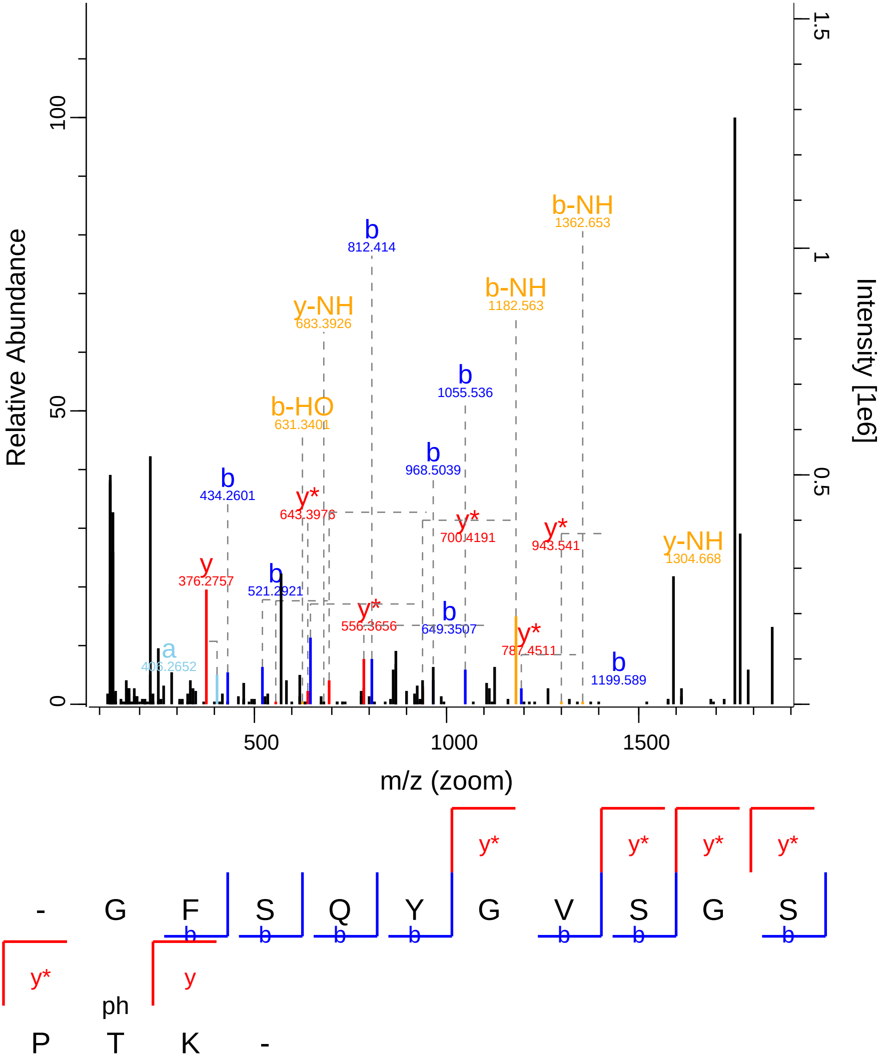

Raw File	Scan	Method	Score	m/z	Gene names
RO-QC-160121_ZackD_SS_Set_01_Fractio	21705	FTMS; HC	40.5	521.53	PRSS55

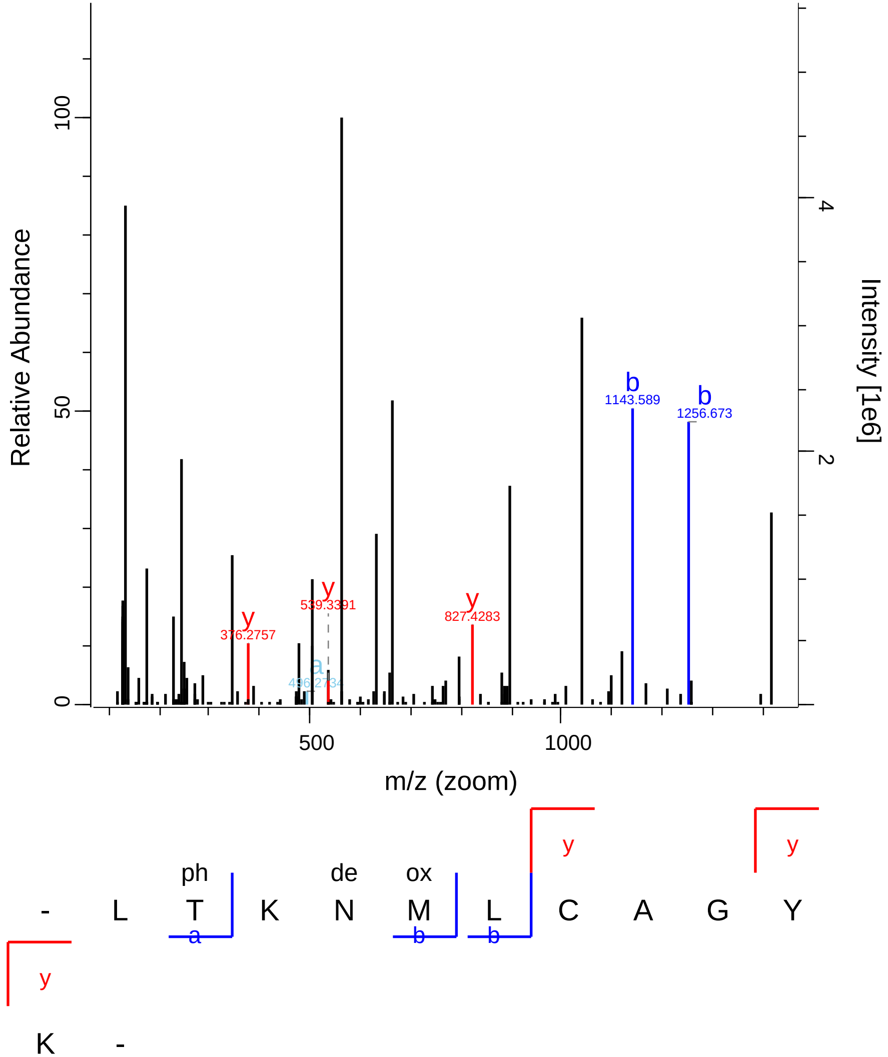

Raw File	Scan	Method	Score	m/z	Gene names
RO-QC-160121_ZackD_SS_Set_01_Fractic	22170	FTMS; HC	171.26	1285.11	MAP1B

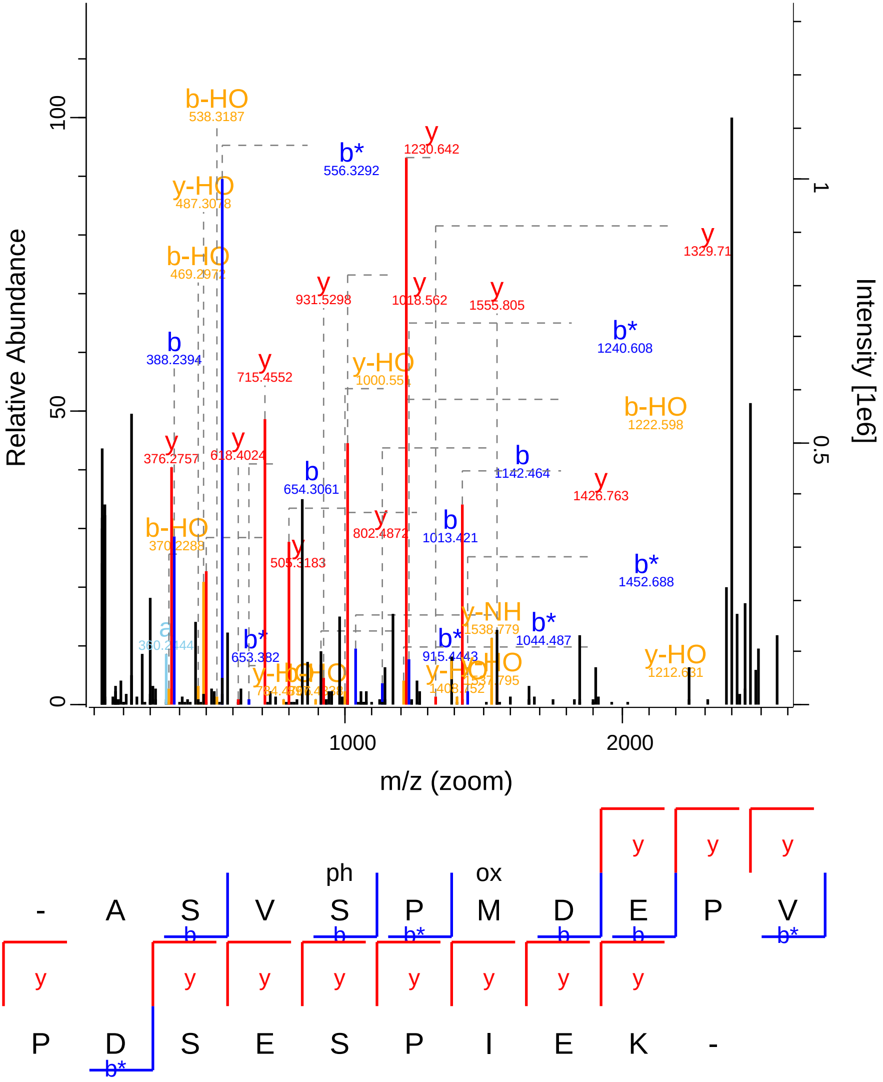

Raw File	Scan	Method	Score	m/z	Gene names
RO-QC-160121_ZackD_SS_Set_01_Fractic	22833	FTMS; HC	61.65	1016.51	CASC4

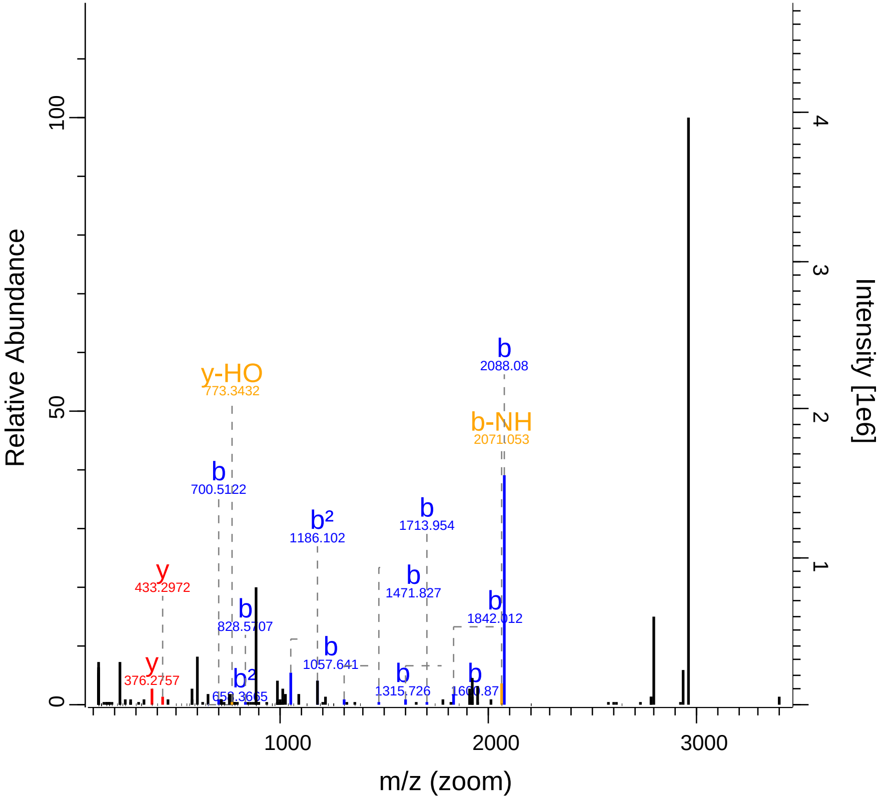

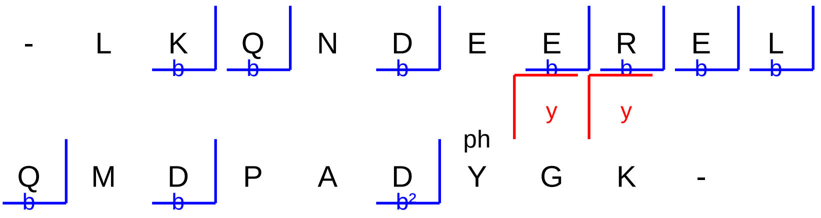

Raw File	Scan	Method	Score	m/z	Gene names
RO-QC-160121_ZackD_SS_Set_01_Fractio	22988	FTMS; HC	42.56	568.56	LSM7

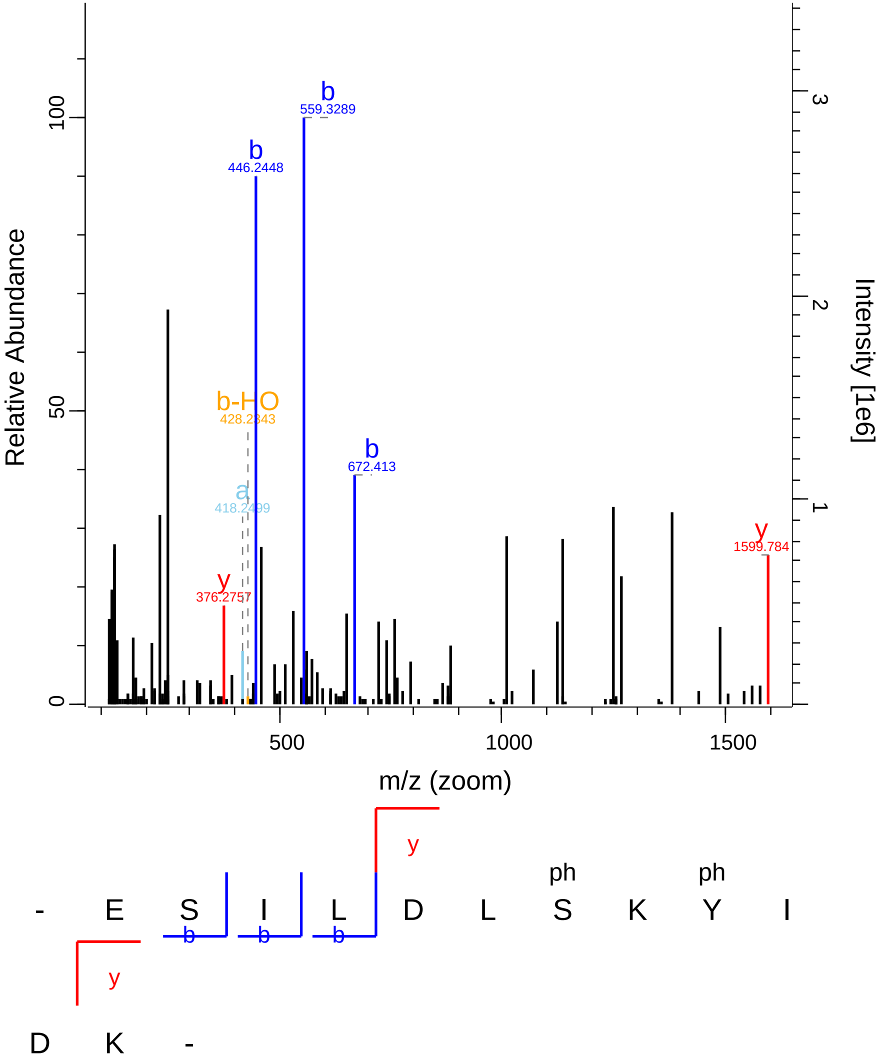

Raw File	Scan	Method	Score	m/z	Gene names
RO-QC-160121_ZackD_SS_Set_01_Fractio	23377	FTMS; HC	78.76	615.33	GPRC5A

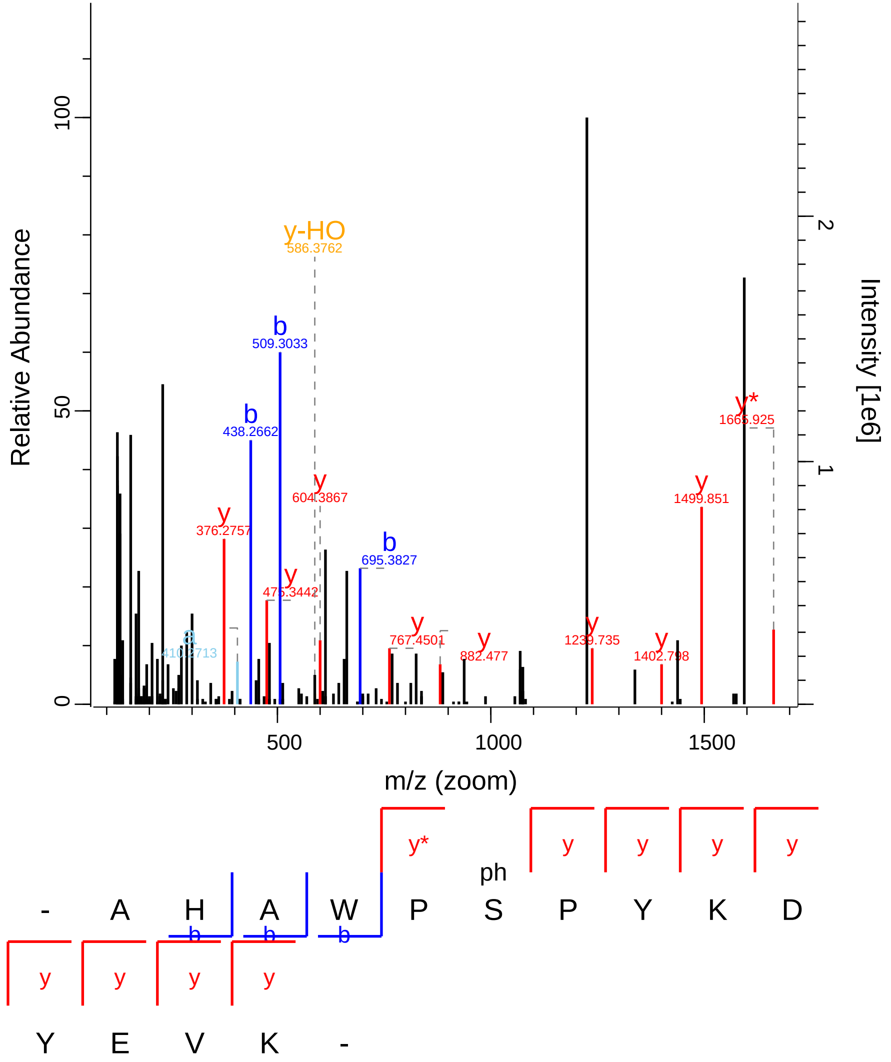

Raw File	Scan	Method	Score	m/z	Gene names
RO-QC-160121_ZackD_SS_Set_01_Fractio	23456	FTMS; HC	83.76	777.42	TBC1D15

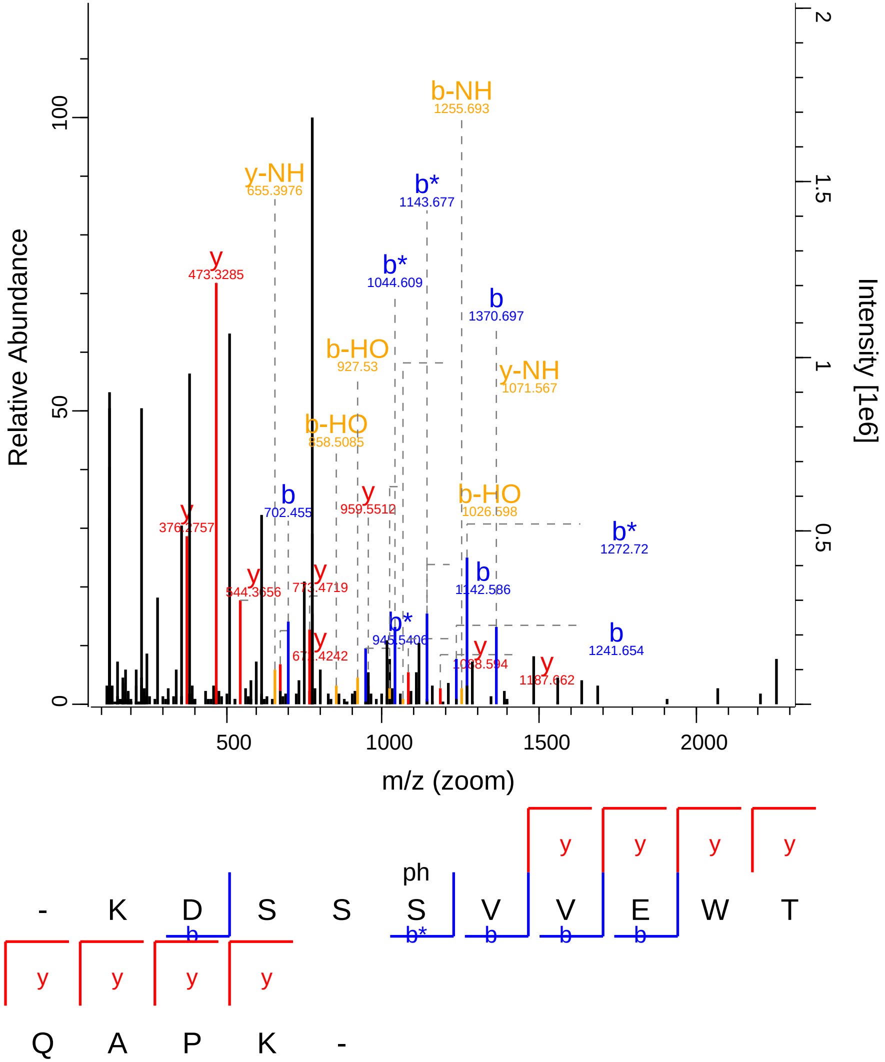

Raw File	Scan	Method	Score	m/z	Gene names
RO-QC-160121_ZackD_SS_Set_01_Fractio	23814	FTMS; HC	83.36	562.32	CTNND1

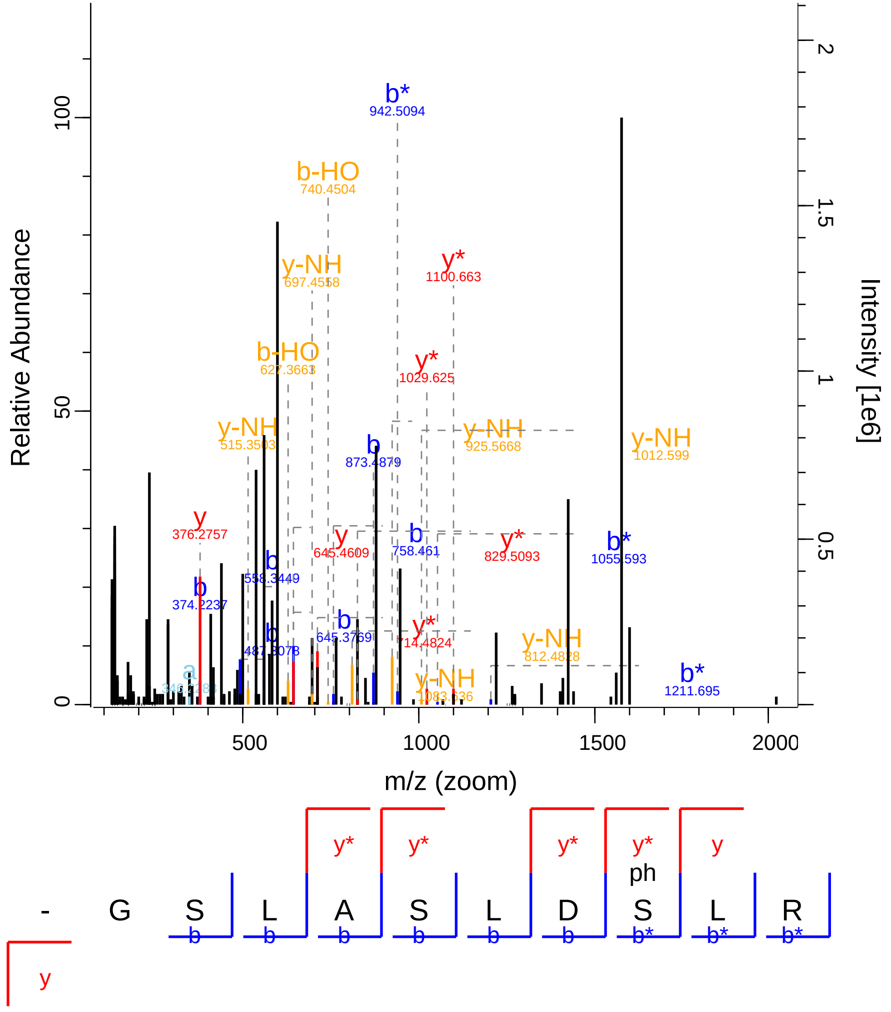

Raw File	Scan	Method	Score	m/z	Gene names
RO-QC-160121_ZackD_SS_Set_01_Fractio	25779	FTMS; HC	111.79	803.19	ADD3

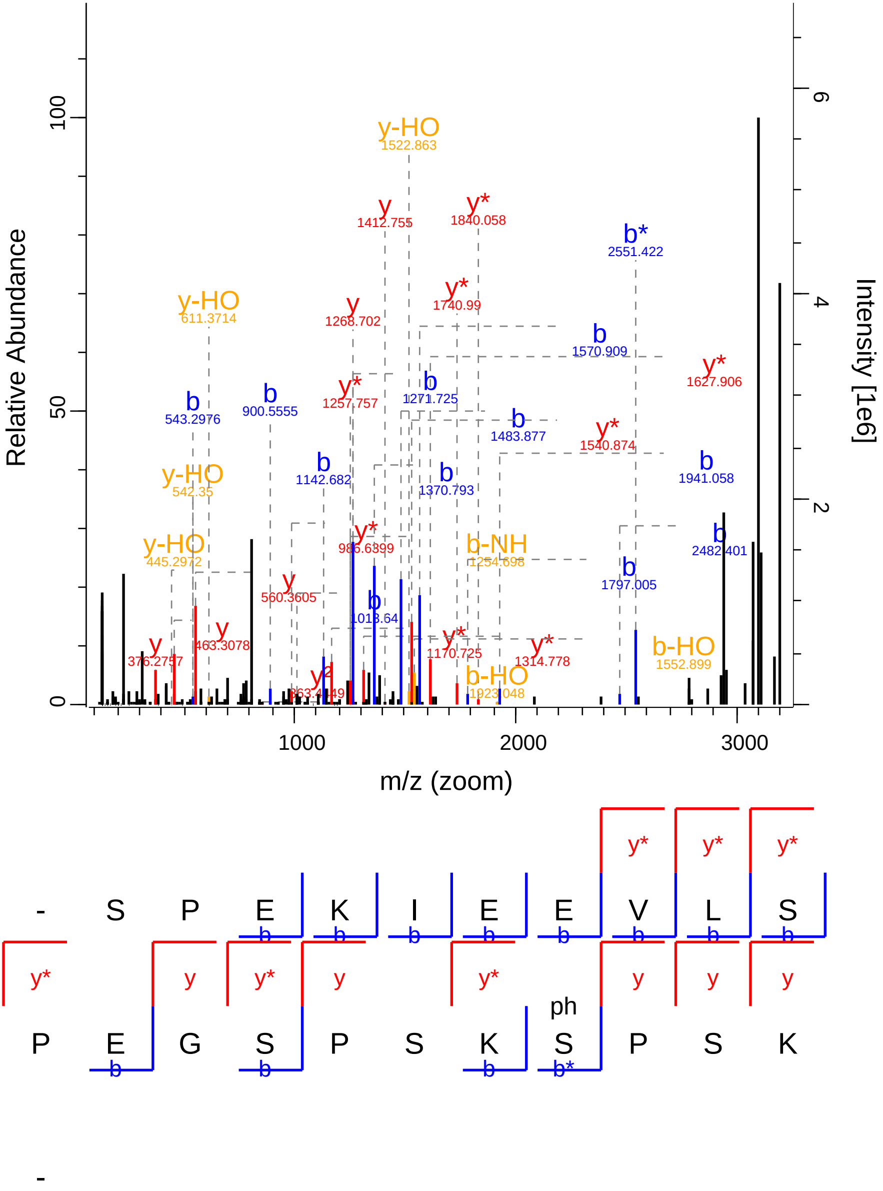

Raw File	Scan	Method	Score	m/z	Gene names
RO-QC-160121_ZackD_SS_Set_01_Fractio	26057	FTMS; HC	75.76	935.46	CEBPB

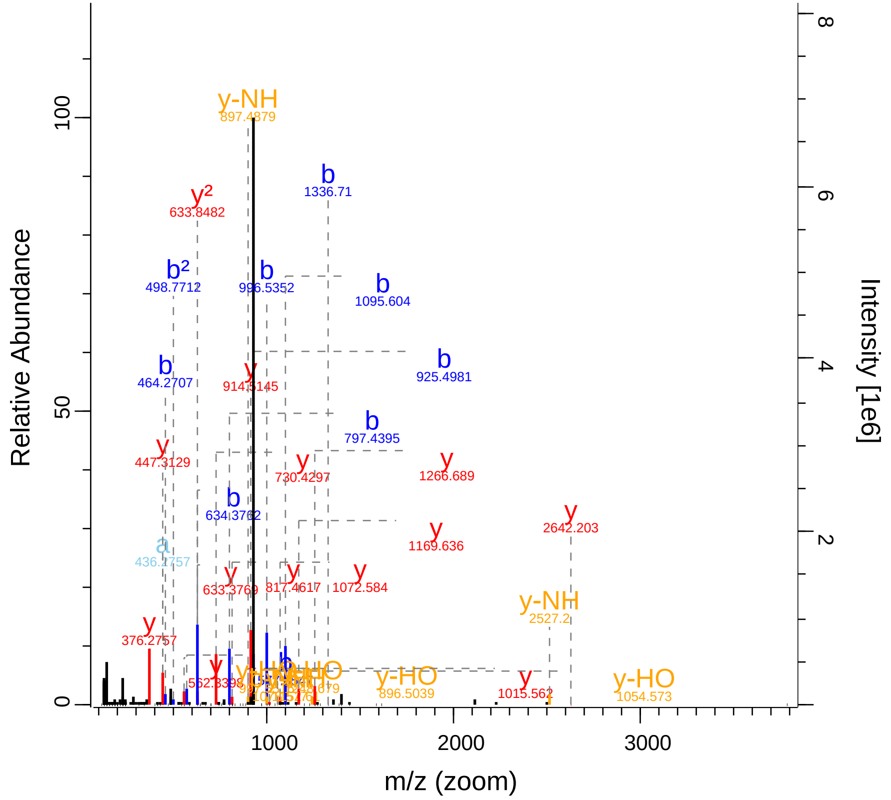

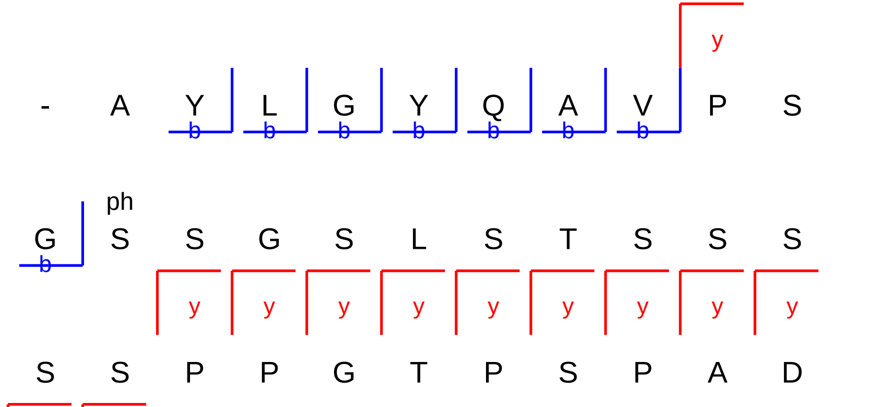

Raw File	Scan	Method	Score	m/z	Gene names
RO-QC-160121_ZackD_SS_Set_01_Fractio	26883	FTMS; HC	156.87	859.07	KPNA3

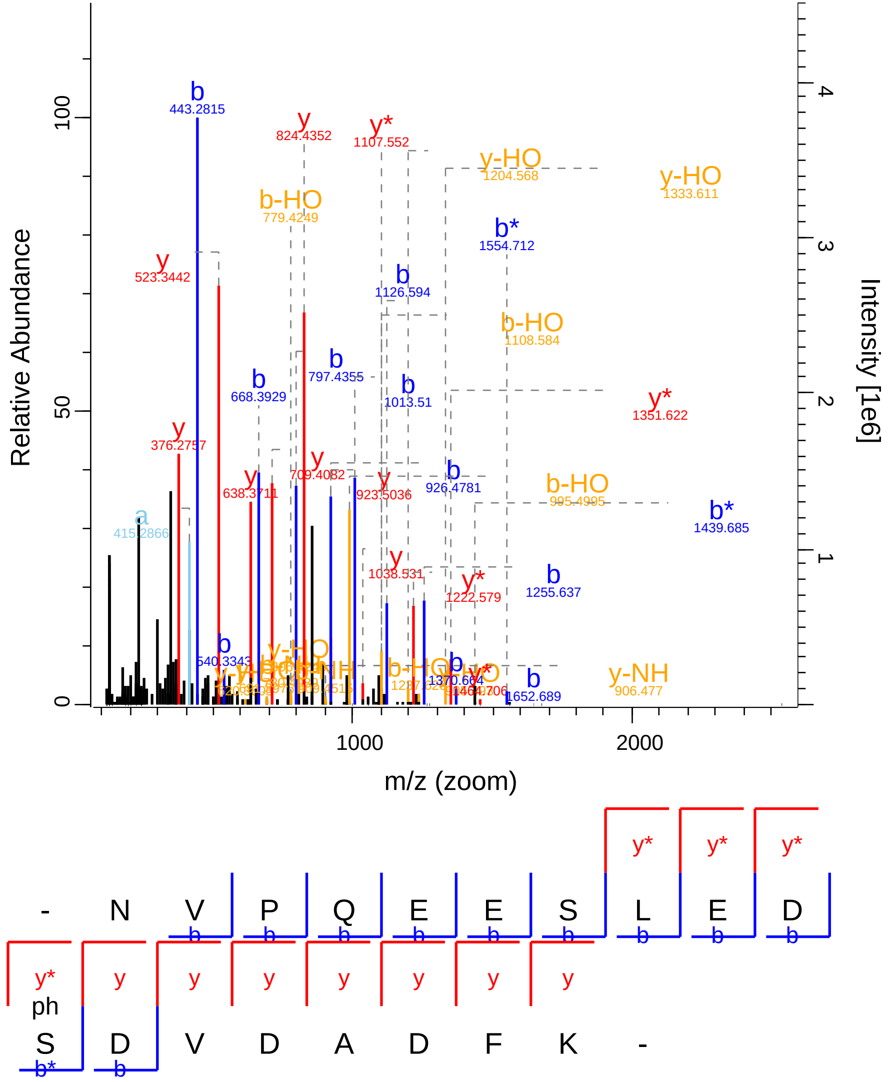

Raw File	Scan	Method	Score	m/z	Gene names
RO-QC-160121_ZackD_SS_Set_01_Fractic	27118	FTMS; HC	146.5	1095.81	STK39

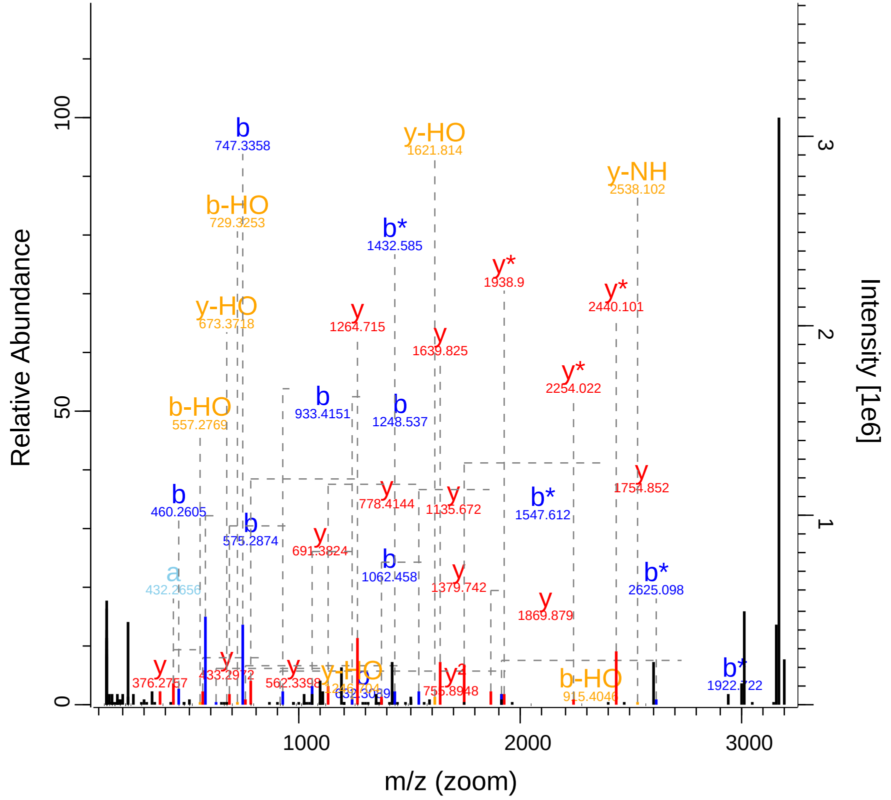

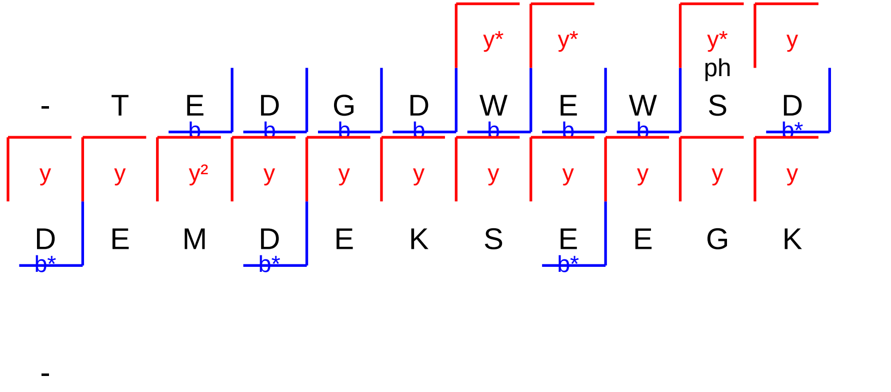

Raw File	Scan	Method	Score	m/z	Gene names
RO-QC-160121_ZackD_SS_Set_01_Fractio	27121	FTMS; HC	41.64	644.59	EDF1

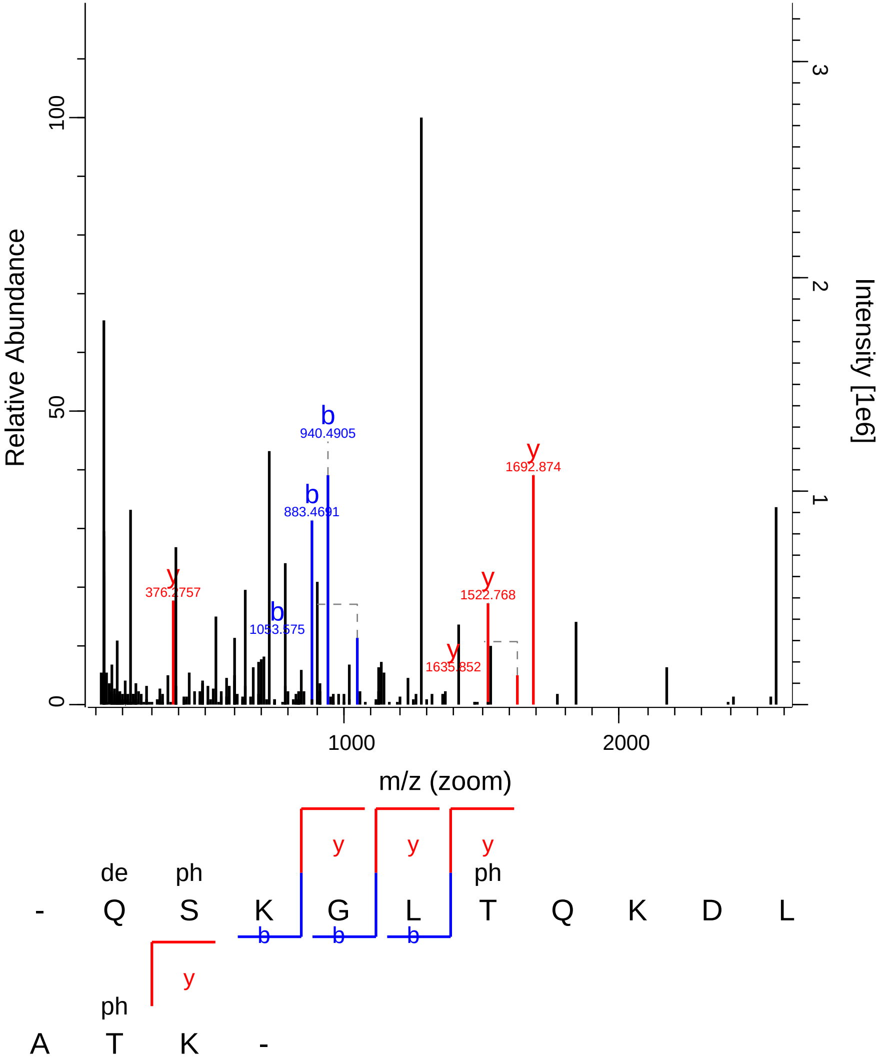

Raw File	Scan	Method	Score	m/z	Gene names
RO-QC-160121_ZackD_SS_Set_01_Fractio	28122	FTMS; HC	154.77	1034.49	TP53BP1

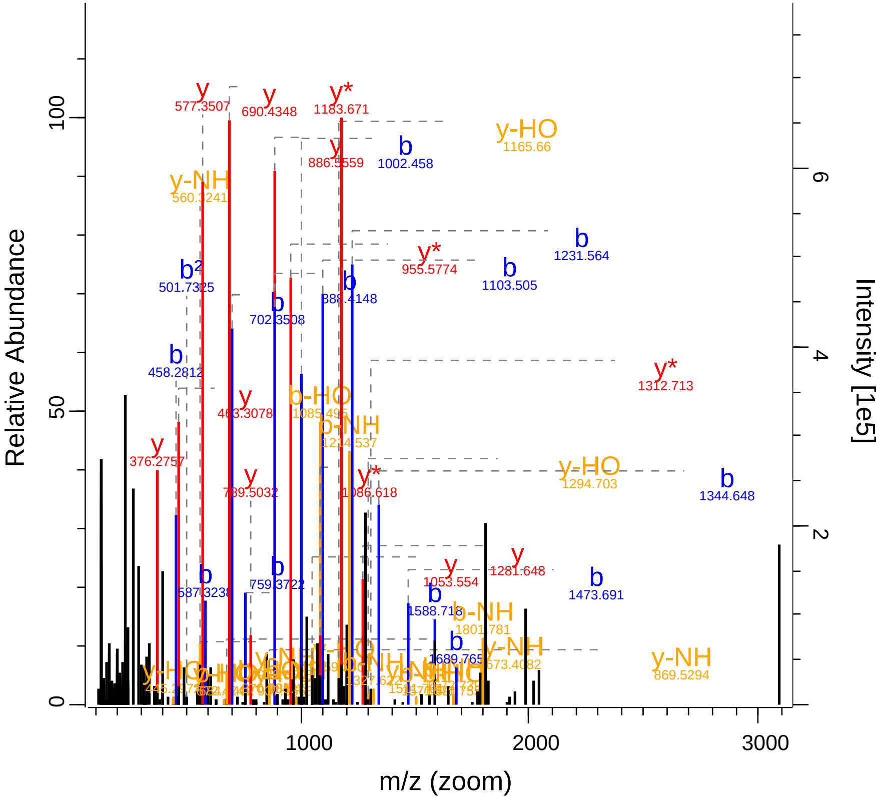

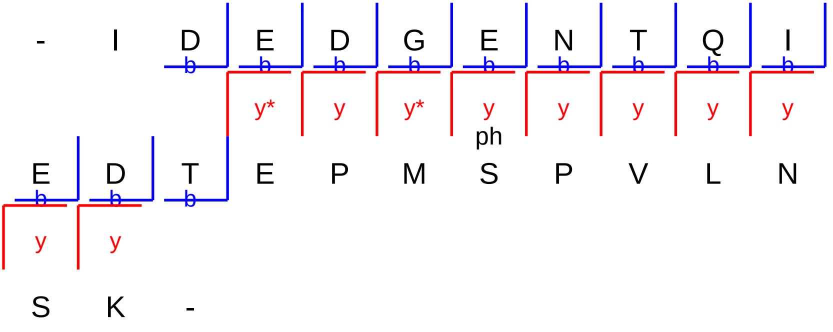

Raw File	Scan	Method	Score	m/z	Gene names
RO-QC-160121_ZackD_SS_Set_01_Fractic	28717	FTMS; HC	56.01	1026.49	SIRPB2

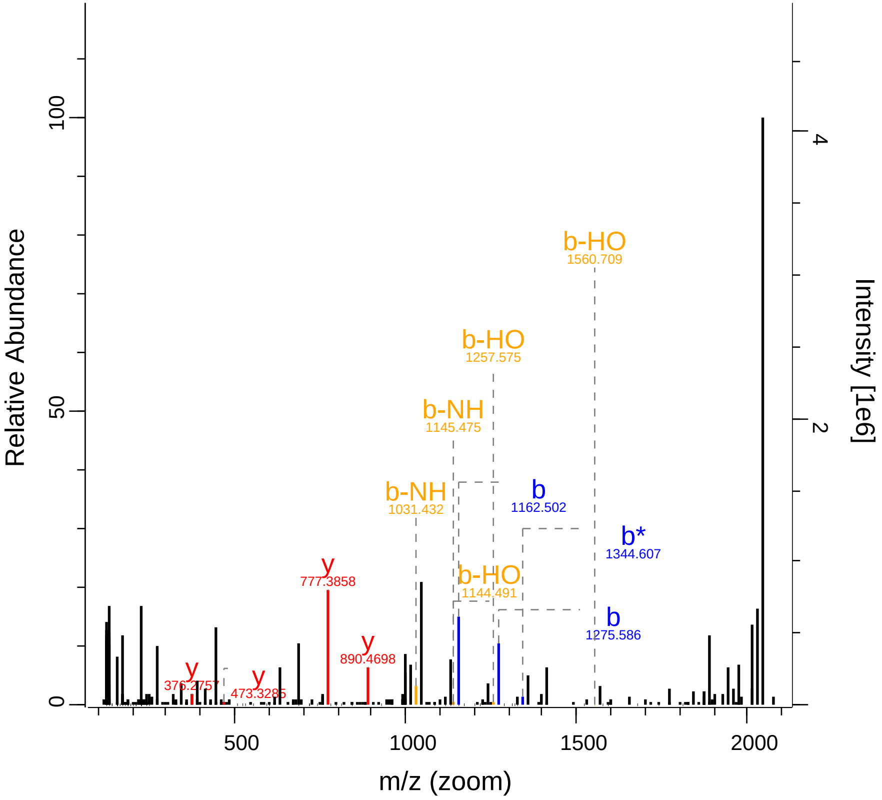

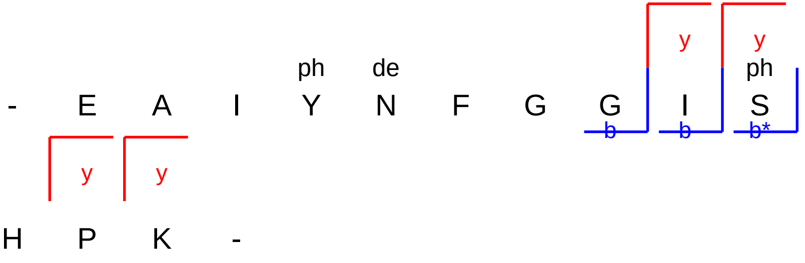

Raw File	Scan	Method	Score	m/z	Gene names
RO-QC-160121_ZackD_SS_Set_01_Fractio	29675	FTMS; HC	120.85	1012.11	EHD2

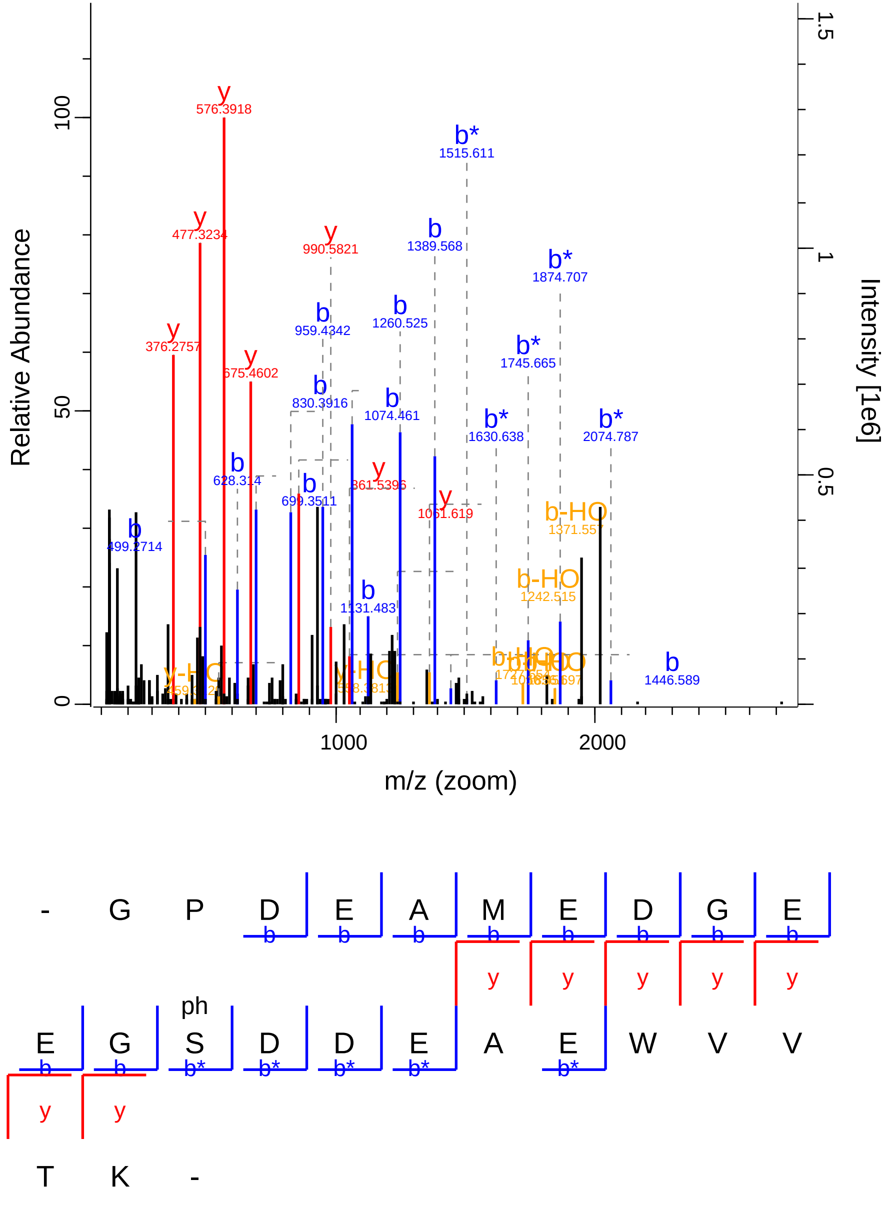

Raw File	Scan	Method	Score	m/z	Gene names
RO-QC-160121_ZackD_SS_Set_01_F	3111	FTMS; H	159.7	920.4	RPS6KA3;RPS6KA6;RP

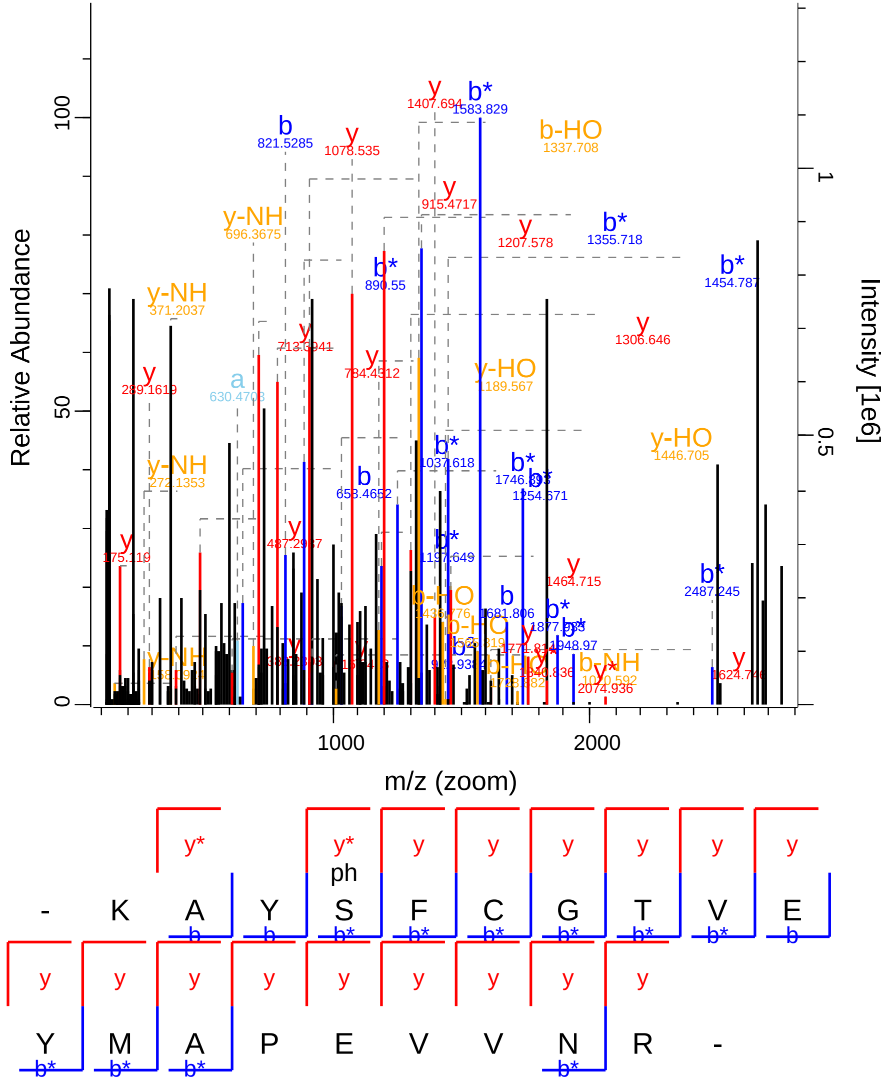

Raw File	Scan	Method	Score	m/z	Gene names
RO-QC-160121_ZackD_SS_Set_01_Fractic	31827	FTMS; HC	100.19	1104.32	TPD52

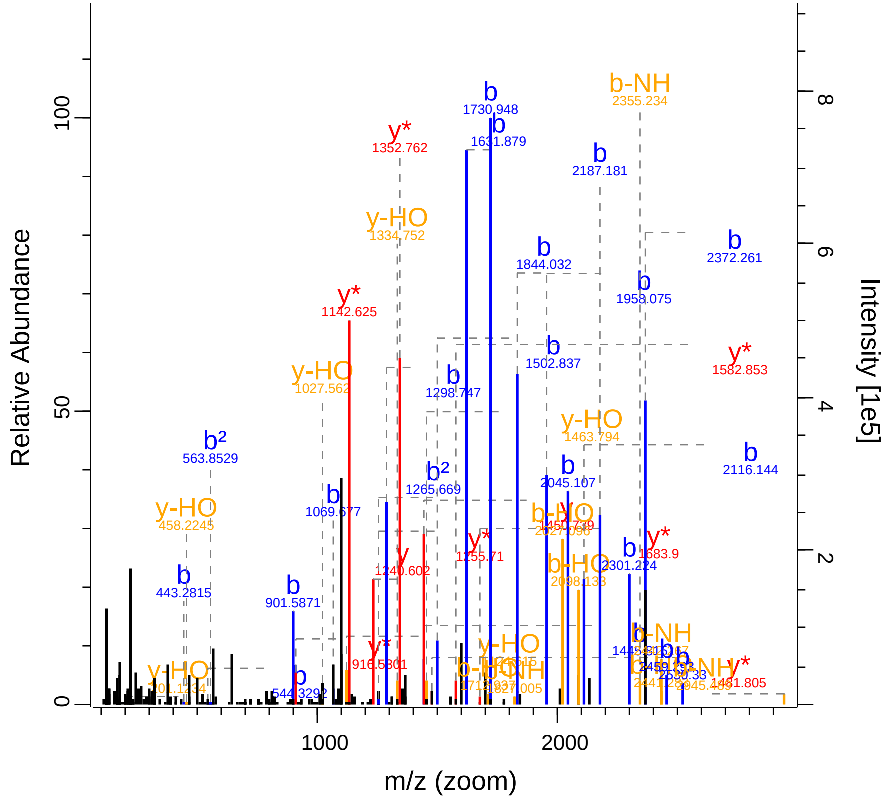

- V G G T K P A G G D
b b b b b² b

F G E V L N S A A N A
b b b b b b b b b b b
y* y* y* y y* y y*

S A T T T E P L P E K
b b

Raw File	Scan	Method	Score	m/z	Gene names
RO-QC-160121_ZackD_SS_Set_01_Fractio	33488	FTMS; HC	40.15	581.92	NLRC5

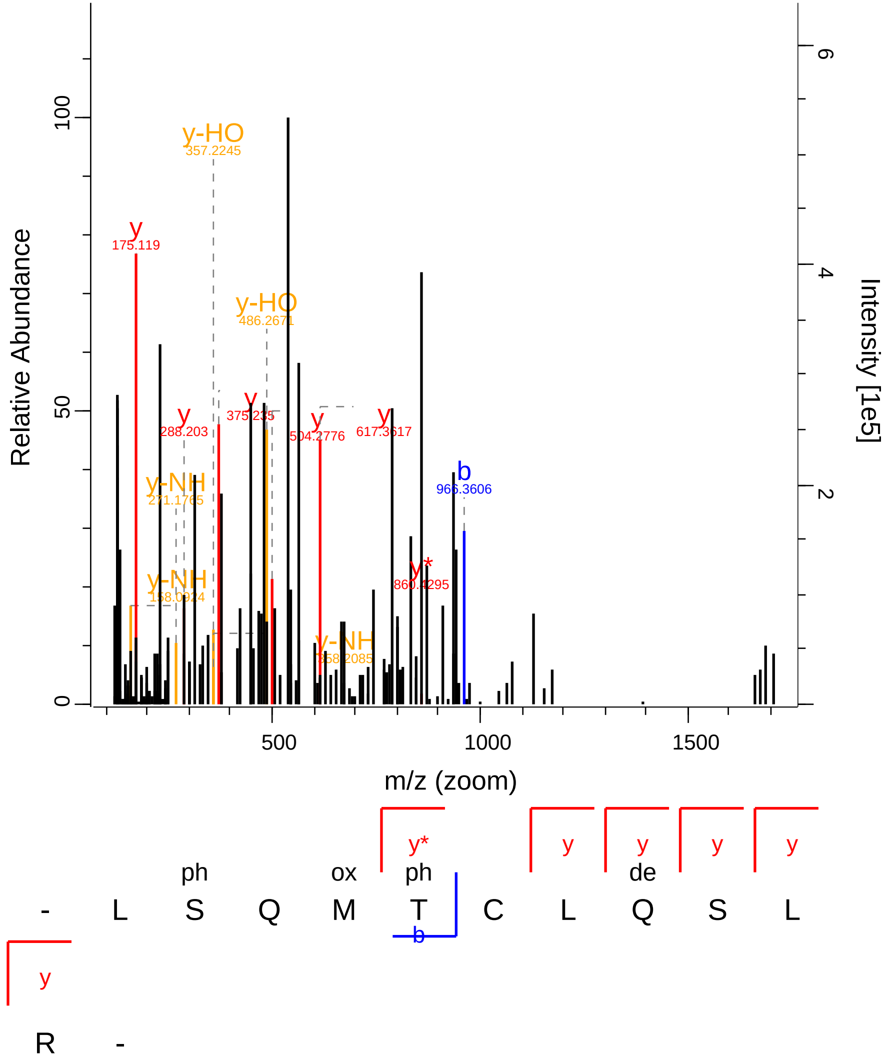

Raw File	Scan	Method	Score	m/z	Gene names
RO-QC-160121_ZackD_SS_Set_01_Fractio	35822	FTMS; HC	107.75	929.72	PPA2

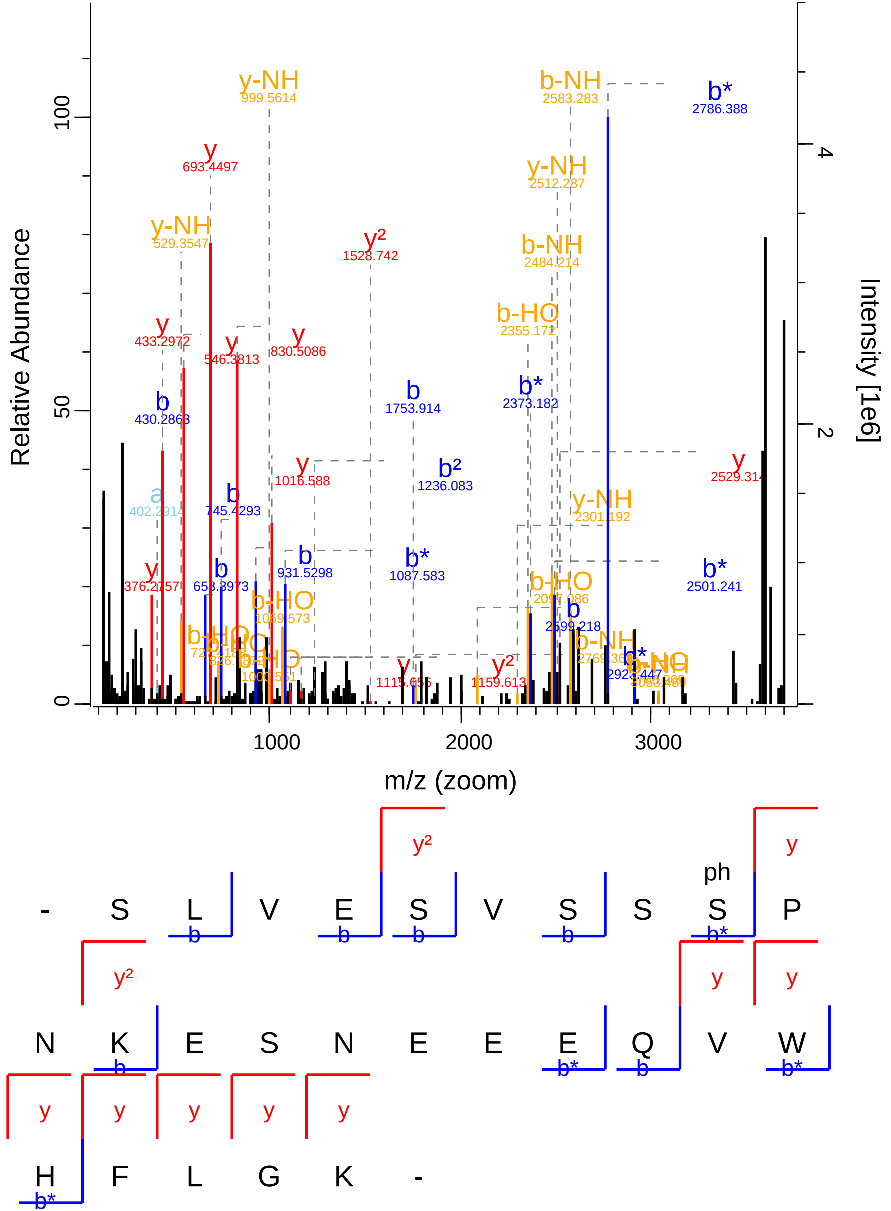

Raw File	Scan	Method	Score	m/z	Gene names
RO-QC-160121_ZackD_SS_Set_01_Fractic	36357	FTMS; HC	89.55	1230.64	NEK10

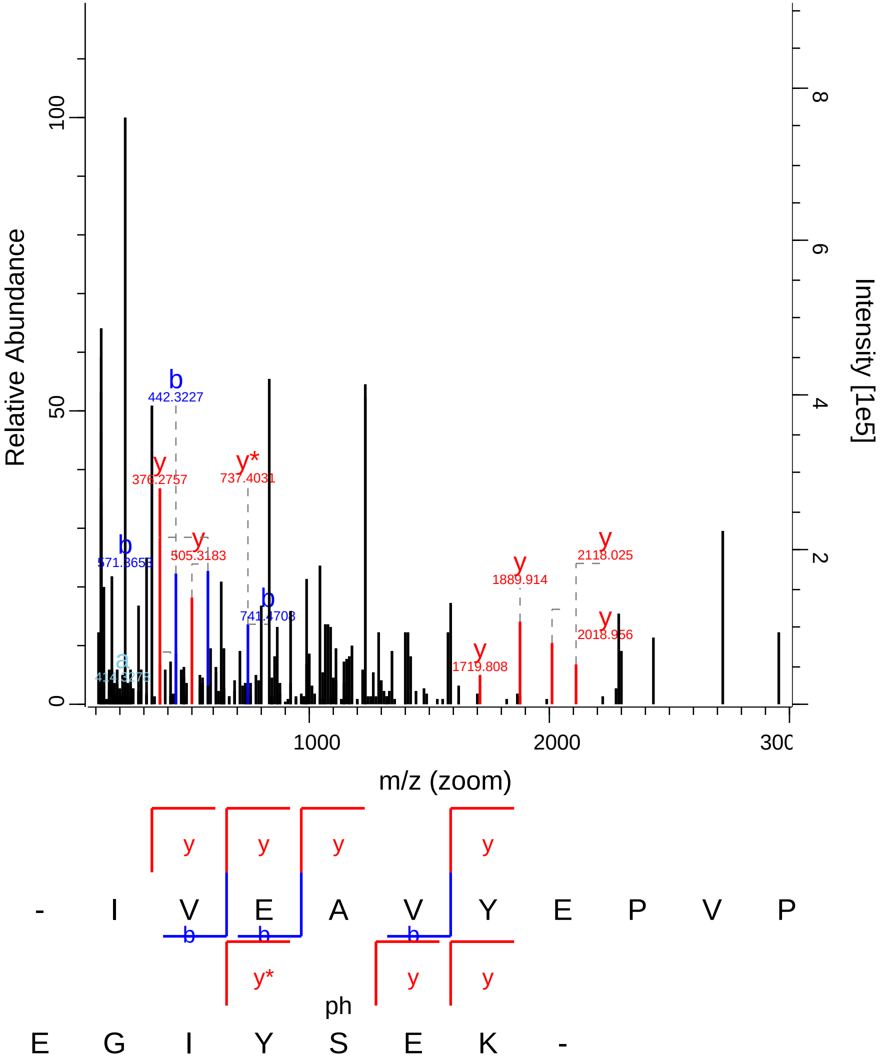

Raw File	Scan	Method	Score	m/z	Gene names
RO-QC-160121_ZackD_SS_Set_01_Fractic	36527	FTMS; HC	134.14	1065.54	PAK4

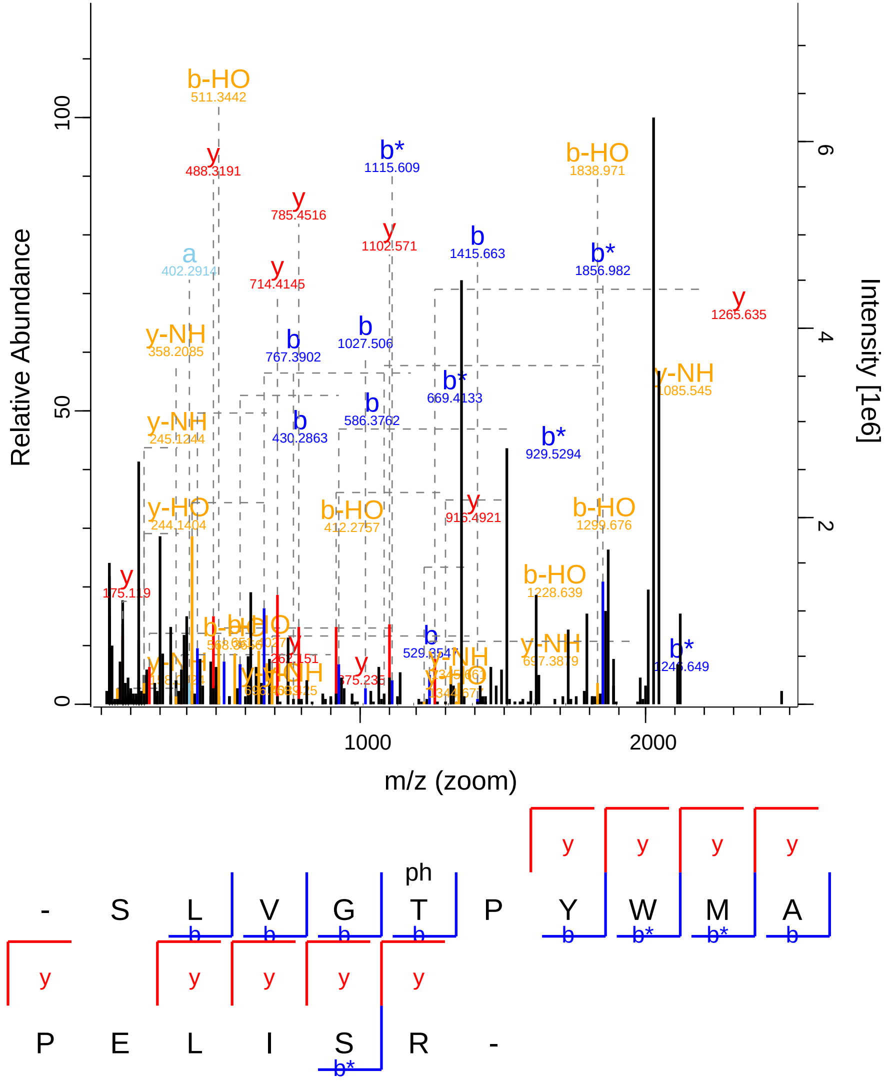

Raw File	Scan	Method	Score	m/z	Gene names
RO-QC-160121_ZackD_SS_Set_01_Fractio	36550	FTMS; HC	65.28	837.93	DYNC1I2

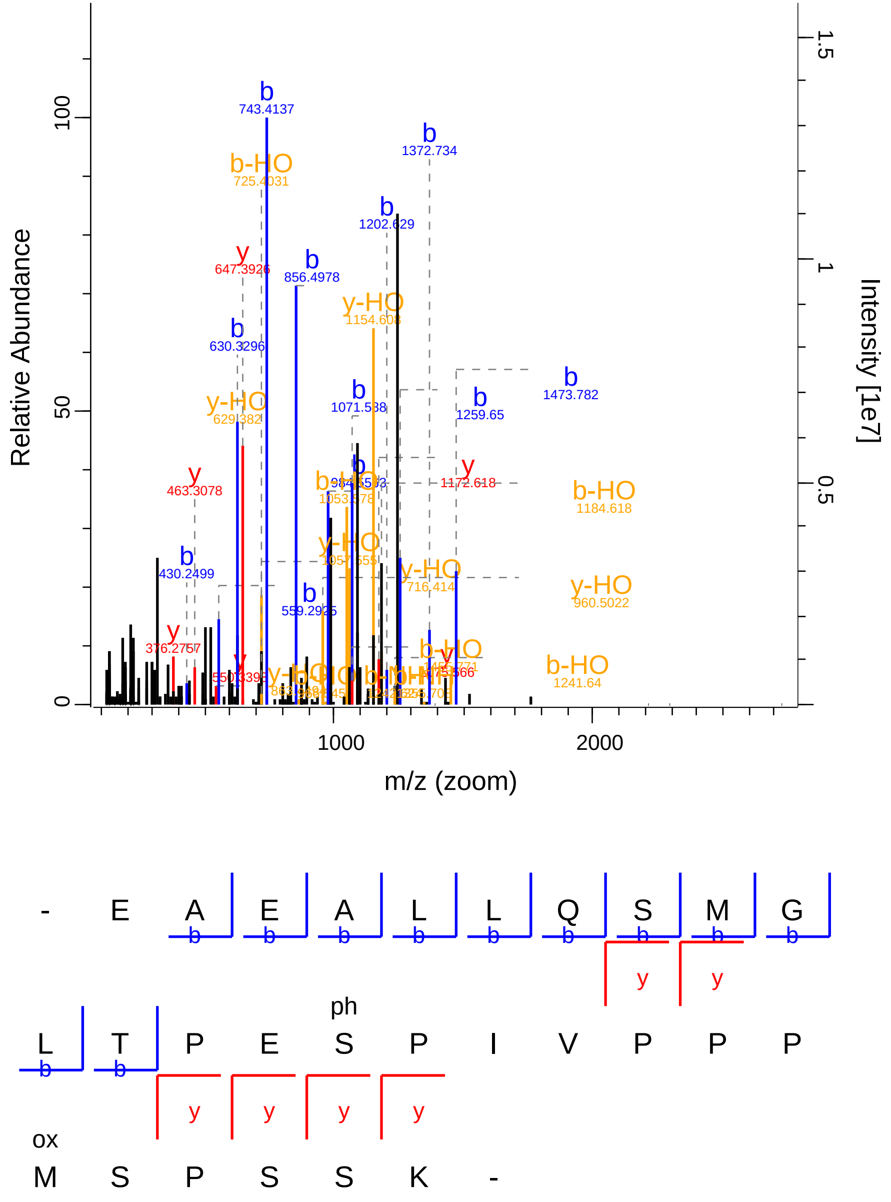

Raw File	Scan	Method	Score	m/z	Gene names
RO-QC-160121_ZackD_SS_Set_01_Fractior	3665	FTMS; HCI	51.21	479.73	SLC8A3

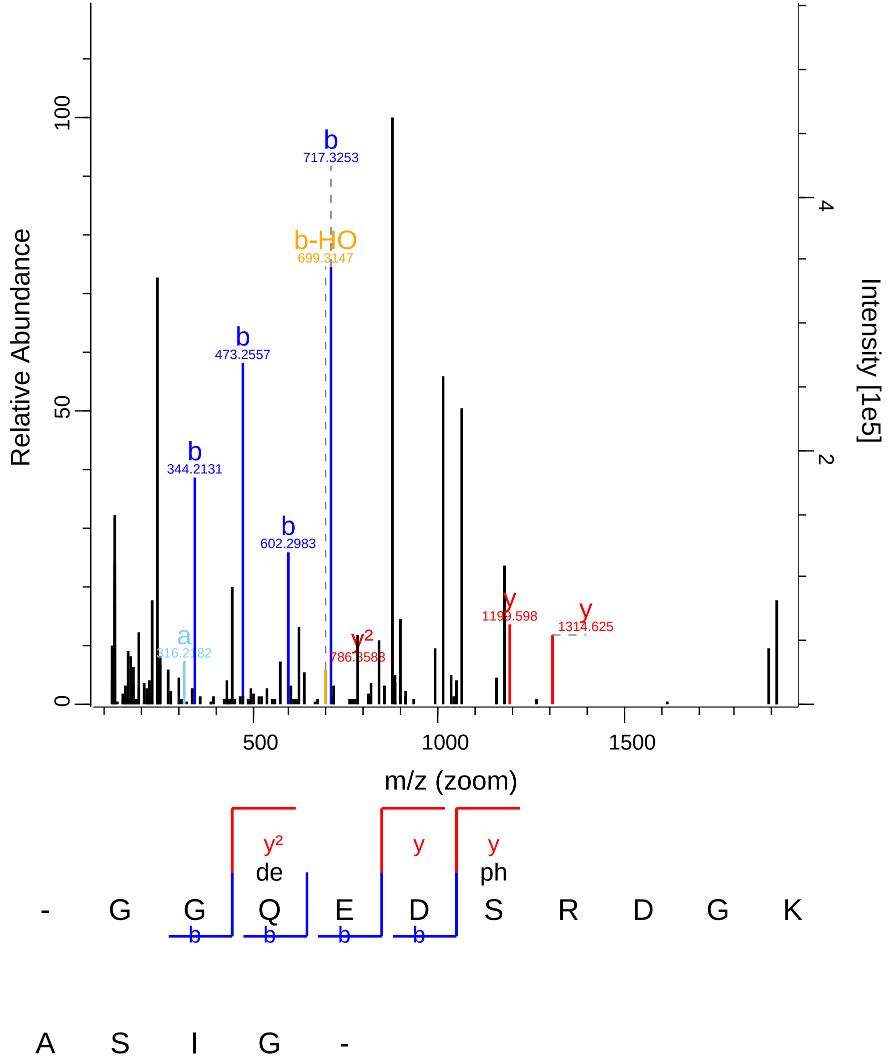

Raw File	Scan	Method	Score	m/z	Gene names
RO-QC-160121_ZackD_SS_Set_01_Fractior	4761	FTMS; HCI	45.08	811.13	MYCBP2

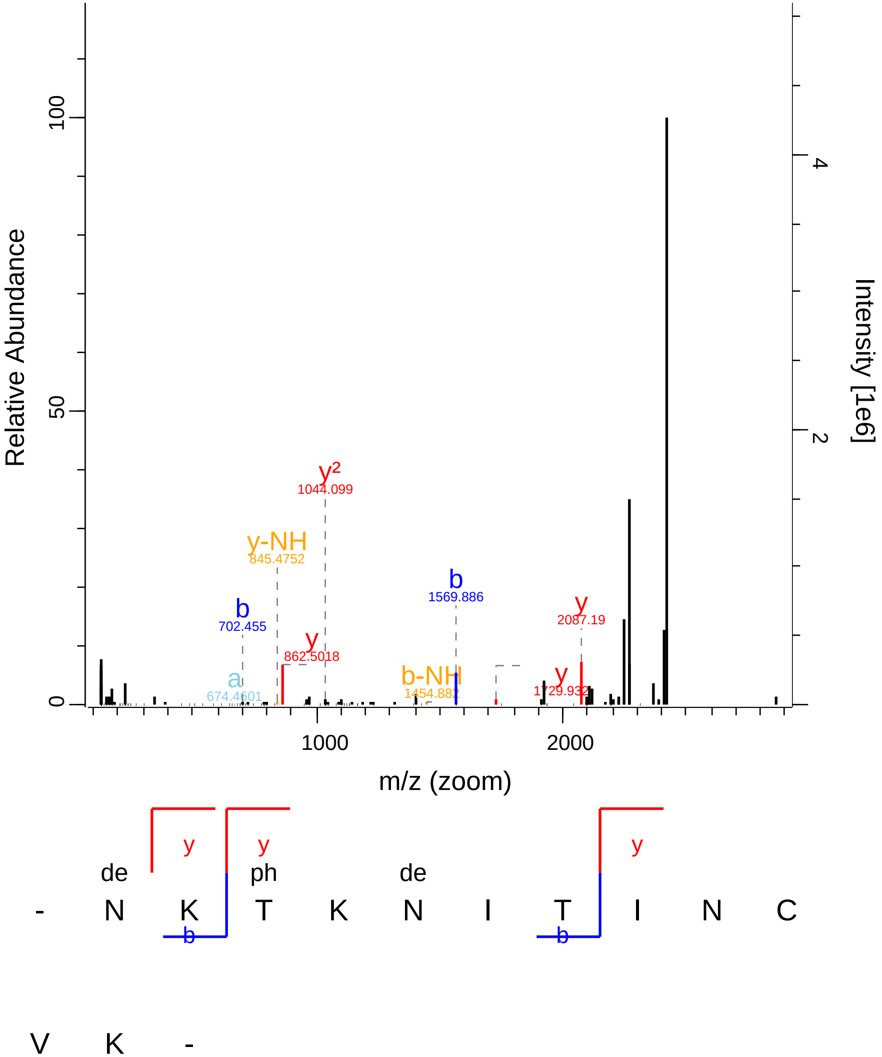

Raw File	Scan	Method	Score	m/z	Gene names
RO-QC-160121_ZackD_SS_Set_01_Fractior	5422	FTMS; HCI	41.98	786.39	HAS1

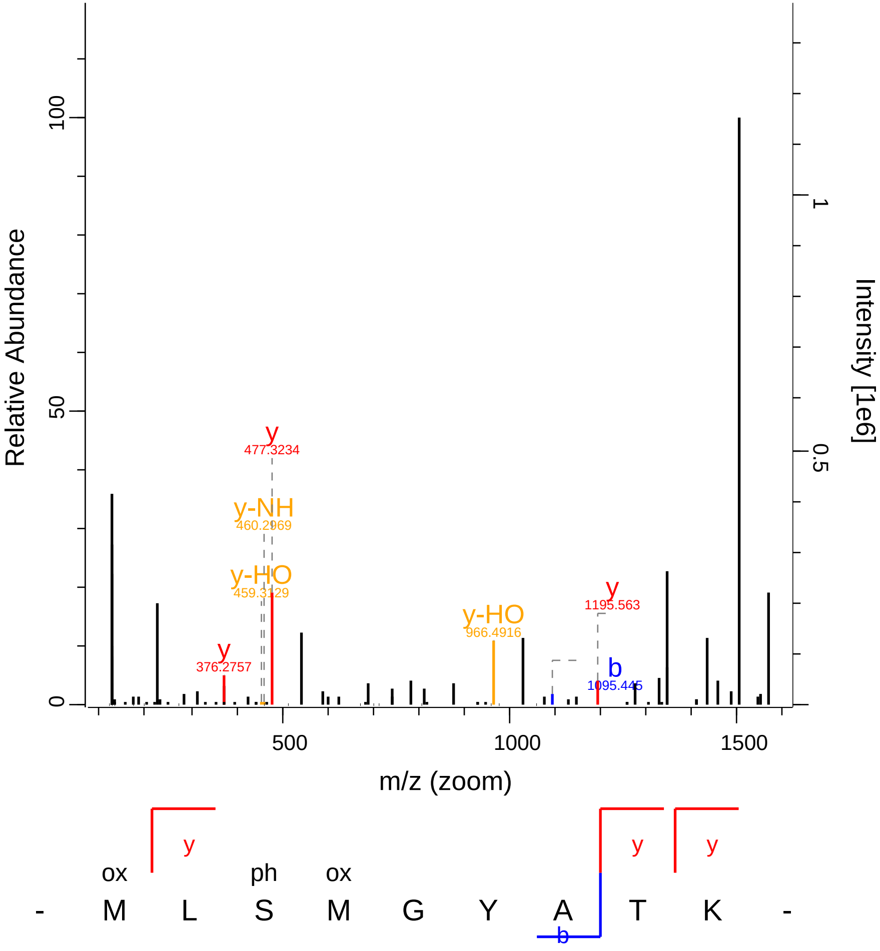

Raw File	Scan	Method	Score	m/z	Gene names
RO-QC-160121_ZackD_SS_Set_01_Fractior	5831	FTMS; HCI	78.24	646.98	C18orf25

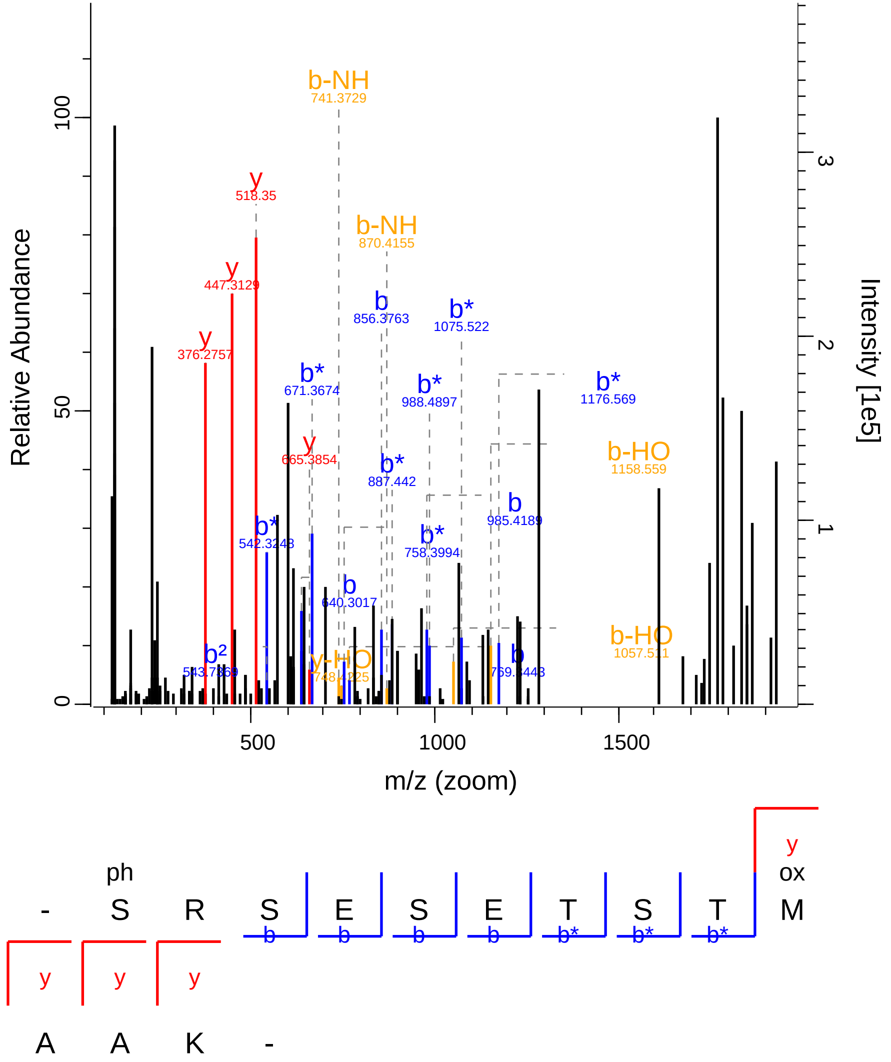

Raw File	Scan	Method	Score	m/z	Gene names
RO-QC-160121_ZackD_SS_Set_01_Fractio	7000	FTMS; HC	128.81	782.87	SRRM2

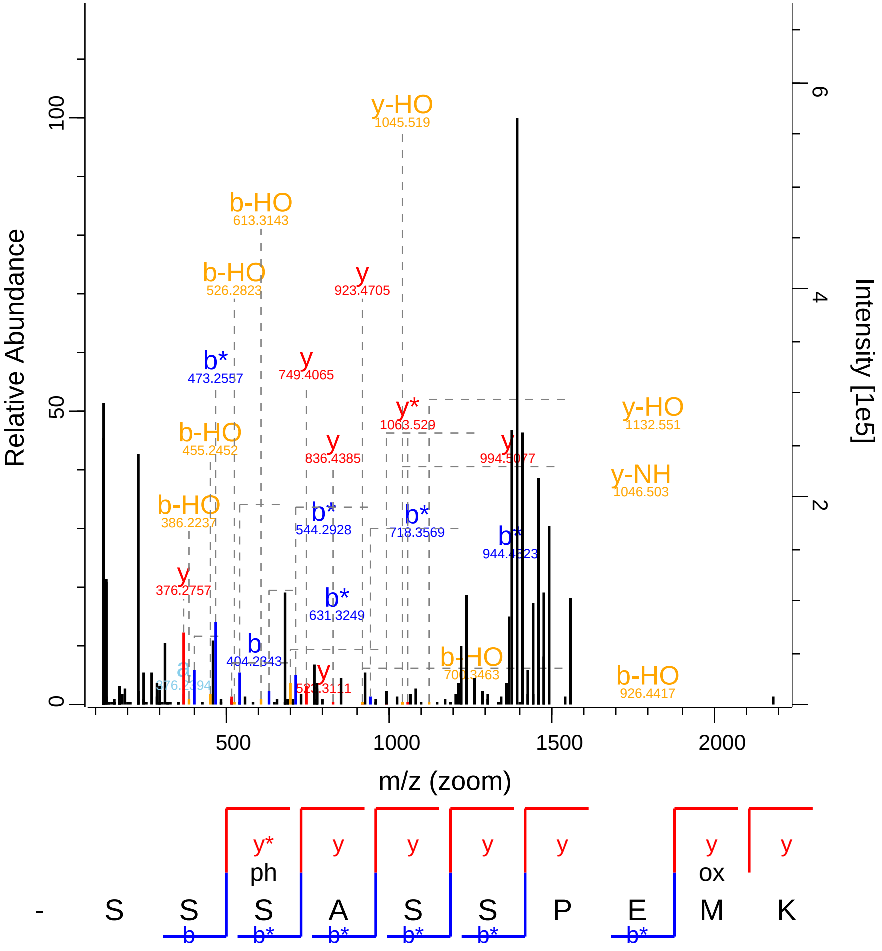

Raw File	Scan	Method	Score	m/z	Gene names
RO-QC-160121_ZackD_SS_Set_01_Fractior	7384	FTMS; HCI	81.45	605.67	CCDC86

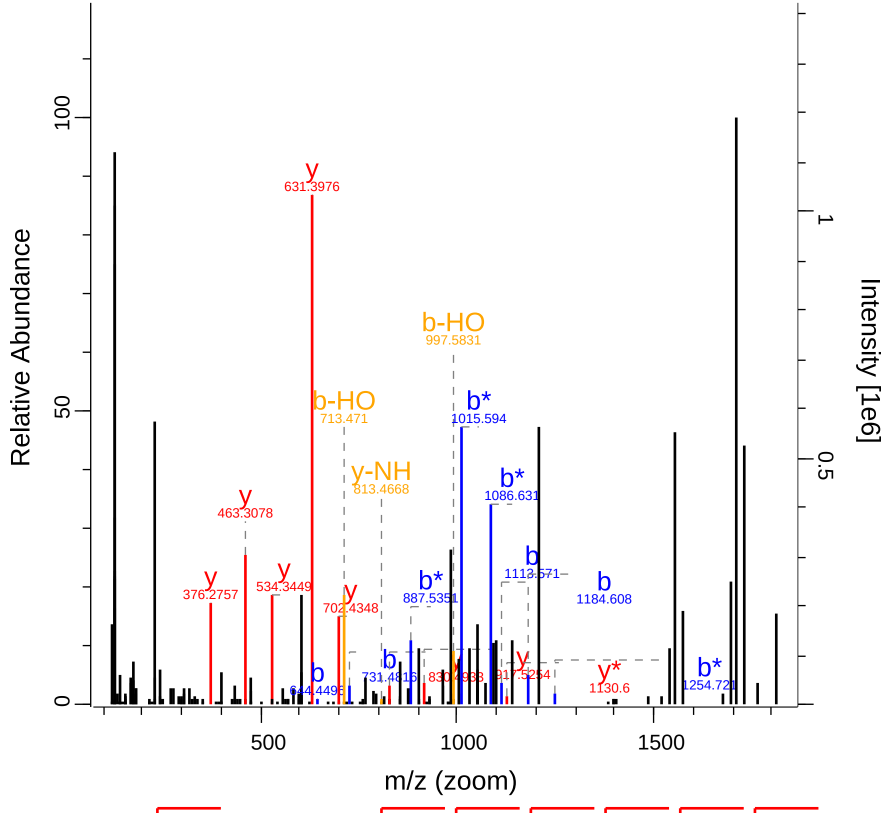

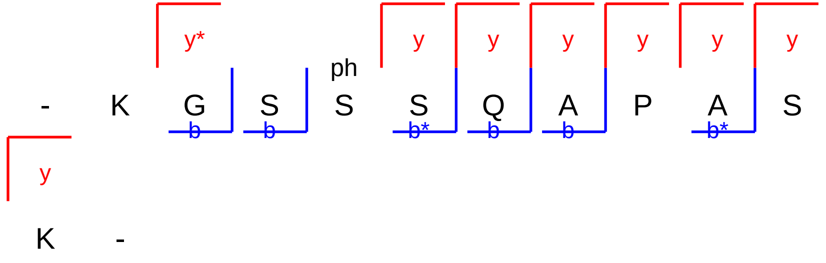

Raw File	Scan	Method	Score	m/z	Gene names
RO-QC-160121_ZackD_SS_Set_01_Fractio	7387	FTMS; HC	124.43	1110.52	EIF4B

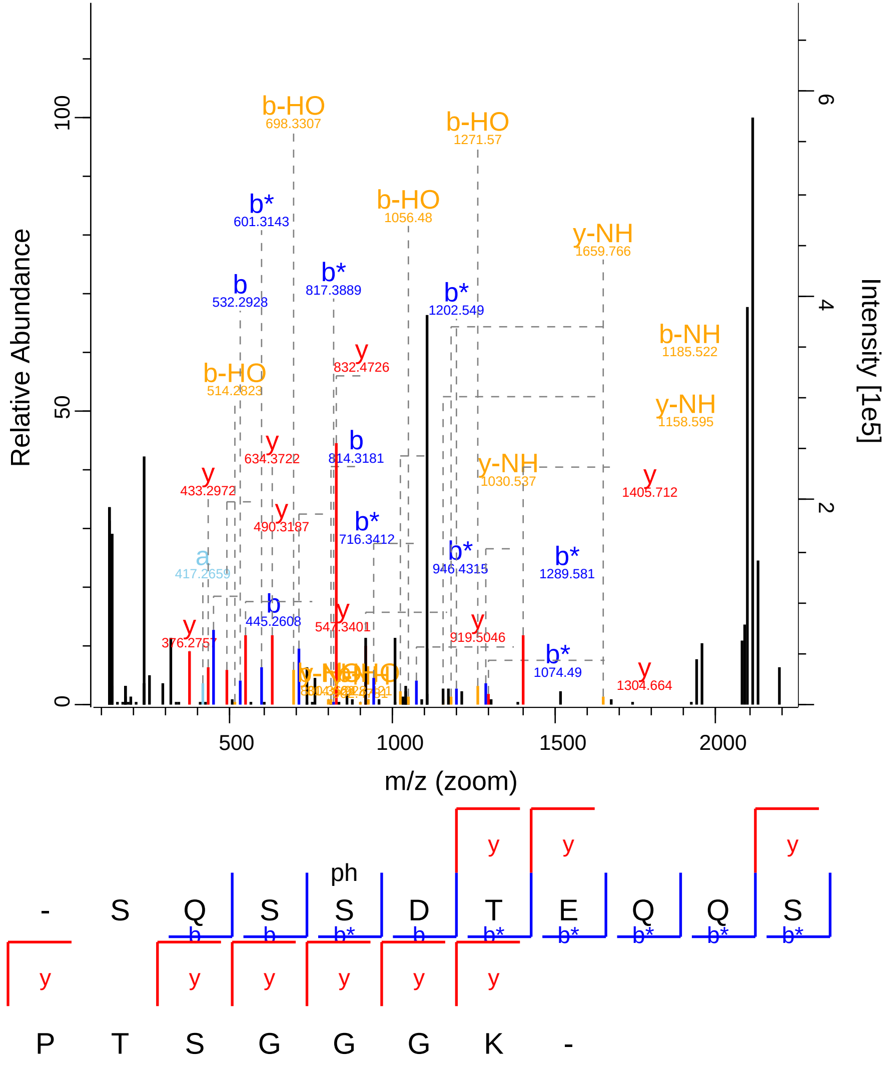

Raw File	Scan	Method	Score	m/z	Gene names
RO-QC-160121_ZackD_SS_Set_01_Fractior	7713	FTMS; HCI	65.11	648.36	SLTM

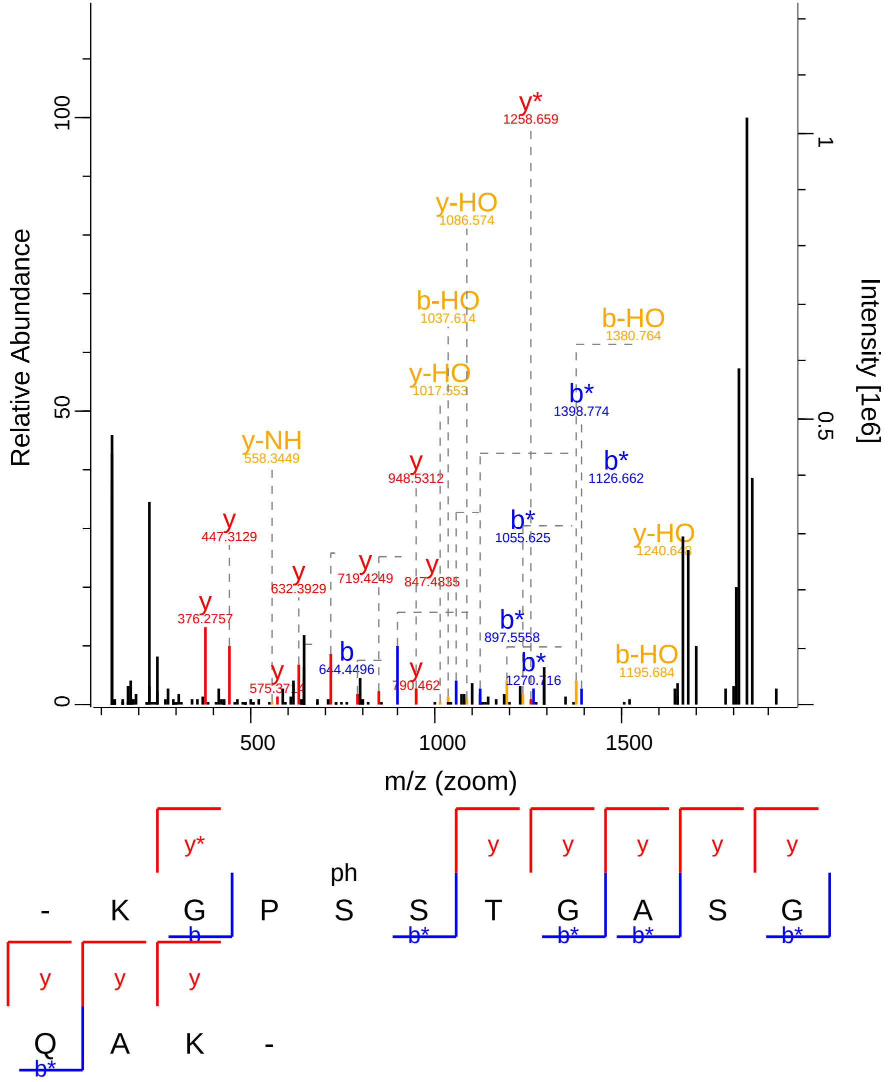

Raw File	Scan	Method	Score	m/z	Gene names
RO-QC-160121_ZackD_SS_Set_01_Fractio	10414	FTMS; HC	71.56	817.44	PRRC2A

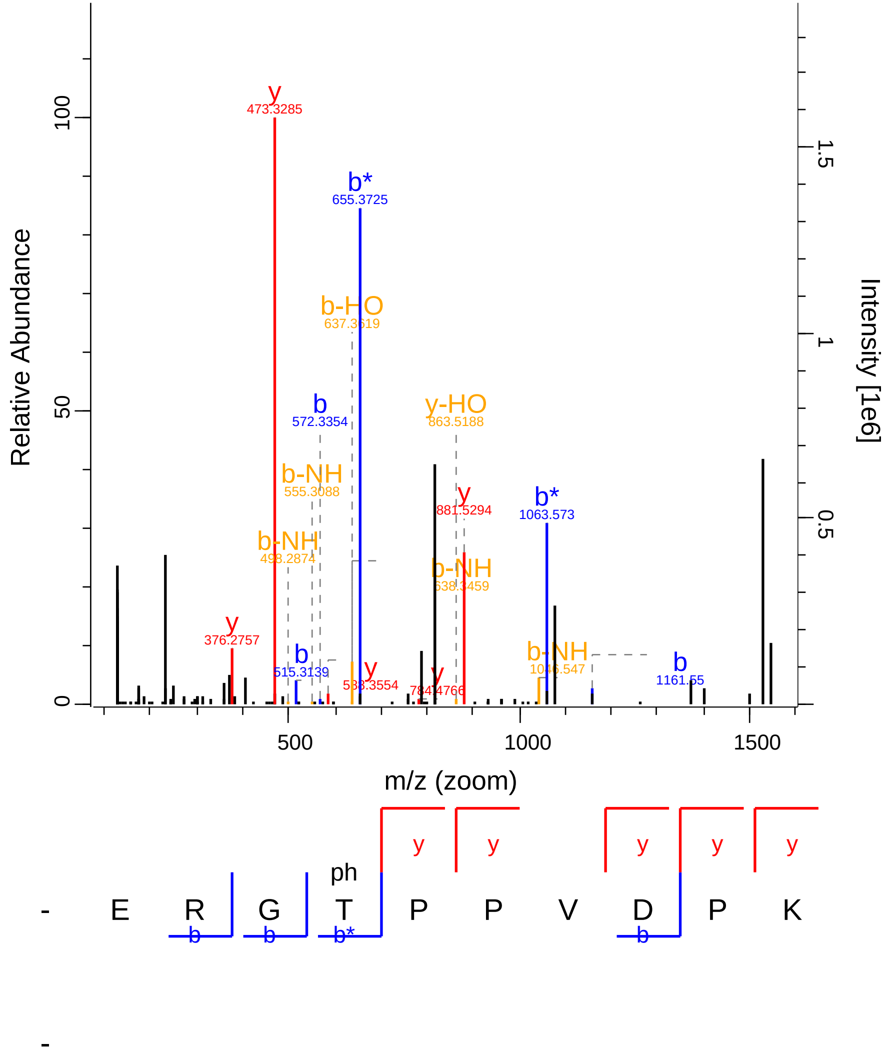

Raw File	Scan	Method	Score	m/z	Gene names
RO-QC-160121_ZackD_SS_Set_01_Fractio	10501	FTMS; HC	104.44	711.74	CC2D1A

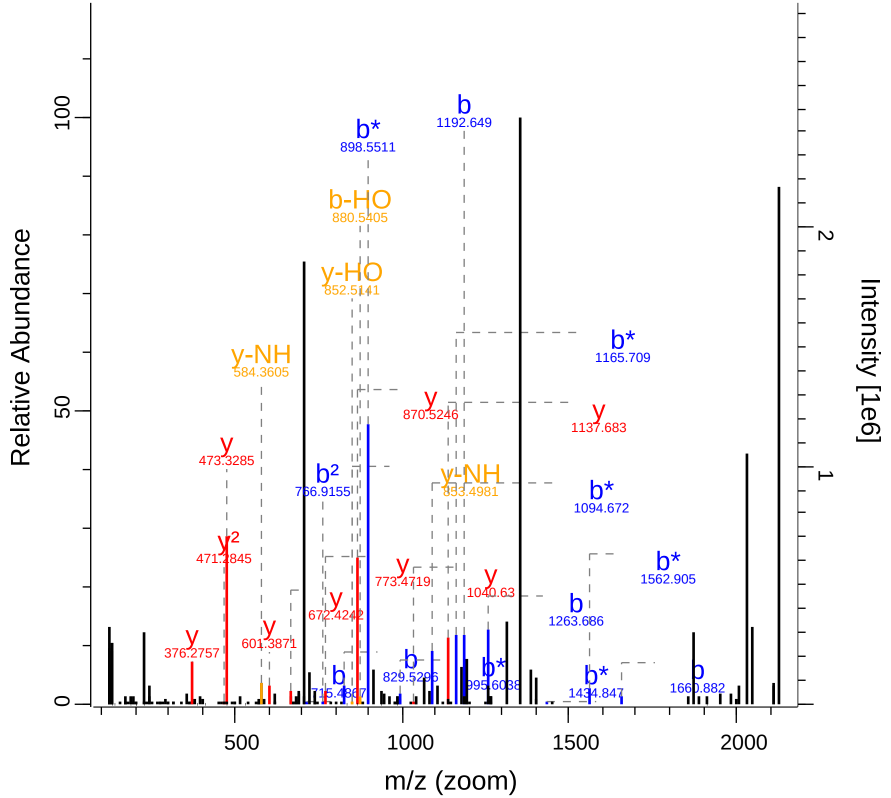

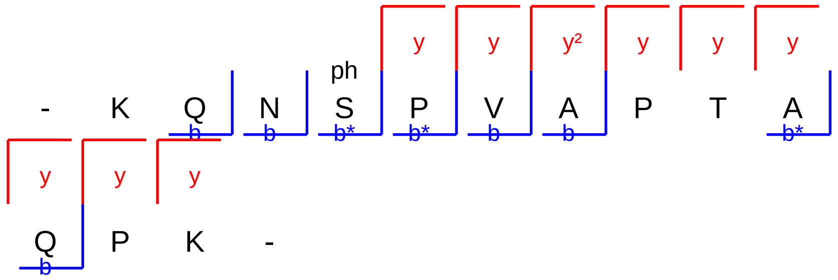

Raw File	Scan	Method	Score	m/z	Gene names
RO-QC-160121_ZackD_SS_Set_01_Fractior	11460	FTMS; HCI	44.45	761.4	ZNF490

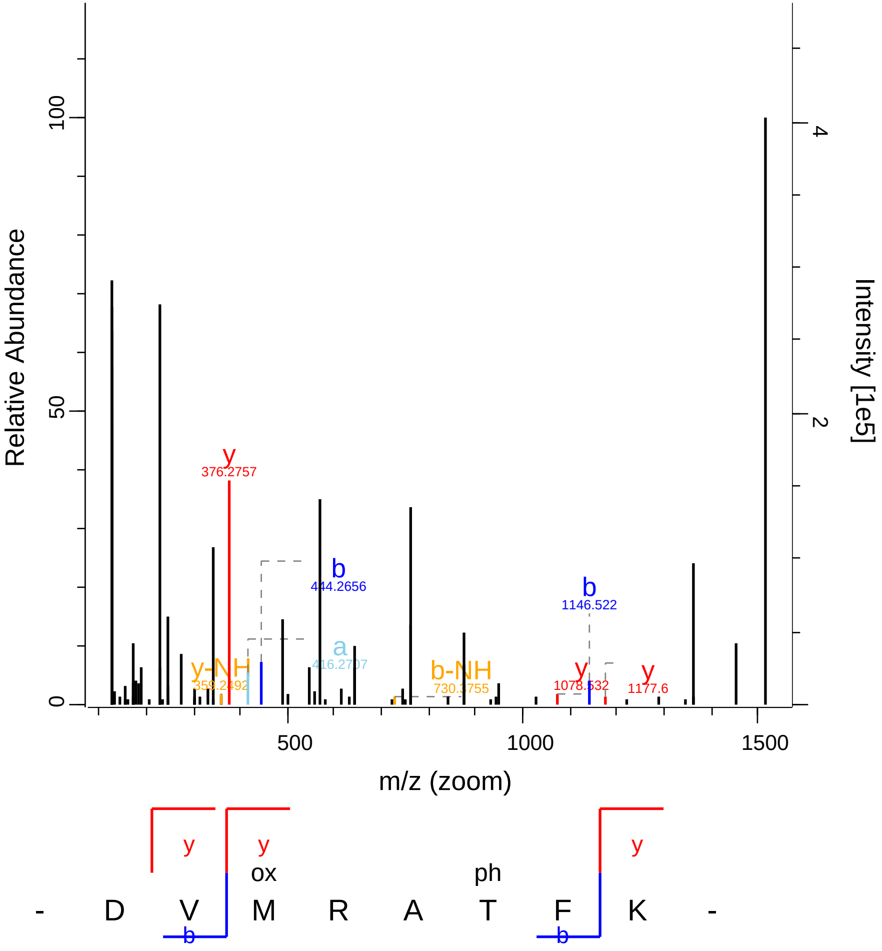

Raw File	Scan	Method	Score	m/z	Gene names
RO-QC-160121_ZackD_SS_Set_01_Fractio	11663	FTMS; HC	98.82	741.71	PI4KB

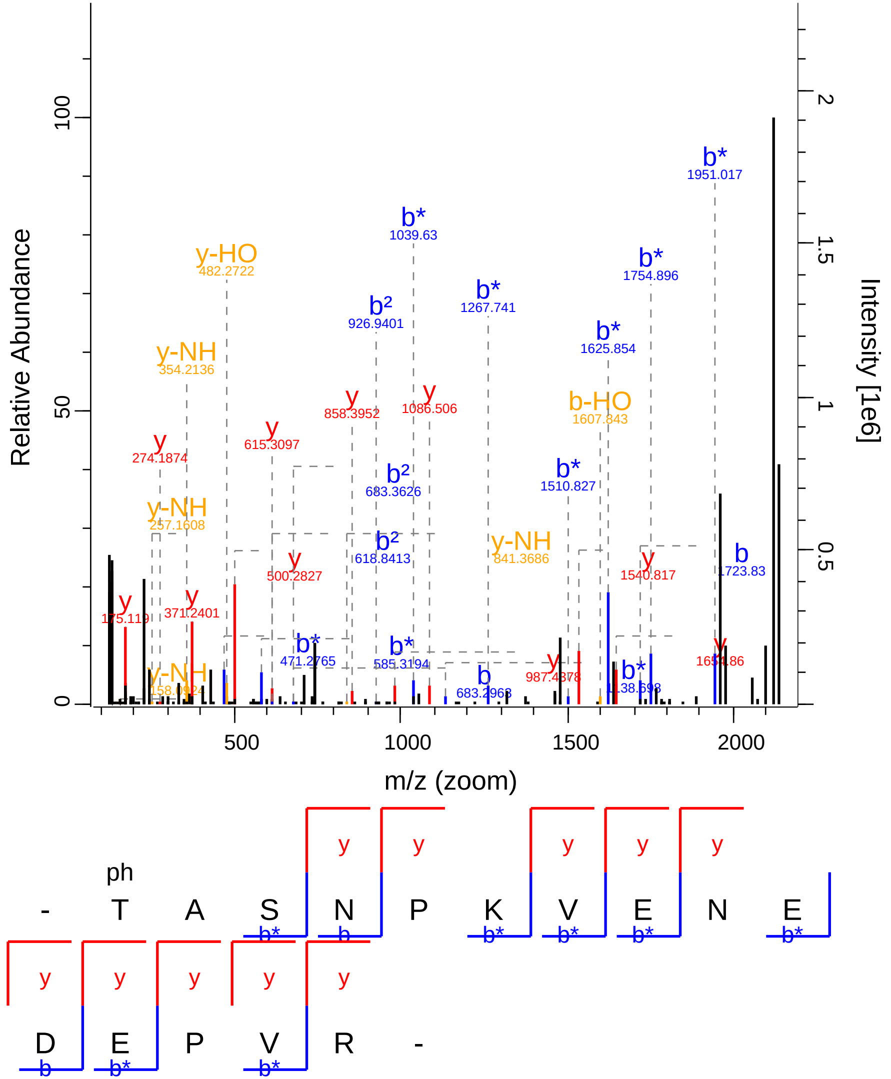

Raw File	Scan	Method	Score	m/z	Gene names
RO-QC-160121_ZackD_SS_Set_01_Fractio	11792	FTMS; HC	135.13	919.48	HNRNPA1

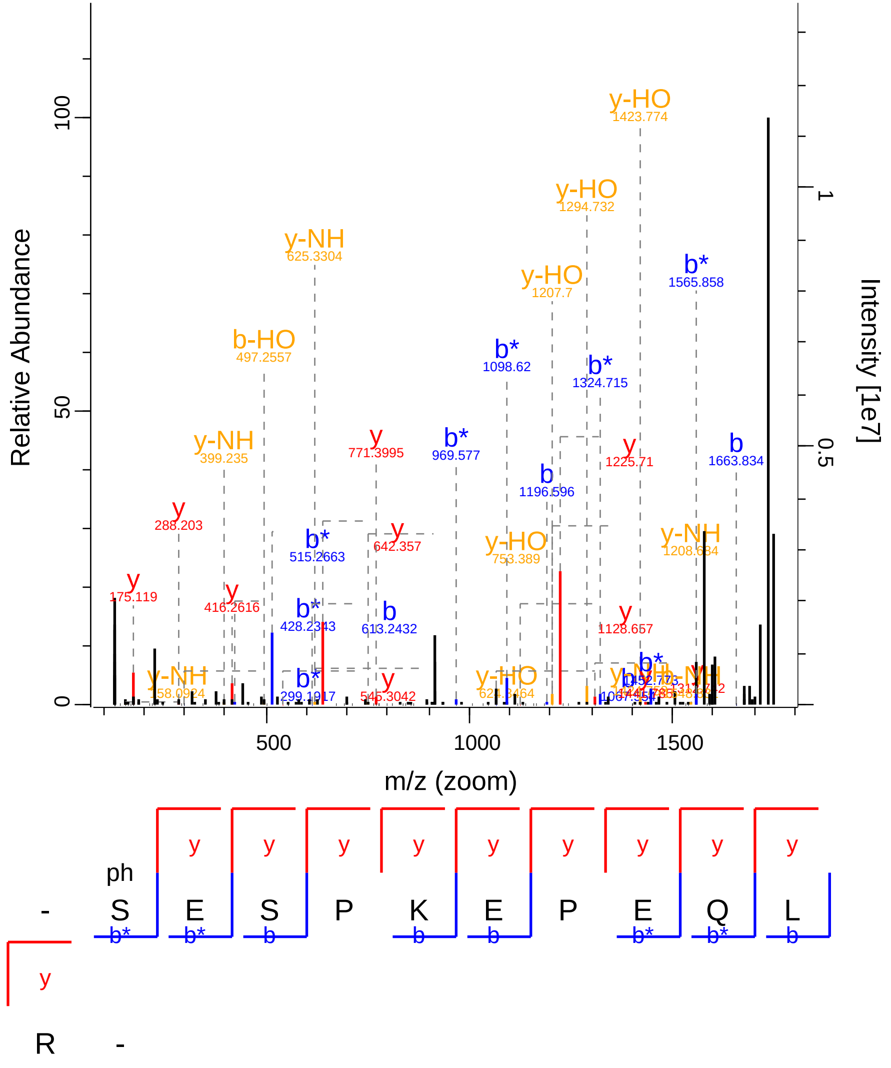

Raw File	Scan	Method	Score	m/z	Gene names
RO-QC-160121_ZackD_SS_Set_01_Fractio	13830	FTMS; HC	85.93	819.46	XRCC1

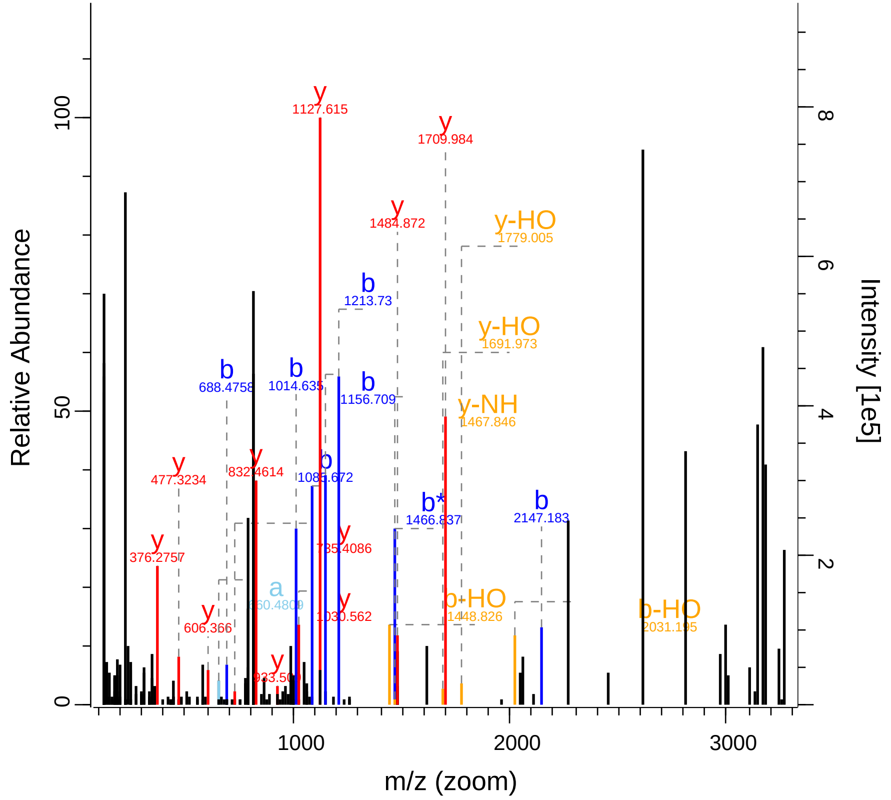

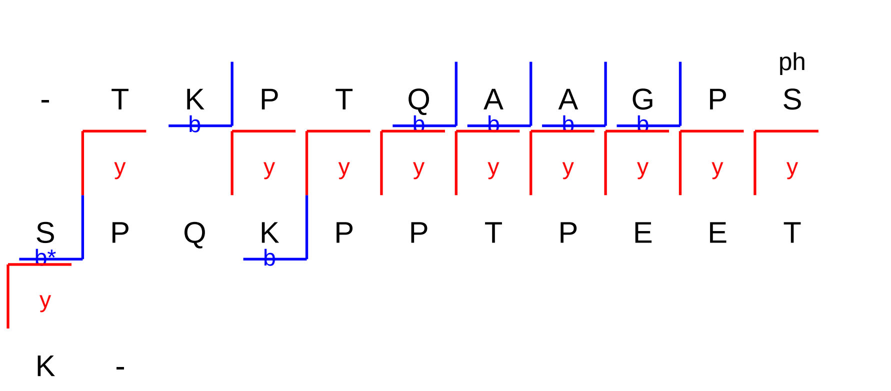

Raw File	Scan	Method	Score	m/z	Gene names
RO-QC-160121_ZackD_SS_Set_01_Fractio	15026	FTMS; HC	58.68	744.04	CNTLN

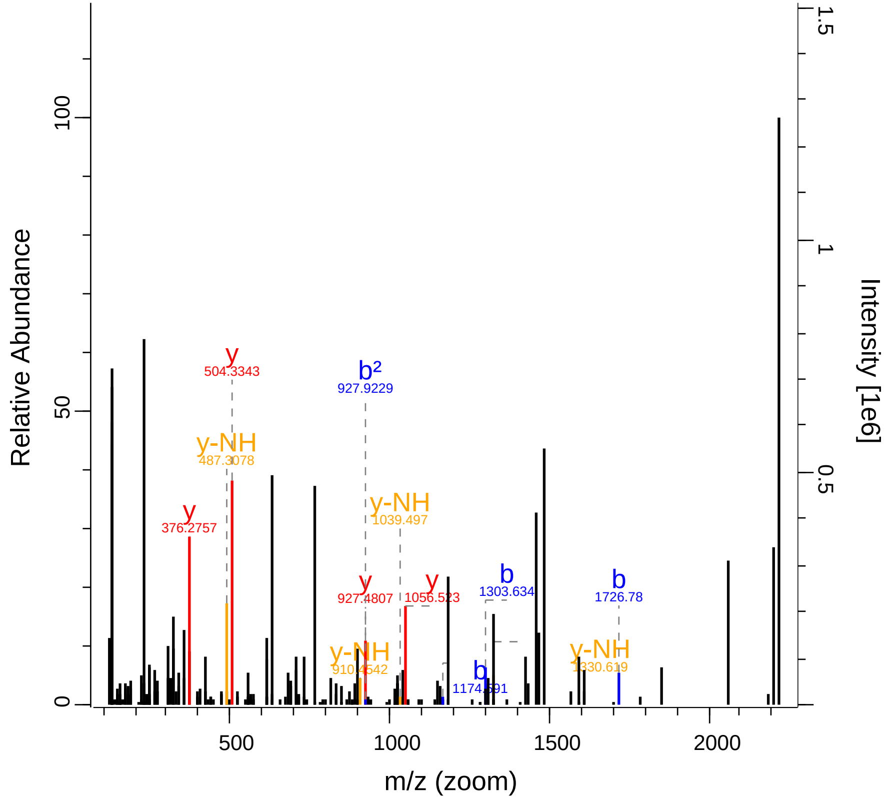

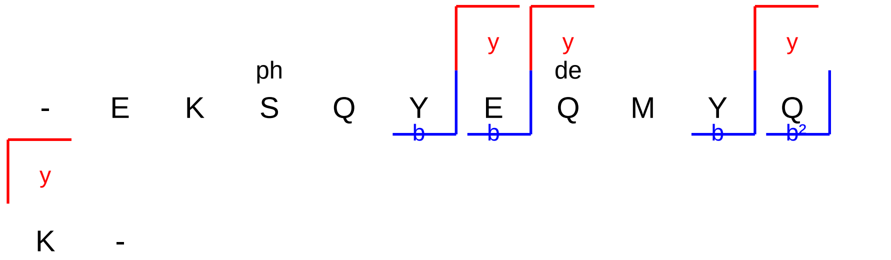

Raw File	Scan	Method	Score	m/z	Gene names
RO-QC-160121_ZackD_SS_Set_01_Fractio	15436	FTMS; HC	124.3	956.48	PCIF1

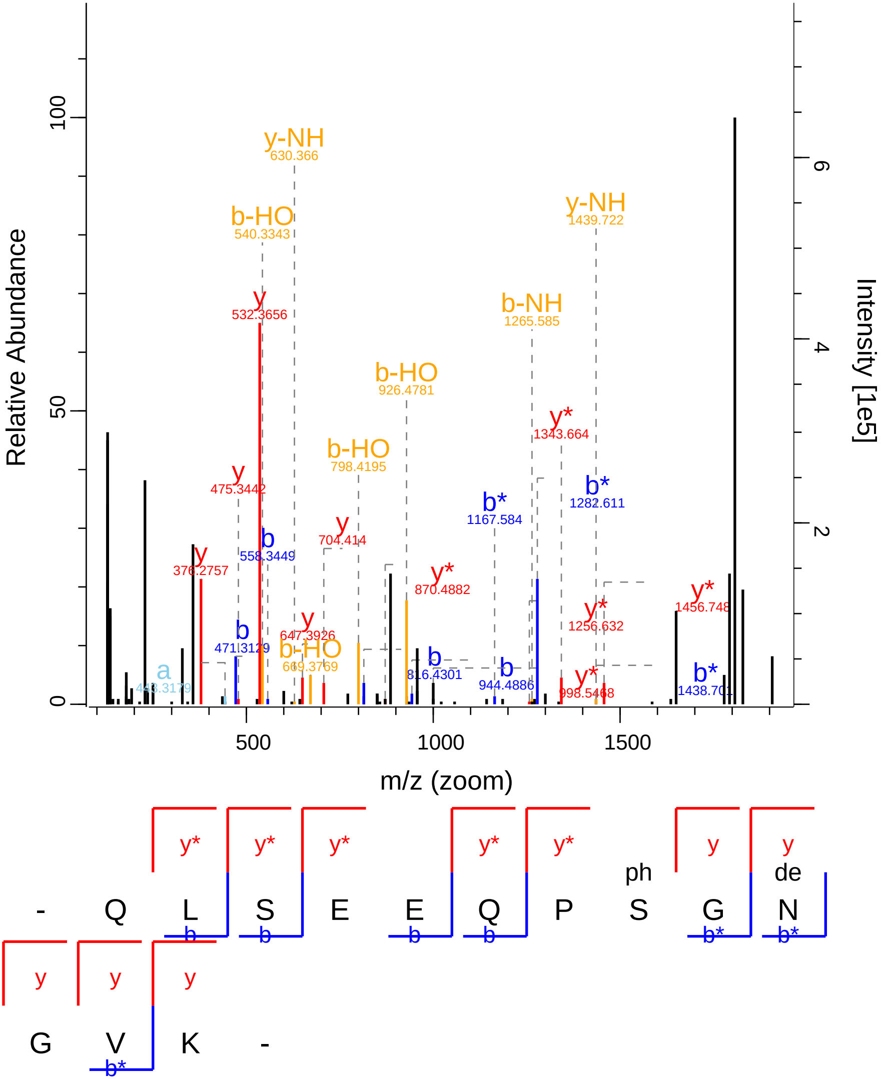

Raw File	Scan	Method	Score	m/z	Gene names
RO-QC-160121_ZackD_SS_Set_01_Fractic	16678	FTMS; HC	141.6	1281.66	HIST1H1B

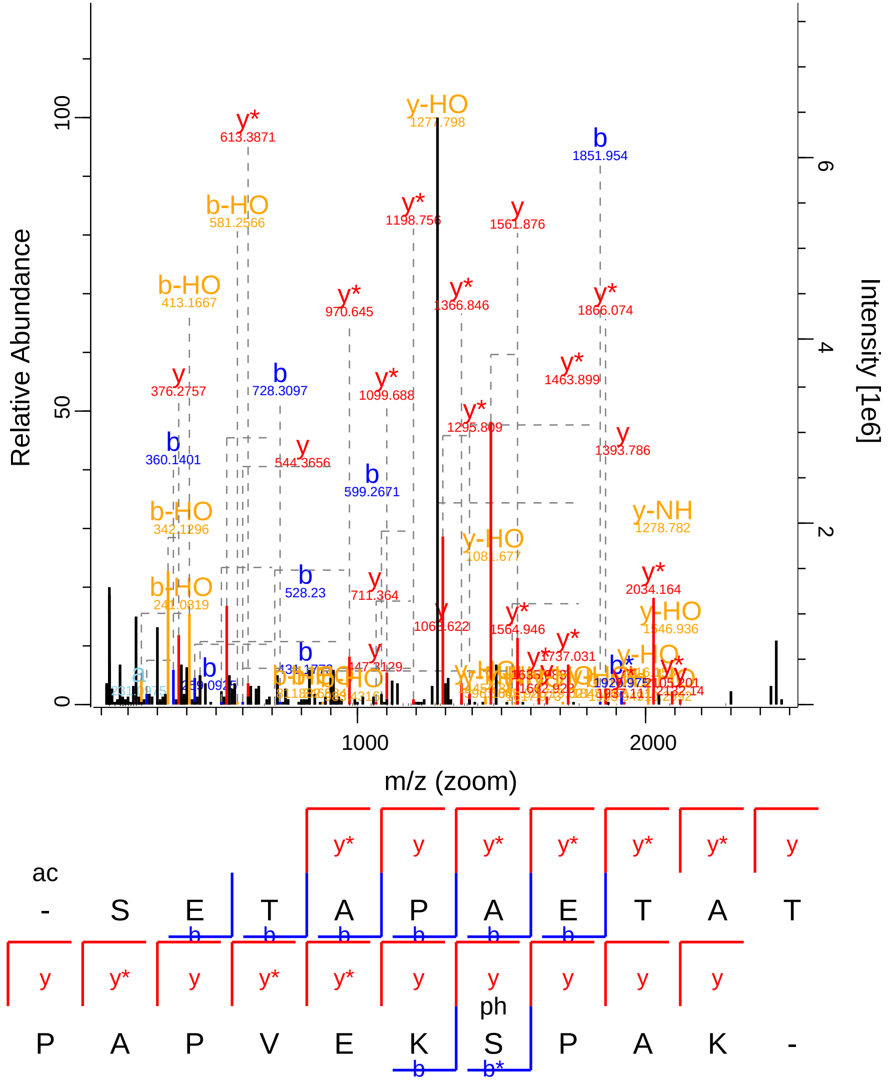

Raw File	Scan	Method	Score	m/z	Gene names
RO-QC-160121_ZackD_SS_Set_01_Fractio	17314	FTMS; HC	117.09	846.44	BRD3

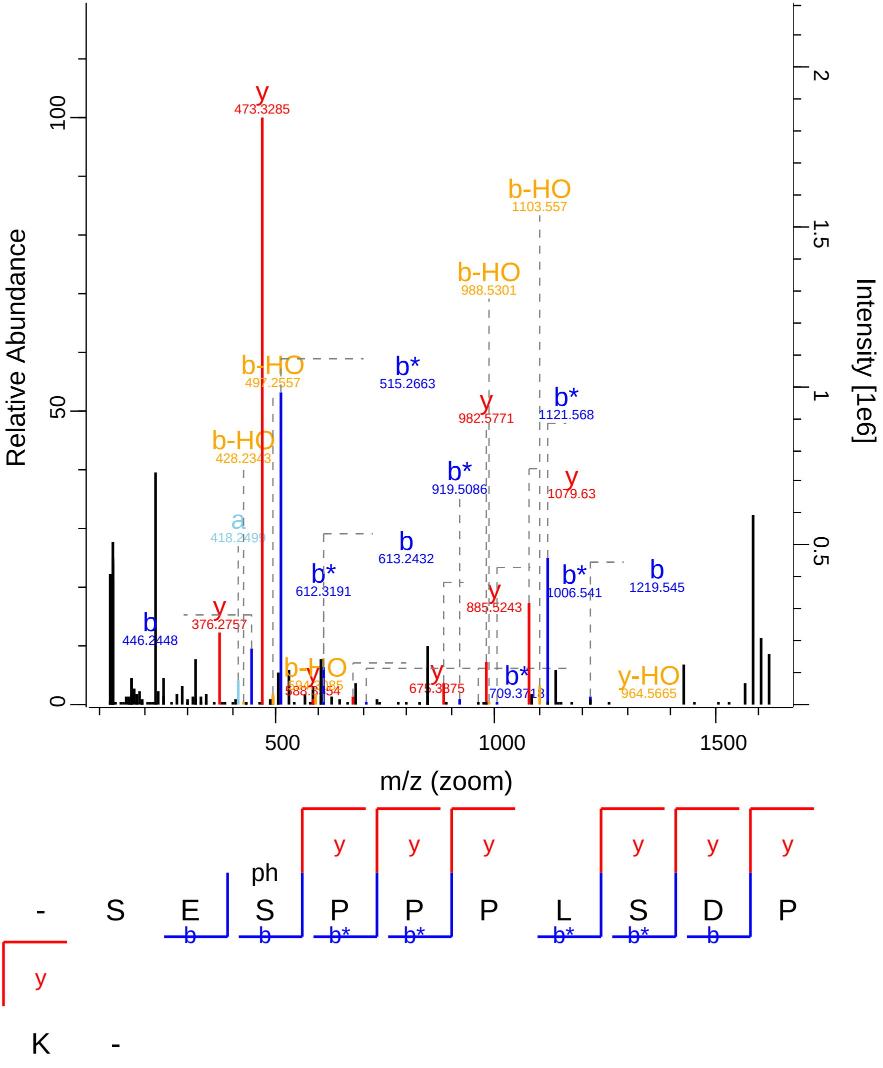

Raw File	Scan	Method	Score	m/z	Gene names
RO-QC-160121_ZackD_SS_Set_01_Fractio	17980	FTMS; HC	132.79	975.42	EIF5B

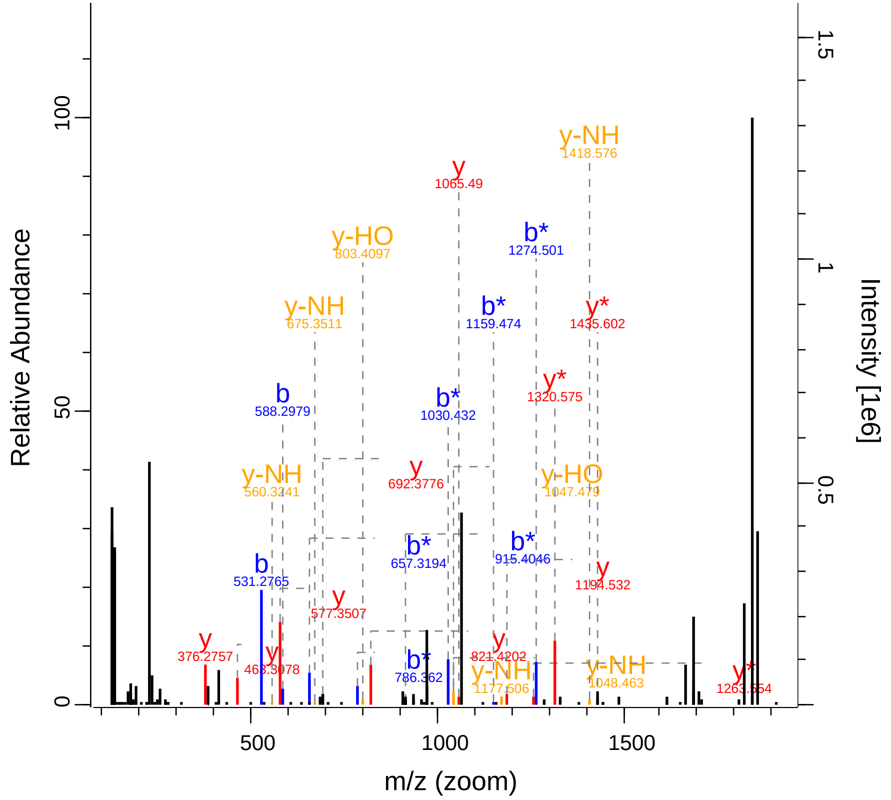

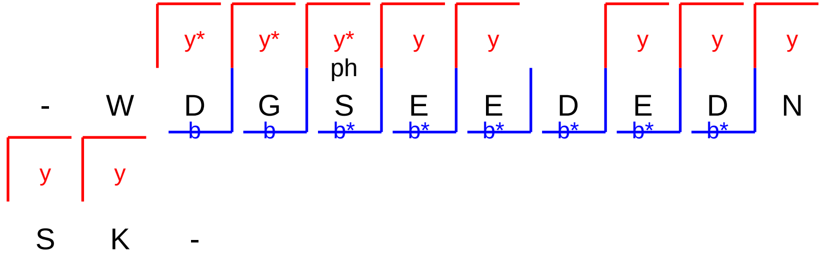

Raw File	Scan	Method	Score	m/z	Gene names
RO-QC-160121_ZackD_SS_Set_01_Fractio	18580	FTMS; HC	134.49	692.86	HSPB1

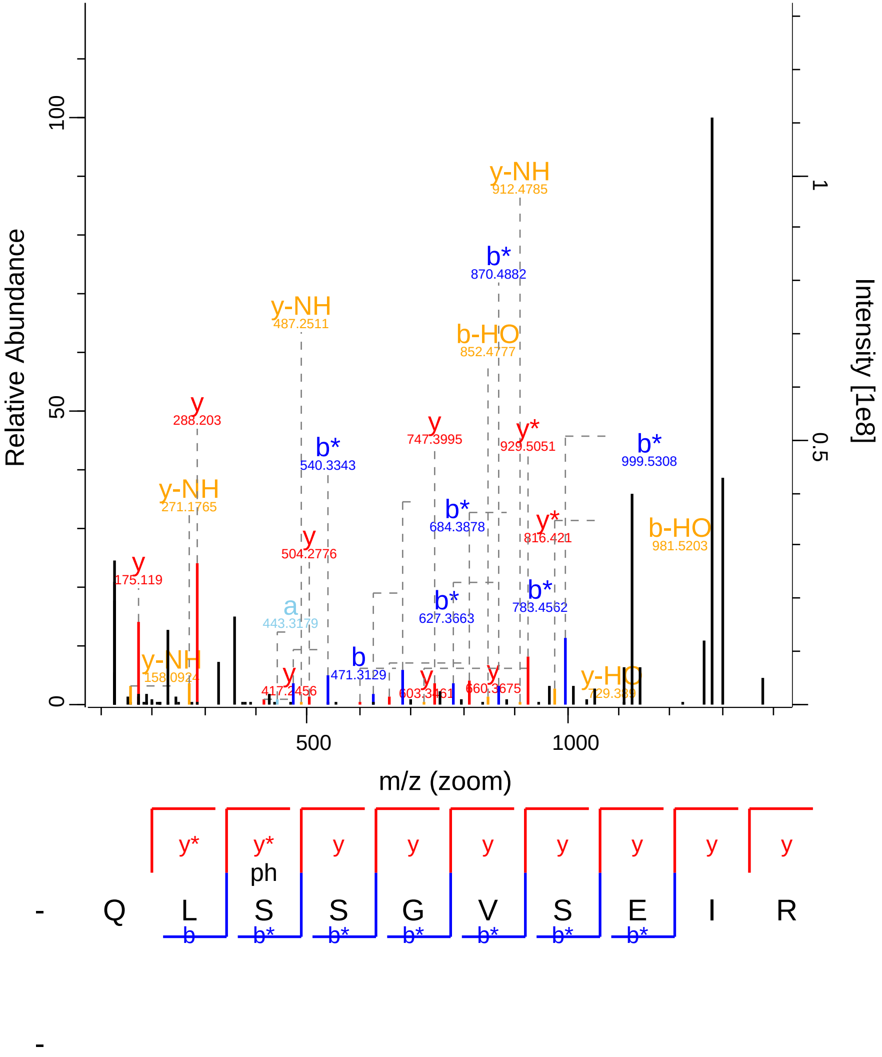

Raw File	Scan	Method	Score	m/z	Gene names
RO-QC-160121_ZackD_SS_Set_01_Fractic	19103	FTMS; HC	45.66	1173.57	LRRC28

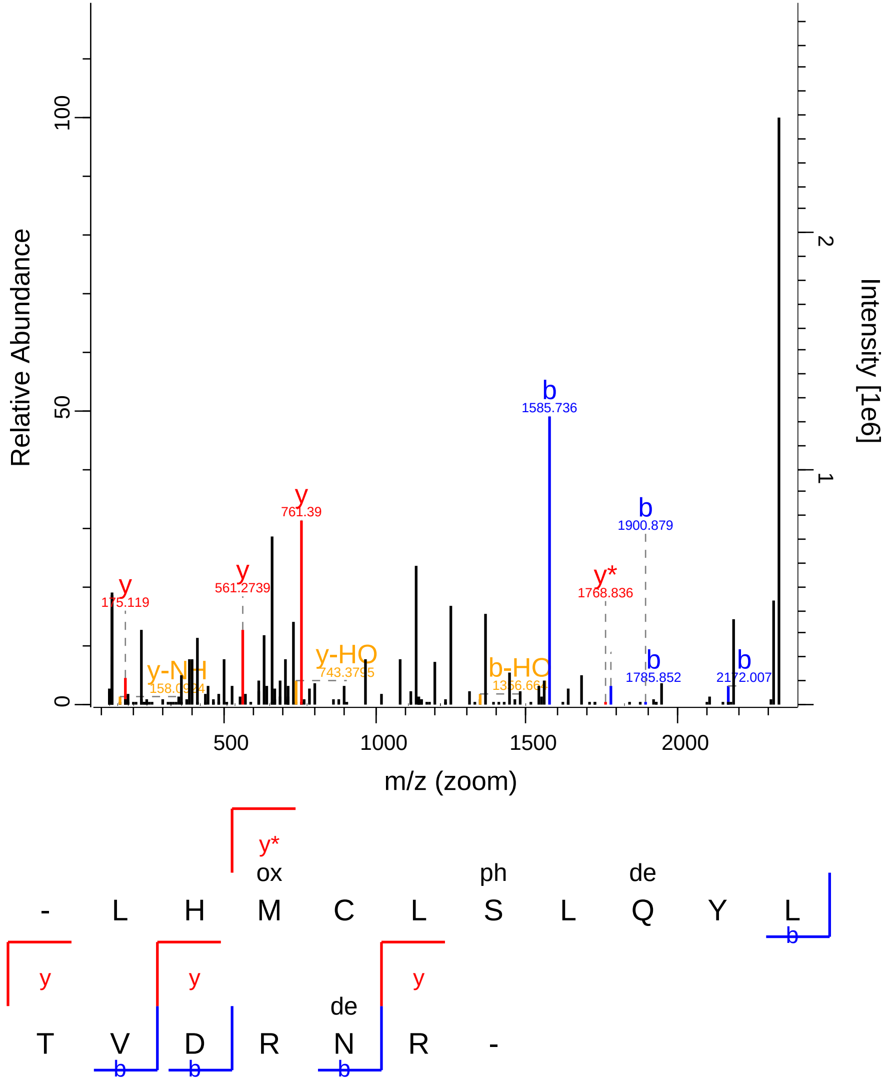

Raw File	Scan	Method	Score	m/z	Gene names
RO-QC-160121_ZackD_SS_Set_01_Fractio	20831	FTMS; HC	66.06	1235.1	HELT

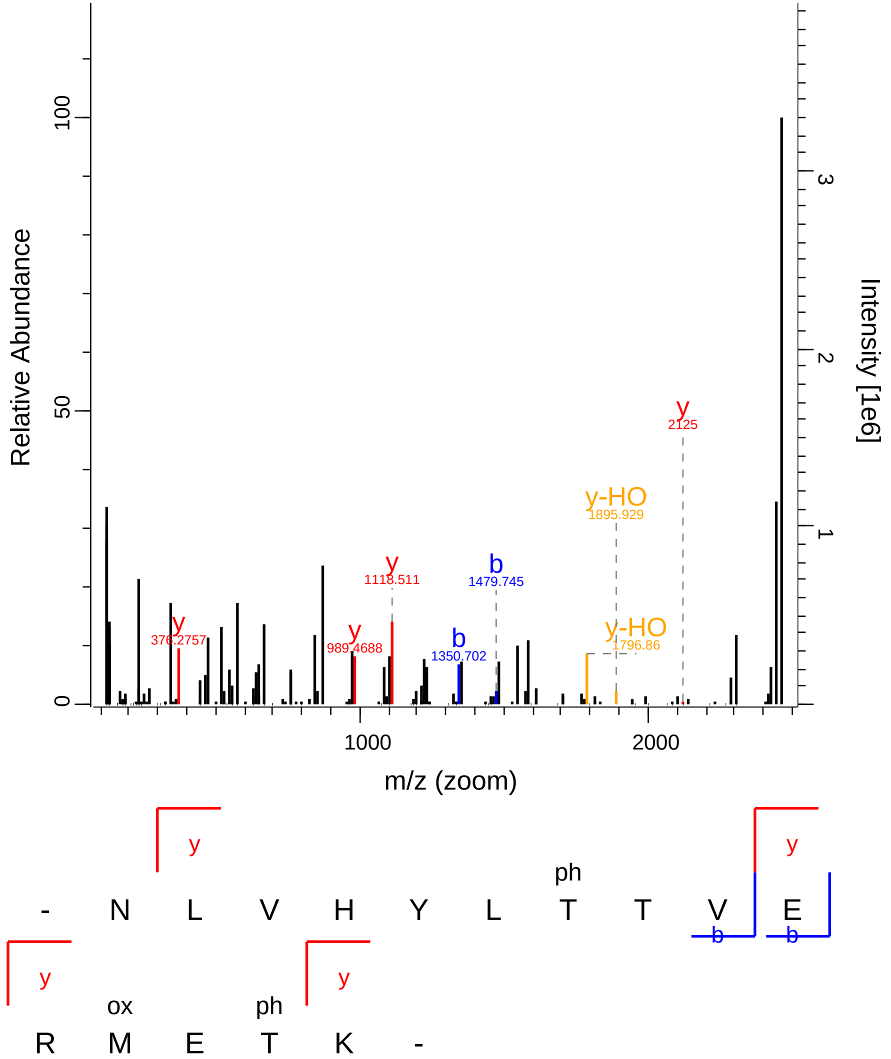

Raw File	Scan	Method	Score	m/z	Gene names
RO-QC-160121_ZackD_SS_Set_01_Fractio	20872	FTMS; HC	47.07	689.86	CNKSR1

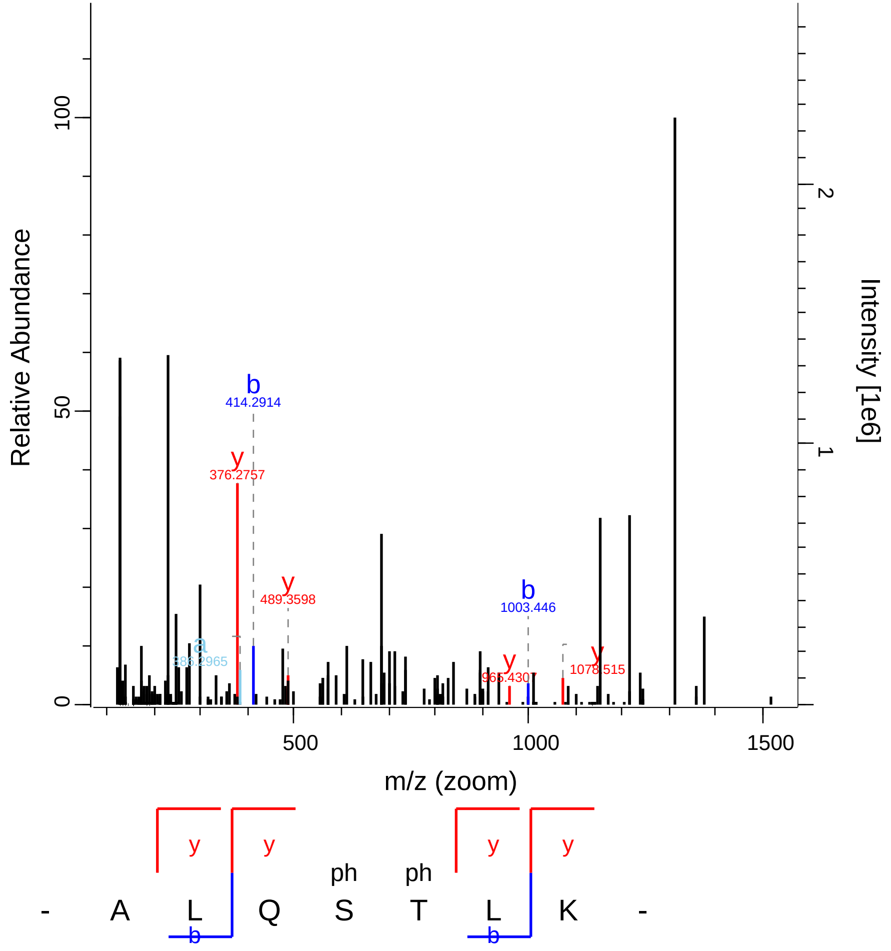

Raw File	Scan	Method	Score	m/z	Gene names
RO-QC-160121_ZackD_SS_Set_01_Fractio	21184	FTMS; HC	43	471.91	NKPD1

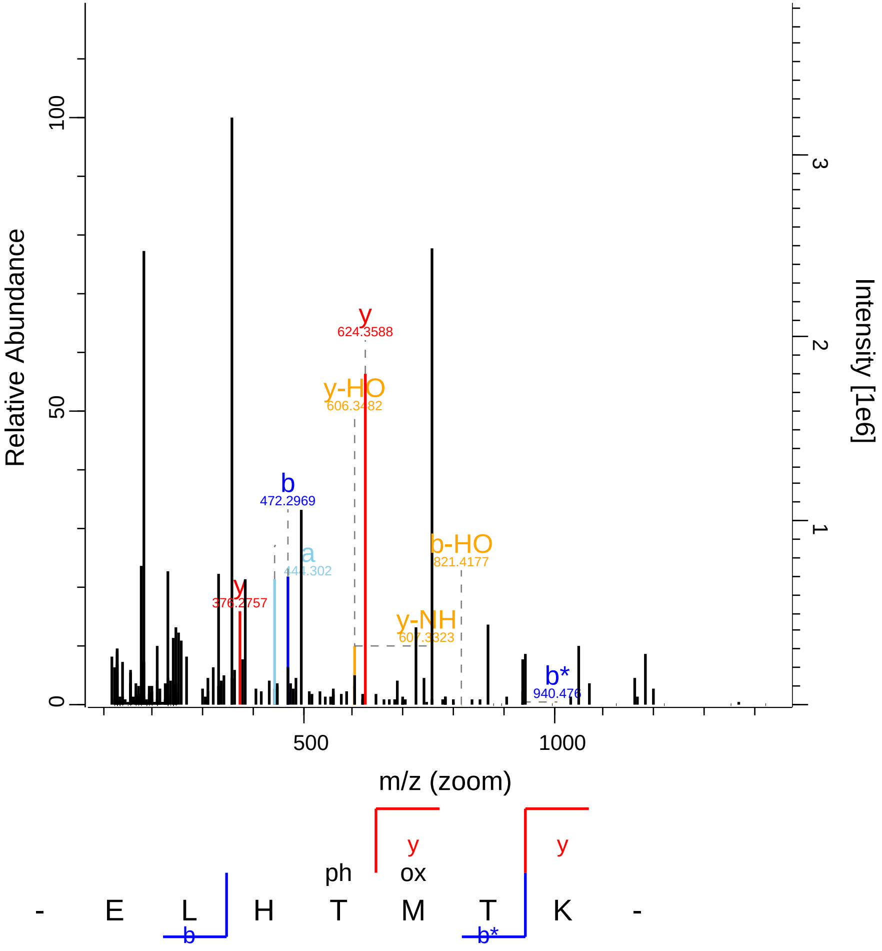

Raw File	Scan	Method	Score	m/z	Gene names
RO-QC-160121_ZackD_SS_Set_01_Fractio	21416	FTMS; HC	178.43	887.72	FBXW9

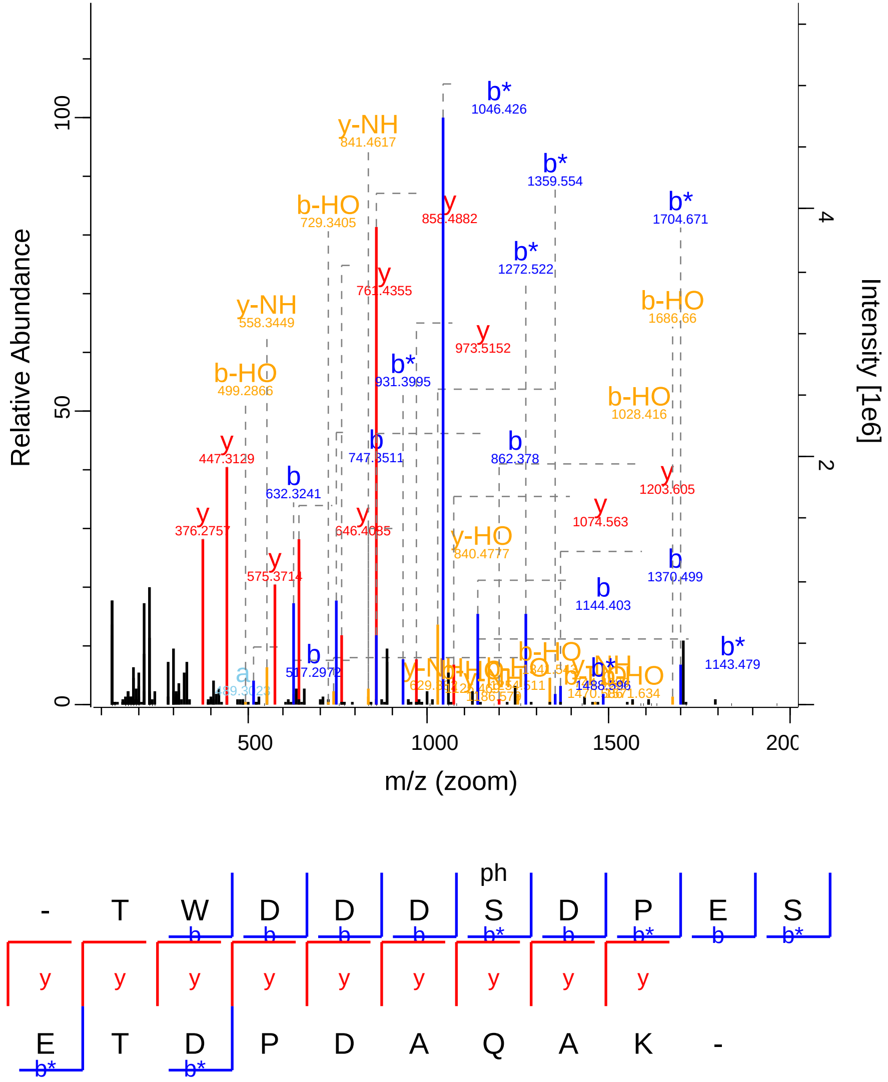

Raw File	Scan	Method	Score	m/z	Gene names
RO-QC-160121_ZackD_SS_Set_01_Fractio	21560	FTMS; HC	107.08	867.49	ZYX

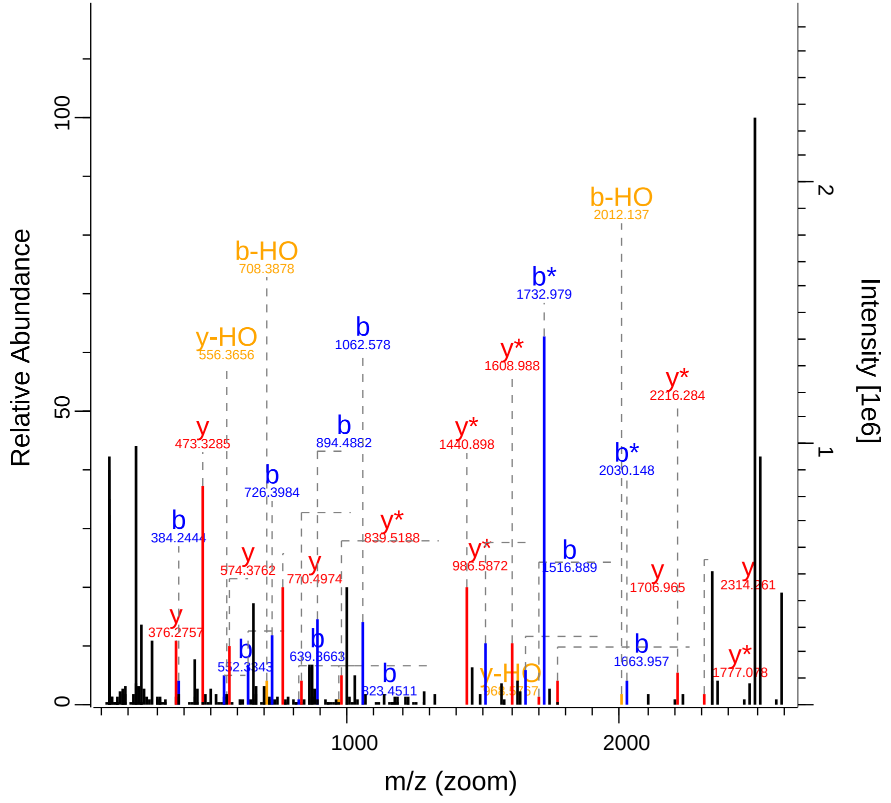

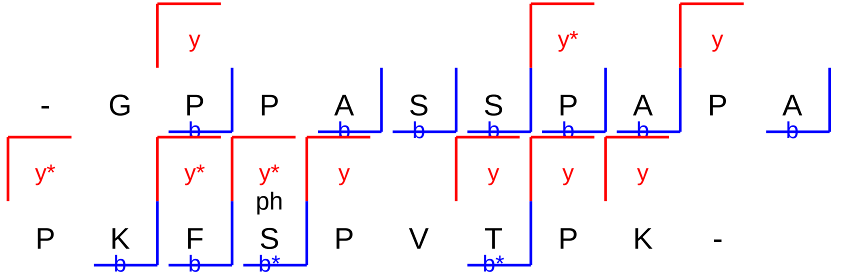

Raw File	Scan	Method	Score	m/z	Gene names
RO-QC-160121_ZackD_SS_Set_01_Fractio	22835	FTMS; HC	60.81	890.42	CCDC132

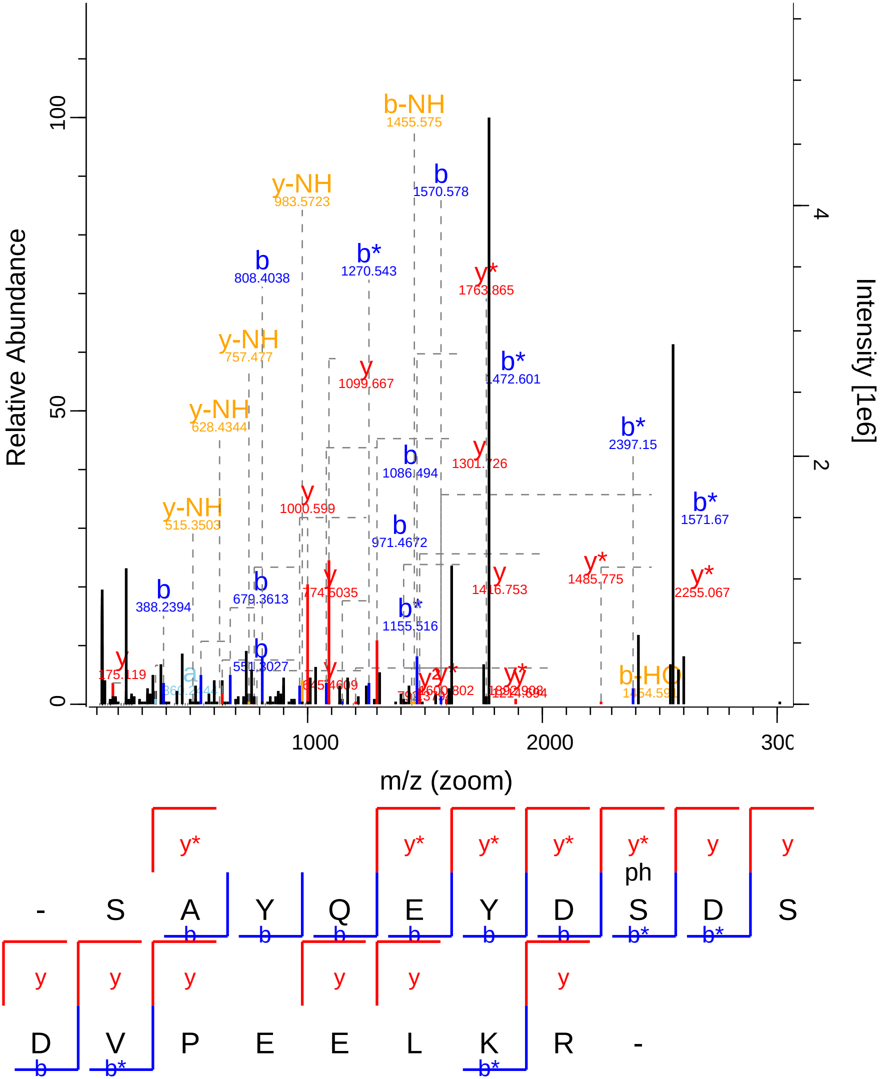

Raw File	Scan	Method	Score	m/z	Gene names
RO-QC-160121_ZackD_SS_Set_01_Fractio	24846	FTMS; HC	99.83	563.64	CDK18

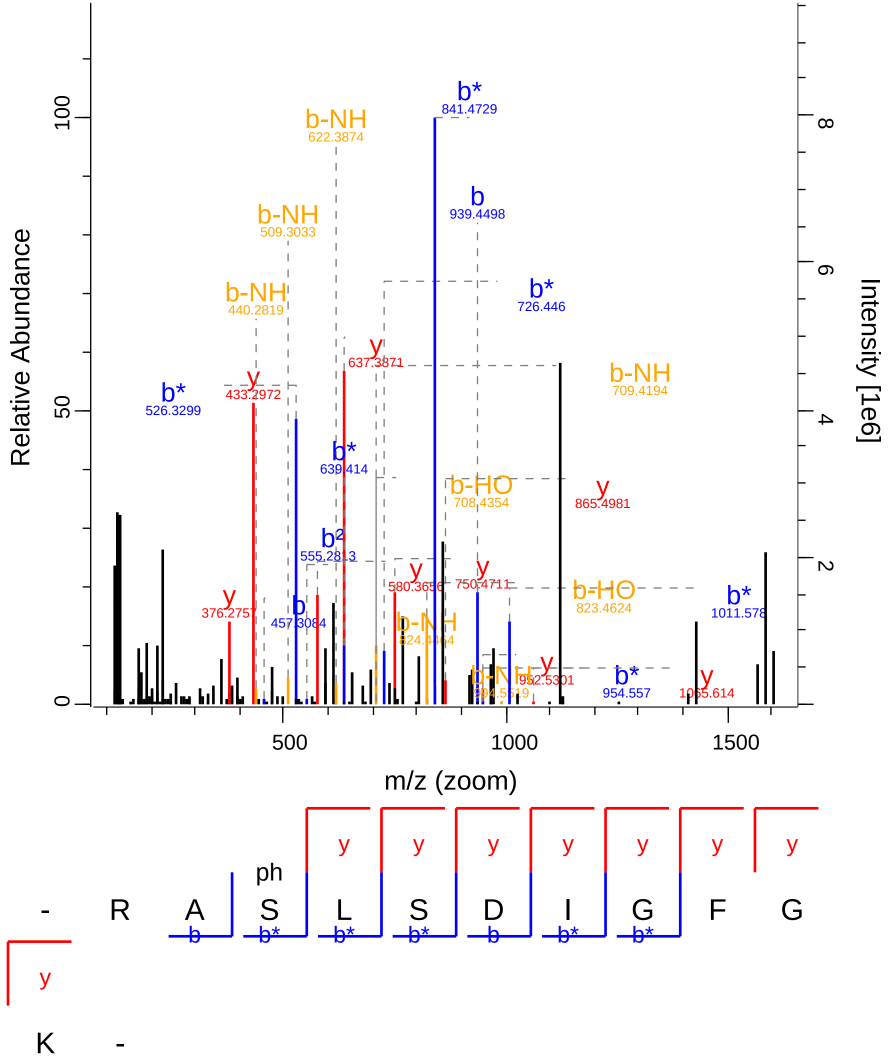

Raw File	Scan	Method	Score	m/z	Gene names
RO-QC-160121_ZackD_SS_Set_01_Fractio	26651	FTMS; HC	100	967.44	MAP1A

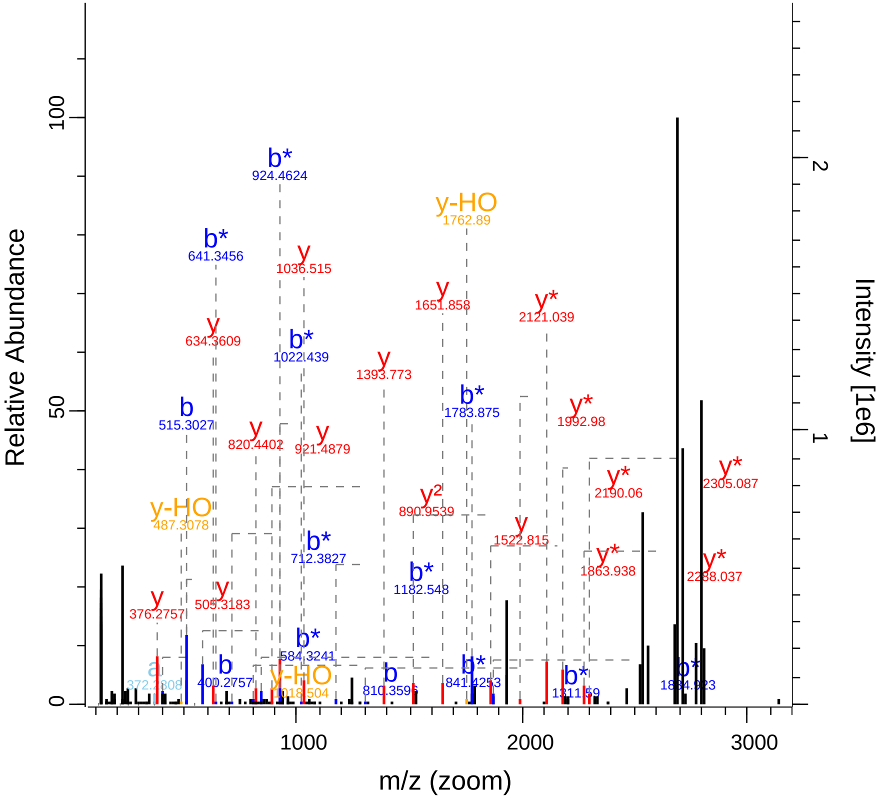

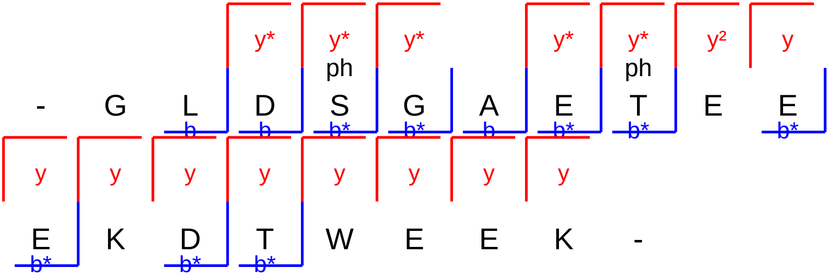

Raw File	Scan	Method	Score	m/z	Gene names
RO-QC-160121_ZackD_SS_Set_01_Fractio	26674	FTMS; HC	49.5	1164.1	KCNQ1

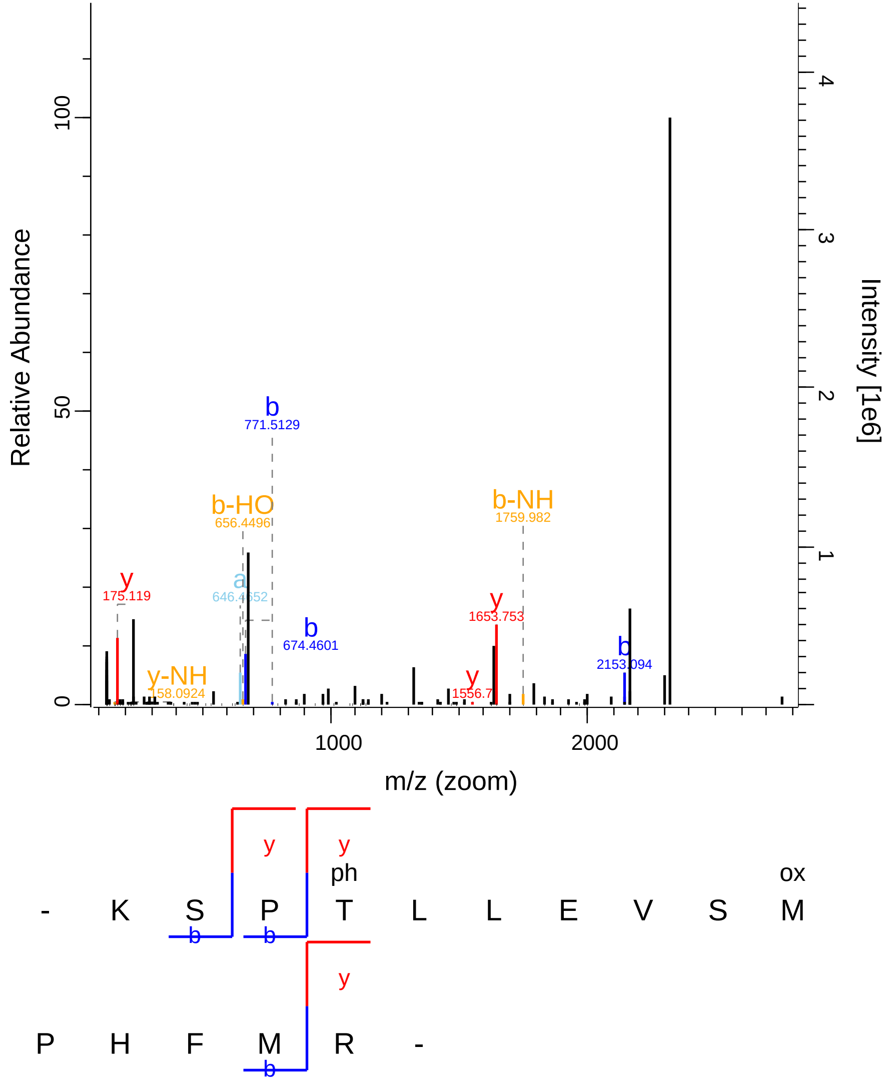

Raw File	Scan	Method	Score	m/z	Gene names
RO-QC-160121_ZackD_SS_Set_01_Fractio	27451	FTMS; HC	45.86	694.63	PCNT

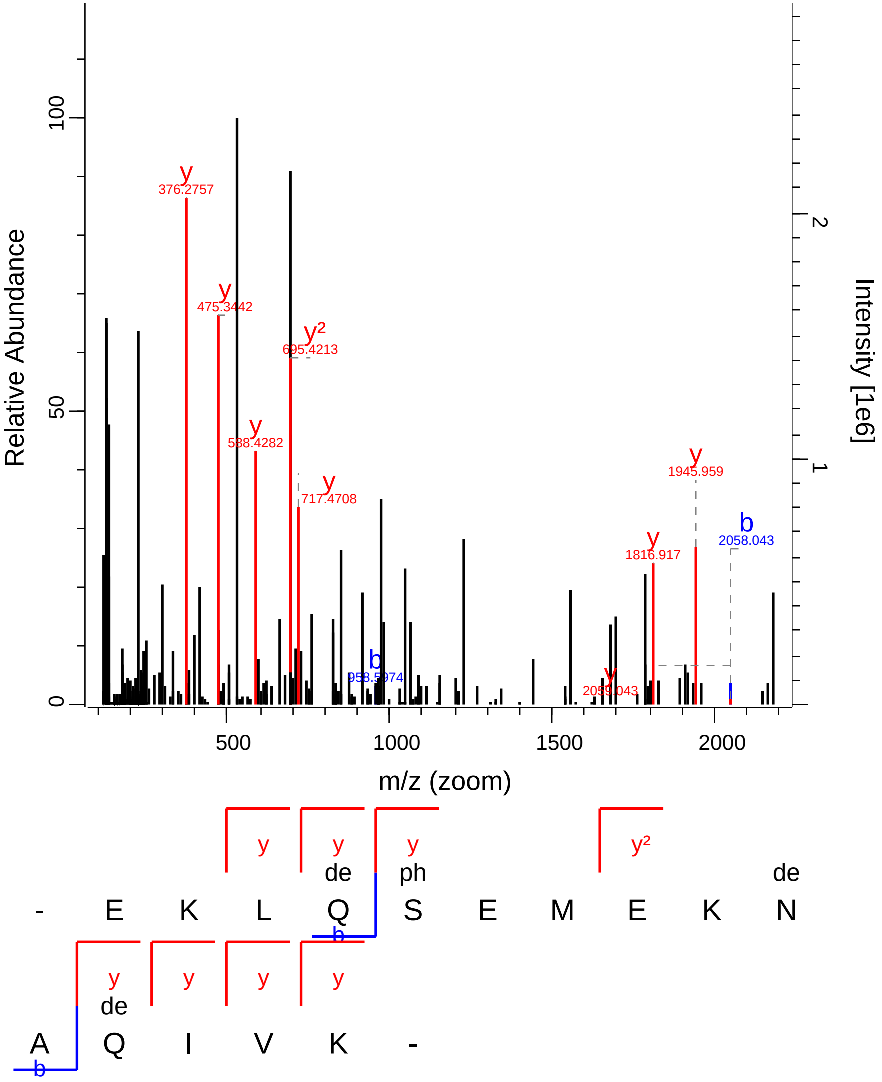

Raw File	Scan	Method	Score	m/z	Gene names
RO-QC-160121_ZackD_SS_Set_01_Fractic	27781	FTMS; HC	68.17	1001.02	TERF2IP

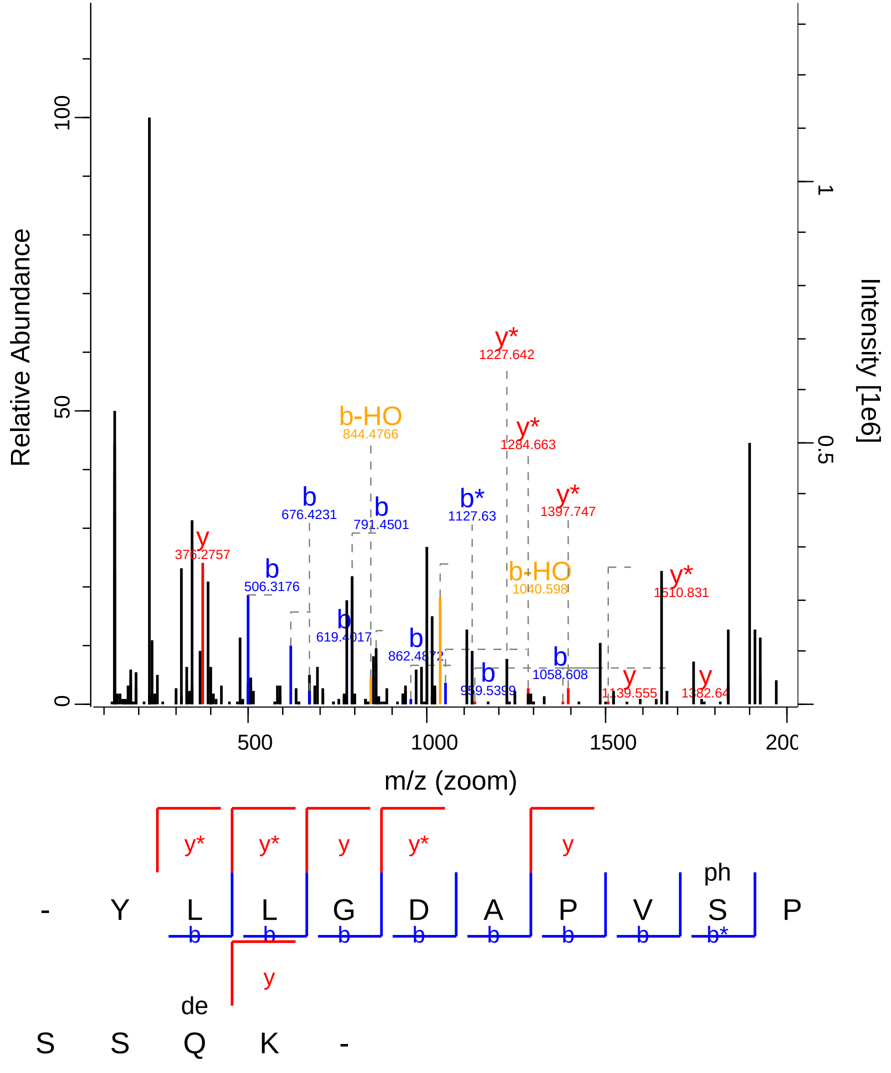

Raw File	Scan	Method	Score	m/z	Gene names
RO-QC-160121_ZackD_SS_Set_01_Fractio	27915	FTMS; HC	188.44	1261.08	MAP1B

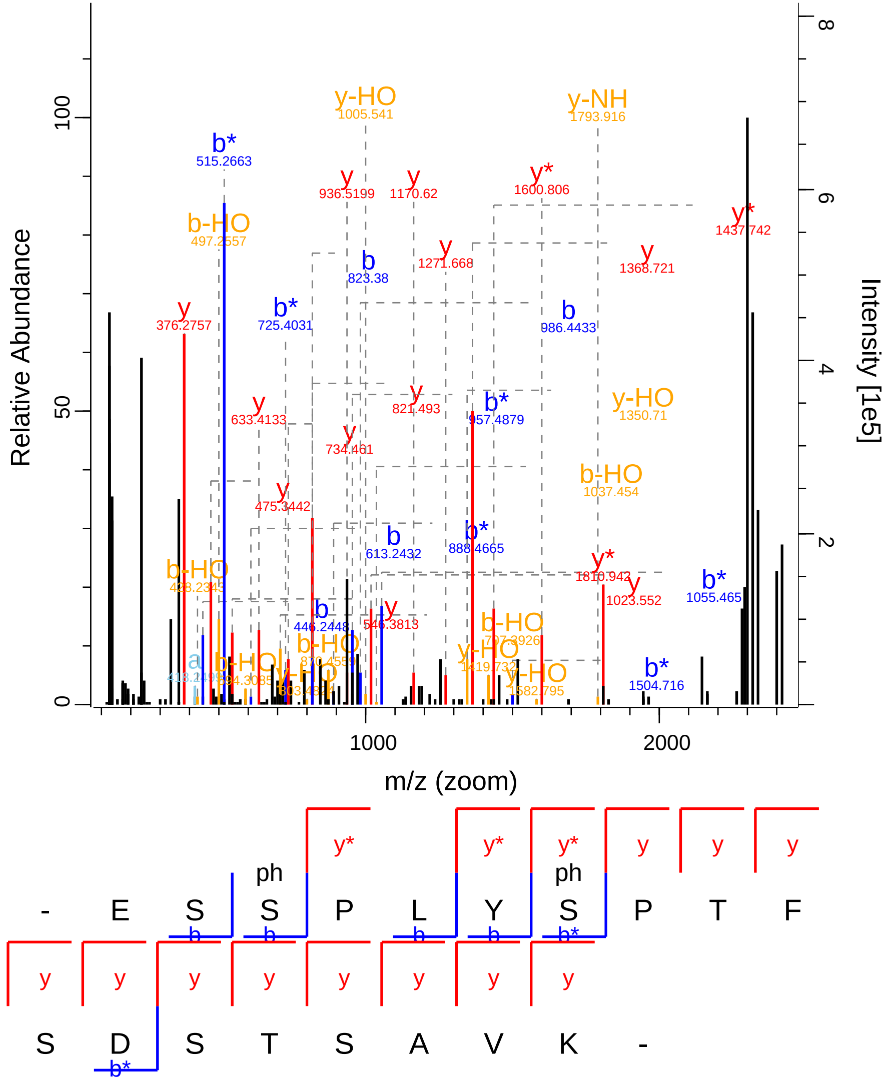

Raw File	Scan	Method	Score	m/z	Gene names
RO-QC-160121_ZackD_SS_Set_01_Fractio	28372	FTMS; HC	170.79	848.97	KLC1

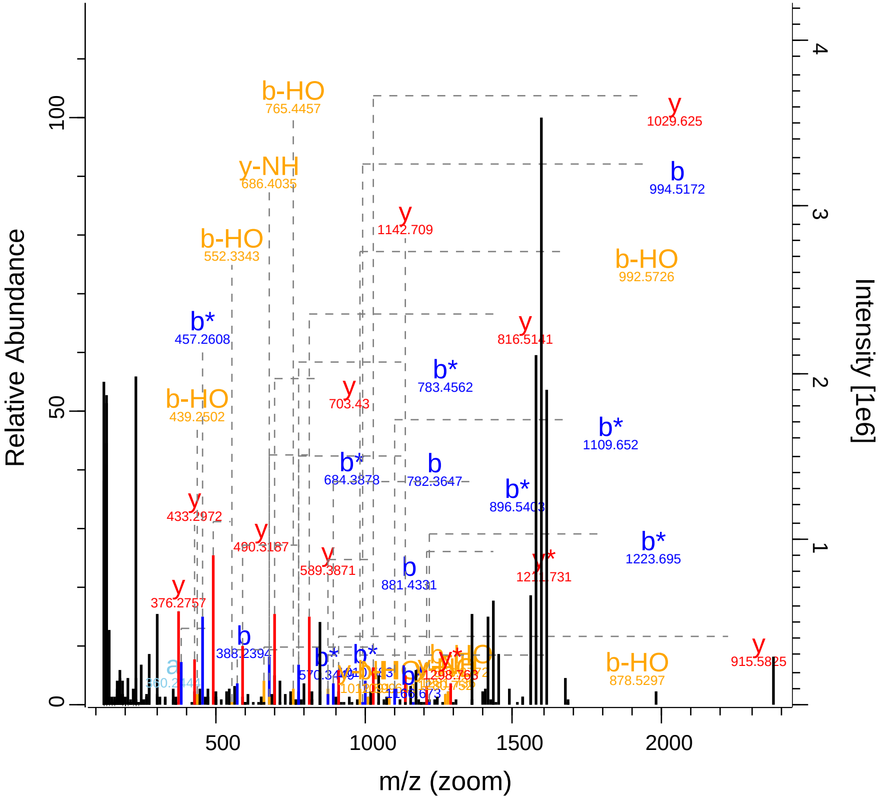

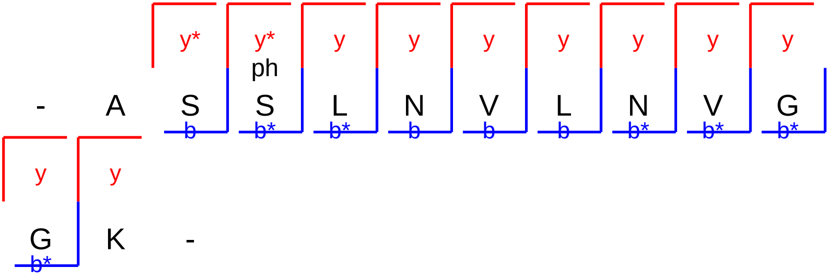

Raw File	Scan	Method	Score	m/z	Gene names
RO-QC-160121_ZackD_SS_Set_01_Fractio	28880	FTMS; HC	105.92	1100.5	MAP1B

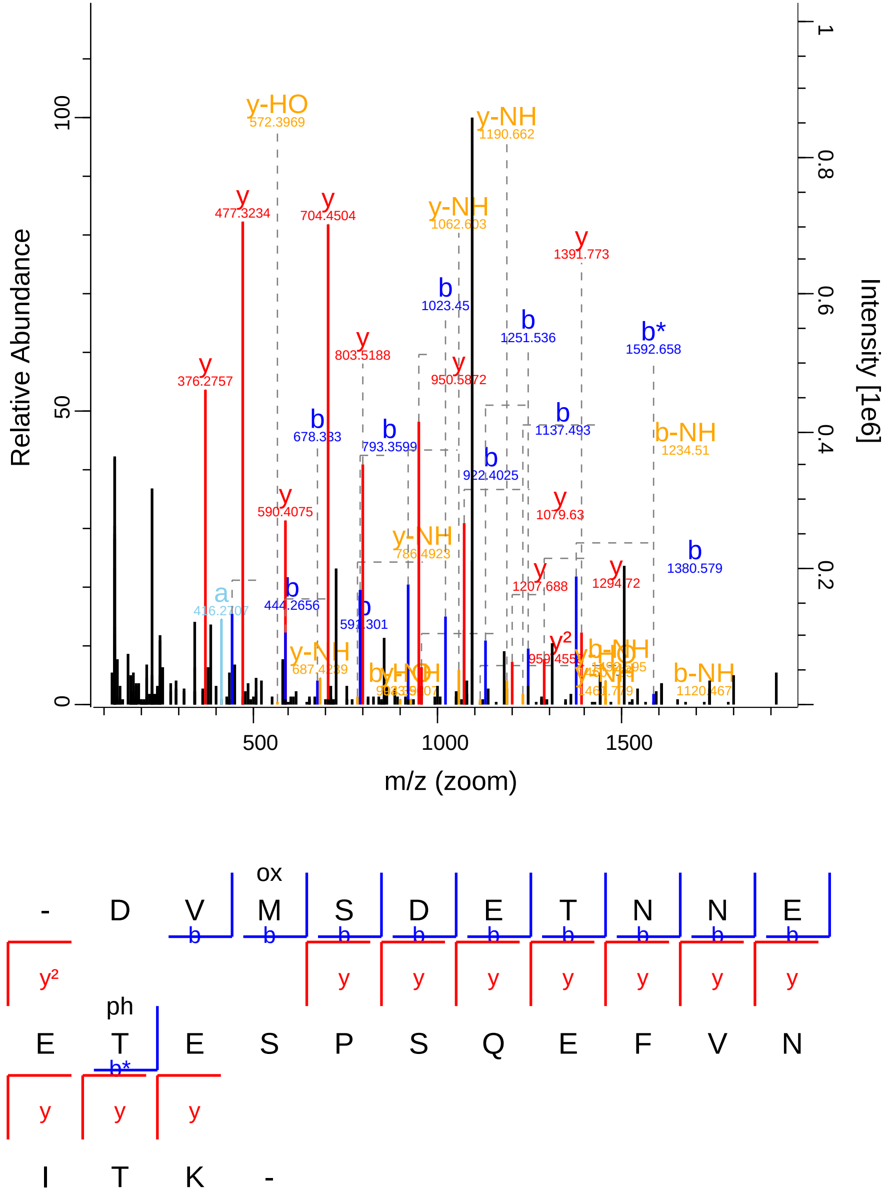

Raw File	Scan	Method	Score	m/z	Gene names
RO-QC-160121_ZackD_SS_Set_01_Fractio	29869	FTMS; HC	43.2	912.72	DNTTIP2

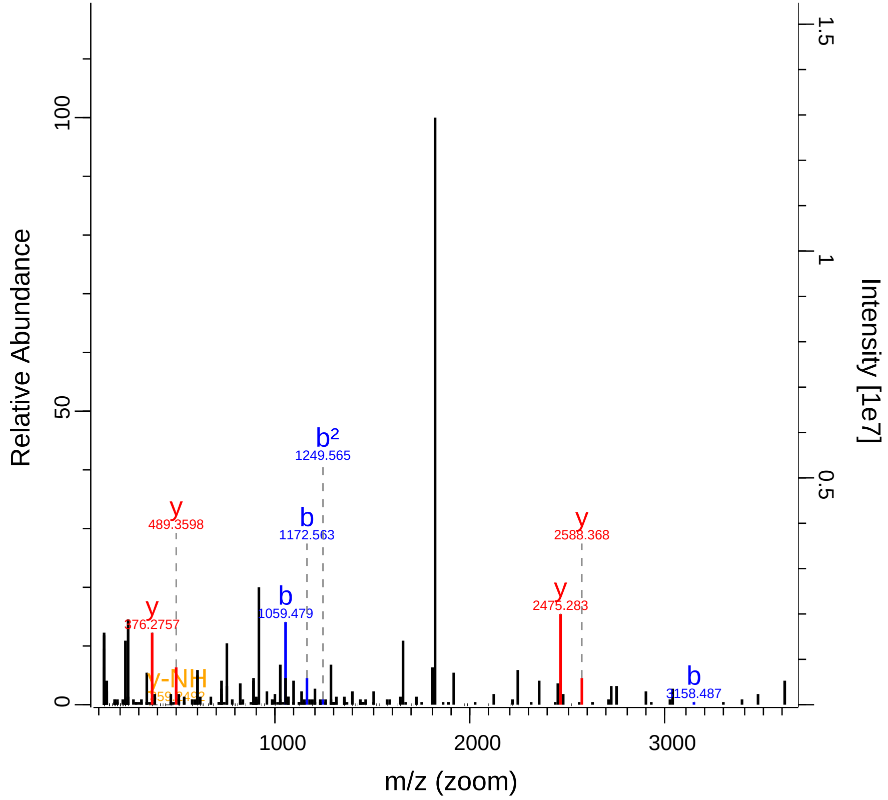

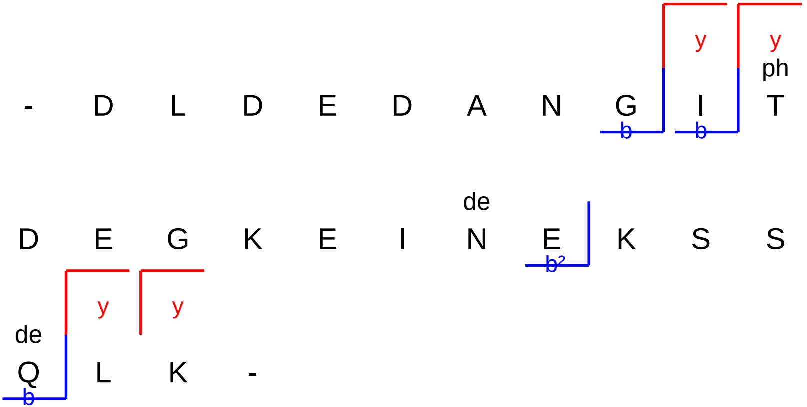

Raw File	Scan	Method	Score	m/z	Gene names
RO-QC-160121_ZackD_SS_Set_01_Fractio	31594	FTMS; HC	61.37	784.37	AHNAK

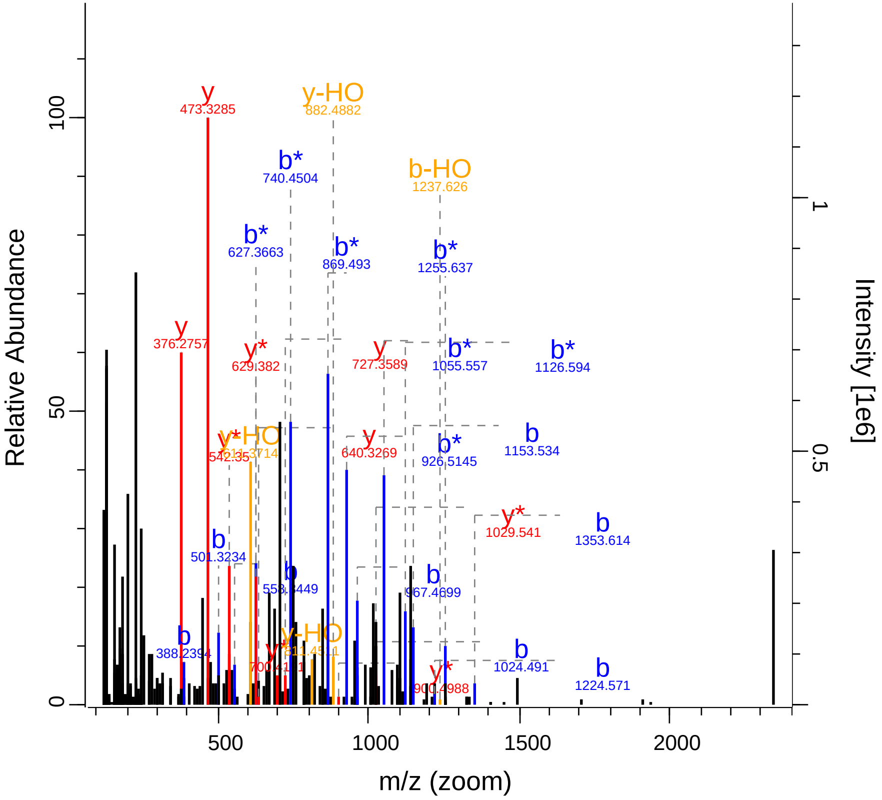

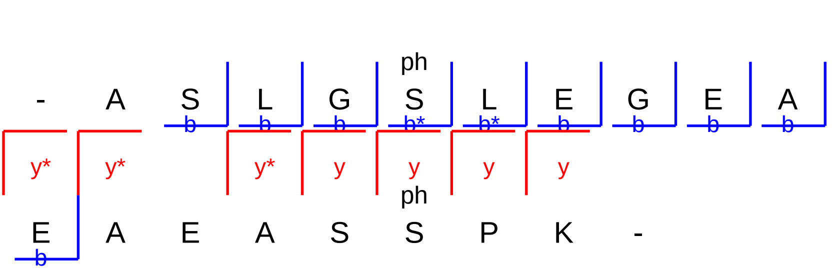

Raw File	Scan	Method	Score	m/z	Gene names
RO-QC-160121_ZackD_SS_Set_01_Fractic	32023	FTMS; HC	41.73	1303.26	DMKN

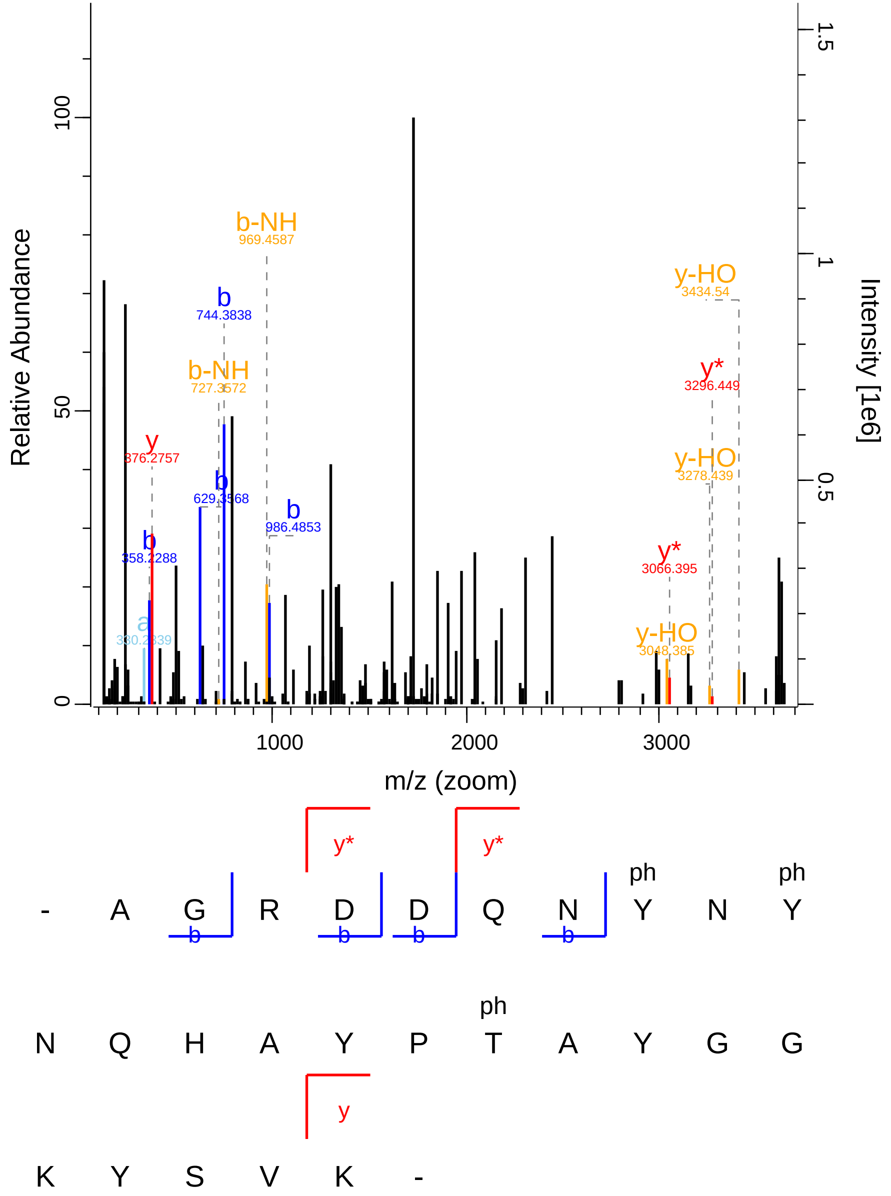

Raw File	Scan	Method	Score	m/z	Gene names
RO-QC-160121_ZackD_SS_Set_01_Fractic	32440	FTMS; HC	82.71	1325.62	AHNAK

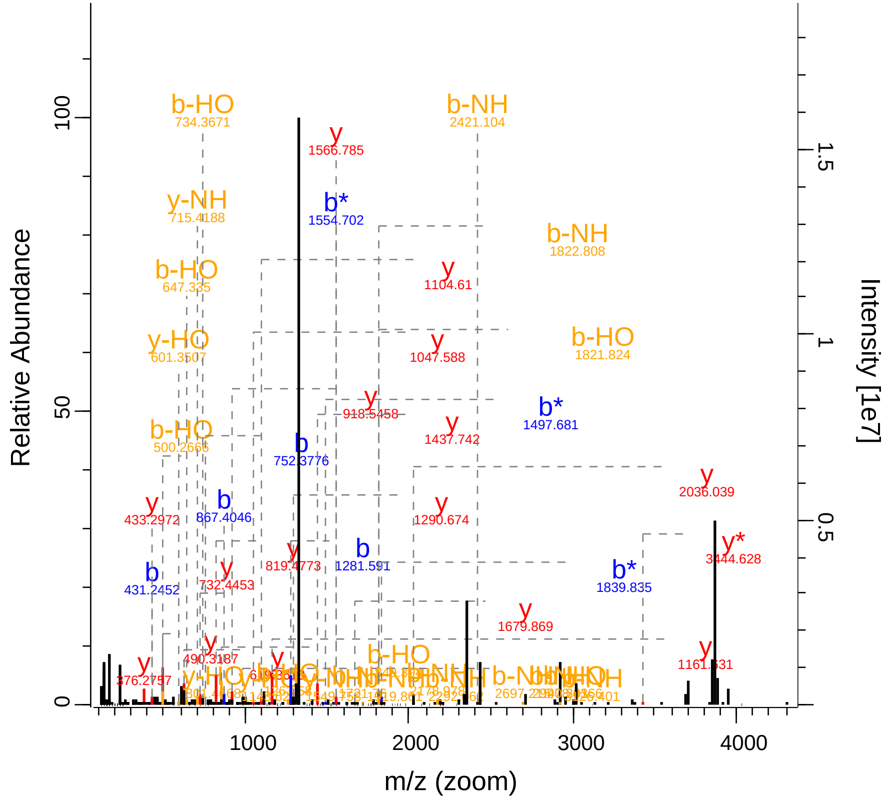

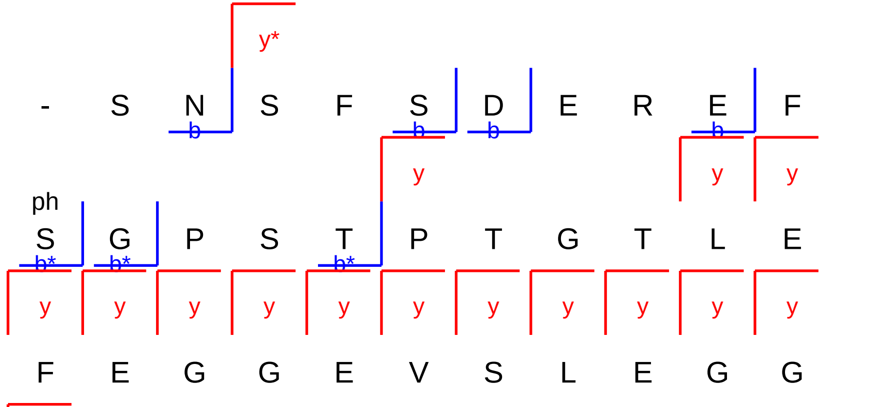

Raw File	Scan	Method	Score	m/z	Gene names
RO-QC-160121_ZackD_SS_Set_01_Fractio	33890	FTMS; HC	47.4	1069.9	C2CD4C

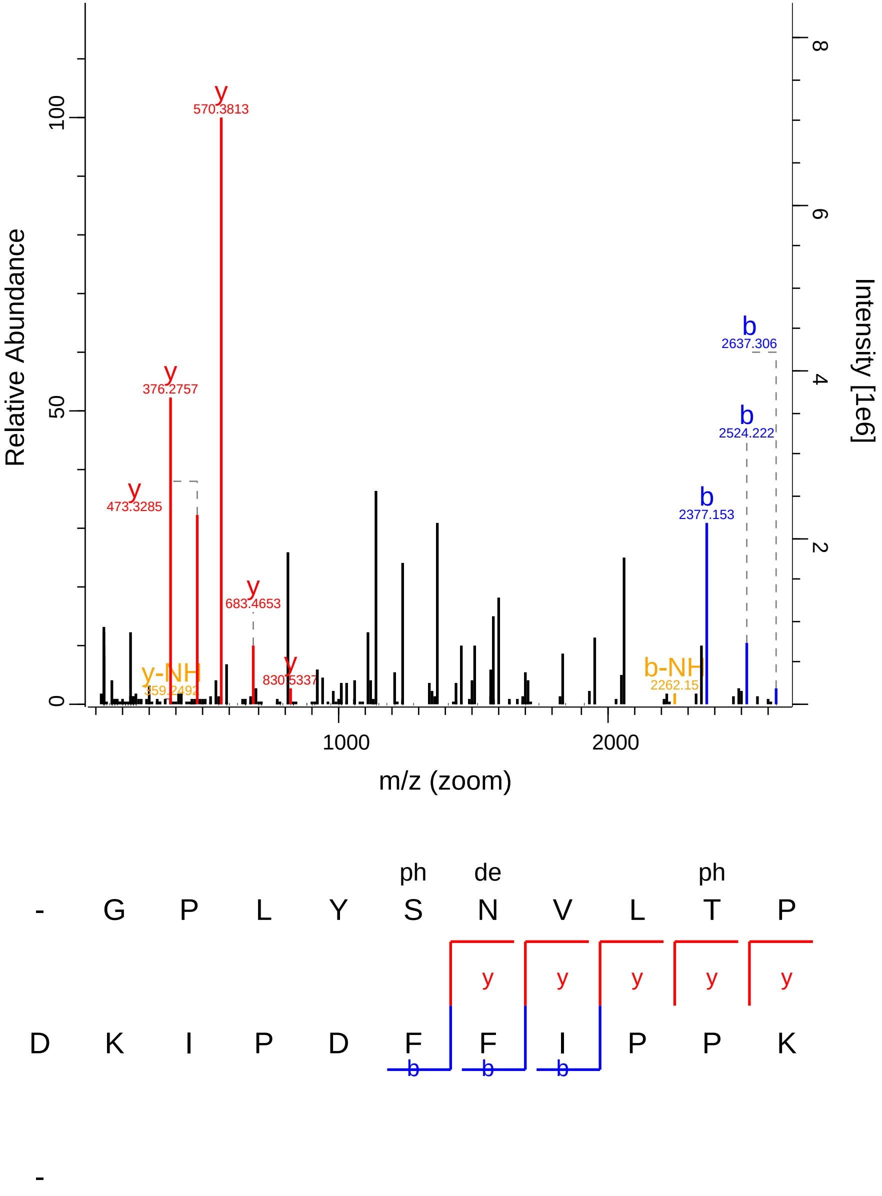

Raw File	Scan	Method	Score	m/z	Gene names
RO-QC-160121_ZackD_SS_Set_01_Fractic	34389	FTMS; HC	49.25	1007.18	ATP6V0D1

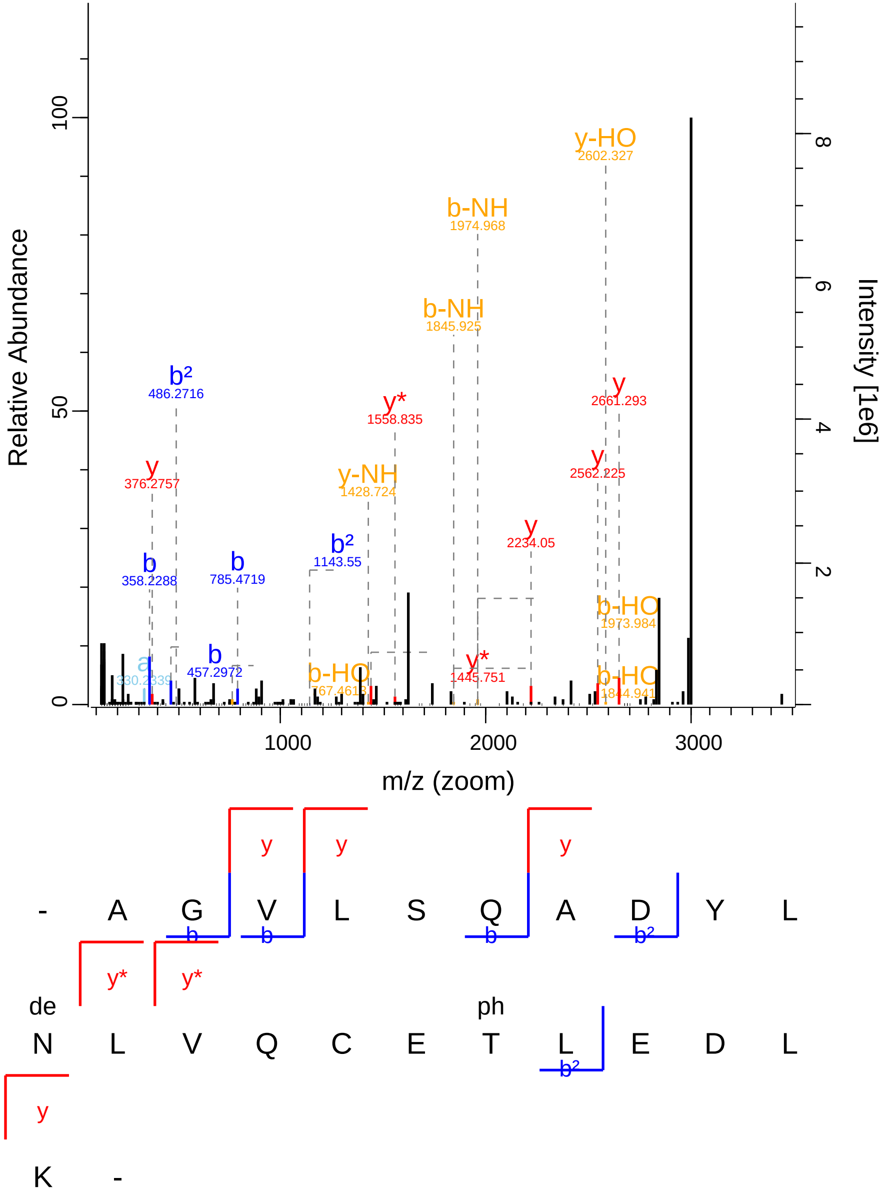

Raw File	Scan	Method	Score	m/z	Gene names
RO-QC-160121_ZackD_SS_Set_01_Fractic	34408	FTMS; HC	105.03	1011.14	GJA1

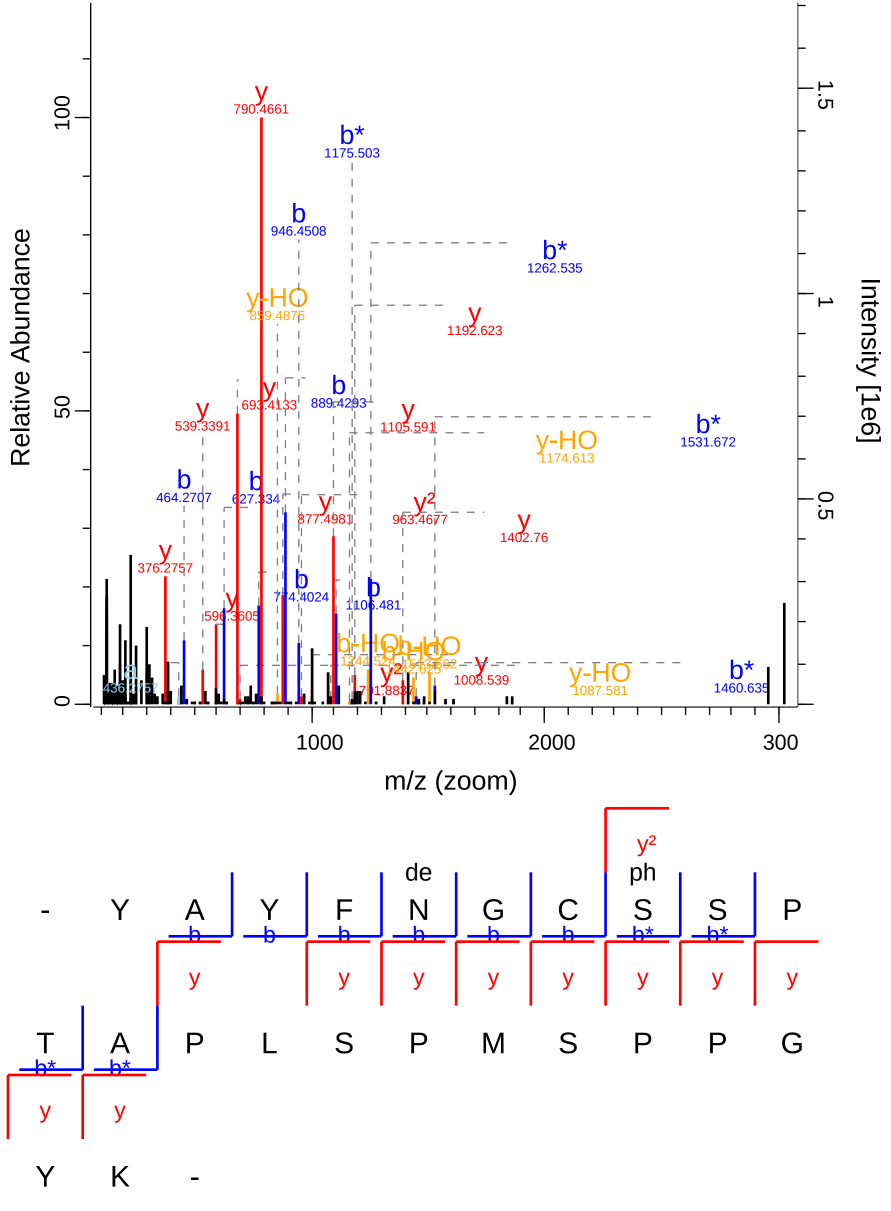

Raw File	Scan	Method	Score	m/z	Gene names
RO-QC-160121_ZackD_SS_Set_01_Fractio	35259	FTMS; HC	70.45	904.42	RRAGC

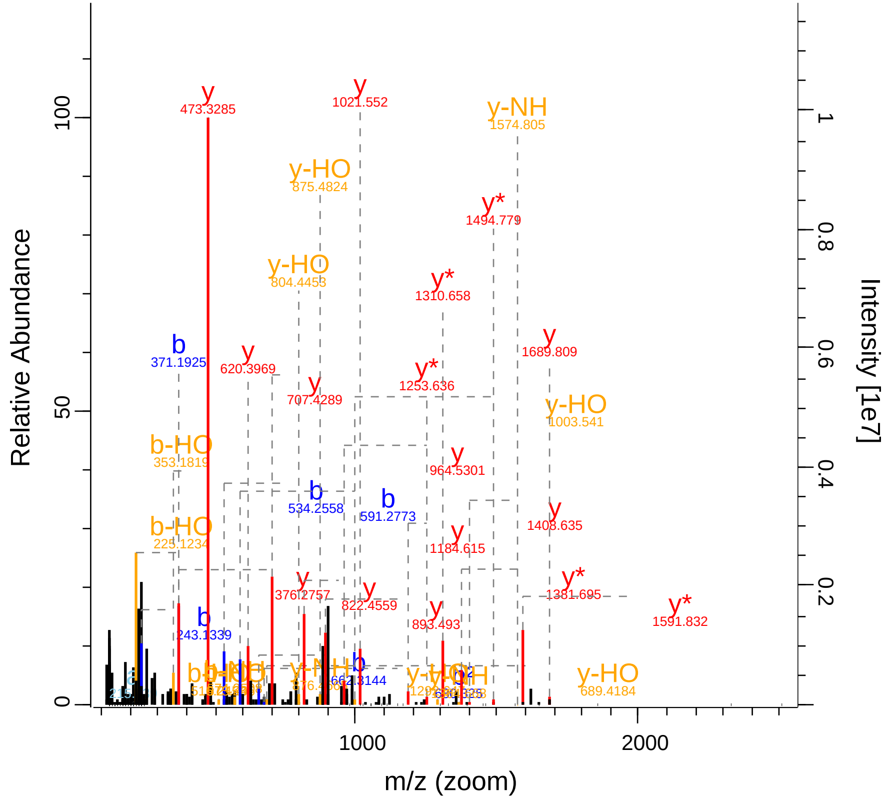

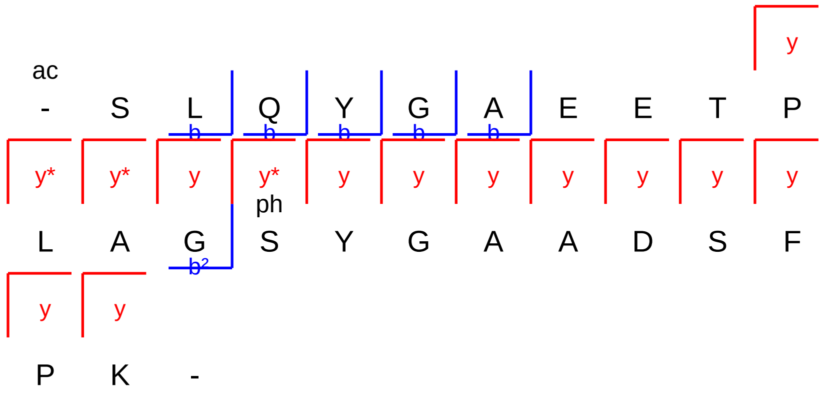

Raw File	Scan	Method	Score	m/z	Gene names
RO-QC-160121_ZackD_SS_Set_01_Fractio	36756	FTMS; HC	131.62	671.85	GOLGA4

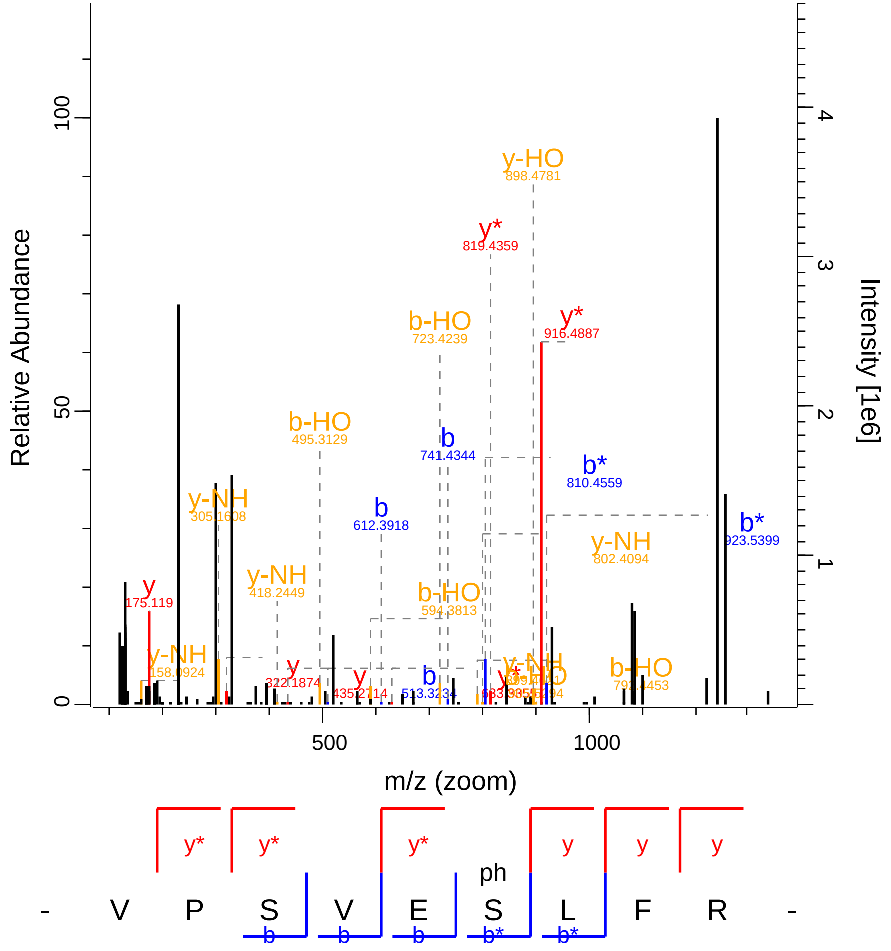

Raw File	Scan	Method	Score	m/z	Gene names
RO-QC-160121_ZackD_SS_Set_01_Fractio	37820	FTMS; HC	103.71	1106.91	MAP1B

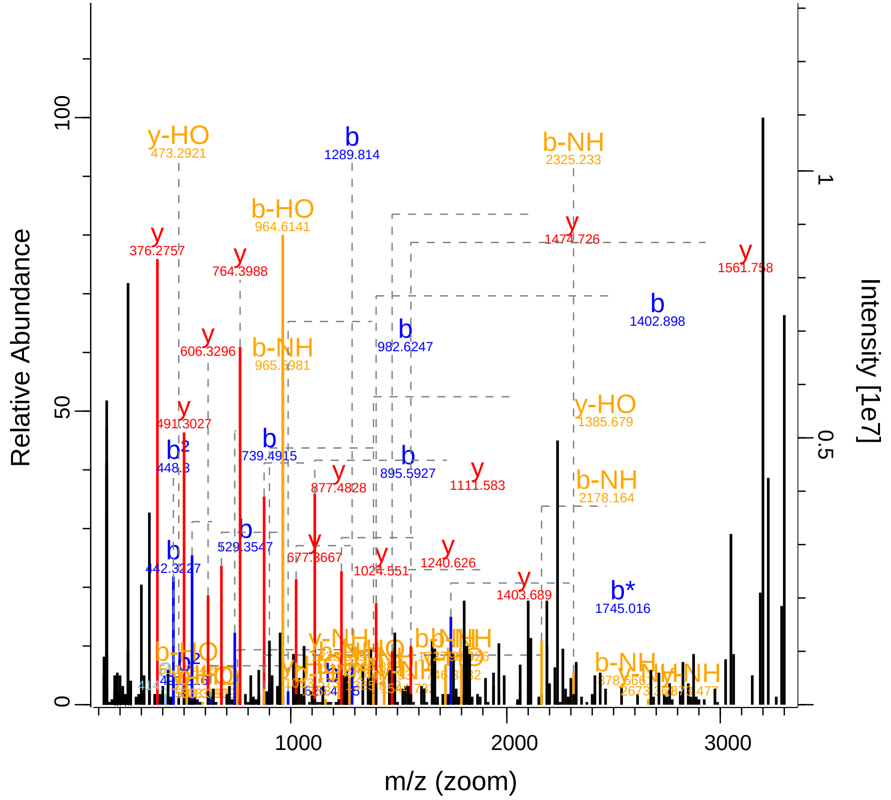

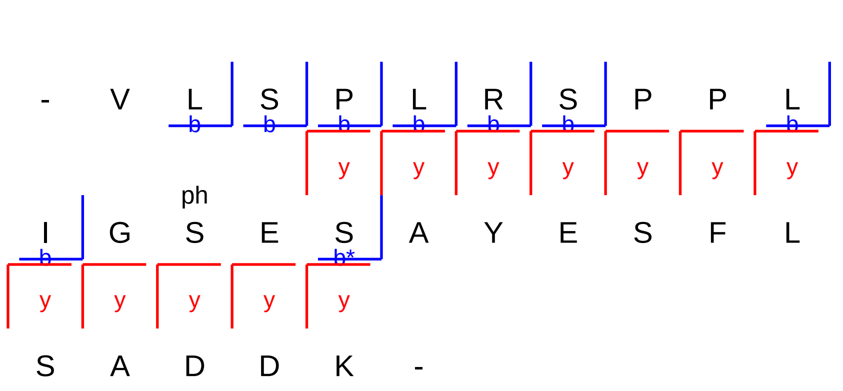

Raw File	Scan	Method	Score	m/z	Gene names
RO-QC-160121_ZackD_SS_Set_01_Fractic	38199	FTMS; HC	61.35	1146.57	CHD8

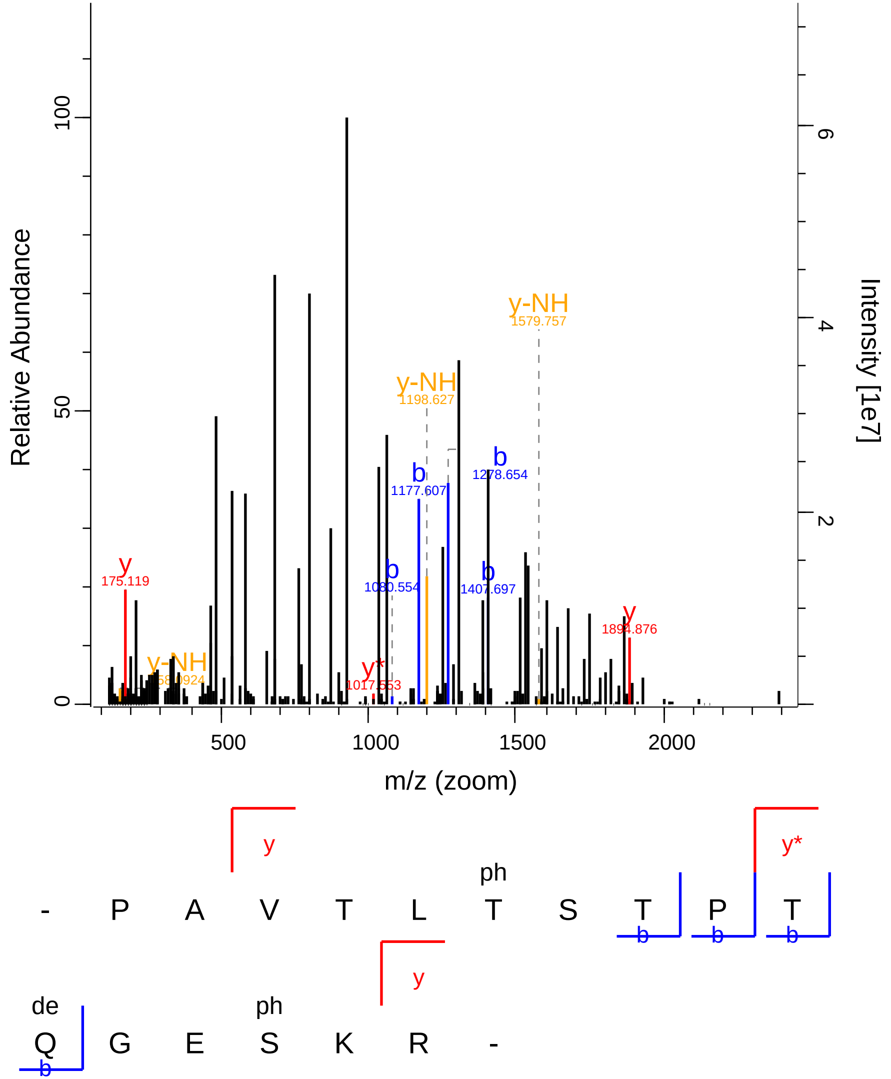

Raw File	Scan	Method	Score	m/z	Gene names
RO-QC-160121_ZackD_SS_Set_01_Fractio	38318	FTMS; HC	166.49	821.44	USP24

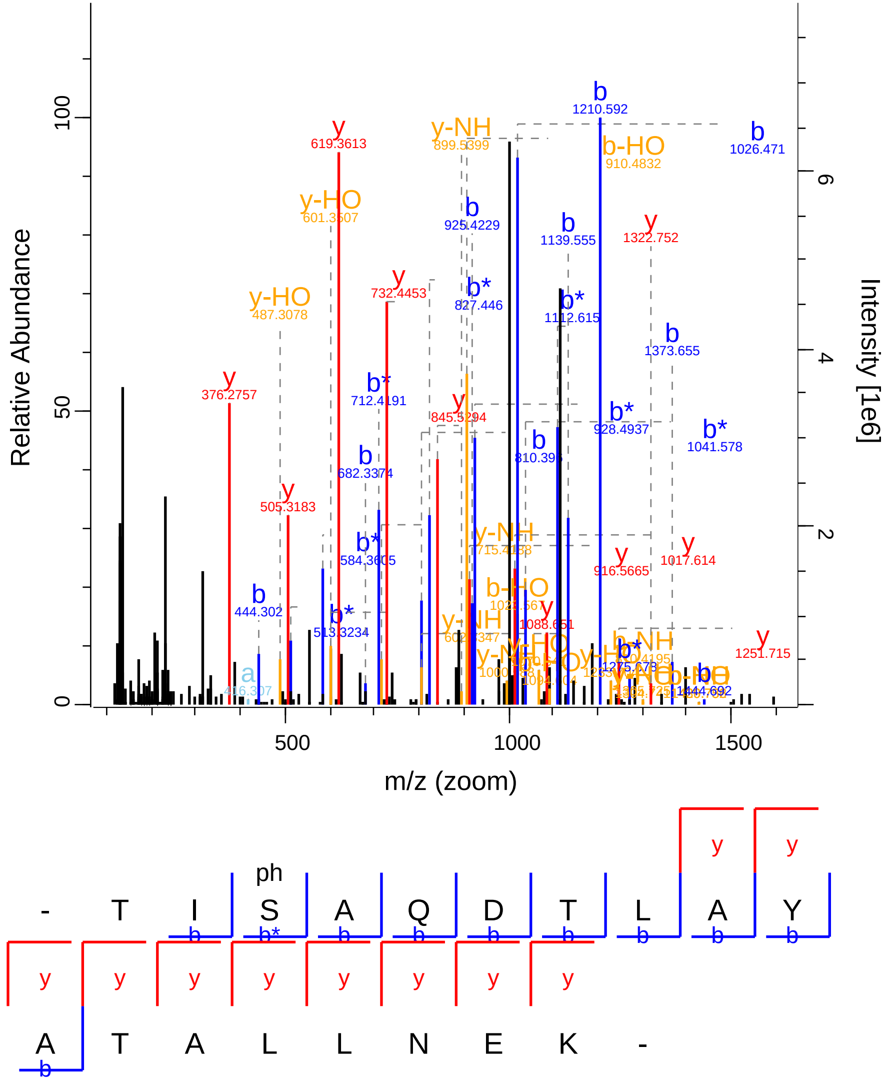

Raw File	Scan	Method	Score	m/z	Gene names
RO-QC-160121_ZackD_SS_Set_01_Fr	3845	FTMS; H(	190.63	911.15	PRKACA;PRKACG;PF

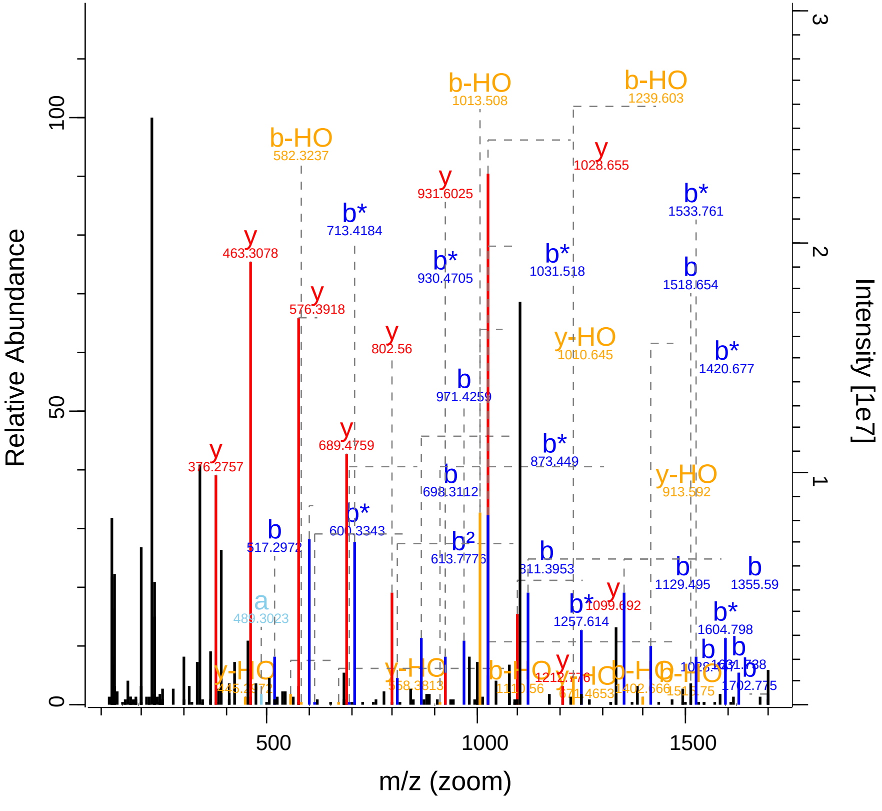

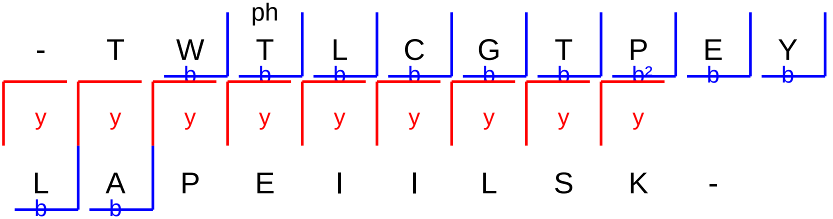

Raw File	Scan	Method	Score	m/z	Gene names
RO-QC-160121_ZackD_SS_Set_01_Fractio	38492	FTMS; HC	57.35	1284.7	BBS7

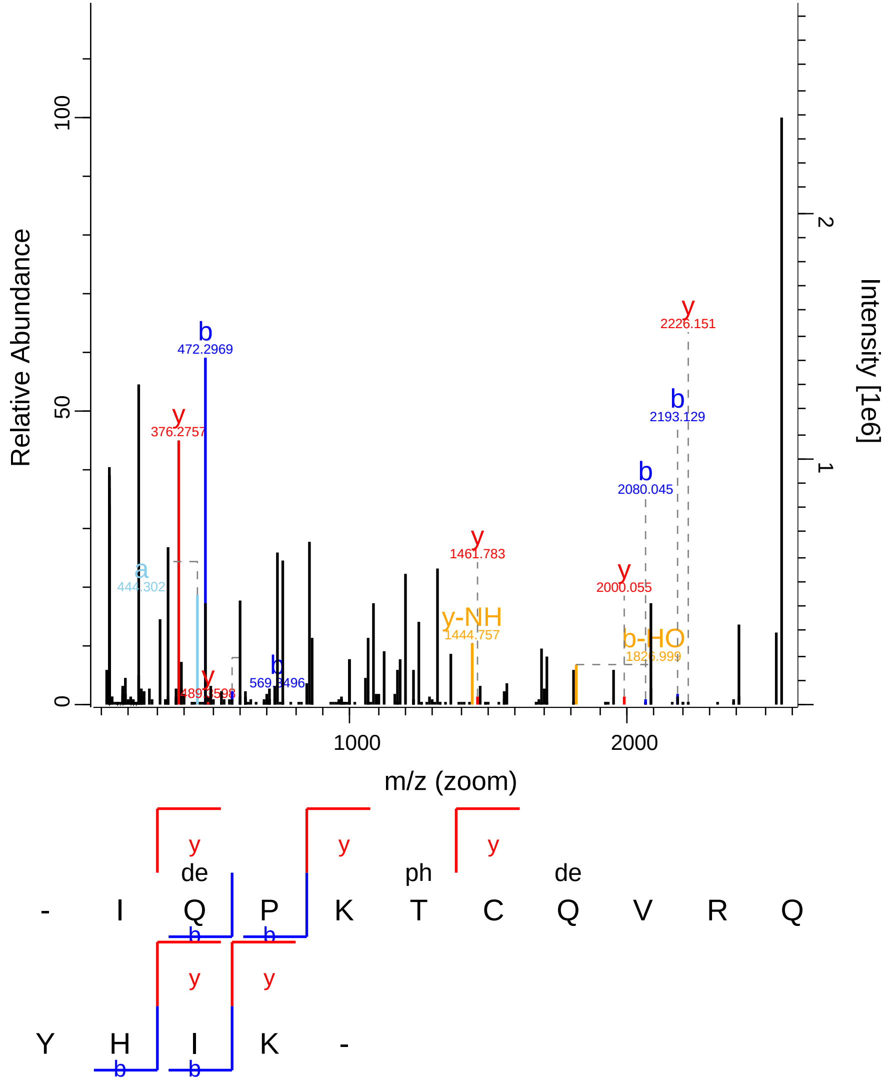

Raw File	Scan	Method	Score	m/z	Gene names
RO-QC-160121_ZackD_SS_Set_01_Fractio	38938	FTMS; HC	52.68	733.38	SCN10A

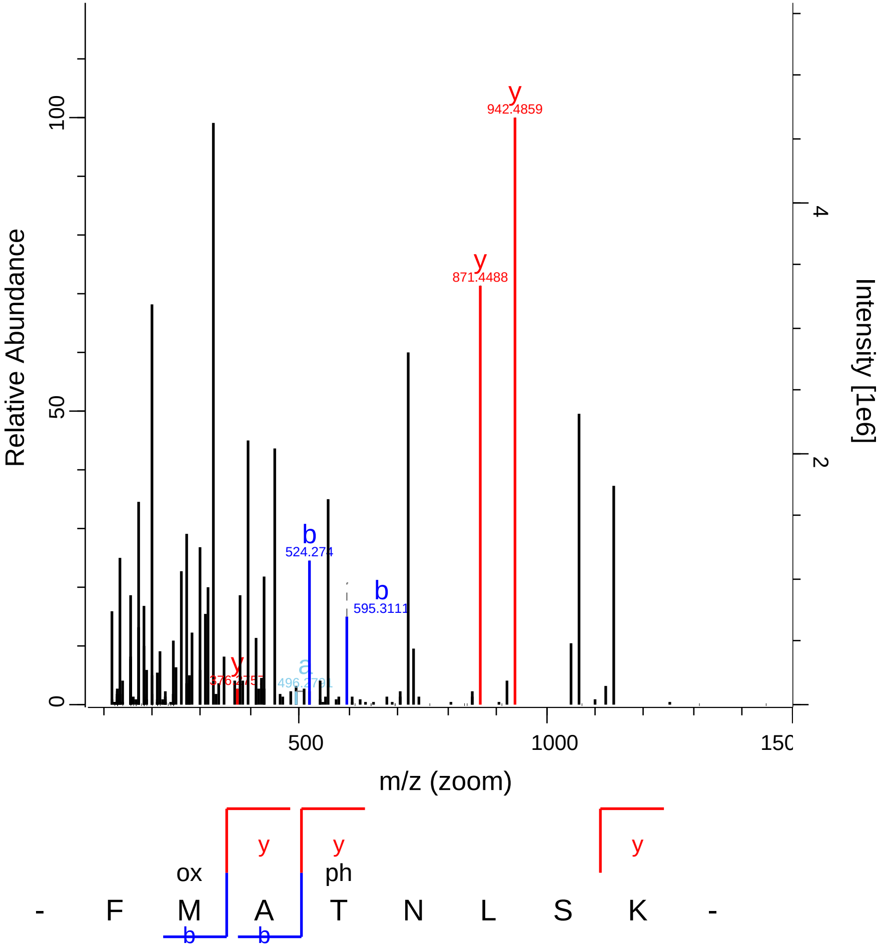

Raw File	Scan	Method	Score	m/z	Gene names
RO-QC-160121_ZackD_SS_Set_01_Fractior	4594	FTMS; HCI	60.03	587.04	PRRC2C

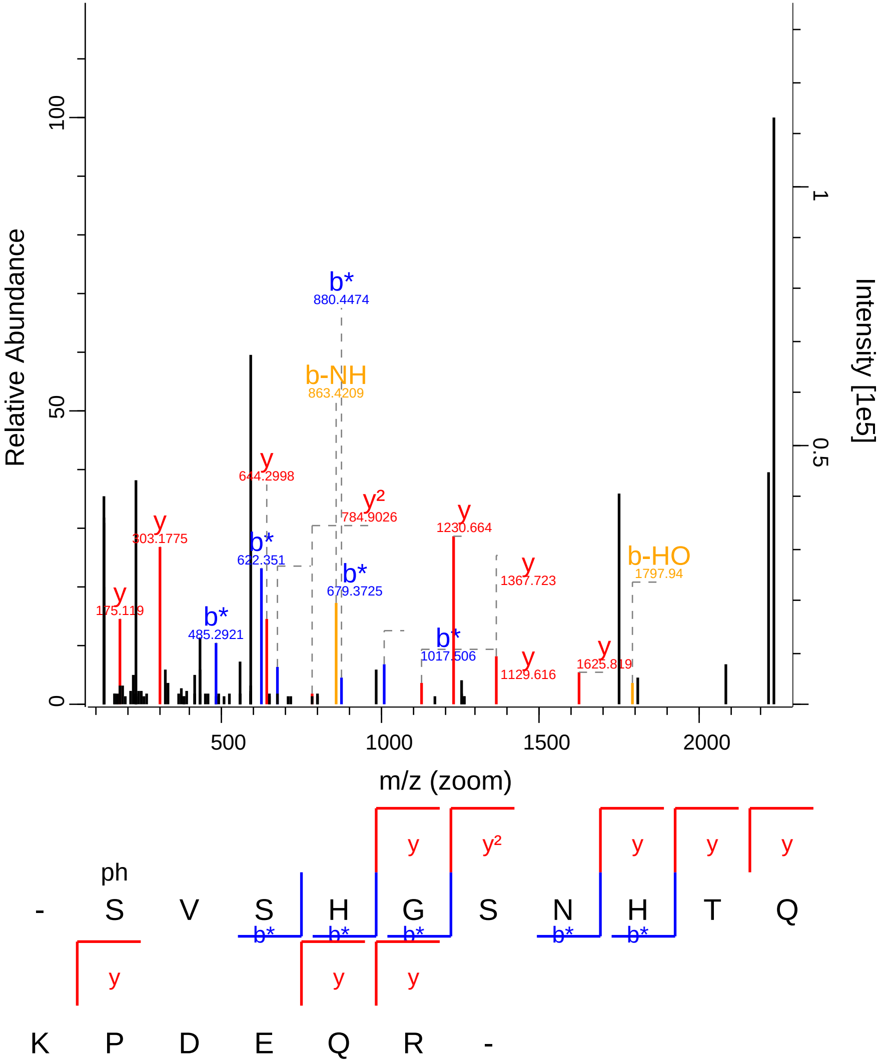

Raw File	Scan	Method	Score	m/z	Gene names
RO-QC-160121_ZackD_SS_Set_01_Fractior	5311	FTMS; HCI	51.01	351.19	PRC1

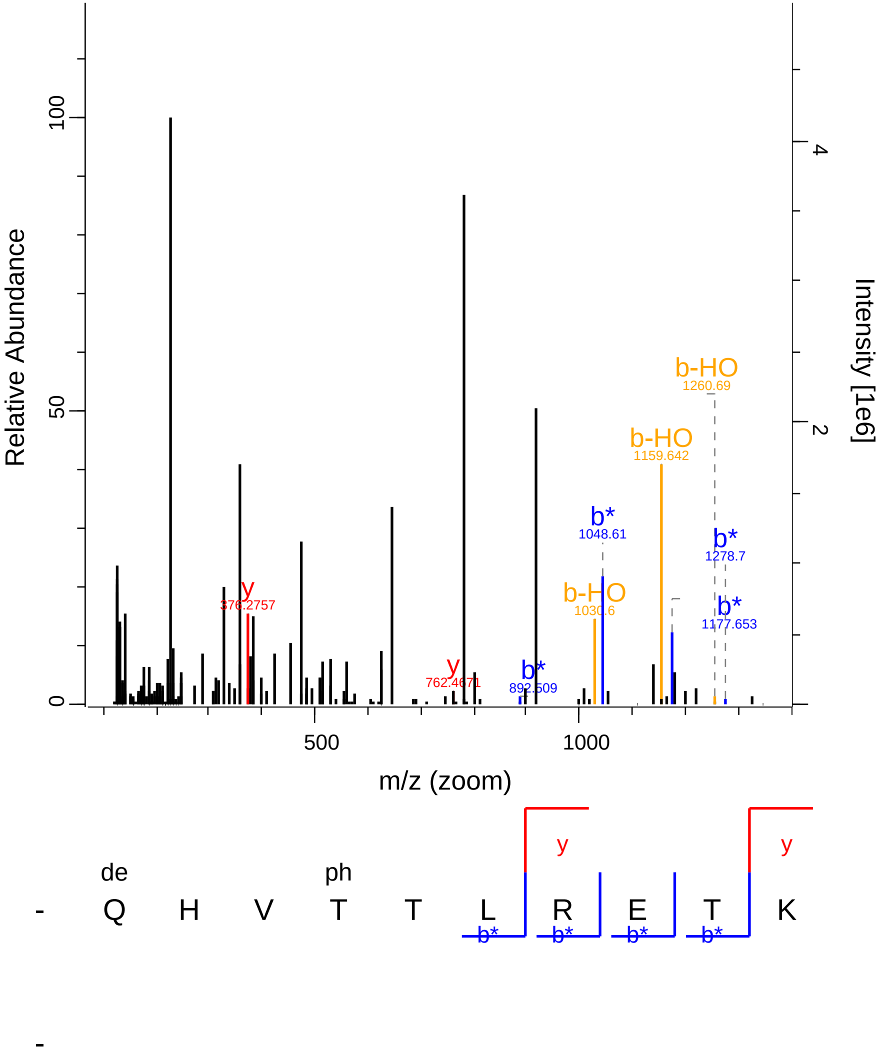

Raw File	Scan	Method	Score	m/z	Gene names
RO-QC-160121_ZackD_SS_Set_01_Fractioı	6494	FTMS; HC	135.56	729.87	SRRM2

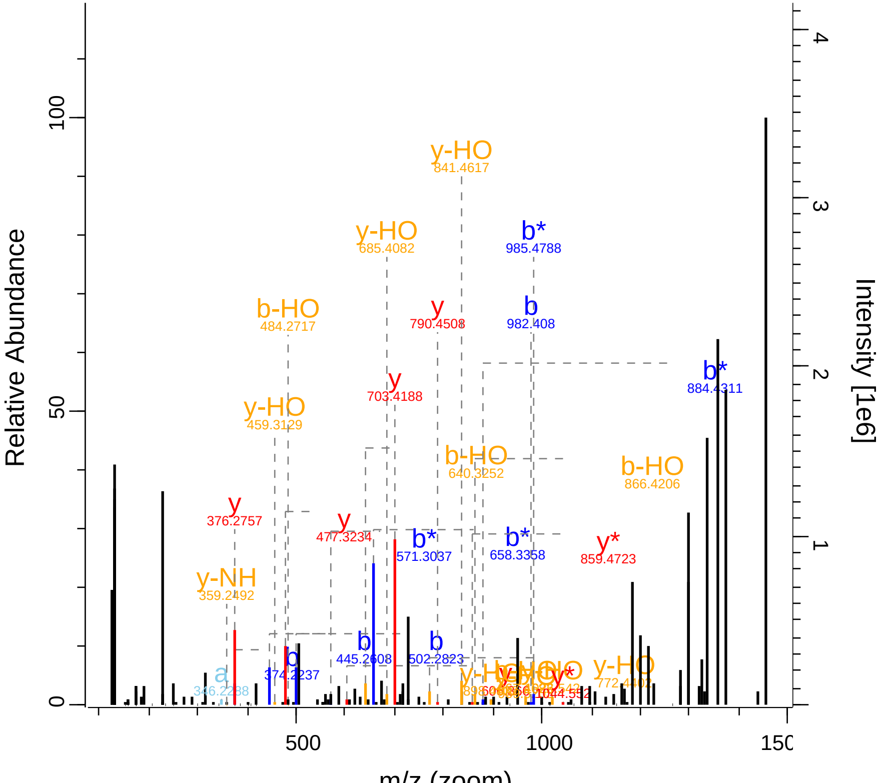

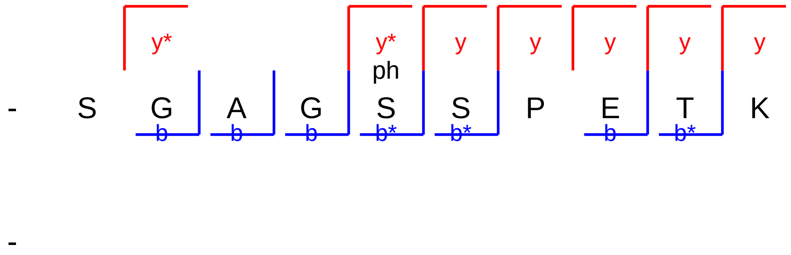

Raw File	Scan	Method	Score	m/z	Gene names
RO-QC-160121_ZackD_SS_Set_01_Fractior	6509	FTMS; HCI	83.05	544.26	GTF2F2

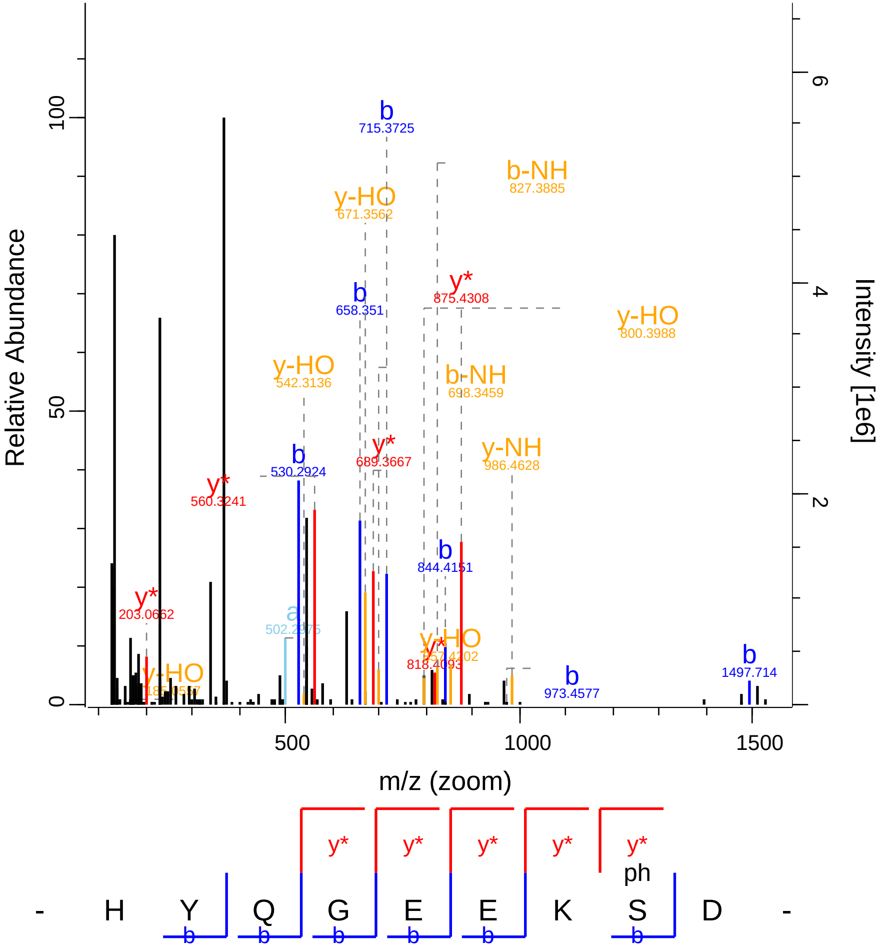

Raw File	Scan	Method	Score	m/z	Gene names
RO-QC-160121_ZackD_SS_Set_01_Fractio[illegible]	6924	FTMS; HC	131.46	740.38	LIMA1

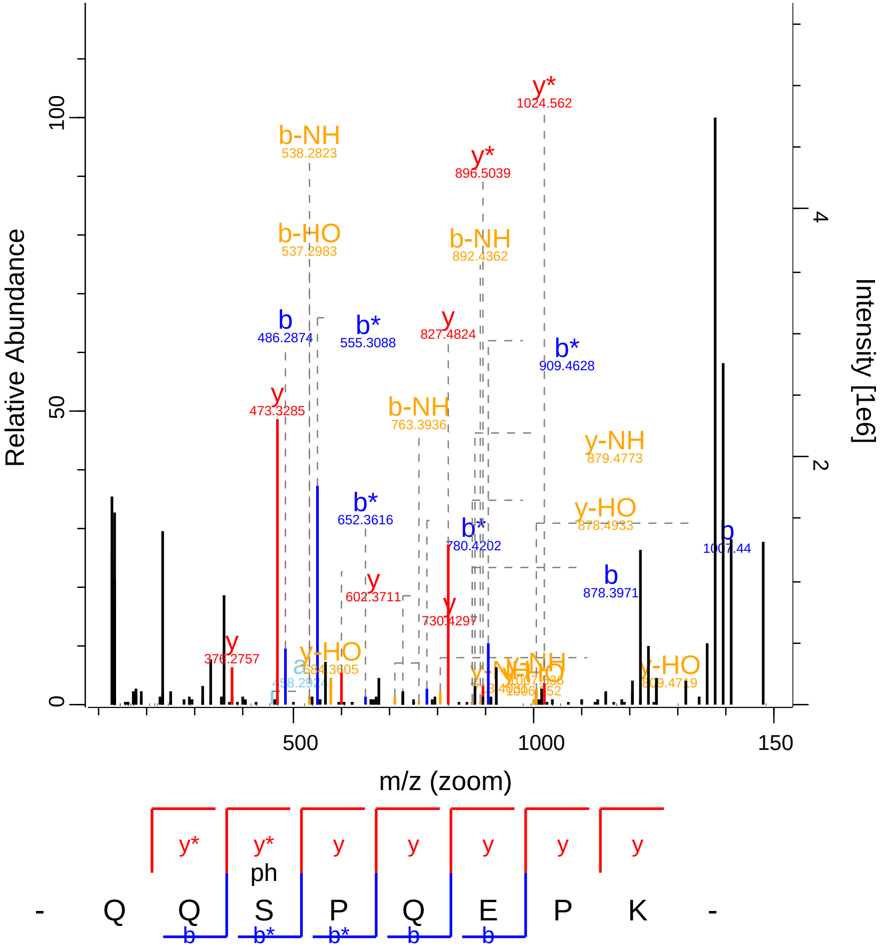

Raw File	Scan	Method	Score	m/z	Gene names
RO-QC-160121_ZackD_SS_Set_01_Fractio	7163	FTMS; HC	113.77	886.45	ZC3H13

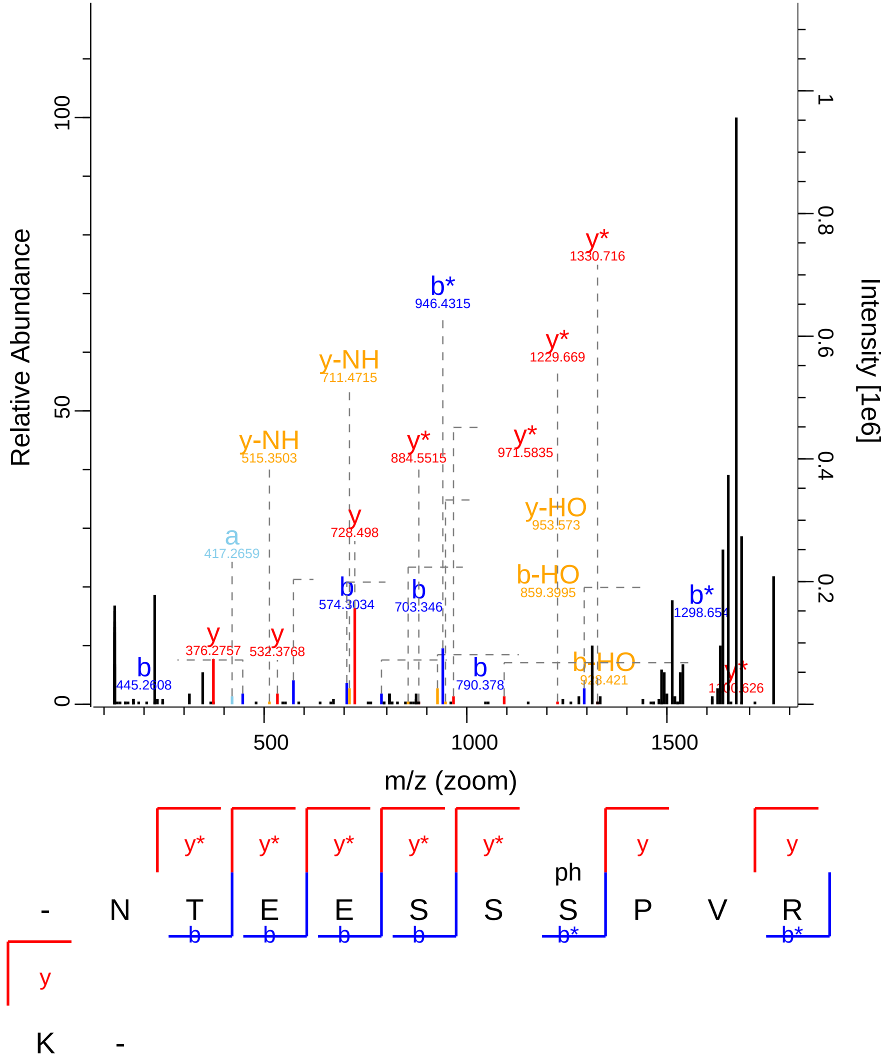

Raw File	Scan	Method	Score	m/z	Gene names
RO-QC-160121_ZackD_SS_Set_01_Fractio	8758	FTMS; HC	147.26	813.91	SF3B1

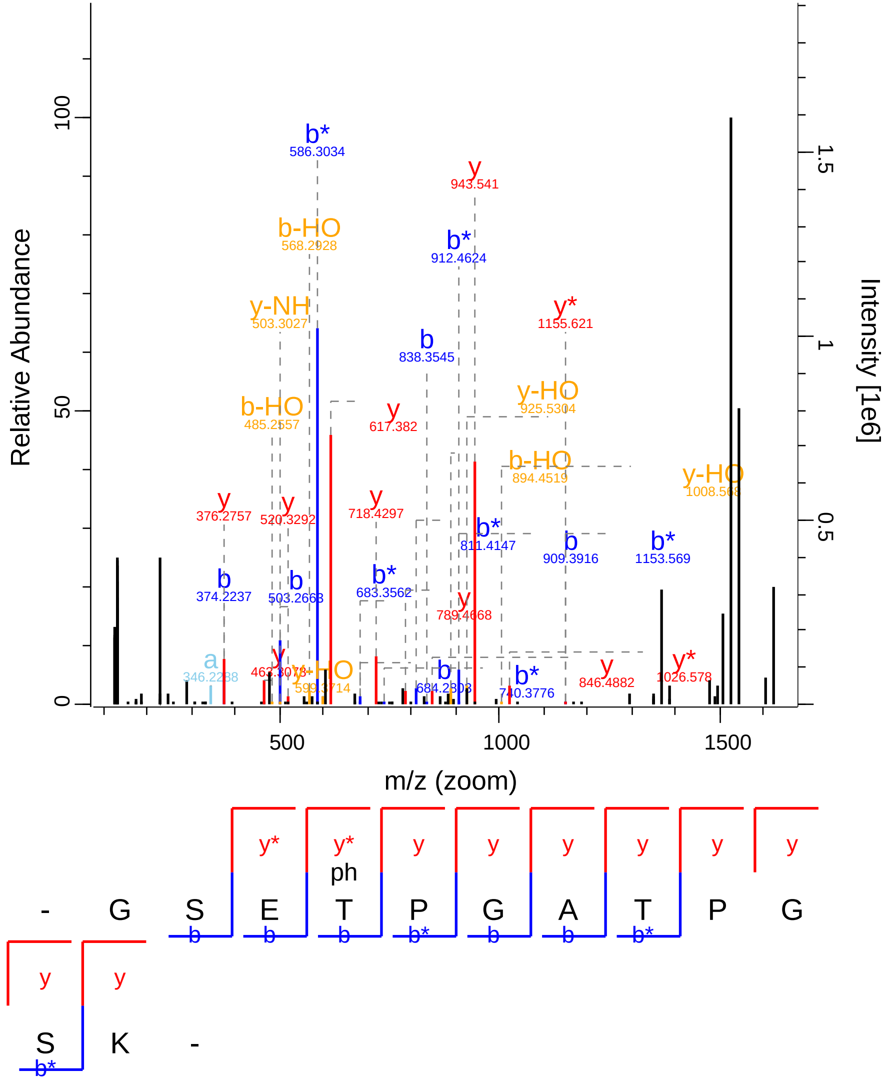

Raw File	Scan	Method	Score	m/z	Gene names
RO-QC-160121_ZackD_SS_Set_01_Fractior	8836	FTMS; HCI	50.87	772.61	UBAP2L

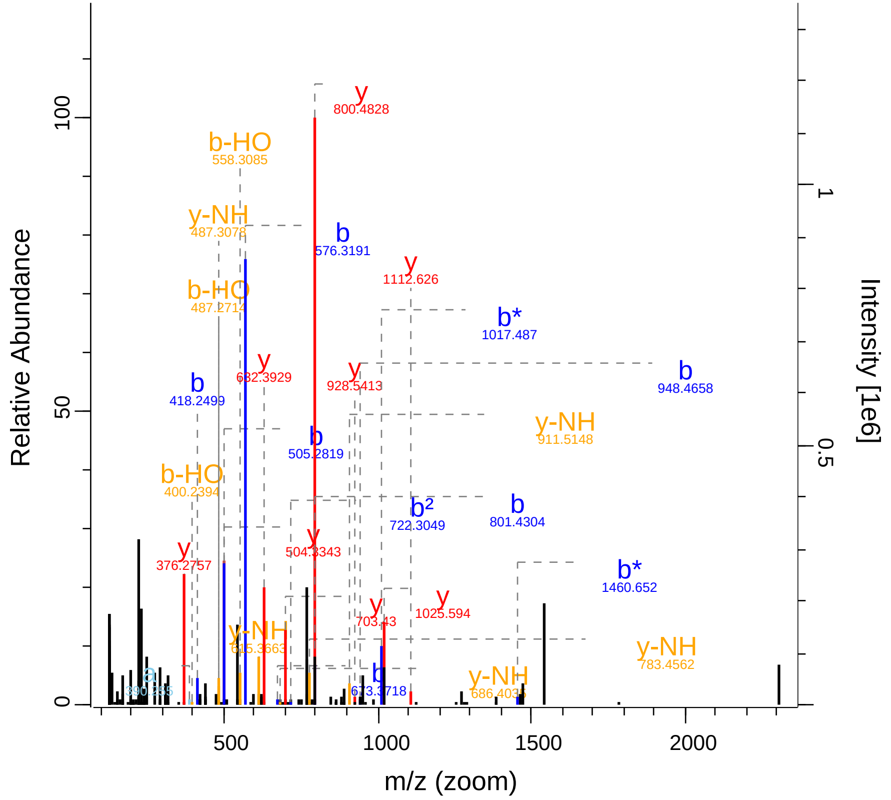

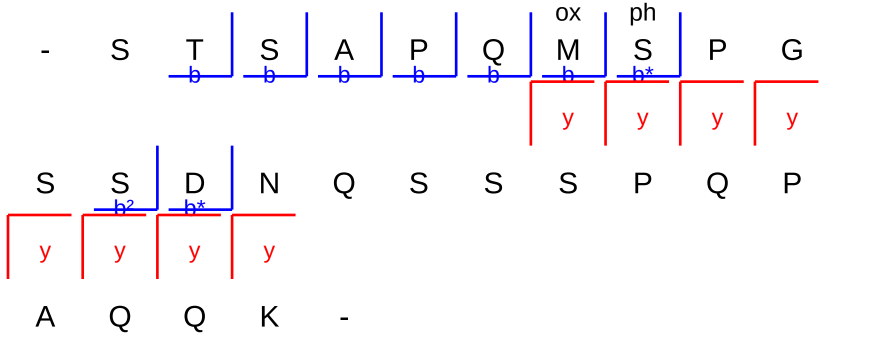

Raw File	Scan	Method	Score	m/z	Gene names
RO-QC-160121_ZackD_SS_Set_01_Fractic	10452	FTMS; HC	90.63	1128.65	ZNF559

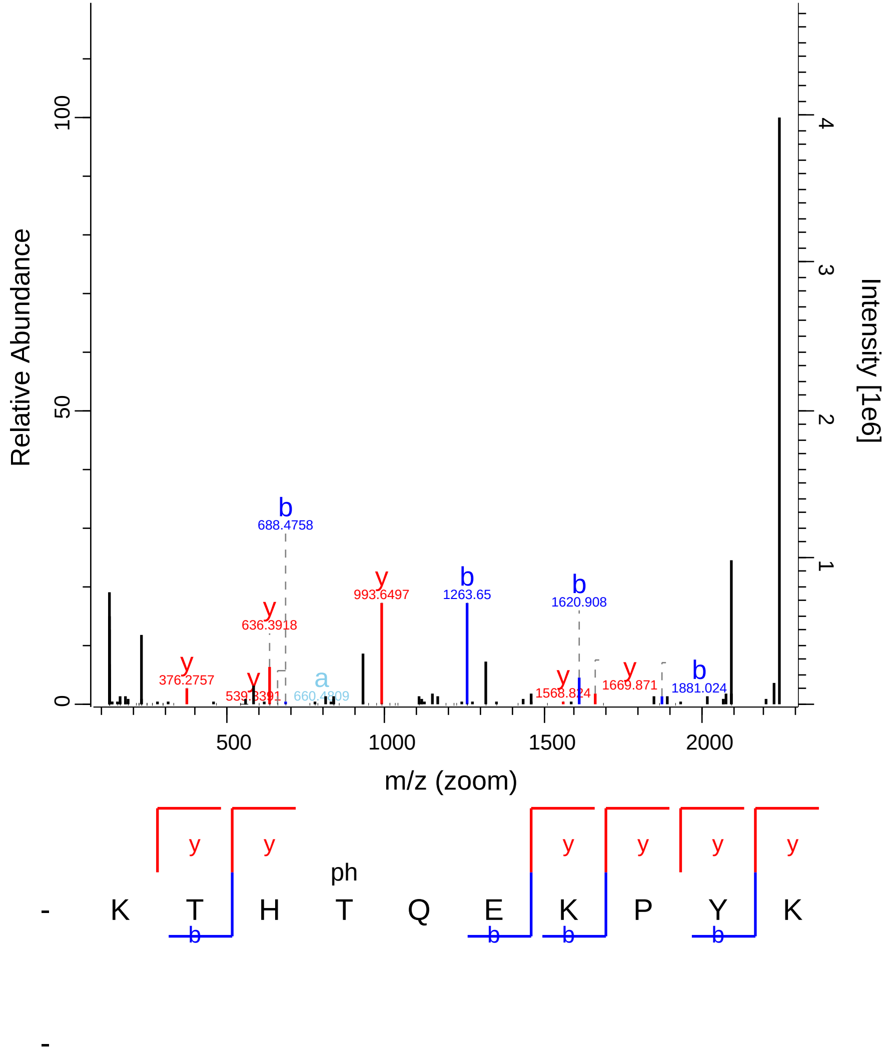

Raw File	Scan	Method	Score	m/z	Gene names
RO-QC-160121_ZackD_SS_Set_01_Fractio	10551	FTMS; HC	59.76	564.83	ZNF559

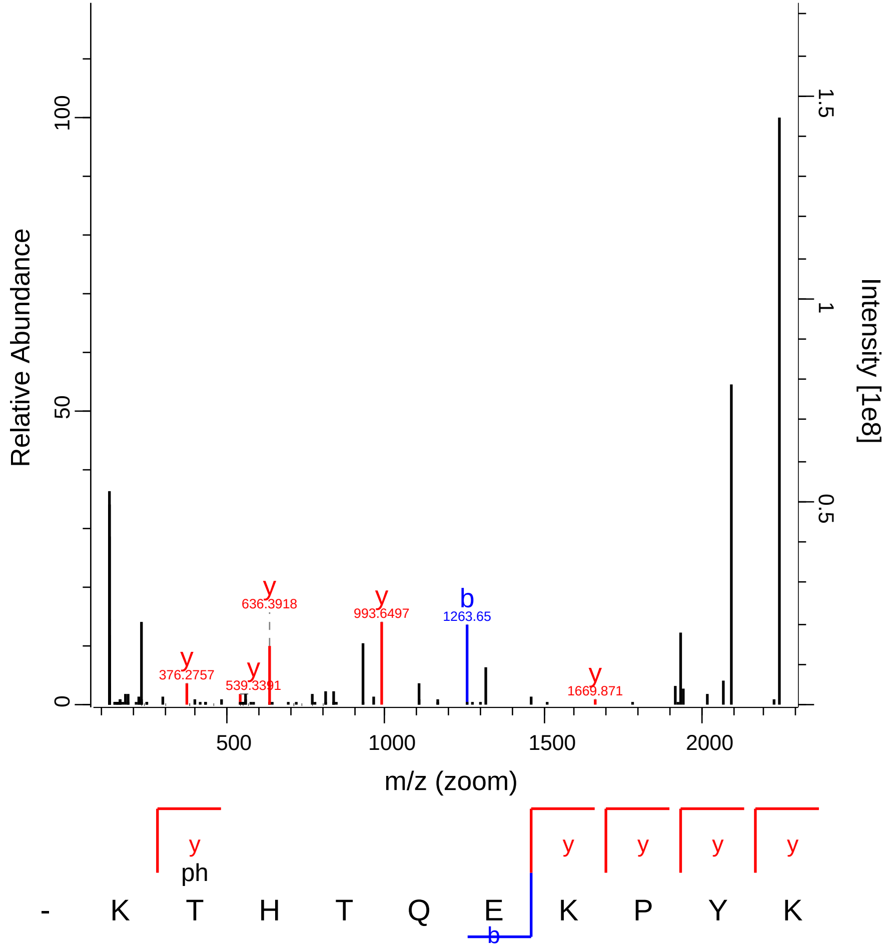

-

Raw File	Scan	Method	Score	m/z	Gene names
RO-QC-160121_ZackD_SS_Set_01_Fractio	10603	FTMS; HC	91.09	805.98	GTF3C2

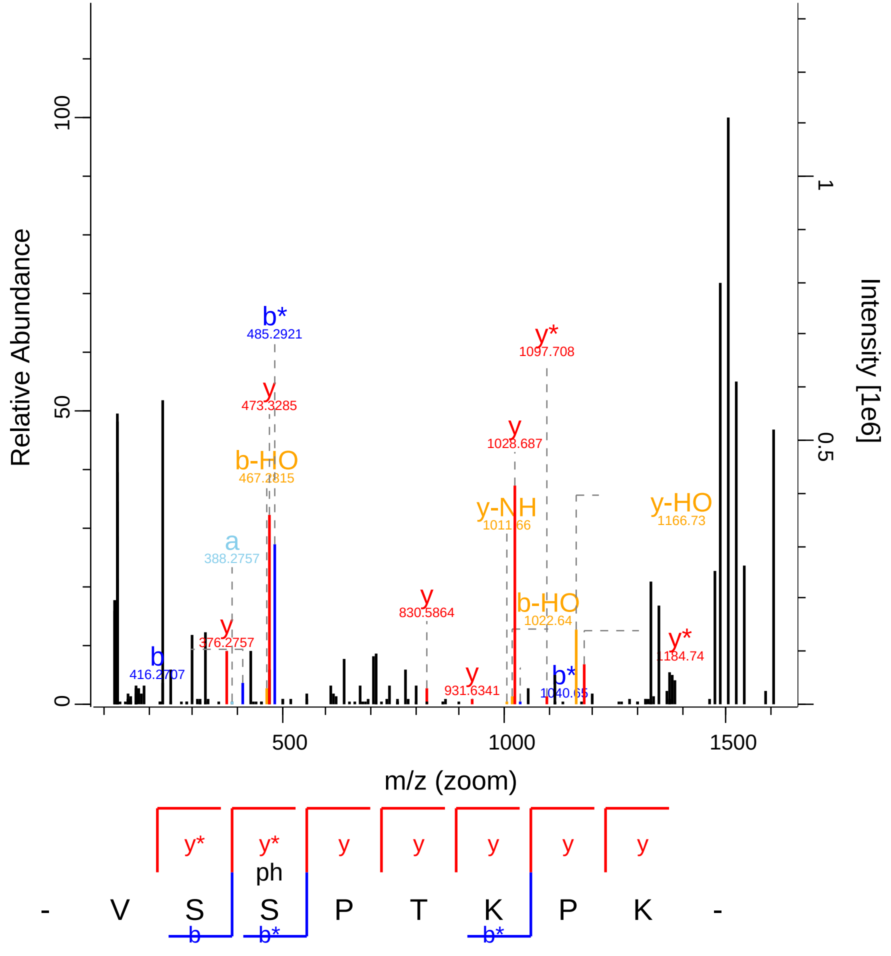

Raw File	Scan	Method	Score	m/z	Gene names
RO-QC-160121_ZackD_SS_Set_01_Fractio	11114	FTMS; HC	143	843.42	SON

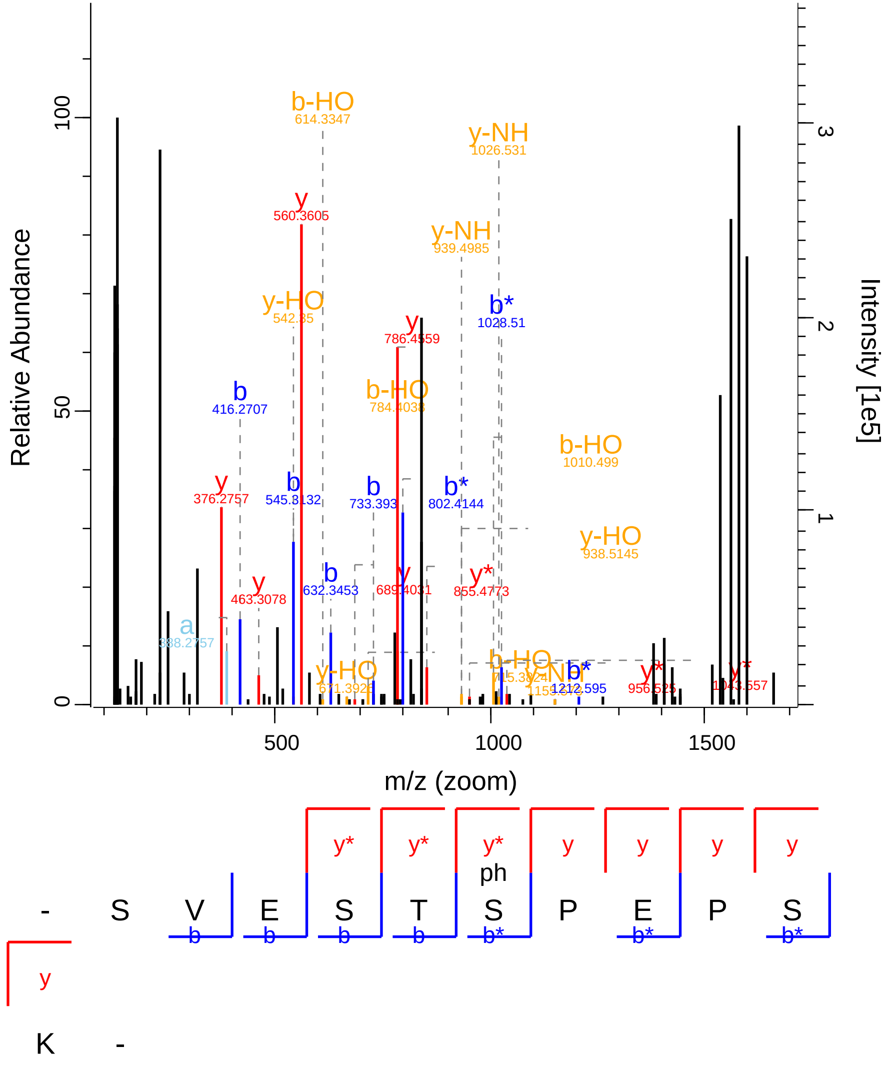

Raw File	Scan	Method	Score	m/z	Gene names
RO-QC-160121_ZackD_SS_Set_01_Fractio	11612	FTMS; HC	249.32	744.11	CANX

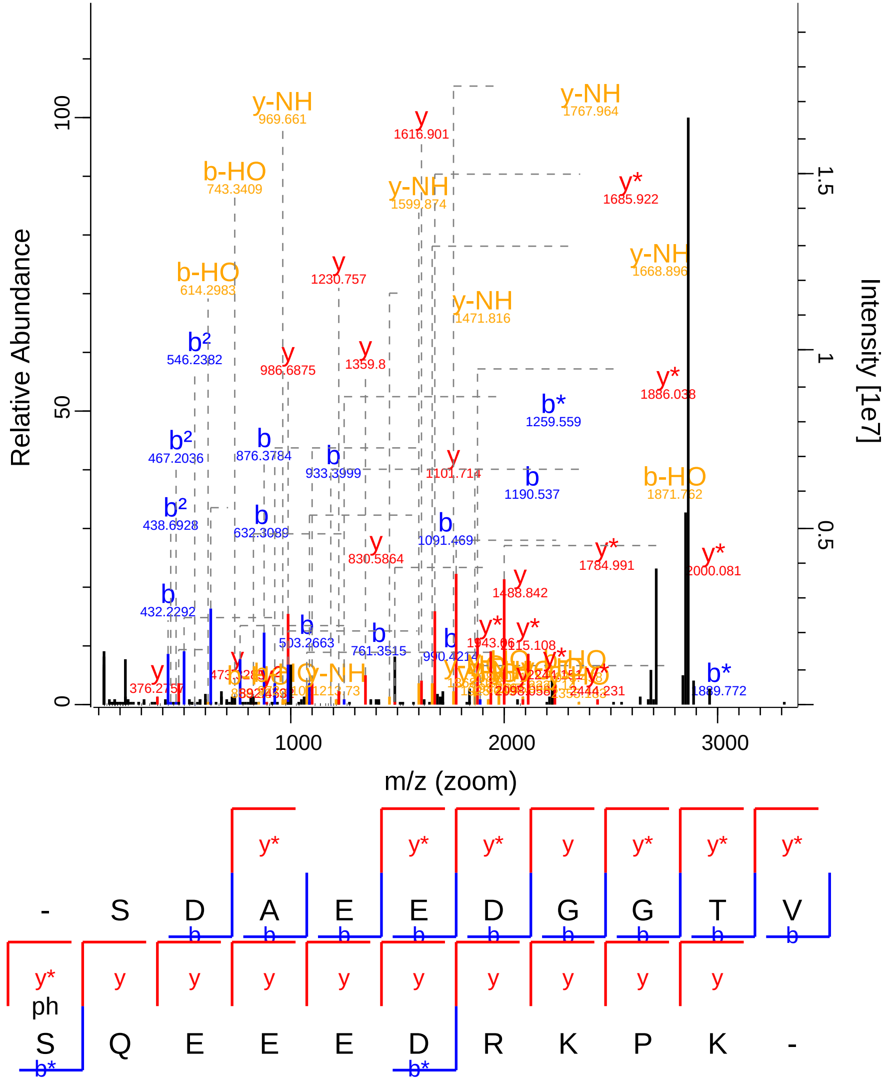

Raw File	Scan	Method	Score	m/z	Gene names
RO-QC-160121_ZackD_SS_Set_01_Fractio	11803	FTMS; HC	120.96	782.88	WWC1

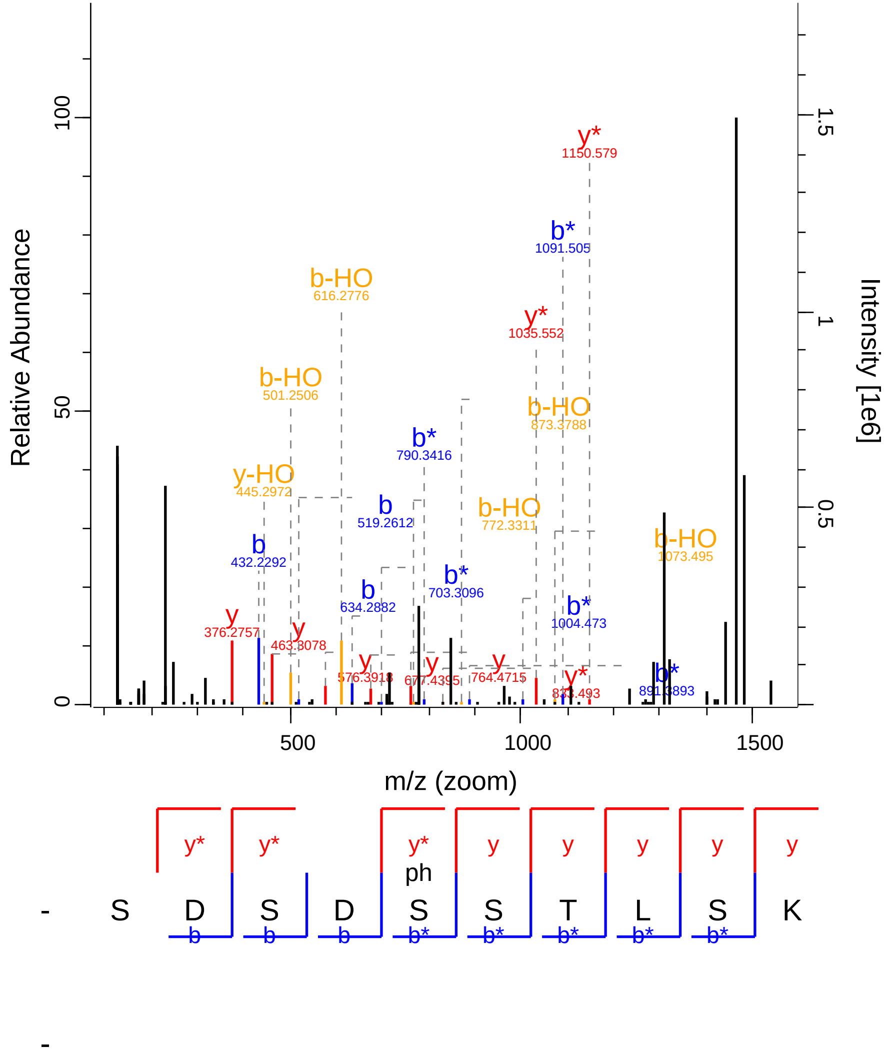

Raw File	Scan Method	Score	m/z	Gene names
RO-QC-160121_ZackD_SS_Set_0: 1213	ra FTMS; H	68.42	913.9	ACTB;POTEF;ACTG1;POTEI;P

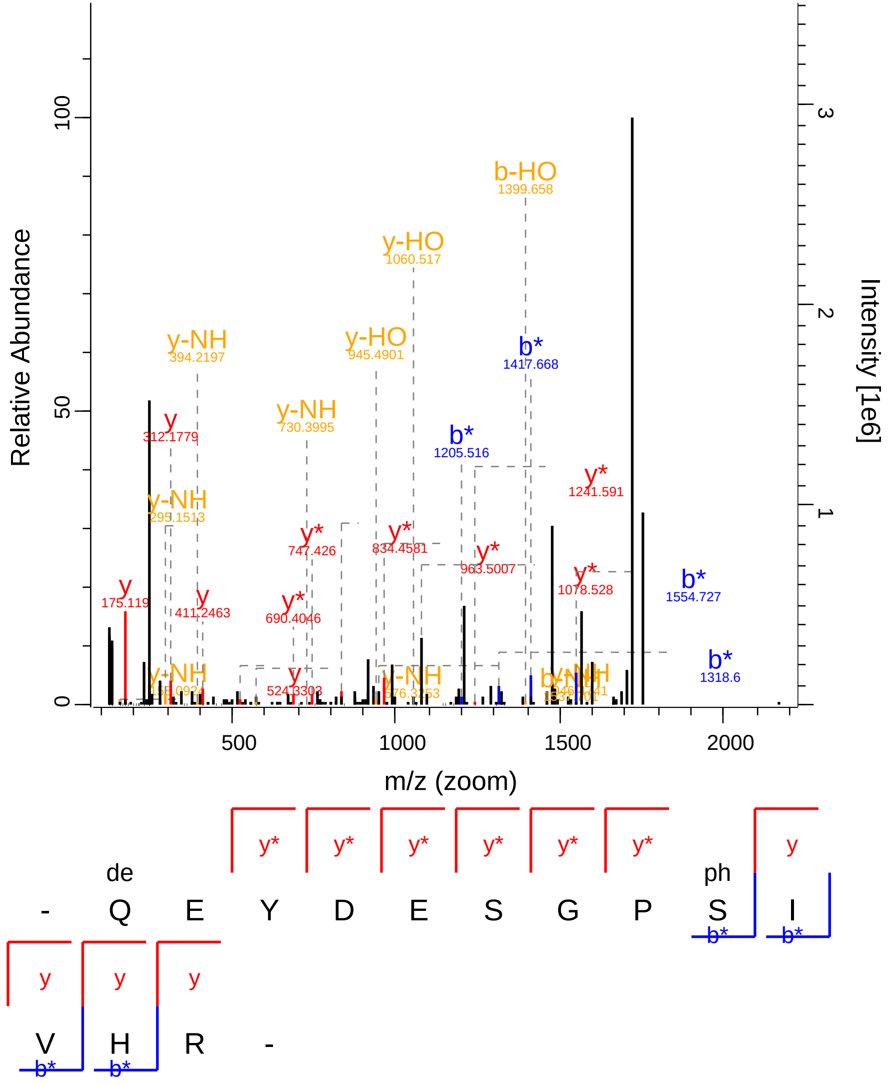

Raw File	Scan	Method	Score	m/z	Gene names
RO-QC-160121_ZackD_SS_Set_01_Fractio	12569	FTMS; HC	51.77	575.34	SRSF11

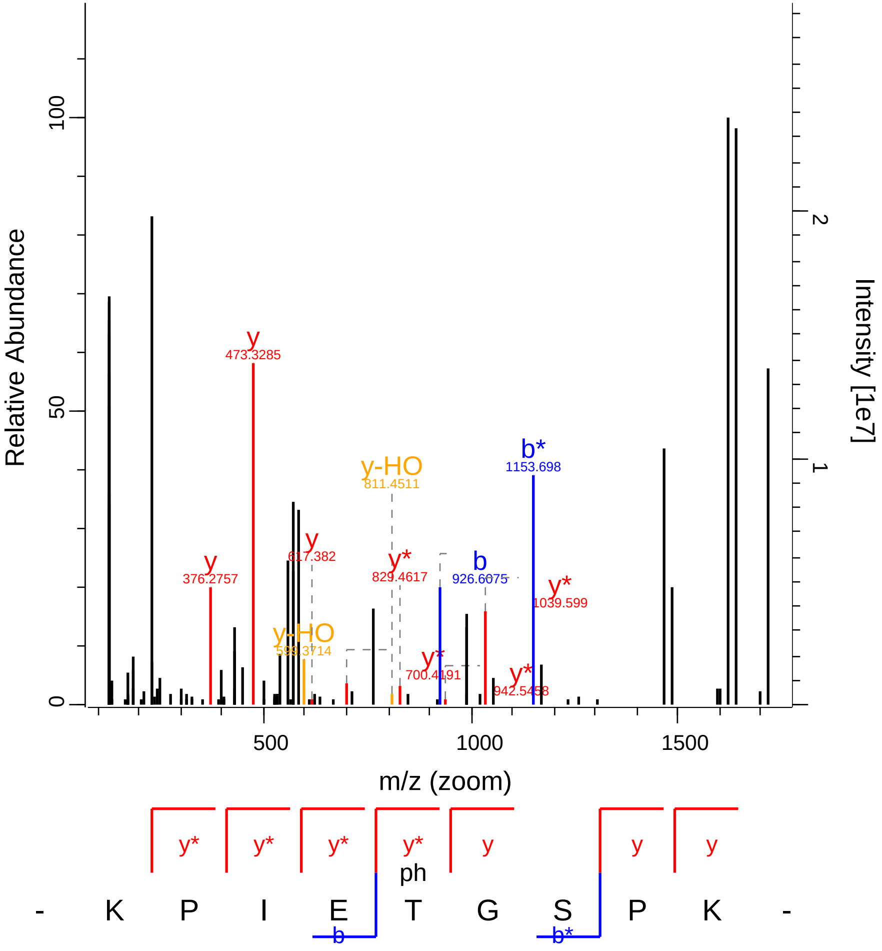

Raw File	Scan	Method	Score	m/z	Gene names
RO-QC-160121_ZackD_SS_Set_01_Fractio	13633	FTMS; HC	74.96	556.33	SCAMP3

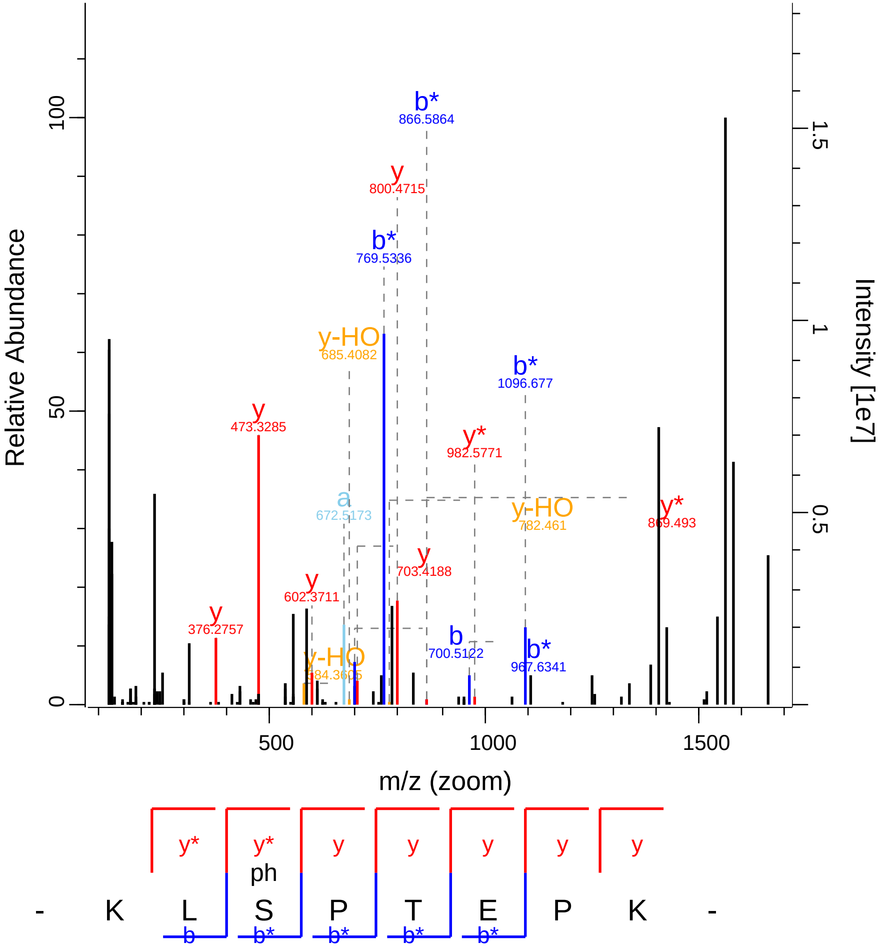

Raw File	Scan	Method	Score	m/z	Gene names
RO-QC-160121_ZackD_SS_Set_01_Fractio	14261	FTMS; HC	51.85	880.48	CEP350

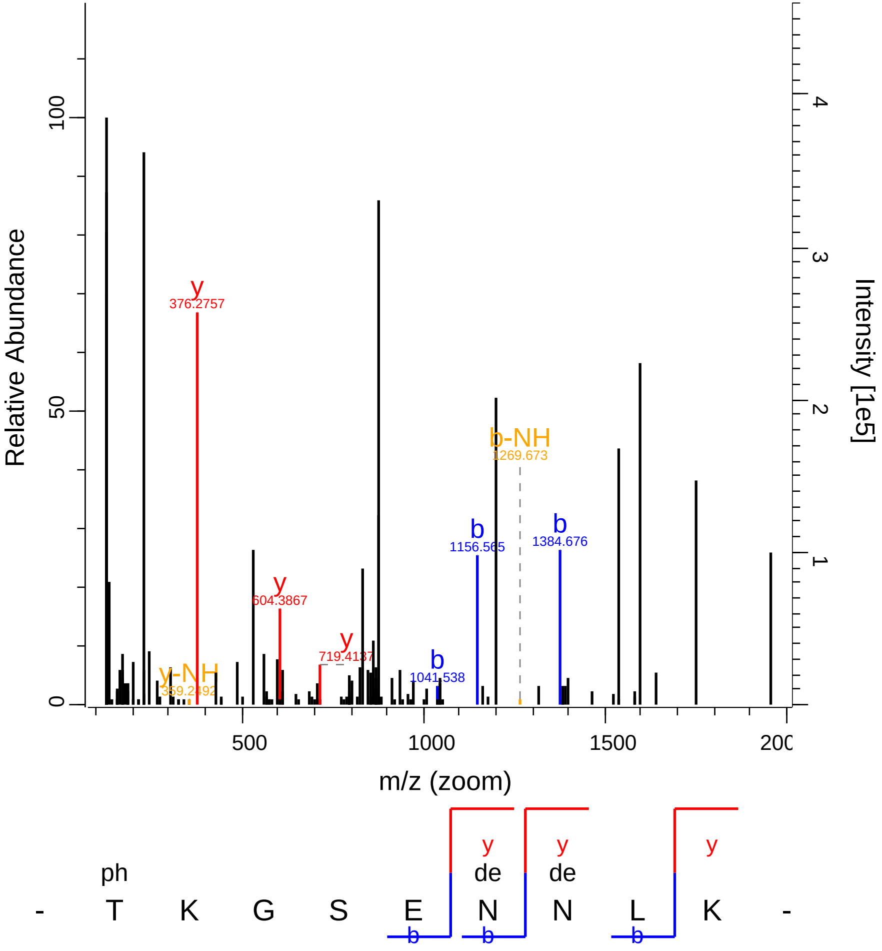

Raw File	Scan	Method	Score	m/z	Gene names
RO-QC-160121_ZackD_SS_Set_01_Fractio	14927	FTMS; HC	90.32	769.03	CASK

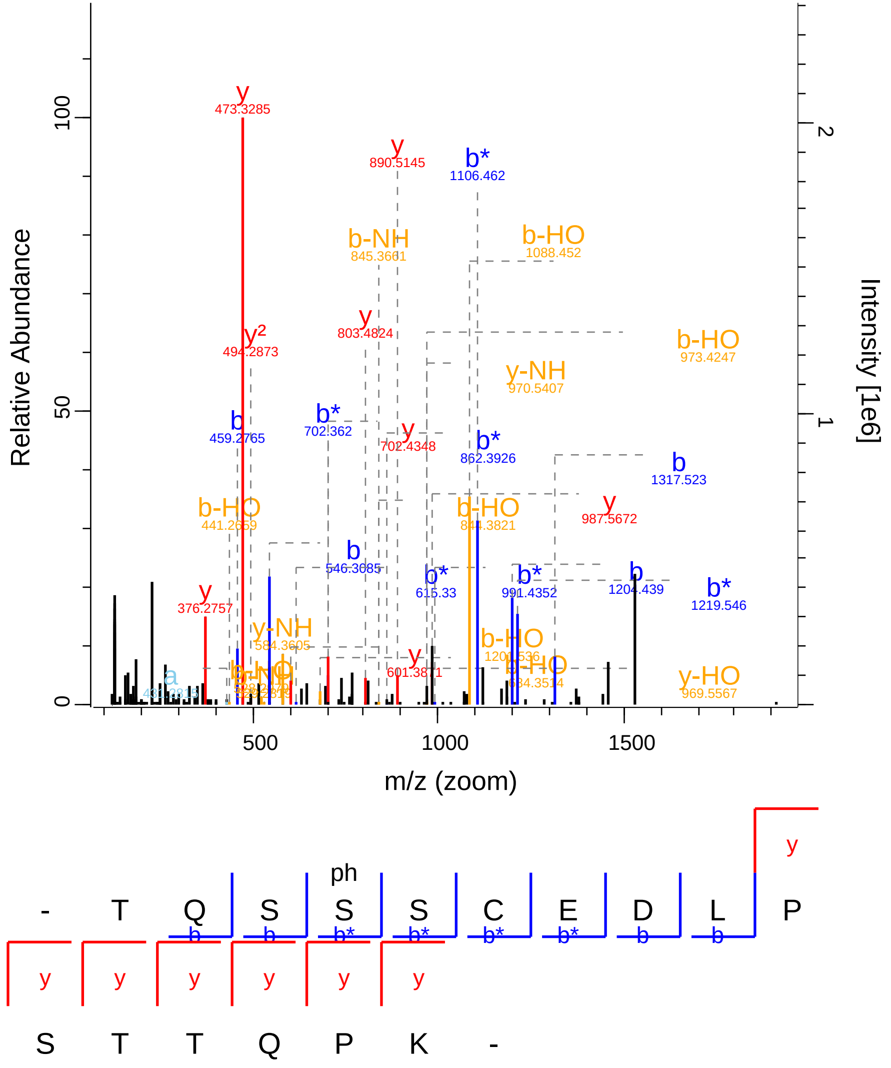

Raw File	Scan	Method	Score	m/z	Gene names
RO-QC-160121_ZackD_SS_Set_01_Fractic	16632	FTMS; HC	89.63	1242.66	HIST1H1E

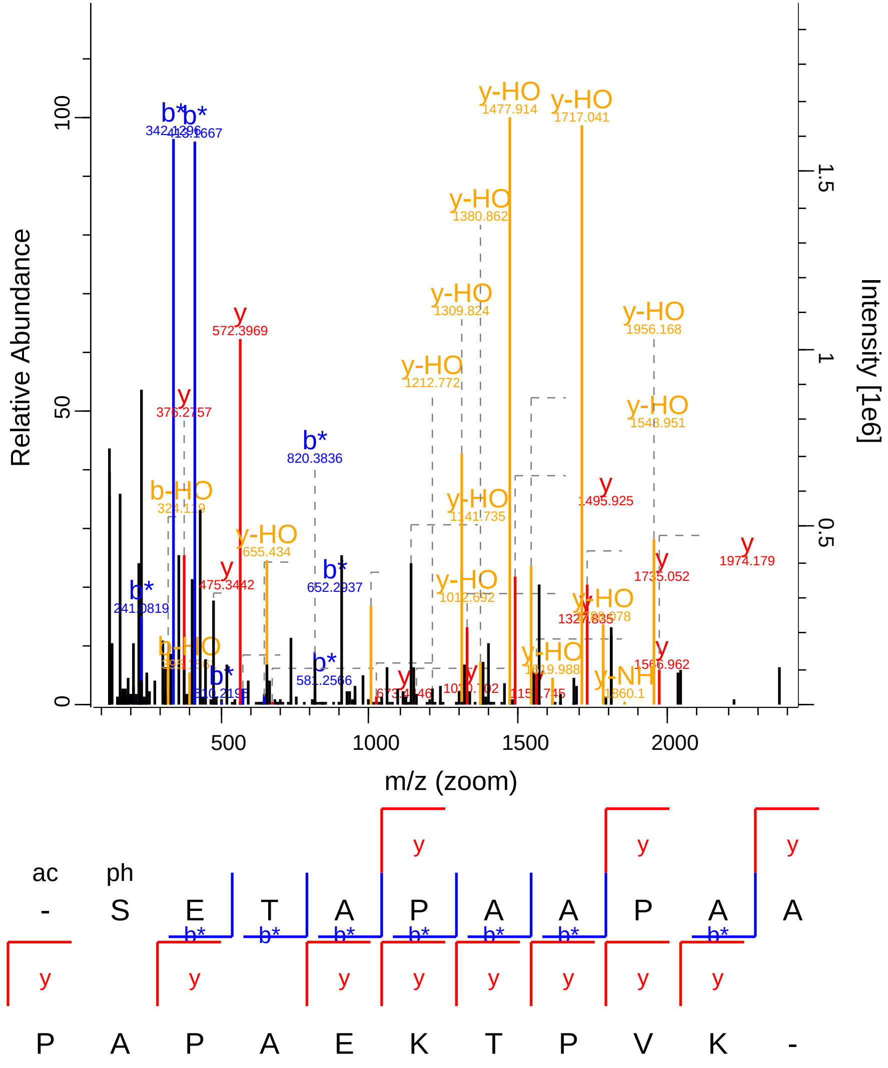

Raw File	Scan	Method	Score	m/z	Gene names
RO-QC-160121_ZackD_SS_Set_01_Fractio	16781	FTMS; HC	43.79	991.19	RBL1

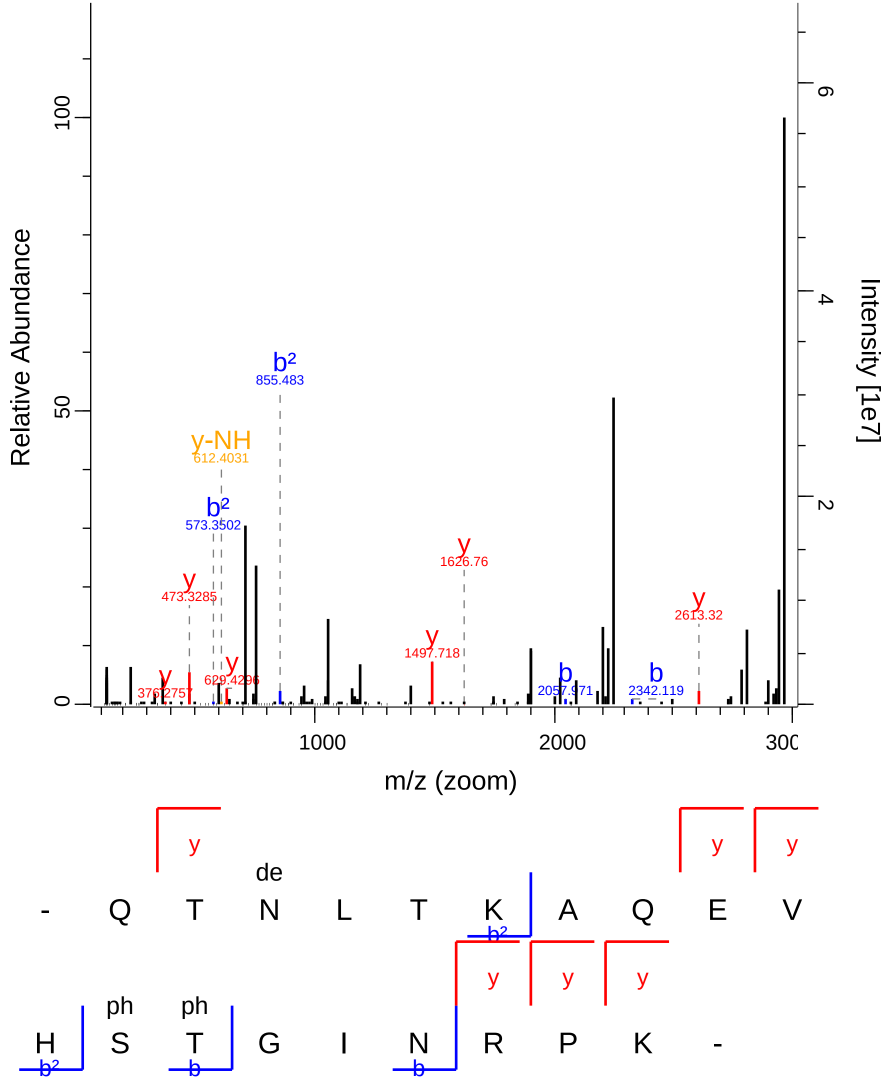

Raw File	Scan	Method	Score	m/z	Gene names
RO-QC-160121_ZackD_SS_Set_01_Fractio	17621	FTMS; HC	43.96	796.11	TP53BP1

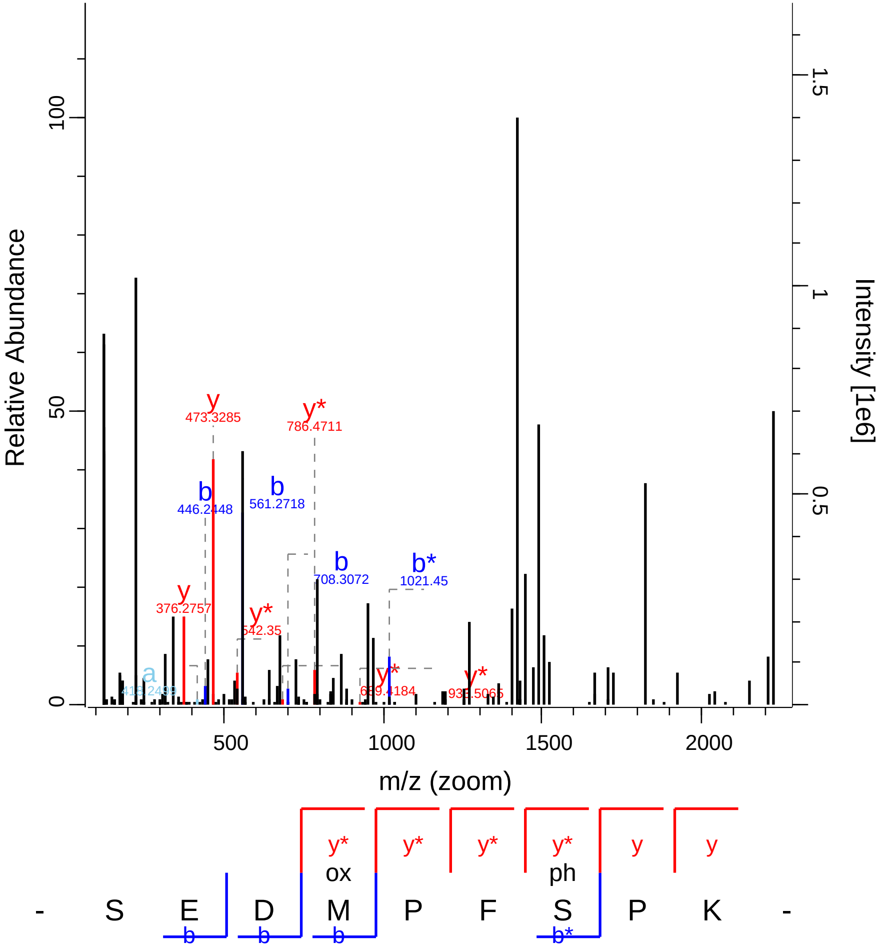

Raw File	Scan	Method	Score	m/z	Gene names
RO-QC-160121_ZackD_SS_Set_01_Fractic	18809	FTMS; HC	89.31	1236.59	MAP1B

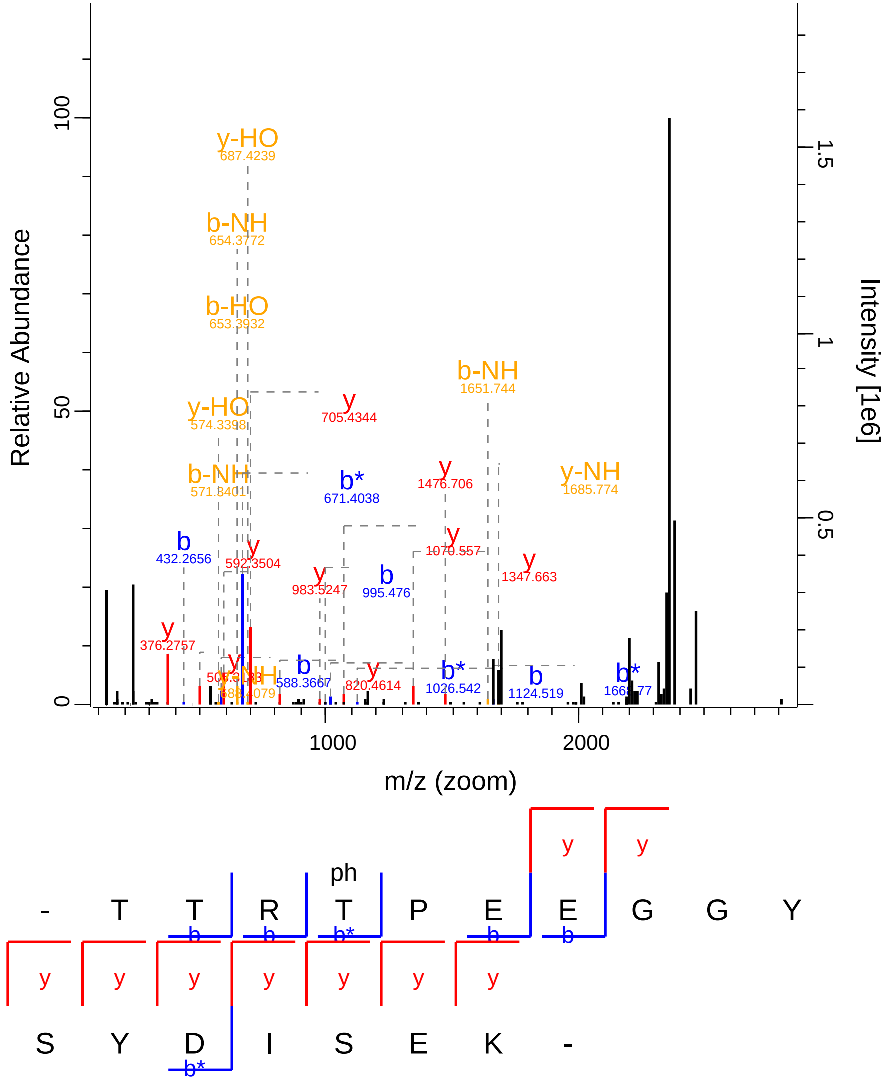

Raw File	Scan	Method	Score	m/z	Gene names
RO-QC-160121_ZackD_SS_Set_01_Fractio	18896	FTMS; HC	126.99	715.17	PGRMC2

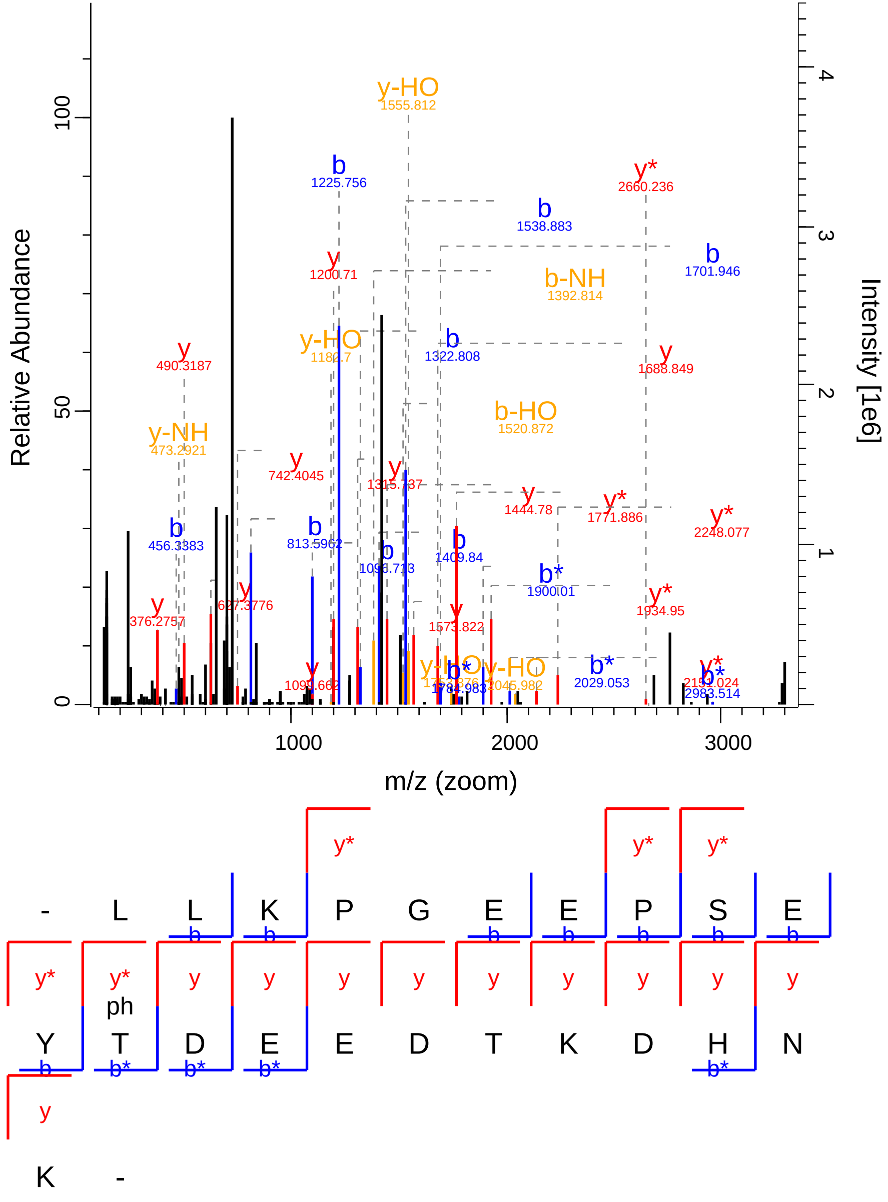

Raw File	Scan	Method	Score	m/z	Gene names
RO-QC-160121_ZackD_SS_Set_01_Fractio	18934	FTMS; HC	76.2	893.71	PGRMC2

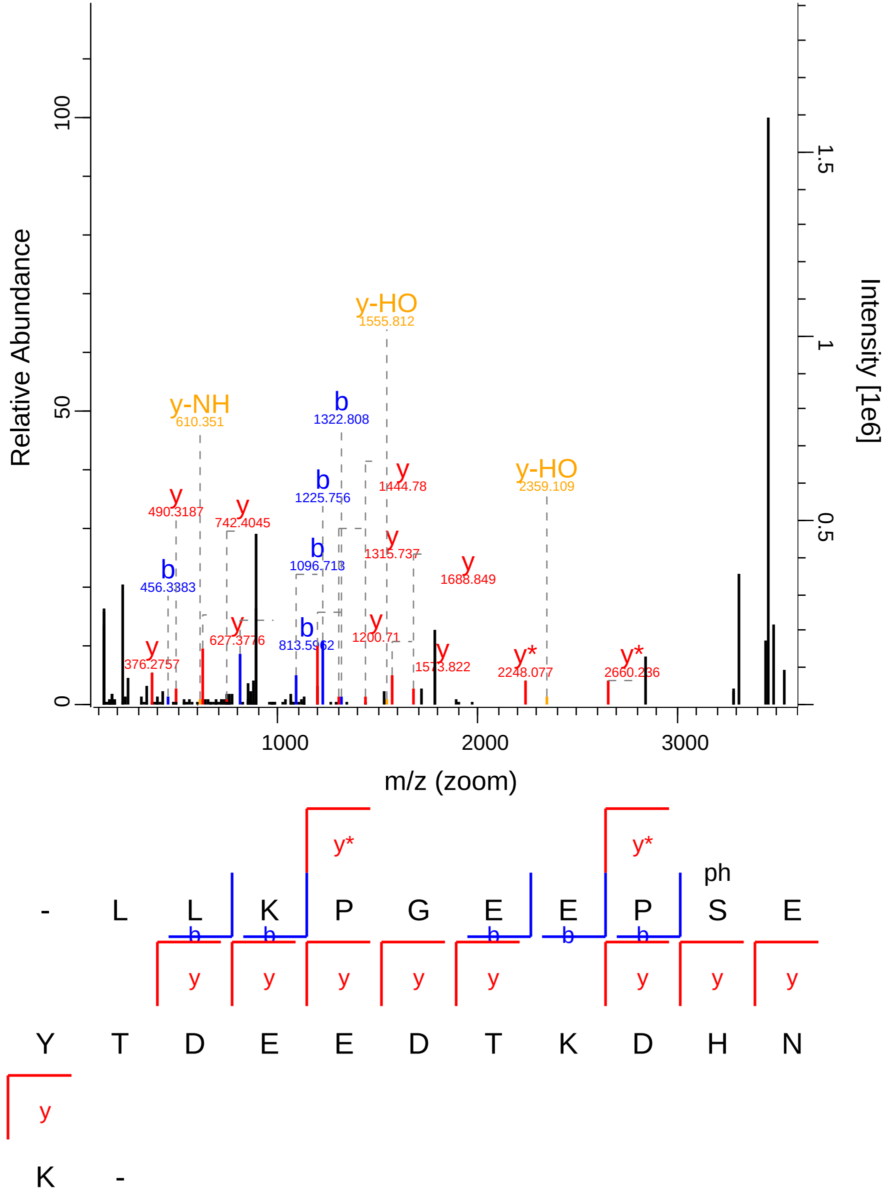

Raw File	Scan	Method	Score	m/z	Gene names
RO-QC-160121_ZackD_SS_Set_01_Fractior	20034	FTMS; HCl	79.24	943.2	PCDH7

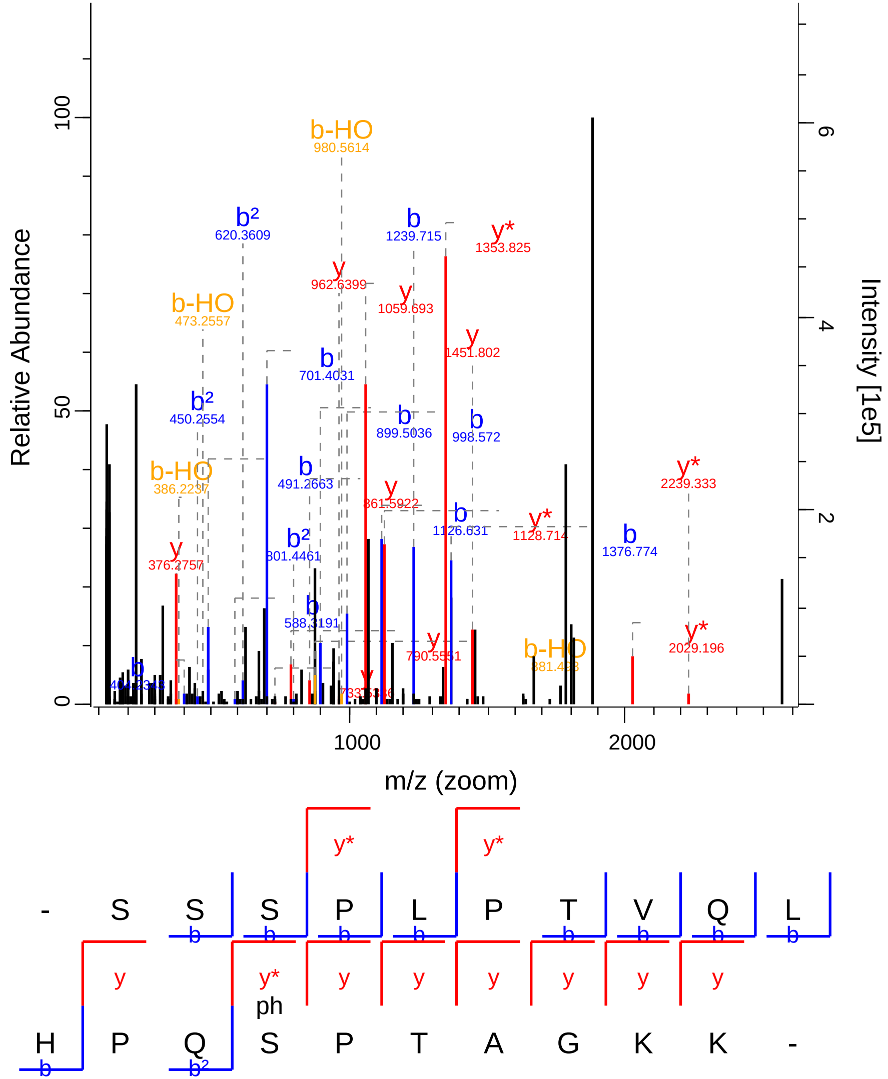

Raw File	Scan	Method	Score	m/z	Gene names
RO-QC-160121_ZackD_SS_Set_01_Fractio	20506	FTMS; HC	206.17	900.93	AP3B1

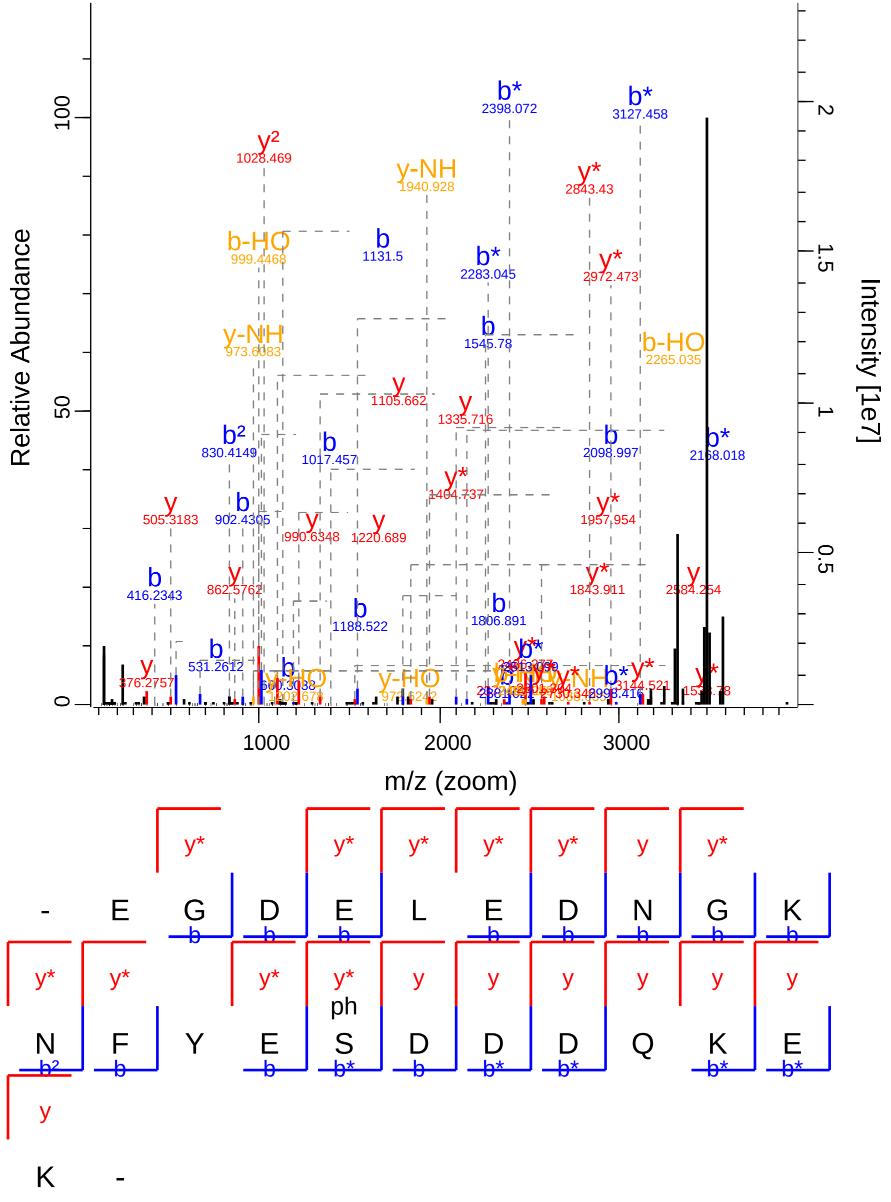

Raw File	Scan	Method	Score	m/z	Gene names
RO-QC-160121_ZackD_SS_Set_01_Fractio	21111	FTMS; HC	43.7	555.32	ABCD2

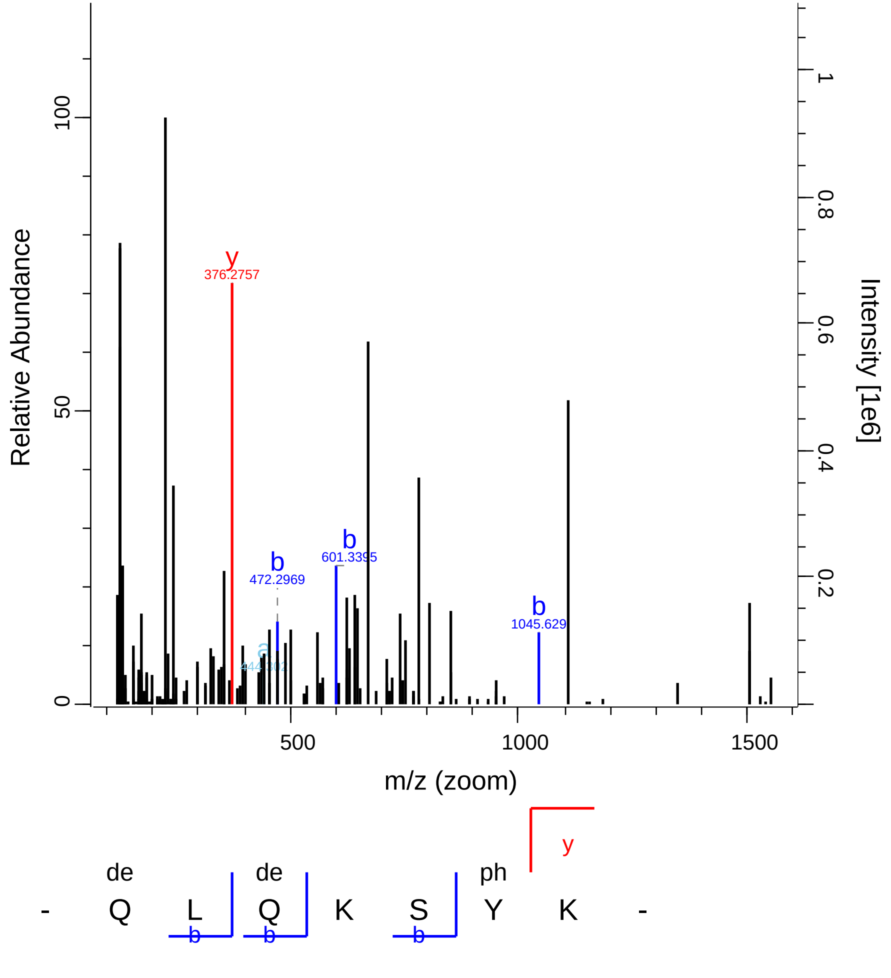

Raw File	Scan	Method	Score	m/z	Gene names
RO-QC-160121_ZackD_SS_Set_01_Fractio	21218	FTMS; HC	64.52	636.34	PRKCB

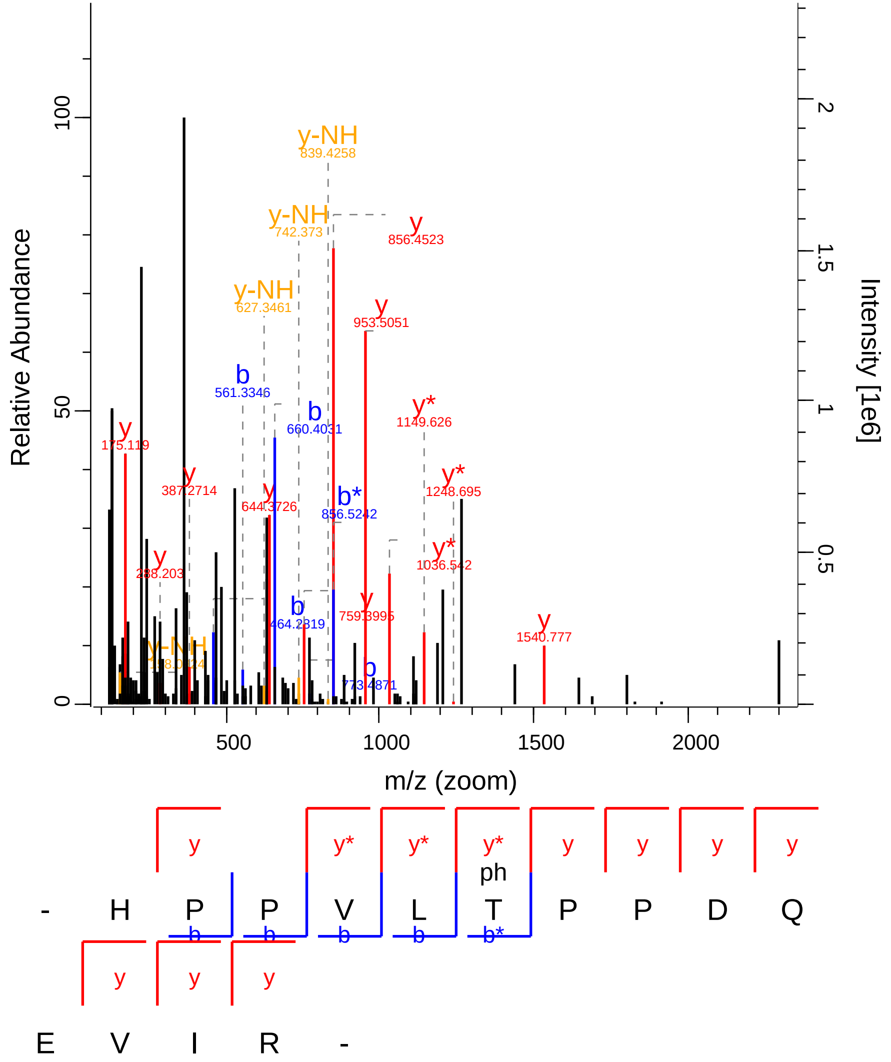

Raw File	Scan	Method	Score	m/z	Gene names
RO-QC-160121_ZackD_SS_Set_01_Fractio	22746	FTMS; HC	105	980.47	CEP170

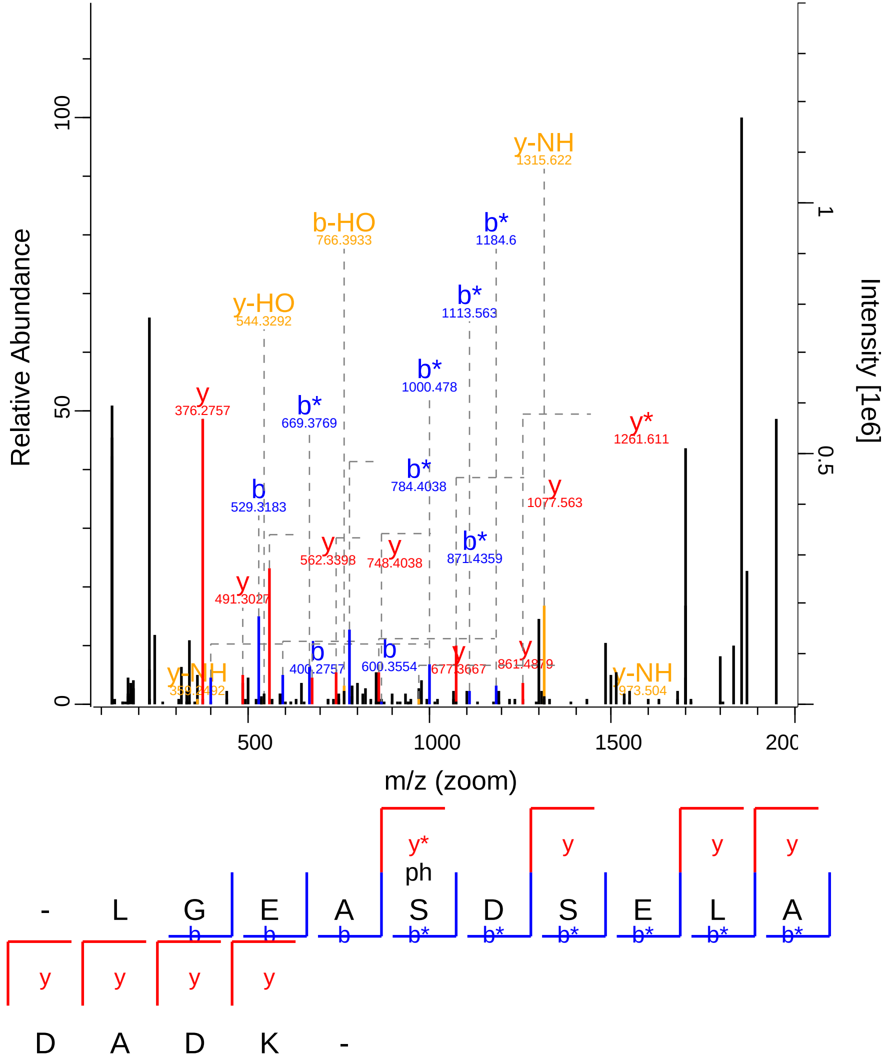

Raw File	Scan	Method	Score	m/z	Gene names
RO-QC-160121_ZackD_SS_Set_01_Fractio	24292	FTMS; HC	58.63	916.99	TSHZ2

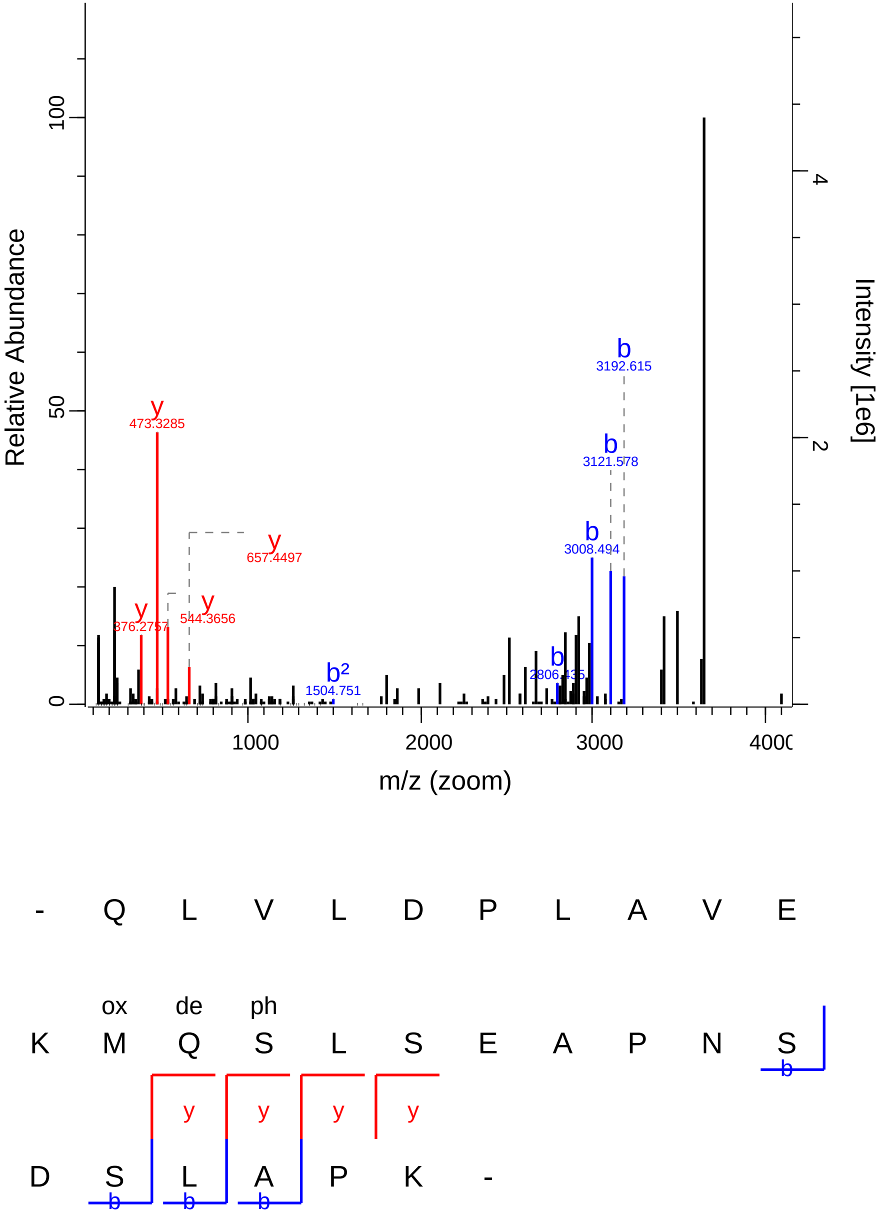

Raw File	Scan	Method	Score	m/z	Gene names
RO-QC-160121_ZackD_SS_Set_01_F	24966	FTMS; H	50.83	699.08	HIST1H1E;HIST1H1C;HIS

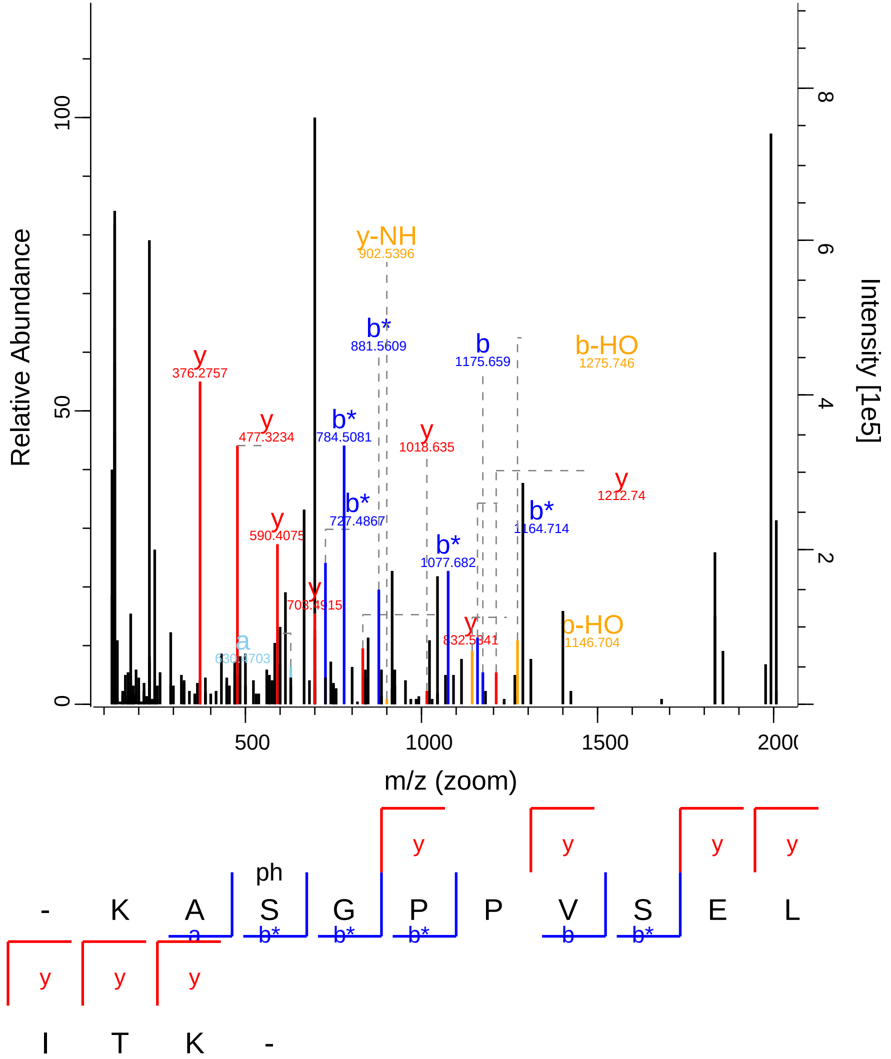

Raw File	Scan	Method	Score	m/z	Gene names
RO-QC-160121_ZackD_SS_Set_01_Fractic	26209	FTMS; HC	138.42	1005.54	RPS3

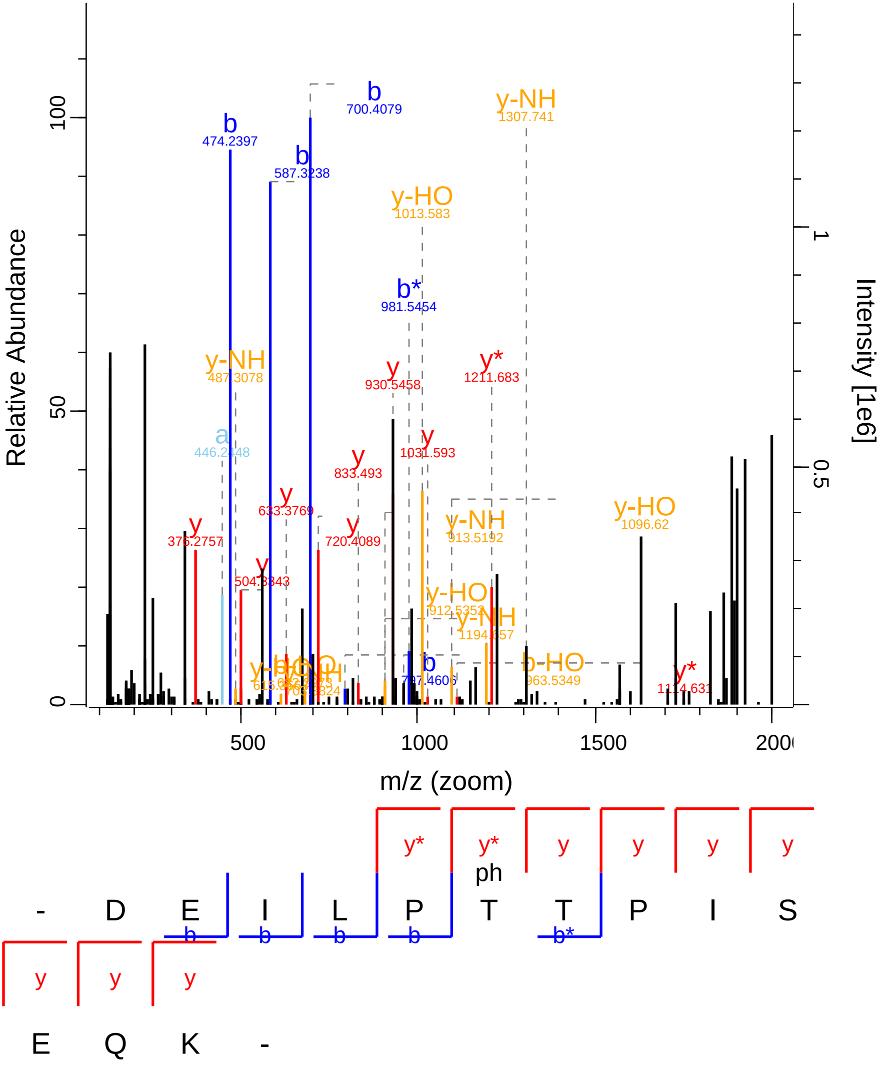

Raw File	Scan	Method	Score	m/z	Gene names
RO-QC-160121_ZackD_SS_Set_01_Fractio	26227	FTMS; HC	89.25	898.48	ABL2

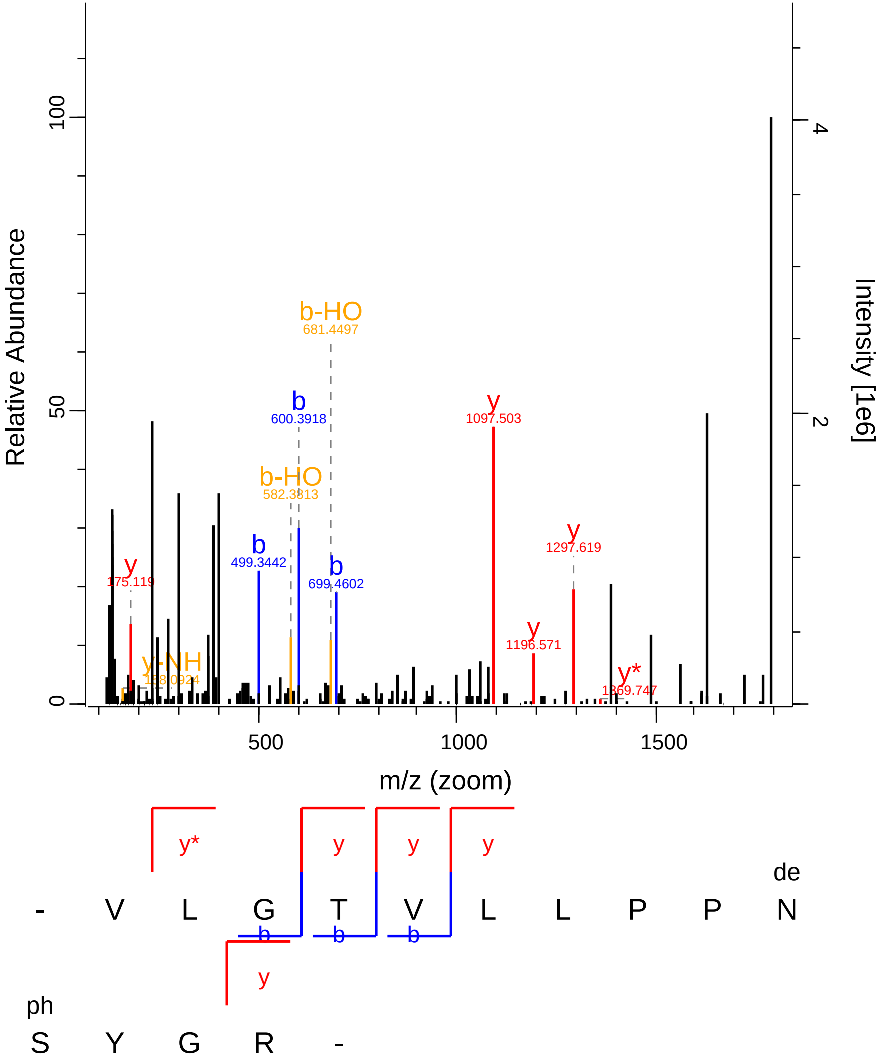

Raw File	Scan	Method	Score	m/z	Gene names
RO-QC-160121_ZackD_SS_Set_01_Fractic	26283	FTMS; HC	104.52	1141.51	EDC4

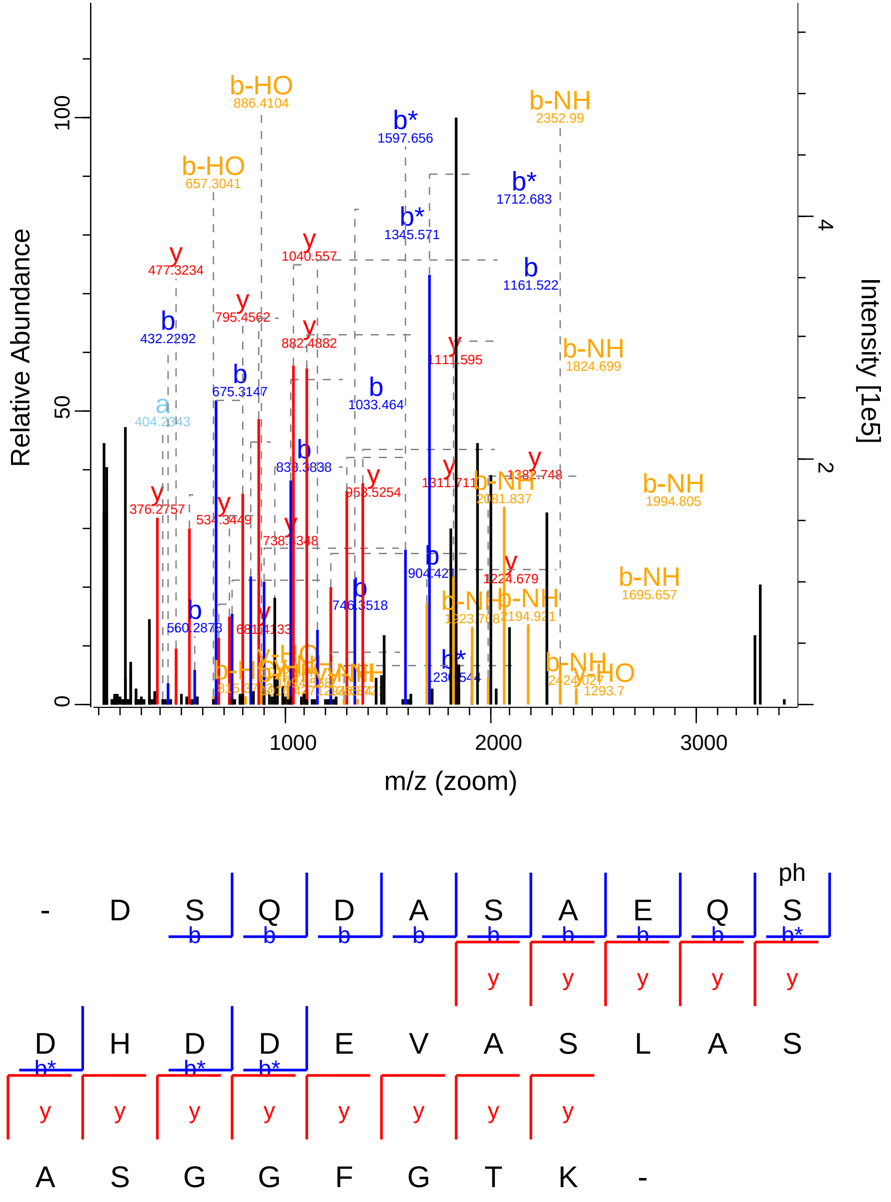

Raw File	Scan	Method	Score	m/z	Gene names
RO-QC-160121_ZackD_SS_Set_01_Fractio	26552	FTMS; HC	84.14	613.03	NUFIP2

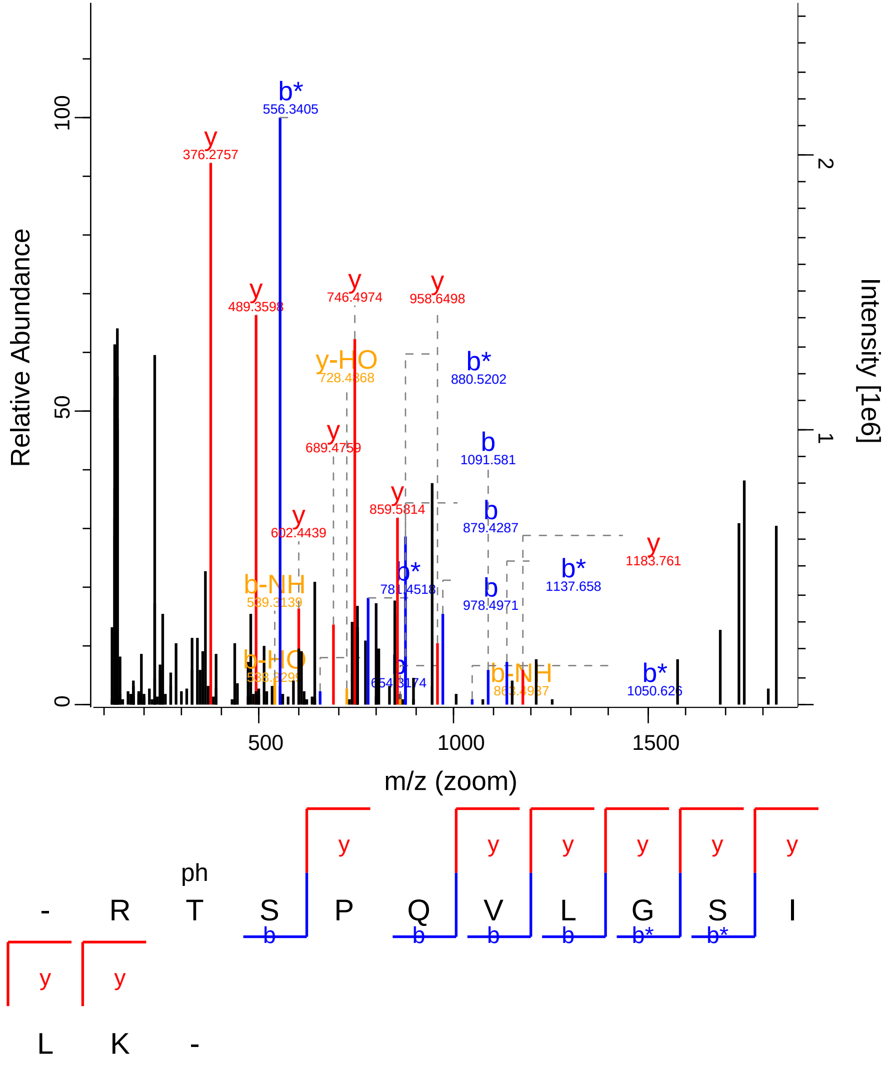

Raw File	Scan	Method	Score	m/z	Gene names
RO-QC-160121_ZackD_SS_Set_01_Fractic	26993	FTMS; HC	70.53	1388.22	COL4A1

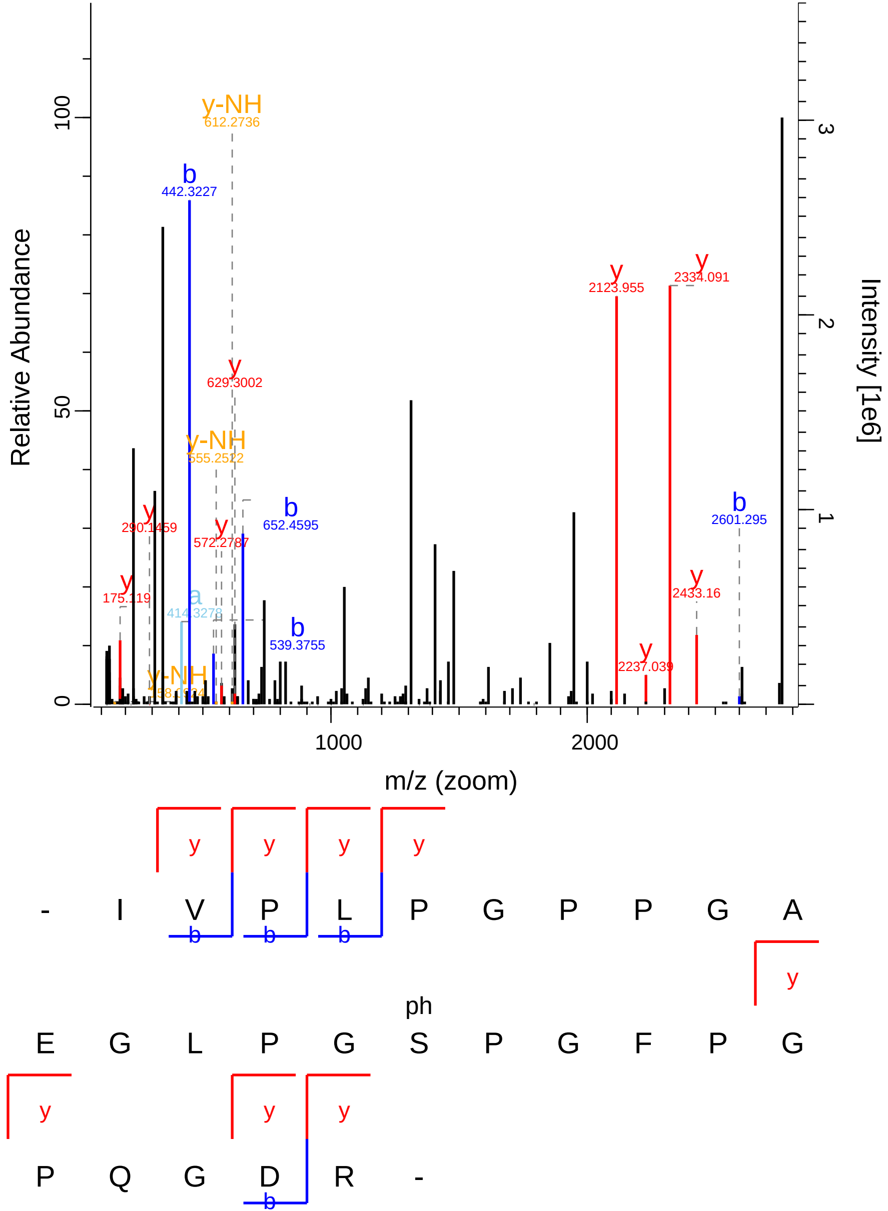

Raw File	Scan	Method	Score	m/z	Gene names
RO-QC-160121_ZackD_SS_Set_01_Fractio	27133	FTMS; HC	86.09	405.21	SCTR

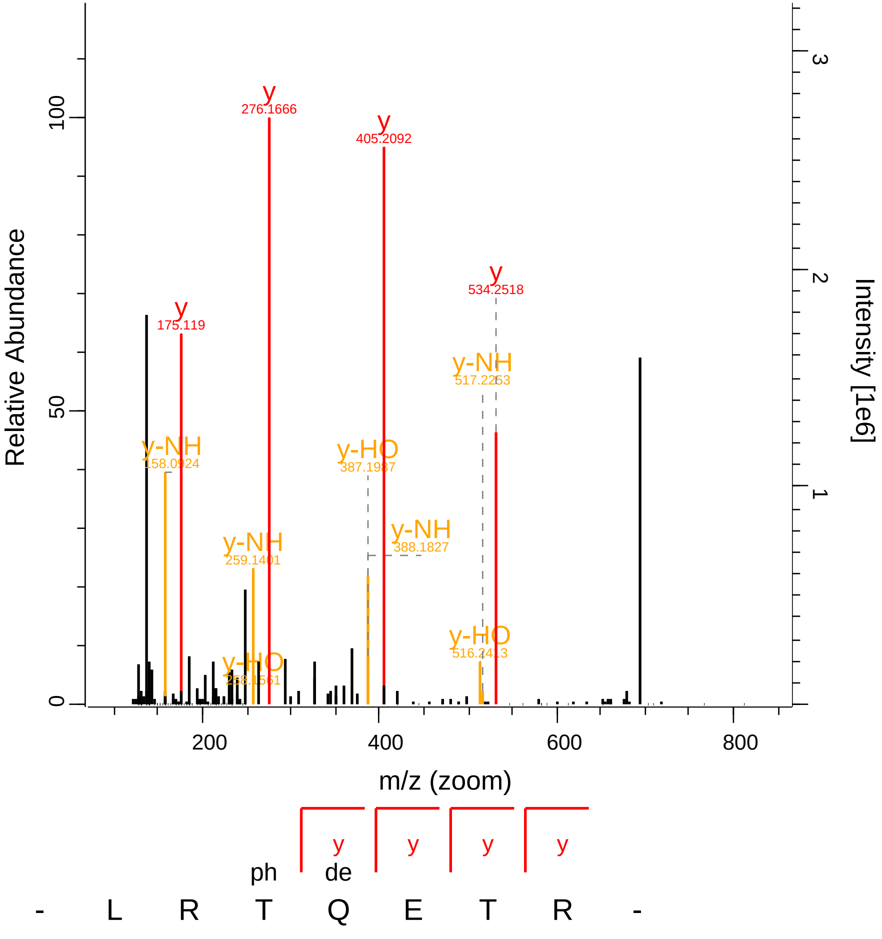

Raw File	Scan	Method	Score	m/z	Gene names
RO-QC-160121_ZackD_SS_Set_01_Fractic	28092	FTMS; HC	66.53	1499.21	RTN4

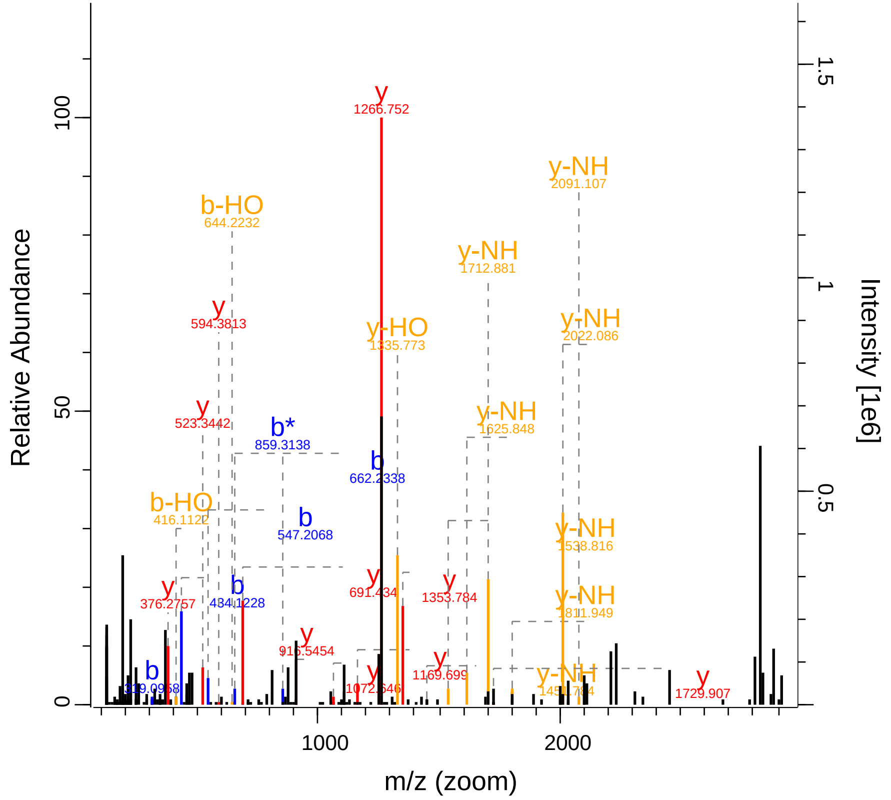

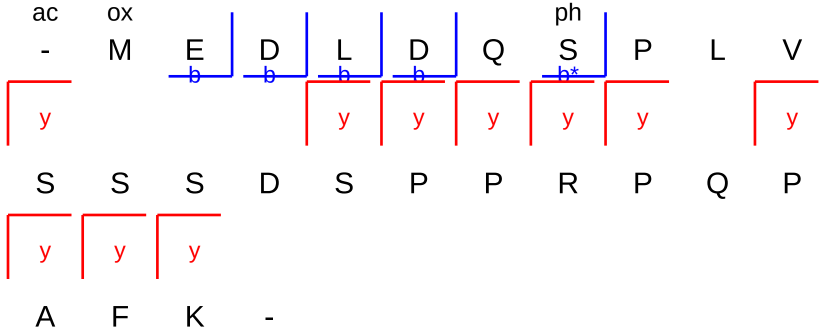

Raw File	Scan	Method	Score	m/z	Gene names
RO-QC-160121_ZackD_SS_Set_01_Fractio	28561	FTMS; HC	41.91	873.76	N4BP2

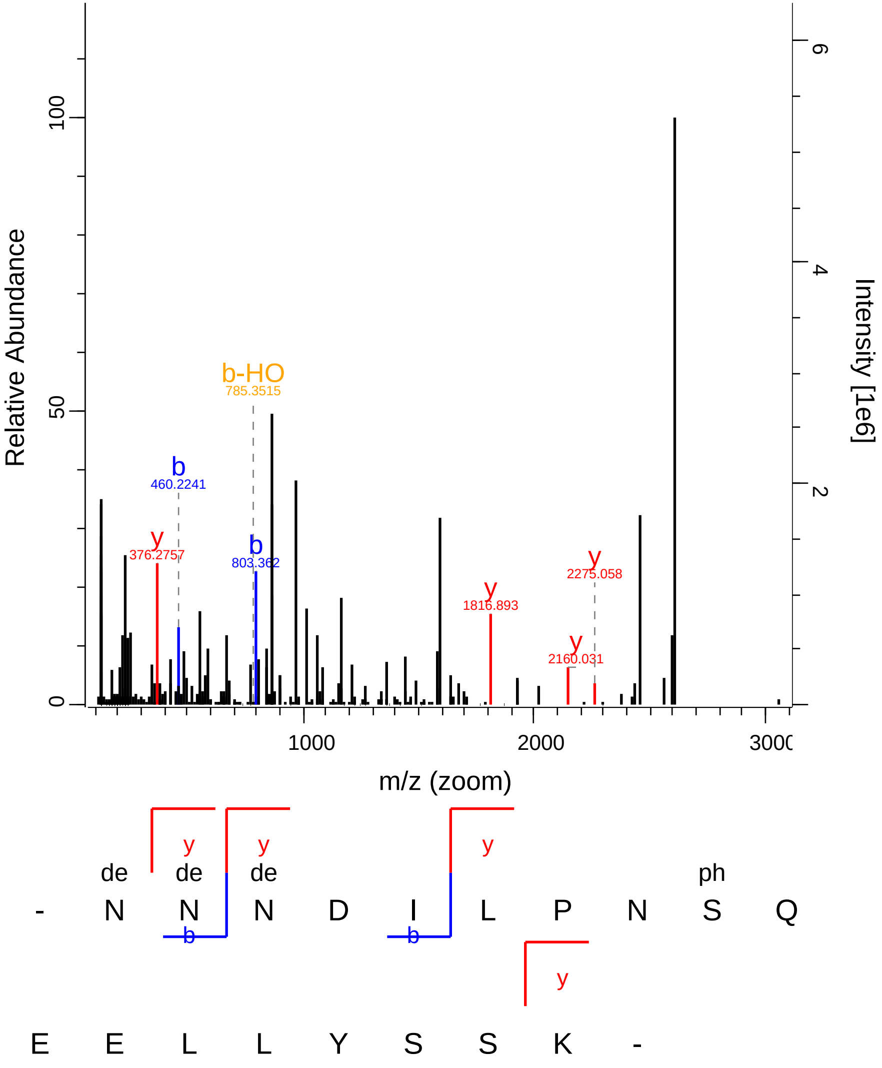

Raw File	Scan	Method	Score	m/z	Gene names
RO-QC-160121_ZackD_SS_Set_01_Fractio	29216	FTMS; HC	150.45	1013.17	SLC2A1

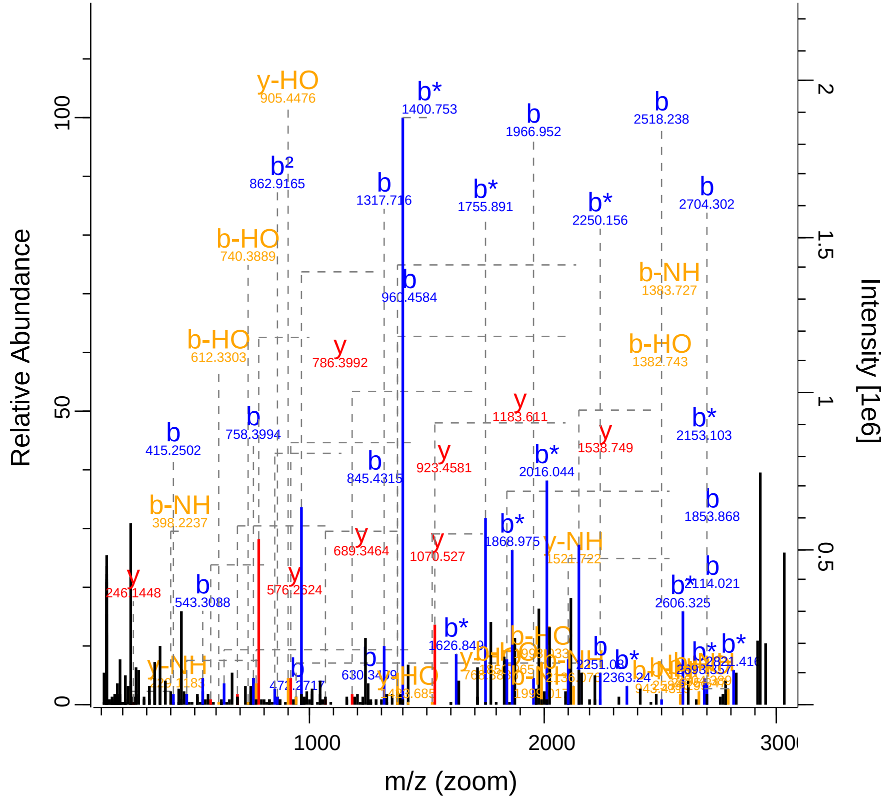

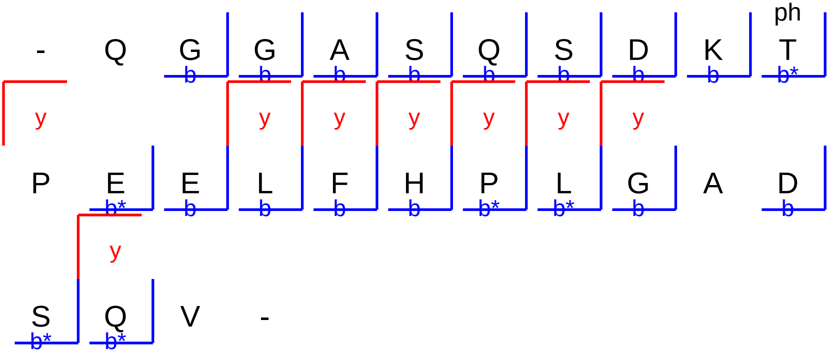

Raw File	Scan	Method	Score	m/z	Gene names
RO-QC-160121_ZackD_SS_Set_01_Fractio	29436	FTMS; HC	41.64	732.38	SLC22A7

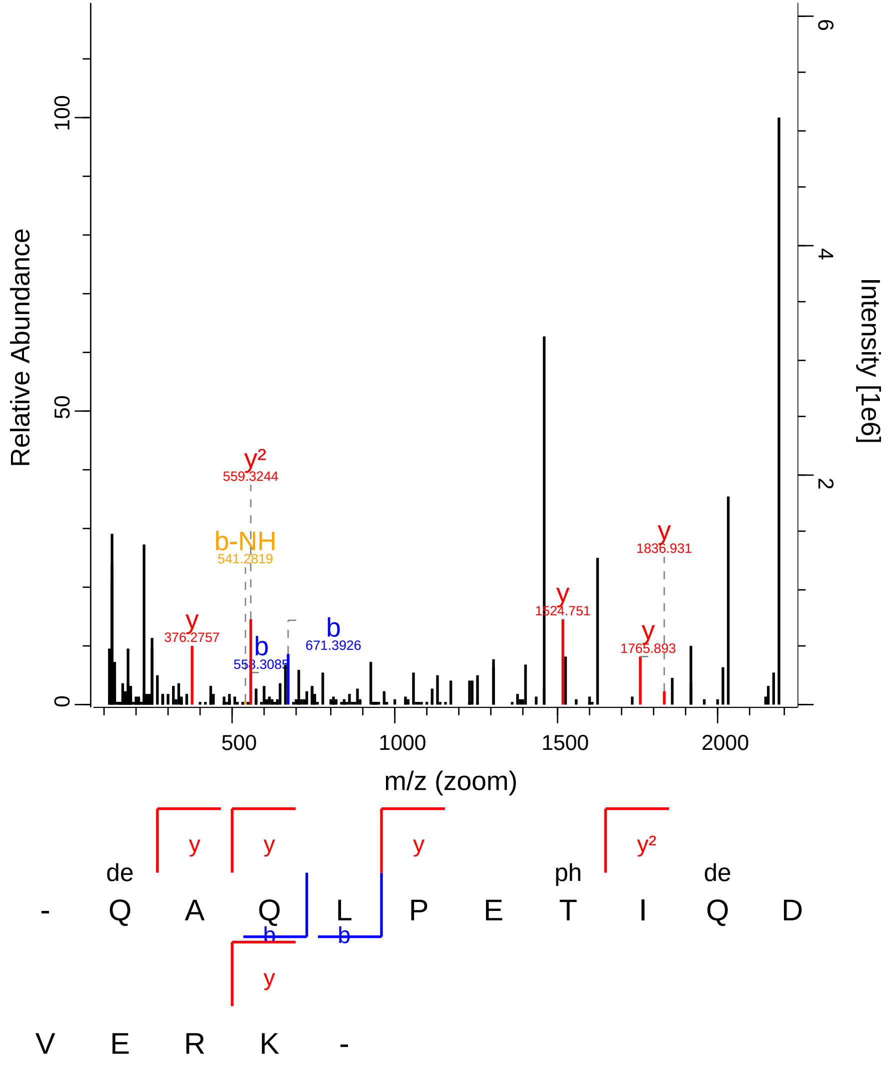

Raw File	Scan	Method	Score	m/z	Gene names
RO-QC-160121_ZackD_SS_Set_01_Fractio	29440	FTMS; HC	216.48	1053.21	CCNY

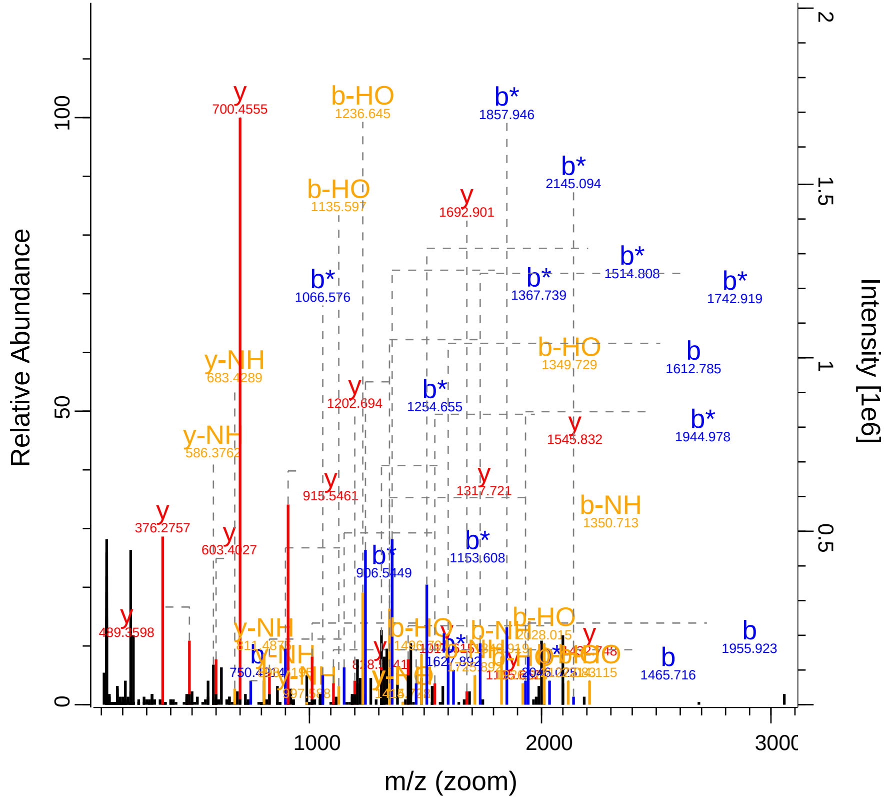

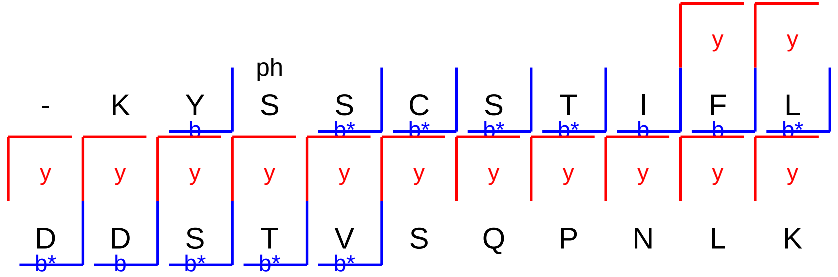

-

Raw File	Scan	Method	Score	m/z	Gene names
RO-QC-160121_ZackD_SS_Set_01_Fractio	30470	FTMS; HC	73.29	643.87	SYNPO

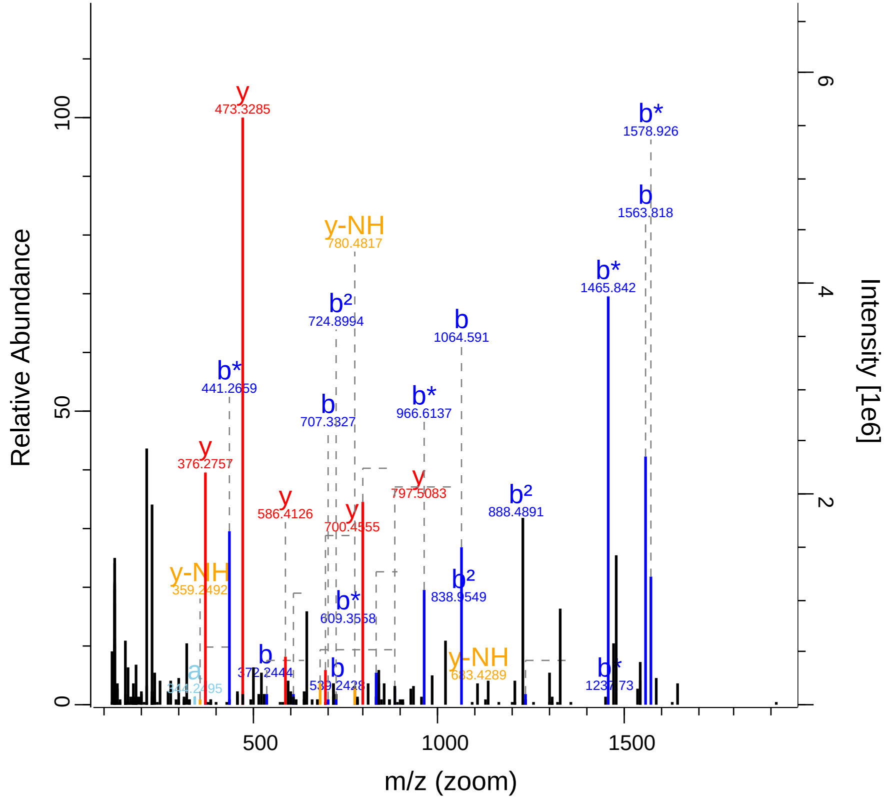

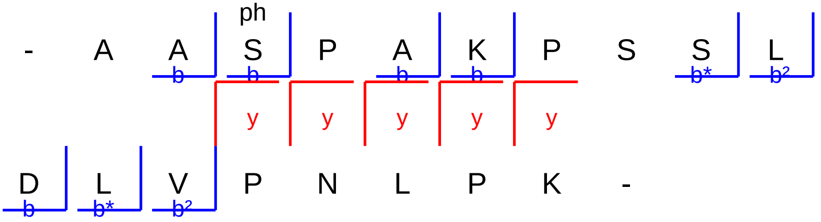

Raw File	Scan	Method	Score	m/z	Gene names
RO-QC-160121_ZackD_SS_Set_01_Fractic	30886	FTMS; HC	159.56	1094.83	MAP1B

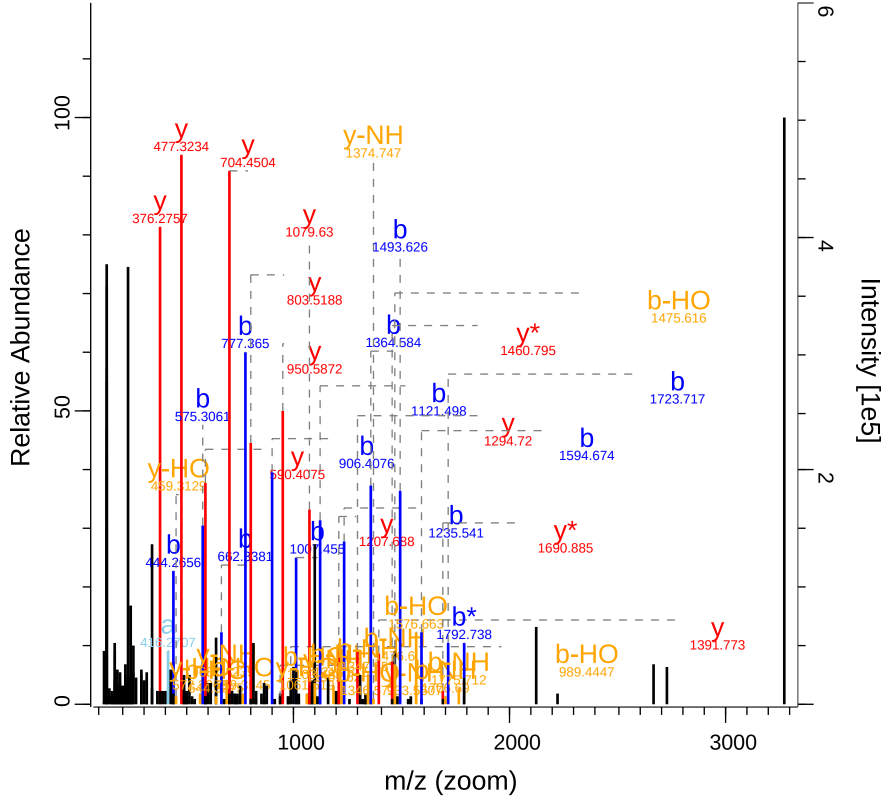

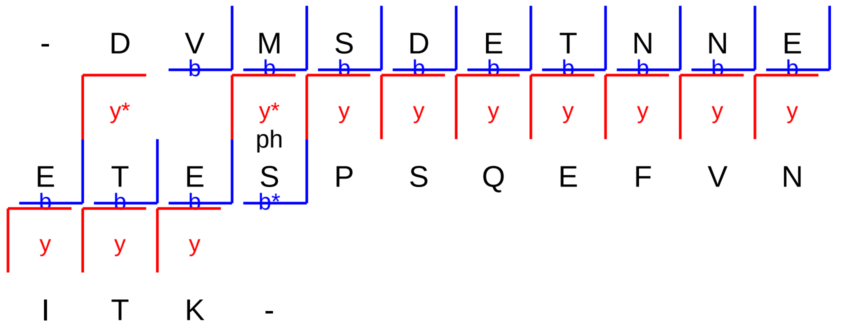

Raw File	Scan	Method	Score	m/z	Gene names
RO-QC-160121_ZackD_SS_Set_01_Fractio	31047	FTMS; HC	96.88	889.98	FAM129B

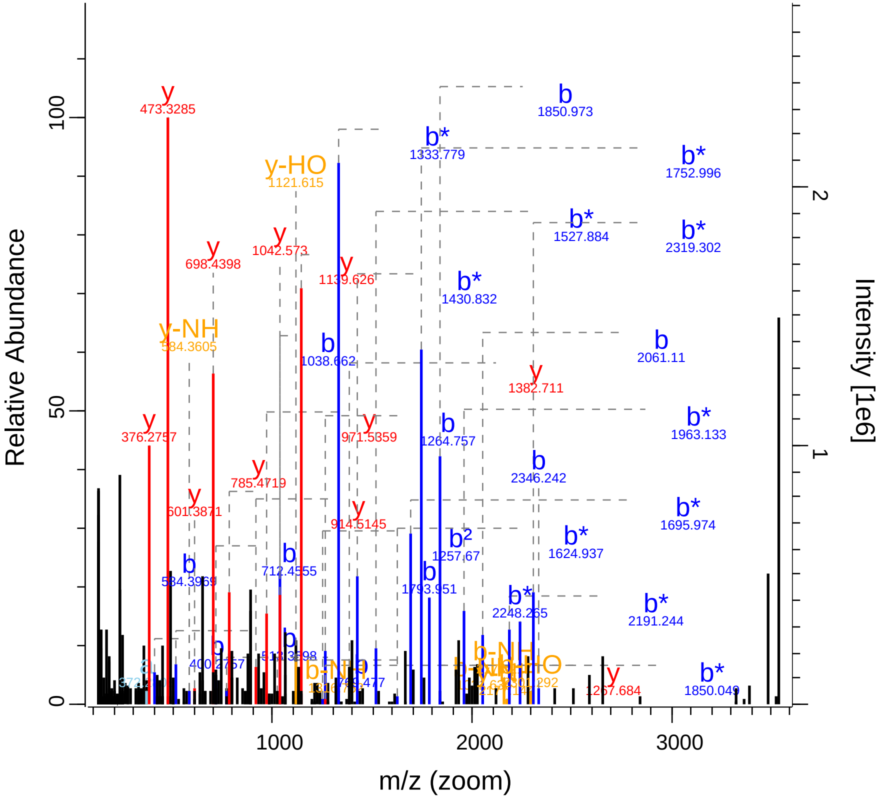

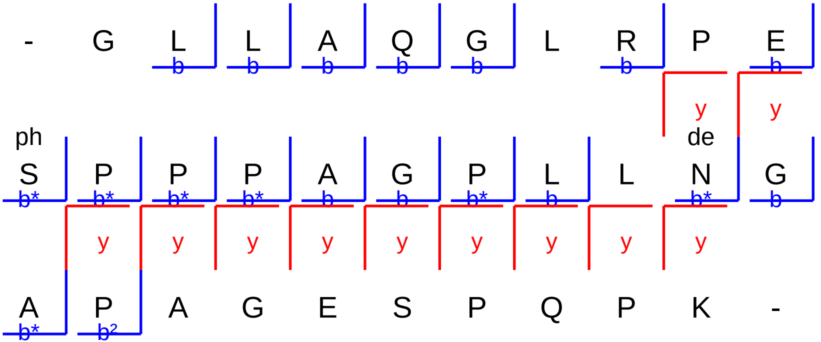

Raw File	Scan	Method	Score	m/z	Gene names
RO-QC-160121_ZackD_SS_Set_01_Fractio	31239	FTMS; HC	135.86	874.39	AQP1

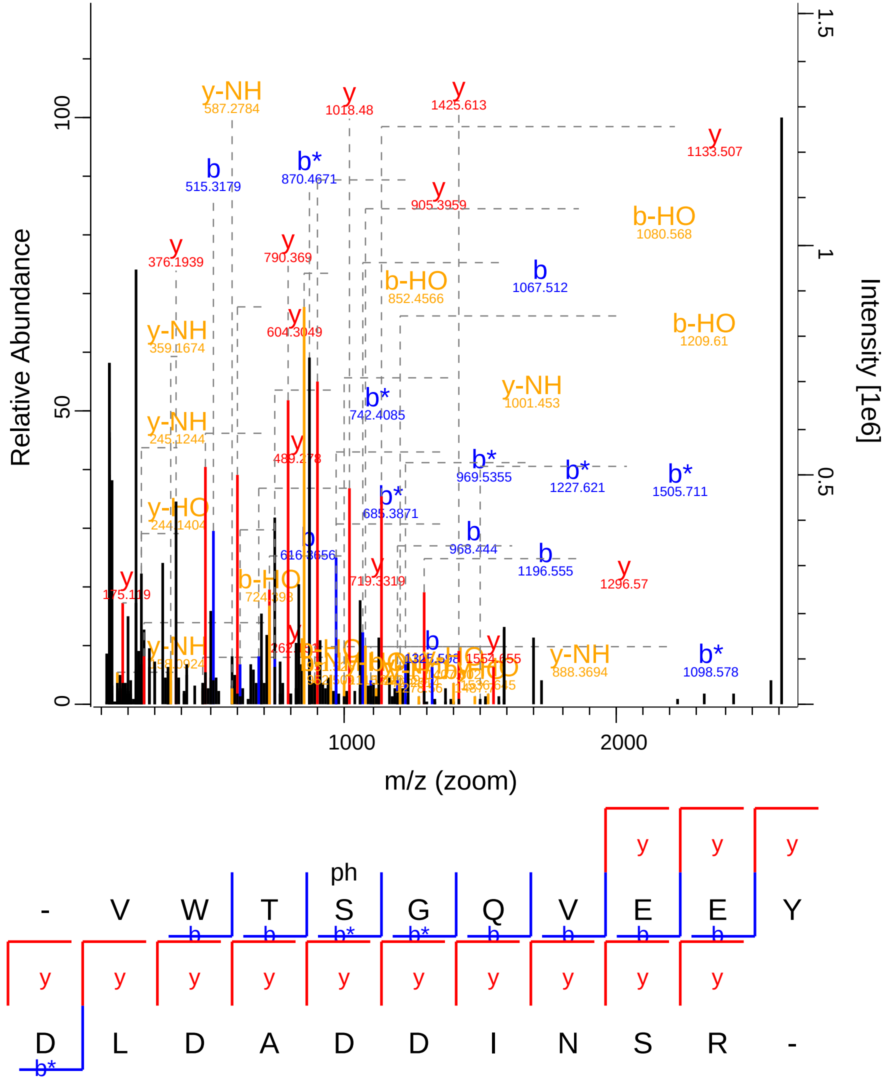

Raw File	Scan	Method	Score	m/z	Gene names
RO-QC-160121_ZackD_SS_Set_01_Fractio	32008	FTMS; HC	211.77	1325.29	AHNAK

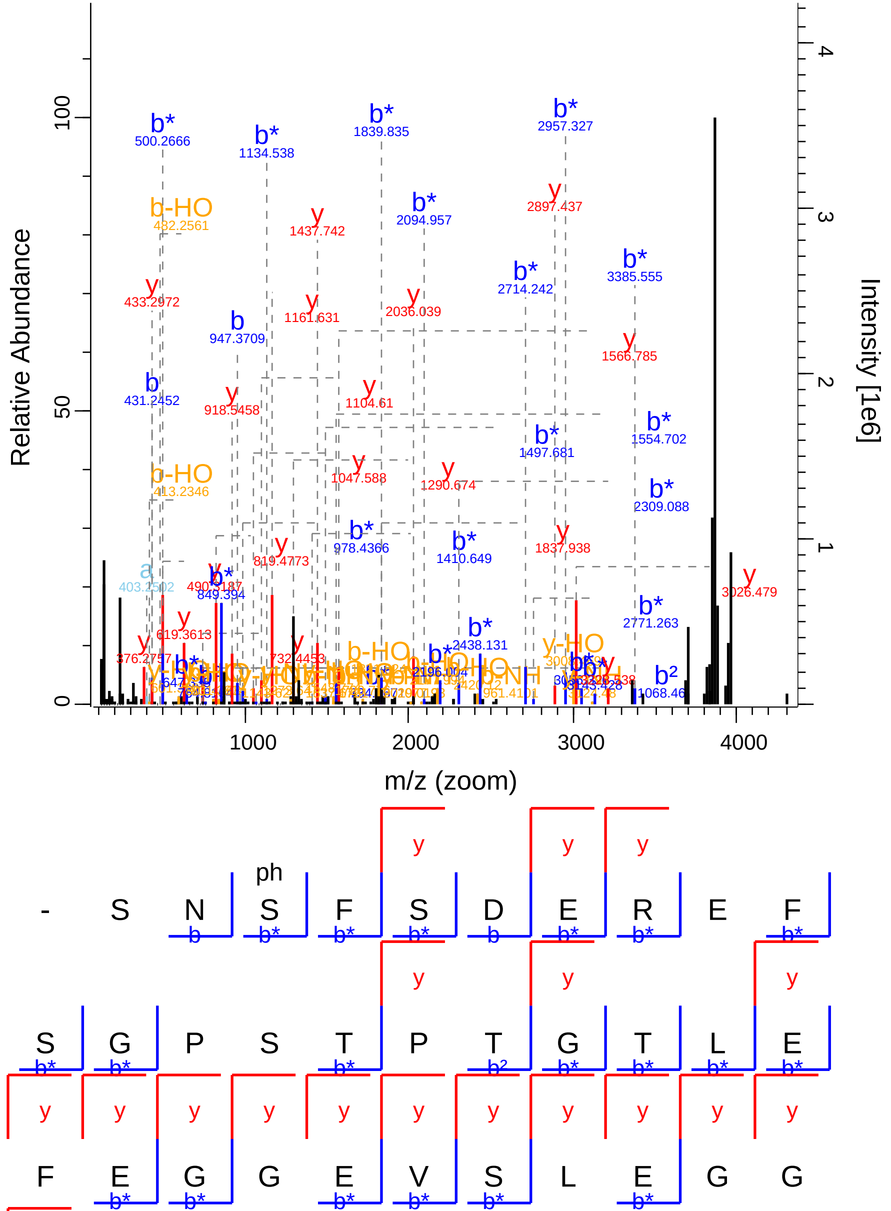

Raw File	Scan	Method	Score	m/z	Gene names
RO-QC-160121_ZackD_SS_Set_01_Fractio	32049	FTMS; HC	122.06	1017.49	SQSTM1

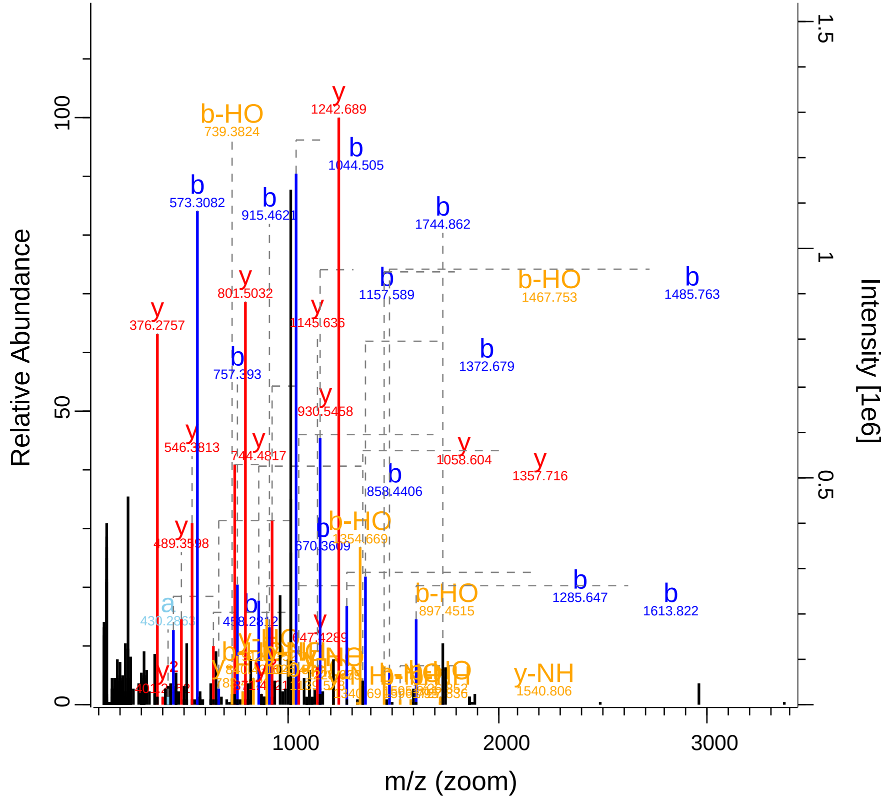

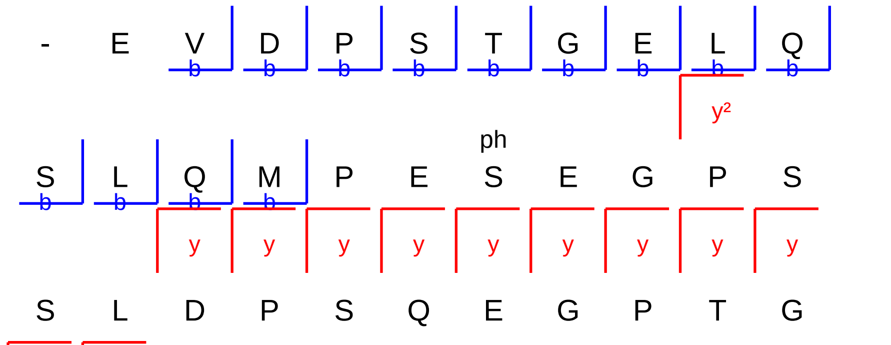

Raw File	Scan	Method	Score	m/z	Gene names
RO-QC-160121_ZackD_SS_Set_01_Fractio	32295	FTMS; HC	43.73	847.24	SYNE1

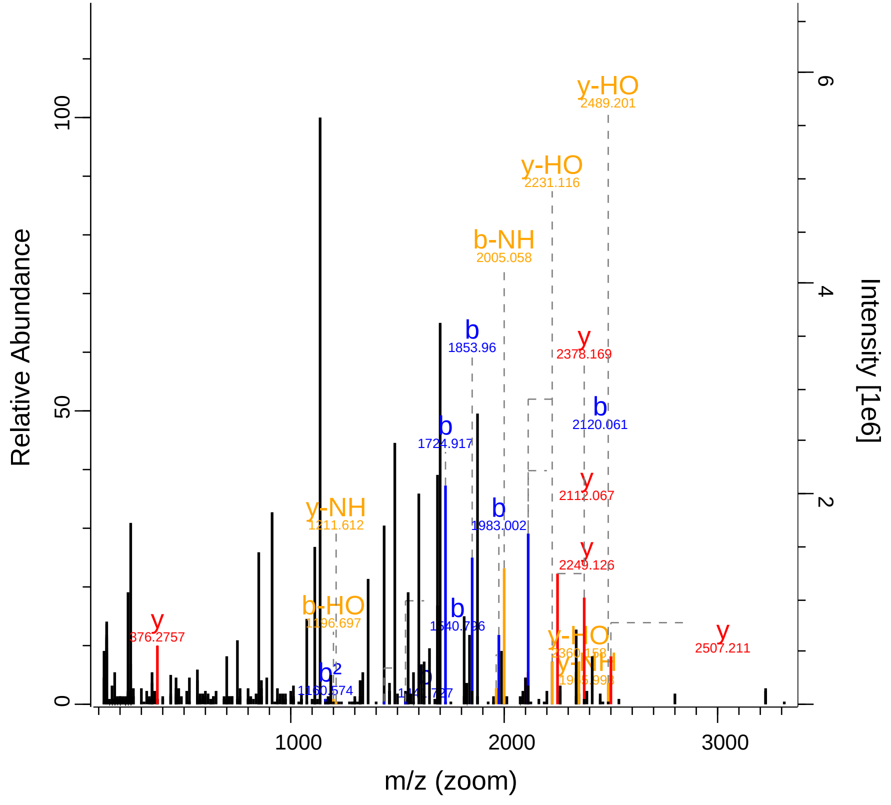

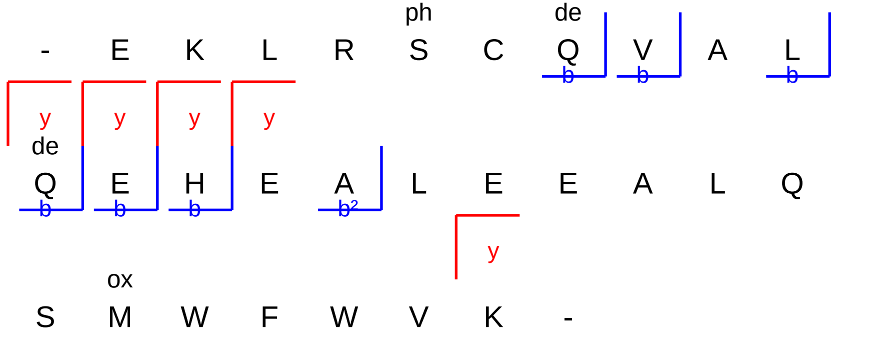

Raw File	Scan	Method	Score	m/z	Gene names
RO-QC-160121_ZackD_SS_Set_01_Fractio	32810	FTMS; HC	141.78	994.14	RTN4

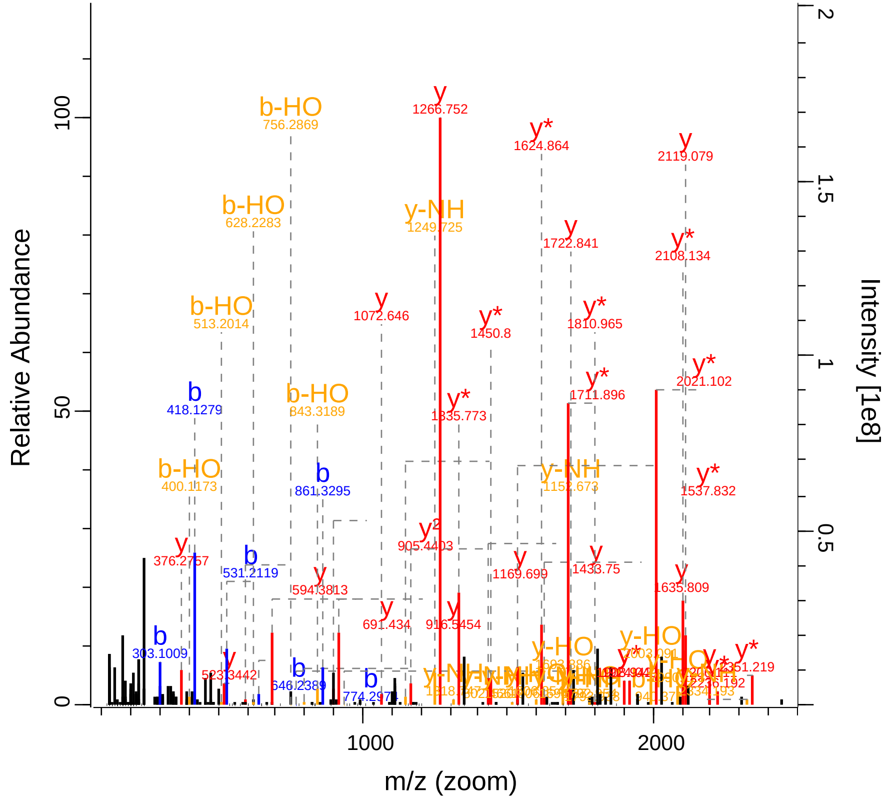

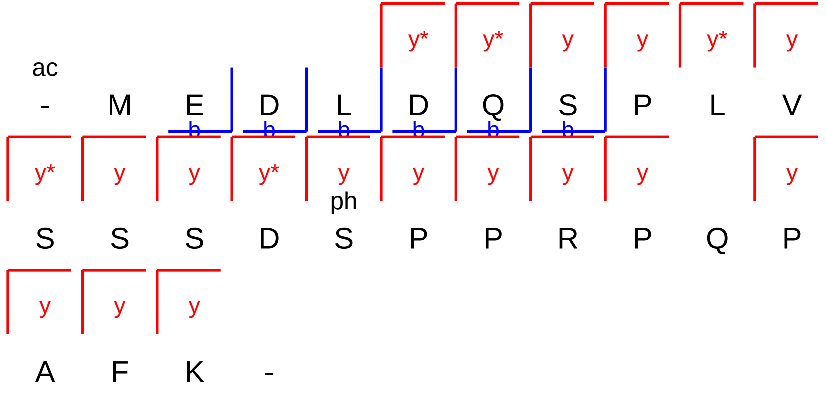

Raw File	Scan	Method	Score	m/z	Gene names
RO-QC-160121_ZackD_SS_Set_01_Fractic	32899	FTMS; HC	216.04	1490.71	RTN4

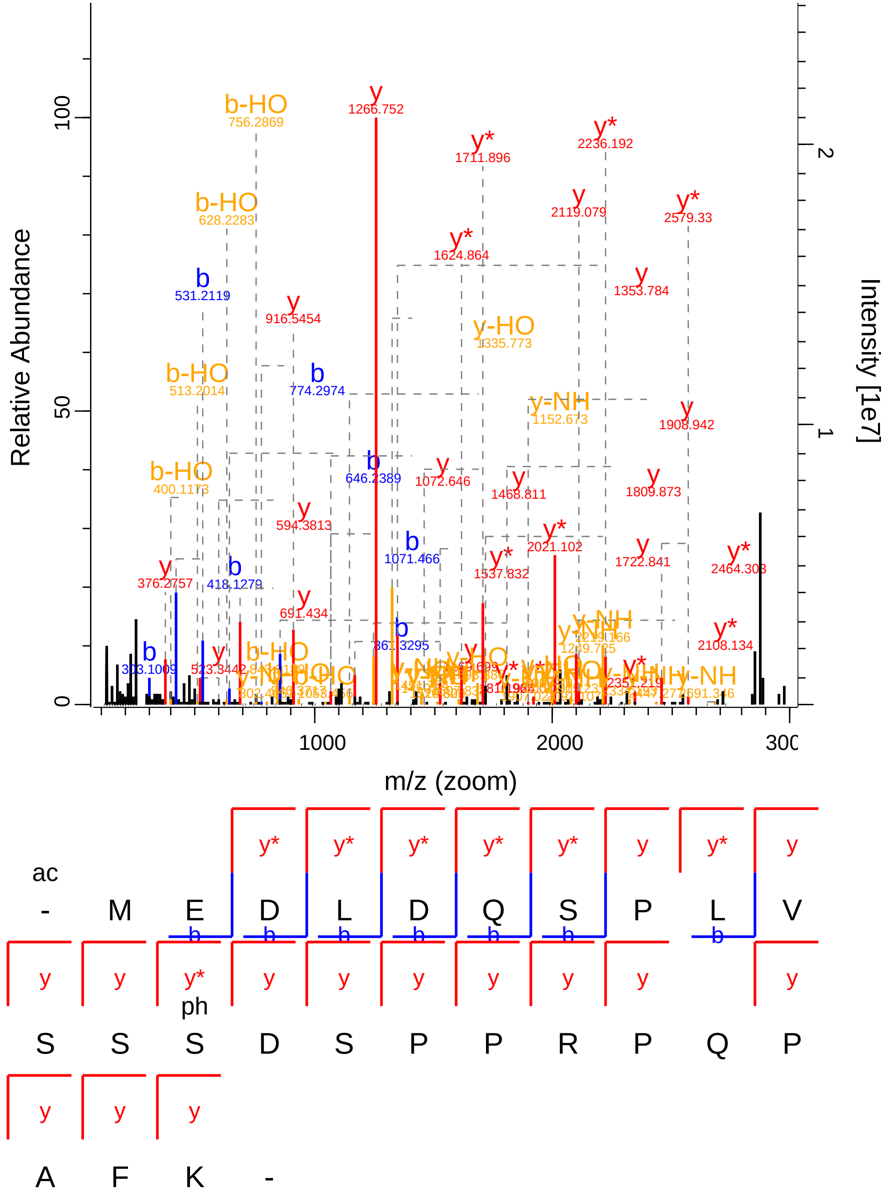

Raw File	Scan	Method	Score	m/z	Gene names
RO-QC-160121_ZackD_SS_Set_01_Fractio	33492	FTMS; HC	118.28	1271.15	DGKG

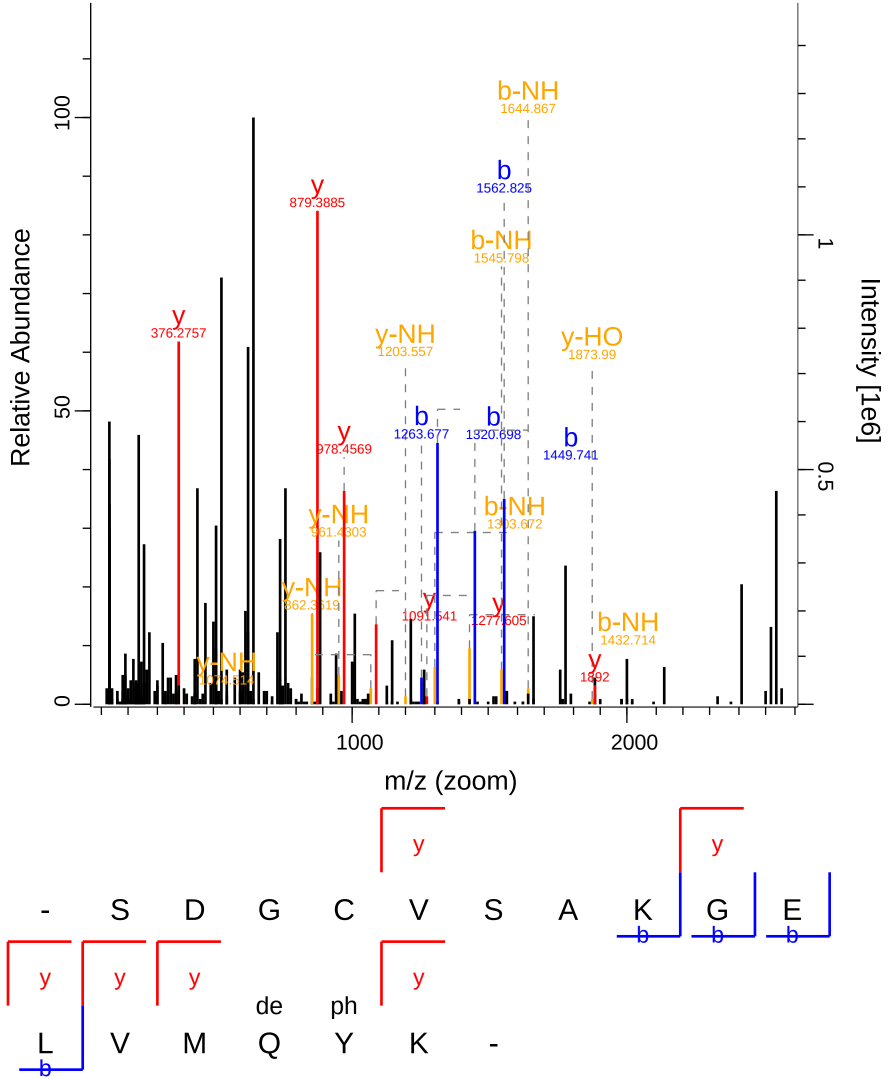

Raw File	Scan	Method	Score	m/z	Gene names
RO-QC-160121_ZackD_SS_Set_01_Fraction	35159	FTMS; HCI	40.5	1086	SMC3

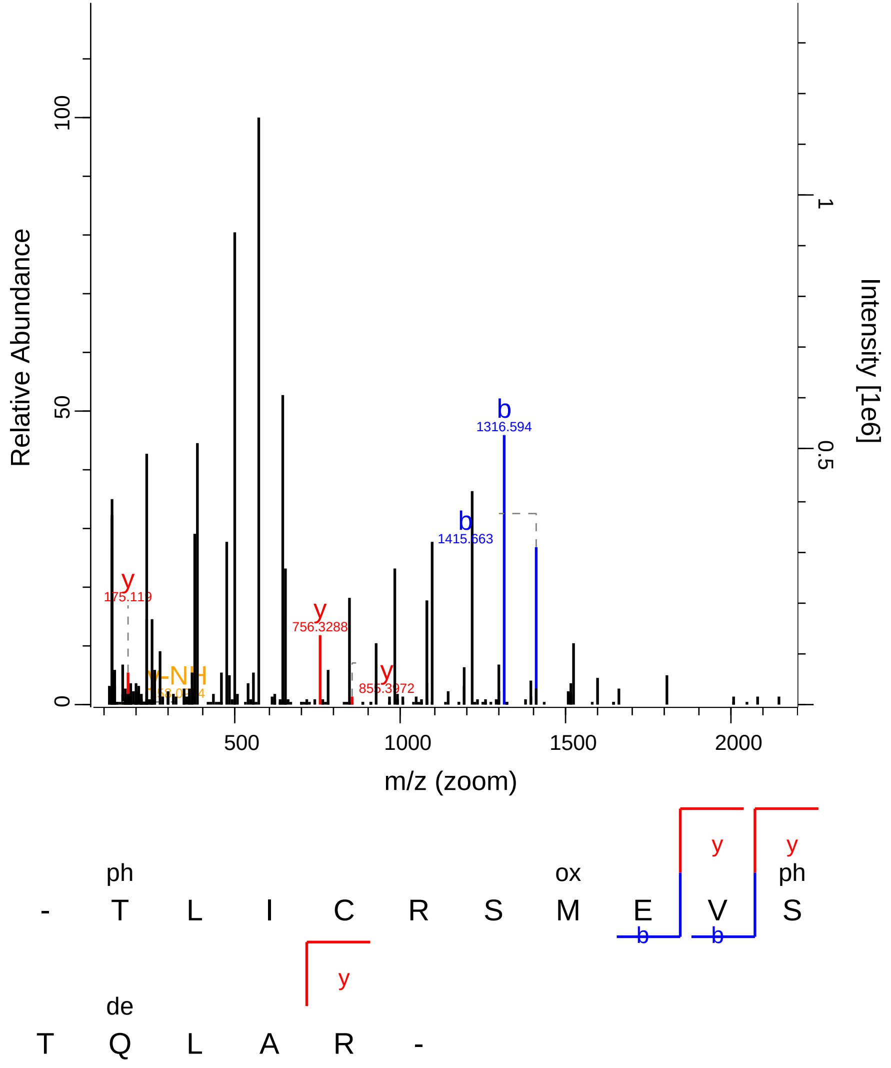

Raw File	Scan	Method	Score	m/z	Gene names
RO-QC-160121_ZackD_SS_Set_01_Fractio	36083	FTMS; HC	96.1	765.38	AKAP2

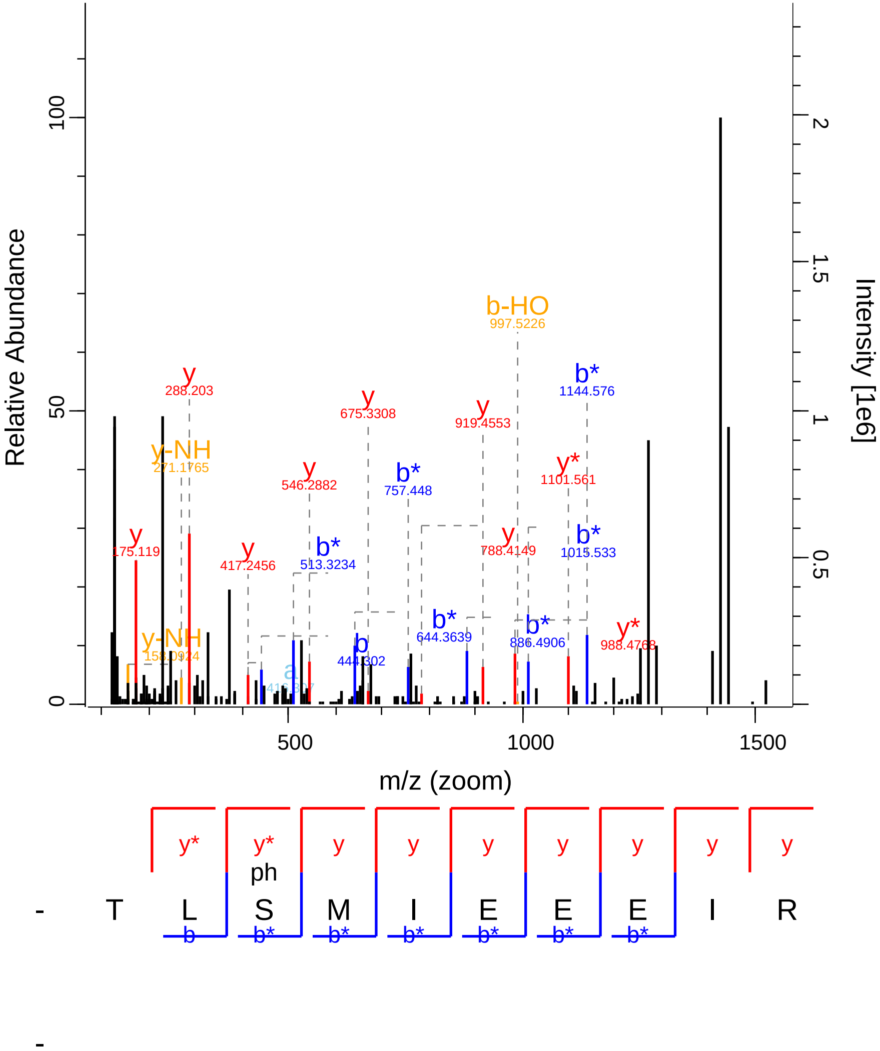

Raw File	Scan	Method	Score	m/z	Gene names
RO-QC-160121_ZackD_SS_Set_01_Fractic	36146	FTMS; HC	43.29	1023.04	SMG1

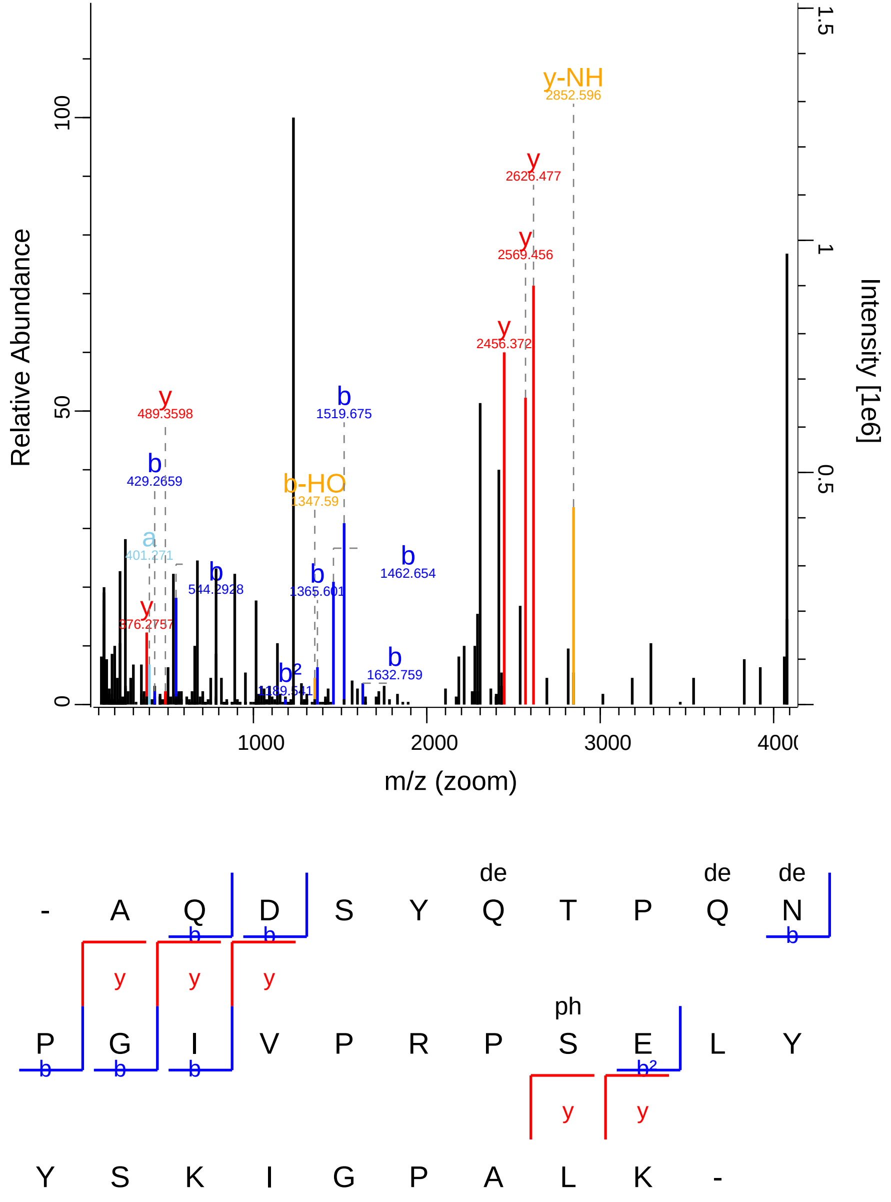

Raw File	Scan	Method	Score	m/z	Gene names
RO-QC-160121_ZackD_SS_Set_01_Fractio	36729	FTMS; HC	40.02	860.81	GNPNAT1

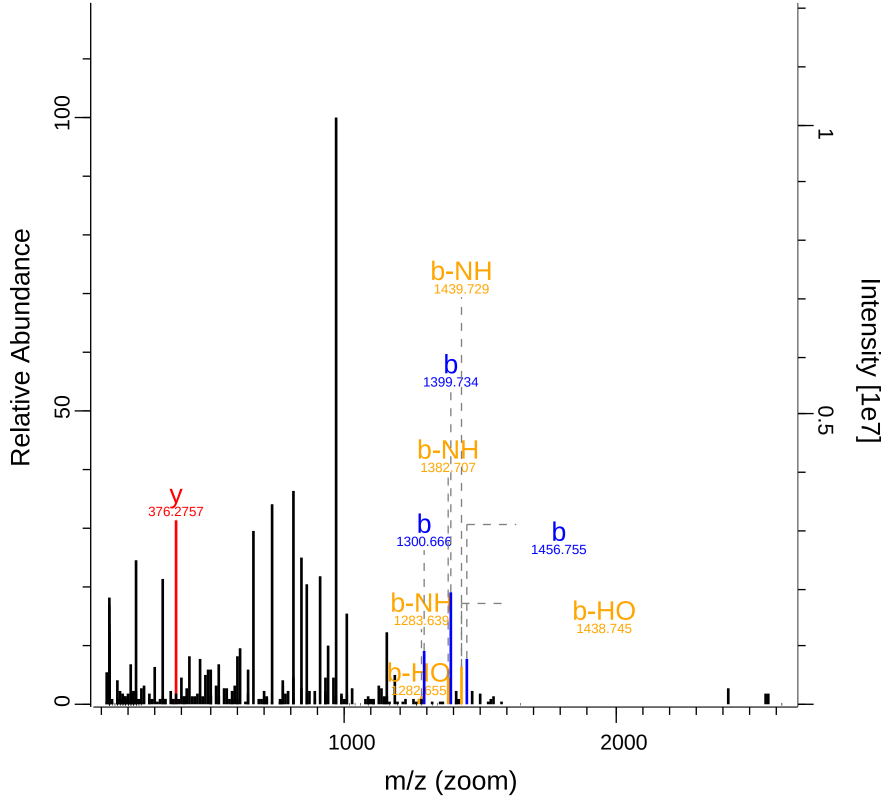

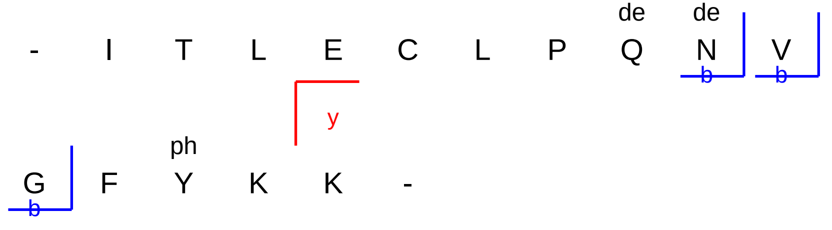

Raw File	Scan	Method	Score	m/z	Gene names
RO-QC-160121_ZackD_SS_Set_01_Fractio	38250	FTMS; HC	161.55	1381.12	TNKS1BP1

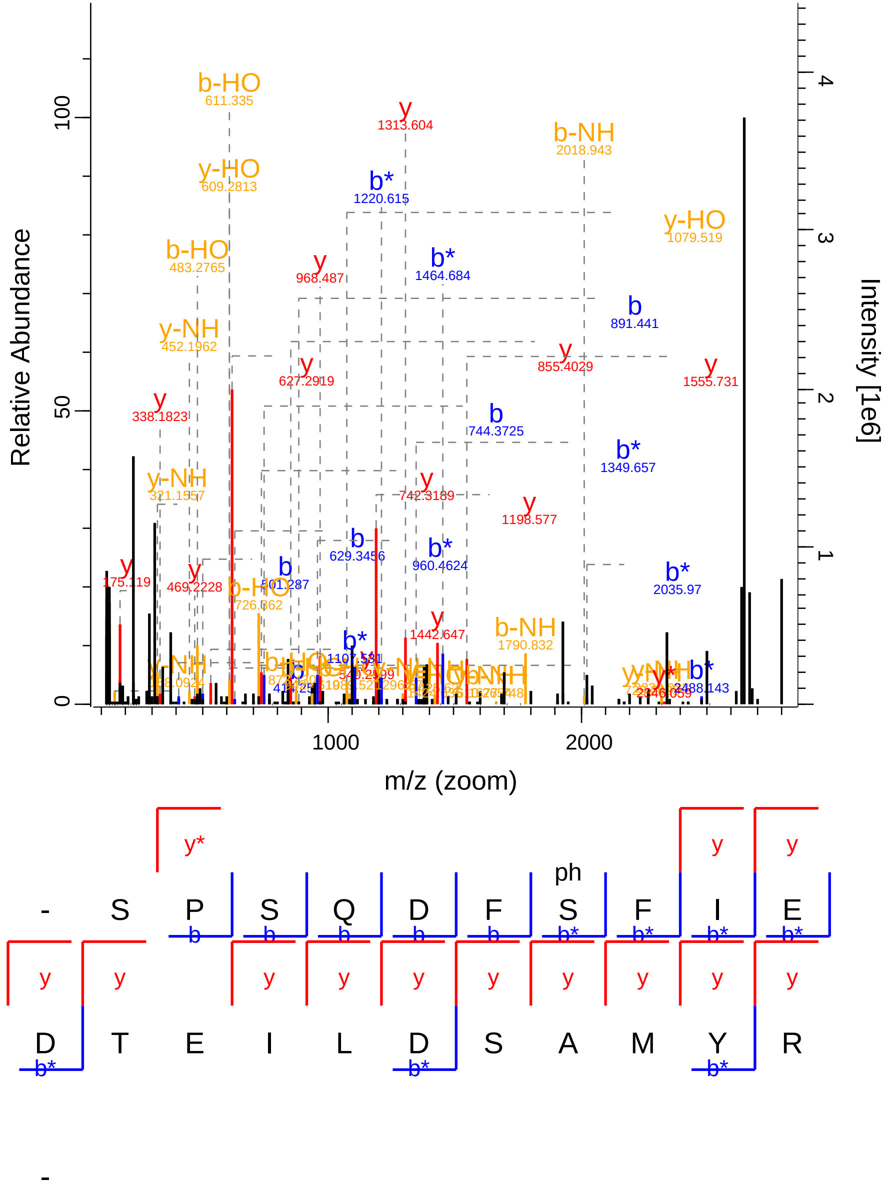

Raw File	Scan	Method	Score	m/z	Gene names
RO-QC-160121_ZackD_SS_Set_01_Fractior	38507	FTMS; HCI	40.96	998.5	ITGB6

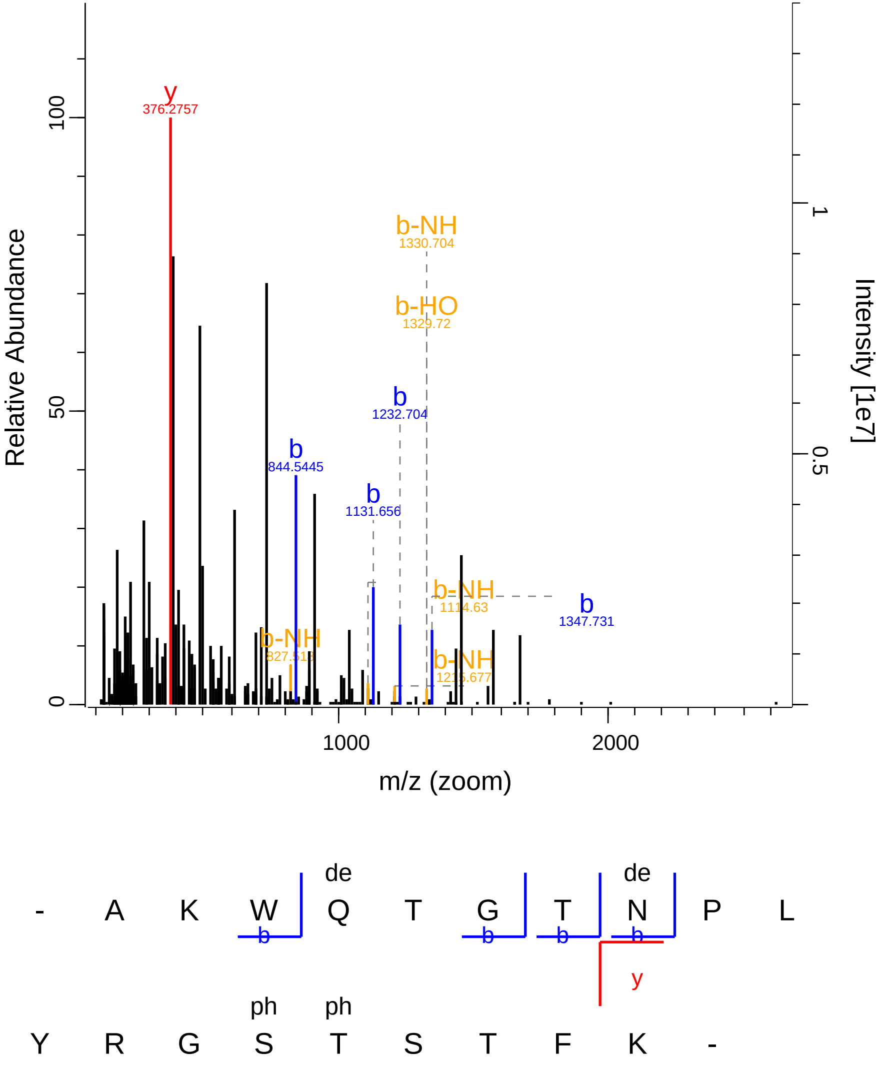

Raw File	Scan	Method	Score	m/z	Gene names
RO-QC-160121_ZackD_SS_Set_01_Fractio	38677	FTMS; HC	153.72	740.39	PSME3

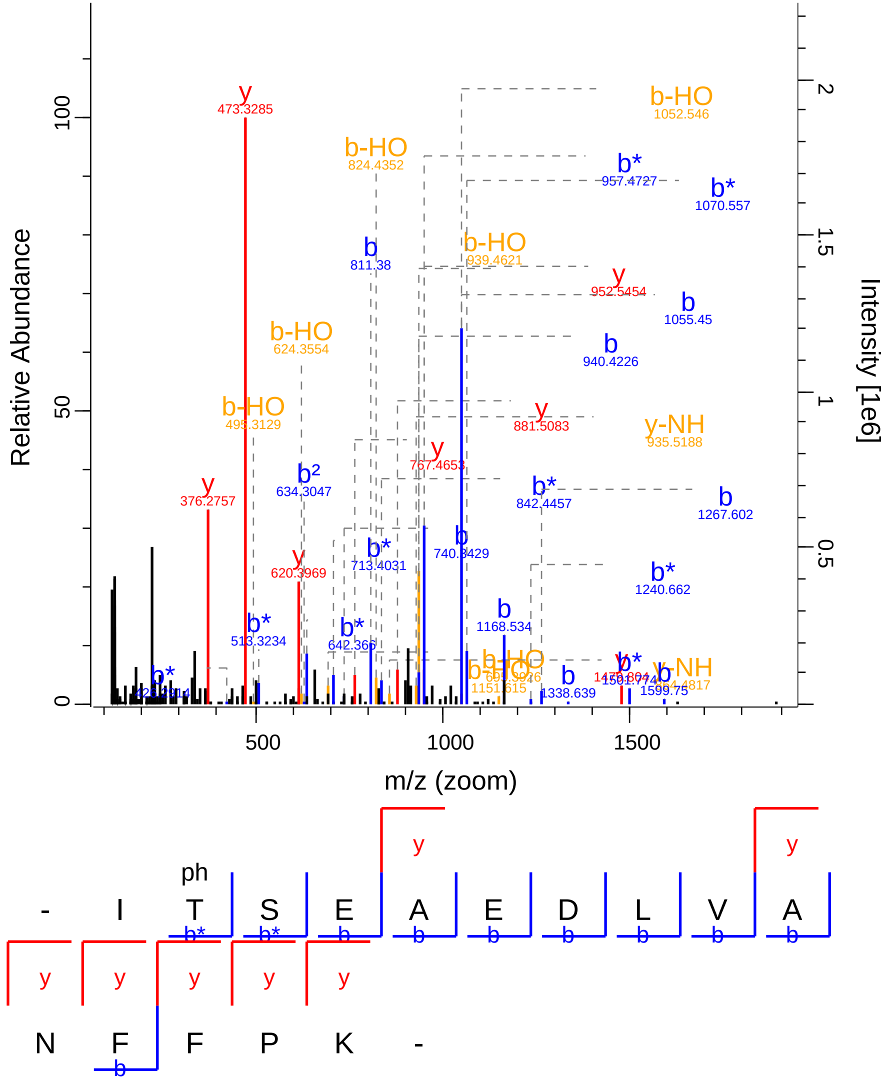

Raw File	Scan	Method	Score	m/z	Gene names
RO-QC-160121_ZackD_SS_Set_01_Fractic	38915	FTMS; HC	53.38	1476.74	DIP2C

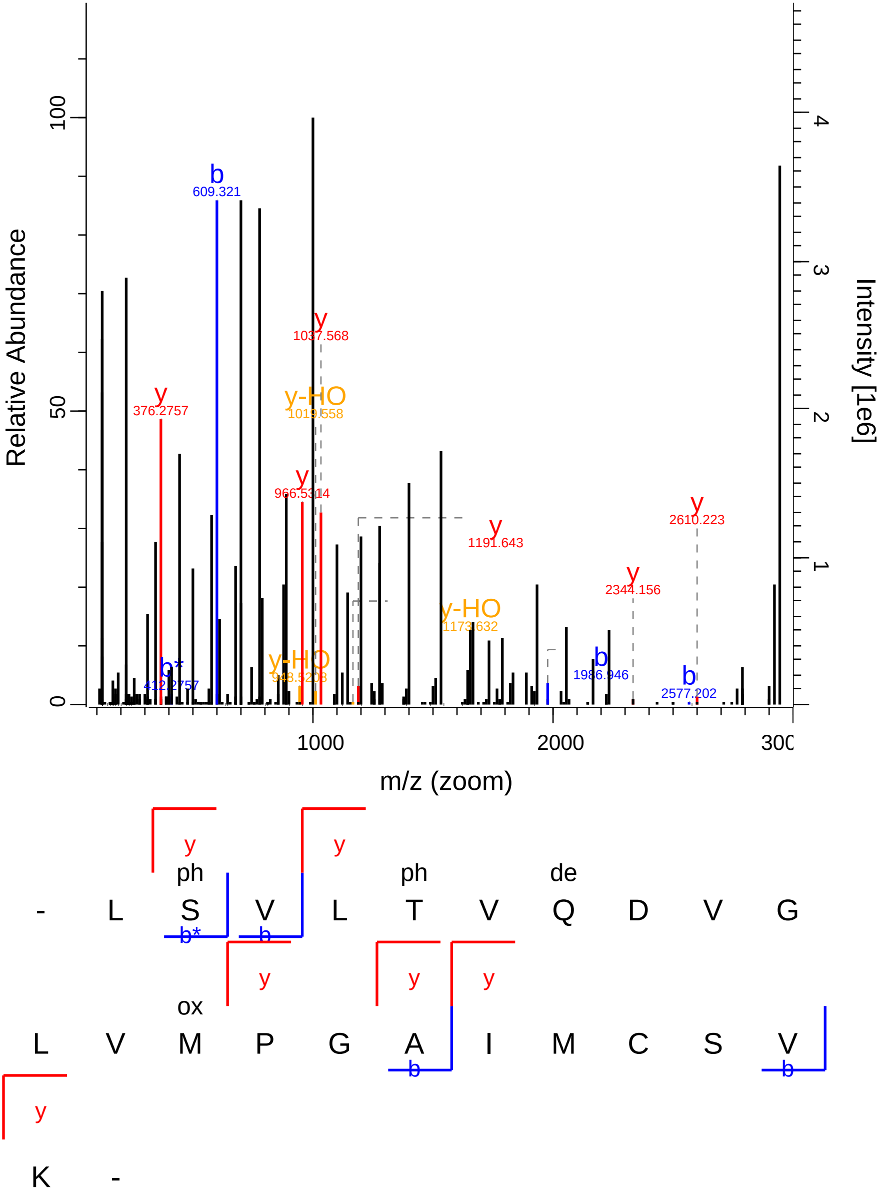

Raw File	Scan	Method	Score	m/z	Gene names
RO-QC-160121_ZackD_SS_Set_01_Fractior	5604	FTMS; HCI	45.7	791.08	NPM1

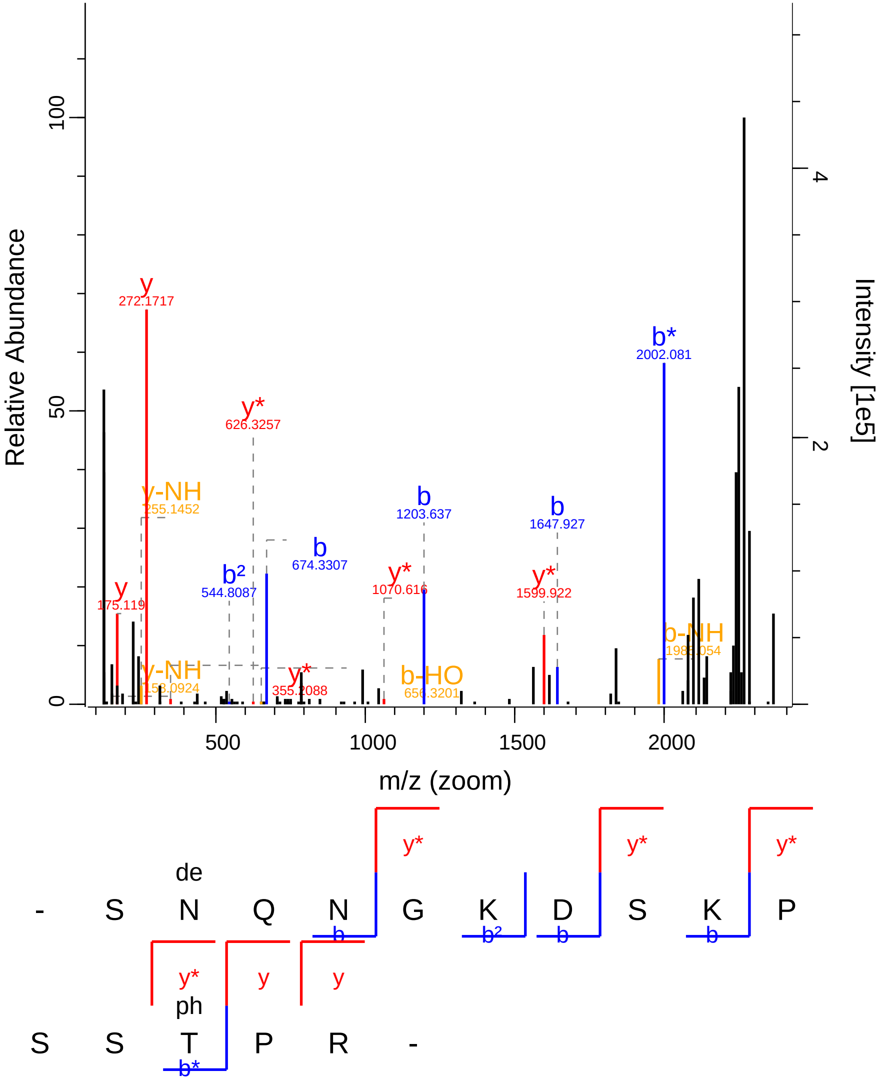

Raw File	Scan	Method	Score	m/z	Gene names
RO-QC-160121_ZackD_SS_Set_01_Fractio	6235	FTMS; HC	113.35	474.77	INTS1

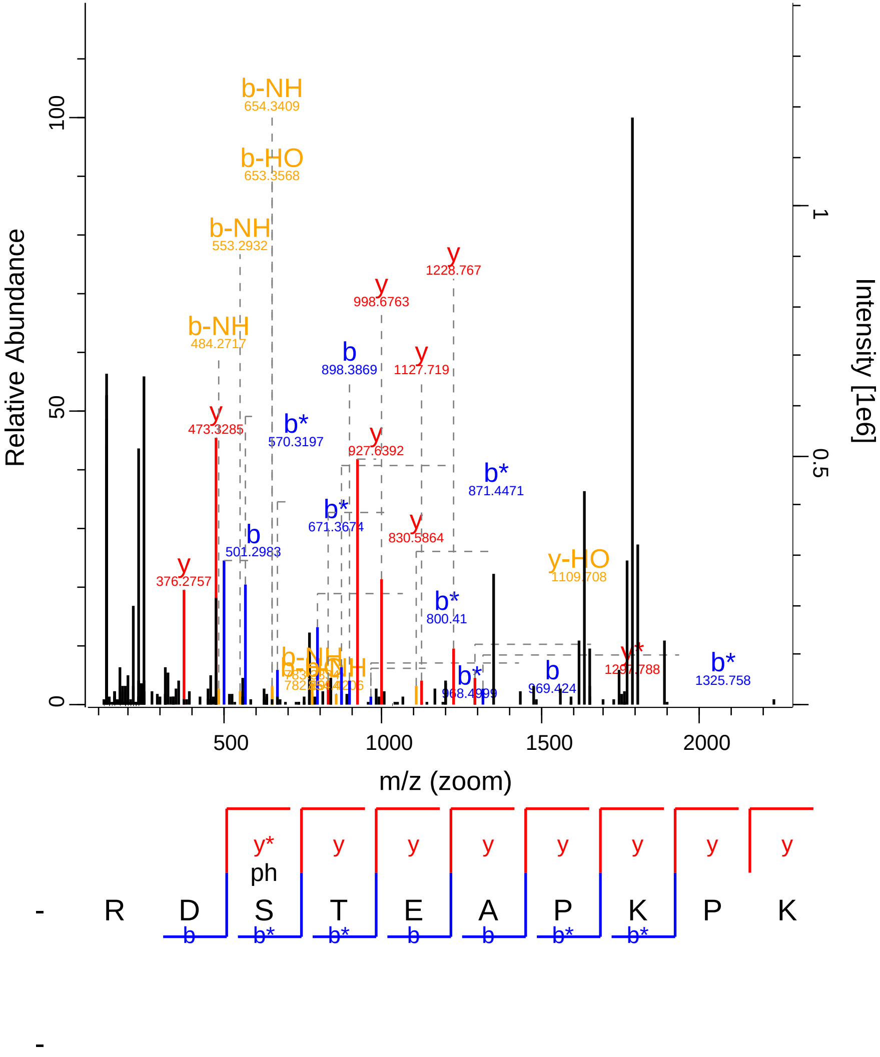

Raw File	Scan	Method	Score	m/z	Gene names
RO-QC-160121_ZackD_SS_Set_01_Fractio	6611	FTMS; HC	103.39	773.09	HTATSF1

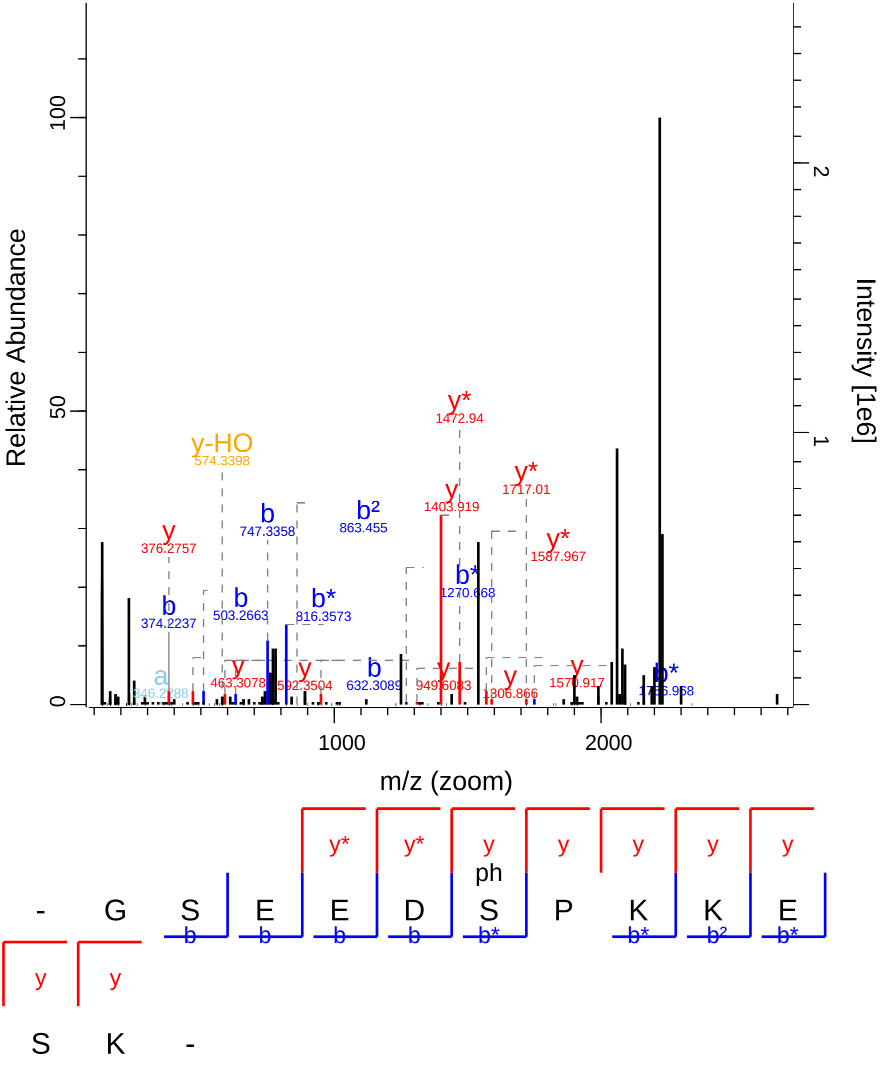

Raw File	Scan	Method	Score	m/z	Gene names
RO-QC-160121_ZackD_SS_Set_01_Fractio[illegible]	7305	FTMS; HC	108.75	741.05	CBX3

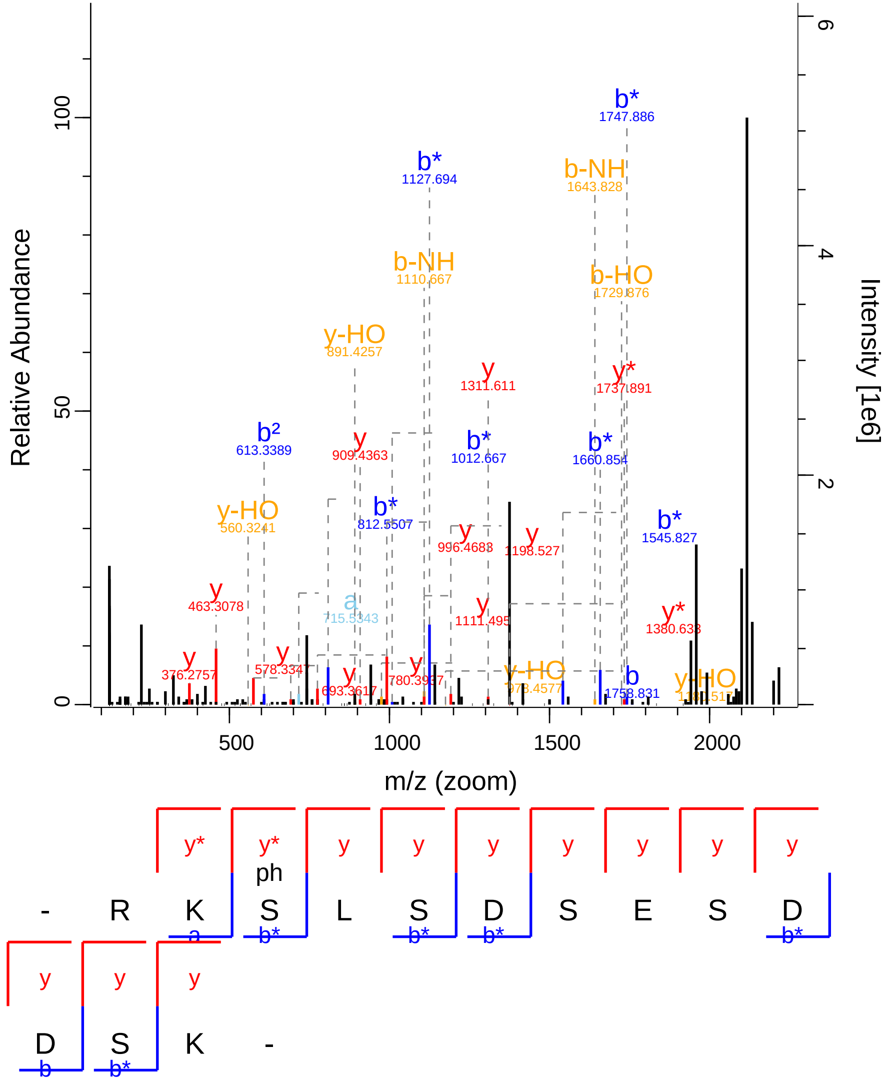

Raw File	Scan	Method	Score	m/z	Gene names
RO-QC-160121_ZackD_SS_Set_01_Fractio[illegible]	7328	FTMS; HC	122.33	877.49	SEC62

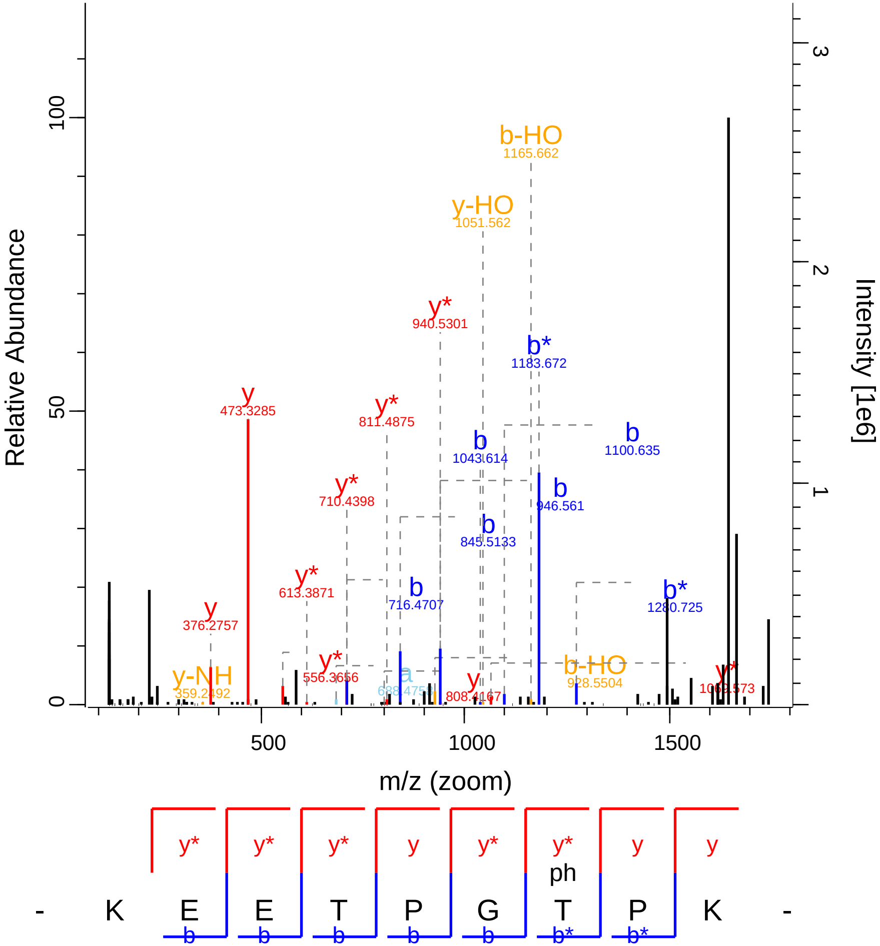

Raw File	Scan	Method	Score	m/z	Gene names
RO-QC-160121_ZackD_SS_Set_01_Fractior	7330	FTMS; HCI	50.68	714.07	SRRM1

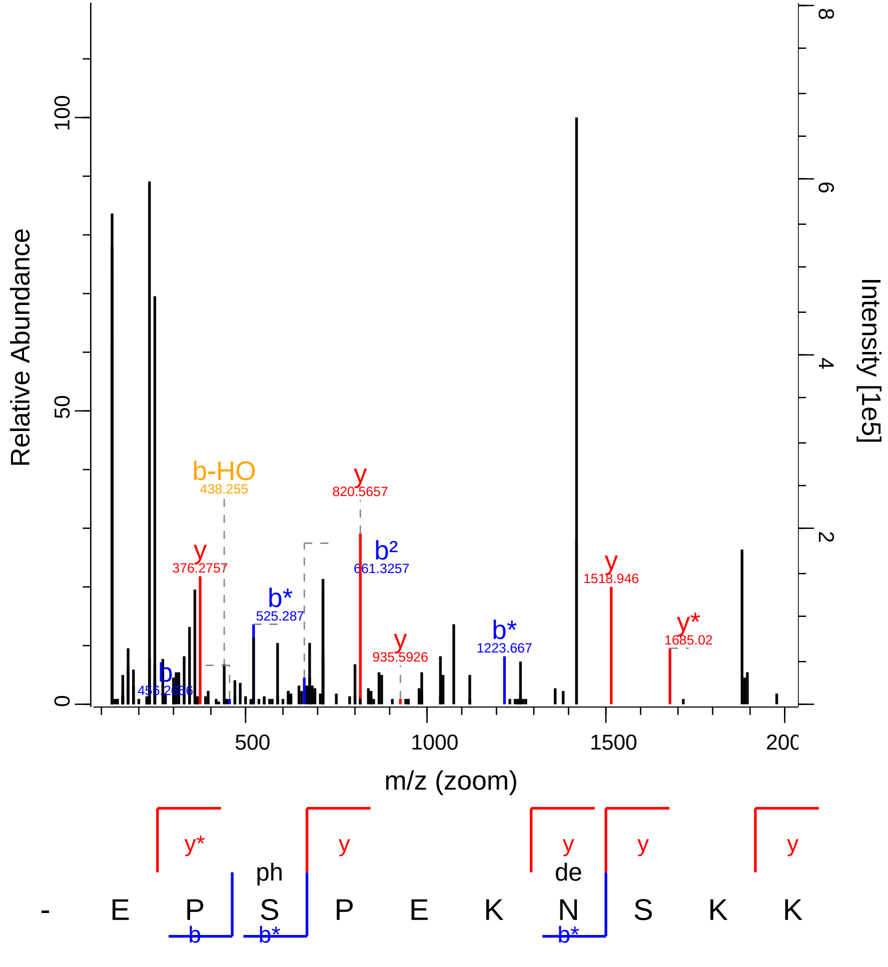

Raw File	Scan	Method	Score	m/z	Gene names
RO-QC-160121_ZackD_SS_Set_01_Fractio	11233	FTMS; HC	58.37	661.36	CAD

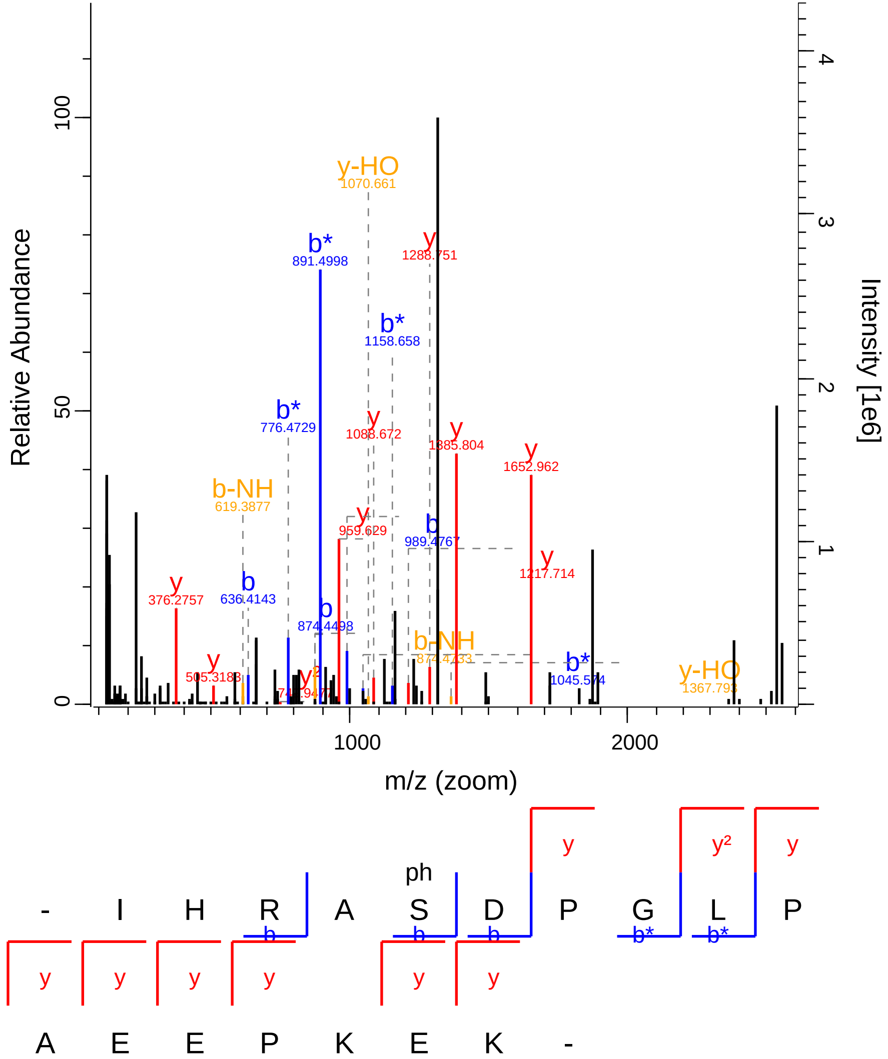

Raw File	Scan	Method	Score	m/z	Gene names
RO-QC-160121_ZackD_SS_Set_01_Fractio	11630	FTMS; HC	102.48	610.72	NUCKS1

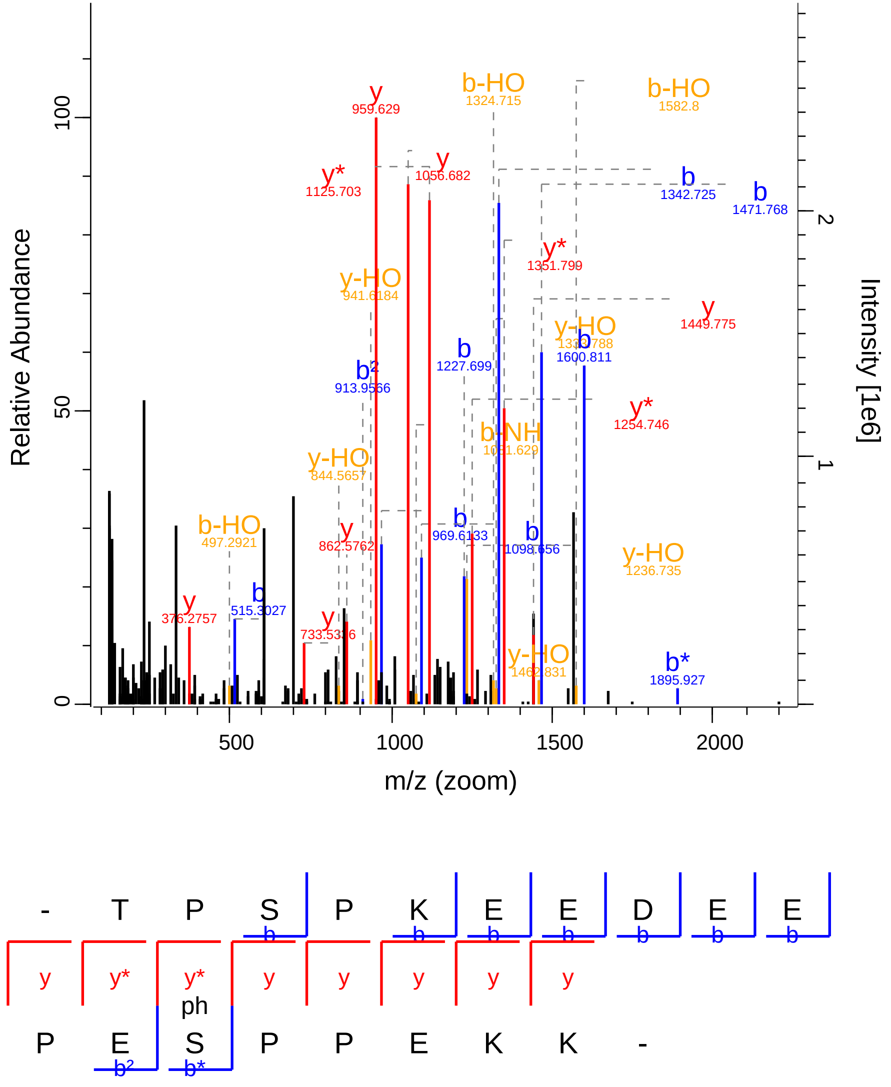

Raw File	Scan	Method	Score	m/z
RO-QC-160121_ZackD_SS_Set_01_Fraction_7	12214	FTMS; HCD	53.47	755.37

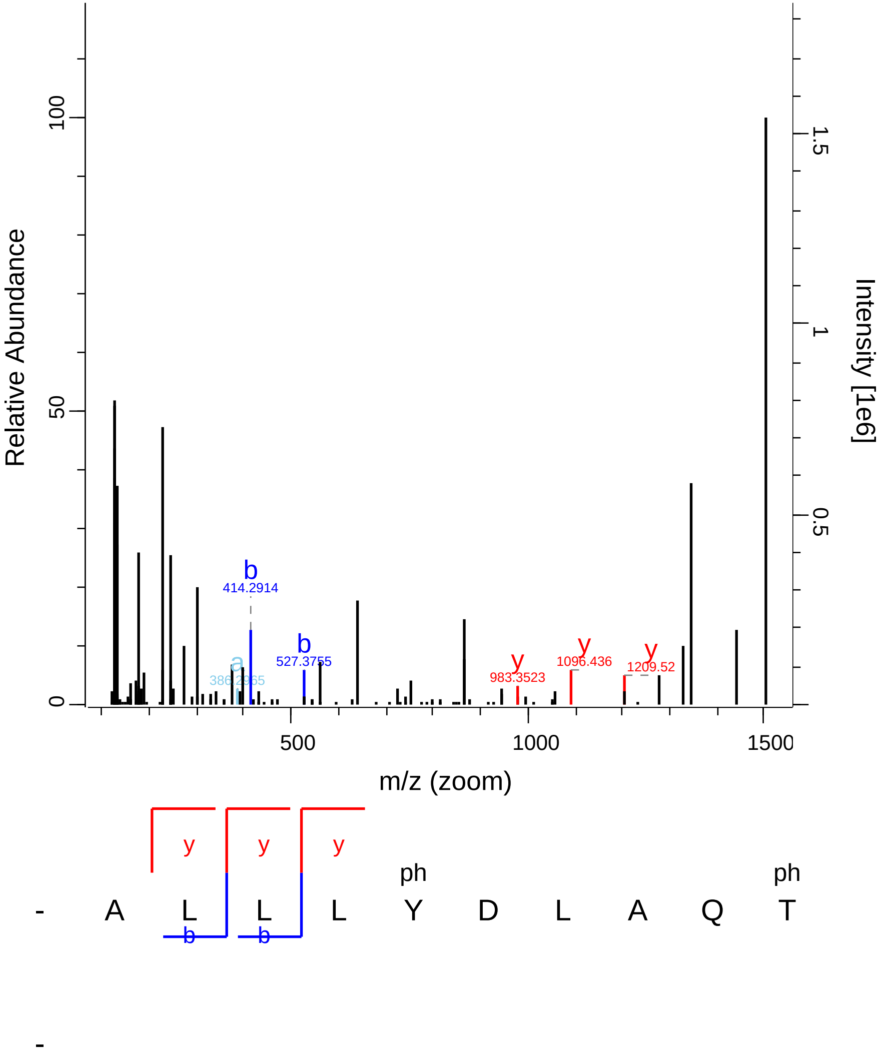

Raw File	Scan	Method	Score	m/z	Gene names
RO-QC-160121_ZackD_SS_Set_01_Fractio	12508	FTMS; HC	105.39	671.37	ADD1

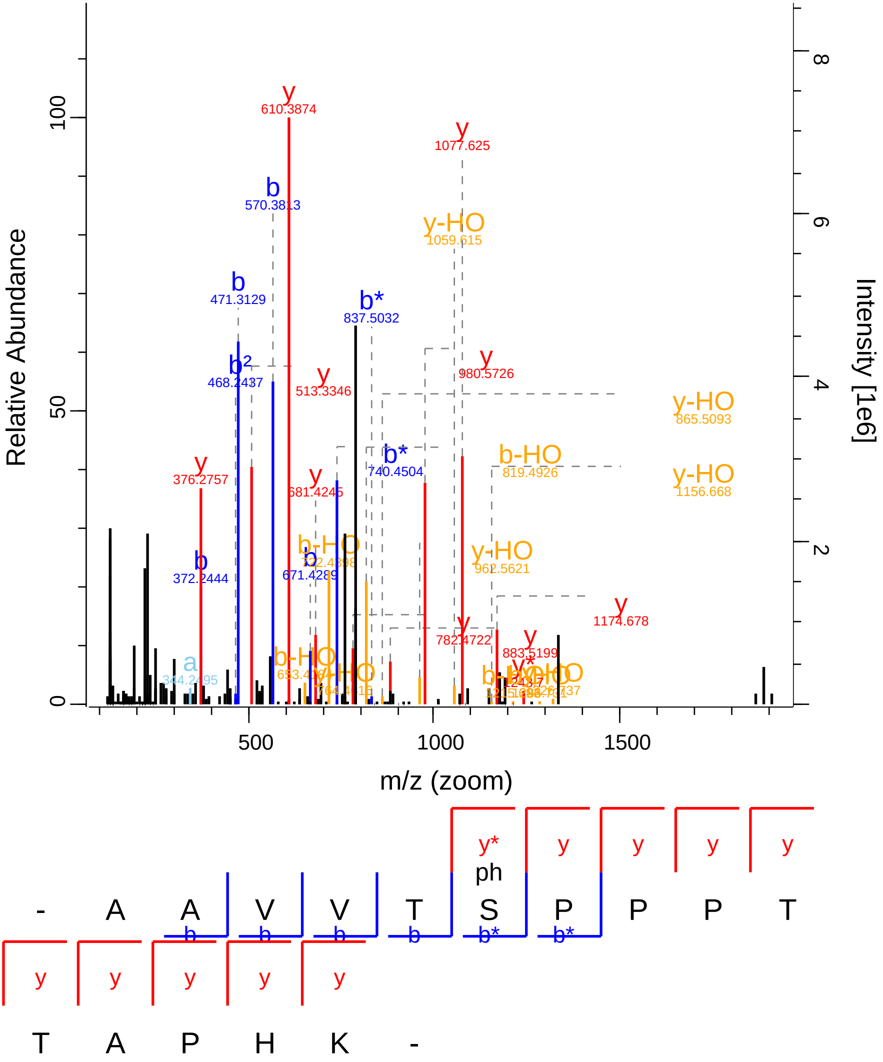

Raw File	Scan	Method	Score	m/z	Gene names
RO-QC-160121_ZackD_SS_Set_01_Fractio	13594	FTMS; HC	61.96	377.21	ZNF224

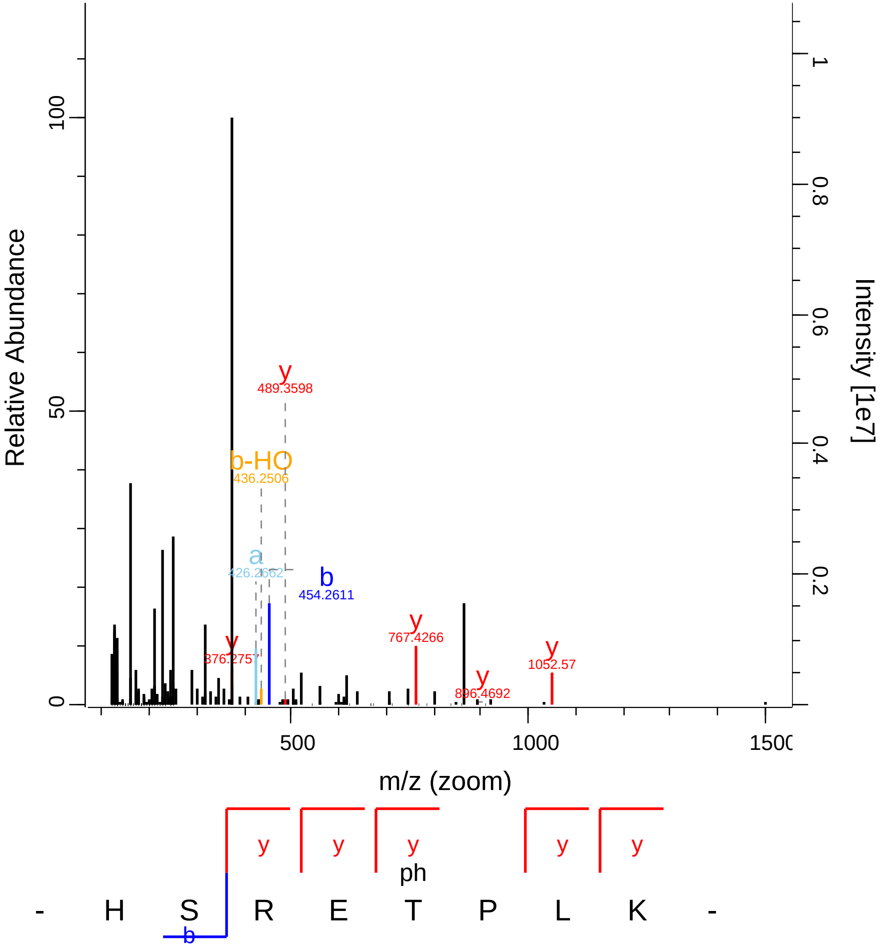

Raw File	Scan	Method	Score	m/z	Gene names
RO-QC-160121_ZackD_SS_Set_01_Fractio	15956	FTMS; HC	69.12	823.45	LAYN

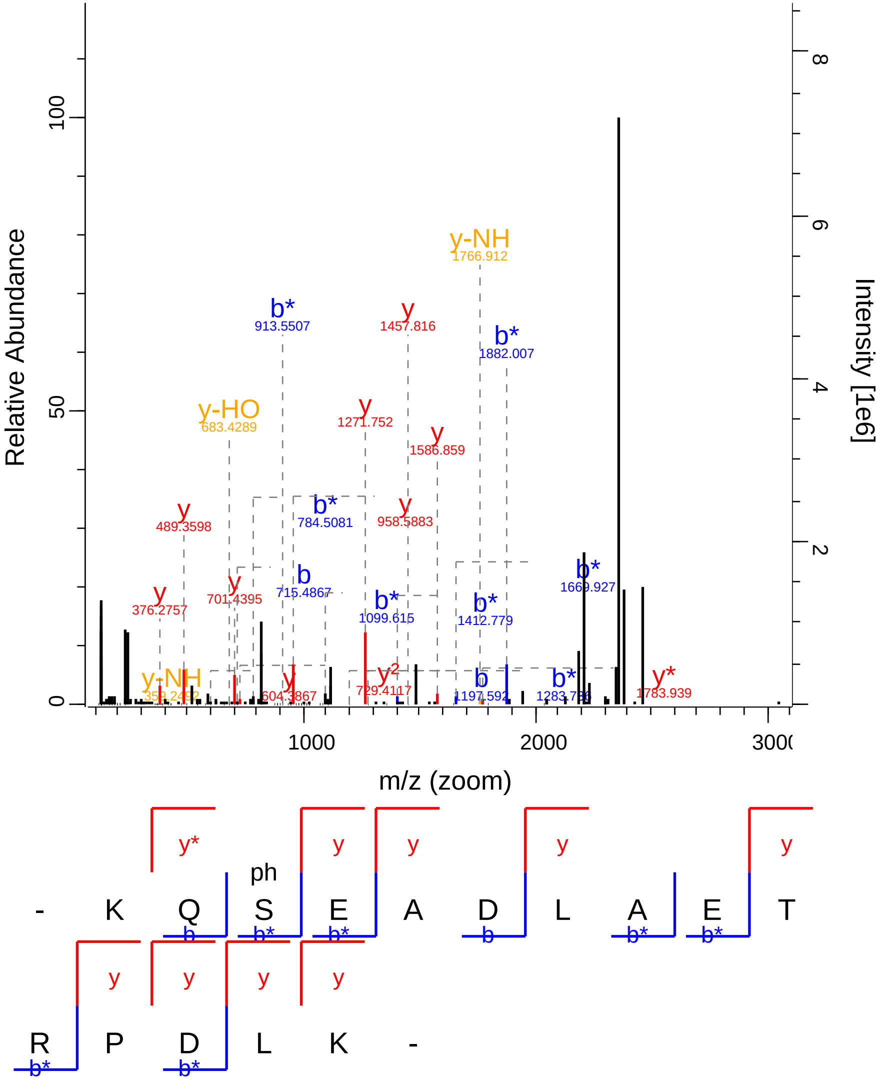

Raw File	Scan	Method	Score	m/z	Gene names
RO-QC-160121_ZackD_SS_Set_01_Fractio	18208	FTMS; HC	68.66	971.02	KSR2

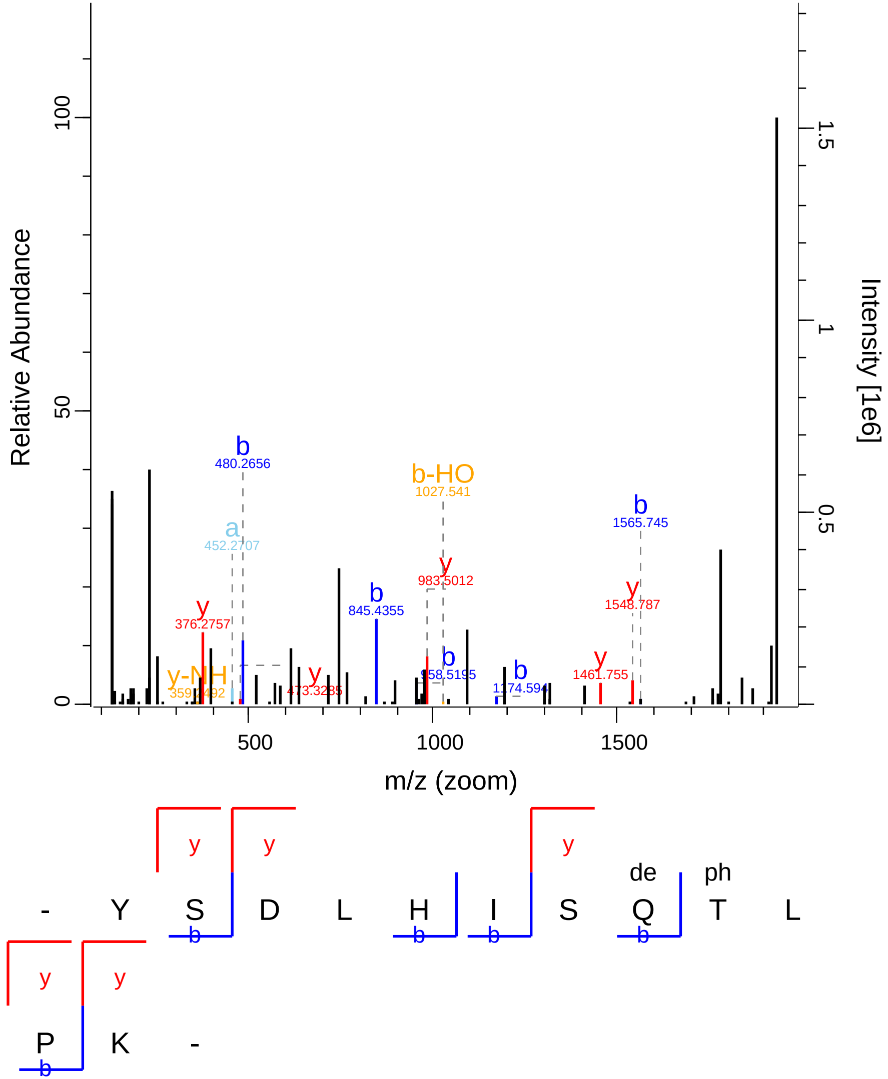

Raw File	Scan	Method	Score	m/z	Gene names
RO-QC-160121_ZackD_SS_Set_01_Fractio	18472	FTMS; HC	76.82	635.85	MAP4

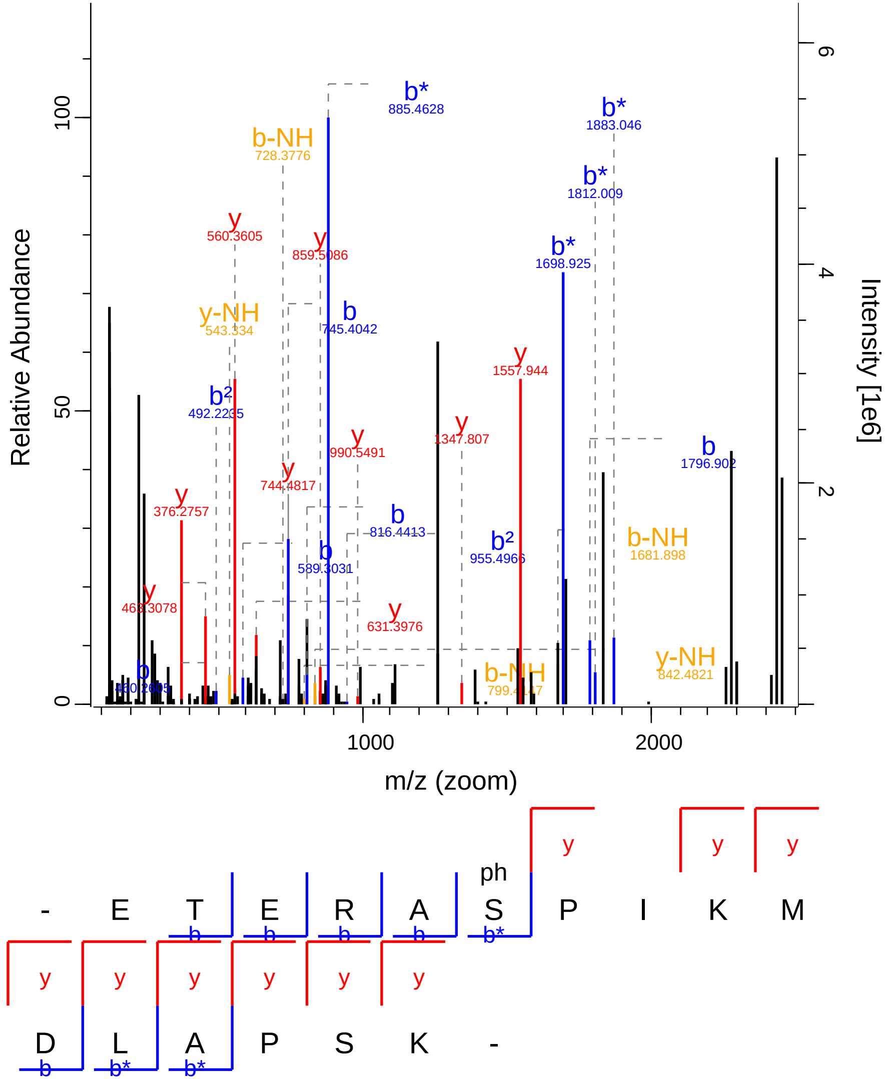

Raw File	Scan	Method	Score	m/z	Gene names
RO-QC-160121_ZackD_SS_Set_01_Fractio	18551	FTMS; HC	84.93	833.78	UBAP2L

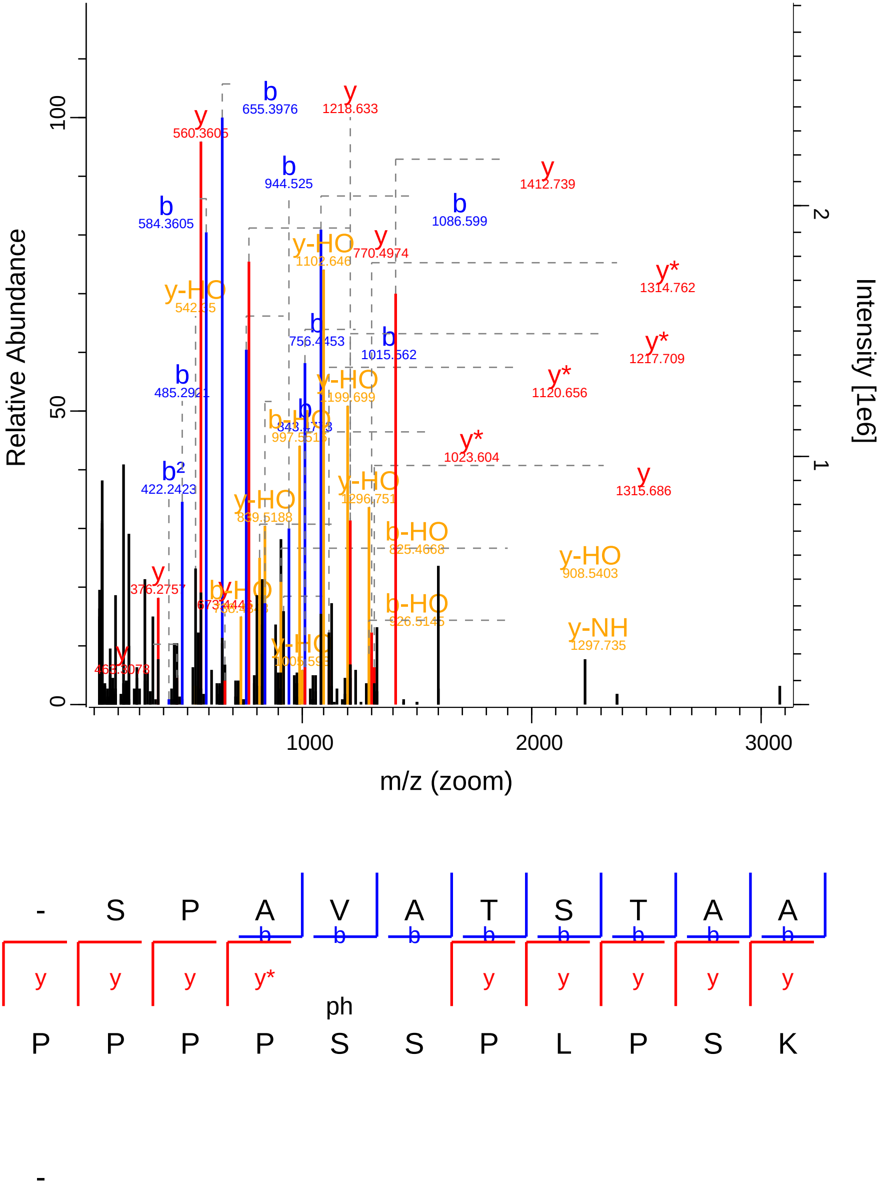

Raw File	Scan	Method	Score	m/z	Gene names
RO-QC-160121_ZackD_SS_Set_01_Fractio	20310	FTMS; HC	88.94	752.74	SORBS2

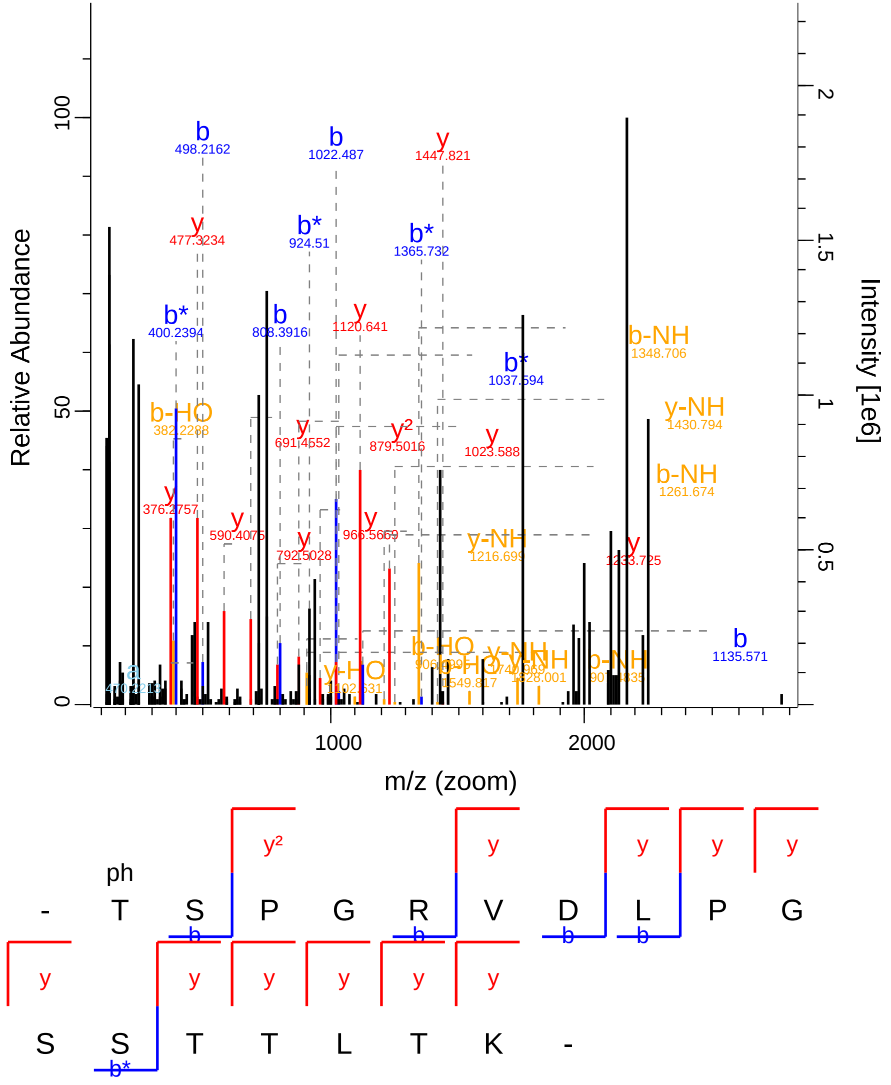

Raw File	Scan	Method	Score	m/z	Gene names
RO-QC-160121_ZackD_SS_Set_01_Fractio	20350	FTMS; HC	206.46	869.43	VIM

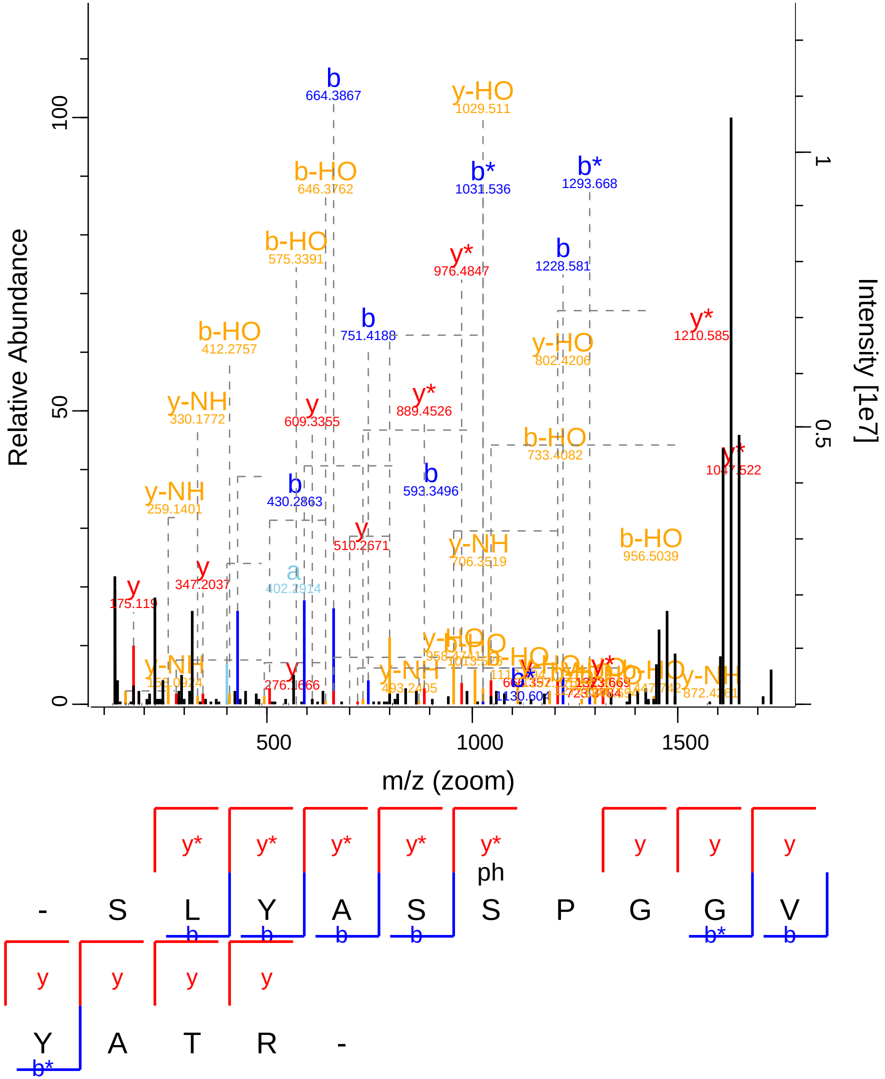

Raw File	Scan	Method	Score	m/z	Gene names
RO-QC-160121_ZackD_SS_Set_01_Fractic	20382	FTMS; HC	76.14	1134.08	SPTLC3

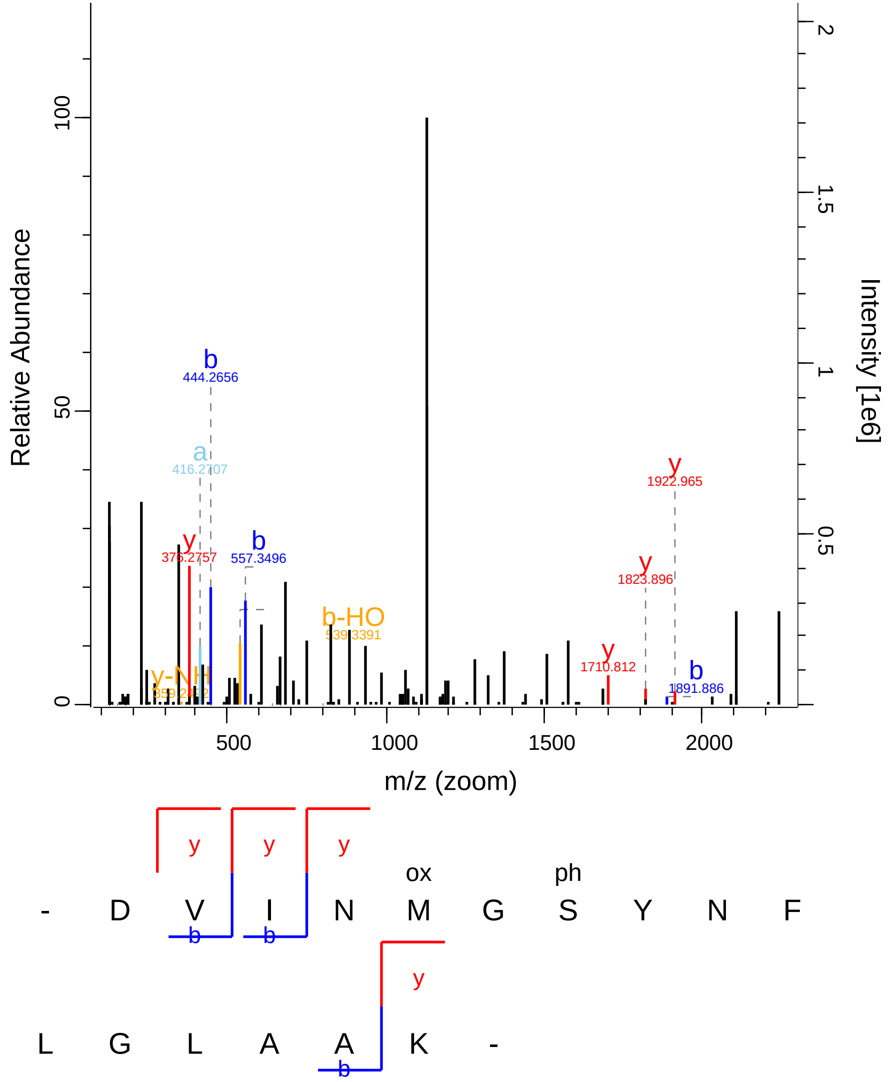

Raw File	Scan	Method	Score	m/z	Gene names
RO-QC-160121_ZackD_SS_Set_01_Fractio	20383	FTMS; HC	41.19	829.56	RECQL5

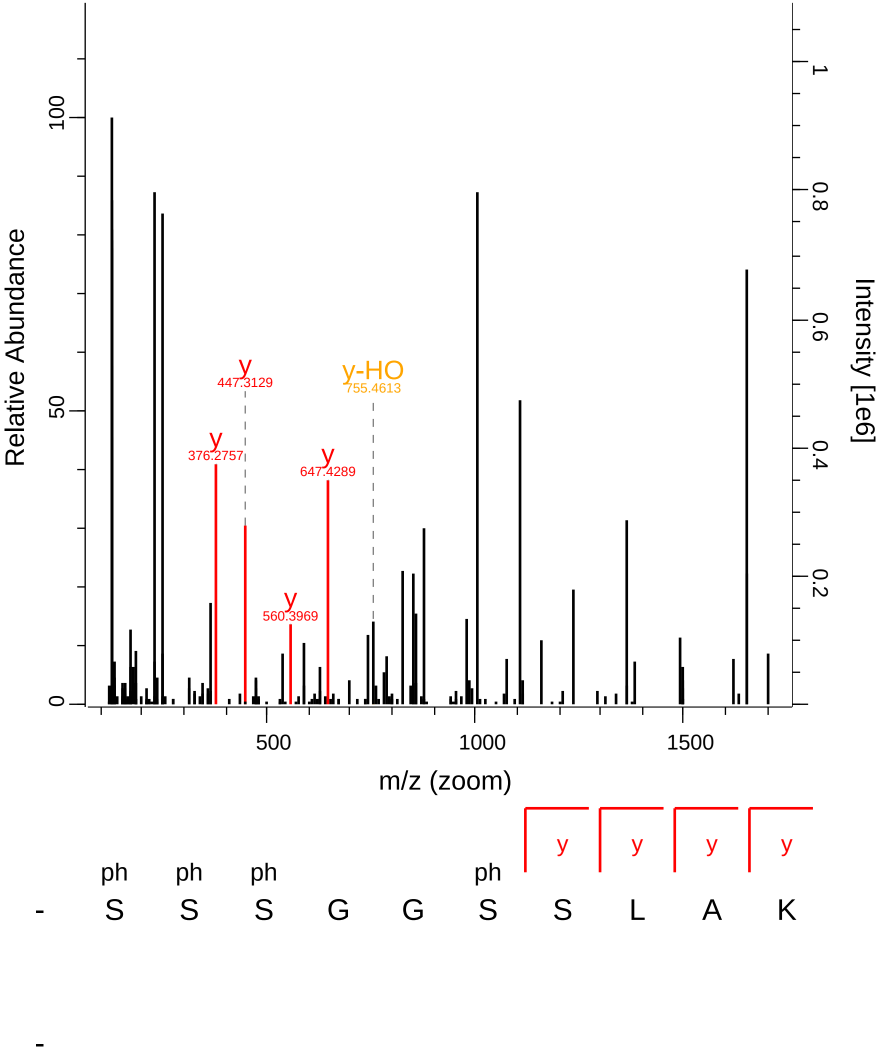

Raw File	Scan	Method	Score	m/z	Gene names
RO-QC-160121_ZackD_SS_Set_01_Fractic	21866	FTMS; HC	64.65	1154.63	PRC1

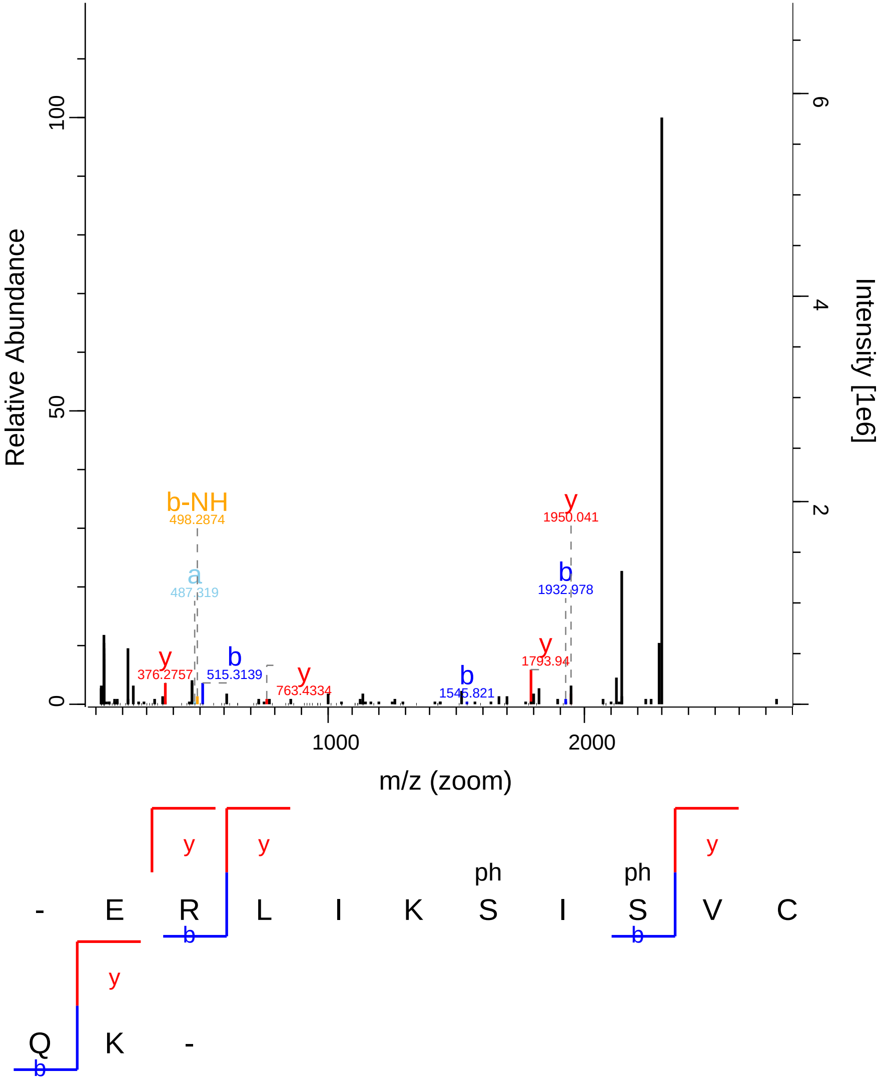

Raw File	Scan	Method	Score	m/z	Gene names
RO-QC-160121_ZackD_SS_Set_01_Fractio	22651	FTMS; HC	191.01	981.03	MAP1B

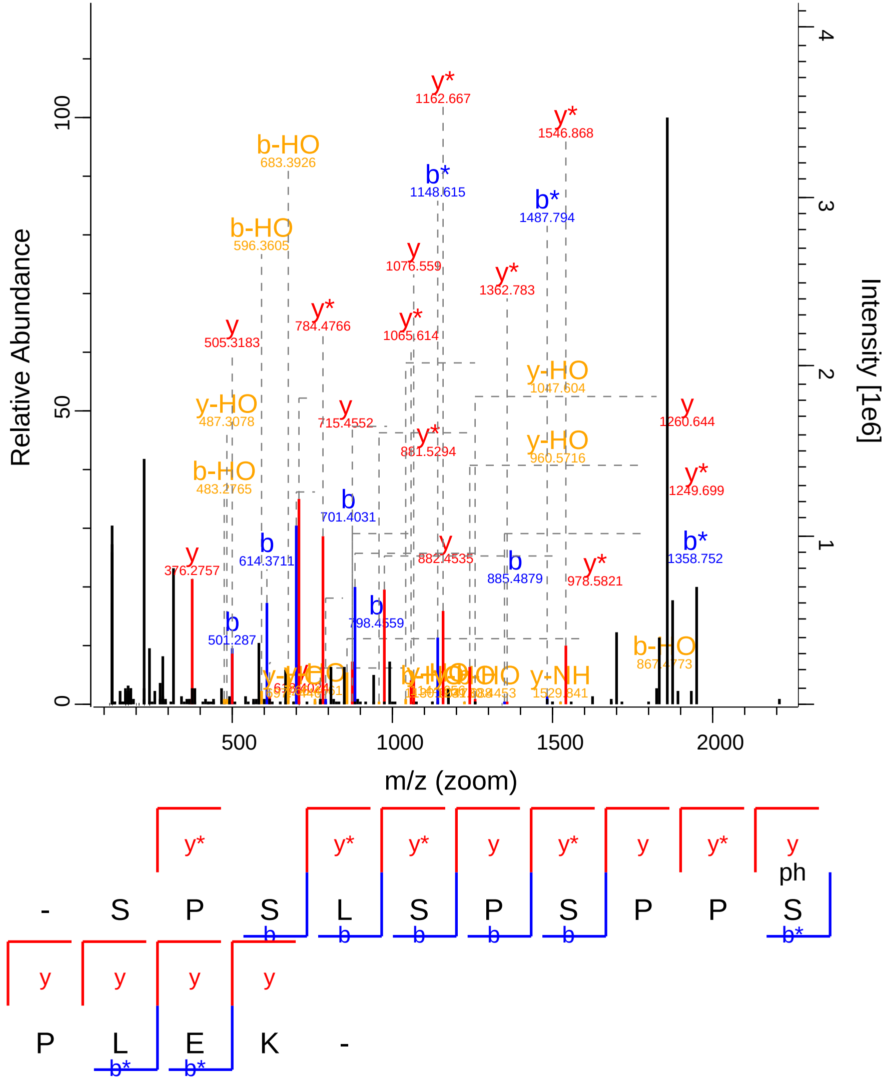

Raw File	Scan	Method	Score	m/z	Gene names
RO-QC-160121_ZackD_SS_Set_01_Fractic	24456	FTMS; HC	50.56	1198.58	CCDC110

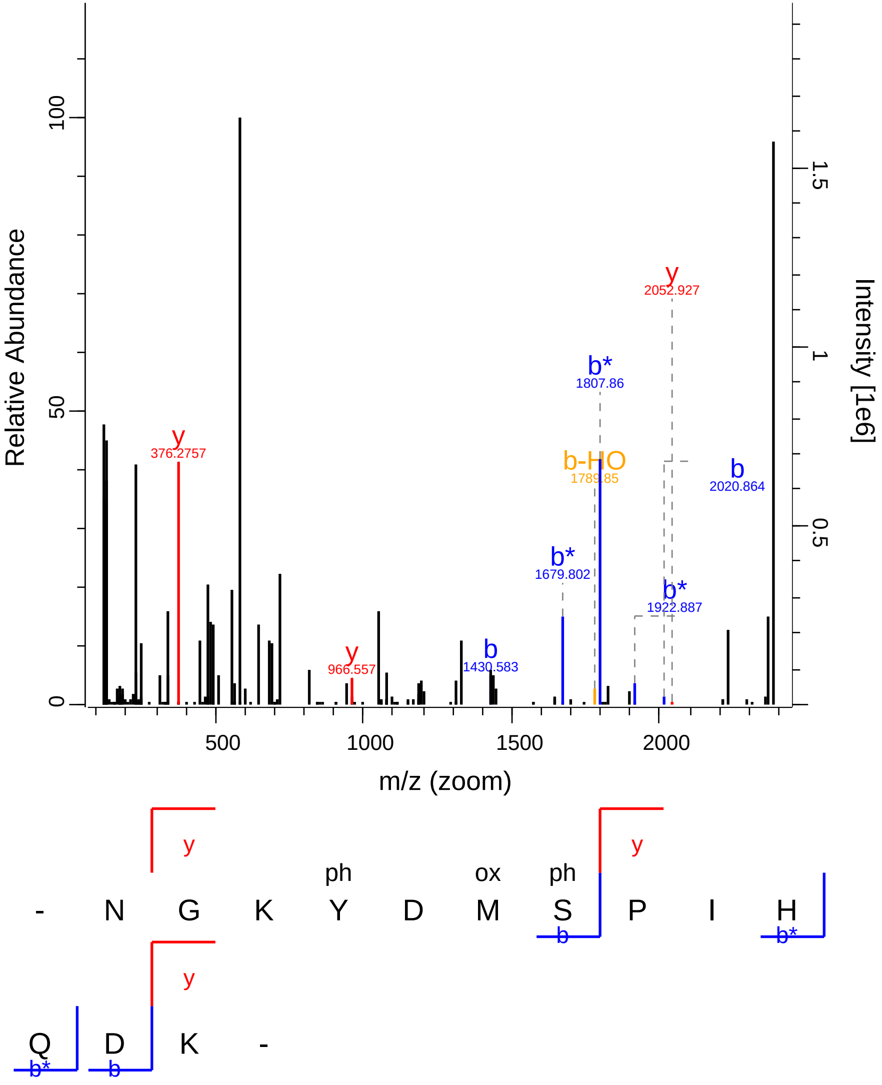

Raw File	Scan	Method	Score	m/z	Gene names
RO-QC-160121_ZackD_SS_Set_01_Fractio	27271	FTMS; HC	40.43	873.69	TTN

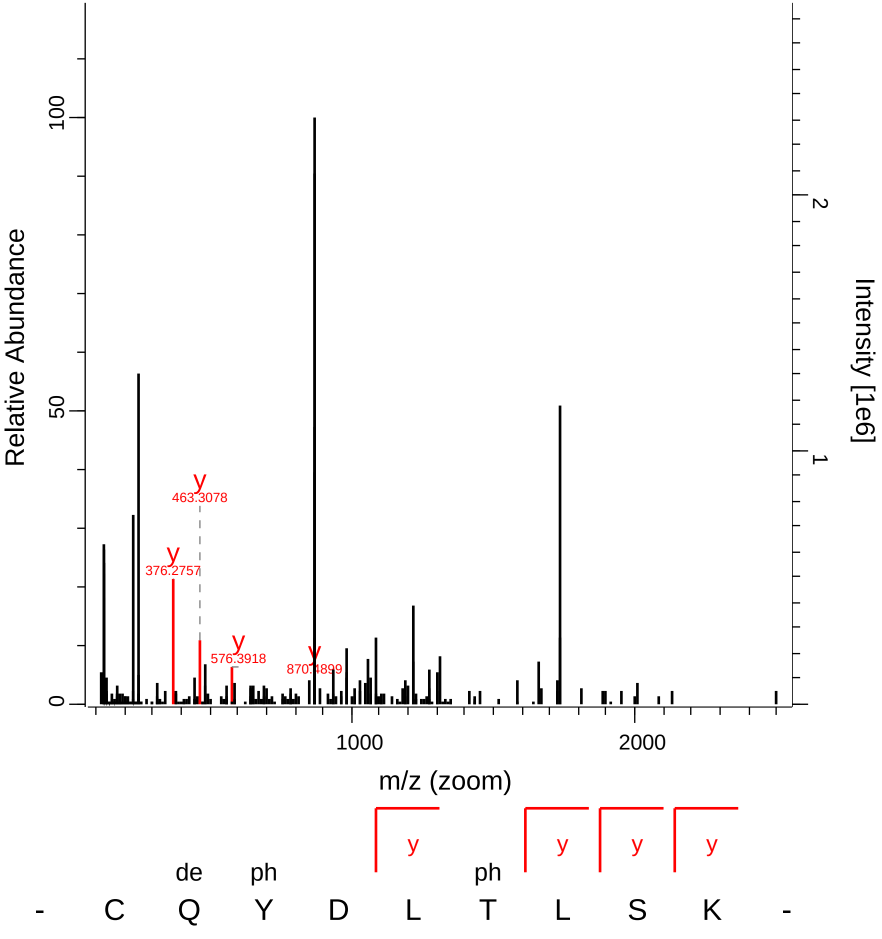

Raw File	Scan	Method	Score	m/z	Gene names
RO-QC-160121_ZackD_SS_Set_01_Fractic	27986	FTMS; HC	54.61	1075.59	DRAXIN

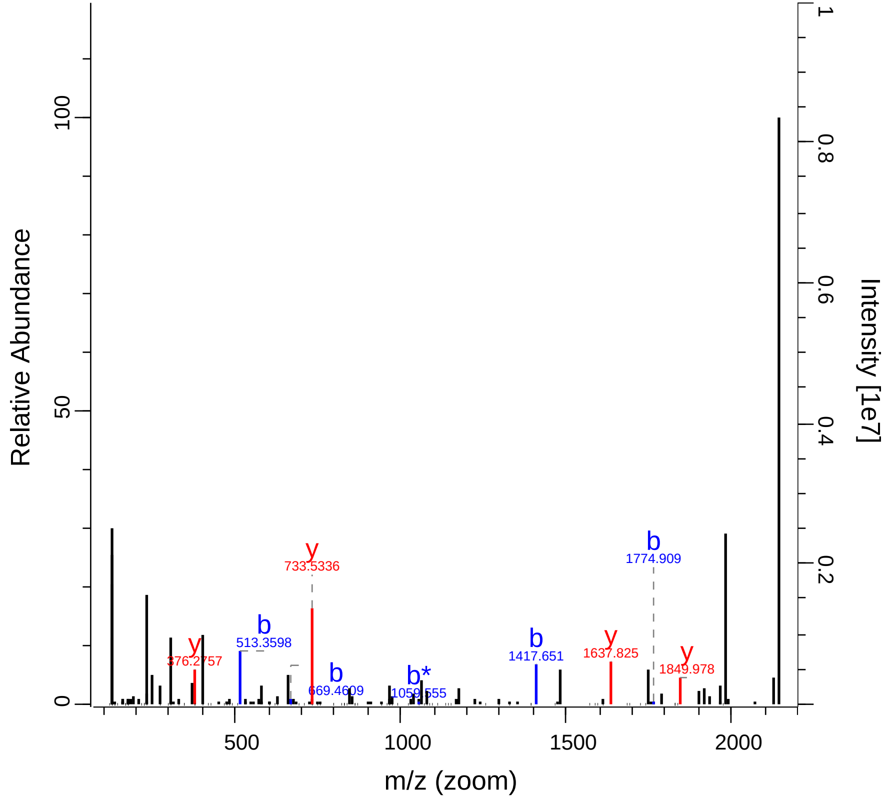

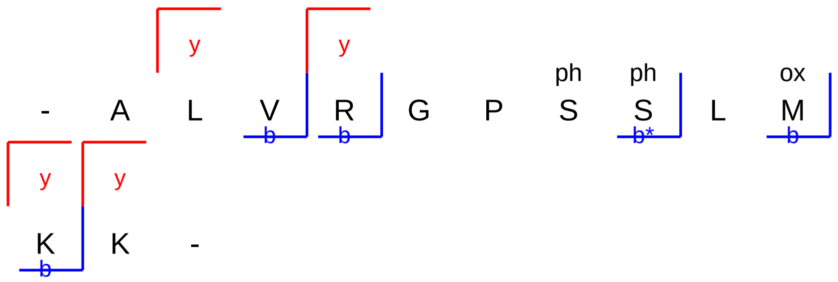

Raw File	Scan	Method	Score	m/z	Gene names
RO-QC-160121_ZackD_SS_Set_01_Fractio	28567	FTMS; HC	64.44	1253.7	KIF15

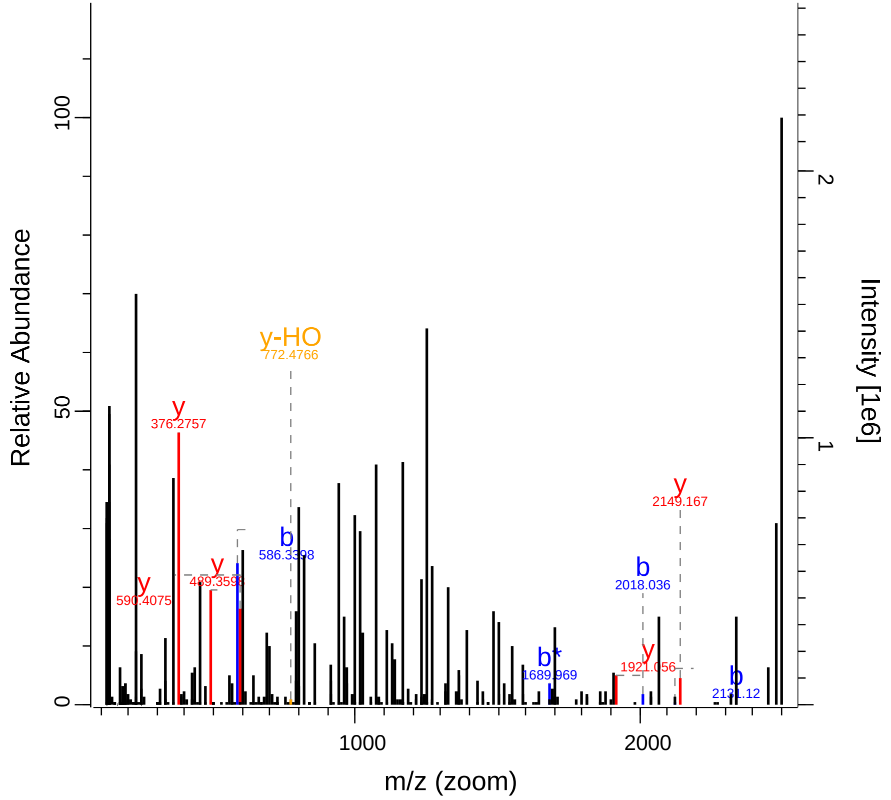

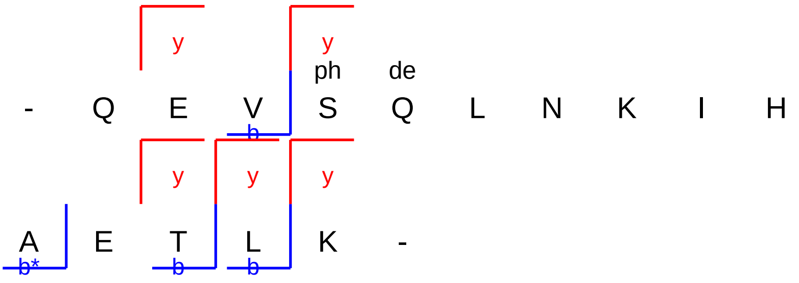

Raw File	Scan	Method	Score	m/z	Gene names
RO-QC-160121_ZackD_SS_Set_01_Fractic	28874	FTMS; HC	50.3	1740.91	POU6F2

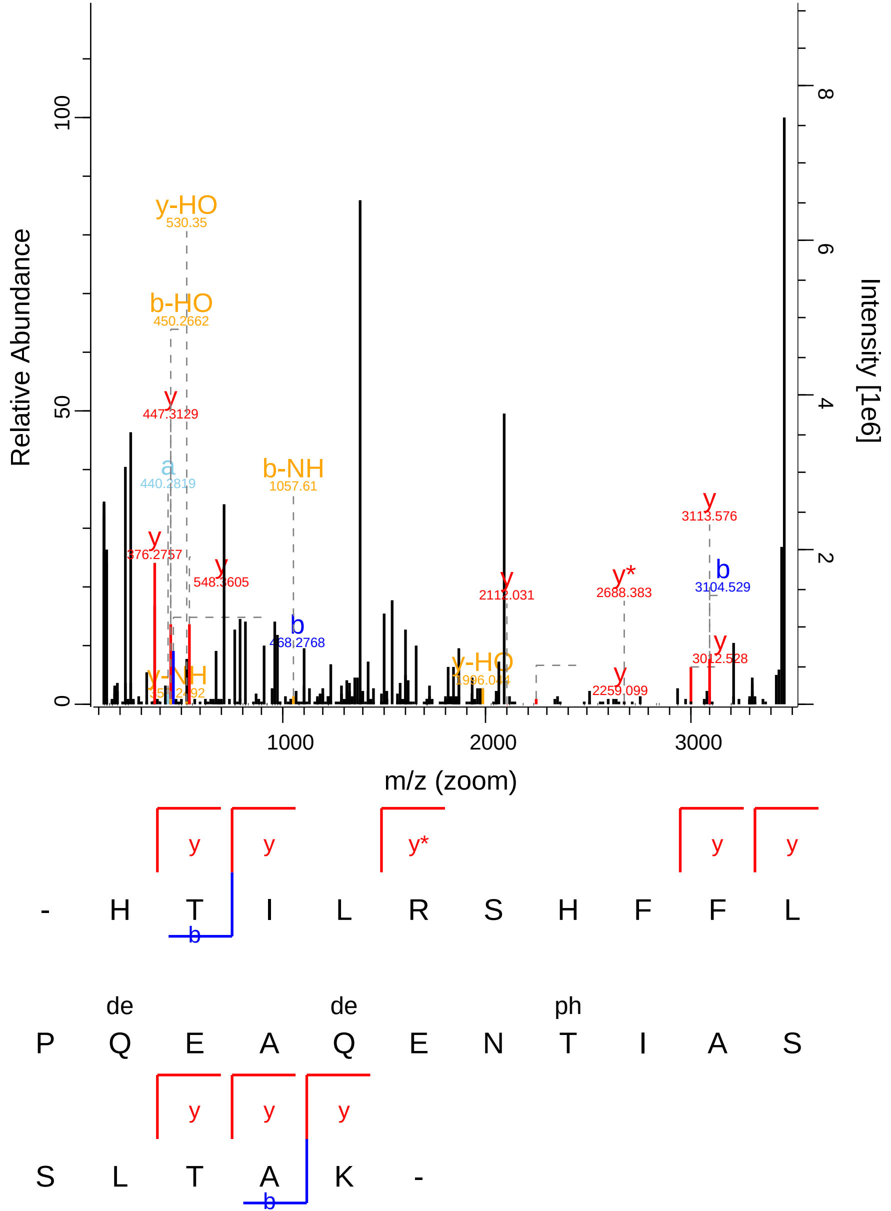

Raw File	Scan	Method	Score	m/z	Gene names
RO-QC-160121_ZackD_SS_Set_01_Fractic	29871	FTMS; HC	55.45	1136.05	LGI4

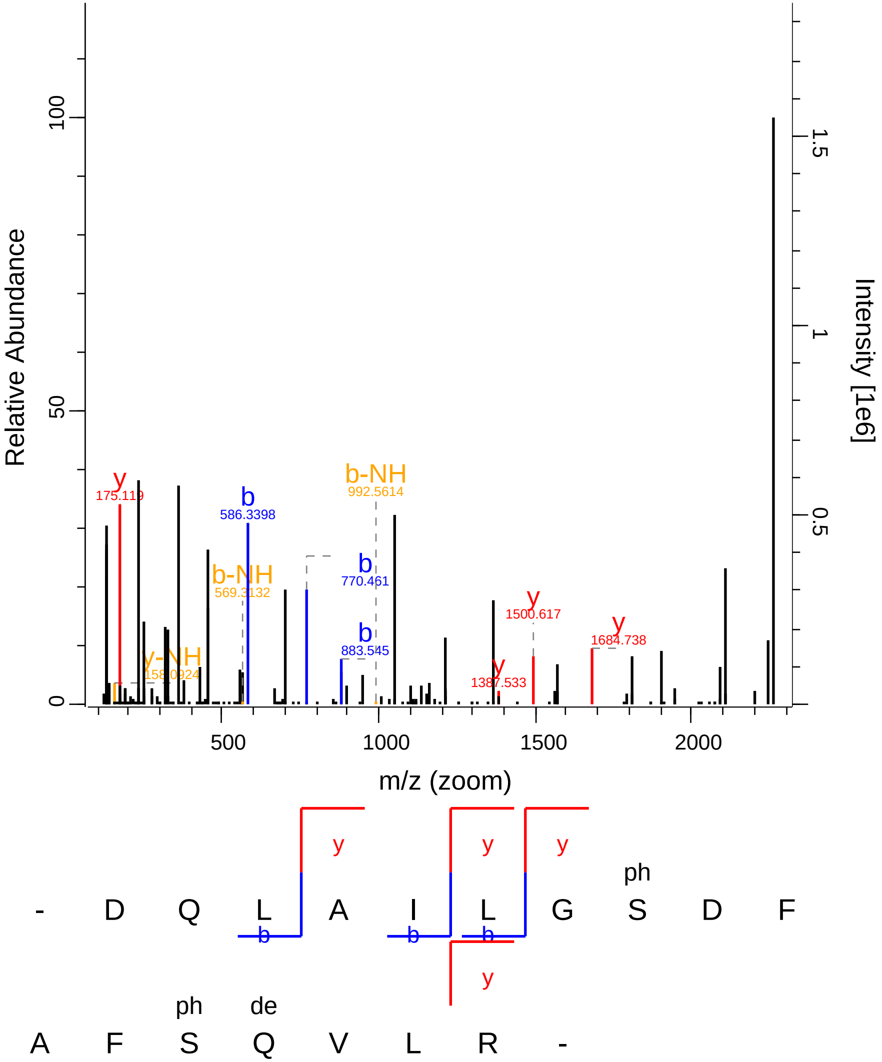

Raw File	Scan	Method	Score	m/z	Gene names
RO-QC-160121_ZackD_SS_Set_01_Fractic	30503	FTMS; HC	115.33	1104.57	VIM

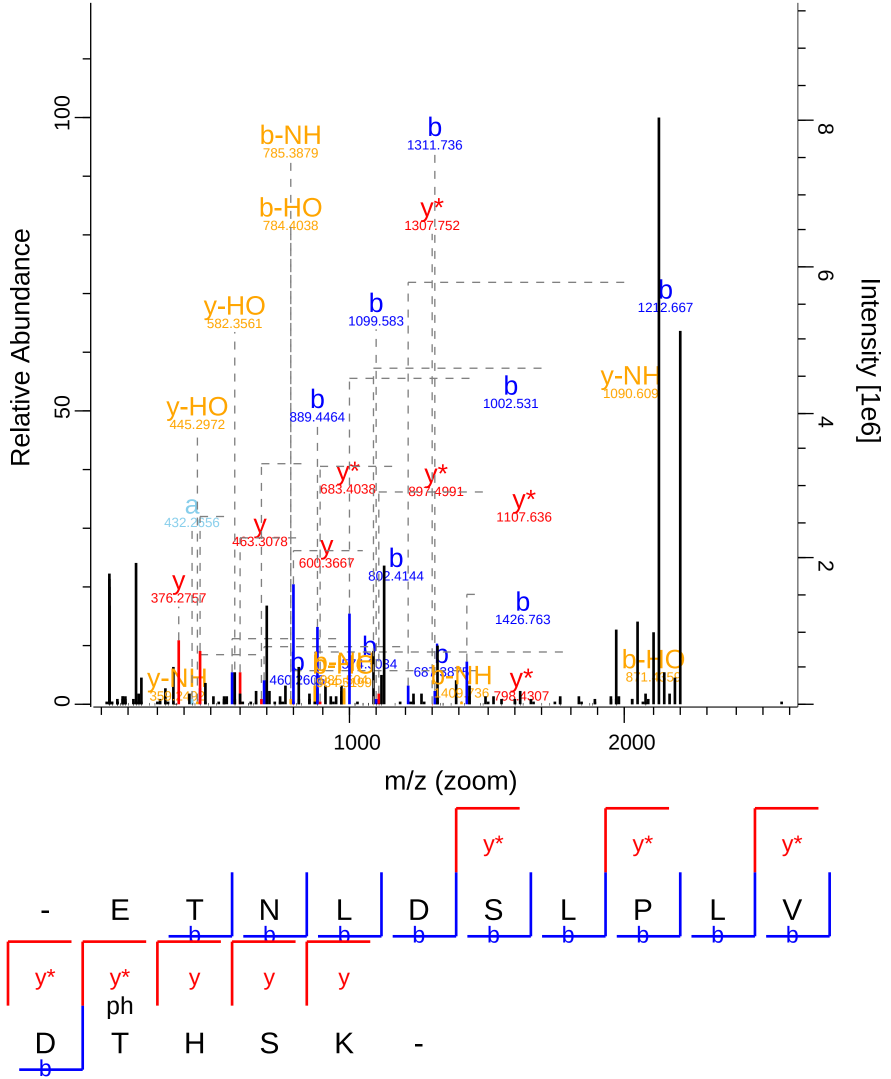

Raw File	Scan	Method	Score	m/z	Gene names
RO-QC-160121_ZackD_SS_Set_01_Fractic	32362	FTMS; HC	70.94	1318.66	NEUROD4

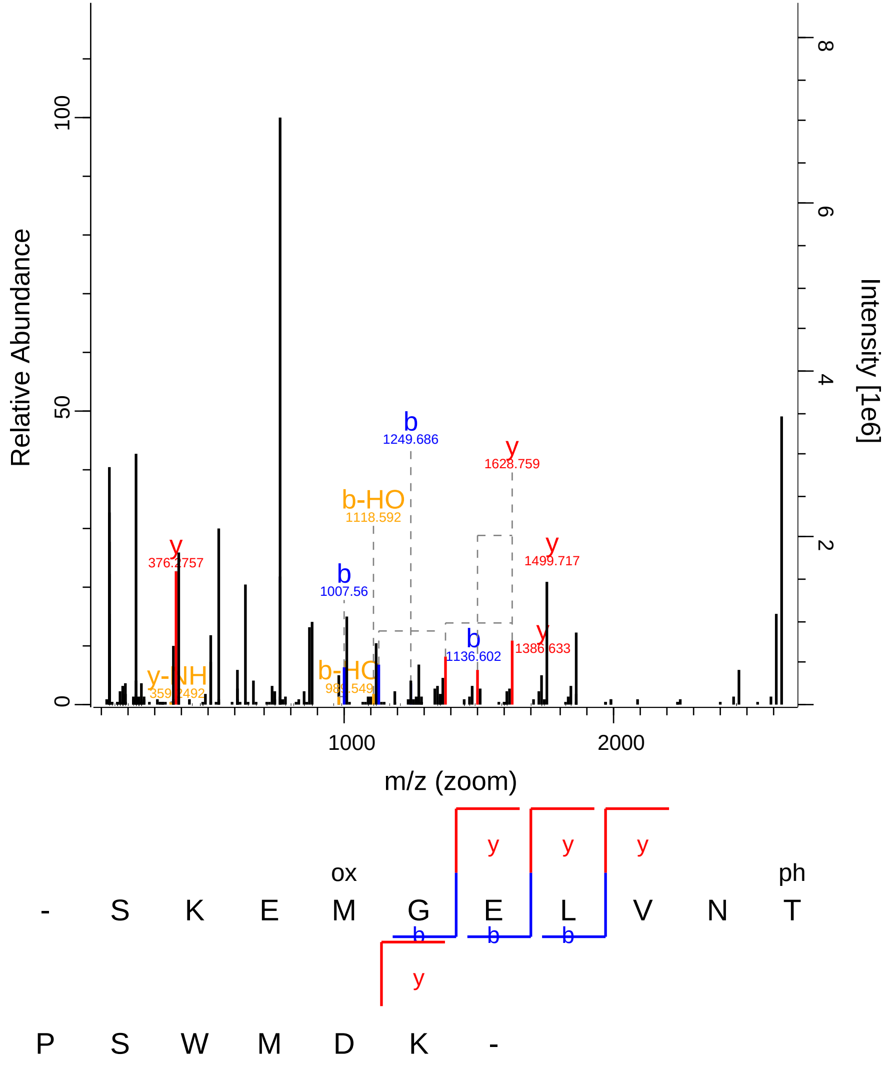

Raw File	Scan	Method	Score	m/z	Gene names
RO-QC-160121_ZackD_SS_Set_01_Fractio	32422	FTMS; HC	41.53	872.71	DDX50

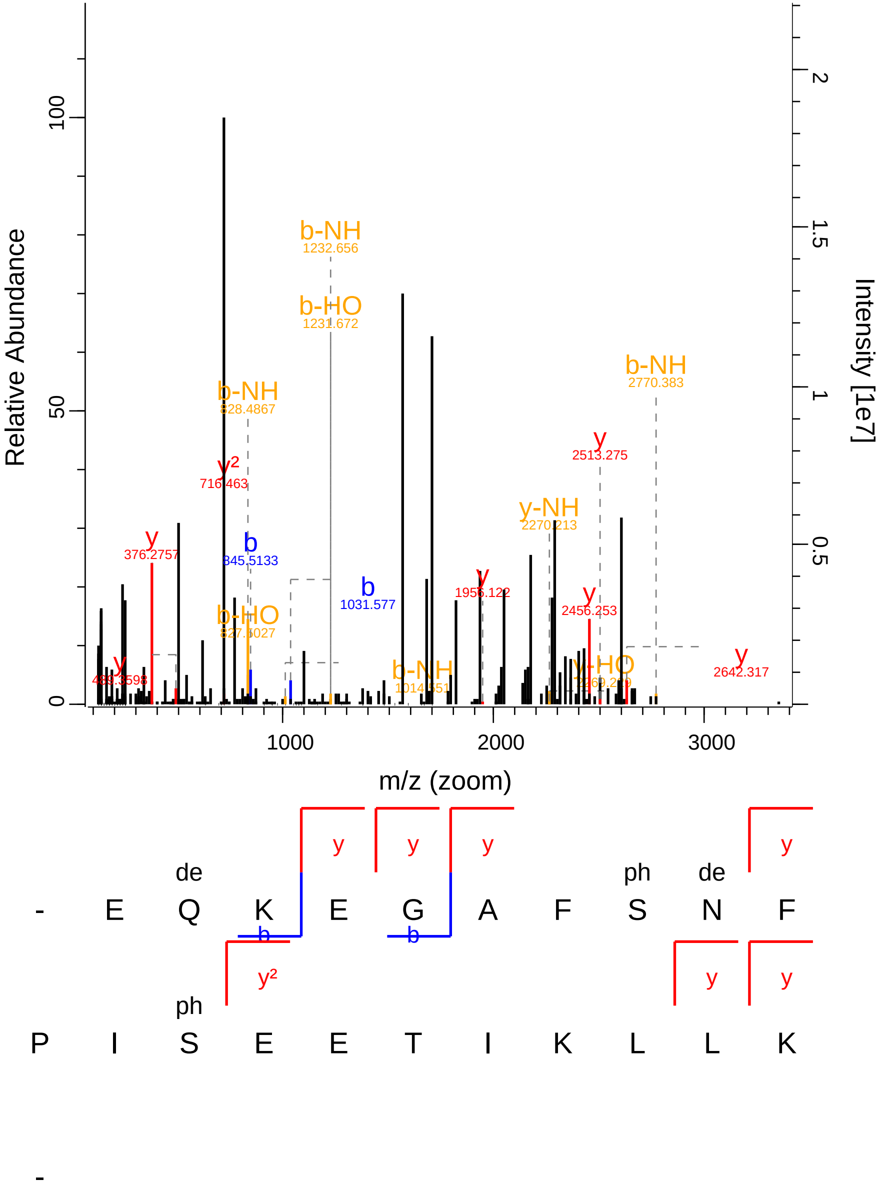

Raw File	Scan	Method	Score	m/z	Gene names
RO-QC-160121_ZackD_SS_Set_01_Fractic	34832	FTMS; HC	50.03	1155.63	EBF2

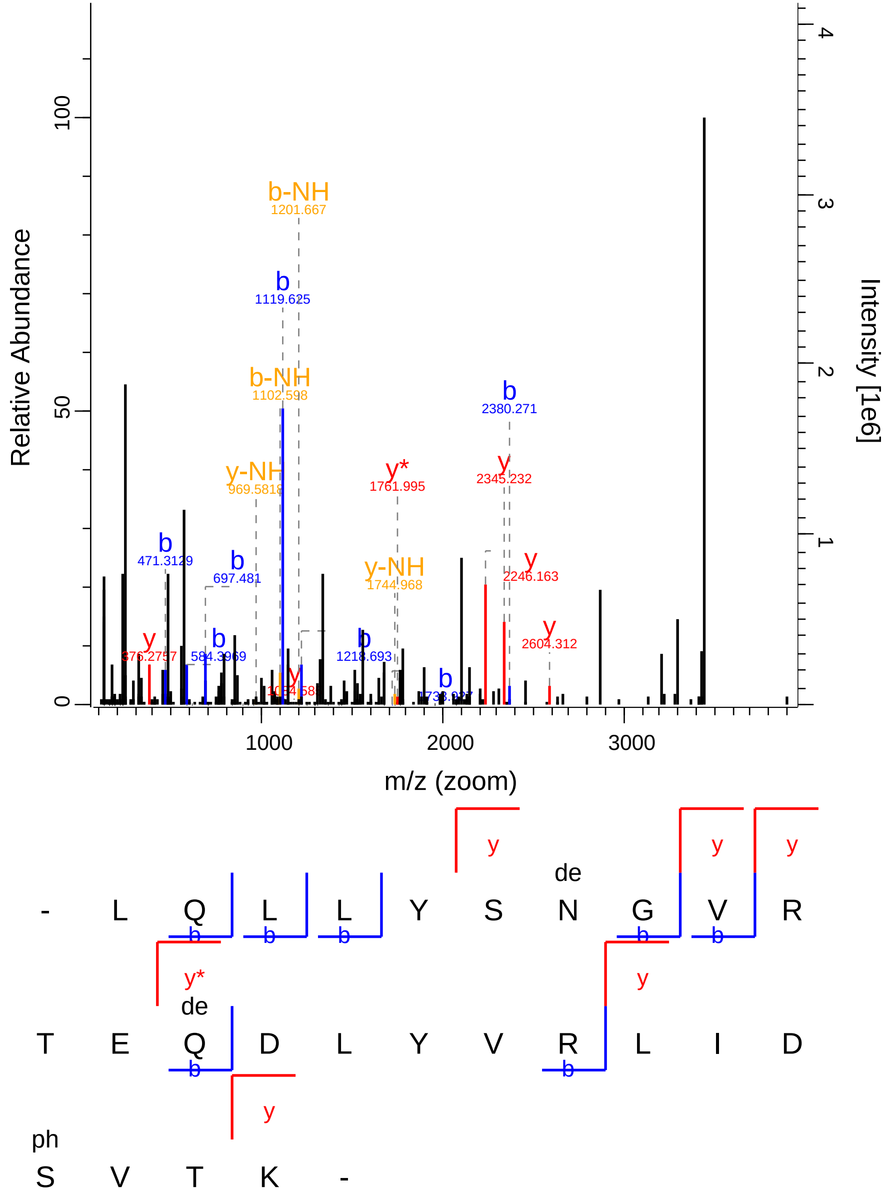

Raw File	Scan	Method	Score	m/z	Gene names
RO-QC-160121_ZackD_SS_Set_01_Fractic	35363	FTMS; HC	84.64	1114.55	TP53BP1

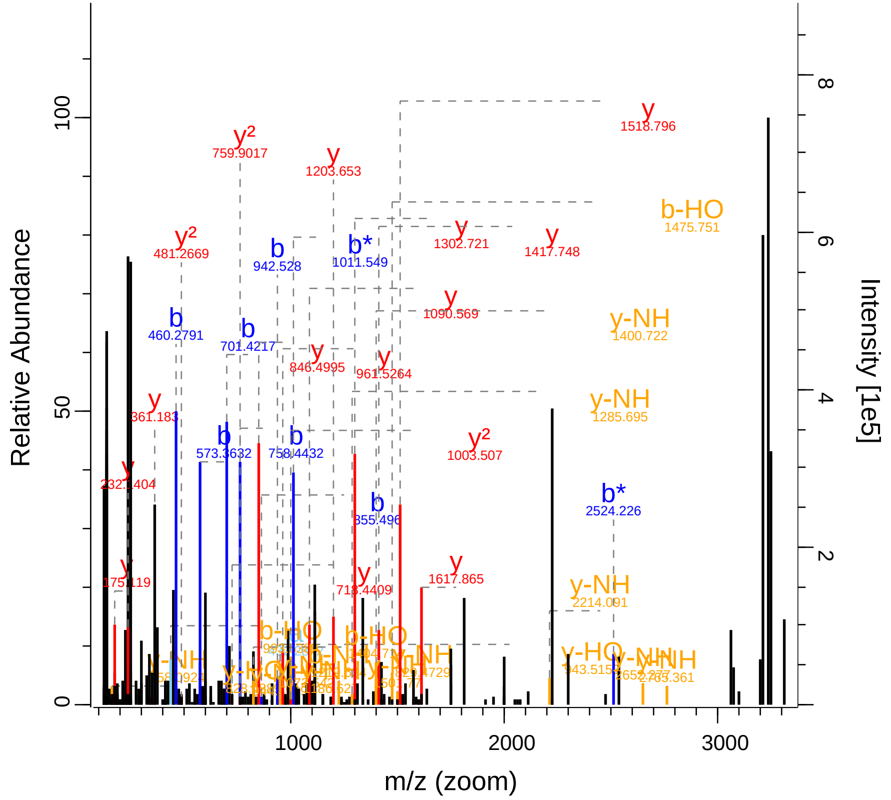

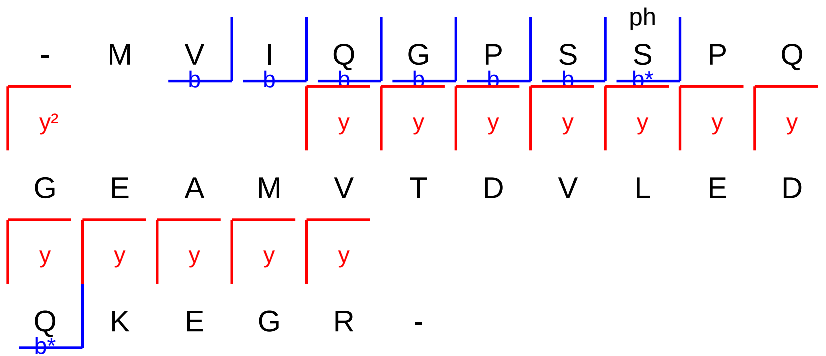

Raw File	Scan	Method	Score	m/z	Gene names
RO-QC-160121_ZackD_SS_Set_01_Fractic	35736	FTMS; HC	65.5	1045.09	EED

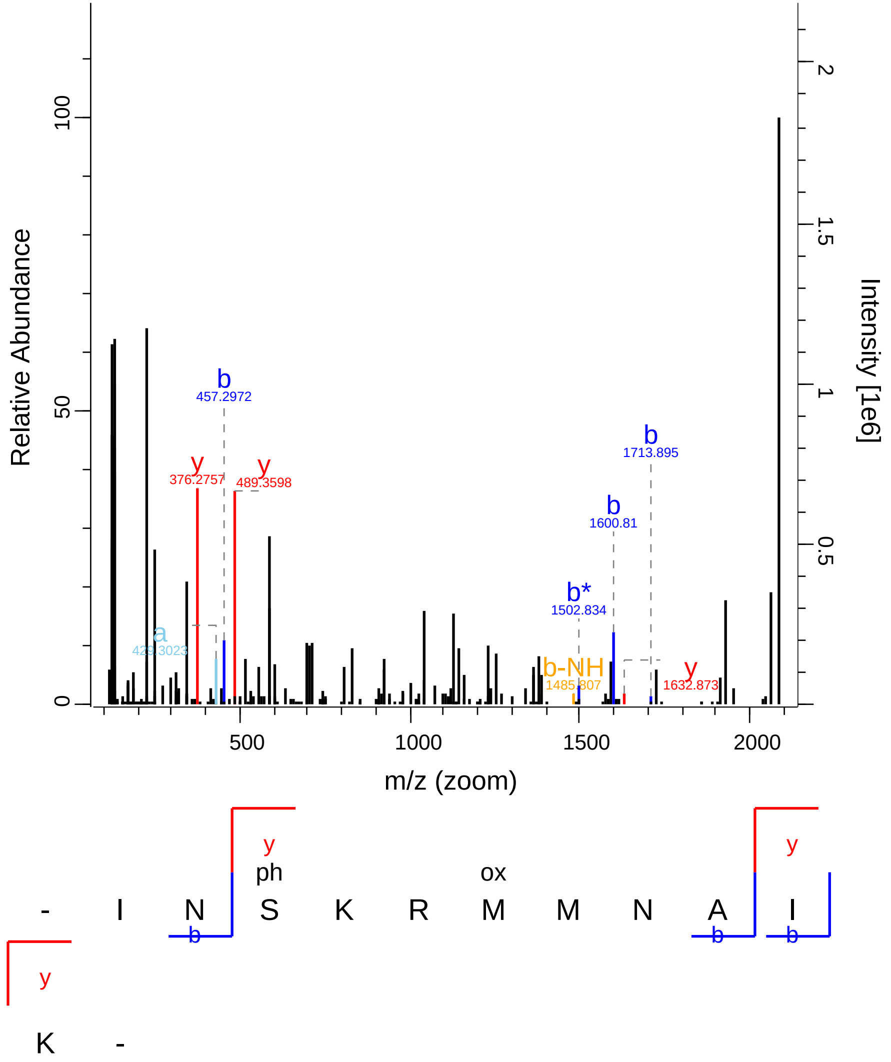

Raw File	Scan	Method	Score	m/z	Gene names
RO-QC-160121_ZackD_SS_Set_01_Fractic	36809	FTMS; HC	76.14	1193.18	MGEA5

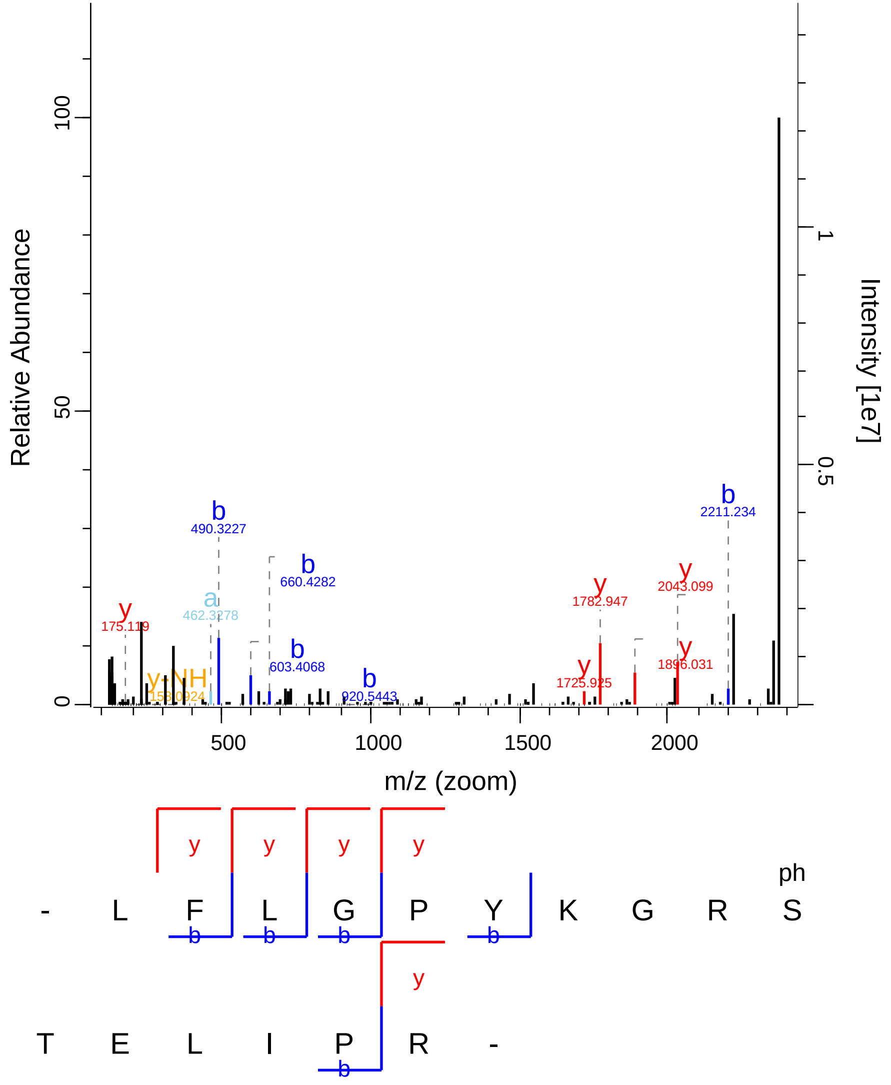

Raw File	Scan	Method	Score	m/z	Gene names
RO-QC-160121_ZackD_SS_Set_01_Fractic	37315	FTMS; HC	84	1305.63	SSR1

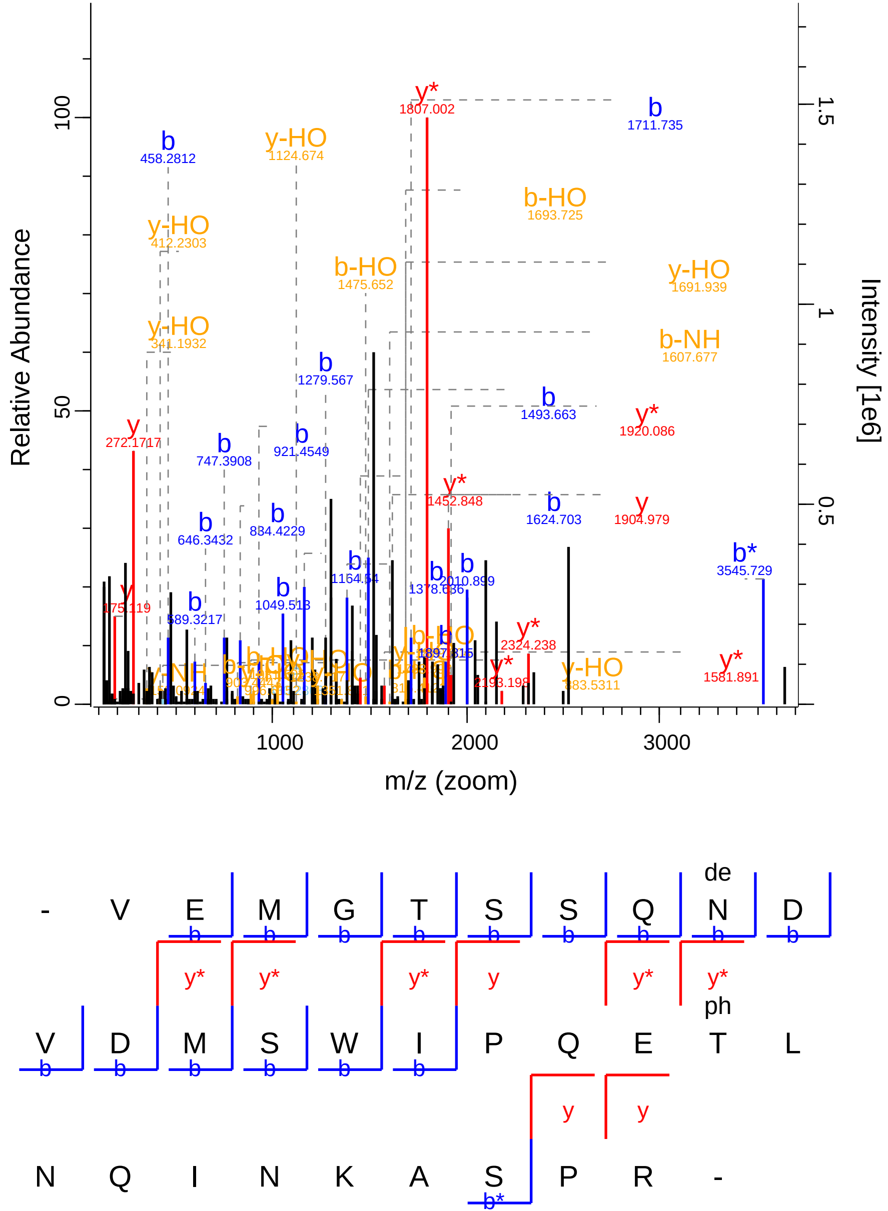

Raw File	Scan	Method	Score	m/z	Gene names
RO-QC-160121_ZackD_SS_Set_01_Fractic	38359	FTMS; HC	194.01	1117.09	CDR2

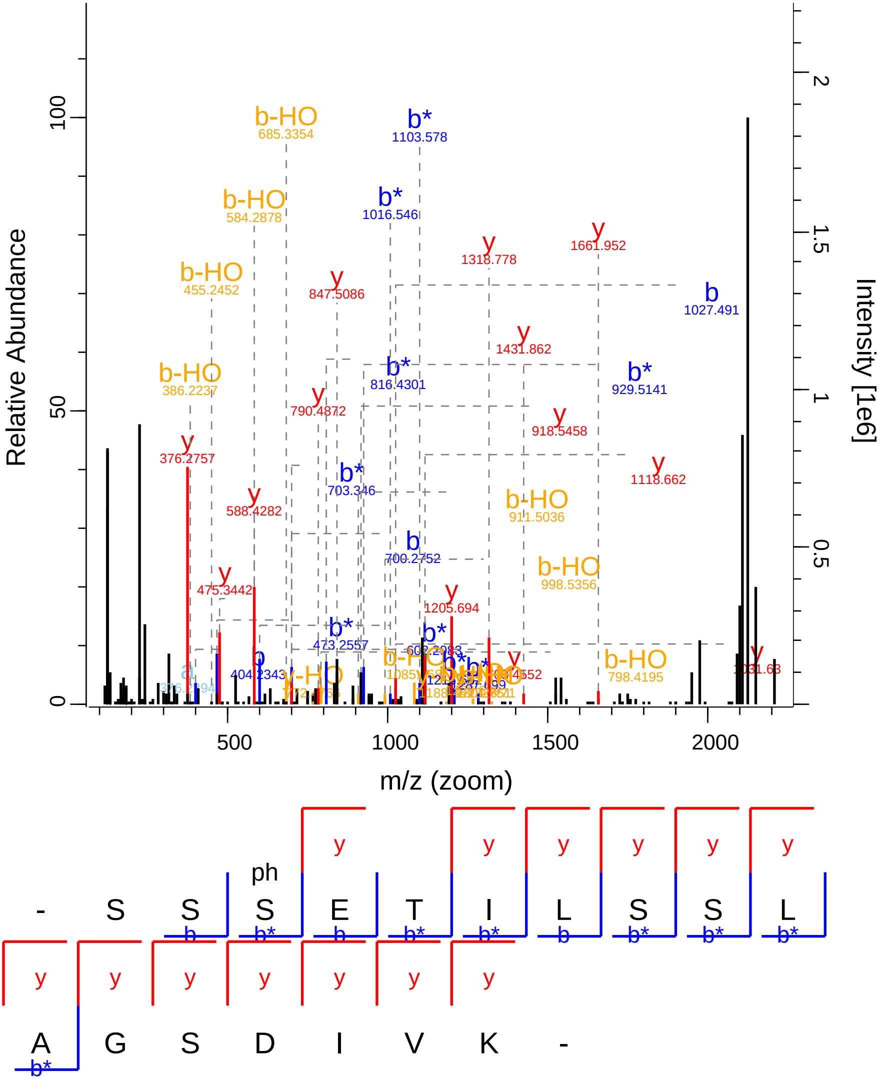

Raw File	Scan	Method	Score	m/z	Gene names
RO-QC-160121_ZackD_SS_Set_01_Fractio	39255	FTMS; HC	50.94	995.54	ANXA1

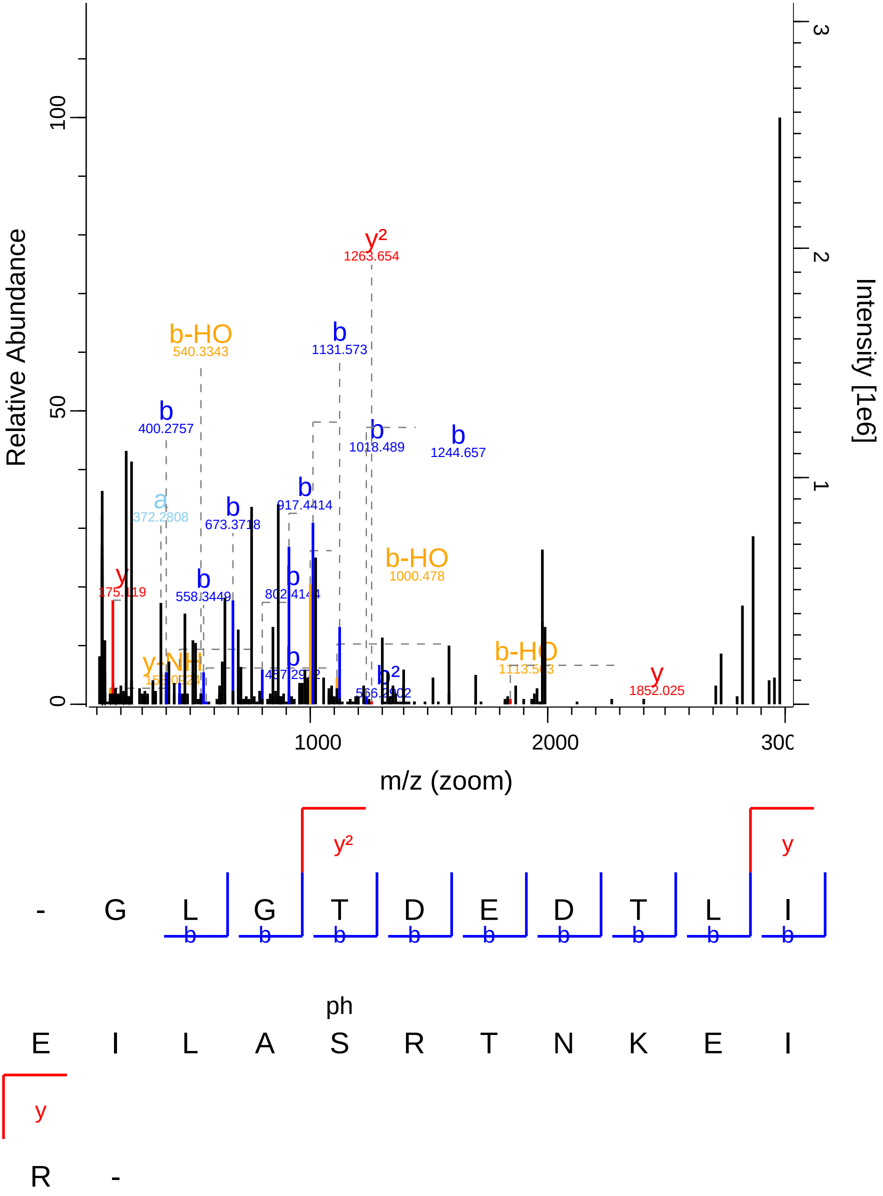

Raw File	Scan	Method	Score	m/z	Gene names
RO-QC-160121_ZackD_SS_Set_01_Fractic	39269	FTMS; HC	53.95	1570.28	POFUT2

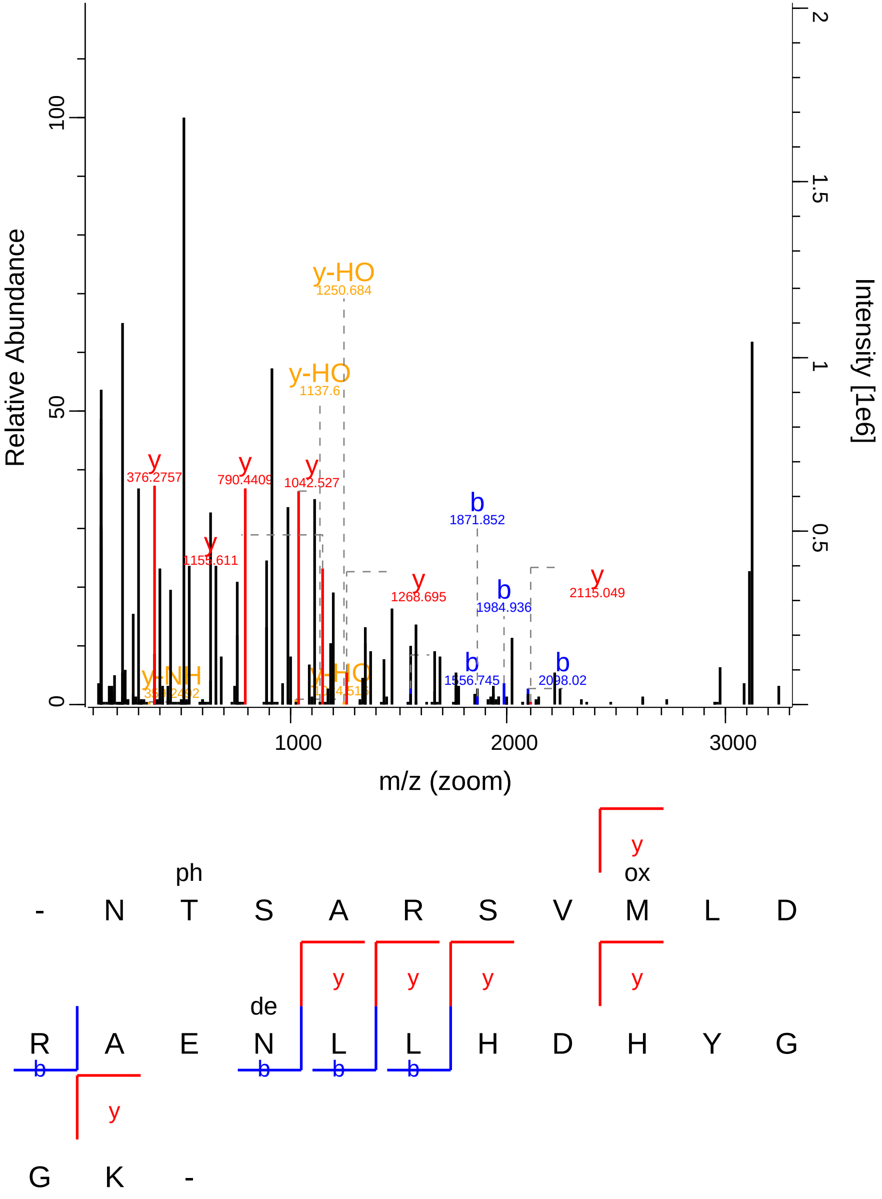

Raw File	Scan	Method	Score	m/z	Gene names
RO-QC-160121_ZackD_SS_Set_01_Fractio	41442	FTMS; HC	75.31	893.96	TINAG

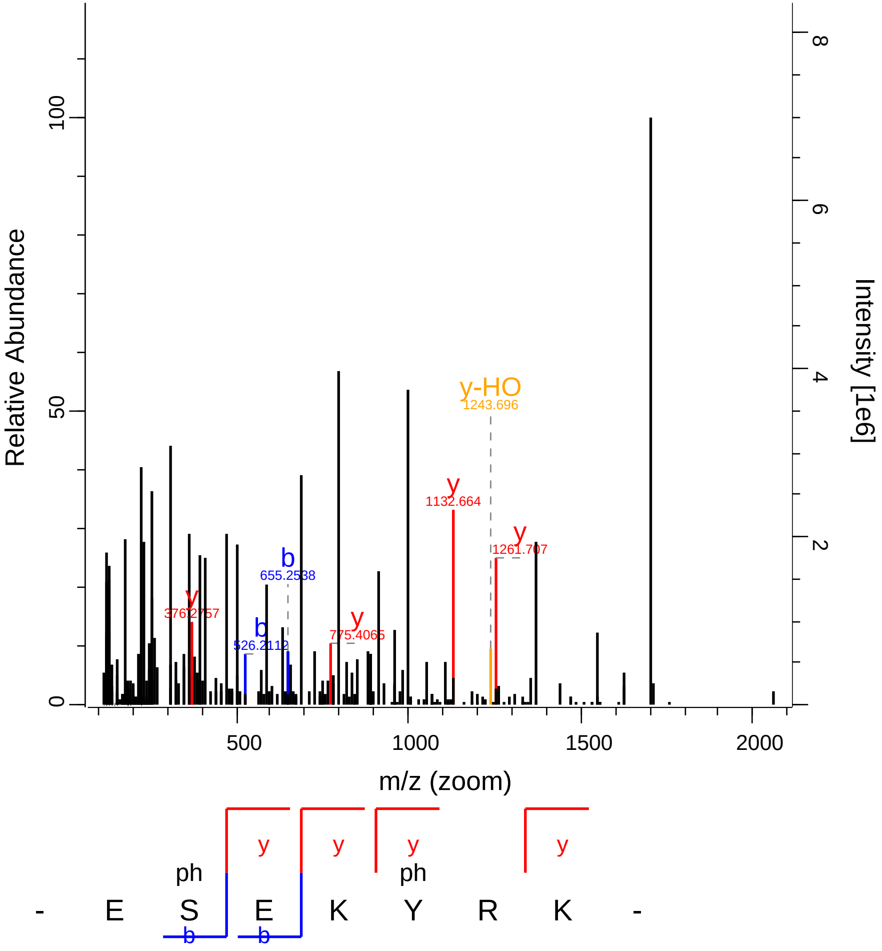

Raw File	Scan	Method	Score	m/z	Gene names
RO-QC-160121_ZackD_SS_Set_01_Fractior	5868	FTMS; HCI	59.9	506.78	NIPBL

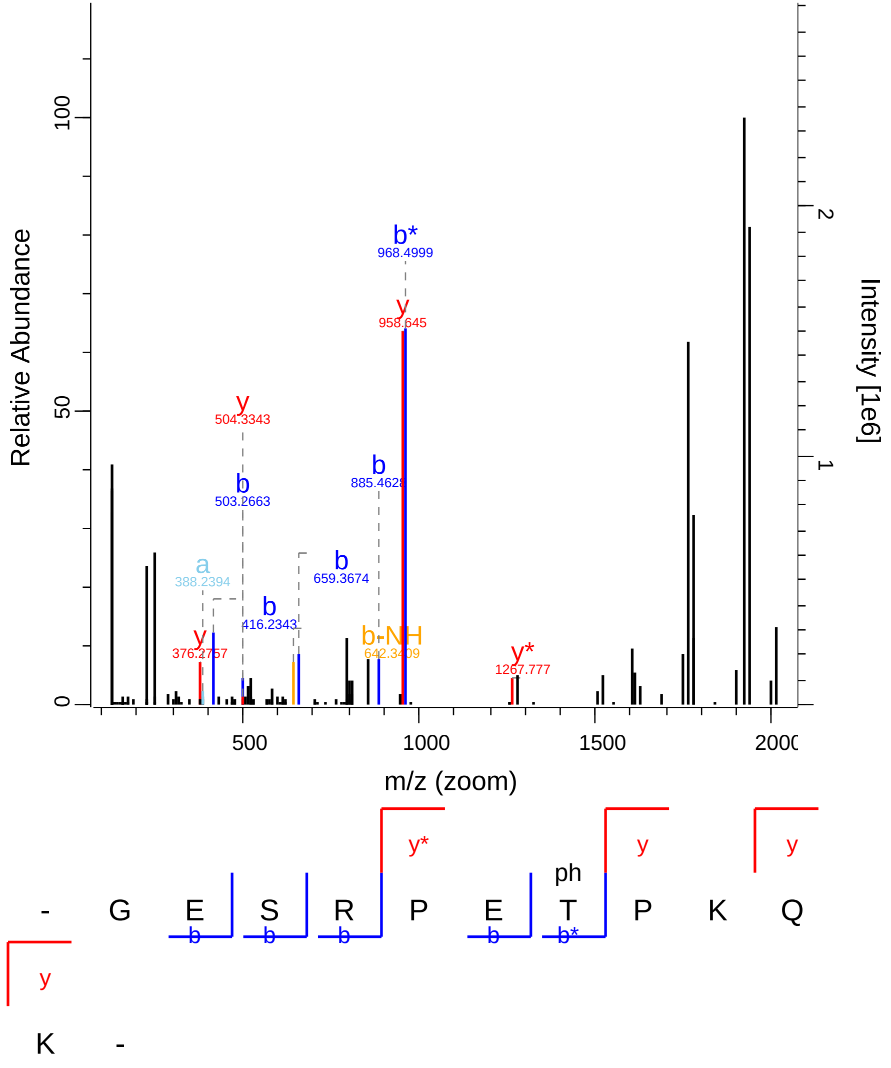

Raw File	Scan	Method	Score	m/z	Gene names
RO-QC-160121_ZackD_SS_Set_01_Fraction	6082	FTMS; HCI	71.69	594.3	NOC2L

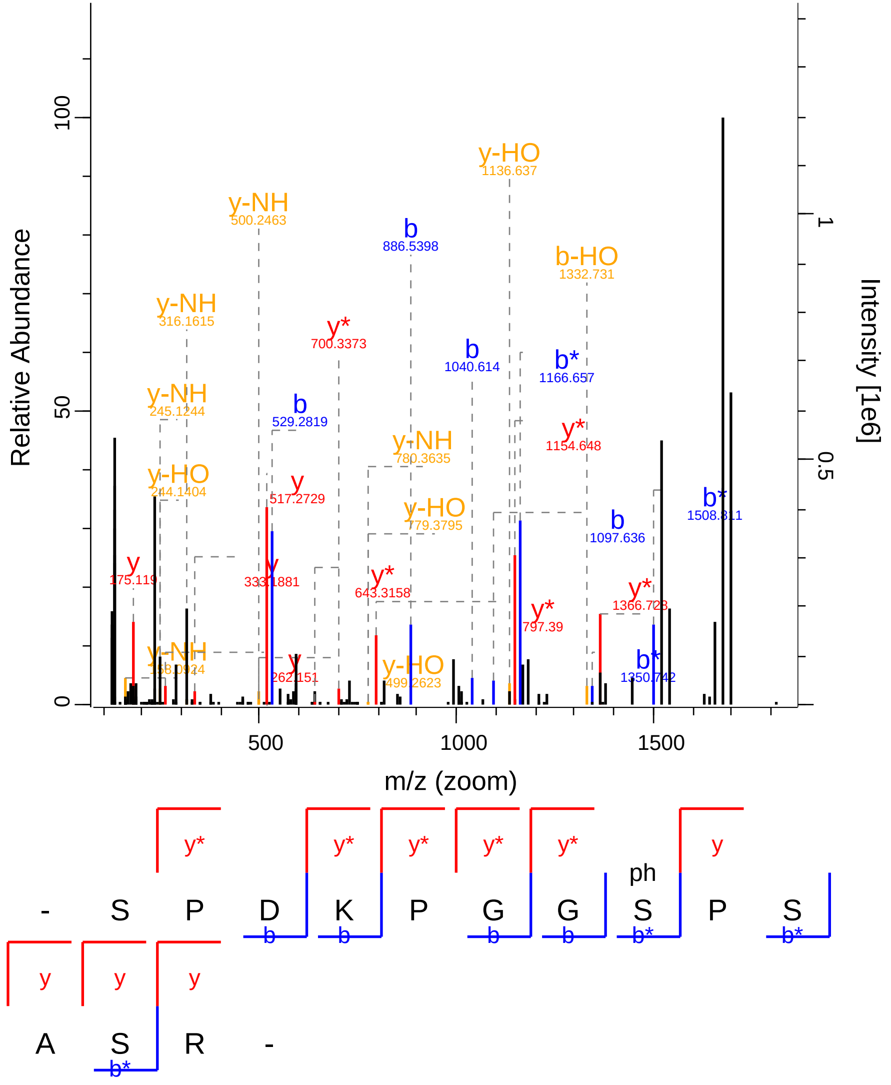

Raw File	Scan	Method	Score	m/z	Gene names
RO-QC-160121_ZackD_SS_Set_01_Fractior	6241	FTMS; HCI	50.35	622.32	PECAM1

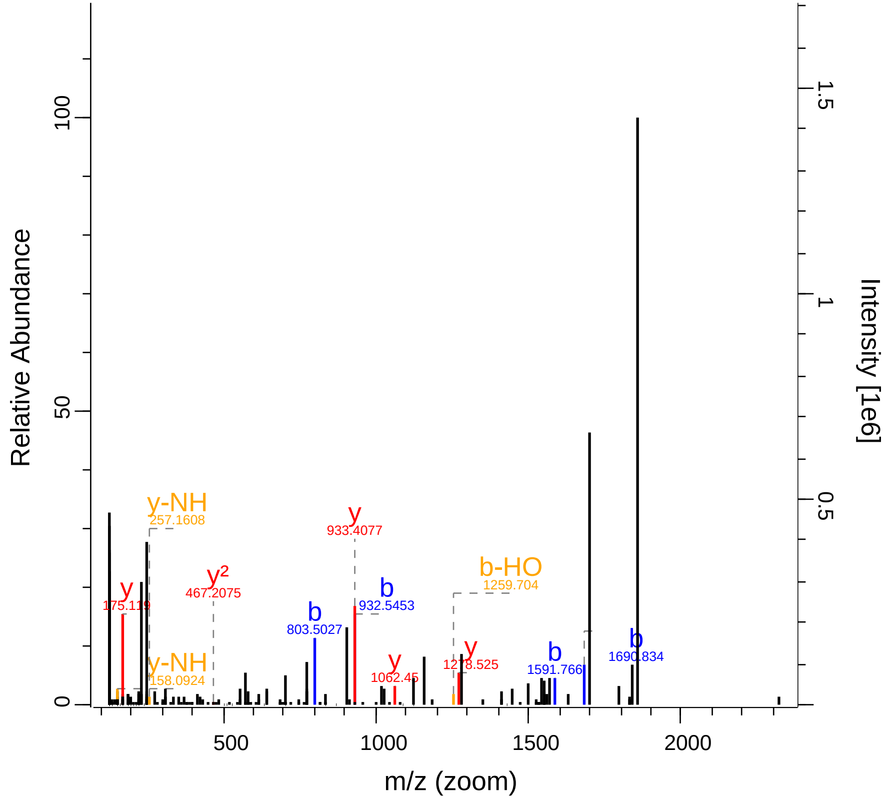

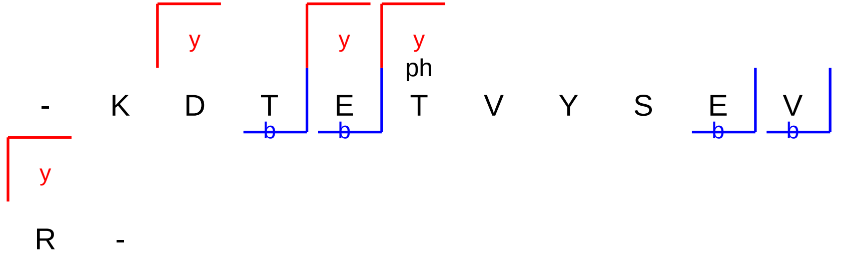

Raw File	Scan	Method	Score	m/z	Gene names
RO-QC-160121_ZackD_SS_Set_01_Fractio	6289	FTMS; HC	102.87	854.41	BMI1

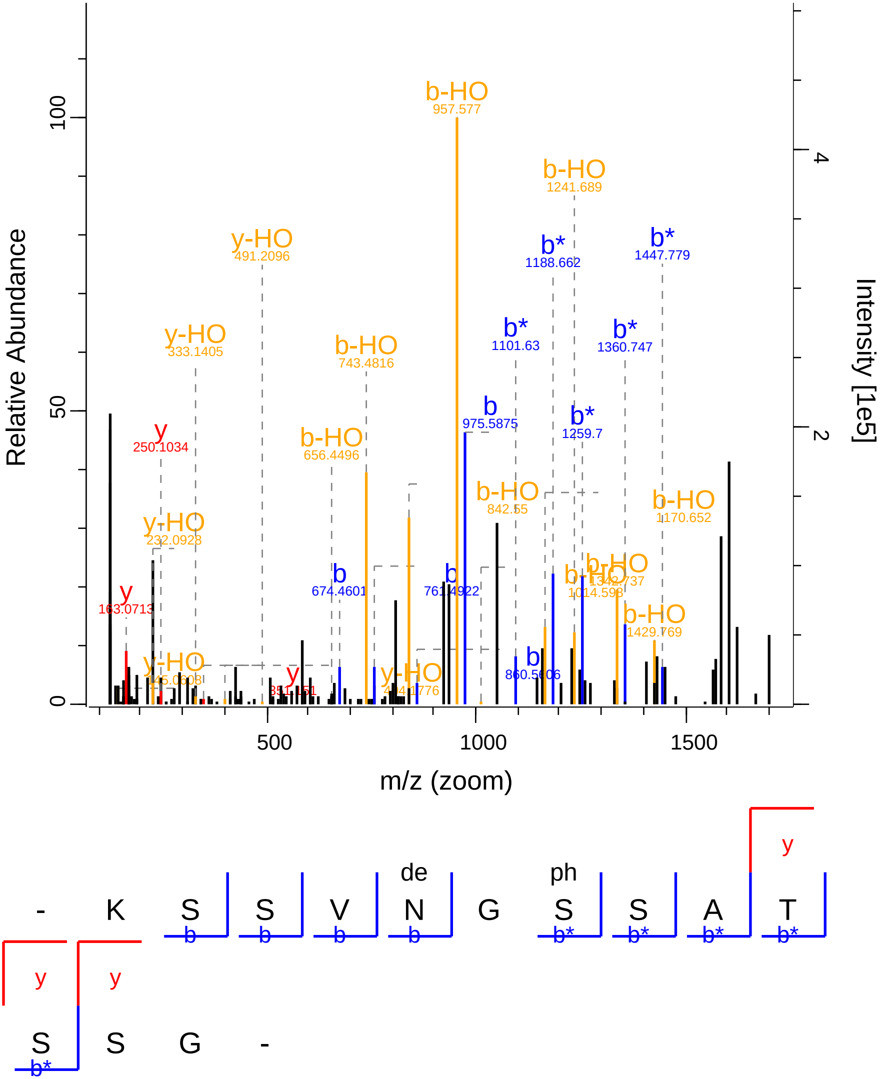

Raw File	Scan	Method	Score	m/z	Gene names
RO-QC-160121_ZackD_SS_Set_01_Fractio	6520	FTMS; HC	101.43	854.42	BMI1

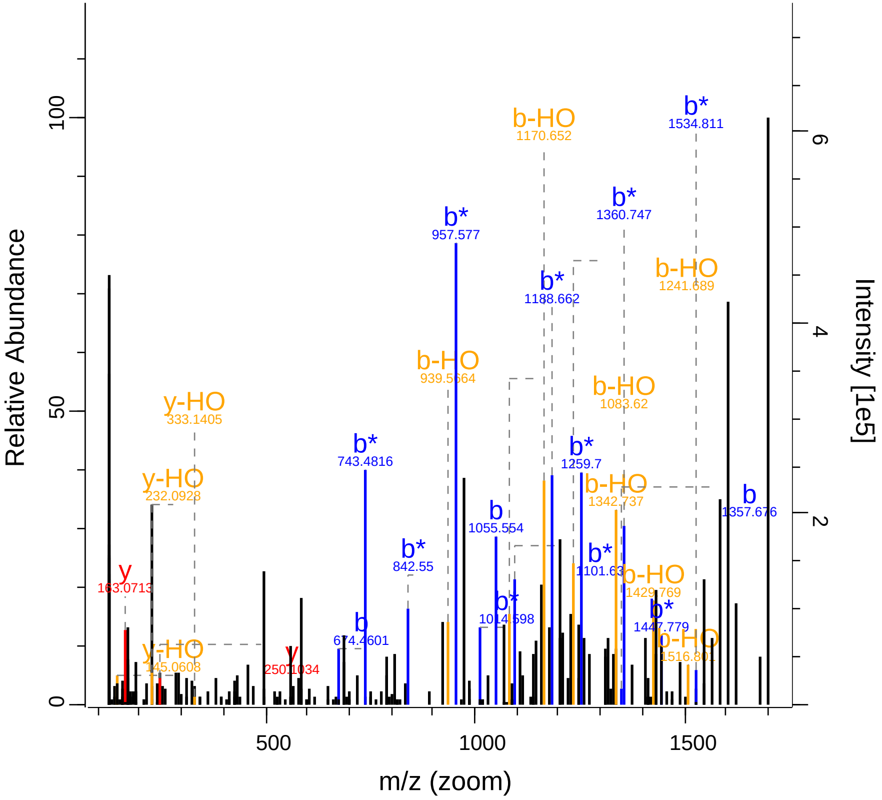

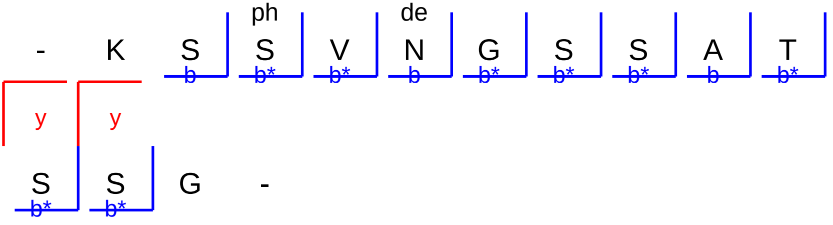

Raw File	Scan	Method	Score	m/z	Gene names
RO-QC-160121_ZackD_SS_Set_01_Fraction_	6589	FTMS; HCI	75.67	663	RPS6

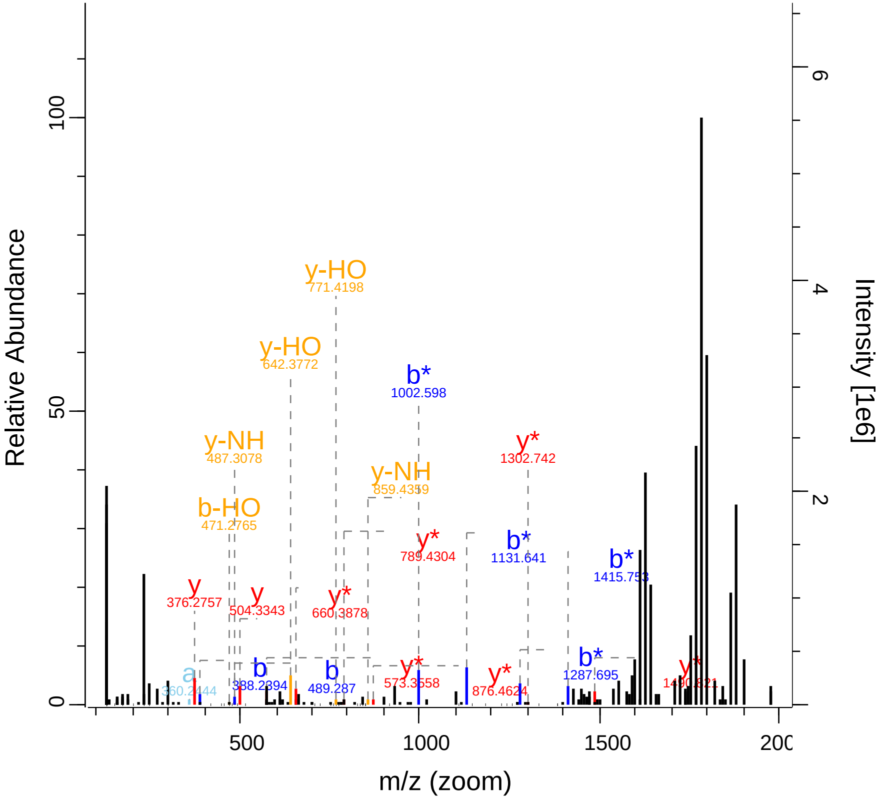

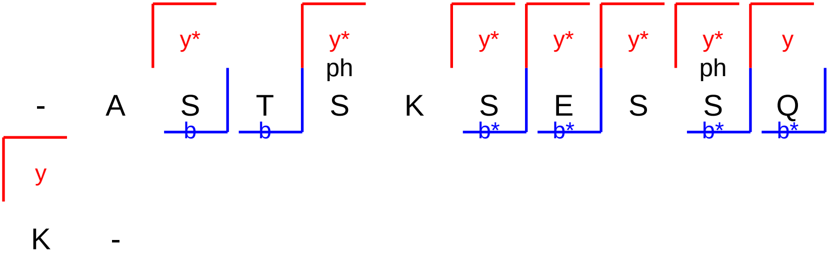

Raw File	Scan	Method	Score	m/z	Gene names
RO-QC-160121_ZackD_SS_Set_01_Fractior	6651	FTMS; HCI	65.4	993.99	RPS6

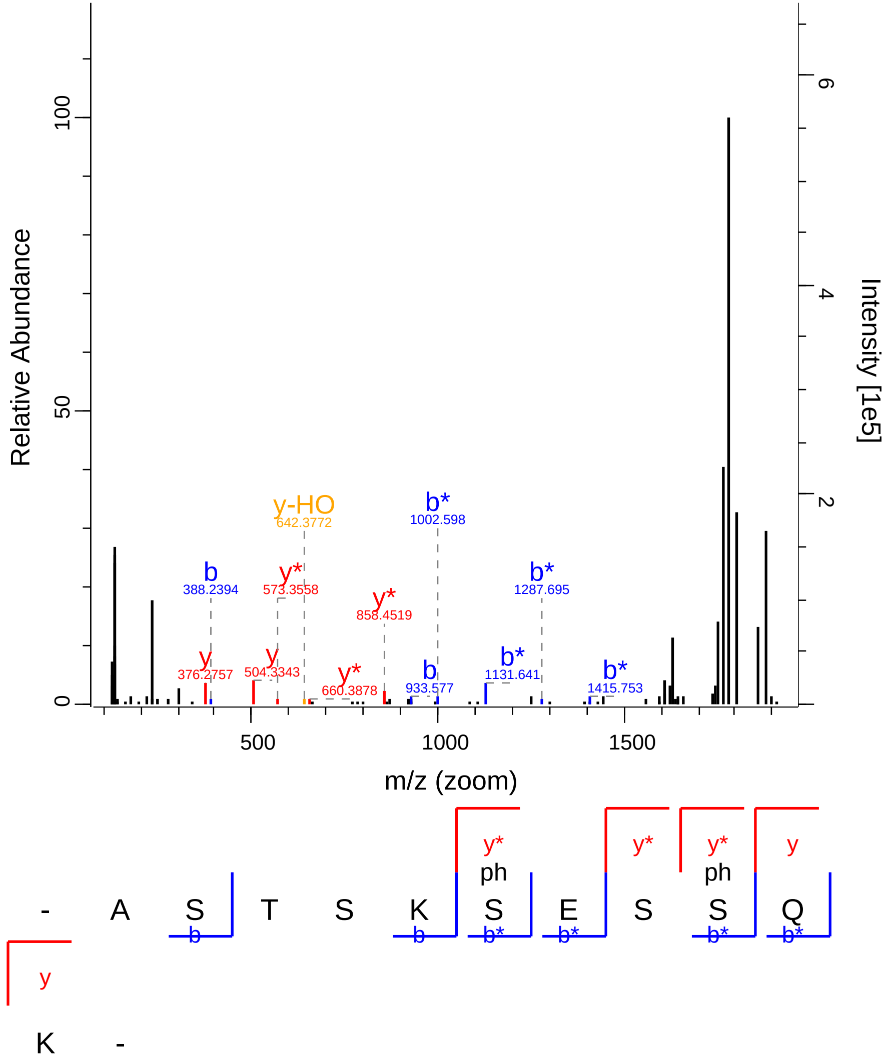

Raw File	Scan	Method	Score	m/z	Gene names
RO-QC-160121_ZackD_SS_Set_01_Fractior	6841	FTMS; HCI	89.46	597.99	SEPT9

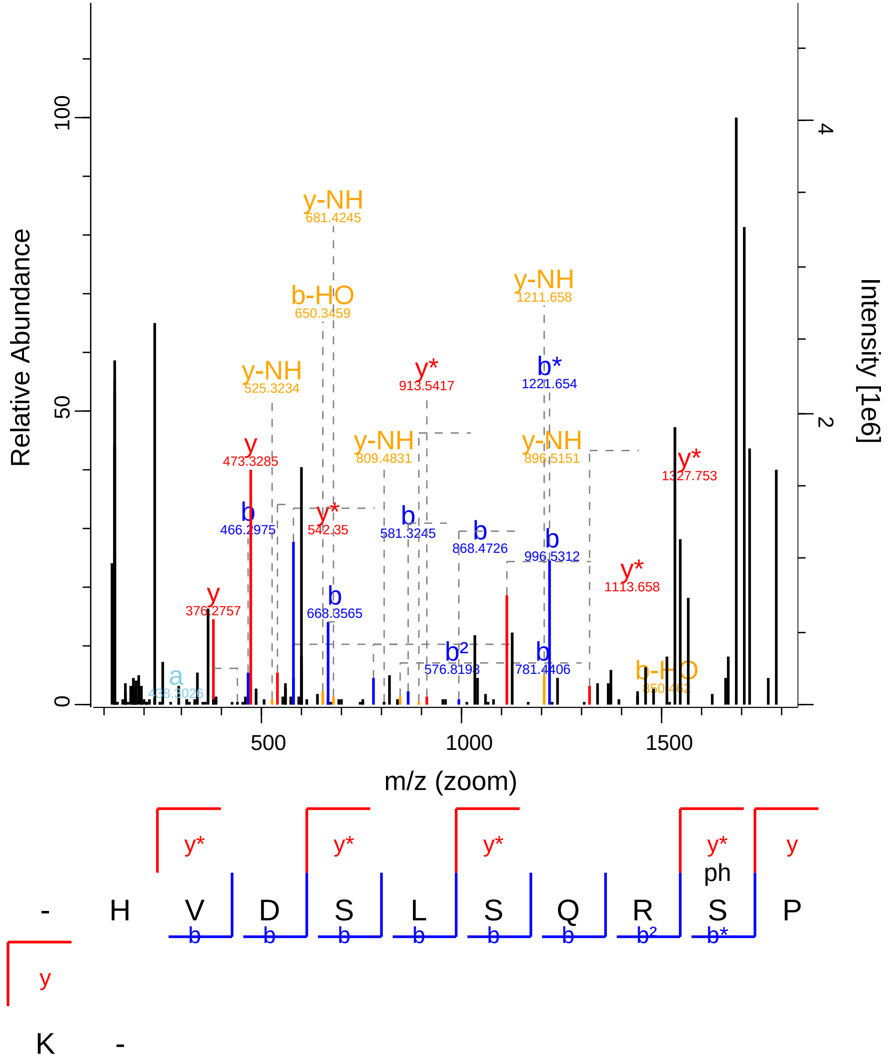

Raw File	Scan	Method	Score	m/z	Gene names
RO-QC-160121_ZackD_SS_Set_01_Fractior	7499	FTMS; HCl	66.26	394.98	MYBPC3

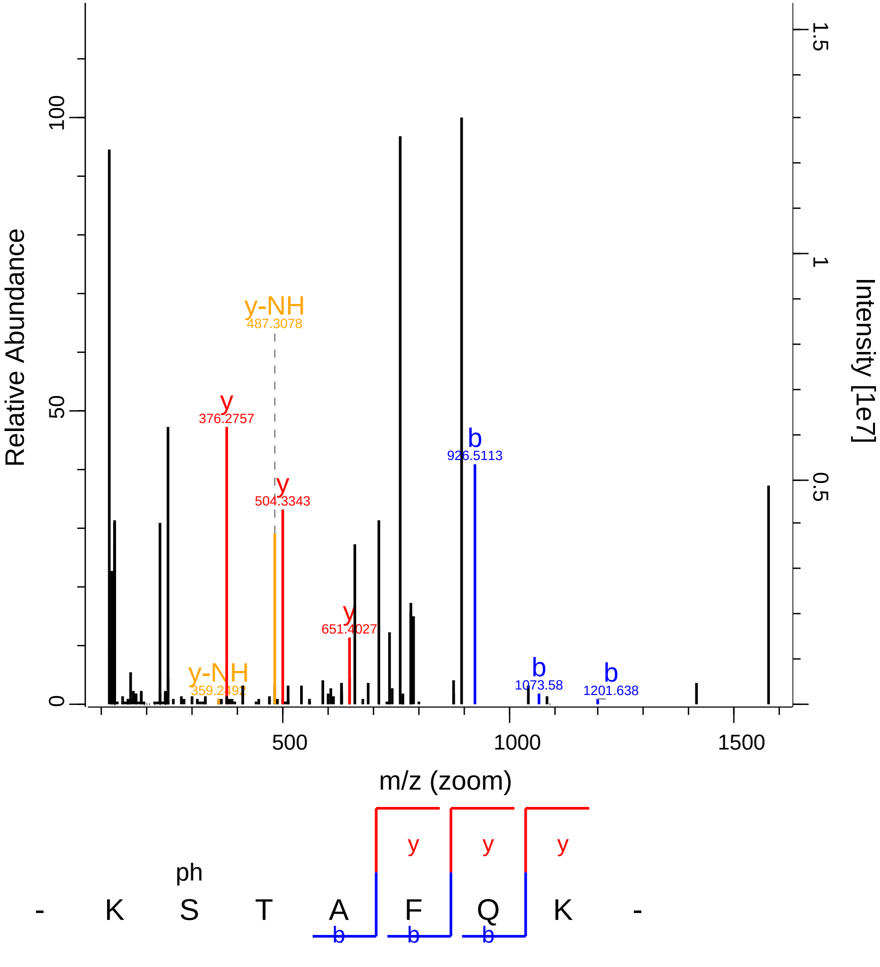

Raw File	Scan	Method	Score	m/z
RO-QC-160121_ZackD_SS_Set_01_Fraction_8	11592	FTMS; HCD	42.6	835.41

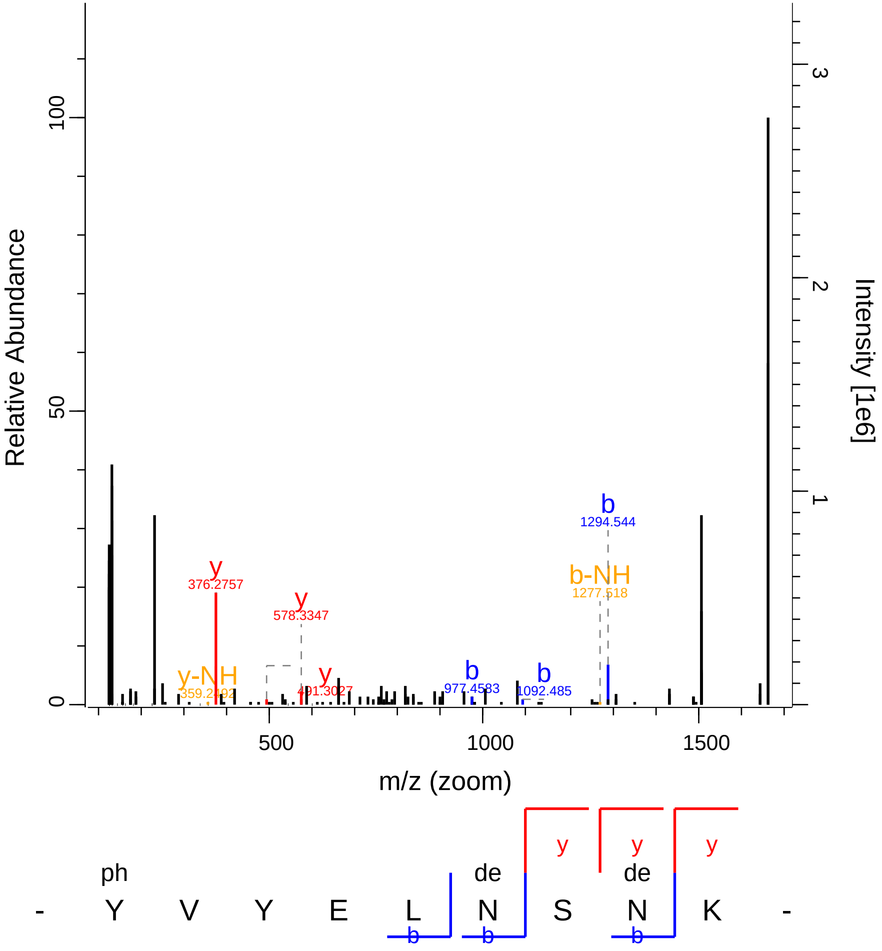

Raw File	Scan	Method	Score	m/z	Gene names
RO-QC-160121_ZackD_SS_Set_01_Fractio	12482	FTMS; HC	42.74	1195.6	SPATA31D1

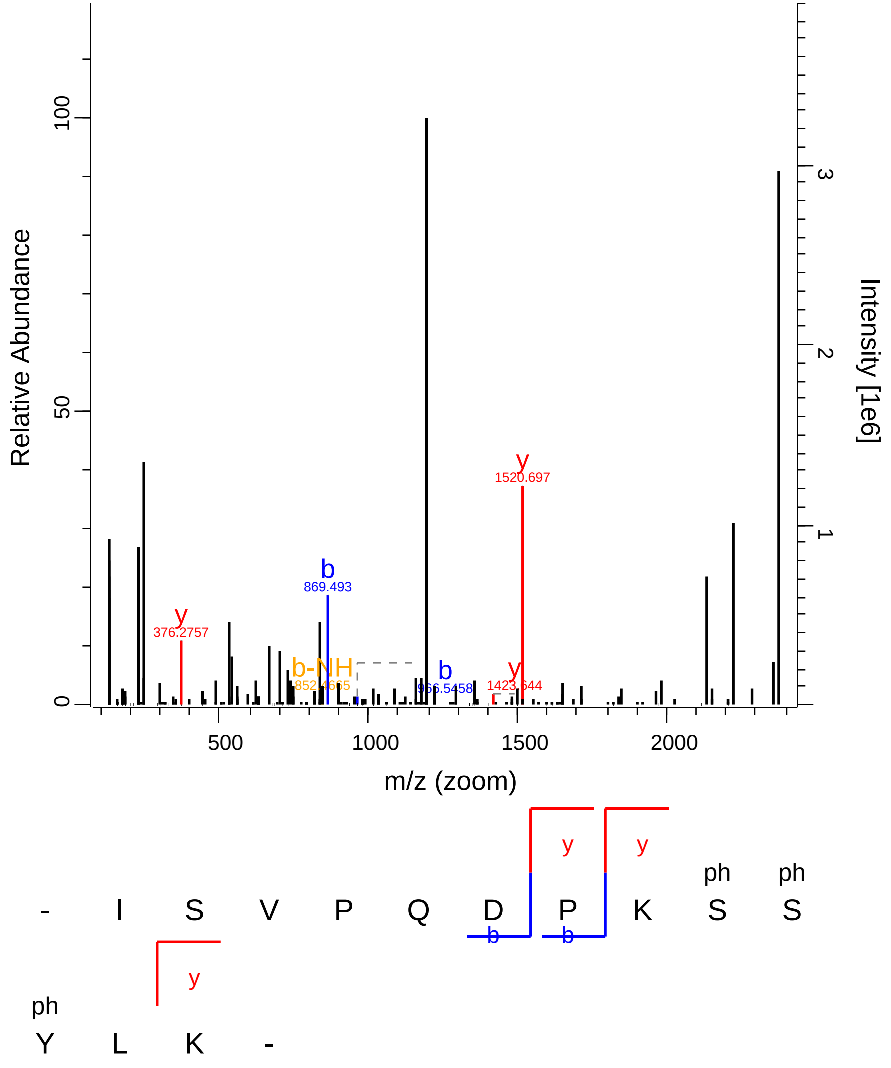

Raw File	Scan	Method	Score	m/z	Gene names
RO-QC-160121_ZackD_SS_Set_01_Fractio	13418	FTMS; HC	123.2	729.32	SORBS2

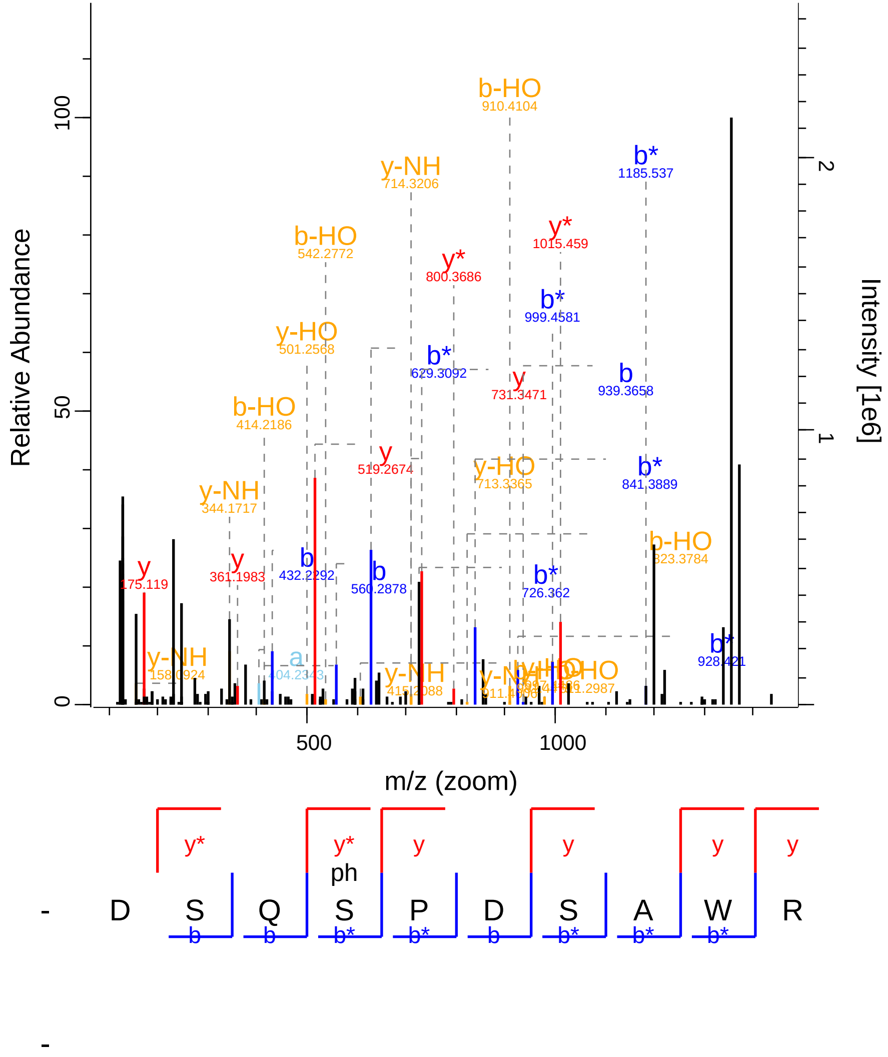

Raw File	Scan	Method	Score	m/z	Gene names
RO-QC-160121_ZackD_SS_Set_01_Fractio	13919	FTMS; HC	70.92	854.46	CARD14

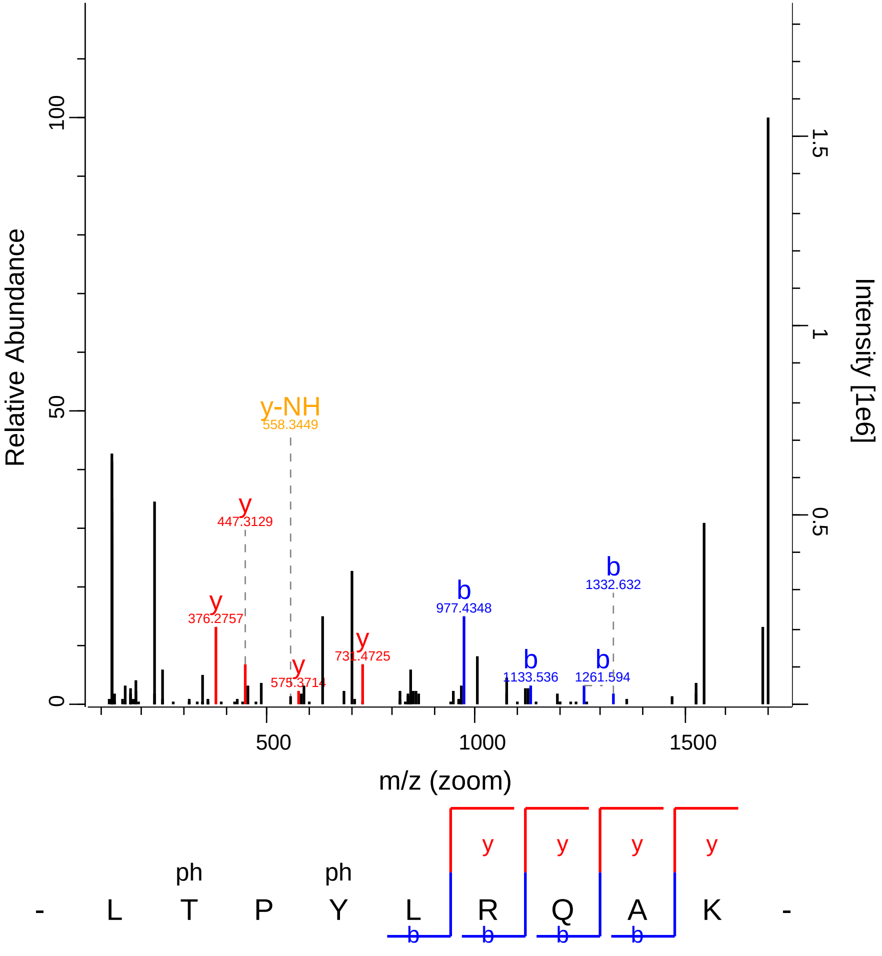

Raw File	Scan	Method	Score	m/z	Gene names
RO-QC-160121_ZackD_SS_Set_01_Fractic	14102	FTMS; HC	45.88	1211.59	LARP4

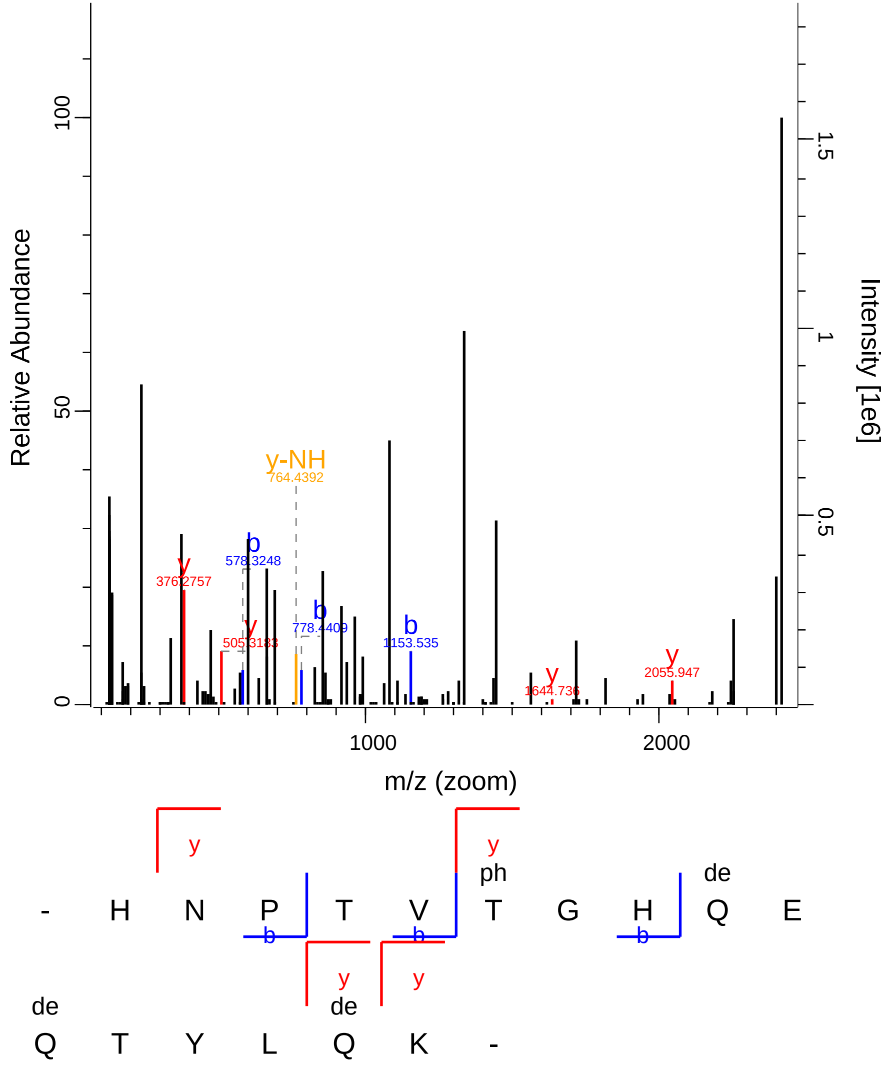

Raw File	Scan	Method	Score	m/z	Gene names
RO-QC-160121_ZackD_SS_Set_01_Fractio	14674	FTMS; HC	59.9	470.07	WDR31

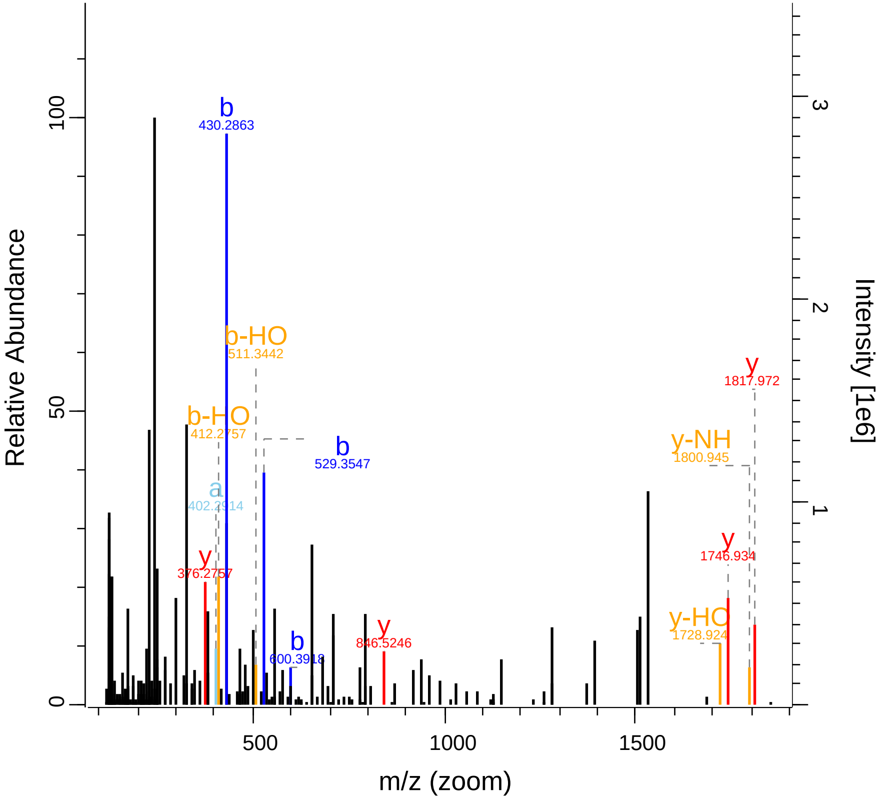

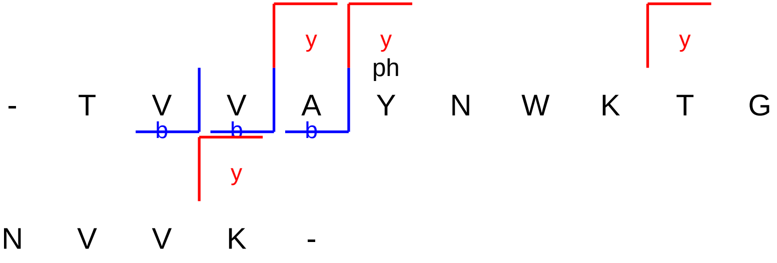

Raw File	Scan	Method	Score	m/z	Gene names
RO-QC-160121_ZackD_SS_Set_01_Fractio	14993	FTMS; HC	136.1	853.09	TRAM1

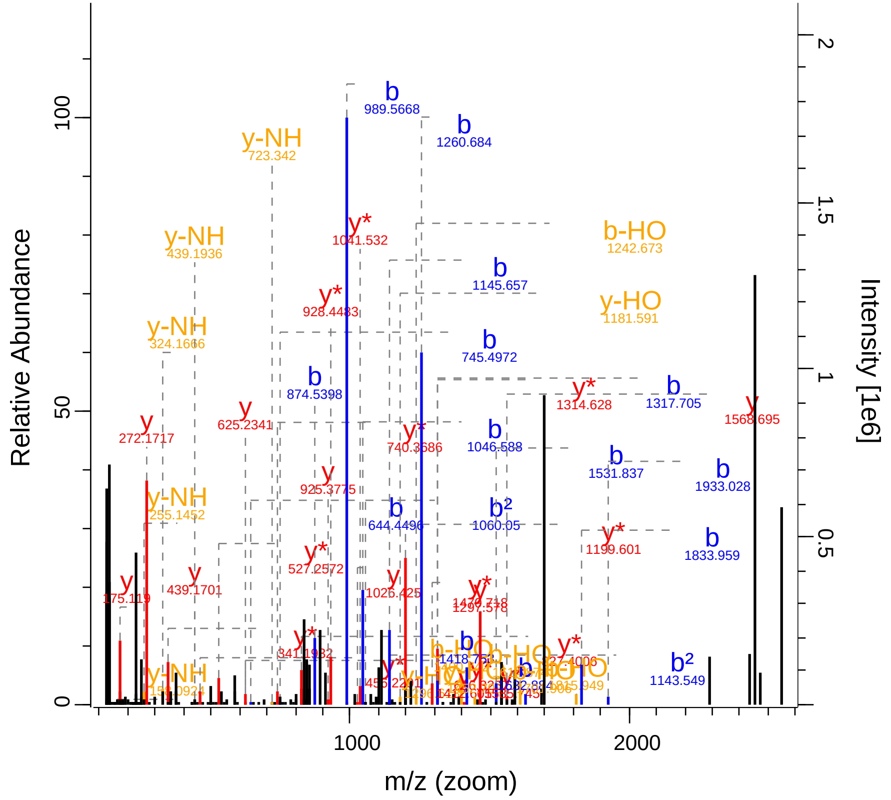

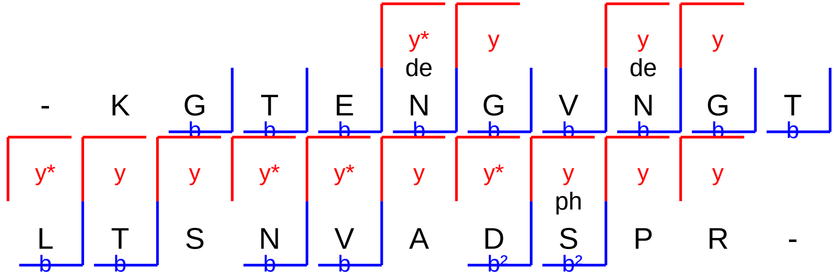

Raw File	Scan	Method	Score	m/z	Gene names
RO-QC-160121_ZackD_SS_Set_01_Fractio	15049	FTMS; HC	70.33	787.82	MAP1B

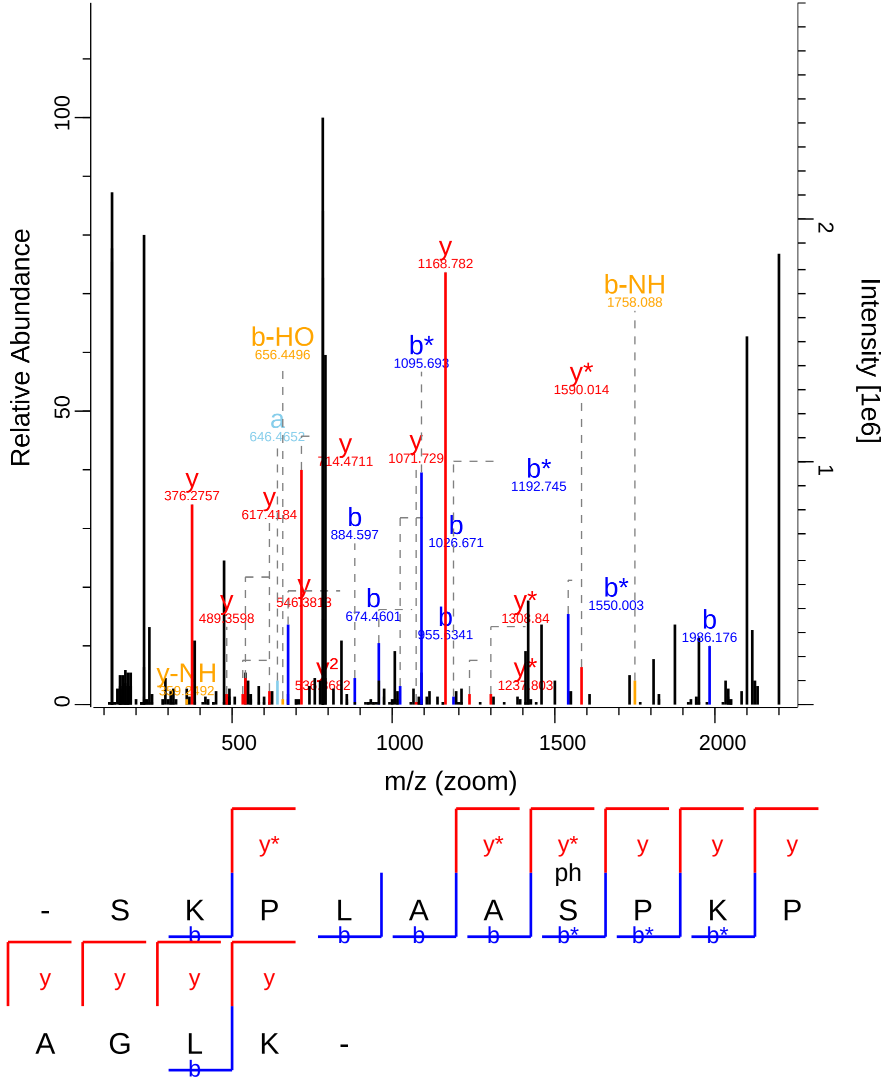

Raw File	Scan	Method	Score	m/z	Gene names
RO-QC-160121_ZackD_SS_Set_01_Fractio	15876	FTMS; HC	95.04	988.15	KIF21A

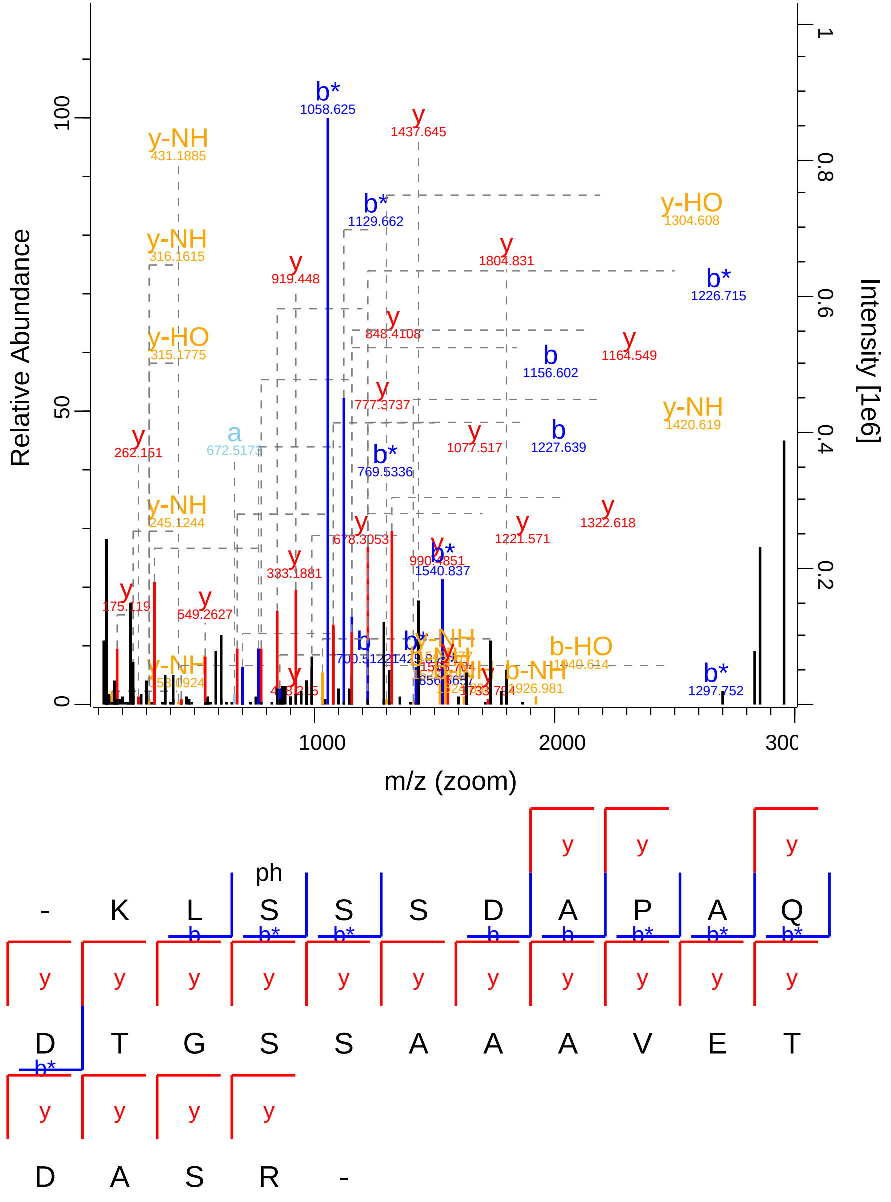

Raw File	Scan	Method	Score	m/z	Gene names
RO-QC-160121_ZackD_SS_Set_01_Fractio	16909	FTMS; HC	62.82	965.03	C2CD5

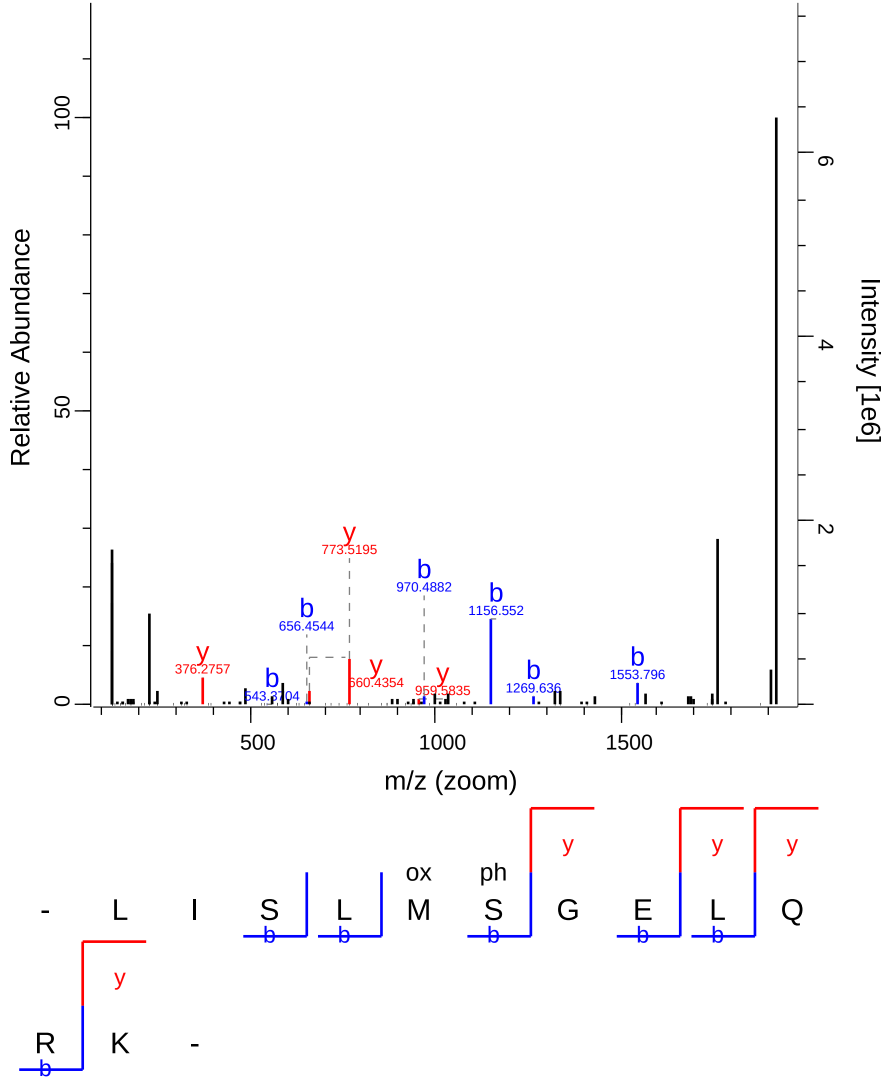

Raw File	Scan	Method	Score	m/z	Gene names
RO-QC-160121_ZackD_SS_Set_01_Fractic	18118	FTMS; HC	58.98	1116.62	NUP107

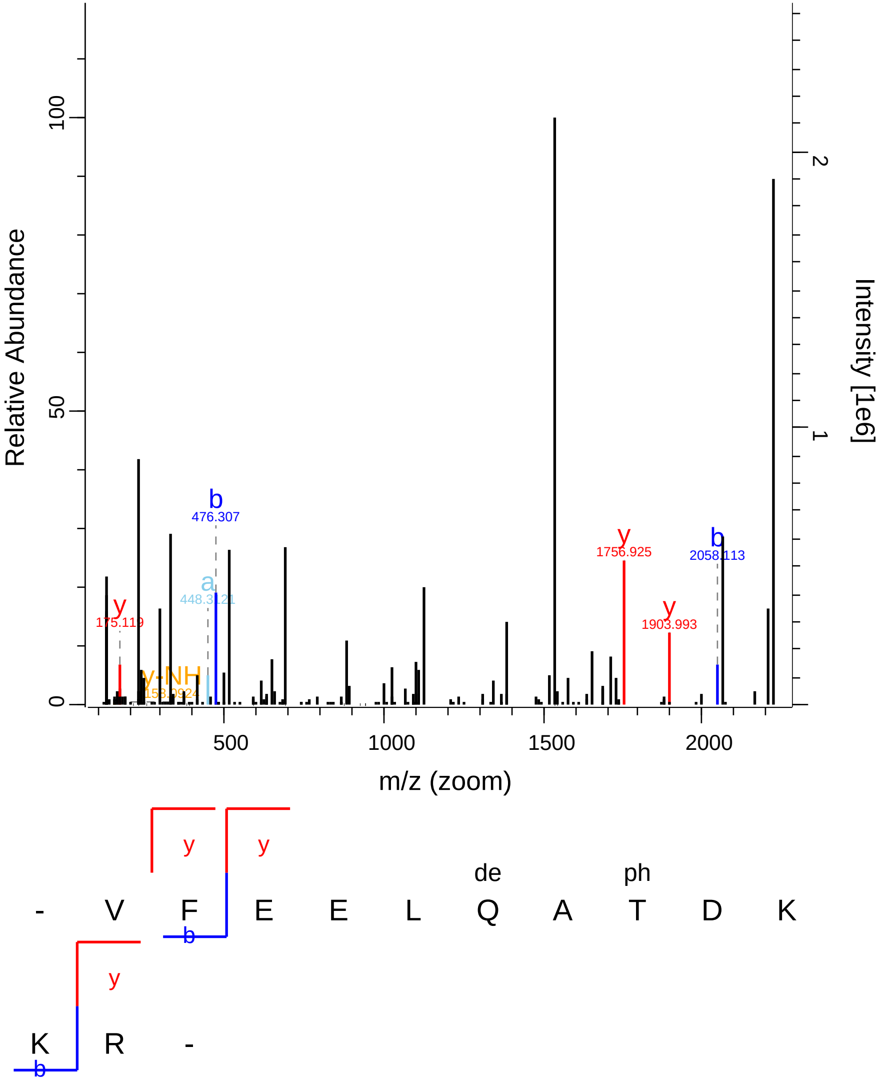

Raw File	Scan	Method	Score	m/z	Gene names
RO-QC-160121_ZackD_SS_Set_01_Fractio	18199	FTMS; HC	54.28	574.65	USP24

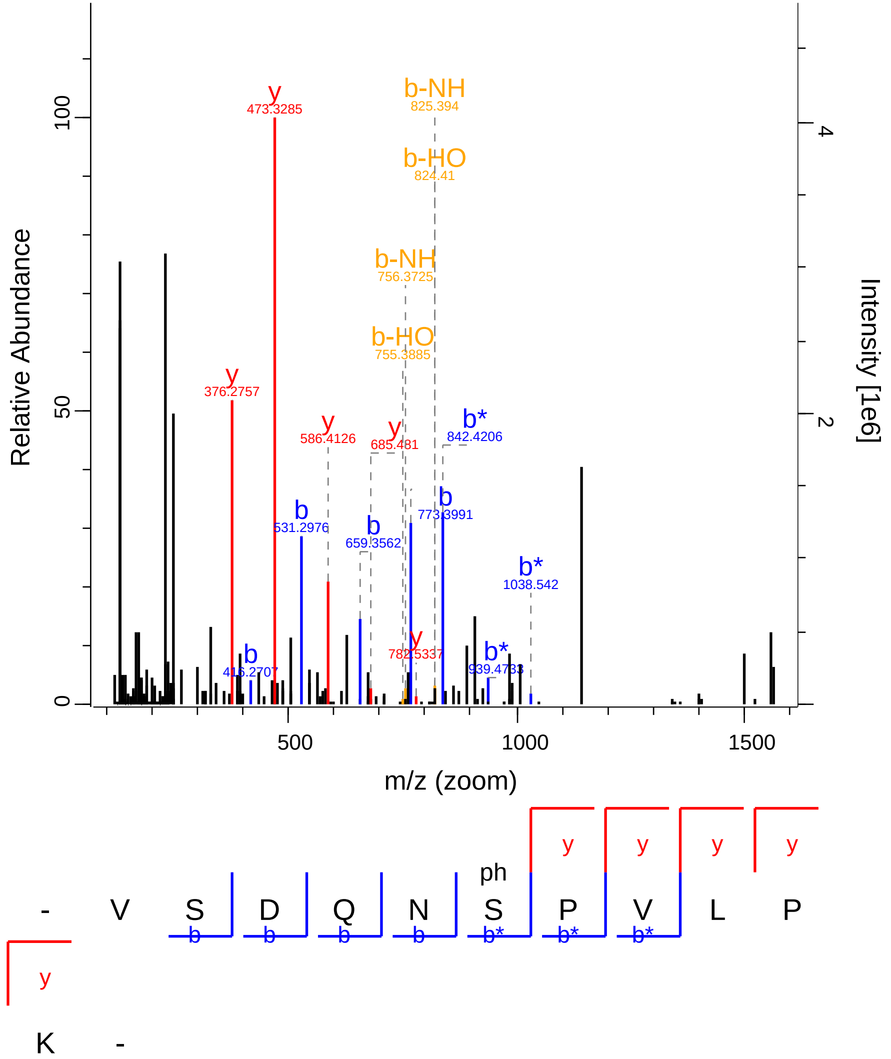

Raw File	Scan	Method	Score	m/z	Gene names
RO-QC-160121_ZackD_SS_Set_01_Fractic	18906	FTMS; HC	48.4	1155.12	DHX8

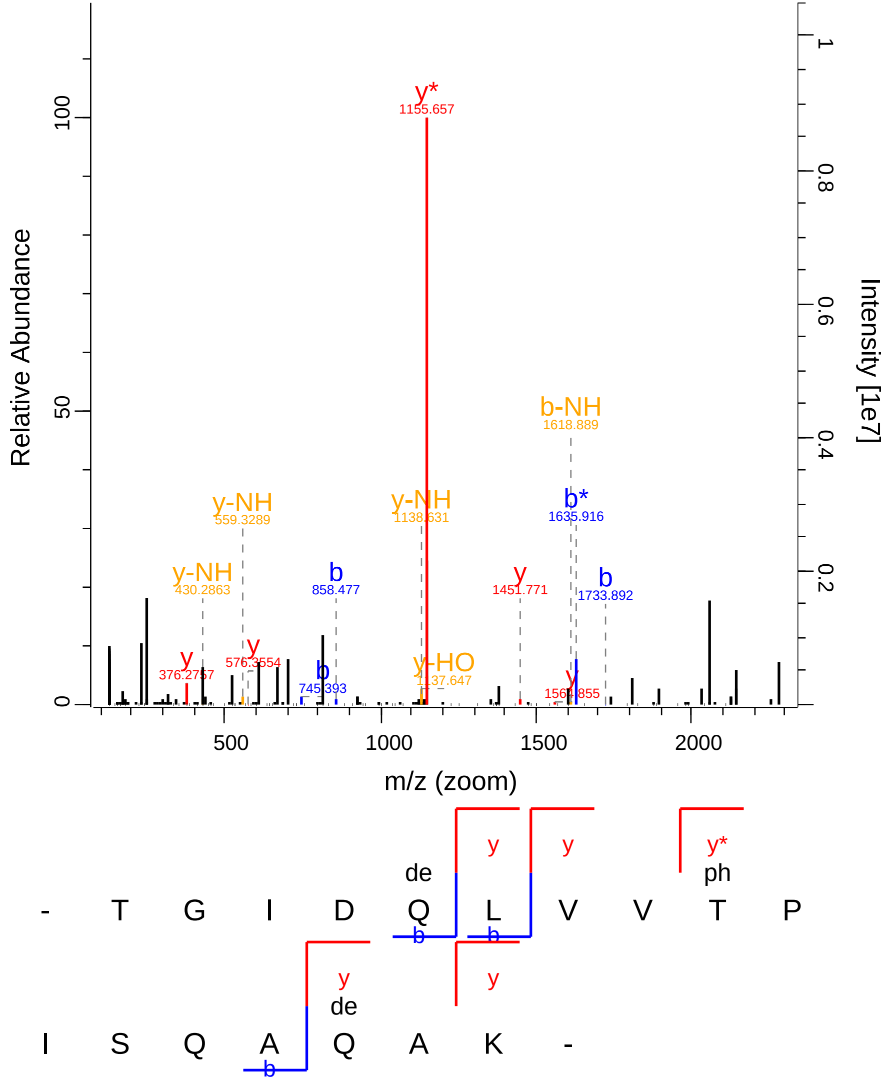

Raw File	Scan	Method	Score	m/z	Gene names
RO-QC-160121_ZackD_SS_Set_01_Fractic	18978	FTMS; HC	64.3	1078.54	ADCY9

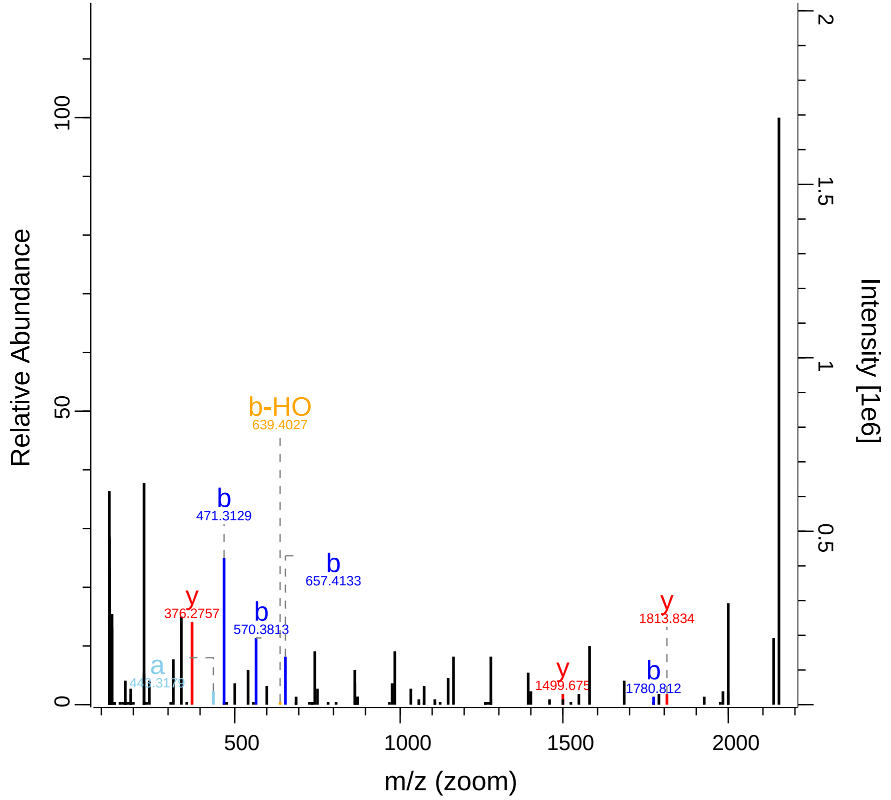

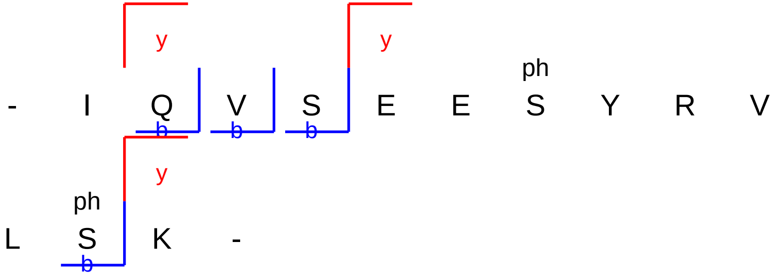

Raw File	Scan	Method	Score	m/z	Gene names
RO-QC-160121_ZackD_SS_Set_01_Fractio	19265	FTMS; HC	40.08	869.18	VLDLR

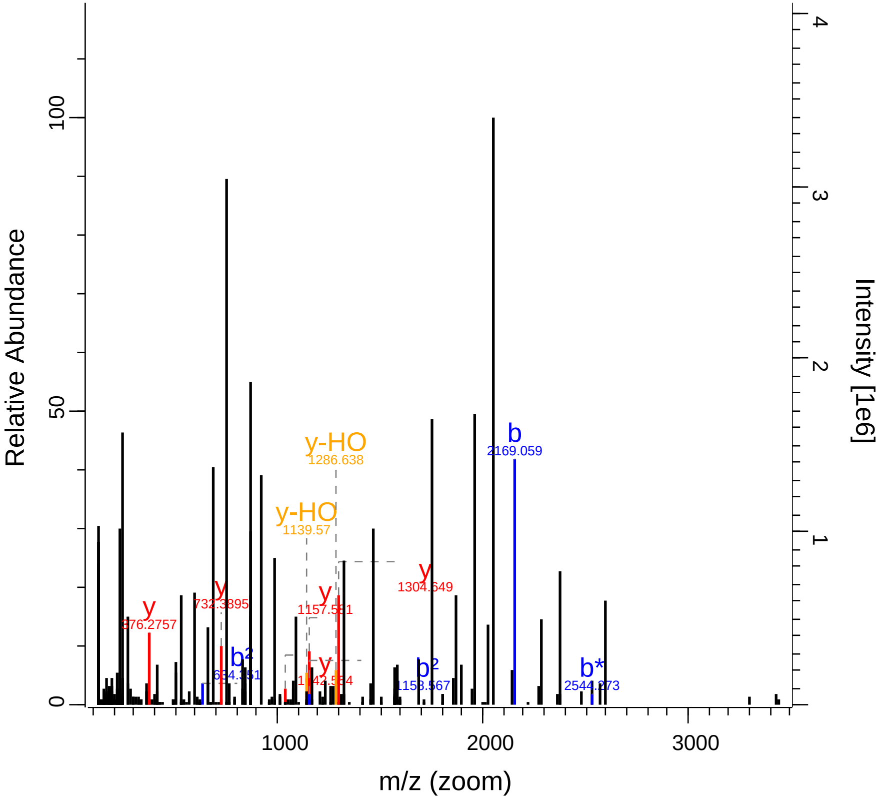

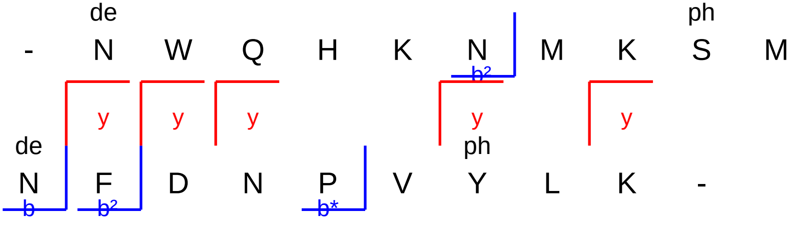

Raw File	Scan	Method	Score	m/z	Gene names
RO-QC-160121_ZackD_SS_Set_01_Fractic	19338	FTMS; HC	48.74	1082.04	ELAVL1

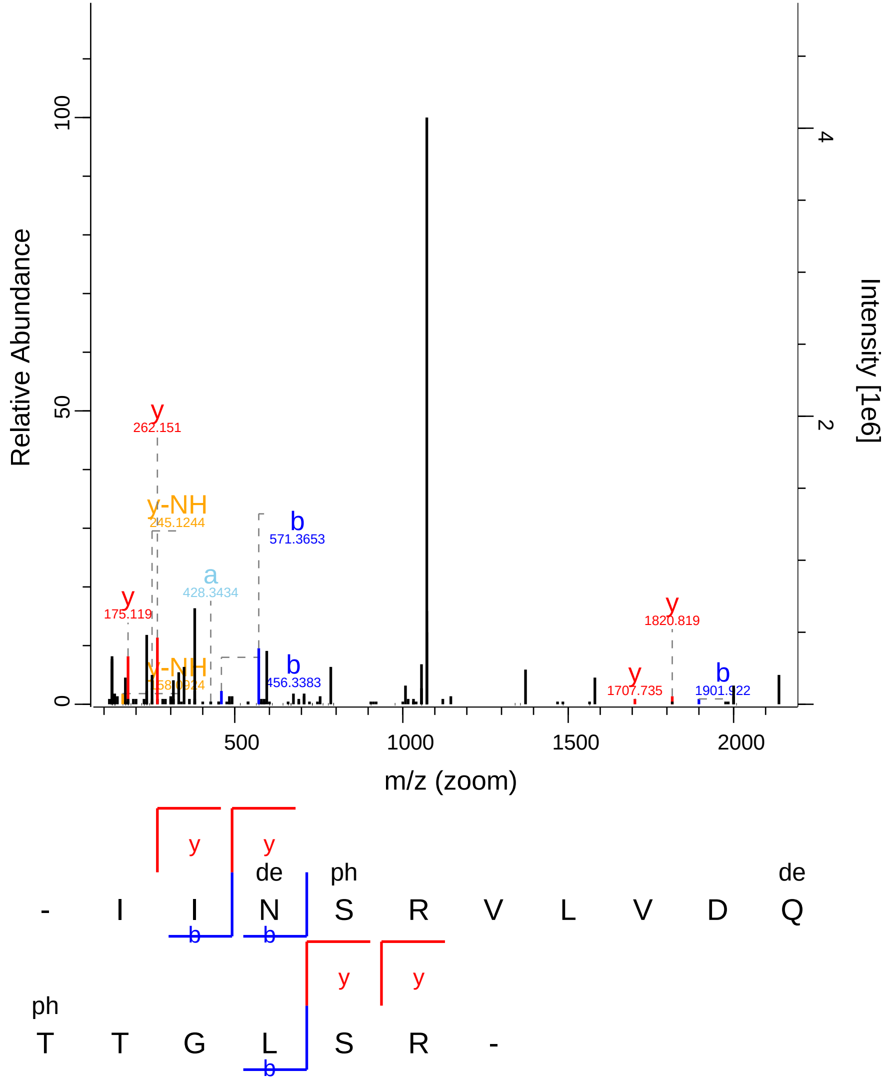

Raw File	Scan	Method	Score	m/z	Gene names
RO-QC-160121_ZackD_SS_Set_01_Fractio	20152	FTMS; HC	55.53	825.42	CELSR3

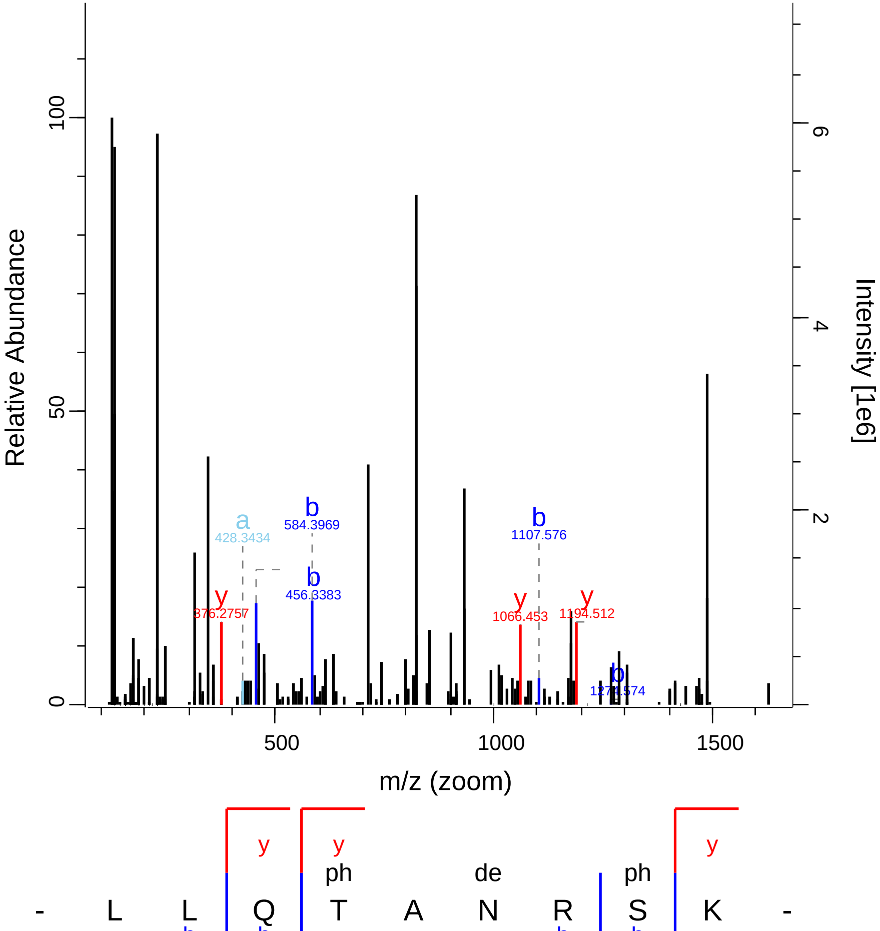

Raw File	Scan	Method	Score	m/z	Gene names
RO-QC-160121_ZackD_SS_Set_01_Fractio	20579	FTMS; HC	214.34	976.49	AHNAK

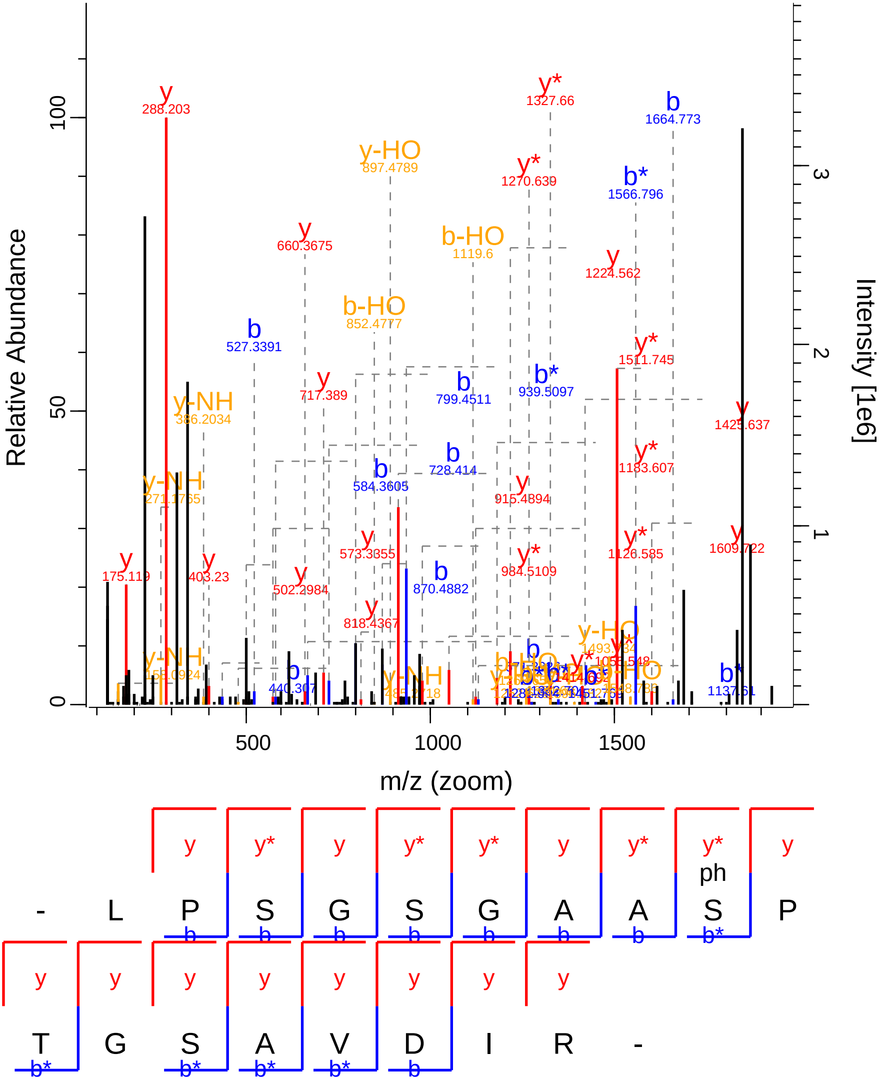

Raw File	Scan	Method	Score	m/z	Gene names
RO-QC-160121_ZackD_SS_Set_01_Fractio	20909	FTMS; HC	65.25	810.91	SRRM2

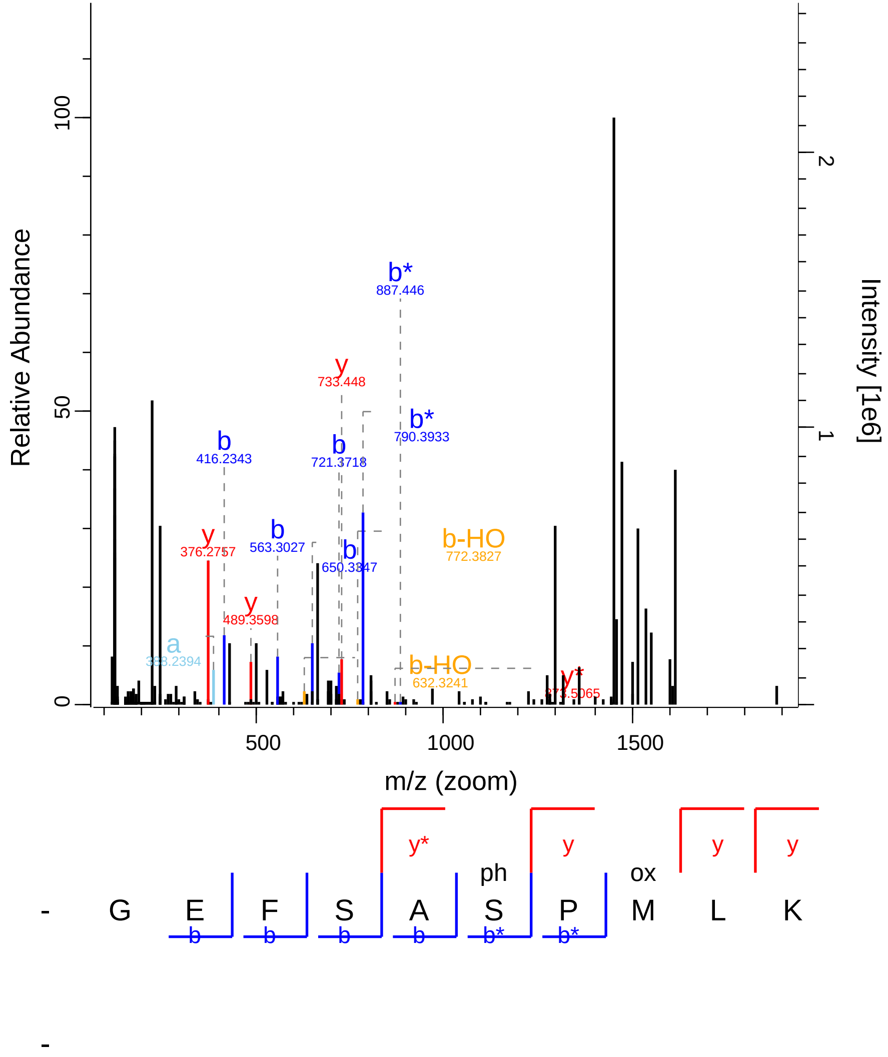

Raw File	Scan	Method	Score	m/z	Gene names
RO-QC-160121_ZackD_SS_Set_01_Fractio	21855	FTMS; HC	71.55	702.73	CHM

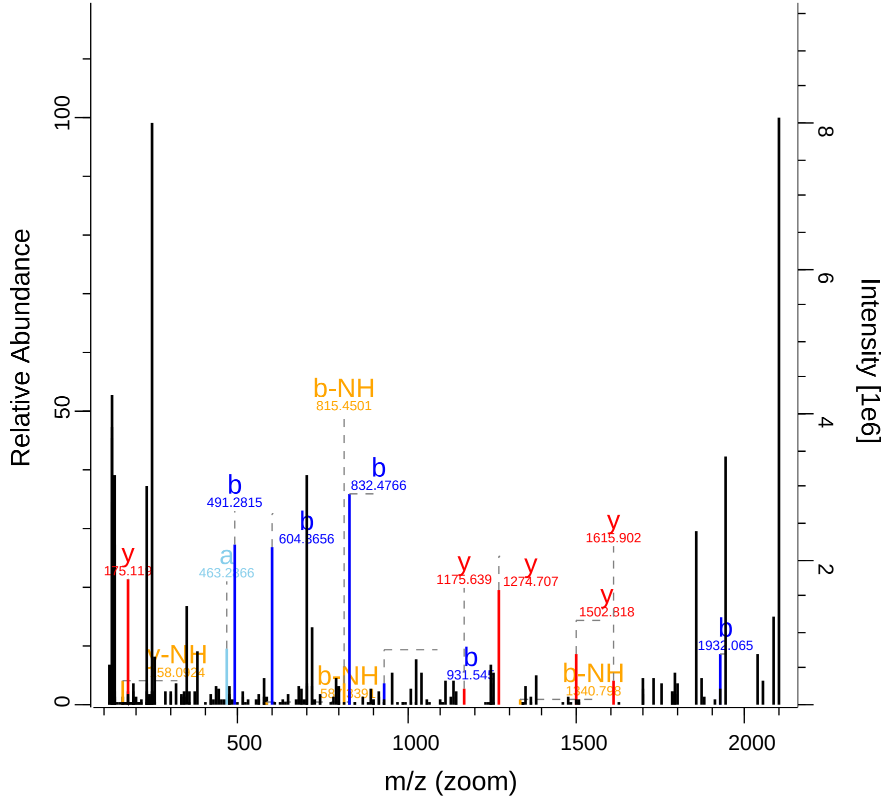

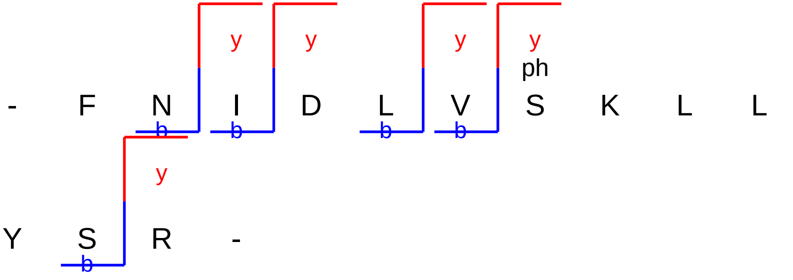

Raw File	Scan	Method	Score	m/z	Gene names
RO-QC-160121_ZackD_SS_Set_01_Fractio	22840	FTMS; HC	78.1	1080.6	CHM

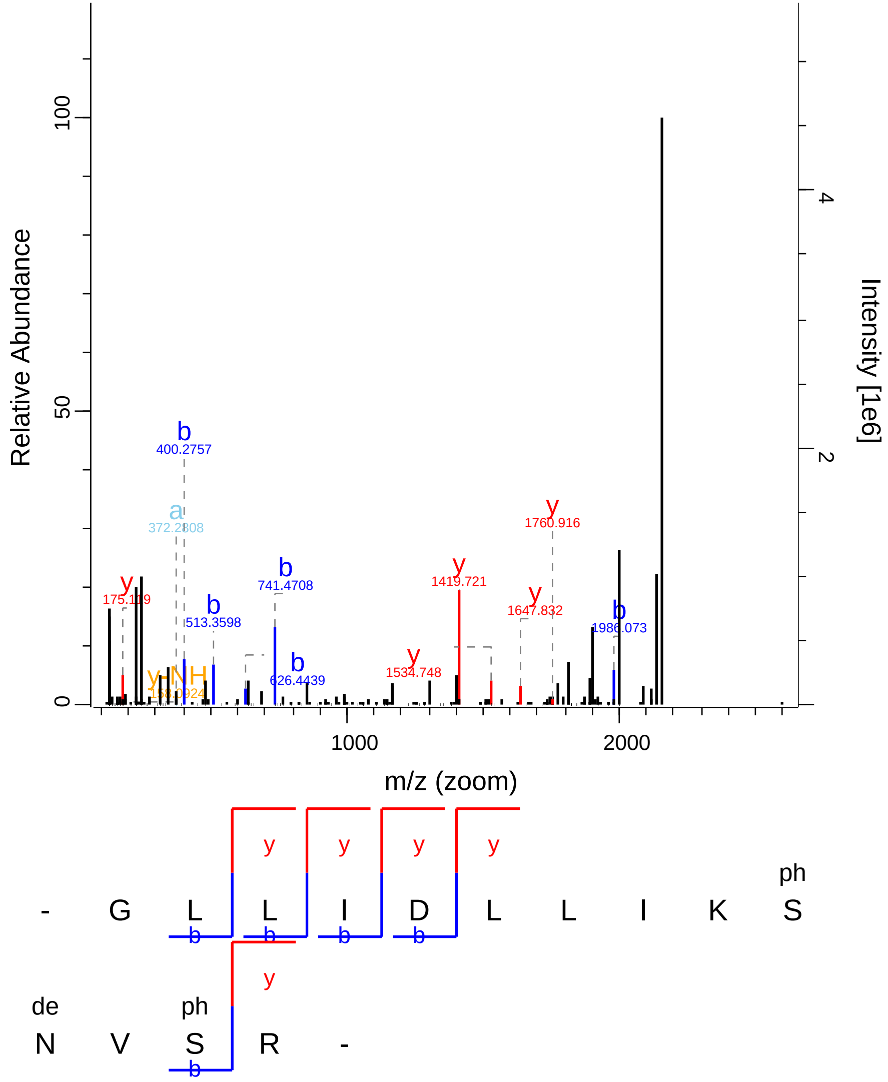

Raw File	Scan	Method	Score	m/z	Gene names
RO-QC-160121_ZackD_SS_Set_01_Fractic	22971	FTMS; HC	47.56	1016.01	INPP5D

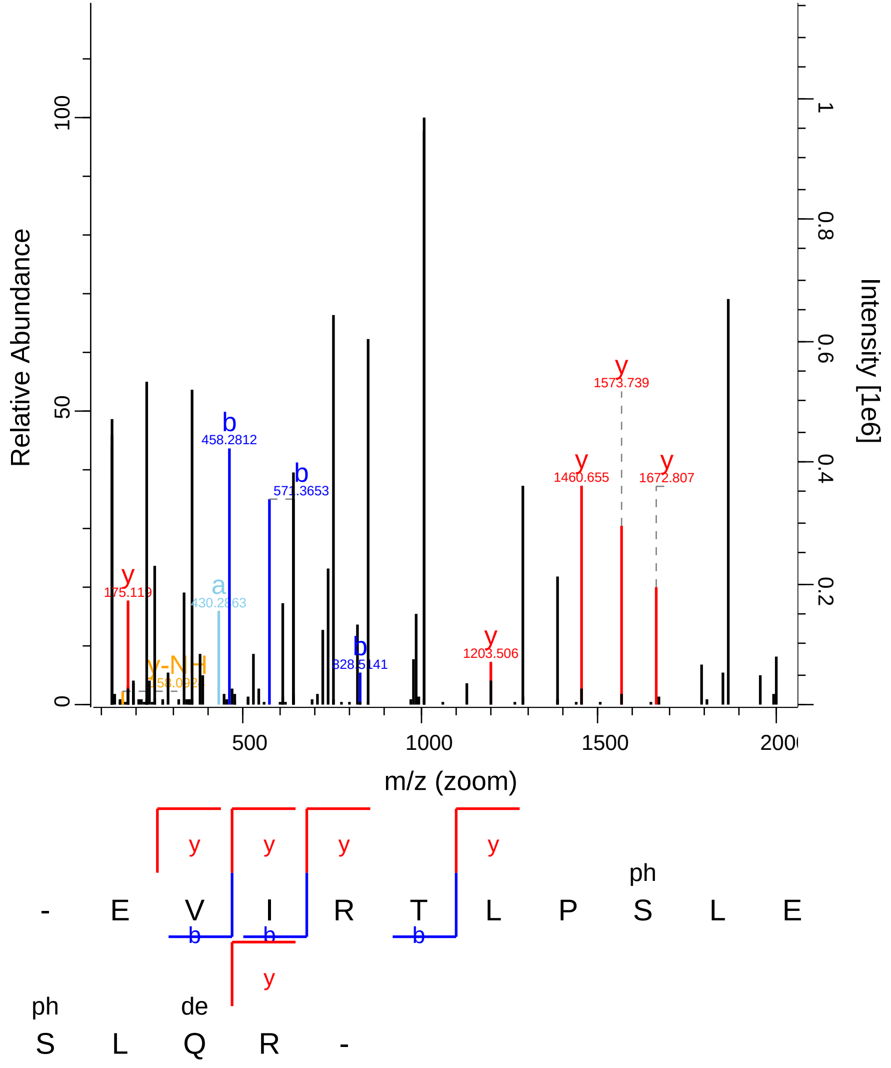

Raw File	Scan	Method	Score	m/z	Gene names
RO-QC-160121_ZackD_SS_Set_01_Fractic	24616	FTMS; HC	146.2	1086.05	PEBP1

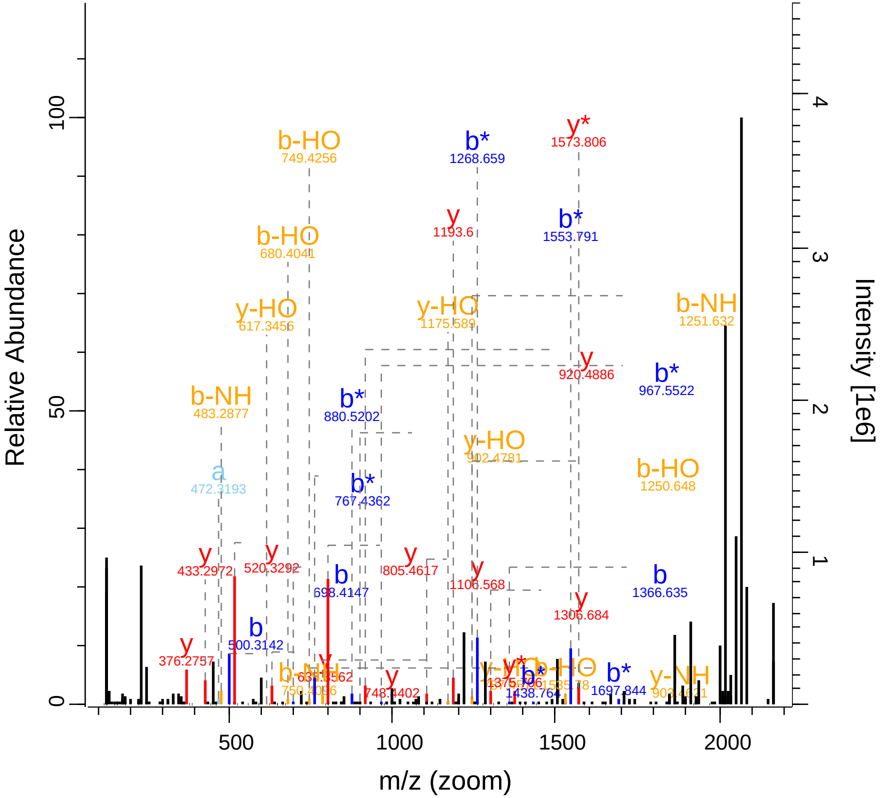

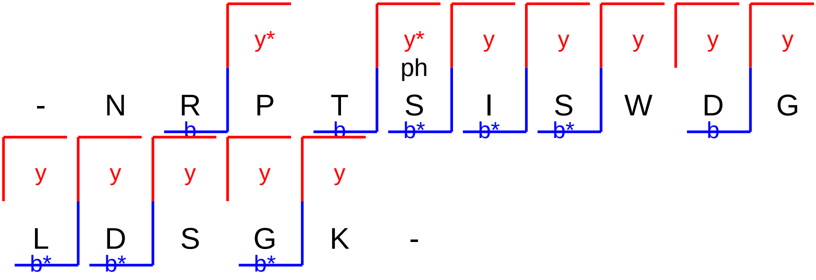

Raw File	Scan	Method	Score	m/z	Gene names
RO-QC-160121_ZackD_SS_Set_01_Fractio	26029	FTMS; HC	41.98	809.41	ZDBF2

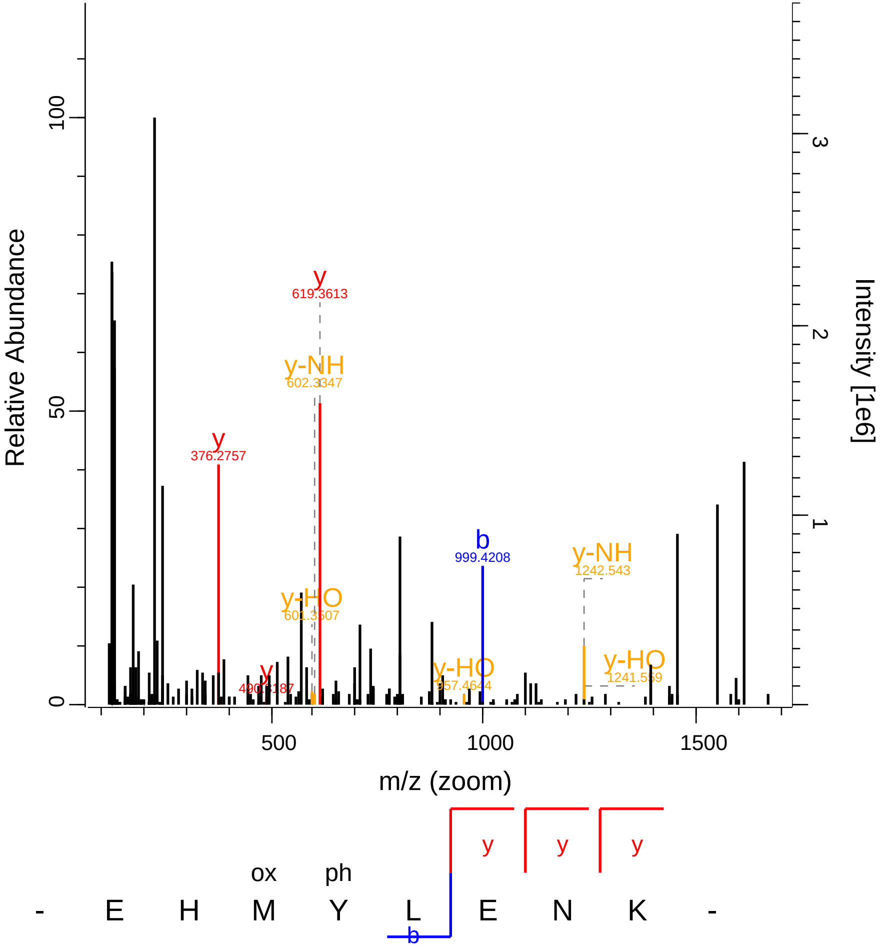

Raw File	Scan	Method	Score	m/z	Gene names
RO-QC-160121_ZackD_SS_Set_01_Fractior	26230	FTMS; HCI	54.6	948.2	PIBF1

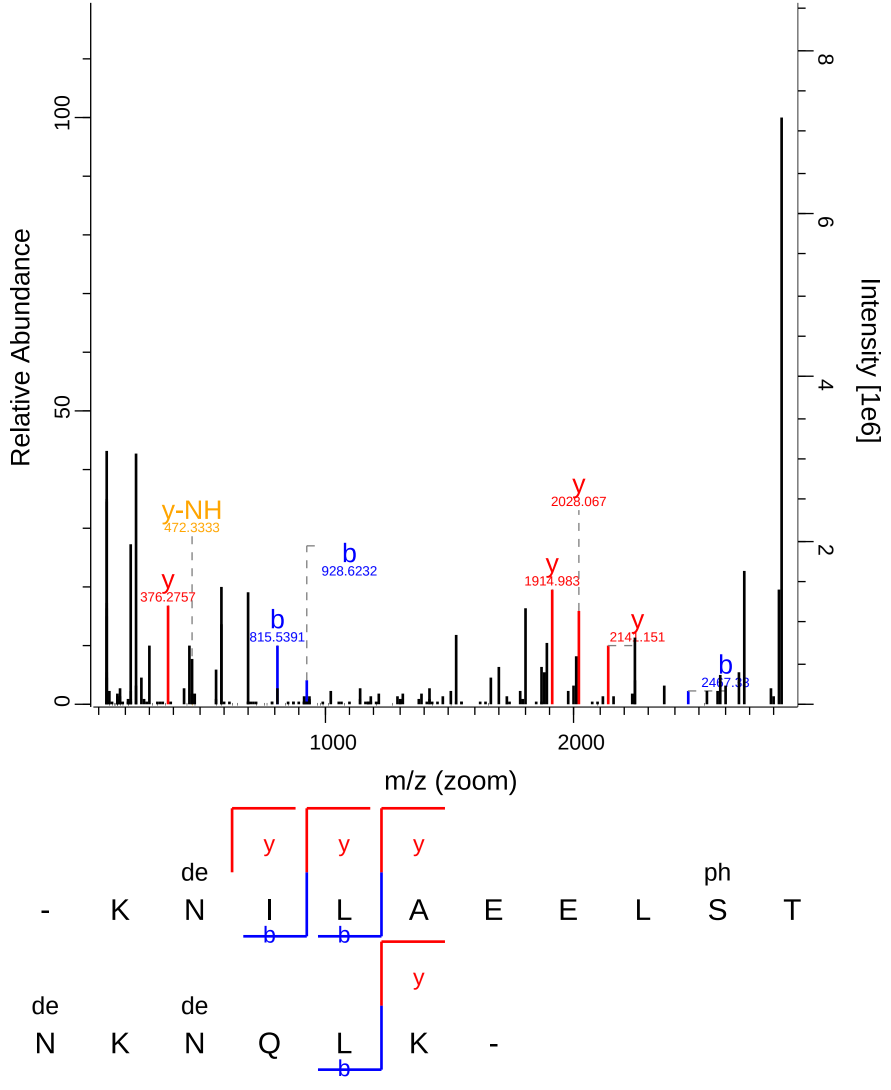

Raw File	Scan	Method	Score	m/z	Gene names
RO-QC-160121_ZackD_SS_Set_01_Fractic	26990	FTMS; HC	56.2	1116.63	CNGA2

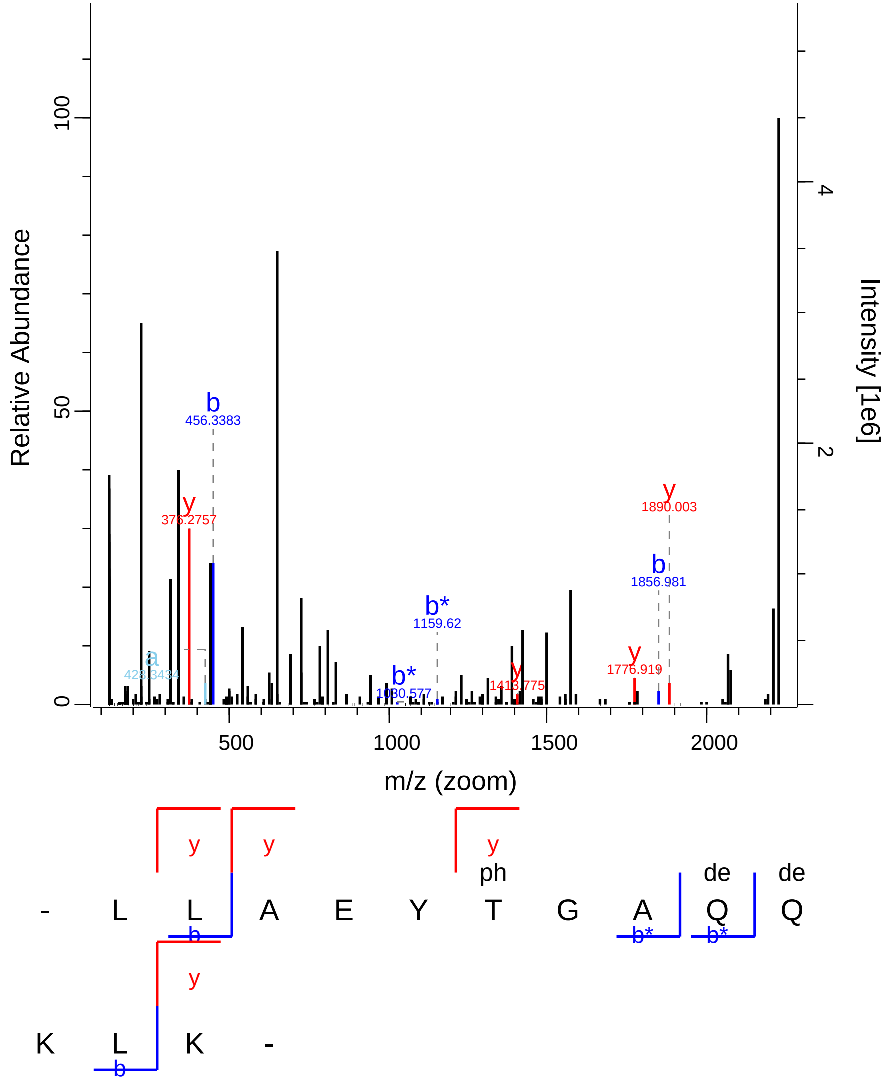

Raw File	Scan	Method	Score	m/z	Gene names
RO-QC-160121_ZackD_SS_Set_01_Fractic	27525	FTMS; HC	64.22	1283.22	CYB561D2

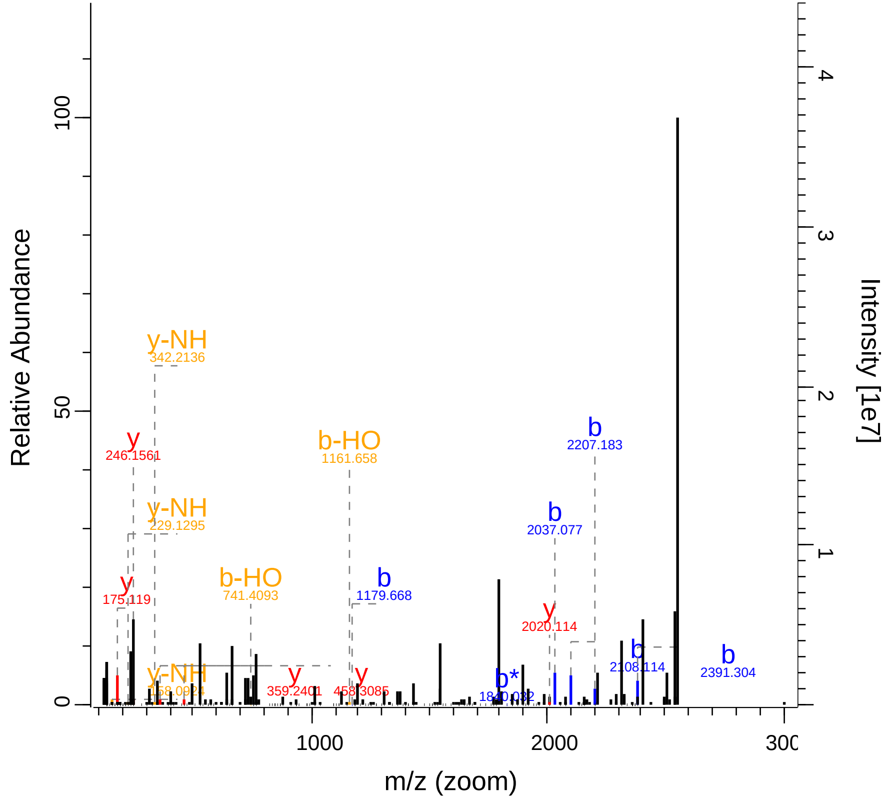

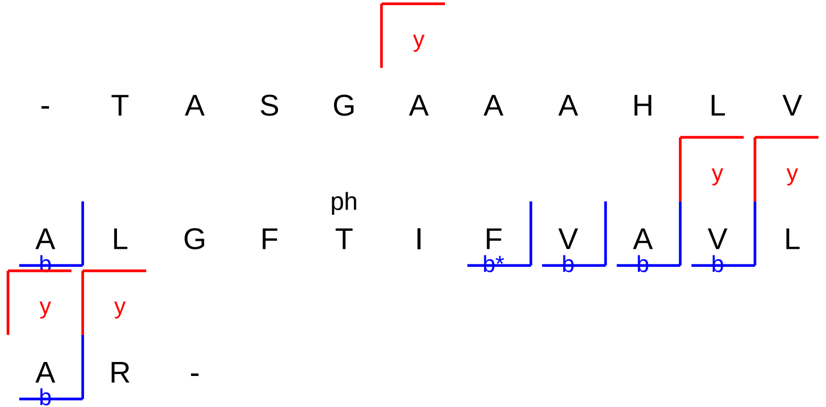

Raw File	Scan	Method	Score	m/z	Gene names
RO-QC-160121_ZackD_SS_Set_01_Fractio	29113	FTMS; HC	199.36	1430.7	LRRC47

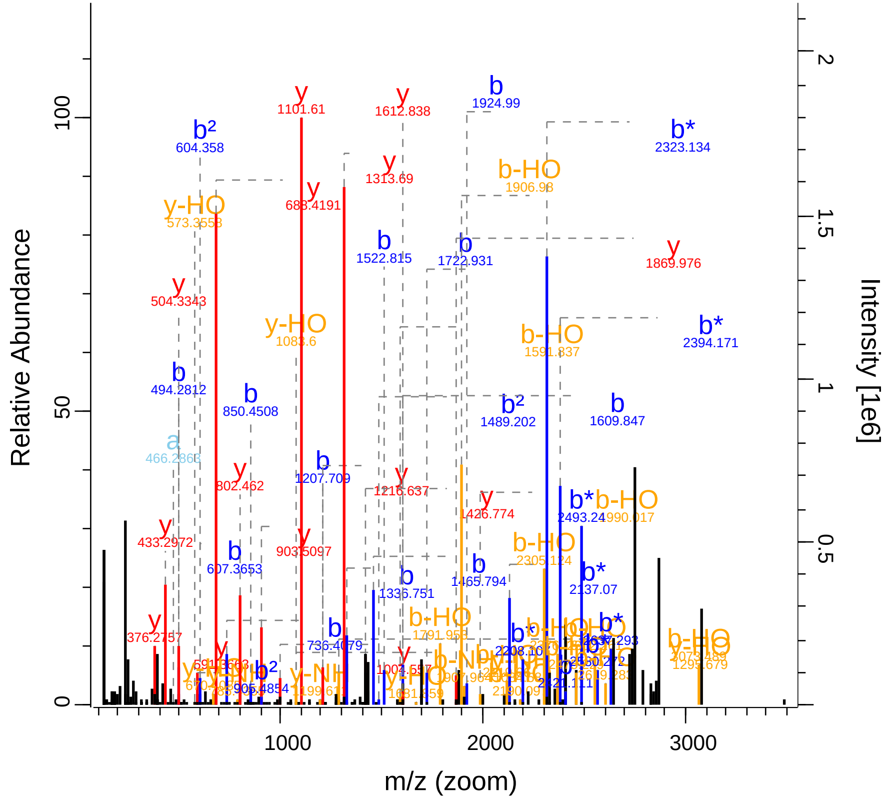

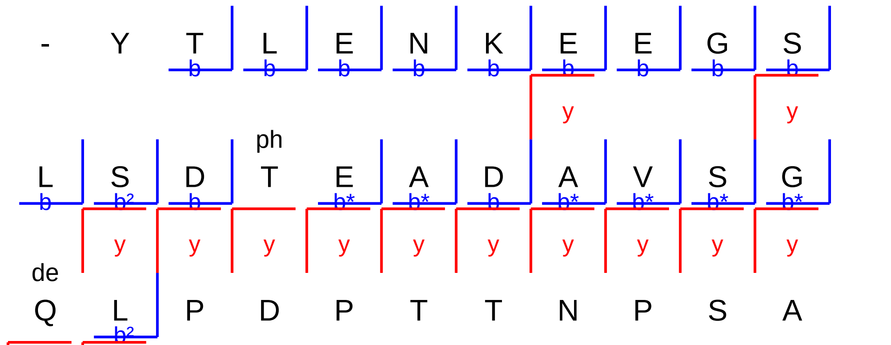

Raw File	Scan	Method	Score	m/z	Gene names
RO-QC-160121_ZackD_SS_Set_01_Fractic	29594	FTMS; HC	66.6	1210.63	ANK1

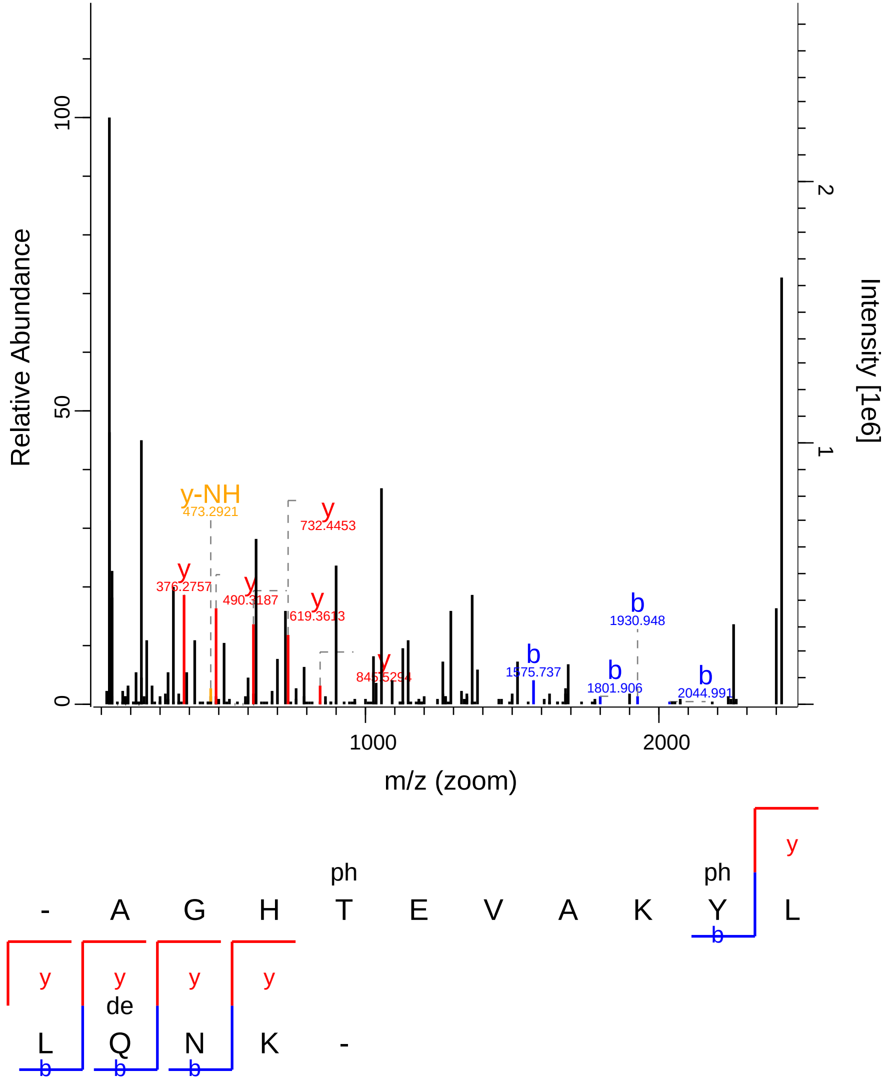

Raw File	Scan	Method	Score	m/z	Gene names
RO-QC-160121_ZackD_SS_Set_01_Fractic	29933	FTMS; HC	55.55	1048.47	C5orf42

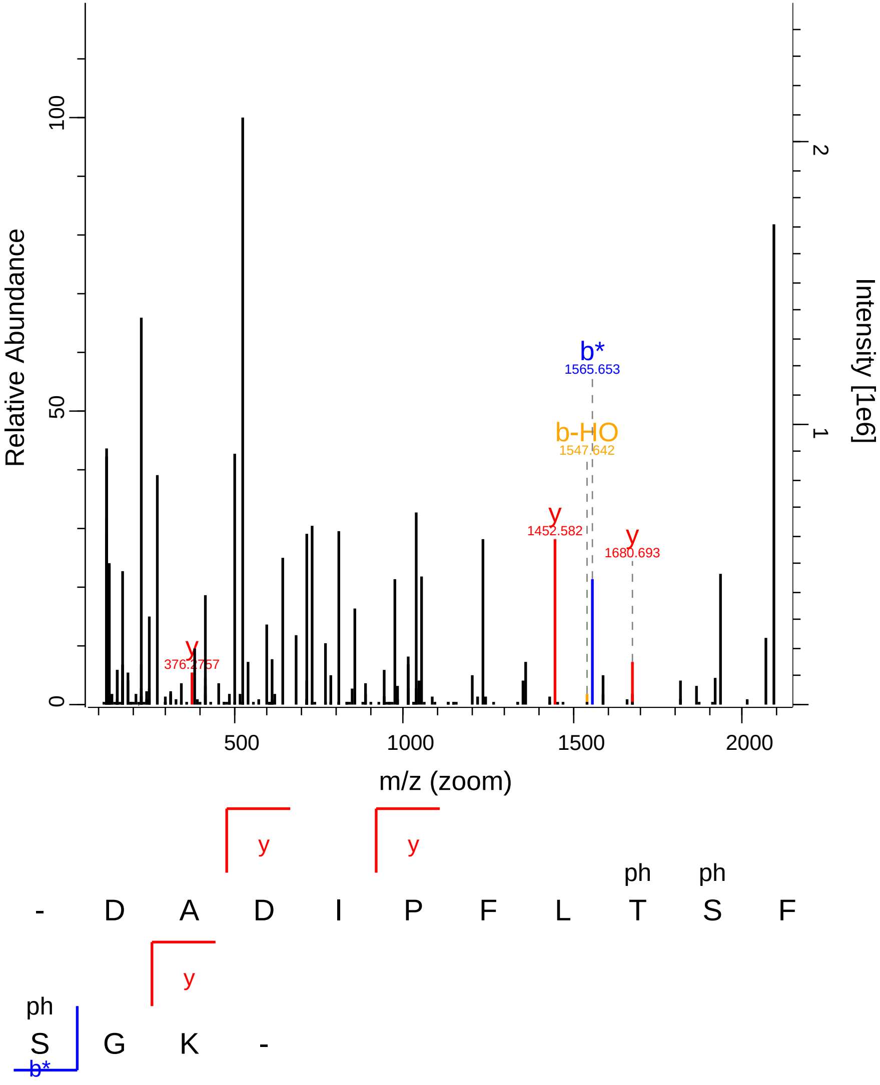

Raw File	Scan	Method	Score	m/z	Gene names
RO-QC-160121_ZackD_SS_Set_01_Fractio	30406	FTMS; HC	61.45	828.71	EIF4G1

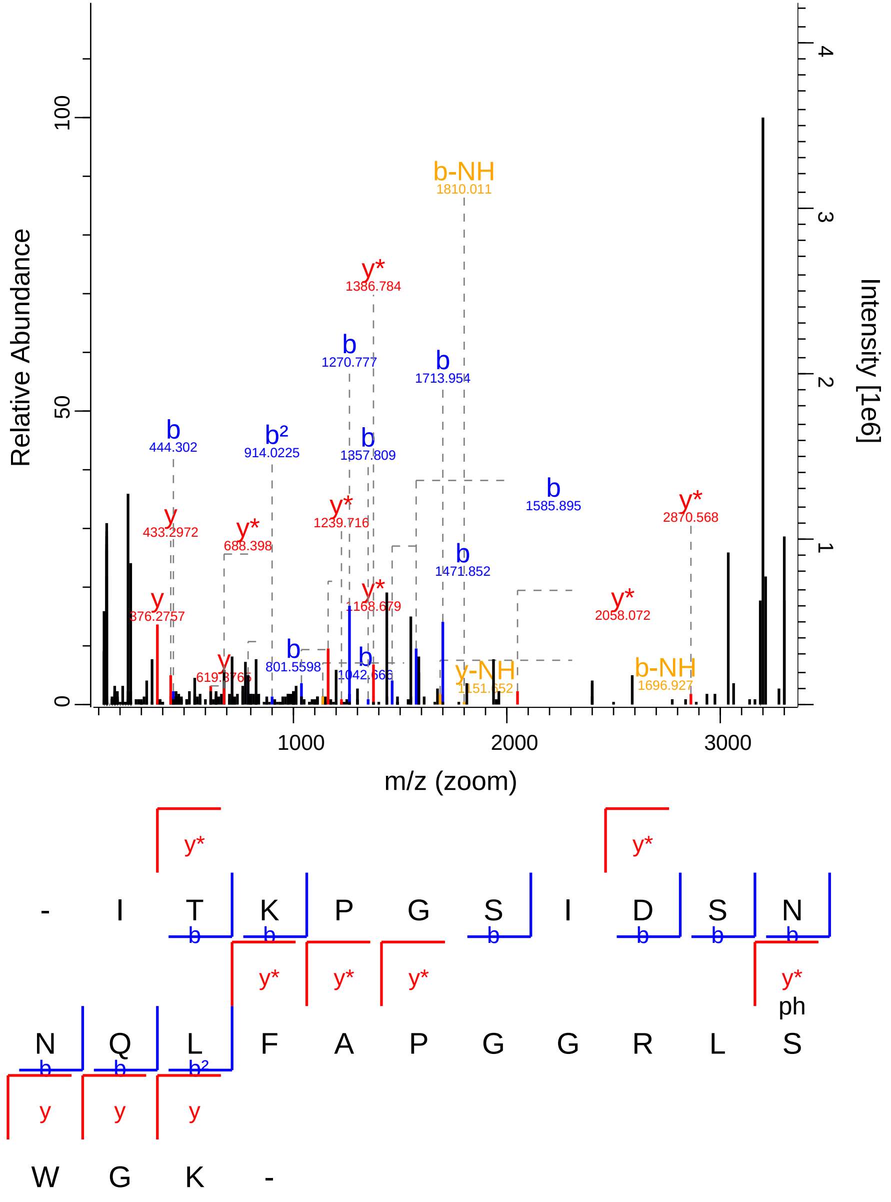

Raw File	Scan	Method	Score	m/z	Gene names
RO-QC-160121_ZackD_SS_Set_01_Fractic	32185	FTMS; HC	247.14	1032.01	GFPT1

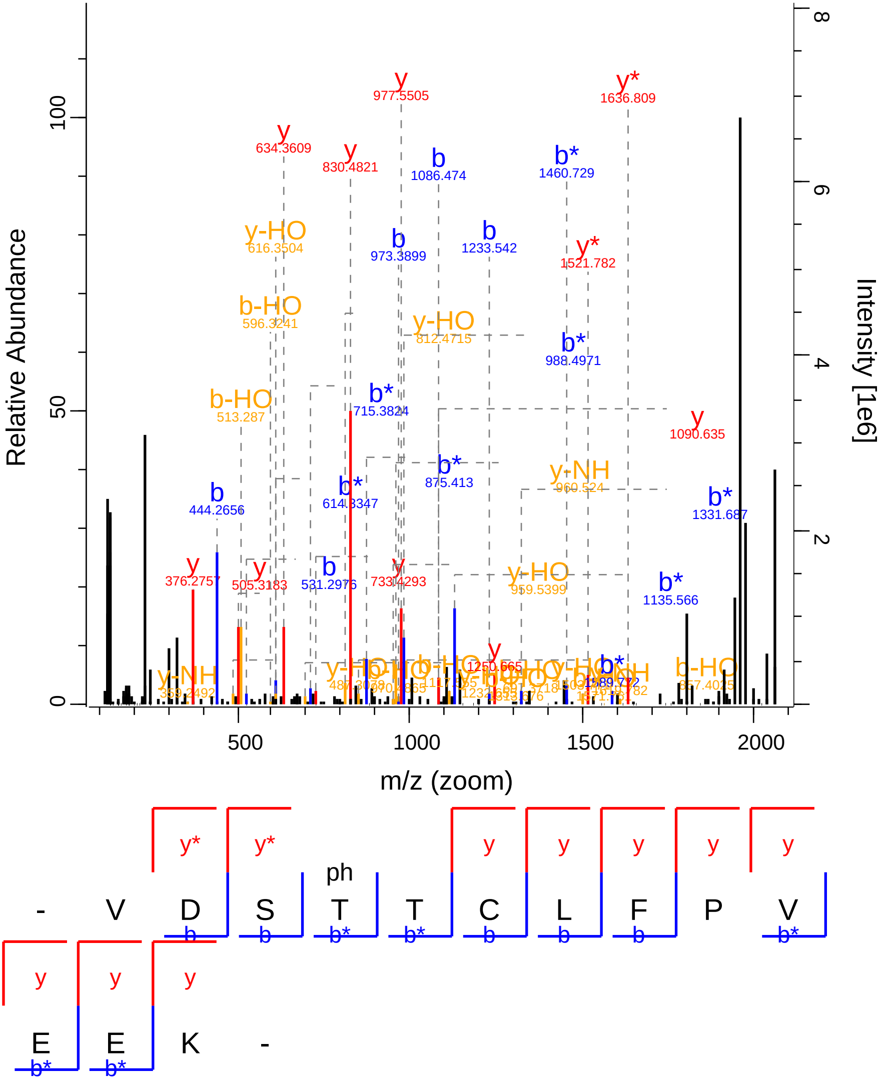

Raw File	Scan	Method	Score	m/z	Gene names
RO-QC-160121_ZackD_SS_Set_01_Fractic	33181	FTMS; HC	47.67	1463.77	ST6GALNAC5

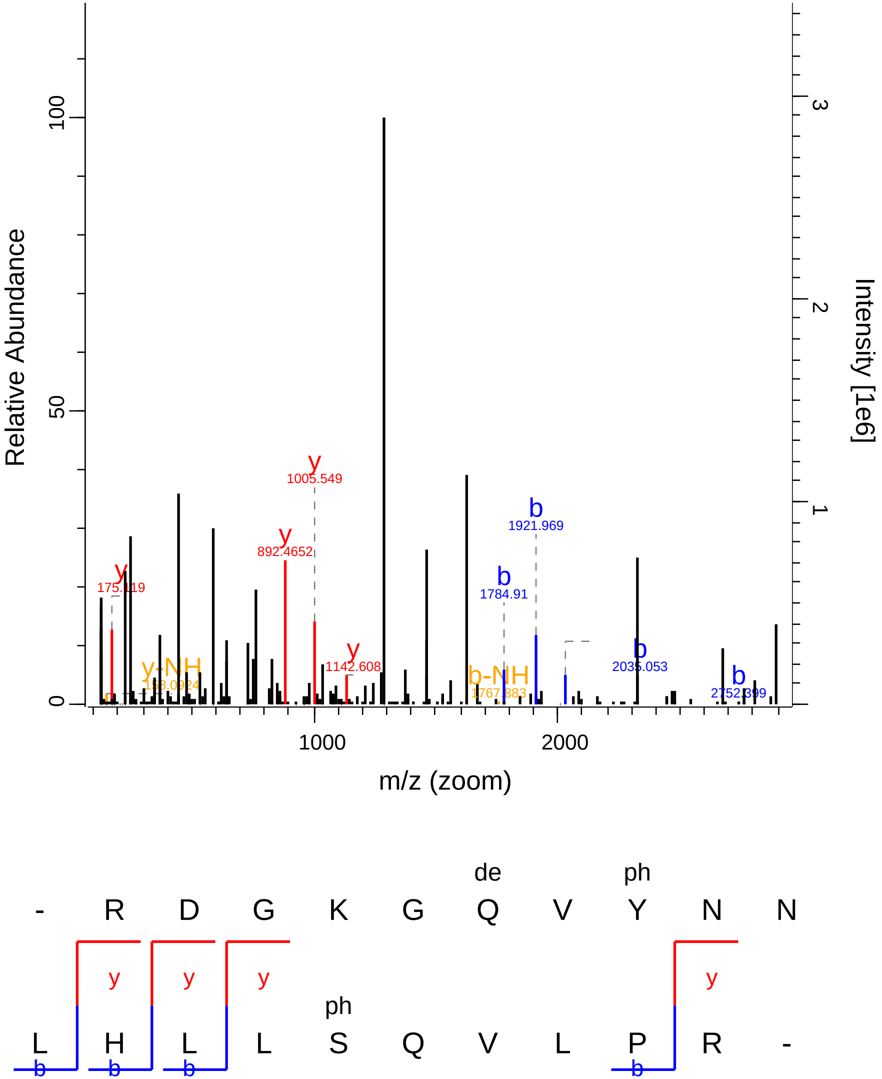

Raw File	Scan	Method	Score	m/z	Gene names
RO-QC-160121_ZackD_SS_Set_01_Fractic	33217	FTMS; HC	91.94	1315.12	ZNF540

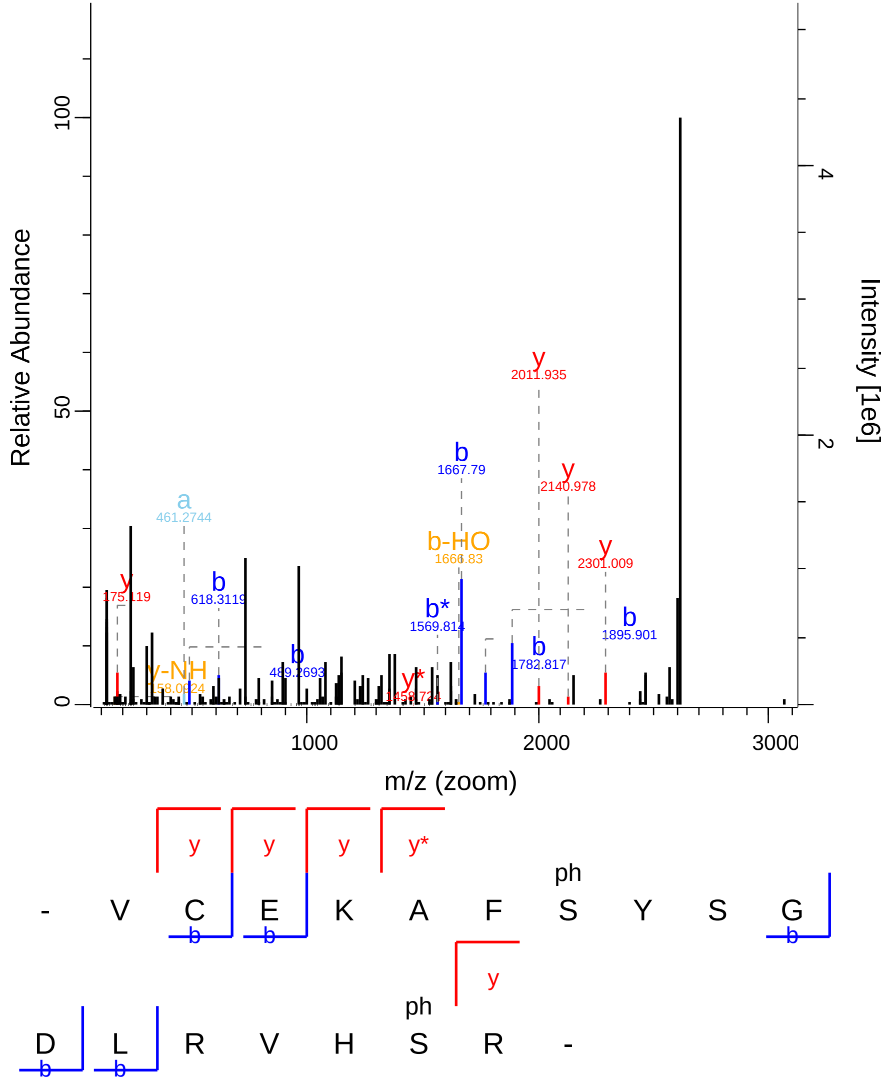

Raw File	Scan	Method	Score	m/z	Gene names
RO-QC-160121_ZackD_SS_Set_01_Fractic	33469	FTMS; HC	57.35	1225.08	ULK3

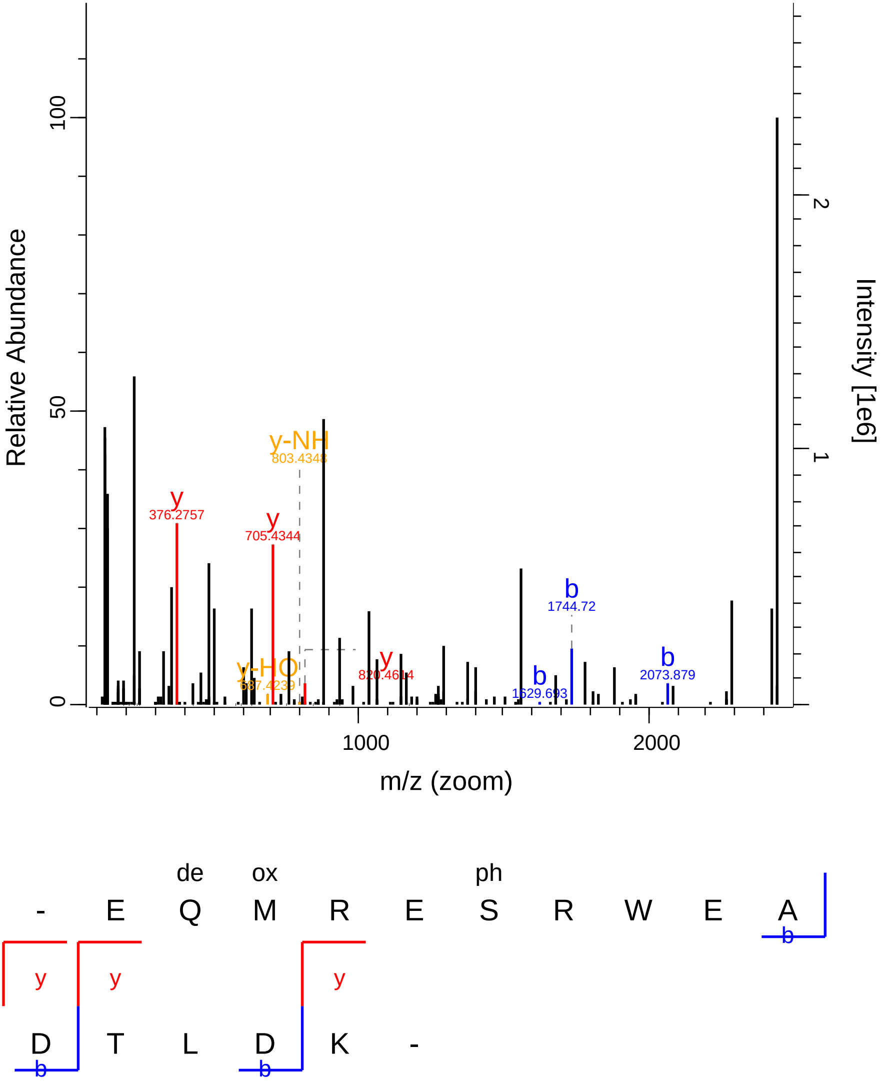

Raw File	Scan	Method	Score	m/z	Gene names
RO-QC-160121_ZackD_SS_Set_01_Fractio	33632	FTMS; HC	61.38	586.08	BCLAF1

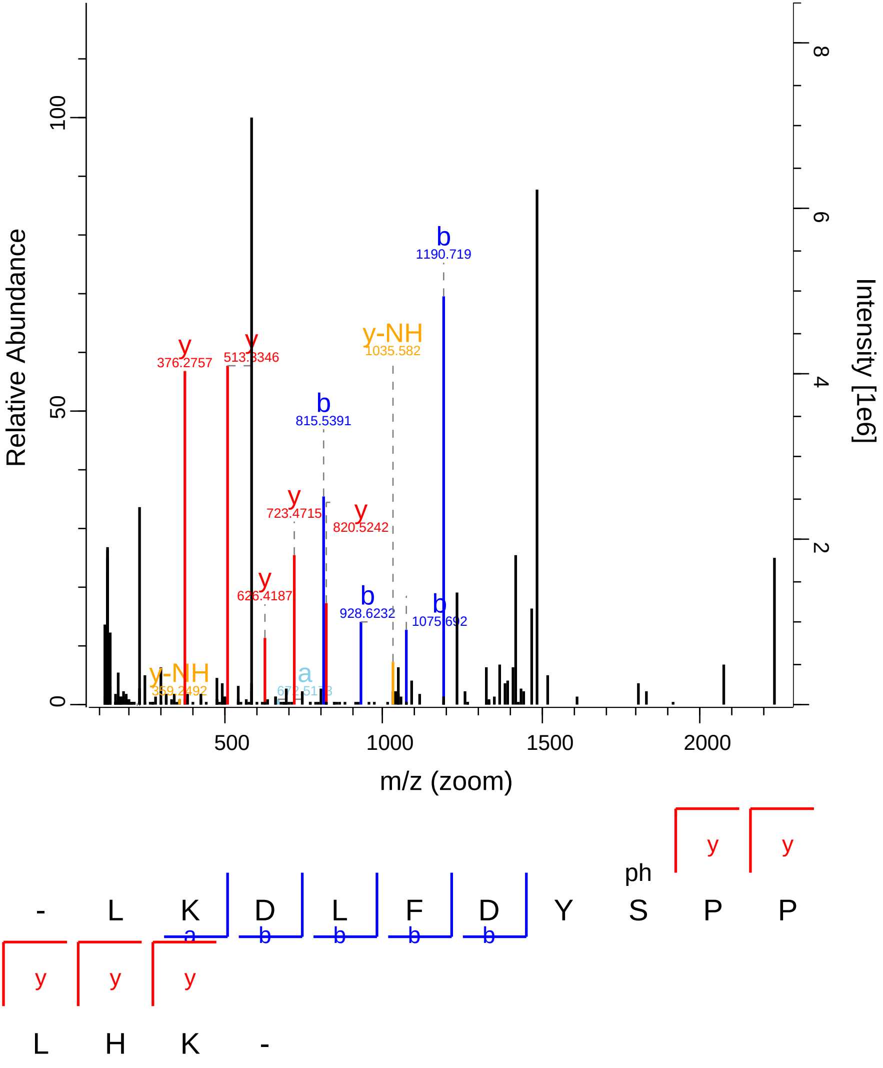

Raw File	Scan	Method	Score	m/z	Gene names
RO-QC-160121_ZackD_SS_Set_01_Fractior	35300	FTMS; HCl	123.67	538.3	SKIV2L

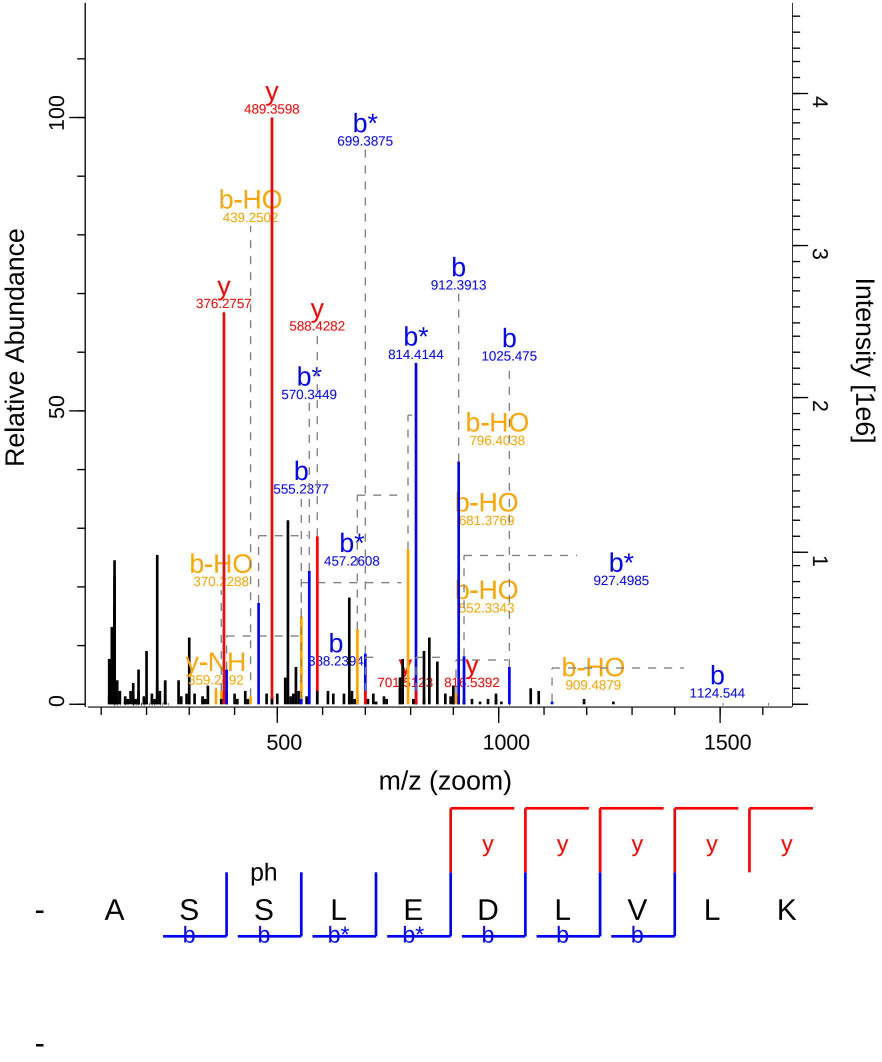

Raw File	Scan	Method	Score	m/z	Gene names
RO-QC-160121_ZackD_SS_Set_01_Fractio	36396	FTMS; HC	185.95	895.45	PLEC

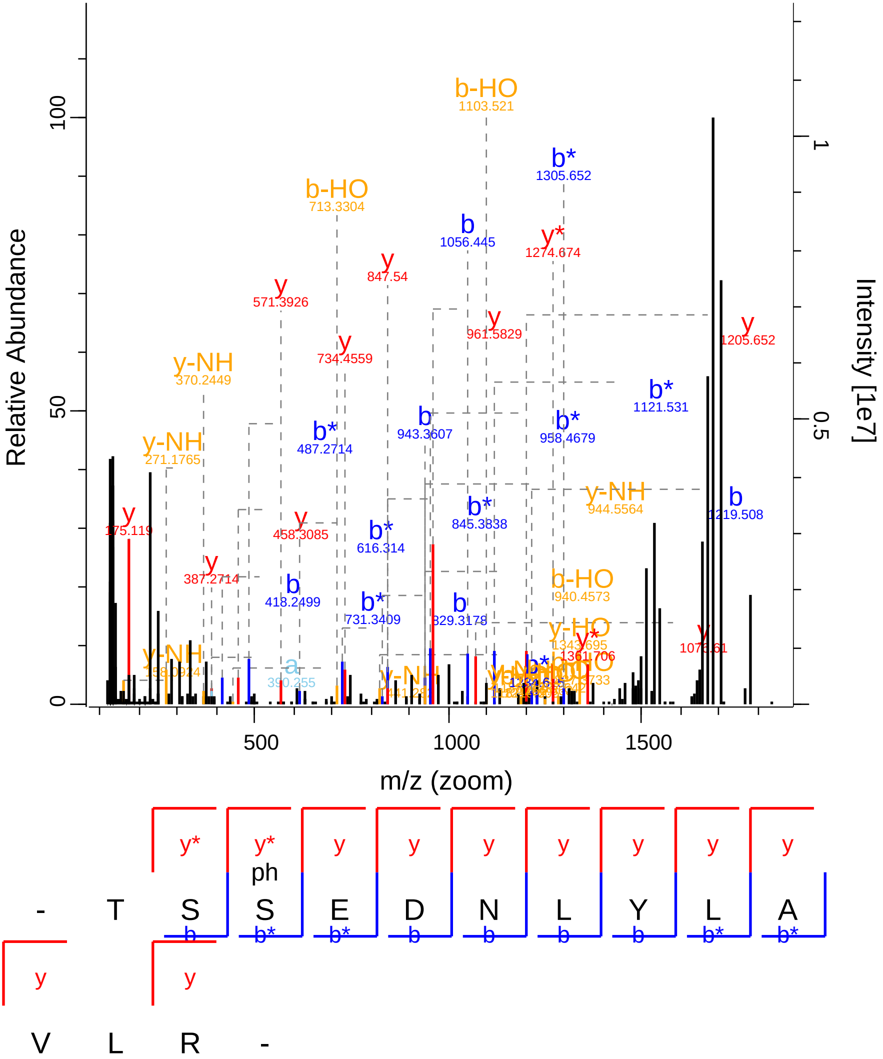

Raw File	Scan	Method	Score	m/z	Gene names
RO-QC-160121_ZackD_SS_Set_01_Fractic	36837	FTMS; HC	64.88	1195.63	HMGXB4

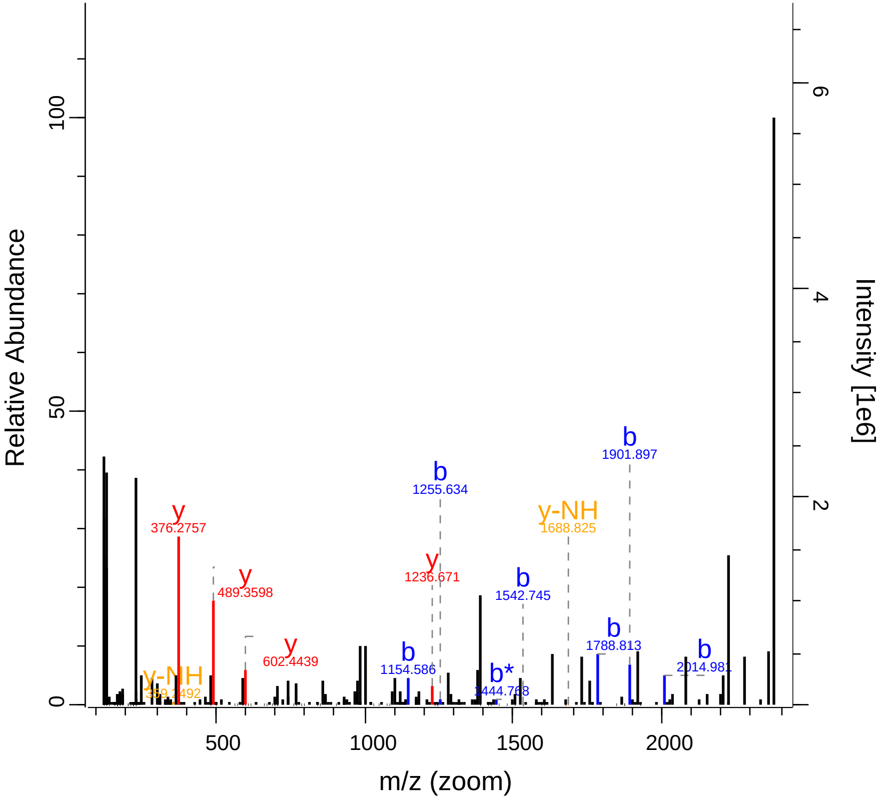

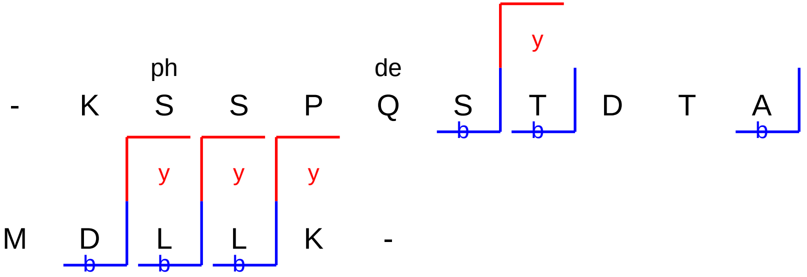

Raw File	Scan	Method	Score	m/z	Gene names
RO-QC-160121_ZackD_SS_Set_01_Fractio	37051	FTMS; HC	52.25	808.15	AKT3

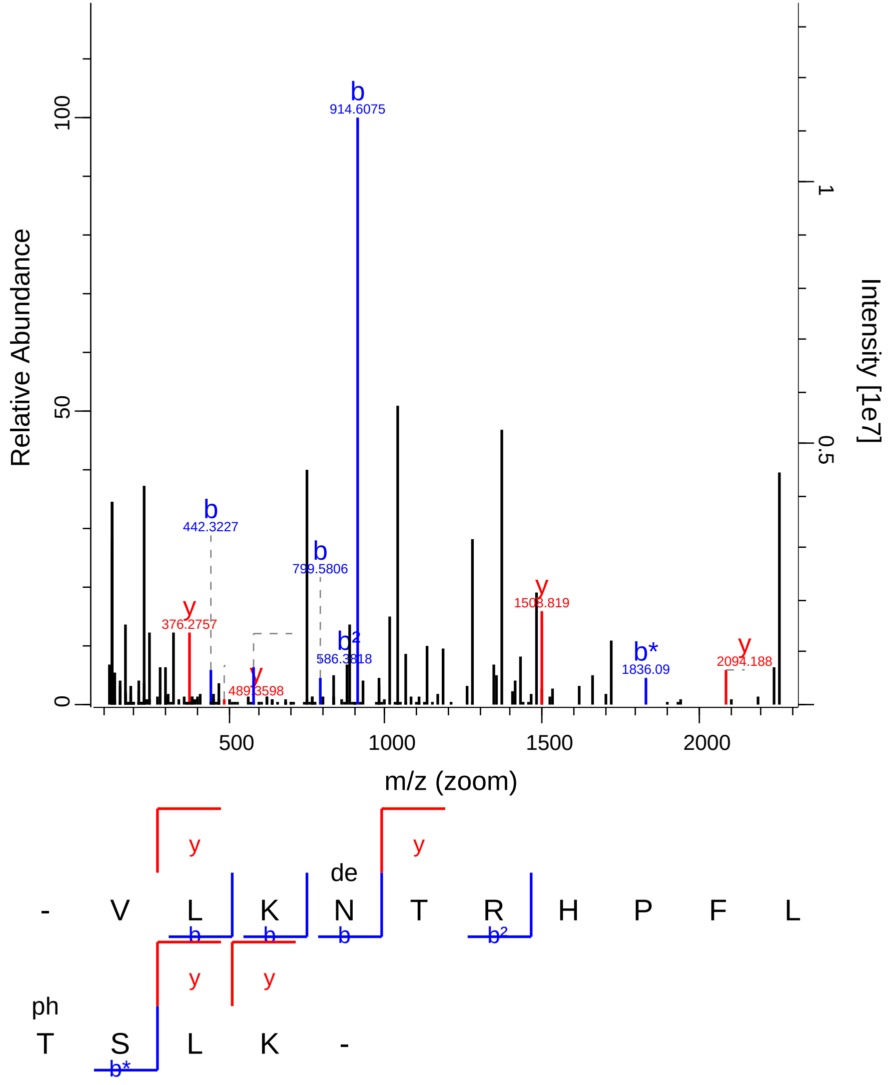

Raw File	Scan	Method	Score	m/z	Gene names
RO-QC-160121_ZackD_SS_Set_01_Fractic	37661	FTMS; HC	50.8	1070.04	REPIN1

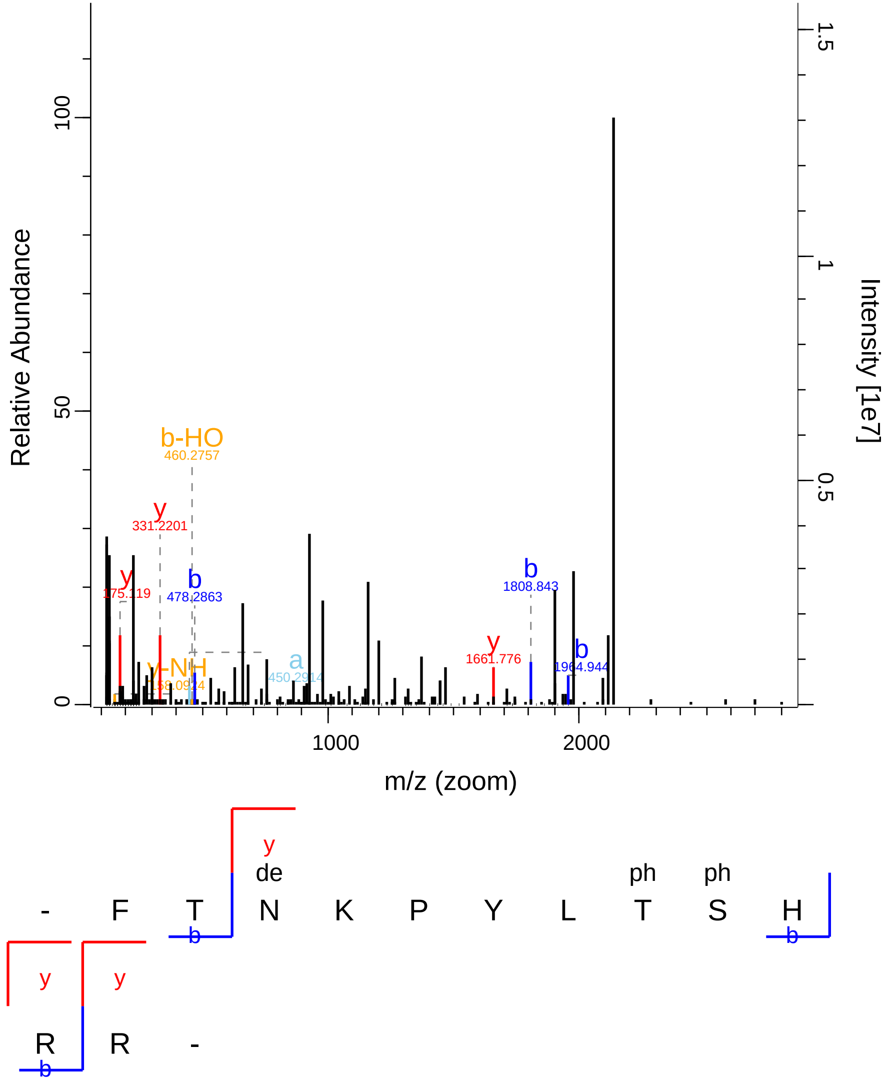

Raw File	Scan	Method	Score	m/z	Gene names
RO-QC-160121_ZackD_SS_Set_01_Fractic	39487	FTMS; HC	44.82	1107.23	FANCI

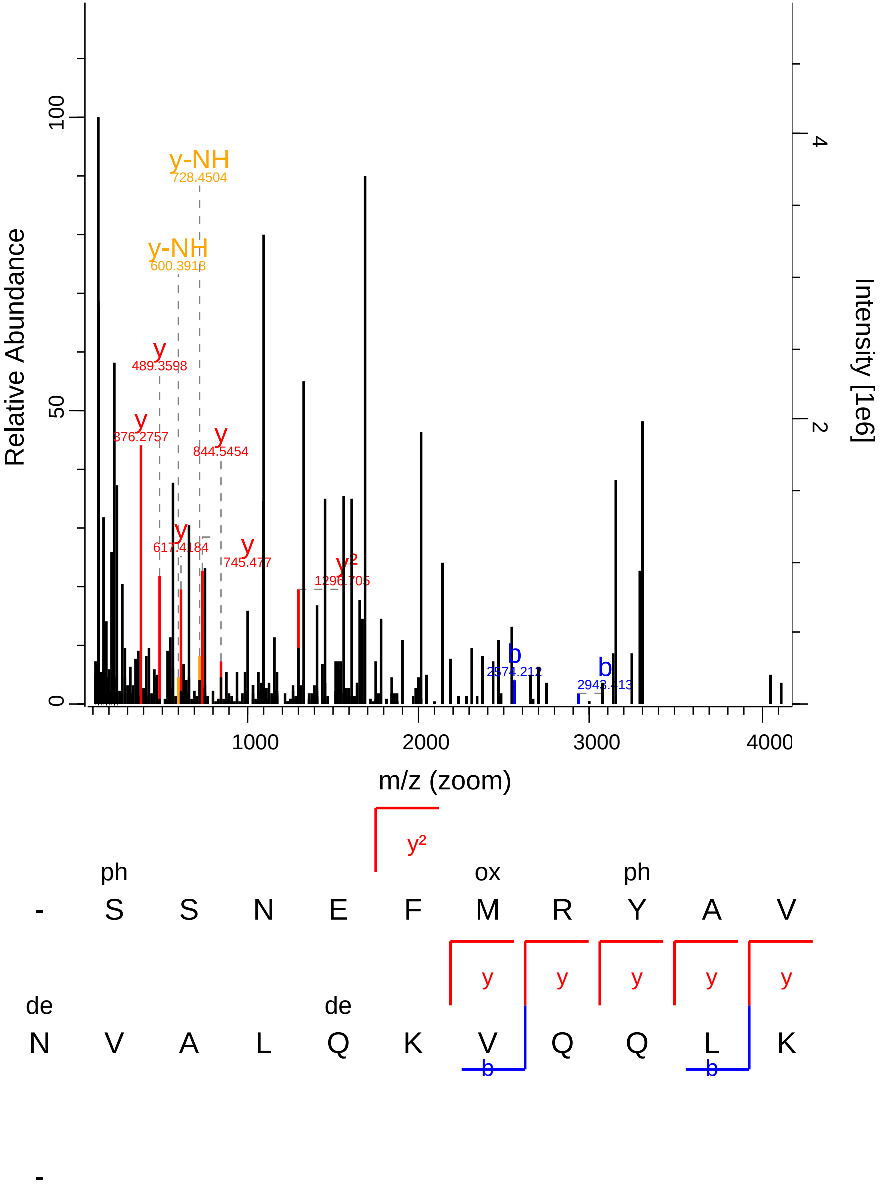

Raw File	Scan	Method	Score	m/z	Gene names
RO-QC-160121_ZackD_SS_Set_01_Fraction	40420	FTMS; HCI	70.92	917	ITGA9

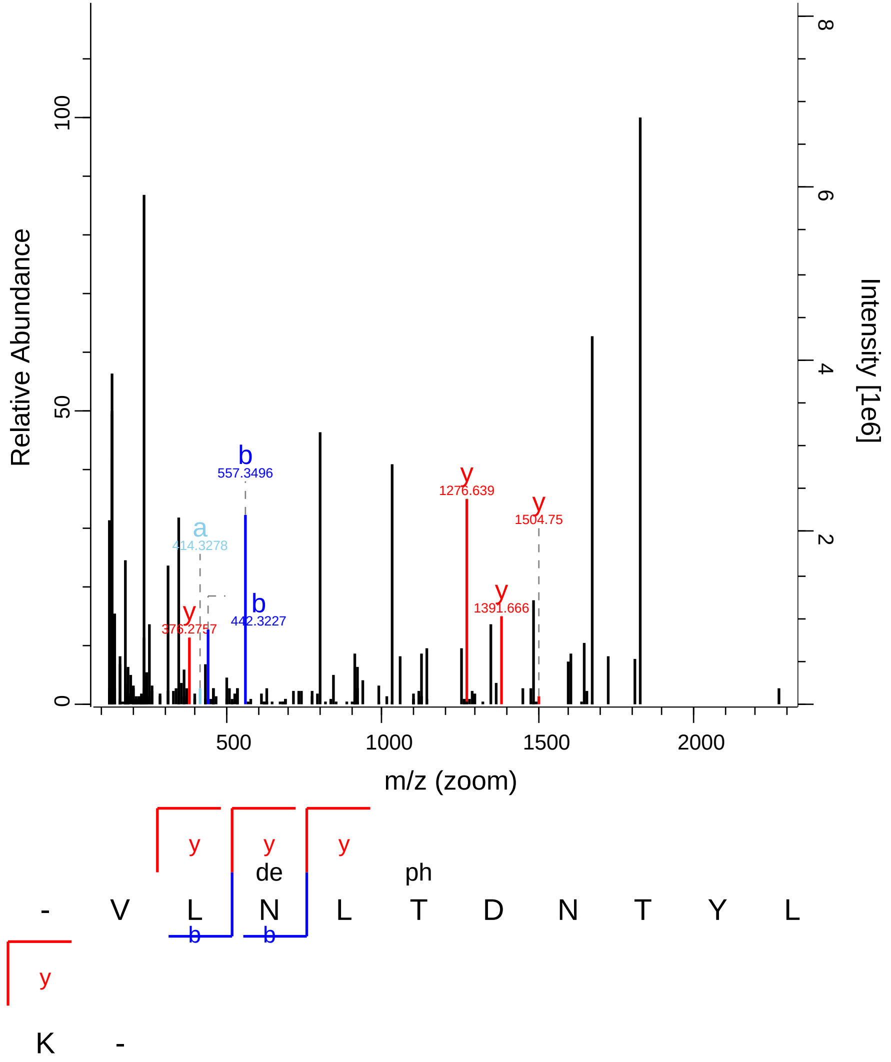

Raw File	Scan	Method	Score	m/z	Gene names
RO-QC-160121_ZackD_SS_Set_01_Fractior	5192	FTMS; HCI	98.94	427.99	PRPF38B

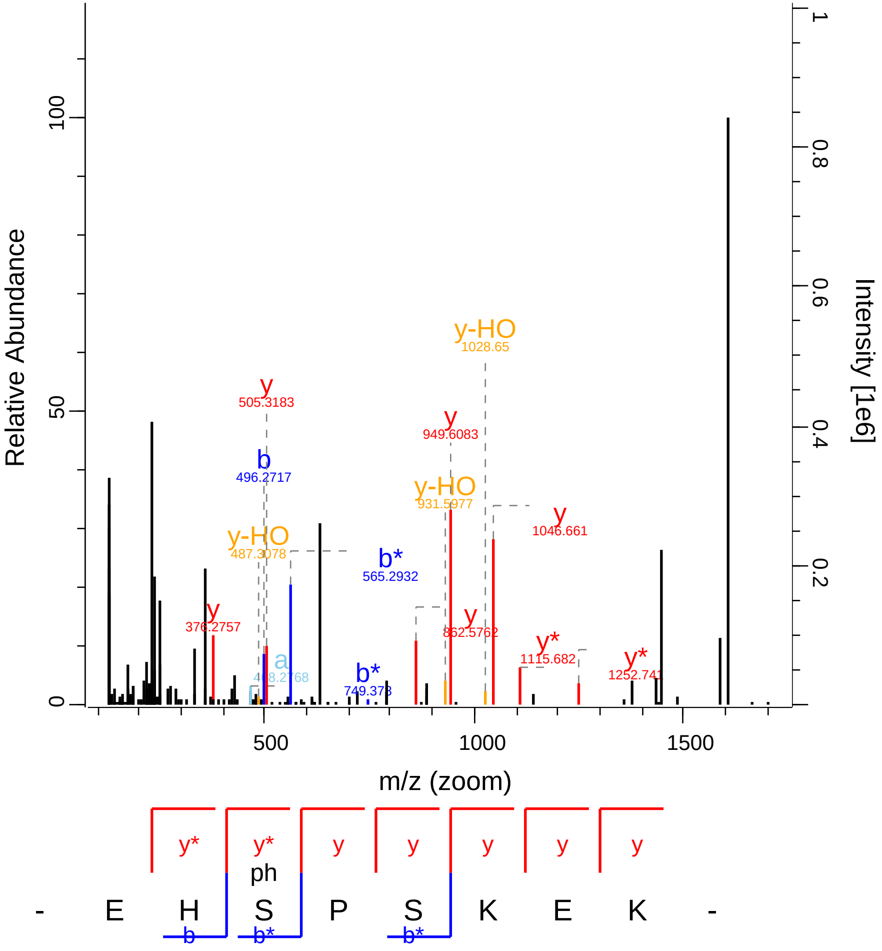

Raw File	Scan	Method	Score	m/z	Gene names
RO-QC-160121_ZackD_SS_Set_01_Fractior	6439	FTMS; HCI	43.54	585.81	ASCC1

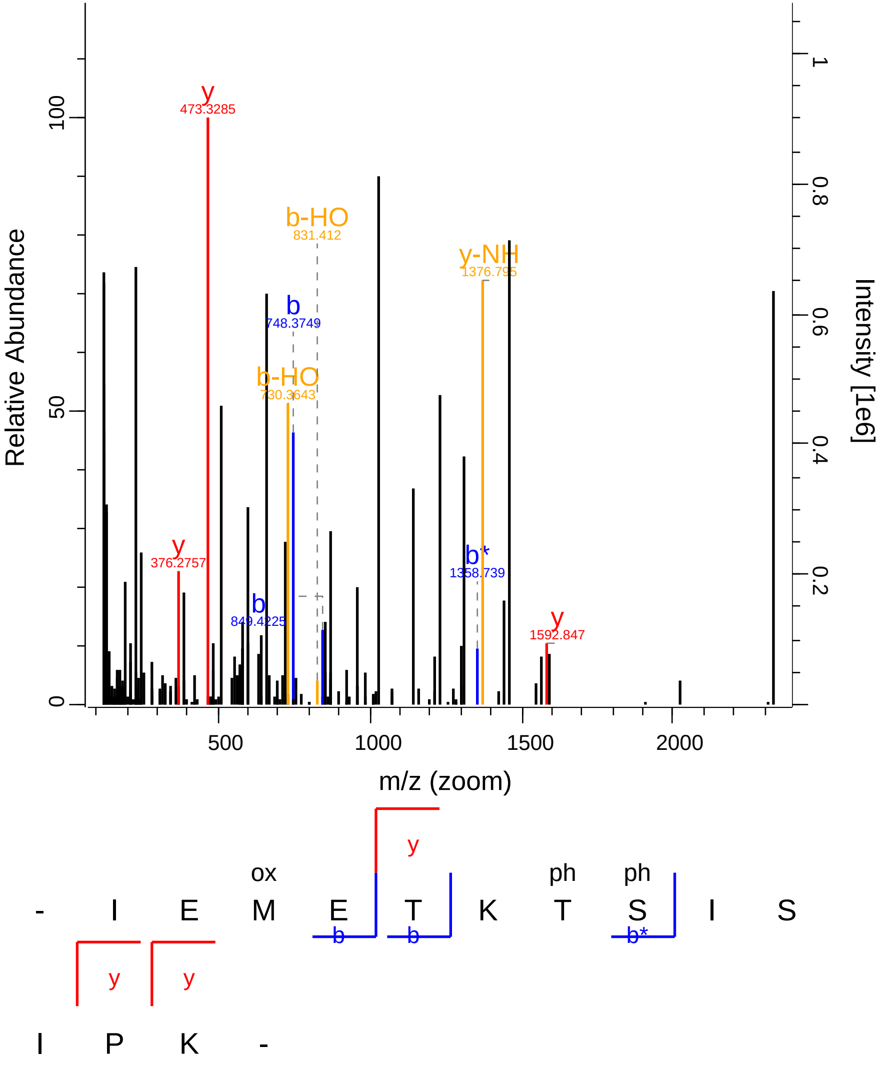

Raw File	Scan	Method	Score	m/z	Gene names
RO-QC-160121_ZackD_SS_Set_01_Fractio	7417	FTMS; HC	130.83	837.39	HNRNPD

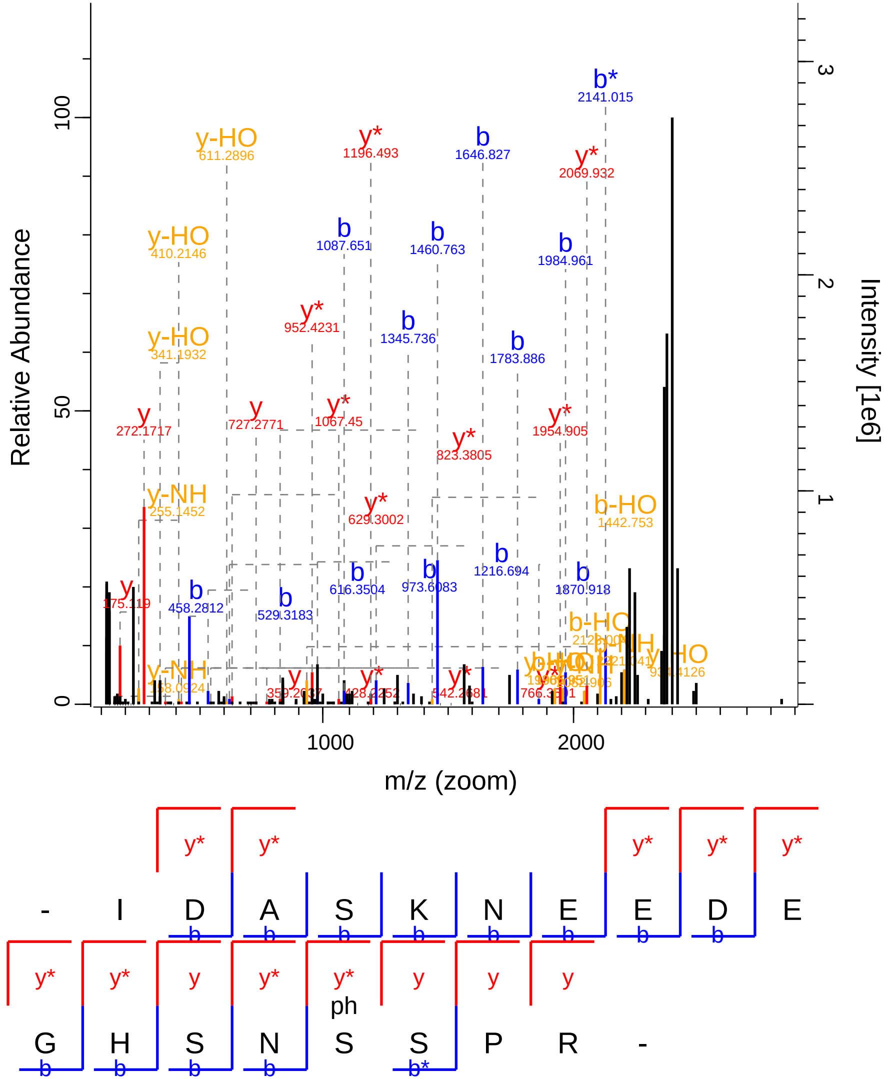

Raw File	Scan	Method	Score	m/z	Gene names
RO-QC-160121_ZackD_SS_Set_01_Fractior	8210	FTMS; HCI	56.26	920.99	FES

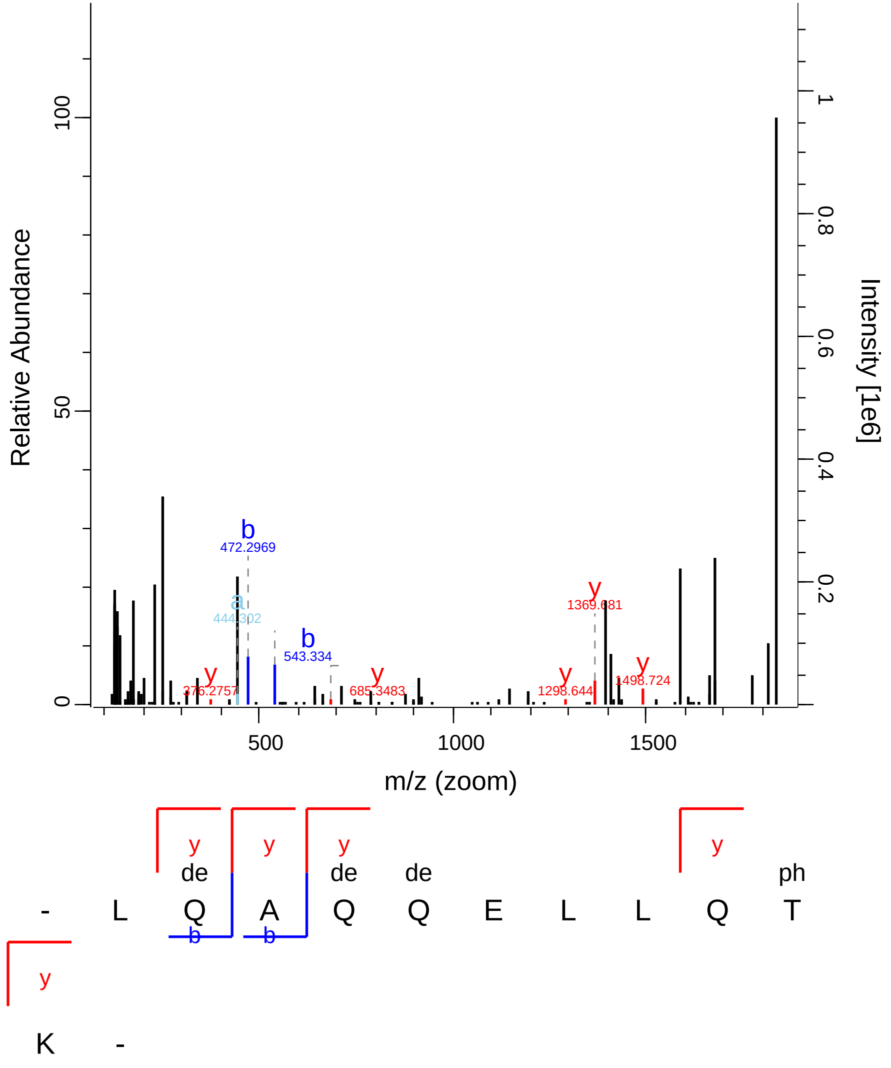

Raw File	Scan	Method	Score	m/z	Gene names
RO-QC-160121_ZackD_SS_Set_01_Fractio	9356	FTMS; HC	162.38	807.94	ZYX

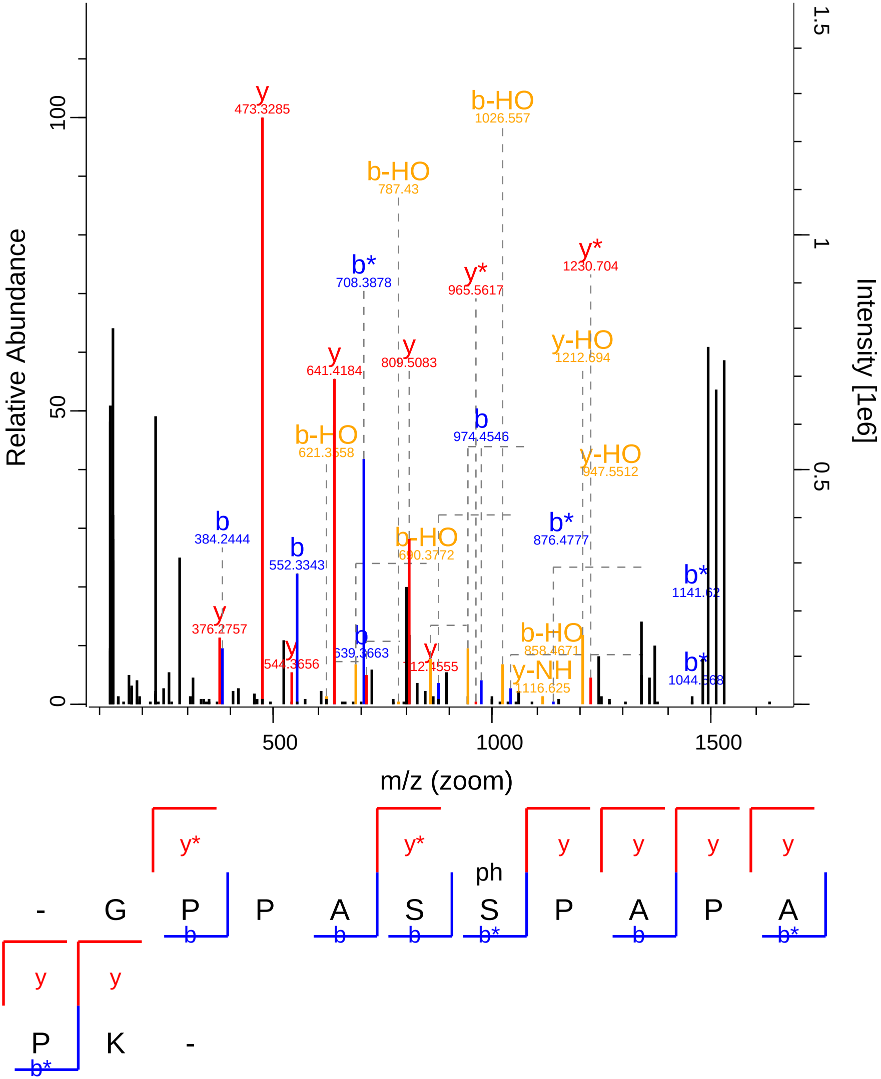

Raw File	Scan	Method	Score	m/z	Gene names
RO-QC-160121_ZackD_SS_Set_01_Fractio	10624	FTMS; HC	66.87	755.73	MYO9B

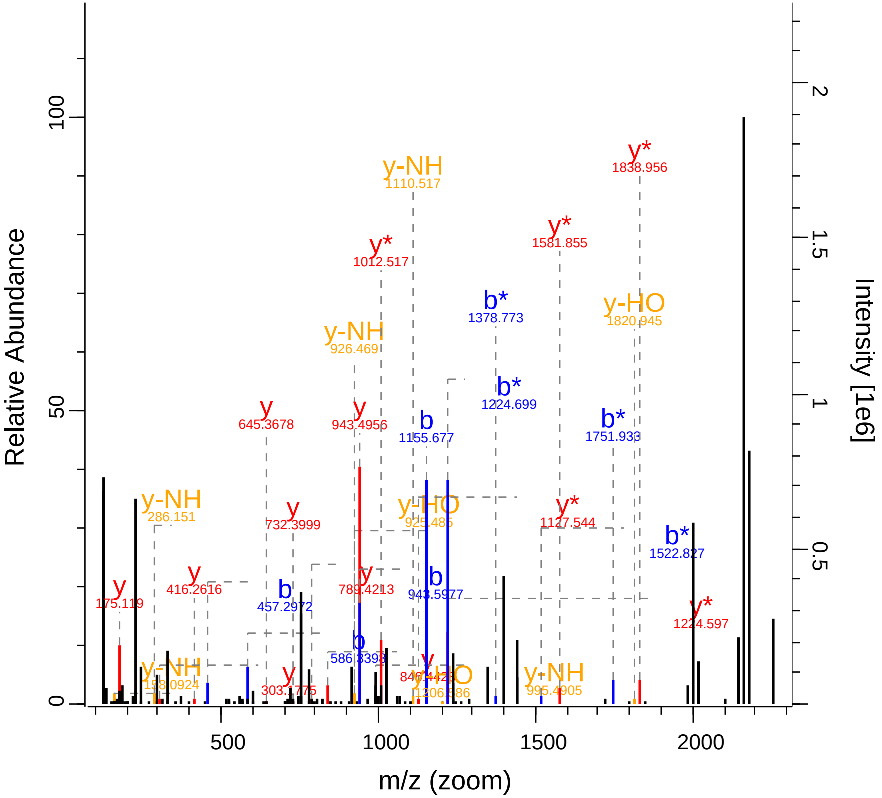

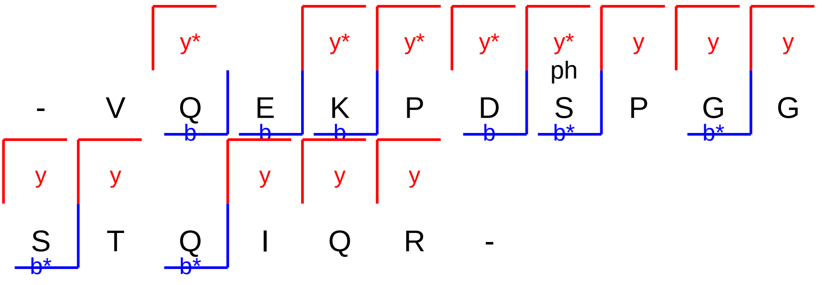

Raw File	Scan	Method	Score	m/z	Gene names
RO-QC-160121_ZackD_SS_Set_01_Fractio	10654	FTMS; HC	115.57	739.38	AFAP1

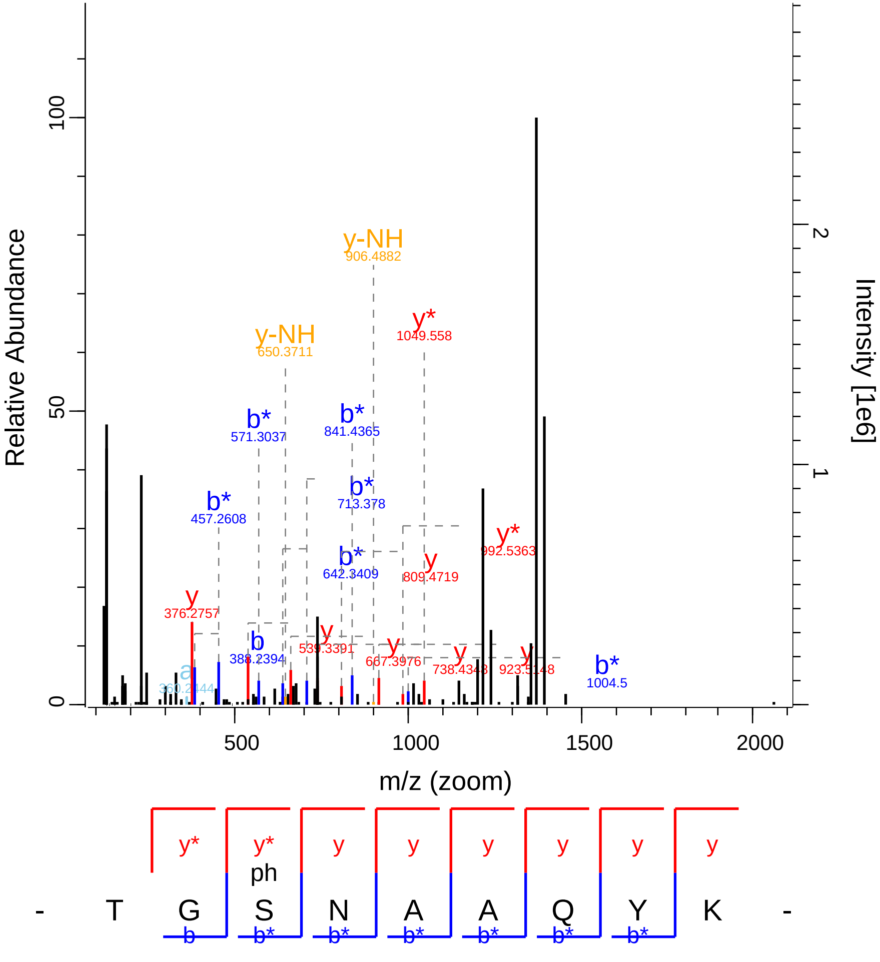

Raw File	Scan	Method	Score	m/z	Gene names
RO-QC-160121_ZackD_SS_Set_01_Fractio	11140	FTMS; HC	109.84	732.41	HNRNPA1

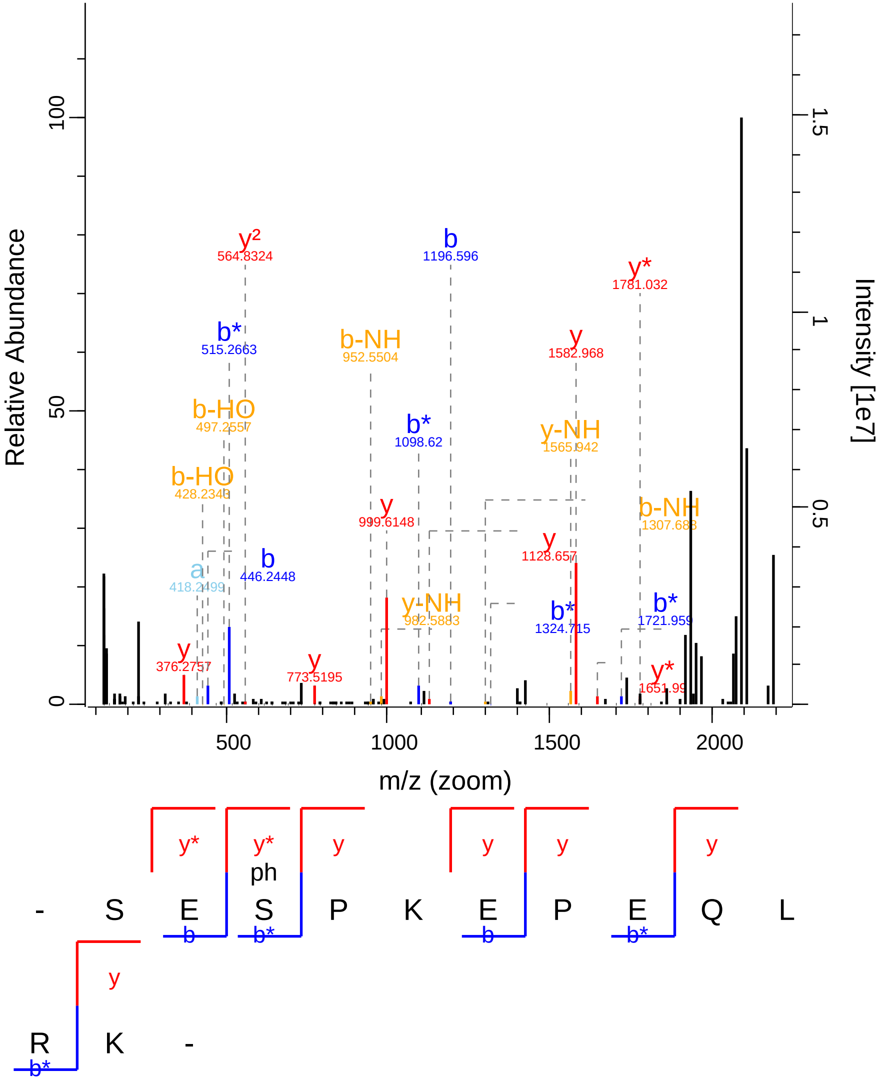

Raw File	Scan	Method	Score	m/z	Gene names
RO-QC-160121_ZackD_SS_Set_01_Fractio	13132	FTMS; HC	103.34	814.42	LIMA1

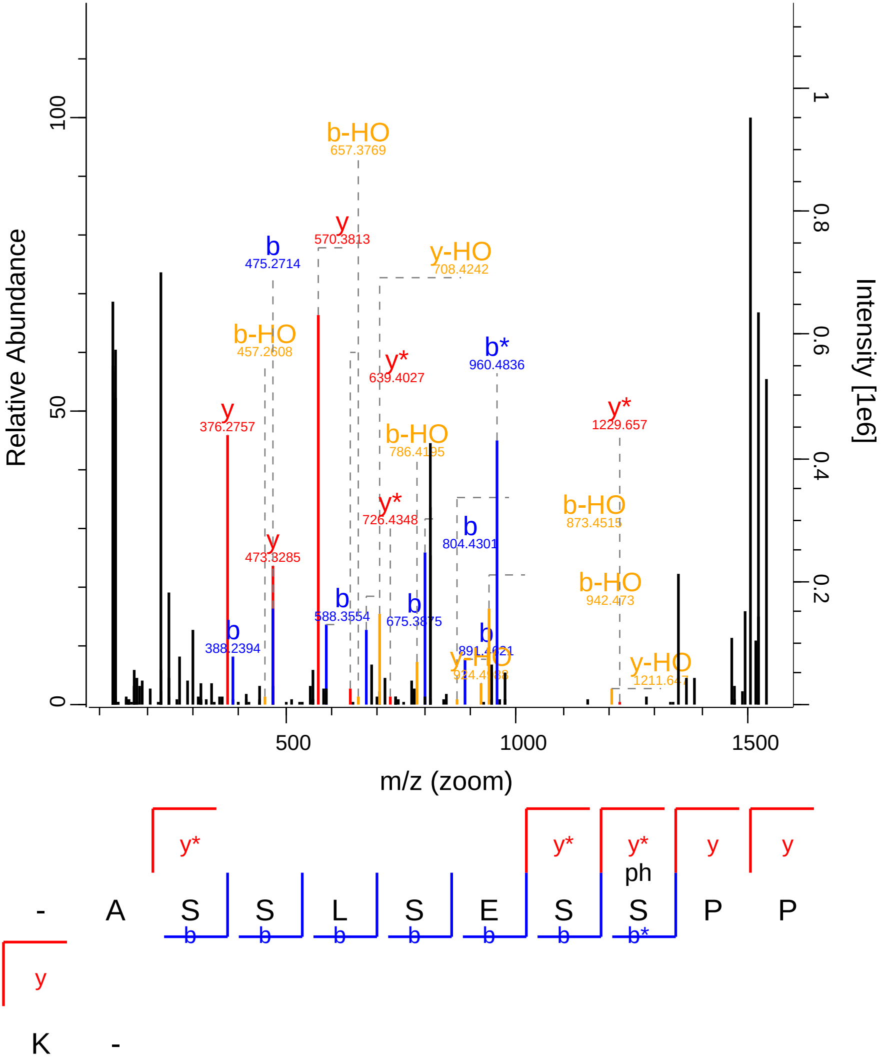

Raw File	Scan	Method	Score	m/z	Gene names
RO-QC-160121_ZackD_SS_Set_01_Fractior	13915	FTMS; HCI	51.01	943.4	PDCD5

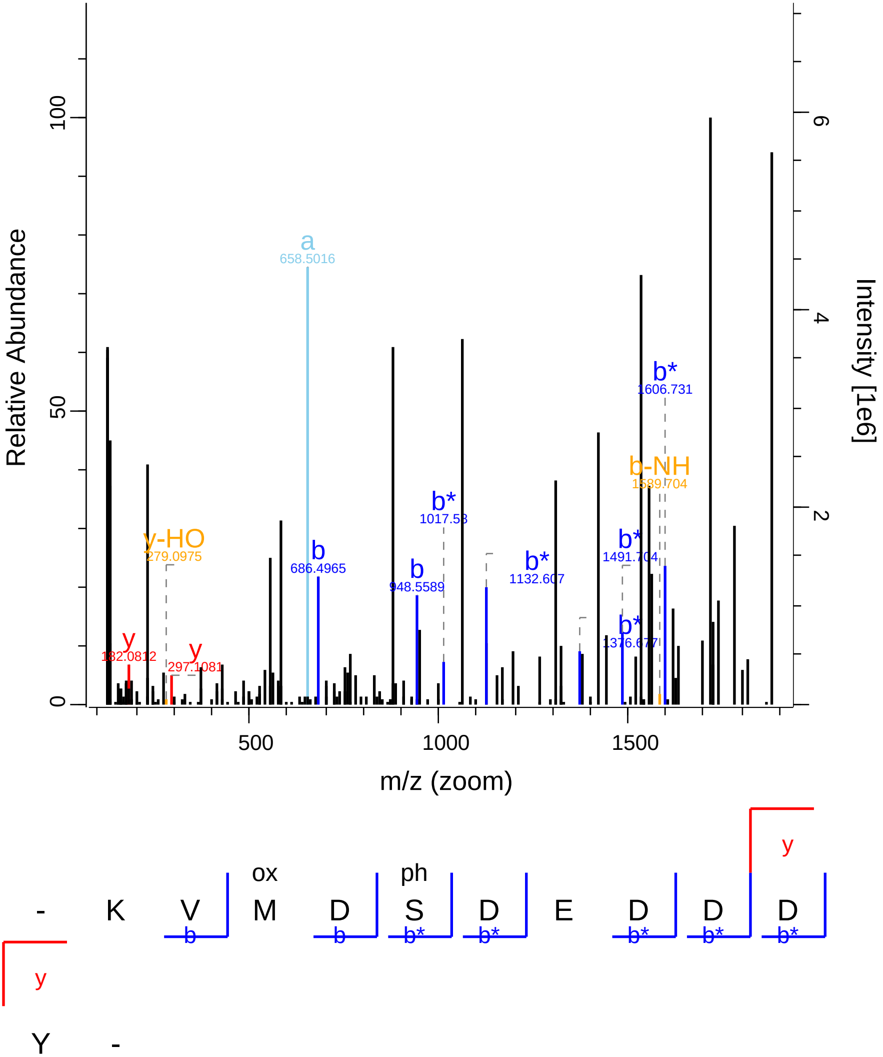

Raw File	Scan	Method	Score	m/z	Gene names
RO-QC-160121_ZackD_SS_Set_01_Fractio	14339	FTMS; HC	74.65	484.04	RASSF8

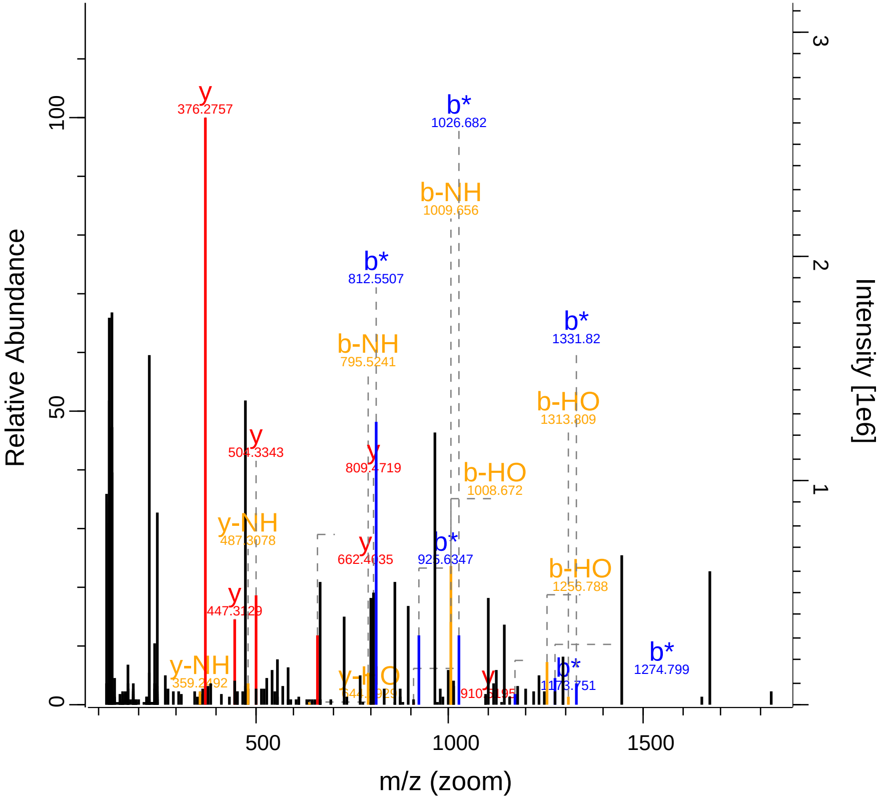

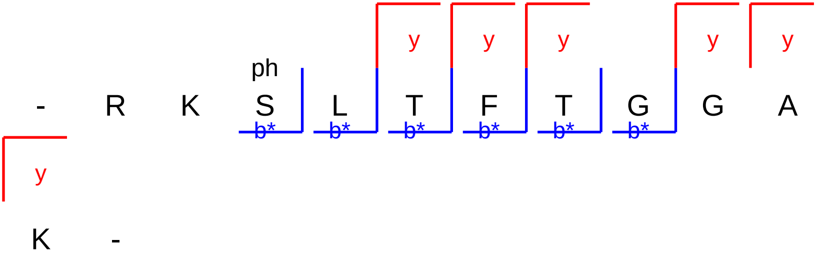

Raw File	Scan	Method	Score	m/z	Gene names
RO-QC-160121_ZackD_SS_Set_01_Fractio	15510	FTMS; HC	137.35	793.65	AKAP12

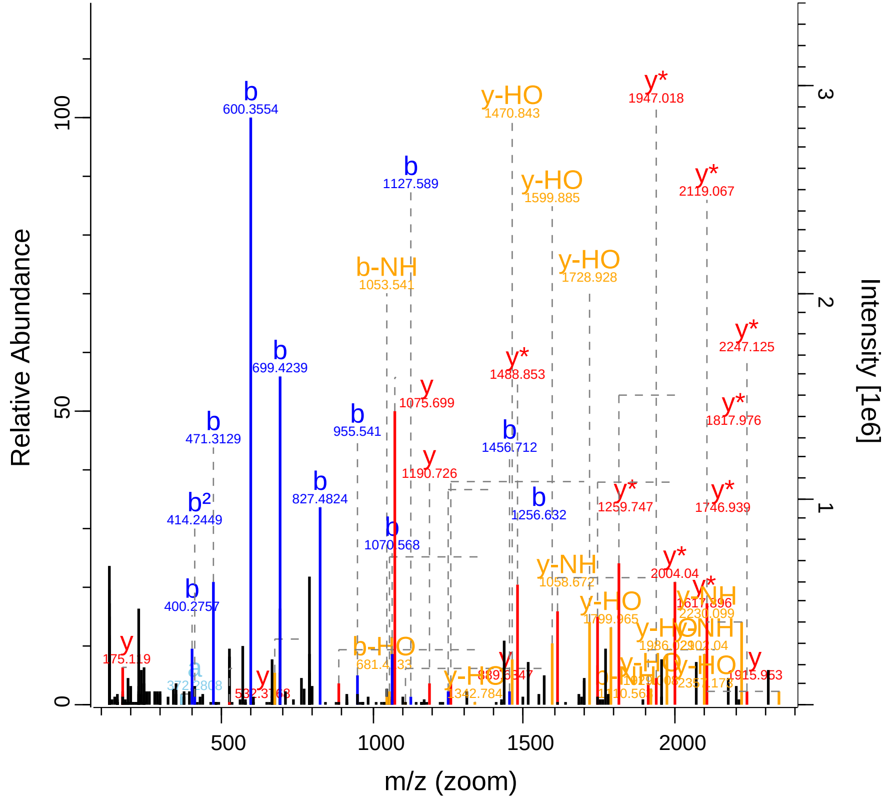

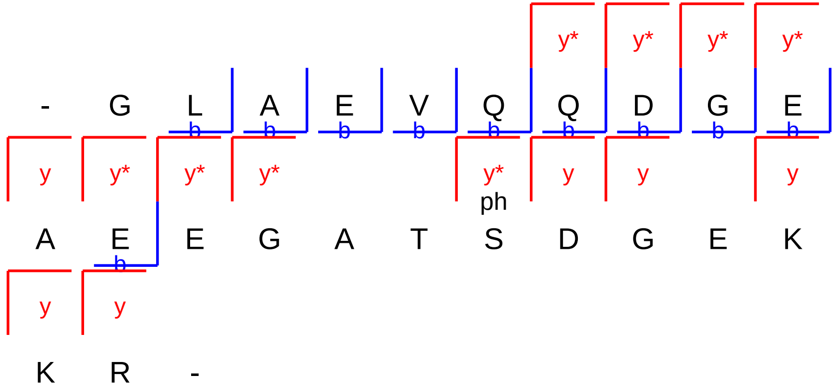

Raw File	Scan	Method	Score	m/z	Gene names
RO-QC-160121_ZackD_SS_Set_01_Fractio	16392	FTMS; HC	61.22	813.08	MYRIP

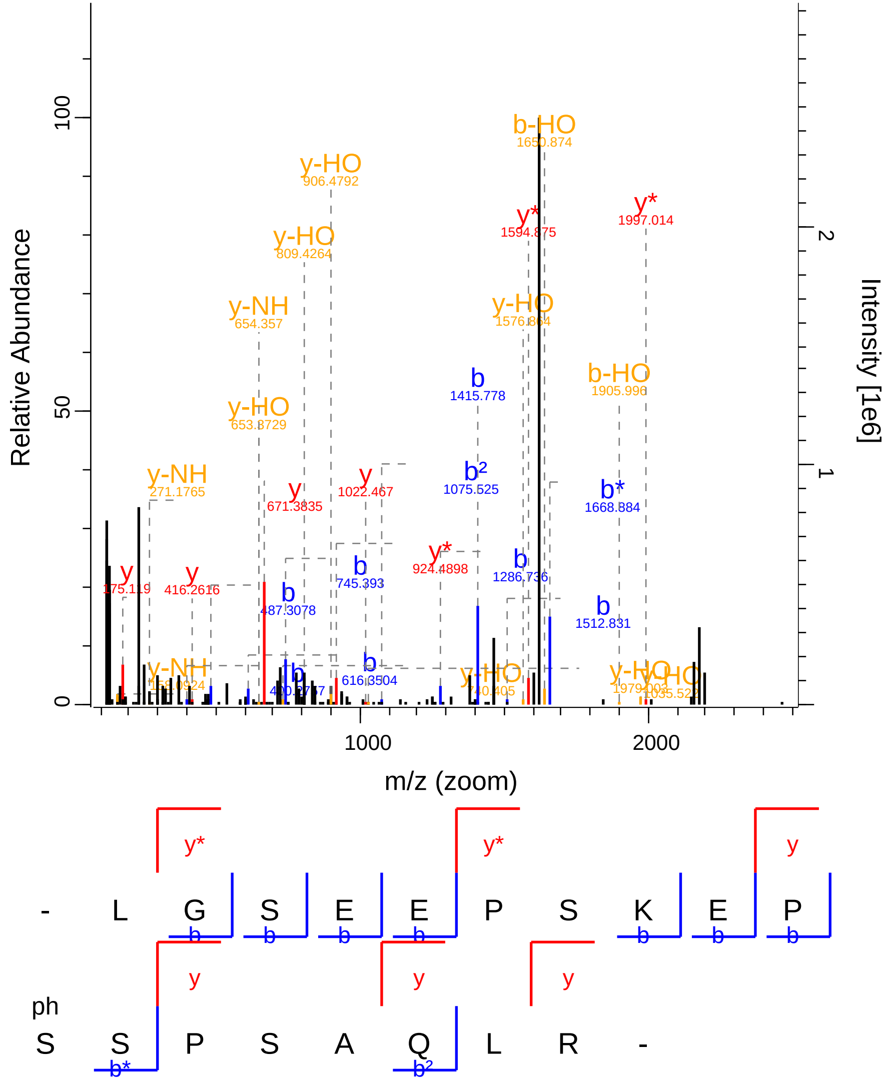

Raw File	Scan	Method	Score	m/z	Gene names
RO-QC-160121_ZackD_SS_Set_01_Fractio	17179	FTMS; HC	44.09	670.74	HNRNPF

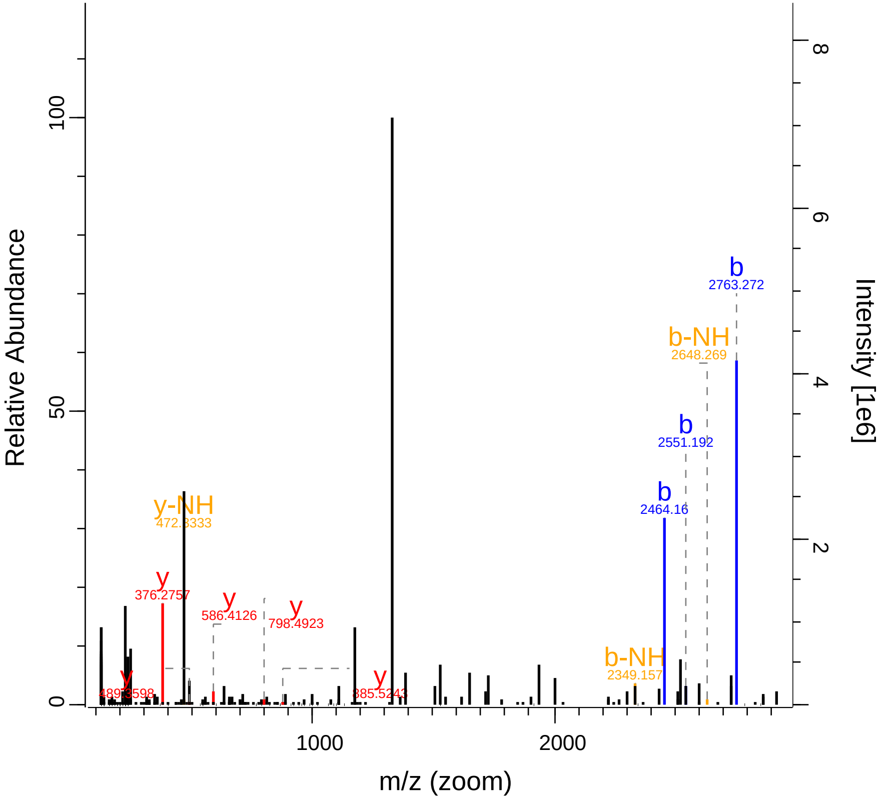

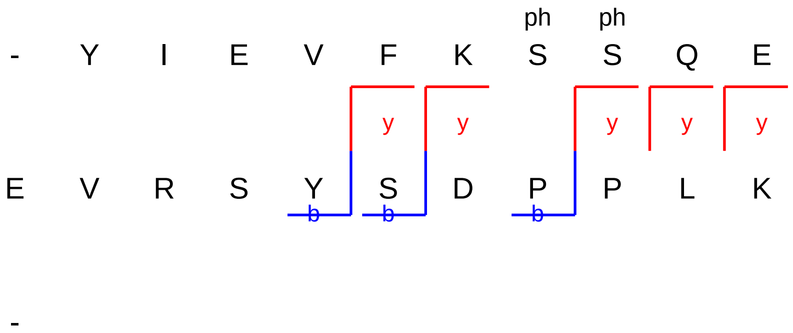

Raw File	Scan	Method	Score	m/z	Gene names
RO-QC-160121_ZackD_SS_Set_01_Fractio	17270	FTMS; HC	120.14	824.96	PLEC

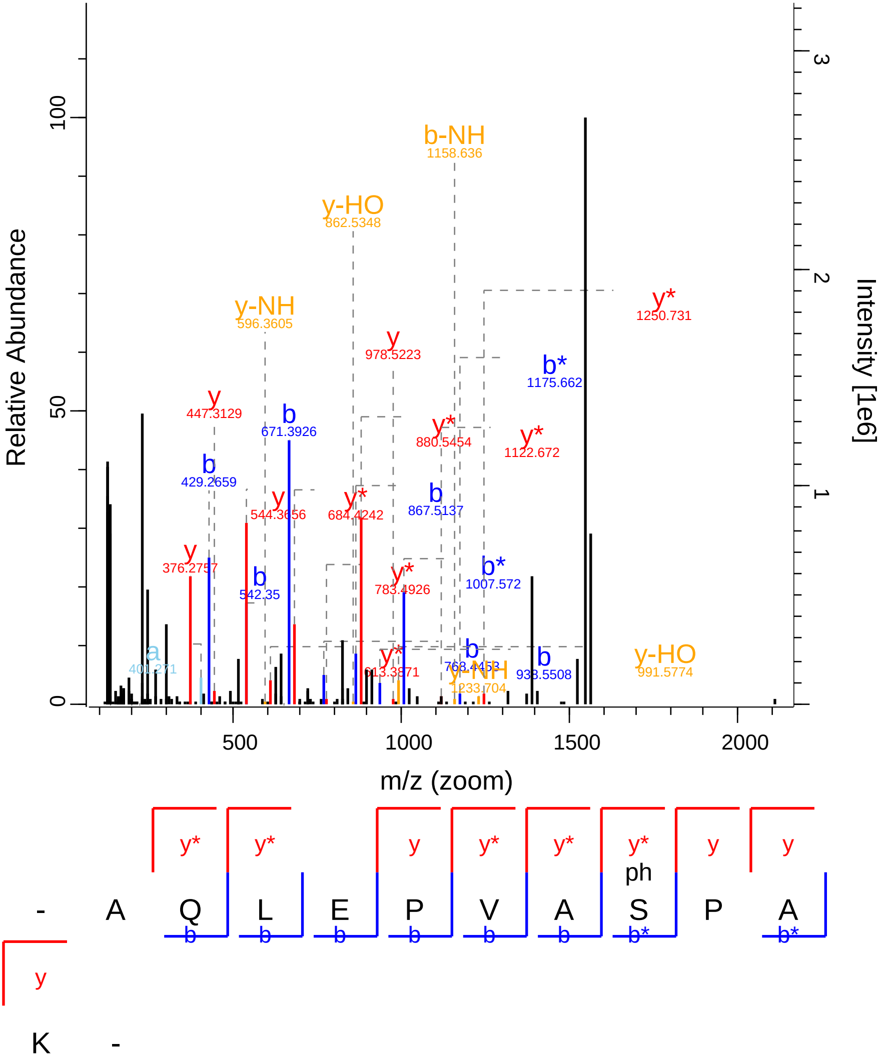

Raw File	Scan	Method	Score	m/z	Gene names
RO-QC-160121_ZackD_SS_Set_01_Fractio	17747	FTMS; HC	83.88	797.68	SLC16A1

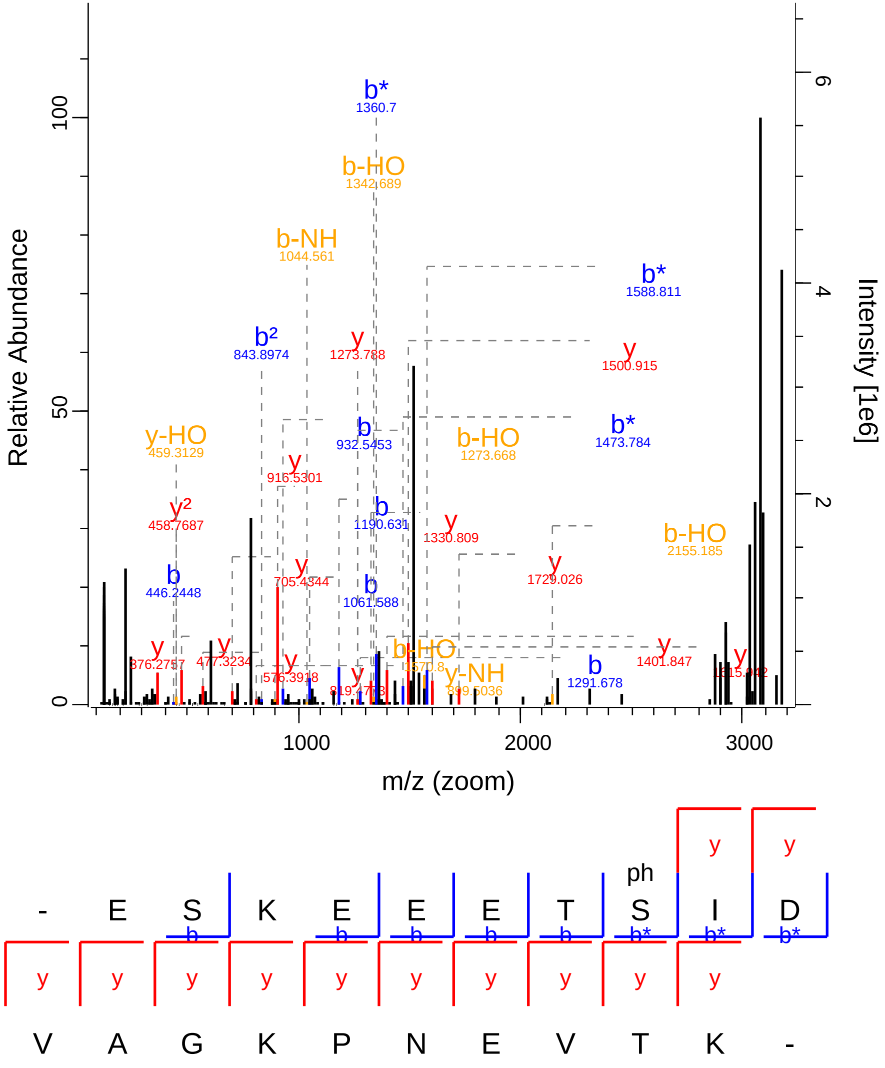

Raw File	Scan	Method	Score	m/z	Gene names
RO-QC-160121_ZackD_SS_Set_01_Fractio	18714	FTMS; HC	40.28	783.42	CTNND2

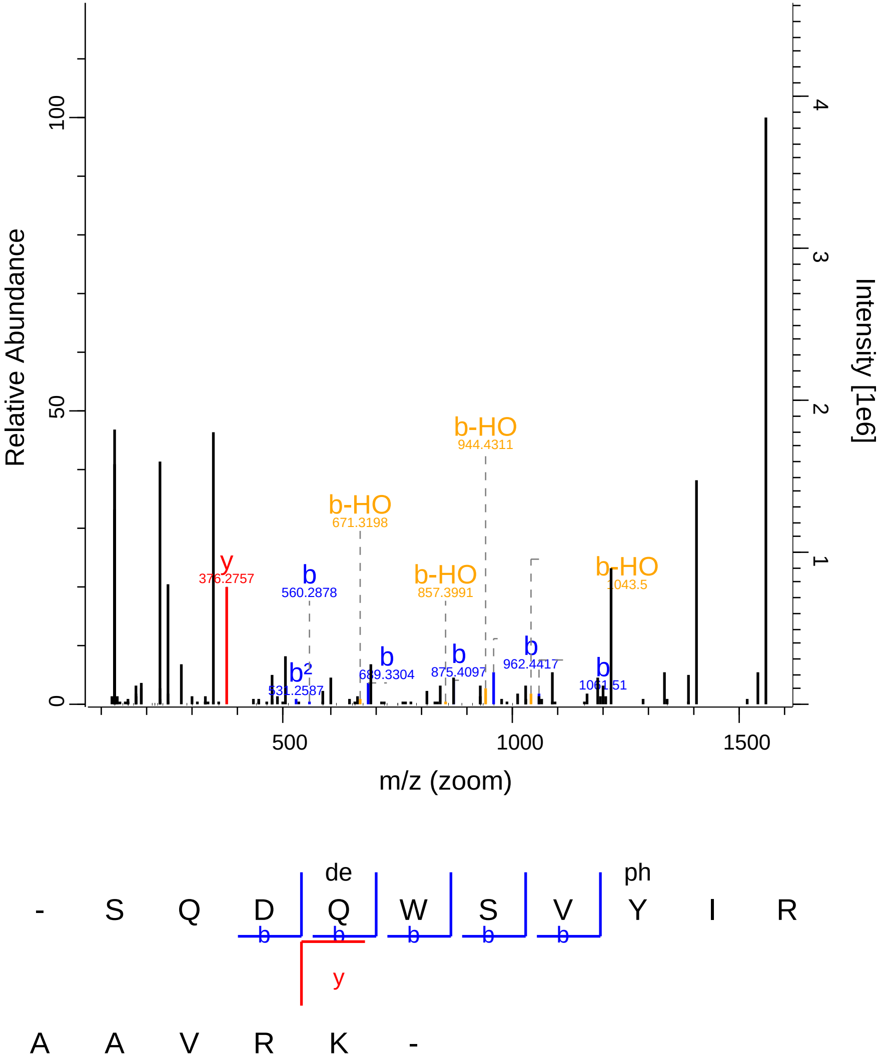

Raw File	Scan	Method	Score	m/z	Gene names
RO-QC-160121_ZackD_SS_Set_01_Fractio	19153	FTMS; HC	40	890.94	EIF4G2

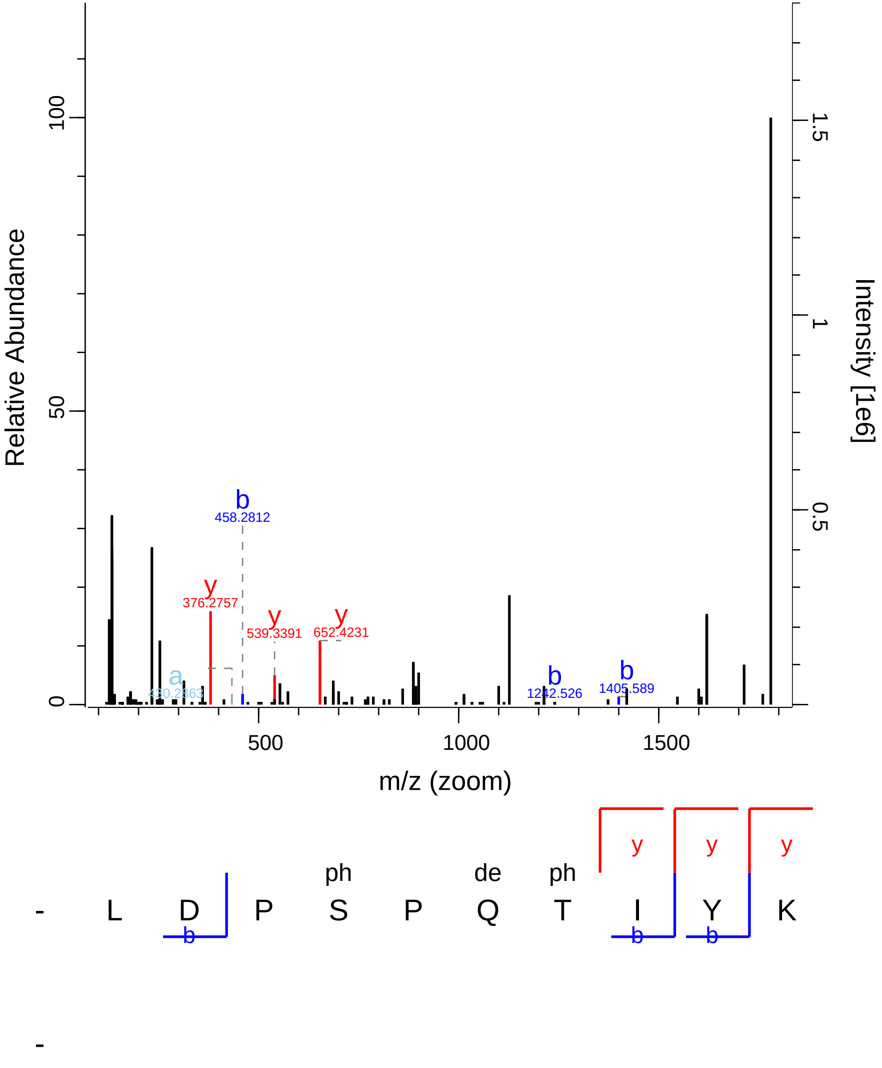

Raw File	Scan	Method	Score	m/z	Gene names
RO-QC-160121_ZackD_SS_Set_01_Fractio	19563	FTMS; HC	148.85	1061.1	SRRM1

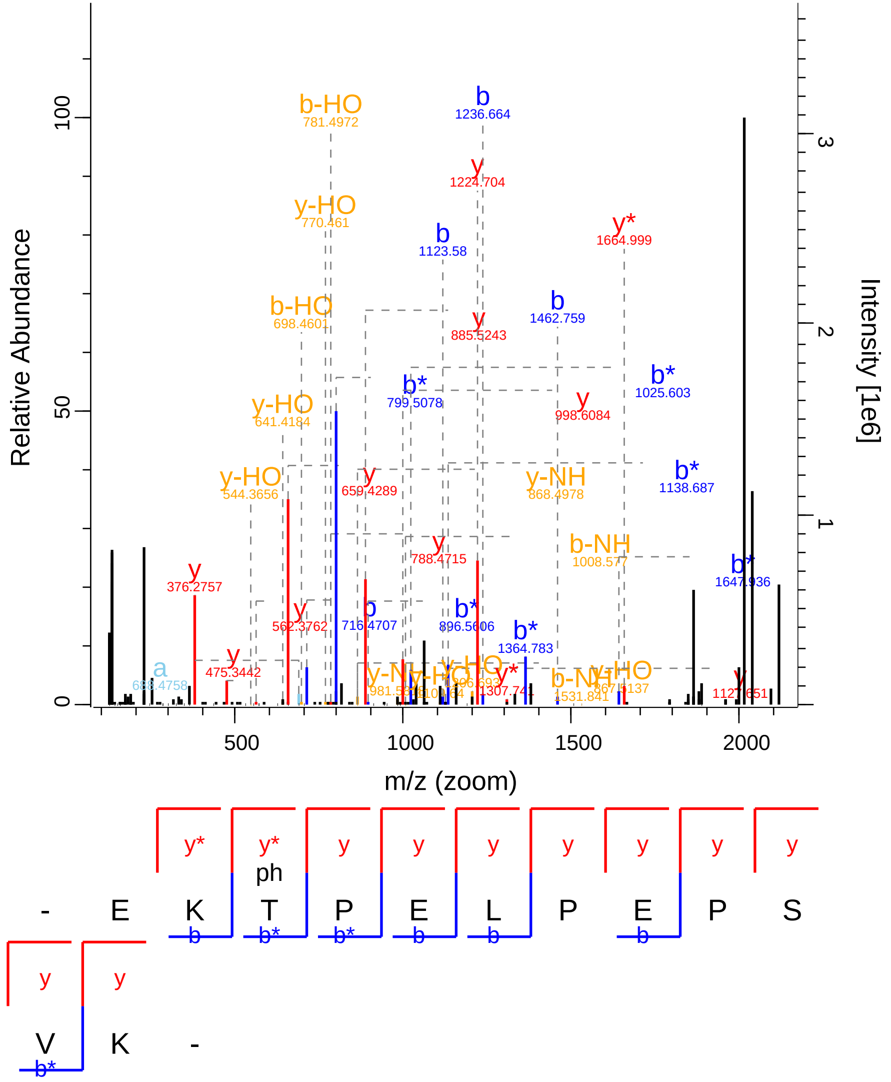

Raw File	Scan	Method	Score	m/z
RO-QC-160121_ZackD_SS_Set_01_Fraction_9	20183	FTMS; HCD	51.93	500.6

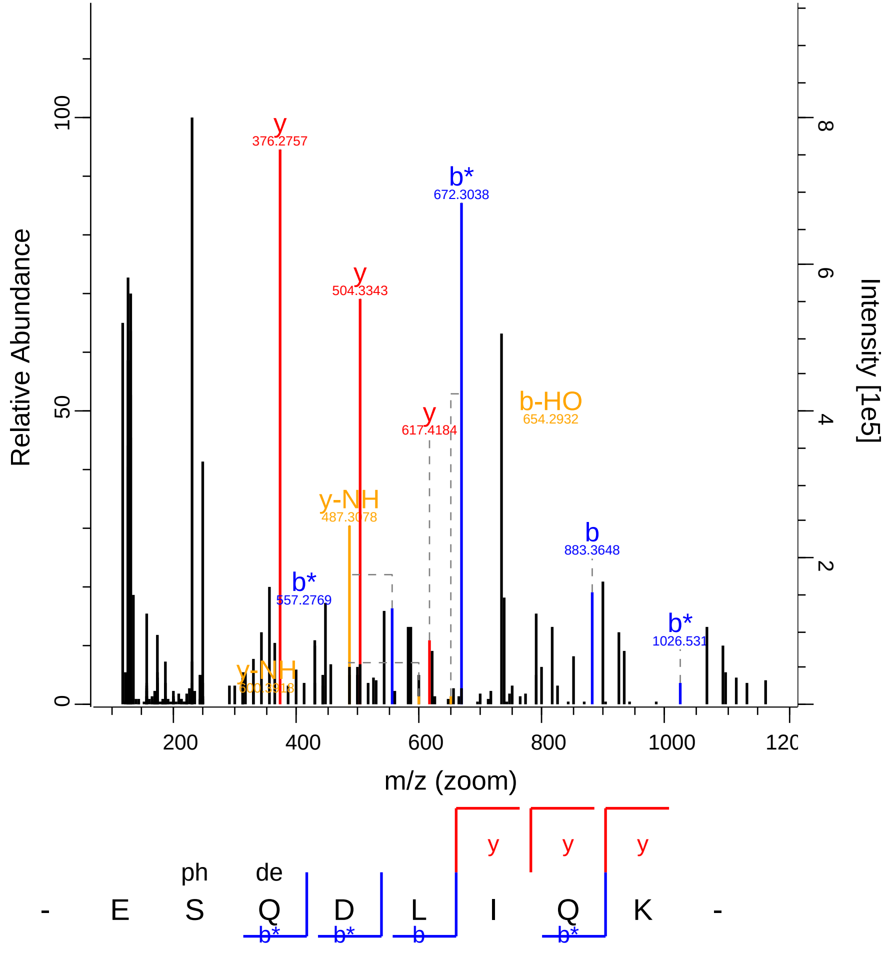

Raw File	Scan	Method	Score	m/z	Gene names
RO-QC-160121_ZackD_SS_Set_01_Fractio	20466	FTMS; HC	42.81	717.36	VPS13B

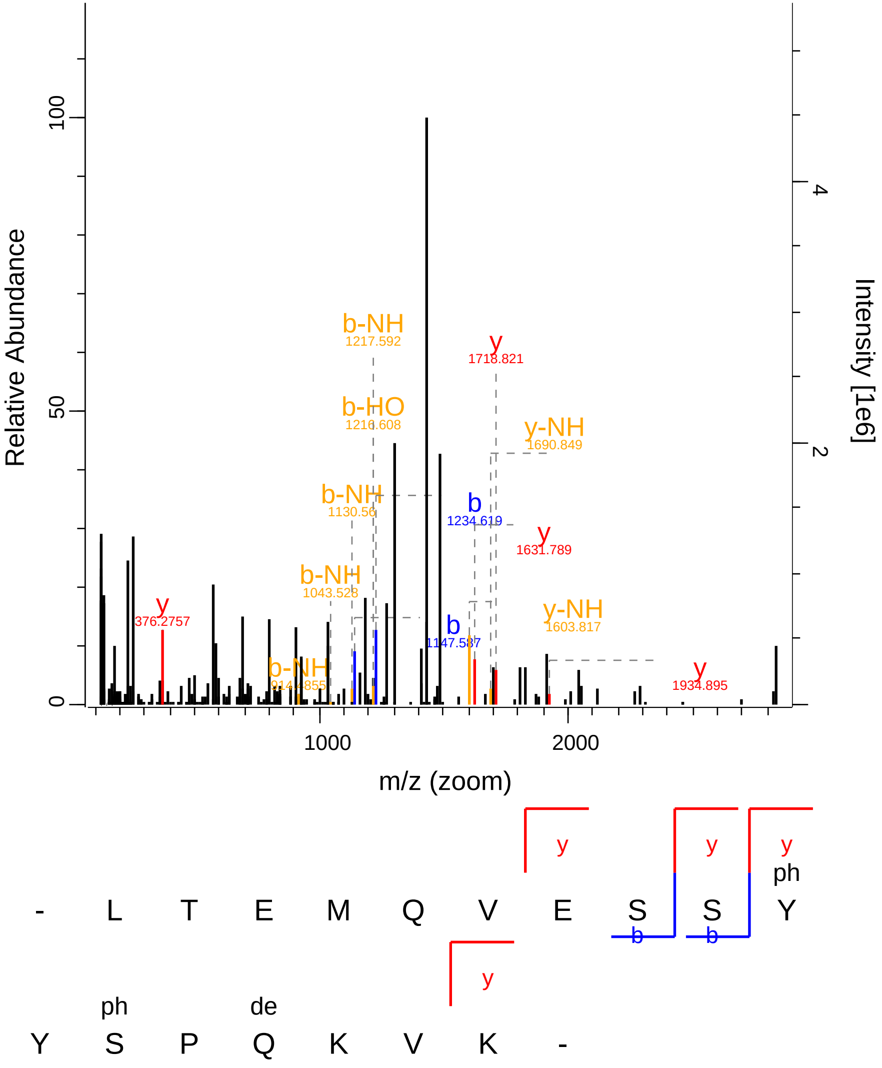

Raw File	Scan	Method	Score	m/z	Gene names
RO-QC-160121_ZackD_SS_Set_01_Fractic	22804	FTMS; HC	52.93	1046.56	ZFHX3

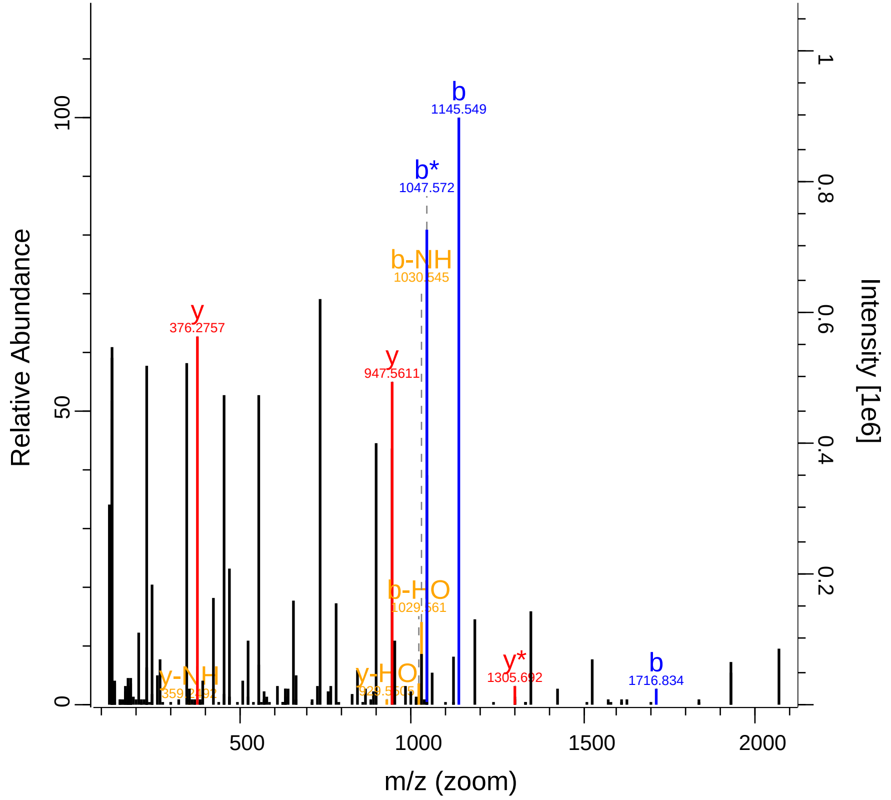

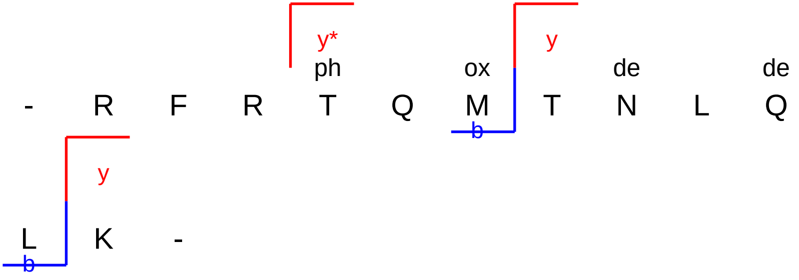

Raw File	Scan	Method	Score	m/z	Gene names
RO-QC-160121_ZackD_SS_Set_01_Fractic	23306	FTMS; HC	54.78	1219.65	CELA2A

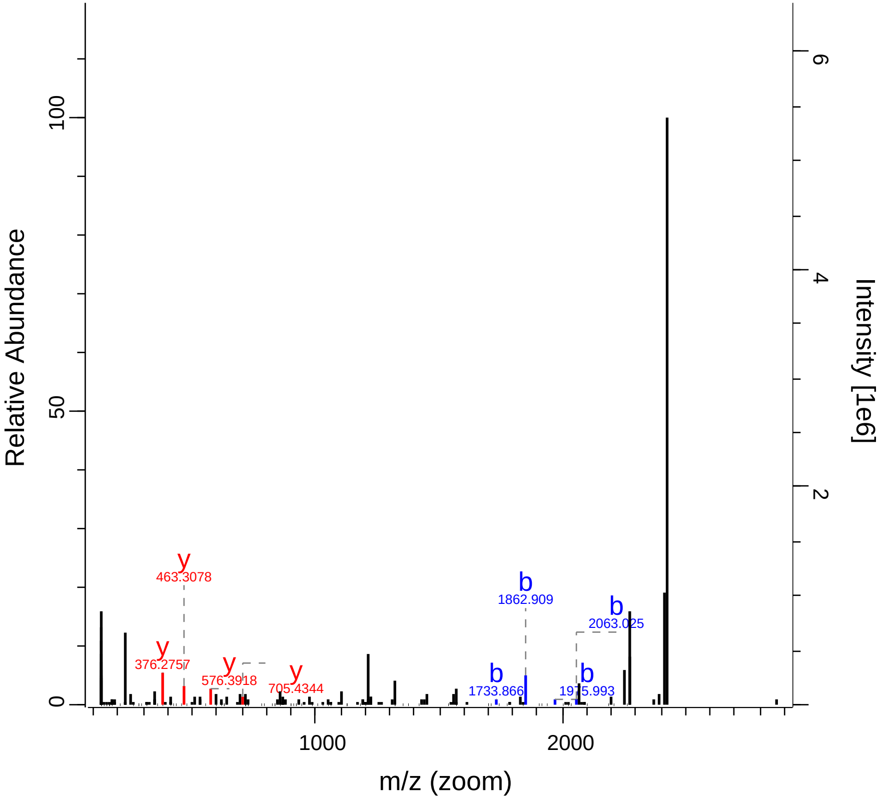

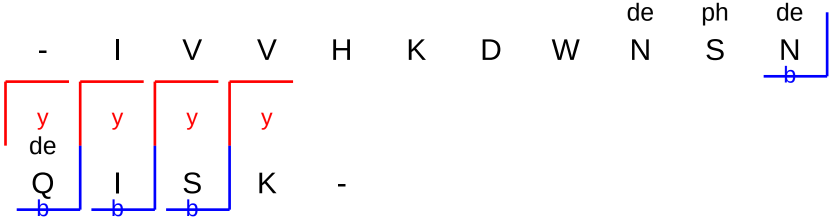

Raw File	Scan	Method	Score	m/z	Gene names
RO-QC-160121_ZackD_SS_Set_01_Fractio	24080	FTMS; HC	205.9	992.46	SEPT2

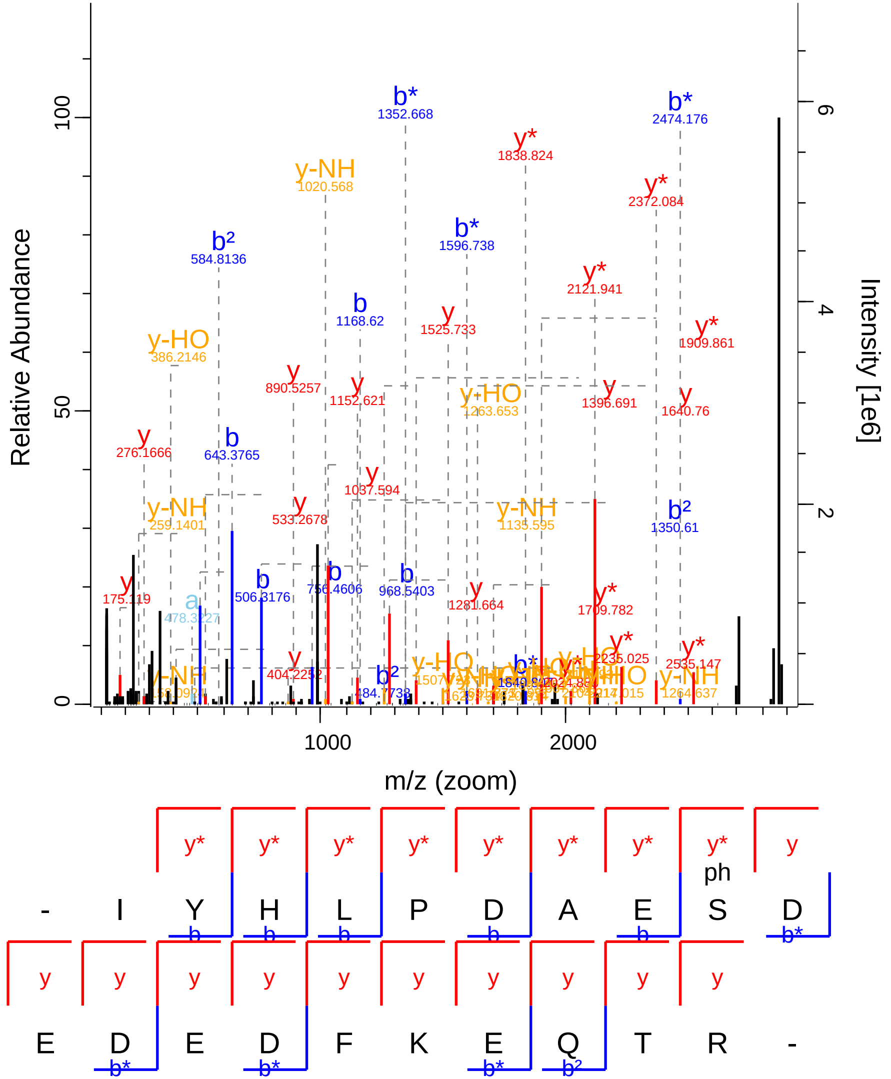

Raw File	Scan	Method	Score	m/z	Gene names
RO-QC-160121_ZackD_SS_Set_01_Fractior	24120	FTMS; HCI	40.8	528.9	MYH15

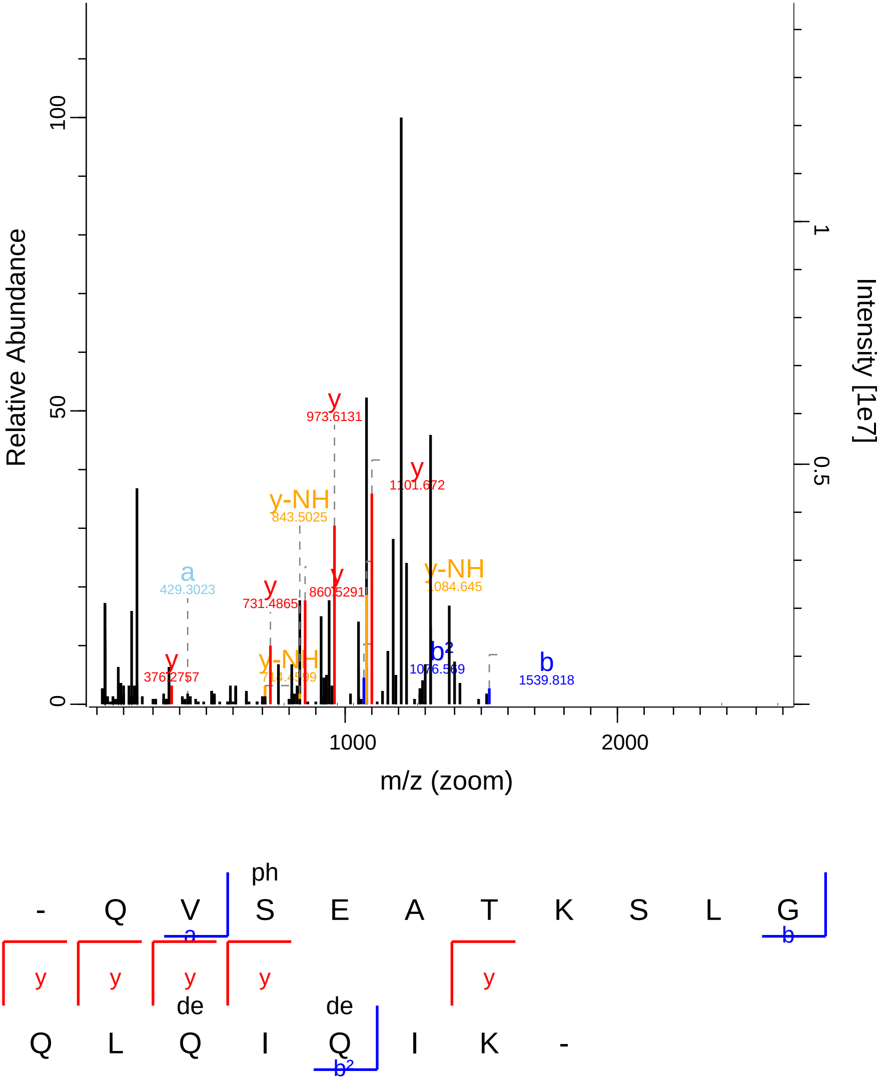

Raw File	Scan	Method	Score	m/z	Gene names
RO-QC-160121_ZackD_SS_Set_01_Fractic	27234	FTMS; HC	46.61	1237.69	LRRC66

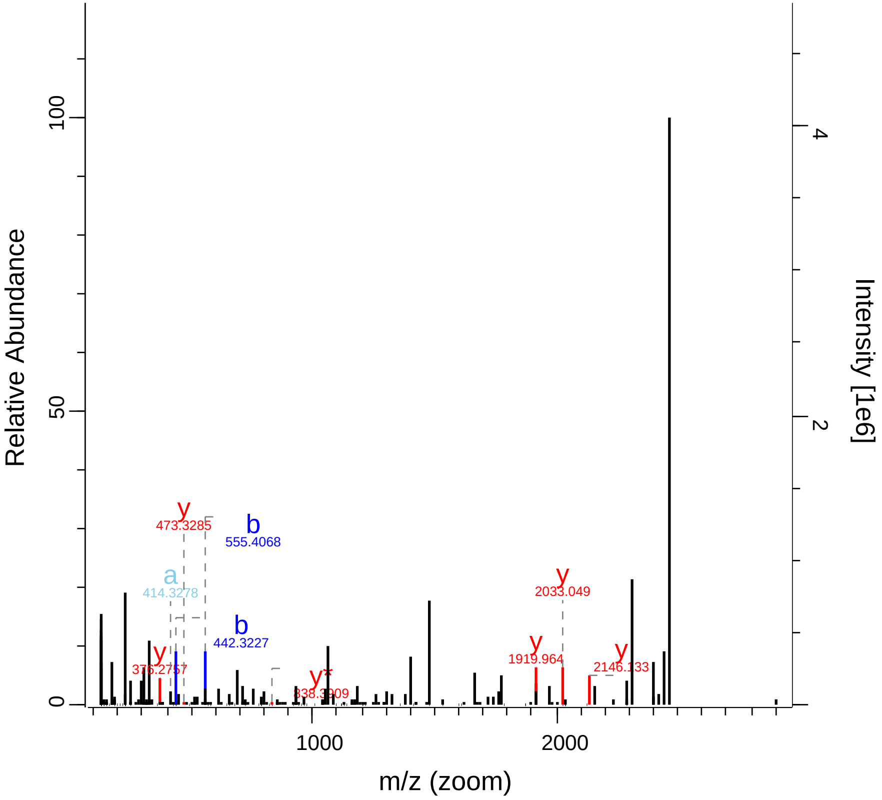

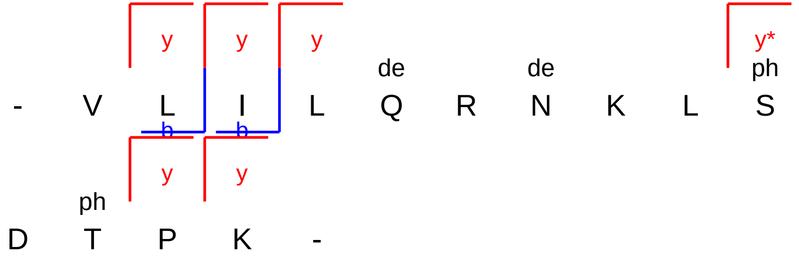

Raw File	Scan	Method	Score	m/z	Gene names
RO-QC-160121_ZackD_SS_Set_01_Fractic	27377	FTMS; HC	60.16	1221.17	CYP4F12

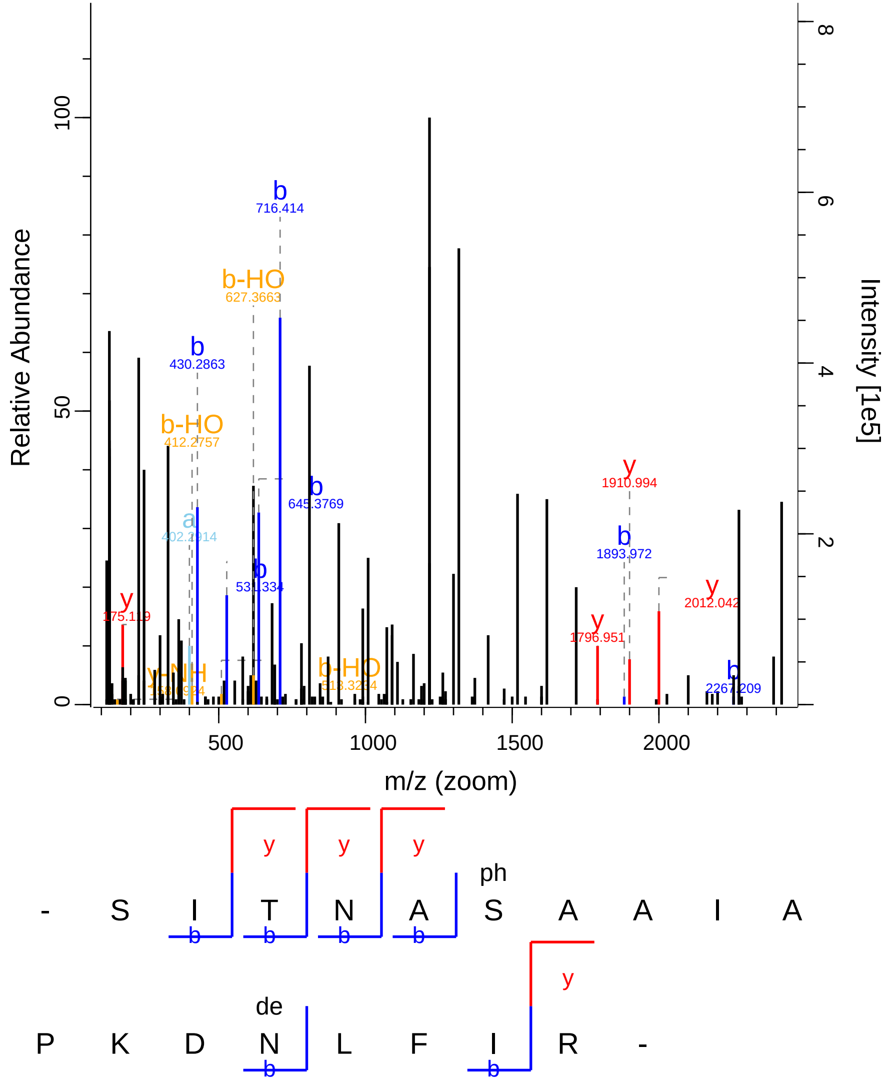

Raw File	Scan	Method	Score	m/z	Gene names
RO-QC-160121_ZackD_SS_Set_01_Fractic	28306	FTMS; HC	50.29	1194.13	TCERG1L

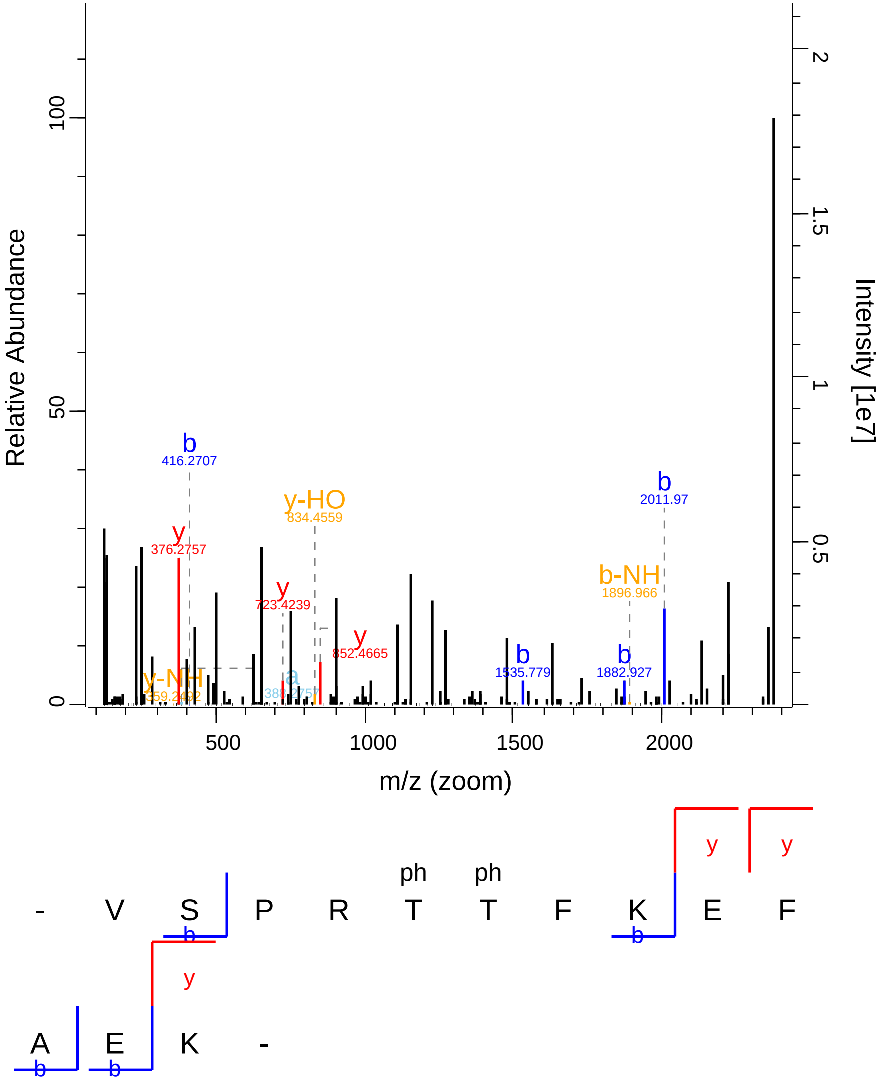

Raw File	Scan	Method	Score	m/z	Gene names
RO-QC-160121_ZackD_SS_Set_01_Fractic	29683	FTMS; HC	243.06	1254.16	LIPE

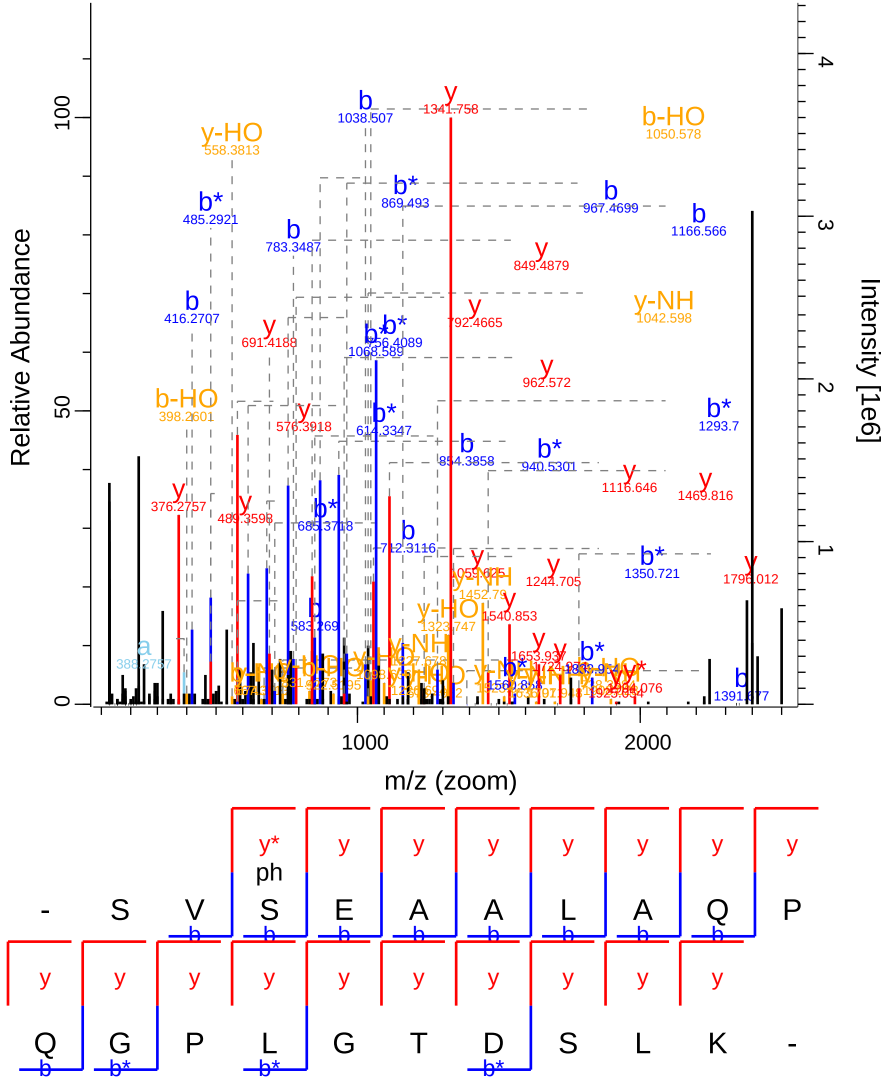

Raw File	Scan	Method	Score	m/z	Gene names
RO-QC-160121_ZackD_SS_Set_01_Fractic	29863	FTMS; HC	56.09	1041.06	TICRR

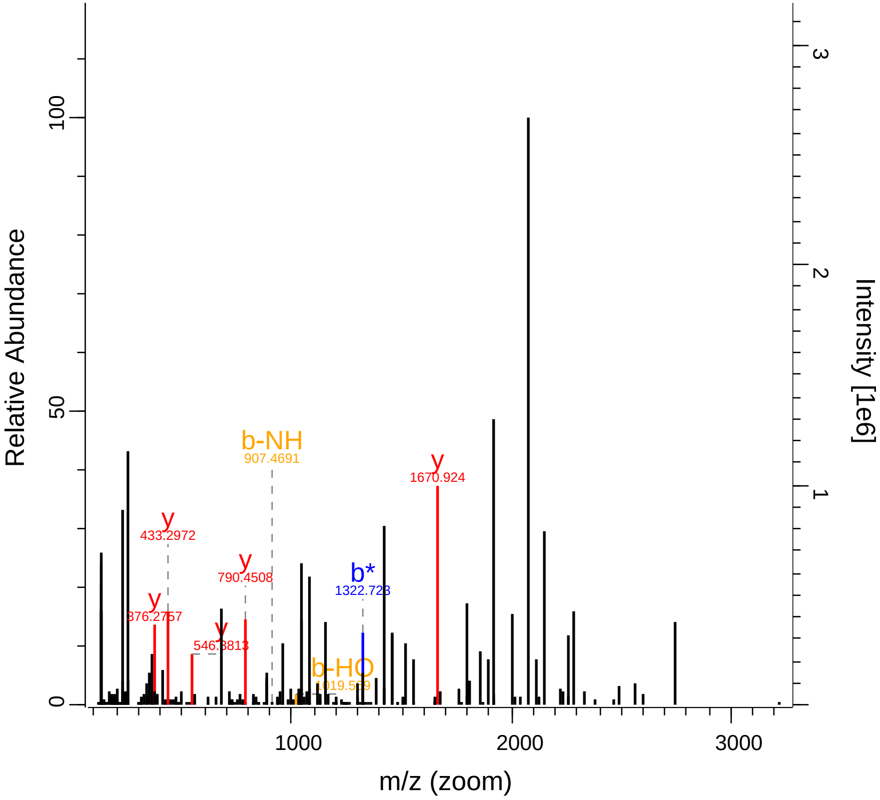

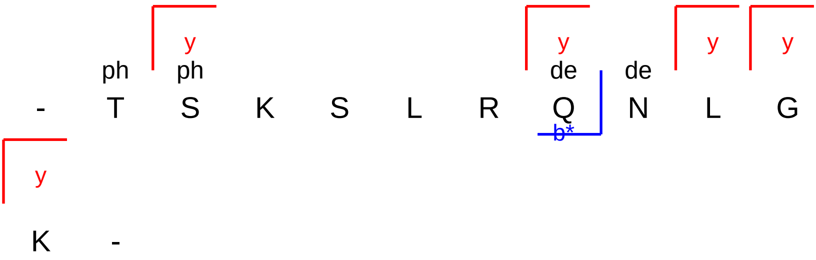

Raw File	Scan	Method	Score	m/z	Gene names
RO-QC-160121_ZackD_SS_Set_01_Fractio	30567	FTMS; HC	52.61	899.13	CEMIP

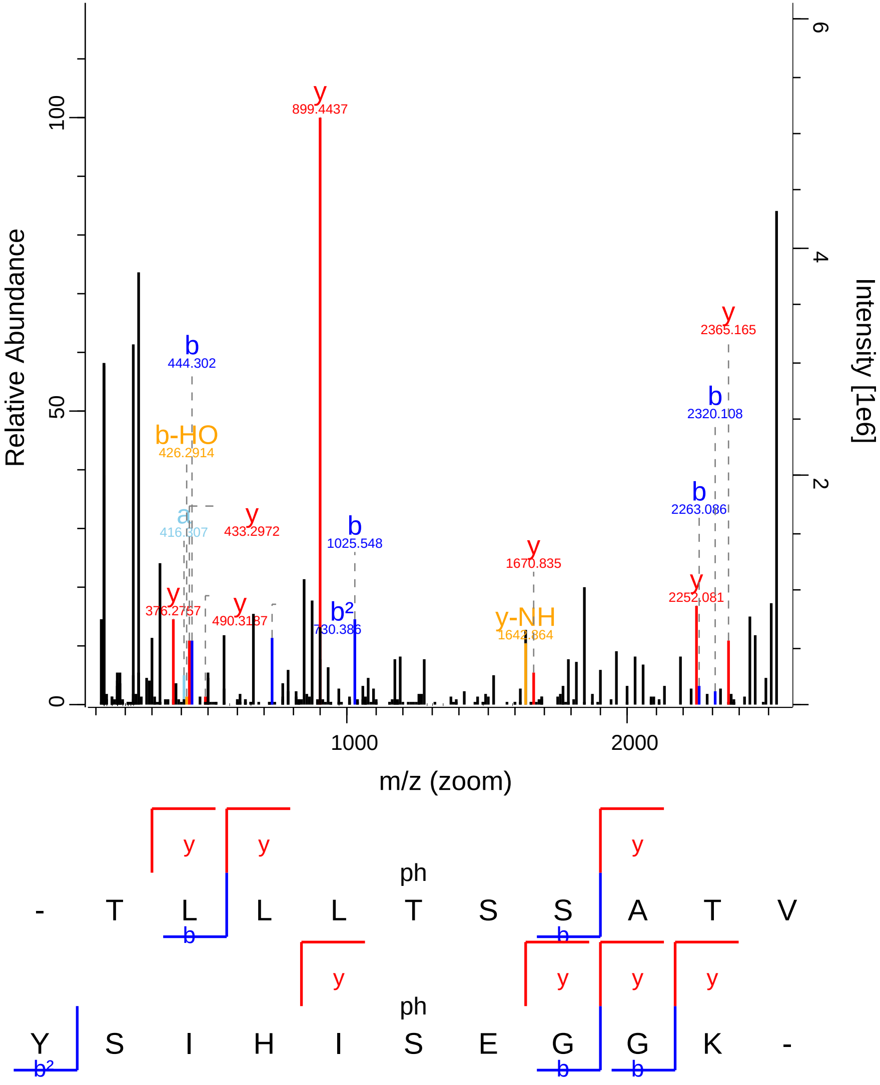

Raw File	Scan	Method	Score	m/z	Gene names
RO-QC-160121_ZackD_SS_Set_01_Fractio	31294	FTMS; HC	89.05	1133.6	CEP152

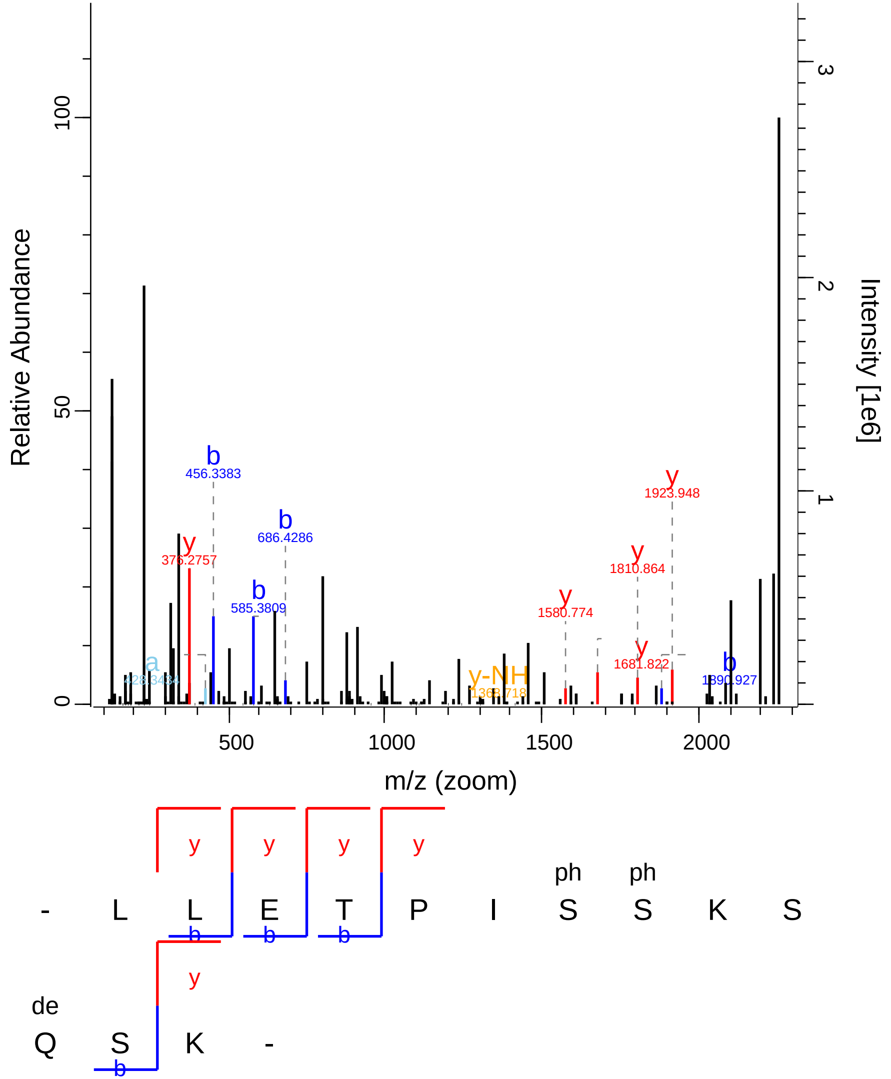

Raw File	Scan	Method	Score	m/z	Gene names
RO-QC-160121_ZackD_SS_Set_01_Fractic	32480	FTMS; HC	48.66	1446.27	CACNA1S

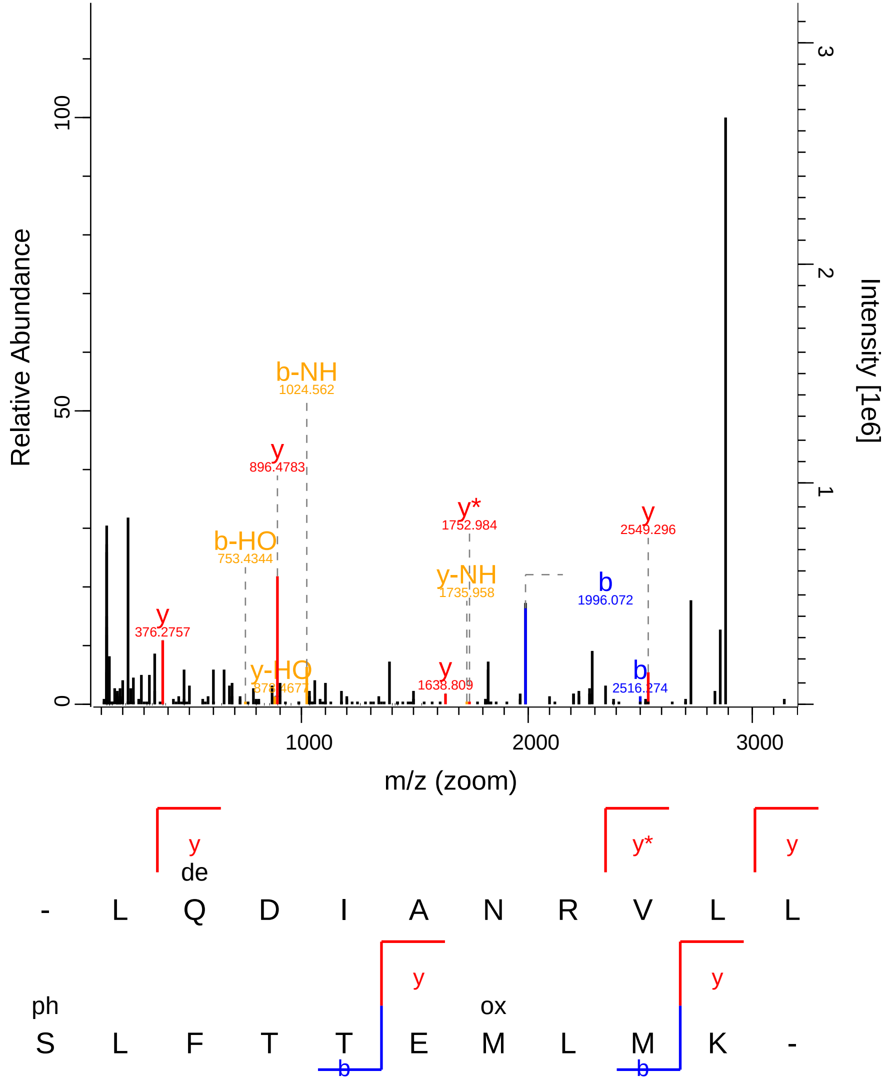

Raw File	Scan	Method	Score	m/z	Gene names
RO-QC-160121_ZackD_SS_Set_01_Fractior	35601	FTMS; HCI	40.18	976.5	ENAM

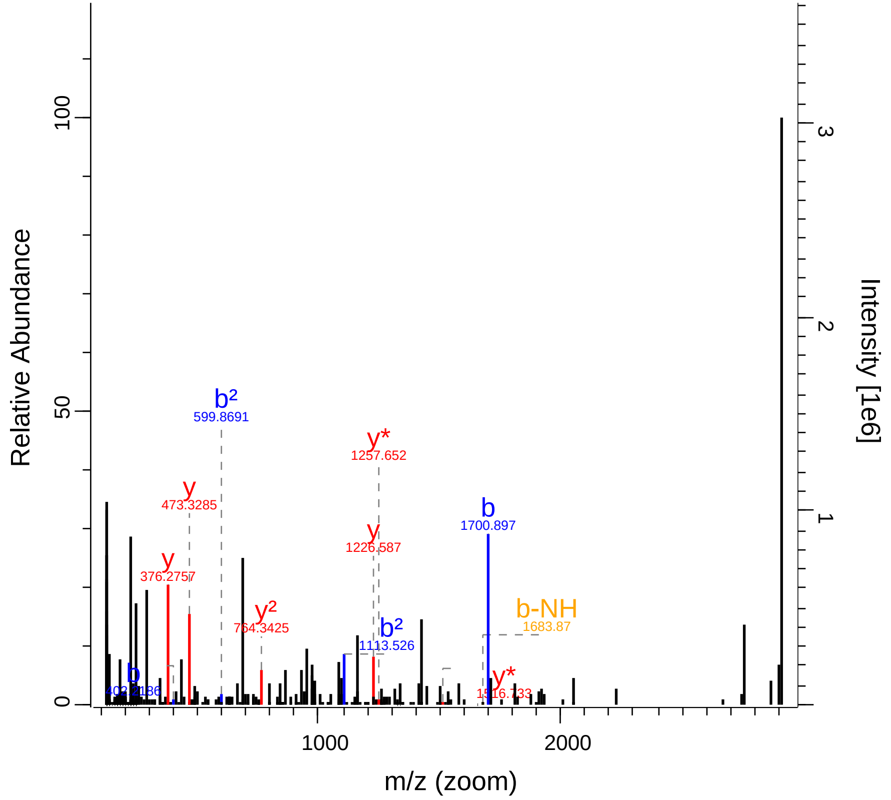

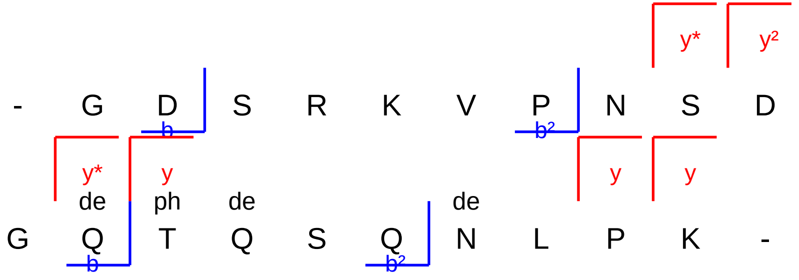

Raw File	Scan	Method	Score	m/z	Gene names
RO-QC-160121_ZackD_SS_Set_01_Fractic	37749	FTMS; HC	48.66	1243.32	ZNF300

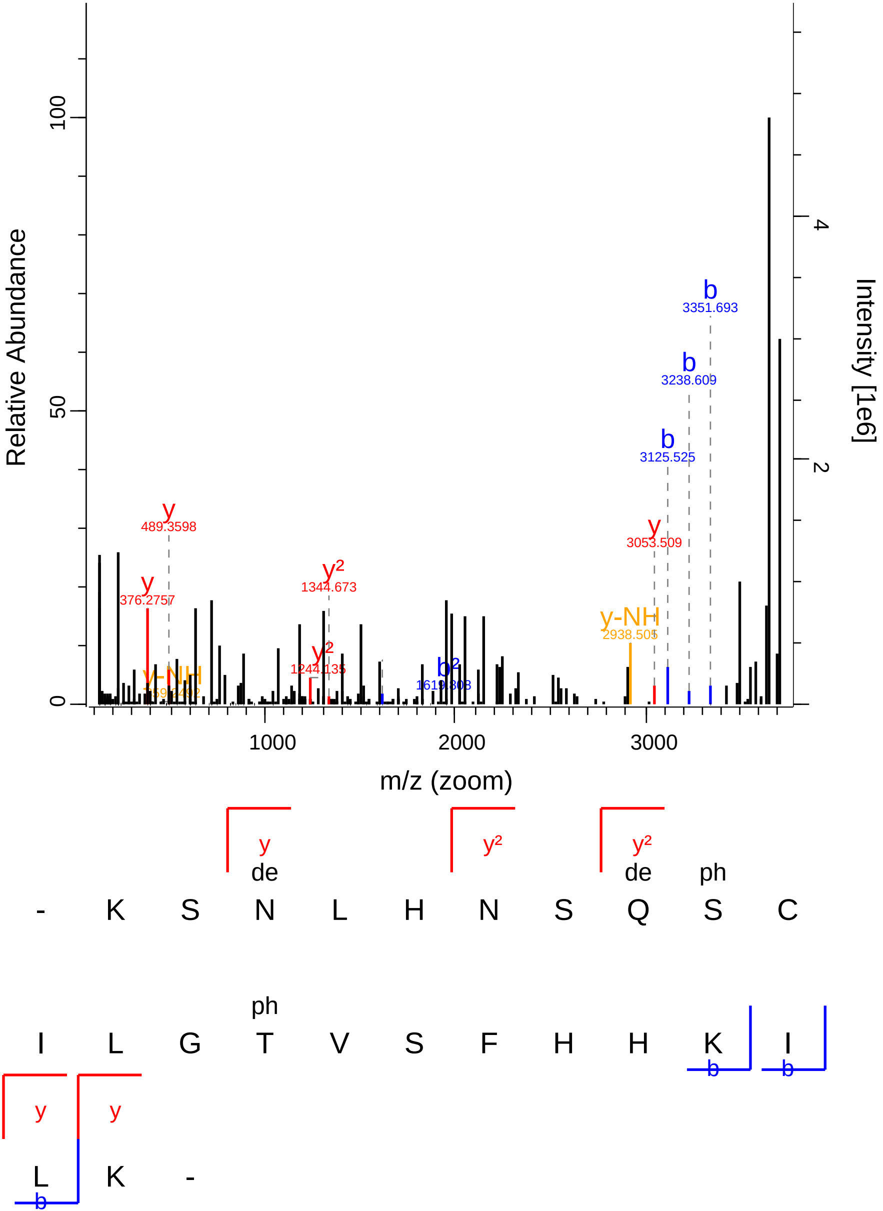

Raw File	Scan	Method	Score	m/z	Gene names
RO-QC-160121_ZackD_SS_Set_01_Fractic	40211	FTMS; HC	51.73	1233.16	RASA1

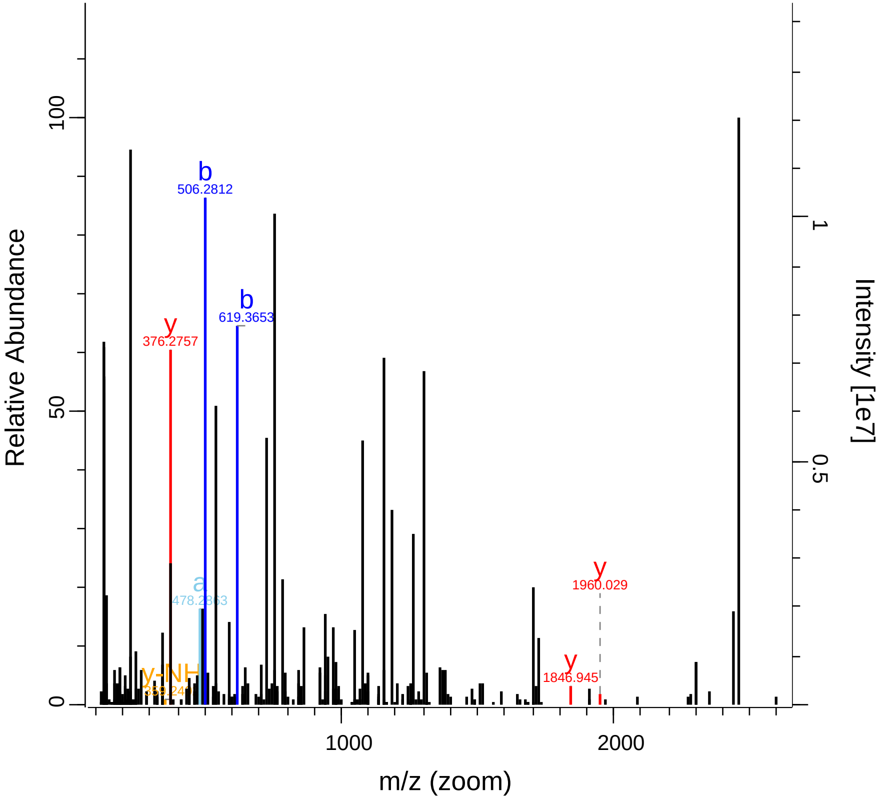

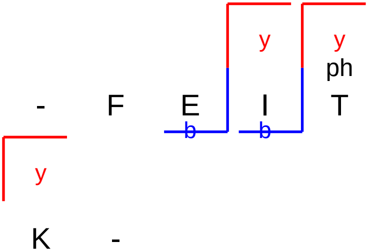

Raw File	Scan	Method	Score	m/z	Gene names
RO-QC-160121_ZackD_SS_Set_01_Fractic	40359	FTMS; HC	40.76	1047.21	BBS7

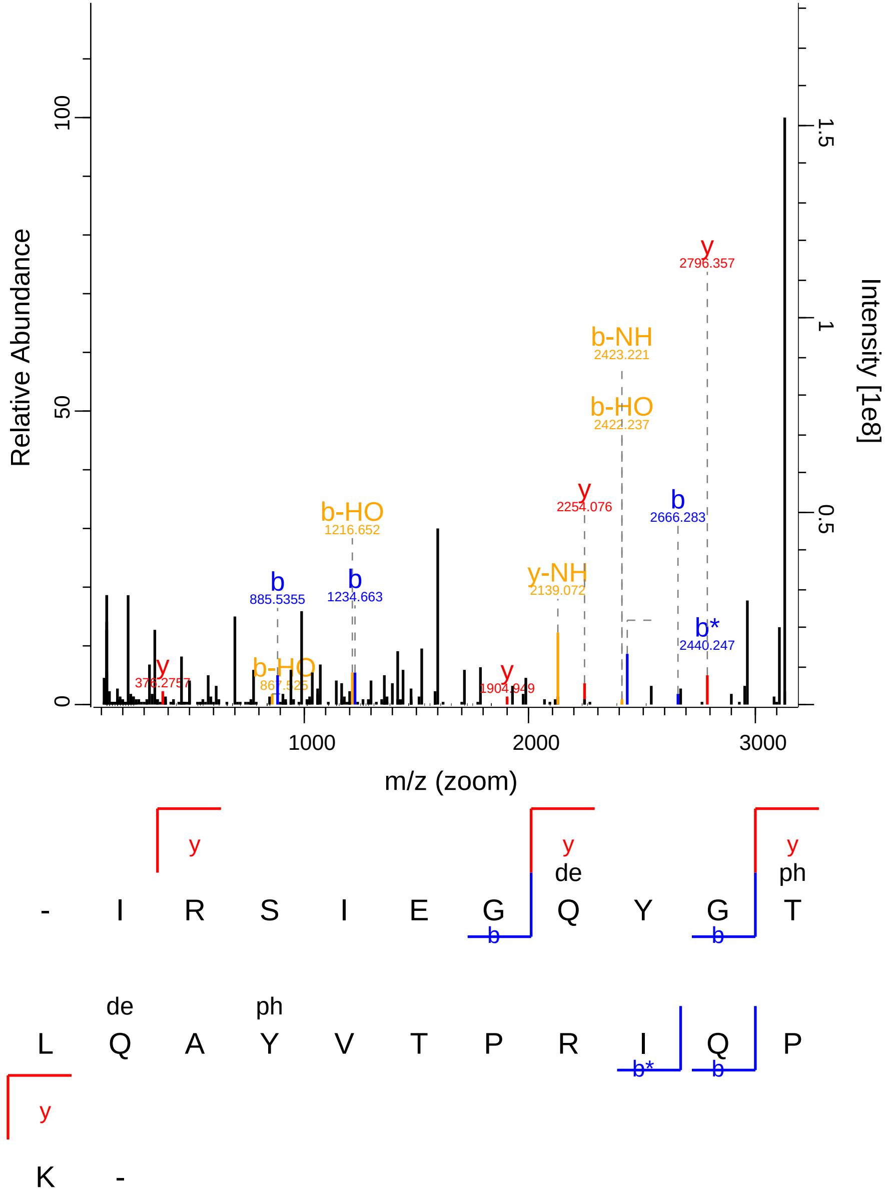

Raw File	Scan	Method	Score	m/z	Gene names
RO-QC-160121_ZackD_SS_Set_01_Fractio	41715	FTMS; HC	47.99	640.03	PIGR

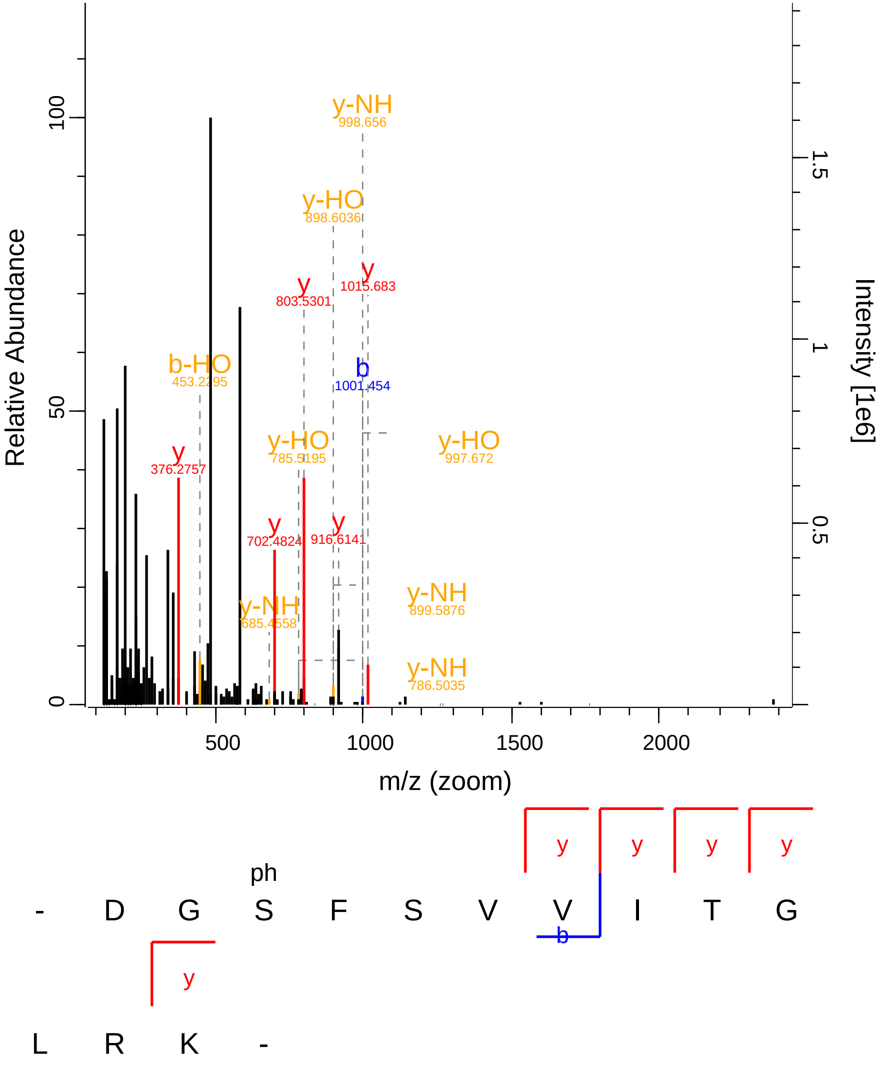

Raw File	Scan	Method	Score	m/z	Gene names
RO-QC-160121_ZackD_SS_Set_01_Fraction	4411	FTMS; HC	94.45	505.56	RBM15B

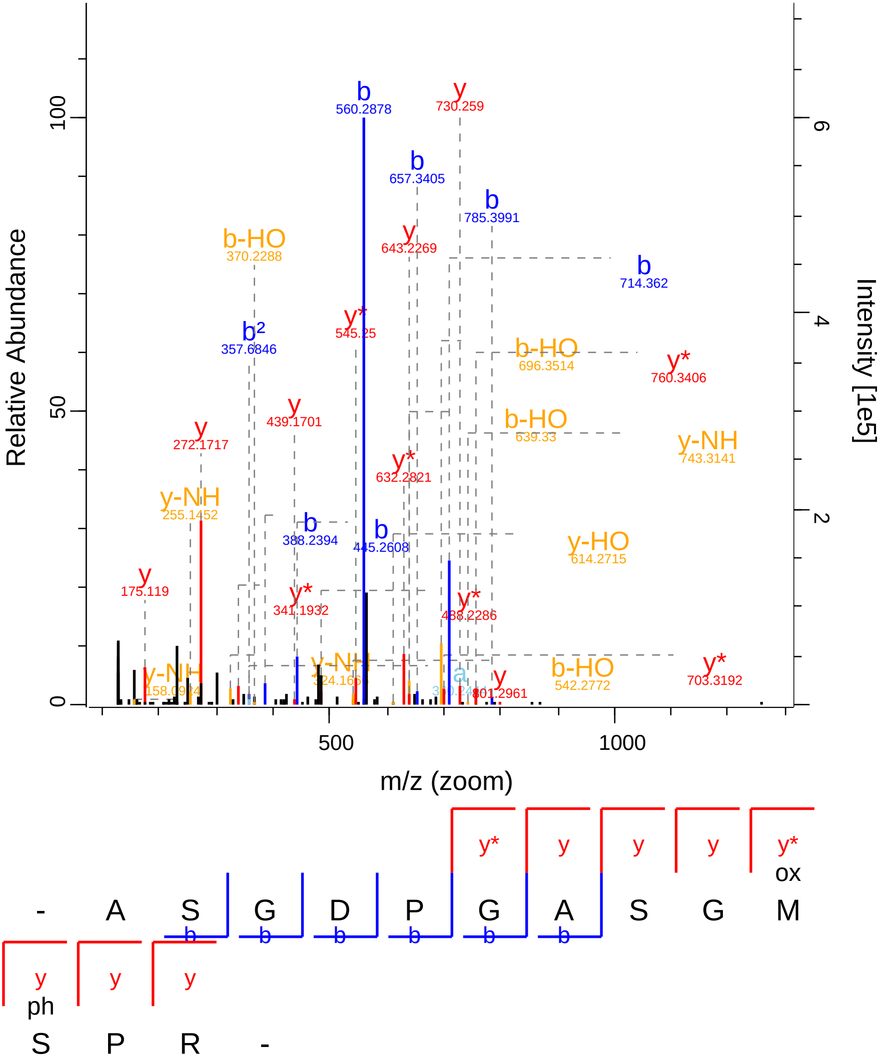

Raw File	Scan	Method	Score	m/z	Gene names
RO-QC-160121_ZackD_SS_Set_01_Fraction	4511	FTMS; HC	107.45	721.84	ENAH

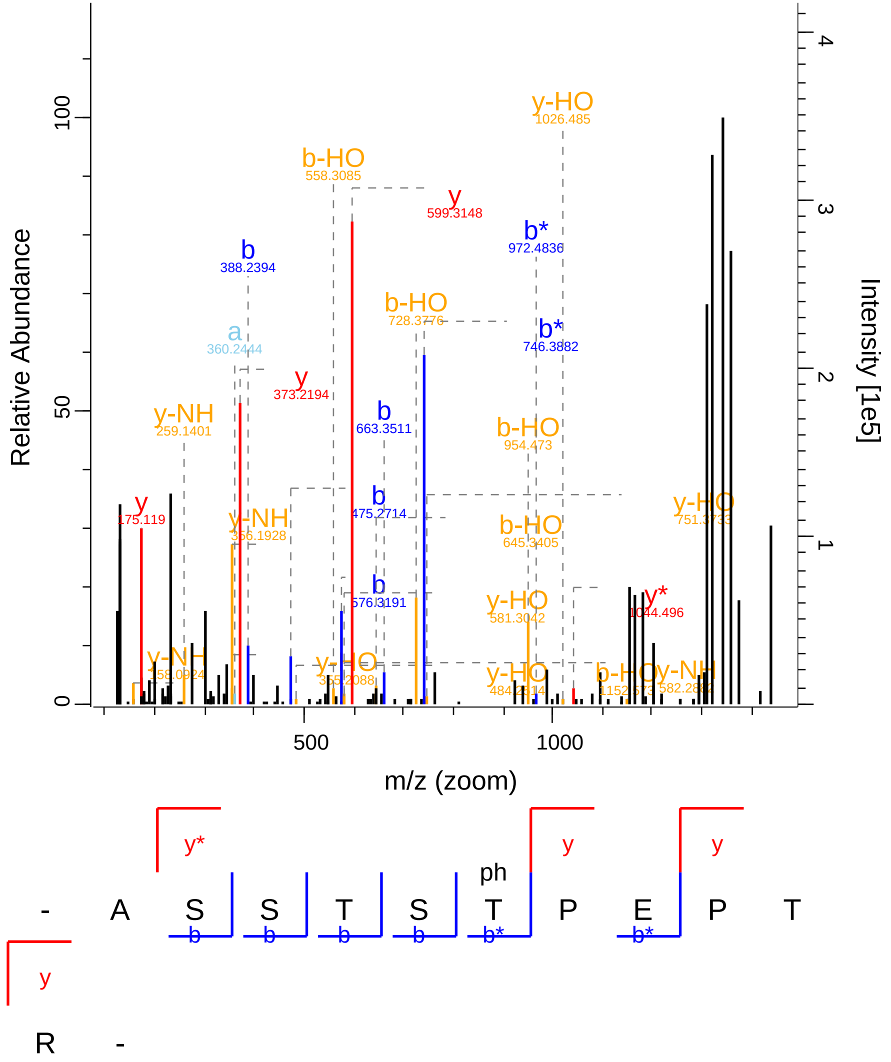

Raw File	Scan	Method	Score	m/z	Gene names
RO-QC-160121_ZackD_SS_Set_01_Fraction	5508	FTMS; HC	44.39	834.41	B3GALT2

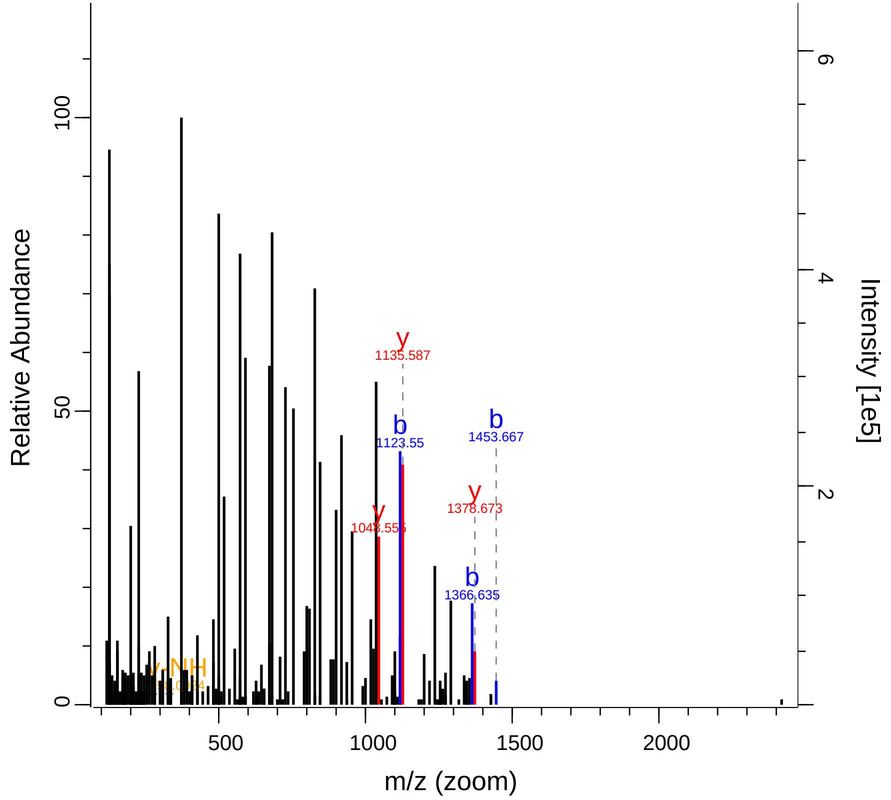

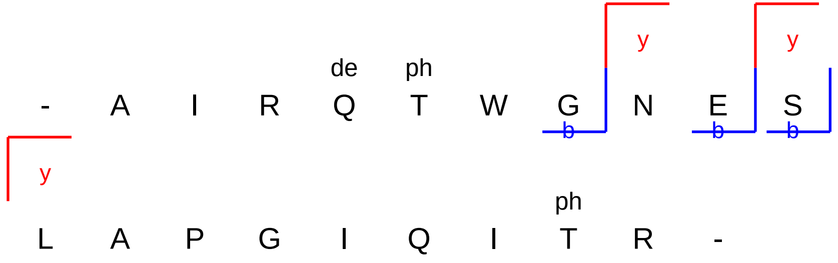

Raw File	Scan	Method	Score	m/z	Gene names
RO-QC-160121_ZackD_SS_Set_01_Fraction	5865	FTMS; HC	54.55	440.52	C15orf41

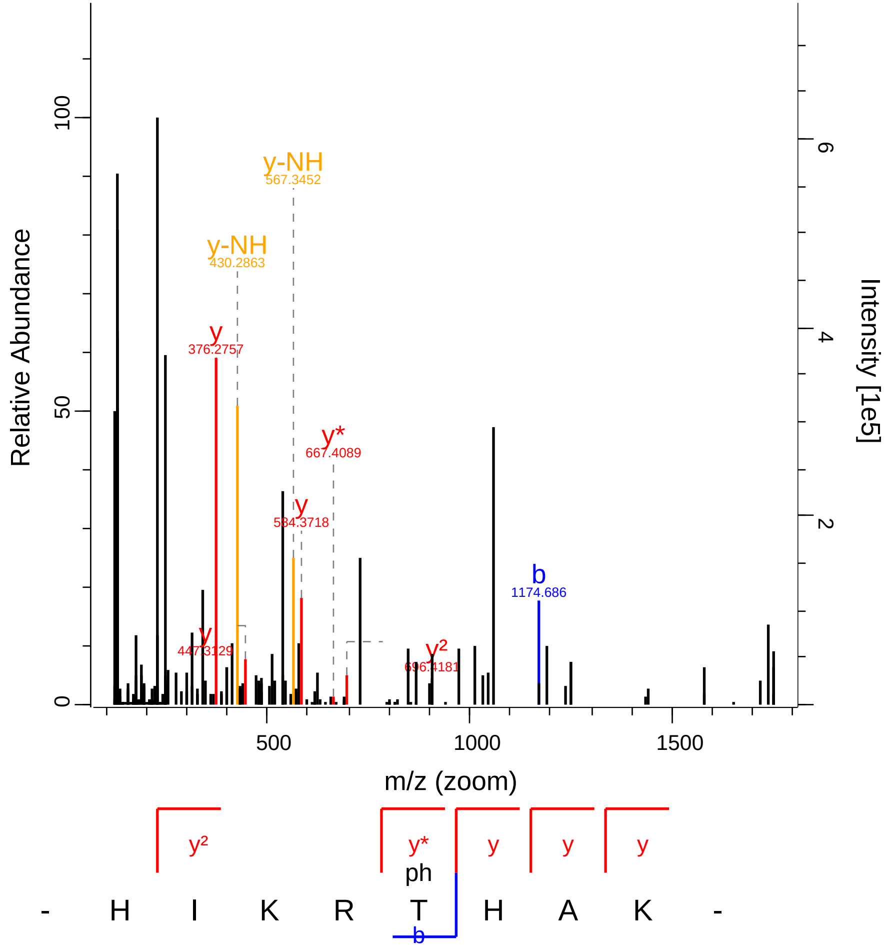

Raw File	Scan	Method	Score	m/z	Gene names
RO-QC-160121_ZackD_SS_Set_01_Fraction	7241	FTMS; HC	101.67	677.64	ADD1

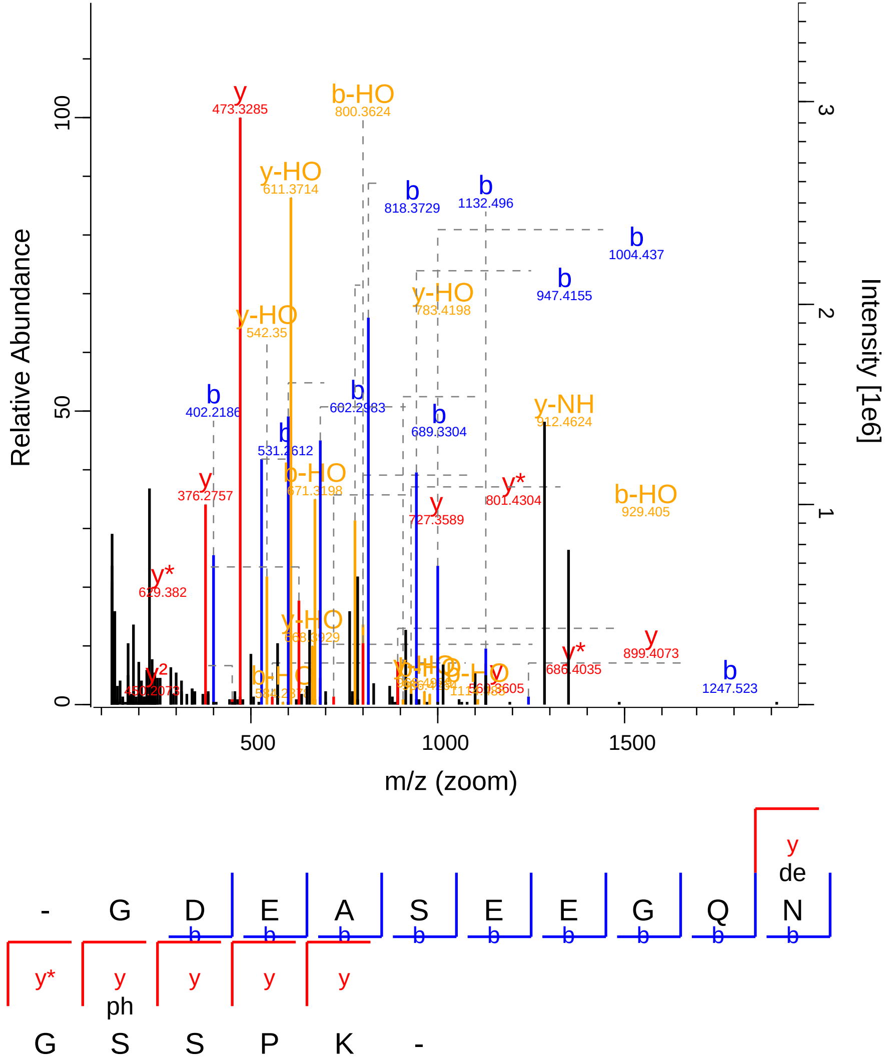

Raw File	Scan	Method	Score	m/z	Gene names
RO-QC-160121_ZackD_SS_Set_01_Fraction	7650	FTMS; HC	113.61	817.44	SPTBN1

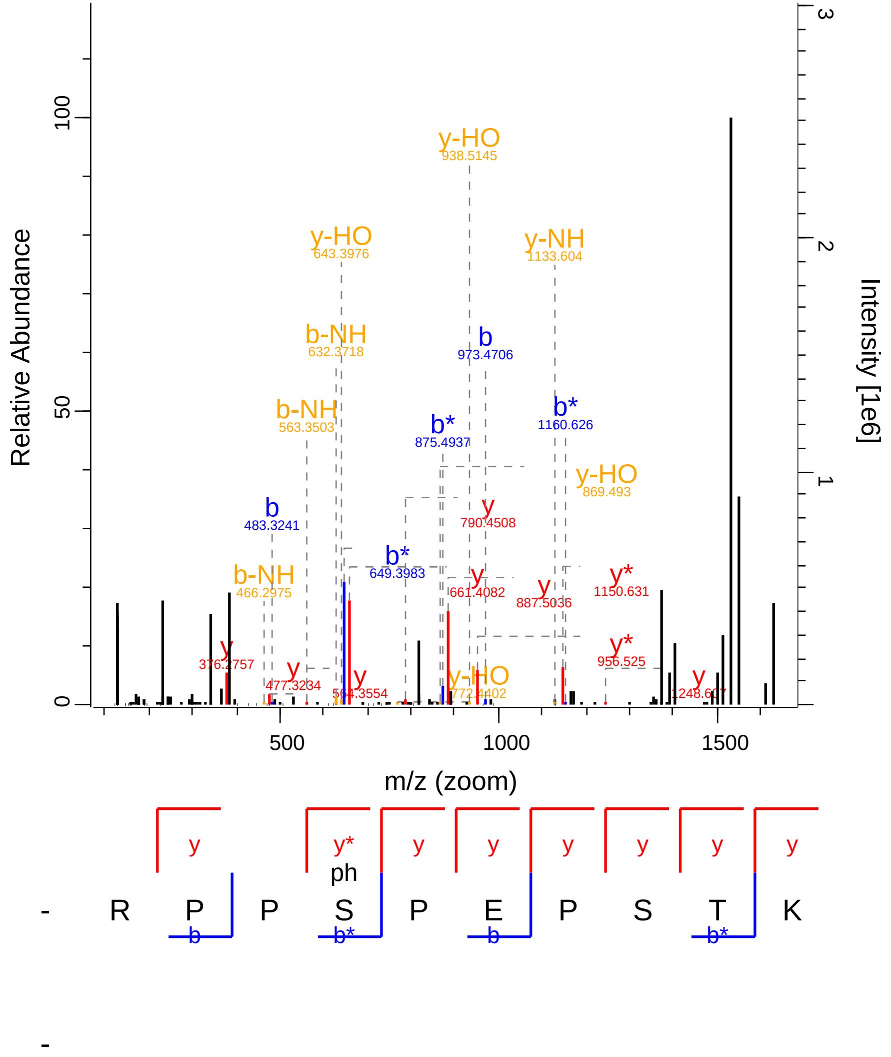

Raw File	Scan	Method	Score	m/z	Gene names
RO-QC-160121_ZackD_SS_Set_01_Fraction	8132	FTMS; HC	49.93	679.82	KPNA5

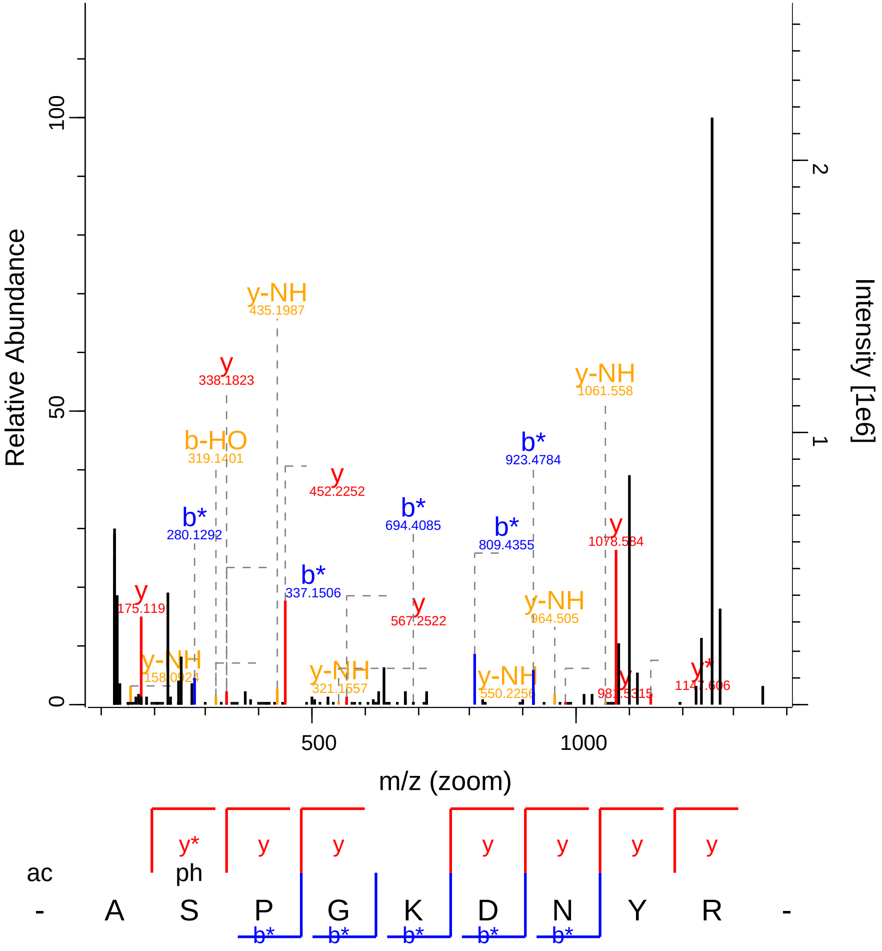

Raw File	Scan	Method	Score	m/z	Gene names
RO-QC-160121_ZackD_SS_Set_01_Fractio	9324	FTMS; HC	207.79	1212.17	TCEA1

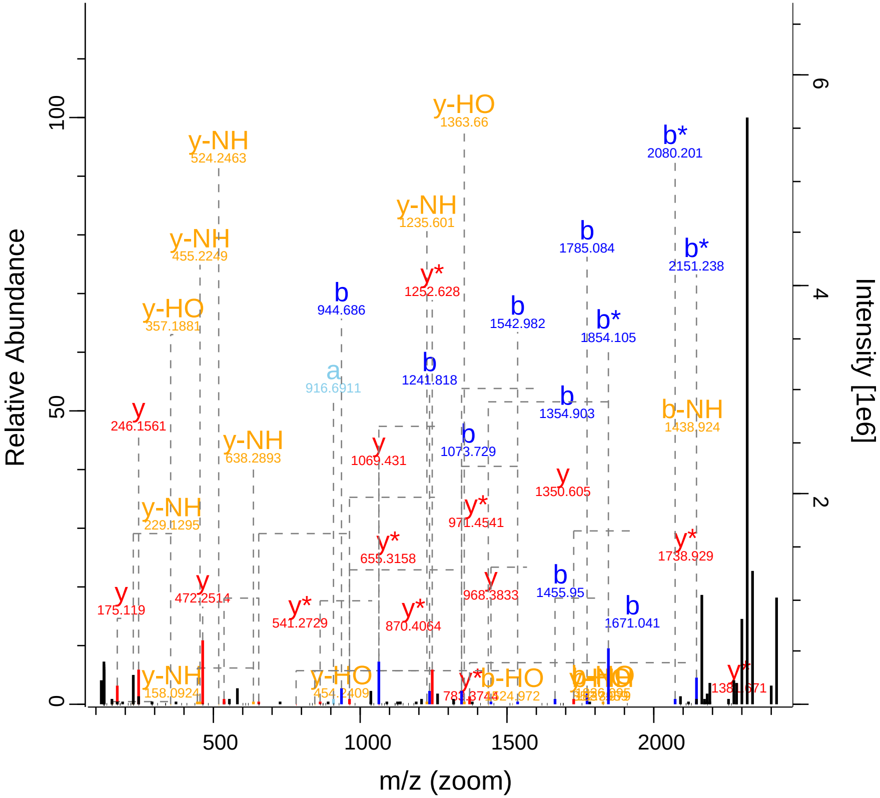

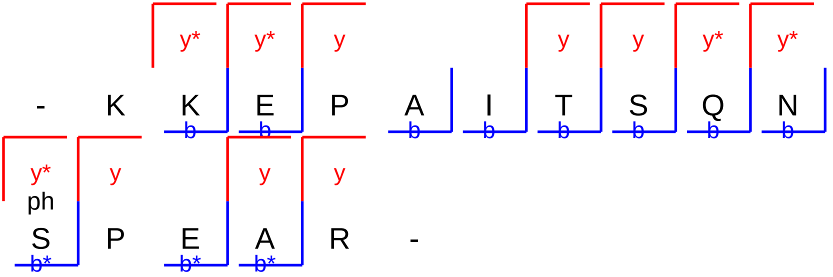

Raw File	Scan	Method	Score	m/z	Gene names
RO-QC-160121_ZackD_SS_Set_01_Fraction_	9843	FTMS; HC	58.58	873.79	CBX1

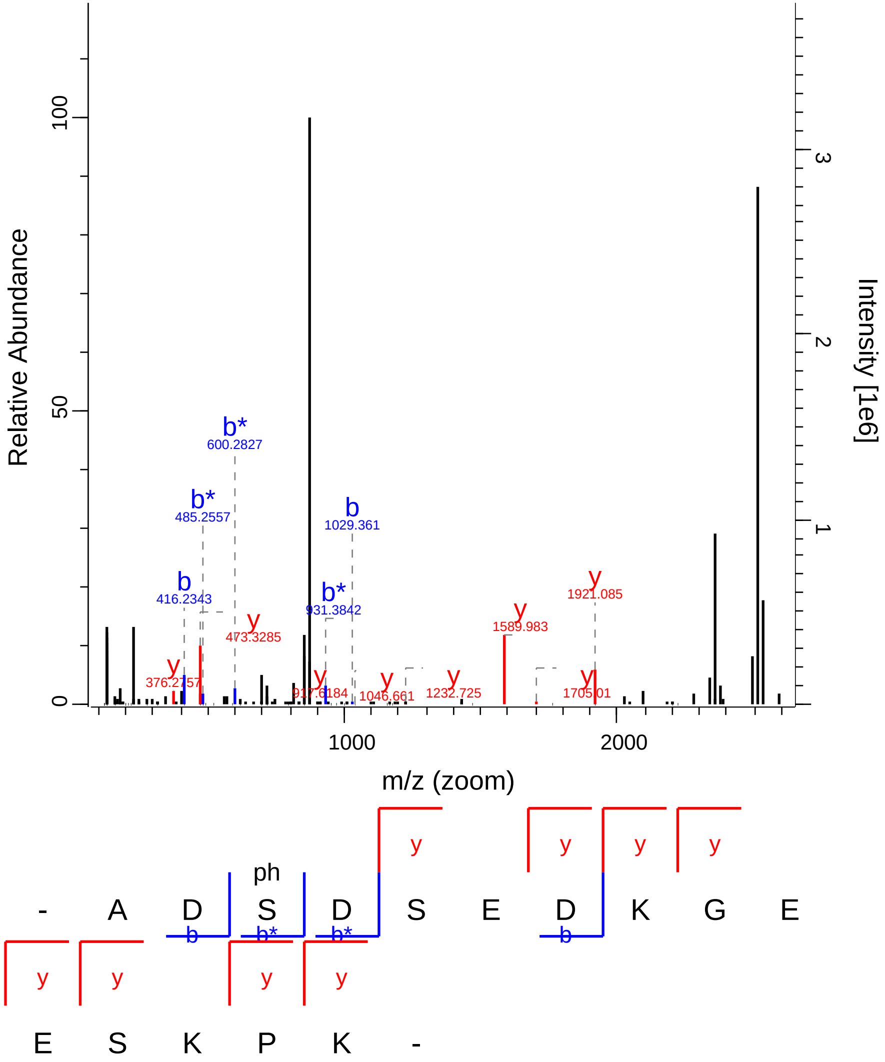

Raw File	Scan	Method	Score	m/z	Gene names
RO-QC-160121_ZackD_SS_Set_01_Fraction_	11987	FTMS; HC	105.46	936	TSPAN10

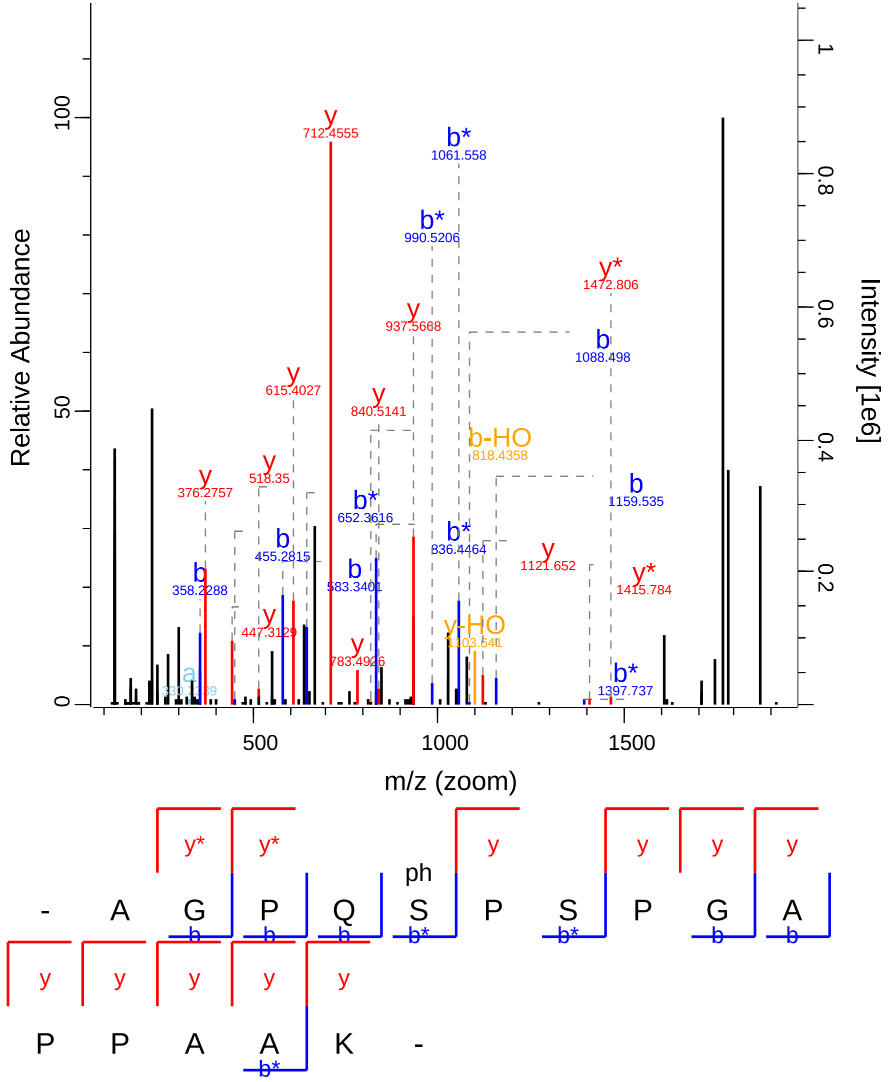

Raw File	Scan	Method	Score	m/z	Gene names
RO-QC-160121_ZackD_SS_Set_01_Fractior	13612	FTMS; HC	65.25	715.38	TMIGD3

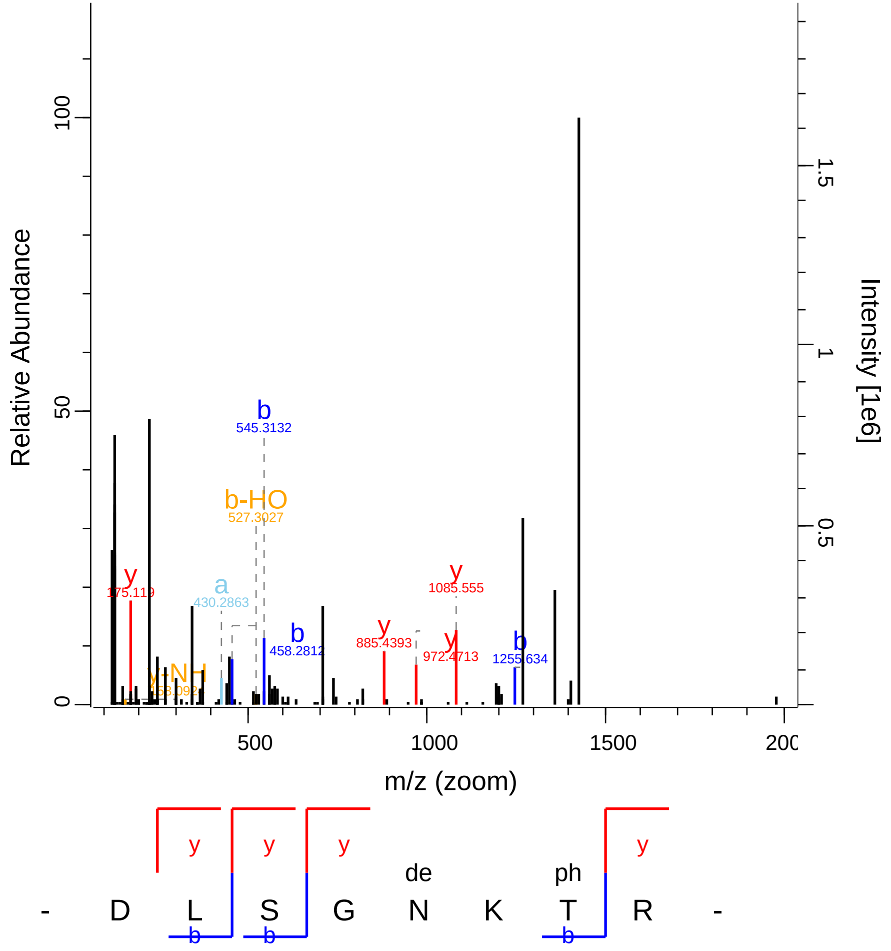

Raw File	Scan	Method	Score	m/z	Gene names
RO-QC-160121_ZackD_SS_Set_01_Fractio[illegible]	14134	FTMS; HC	131.32	813.42	NMT1

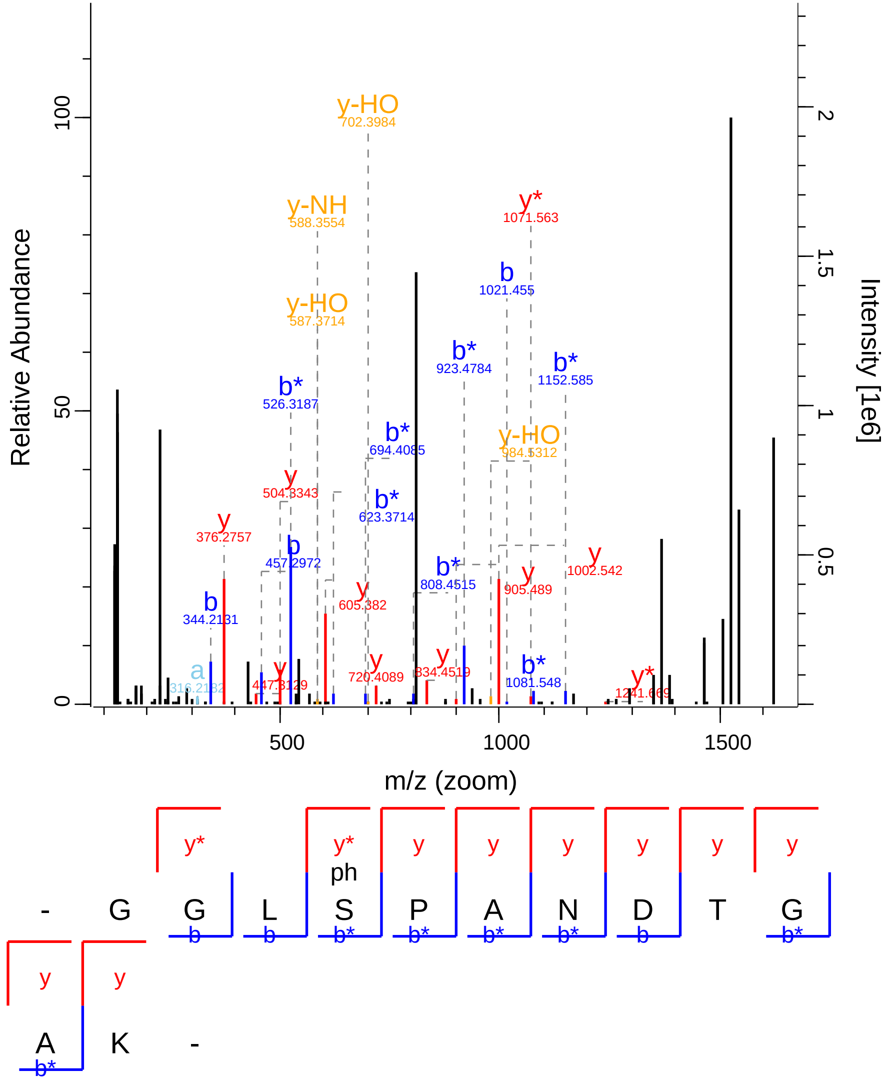

Raw File	Scan	Method	Score	m/z	Gene names
RO-QC-160121_ZackD_SS_Set_01_Fractio	16750	FTMS; HC	78.23	1120.58	PLEKHA6

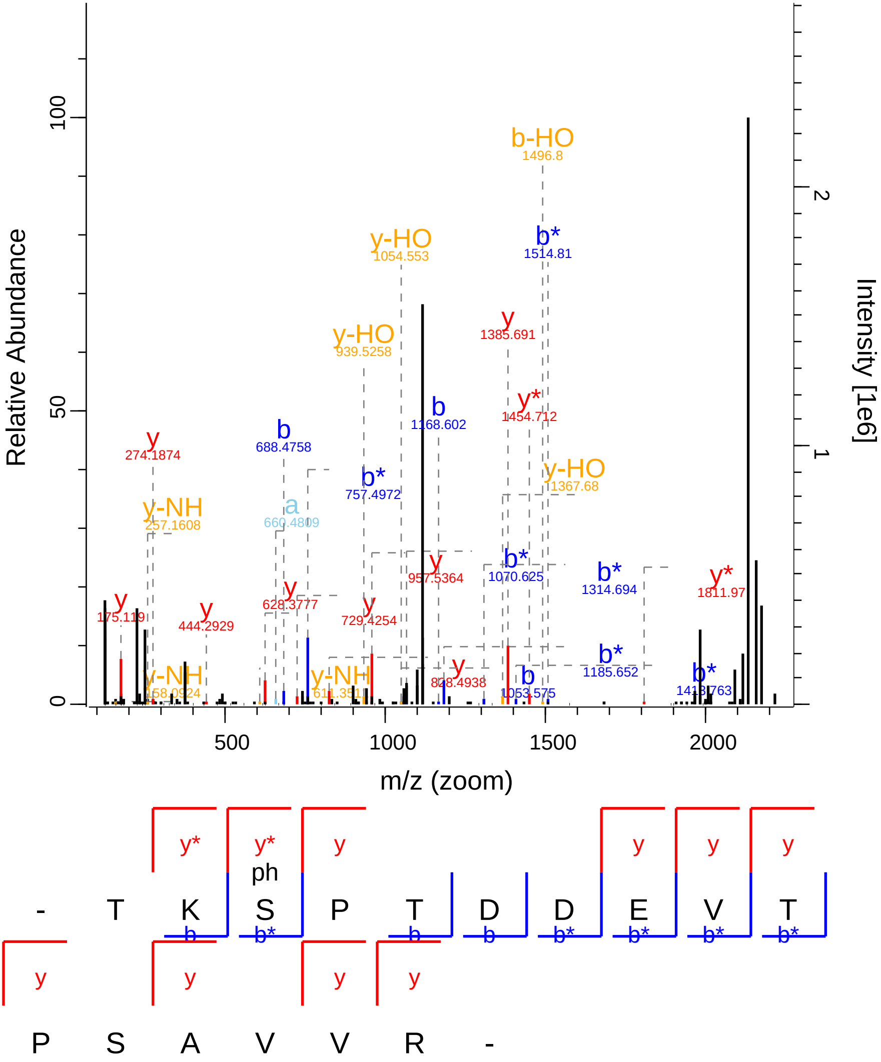

Raw File	Scan	Method	Score	m/z	Gene names
RO-QC-160121_ZackD_SS_Set_01_Fractior	17086	FTMS; HC	44.19	711.05	KIAA2018

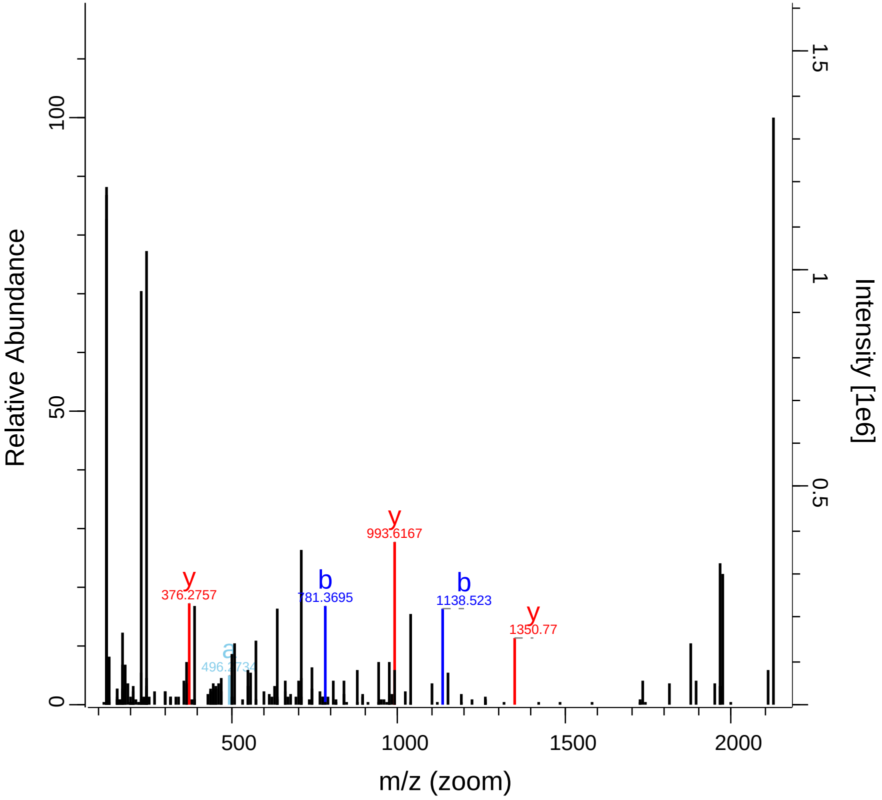

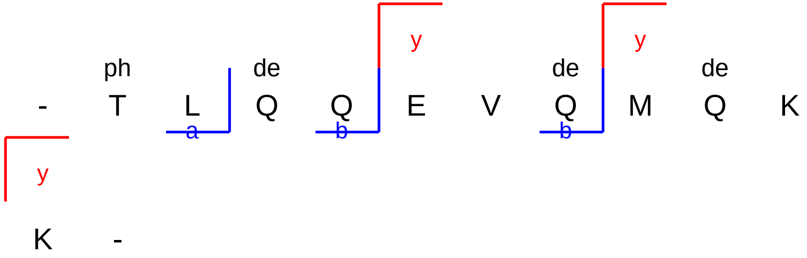

Raw File	Scan	Method	Score	m/z	Gene names
RO-QC-160121_ZackD_SS_Set_01_Fractior	17774	FTMS; HC	224.5	936.48	AHNAK

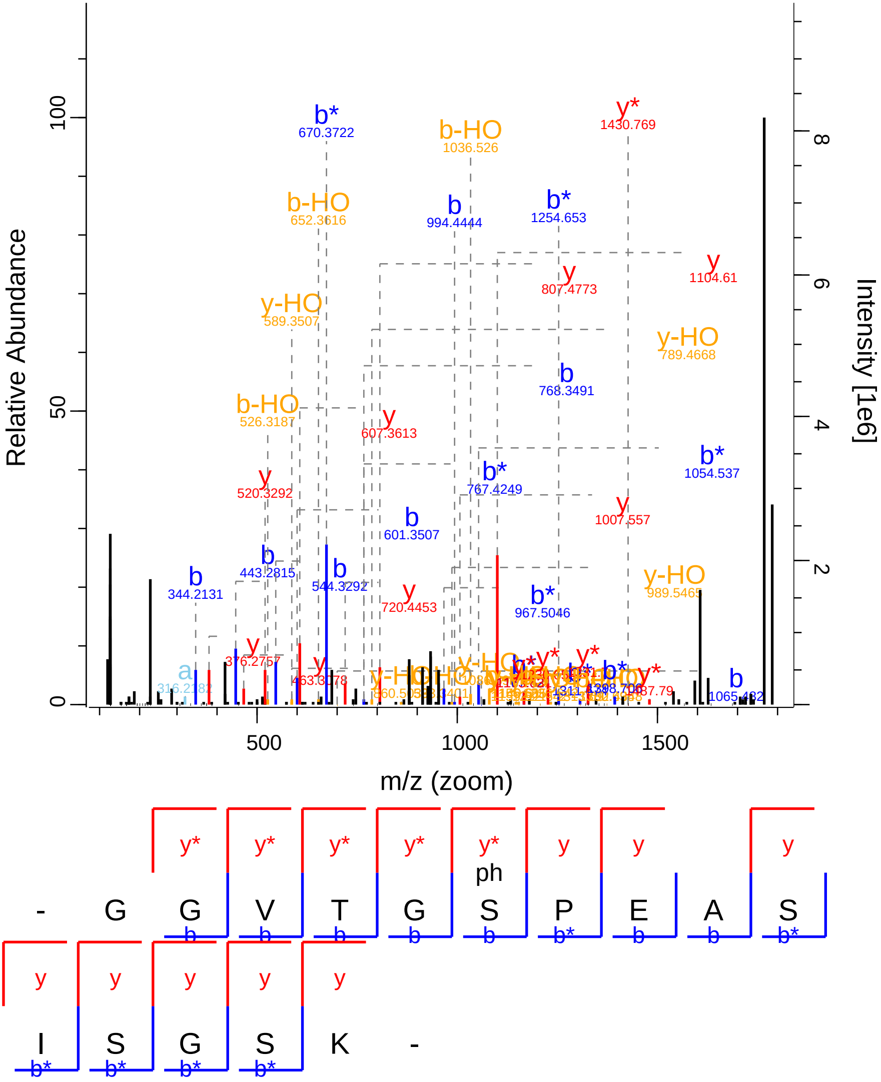

Raw File	Scan	Method	Score	m/z	Gene names
RO-QC-160121_ZackD_SS_Set_01_Fractioı	17871	FTMS; HC	105.17	726.11	SRRM2

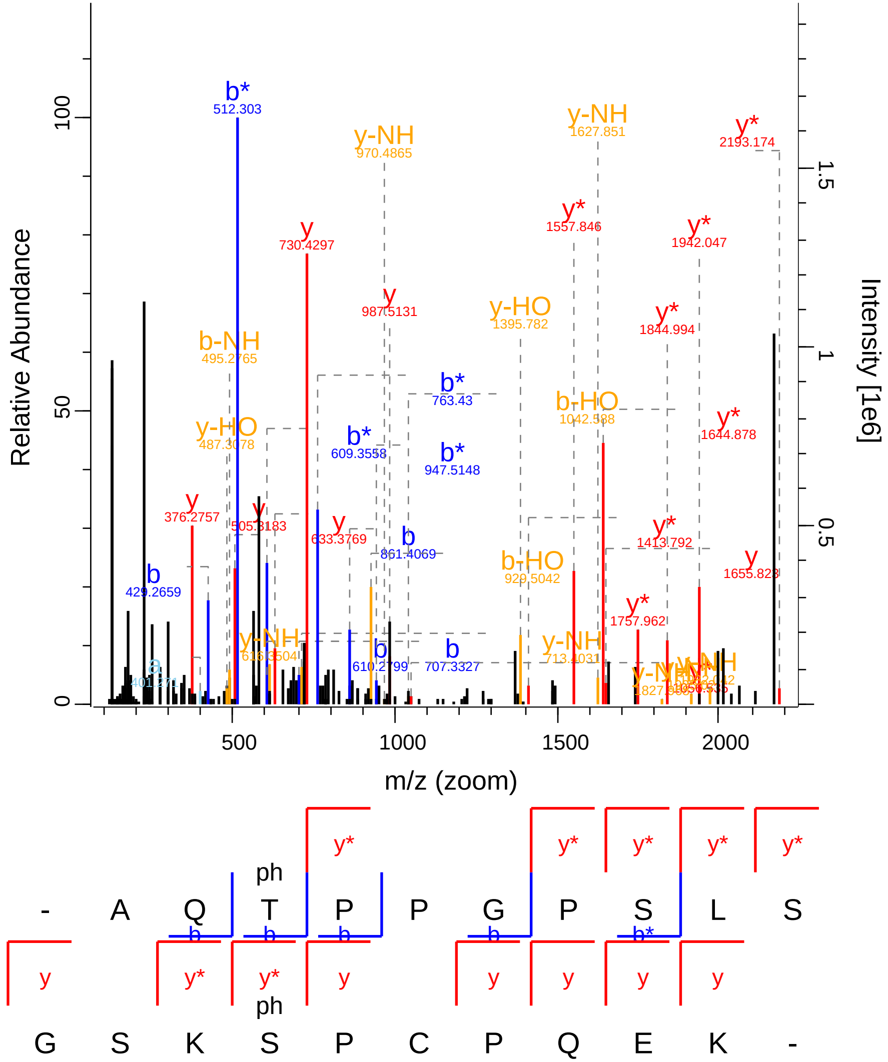

Raw File	Scan	Method	Score	m/z	Gene names
RO-QC-160121_ZackD_SS_Set_01_Fractior	22474	FTMS; HC	102.48	895.47	ARHGAP5

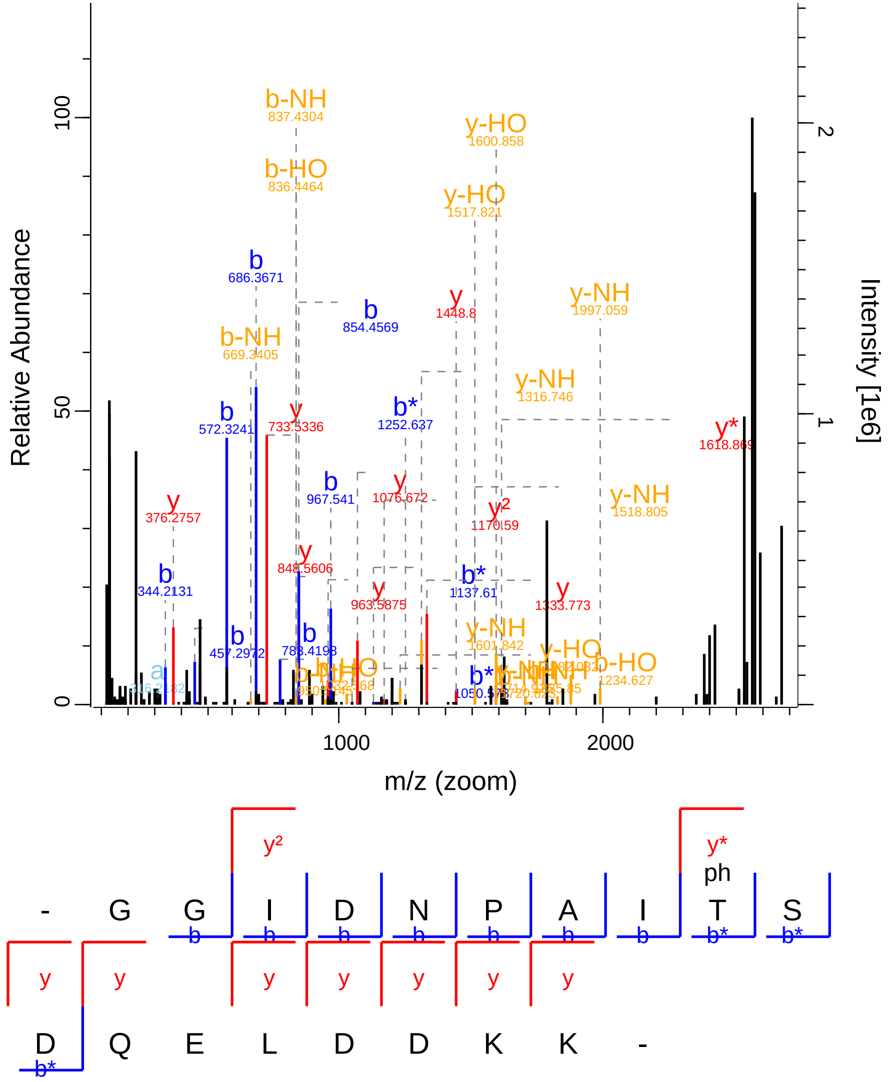

Raw File	Scan	Method	Score	m/z	Gene names
RO-QC-160121_ZackD_SS_Set_01_Fractior	22519	FTMS; HC	96.72	671.85	ARHGAP5

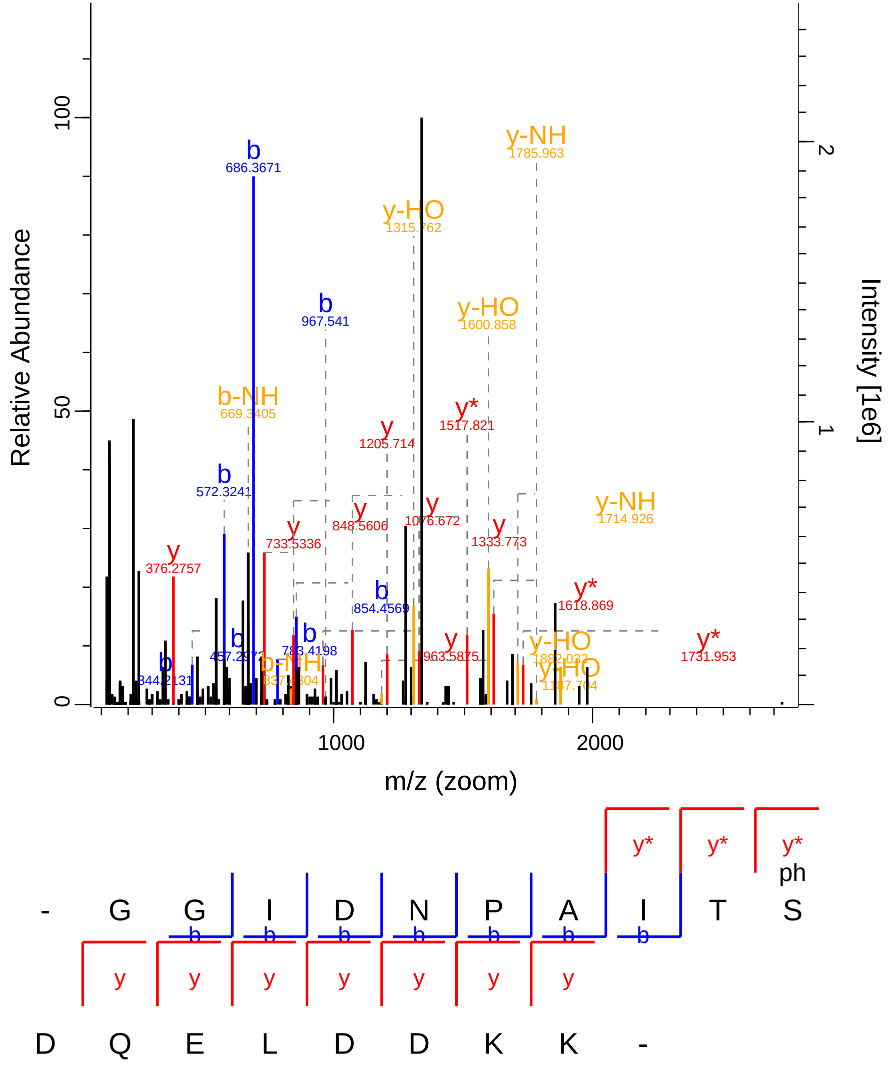

Raw File	Scan	Method	Score	m/z	Gene names
RO-QC-160121_ZackD_SS_Set_01_Fractior	23523	FTMS; HC	69.44	951.81	GORASP2

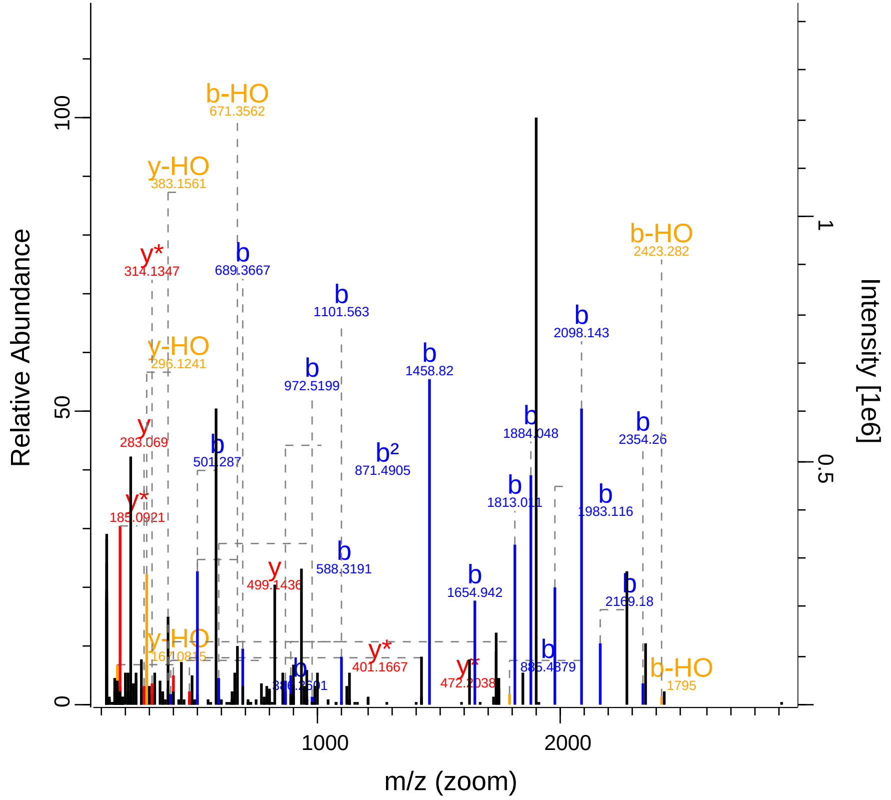

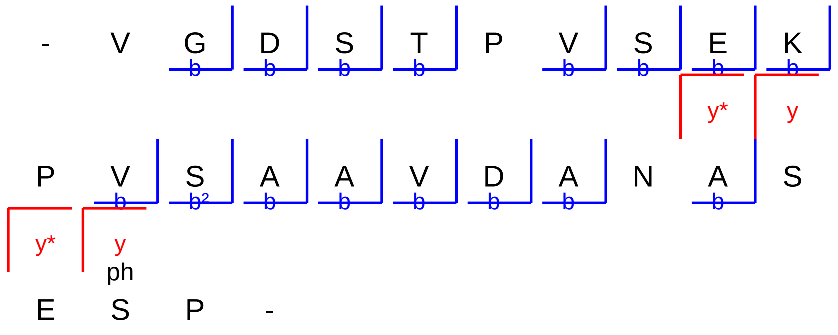

Raw File	Scan	Method	Score	m/z	Gene names
RO-QC-160121_ZackD_SS_Set_01_Fractio	23829	FTMS; HC	94.2	1127.24	HDGF

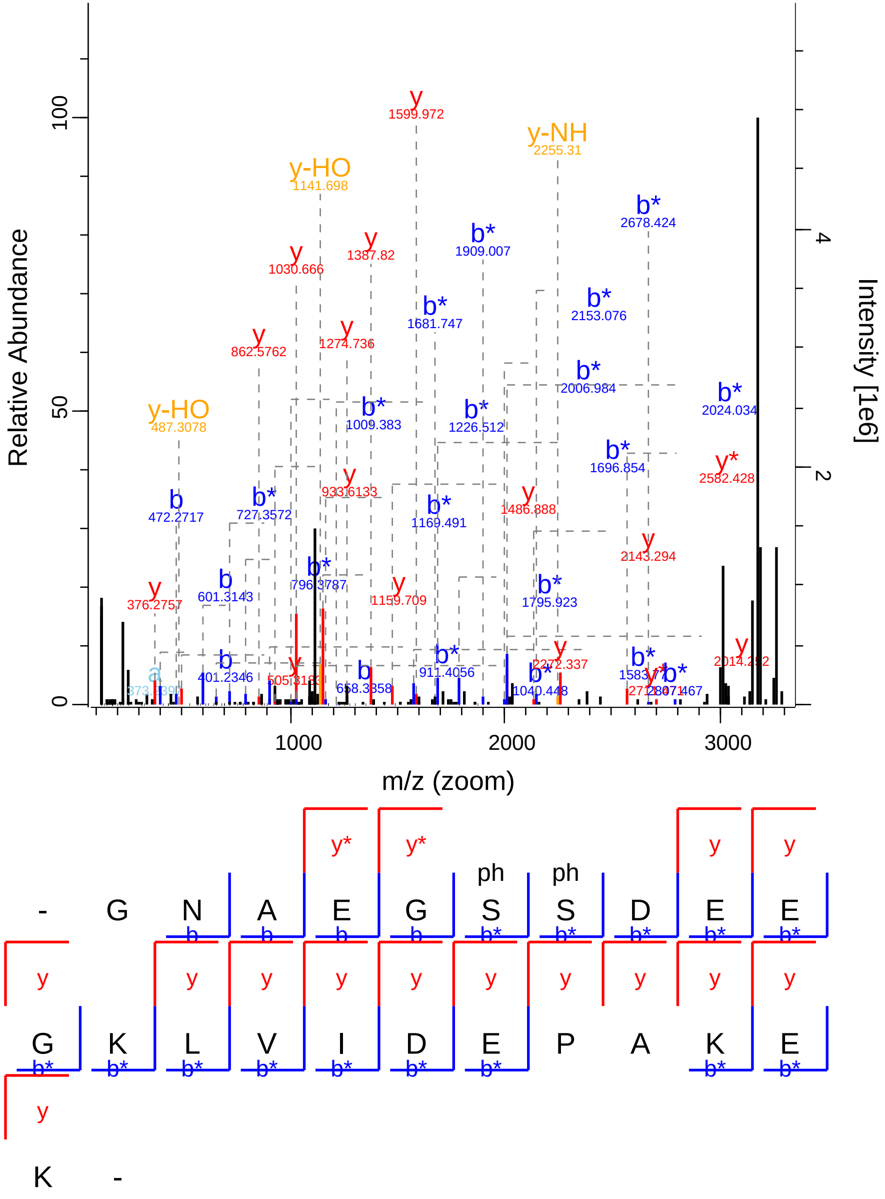

Raw File	Scan	Method	Score	m/z	Gene names
RO-QC-160121_ZackD_SS_Set_01_Fractior	25288	FTMS; HC	45.86	611.56	HOXD8

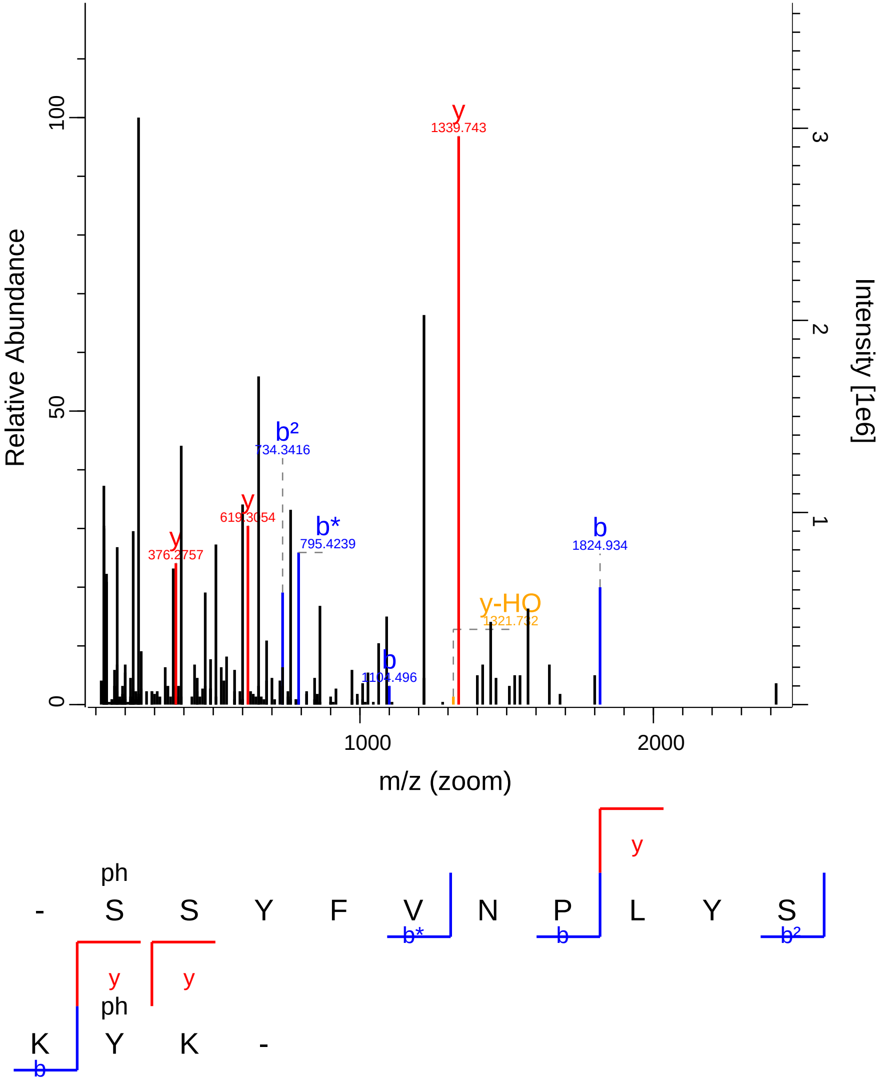

Raw File	Scan	Method	Score	m/z	Gene names
RO-QC-160121_ZackD_SS_Set_01_Fractior	27539	FTMS; HC	77.51	776.62	G3BP2

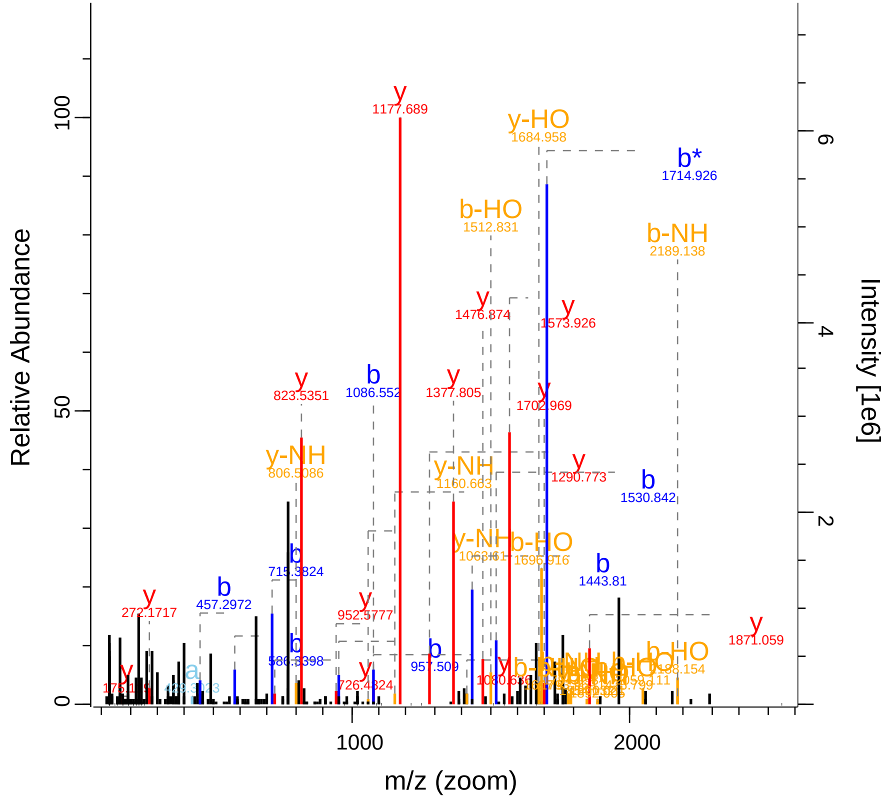

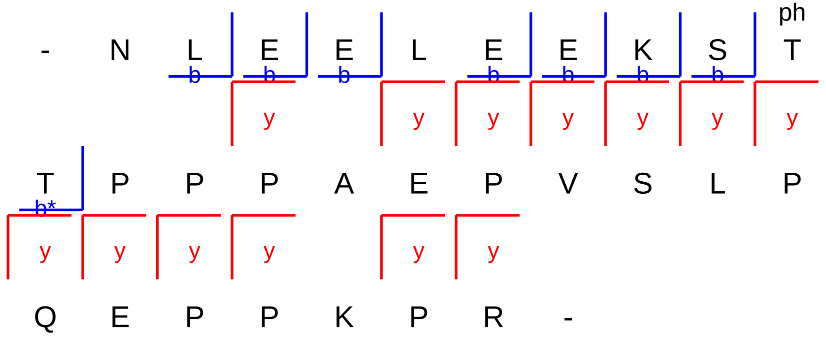

Raw File	Scan	Method	Score	m/z	Gene names
RO-QC-160121_ZackD_SS_Set_01_Fractio	27545	FTMS; HC	107.74	1080.23	SPAG9

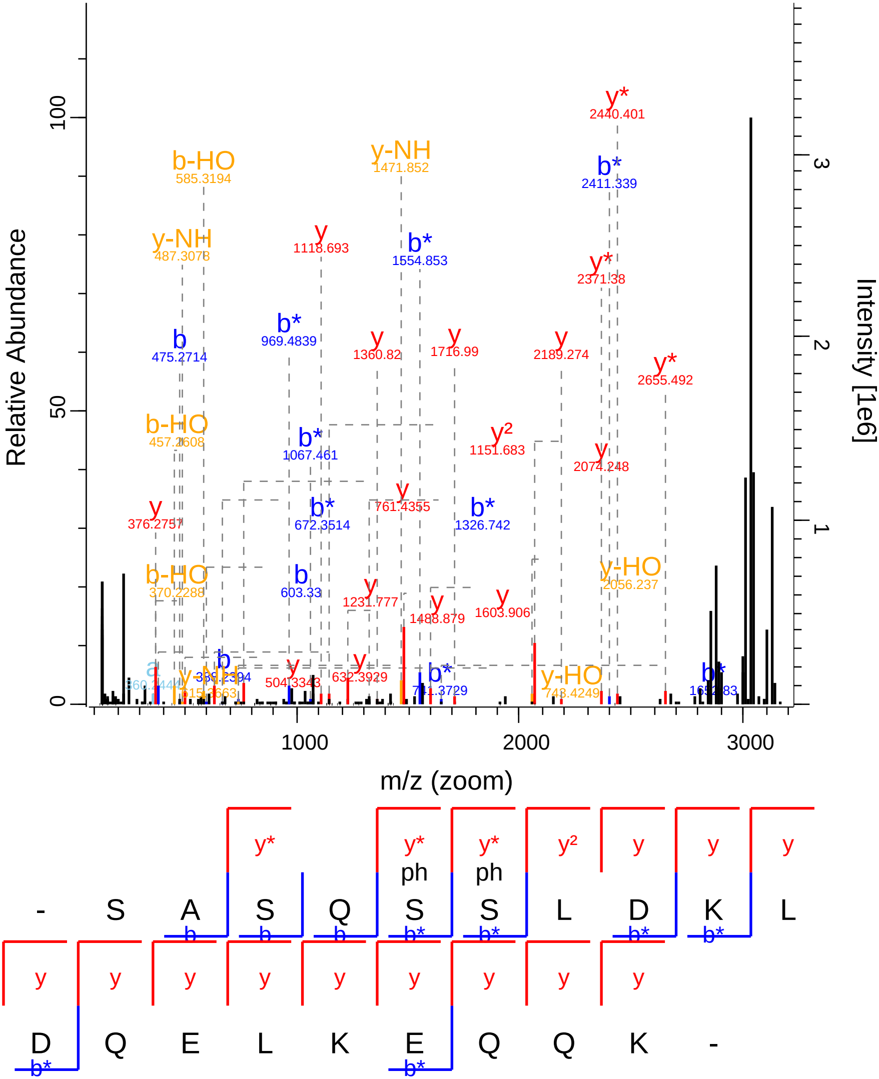

Raw File	Scan	Method	Score	m/z	Gene names
RO-QC-160121_ZackD_SS_Set_01_Fractior	29831	FTMS; HC	52.03	752.91	PSAP

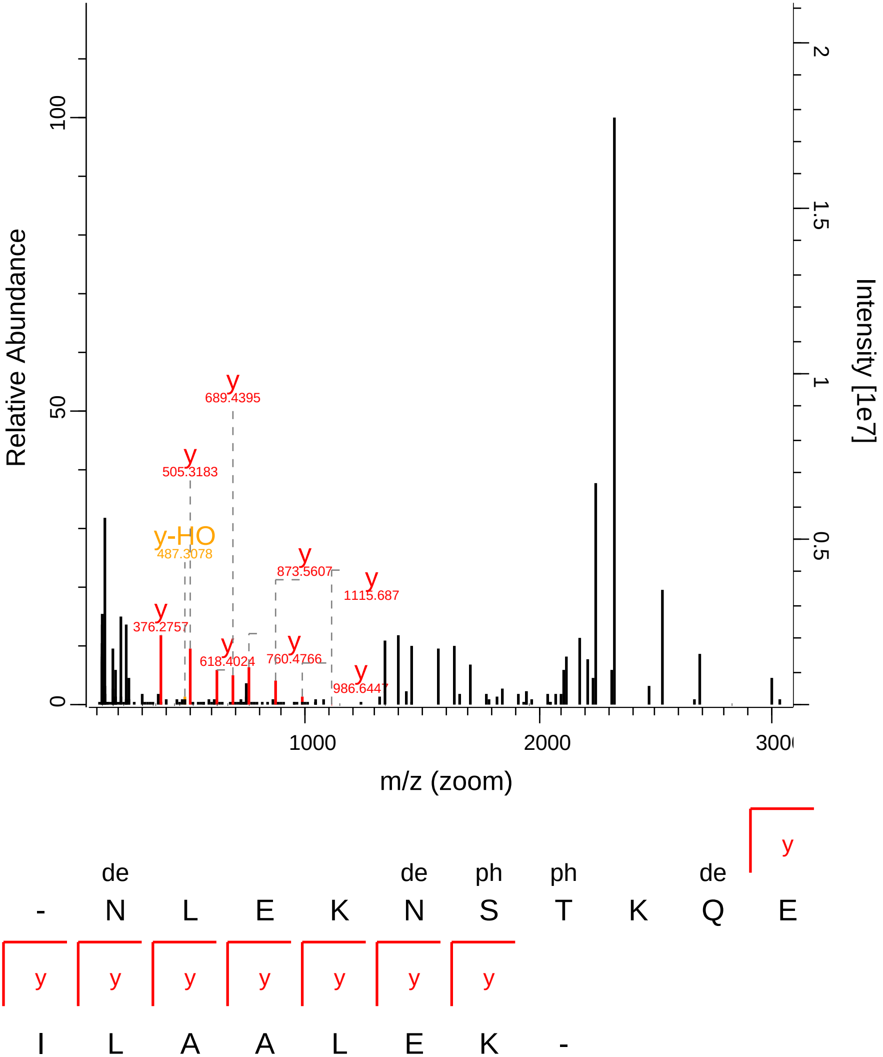

Raw File	Scan	Method	Score	m/z	Gene names
RO-QC-160121_ZackD_SS_Set_01_Fractio	30405	FTMS; HC	233.49	1127.9	ARMC10

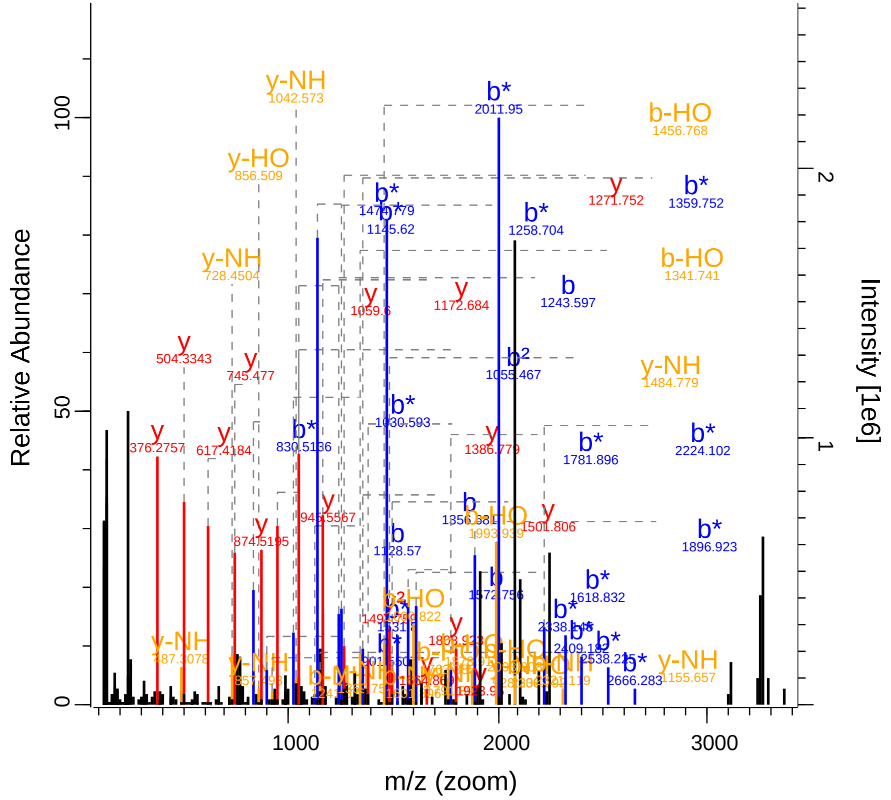

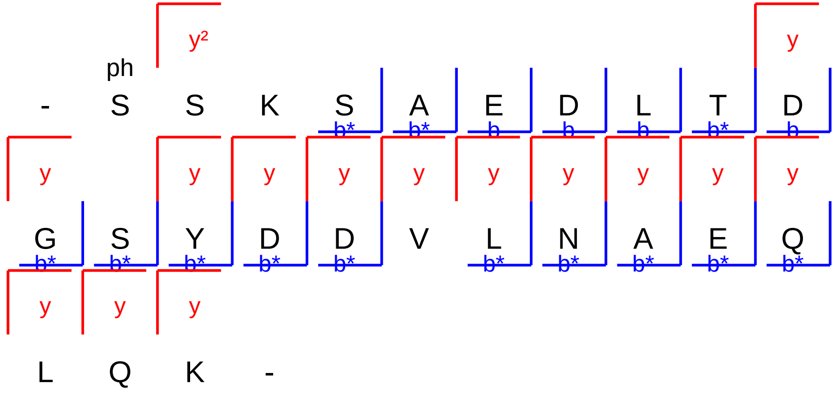

Raw File	Scan	Method	Score	m/z	Gene names
RO-QC-160121_ZackD_SS_Set_01_Fractior	30657	FTMS; HC	40.58	571.09	VPS13D

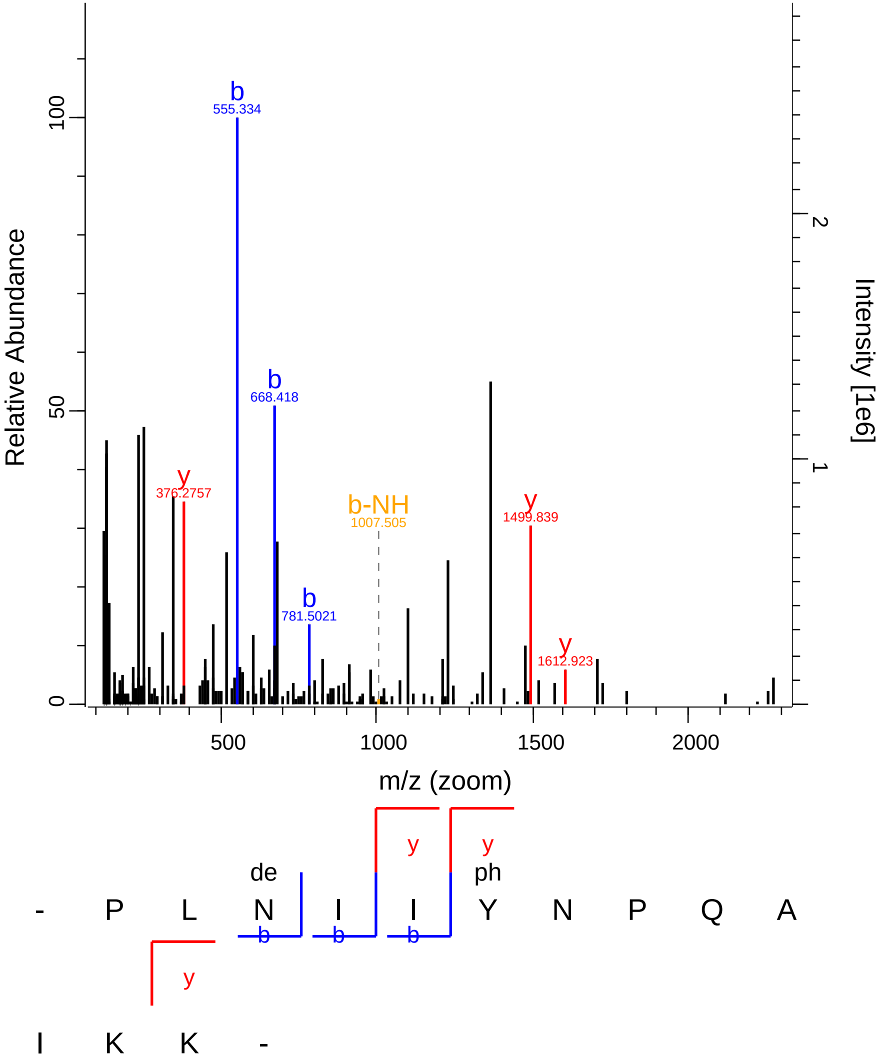

Raw File	Scan	Method	Score	m/z	Gene names
RO-QC-160121_ZackD_SS_Set_01_Fractio	33394	FTMS; HC	128.68	1015.53	VIM

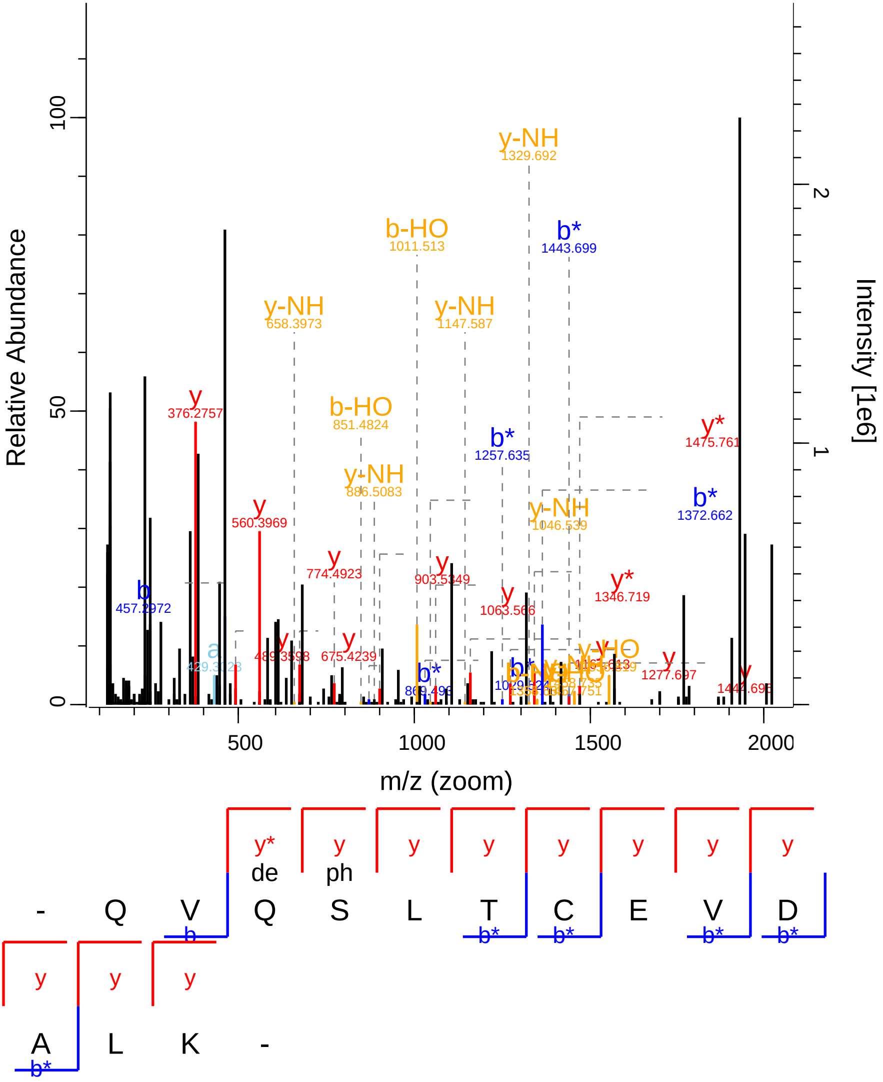

Raw File	Scan	Method	Score	m/z	Gene names
RO-QC-160121_ZackD_SS_Set_01_Fractio	34539	FTMS; HC	205.62	1411.26	MAP1B

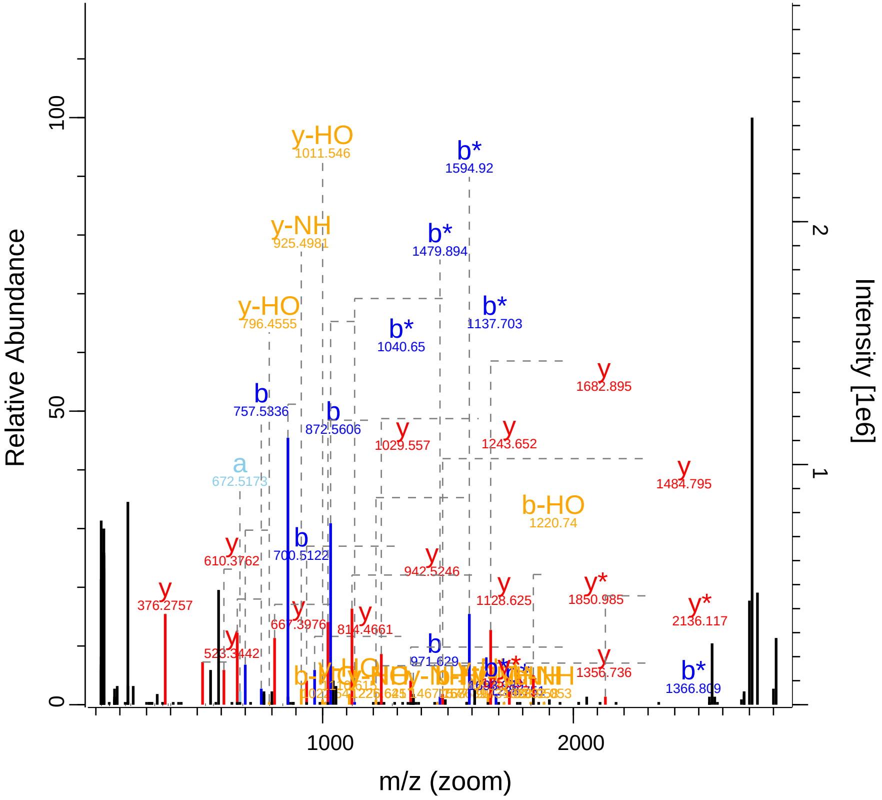

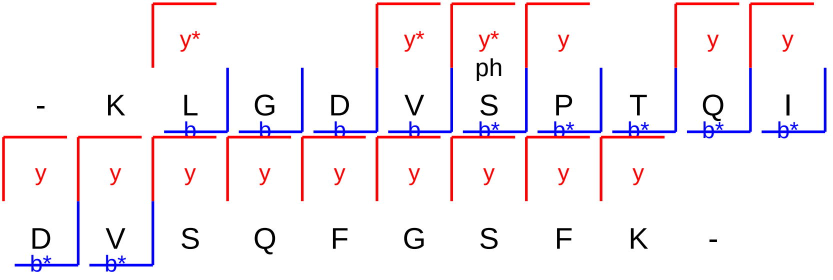

Raw File	Scan	Method	Score	m/z	Gene names
RO-QC-160121_ZackD_SS_Set_01_Fractior	35548	FTMS; HC	47.82	976.99	ENC1

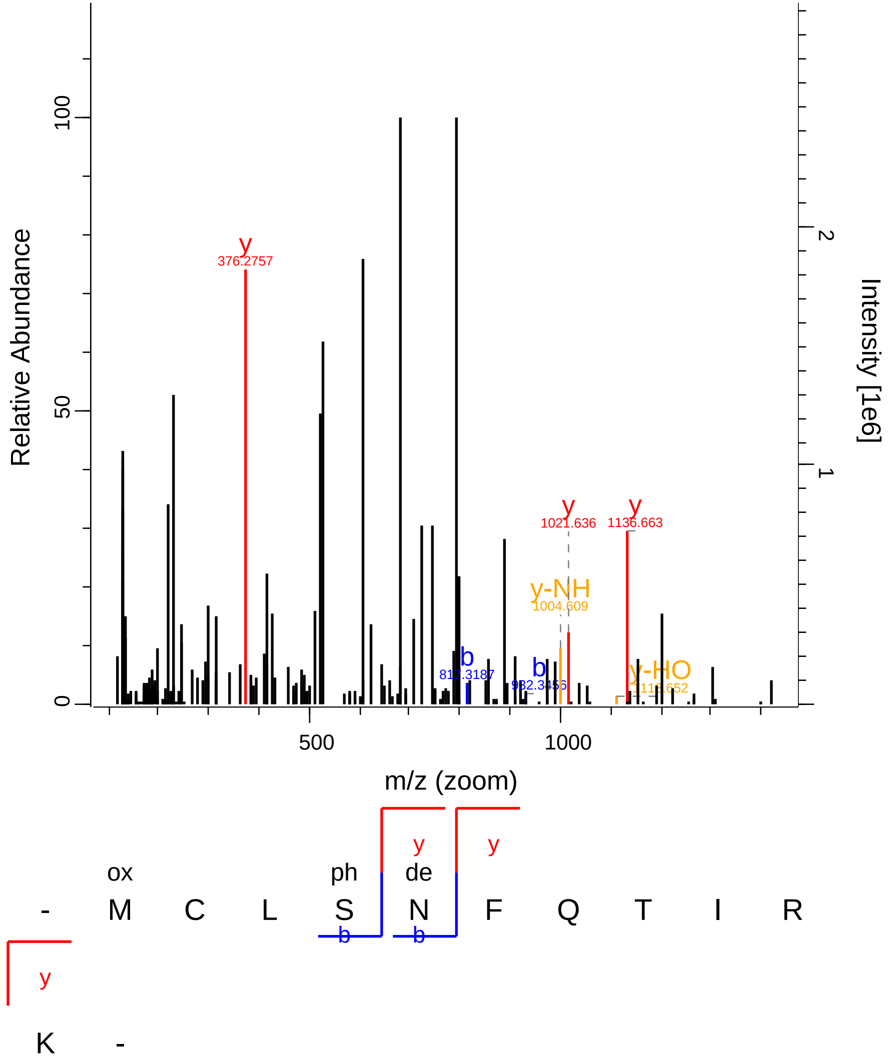

Raw File	Scan	Method	Score	m/z	Gene names
RO-QC-160121_ZackD_SS_Set_01_Fractic	36186	FTMS; HC	199.29	906.42	FAM21A;FAM21

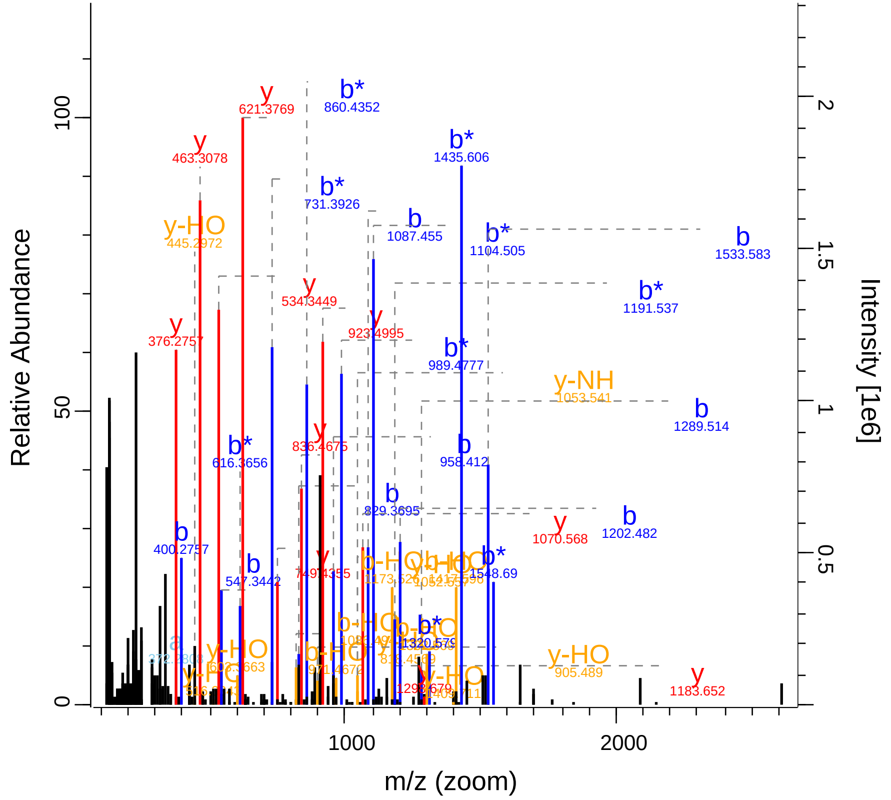

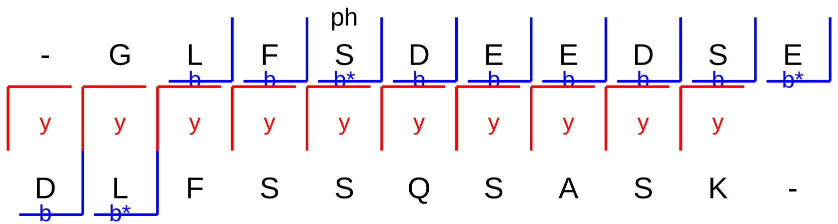

Raw File	Scan	Method	Score	m/z
RO-QC-160121_ZackD_SS_Set_01_Fraction_10	36822	FTMS; HCD	56.43	881.92

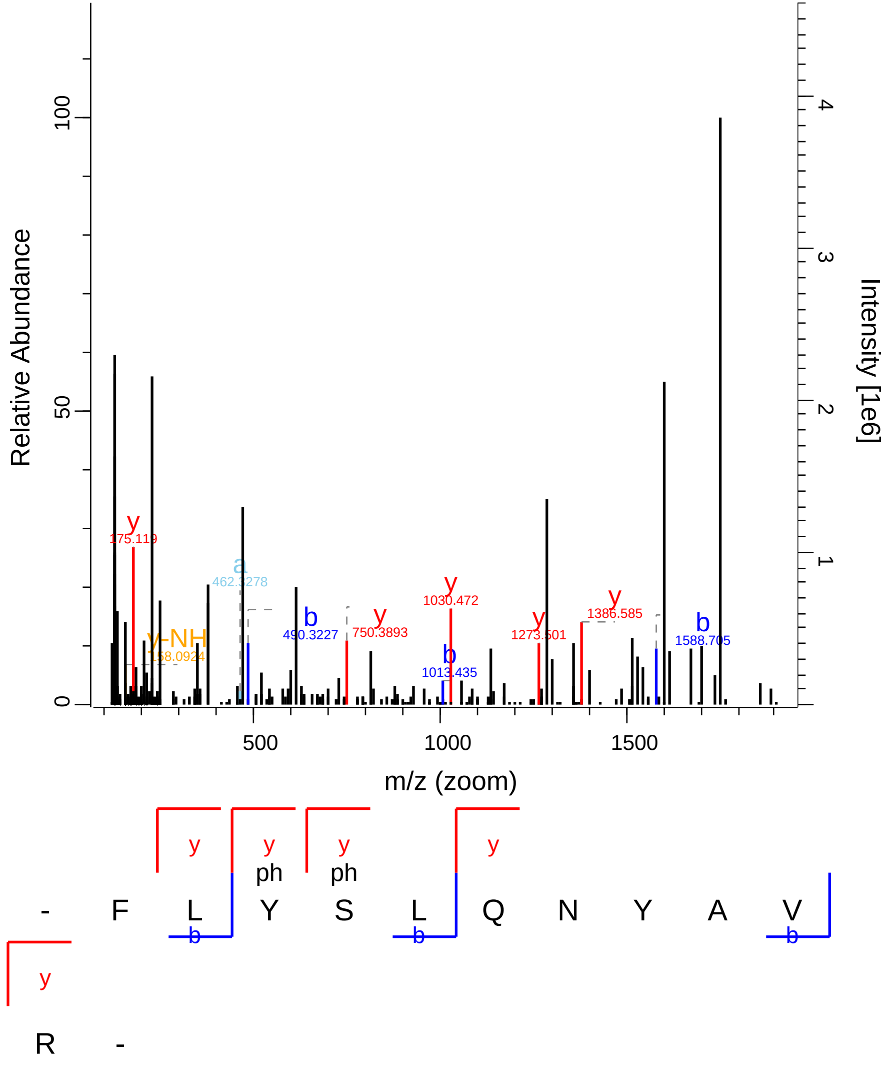

Raw File	Scan	Method	Score	m/z	Gene names
RO-QC-160121_ZackD_SS_Set_01_Fractio	36966	FTMS; HC	113.62	1016.08	EPHA7

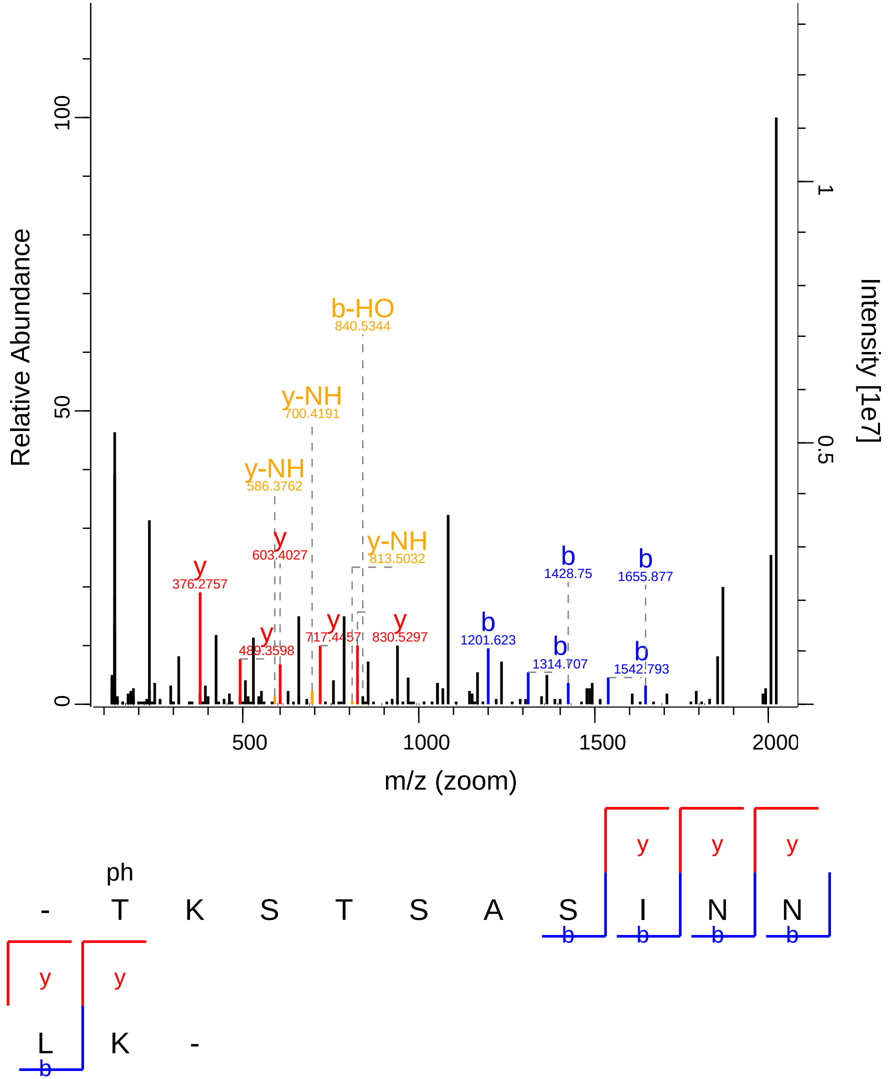

Raw File	Scan	Method	Score	m/z	Gene names
RO-QC-160121_ZackD_SS_Set_01_Fractior	37434	FTMS; HC	41.73	864.84	SCAPER

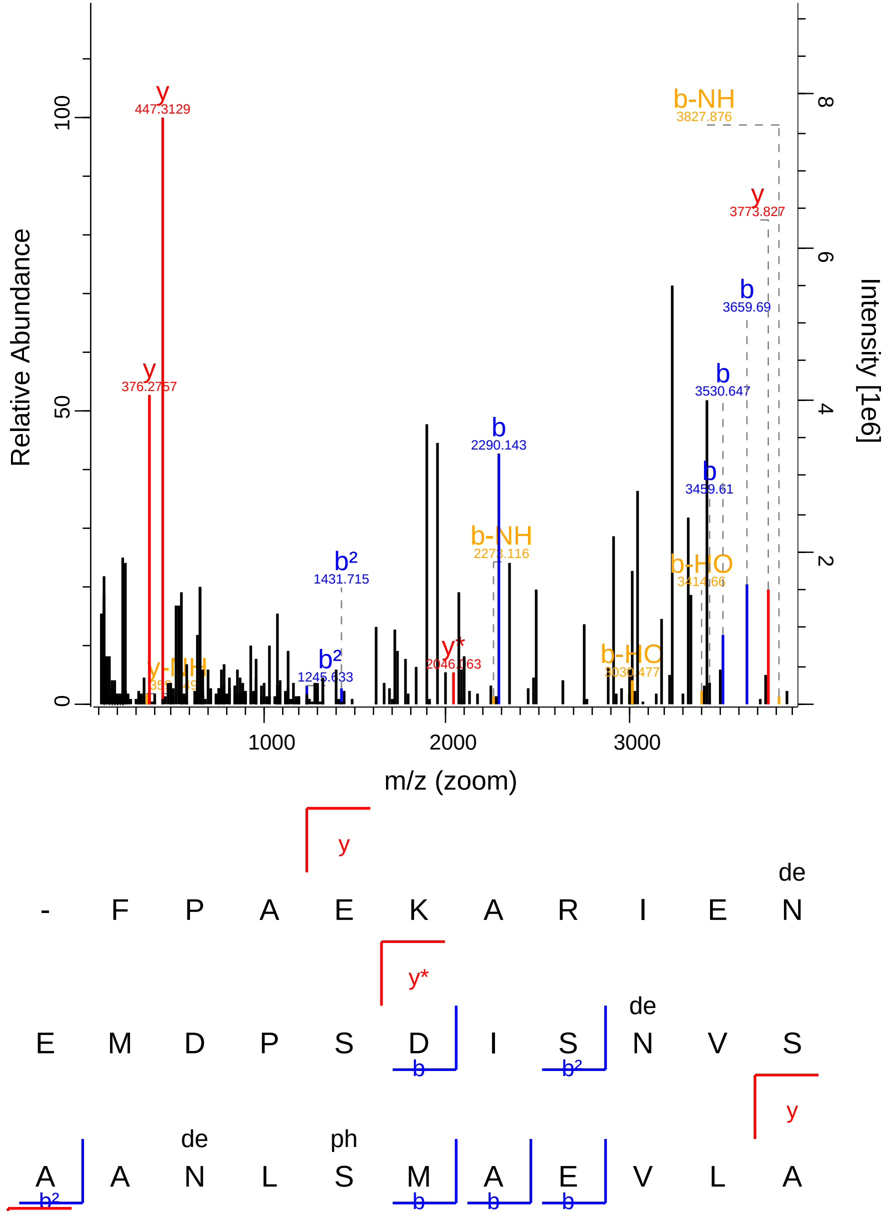

Raw File	Scan	Method	Score	m/z	Gene names
RO-QC-160121_ZackD_SS_Set_01_Fractior	38518	FTMS; HC	58.92	924.79	YAP1

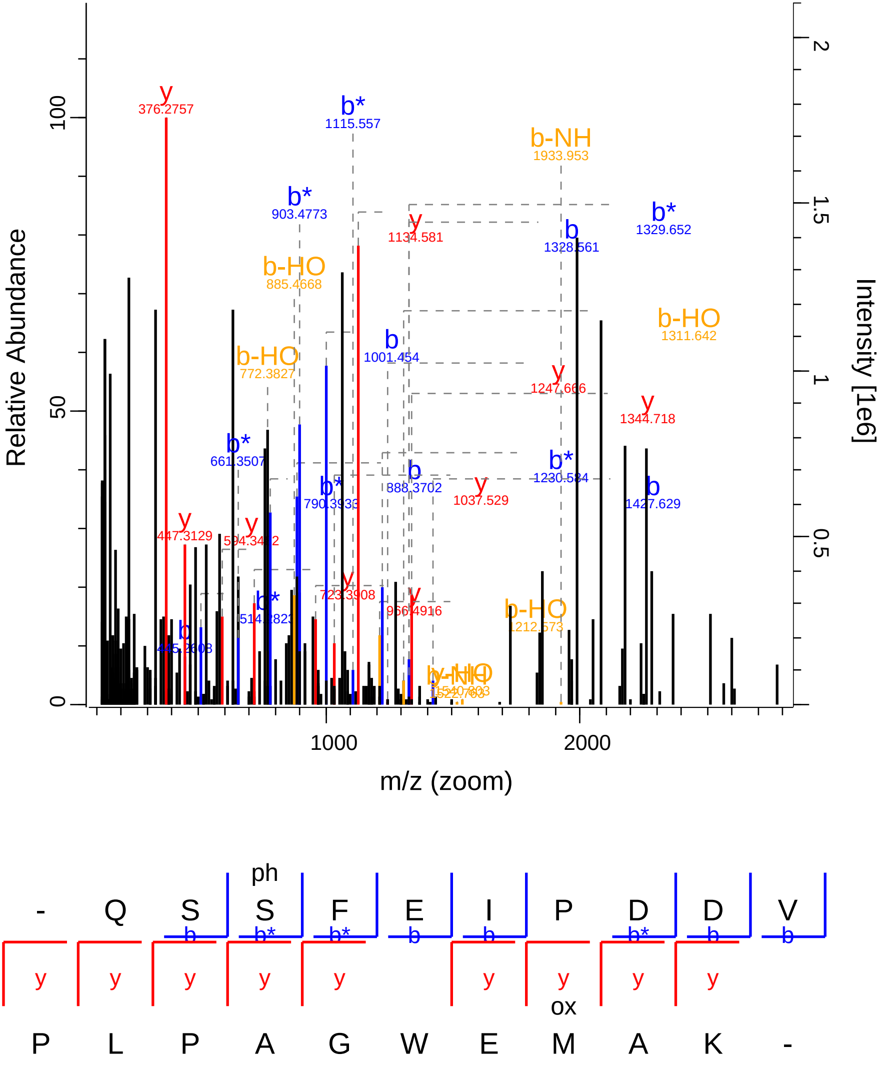

Raw File	Scan	Method	Score	m/z	Gene names
RO-QC-160121_ZackD_SS_Set_01_Fractio	38903	FTMS; HC	50.23	1076.26	CCNL1

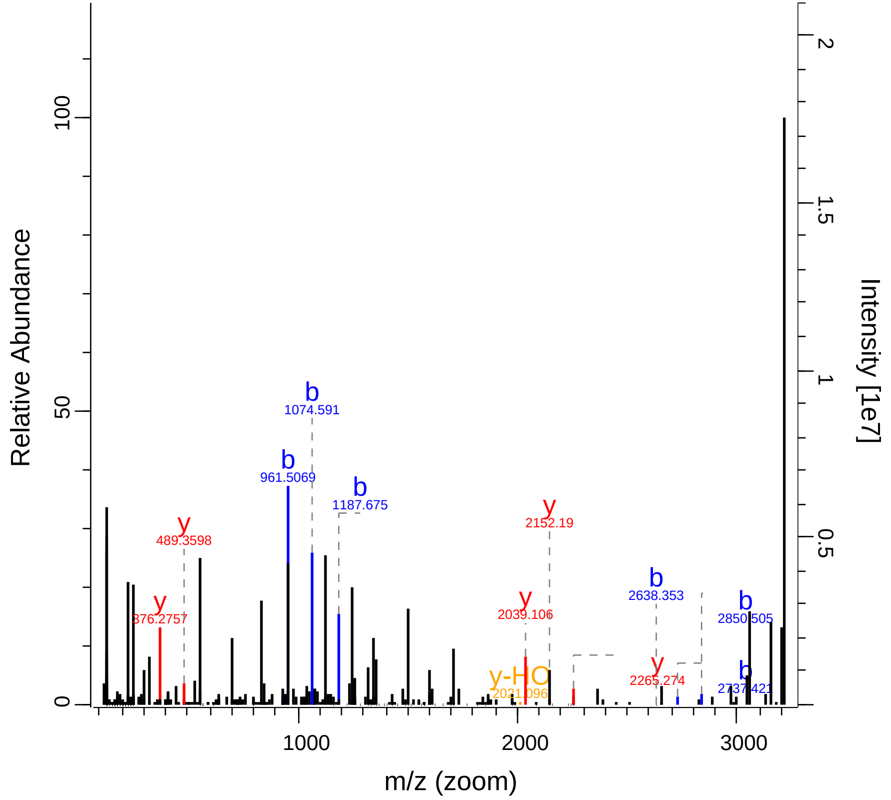

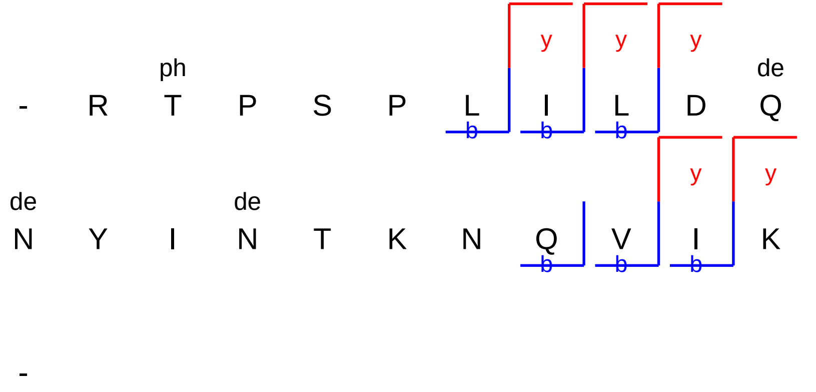

Raw File	Scan	Method	Score	m/z	Gene names
RO-QC-160121_ZackD_SS_Set_01_Fractio	40182	FTMS; HC	94.69	1017.52	FREM1

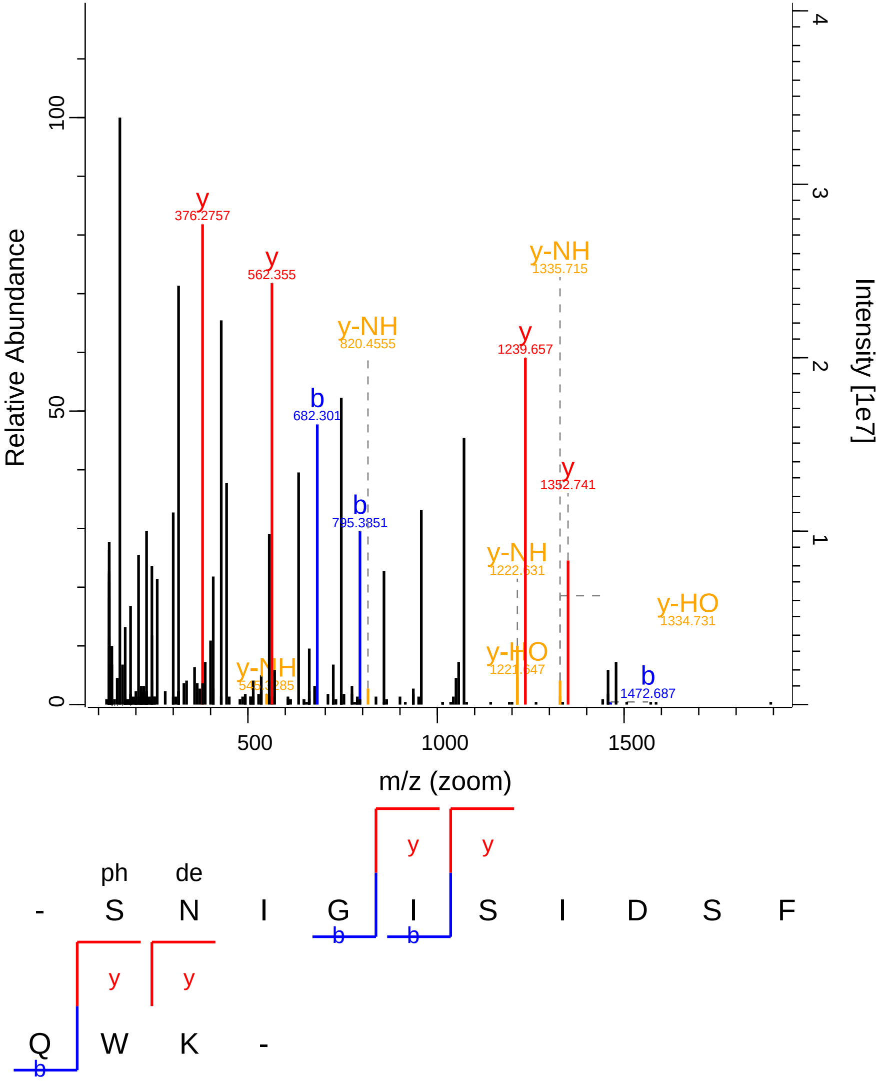

Raw File	Scan	Method	Score	m/z	Gene names
RO-QC-160121_ZackD_SS_Set_01_Fractio	40908	FTMS; HC	61.38	1440.68	TCF7L1

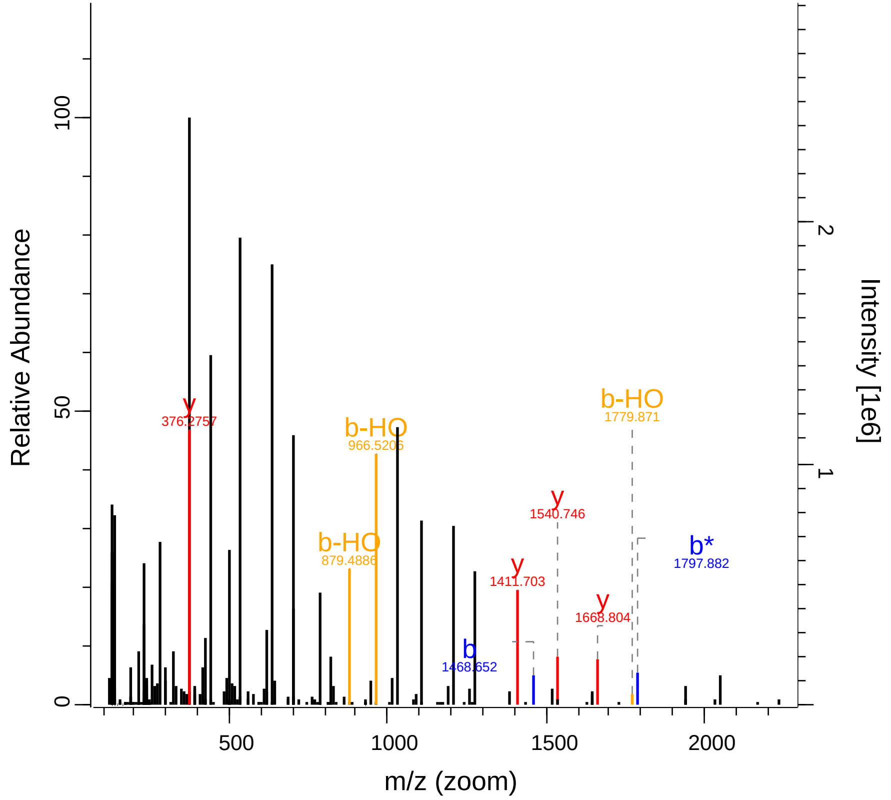

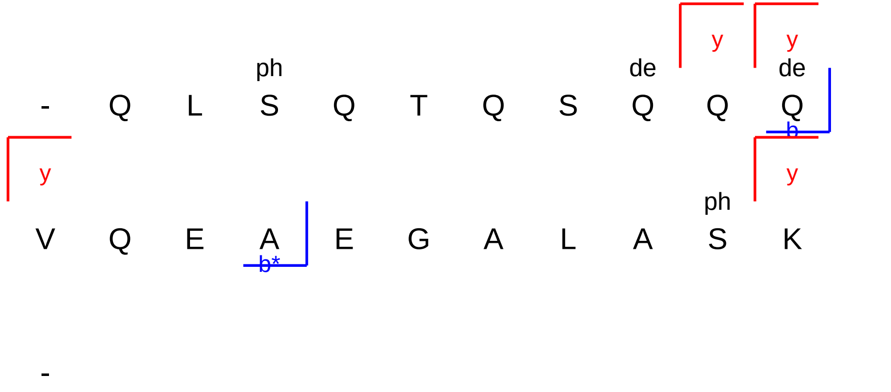

Raw File	Scan	Method	Score	m/z	Gene names
RO-QC-160121_ZackD_SS_Set_01_Fraction	4891	FTMS; HC	43.38	552.31	PADI4

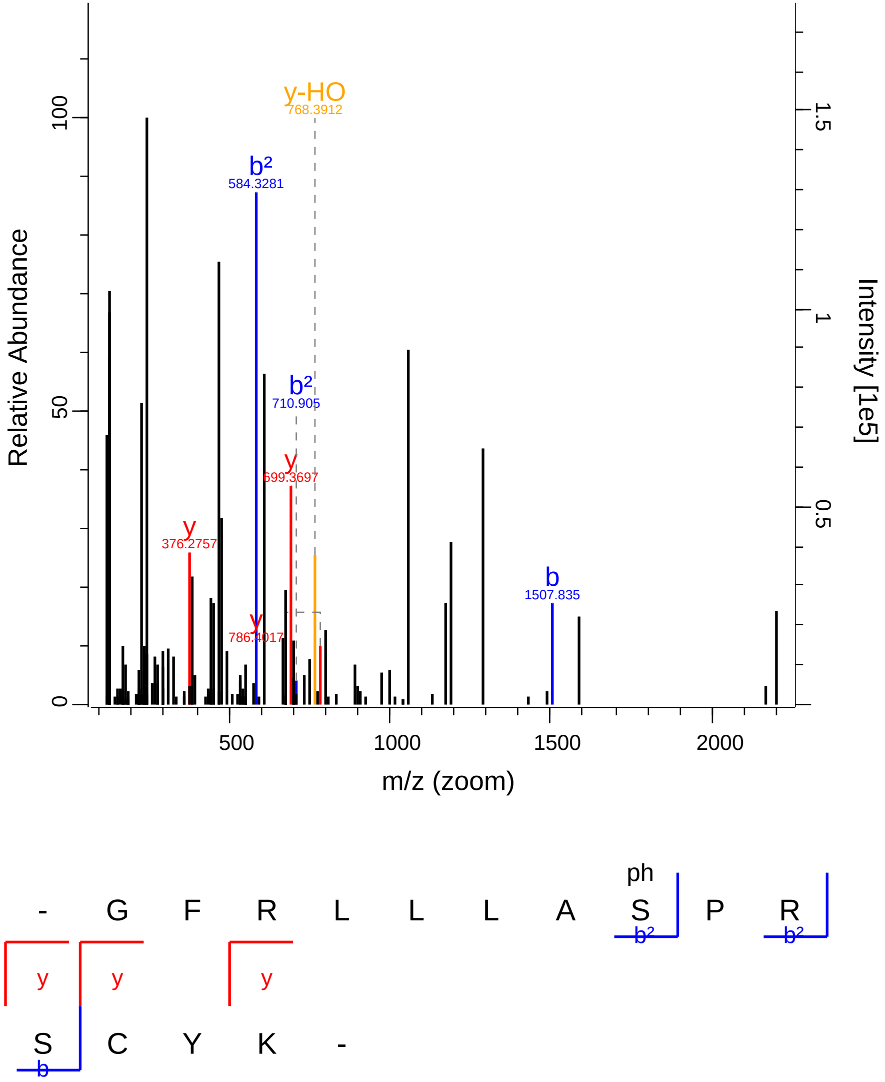

Raw File	Scan	Method	Score	m/z	Gene names
RO-QC-160121_ZackD_SS_Set_01_Fraction	4940	FTMS; HC	92.26	686.34	LMNA

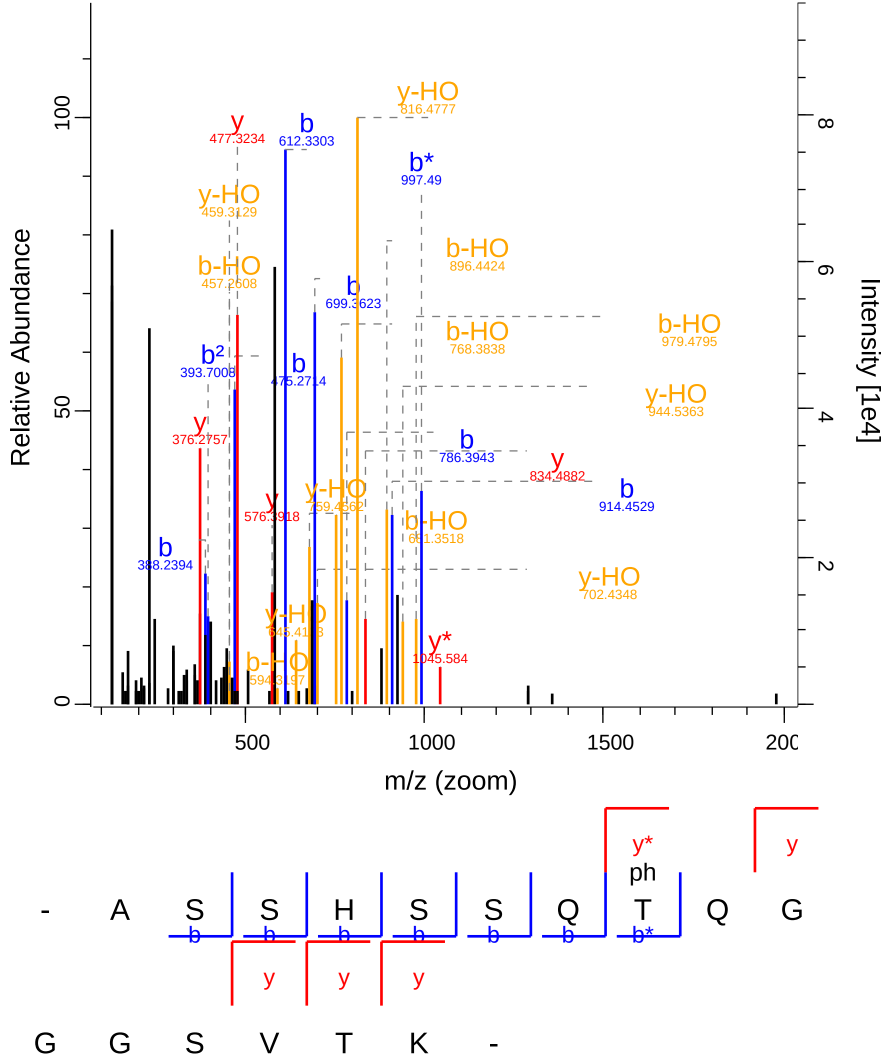

Raw File	Scan	Method	Score	m/z	Gene names
RO-QC-160121_ZackD_SS_Set_01_Fraction	5131	FTMS; HC	58.51	512.93	TNIK

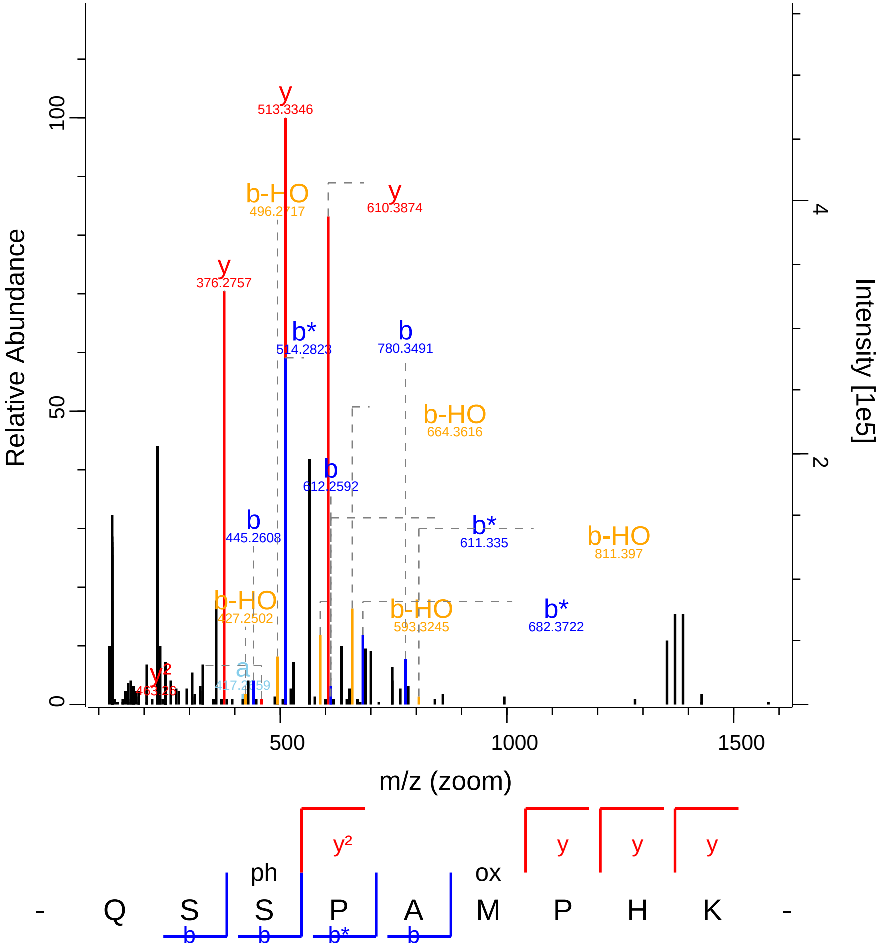

Raw File	Scan	Method	Score	m/z	Gene names
RO-QC-160121_ZackD_SS_Set_01_Fraction	5512	FTMS; HC	253.79	781.33	CDS2

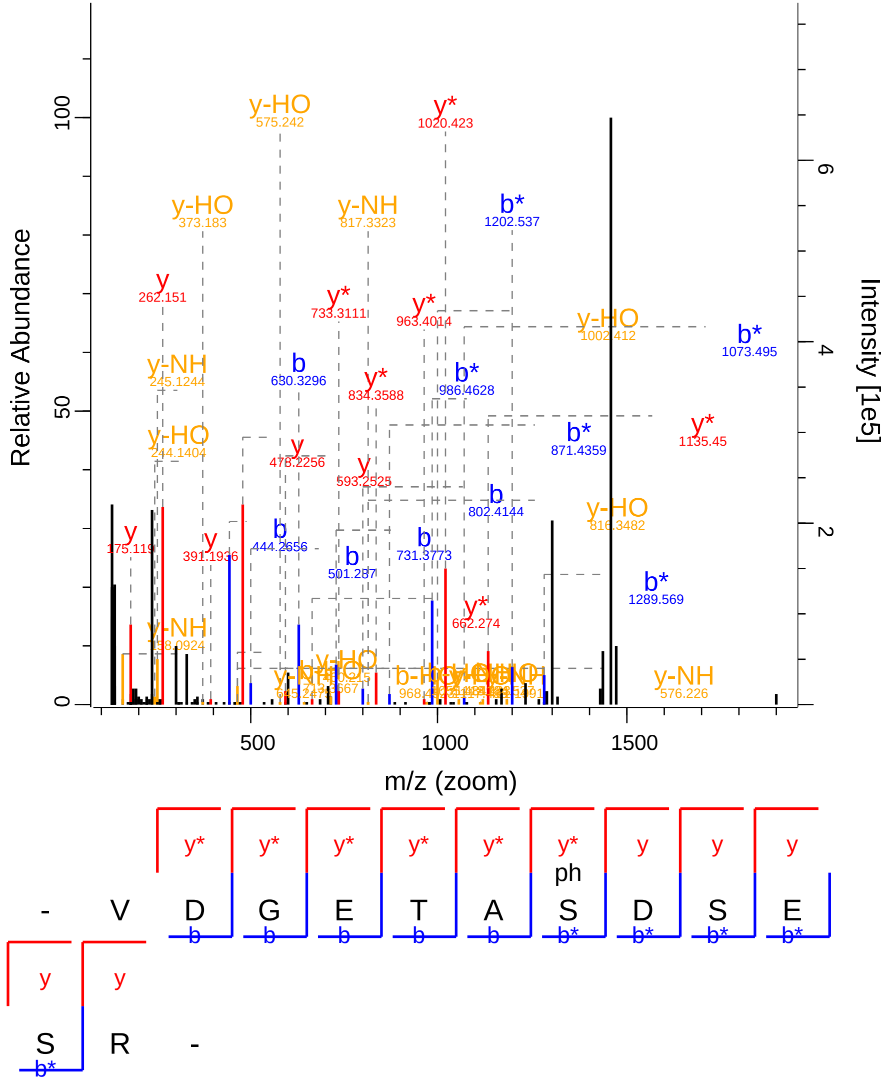

Raw File	Scan	Method	Score	m/z	Gene names
RO-QC-160121_ZackD_SS_Set_01_Fraction	5606	FTMS; HC	121.68	807.42	GTF2I

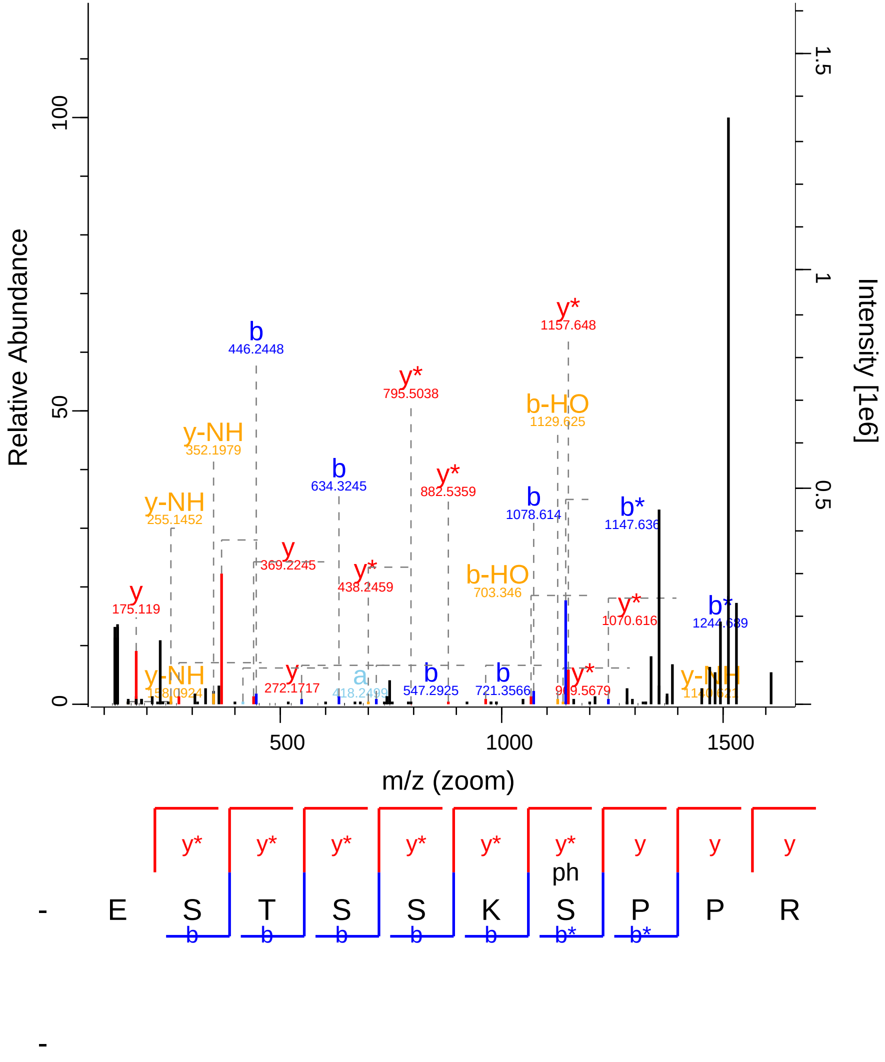

Raw File	Scan	Method	Score	m/z	Gene names
RO-QC-160121_ZackD_SS_Set_01_Fraction	5777	FTMS; HC	138.45	568.29	ABCC1

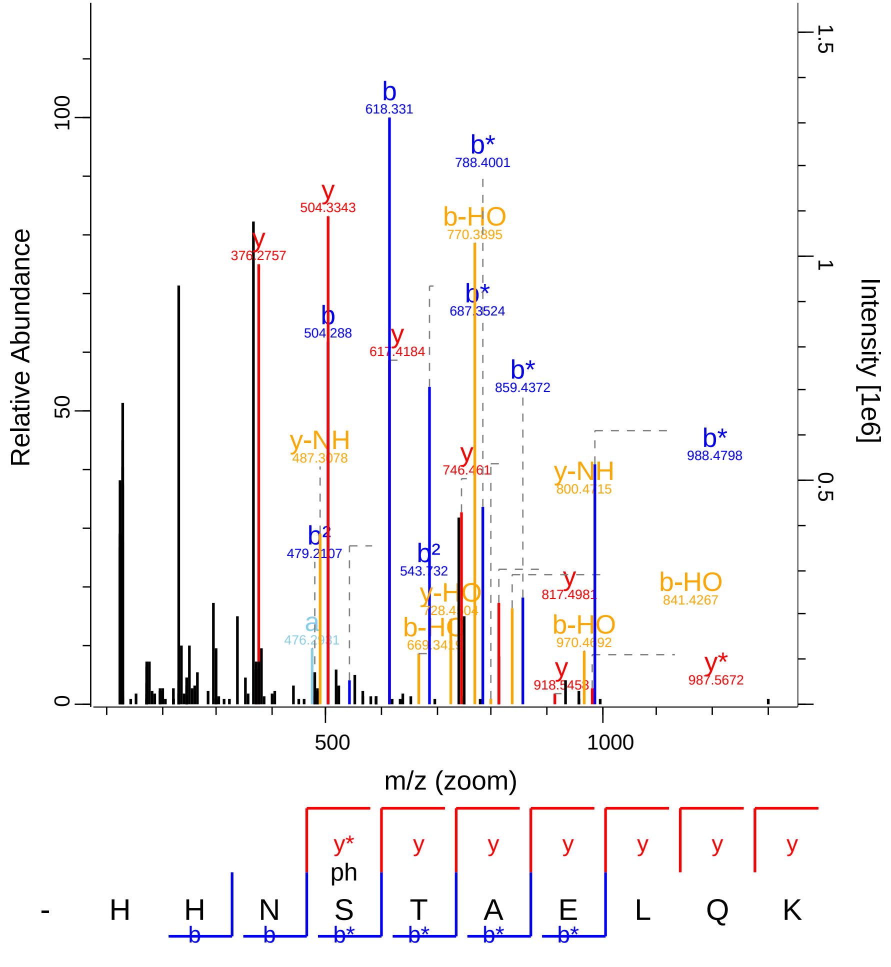

Raw File	Scan	Method	Score	m/z	Gene names
RO-QC-160121_ZackD_SS_Set_01_Fraction_	5861	FTMS; HC	46.43	540.93	FAM65A

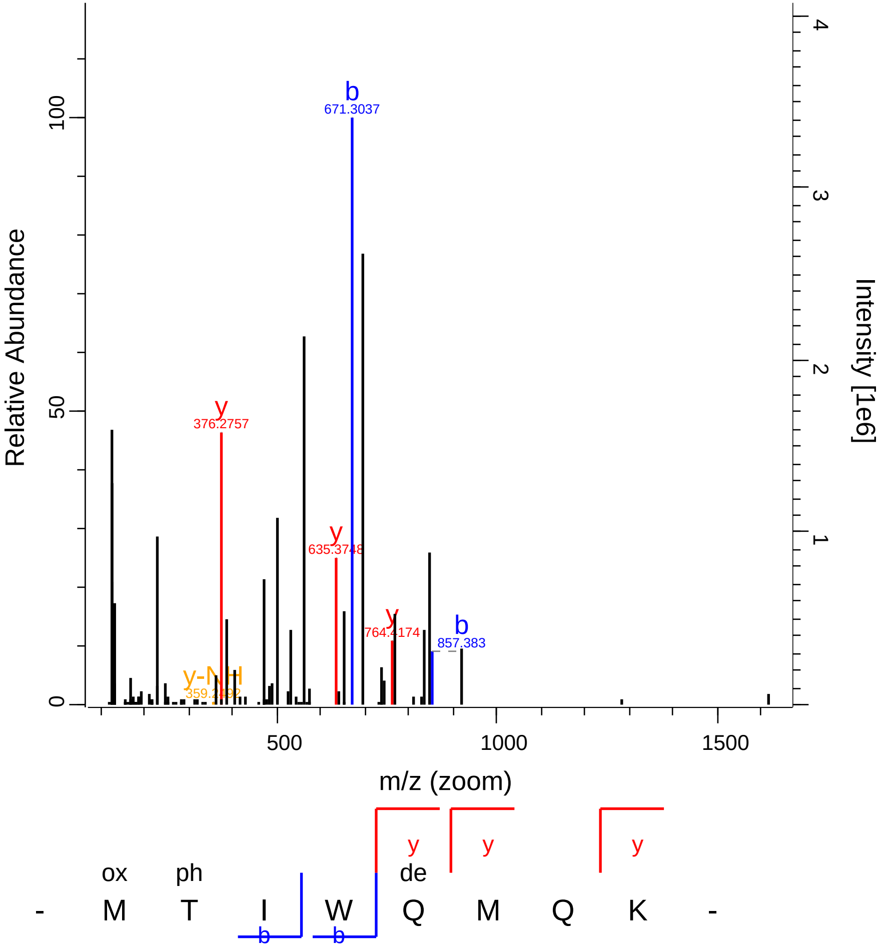

Raw File	Scan	Method	Score	m/z	Gene names
RO-QC-160121_ZackD_SS_Set_01_Fraction_	6346	FTMS; HC	106.58	629.8	RABGEF1

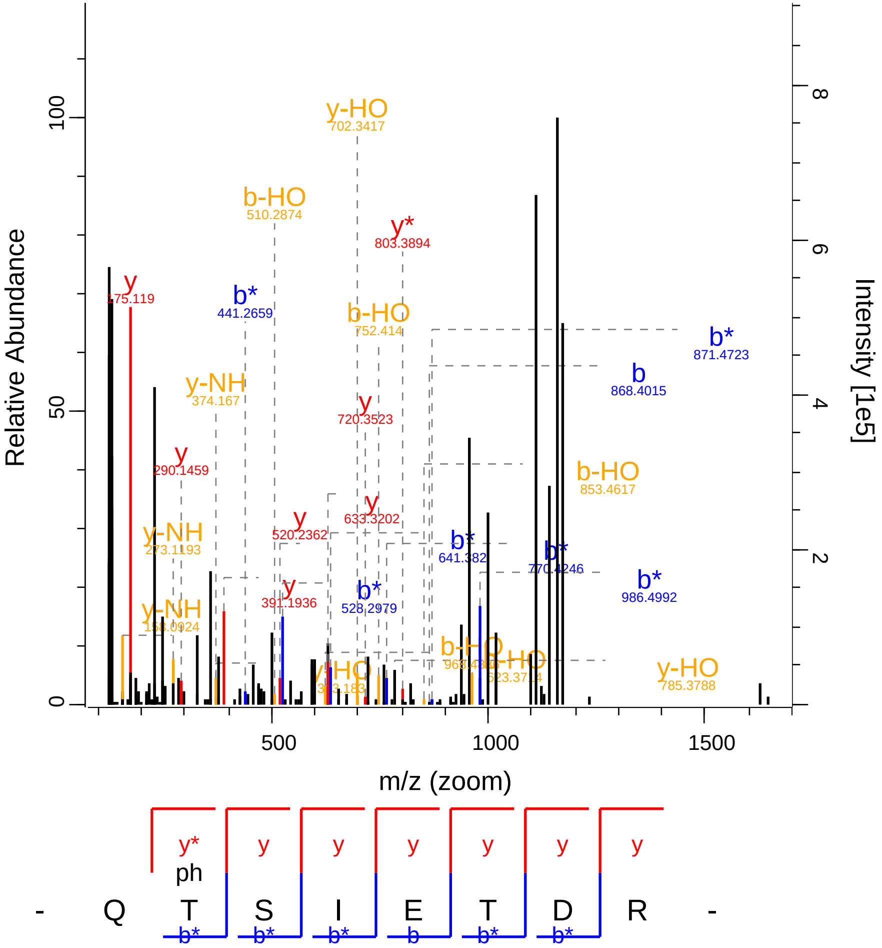

Raw File	Scan	Method	Score	m/z
RO-QC-160121_ZackD_SS_Set_01_Fraction_11	8021	FTMS; HCD	52.4	795.4

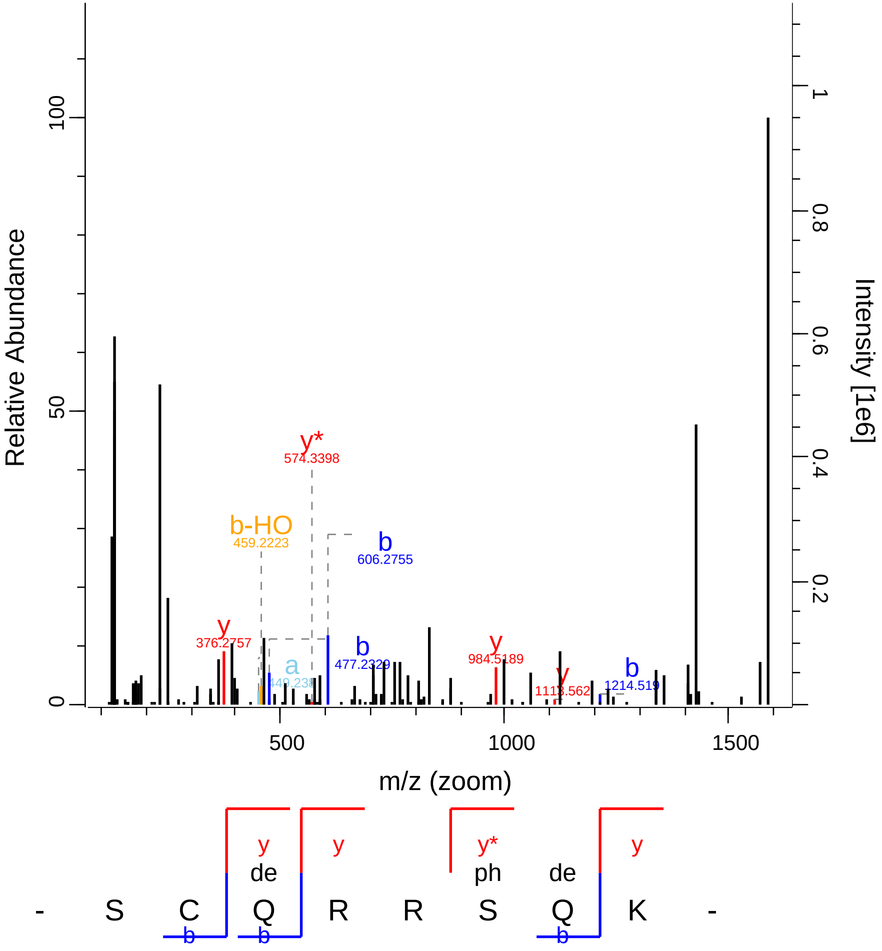

Raw File	Scan	Method	Score	m/z	Gene names
RO-QC-160121_ZackD_SS_Set_01_Fraction	8386	FTMS; HC	45.14	751.89	LRRC74A

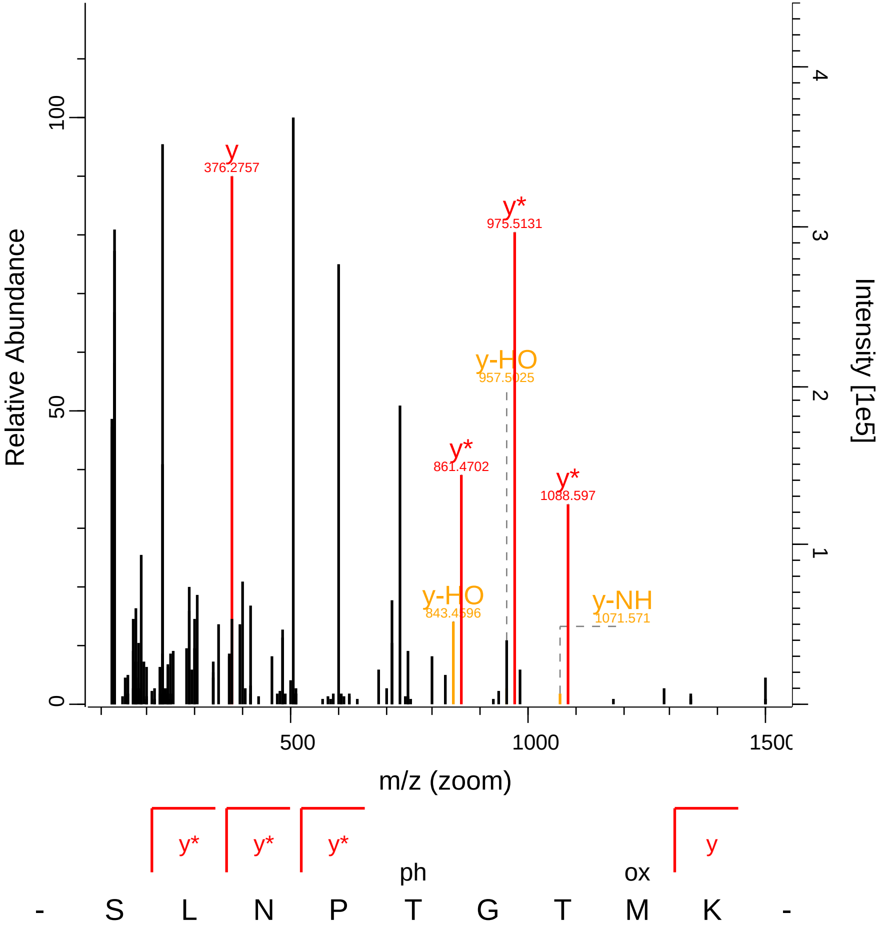

Raw File	Scan	Method	Score	m/z	Gene names
RO-QC-160121_ZackD_SS_Set_01_Fraction	9084	FTMS; HC	139.32	641.32	LMNA

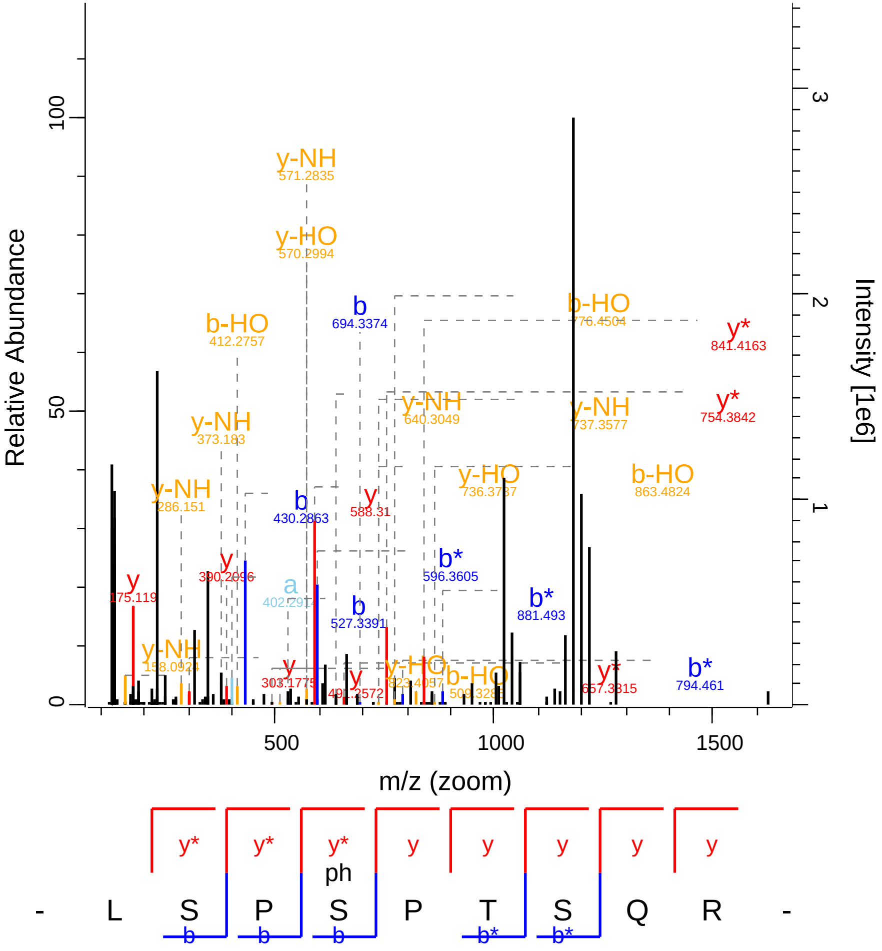

Raw File	Scan	Method	Score	m/z	Gene names
RO-QC-160121_ZackD_SS_Set_01_Fractior	10283	FTMS; HC	106.67	841.95	ACIN1

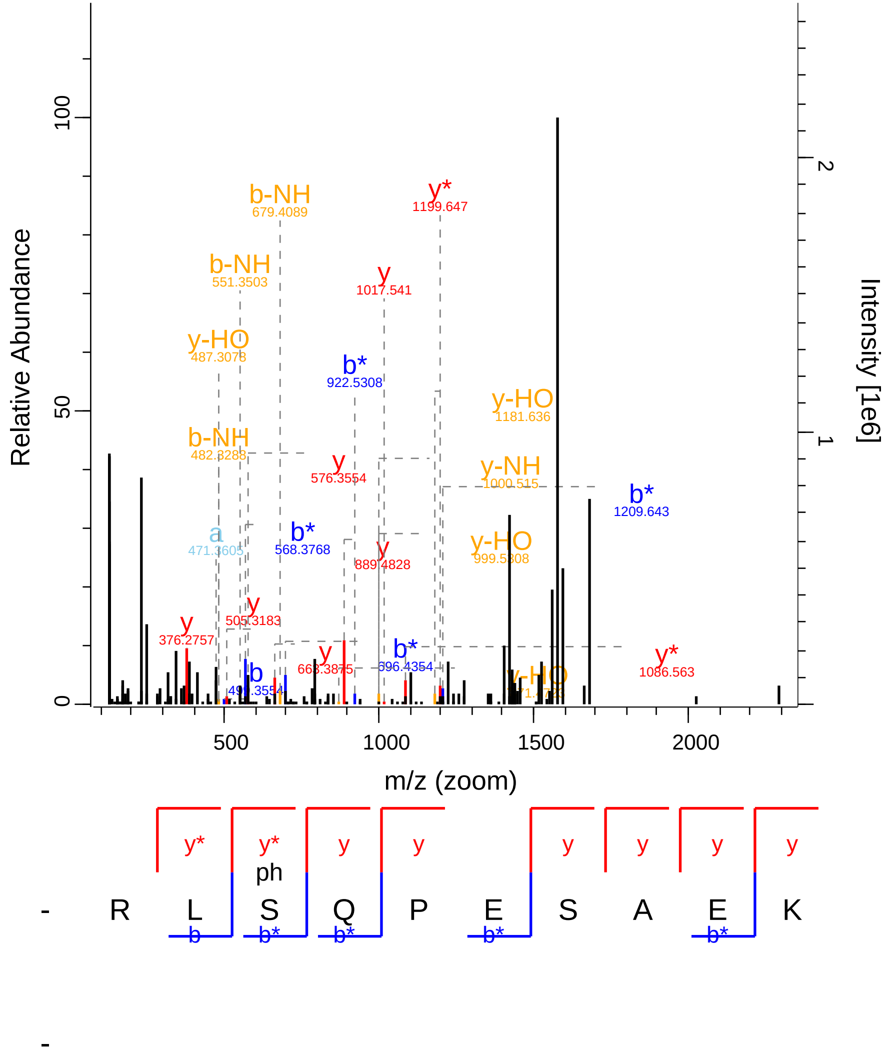

Raw File	Scan	Method	Score	m/z	Gene names
RO-QC-160121_ZackD_SS_Set_01_Fractior	11015	FTMS; HC	42.21	476.47	C6orf165

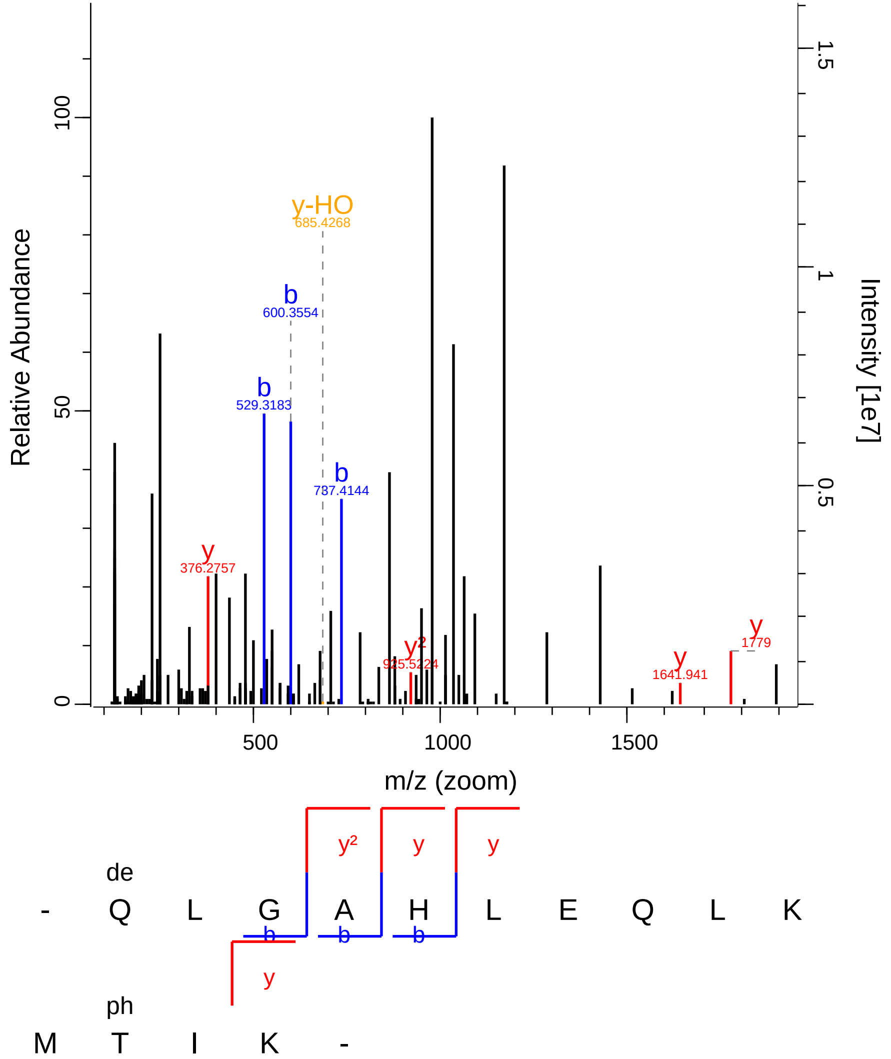

Raw File	Scan	Method	Score	m/z	Gene names
RO-QC-160121_ZackD_SS_Set_01_Fractio	13465	FTMS; HC	102.58	699.69	DUT

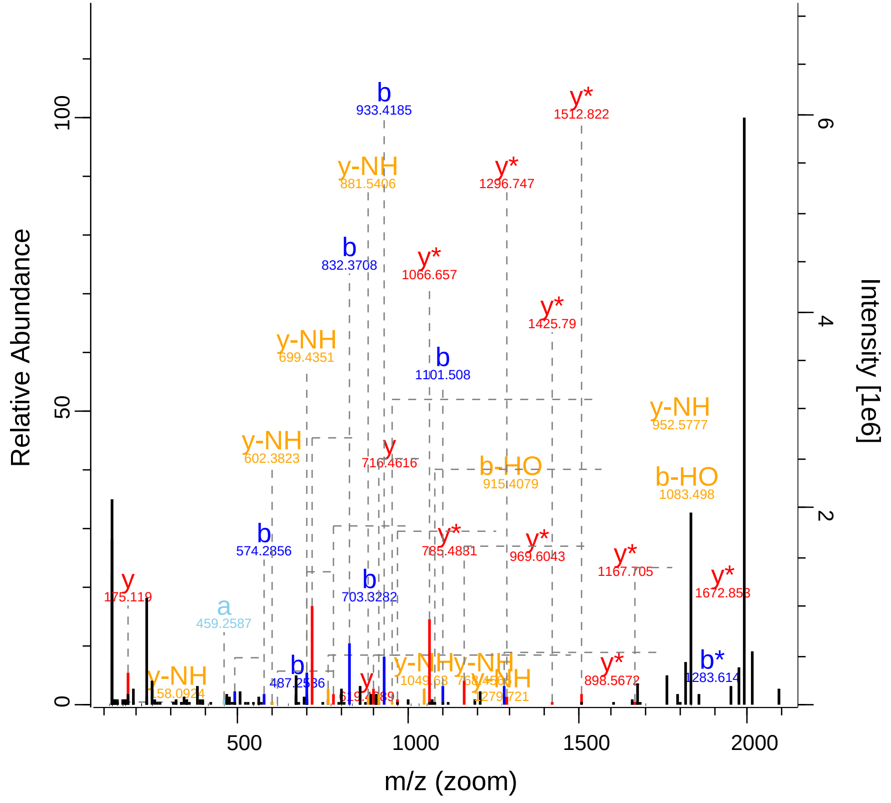

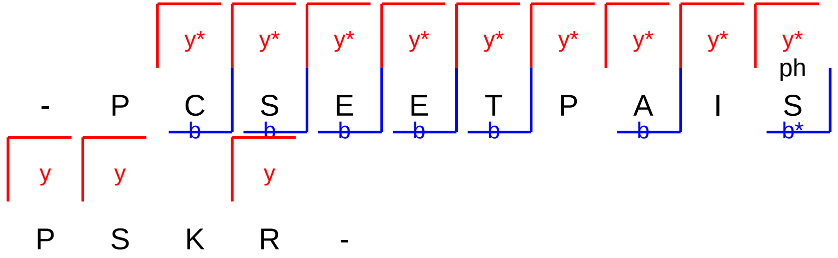

Raw File	Scan	Method	Score	m/z	Gene names
RO-QC-160121_ZackD_SS_Set_01_Fractior	13952	FTMS; HC	77.22	638.67	CLASP2

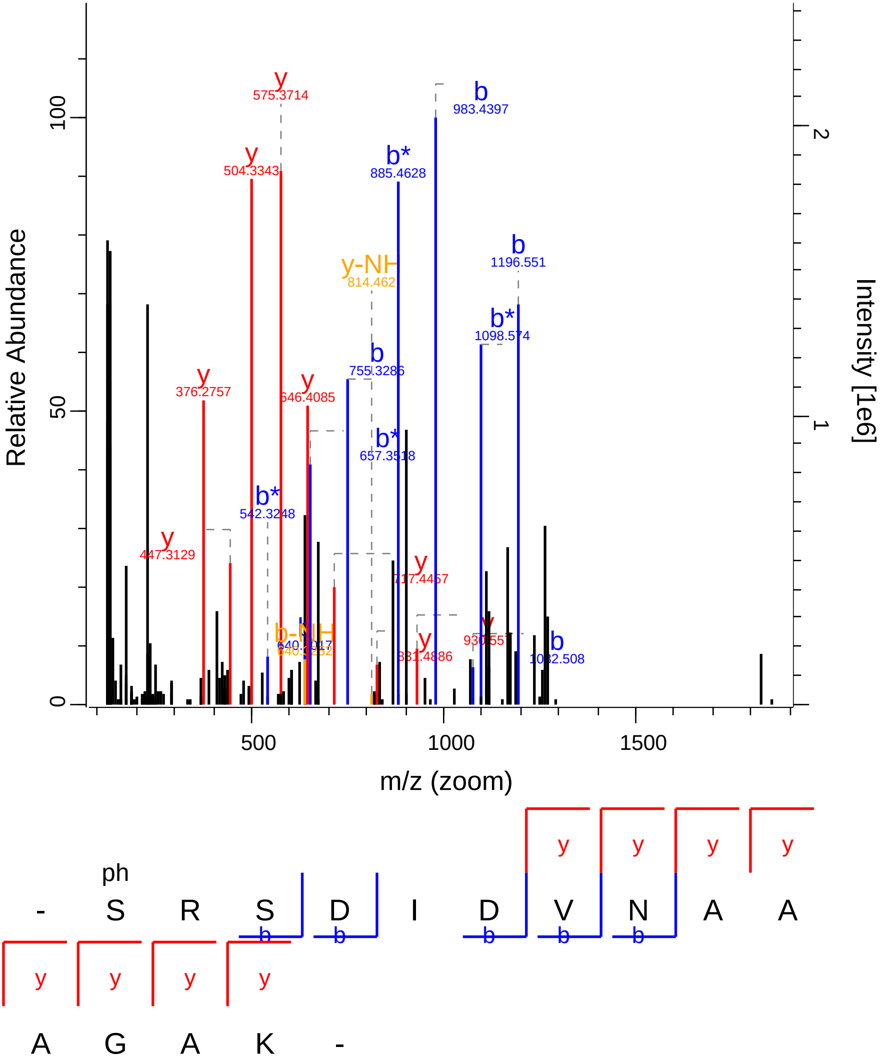

Raw File	Scan	Method	Score	m/z	Gene names
RO-QC-160121_ZackD_SS_Set_01_Fractio	14064	FTMS; HC	122.73	850.93	EP300

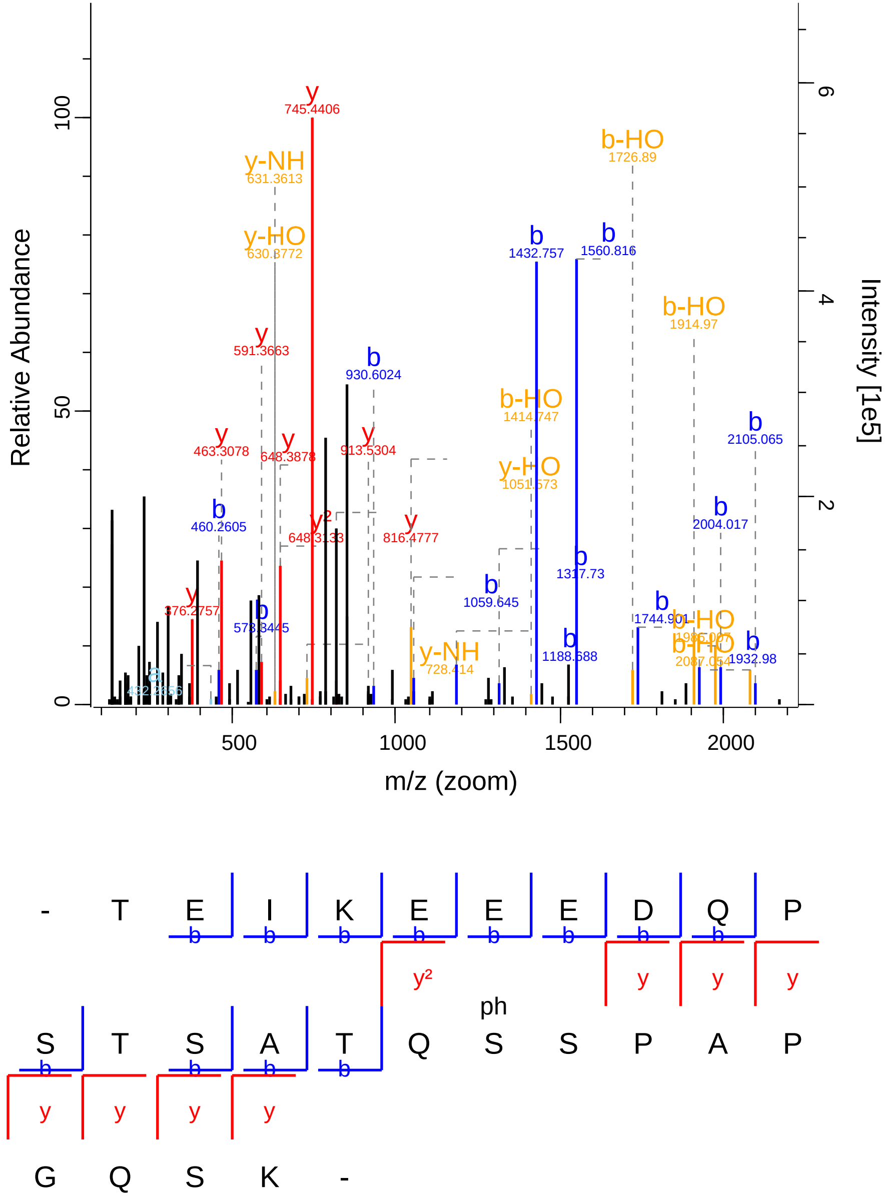

Raw File	Scan	Method	Score	m/z	Gene names
RO-QC-160121_ZackD_SS_Set_01_Fractior	14160	FTMS; HC	67.36	716.37	HSP90AB1

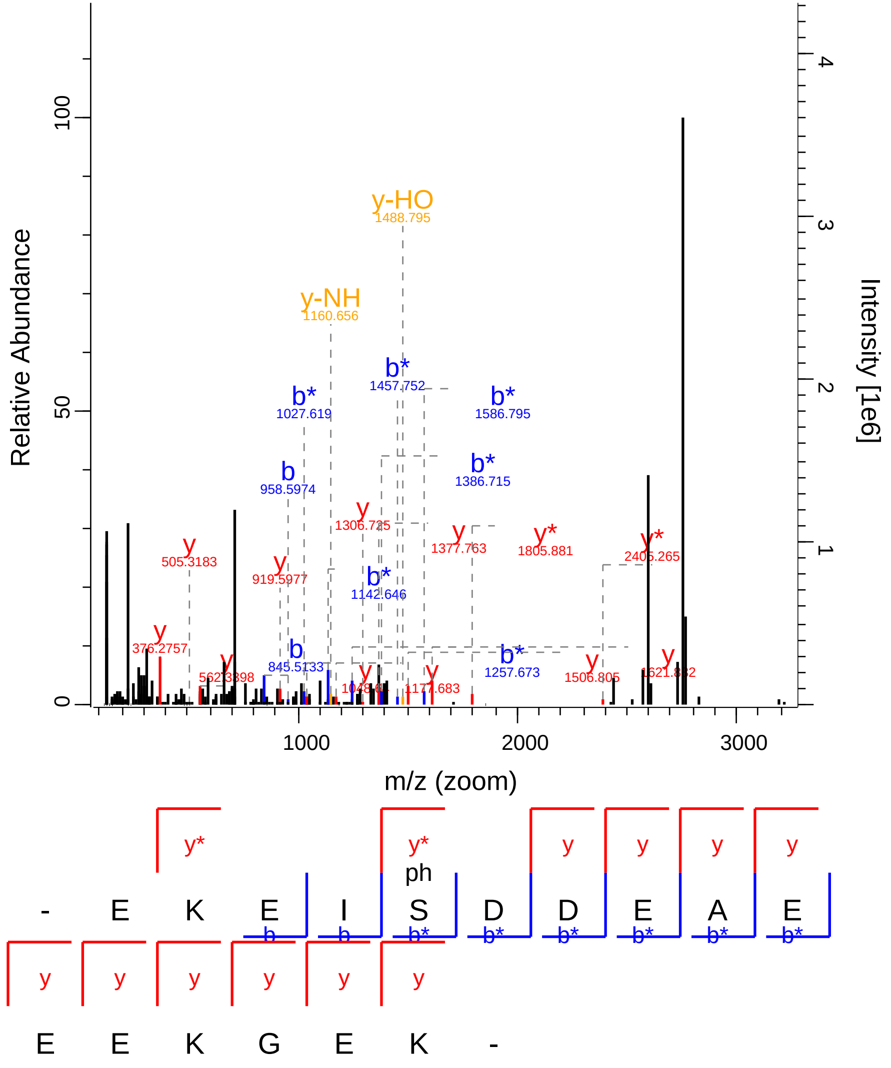

Raw File	Scan	Method	Score	m/z	Gene names
RO-QC-160121_ZackD_SS_Set_01_Fractior	14213	FTMS; HC	77.15	788.47	NOP2

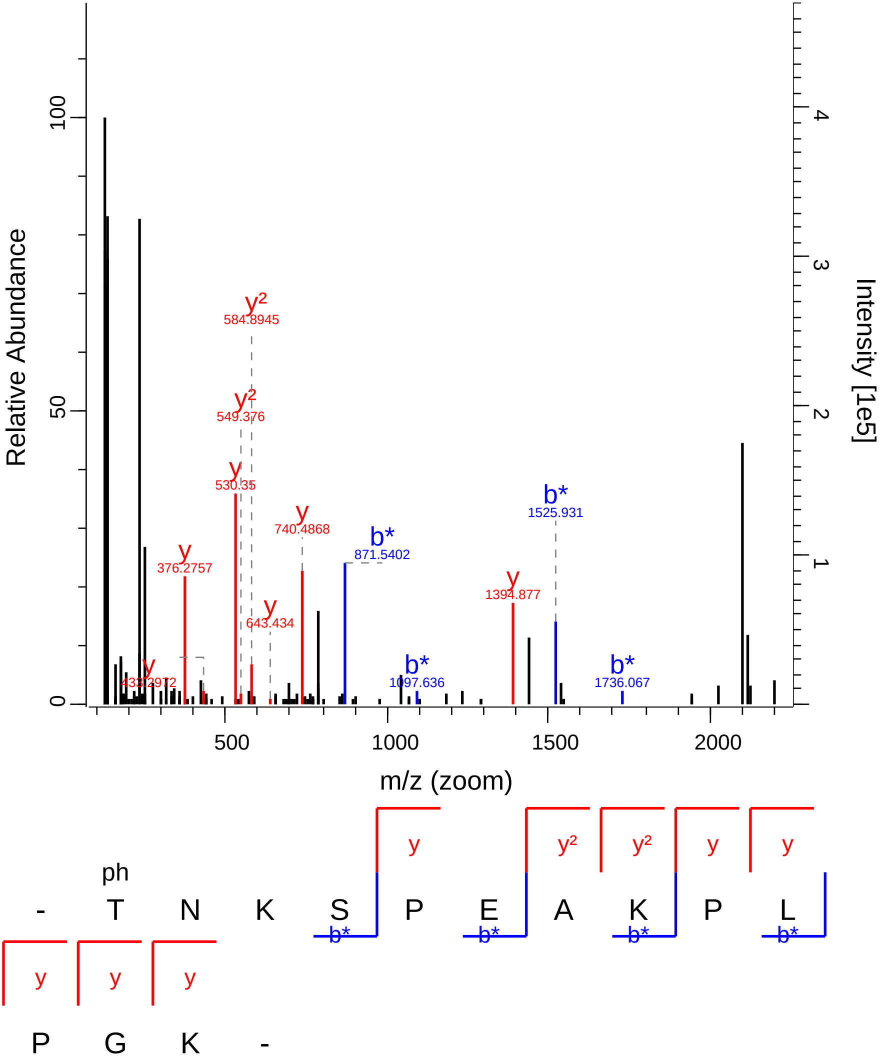

Raw File	Scan	Method	Score	m/z	Gene names
RO-QC-160121_ZackD_SS_Set_01_Fractior	16560	FTMS; HC	85.76	659.84	IRS2

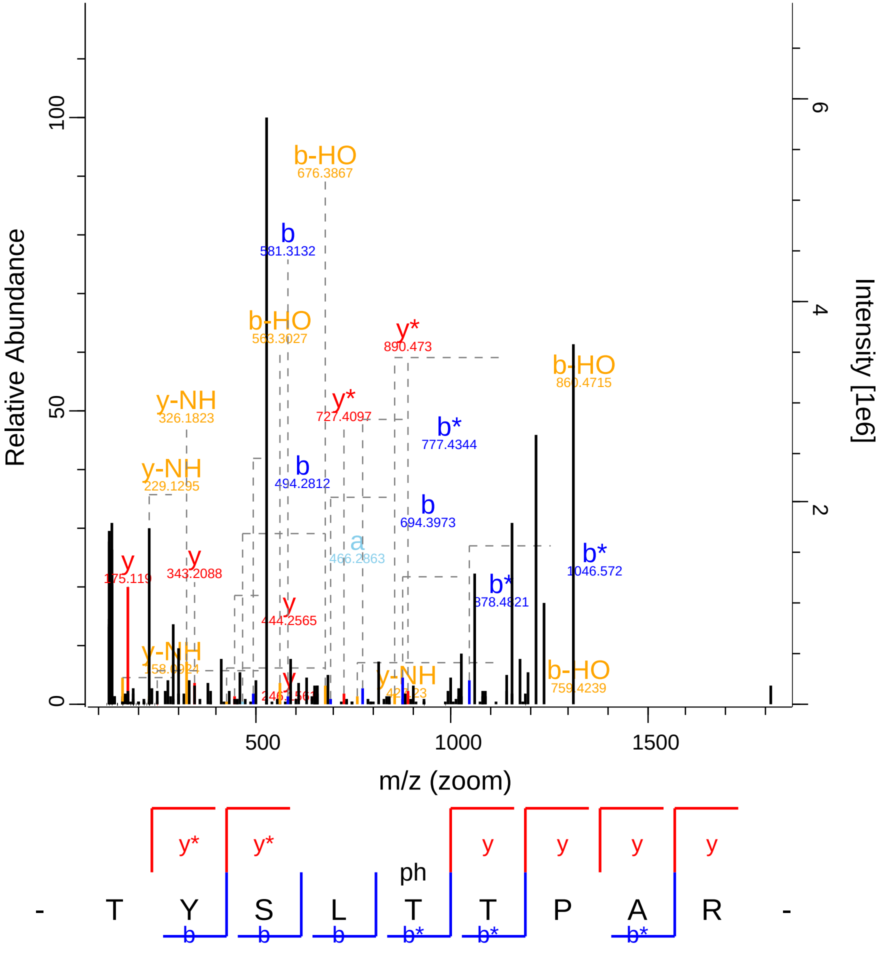

Raw File	Scan	Method	Score	m/z	Gene names
RO-QC-160121_ZackD_SS_Set_01_Fractior	16693	FTMS; HC	69.52	661.36	NRD1

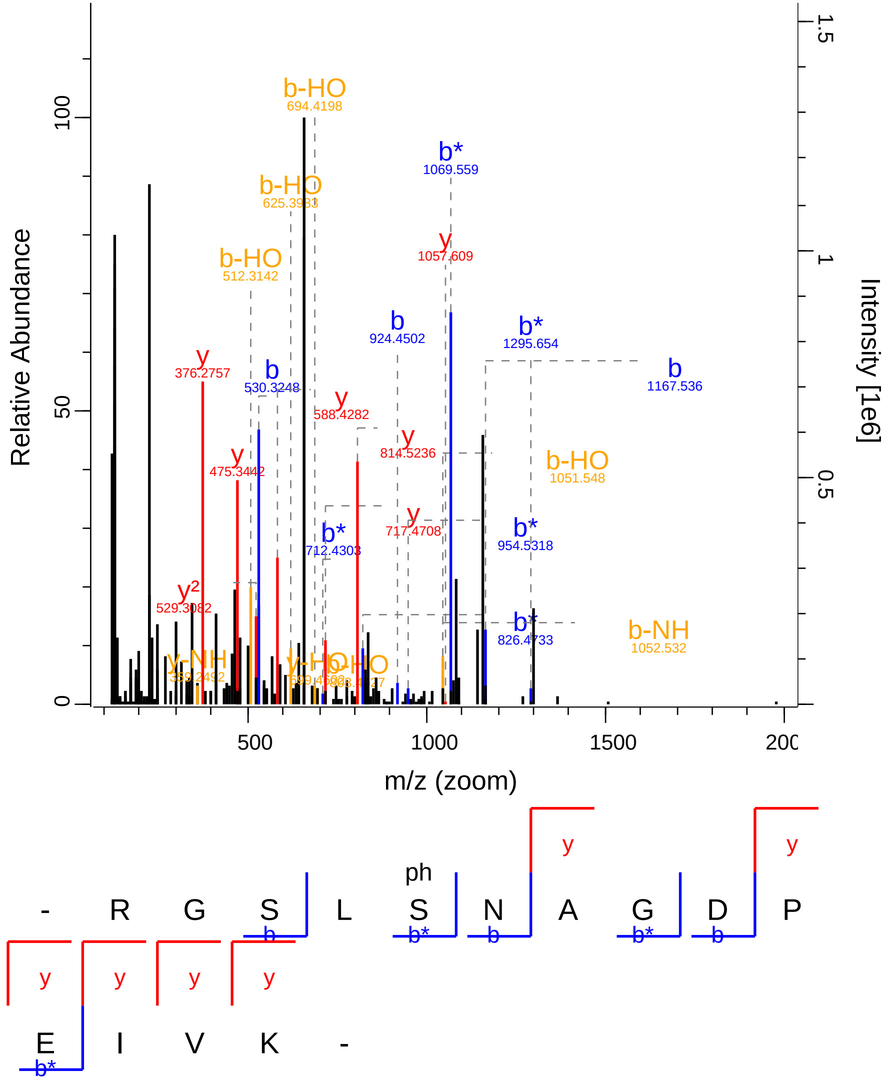

Raw File	Scan	Method	Score	m/z	Gene names
RO-QC-160121_ZackD_SS_Set_01_Fractior	17232	FTMS; HC	71.02	876.96	DAP

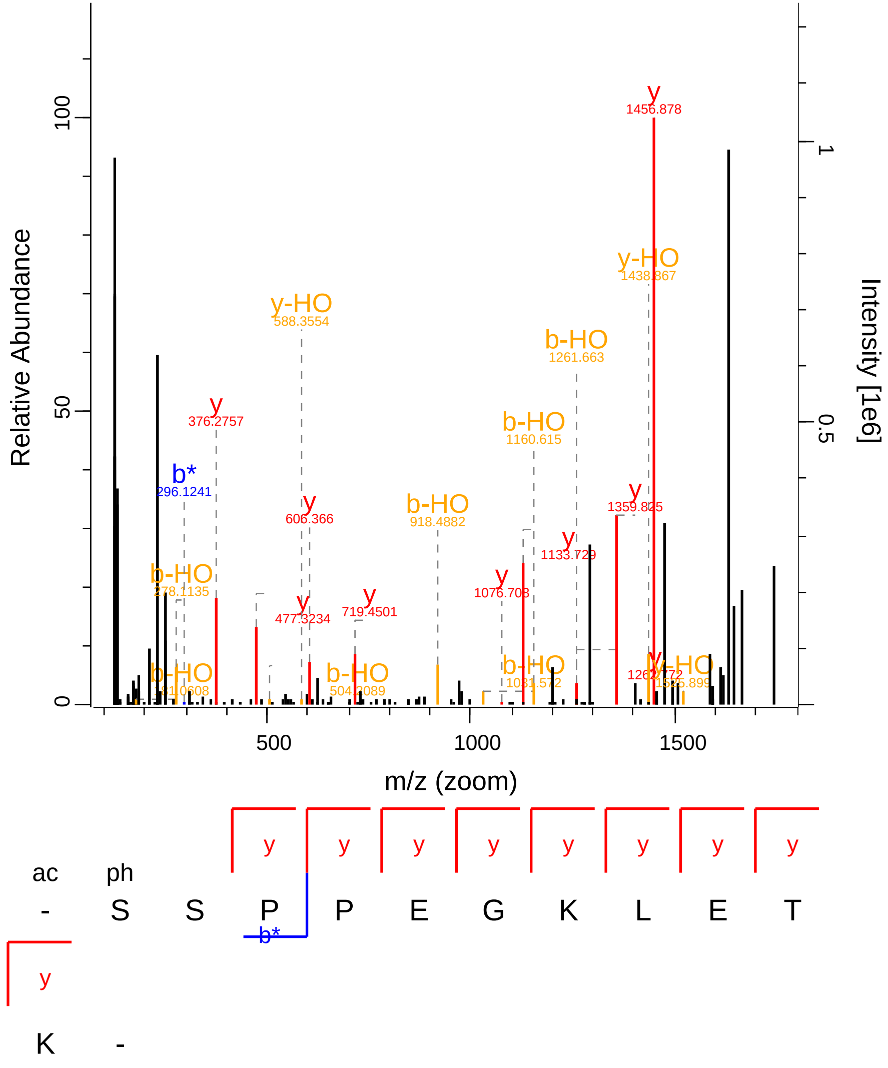

Raw File	Scan	Method	Score	m/z	Gene names
RO-QC-160121_ZackD_SS_Set_01_Fractic	17942	FTMS; HC	44.46	786.76	FCGR3B;FCGR:

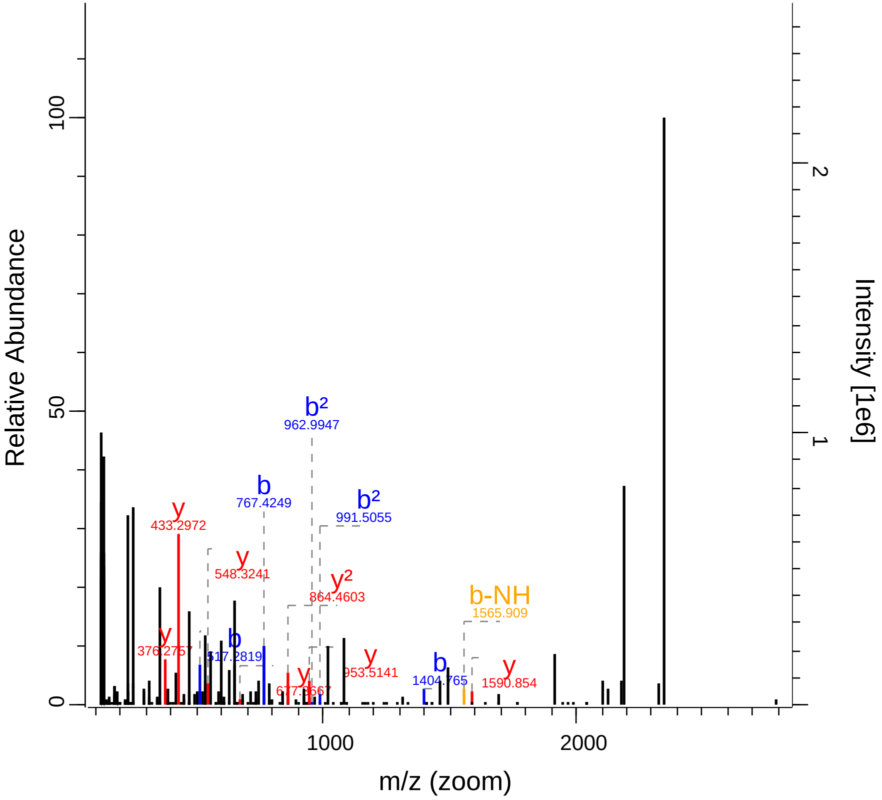

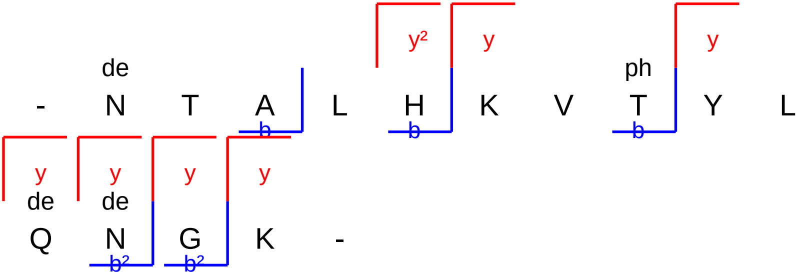

Raw File	Scan	Method	Score	m/z	Gene names
RO-QC-160121_ZackD_SS_Set_01_Fractioı	19103	FTMS; HC	136.81	793.42	FCHO2

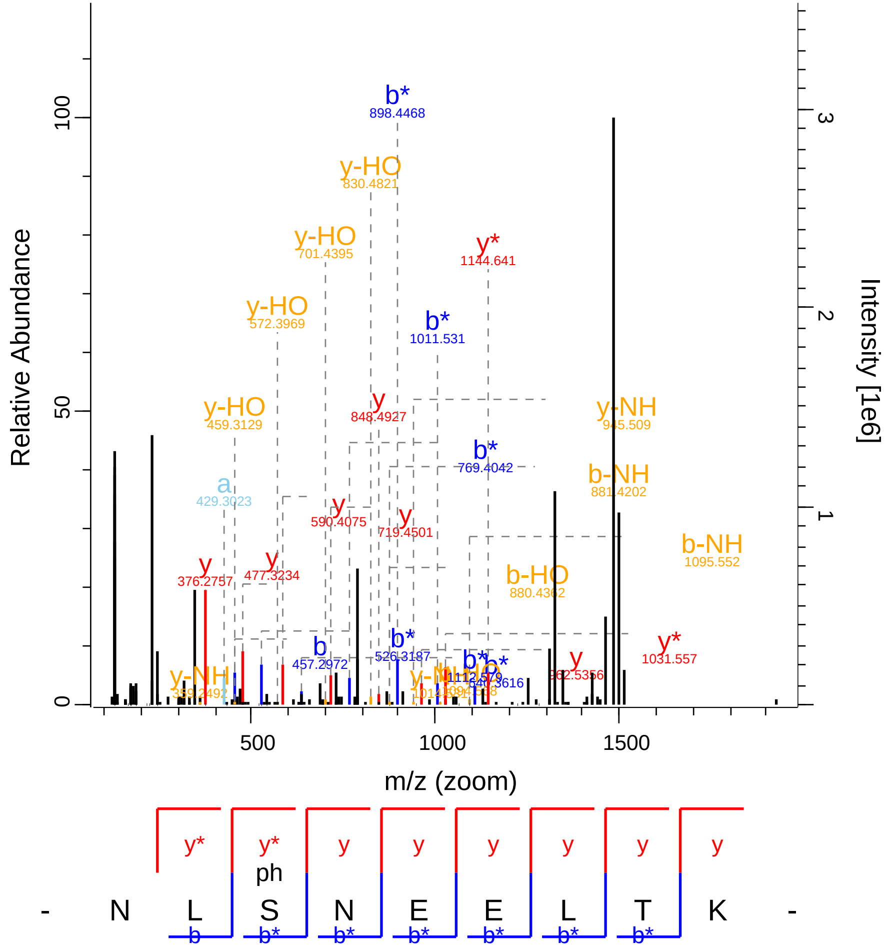

Raw File	Scan	Method	Score	m/z	Gene names
RO-QC-160121_ZackD_SS_Set_01_Fractioı	19377	FTMS; HC	141.88	764.41	TEX2

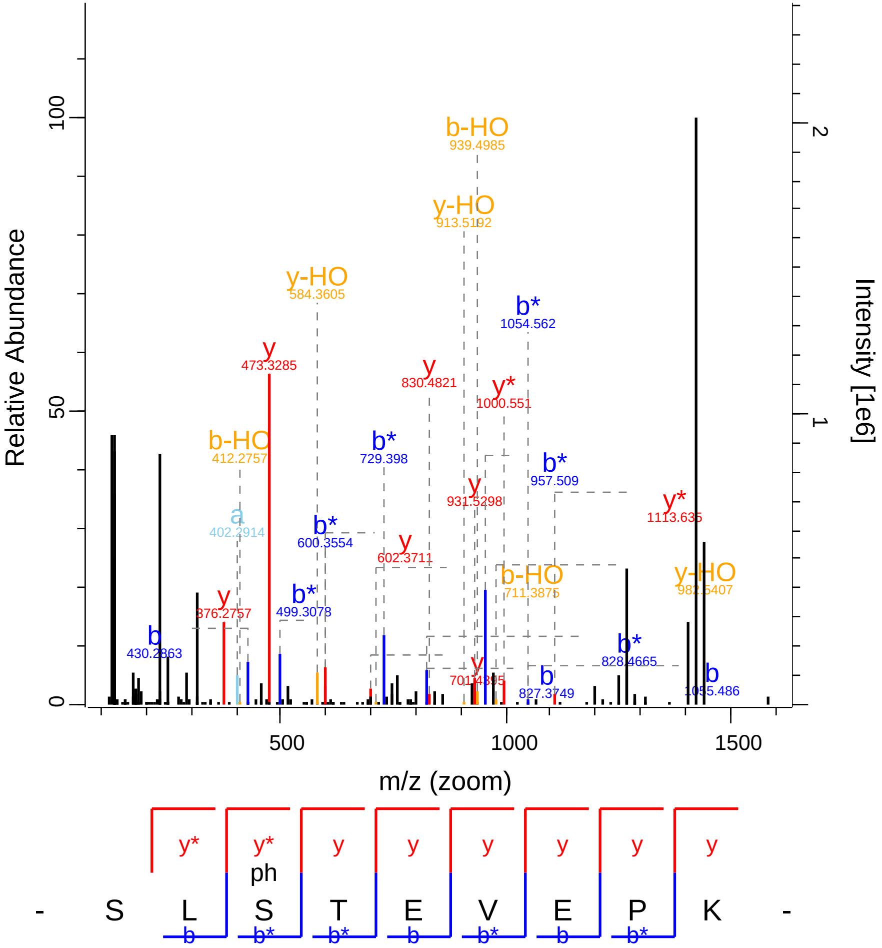

Raw File	Scan	Method	Score	m/z	Gene names
RO-QC-160121_ZackD_SS_Set_01_Fractior	19602	FTMS; HC	89.8	839.41	STX7

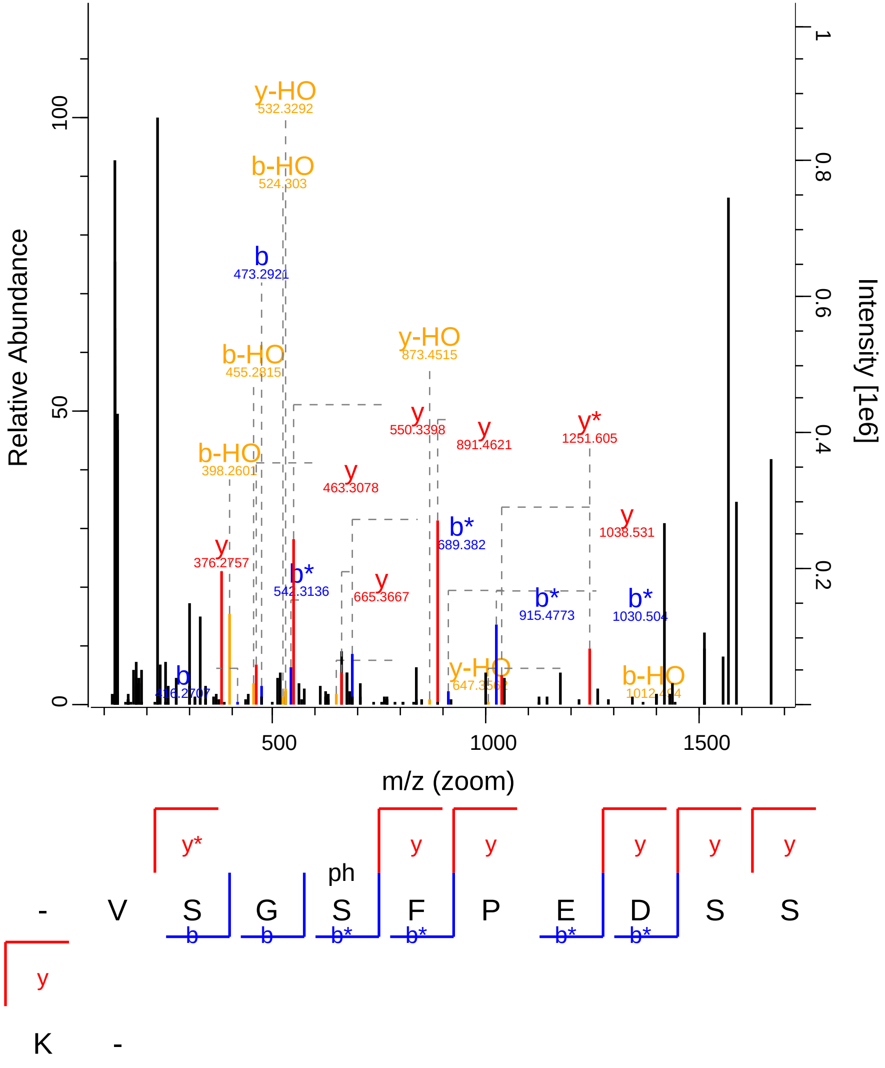

Raw File	Scan	Method	Score	m/z	Gene names
RO-QC-160121_ZackD_SS_Set_01_Fractior	21005	FTMS; HC	58.72	749.92	STX12

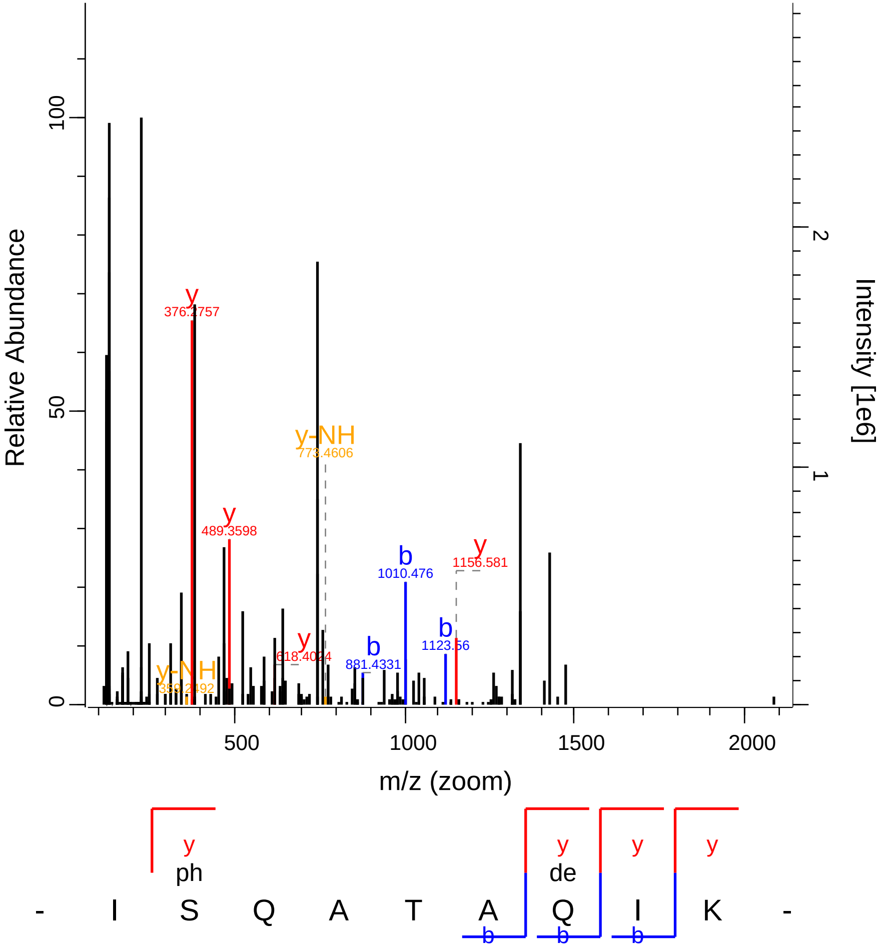

Raw File	Scan	Method	Score	m/z	Gene names
RO-QC-160121_ZackD_SS_Set_01_Fractior	22783	FTMS; HC	77.67	806.45	GSDMA

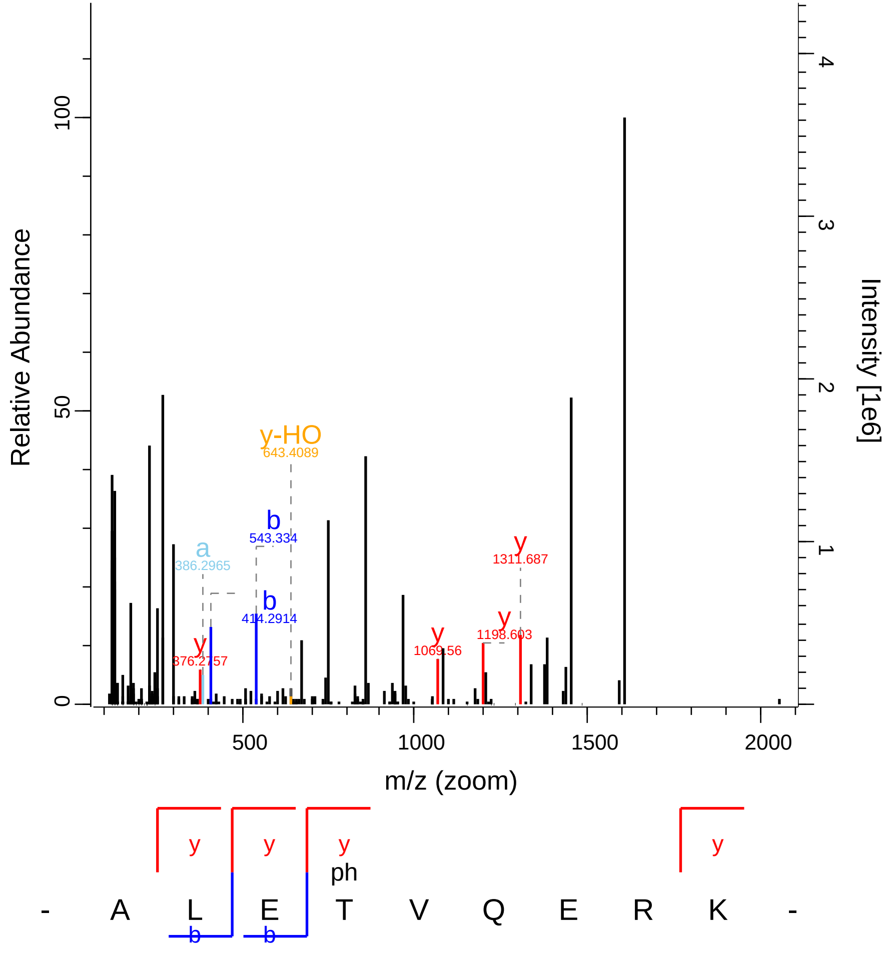

Raw File	Scan	Method	Score	m/z	Gene names
RO-QC-160121_ZackD_SS_Set_01_Fractio	25637	FTMS; HC	174.71	747.38	PGM2

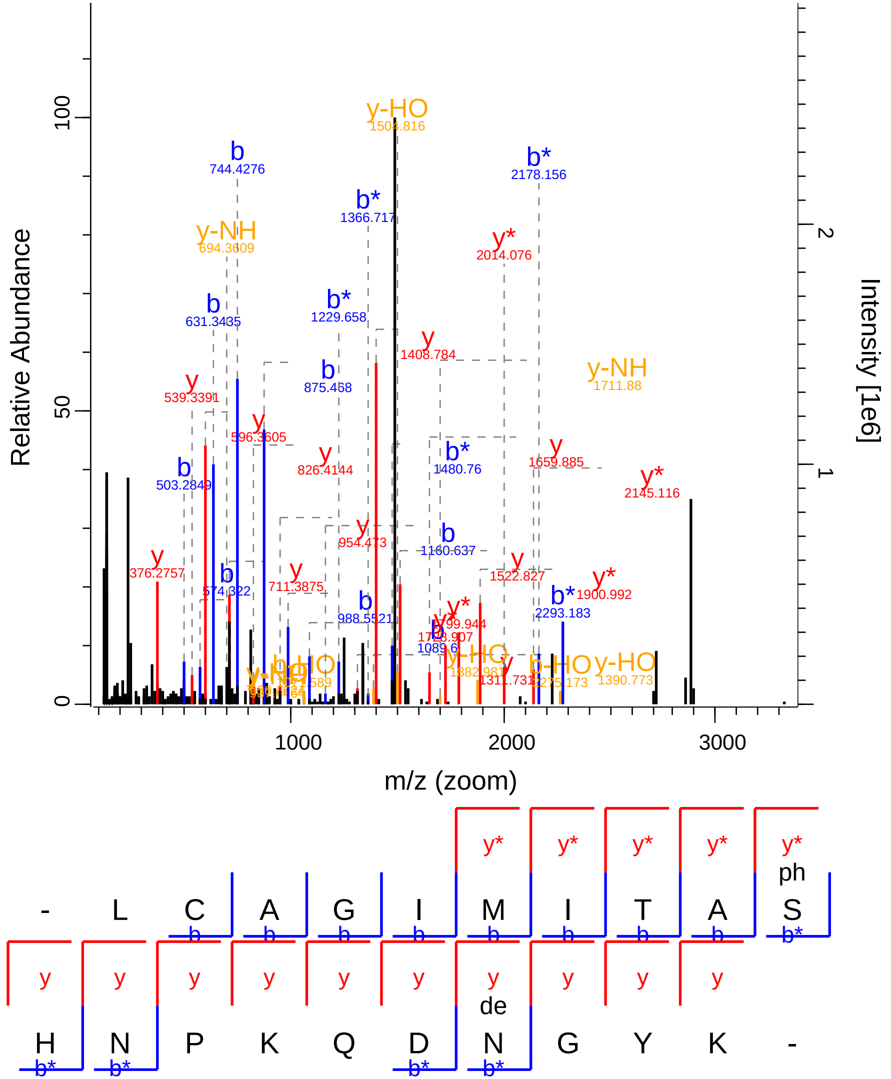

Raw File	Scan	Method	Score	m/z	Gene names
RO-QC-160121_ZackD_SS_Set_01_Fractior	25863	FTMS; HC	100.45	642.37	RASSF8

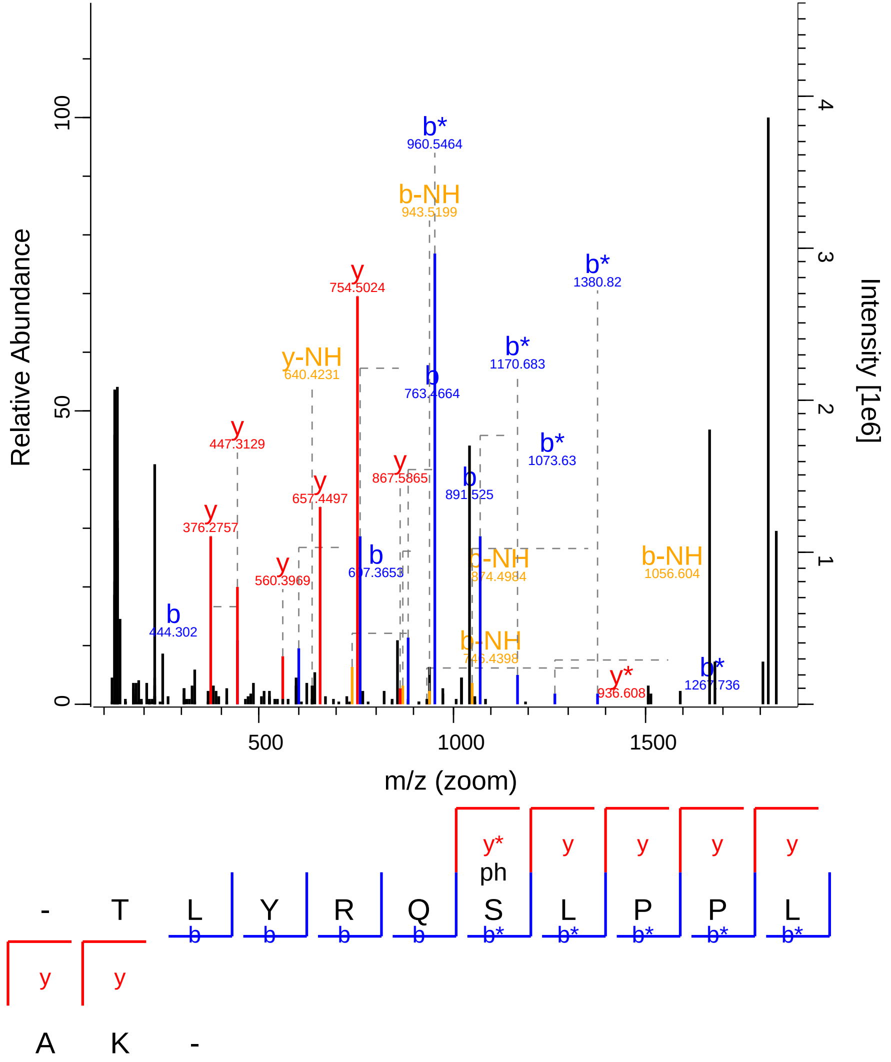

Raw File	Scan	Method	Score	m/z
RO-QC-160121_ZackD_SS_Set_01_Fraction_11	26500	FTMS; HCD	44.3	533.54

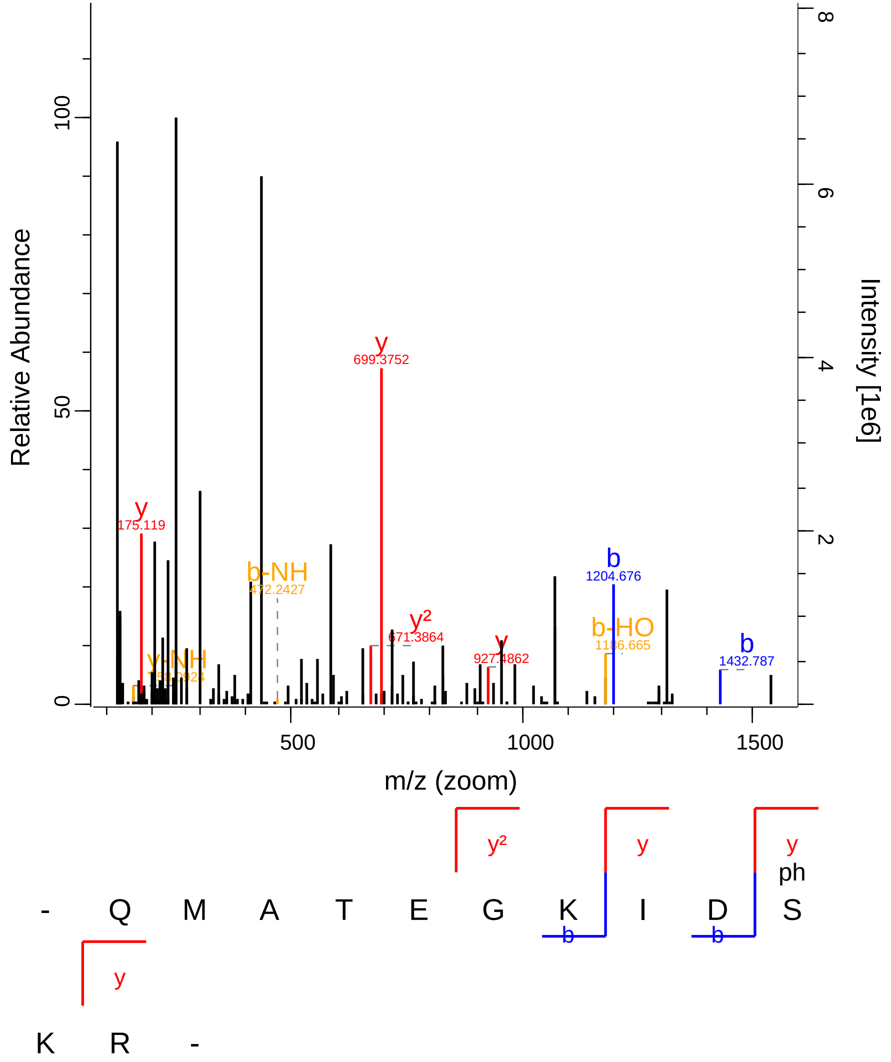

Raw File	Scan	Method	Score	m/z	Gene names
RO-QC-160121_ZackD_SS_Set_01_Fractior	27328	FTMS; HC	68.42	841.97	PSMA7;PSMA8

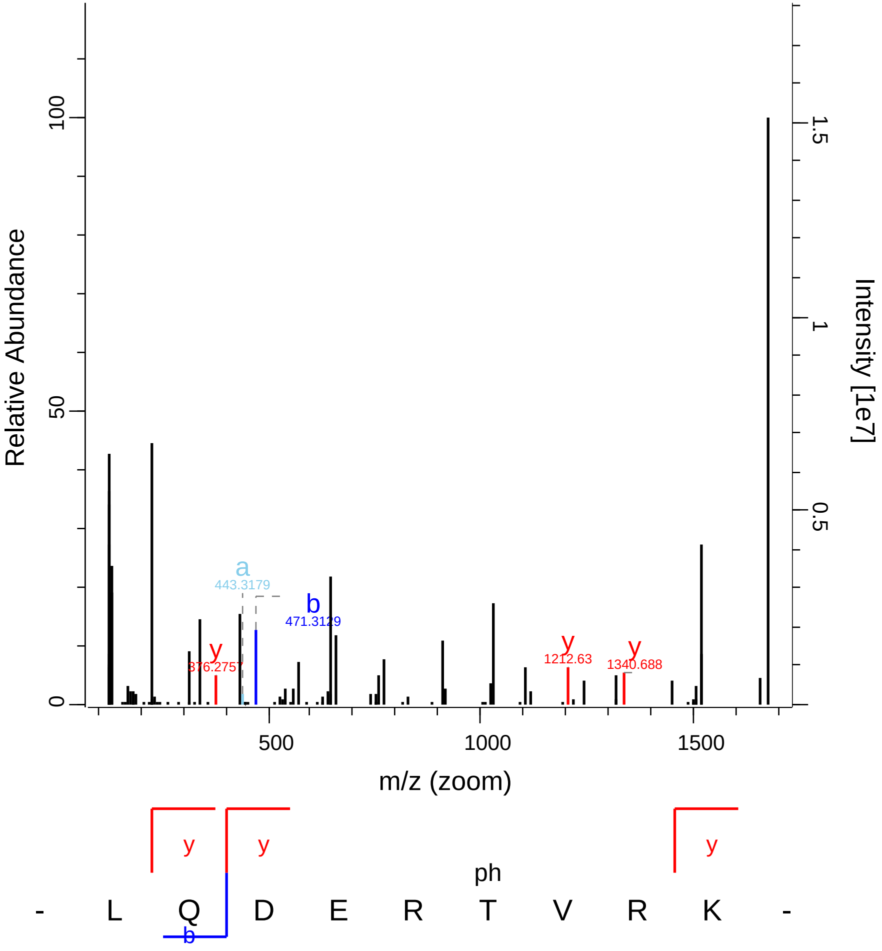

Raw File	Scan	Method	Score	m/z	Gene names
RO-QC-160121_ZackD_SS_Set_01_Fractio	27592	FTMS; HC	180.48	923.71	RAB11FIP1

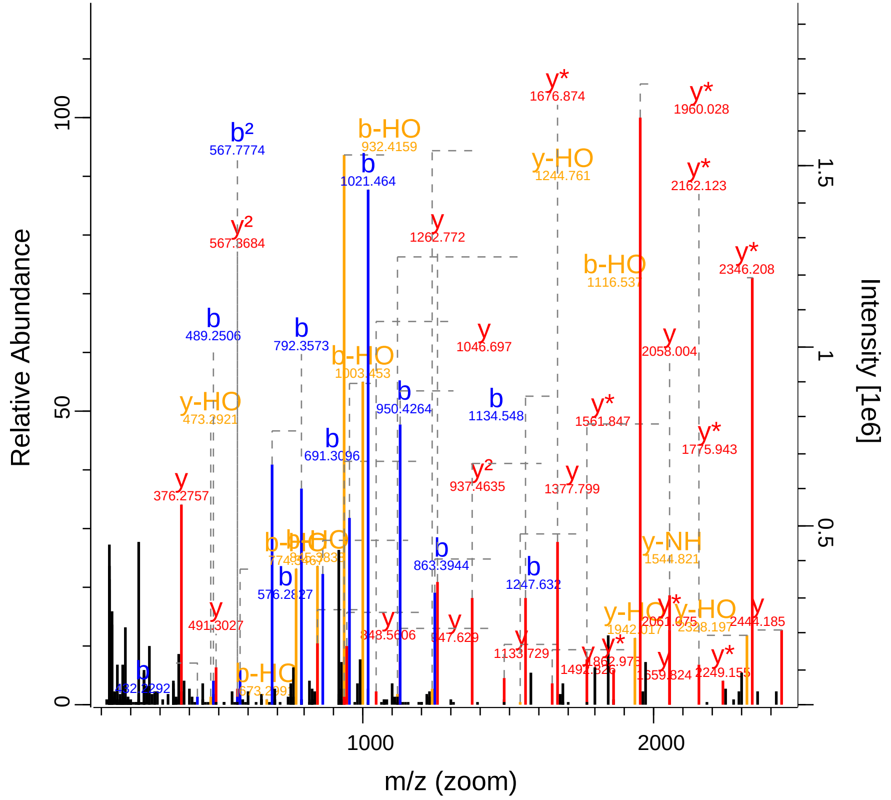

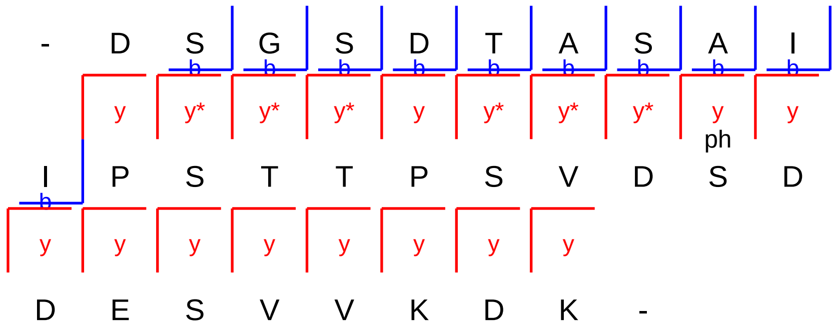

Raw File	Scan	Method	Score	m/z	Gene names
RO-QC-160121_ZackD_SS_Set_01_Fractior	28583	FTMS; HC	75.77	758.04	AHNAK

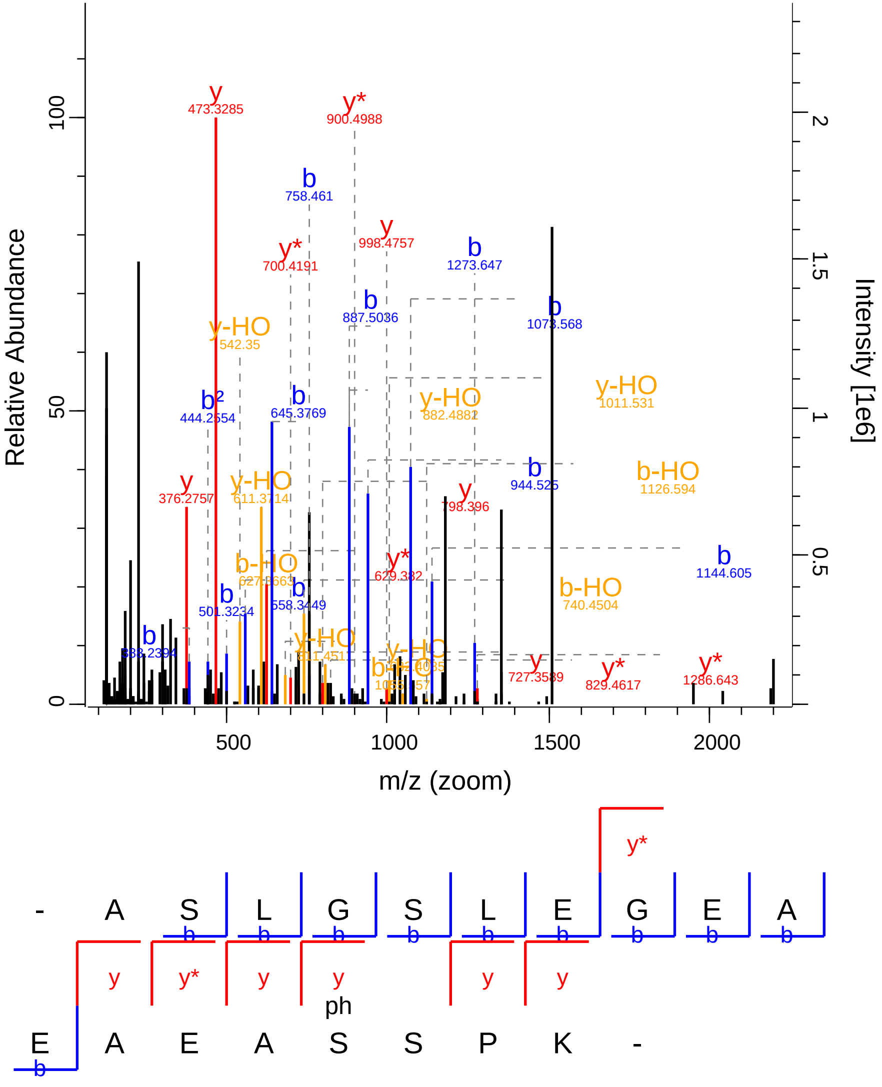

Raw File	Scan	Method	Score	m/z	Gene names
RO-QC-160121_ZackD_SS_Set_01_Fractio	29381	FTMS; HC	148.24	736.38	VIM

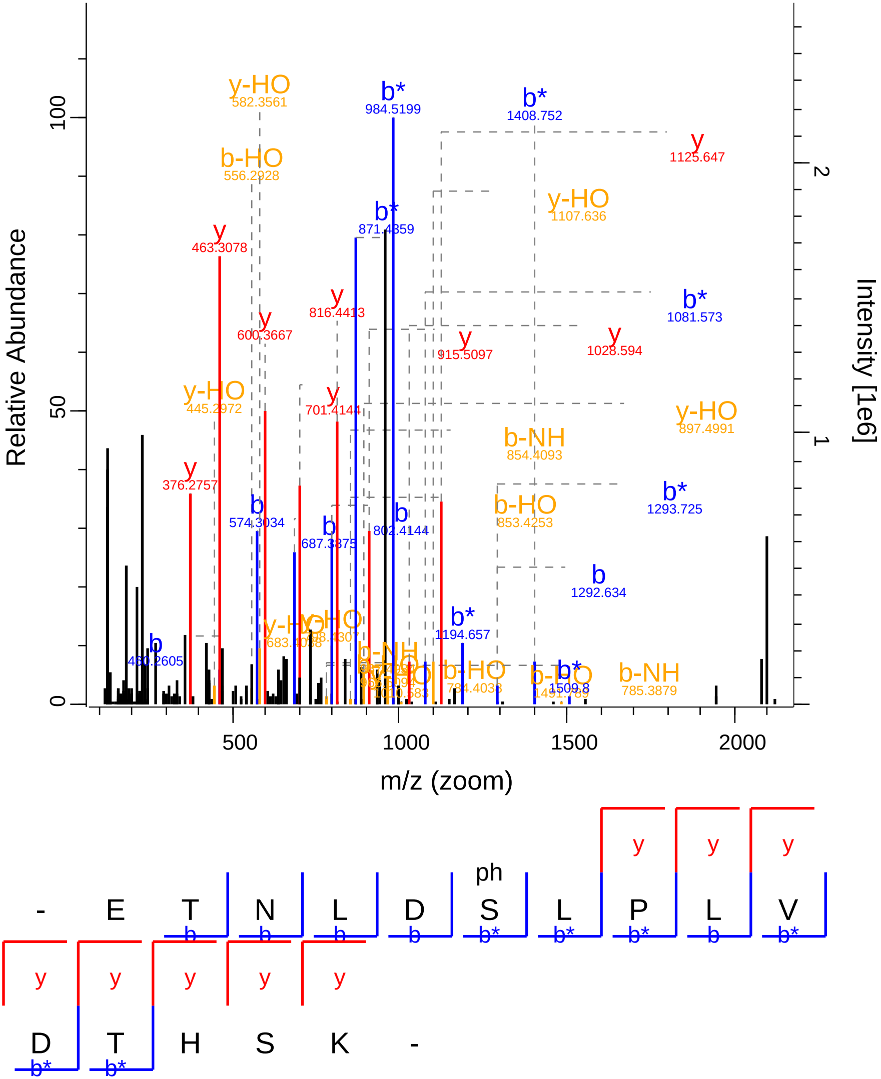

Raw File	Scan	Method	Score	m/z	Gene names
RO-QC-160121_ZackD_SS_Set_01_Fractio	29438	FTMS; HC	73.34	1001.52	METTL3

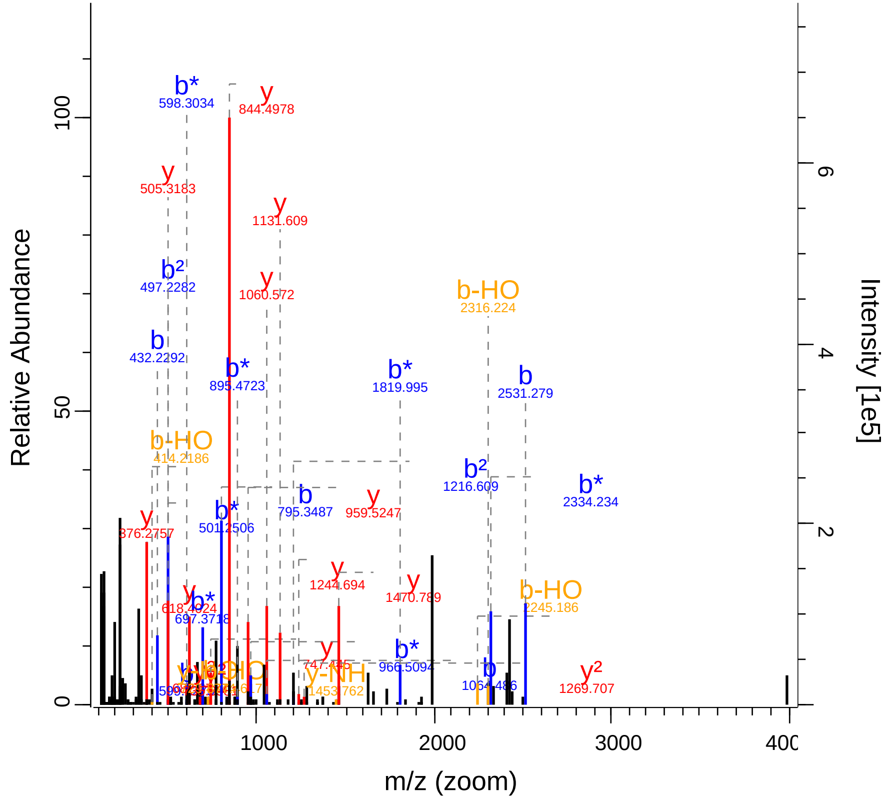

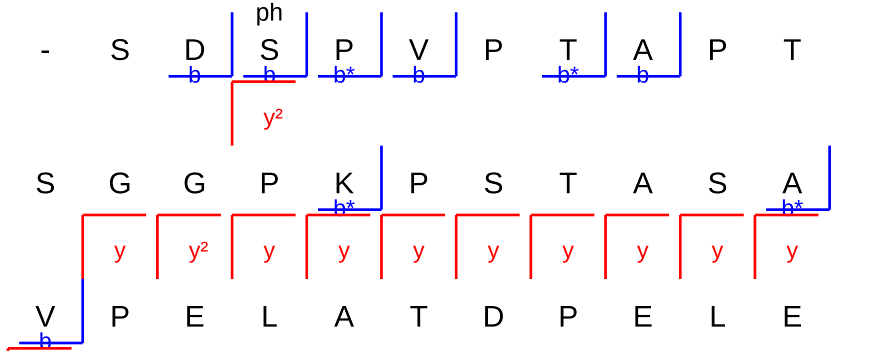

Raw File	Scan	Method	Score	m/z	Gene names
RO-QC-160121_ZackD_SS_Set_01_Fractior	30760	FTMS; HC	78.33	991.06	OGN

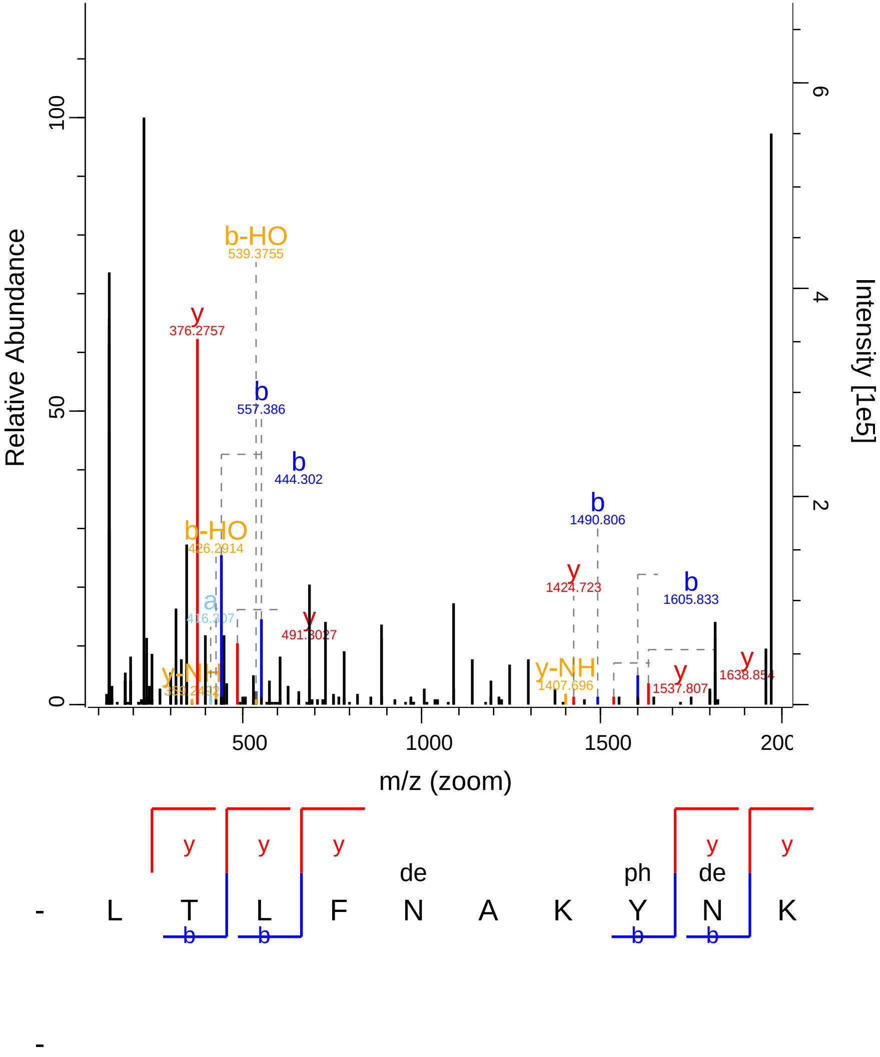

Raw File	Scan	Method	Score	m/z	Gene names
RO-QC-160121_ZackD_SS_Set_01_Fractio	31655	FTMS; HC	103.07	1013.17	SLC2A1

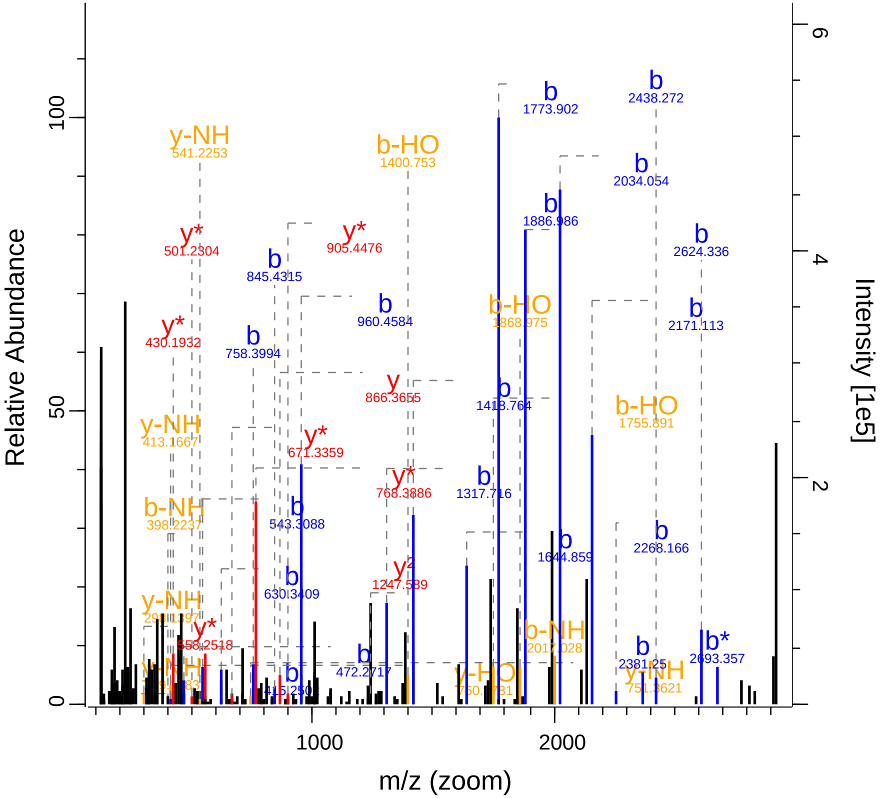

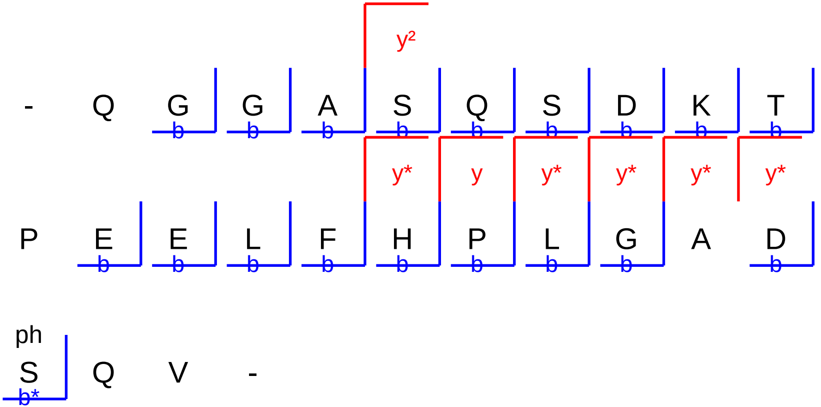

Raw File	Scan	Method	Score	m/z	Gene names
RO-QC-160121_ZackD_SS_Set_01_Fractio	31751	FTMS; HC	65.05	1015.42	CTNND1

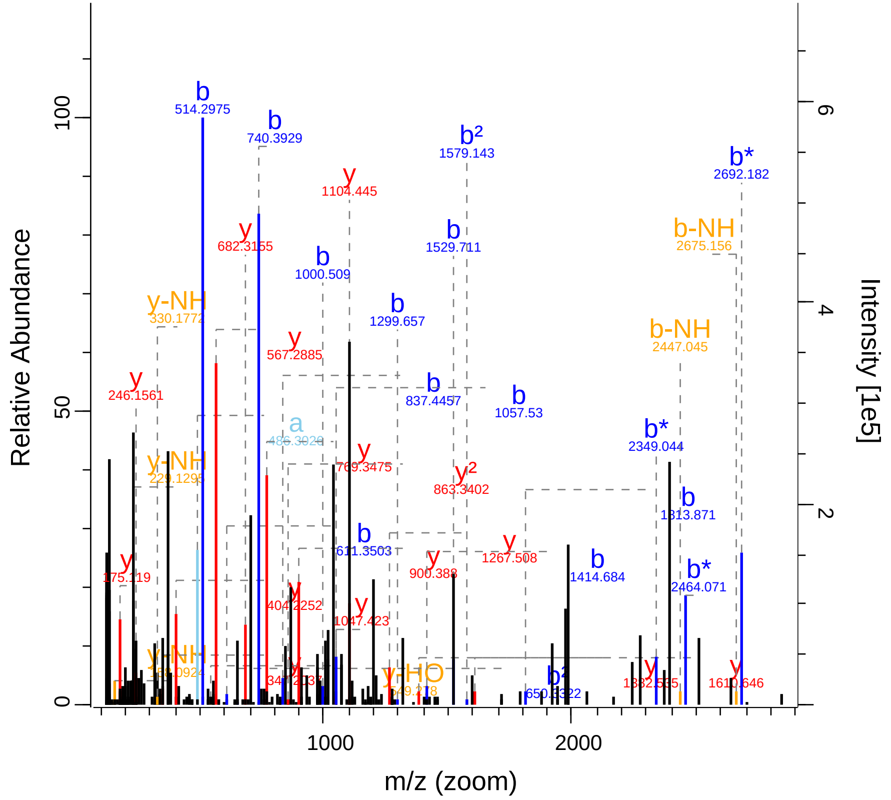

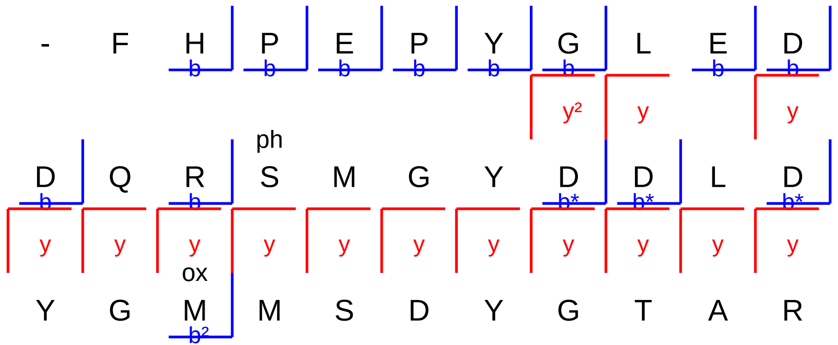

Raw File	Scan	Method	Score	m/z	Gene names
RO-QC-160121_ZackD_SS_Set_01_Fractioı	32105	FTMS; HC	141.99	920.97	GIT1

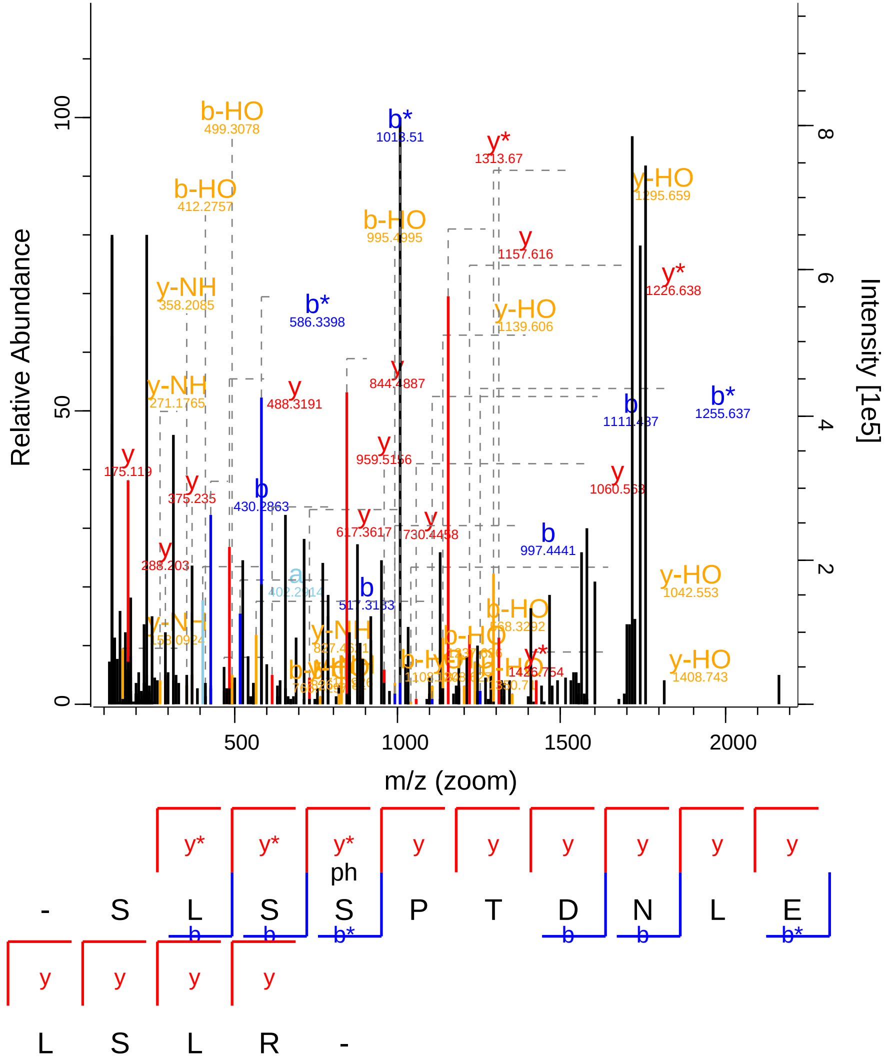

Raw File	Scan	Method	Score	m/z	Gene names
RO-QC-160121_ZackD_SS_Set_01_Fractio	33050	FTMS; HC	46	1016.55	TMTC3

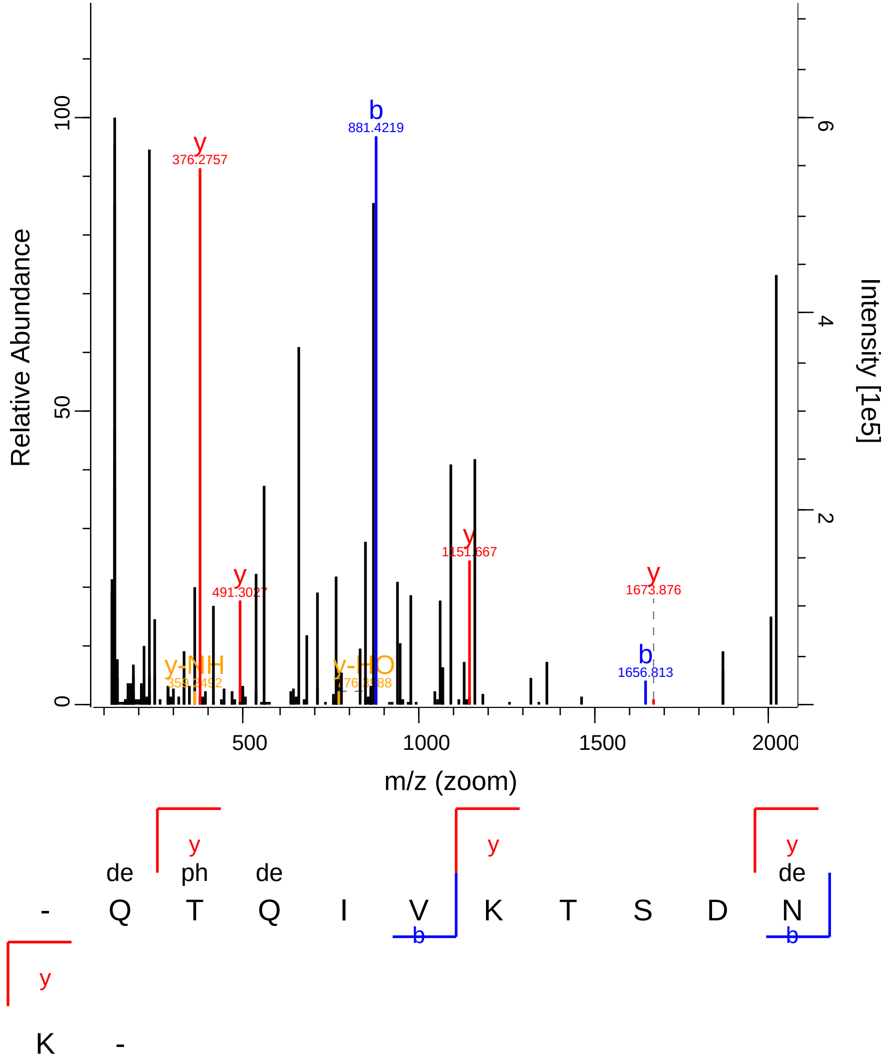

Raw File	Scan	Method	Score	m/z	Gene names
RO-QC-160121_ZackD_SS_Set_01_Fractior	33931	FTMS; HC	64.88	720.09	OFD1

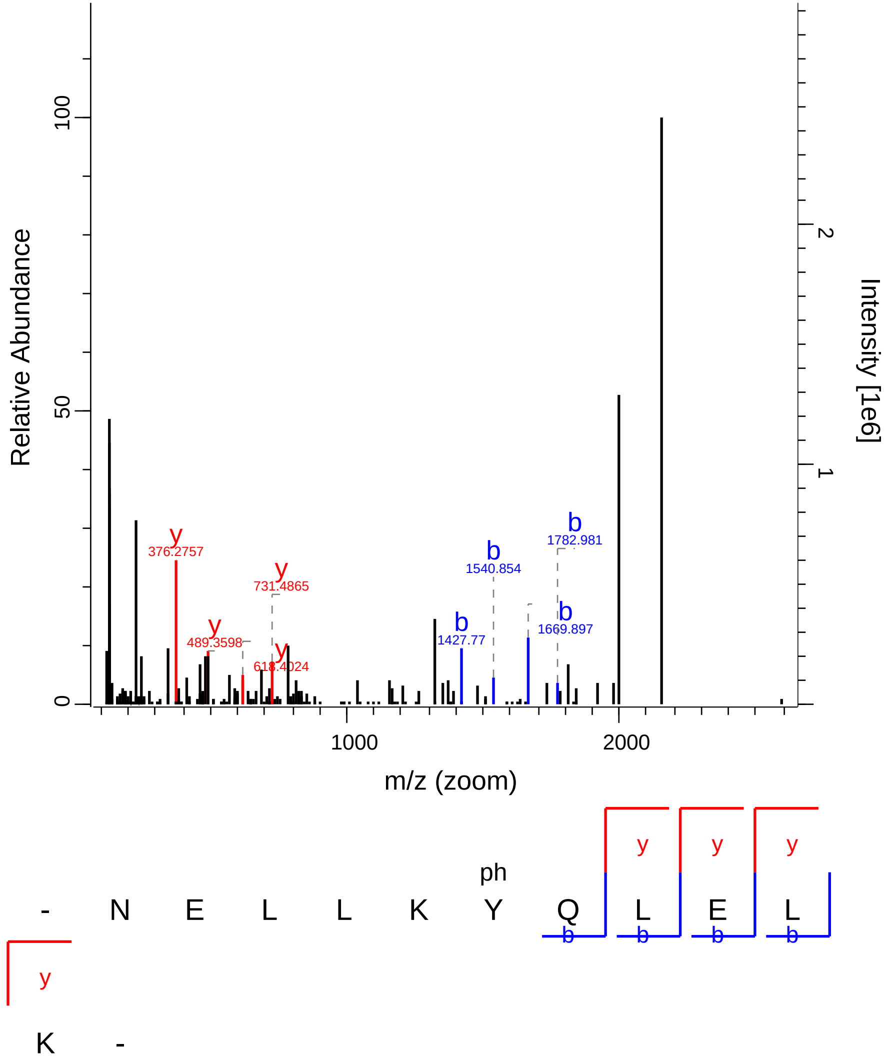

Raw File	Scan	Method	Score	m/z	Gene names
RO-QC-160121_ZackD_SS_Set_01_Fractio	34404	FTMS; HC	108.09	1078.81	ADD1

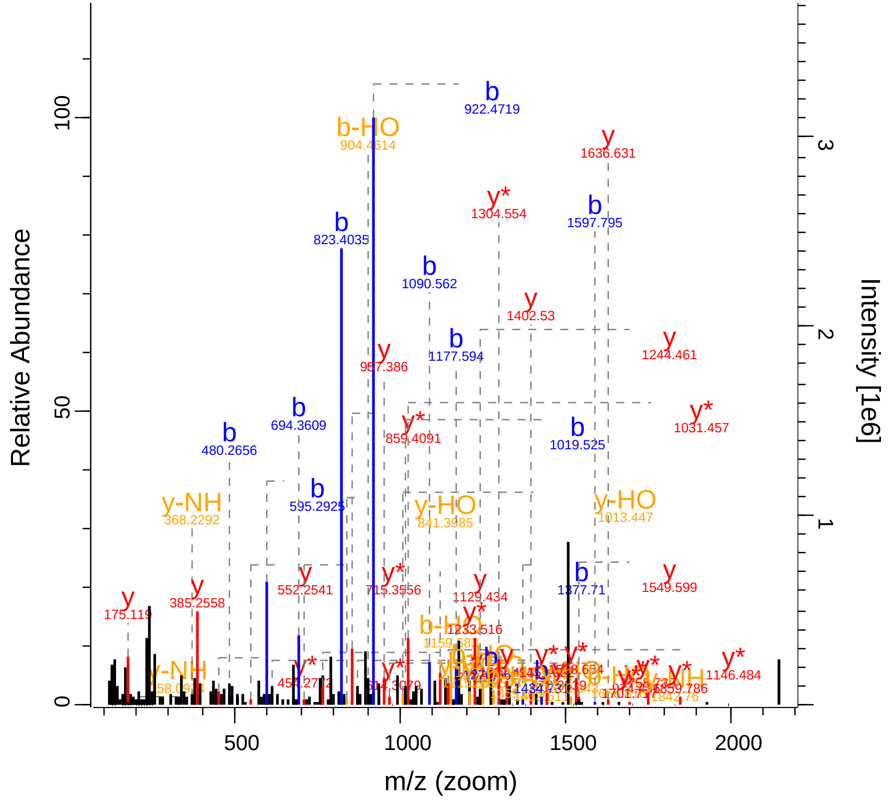

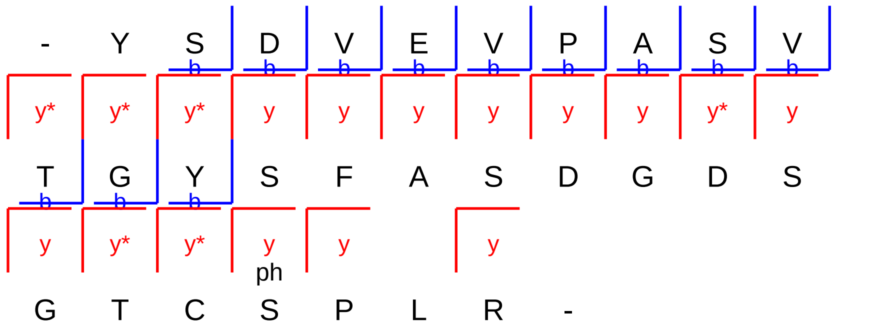

Raw File	Scan	Method	Score	m/z	Gene names
RO-QC-160121_ZackD_SS_Set_01_Fractior	34511	FTMS; HC	69.38	829.96	PLEC

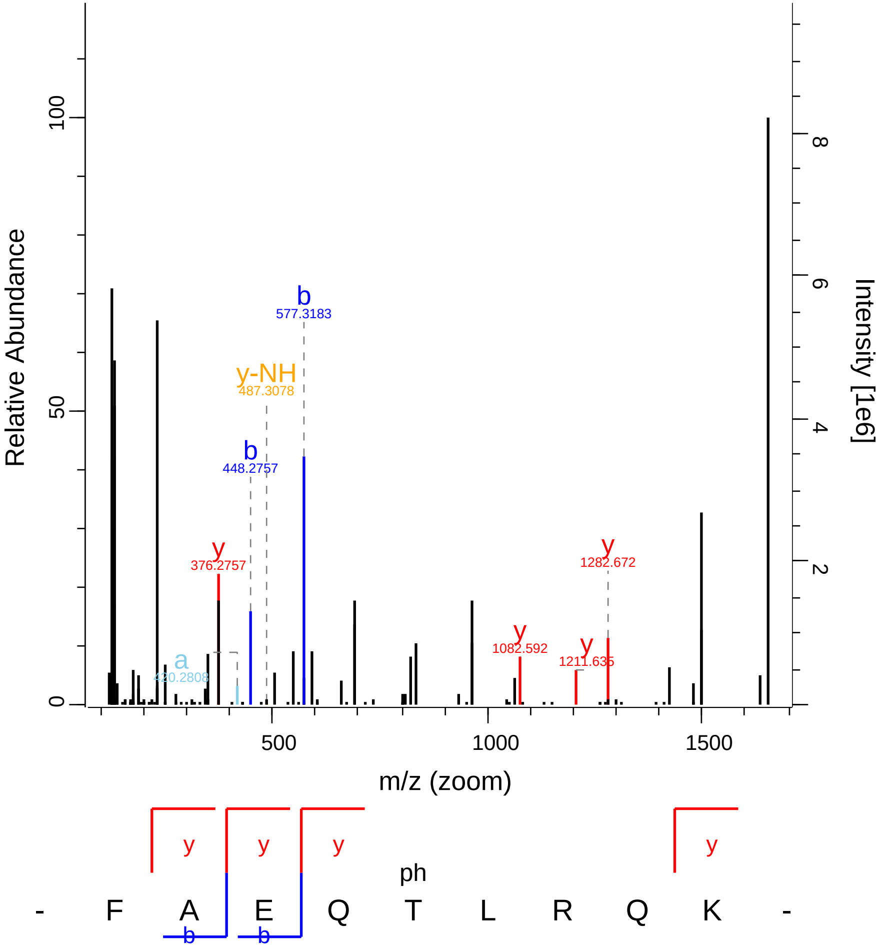

Raw File	Scan	Method	Score	m/z	Gene names
RO-QC-160121_ZackD_SS_Set_01_Fractio	35146	FTMS; HC	298.33	1080.75	NAP1L4

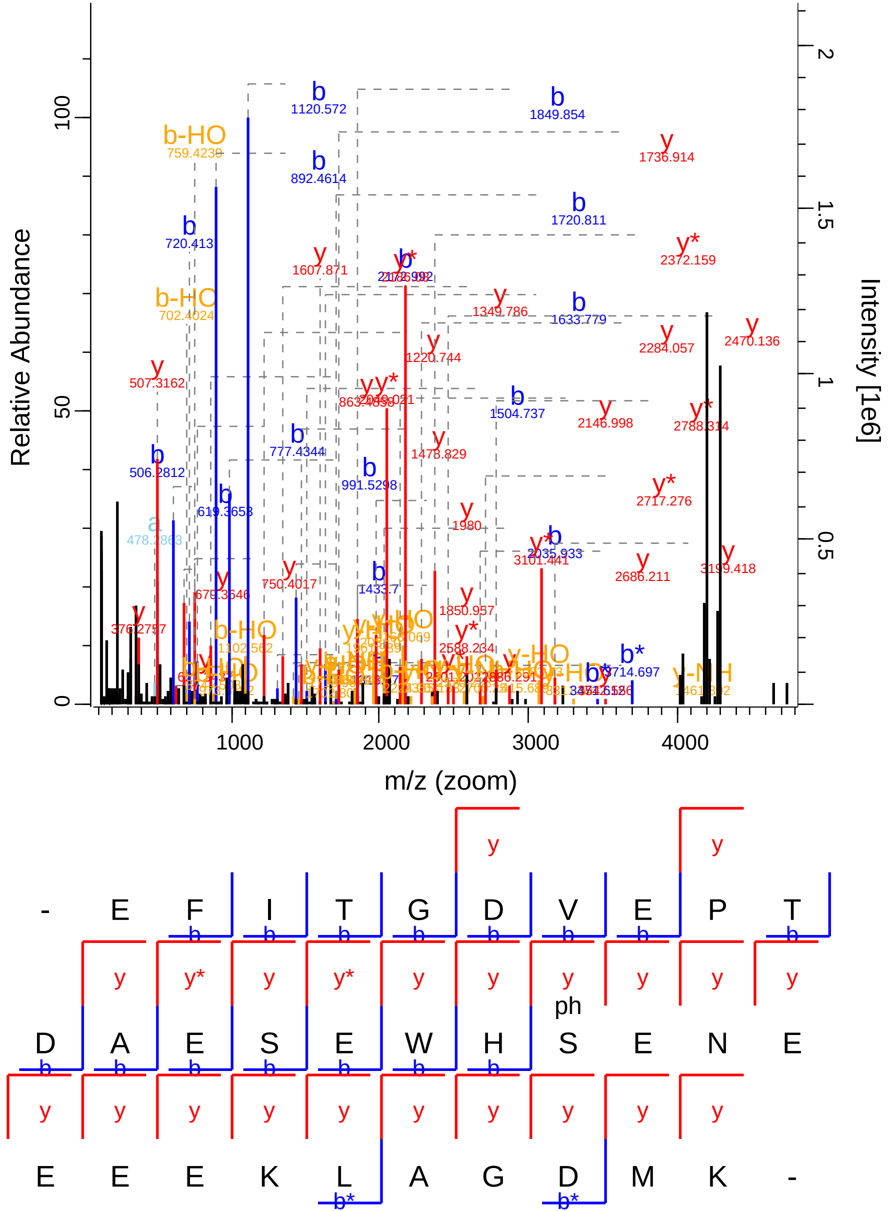

Raw File	Scan	Method	Score	m/z	Gene names
RO-QC-160121_ZackD_SS_Set_01_Fractior	35307	FTMS; HC	51.73	950.93	RTF1

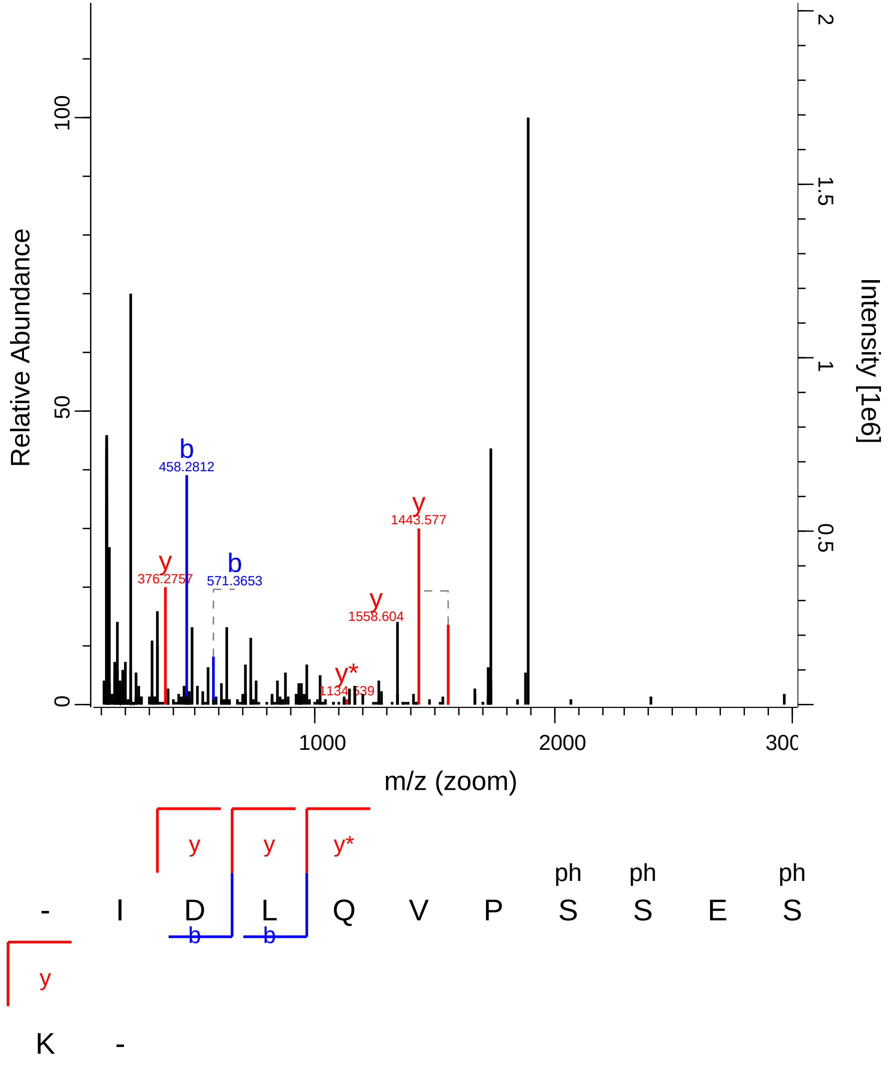

Raw File	Scan	Method	Score	m/z	Gene names
RO-QC-160121_ZackD_SS_Set_01_Fractior	36023	FTMS; HC	43.16	837.23	TDRD10

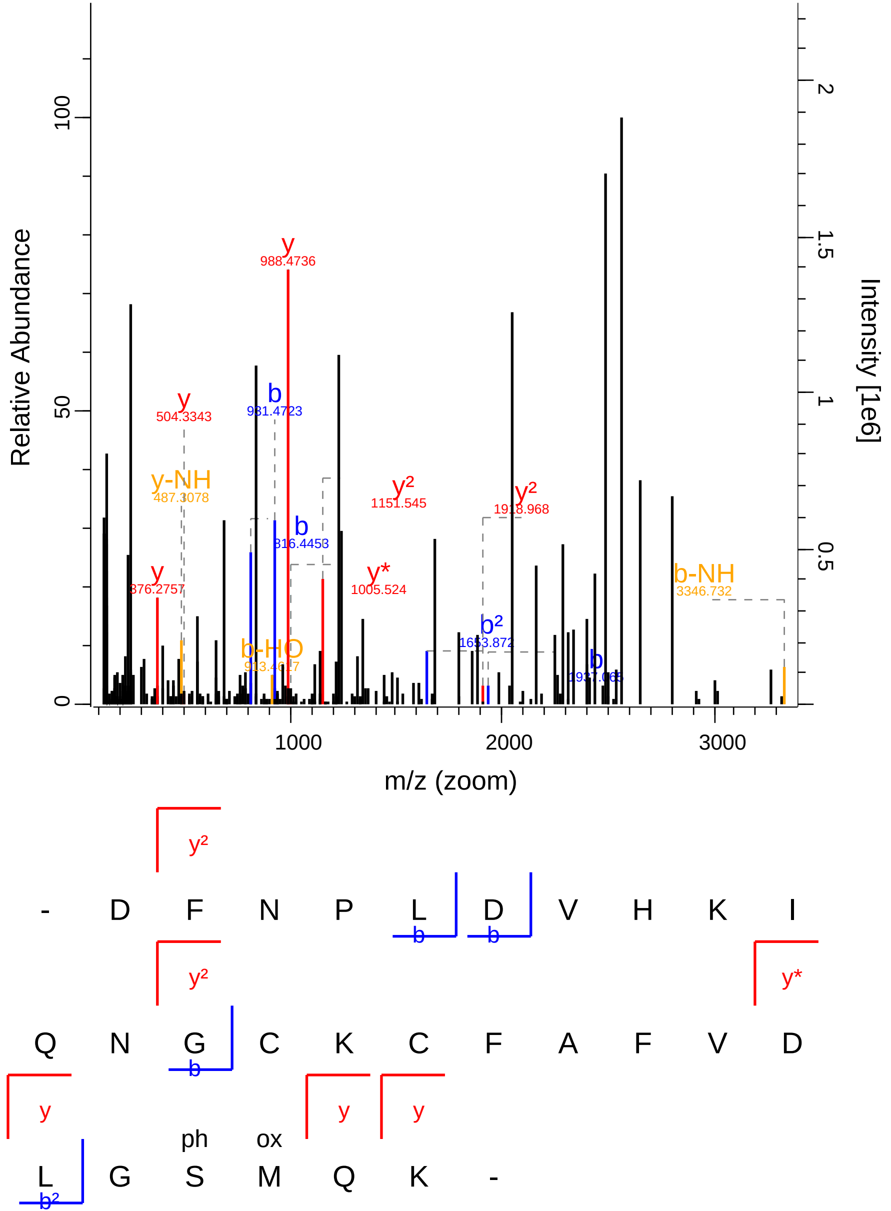

Raw File	Scan	Method	Score	m/z	Gene names
RO-QC-160121_ZackD_SS_Set_01_Fractio	36269	FTMS; HC	66.92	1150.88	CIR1

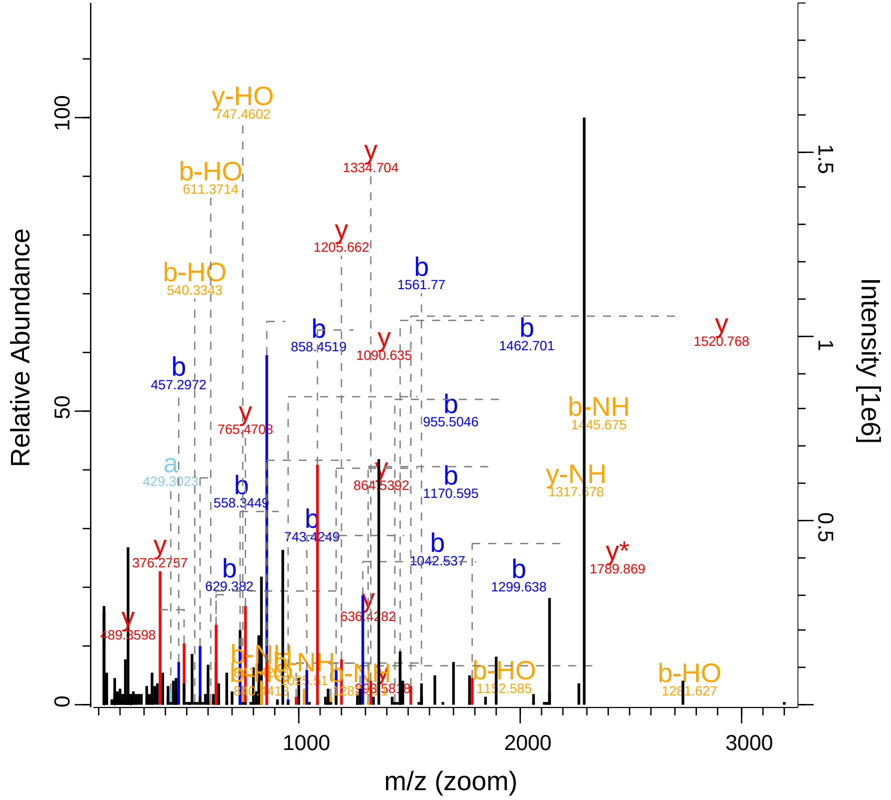

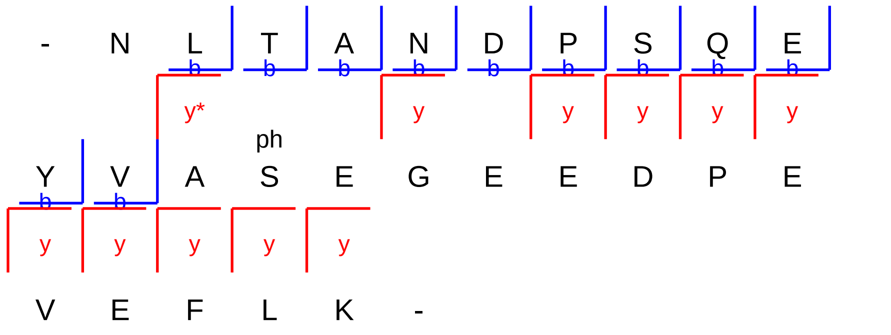

Raw File	Scan	Method	Score	m/z	Gene names
RO-QC-160121_ZackD_SS_Set_01_Fractior	37682	FTMS; HC	40.75	793.07	CEP295

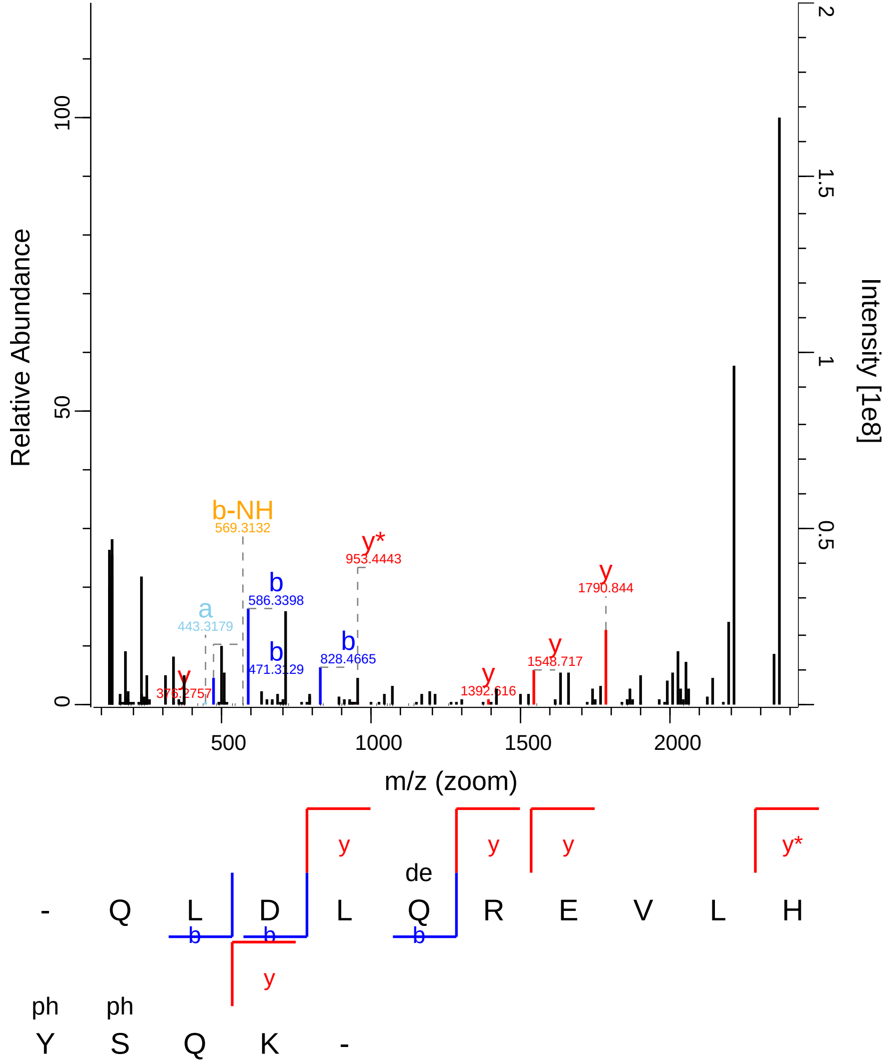

Raw File	Scan	Method	Score	m/z	Gene names
RO-QC-160121_ZackD_SS_Set_01_Fractio	37879	FTMS; HC	70.94	1313.69	HELZ2

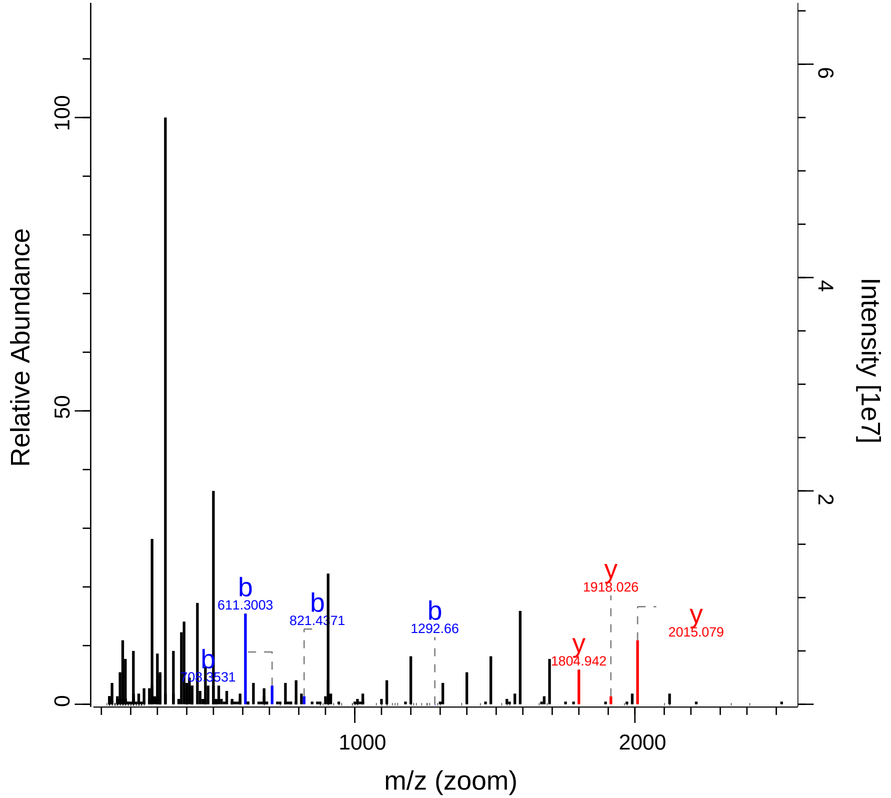

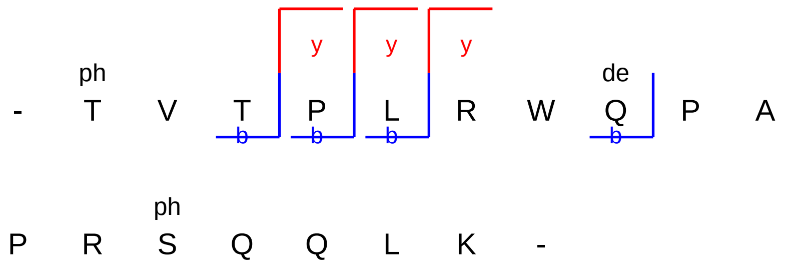

Raw File	Scan	Method	Score	m/z	Gene names
RO-QC-160121_ZackD_SS_Set_01_Fraction	4711	FTMS; HC	151.26	732.84	RFTN1

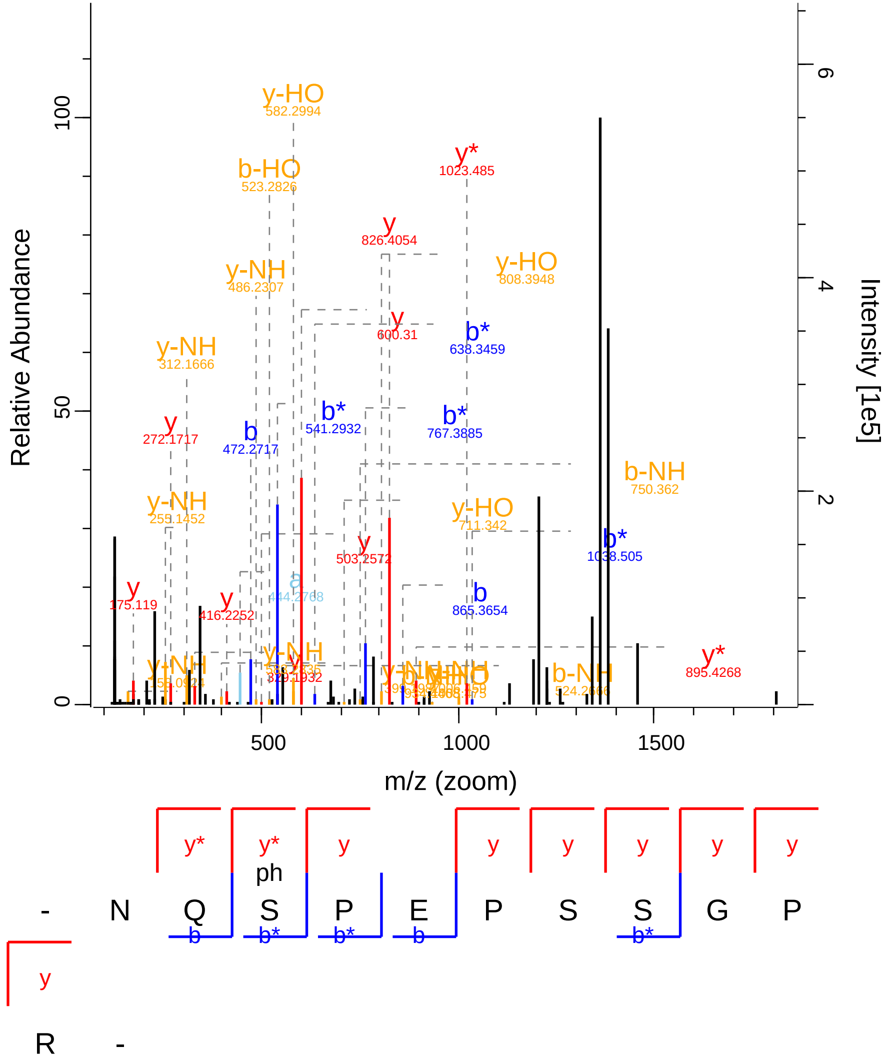

Raw File	Scan	Method	Score	m/z	Gene names
RO-QC-160121_ZackD_SS_Set_01_Fraction	6195	FTMS; HC	60.91	972.99	CDCA7

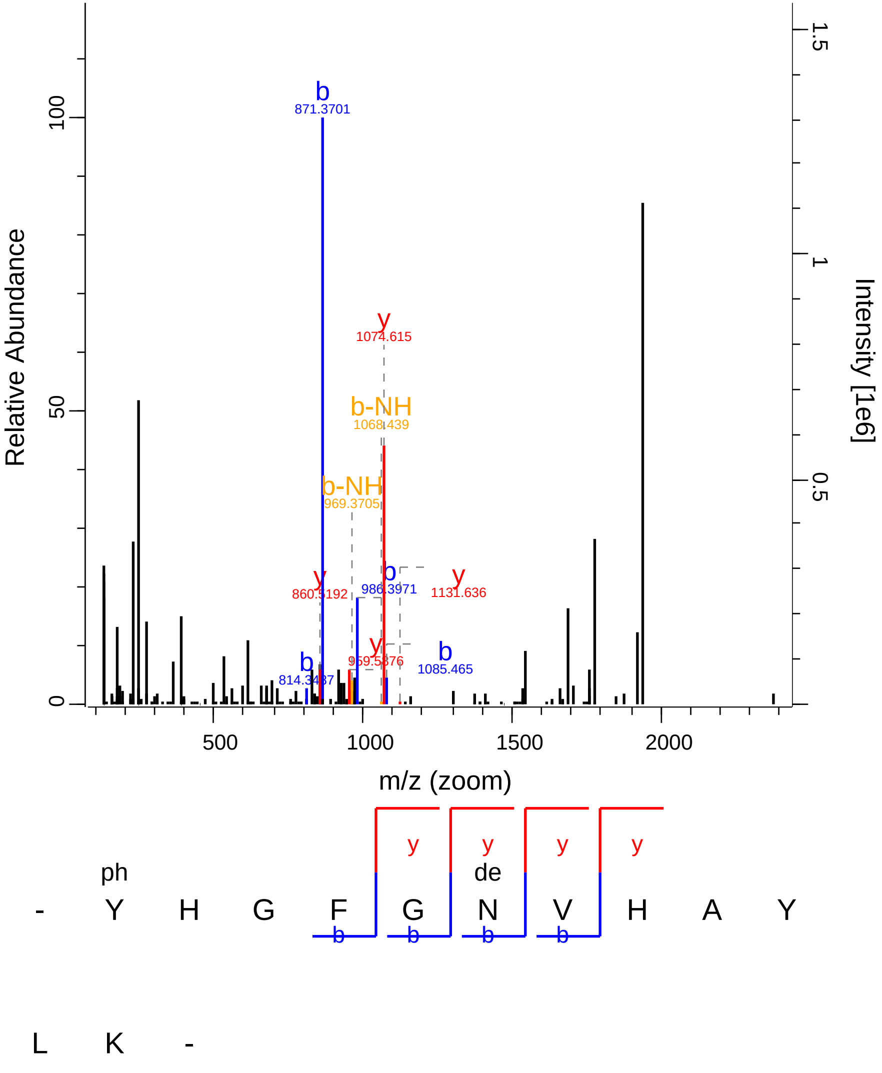

Raw File	Scan	Method	Score	m/z	Gene names
RO-QC-160121_ZackD_SS_Set_01_Fraction	6541	FTMS; HC	67.08	469.78	THRAP3

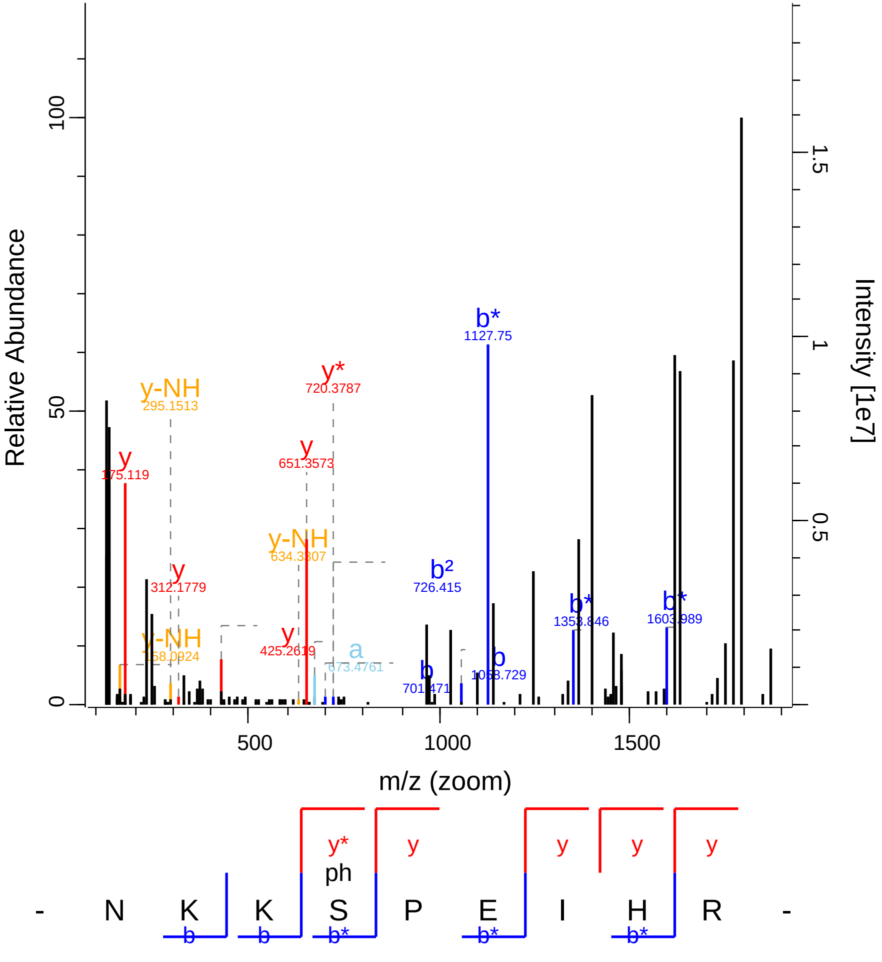

Raw File	Scan	Method	Score	m/z	Gene names
RO-QC-160121_ZackD_SS_Set_01_Fraction	8440	FTMS; HC	112.63	618.31	SRRM2

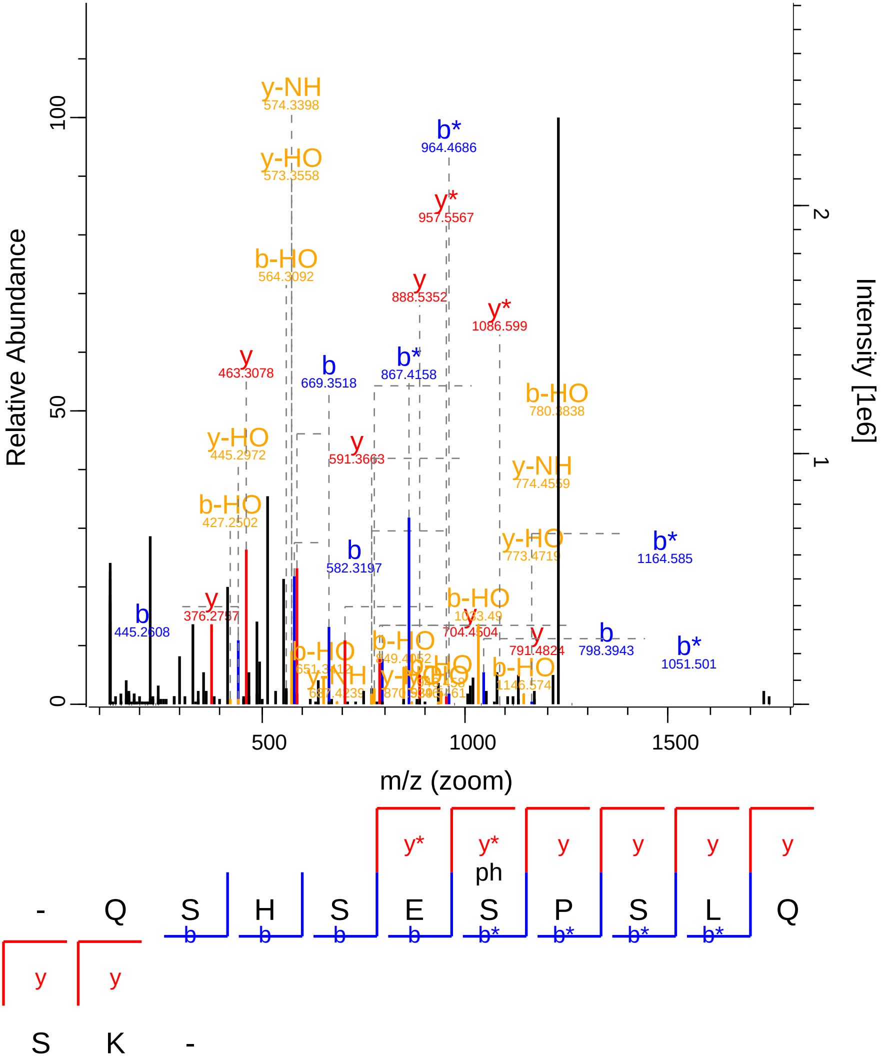

Raw File	Scan	Method	Score	m/z	Gene names
RO-QC-160121_ZackD_SS_Set_01_Fractioı	10529	FTMS; HC	126.02	659.08	ACIN1

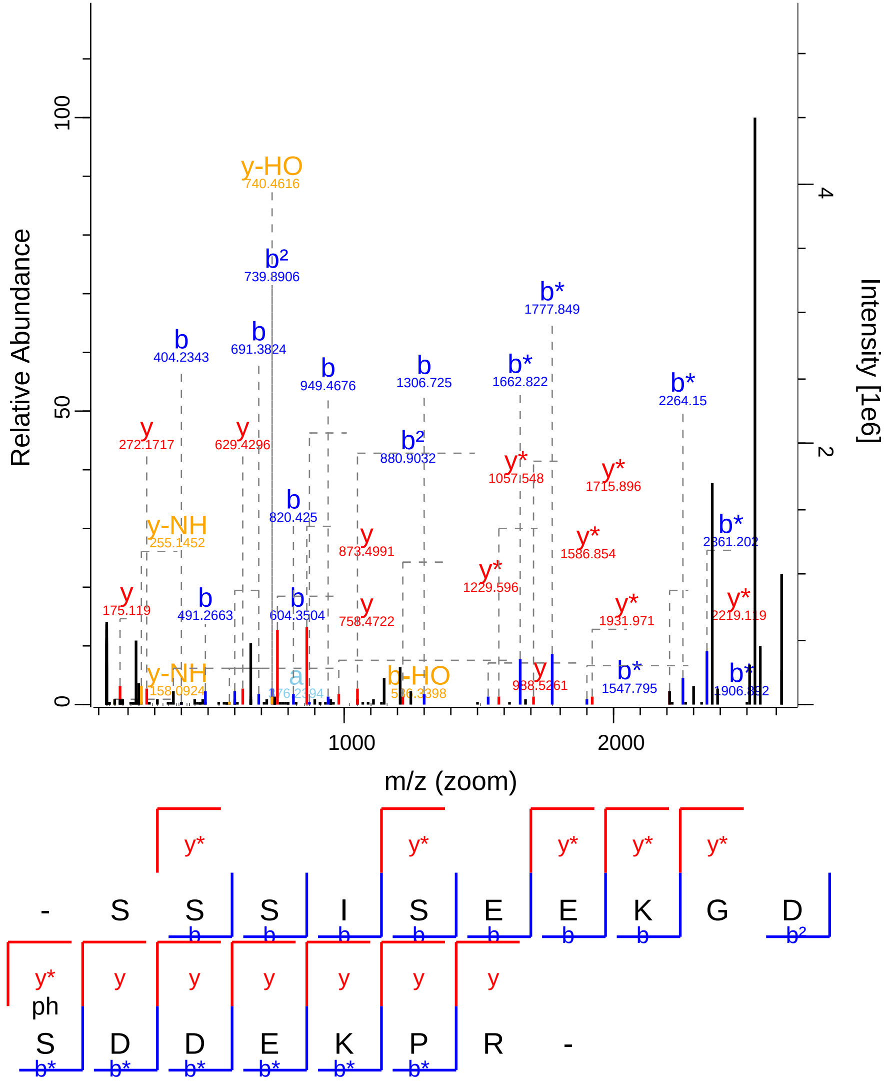

Raw File	Scan	Method	Score	m/z	Gene names
RO-QC-160121_ZackD_SS_Set_01_Fractior	11976	FTMS; HC	56.56	915.99	TRIM36

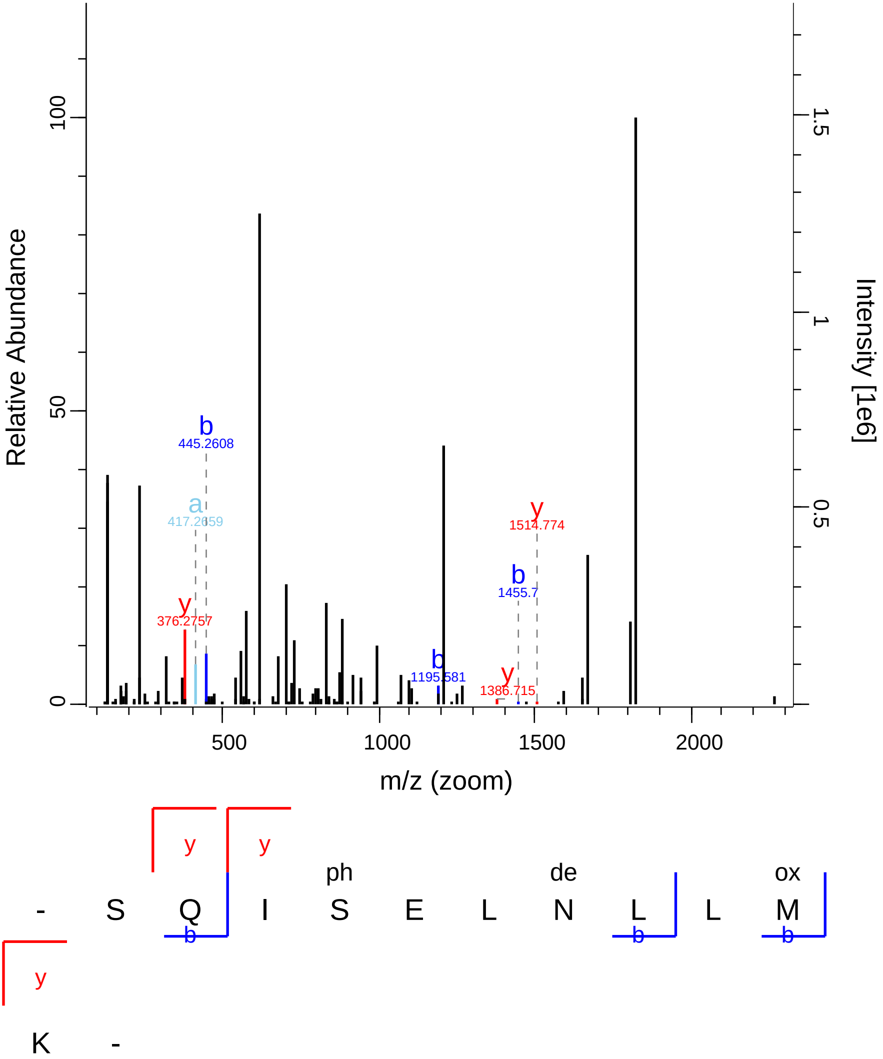

Raw File	Scan	Method	Score	m/z	Gene names
RO-QC-160121_ZackD_SS_Set_01_Fractior	12833	FTMS; HC	141	946.48	ADD1

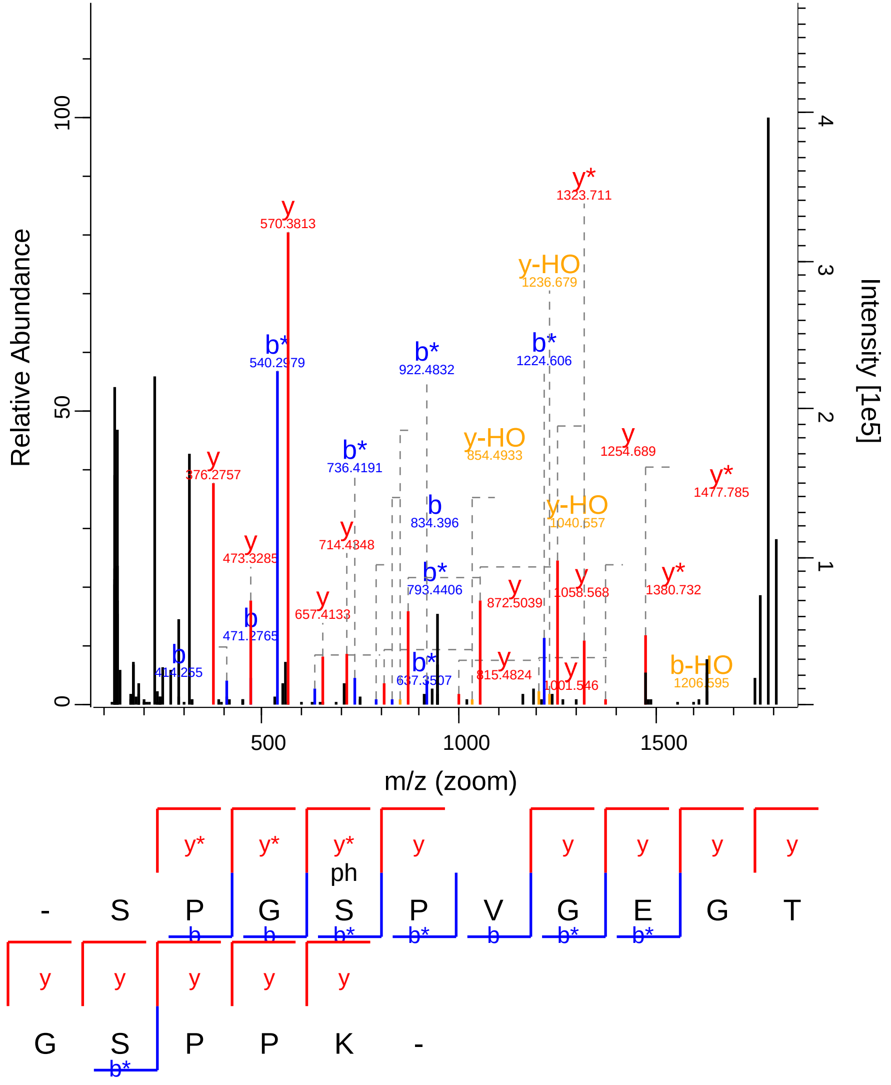

Raw File	Scan	Method	Score	m/z	Gene names
RO-QC-160121_ZackD_SS_Set_01_Fractior	14004	FTMS; HC	108.59	927.49	SPECC1

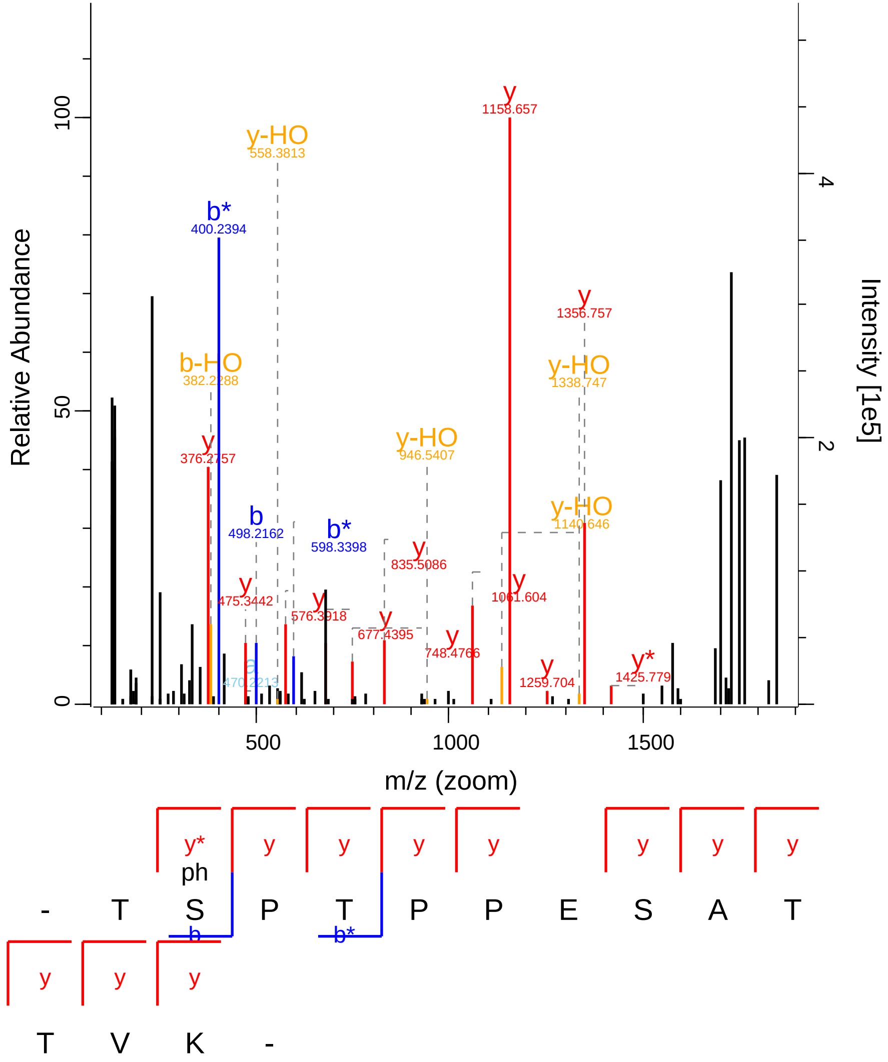

Raw File	Scan	Method	Score	m/z	Gene names
RO-QC-160121_ZackD_SS_Set_01_Fractio	15373	FTMS; HC	98.51	1119.53	SLC4A7

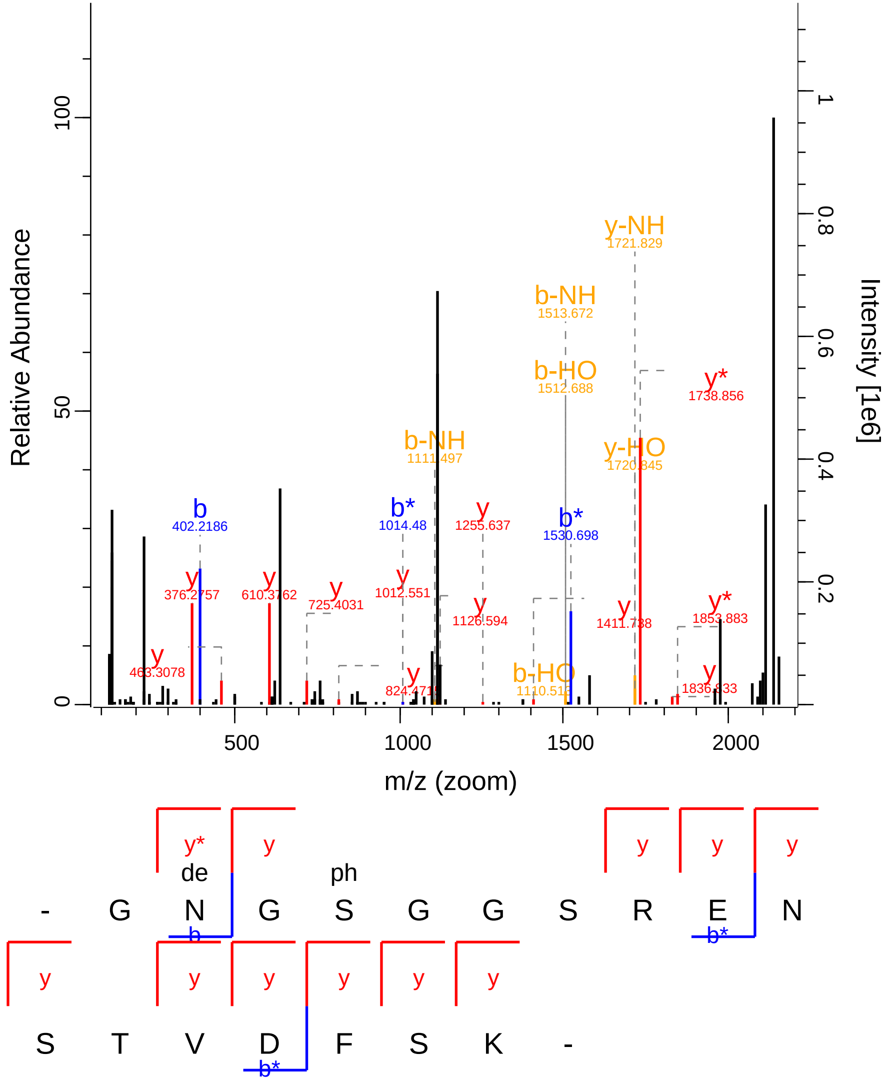

Raw File	Scan	Method	Score	m/z	Gene names
RO-QC-160121_ZackD_SS_Set_01_Fractio	15667	FTMS; HC	108.15	991.46	GSK3A

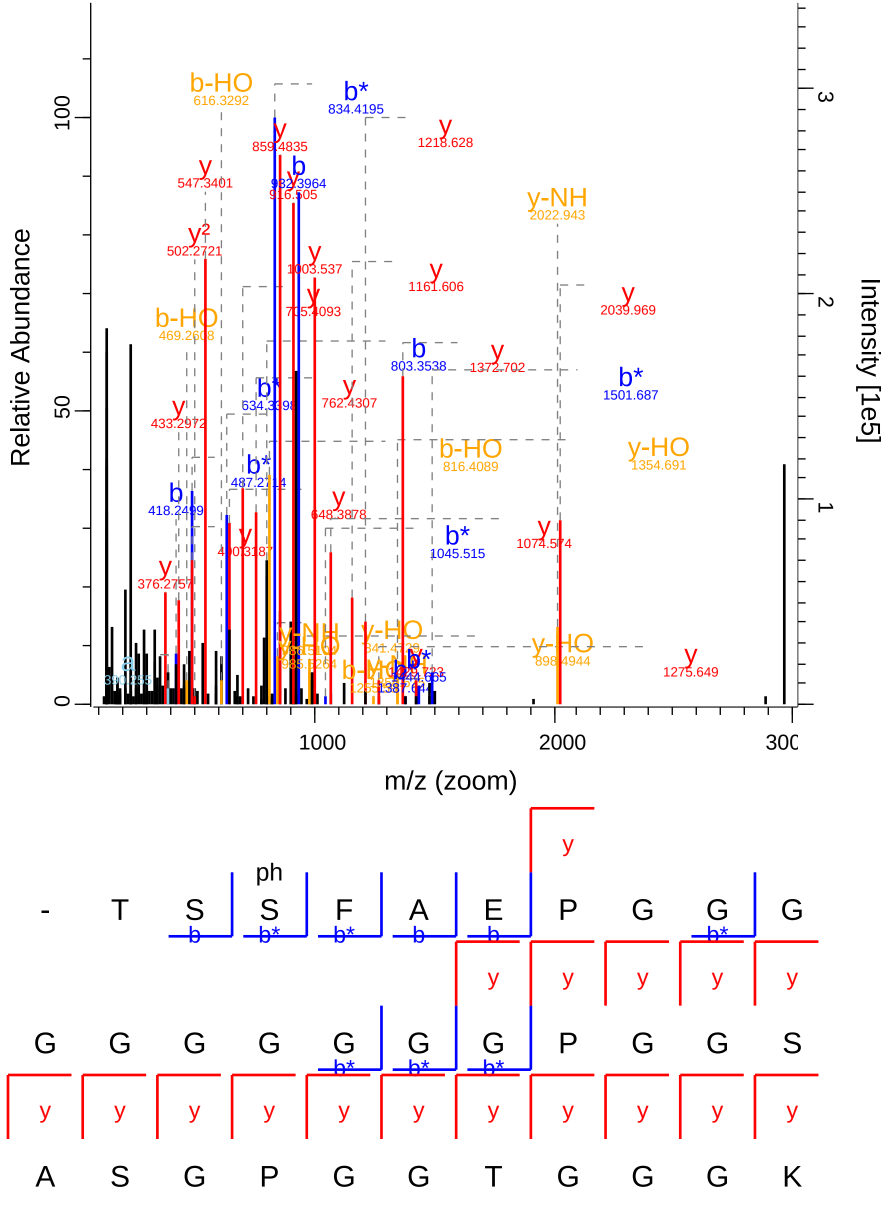

Raw File	Scan	Method	Score	m/z	Gene names
RO-QC-160121_ZackD_SS_Set_01_Fractio	16302	FTMS; HC	146.89	855.76	ZMYM4

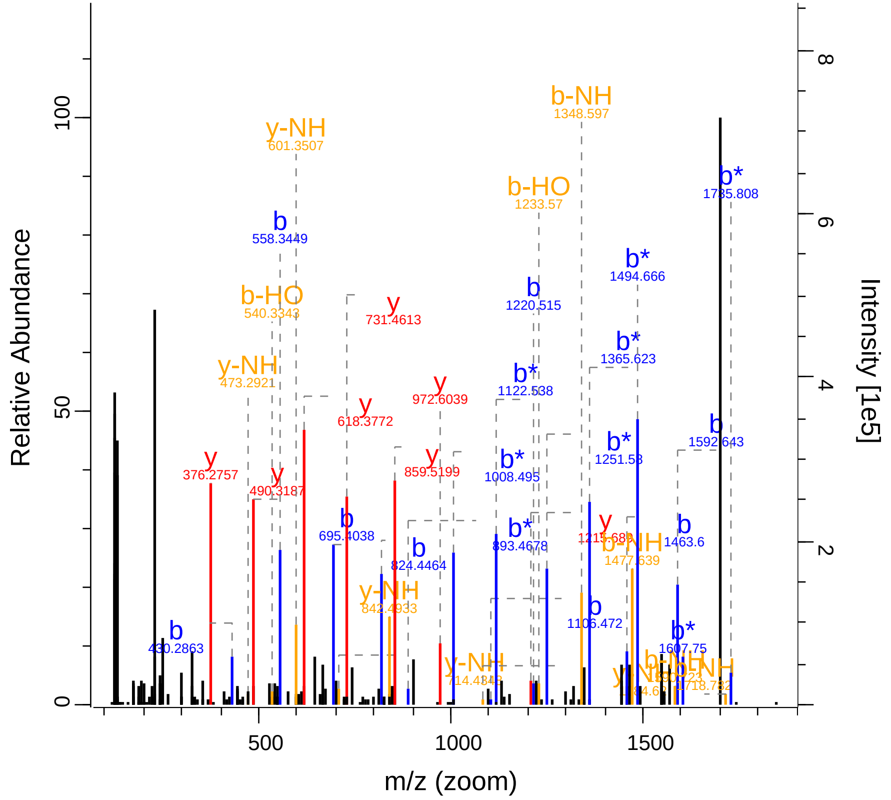

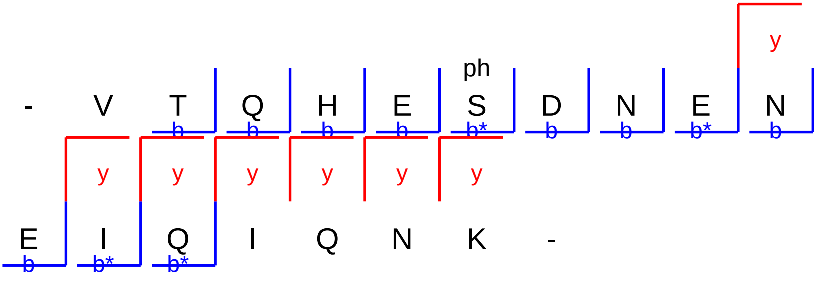

Raw File	Scan	Method	Score	m/z	Gene names
RO-QC-160121_ZackD_SS_Set_01_Fractio	17059	FTMS; HC	164.97	794.89	MTDH

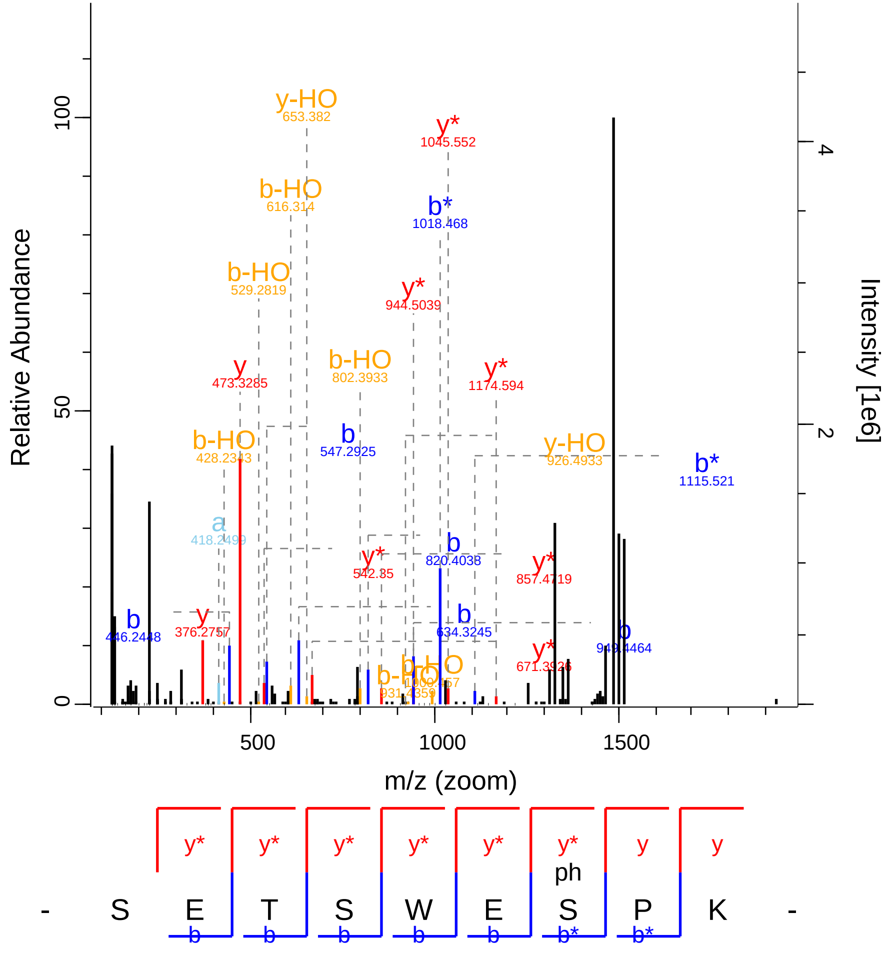

Raw File	Scan	Method	Score	m/z	Gene names
RO-QC-160121_ZackD_SS_Set_01_Fractior	17212	FTMS; HC	96.14	876.96	DAP

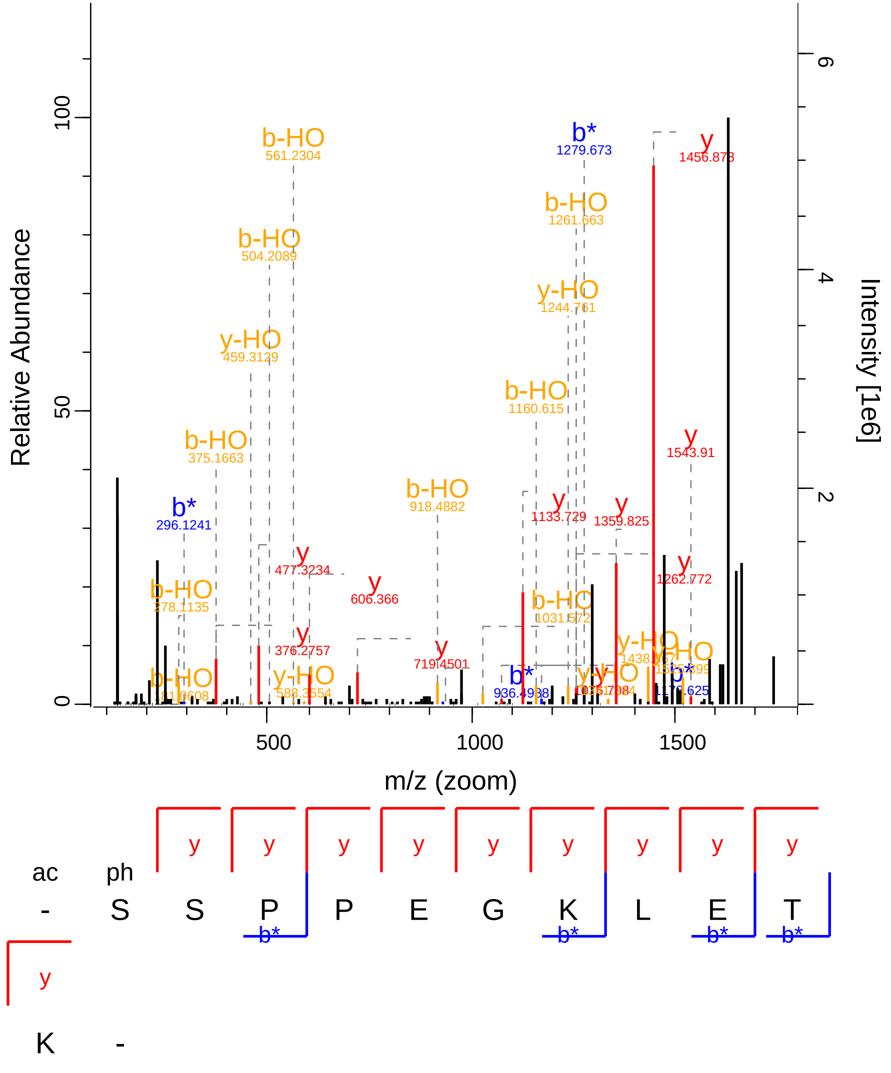

Raw File	Scan	Method	Score	m/z	Gene names
RO-QC-160121_ZackD_SS_Set_01_Fraction	18509	FTMS; HC	132.69	828.9	TMPO

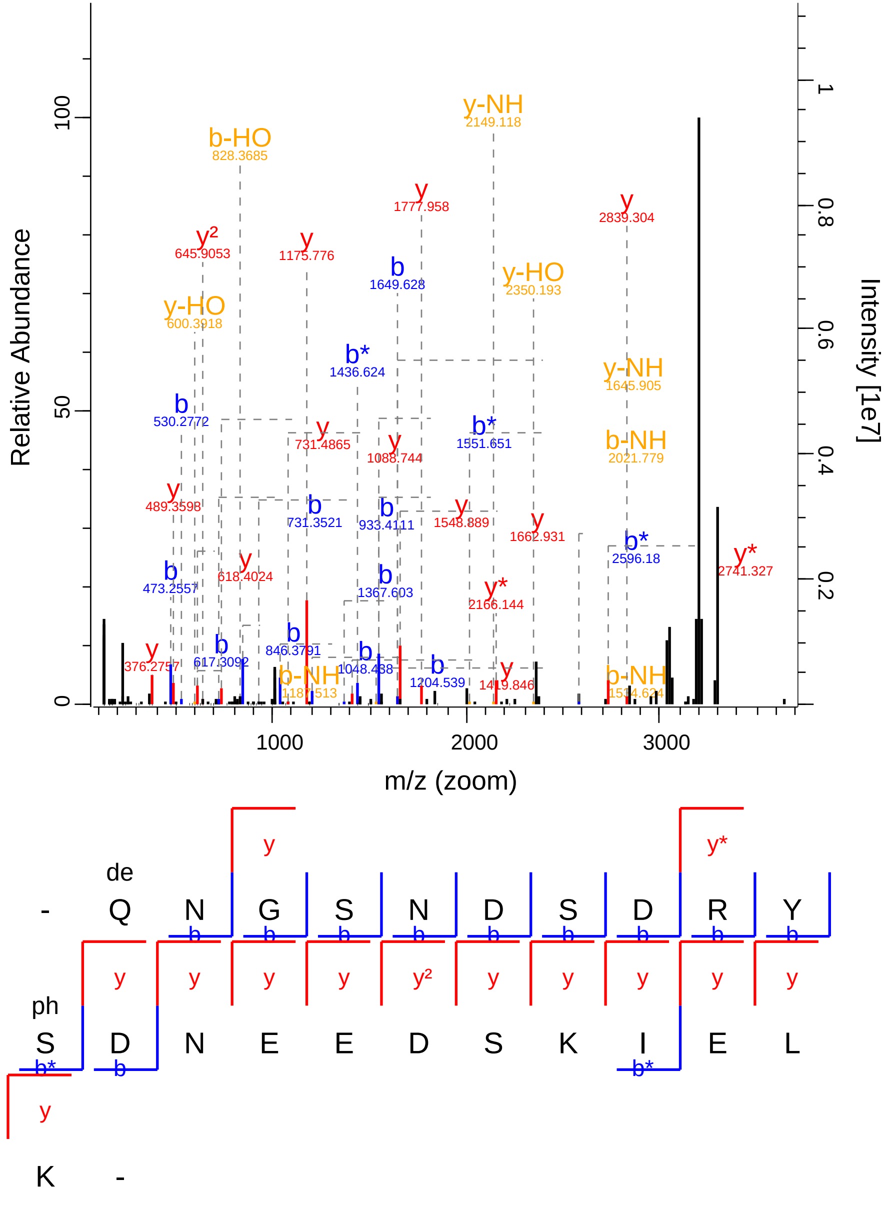

Raw File	Scan	Method	Score	m/z	Gene names
RO-QC-160121_ZackD_SS_Set_01_Fractio	18990	FTMS; HC	107.39	763.86	FLNA

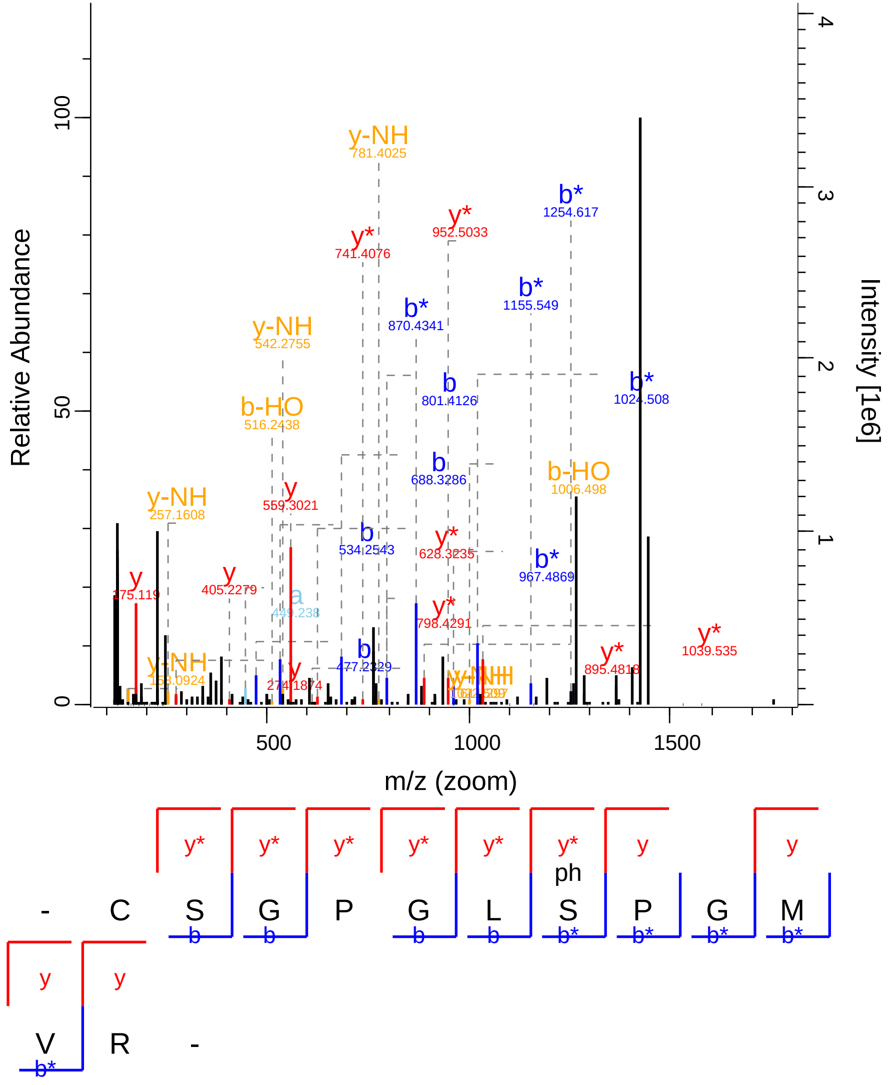

Raw File	Scan	Method	Score	m/z	Gene names
RO-QC-160121_ZackD_SS_Set_01_Fraction	22292	FTMS; HC	118.29	813.7	CD44

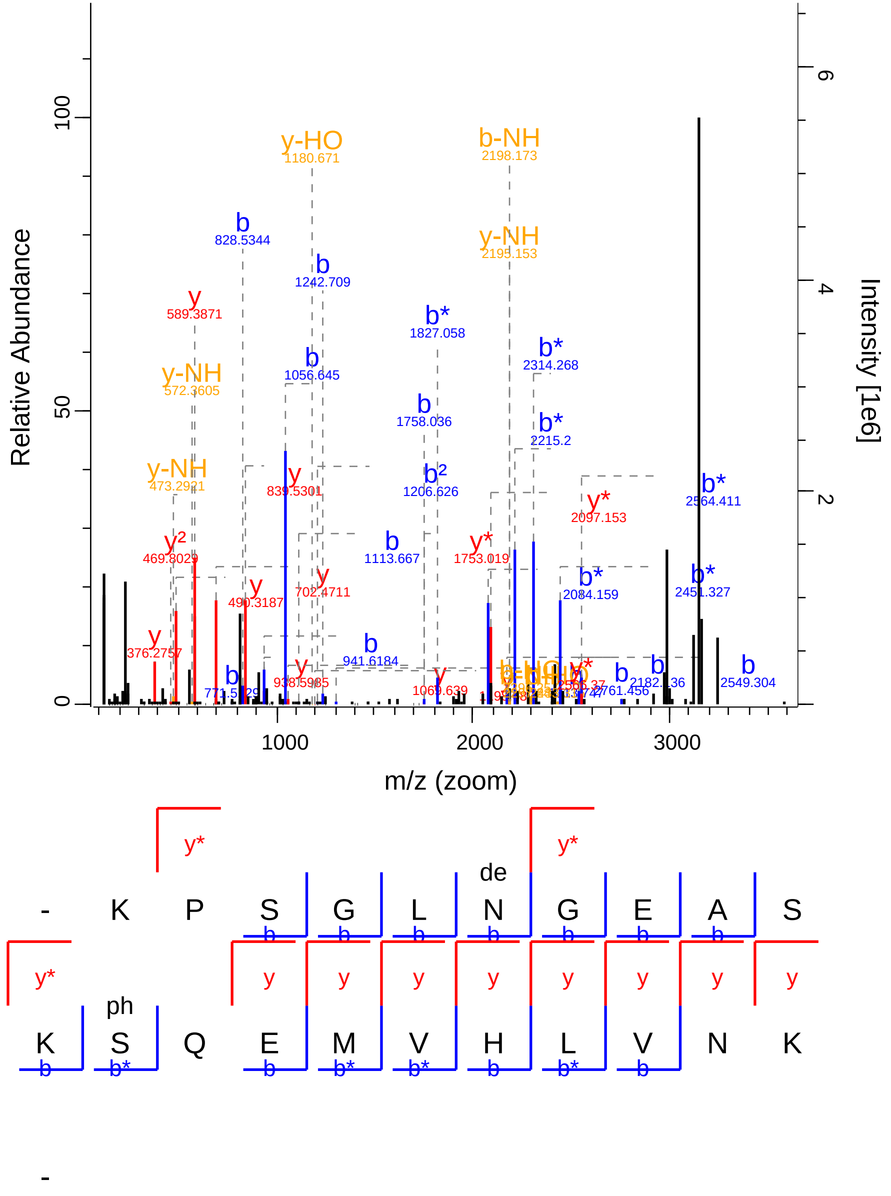

Raw File	Scan	Method	Score	m/z	Gene names
RO-QC-160121_ZackD_SS_Set_01_Fractio	26216	FTMS; HC	48.09	1049.52	ZNF107

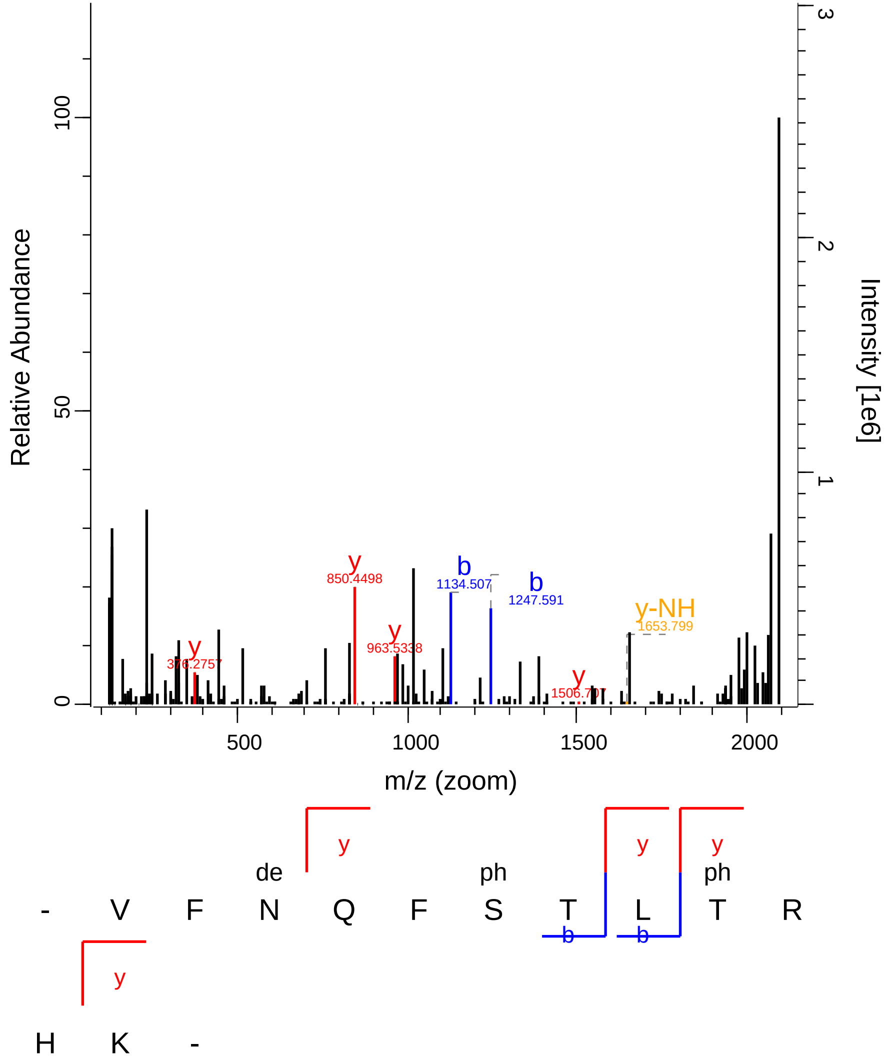

Raw File	Scan	Method	Score	m/z	Gene names
RO-QC-160121_ZackD_SS_Set_01_Fractior	26304	FTMS; HC	69.34	483.93	ZNF770

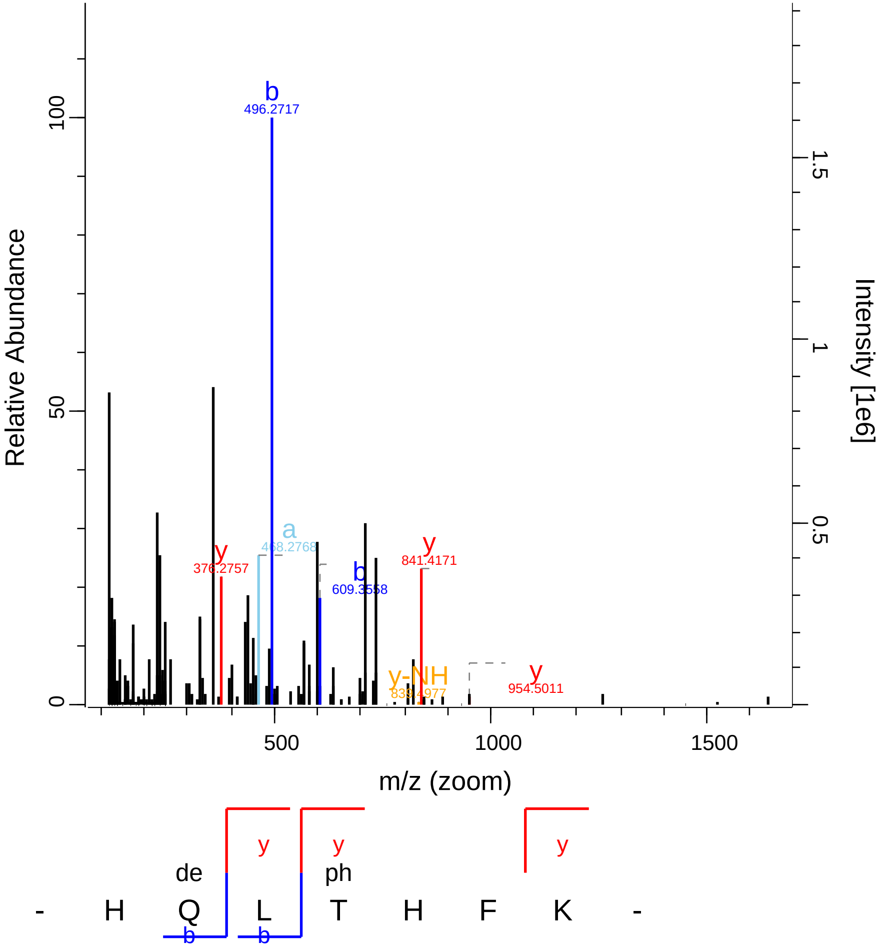

Raw File	Scan	Method	Score	m/z	Gene names
RO-QC-160121_ZackD_SS_Set_01_Fractio	26729	FTMS; HC	141.97	1133.13	PPP1R12A

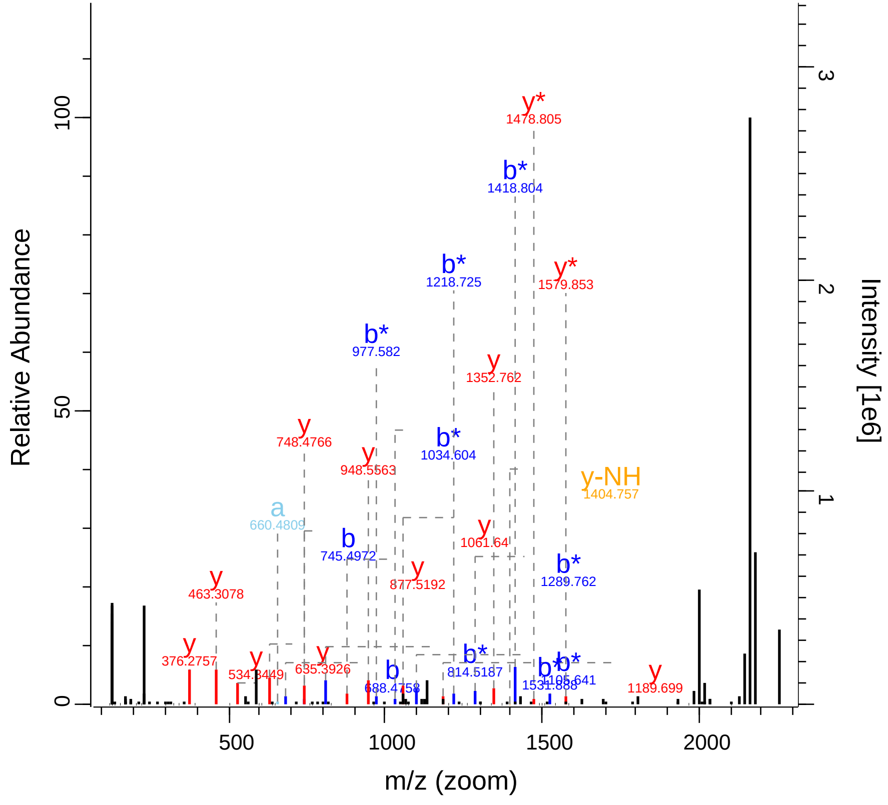

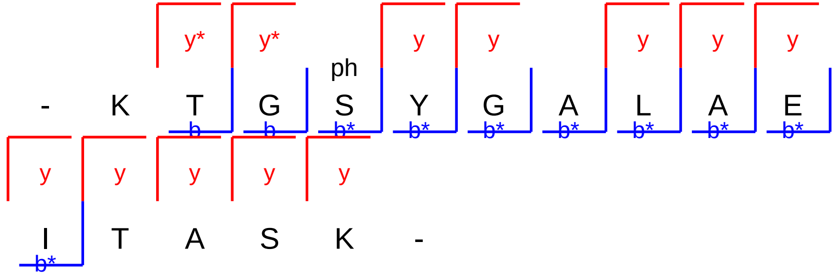

Raw File	Scan	Method	Score	m/z	Gene names
RO-QC-160121_ZackD_SS_Set_01_Fractio	26969	FTMS; HC	53.68	1165.14	FHOD3

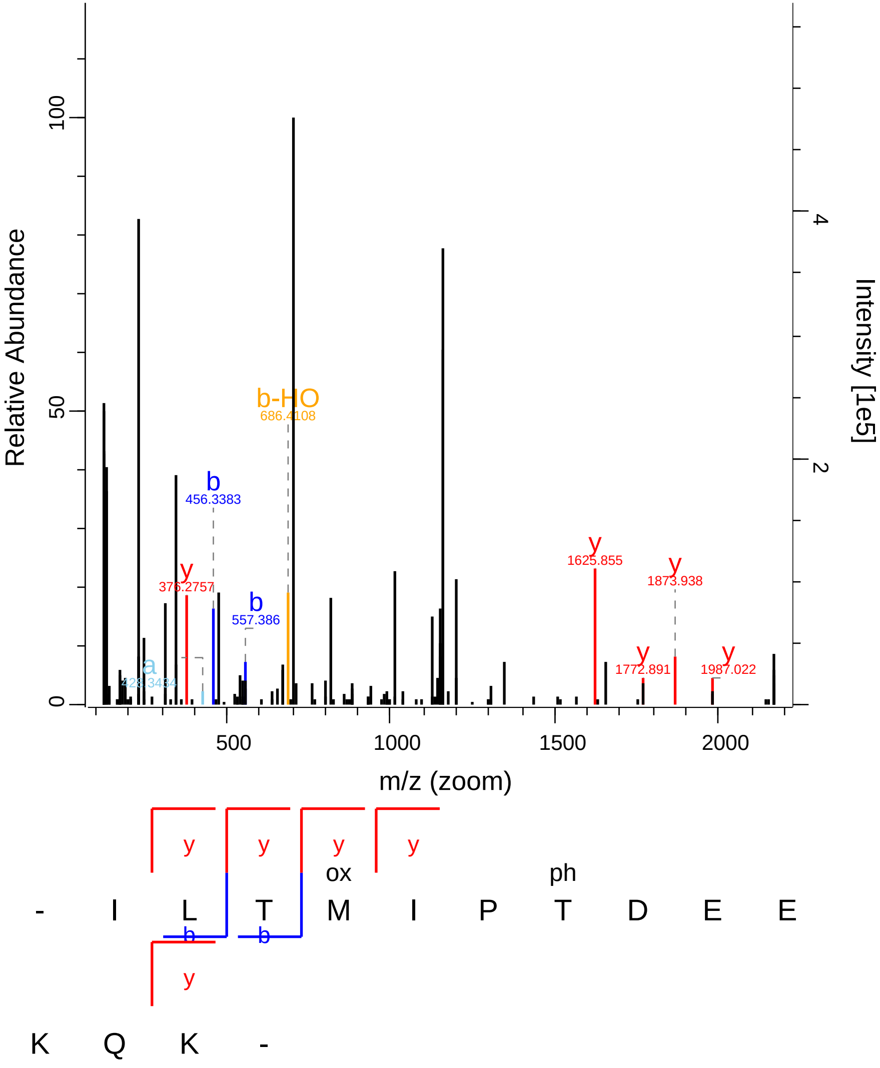

Raw File	Scan	Method	Score	m/z	Gene names
RO-QC-160121_ZackD_SS_Set_01_Fraction	27045	FTMS; HC	67.43	708.9	SLC6A15

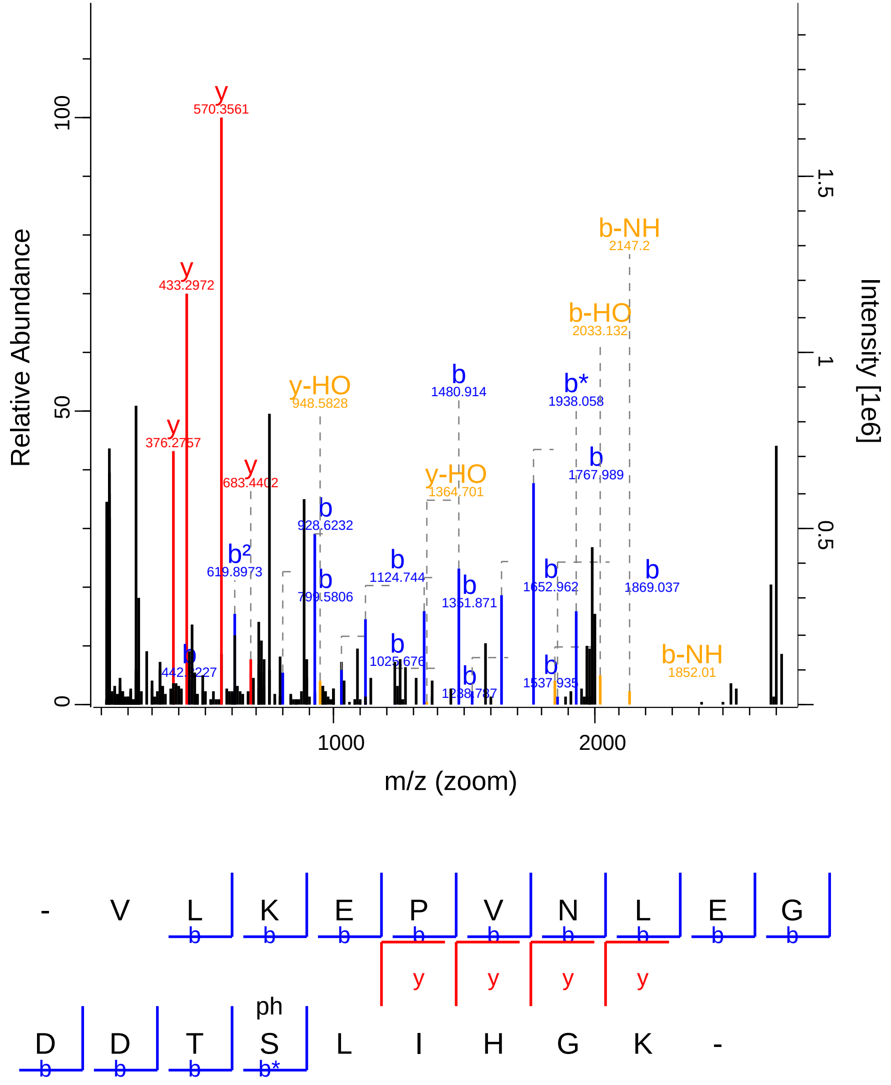

Raw File	Scan	Method	Score	m/z	Gene names
RO-QC-160121_ZackD_SS_Set_01_Fractio	28729	FTMS; HC	52.79	1379.74	MAMDC2

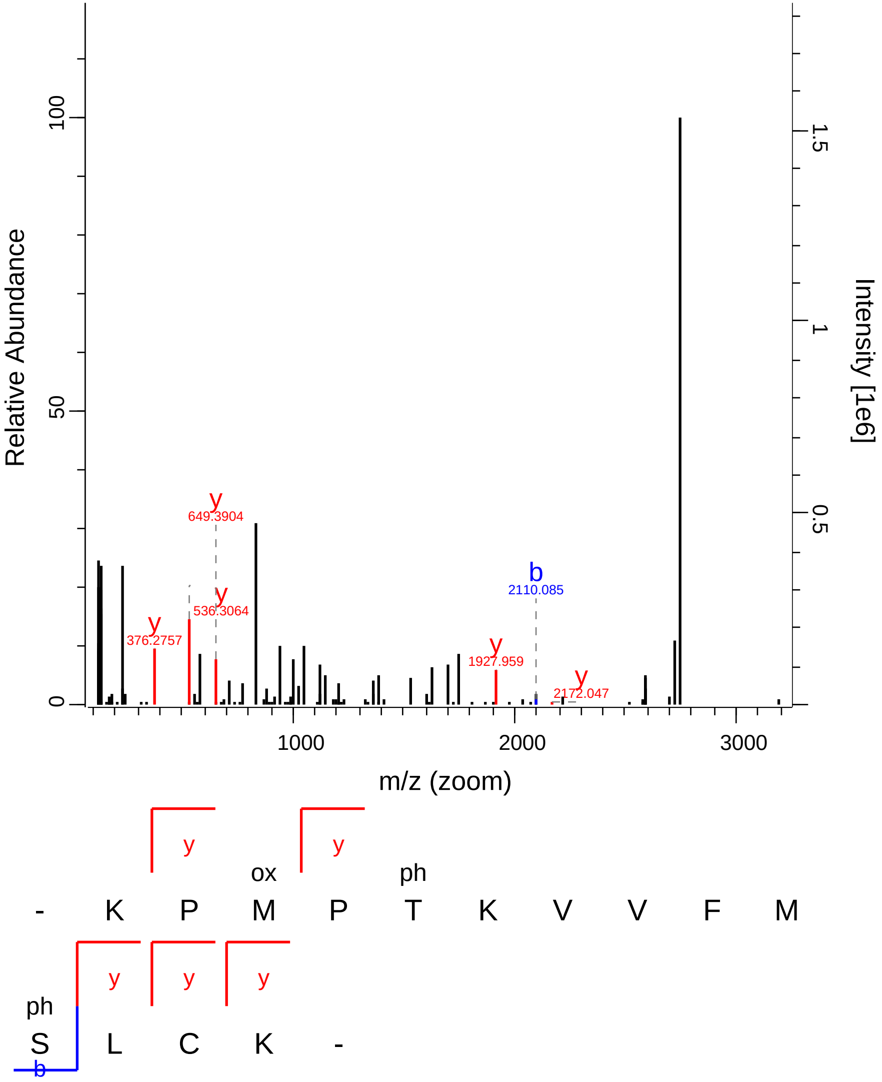

Raw File	Scan	Method	Score	m/z	Gene names
RO-QC-160121_ZackD_SS_Set_01_Fractior	28789	FTMS; HC	85.08	577.66	CDK16

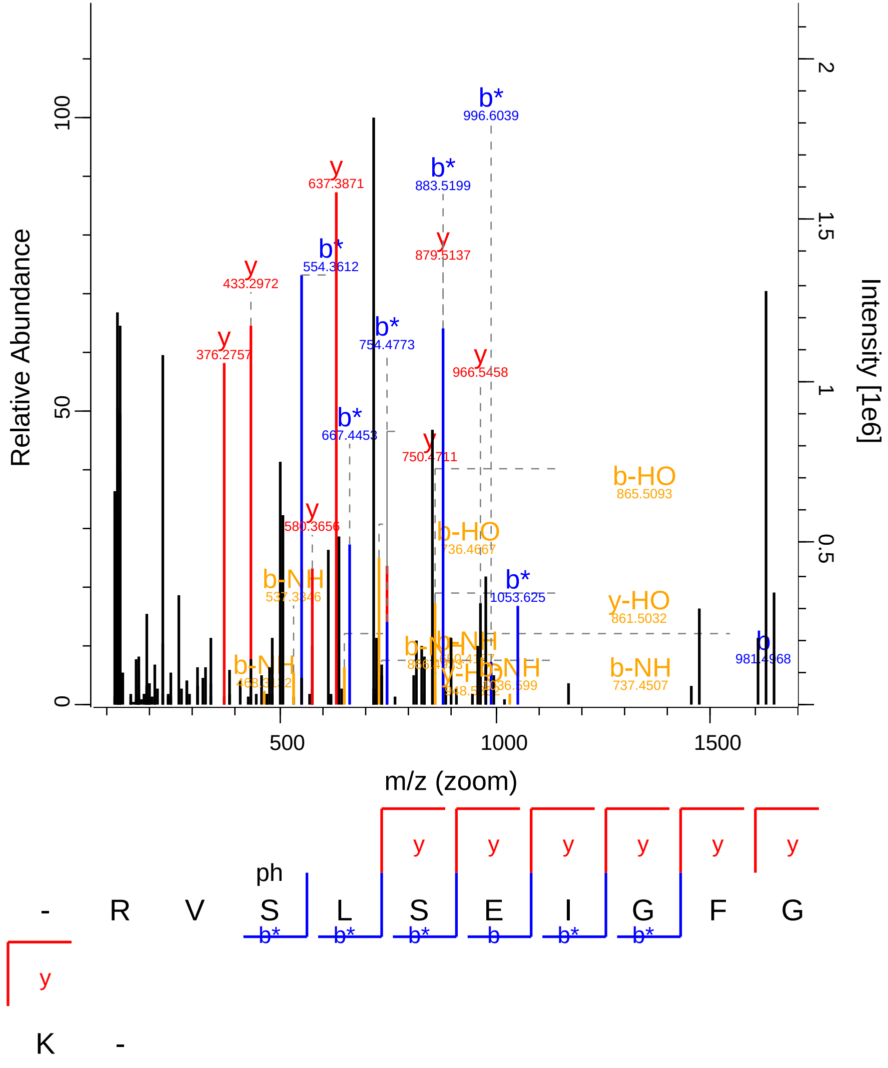

Raw File	Scan	Method	Score	m/z	Gene names
RO-QC-160121_ZackD_SS_Set_01_Fractio	29020	FTMS; HC	252.69	1290.28	TRIM28

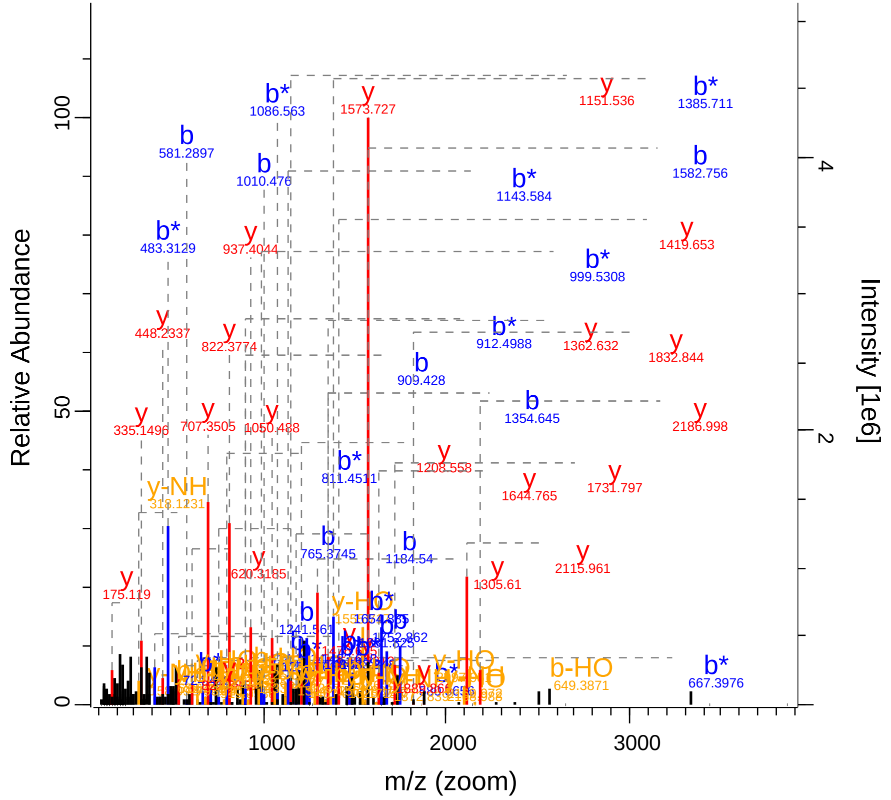

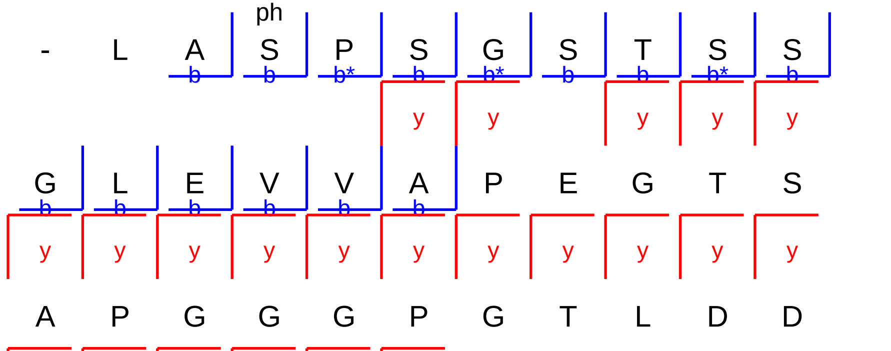

Raw File	Scan	Method	Score	m/z	Gene names
RO-QC-160121_ZackD_SS_Set_01_Fractio	30103	FTMS; HC	263.45	1248.58	SLC12A4

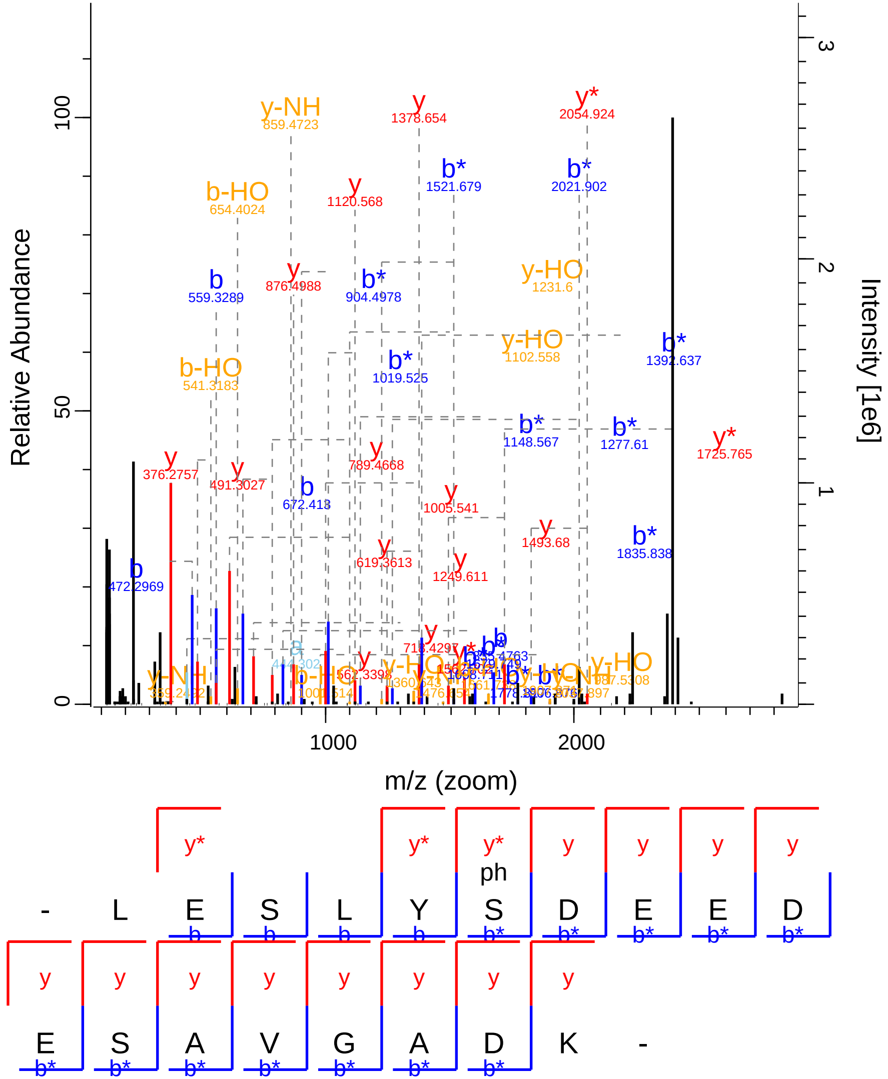

Raw File	Scan	Method	Score	m/z	Gene names
RO-QC-160121_ZackD_SS_Set_01_Fractior	30116	FTMS; HC	90.39	775.44	AHNAK

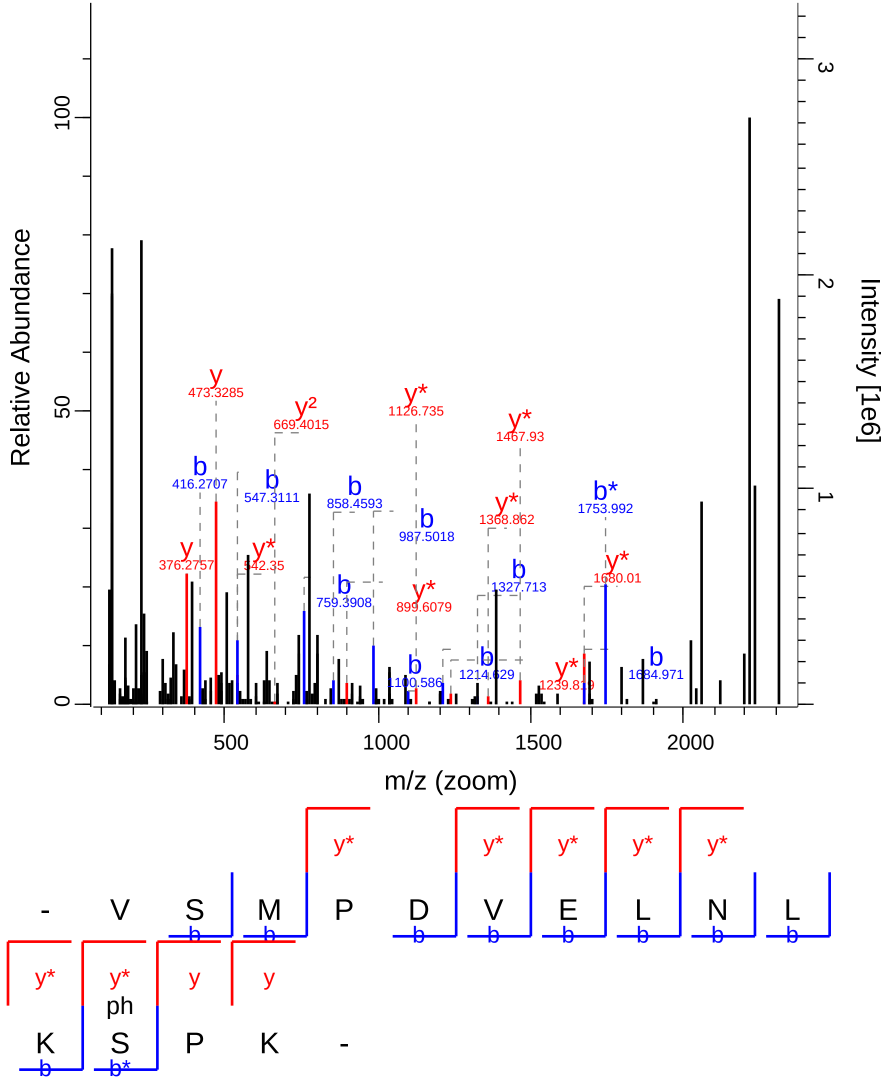

Raw File	Scan	Method	Score	m/z	Gene names
RO-QC-160121_ZackD_SS_Set_01_Fractior	30314	FTMS; HC	49.12	842.16	SIRT7

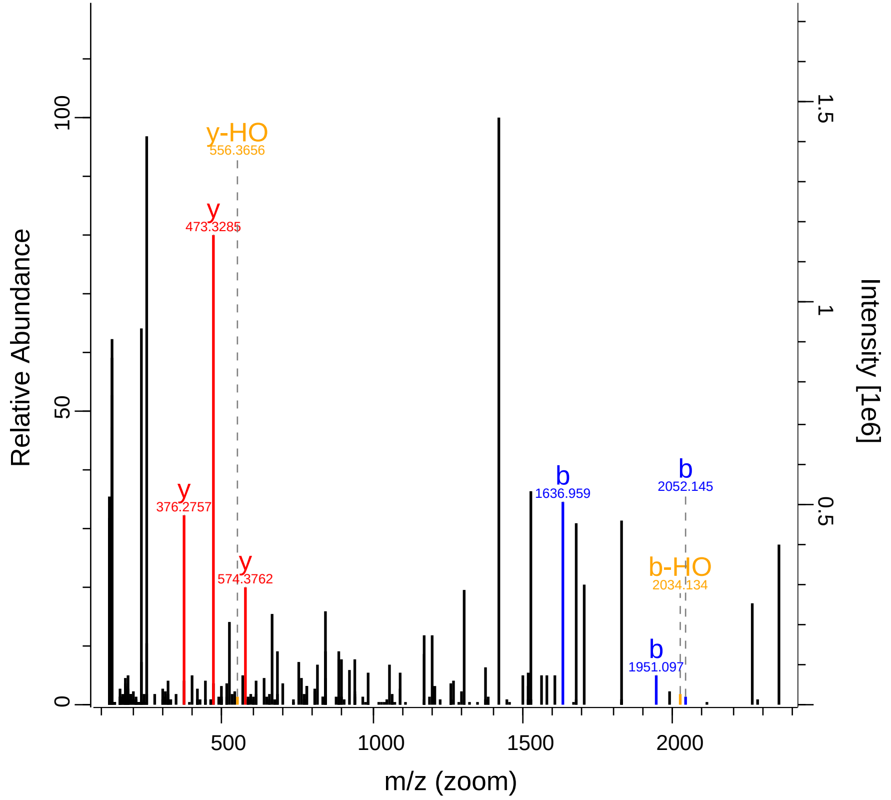

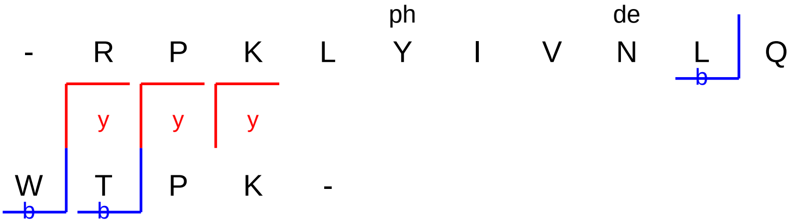

Raw File	Scan	Method	Score	m/z	Gene names
RO-QC-160121_ZackD_SS_Set_01_Fractior	31820	FTMS; HC	73.11	941.17	RUFY1

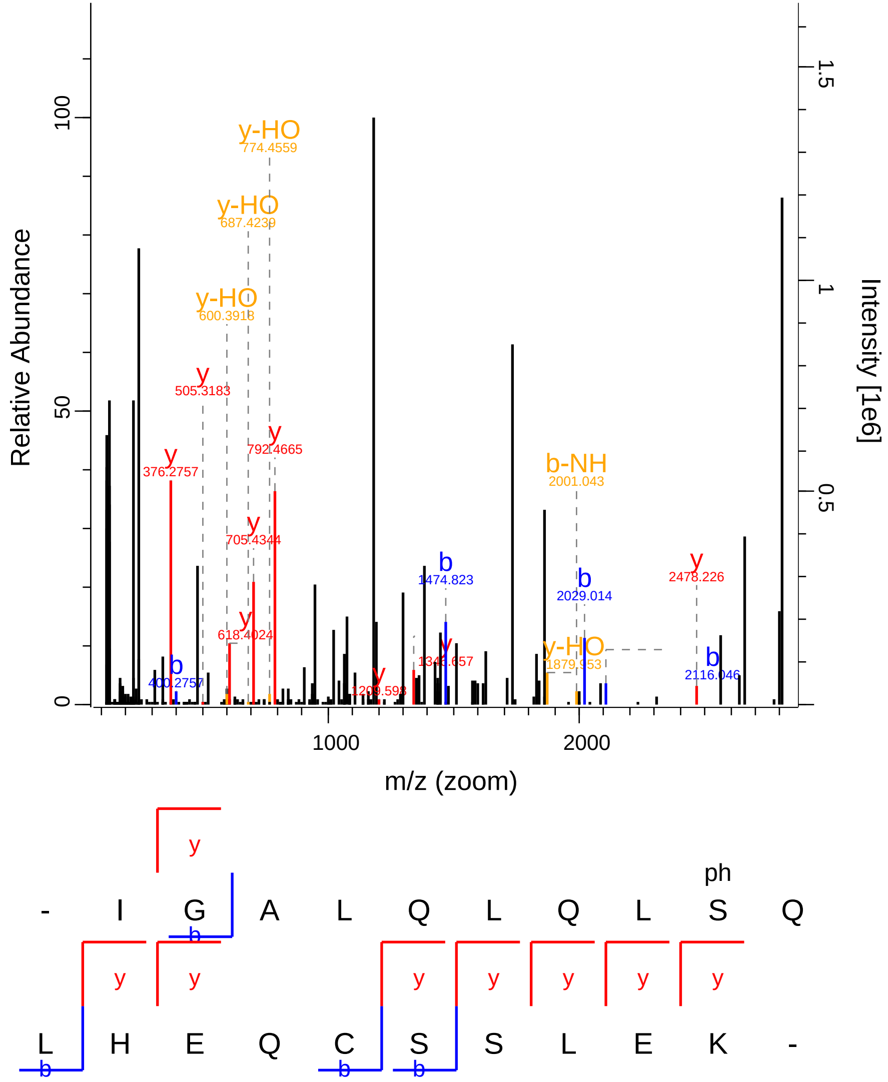

Raw File	Scan	Method	Score	m/z	Gene names
RO-QC-160121_ZackD_SS_Set_01_Fractio	33592	FTMS; HC	132.35	1004.15	MTSS1L

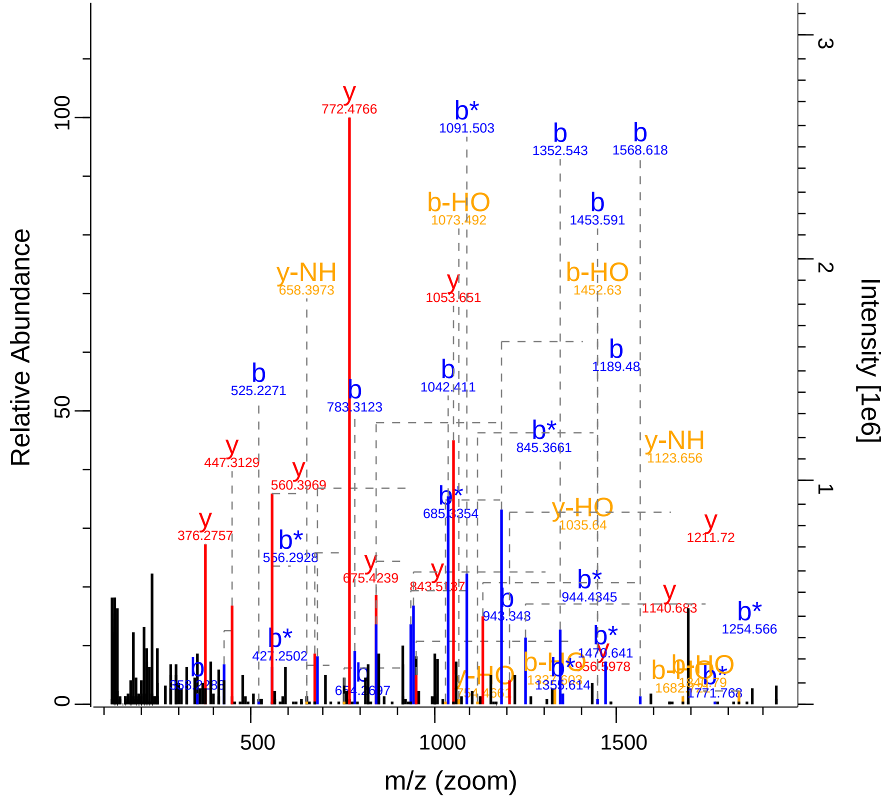

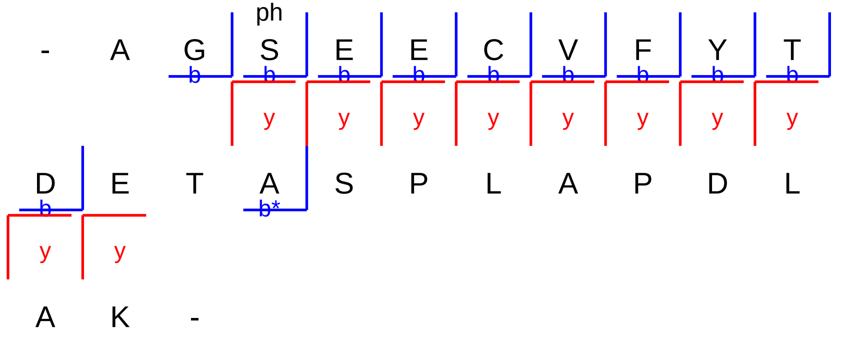

Raw File	Scan	Method	Score	m/z	Gene names
RO-QC-160121_ZackD_SS_Set_01_Fractio	34378	FTMS; HC	41.89	1041.25	MOCS1

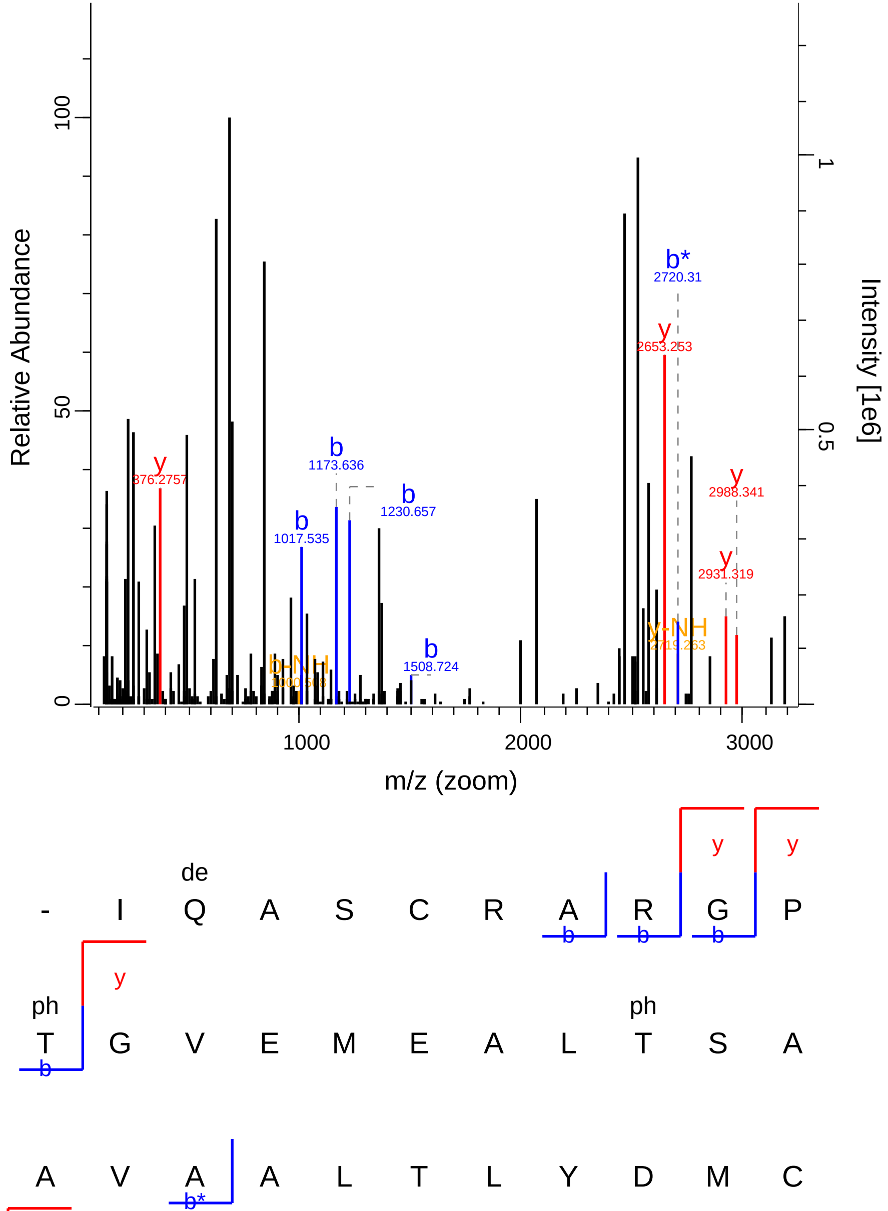

Raw File	Scan	Method	Score	m/z
RO-QC-160121_ZackD_SS_Set_01_Fraction_12	35845	FTMS; HCD	63.57	1070.09

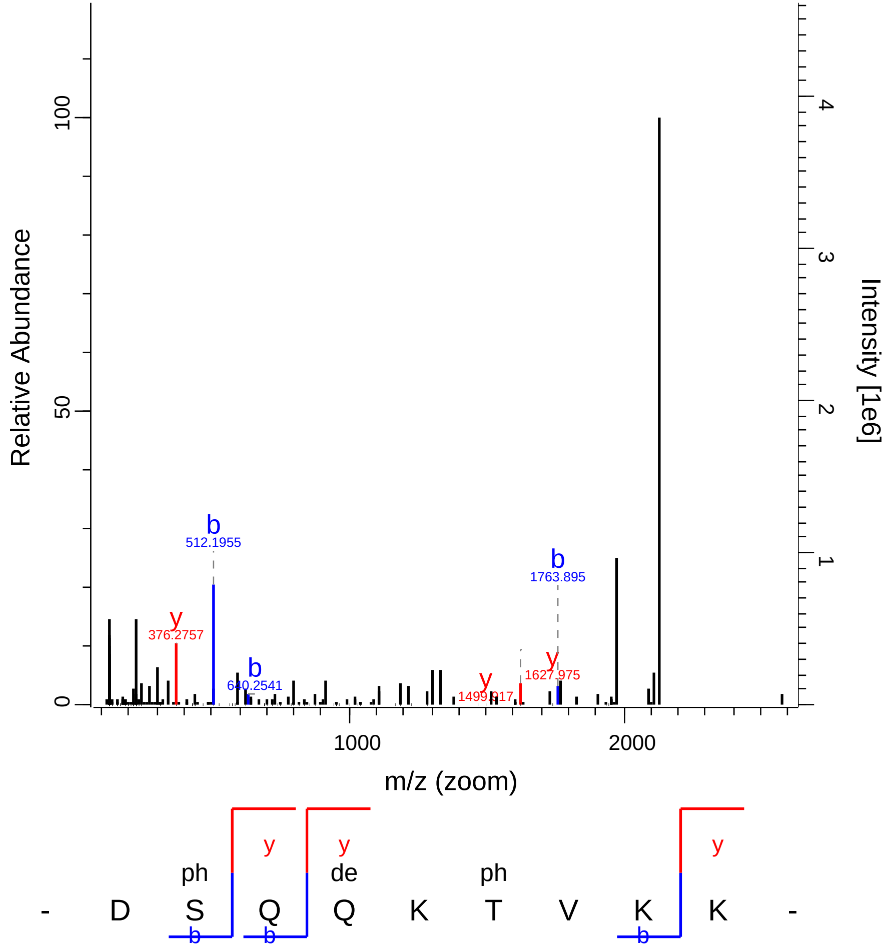

Raw File	Scan	Method	Score	m/z	Gene names
RO-QC-160121_ZackD_SS_Set_01_Fractior	37707	FTMS; HC	91.58	1117.1	RPS13

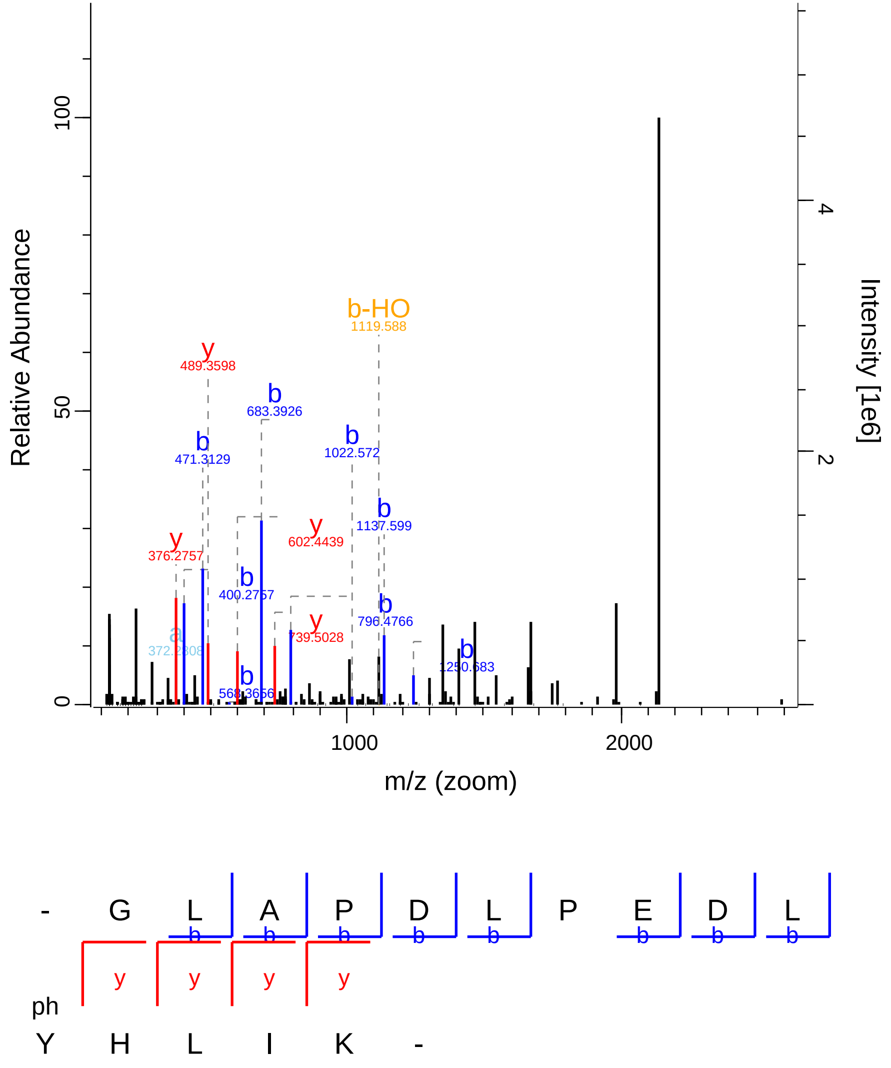

Raw File	Scan	Method	Score	m/z	Gene names
RO-QC-160121_ZackD_SS_Set_01_Fractior	37851	FTMS; HC	62.84	723.39	WWC2

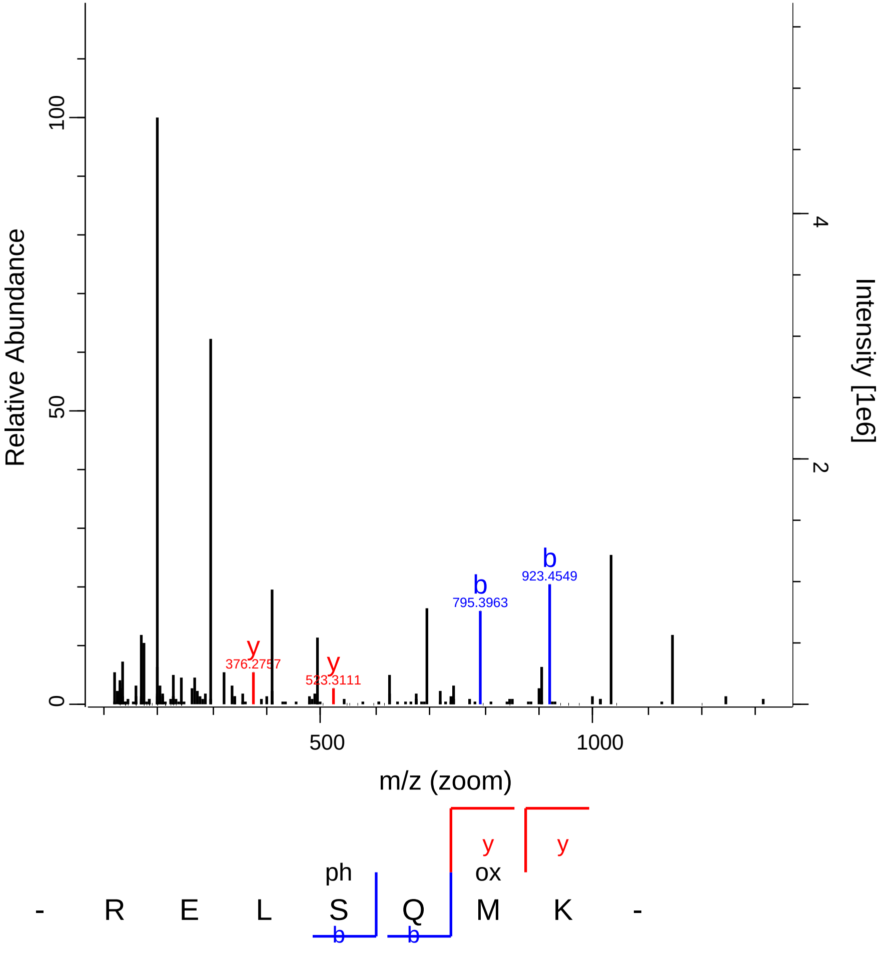

Raw File	Scan	Method	Score	m/z	Gene names
RO-QC-160121_ZackD_SS_Set_01_Fractior	37992	FTMS; HC	73.78	969.53	RPS7

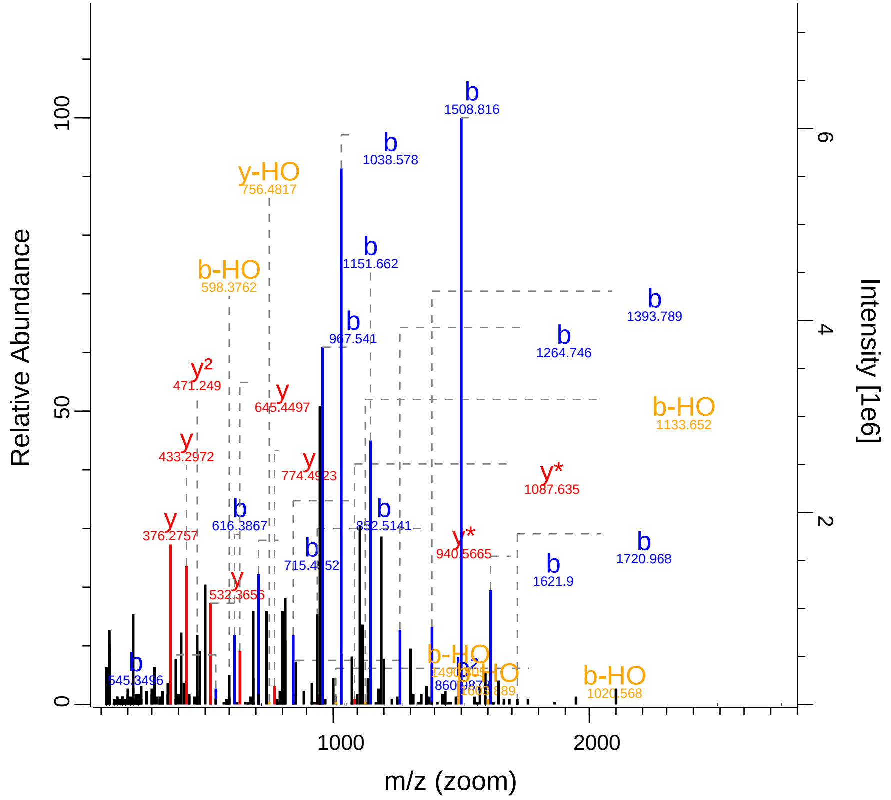

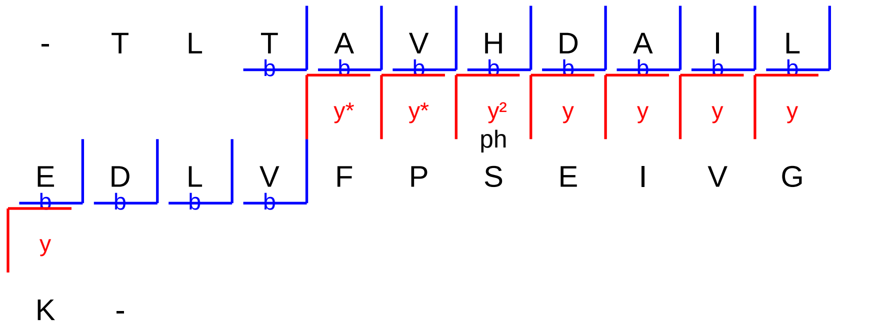

Raw File	Scan	Method	Score	m/z	Gene names
RO-QC-160121_ZackD_SS_Set_02_Fraction	4706	FTMS; HCI	132.25	637.3	TANC2

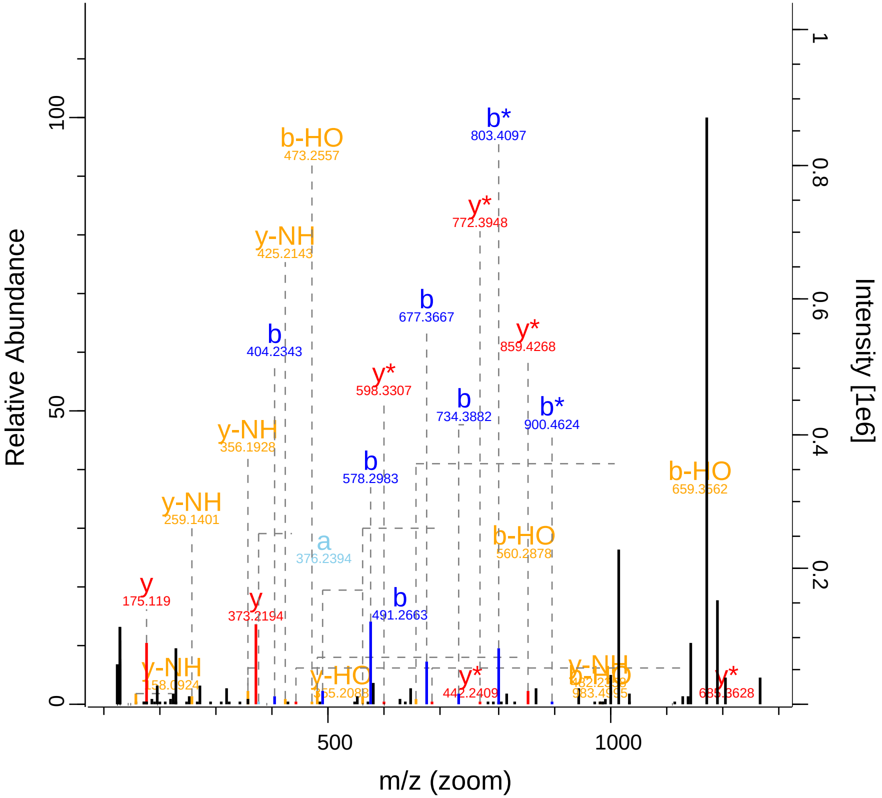

- S S S S V G S P T R

ph

-

Raw File	Scan	Method	Score	m/z	Gene names
RO-QC-160121_ZackD_SS_Set_02_Fractioı	5464	FTMS; HC	132.44	697.64	CEP350

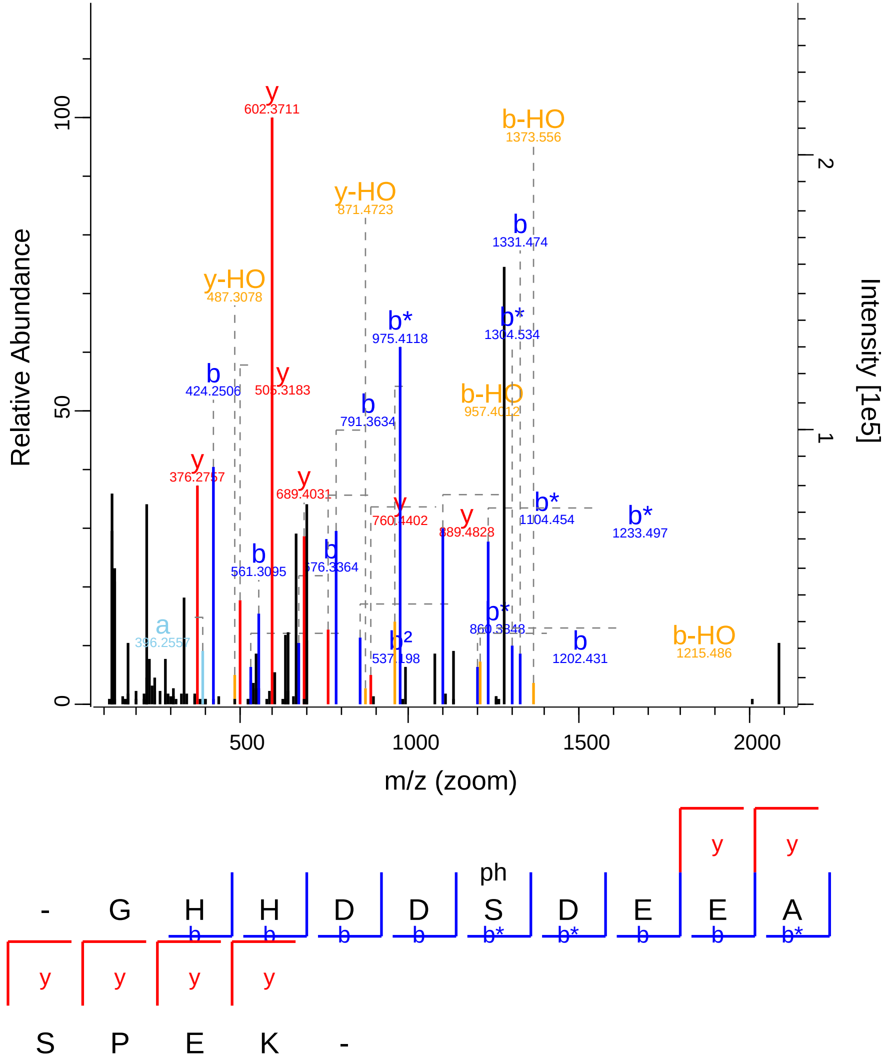

Raw File	Scan	Method	Score	m/z	Gene names
RO-QC-160121_ZackD_SS_Set_02_Fractior	5777	FTMS; HCl	67.23	659.02	RNPS1

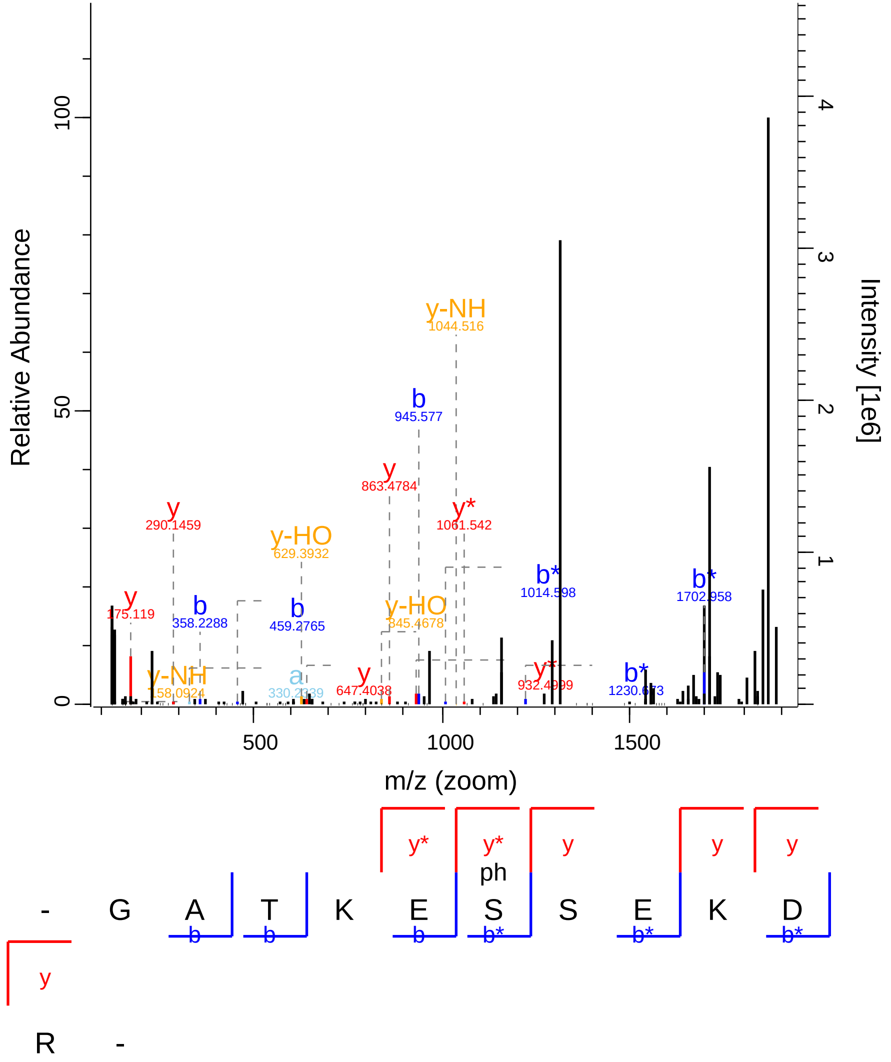

Raw File	Scan	Method	Score	m/z	Gene names
RO-QC-160121_ZackD_SS_Set_02_Fractior	6201	FTMS; HCl	47.26	485.53	SRSF4

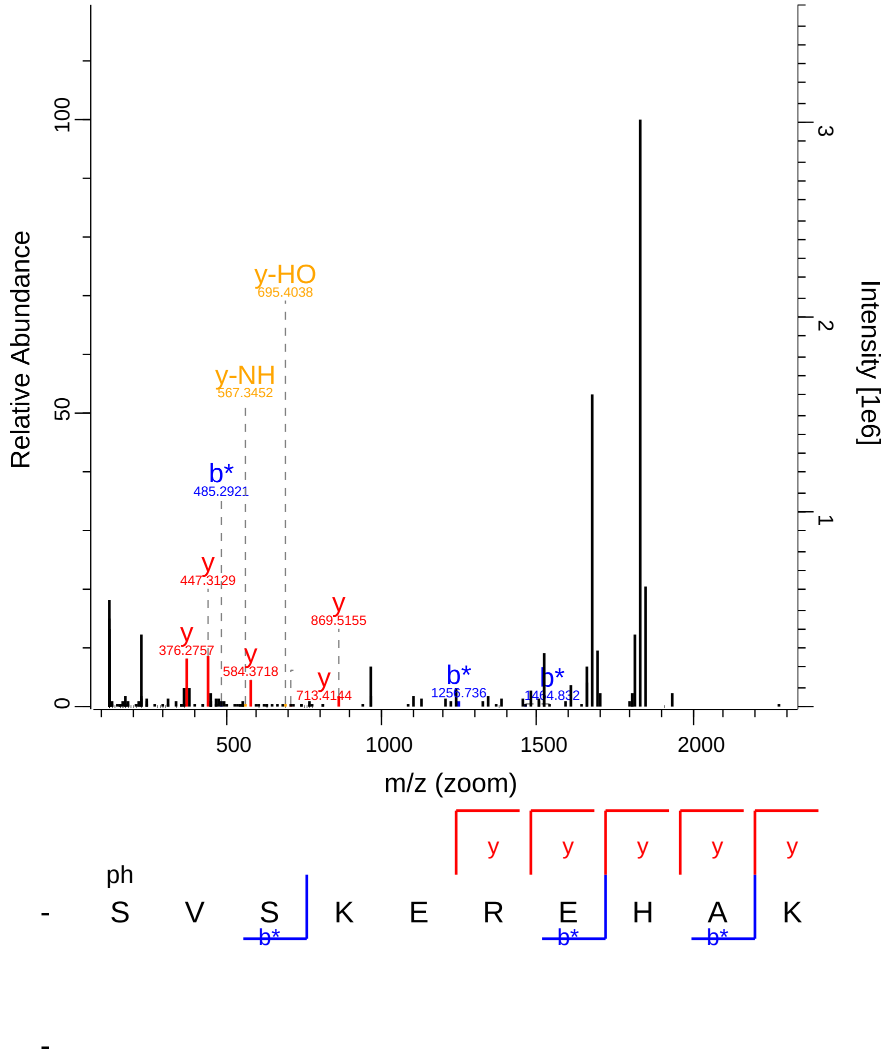

Raw File	Scan	Method	Score	m/z	Gene names
RO-QC-160121_ZackD_SS_Set_02_Fractior	6281	FTMS; HCI	60.6	613.36	NIPBL

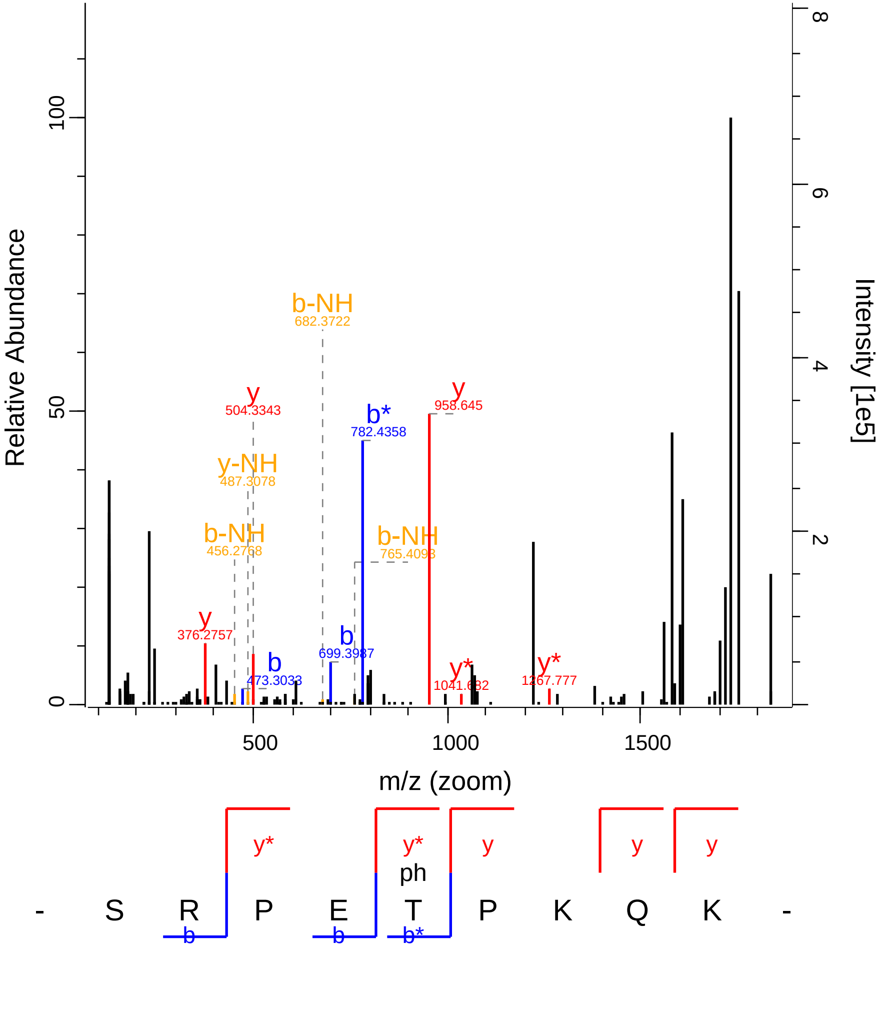

Raw File	Scan	Method	Score	m/z	Gene names
RO-QC-160121_ZackD_SS_Set_02_Fractio	6298	FTMS; HC	116.24	772.34	ZMYM4

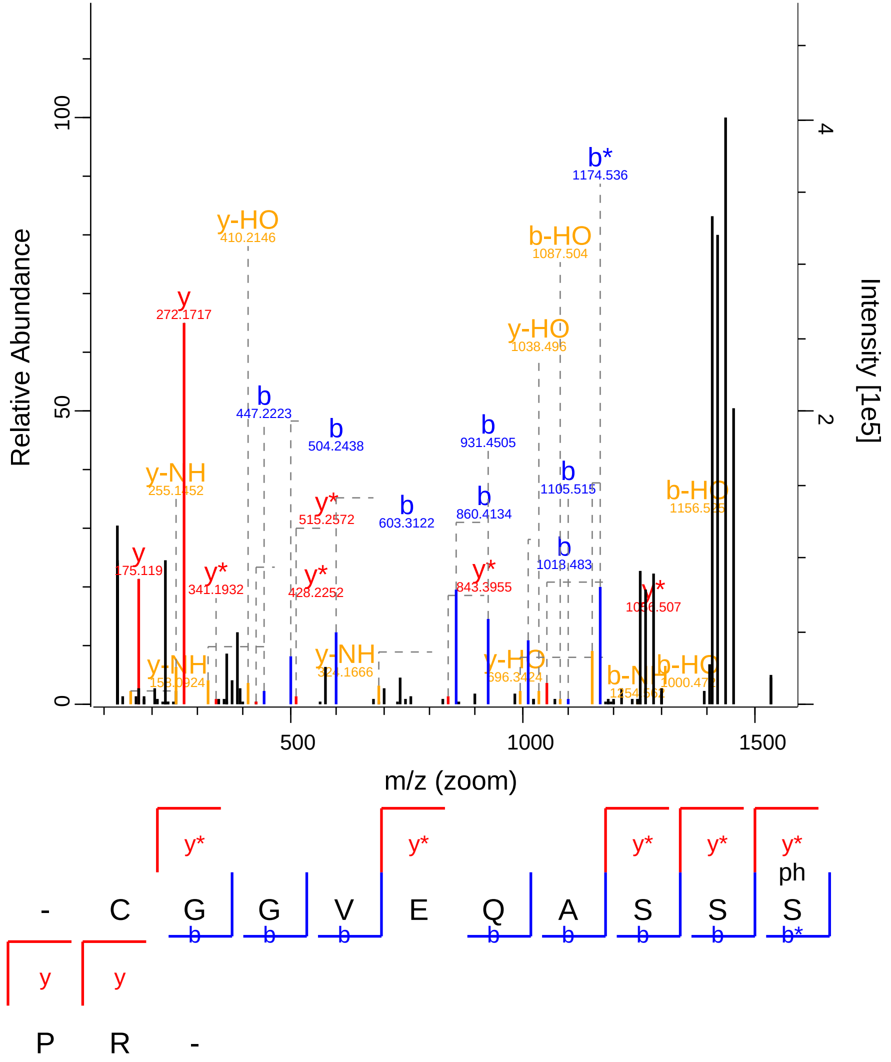

Raw File	Scan	Method	Score	m/z	Gene names
RO-QC-160121_ZackD_SS_Set_02_Fractior	6855	FTMS; HC	131.44	692.29	FTH1

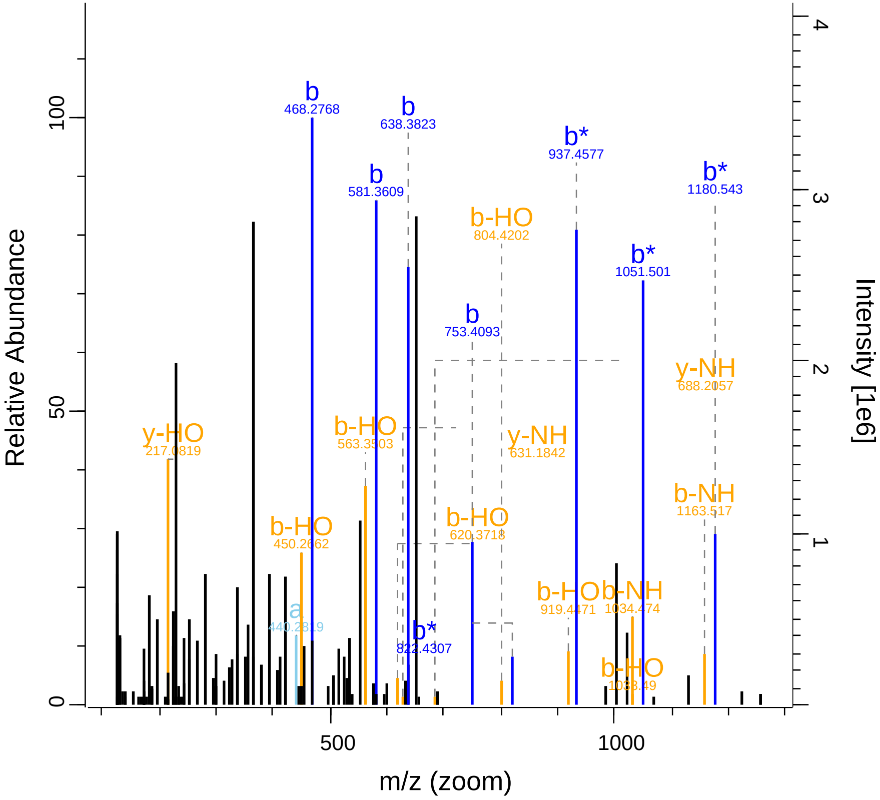

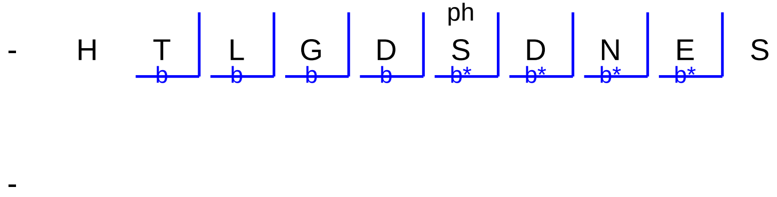

Raw File	Scan	Method	Score	m/z	Gene names
RO-QC-160121_ZackD_SS_Set_02_Fractior	7021	FTMS; HCI	87.9	610.78	KRT8

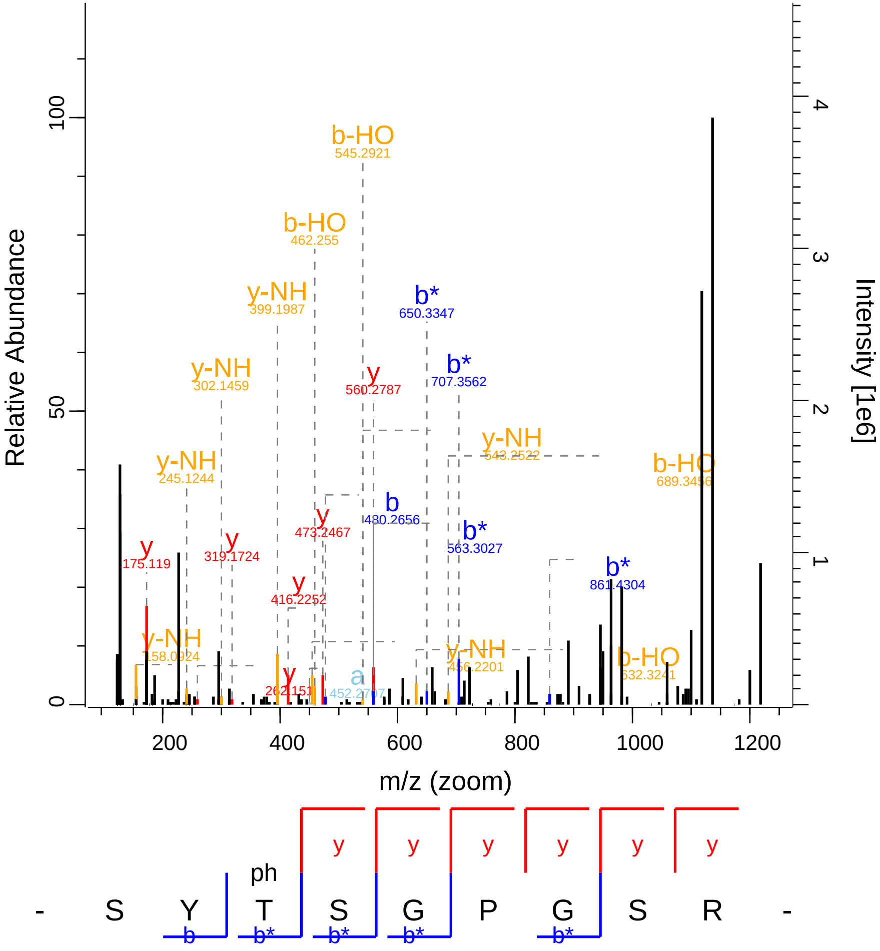

Raw File	Scan	Method	Score	m/z	Gene names
RO-QC-160121_ZackD_SS_Set_02_Fractior	8527	FTMS; HCI	63.6	595.67	CEND1

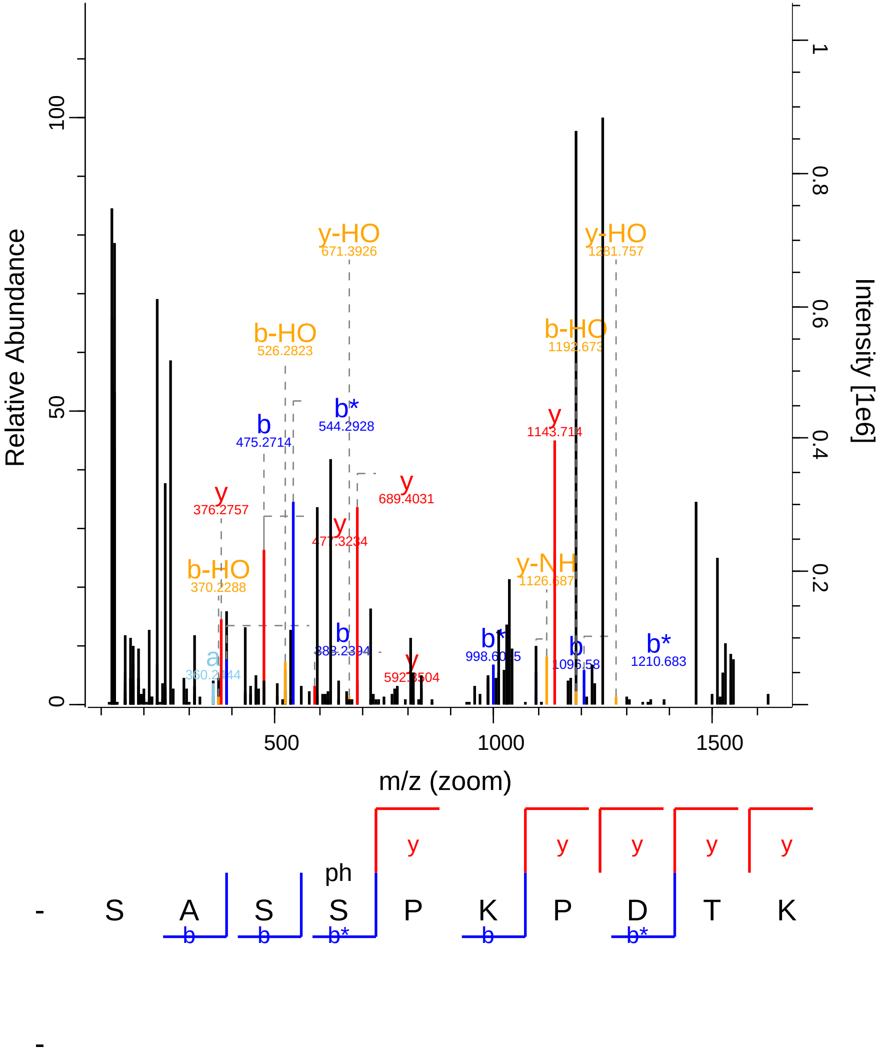

Raw File	Scan	Method	Score	m/z	Gene names
RO-QC-160121_ZackD_SS_Set_02_Fractio	10894	FTMS; HC	47.26	996.96	ANO3

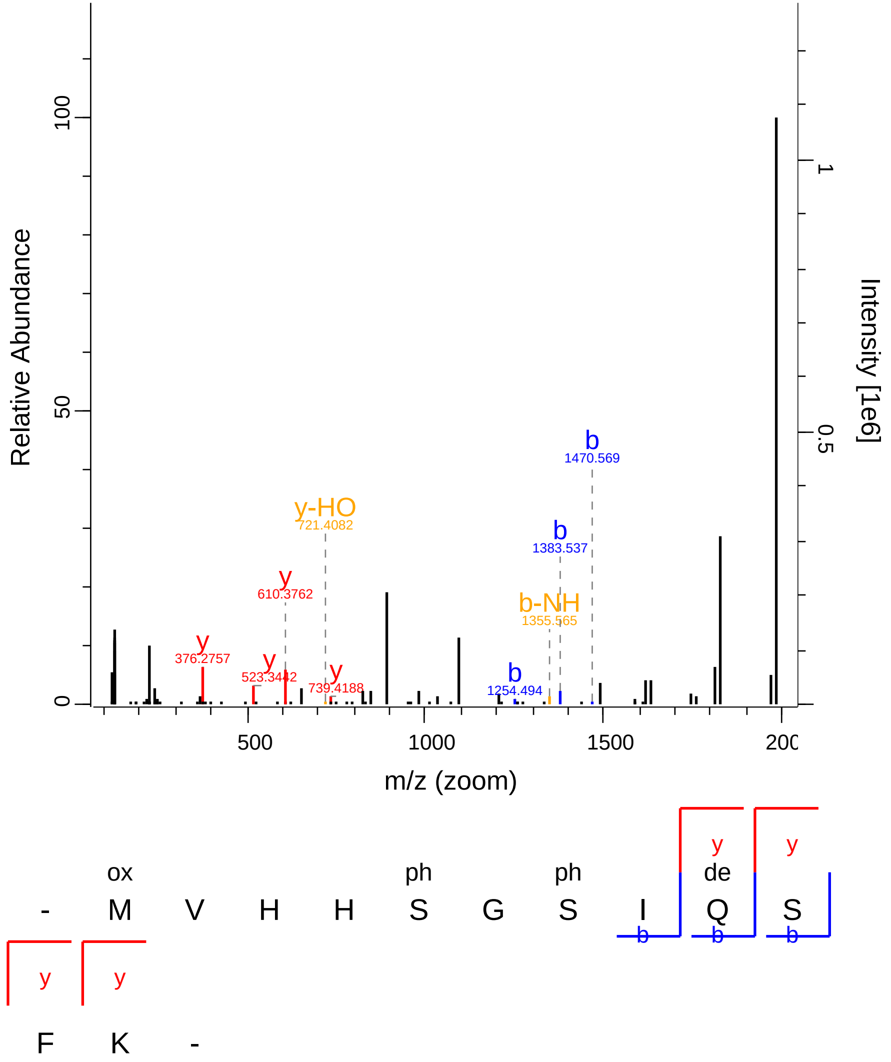

Raw File	Scan	Method	Score	m/z	Gene names
RO-QC-160121_ZackD_SS_Set_02_Fractior	11403	FTMS; HCI	67.99	782.4	H2AFX

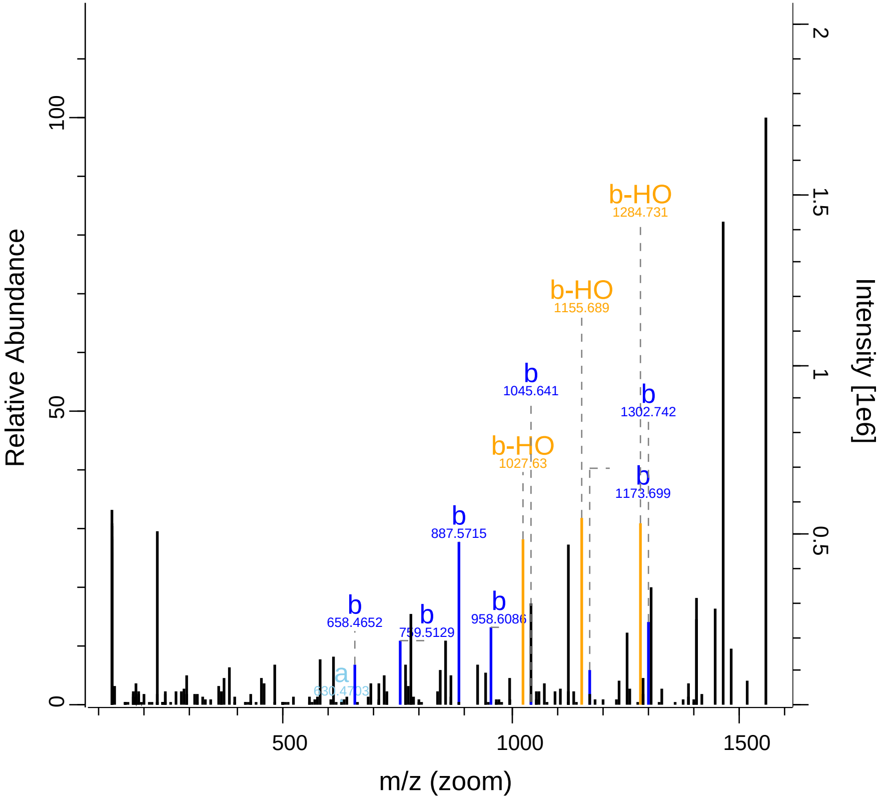

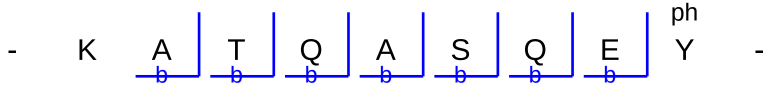

Raw File	Scan	Method	Score	m/z	Gene names
RO-QC-160121_ZackD_SS_Set_02_Fractio	11944	FTMS; HC	120.55	777.39	CHD4

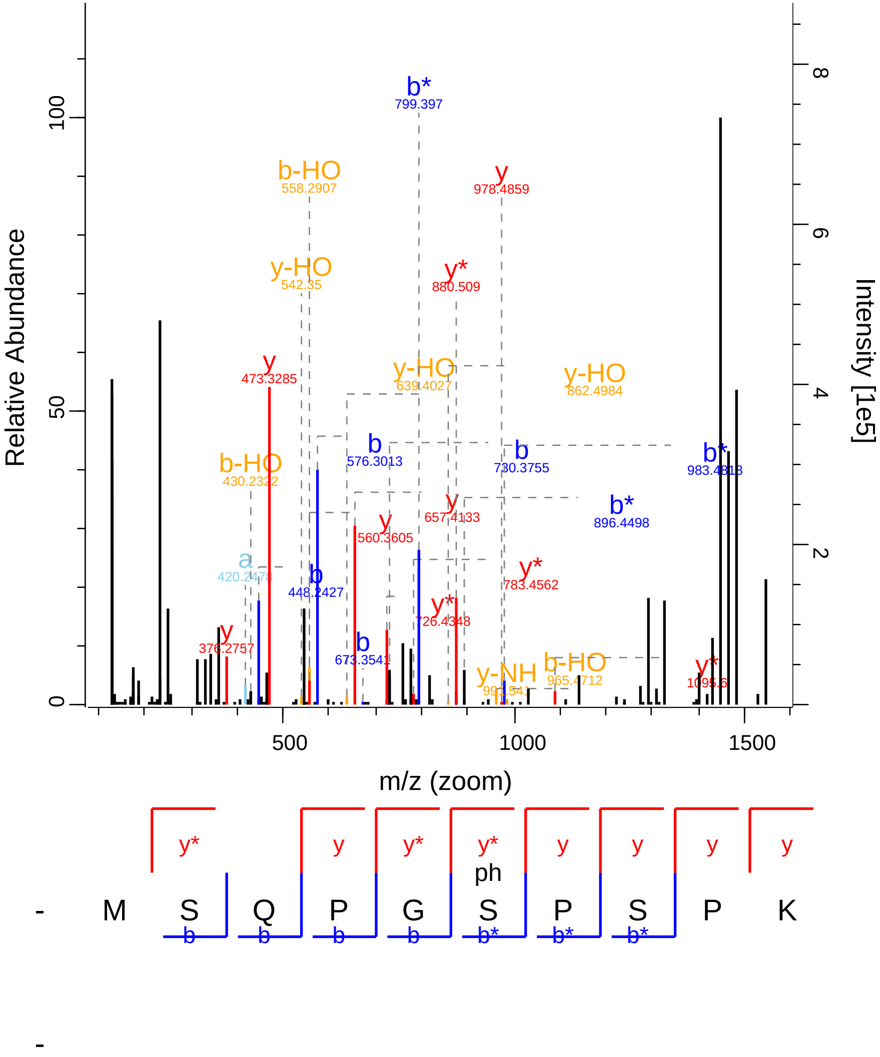

Raw File	Scan	Method	Score	m/z	Gene names
RO-QC-160121_ZackD_SS_Set_02_Fractio	12024	FTMS; HC	52.79	639.37	CCP110

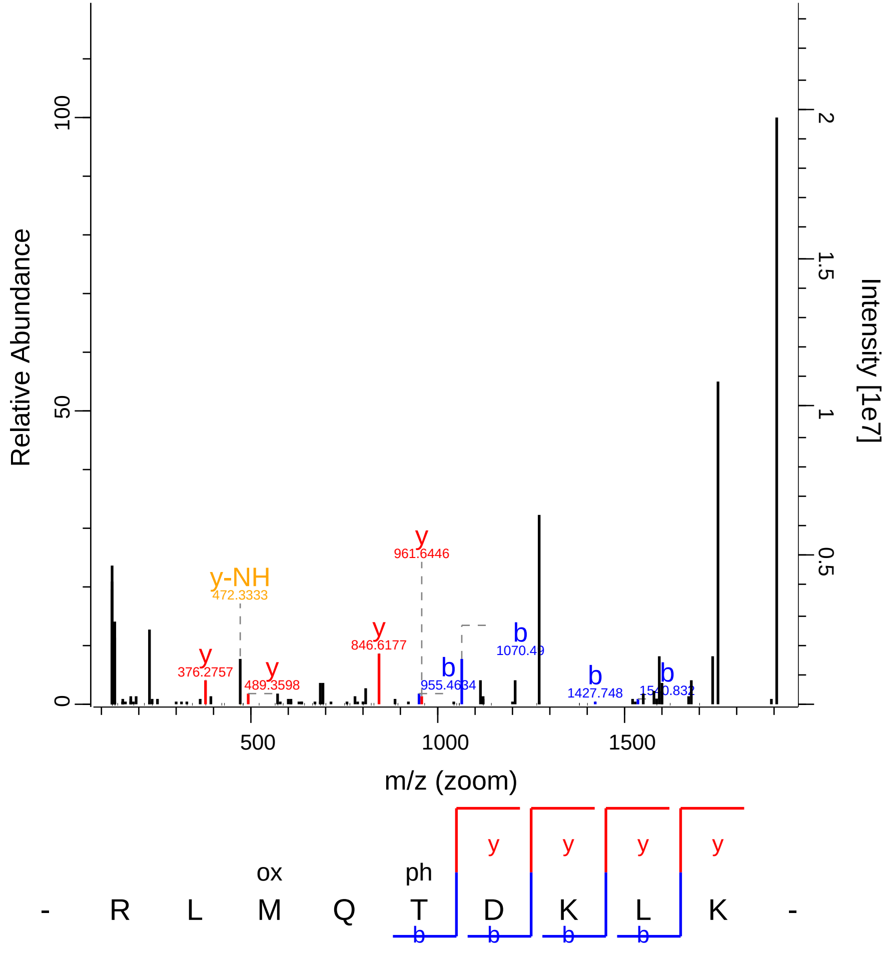

Raw File	Scan	Method	Score	m/z	Gene names
RO-QC-160121_ZackD_SS_Set_02_Fractio	12047	FTMS; HC	80.28	812.09	SRSF11

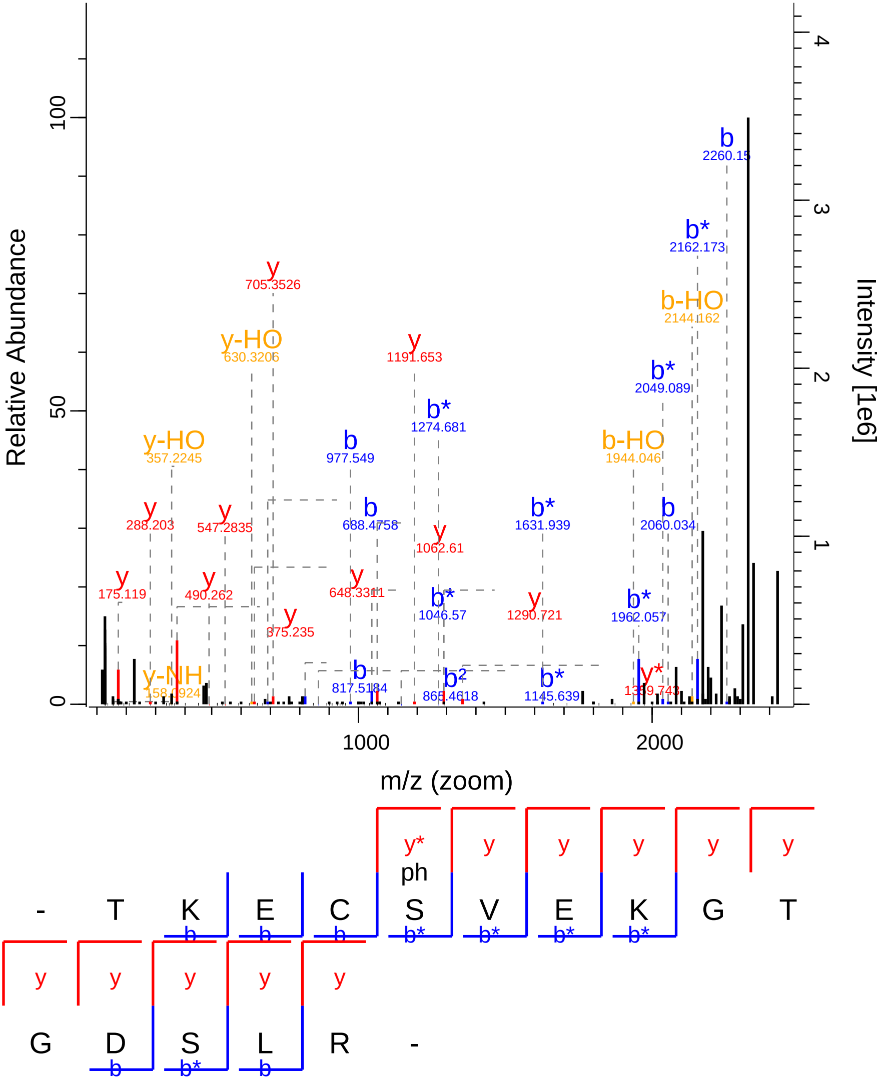

Raw File	Scan	Method	Score	m/z	Gene names
RO-QC-160121_ZackD_SS_Set_02_Fractio	12346	FTMS; HC	86.55	860.09	GTF3C1

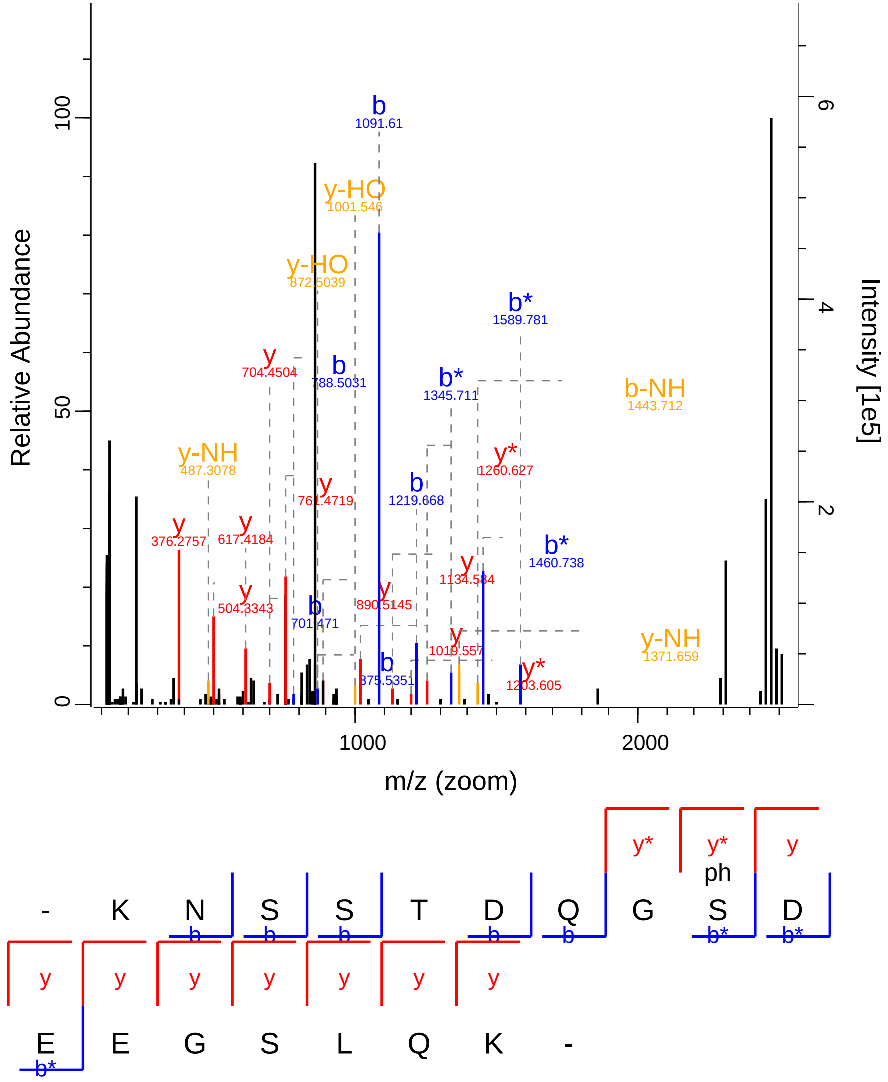

Raw File	Scan	Method	Score	m/z	Gene names
RO-QC-160121_ZackD_SS_Set_02_Fractio	12414	FTMS; HC	221.66	838.38	MYRIP

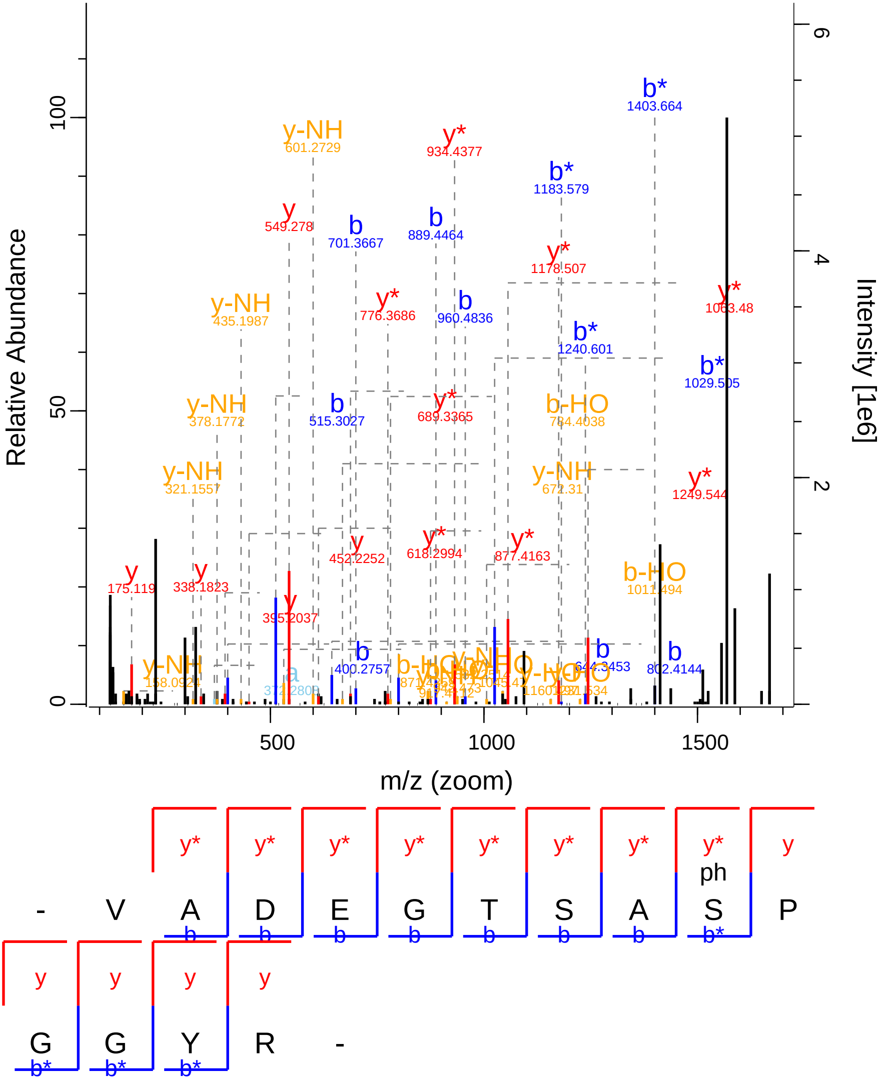

Raw File	Scan	Method	Score	m/z	Gene names
RO-QC-160121_ZackD_SS_Set_02_Fractior	12517	FTMS; HCI	231.74	878.9	FNBP4

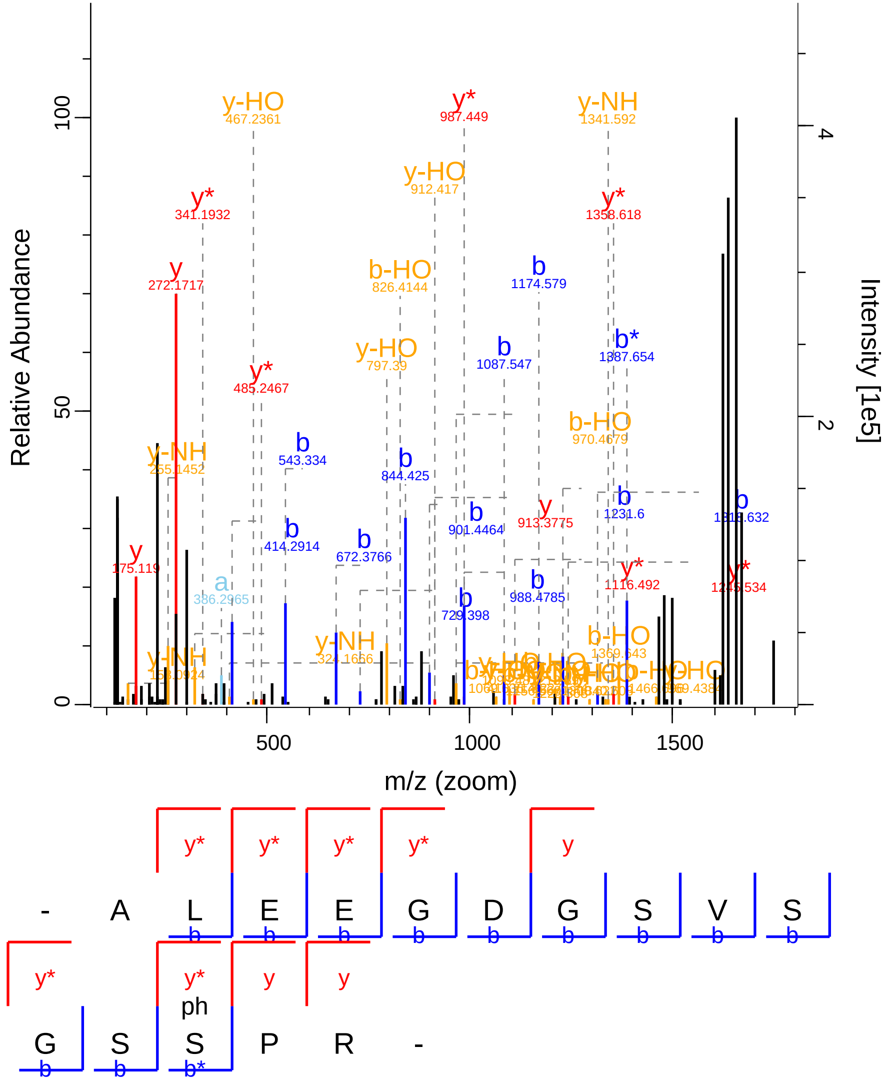

Raw File	Scan	Method	Score	m/z	Gene names
RO-QC-160121_ZackD_SS_Set_02_Fractio	12762	FTMS; HC	114.4	779.39	SRRM2

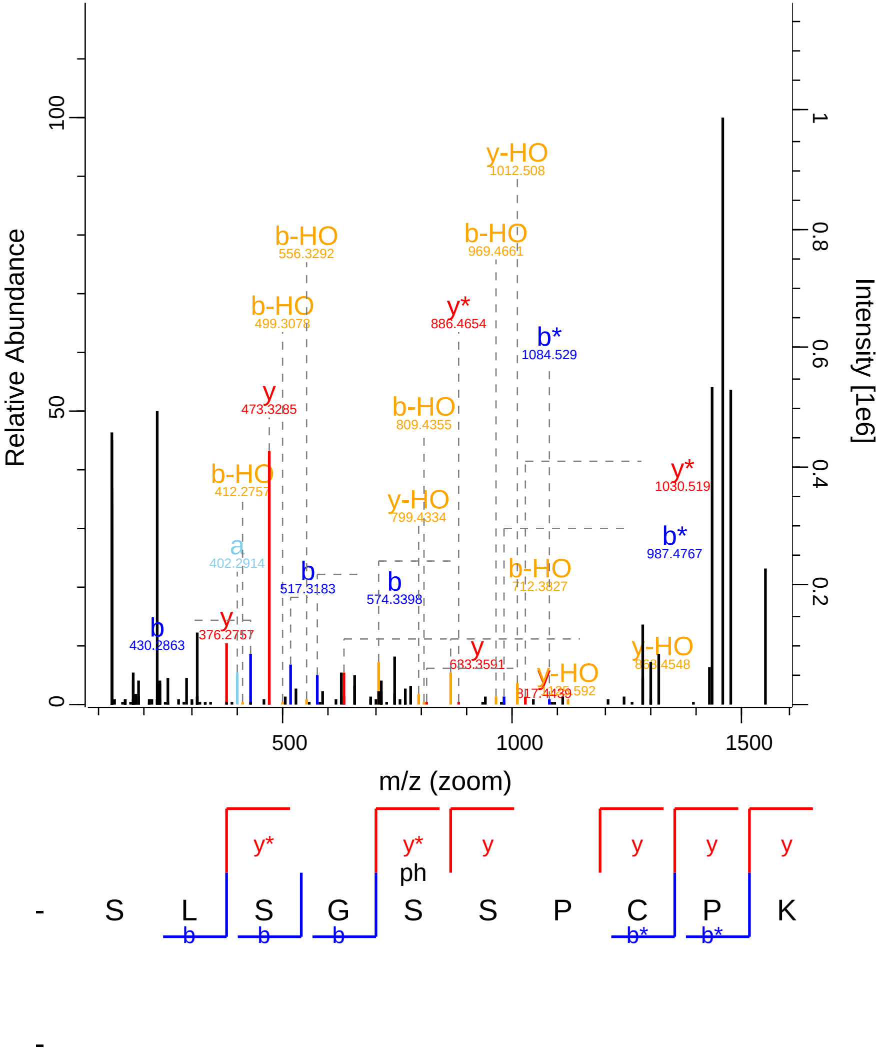

Raw File	Scan	Method	Score	m/z	Gene names
RO-QC-160121_ZackD_SS_Set_02_Fractio	12981	FTMS; HC	90.05	835.97	ARHGEF7

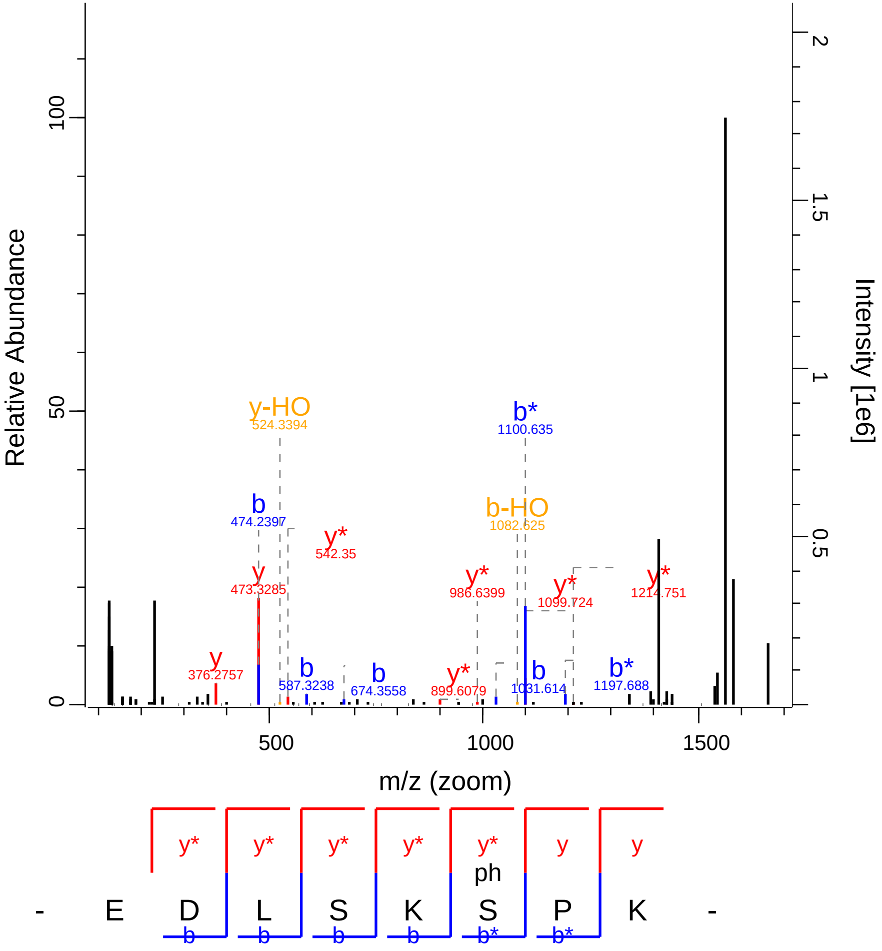

Raw File	Scan	Method	Score	m/z	Gene names
RO-QC-160121_ZackD_SS_Set_02_Fractio	14048	FTMS; HC	94.2	715.38	LEO1

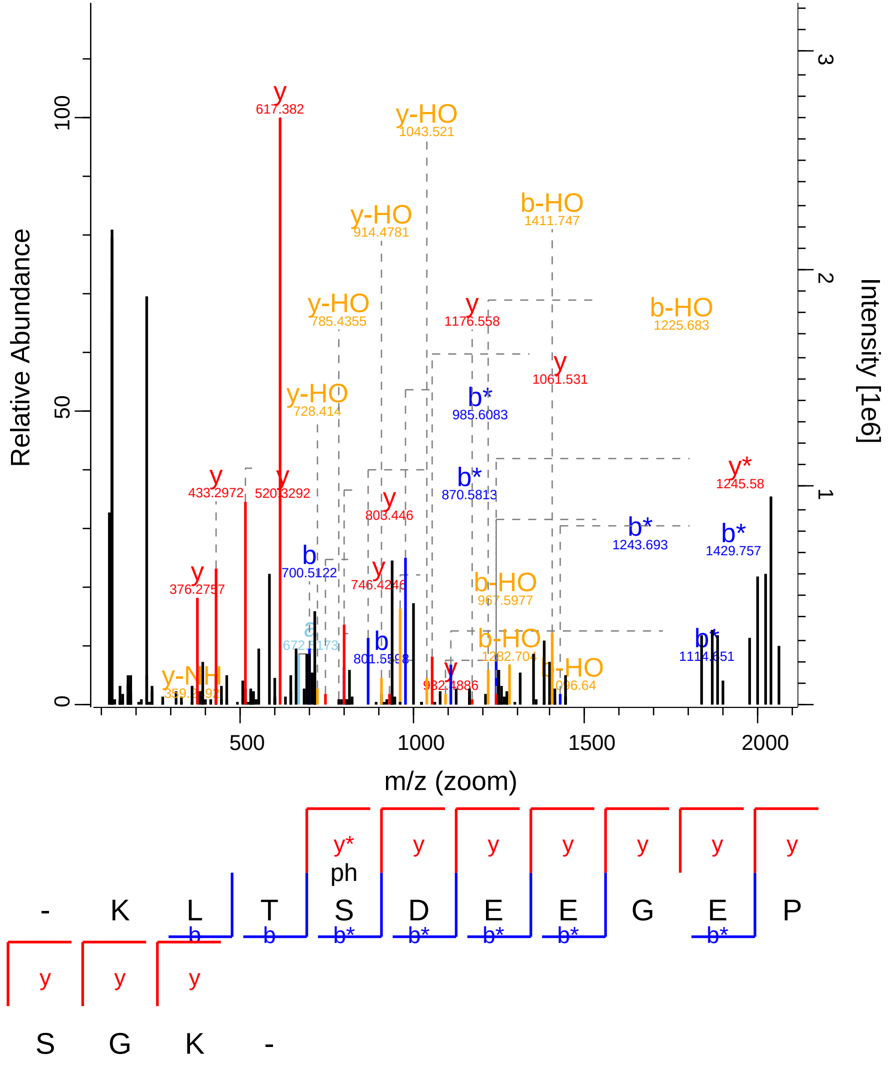

Raw File	Scan	Method	Score	m/z	Gene names
RO-QC-160121_ZackD_SS_Set_02_Fractio	14177	FTMS; HC	139.64	835.39	PPAN

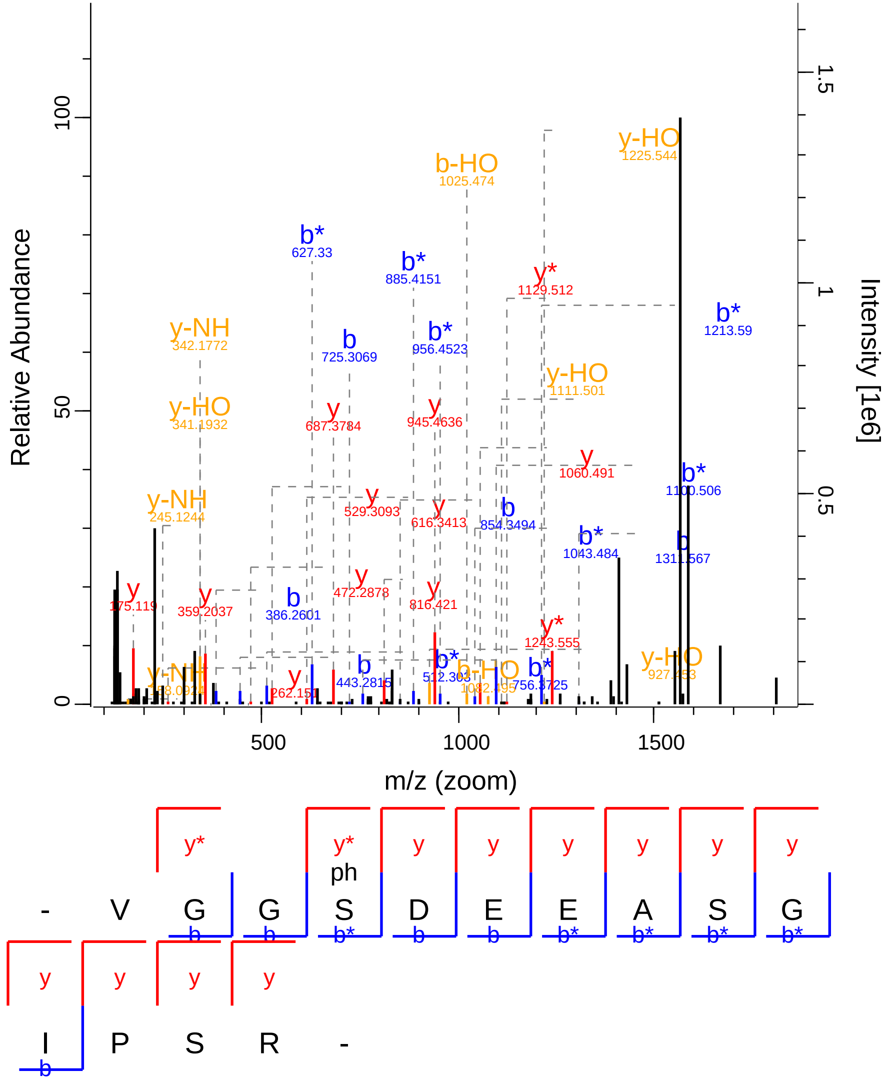

Raw File	Scan	Method	Score	m/z	Gene names
RO-QC-160121_ZackD_SS_Set_02_Fractio	14398	FTMS; HC	164.55	1033.2	AKAP2

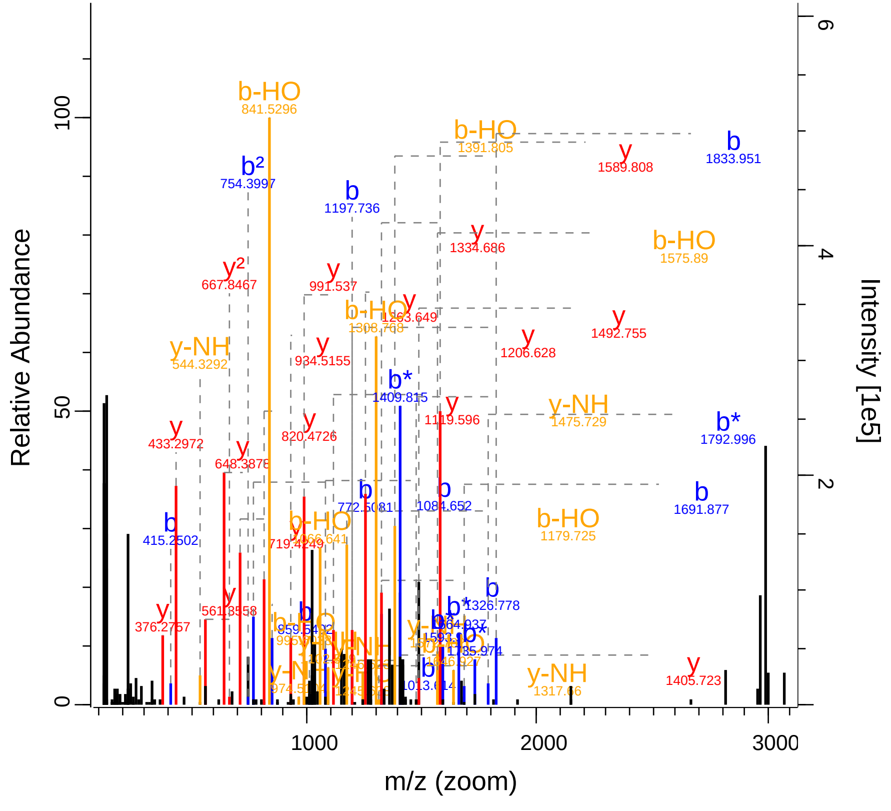

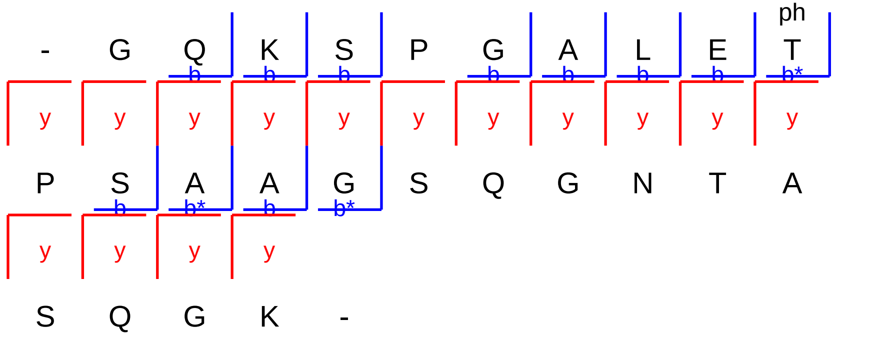

Raw File	Scan	Method	Score	m/z	Gene names
RO-QC-160121_ZackD_SS_Set_02_Fractio	15403	FTMS; HC	97.73	764.93	WRNIP1

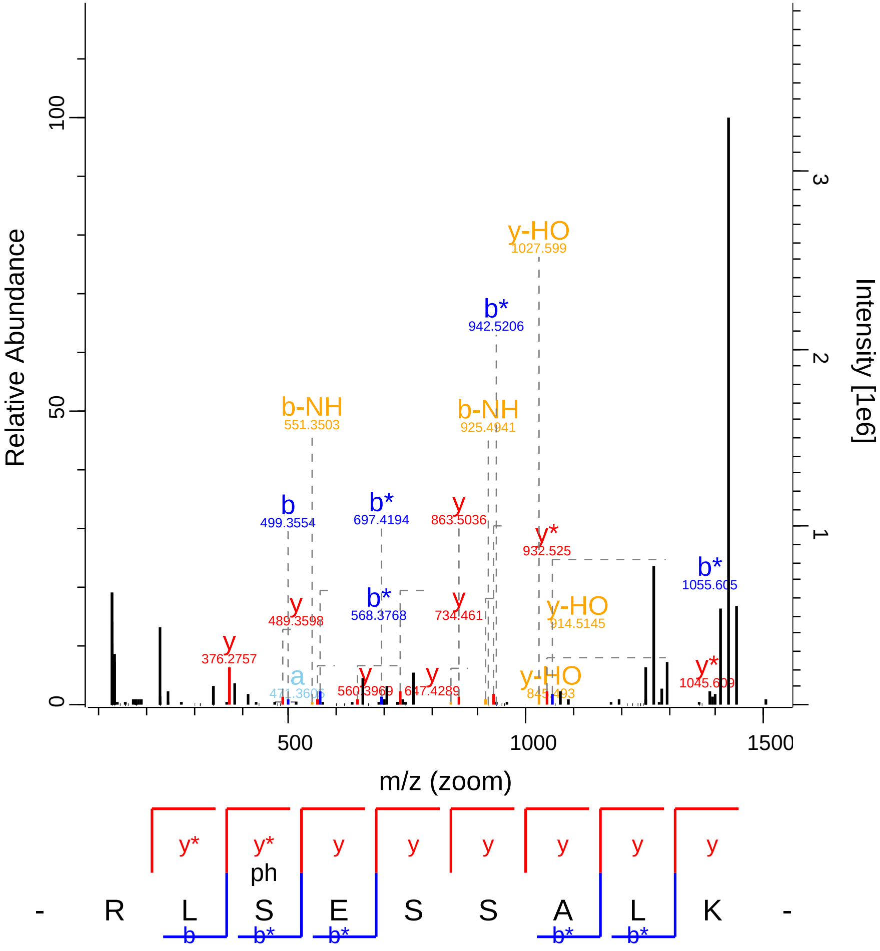

Raw File	Scan	Method	Score	m/z	Gene names
RO-QC-160121_ZackD_SS_Set_02_Fractio	15732	FTMS; HC	59.21	462.26	RPL4

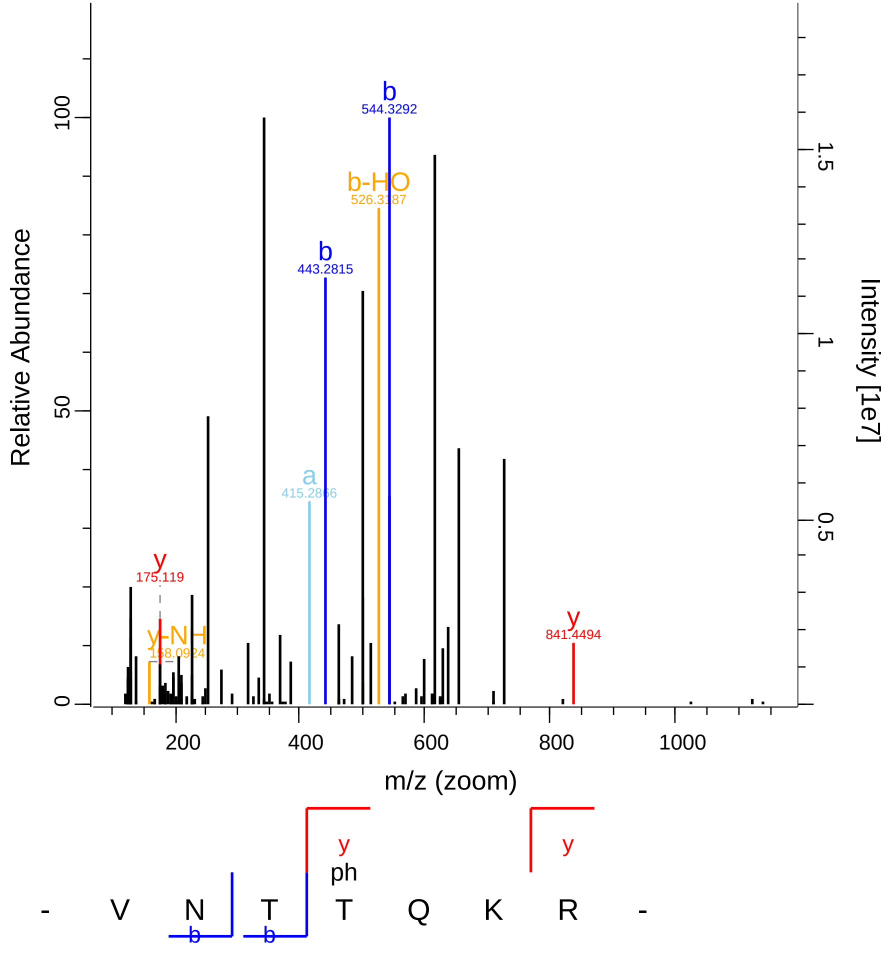

Raw File	Scan	Method	Score	m/z	Gene names
RO-QC-160121_ZackD_SS_Set_02_Fractio	16106	FTMS; HC	71.43	941.16	SRRM2

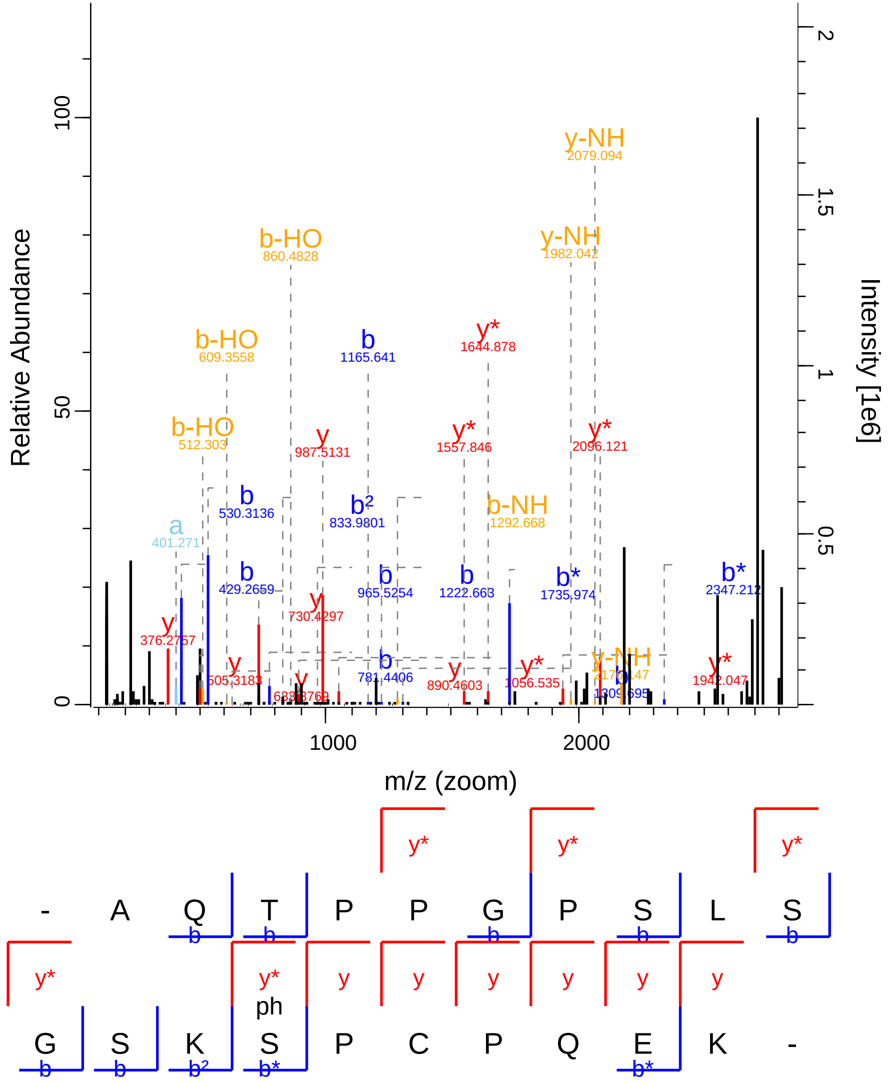

Raw File	Scan	Method	Score	m/z	Gene names
RO-QC-160121_ZackD_SS_Set_02_Fractio	16114	FTMS; HC	107.17	706.12	SRRM2

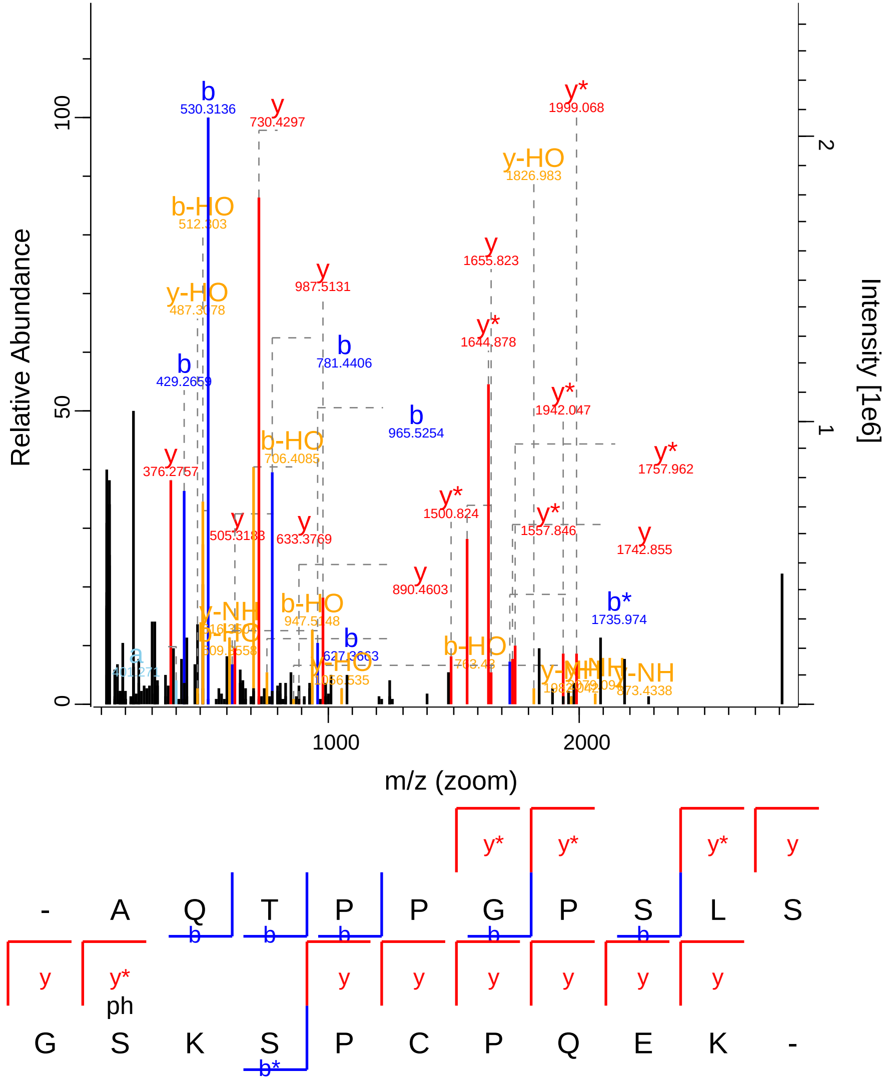

Raw File	Scan	Method	Score	m/z	Gene names
RO-QC-160121_ZackD_SS_Set_02_Fractior	16407	FTMS; HCI	69.4	641.1	ACLY

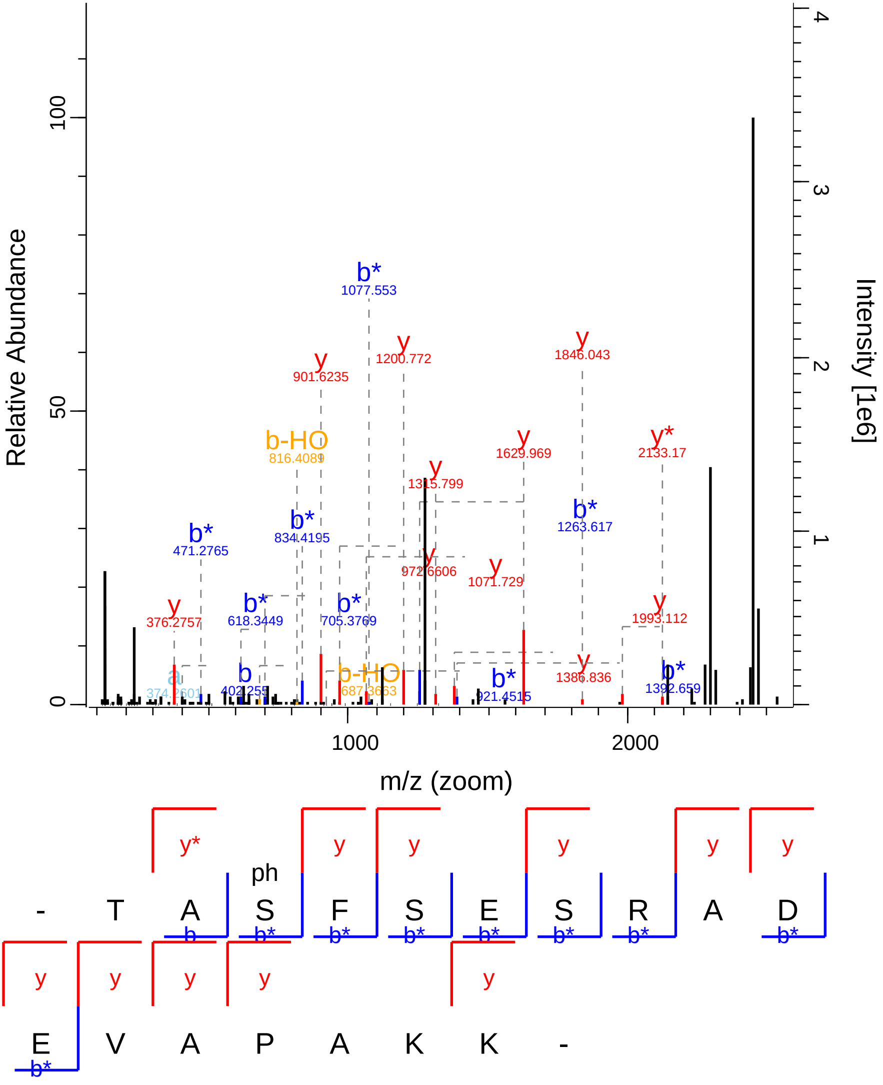

Raw File	Scan	Method	Score	m/z	Gene names
RO-QC-160121_ZackD_SS_Set_02_Fraction	16512	FTMS; HCI	87.57	644	DBNL

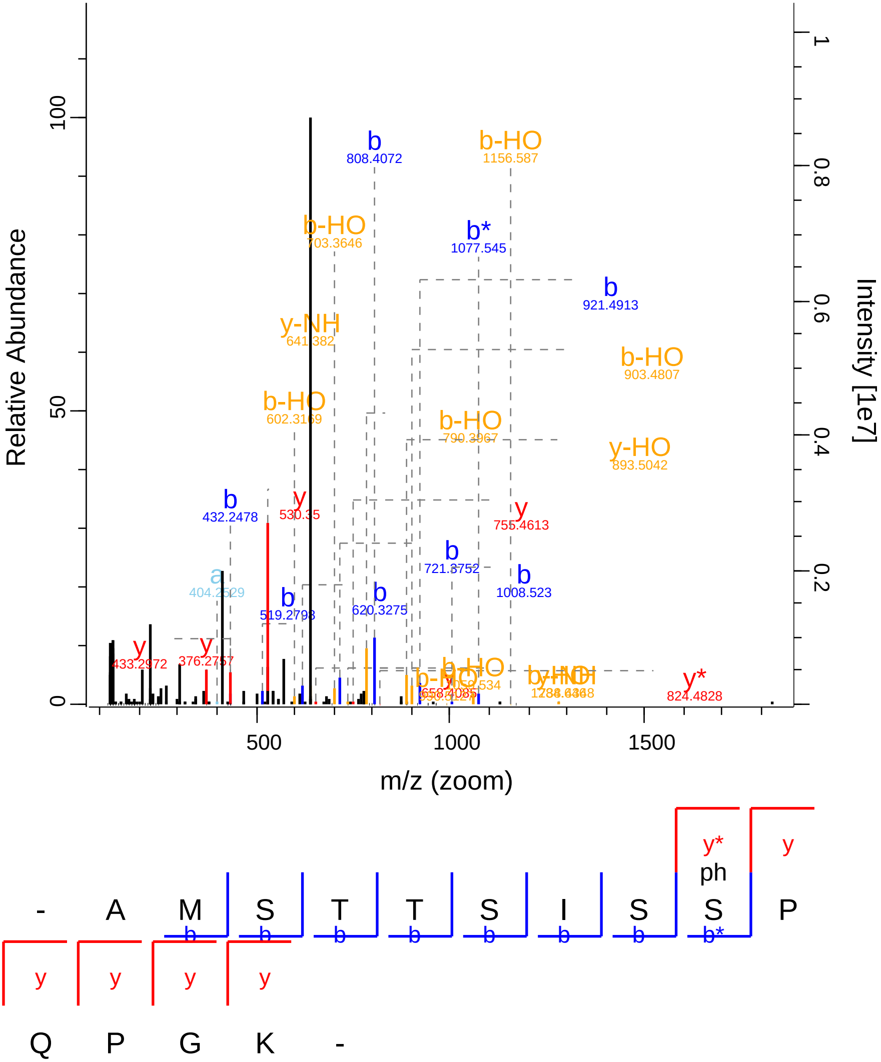

Raw File	Scan	Method	Score	m/z	Gene names
RO-QC-160121_ZackD_SS_Set_02_Fractio	16596	FTMS; HC	114.46	755.41	IRF2BPL

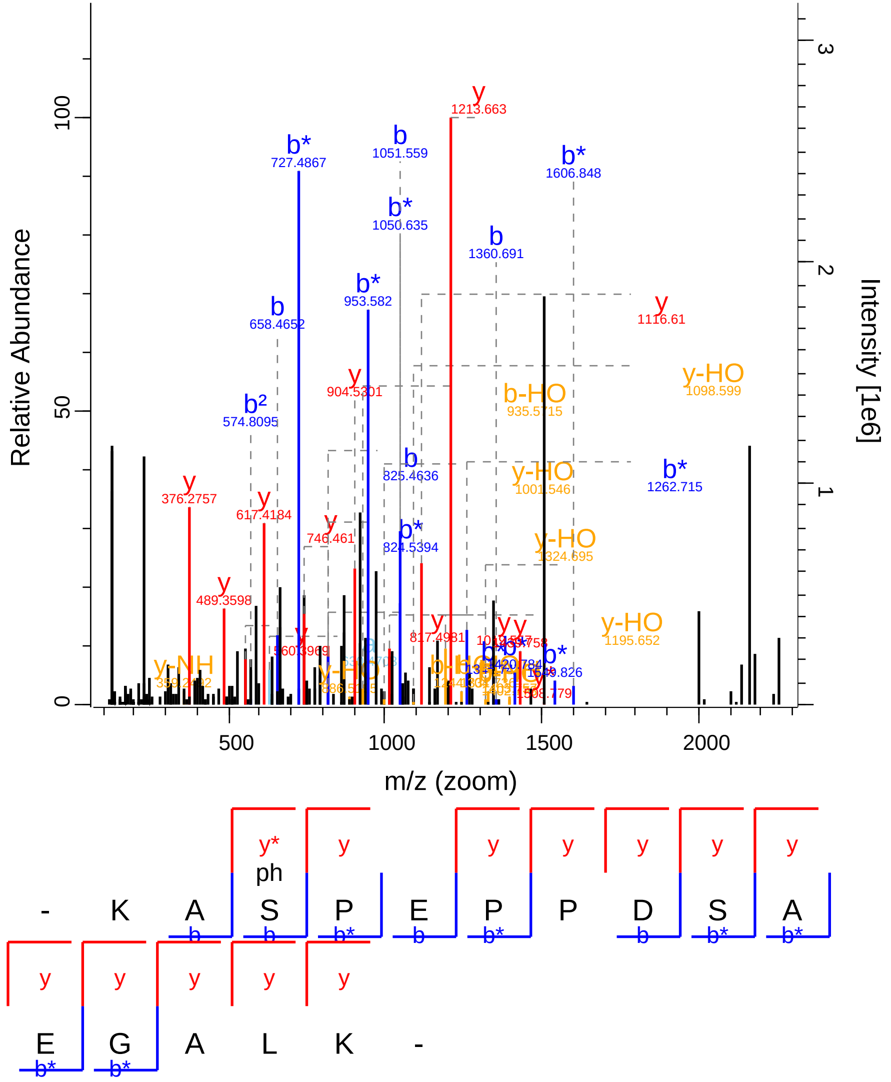

Raw File	Scan	Method	Score	m/z	Gene names
RO-QC-160121_ZackD_SS_Set_02_Fractio	16778	FTMS; HC	63.73	715.41	FANCB

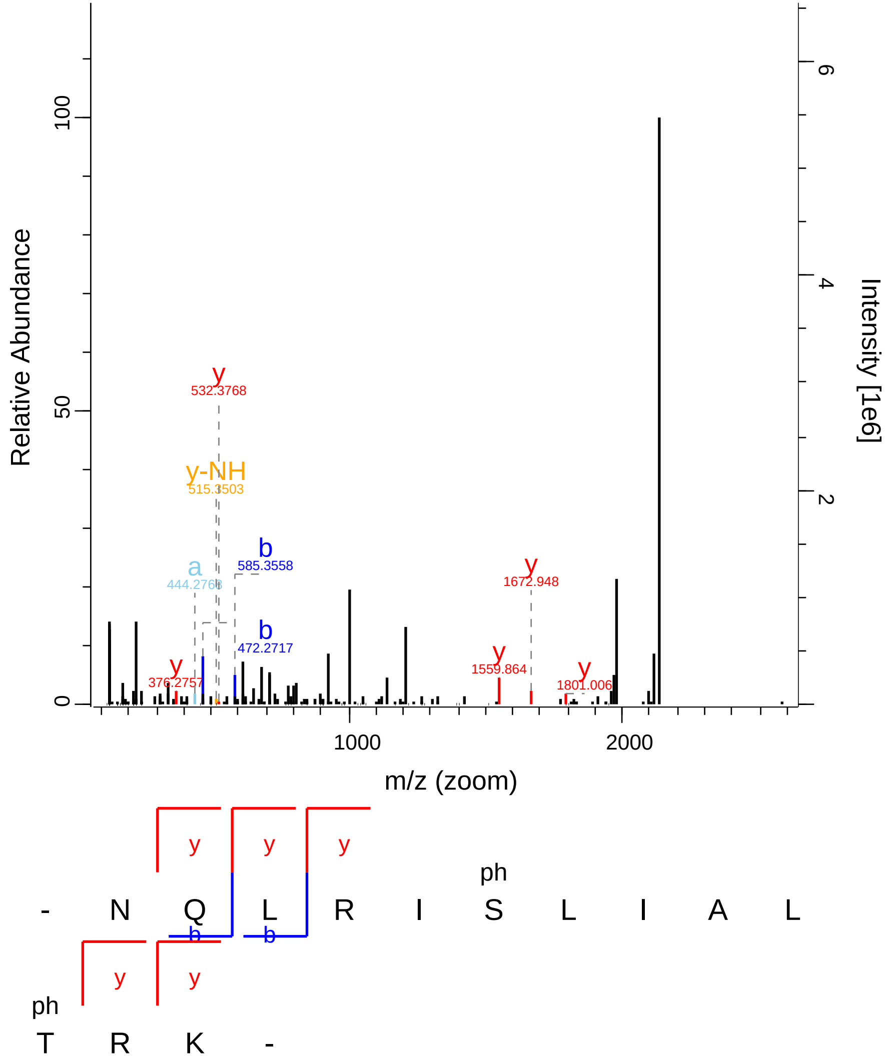

Raw File	Scan	Method	Score	m/z	Gene names
RO-QC-160121_ZackD_SS_Set_02_Fractio	16843	FTMS; HC	53.28	772.06	PCNP

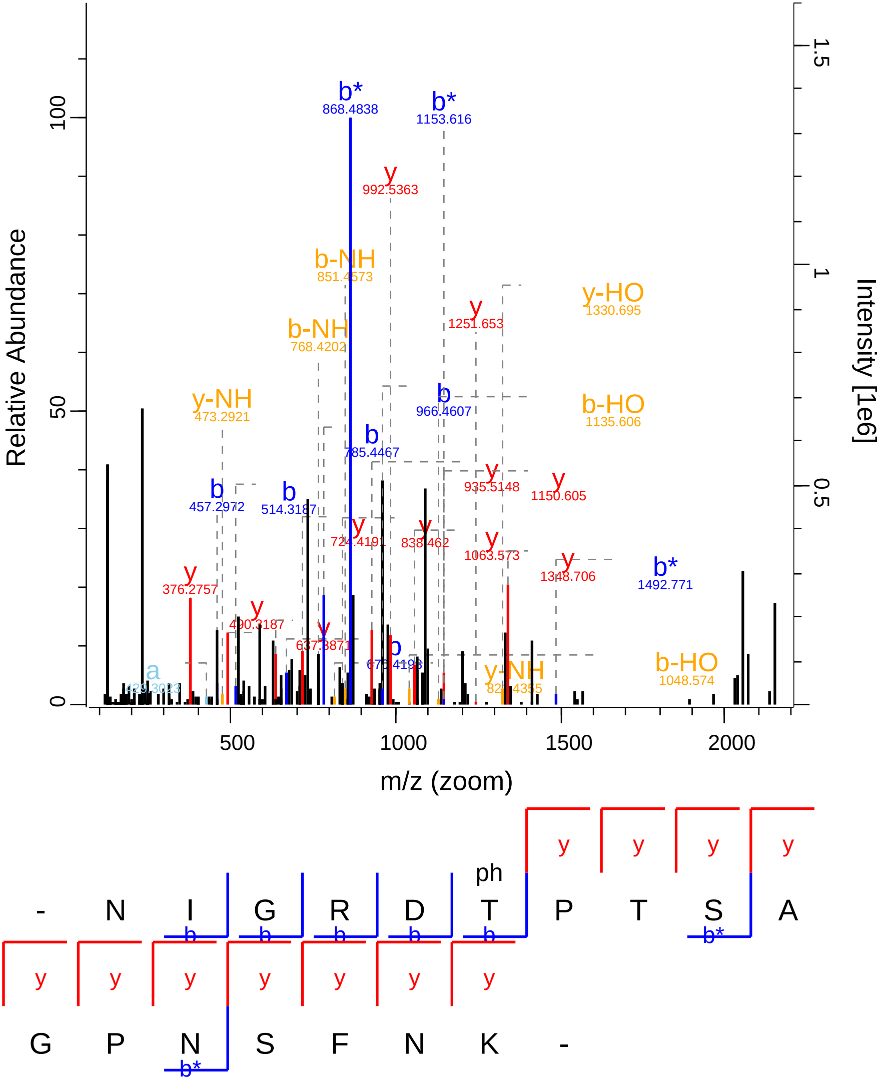

Raw File	Scan	Method	Score	m/z	Gene names
RO-QC-160121_ZackD_SS_Set_02_Fractic	17211	FTMS; HC	122.34	1306.18	SRRM1

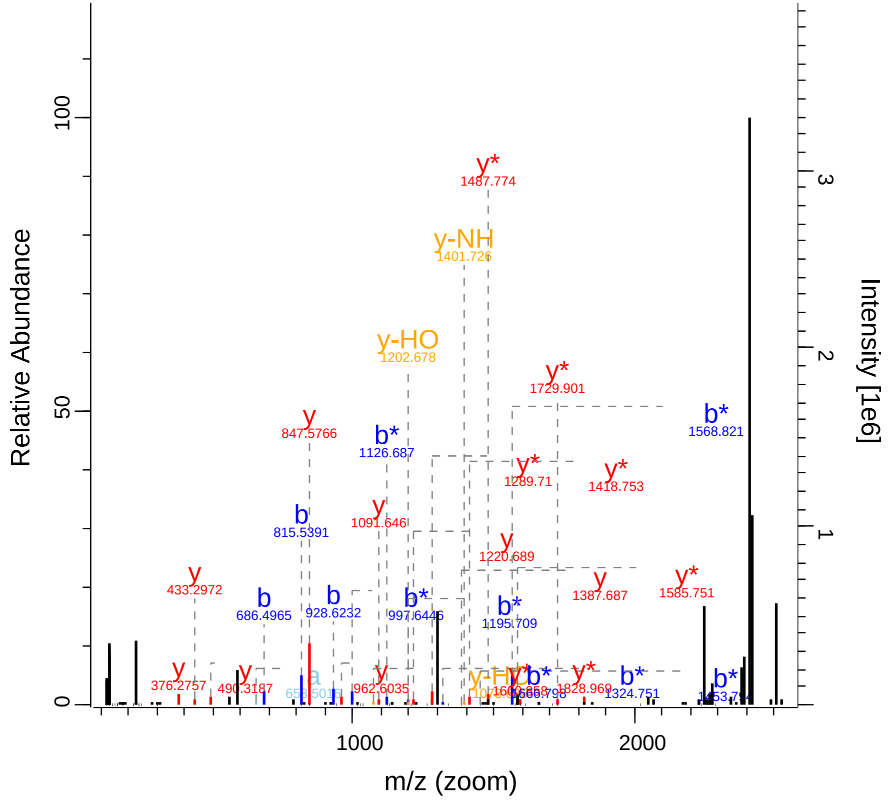

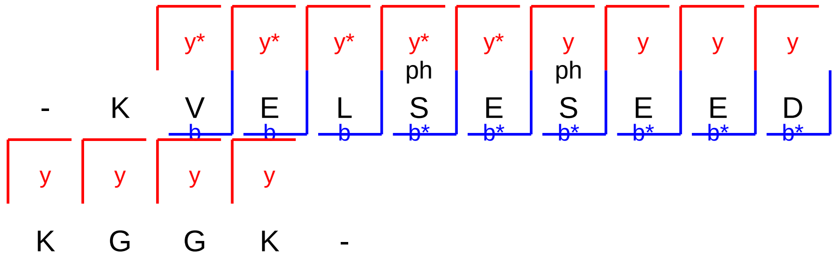

Raw File	Scan	Method	Score	m/z	Gene names
RO-QC-160121_ZackD_SS_Set_02_Fractic	18271	FTMS; HC	188.59	1042.56	SEPT7

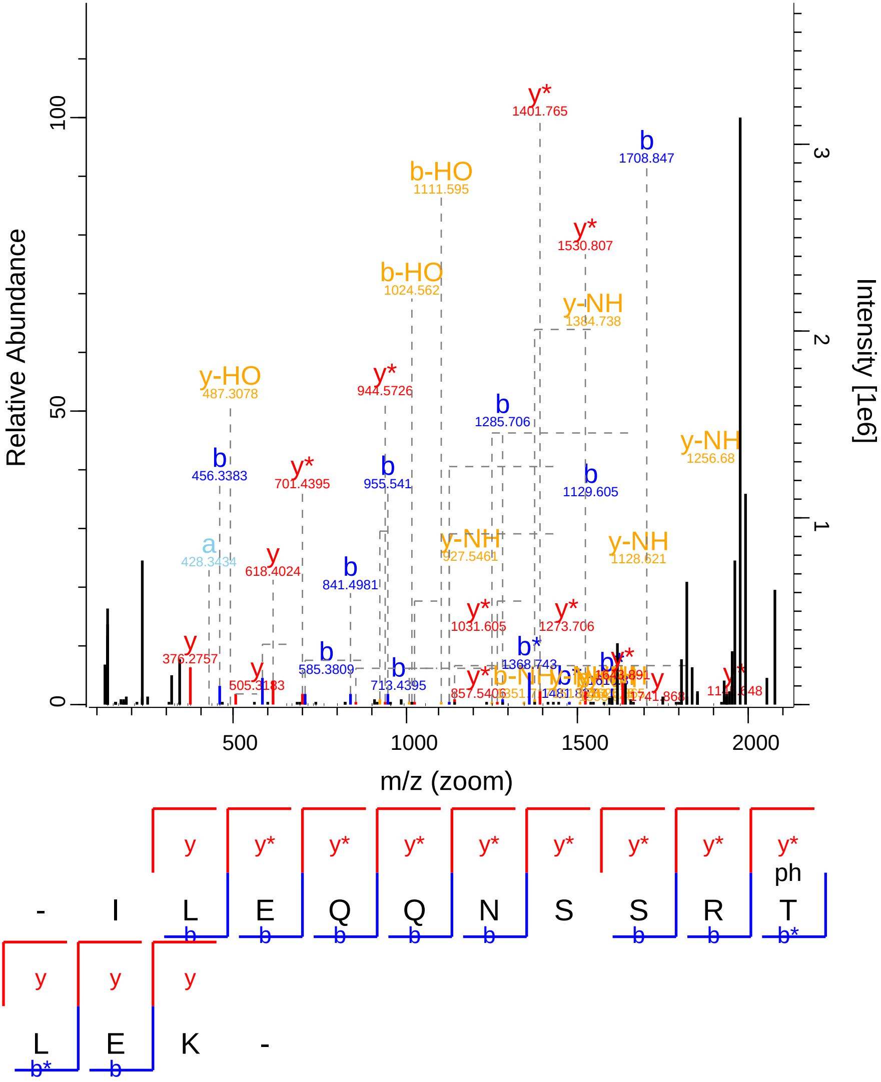

Raw File	Scan	Method	Score	m/z	Gene names
RO-QC-160121_ZackD_SS_Set_02_Fractic	18586	FTMS; HC	46.35	1164.61	PATL2

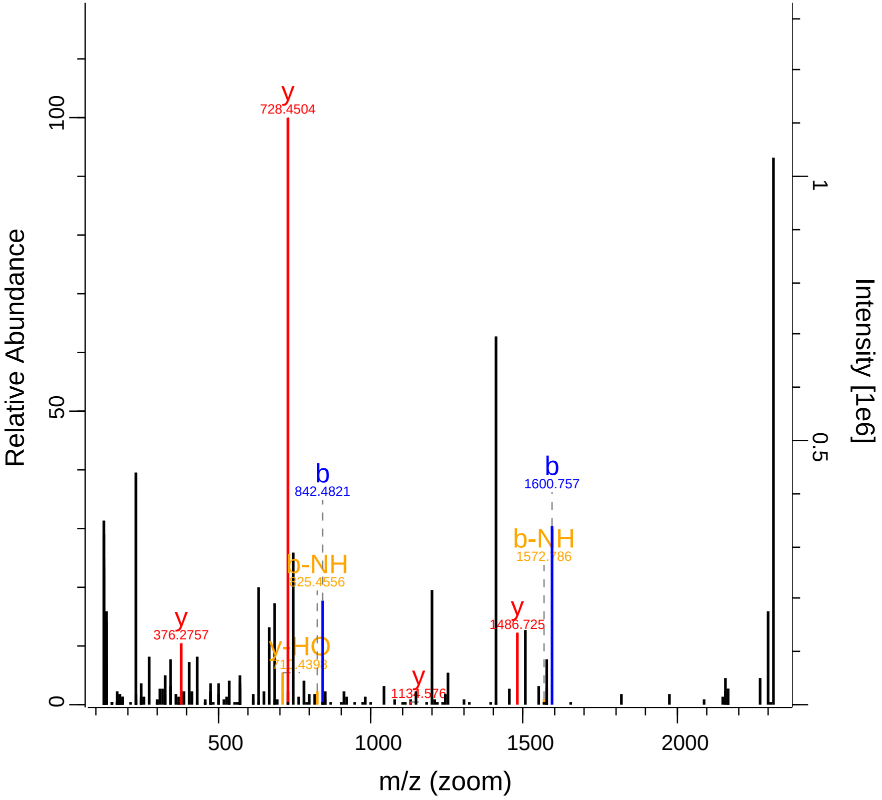

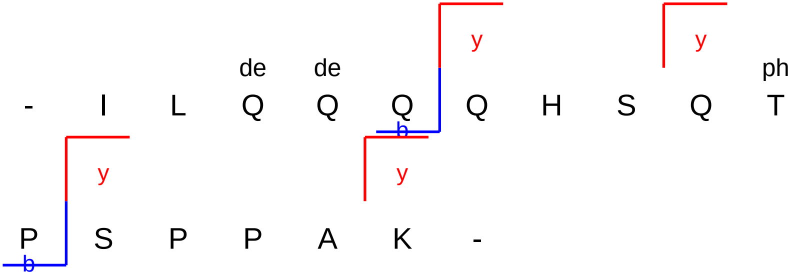

Raw File	Scan	Method	Score	m/z	Gene names
RO-QC-160121_ZackD_SS_Set_02_Fractio	19007	FTMS; HC	106.32	842.93	DOCK7

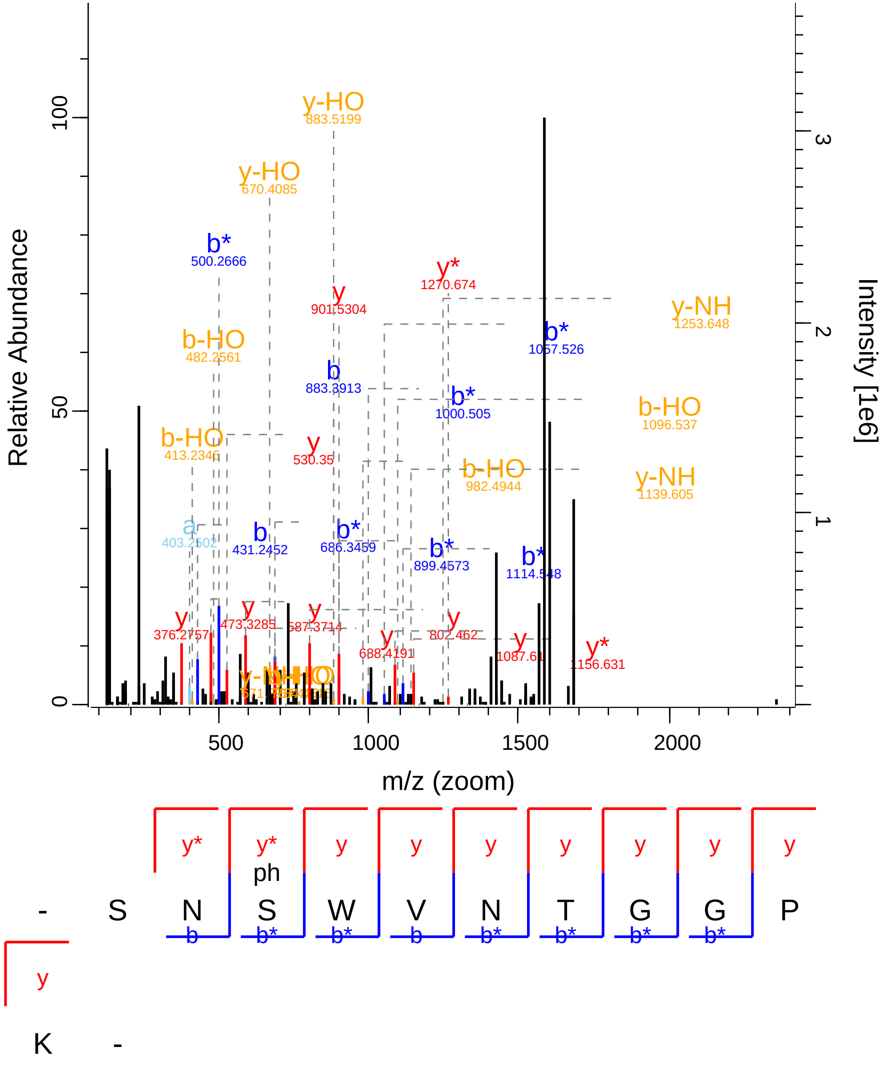

Raw File	Scan	Method	Score	m/z	Gene names
RO-QC-160121_ZackD_SS_Set_02_Fractio	19103	FTMS; HC	104.44	732.06	BNIP2

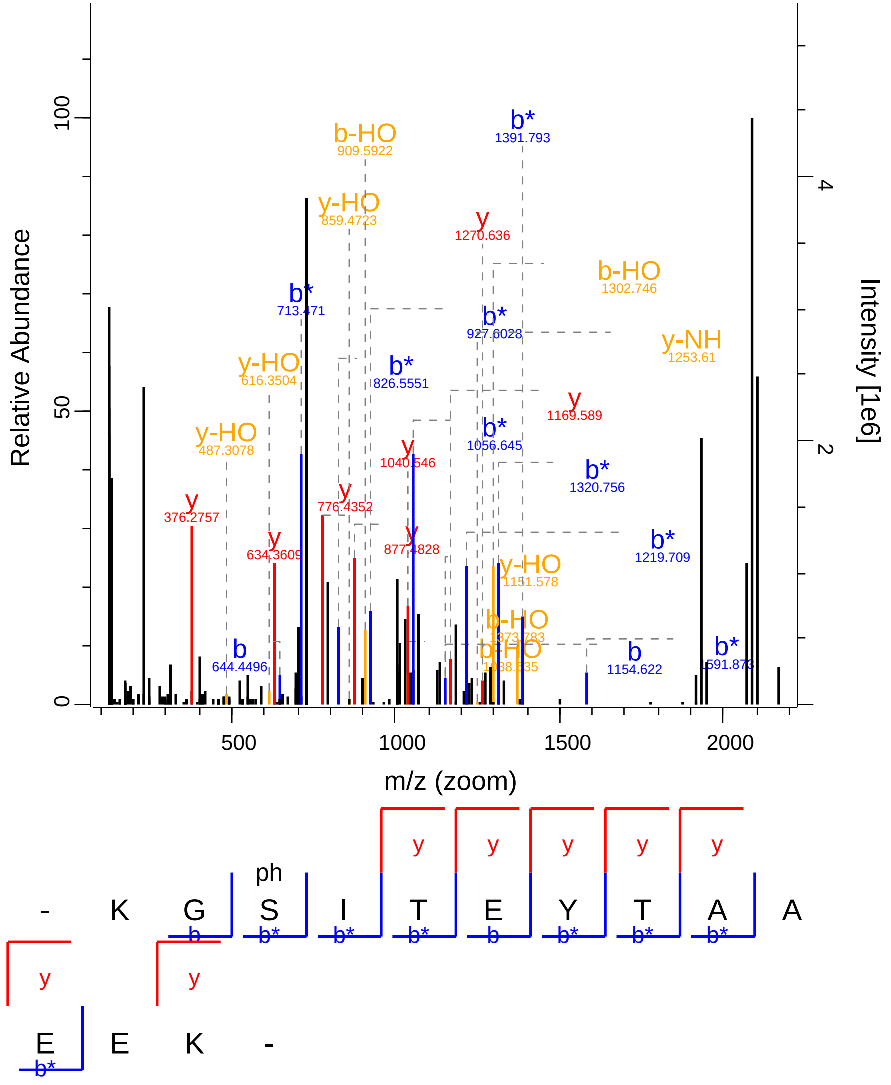

Raw File	Scan	Method	Score	m/z	Gene names
RO-QC-160121_ZackD_SS_Set_02_Fractio	19206	FTMS; HC	66.61	903.81	AKAP12

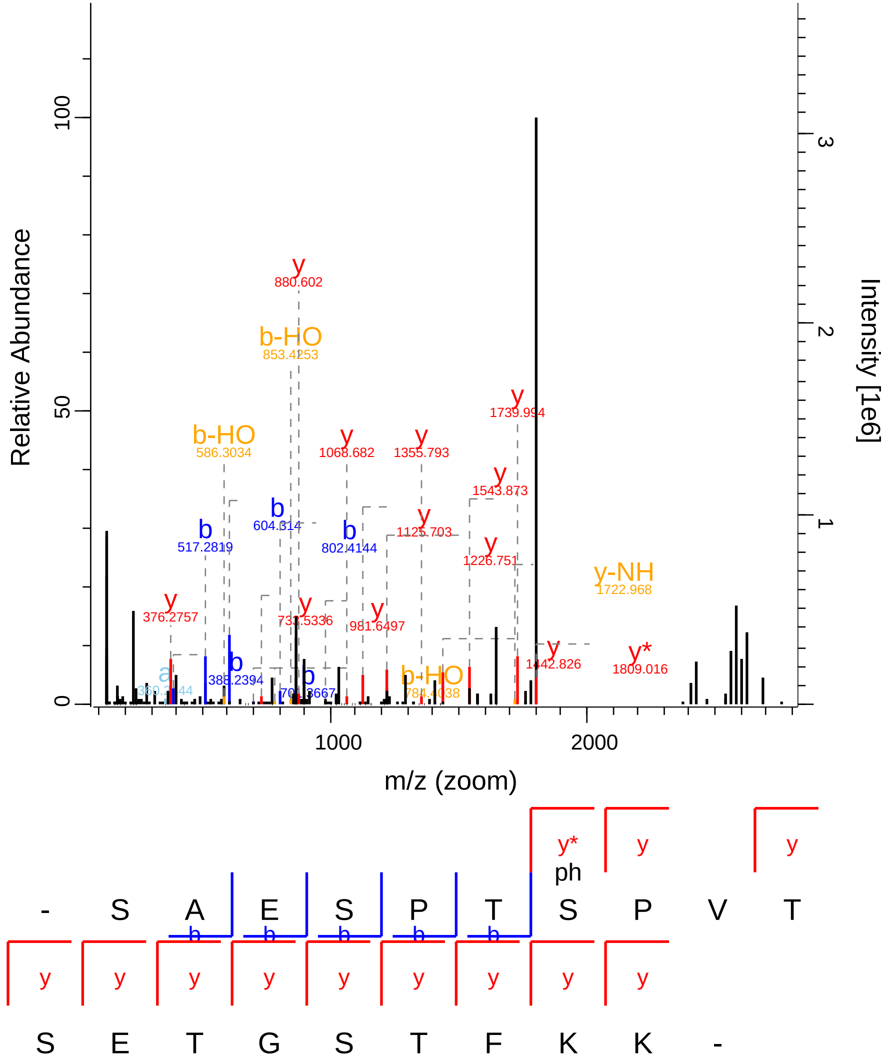

Raw File	Scan	Method	Score	m/z	Gene names
RO-QC-160121_ZackD_SS_Set_02_Fractio	19387	FTMS; HC	137.72	701.35	EIF5B

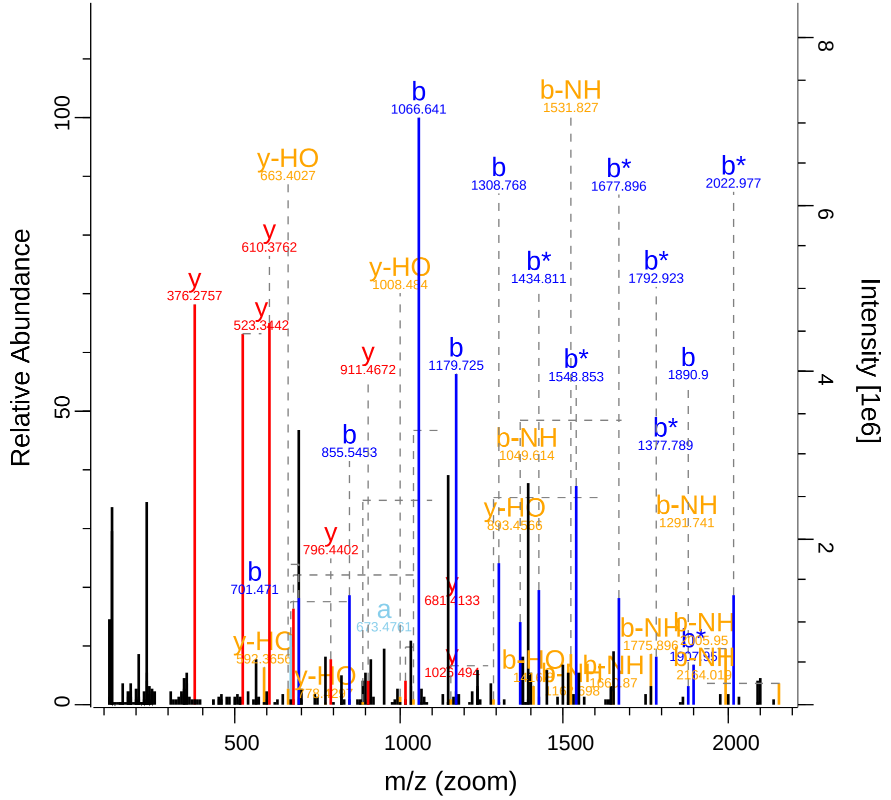

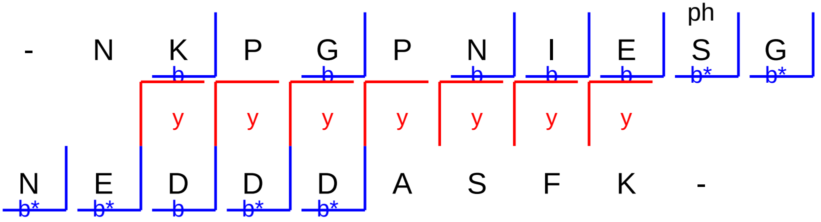

Raw File	Scan	Method	Score	m/z	Gene names
RO-QC-160121_ZackD_SS_Set_02_Fractio	19498	FTMS; HC	56.73	953.01	MYO1A

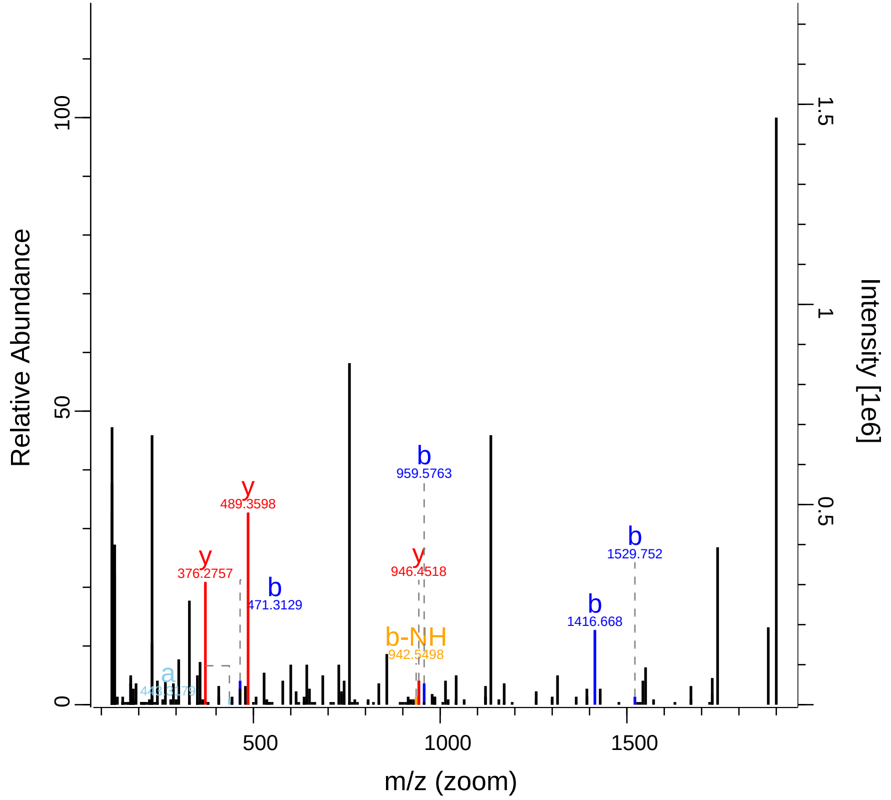

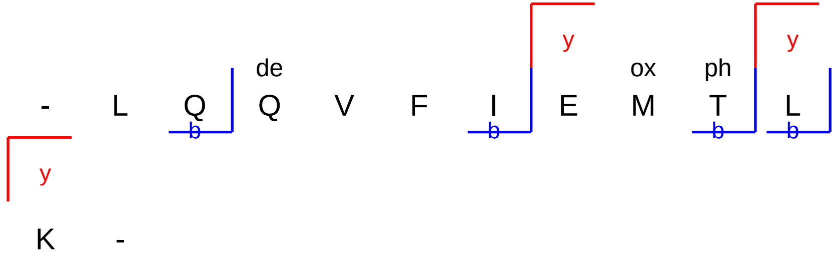

Raw File	Scan	Method	Score	m/z	Gene names
RO-QC-160121_ZackD_SS_Set_02_Fractio	20141	FTMS; HC	72.03	810.09	MAF1

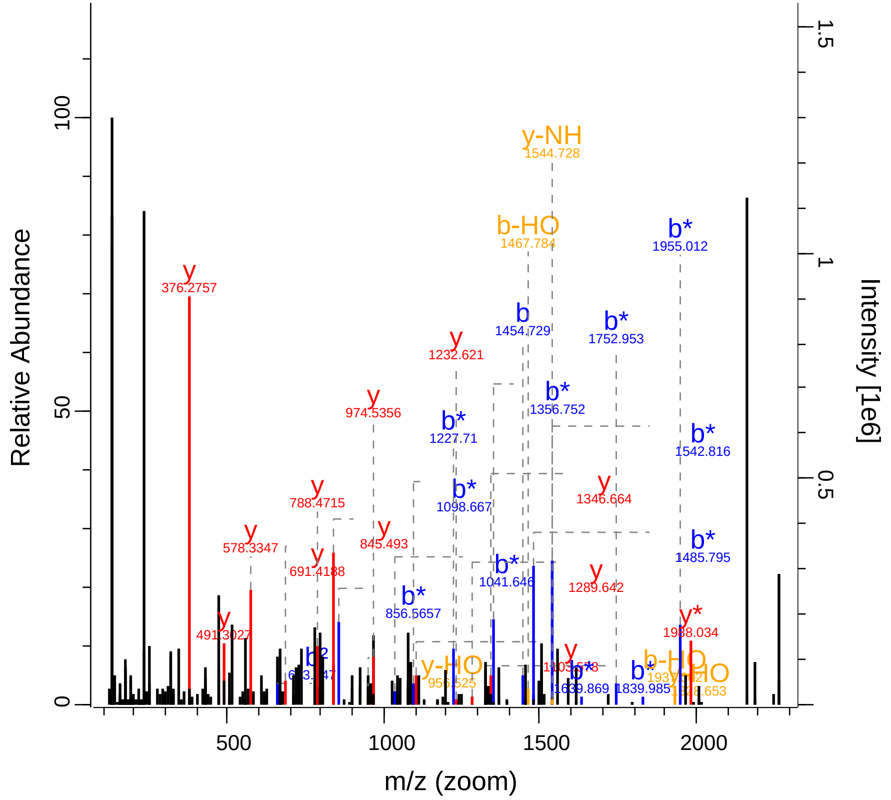

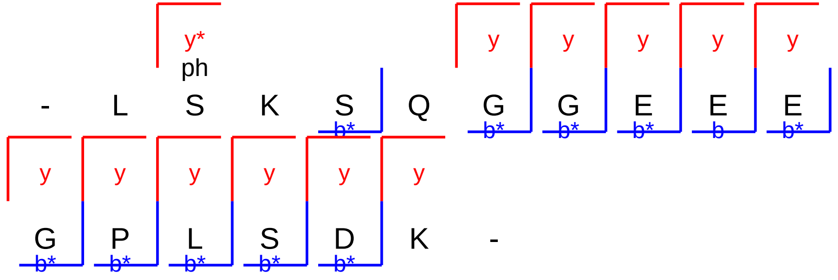

Raw File	Scan	Method	Score	m/z	Gene names
RO-QC-160121_ZackD_SS_Set_02_Fractio	20225	FTMS; HC	53.48	864.47	TP53BP1

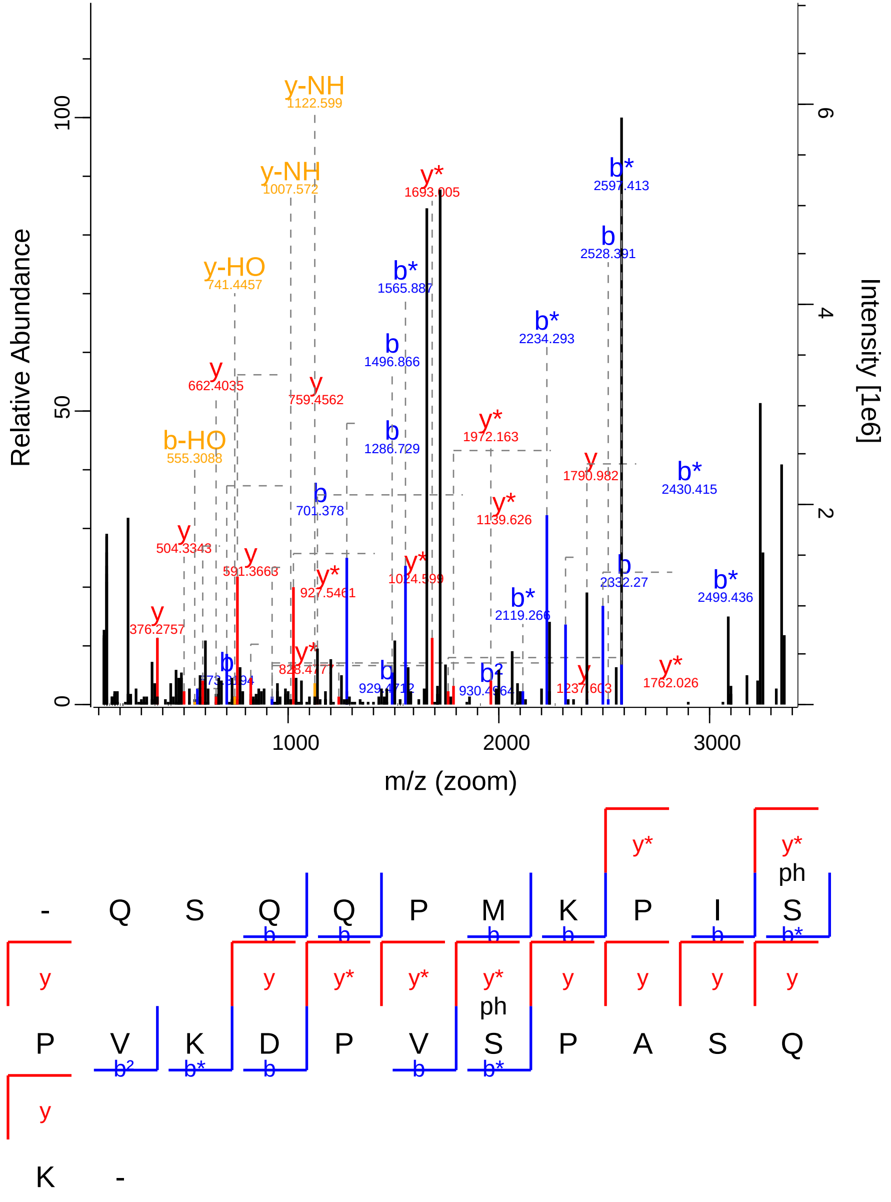

Raw File	Scan	Method	Score	m/z	Gene names
RO-QC-160121_ZackD_SS_Set_02_Fractio	20994	FTMS; HC	153.39	1067.58	MICALL1

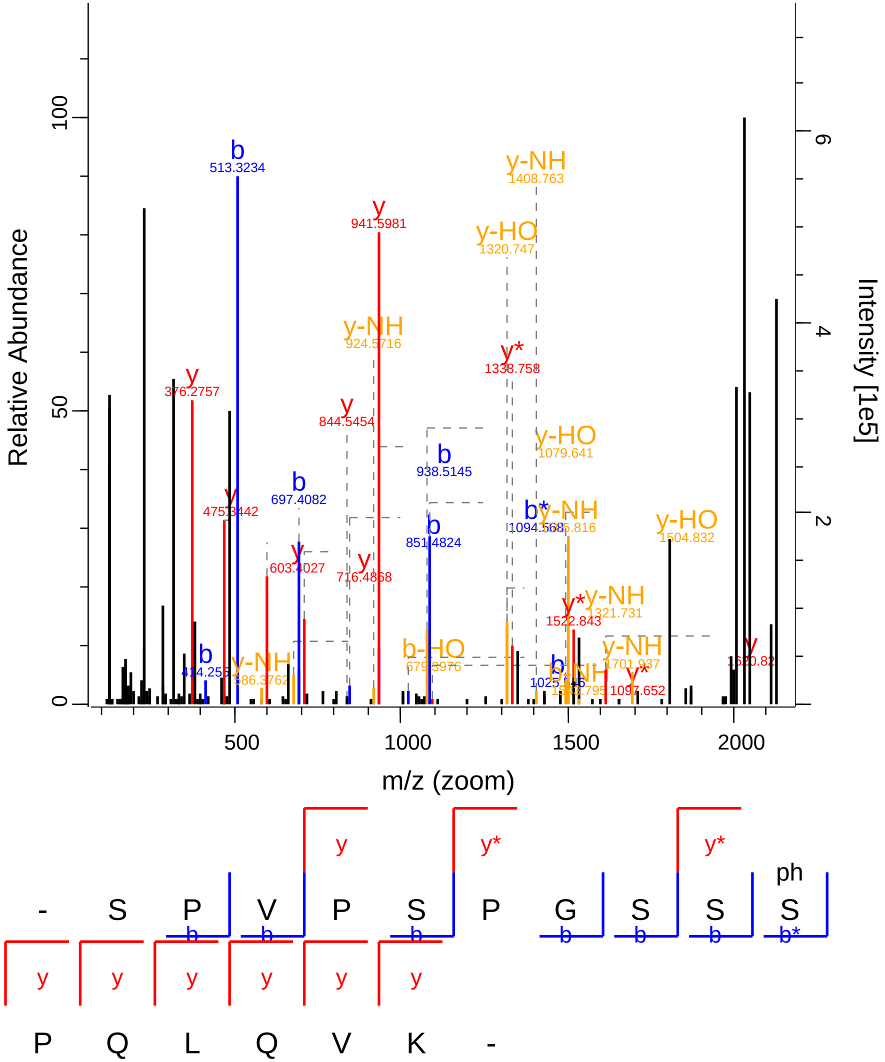

Raw File	Scan	Method	Score	m/z	Gene names
RO-QC-160121_ZackD_SS_Set_02_Fractio	21140	FTMS; HC	79.01	711.72	MICALL1

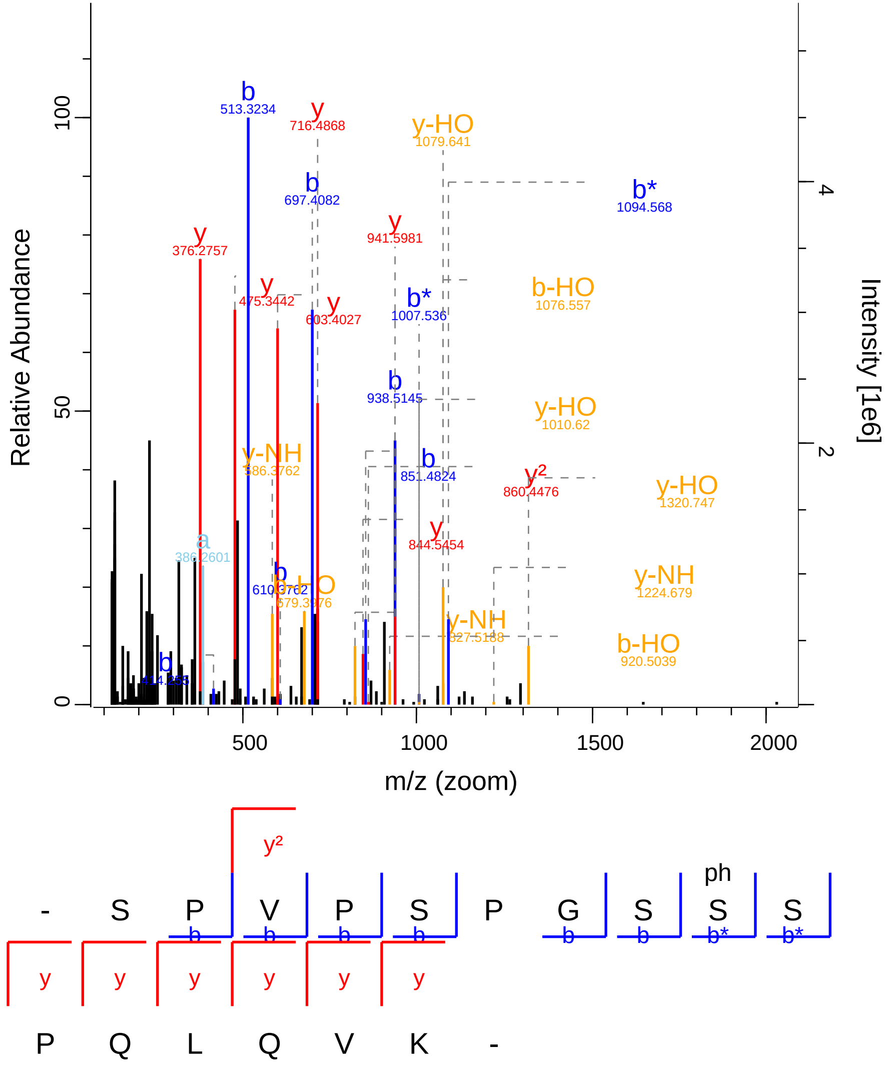

Raw File	Scan	Method	Score	m/z	Gene names
RO-QC-160121_ZackD_SS_Set_02_Fractic	21595	FTMS; HC	261.26	1097.56	AHNAK

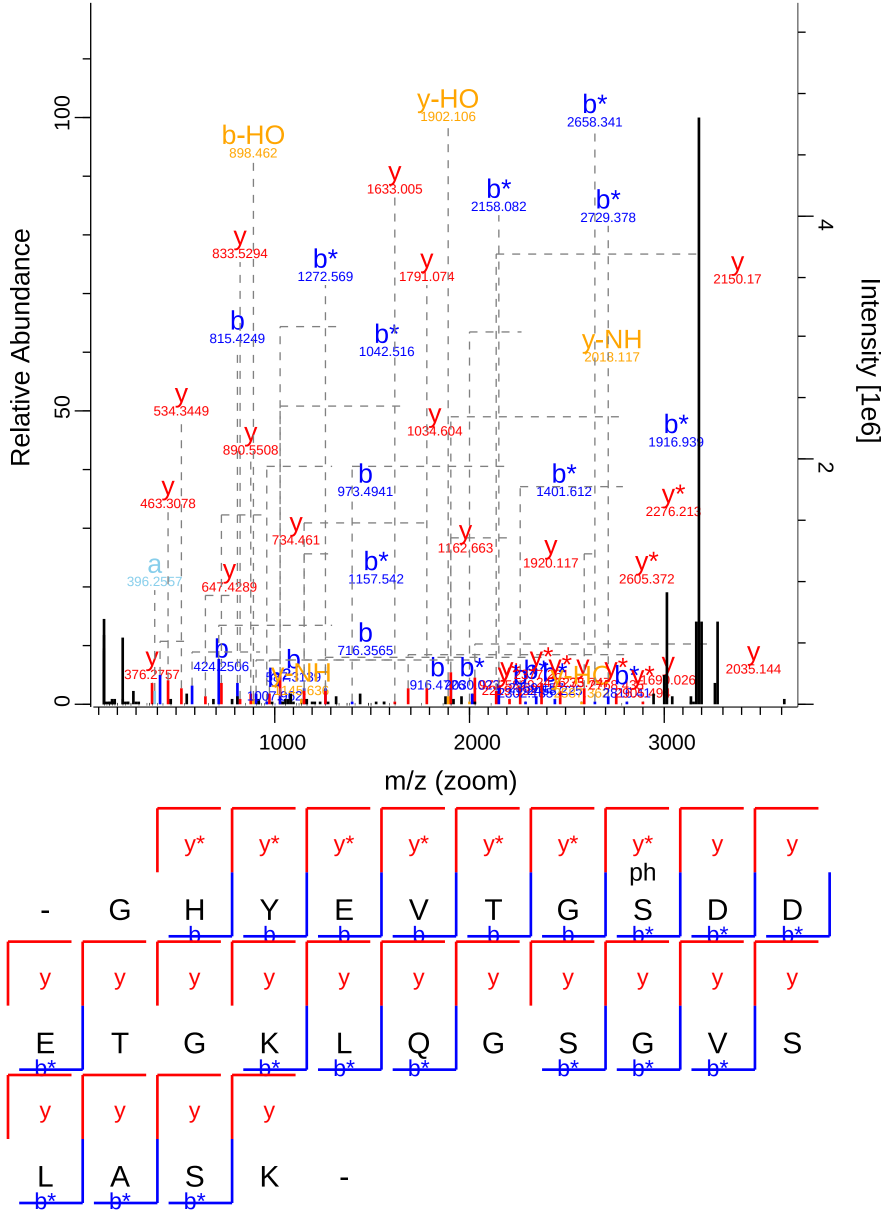

Raw File	Scan	Method	Score	m/z	Gene names
RO-QC-160121_ZackD_SS_Set_02_Fractio	21831	FTMS; HC	81.62	827.81	LARP1

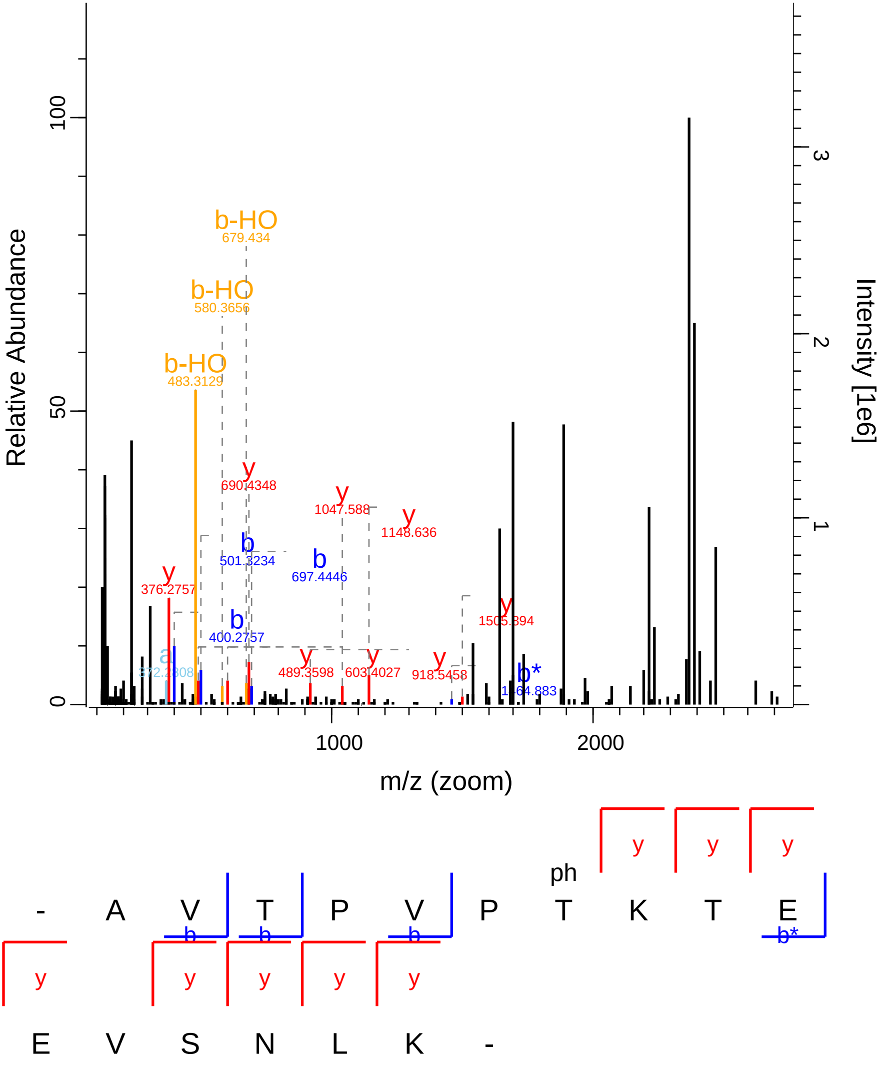

Raw File	Scan	Method	Score	m/z
RO-QC-160121_ZackD_SS_Set_02_Fraction_1	21849	FTMS; HCD	95.1	892.47

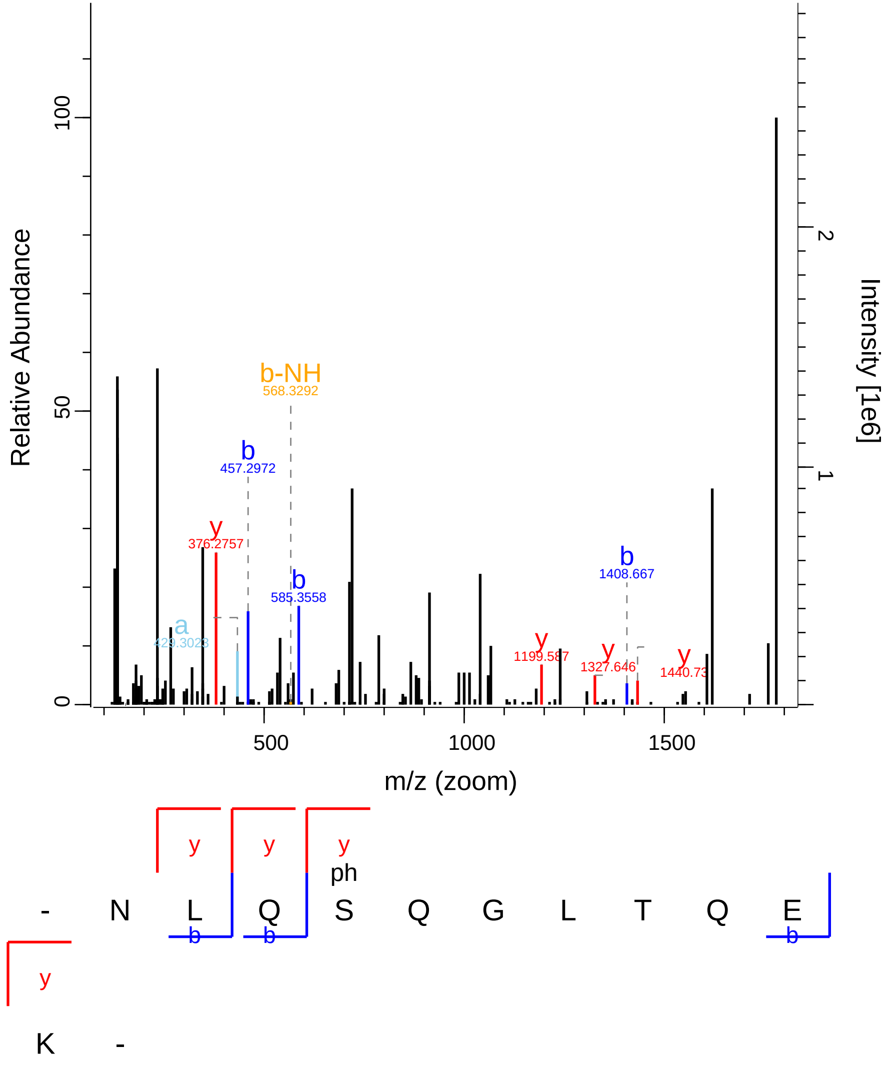

Raw File	Scan	Method	Score	m/z	Gene names
RO-QC-160121_ZackD_SS_Set_02_Fractio	22098	FTMS; HC	47.99	597.28	ERMP1

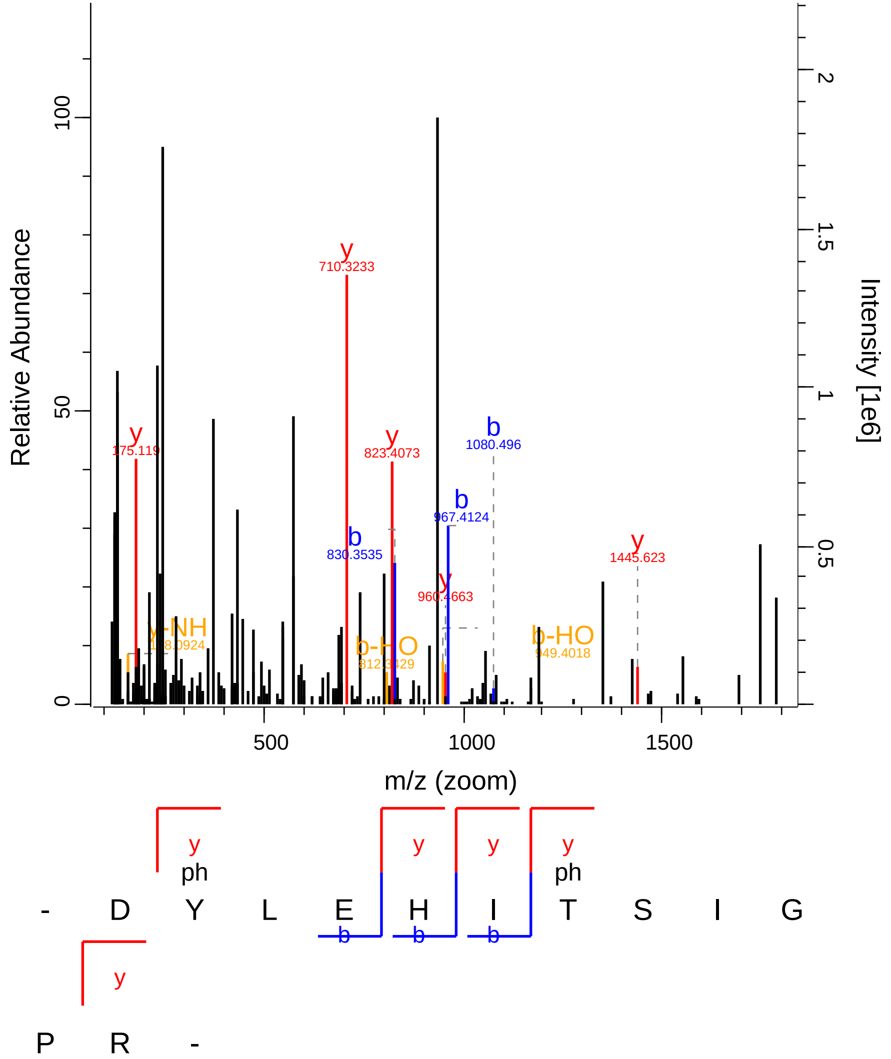

Raw File	Scan	Method	Score	m/z	Gene names
RO-QC-160121_ZackD_SS_Set_02_Fractio	23365	FTMS; HC	136.63	787.12	PCBP1

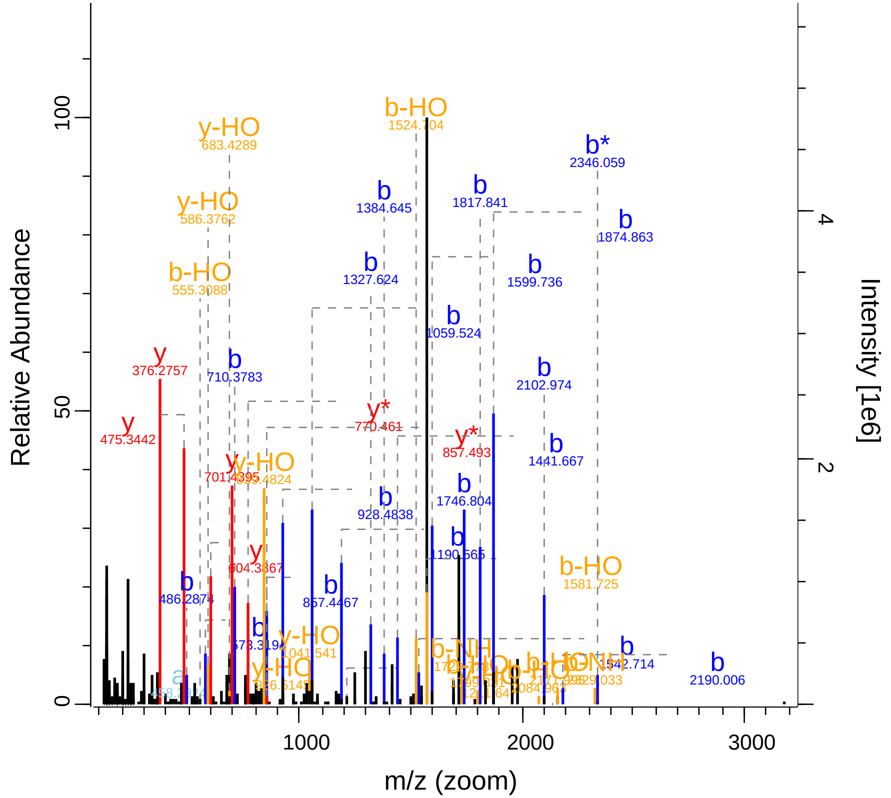

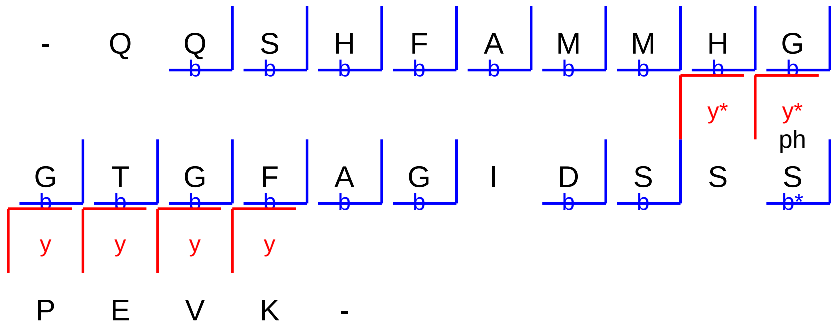

Raw File	Scan	Method	Score	m/z	Gene names
RO-QC-160121_ZackD_SS_Set_02_Fracti	23584	FTMS; HC	88.14	862.96	CDK1;CDK2;CDK

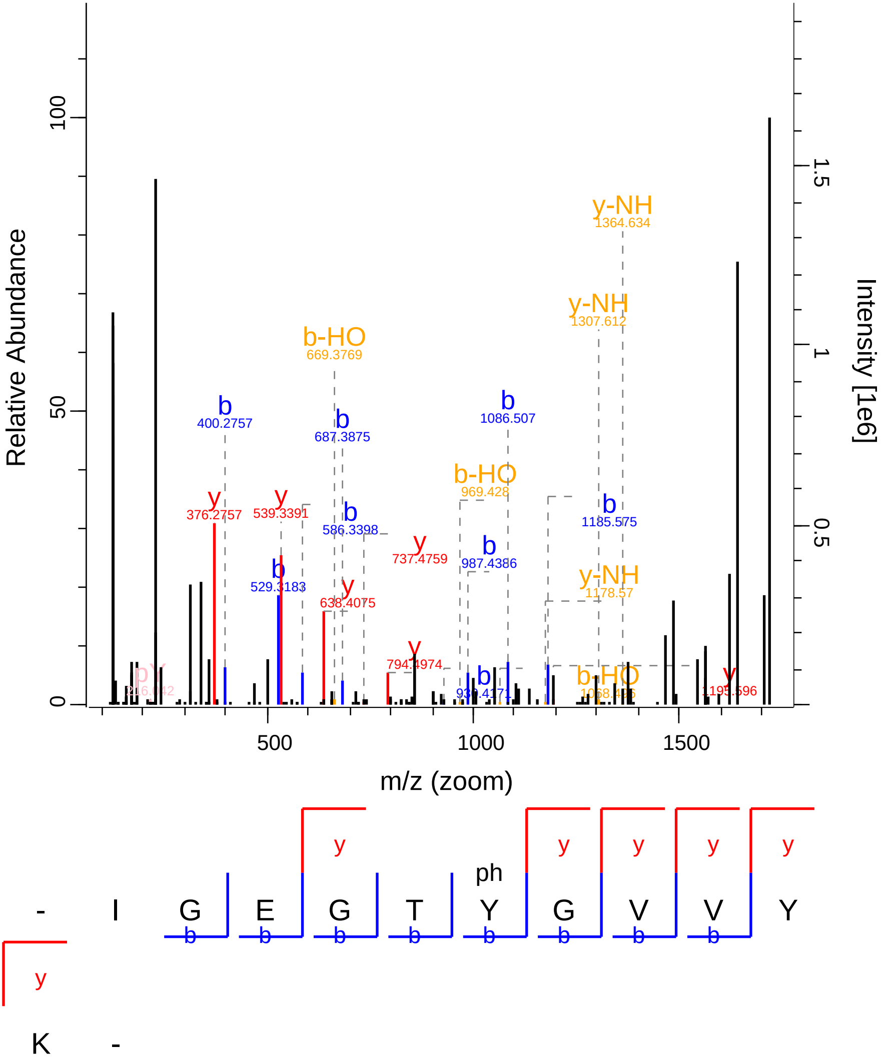

Raw File	Scan	Method	Score	m/z	Gene names
RO-QC-160121_ZackD_SS_Set_02_Fractio	23816	FTMS; HC	132.51	1065.58	MAP4

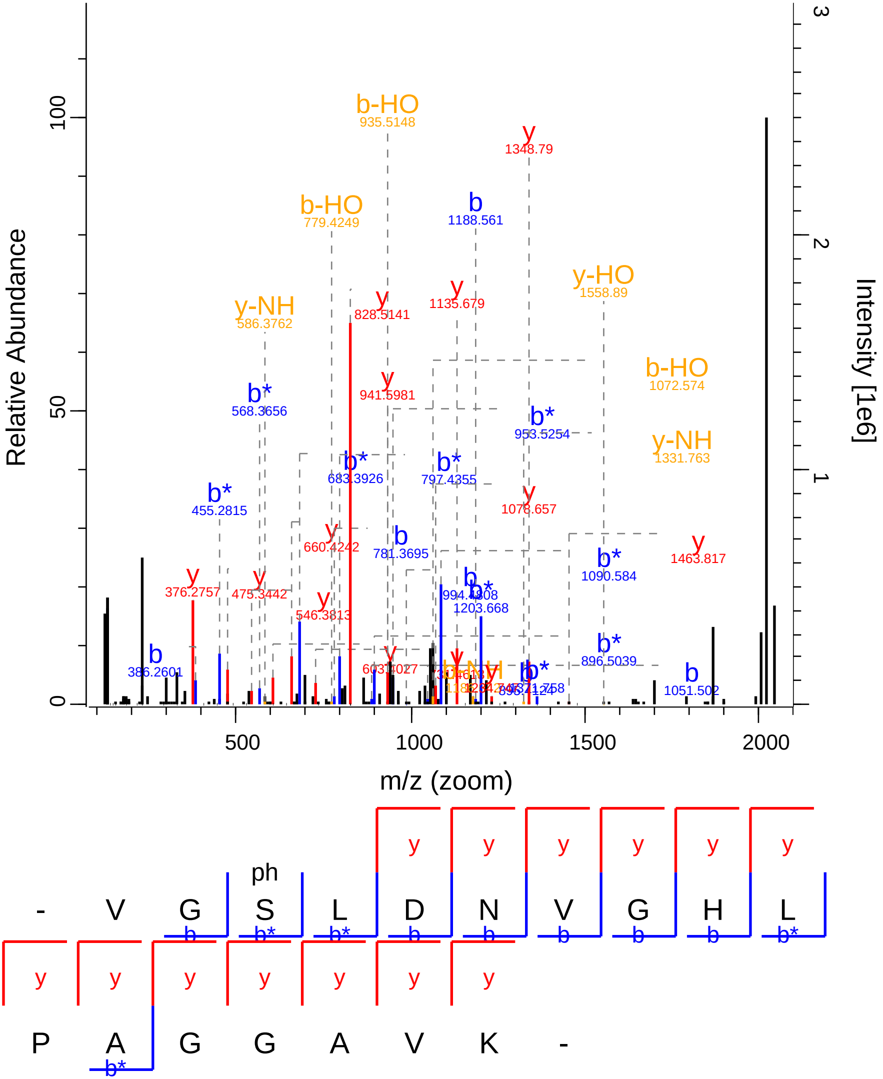

Raw File	Scan	Method	Score	m/z	Gene names
RO-QC-160121_ZackD_SS_Set_02_Fractio	24572	FTMS; HC	84.17	824.77	PNN

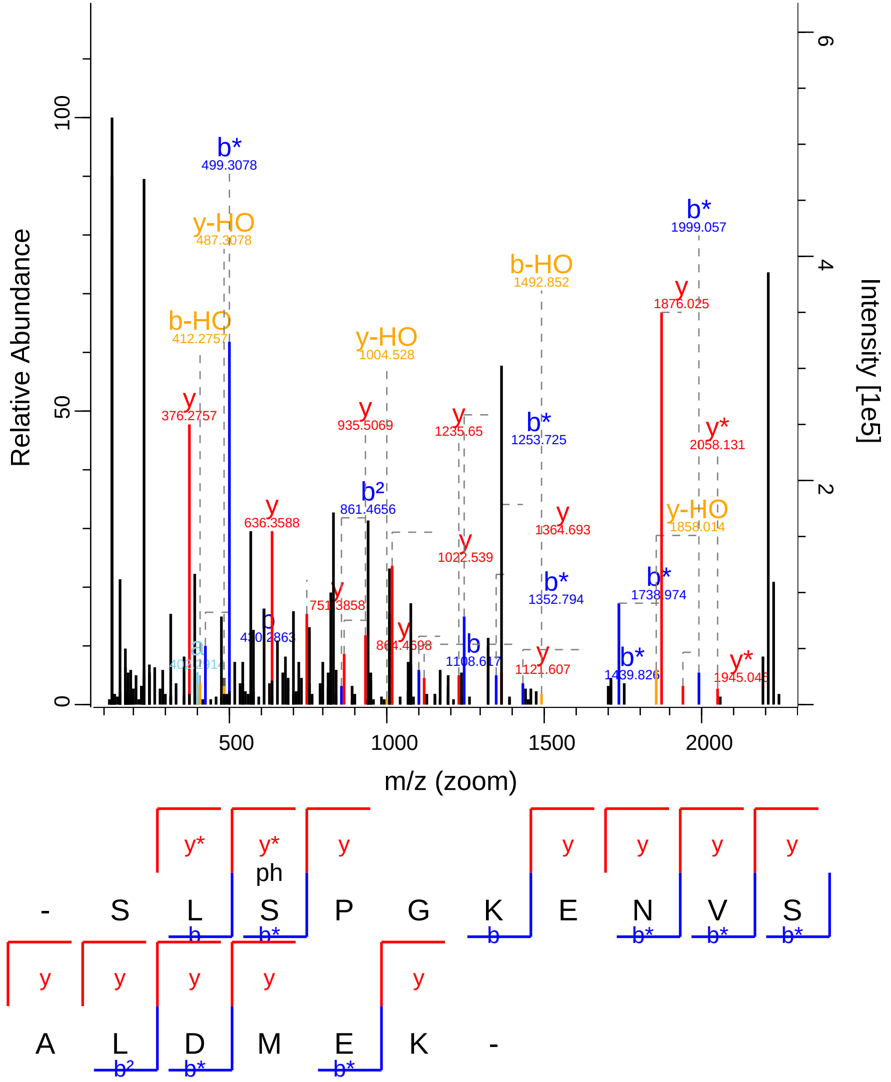

Raw File	Scan	Method	Score	m/z	Gene names
RO-QC-160121_ZackD_SS_Set_02_Fractio	25477	FTMS; HC	125.39	896.78	NOB1

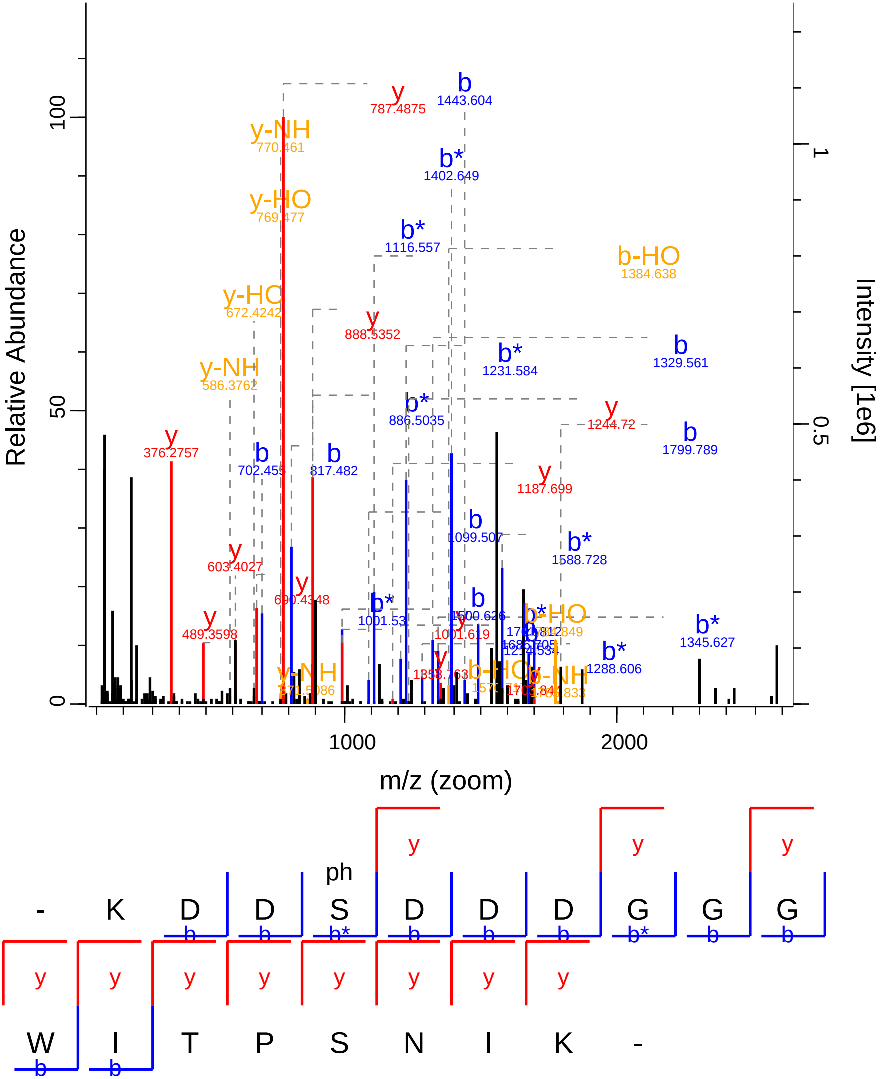

Raw File	Scan	Method	Score	m/z	Gene names
RO-QC-160121_ZackD_SS_Set_02_Fractio	25973	FTMS; HC	41.54	414.22	MACF1

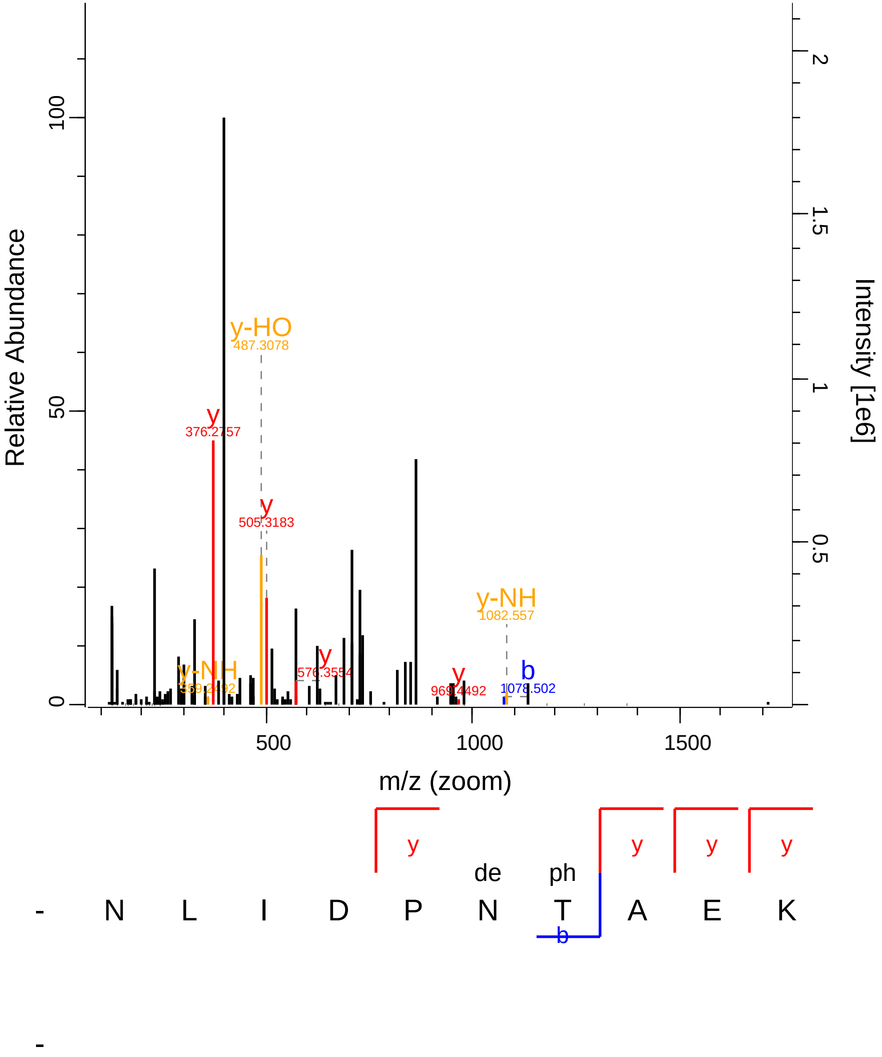

Raw File	Scan	Method	Score	m/z	Gene names
RO-QC-160121_ZackD_SS_Set_02_Fractio	26534	FTMS; HC	83.05	602.63	INA

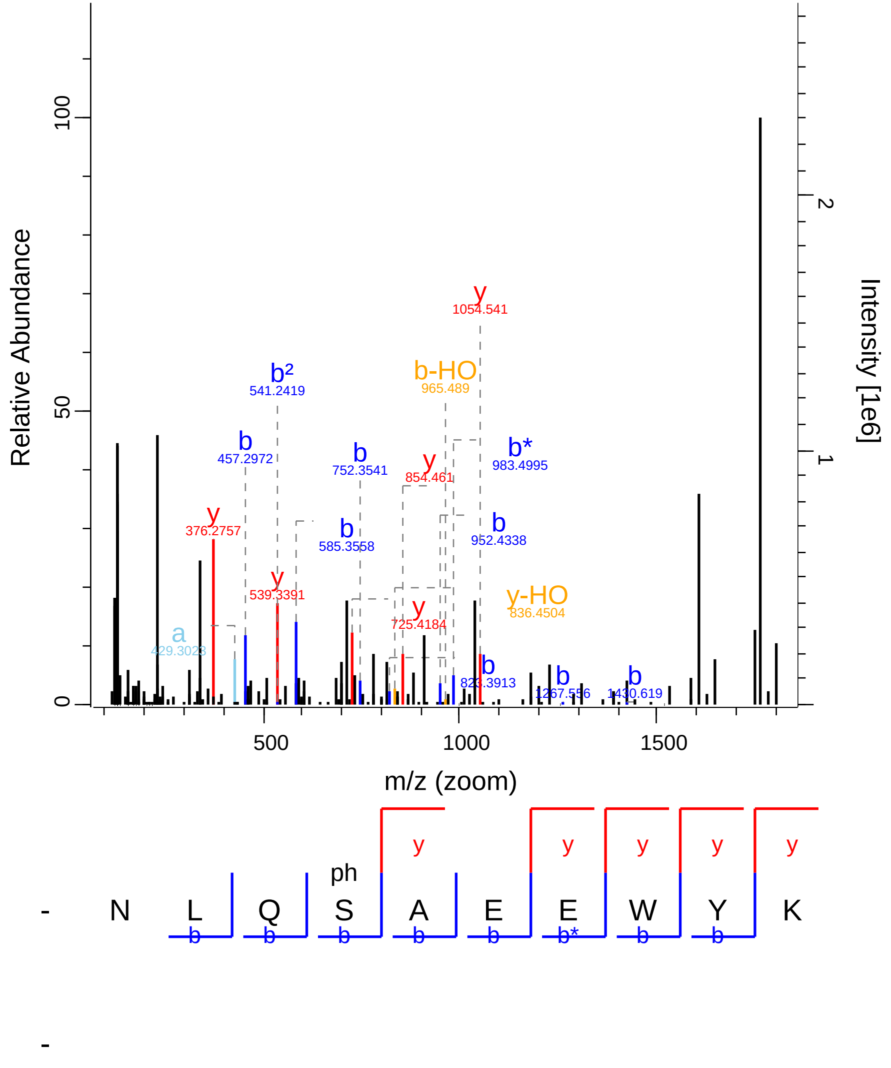

Raw File	Scan	Method	Score	m/z	Gene names
RO-QC-160121_ZackD_SS_Set_02_Fractior	27531	FTMS; HCI	106.13	583.3	PHIP

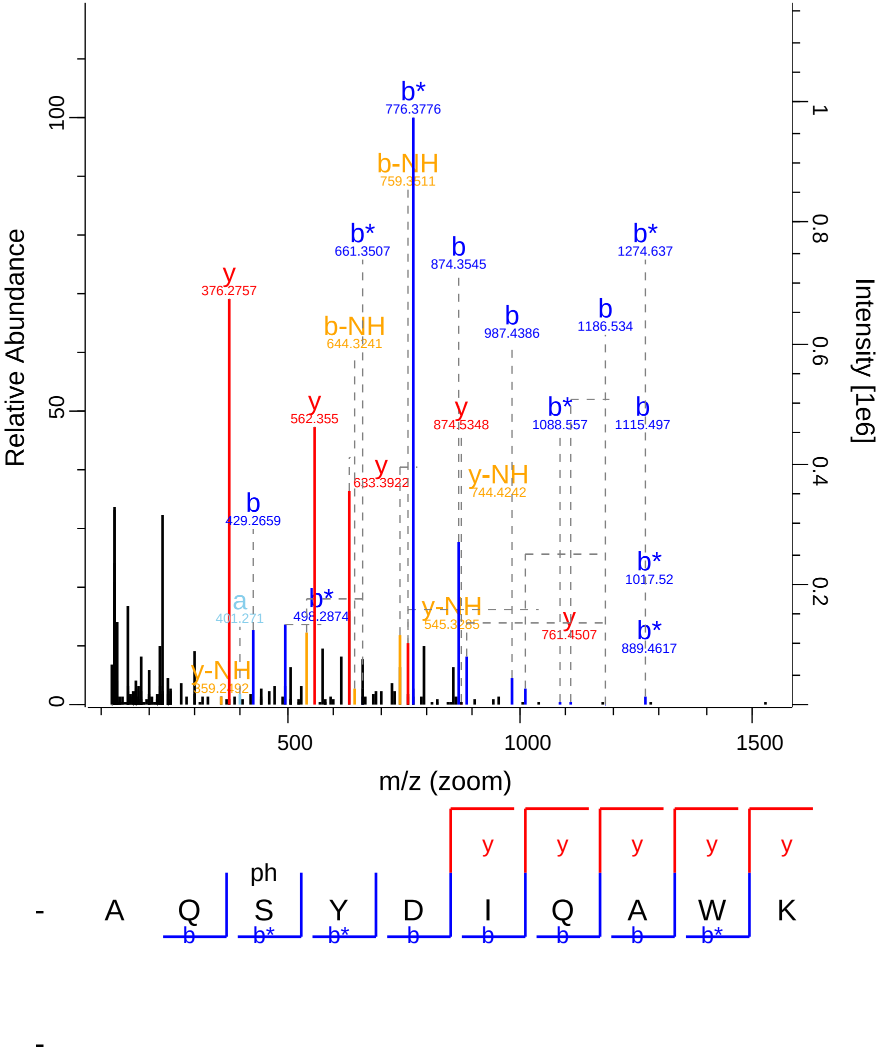

Raw File	Scan	Method	Score	m/z	Gene names
RO-QC-160121_ZackD_SS_Set_02_Fractic	28126	FTMS; HC	93.36	1031.03	UBA1

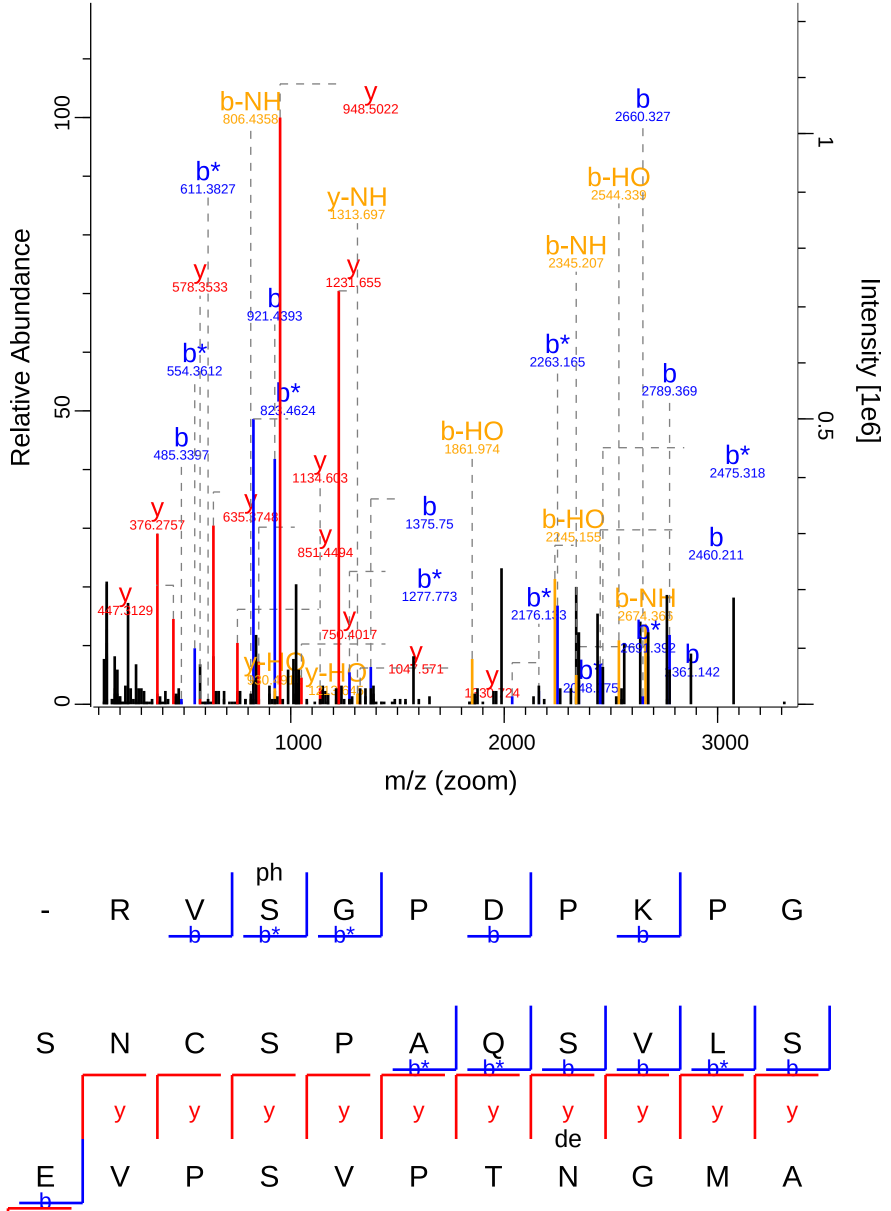

Raw File	Scan	Method	Score	m/z
RO-QC-160121_ZackD_SS_Set_02_Fraction_1	28137	FTMS; HCD	71.18	1262.15

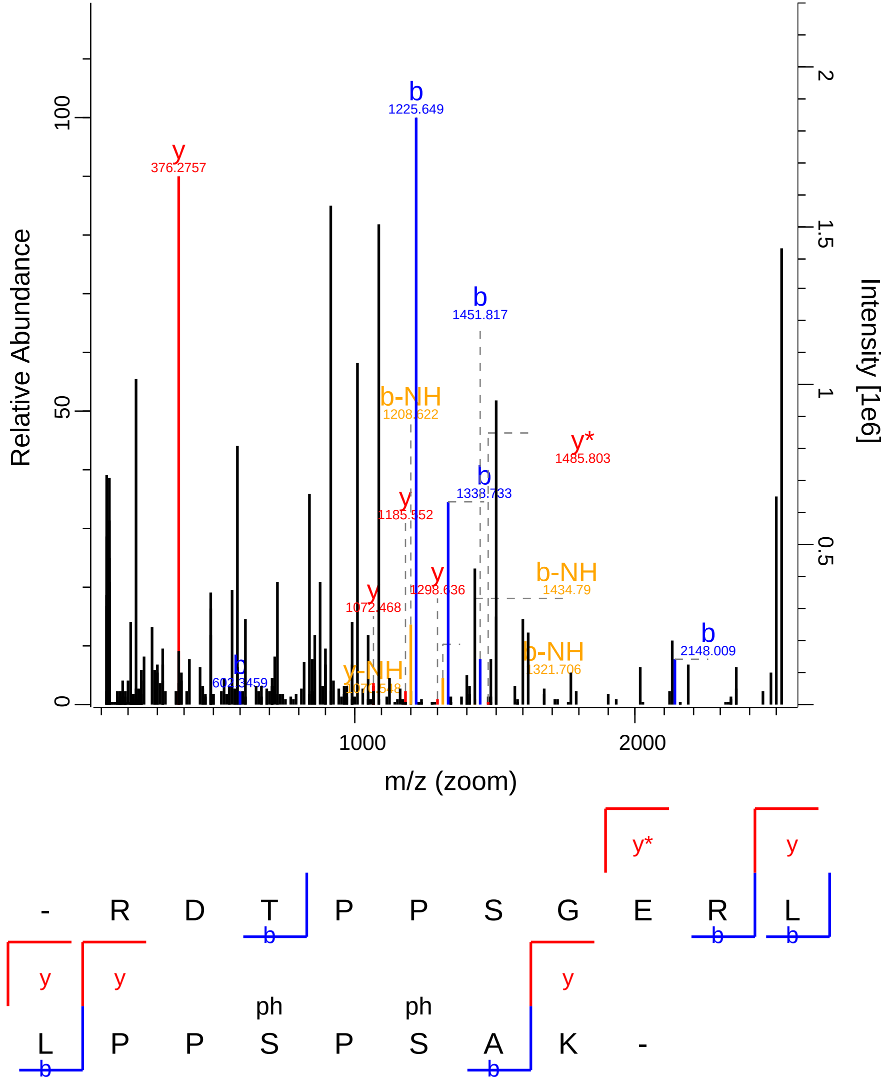

Raw File	Scan	Method	Score	m/z	Gene names
RO-QC-160121_ZackD_SS_Set_02_Fractio	28479	FTMS; HC	51.21	843.44	AKAP9

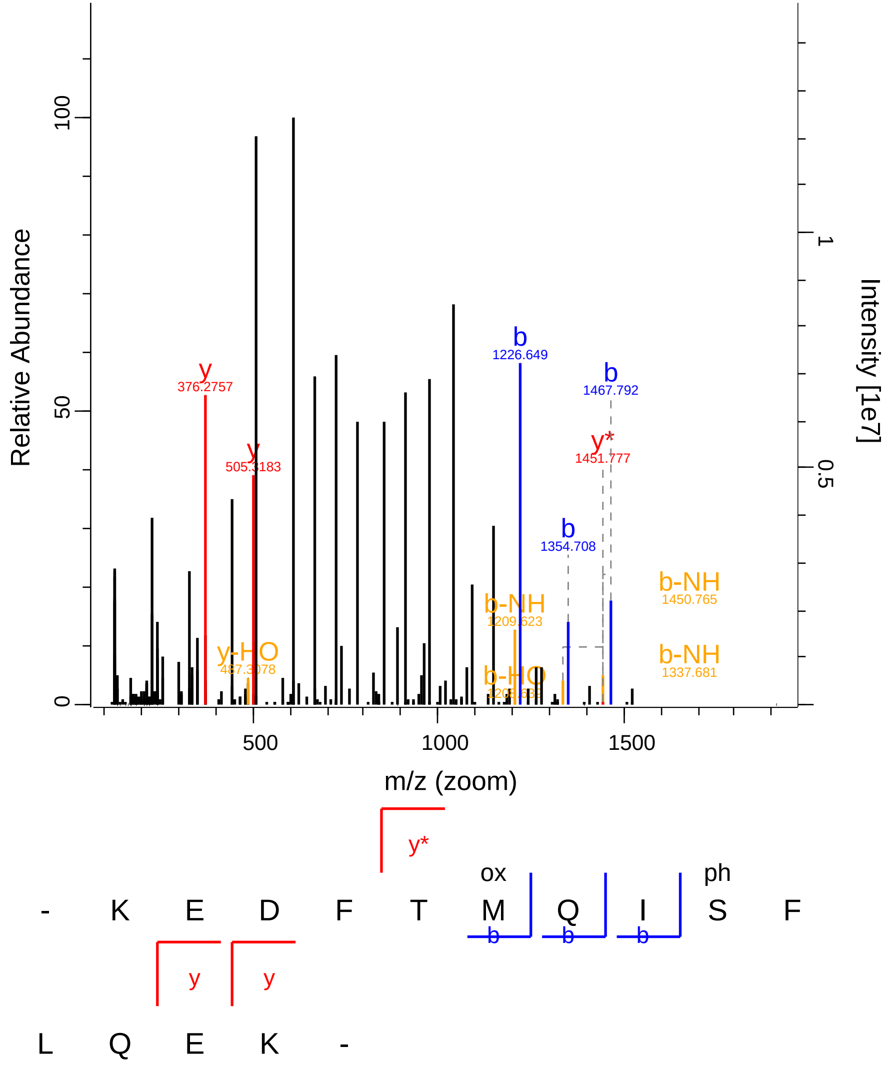

Raw File	Scan	Method	Score	m/z	Gene names
RO-QC-160121_ZackD_SS_Set_02_Fractio	28708	FTMS; HC	140.35	807.45	PSIP1

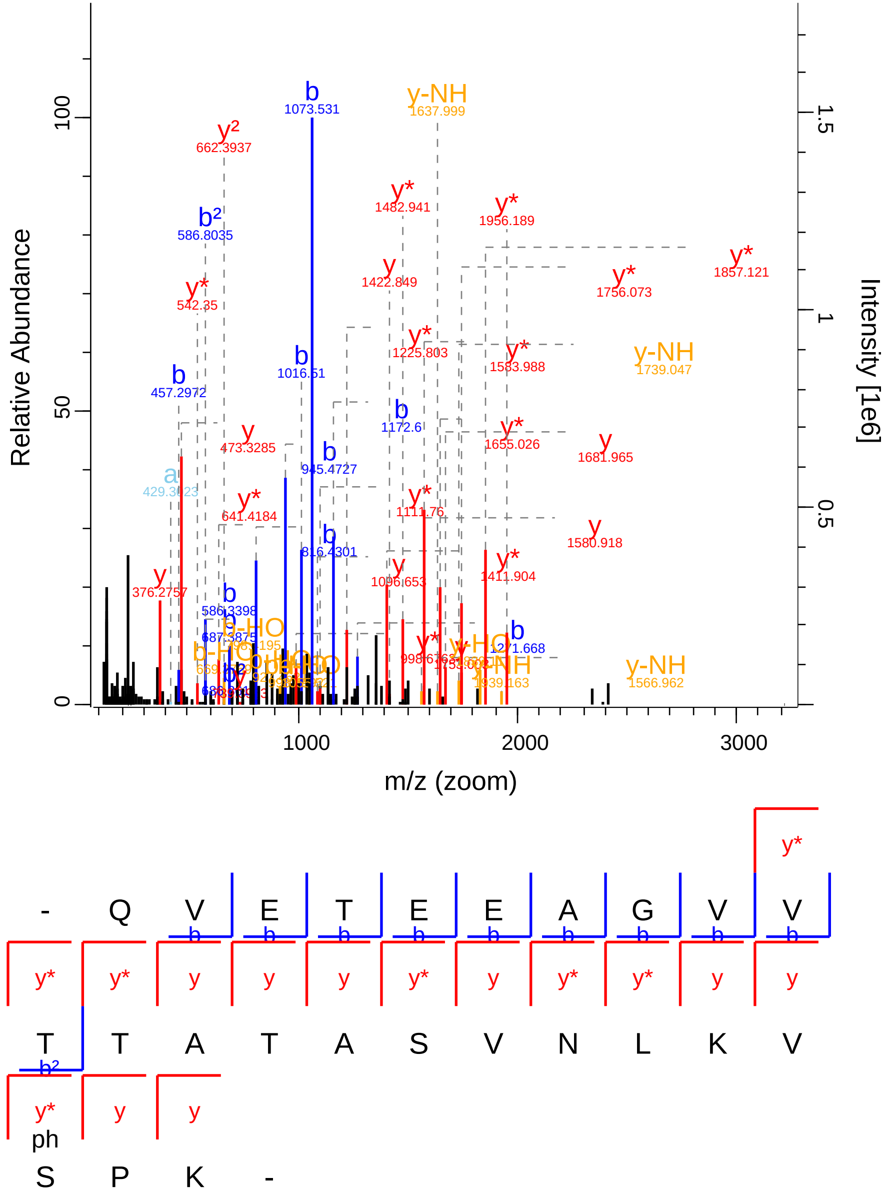

Raw File	Scan	Method	Score	m/z	Gene names
RO-QC-160121_ZackD_SS_Set_02_Fractio	28898	FTMS; HC	105.39	636.34	PPP1R12A

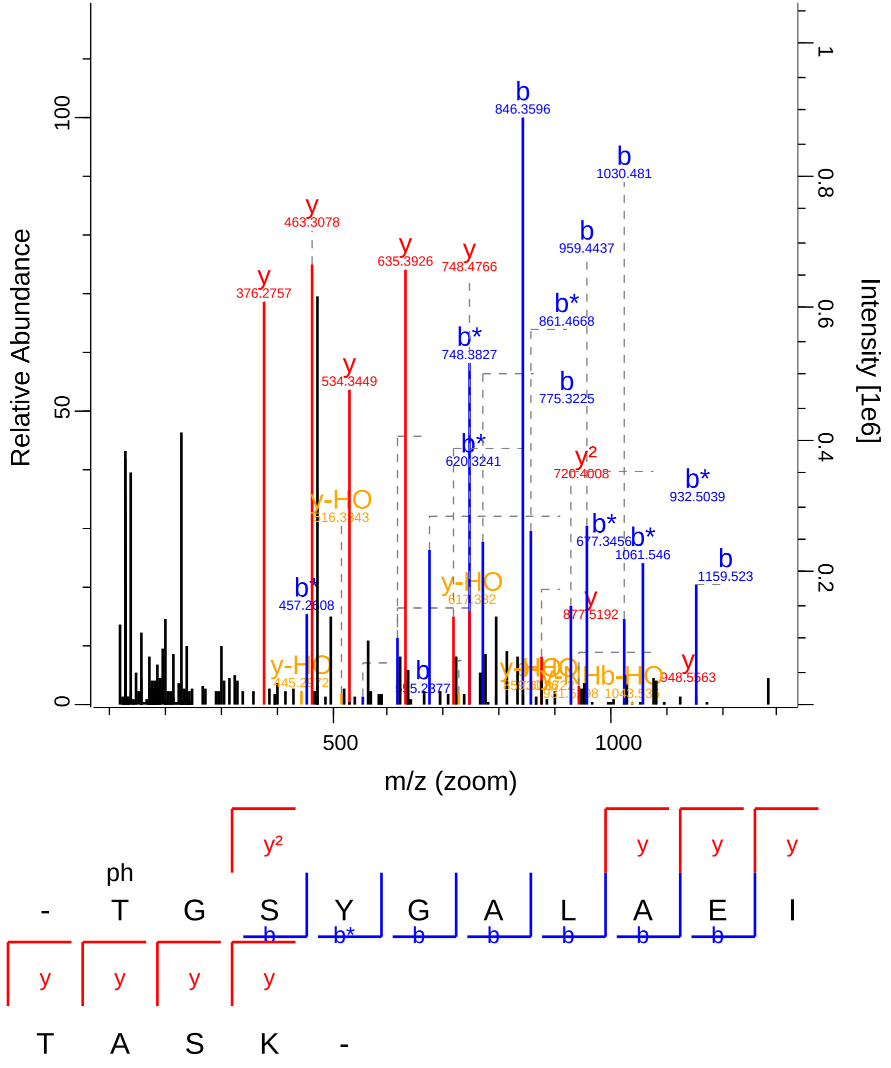

Raw File	Scan	Method	Score	m/z	Gene names
RO-QC-160121_ZackD_SS_Set_02_Fractio	29975	FTMS; HC	46.31	876.94	TUBA8

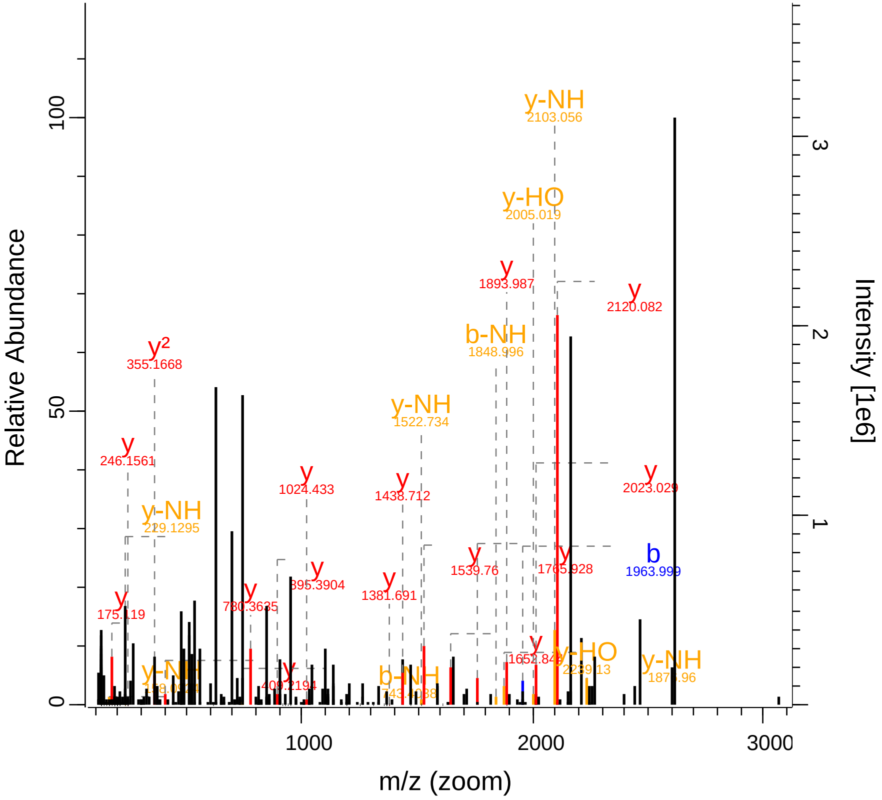

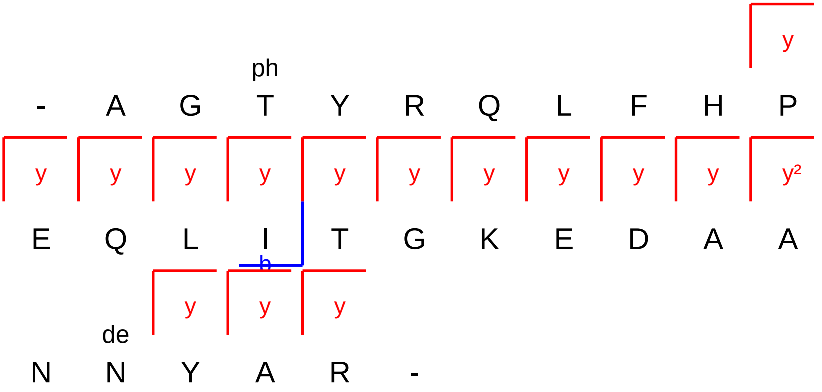

Raw File	Scan	Method	Score	m/z	Gene names
RO-QC-160121_ZackD_SS_Set_02_Fractio	30425	FTMS; HC	84.16	902.48	MAP1A

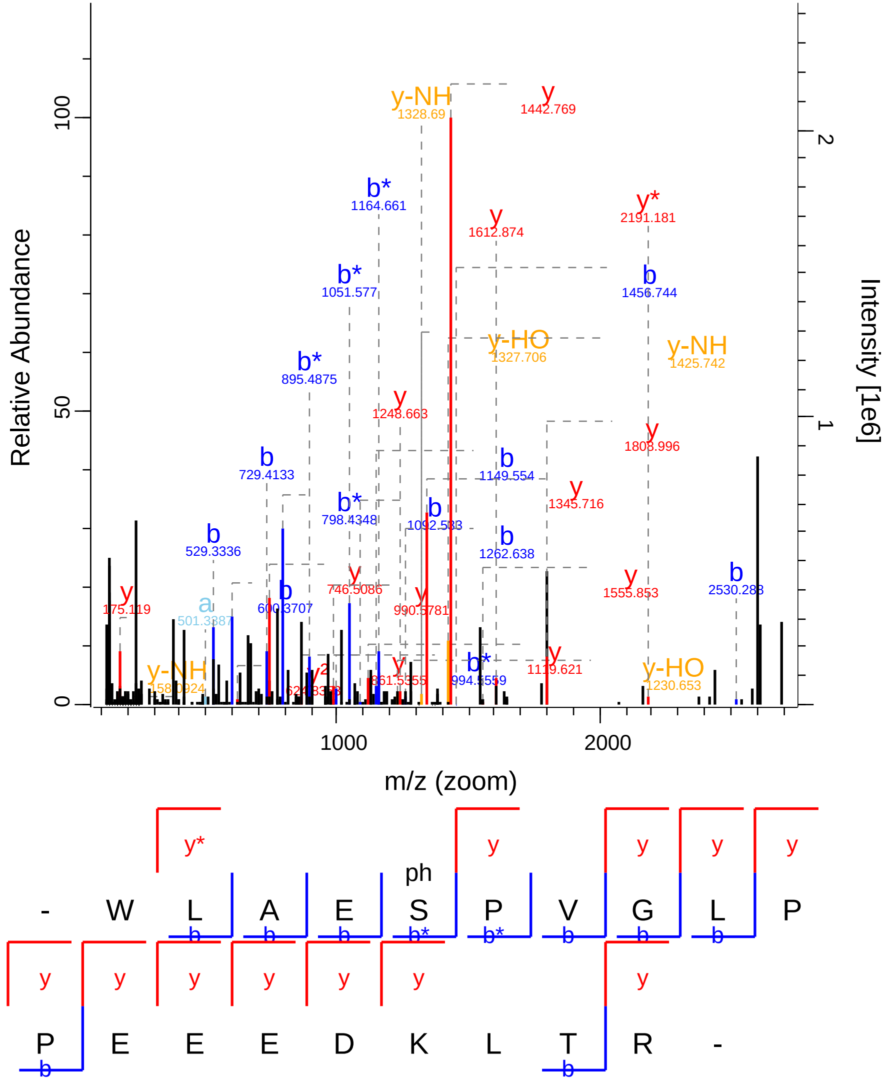

Raw File	Scan	Method	Score	m/z	Gene names
RO-QC-160121_ZackD_SS_Set_02_Fractic	30957	FTMS; HC	93.97	1005.74	OXSR1

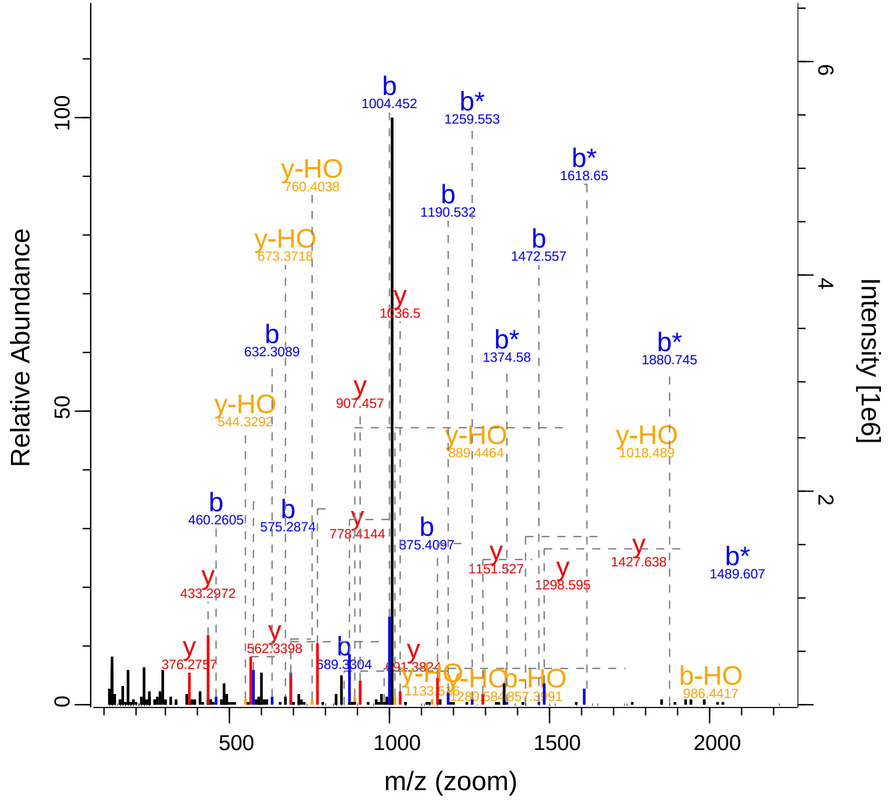

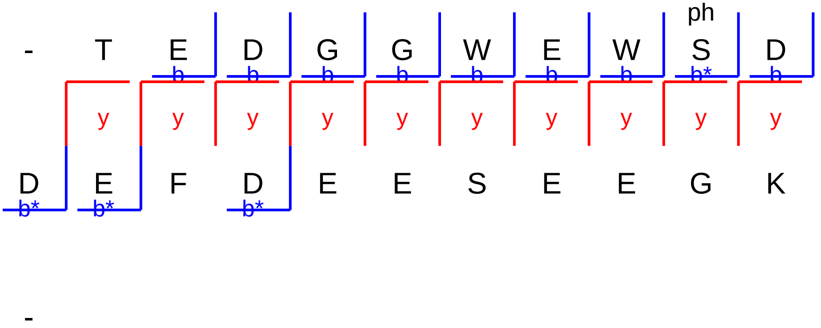

Raw File	Scan	Method	Score	m/z	Gene names
RO-QC-160121_ZackD_SS_Set_02_Fractio	31407	FTMS; HC	137.09	732.36	G3BP1

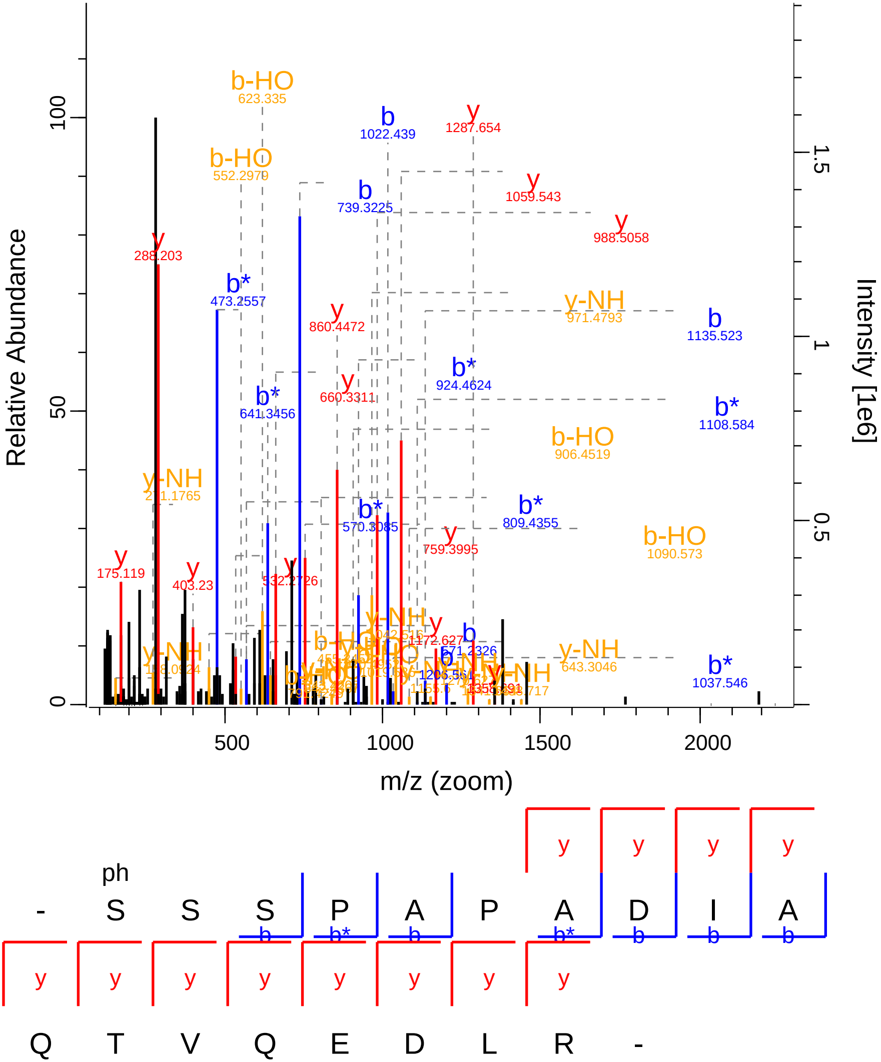

Raw File	Scan	Method	Score	m/z	Gene names
RO-QC-160121_ZackD_SS_Set_02_Fractio	32025	FTMS; HC	108.1	1033.5	PLEC

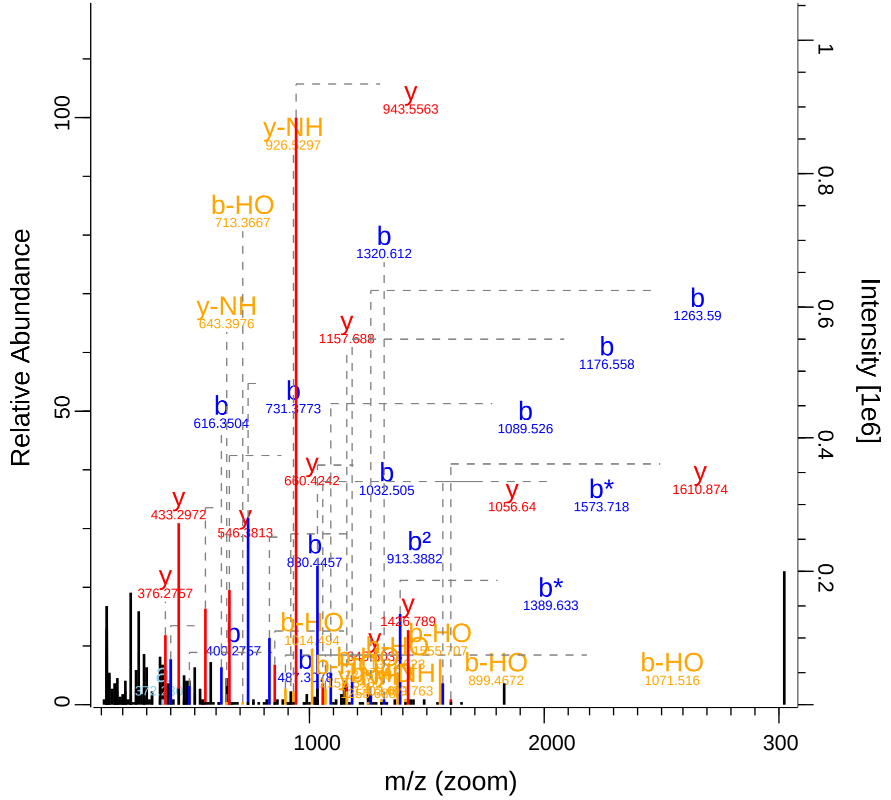

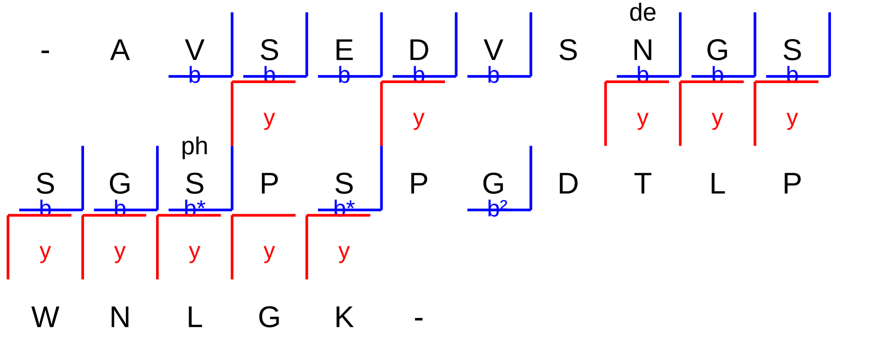

Raw File	Scan	Method	Score	m/z	Gene names
RO-QC-160121_ZackD_SS_Set_02_Fractic	33115	FTMS; HC	139.75	1305.97	SSR1

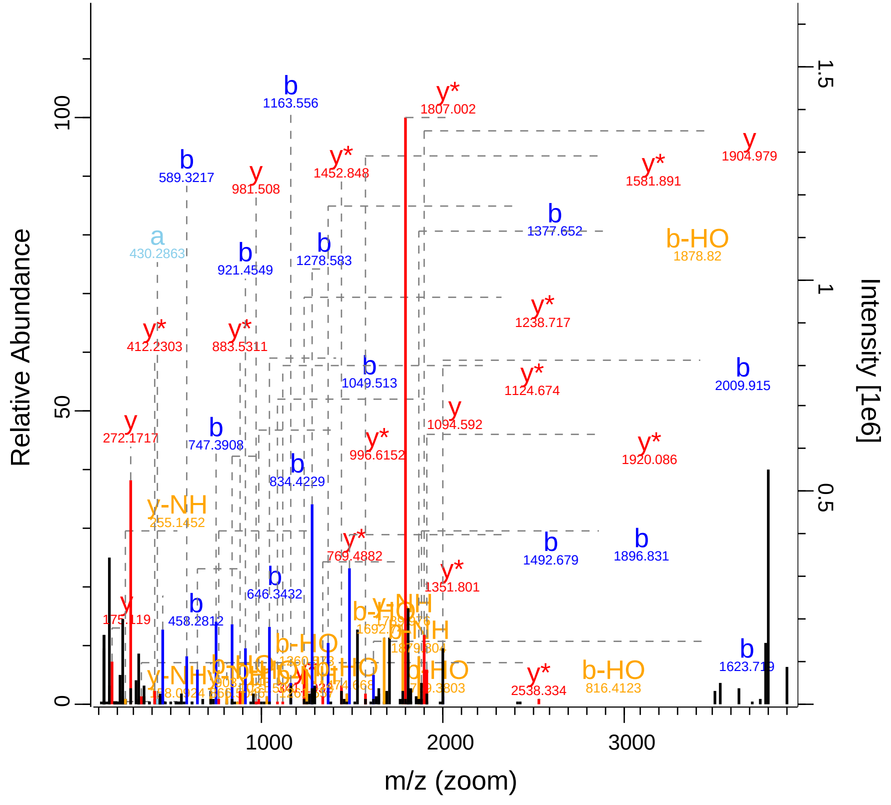

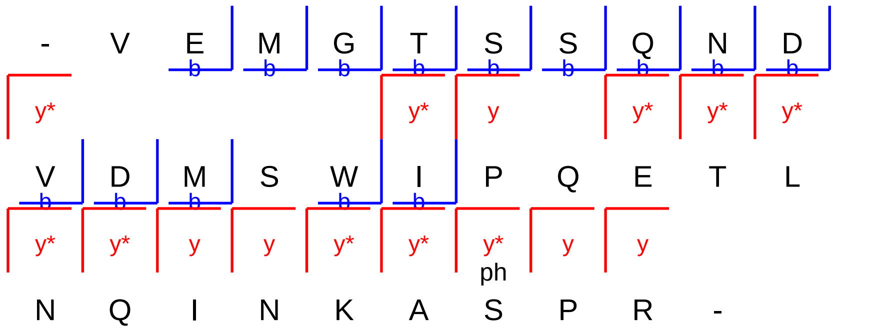

Raw File	Scan	Method	Score	m/z	Gene names
RO-QC-160121_ZackD_SS_Set_02_Fractio	33165	FTMS; HC	91.11	819.93	MYRIP

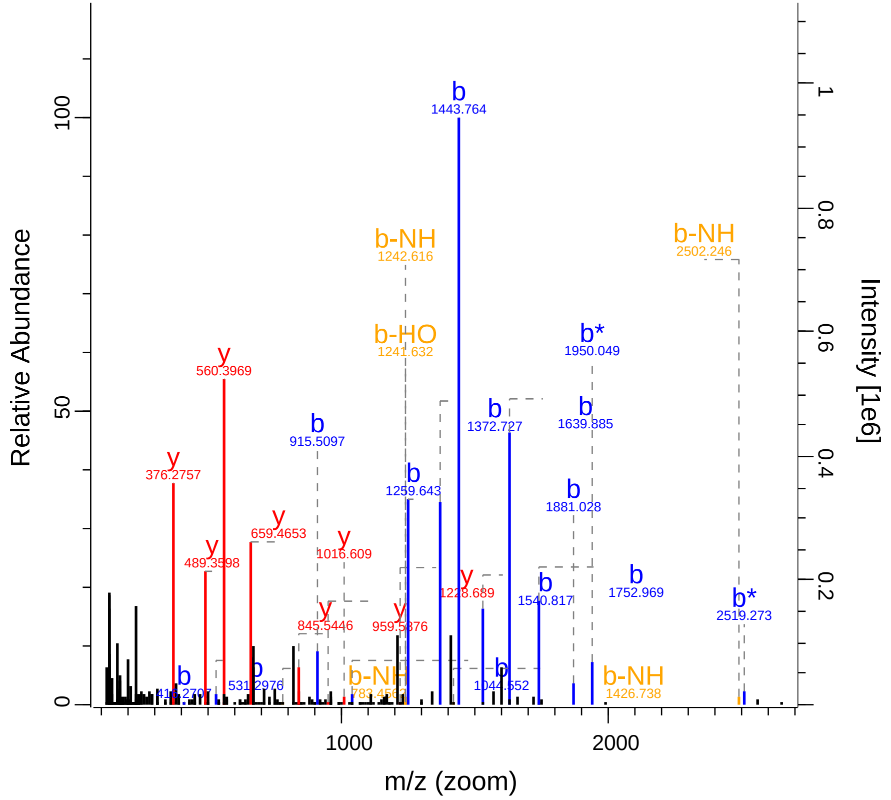

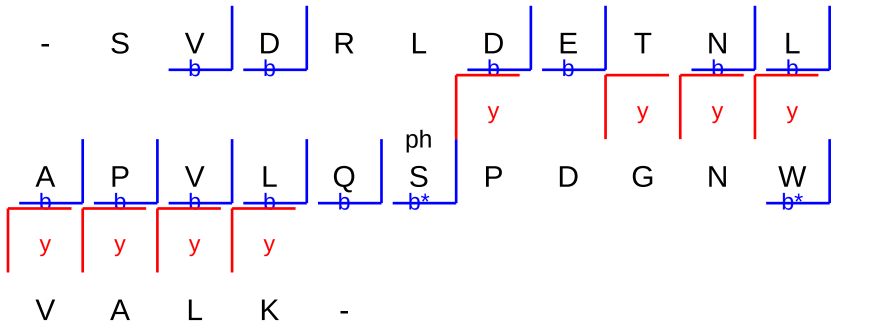

Raw File	Scan	Method	Score	m/z
RO-QC-160121_ZackD_SS_Set_02_Fraction_1	33277	FTMS; HCD	73.91	800.79

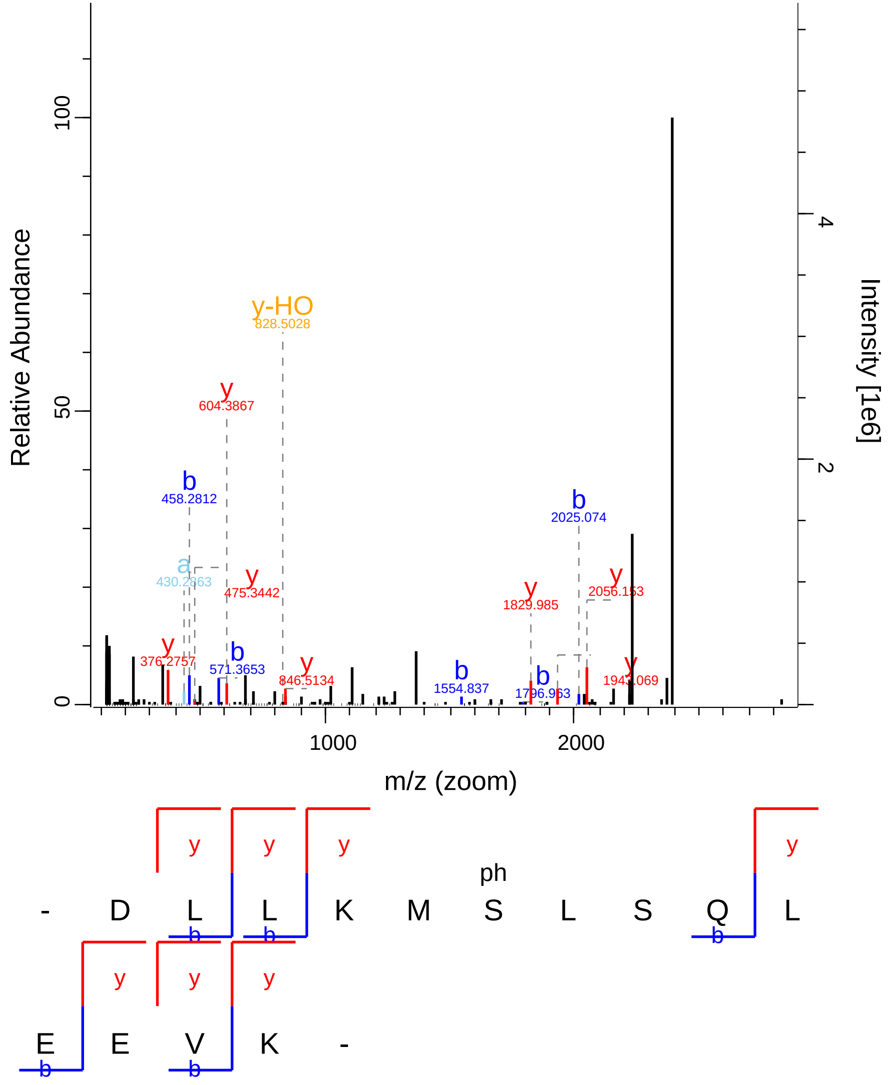

Raw File	Scan	Method	Score	m/z	Gene names
RO-QC-160121_ZackD_SS_Set_02_Fractic	33562	FTMS; HC	133.62	1067.51	SCAF1

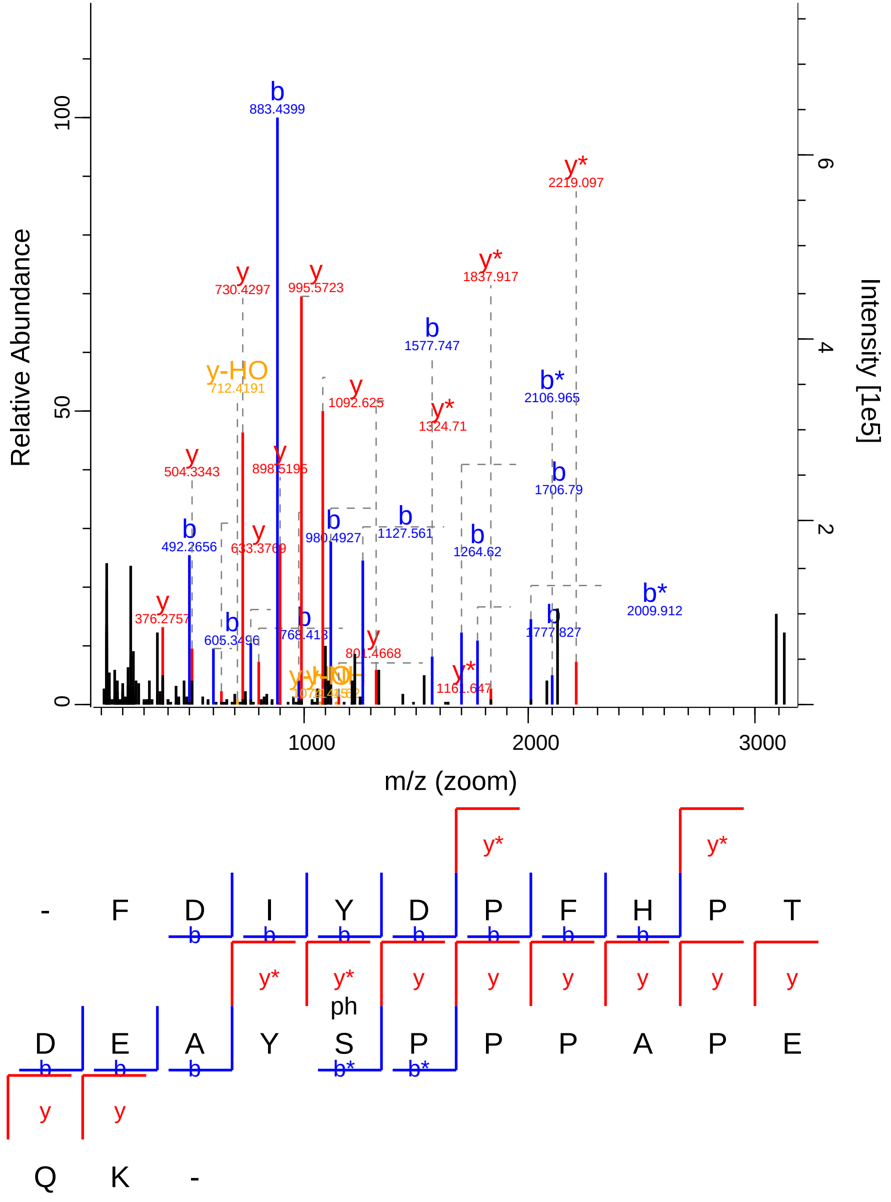

Raw File	Scan	Method	Score	m/z	Gene names
RO-QC-160121_ZackD_SS_Set_02_Fractio	33621	FTMS; HC	132.84	921.49	DMXL1

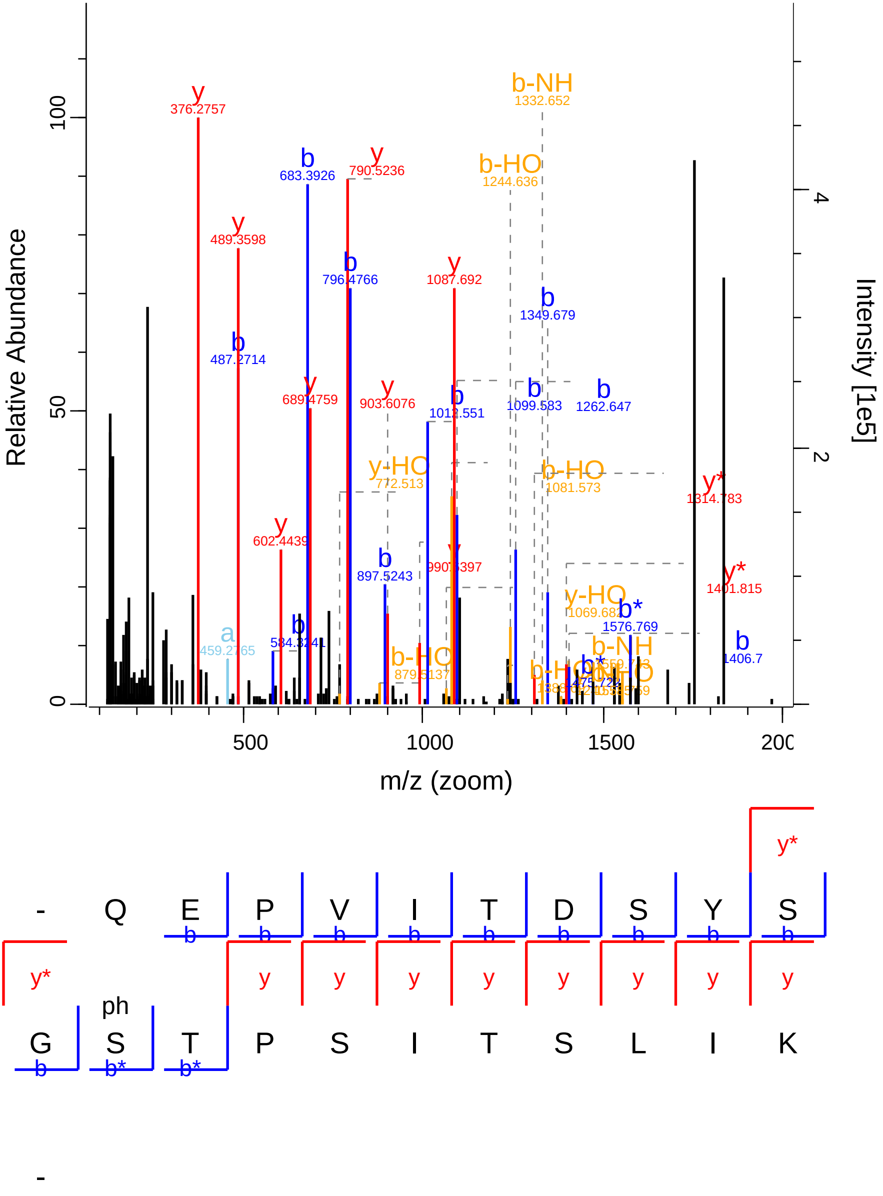

Raw File	Scan	Method	Score	m/z	Gene names
RO-QC-160121_ZackD_SS_Set_02_Fractio	33827	FTMS; HC	75.38	522.29	WASL

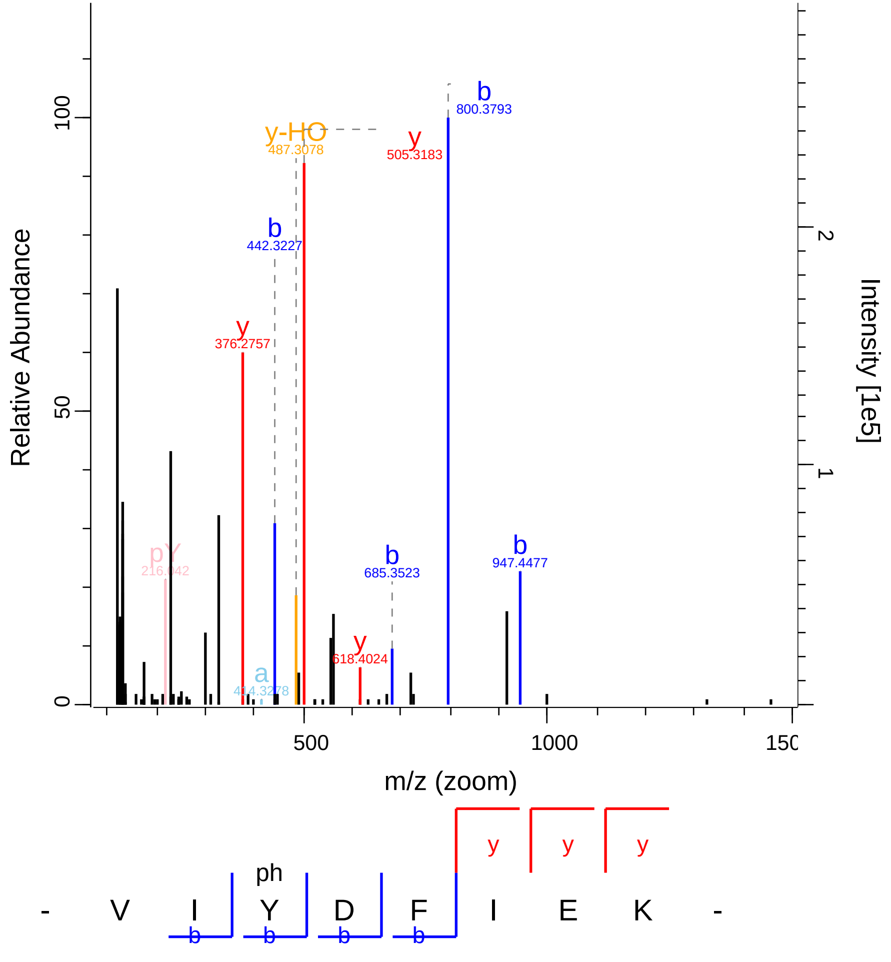

Raw File	Scan	Method	Score	m/z	Gene names
RO-QC-160121_ZackD_SS_Set_02_Fractio	34387	FTMS; HC	149.57	903.41	CRYAB

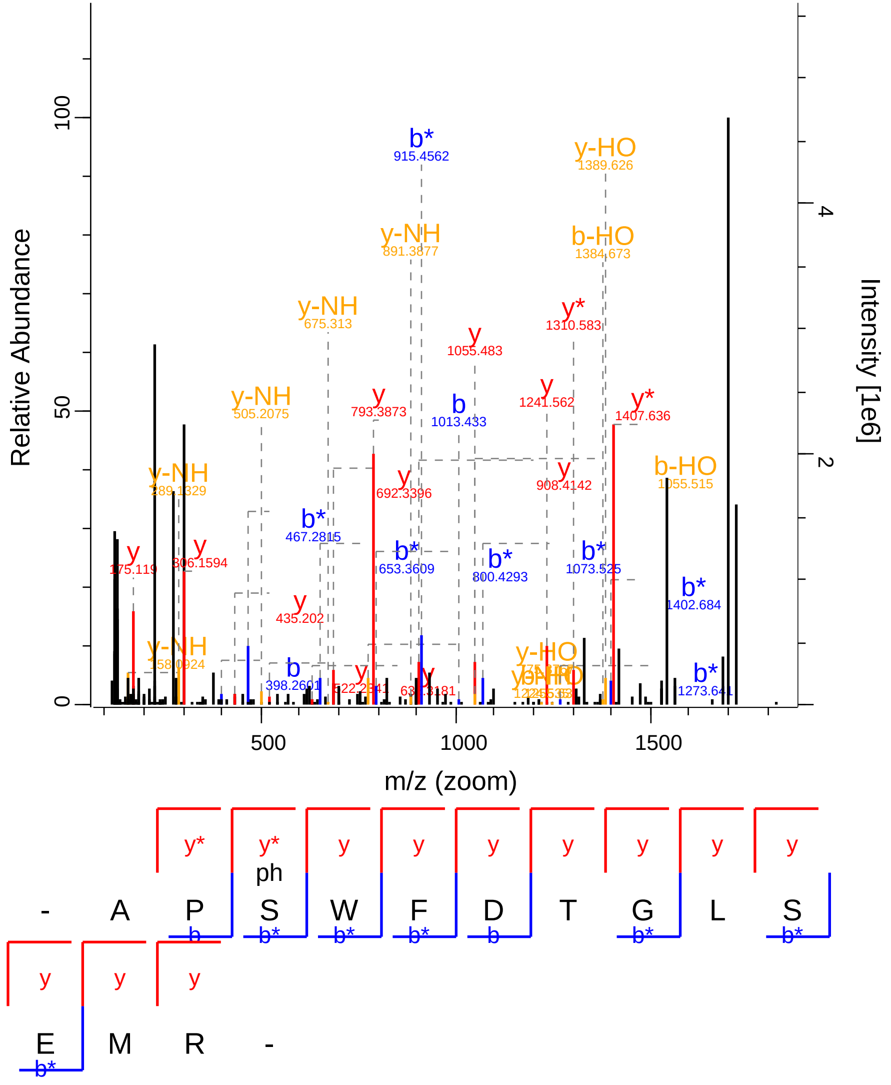

Raw File	Scan	Method	Score	m/z	Gene names
RO-QC-160121_ZackD_SS_Set_02_Fractio	34468	FTMS; HC	63.11	729.88	GPRASP2

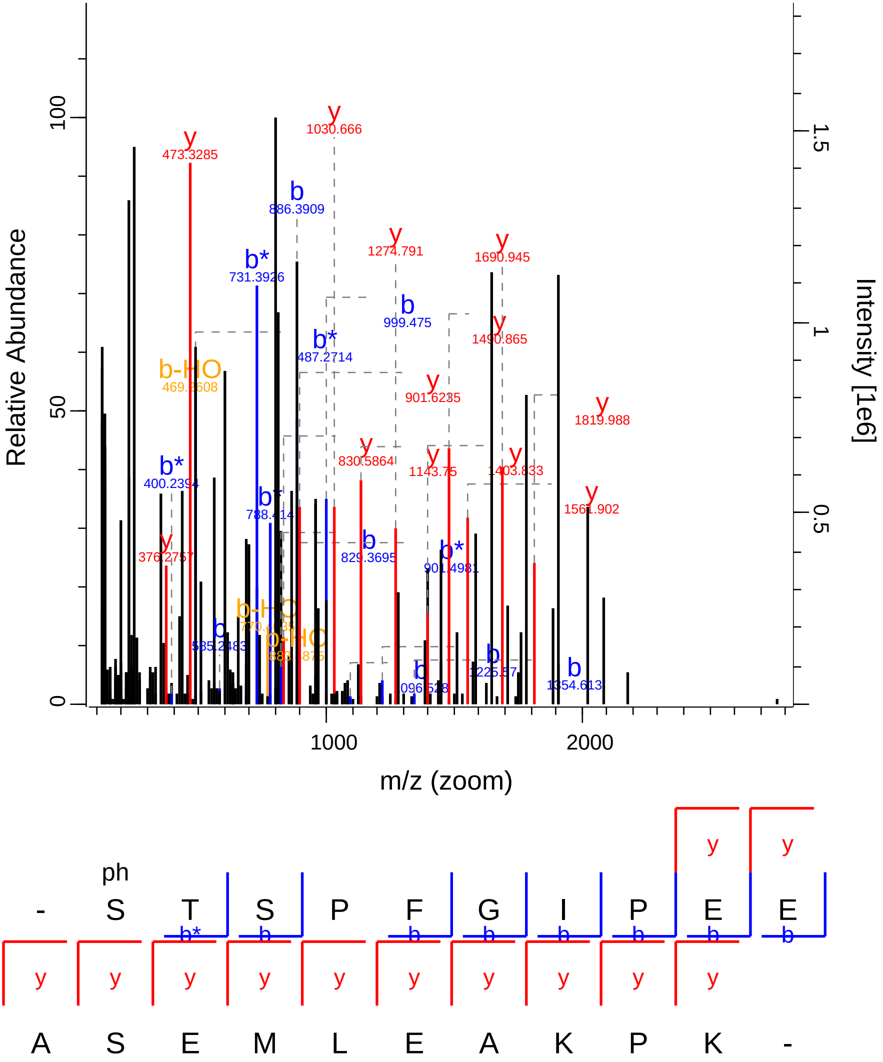

Raw File	Scan	Method	Score	m/z	Gene names
RO-QC-160121_ZackD_SS_Set_02_Fractio	34815	FTMS; HC	152.5	926.47	PRKCQ

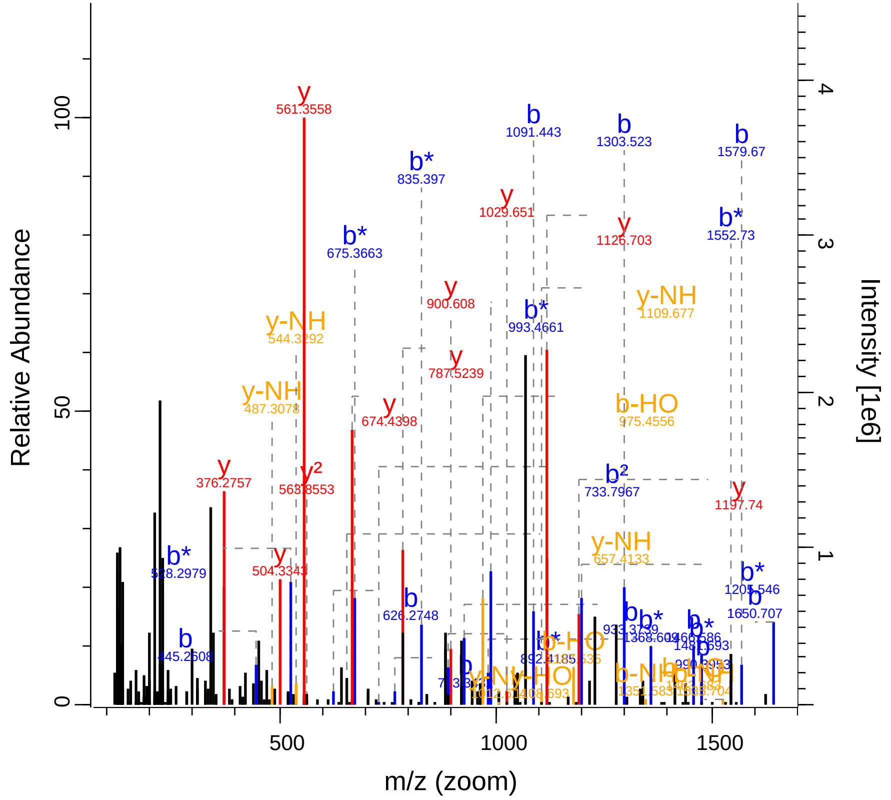

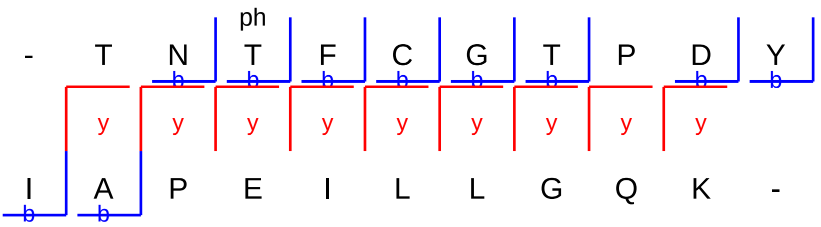

Raw File	Scan	Method	Score	m/z	Gene names
RO-QC-160121_ZackD_SS_Set_02_Fractio	35403	FTMS; HC	55.72	836.96	GLS2

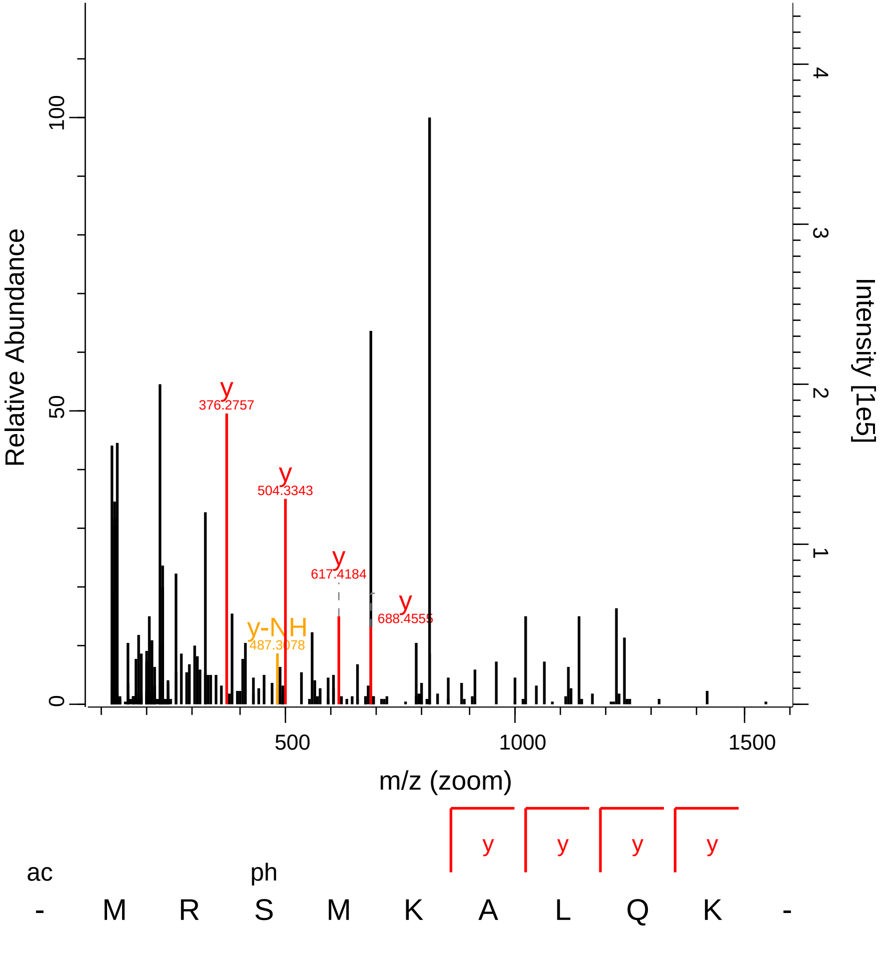

Raw File	Scan	Method	Score	m/z	Gene names
RO-QC-160121_ZackD_SS_Set_02_Fractior	5929	FTMS; HCI	94.69	698.84	ATXN2L

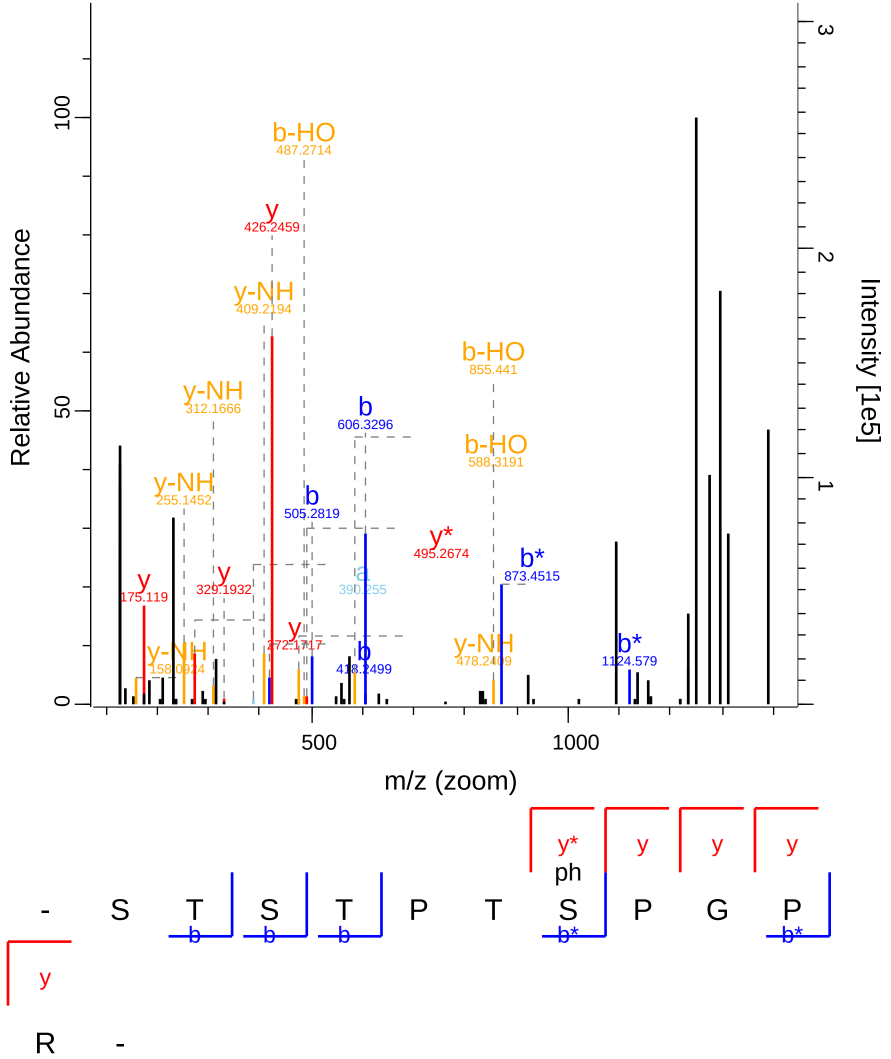

Raw File	Scan	Method	Score	m/z	Gene names
RO-QC-160121_ZackD_SS_Set_02_Fractior	6092	FTMS; HCI	78.53	638.35	HMGN3

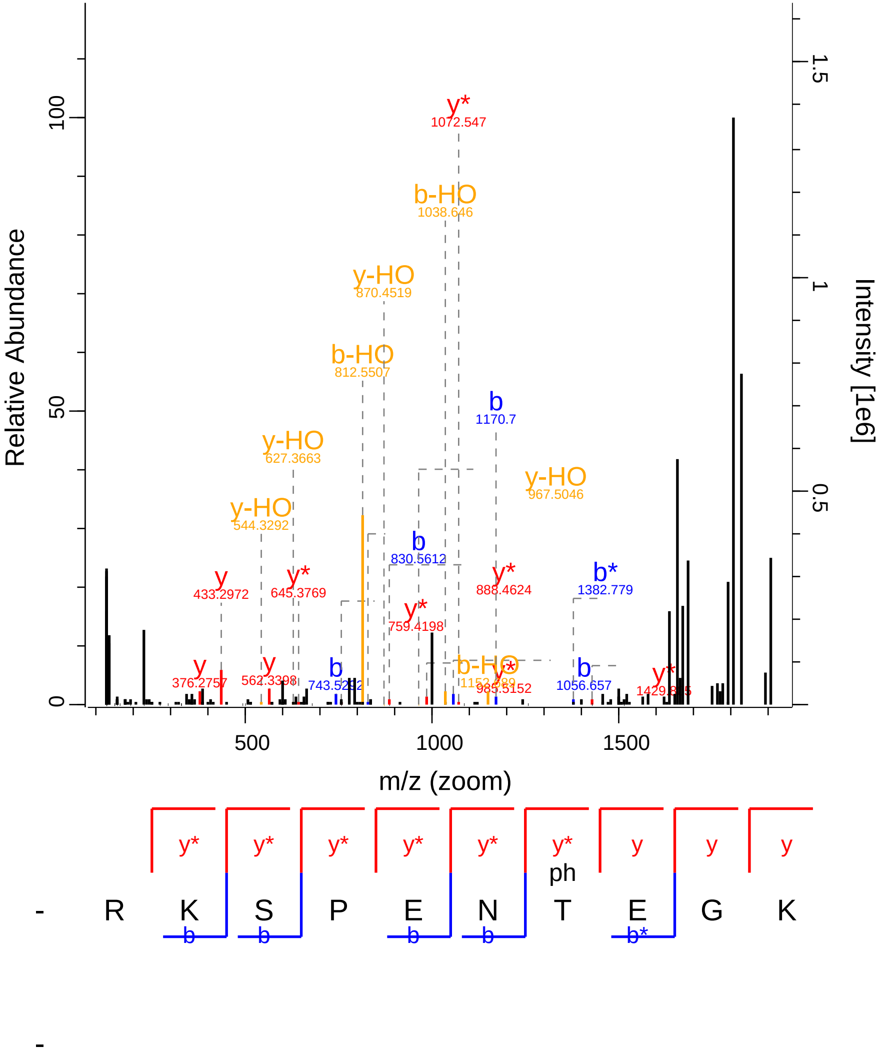

Raw File	Scan	Method	Score	m/z	Gene names
RO-QC-160121_ZackD_SS_Set_02_Fractior	6286	FTMS; HCI	97.63	653.32	CHAMP1

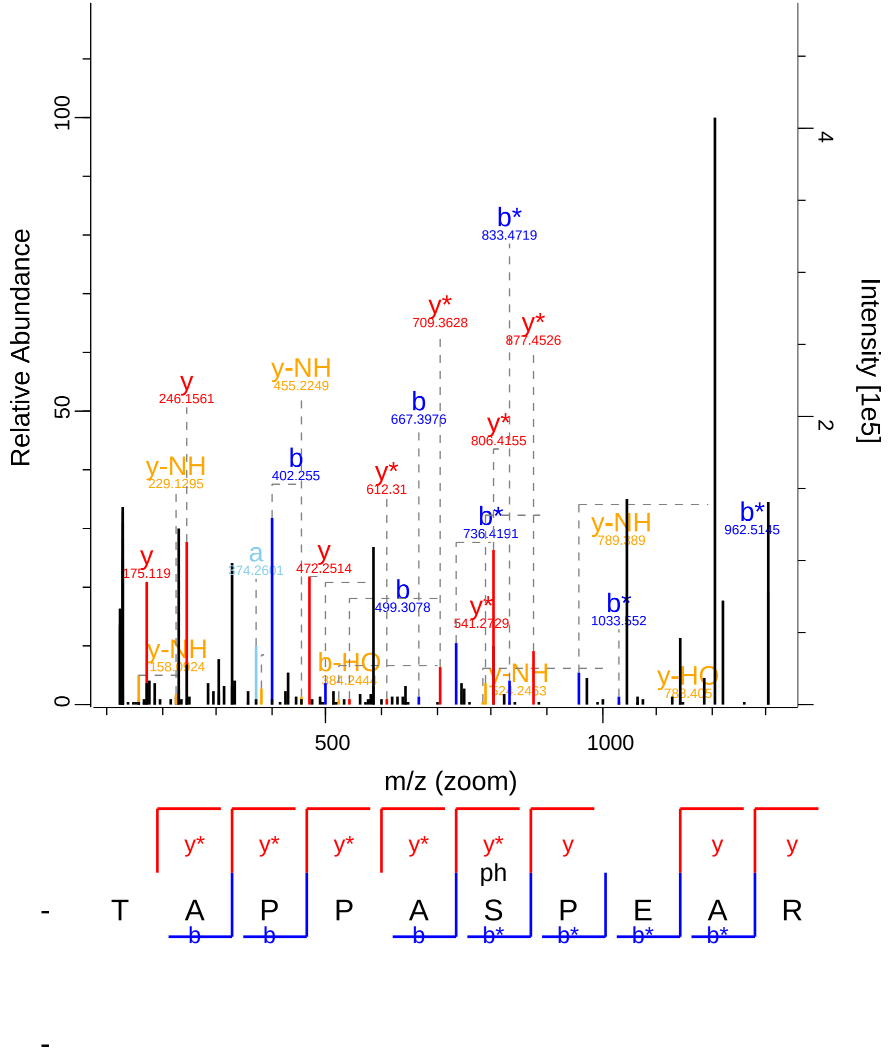

Raw File	Scan	Method	Score	m/z	Gene names
RO-QC-160121_ZackD_SS_Set_02_Fractior	6554	FTMS; HCI	83.25	713.67	SIPA1L3

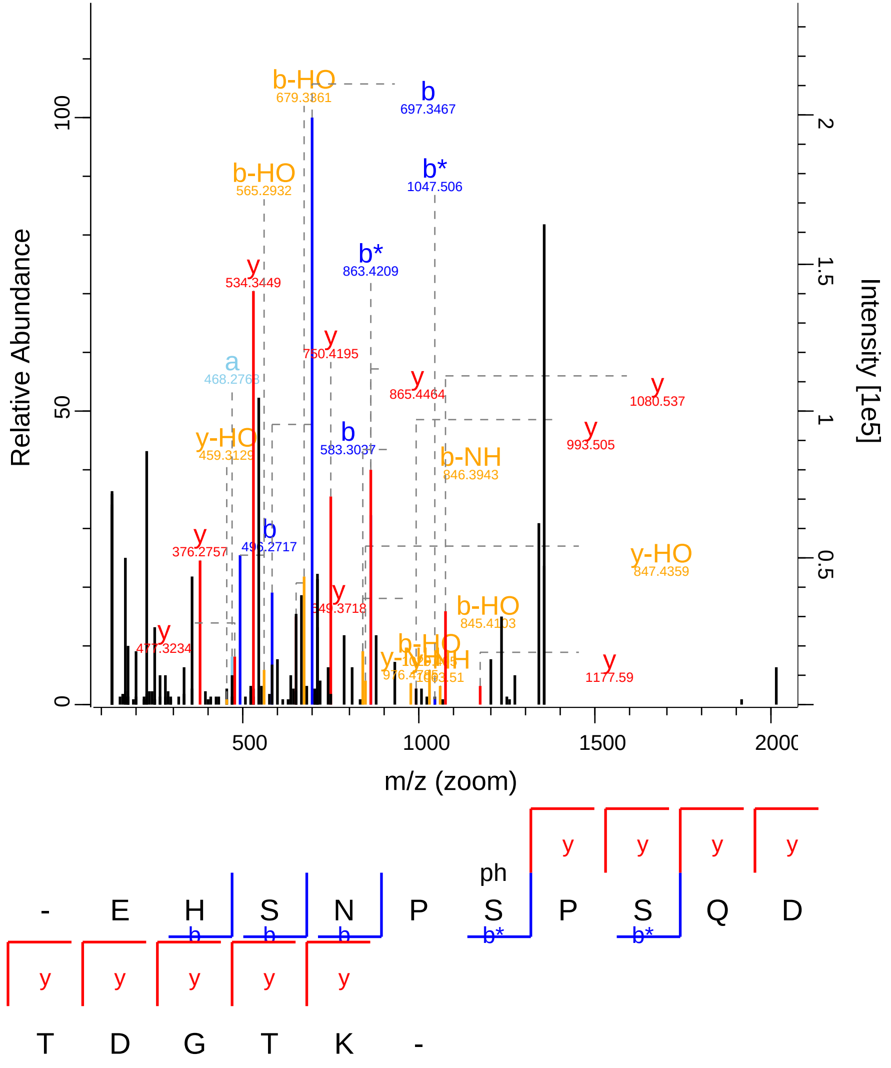

Raw File	Scan	Method	Score	m/z	Gene names
RO-QC-160121_ZackD_SS_Set_02_Fractio	6983	FTMS; HC	239.87	1041.96	SH3KBP1

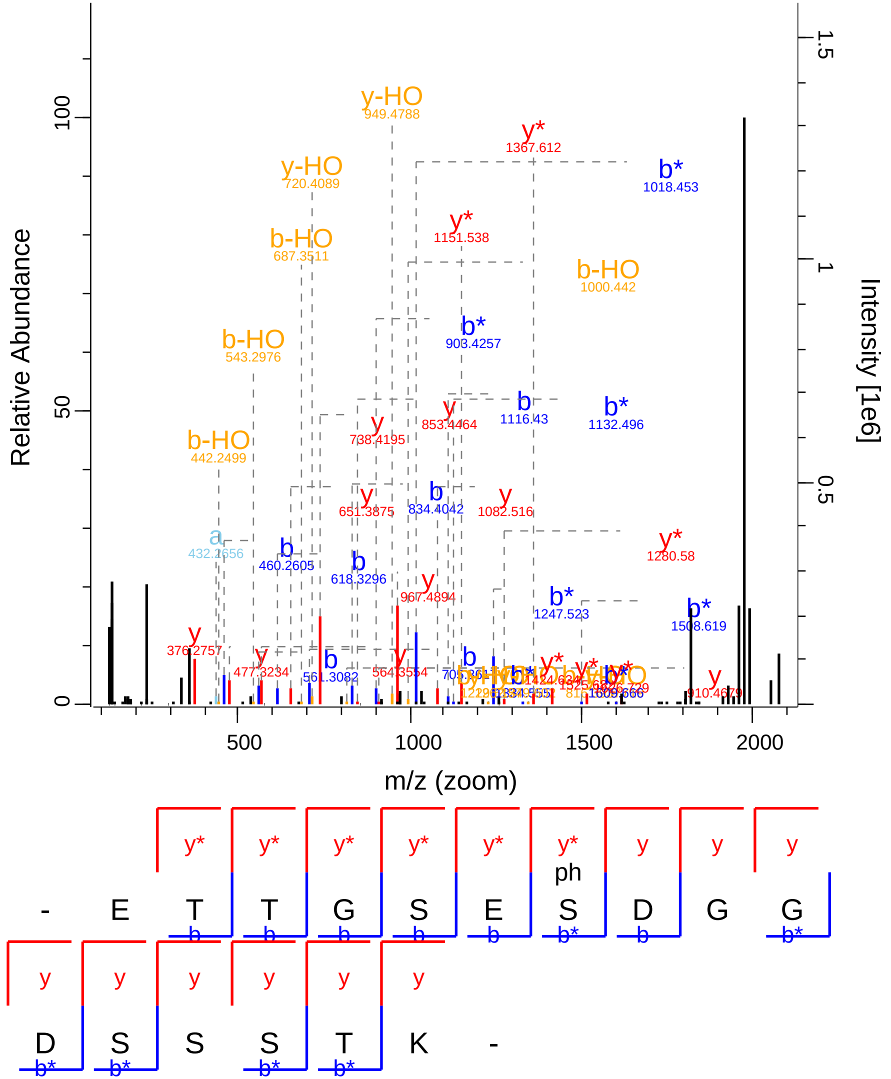

Raw File	Scan	Method	Score	m/z	Gene names
RO-QC-160121_ZackD_SS_Set_02_Fractior	7459	FTMS; HC	119.39	773.37	PRKAA1

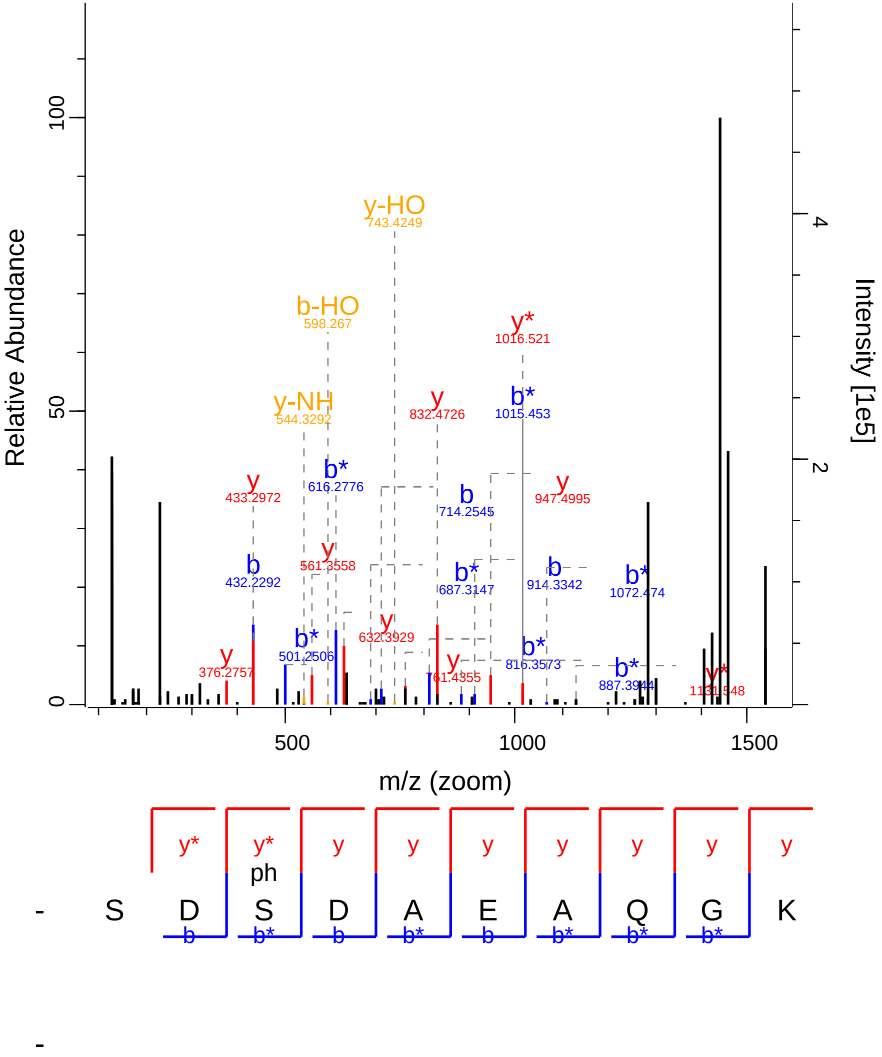

Raw File	Scan	Method	Score	m/z	Gene names
RO-QC-160121_ZackD_SS_Set_02_Fractior	7825	FTMS; HCI	57.41	586.32	RRBP1

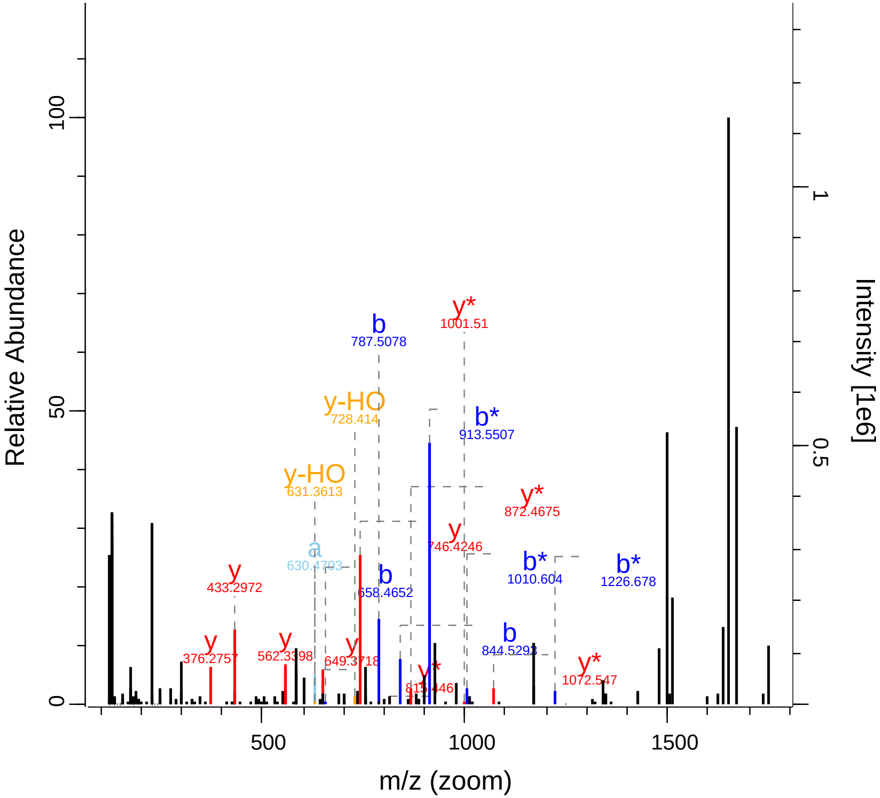

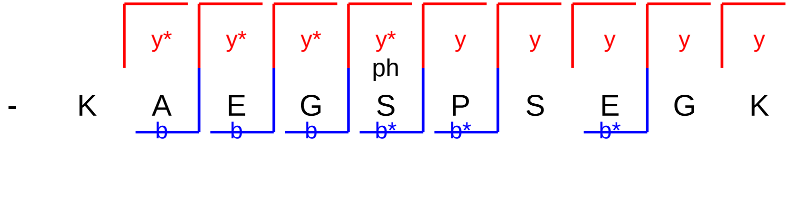

Raw File	Scan	Method	Score	m/z	Gene names
RO-QC-160121_ZackD_SS_Set_02_Fractior	8578	FTMS; HCI	58.83	717.06	TPPP

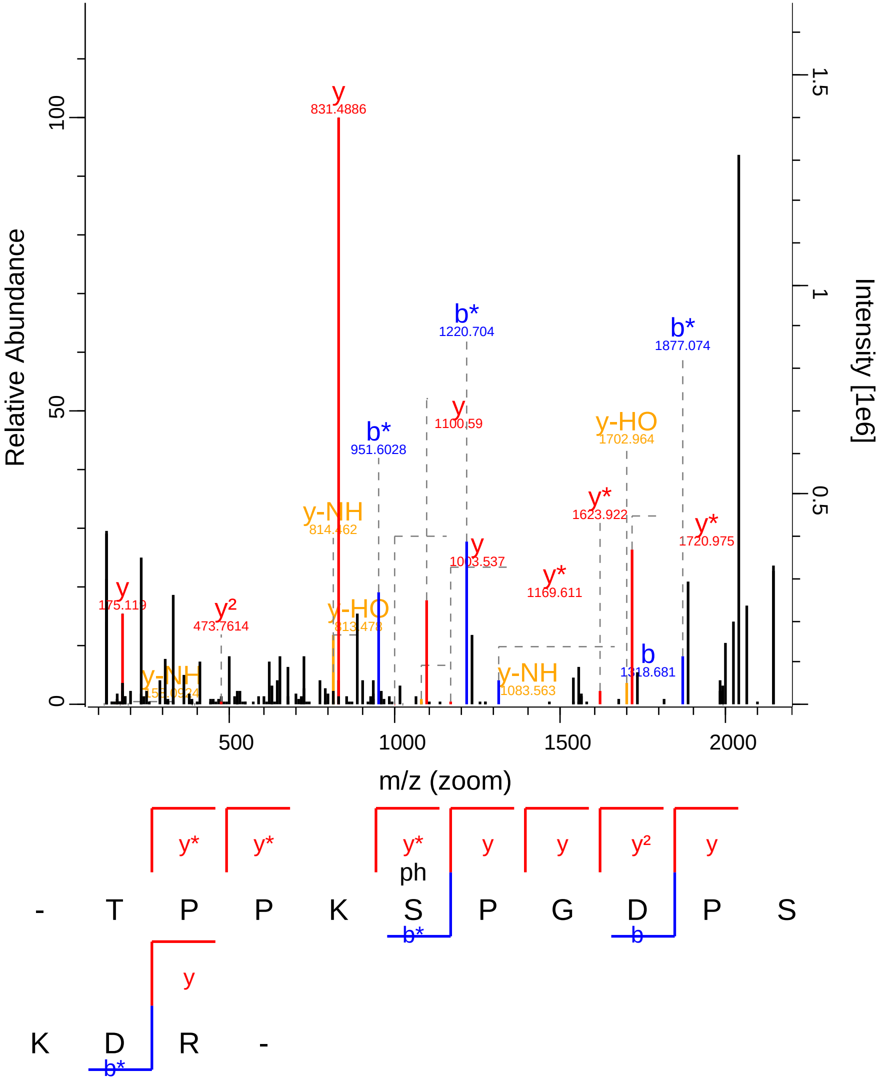

Raw File	Scan	Method	Score	m/z	Gene names
RO-QC-160121_ZackD_SS_Set_02_Fractior	9132	FTMS; HCI	73.46	741.07	HMGA2

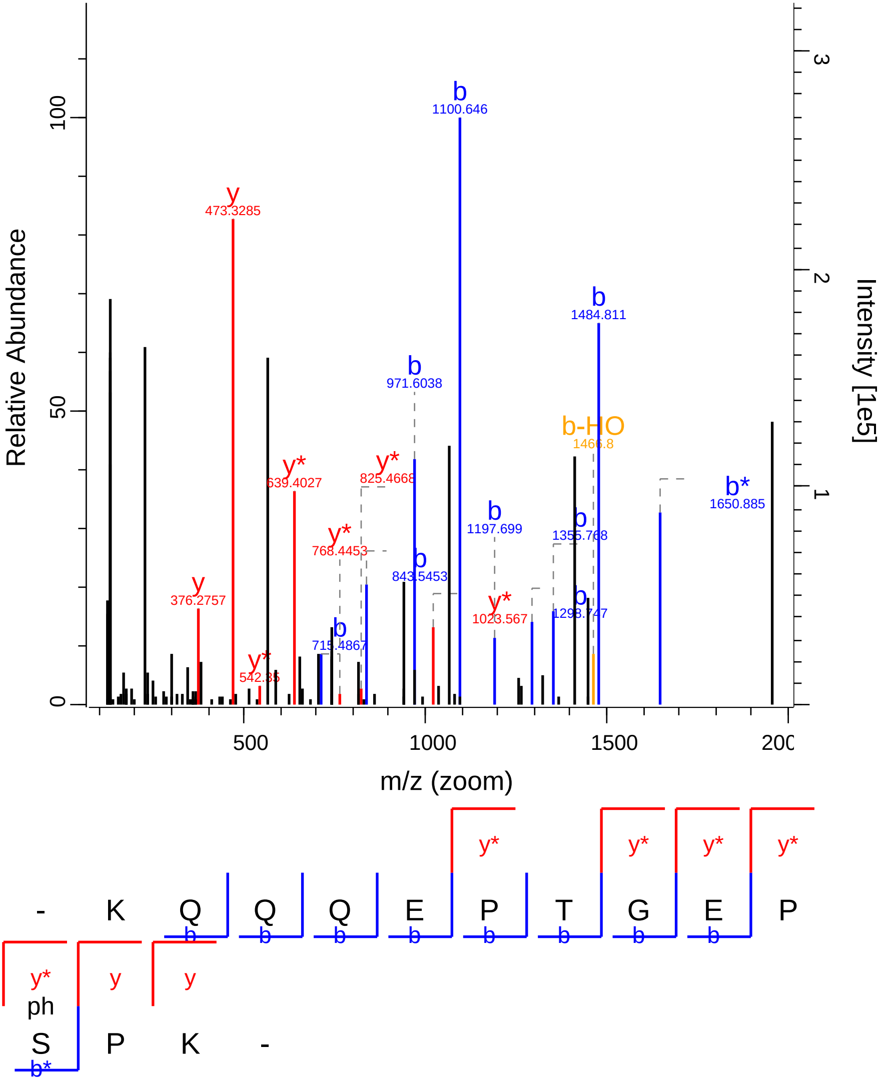

Raw File	Scan	Method	Score	m/z	Gene names
RO-QC-160121_ZackD_SS_Set_02_Fractior	9457	FTMS; HCI	50.08	554.31	HP1BP3

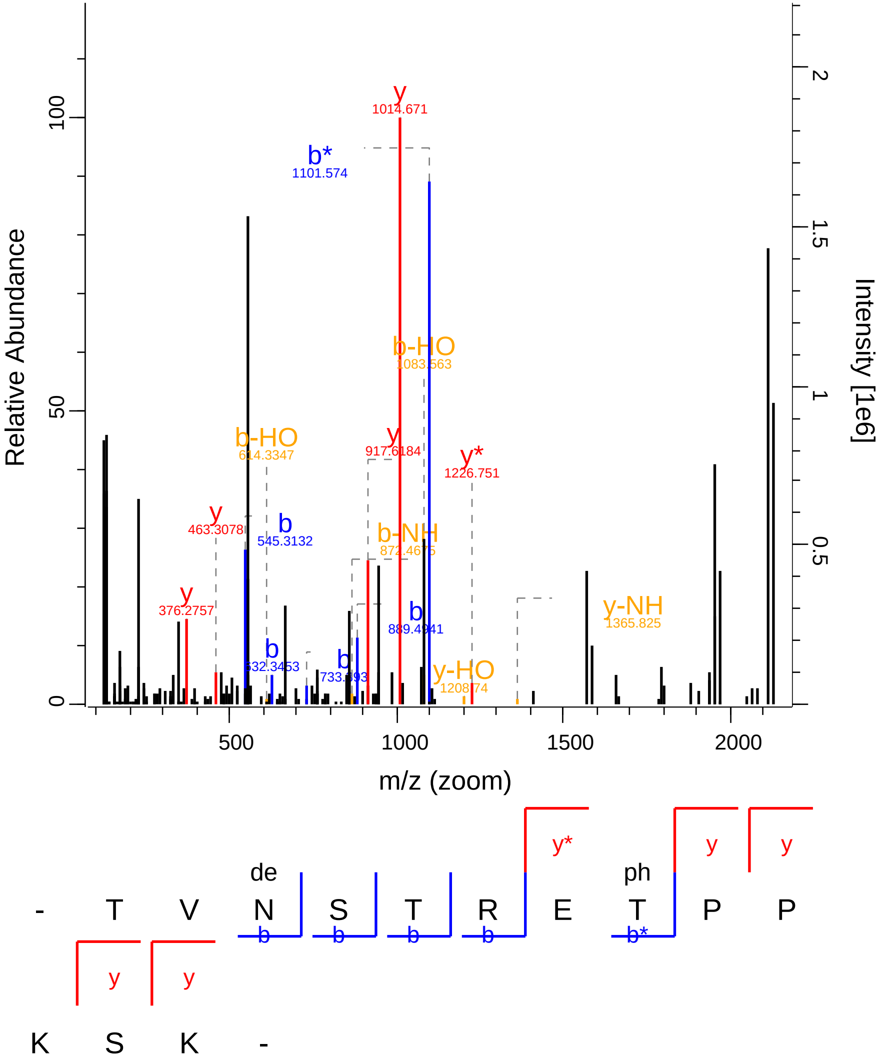

Raw File	Scan	Method	Score	m/z	Gene names
RO-QC-160121_ZackD_SS_Set_02_Fractio	10440	FTMS; HC	50.28	906.92	CC2D2B

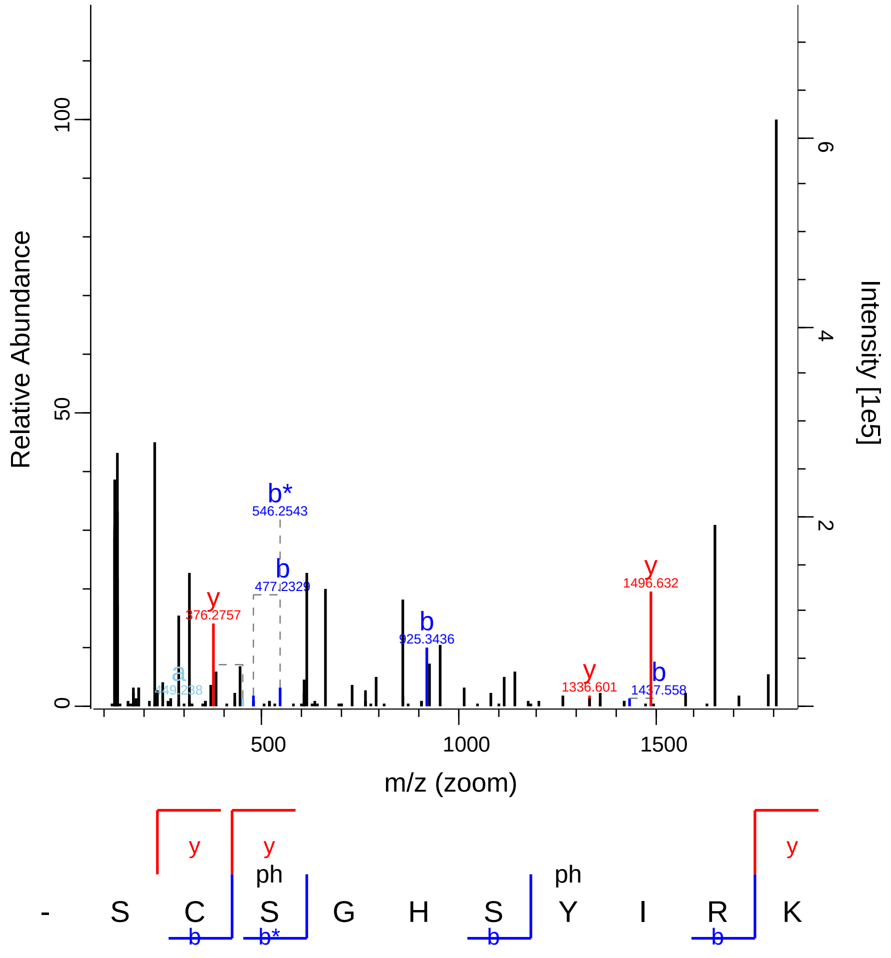

-

Raw File	Scan	Method	Score	m/z	Gene names
RO-QC-160121_ZackD_SS_Set_02_Fractio	10627	FTMS; HC	40.59	646.31	ATF7IP2

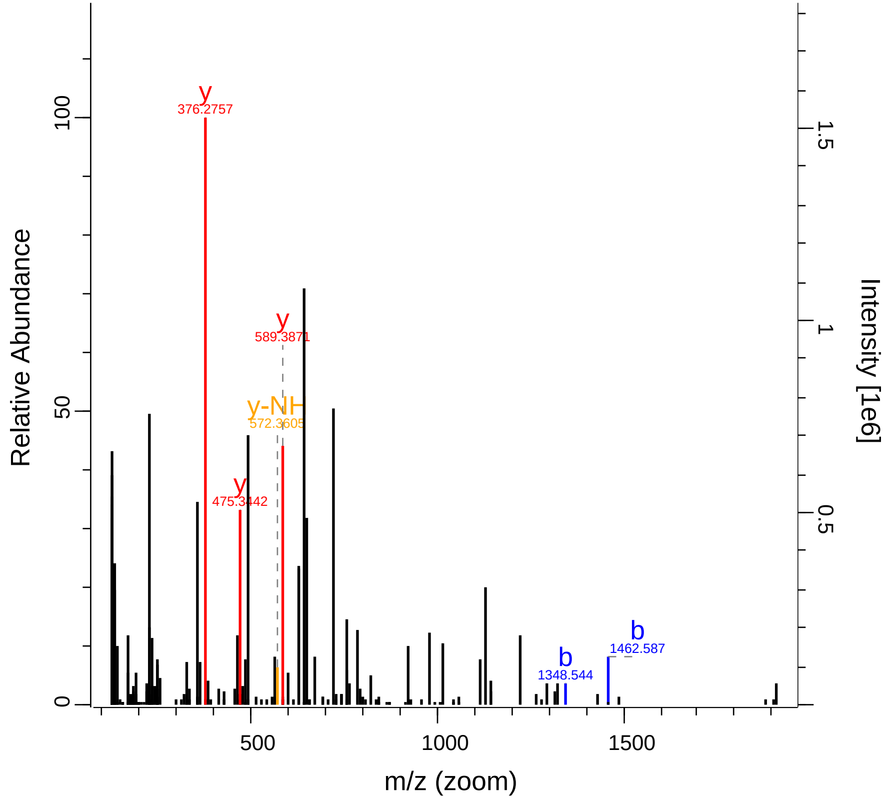

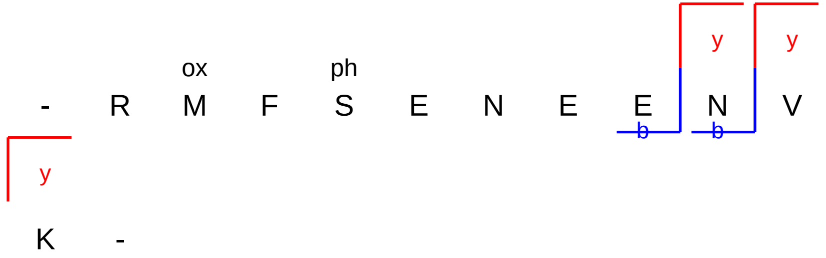

Raw File	Scan	Method	Score	m/z	Gene names
RO-QC-160121_ZackD_SS_Set_02_Fract	11112	FTMS; HC	135.09	665.3	HNRNPH1;HNRNP

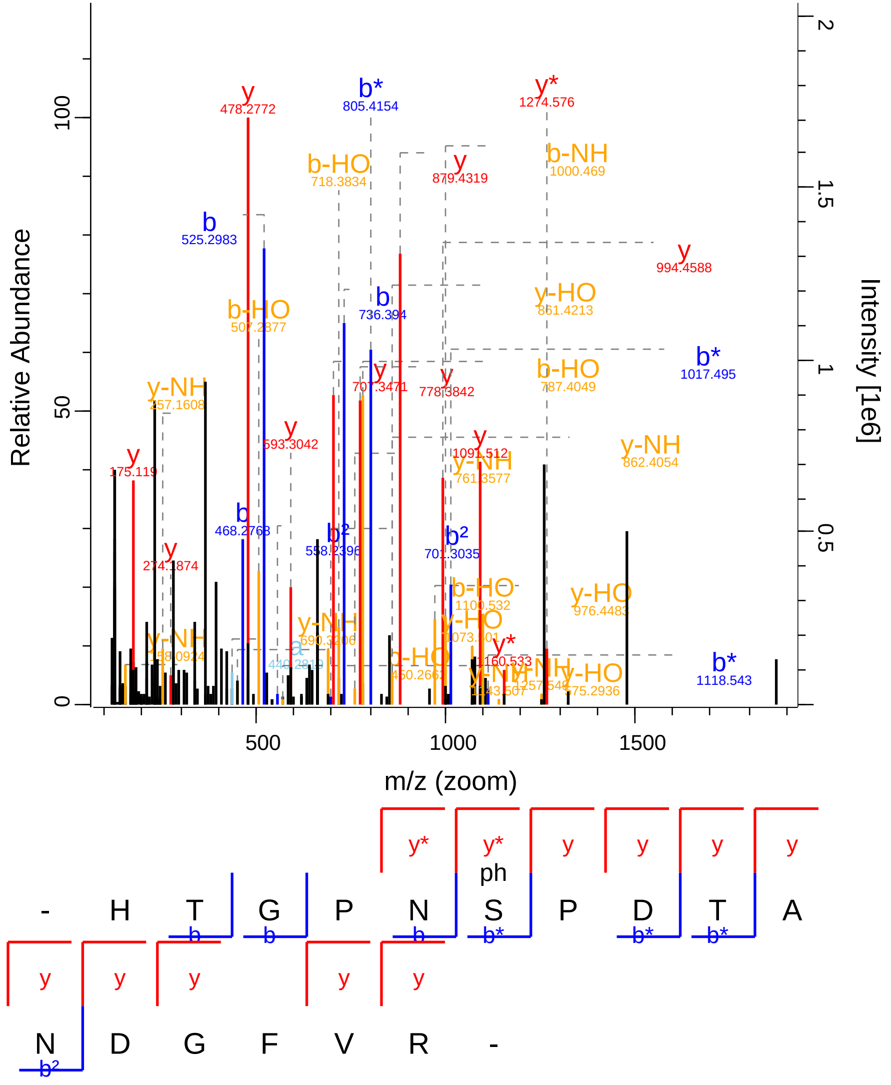

Raw File	Scan	Method	Score	m/z	Gene names
RO-QC-160121_ZackD_SS_Set_02_Fractio	11197	FTMS; HC	55.75	680.72	MAP1B

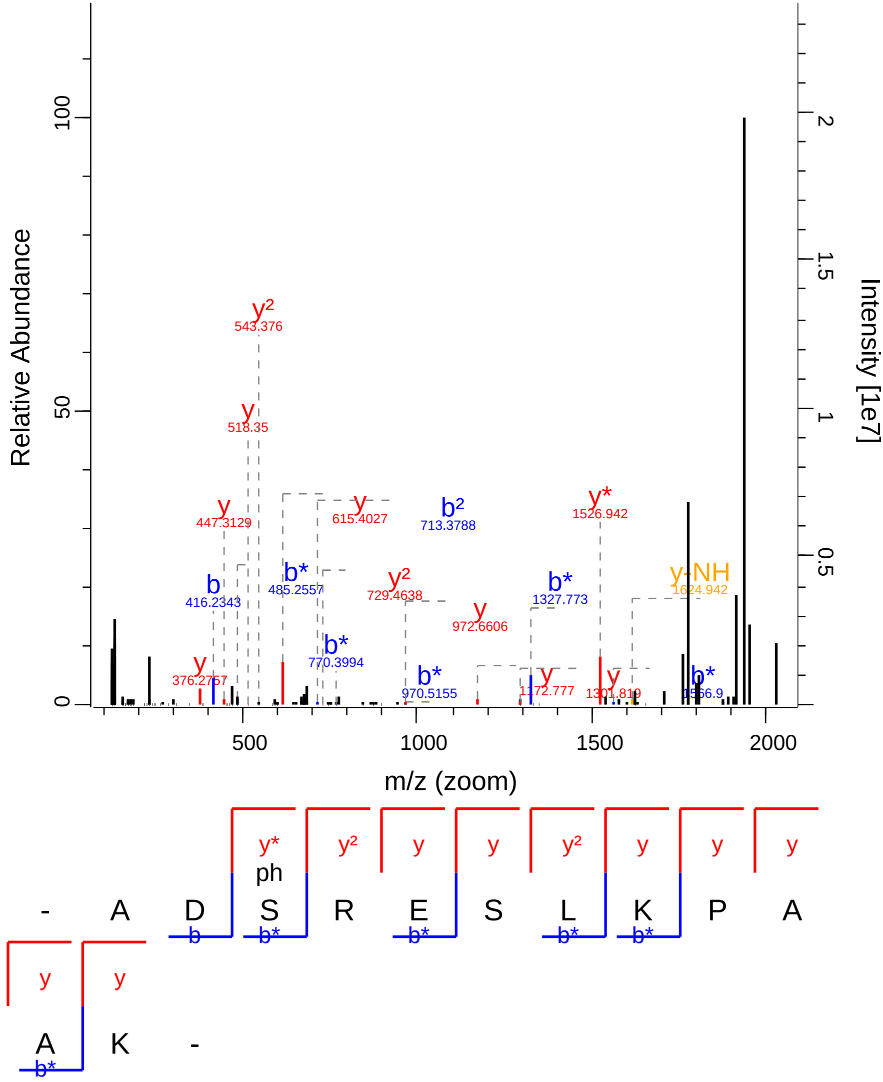

Raw File	Scan	Method	Score	m/z	Gene names
RO-QC-160121_ZackD_SS_Set_02_Fractio	11825	FTMS; HC	120.63	505.07	MATR3

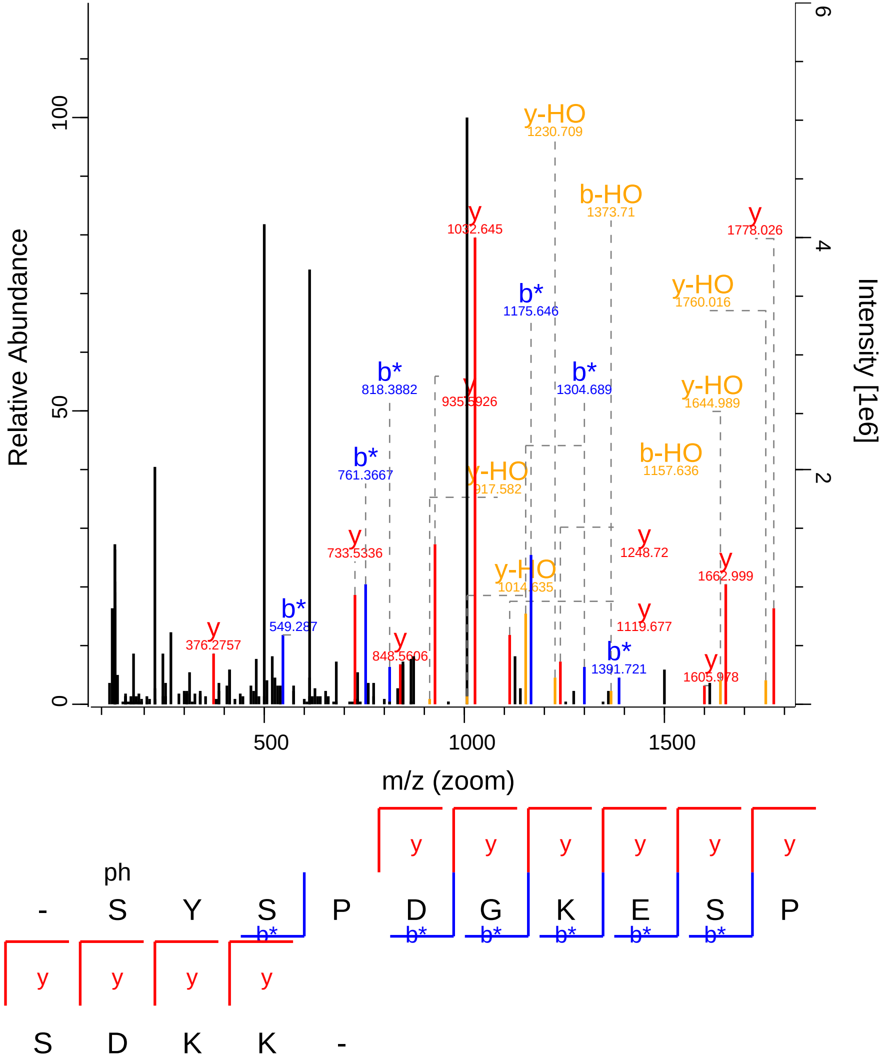

Raw File	Scan	Method	Score	m/z	Gene names
RO-QC-160121_ZackD_SS_Set_02_Fractio	11961	FTMS; HC	139	814.04	UTP18

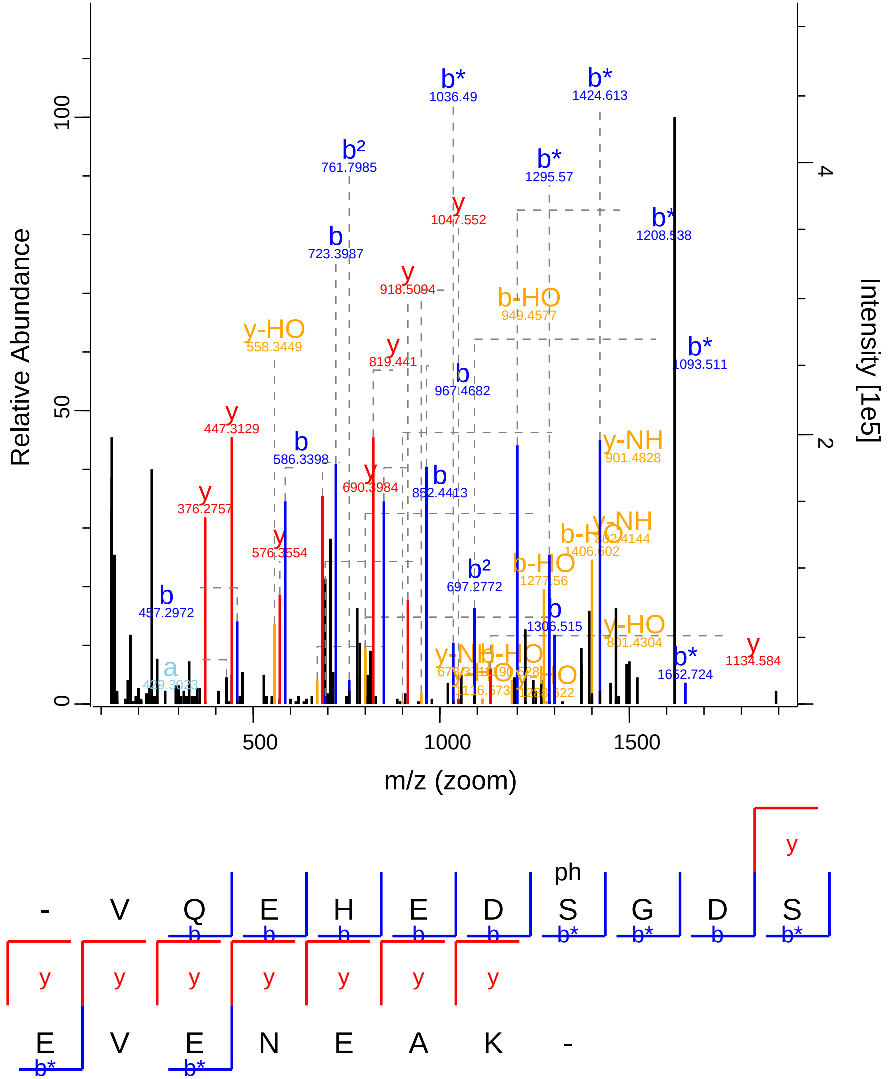

Raw File	Scan	Method	Score	m/z	Gene names
RO-QC-160121_ZackD_SS_Set_02_Fractio	12082	FTMS; HC	60.96	584.79	HNRNPU

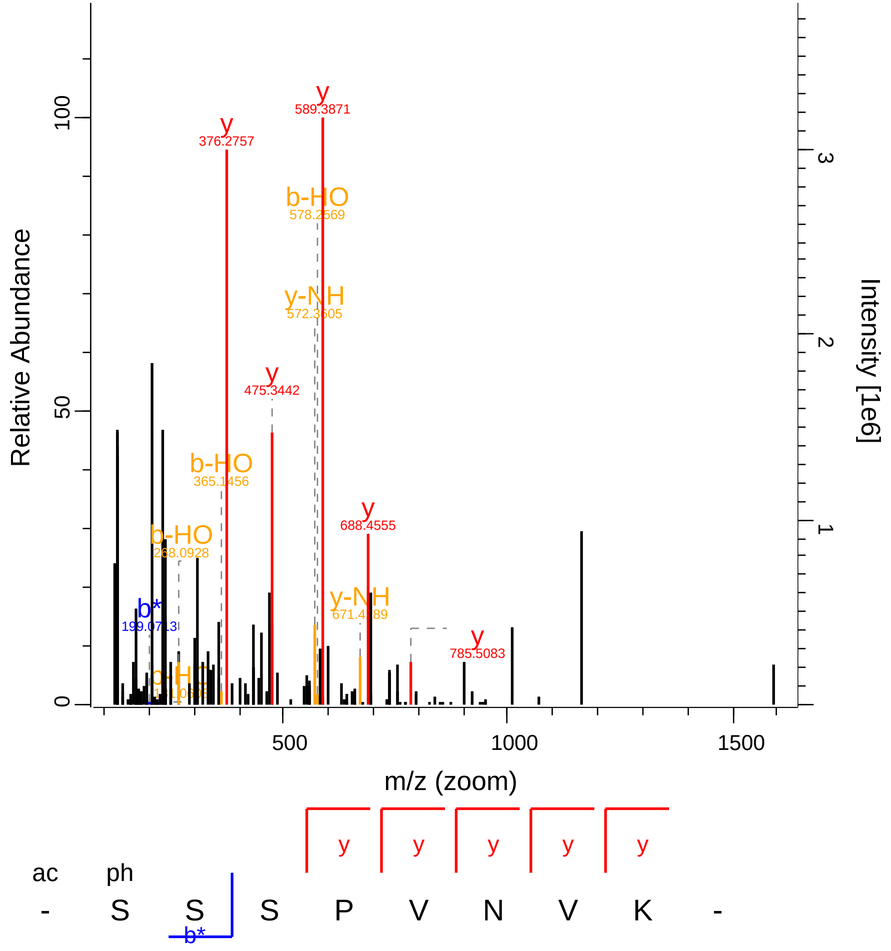

Raw File	Scan	Method	Score	m/z	Gene names
RO-QC-160121_ZackD_SS_Set_02_Fractio	12192	FTMS; HC	147.54	840.13	VPS4B

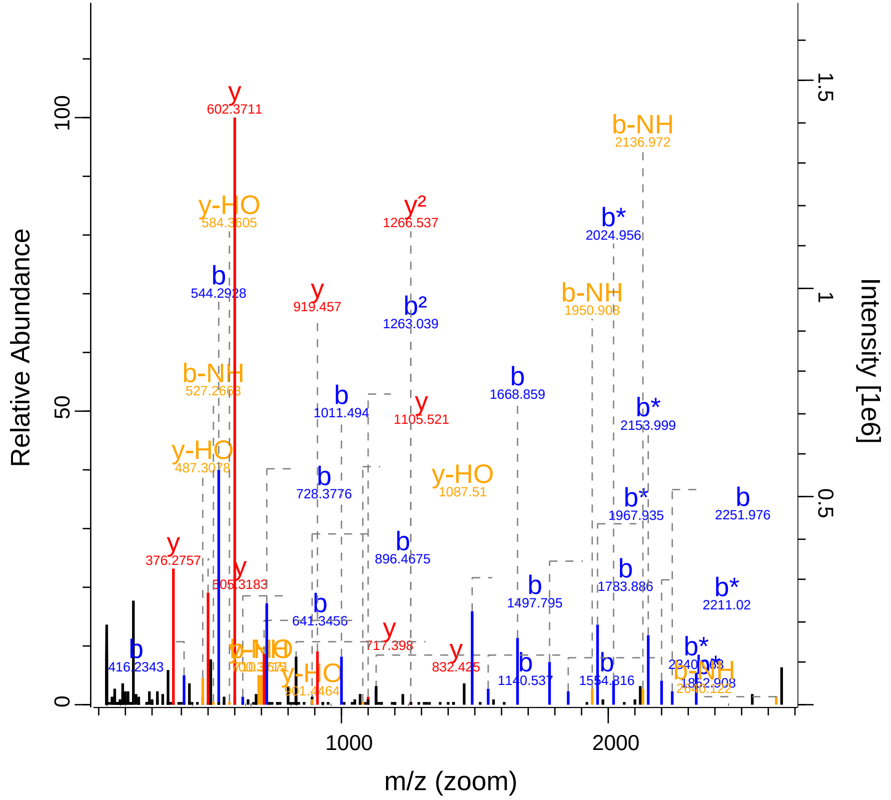

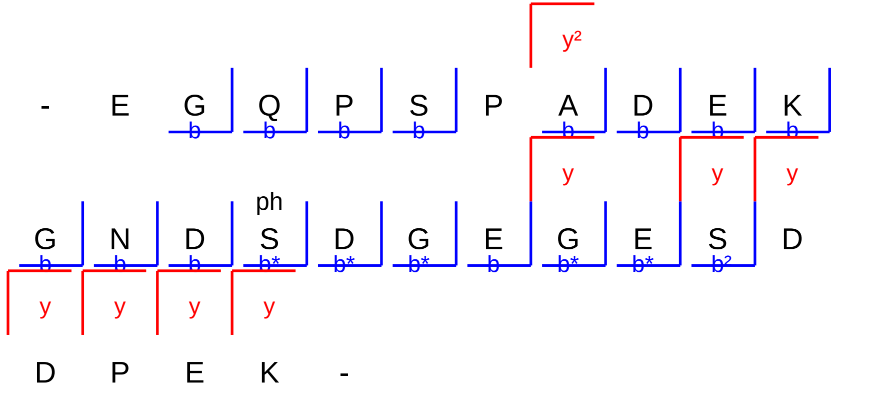

Raw File	Scan	Method	Score	m/z	Gene names
RO-QC-160121_ZackD_SS_Set_02_Fractior	12285	FTMS; HCI	83.24	452.5	ARHGEF12

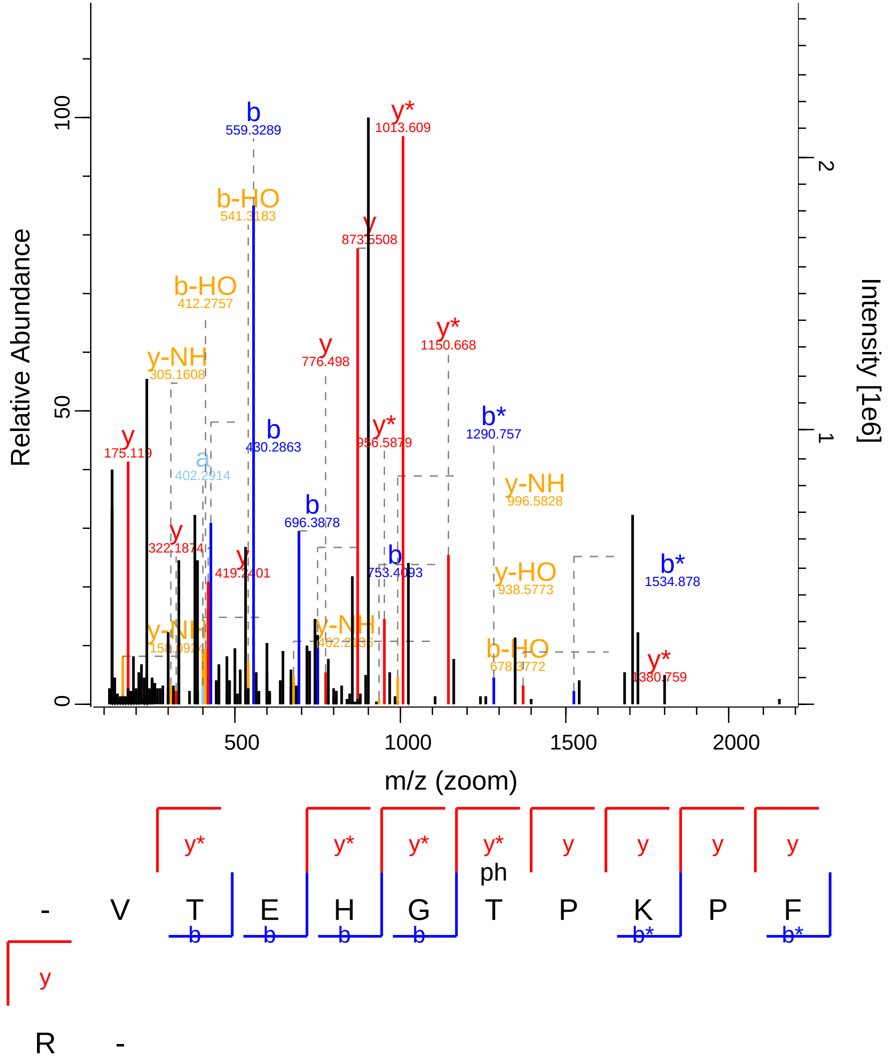

Raw File	Scan	Method	Score	m/z	Gene names
RO-QC-160121_ZackD_SS_Set_02_Fractior	12794	FTMS; HCI	108.15	647.8	TNKS1BP1

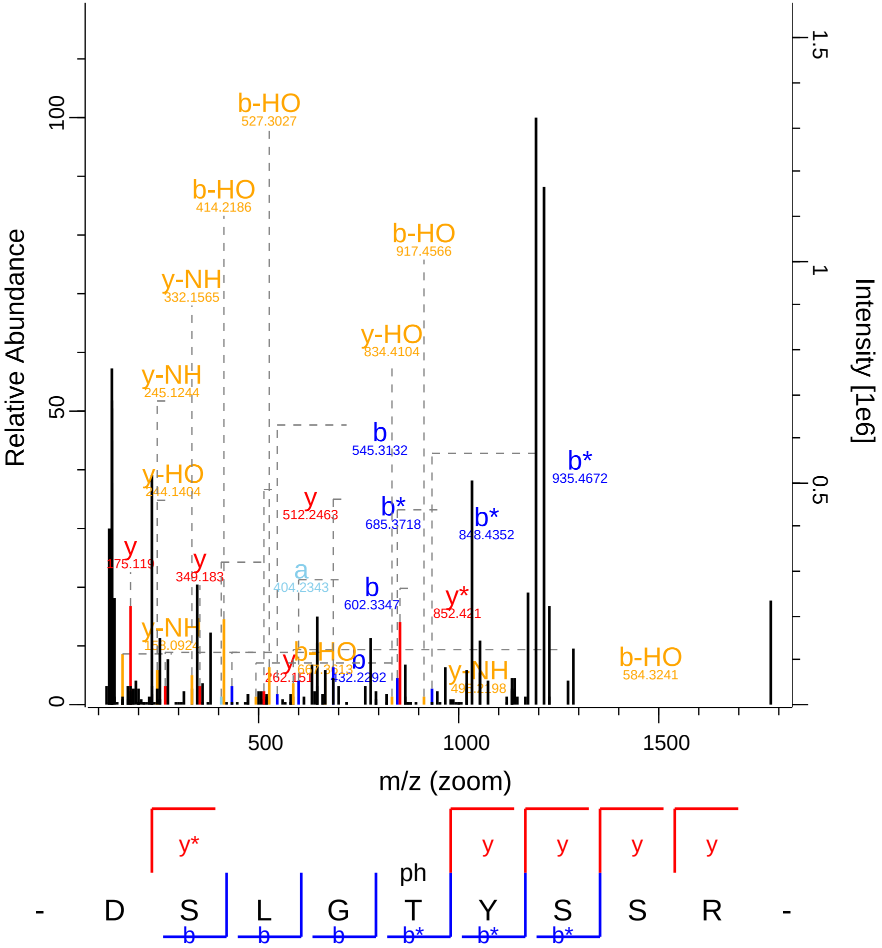

Raw File	Scan	Method	Score	m/z	Gene names
RO-QC-160121_ZackD_SS_Set_02_Fractio	12975	FTMS; HC	151.07	768.87	PCDH7

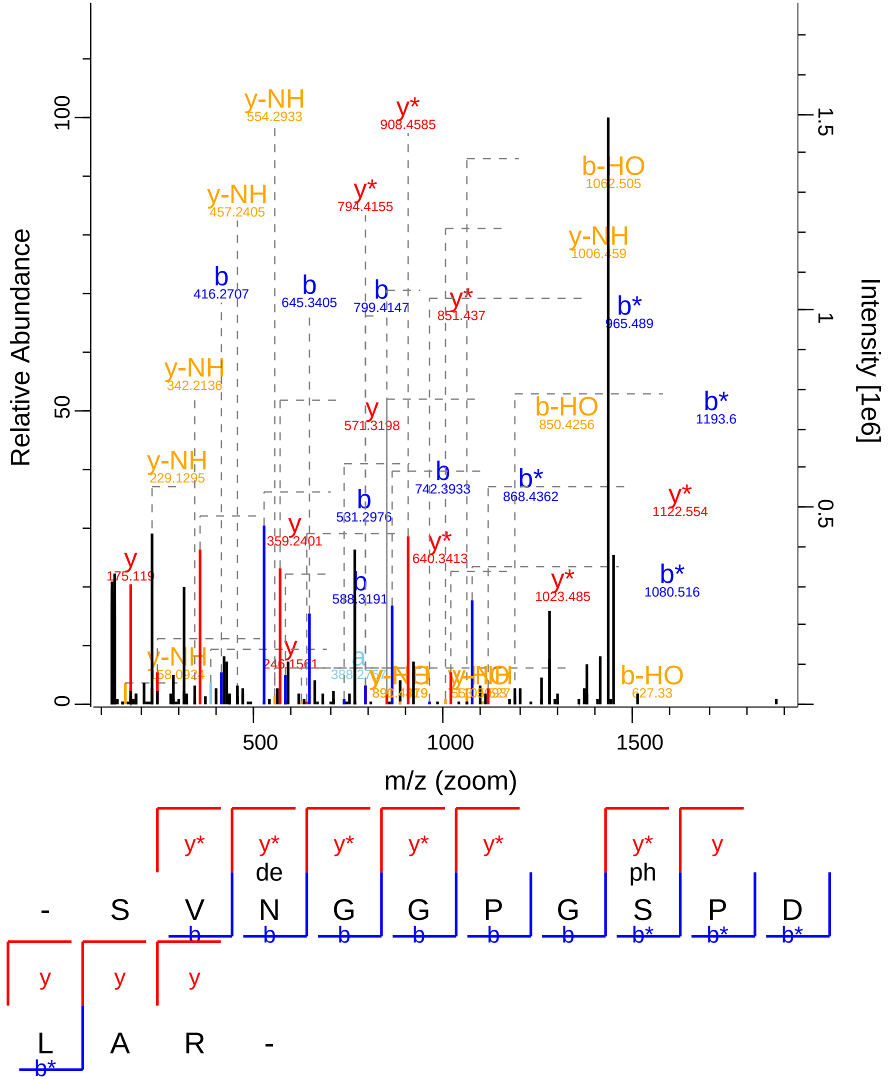

Raw File	Scan	Method	Score	m/z	Gene names
RO-QC-160121_ZackD_SS_Set_02_Fractio	14061	FTMS; HC	45.72	411.99	RBM39

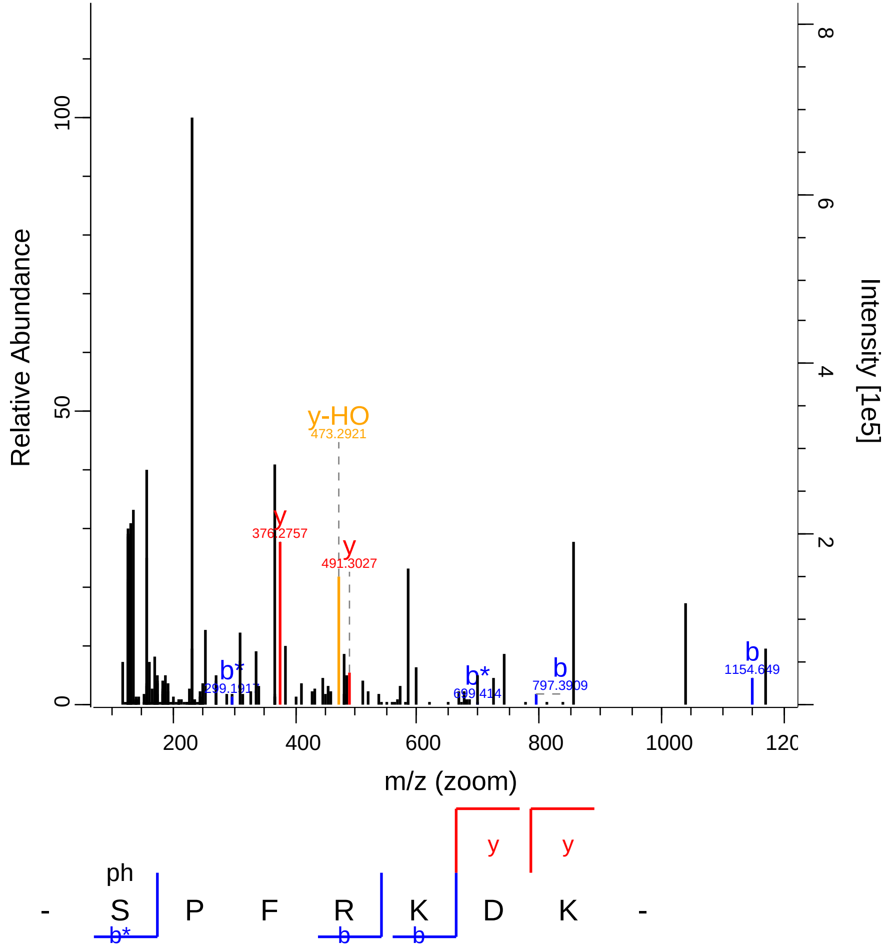

Raw File	Scan	Method	Score	m/z	Gene names
RO-QC-160121_ZackD_SS_Set_02_Fractio	14162	FTMS; HC	121.63	781.42	TP53BP1

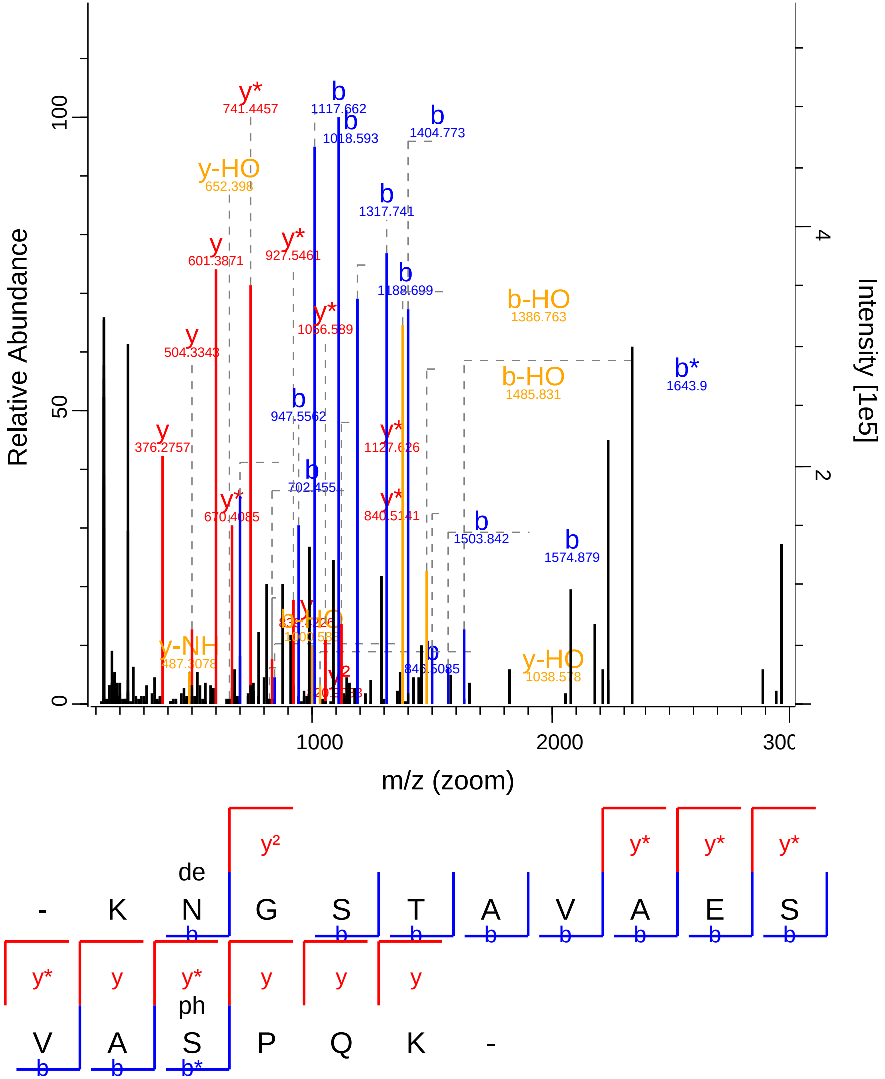

Raw File	Scan	Method	Score	m/z	Gene names
RO-QC-160121_ZackD_SS_Set_02_Fractio	14178	FTMS; HC	140.35	867.43	BASP1

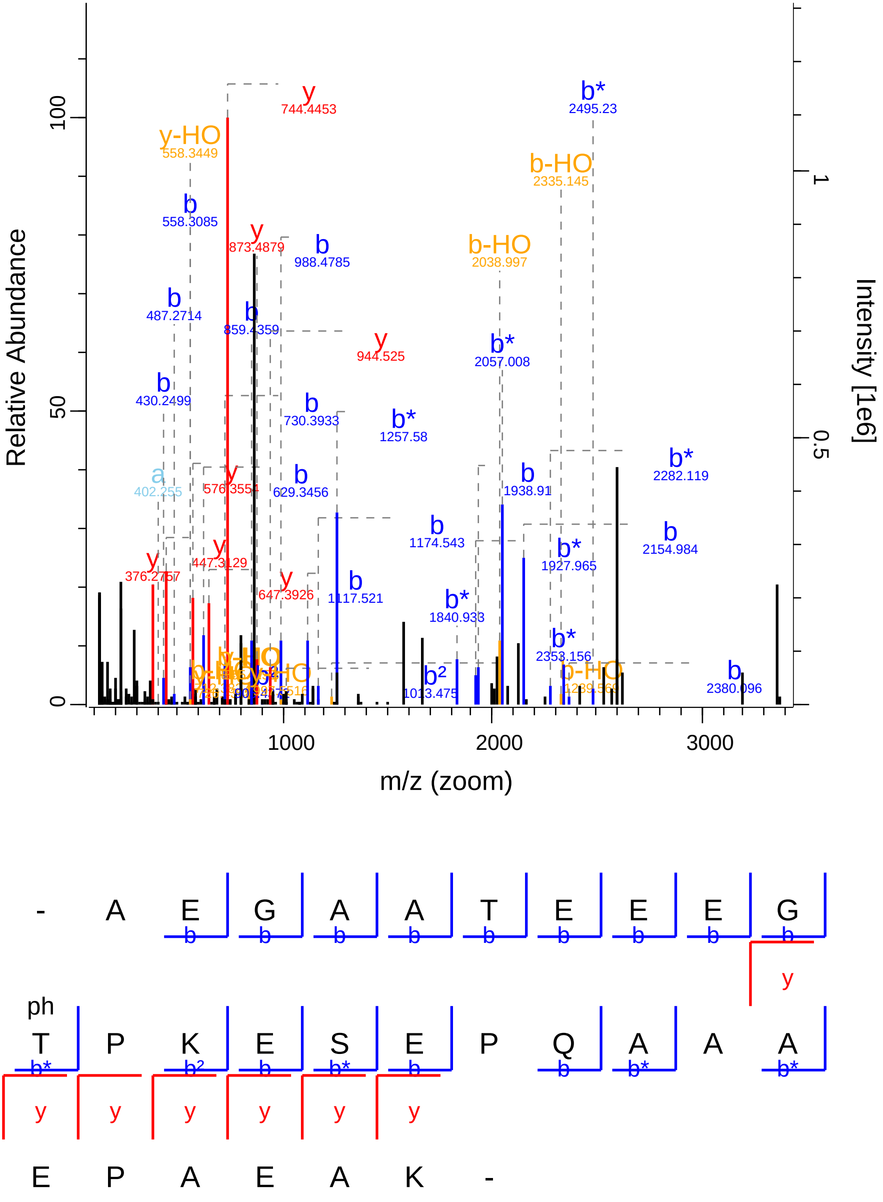

Raw File	Scan	Method	Score	m/z	Gene names
RO-QC-160121_ZackD_SS_Set_02_Fractio	14666	FTMS; HC	45.43	576.52	TUFT1

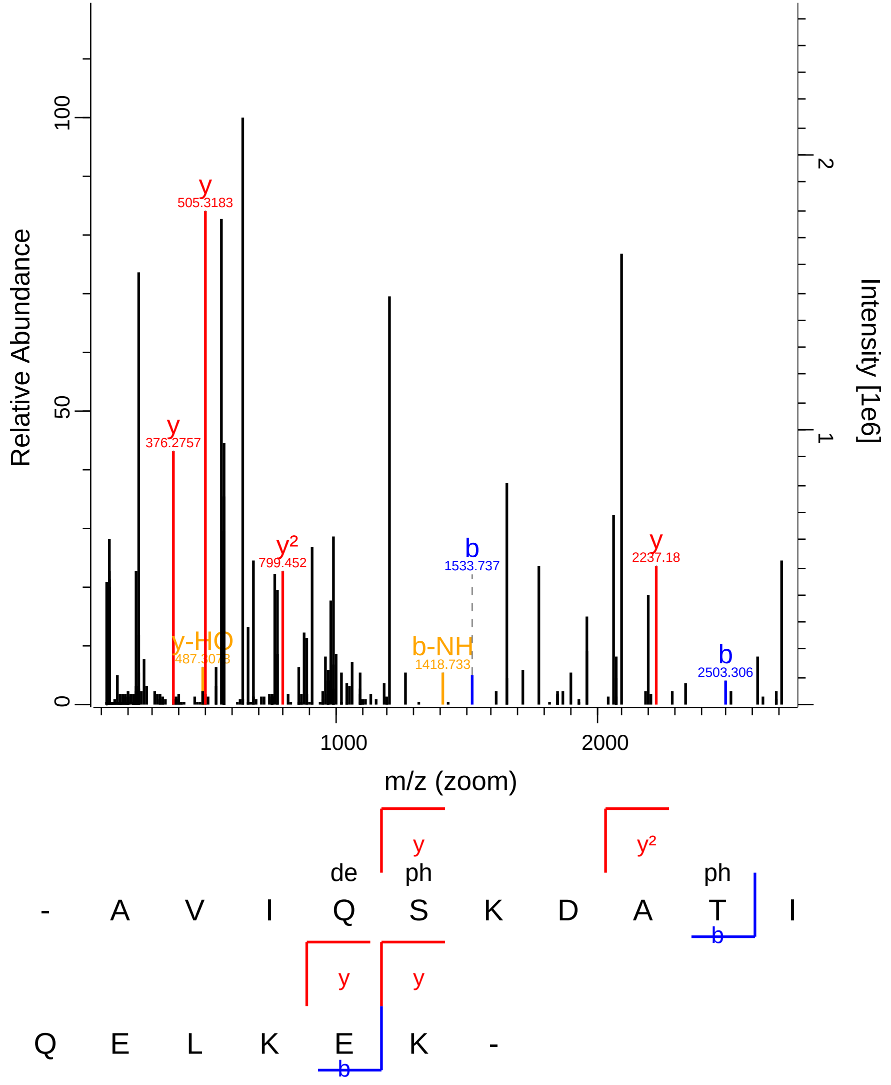

Raw File	Scan	Method	Score	m/z	Gene names
RO-QC-160121_ZackD_SS_Set_02_Fractior	15355	FTMS; HCI	67.14	514.8	CALD1

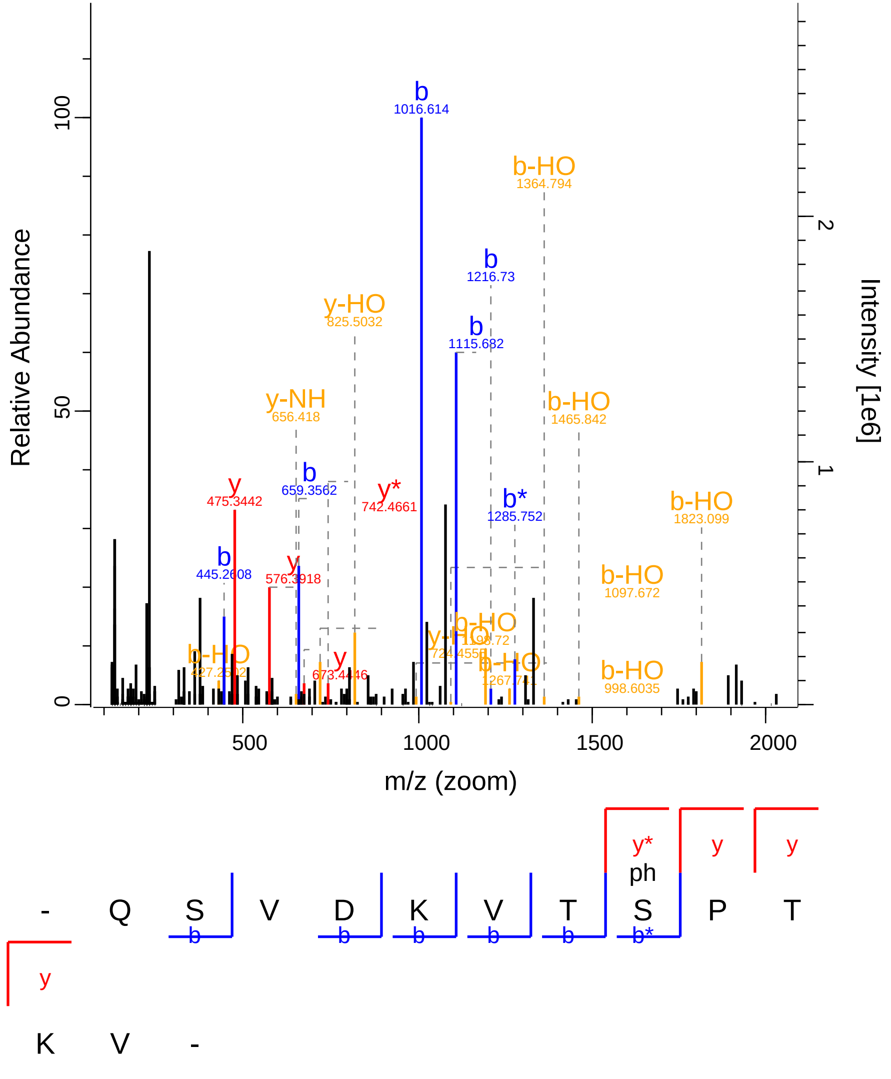

Raw File	Scan	Method	Score	m/z	Gene names
RO-QC-160121_ZackD_SS_Set_02_Fractio	15841	FTMS; HC	84.52	852.45	CAMK2D

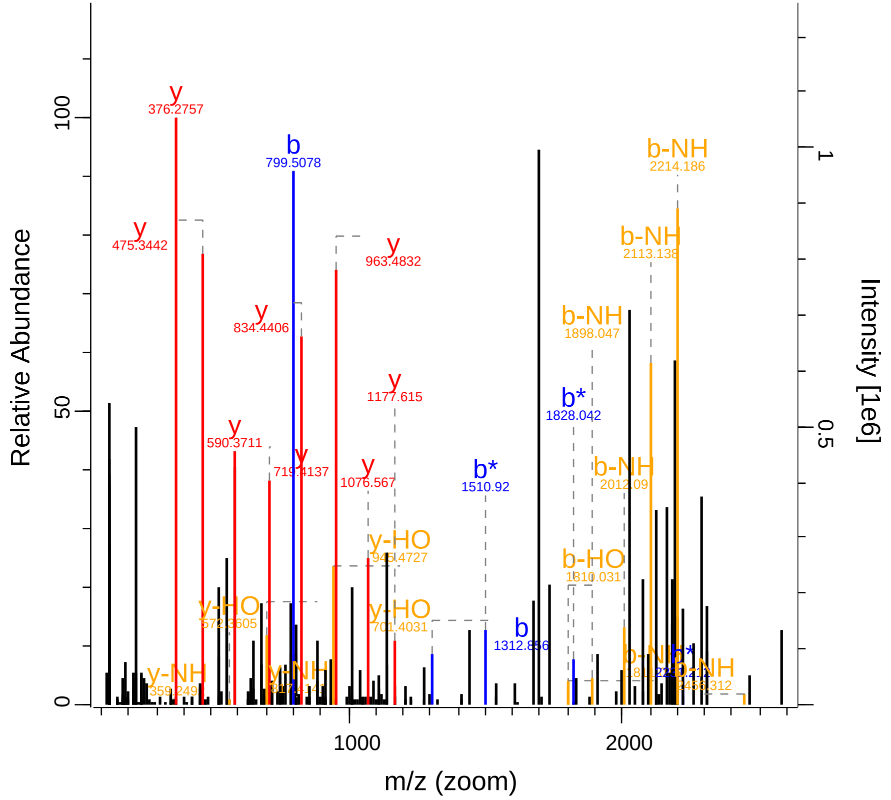

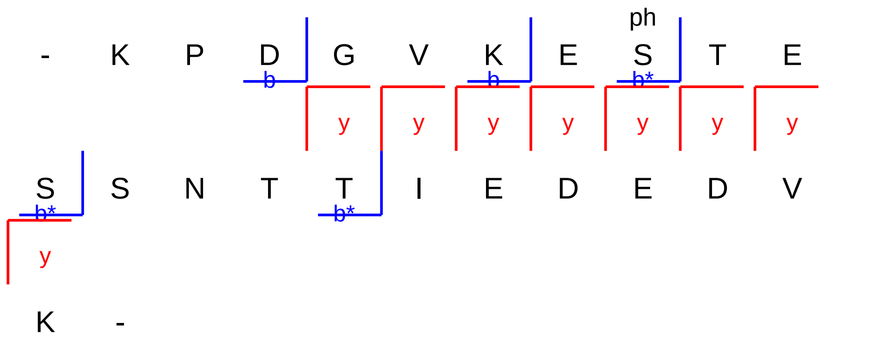

Raw File	Scan	Method	Score	m/z	Gene names
RO-QC-160121_ZackD_SS_Set_02_Fractio	16148	FTMS; HC	139.39	605.82	ZC3H18

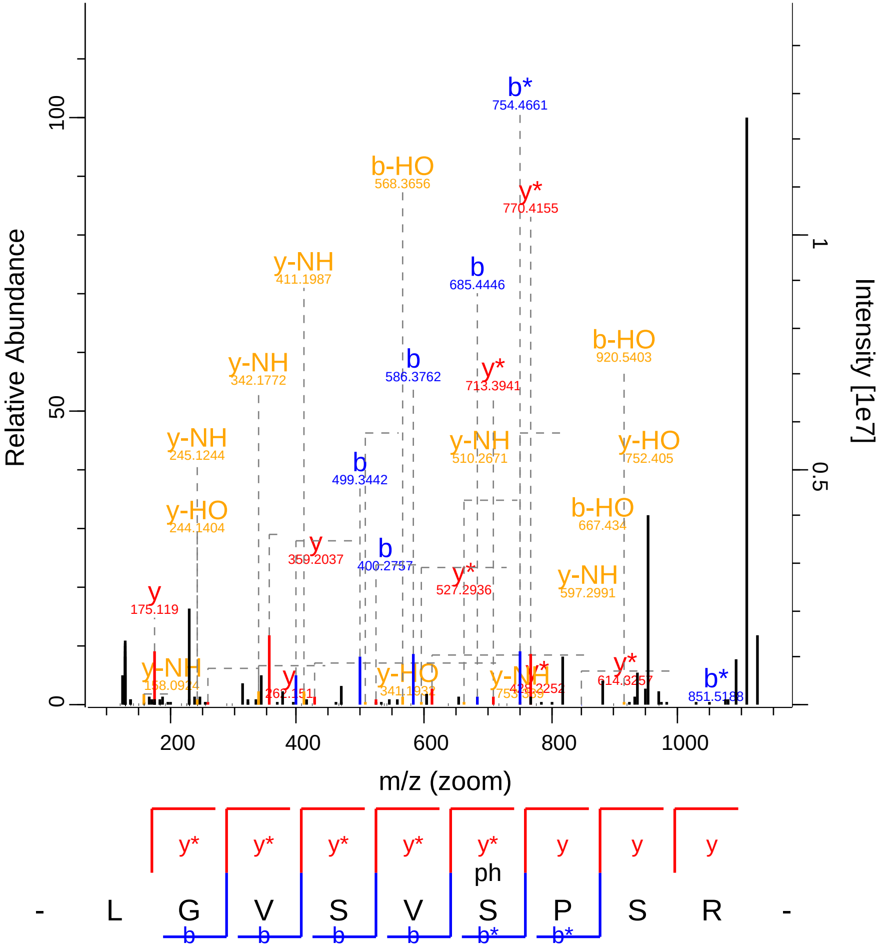

Raw File	Scan	Method	Score	m/z	Gene names
RO-QC-160121_ZackD_SS_Set_02_Fractio	16228	FTMS; HC	88.73	549.06	HDGF

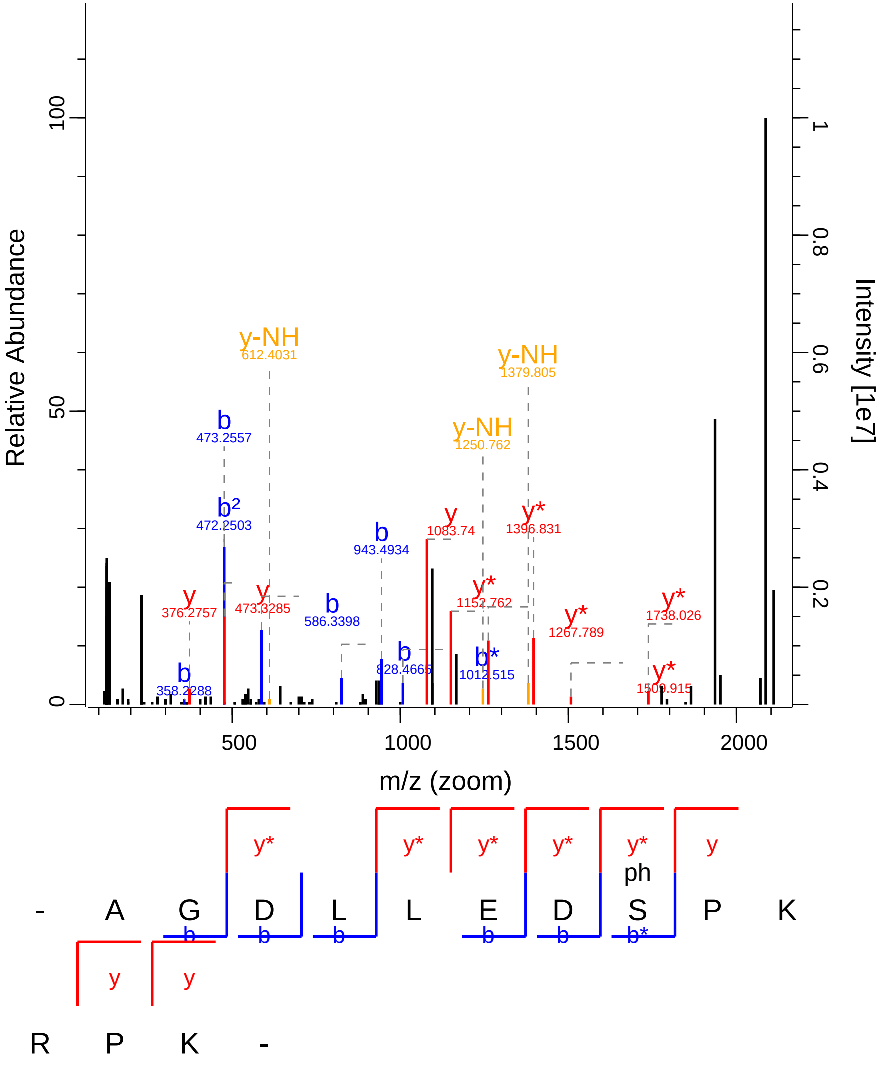

Raw File	Scan	Method	Score	m/z	Gene names
RO-QC-160121_ZackD_SS_Set_02_Fractio	16252	FTMS; HC	52.4	670.81	TOMM70A

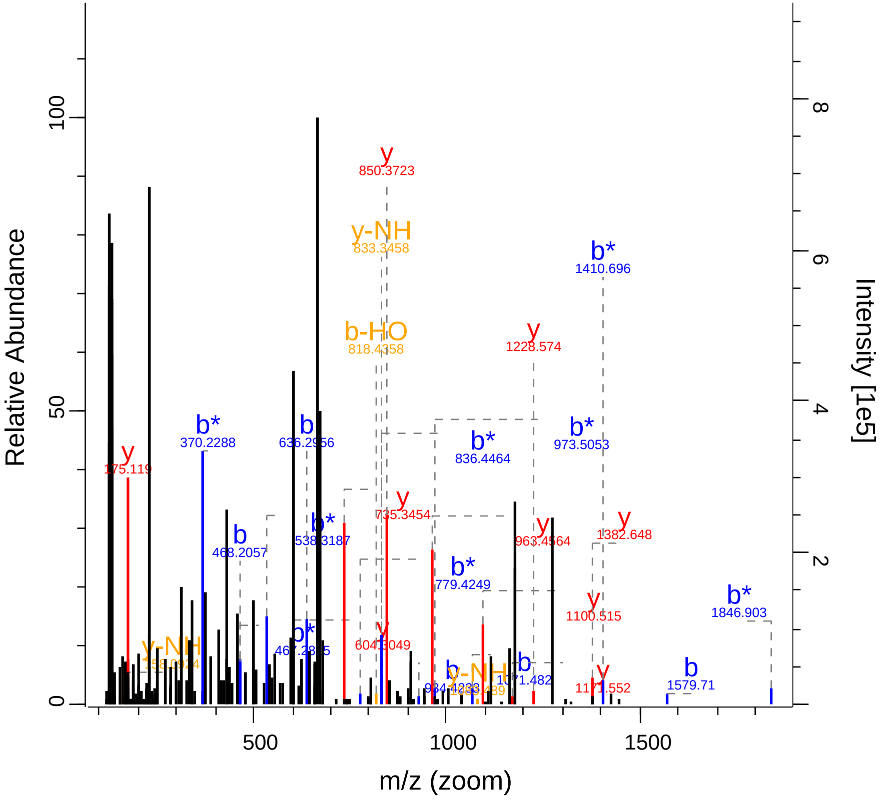

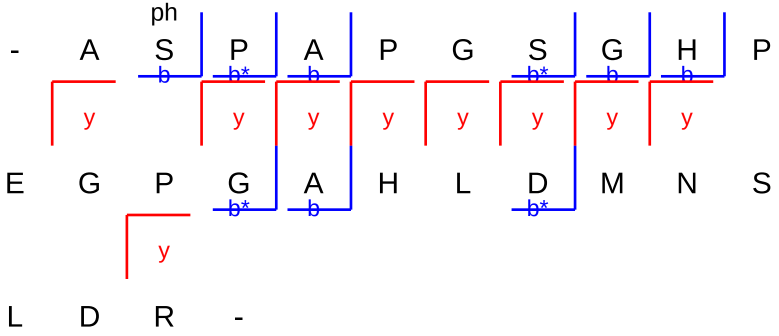

Raw File	Scan	Method	Score	m/z	Gene names
RO-QC-160121_ZackD_SS_Set_02_Fractio	16552	FTMS; HC	95.81	594.56	SRRM2

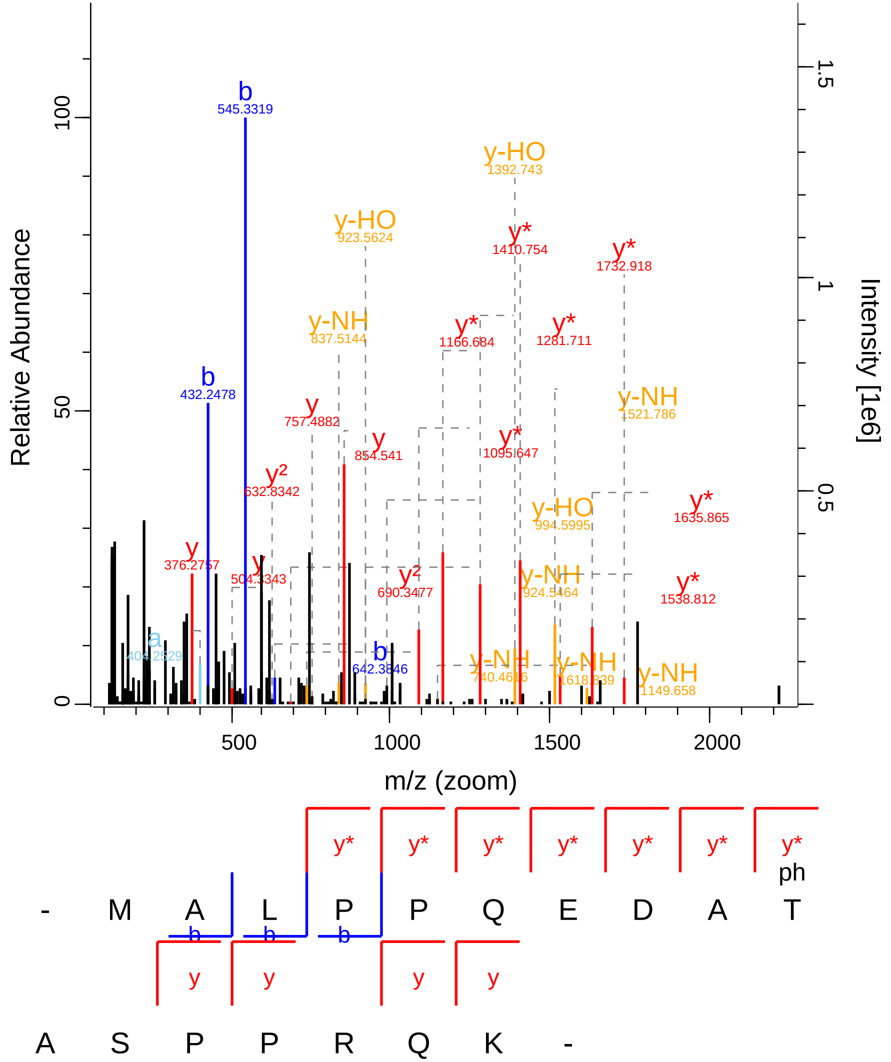

Raw File	Scan	Method	Score	m/z	Gene names
RO-QC-160121_ZackD_SS_Set_02_Fractio	16739	FTMS; HC	51.27	923.01	CEP350

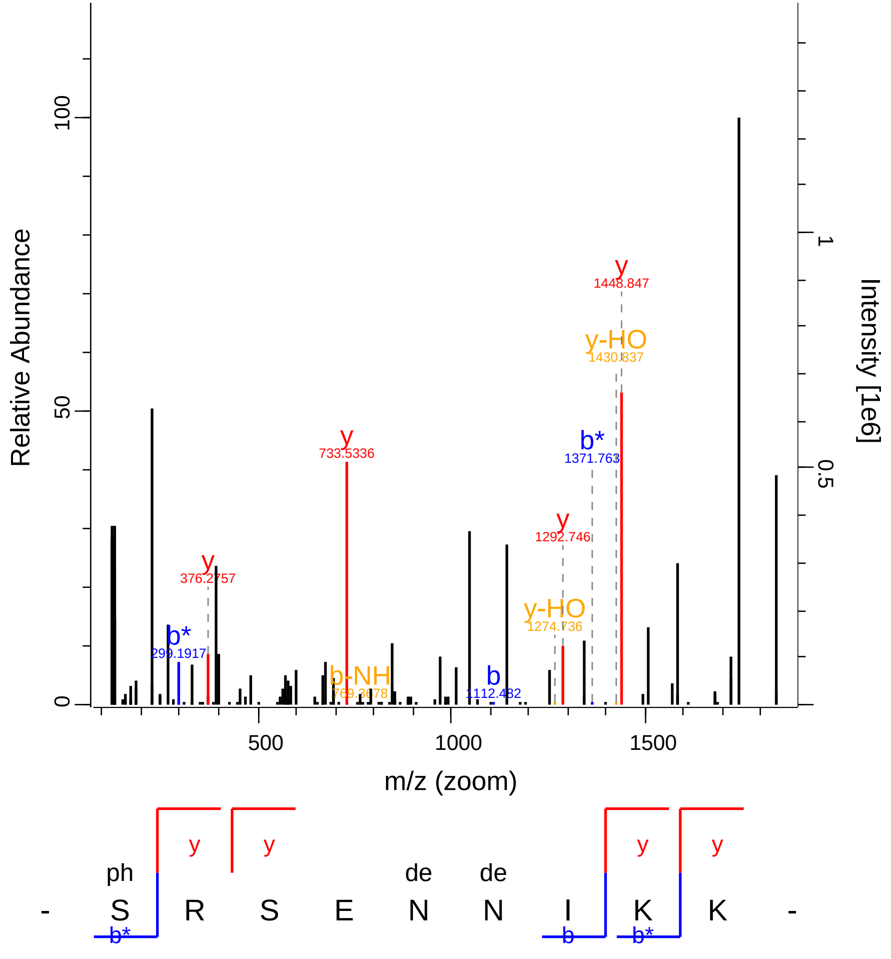

Raw File	Scan	Method	Score	m/z	Gene names
RO-QC-160121_ZackD_SS_Set_02_Fractio	17411	FTMS; HC	66.64	800.03	ABCA2

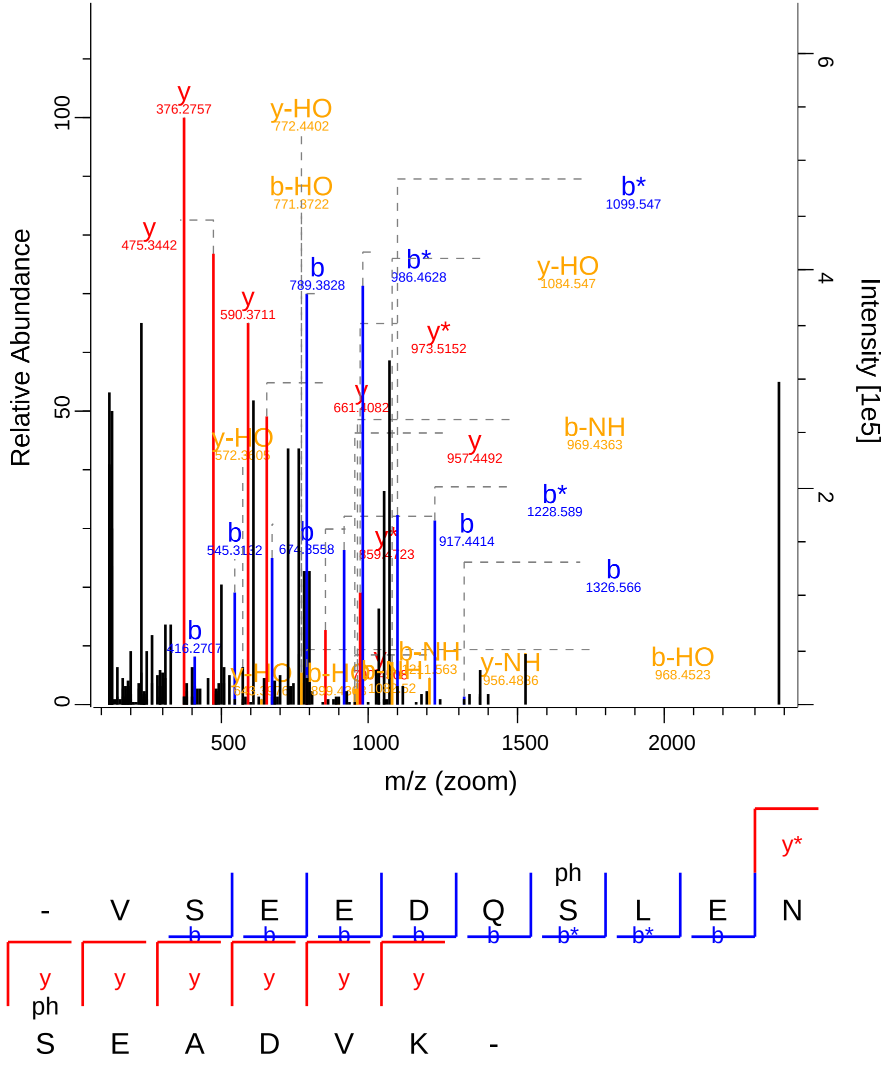

Raw File	Scan	Method	Score	m/z	Gene names
RO-QC-160121_ZackD_SS_Set_02_Fractior	17568	FTMS; HCI	79.01	637.7	WDR77

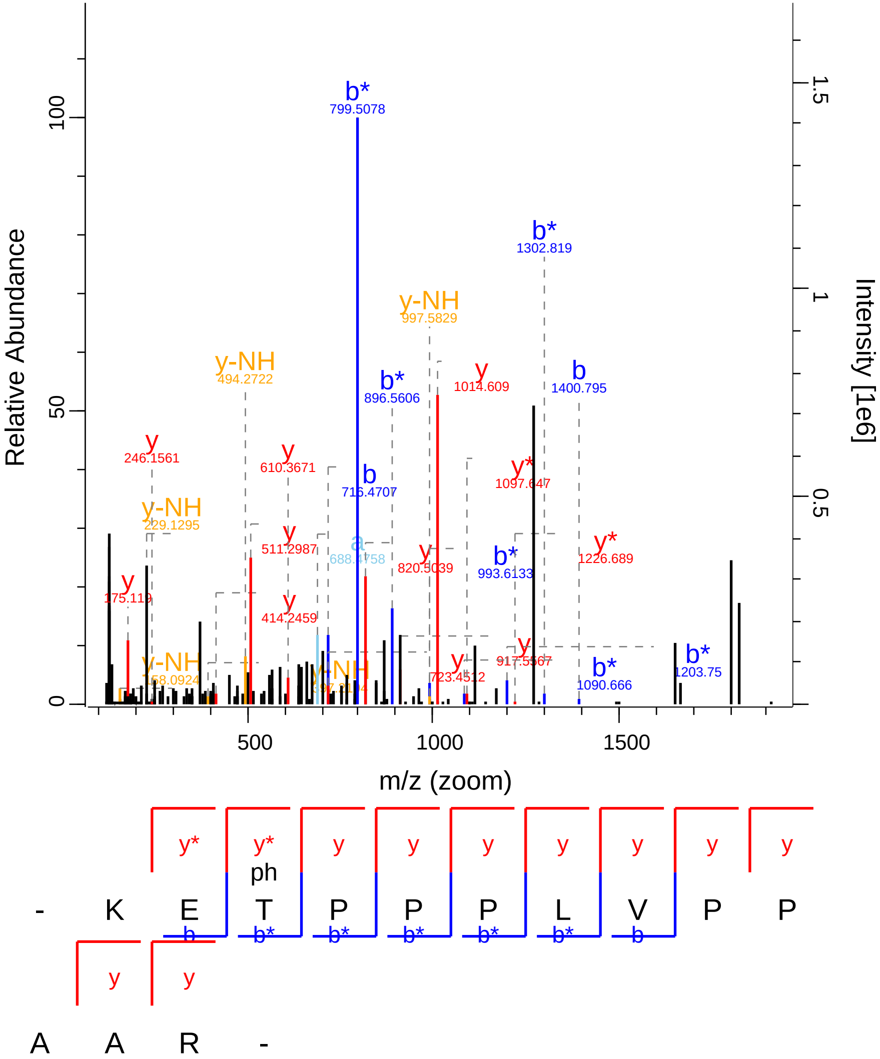

Raw File	Scan	Method	Score	m/z	Gene names
RO-QC-160121_ZackD_SS_Set_02_Fractic	17855	FTMS; HC	77.67	906.52	ZBTB47;ZNF652

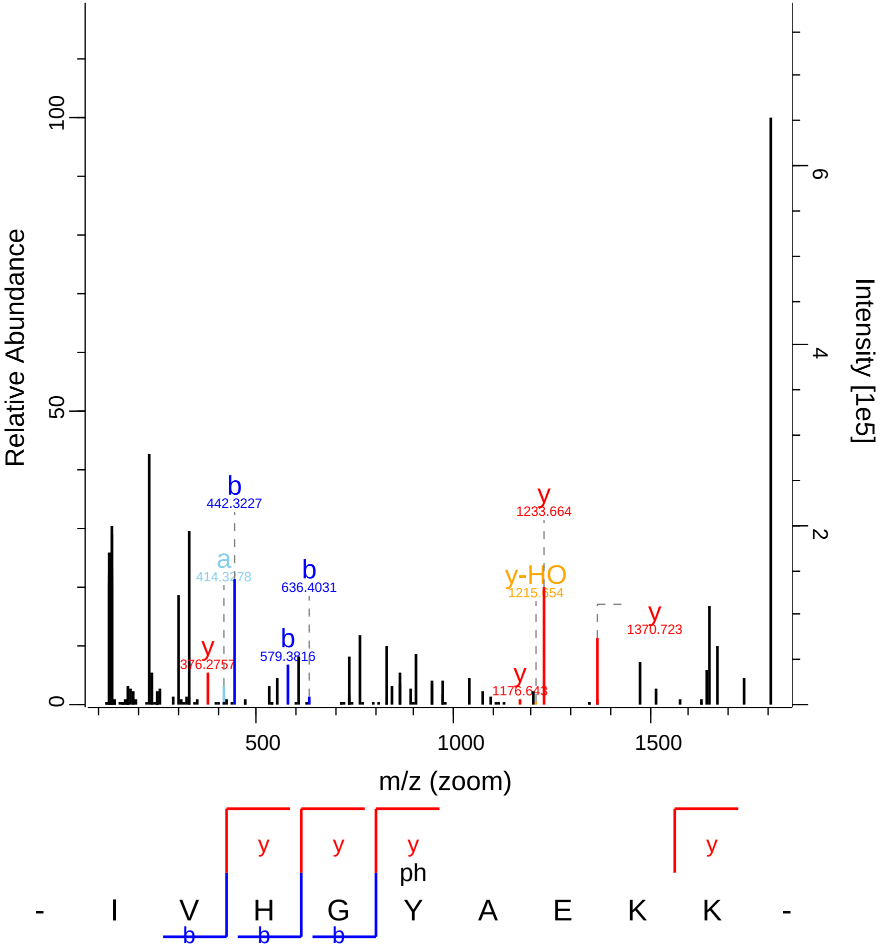

Raw File	Scan	Method	Score	m/z	Gene names
RO-QC-160121_ZackD_SS_Set_02_Fractio	17948	FTMS; HC	123.55	734.35	FAM134A

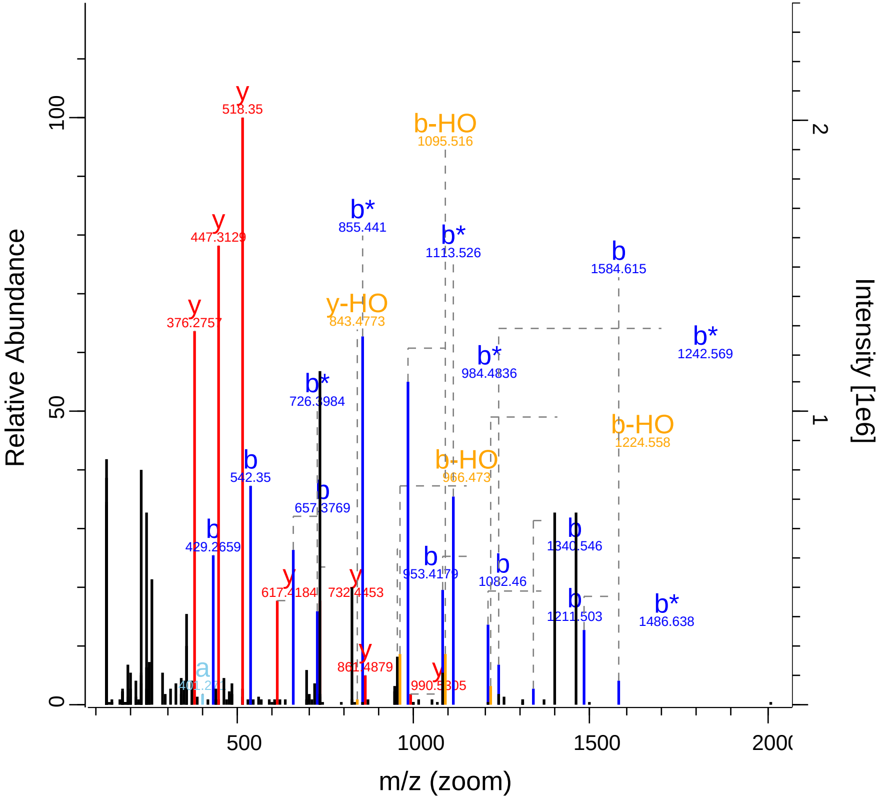

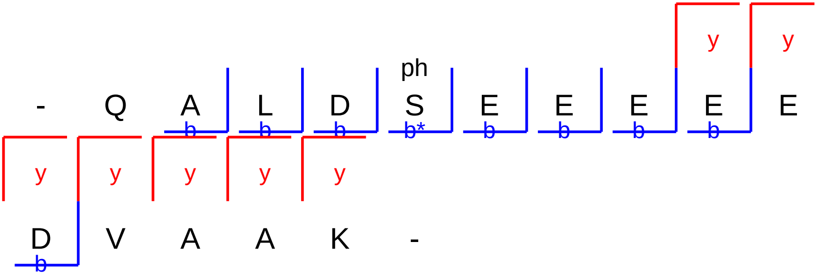

Raw File	Scan	Method	Score	m/z	Gene names
RO-QC-160121_ZackD_SS_Set_02_Fractio	17955	FTMS; HC	49.22	903.81	AKAP12

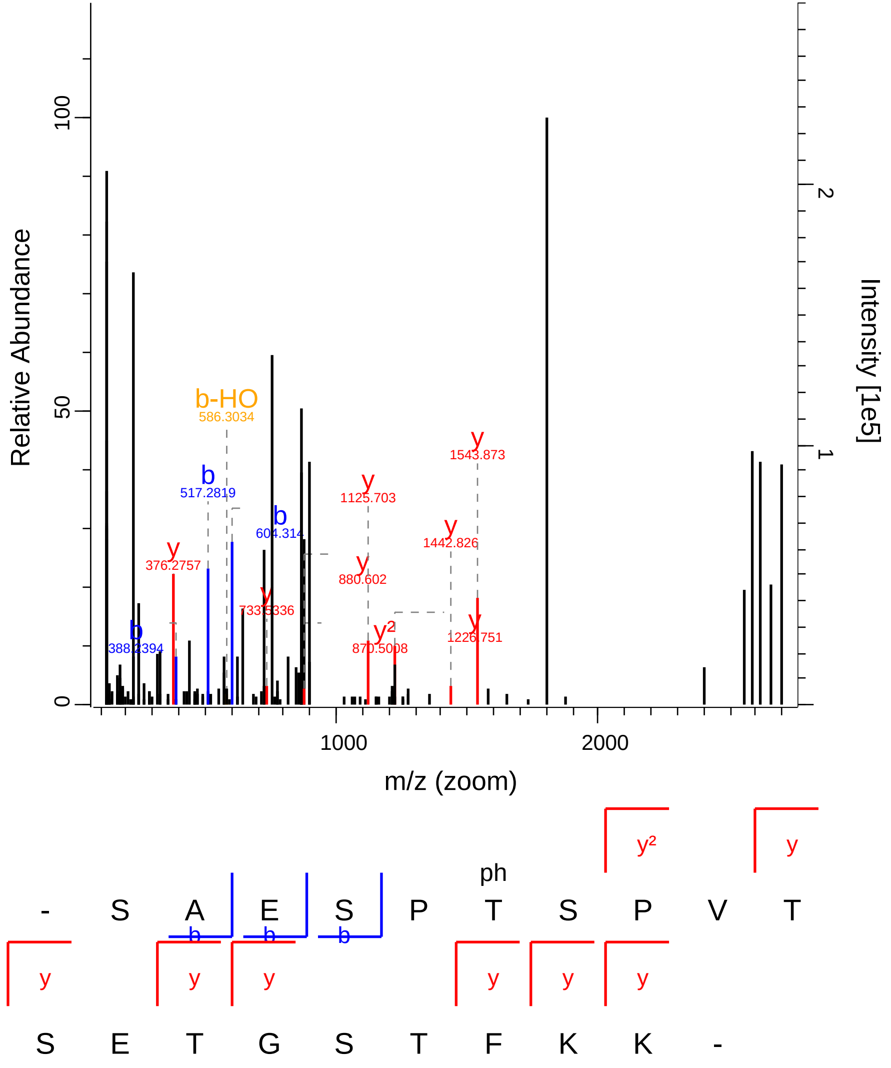

Raw File	Scan	Method	Score	m/z	Gene names
RO-QC-160121_ZackD_SS_Set_02_Fractic	18689	FTMS; HC	70.15	1152.54	MARCKS

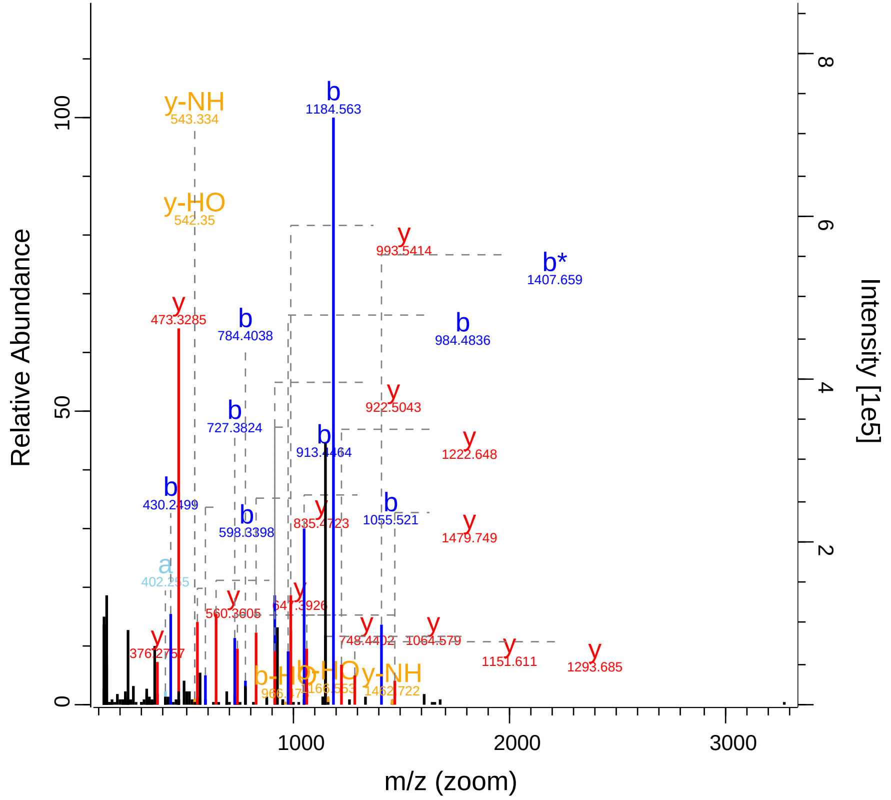

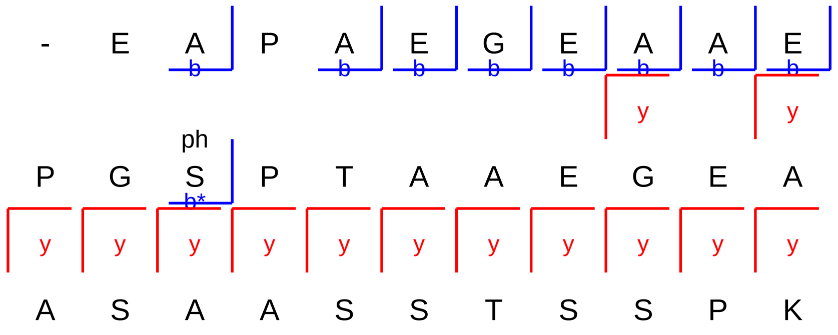

Raw File	Scan	Method	Score	m/z	Gene names
RO-QC-160121_ZackD_SS_Set_02_Fractio	18785	FTMS; HC	53.07	691.73	MARCKS

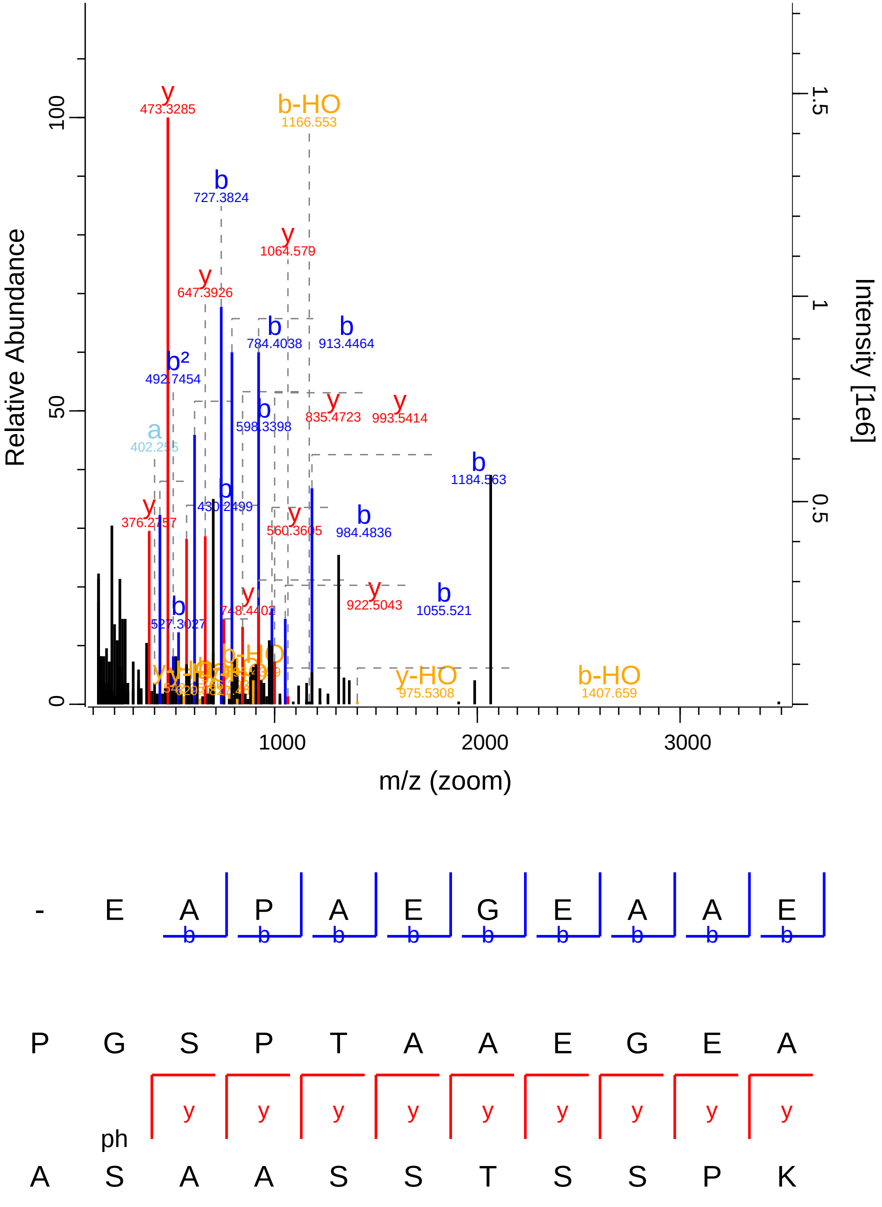

Raw File	Scan	Method	Score	m/z	Gene names
RO-QC-160121_ZackD_SS_Set_02_Fractio	19215	FTMS; HC	40.55	616.07	GRM4

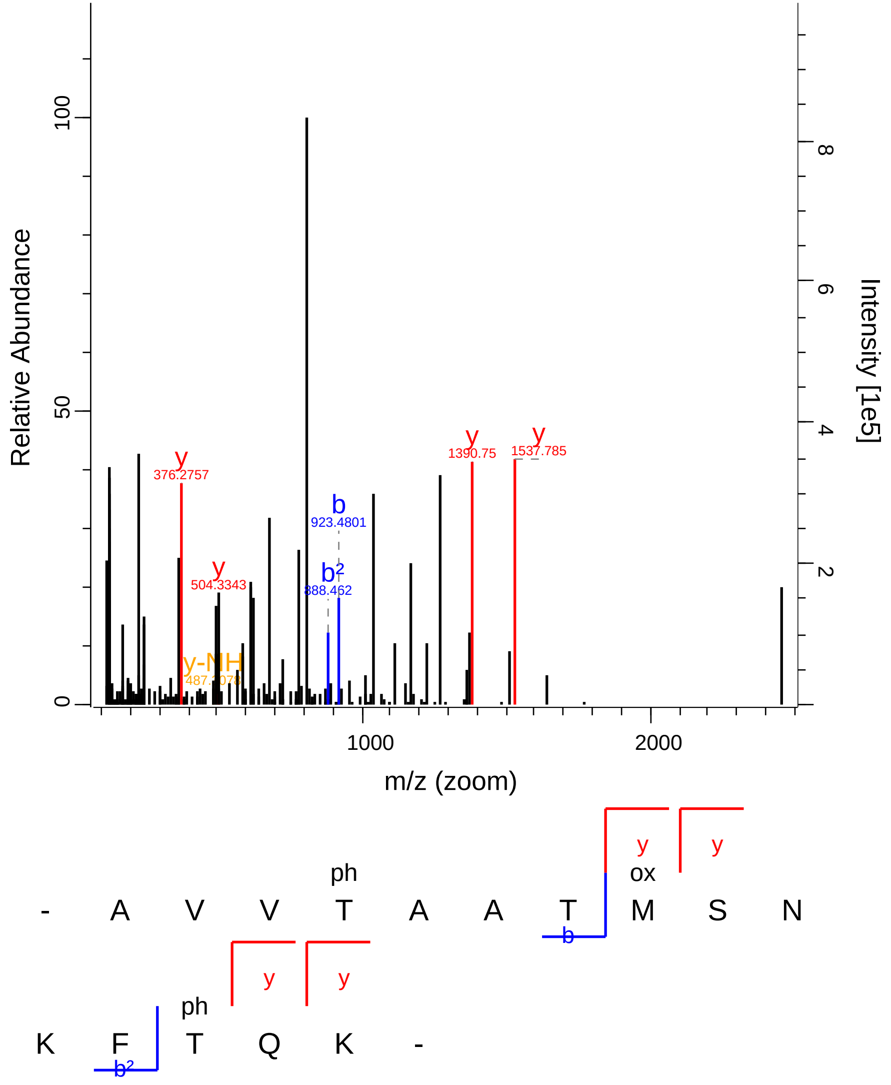

Raw File	Scan	Method	Score	m/z	Gene names
RO-QC-160121_ZackD_SS_Set_02_Fractio	19453	FTMS; HC	117.99	996.45	GOLGA4

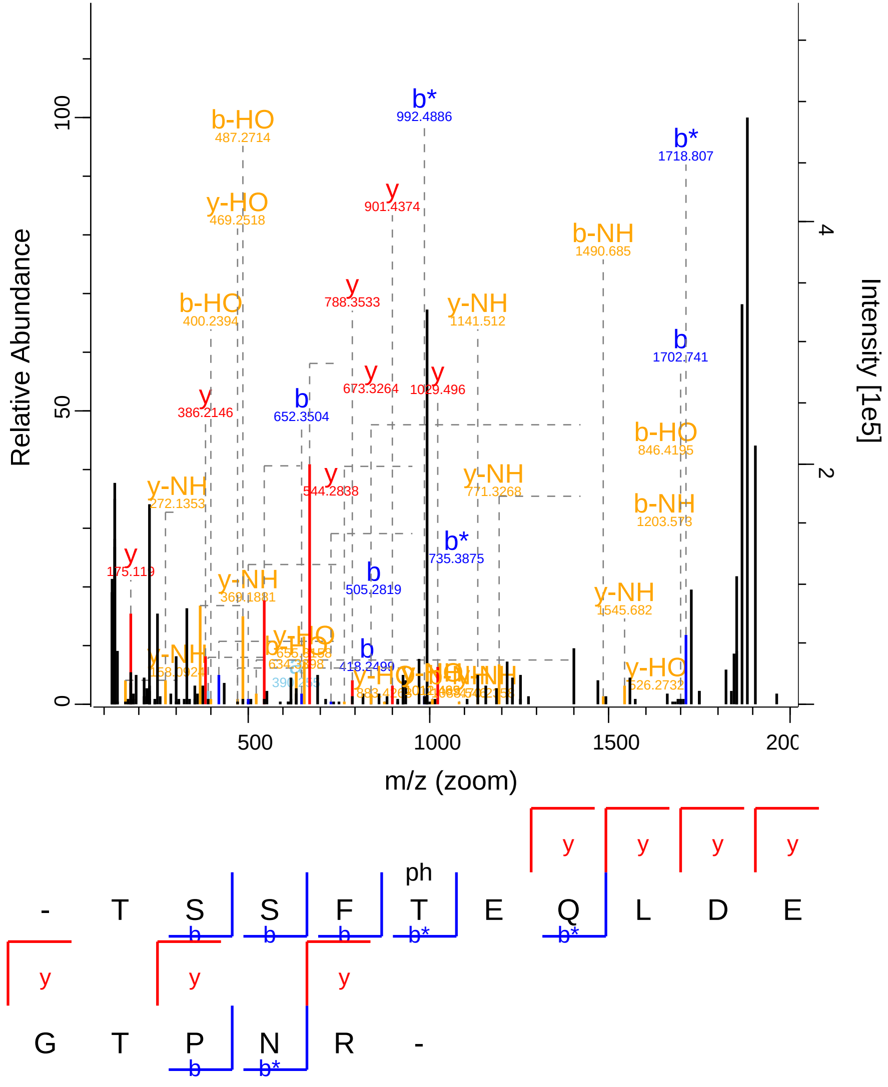

Raw File	Scan	Method	Score	m/z	Gene names
RO-QC-160121_ZackD_SS_Set_02_Fractio	19462	FTMS; HC	111.66	815.04	CBX5

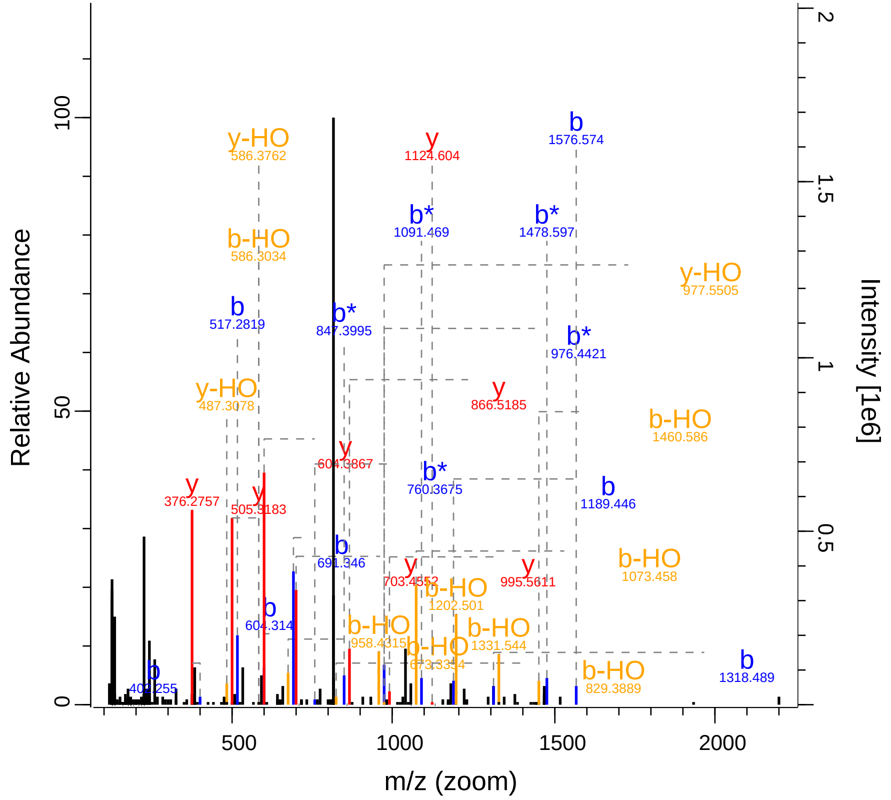

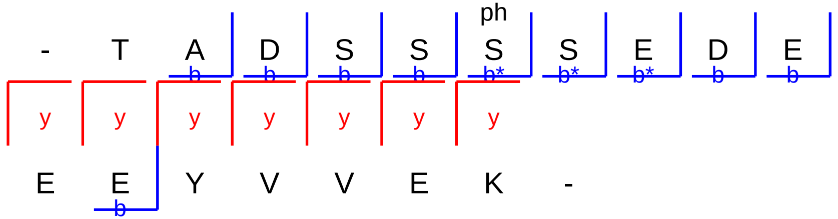

Raw File	Scan	Method	Score	m/z	Gene names
RO-QC-160121_ZackD_SS_Set_02_Fractio	19743	FTMS; HC	105.04	883.73	MAP1B

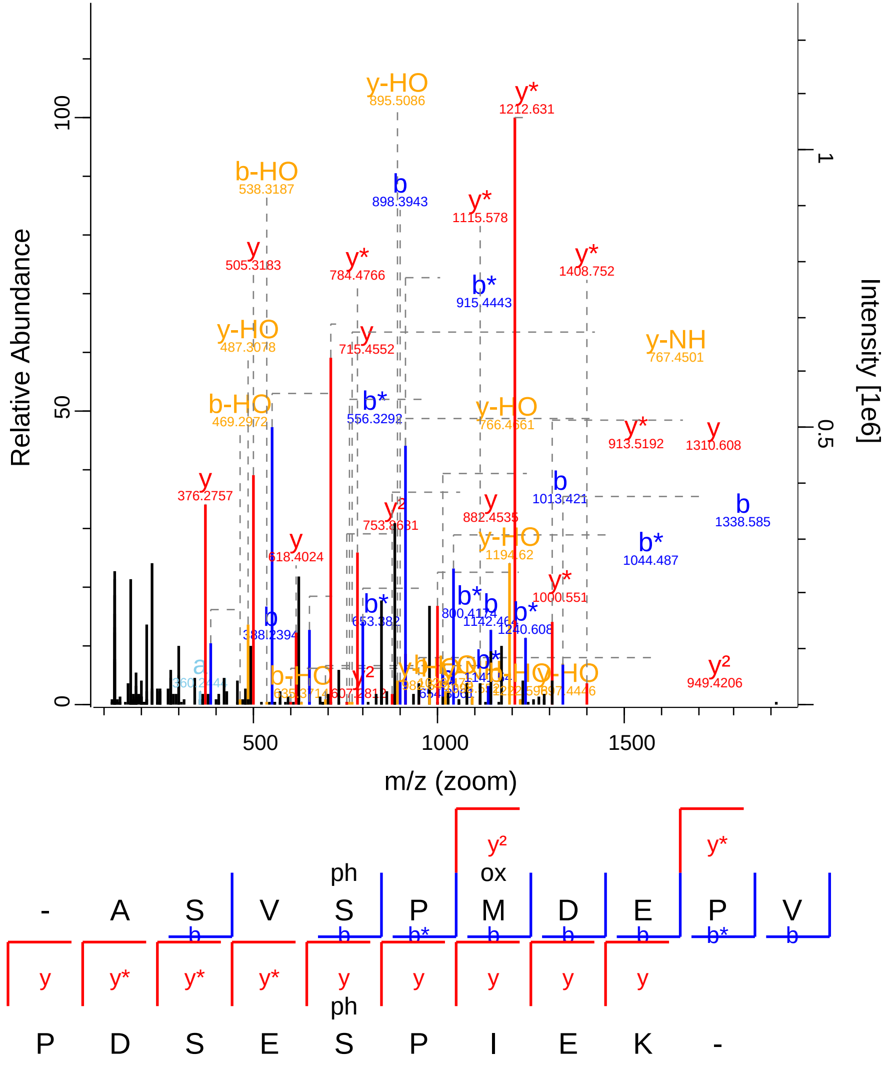

Raw File	Scan	Method	Score	m/z	Gene names
RO-QC-160121_ZackD_SS_Set_02_Fractio	20365	FTMS; HC	114.91	1052.53	RASAL2

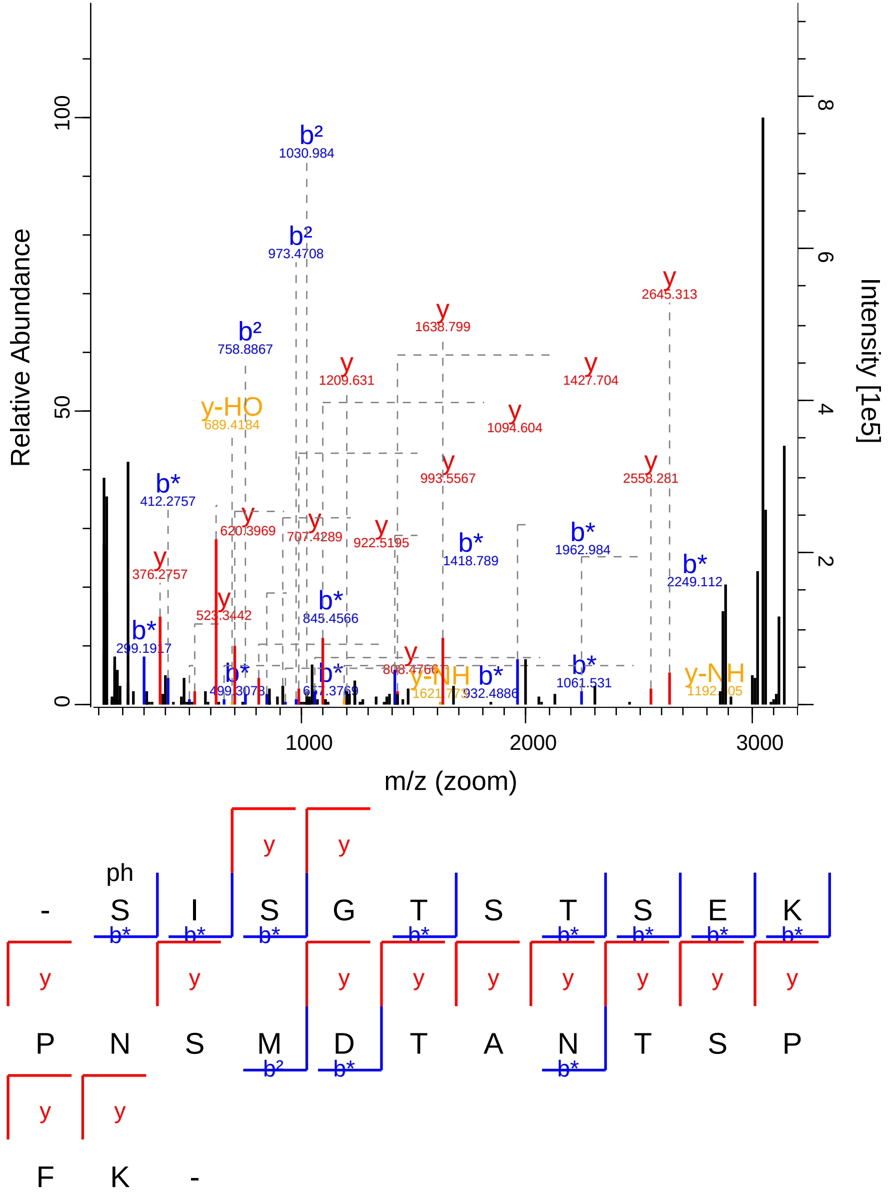

Raw File	Scan	Method	Score	m/z	Gene names
RO-QC-160121_ZackD_SS_Set_02_Fract	20458	FTMS; HC	79.69	528.94	ARHGEF7;ARHGE

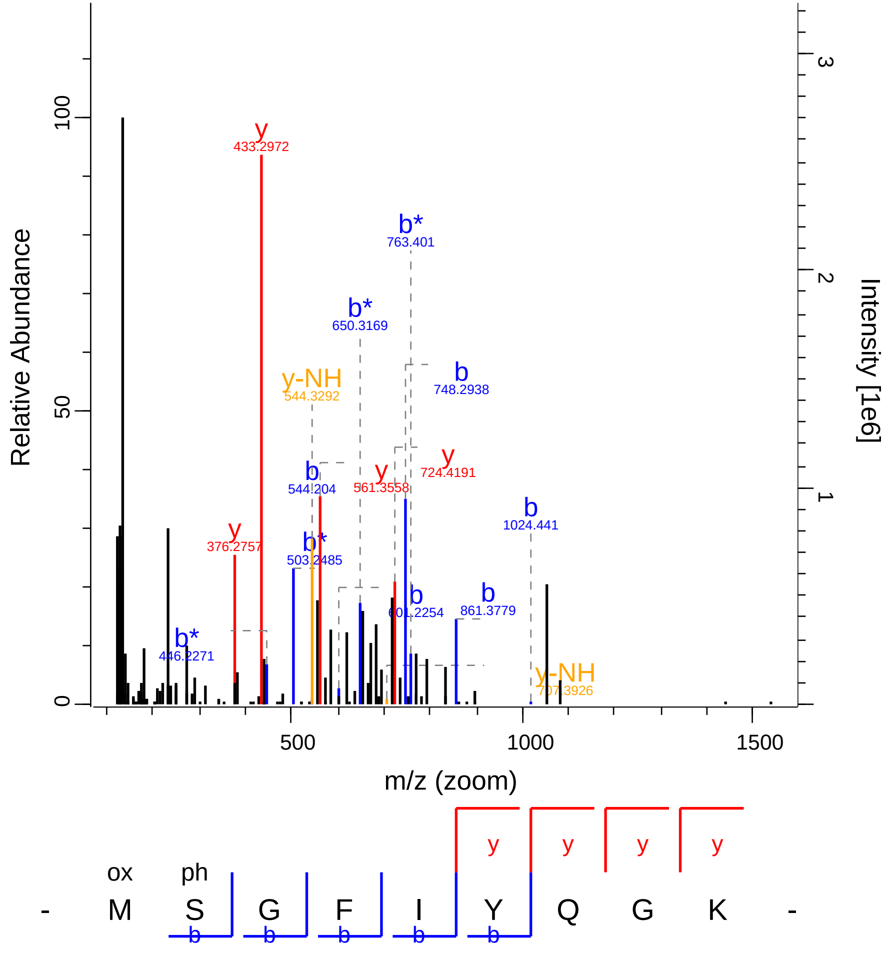

Raw File	Scan	Method	Score	m/z	Gene names
RO-QC-160121_ZackD_SS_Set_02_Fractio	21104	FTMS; HC	101.49	787.75	TEX2

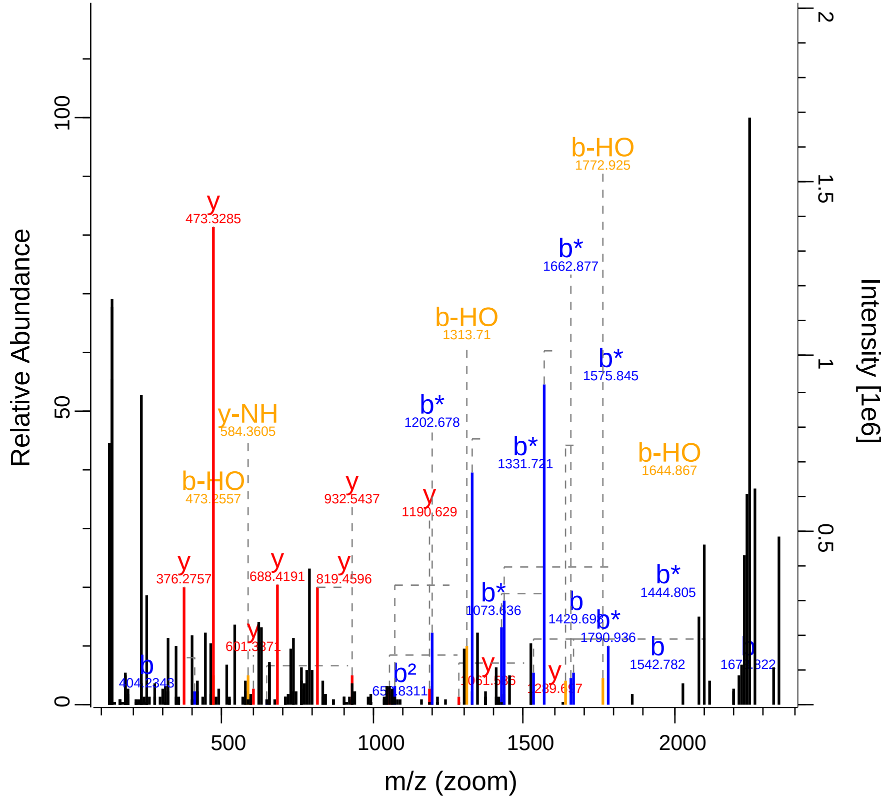

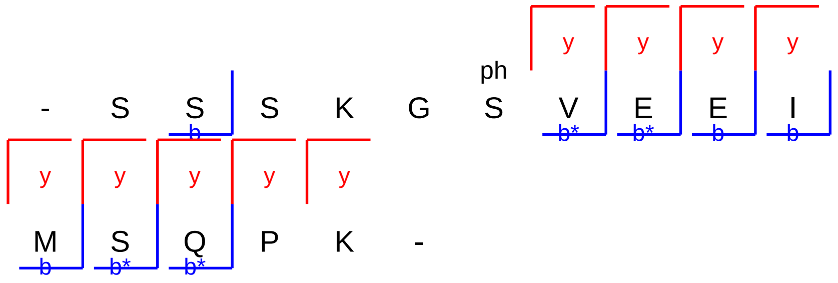

Raw File	Scan	Method	Score	m/z	Gene names
RO-QC-160121_ZackD_SS_Set_02_Fractio	21117	FTMS; HC	96.92	591.06	TEX2

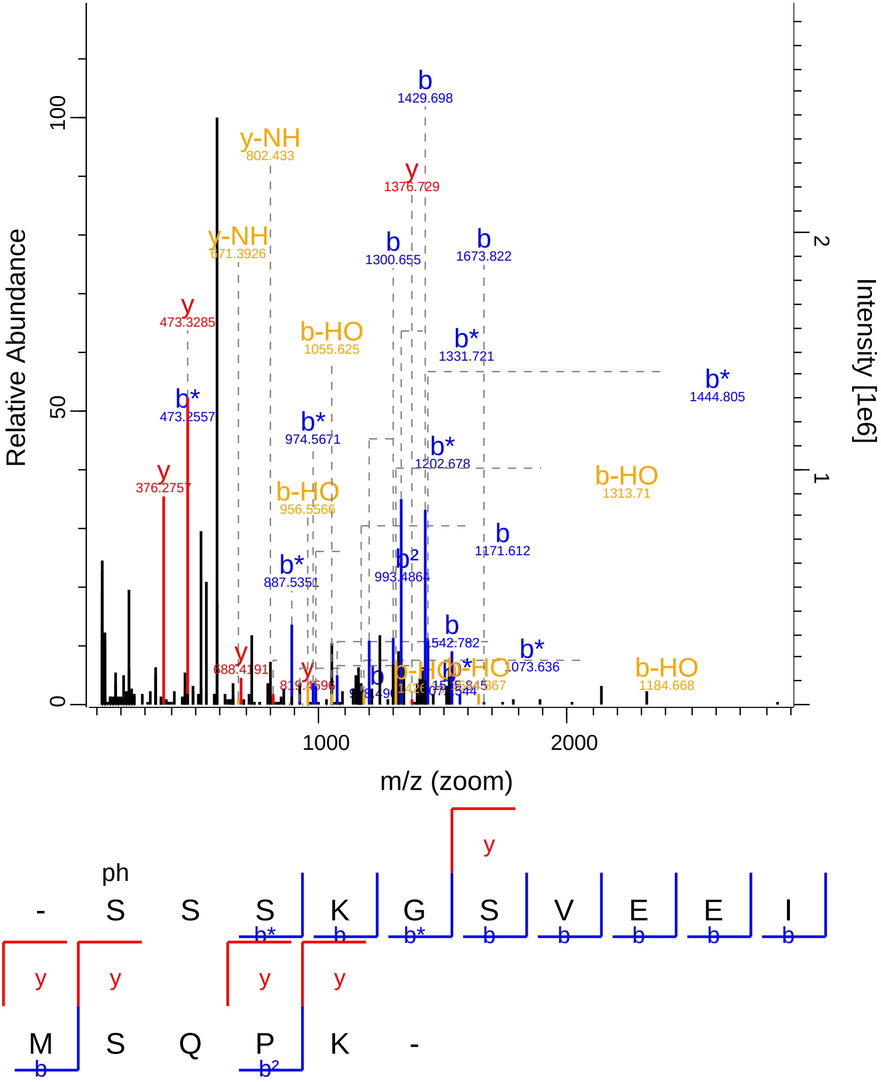

Raw File	Scan	Method	Score	m/z	Gene names
RO-QC-160121_ZackD_SS_Set_02_Fractio	21167	FTMS; HC	40.38	814.65	AZI2

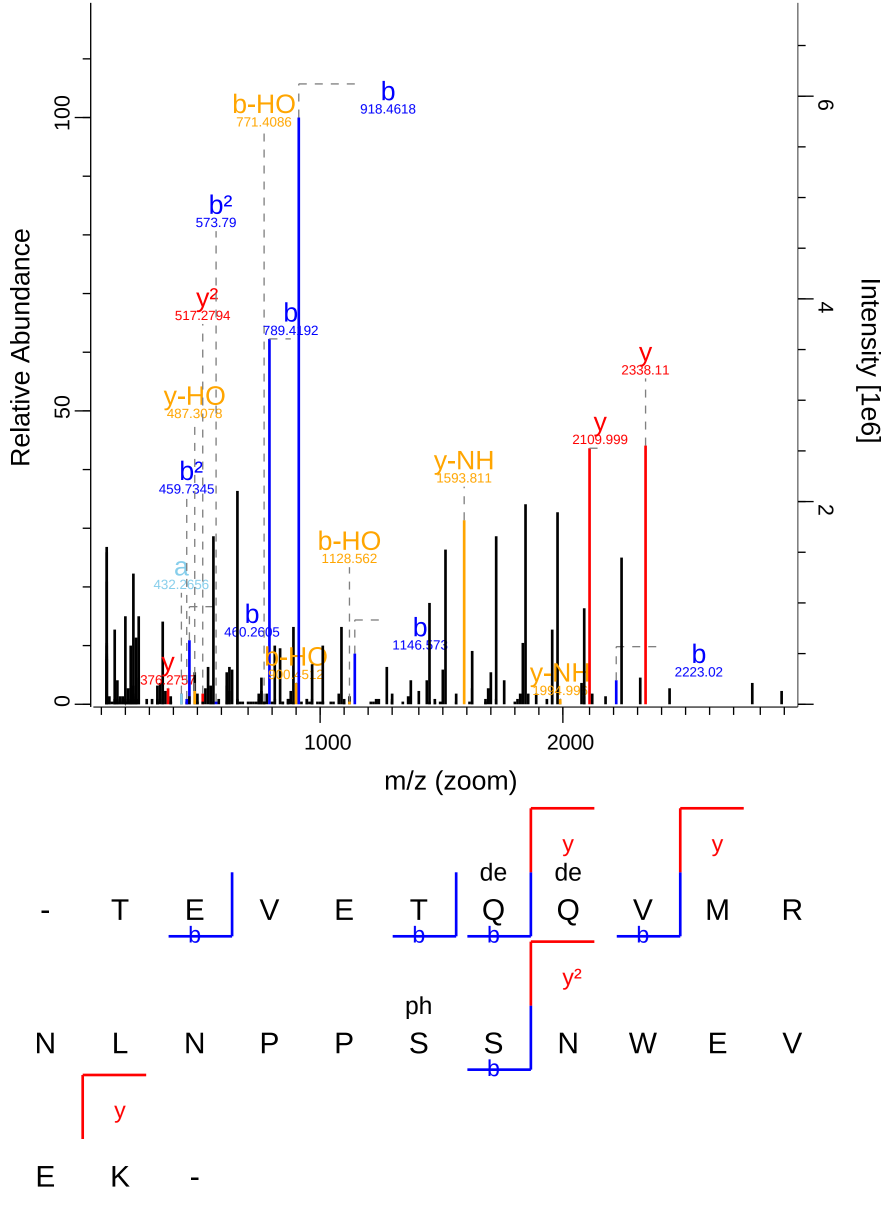

Raw File	Scan	Method	Score	m/z	Gene names
RO-QC-160121_ZackD_SS_Set_02_Fractio	21199	FTMS; HC	58.32	820.45	SIK3

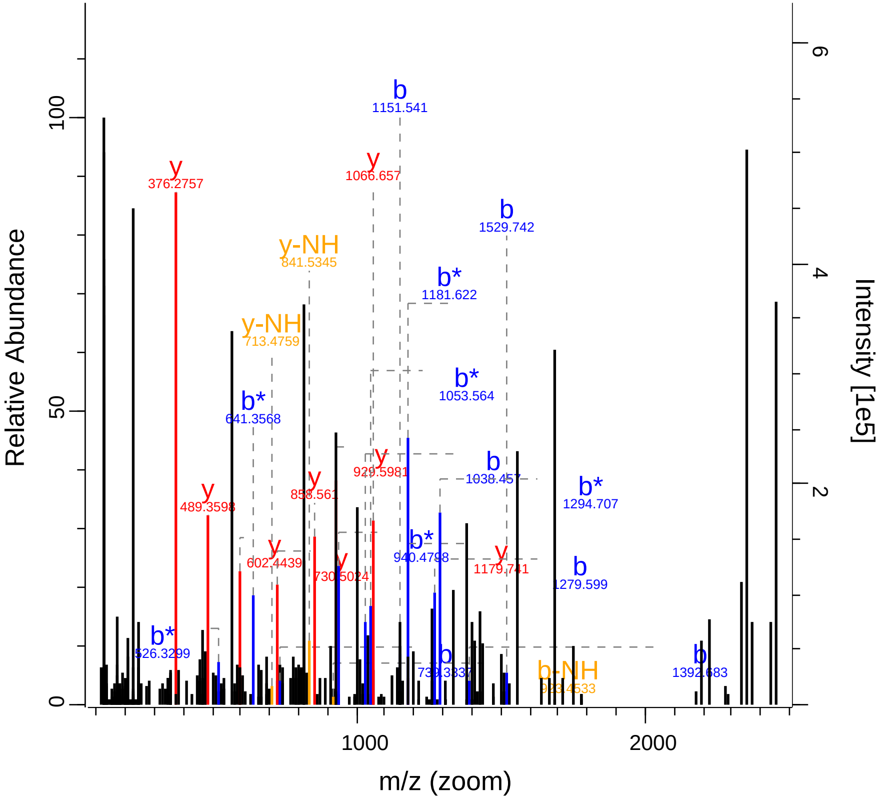

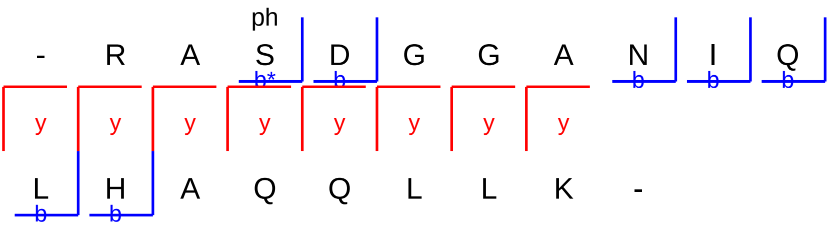

Raw File	Scan	Method	Score	m/z	Gene names
RO-QC-160121_ZackD_SS_Set_02_Fractio	21200	FTMS; HC	40.04	857.87	ERVK-16

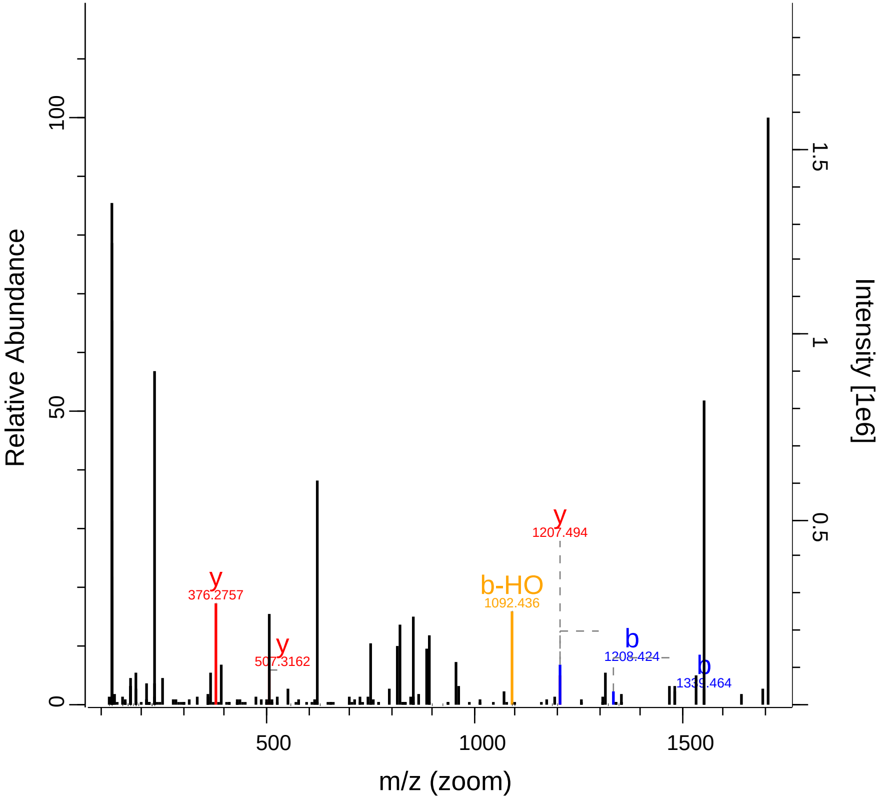

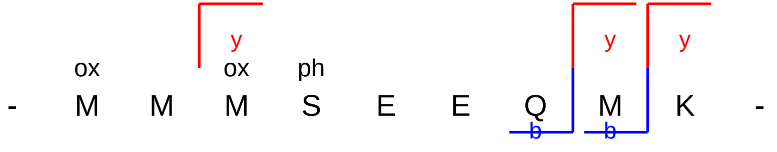

Raw File	Scan	Method	Score	m/z	Gene names
RO-QC-160121_ZackD_SS_Set_02_Fractio	21236	FTMS; HC	163.18	1016.11	STK3

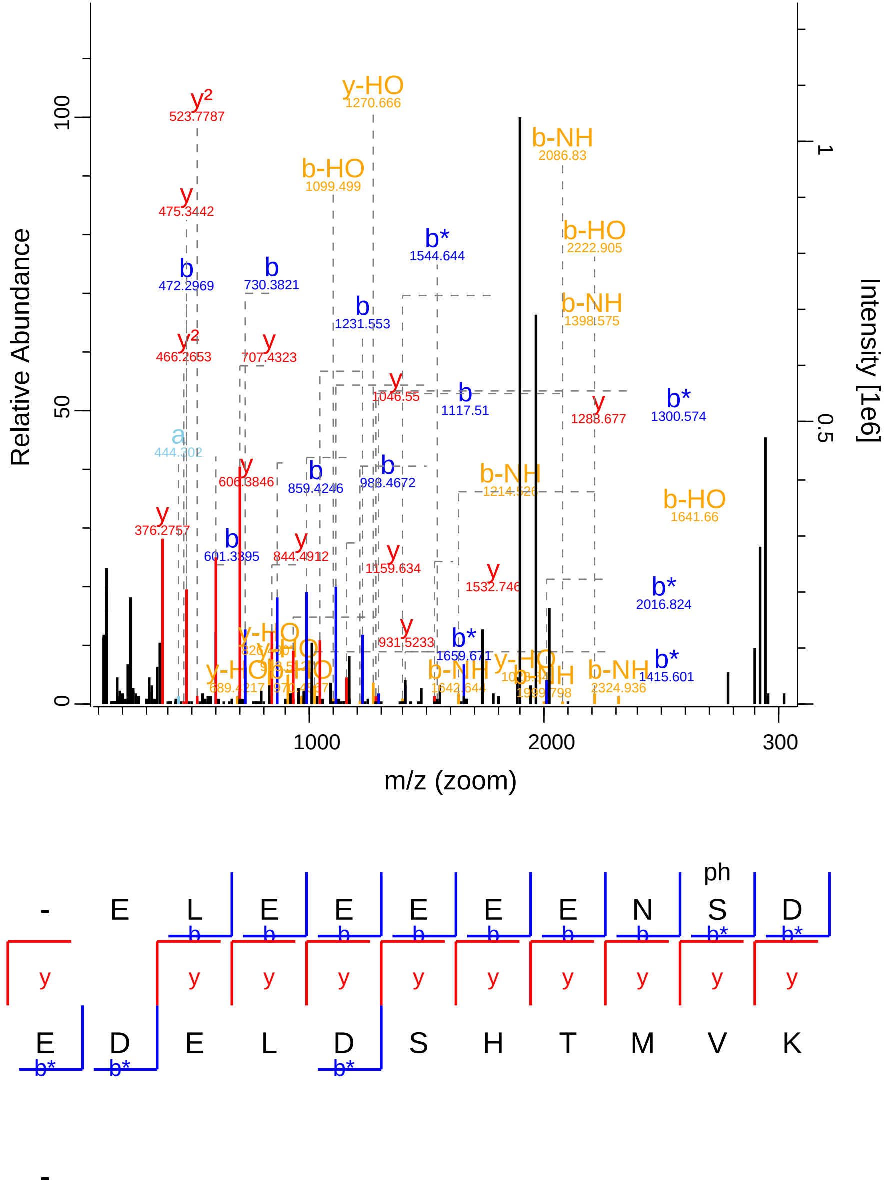

Raw File	Scan	Method	Score	m/z	Gene names
RO-QC-160121_ZackD_SS_Set_02_Fractio	21507	FTMS; HC	100.28	877.12	ITSN1

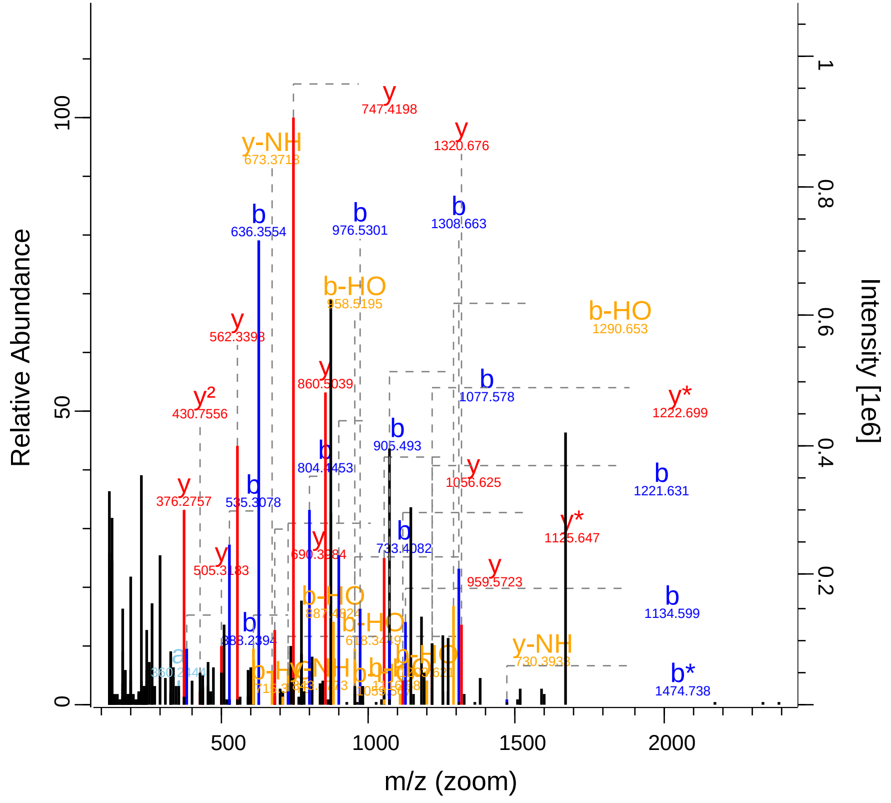

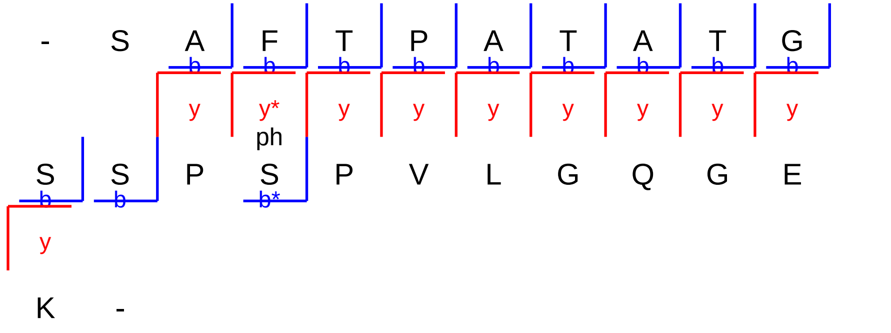

Raw File	Scan	Method	Score	m/z	Gene names
RO-QC-160121_ZackD_SS_Set_02_Fractio	21878	FTMS; HC	86.5	624.34	ALDOA

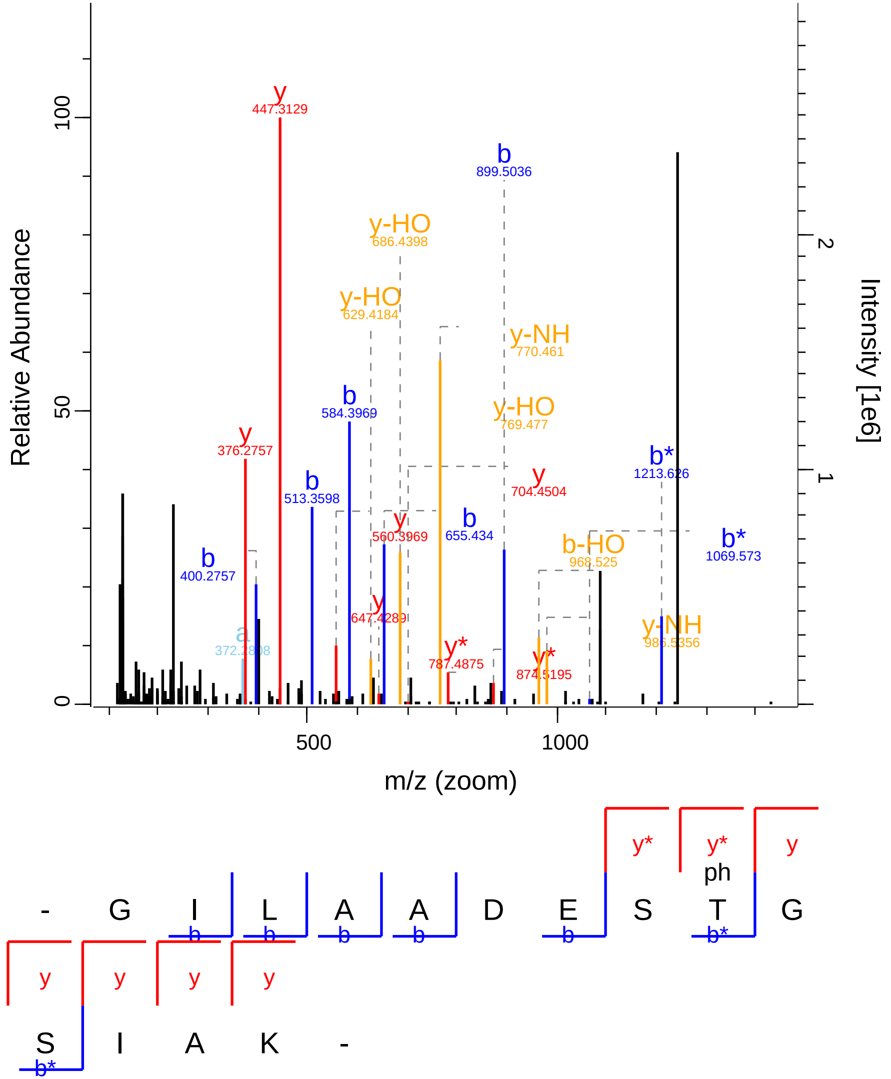

Raw File	Scan	Method	Score	m/z	Gene names
RO-QC-160121_ZackD_SS_Set_02_Fractio	22046	FTMS; HC	50.3	652.36	KIF5C

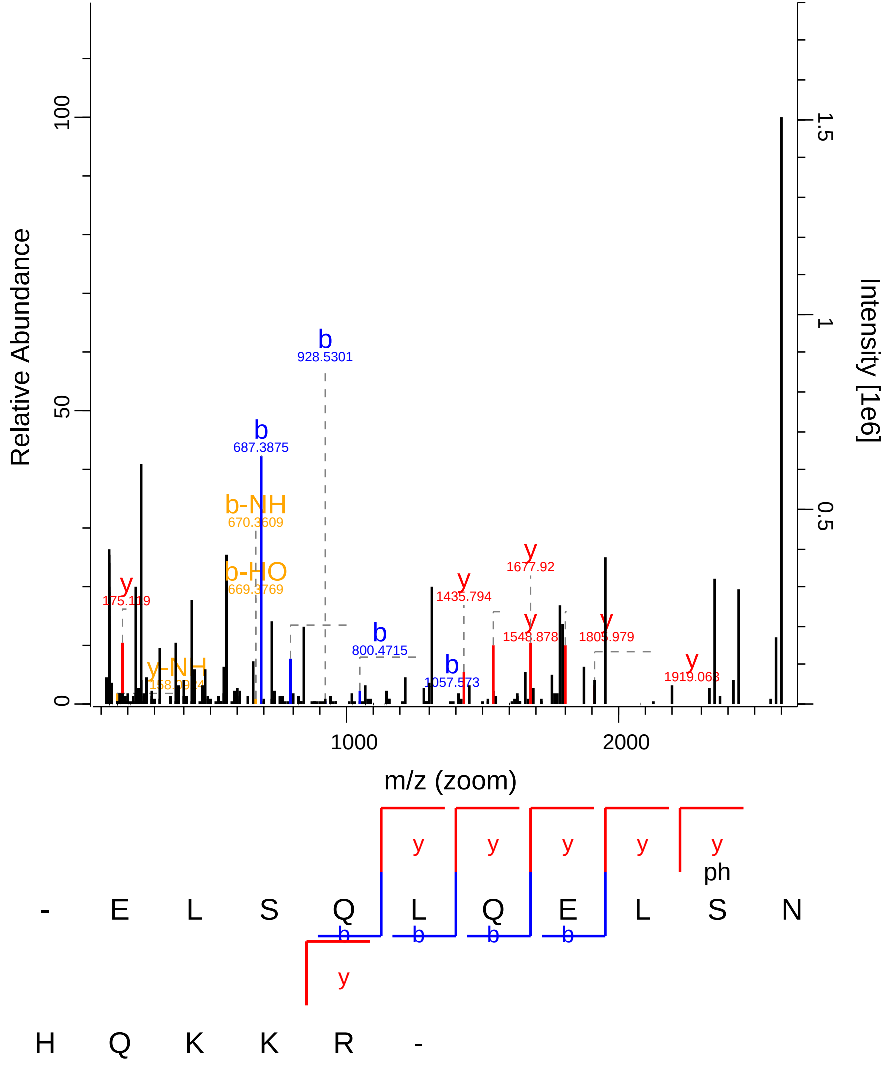

Raw File	Scan	Method	Score	m/z	Gene names
RO-QC-160121_ZackD_SS_Set_02_Fractio	22210	FTMS; HC	96.34	840.36	MCM3

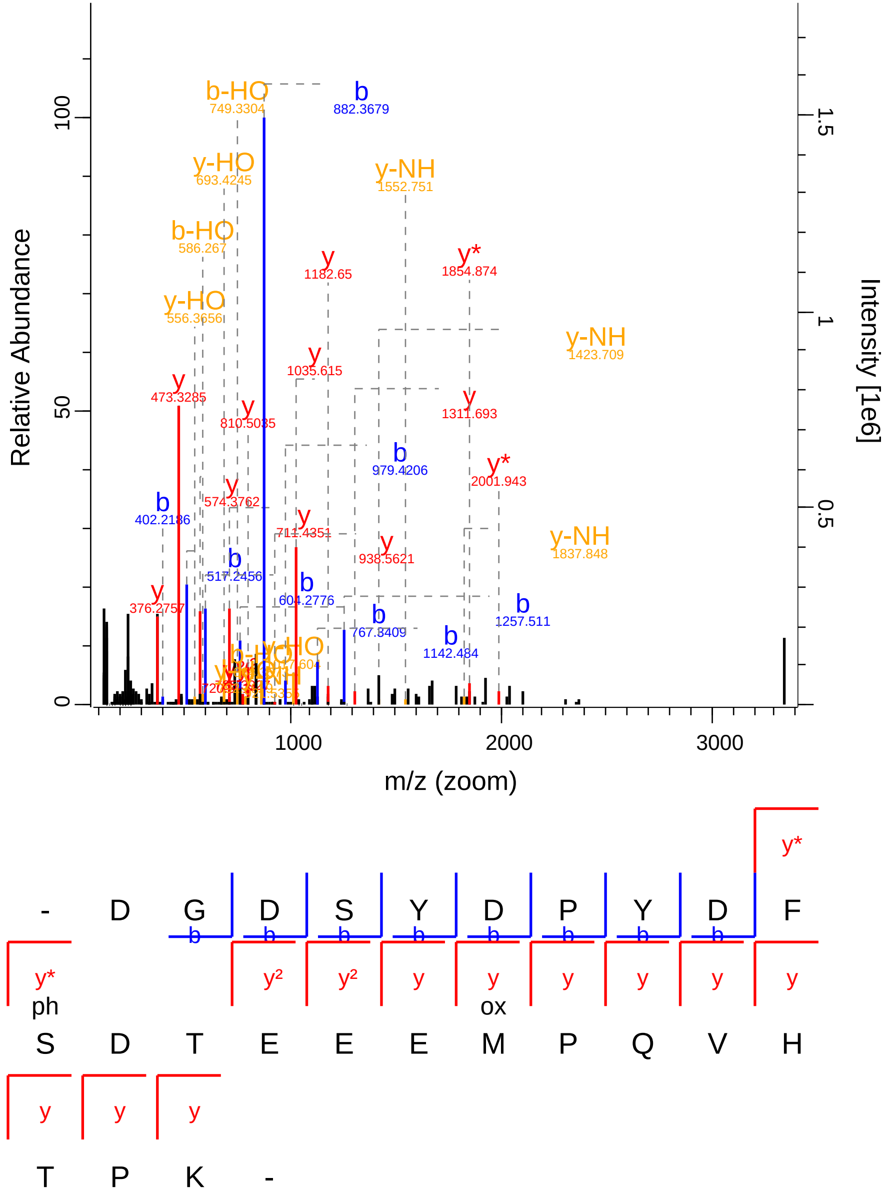

Raw File	Scan	Method	Score	m/z	Gene names
RO-QC-160121_ZackD_SS_Set_02_Fractio	22421	FTMS; HC	75.65	835.41	KIF23

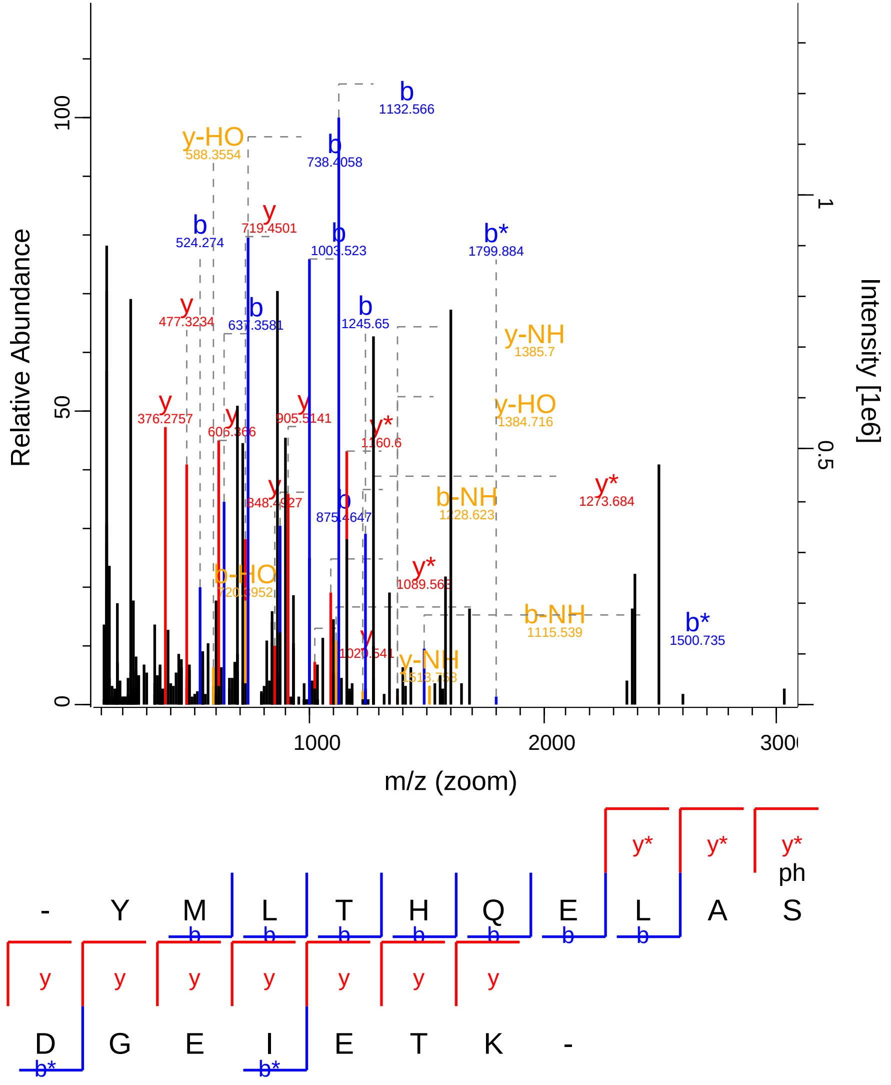

Raw File	Scan	Method	Score	m/z	Gene names
RO-QC-160121_ZackD_SS_Set_02_Fractio	22457	FTMS; HC	47.6	721.69	MOGAT2

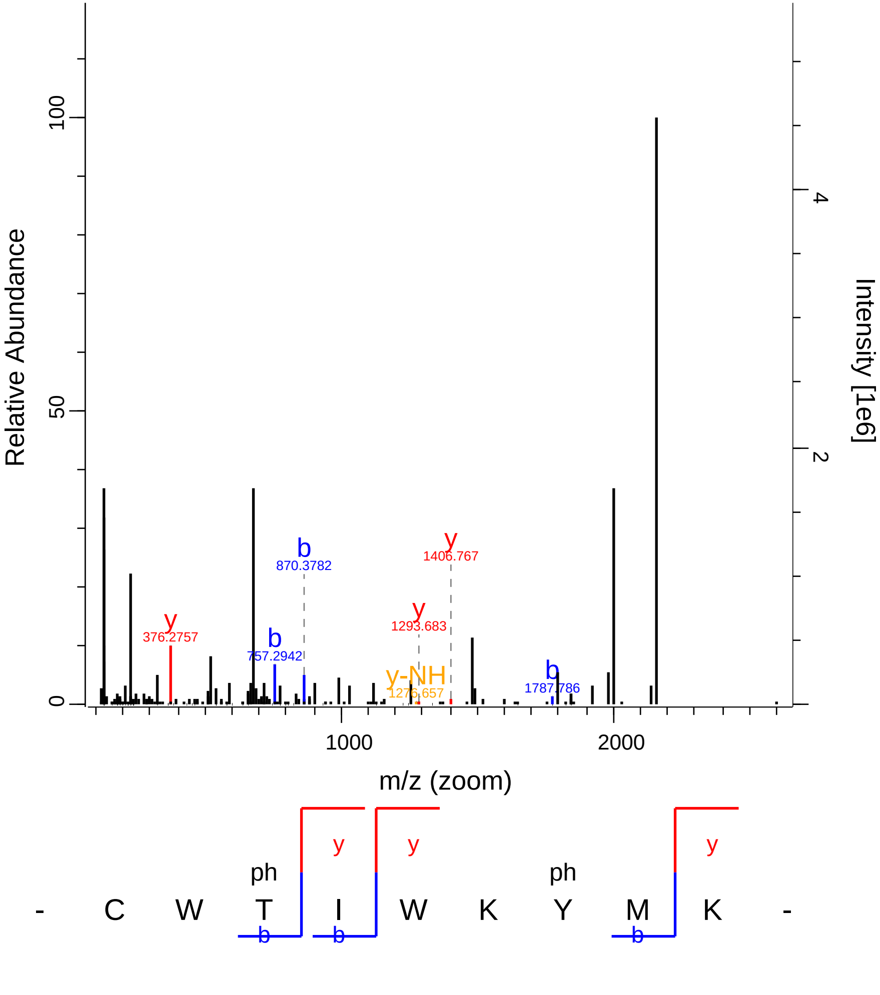

Raw File	Scan	Method	Score	m/z	Gene names
RO-QC-160121_ZackD_SS_Set_02_Fractio	22494	FTMS; HC	101.59	852.78	NAV1

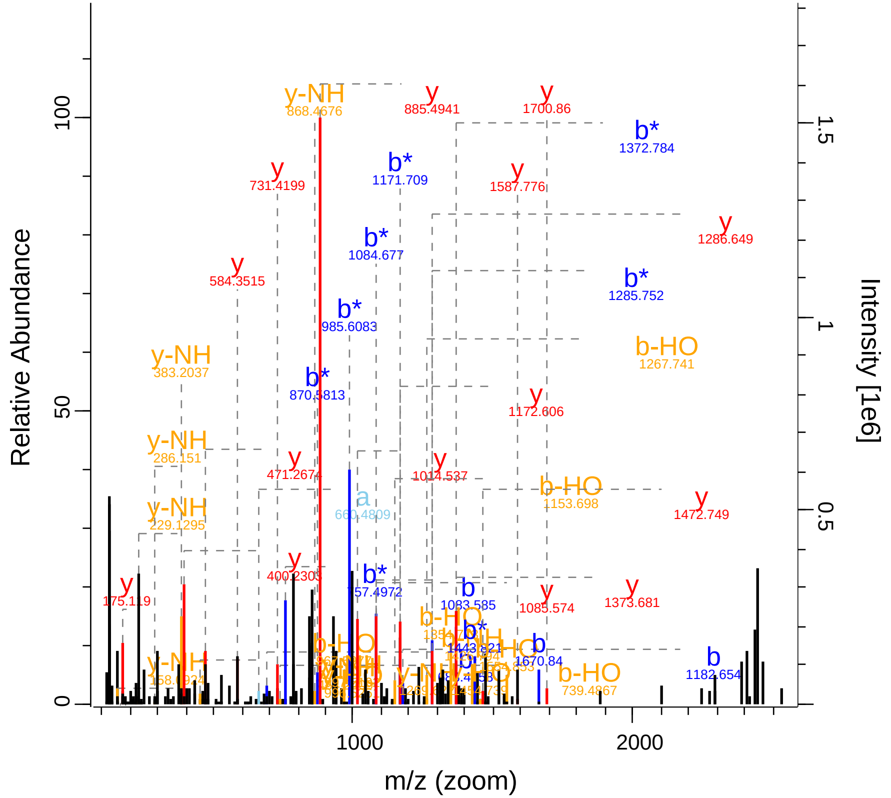

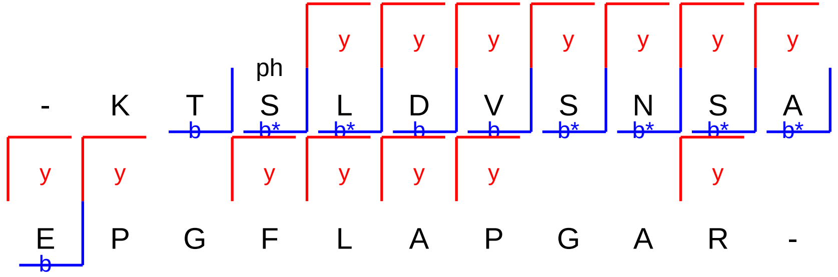

Raw File	Scan	Method	Score	m/z	Gene names
RO-QC-160121_ZackD_SS_Set_02_Fraction	22562	FTMS; HCI	237.32	936	ALDOA

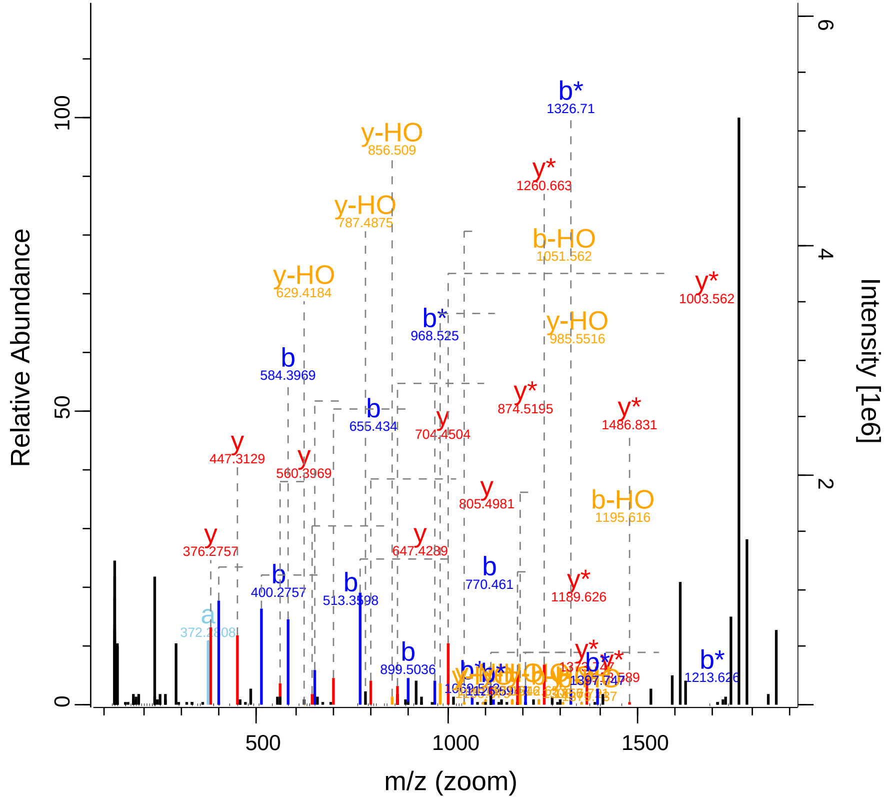

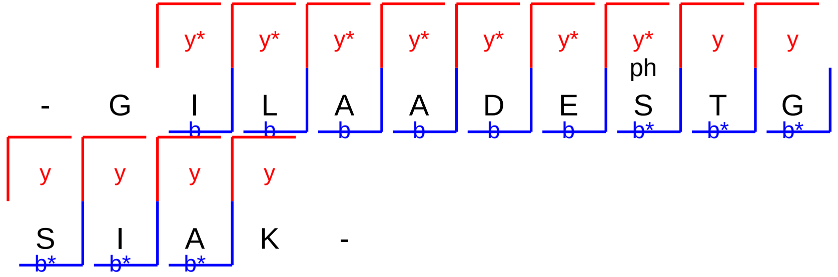

Raw File	Scan	Method	Score	m/z	Gene names
RO-QC-160121_ZackD_SS_Set_02_Fractio	23147	FTMS; HC	273.81	1065.25	MYRIP

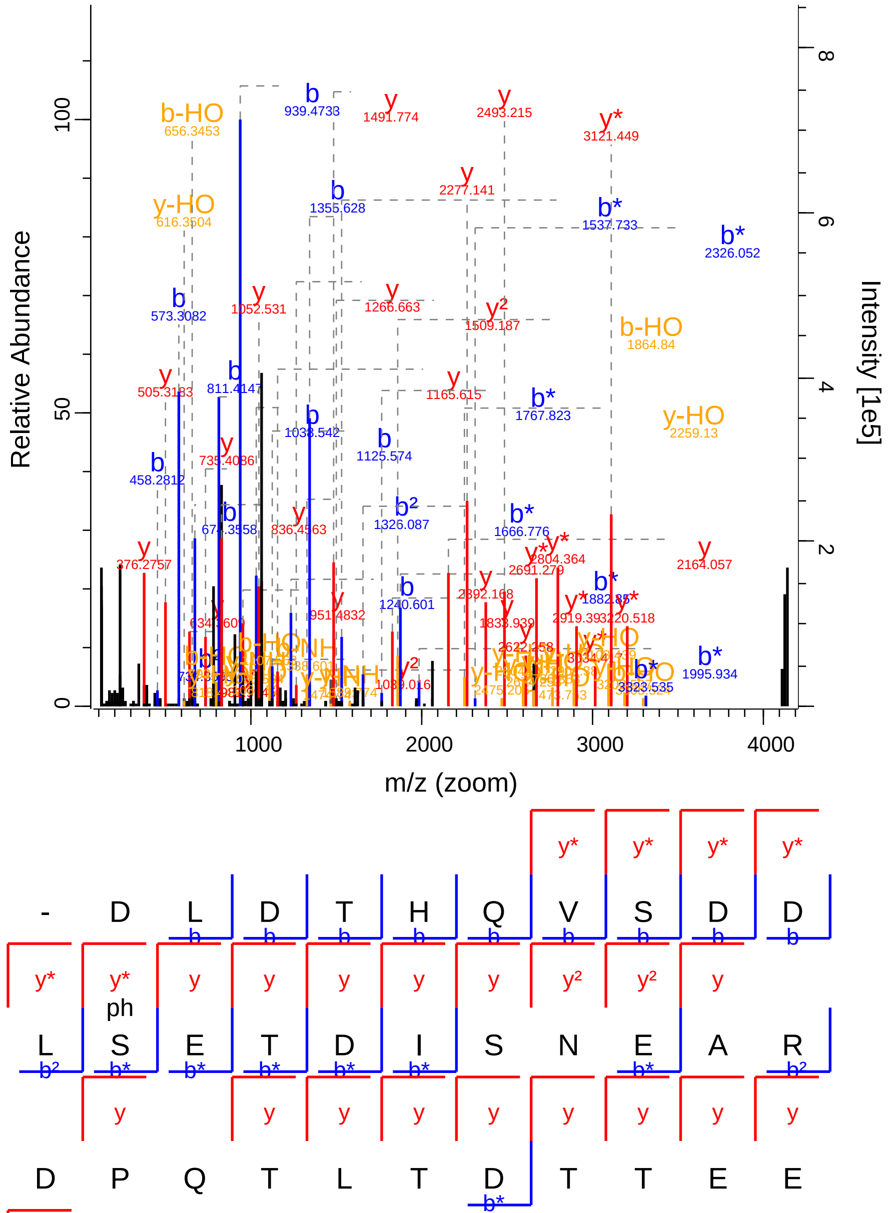

Raw File	Scan	Method	Score	m/z	Gene names
RO-QC-160121_ZackD_SS_Set_02_Fractio	23541	FTMS; HC	52.45	753.92	LIMK2

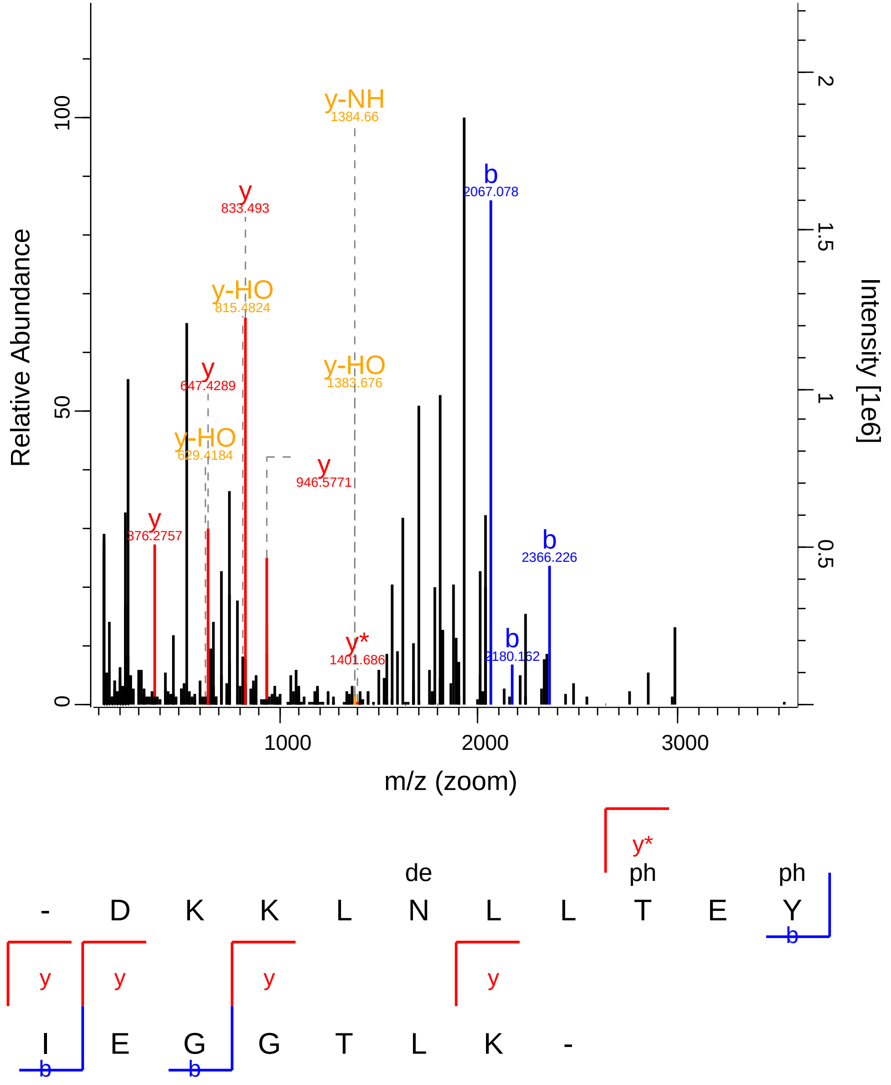

Raw File	Scan	Method	Score	m/z	Gene names
RO-QC-160121_ZackD_SS_Set_02_Fractio	23607	FTMS; HCI	135.04	849.1	NFIC

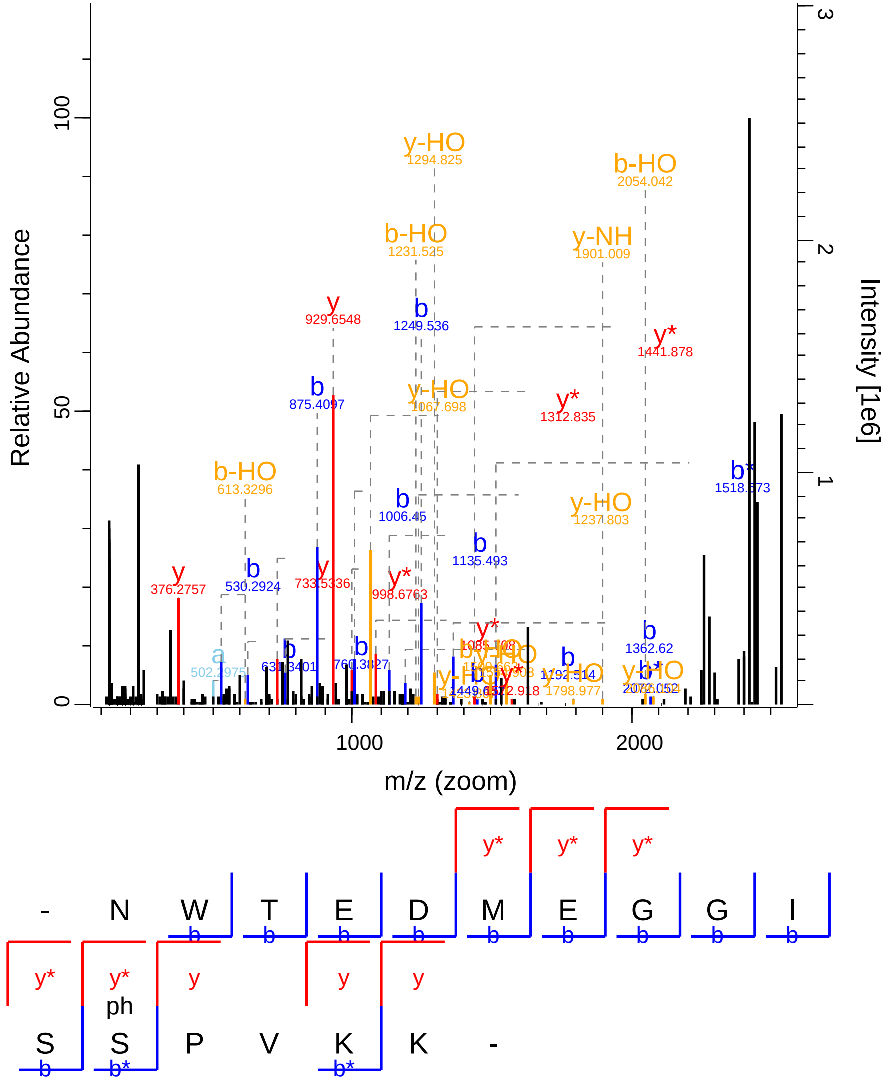

Raw File	Scan	Method	Score	m/z	Gene names
RO-QC-160121_ZackD_SS_Set_02_Fractio	23627	FTMS; HC	112.82	637.33	NFIC

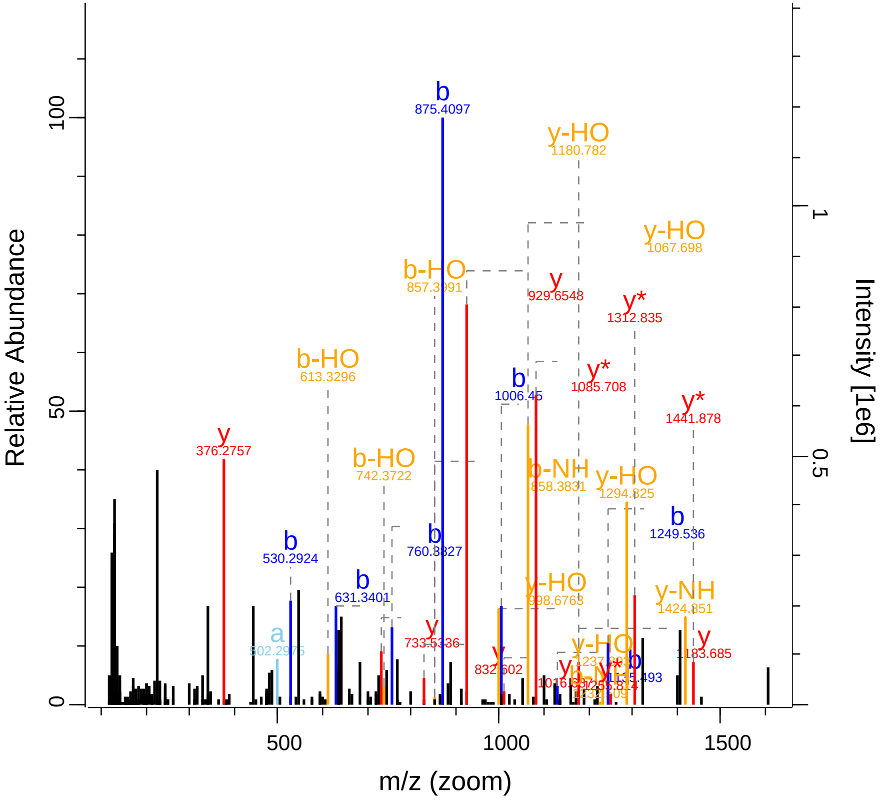

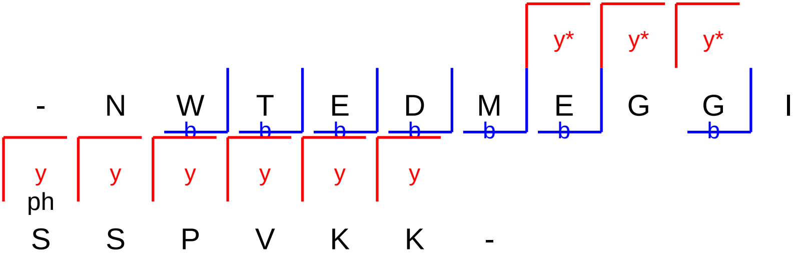

Raw File	Scan	Method	Score	m/z	Gene names
RO-QC-160121_ZackD_SS_Set_02_Fractio	24106	FTMS; HC	120.33	903.82	HS1BP3

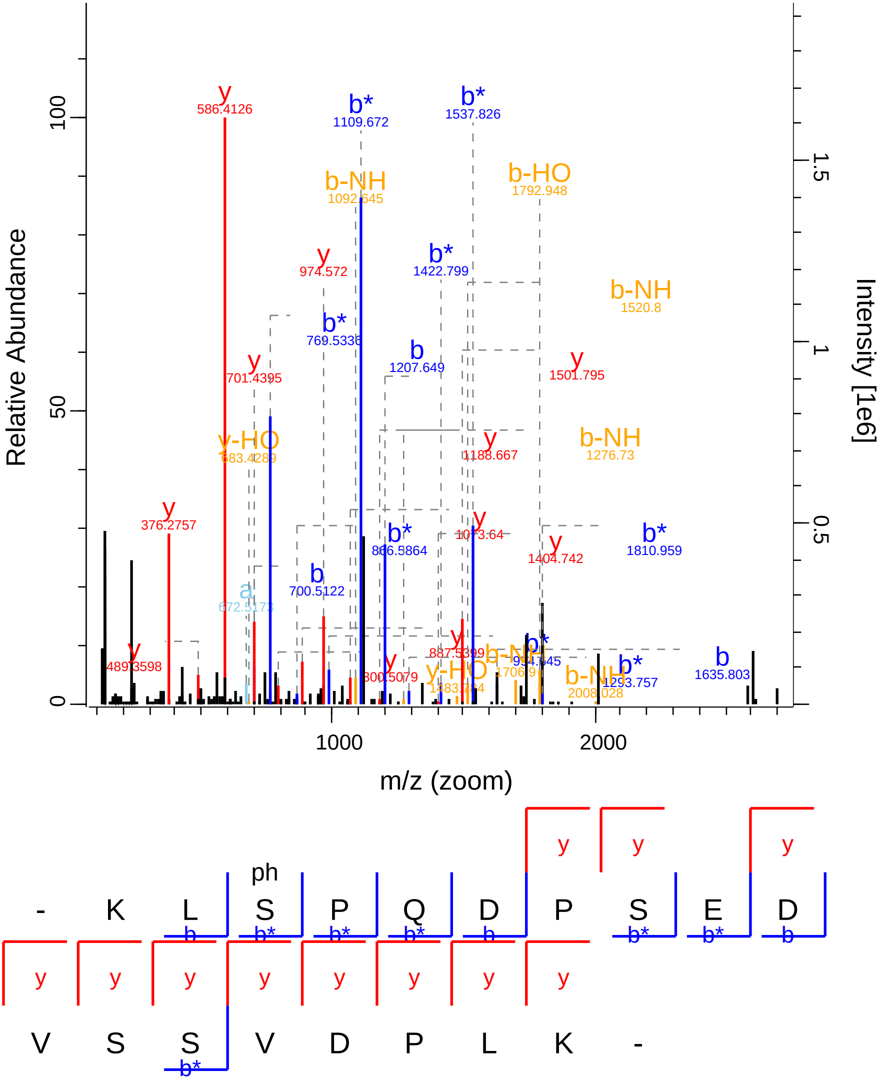

Raw File	Scan	Method	Score	m/z	Gene names
RO-QC-160121_ZackD_SS_Set_02_Fractio[illegible]	24174	FTMS; HC	59.87	678.37	HS1BP3

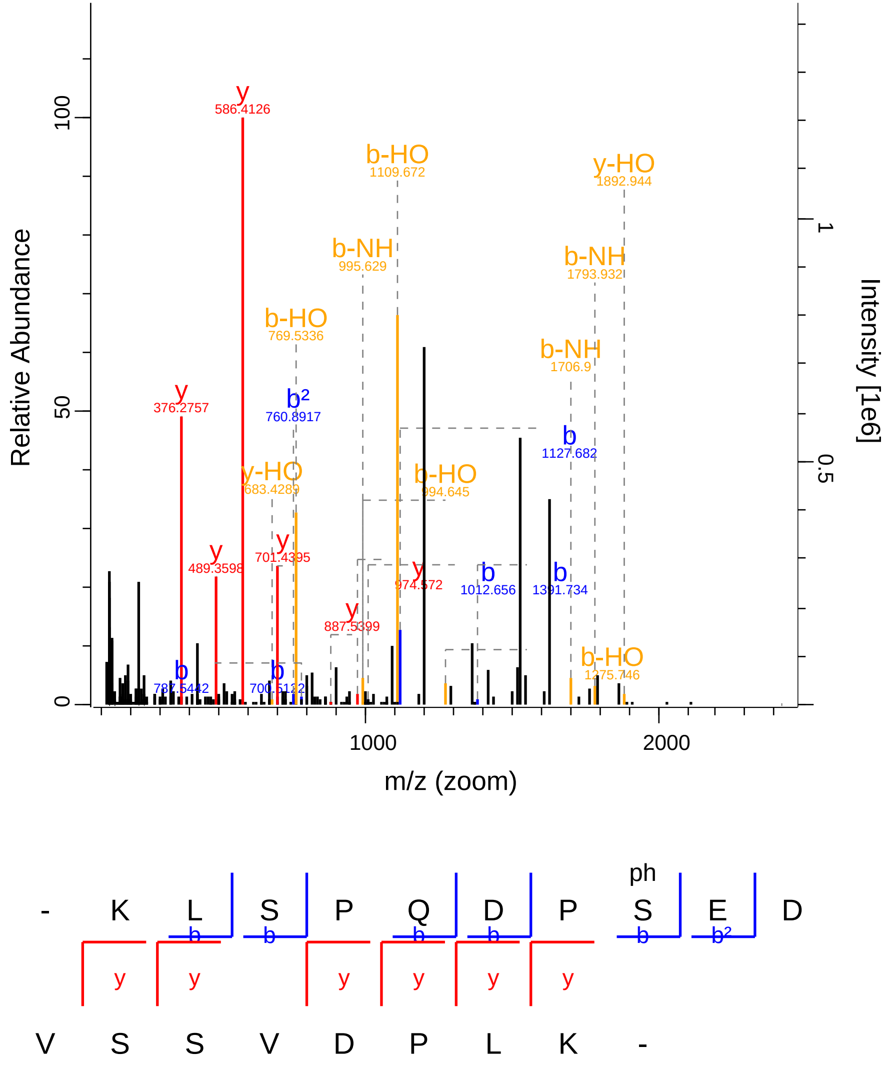

Raw File	Scan	Method	Score	m/z	Gene names
RO-QC-160121_ZackD_SS_Set_02_Fractio	24180	FTMS; HC	167.69	695.69	BCL2L13

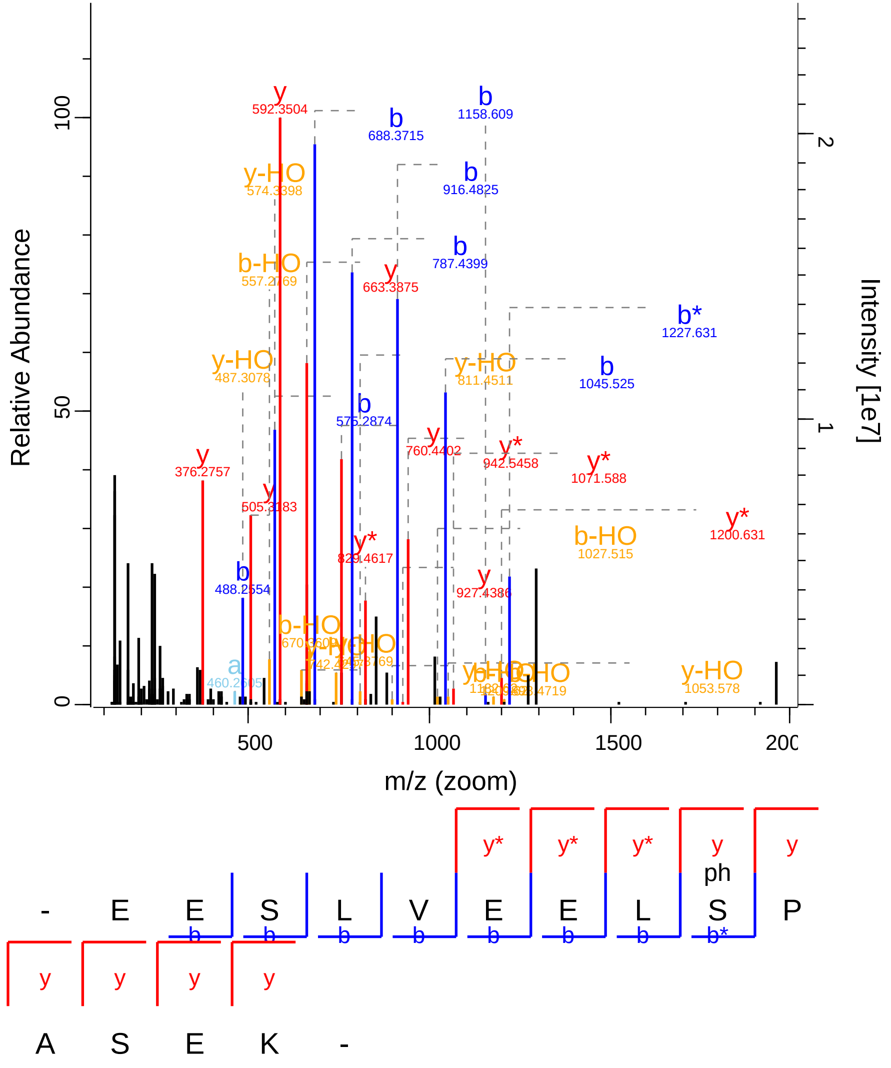

Raw File	Scan	Method	Score	m/z	Gene names
RO-QC-160121_ZackD_SS_Set_02_Fractio	24512	FTMS; HC	100.35	579.82	MAP4

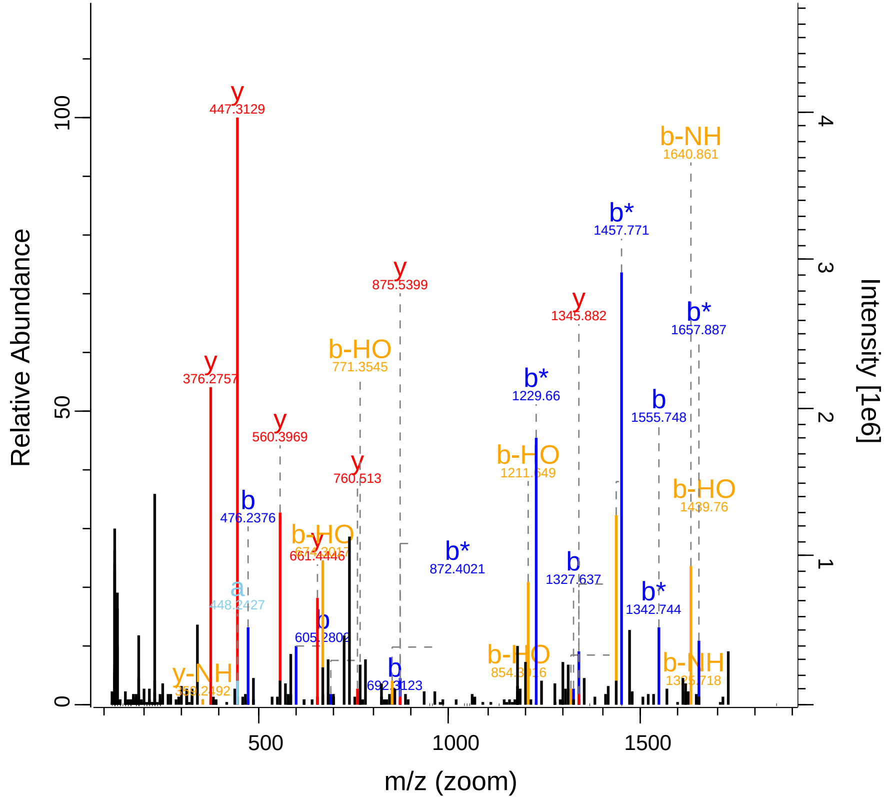

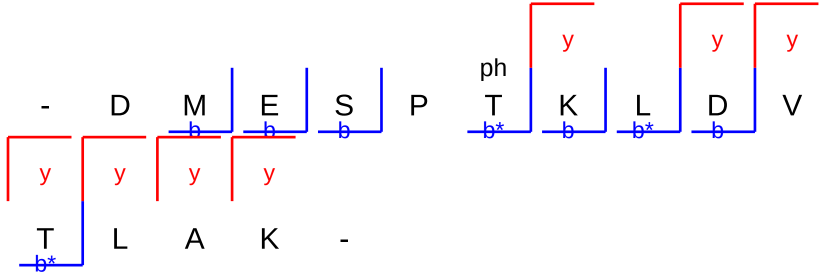

Raw File	Scan	Method	Score	m/z	Gene names
RO-QC-160121_ZackD_SS_Set_02_Fractic	24568	FTMS; HC	76.82	1057.16	MPP2

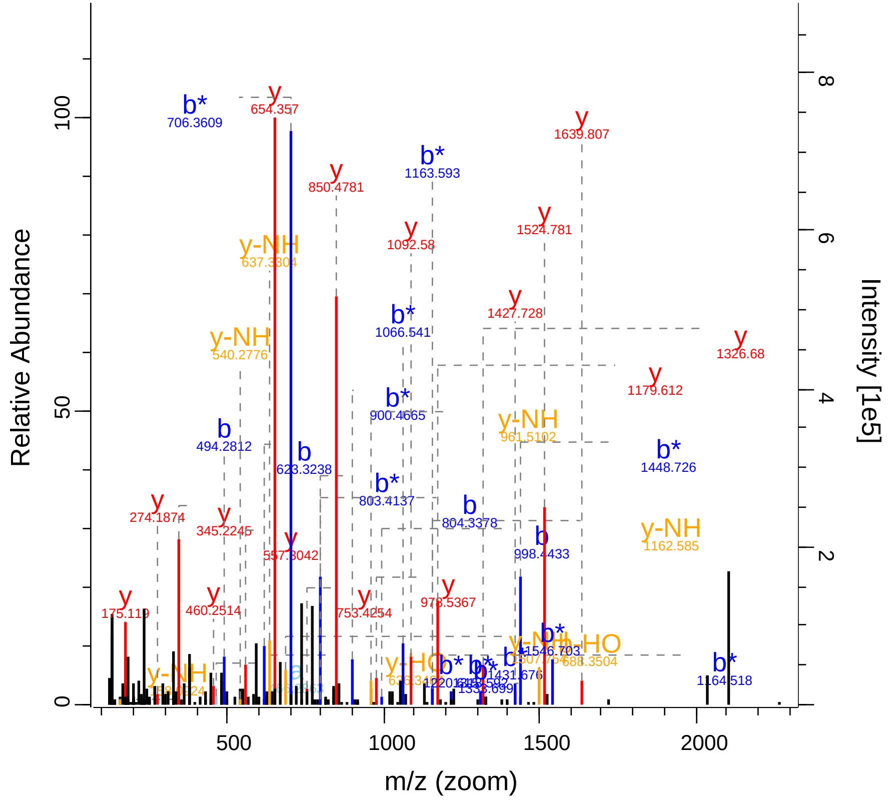

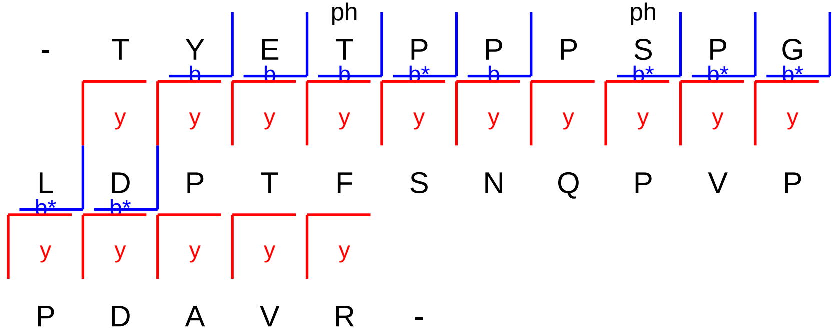

Raw File	Scan	Method	Score	m/z	Gene names
RO-QC-160121_ZackD_SS_Set_02_Fractic	24608	FTMS; HC	121.3	1025.82	GTF3C4

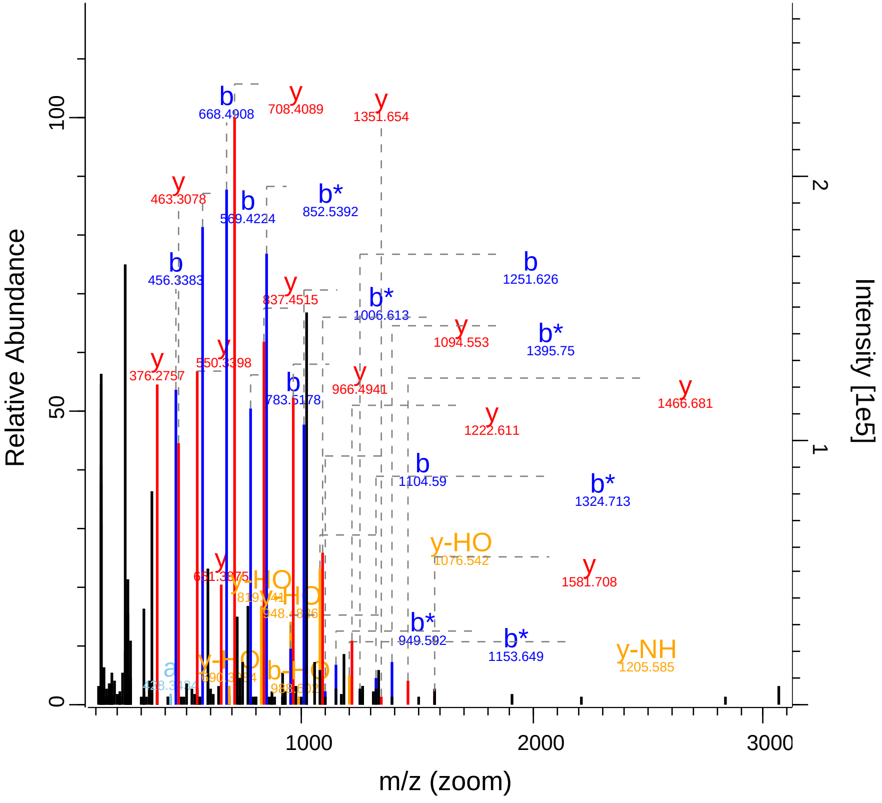

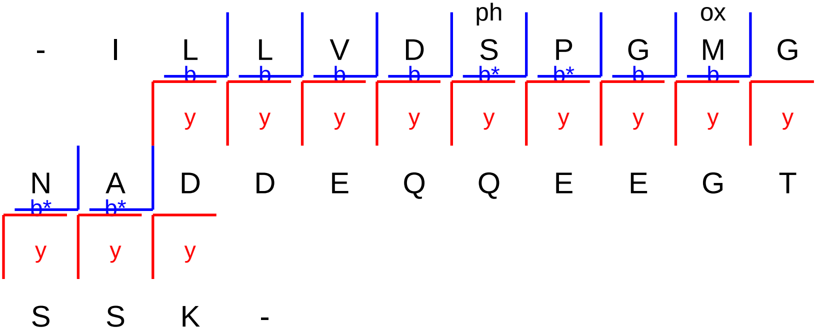

Raw File	Scan	Method	Score	m/z	Gene names
RO-QC-160121_ZackD_SS_Set_02_Fractic	24821	FTMS; HC	69.35	1035.56	GPR98

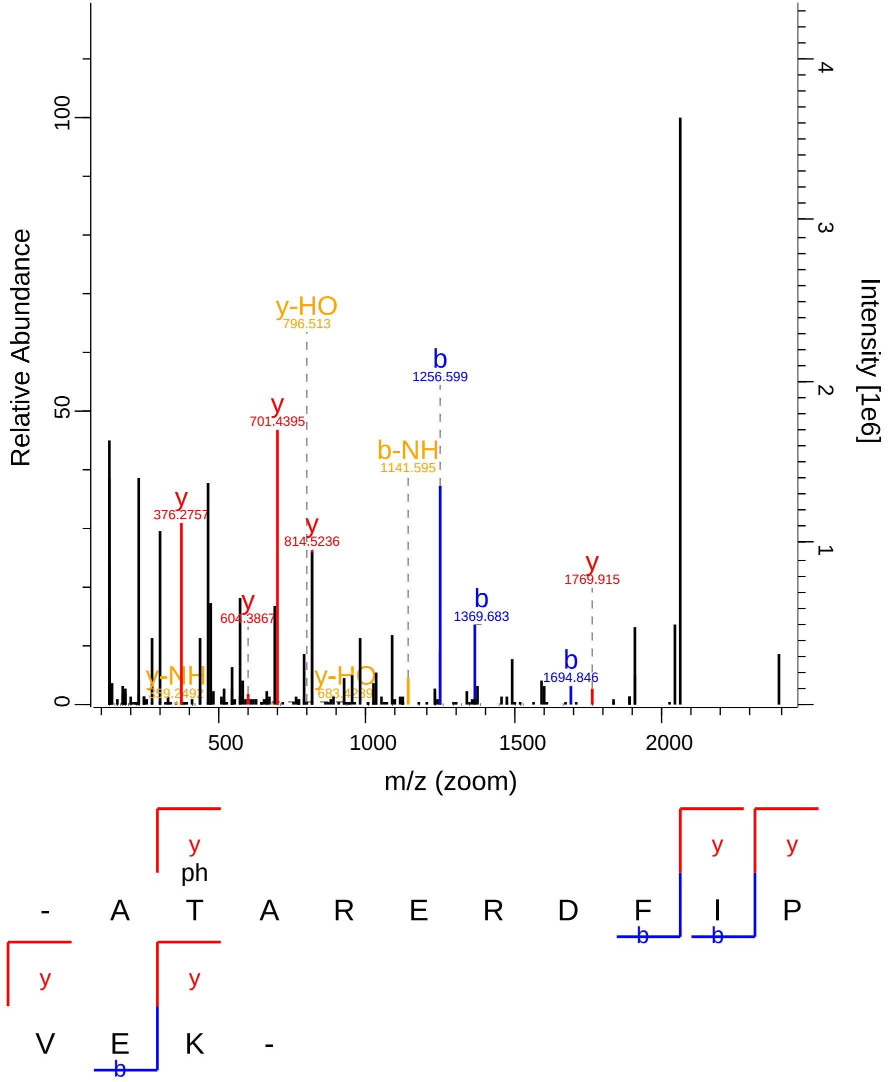

Raw File	Scan	Method	Score	m/z	Gene names
RO-QC-160121_ZackD_SS_Set_02_Fractio	24845	FTMS; HC	49.81	891.47	PRMT8

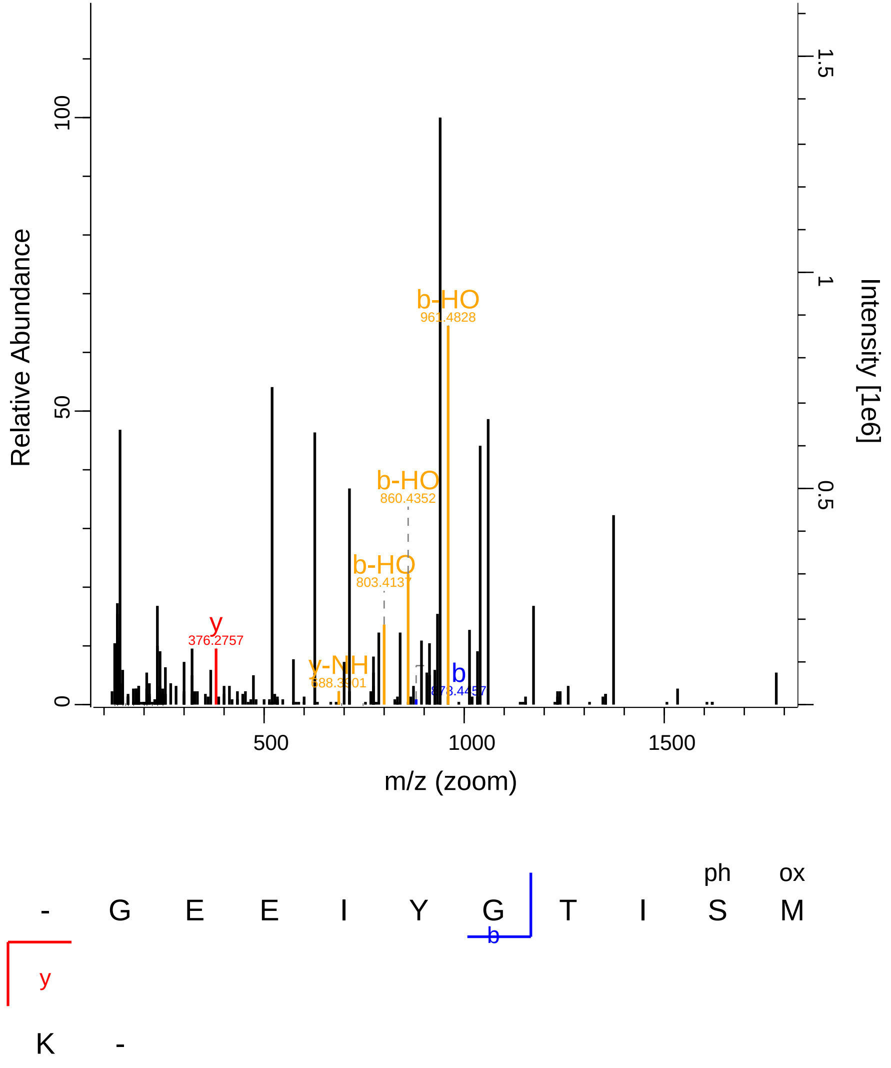

Raw File	Scan	Method	Score	m/z	Gene names
RO-QC-160121_ZackD_SS_Set_02_Fractio	24876	FTMS; HC	48.48	612.31	TSNAX

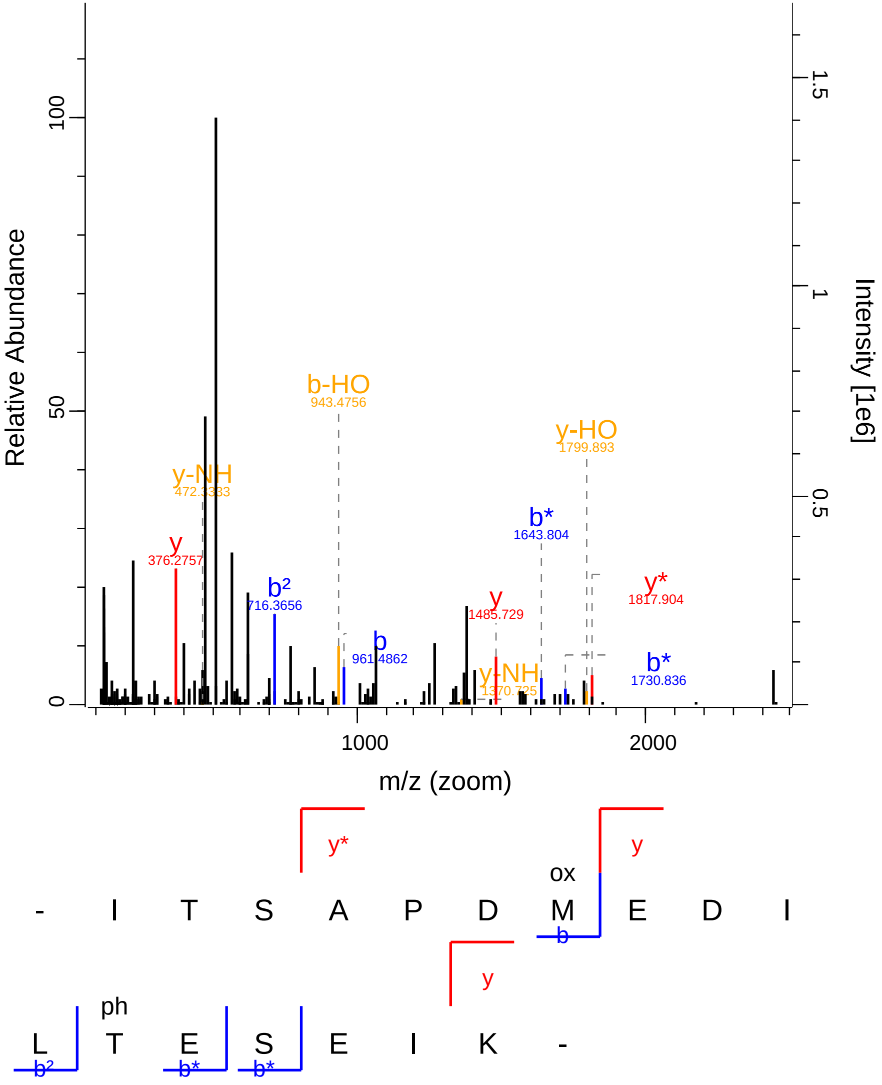

Raw File	Scan	Method	Score	m/z	Gene names
RO-QC-160121_ZackD_SS_Set_02_Fractio	26240	FTMS; HC	40.38	923.47	DDX31

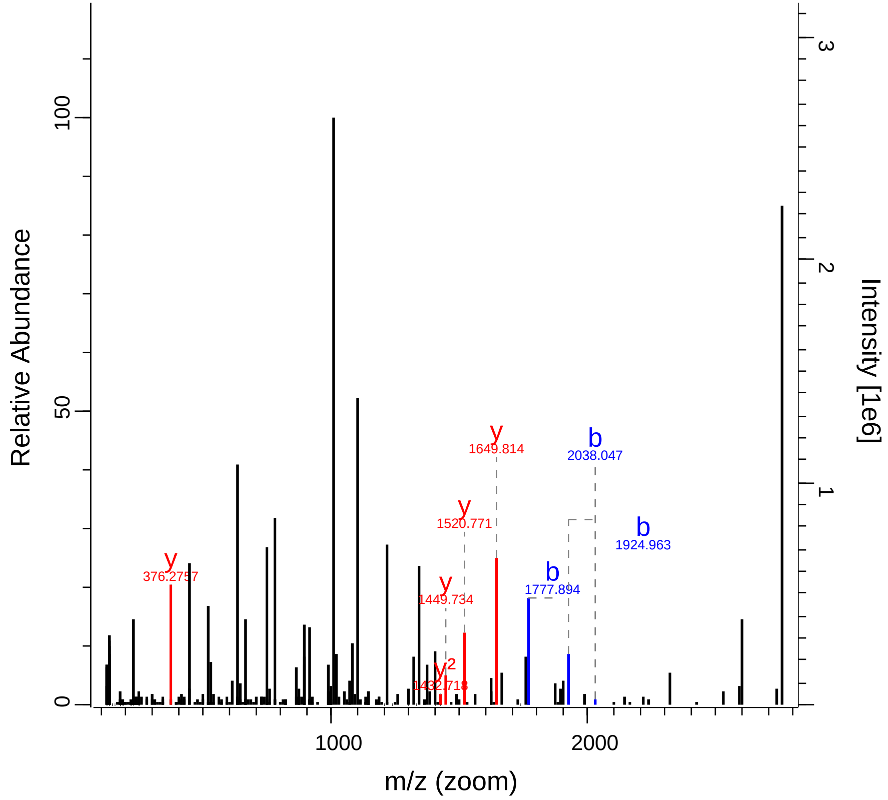

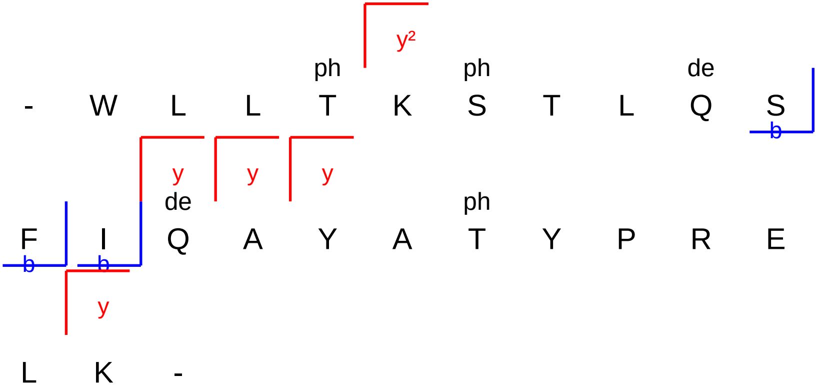

Raw File	Scan	Method	Score	m/z	Gene names
RO-QC-160121_ZackD_SS_Set_02_Fractio	26443	FTMS; HC	171.11	854.47	SSFA2

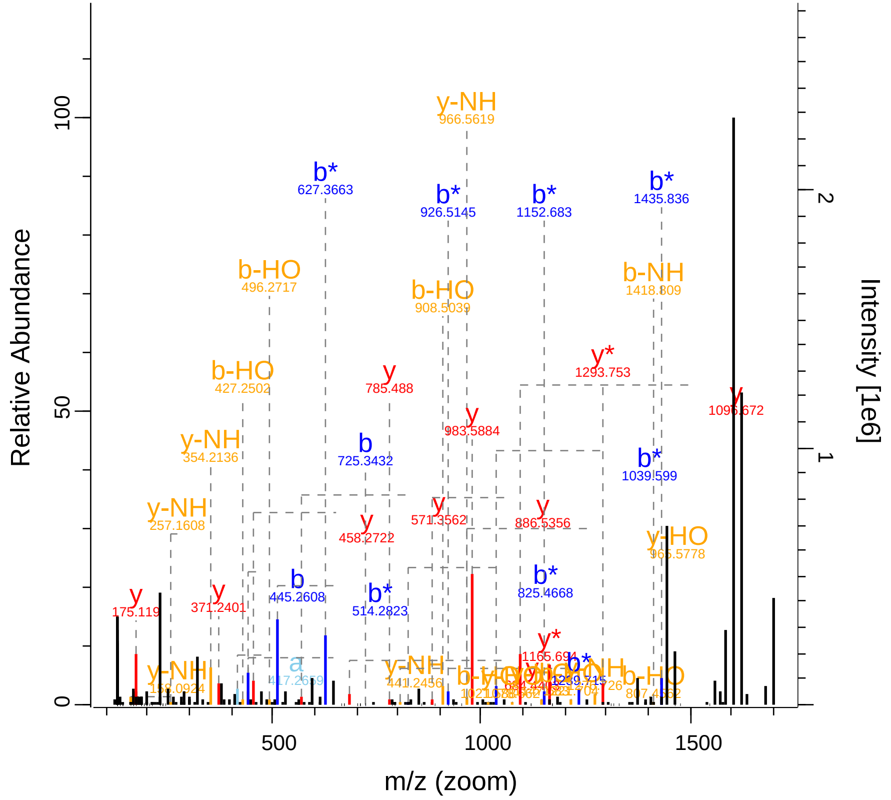

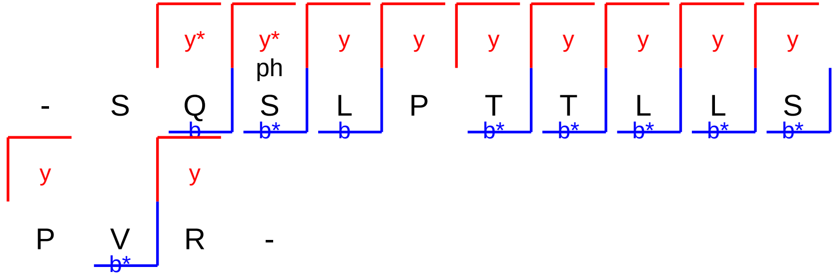

Raw File	Scan	Method	Score	m/z	Gene names
RO-QC-160121_ZackD_SS_Set_02_Fractio	27992	FTMS; HC	112.8	757.91	MRPS36

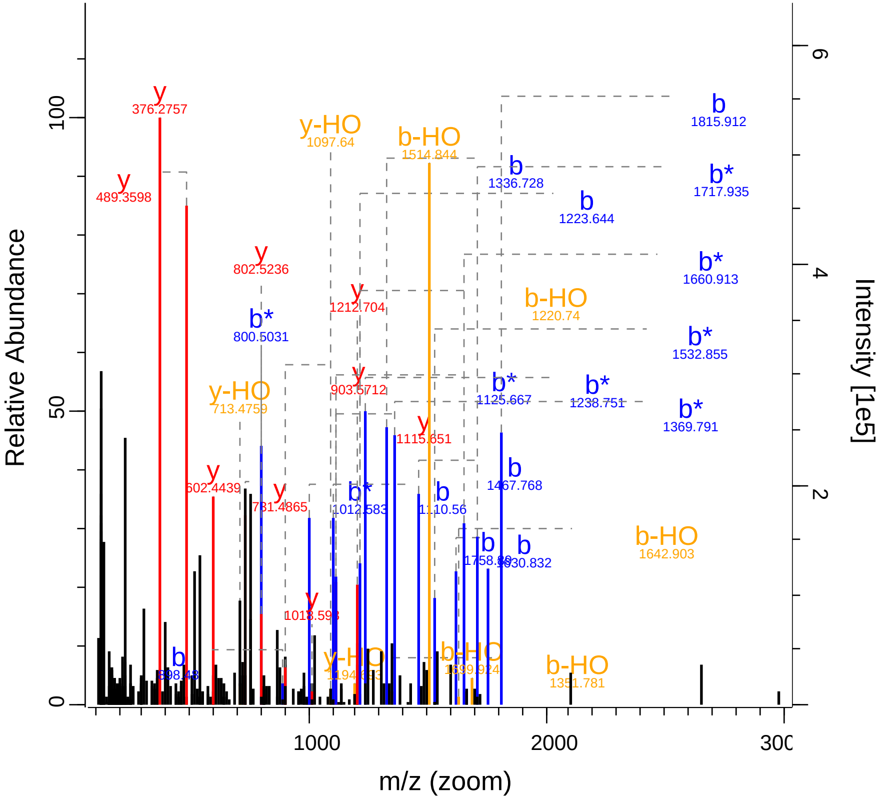

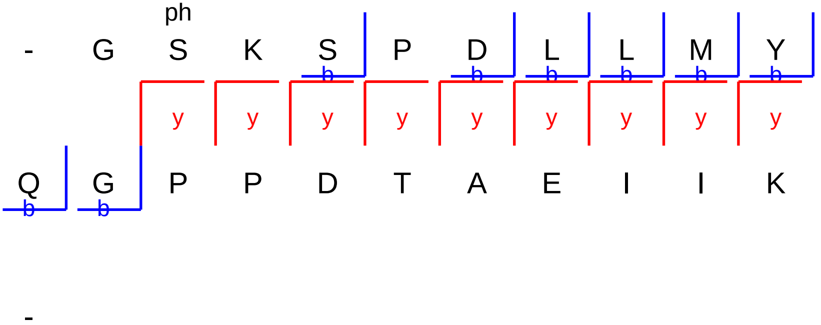

Raw File	Scan	Method	Score	m/z	Gene names
RO-QC-160121_ZackD_SS_Set_02_Fractio	28380	FTMS; HC	117.88	982.45	EIF3J

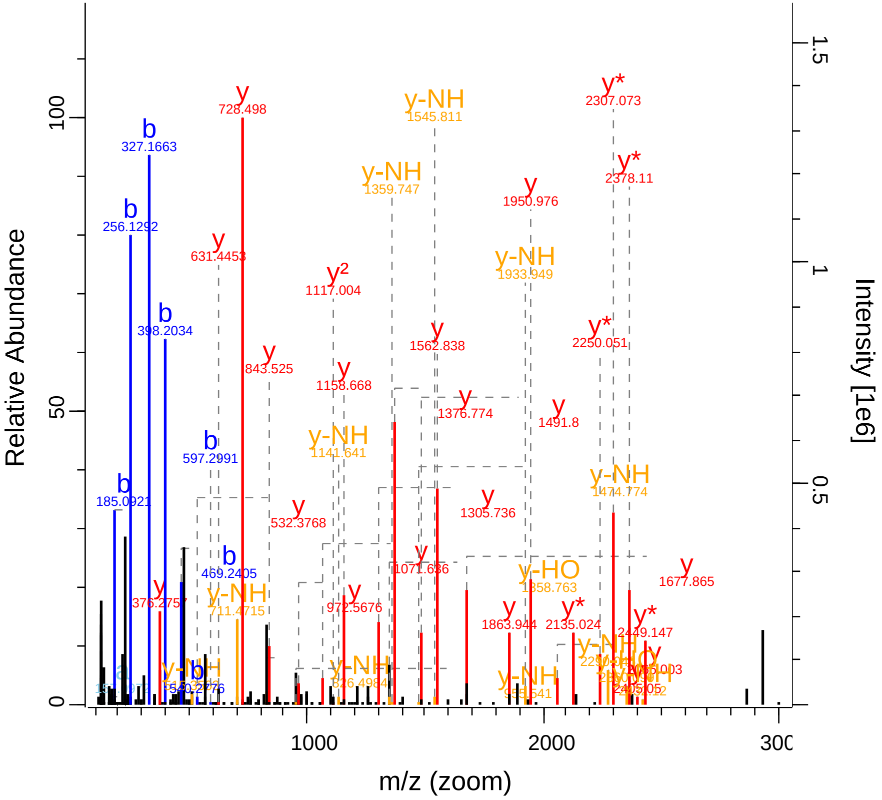

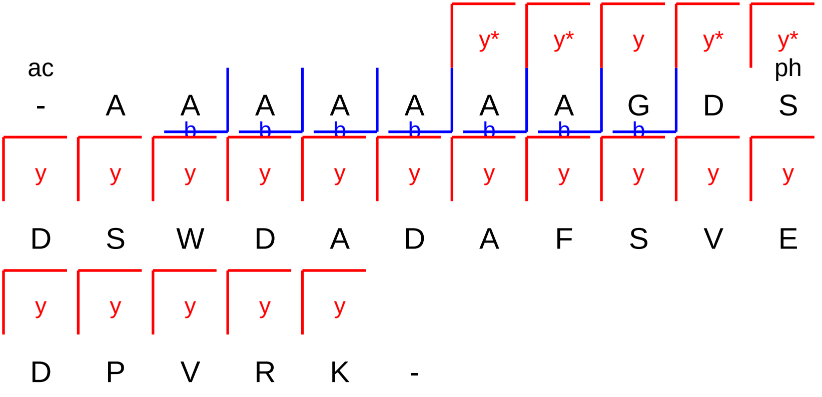

Raw File	Scan	Method	Score	m/z	Gene names
RO-QC-160121_ZackD_SS_Set_02_Fractio	28940	FTMS; HC	51.61	714.88	EHD1

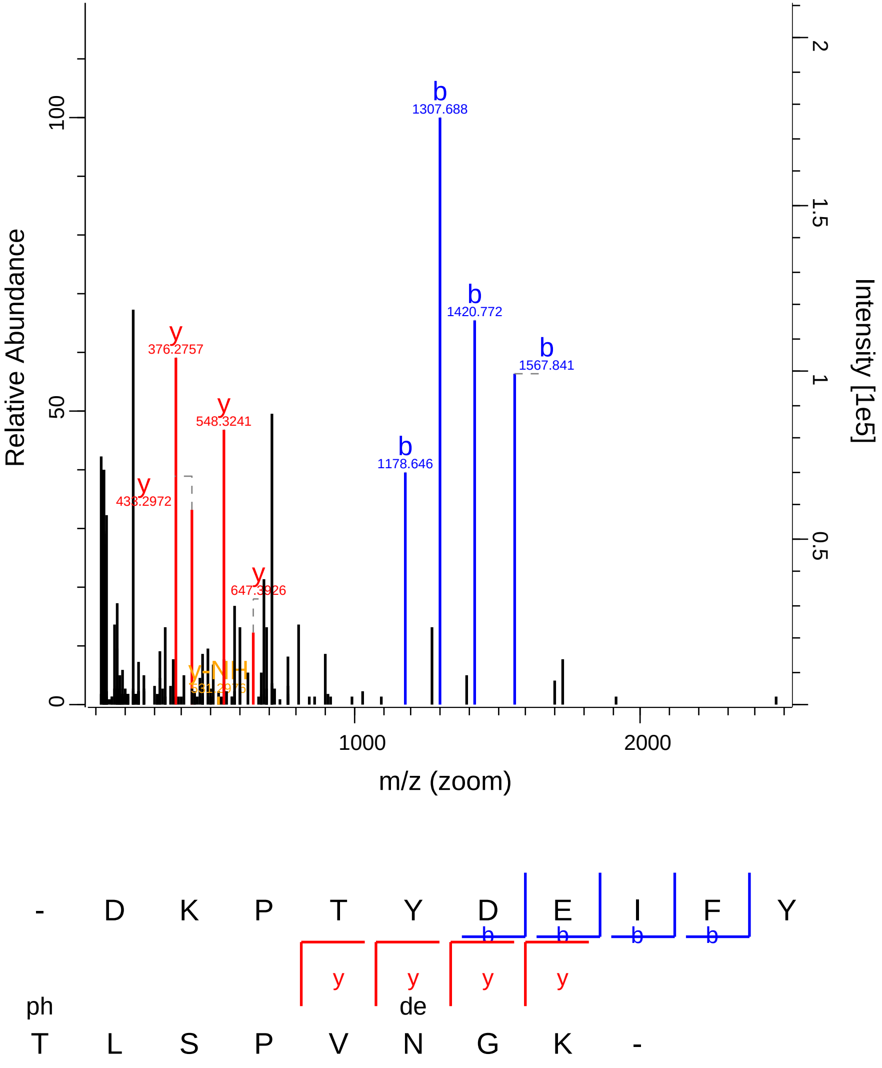

Raw File	Scan	Method	Score	m/z	Gene names
RO-QC-160121_ZackD_SS_Set_02_Frac 29663		FTMS; HC	63.75	994.02	FAM120A;C9orf129

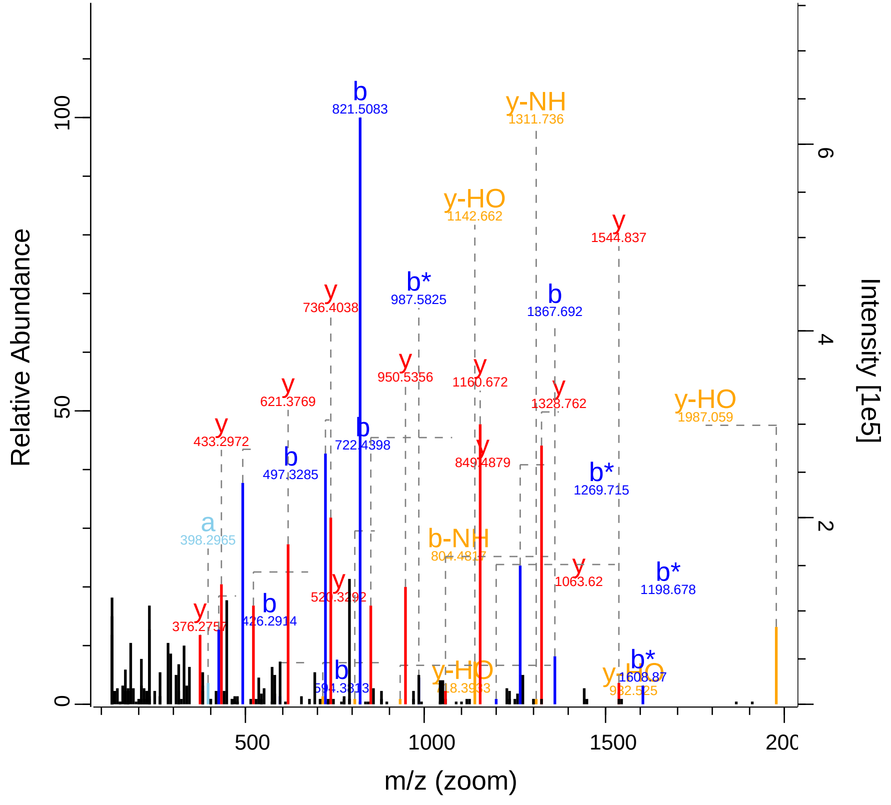

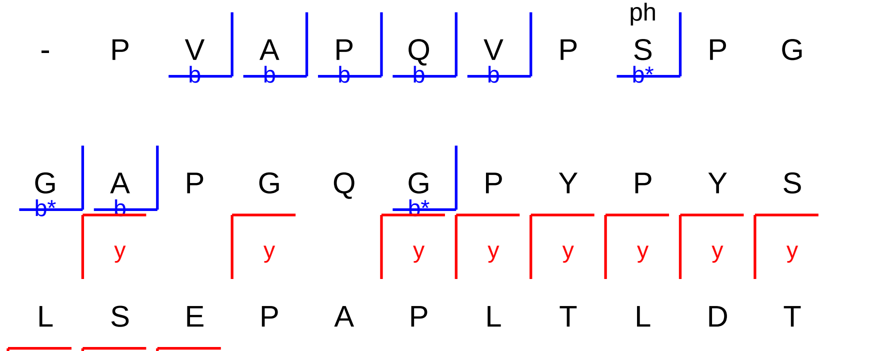

Raw File	Scan	Method	Score	m/z	Gene names
RO-QC-160121_ZackD_SS_Set_02_Fractio	30793	FTMS; HC	115.31	682.02	PLEKHA5

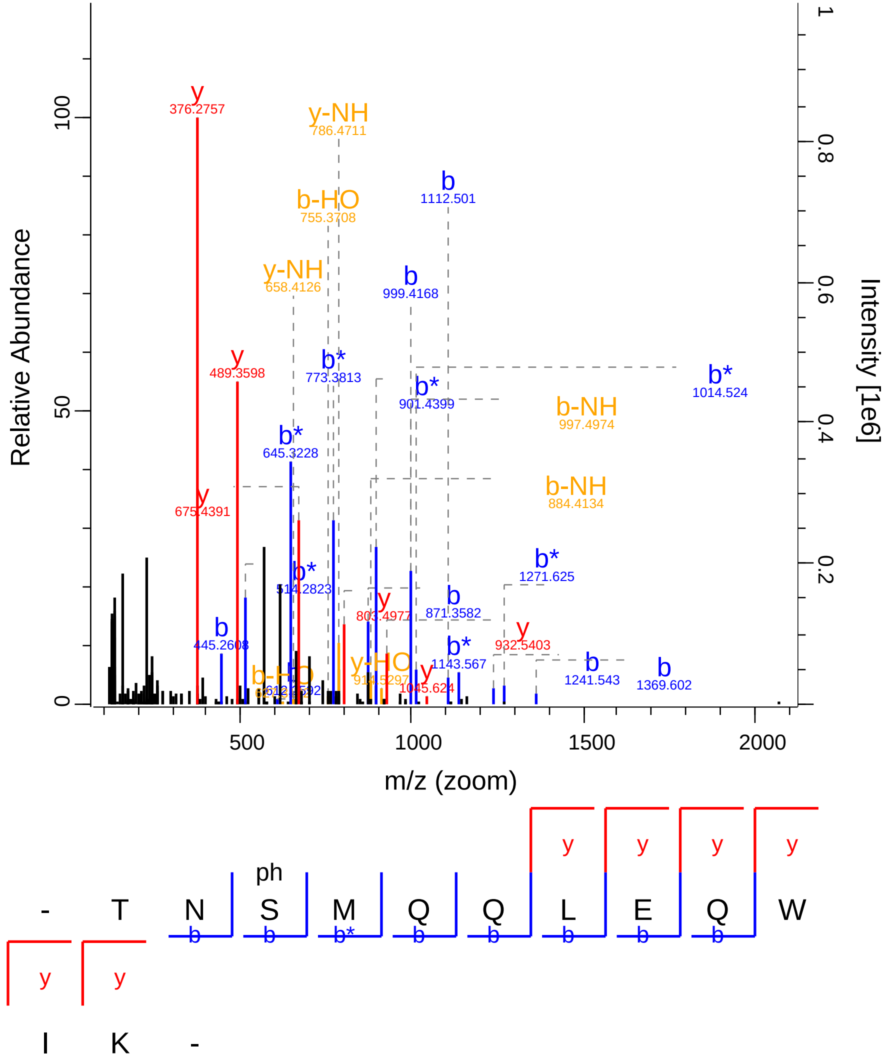

Raw File	Scan	Method	Score	m/z	Gene names
RO-QC-160121_ZackD_SS_Set_02_Fractio	30841	FTMS; HC	56.23	938.96	XPO6

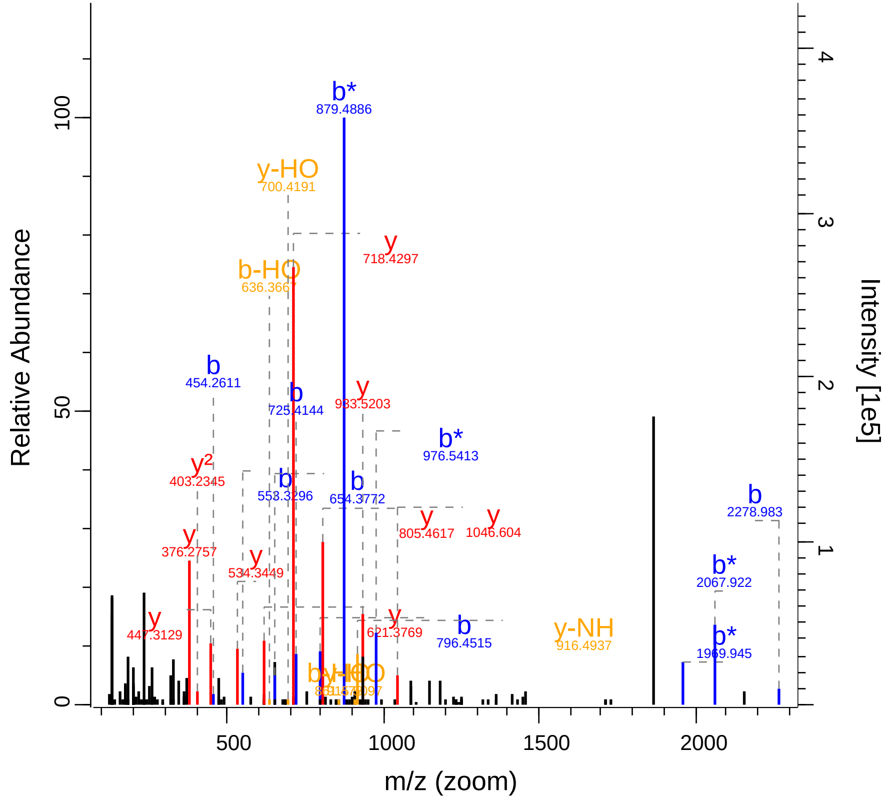

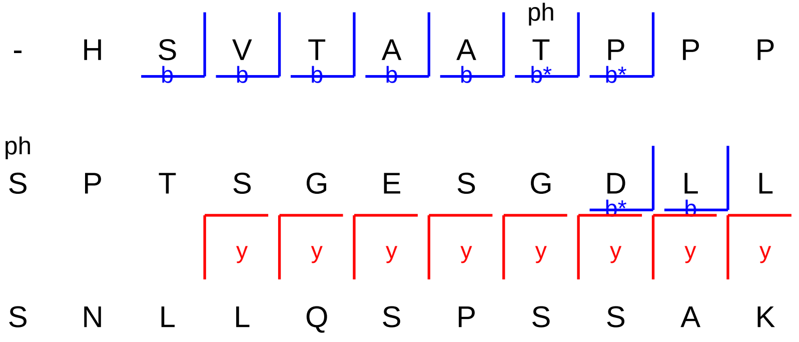

Raw File	Scan	Method	Score	m/z	Gene names
RO-QC-160121_ZackD_SS_Set_02_Fractio	31732	FTMS; HC	64.09	818.45	SMAD2

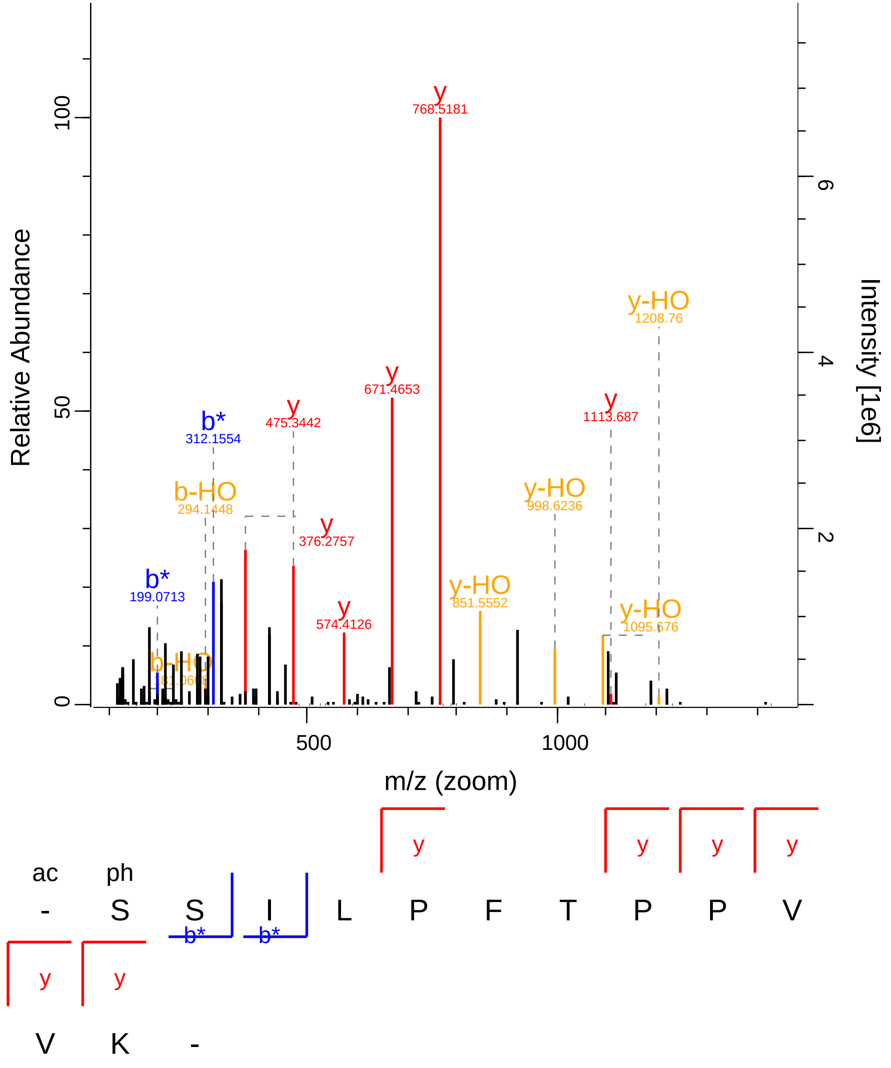

Raw File	Scan	Method	Score	m/z	Gene names
RO-QC-160121_ZackD_SS_Set_02_Fractior	4045	FTMS; HCI	67.46	466.01	BMP2K

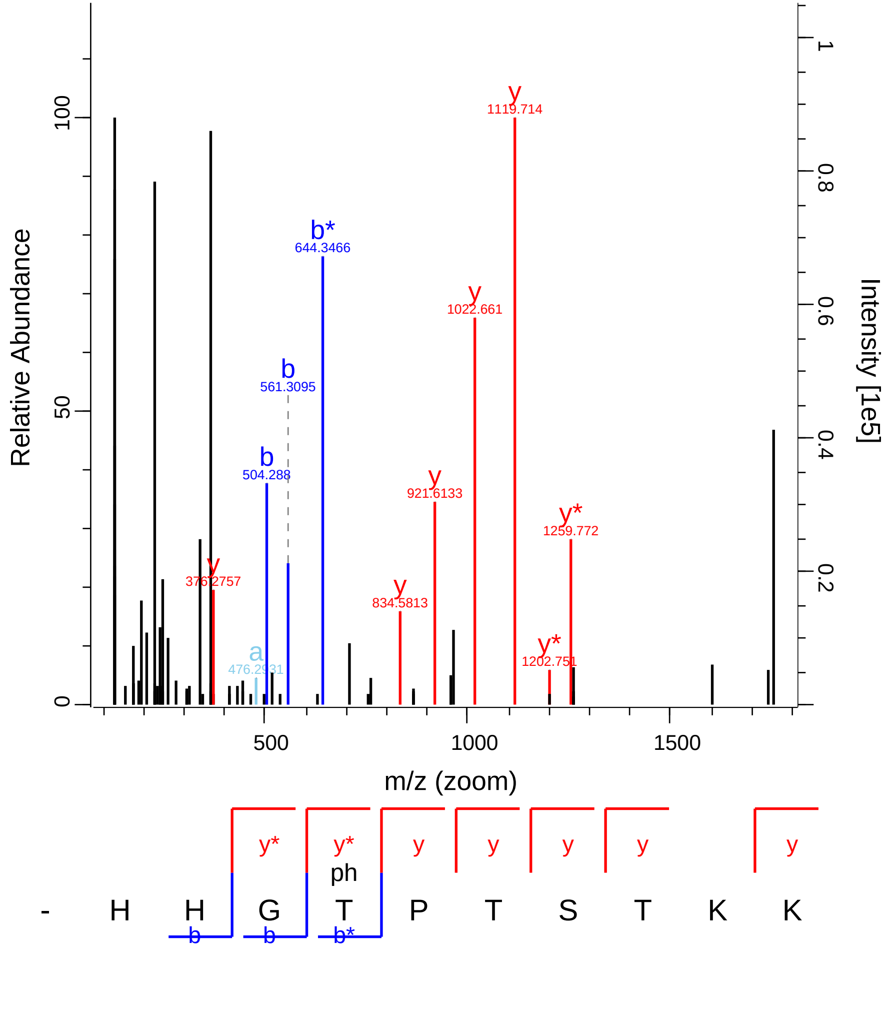

Raw File	Scan	Method	Score	m/z	Gene names
RO-QC-160121_ZackD_SS_Set_02_Fractior	5411	FTMS; HCI	60.92	690.72	PDS5A

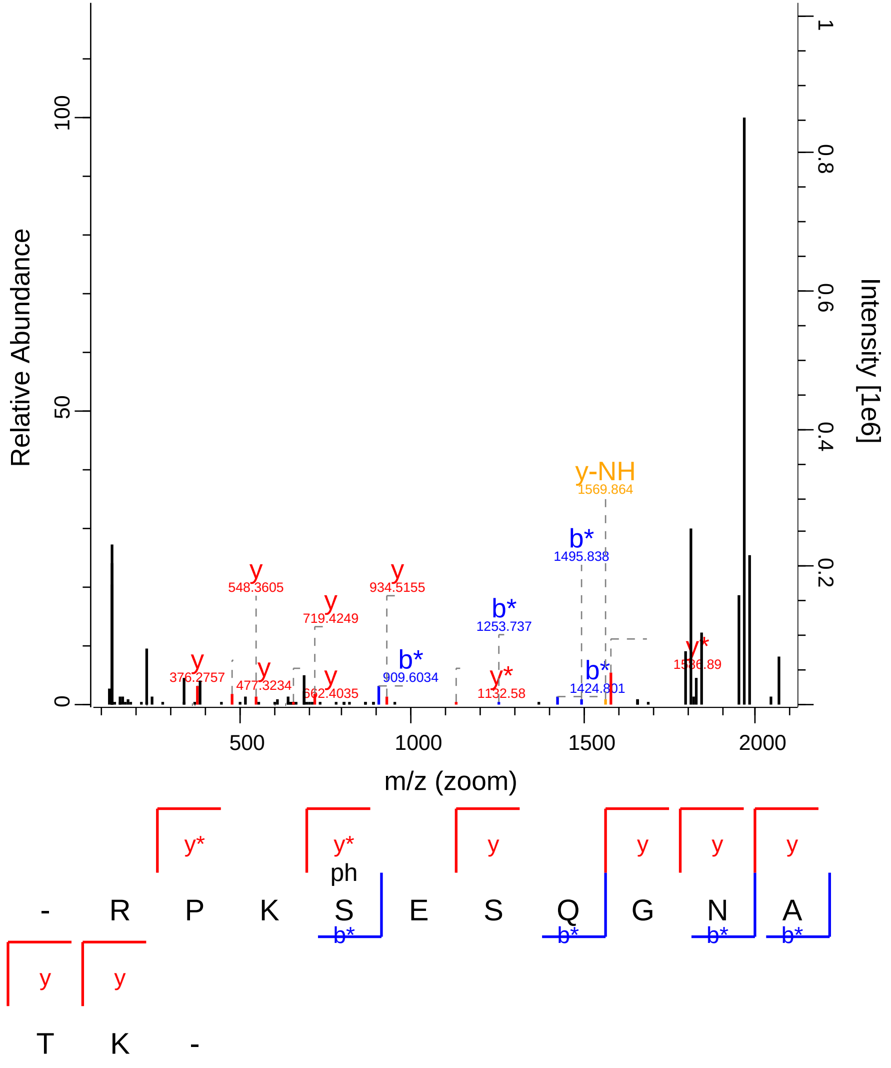

Raw File	Scan	Method	Score	m/z	Gene names
RO-QC-160121_ZackD_SS_Set_02_Fraction_	5480	FTMS; HCI	103.22	430	ZNF638

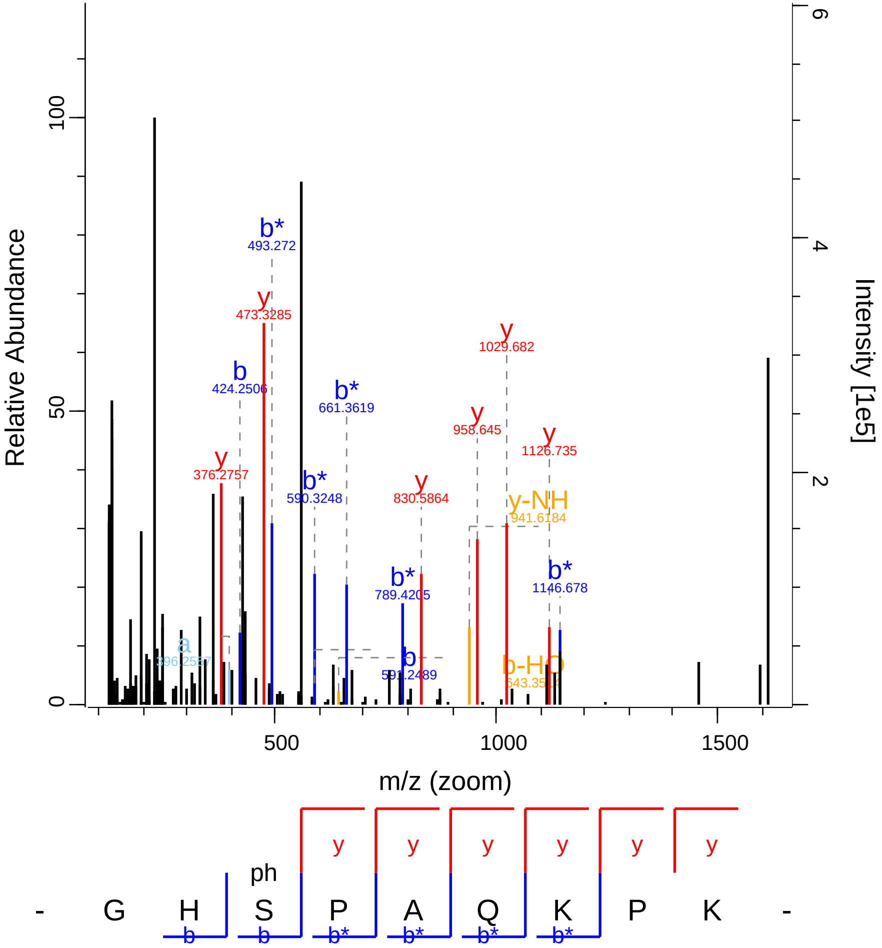

Raw File	Scan	Method	Score	m/z	Gene names
RO-QC-160121_ZackD_SS_Set_02_Fractior	5504	FTMS; HCI	90.97	934.77	LDB1

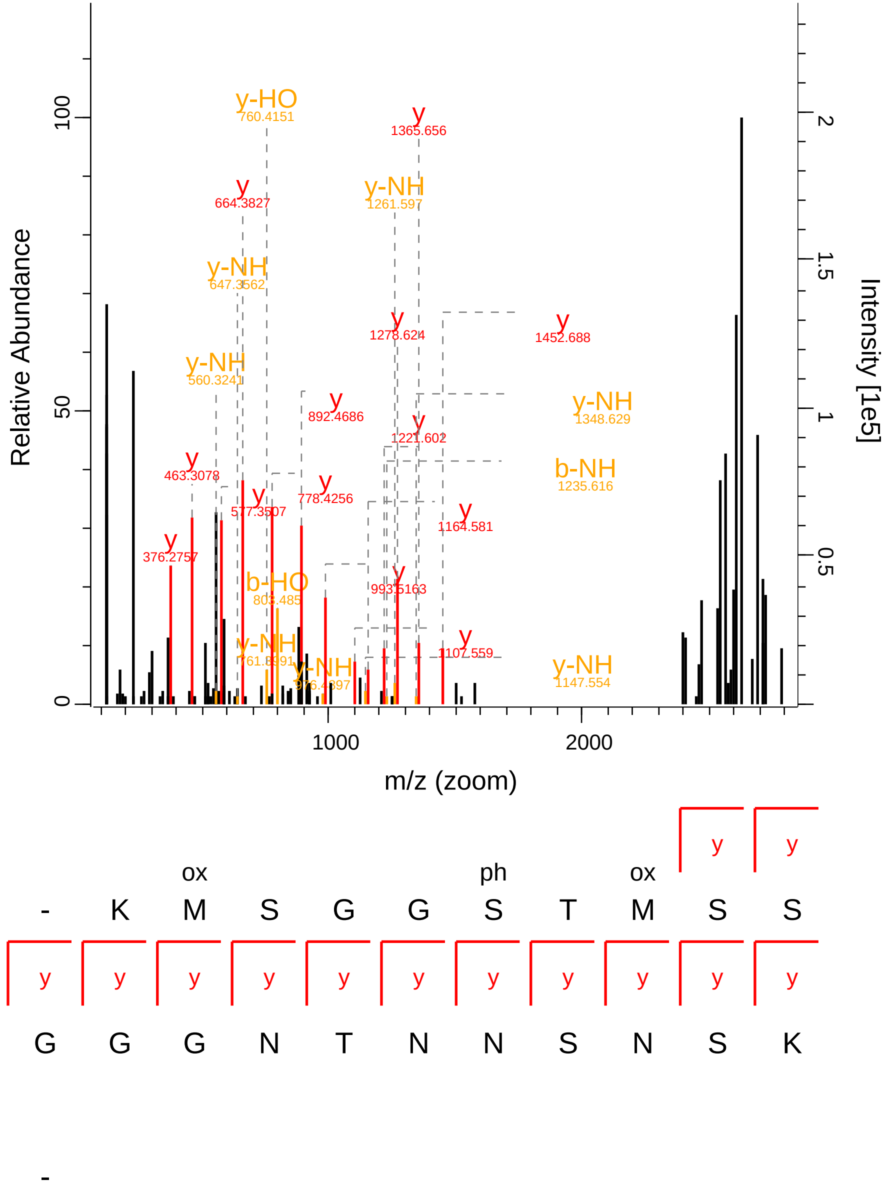

Raw File	Scan	Method	Score	m/z	Gene names
RO-QC-160121_ZackD_SS_Set_02_Fractio	6627	FTMS; HC	153.06	686.06	NUCKS1

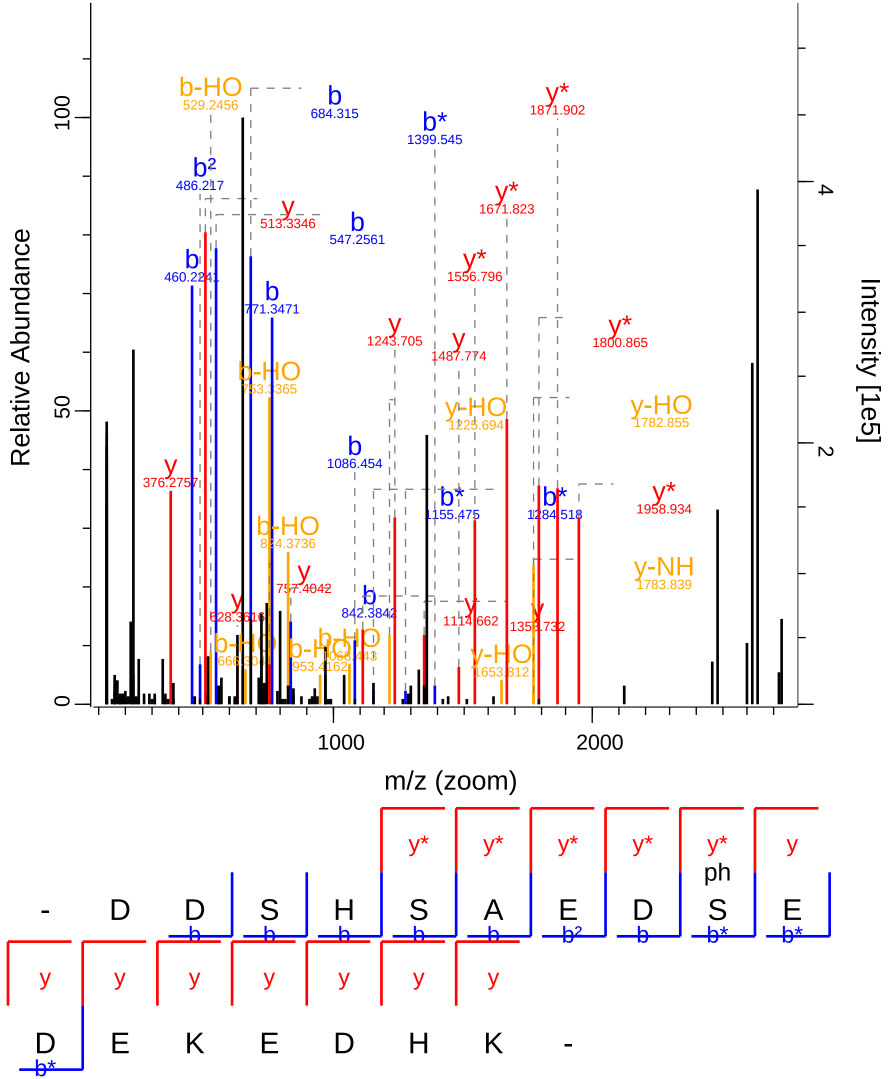

Raw File	Scan	Method	Score	m/z	Gene names
RO-QC-160121_ZackD_SS_Set_02_Fraction	9640	FTMS; HCI	99.16	853.9	SF3B1

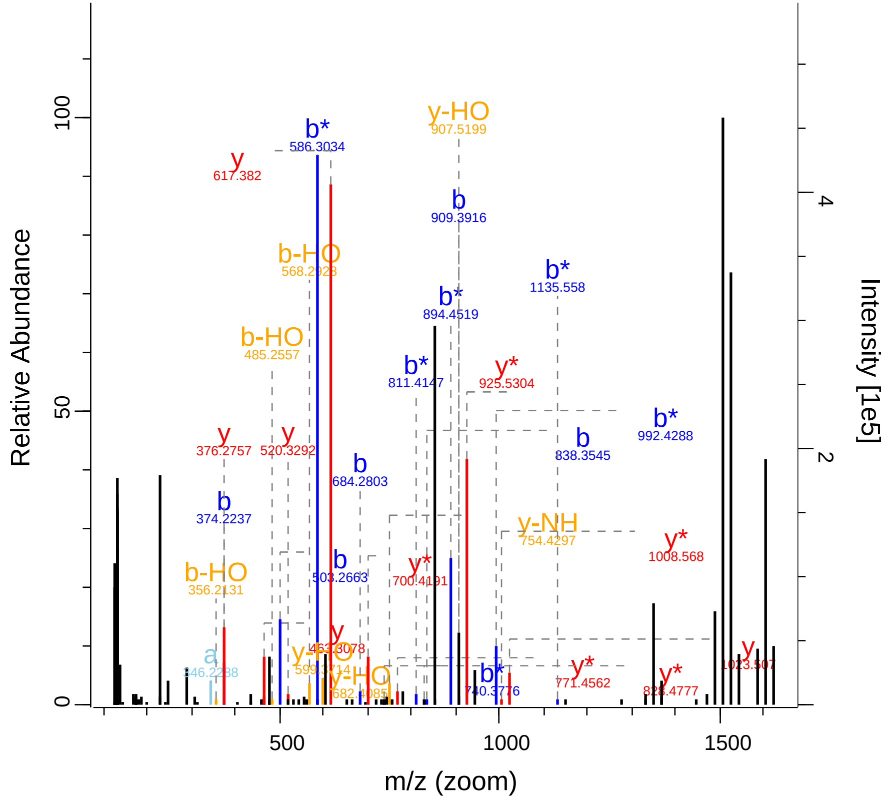

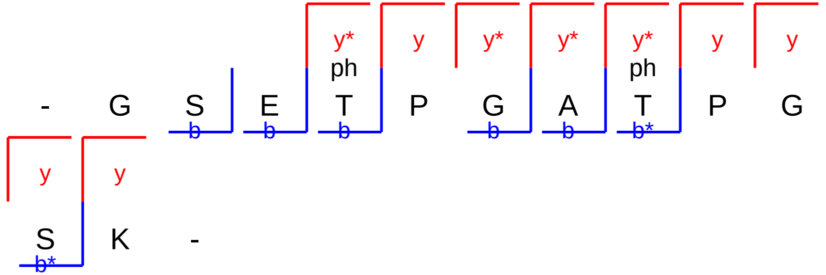

Raw File	Scan	Method	Score	m/z	Gene names
RO-QC-160121_ZackD_SS_Set_02_Fractior	9912	FTMS; HCI	42.82	584.67	OR2AE1

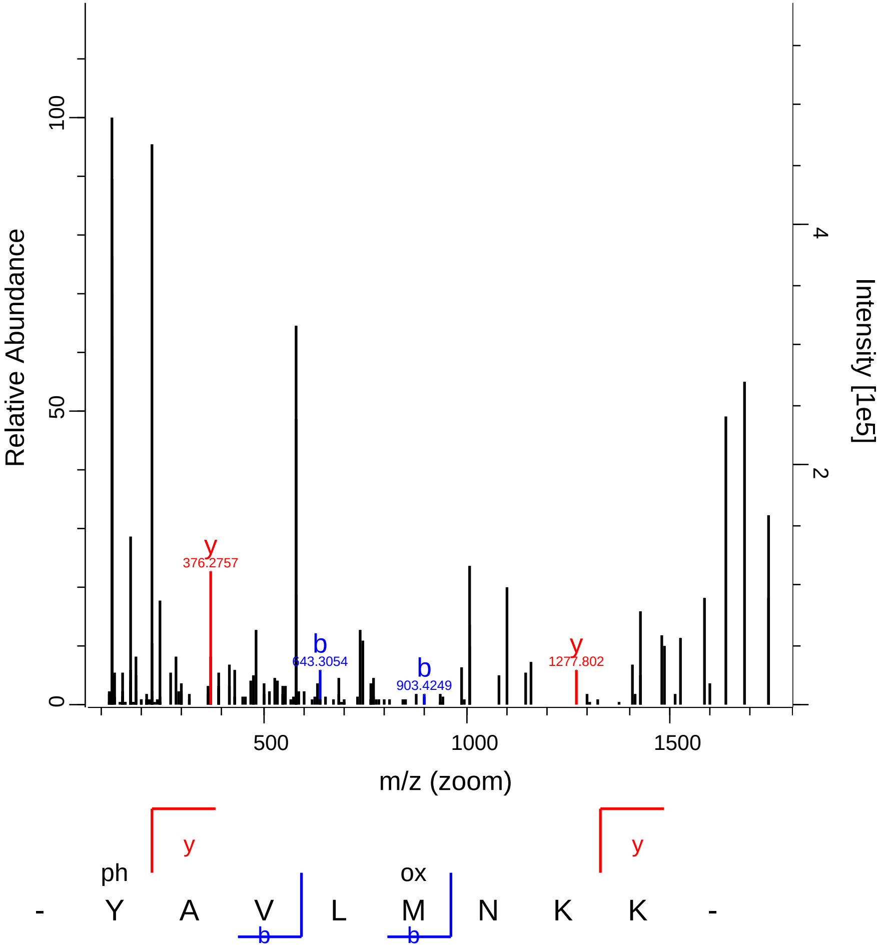

Raw File	Scan	Method	Score	m/z	Gene names
RO-QC-160121_ZackD_SS_Set_02_Fractior	9938	FTMS; HC	113.59	834.91	LMNA

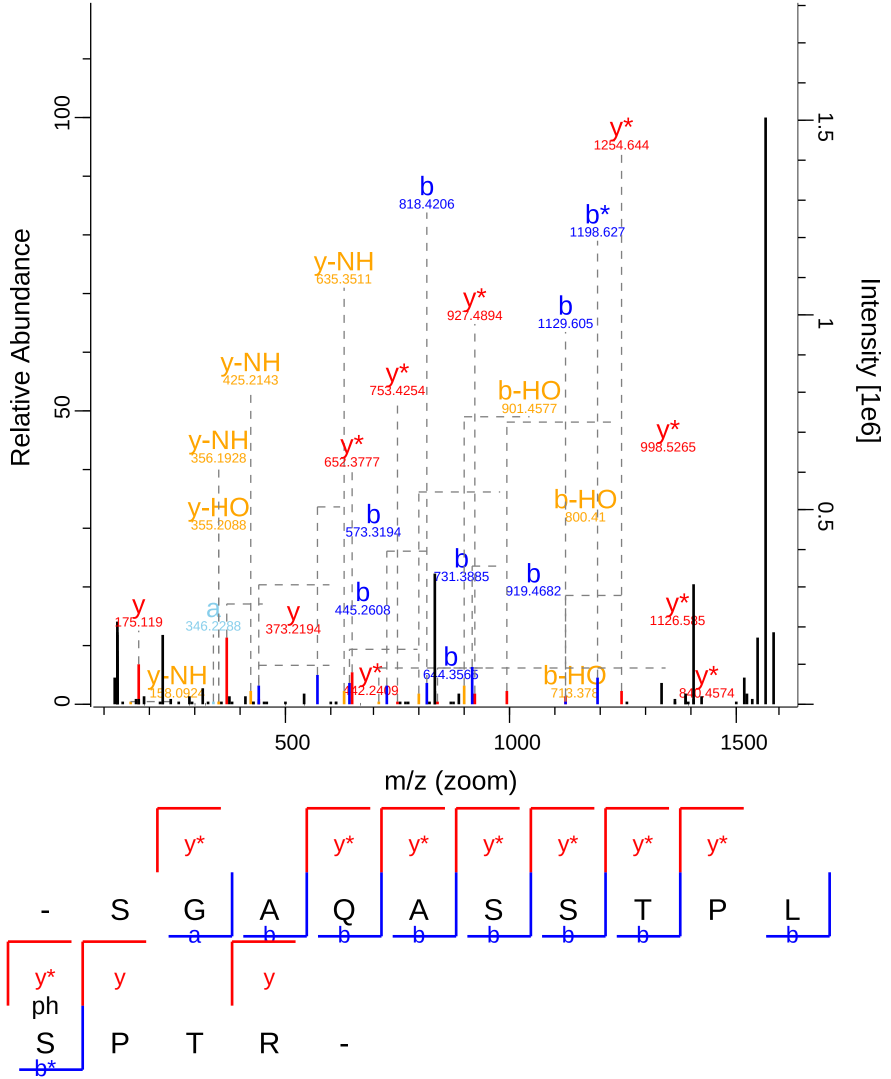

Raw File	Scan	Method	Score	m/z	Gene names
RO-QC-160121_ZackD_SS_Set_02_Fractio	10005	FTMS; HC	59.86	707.72	PRRC2A

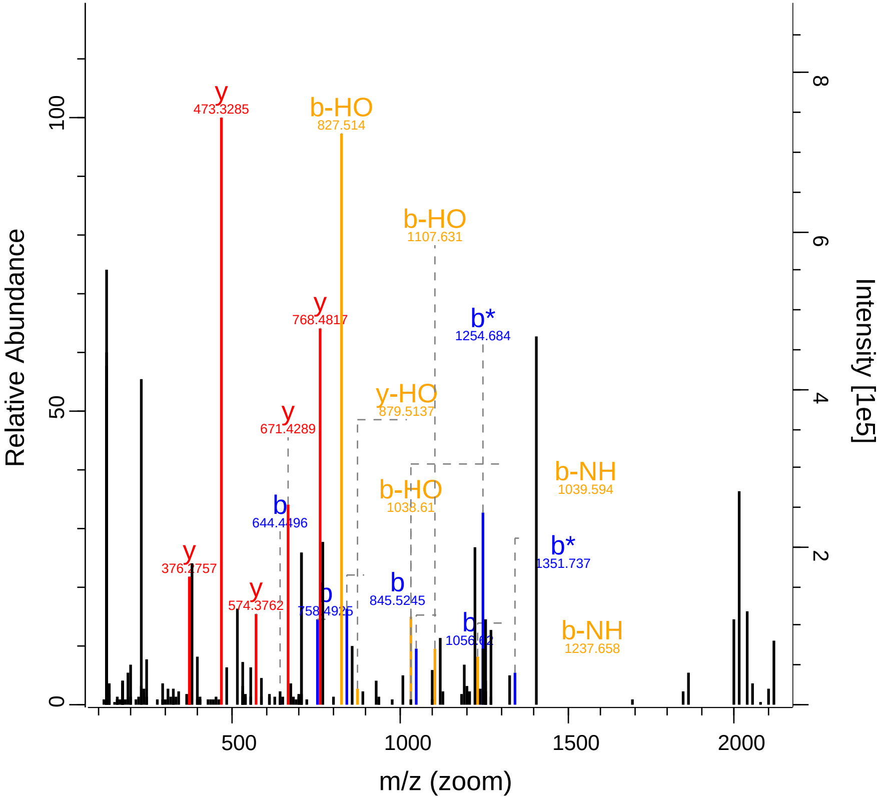

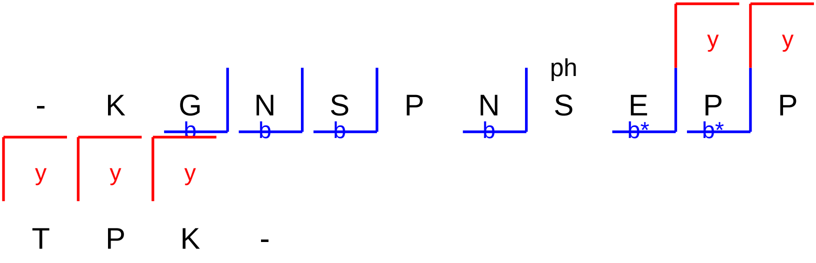

Raw File	Scan	Method	Score	m/z	Gene names
RO-QC-160121_ZackD_SS_Set_02_Fractio	10506	FTMS; HC	133.45	657.67	EPB41L1

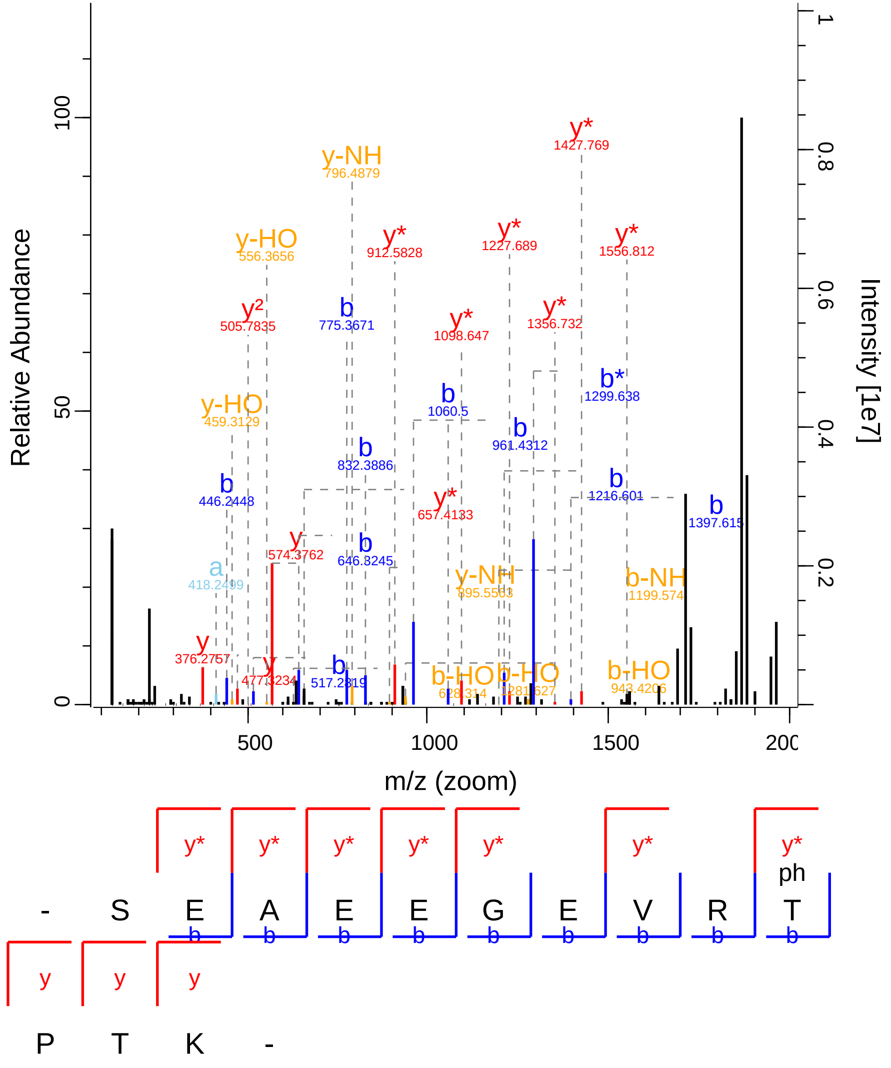

Raw File	Scan	Method	Score	m/z	Gene names
RO-QC-160121_ZackD_SS_Set_02_Fractio	10900	FTMS; HC	76.3	673.35	EGLN1

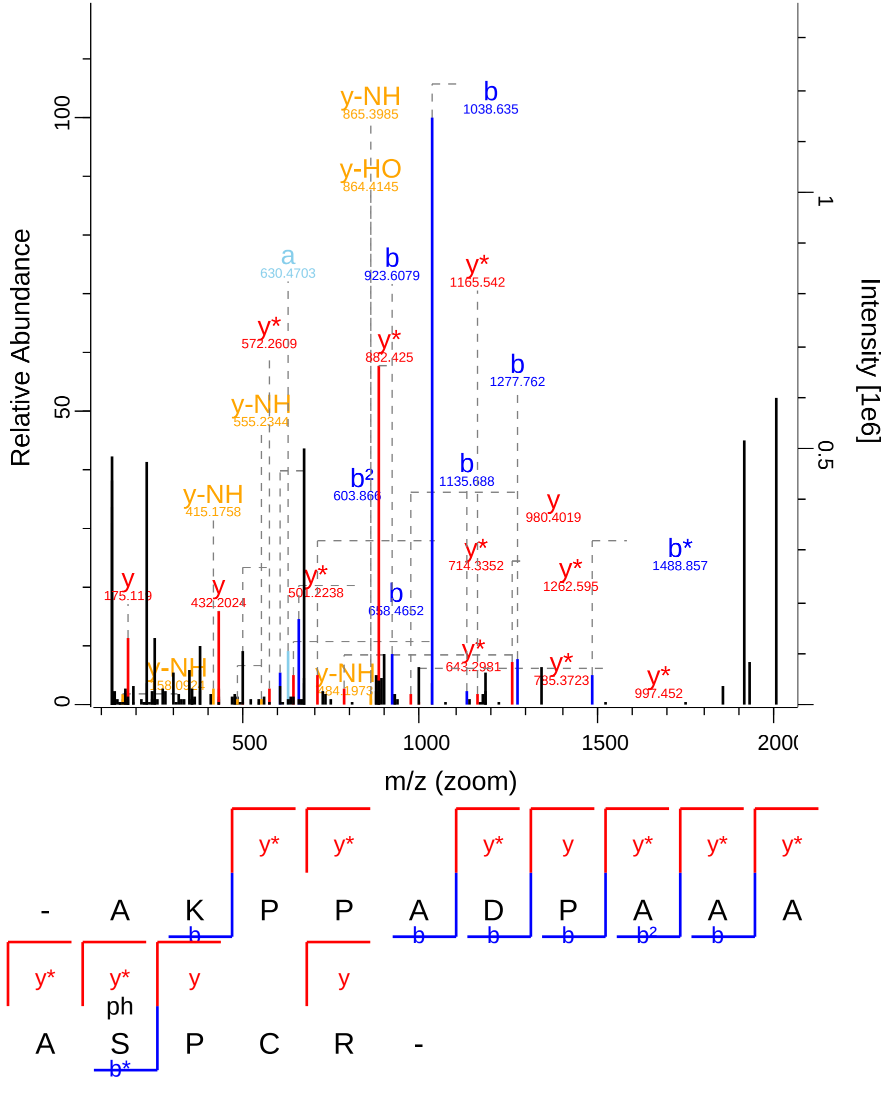

Raw File	Scan	Method	Score	m/z	Gene names
RO-QC-160121_ZackD_SS_Set_02_Fractio	11057	FTMS; HC	117.41	744.85	FNBP1

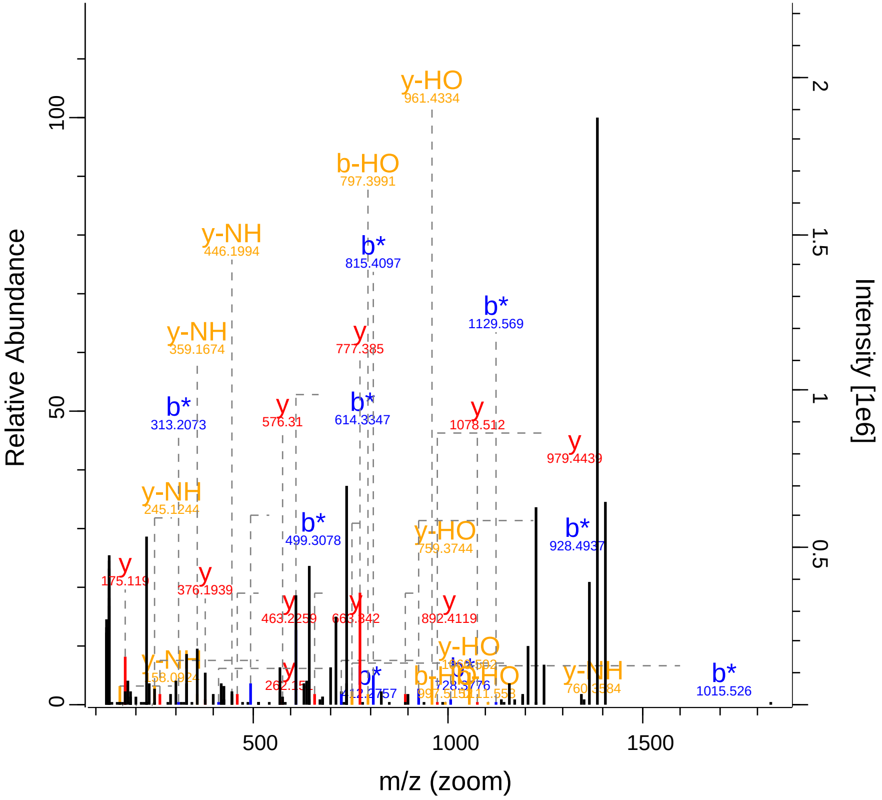

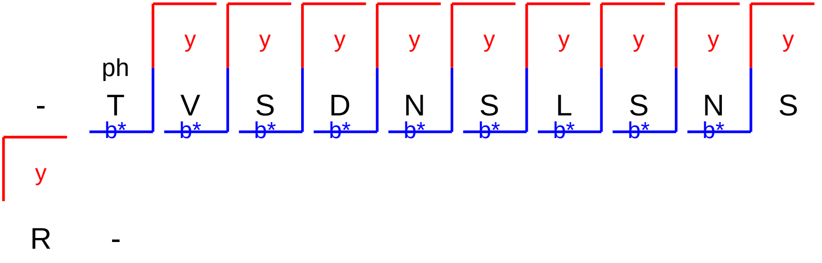

Raw File	Scan	Method	Score	m/z	Gene names
RO-QC-160121_ZackD_SS_Set_02_Fractio	12621	FTMS; HC	63.57	898.74	CDK12

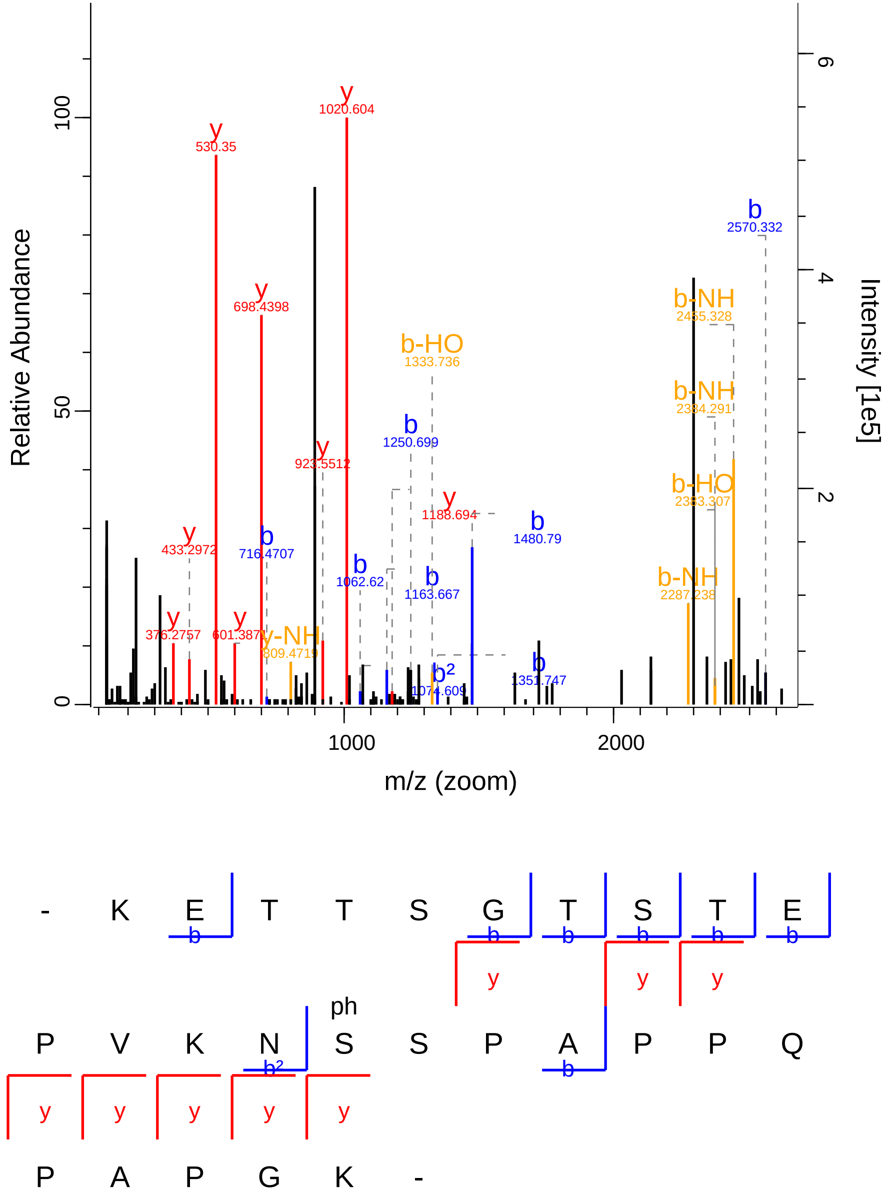

Raw File	Scan	Method	Score	m/z	Gene names
RO-QC-160121_ZackD_SS_Set_02_Fractio	12776	FTMS; HC	93.76	801.12	MAP1B

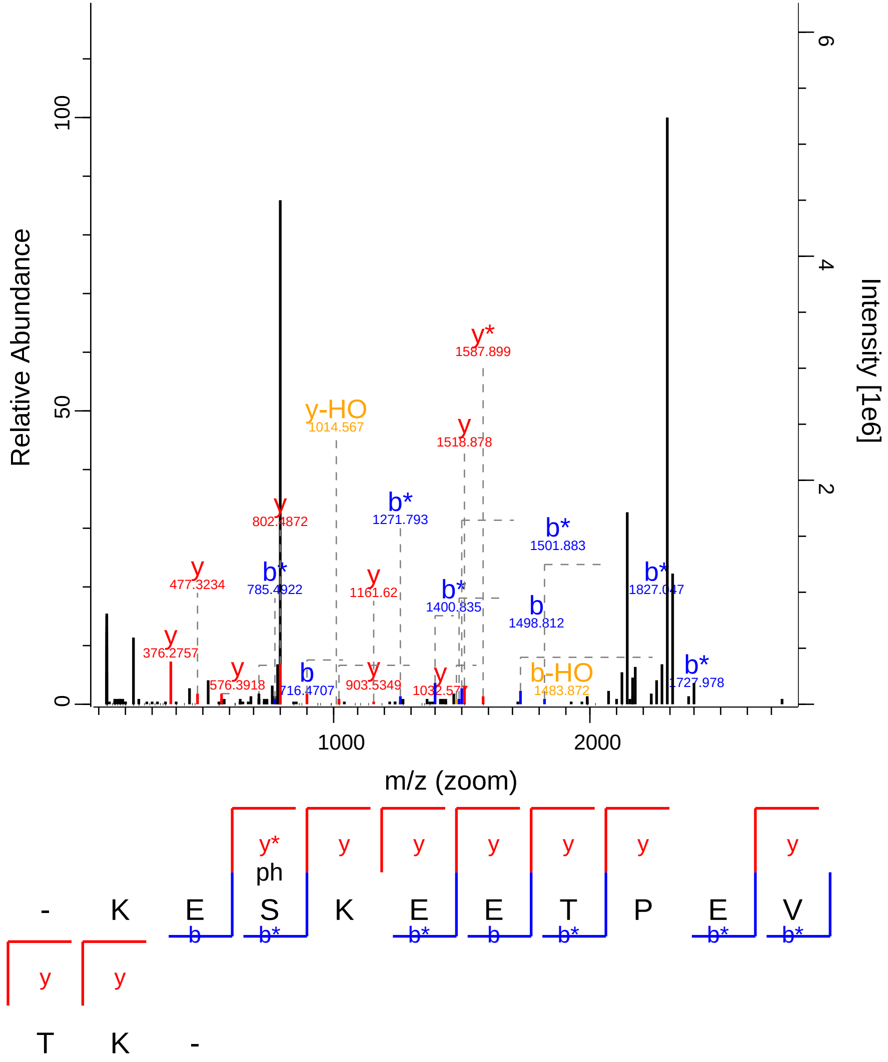

Raw File	Scan	Method	Score	m/z	Gene names
RO-QC-160121_ZackD_SS_Set_02_Fractio	12903	FTMS; HC	50.91	629.35	SLC7A14

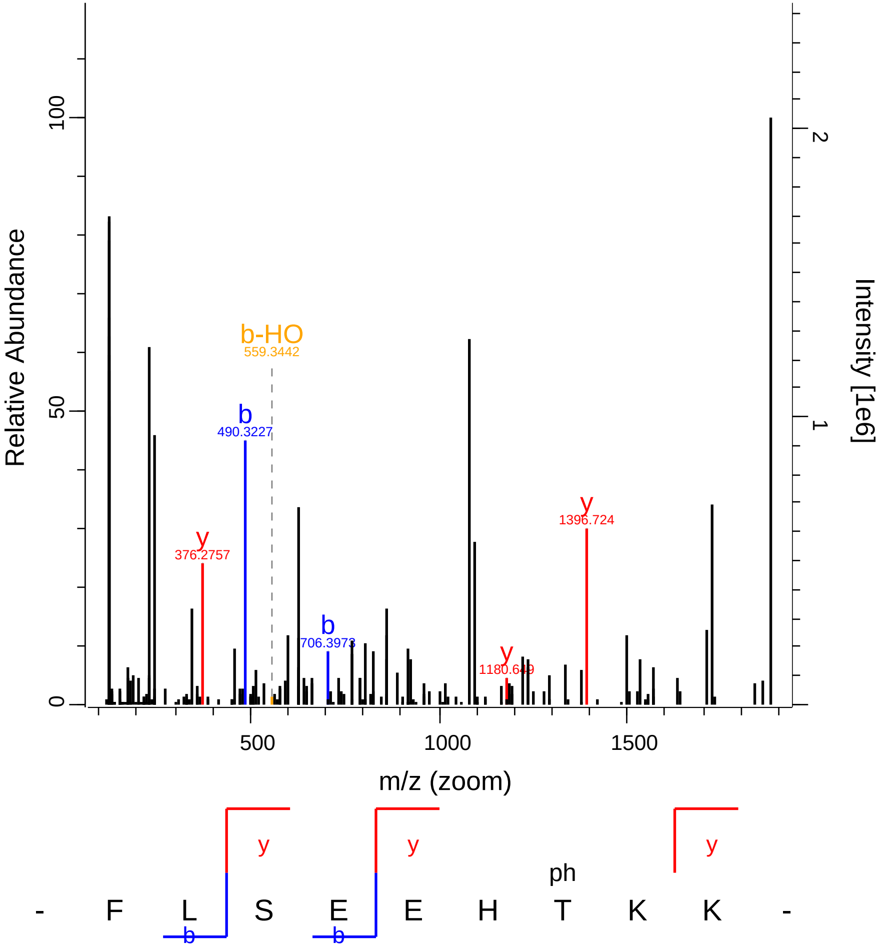

Raw File	Scan	Method	Score	m/z	Gene names
RO-QC-160121_ZackD_SS_Set_02_Fractio	14459	FTMS; HC	109.85	815.71	CDC42EP4

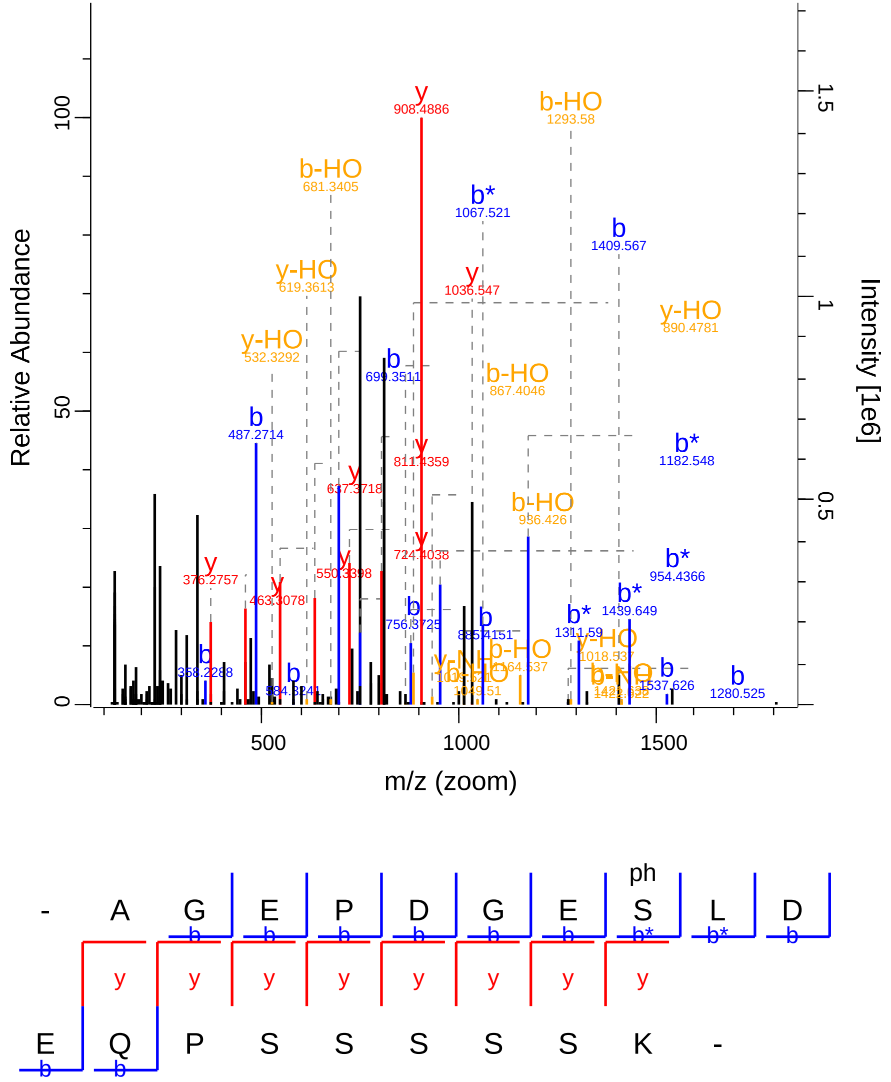

Raw File	Scan	Method	Score	m/z	Gene names
RO-QC-160121_ZackD_SS_Set_02_Fractio	15185	FTMS; HC	66.27	403.98	BPIFB4

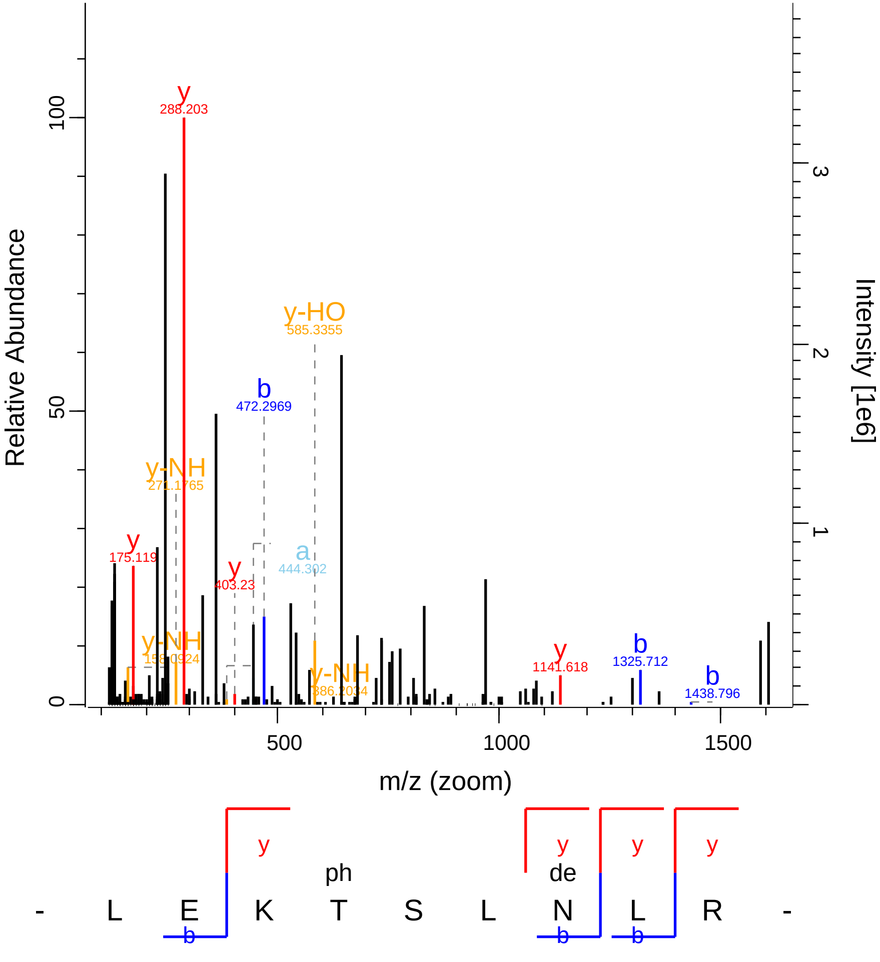

Raw File	Scan	Method	Score	m/z	Gene names
RO-QC-160121_ZackD_SS_Set_02_Fractio	15359	FTMS; HC	40.07	704.33	TBX19

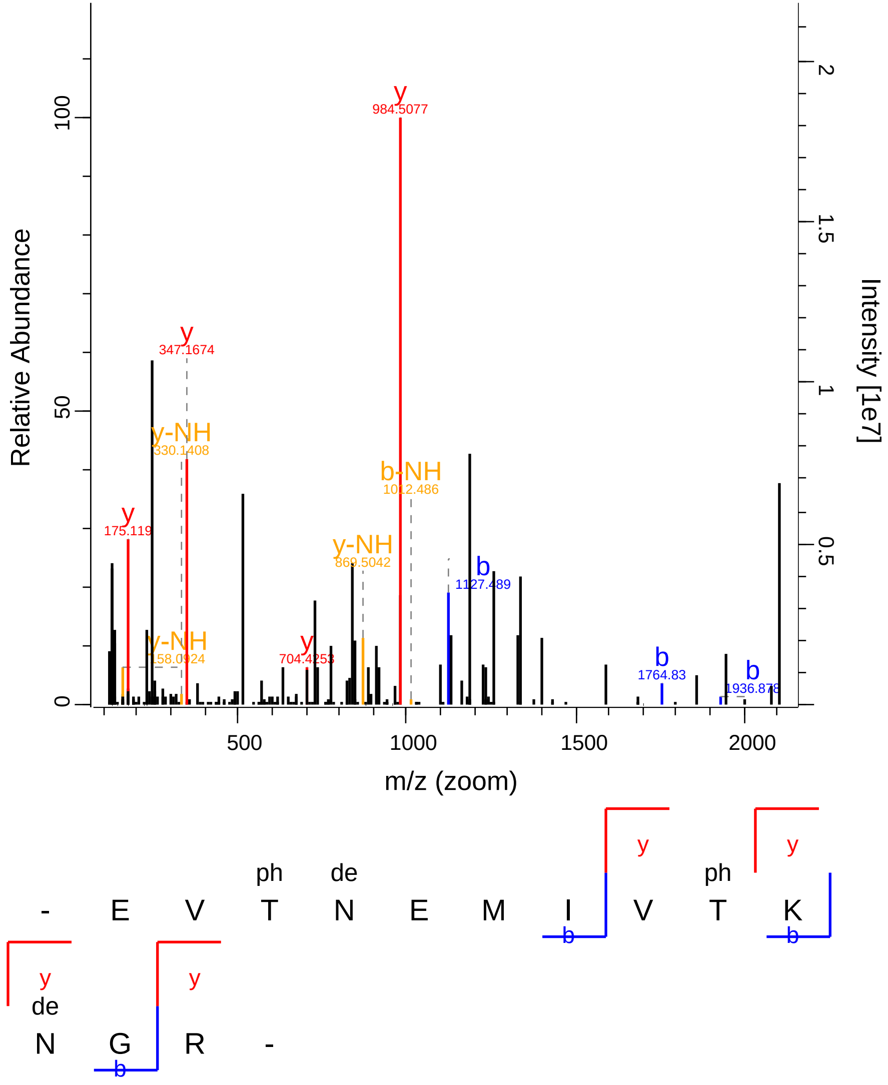

Raw File	Scan	Method	Score	m/z	Gene names
RO-QC-160121_ZackD_SS_Set_02_Fractio	15429	FTMS; HC	128.01	871.43	IRS2

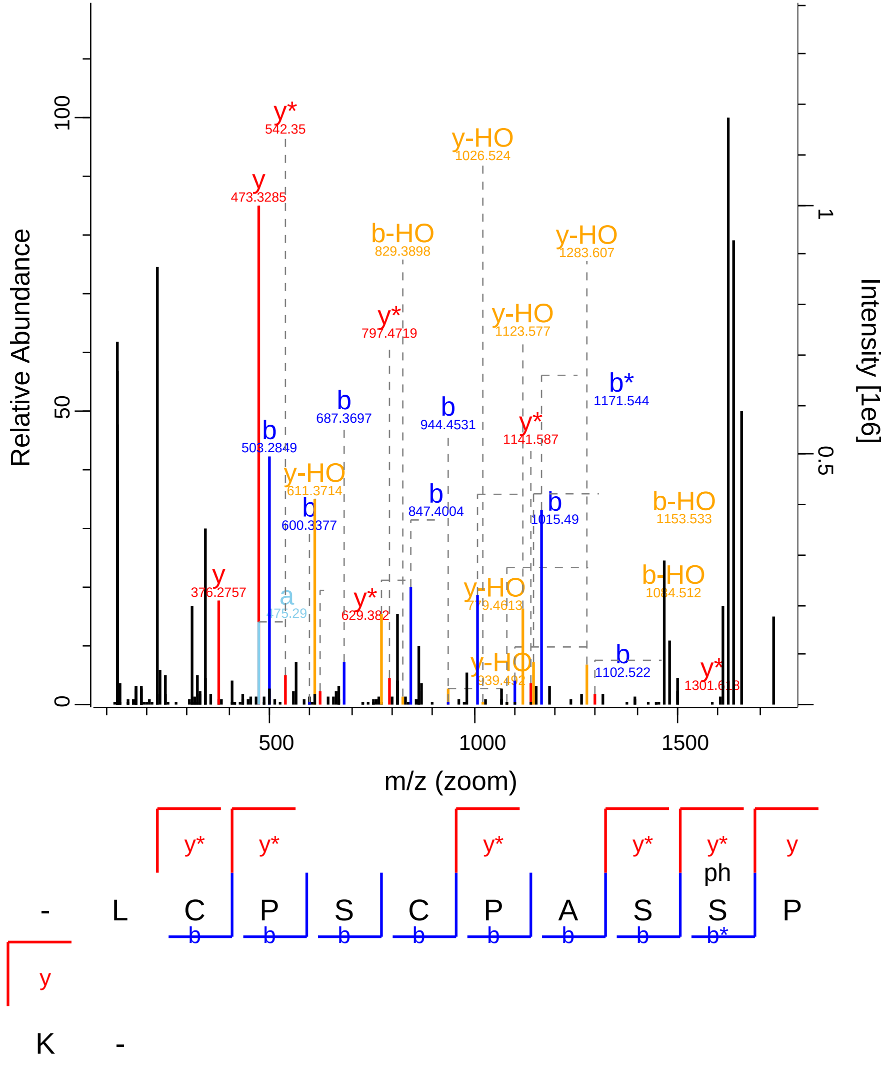

Raw File	Scan	Method	Score	m/z	Gene names
RO-QC-160121_ZackD_SS_Set_02_Fractio	15510	FTMS; HC	48	417.96	HOOK2

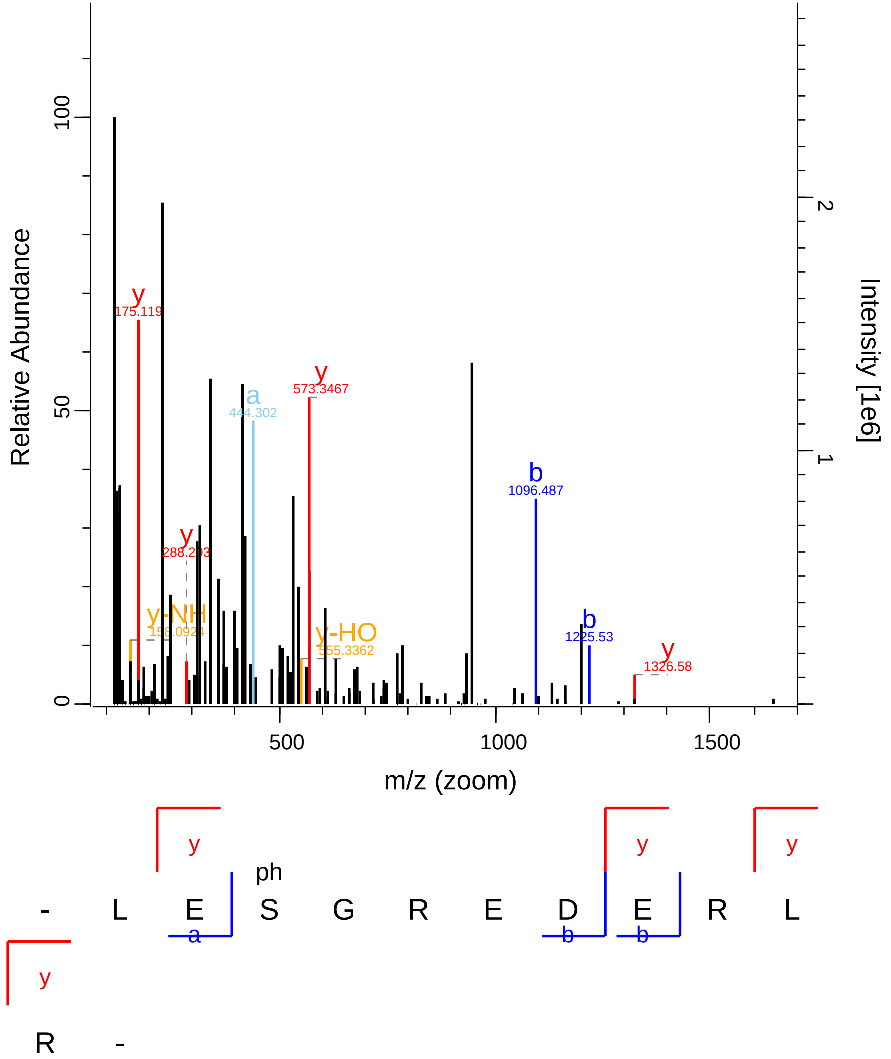

Raw File	Scan	Method	Score	m/z	Gene names
RO-QC-160121_ZackD_SS_Set_02_Fractio	15749	FTMS; HC	73.45	605.33	ABI2

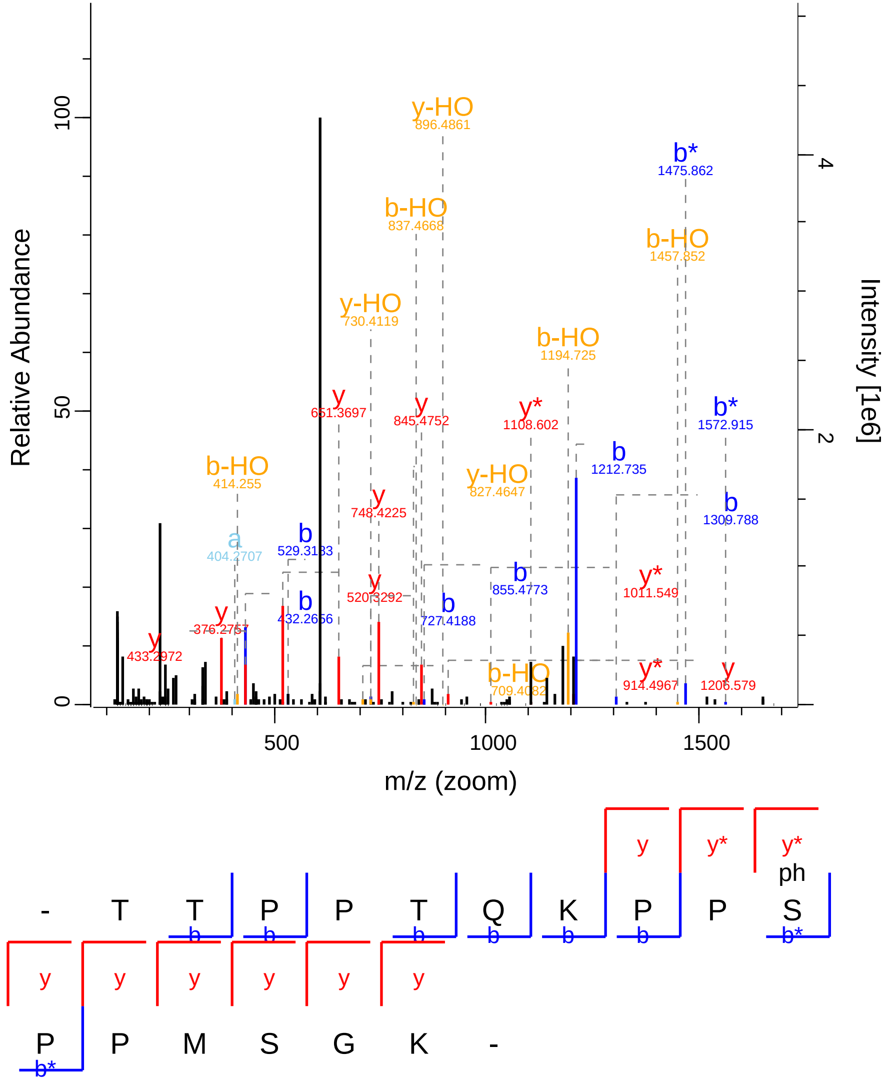

Raw File	Scan	Method	Score	m/z	Gene names
RO-QC-160121_ZackD_SS_Set_02_Fractio	16051	FTMS; HC	75.55	923.15	ZRANB2

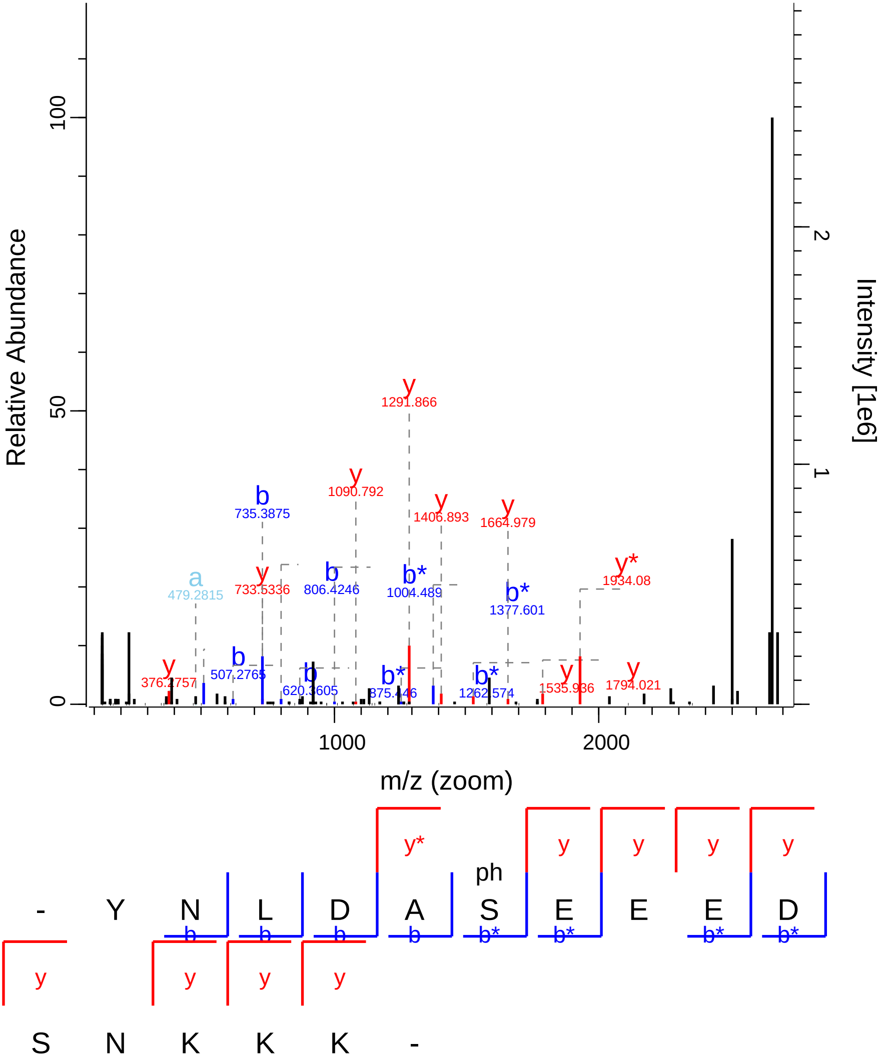

Raw File	Scan	Method	Score	m/z	Gene names
RO-QC-160121_ZackD_SS_Set_02_Fractic	16667	FTMS; HC	138.35	1002.49	HMGA1

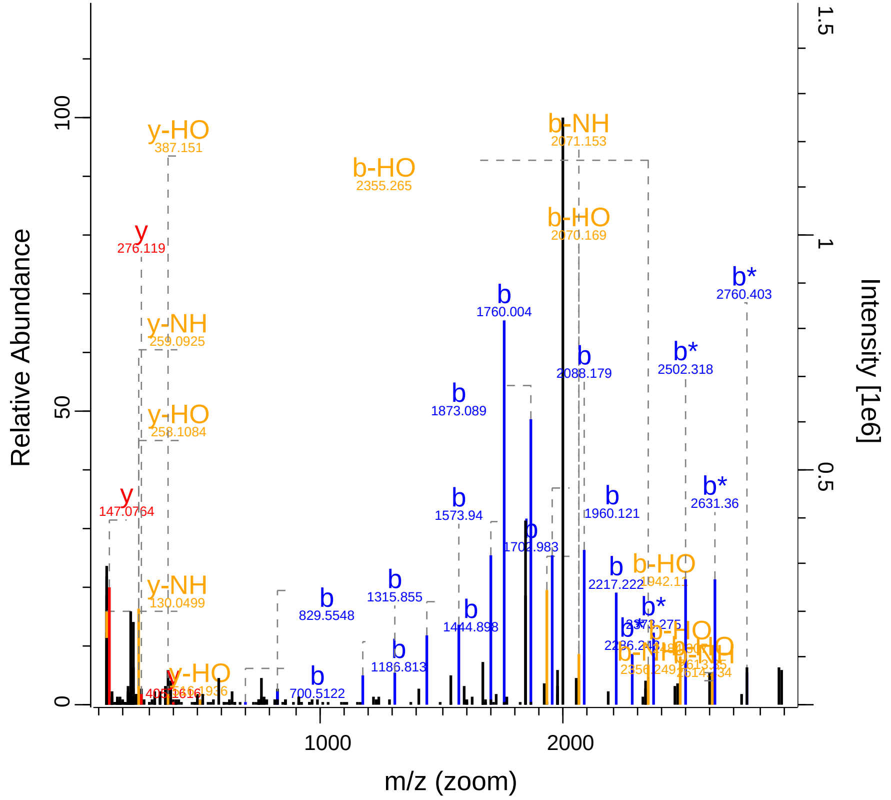

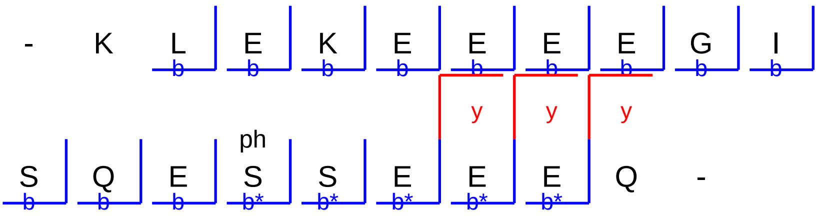

Raw File	Scan	Method	Score	m/z	Gene names
RO-QC-160121_ZackD_SS_Set_02_Fractio	16751	FTMS; HC	76.48	853.43	TERF1

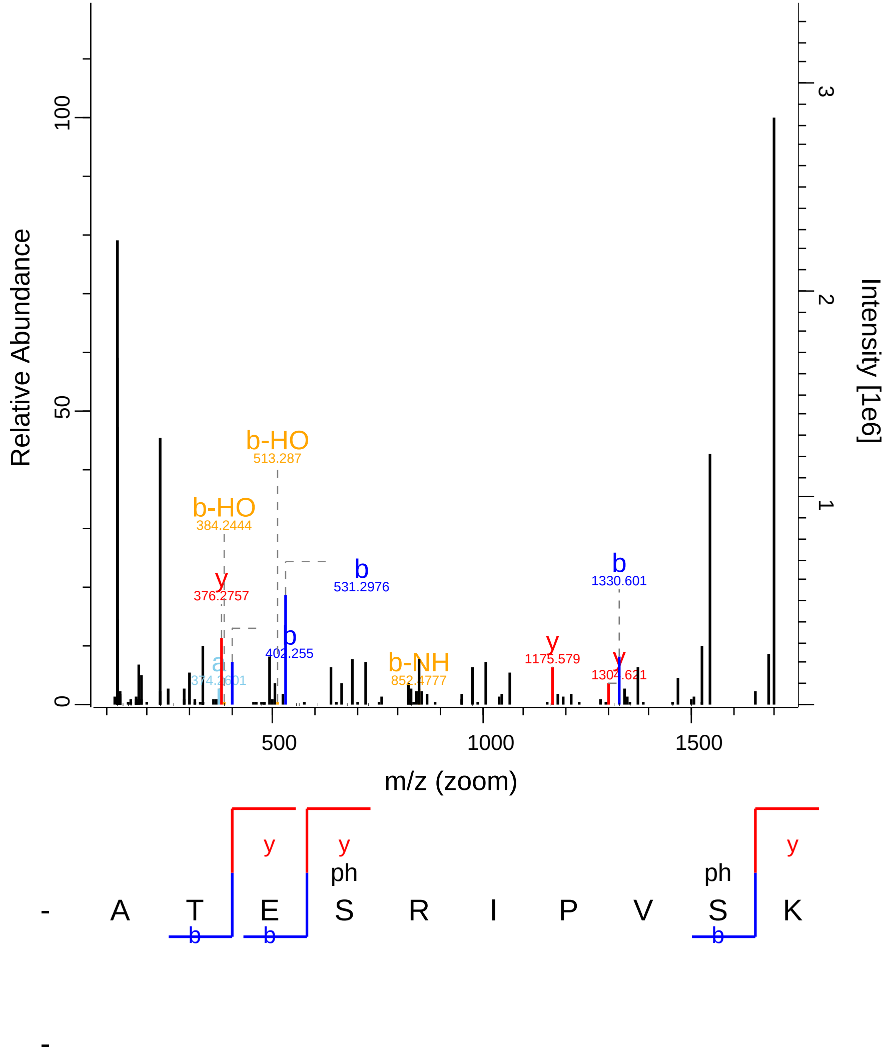

Raw File	Scan	Method	Score	m/z	Gene names
RO-QC-160121_ZackD_SS_Set_02_Fractio	16820	FTMS; HC	99.97	871.51	EIF4ENIF1

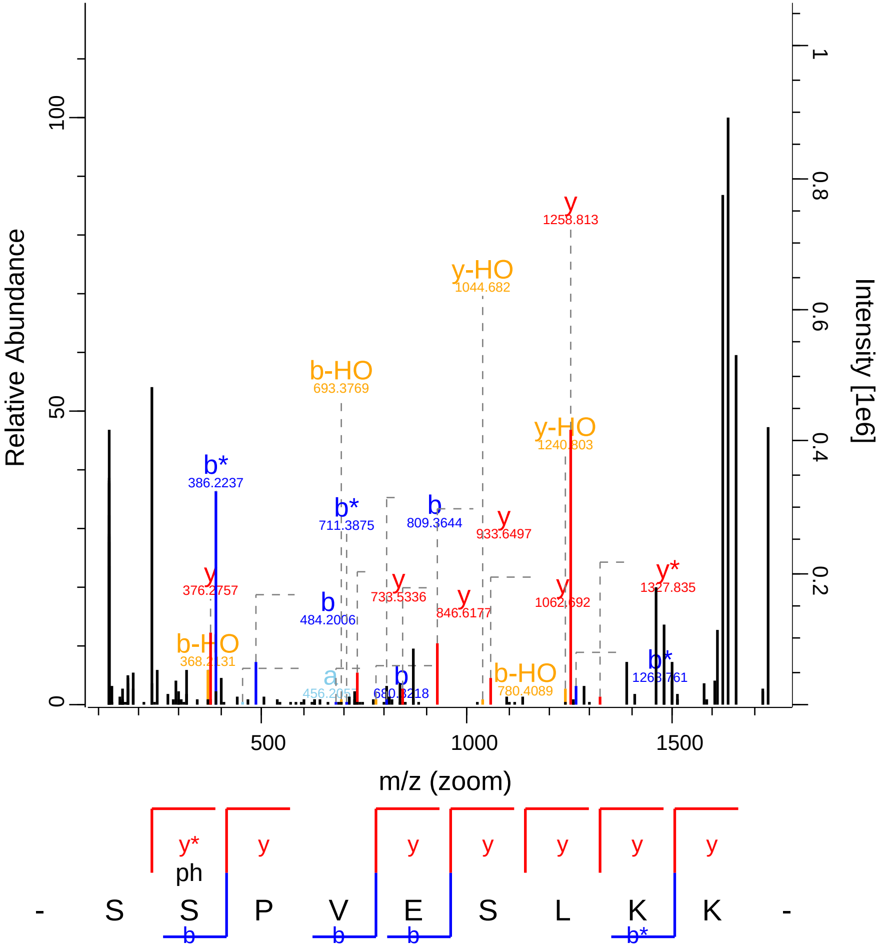

Raw File	Scan	Method	Score	m/z	Gene names
RO-QC-160121_ZackD_SS_Set_02_Fractio	17431	FTMS; HC	73.04	898.13	PALM

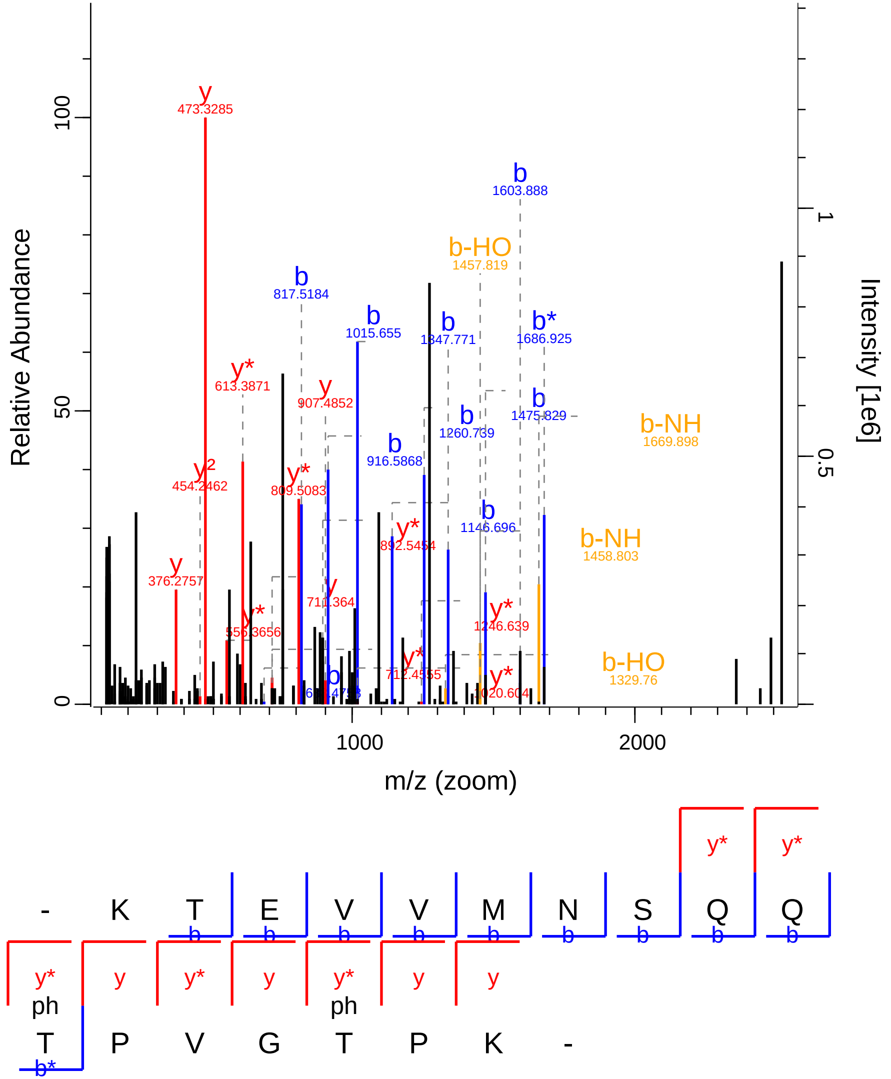

Raw File	Scan	Method	Score	m/z	Gene names
RO-QC-160121_ZackD_SS_Set_02_Fractic	17502	FTMS; HC	79.47	1213.12	DYNC1LI1

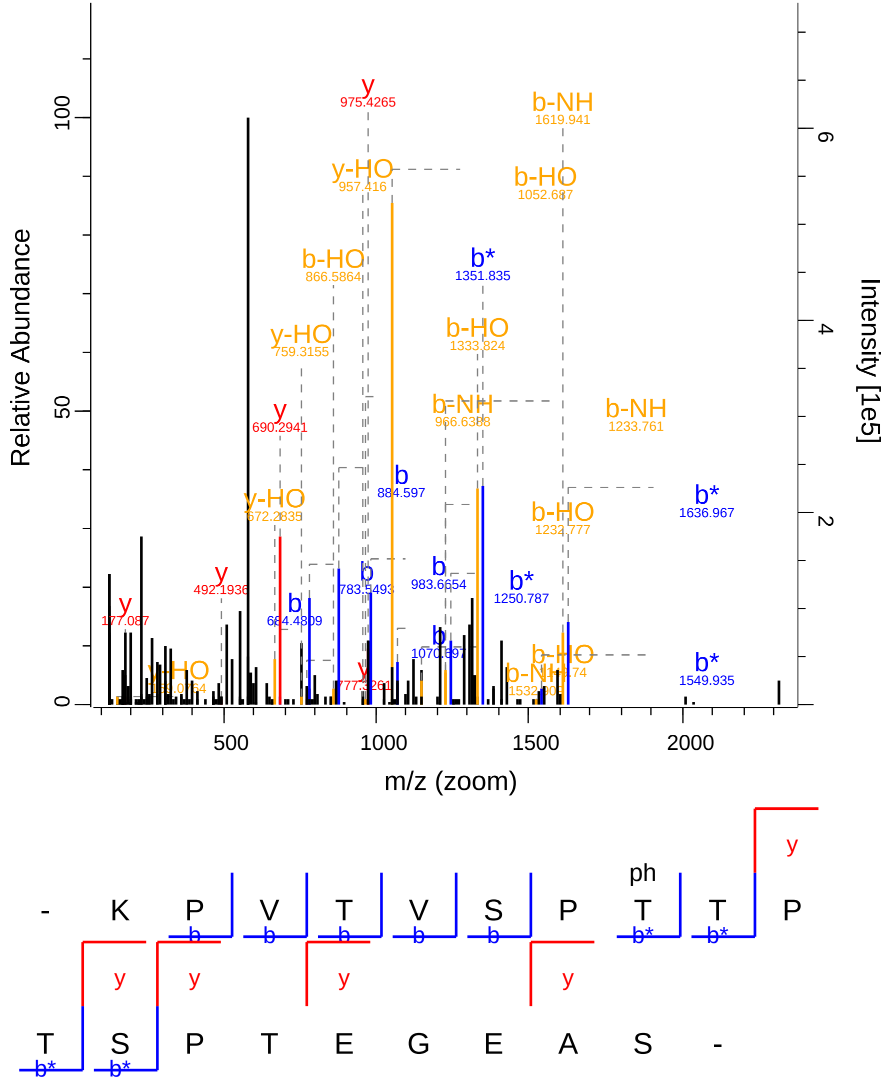

Raw File	Scan	Method	Score	m/z	Gene names
RO-QC-160121_ZackD_SS_Set_02_Fractio	18432	FTMS; HC	44.26	900.48	BCL2L13

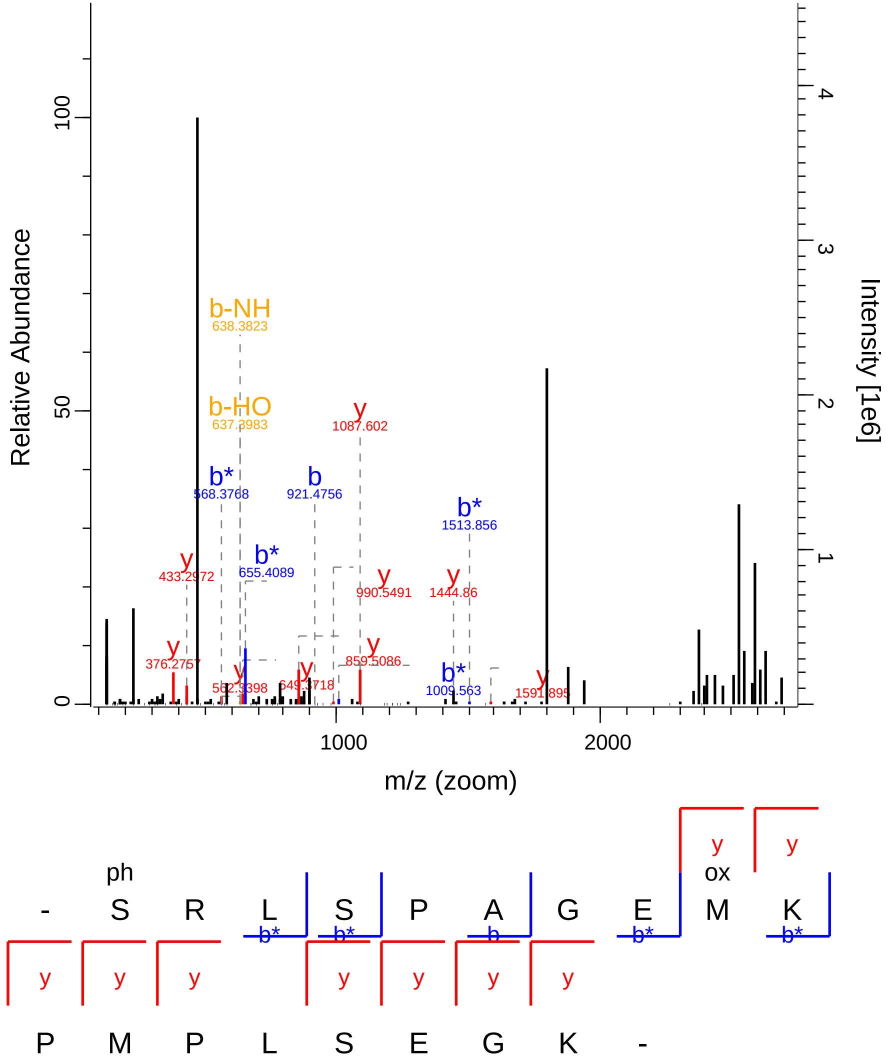

Raw File	Scan	Method	Score	m/z	Gene names
RO-QC-160121_ZackD_SS_Set_02_Fractio	18662	FTMS; HC	84.21	974.04	BBS1

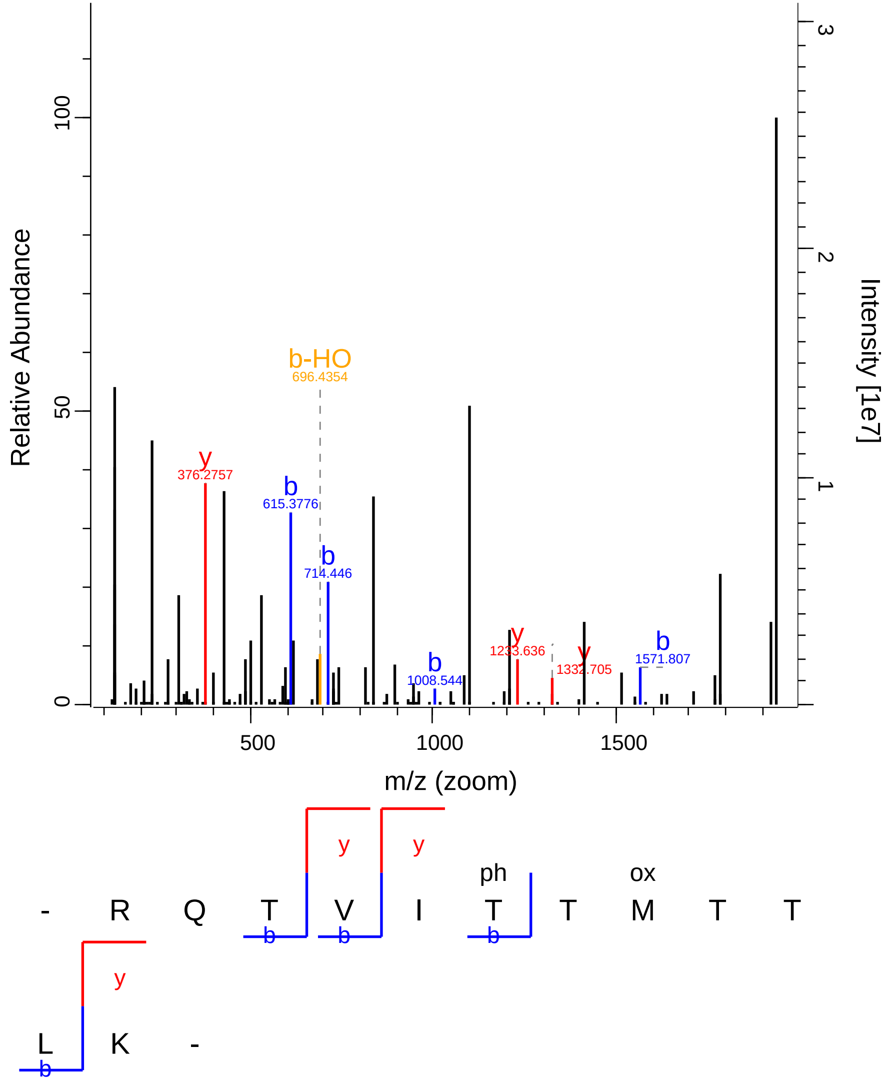

Raw File	Scan	Method	Score	m/z	Gene names
RO-QC-160121_ZackD_SS_Set_02_Fractio	19073	FTMS; HC	138.25	762.93	PDCD4

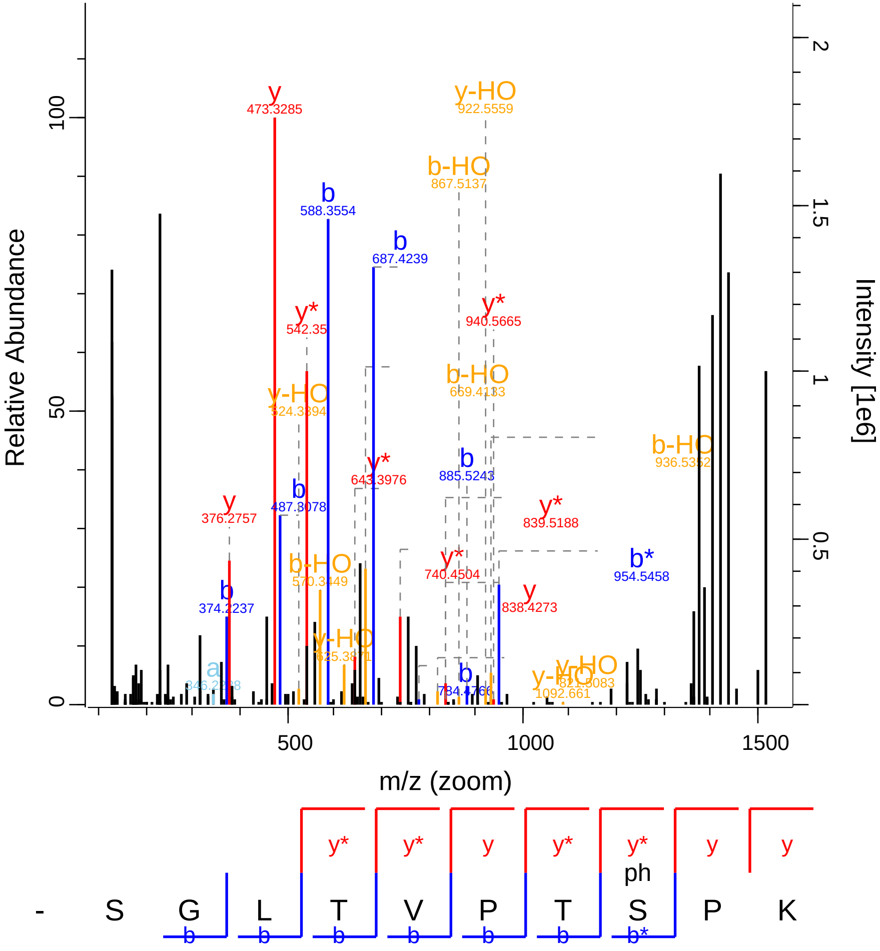

-

Raw File	Scan	Method	Score	m/z	Gene names
RO-QC-160121_ZackD_SS_Set_02_Fractio	19523	FTMS; HC	162.07	996.22	PAK4

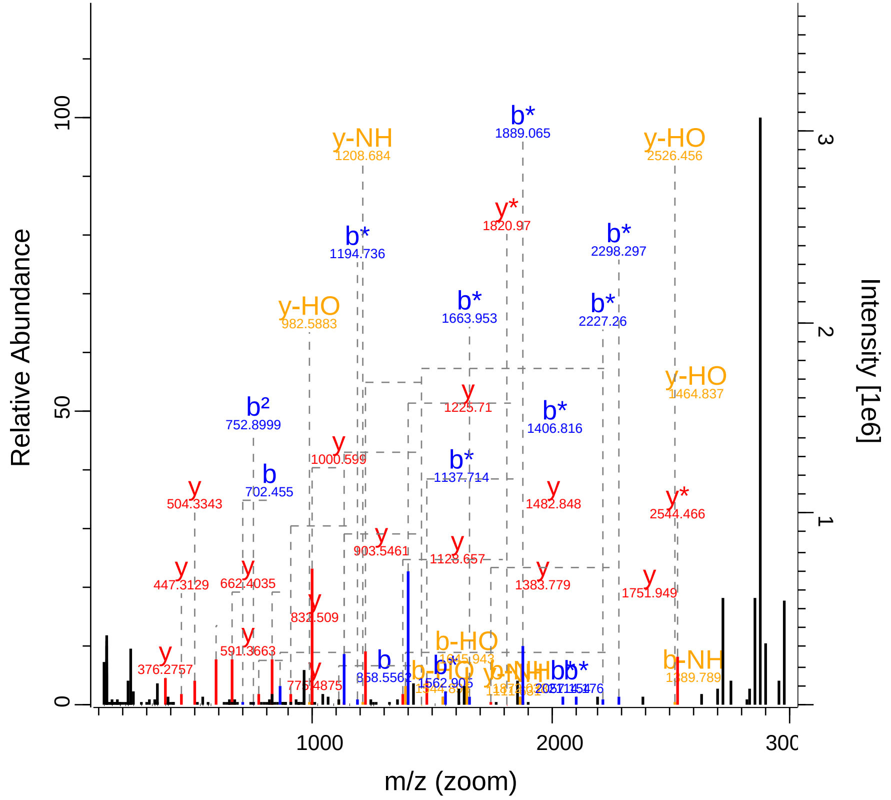

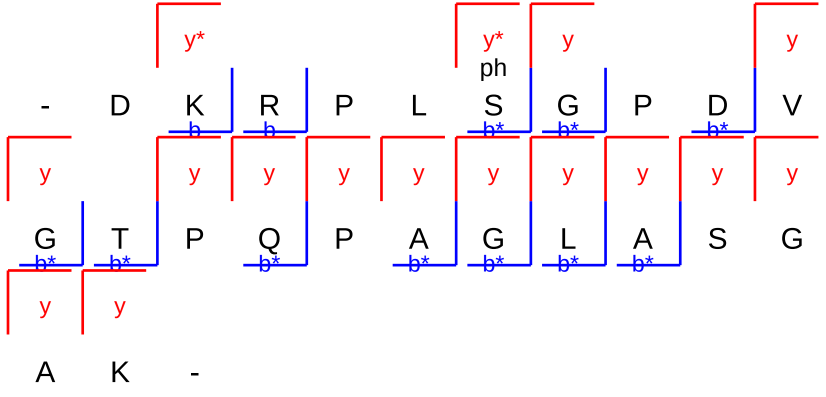

Raw File	Scan	Method	Score	m/z	Gene names
RO-QC-160121_ZackD_SS_Set_02_Fractio	19597	FTMS; HC	73.02	794.45	TRPV5

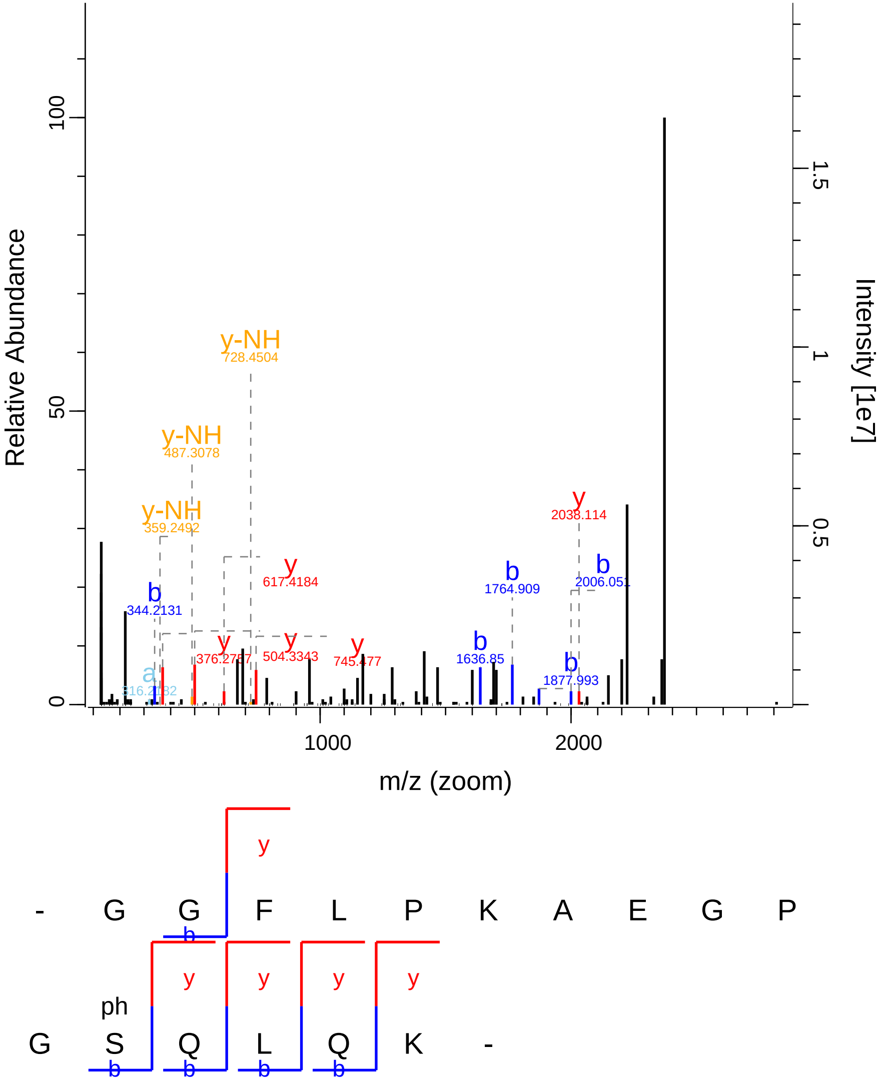

Raw File	Scan	Method	Score	m/z	Gene names
RO-QC-160121_ZackD_SS_Set_02_Fractio	19693	FTMS; HC	125.51	773.03	ABCA2

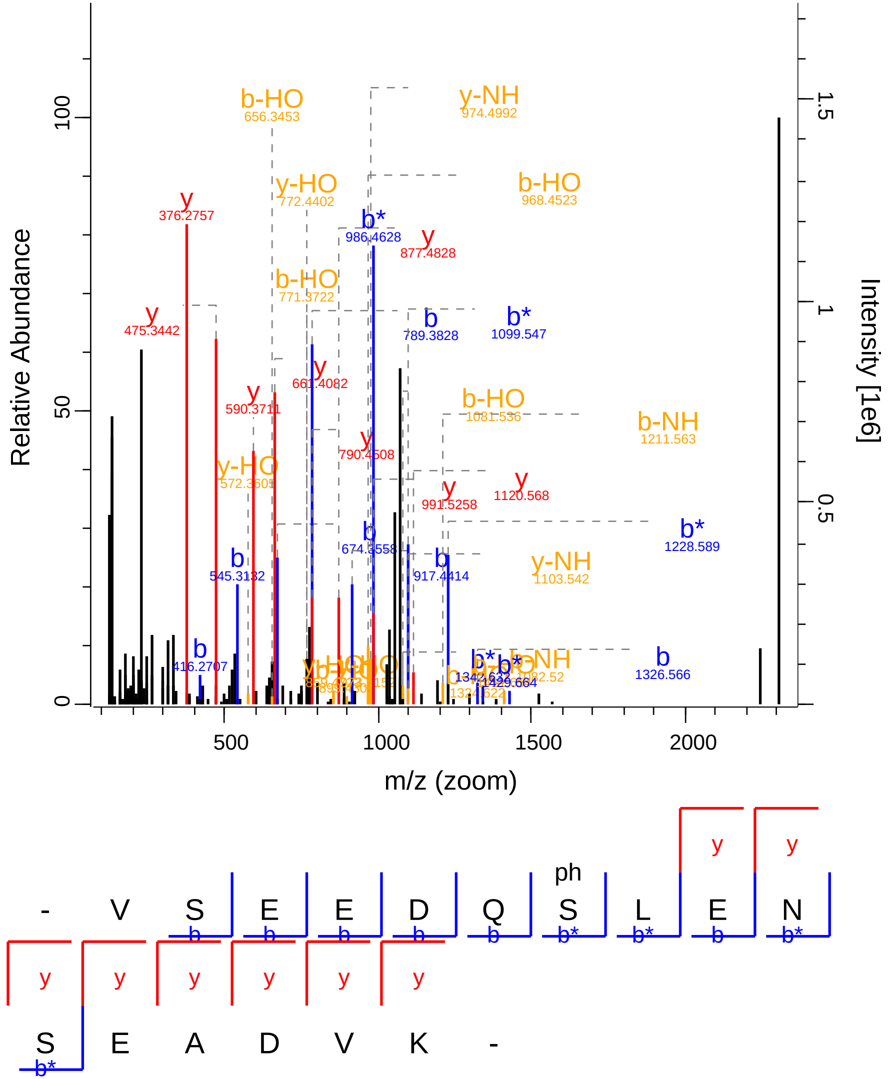

Raw File	Scan	Method	Score	m/z	Gene names
RO-QC-160121_ZackD_SS_Set_02_Fractio	20020	FTMS; HC	92.29	863.78	MAP1B

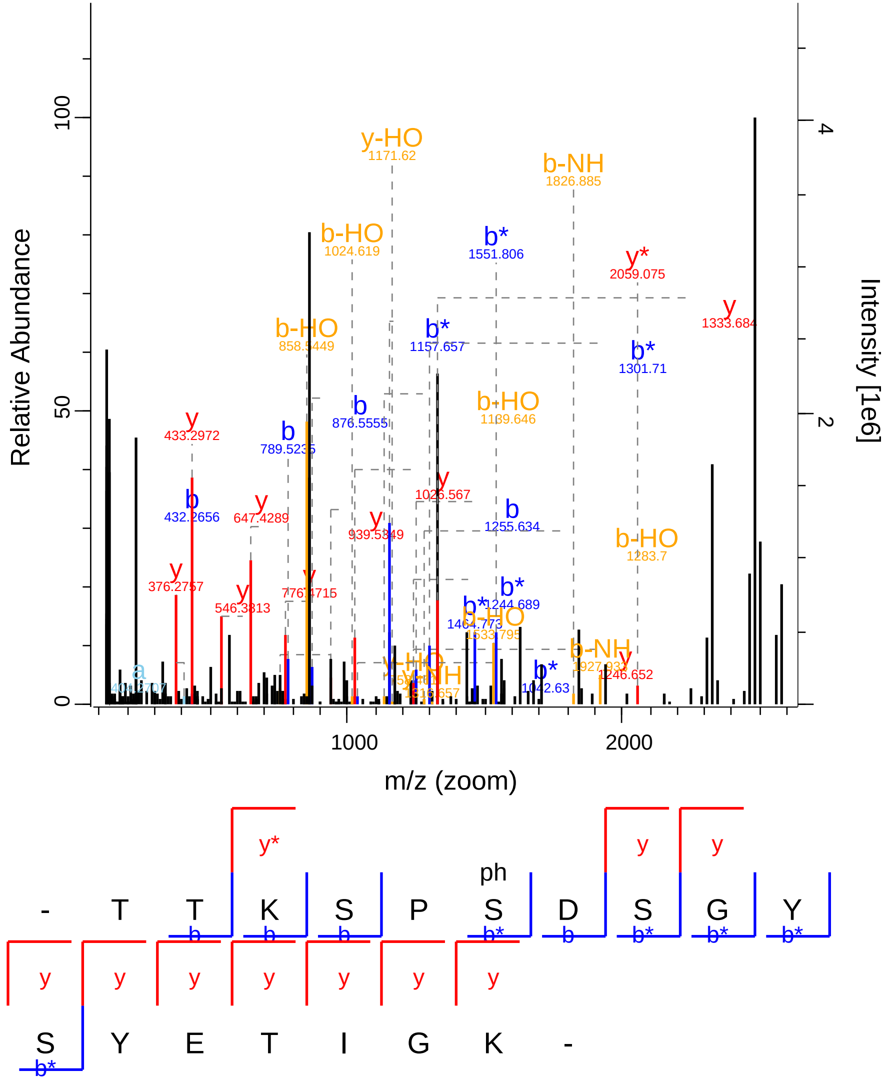

Raw File	Scan	Method	Score	m/z	Gene names
RO-QC-160121_ZackD_SS_Set_02_Fractio	20246	FTMS; HC	47.75	796.66	OR51F2

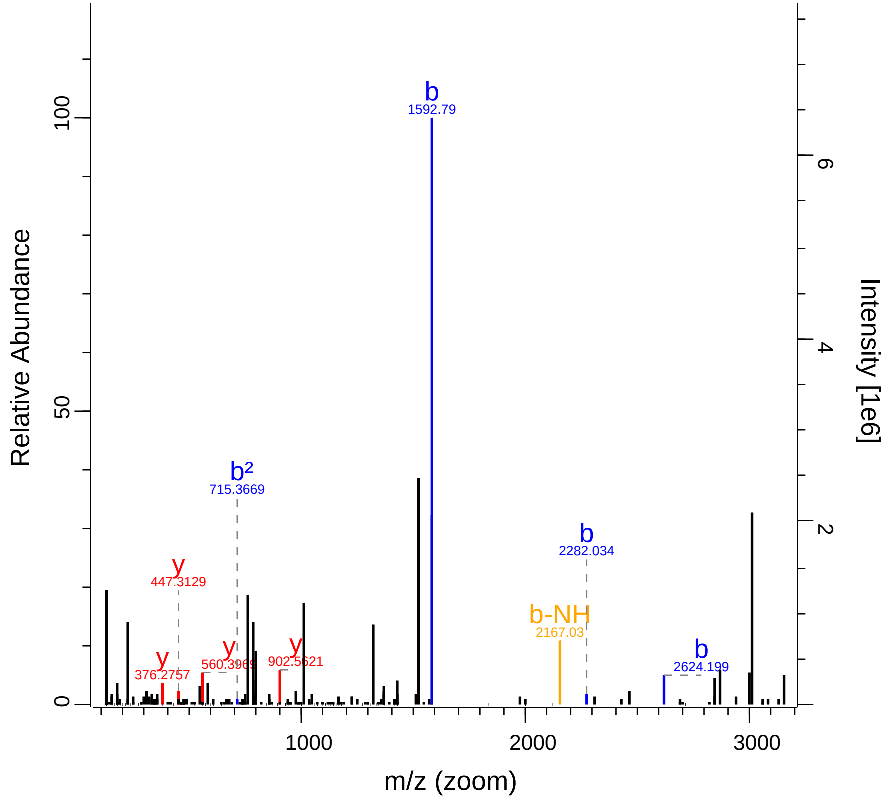

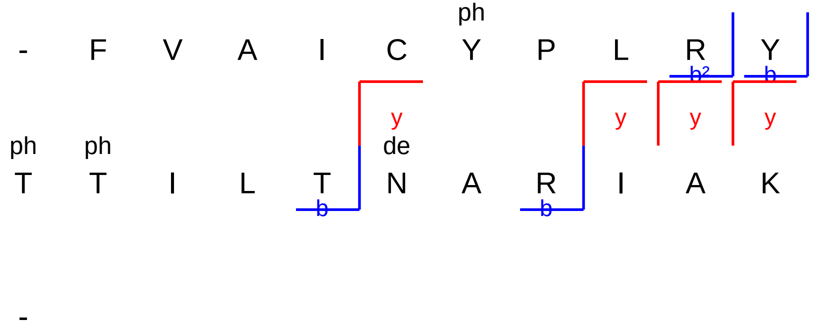

Raw File	Scan	Method	Score	m/z	Gene names
RO-QC-160121_ZackD_SS_Set_02_Fractio	21333	FTMS; HC	150.12	1041.6	MAP1B

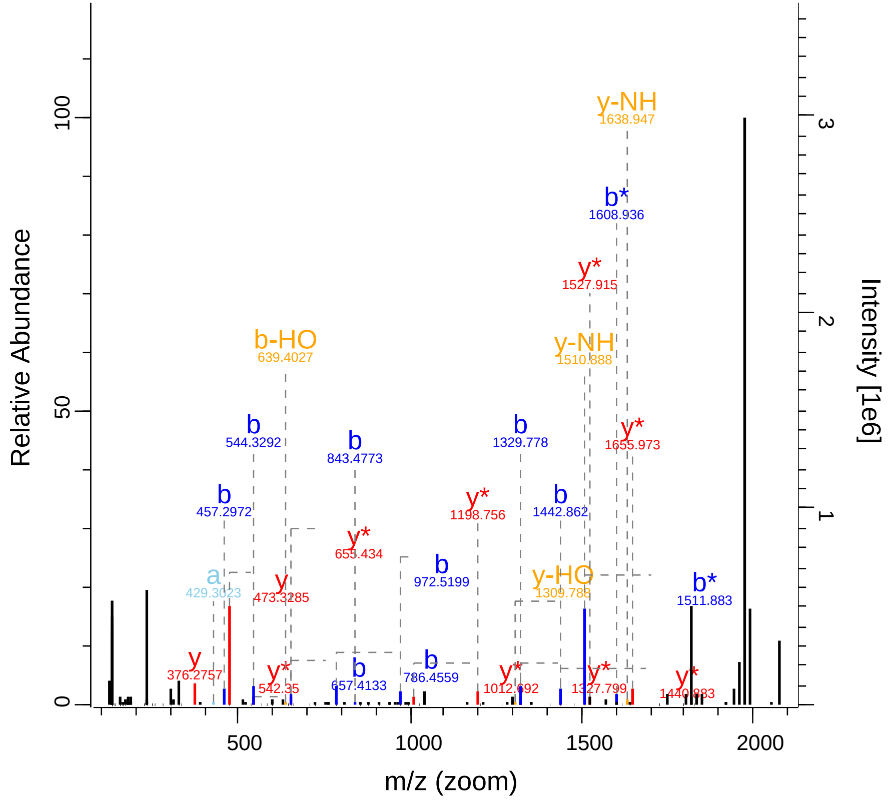

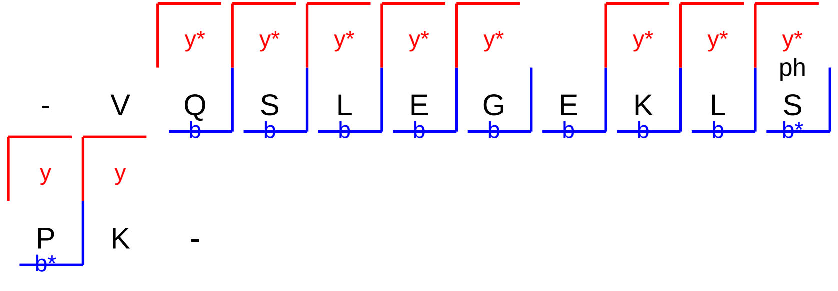

Raw File	Scan	Method	Score	m/z	Gene names
RO-QC-160121_ZackD_SS_Set_02_Fractio	21482	FTMS; HC	114.86	752.89	MAP7

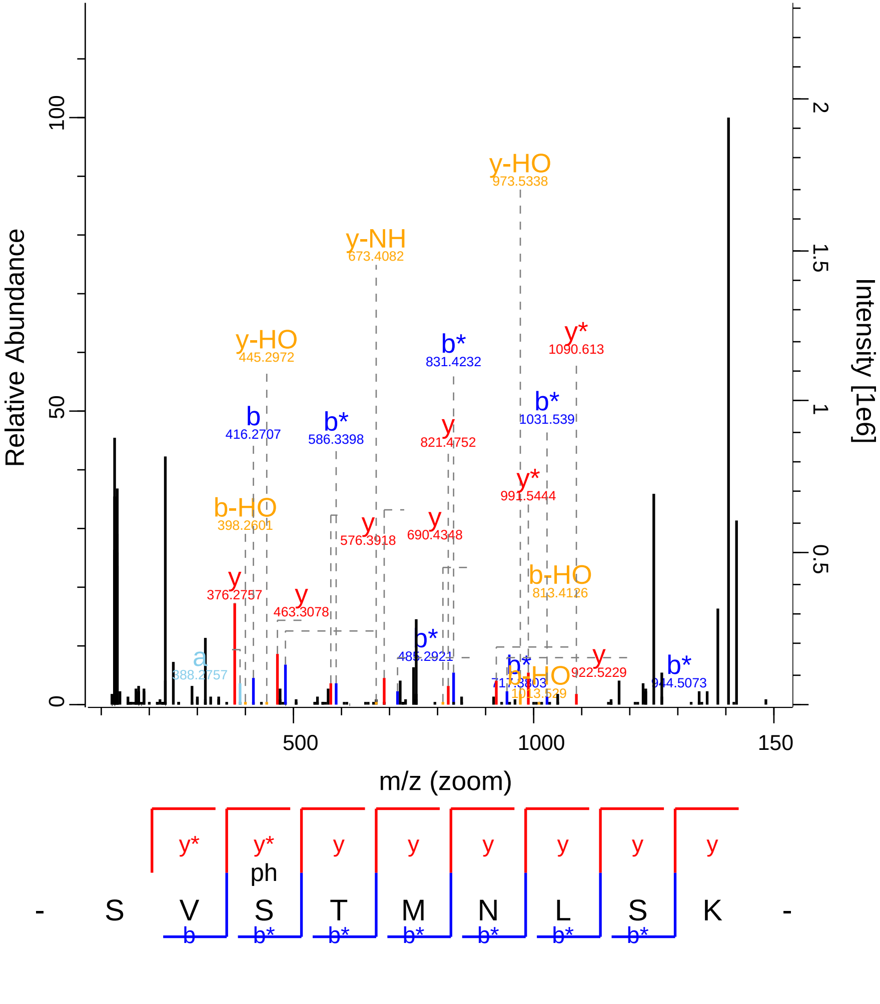

Raw File	Scan	Method	Score	m/z	Gene names
RO-QC-160121_ZackD_SS_Set_02_Fractio	22439	FTMS; HC	54.05	878.47	PPFIBP2

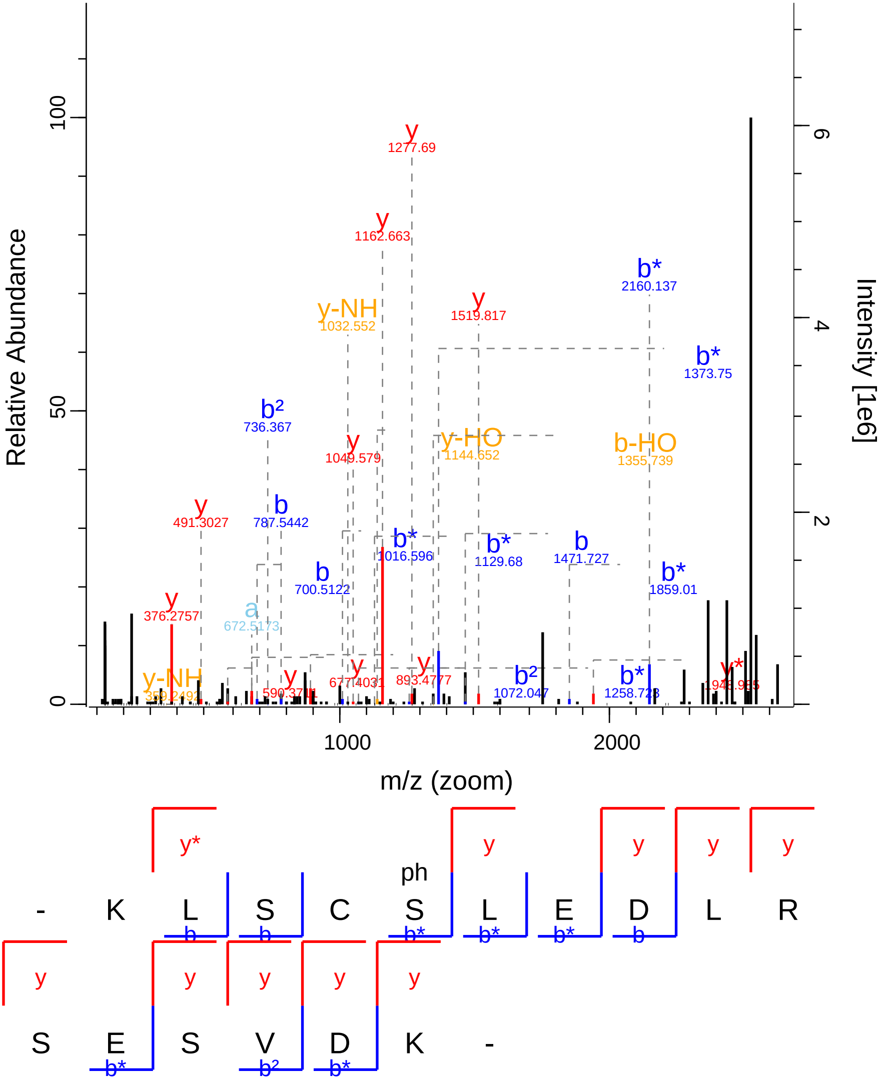

Raw File	Scan	Method	Score	m/z	Gene names
RO-QC-160121_ZackD_SS_Set_02_Fractio	22539	FTMS; HC	174.05	928.44	NCCRP1

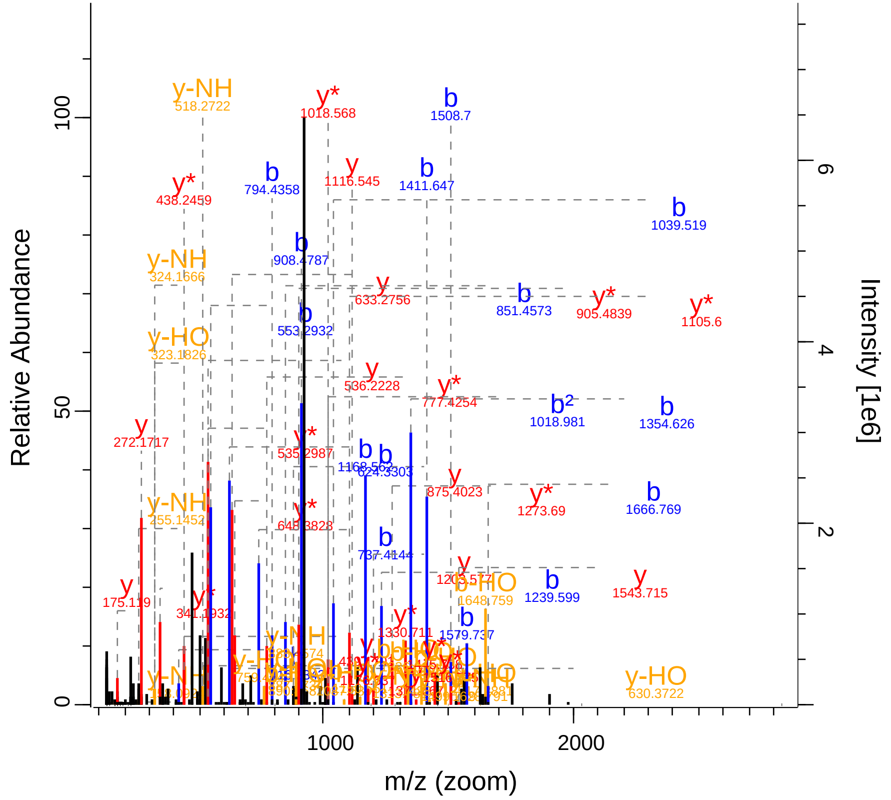

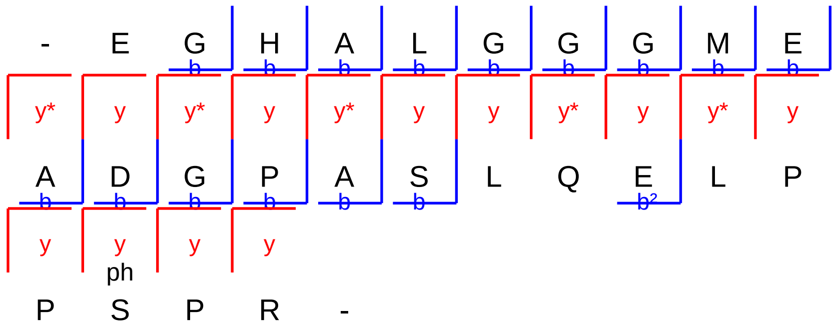

Raw File	Scan	Method	Score	m/z	Gene names
RO-QC-160121_ZackD_SS_Set_02_Fraction	22965	FTMS; HCI	101.64	829	TRDC

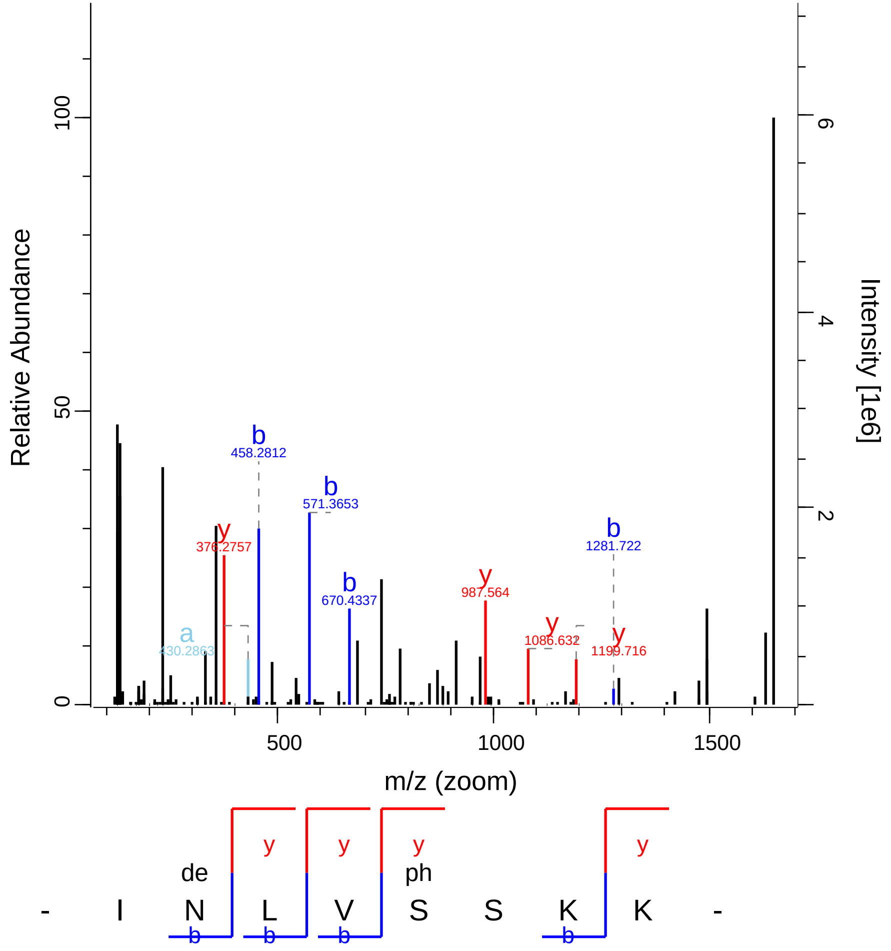

Raw File	Scan	Method	Score	m/z	Gene names
RO-QC-160121_ZackD_SS_Set_02_Fractio	24124	FTMS; HC	42.56	739.04	TRPM6

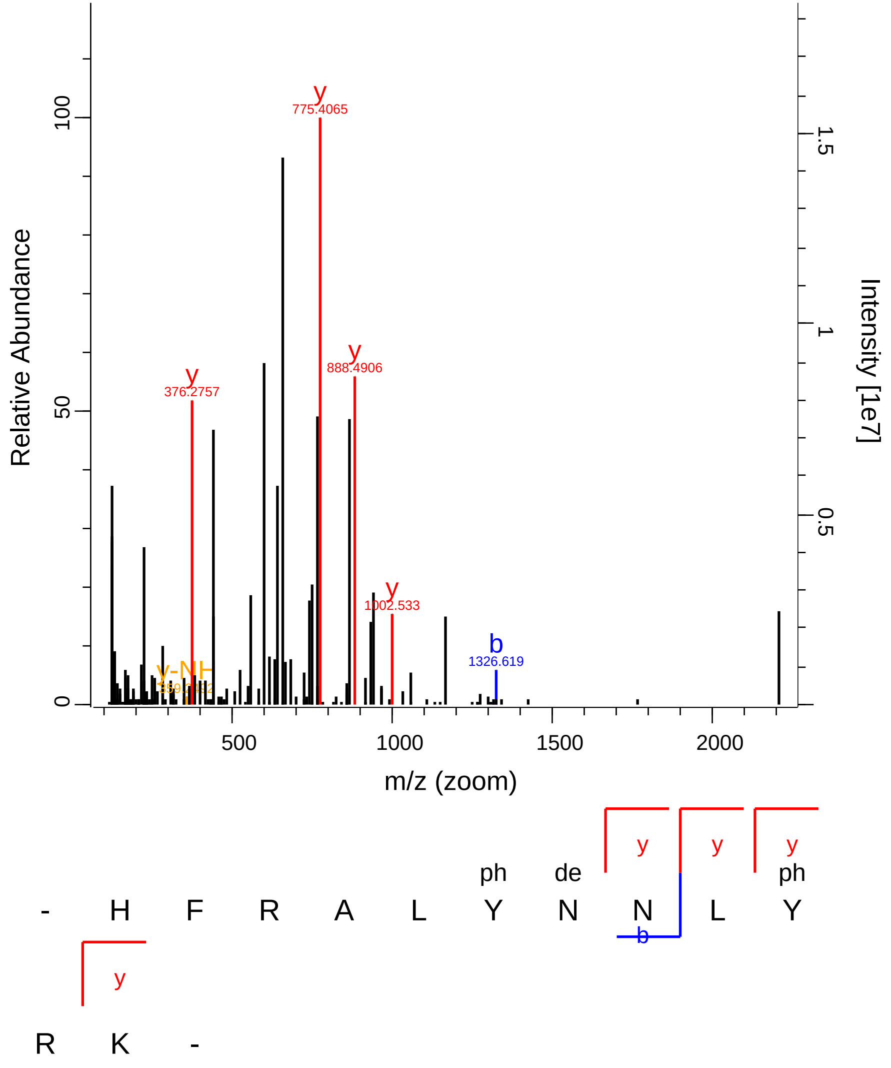

Raw File	Scan	Method	Score	m/z	Gene names
RO-QC-160121_ZackD_SS_Set_02_Fractio	24333	FTMS; HC	153.83	866.81	SPTBN1

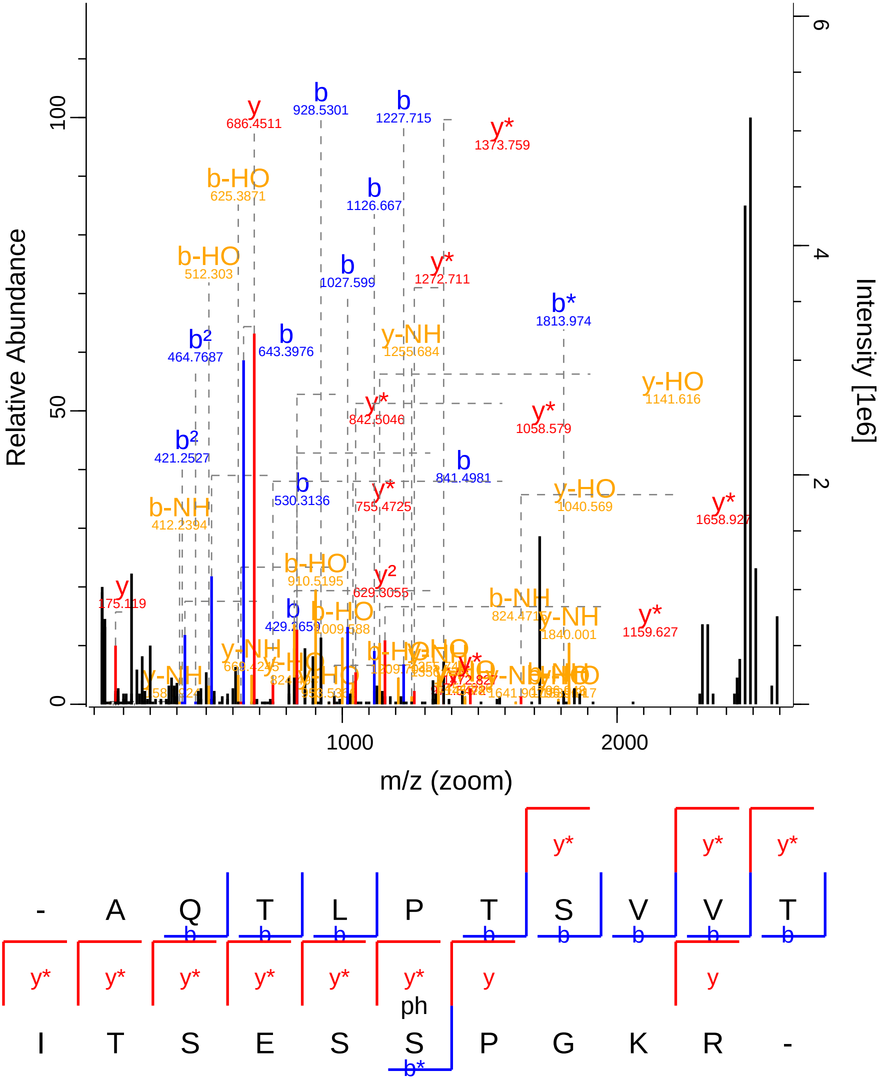

Raw File	Scan	Method	Score	m/z	Gene names
RO-QC-160121_ZackD_SS_Set_02_Fractio	25735	FTMS; HC	111.46	858.44	ERC2

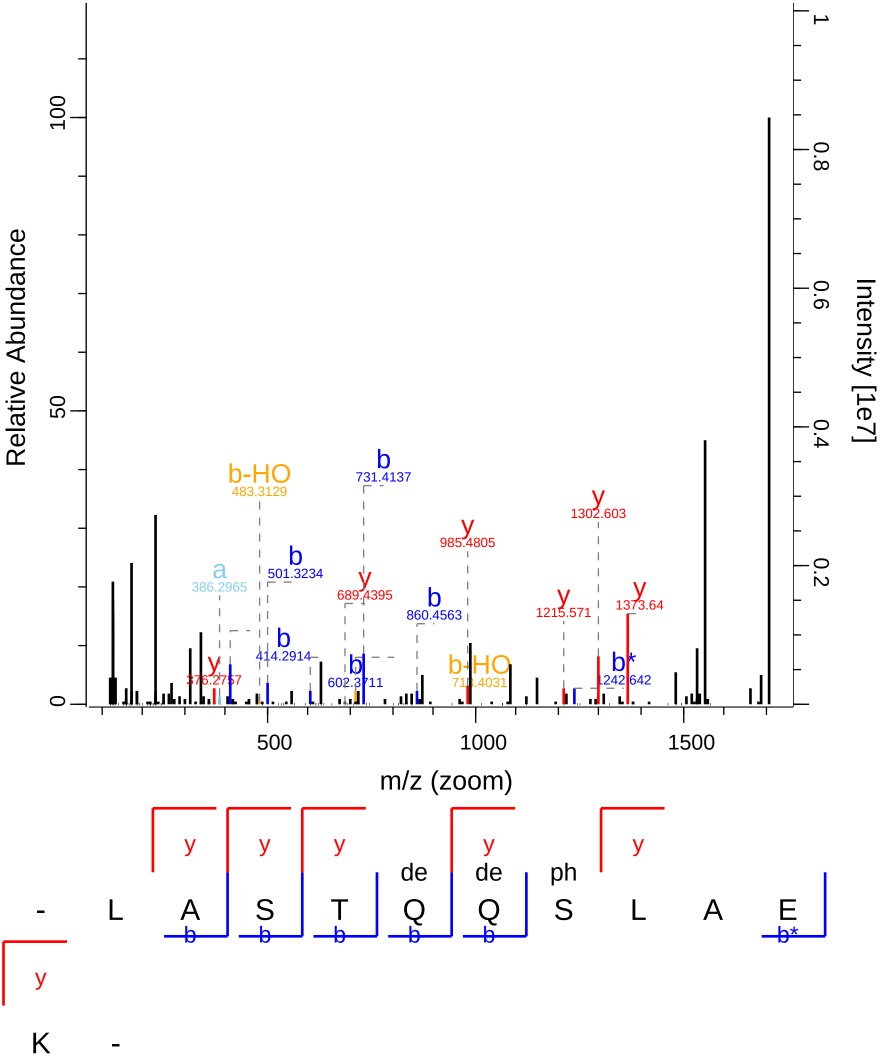

Raw File	Scan	Method	Score	m/z	Gene names
RO-QC-160121_ZackD_SS_Set_02_Fraction	26247	FTMS; HCI	75.29	1010	FTMT

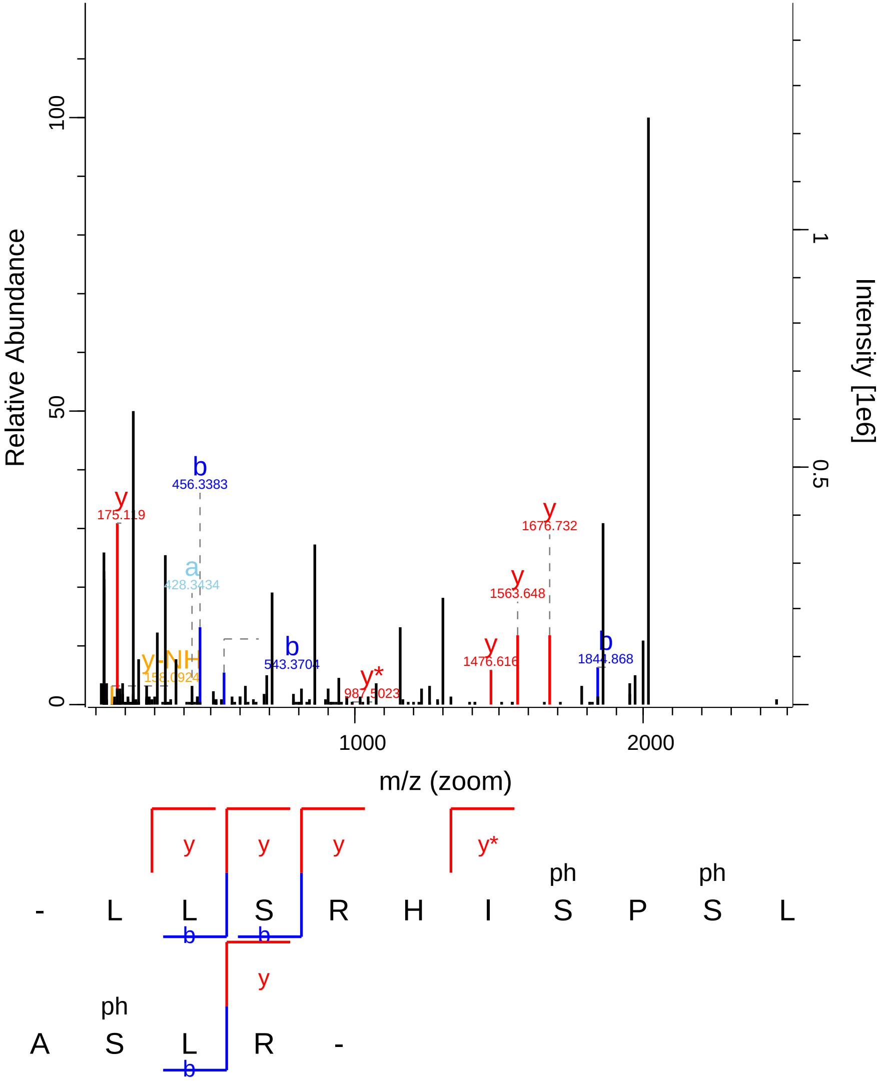

Raw File	Scan	Method	Score	m/z	Gene names
RO-QC-160121_ZackD_SS_Set_02_Fractior	26766	FTMS; HCI	99.09	706.4	EIF4G2

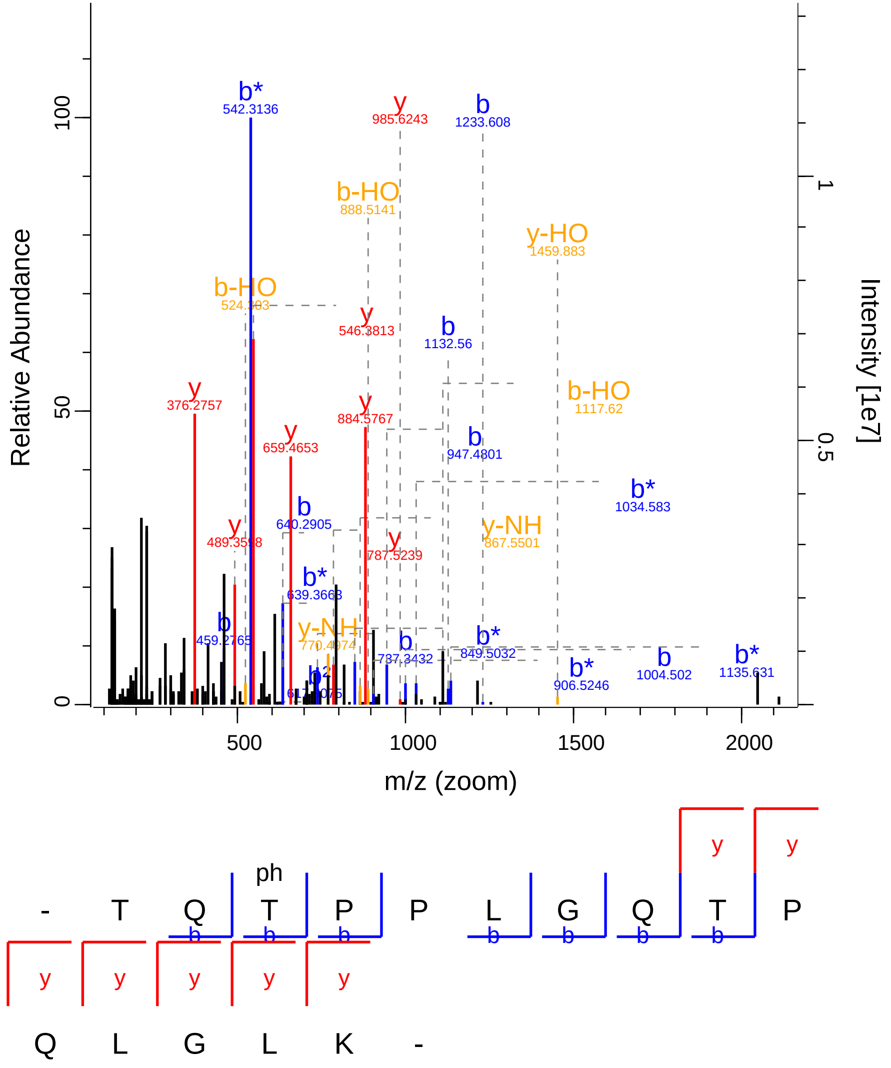

Raw File	Scan	Method	Score	m/z	Gene names
RO-QC-160121_ZackD_SS_Set_02_Fractio	26782	FTMS; HC	71.09	578.08	STMN1

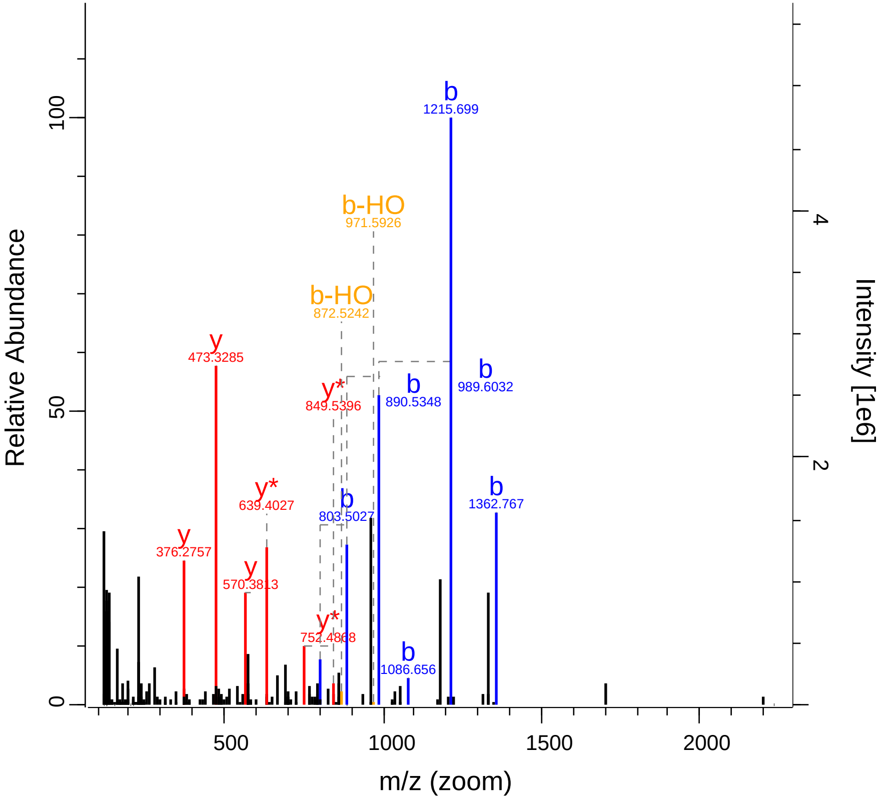

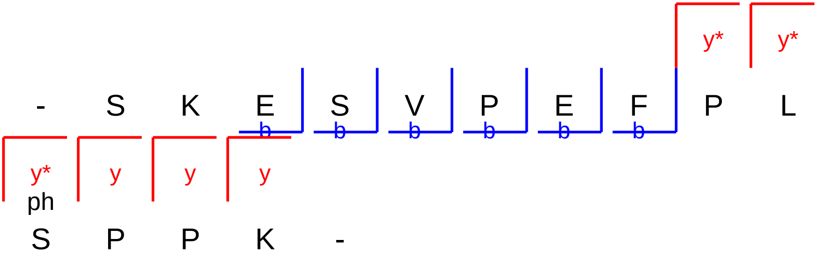

Raw File	Scan	Method	Score	m/z	Gene names
RO-QC-160121_ZackD_SS_Set_02_Fractic	27328	FTMS; HC	63.93	1086.54	NOP56

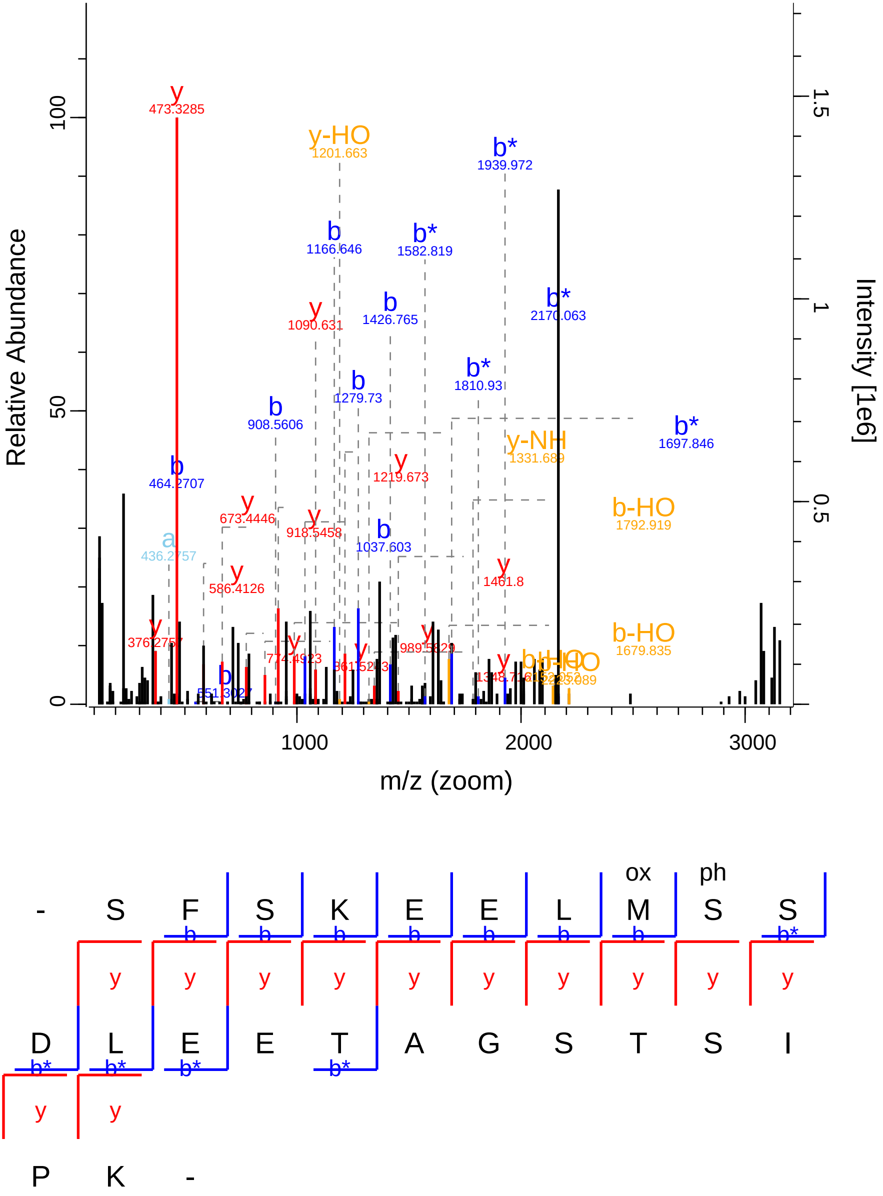

Raw File	Scan	Method	Score	m/z	Gene names
RO-QC-160121_ZackD_SS_Set_02_Fractic	27361	FTMS; HC	52.28	1095.03	ZNF777

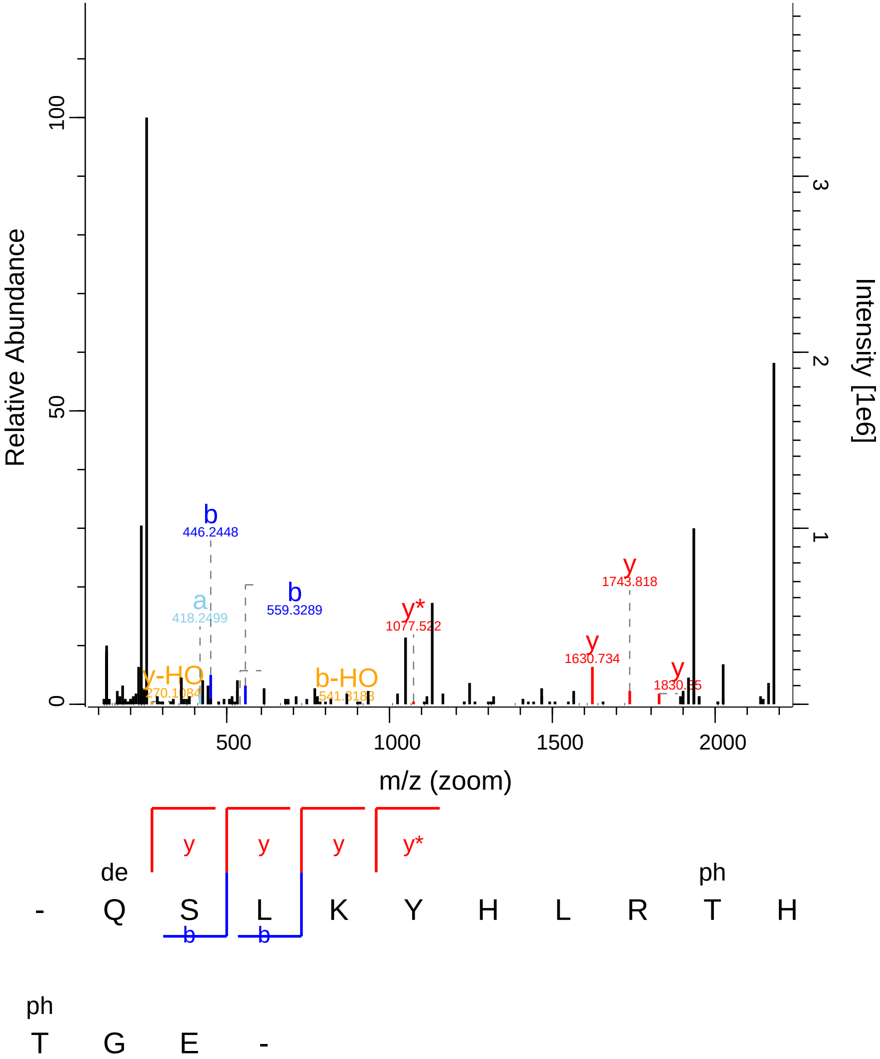

Raw File	Scan	Method	Score	m/z	Gene names
RO-QC-160121_ZackD_SS_Set_02_Fractio	27417	FTMS; HC	44.2	613.56	INADL

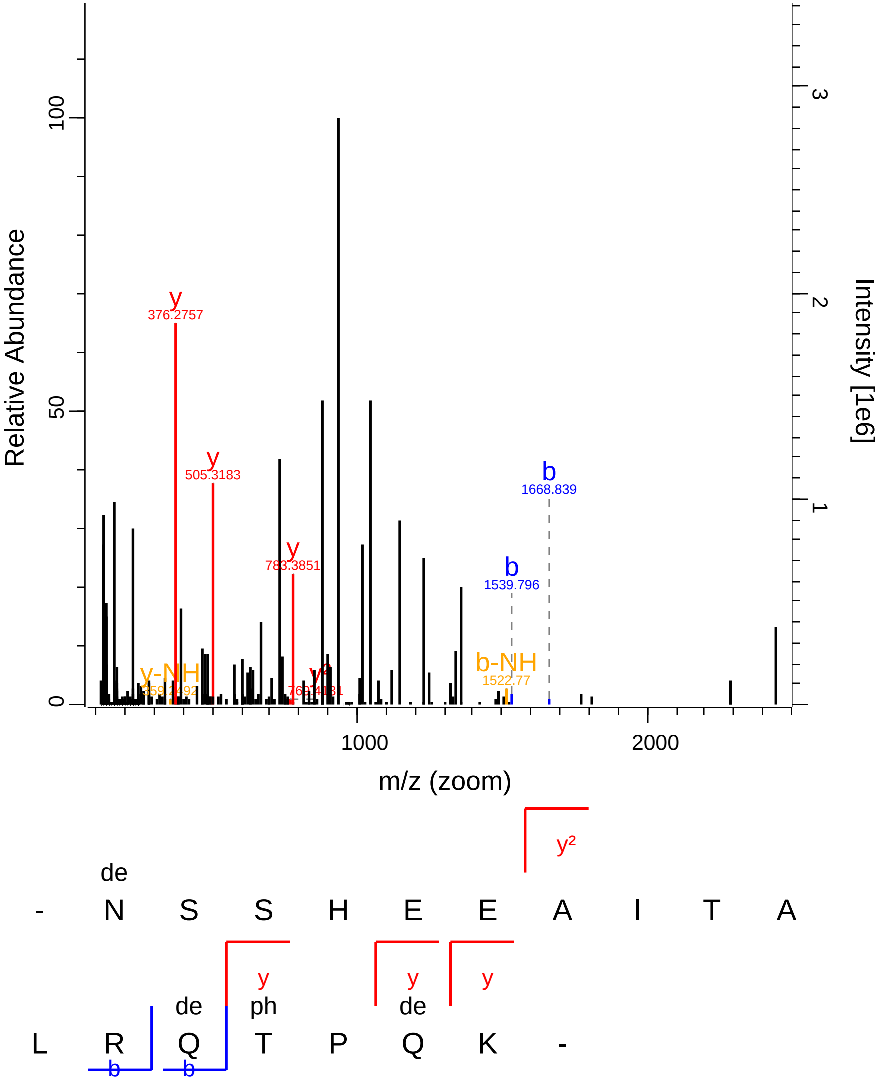

Raw File	Scan	Method	Score	m/z	Gene names
RO-QC-160121_ZackD_SS_Set_02_Fractio	27491	FTMS; HC	103.63	898.13	PGM5

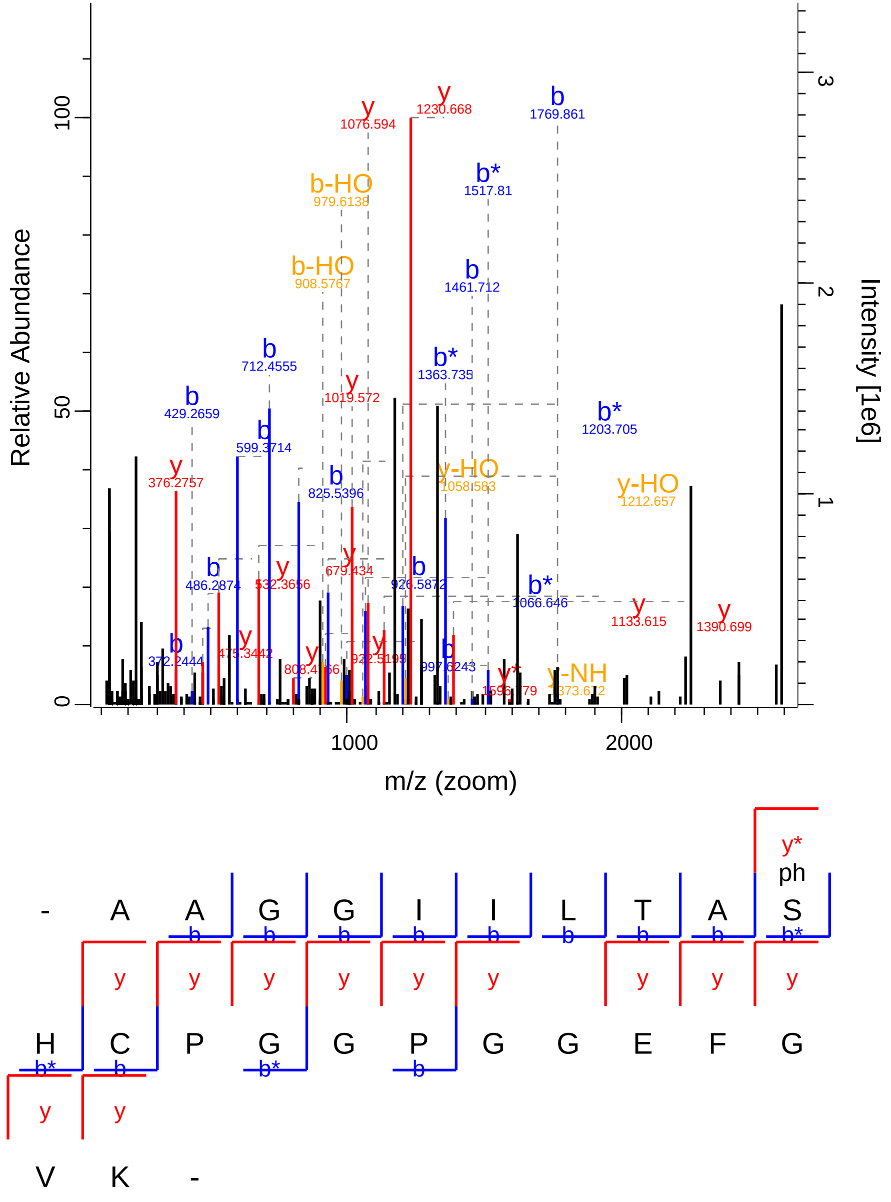

Raw File	Scan	Method	Score	m/z	Gene names
RO-QC-160121_ZackD_SS_Set_02_Fractic	27640	FTMS; HC	50.11	1226.12	LRRK1

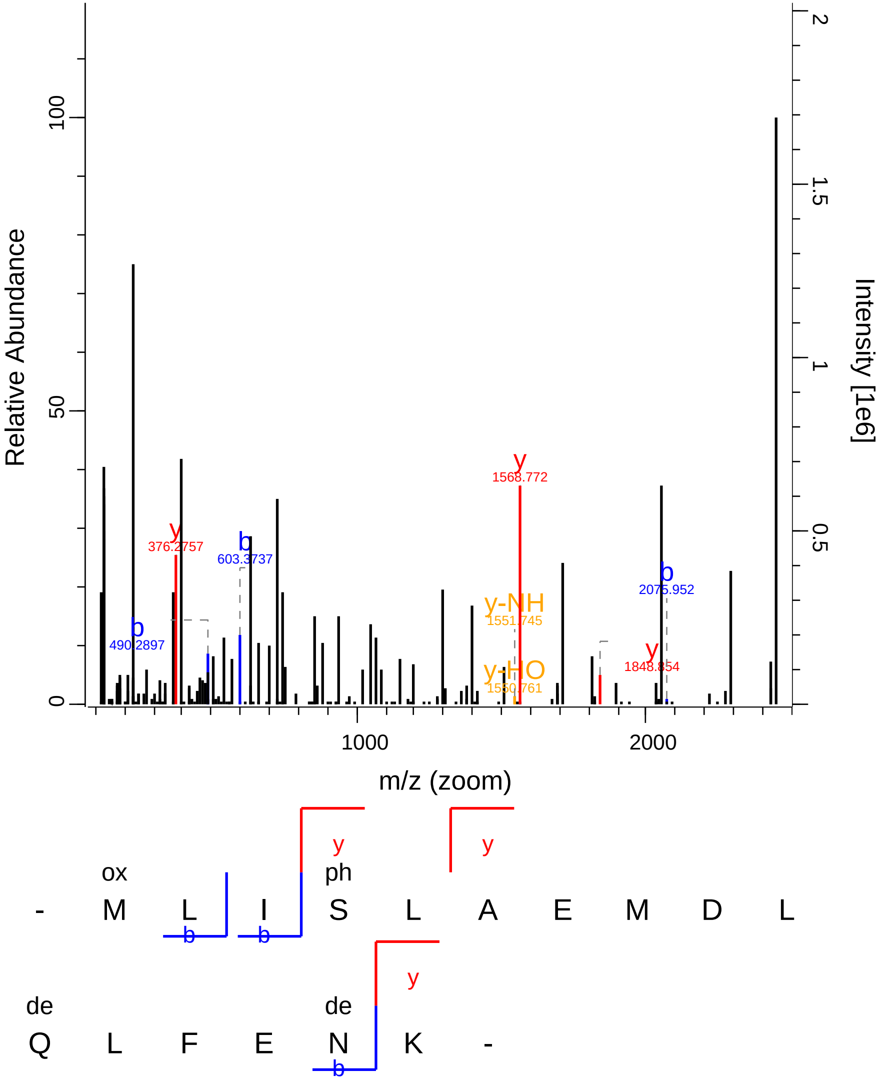

Raw File	Scan	Method	Score	m/z	Gene names
RO-QC-160121_ZackD_SS_Set_02_Fractio[illegible]	29006	FTMS; HC	47.85	908.96	CCDC180

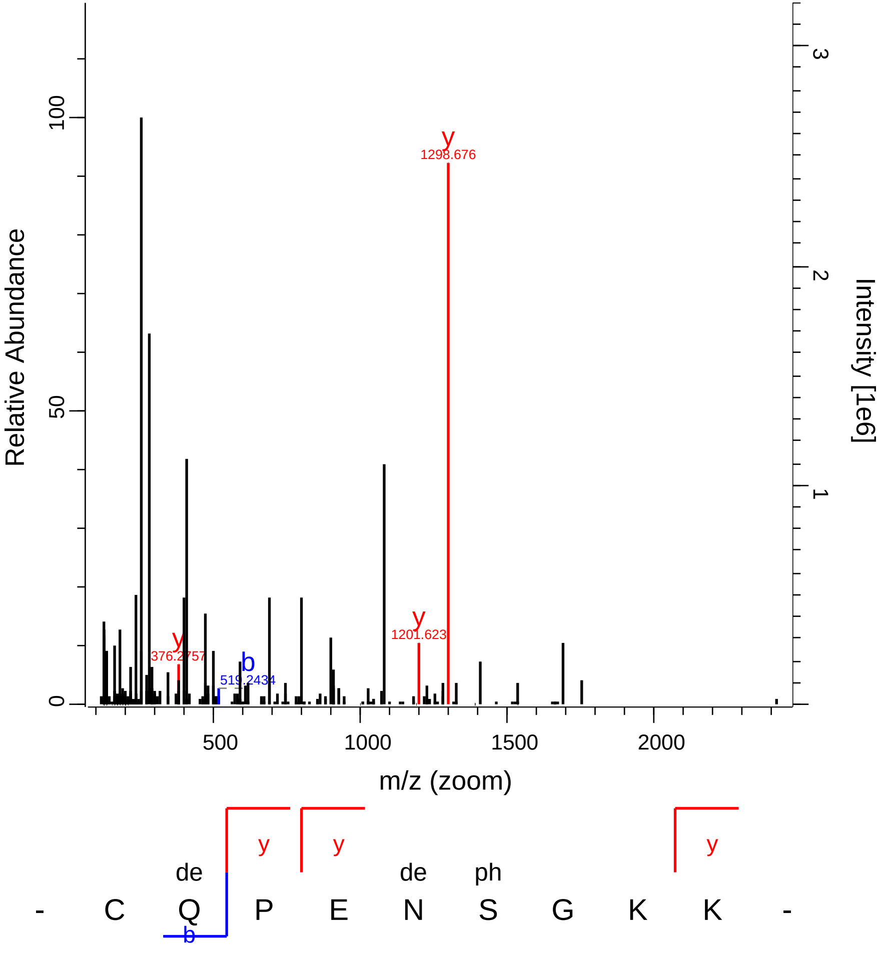

Raw File	Scan	Method	Score	m/z	Gene names
RO-QC-160121_ZackD_SS_Set_02_Fractio	29366	FTMS; HC	46.16	495.44	ACTBL2

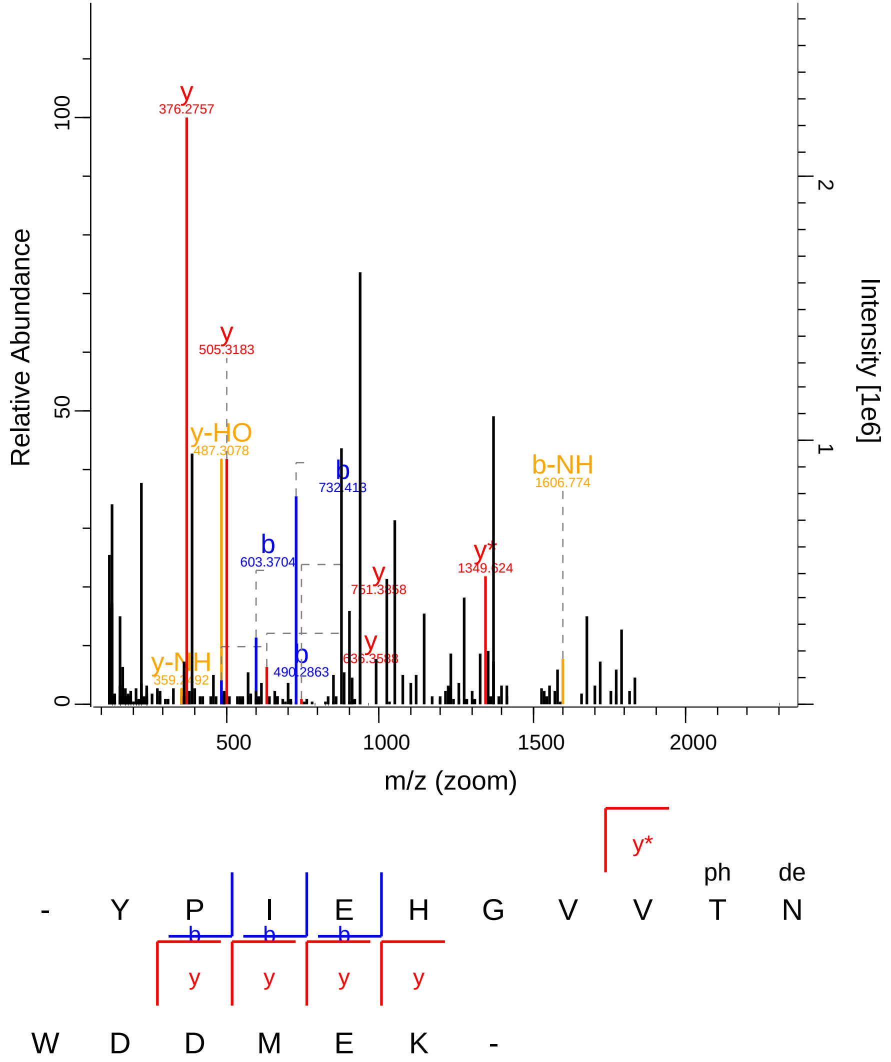

Raw File	Scan	Method	Score	m/z	Gene names
RO-QC-160121_ZackD_SS_Set_02_Fractio	30955	FTMS; HC	112.15	941.76	HDAC4

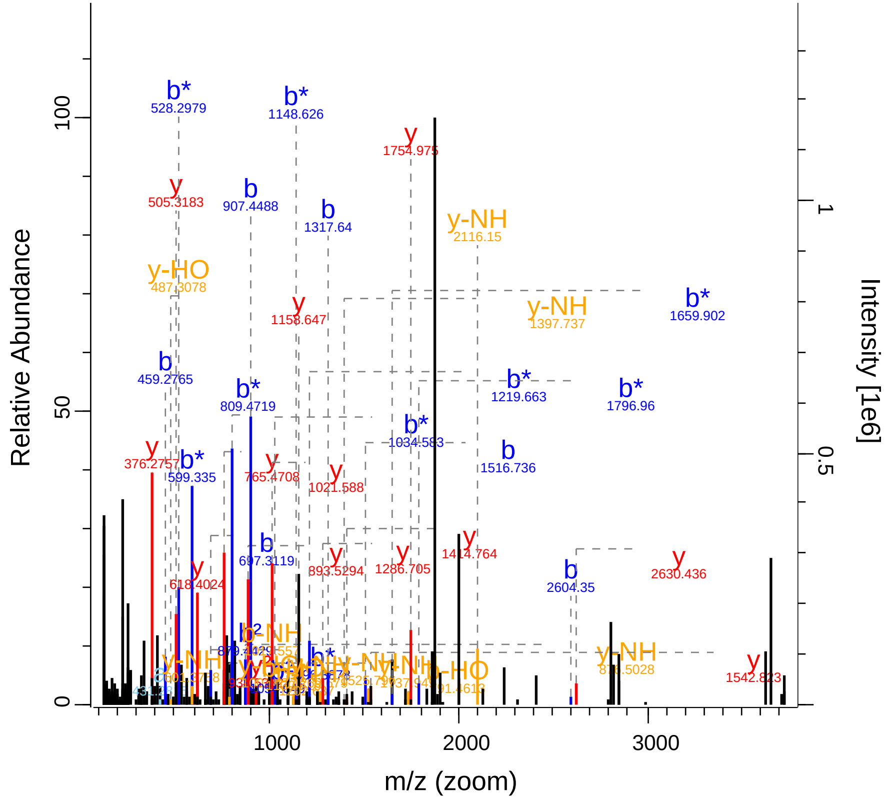

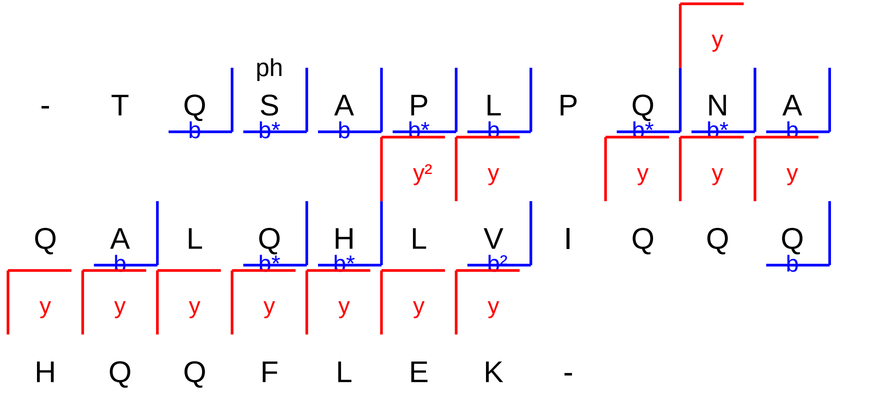

Raw File	Scan	Method	Score	m/z	Gene names
RO-QC-160121_ZackD_SS_Set_02_Fractic	31007	FTMS; HC	41.76	1046.18	ALPK3

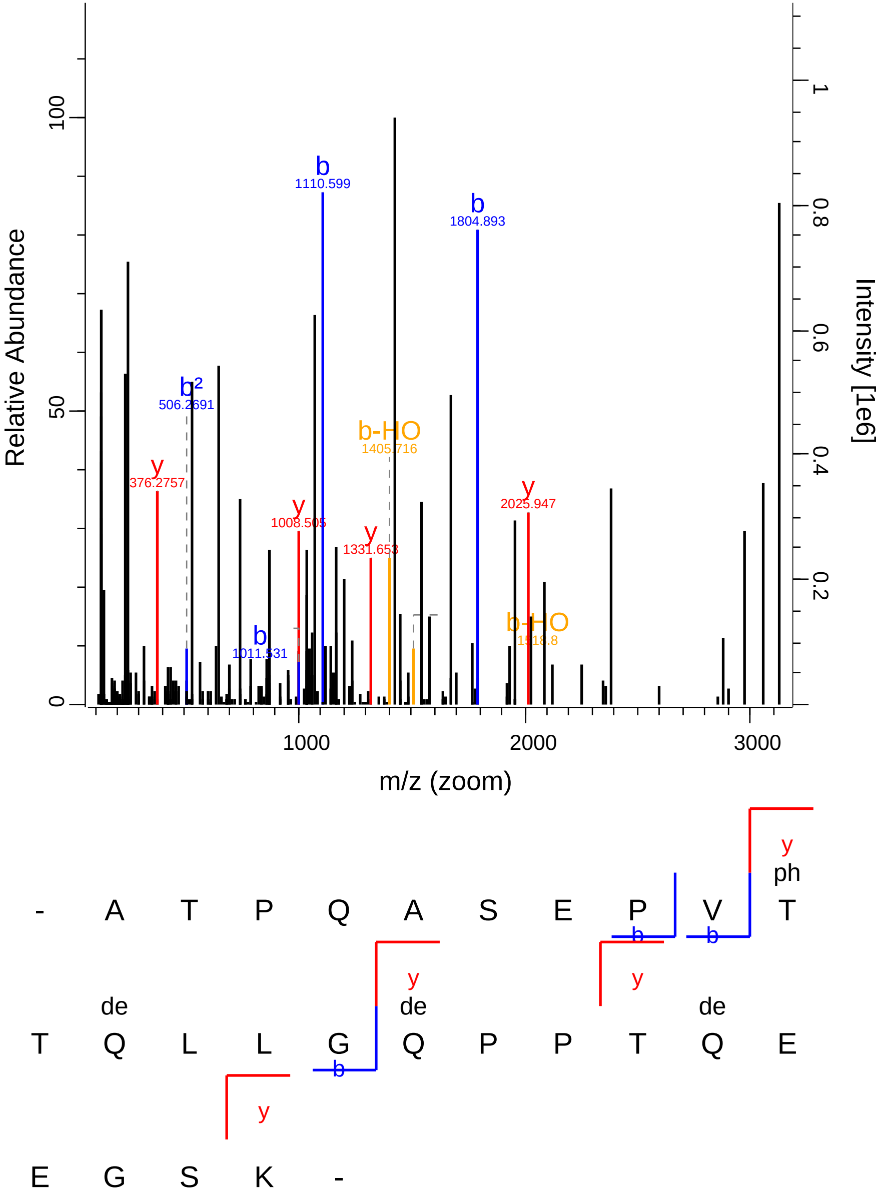

Raw File	Scan	Method	Score	m/z	Gene names
RO-QC-160121_ZackD_SS_Set_02_Fractio	32382	FTMS; HC	87.18	608.97	MTFR1L

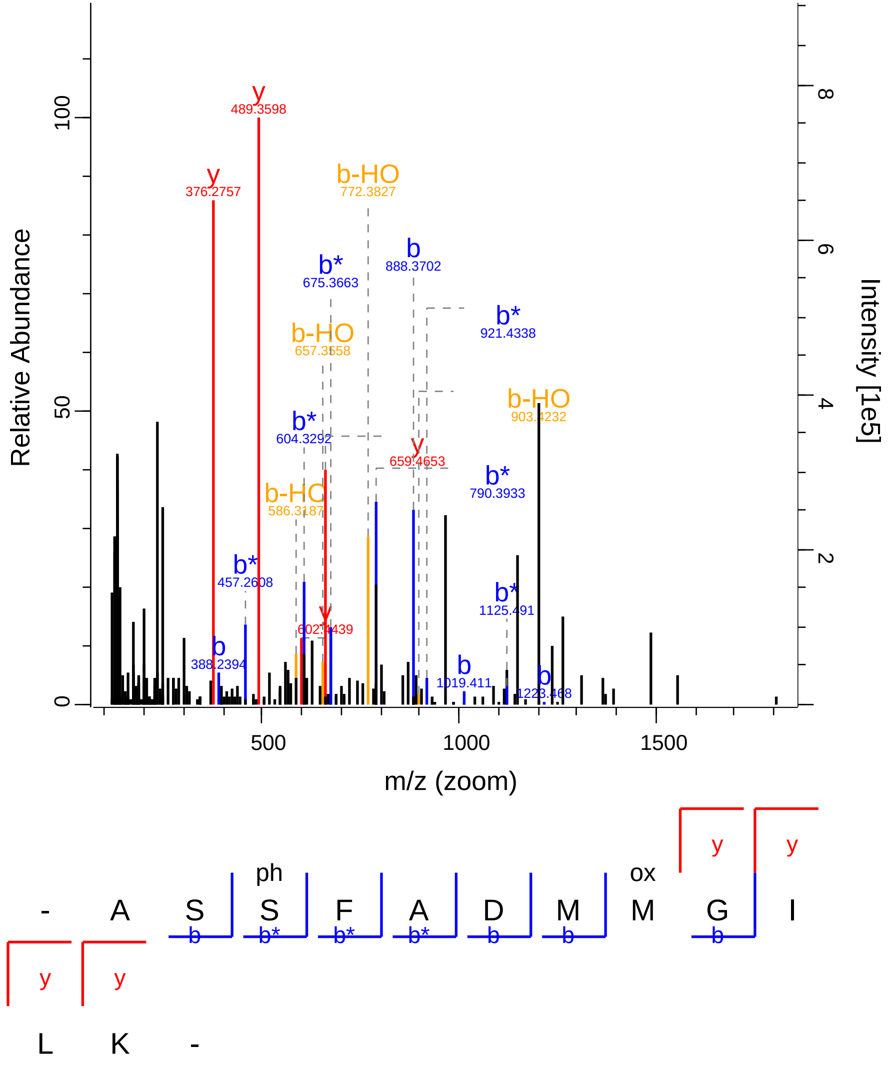

Raw File	Scan	Method	Score	m/z	Gene names
RO-QC-160121_ZackD_SS_Set_02_Fractio	32742	FTMS; HC	41.54	680.34	NPHS2

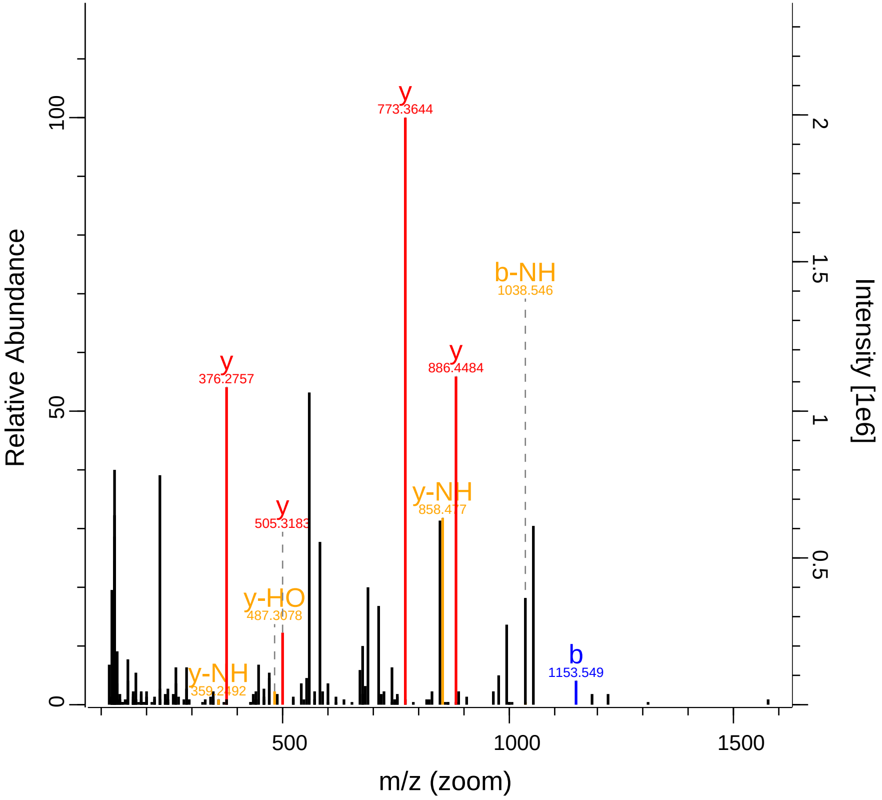

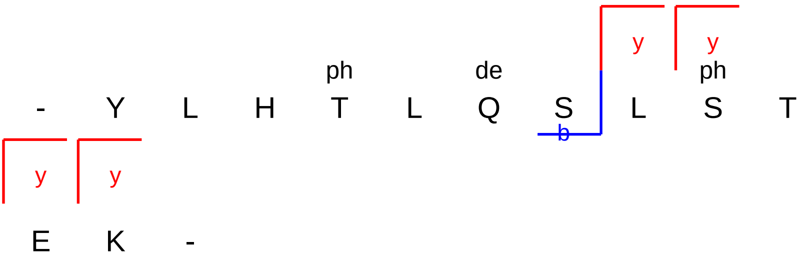

Raw File	Scan	Method	Score	m/z	Gene names
RO-QC-160121_ZackD_SS_Set_02_Fractio	32882	FTMS; HC	159.69	1014.53	AHNAK

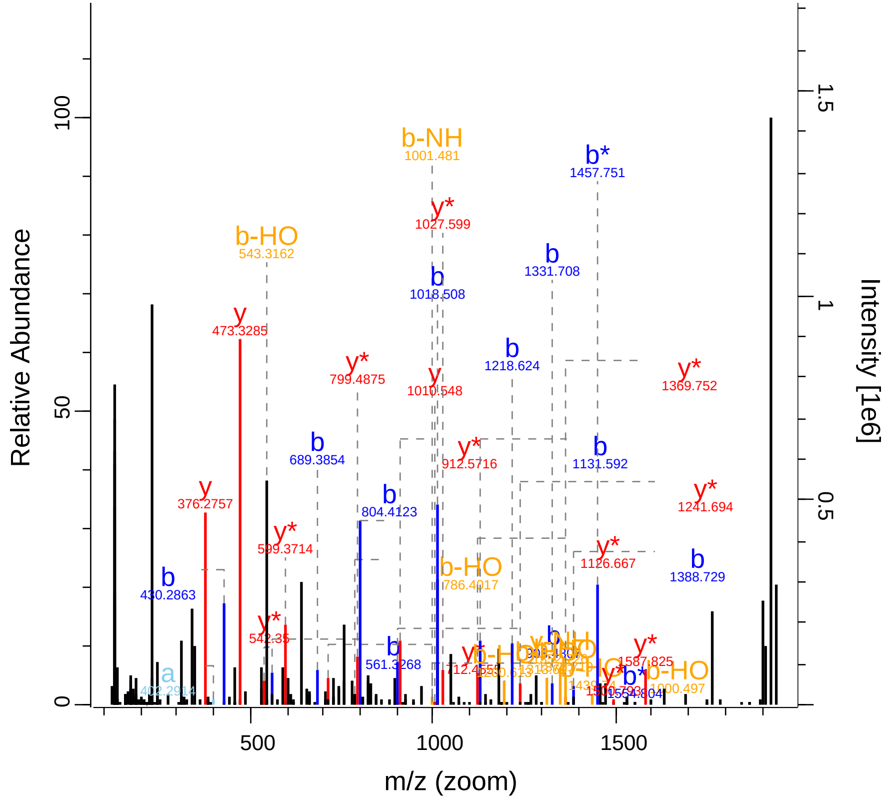

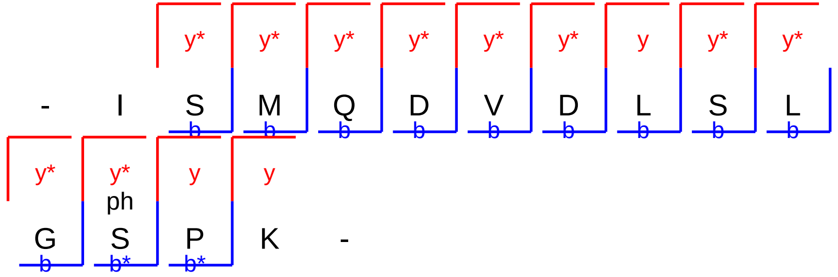

Raw File	Scan	Method	Score	m/z	Gene names
RO-QC-160121_ZackD_SS_Set_02_Fractio	33606	FTMS; HC	79.47	808.42	SRRM2

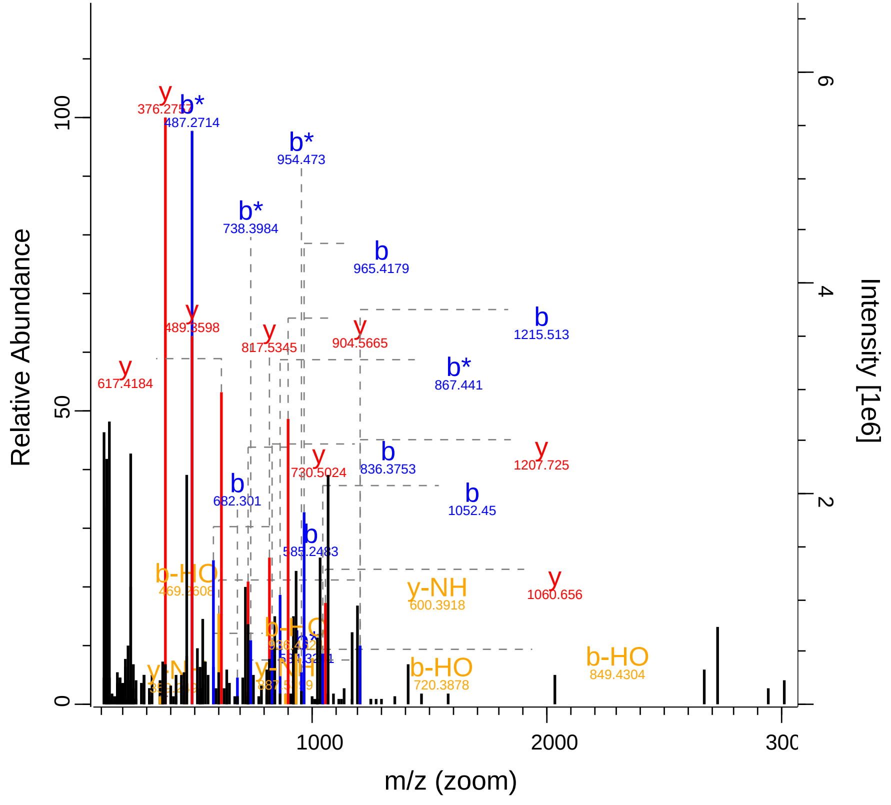

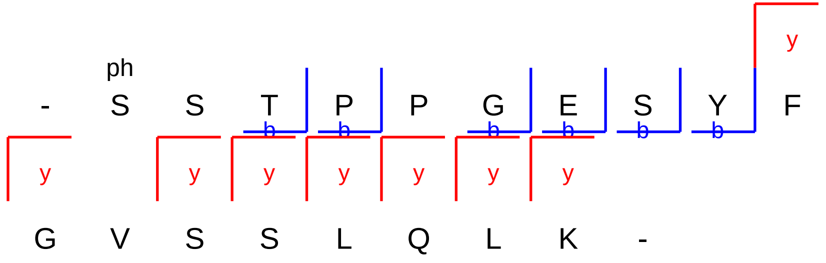

Raw File	Scan	Method	Score	m/z	Gene names
RO-QC-160121_ZackD_SS_Set_02_Fractio	33645	FTMS; HC	117.79	1212.12	SRRM2

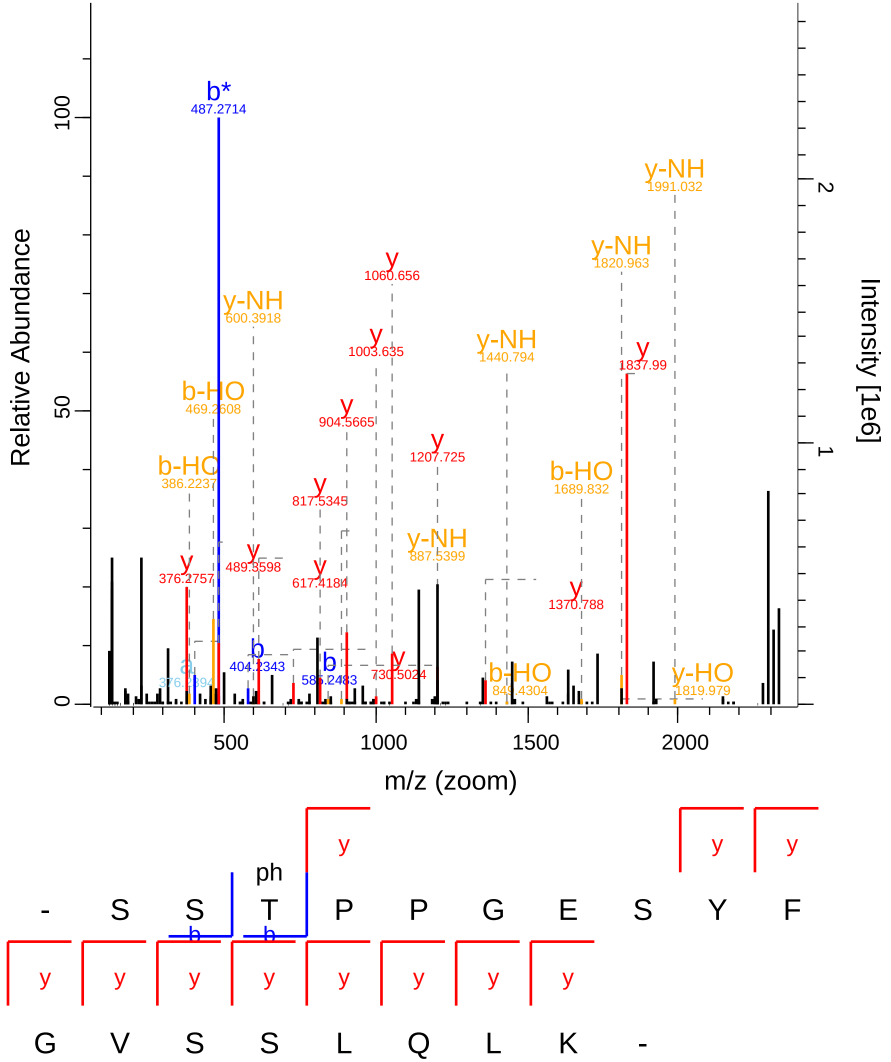

Raw File	Scan	Method	Score	m/z	Gene names
RO-QC-160121_ZackD_SS_Set_02_Fractio	34217	FTMS; HC	82.01	720.38	ANAPC1

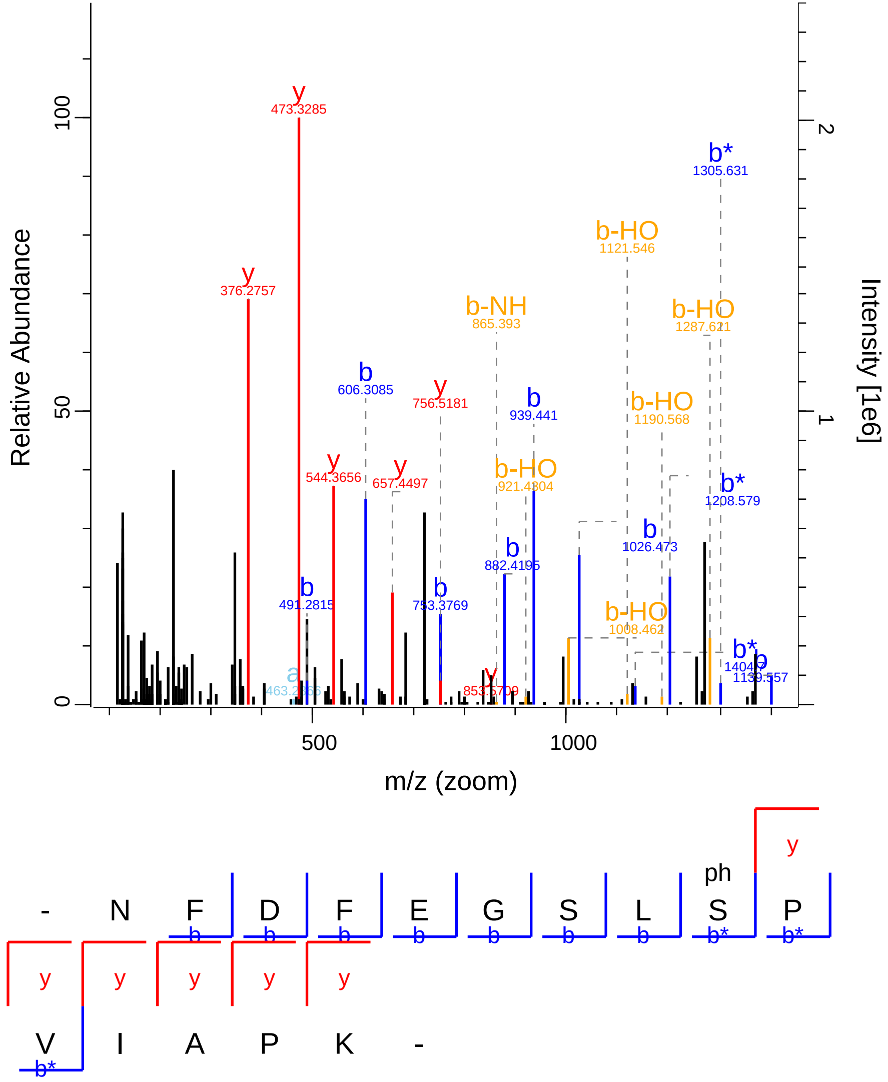

Raw File	Scan	Method	Score	m/z	Gene names
RO-QC-160121_ZackD_SS_Set_02_Fraction_	34437	FTMS; HCI	44.76	638	BCAR3

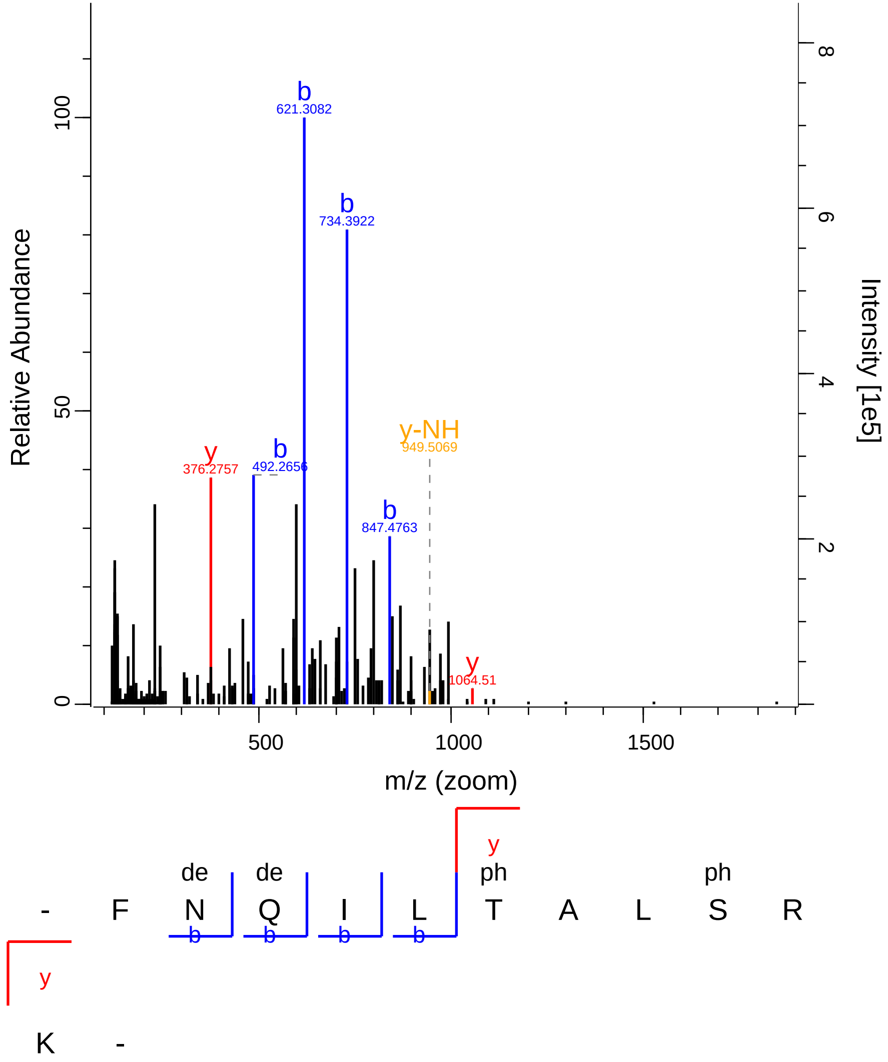

Raw File	Scan	Method	Score	m/z	Gene names
RO-QC-160121_ZackD_SS_Set_02_Fractic	34732	FTMS; HC	42.71	1122.55	KALRN

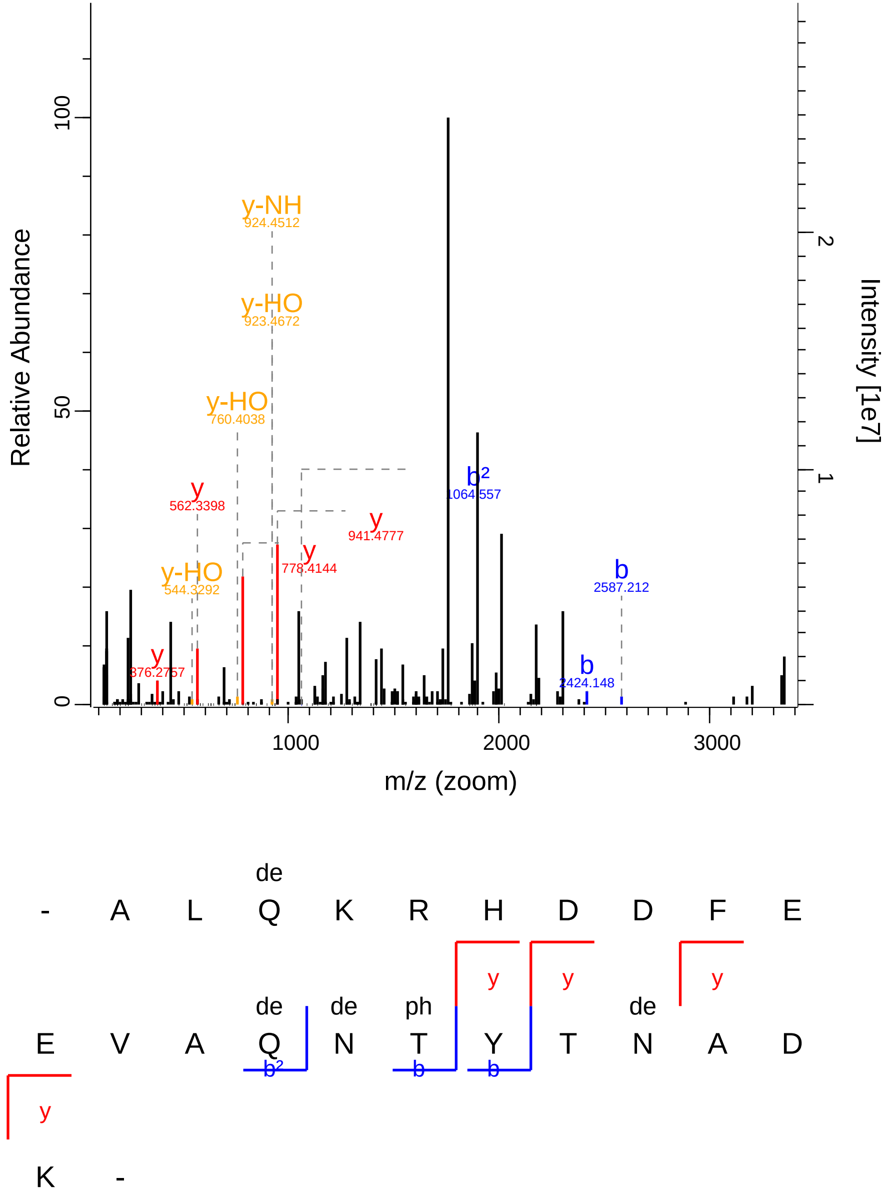

Raw File	Scan	Method	Score	m/z	Gene names
RO-QC-160121_ZackD_SS_Set_02_Fractio	35654	FTMS; HC	124.09	1092.06	FERMT2

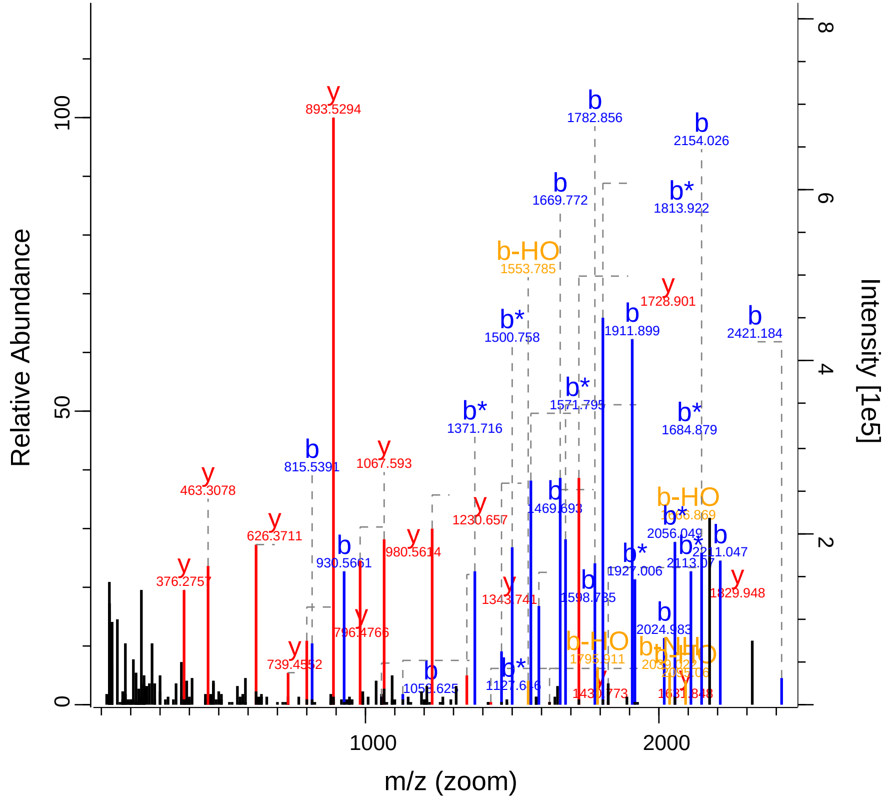

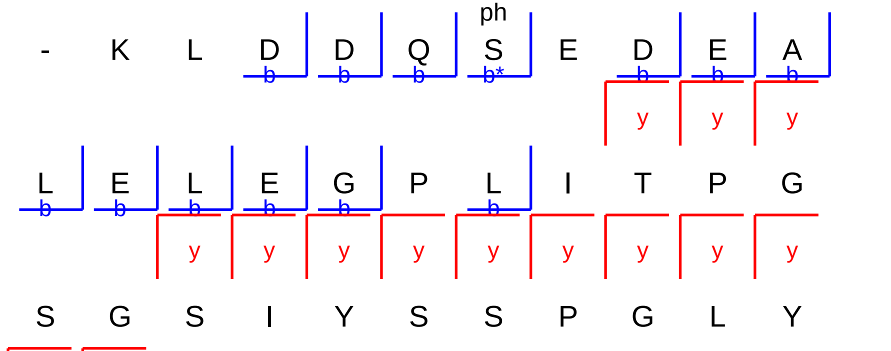

Raw File	Scan	Method	Score	m/z	Gene names
RO-QC-160121_ZackD_SS_Set_02_Fractic	35885	FTMS; HC	94.31	1145.64	SLC35A5

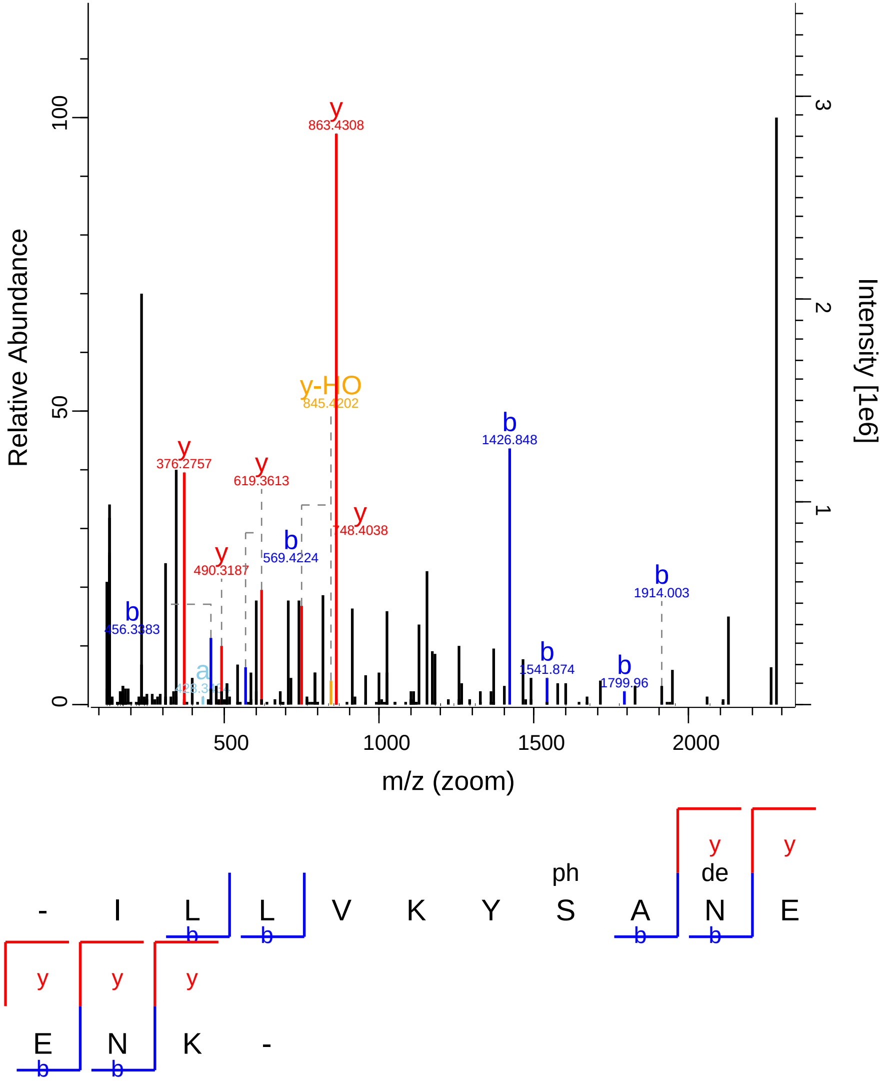

Raw File	Scan	Method	Score	m/z	Gene names
RO-QC-160121_ZackD_SS_Set_02_Fractio	36687	FTMS; HC	42.65	884.75	ANKRD26

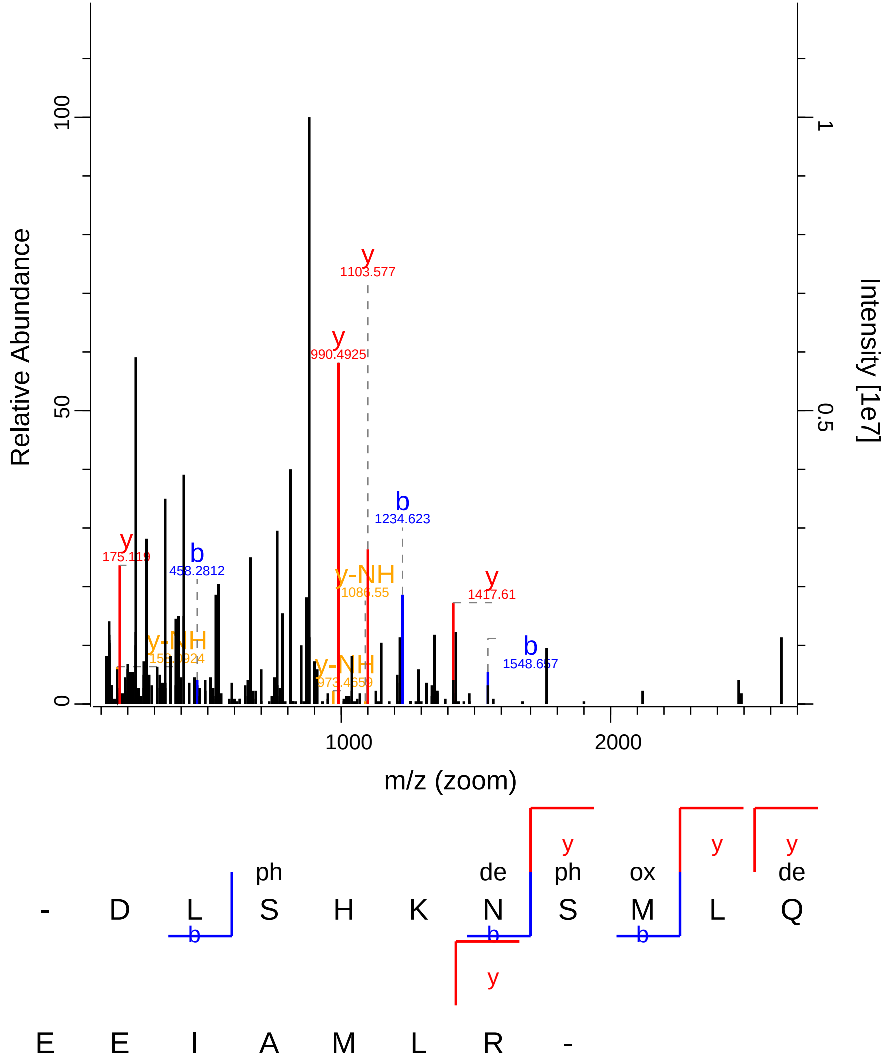

Raw File	Scan	Method	Score	m/z	Gene names
RO-QC-160121_ZackD_SS_Set_02_Fractio	36833	FTMS; HC	41.64	703.67	AHNAK

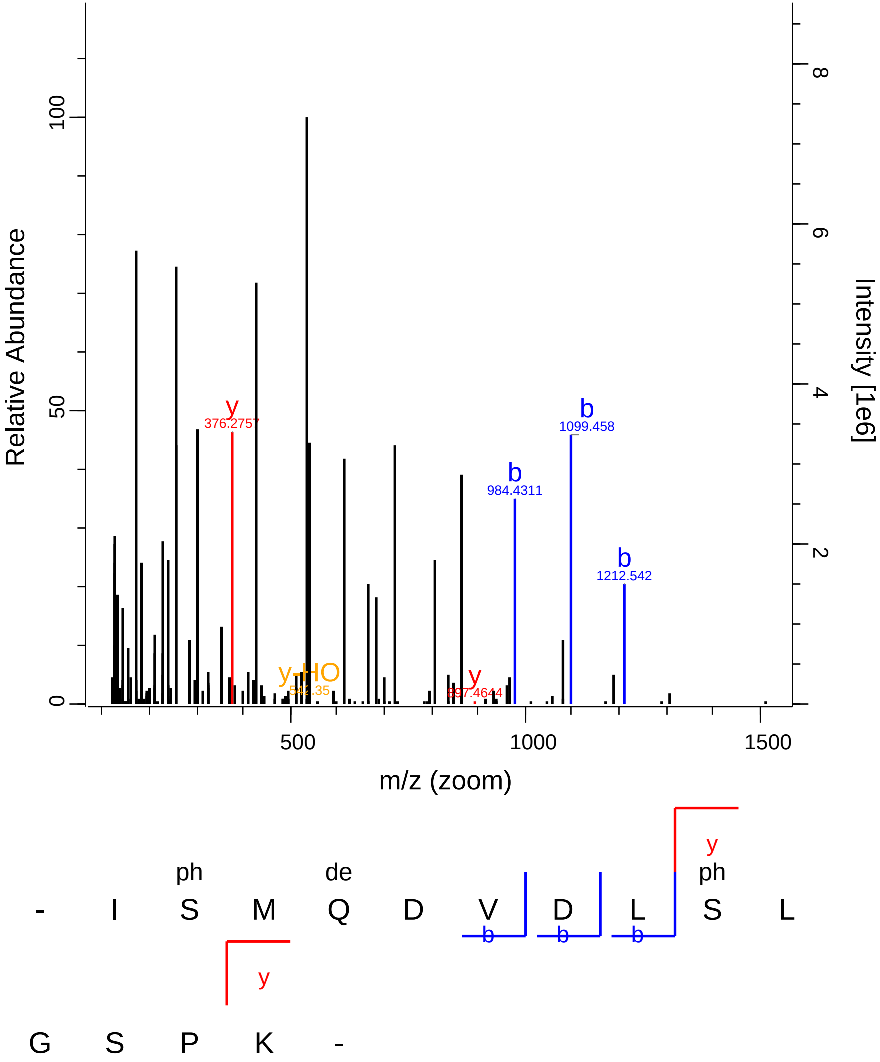

Raw File	Scan	Method	Score	m/z	Gene names
RO-QC-160121_ZackD_SS_Set_02_Fractio	37014	FTMS; HC	47.29	825.96	SMAD3

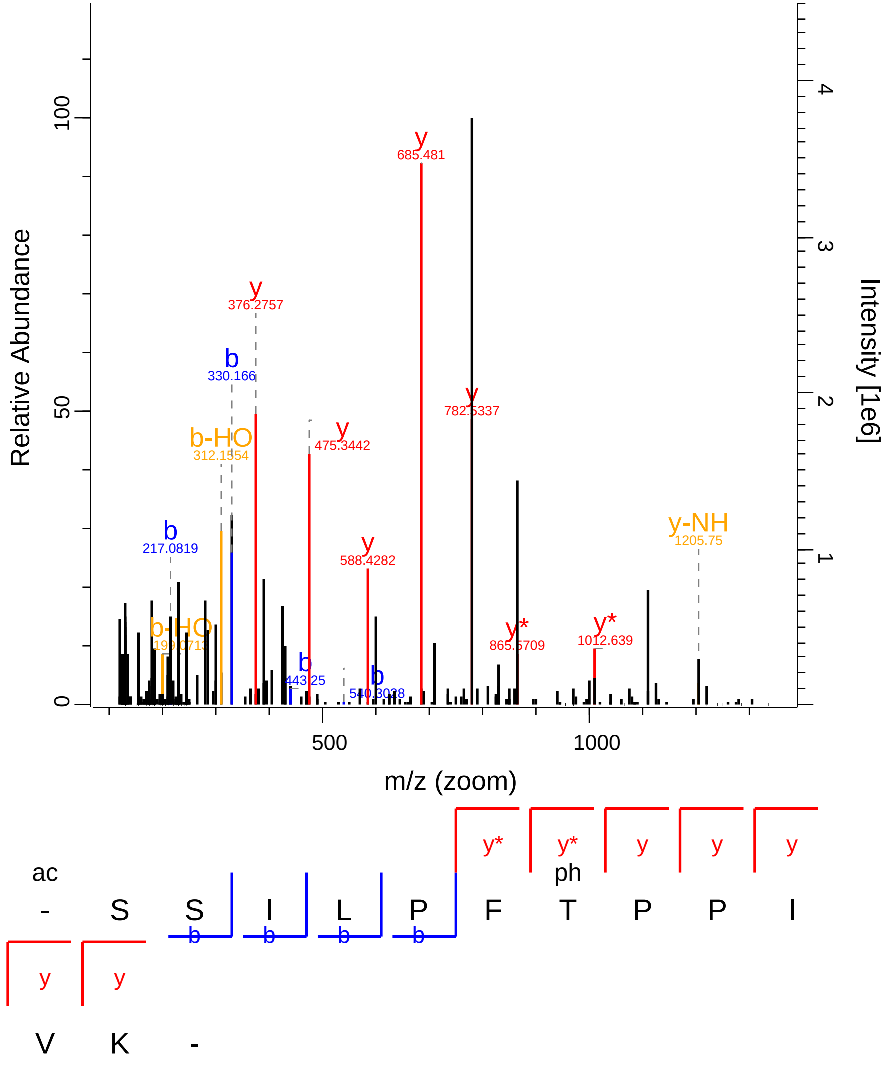

Raw File	Scan	Method	Score	m/z	Gene names
RO-QC-160121_ZackD_SS_Set_02_Fractio	37175	FTMS; HC	55.26	698.05	YWHAE

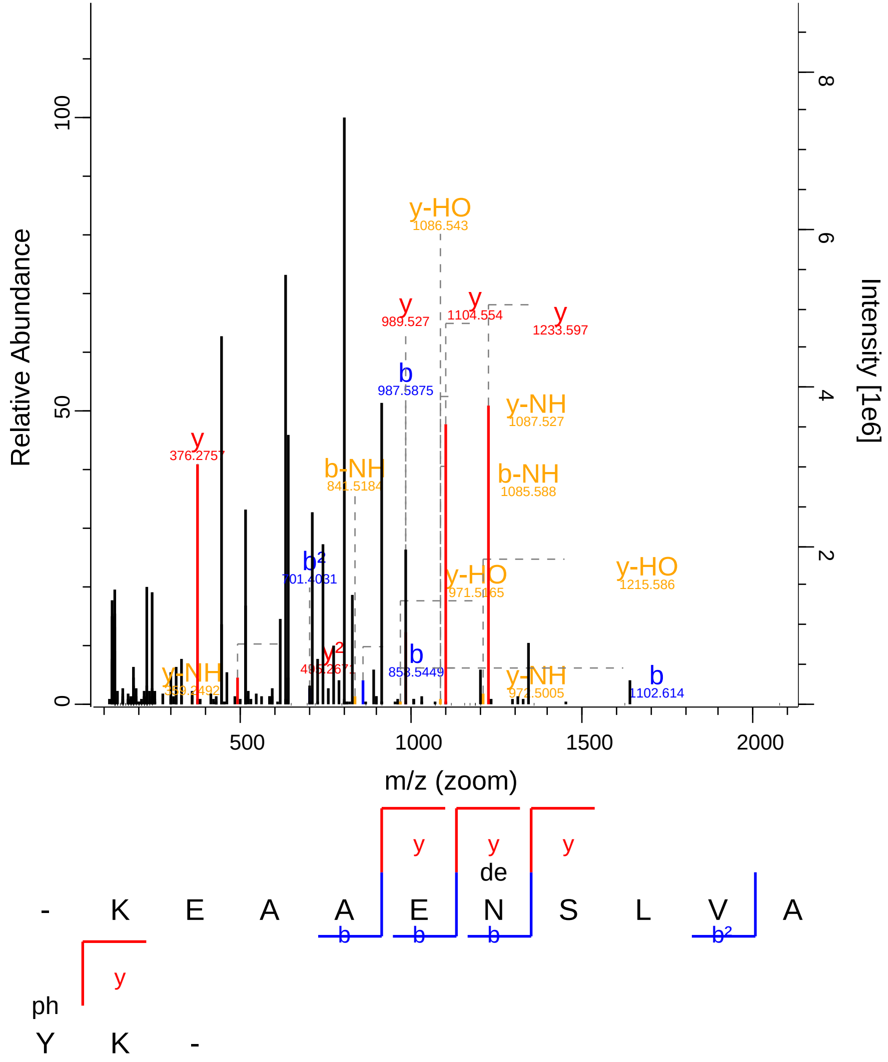

Raw File	Scan	Method	Score	m/z	Gene names
RO-QC-160121_ZackD_SS_Set_02_Fra	37422	FTMS; HC	74.44	791.88	HDAC1;HDAC2;HDA

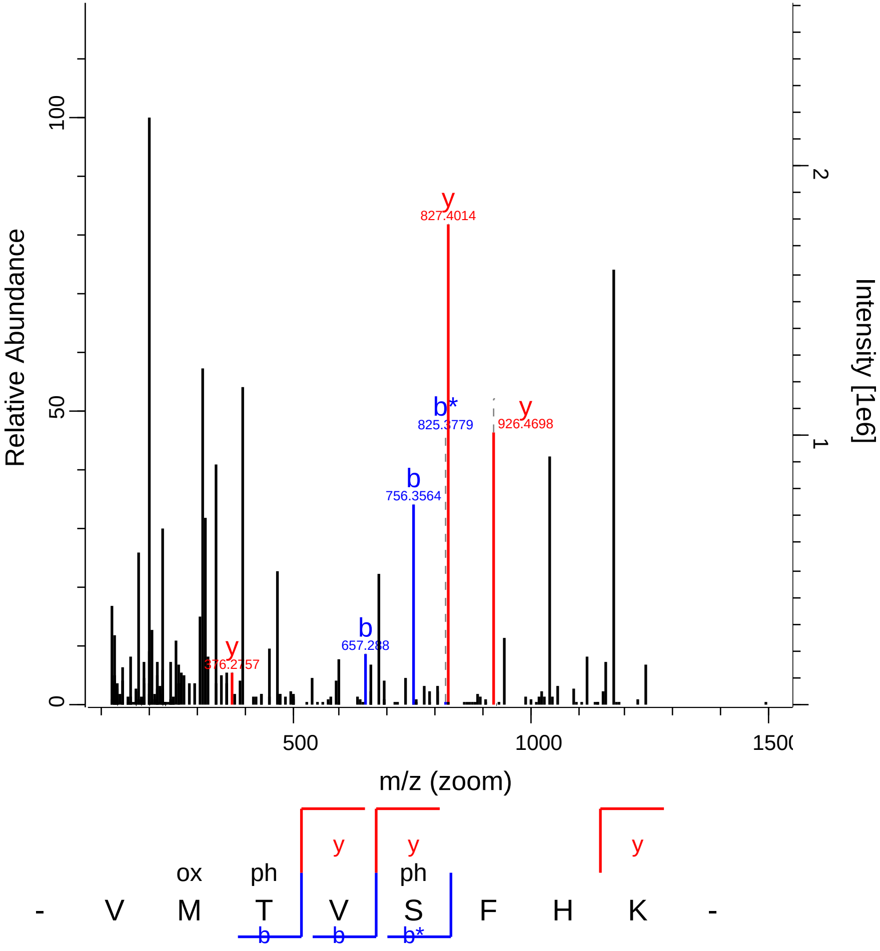

Raw File	Scan	Method	Score	m/z	Gene names
RO-QC-160121_ZackD_SS_Set_02_Fractio	5277	FTMS; HC	113.54	749.58	AES

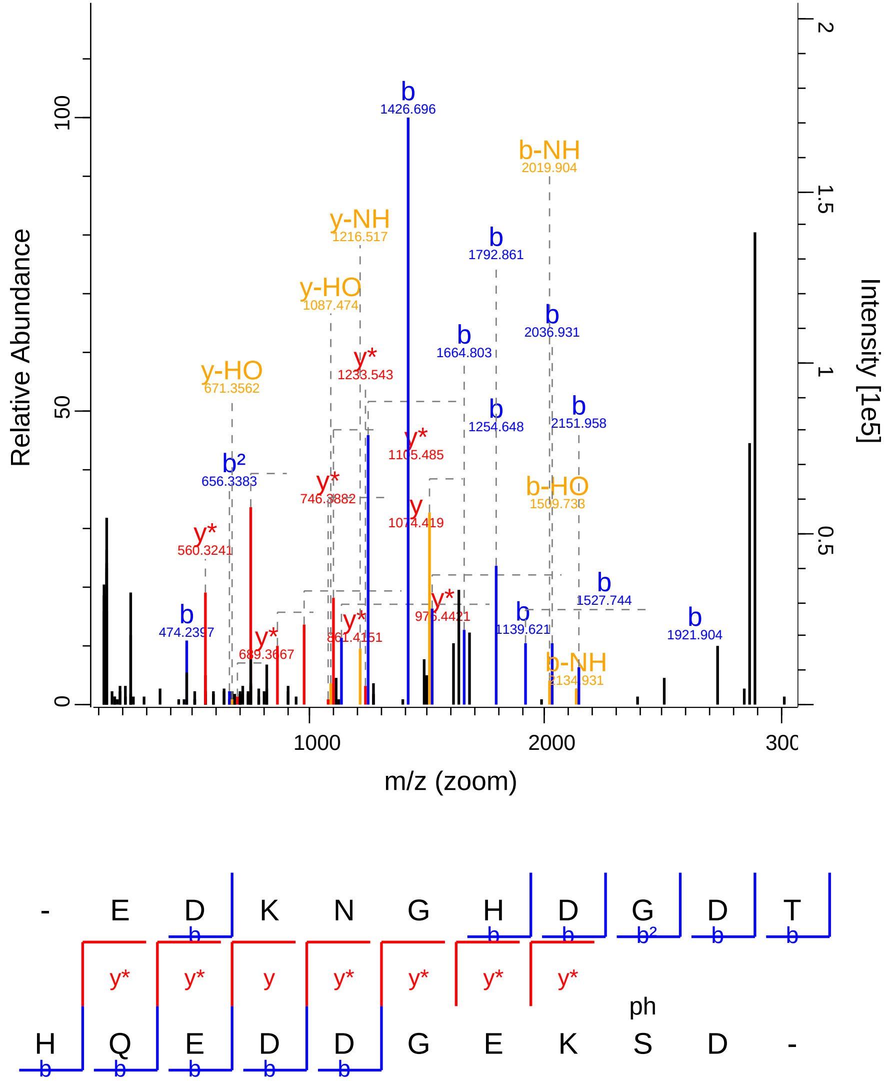

Raw File	Scan	Method	Score	m/z	Gene names
RO-QC-160121_ZackD_SS_Set_02_Fraction	5638	FTMS; HCI	52.79	440.5	FAM111B

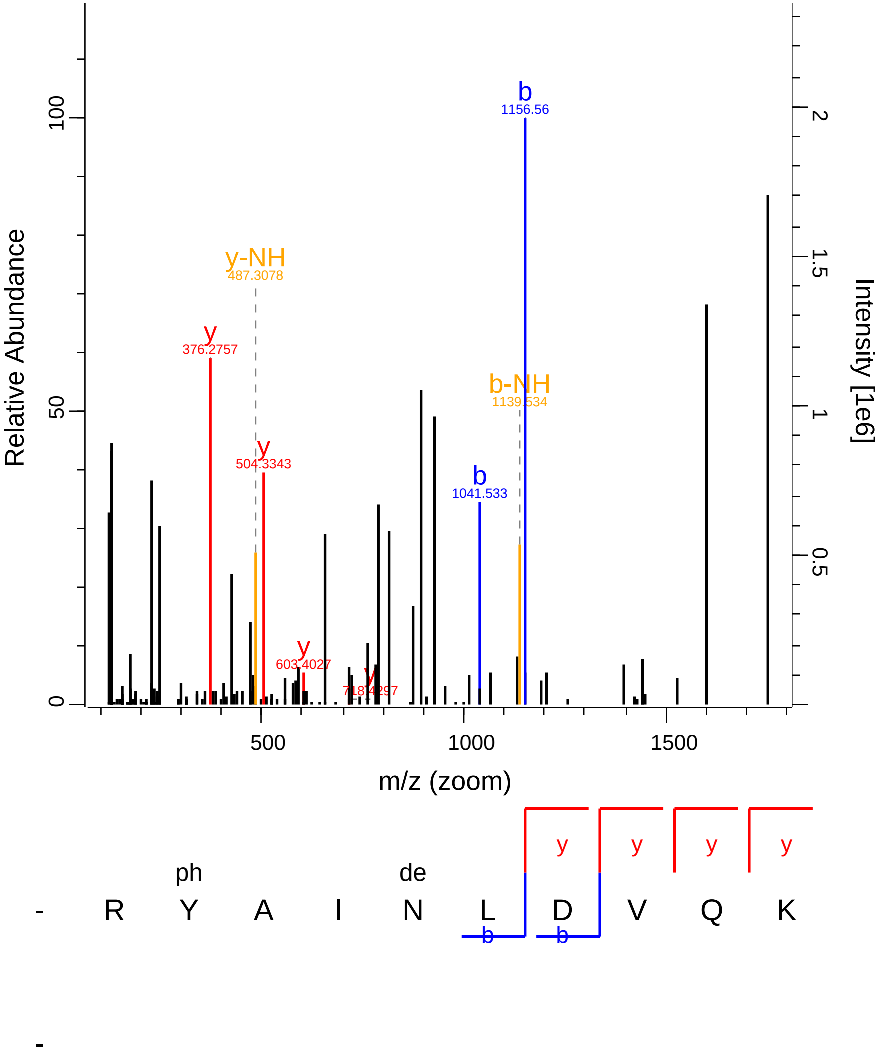

Raw File	Scan	Method	Score	m/z	Gene names
RO-QC-160121_ZackD_SS_Set_02_Fractio	5883	FTMS; HC	135.24	633.32	SRPK2

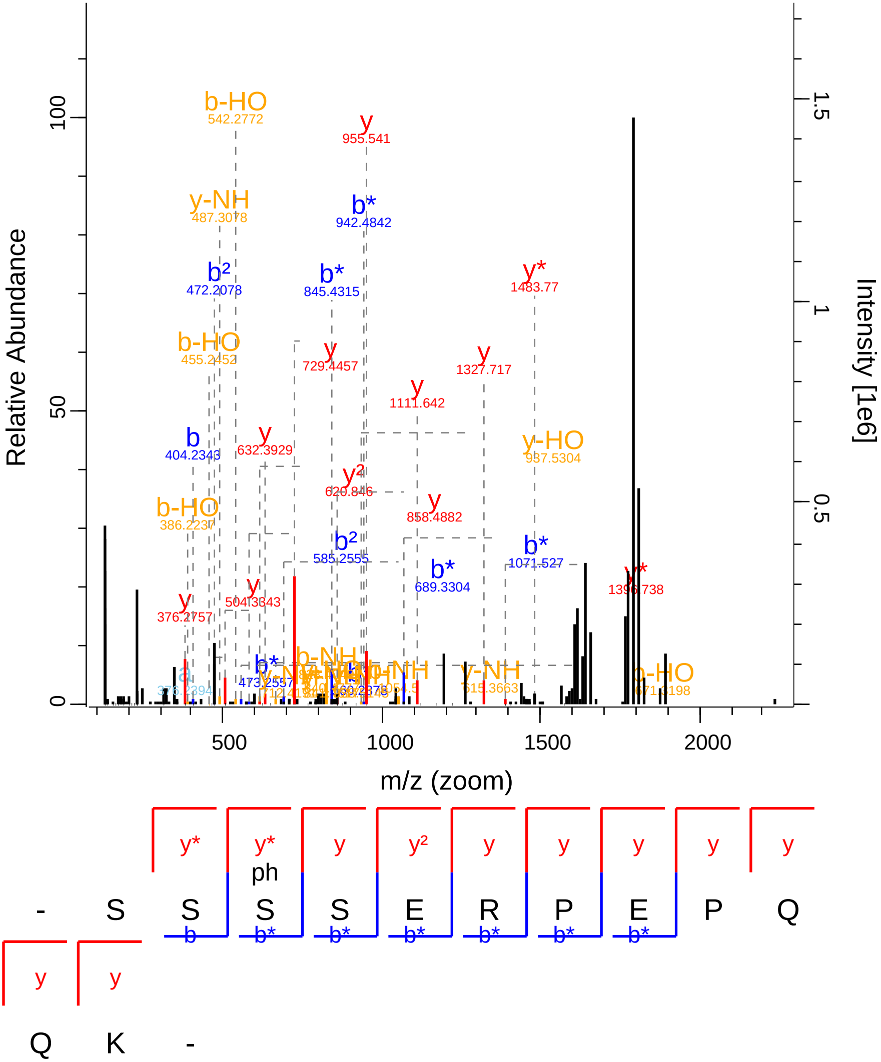

Raw File	Scan	Method	Score	m/z	Gene names
RO-QC-160121_ZackD_SS_Set_02_Fractio[illegible]	6545	FTMS; HC	116.65	740.68	EIF4B

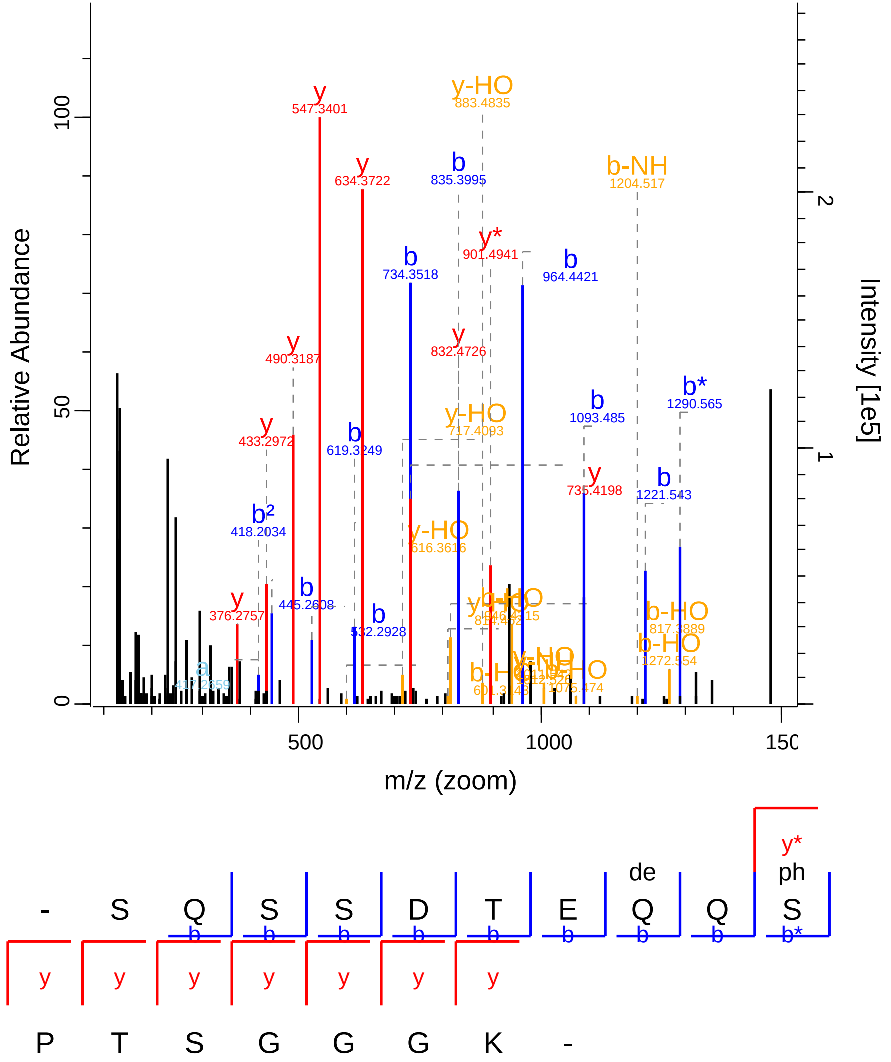

Raw File	Scan	Method	Score	m/z	Gene names
RO-QC-160121_ZackD_SS_Set_02_Fractior	6698	FTMS; HCI	78.1	453.51	AHCTF1

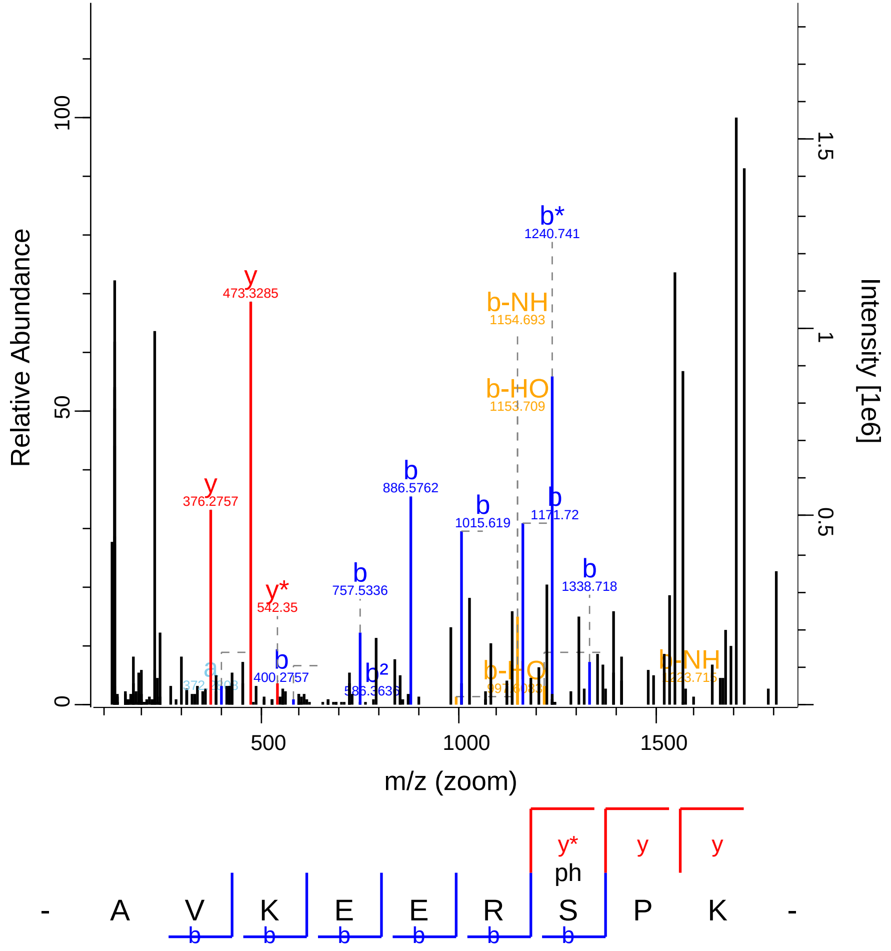

Raw File	Scan	Method	Score	m/z	Gene names
RO-QC-160121_ZackD_SS_Set_02_Fractior	7506	FTMS; HCI	66	716.07	SRSF4

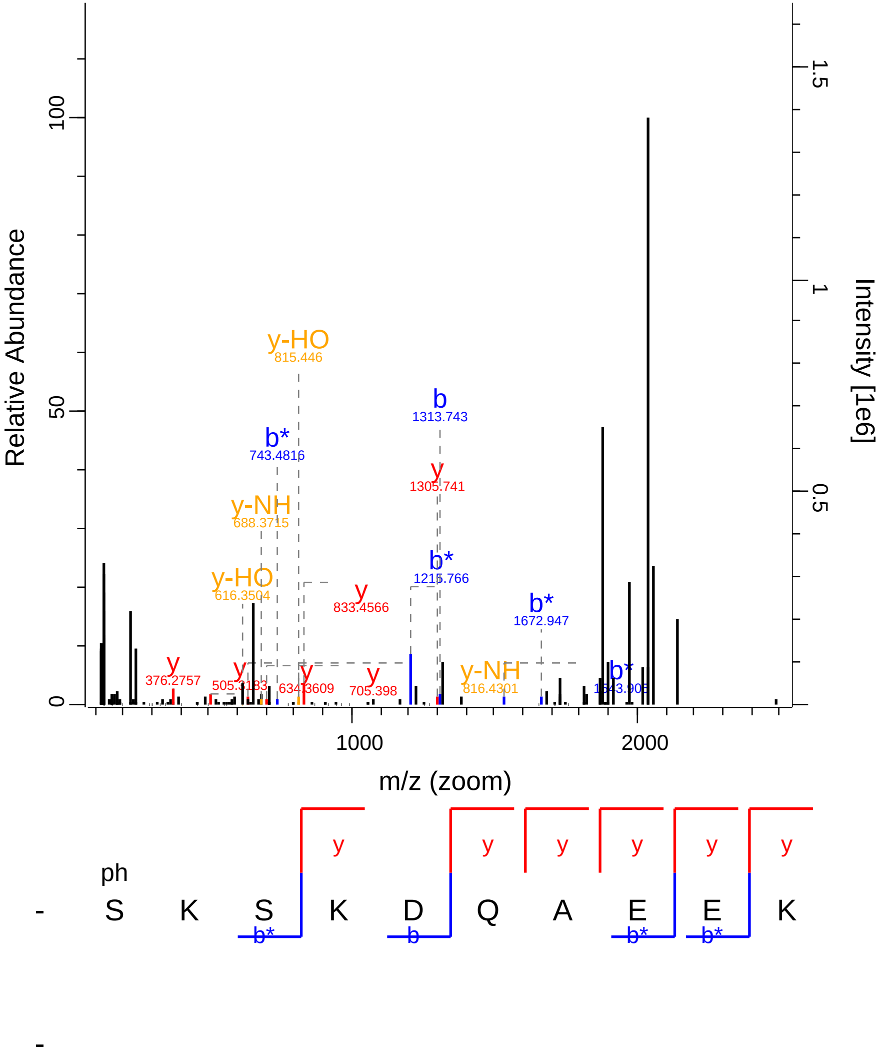

Raw File	Scan	Method	Score	m/z	Gene names
RO-QC-160121_ZackD_SS_Set_02_Fractior	8740	FTMS; HCI	78.32	666.06	PPIG

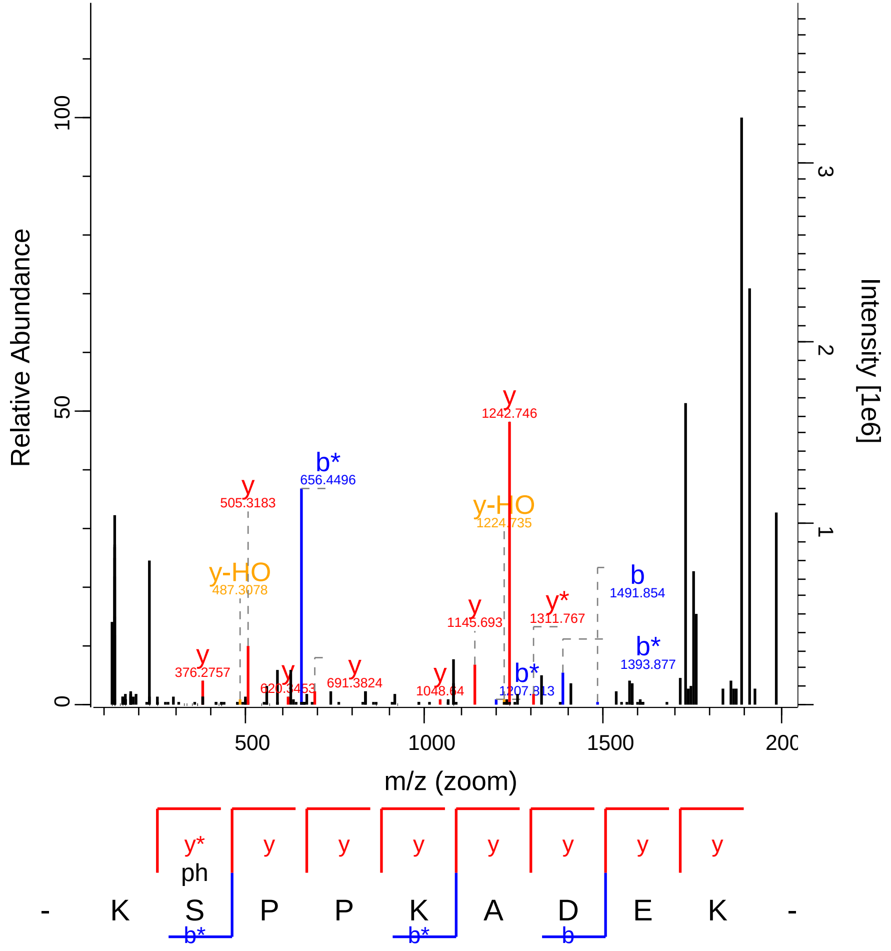

Raw File	Scan	Method	Score	m/z	Gene names
RO-QC-160121_ZackD_SS_Set_02_Fractic	10982	FTMS; HC	150.49	1023.56	PPIG

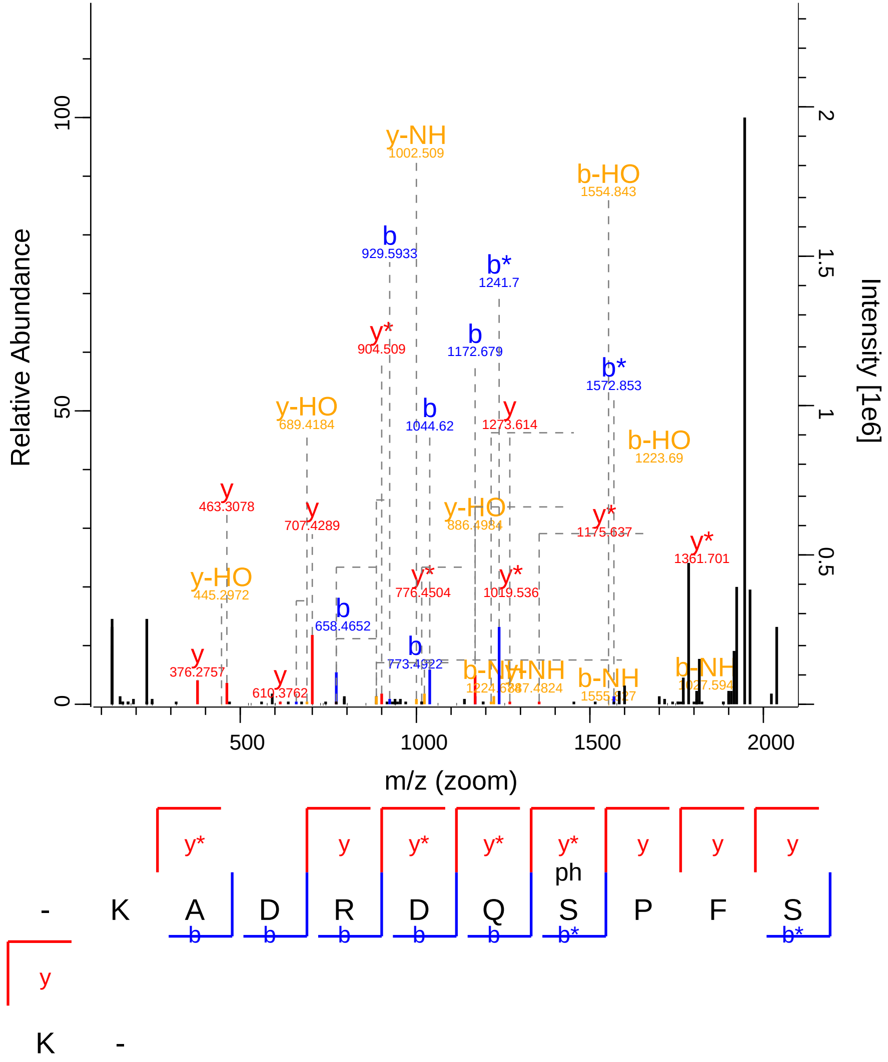

Raw File	Scan	Method	Score	m/z	Gene names
RO-QC-160121_ZackD_SS_Set_02_Fractio	12361	FTMS; HC	63.15	365.56	DIAPH2

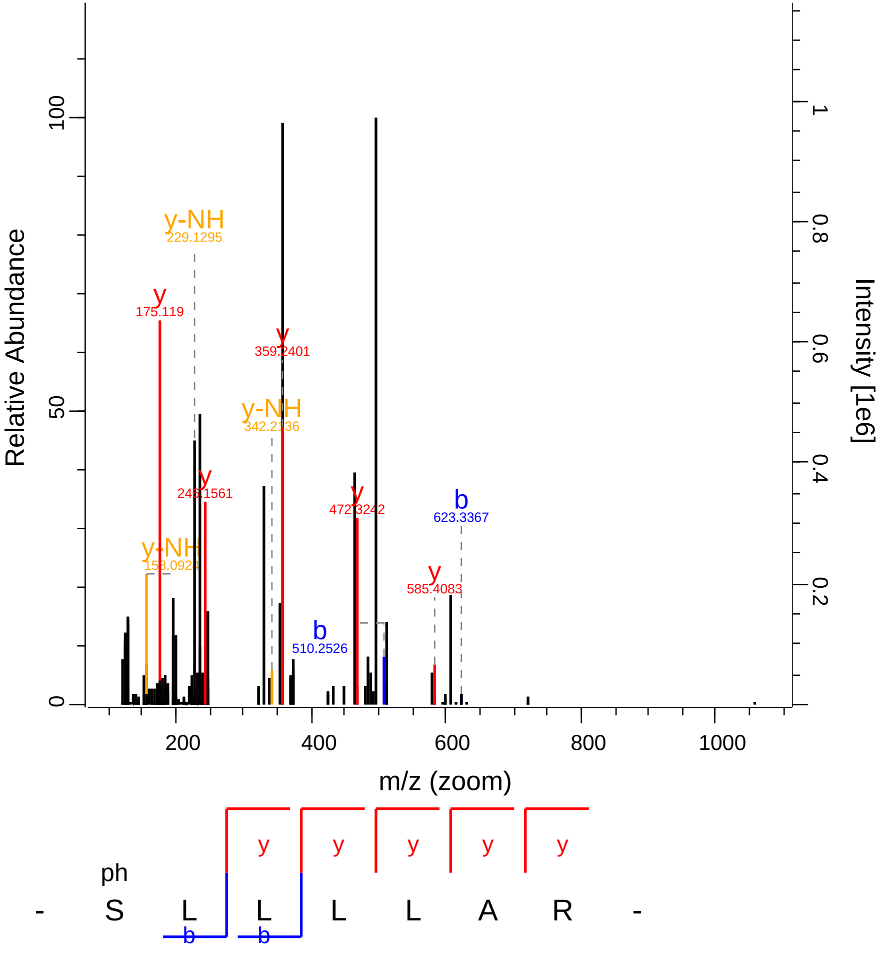

Raw File	Scan	Method	Score	m/z	Gene names
RO-QC-160121_ZackD_SS_Set_02_Fractio	12650	FTMS; HC	84.36	915.53	MAP4

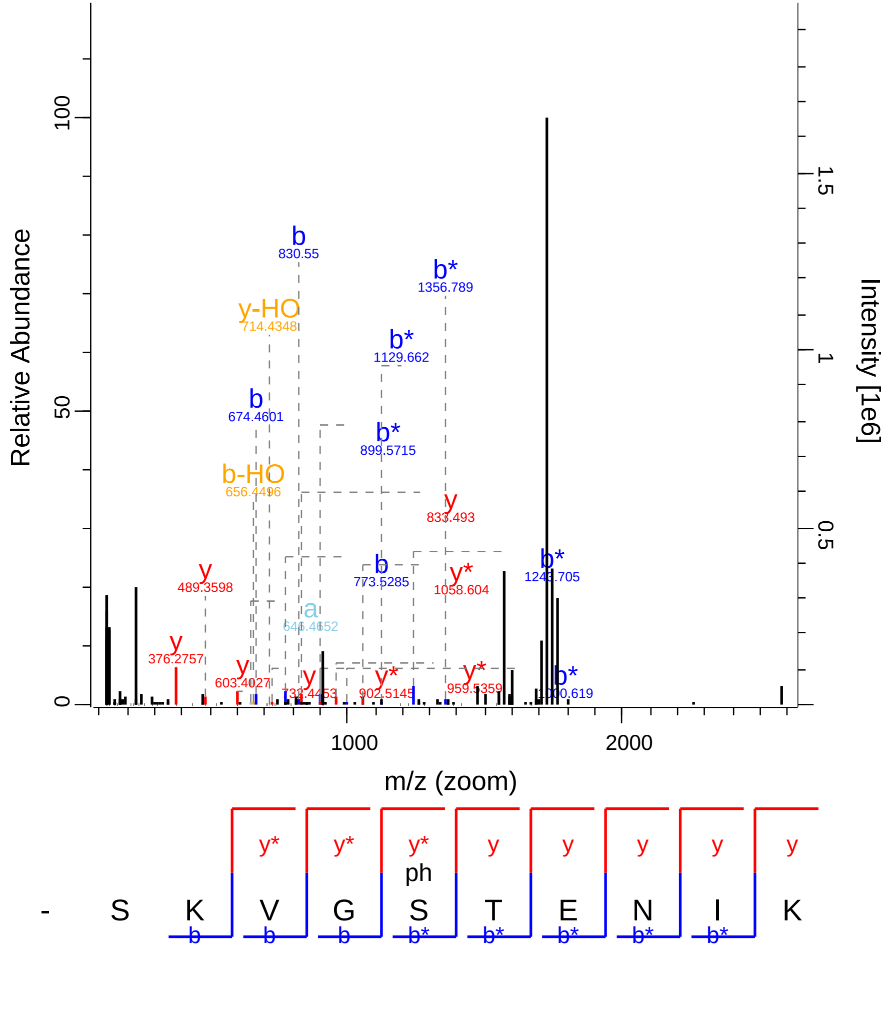

Raw File	Scan	Method	Score	m/z	Gene names
RO-QC-160121_ZackD_SS_Set_02_Fractioı	12670	FTMS; HCI	129.24	819.2	XRCC1

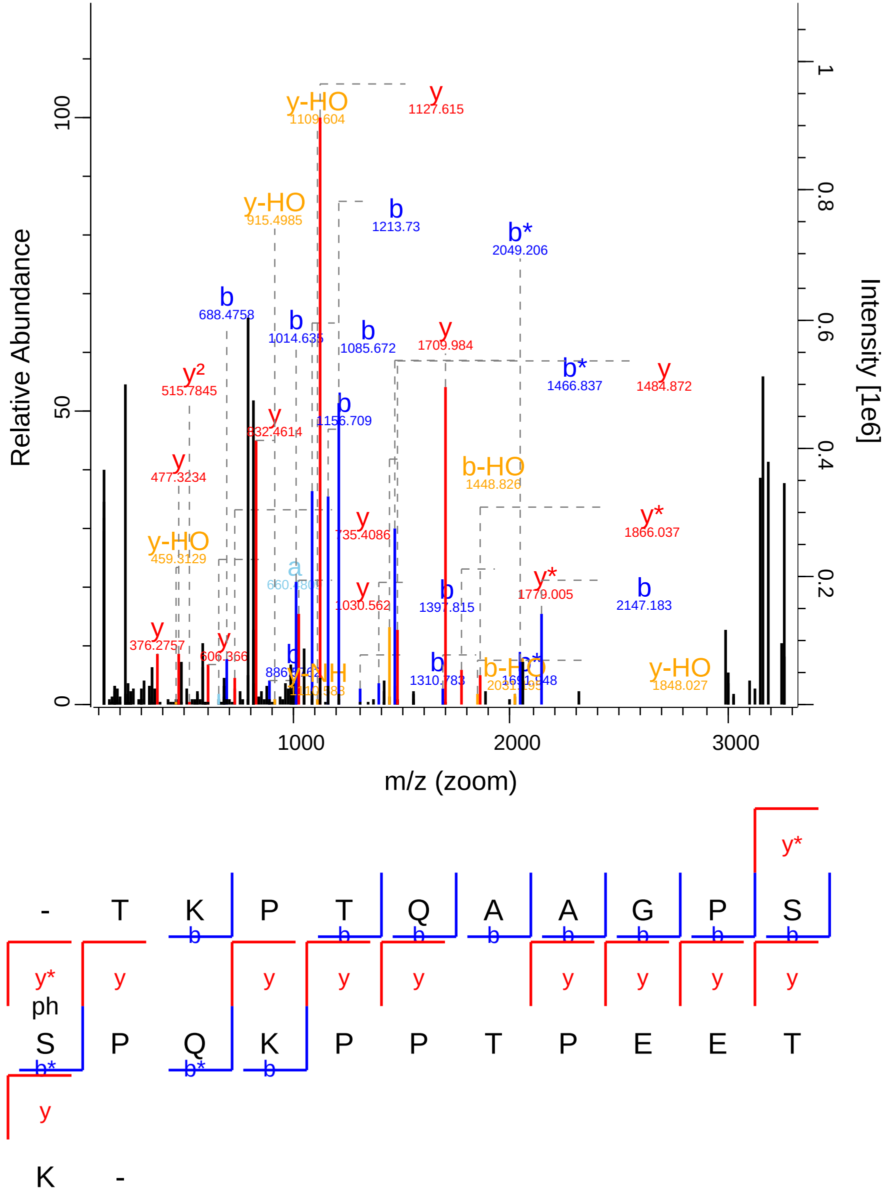

Raw File	Scan	Method	Score	m/z	Gene names
RO-QC-160121_ZackD_SS_Set_02_Fractio	12920	FTMS; HC	42.81	682.35	RRAS2

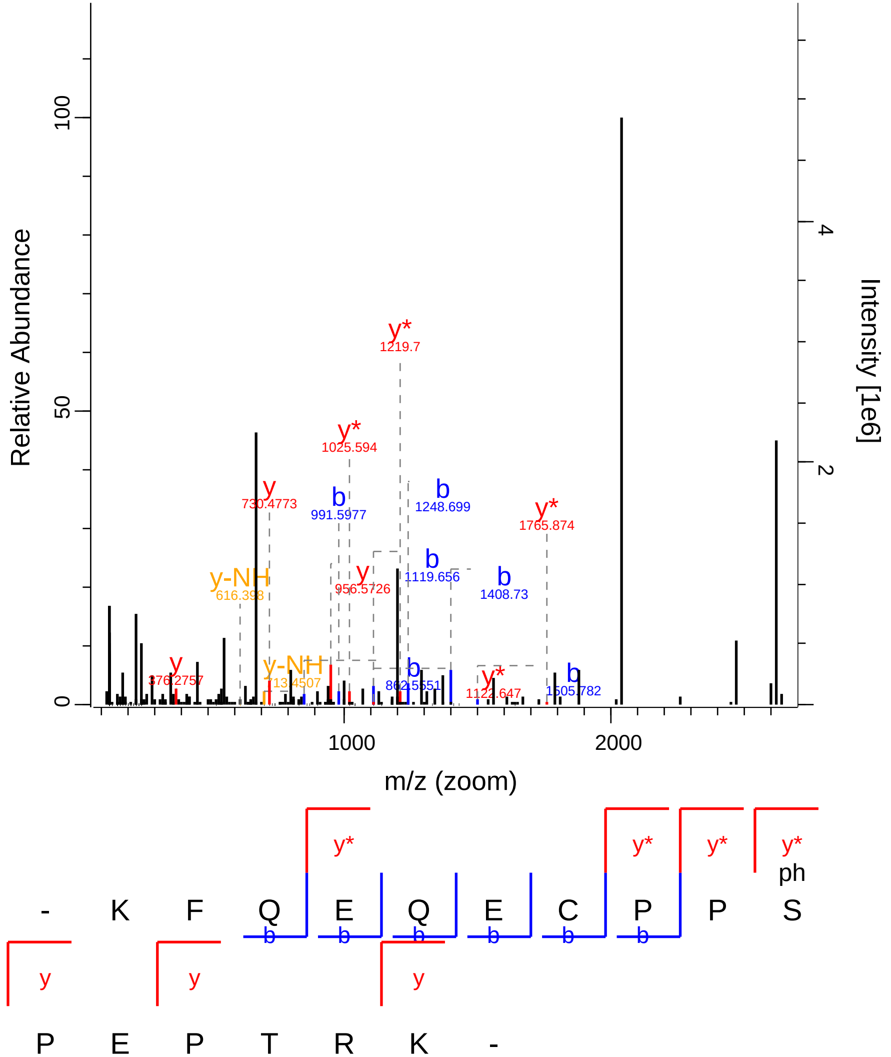

Raw File	Scan	Method	Score	m/z	Gene names
RO-QC-160121_ZackD_SS_Set_02_Fractio	13608	FTMS; HC	95.35	824.39	NDRG1

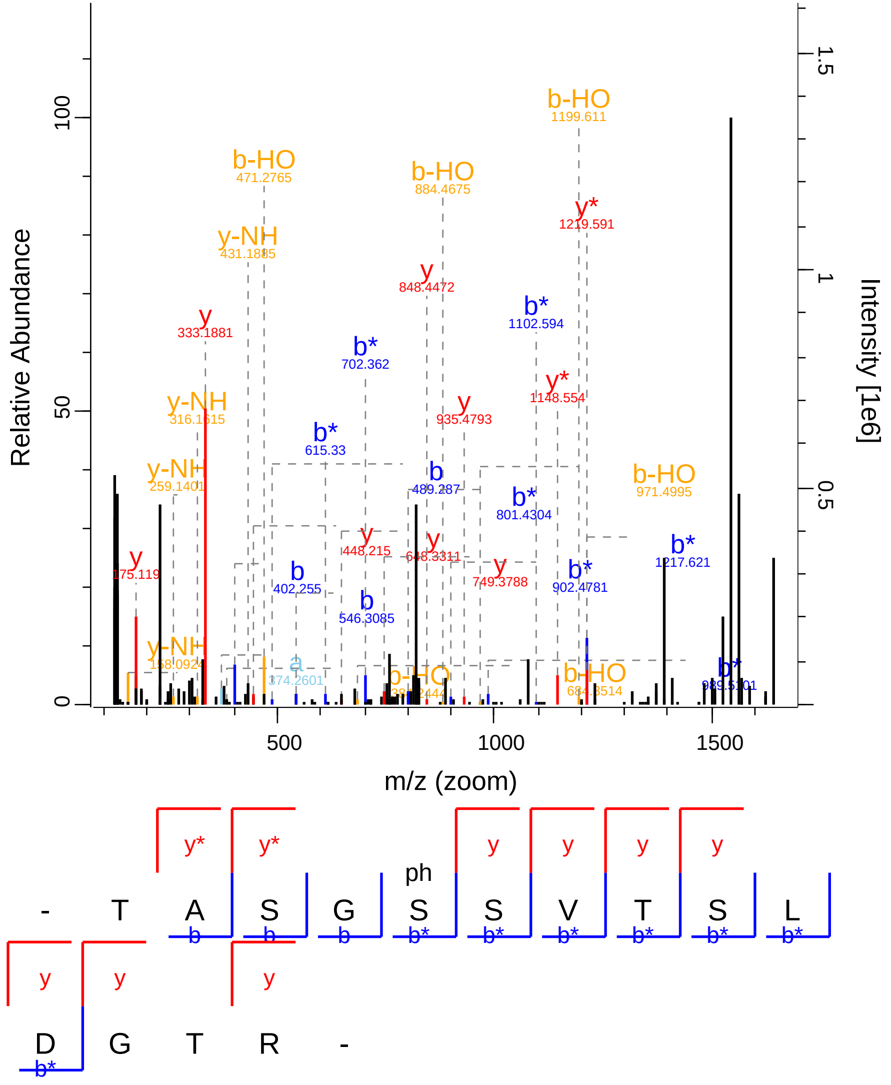

Raw File	Scan	Method	Score	m/z	Gene names
RO-QC-160121_ZackD_SS_Set_02_Fractio	15193	FTMS; HC	50.54	905.48	BCL2L13

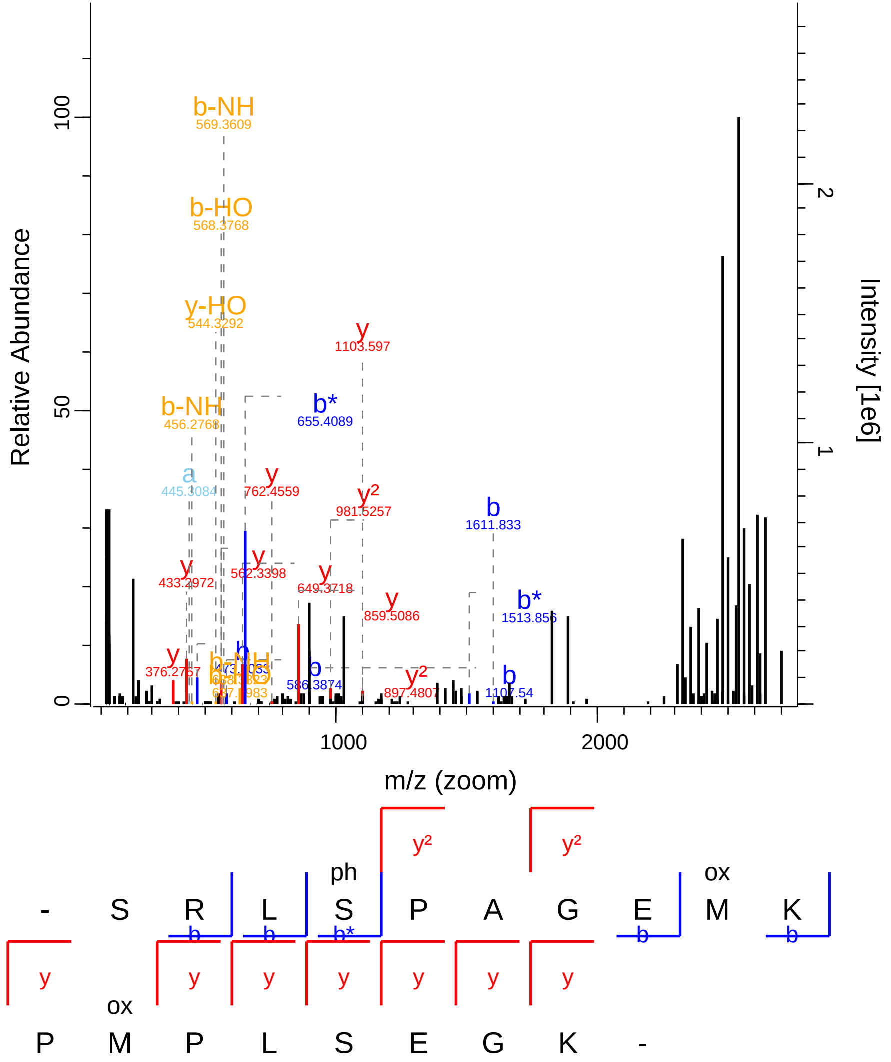

Raw File	Scan	Method	Score	m/z	Gene names
RO-QC-160121_ZackD_SS_Set_02_Fracti	15194	FTMS; HC	157.37	1043.53	LIMA1

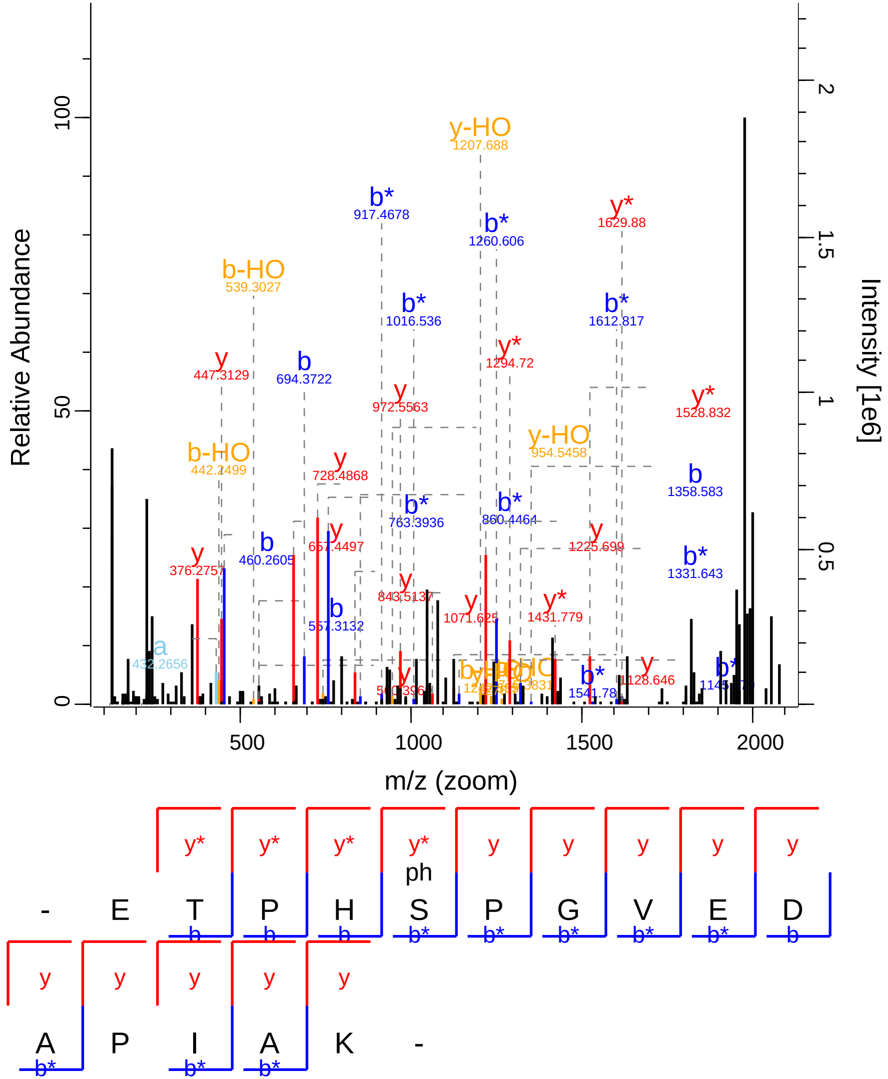

Raw File	Scan	Method	Score	m/z	Gene names
RO-QC-160121_ZackD_SS_Set_02_Fractio	15839	FTMS; HC	120.15	805.73	PDS5B

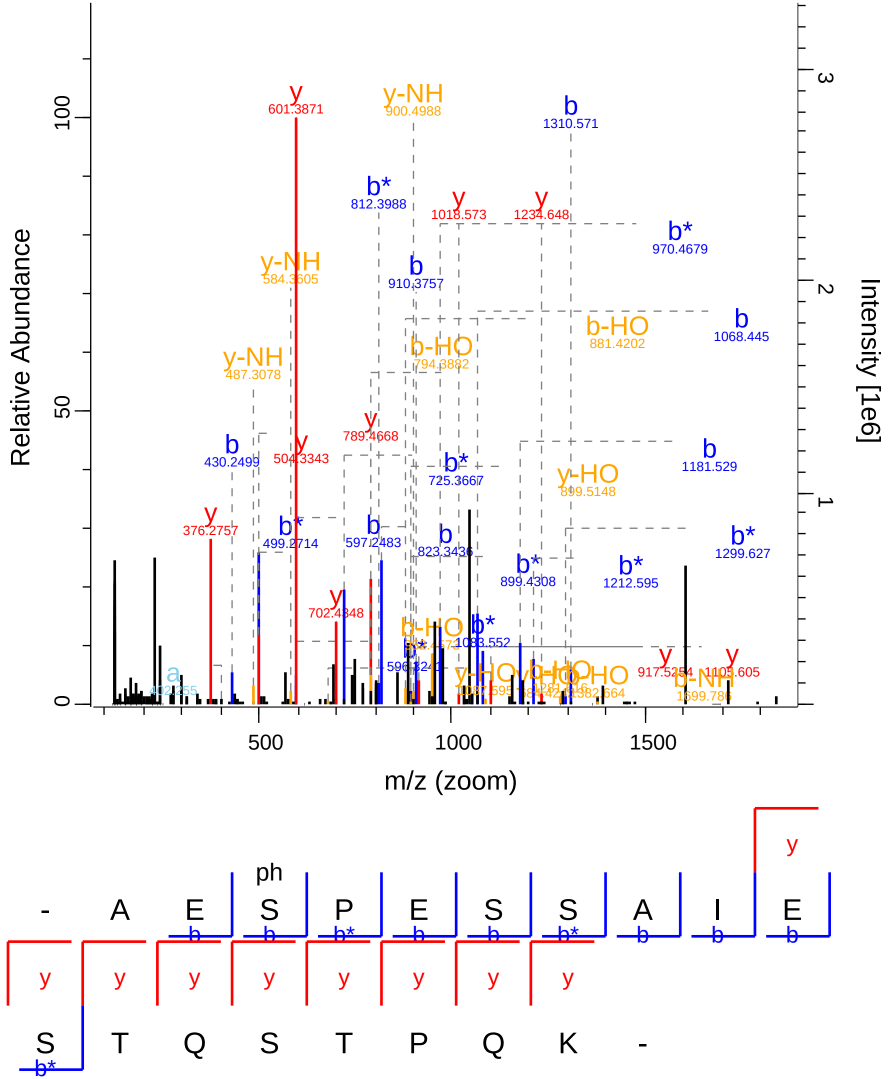

Raw File	Scan	Method	Score	m/z	Gene names
RO-QC-160121_ZackD_SS_Set_02_Fractic	15905	FTMS; HC	265.37	1208.09	PDS5B

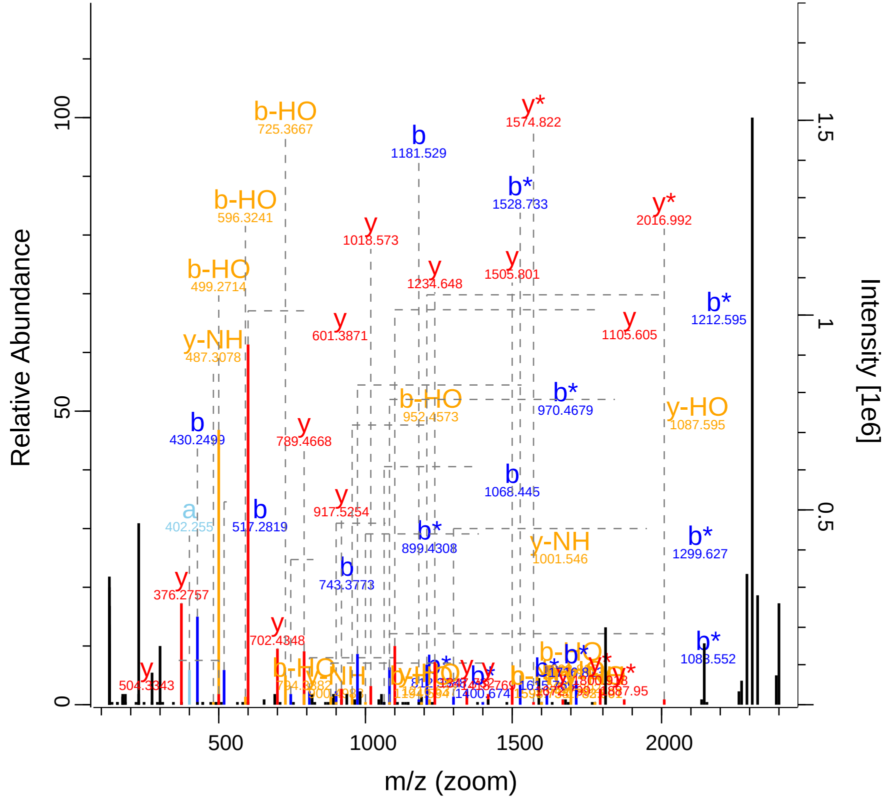

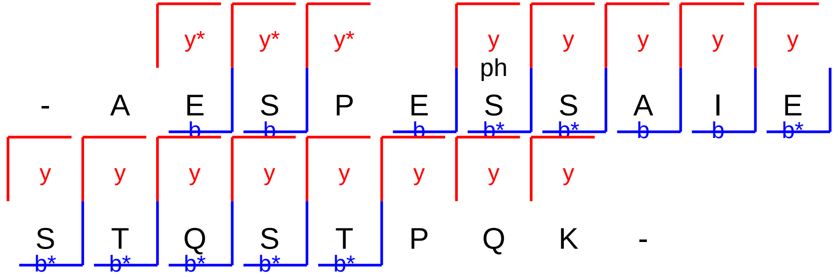

Raw File	Scan	Method	Score	m/z	Gene names
RO-QC-160121_ZackD_SS_Set_02_Fractio	16609	FTMS; HC	122.74	773.42	EXOC1

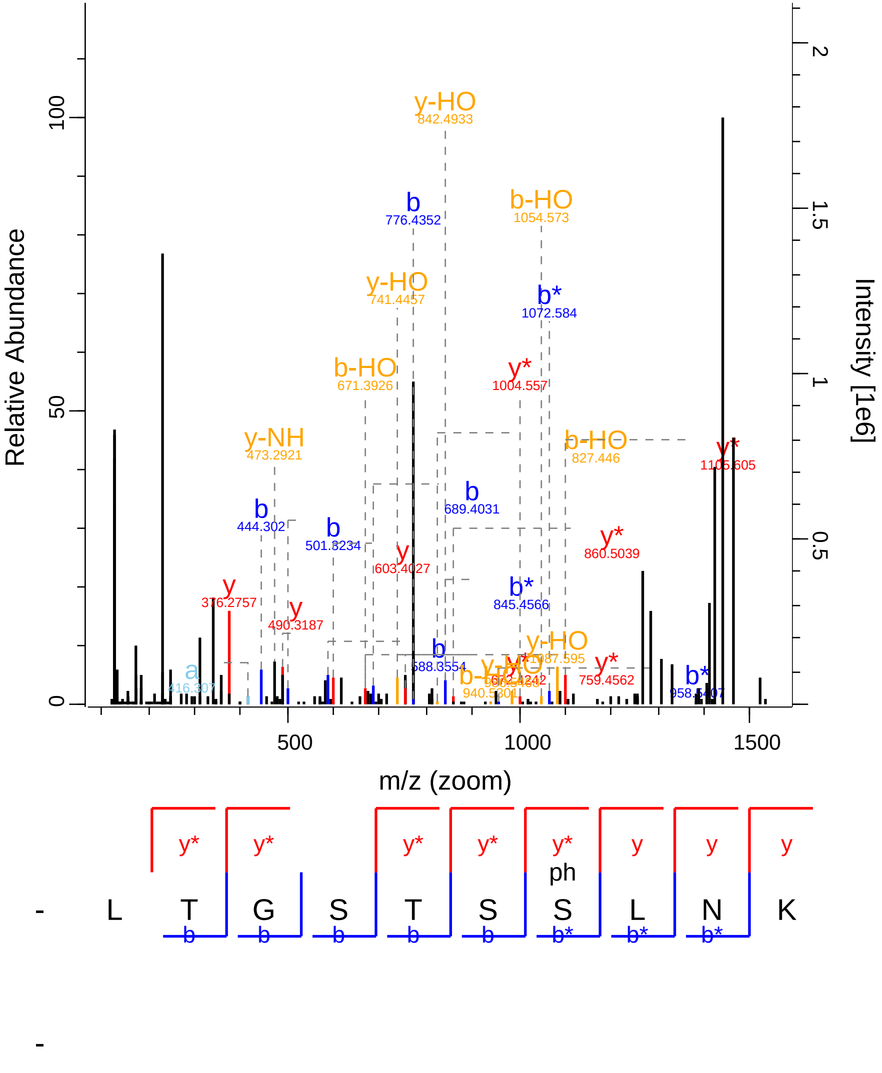

Raw File	Scan	Method	Score	m/z	Gene names
RO-QC-160121_ZackD_SS_Set_02_Fractio	17694	FTMS; HC	71.56	774.78	MEPCE

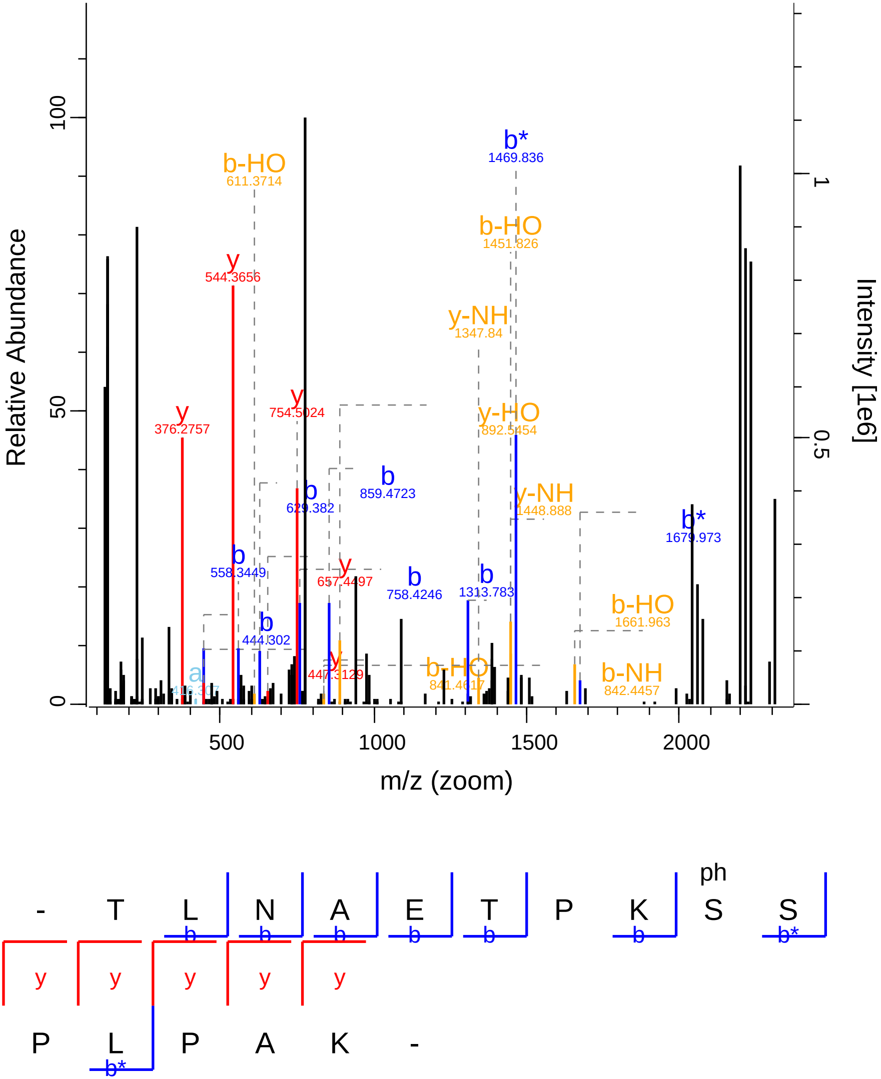

Raw File	Scan	Method	Score	m/z	Gene names
RO-QC-160121_ZackD_SS_Set_02_Fractio	17947	FTMS; HC	164.15	1085.6	TJP1

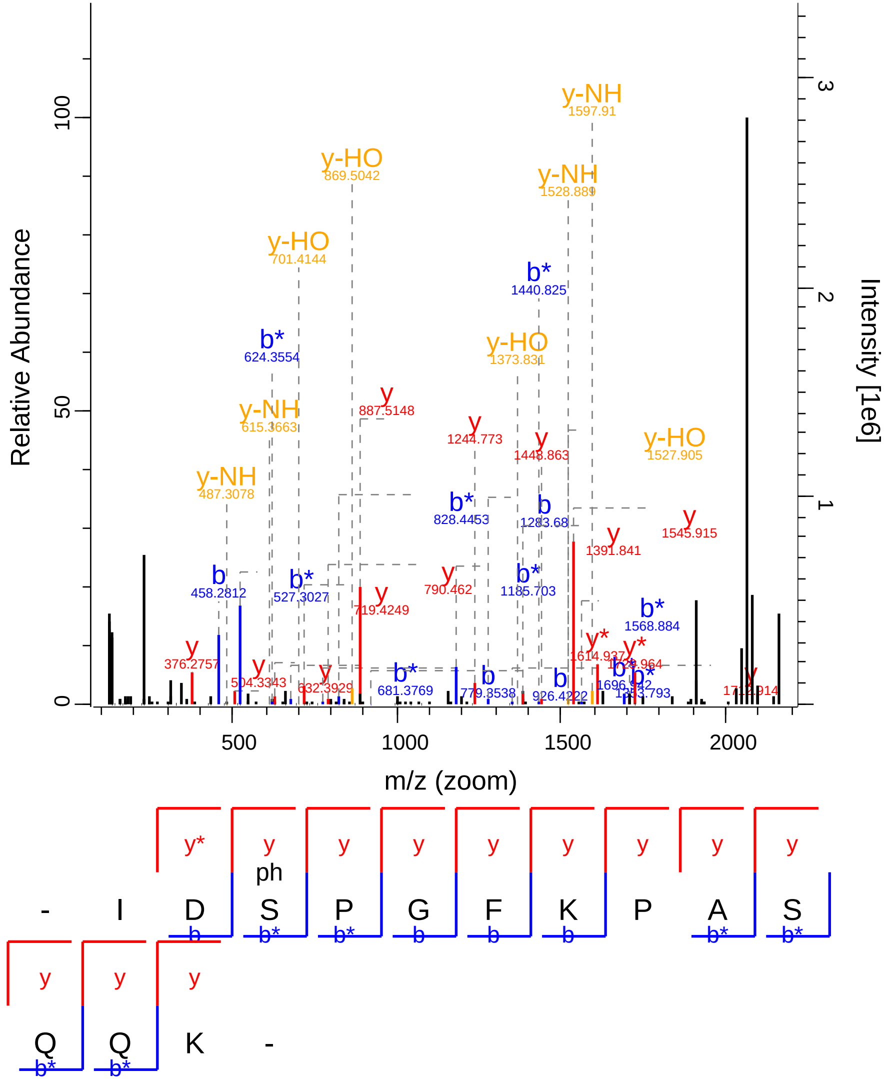

Raw File	Scan	Method	Score	m/z	Gene names
RO-QC-160121_ZackD_SS_Set_02_Fractio	18737	FTMS; HC	94.55	727.37	CCNY

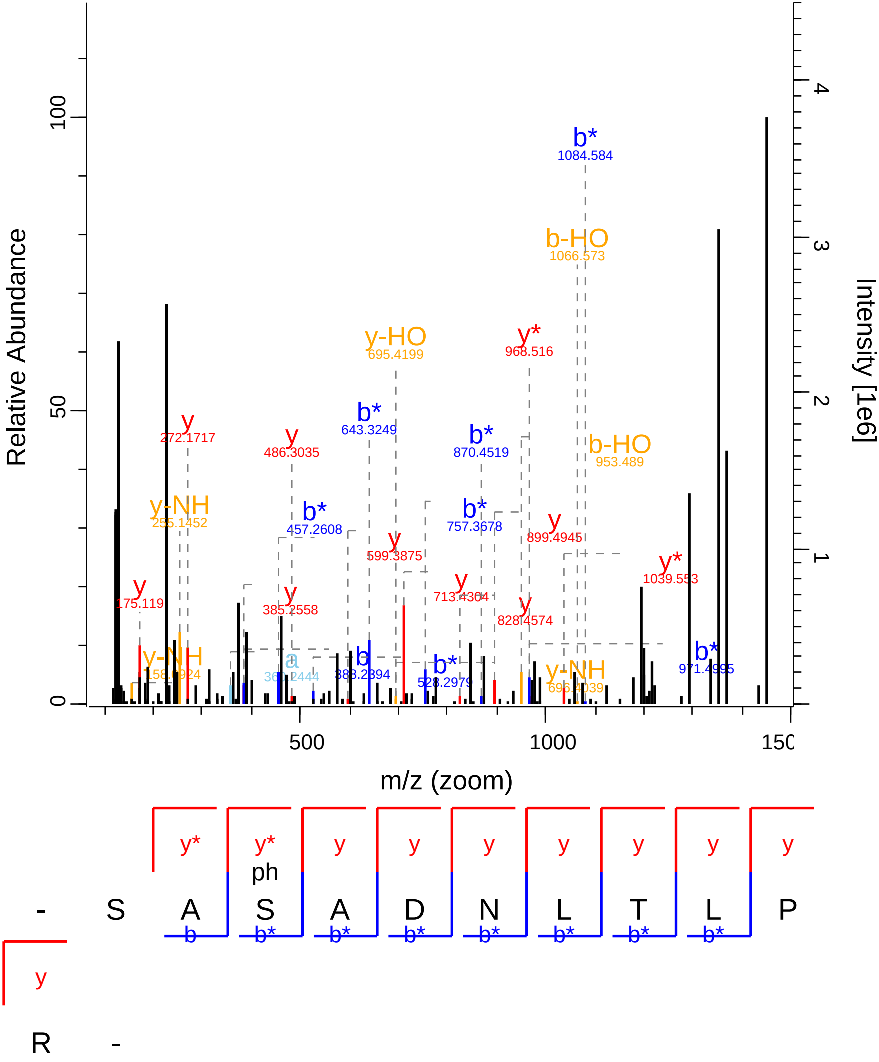

Raw File	Scan	Method	Score	m/z	Gene names
RO-QC-160121_ZackD_SS_Set_02_Fractio	18845	FTMS; HC	48.44	594.62	EXOC3L1

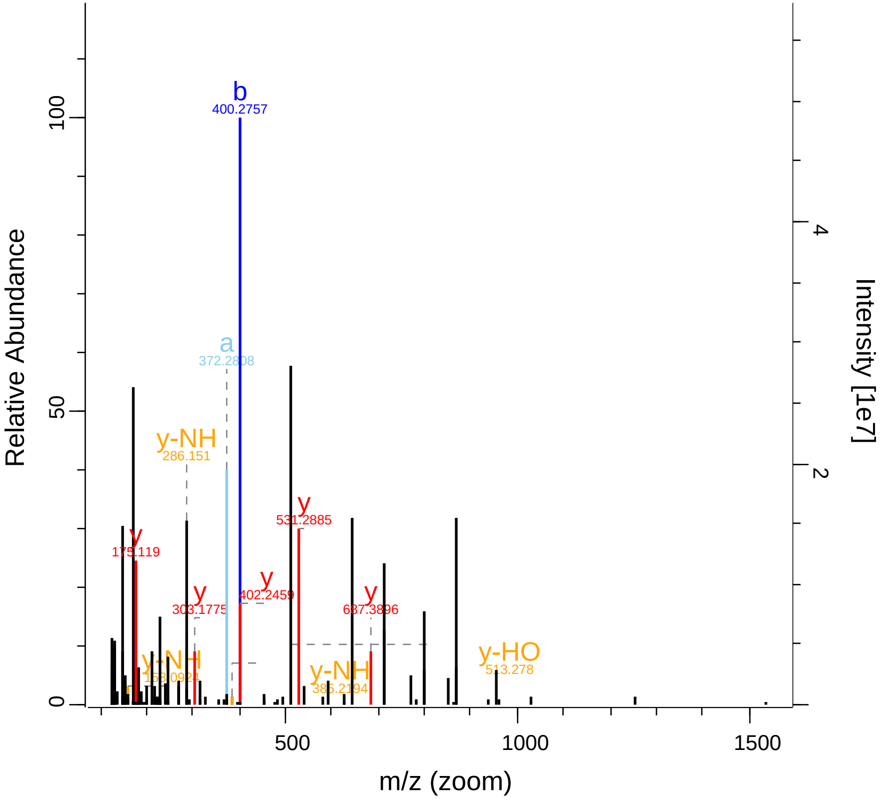

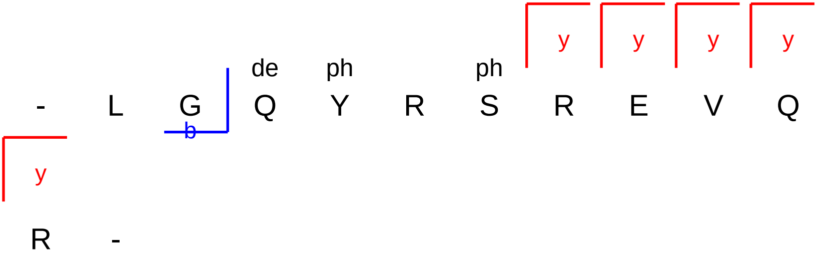

Raw File	Scan	Method	Score	m/z	Gene names
RO-QC-160121_ZackD_SS_Set_02_Fractio	19283	FTMS; HC	114.76	735.38	PRPH

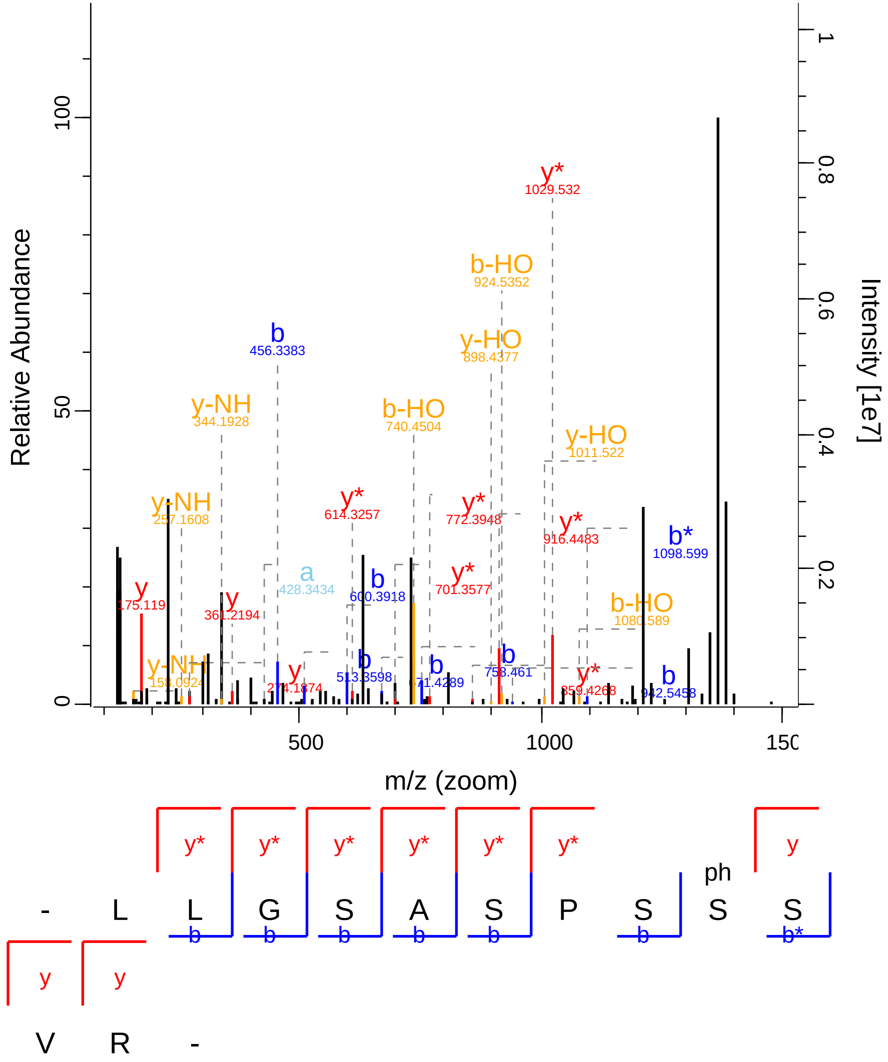

Raw File	Scan	Method	Score	m/z	Gene names
RO-QC-160121_ZackD_SS_Set_02_Fractio	20020	FTMS; HC	55.59	805.08	STIM2

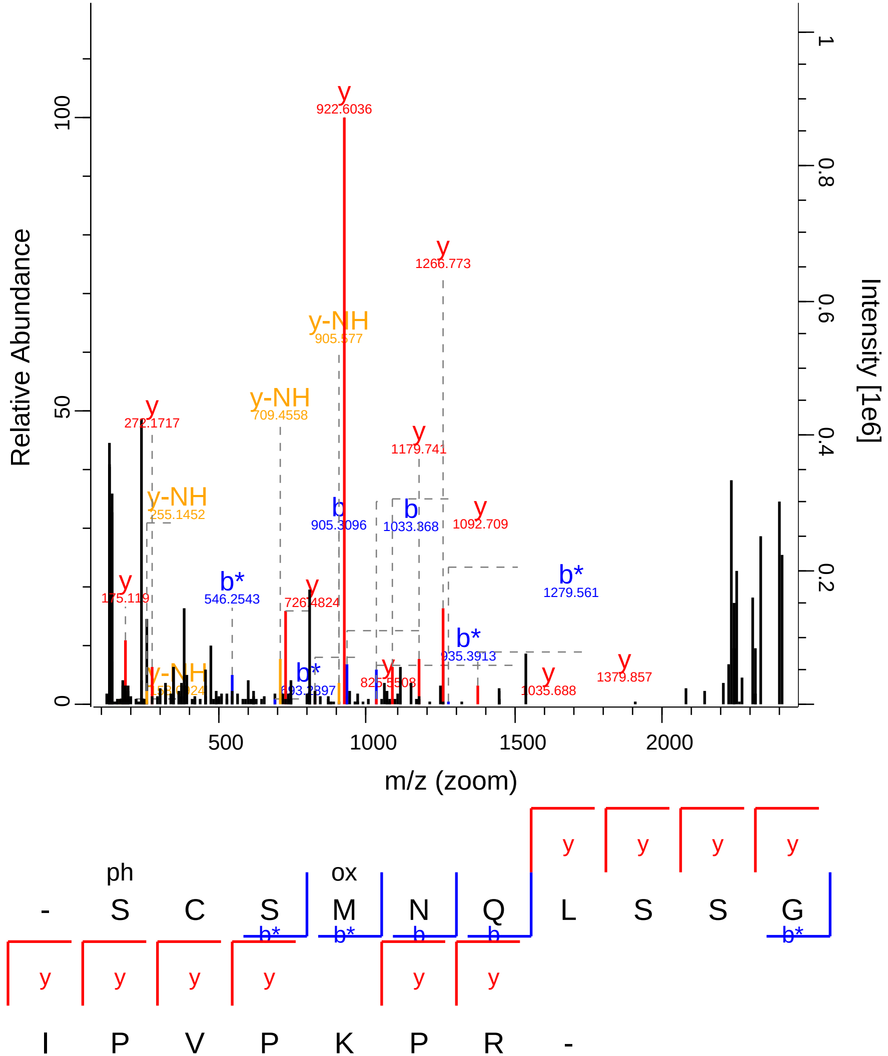

Raw File	Scan	Method	Score	m/z	Gene names
RO-QC-160121_ZackD_SS_Set_02_Fractio	20082	FTMS; HC	76.42	835.77	LIPE

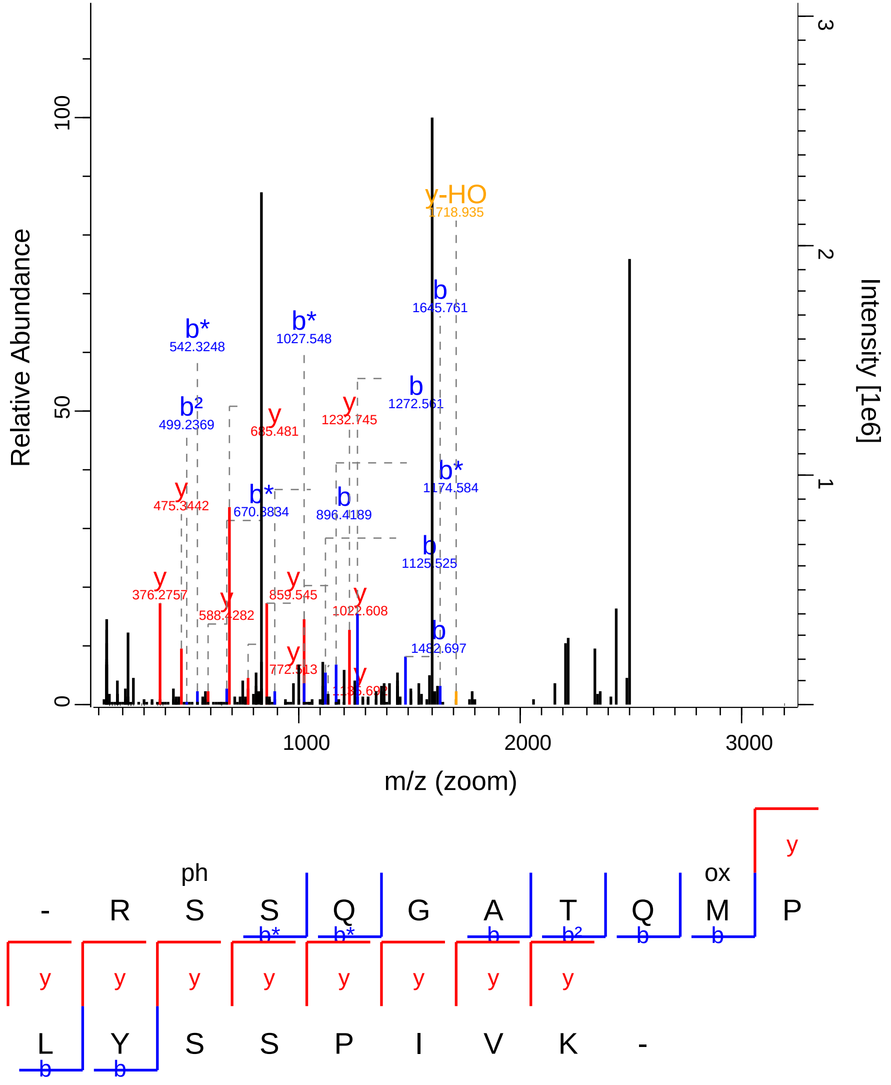

Raw File	Scan	Method	Score	m/z	Gene names
RO-QC-160121_ZackD_SS_Set_02_Fractic	20635	FTMS; HC	64.04	1029.98	ZNF215

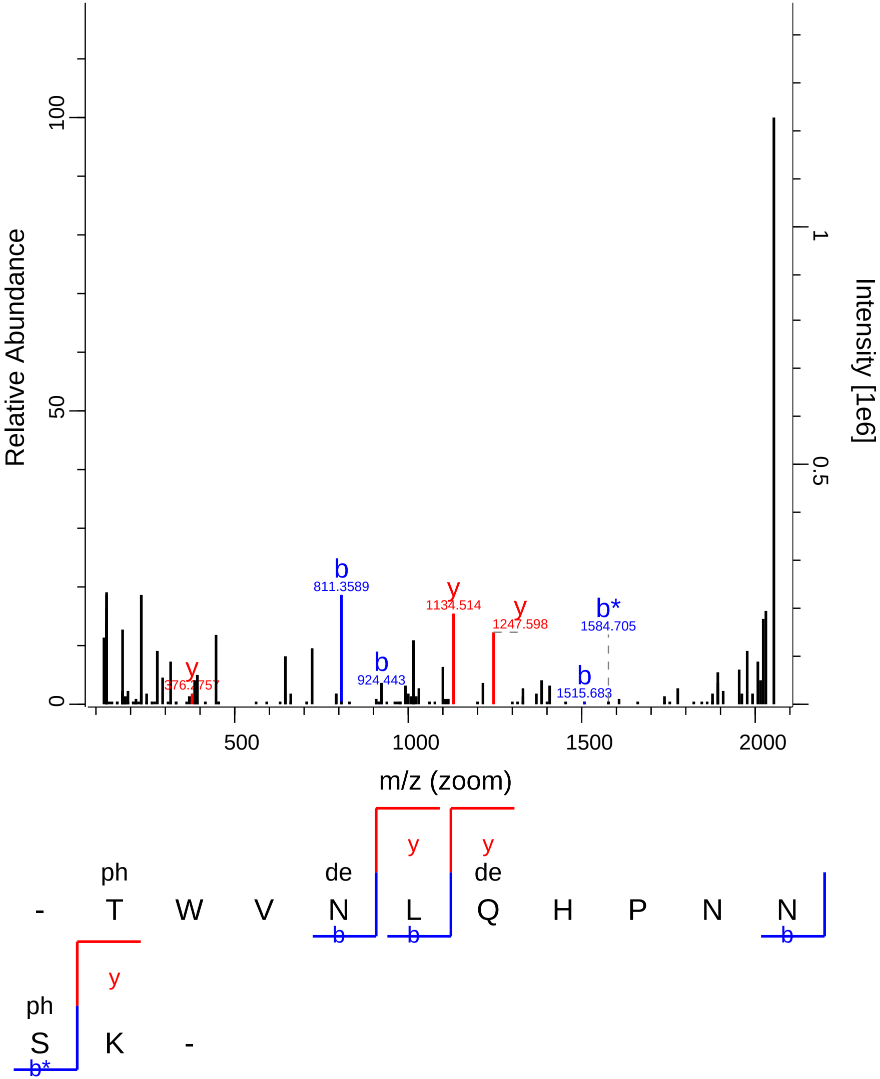

Raw File	Scan	Method	Score	m/z	Gene names
RO-QC-160121_ZackD_SS_Set_02_Fractio	21177	FTMS; HC	149.44	966.77	AKAP2

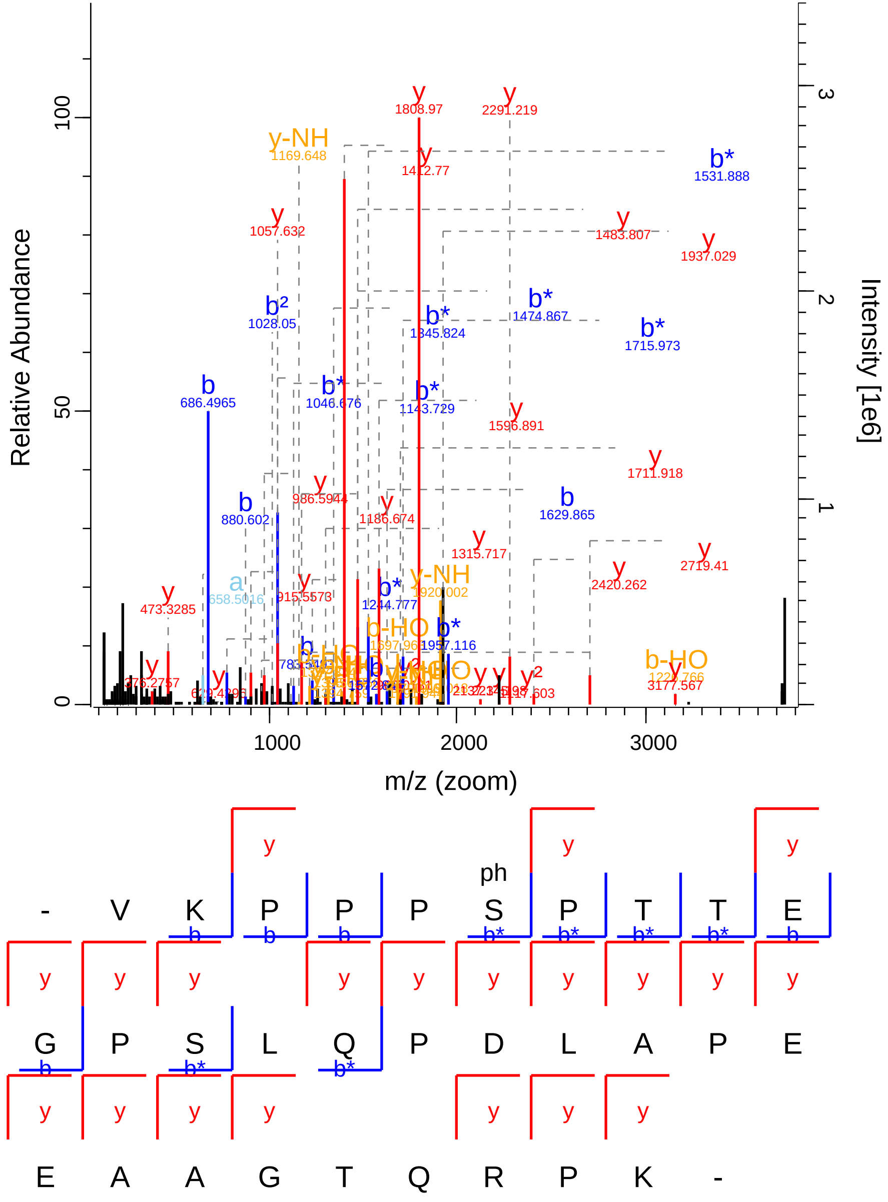

Raw File	Scan	Method	Score	m/z	Gene names
RO-QC-160121_ZackD_SS_Set_02_Fractio	21490	FTMS; HC	42.56	559.06	LRRN1

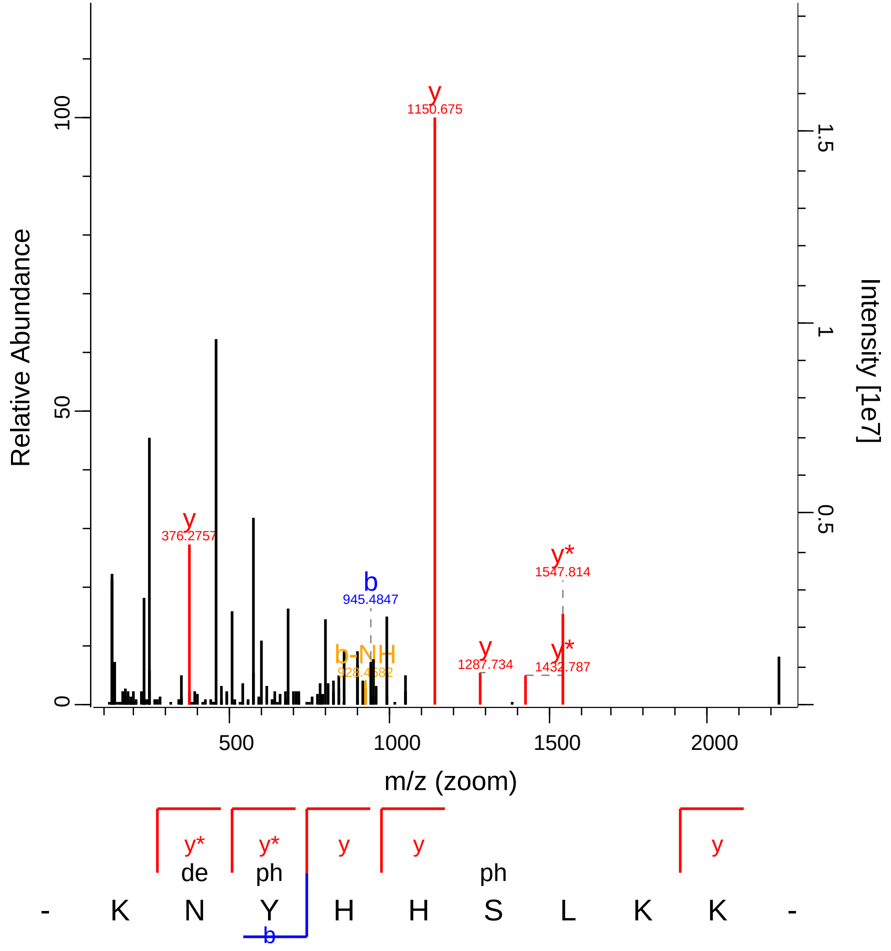

Raw File	Scan	Method	Score	m/z	Gene names
RO-QC-160121_ZackD_SS_Set_02_Fractio	21589	FTMS; HC	41.05	562.56	CYP17A1

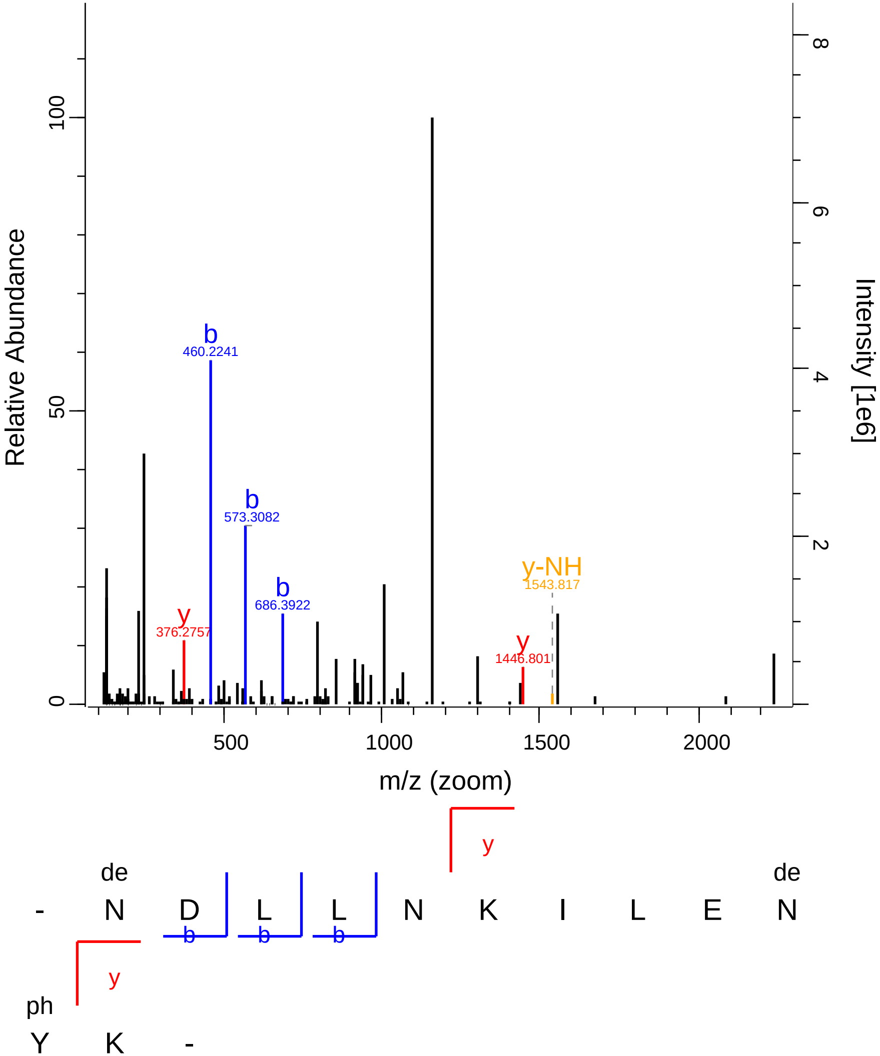

Raw File	Scan	Method	Score	m/z	Gene names
RO-QC-160121_ZackD_SS_Set_02_Fractic	23710	FTMS; HC	65.5	1028.49	PMPCB

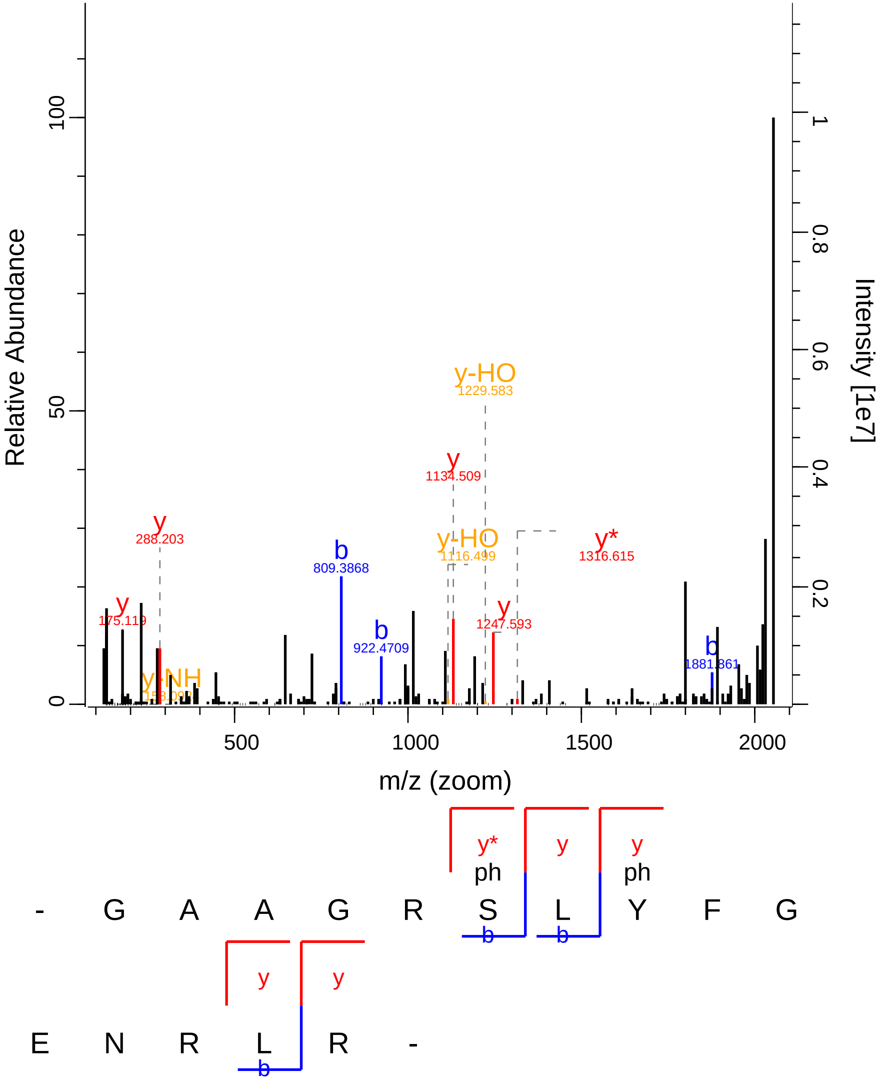

Raw File	Scan	Method	Score	m/z	Gene names
RO-QC-160121_ZackD_SS_Set_02_Fractio	23733	FTMS; HC	98.03	727.38	CFL1

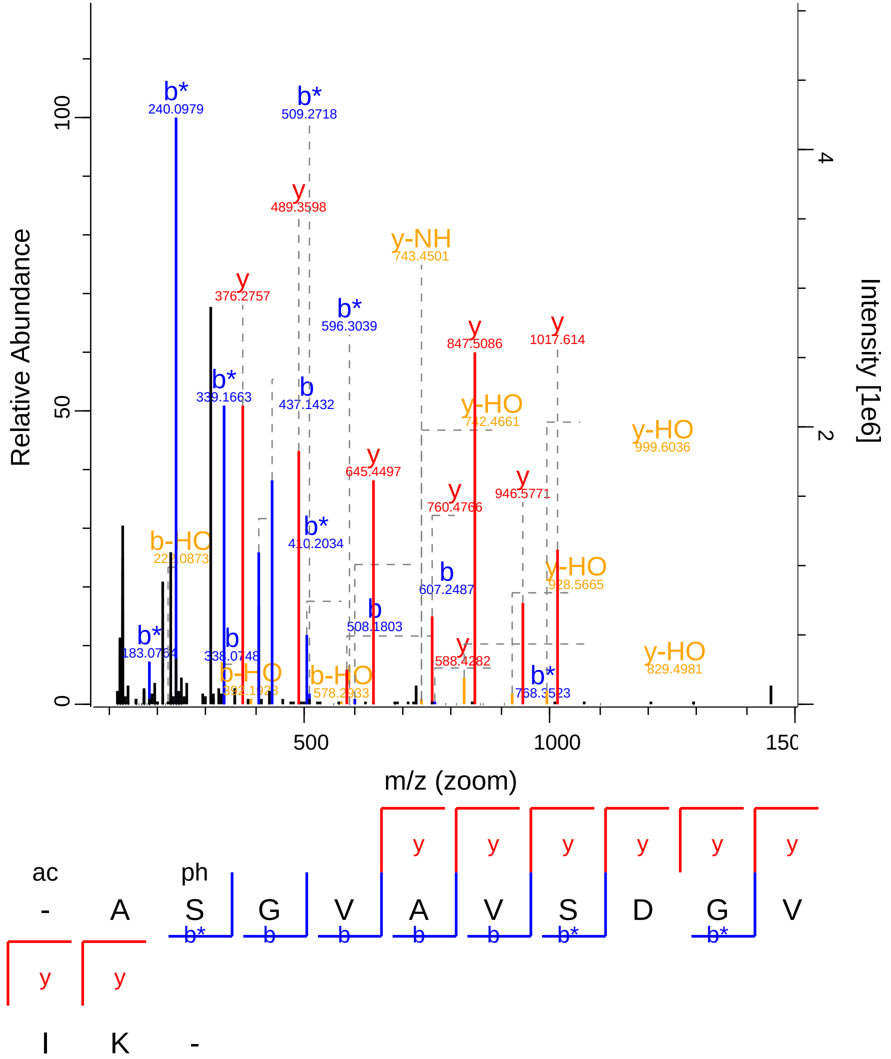

Raw File	Scan	Method	Score	m/z	Gene names
RO-QC-160121_ZackD_SS_Set_02_Fractic	24358	FTMS; HC	50.88	1026.83	FNDC1

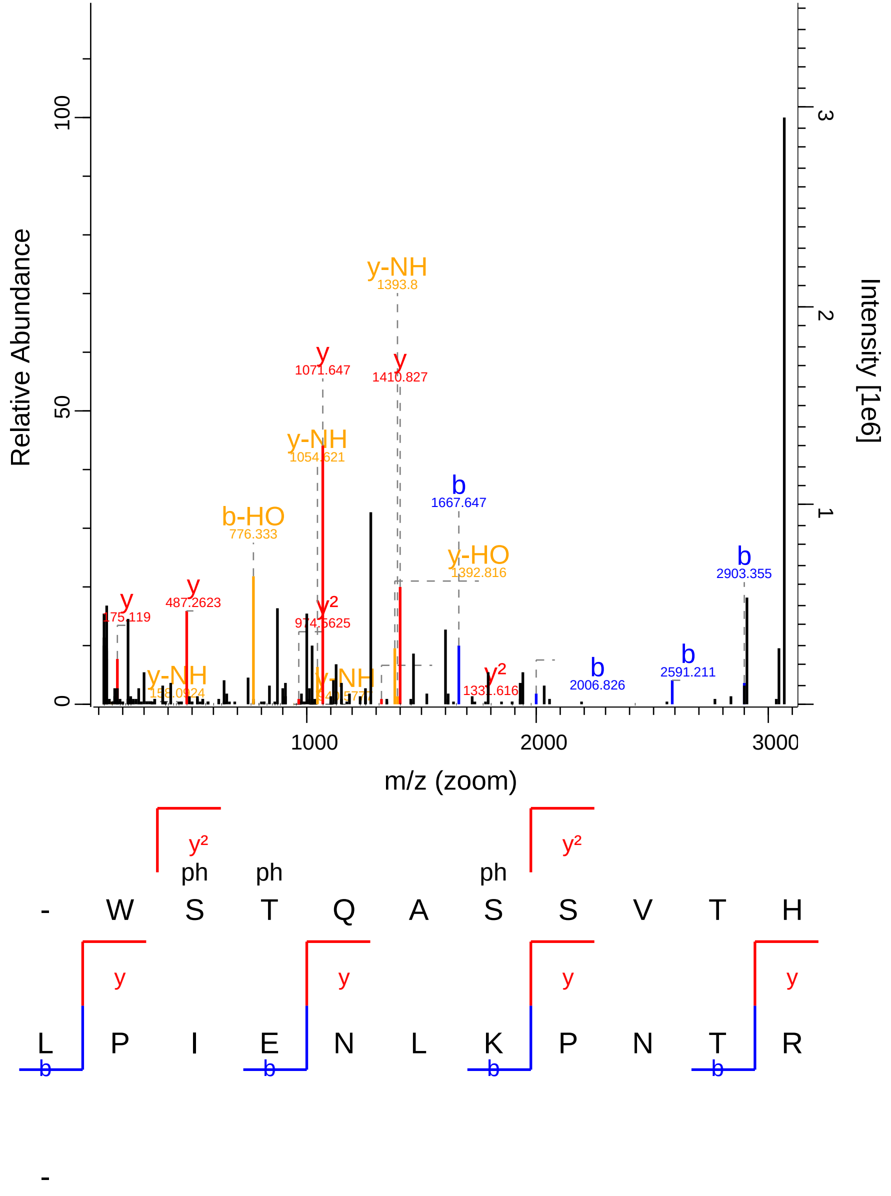

Raw File	Scan	Method	Score	m/z	Gene names
RO-QC-160121_ZackD_SS_Set_02_Fractio	24588	FTMS; HC	84.95	803.19	ADD3

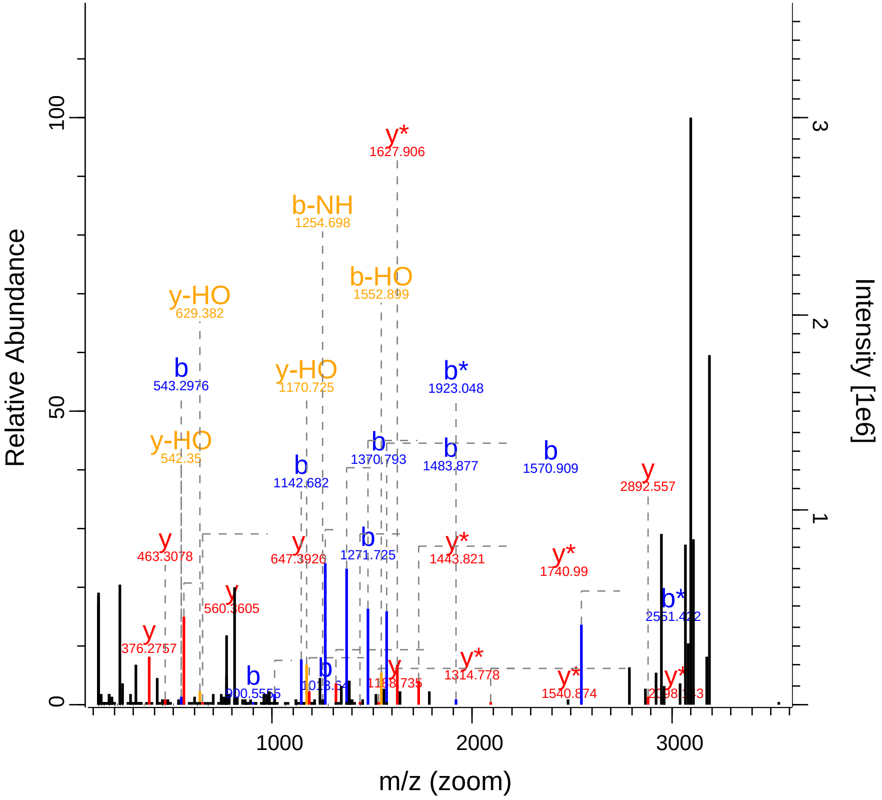

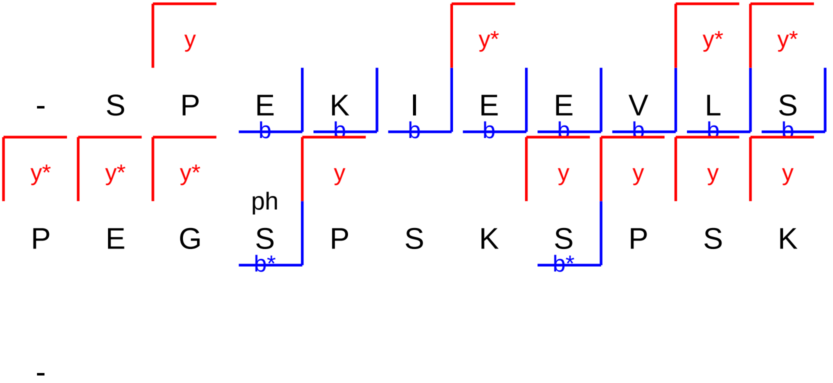

Raw File	Scan	Method	Score	m/z	Gene names
RO-QC-160121_ZackD_SS_Set_02_Fractio	28213	FTMS; HC	46.13	737.86	TRIL

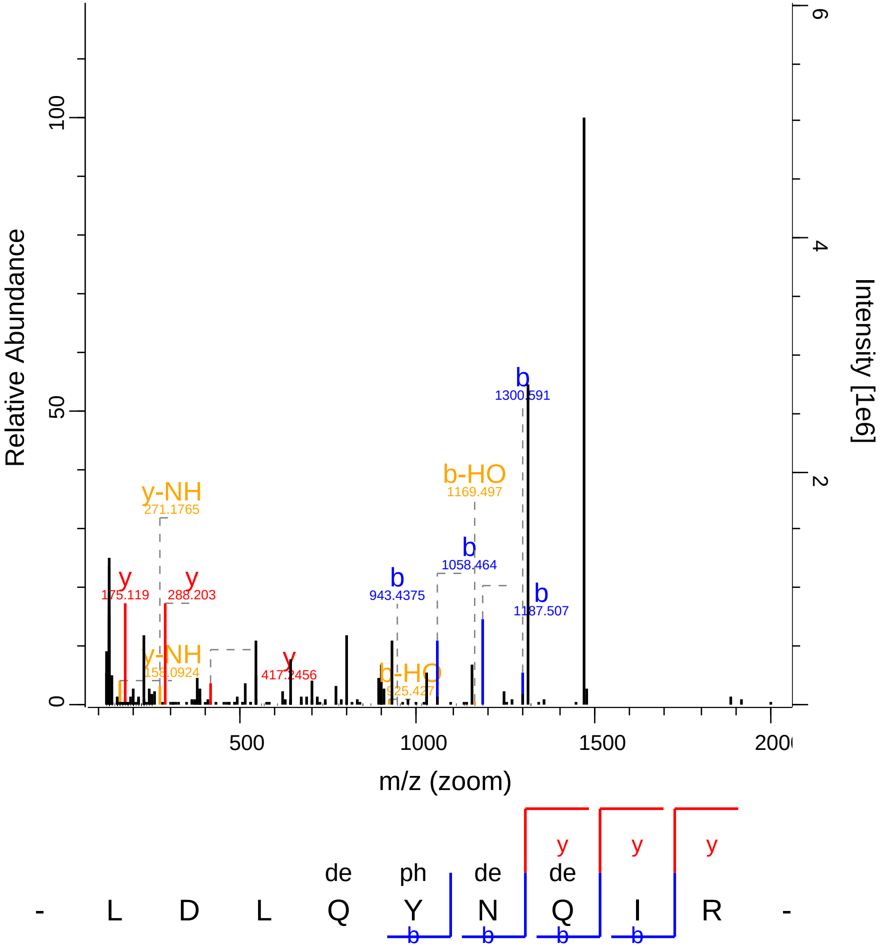

Raw File	Scan	Method	Score	m/z	Gene names
RO-QC-160121_ZackD_SS_Set_02_Fractio	28877	FTMS; HC	44.16	815.69	SLITRK4

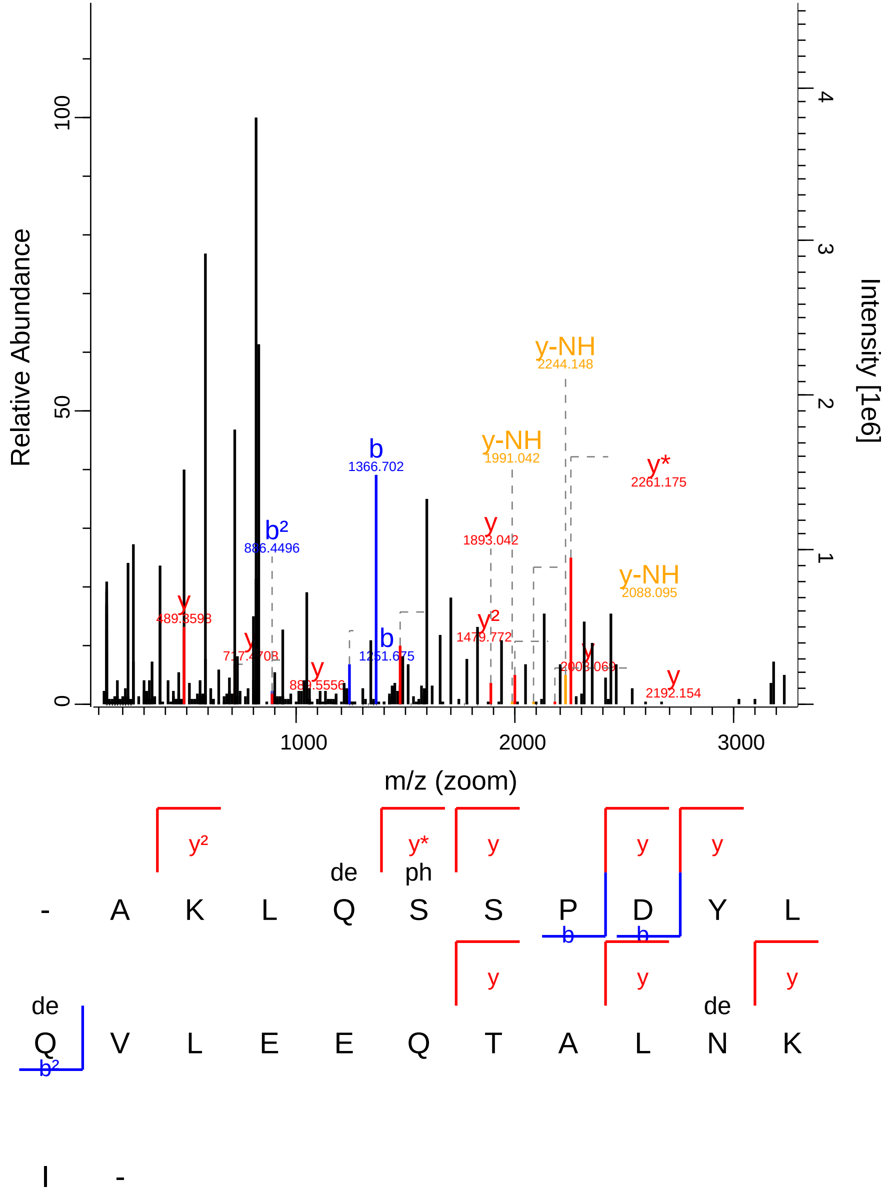

Raw File	Scan	Method	Score	m/z	Gene names
RO-QC-160121_ZackD_SS_Set_02_Fractio	29805	FTMS; HC	56.26	972.51	TUBGCP2

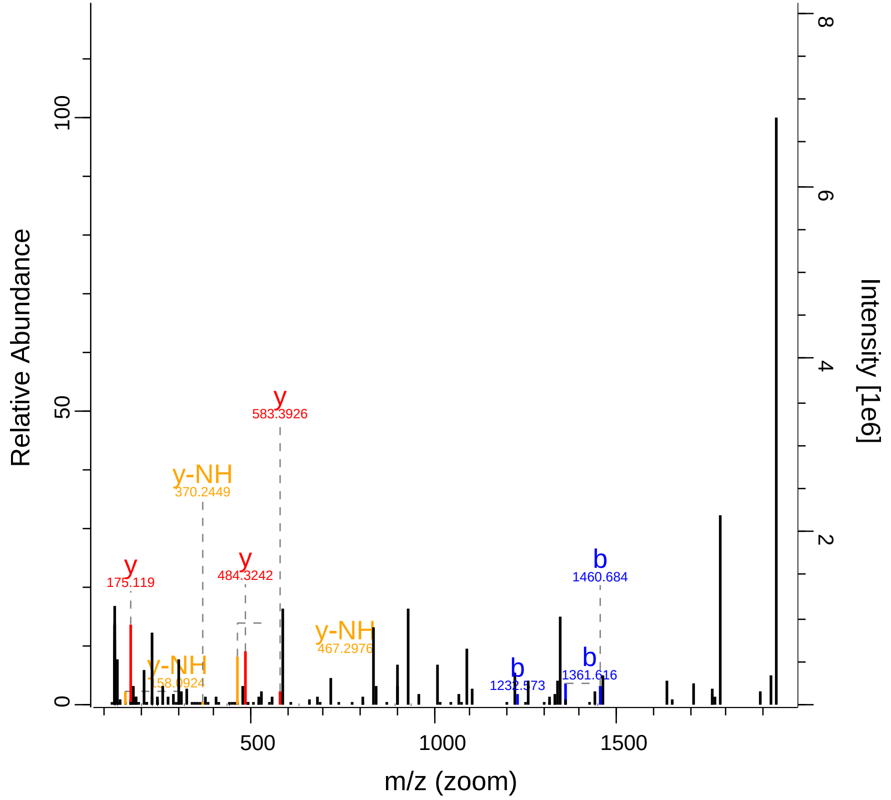

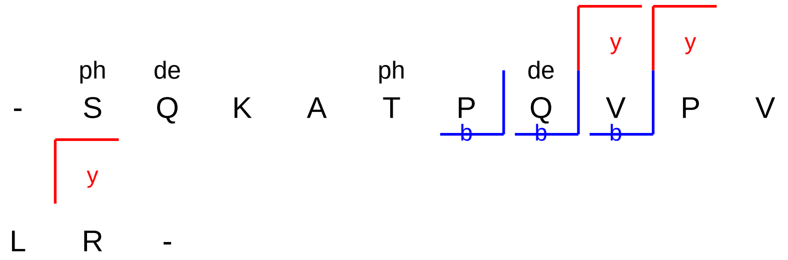

Raw File	Scan	Method	Score	m/z	Gene names
RO-QC-160121_ZackD_SS_Set_02_Fractic	30151	FTMS; HC	53.24	1125.06	CLEC12A

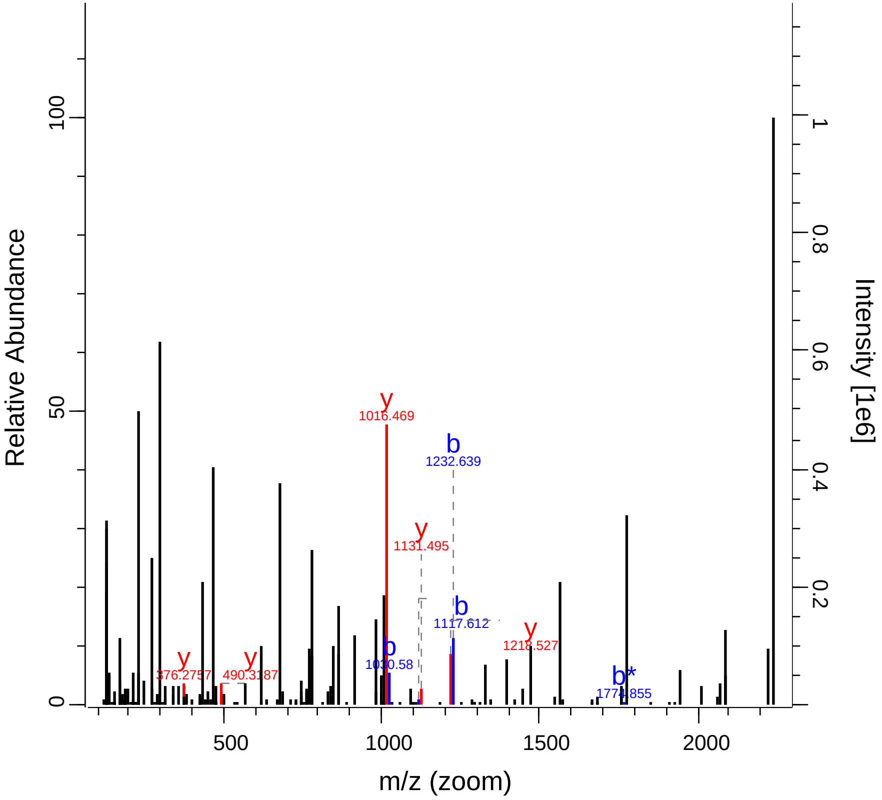

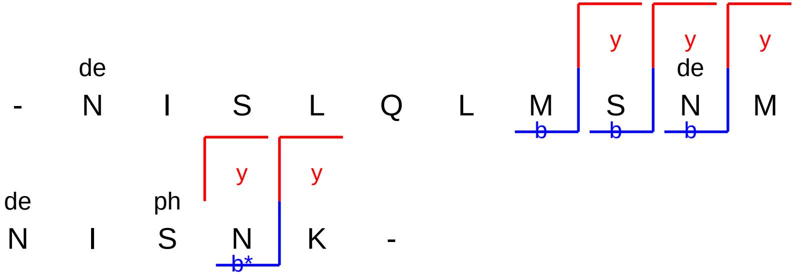

Raw File	Scan	Method	Score	m/z	Gene names
RO-QC-160121_ZackD_SS_Set_02_Fractio	30270	FTMS; HC	43.08	620.65	SLFN13

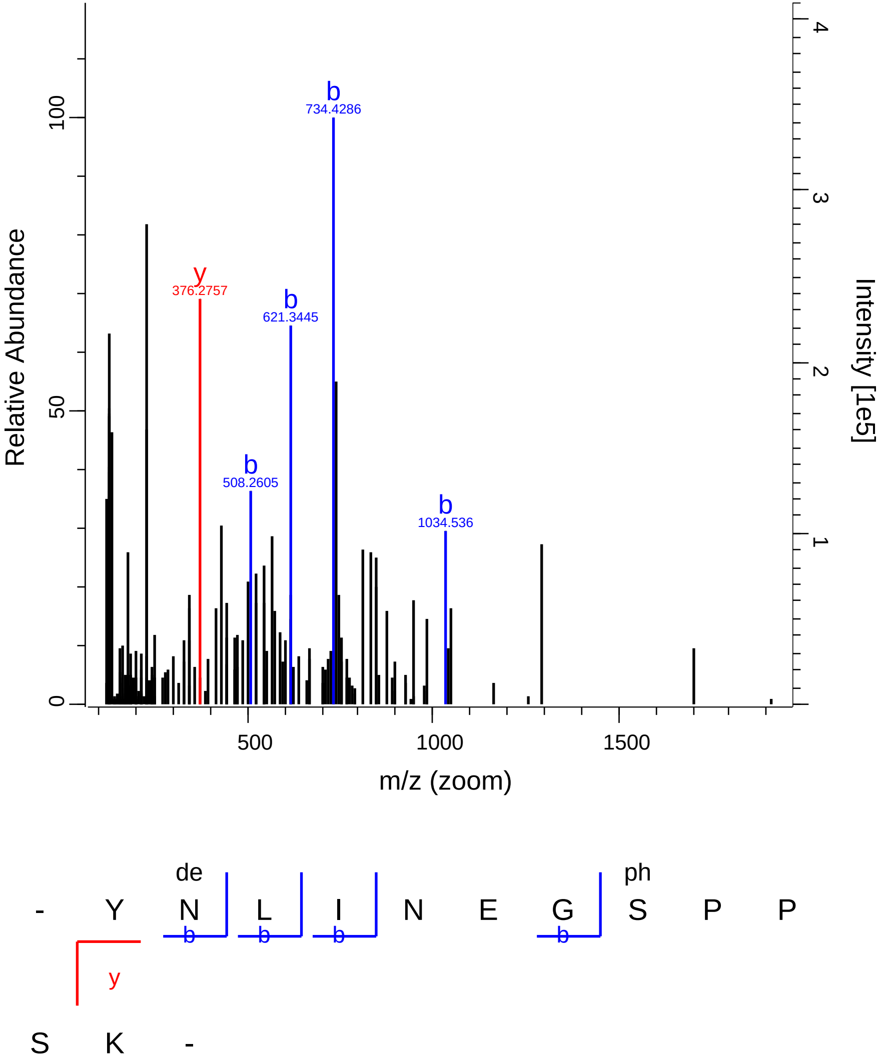

Raw File	Scan	Method	Score	m/z	Gene names
RO-QC-160121_ZackD_SS_Set_02_Fractic	30438	FTMS; HC	74.44	1122.57	DYNC1I2

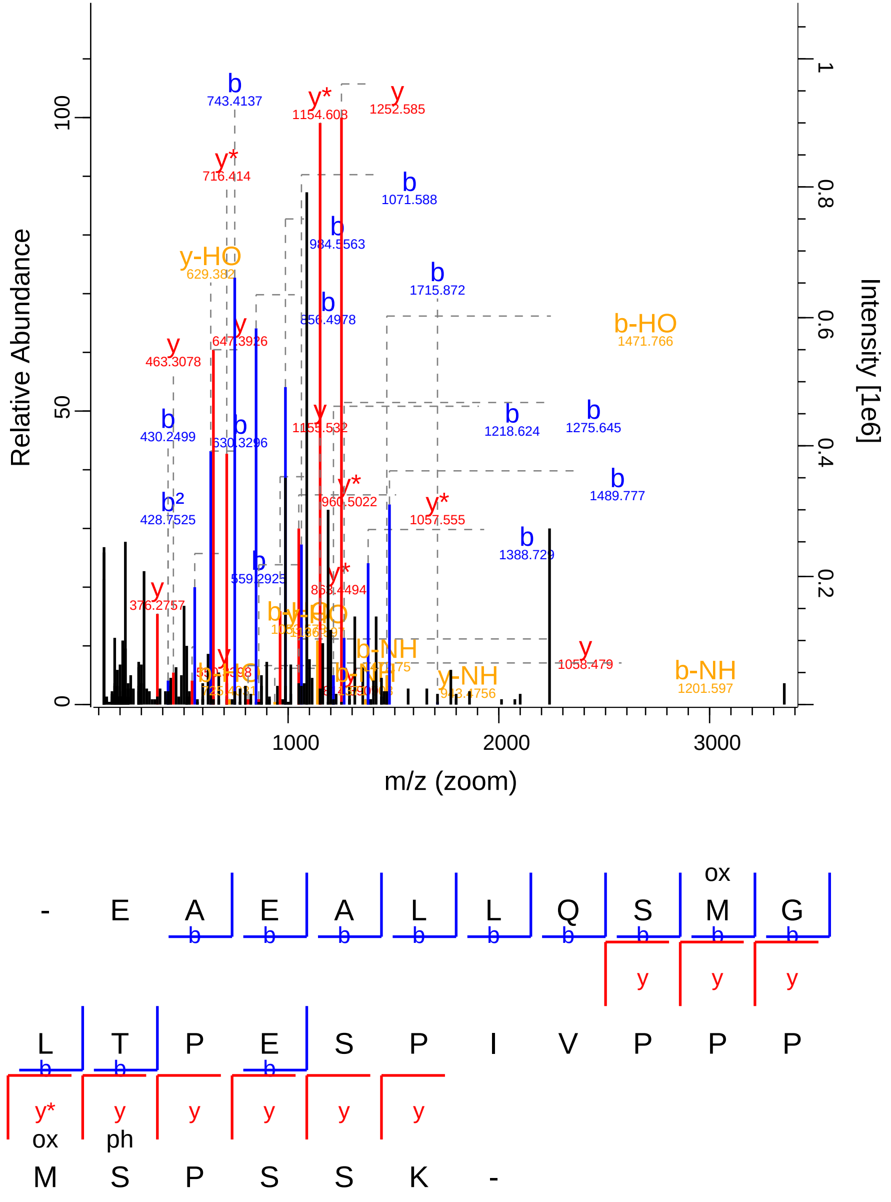

Raw File	Scan	Method	Score	m/z	Gene names
RO-QC-160121_ZackD_SS_Set_02_Fractio	30706	FTMS; HC	136.63	954.51	RANBP2

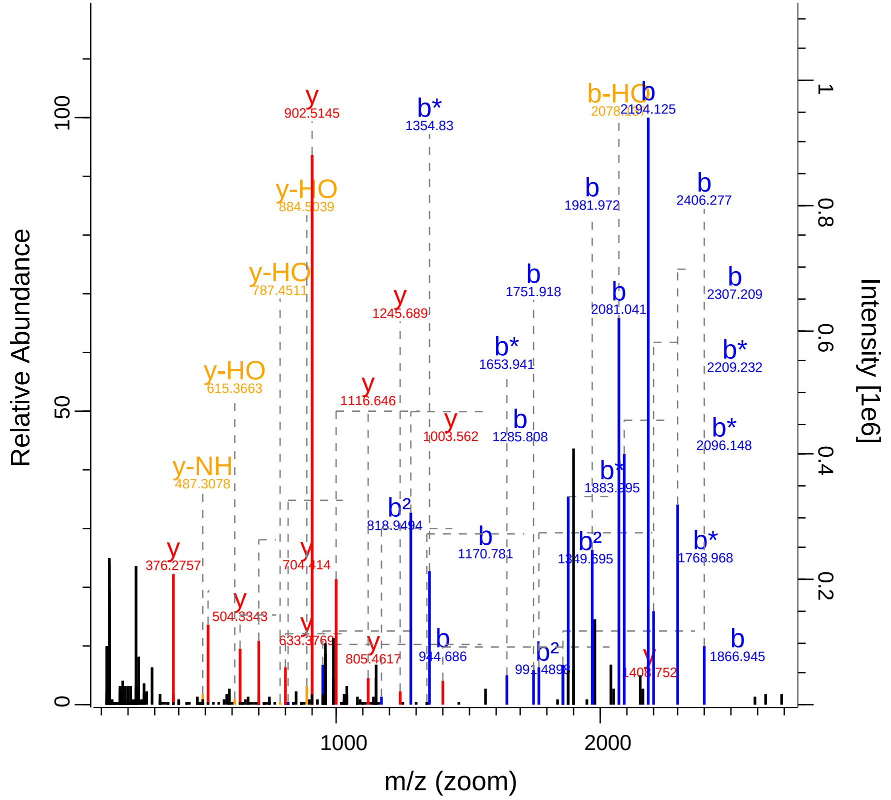

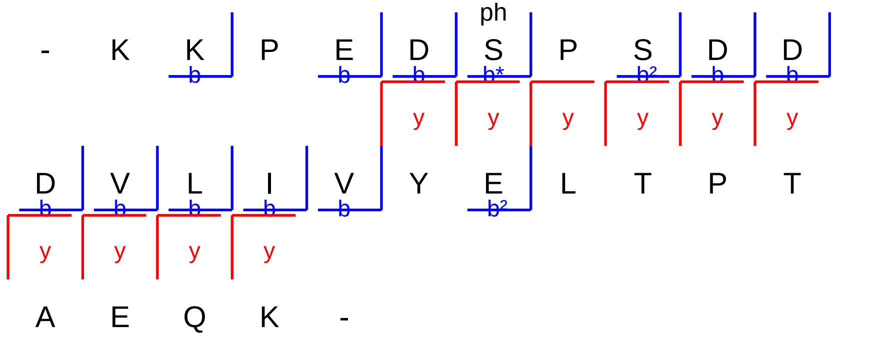

Raw File	Scan	Method	Score	m/z	Gene names
RO-QC-160121_ZackD_SS_Set_02_Fractic	30872	FTMS; HC	93.77	1381.13	LGALS3BP

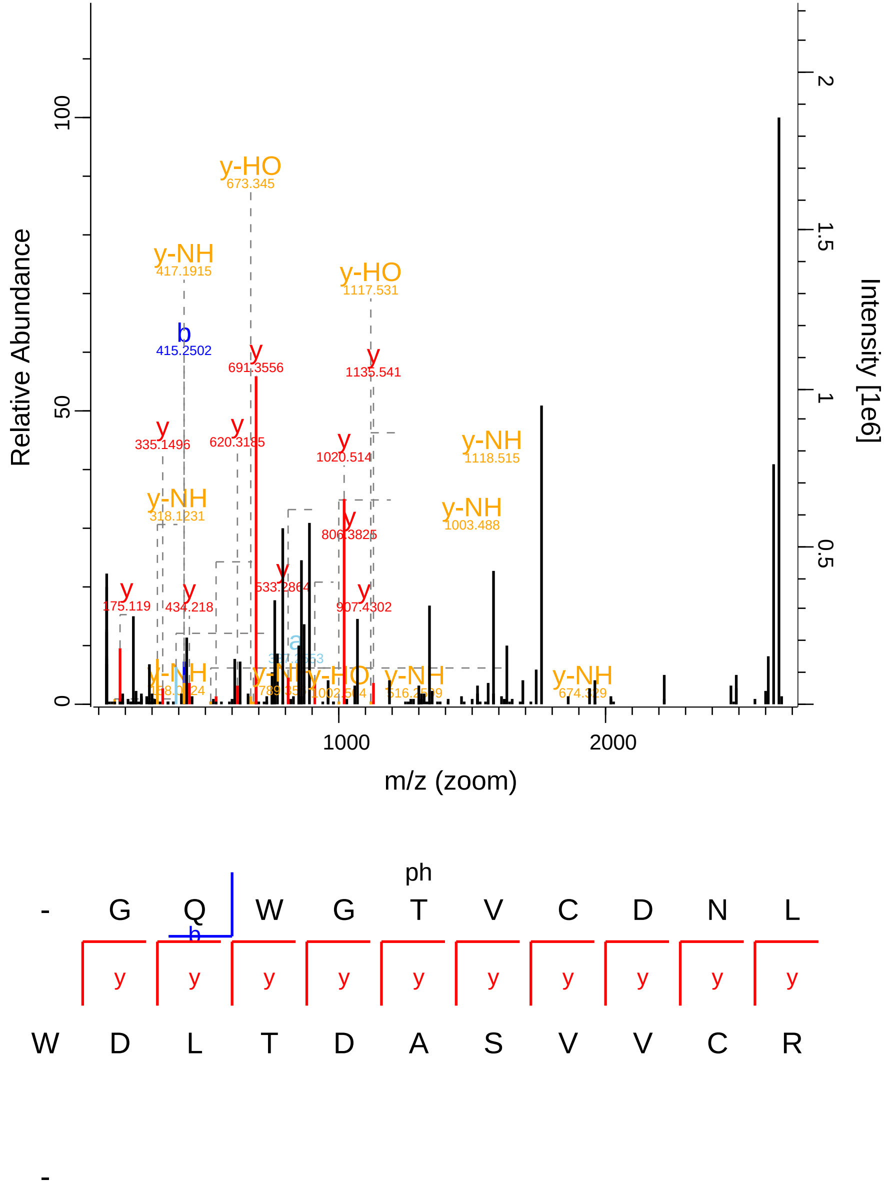

Raw File	Scan	Method	Score	m/z	Gene names
RO-QC-160121_ZackD_SS_Set_02_Fractio	31519	FTMS; HC	45.37	919.13	FOXC2

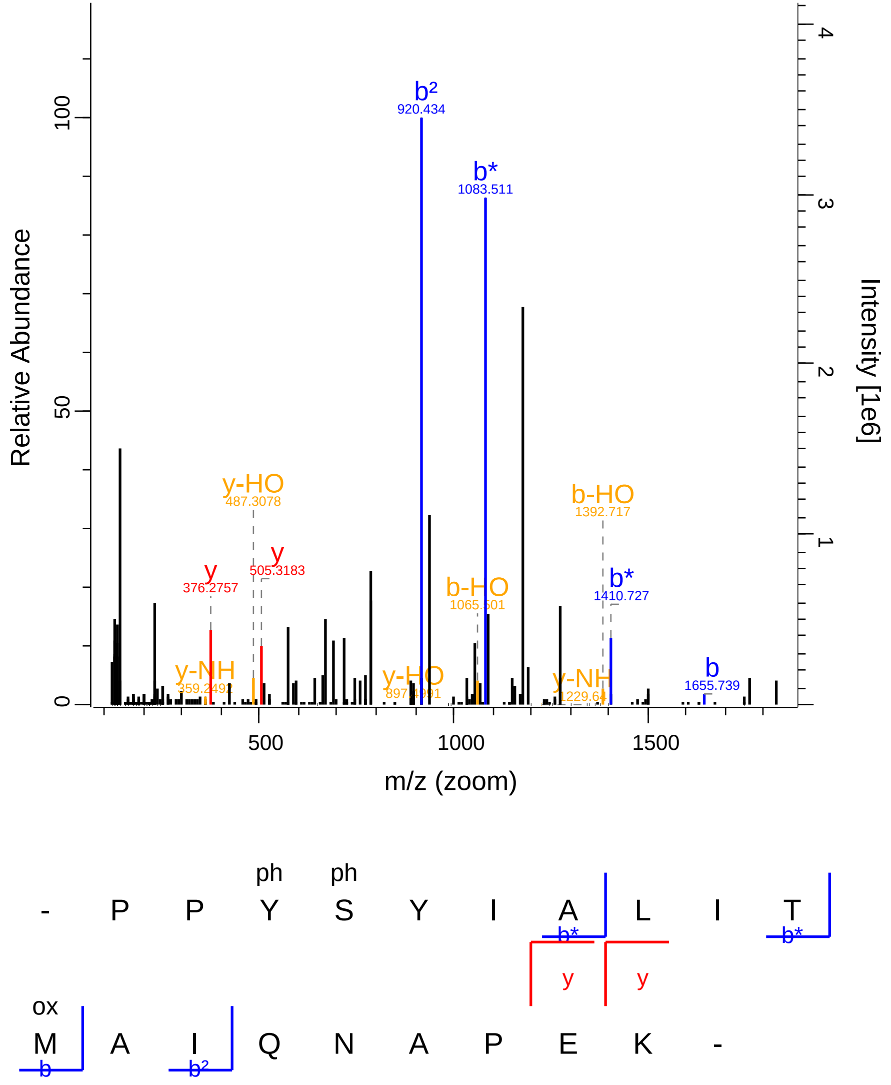

Raw File	Scan	Method	Score	m/z	Gene names
RO-QC-160121_ZackD_SS_Set_02_Fractio	32403	FTMS; HC	83.63	655.02	SH3BP4

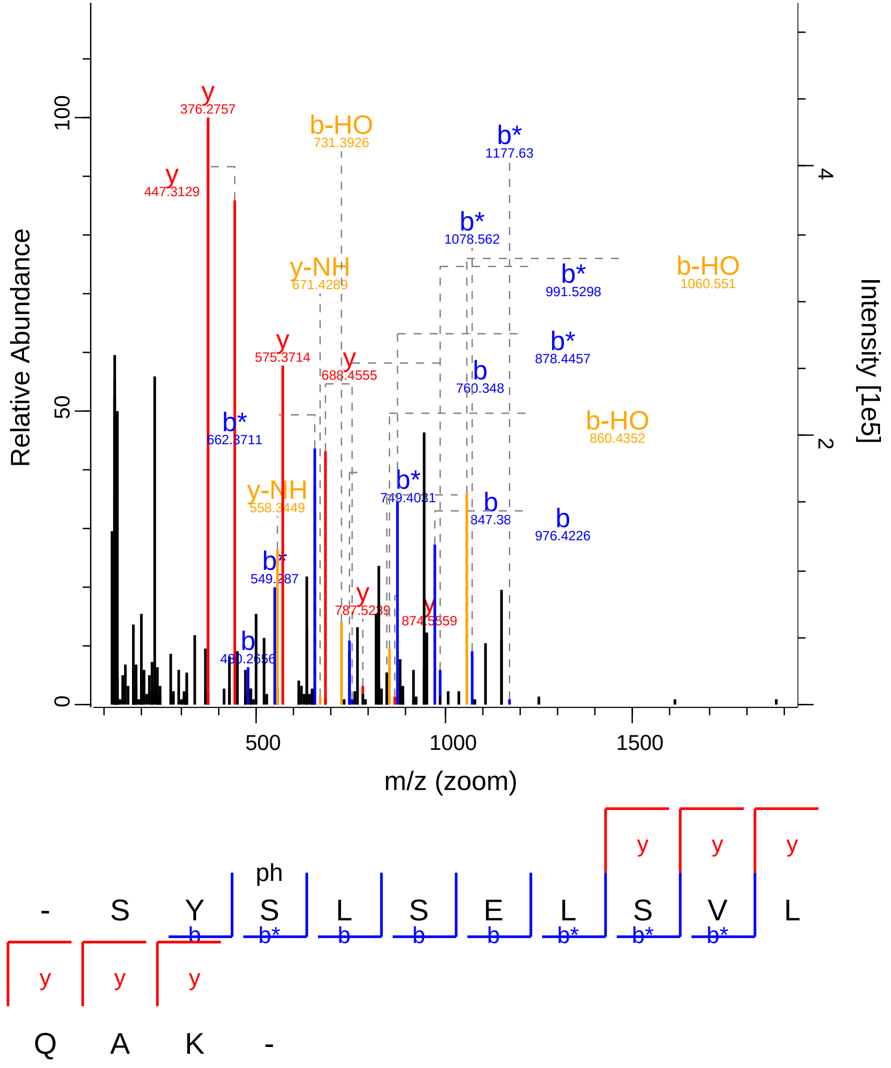

Raw File	Scan	Method	Score	m/z	Gene names
RO-QC-160121_ZackD_SS_Set_02_Fractic	32693	FTMS; HC	75.22	1097.84	LGMN

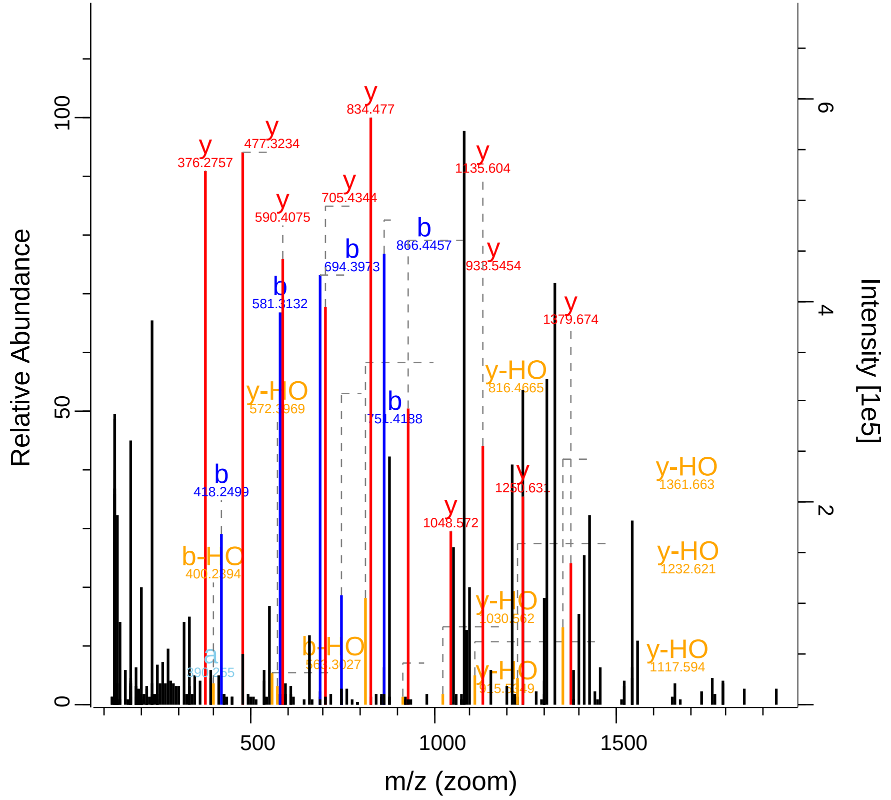

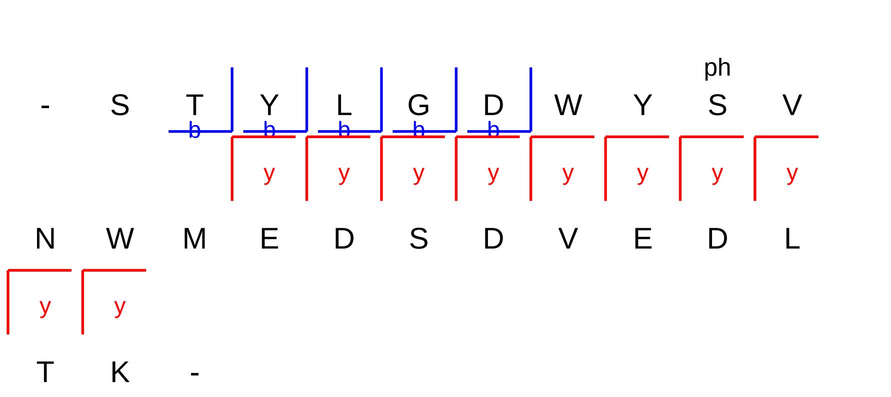

Raw File	Scan	Method	Score	m/z	Gene names
RO-QC-160121_ZackD_SS_Set_02_Fractic	33141	FTMS; HC	78.66	1083.09	RSL1D1

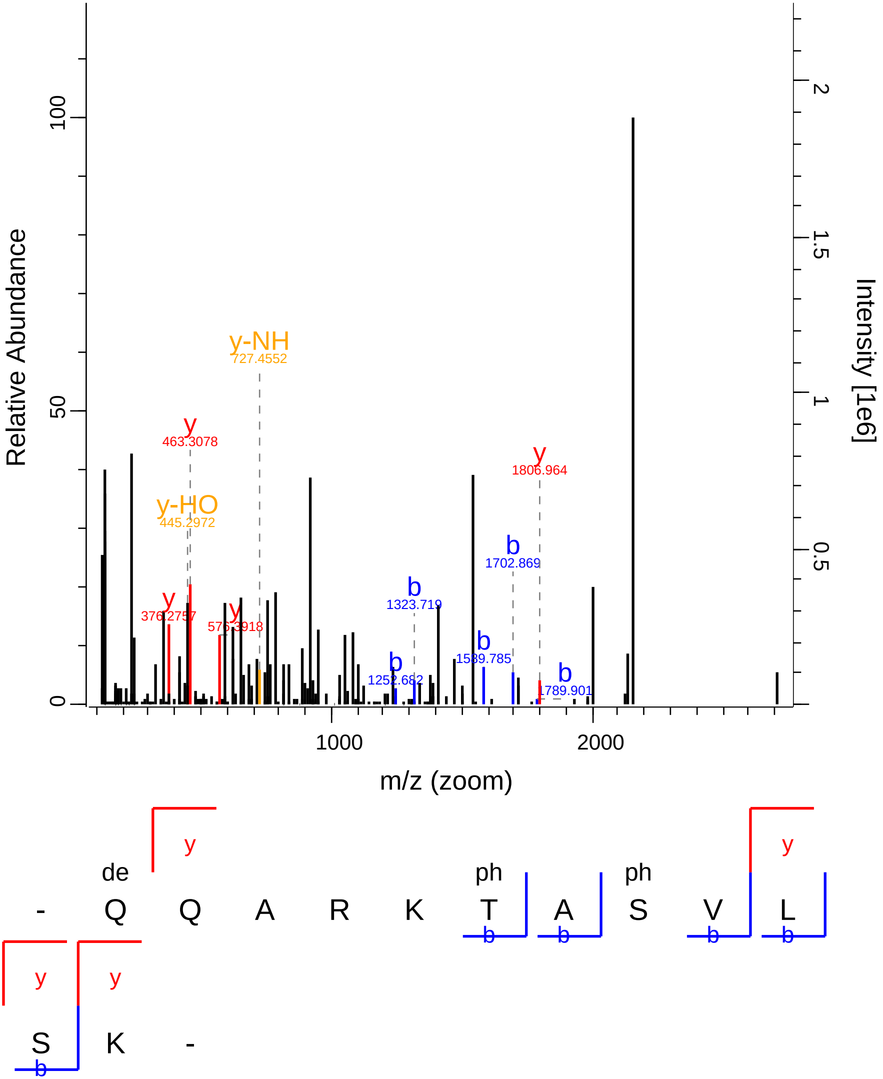

Raw File	Scan	Method	Score	m/z	Gene names
RO-QC-160121_ZackD_SS_Set_02_Fractio	33615	FTMS; HC	63.15	768.11	VIM

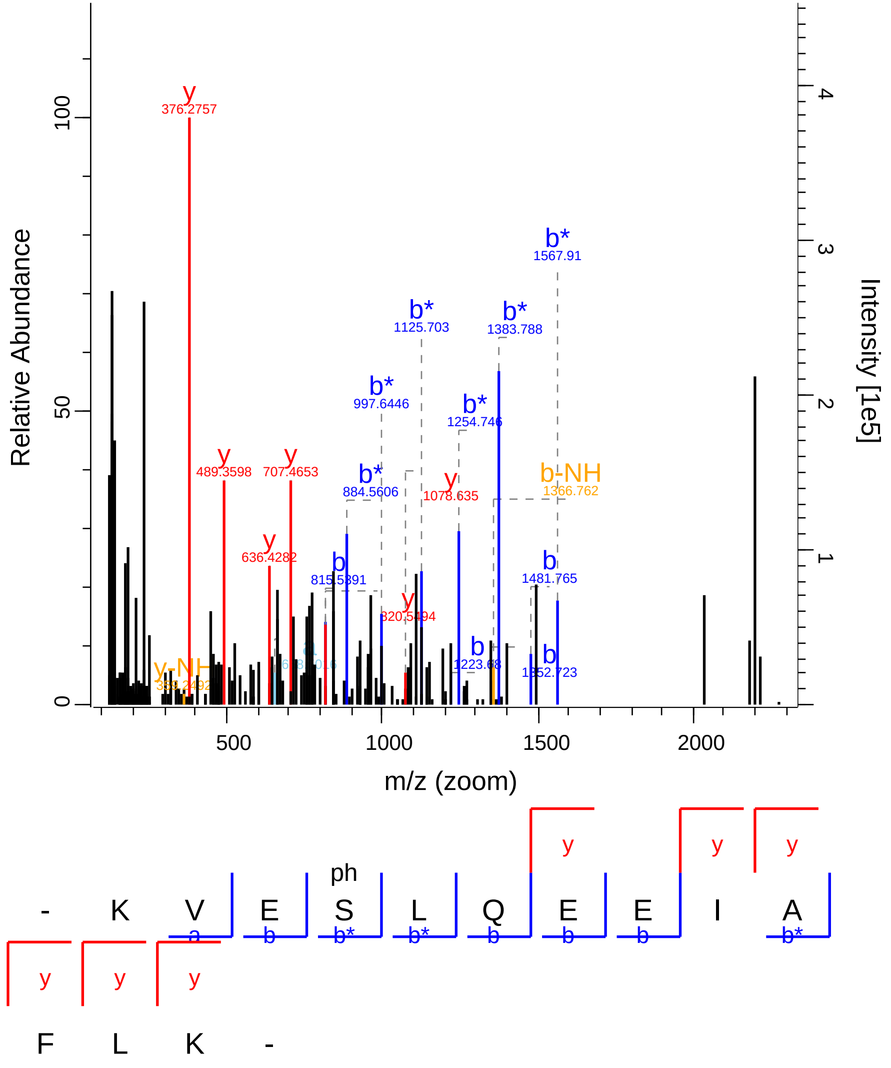

Raw File	Scan	Method	Score	m/z	Gene names
RO-QC-160121_ZackD_SS_Set_02_Fra	34083	FTMS; HC	71.14	882.77	MYL12B;MYL12A;MY

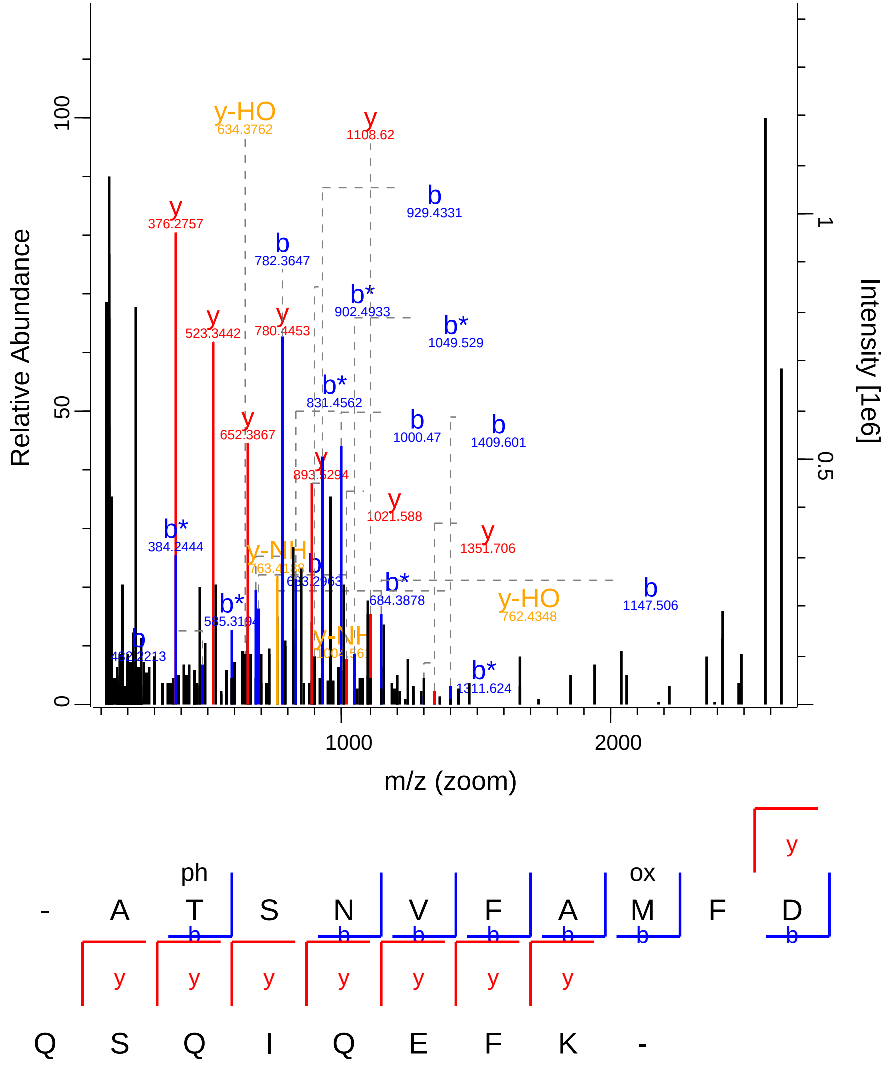

Raw File	Scan	Method	Score	m/z	Gene names
RO-QC-160121_ZackD_SS_Set_02_Fractio	34851	FTMS; HC	177.78	1034.58	NELFB

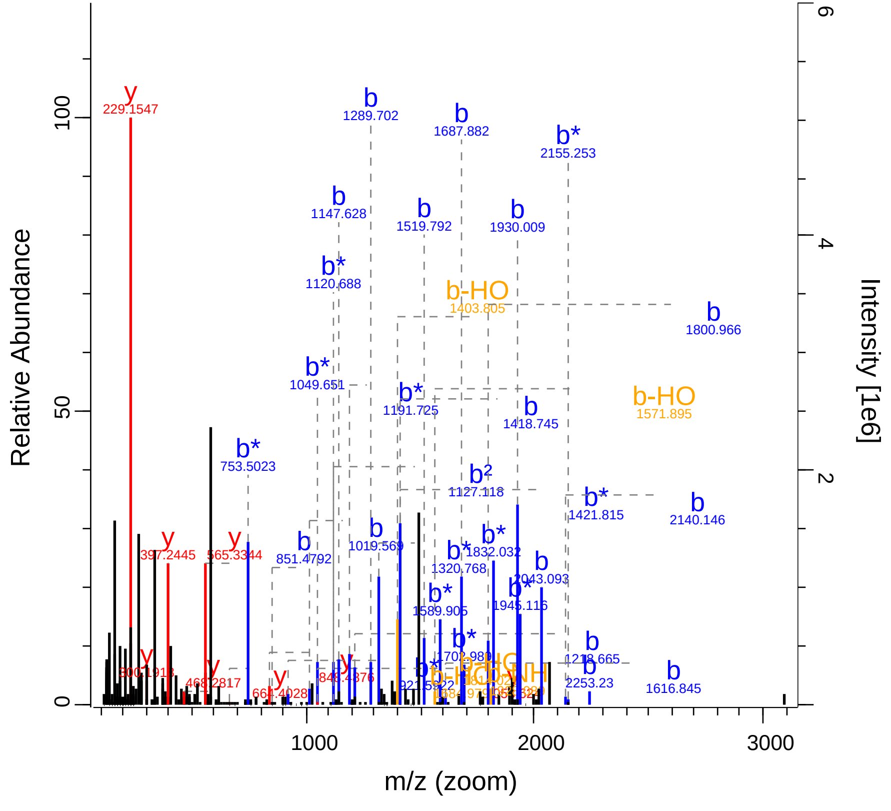

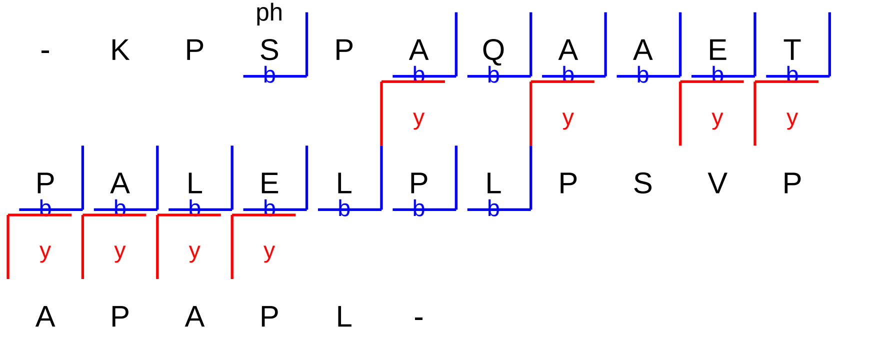

Raw File	Scan	Method	Score	m/z	Gene names
RO-QC-160121_ZackD_SS_Set_02_Fractio	35035	FTMS; HC	48.88	904.84	PKHD1L1

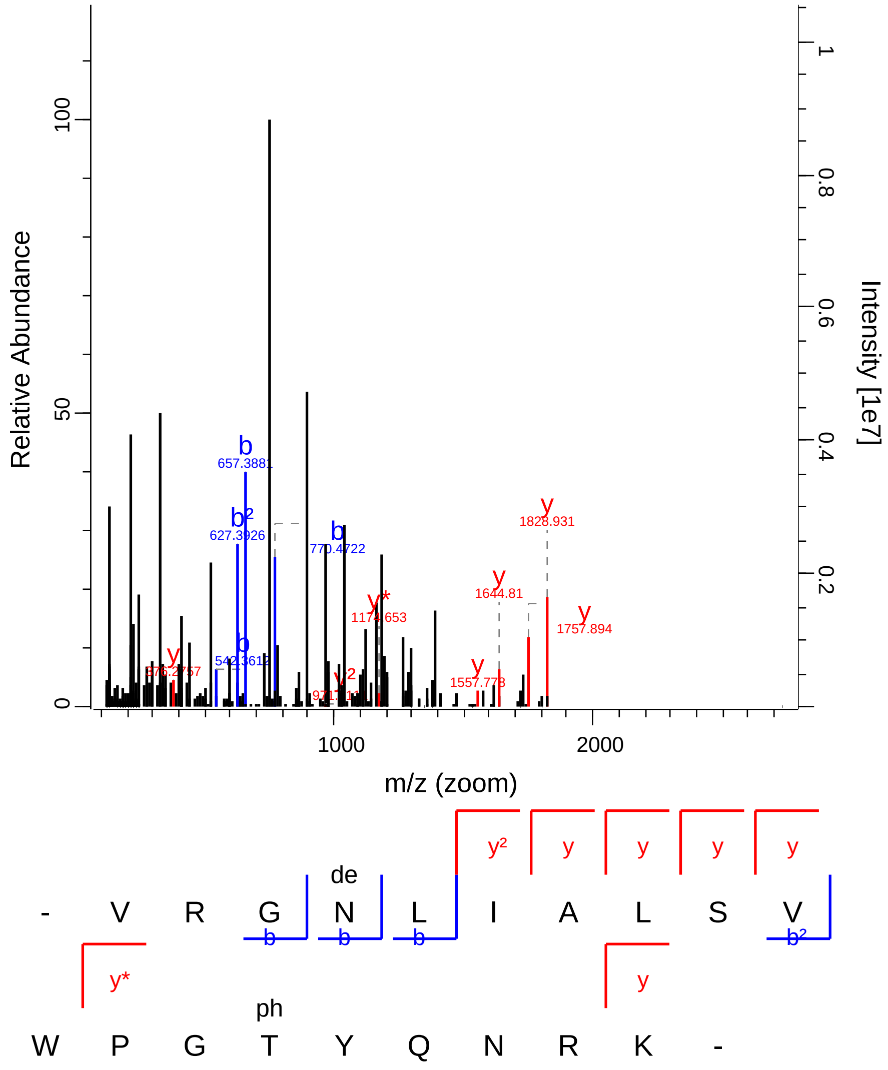

Raw File	Scan	Method	Score	m/z	Gene names
RO-QC-160121_ZackD_SS_Set_02_Fractior	35068	FTMS; HCI	76.26	684.4	ATR

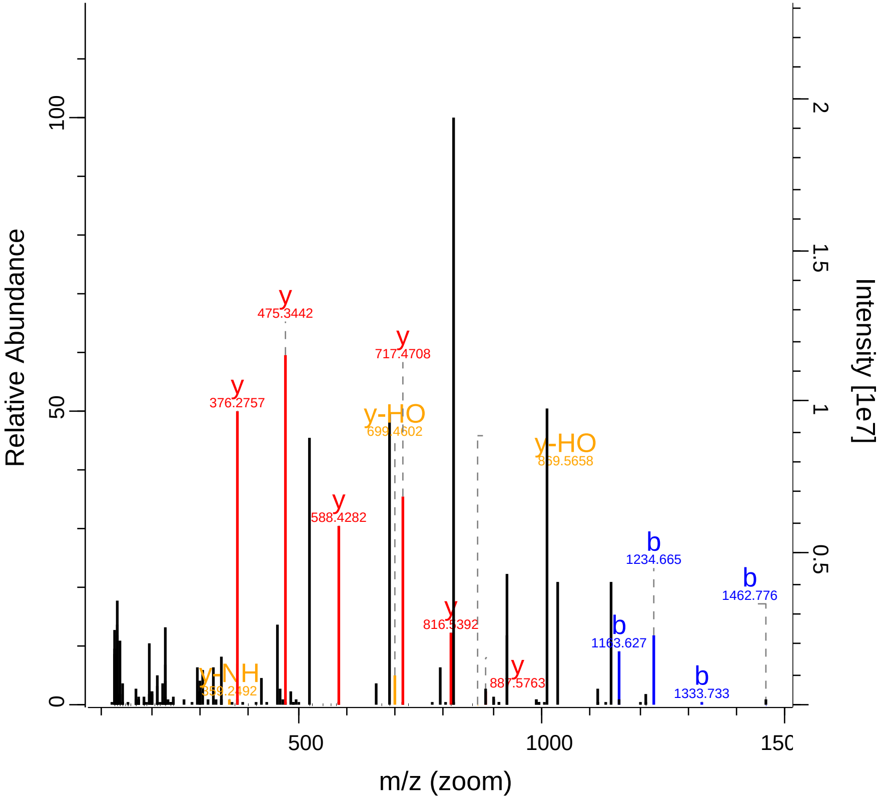

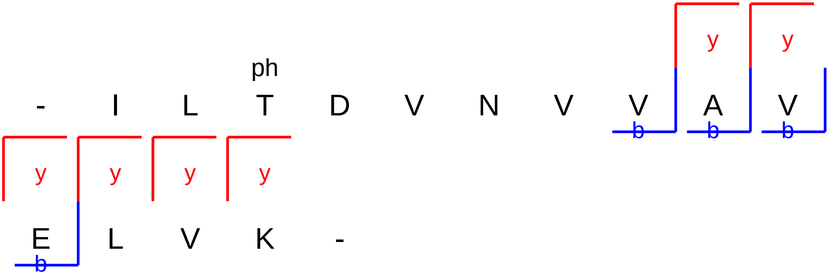

Raw File	Scan	Method	Score	m/z	Gene names
RO-QC-160121_ZackD_SS_Set_02_Fractio	35076	FTMS; HC	105.79	627.31	YAP1

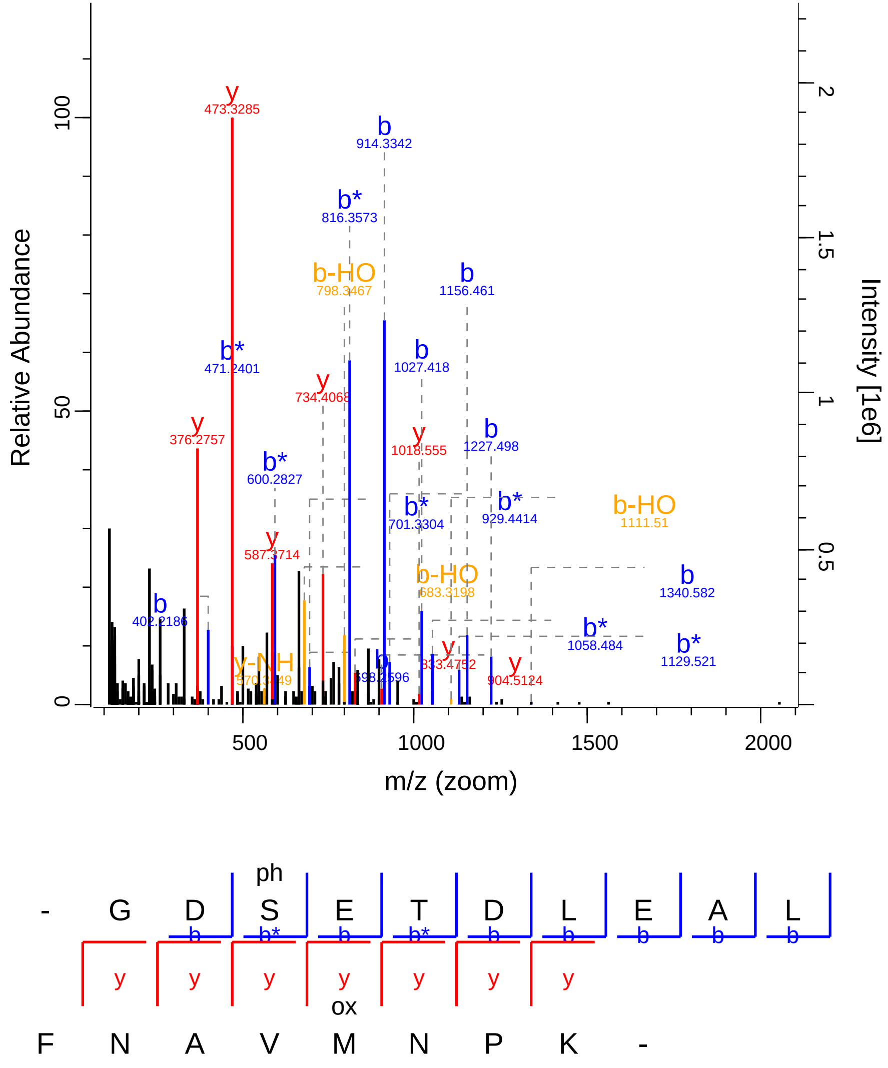

Raw File	Scan	Method	Score	m/z	Gene names
RO-QC-160121_ZackD_SS_Set_02_Fractio	35363	FTMS; HC	80.24	805.94	CCT2

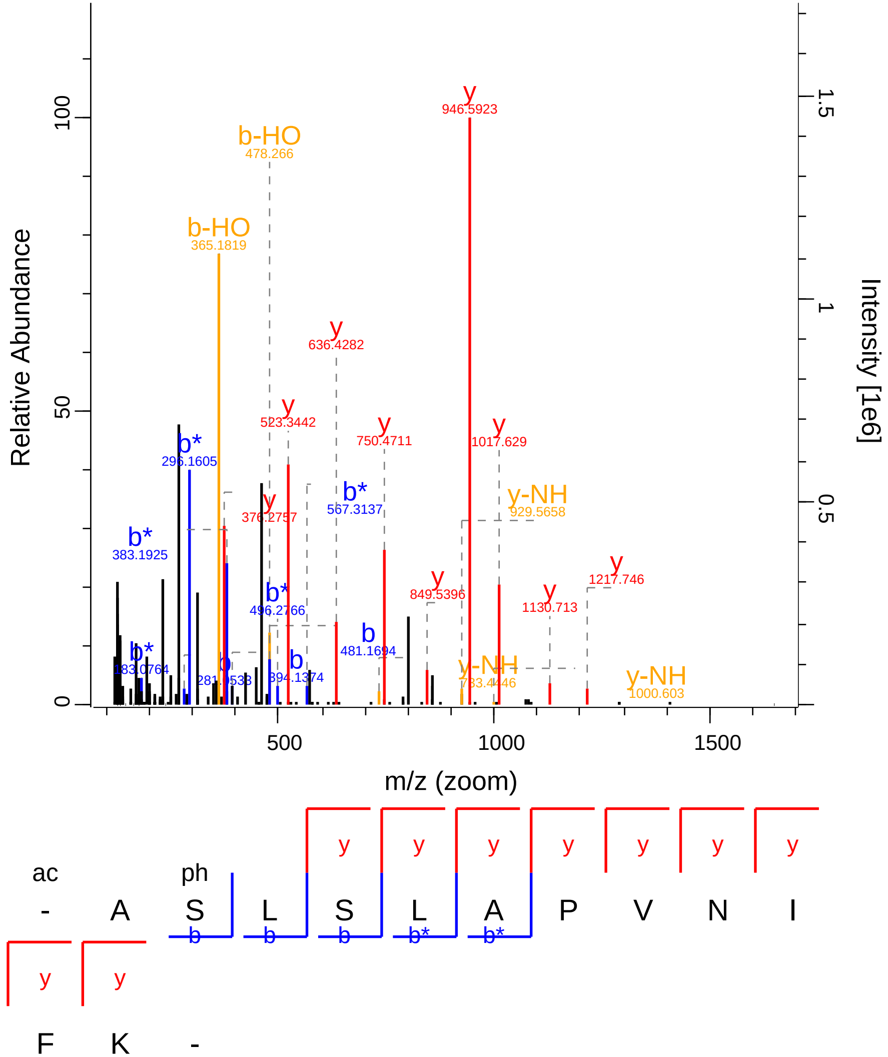

Raw File	Scan	Method	Score	m/z	Gene names
RO-QC-160121_ZackD_SS_Set_02_Fractior	4844	FTMS; HCI	80.74	673.67	APC

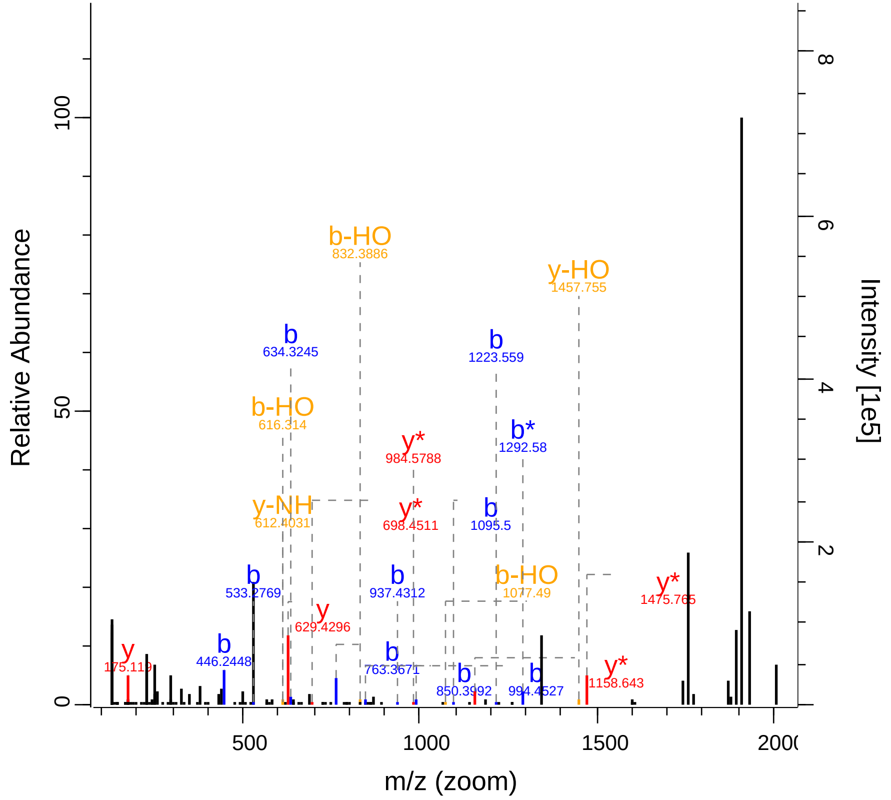

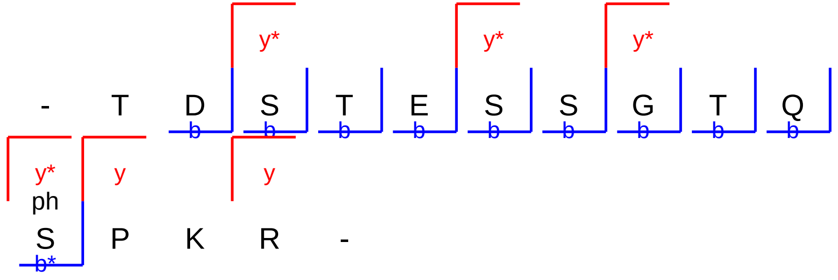

Raw File	Scan	Method	Score	m/z	Gene names
RO-QC-160121_ZackD_SS_Set_02_Fractior	4980	FTMS; HCI	67.46	628.02	RRBP1

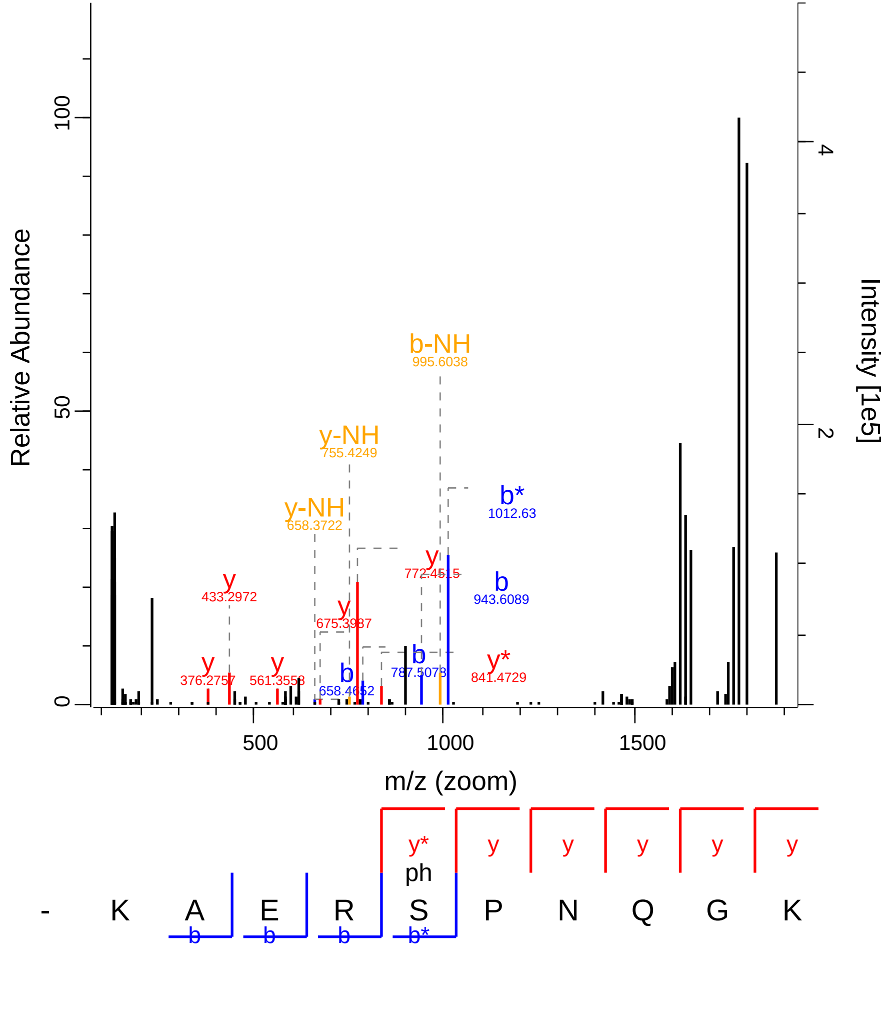

Raw File	Scan	Method	Score	m/z	Gene names
RO-QC-160121_ZackD_SS_Set_02_Fractio	6471	FTMS; HC	103.26	666.01	SRSF11

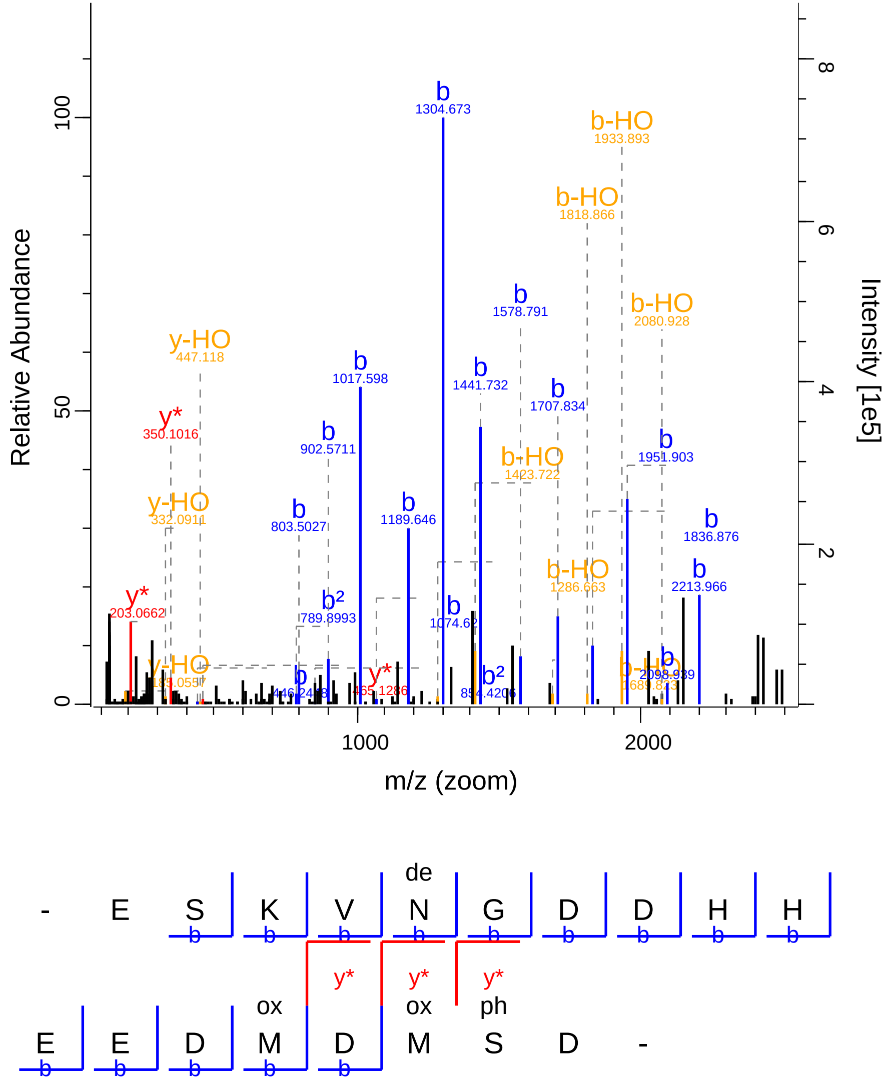

Raw File	Scan	Method	Score	m/z	Gene names
RO-QC-160121_ZackD_SS_Set_02_Fractior	6507	FTMS; HCI	71.08	711.69	LZTS3

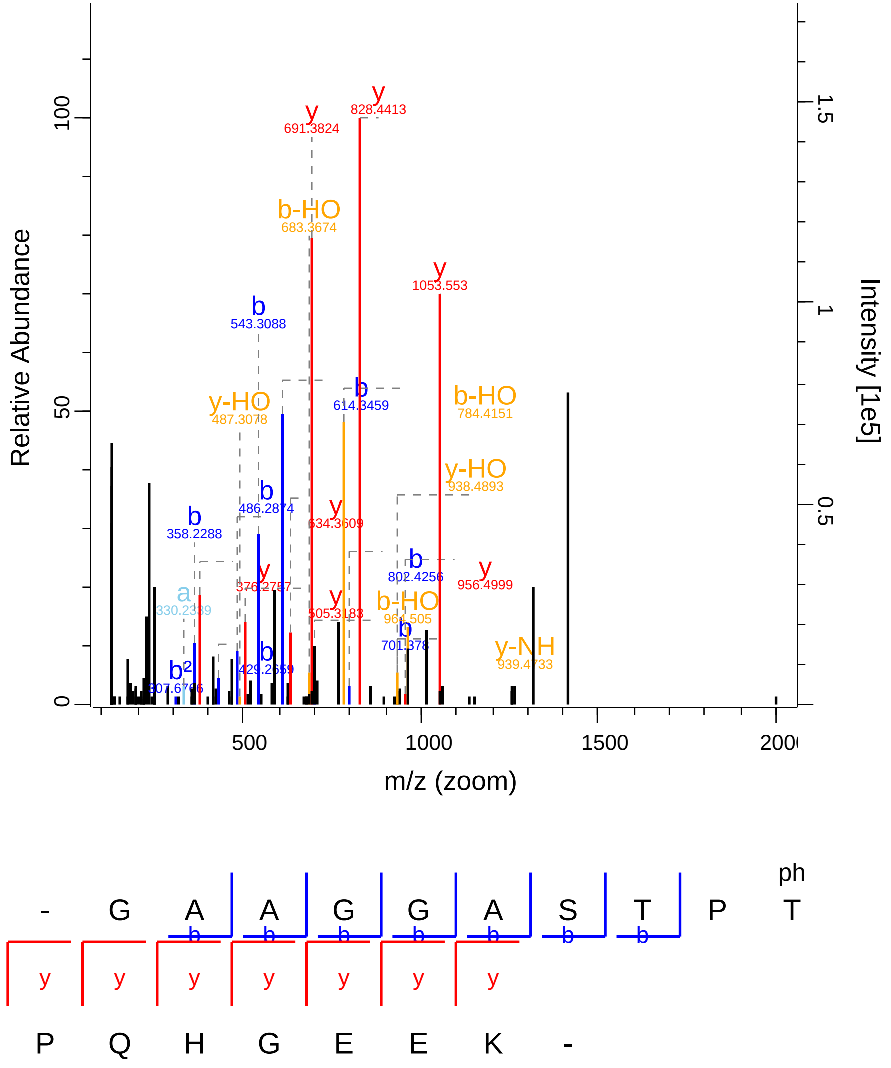

Raw File	Scan	Method	Score	m/z	Gene names
RO-QC-160121_ZackD_SS_Set_02_Fraction	6536	FTMS; HCI	59.51	527.8	SAP130

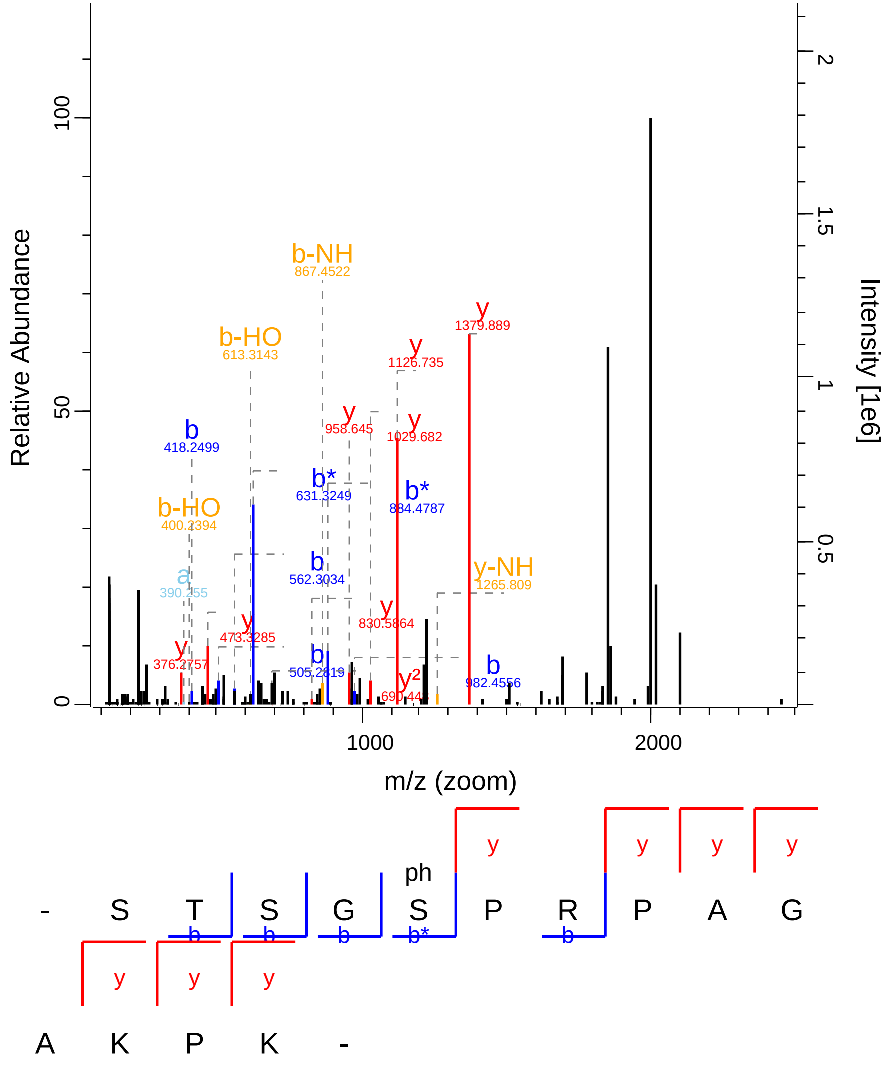

Raw File	Scan	Method	Score	m/z	Gene names
RO-QC-160121_ZackD_SS_Set_02_Fractior	6969	FTMS; HCI	78.08	605.67	CCDC86

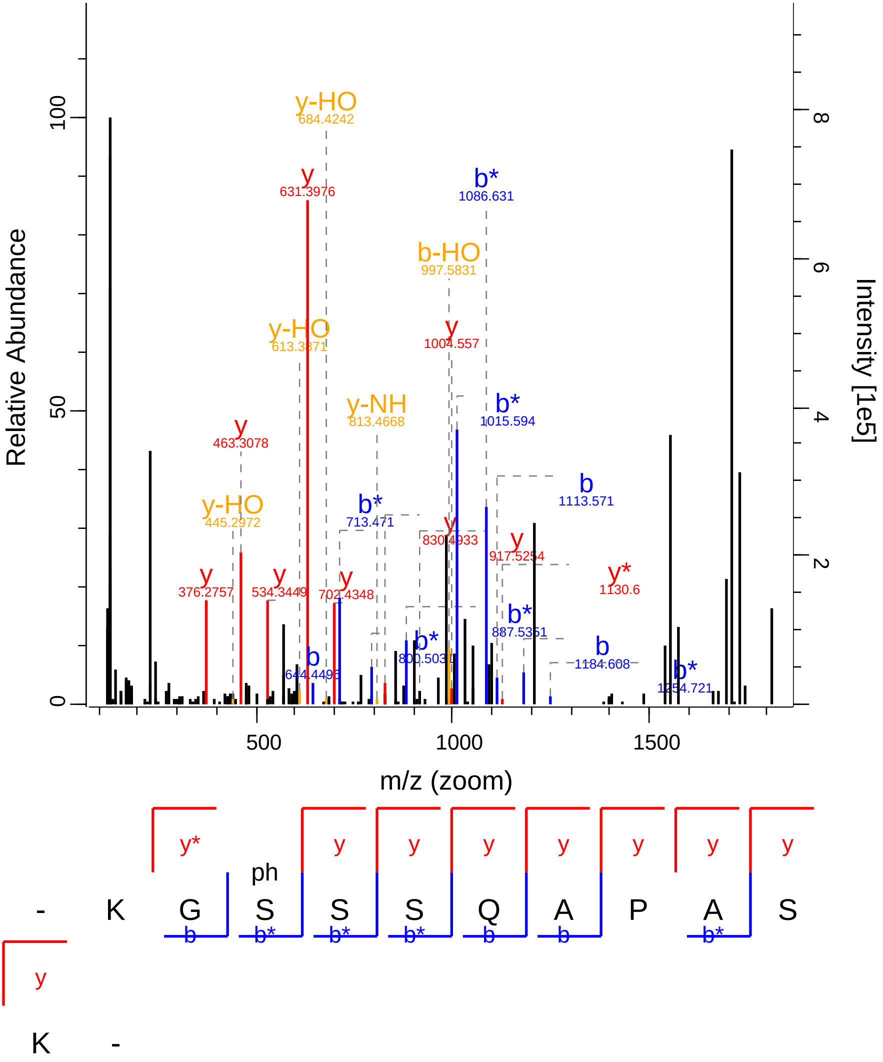

Raw File	Scan	Method	Score	m/z	Gene names
RO-QC-160121_ZackD_SS_Set_02_Fraction_	7067	FTMS; HCI	87.79	713	LMNA

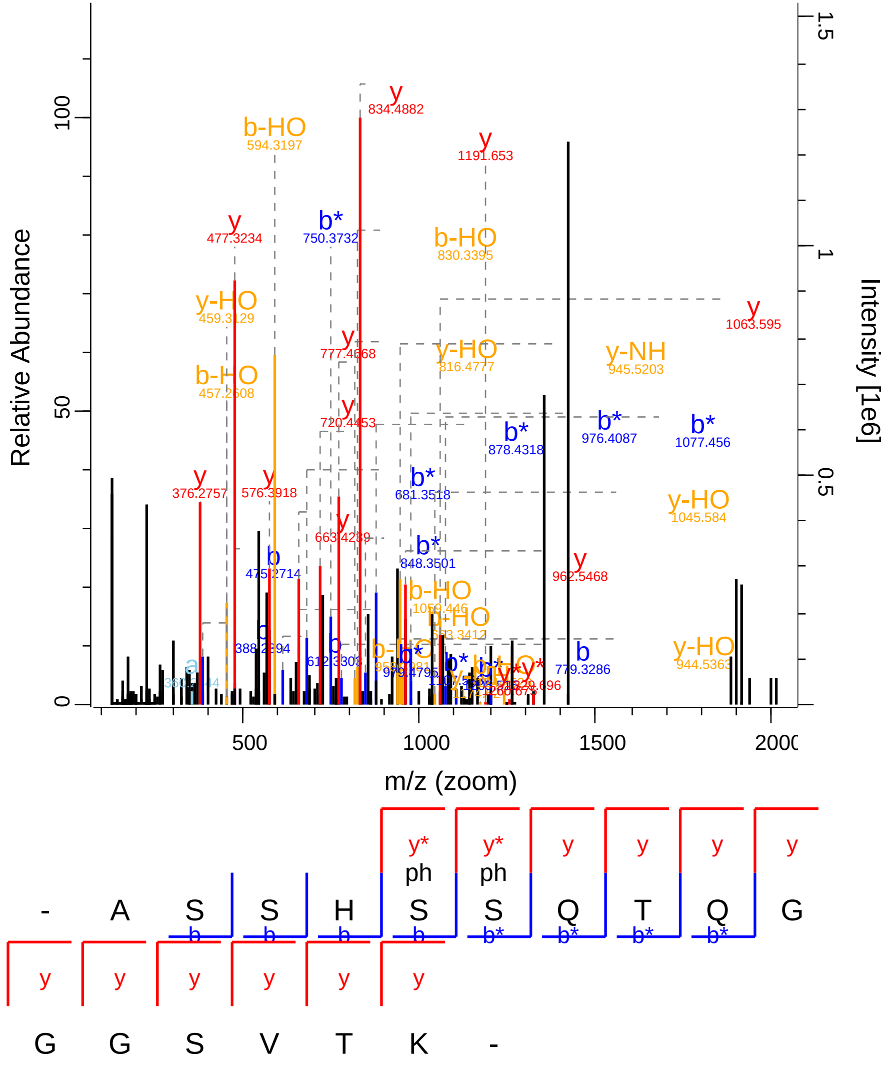

Raw File	Scan	Method	Score	m/z	Gene names
RO-QC-160121_ZackD_SS_Set_02_Fractio	7098	FTMS; HC	138.73	1068.99	LMNA

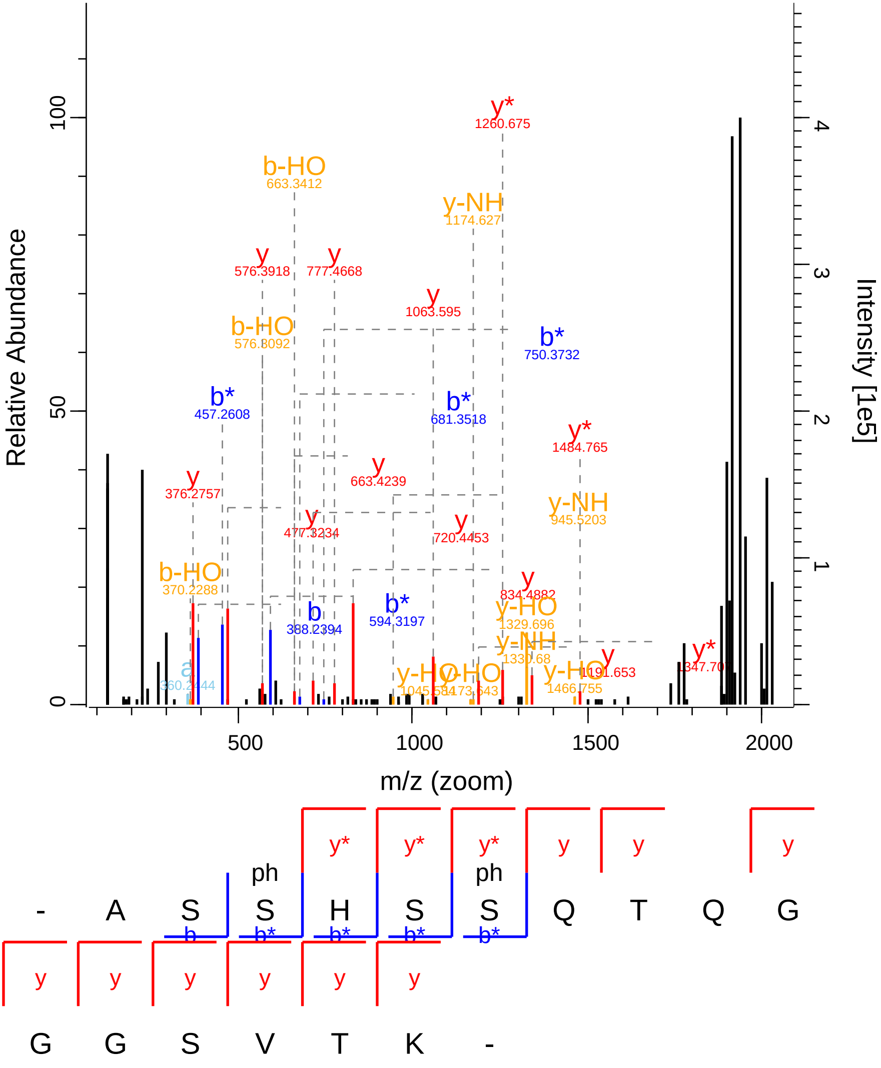

Raw File	Scan	Method	Score	m/z	Gene names
RO-QC-160121_ZackD_SS_Set_02_Fractior	7227	FTMS; HCI	50.08	648.36	SLTM

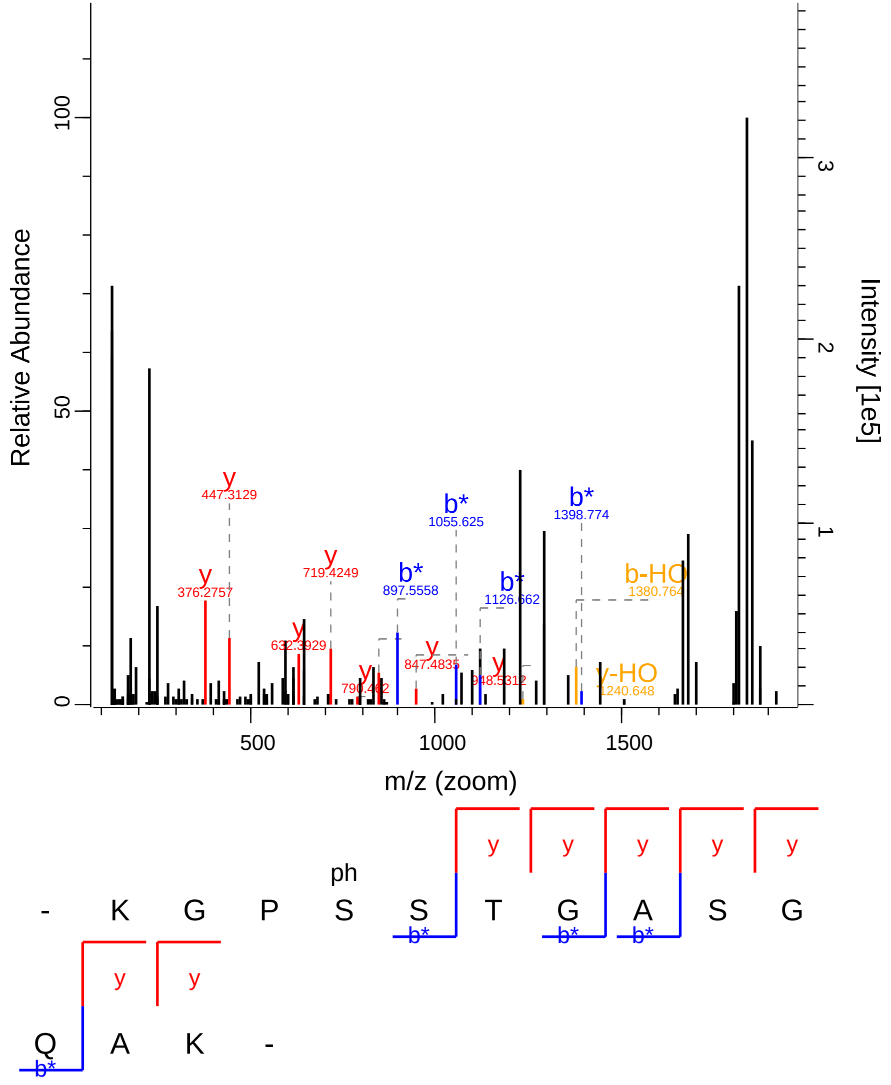

Raw File	Scan	Method	Score	m/z	Gene names
RO-QC-160121_ZackD_SS_Set_02_Fractior	7329	FTMS; HCI	85.32	722.86	SLC6A13

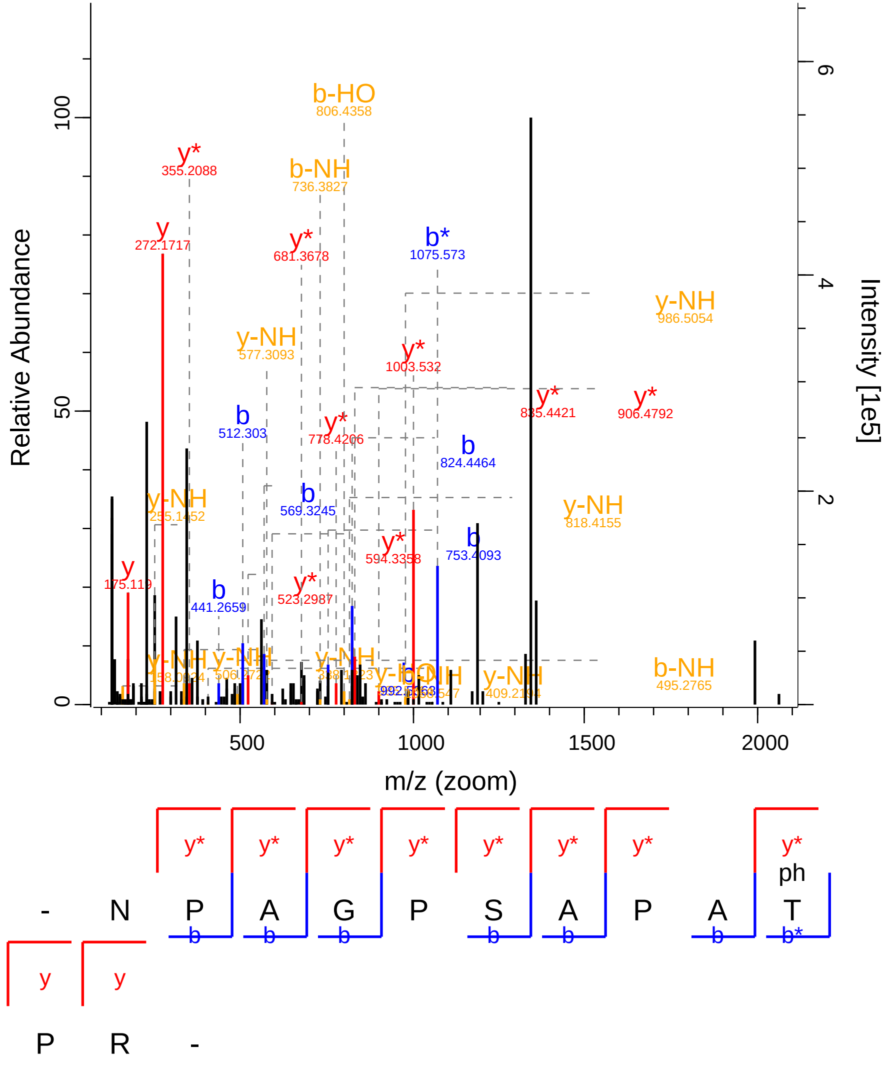

Raw File	Scan	Method	Score	m/z	Gene names
RO-QC-160121_ZackD_SS_Set_02_Fractior	8949	FTMS; HCI	84.87	757.74	ADD1

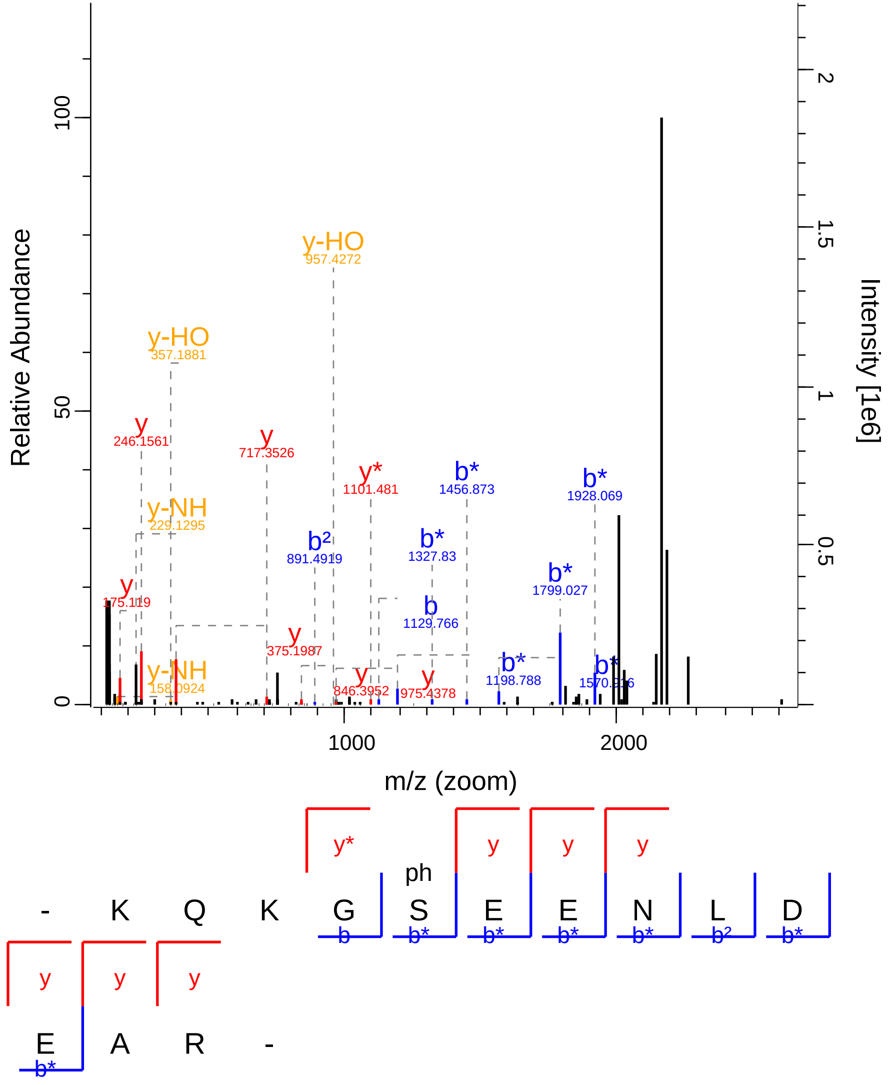

Raw File	Scan	Method	Score	m/z	Gene names
RO-QC-160121_ZackD_SS_Set_02_Fractioı	10674	FTMS; HCI	102.94	931.2	CLCC1

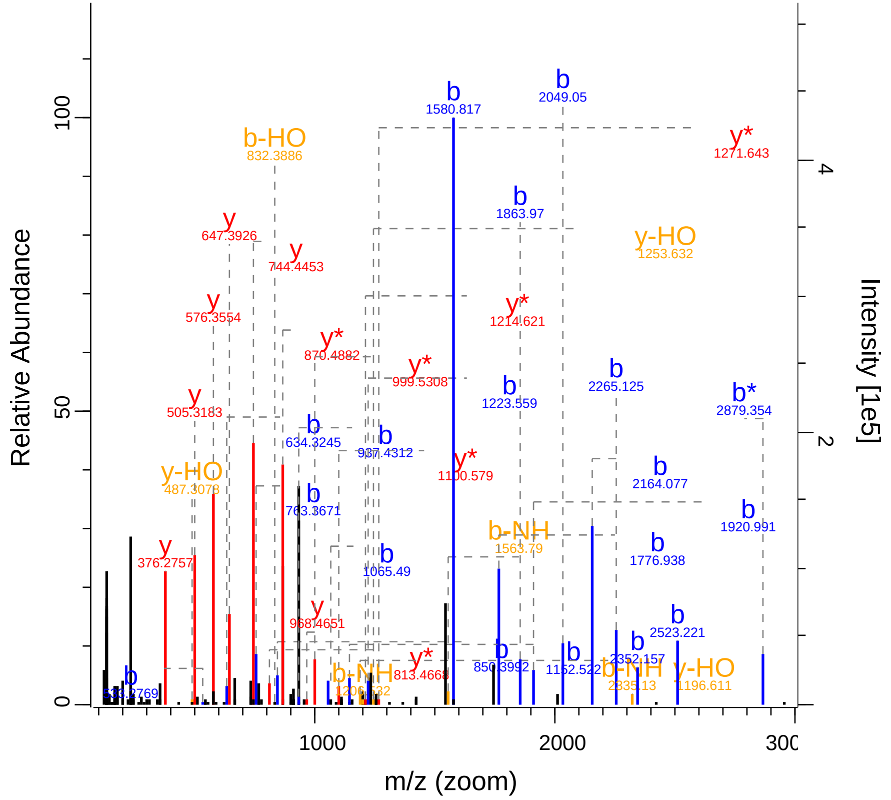

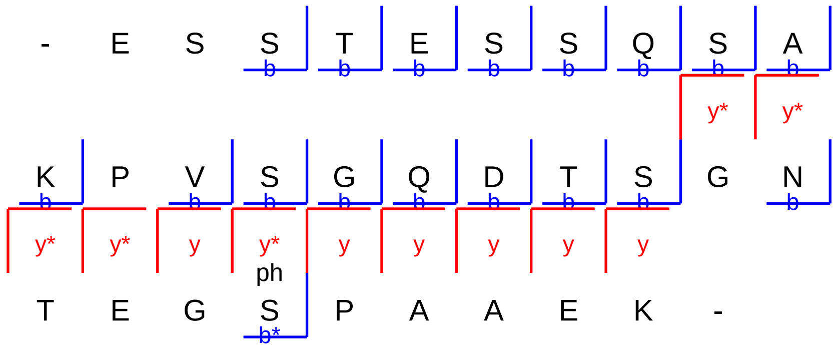

Raw File	Scan	Method	Score	m/z	Gene names
RO-QC-160121_ZackD_SS_Set_02_Fractio	10853	FTMS; HC	104.84	741.71	PI4KB

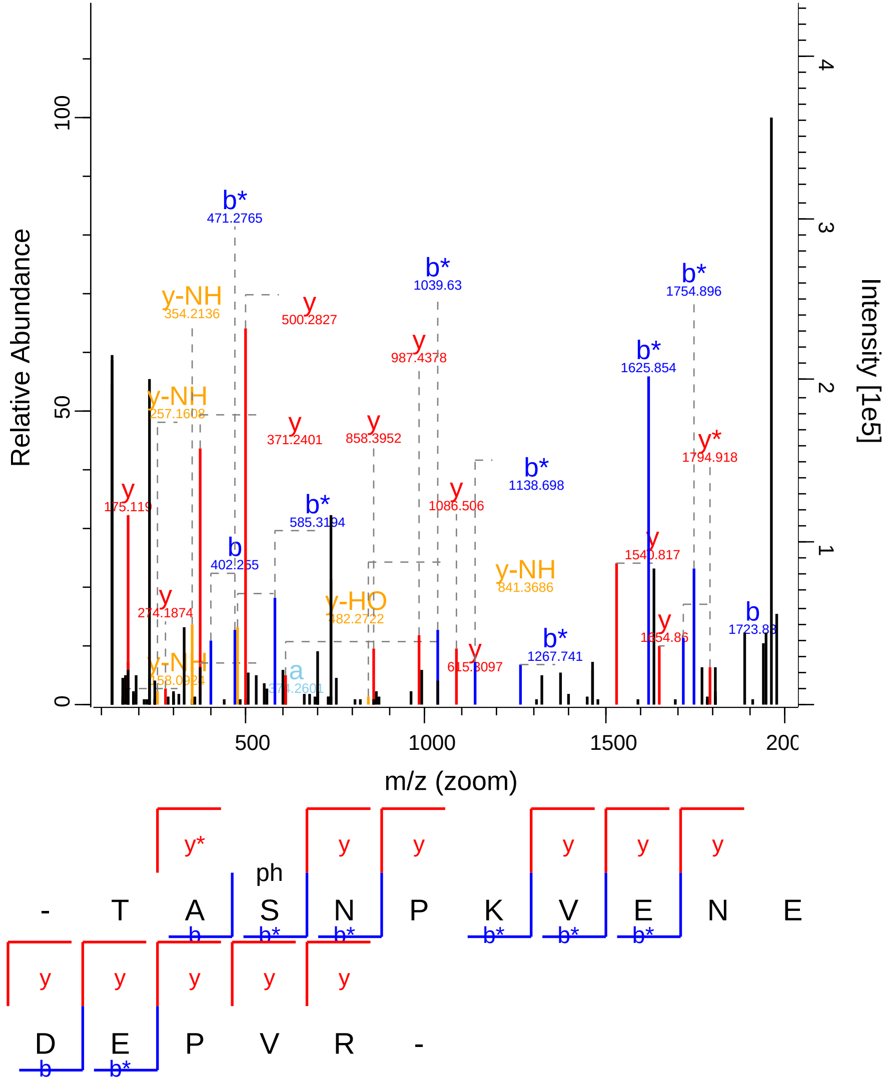

Raw File	Scan	Method	Score	m/z	Gene names
RO-QC-160121_ZackD_SS_Set_02_Fractio	11243	FTMS; HC	42.81	717.36	TRAF1

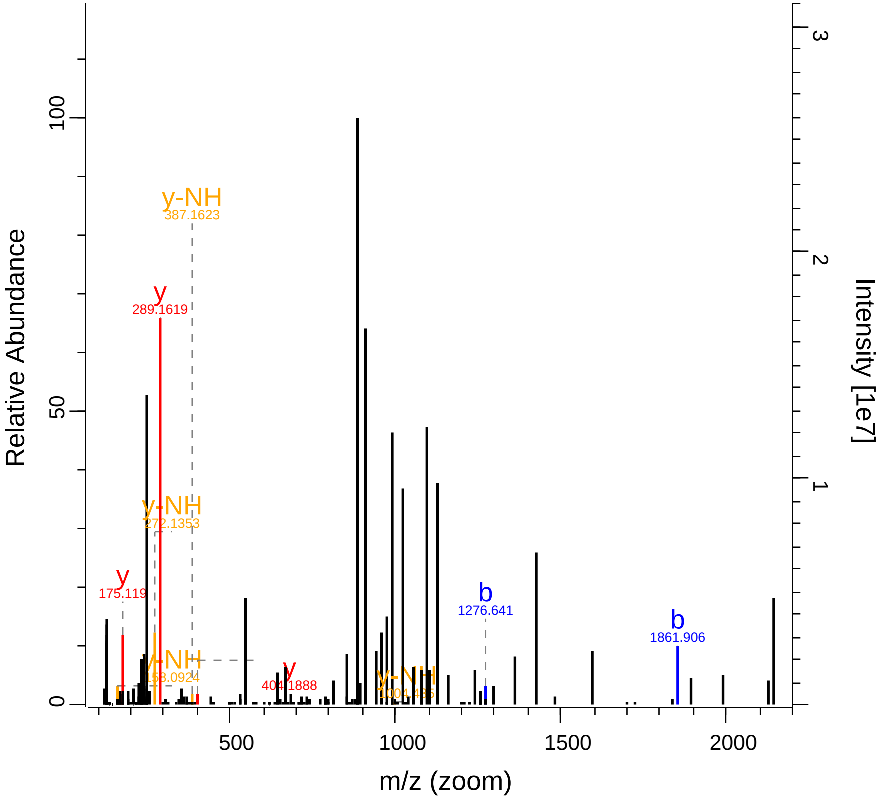

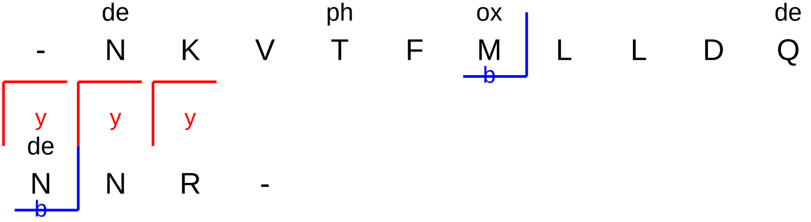

Raw File	Scan	Method	Score	m/z	Gene names
RO-QC-160121_ZackD_SS_Set_02_Fractio	11597	FTMS; HC	93.27	858.05	NUCKS1

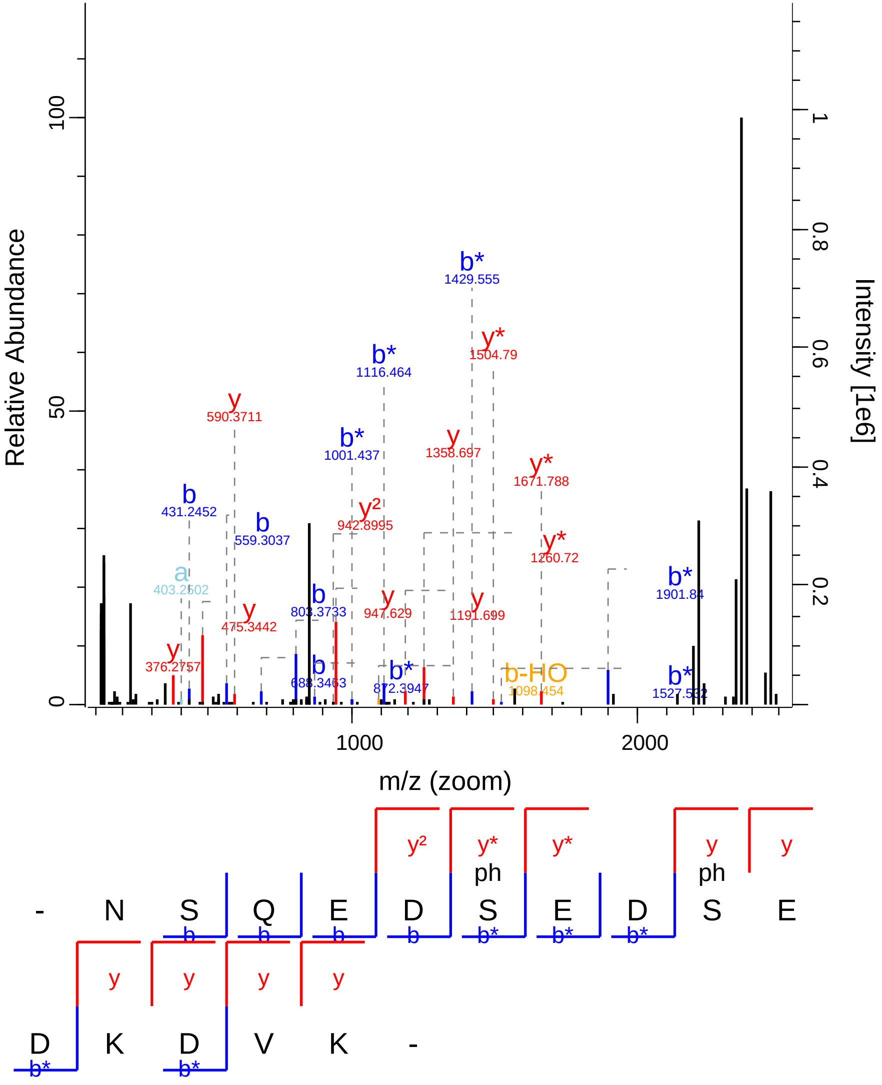

Raw File	Scan	Method	Score	m/z	Gene names
RO-QC-160121_ZackD_SS_Set_02_Fraction	11637	FTMS; HCI	81.67	728	NUFIP2

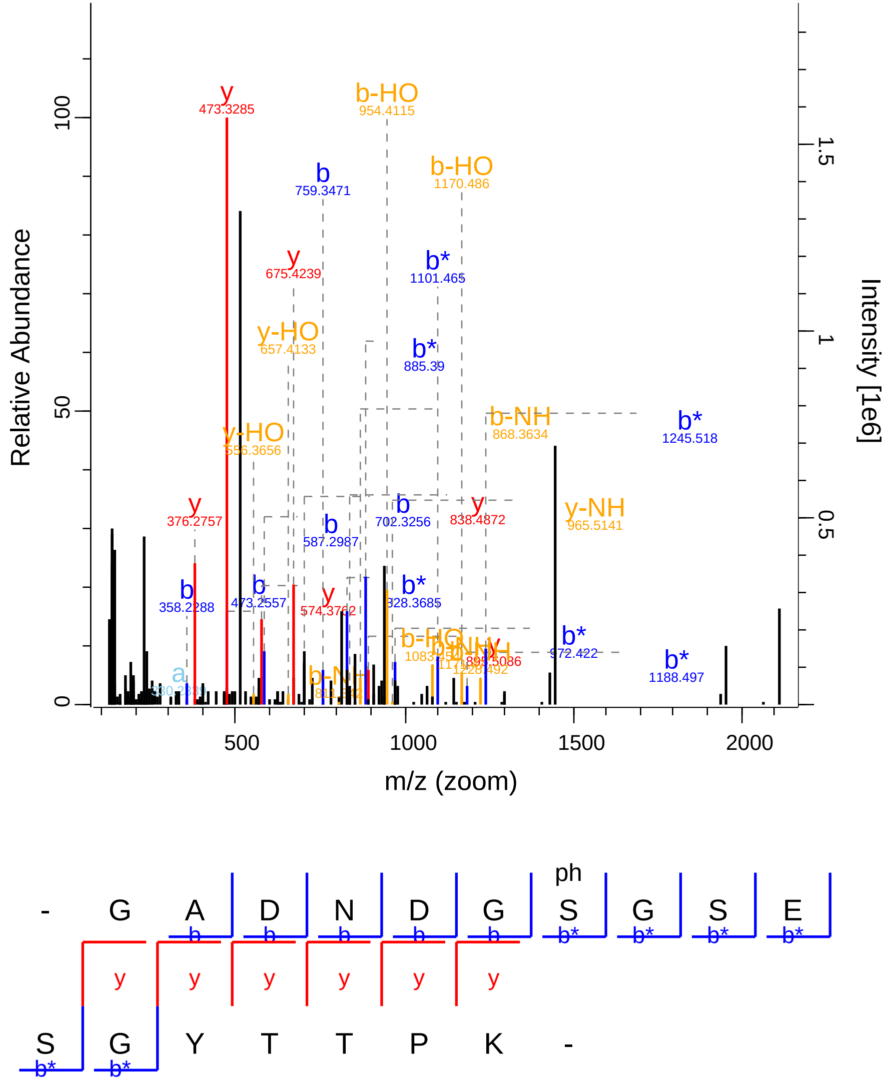

Raw File	Scan	Method	Score	m/z	Gene names
RO-QC-160121_ZackD_SS_Set_02_Fractio	11639	FTMS; HC	41.82	519.04	SIPA1L1

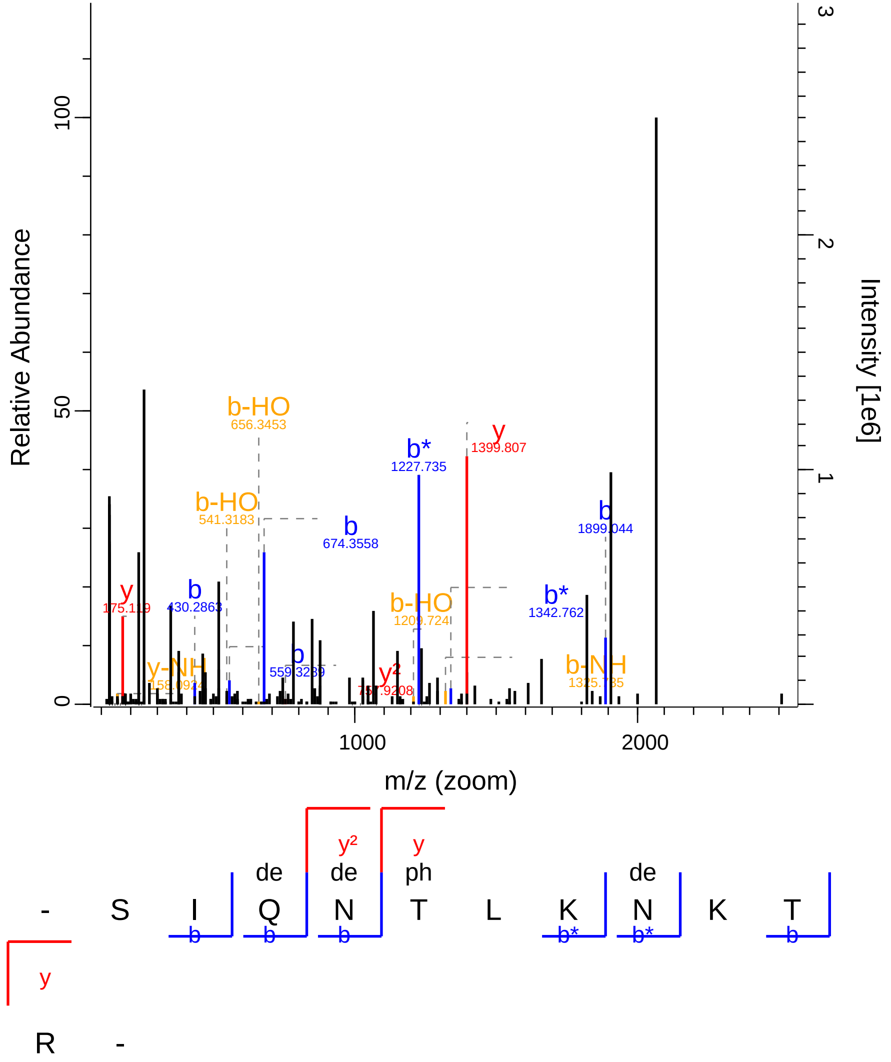

Raw File	Scan	Method	Score	m/z	Gene names
RO-QC-160121_ZackD_SS_Set_02_Fractic	13888	FTMS; HC	50.06	1077.48	TYRP1

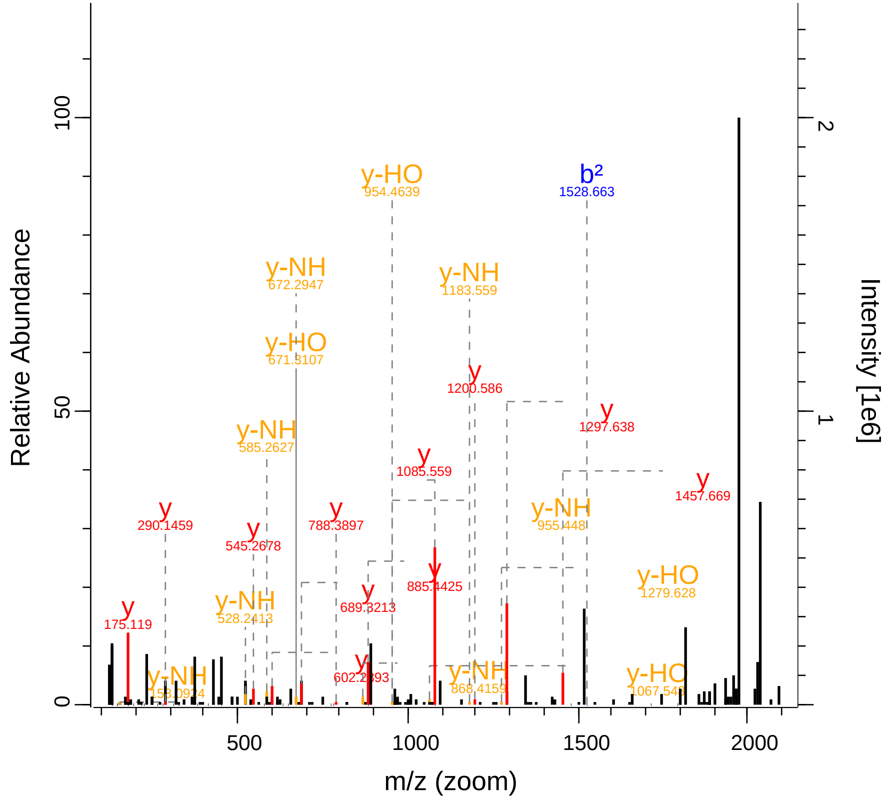

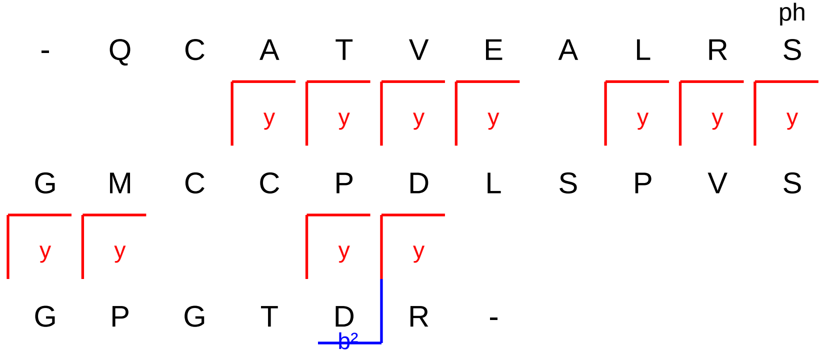

Raw File	Scan	Method	Score	m/z	Gene names
RO-QC-160121_ZackD_SS_Set_02_Fractio	14316	FTMS; HC	86.08	873.79	THRAP3

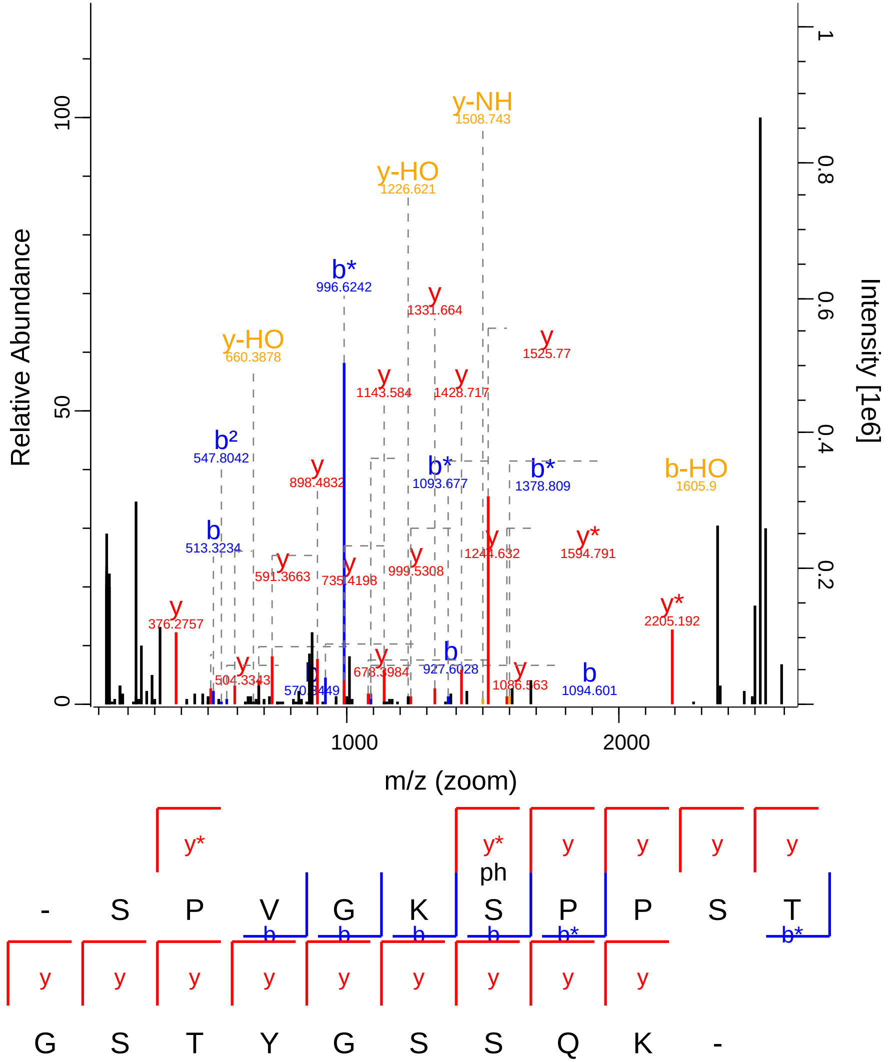

Raw File	Scan	Method	Score	m/z	Gene names
RO-QC-160121_ZackD_SS_Set_02_Fractio	14694	FTMS; HC	50.19	633.71	KTN1

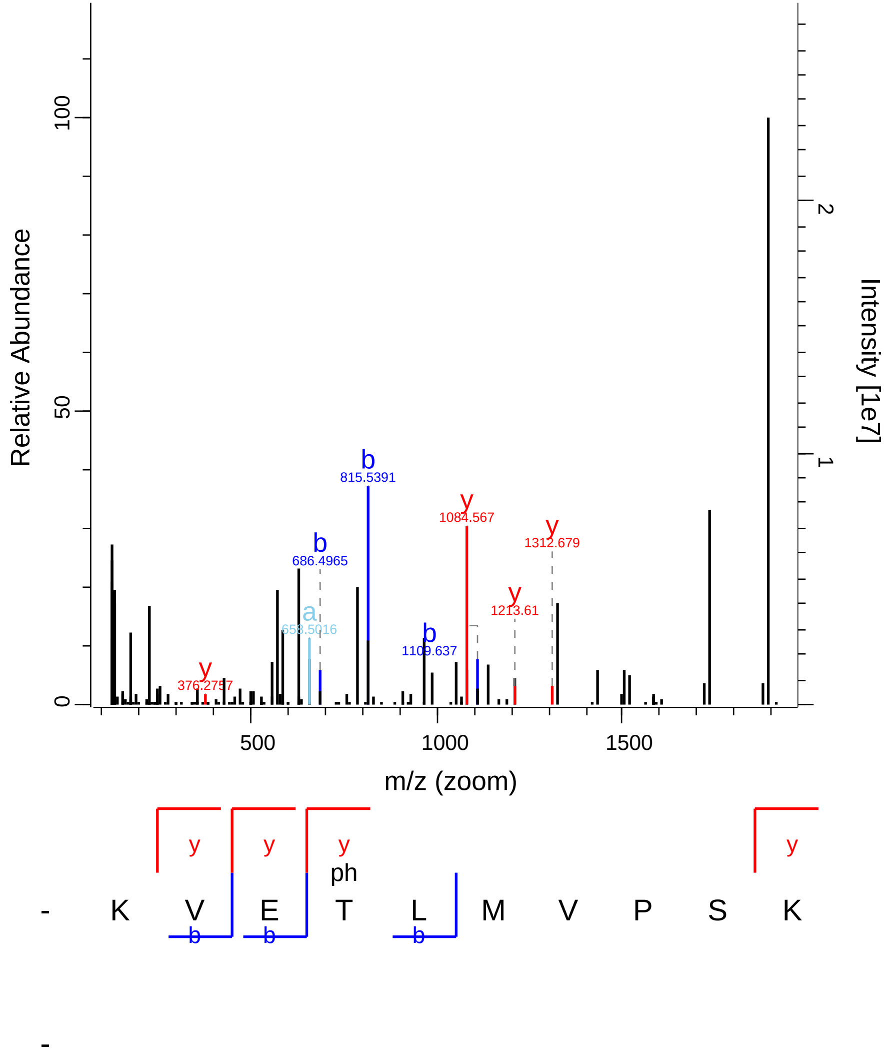

Raw File	Scan	Method	Score	m/z	Gene names
RO-QC-160121_ZackD_SS_Set_02_Fractic	15411	FTMS; HC	134.21	1281.66	HIST1H1B

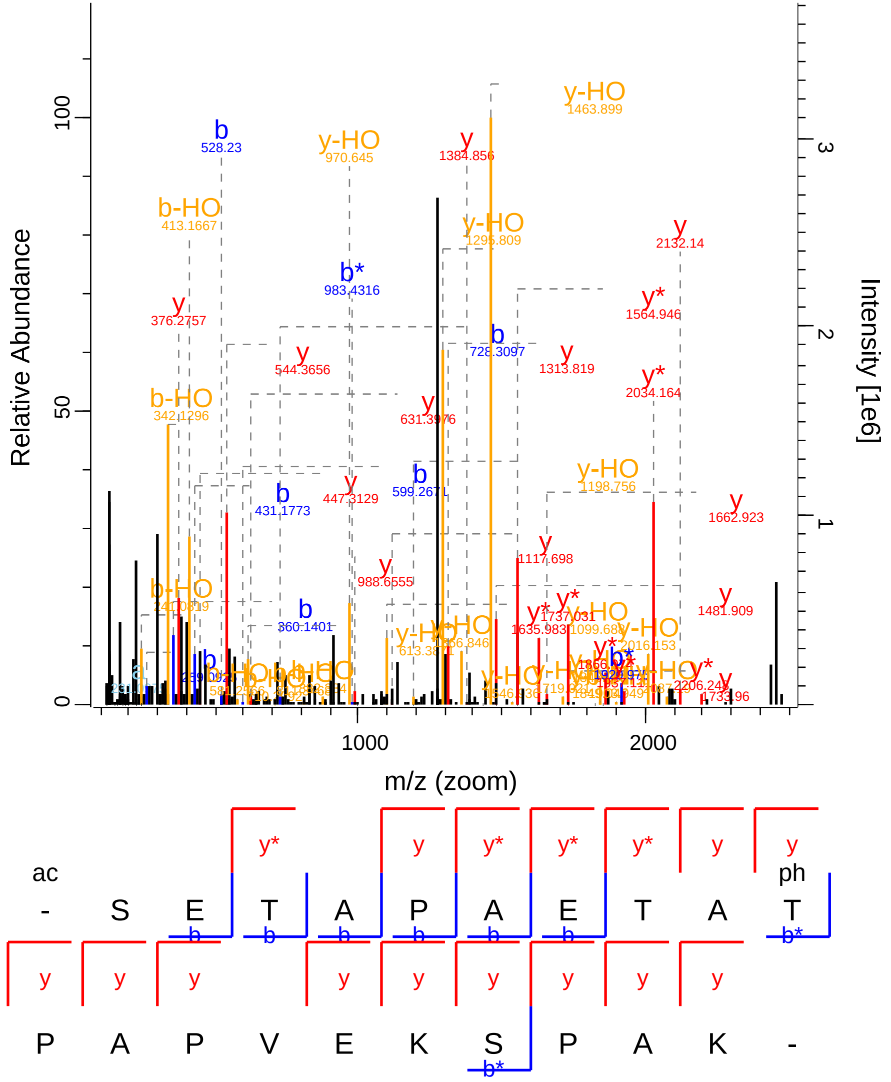

Raw File	Scan	Method	Score	m/z	Gene names
RO-QC-160121_ZackD_SS_Set_02_Fractic	16079	FTMS; HC	84.41	1212.62	DYNC1LI1

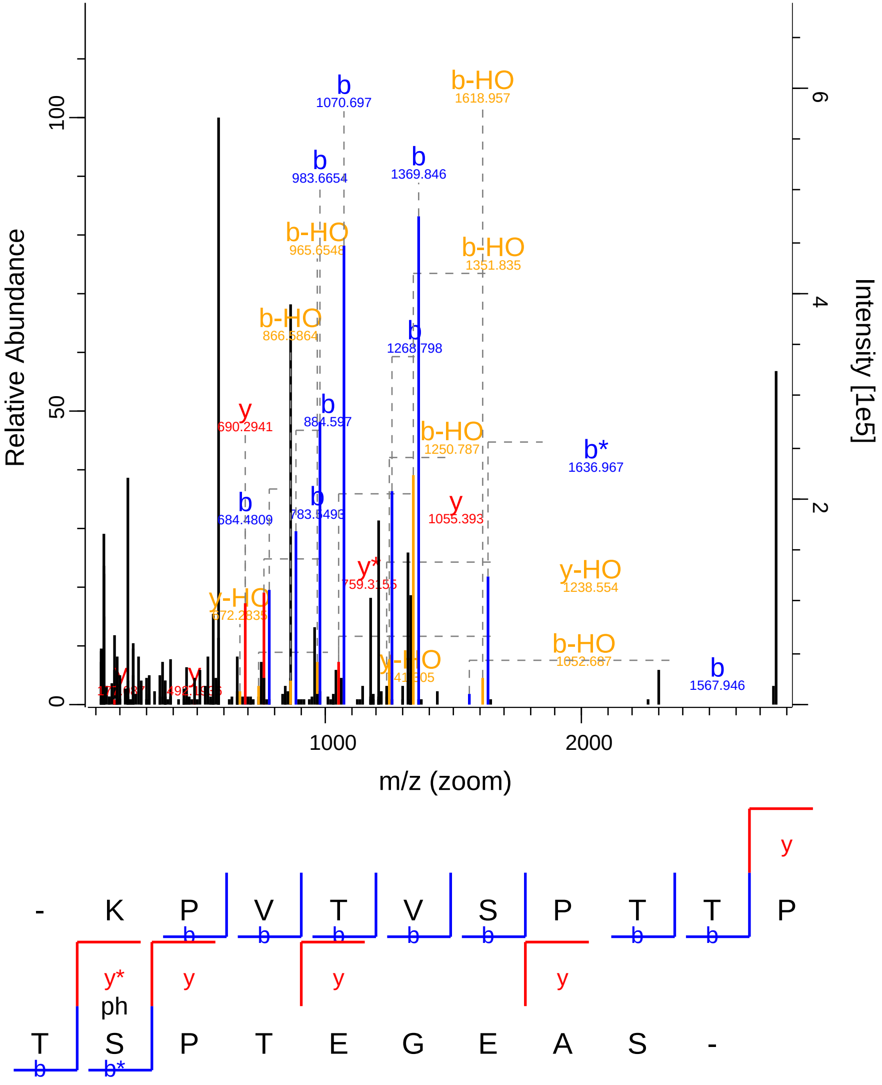

Raw File	Scan	Method	Score	m/z	Gene names
RO-QC-160121_ZackD_SS_Set_02_Fractio	16601	FTMS; HC	117.21	939.81	PNKP

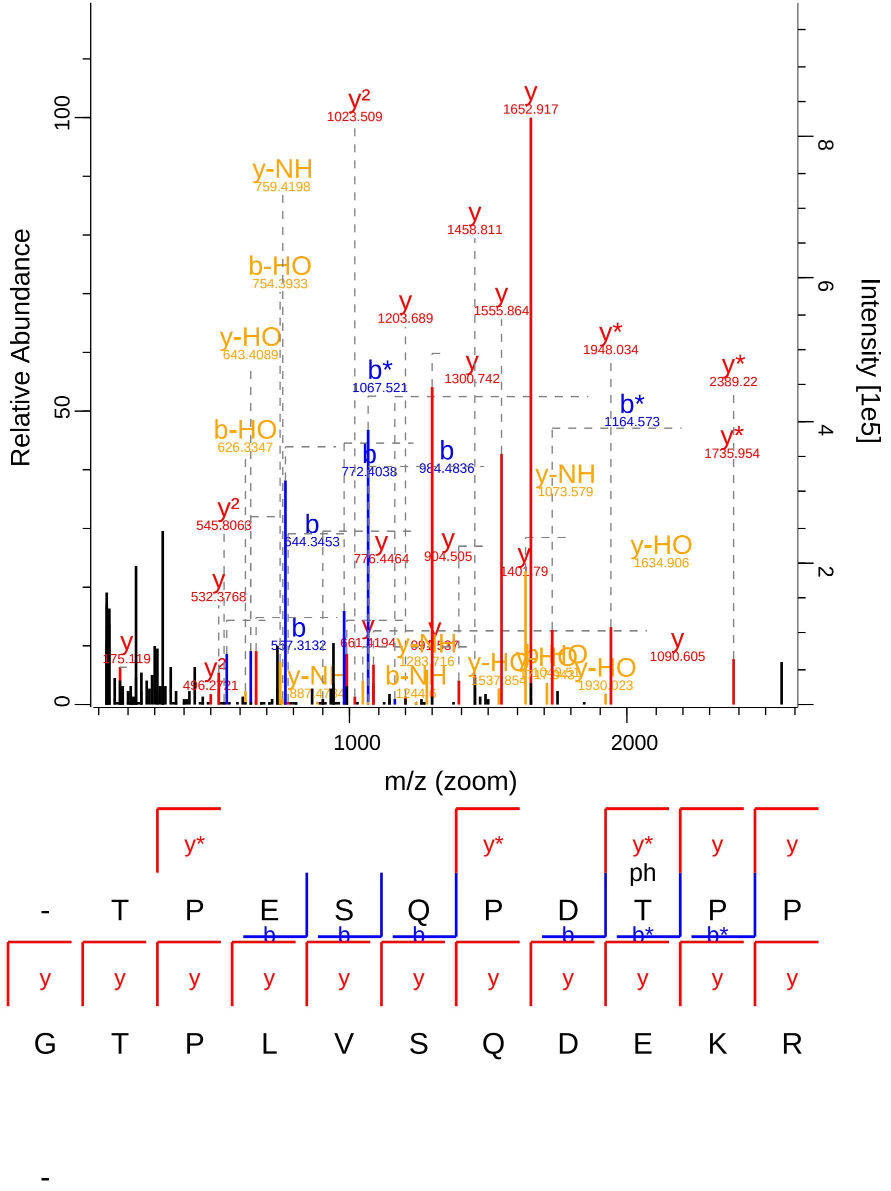

Raw File	Scan	Method	Score	m/z	Gene names
RO-QC-160121_ZackD_SS_Set_02_Fractio	17297	FTMS; HC	128.85	692.86	HSPB1

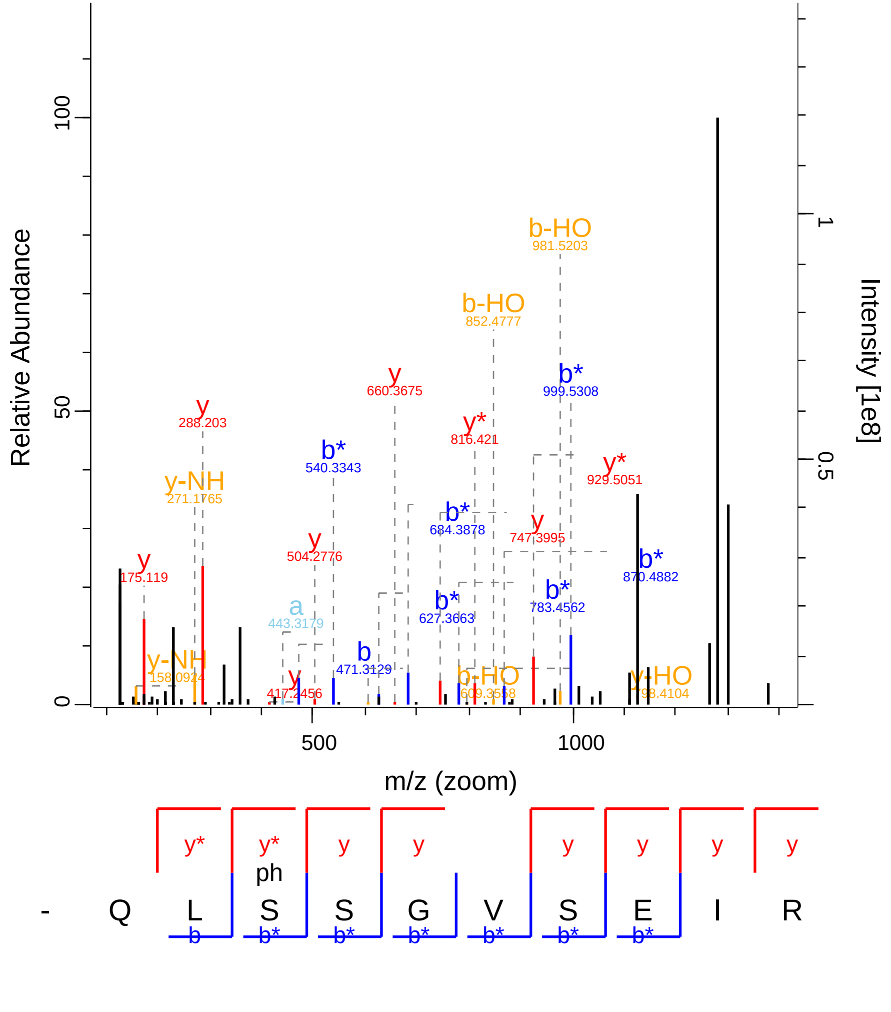

Raw File	Scan	Method	Score	m/z	Gene names
RO-QC-160121_ZackD_SS_Set_02_Fractior	17487	FTMS; HCI	87.36	832.9	PDE4D;PDE4E

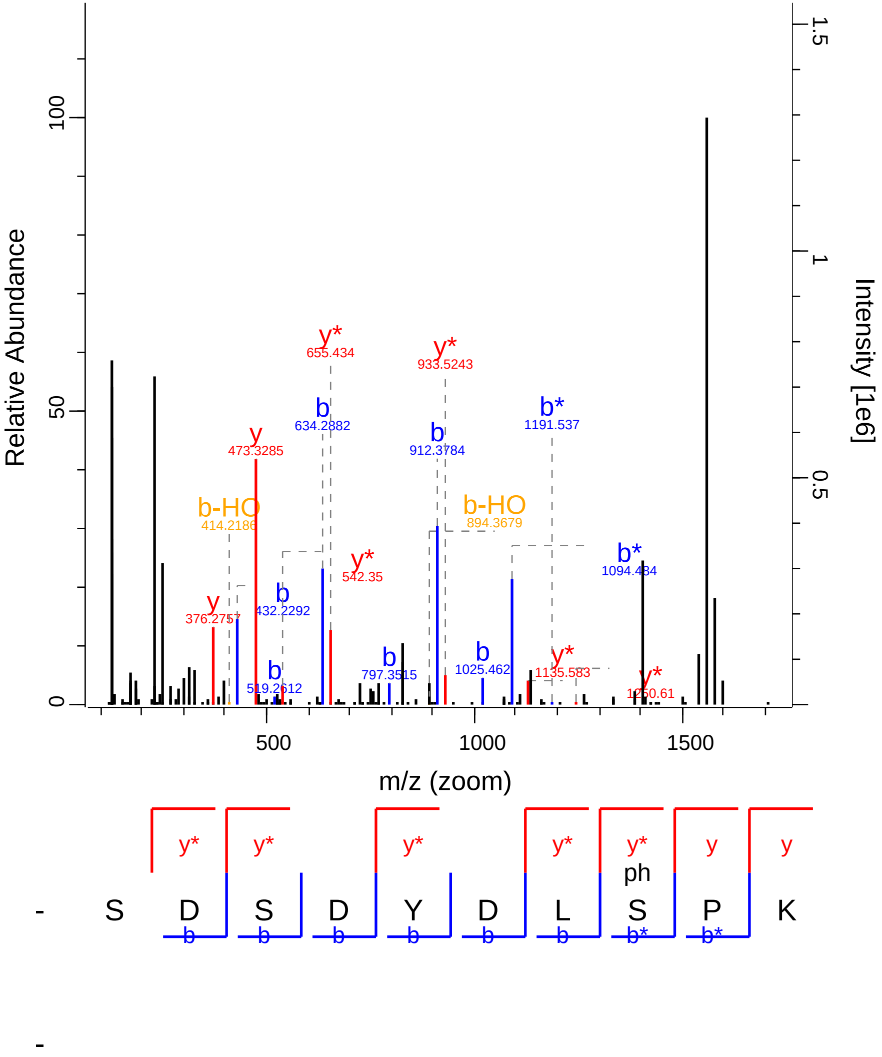

Raw File	Scan	Method	Score	m/z	Gene names
RO-QC-160121_ZackD_SS_Set_02_Fractio	17527	FTMS; HC	80.24	677.86	DOCK7

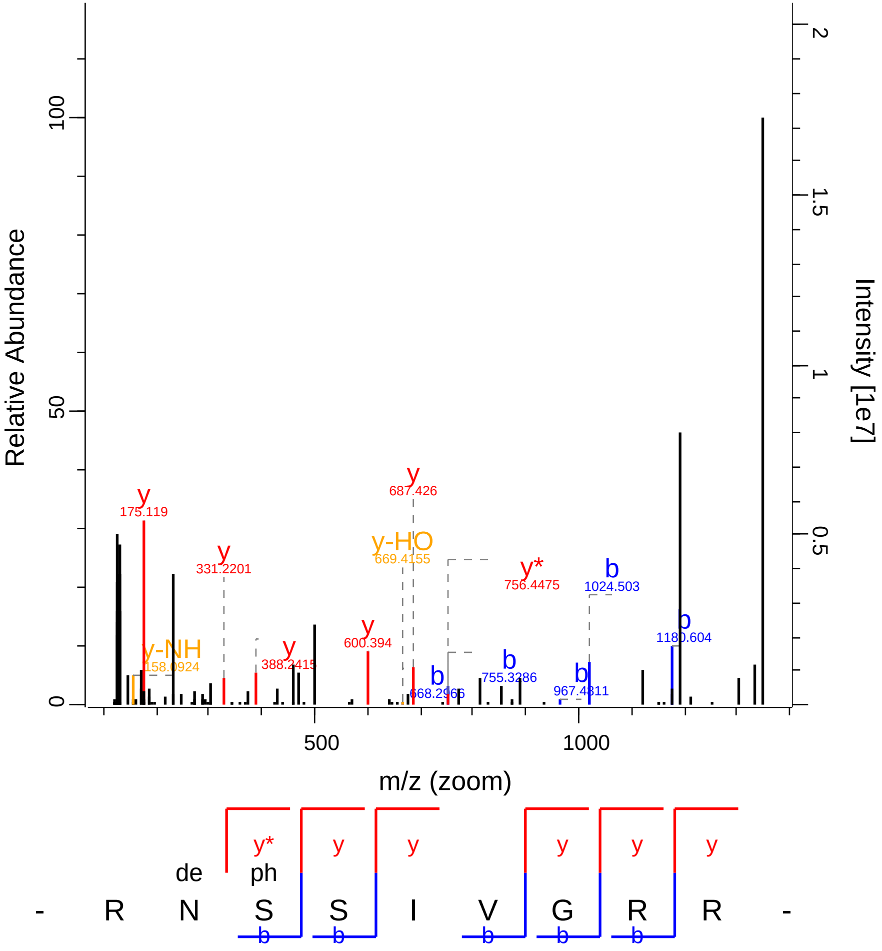

Raw File	Scan	Method	Score	m/z	Gene names
RO-QC-160121_ZackD_SS_Set_02_Fractio	19142	FTMS; HC	86.79	831.42	CDK19

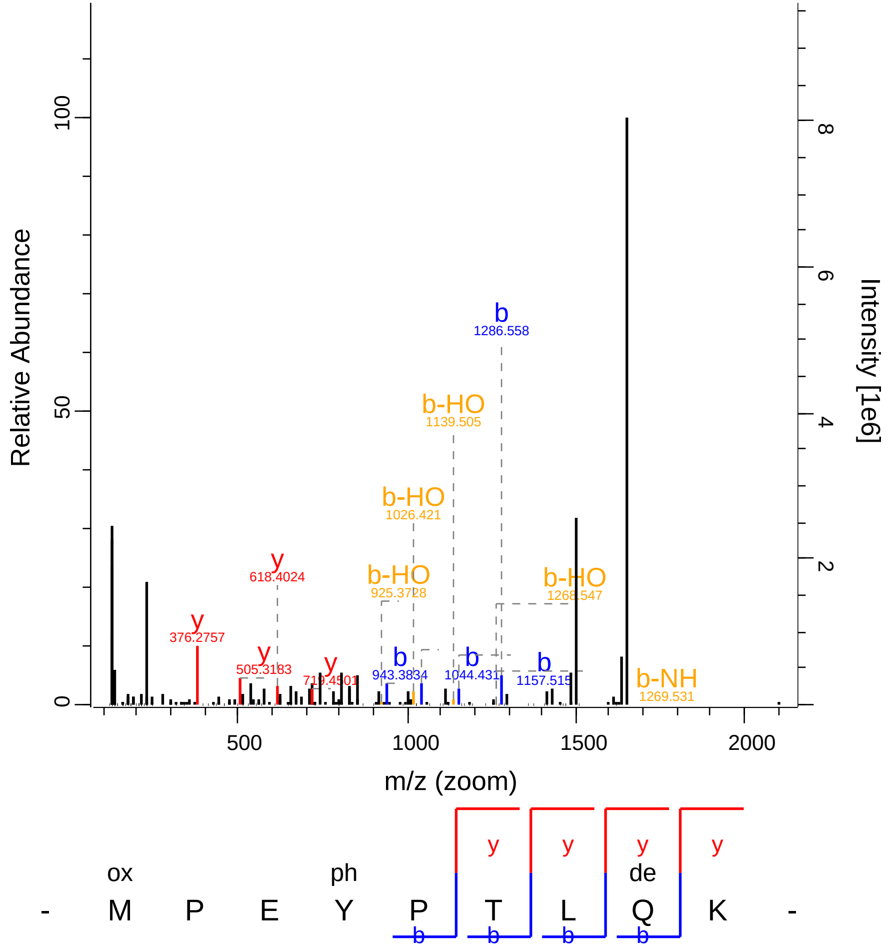

Raw File	Scan	Method	Score	m/z	Gene names
RO-QC-160121_ZackD_SS_Set_02_Fractio	20329	FTMS; HC	172.18	1018.46	MYH11

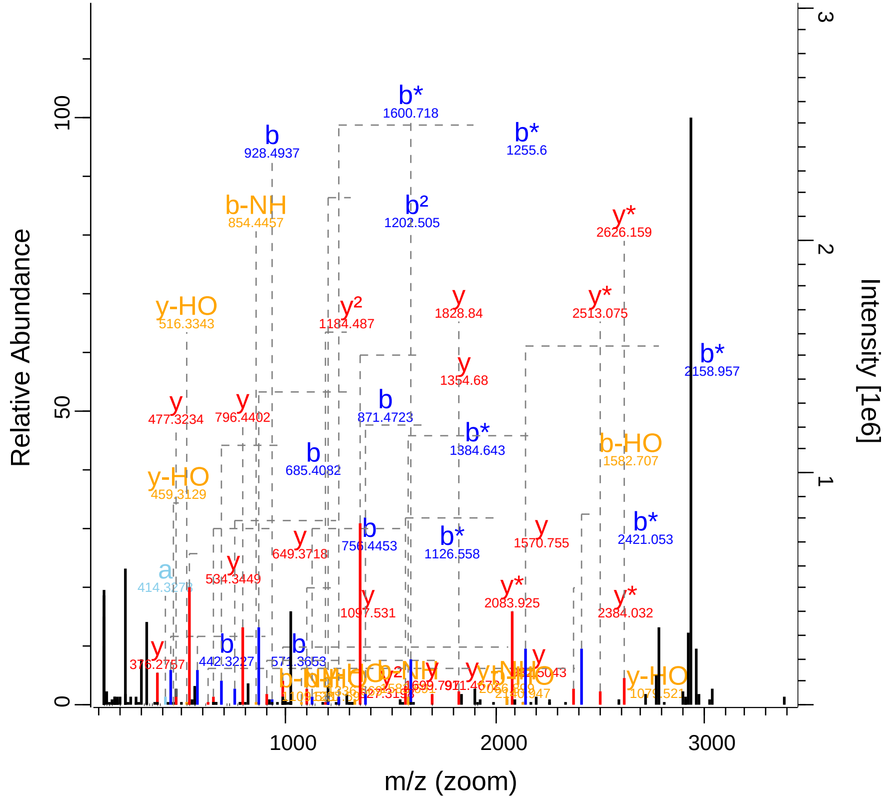

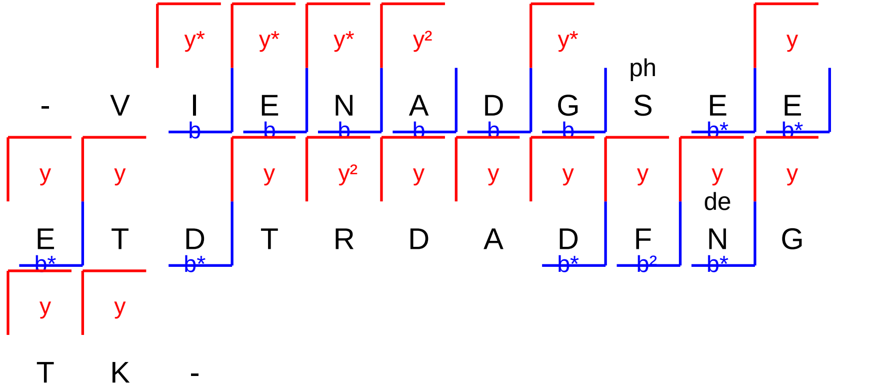

Raw File	Scan	Method	Score	m/z	Gene names
RO-QC-160121_ZackD_SS_Set_02_Fractio	20618	FTMS; HC	66.28	907.08	MAP1B

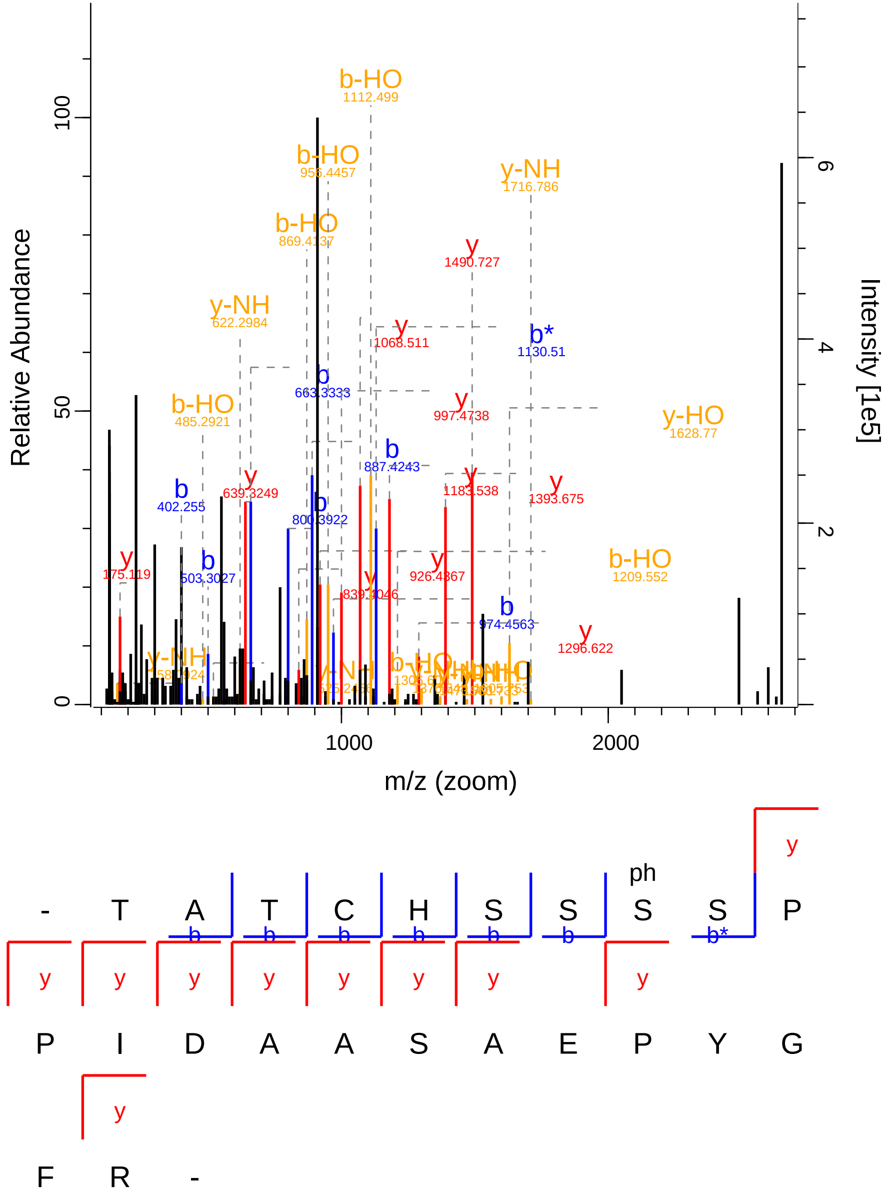

Raw File	Scan	Method	Score	m/z	Gene names
RO-QC-160121_ZackD_SS_Set_02_Fractio	21010	FTMS; HC	87.16	614.29	MAPK1

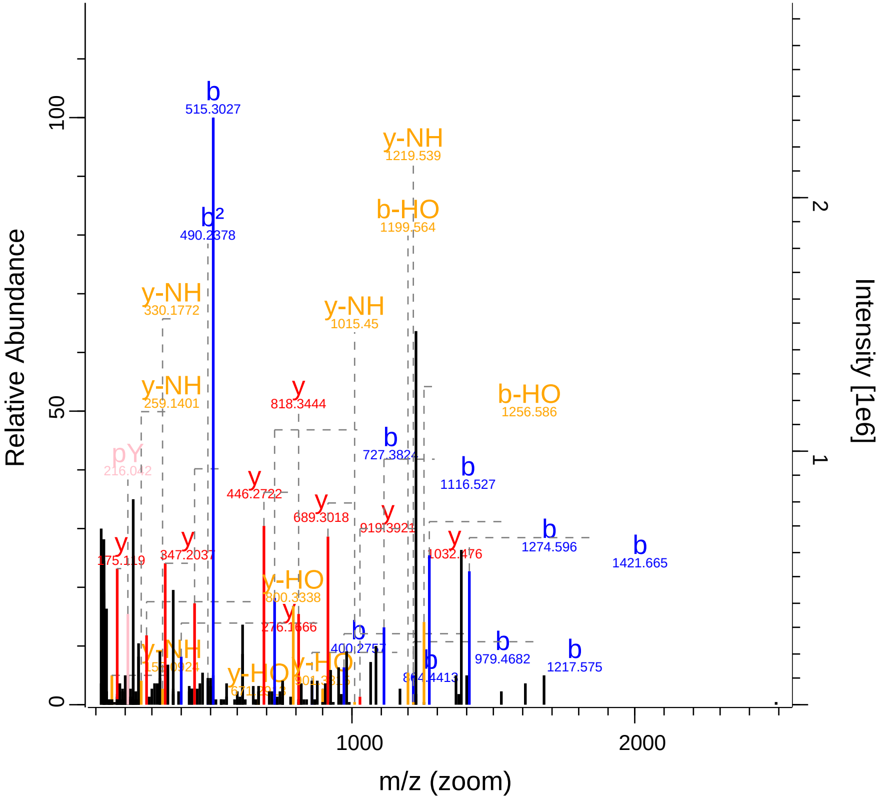

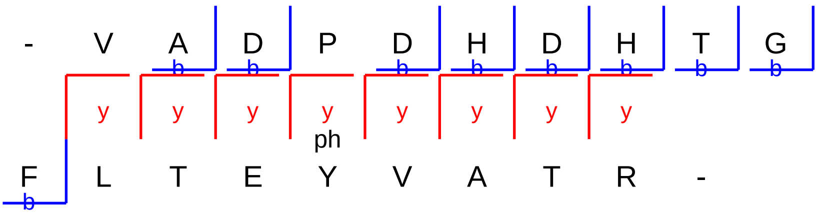

Raw File	Scan	Method	Score	m/z	Gene names
RO-QC-160121_ZackD_SS_Set_02_Fractio	22086	FTMS; HC	64.04	896.45	LINC01387

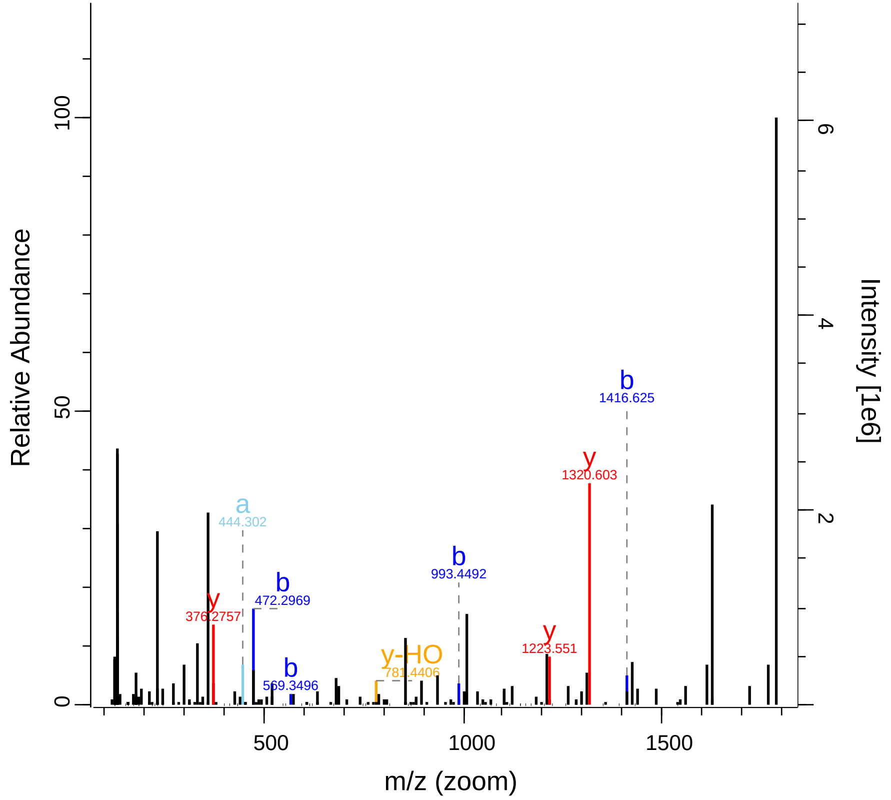

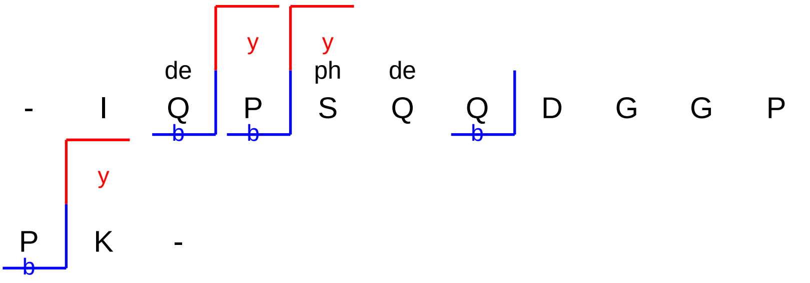

Raw File	Scan	Method	Score	m/z	Gene names
RO-QC-160121_ZackD_SS_Set_02_Fractio	23925	FTMS; HC	80.79	857.88	GJA1

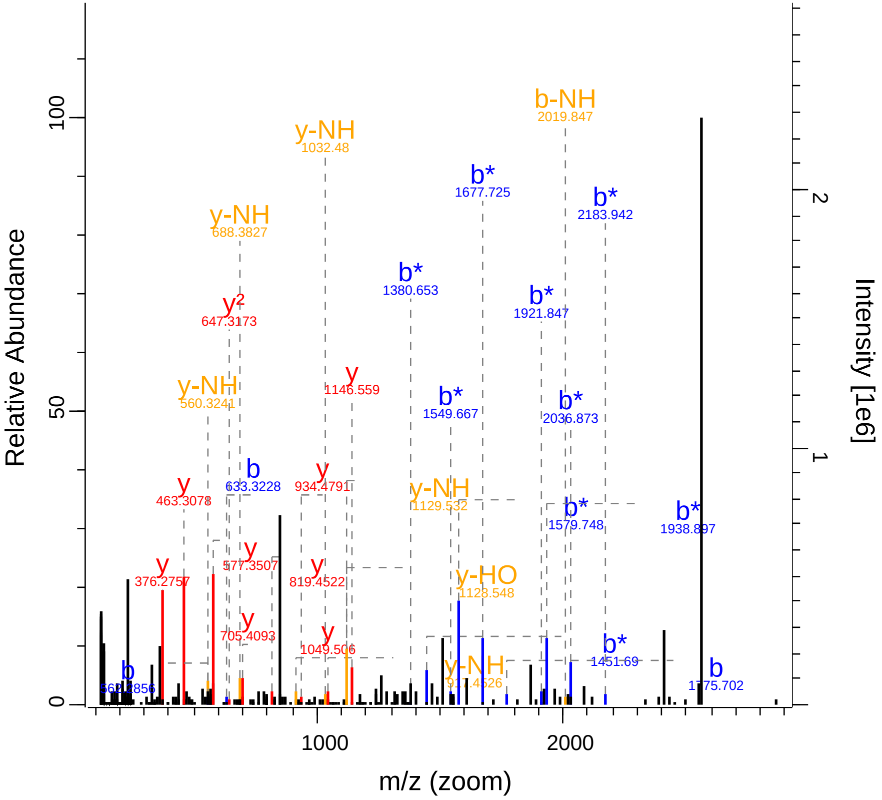

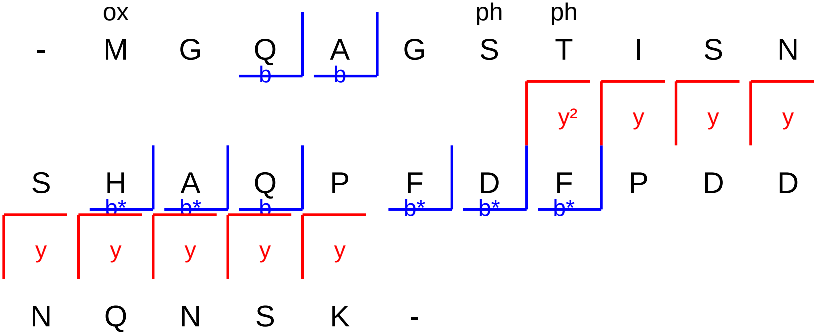

Raw File	Scan	Method	Score	m/z	Gene names
RO-QC-160121_ZackD_SS_Set_02_Fractio	24187	FTMS; HC	198.95	1174.92	FASN

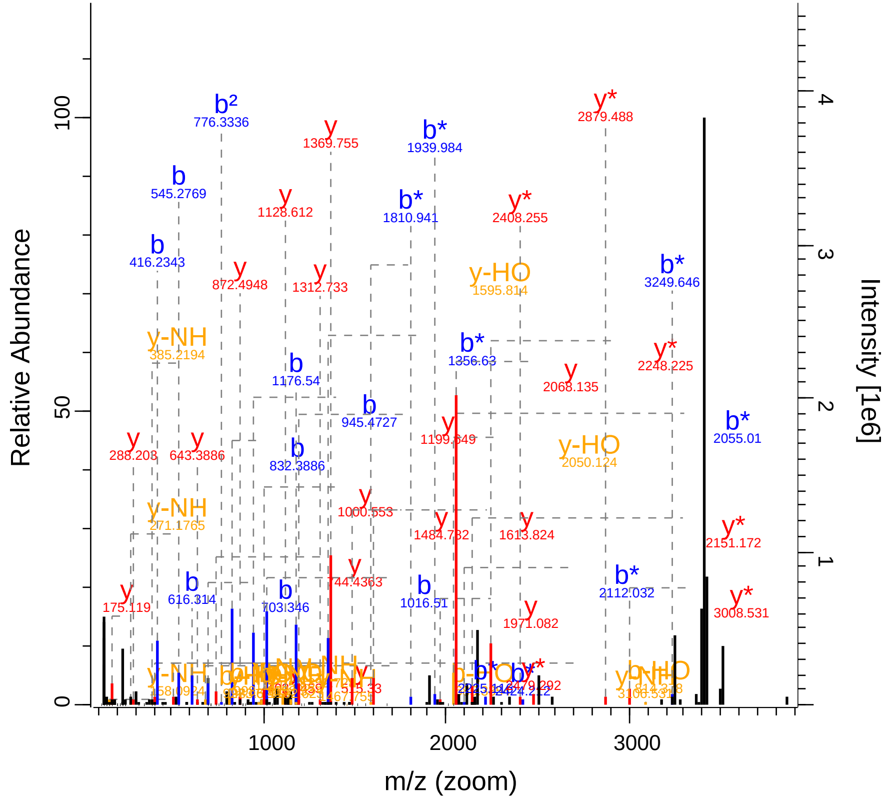

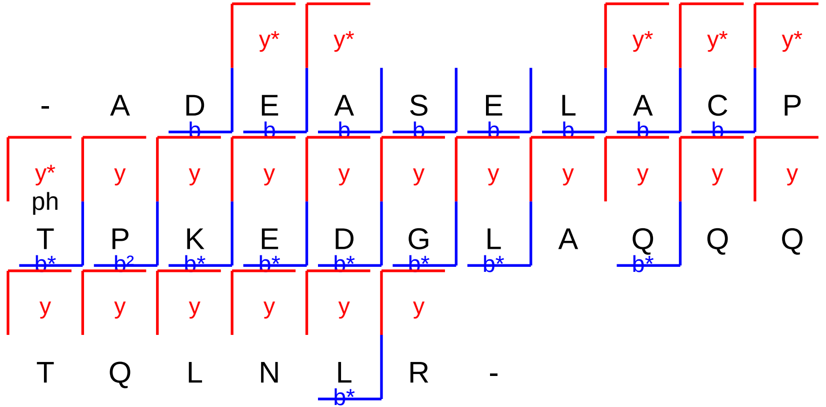

Raw File	Scan	Method	Score	m/z	Gene names
RO-QC-160121_ZackD_SS_Set_02_Fractio	26482	FTMS; HC	57.02	672.37	TOR1AIP1

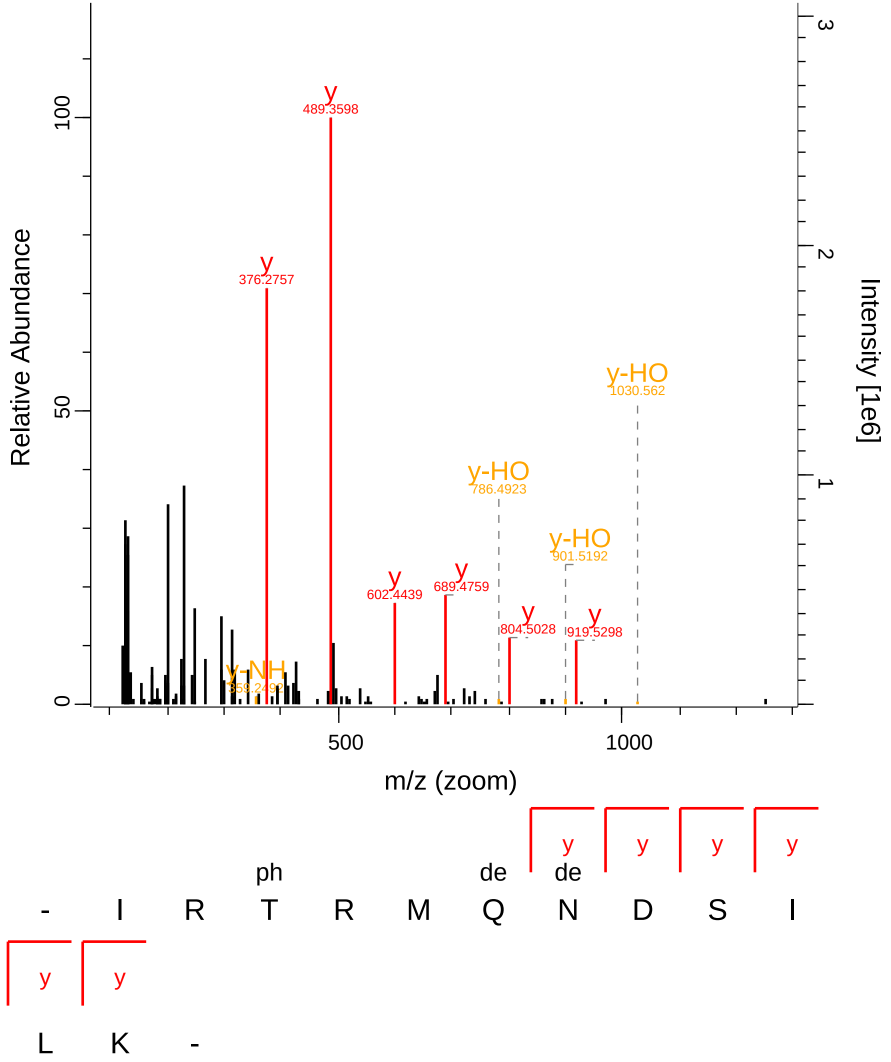

Raw File	Scan	Method	Score	m/z	Gene names
RO-QC-160121_ZackD_SS_Set_02_Fractio	26508	FTMS; HC	94.4	623.82	FLNC

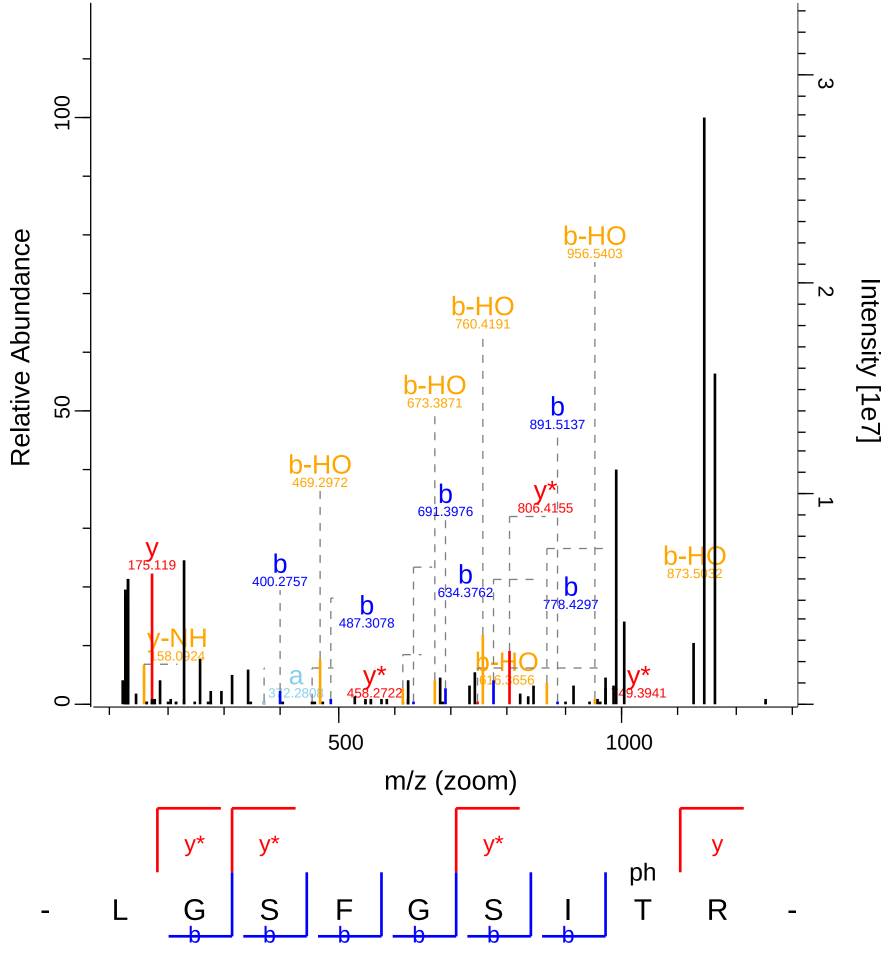

Raw File	Scan	Method	Score	m/z	Gene names
RO-QC-160121_ZackD_SS_Set_02_Fractic	26557	FTMS; HC	91.64	1498.71	RTN4

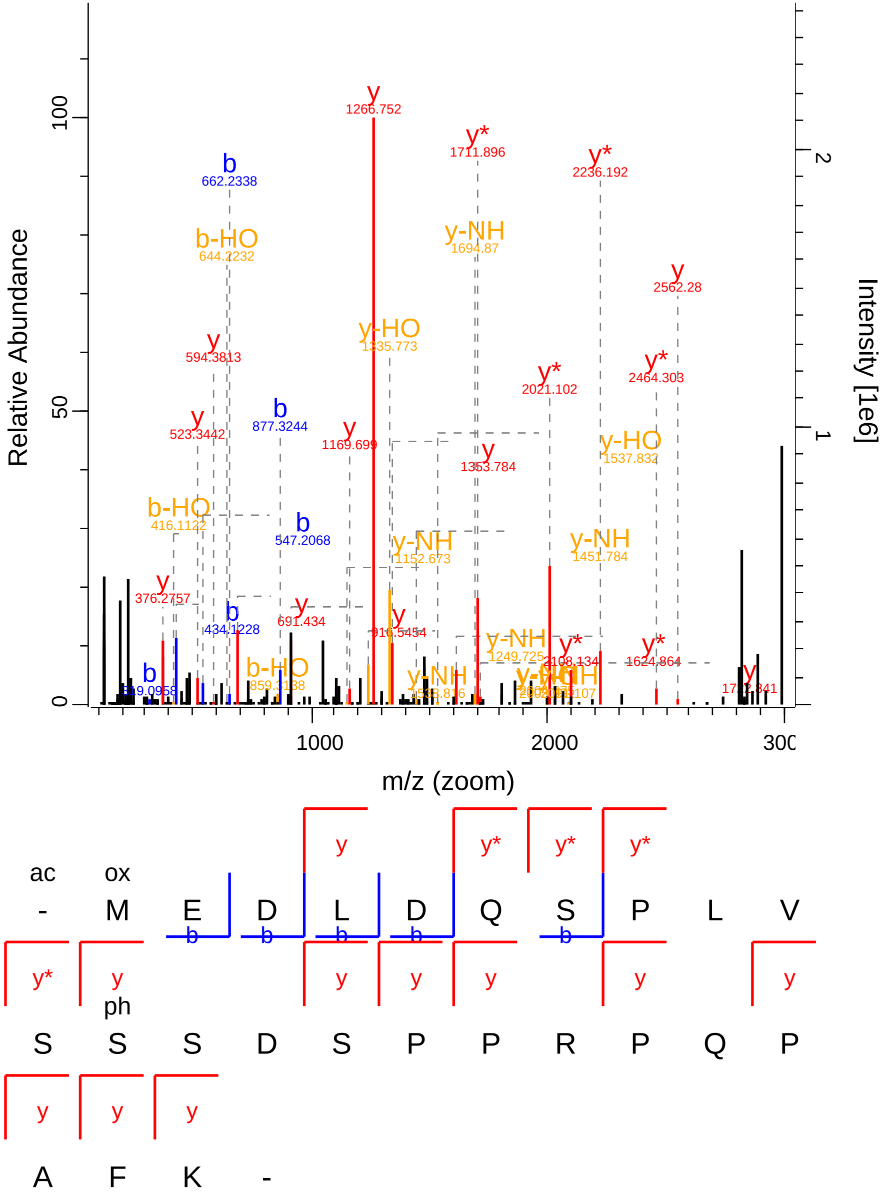

Raw File	Scan	Method	Score	m/z	Gene names
RO-QC-160121_ZackD_SS_Set_02_Fract	27046	FTMS; HC	45.68	904.45	AGO3;AGO1;AGO

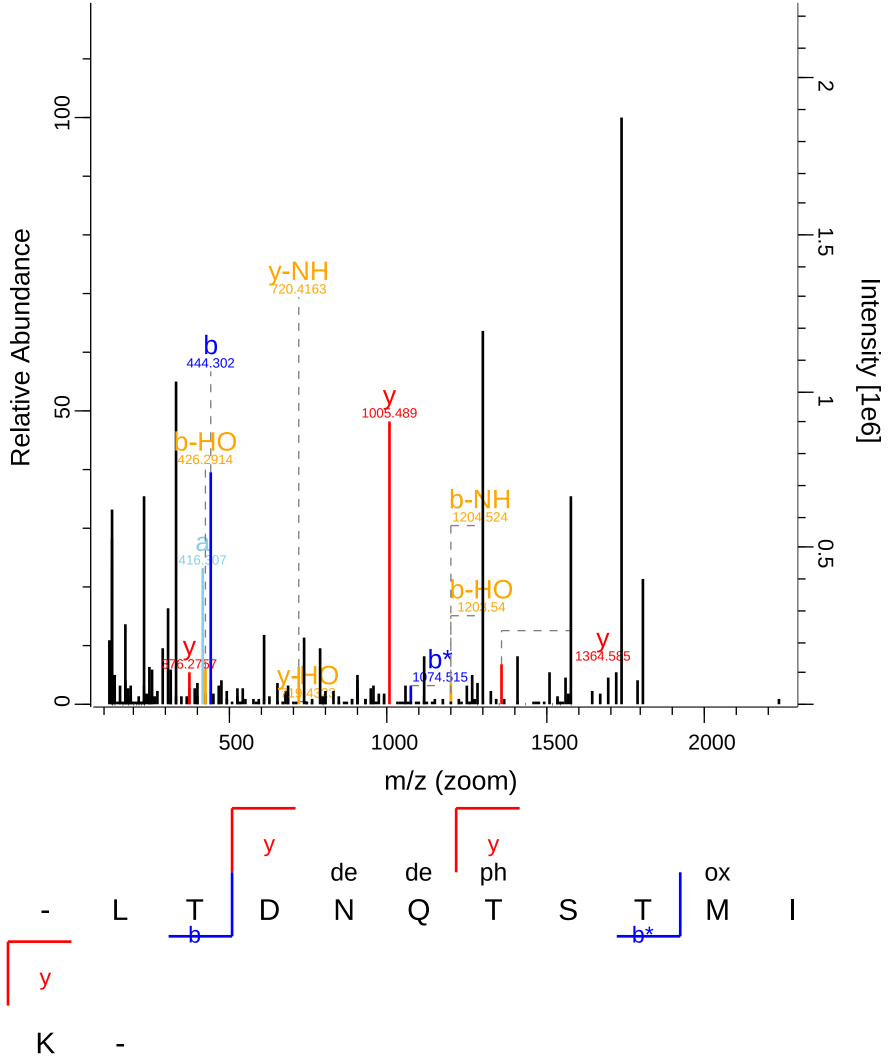

Raw File	Scan	Method	Score	m/z	Gene names
RO-QC-160121_ZackD_SS_Set_02_Fractio	28132	FTMS; HC	91.66	810.43	PAK2

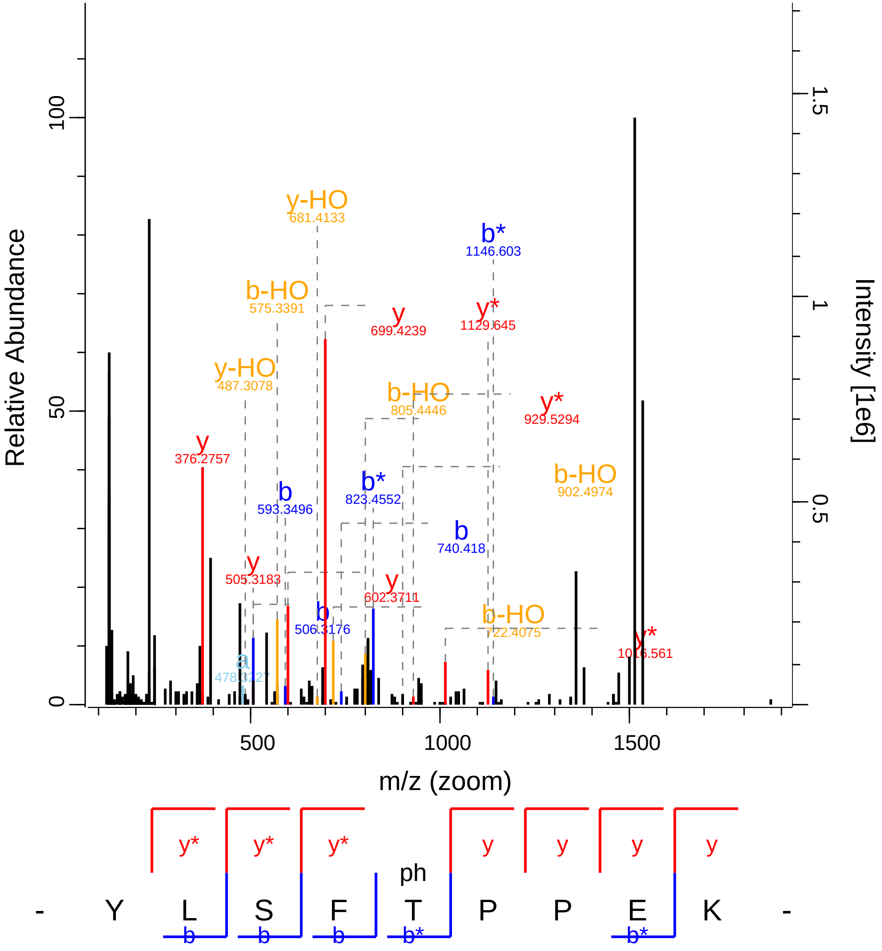

Raw File	Scan	Method	Score	m/z	Gene names
RO-QC-160121_ZackD_SS_Set_02_Fractio	28232	FTMS; HC	83.36	730.15	EBAG9

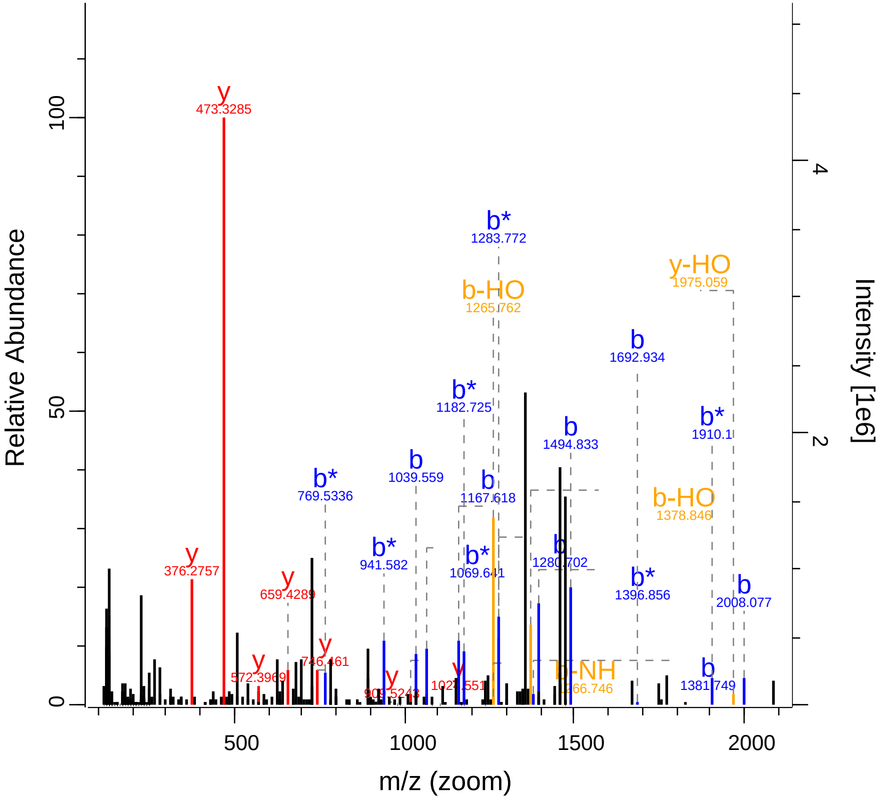

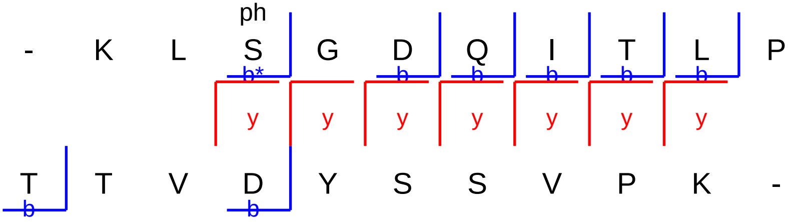

Raw File	Scan	Method	Score	m/z	Gene names
RO-QC-160121_ZackD_SS_Set_02_Fractio	28419	FTMS; HC	85.29	884.47	USP21

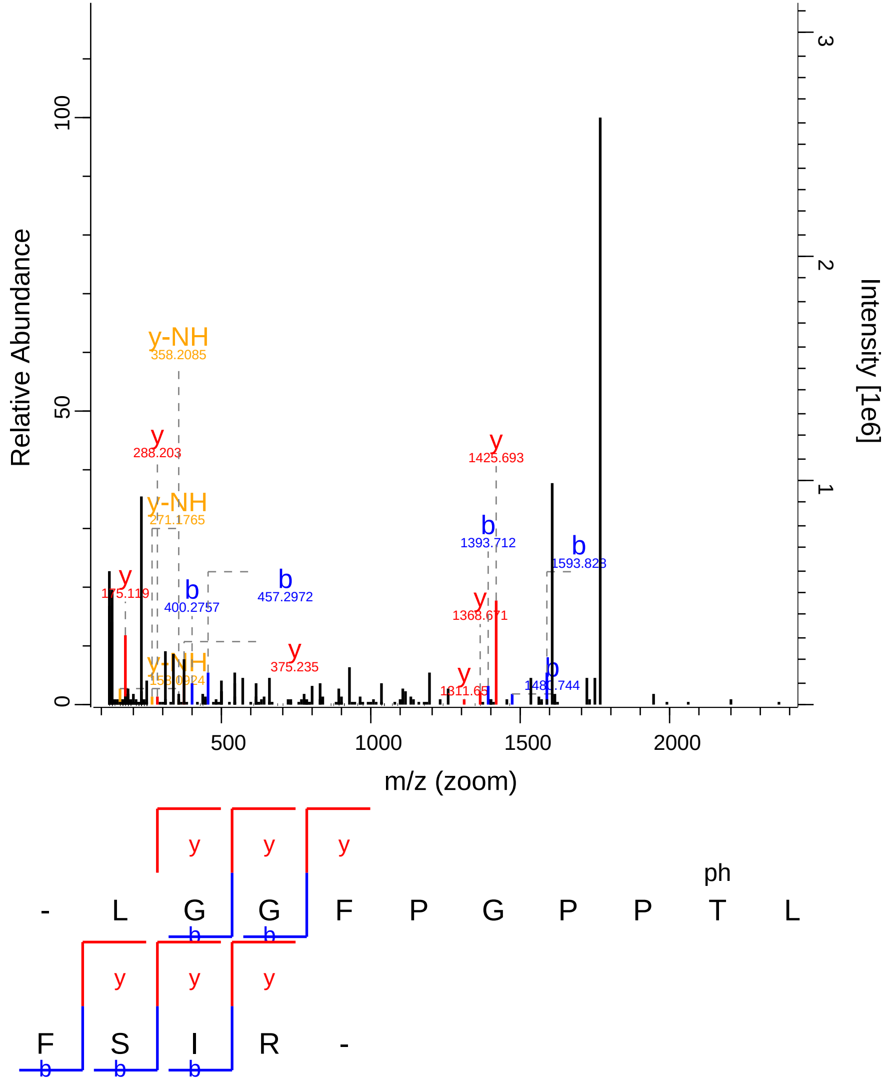

Raw File	Scan	Method	Score	m/z	Gene names
RO-QC-160121_ZackD_SS_Set_02_Fractio	28642	FTMS; HC	128.67	781.74	EGFR

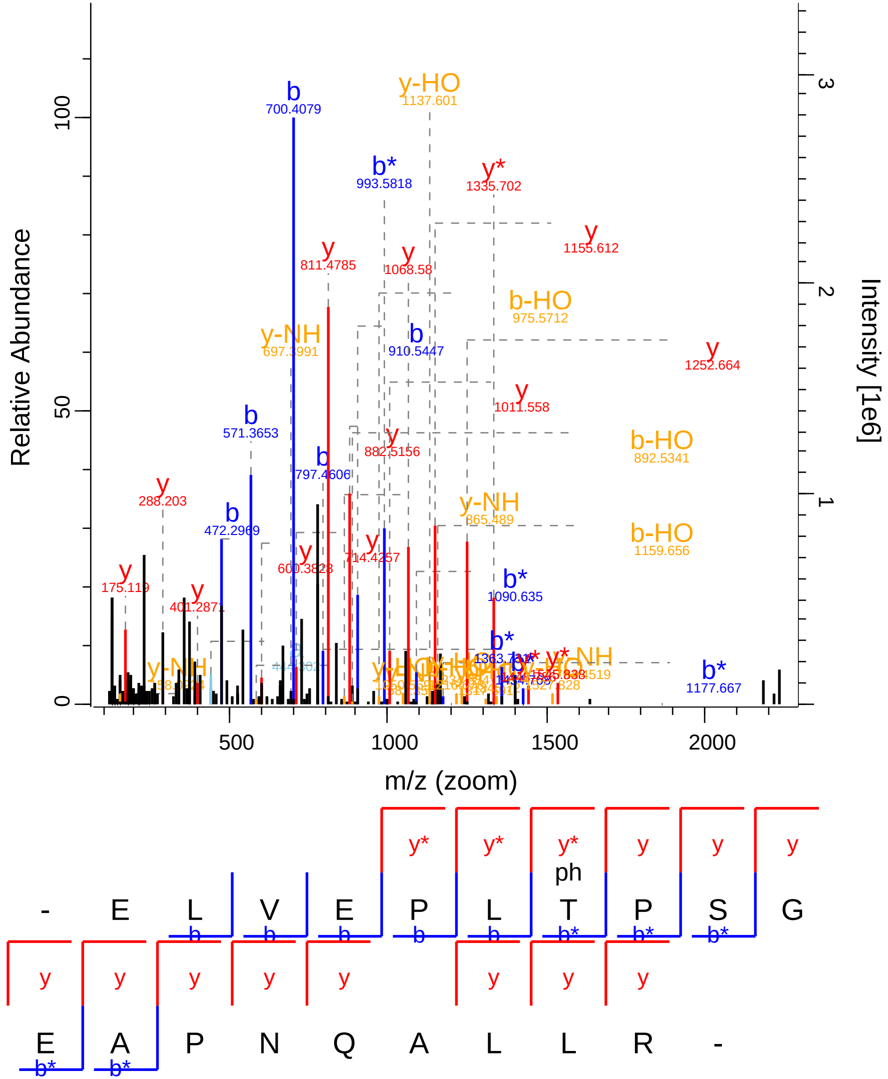

Raw File	Scan	Method	Score	m/z	Gene names
RO-QC-160121_ZackD_SS_Set_02_Fractior	29015	FTMS; HCI	50.15	629.6	KIAA1328

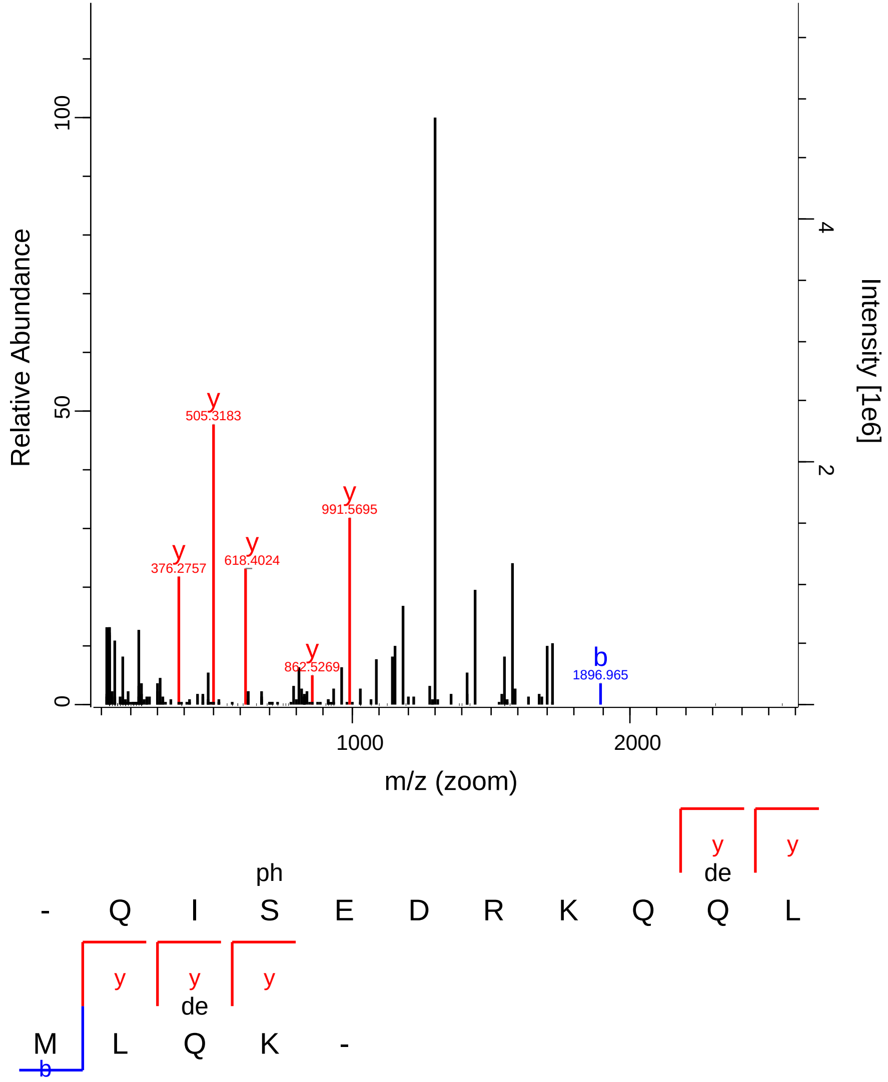

Raw File	Scan	Method	Score	m/z	Gene names
RO-QC-160121_ZackD_SS_Set_02_Fractior	29626	FTMS; HCI	213.41	977.7	MAP1B

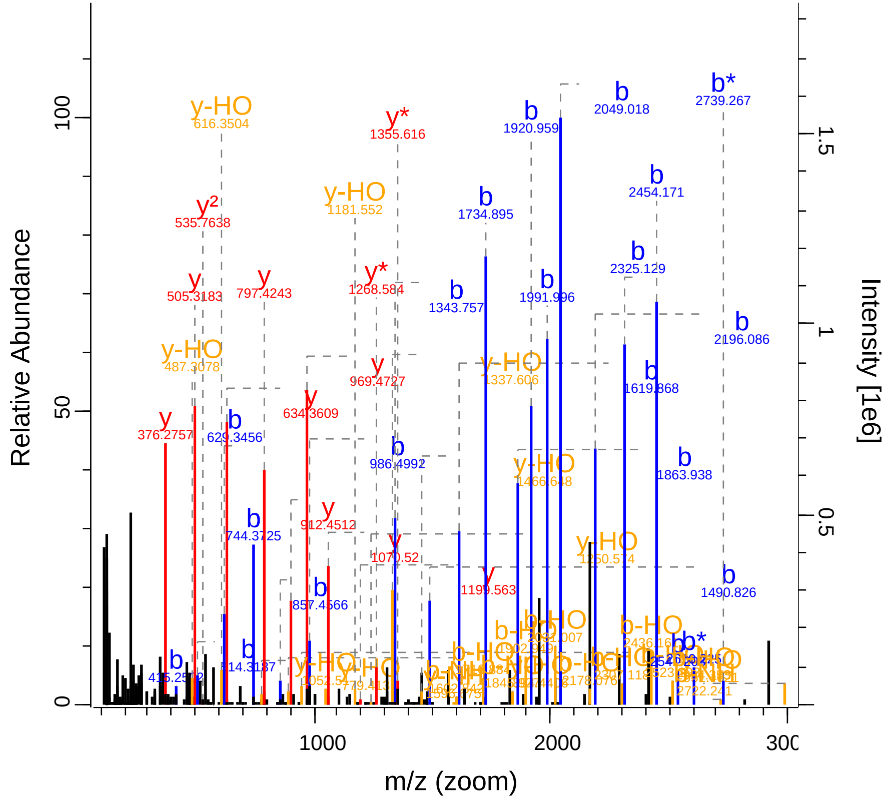

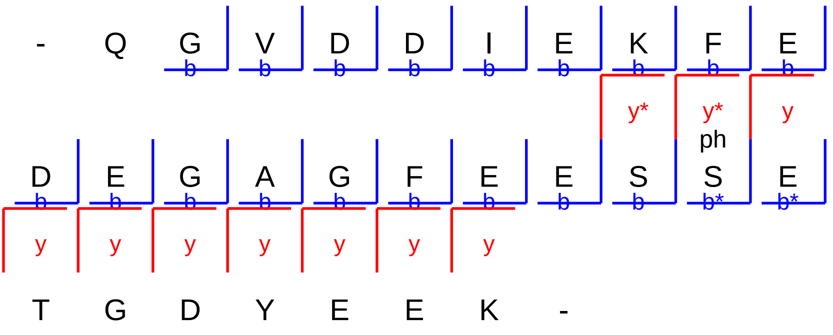

Raw File	Scan	Method	Score	m/z	Gene names
RO-QC-160121_ZackD_SS_Set_02_Fractio	29658	FTMS; HC	43.54	730.73	MAGI1

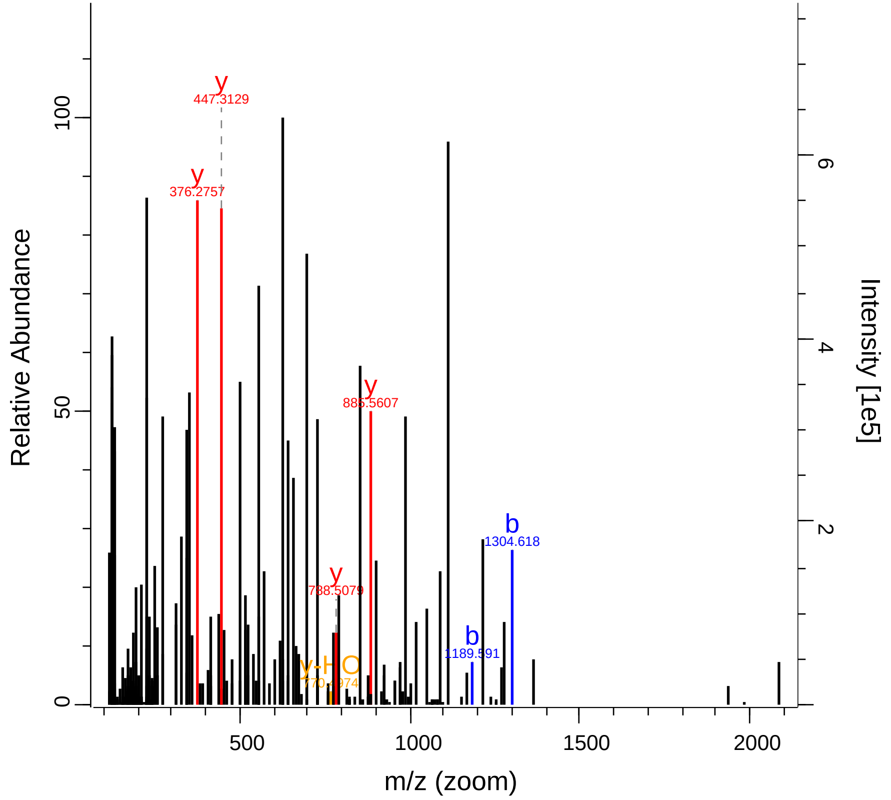

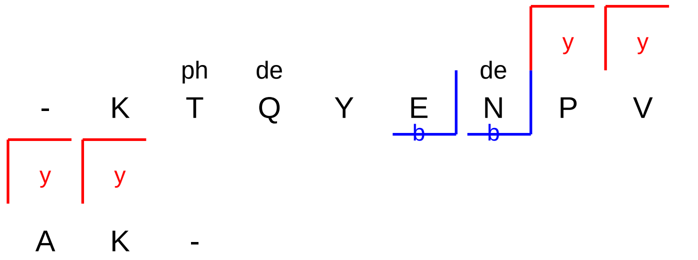

Raw File	Scan	Method	Score	m/z	Gene names
RO-QC-160121_ZackD_SS_Set_02_Fractio	29714	FTMS; HC	48.29	680.59	GCN1L1

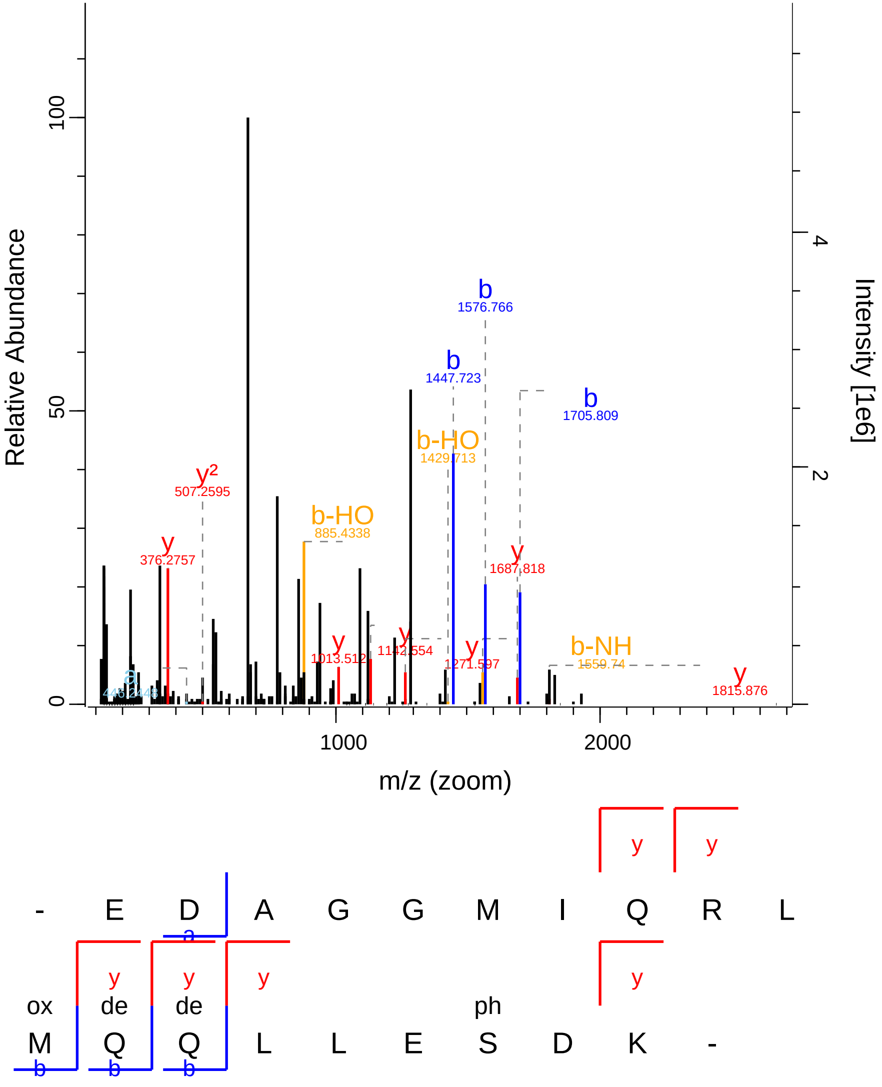

Raw File	Scan	Method	Score	m/z	Gene names
RO-QC-160121_ZackD_SS_Set_02_Fractic	29898	FTMS; HC	216.54	1325.29	AHNAK

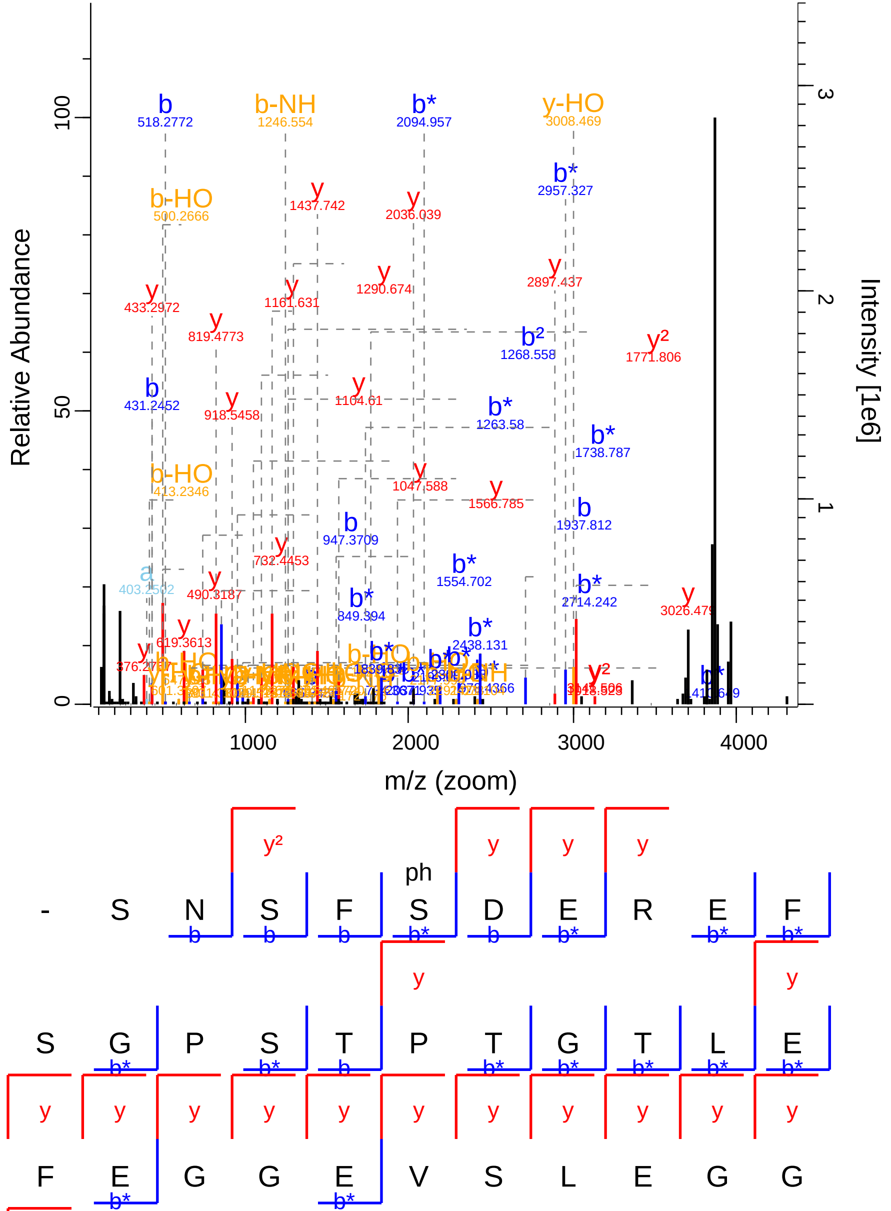

Raw File	Scan	Method	Score	m/z	Gene names
RO-QC-160121_ZackD_SS_Set_02_Fractio	29905	FTMS; HC	153.89	994.47	AHNAK

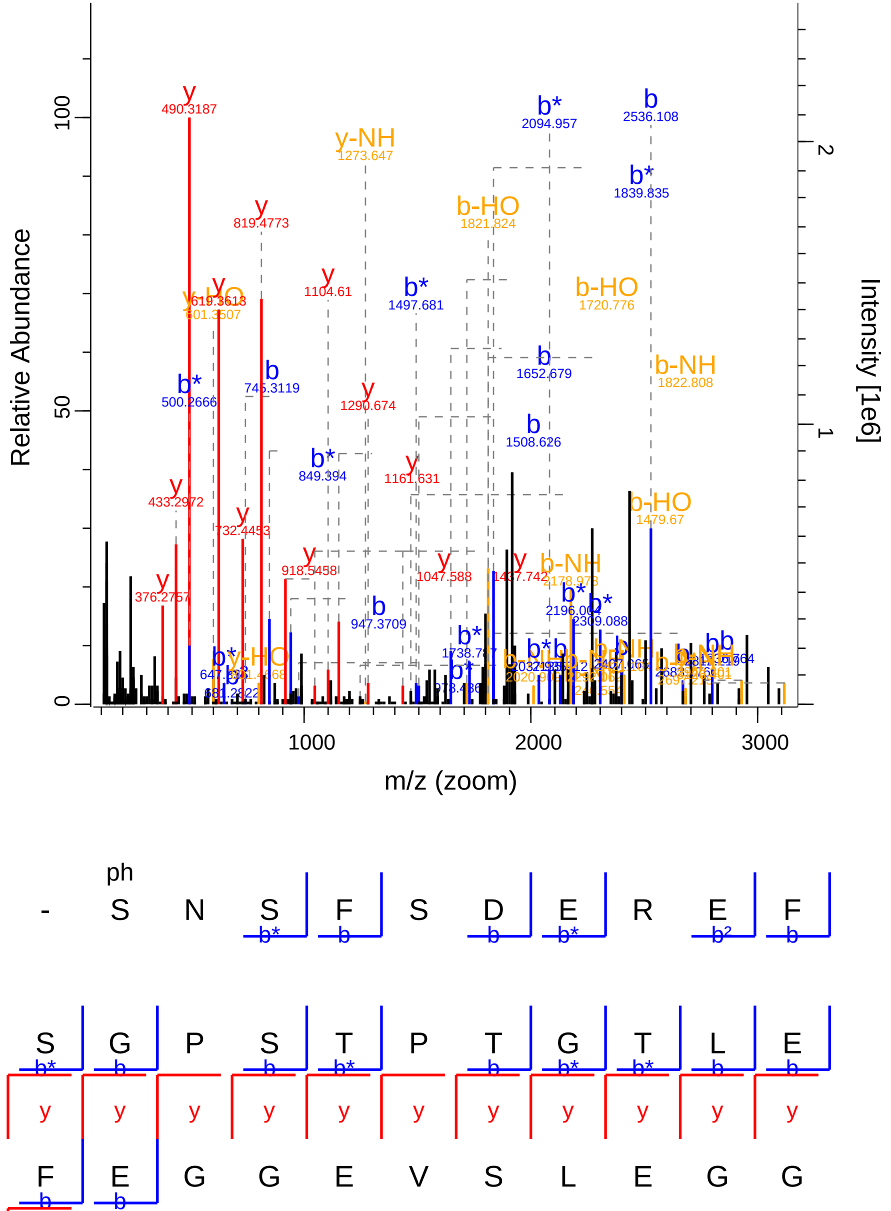

Raw File	Scan	Method	Score	m/z	Gene names
RO-QC-160121_ZackD_SS_Set_02_Fractio	30642	FTMS; HC	60.44	963.54	OR7A10

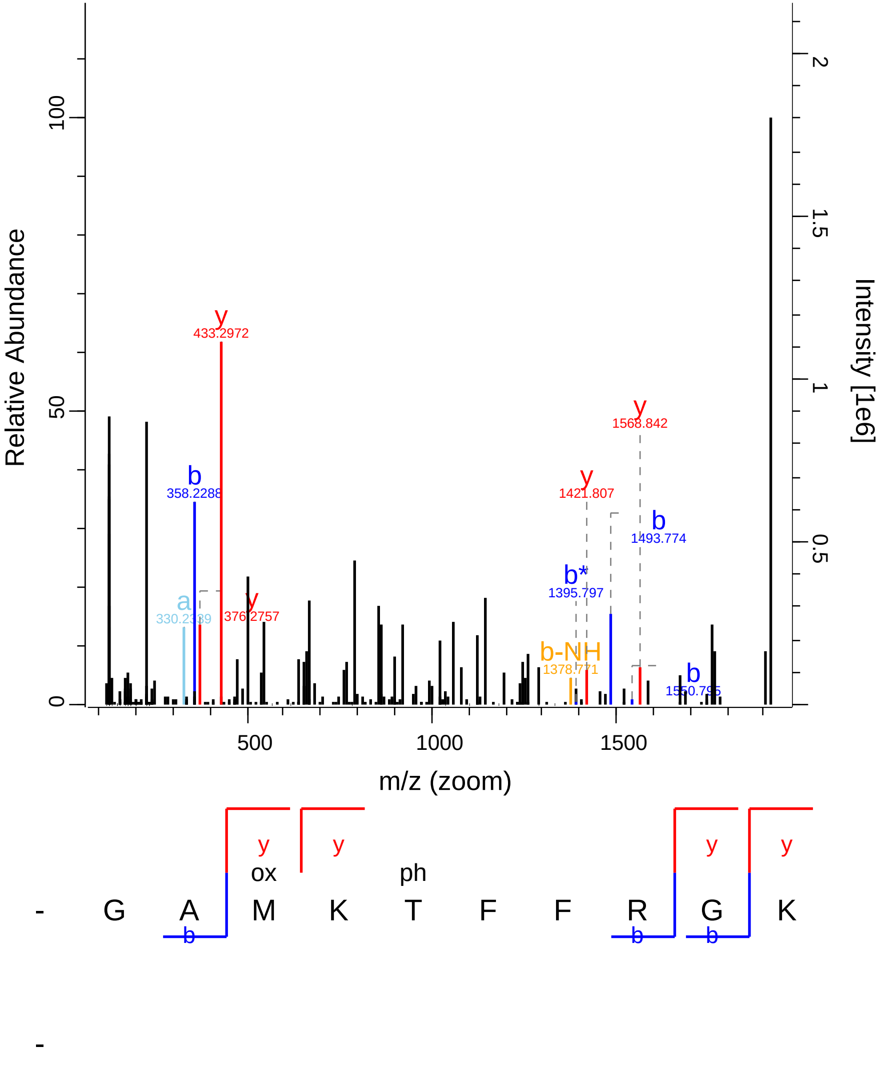

Raw File	Scan	Method	Score	m/z	Gene names
RO-QC-160121_ZackD_SS_Set_02_Fractio	30936	FTMS; HC	123.99	1038.14	WAPAL

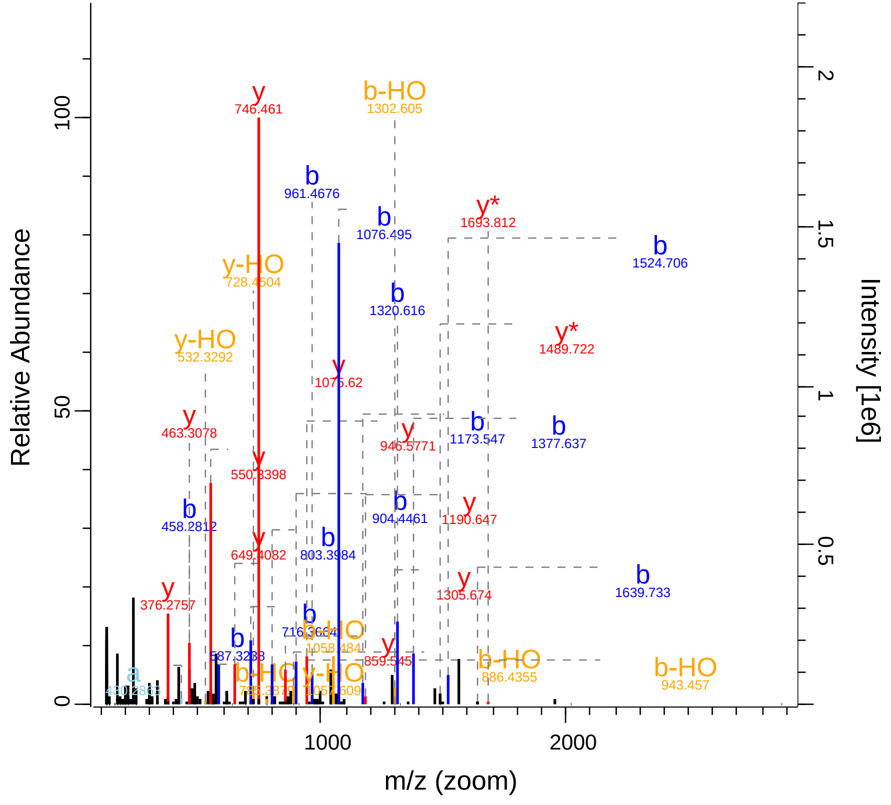

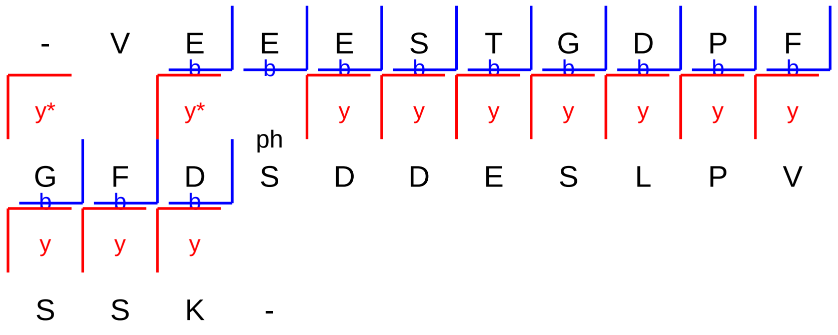

Raw File	Scan	Method	Score	m/z	Gene names
RO-QC-160121_ZackD_SS_Set_02_Fractic	30990	FTMS; HC	73.98	1172.14	TFPI2

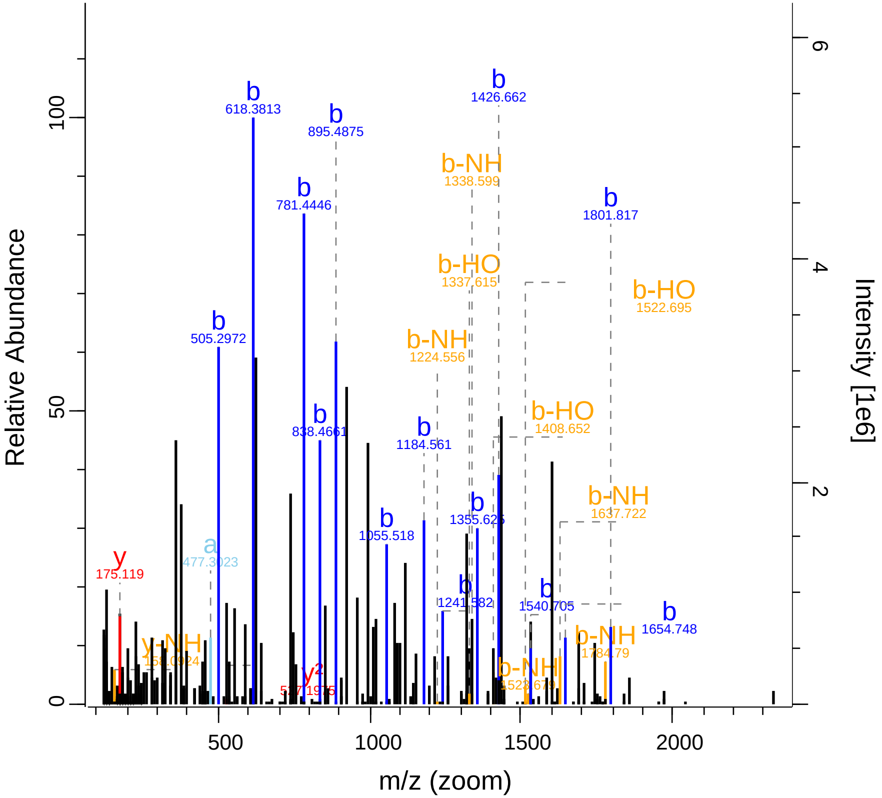

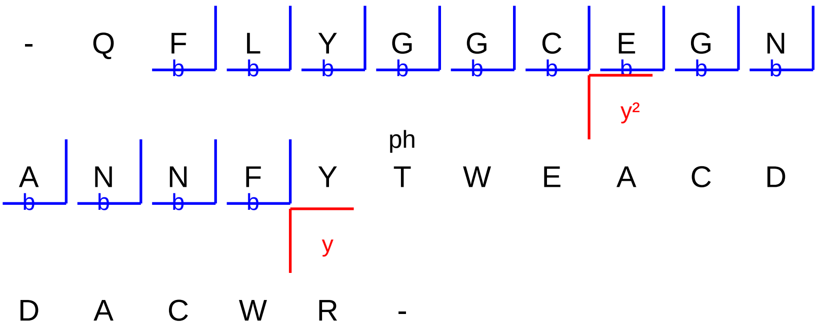

Raw File	Scan	Method	Score	m/z	Gene names
RO-QC-160121_ZackD_SS_Set_02_Fractic	31004	FTMS; HC	61.65	1160.56	SGOL2

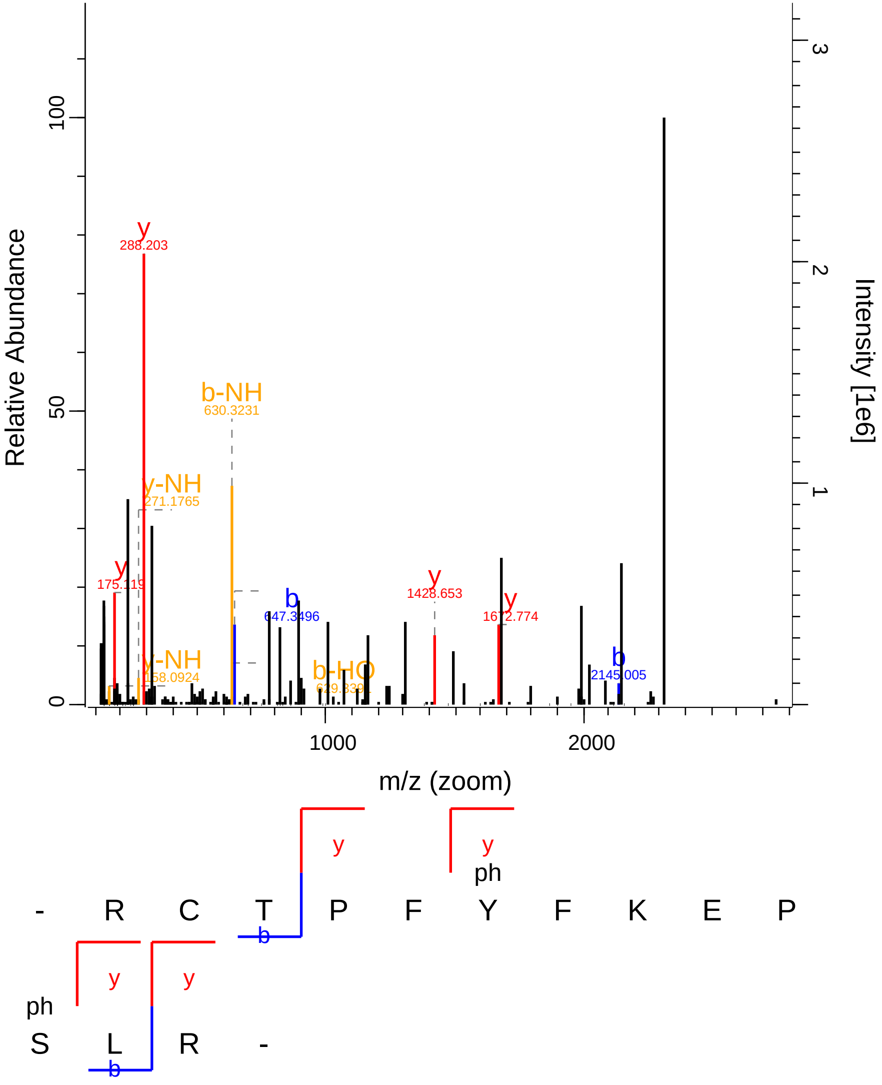

Raw File	Scan	Method	Score	m/z	Gene names
RO-QC-160121_ZackD_SS_Set_02_Fractio	32105	FTMS; HC	72.94	657.37	CD96

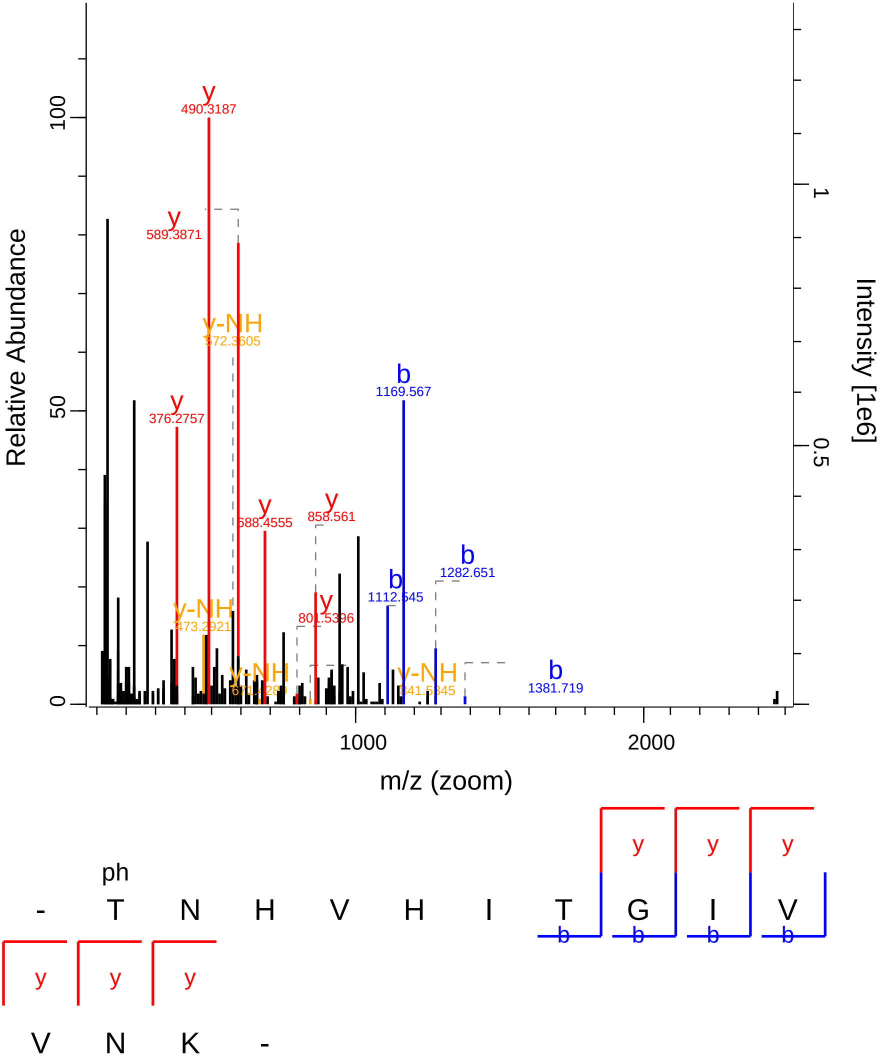

Raw File	Scan	Method	Score	m/z	Gene names
RO-QC-160121_ZackD_SS_Set_02_Fractic	32200	FTMS; HC	58.08	1004.21	SMARCC2

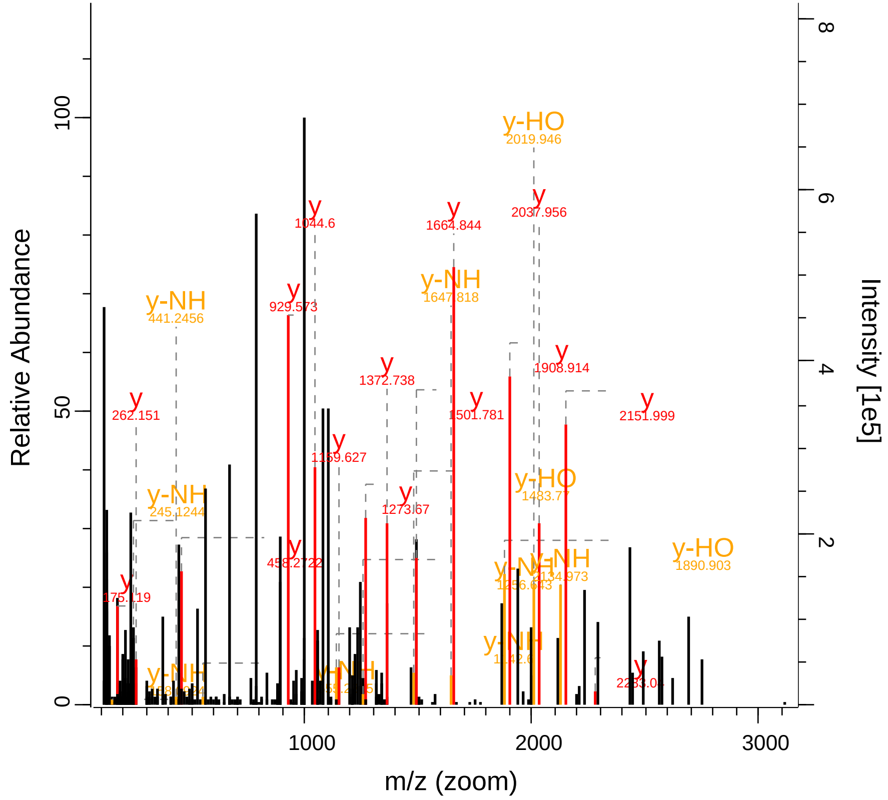

ph

-	W	I	L	D	T	D	T	F	N	E
	y	y	y	y		y	y	y	y	y
W	M	N	E	E	D	Y	E	V	N	D
y	y		y		y	y				
D	K	N	P	V	S	R	-			

Raw File	Scan	Method	Score	m/z	Gene names
RO-QC-160121_ZackD_SS_Set_02_Fractio	33675	FTMS; HC	113.93	671.85	GOLGA4

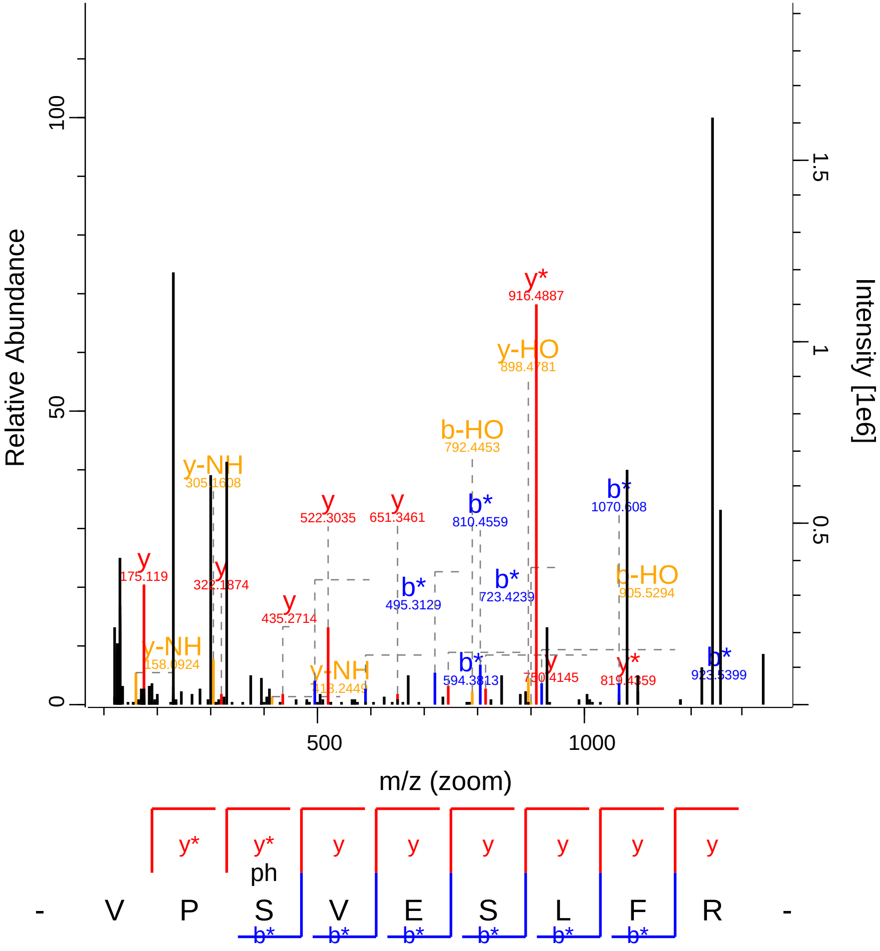

Raw File	Scan	Method	Score	m/z	Gene names
RO-QC-160121_ZackD_SS_Set_02_Fractio	33965	FTMS; HC	62.7	738.66	CLASP2

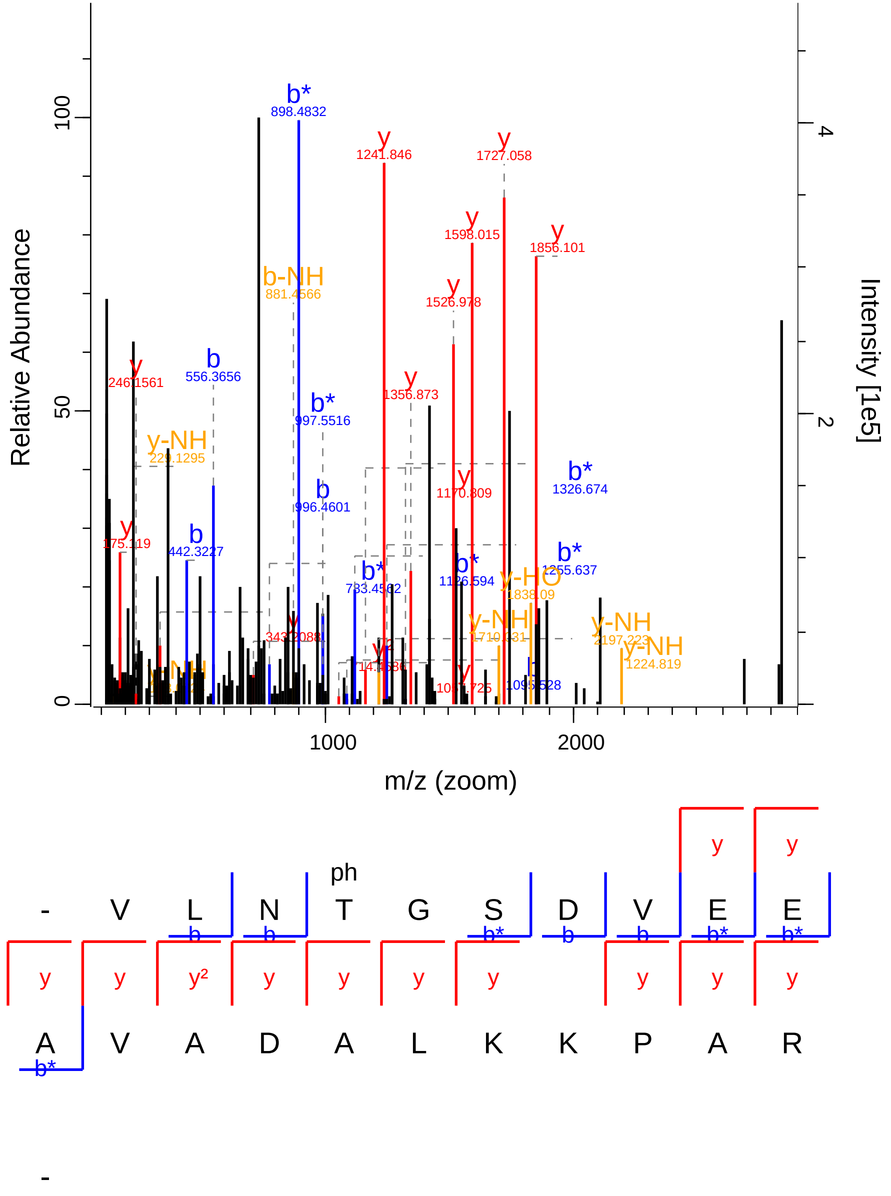

Raw File	Scan	Method	Score	m/z	Gene names
RO-QC-160121_ZackD_SS_Set_02_Fractio	34258	FTMS; HC	73.21	895.03	PPEF1

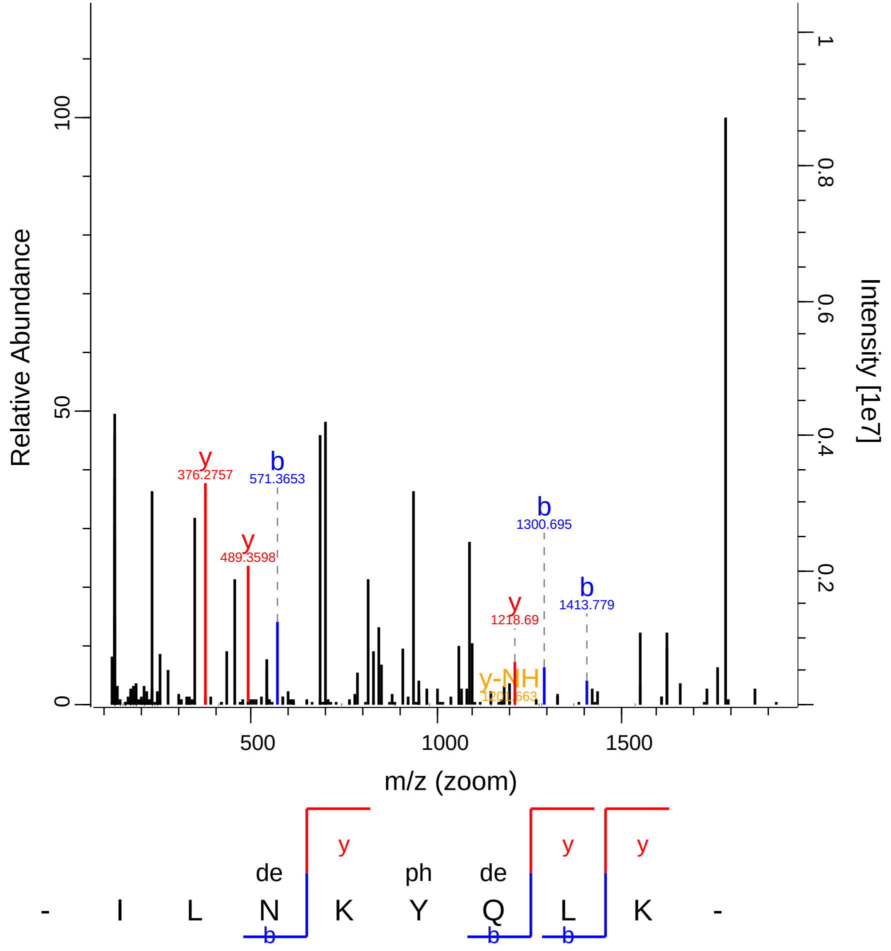

Raw File	Scan	Method	Score	m/z	Gene names
RO-QC-160121_ZackD_SS_Set_02_Fractio	34387	FTMS; HC	122.37	947.49	CAST

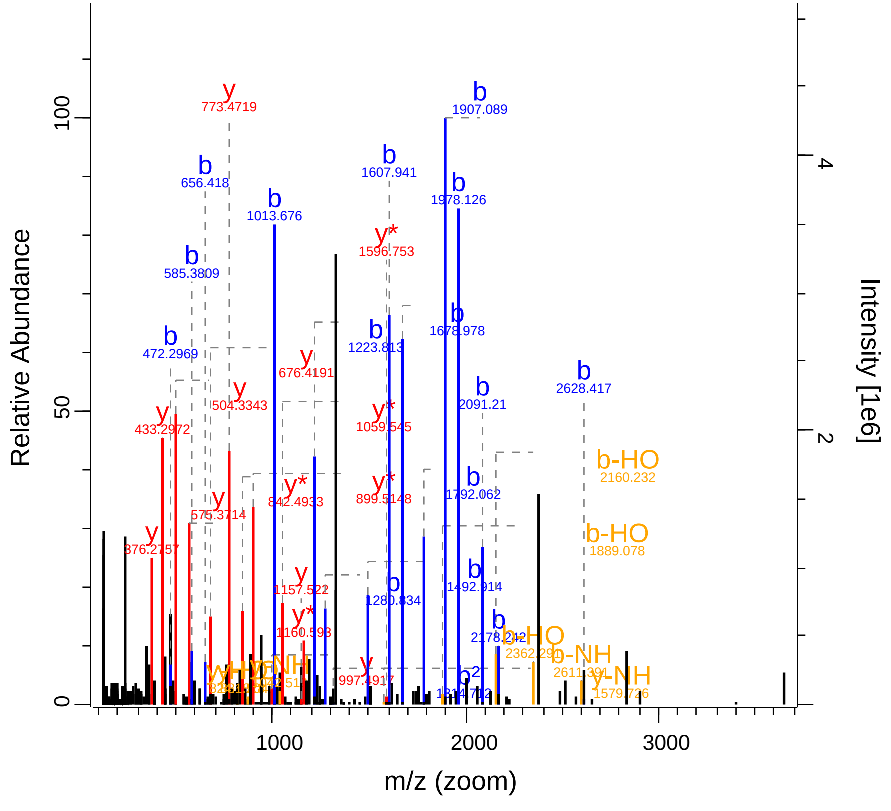

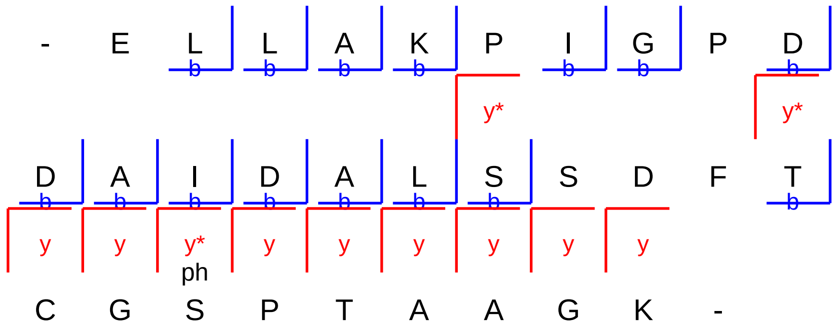

Raw File	Scan	Method	Score	m/z	Gene names
RO-QC-160121_ZackD_SS_Set_02_Fractio	34410	FTMS; HC	336.21	1106.58	MAP1B

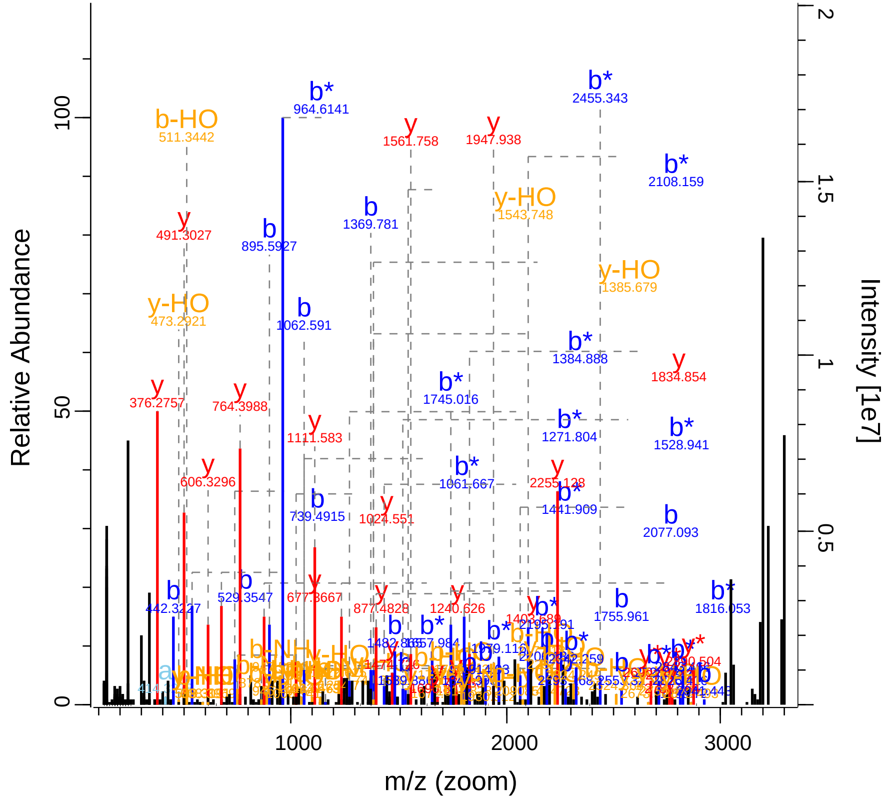

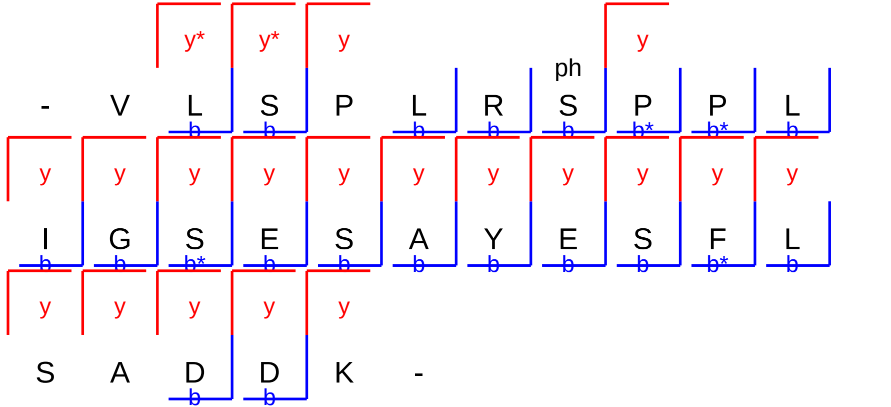

Raw File	Scan	Method	Score	m/z	Gene names
RO-QC-160121_ZackD_SS_Set_02_Fraction	4519	FTMS; HCI	51.26	536.8	MAPRE2

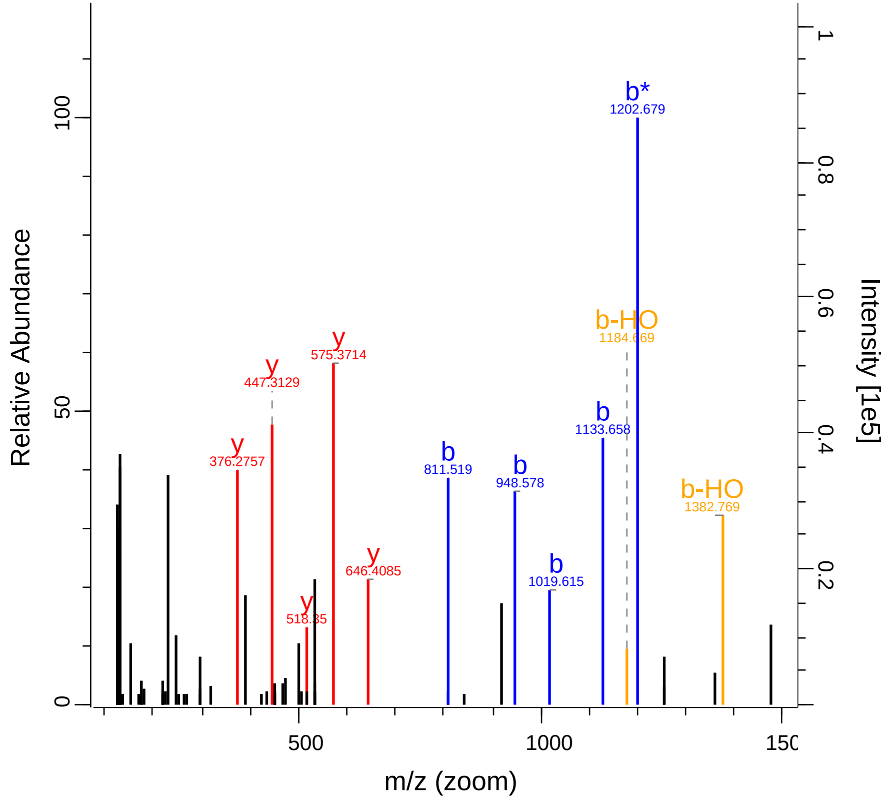

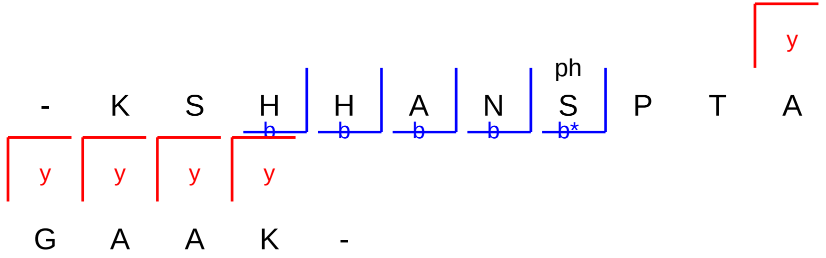

Raw File	Scan	Method	Score	m/z	Gene names
RO-QC-160121_ZackD_SS_Set_02_Fractior	6226	FTMS; HCI	82.42	720.37	RRBP1

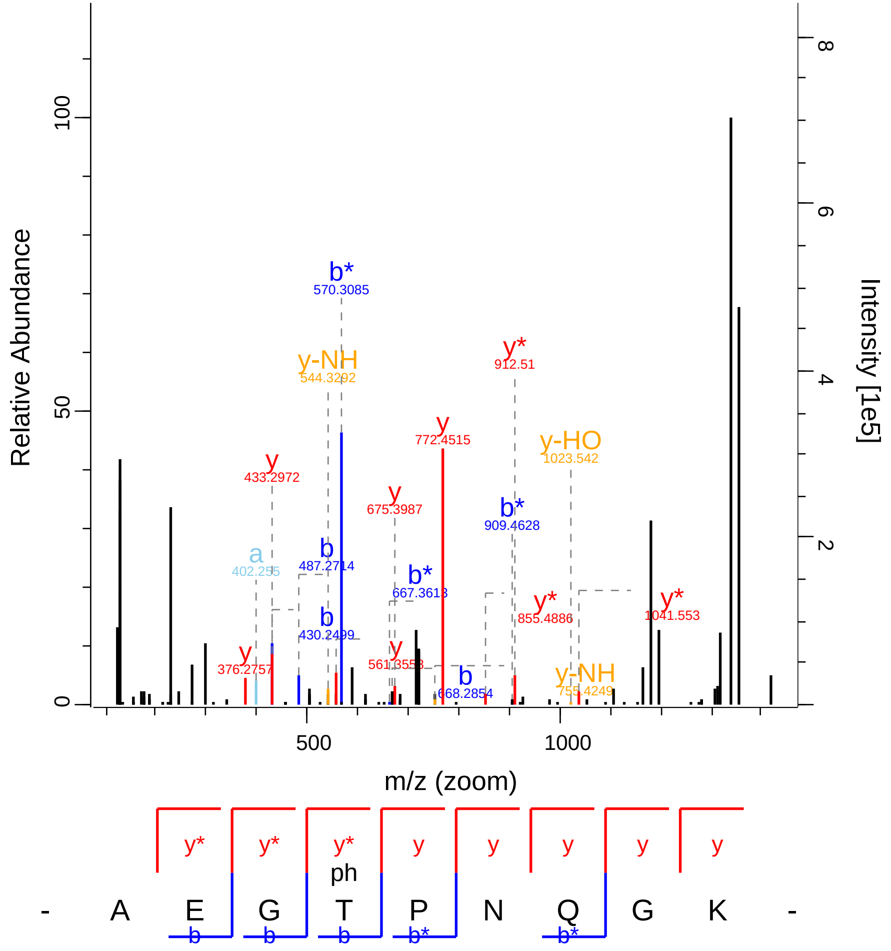

Raw File	Scan	Method	Score	m/z	Gene names
RO-QC-160121_ZackD_SS_Set_02_Fractioı	7044	FTMS; HC	120.57	886.45	ZC3H13

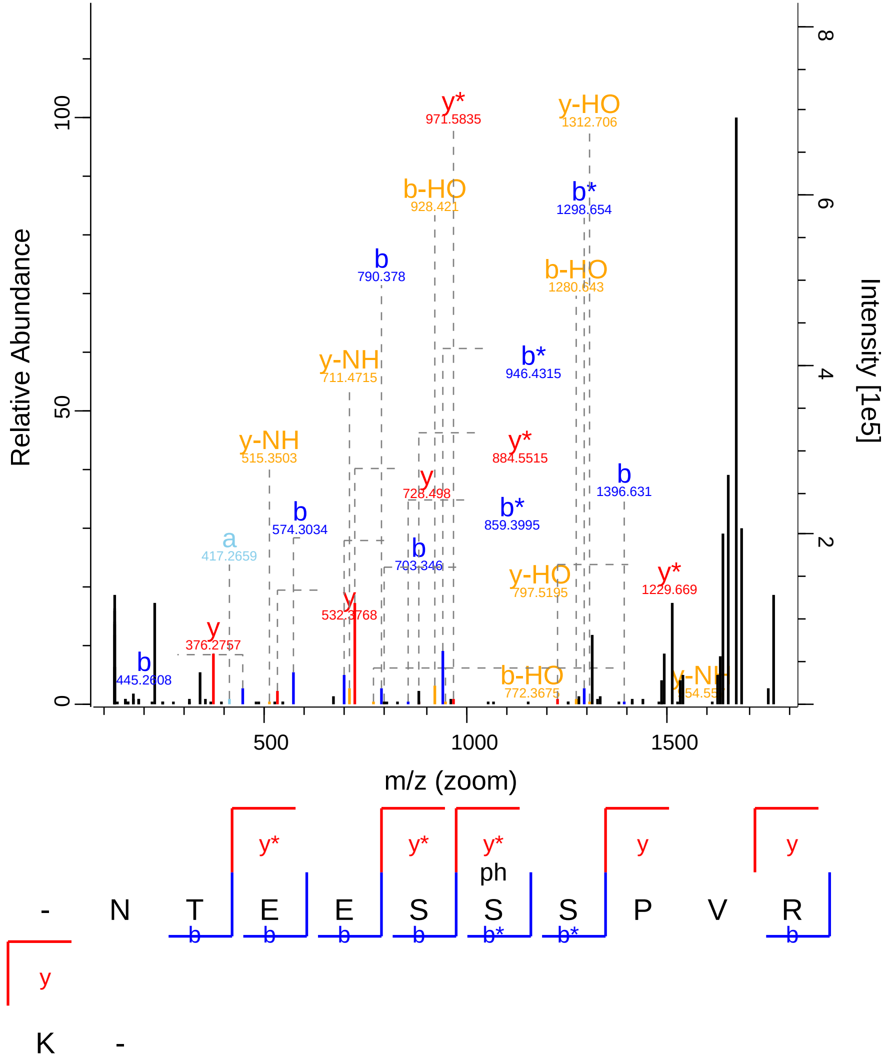

Raw File	Scan	Method	Score	m/z	Gene names
RO-QC-160121_ZackD_SS_Set_02_Fractio	8672	FTMS; HC	113.15	1029.48	UBAP2L

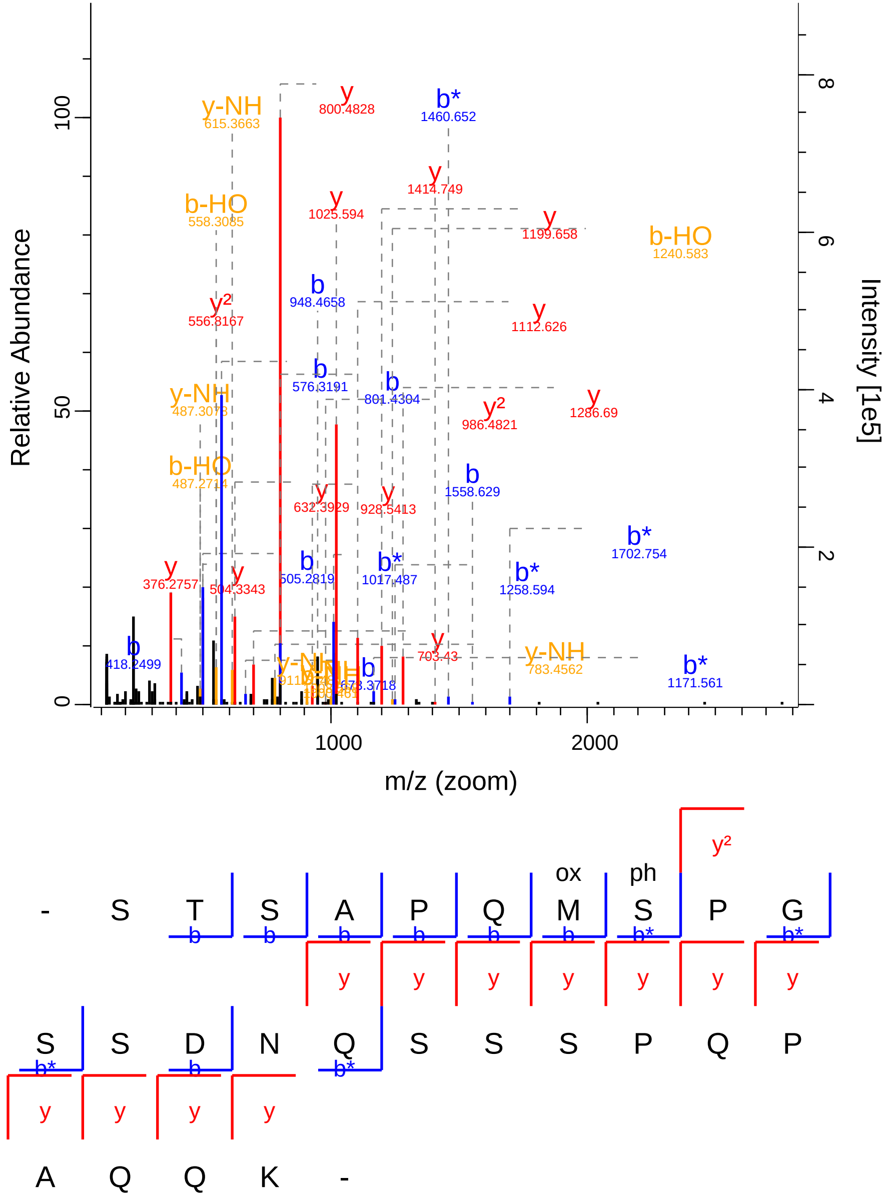

Raw File	Scan	Method	Score	m/z	Gene names
RO-QC-160121_ZackD_SS_Set_02_Fractio	10440	FTMS; HC	91.87	805.98	GTF3C2

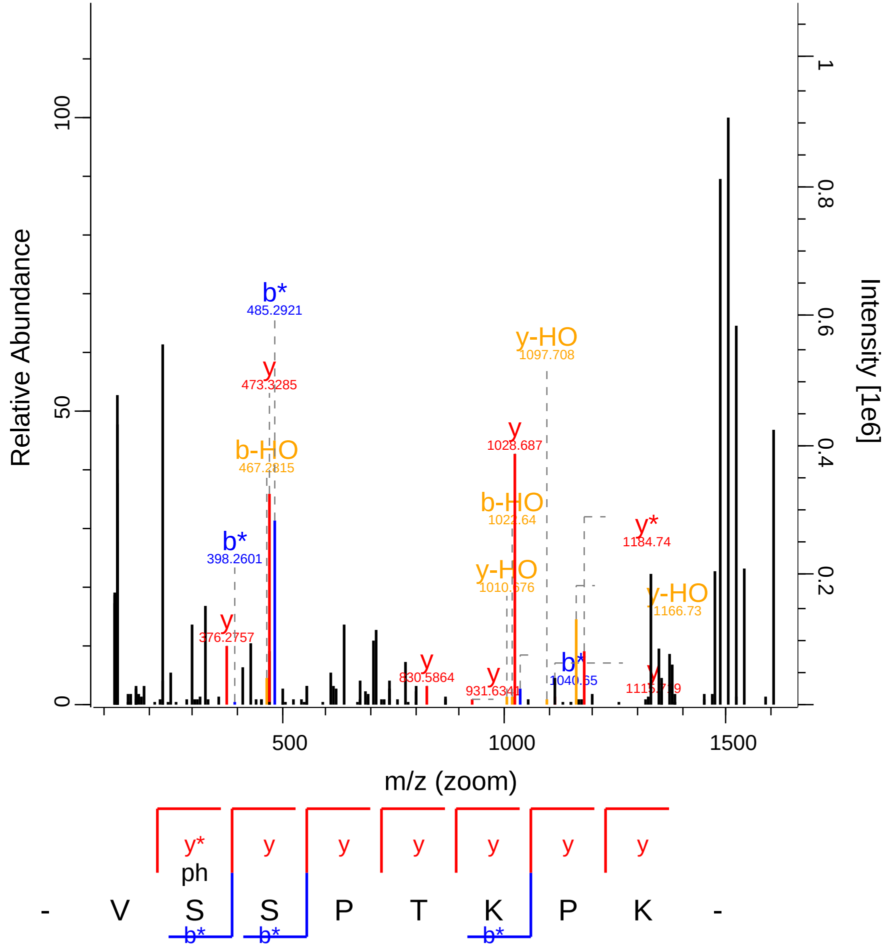

Raw File	Scan	Method	Score	m/z	Gene names
RO-QC-160121_ZackD_SS_Set_02_Fractio	10648	FTMS; HC	91.91	911.53	MICALL1

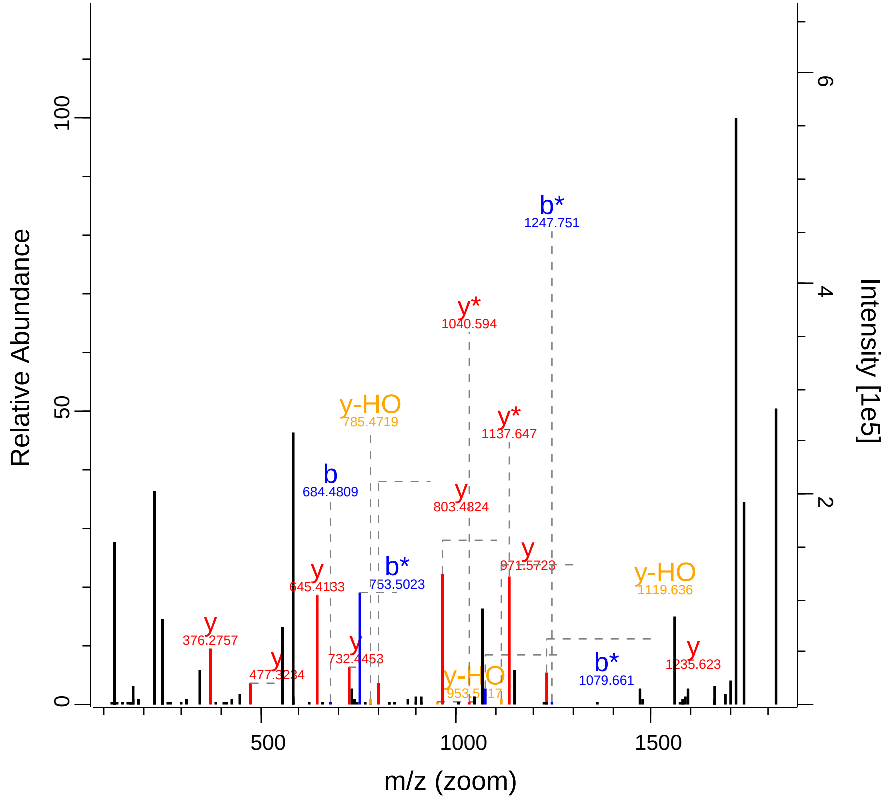

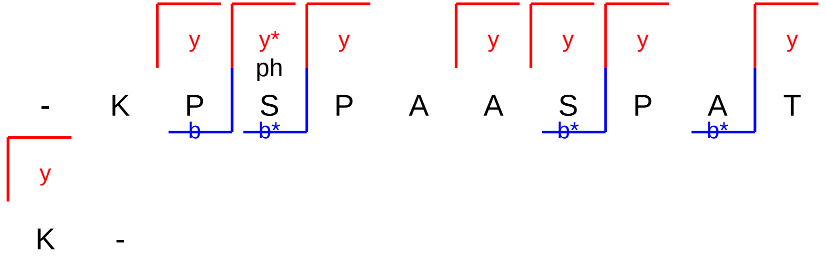

Raw File	Scan	Method	Score	m/z	Gene names
RO-QC-160121_ZackD_SS_Set_02_Fractio	10675	FTMS; HC	164.65	843.42	SON

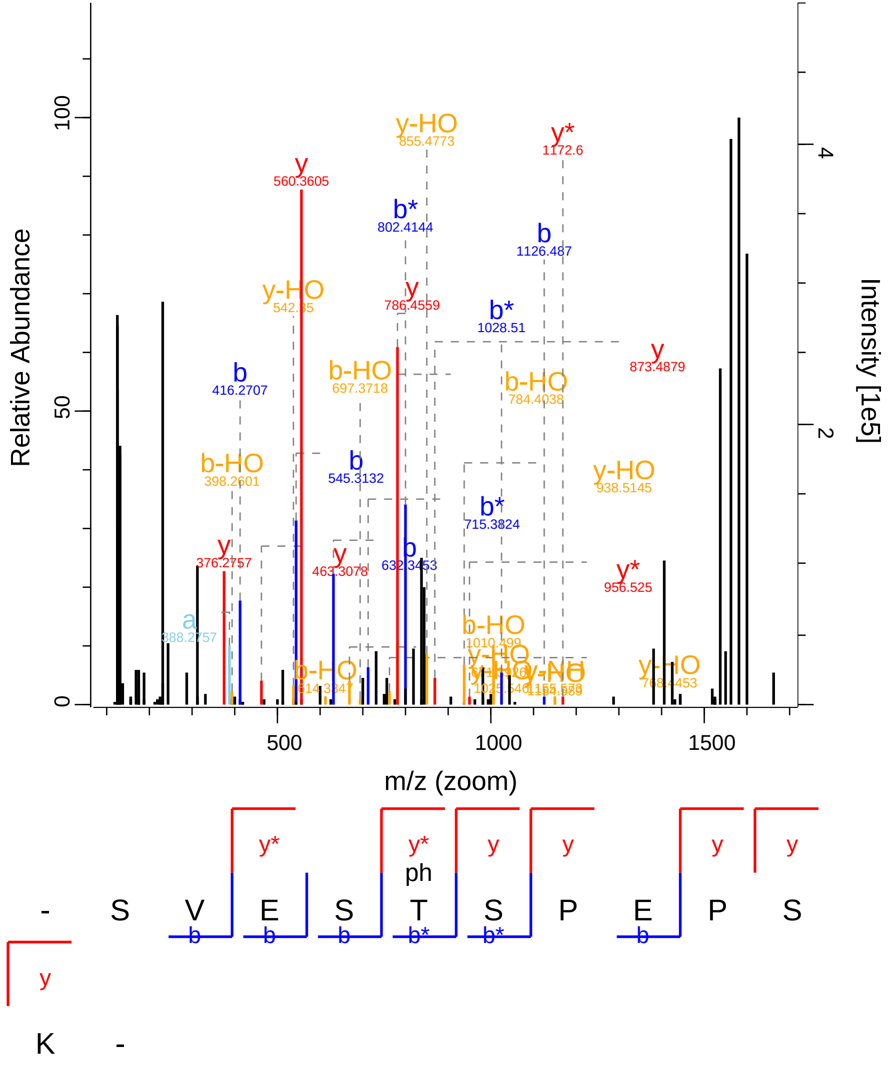

Raw File	Scan	Method	Score	m/z	Gene names
RO-QC-160121_ZackD_SS_Set_02_Fractio	10704	FTMS; HC	133.78	624.05	NUCKS1

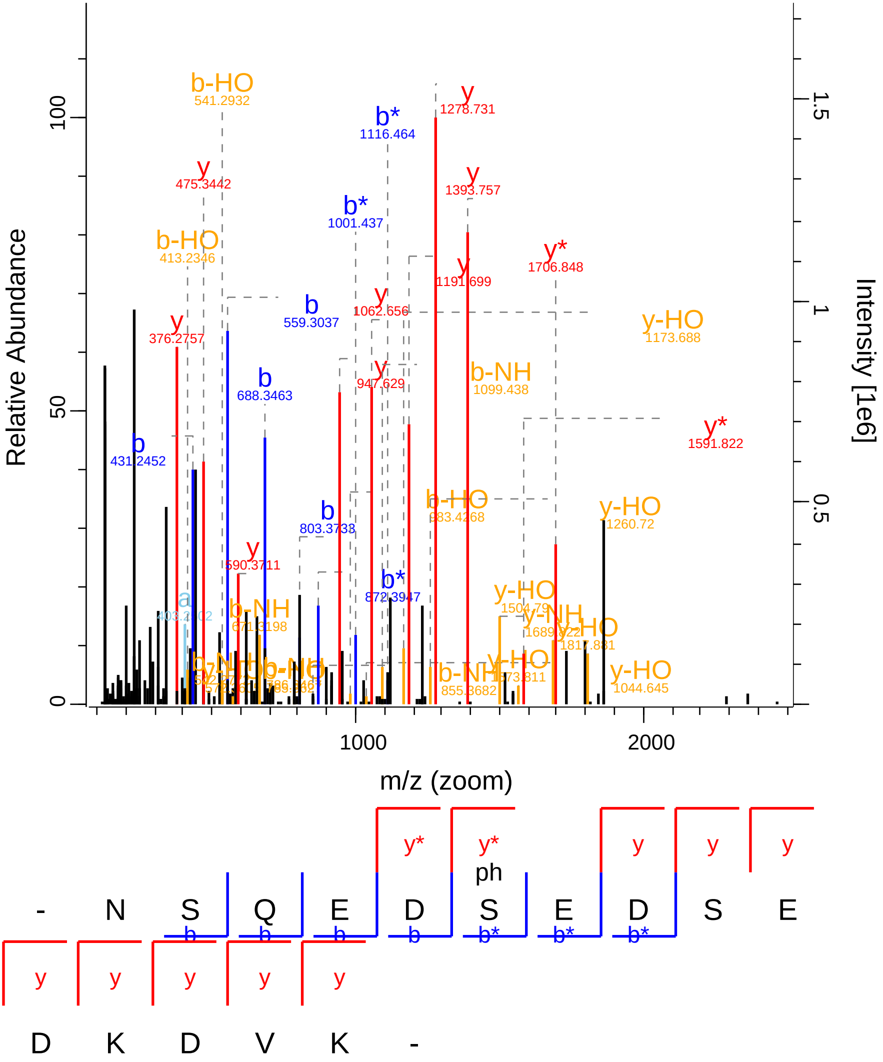

Raw File	Scan	Method	Score	m/z	Gene names
RO-QC-160121_ZackD_SS_Set_02_Fracti	11268	FTMS; HC	221.78	1114.54	EDC3

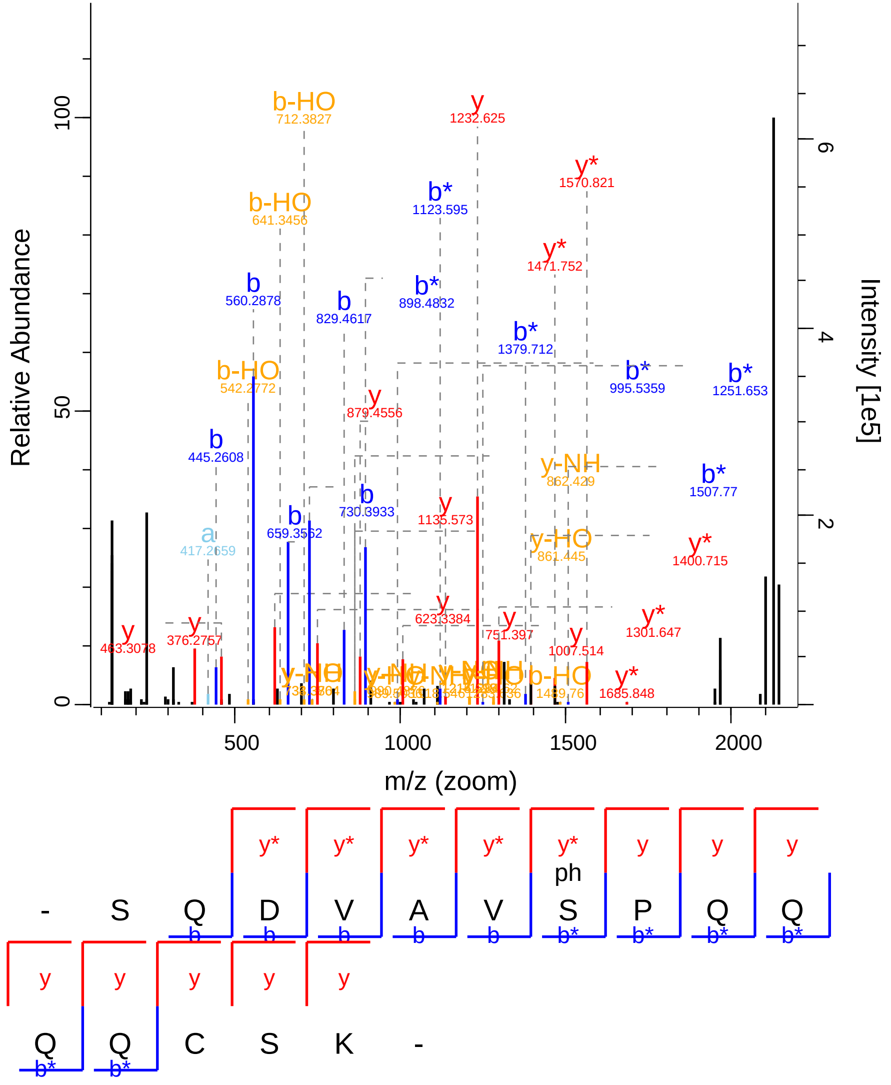

Raw File	Scan	Method	Score	m/z	Gene names
RO-QC-160121_ZackD_SS_Set_02_Fractio	11341	FTMS; HC	95.48	445.89	CTNND1

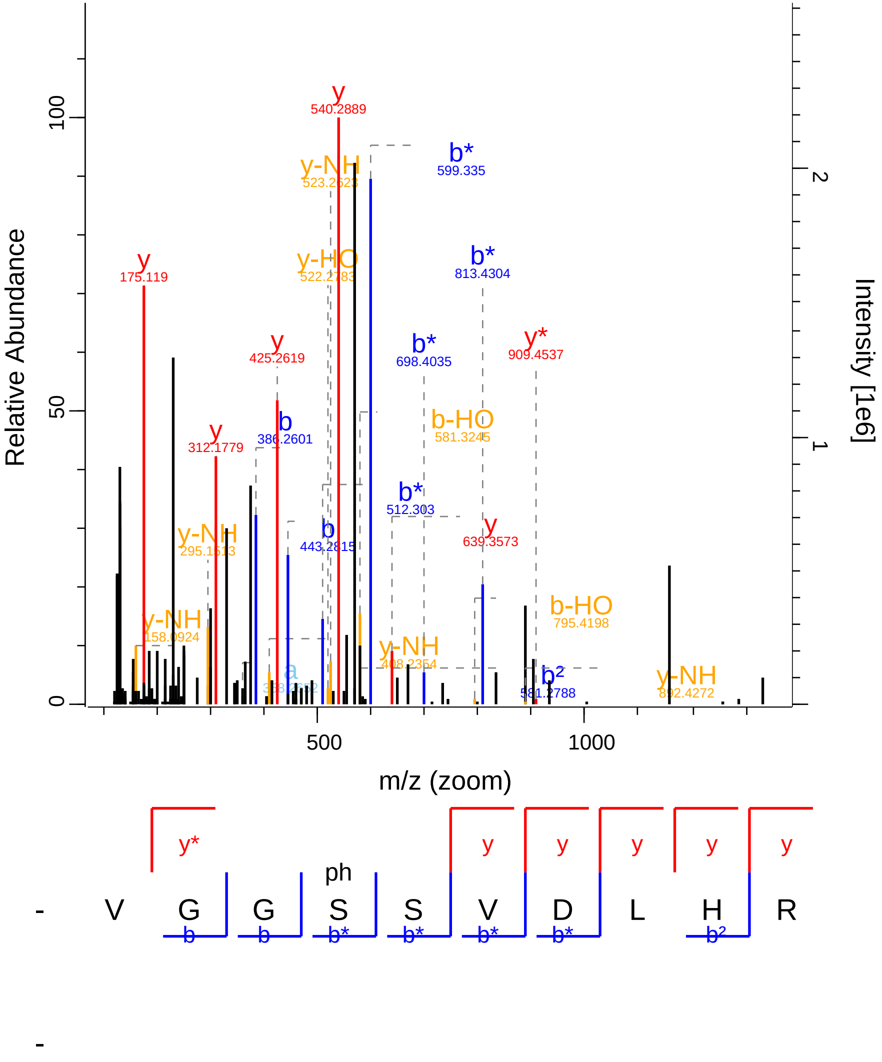

Raw File	Scan	Method	Score	m/z	Gene names
RO-QC-160121_ZackD_SS_Set_02_Fractio	11631	FTMS; HC	151.79	782.88	WWC1

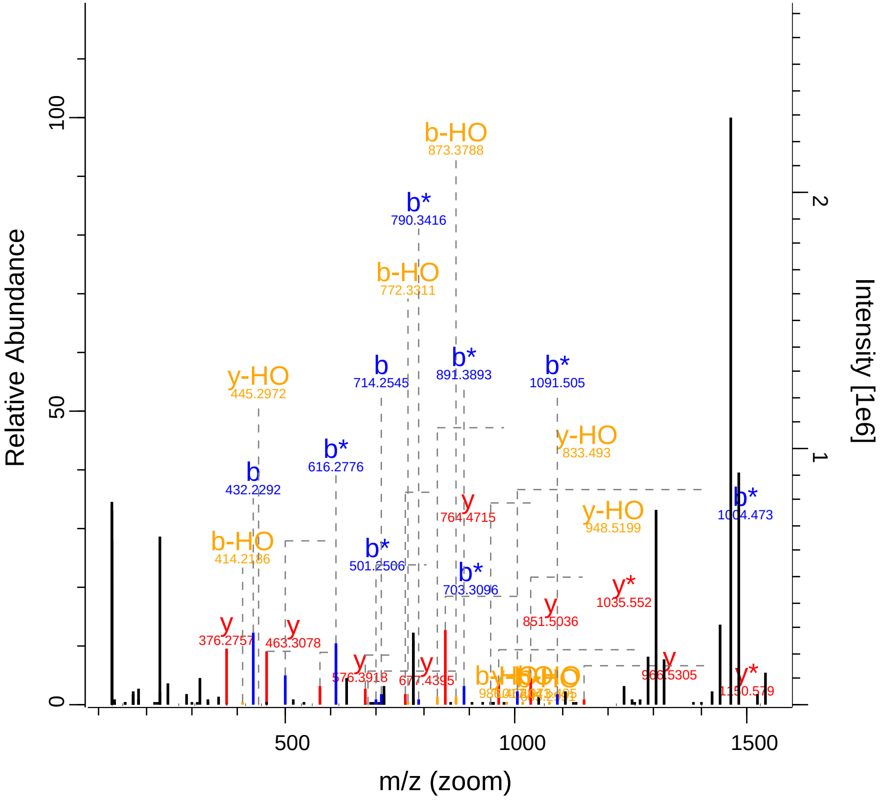

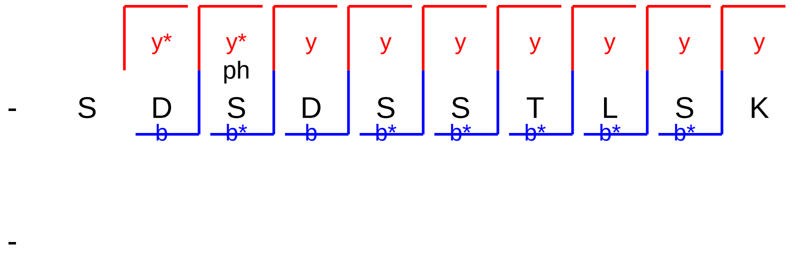

Raw File	Scan	Method	Score	m/z	Gene names
RO-QC-160121_ZackD_SS_Set_02_Fractio	11988	FTMS; HC	97.28	721.02	HSP90AA1

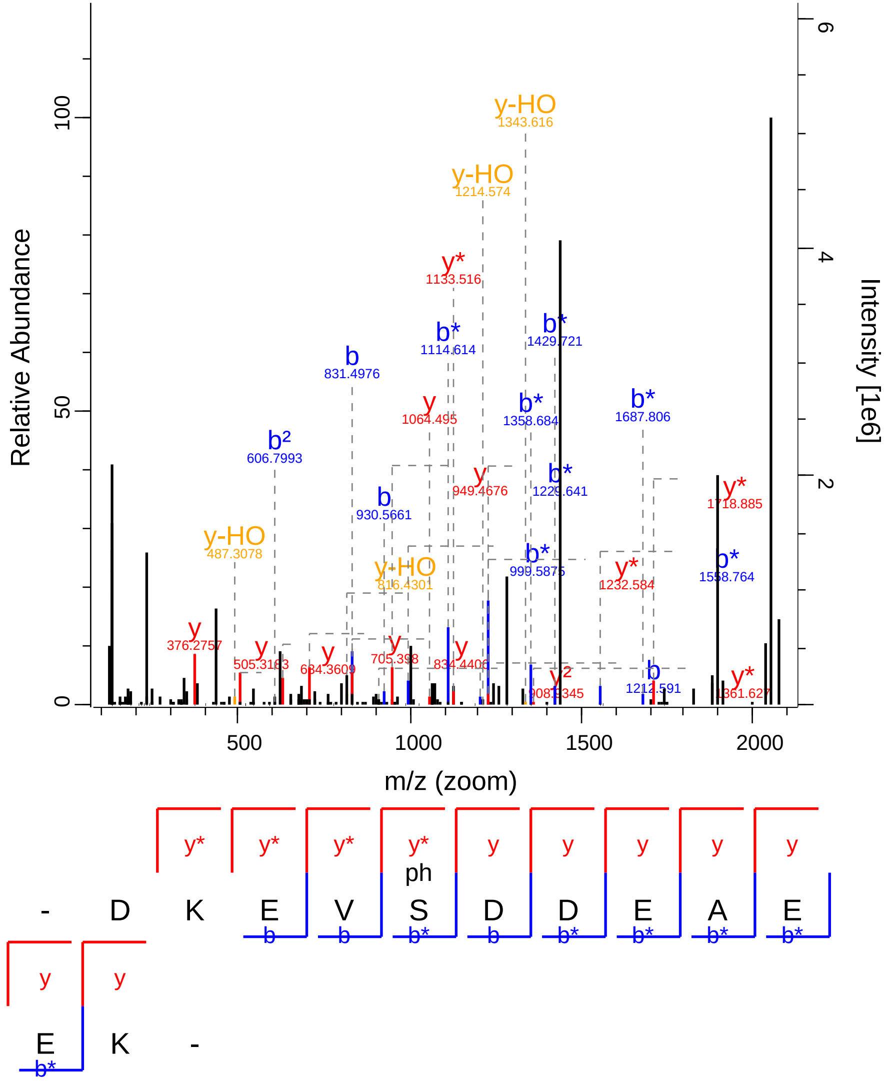

Raw File	Scan	Method	Score	m/z	Gene names
RO-QC-160121_ZackD_SS_Set_02_Fractio	12177	FTMS; HC	119.68	716.35	KLC1

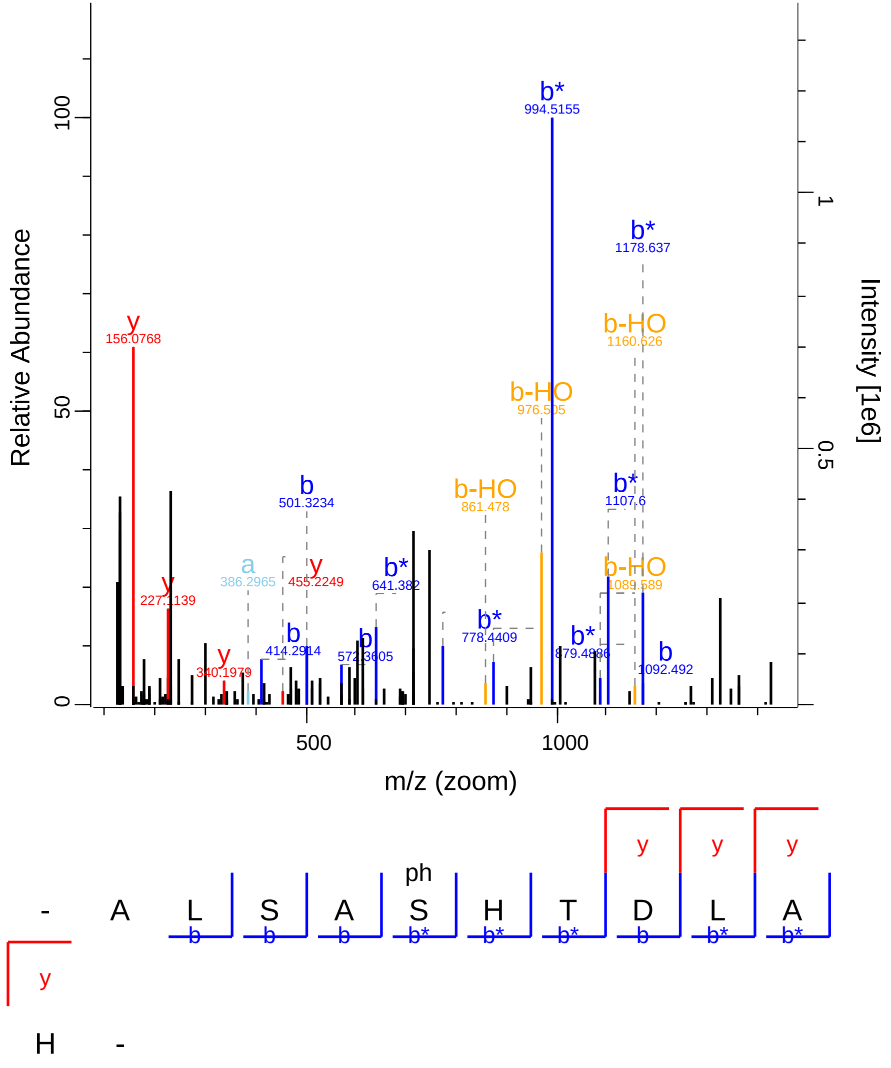

Raw File	Scan	Method	Score	m/z	Gene names
RO-QC-160121_ZackD_SS_Set_02_Fractior	12525	FTMS; HCI	85.52	862.5	SRSF11

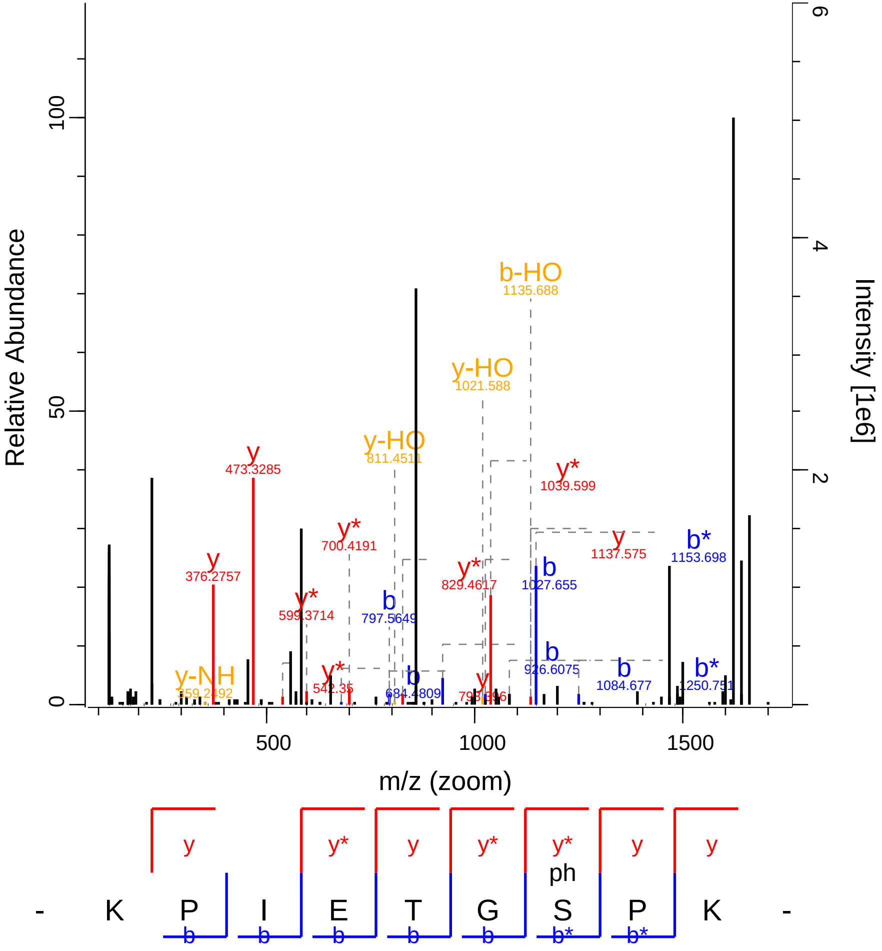

Raw File	Scan	Method	Score	m/z	Gene names
RO-QC-160121_ZackD_SS_Set_02_Fractio	12681	FTMS; HC	149.18	734.63	KTN1

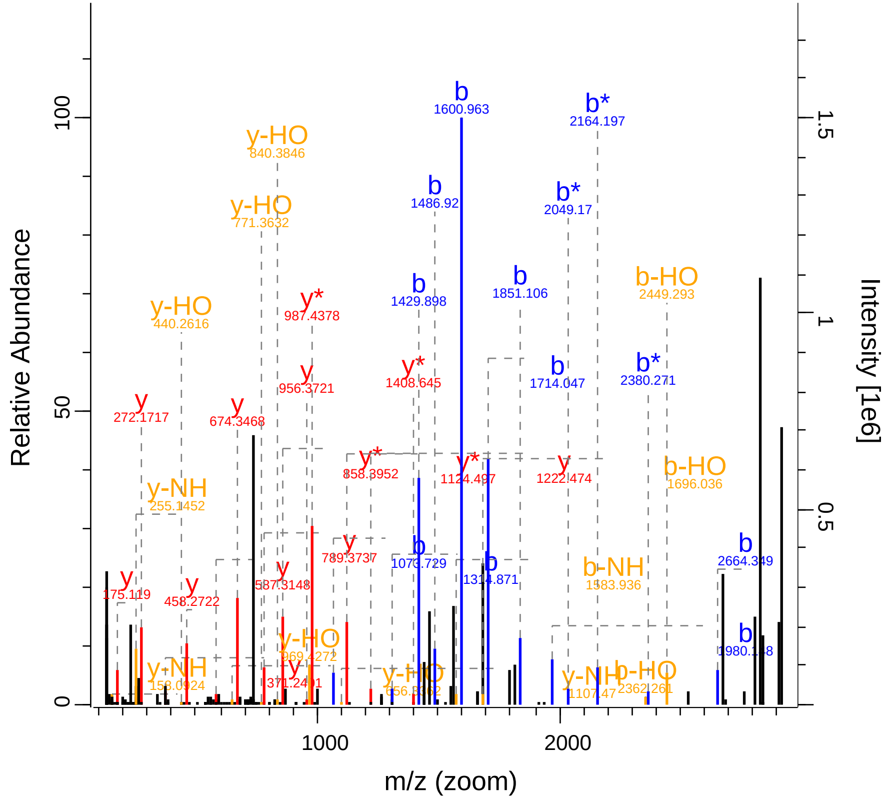

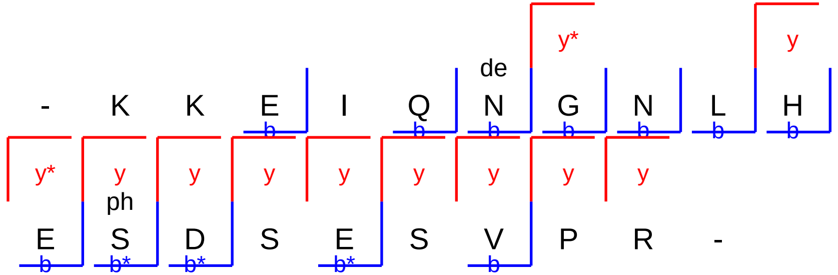

Raw File	Scan	Method	Score	m/z	Gene names
RO-QC-160121_ZackD_SS_Set_02_Fractior	13344	FTMS; HCI	163.18	903.4	SSB

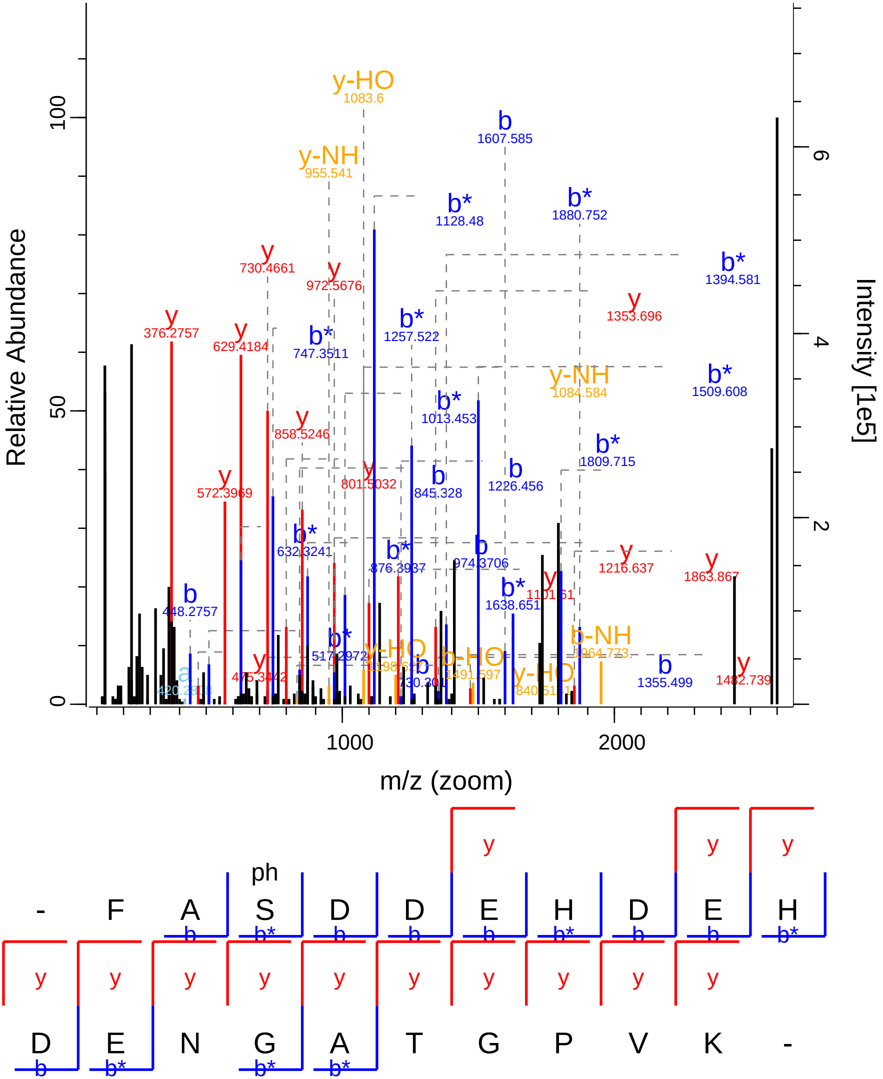

Raw File	Scan	Method	Score	m/z	Gene names
RO-QC-160121_ZackD_SS_Set_02_Fractio	13347	FTMS; HC	119.68	833.99	SCAMP3

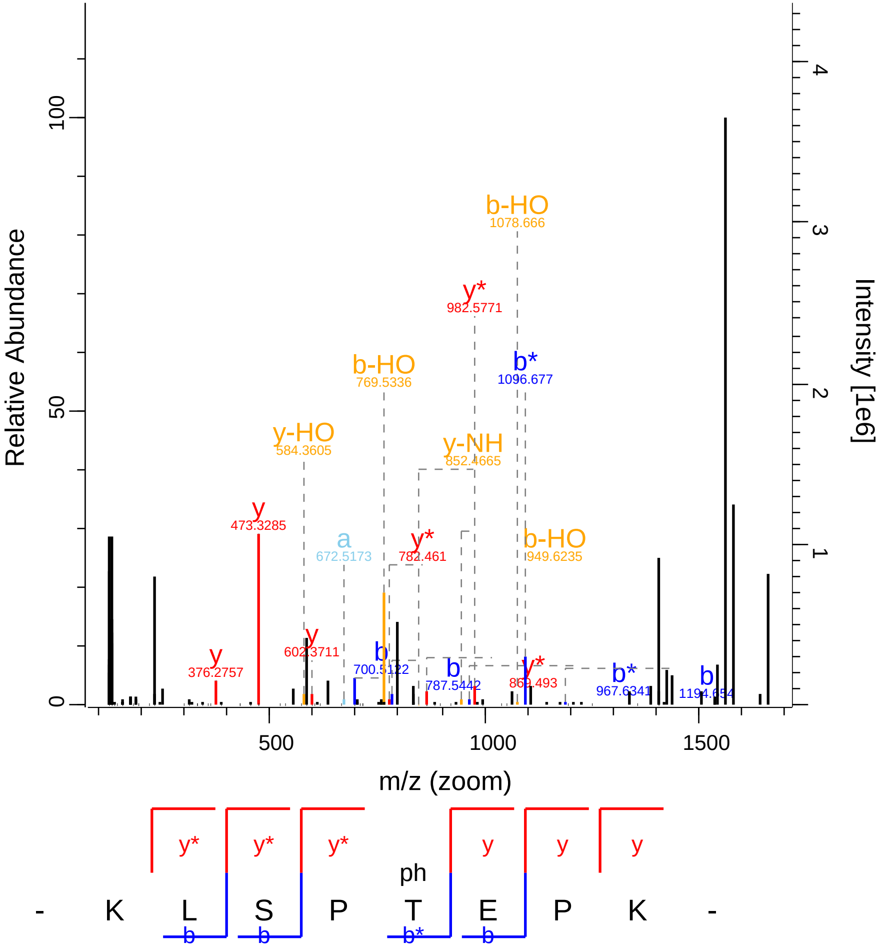

Raw File	Scan	Method	Score	m/z	Gene names
RO-QC-160121_ZackD_SS_Set_02_Fractio	13537	FTMS; HC	202.34	1136.58	SQSTM1

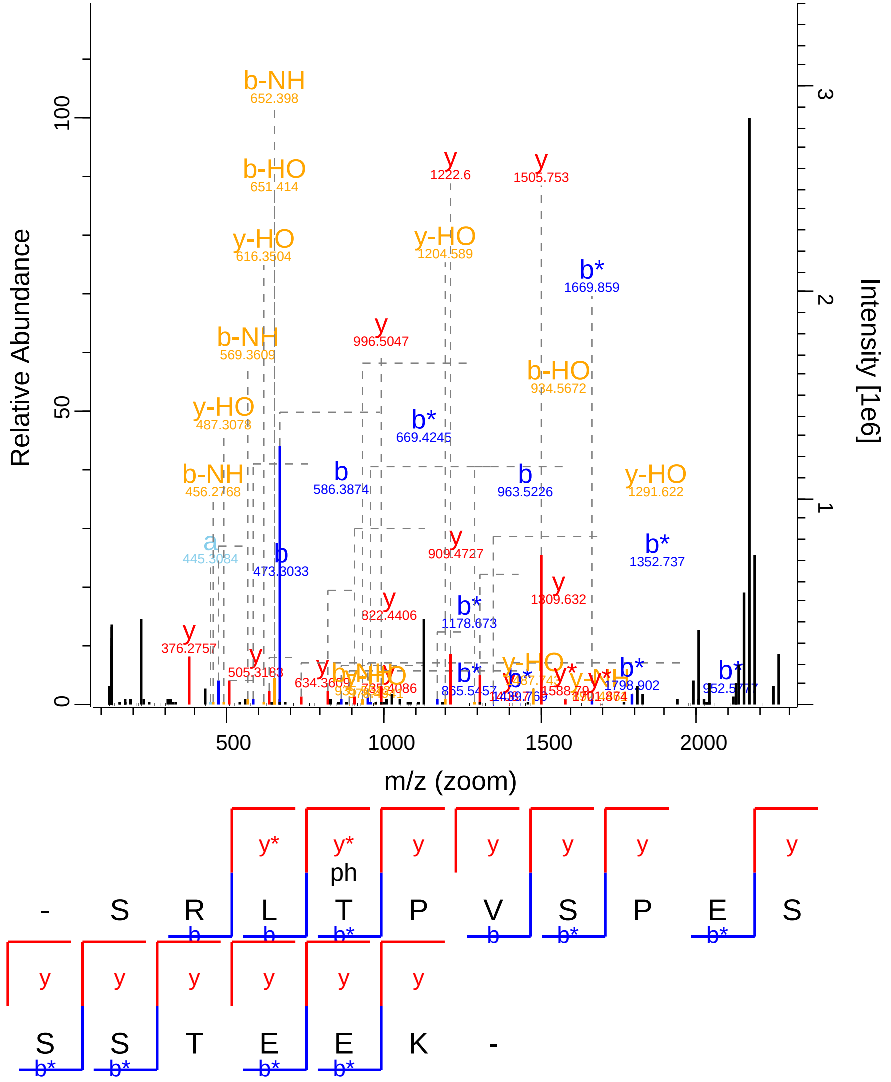

Raw File	Scan	Method	Score	m/z	Gene names
RO-QC-160121_ZackD_SS_Set_02_Fractio	13831	FTMS; HC	103.61	737.64	PSMA3

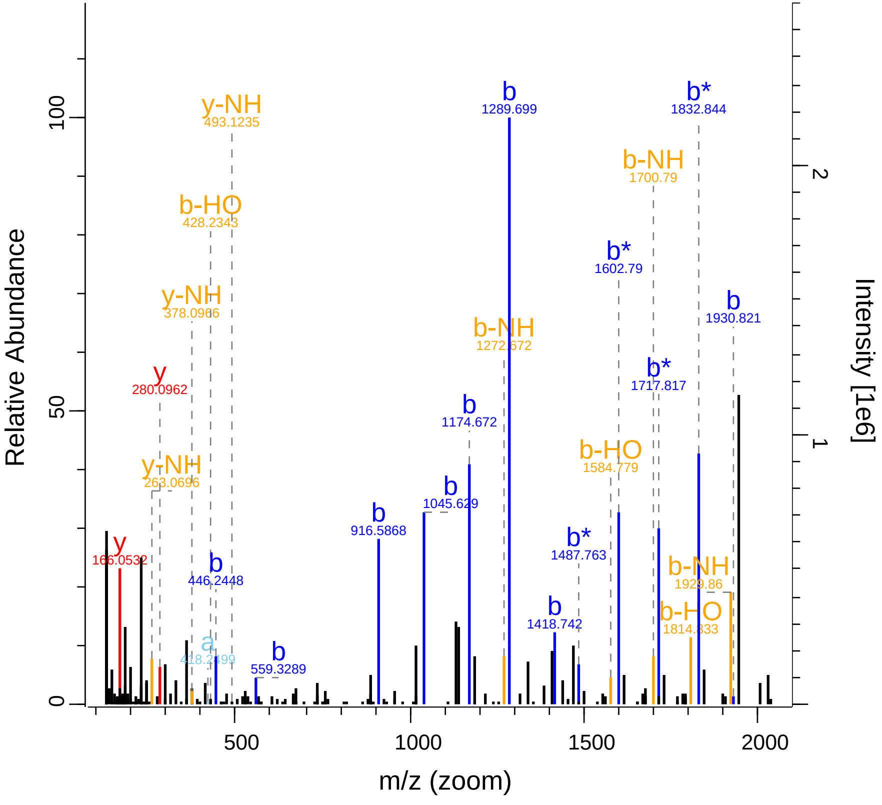

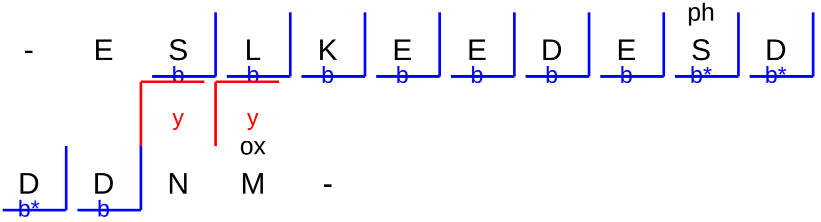

Raw File	Scan	Method	Score	m/z	Gene names
RO-QC-160121_ZackD_SS_Set_02_Fraction	14347	FTMS; HCI	58.98	899	CNTRL

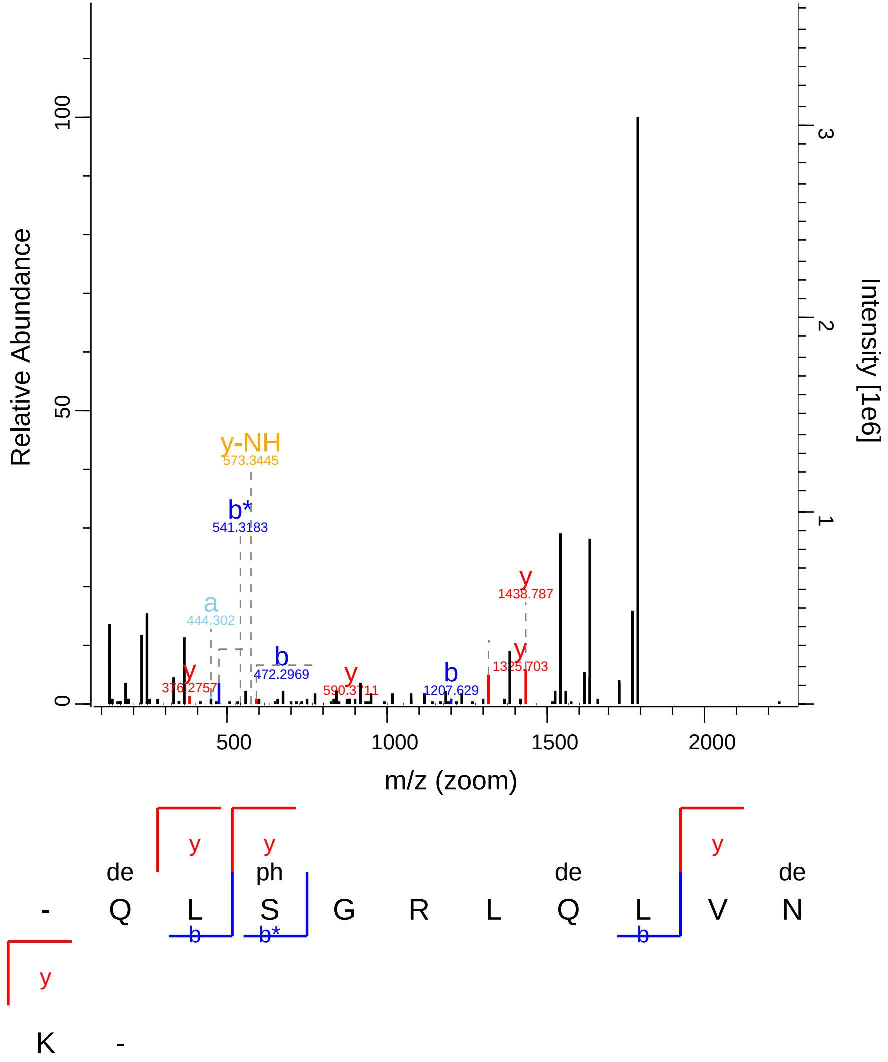

Raw File	Scan	Method	Score	m/z	Gene names
RO-QC-160121_ZackD_SS_Set_02_Fractic	14680	FTMS; HC	233.49	1152.55	CASK

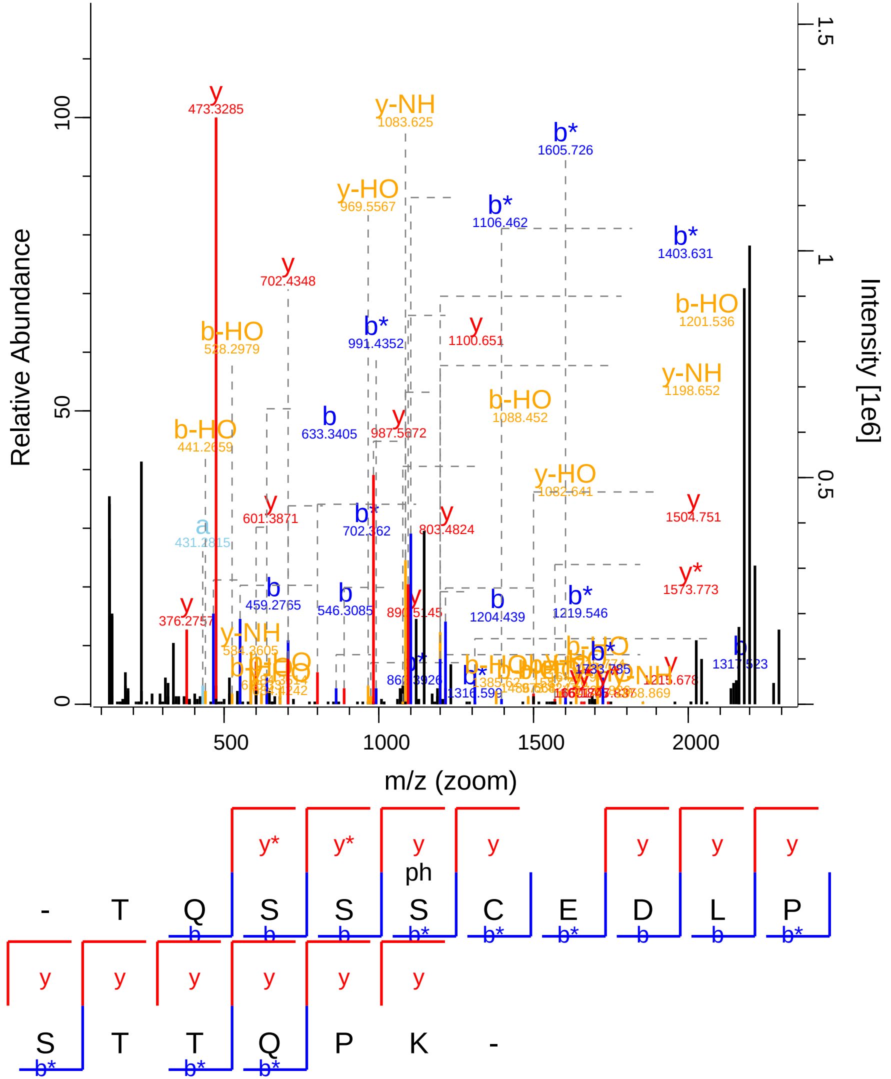

Raw File	Scan	Method	Score	m/z	Gene names
RO-QC-160121_ZackD_SS_Set_02_Fractio	15617	FTMS; HC	40.99	448.59	DDX23

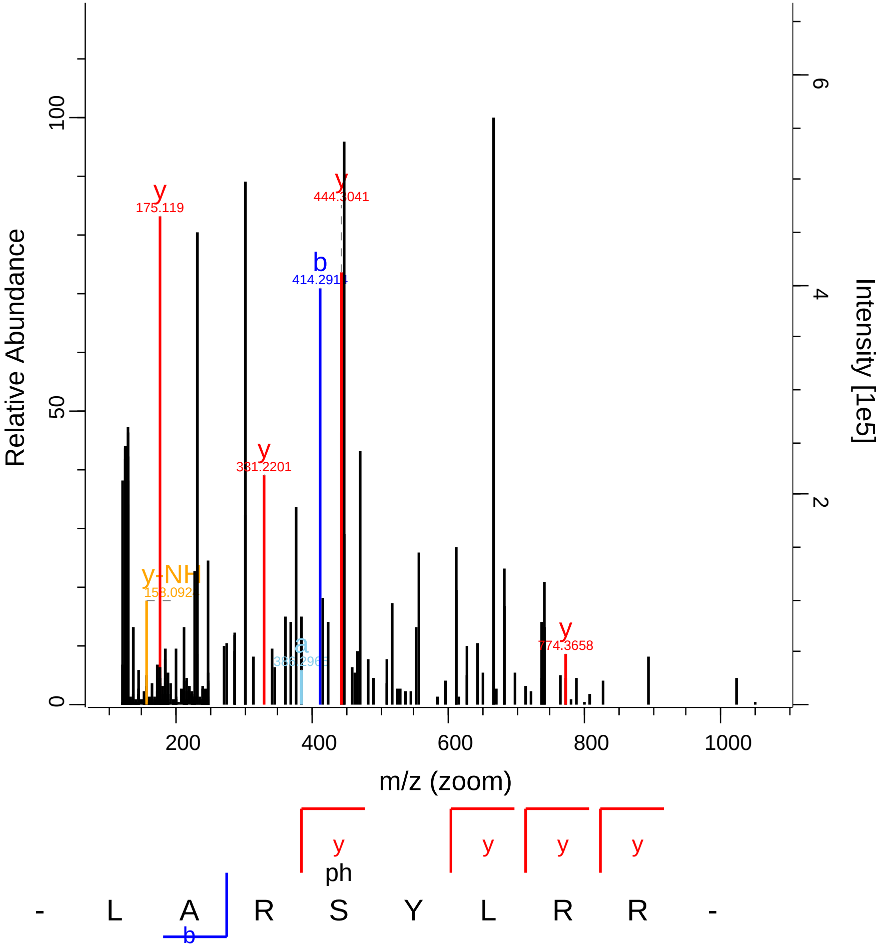

Raw File	Scan	Method	Score	m/z	Gene names
RO-QC-160121_ZackD_SS_Set_02_Fractio	15632	FTMS; HC	117.2	975.44	KIF13A

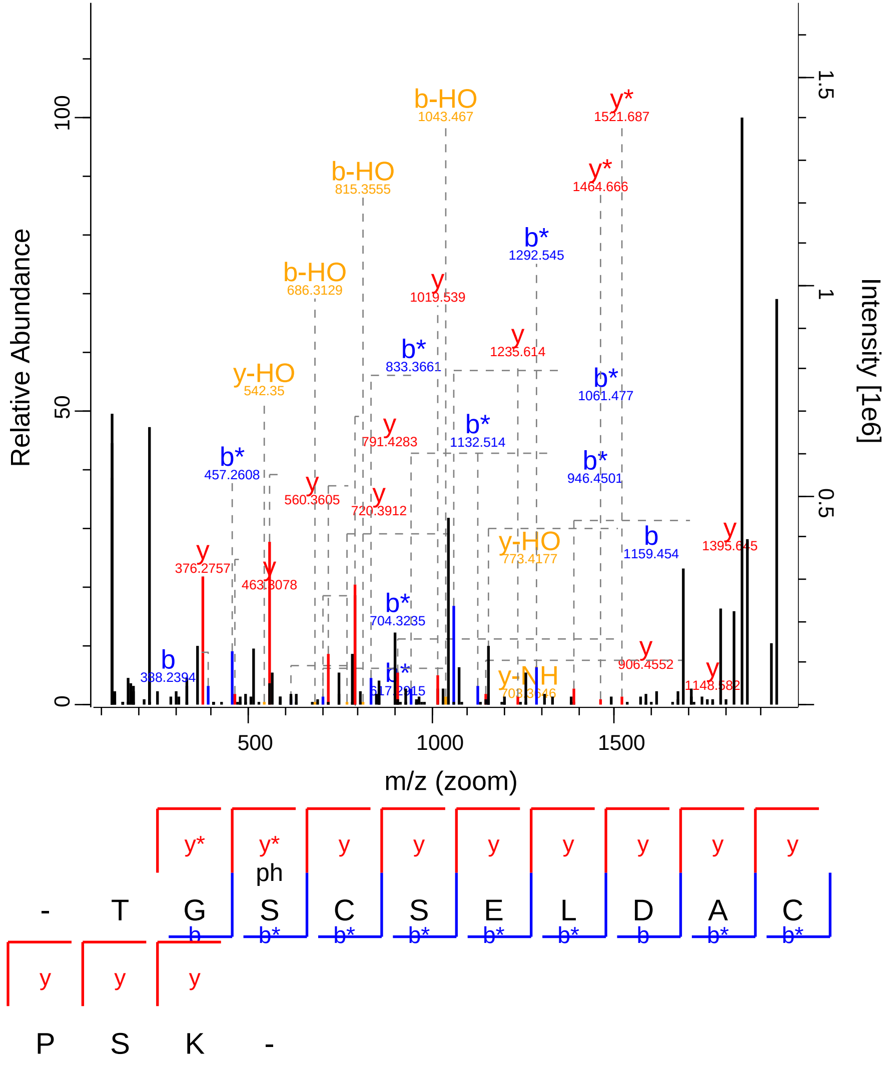

Raw File	Scan	Method	Score	m/z	Gene names
RO-QC-160121_ZackD_SS_Set_02_Fractio	15690	FTMS; HC	91.72	811.03	NUP98

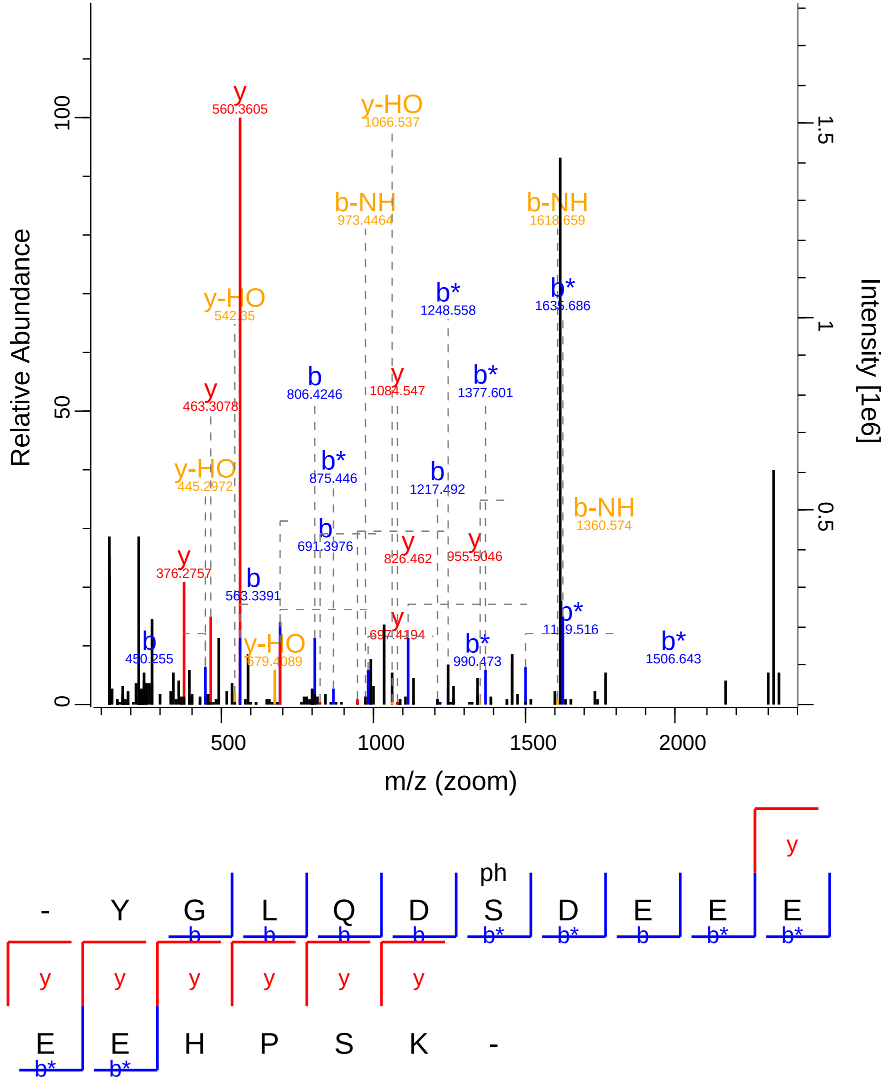

Raw File	Scan	Method	Score	m/z	Gene names
RO-QC-160121_ZackD_SS_Set_02_Fractio	16006	FTMS; HC	194.77	858.91	STT3B

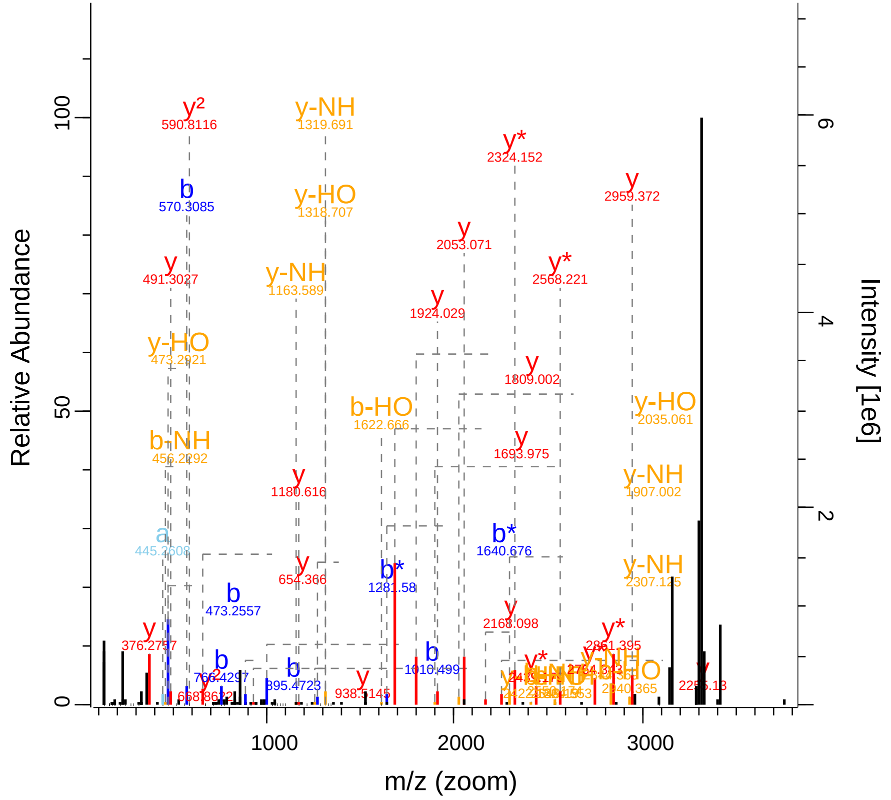

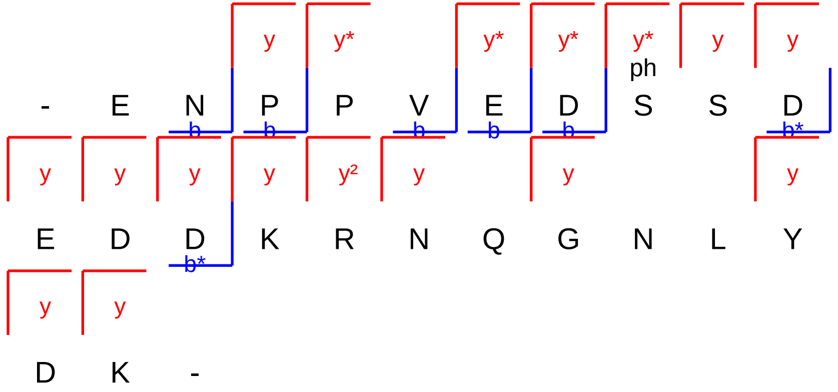

Raw File	Scan	Method	Score	m/z	Gene names
RO-QC-160121_ZackD_SS_Set_02_Fractio	16254	FTMS; HC	67.73	572.82	CCDC173

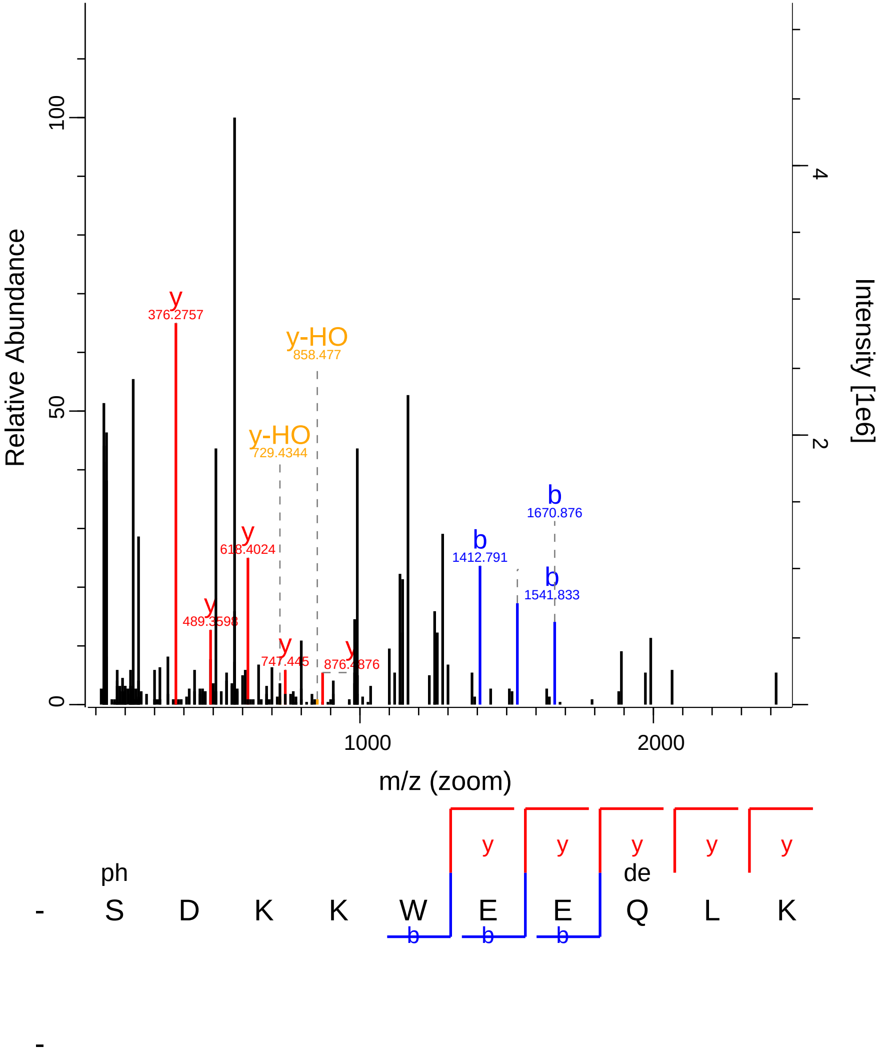

Raw File	Scan	Method	Score	m/z
RO-QC-160121_ZackD_SS_Set_02_Fraction_6	17011	FTMS; HCD	42.79	748.75

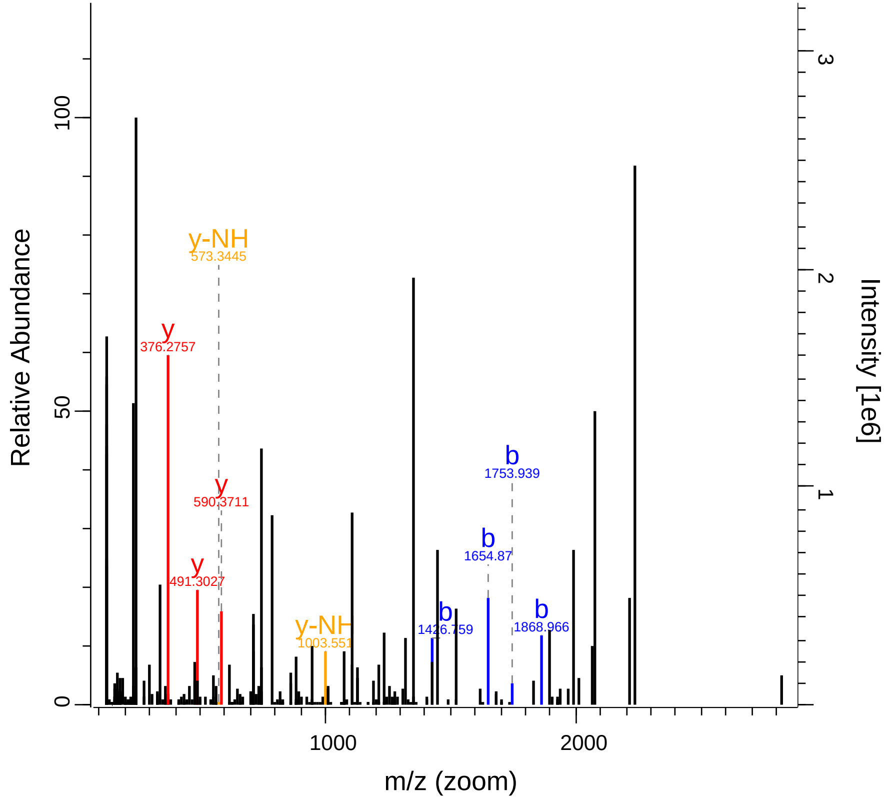

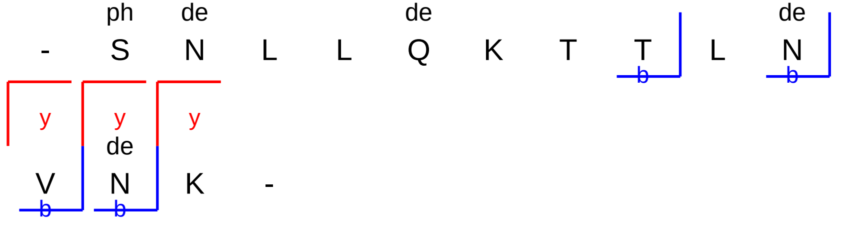

Raw File	Scan	Method	Score	m/z	Gene names
RO-QC-160121_ZackD_SS_Set_02_Fractic	17989	FTMS; HC	208.43	1258.15	PRKAR1A

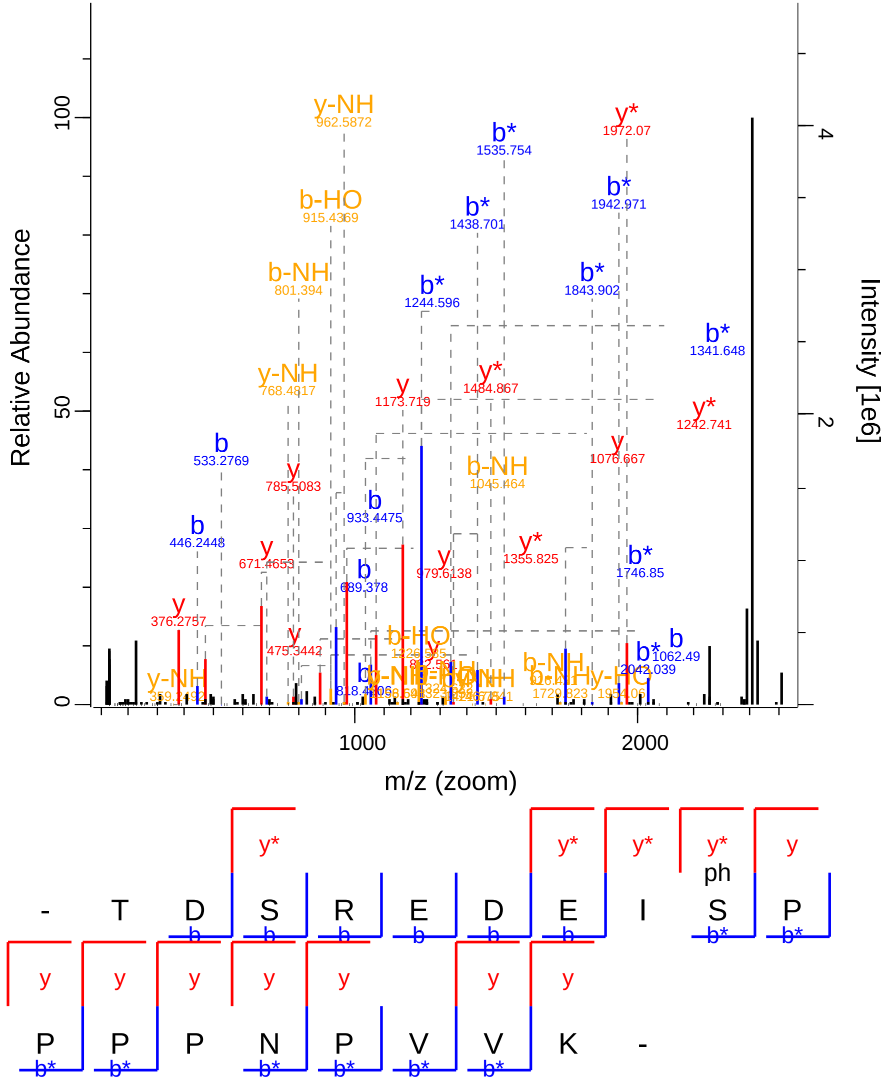

Raw File	Scan	Method	Score	m/z	Gene names
RO-QC-160121_ZackD_SS_Set_02_Fractior	18271	FTMS; HCI	136.35	1033	PLEC

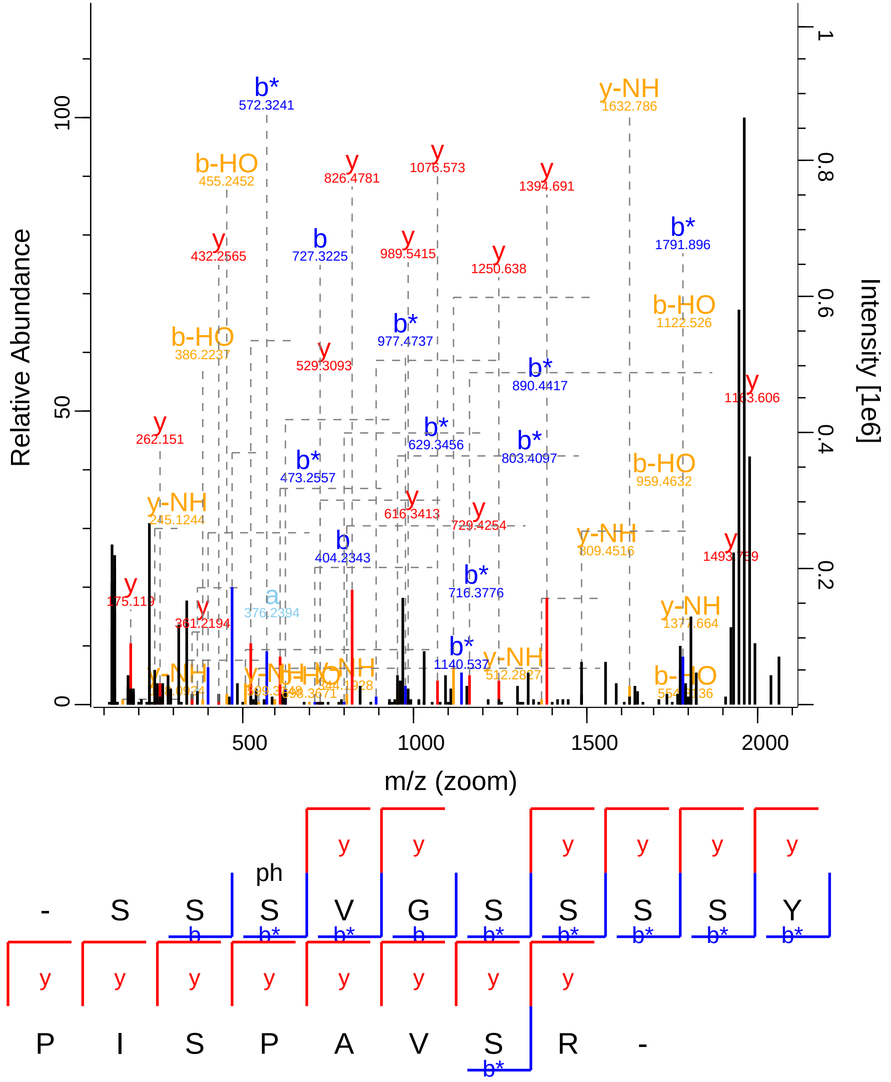

Raw File	Scan	Method	Score	m/z	Gene names
RO-QC-160121_ZackD_SS_Set_02_Fractio	18443	FTMS; HC	161.25	830.41	HSP90AA1

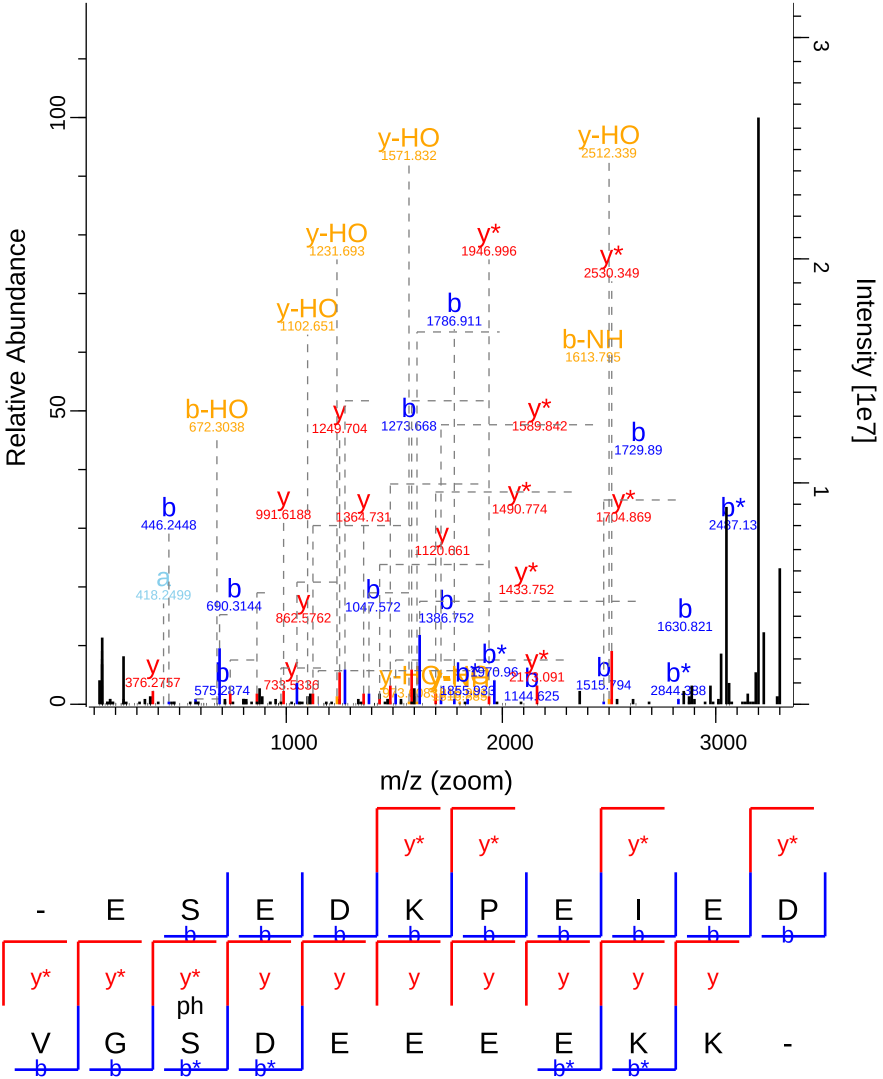

Raw File	Scan	Method	Score	m/z	Gene names
RO-QC-160121_ZackD_SS_Set_02_Fractio	18643	FTMS; HC	53.97	748.05	SCAF11

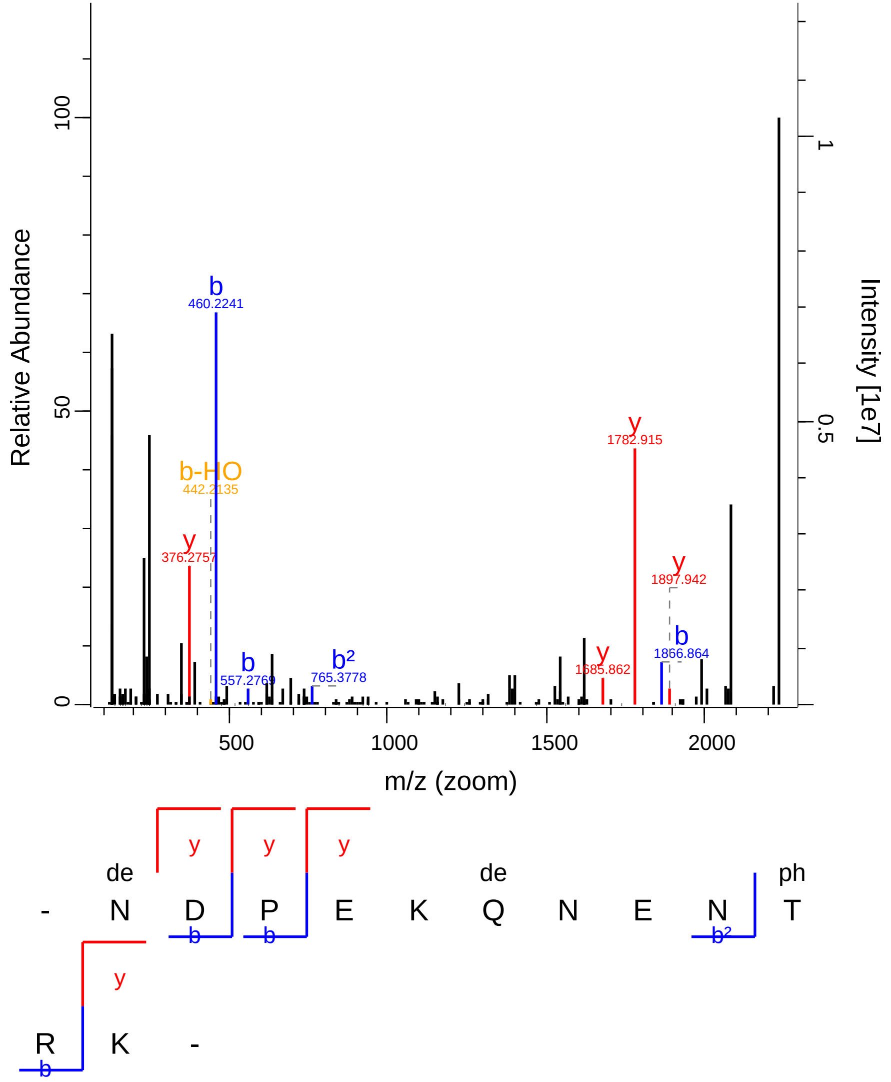

Raw File	Scan	Method	Score	m/z	Gene names
RO-QC-160121_ZackD_SS_Set_02_Fractior	19143	FTMS; HCI	83.4	726.4	NOP56

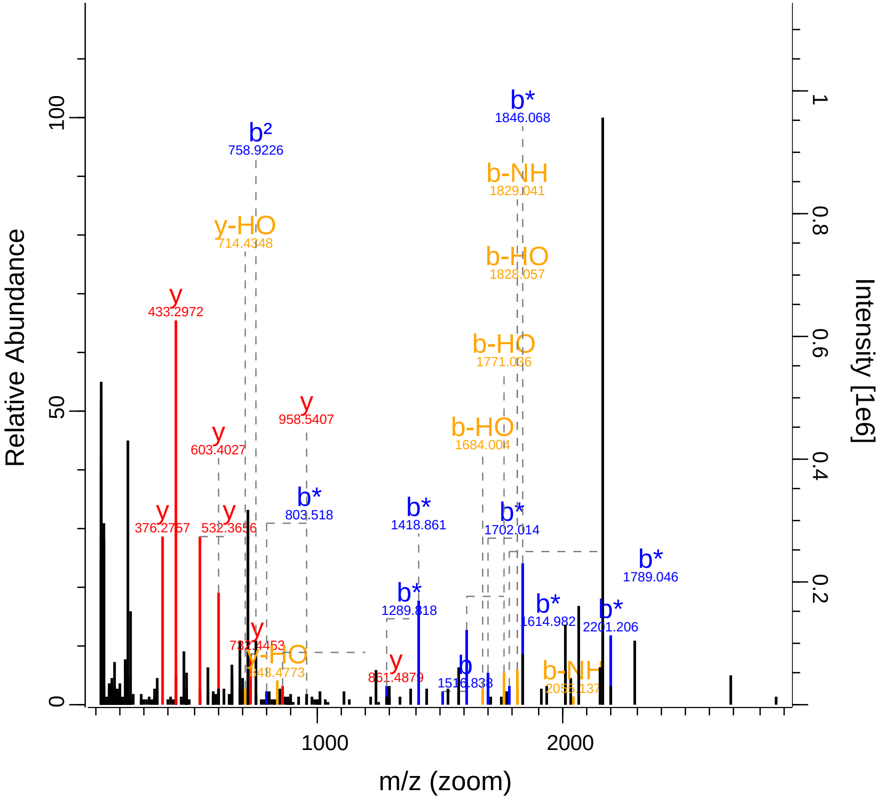

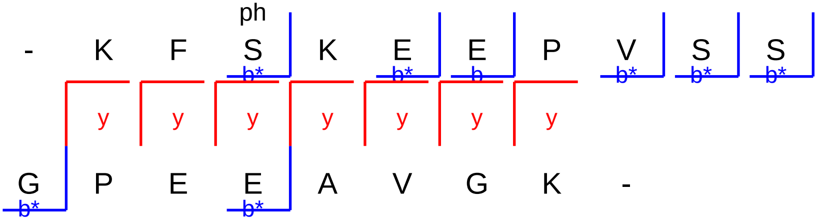

Raw File	Scan	Method	Score	m/z	Gene names
RO-QC-160121_ZackD_SS_Set_02_Fractio	19169	FTMS; HC	83.6	923.76	EIF5B

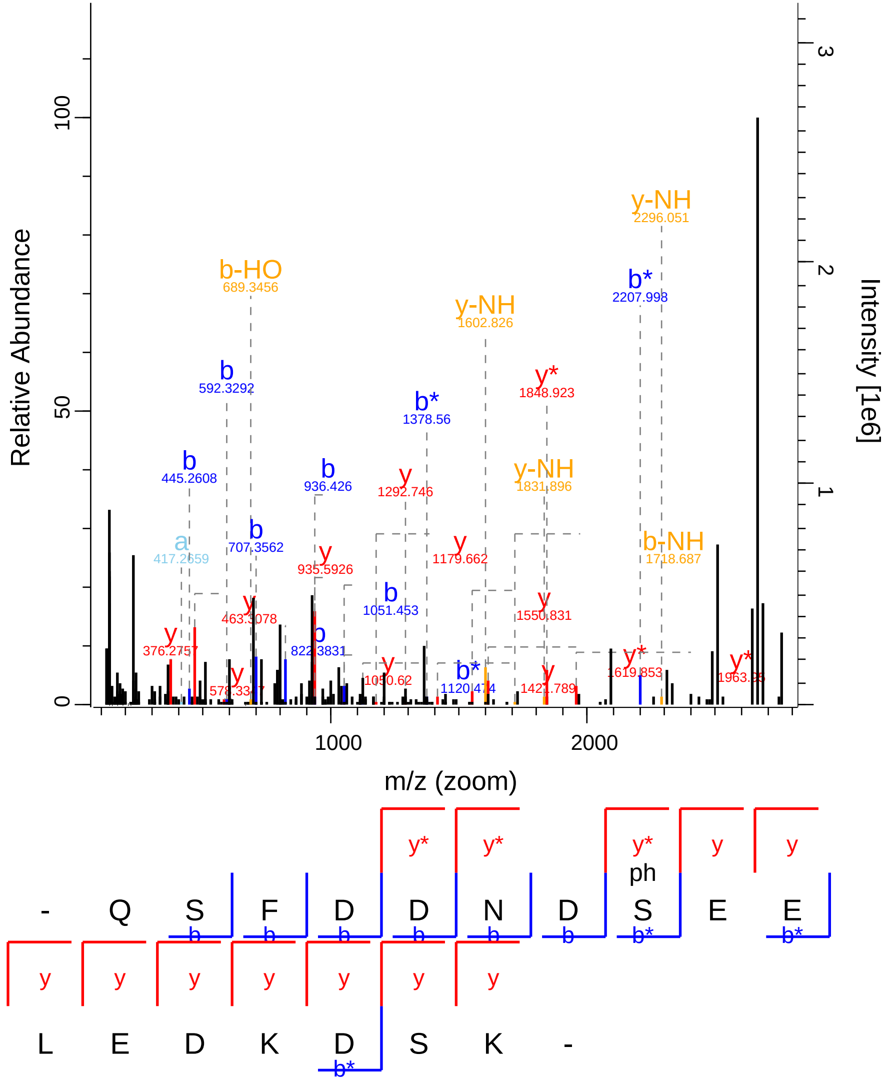

Raw File	Scan	Method	Score	m/z	Gene names
RO-QC-160121_ZackD_SS_Set_02_Fractio	19320	FTMS; HC	46.43	817.77	RTTN

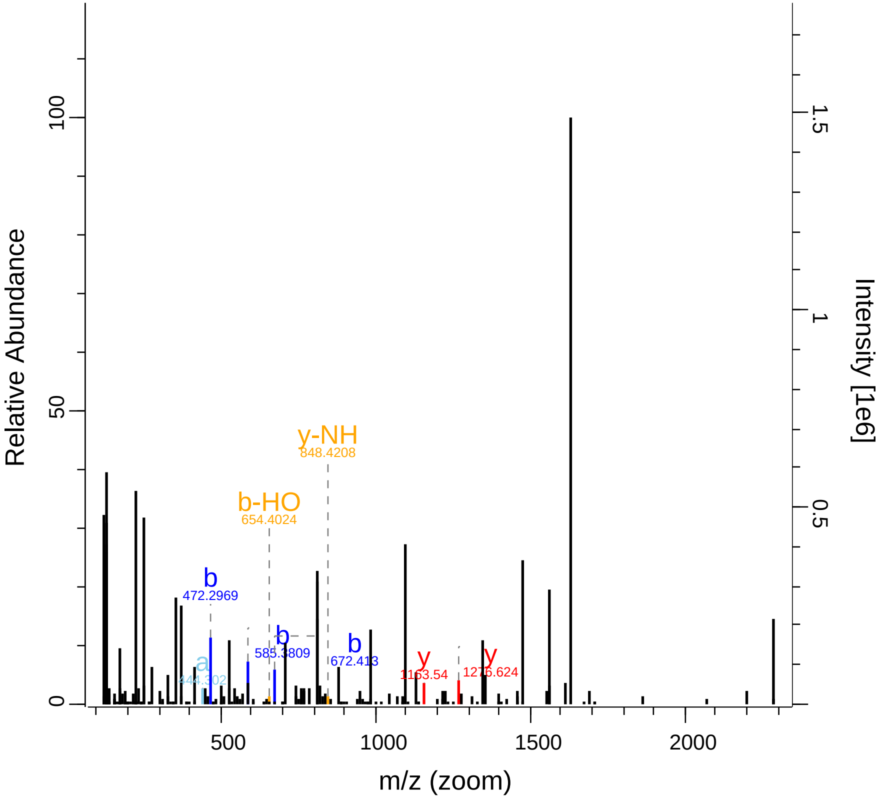

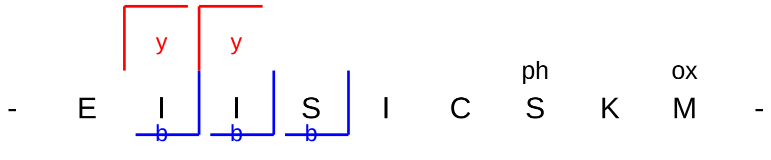

Raw File	Scan	Method	Score	m/z	Gene names
RO-QC-160121_ZackD_SS_Set_02_Fractic	19359	FTMS; HC	52.55	1056.53	SNRPA

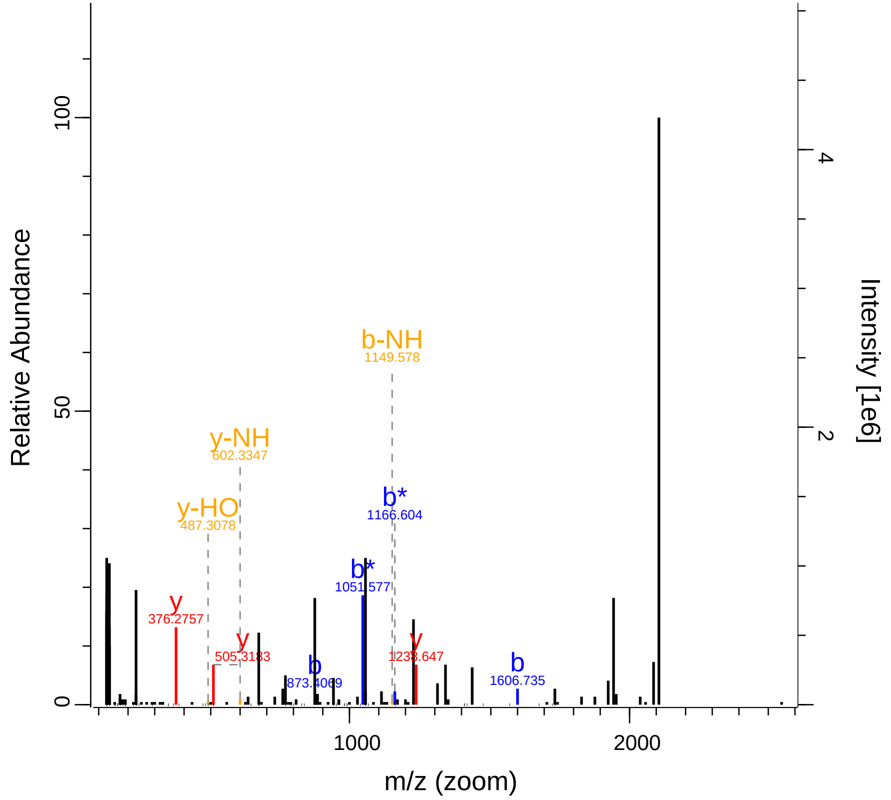

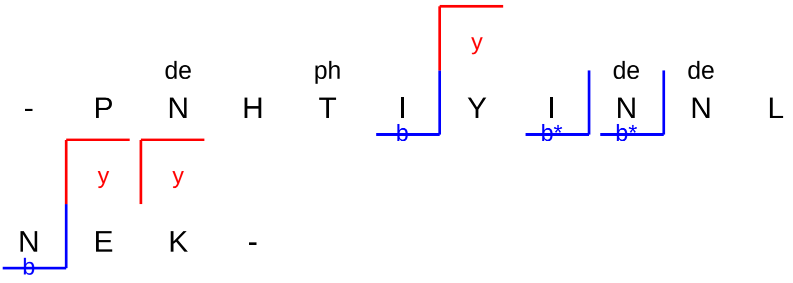

Raw File	Scan	Method	Score	m/z	Gene names
RO-QC-160121_ZackD_SS_Set_02_Fractio	19460	FTMS; HC	50.56	755.07	GIMAP7

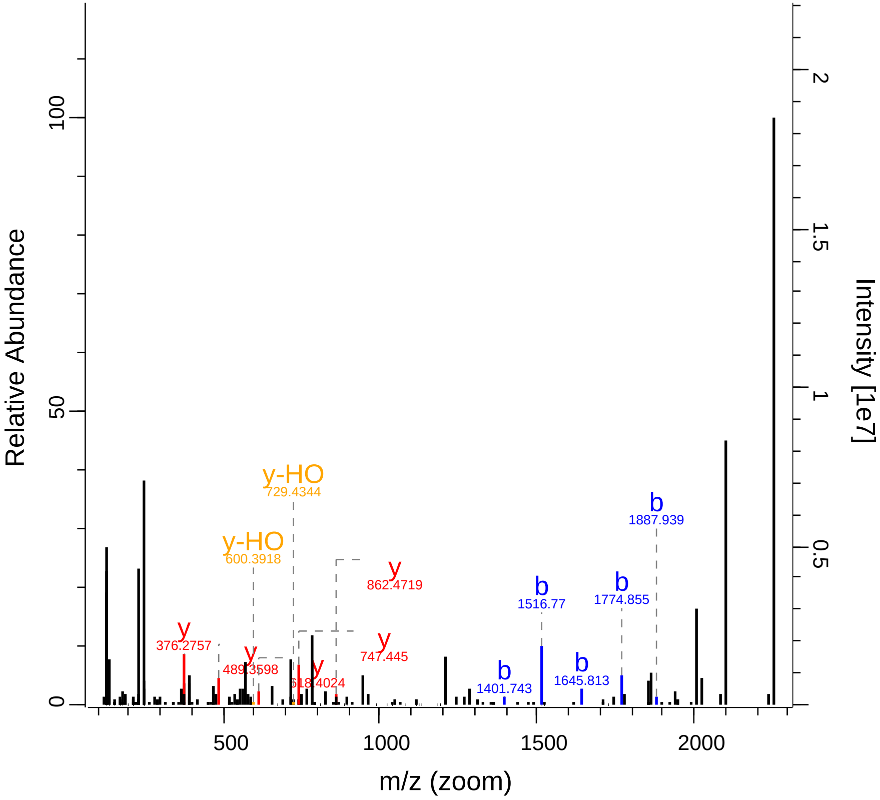

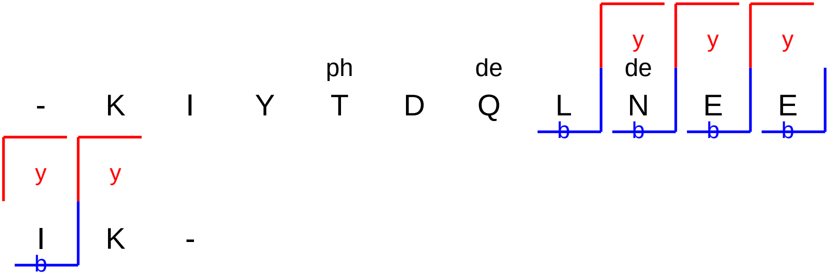

Raw File	Scan	Method	Score	m/z	Gene names
RO-QC-160121_ZackD_SS_Set_02_Fractio	21748	FTMS; HC	84.53	980.47	MAP1B

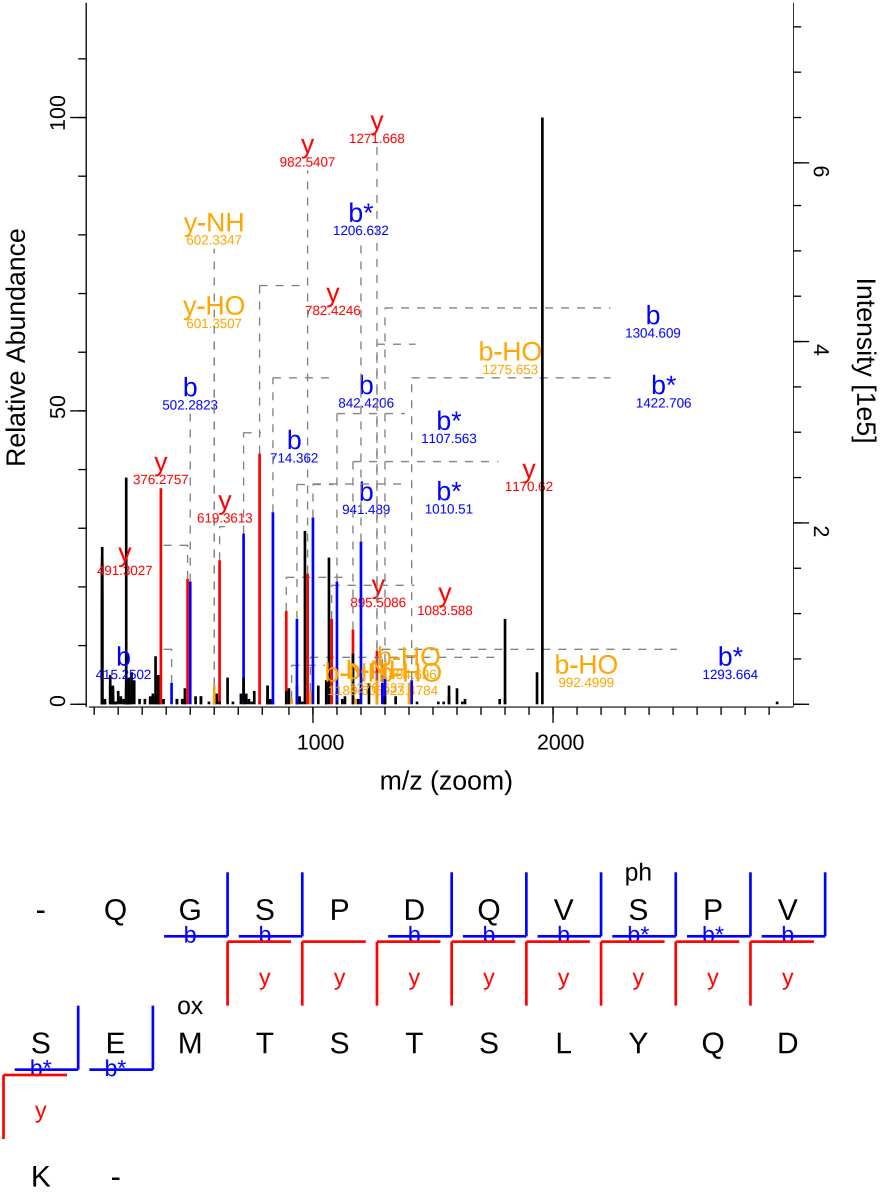

Raw File	Scan	Method	Score	m/z	Gene names
RO-QC-160121_ZackD_SS_Set_02_Fractio	25432	FTMS; HC	123.36	904.76	SRRM1

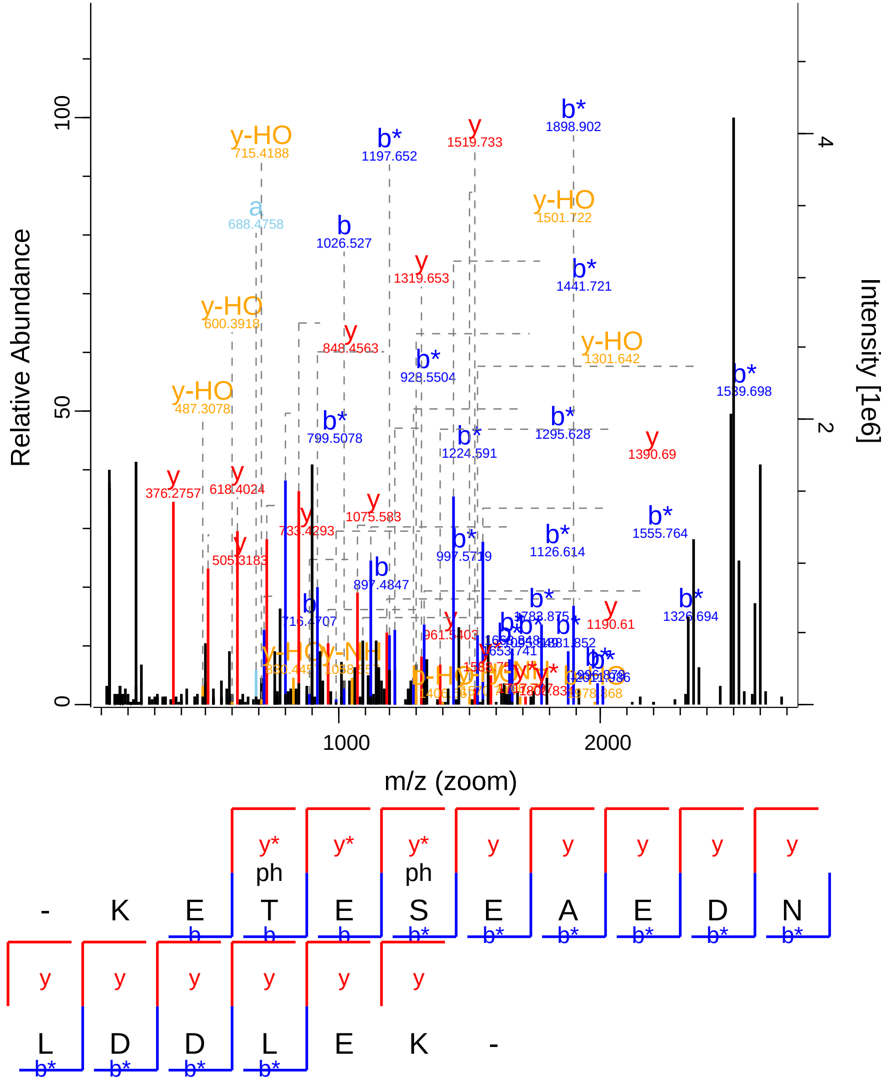

Raw File	Scan	Method	Score	m/z	Gene names
RO-QC-160121_ZackD_SS_Set_02_Fractio	25526	FTMS; HC	80.63	670.36	RPS3

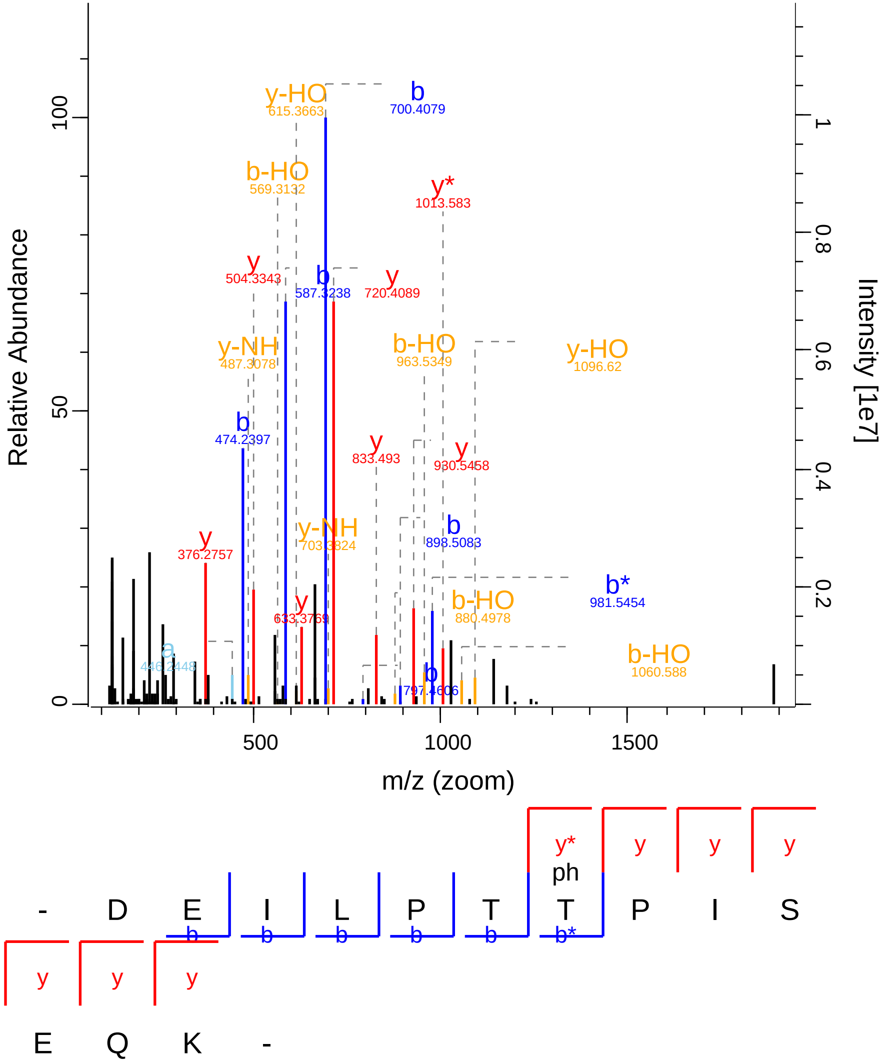

Raw File	Scan	Method	Score	m/z	Gene names
RO-QC-160121_ZackD_SS_Set_02_Fractio	25980	FTMS; HC	148.44	840.42	USP8

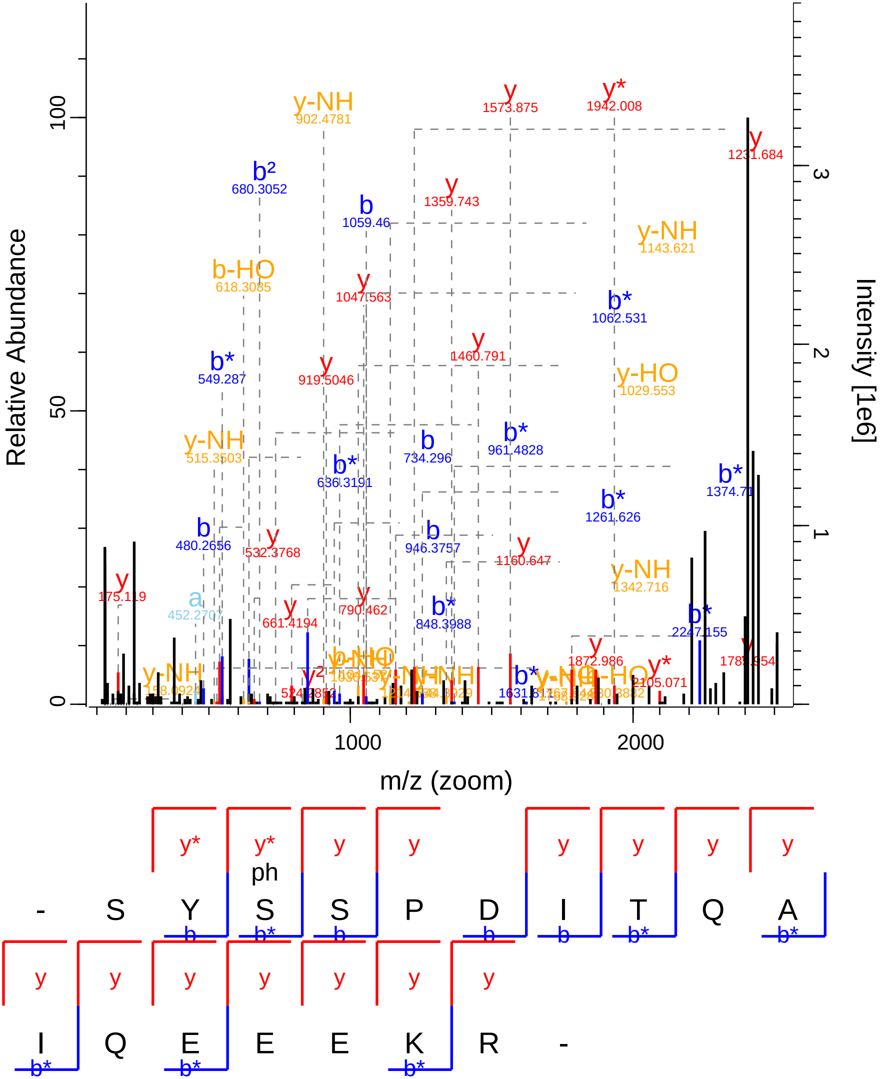

Raw File	Scan	Method	Score	m/z	Gene names
RO-QC-160121_ZackD_SS_Set_02_Fractior	27590	FTMS; HCI	178.28	904.7	PRPSAP1

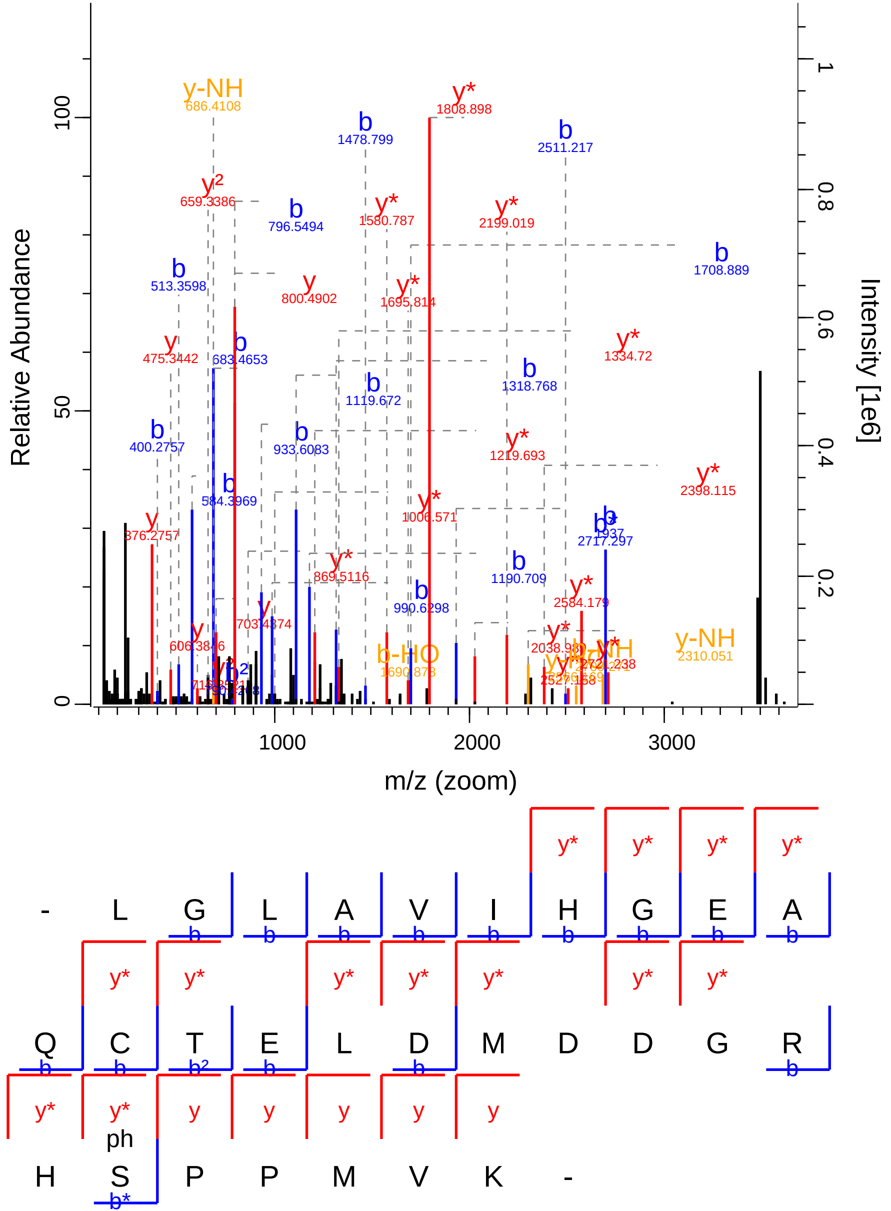

Raw File	Scan	Method	Score	m/z	Gene names
RO-QC-160121_ZackD_SS_Set_02_Fractic	28564	FTMS; HC	185.7	1053.55	CCNY

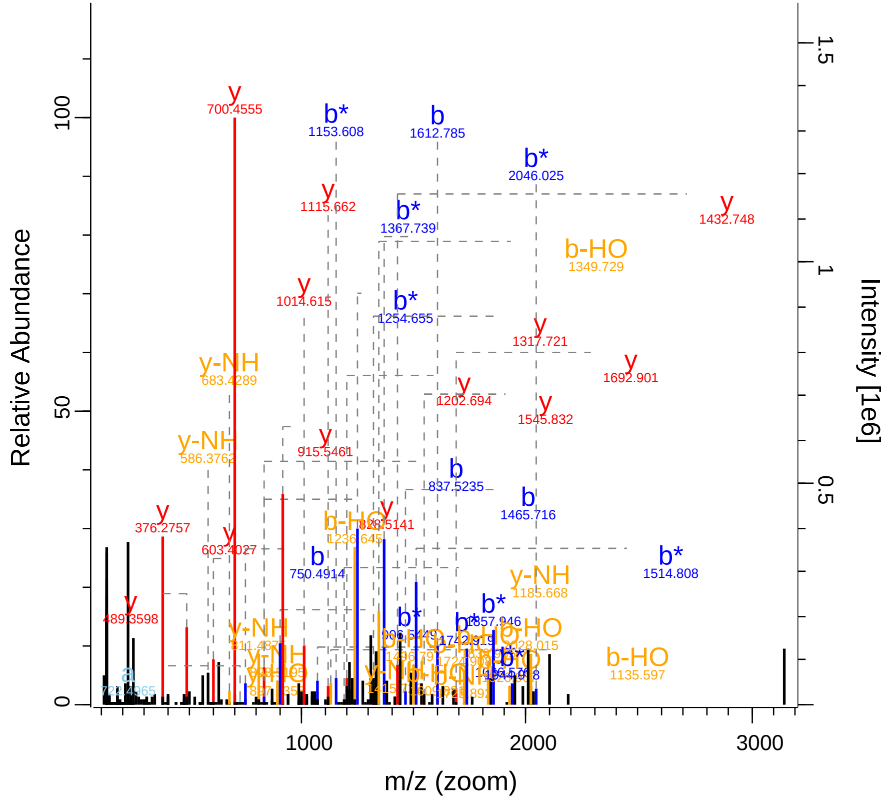

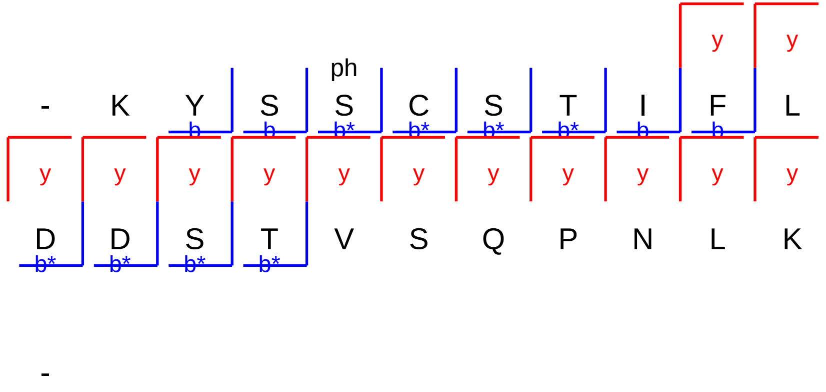

Raw File	Scan	Method	Score	m/z	Gene names
RO-QC-160121_ZackD_SS_Set_02_Fractio	28994	FTMS; HC	84.5	605.34	DHX29

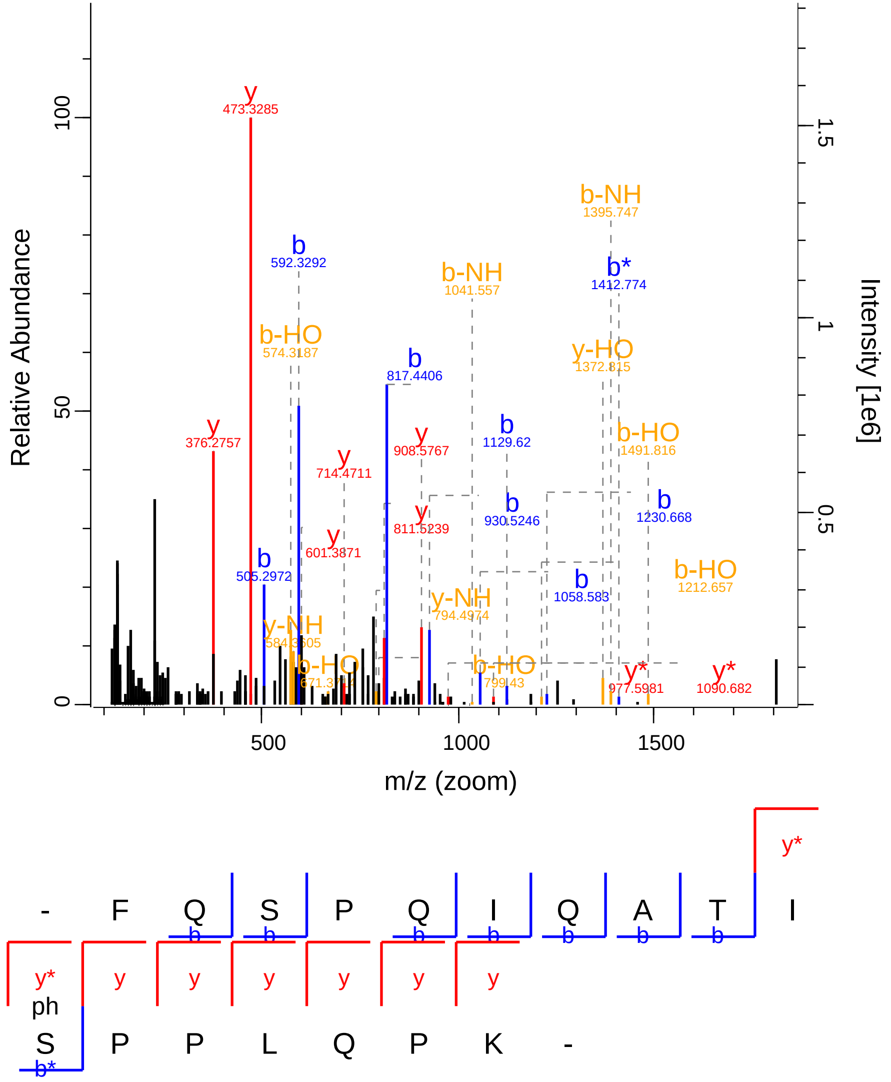

Raw File	Scan	Method	Score	m/z	Gene names
RO-QC-160121_ZackD_SS_Set_02_Fractio	29693	FTMS; HC	42.57	817.45	FAM73B

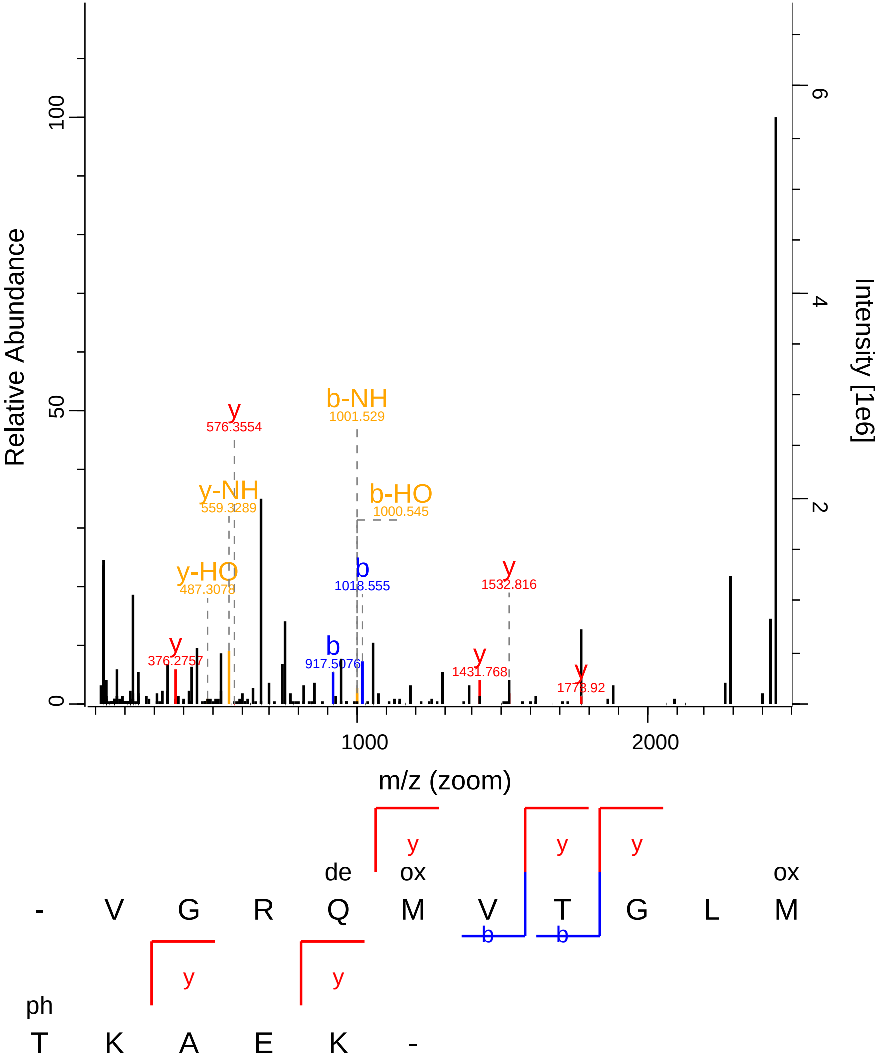

Raw File	Scan	Method	Score	m/z	Gene names
RO-QC-160121_ZackD_SS_Set_02_Fractic	30133	FTMS; HC	44.81	1295.98	DCP1A

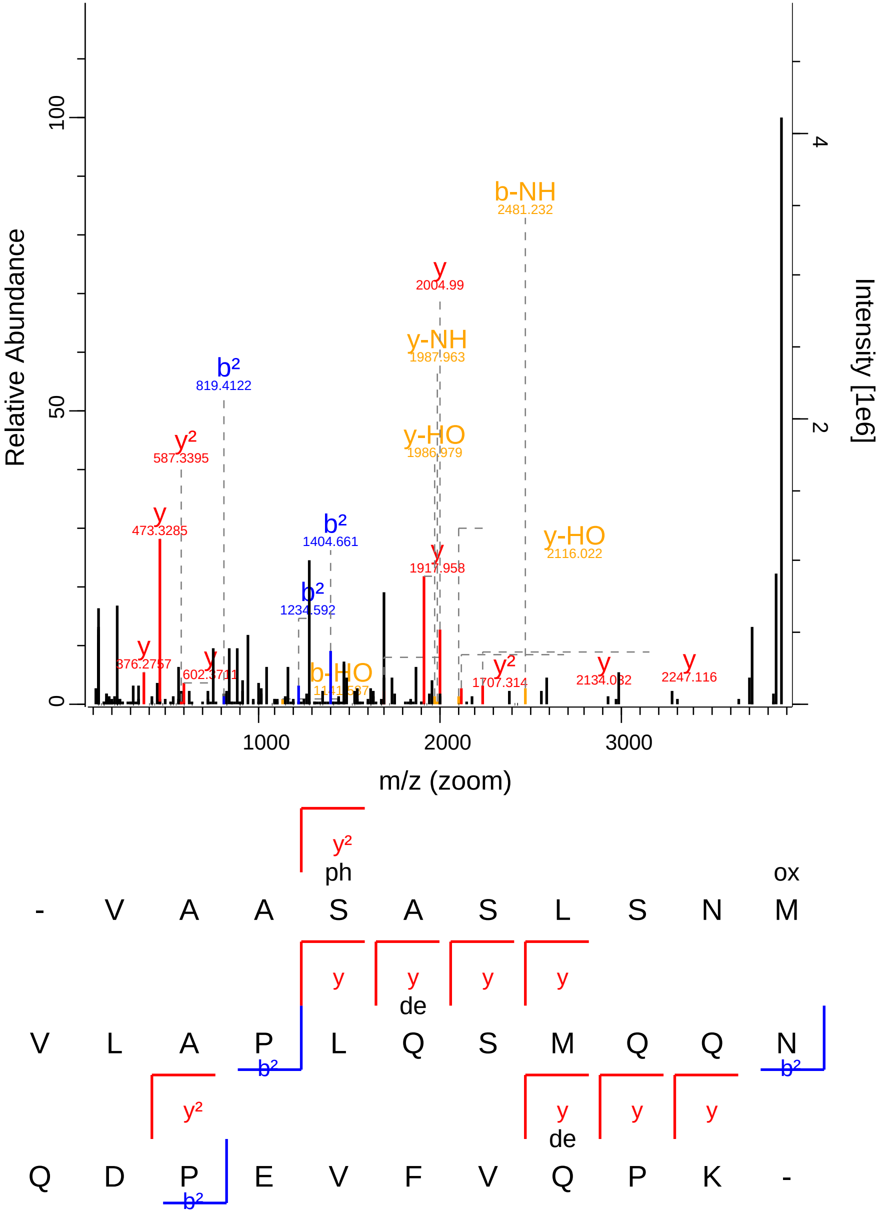

Raw File	Scan	Method	Score	m/z	Gene names
RO-QC-160121_ZackD_SS_Set_02_Fractic	30176	FTMS; HC	190.6	1067.84	BCLAF1

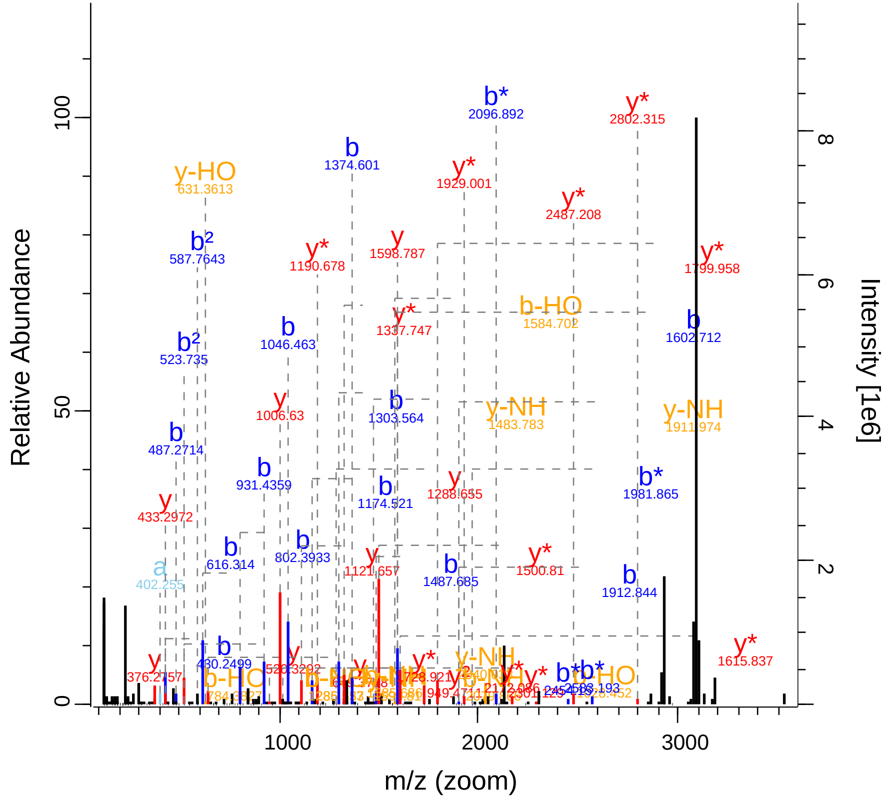

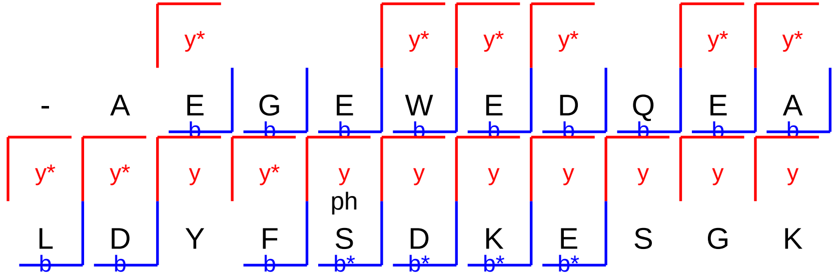

-

Raw File	Scan	Method	Score	m/z	Gene names
RO-QC-160121_ZackD_SS_Set_02_Fractic	30964	FTMS; HC	154.02	1017.24	SQSTM1

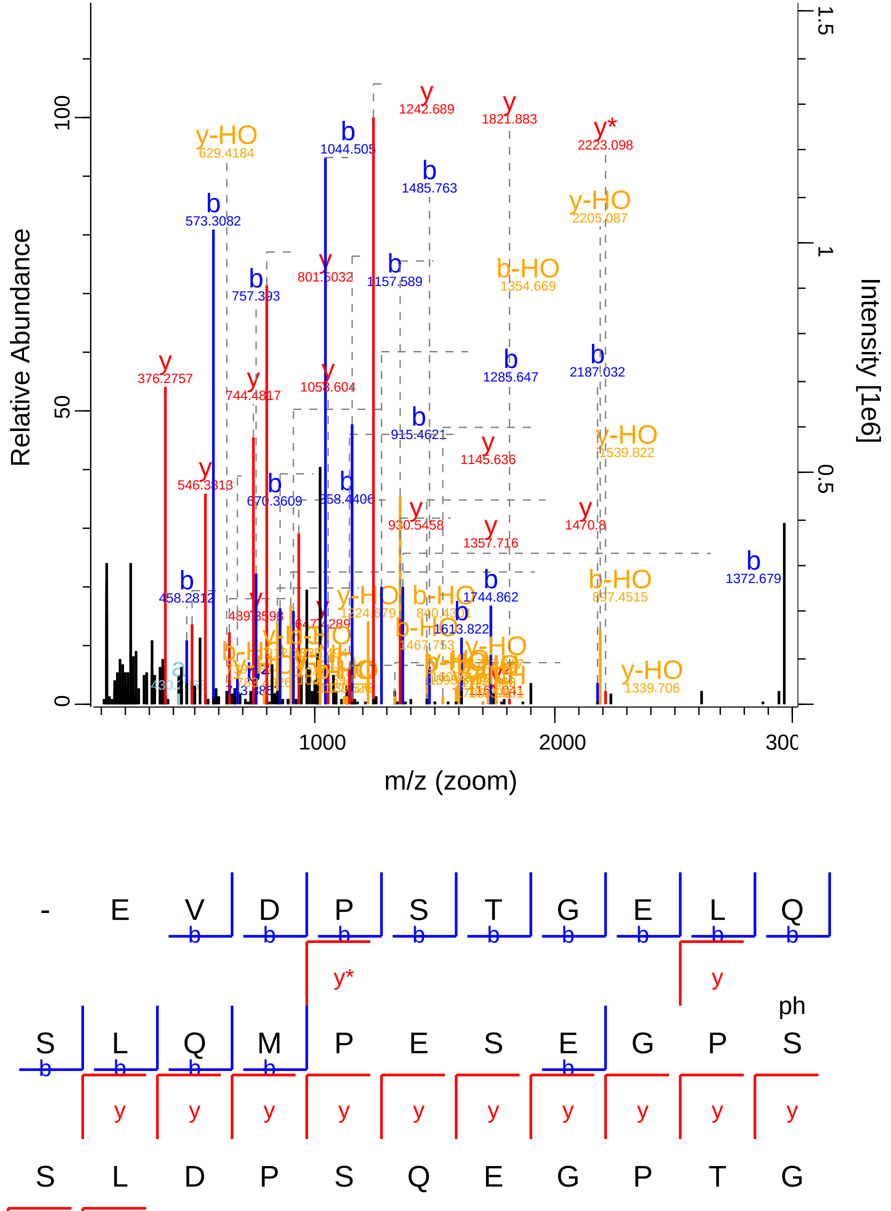

Raw File	Scan	Method	Score	m/z	Gene names
RO-QC-160121_ZackD_SS_Set_02_Fractio	31201	FTMS; HC	40.01	862.22	CCDC150

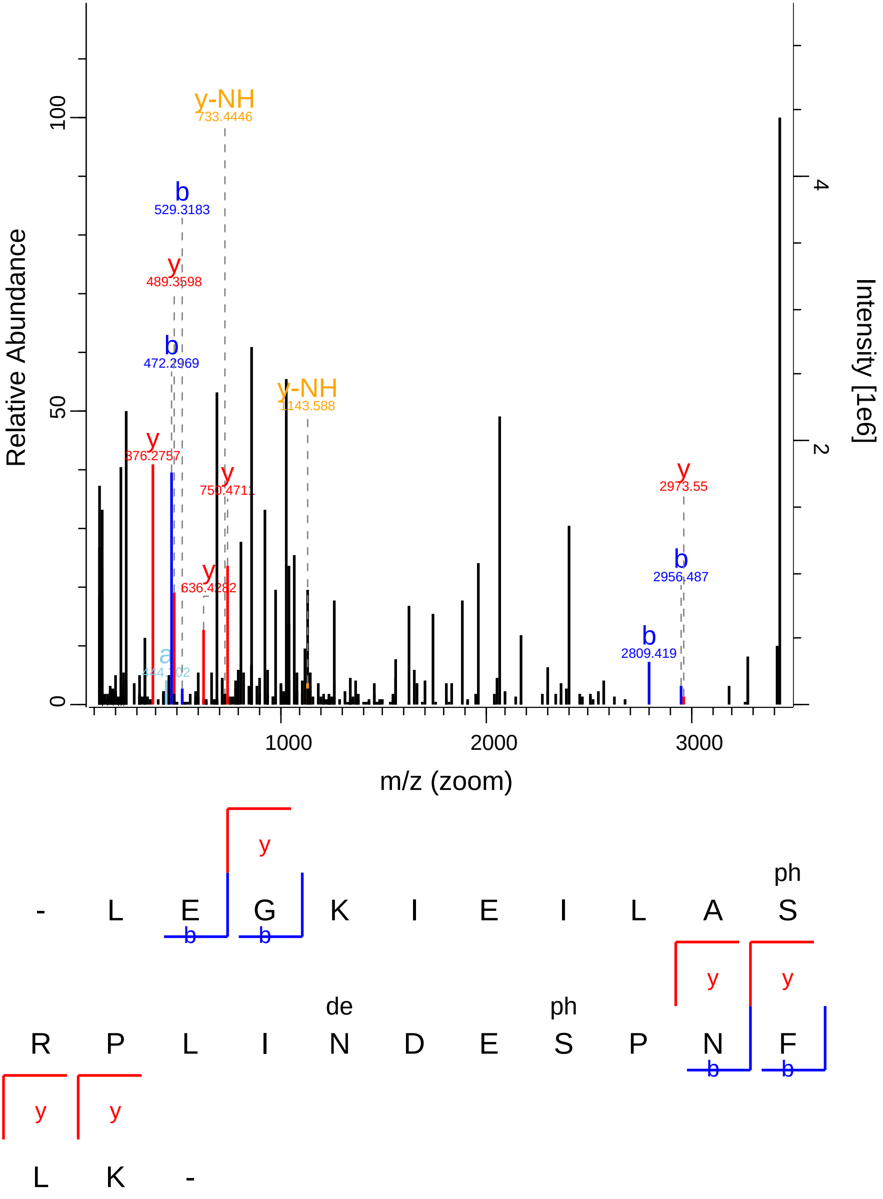

Raw File	Scan	Method	Score	m/z
RO-QC-160121_ZackD_SS_Set_02_Fraction_6	31596	FTMS; HCD	47.82	529.64

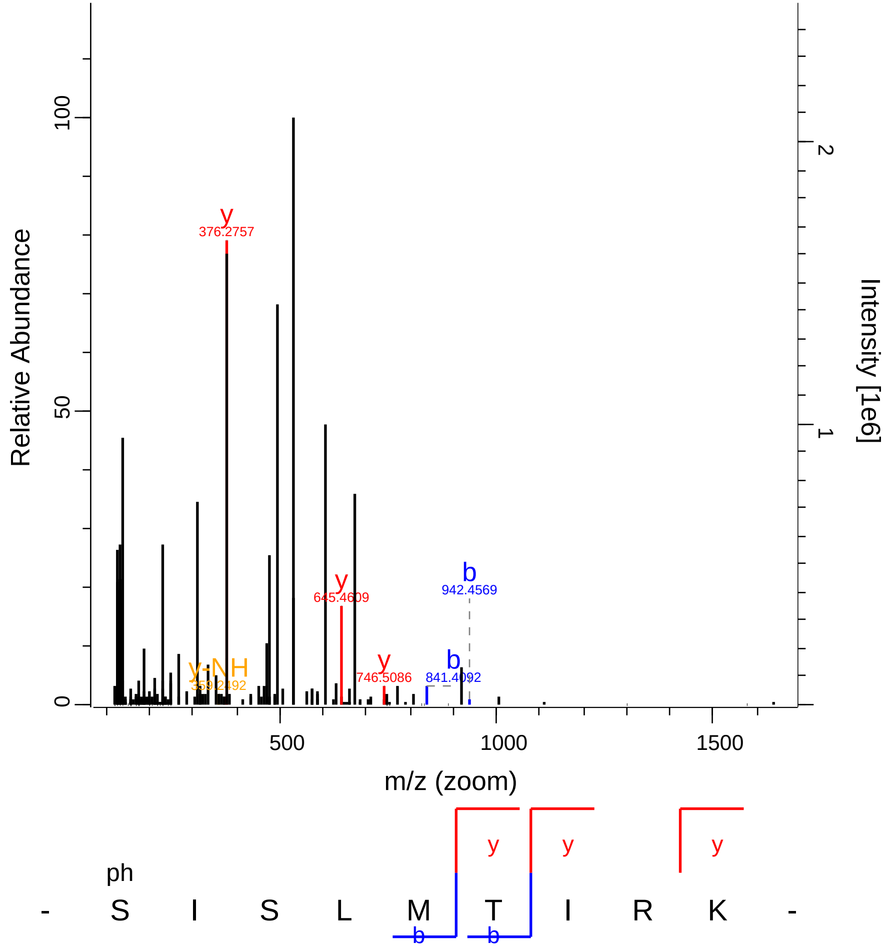

Raw File	Scan	Method	Score	m/z	Gene names
RO-QC-160121_ZackD_SS_Set_02_Fractic	32197	FTMS; HC	65.38	1006.47	PODXL

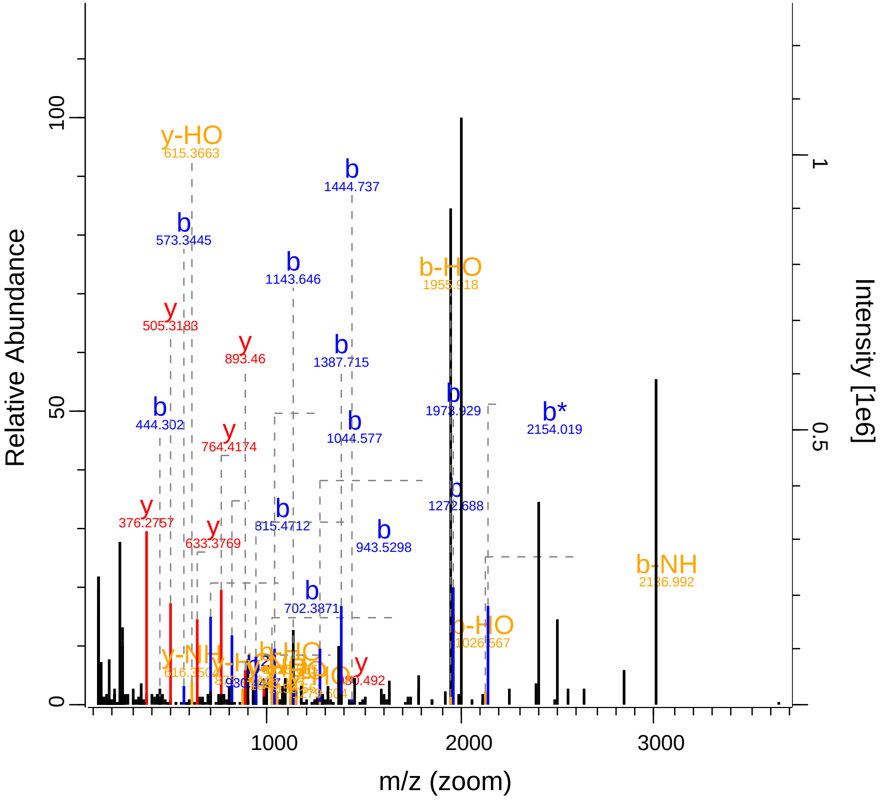

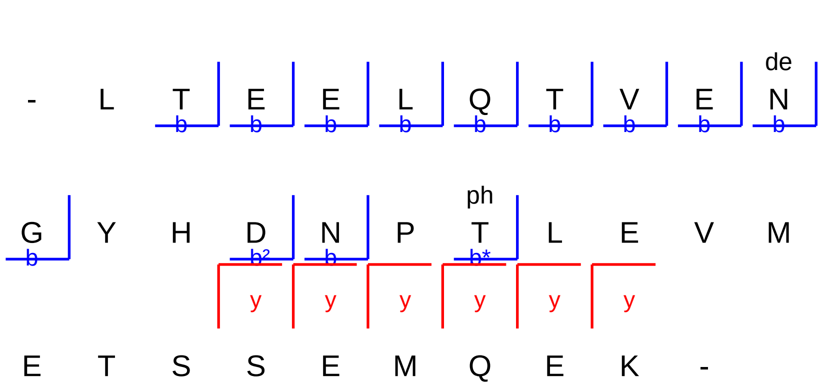

Raw File	Scan	Method	Score	m/z	Gene names
RO-QC-160121_ZackD_SS_Set_02_Fractior	32736	FTMS; HCI	45.26	754.9	CFTR

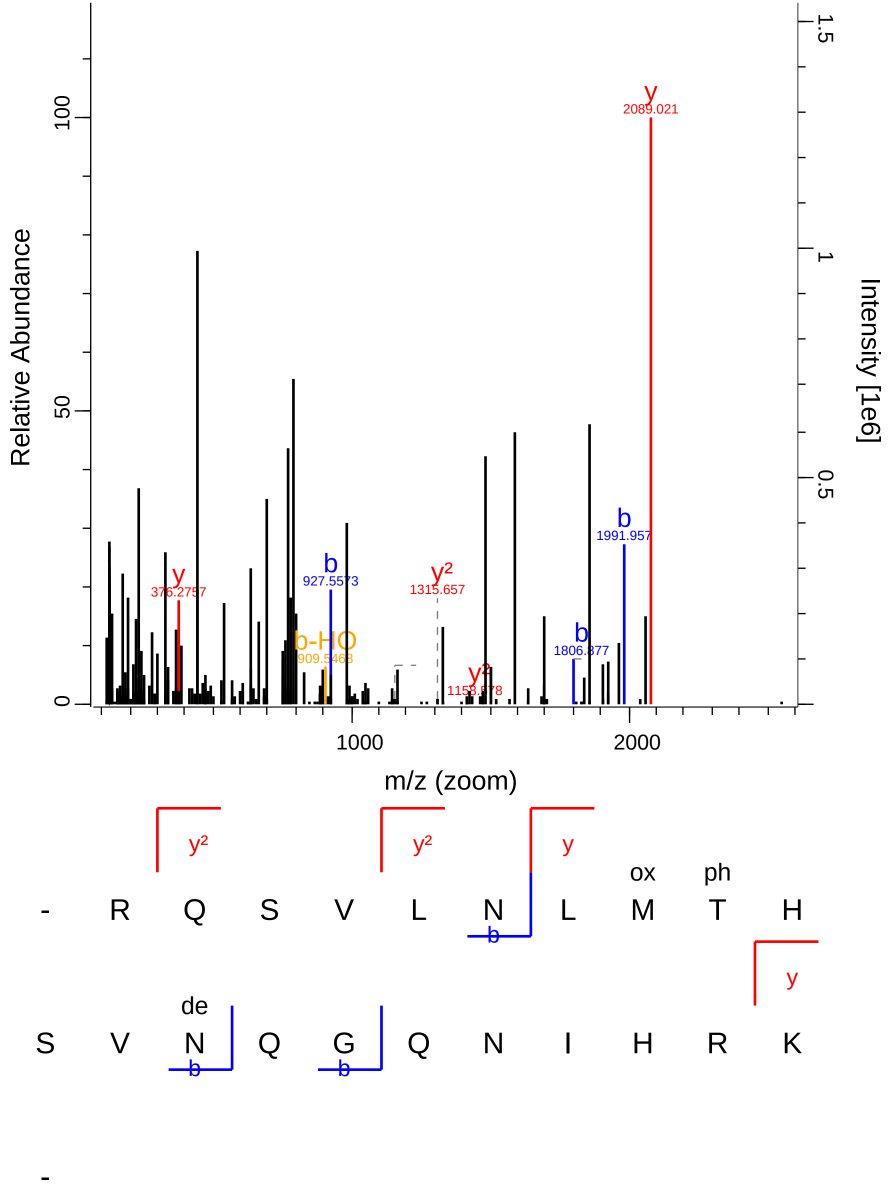

Raw File	Scan	Method	Score	m/z	Gene names
RO-QC-160121_ZackD_SS_Set_02_Fractio	32903	FTMS; HC	48.5	851.95	LMOD1

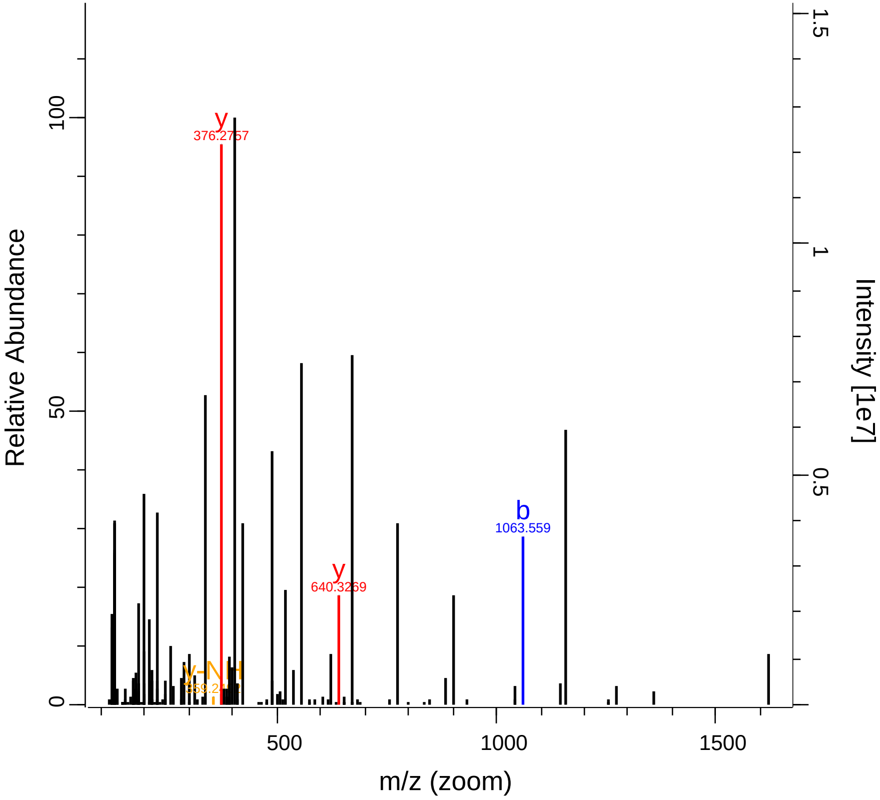

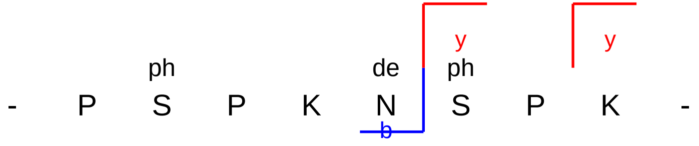

Raw File	Scan	Method	Score	m/z	Gene names
RO-QC-160121_ZackD_SS_Set_02_Fractior	33006	FTMS; HCI	40.24	908.8	CYP4F12

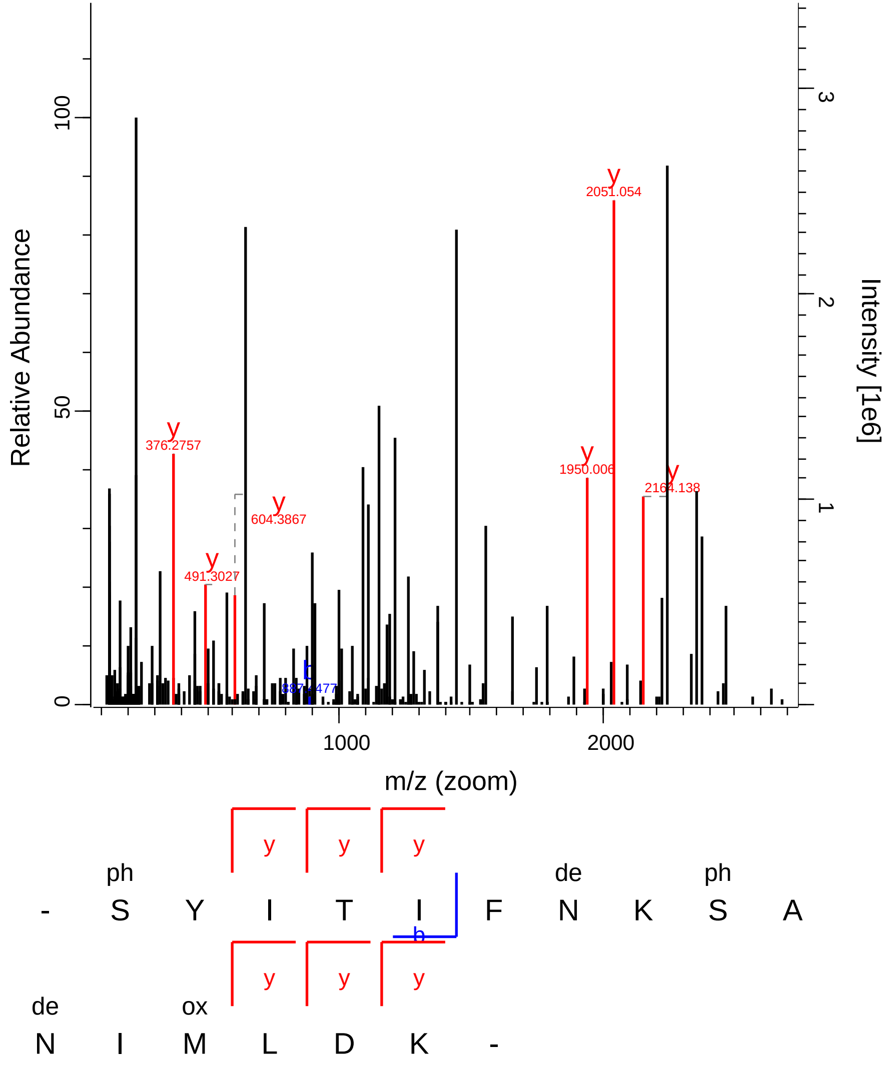

Raw File	Scan	Method	Score	m/z	Gene names
RO-QC-160121_ZackD_SS_Set_02_Fractio	34931	FTMS; HC	43.2	848.44	MYO1D

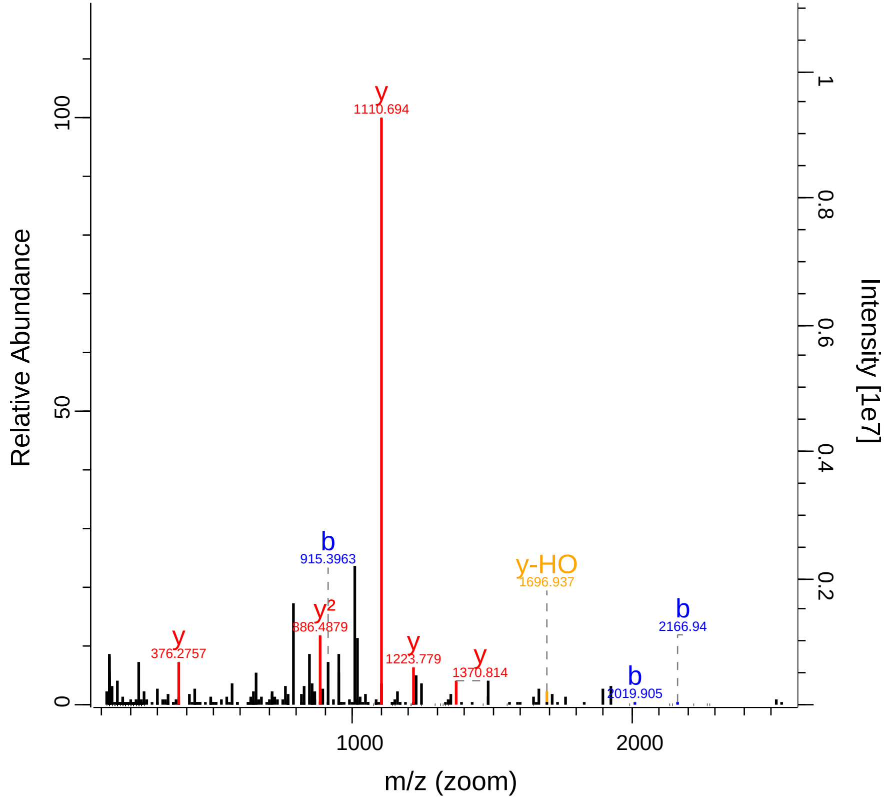

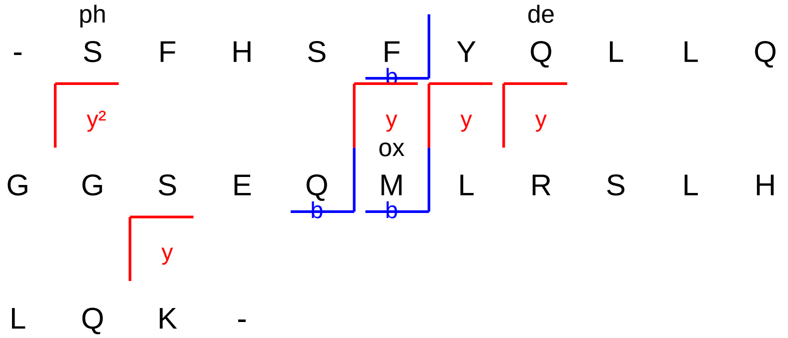

Raw File	Scan	Method	Score	m/z	Gene names
RO-QC-160121_ZackD_SS_Set_02_Fractic	35005	FTMS; HC	45.55	1243.66	LPPR5

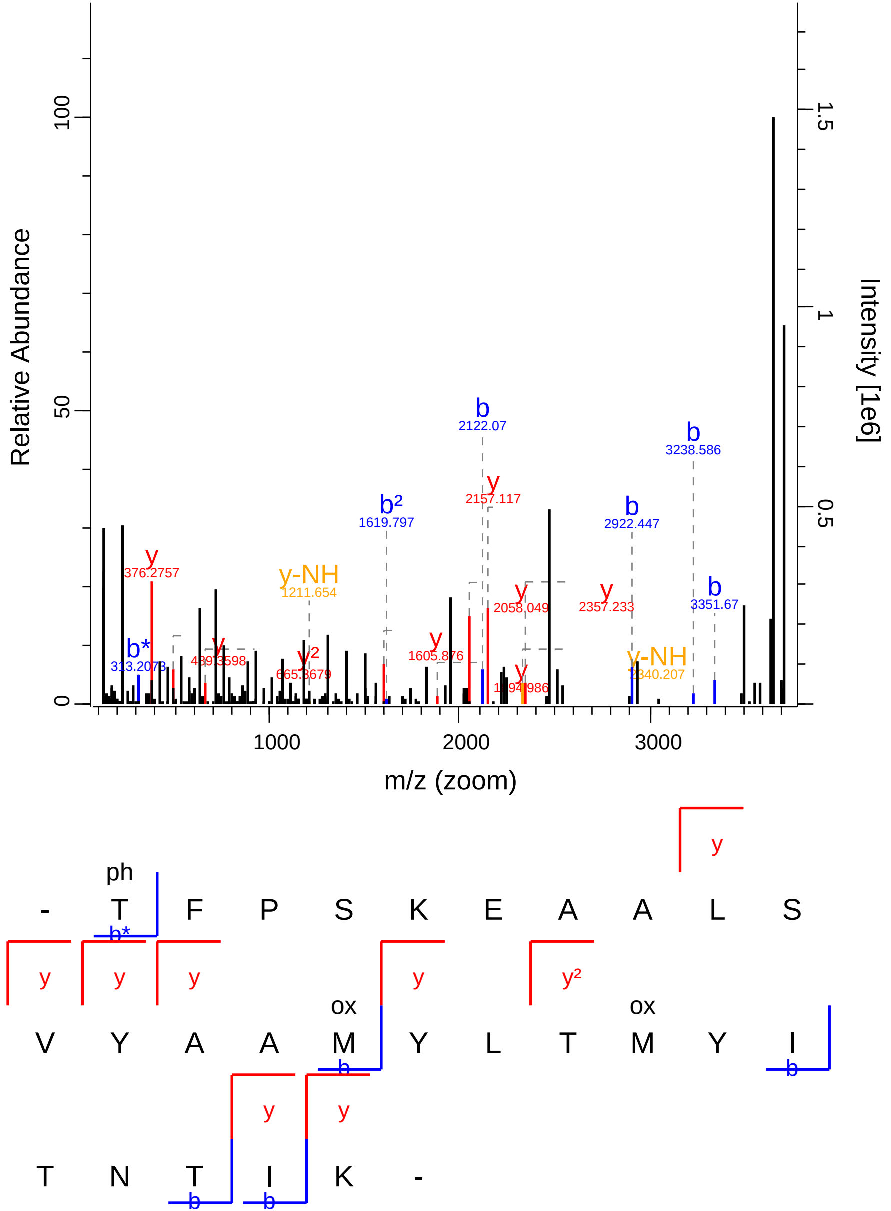

Raw File	Scan	Method	Score	m/z	Gene names
RO-QC-160121_ZackD_SS_Set_02_Fractio	35354	FTMS; HC	53.17	577.66	CABIN1

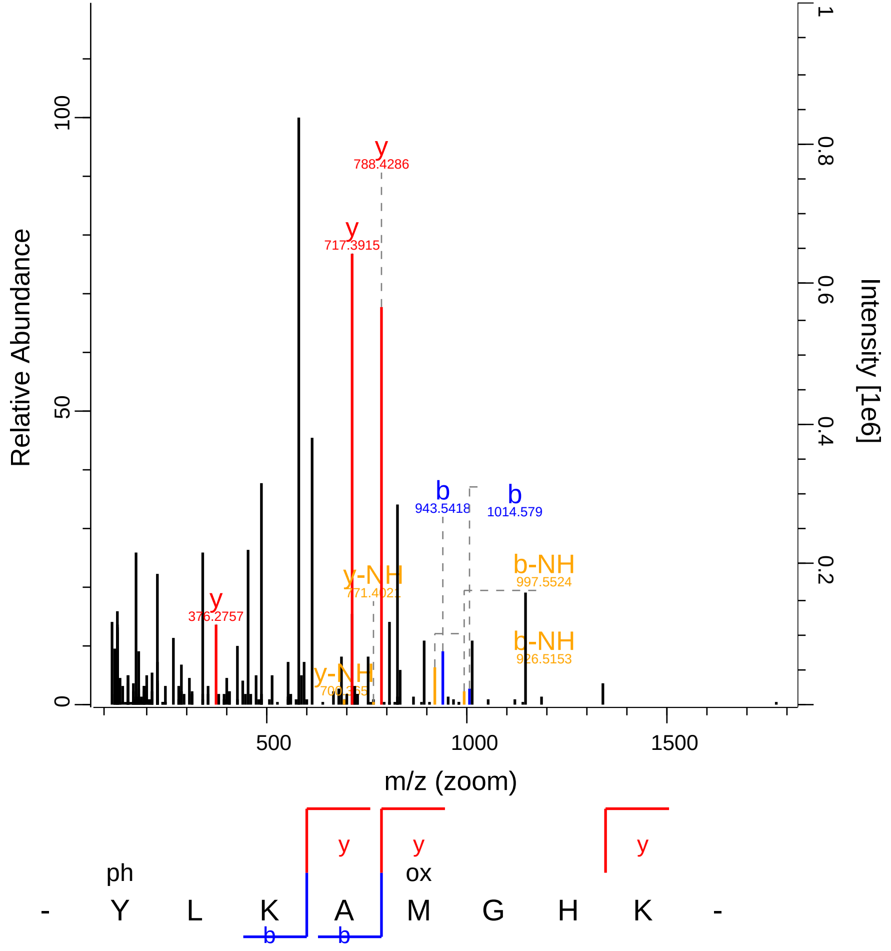

Raw File	Scan	Method	Score	m/z	Gene names
RO-QC-160121_ZackD_SS_Set_02_Fractio	35922	FTMS; HC	105.79	782.08	DNAJC5

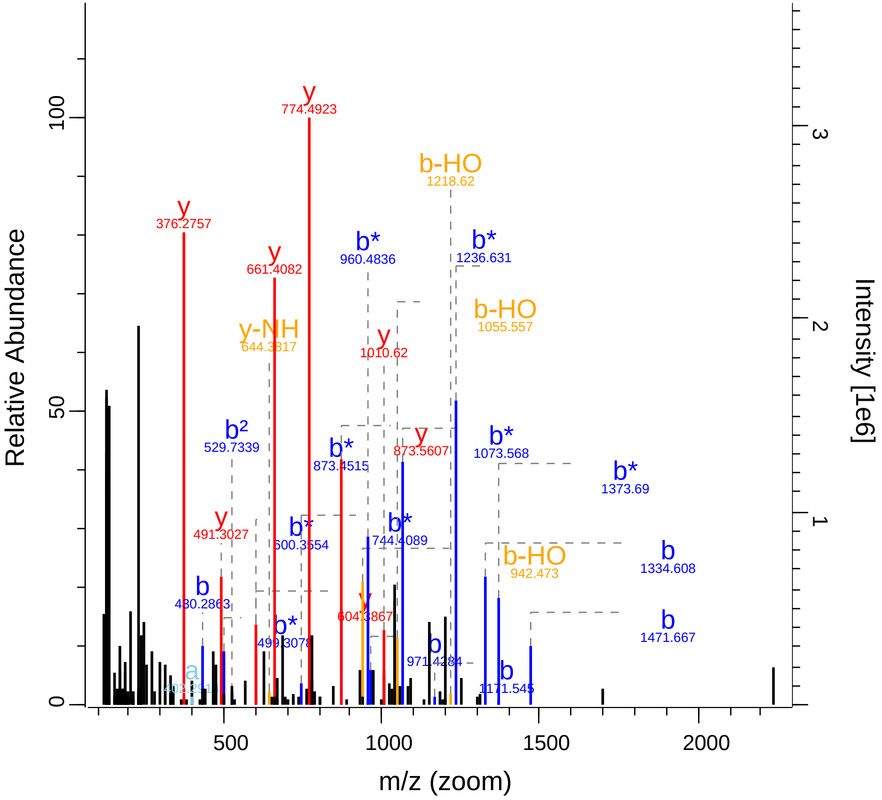

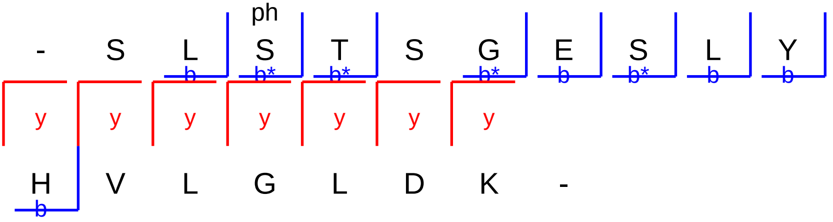

Raw File	Scan	Method	Score	m/z	Gene names
RO-QC-160121_ZackD_SS_Set_02_Fractio	36599	FTMS; HC	116.73	860.44	PRSS58

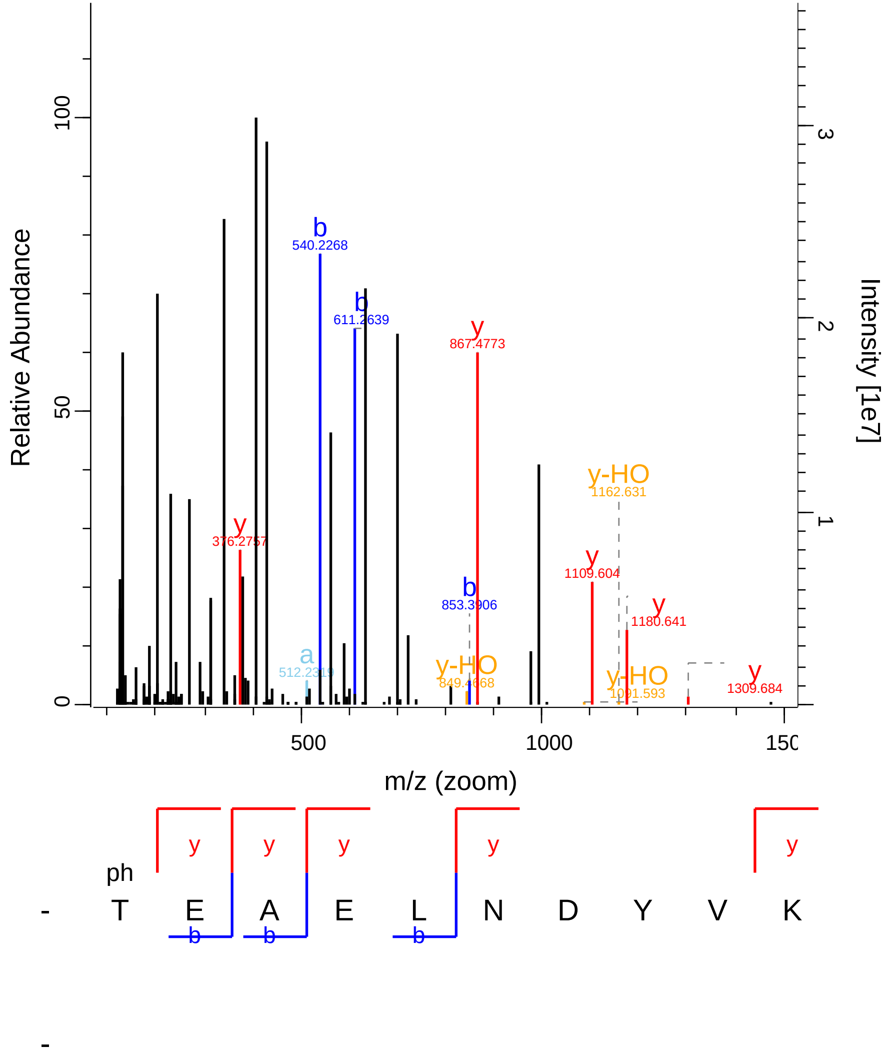

Raw File	Scan	Method	Score	m/z	Gene names
RO-QC-160121_ZackD_SS_Set_02_Fractio[illegible]	36717	FTMS; HC[illegible]	129.46	754.4	VPRBP

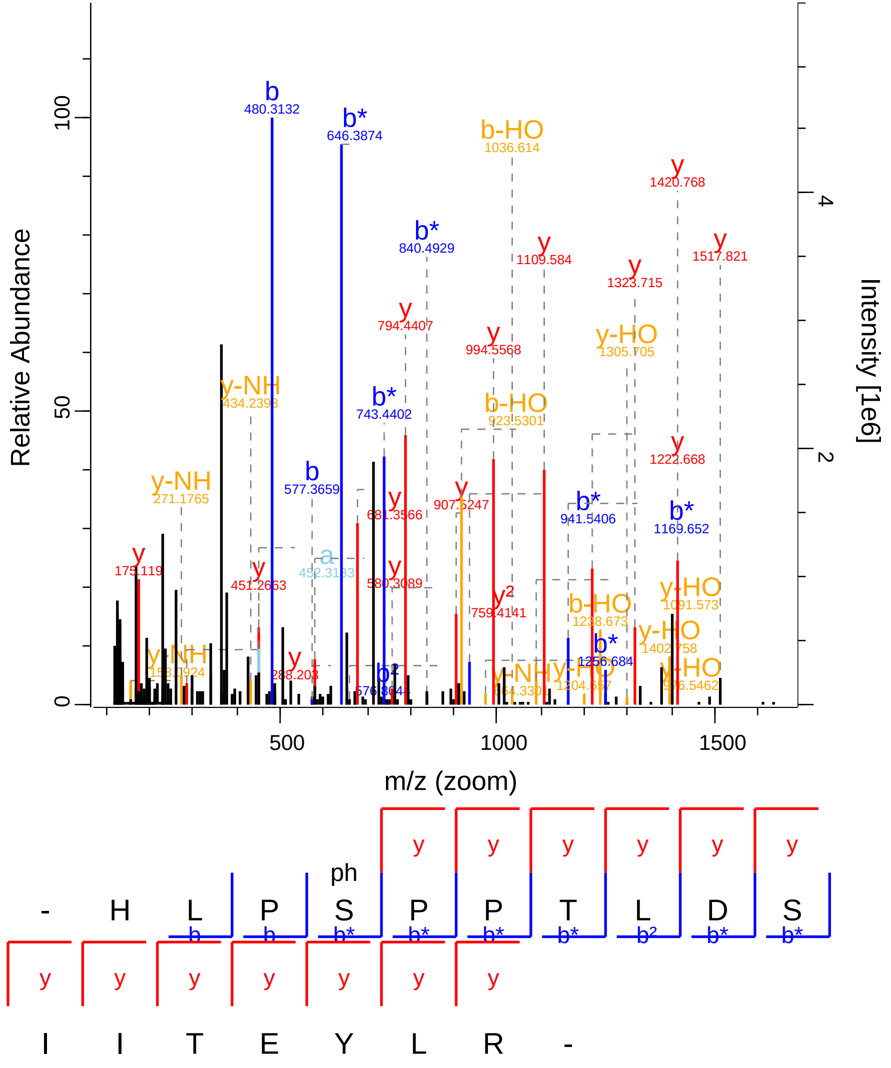

Raw File	Scan	Method	Score	m/z	Gene names
RO-QC-160121_ZackD_SS_Set_02_Fractio	36744	FTMS; HC	46.43	890.94	MIEF2

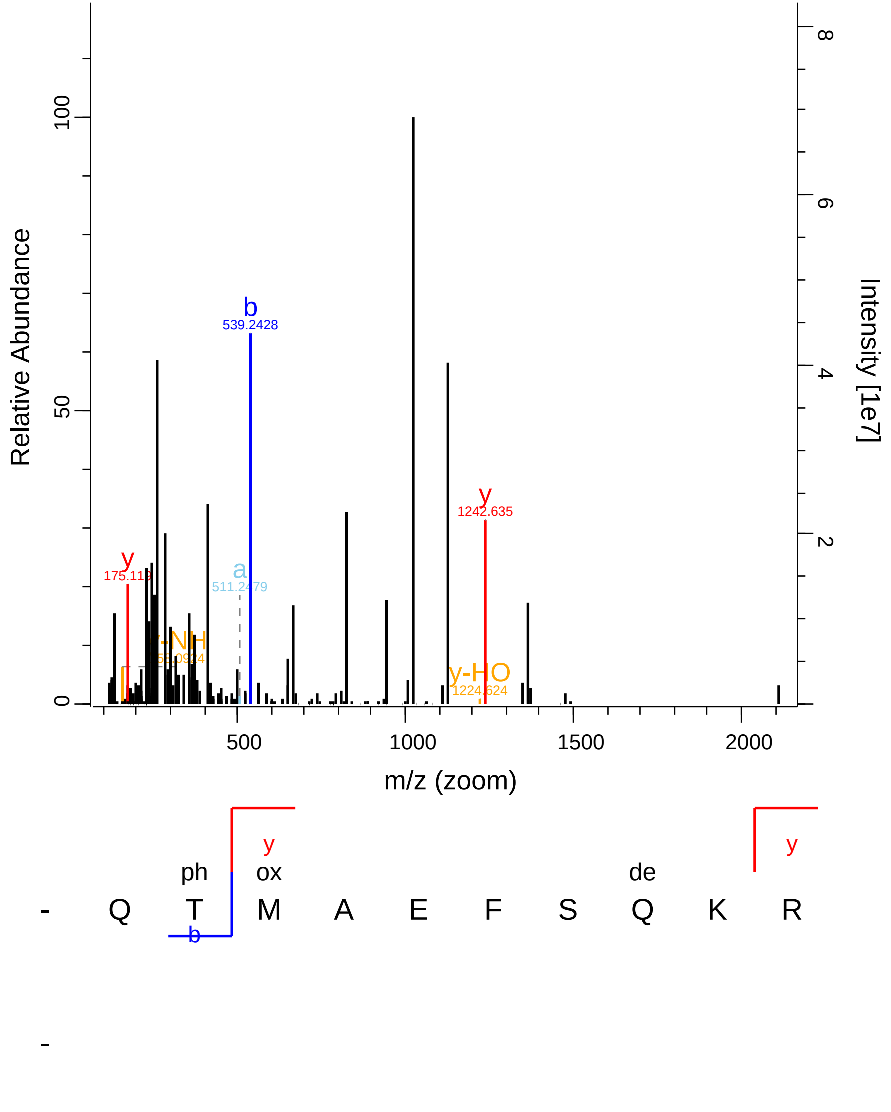

Raw File	Scan	Method	Score	m/z	Gene names
RO-QC-160121_ZackD_SS_Set_02_Fractio	36811	FTMS; HC	185.7	921.09	TNKS1BP1

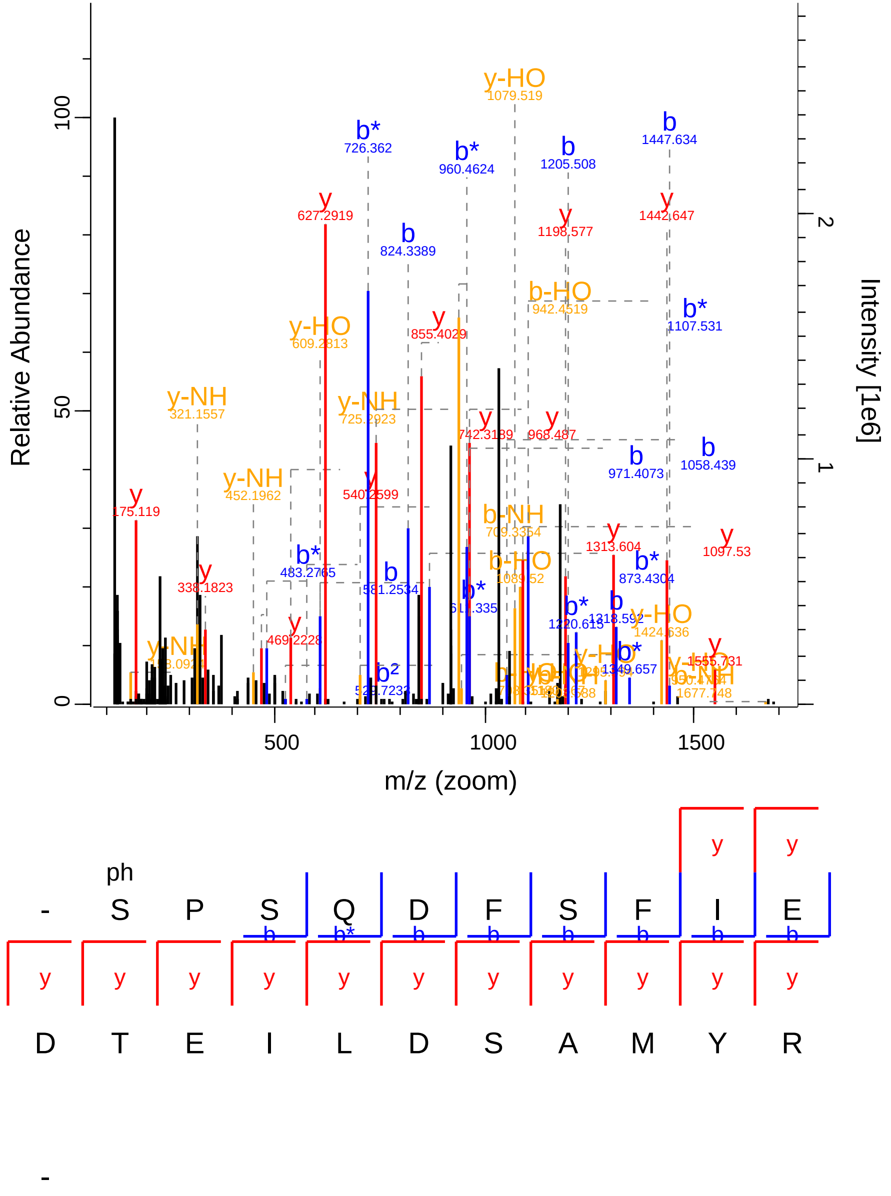

Raw File	Scan	Method	Score	m/z	Gene names
RO-QC-160121_ZackD_SS_Set_02_Fractior	37030	FTMS; HCI	40.96	998.5	ITGB6

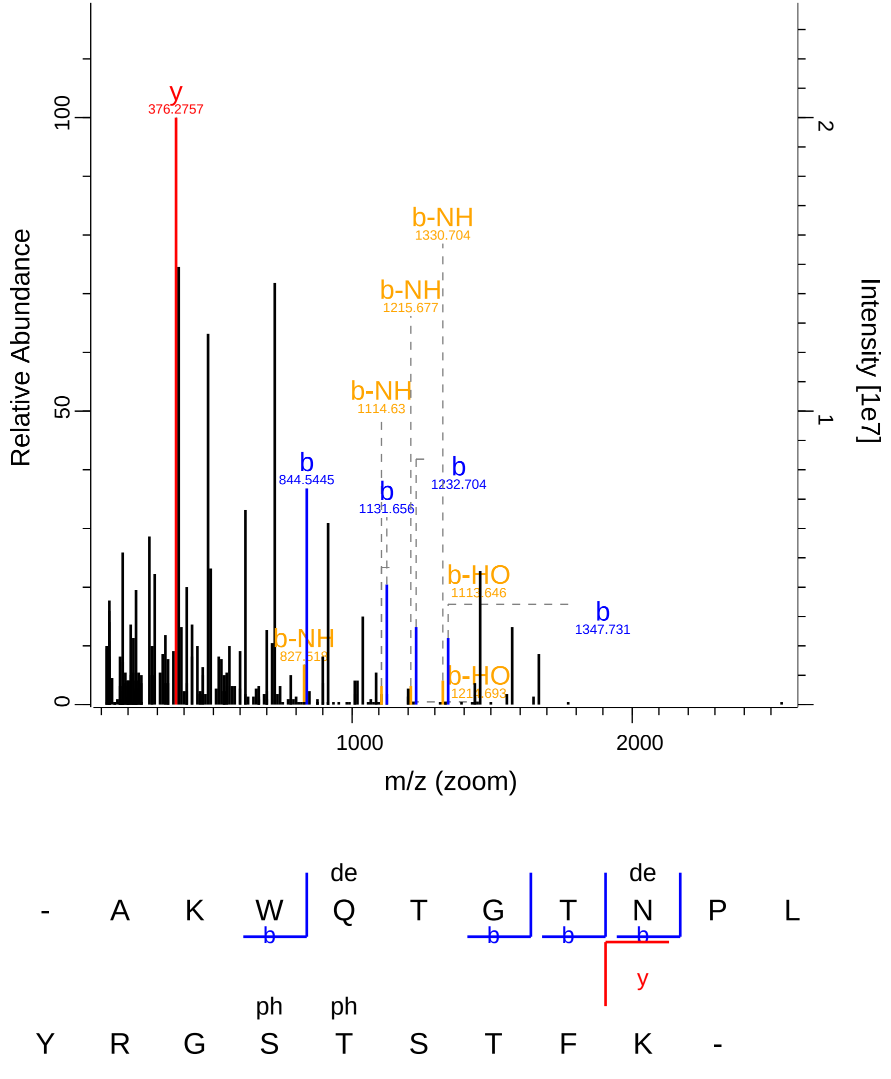

Raw File	Scan	Method	Score	m/z	Gene names
RO-QC-160121_ZackD_SS_Set_02_Fractio	5051	FTMS; HC	121.79	677.03	FLII

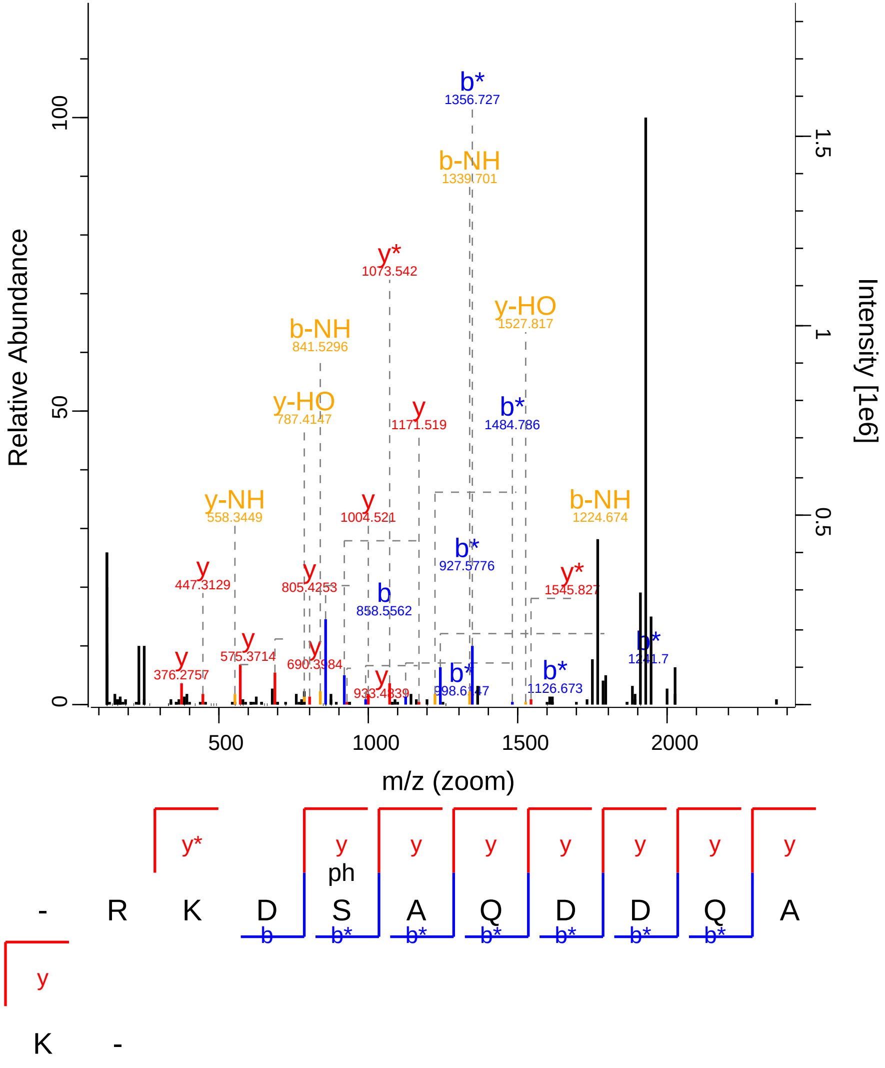

Raw File	Scan	Method	Score	m/z	Gene names
RO-QC-160121_ZackD_SS_Set_02_Fractior	5729	FTMS; HCI	44.93	593.81	NPM1

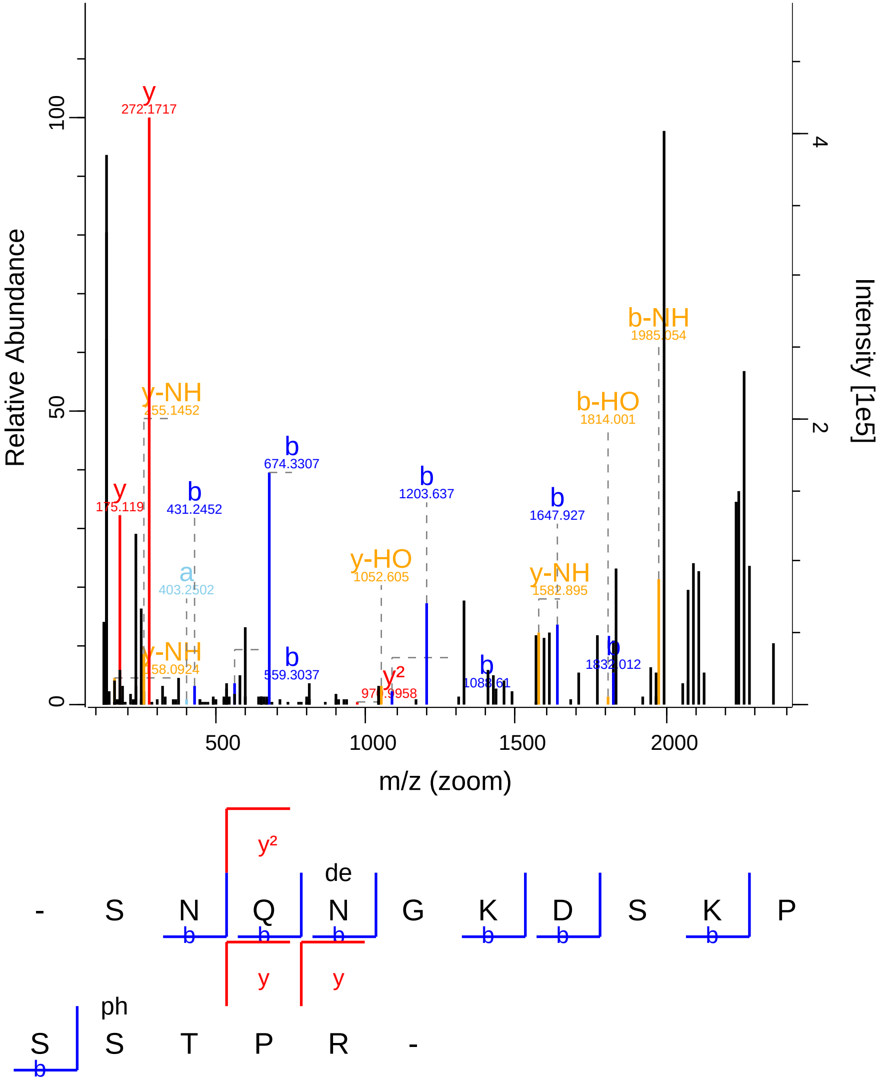

Raw File	Scan	Method	Score	m/z	Gene names
RO-QC-160121_ZackD_SS_Set_02_Fractior	6031	FTMS; HCI	86.8	594.99	RRBP1

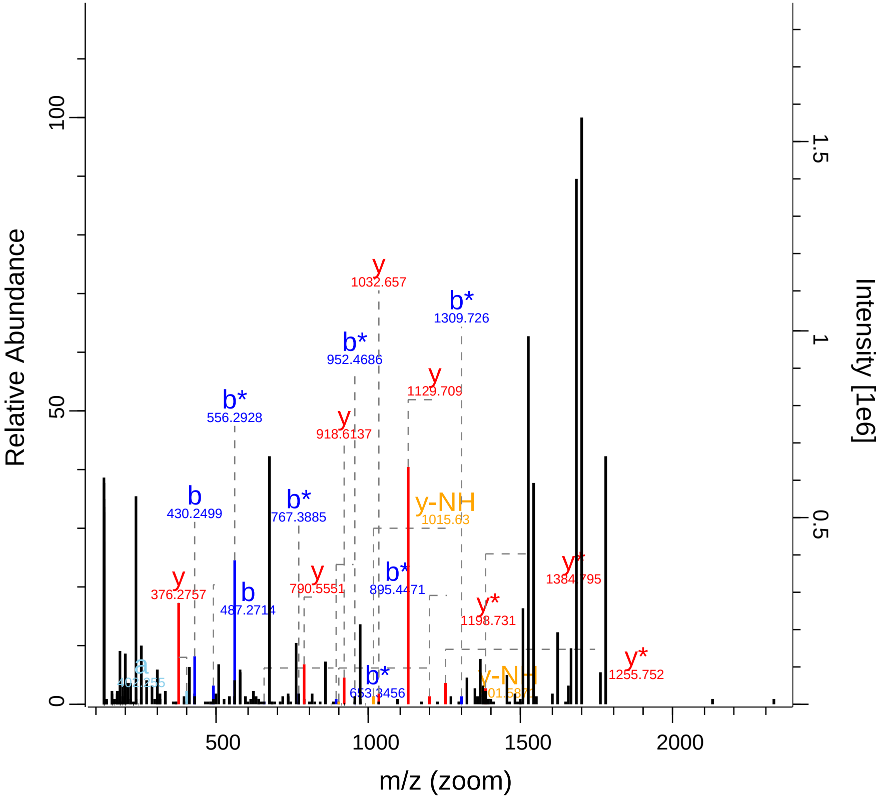

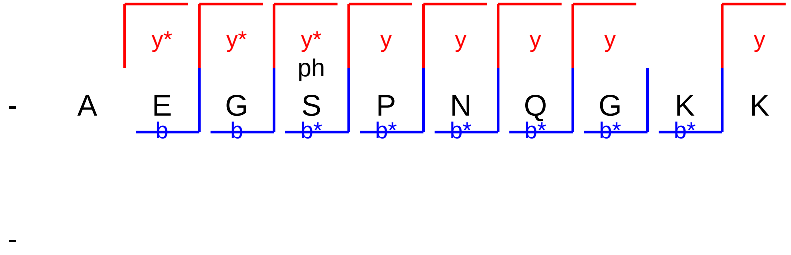

Raw File	Scan	Method	Score	m/z	Gene names
RO-QC-160121_ZackD_SS_Set_02_Fractior	6077	FTMS; HCI	69.15	551.63	PML

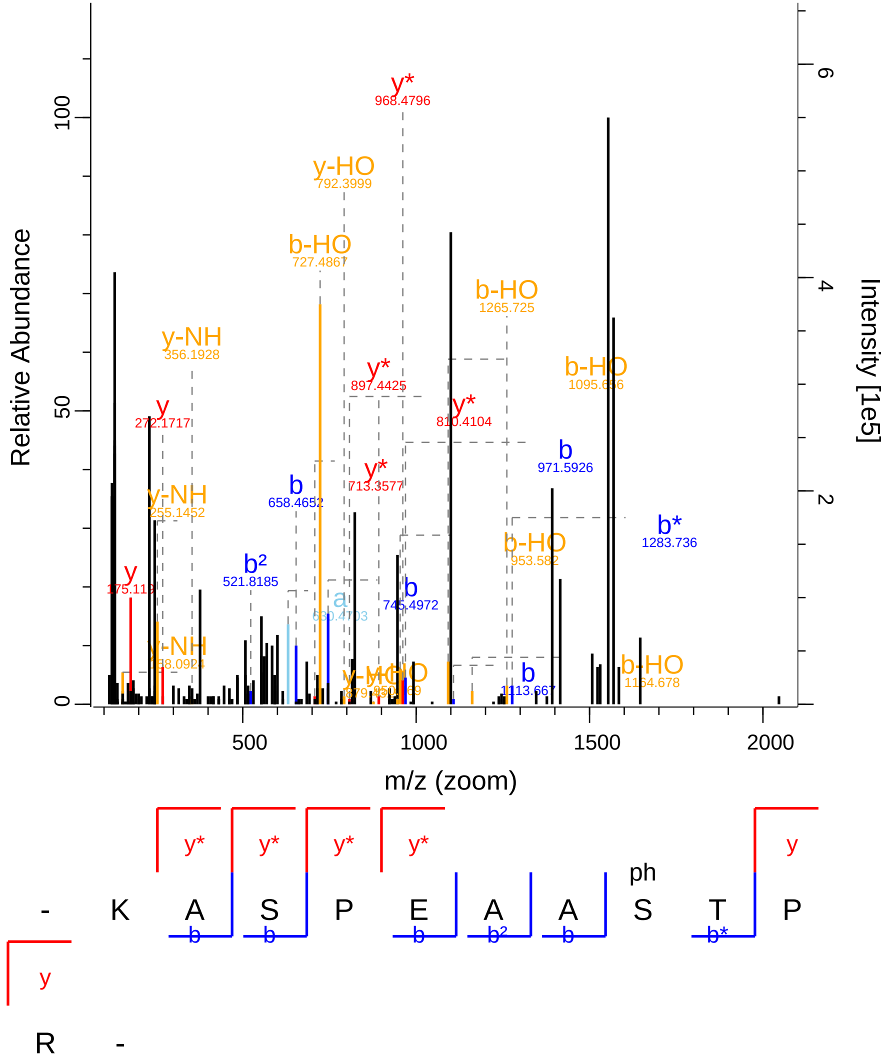

Raw File	Scan	Method	Score	m/z	Gene names
RO-QC-160121_ZackD_SS_Set_02_Fractior	7327	FTMS; HCI	54.61	393.63	FIG4

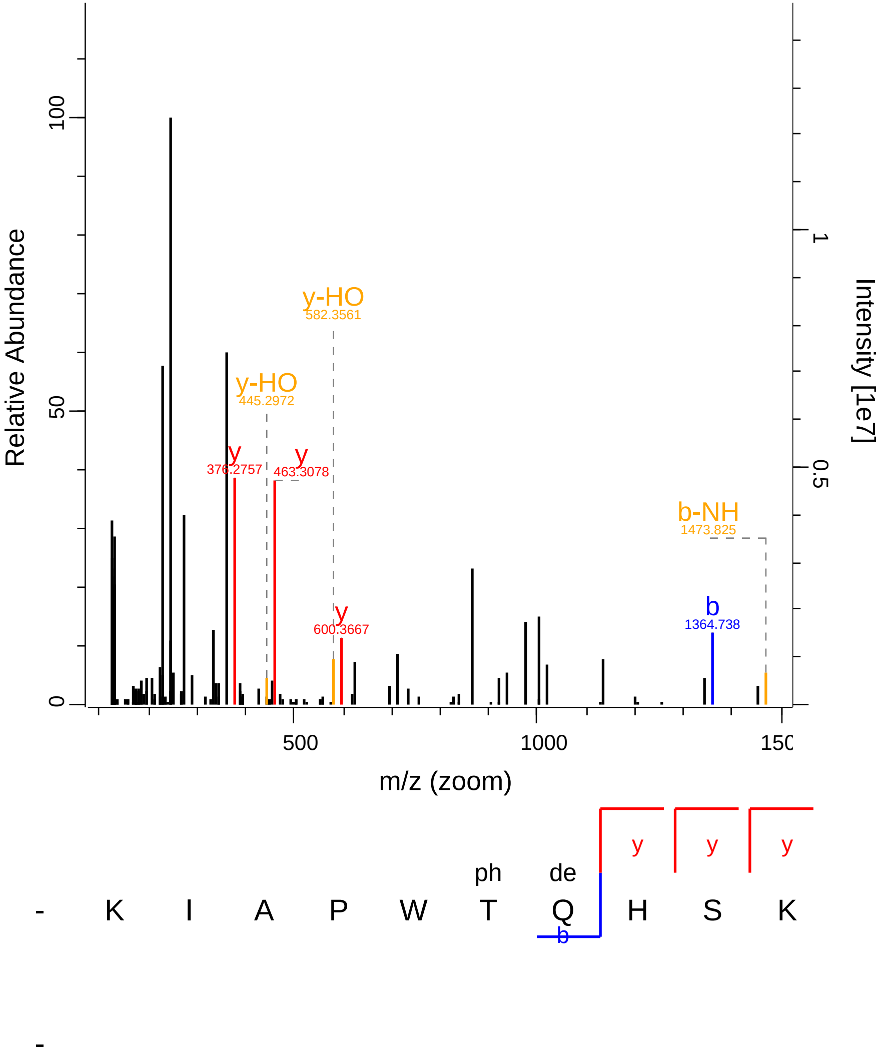

Raw File	Scan	Method	Score	m/z	Gene names
RO-QC-160121_ZackD_SS_Set_02_Fractior	7889	FTMS; HCI	57.17	592.67	RBM6

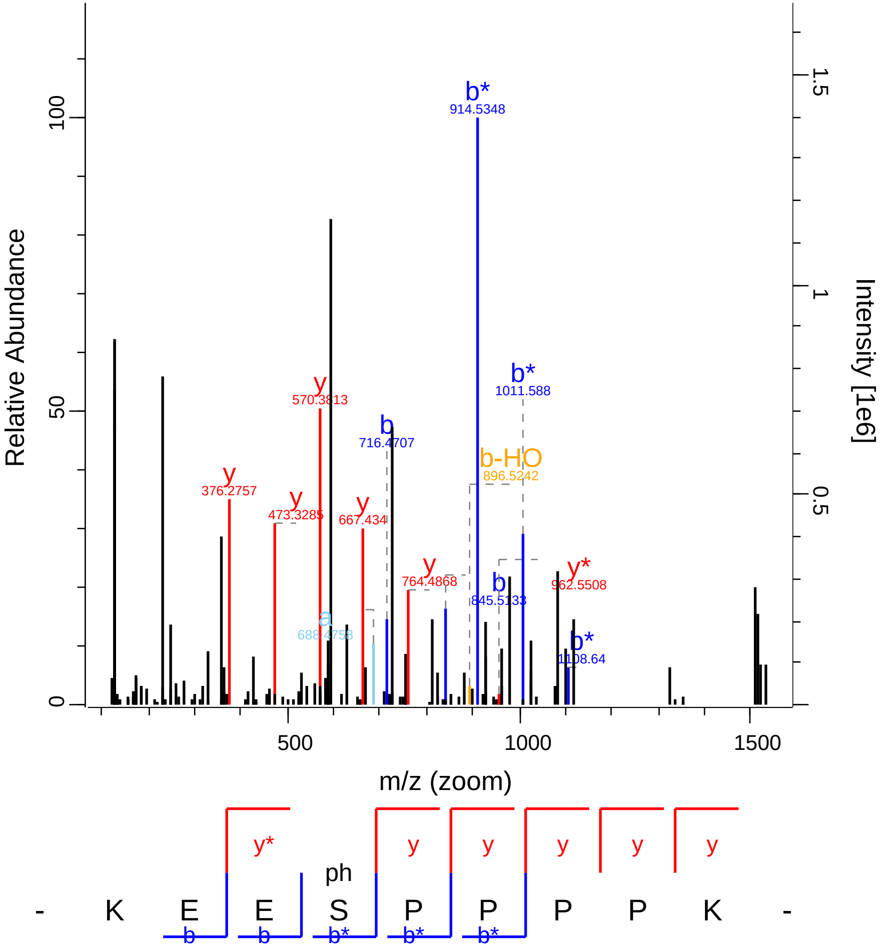

Raw File	Scan	Method	Score	m/z	Gene names
RO-QC-160121_ZackD_SS_Set_02_Fractior	8710	FTMS; HCI	87.24	680.03	IRF2BP1

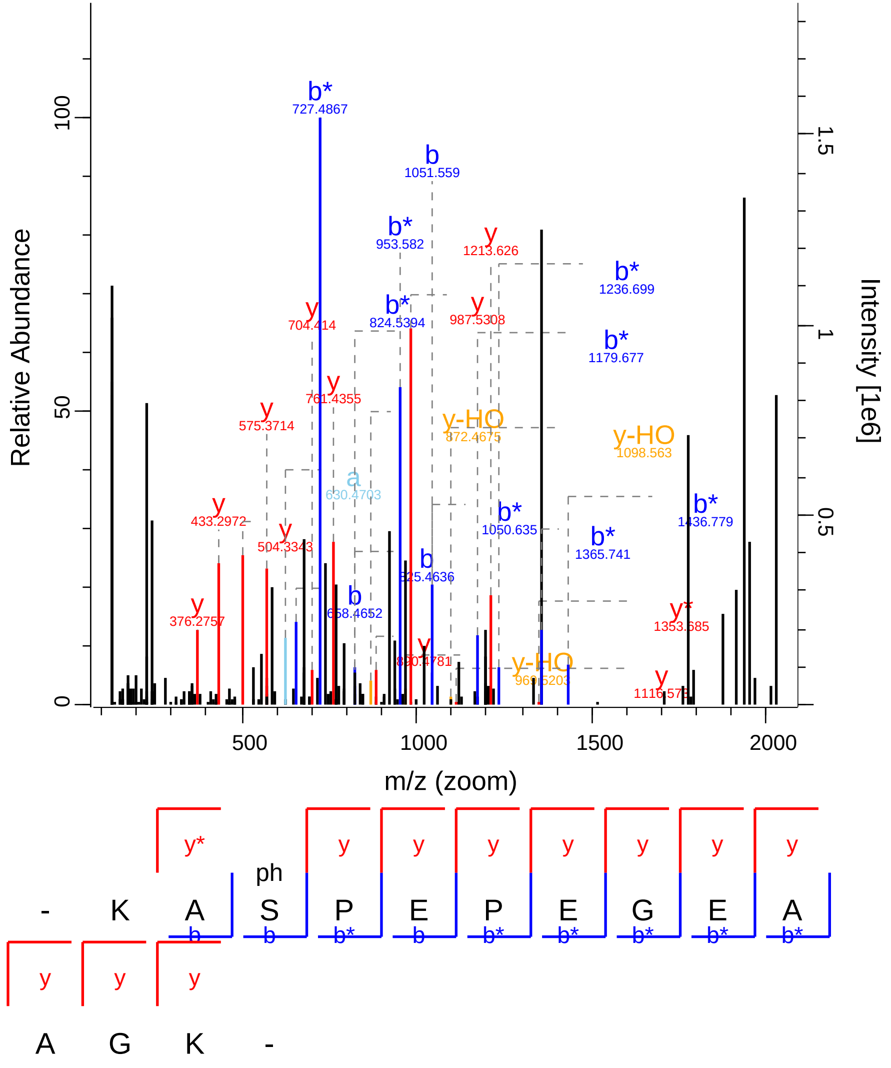

Raw File	Scan	Method	Score	m/z	Gene names
RO-QC-160121_ZackD_SS_Set_02_Fractior	9019	FTMS; HCI	67.52	529.97	SRRM2

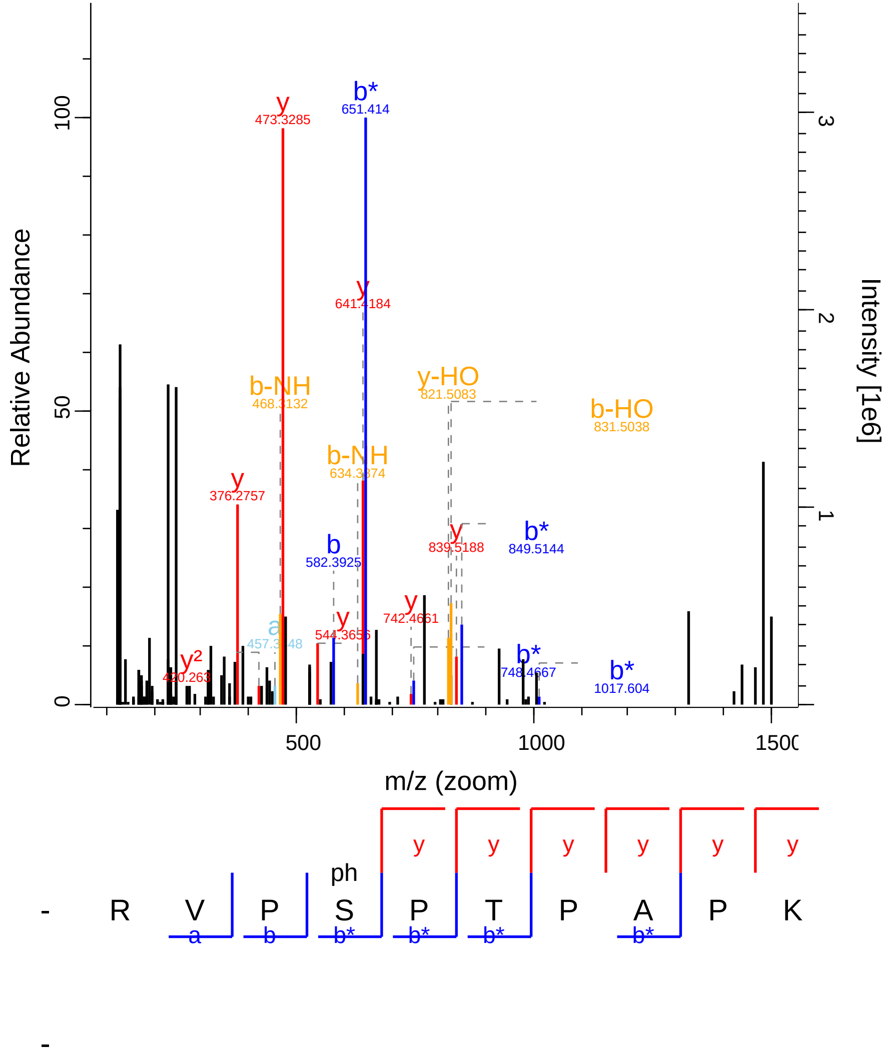

Raw File	Scan	Method	Score	m/z	Gene names
RO-QC-160121_ZackD_SS_Set_02_Fraction	9159	FTMS; HCI	91.46	818.7	IWS1

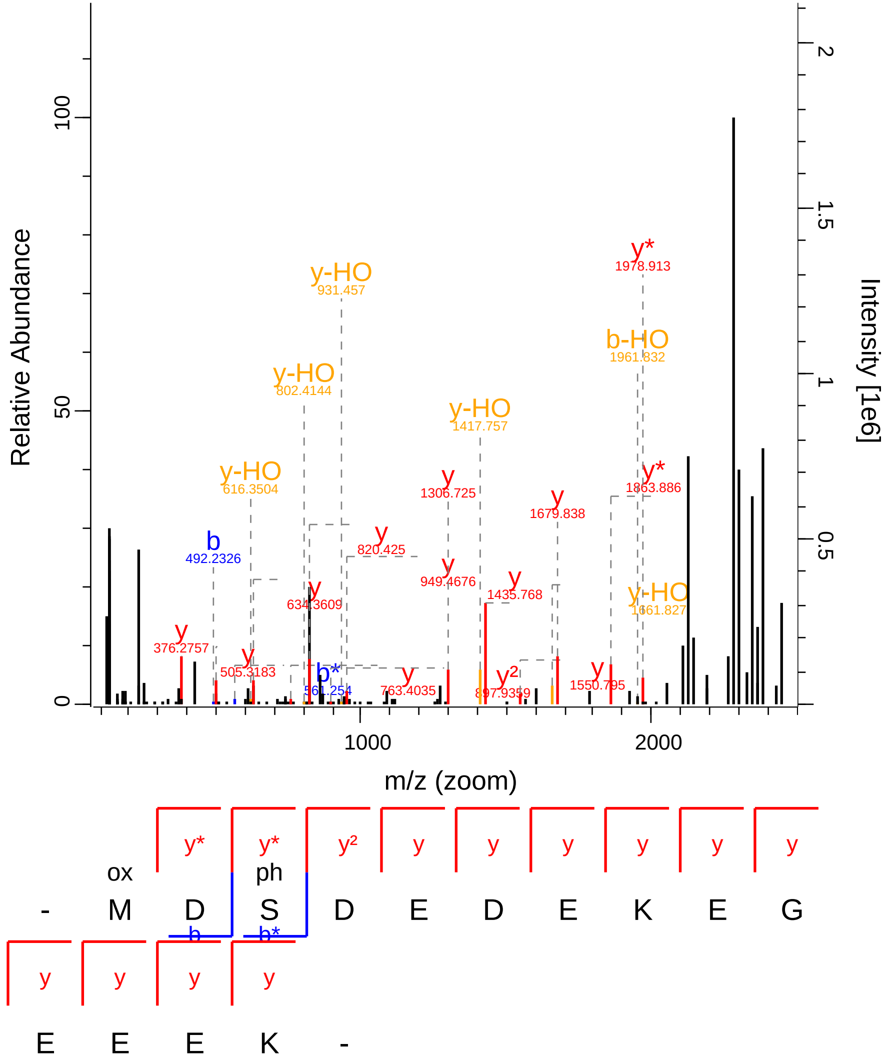

Raw File	Scan	Method	Score	m/z	Gene names
RO-QC-160121_ZackD_SS_Set_02_Fraction	9489	FTMS; HCI	63.49	764.4	LPHN2

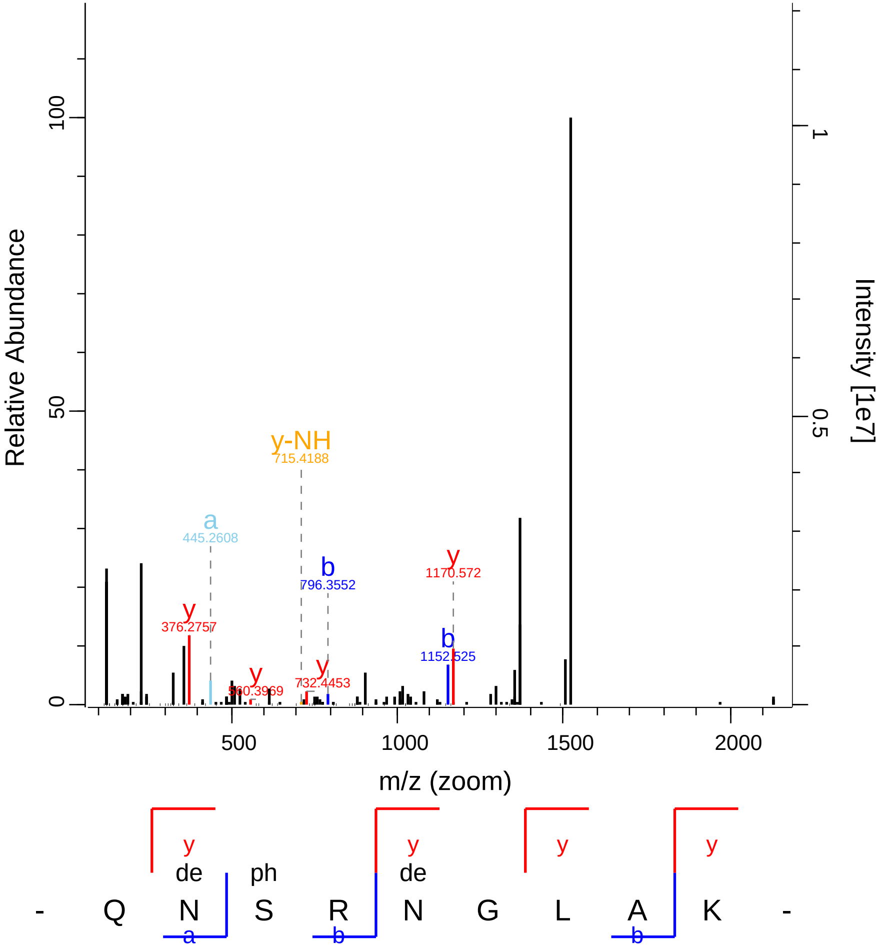

Raw File	Scan	Method	Score	m/z	Gene names
RO-QC-160121_ZackD_SS_Set_02_Fractio	10452	FTMS; HC	41.28	394.84	EFNB1

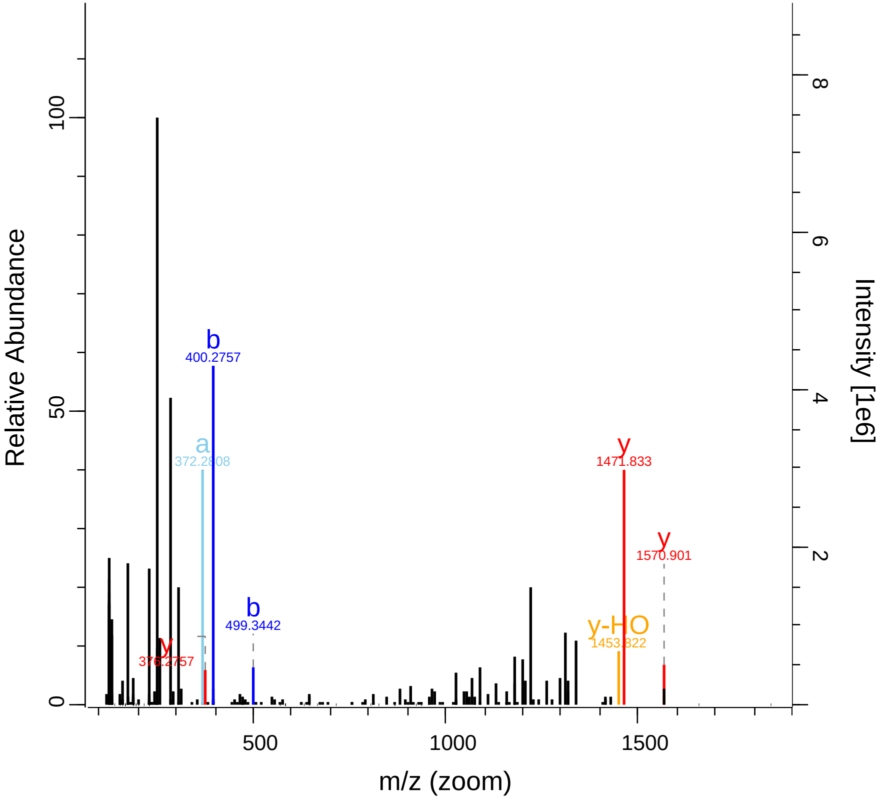

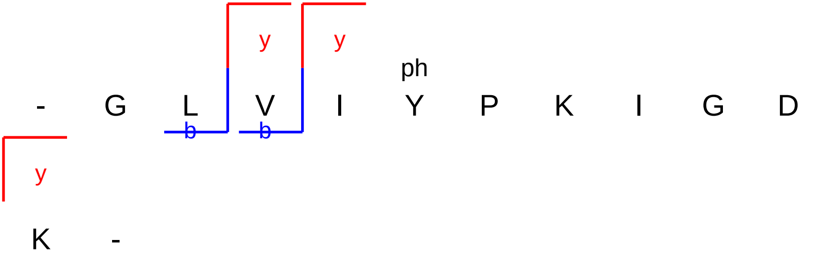

Raw File	Scan	Method	Score	m/z	Gene names
RO-QC-160121_ZackD_SS_Set_02_Fractio	11646	FTMS; HC	108.17	634.5	SSB

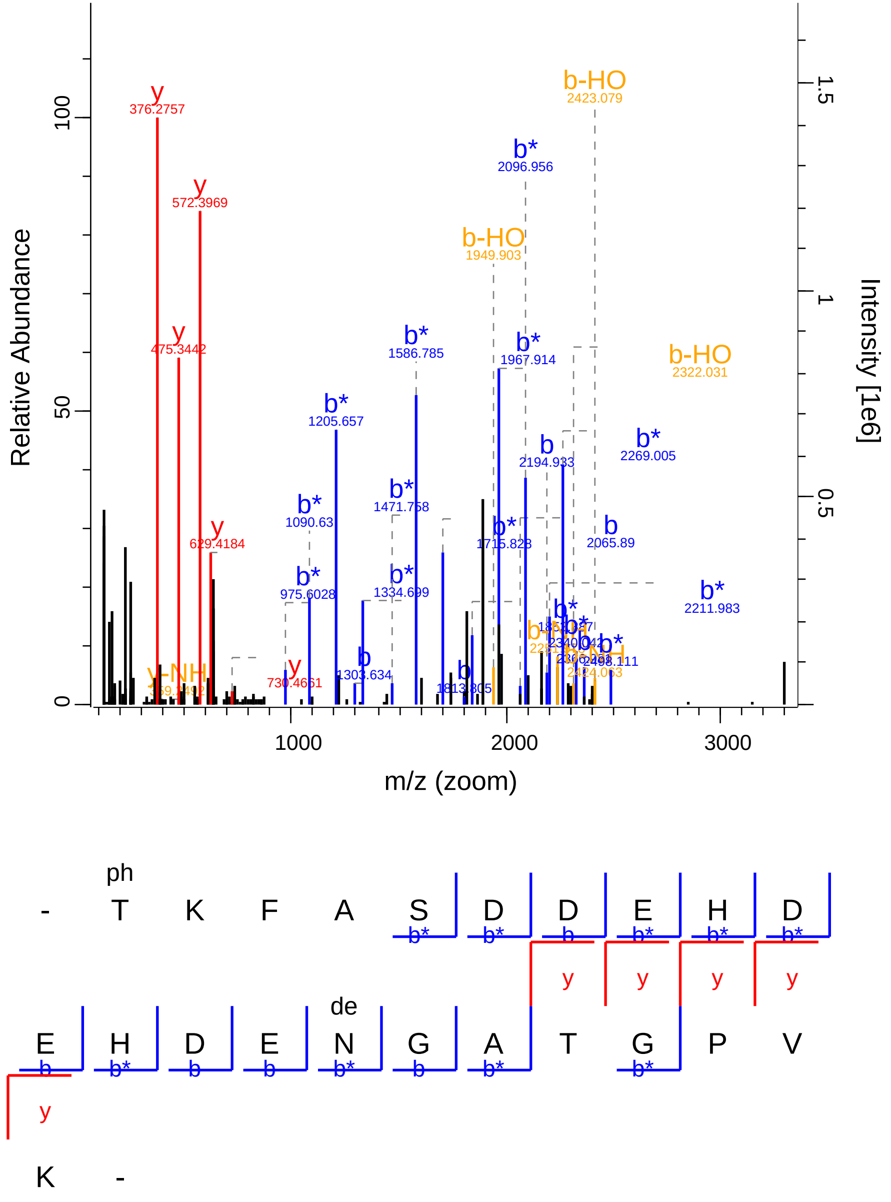

Raw File	Scan	Method	Score	m/z	Gene names
RO-QC-160121_ZackD_SS_Set_02_Fractio	12814	FTMS; HC	67.68	671.71	ADD1

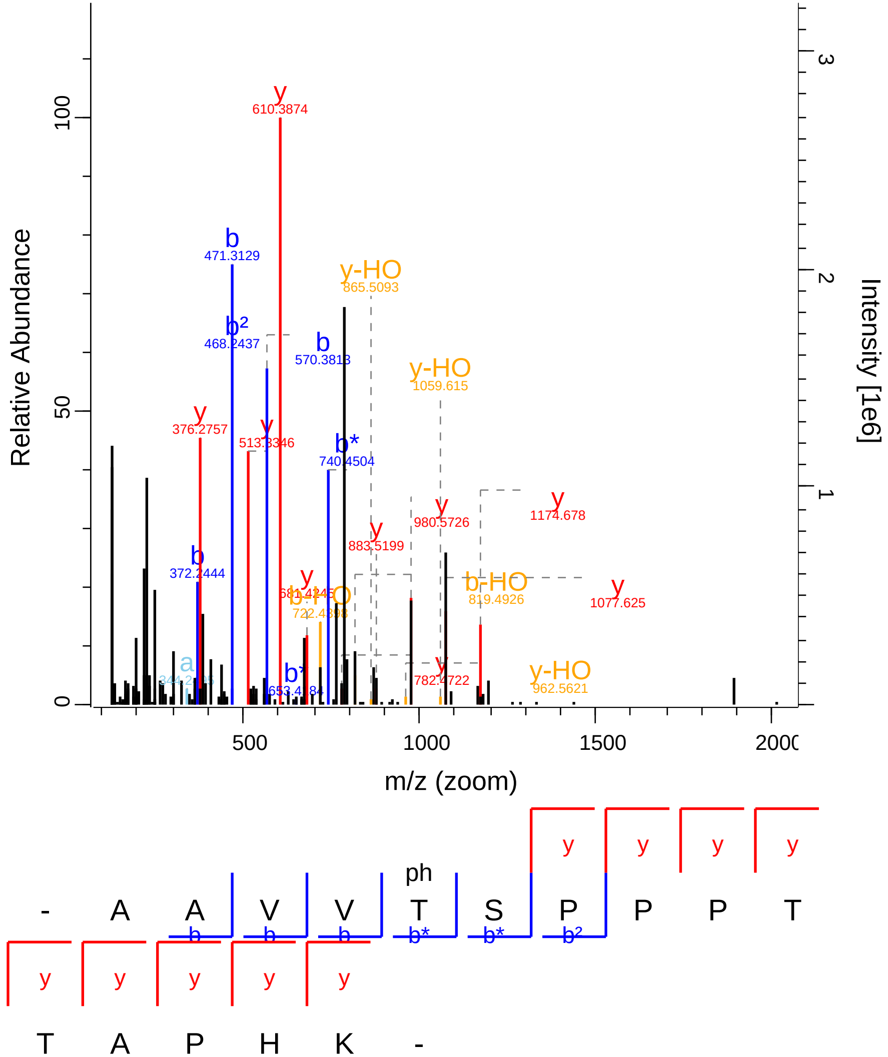

Raw File	Scan	Method	Score	m/z	Gene names
RO-QC-160121_ZackD_SS_Set_02_Fractio	13802	FTMS; HC	87.15	852.89	PRDX6

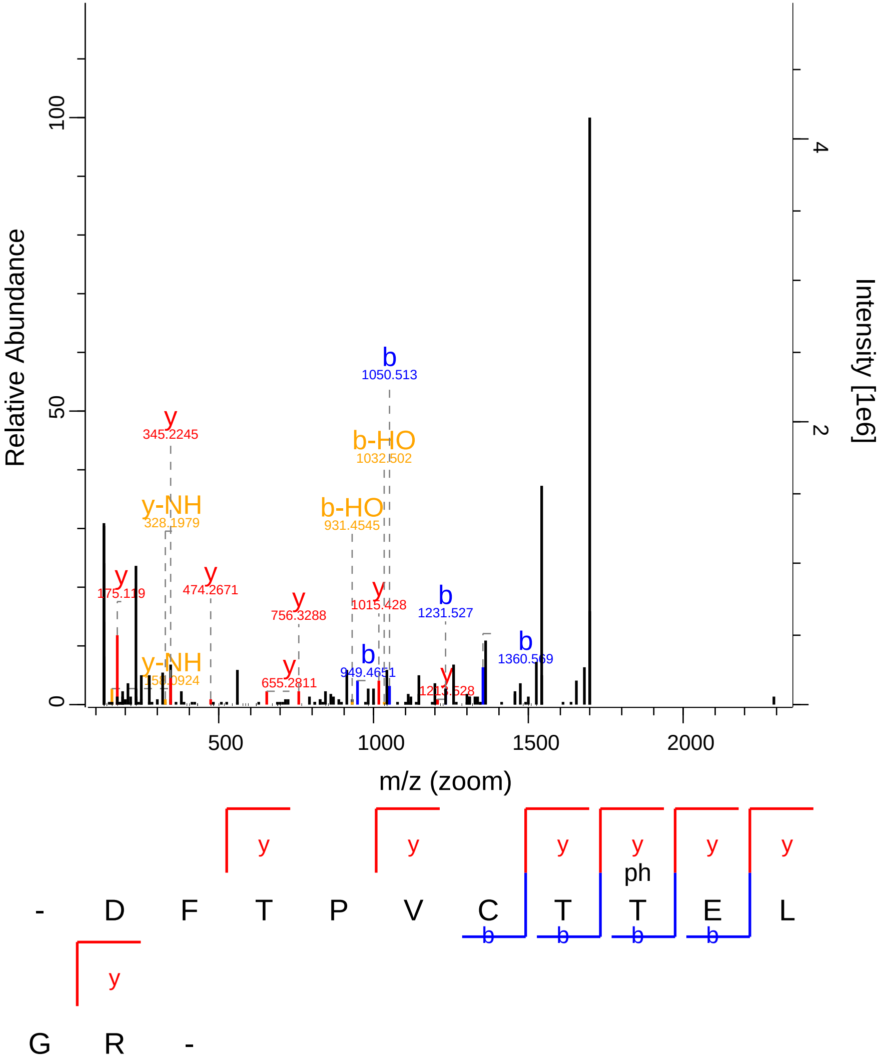

Raw File	Scan	Method	Score	m/z	Gene names
RO-QC-160121_ZackD_SS_Set_02_Fractior	14583	FTMS; HCI	87.31	998.5	DNAH3

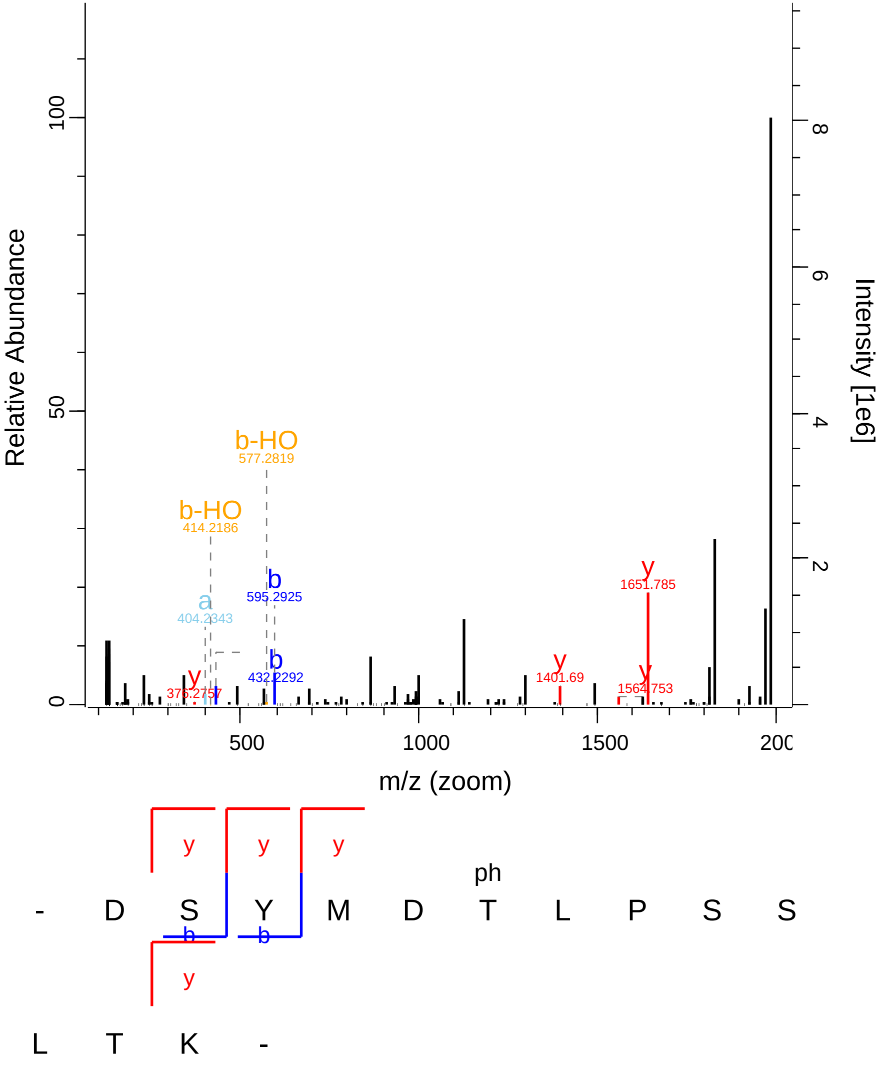

Raw File	Scan	Method	Score	m/z	Gene names
RO-QC-160121_ZackD_SS_Set_02_Fractio	15045	FTMS; HC	45.14	661.75	NCK2

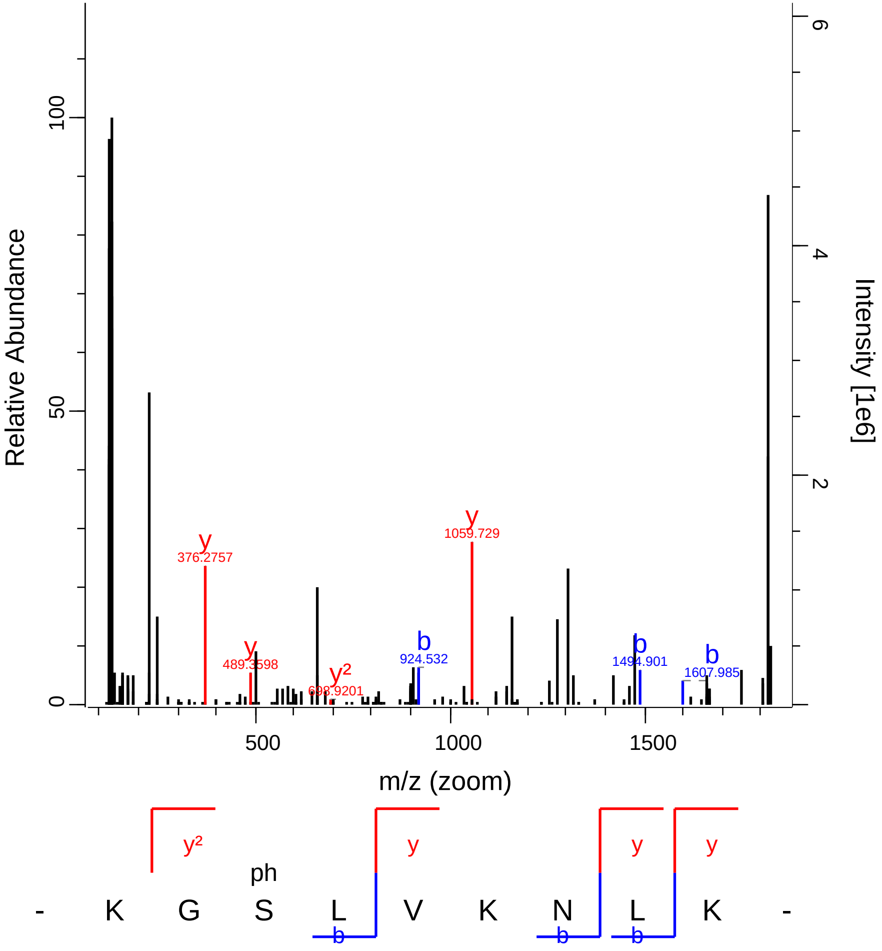

Raw File	Scan	Method	Score	m/z	Gene names
RO-QC-160121_ZackD_SS_Set_02_Fractio	18429	FTMS; HC	54.61	974.53	BBS1

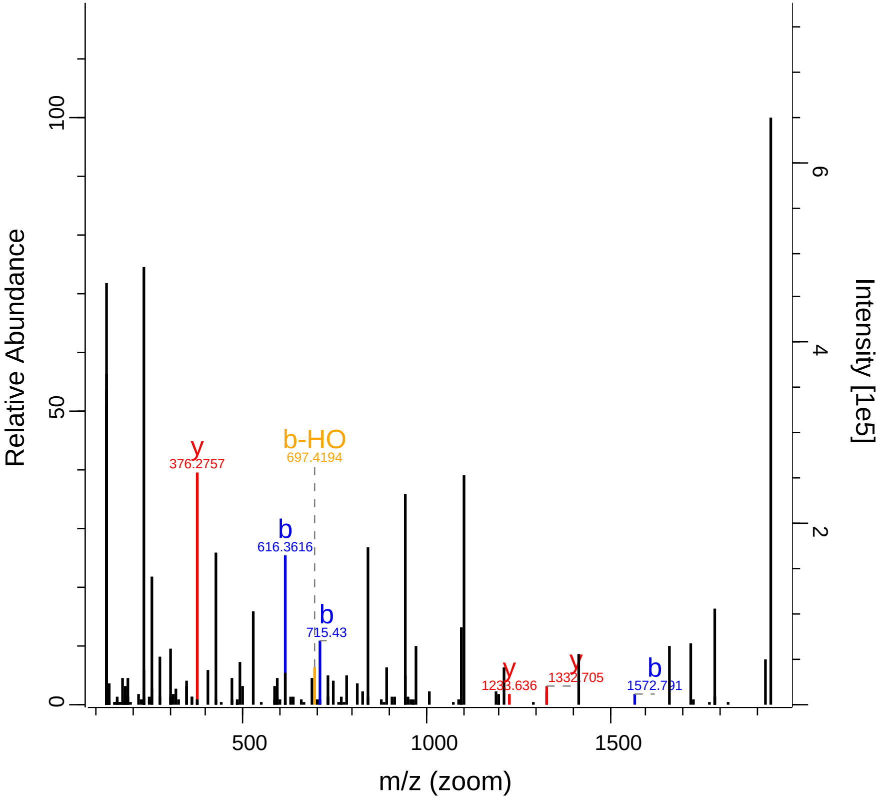

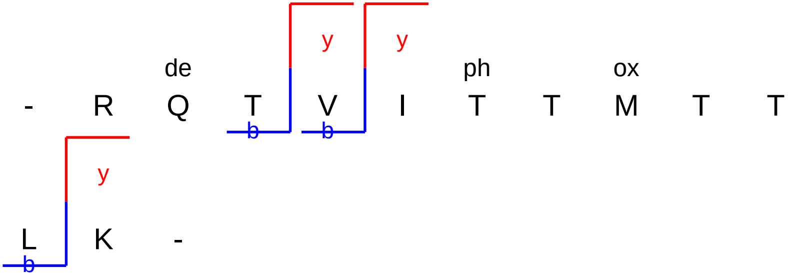

Raw File	Scan	Method	Score	m/z	Gene names
RO-QC-160121_ZackD_SS_Set_02_Fractio	18540	FTMS; HC	43.38	415.26	INTS9

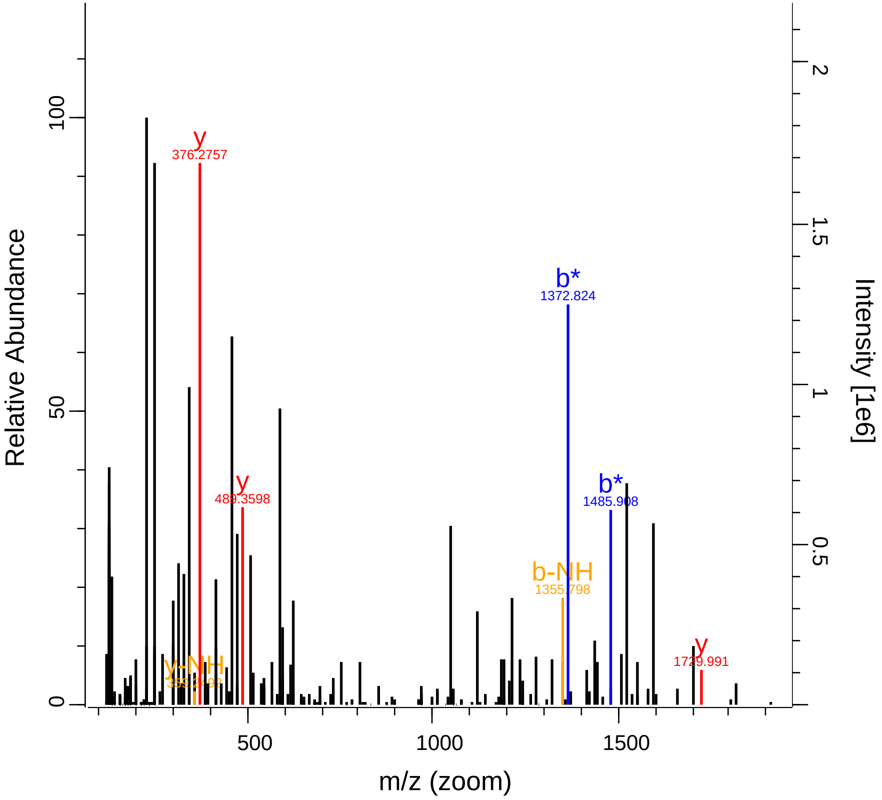

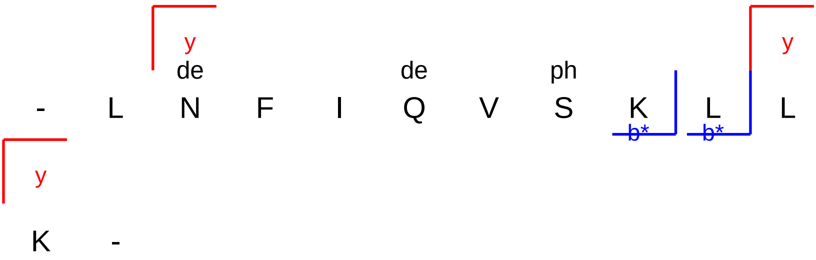

Raw File	Scan	Method	Score	m/z	Gene names
RO-QC-160121_ZackD_SS_Set_02_Fractic	18573	FTMS; HC	149.52	1250.17	UBAP2L

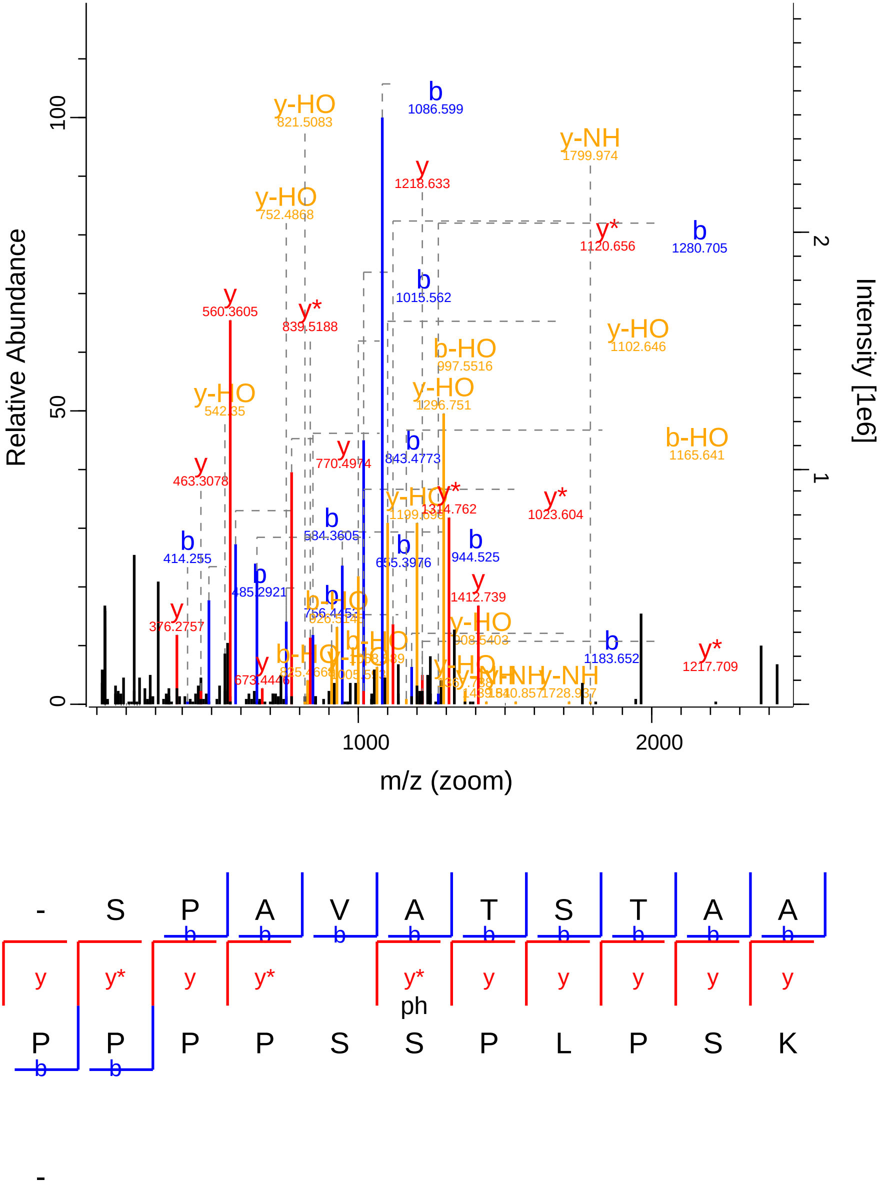

Raw File	Scan	Method	Score	m/z	Gene names
RO-QC-160121_ZackD_SS_Set_02_Fra	194211	FTMS; H(	40.5	989.46	SPATA31C2;SPATA31

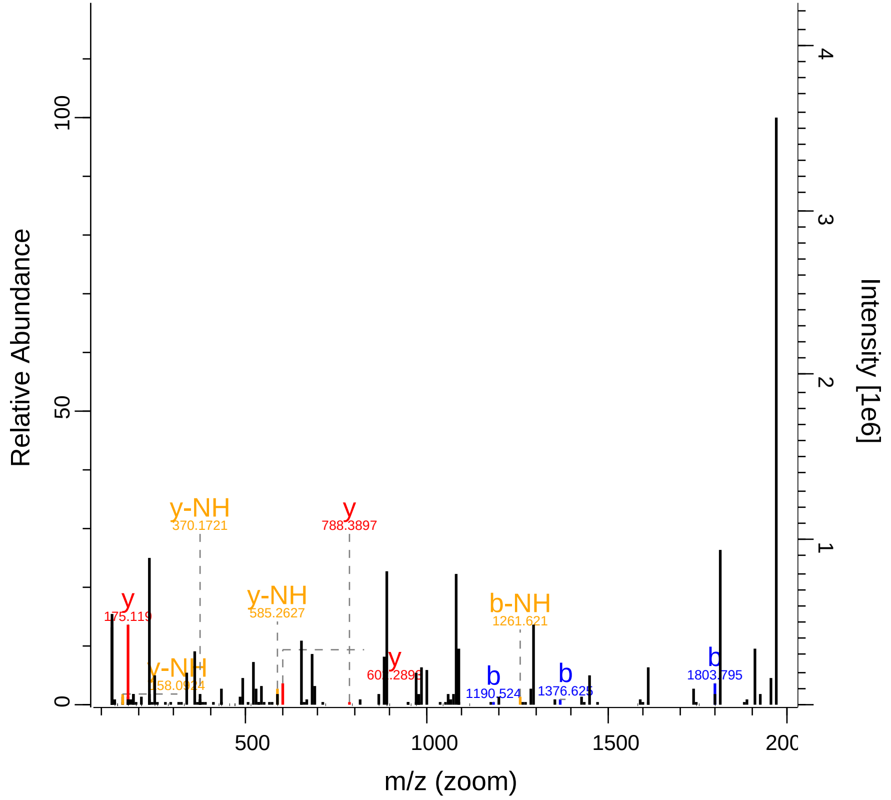

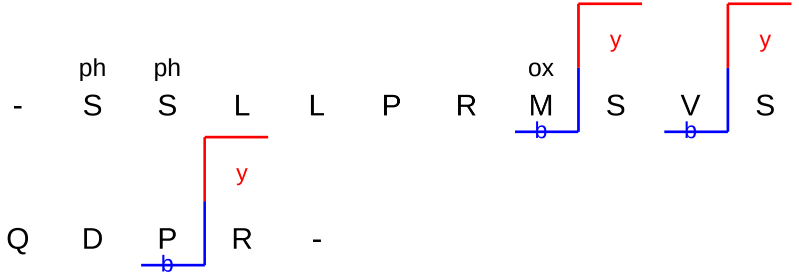

Raw File	Scan	Method	Score	m/z	Gene names
RO-QC-160121_ZackD_SS_Set_02_Fractio	20316	FTMS; HC	204.18	869.42	VIM

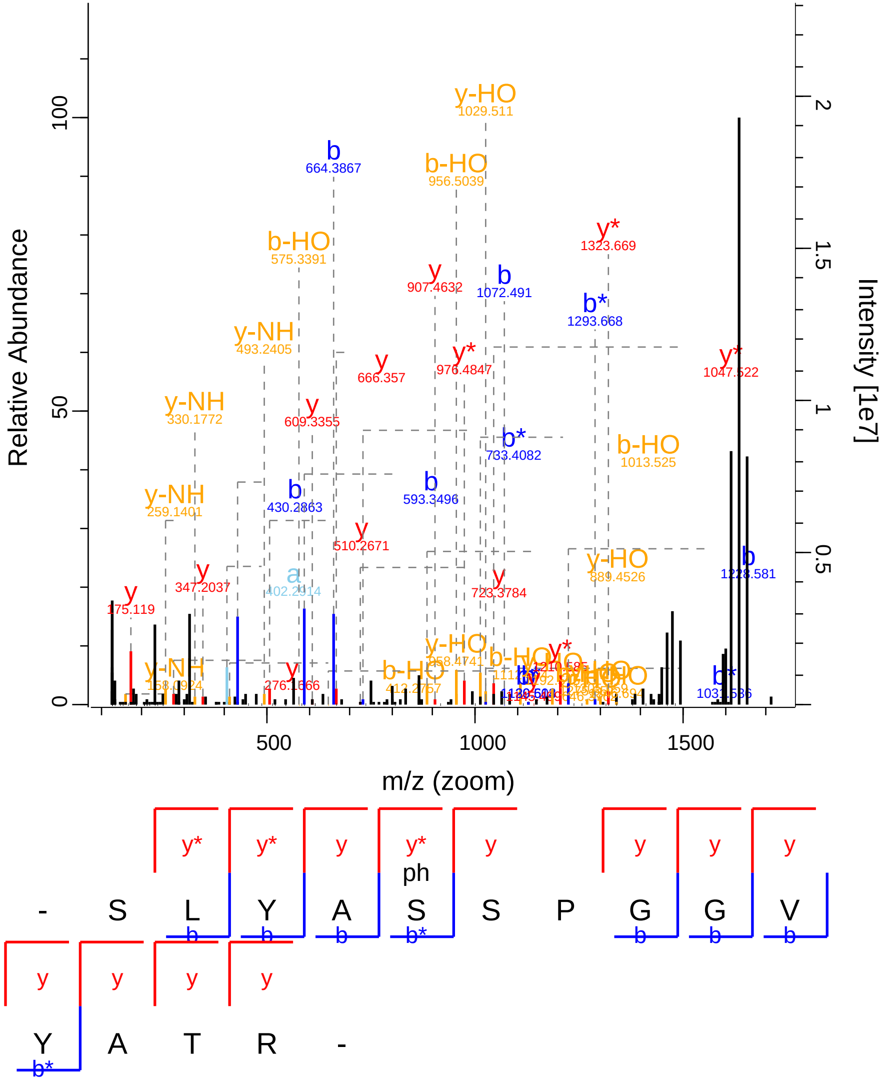

Raw File	Scan	Method	Score	m/z	Gene names
RO-QC-160121_ZackD_SS_Set_02_Fractio	20403	FTMS; HC	128.67	752.41	SORBS2

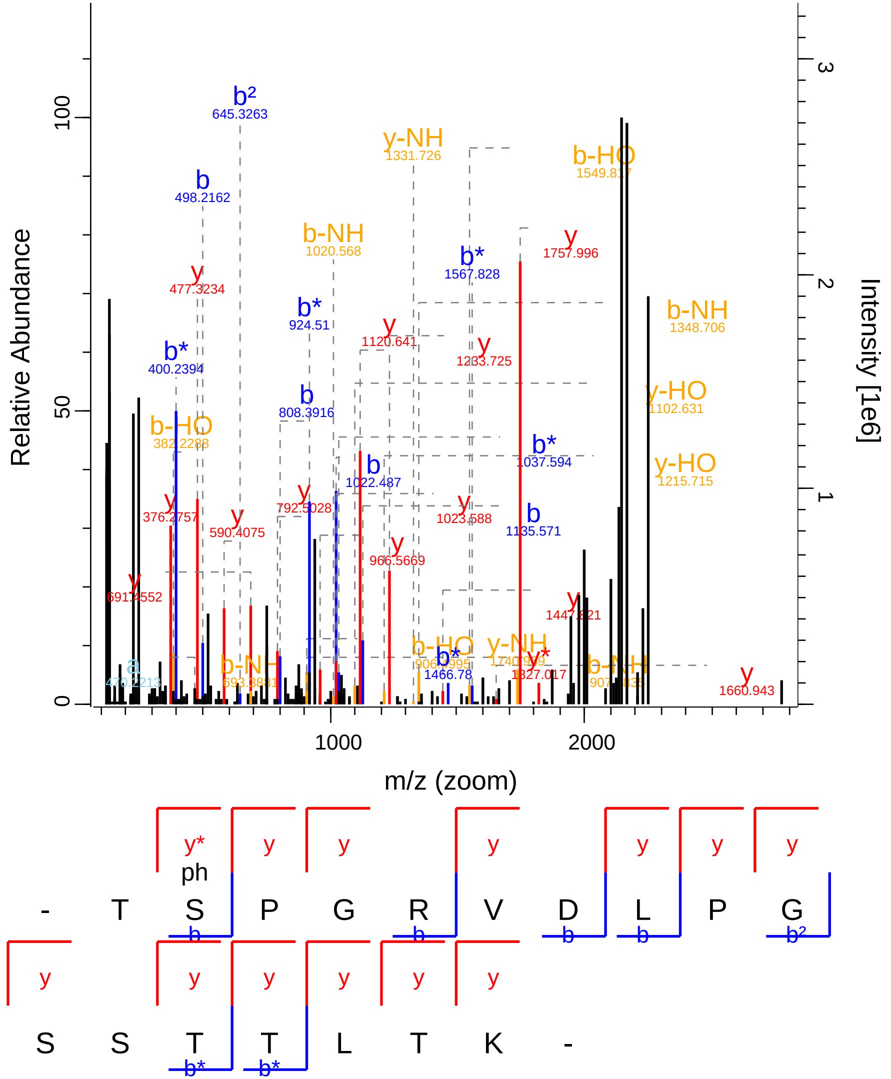

Raw File	Scan	Method	Score	m/z	Gene names
RO-QC-160121_ZackD_SS_Set_02_Fractio	20854	FTMS; HC	51.87	702.07	ZNF518A

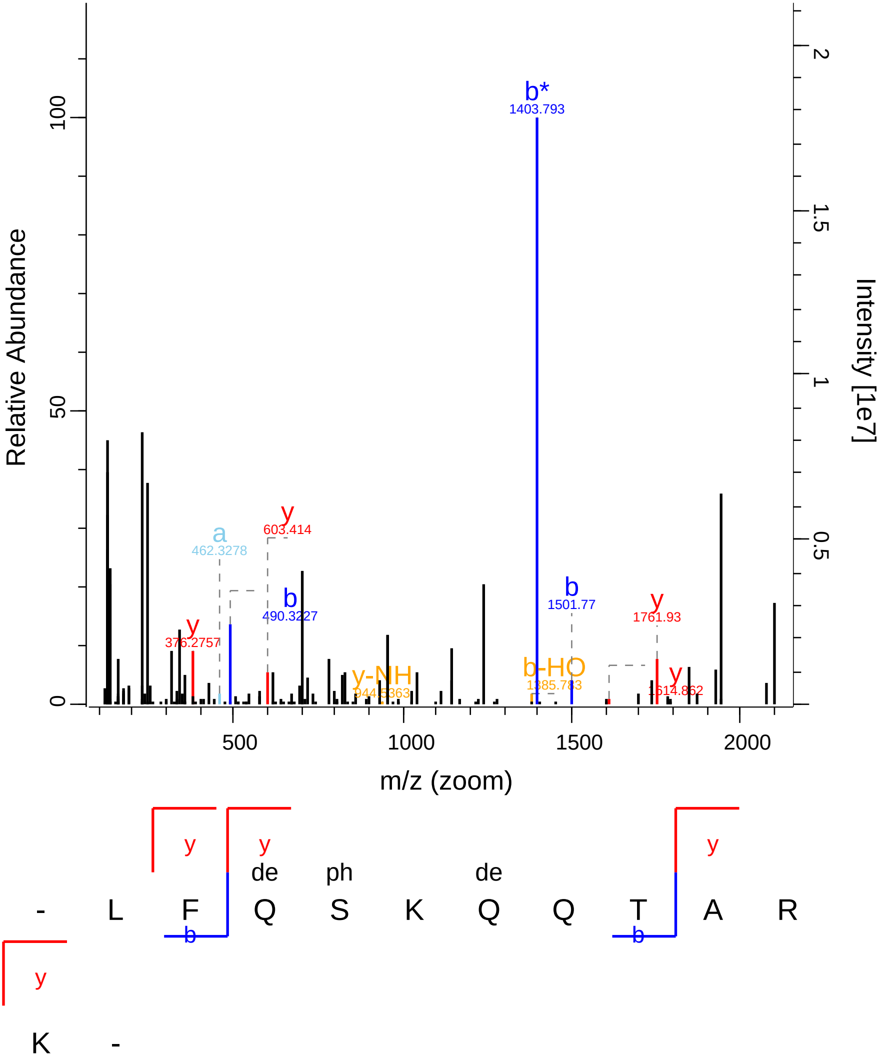

Raw File	Scan	Method	Score	m/z	Gene names
RO-QC-160121_ZackD_SS_Set_02_Fractio	20914	FTMS; HC	40.27	449.26	WAPAL

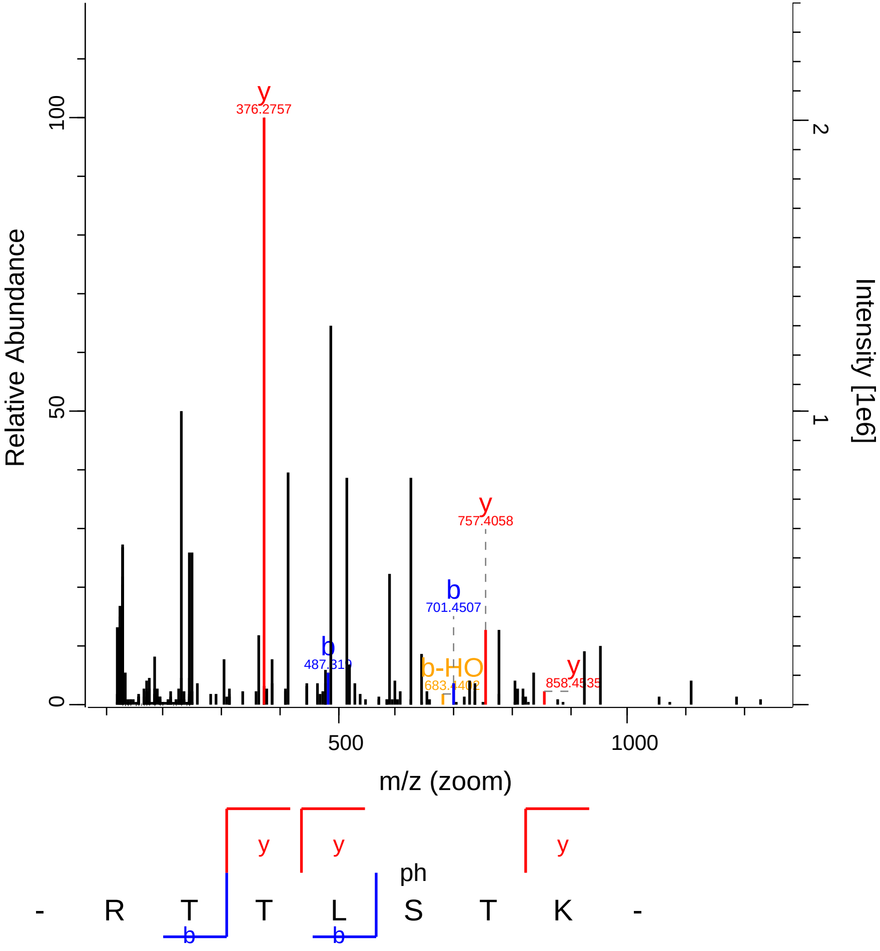

Raw File	Scan	Method	Score	m/z	Gene names
RO-QC-160121_ZackD_SS_Set_02_Fractio	21023	FTMS; HC	93.15	875.77	PGM3

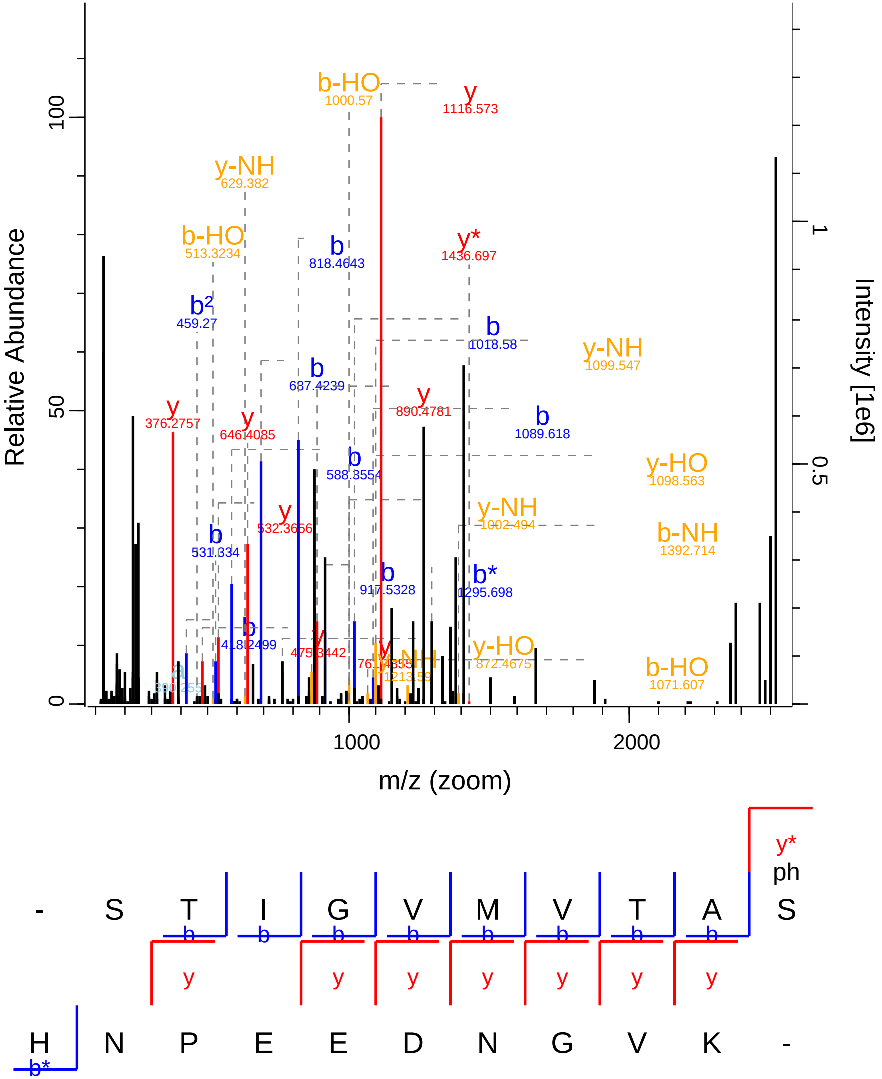

Raw File	Scan	Method	Score	m/z	Gene names
RO-QC-160121_ZackD_SS_Set_02_Fractic	21645	FTMS; HC	62.47	1078.05	NACA2

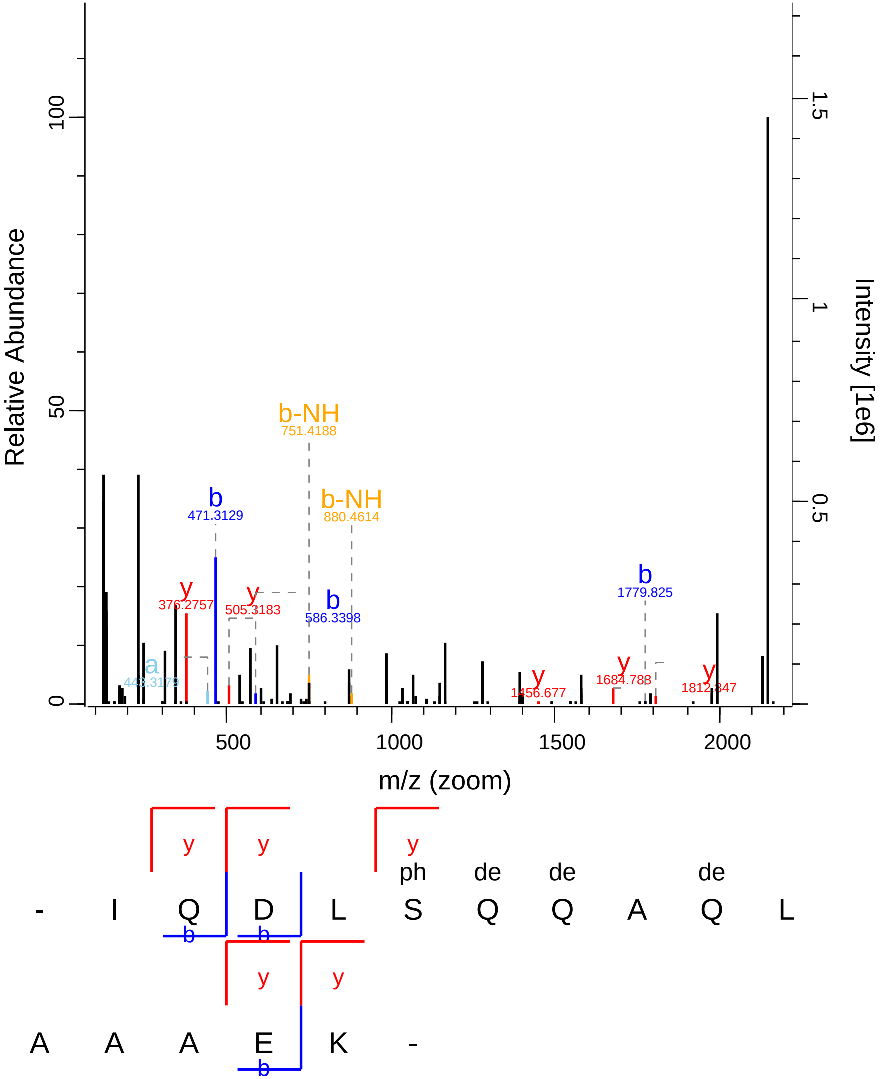

Raw File	Scan	Method	Score	m/z	Gene names
RO-QC-160121_ZackD_SS_Set_02_Fractio	22534	FTMS; HC	203.11	981.02	MAP1B

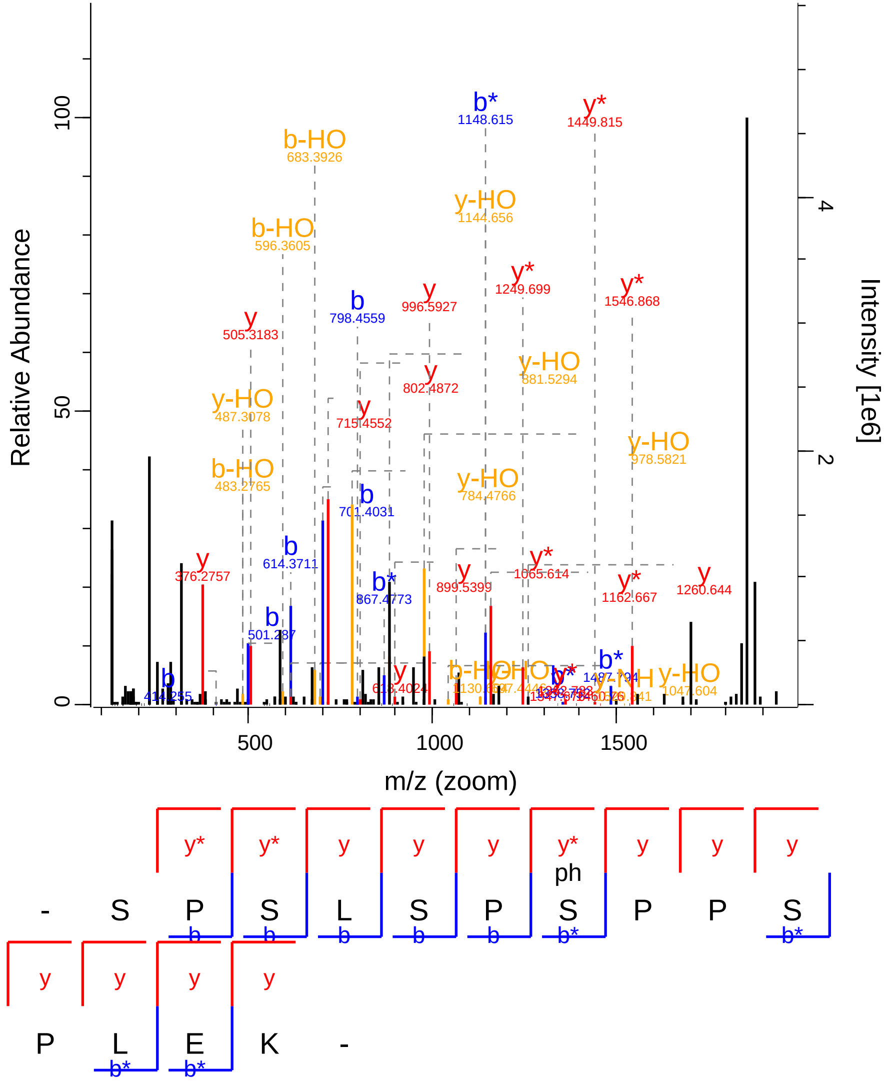

Raw File	Scan	Method	Score	m/z	Gene names
RO-QC-160121_ZackD_SS_Set_02_Fractic	23031	FTMS; HC	71.89	1052.08	DNAH17

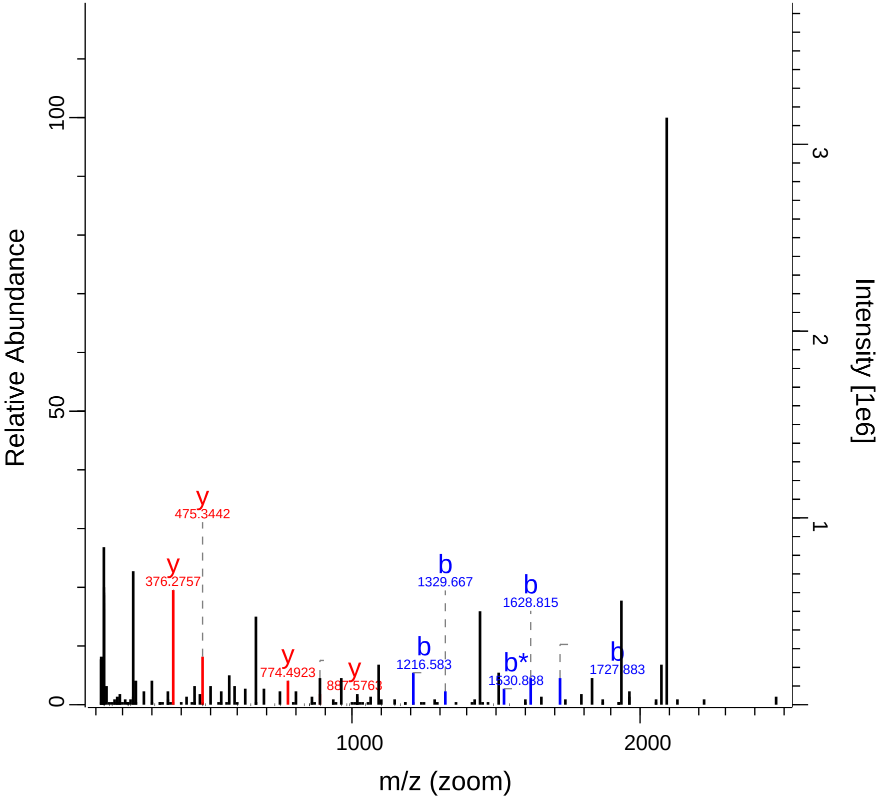

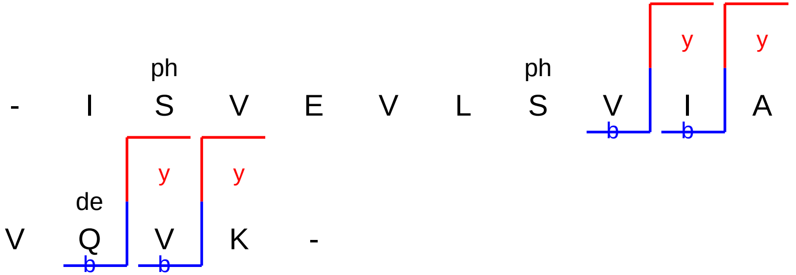

Raw File	Scan	Method	Score	m/z	Gene names
RO-QC-160121_ZackD_SS_Set_02_Fractio	23164	FTMS; HC	56.01	903.45	TOP2B

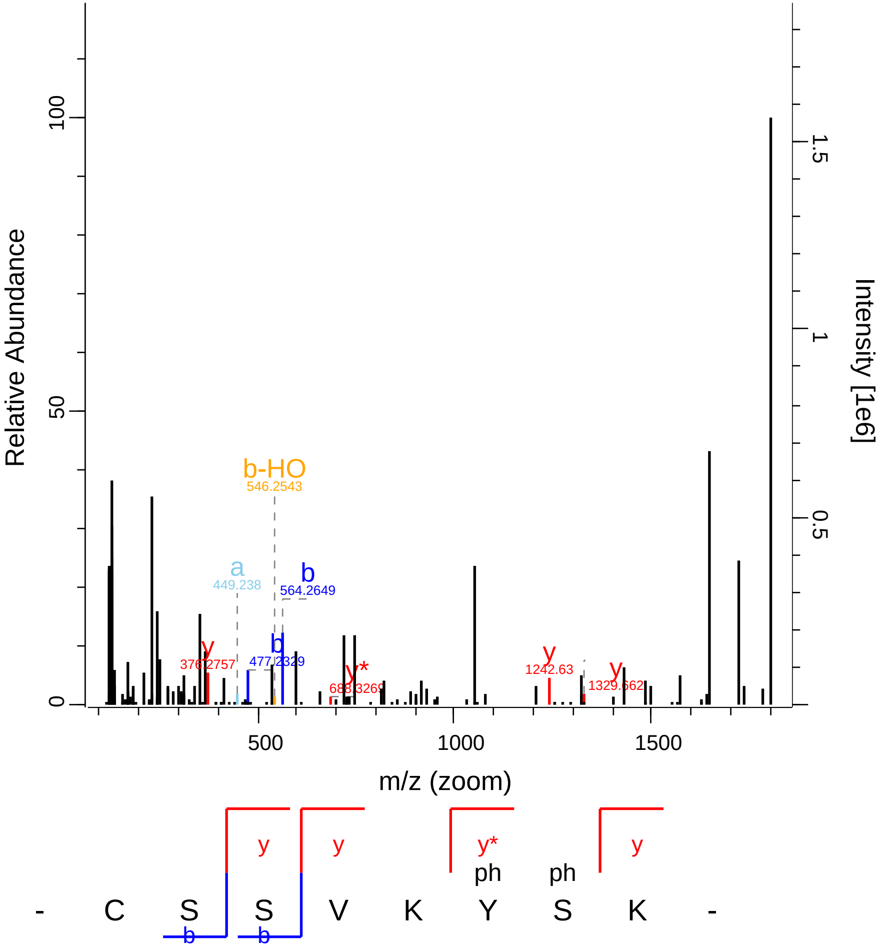

Raw File	Scan	Method	Score	m/z	Gene names
RO-QC-160121_ZackD_SS_Set_02_Fractio	23494	FTMS; HC	44.64	808.71	SCN9A

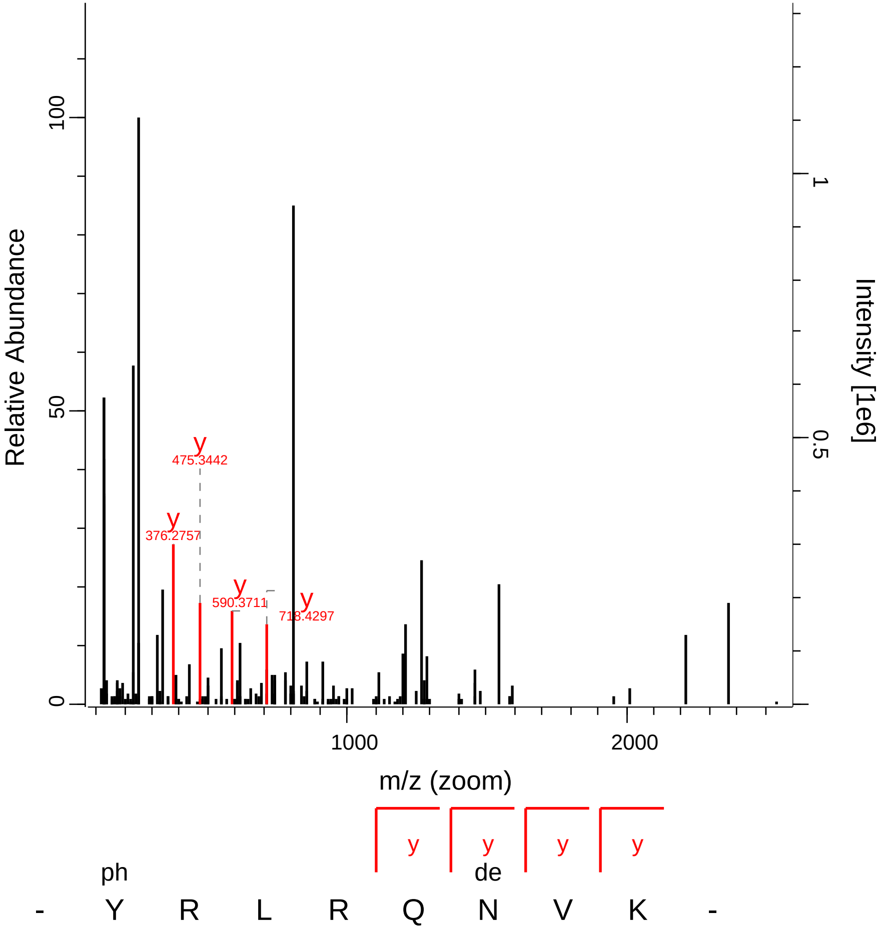

Raw File	Scan	Method	Score	m/z	Gene names
RO-QC-160121_ZackD_SS_Set_02_Fractic	25858	FTMS; HC	52.61	1065.22	CLC

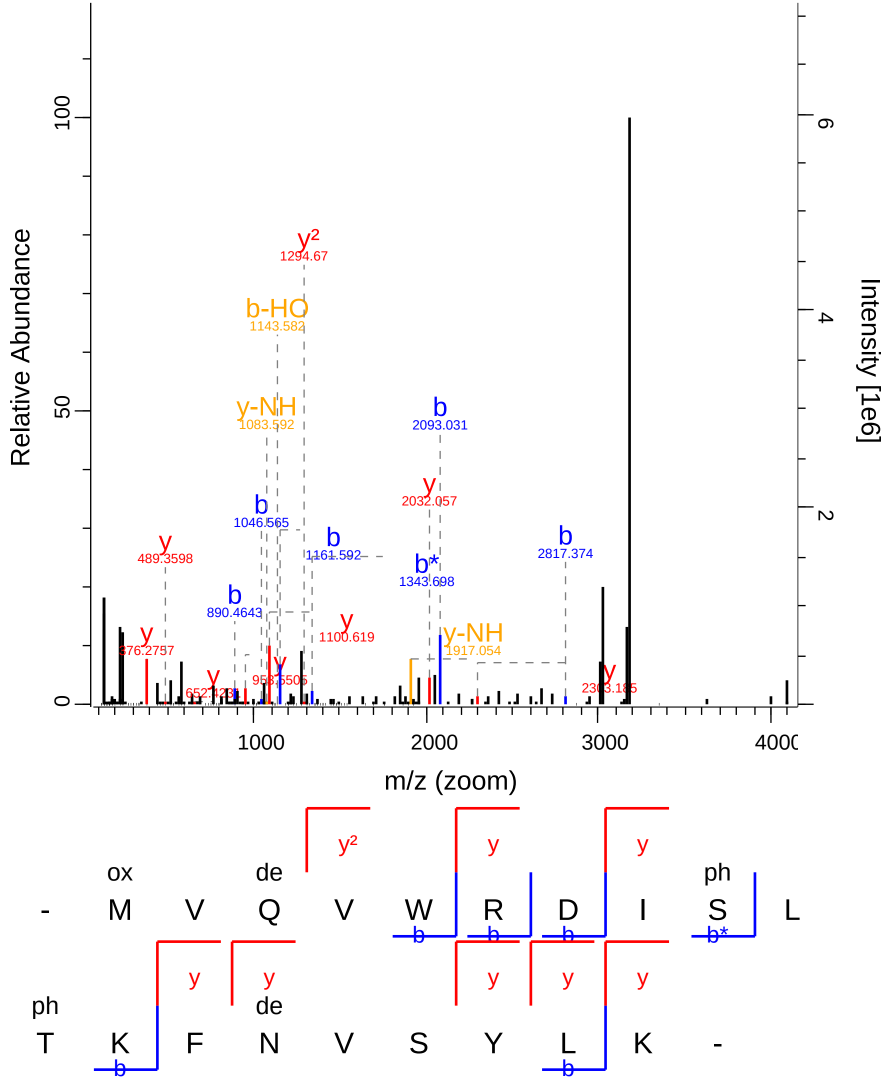

Raw File	Scan	Method	Score	m/z	Gene names
RO-QC-160121_ZackD_SS_Set_02_Fractic	26039	FTMS; HC	178.8	1128.02	MAP1B

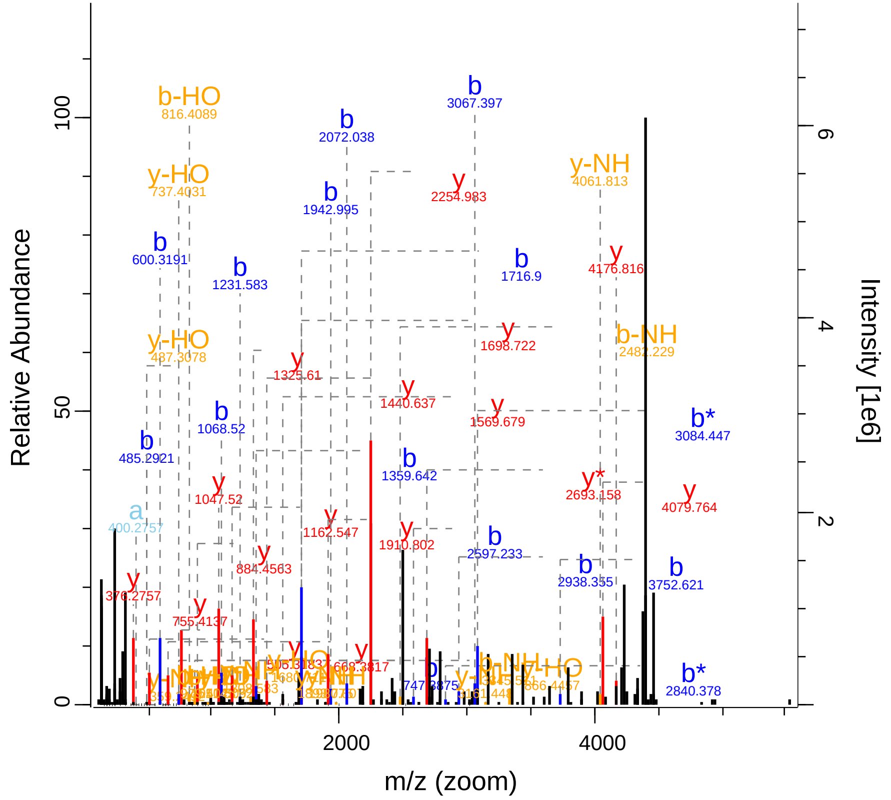

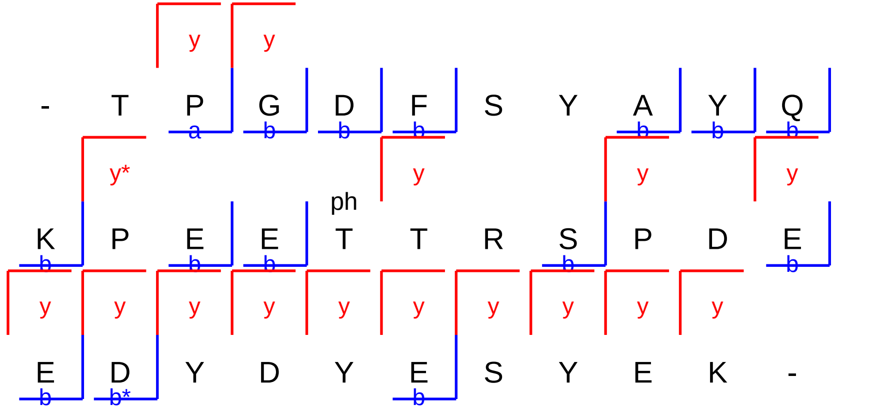

Raw File	Scan	Method	Score	m/z	Gene names
RO-QC-160121_ZackD_SS_Set_02_Fractio	26267	FTMS; HC	49.42	584.33	RMI1

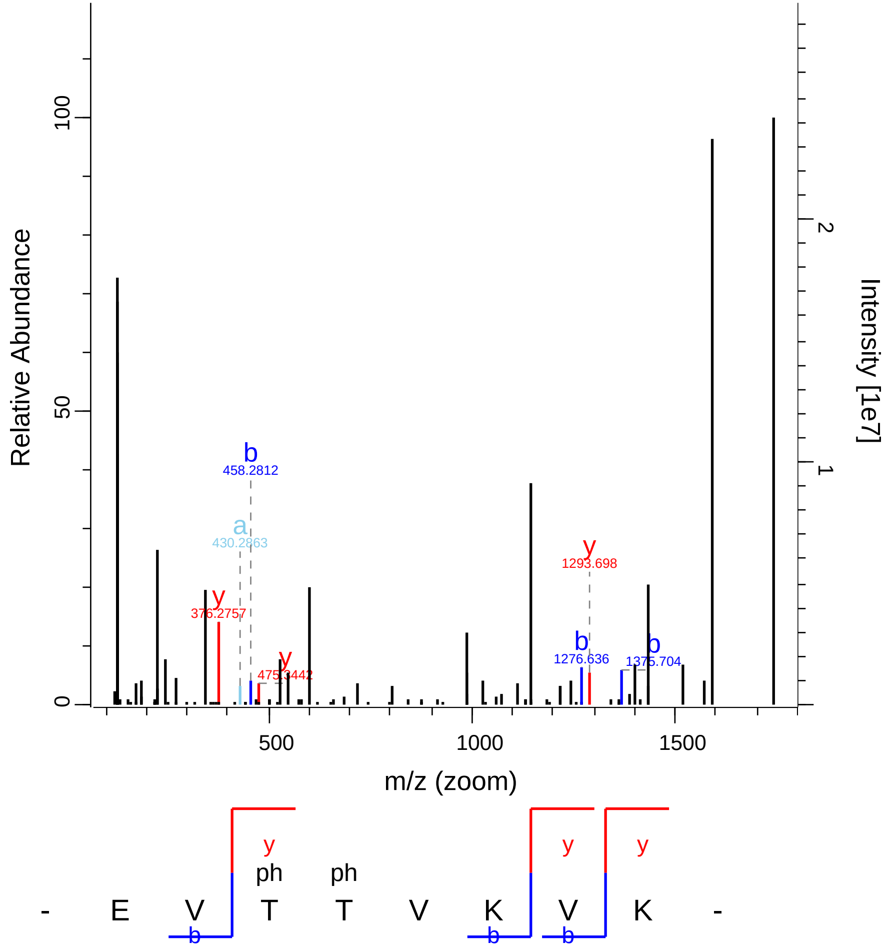

Raw File	Scan	Method	Score	m/z	Gene names
RO-QC-160121_ZackD_SS_Set_02_Fractio	26481	FTMS; HC	56.48	823.44	CHDC2

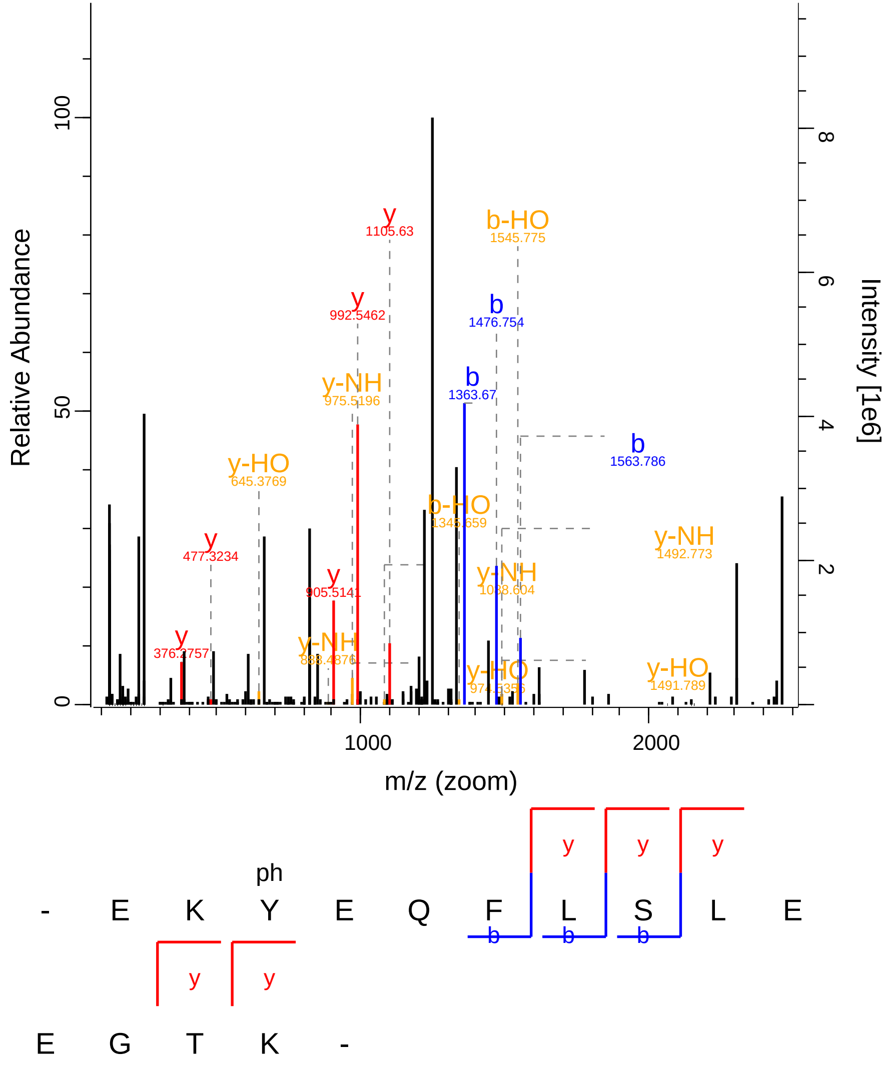

Raw File	Scan	Method	Score	m/z	Gene names
RO-QC-160121_ZackD_SS_Set_02_Fractio	27209	FTMS; HC	40.02	660.12	ABCA13

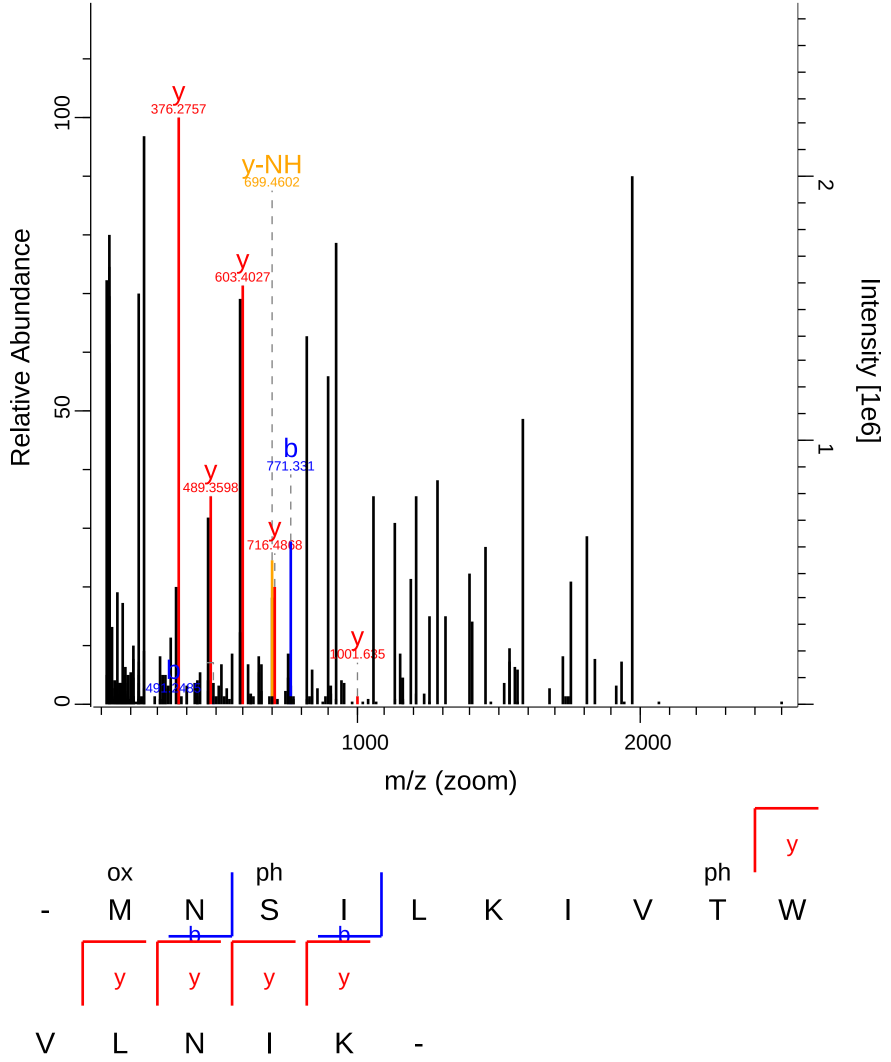

Raw File	Scan	Method	Score	m/z	Gene names
RO-QC-160121_ZackD_SS_Set_02_Fractic	28661	FTMS; HC	63.22	1124.62	FABP2

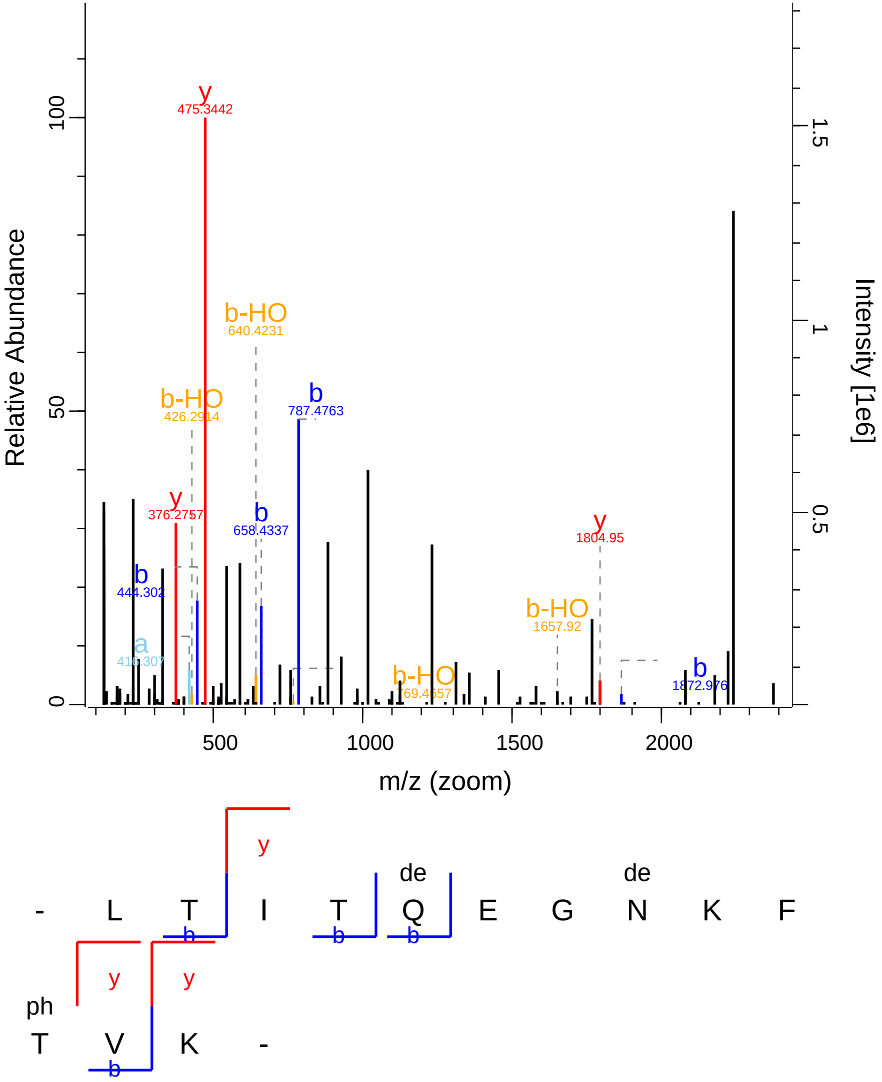

Raw File	Scan	Method	Score	m/z	Gene names
RO-QC-160121_ZackD_SS_Set_02_Fractio	29813	FTMS; HC	200.84	928.71	GPR143

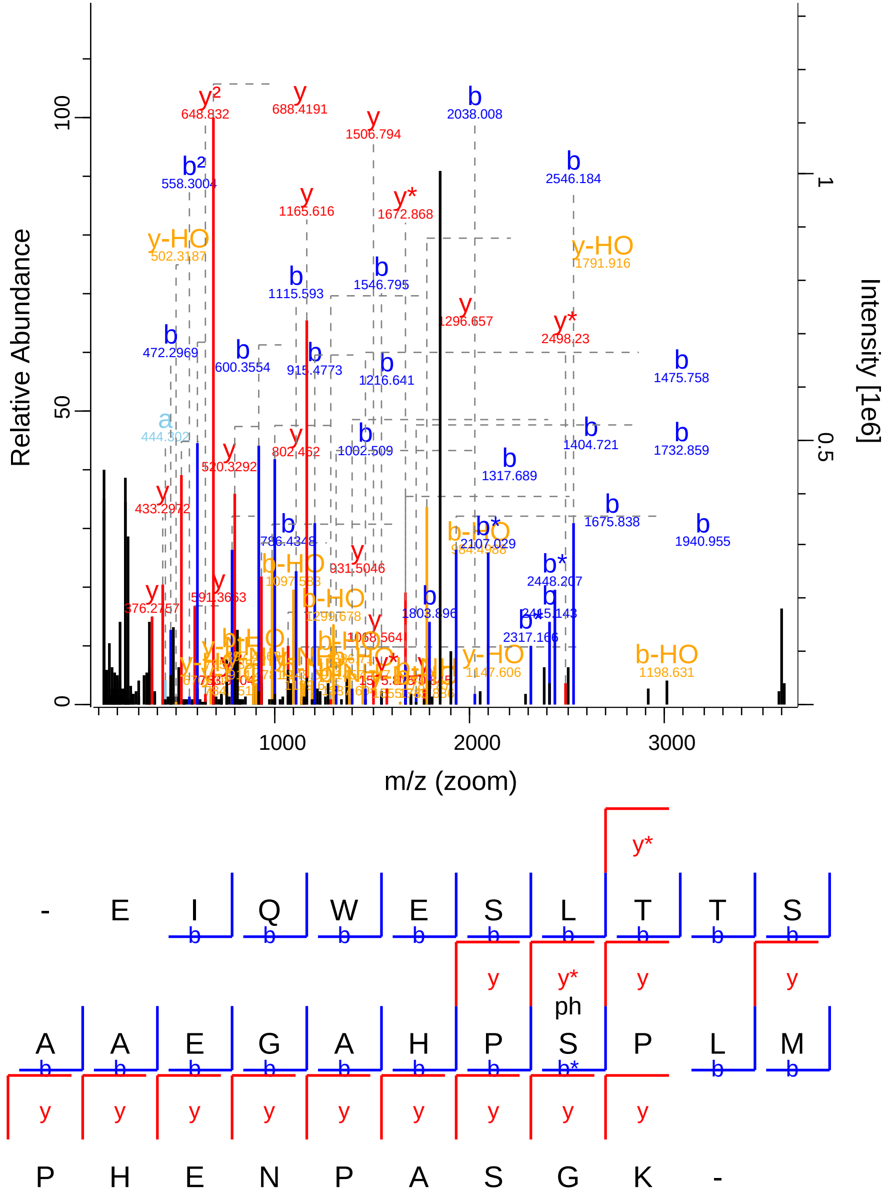

Raw File	Scan	Method	Score	m/z	Gene names
RO-QC-160121_ZackD_SS_Set_02_Fractic	32950	FTMS; HC	190.55	1153.15	AMPD2

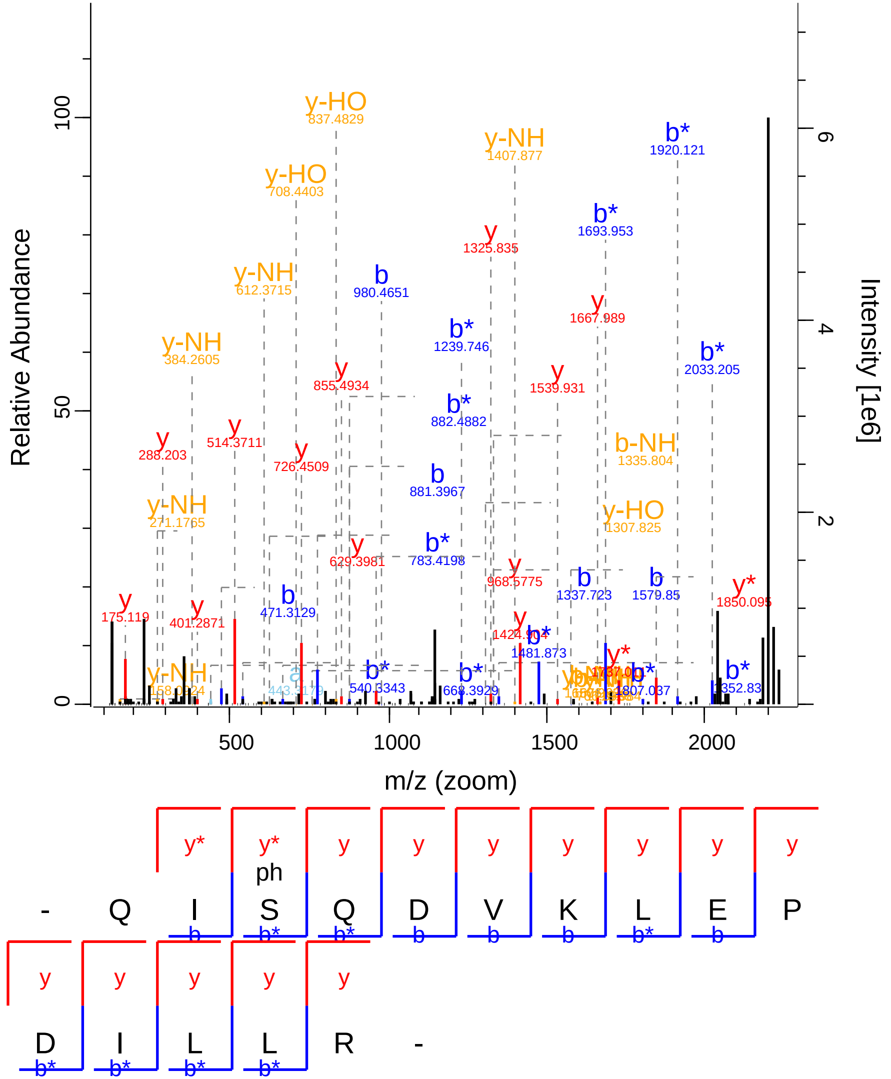

Raw File	Scan	Method	Score	m/z	Gene names
RO-QC-160121_ZackD_SS_Set_02_Fractio	33338	FTMS; HC	49.63	721.38	COX7A2

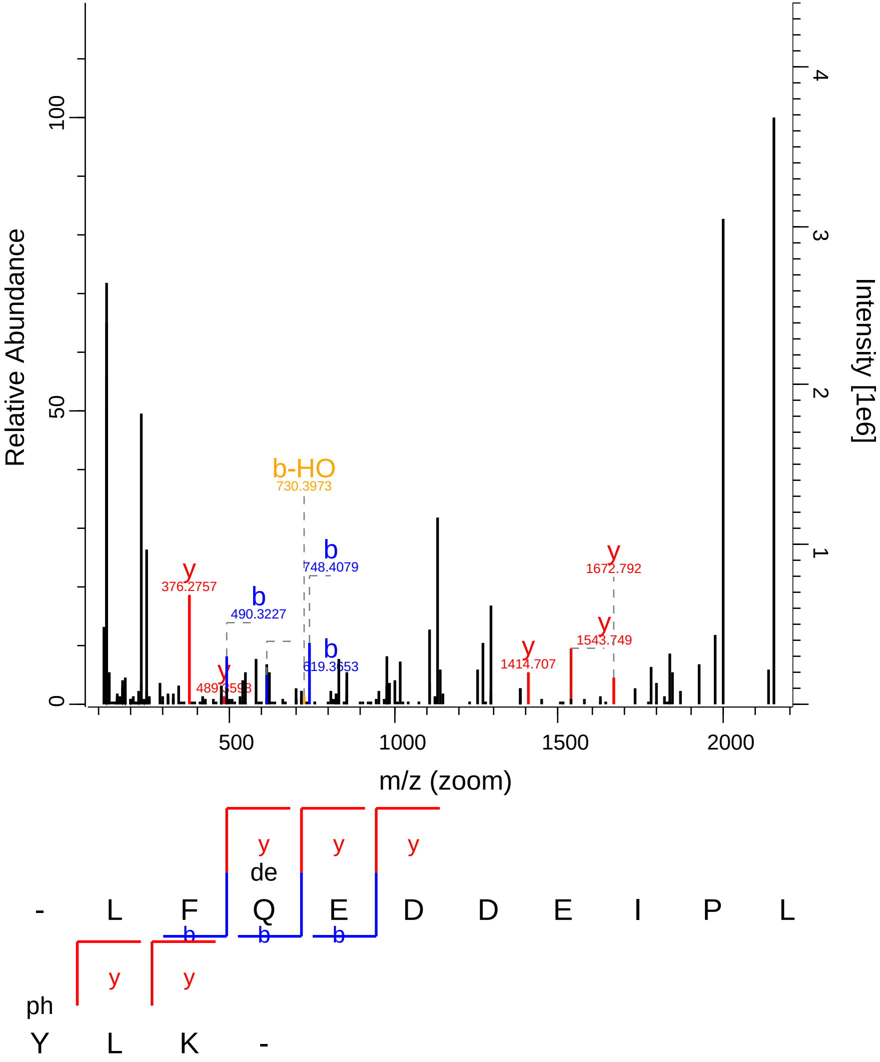

Raw File	Scan	Method	Score	m/z	Gene names
RO-QC-160121_ZackD_SS_Set_02_Fractio	33613	FTMS; HC	48.29	975.53	NCOA1

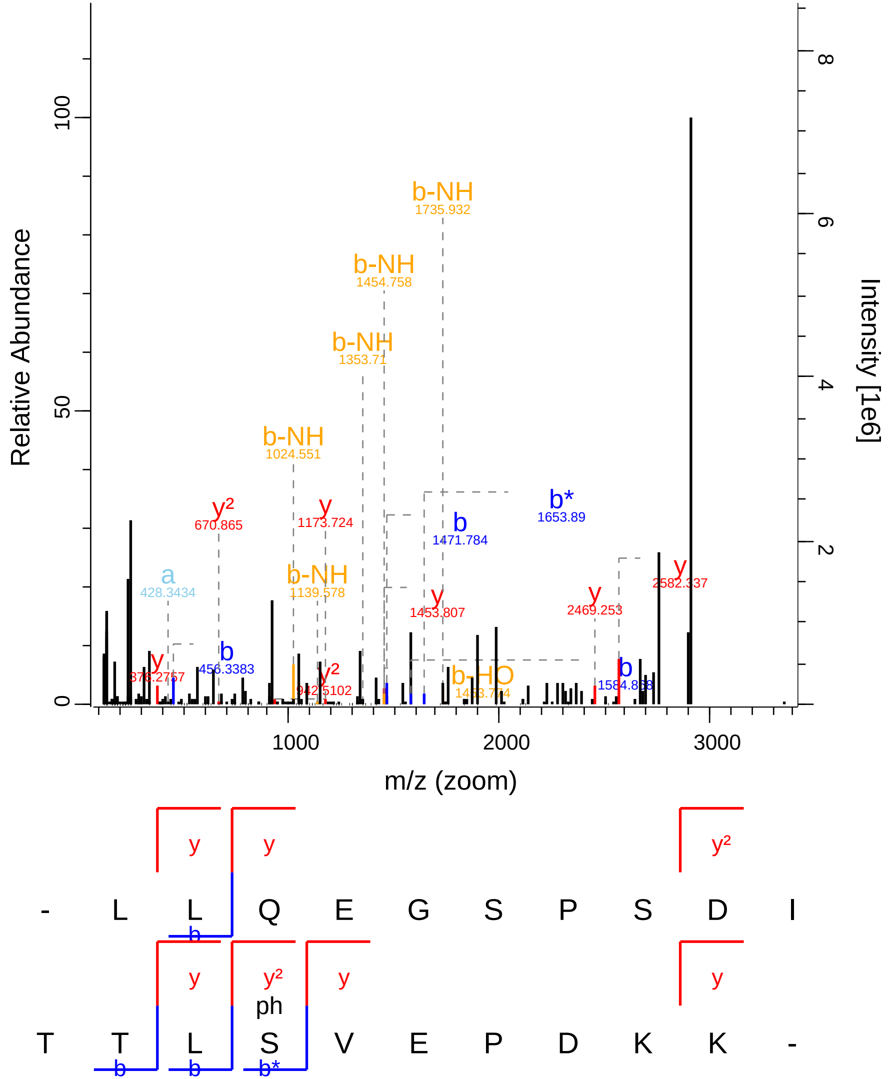

Raw File	Scan	Method	Score	m/z	Gene names
RO-QC-160121_ZackD_SS_Set_02_Fractio	33685	FTMS; HC	198.85	891.67	TOP2B

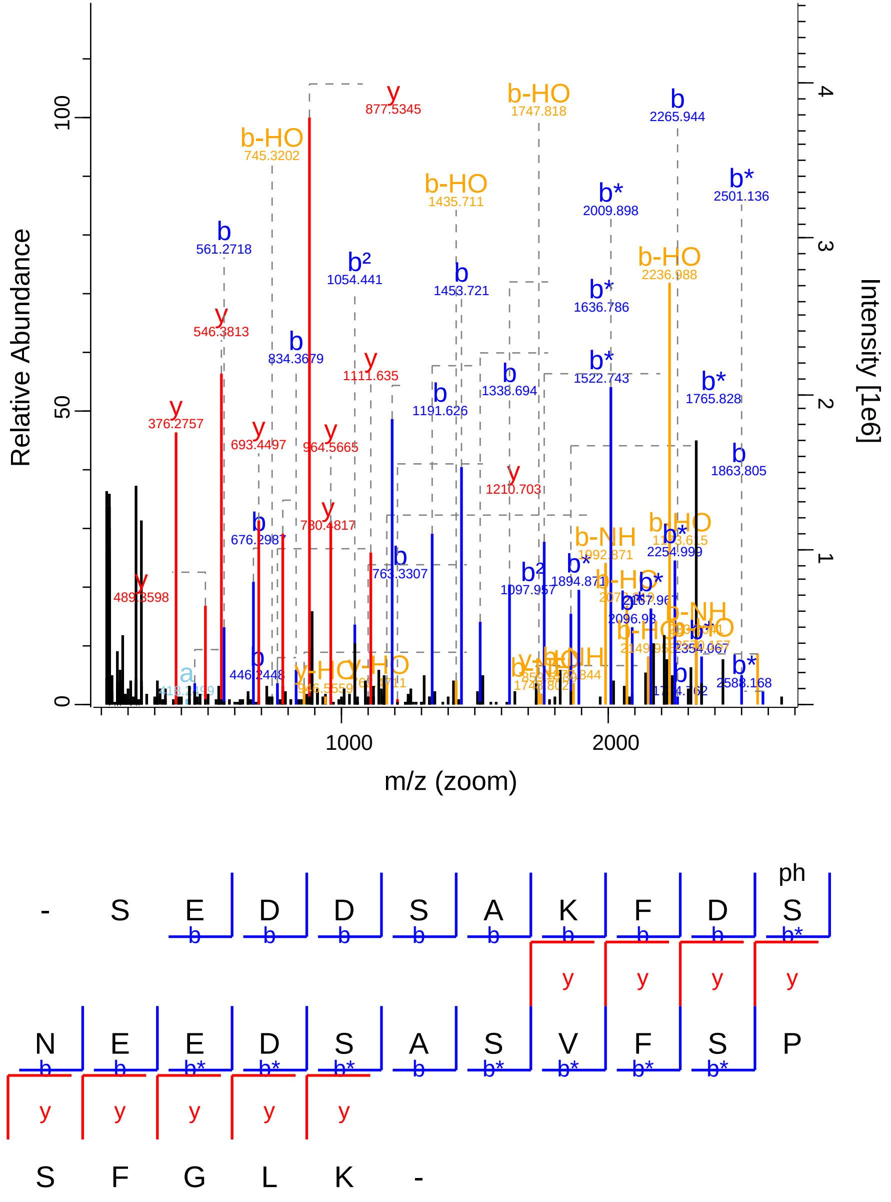

Raw File	Scan	Method	Score	m/z	Gene names
RO-QC-160121_ZackD_SS_Set_02_Fractic	34186	FTMS; HC	50.21	1155.63	EBF2

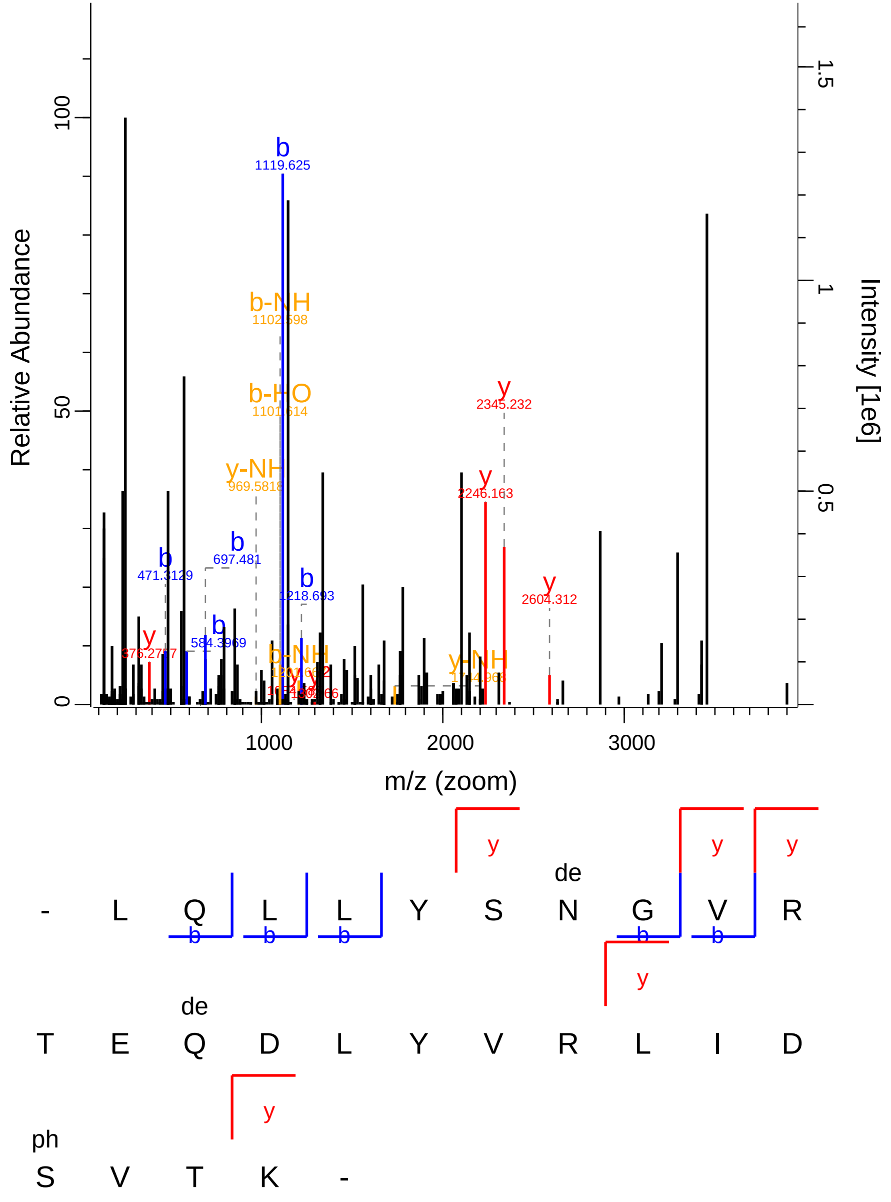

Raw File	Scan	Method	Score	m/z	Gene names
RO-QC-160121_ZackD_SS_Set_02_Fractic	35053	FTMS; HC	64.44	1248.11	KIAA1211L

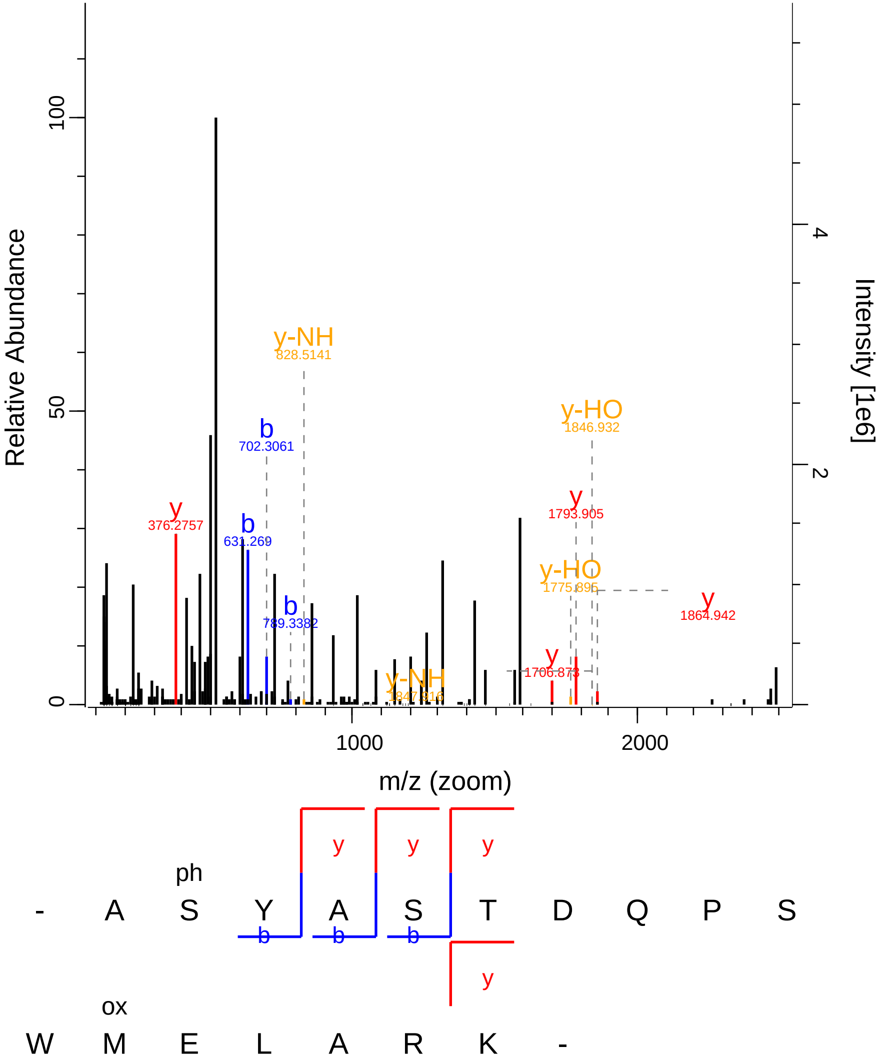

Raw File	Scan	Method	Score	m/z	Gene names
RO-QC-160121_ZackD_SS_Set_02_Fractio	36947	FTMS; HC	40.4	1091.9	MPHOSPH9

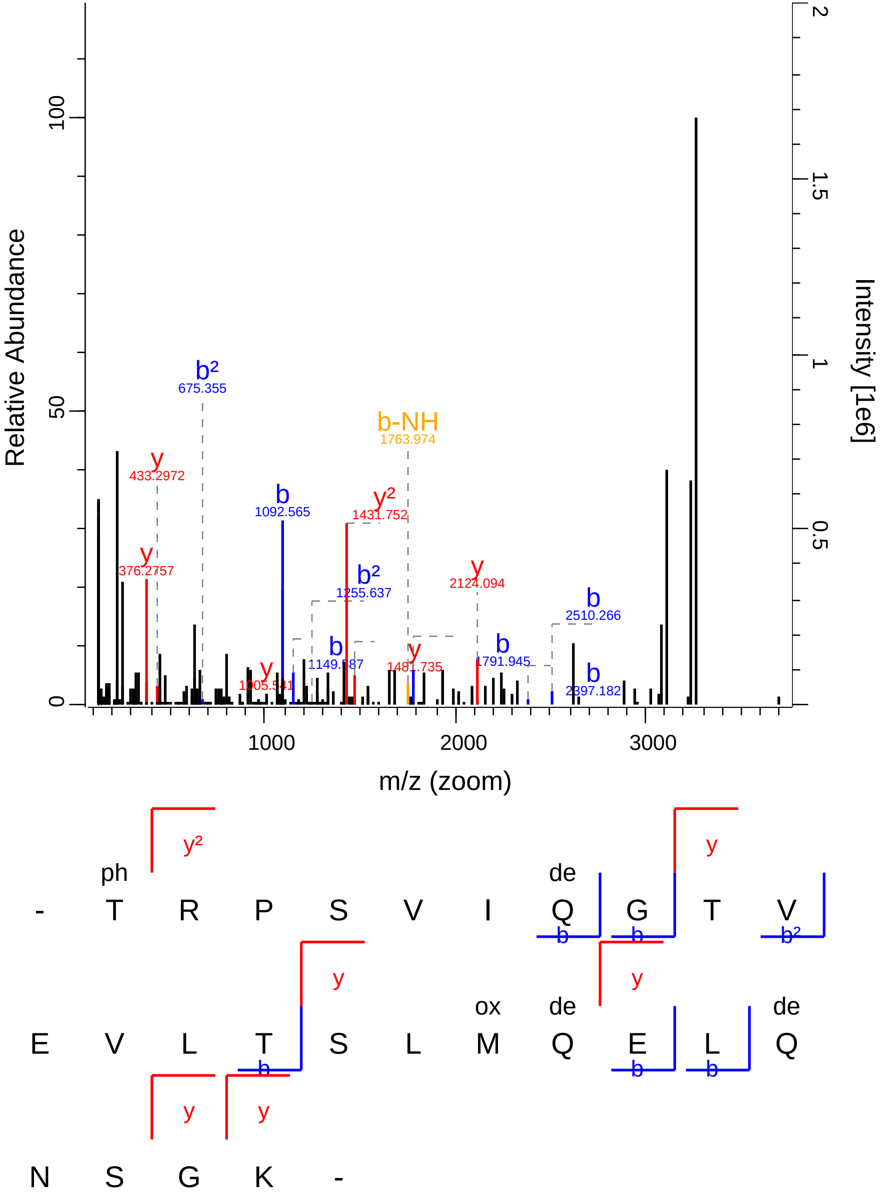

Raw File	Scan	Method	Score	m/z	Gene names
RO-QC-160121_ZackD_SS_Set_02_Fractio	37898	FTMS; HC	102.07	952.73	IGFBP5

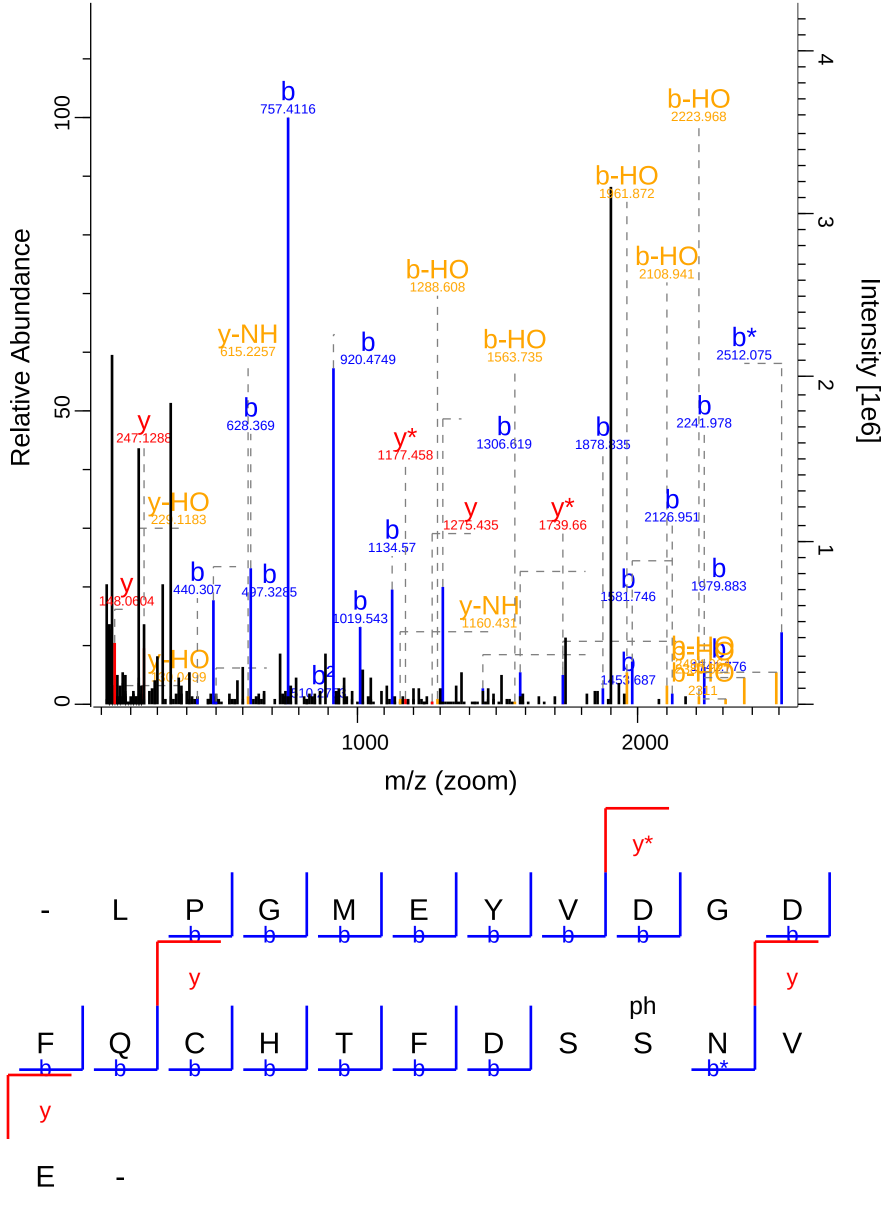

Raw File	Scan	Method	Score	m/z	Gene names
RO-QC-160121_ZackD_SS_Set_02_Fractio	38064	FTMS; HC	132.74	1429.1	IGFBP5

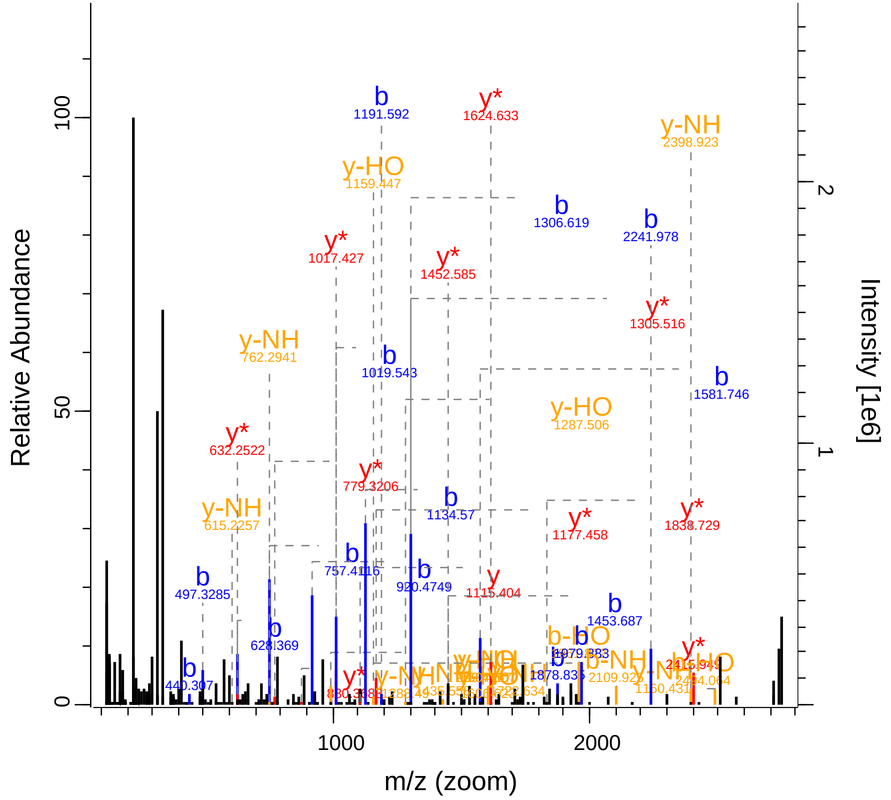

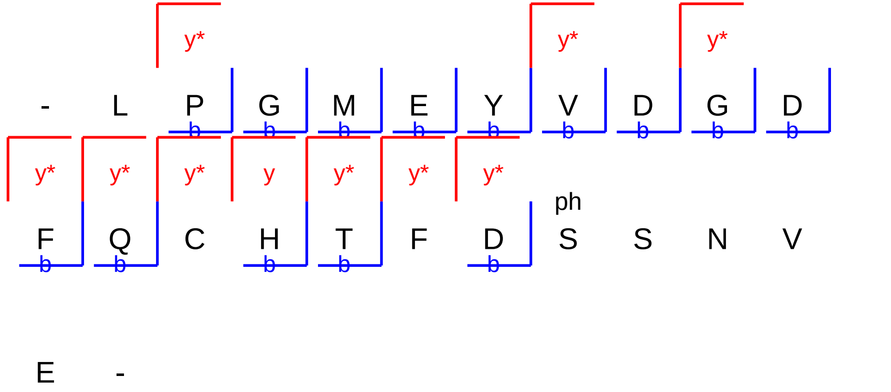

Raw File	Scan	Method	Score	m/z	Gene names
RO-QC-160121_ZackD_SS_Set_02_Fractic	38515	FTMS; HC	43.81	1431.71	RAG1

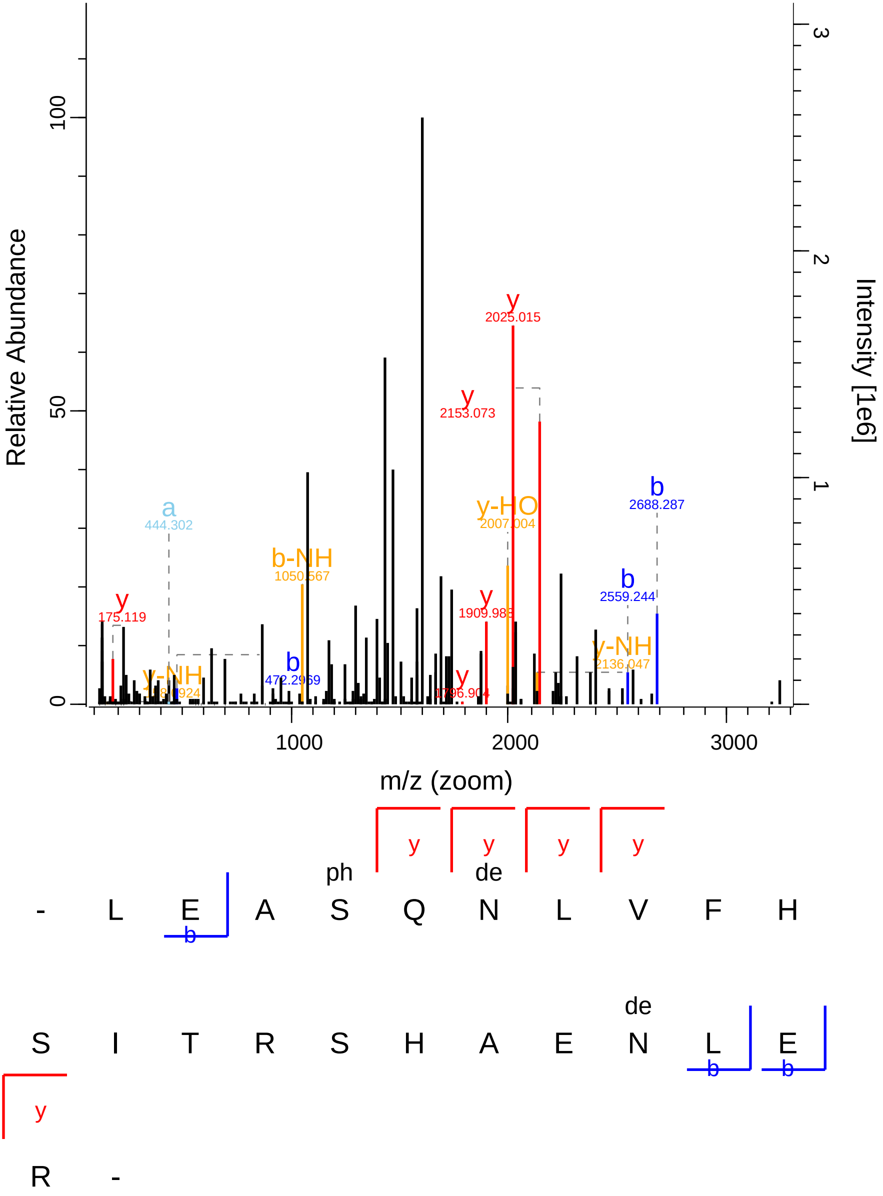

Raw File	Scan	Method	Score	m/z	Gene names
RO-QC-160121_ZackD_SS_Set_02_Fractic	39132	FTMS; HC	55.35	1290.22	SUPT6H

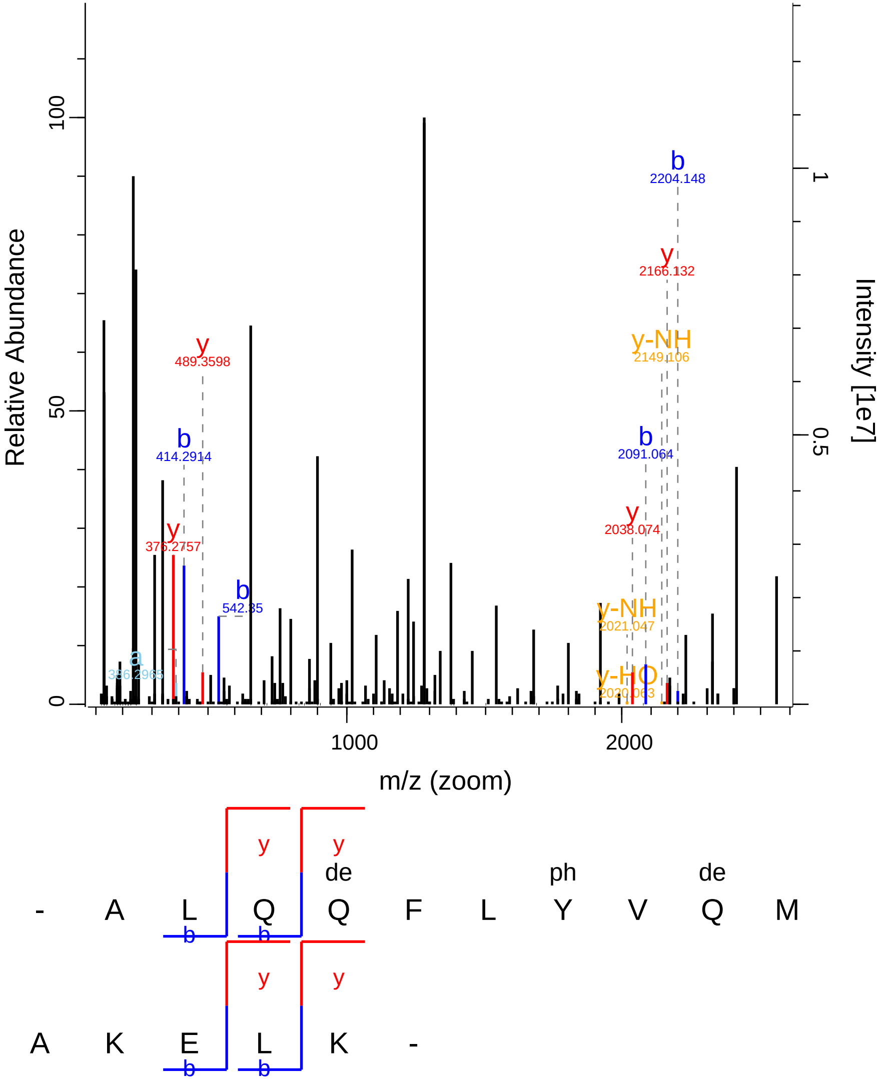

Raw File	Scar	Method	Scor	m/z	Gene names
RO-QC-160121_ZackD_SS_Se	393[illegible]	FTMS[illegible]	82.[illegible]	1015.[illegible]	RBMY1F;RBMY1A1;RBMY1D;RBMY

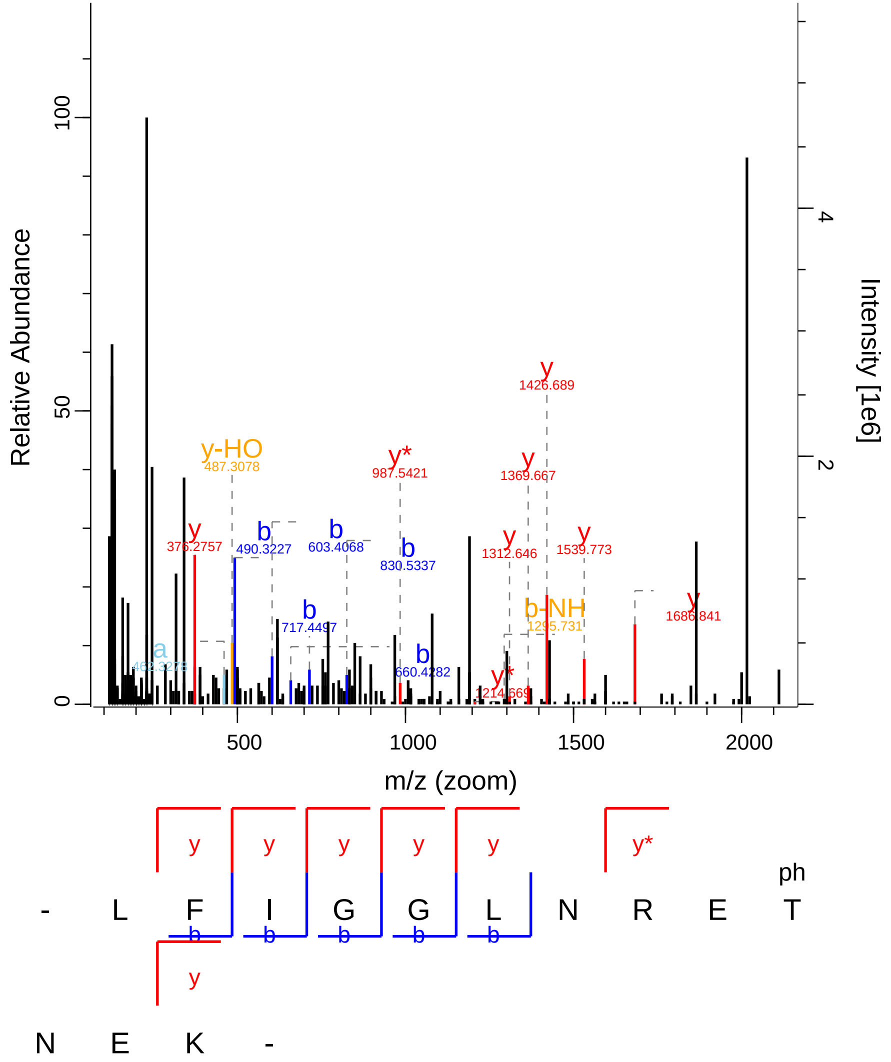

Raw File	Scan	Method	Score	m/z	Gene names
RO-QC-160121_ZackD_SS_Set_02_Fractio	40642	FTMS; HC	43.03	799.92	CENPF

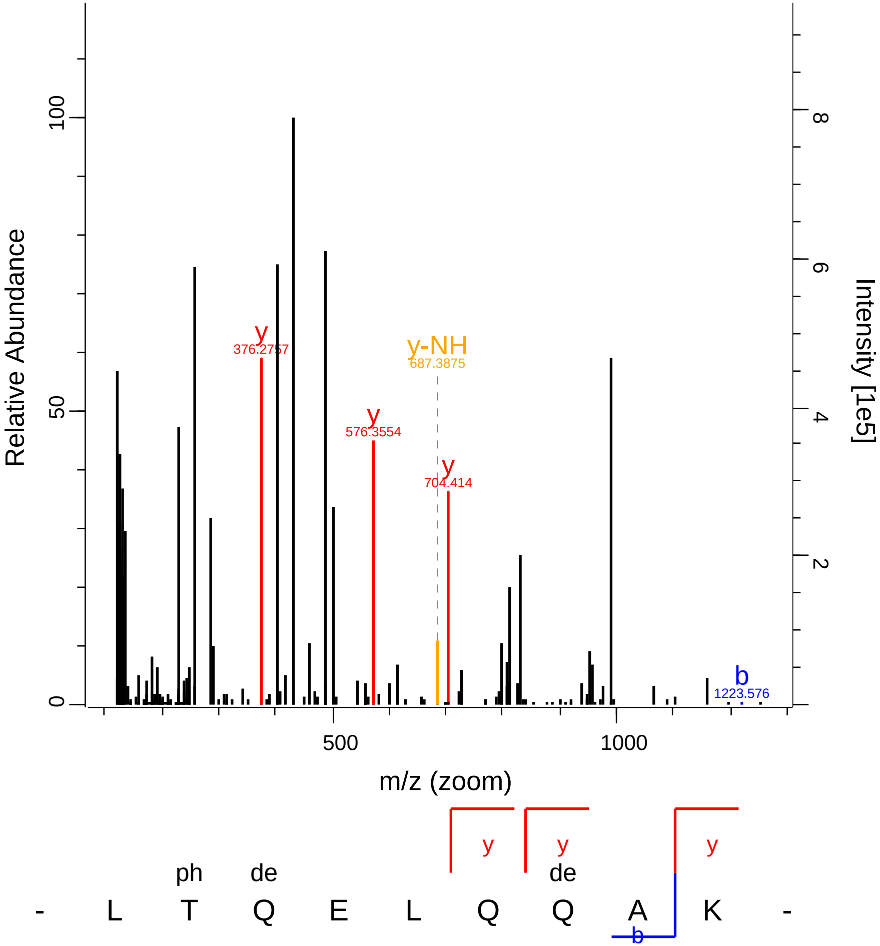

Raw File	Scan	Method	Score	m/z	Gene names
RO-QC-160121_ZackD_SS_Set_02_Fractior	5730	FTMS; HCI	57.8	495.78	NIPBL

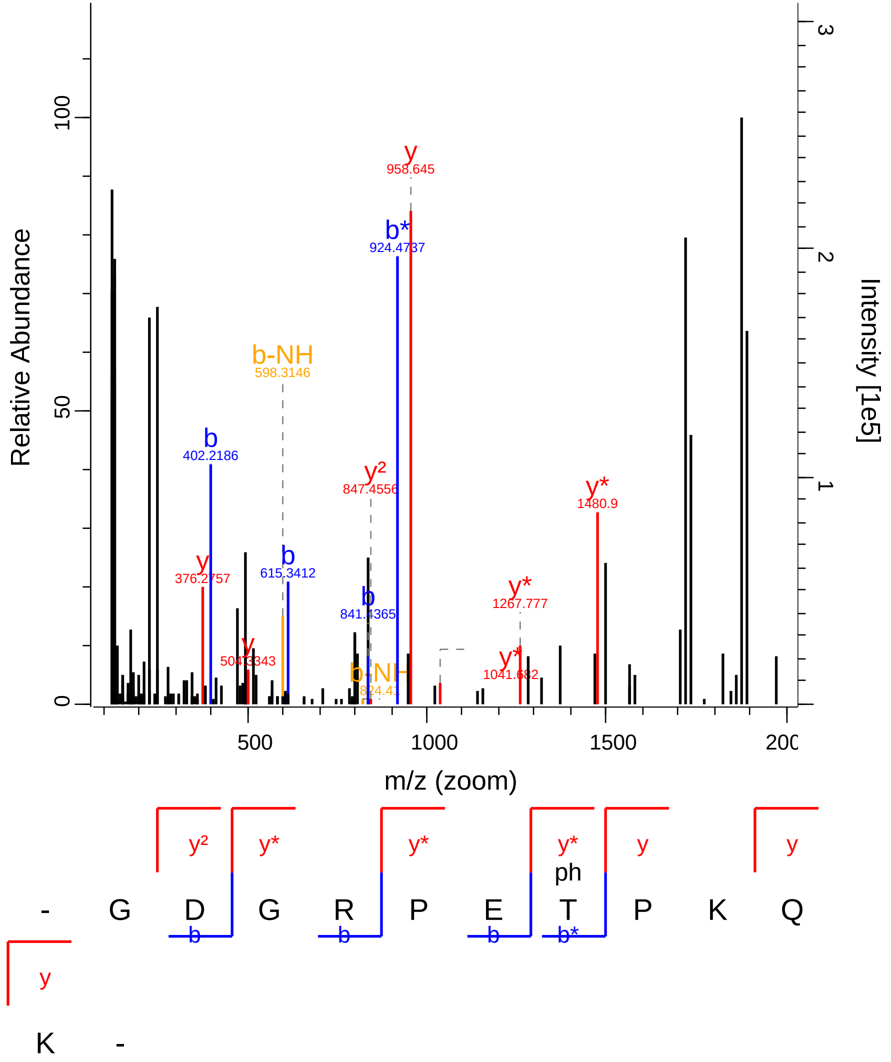

Raw File	Scan	Method	Score	m/z	Gene names
RO-QC-160121_ZackD_SS_Set_02_Fractior	5805	FTMS; HCI	60.63	506.78	NIPBL

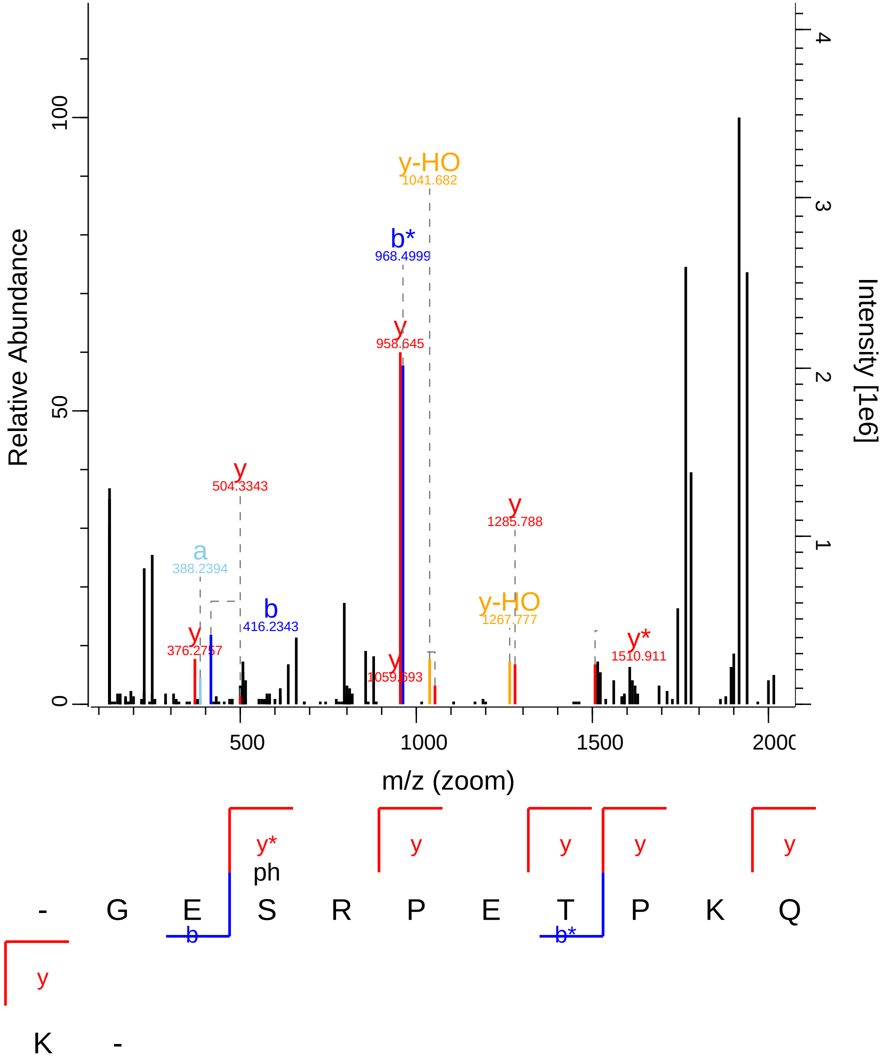

Raw File	Scan	Method	Score	m/z	Gene names
RO-QC-160121_ZackD_SS_Set_02_Fractior	5842	FTMS; HC	120.87	811.94	TCOF1

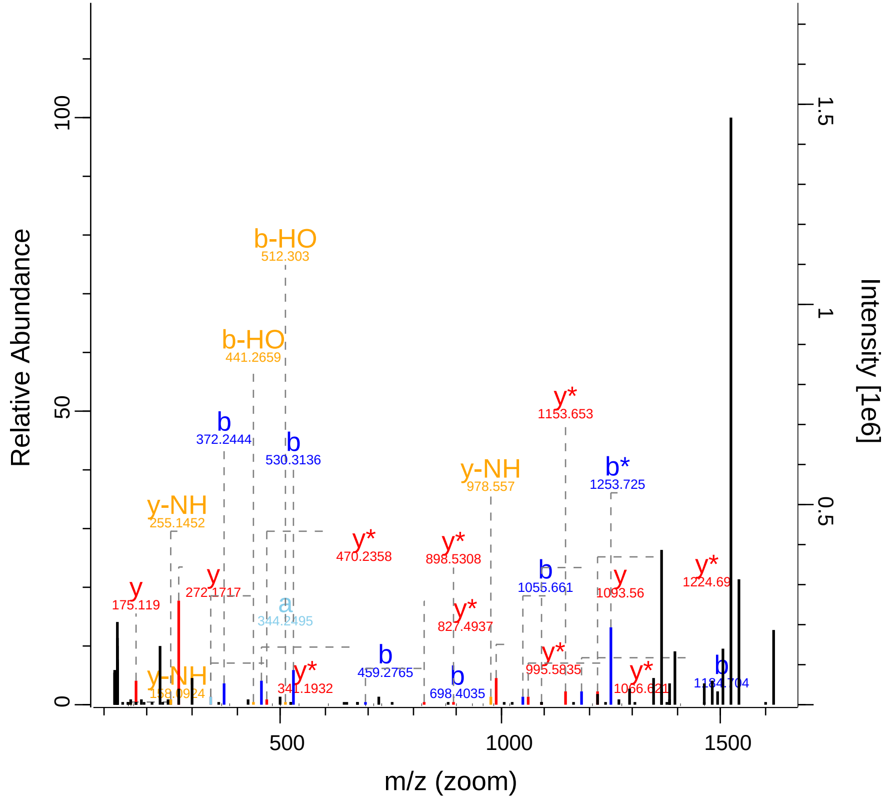

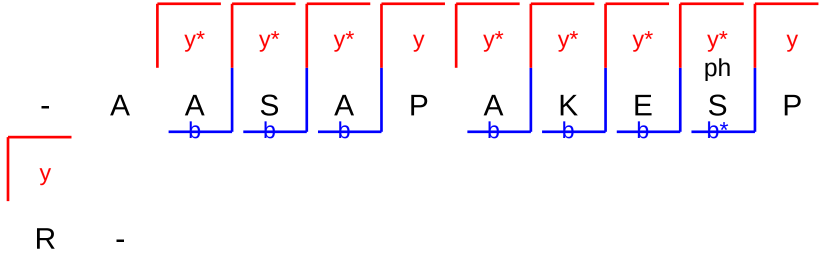

Raw File	Scan	Method	Score	m/z	Gene names
RO-QC-160121_ZackD_SS_Set_02_Fractior	5922	FTMS; HCI	61.41	677.69	U2SURP

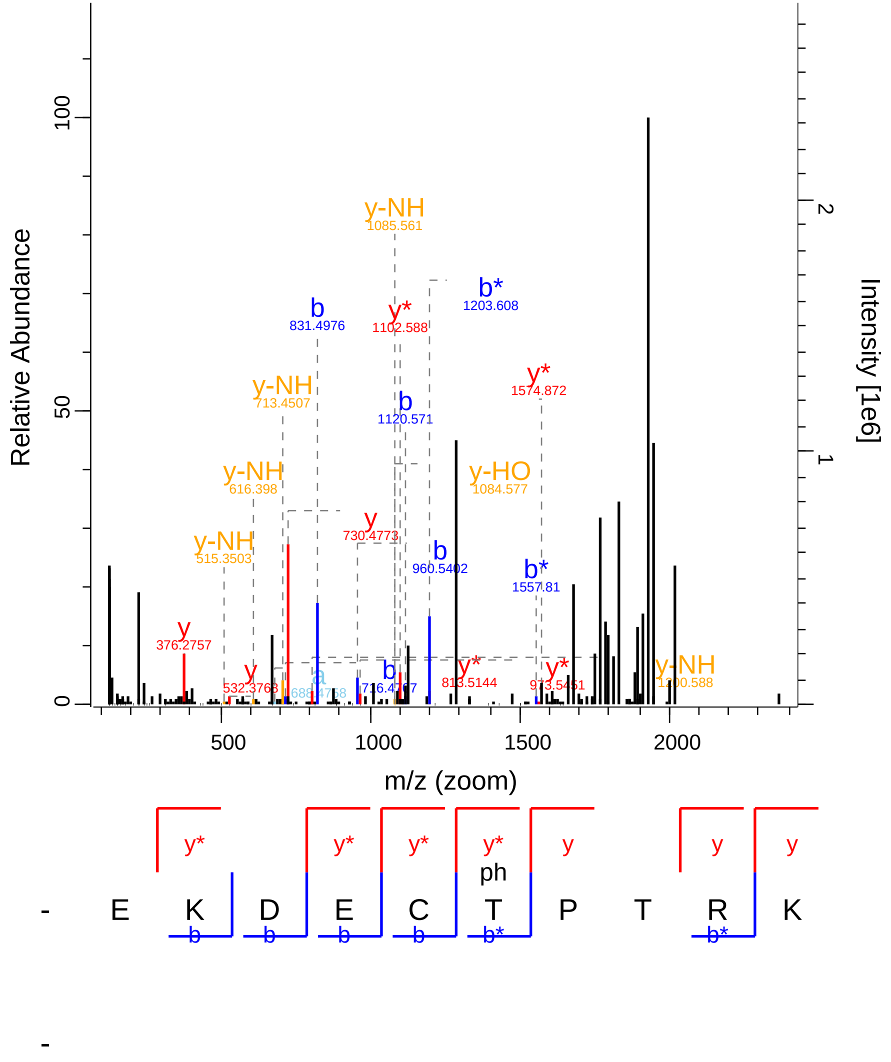

Raw File	Scan	Method	Score	m/z	Gene names
RO-QC-160121_ZackD_SS_Set_02_Fractior	6120	FTMS; HCI	79.01	677.64	ADD1

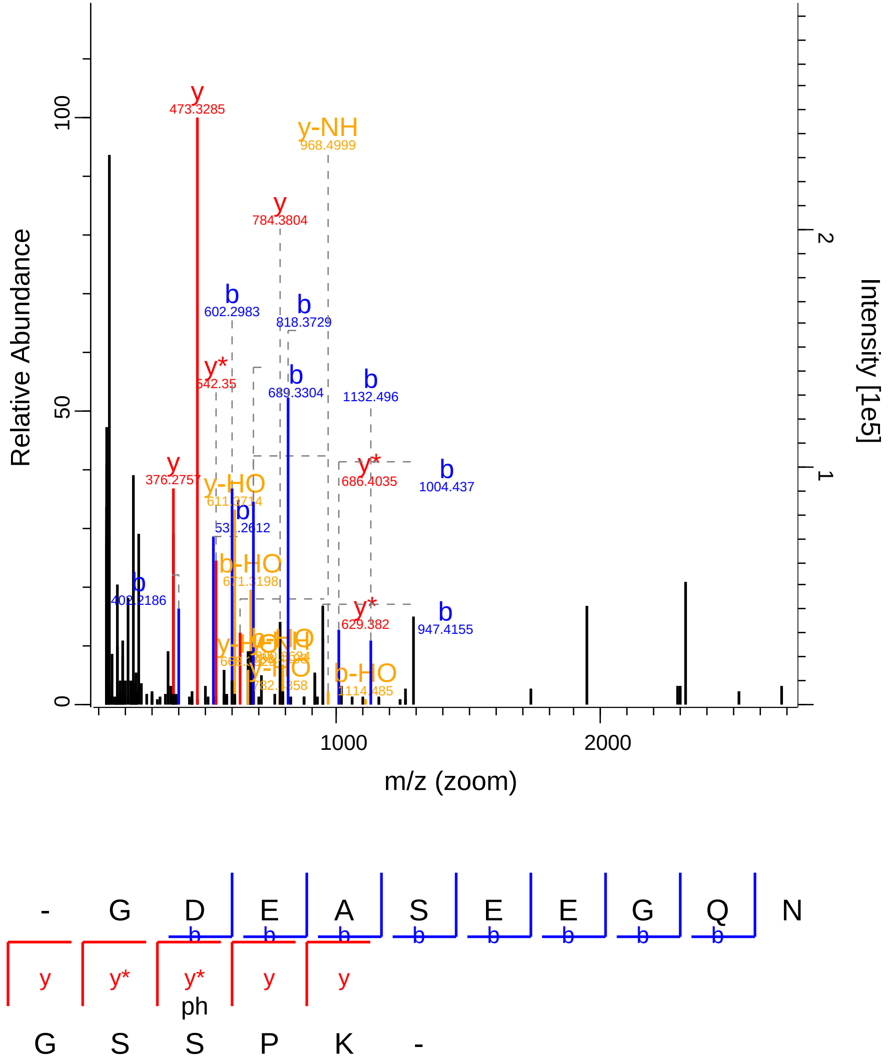

Raw File	Scan	Method	Score	m/z	Gene names
RO-QC-160121_ZackD_SS_Set_02_Fractio[illegible]	6683	FTMS; HC	106.17	854.42	BMI1

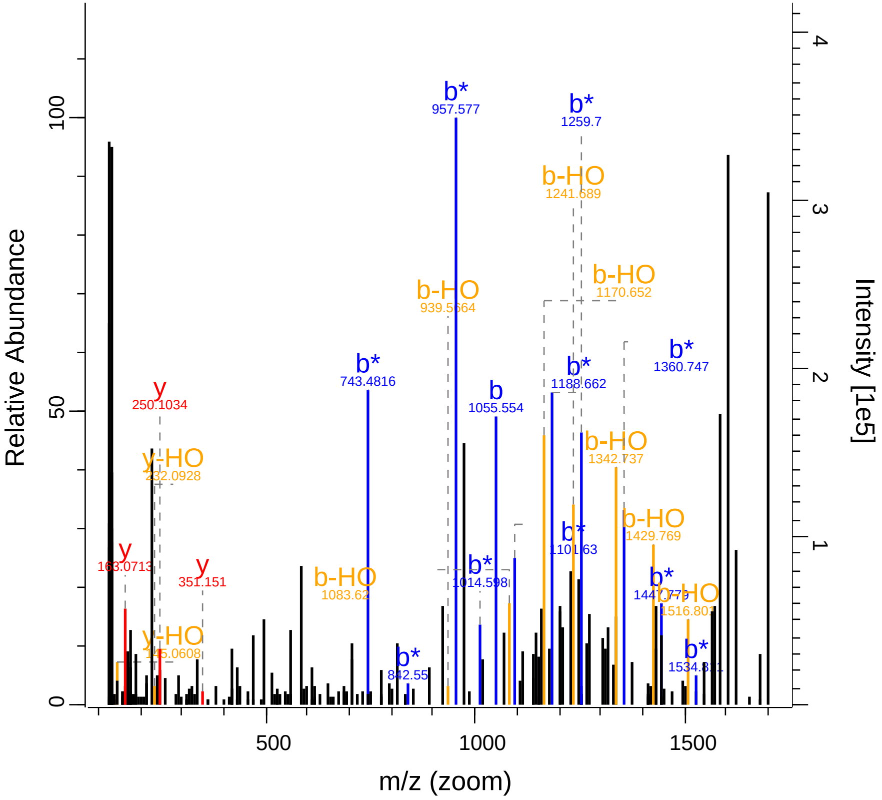

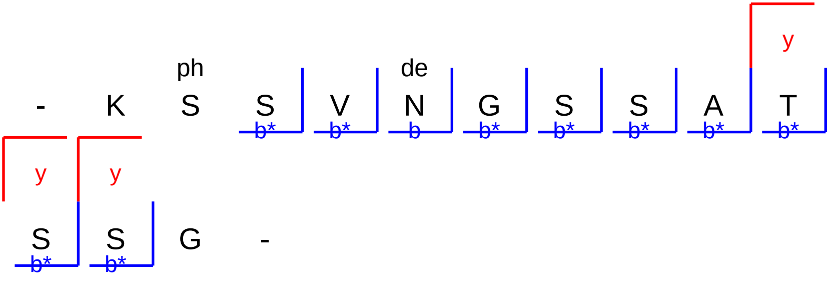

Raw File	Scan	Method	Score	m/z	Gene names
RO-QC-160121_ZackD_SS_Set_02_Fractior	6740	FTMS; HCI	44.76	456.54	HIST1H1E

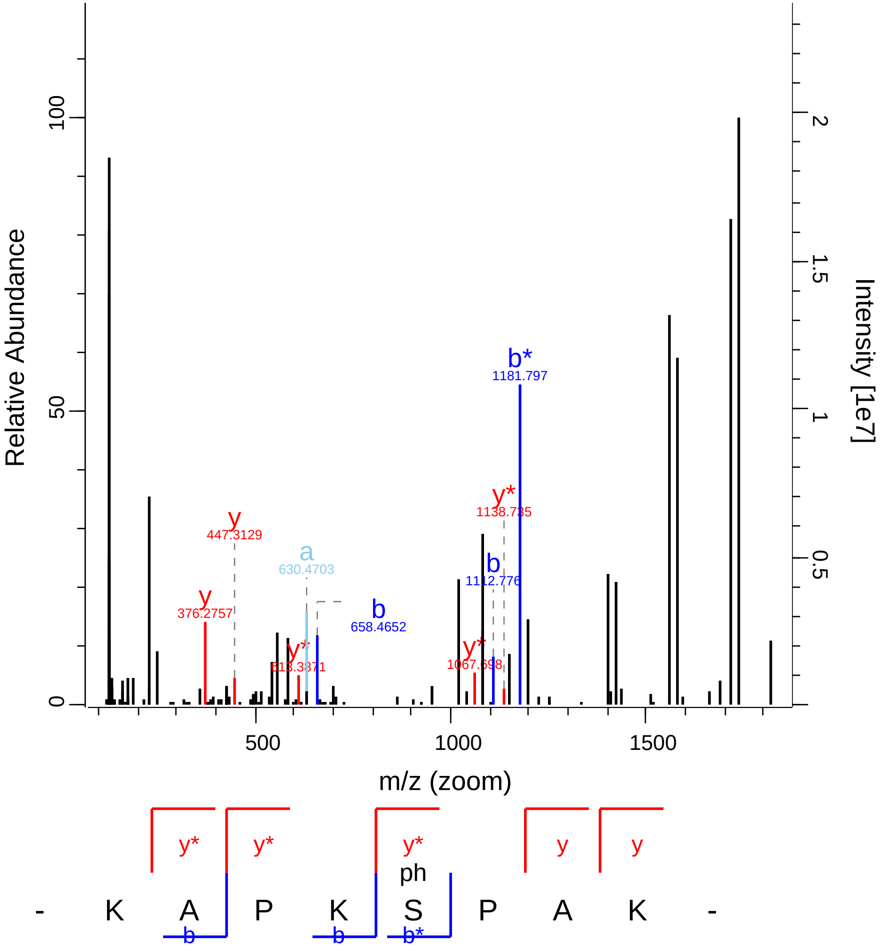

Raw File	Scan	Method	Score	m/z	Gene names
RO-QC-160121_ZackD_SS_Set_02_Fractior	7846	FTMS; HC	110.97	691.37	API5

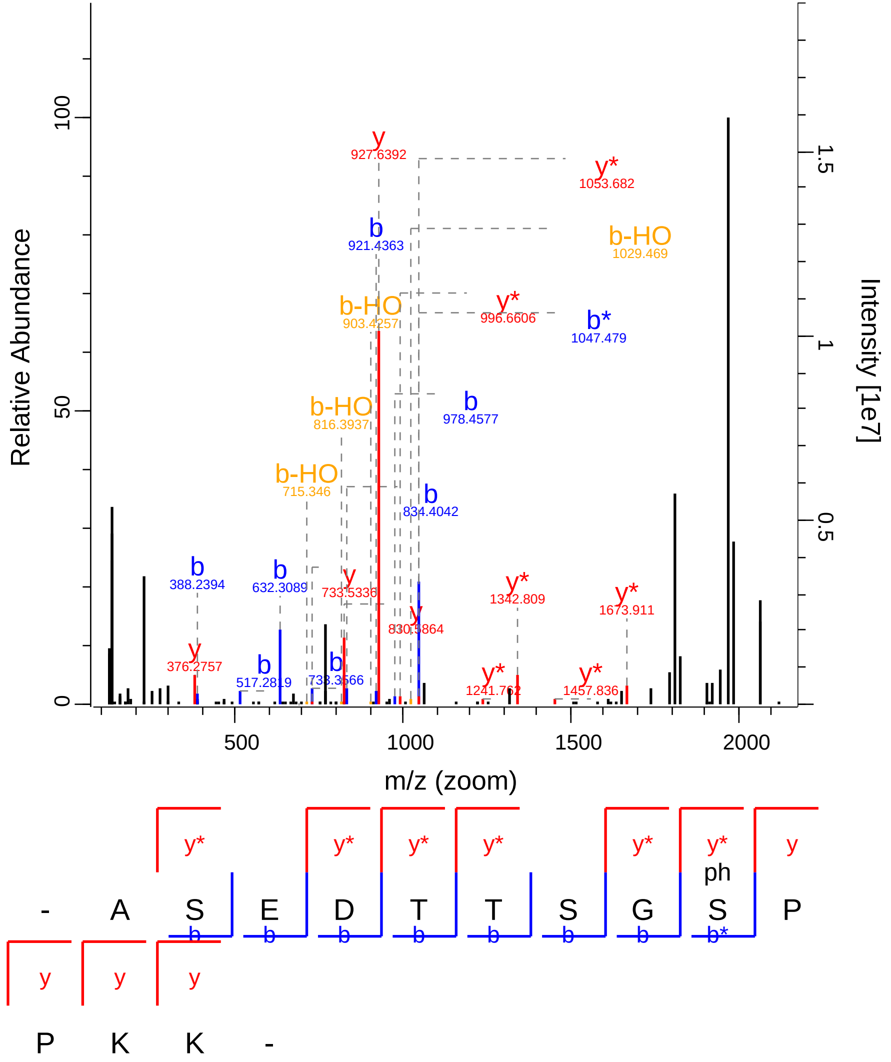

Raw File	Scan	Method	Score	m/z	Gene names
RO-QC-160121_ZackD_SS_Set_02_Fractio	7878	FTMS; HC	106.26	1037.05	API5

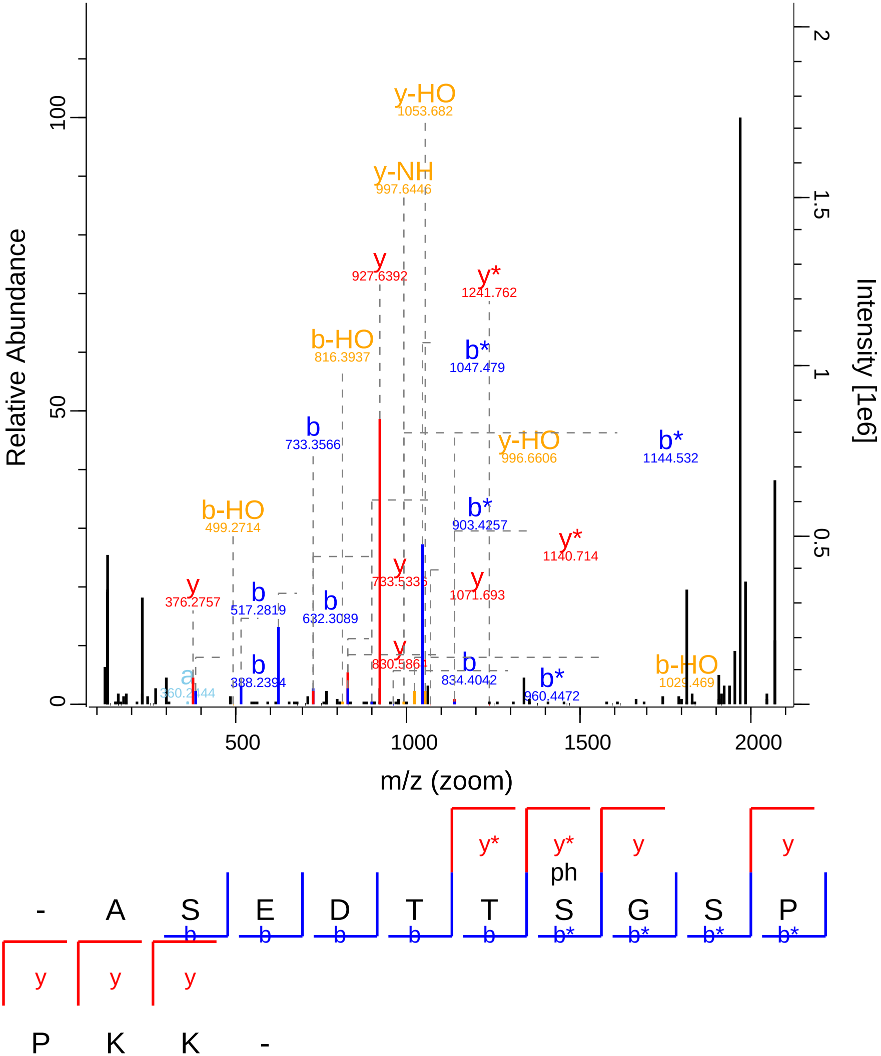

Raw File	Scan	Method	Score	m/z	Gene names
RO-QC-160121_ZackD_SS_Set_02_Fractior	8656	FTMS; HCI	67.96	478.77	LMOD1

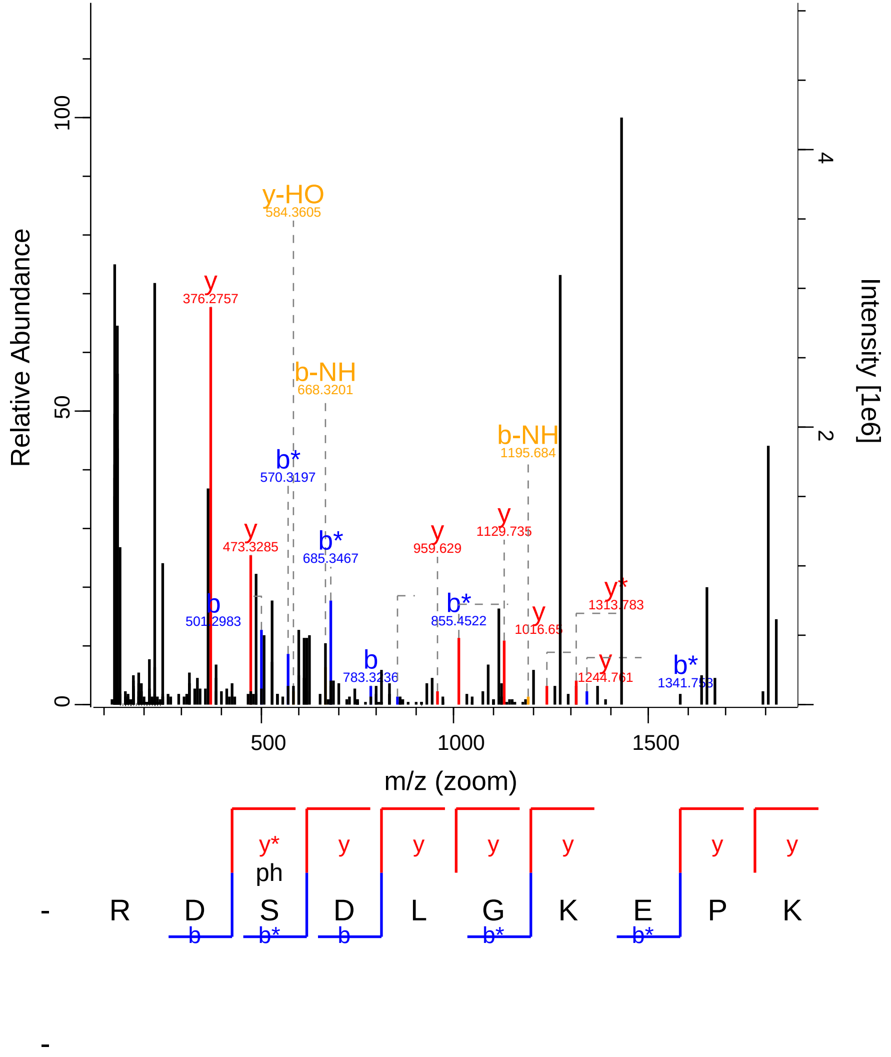

Raw File	Scan	Method	Score	m/z	Gene names
RO-QC-160121_ZackD_SS_Set_02_Fractior	9734	FTMS; HCI	67.99	663.38	RBM20

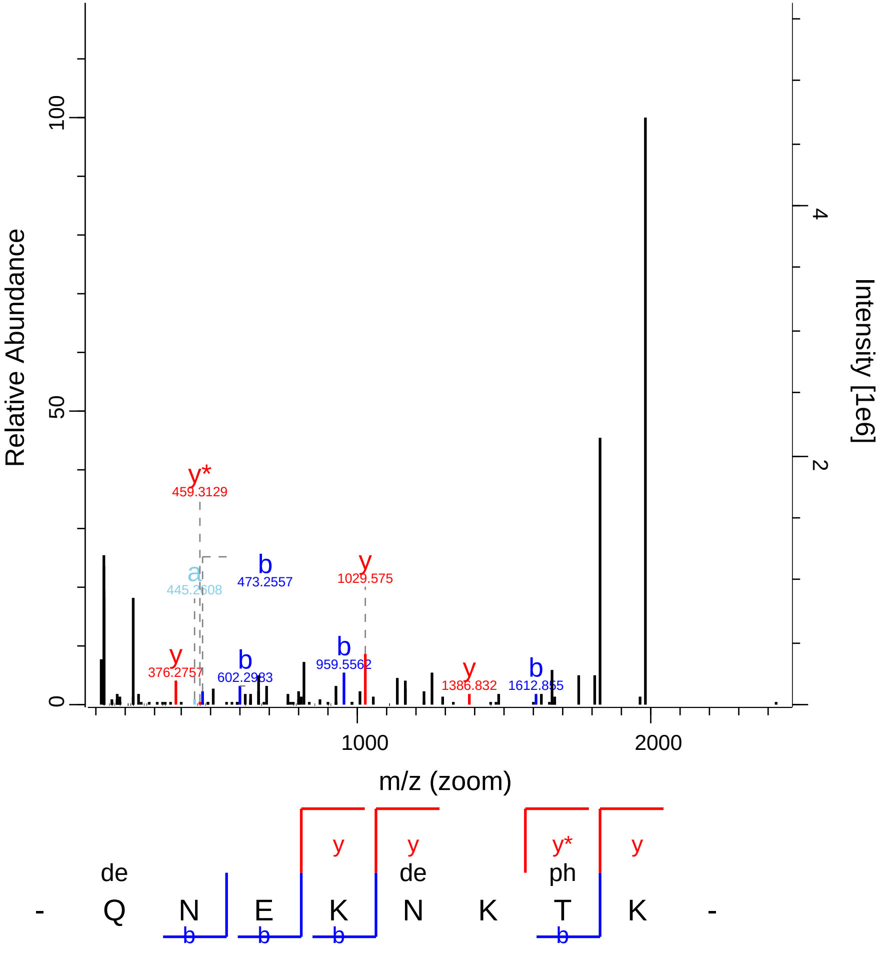

Raw File	Scan	Method	Score	m/z	Gene names
RO-QC-160121_ZackD_SS_Set_02_Fractio	11038	FTMS; HC	140.88	929.22	RAD23B

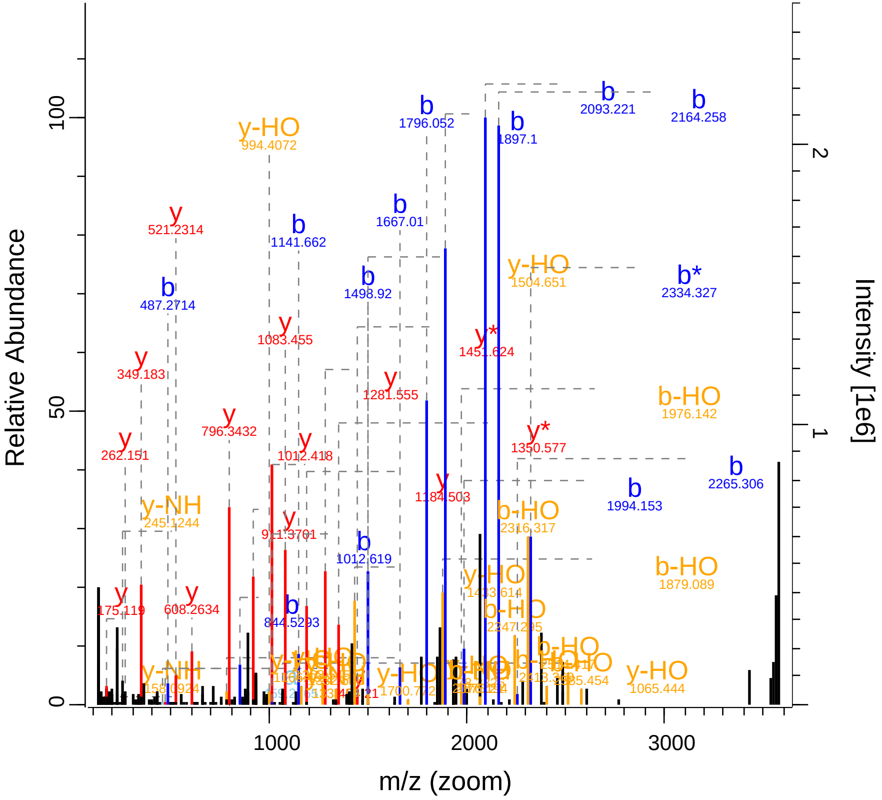

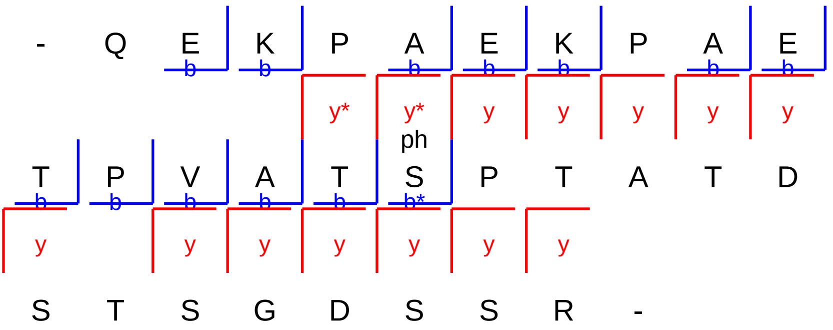

Raw File	Scan	Method	Score	m/z	Gene names
RO-QC-160121_ZackD_SS_Set_02_Fractio	11232	FTMS; HC	111.86	823.96	CCDC86

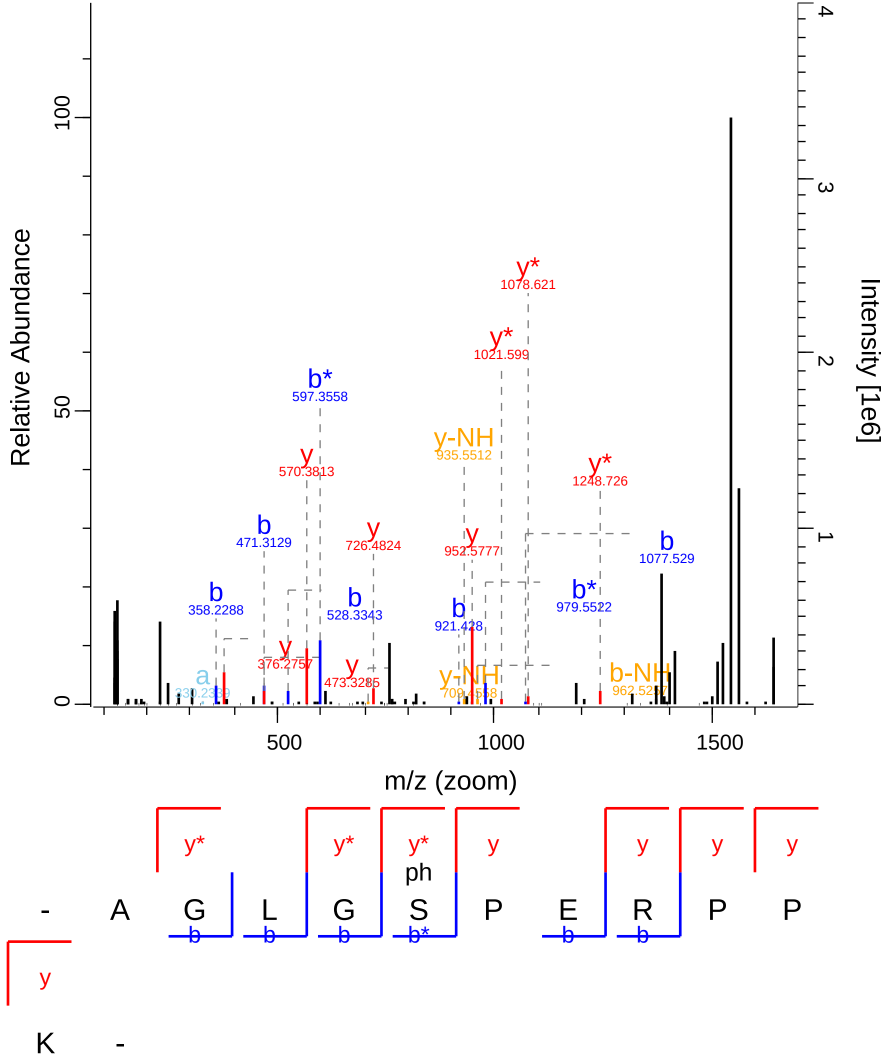

Raw File	Scan	Method	Score	m/z	Gene names
RO-QC-160121_ZackD_SS_Set_02_Fractio	11817	FTMS; HC	137.64	751.63	MYH9

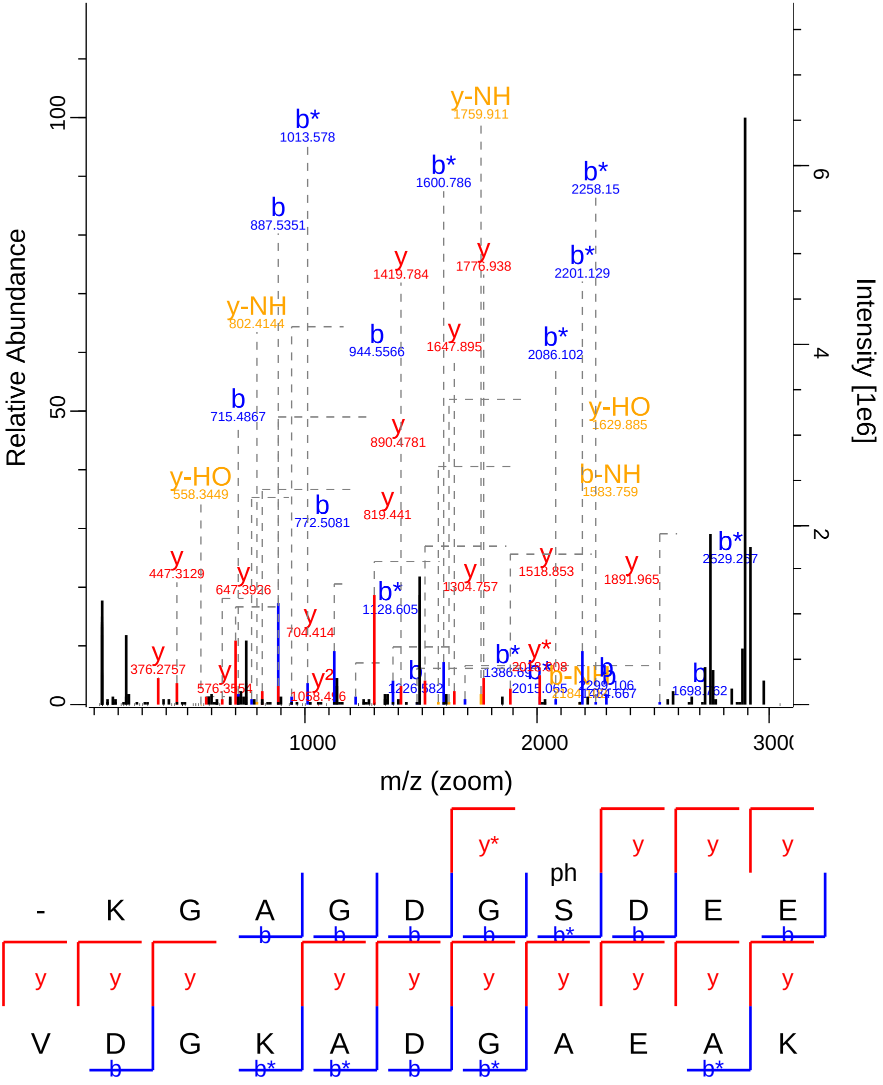

-

Raw File	Scan	Method	Score	m/z	Gene names
RO-QC-160121_ZackD_SS_Set_02_Fractio	12083	FTMS; HC	77.02	651.08	MATR3

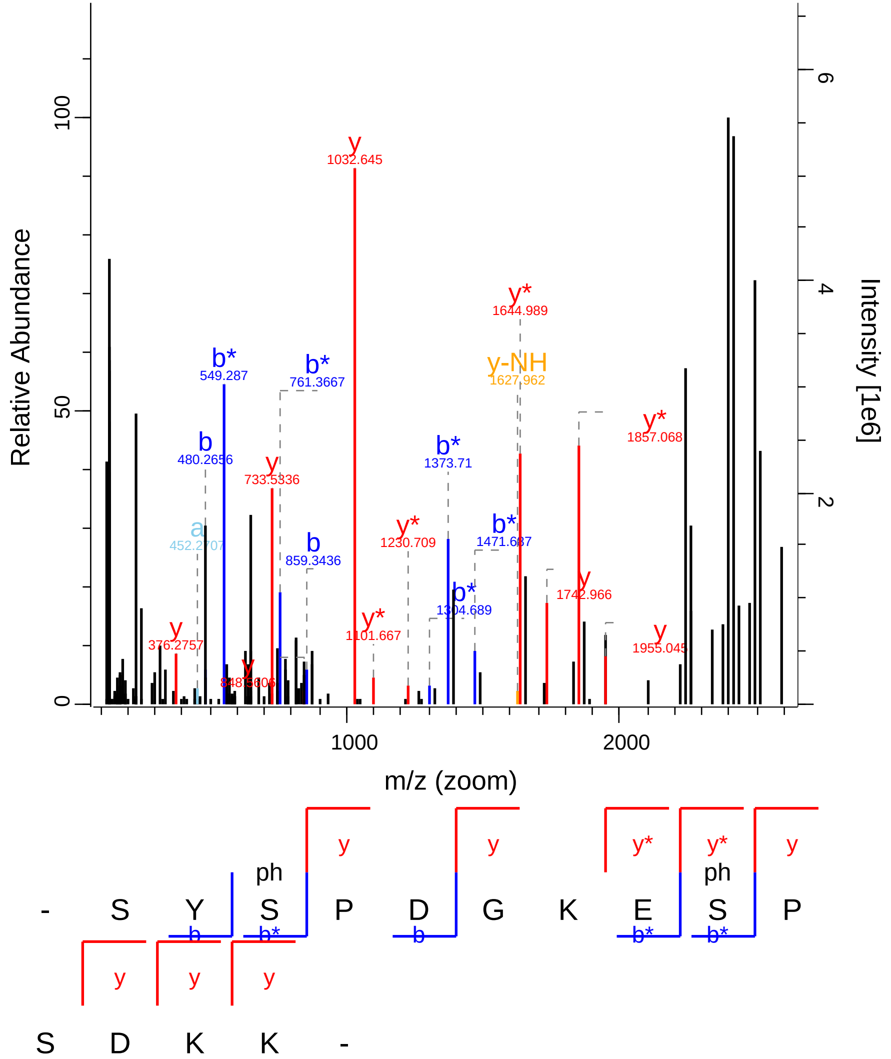

Raw File	Scan	Method	Score	m/z	Gene names
RO-QC-160121_ZackD_SS_Set_02_Fractio	12133	FTMS; HC	104.44	737.06	BCLAF1

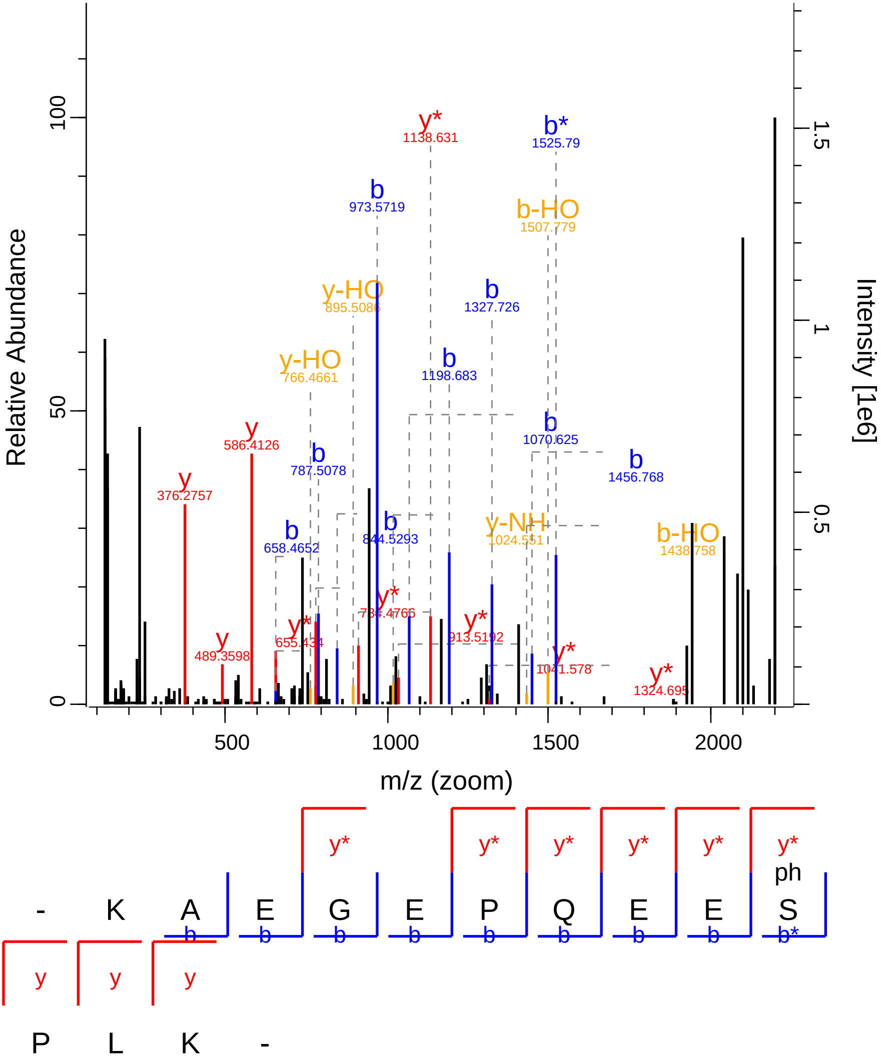

Raw File	Scan	Method	Score	m/z	Gene names
RO-QC-160121_ZackD_SS_Set_02_Fractio	12266	FTMS; HC	124.84	747.74	CALD1

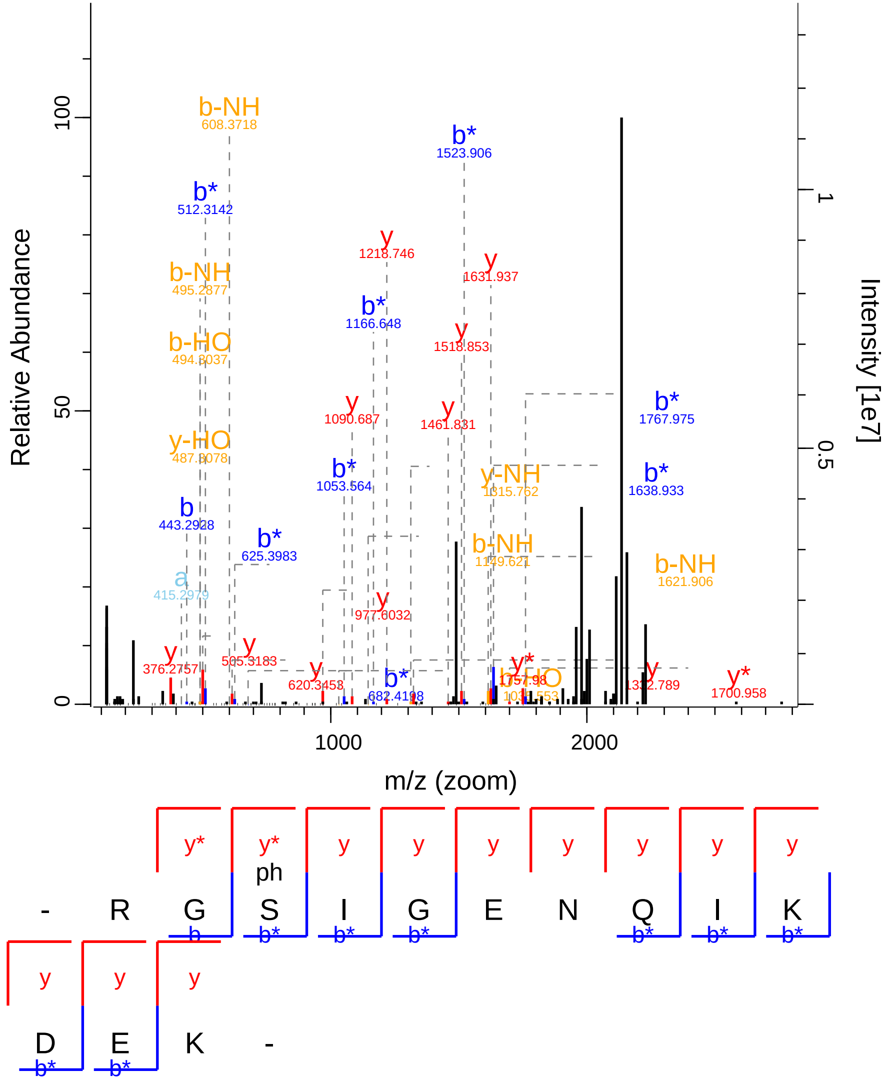

Raw File	Scan	Method	Score	m/z	Gene names
RO-QC-160121_ZackD_SS_Set_02_Fractio	12414	FTMS; HC	71.87	763.43	HNRNPU

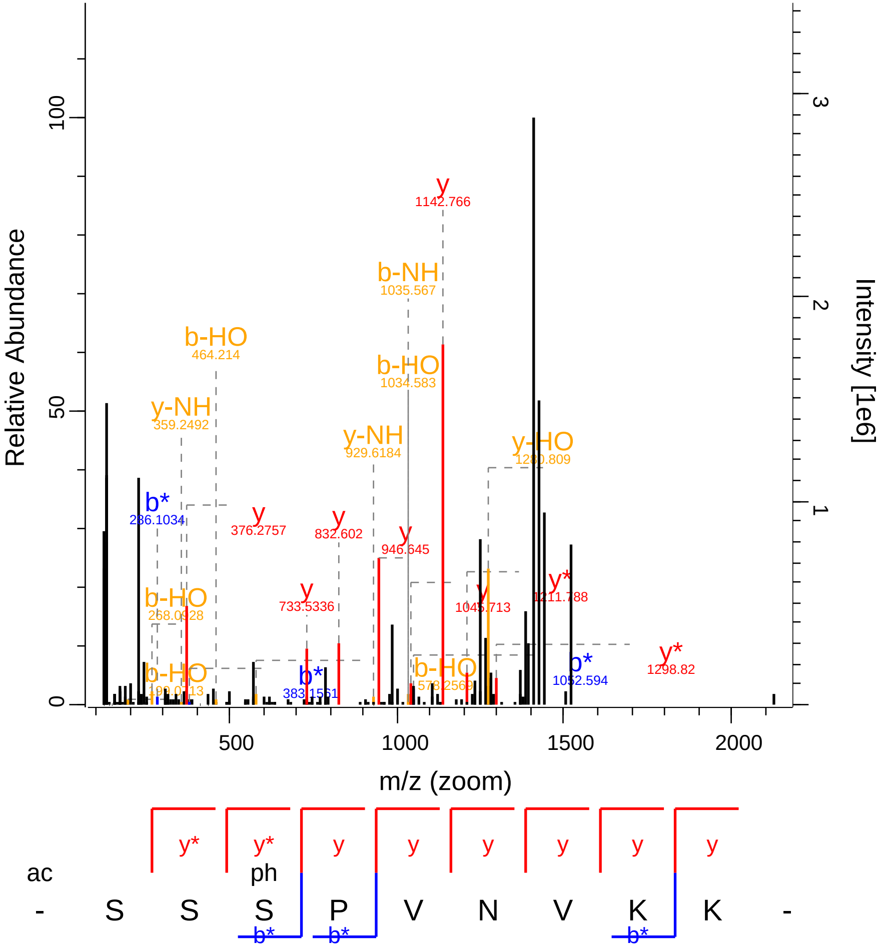

Raw File	Scan	Method	Score	m/z	Gene names
RO-QC-160121_ZackD_SS_Set_02_Fractio	13265	FTMS; HC	51.77	493.51	GABRE

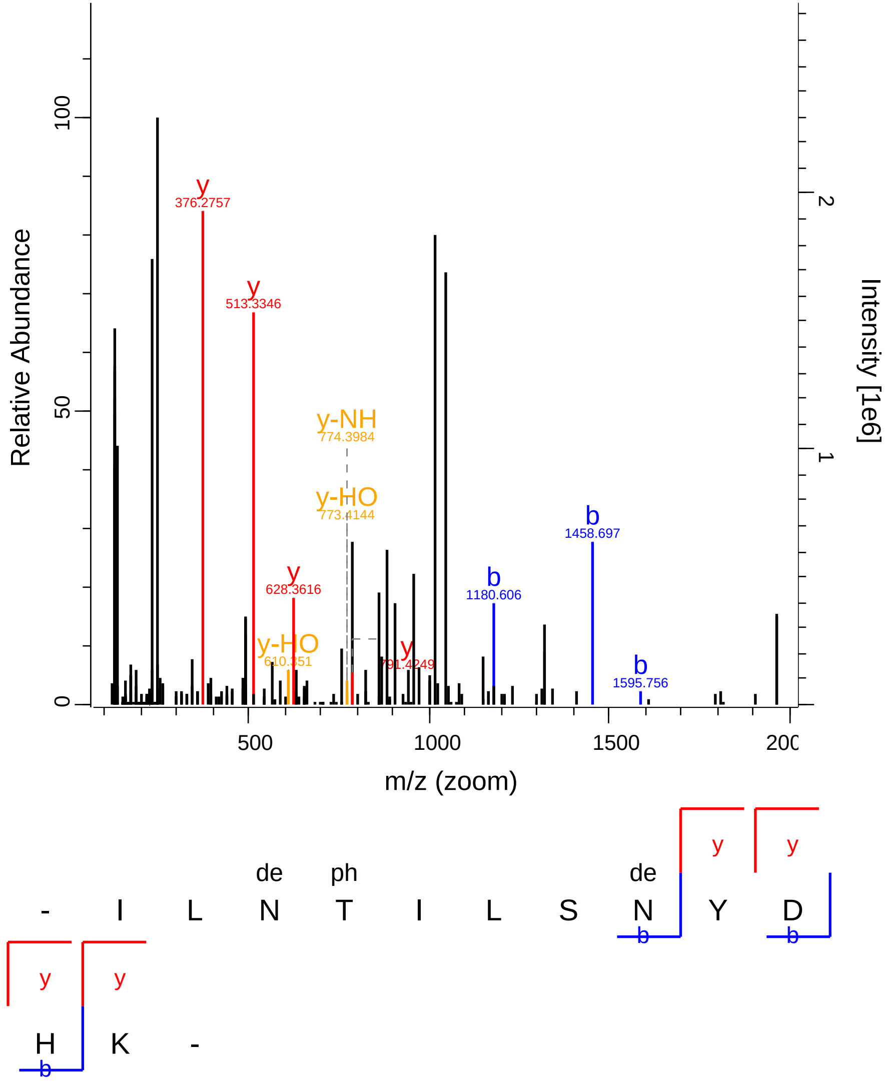

Raw File	Scan	Method	Score	m/z	Gene names
RO-QC-160121_ZackD_SS_Set_02_Fractio	14616	FTMS; HC	80.02	947.86	HIST1H1E

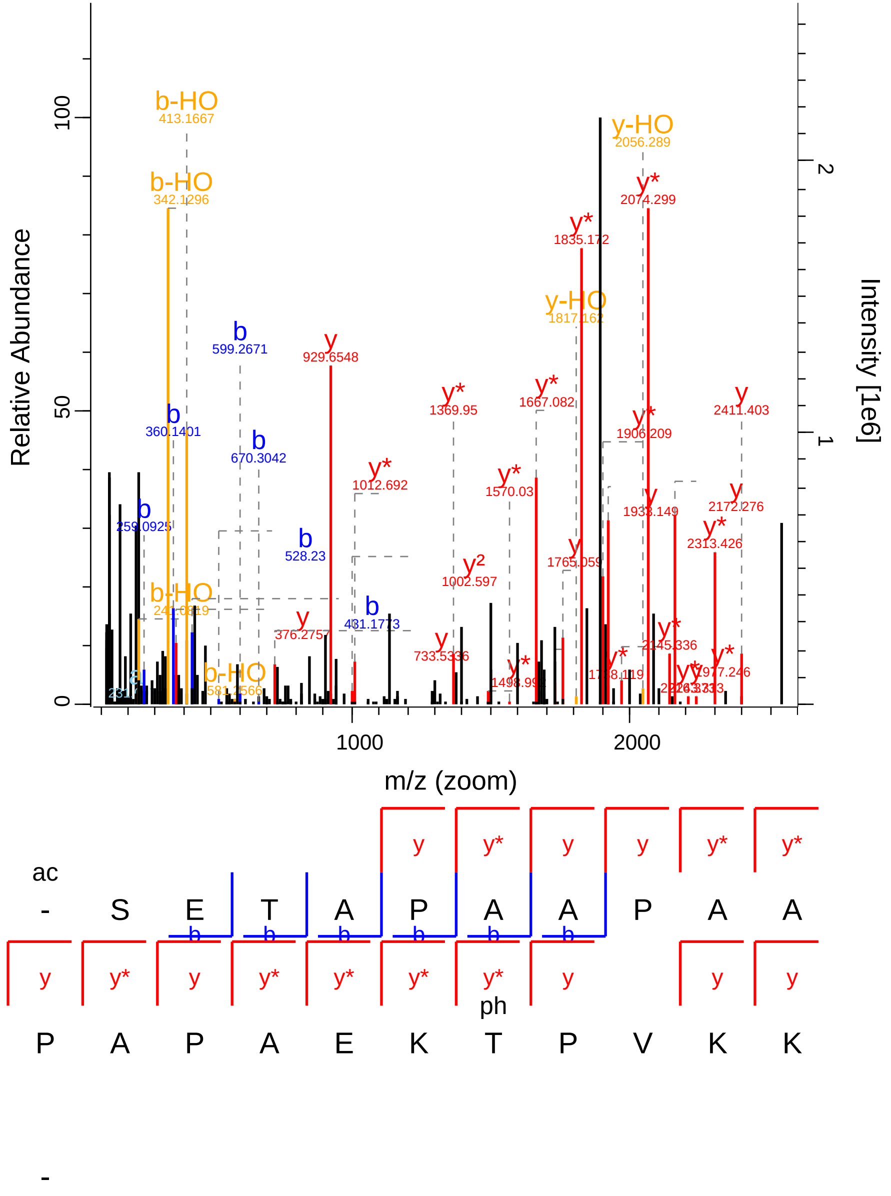

Raw File	Scan	Method	Score	m/z	Gene names
RO-QC-160121_ZackD_SS_Set_02_Fra	1533[illegible]	FTMS; HC	178.75	955.15	HSP90AB1;HSP90A

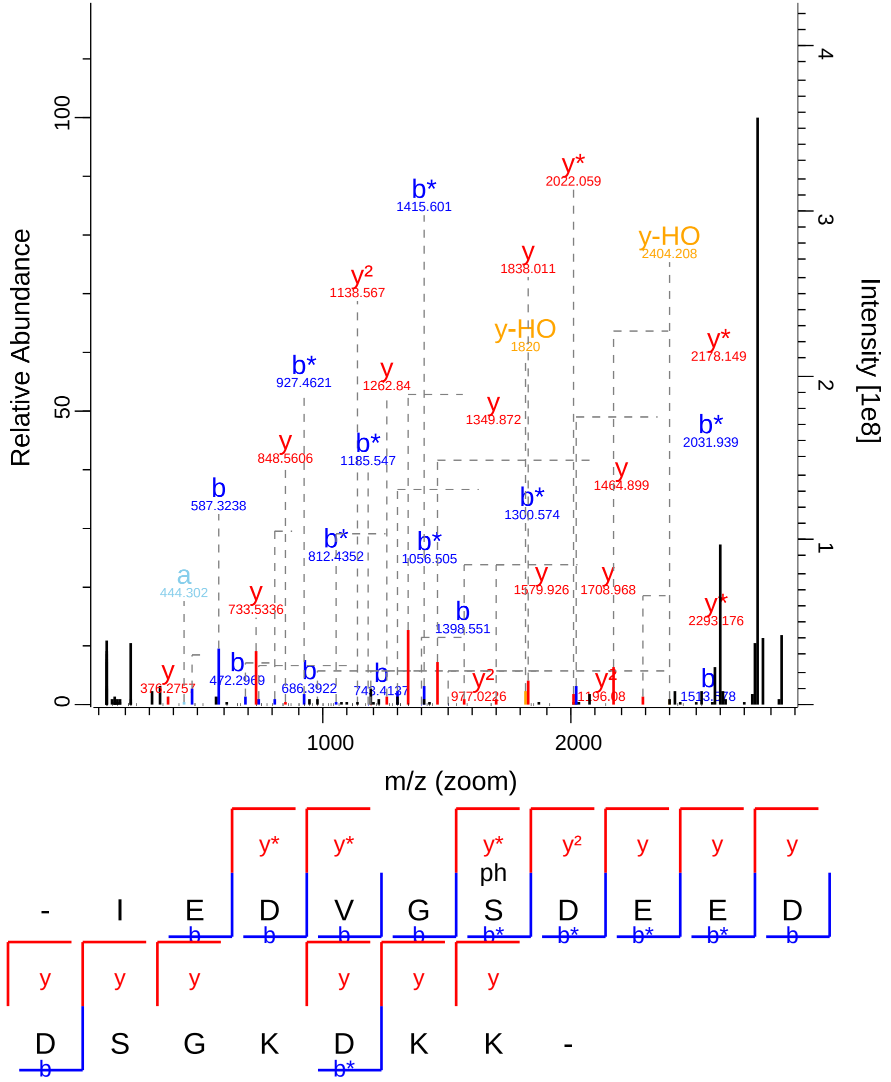

Raw File	Scan	Method	Score	m/z	Gene names
RO-QC-160121_ZackD_SS_Set_02_Fractio	16948	FTMS; HC	81.01	800.14	ZNF326

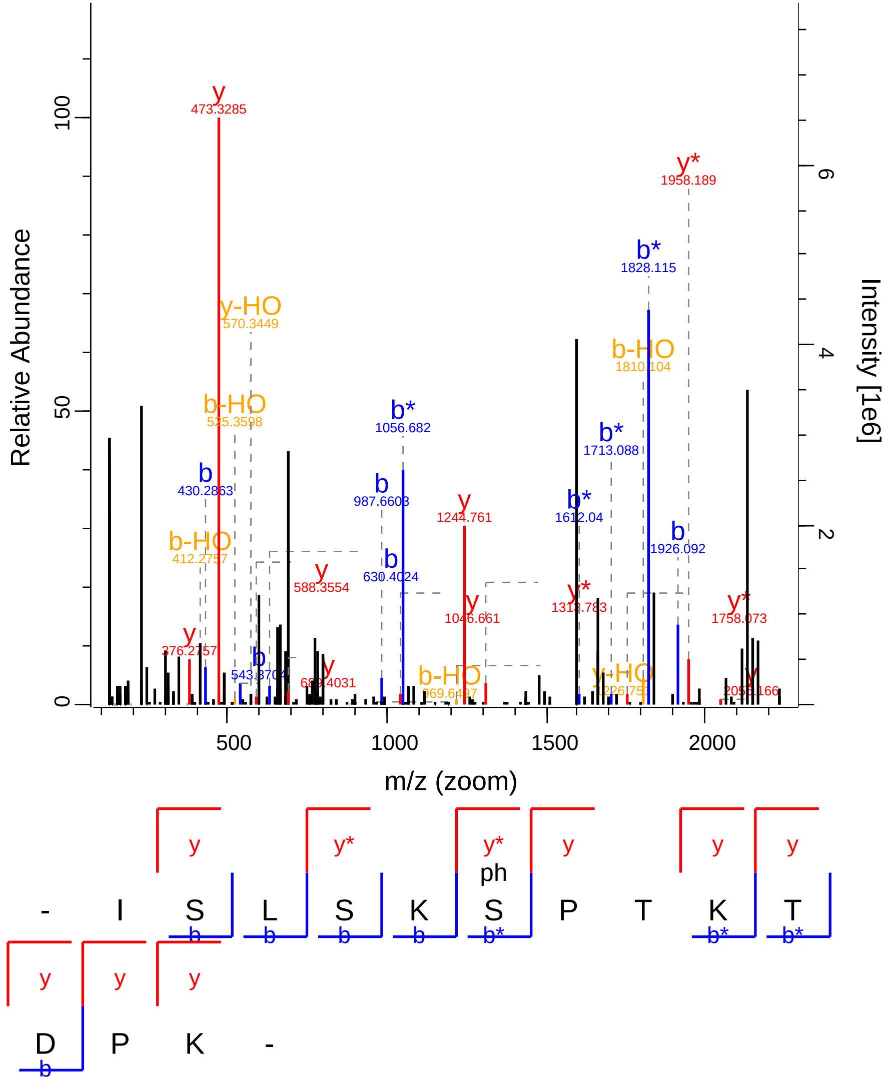

Raw File	Scan	Method	Score	m/z	Gene names
RO-QC-160121_ZackD_SS_Set_02_Fraction	16969	FTMS; HCI	122.08	957	TMX2

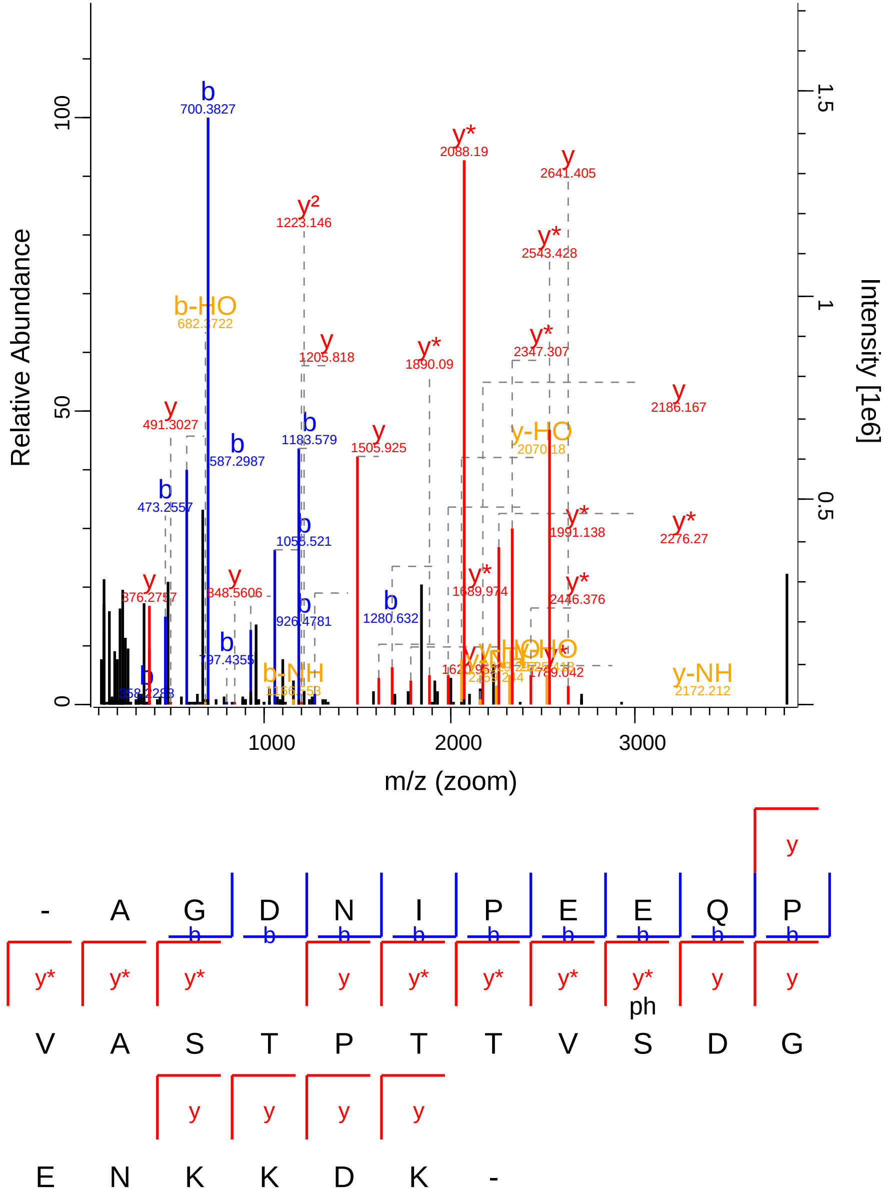

Raw File	Scan	Method	Score	m/z	Gene names
RO-QC-160121_ZackD_SS_Set_02_Fractio	17535	FTMS; HC	51.09	496.55	TFRC

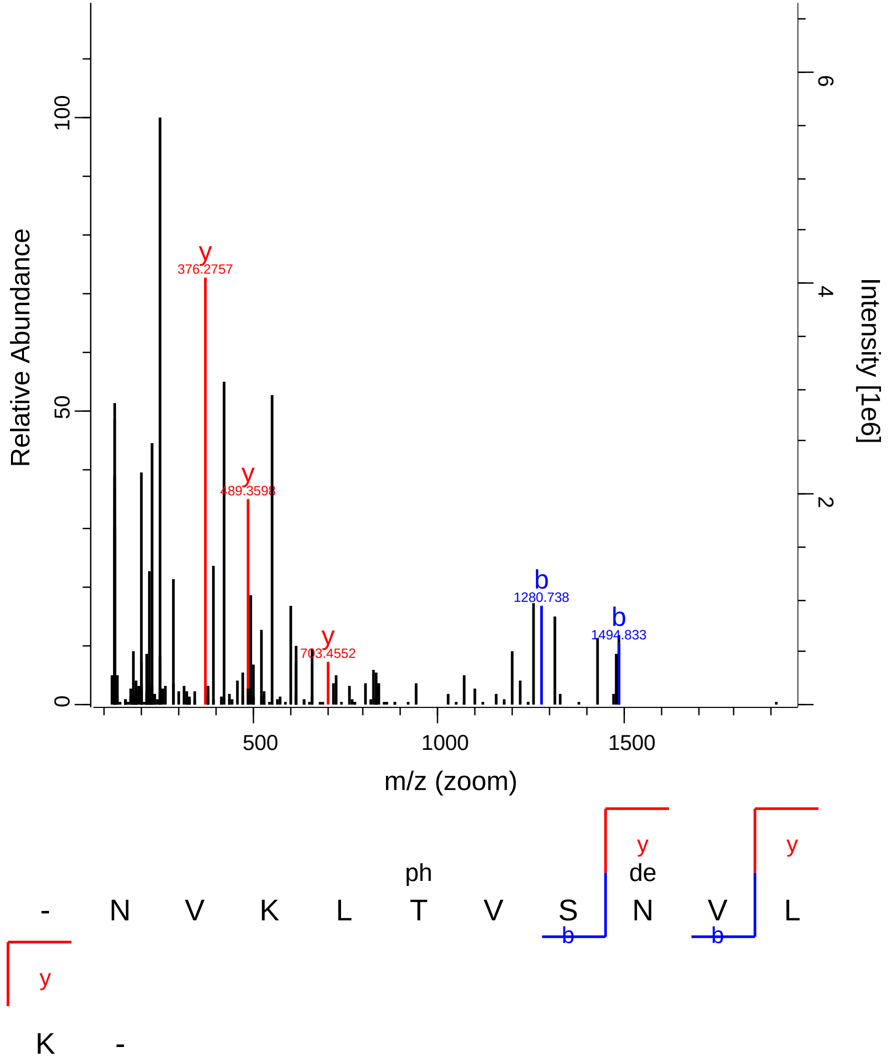

Raw File	Scan	Method	Score	m/z	Gene names
RO-QC-160121_ZackD_SS_Set_02_Fractio	17821	FTMS; HC	139.79	983.88	MAP1B

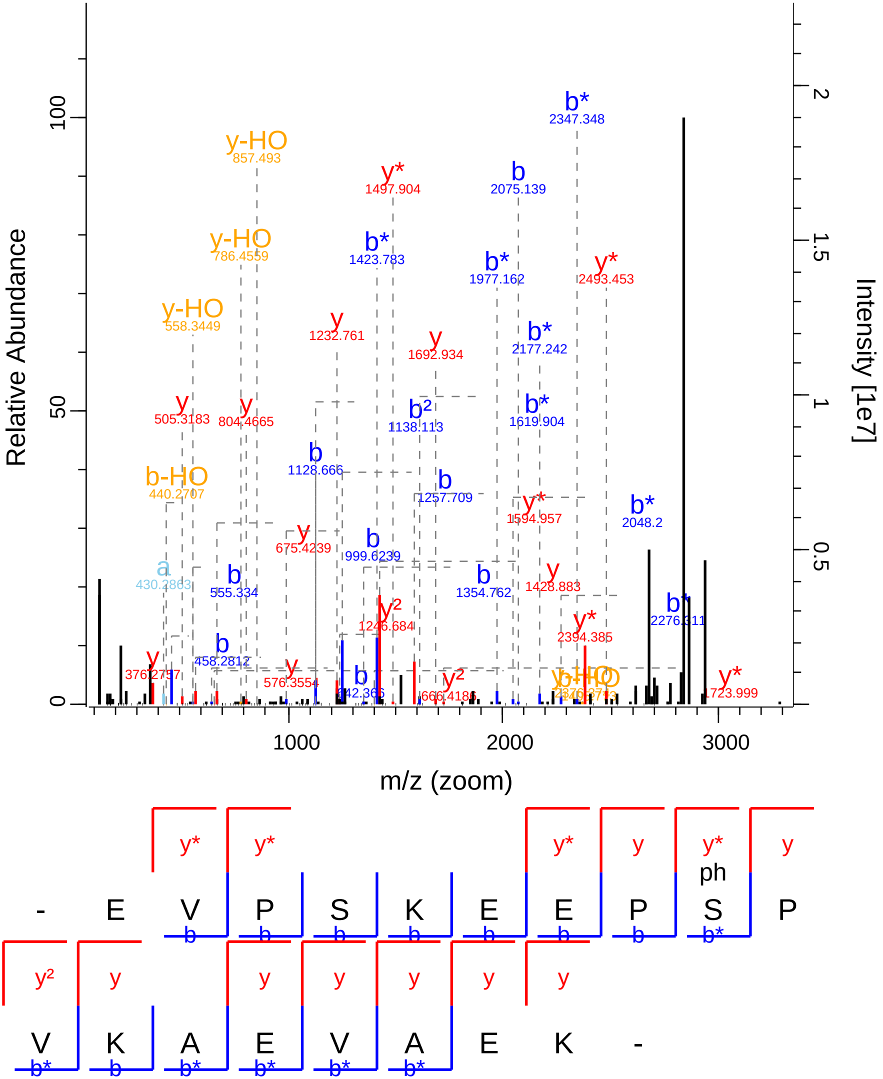

Raw File	Scan	Method	Score	m/z	Gene names
RO-QC-160121_ZackD_SS_Set_02_Fractio	18593	FTMS; HC	101.54	874.45	COL6A3

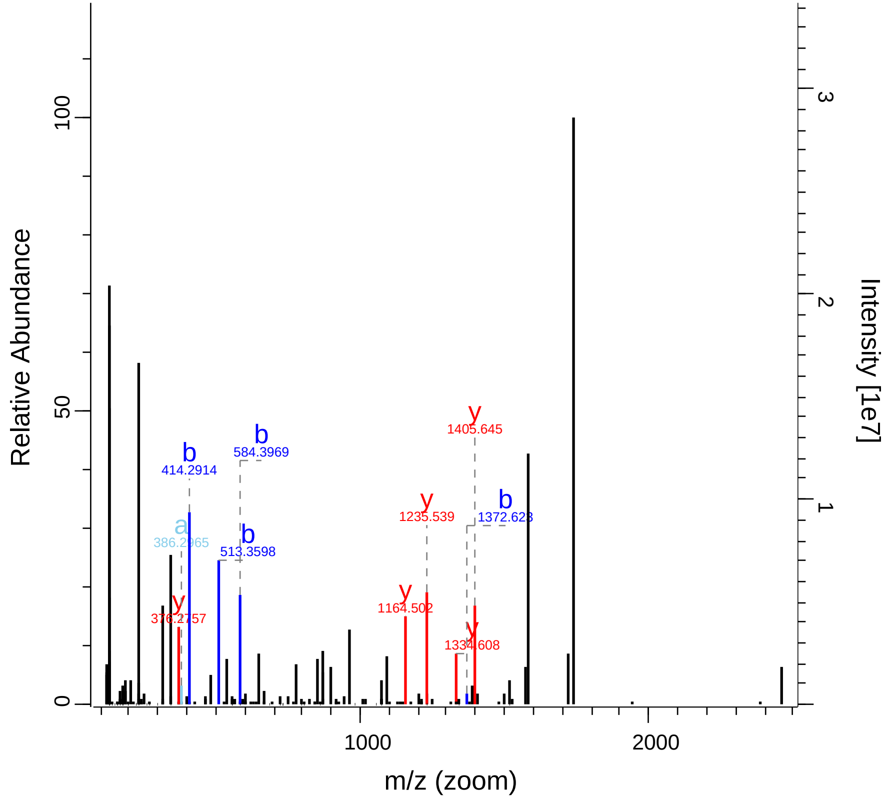

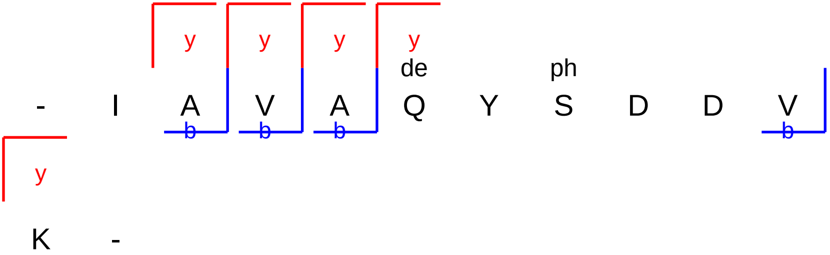

Raw File	Scan	Method	Score	m/z	Gene names
RO-QC-160121_ZackD_SS_Set_02_Fractio	18953	FTMS; HC	157.73	854.46	COPB2

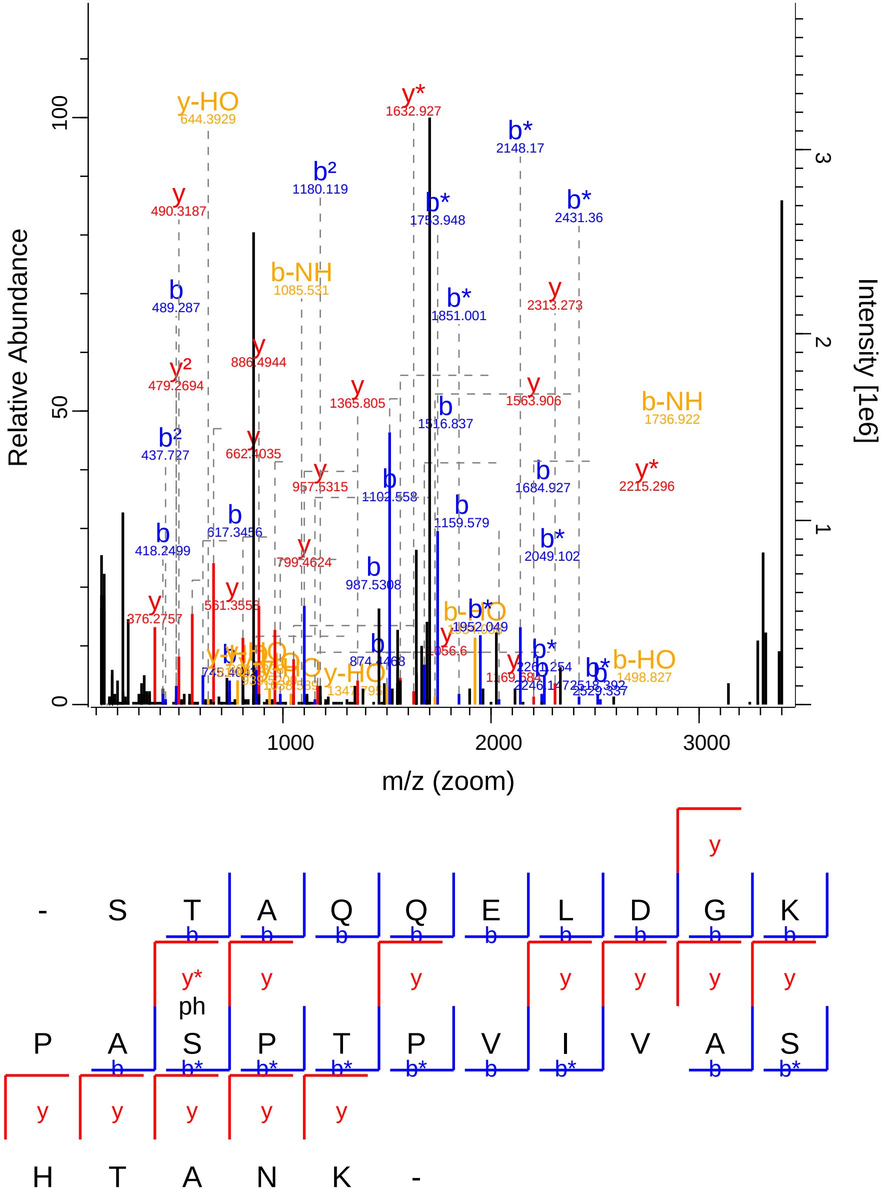

Raw File	Scan	Method	Score	m/z	Gene names
RO-QC-160121_ZackD_SS_Set_02_Fractio	19723	FTMS; HC	90.66	900.47	SYNE2

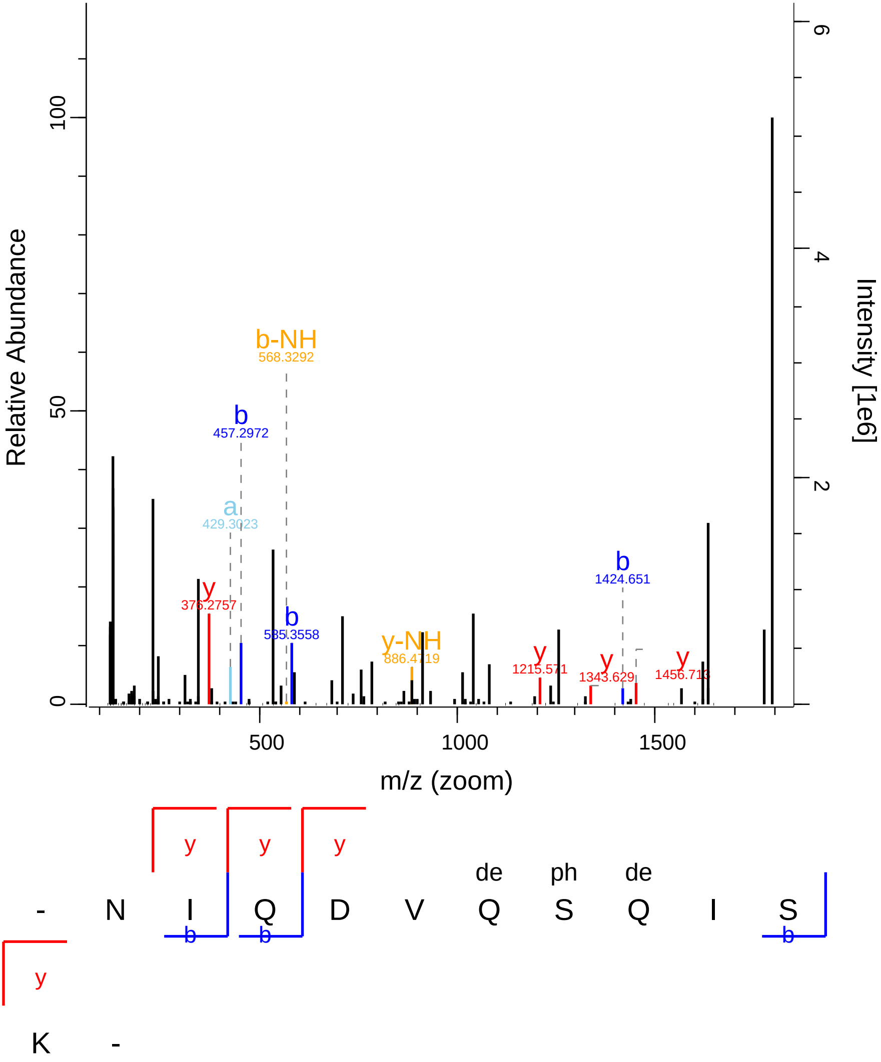

Raw File	Scan	Method	Score	m/z	Gene names
RO-QC-160121_ZackD_SS_Set_02_Fractio	20544	FTMS; HC	43.79	964.13	CTTNBP2

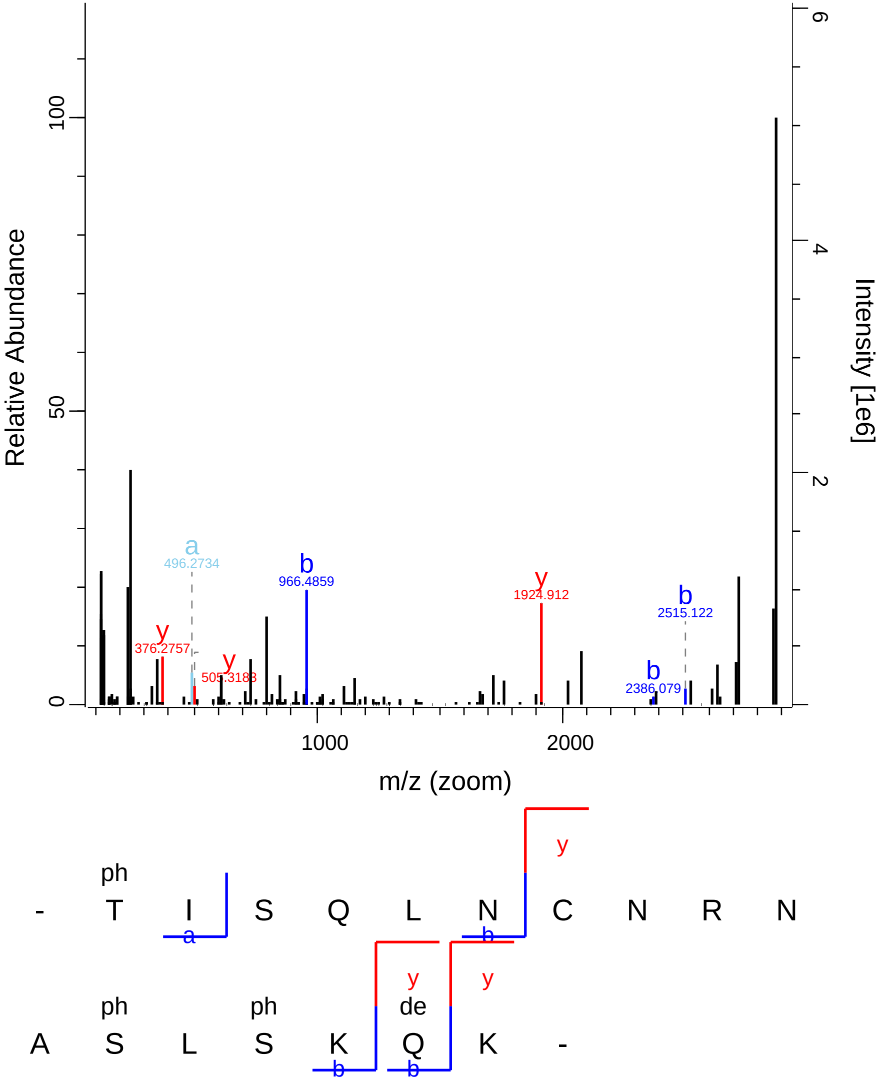

Raw File	Scan	Method	Score	m/z	Gene names
RO-QC-160121_ZackD_SS_Set_02_Fractior	20864	FTMS; HCI	94.69	831.5	C11orf71

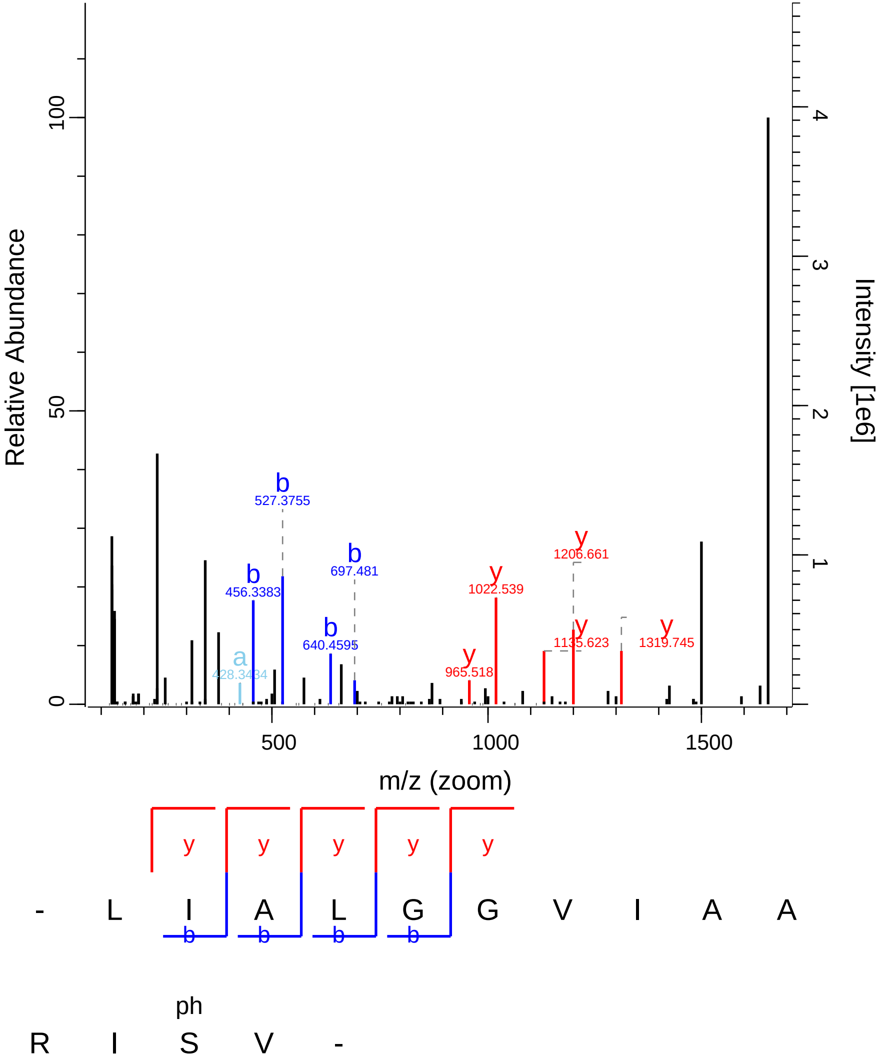

Raw File	Scan	Method	Score	m/z	Gene names
RO-QC-160121_ZackD_SS_Set_02_Fractio	22312	FTMS; HC	49.12	739.08	RIMS1

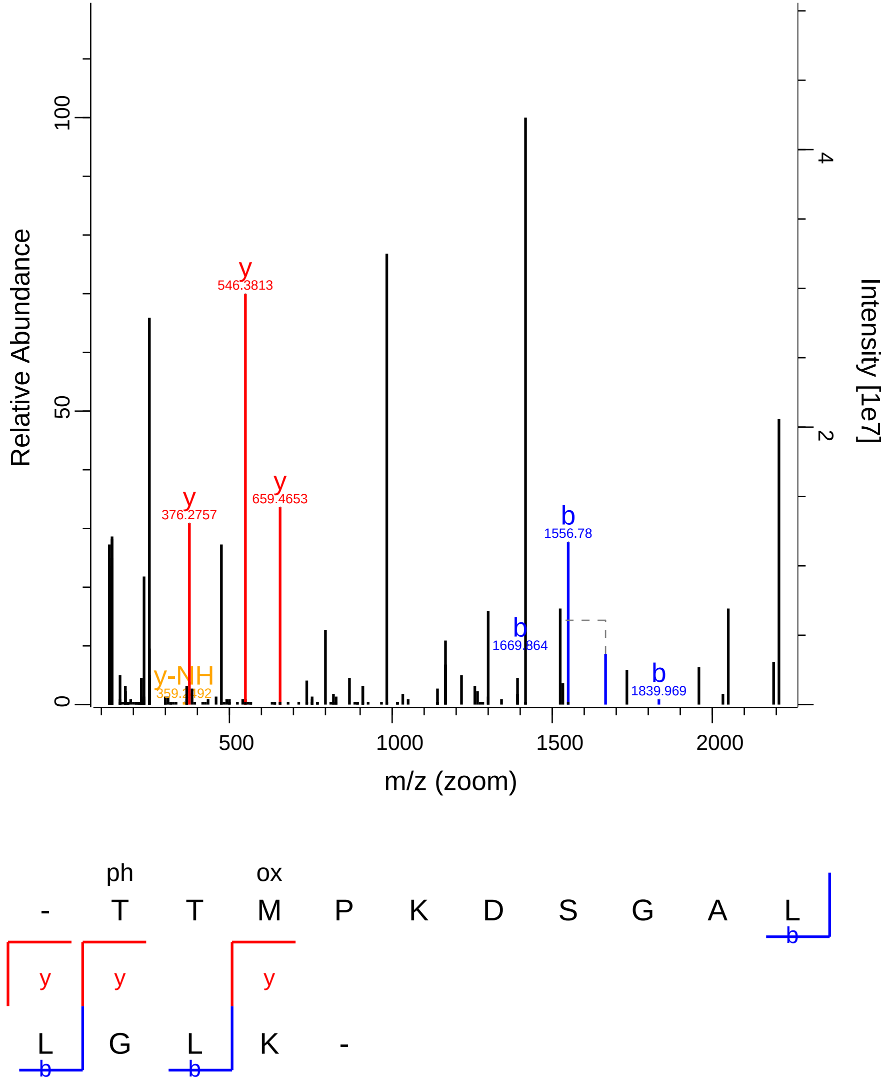

Raw File	Scan	Method	Score	m/z	Gene names
RO-QC-160121_ZackD_SS_Set_02_Fractio	23000	FTMS; HC	57.15	928.72	KIAA1143

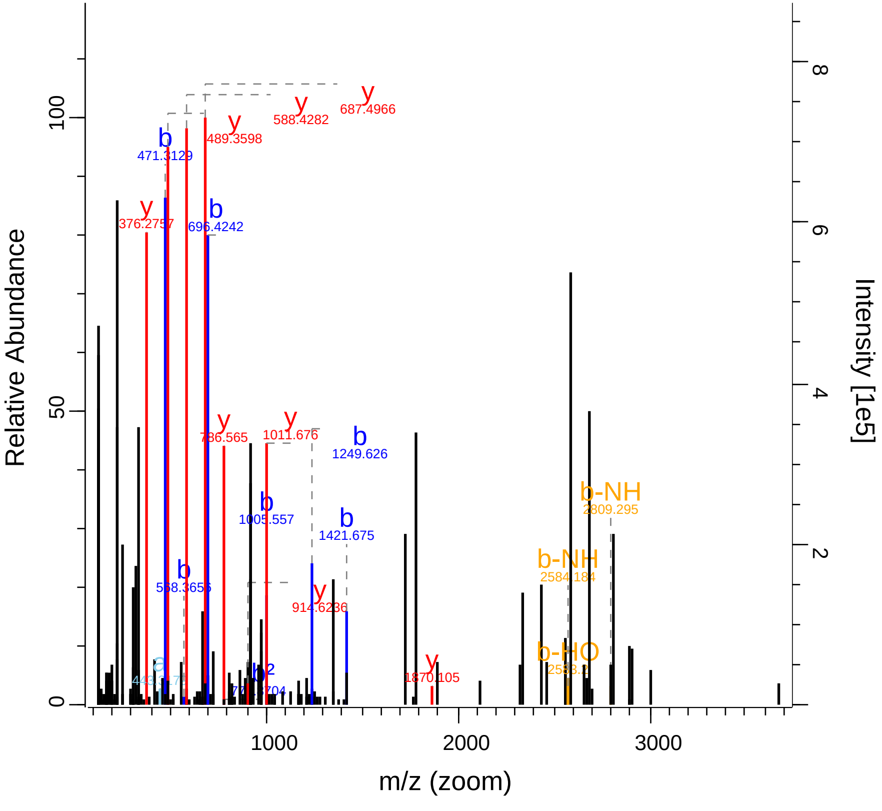

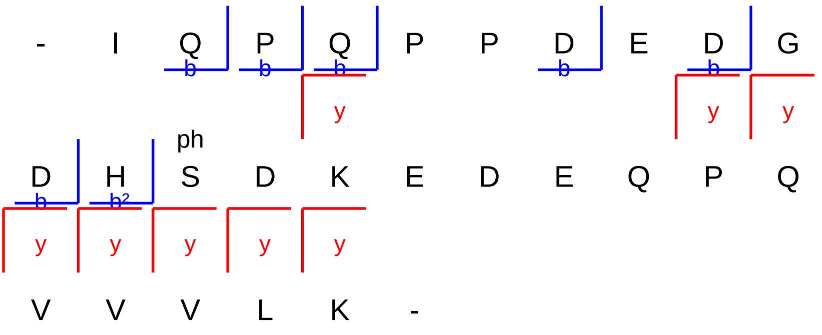

Raw File	Scan	Method	Score	m/z
RO-QC-160121_ZackD_SS_Set_02_Fraction_8	23094	FTMS; HCD	85.53	895.98

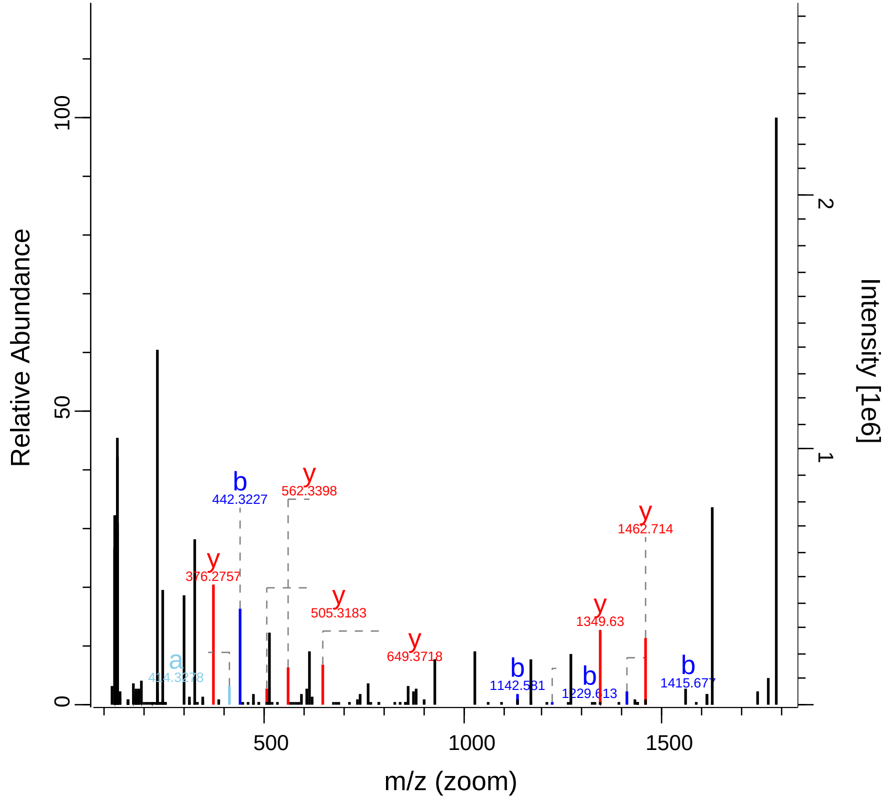

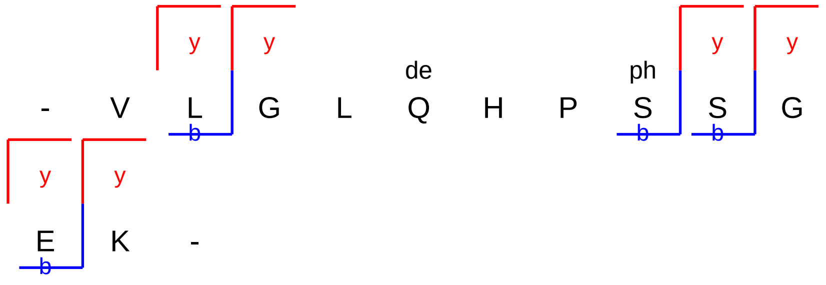

Raw File	Scan	Method	Score	m/z	Gene names
RO-QC-160121_ZackD_SS_Set_02_Fractio	23420	FTMS; HC	63.49	917.01	PTPN22

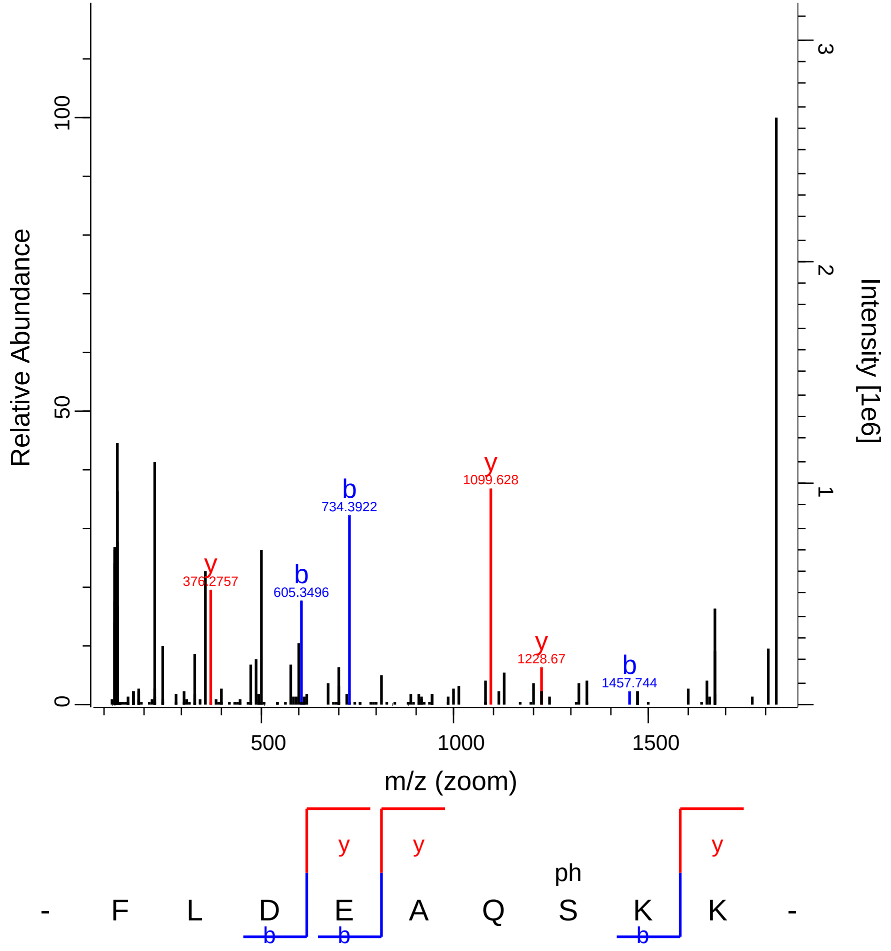

Raw File	Scan	Method	Score	m/z
RO-QC-160121_ZackD_SS_Set_02_Fraction_8	25599	FTMS; HCD	41.21	1000.88

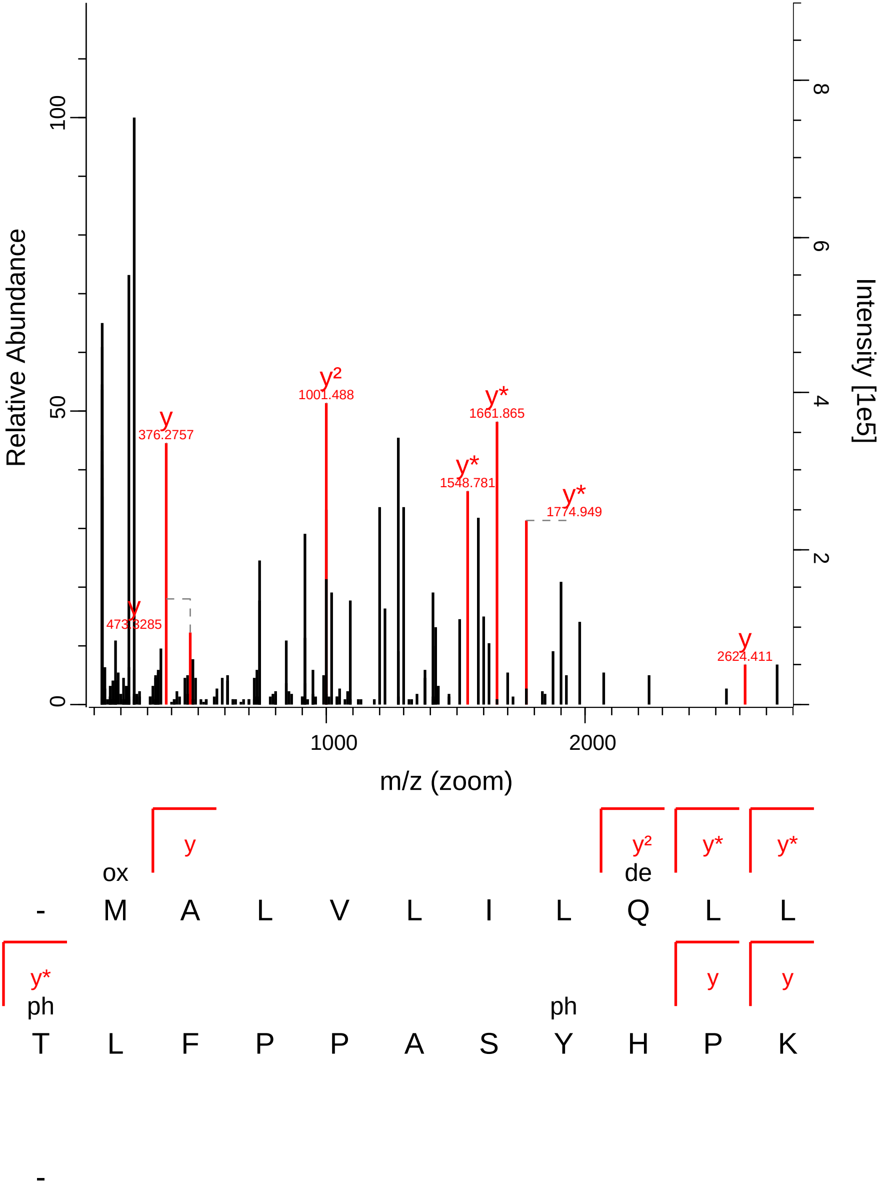

Raw File	Scan	Method	Score	m/z	Gene names
RO-QC-160121_ZackD_SS_Set_02_Fractior	28125	FTMS; HCI	46.75	870.8	CCDC168

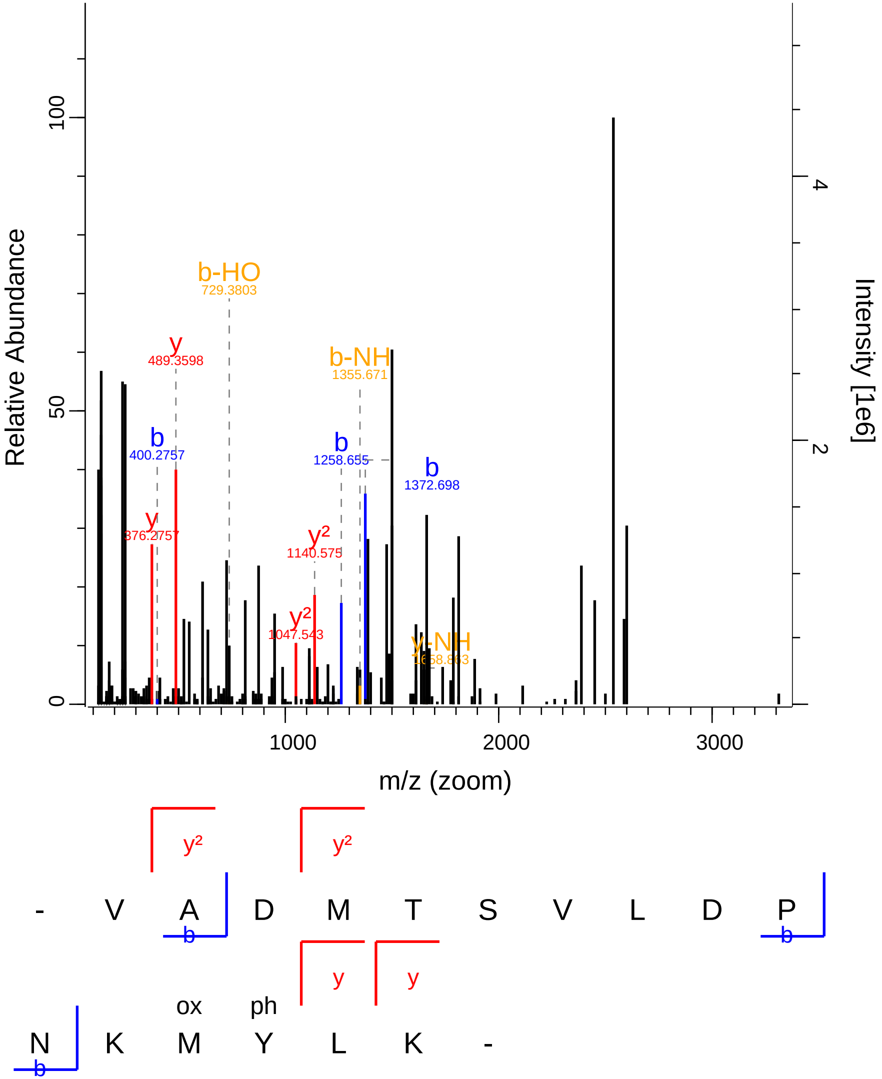

Raw File	Scan	Method	Score	m/z	Gene names
RO-QC-160121_ZackD_SS_Set_02_Fractio	29154	FTMS; HC	195.81	1073.53	LRRC47

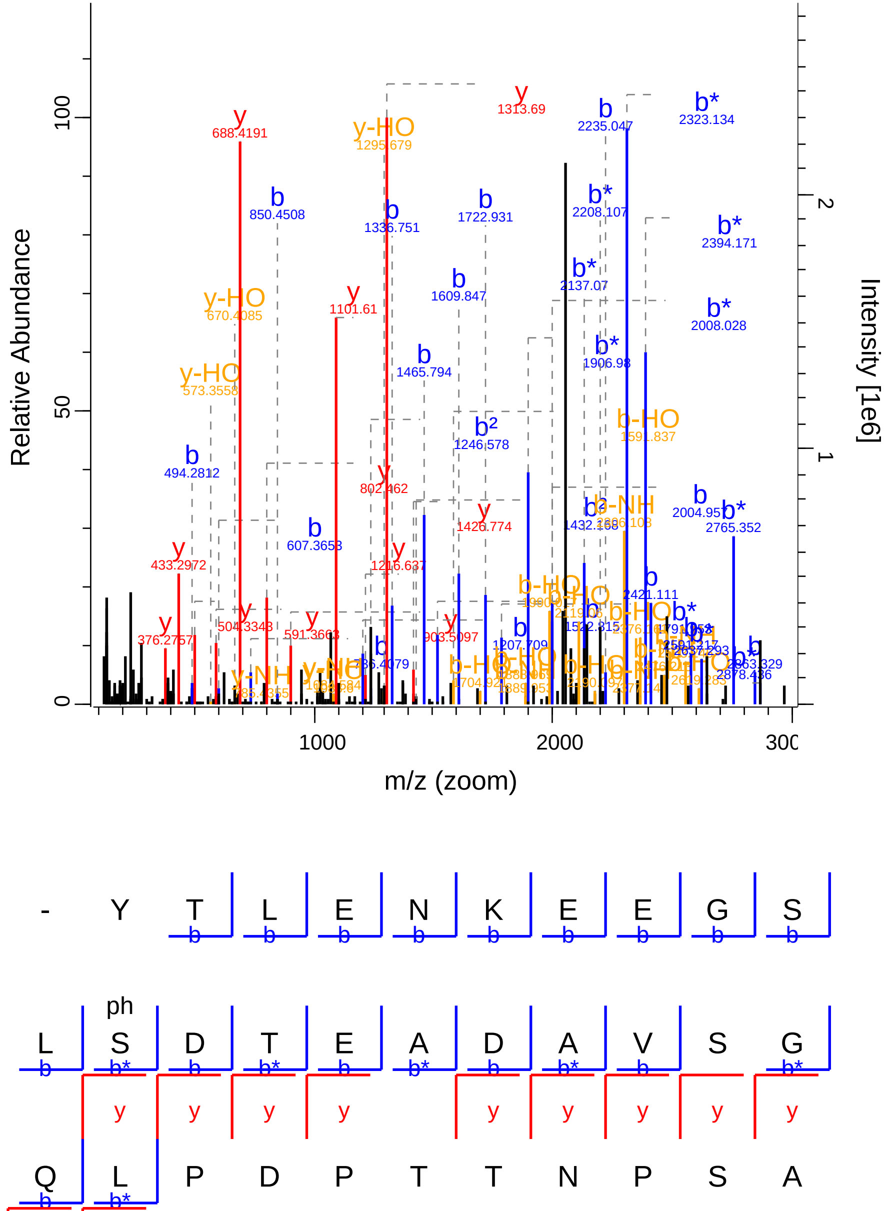

Raw File	Scan	Method	Score	m/z	Gene names
RO-QC-160121_ZackD_SS_Set_02_Fractio	29756	FTMS; HC	107.06	1097.04	ZNF804B

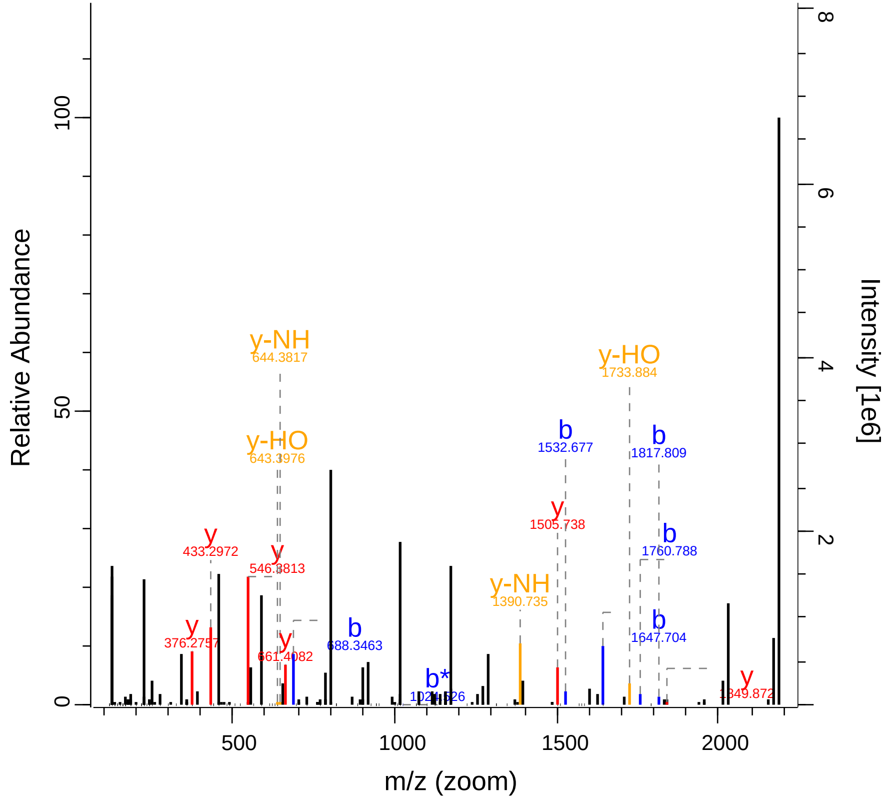

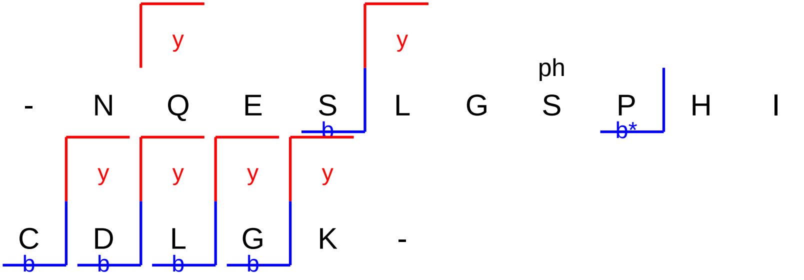

Raw File	Scan	Method	Score	m/z	Gene names
RO-QC-160121_ZackD_SS_Set_02_Fractio	30995	FTMS; HC	83.42	843.46	ABHD5

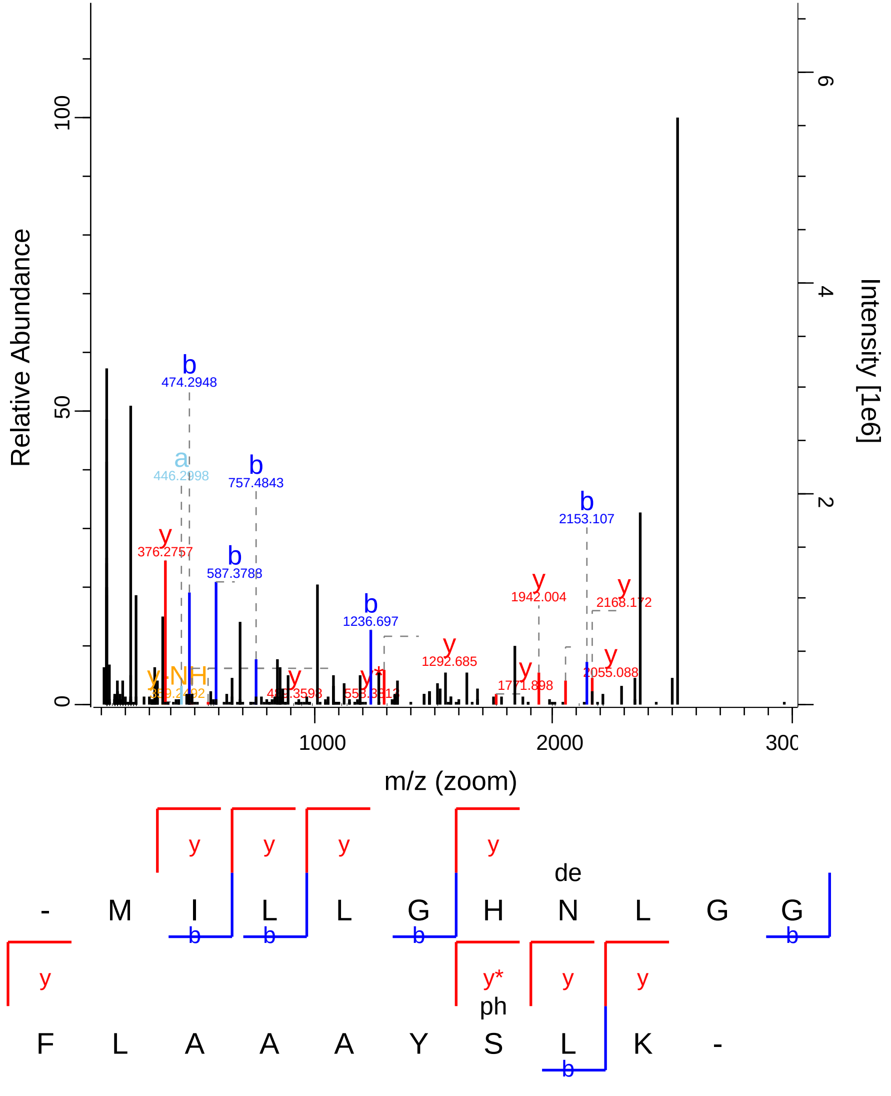

Raw File	Scan	Method	Score	m/z	Gene names
RO-QC-160121_ZackD_SS_Set_02_Fractic	31563	FTMS; HC	148.3	1042.18	CTR9

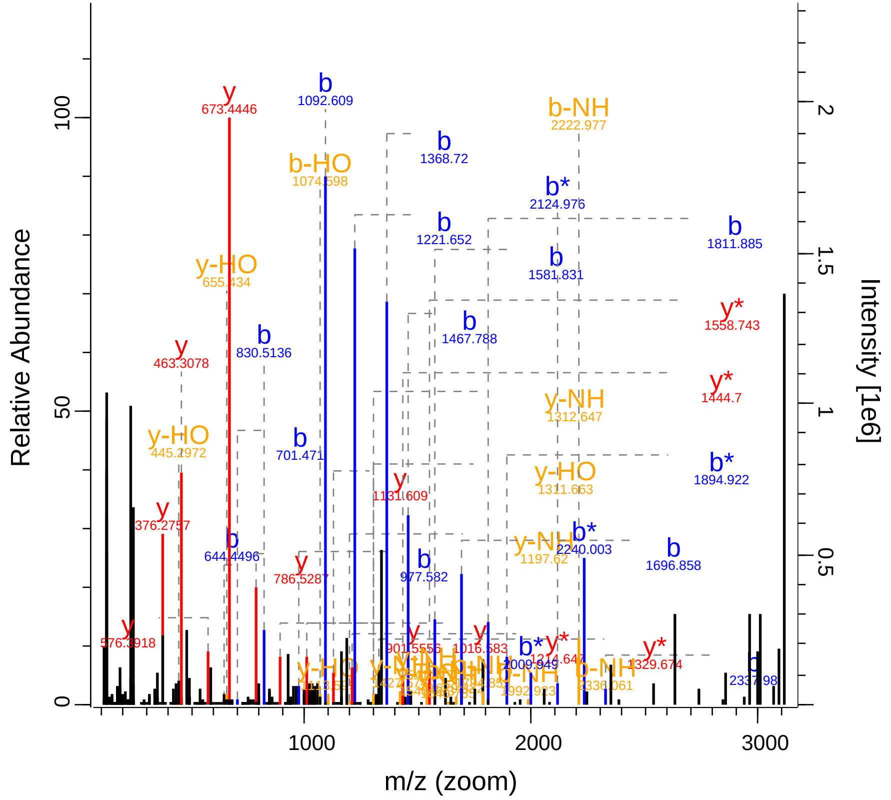

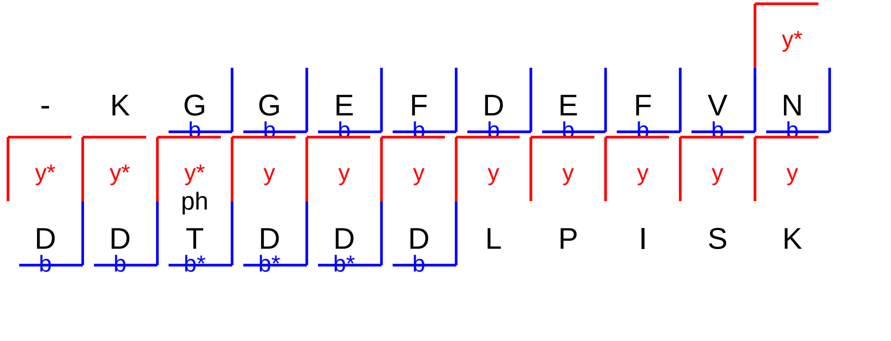

-

Raw File	Scan	Method	Score	m/z	Gene names
RO-QC-160121_ZackD_SS_Set_02_Fractic	31945	FTMS; HC	82.26	1094.08	UTP20

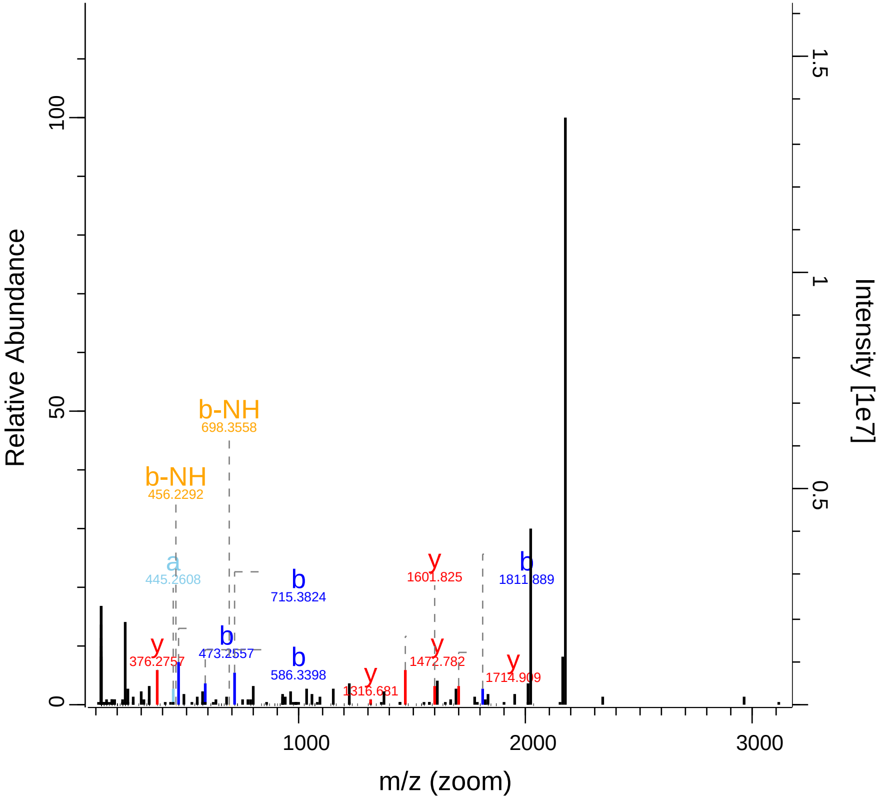

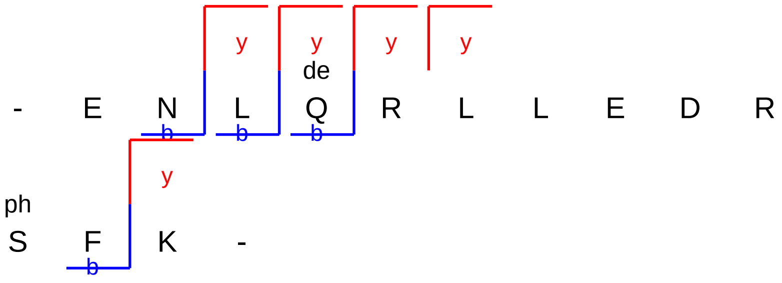

Raw File	Scan	Method	Score	m/z	Gene names
RO-QC-160121_ZackD_SS_Set_02_Fractio	32147	FTMS; HC	96.25	688.34	GFPT1

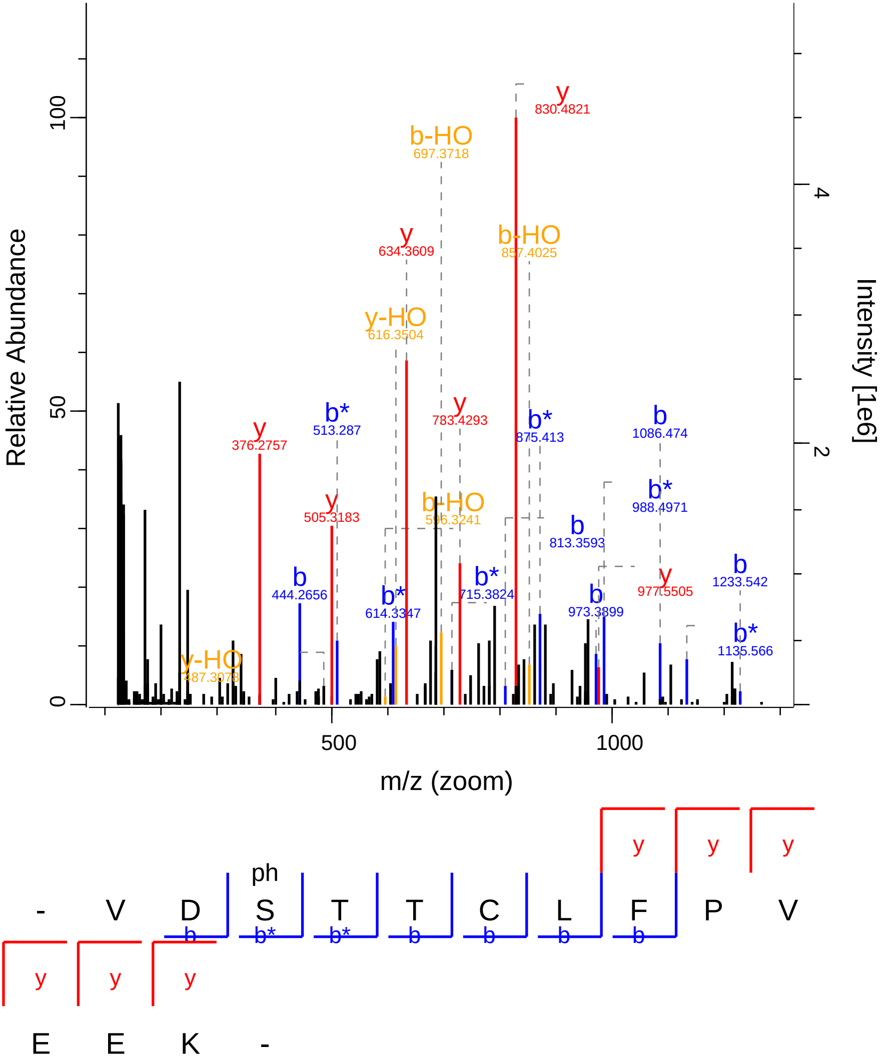

Raw File	Scan	Method	Score	m/z	Gene names
RO-QC-160121_ZackD_SS_Set_02_Fractio	33640	FTMS; HC	72.26	586.08	BCLAF1

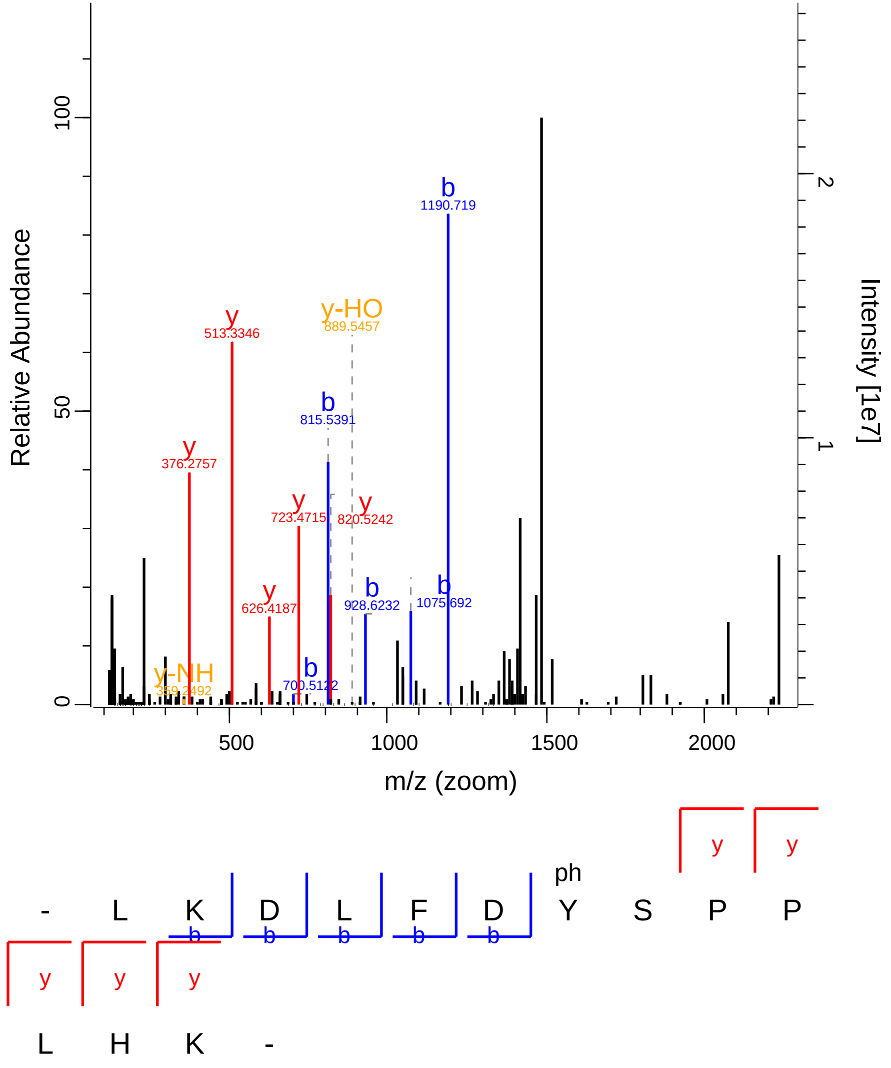

Raw File	Scan	Method	Score	m/z	Gene names
RO-QC-160121_ZackD_SS_Set_02_Fractio	34325	FTMS; HC	46.3	753.07	MAPK4

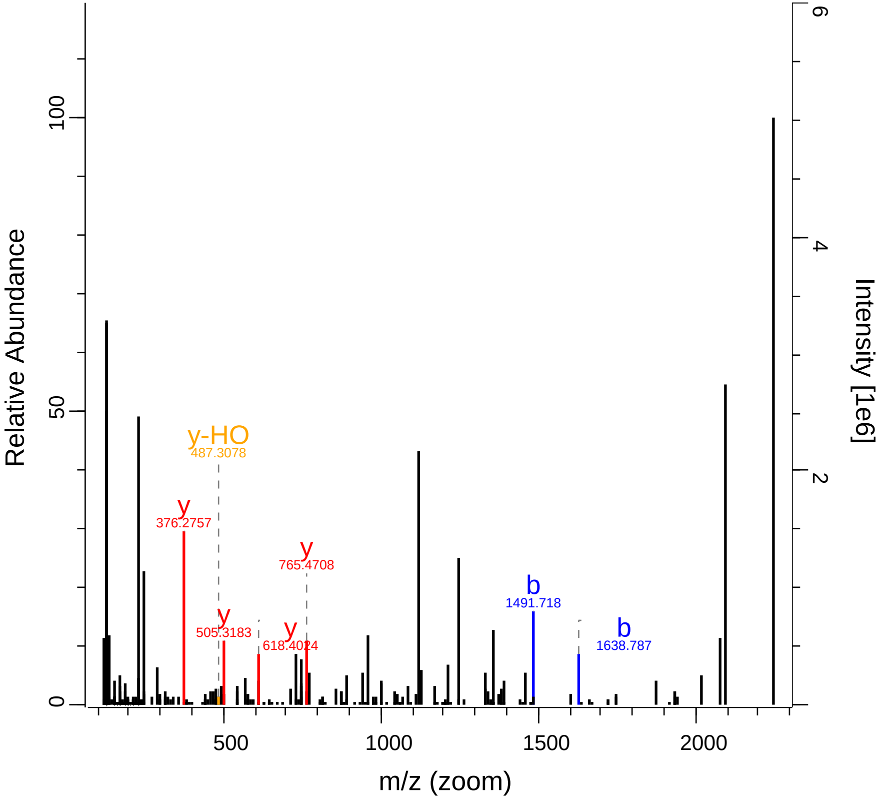

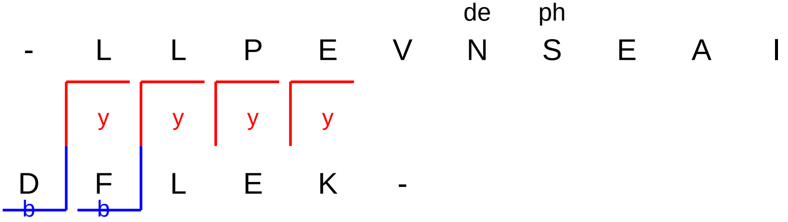

Raw File	Scan	Method	Score	m/z	Gene names
RO-QC-160121_ZackD_SS_Set_02_Fractio	35042	FTMS; HC	78.52	958.03	HDAC7

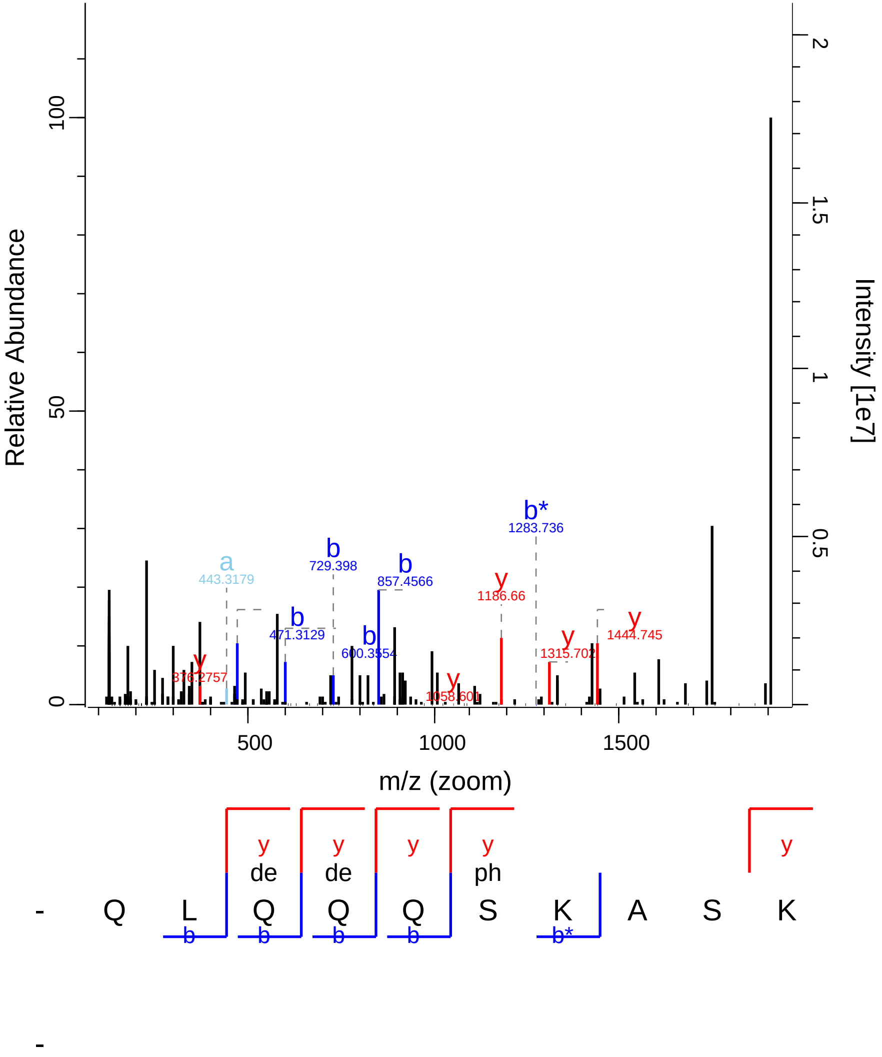

Raw File	Scan	Method	Score	m/z	Gene names
RO-QC-160121_ZackD_SS_Set_02_Fractio	35559	FTMS; HC	157.11	770.79	TPI1

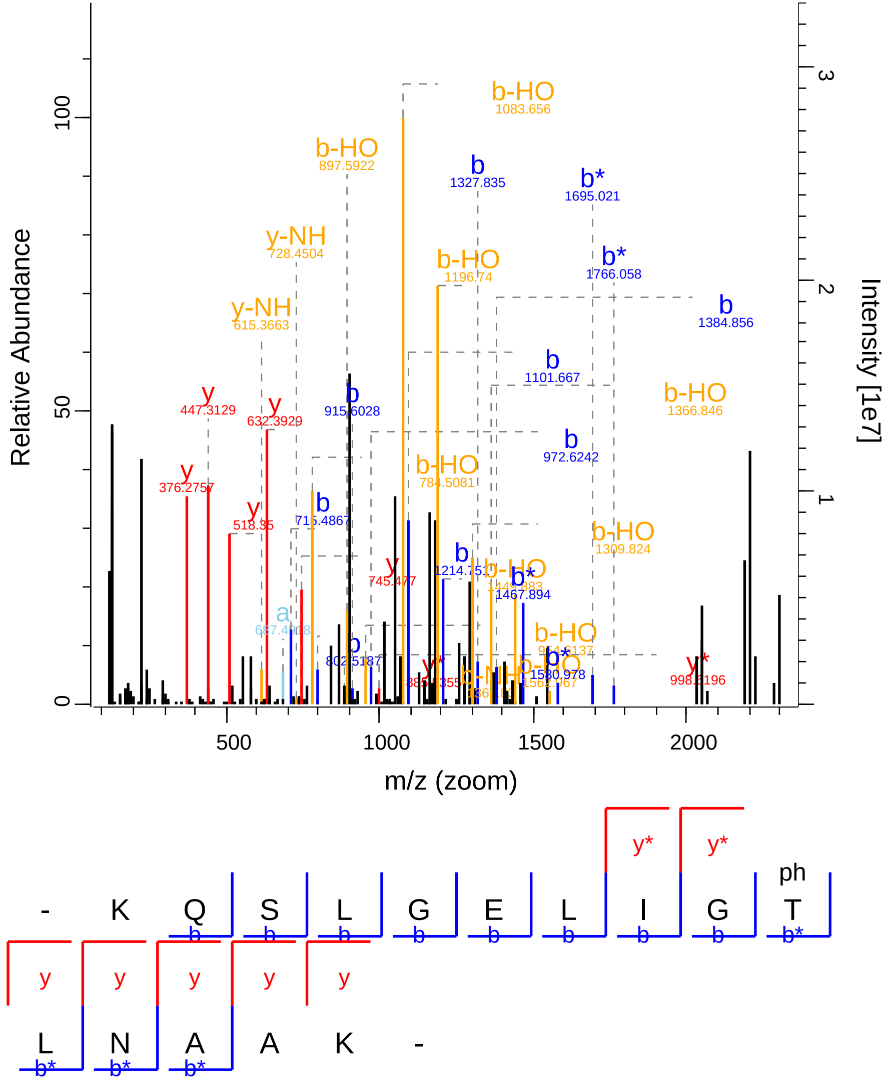

Raw File	Scan	Method	Score	m/z	Gene names
RO-QC-160121_ZackD_SS_Set_02_Fractic	40536	FTMS; HC	52.68	1466.81	GPNMB

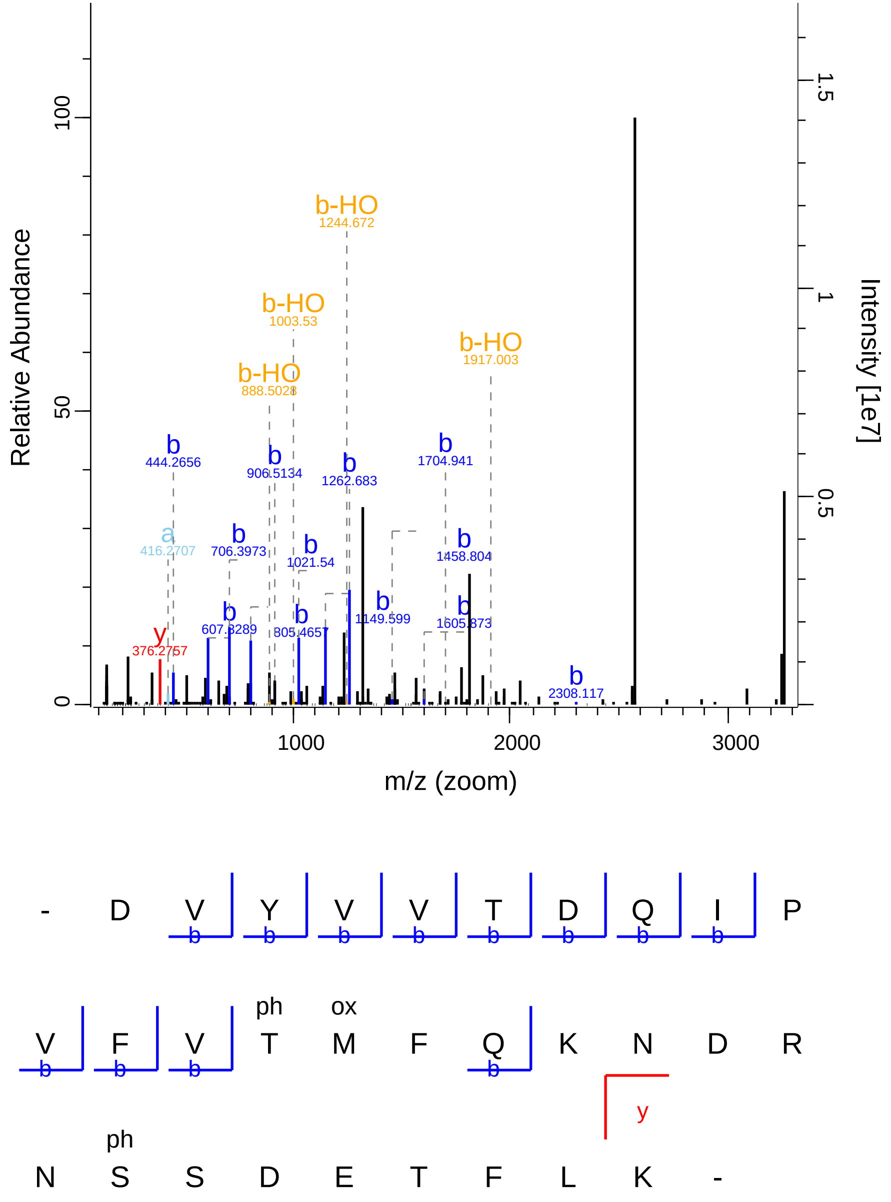

Raw File	Scan	Method	Score	m/z	Gene names
RO-QC-160121_ZackD_SS_Set_02_Fractior	5228	FTMS; HCI	59.18	654.66	GAPVD1

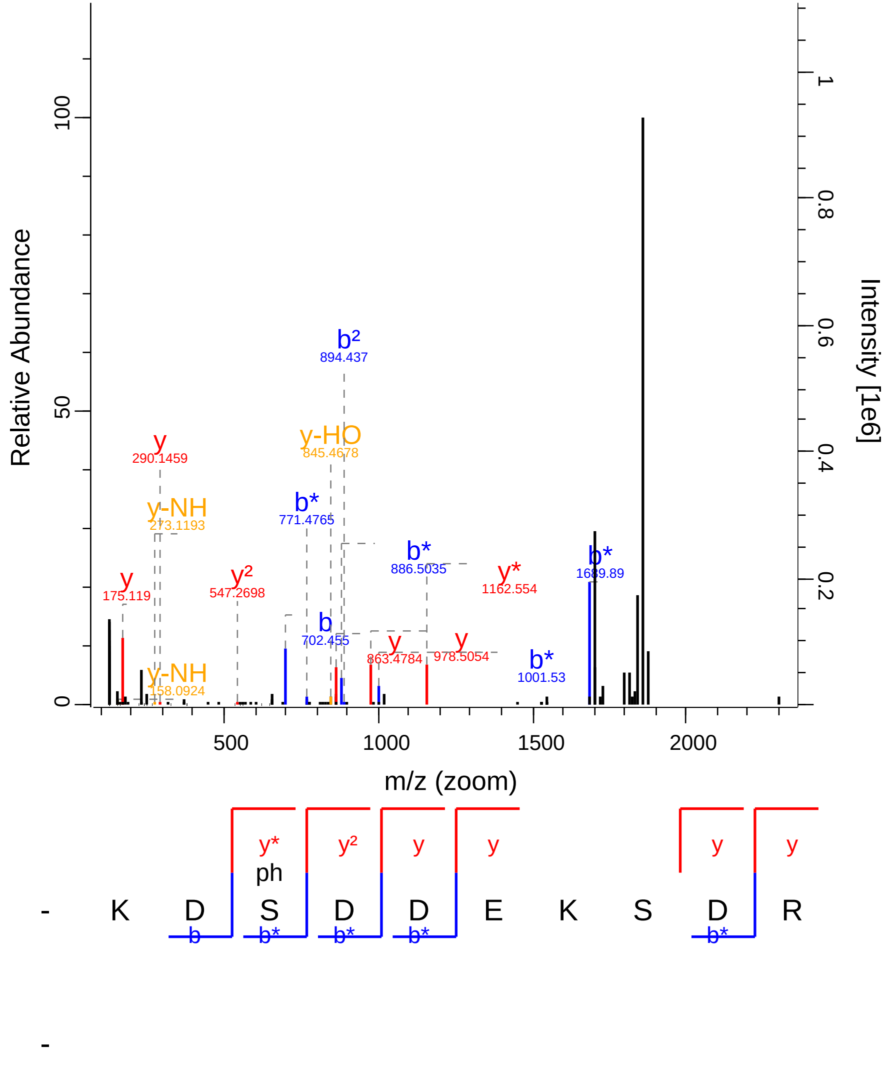

Raw File	Scan	Method	Score	m/z	Gene names
RO-QC-160121_ZackD_SS_Set_02_Fractio	5991	FTMS; HC	116.19	665.36	PAPOLA

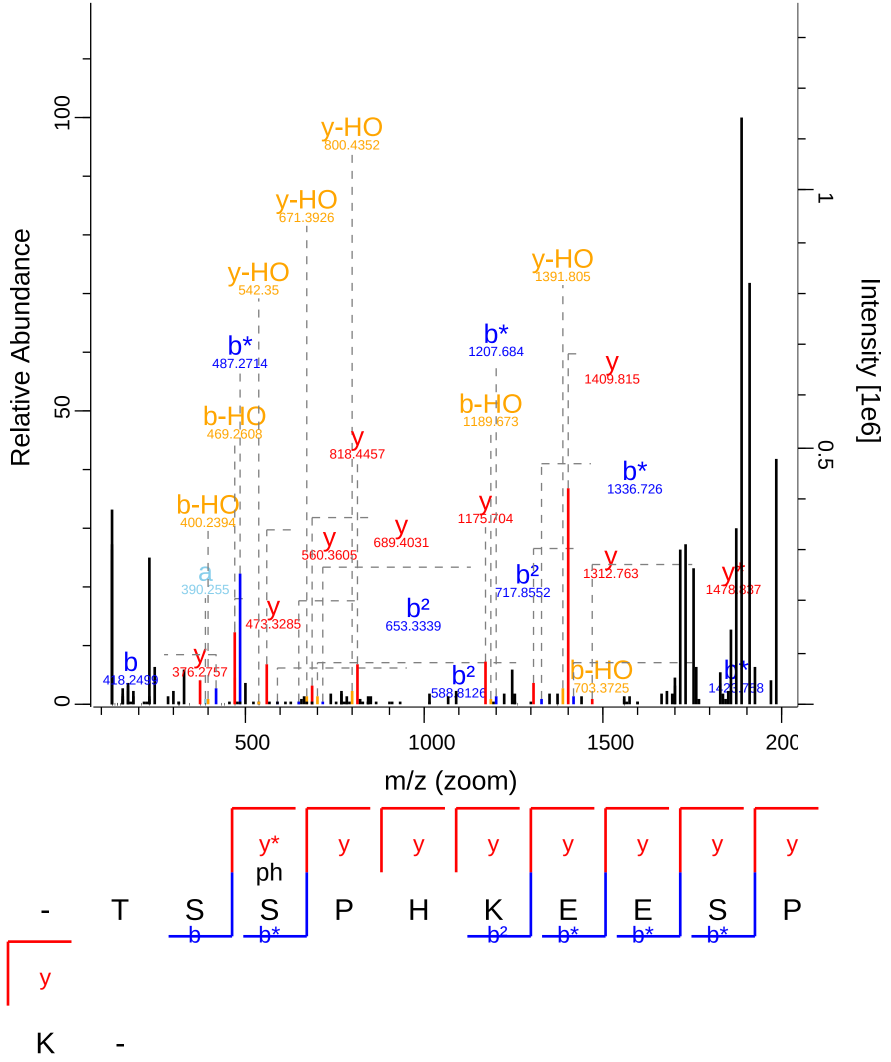

Raw File	Scan	Method	Score	m/z	Gene names
RO-QC-160121_ZackD_SS_Set_02_Fractior	9473	FTMS; HCI	44.72	811.39	STARD9

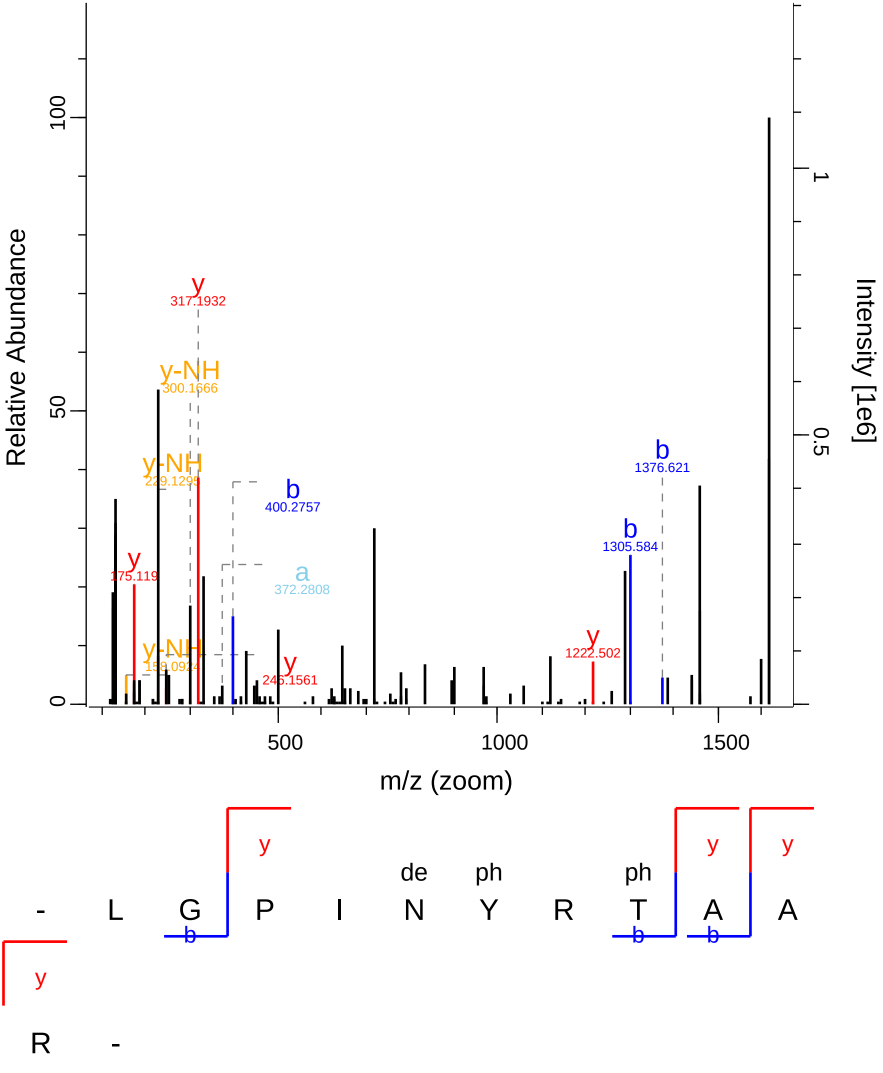

Raw File	Scan	Method	Score	m/z	Gene names
RO-QC-160121_ZackD_SS_Set_02_Fractic	10800	FTMS; HC	51.19	1038.53	USP43

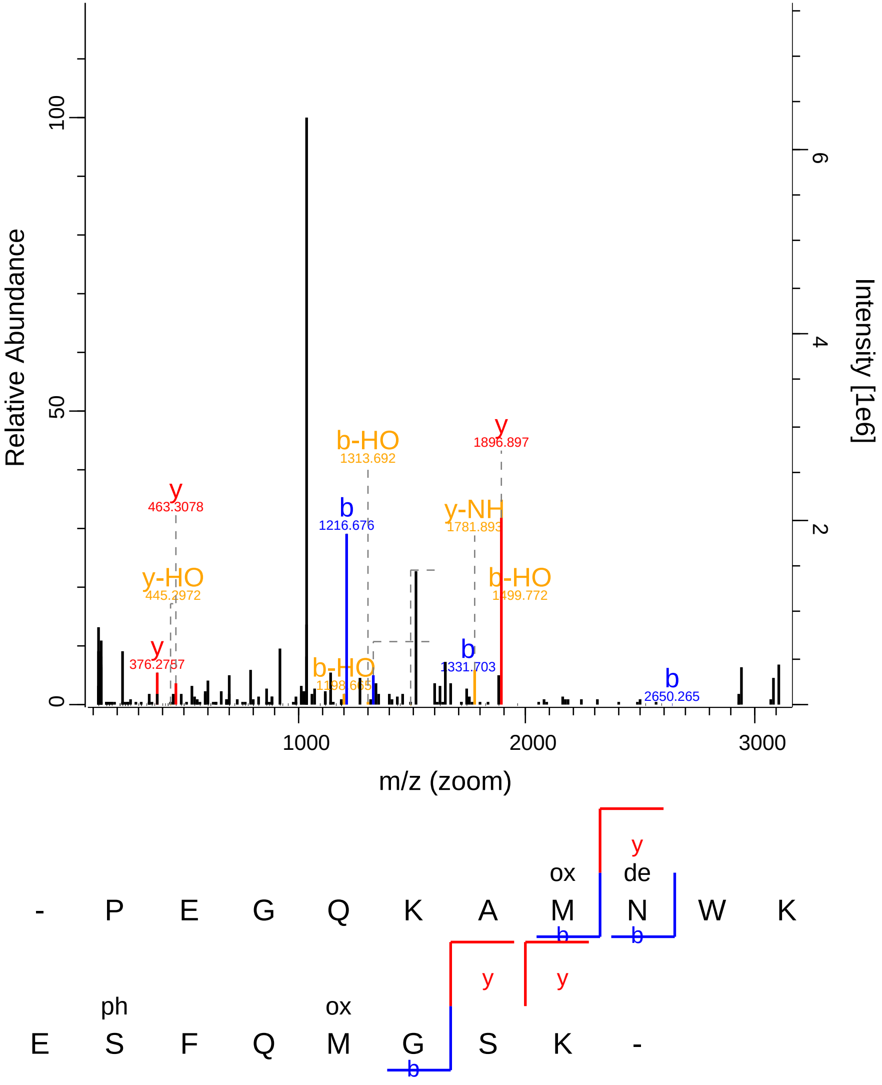

Raw File	Scan	Method	Score	m/z	Gene names
RO-QC-160121_ZackD_SS_Set_02_Fractio	11059	FTMS; HC	55.1	973.49	DBNL

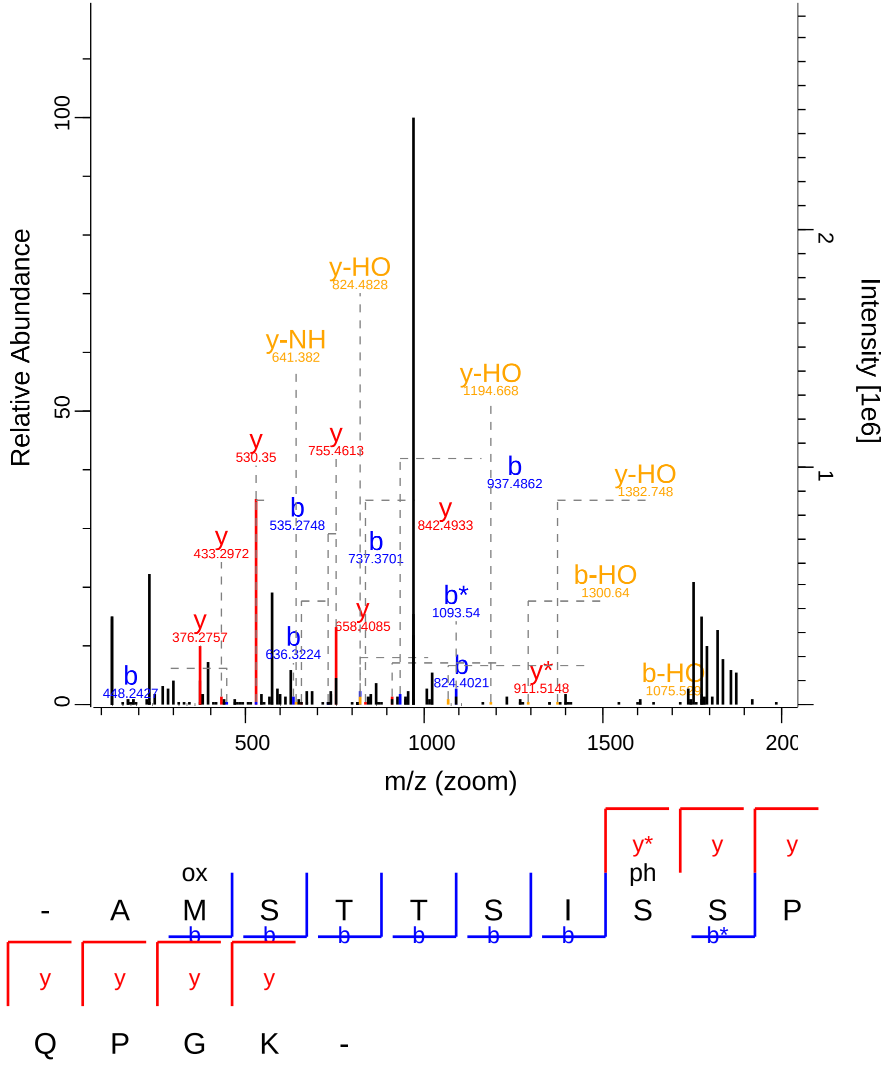

Raw File	Scan	Method	Score	m/z	Gene names
RO-QC-160121_ZackD_SS_Set_02_Fractio	11258	FTMS; HC	89.87	718.03	CBX3

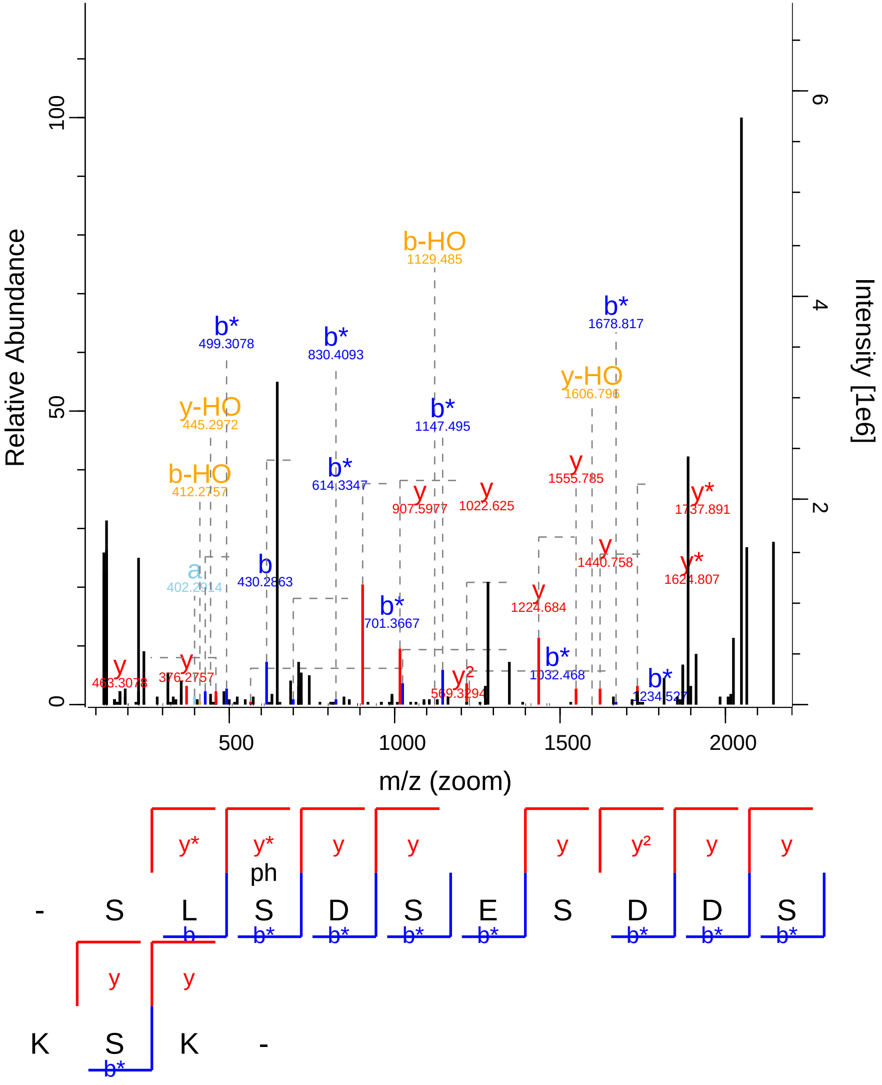

Raw File	Scan	Method	Score	m/z	Gene names
RO-QC-160121_ZackD_SS_Set_02_Fractio	12206	FTMS; HC	90.61	814.42	VCL

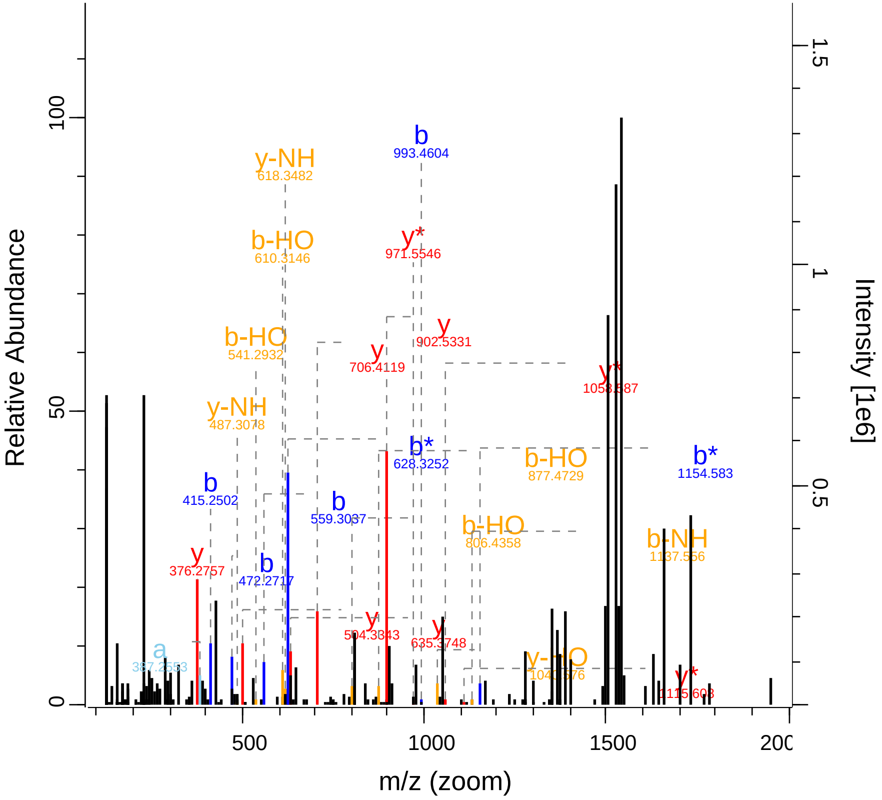

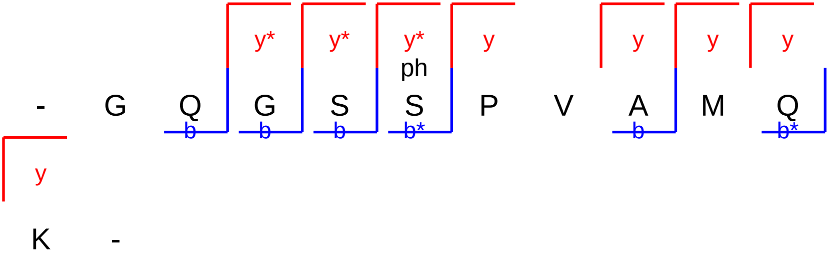

Raw File	Scan	Method	Score	m/z	Gene names
RO-QC-160121_ZackD_SS_Set_02_Fractio	13713	FTMS; HC	92.61	770.91	NAV1

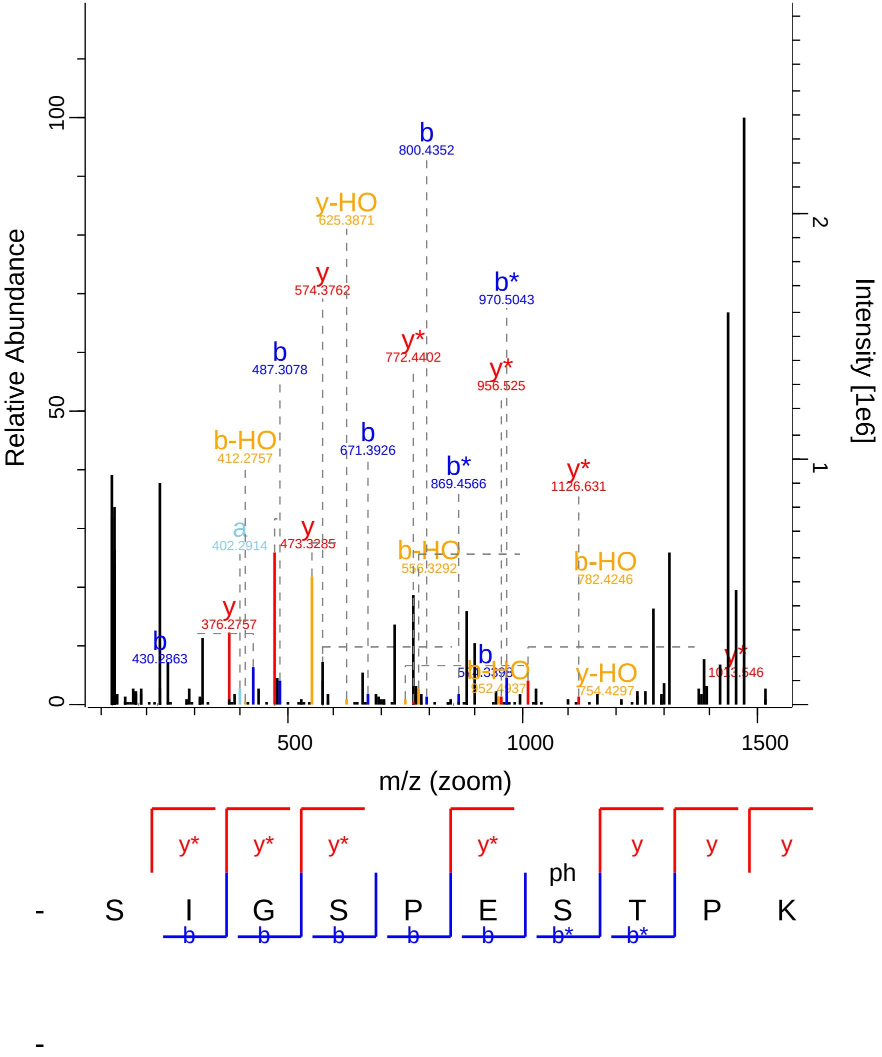

Raw File	Scan	Method	Score	m/z	Gene names
RO-QC-160121_ZackD_SS_Set_02_Fractio	13875	FTMS; HC	99.44	868.44	DKC1

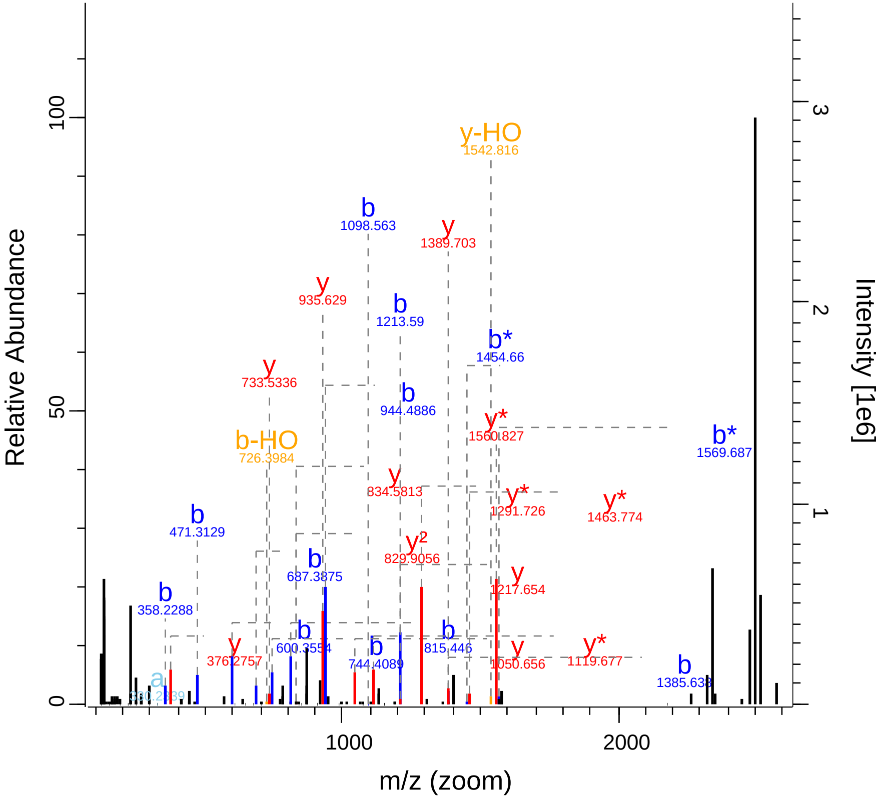

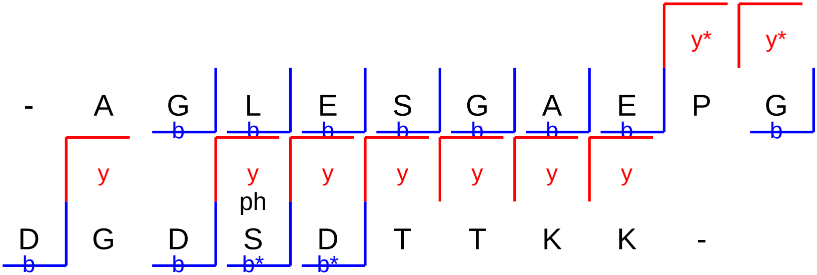

Raw File	Scan	Method	Score	m/z	Gene names
RO-QC-160121_ZackD_SS_Set_02_Fractio	15665	FTMS; HC	58.43	997.48	XPO5

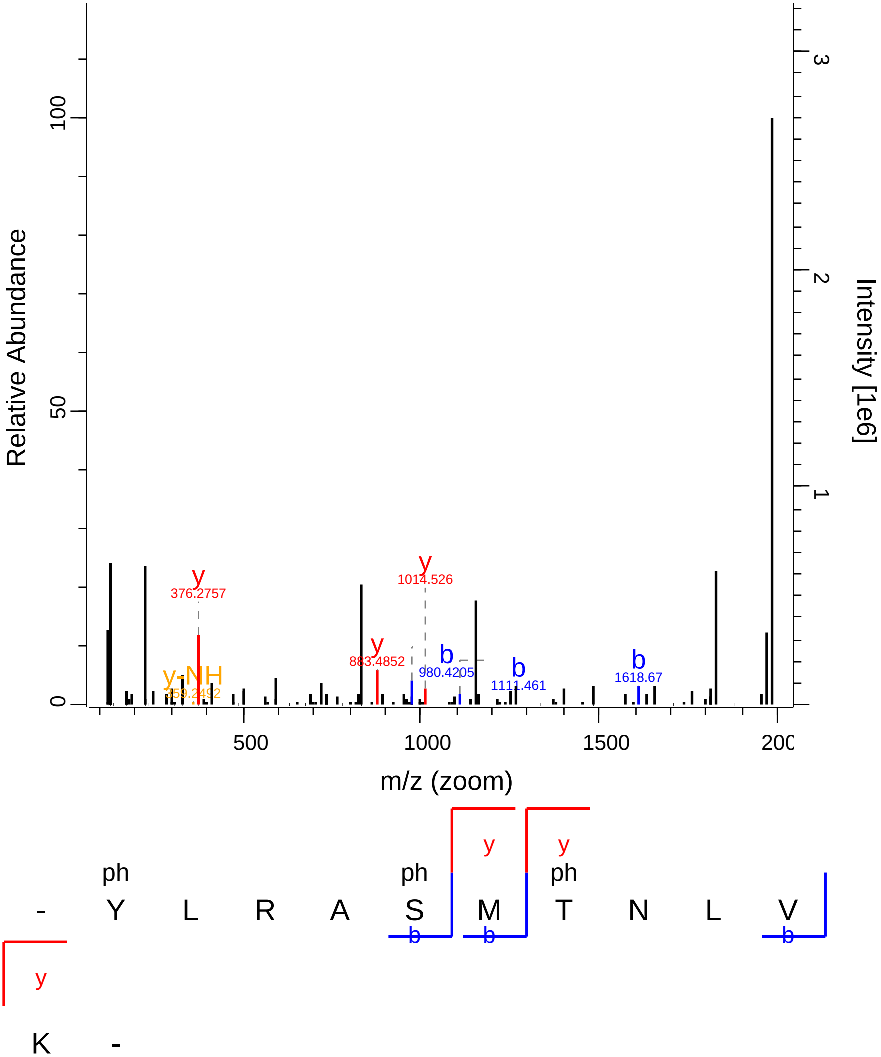

Raw File	Scan	Method	Score	m/z	Gene names
RO-QC-160121_ZackD_SS_Set_02_Fractio	15727	FTMS; HC	106.43	676.86	SEC31A

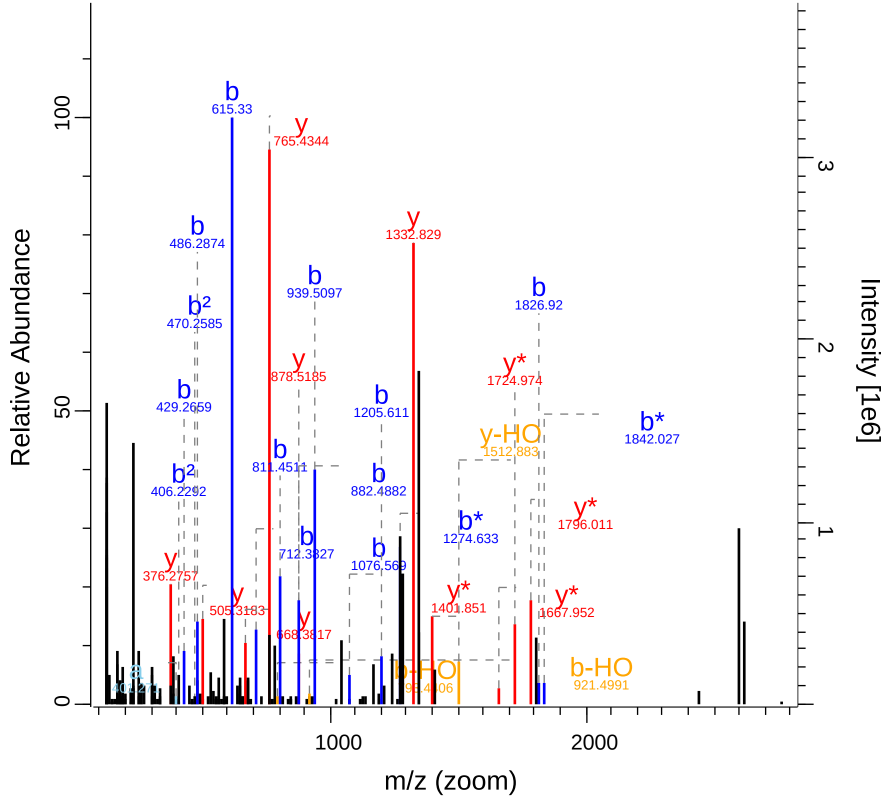

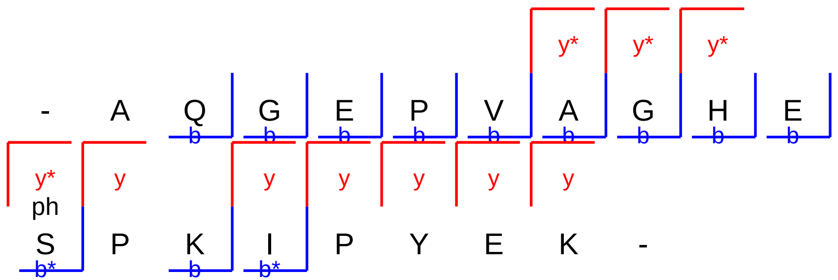

Raw File	Scan	Method	Score	m/z	Gene names
RO-QC-160121_ZackD_SS_Set_02_Fractic	16052	FTMS; HC	233.09	1219.12	MYRIP

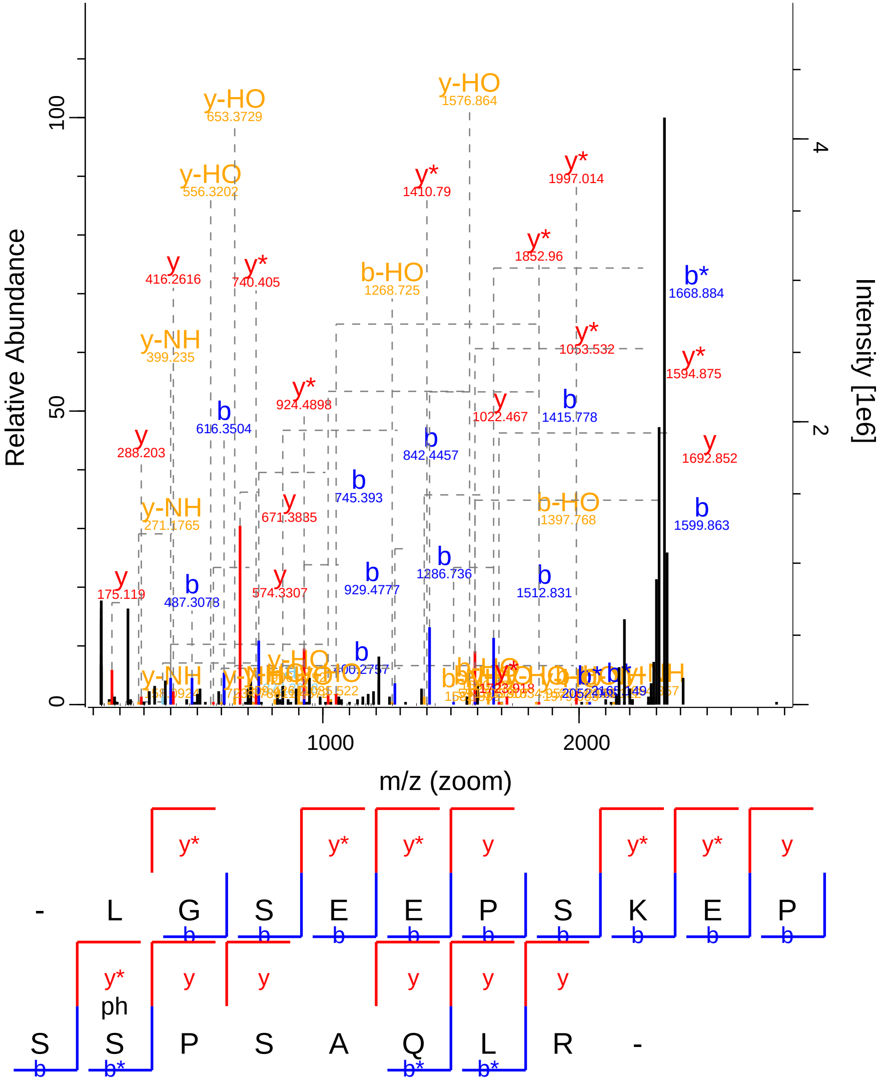

Raw File	Scan	Method	Score	m/z	Gene names
RO-QC-160121_ZackD_SS_Set_02_Fractic	16617	FTMS; HC	180.71	1040.16	SLC9A1

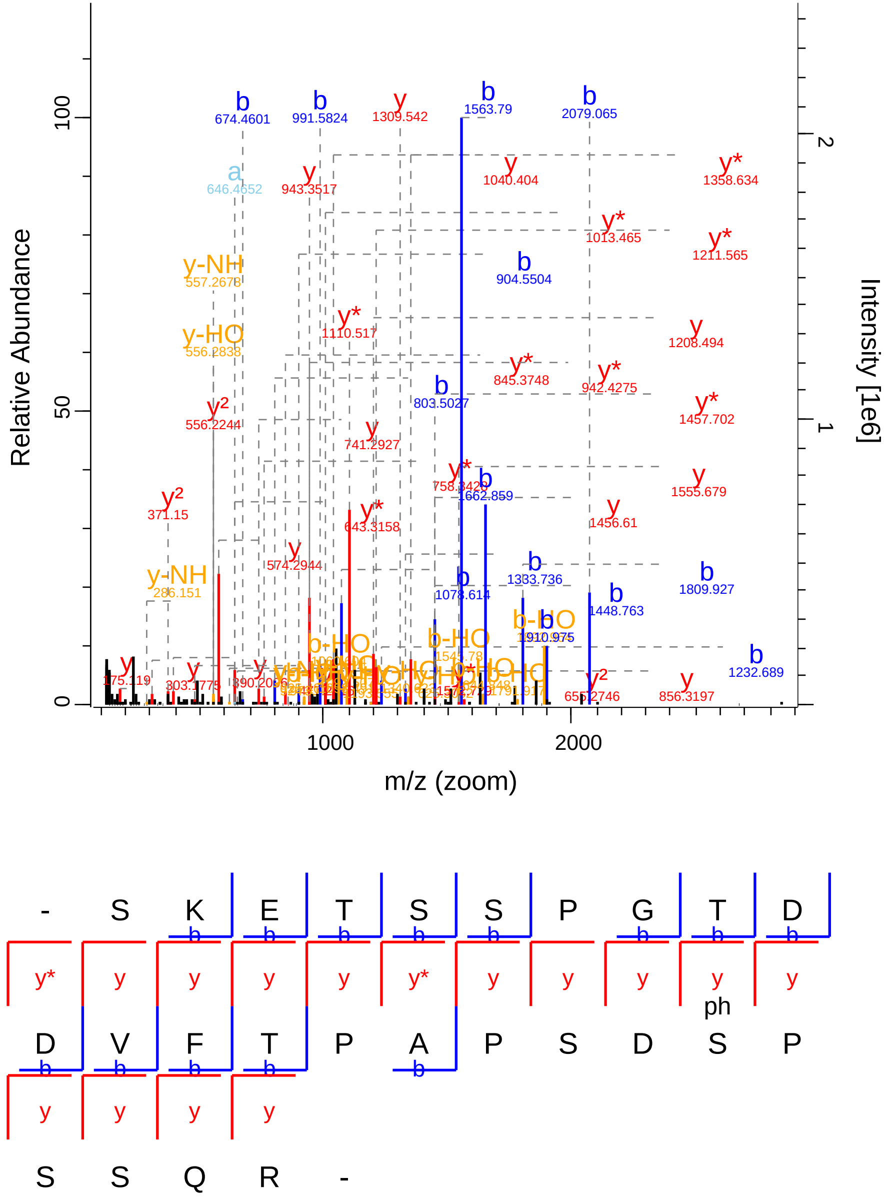

Raw File	Scan	Method	Score	m/z	Gene names
RO-QC-160121_ZackD_SS_Set_02_Fractio	17100	FTMS; HC	47.28	670.74	HNRNPF

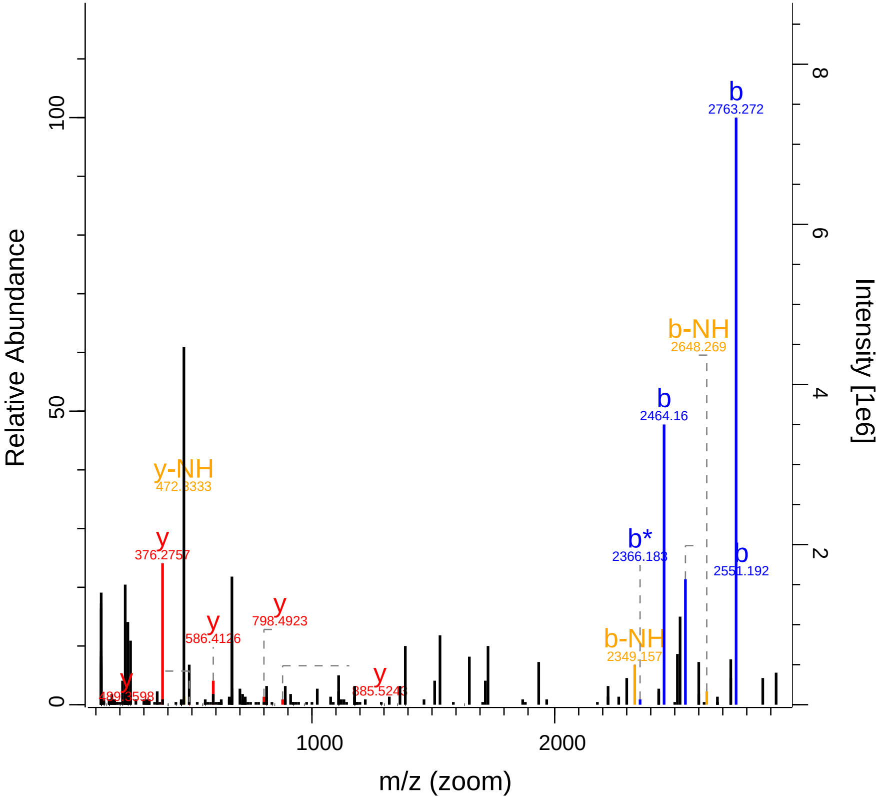

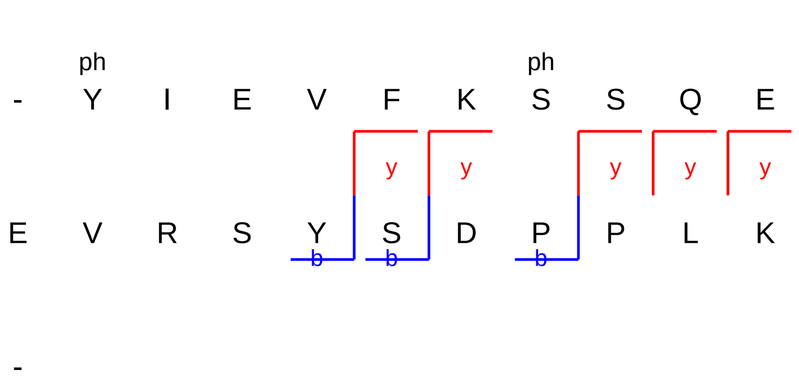

Raw File	Scan	Method	Score	m/z	Gene names
RO-QC-160121_ZackD_SS_Set_02_Fractio	17276	FTMS; HC	48.49	763.65	BIN1

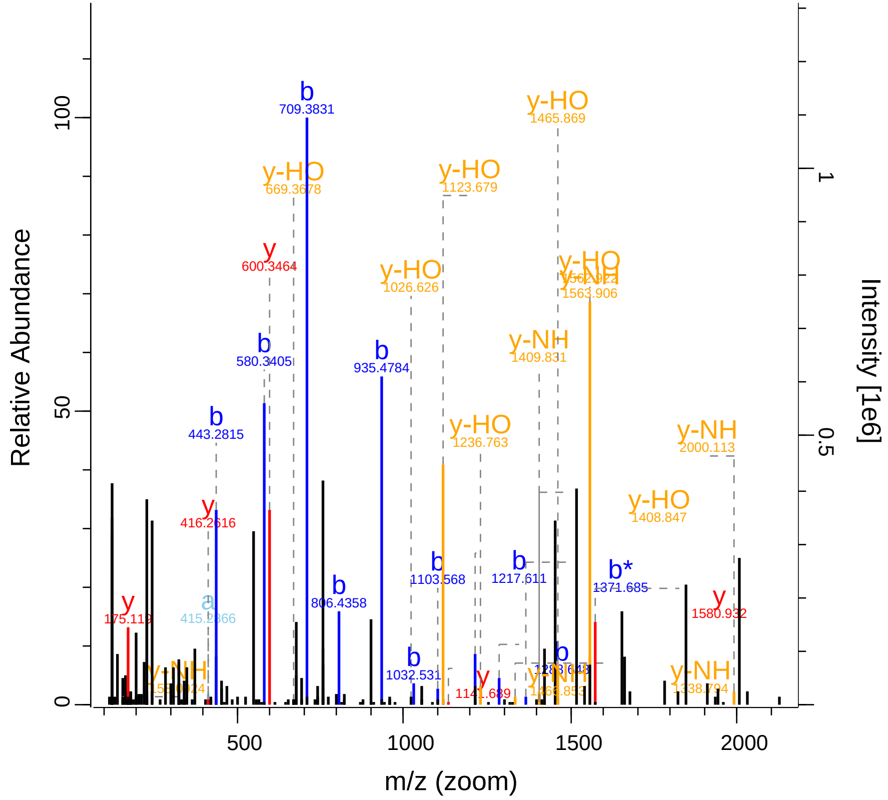

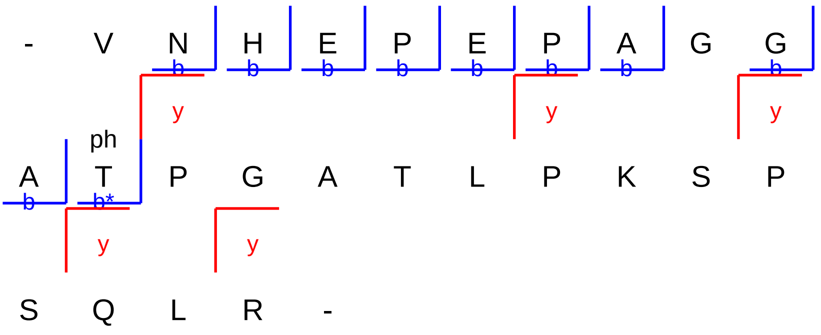

Raw File	Scan	Method	Score	m/z	Gene names
RO-QC-160121_ZackD_SS_Set_02_Fractic	17803	FTMS; HC	76.33	1110.54	TNFRSF10D

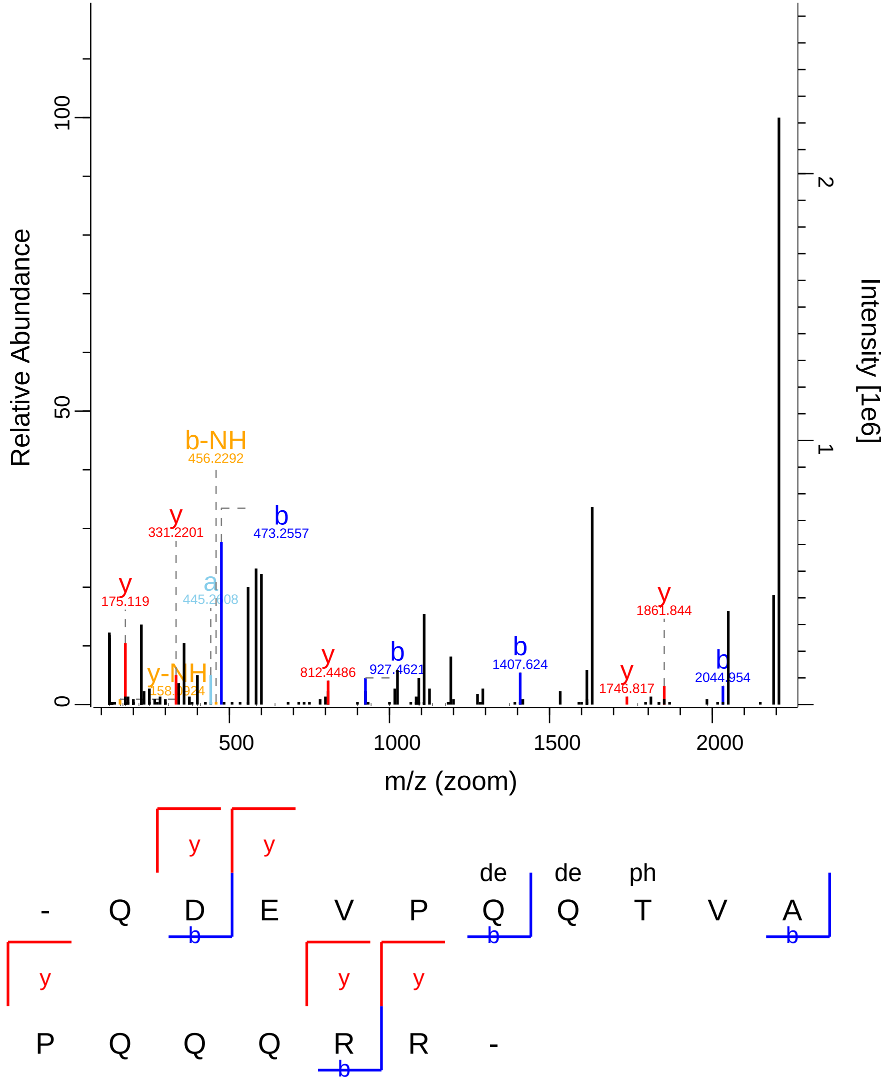

Raw File	Scan	Method	Score	m/z	Gene names
RO-QC-160121_ZackD_SS_Set_02_Fractior	18554	FTMS; HCI	156.94	935.4	PDCD5

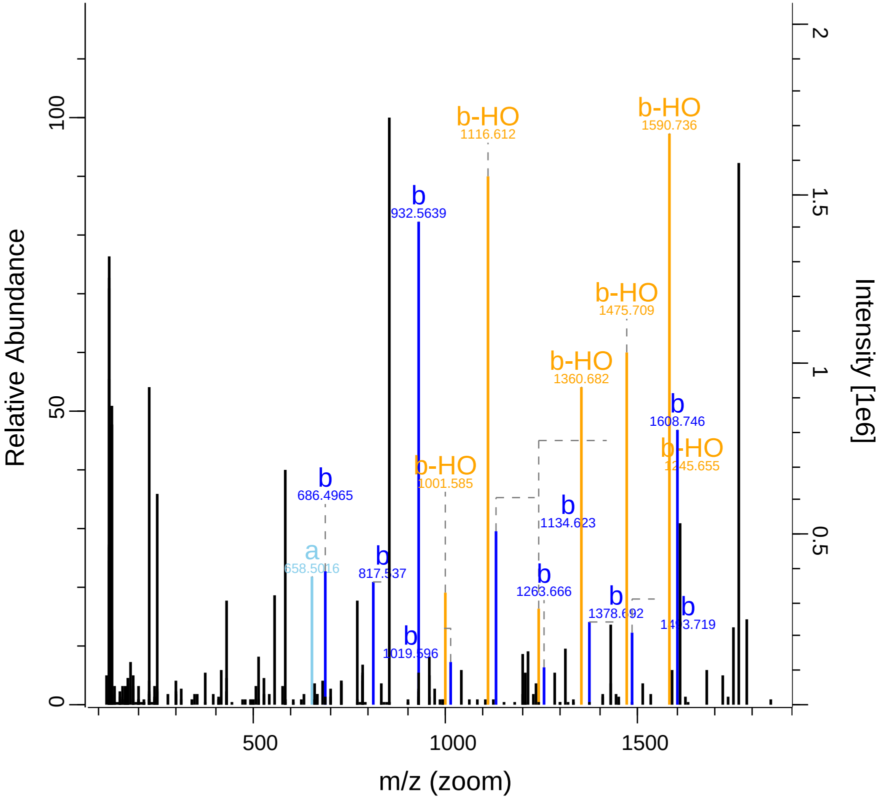

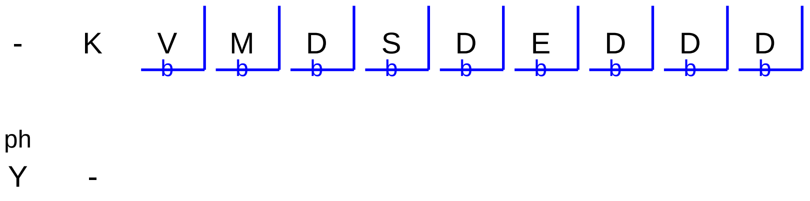

Raw File	Scan	Method	Score	m/z	Gene names
RO-QC-160121_ZackD_SS_Set_02_Fractic	18640	FTMS; HC	104.43	1015.51	SQSTM1

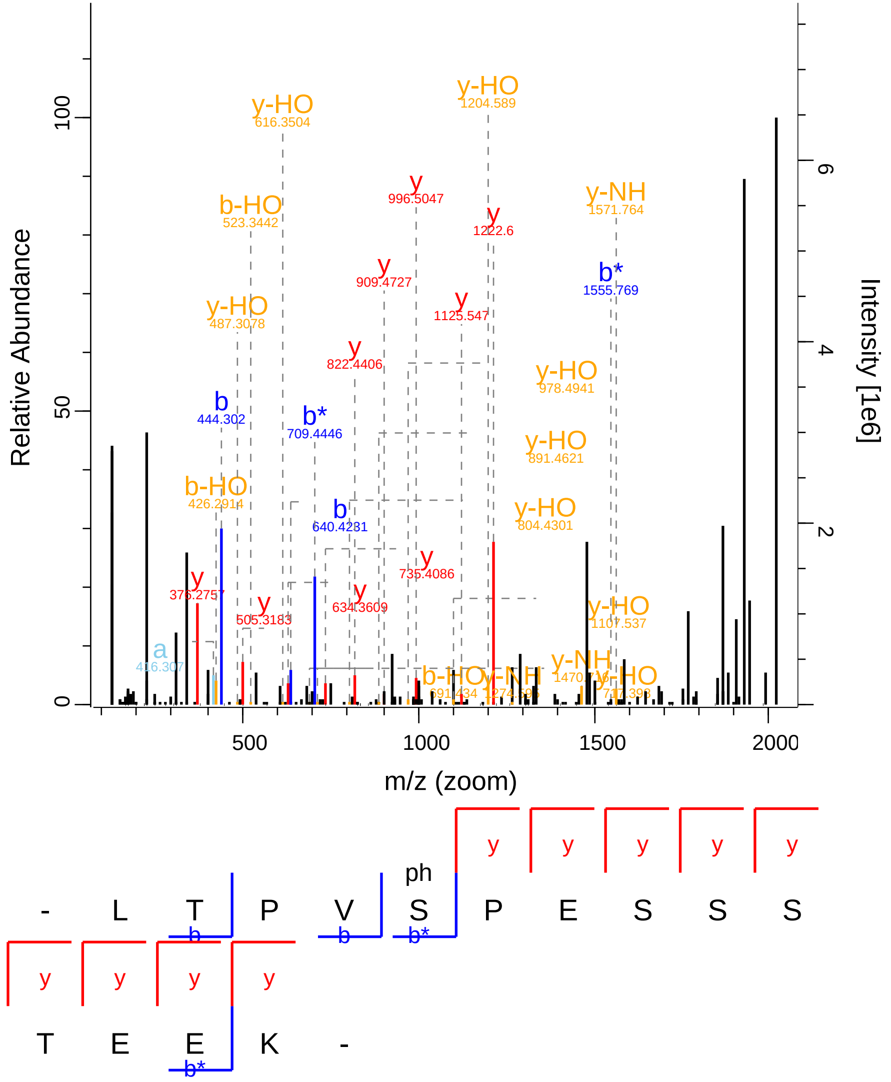

Raw File	Scan	Method	Score	m/z	Gene names
RO-QC-160121_ZackD_SS_Set_02_Fractio	18710	FTMS; HC	43.51	537.56	CCDC60

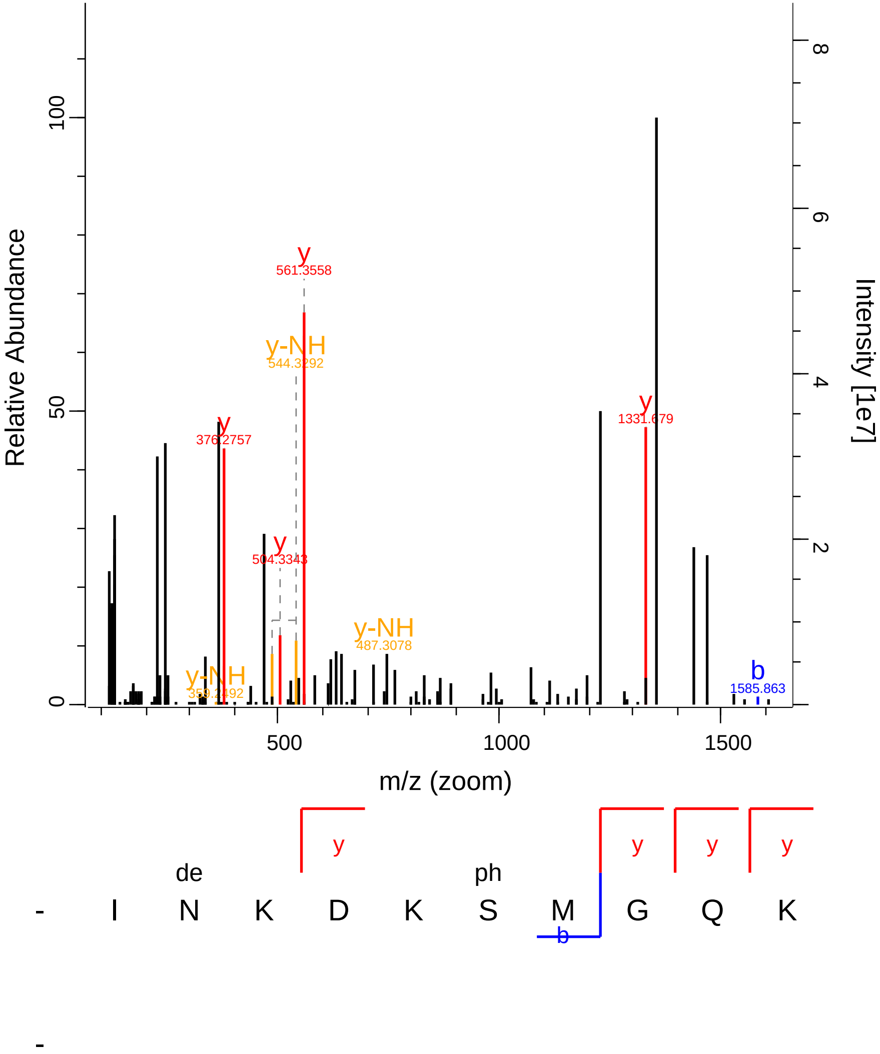

Raw File	Scan	Method	Score	m/z	Gene names
RO-QC-160121_ZackD_SS_Set_02_Fractic	20174	FTMS; HC	76.94	1225.71	RAB11FIP1

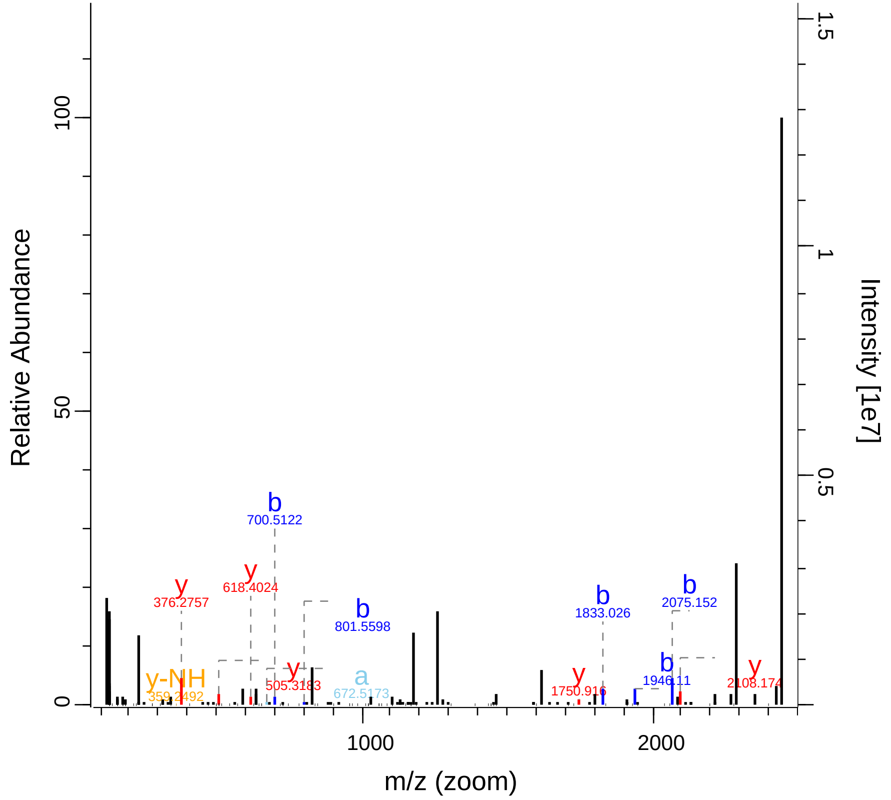

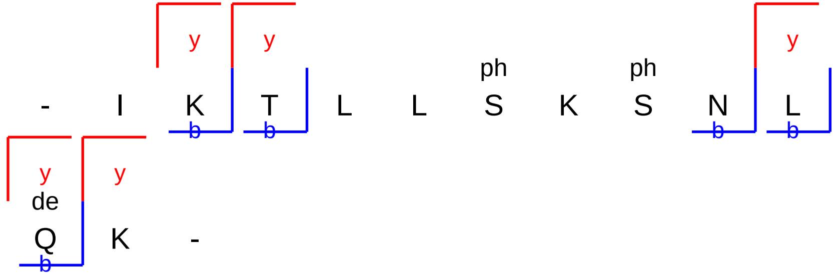

Raw File	Scan	Method	Score	m/z	Gene names
RO-QC-160121_ZackD_SS_Set_02_Fractio	21987	FTMS; HC	86.01	832.98	POSTN

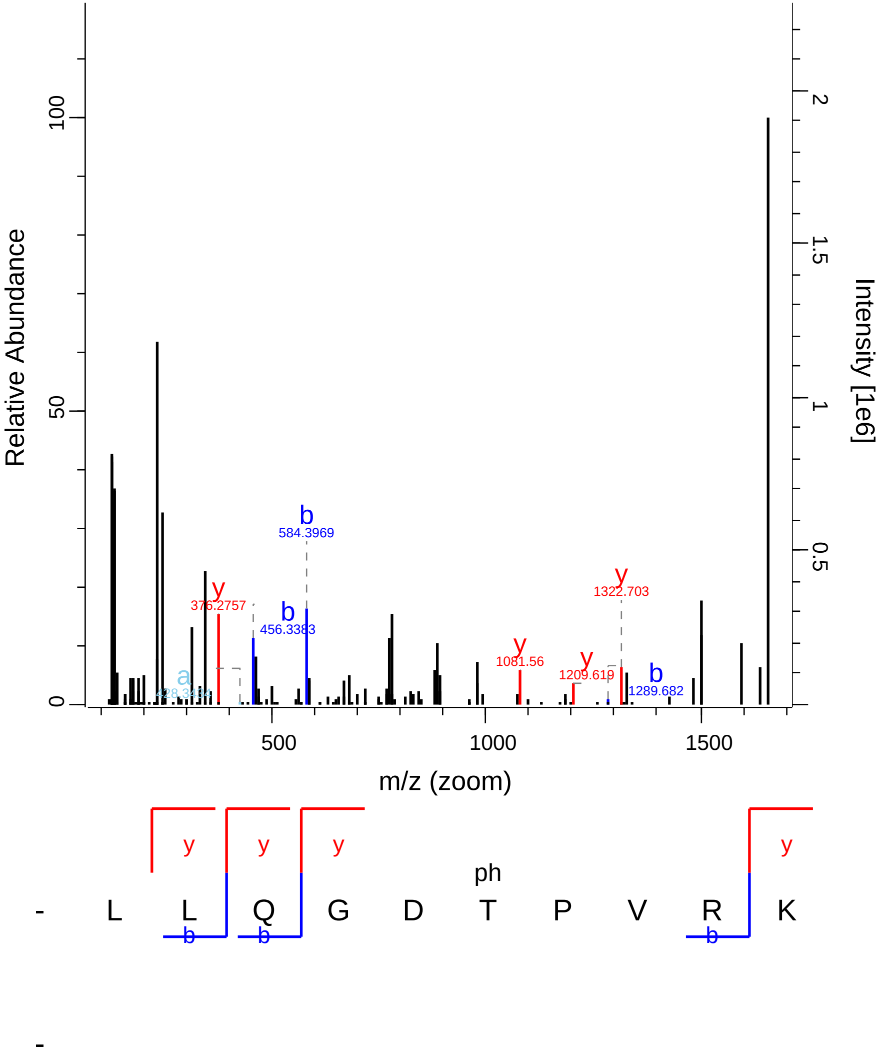

Raw File	Scan	Method	Score	m/z	Gene names
RO-QC-160121_ZackD_SS_Set_02_Fractic	26170	FTMS; HC	60.97	1133.14	BIRC6

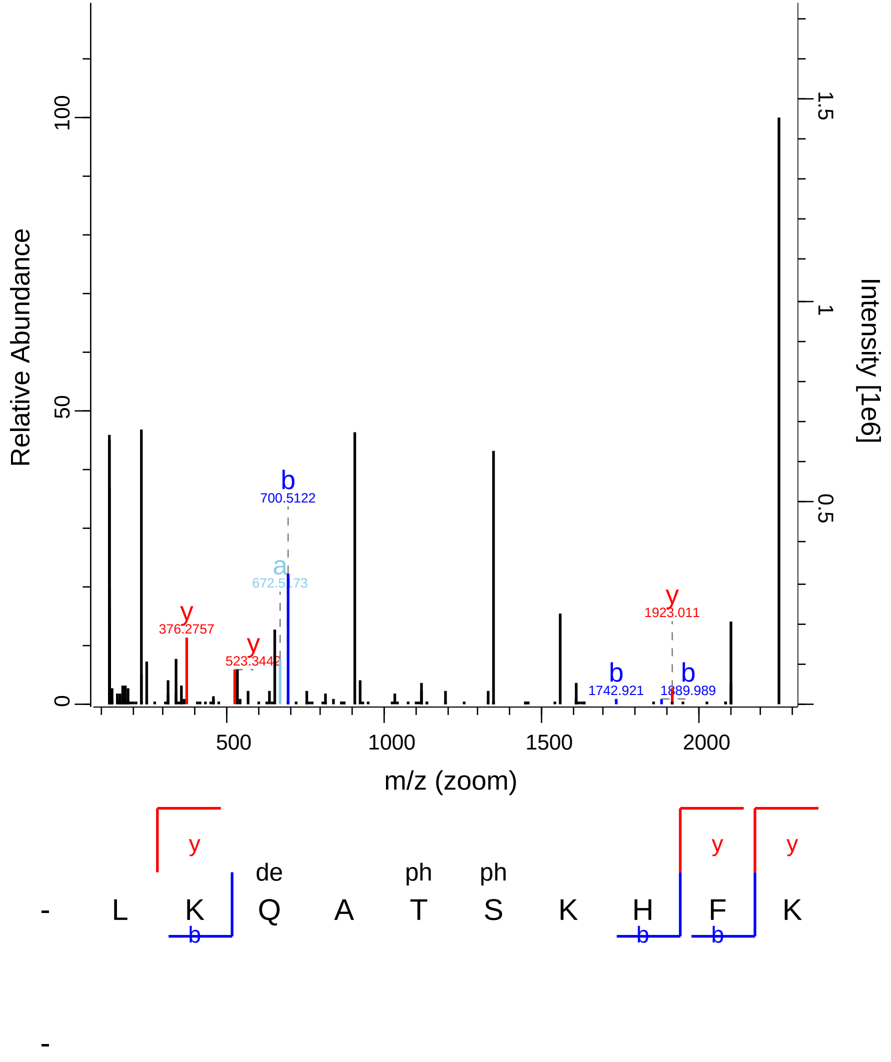

Raw File	Scan	Method	Score	m/z	Gene names
RO-QC-160121_ZackD_SS_Set_02_Fractic	26382	FTMS; HC	81.87	1168.63	BRD4

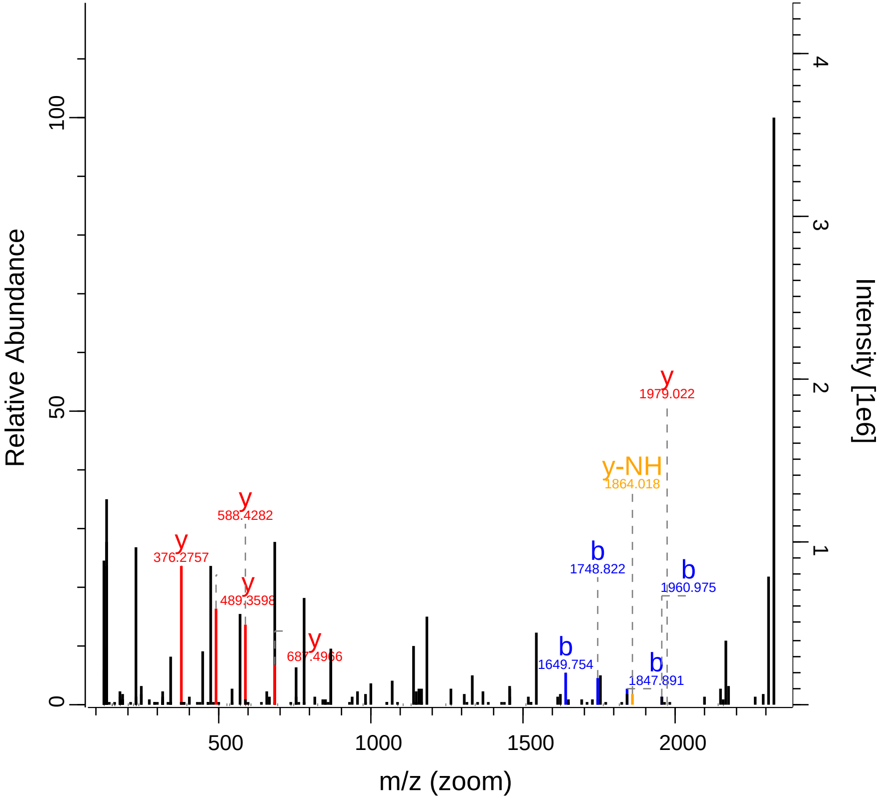

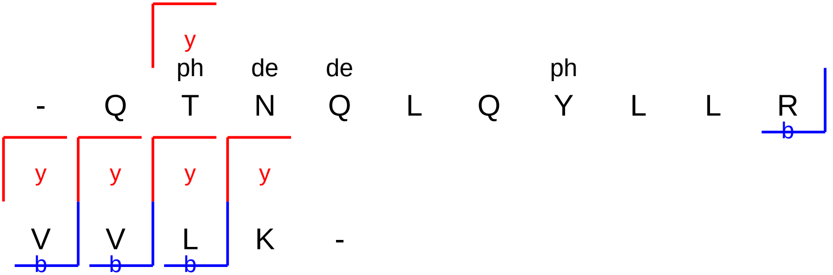

Raw File	Scan	Method	Score	m/z	Gene names
RO-QC-160121_ZackD_SS_Set_02_Fractio	27246	FTMS; HC	117.52	655.85	BCLAF1

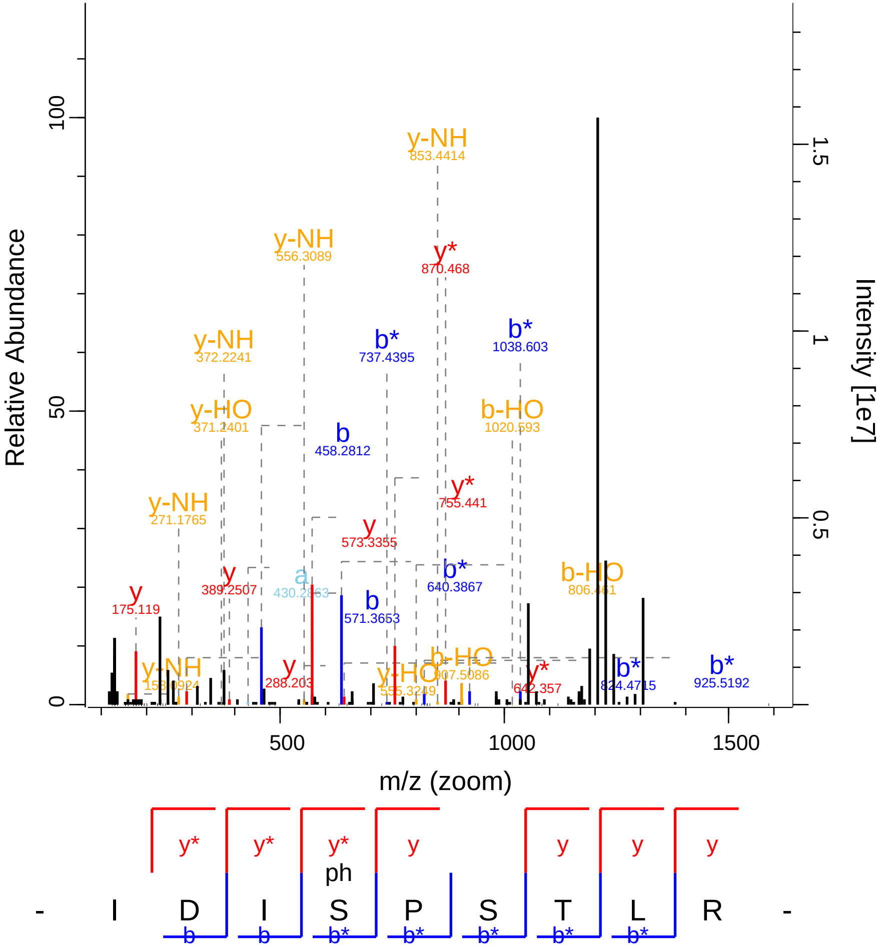

Raw File	Scan	Method	Score	m/z	Gene names
RO-QC-160121_ZackD_SS_Set_02_Fractio	29210	FTMS; HC	52.03	990.86	PMFBP1

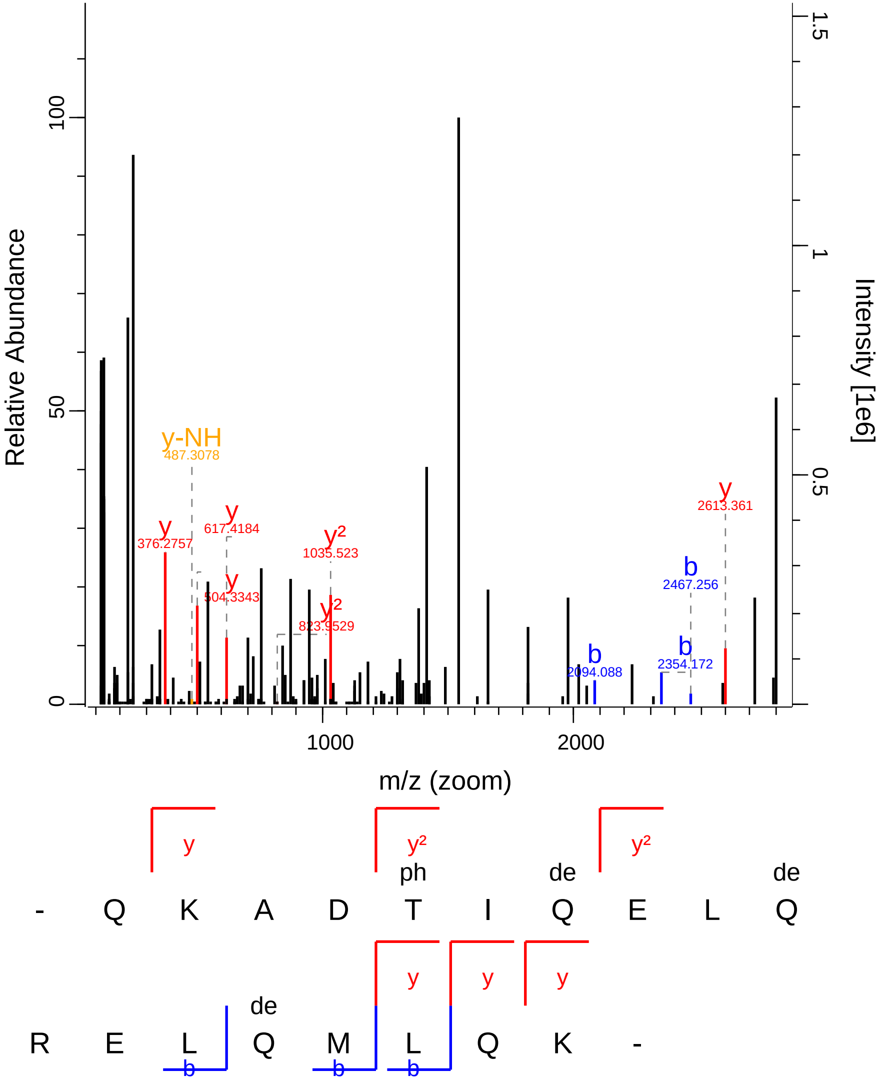

Raw File	Scan	Method	Score	m/z	Gene names
RO-QC-160121_ZackD_SS_Set_02_Fractic	29404	FTMS; HC	65.22	1101.62	RPS6KA5

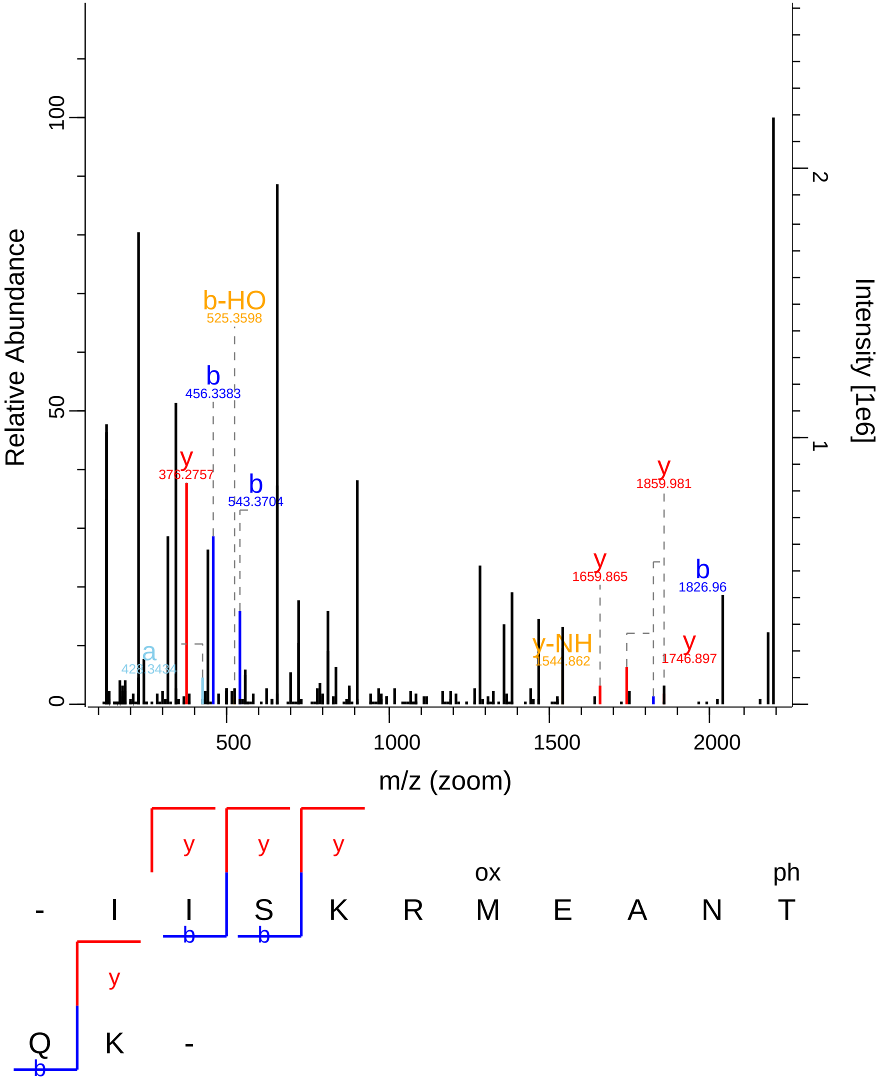

Raw File	Scan	Method	Score	m/z	Gene names
RO-QC-160121_ZackD_SS_Set_02_Fractic	29405	FTMS; HC	69.59	1255.17	CENPF

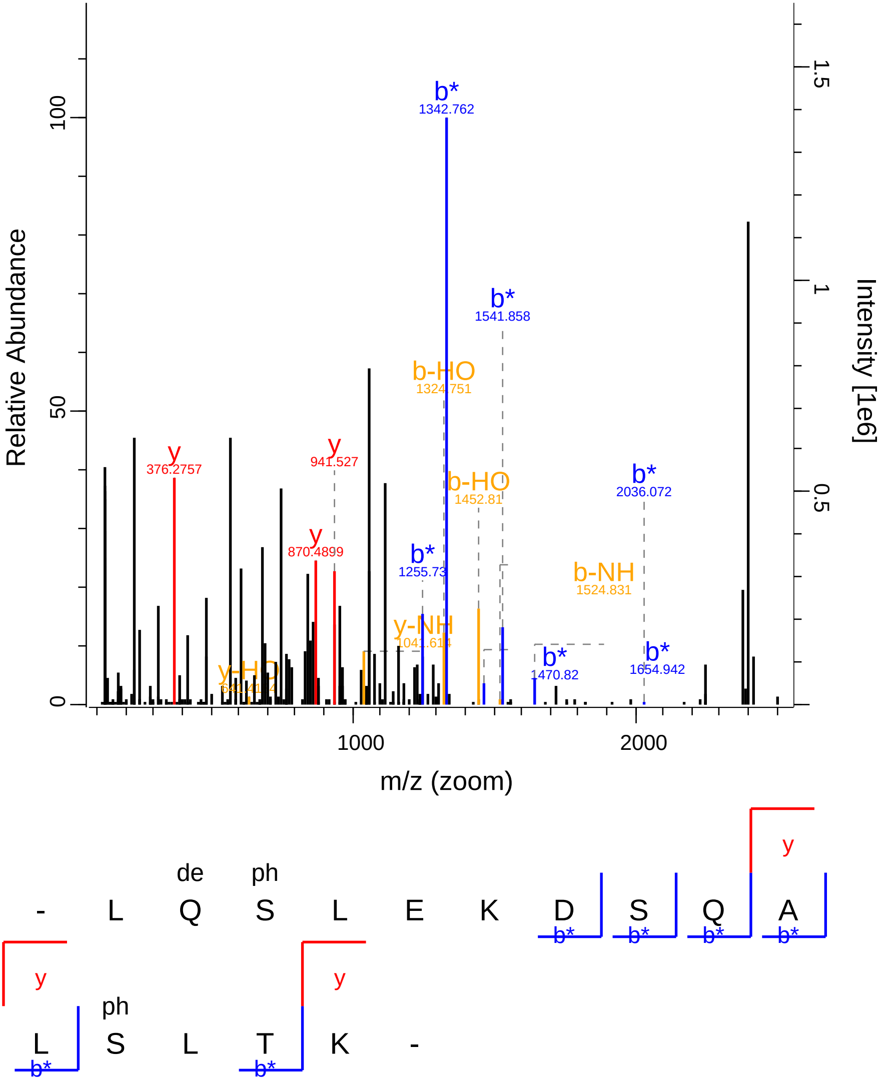

Raw File	Scan	Method	Score	m/z	Gene names
RO-QC-160121_ZackD_SS_Set_02_Fractic	30354	FTMS; HC	64.44	1383.24	CCDC158

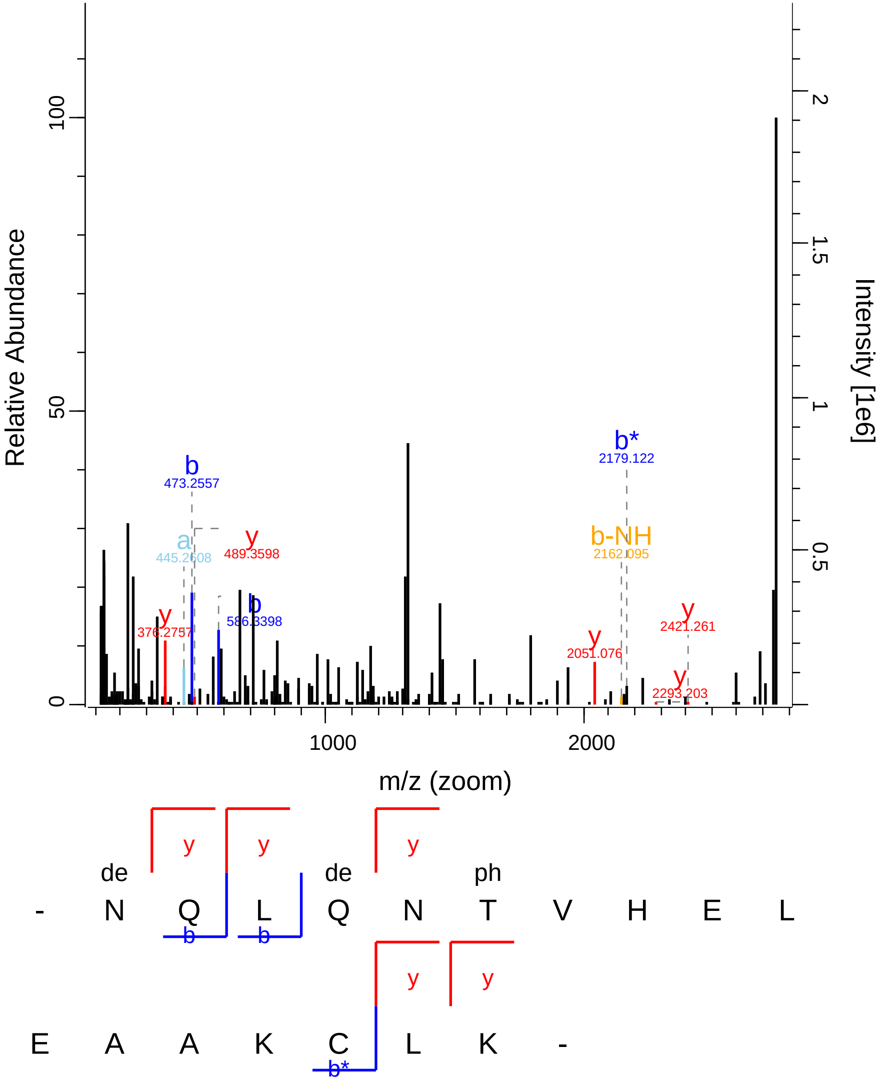

Raw File	Scan	Method	Score	m/z	Gene names
RO-QC-160121_ZackD_SS_Set_02_Fractio	31295	FTMS; HC	49.42	736.36	RRBP1

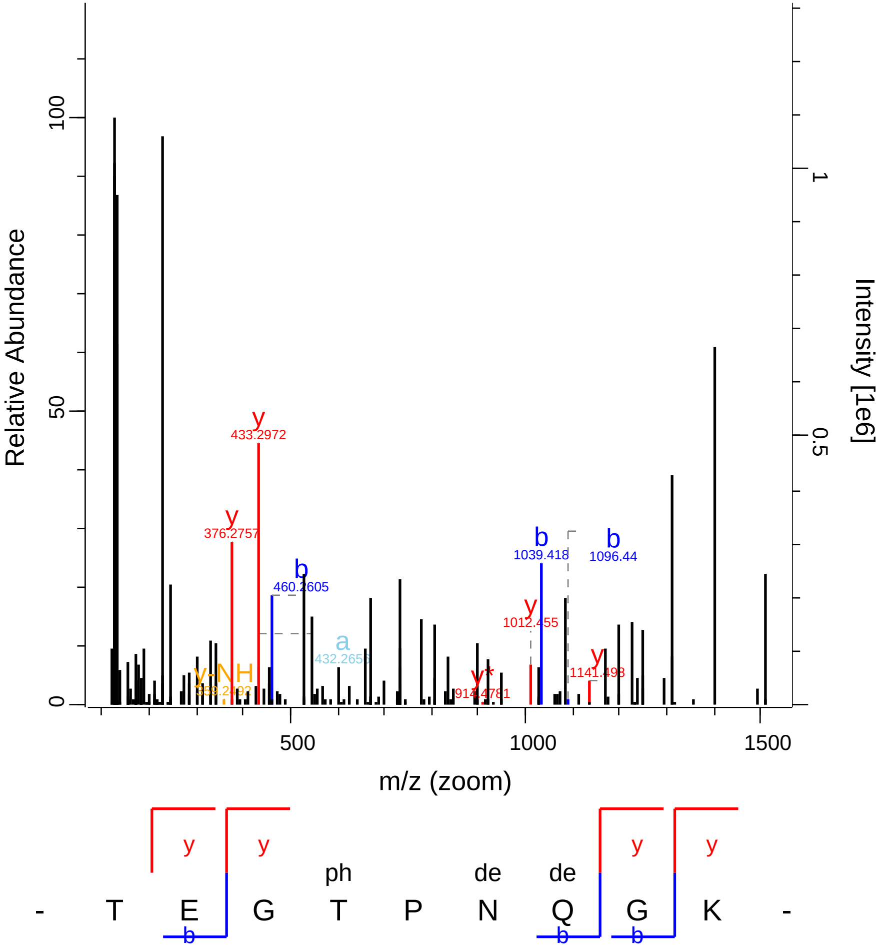

Raw File	Scan	Method	Score	m/z	Gene names
RO-QC-160121_ZackD_SS_Set_02_Fractior	32475	FTMS; HCI	45.7	821.2	MMP1

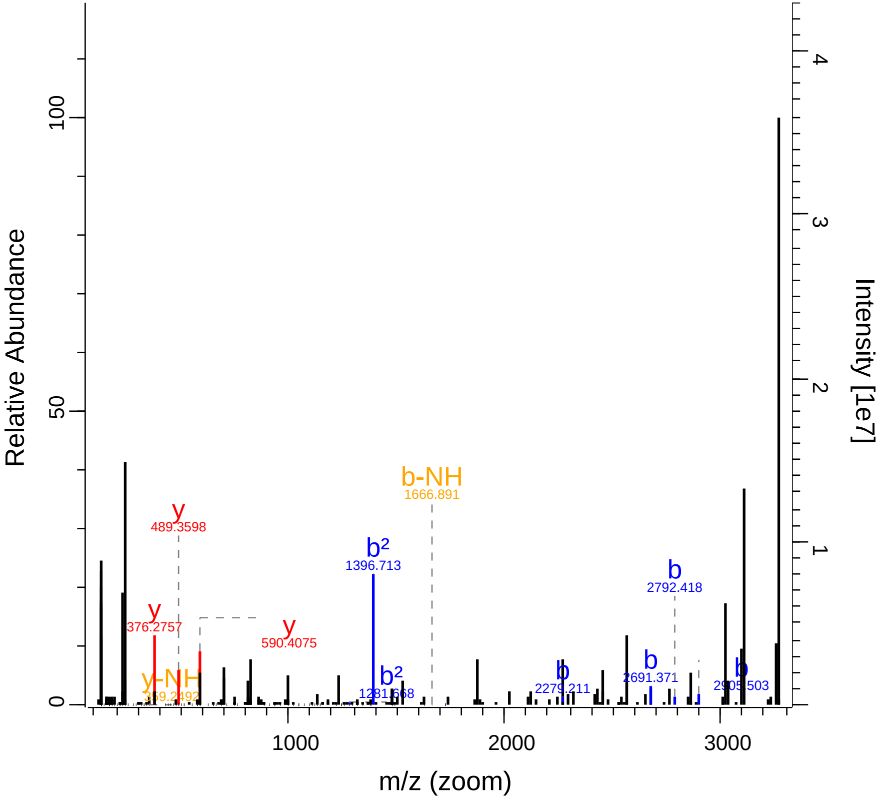

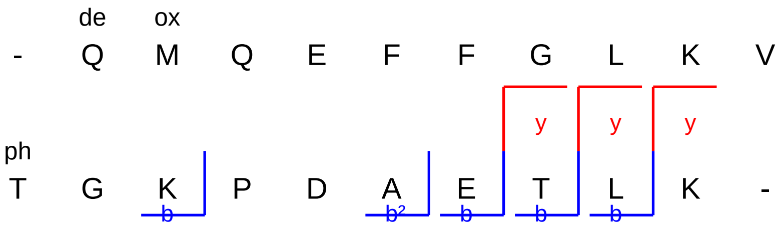

Raw File	Scan	Method	Score	m/z	Gene names
RO-QC-160121_ZackD_SS_Set_02_Fractior	32680	FTMS; HCI	47.2	821.2	TRMT44

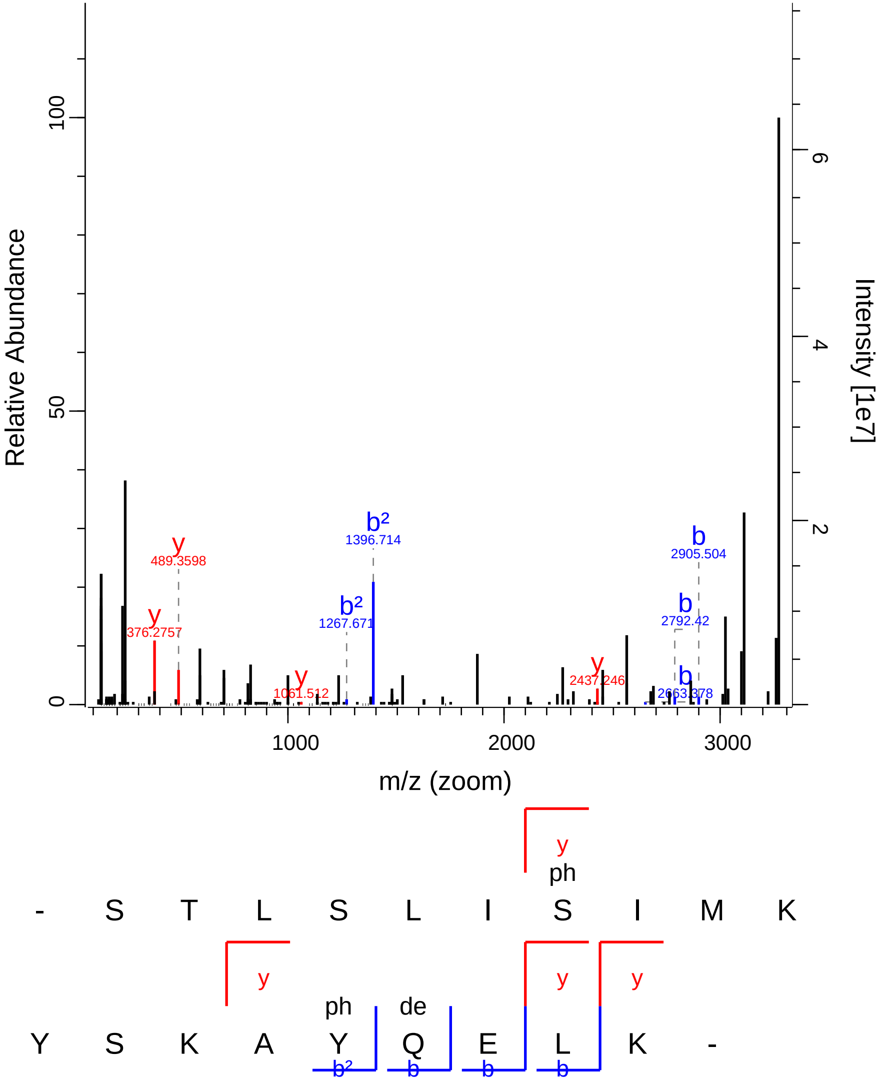

Raw File	Scan	Method	Score	m/z	Gene names
RO-QC-160121_ZackD_SS_Set_02_Fractic	33319	FTMS; HC	177.04	1190.12	RSL1D1

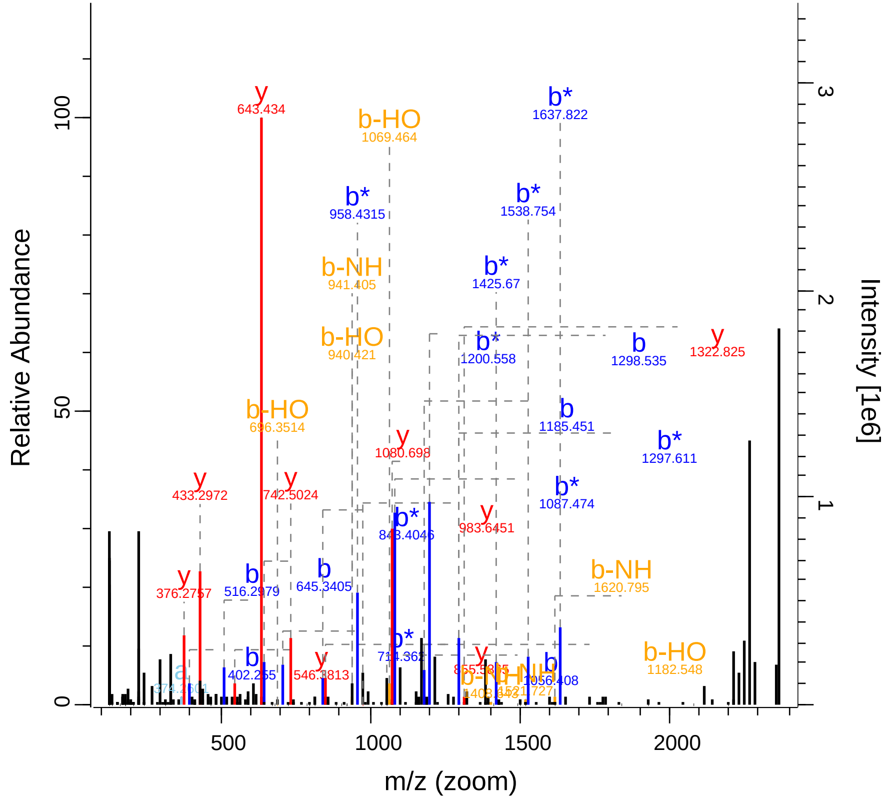

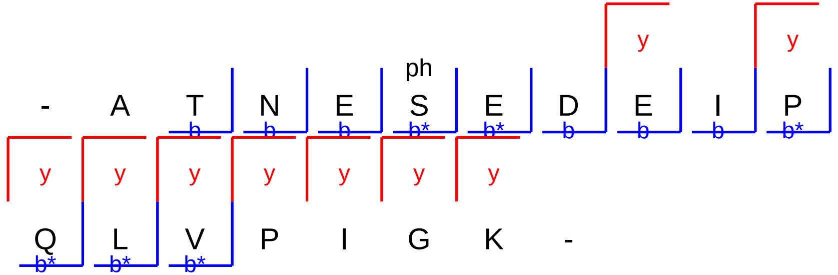

Raw File	Scan	Method	Score	m/z	Gene names
RO-QC-160121_ZackD_SS_Set_02_Fractic	36911	FTMS; HC	80.23	1081.62	C6

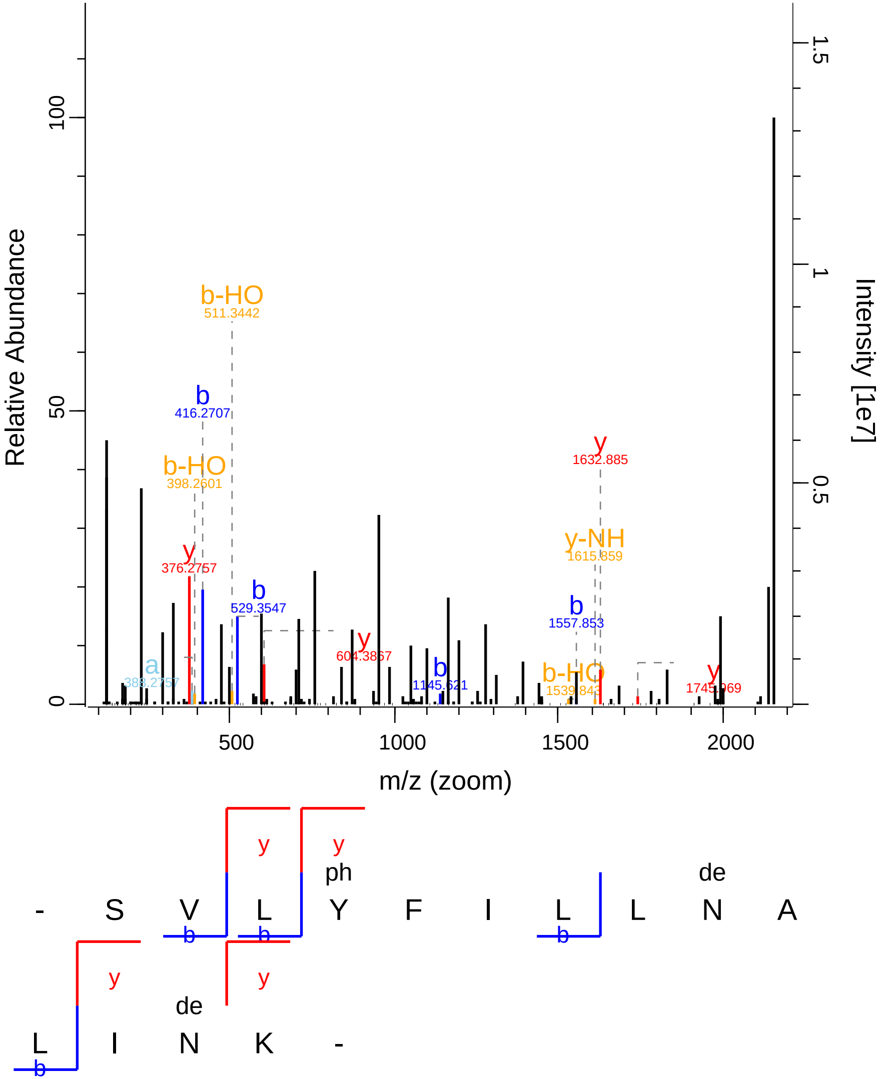

Raw File	Scan	Method	Score	m/z	Gene names
RO-QC-160121_ZackD_SS_Set_02_Fractio	37441	FTMS; HC	53.28	946.88	ERAL1

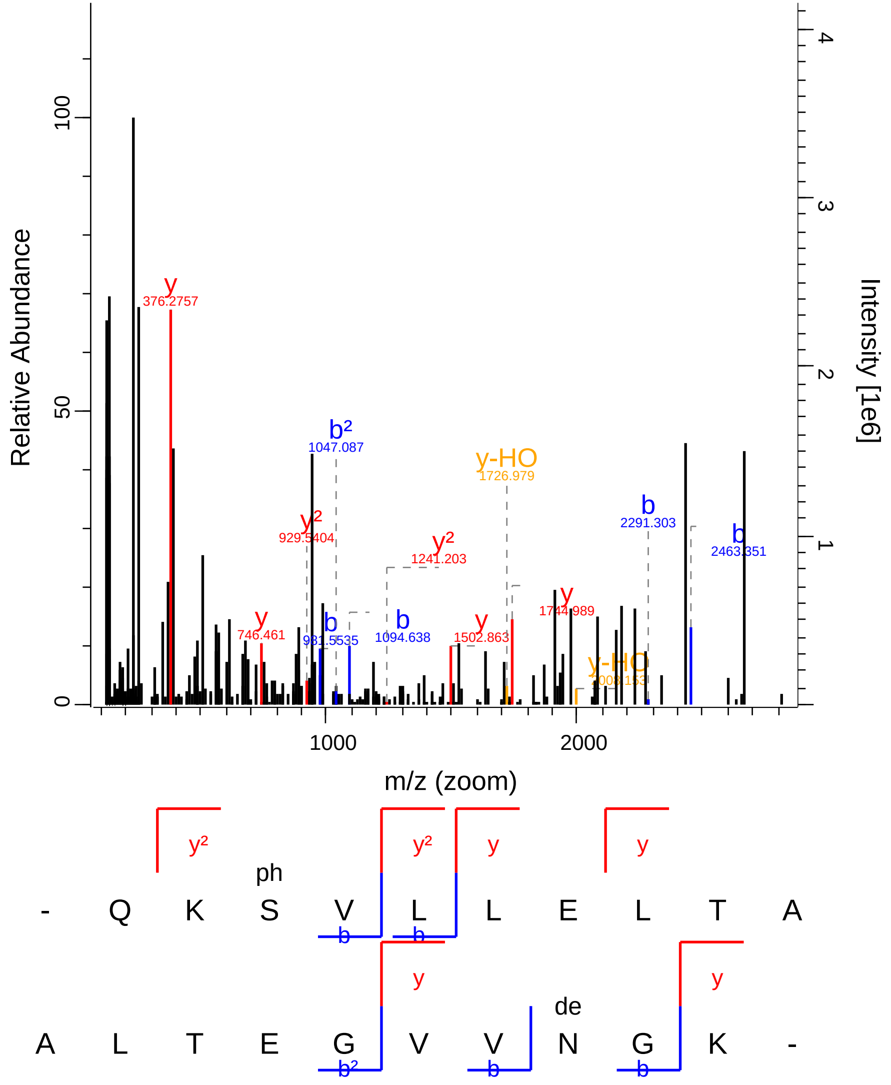

Raw File	Scan	Method	Score	m/z	Gene names
RO-QC-160121_ZackD_SS_Set_02_Fractio	37470	FTMS; HC	111.82	1028.59	ZKSCAN2

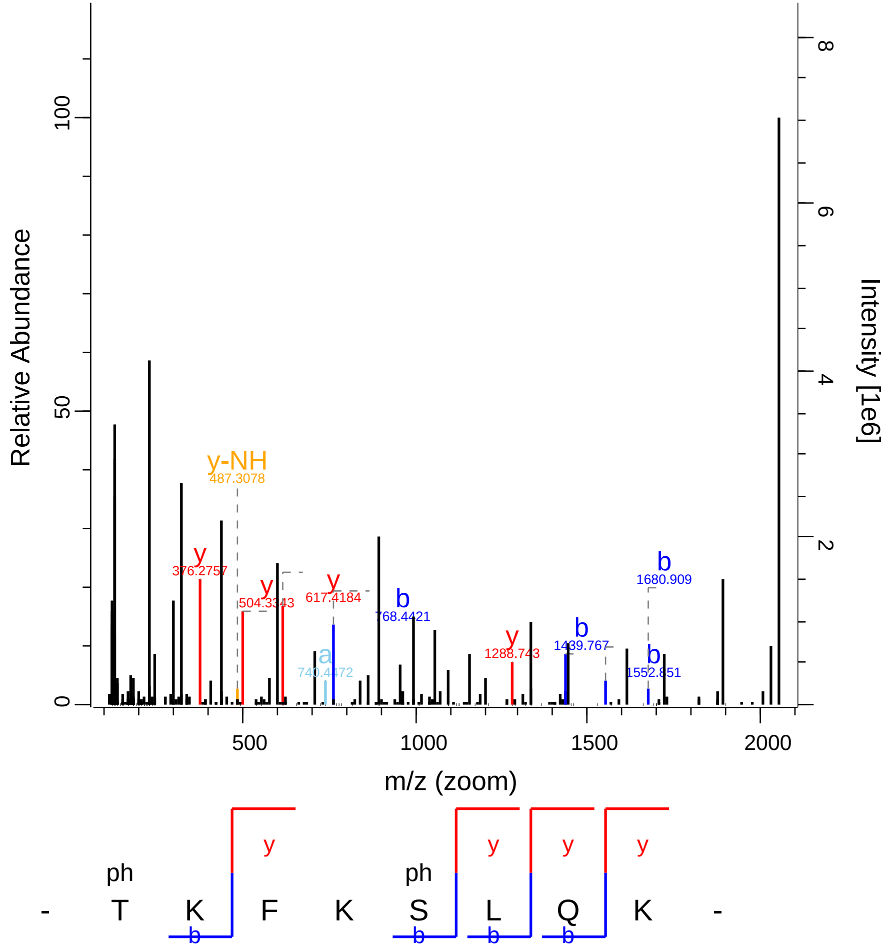

Raw File	Scan	Method	Score	m/z	Gene names
RO-QC-160121_ZackD_SS_Set_02_Fractio	38420	FTMS; HC	48.7	680.87	ASXL3

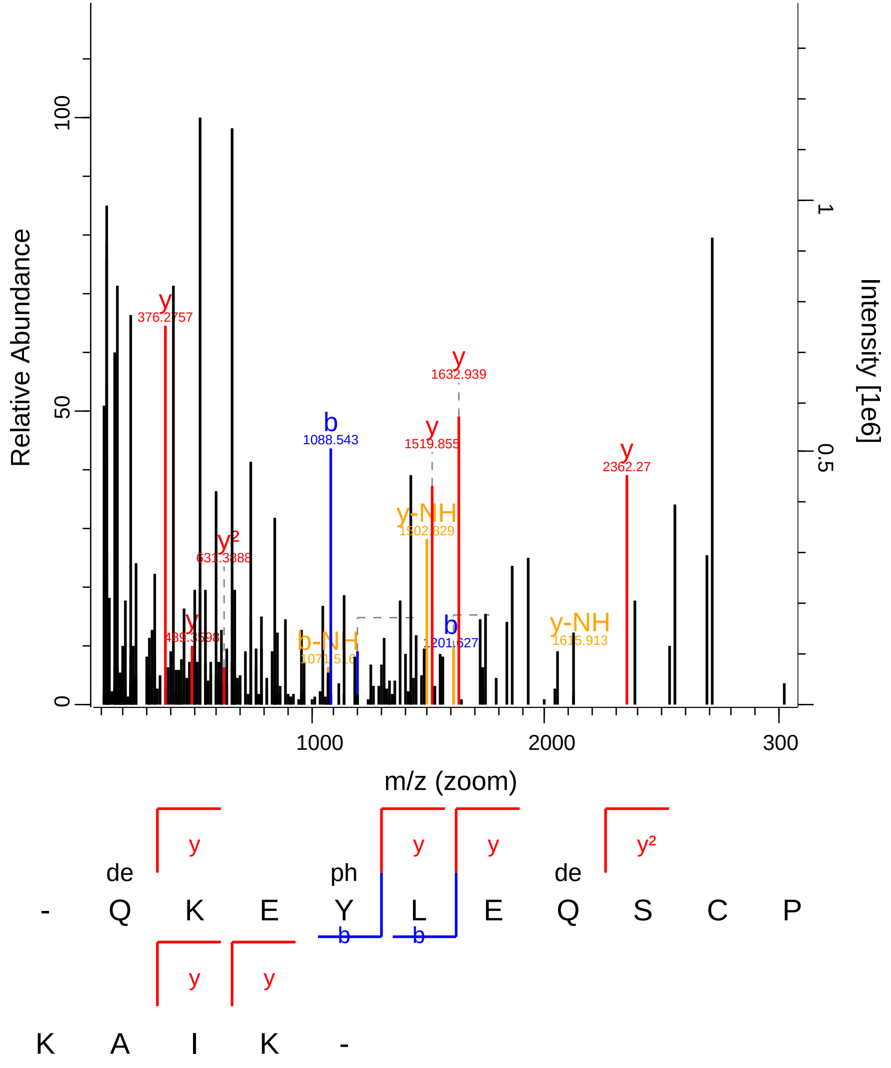

Raw File	Scan	Method	Score	m/z	Gene names
RO-QC-160121_ZackD_SS_Set_02_Fractio	39528	FTMS; HC	48.64	995.93	TRIM24

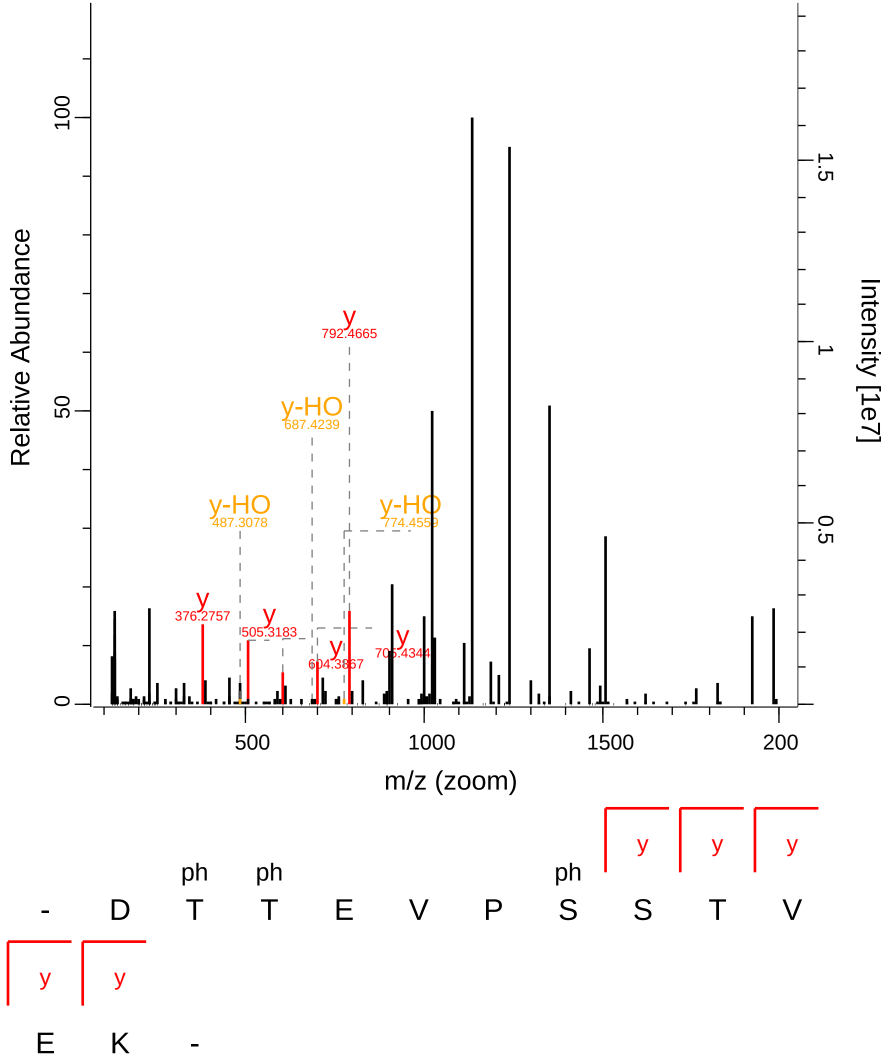

Raw File	Scan	Method	Score	m/z	Gene names
RO-QC-160121_ZackD_SS_Set_02_Fractic	39733	FTMS; HC	48.63	1472.75	UGDH

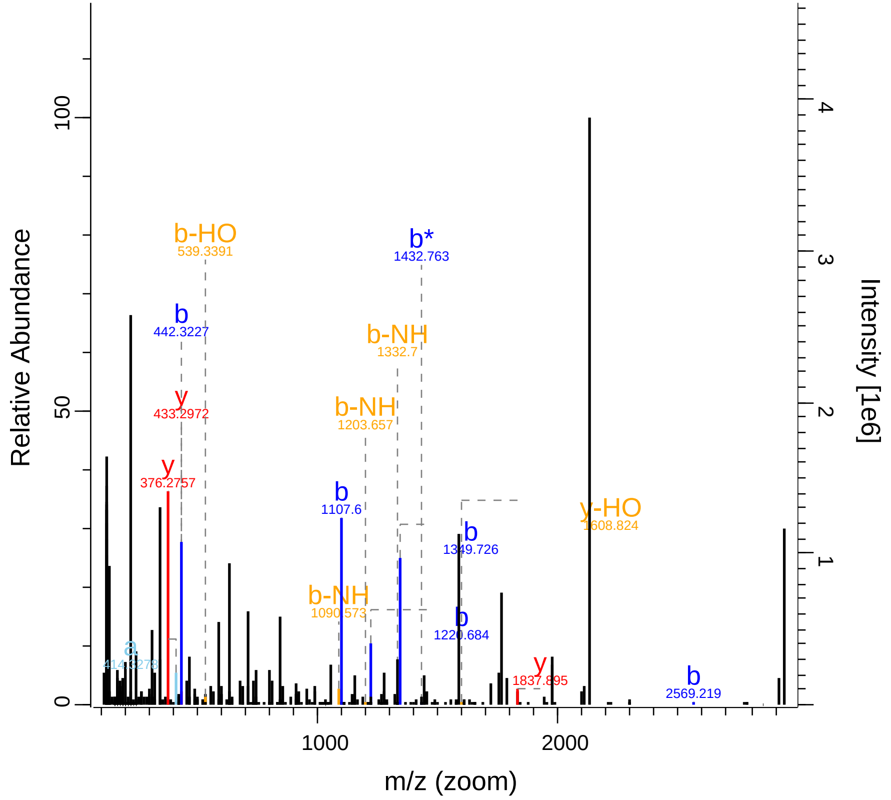

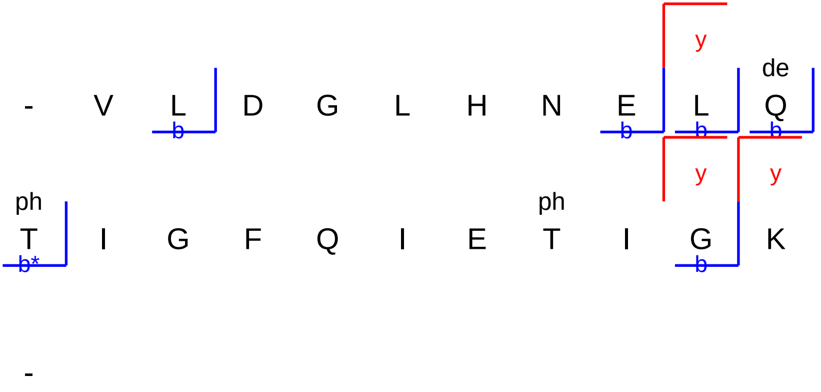

Raw File	Scan	Method	Score	m/z	Gene names
RO-QC-160121_ZackD_SS_Set_02_Fraction	4329	FTMS; HC	92.86	741.82	SORBS3

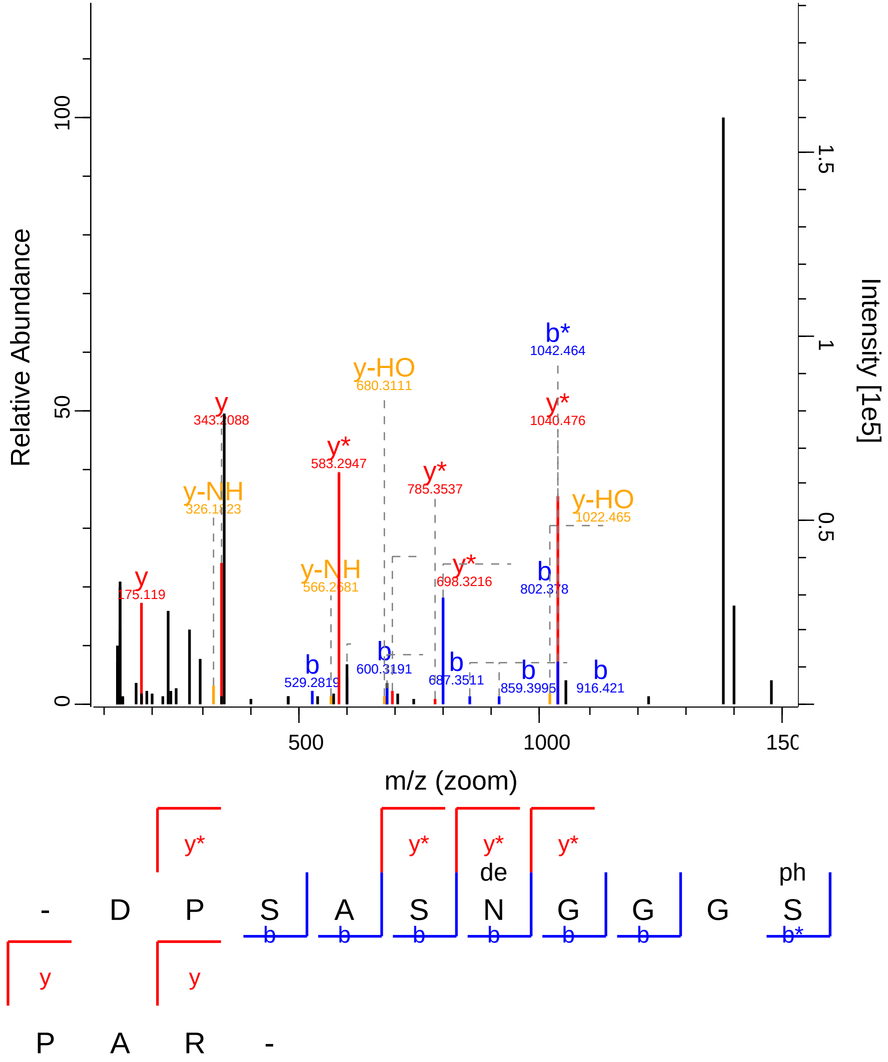

Raw File	Scan	Method	Score	m/z	Gene names
RO-QC-160121_ZackD_SS_Set_02_Fraction	4394	FTMS; HC	47.55	623.28	BCKDK

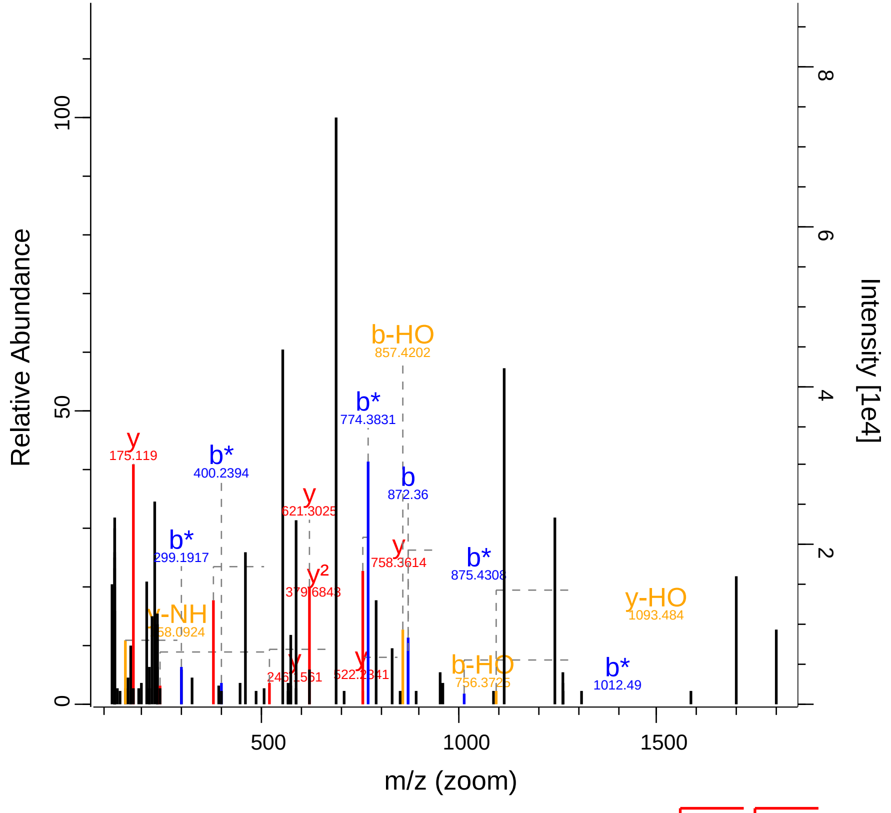

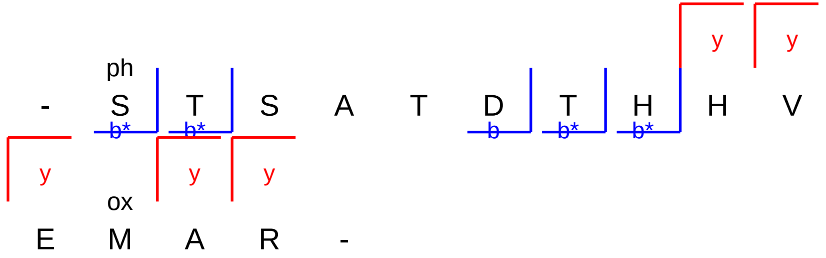

Raw File	Scan	Method	Score	m/z	Gene names
RO-QC-160121_ZackD_SS_Set_02_Fraction	4418	FTMS; HC	51.22	589.62	GYS1

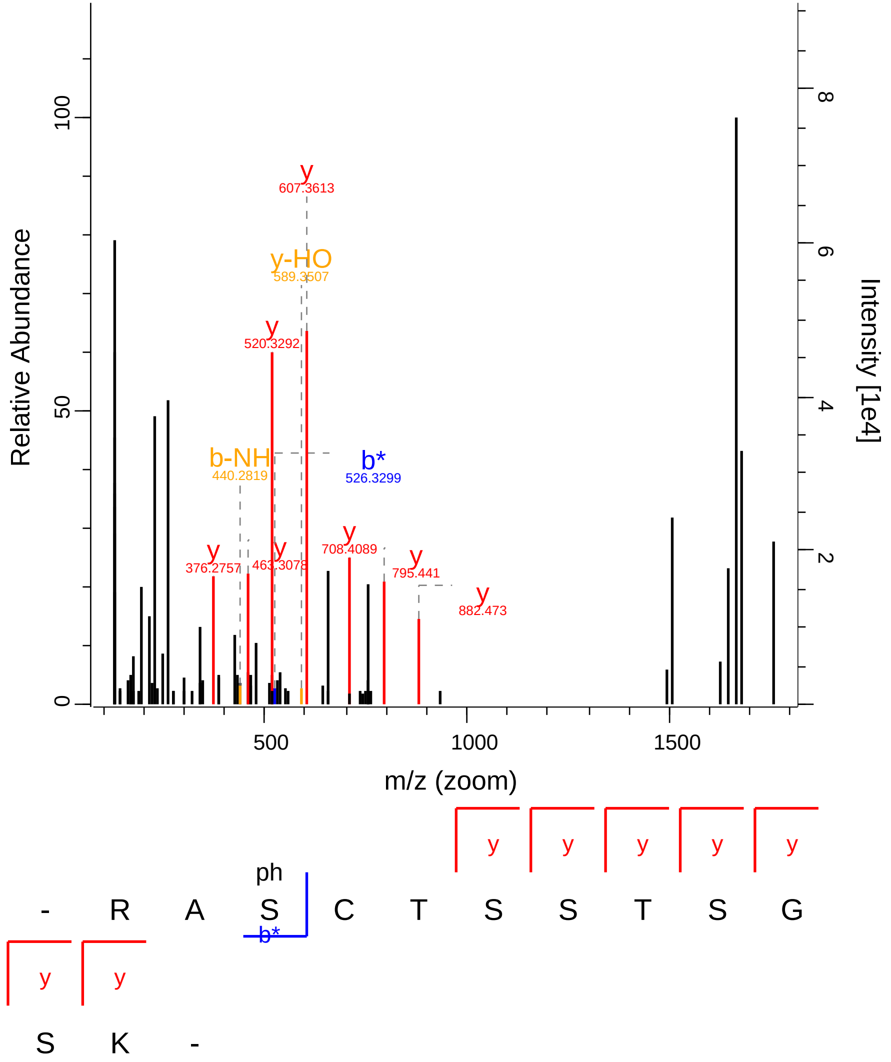

Raw File	Scan	Method	Score	m/z	Gene names
RO-QC-160121_ZackD_SS_Set_02_Fraction	4489	FTMS; HC	81.62	725.81	AAK1

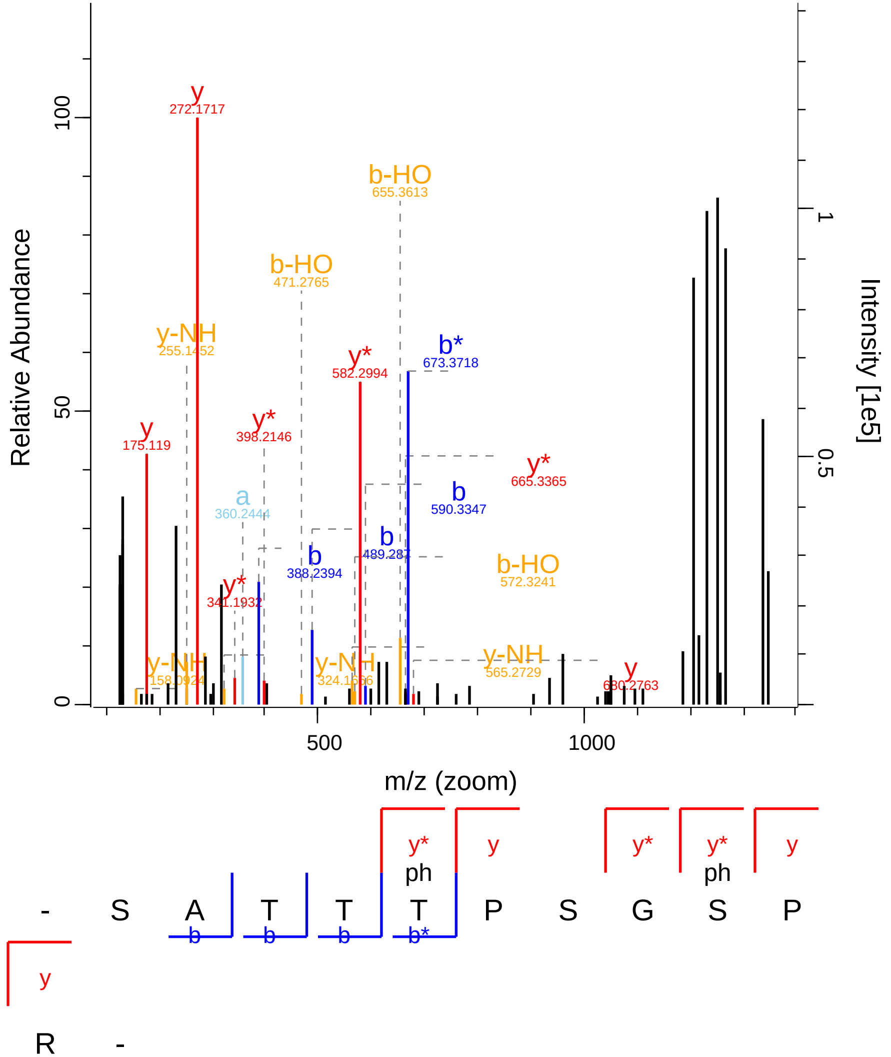

Raw File	Scan	Method	Score	m/z	Gene names
RO-QC-160121_ZackD_SS_Set_02_Fraction	5008	FTMS; HC	105.03	476.88	RBM15B

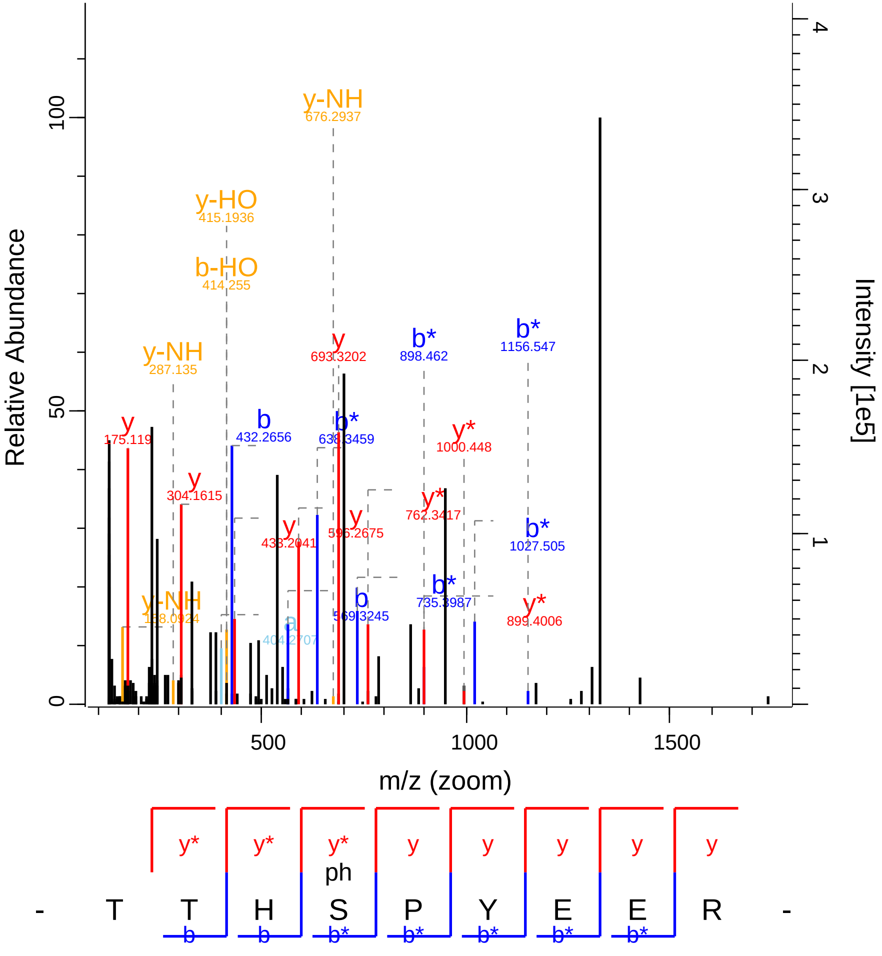

Raw File	Scan	Method	Score	m/z	Gene names
RO-QC-160121_ZackD_SS_Set_02_Fraction	5948	FTMS; HC	133.94	686.34	LMNA

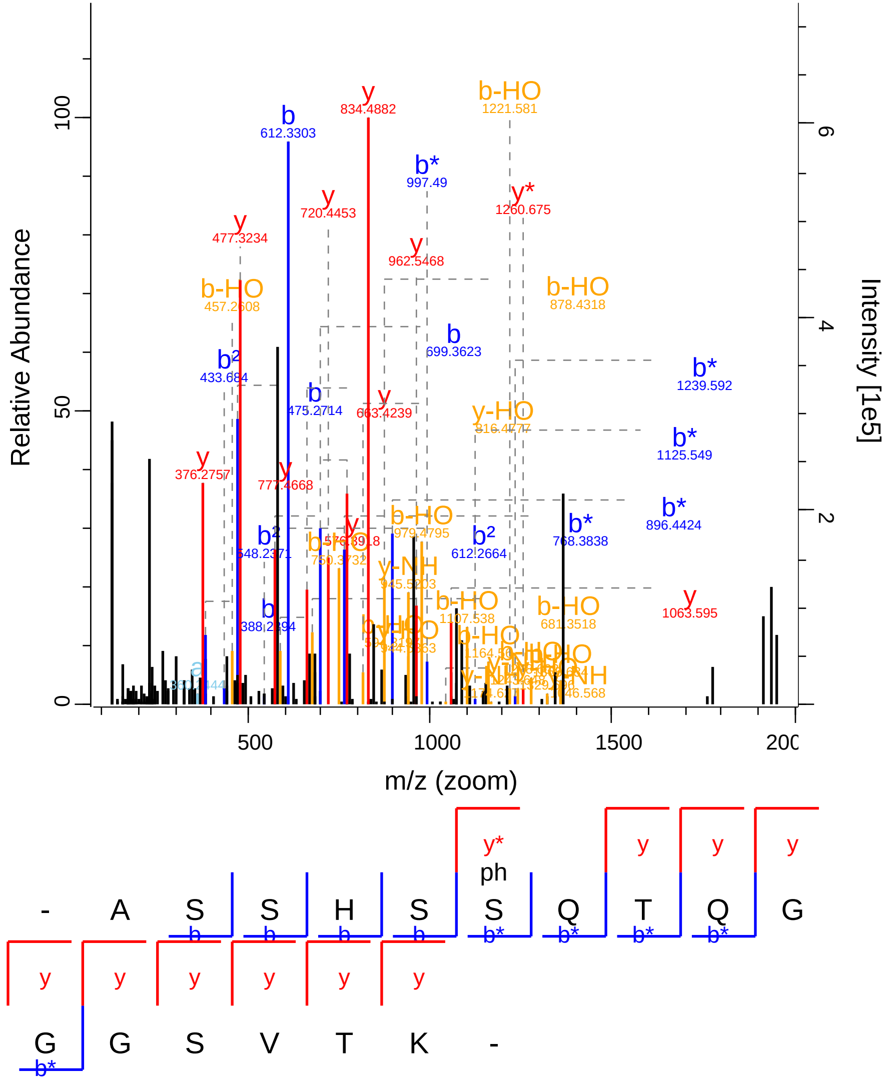

Raw File	Scan	Method	Score	m/z	Gene names
RO-QC-160121_ZackD_SS_Set_02_Fraction	6031	FTMS; HC	207.34	866.93	PRRC2C

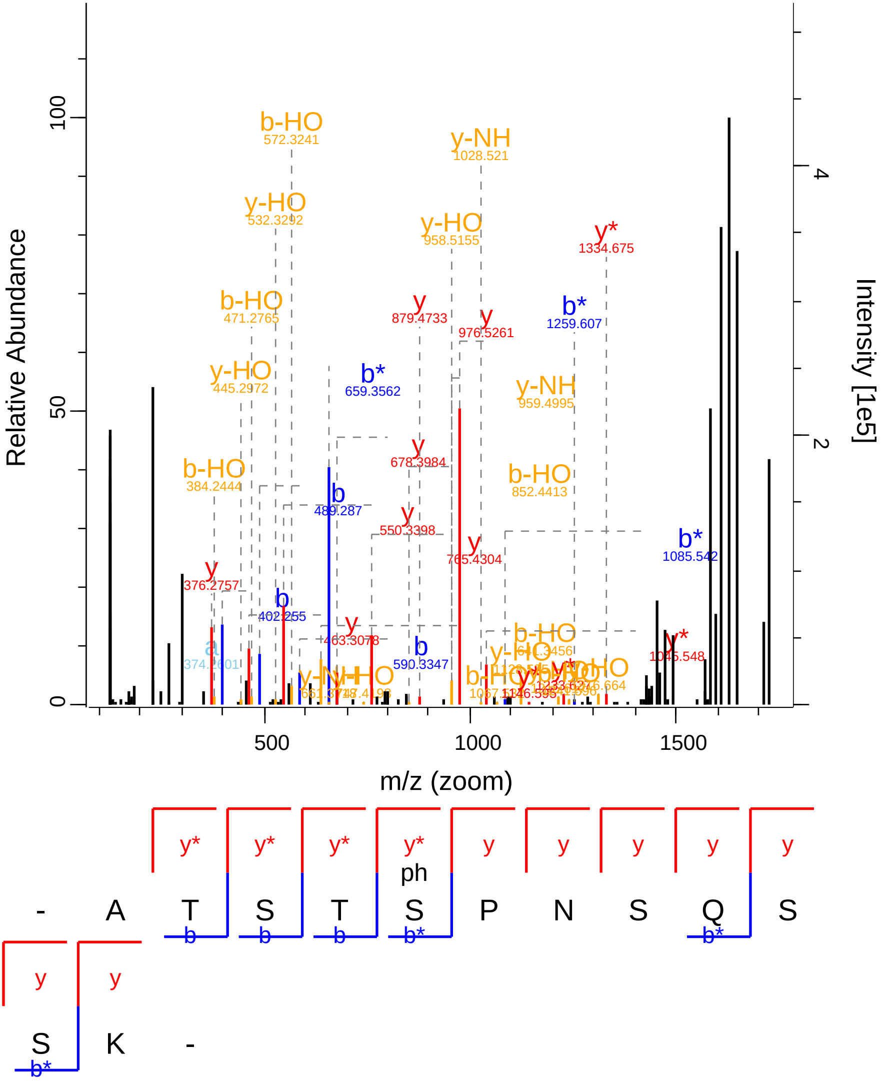

Raw File	Scan	Method	Score	m/z	Gene names
RO-QC-160121_ZackD_SS_Set_02_Fraction	6041	FTMS; HC	64.8	578.29	PRRC2C

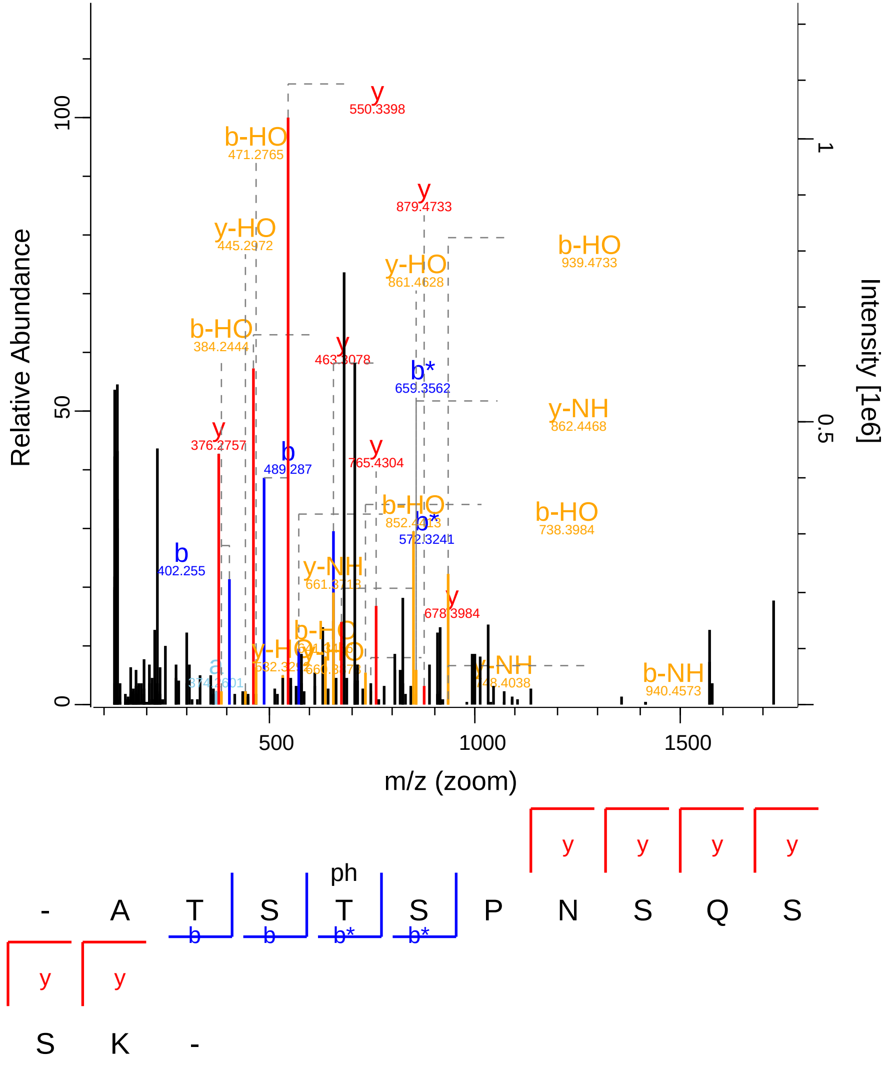

Raw File	Scan	Method	Score	m/z	Gene names
RO-QC-160121_ZackD_SS_Set_02_Fraction	6238	FTMS; HC	127.18	618.28	BCKDK

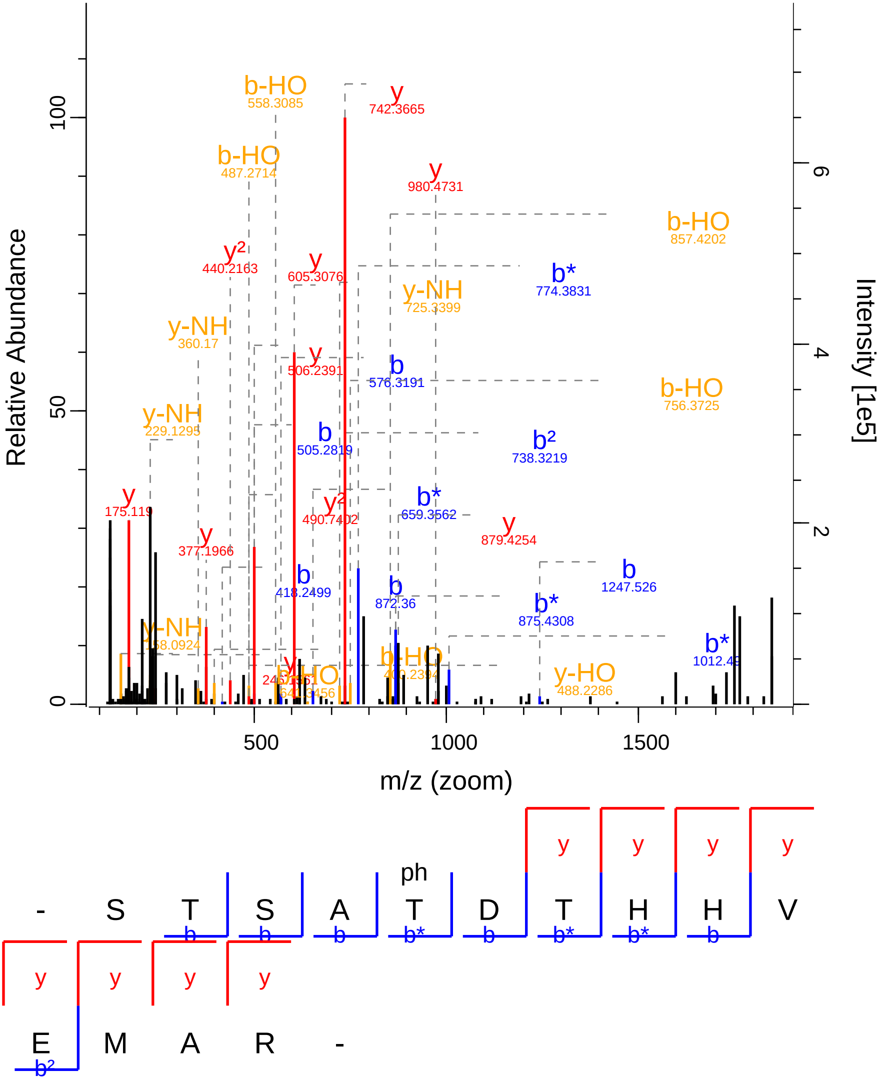

Raw File	Scan	Method	Score	m/z	Gene names
RO-QC-160121_ZackD_SS_Set_02_Fraction	6254	FTMS; HC	213.72	840.06	UFL1

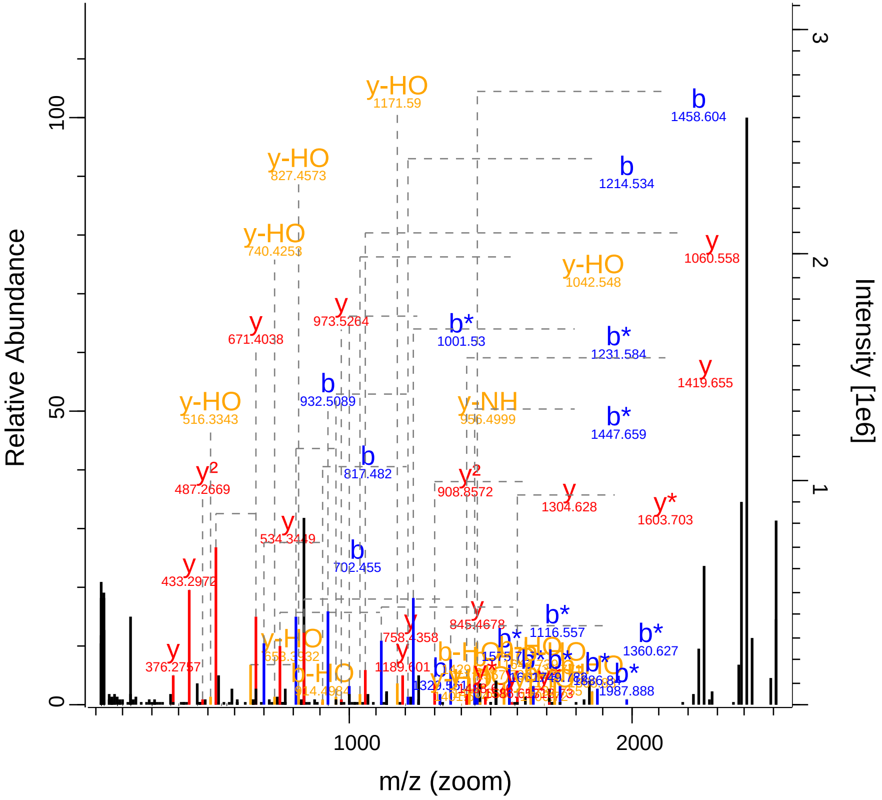

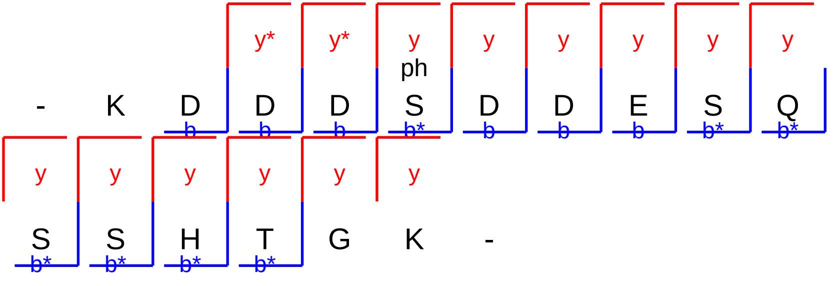

Raw File	Scan	Method	Score	m/z	Gene names
RO-QC-160121_ZackD_SS_Set_02_Fraction	6932	FTMS; HC	131.83	788.91	CAP2

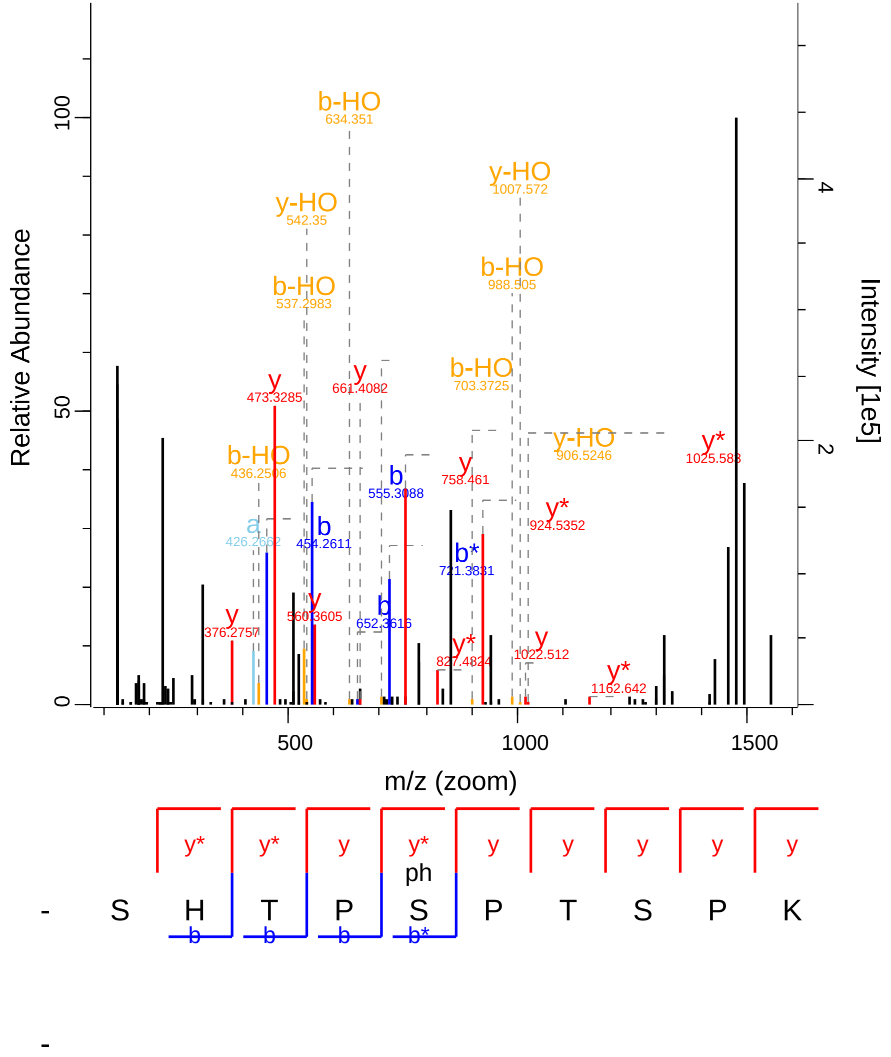

Raw File	Scan	Method	Score	m/z	Gene names
RO-QC-160121_ZackD_SS_Set_02_Fraction	7498	FTMS; HC	119.53	788.91	CAP2

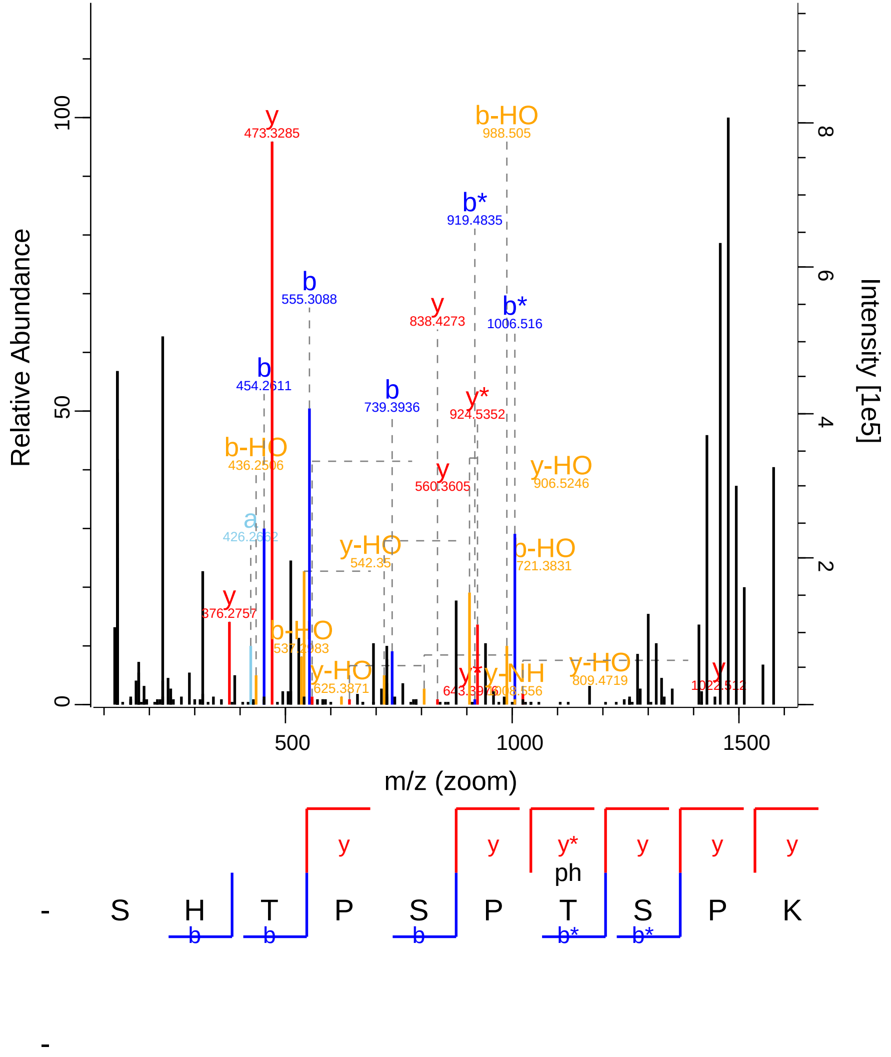

Raw File	Scan	Method	Score	m/z	Gene names
RO-QC-160121_ZackD_SS_Set_02_Fraction	8238	FTMS; HC	75.1	548.64	LRRC1

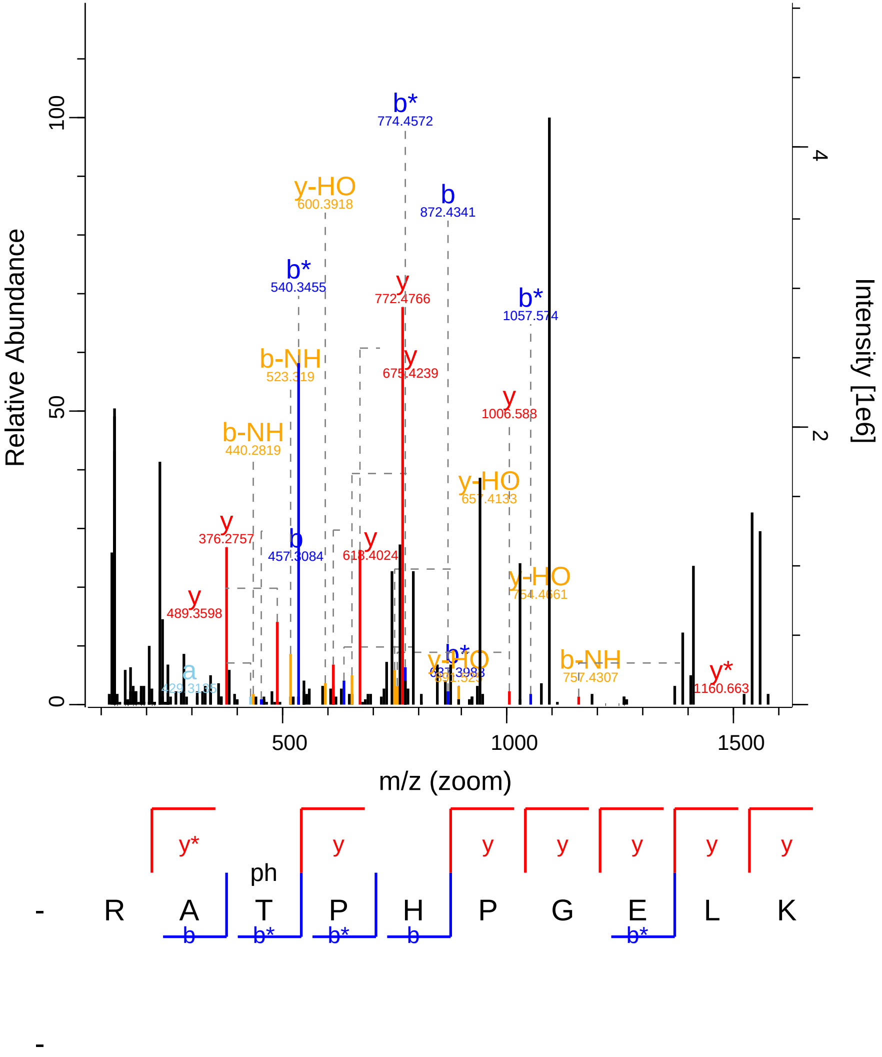

Raw File	Scan	Method	Score	m/z	Gene names
RO-QC-160121_ZackD_SS_Set_02_Fractior	10784	FTMS; HC	55.72	647.05	PRPF4B

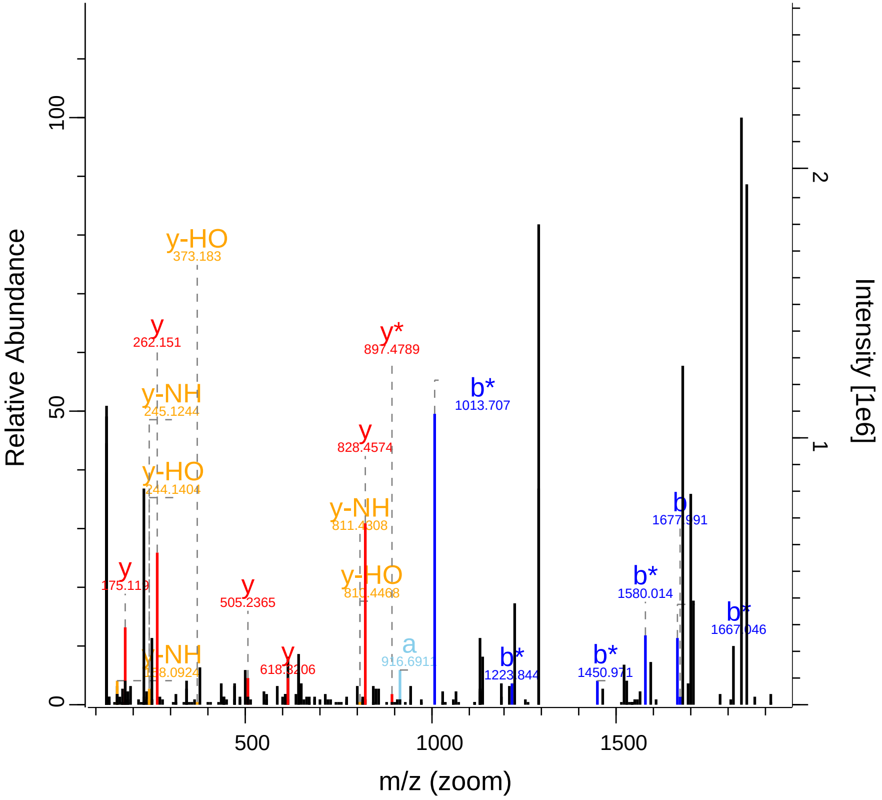

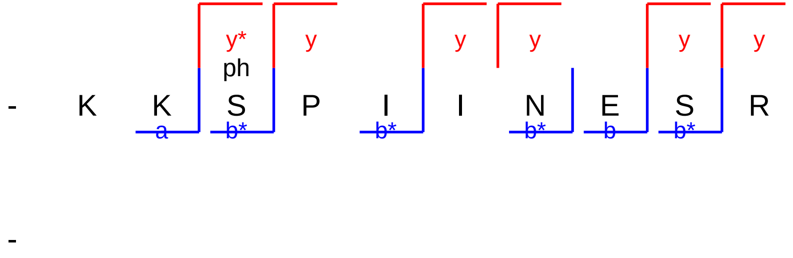

Raw File	Scan	Method	Score	m/z	Gene names
RO-QC-160121_ZackD_SS_Set_02_Fractior	11536	FTMS; HC	157.68	935.99	TSPAN10

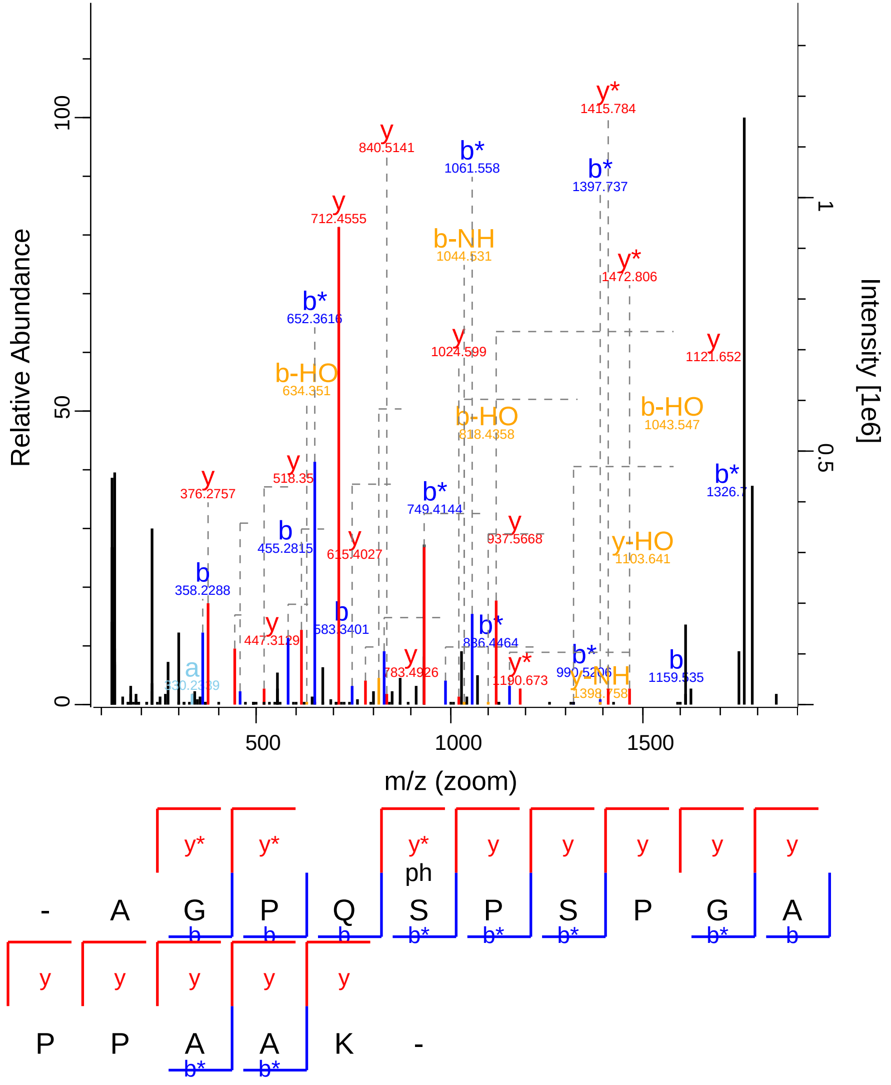

Raw File	Scan	Method	Score	m/z	Gene names
RO-QC-160121_ZackD_SS_Set_02_Fractior	12062	FTMS; HC	106.3	988.01	MLLT4

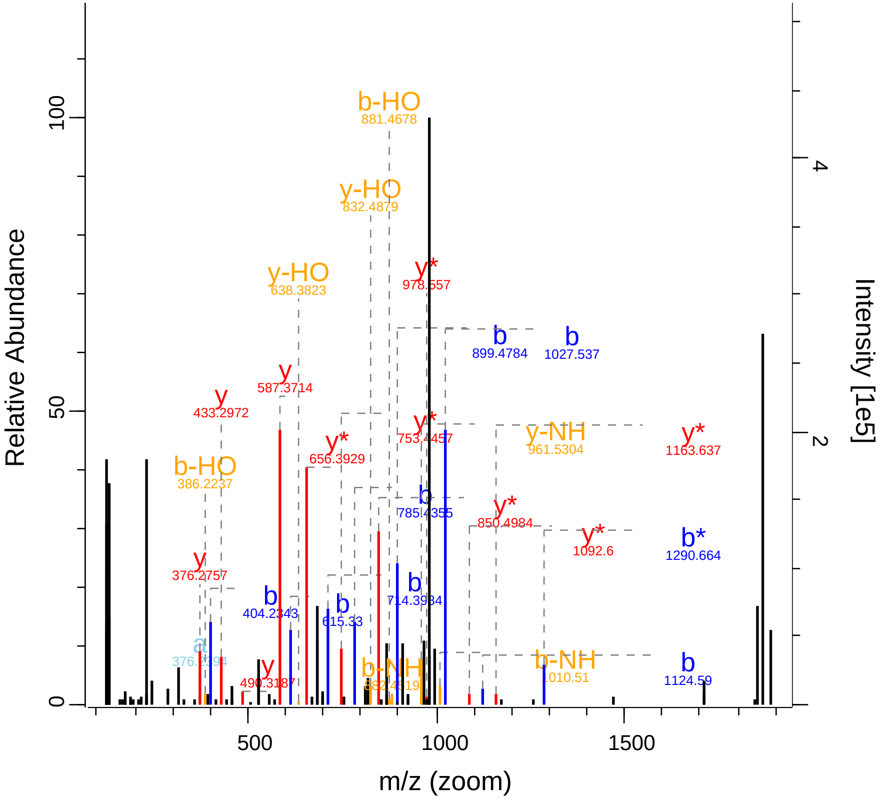

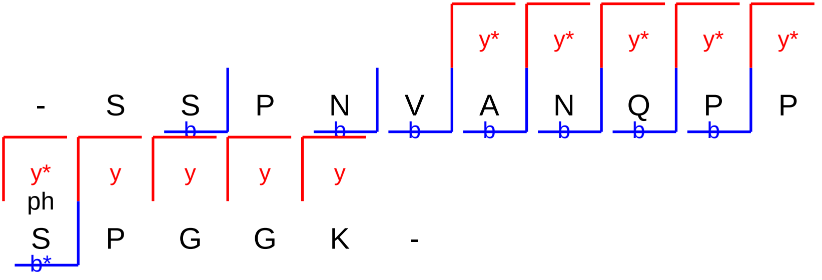

Raw File	Scan	Method	Score	m/z	Gene names
RO-QC-160121_ZackD_SS_Set_02_Fractior	12113	FTMS; HC	63.16	943.04	RP1

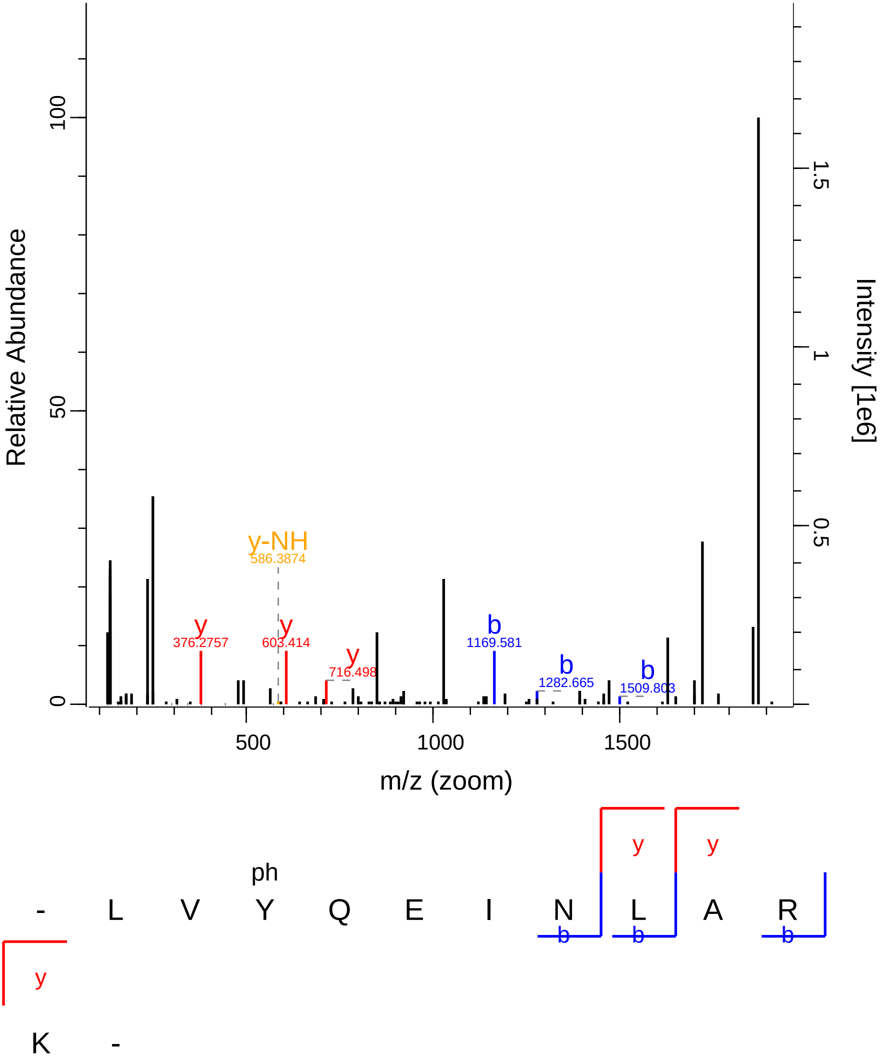

Raw File	Scan	Method	Score	m/z	Gene names
RO-QC-160121_ZackD_SS_Set_02_Fractior	12128	FTMS; HC	130.94	737.38	SRRM2

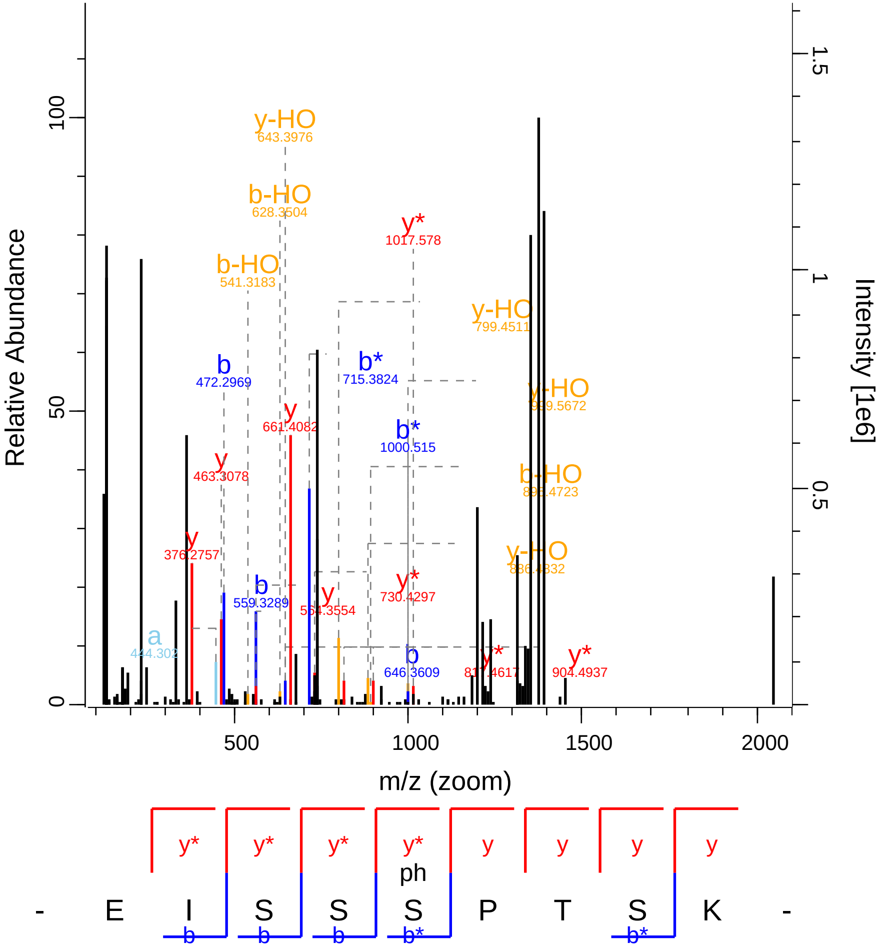

Raw File	Scan	Method	Score	m/z
RO-QC-160121_ZackD_SS_Set_02_Fraction_10	13050	FTMS; HCD	41.09	490.88

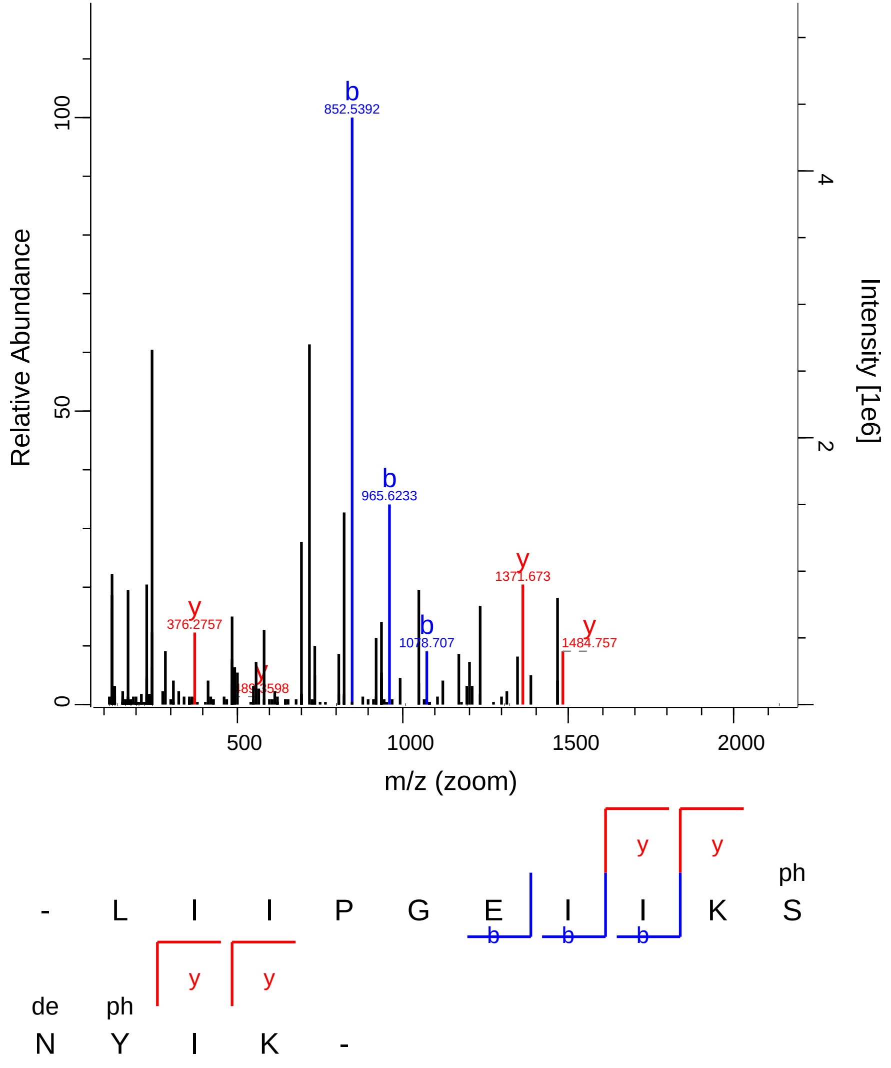

Raw File	Scan	Method	Score	m/z
RO-QC-160121_ZackD_SS_Set_02_Fraction_10	13264	FTMS; HCD	48.74	429.23

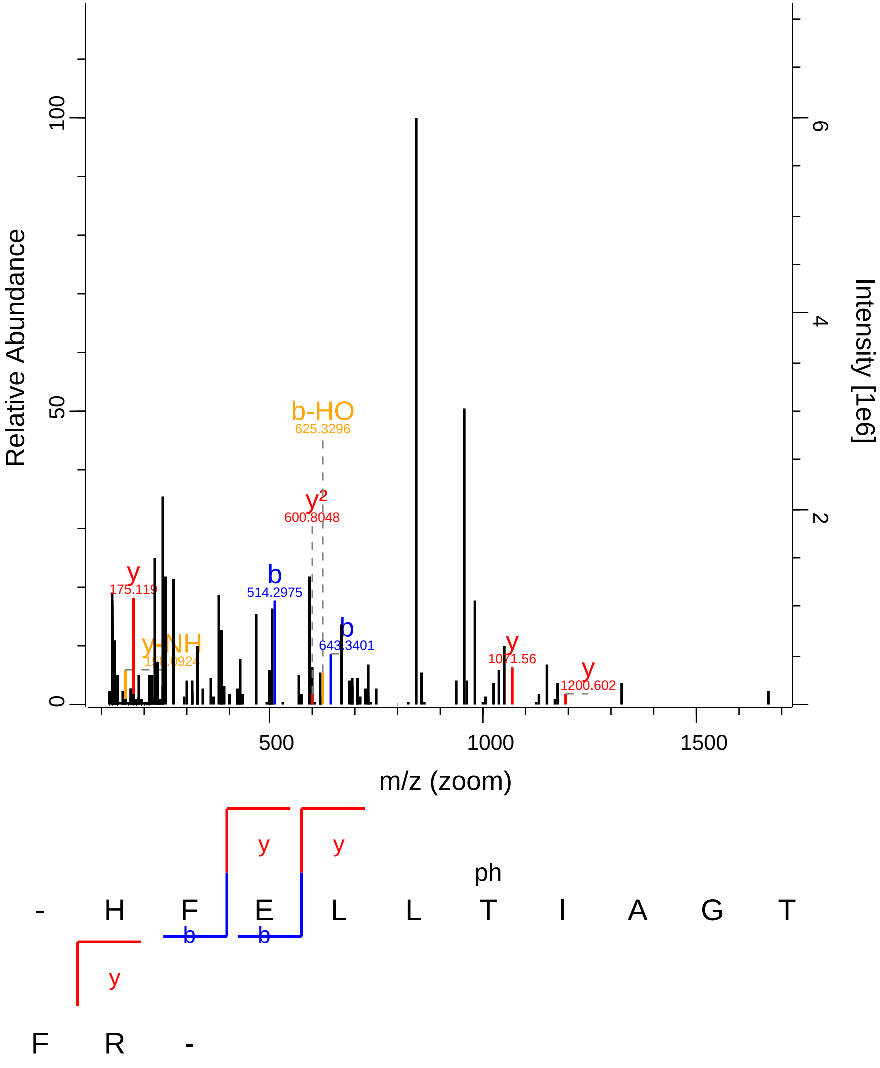

Raw File	Scan	Method	Score	m/z	Gene names
RO-QC-160121_ZackD_SS_Set_02_Fractior	15309	FTMS; HC	98.27	789.05	TRIM25

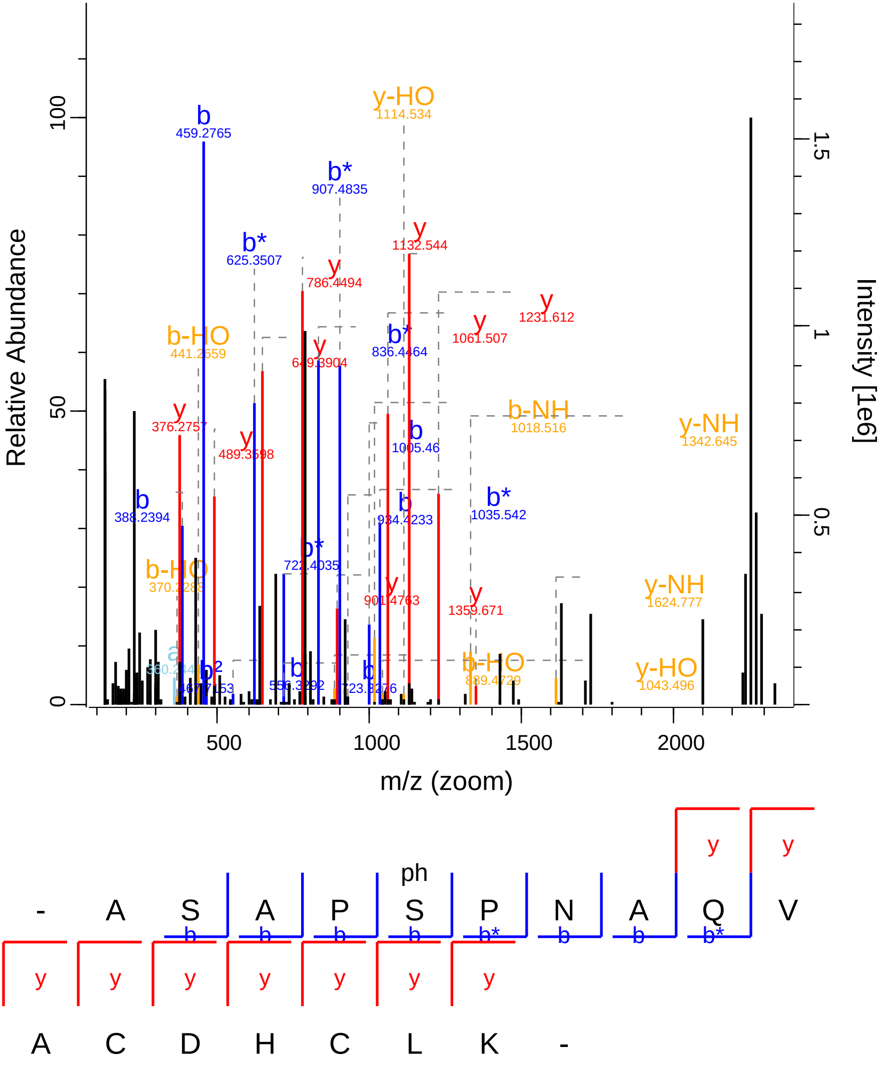

Raw File	Scan	Method	Score	m/z	Gene names
RO-QC-160121_ZackD_SS_Set_02_Fractior	15370	FTMS; HC	101.49	748.12	SRSF11

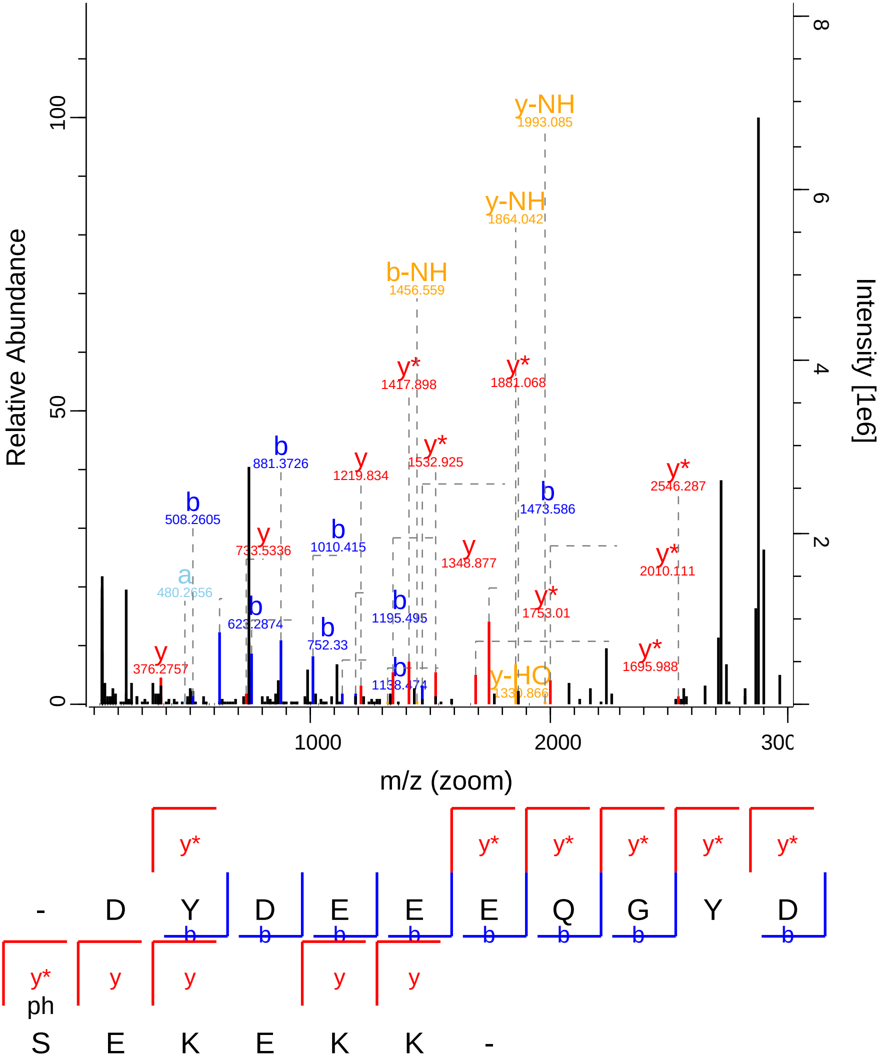

Raw File	Scan	Method	Score	m/z	Gene names
RO-QC-160121_ZackD_SS_Set_02_Fractior	15653	FTMS; HC	42.74	384.03	SMARCA1

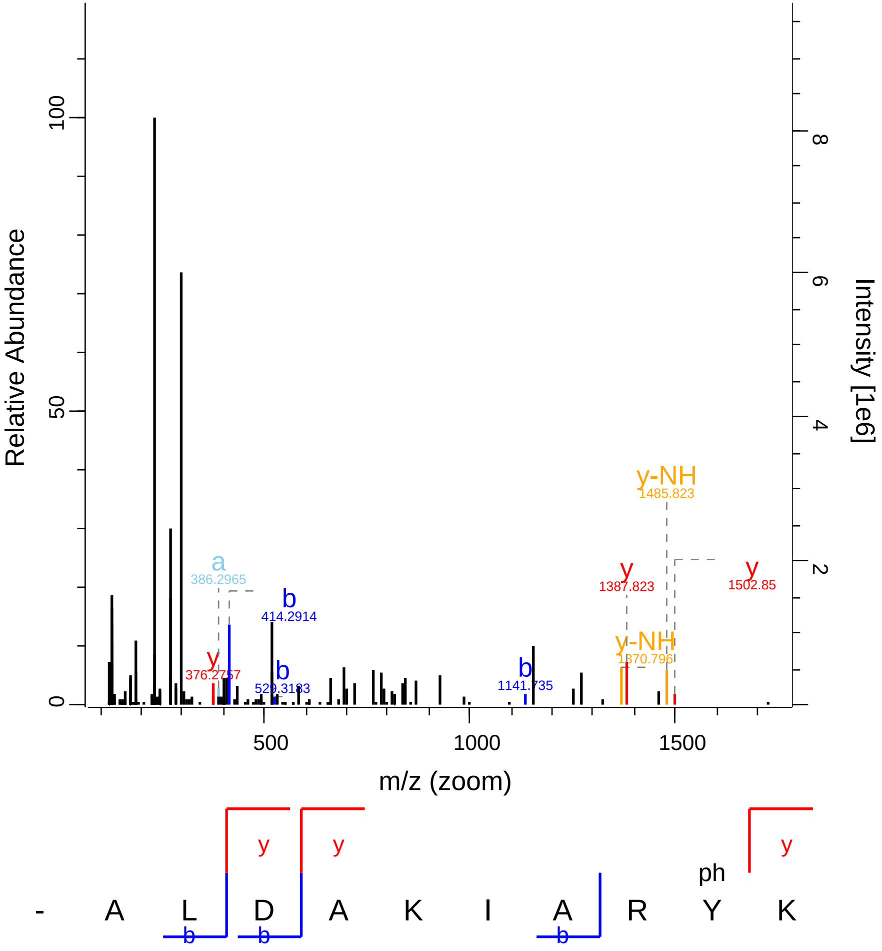

-

Raw File	Scan	Method	Score	m/z	Gene names
RO-QC-160121_ZackD_SS_Set_02_Fractior	16477	FTMS; HC	62.82	649.59	TNIK

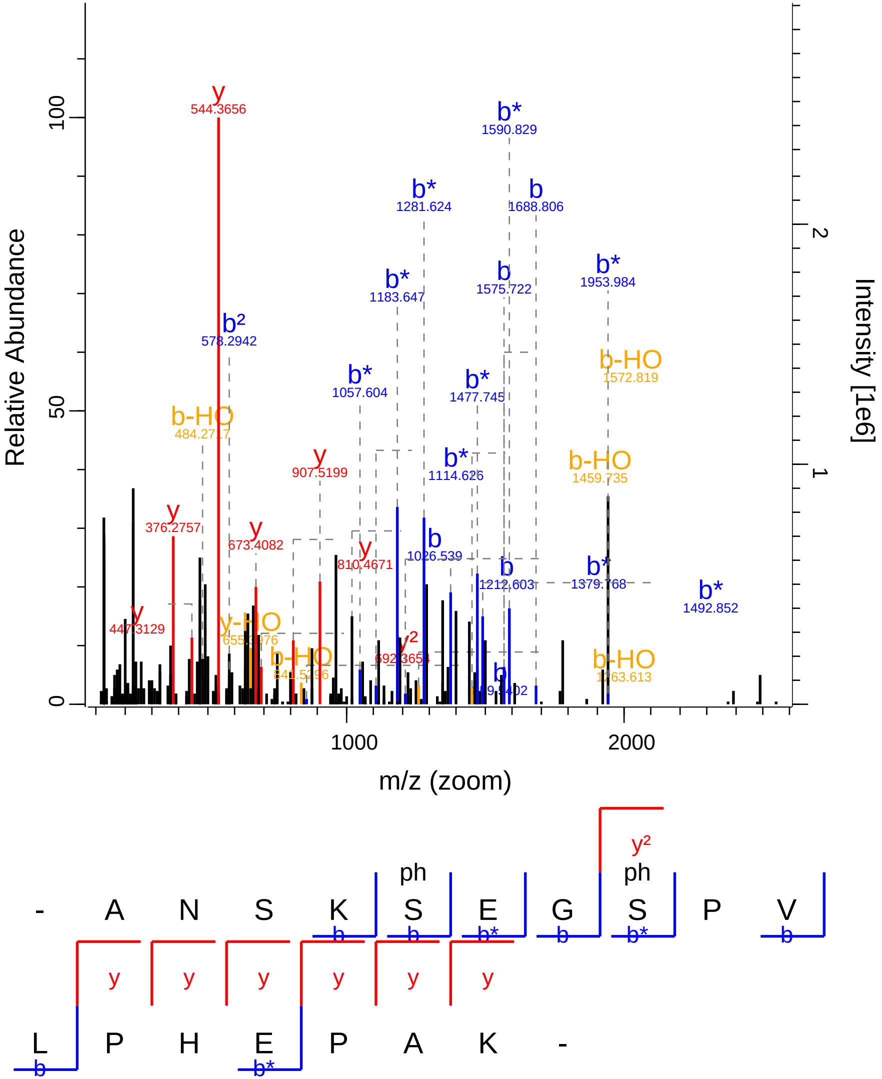

Raw File	Scan	Method	Score	m/z	Gene names
RO-QC-160121_ZackD_SS_Set_02_Fractior	16714	FTMS; HC	52.72	894.97	SYNE2

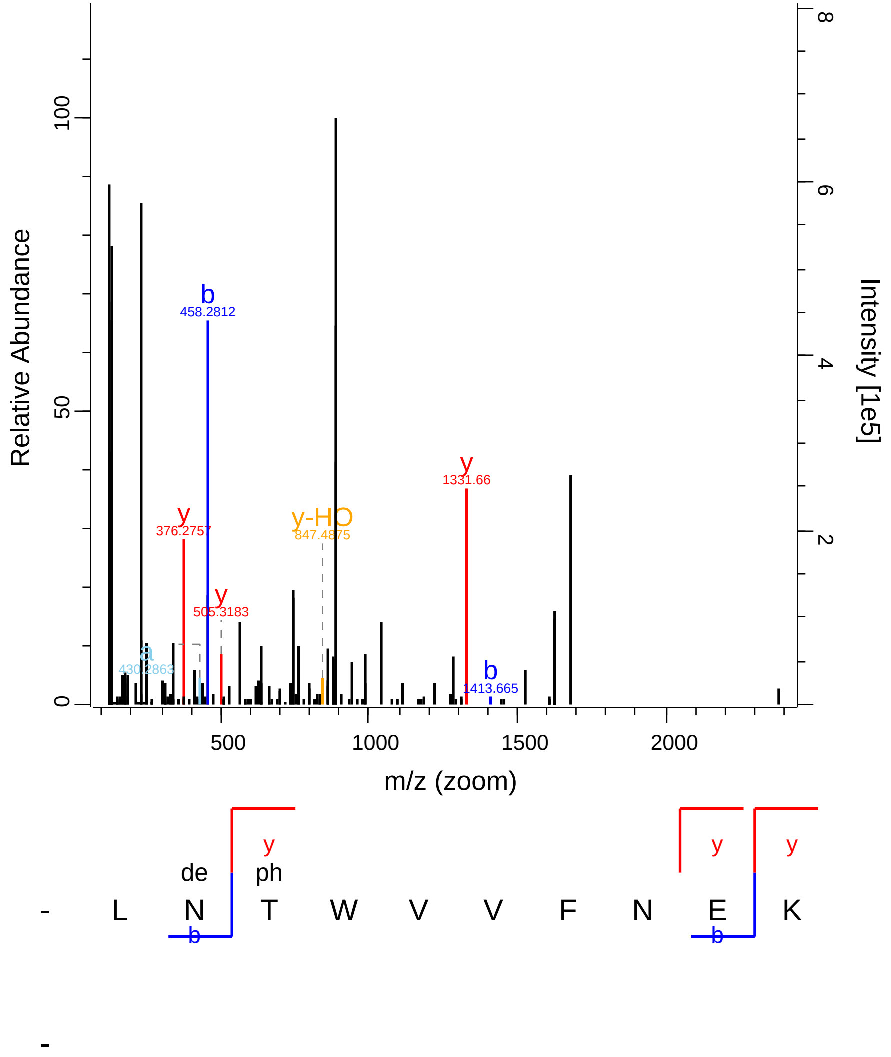

Raw File	Scan	Method	Score	m/z	Gene names
RO-QC-160121_ZackD_SS_Set_02_Fractio	16811	FTMS; HC	194.68	980.53	SCAF8

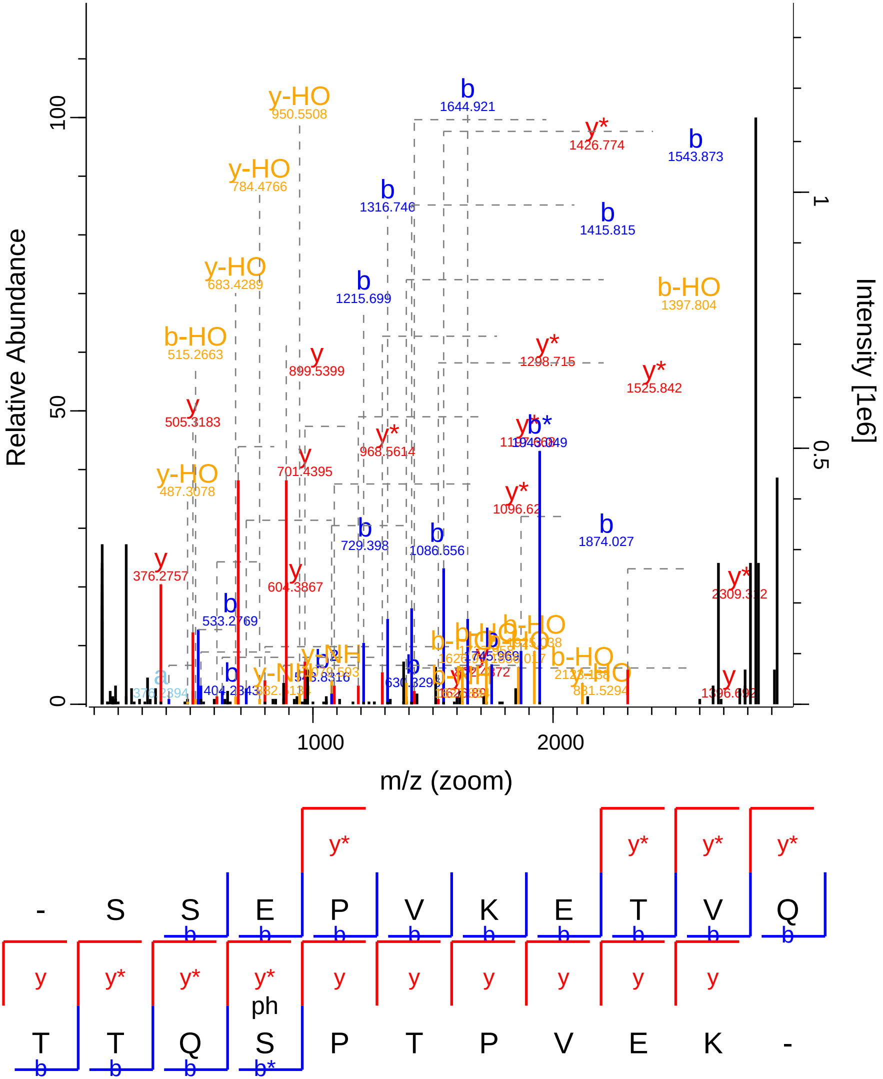

Raw File	Scan	Method	Score	m/z	Gene names
RO-QC-160121_ZackD_SS_Set_02_Fractior	17239	FTMS; HC	46.61	1157.6	JARID2

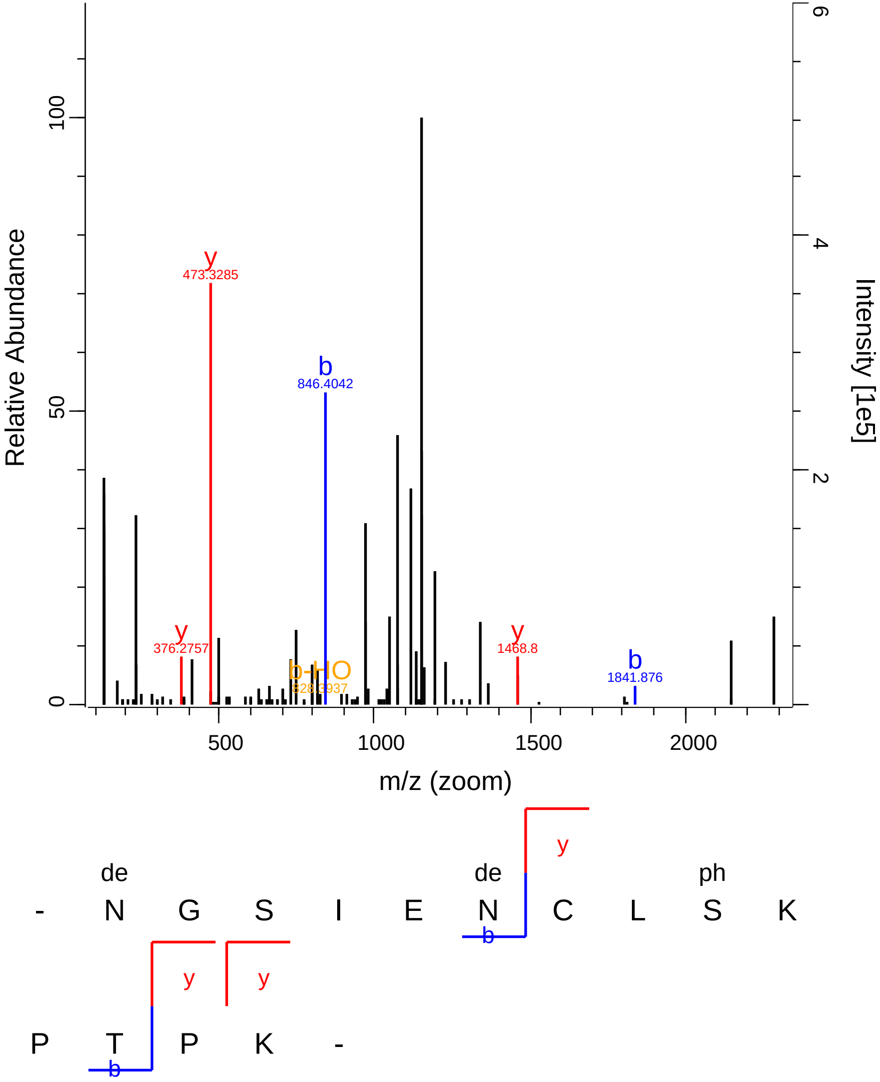

Raw File	Scan	Method	Score	m/z	Gene names
RO-QC-160121_ZackD_SS_Set_02_Fractior	17902	FTMS; HC	69.03	939.51	WAPAL

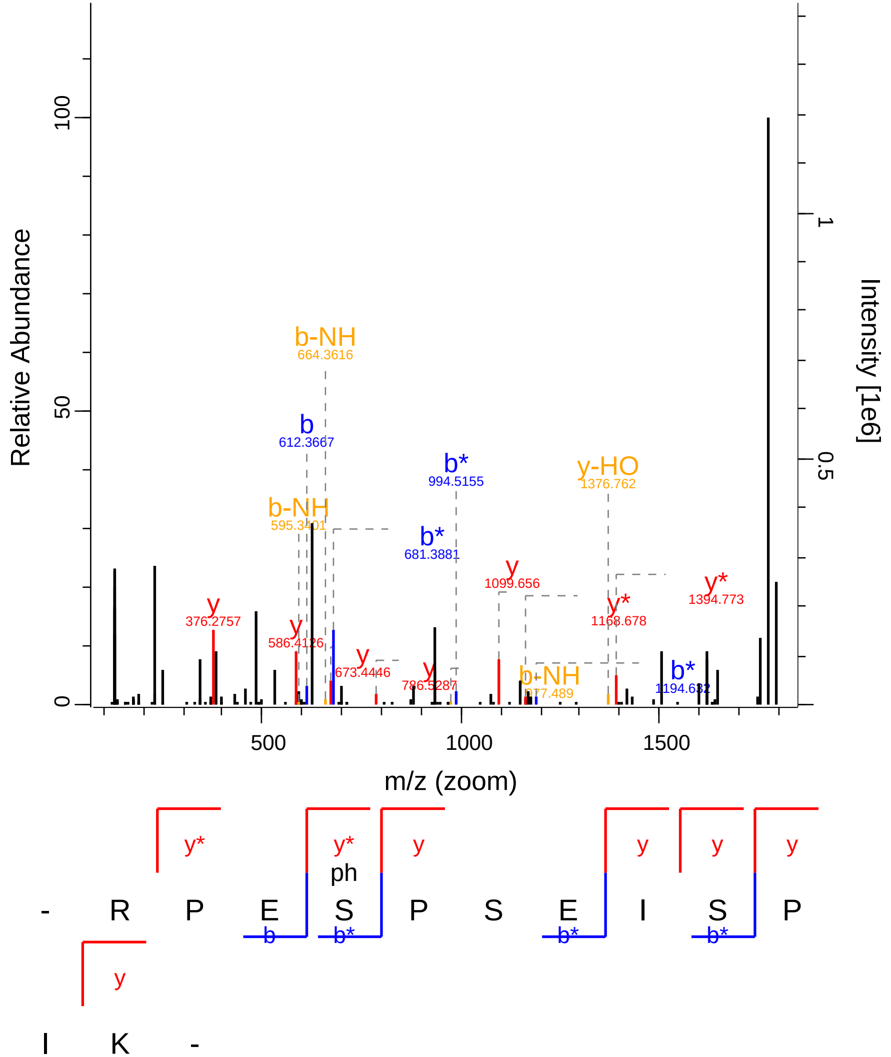

Raw File	Scan	Method	Score	m/z	Gene names
RO-QC-160121_ZackD_SS_Set_02_Fractior	18242	FTMS; HC	122.97	940.48	PUM1

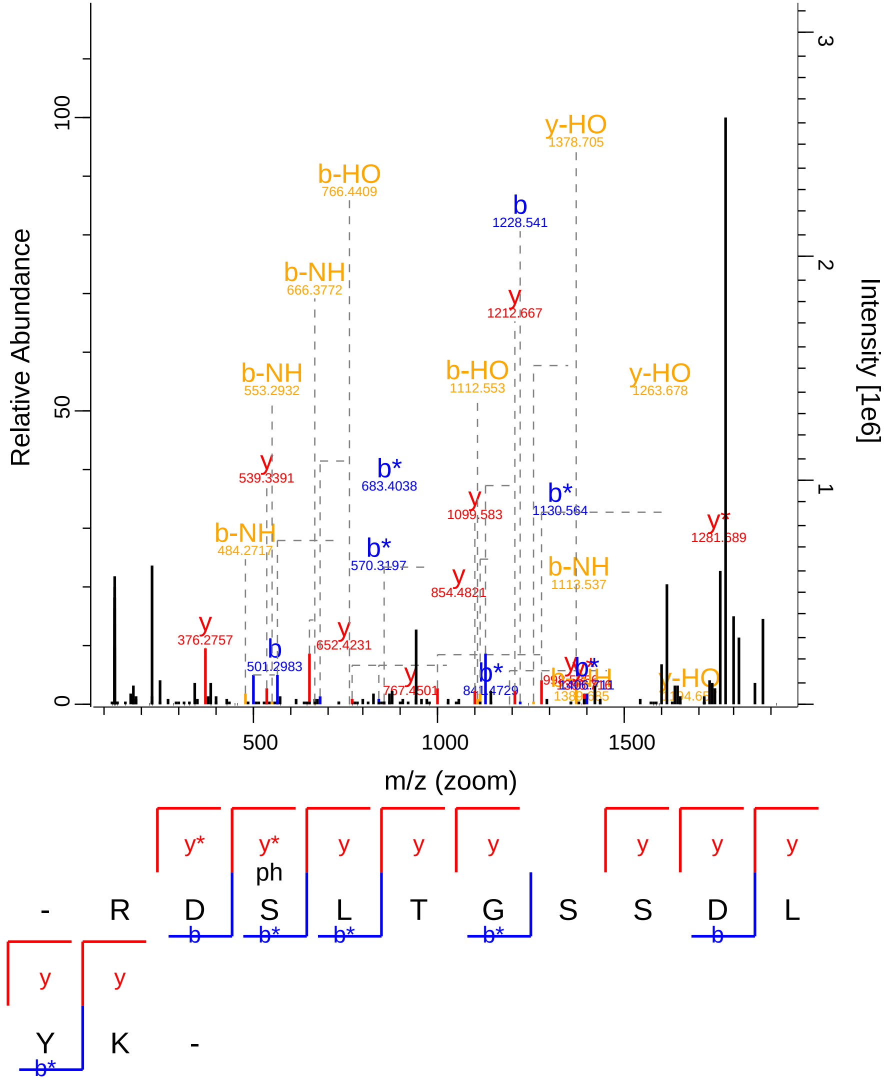

Raw File	Scan	Method	Score	m/z	Gene names
RO-QC-160121_ZackD_SS_Set_02_Fractior	19526	FTMS; HC	96.8	748.36	ARL6IP4

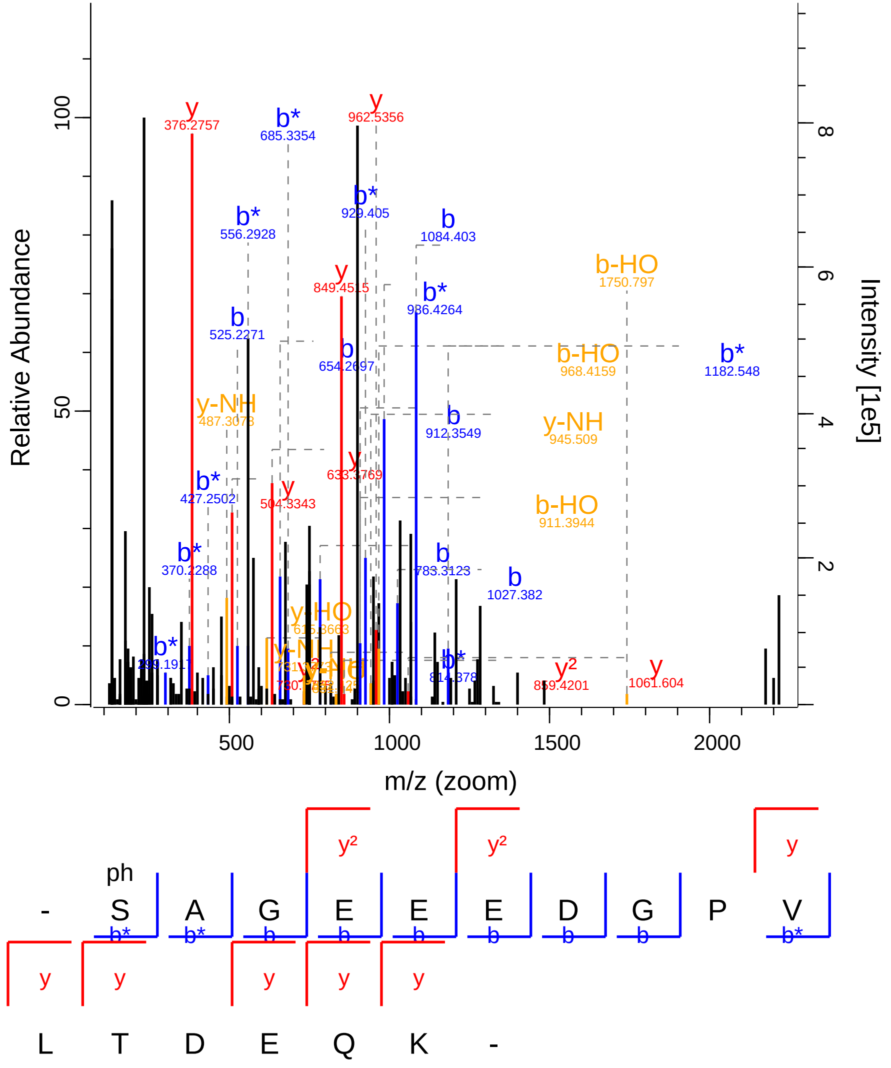

Raw File	Scan	Method	Score	m/z	Gene names
RO-QC-160121_ZackD_SS_Set_02_Fractior	19953	FTMS; HC	43.51	451.25	LIG3

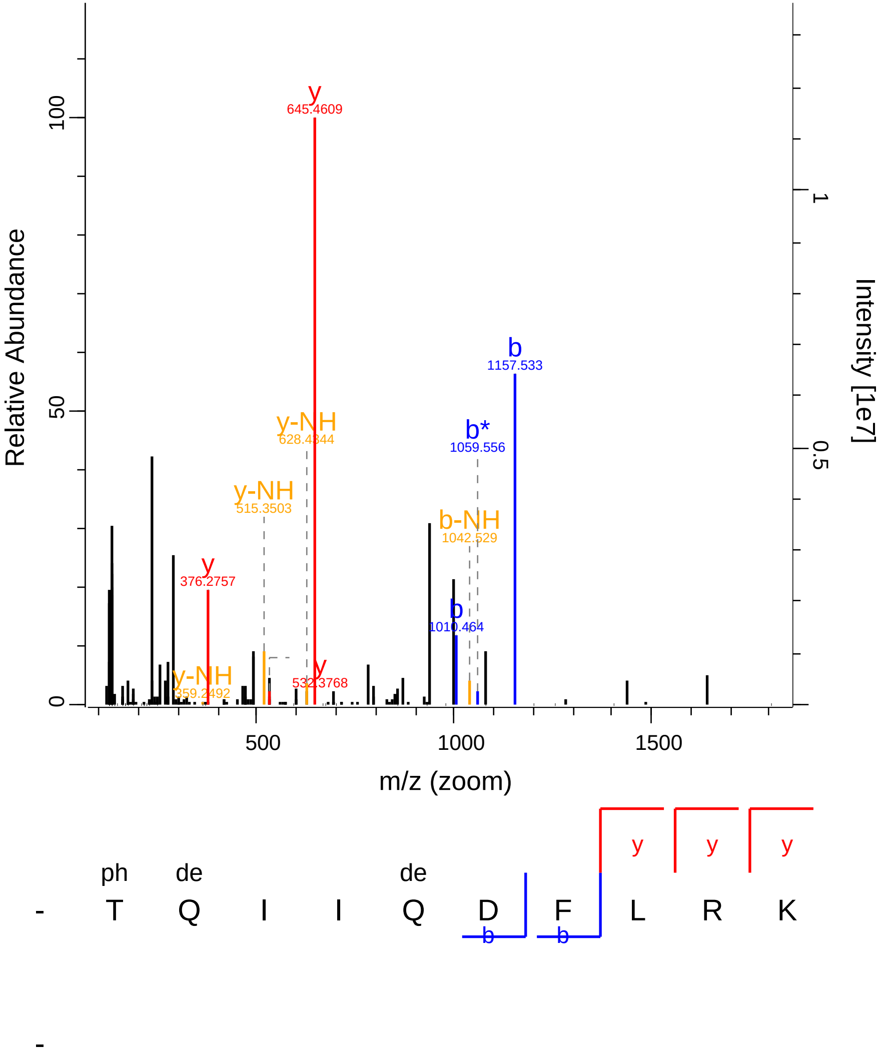

Raw File	Scan	Method	Score	m/z	Gene names
RO-QC-160121_ZackD_SS_Set_02_Fractio	20707	FTMS; HC	239.48	1038.01	PDLIM4

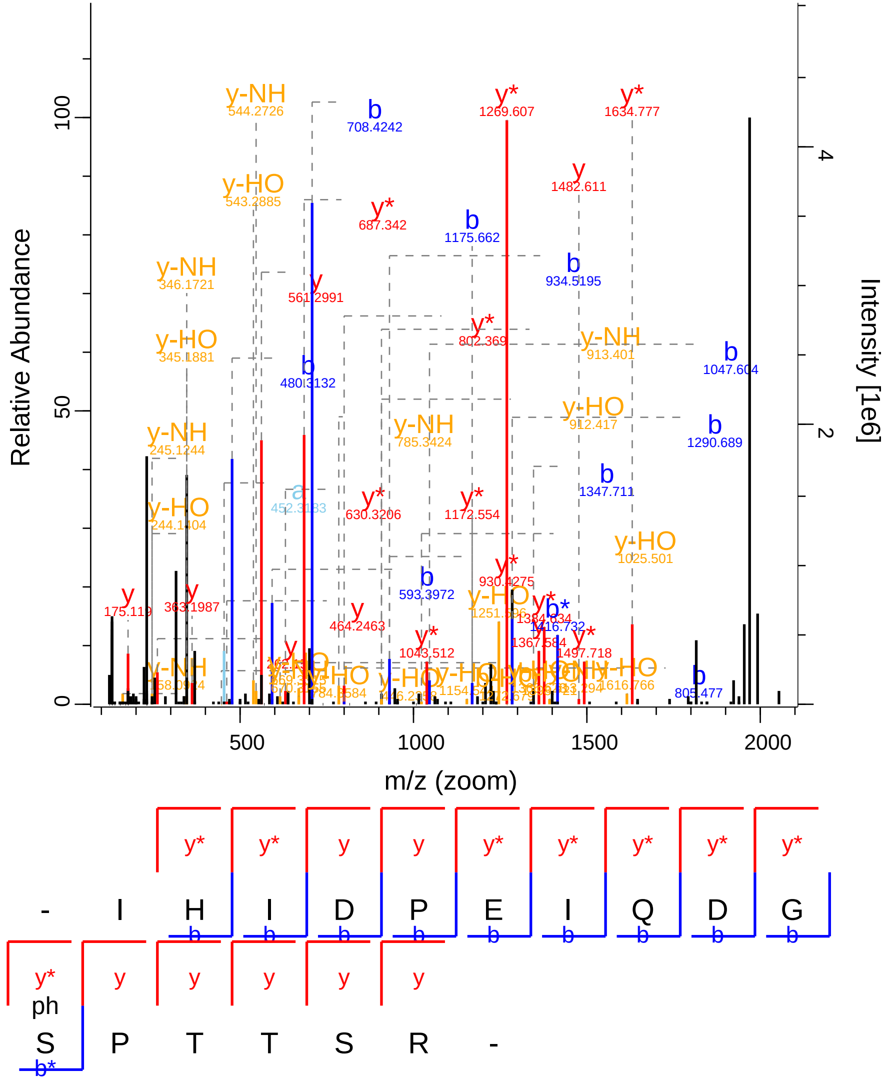

Raw File	Scan	Method	Score	m/z	Gene names
RO-QC-160121_ZackD_SS_Set_02_Fractior	21008	FTMS; HC	62	843.97	NRIP3

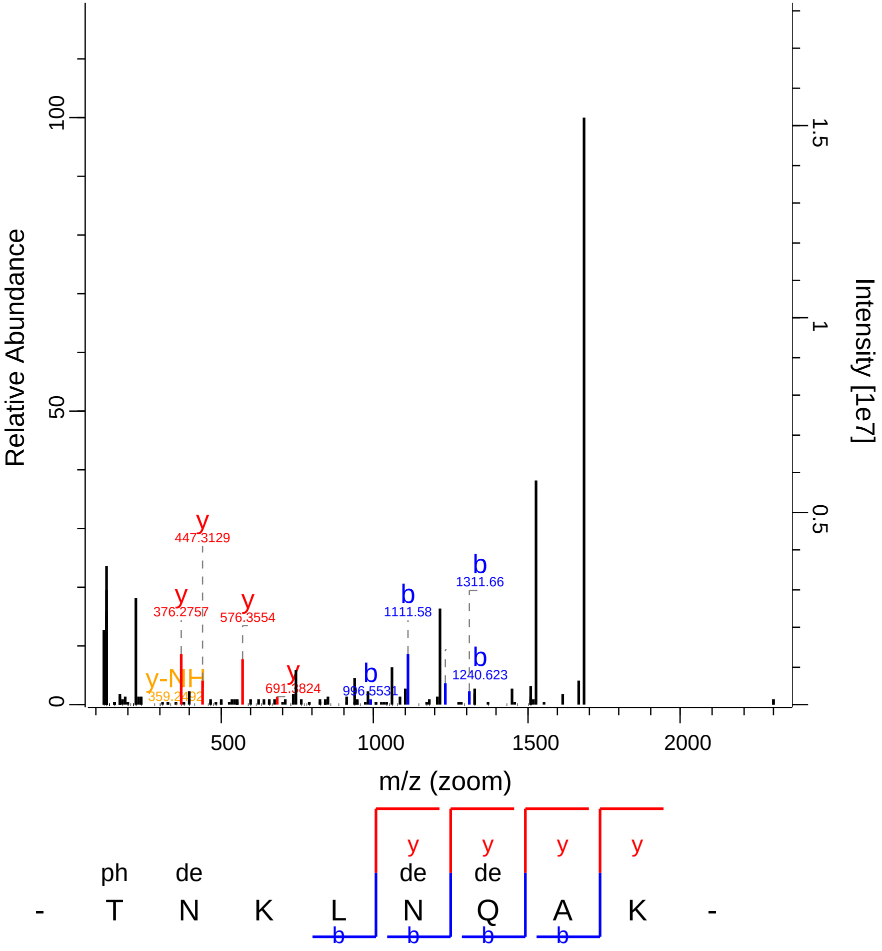

Raw File	Scan	Method	Score	m/z	Gene names
RO-QC-160121_ZackD_SS_Set_02_Fractior	21381	FTMS; HC	86	655.66	LEMD2

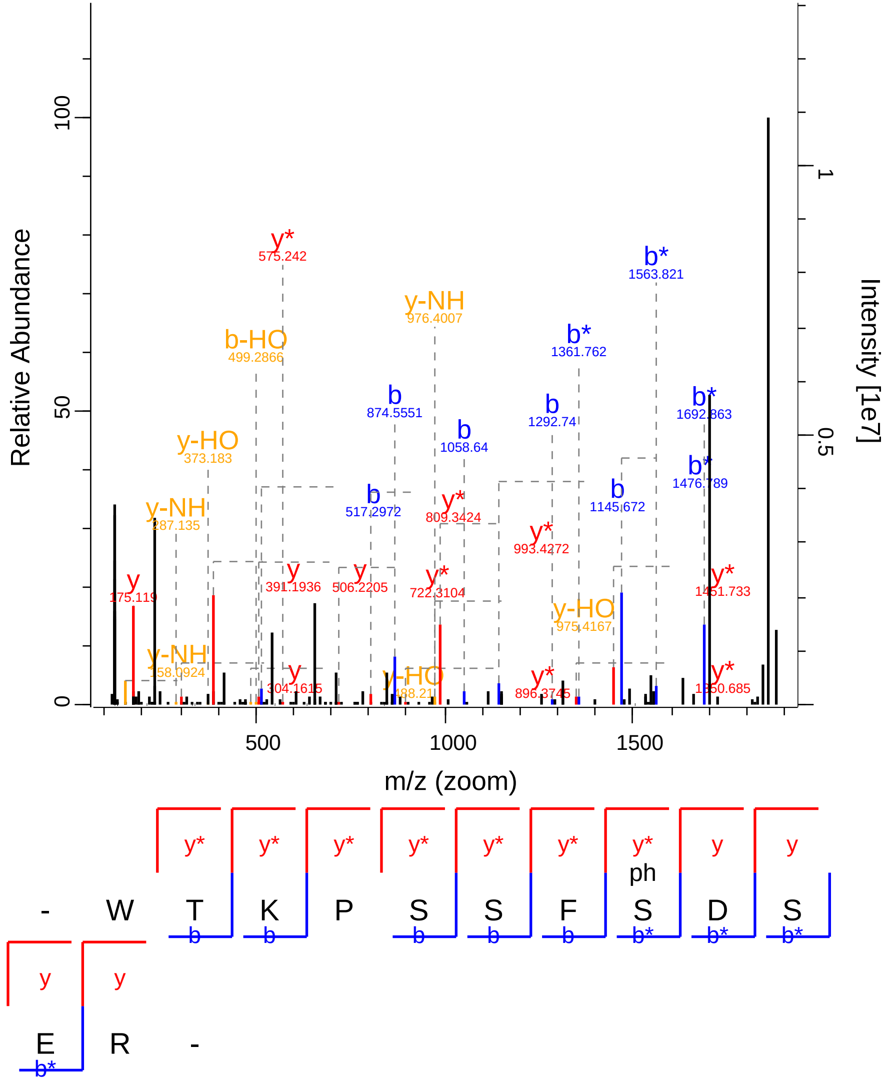

Raw File	Scan	Method	Score	m/z	Gene names
RO-QC-160121_ZackD_SS_Set_02_Fractior	21738	FTMS; HC	53.24	755.05	ADCY10

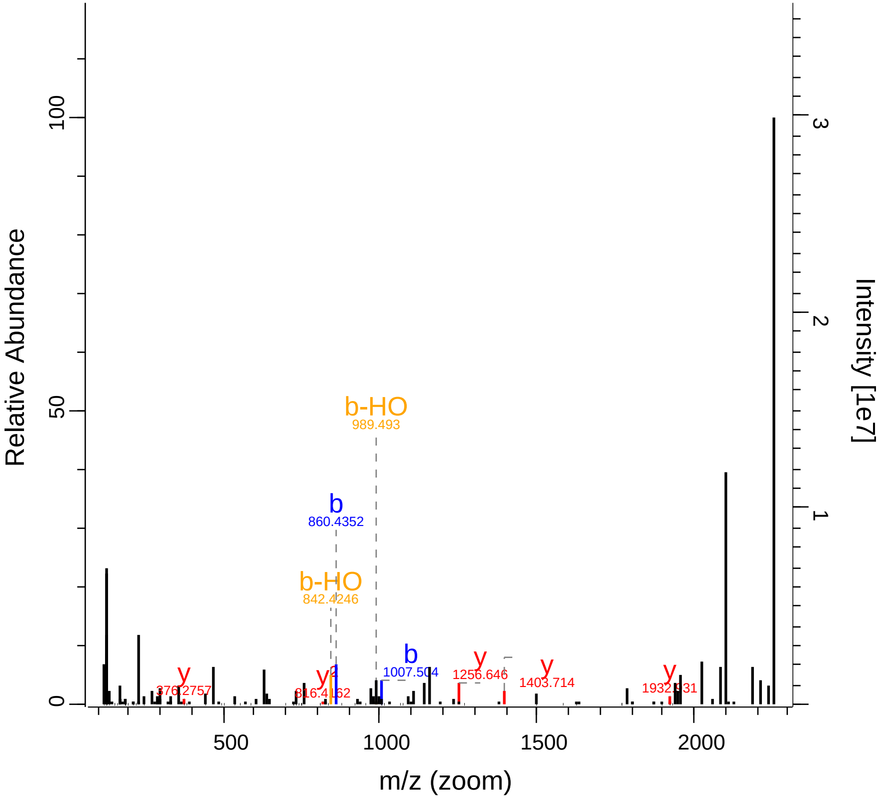

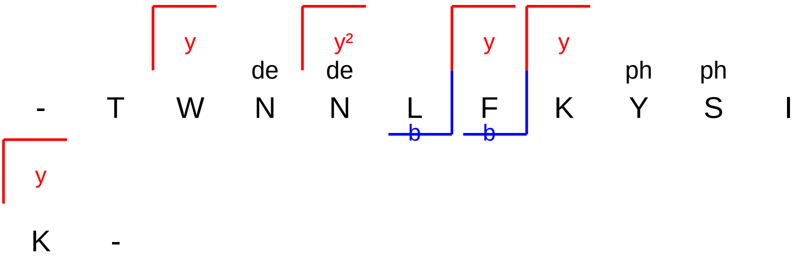

Raw File	Scan	Method	Score	m/z	Gene names
RO-QC-160121_ZackD_SS_Set_02_Fractior	22422	FTMS; HC	71.7	726.41	TPRN

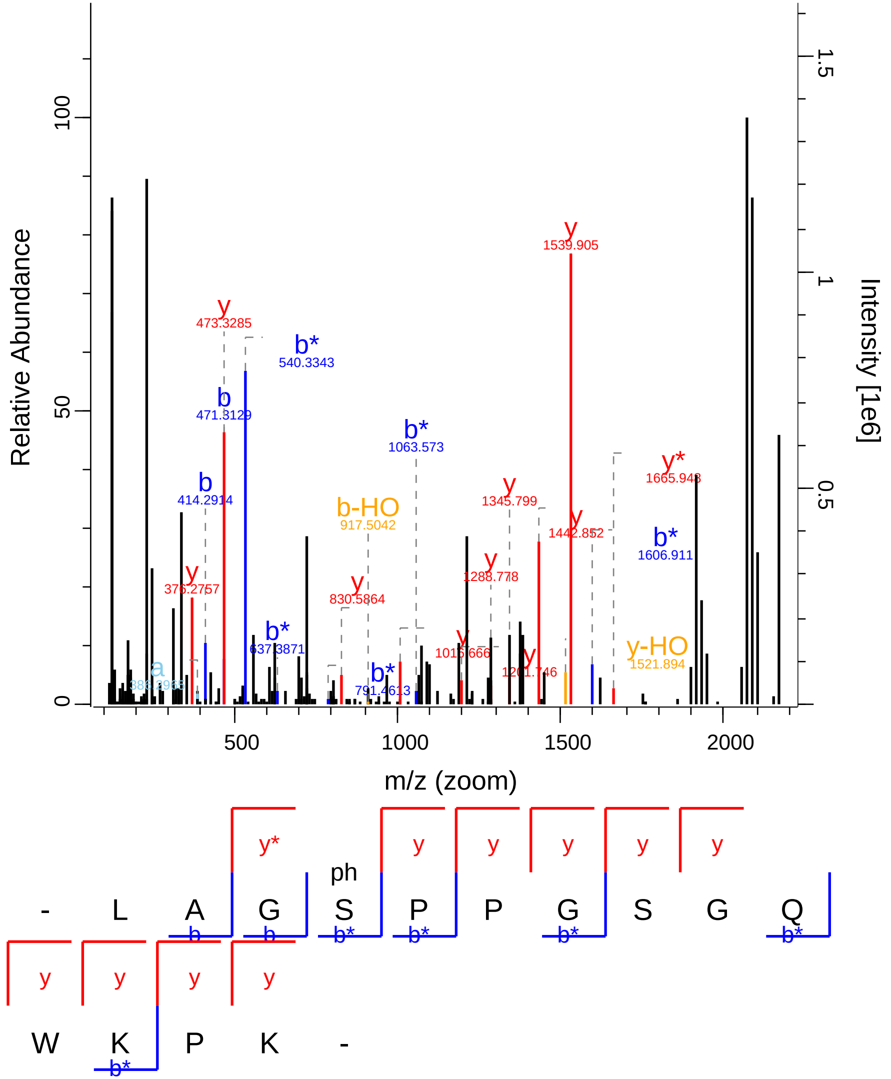

Raw File	Scan	Method	Score	m/z	Gene names
RO-QC-160121_ZackD_SS_Set_02_Fractio	22433	FTMS; HC	198.81	876.94	FARP1

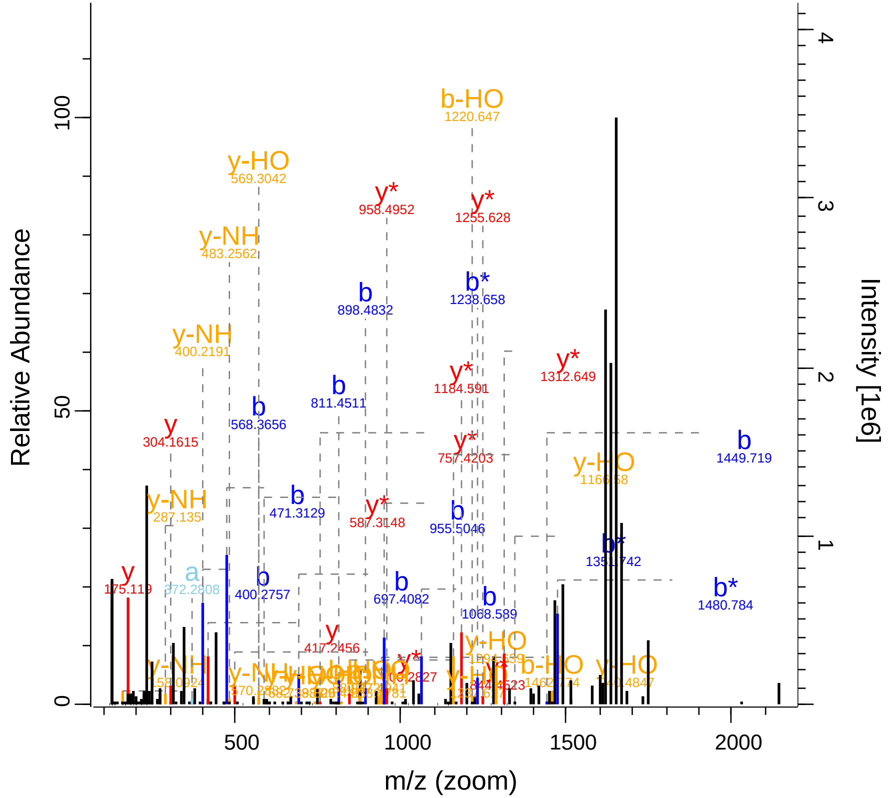

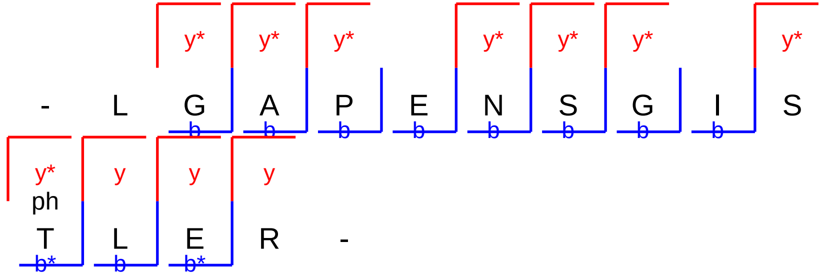

Raw File	Scan	Method	Score	m/z	Gene names
RO-QC-160121_ZackD_SS_Set_02_Fraction	22648	FTMS; HC	131.24	1051	ARFGEF2

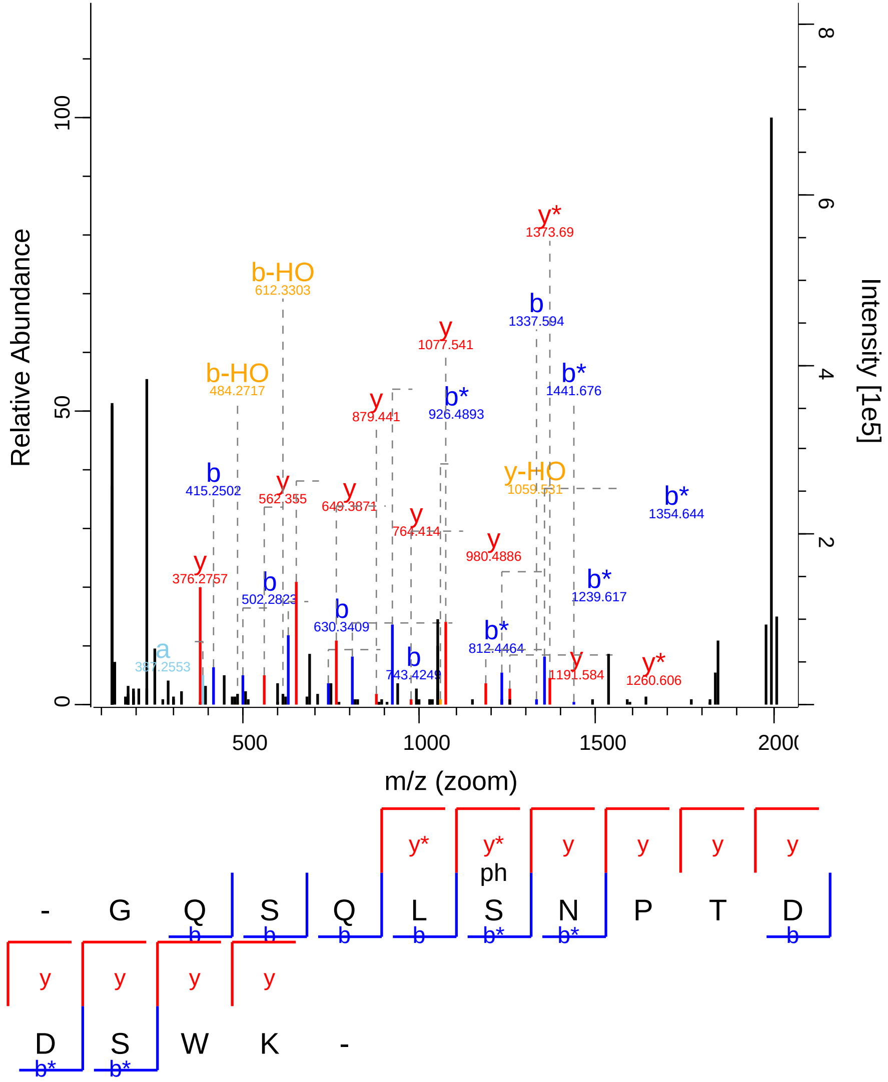

Raw File	Scan	Method	Score	m/z	Gene names
RO-QC-160121_ZackD_SS_Set_02_Fractior	22986	FTMS; HC	46.32	969.97	MAP1B

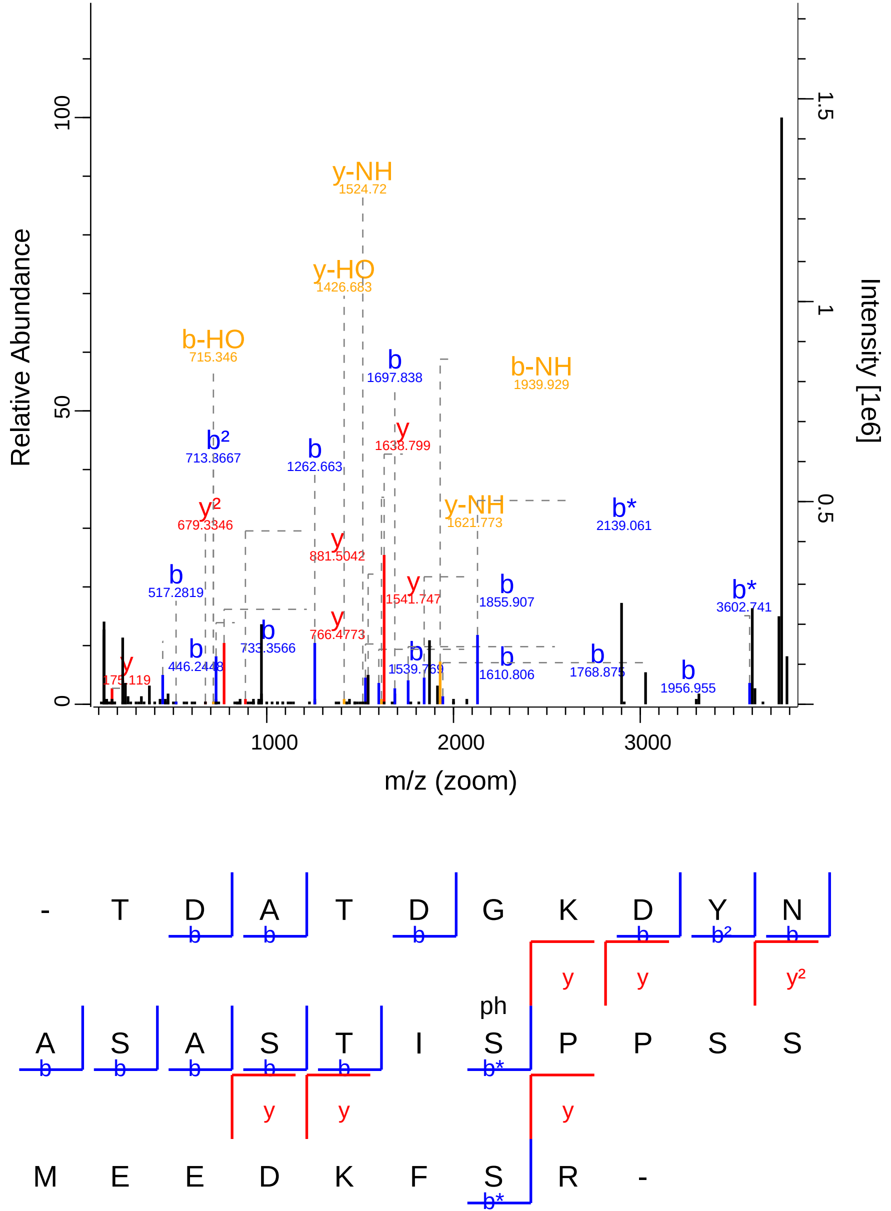

Raw File	Scan	Method	Score	m/z	Gene names
RO-QC-160121_ZackD_SS_Set_02_Fractior	27044	FTMS; HC	56.21	776.42	G3BP2

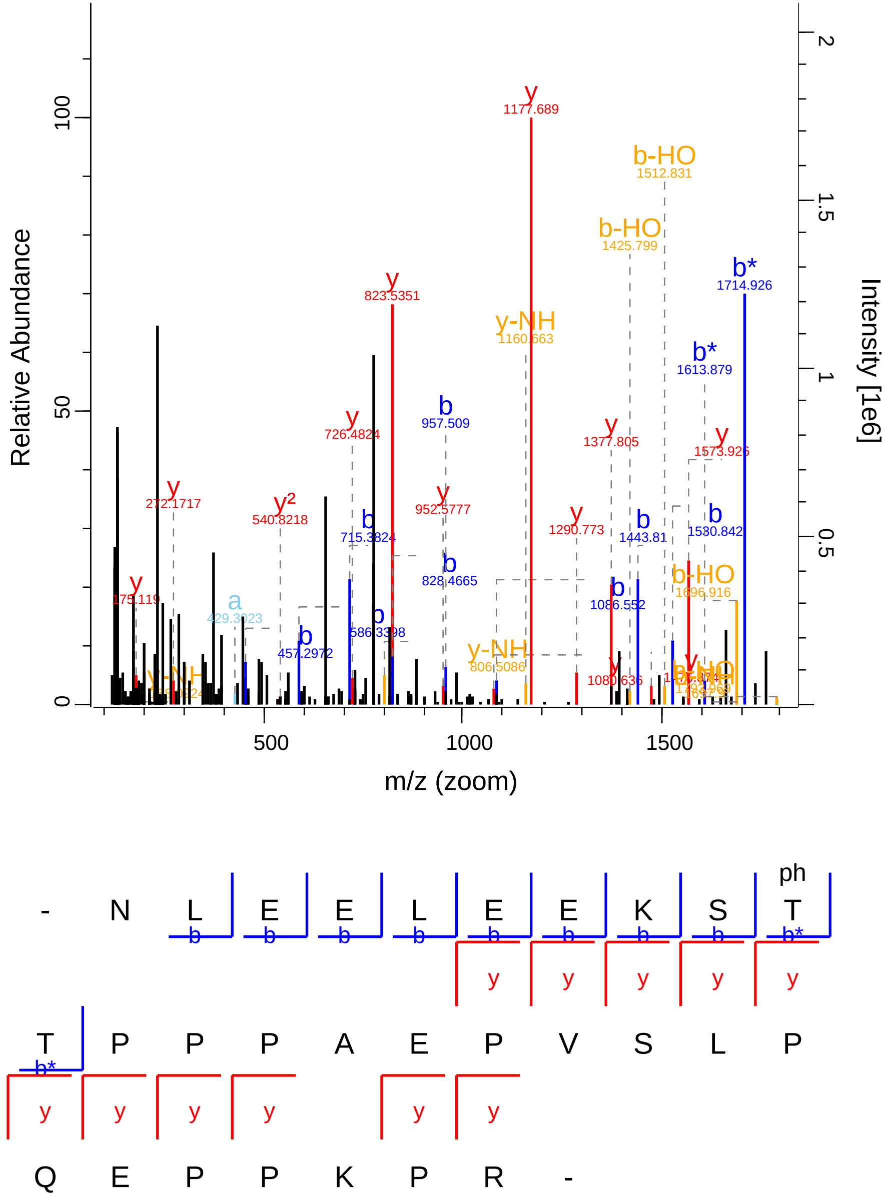

Raw File	Scan	Method	Score	m/z	Gene names
RO-QC-160121_ZackD_SS_Set_02_Fractio	27097	FTMS; HC	90.05	1147.56	SLC8A3

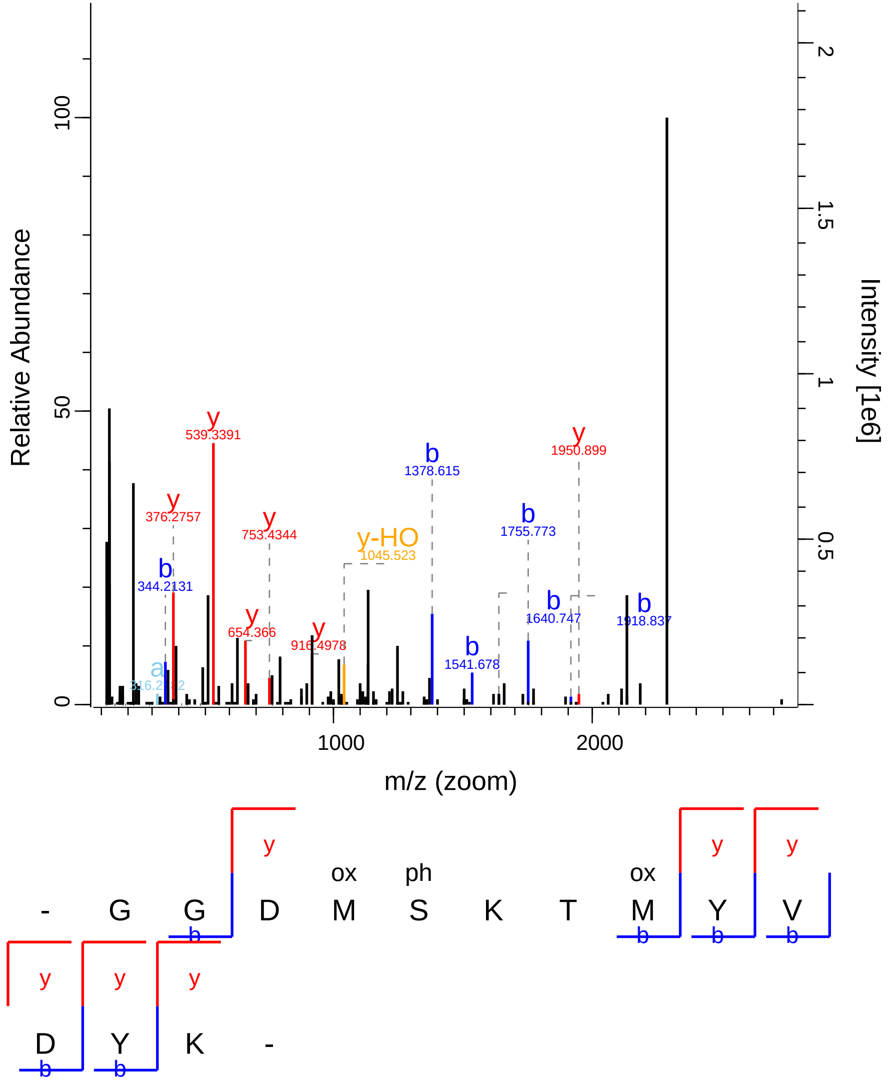

Raw File	Scan	Method	Score	m/z	Gene names
RO-QC-160121_ZackD_SS_Set_02_Fraction	27646	FTMS; HC	125.62	876.1	MYH10

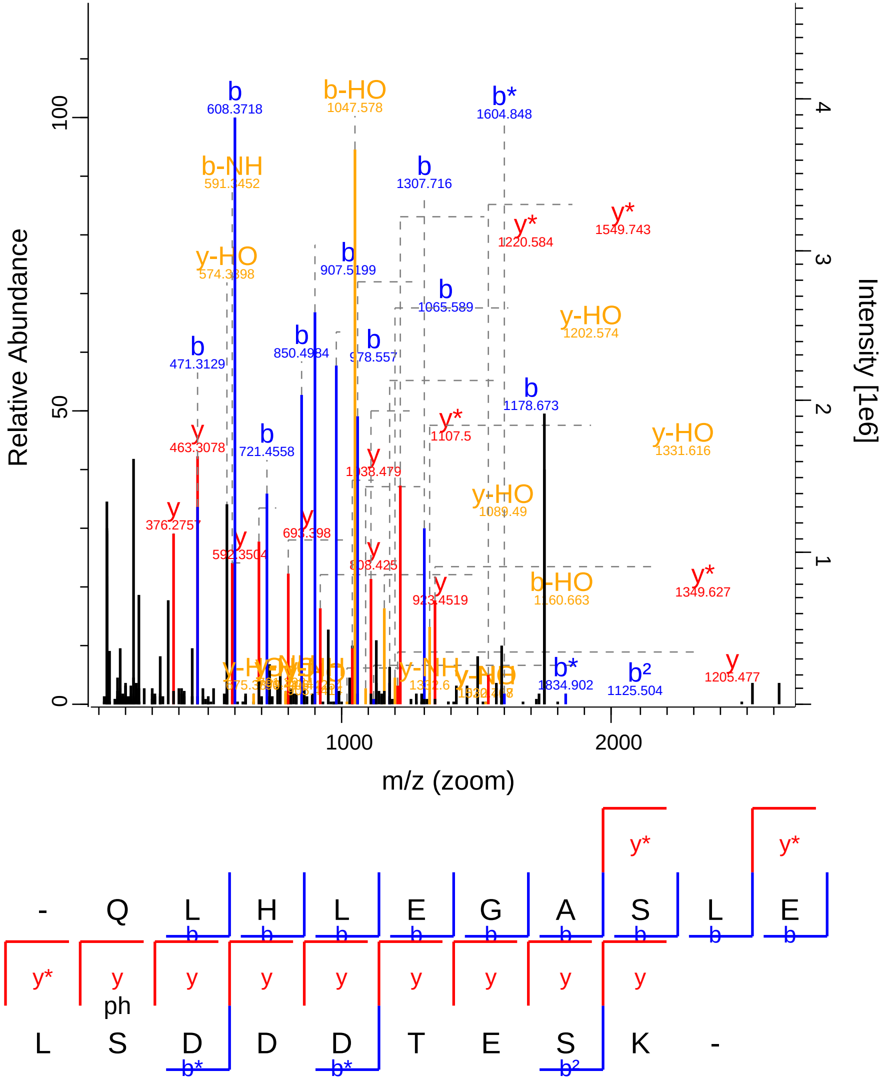

Raw File	Scan	Method	Score	m/z	Gene names
RO-QC-160121_ZackD_SS_Set_02_Fractio	28339	FTMS; HC	77.8	1110.53	DDX42

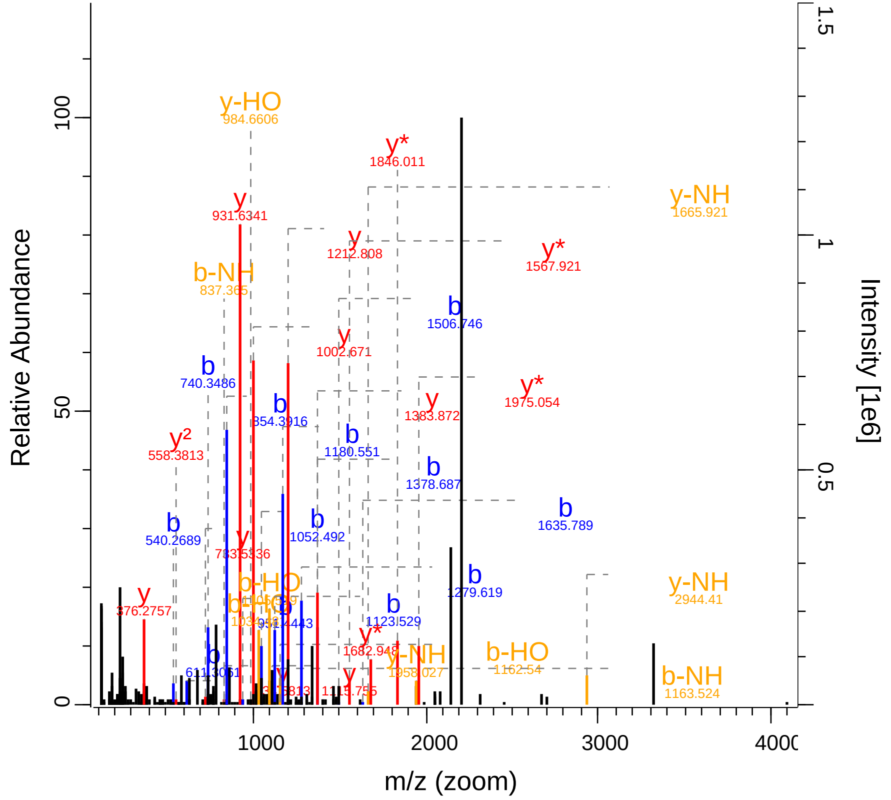

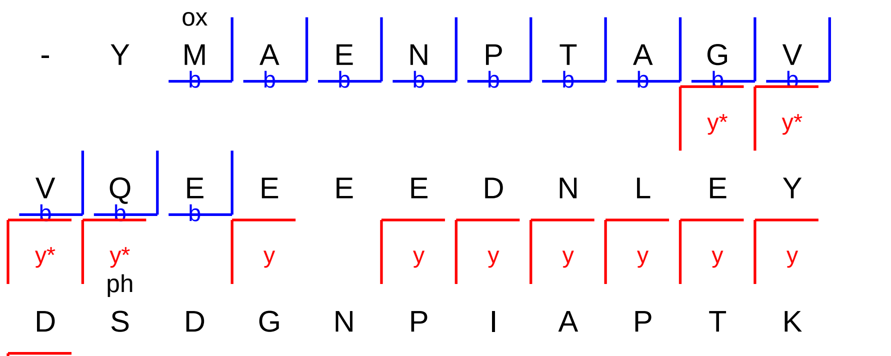

Raw File	Scan	Method	Score	m/z	Gene names
RO-QC-160121_ZackD_SS_Set_02_Fractio	29859	FTMS; HC	281.63	1127.9	ARMC10

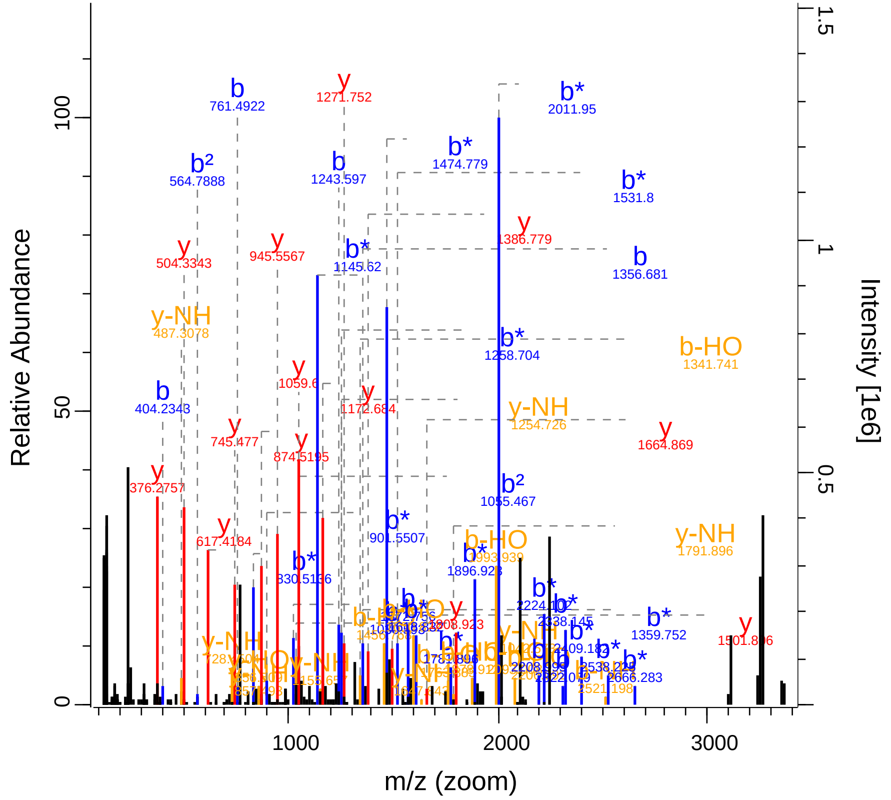

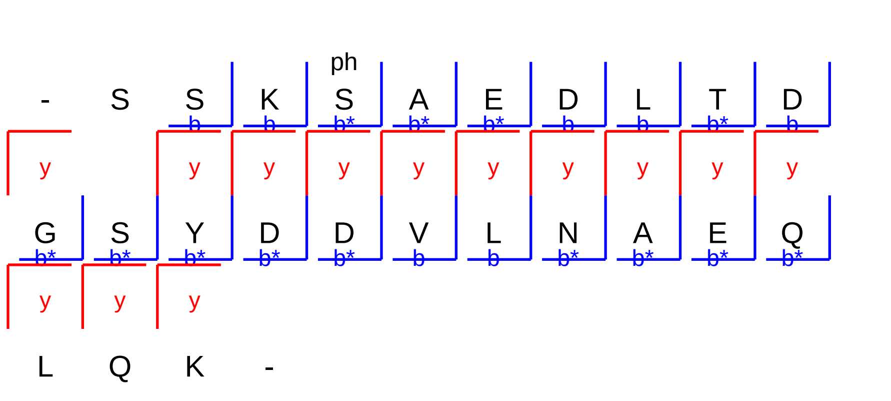

Raw File	Scan	Method	Score	m/z	Gene names
RO-QC-160121_ZackD_SS_Set_02_Fractio	29870	FTMS; HC	211.78	846.18	ARMC10

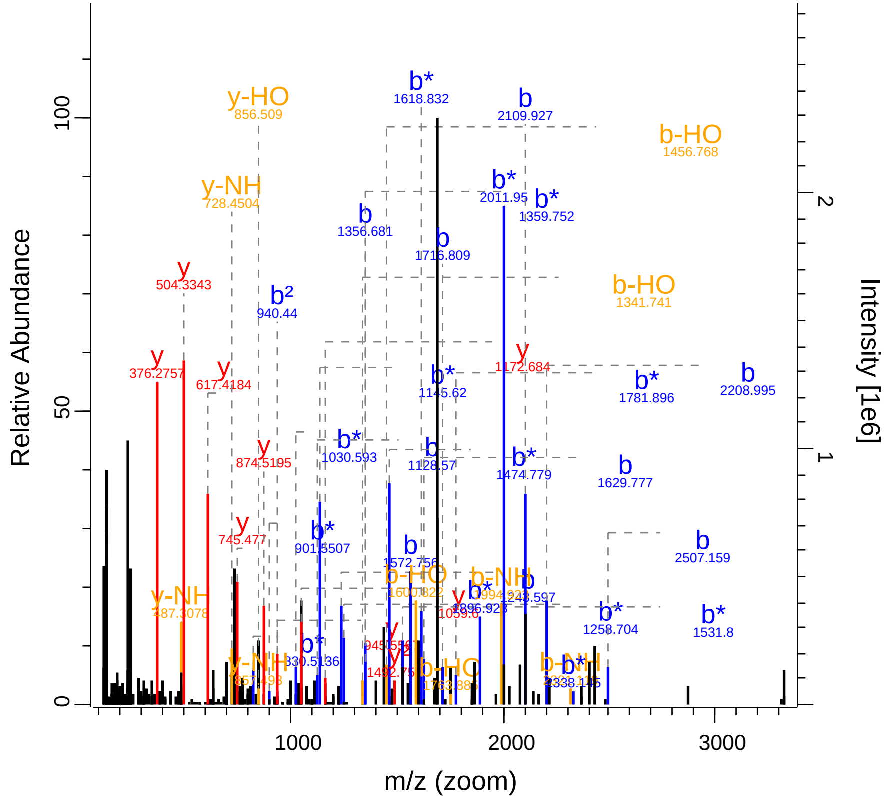

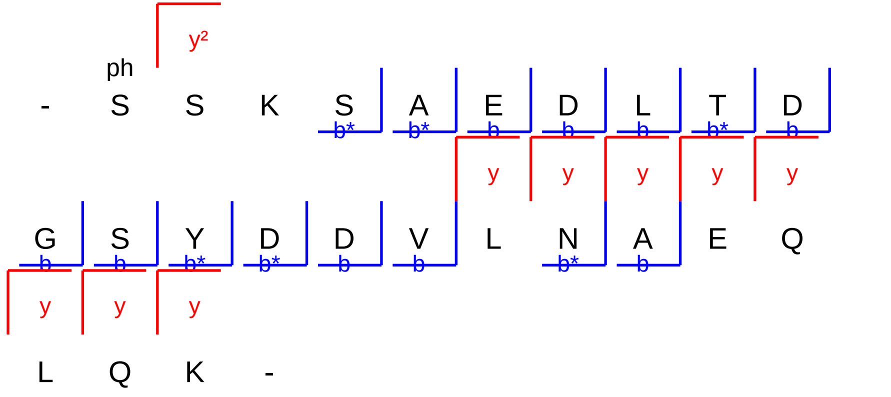

Raw File	Scan	Method	Score	m/z	Gene names
RO-QC-160121_ZackD_SS_Set_02_Fraction	31060	FTMS; HC	42.89	949.2	GPR143

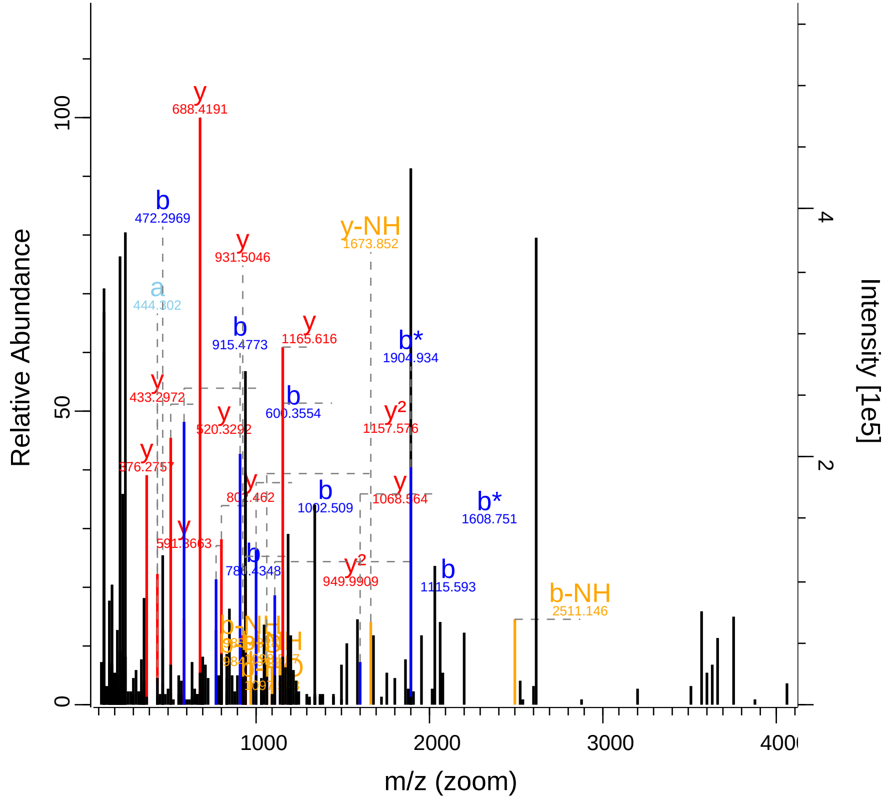

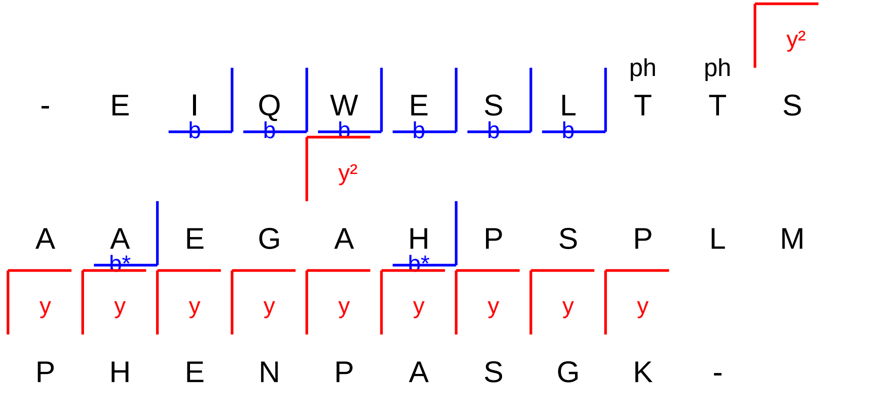

Raw File	Scan	Method	Score	m/z	Gene names
RO-QC-160121_ZackD_SS_Set_02_Fractio	33224	FTMS; HC	42.57	1236.67	BCAS3

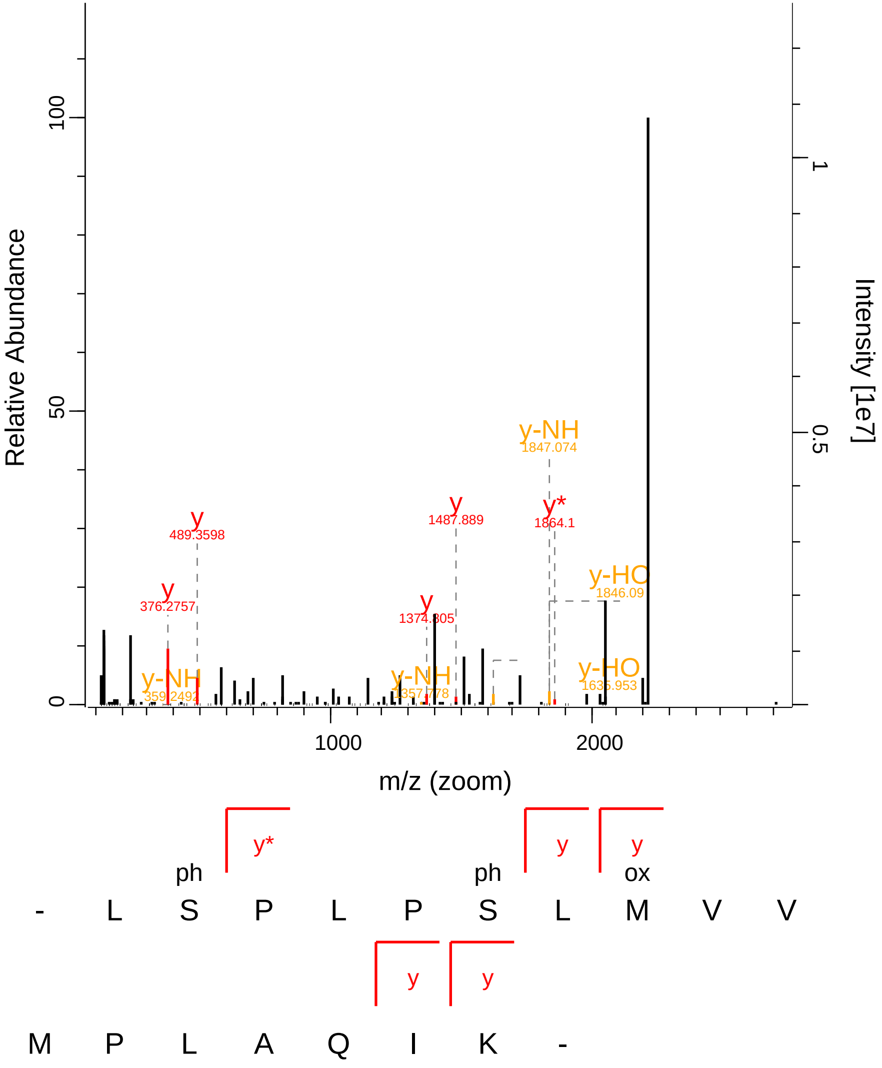

Raw File	Scan	Method	Score	m/z	Gene names
RO-QC-160121_ZackD_SS_Set_02_Fractio	33923	FTMS; HC	150.79	873.42	TNS3

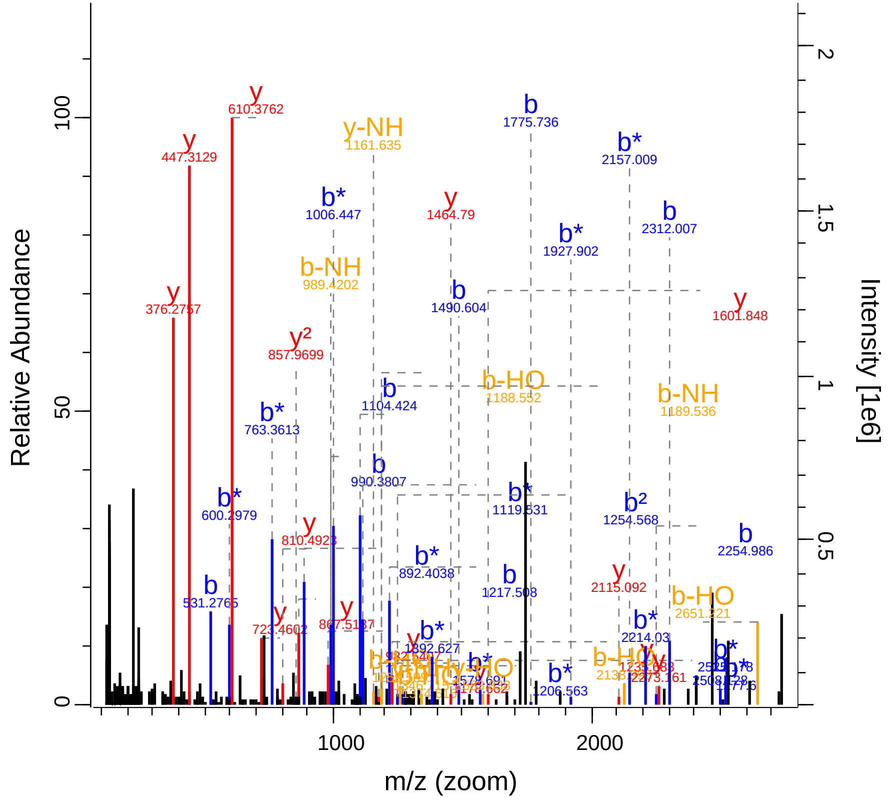

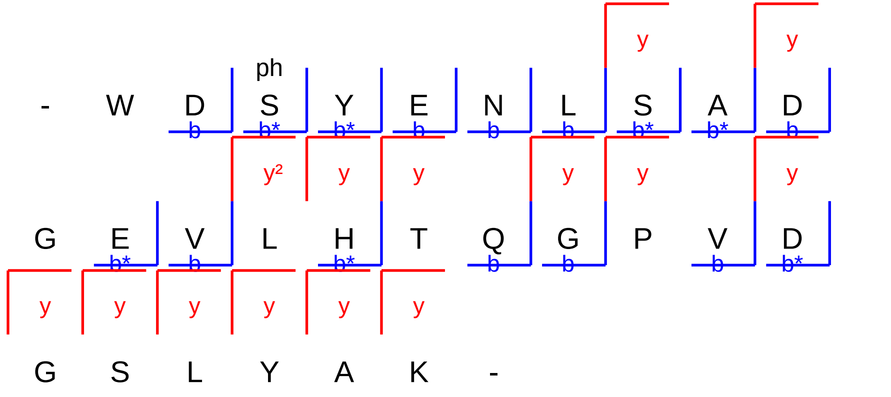

Raw File	Scan	Method	Score	m/z	Gene names
RO-QC-160121_ZackD_SS_Set_02_Fractio	34036	FTMS; HC	58.98	1130.08	KLHL42

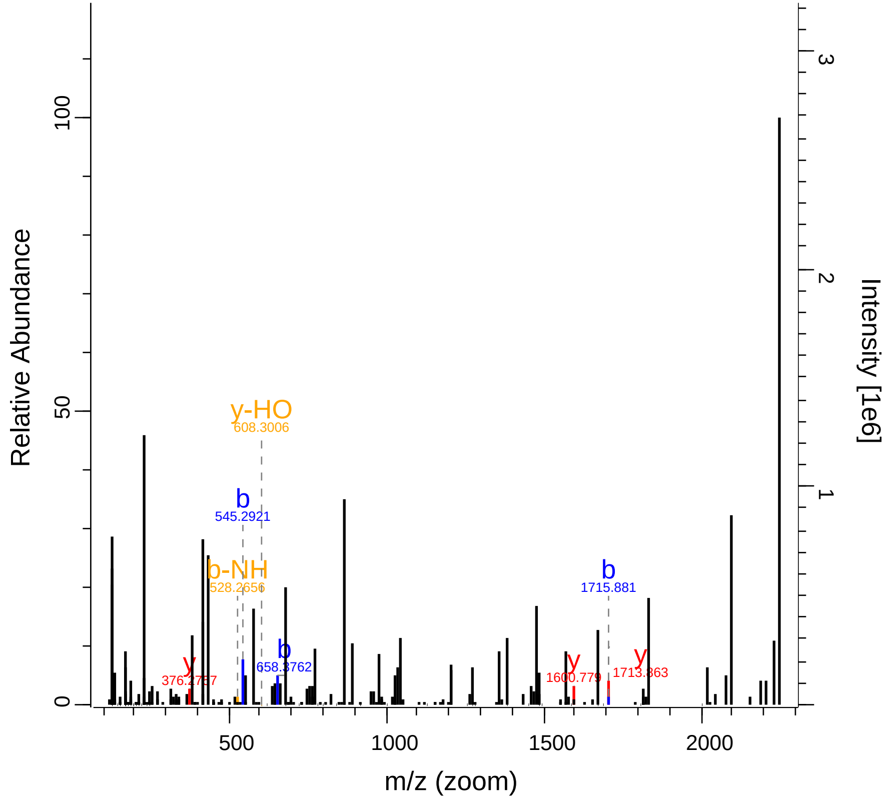

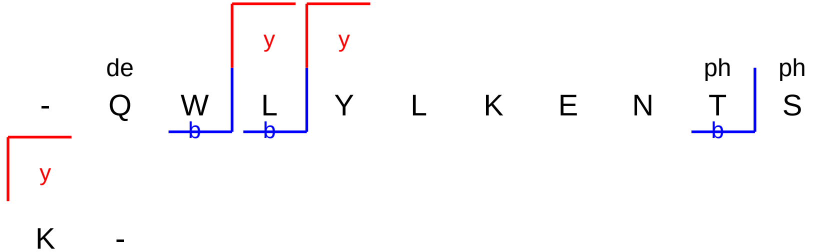

Raw File	Scan	Method	Score	m/z	Gene names
RO-QC-160121_ZackD_SS_Set_02_Fractio	34263	FTMS; HC	40.37	1139.57	ZNF846

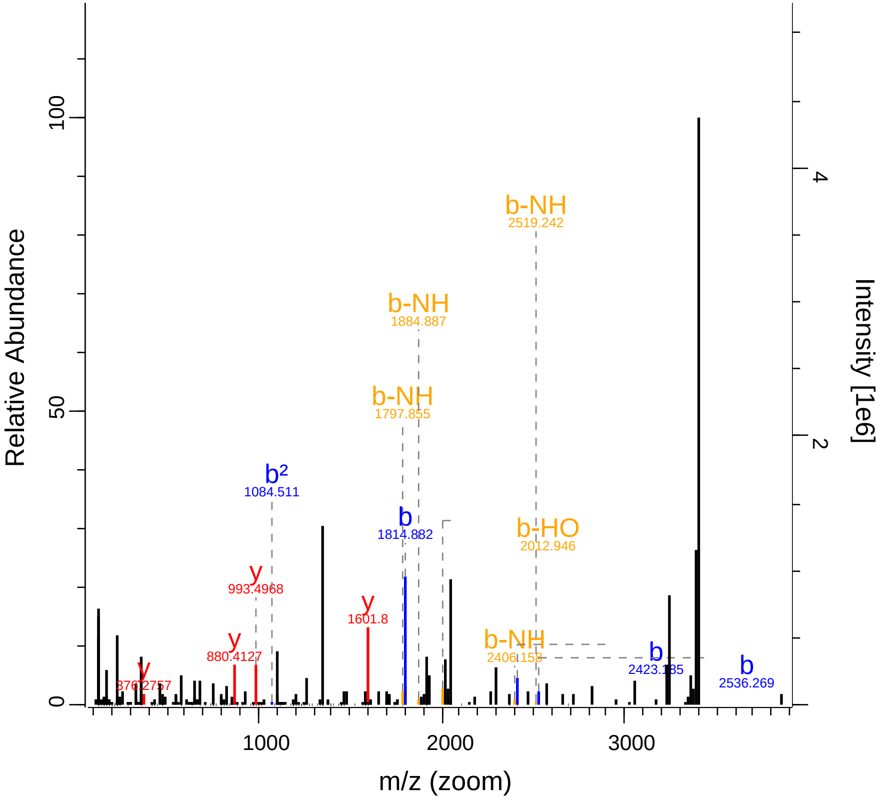

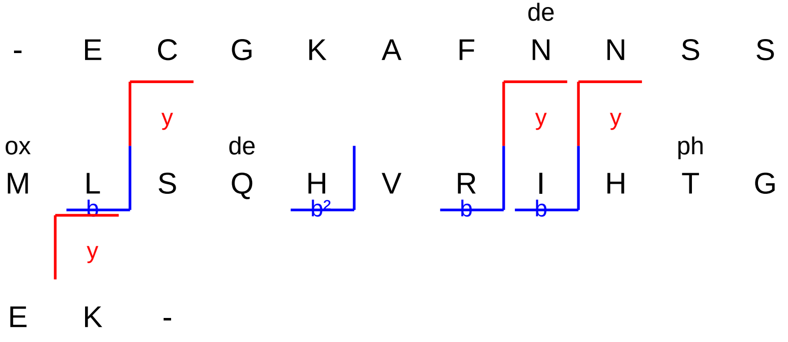

Raw File	Scan	Method	Score	m/z	Gene names
RO-QC-160121_ZackD_SS_Set_02_Fractio	34511	FTMS; HC	47.29	1360.16	CEP295

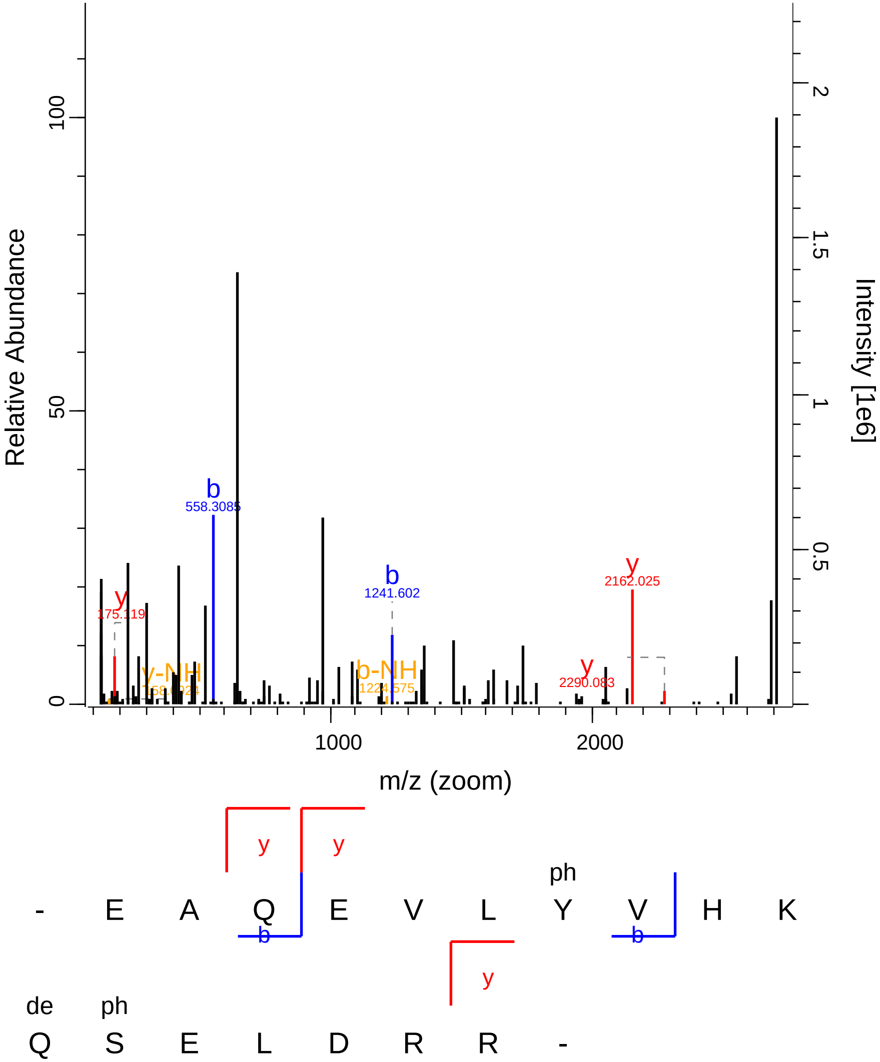

Raw File	Scan	Method	Score	m/z	Gene names
RO-QC-160121_ZackD_SS_Set_02_Fractior	34755	FTMS; HC	63.02	994.49	MAP1S

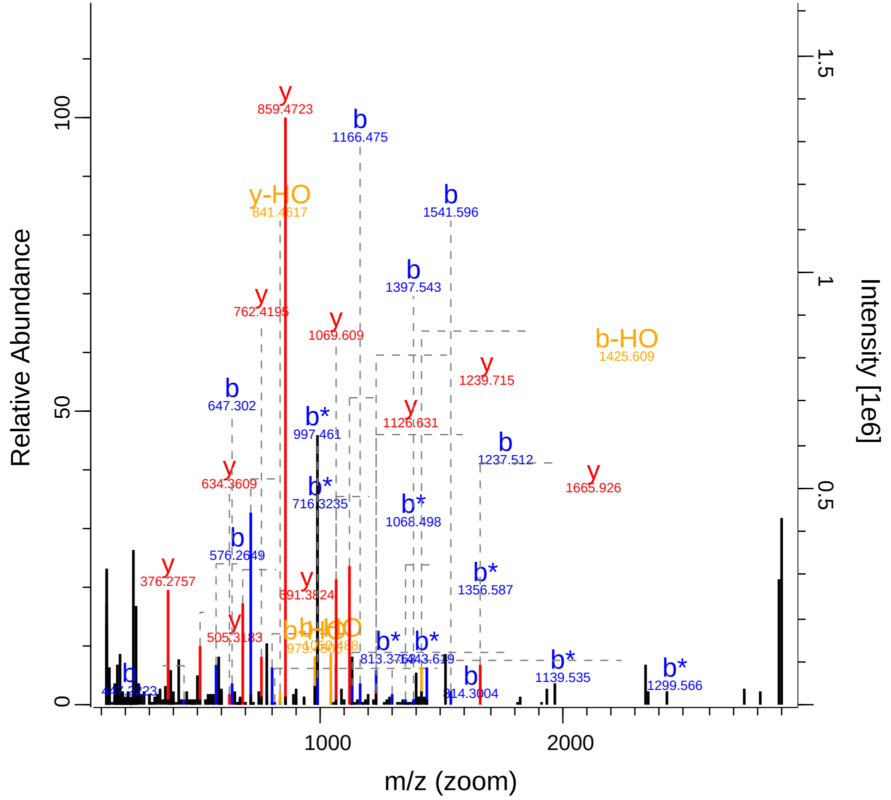

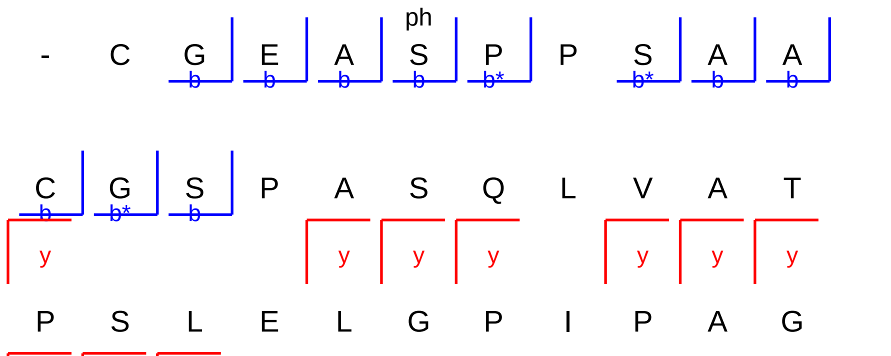

Raw File	Scan	Method	Score	m/z	Gene names
RO-QC-160121_ZackD_SS_Set_02_Fractior	35510	FTMS; HC	43.53	814.42	CCDC7

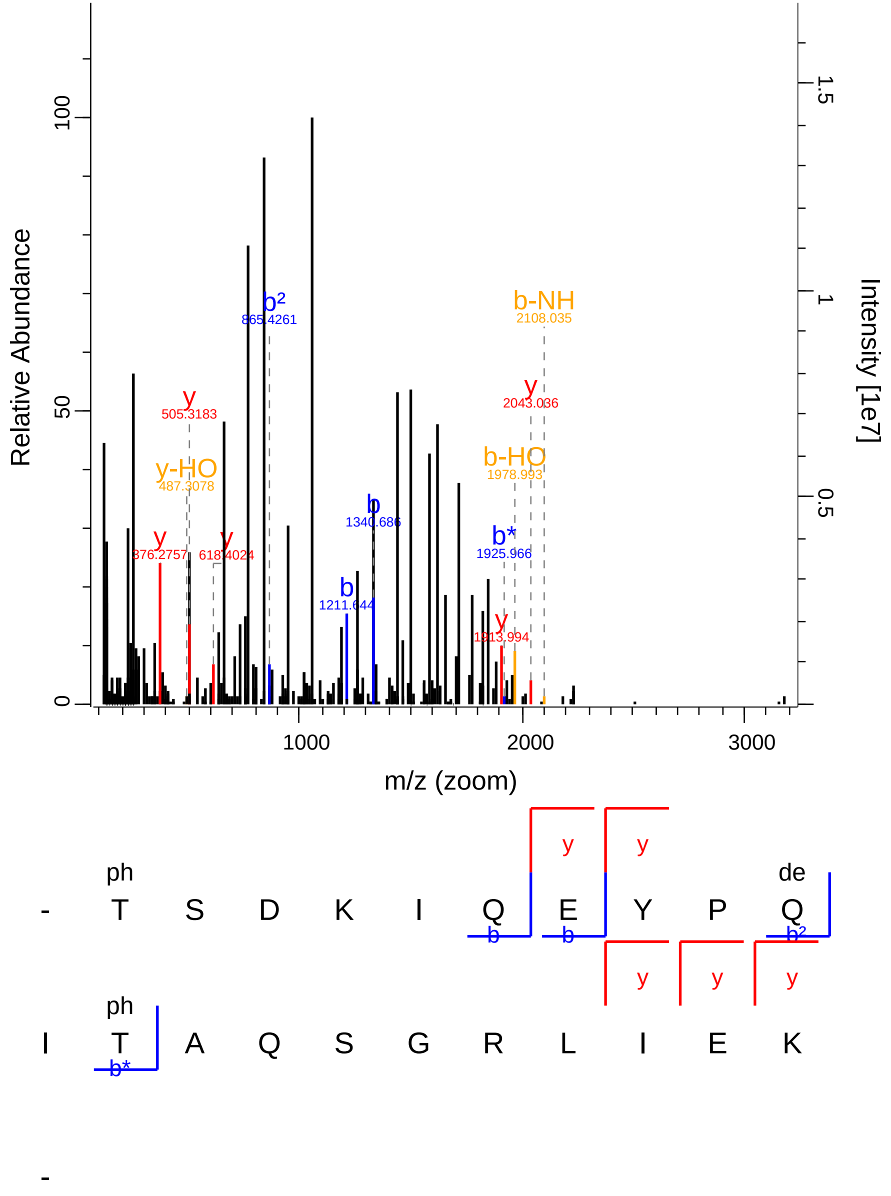

Raw File	Scan	Method	Score	m/z	Gene names
RO-QC-160121_ZackD_SS_Set_02_Fractior	36940	FTMS; HC	118.76	595.99	TMEM192

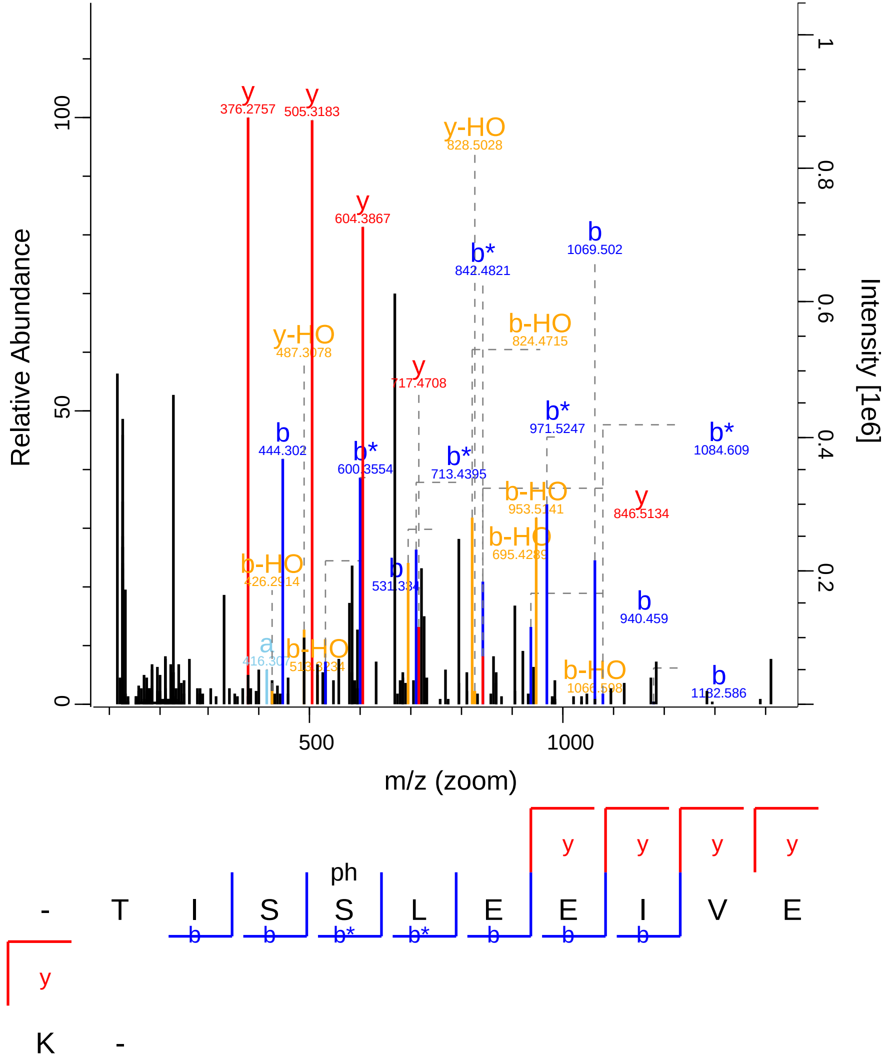

Raw File	Scan	Method	Score	m/z	Gene names
RO-QC-160121_ZackD_SS_Set_02_Fractio	37297	FTMS; HC	160.93	972.48	TTLL12

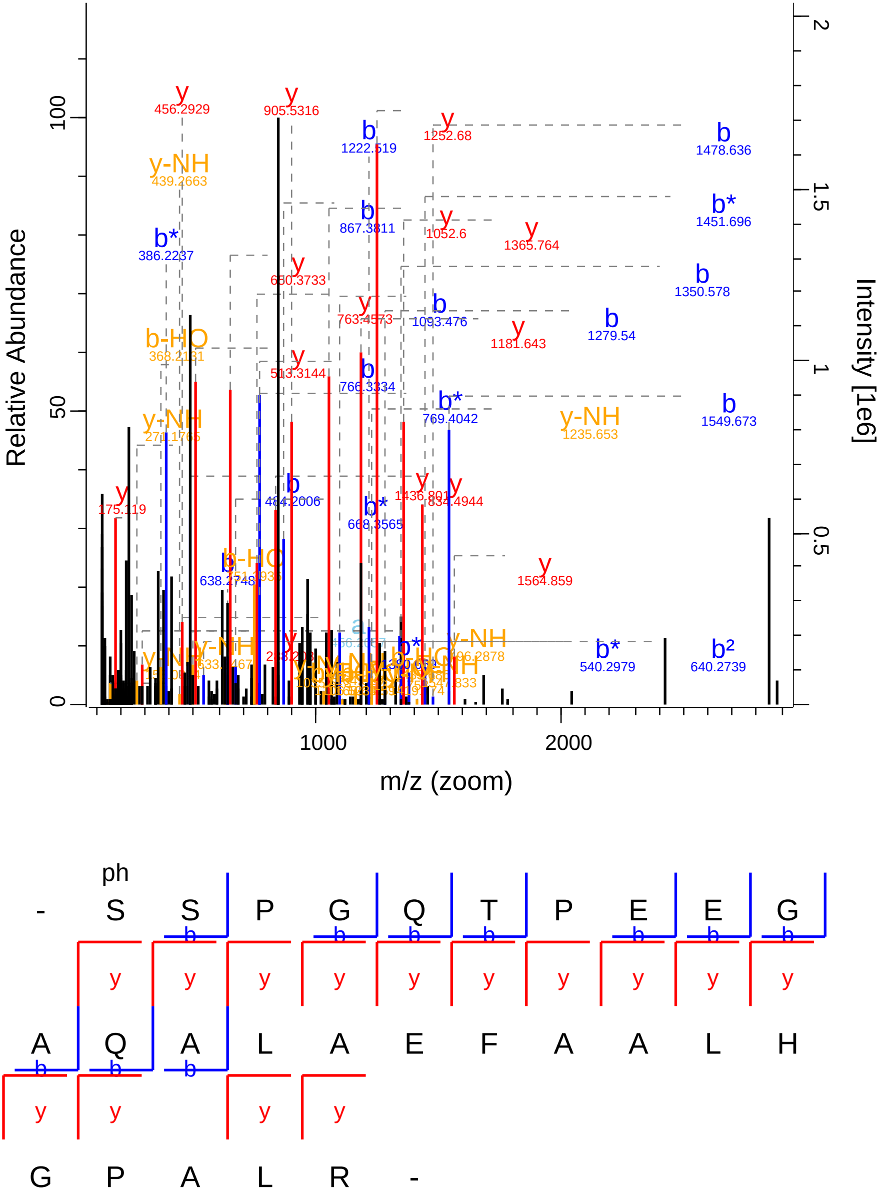

Raw File	Scan	Method	Score	m/z	Gene names
RO-QC-160121_ZackD_SS_Set_02_Fractio	38637	FTMS; HC	102.07	1015.03	COL4A3BP

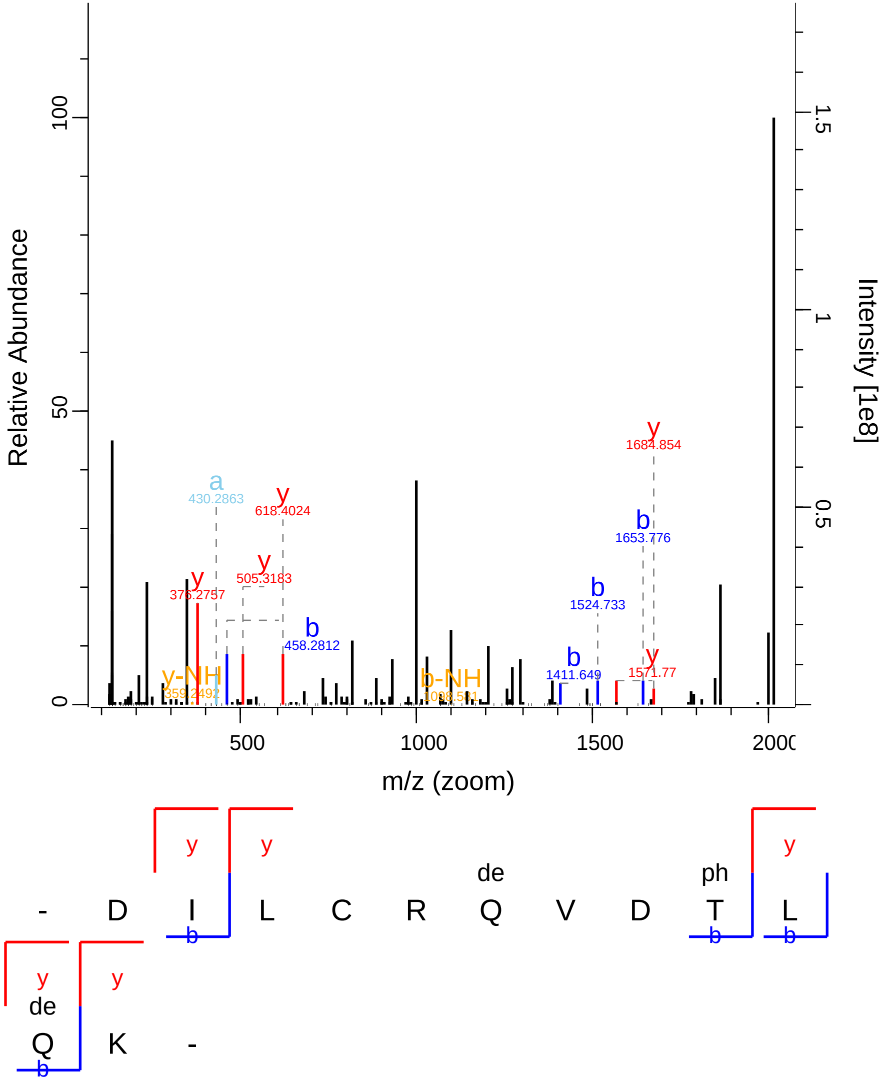

Raw File	Scan	Method	Score	m/z	Gene names
RO-QC-160121_ZackD_SS_Set_02_Fractio	38666	FTMS; HC	222.77	1180.62	TEX2

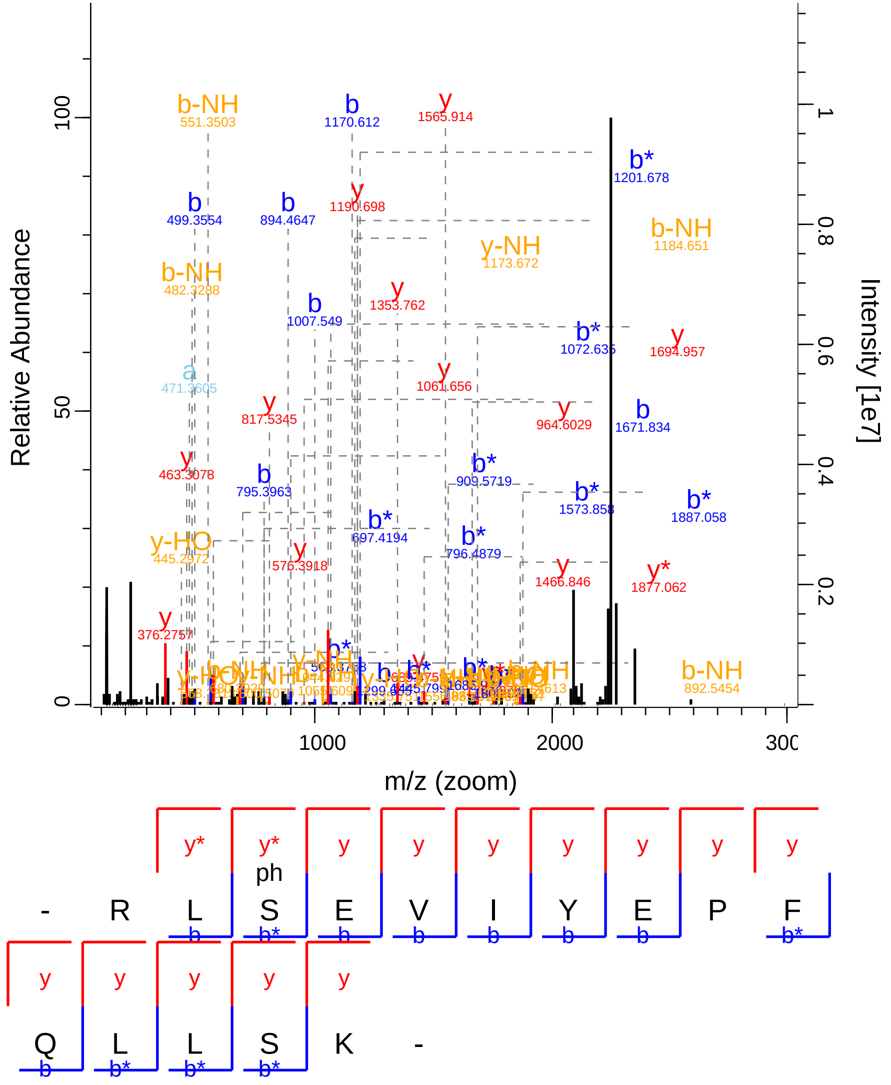

Raw File	Scan	Method	Score	m/z	Gene names
RO-QC-160121_ZackD_SS_Set_02_Fractior	38670	FTMS; HC	46.57	1366.8	ZNF532

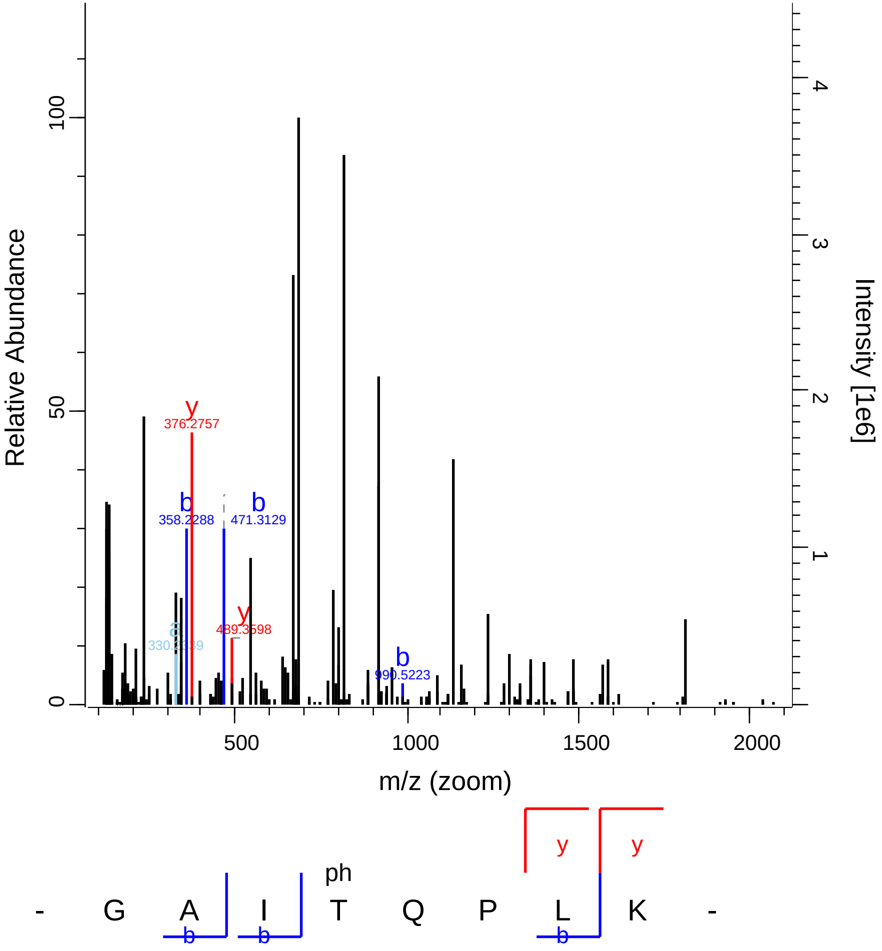

Raw File	Scan	Method	Score	m/z	Gene names
RO-QC-160121_ZackD_SS_Set_02_Fractio	39521	FTMS; HC	46.48	1359.67	DCLK2

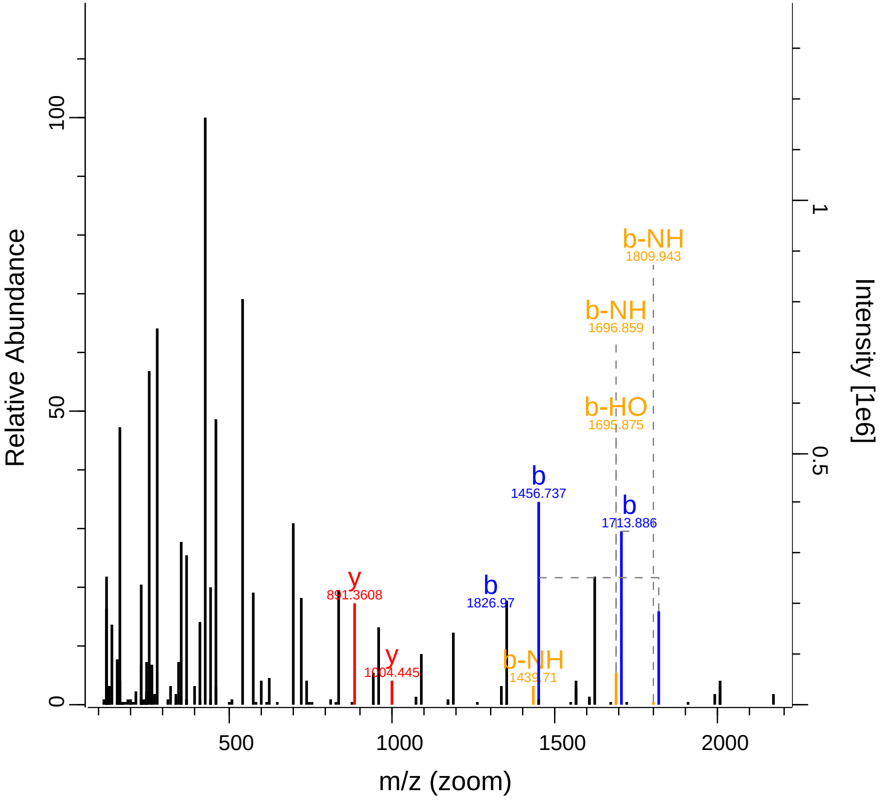

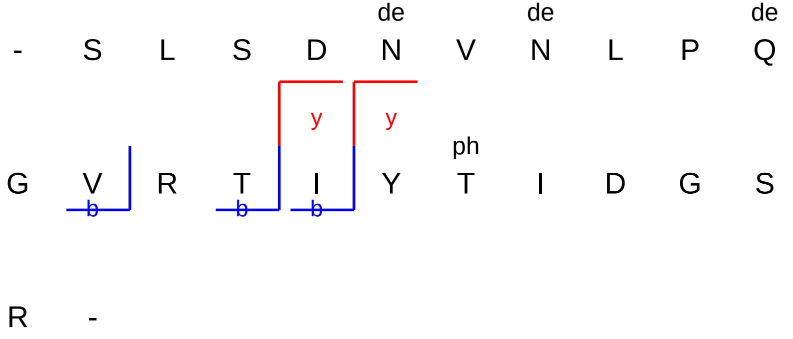

Raw File	Scan	Method	Score	m/z	Gene names
RO-QC-160121_ZackD_SS_Set_02_Fractio	39599	FTMS; HC	56.12	1440.69	TCF7L1

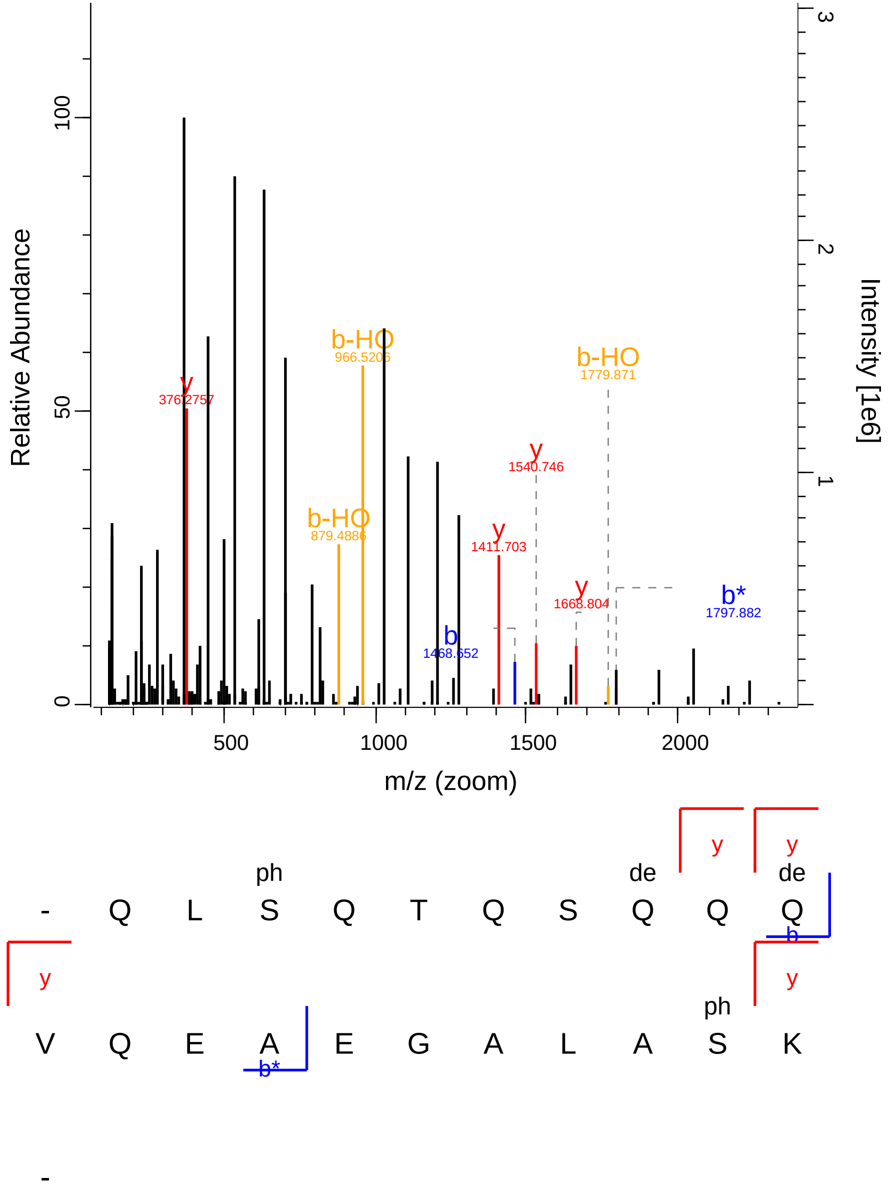

Raw File	Scan	Method	Score	m/z	Gene names
RO-QC-160121_ZackD_SS_Set_02_Fractio	39723	FTMS; HC	181.66	1007.94	ATG4B

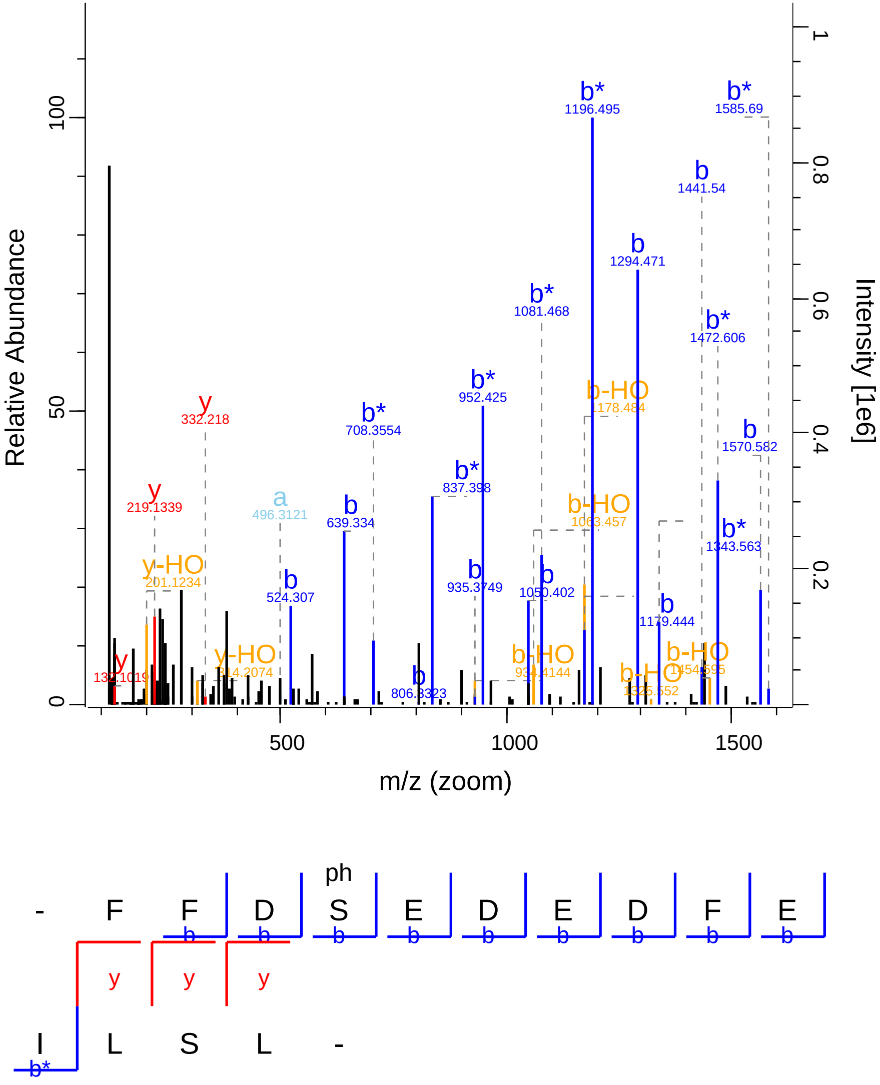

Raw File	Scan	Method	Score	m/z	Gene names
RO-QC-160121_ZackD_SS_Set_02_Fraction	5446	FTMS; HC	98.44	686.67	LMNA

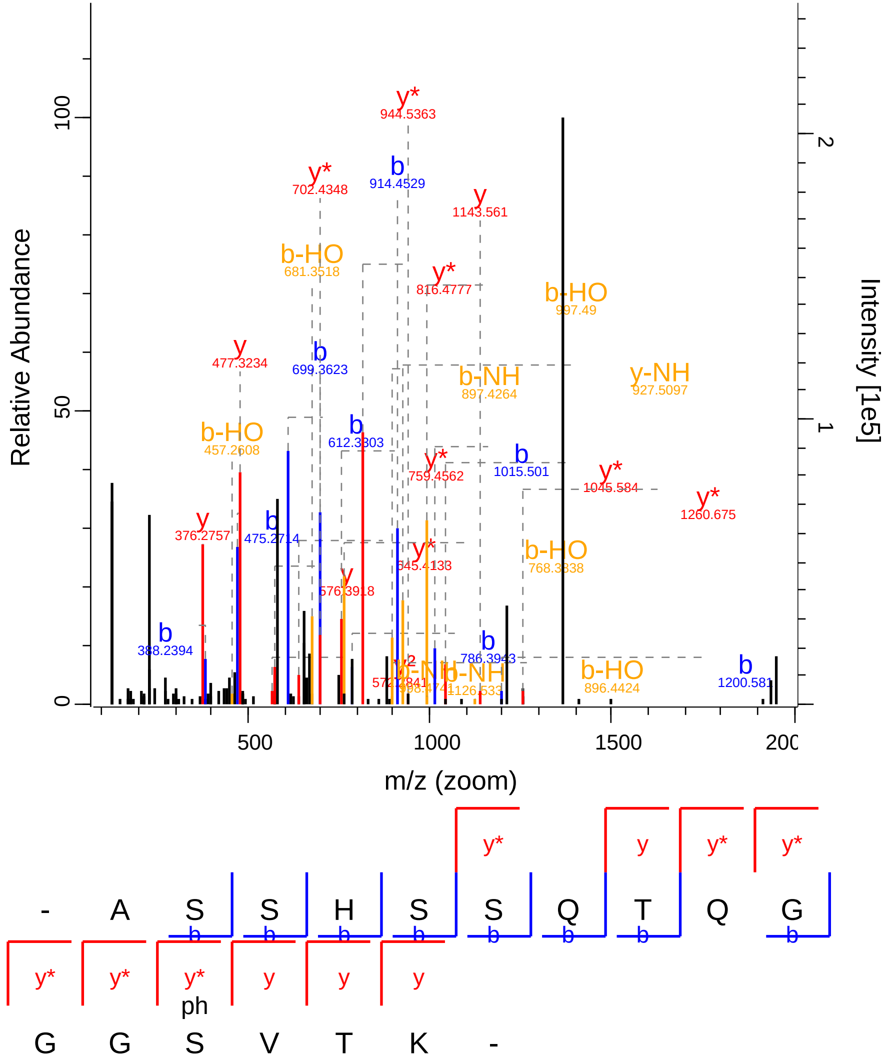

Raw File	Scan	Method	Score	m/z	Gene names
RO-QC-160121_ZackD_SS_Set_02_Fraction	5494	FTMS; HC	110.84	778.91	SNX17

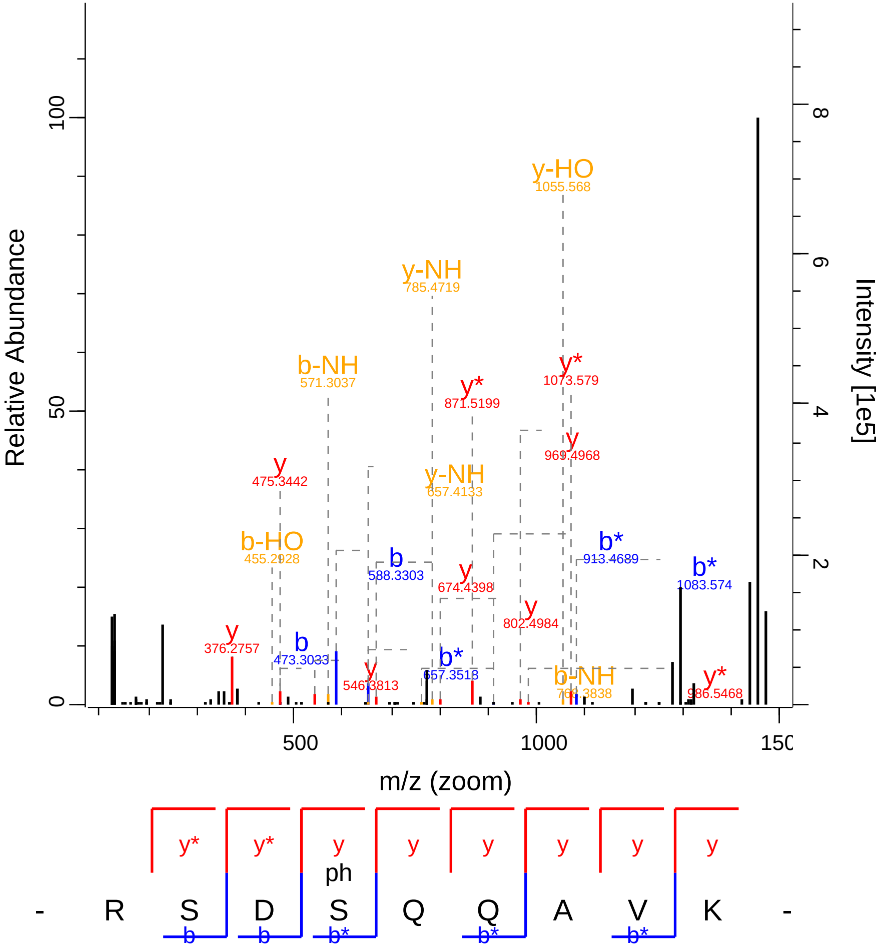

Raw File	Scan	Method	Score	m/z	Gene names
RO-QC-160121_ZackD_SS_Set_02_Fraction	5901	FTMS; HC	120.59	547.62	CAP2

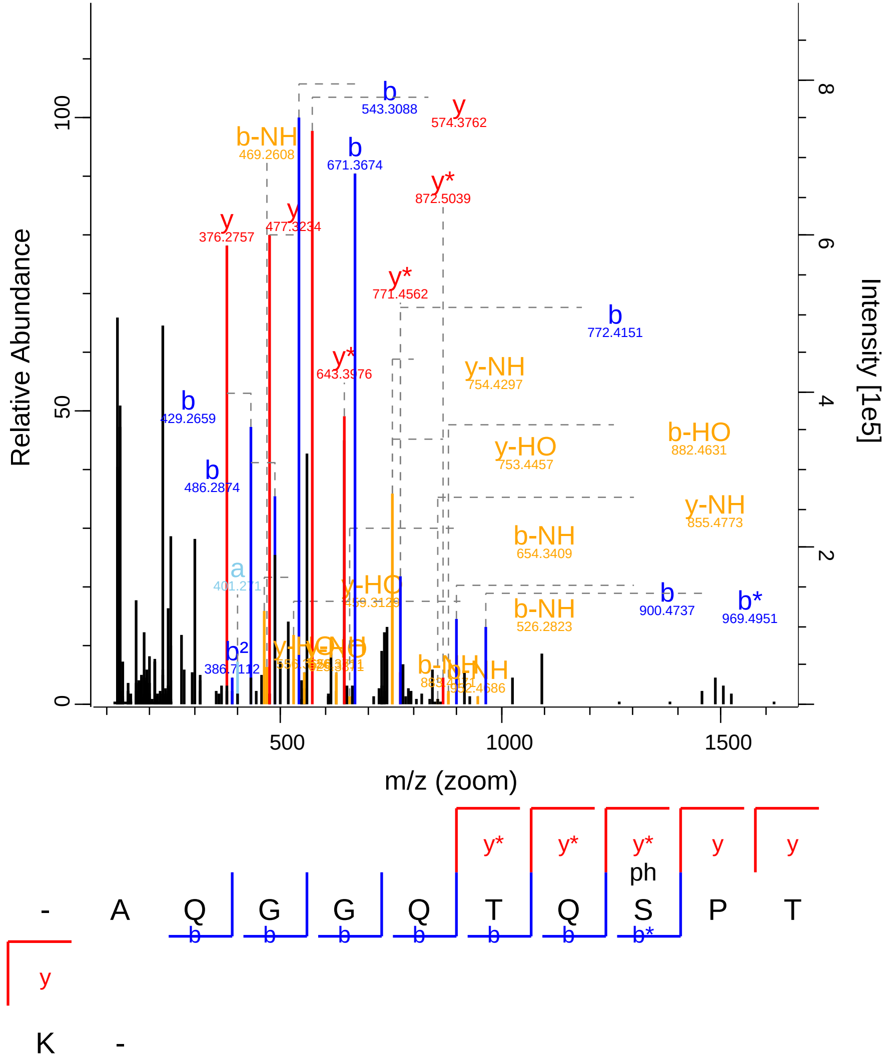

Raw File	Scan	Method	Score	m/z	Gene names
RO-QC-160121_ZackD_SS_Set_02_Fraction_	8194	FTMS; HC	45.16	664.66	KLF2;KLF4

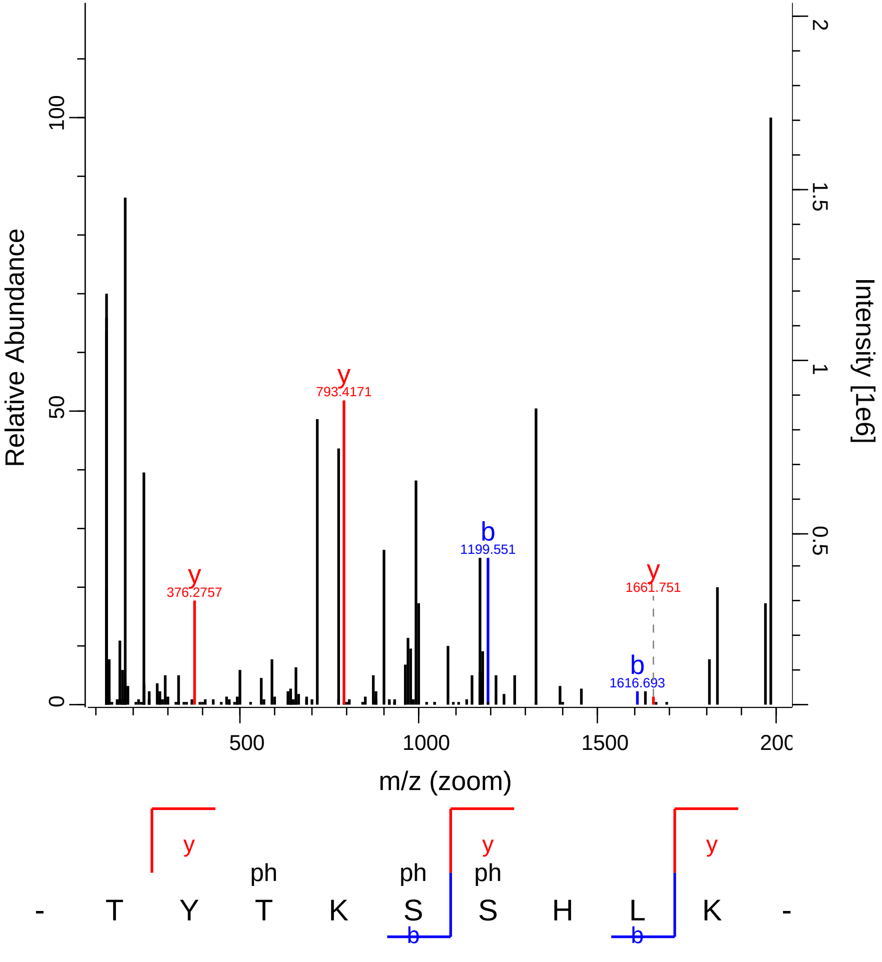

Raw File	Scan	Method	Score	m/z	Gene names
RO-QC-160121_ZackD_SS_Set_02_Fraction	8352	FTMS; HC	93.75	753.71	WBP4

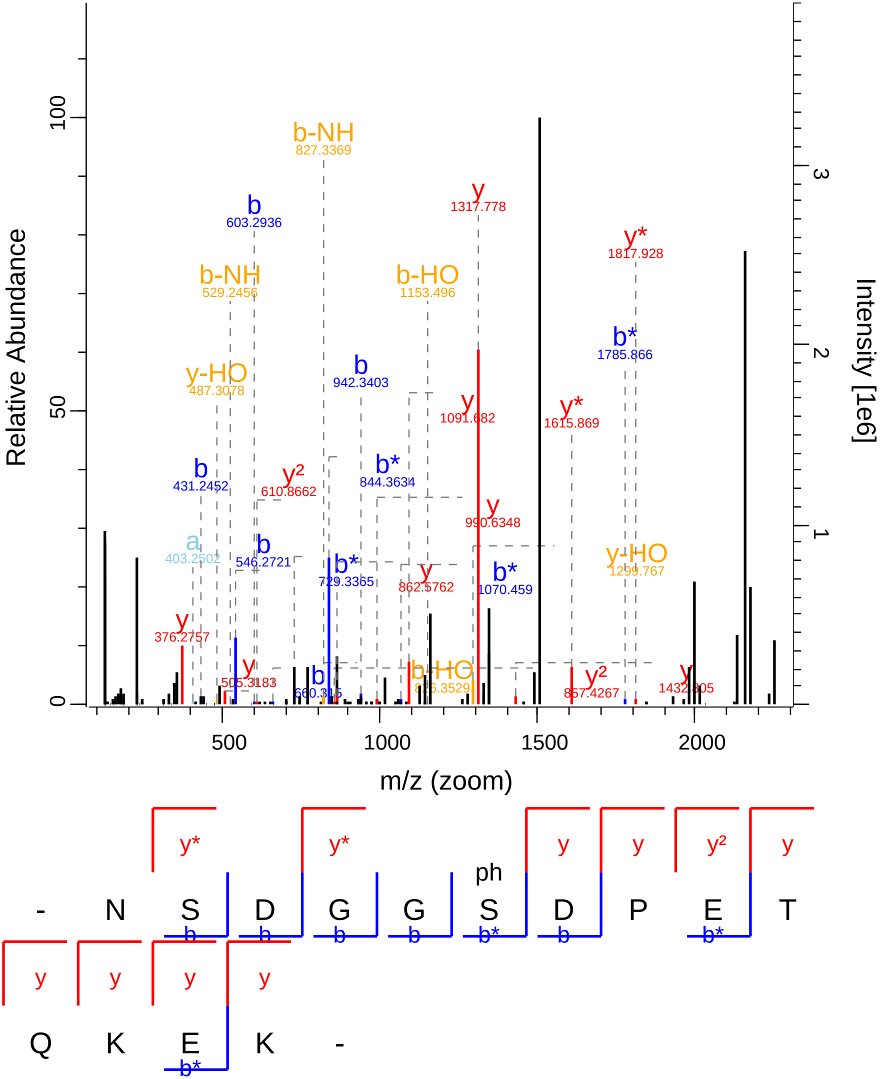

Raw File	Scan	Method	Score	m/z	Gene names
RO-QC-160121_ZackD_SS_Set_02_Fraction_	8857	FTMS; HC	48.42	768.87	PALM

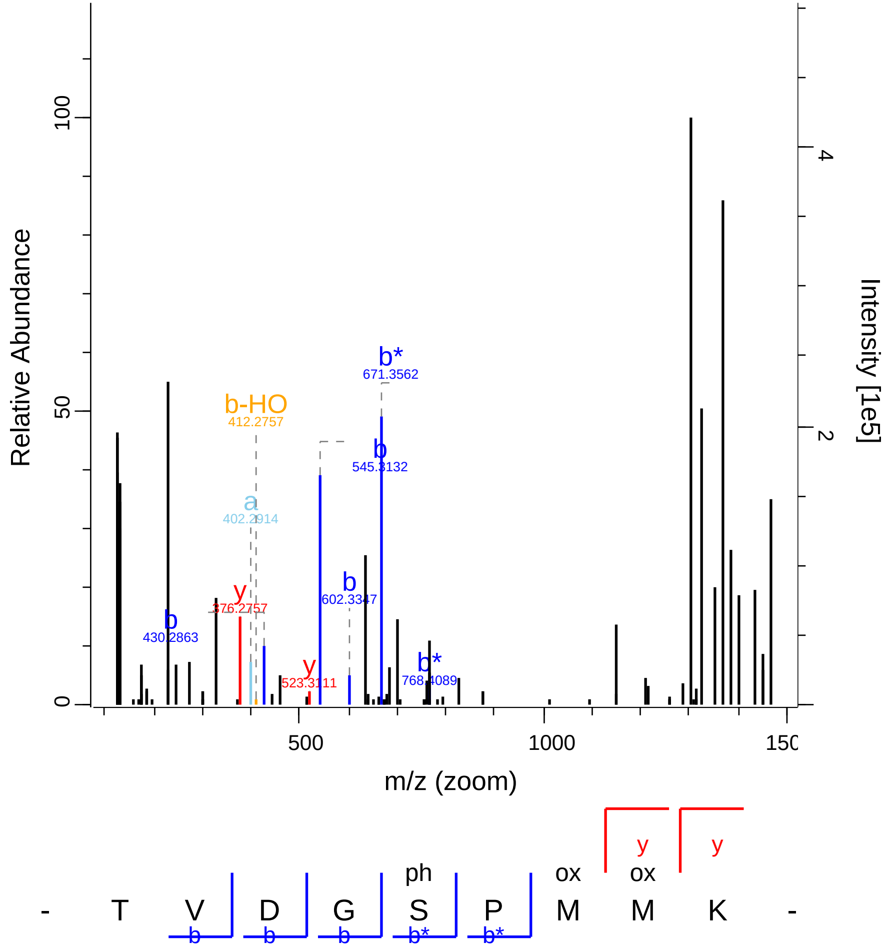

Raw File	Scan	Method	Score	m/z	Gene names
RO-QC-160121_ZackD_SS_Set_02_Fractio	9473	FTMS; HC	245.19	1161.08	HNRNPC

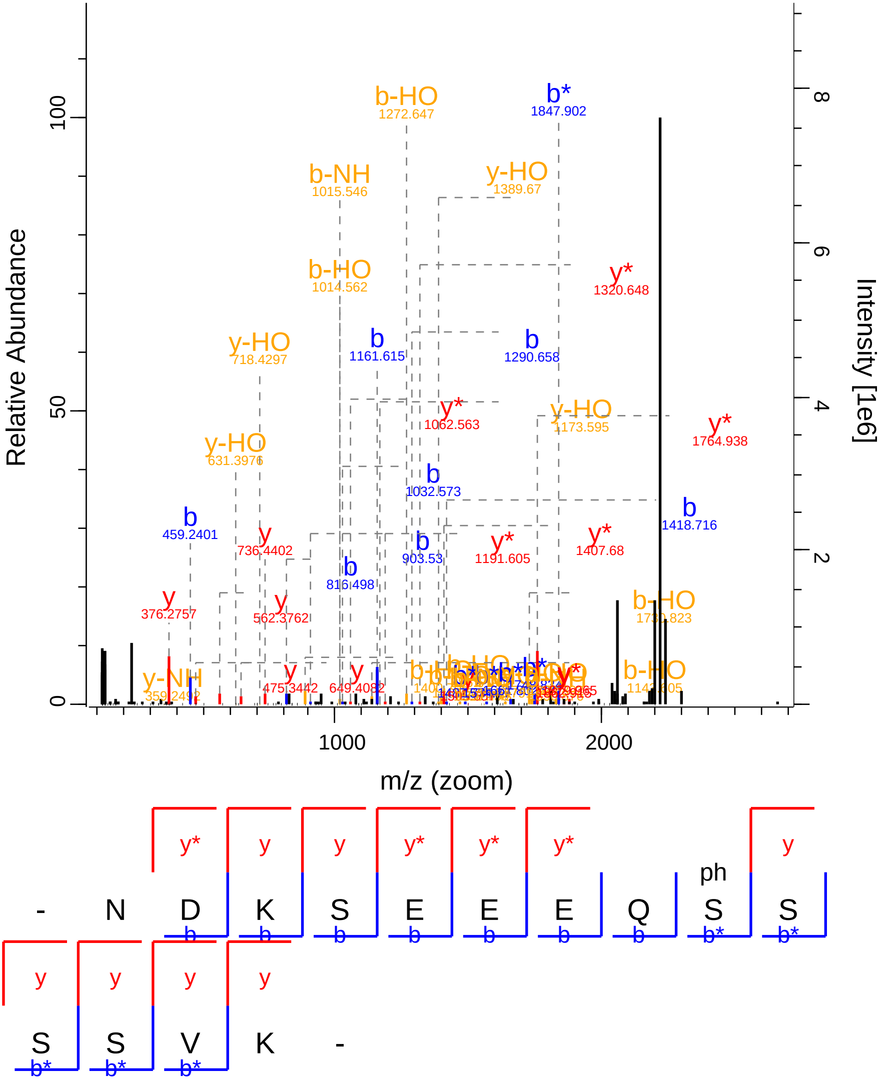

Raw File	Scan	Method	Score	m/z	Gene names
RO-QC-160121_ZackD_SS_Set_02_Fraction	9531	FTMS; HC	106.94	581.04	HNRNPC

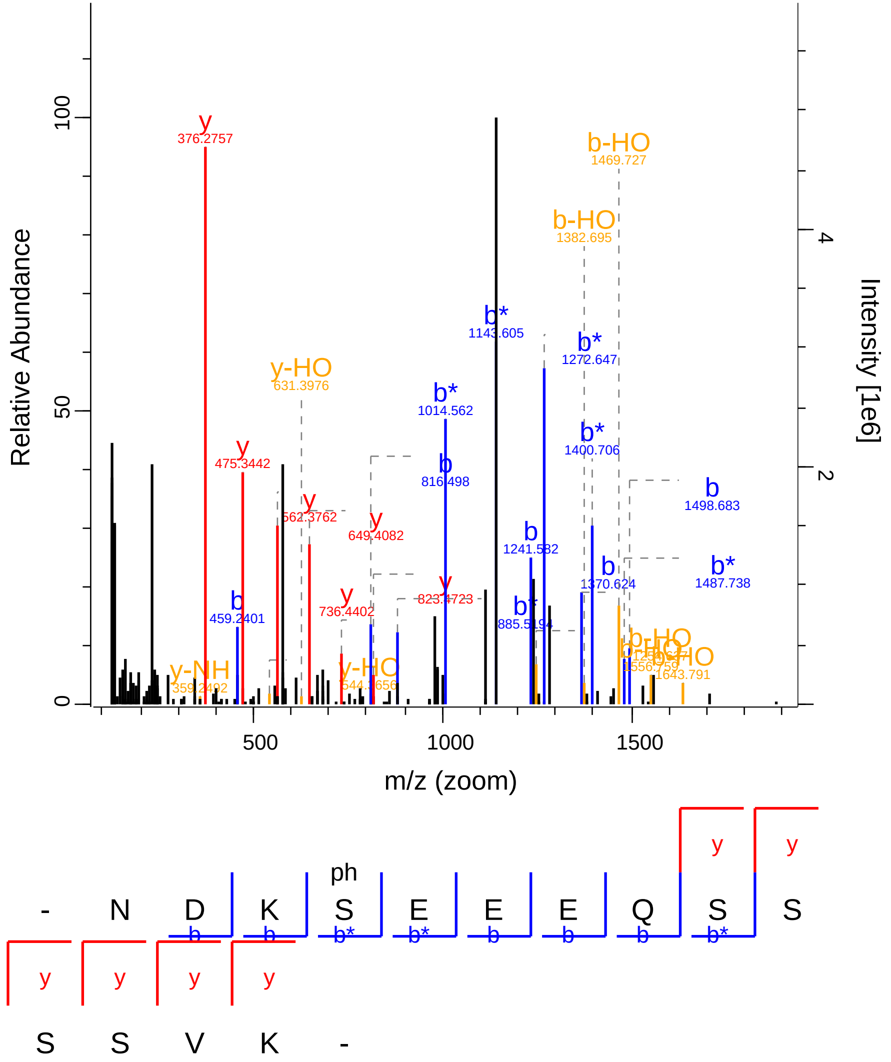

Raw File	Scan	Method	Score	m/z	Gene names
RO-QC-160121_ZackD_SS_Set_02_Fractior	10120	FTMS; HC	88.68	714.38	ATXN2

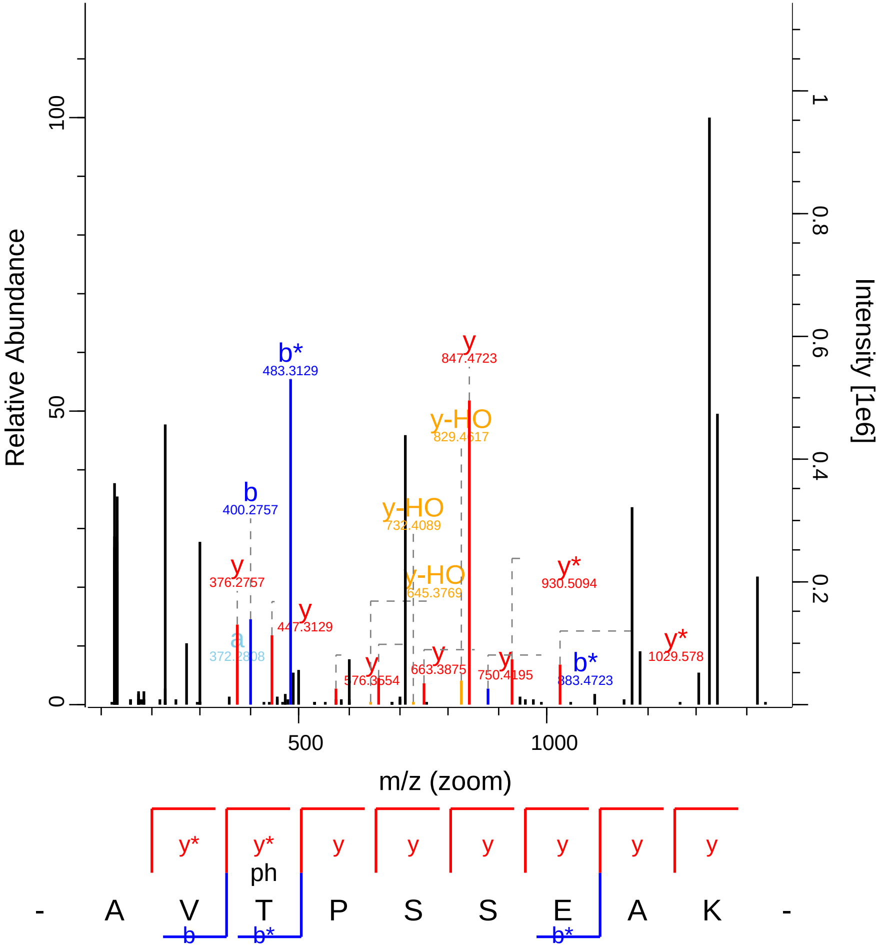

Raw File	Scan	Method	Score	m/z	Gene names
RO-QC-160121_ZackD_SS_Set_02_Fractio	10557	FTMS; HC	127.18	796.44	IGF2BP1

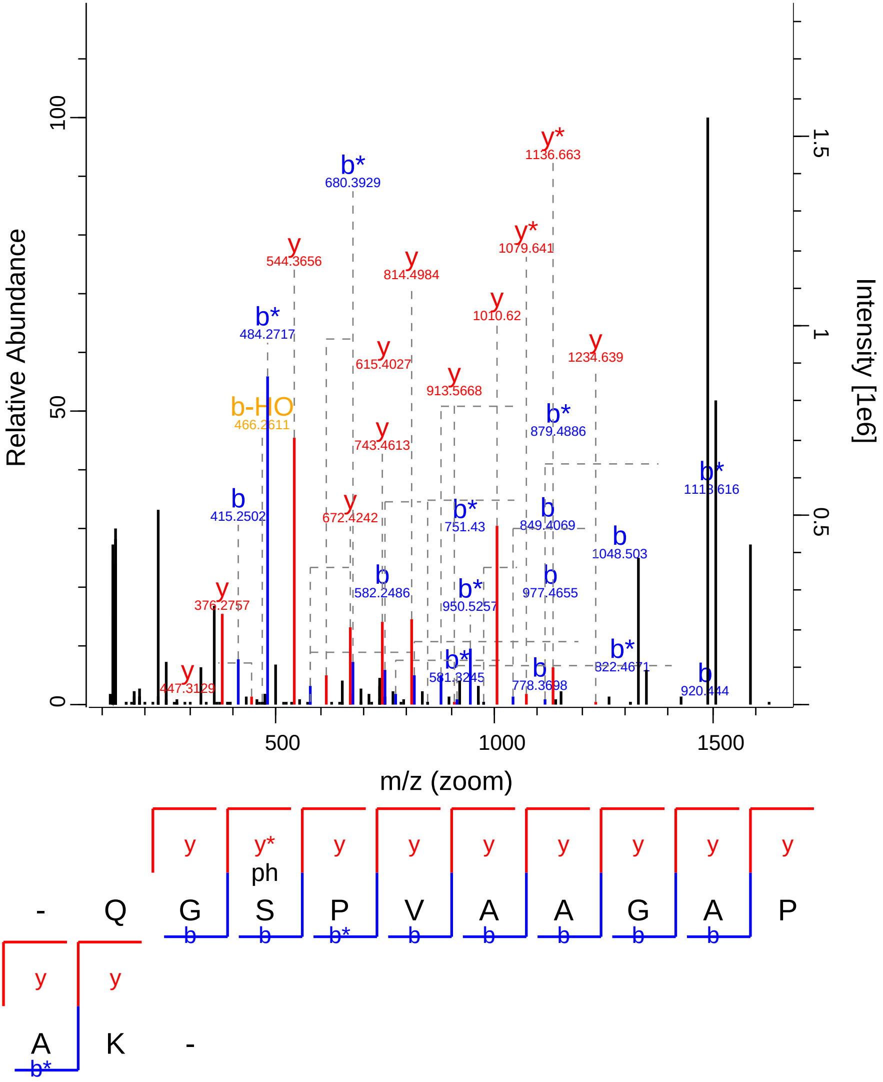

Raw File	Scan	Method	Score	m/z	Gene names
RO-QC-160121_ZackD_SS_Set_02_Fractior	11060	FTMS; HC	56.56	988.51	TNIP1

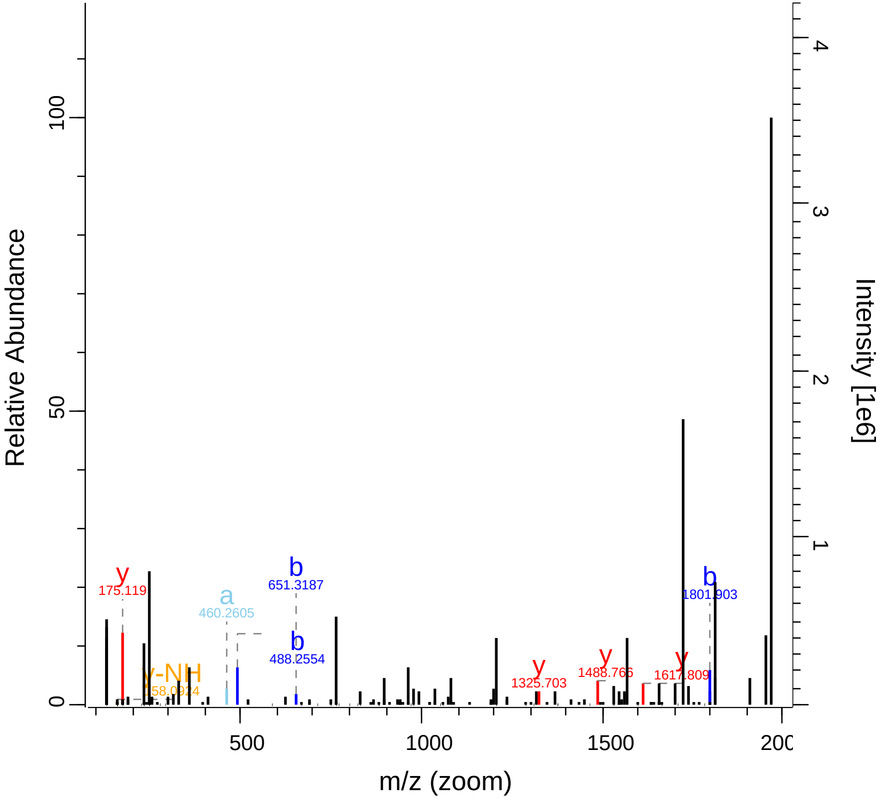

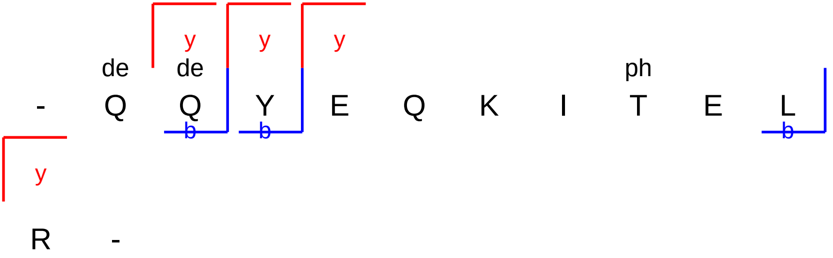

Raw File	Scan	Method	Score	m/z	Gene names
RO-QC-160121_ZackD_SS_Set_02_Fractior	12262	FTMS; HC	88.06	751.39	UHRF1BP1L

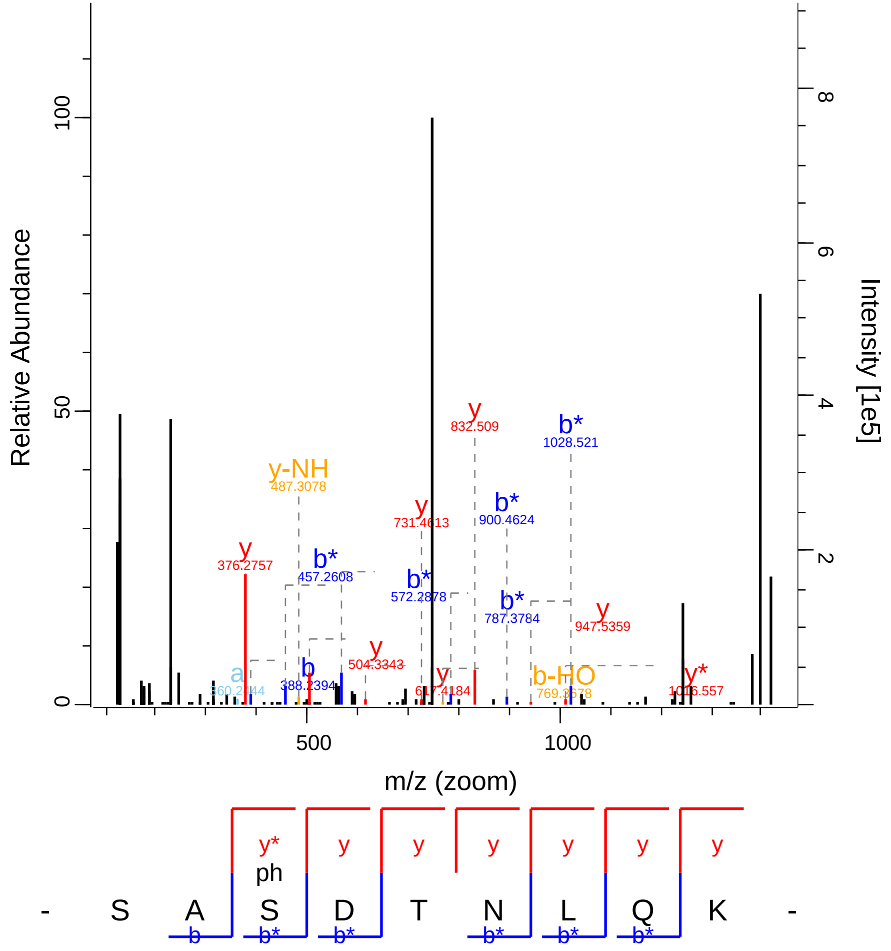

Raw File	Scan	Method	Score	m/z	Gene names
RO-QC-160121_ZackD_SS_Set_02_Fractior	13196	FTMS; HC	82.36	725.07	ZNF350

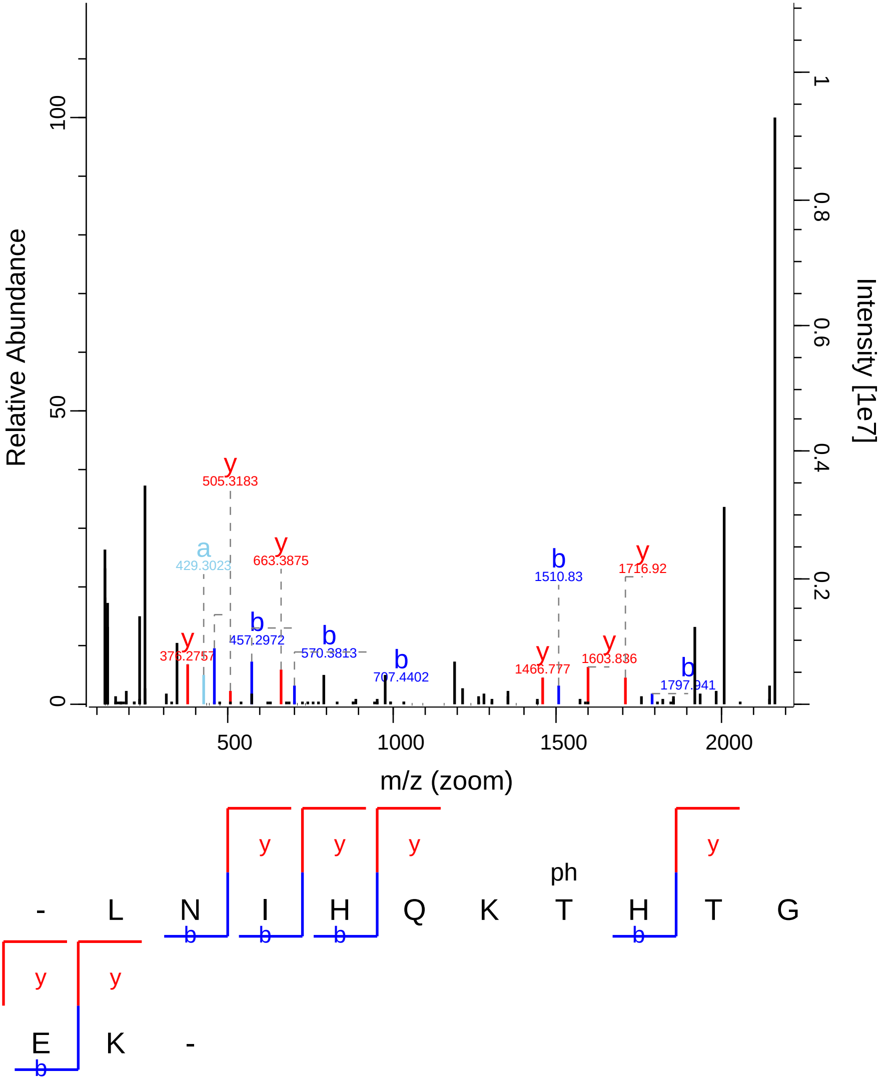

Raw File	Scan	Method	Score	m/z	Gene names
RO-QC-160121_ZackD_SS_Set_02_Fraction_	14026	FTMS; HC	184.01	957	CLASP2

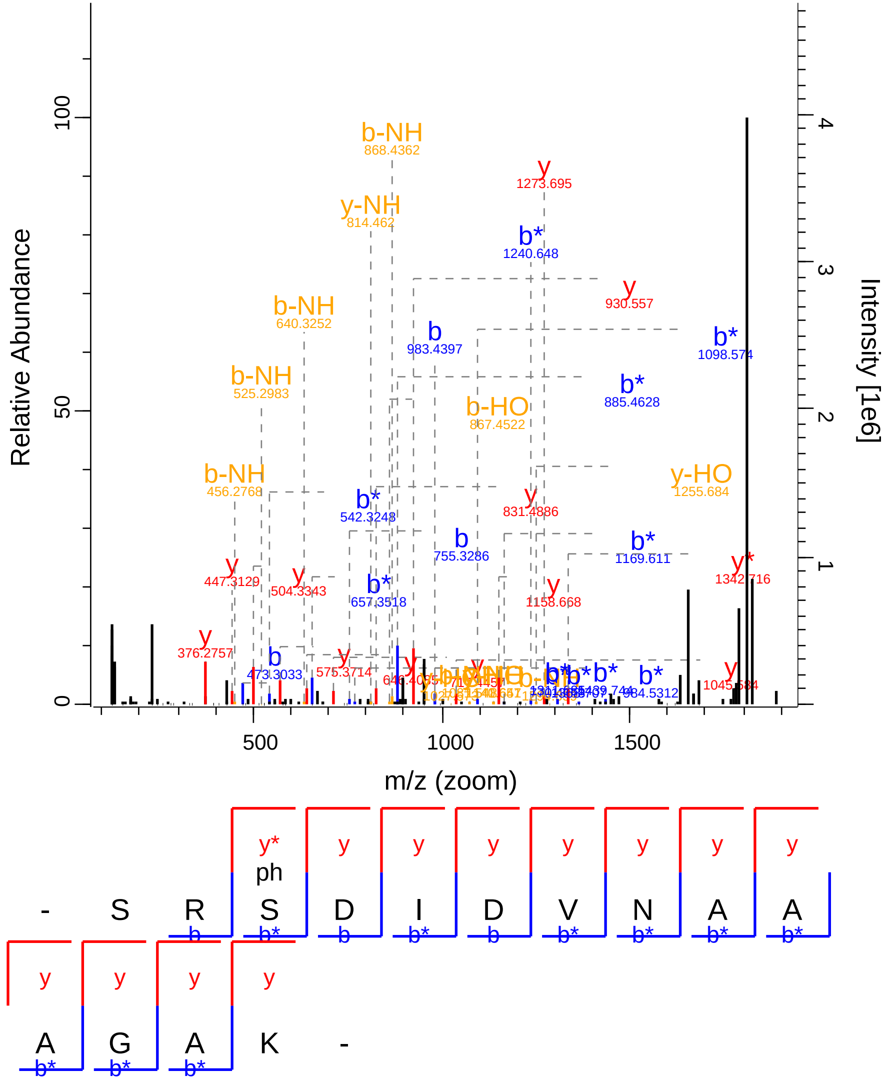

Raw File	Scan	Method	Score	m/z	Gene names
RO-QC-160121_ZackD_SS_Set_02_Fractio	14237	FTMS; HC	125.52	972.01	CLASP1

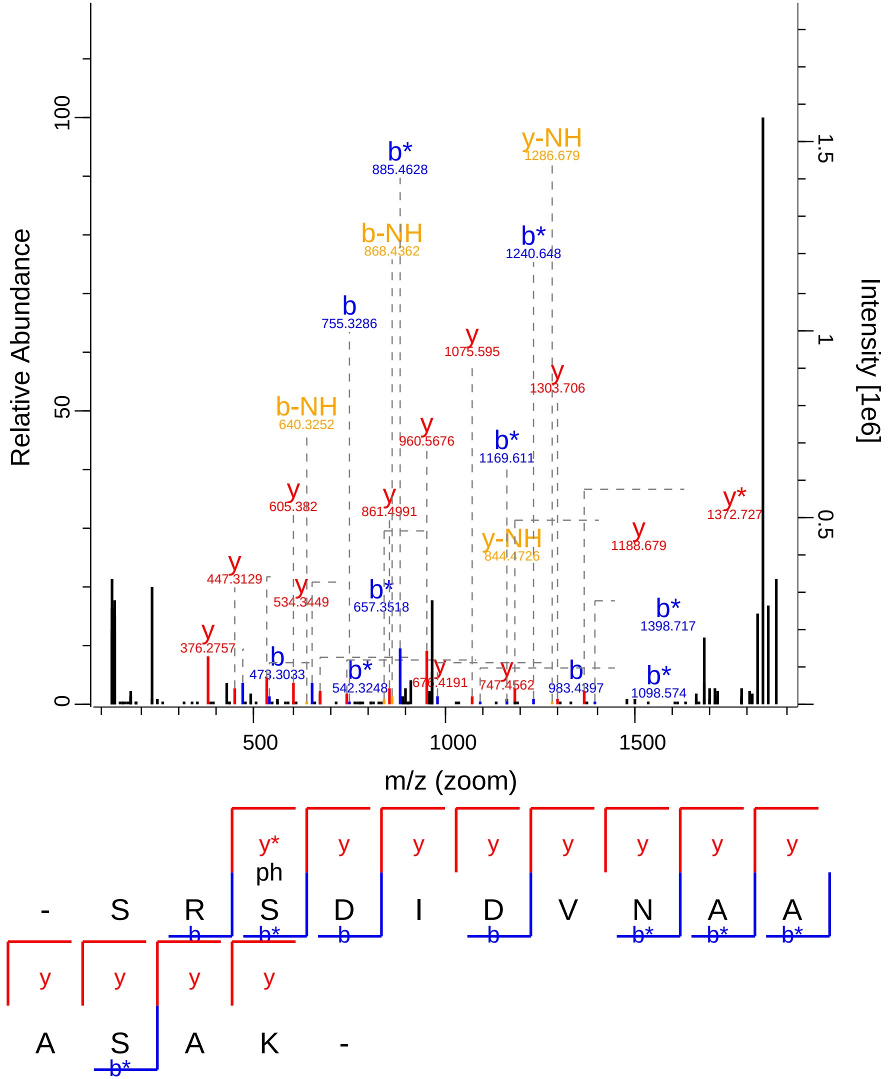

Raw File	Scan	Method	Score	m/z	Gene names
RO-QC-160121_ZackD_SS_Set_02_Fractio	15195	FTMS; HC	146.64	768.15	MTDH

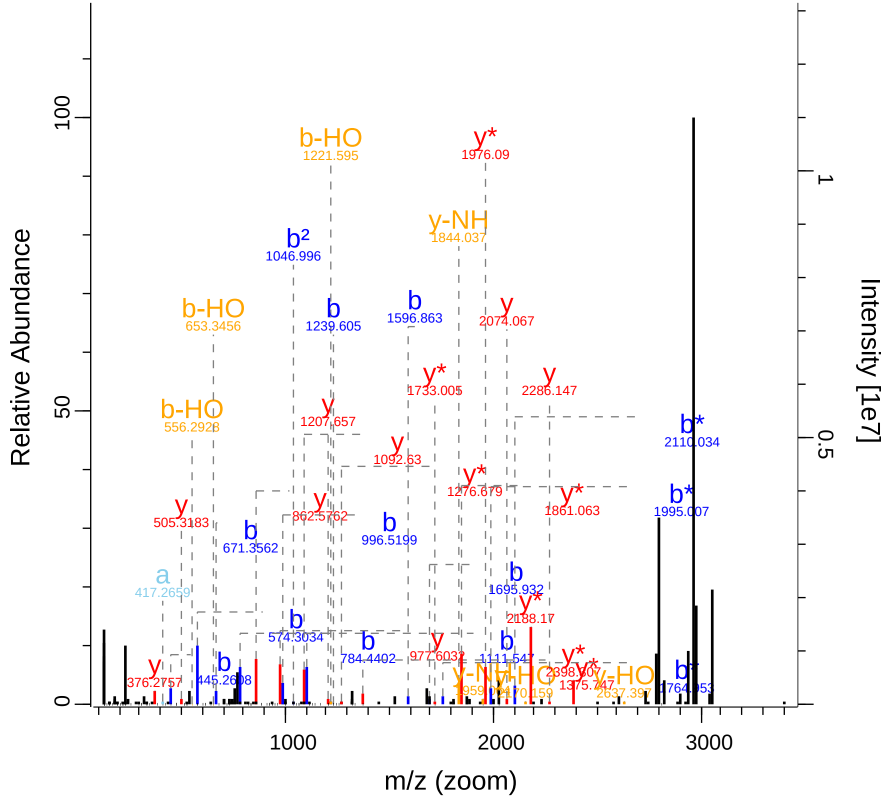

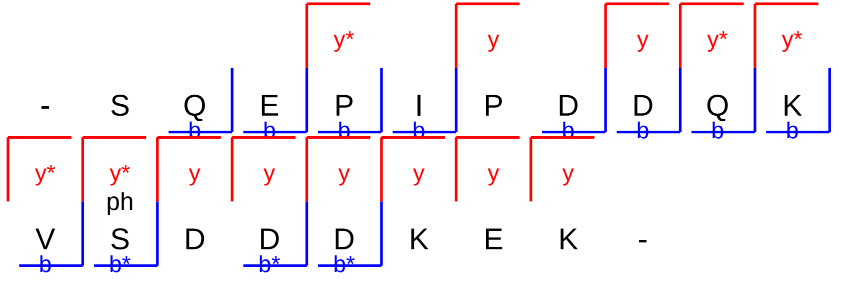

Raw File	Scan	Method	Score	m/z	Gene names
RO-QC-160121_ZackD_SS_Set_02_Fractior	15227	FTMS; HC	74.94	542.34	TMPO

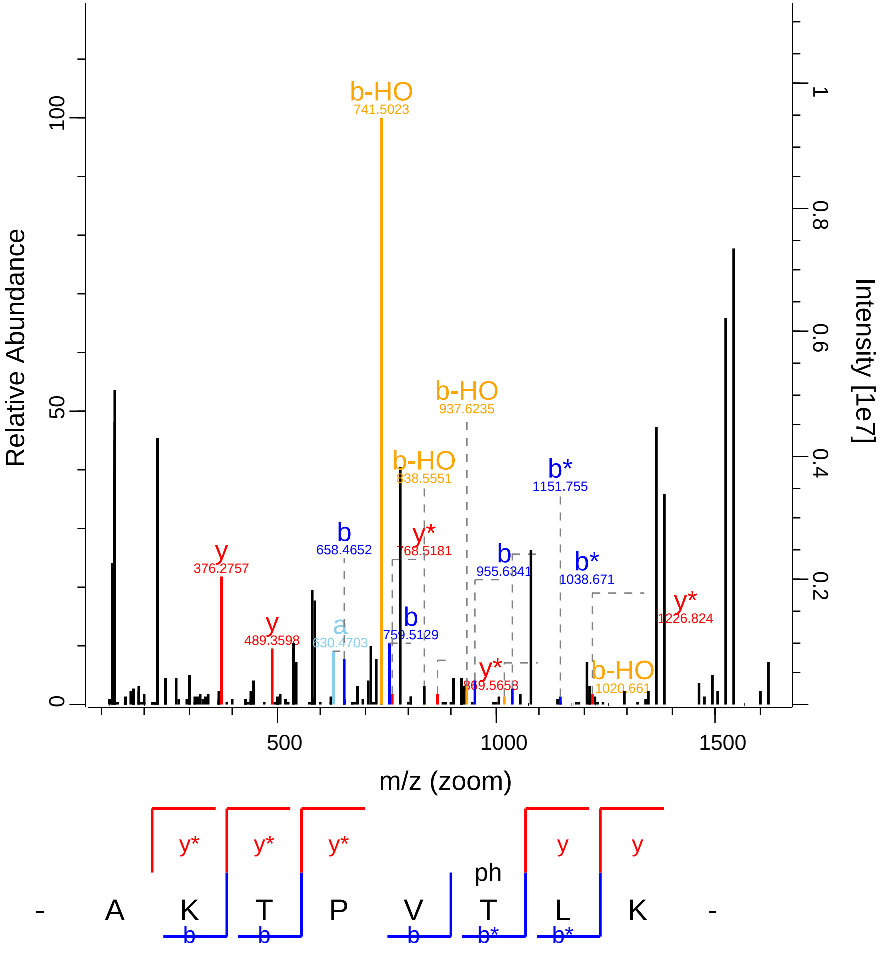

Raw File	Scan	Method	Score	m/z	Gene names
RO-QC-160121_ZackD_SS_Set_02_Fractior	15644	FTMS; HC	101.72	651.72	PLEKHA7

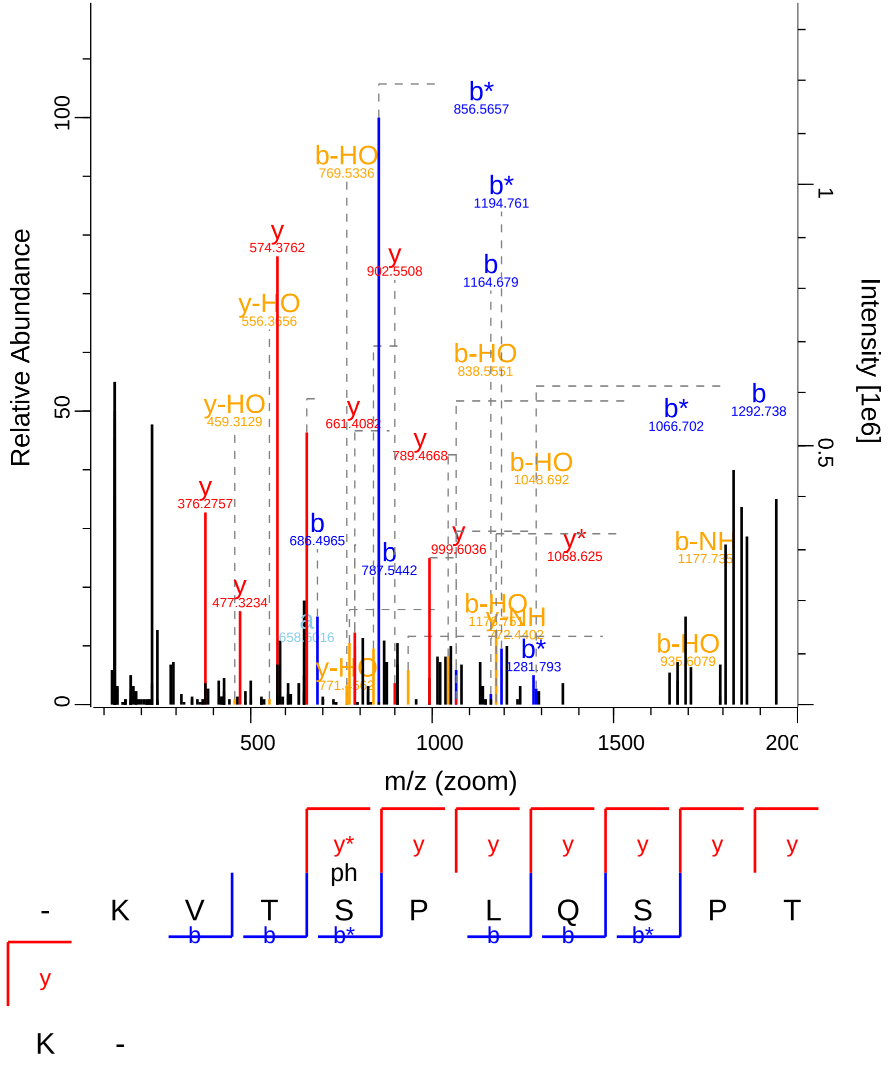

Raw File	Scan	Method	Score	m/z	Gene names
RO-QC-160121_ZackD_SS_Set_02_Fractior	16819	FTMS; HC	92.58	853.11	SUMO1

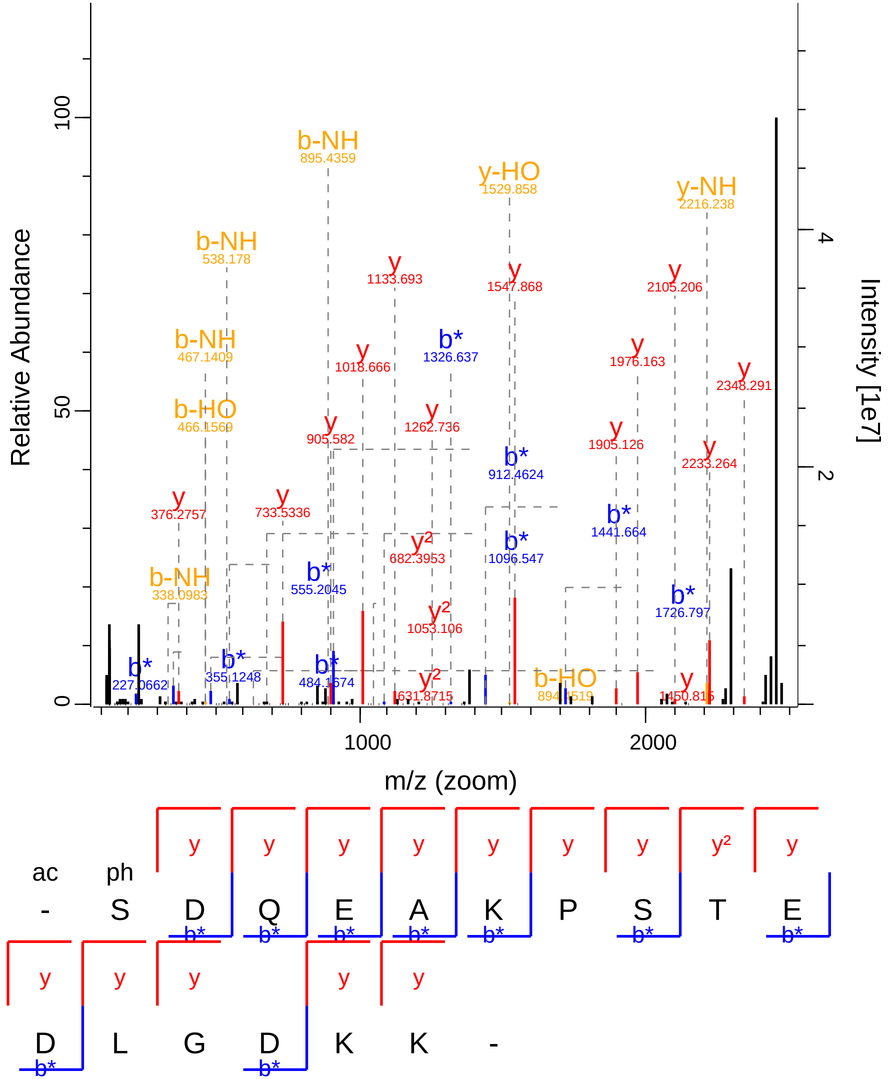

Raw File	Scan	Method	Score	m/z	Gene names
RO-QC-160121_ZackD_SS_Set_02_Fraction	17144	FTMS; HC	65.9	865.9	CYBRD1

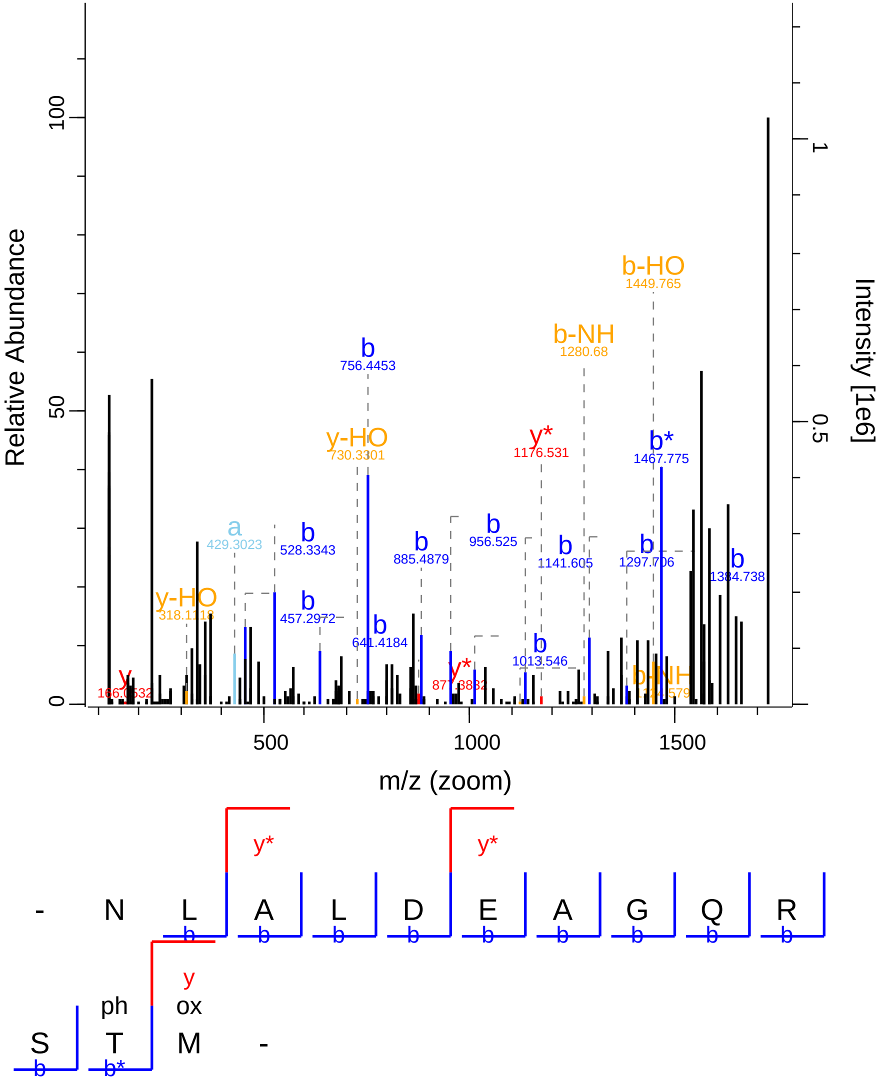

Raw File	Scan	Method	Score	m/z	Gene names
RO-QC-160121_ZackD_SS_Set_02_Fractior	18804	FTMS; HC	46.16	649.04	FTSJ3

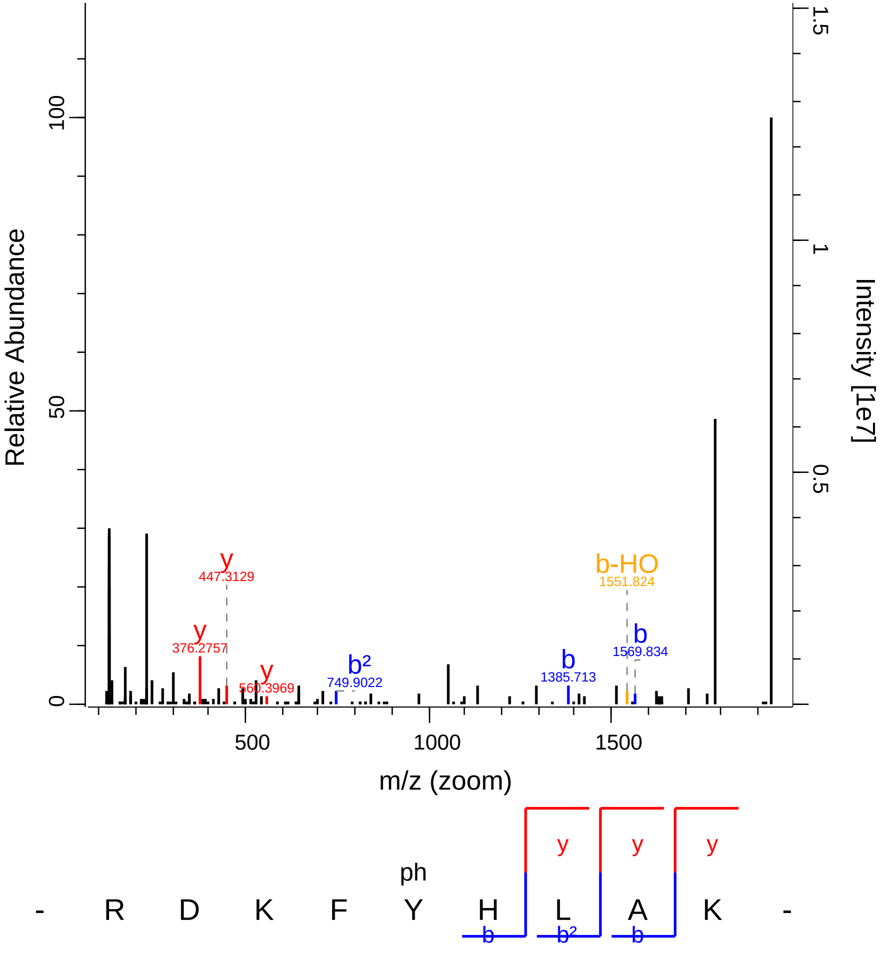

Raw File	Scan	Method	Score	m/z	Gene names
RO-QC-160121_ZackD_SS_Set_02_Fractior	20786	FTMS; HC	87.68	718.69	RBMX;RBMXL

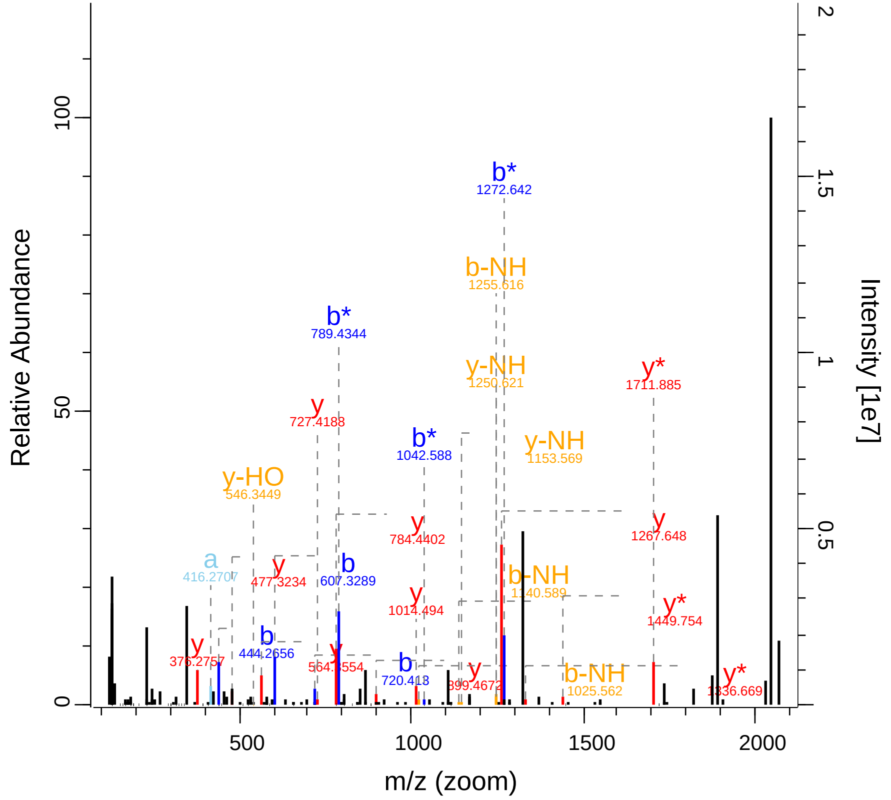

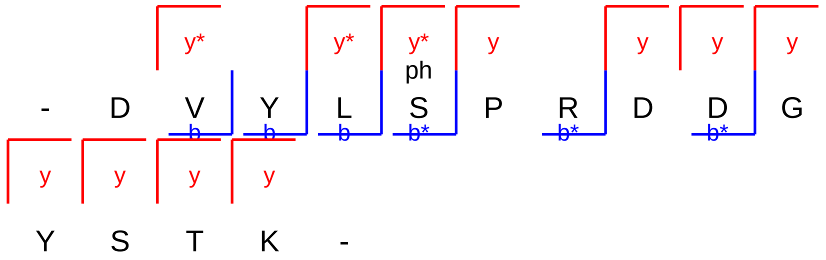

Raw File	Scan	Method	Score	m/z	Gene names
RO-QC-160121_ZackD_SS_Set_02_Fractio	21024	FTMS; HC	184.98	1042.01	MAP1B

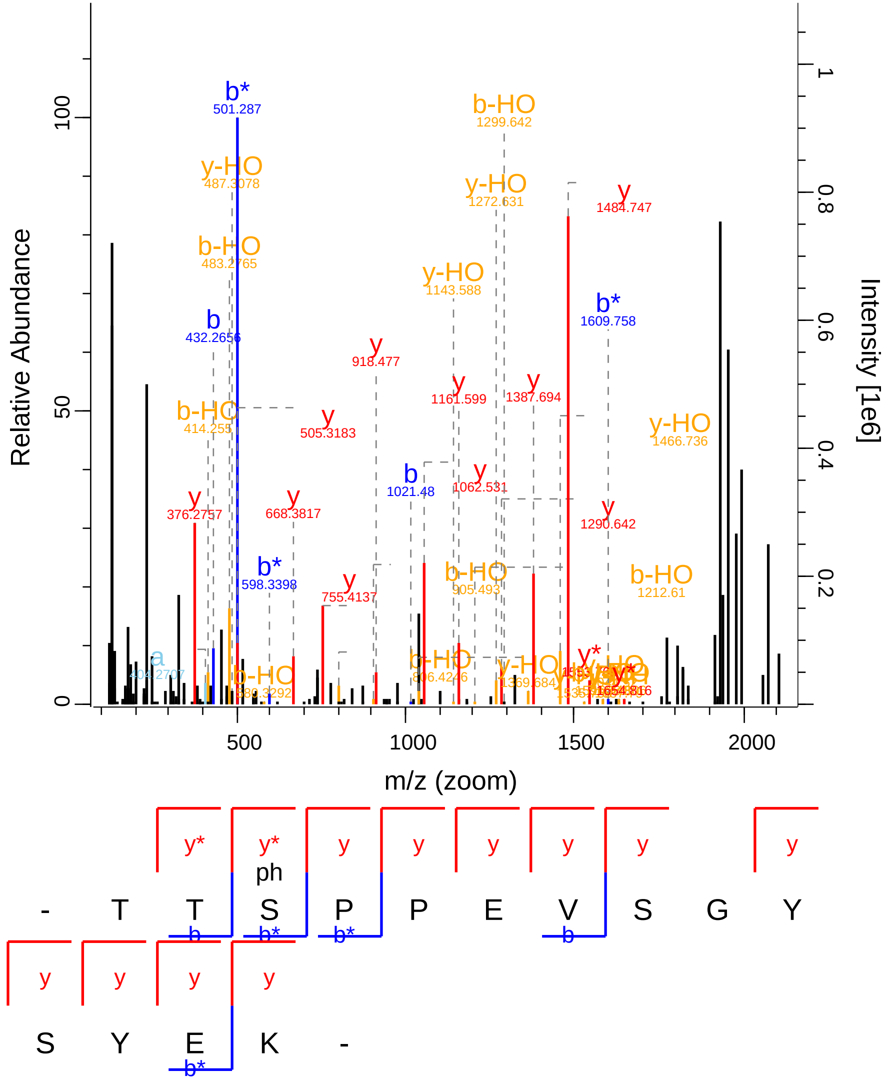

Raw File	Scan	Method	Score	m/z	Gene names
RO-QC-160121_ZackD_SS_Set_02_Fractior	21053	FTMS; HC	152	869.46	WDR44

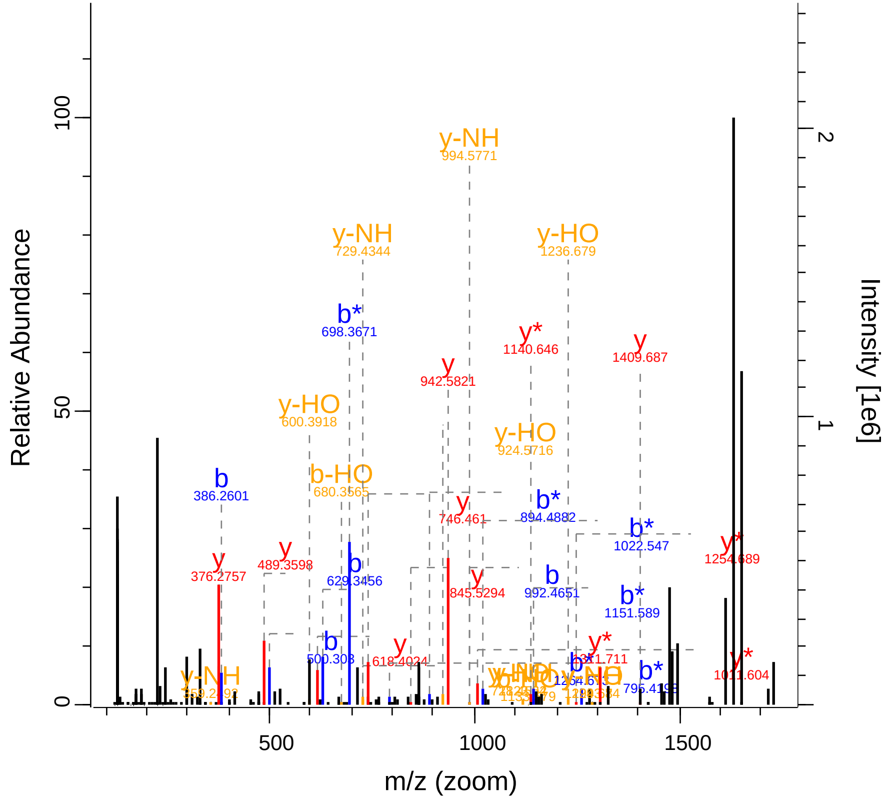

		y	y*	y*	y*	y	y	y	y	y
					ph					
-	V	G	N	E	S	P	V	Q	E	L
		b	b	b	b*	b*	b	b*	b*	b*
y										
K	-									

Raw File	Scan	Method	Score	m/z	Gene names
RO-QC-160121_ZackD_SS_Set_02_Fractio[illegible]	21124	FTMS; HC	117.79	902.36	SORT1

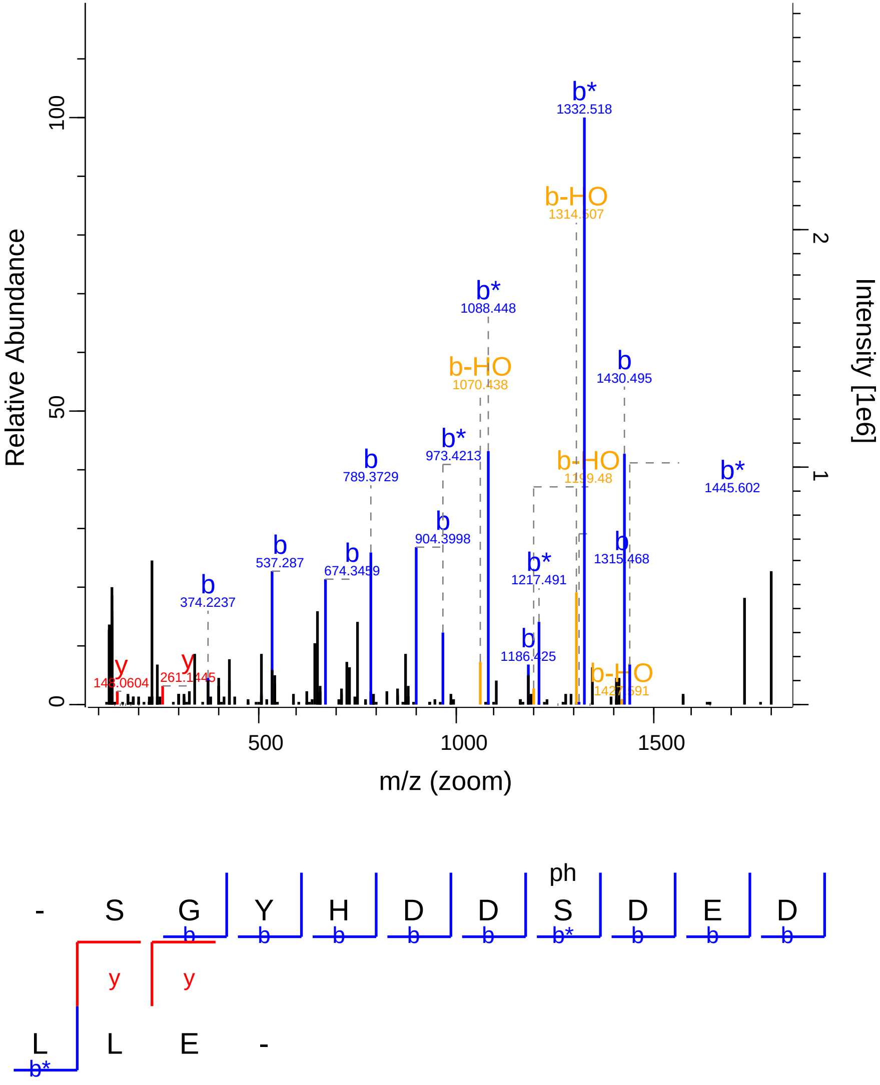

Raw File	Scan	Method	Score	m/z	Gene names
RO-QC-160121_ZackD_SS_Set_02_Fractior	21534	FTMS; HC	44.62	573.33	ORAOV1

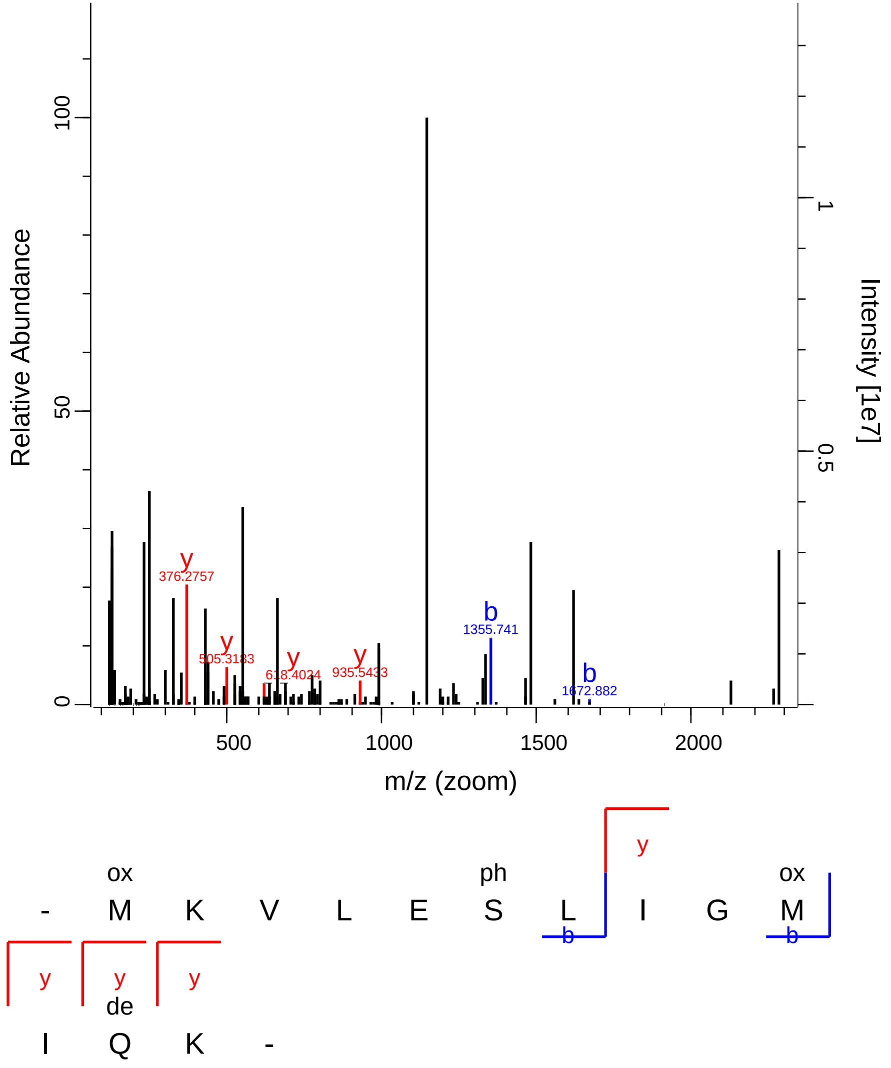

Raw File	Scan	Method	Score	m/z	Gene names
RO-QC-160121_ZackD_SS_Set_02_Fractio	21647	FTMS; HC	290.69	1106.07	EIF3C;EIF3CL

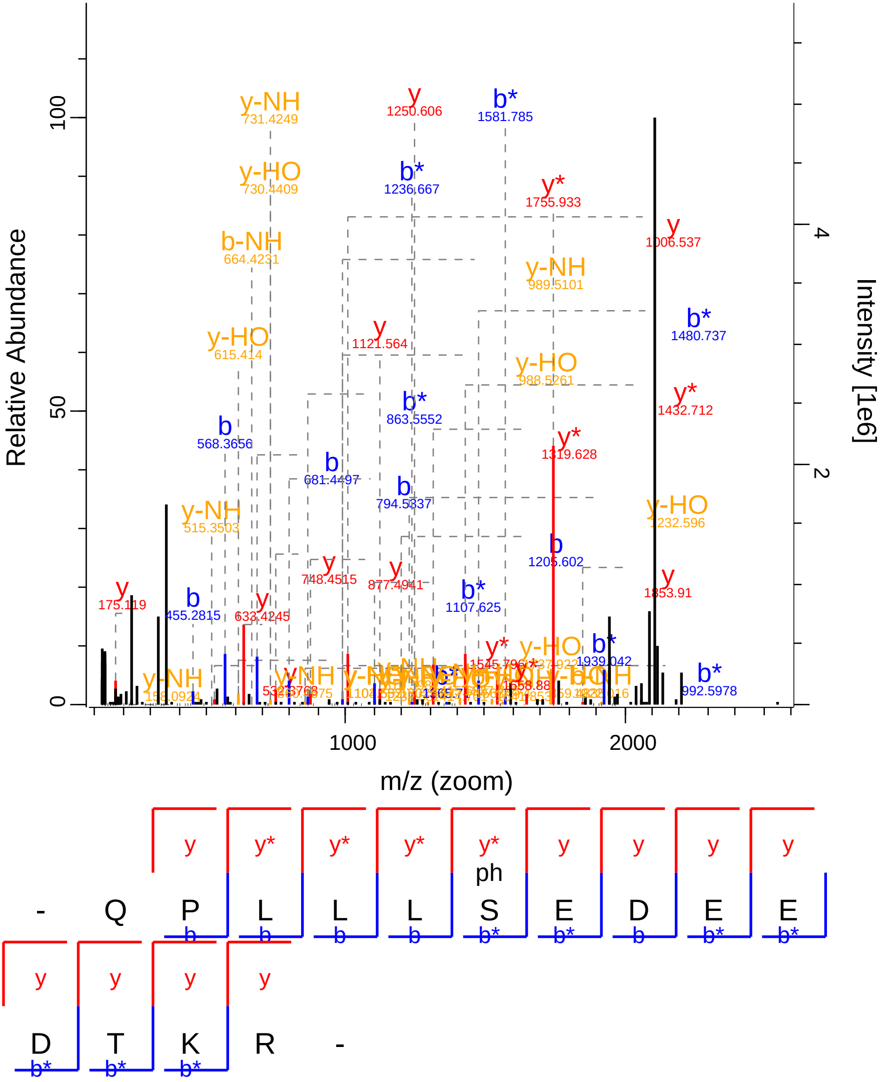

Raw File	Scan	Method	Score	m/z	Gene names
RO-QC-160121_ZackD_SS_Set_02_Fractior	22531	FTMS; HC	83.22	948.22	TFIP11

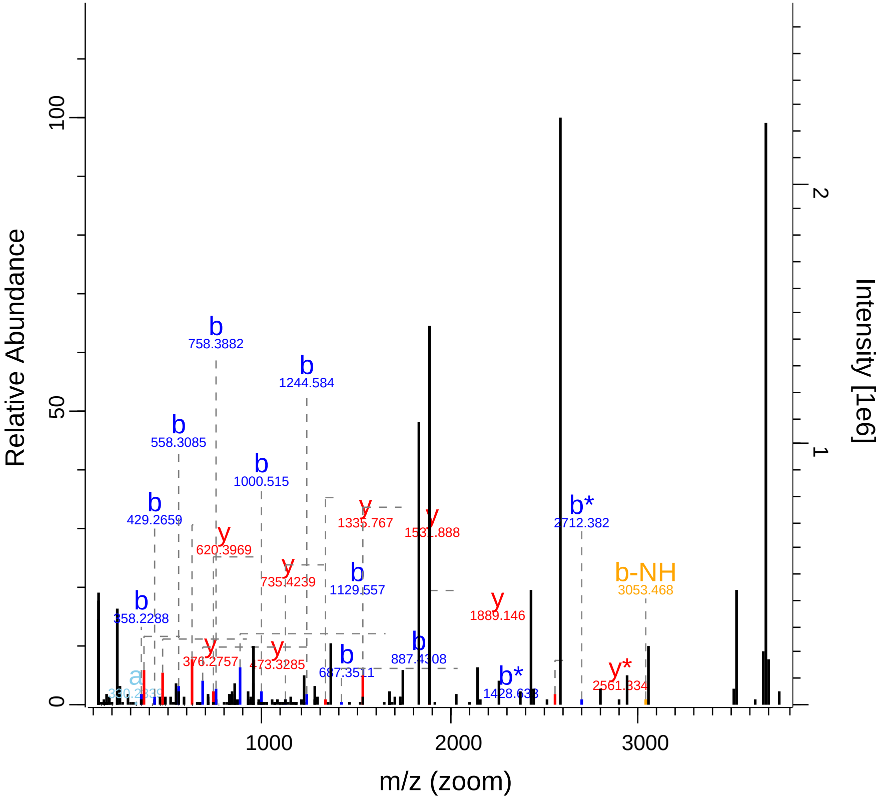

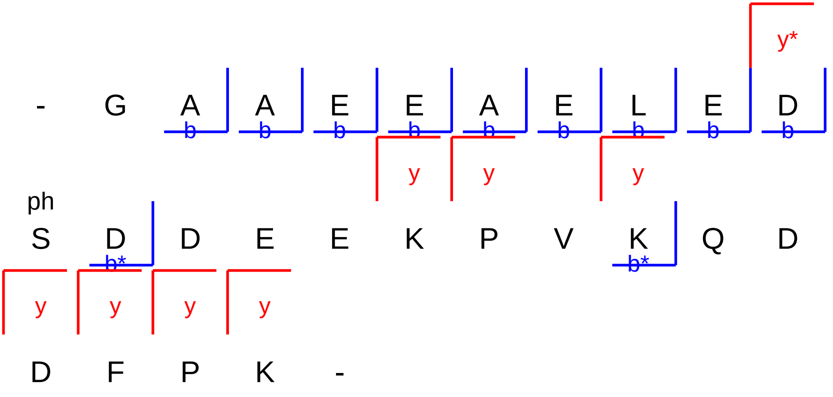

Raw File	Scan	Method	Score	m/z	Gene names
RO-QC-160121_ZackD_SS_Set_02_Fraction	22564	FTMS; HC	115.76	882.7	NAF1

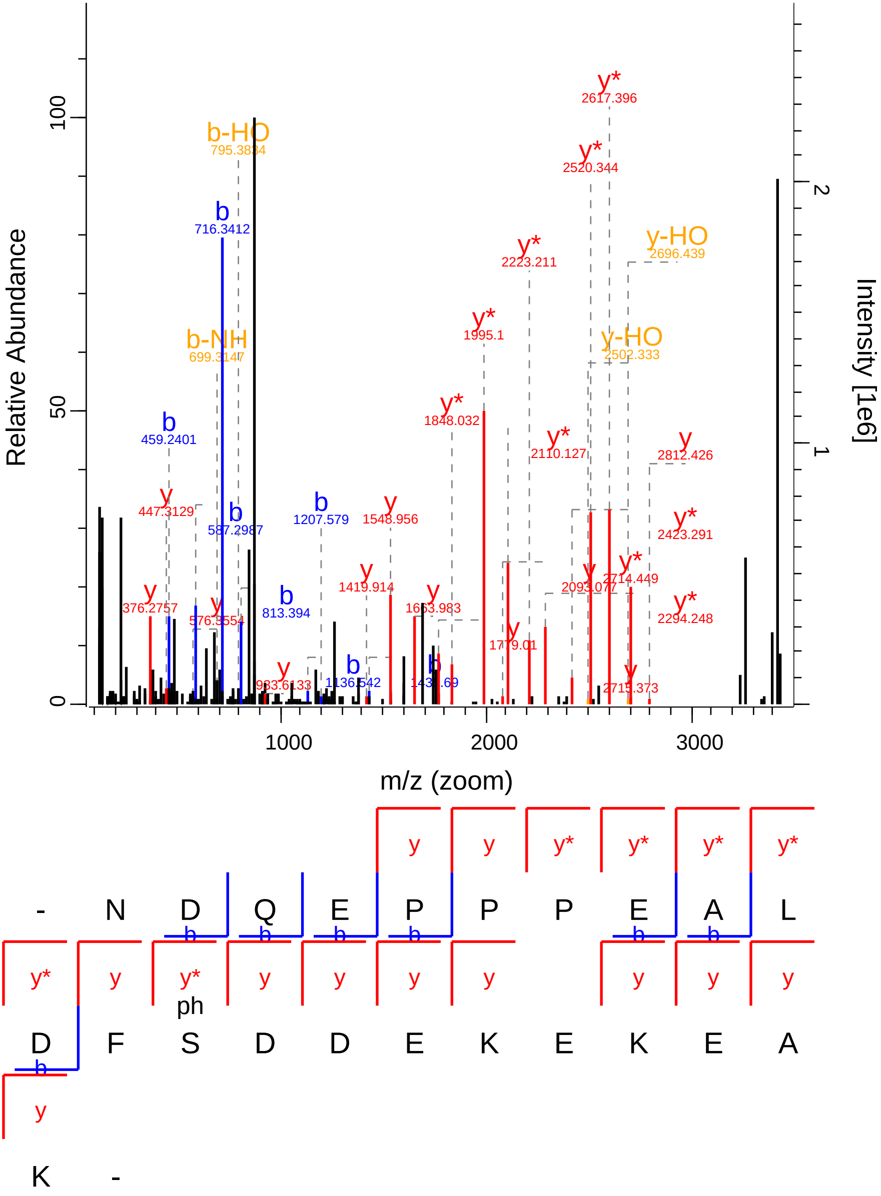

Raw File	Scan	Method	Score	m/z	Gene names
RO-QC-160121_ZackD_SS_Set_02_Fractior	22970	FTMS; HC	91.29	743.38	PIKFYVE

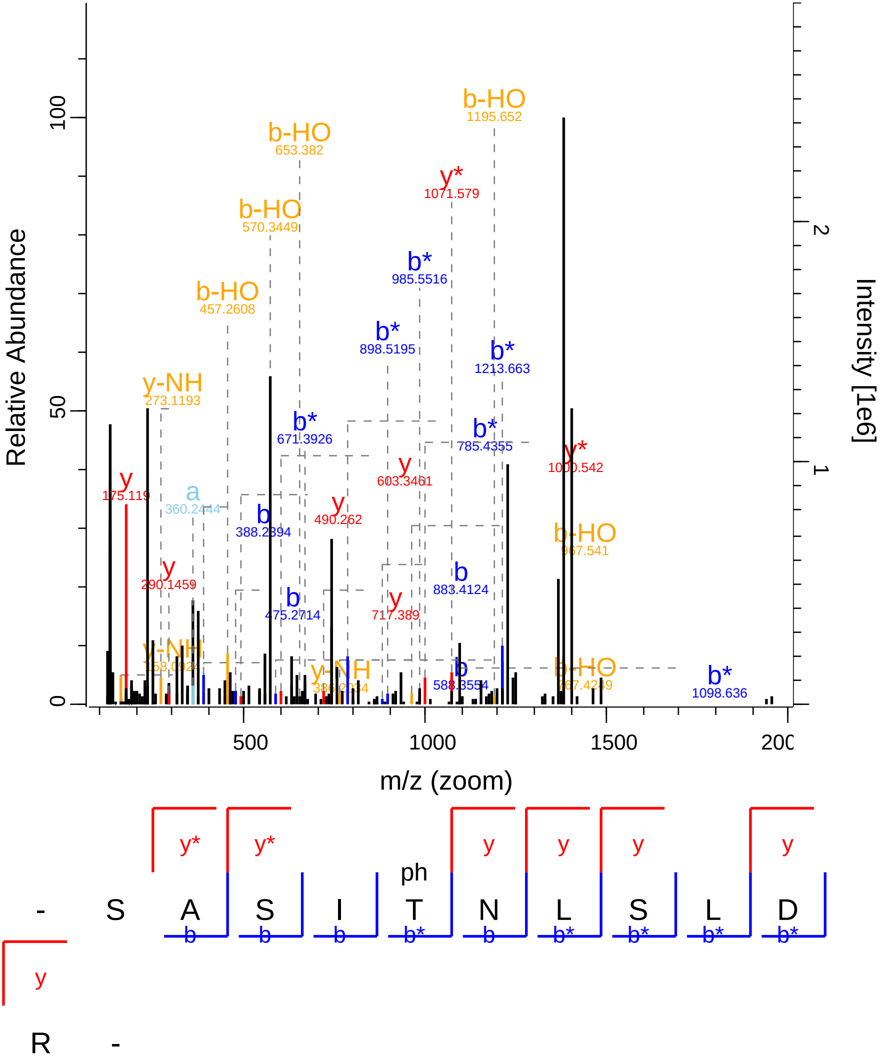

Raw File	Scan	Method	Score	m/z	Gene names
RO-QC-160121_ZackD_SS_Set_02_Fractior	24377	FTMS; HC	48.11	788.76	AARSD1

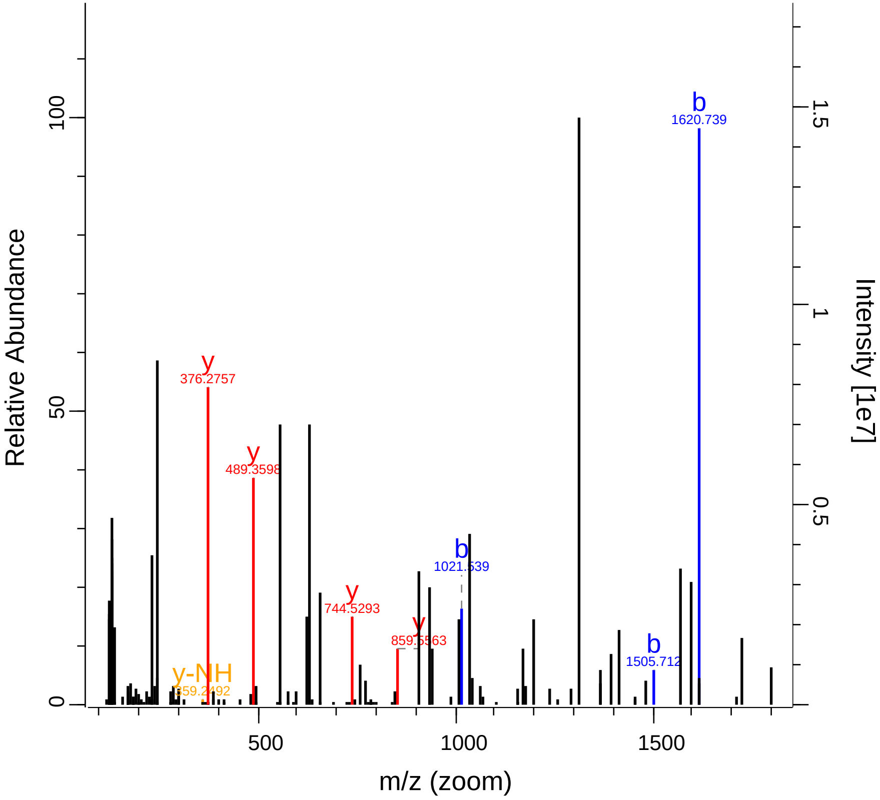

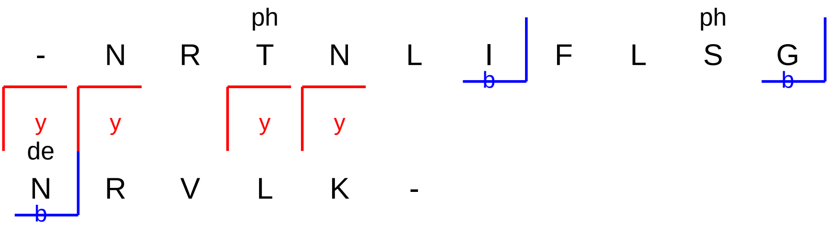

Raw File	Scan	Method	Score	m/z	Gene names
RO-QC-160121_ZackD_SS_Set_02_Fractio	24950	FTMS; HC	145.52	1000.83	UBR4

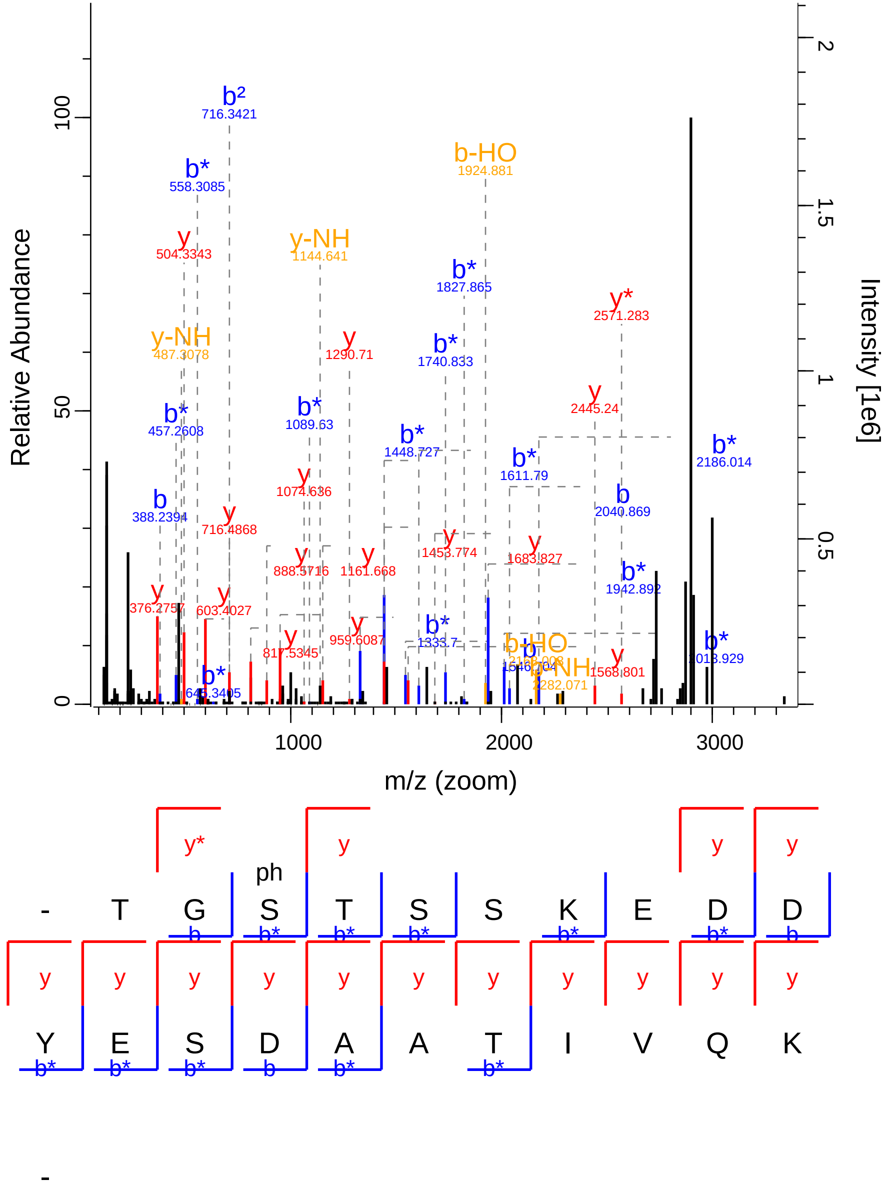

Raw File	Scan	Method	Score	m/z	Gene names
RO-QC-160121_ZackD_SS_Set_02_Fractior	26336	FTMS; HC	319.33	1221.1	MAP1B

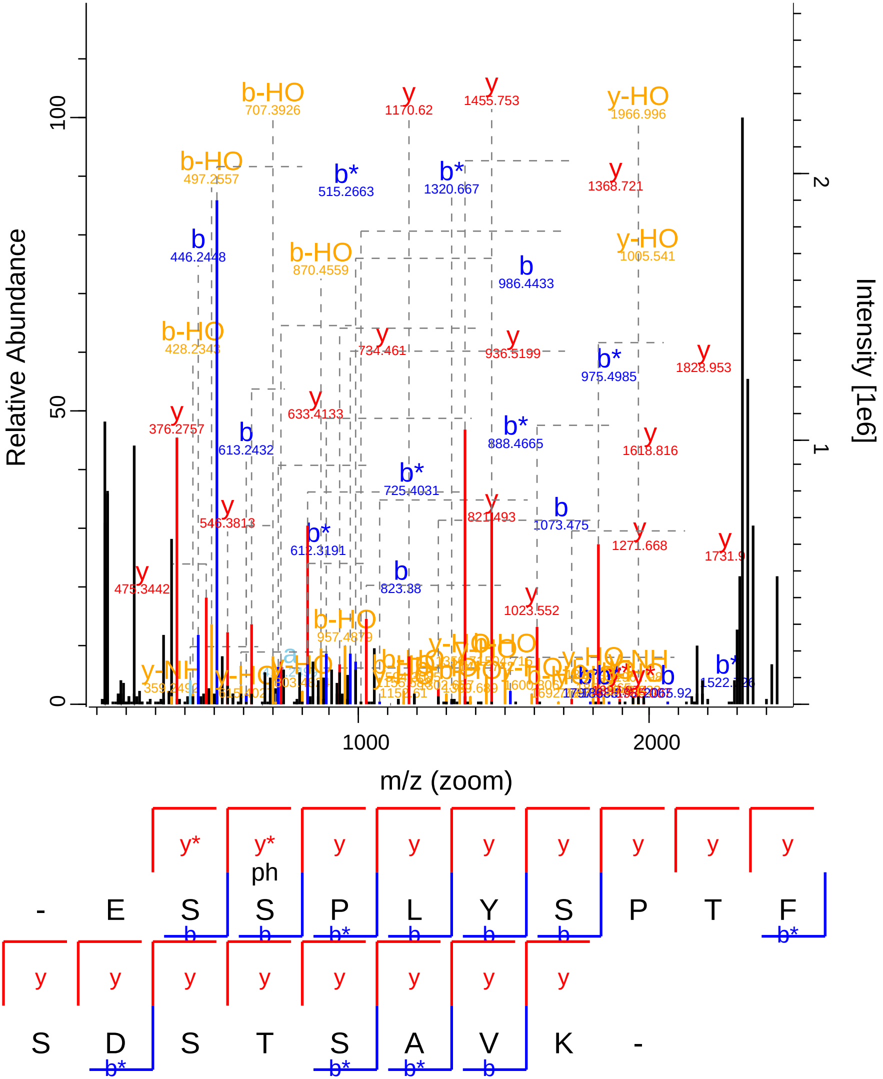

Raw File	Scan	Method	Score	m/z	Gene names
RO-QC-160121_ZackD_SS_Set_02_Fraction	27148	FTMS; HC	70.27	807.2	ZMYND8

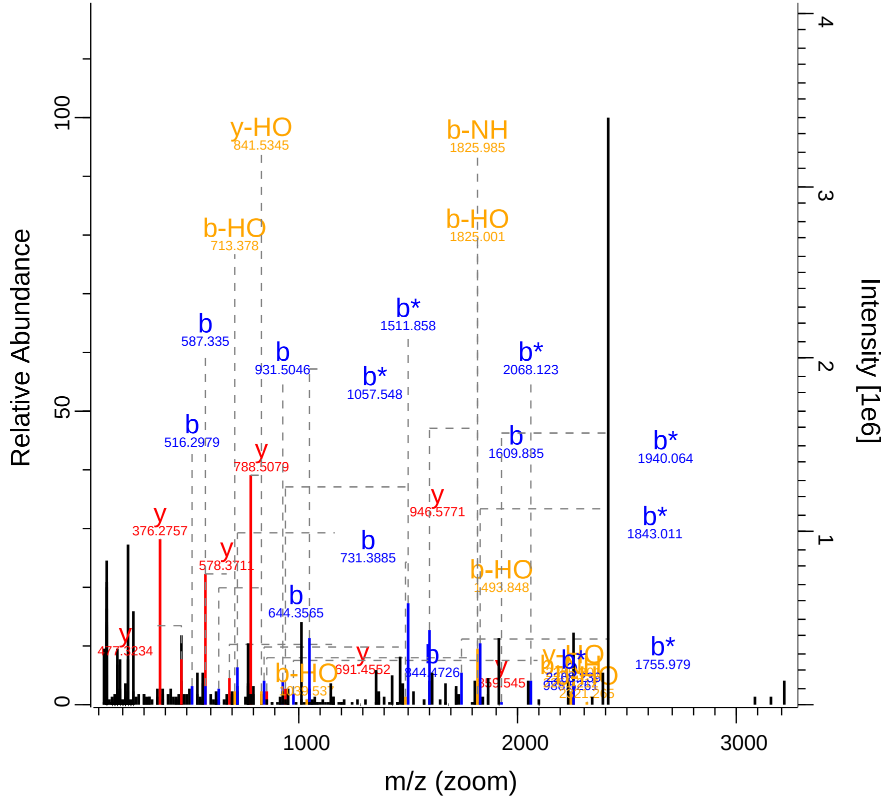

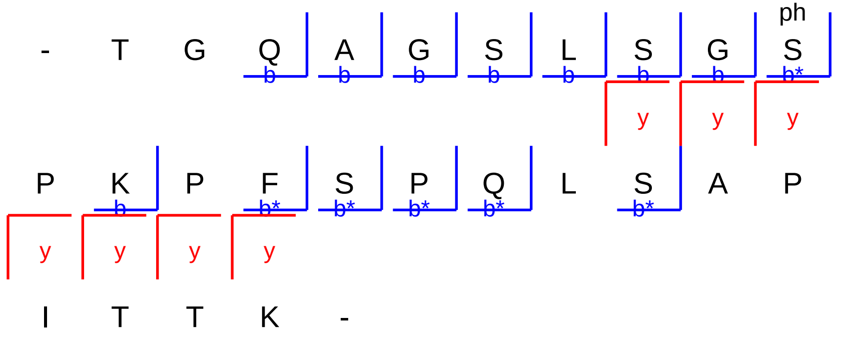

Raw File	Scan	Method	Score	m/z	Gene names
RO-QC-160121_ZackD_SS_Set_02_Fractior	27706	FTMS; HC	79.21	1138.5	GJA1

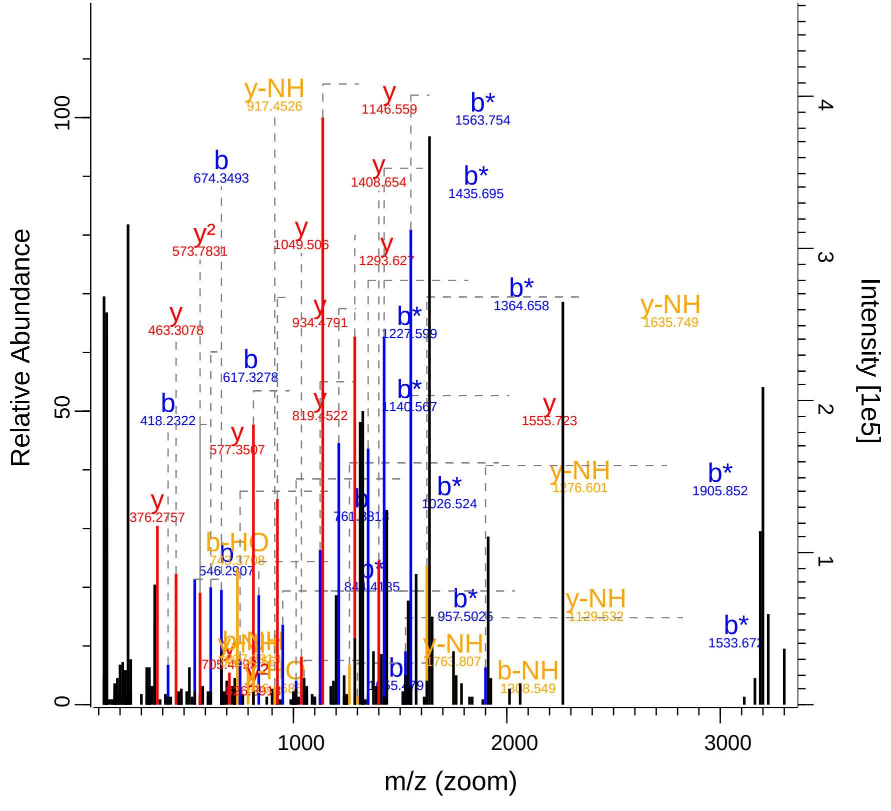

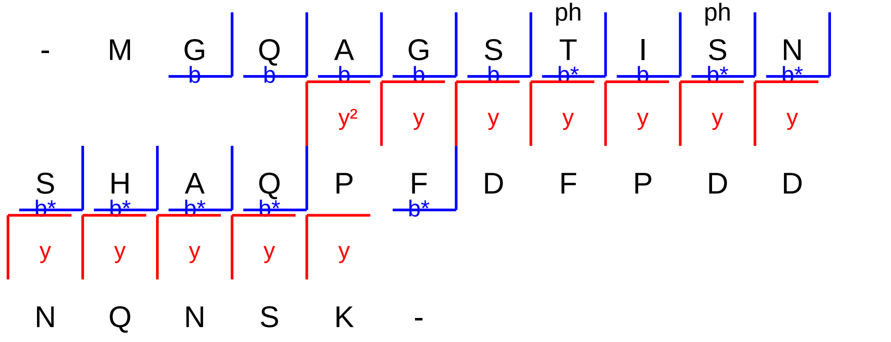

Raw File	Scan	Method	Score	m/z	Gene names
RO-QC-160121_ZackD_SS_Set_02_Fractio	28049	FTMS; HC	139.98	844.93	AHNAK

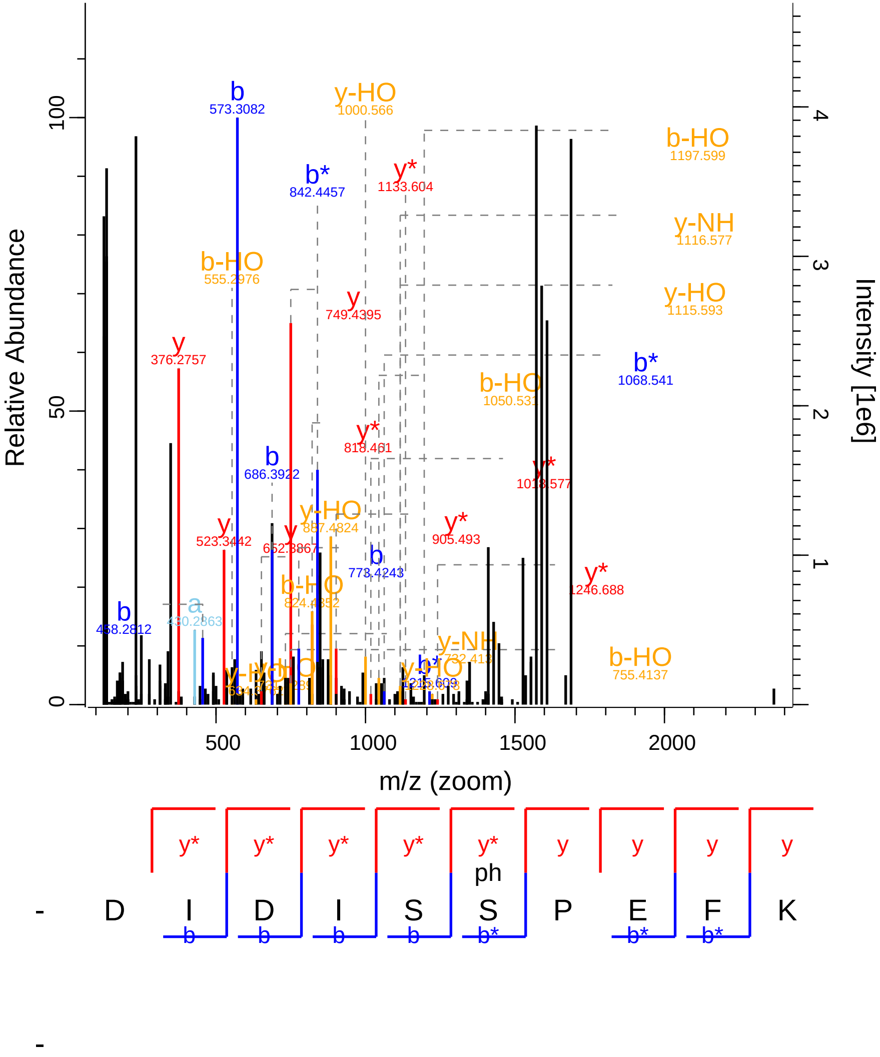

Raw File	Scan	Method	Score	m/z	Gene names
RO-QC-160121_ZackD_SS_Set_02_Fractior	31222	FTMS; HC	162.71	936.17	TNS3

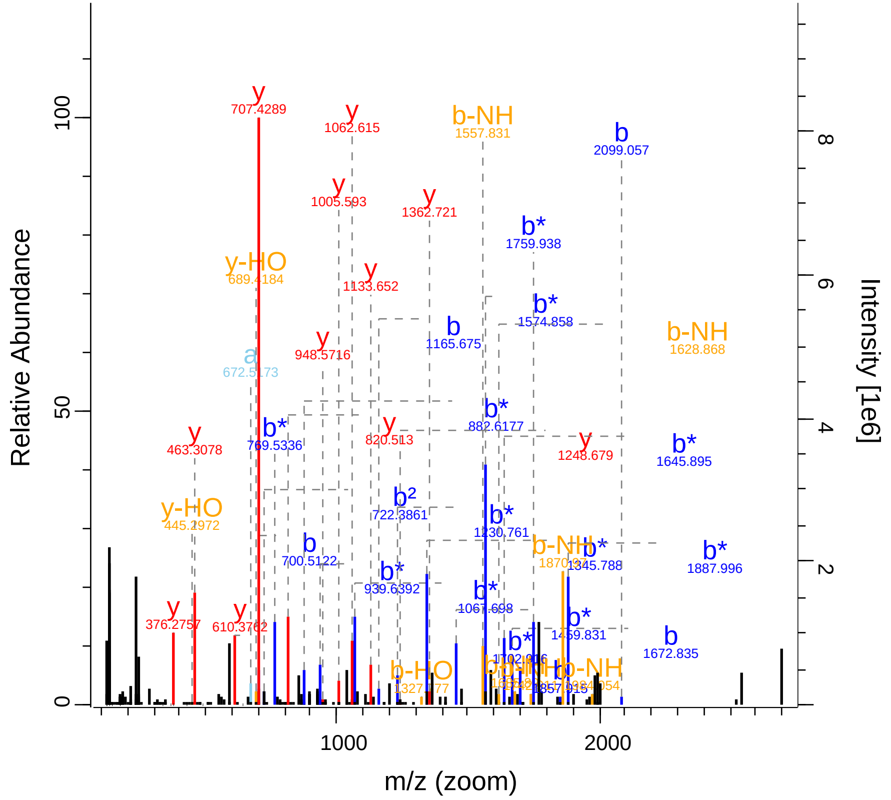

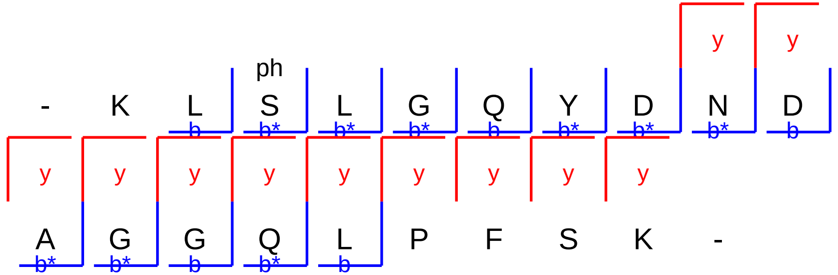

Raw File	Scan	Method	Score	m/z	Gene names
RO-QC-160121_ZackD_SS_Set_02_Fractio	32158	FTMS; HC	65.35	1090.55	APAF1

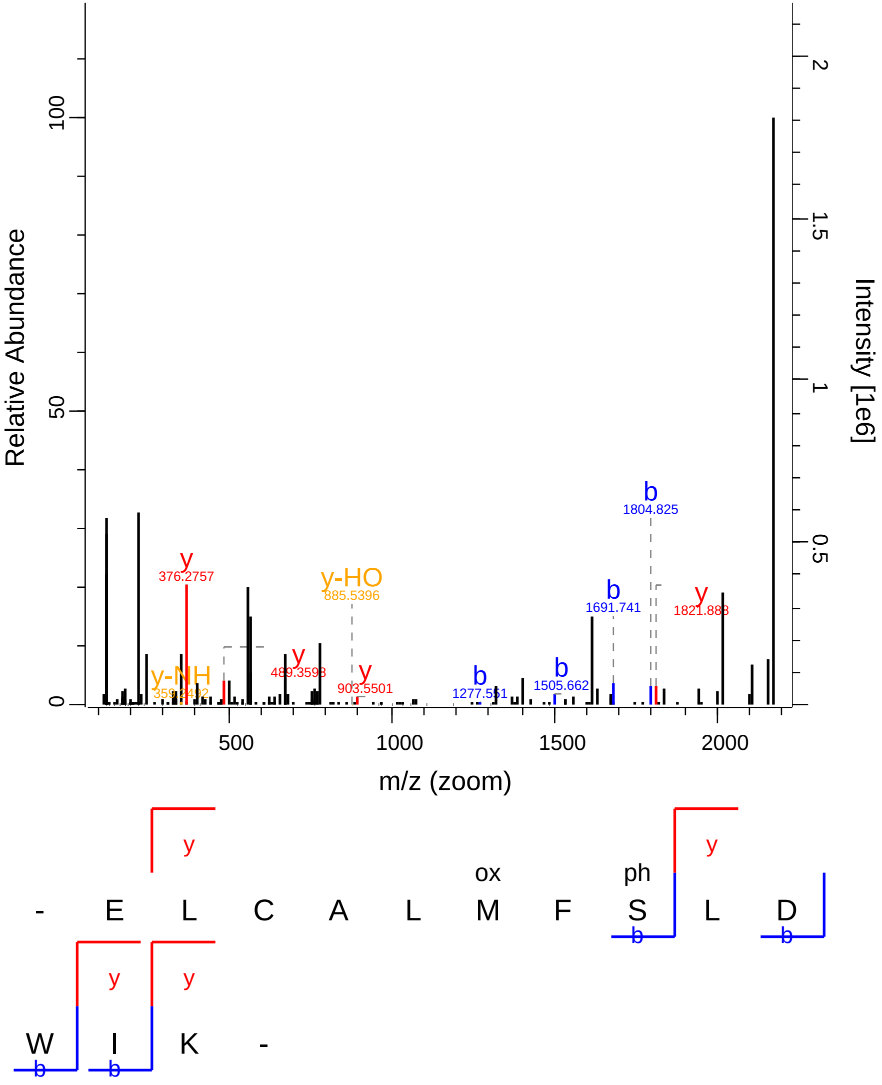

Raw File	Scan	Method	Score	m/z	Gene names
RO-QC-160121_ZackD_SS_Set_02_Fractior	32285	FTMS; HC	118.31	633.37	NSFL1C

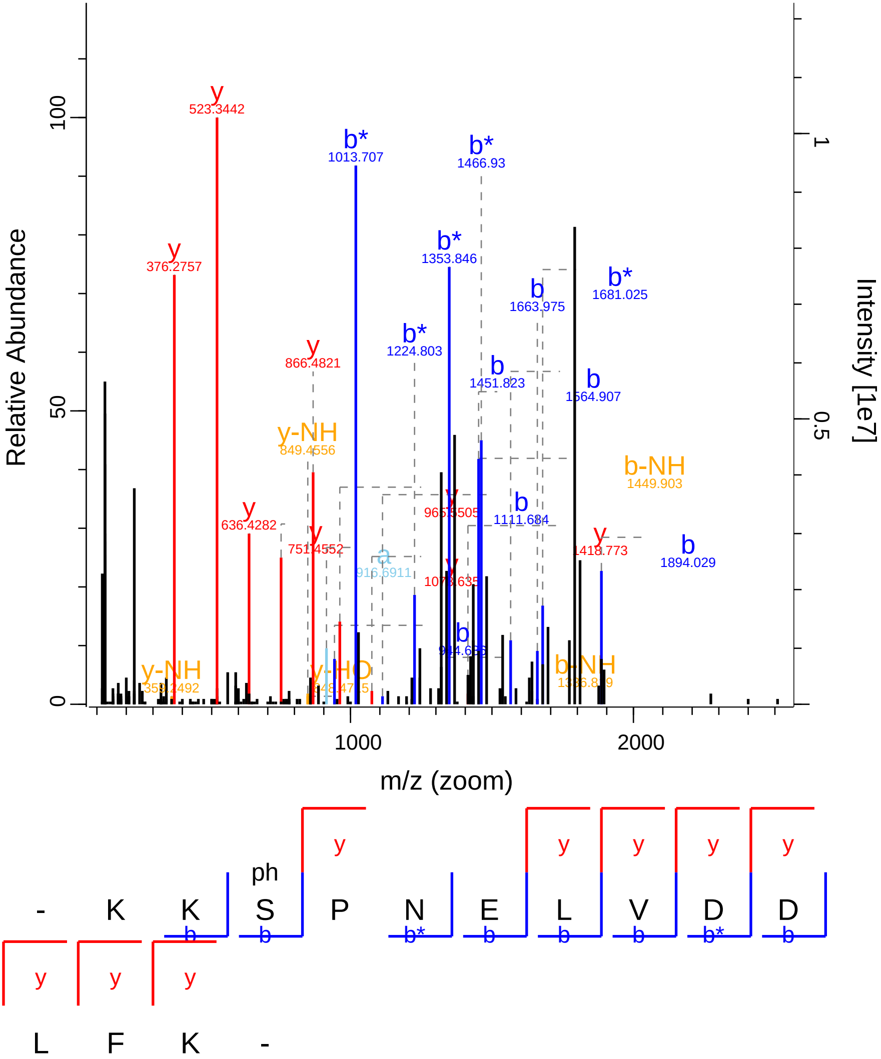

Raw File	Scan	Method	Score	m/z	Gene names
RO-QC-160121_ZackD_SS_Set_02_Fractior	32764	FTMS; HC	40.35	562.97	LMO2

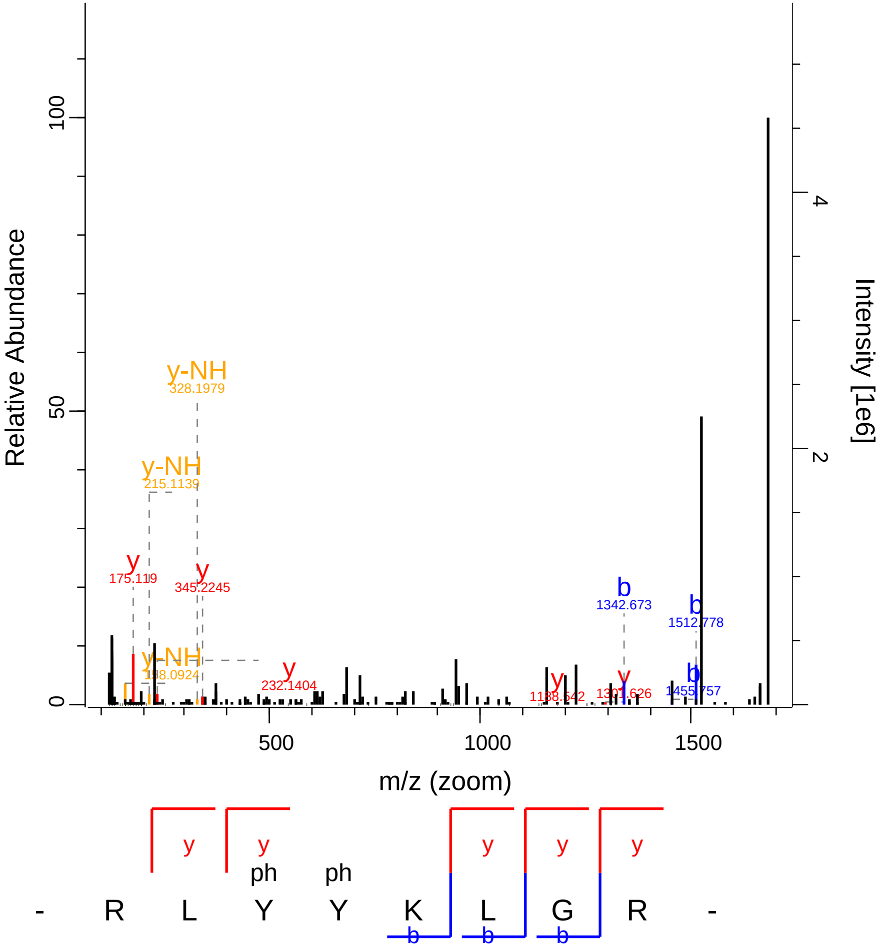

Raw File	Scan	Method	Score	m/z	Gene names
RO-QC-160121_ZackD_SS_Set_02_Fractior	33781	FTMS; HC	73.8	565.65	SCRIB

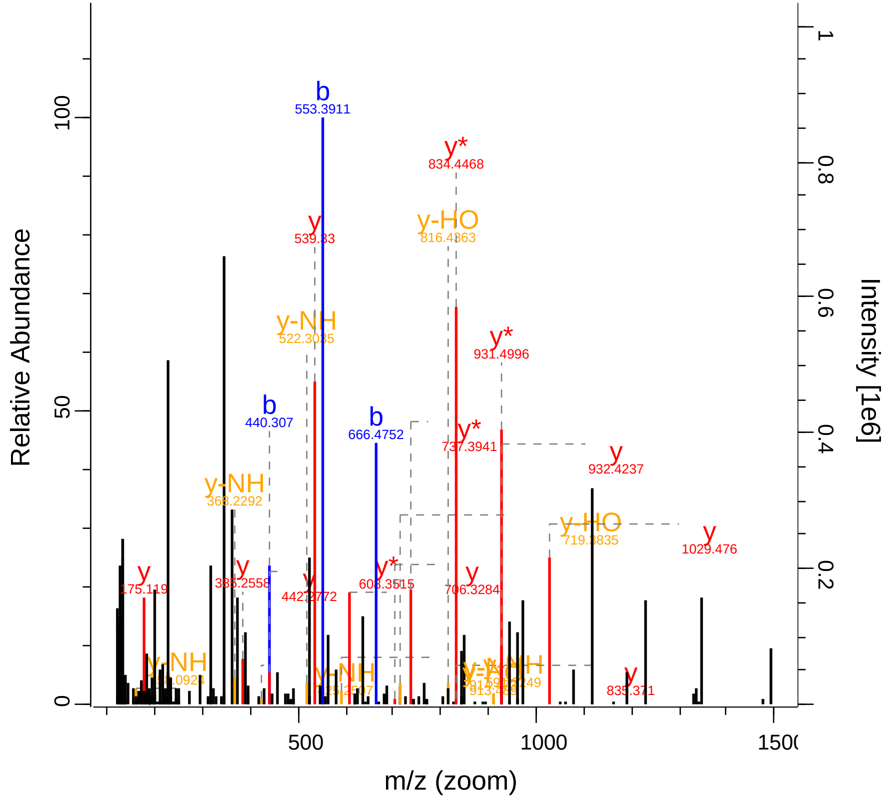

					y	y	y	y	y	y
								ph		
-	L	P	L	L	P	P	E	S	P	G
		b	b	b						
y		y								
P	L	R	-							

Raw File	Scan	Method	Score	m/z	Gene names
RO-QC-160121_ZackD_SS_Set_02_Fractio	35640	FTMS; HC	64.73	1081.54	SLC2A4

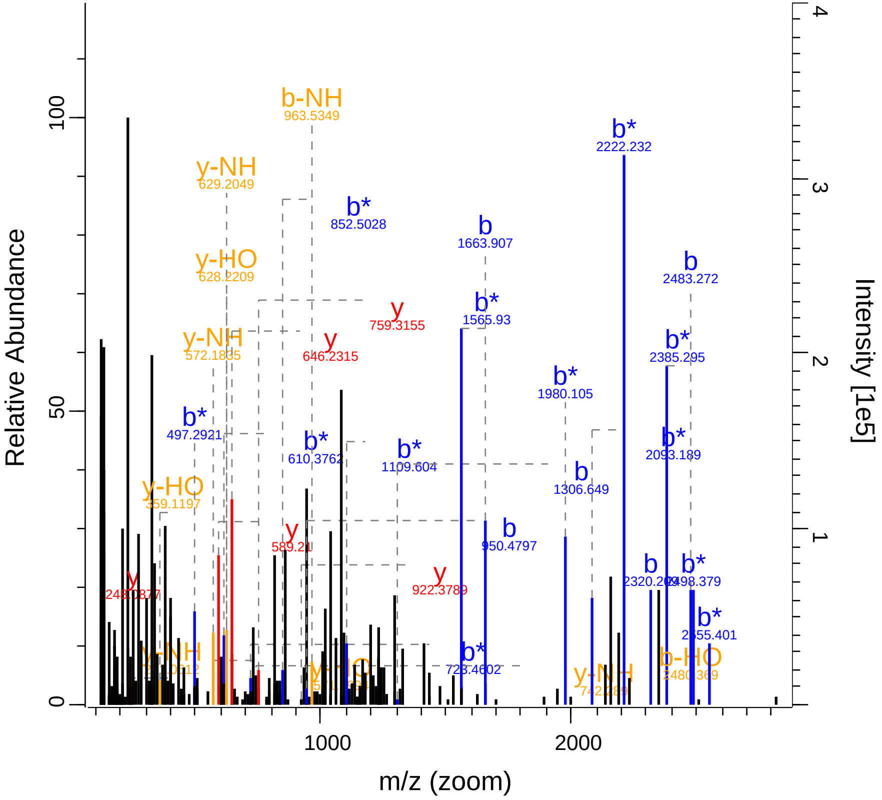

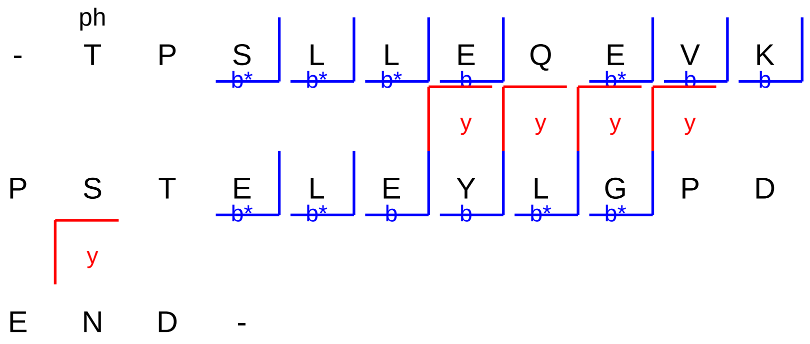

Raw File	Scan	Method	Score	m/z	Gene names
RO-QC-160121_ZackD_SS_Set_02_Fractior	35816	FTMS; HC	126.8	578.59	TPI1

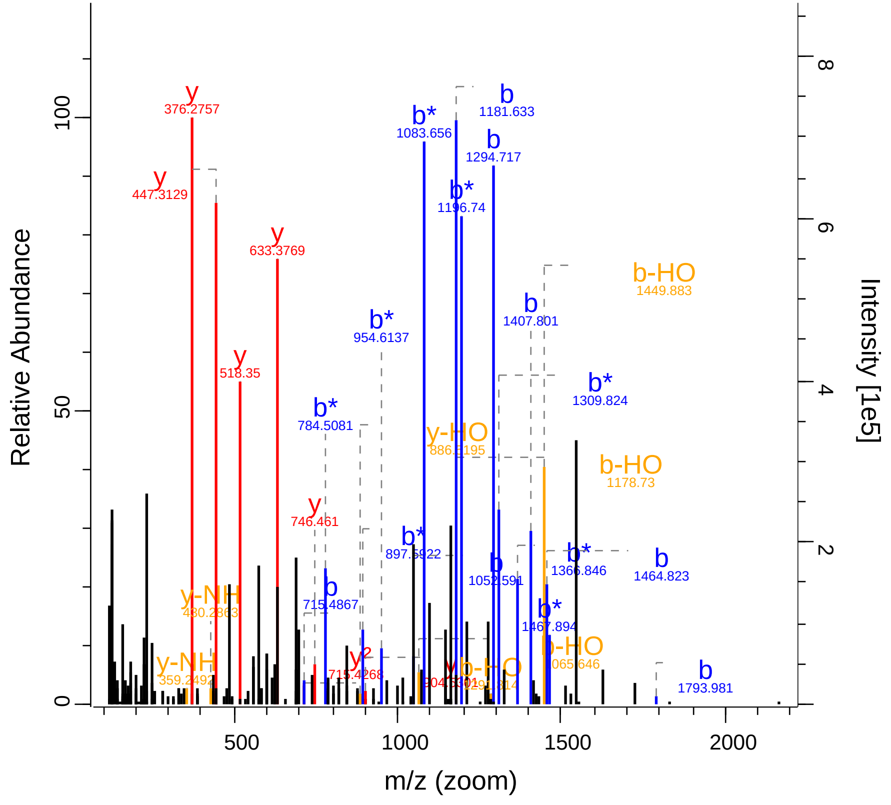

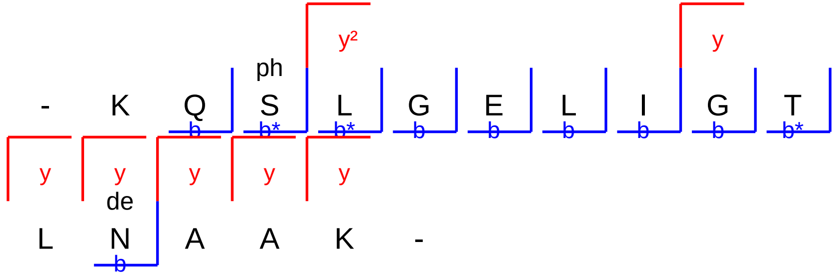

Raw File	Scan	Method	Score	m/z	Gene names
RO-QC-160121_ZackD_SS_Set_02_Fractio	36207	FTMS; HC	45.14	1272.66	ARID2

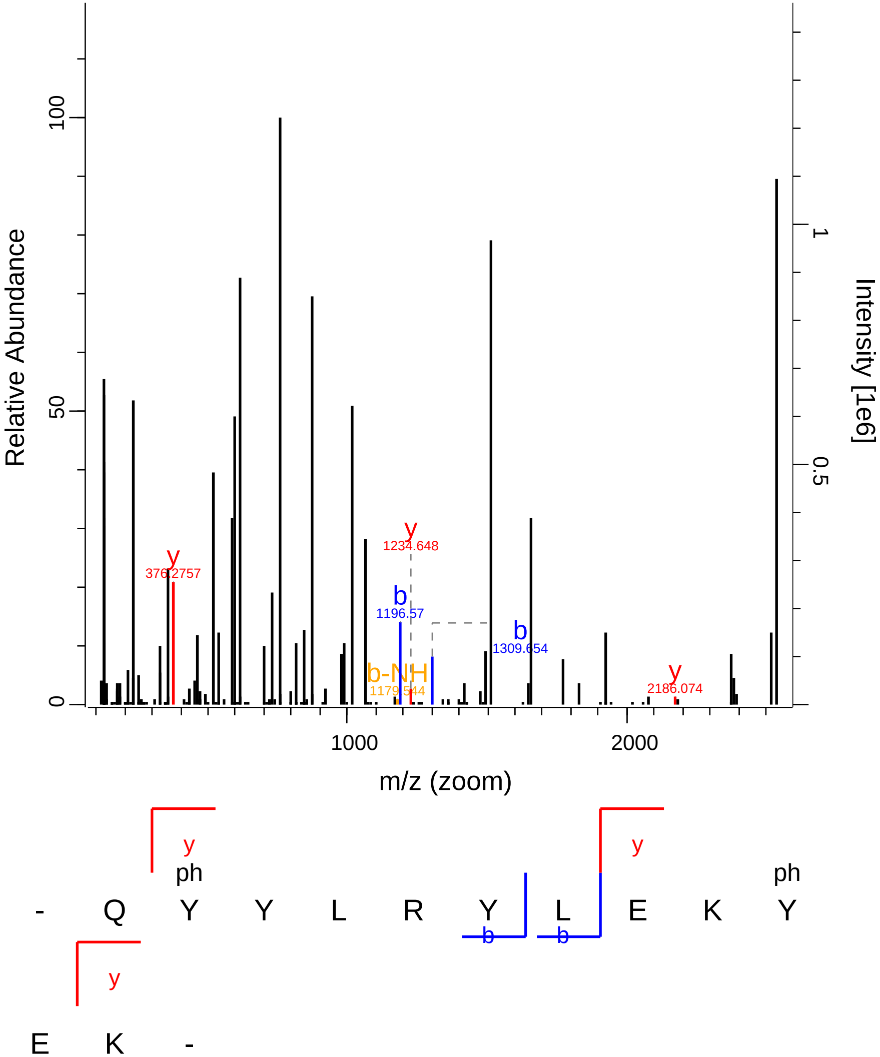

Raw File	Scan	Method	Score	m/z	Gene names
RO-QC-160121_ZackD_SS_Set_02_Fractio	36436	FTMS; HC	109.35	1245.04	RPLP2;RPLP1

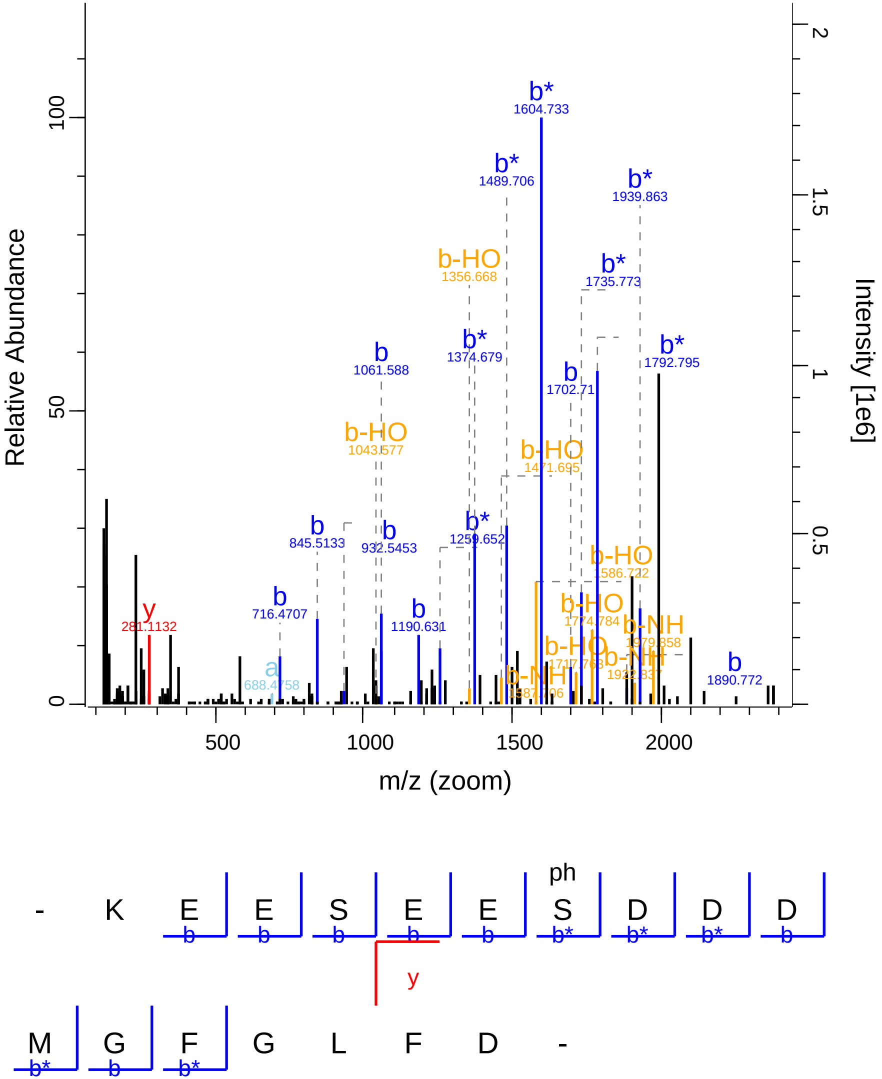

Raw File	Scan	Method	Score	m/z	Gene names
RO-QC-160121_ZackD_SS_Set_02_Fractio	36451	FTMS; HC	50.15	1196.08	DZIP3

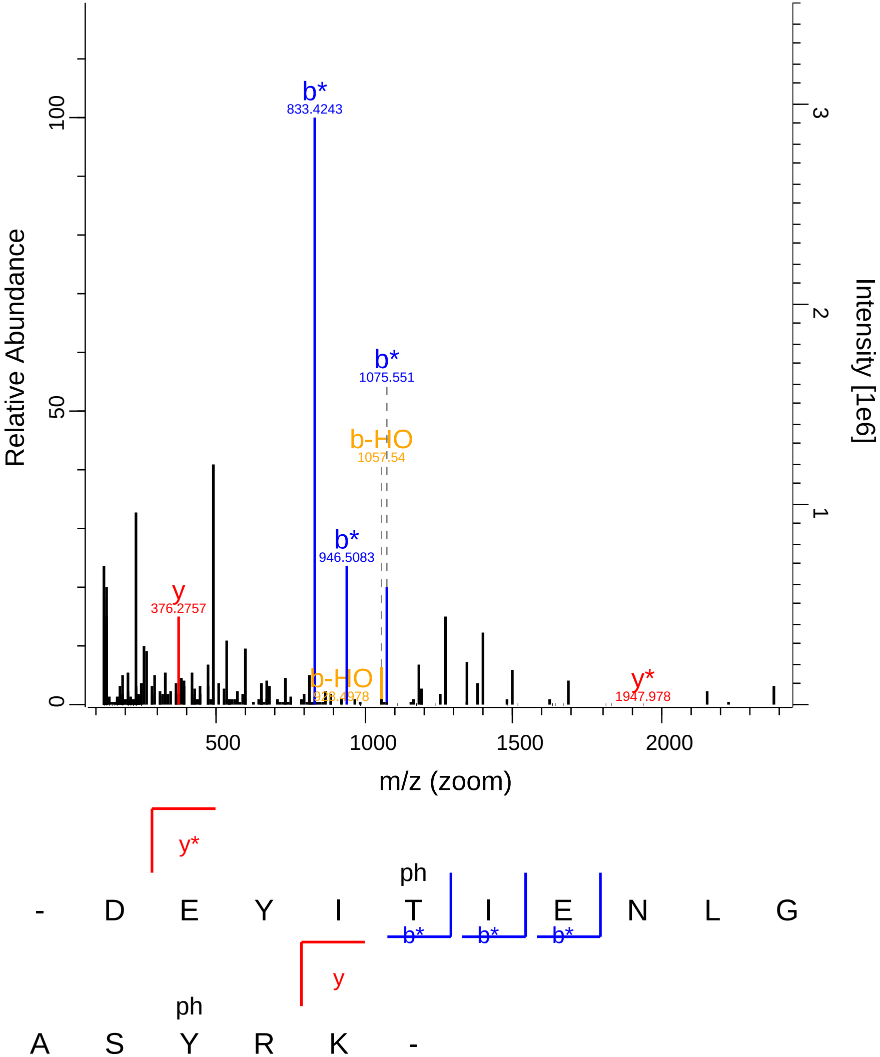

Raw File	Scan	Method	Score	m/z	Gene names
RO-QC-160121_ZackD_SS_Set_02_Fractior	36523	FTMS; HC	78.52	980.04	ST5

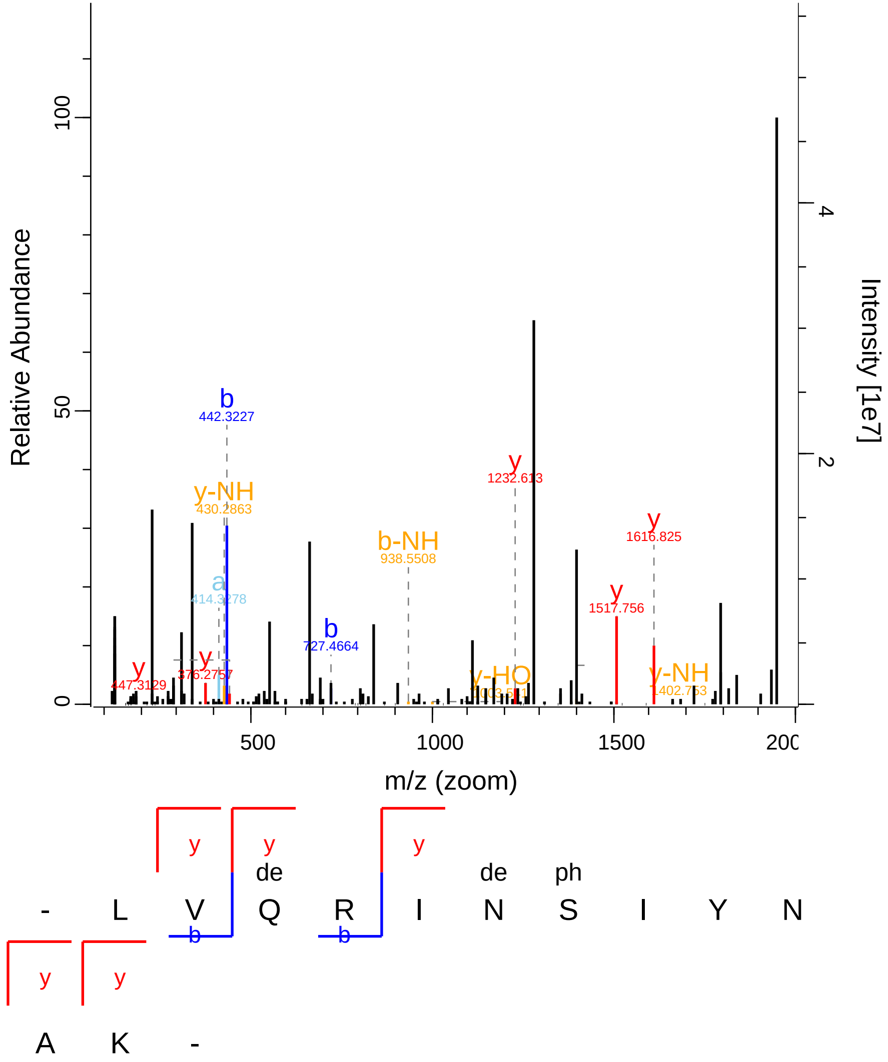

Raw File	Scan	Method	Score	m/z	Gene names
RO-QC-160121_ZackD_SS_Set_02_Fractior	36607	FTMS; HC	46.32	707.86	PTGES2

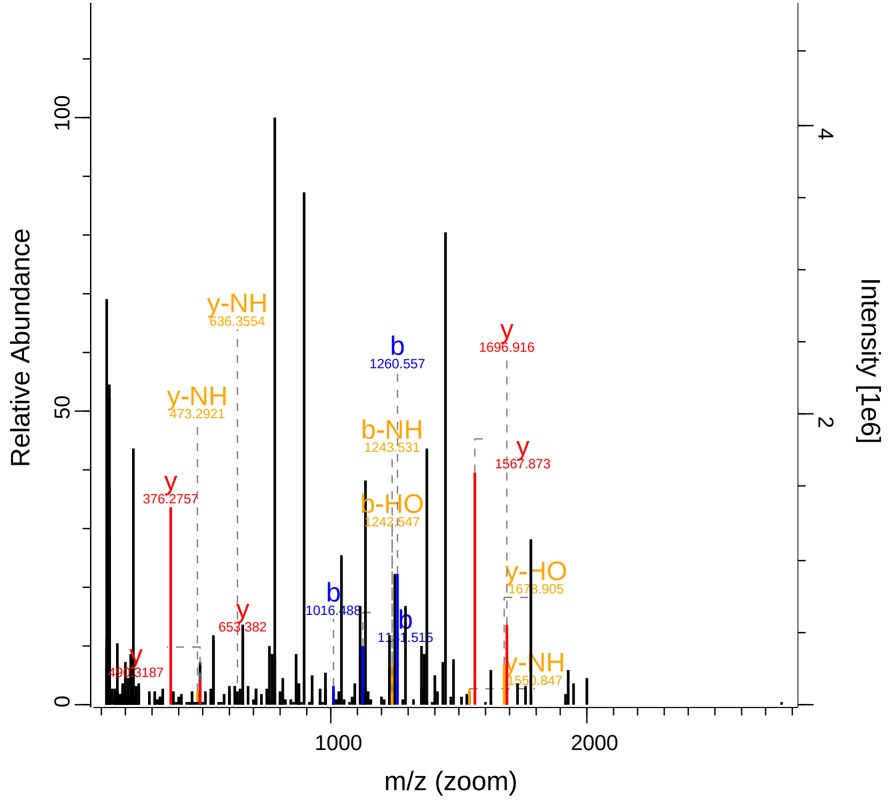

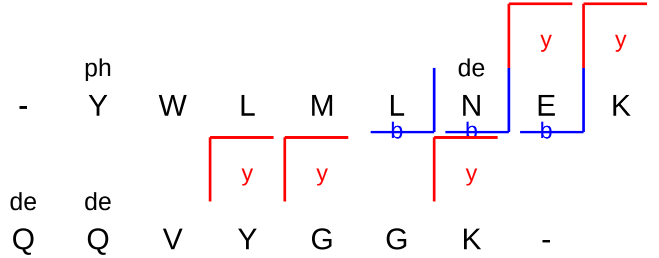

Raw File	Scan	Method	Score	m/z	Gene names
RO-QC-160121_ZackD_SS_Set_02_Fraction	37223	FTMS; HC	83.21	770.1	PGK1

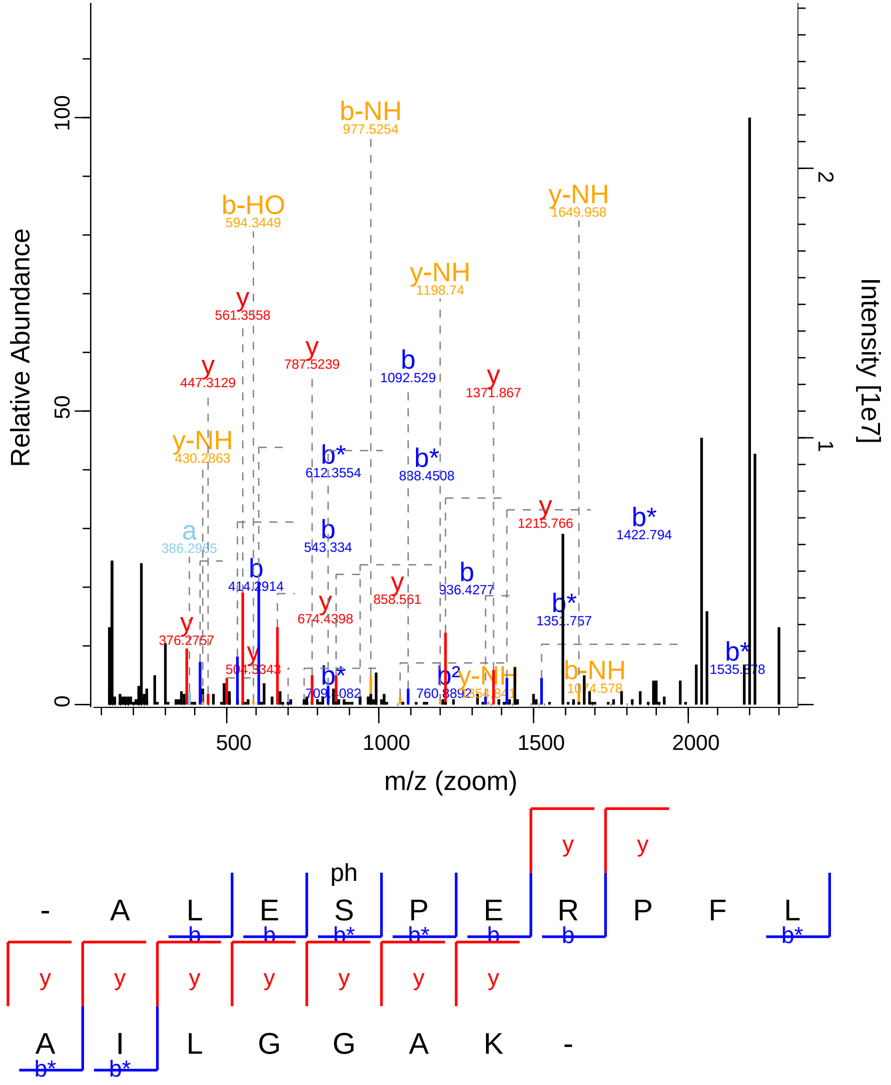

Raw File	Scan	Method	Score	m/z	Gene names
RO-QC-160121_ZackD_SS_Set_02_Fractio	38066	FTMS; HC	44.3	1359.21	RADIL

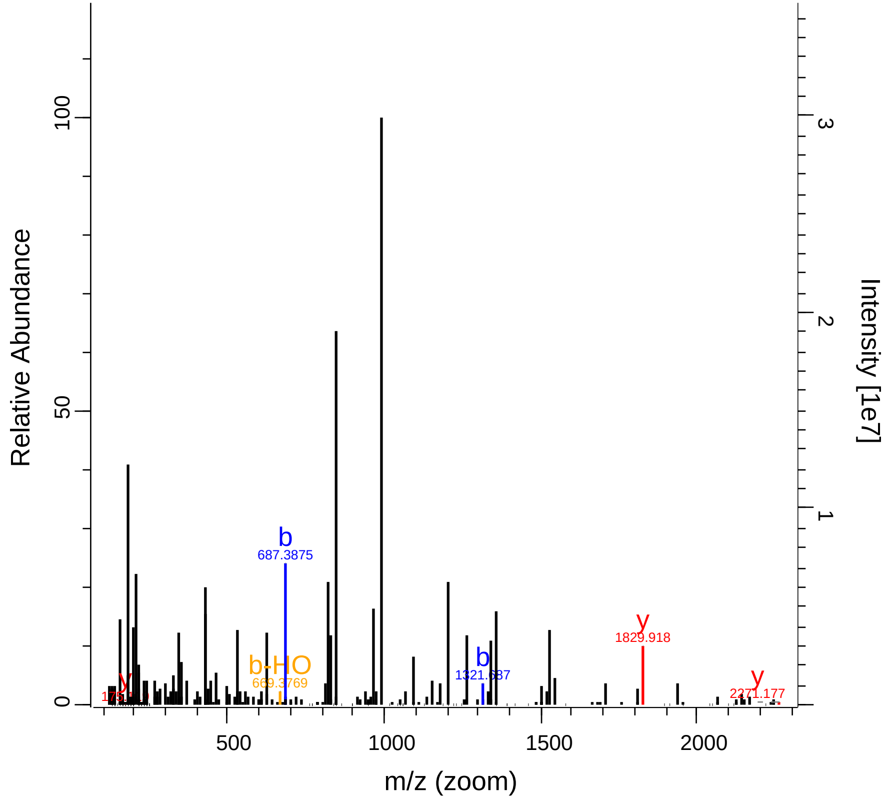

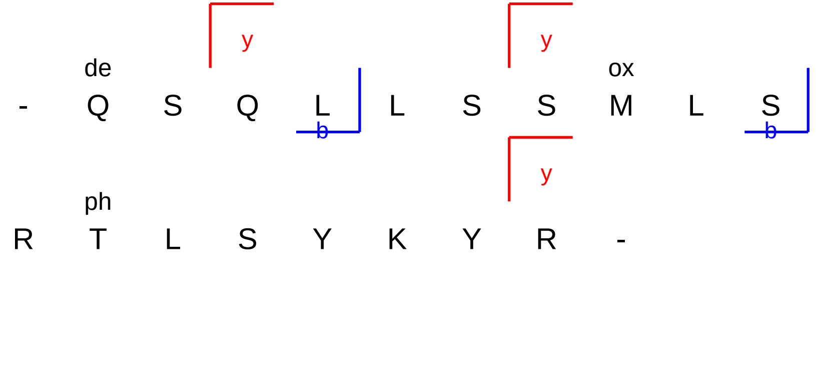

Raw File	Scan	Method	Score	m/z	Gene names
RO-QC-160121_ZackD_SS_Set_02_Fraction	4818	FTMS; HC	56.2	436.25	RBM12

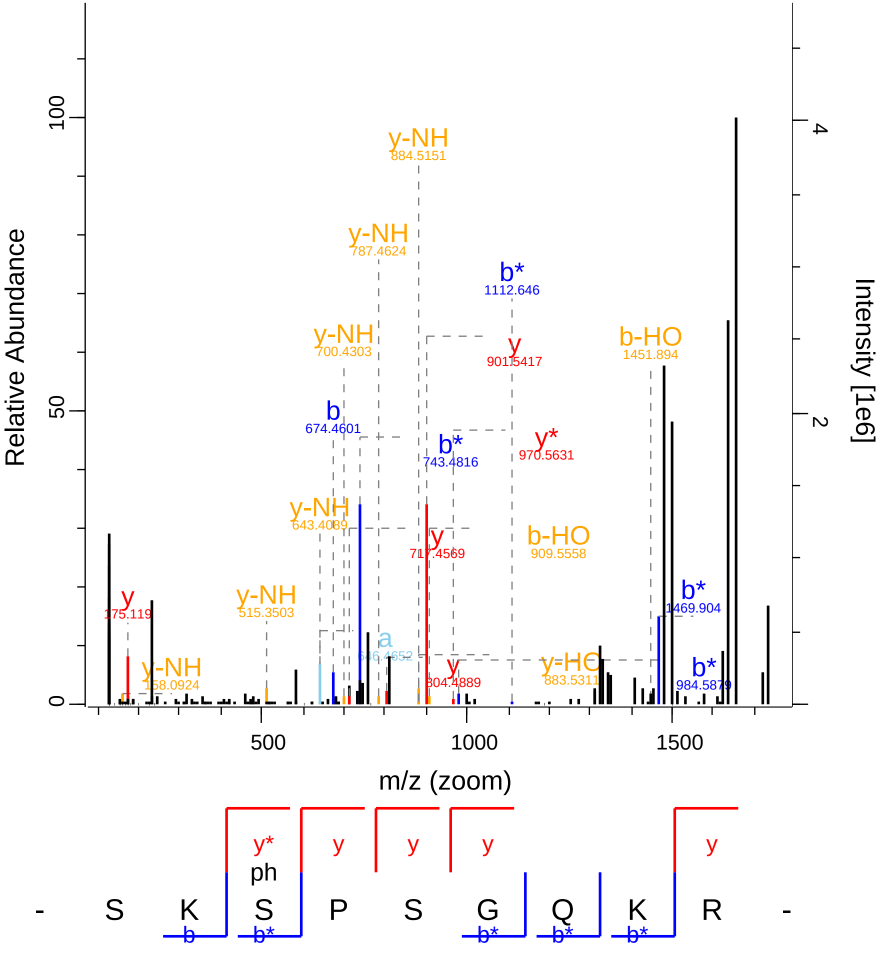

Raw File	Scan	Method	Score	m/z	Gene names
RO-QC-160121_ZackD_SS_Set_02_Fraction	5514	FTMS; HC	82.65	527.61	TNIP1

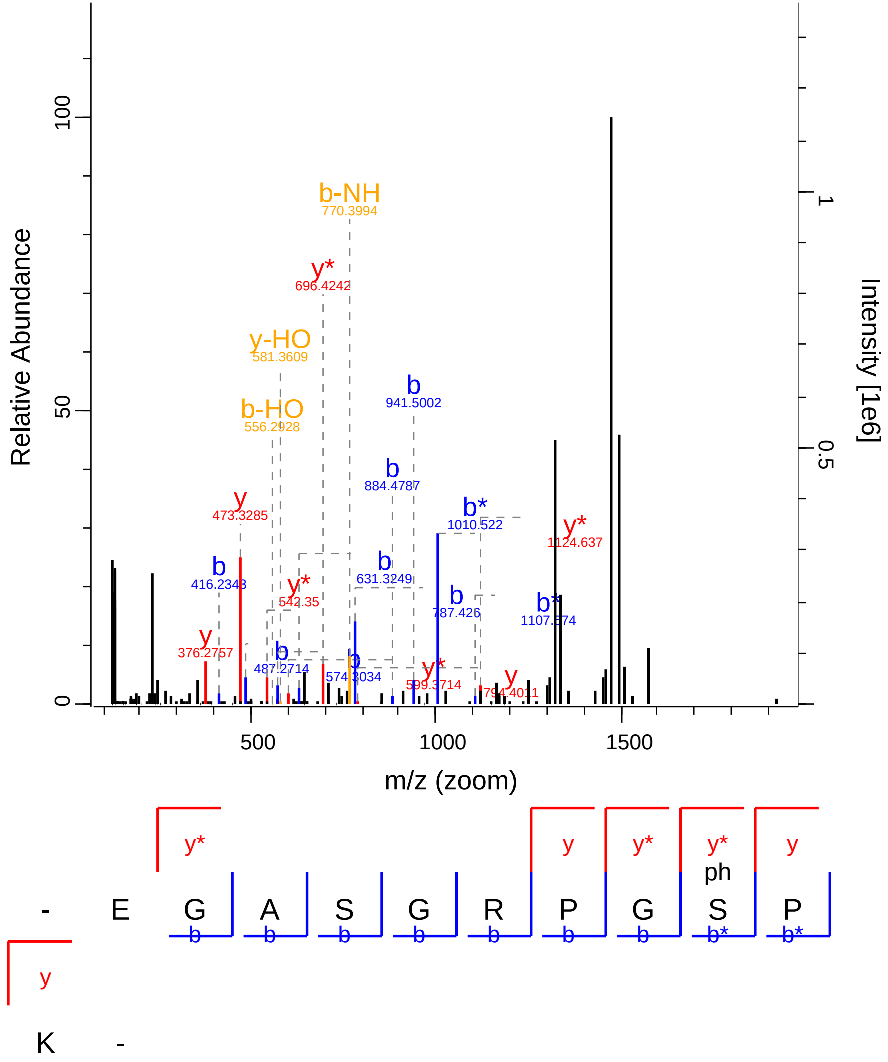

Raw File	Scan	Method	Score	m/z	Gene names
RO-QC-160121_ZackD_SS_Set_02_Fraction_	5673	FTMS; HC	72.65	426.21	RRAGC

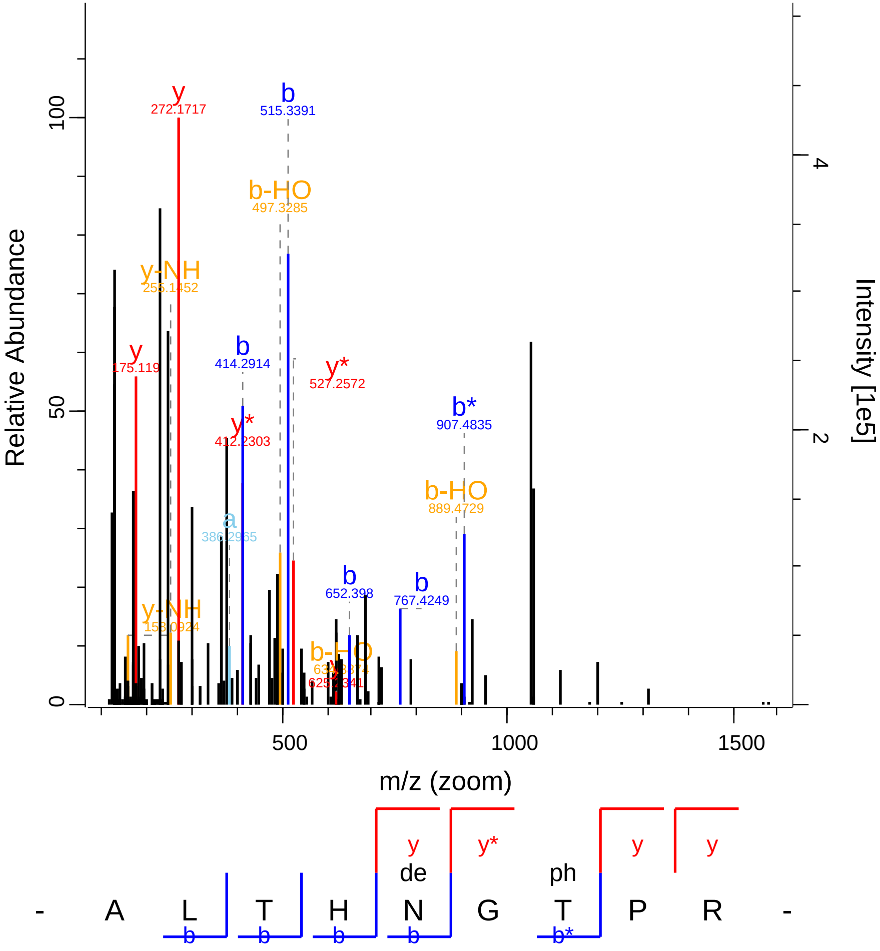

Raw File	Scan	Method	Score	m/z	Gene names
RO-QC-160121_ZackD_SS_Set_02_Fraction	5981	FTMS; HC	41.54	422.44	ZNF836

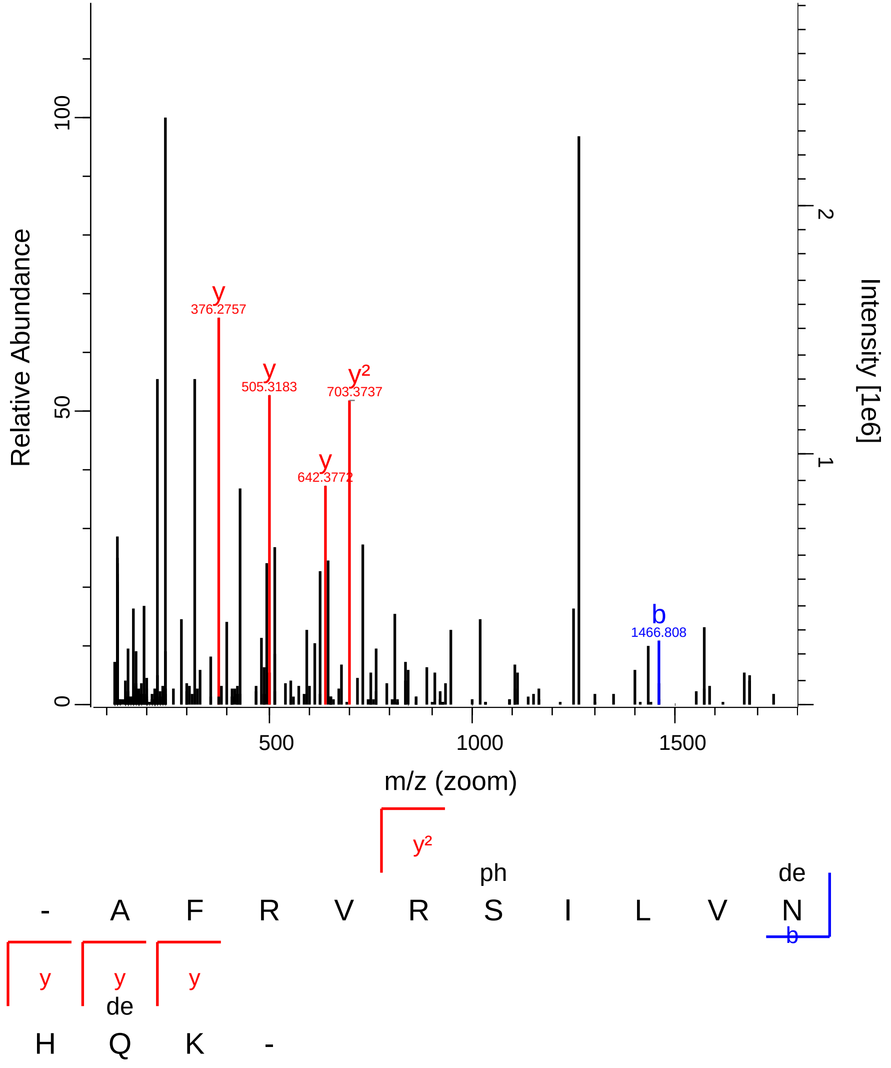

Raw File	Scan	Method	Score	m/z	Gene names
RO-QC-160121_ZackD_SS_Set_02_Fraction_	6322	FTMS; HCI	66.02	717	VAT1L

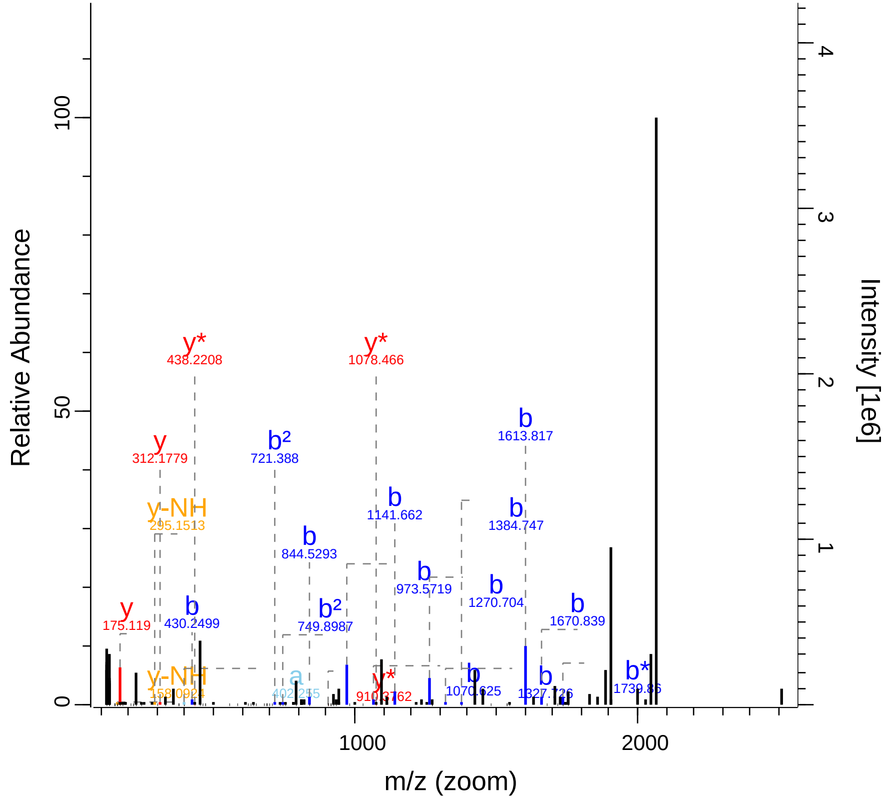

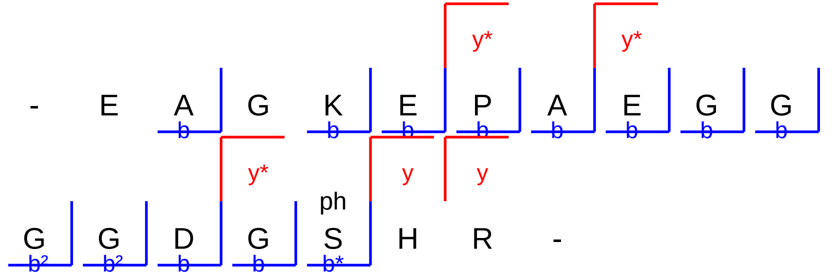

Raw File	Scan	Method	Score	m/z	Gene names
RO-QC-160121_ZackD_SS_Set_02_Fraction	6640	FTMS; HC	50.04	564.79	IGSF21

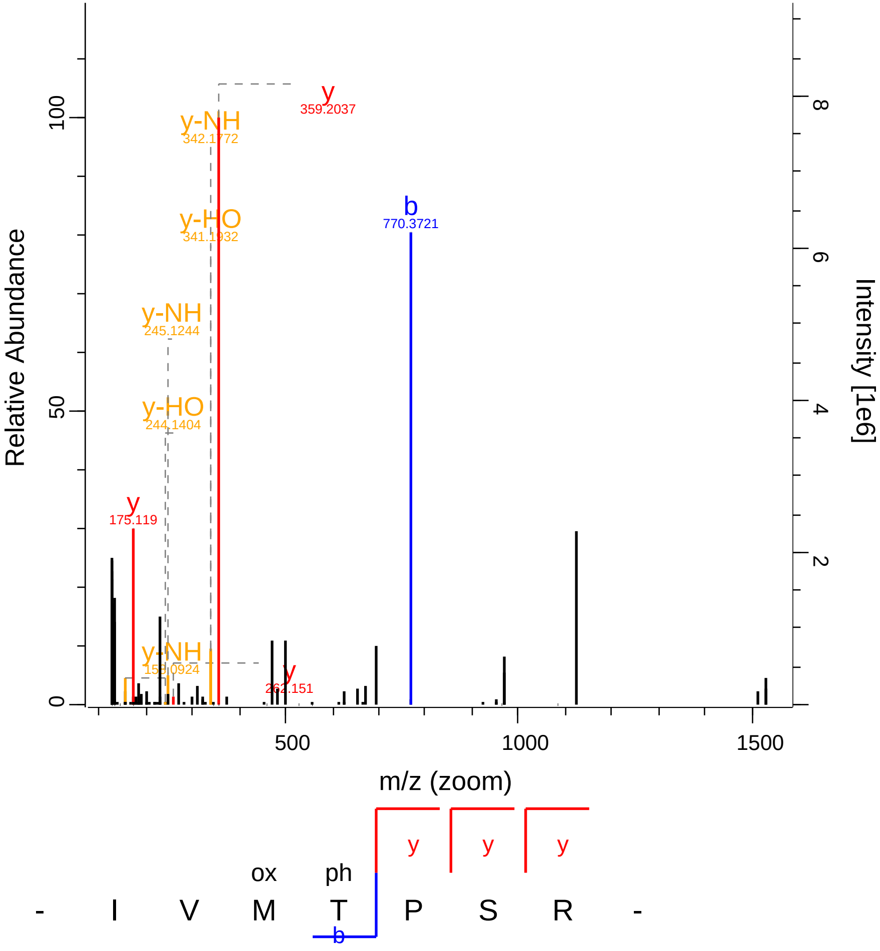

Raw File	Scan	Method	Score	m/z	Gene names
RO-QC-160121_ZackD_SS_Set_02_Fraction[illegible]	6693	FTMS; HC	54.07	889.43	NIFK

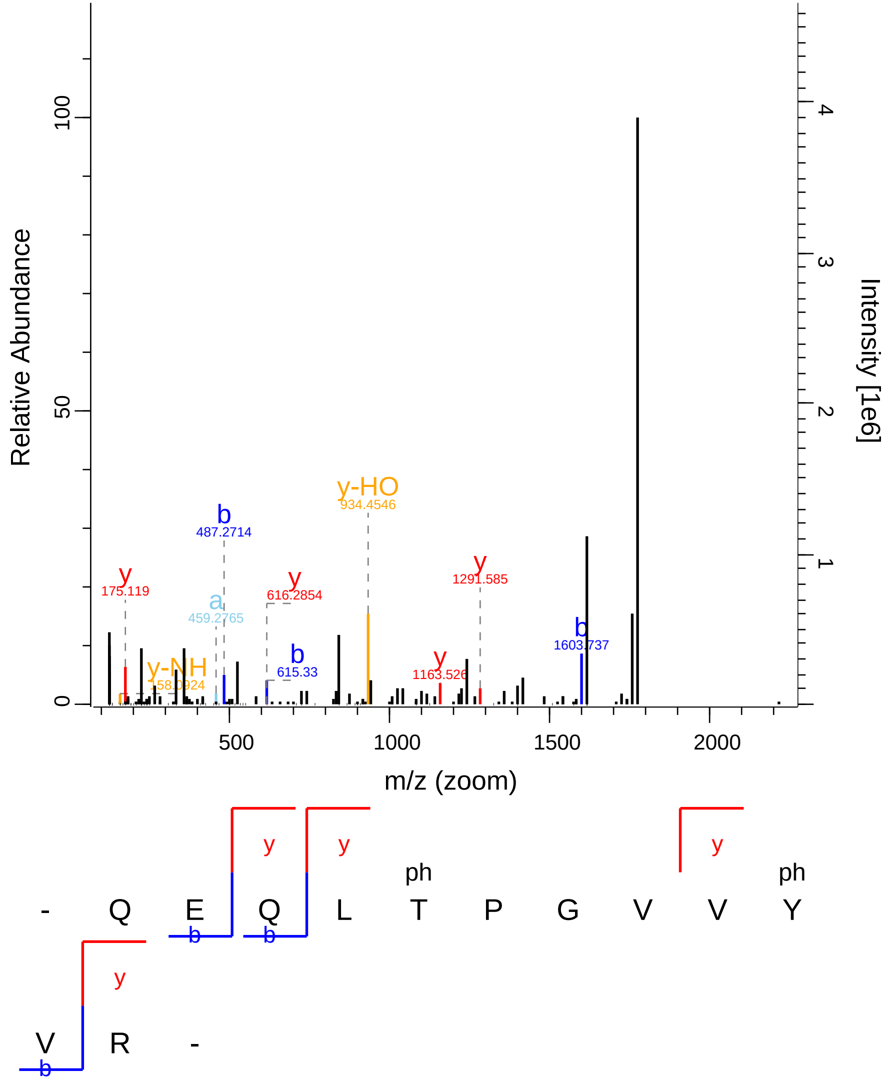

Raw File	Scan	Method	Score	m/z	Gene names
RO-QC-160121_ZackD_SS_Set_02_Fraction	7236	FTMS; HC	115.31	674.66	ERC1

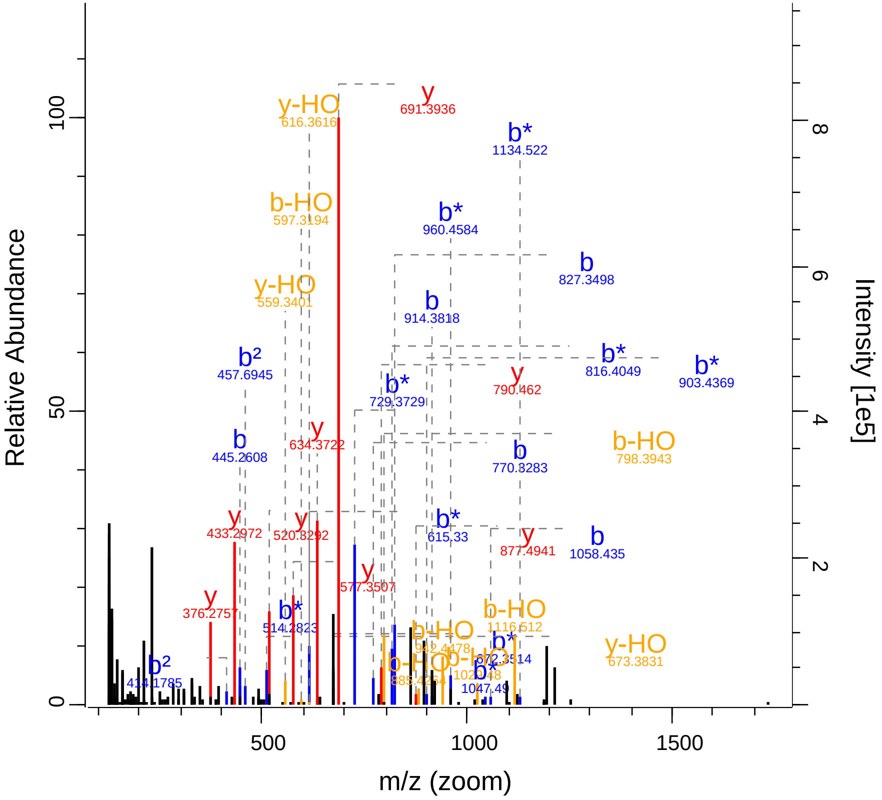

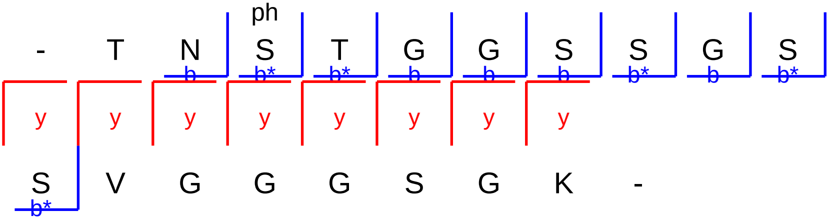

Raw File	Scan	Method	Score	m/z	Gene names
RO-QC-160121_ZackD_SS_Set_02_Fraction	7901	FTMS; HC	138.89	837.92	RALGAPA1

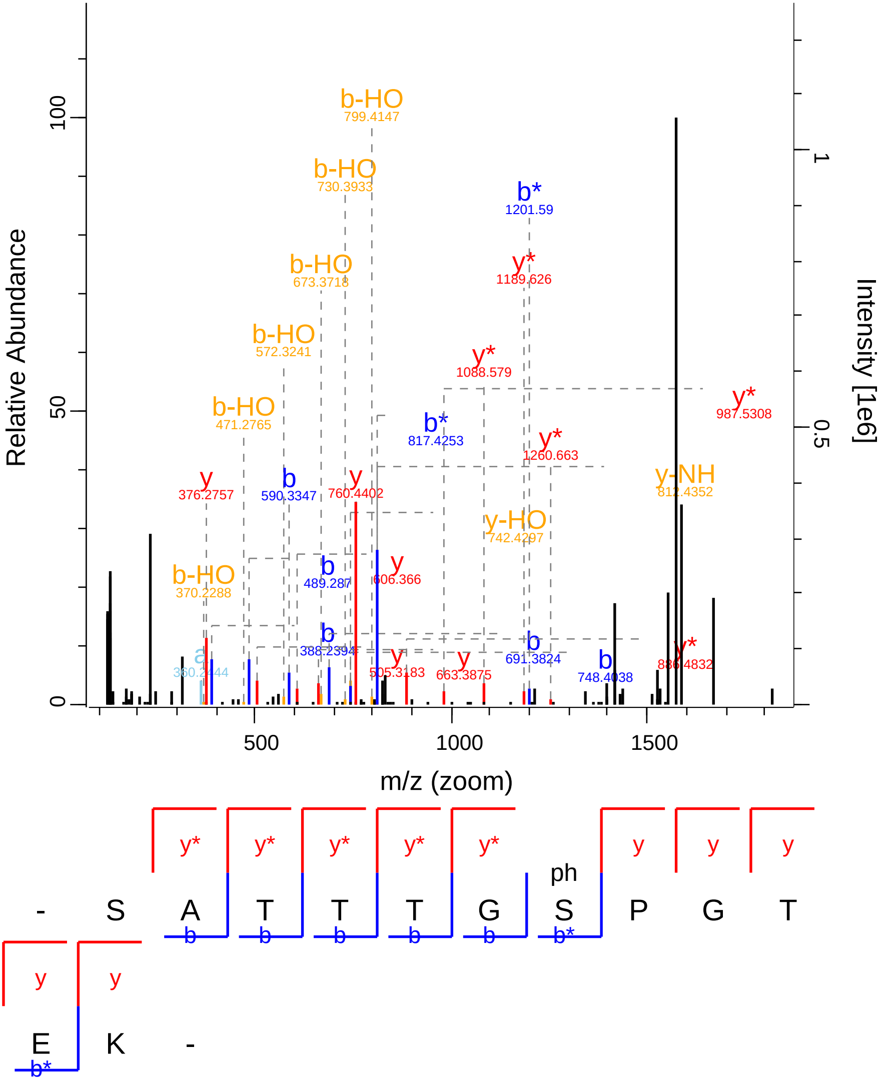

Raw File	Scan	Method	Score	m/z	Gene names
RO-QC-160121_ZackD_SS_Set_02_Fraction	8281	FTMS; HC	167.71	826.92	LBR

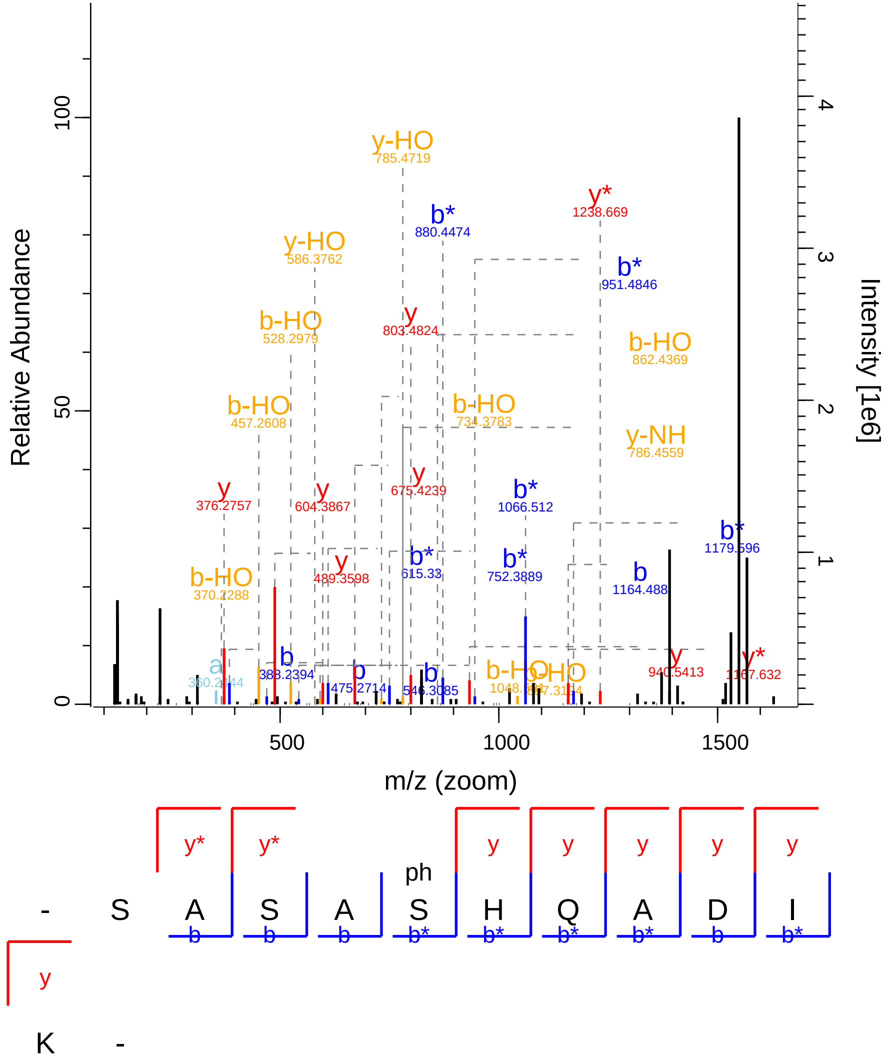

Raw File	Scan	Method	Score	m/z	Gene names
RO-QC-160121_ZackD_SS_Set_02_Fraction	8404	FTMS; HC	105.03	728.88	ASAP1

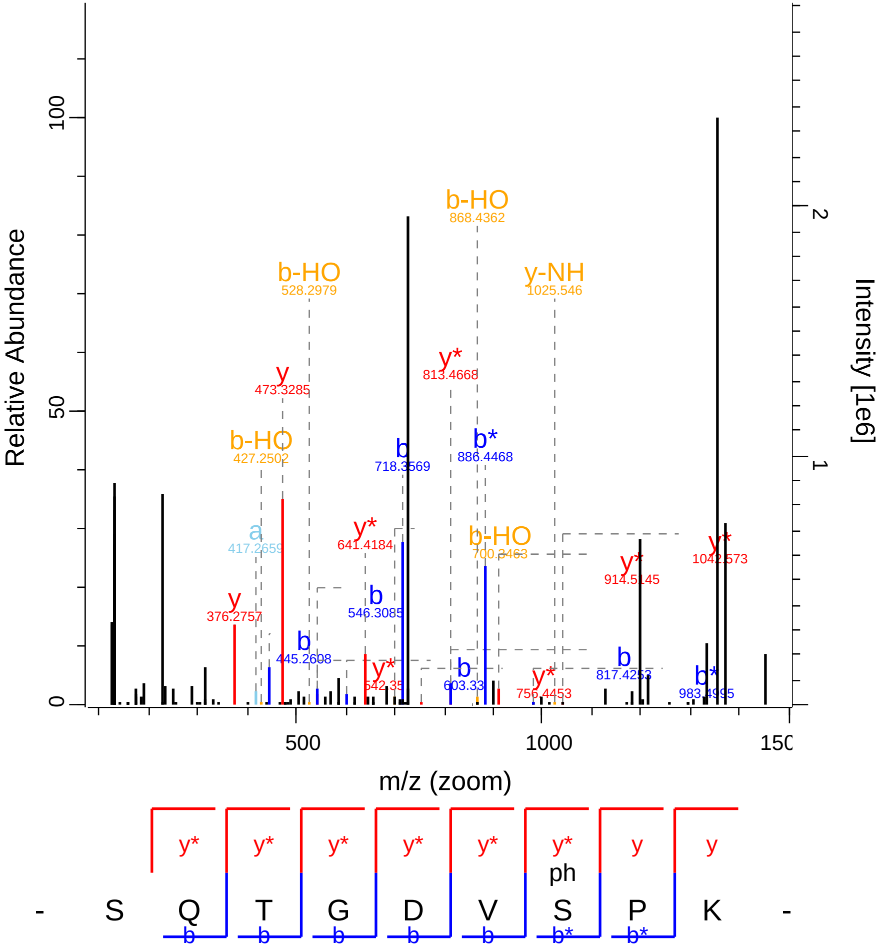

Raw File	Scan	Method	Score	m/z	Gene names
RO-QC-160121_ZackD_SS_Set_02_Fraction	8666	FTMS; HC	72.43	745.03	VPS4A

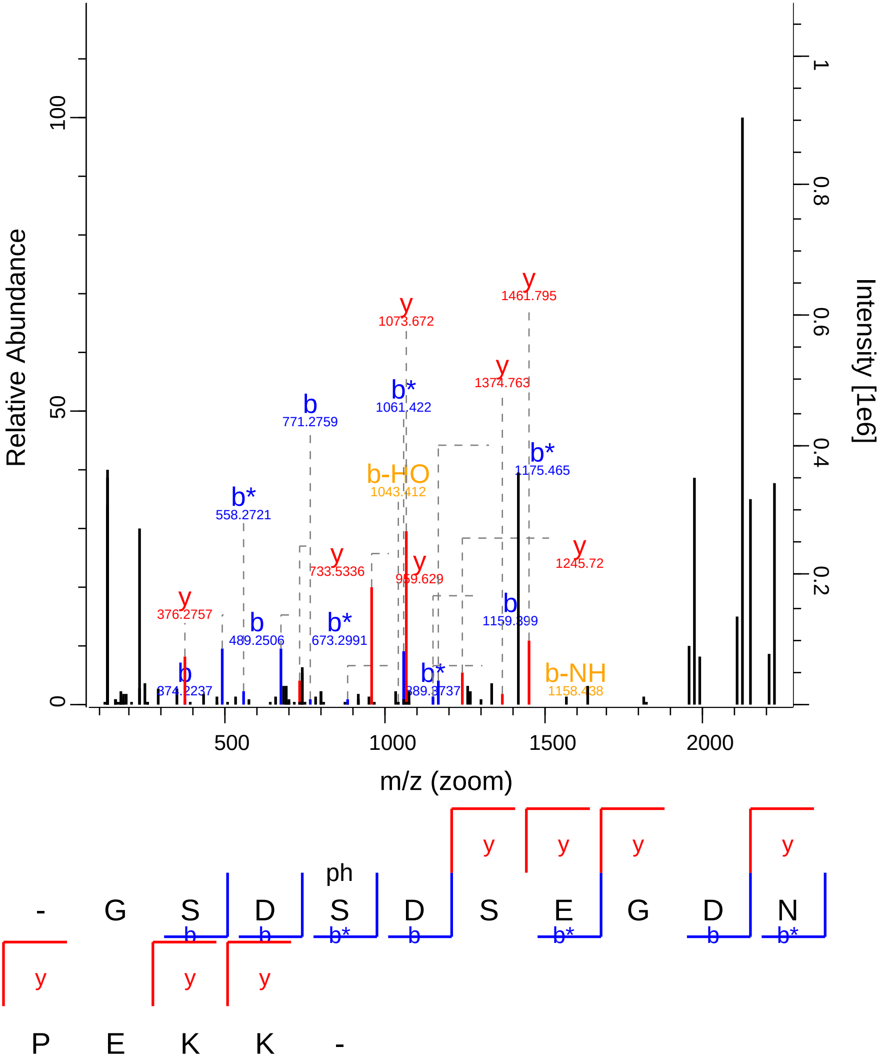

Raw File	Scan	Method	Score	m/z	Gene names
RO-QC-160121_ZackD_SS_Set_02_Fraction	8908	FTMS; HC	77.19	685.39	NSFL1C

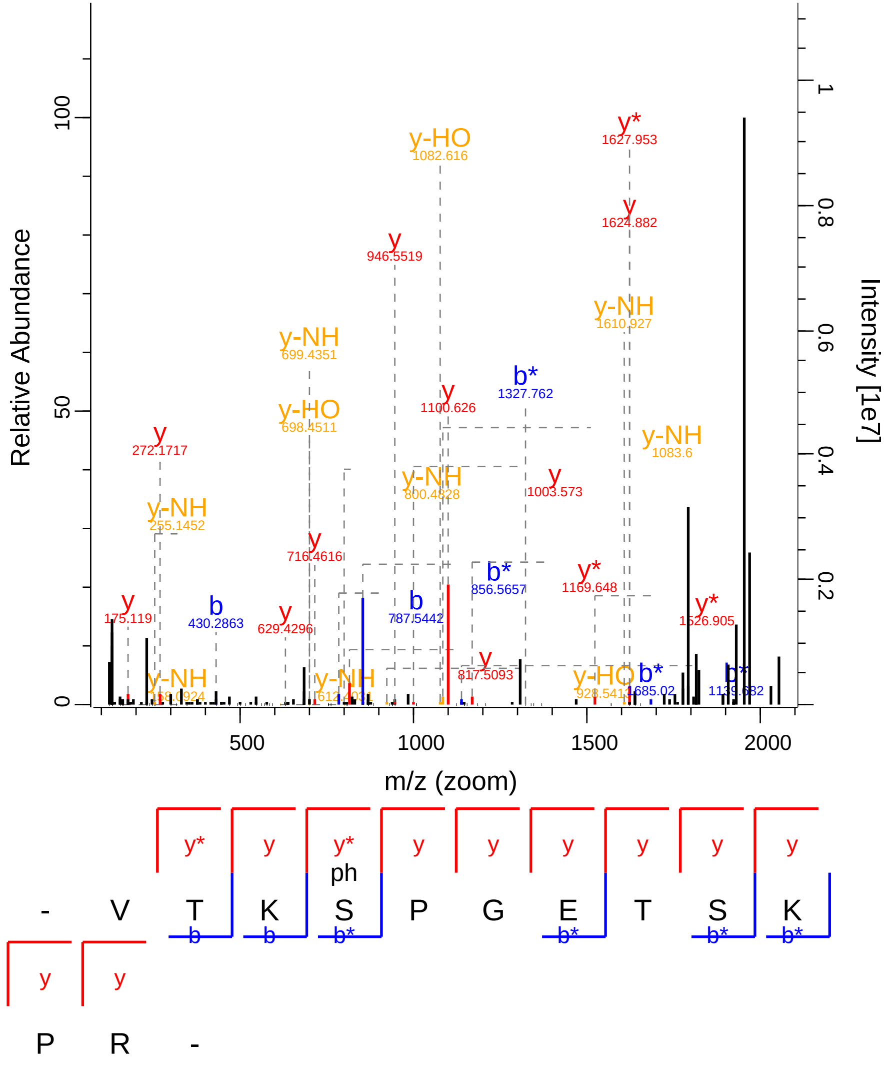

Raw File	Scan	Method	Score	m/z	Gene names
RO-QC-160121_ZackD_SS_Set_02_Fraction	9706	FTMS; HC	114.87	713.84	RSRC2

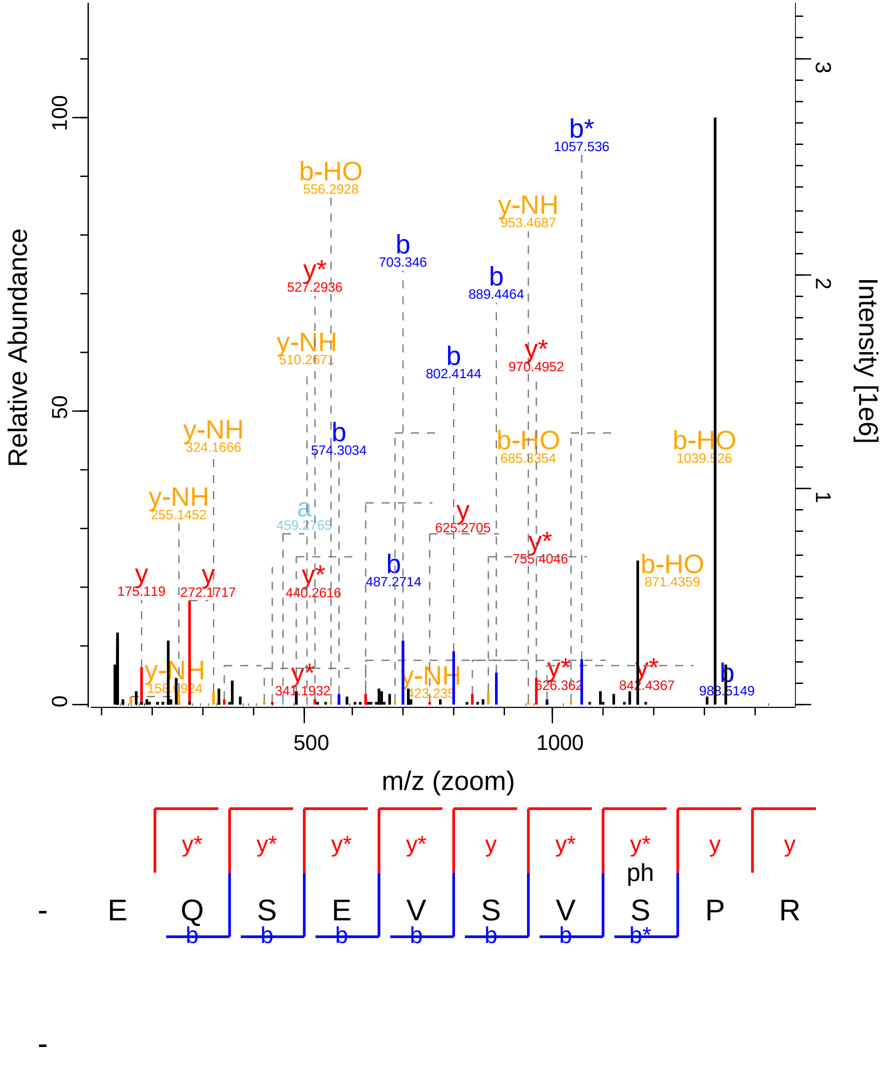

Raw File	Scan	Method	Score	m/z	Gene names
RO-QC-160121_ZackD_SS_Set_02_Fractior	10242	FTMS; HC	82.01	858.08	CTR9

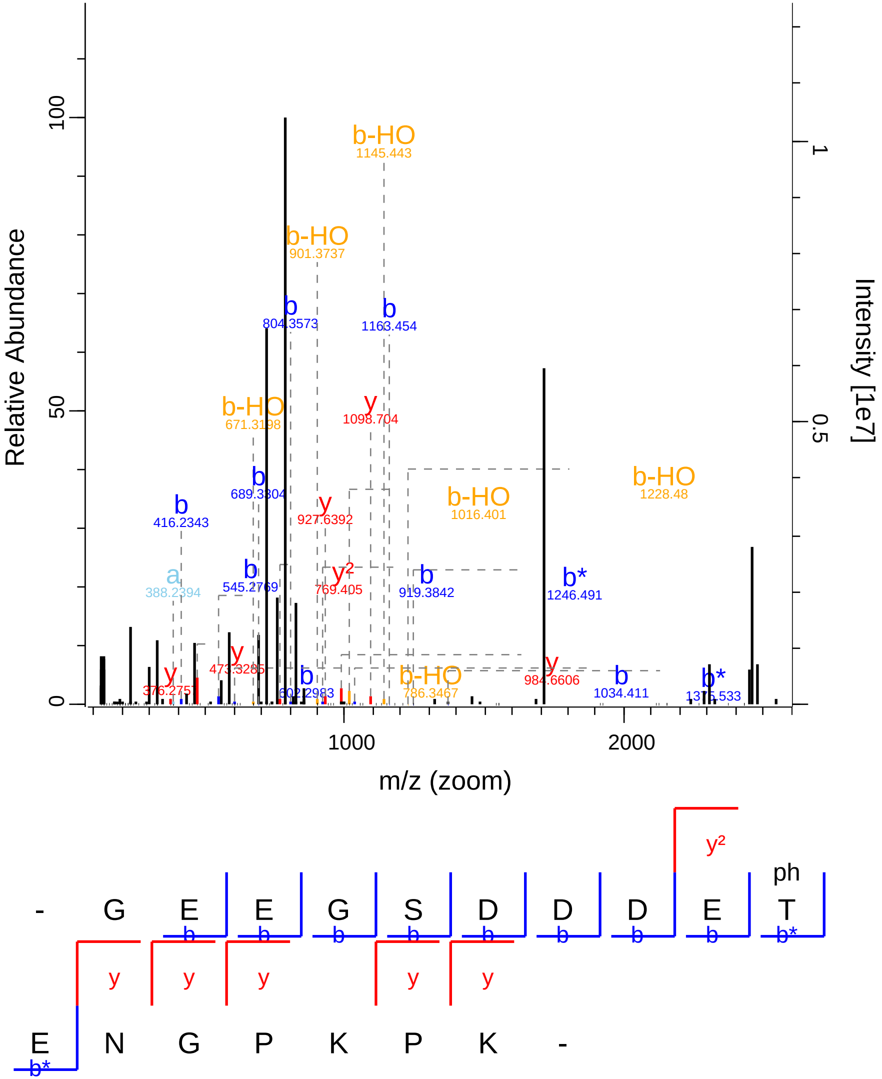

Raw File	Scan	Method	Score	m/z	Gene names
RO-QC-160121_ZackD_SS_Set_02_Fractioi	10289	FTMS; HC	132.79	643.56	CTR9

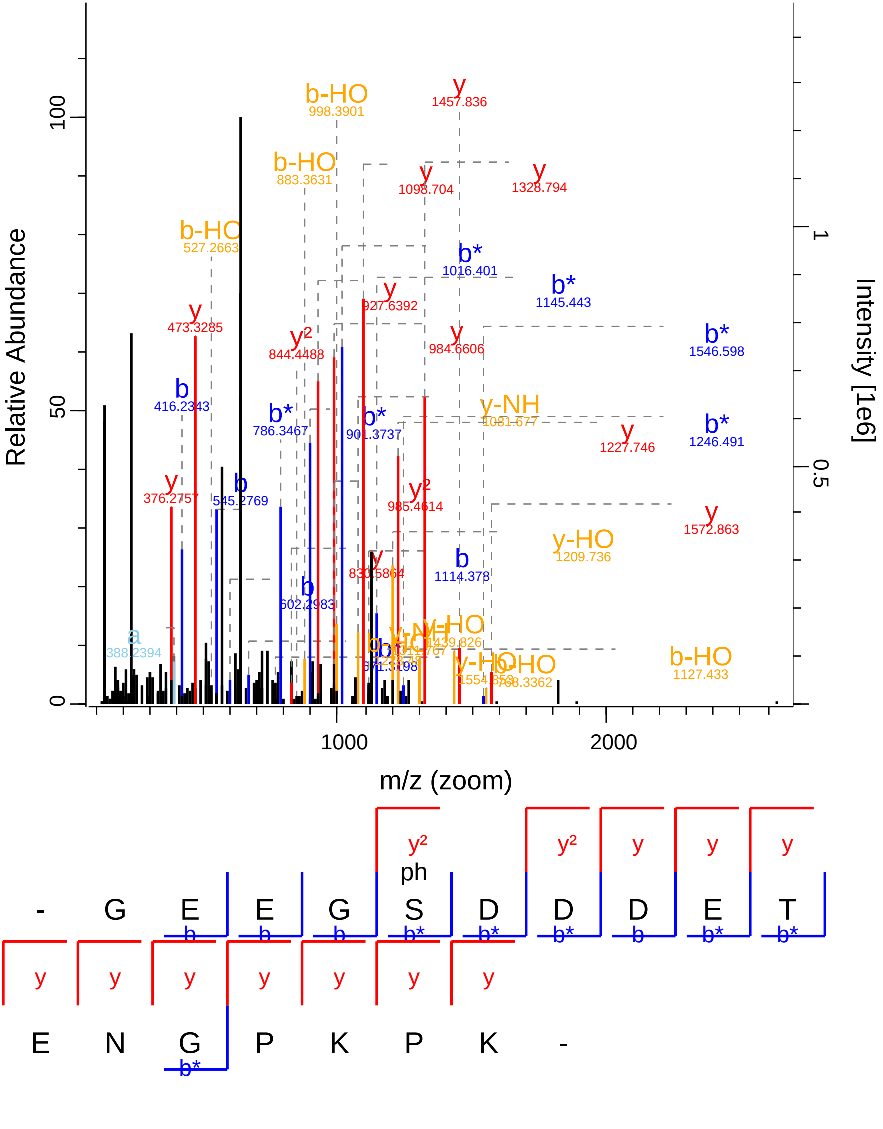

Raw File	Scan	Method	Score	m/z	Gene names
RO-QC-160121_ZackD_SS_Set_02_Fraction	10753	FTMS; HC	103.61	625.1	DDX21

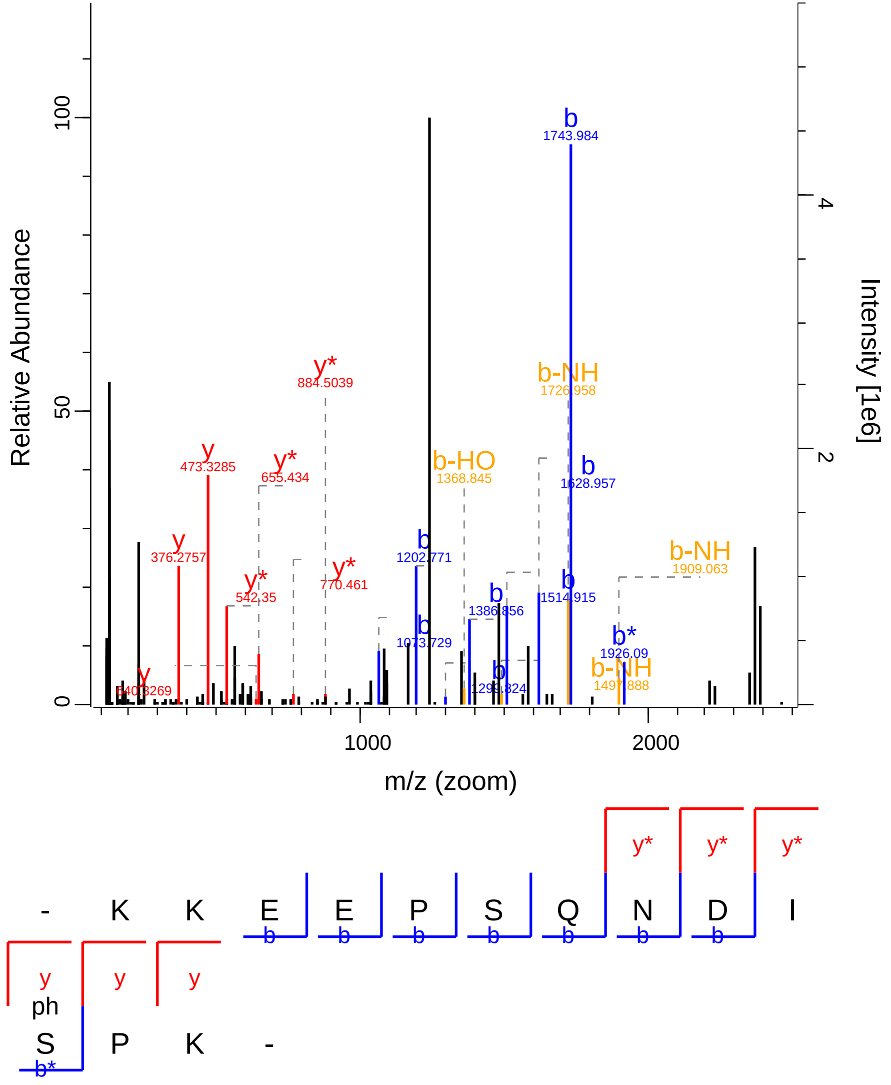

Raw File	Scan	Method	Score	m/z	Gene names
RO-QC-160121_ZackD_SS_Set_02_Fractior	11382	FTMS; HC	56.43	807.97	RBM27

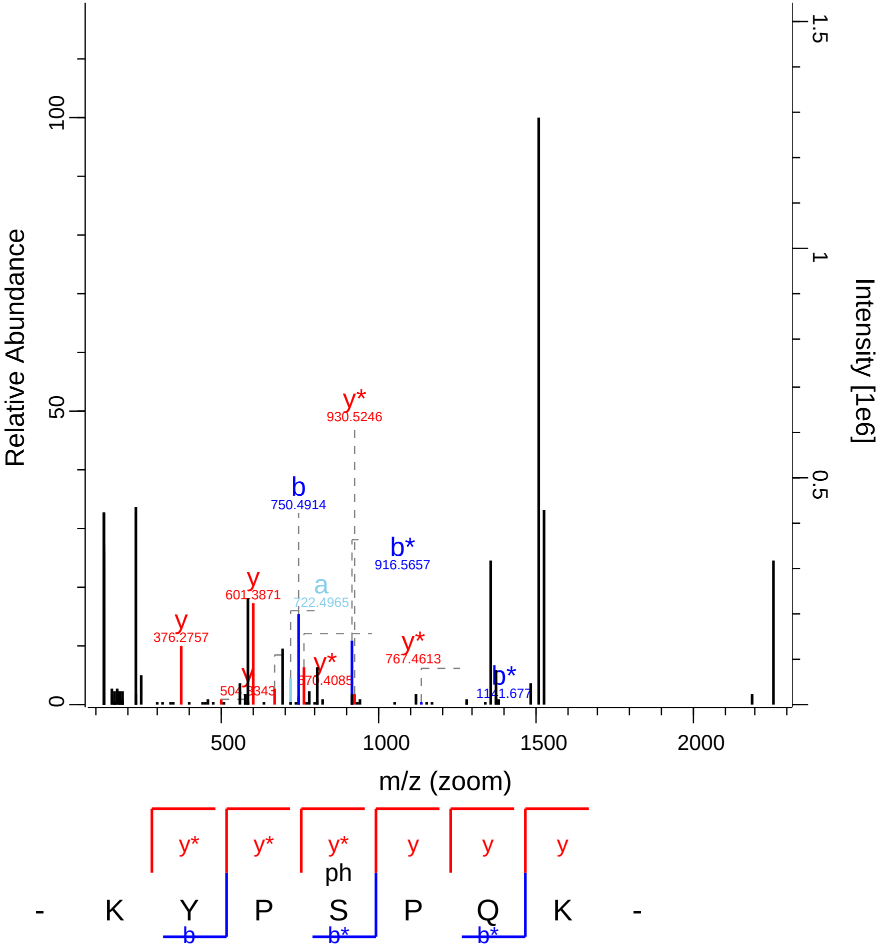

Raw File	Scan	Method	Score	m/z	Gene names
RO-QC-160121_ZackD_SS_Set_02_Fractior	12314	FTMS; HC	49.42	498.23	SRRT

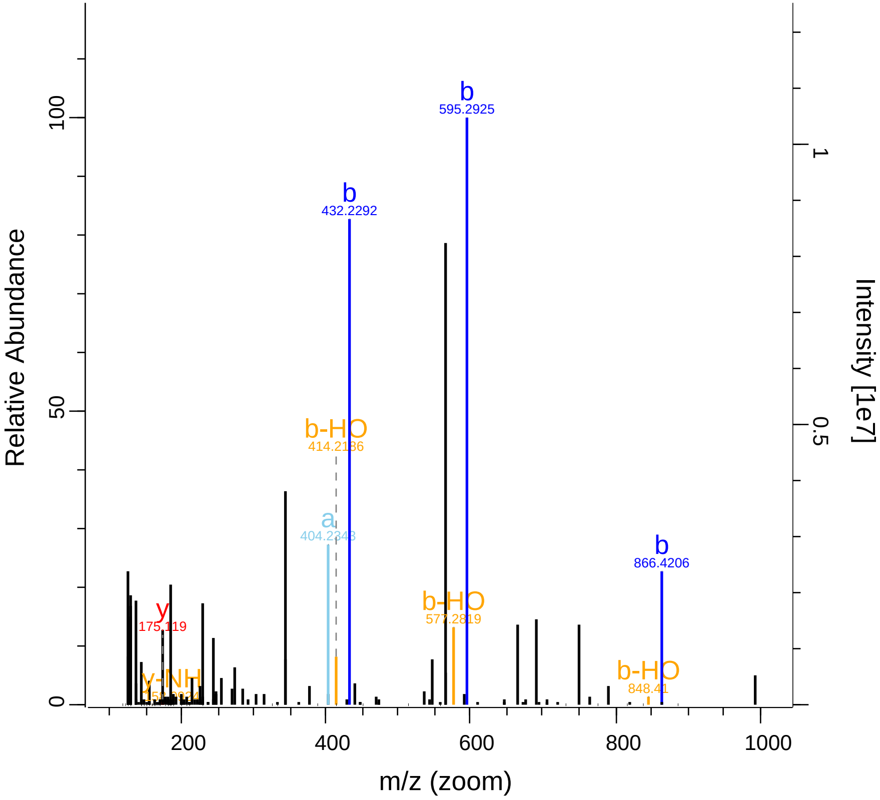

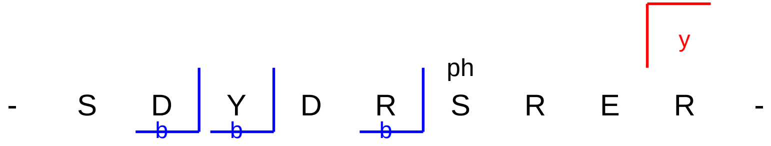

Raw File	Scan	Method	Score	m/z	Gene names
RO-QC-160121_ZackD_SS_Set_02_Fractioı	12382	FTMS; HC	183.86	919.46	TGOLN2

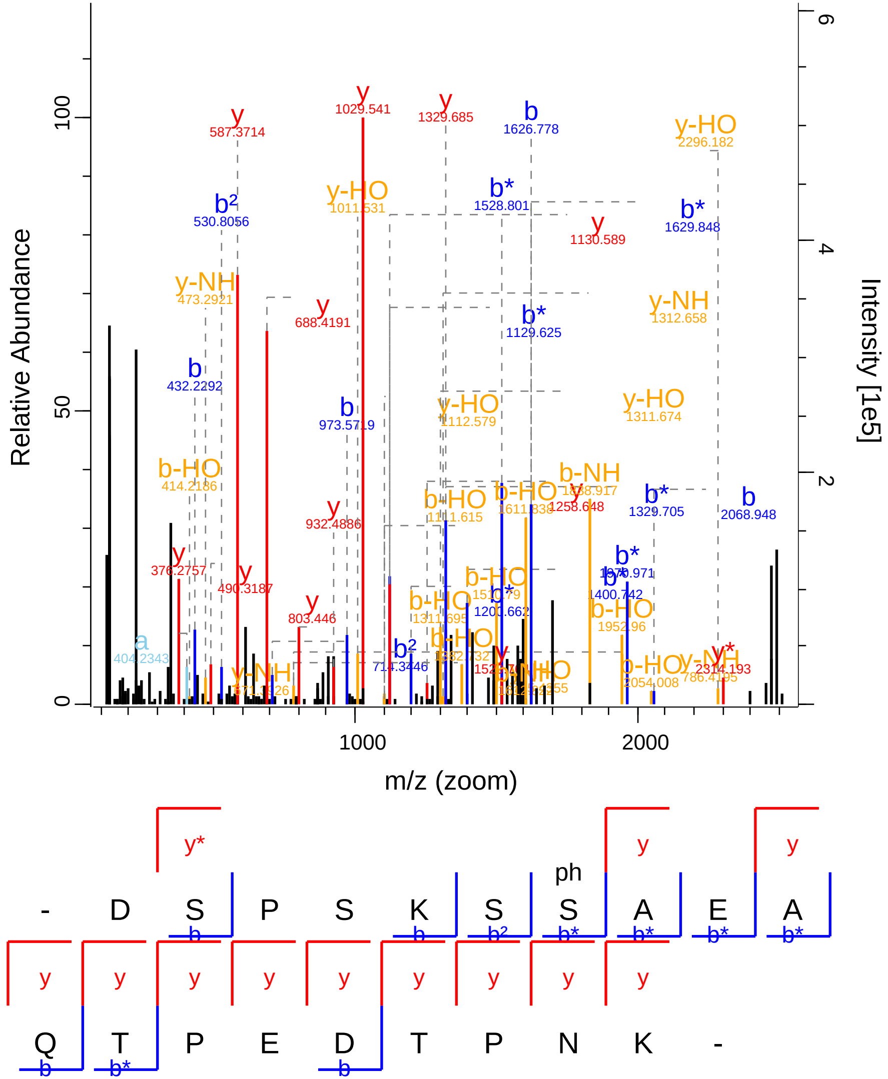

Raw File	Scan	Method	Score	m/z	Gene names
RO-QC-160121_ZackD_SS_Set_02_Fractio	13041	FTMS; HC	184.99	1185.61	SLTM

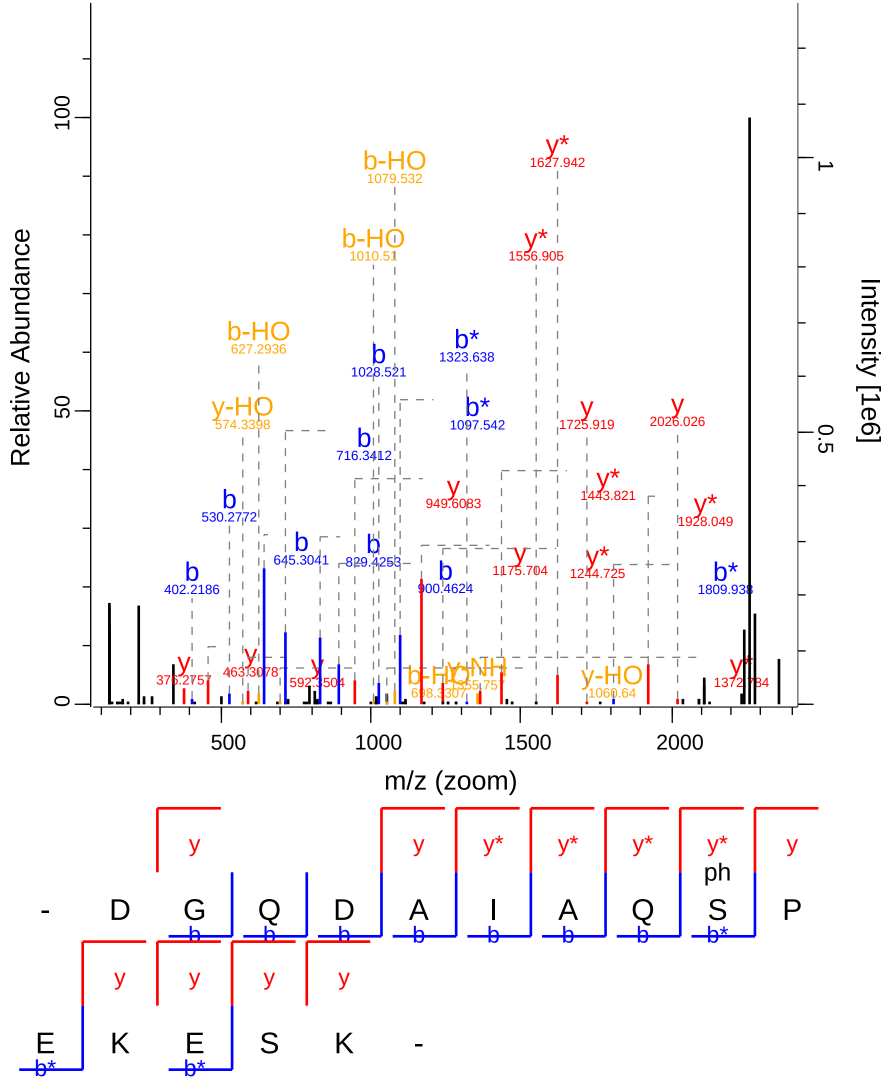

Raw File	Scan	Method	Score	m/z	Gene names
RO-QC-160121_ZackD_SS_Set_02_Fractio	14100	FTMS; HC	119.25	927.49	SPECC1

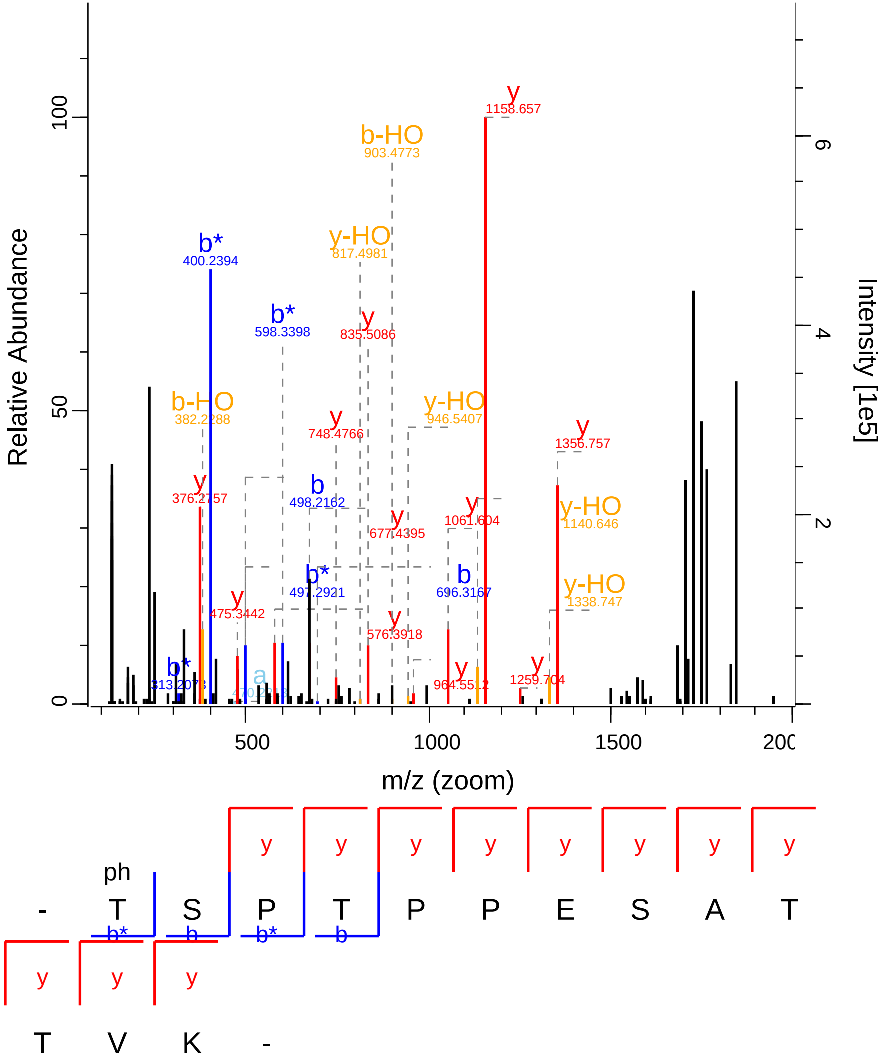

Raw File	Scan	Method	Score	m/z	Gene names
RO-QC-160121_ZackD_SS_Set_02_Fraction	14777	FTMS; HC	106.88	654.8	PDHA1

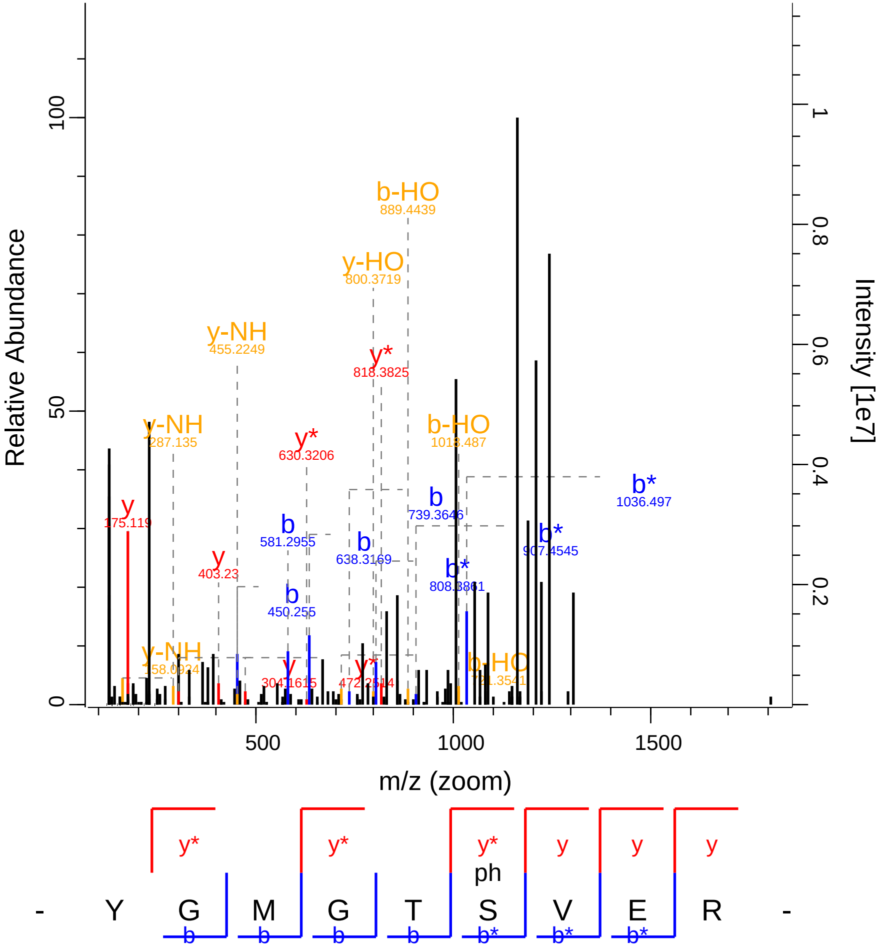

Raw File	Scan	Method	Score	m/z	Gene names
RO-QC-160121_ZackD_SS_Set_02_Fractior	15413	FTMS; HC	54.94	746.69	SLC4A7

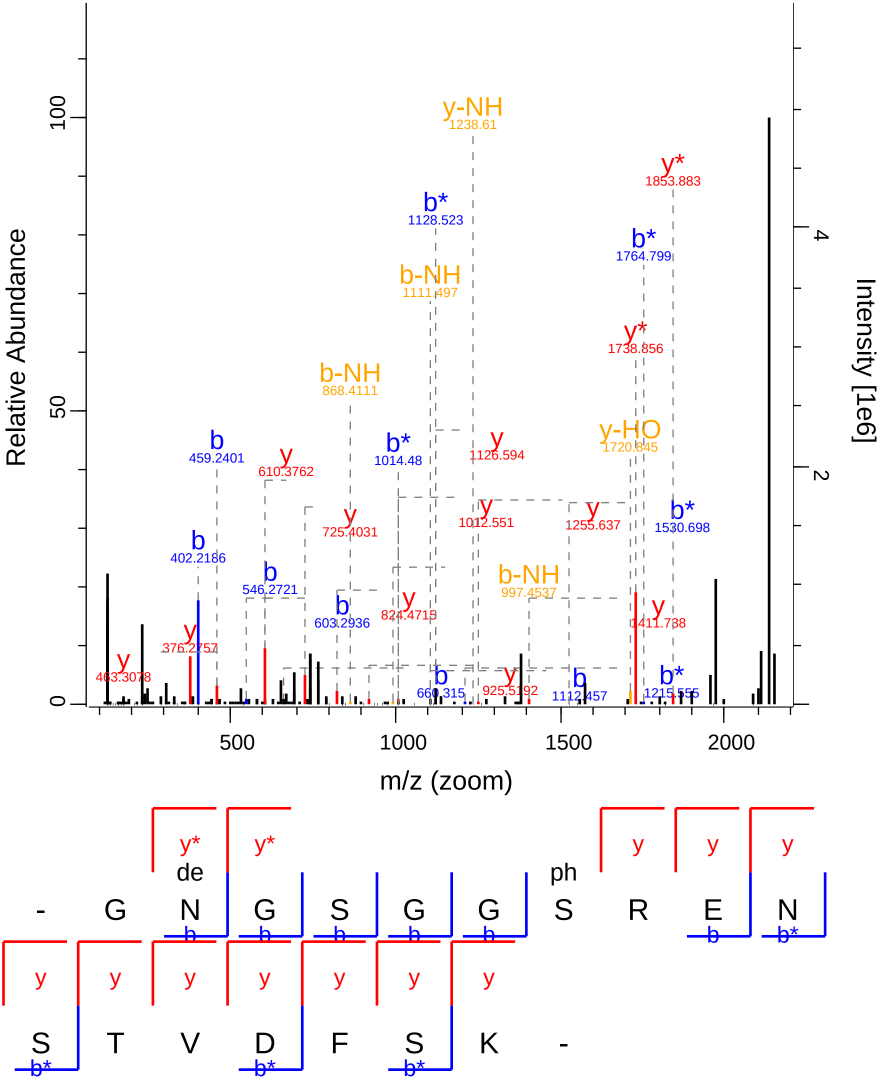

Raw File	Scan	Method	Score	m/z	Gene names
RO-QC-160121_ZackD_SS_Set_02_Fractior	16096	FTMS; HC	86.3	858.91	STT3B

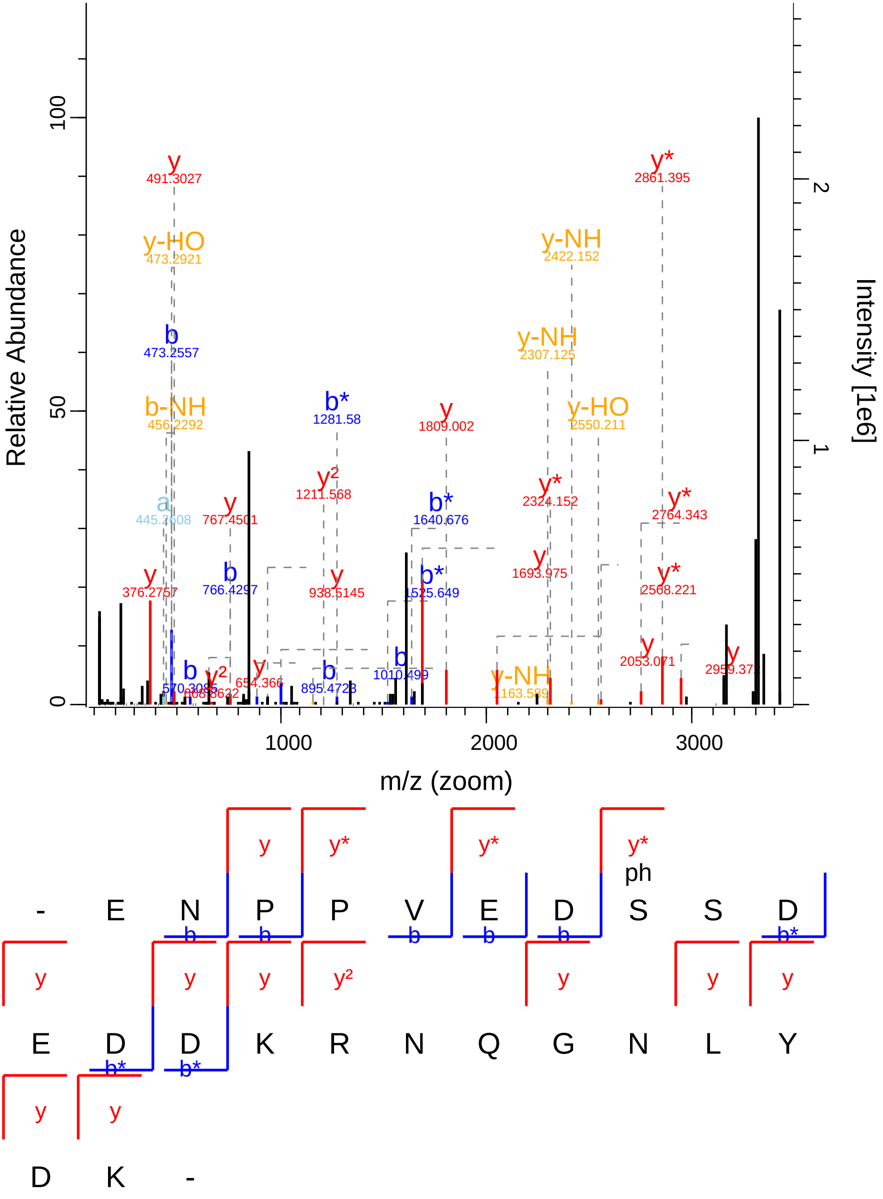

Raw File	Scan	Method	Score	m/z	Gene names
RO-QC-160121_ZackD_SS_Set_02_Fractior	16437	FTMS; HC	106.49	827.09	DDX21

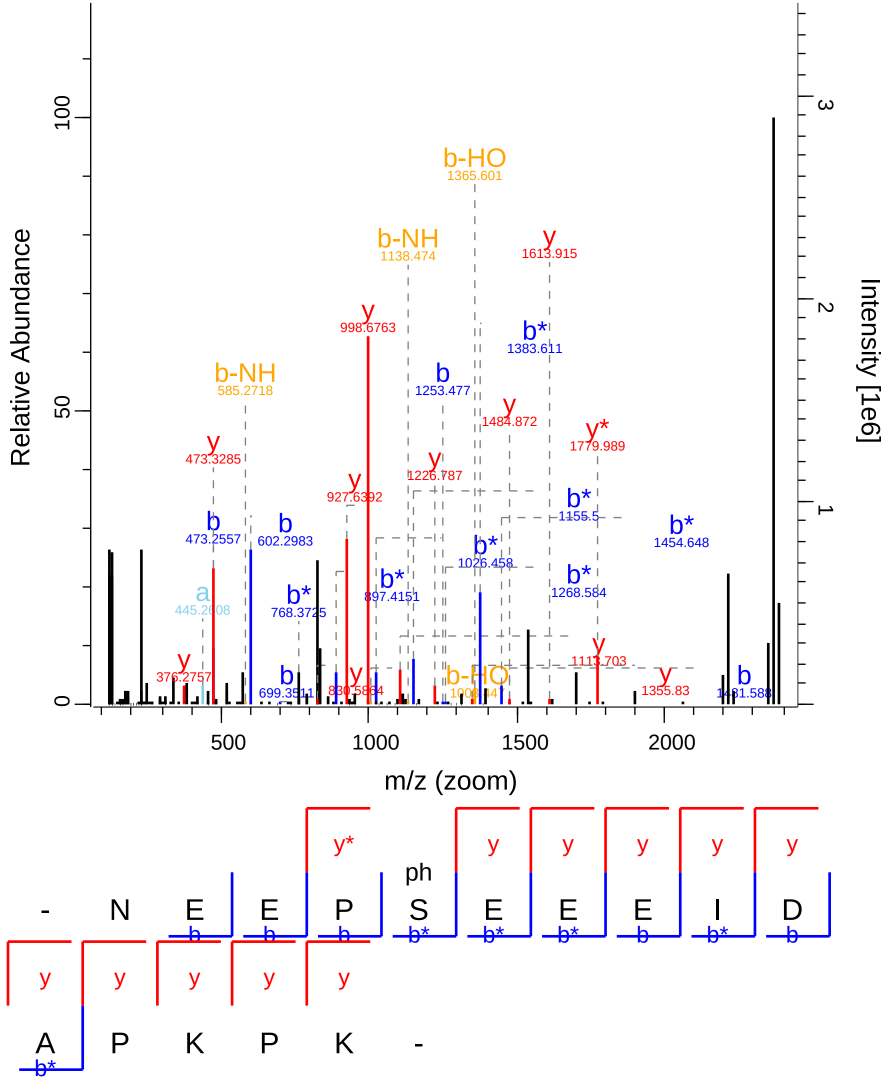

Raw File	Scan	Method	Score	m/z	Gene names
RO-QC-160121_ZackD_SS_Set_02_Fractior	17386	FTMS; HC	136.96	810.92	TLN2

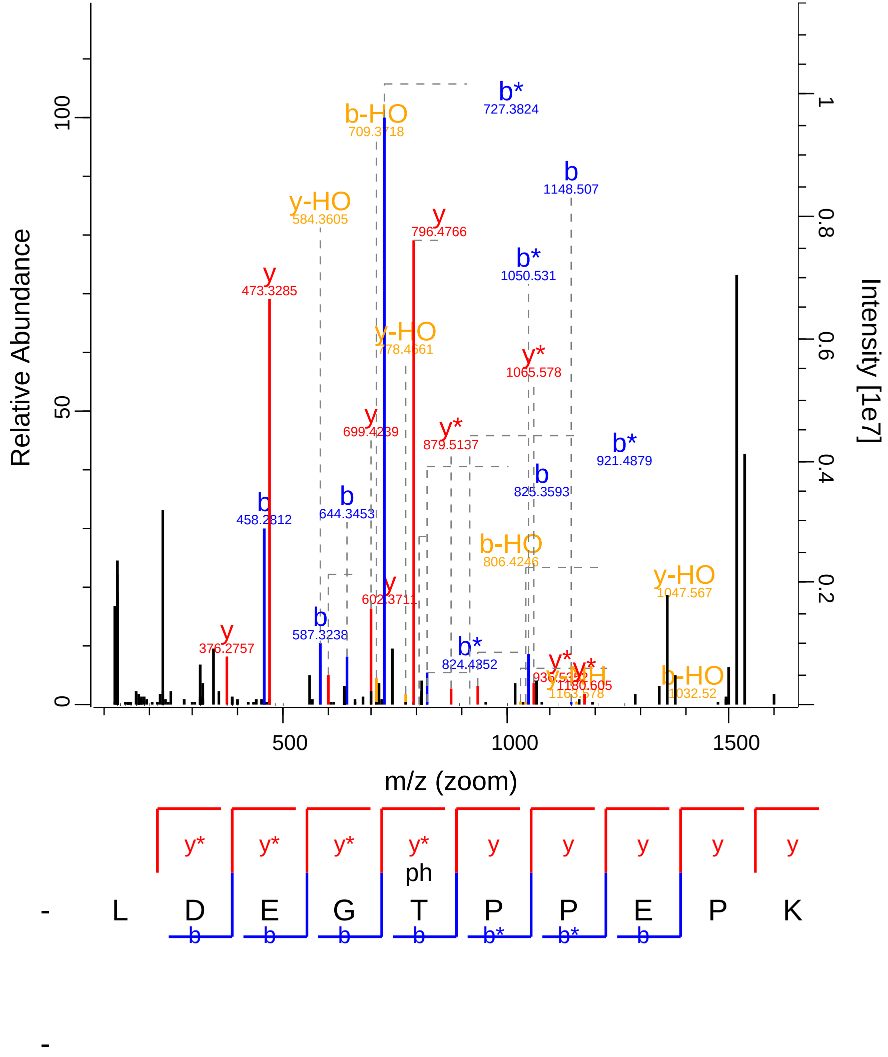

Raw File	Scan	Method	Score	m/z	Gene names
RO-QC-160121_ZackD_SS_Set_02_Fractior	18375	FTMS; HC	44.14	725.37	TTLL6

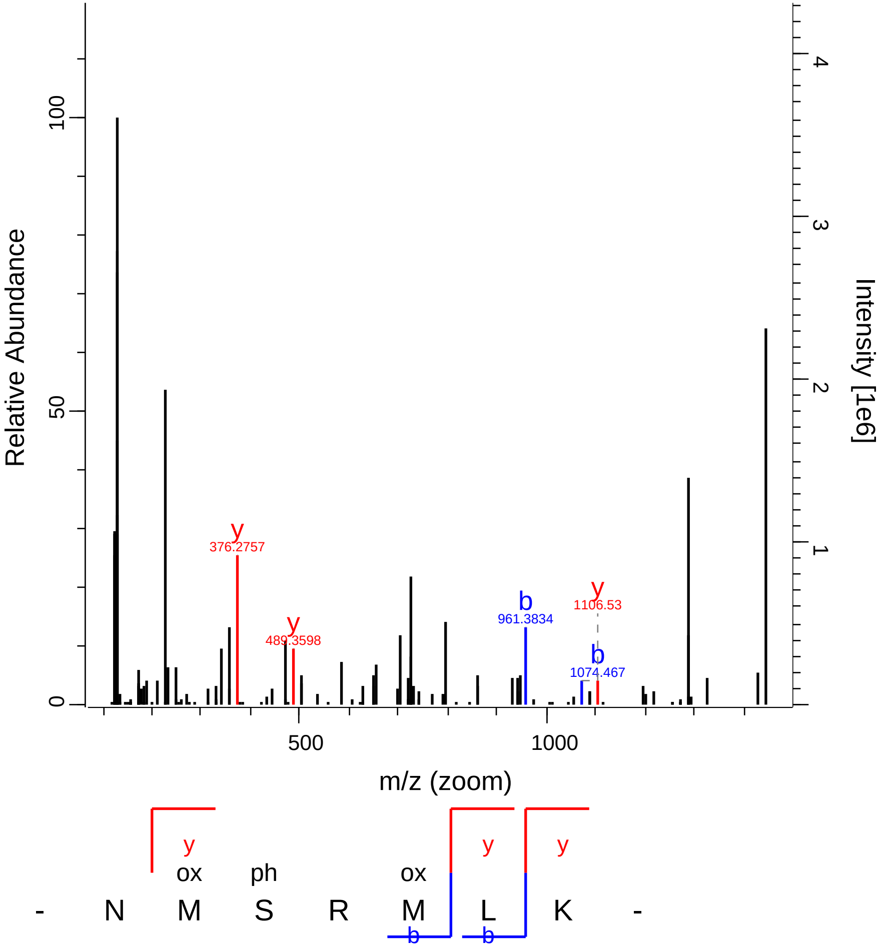

Raw File	Scan	Method	Score	m/z	Gene names
RO-QC-160121_ZackD_SS_Set_02_Fractio	18640	FTMS; HC	125.61	864.95	PALLD

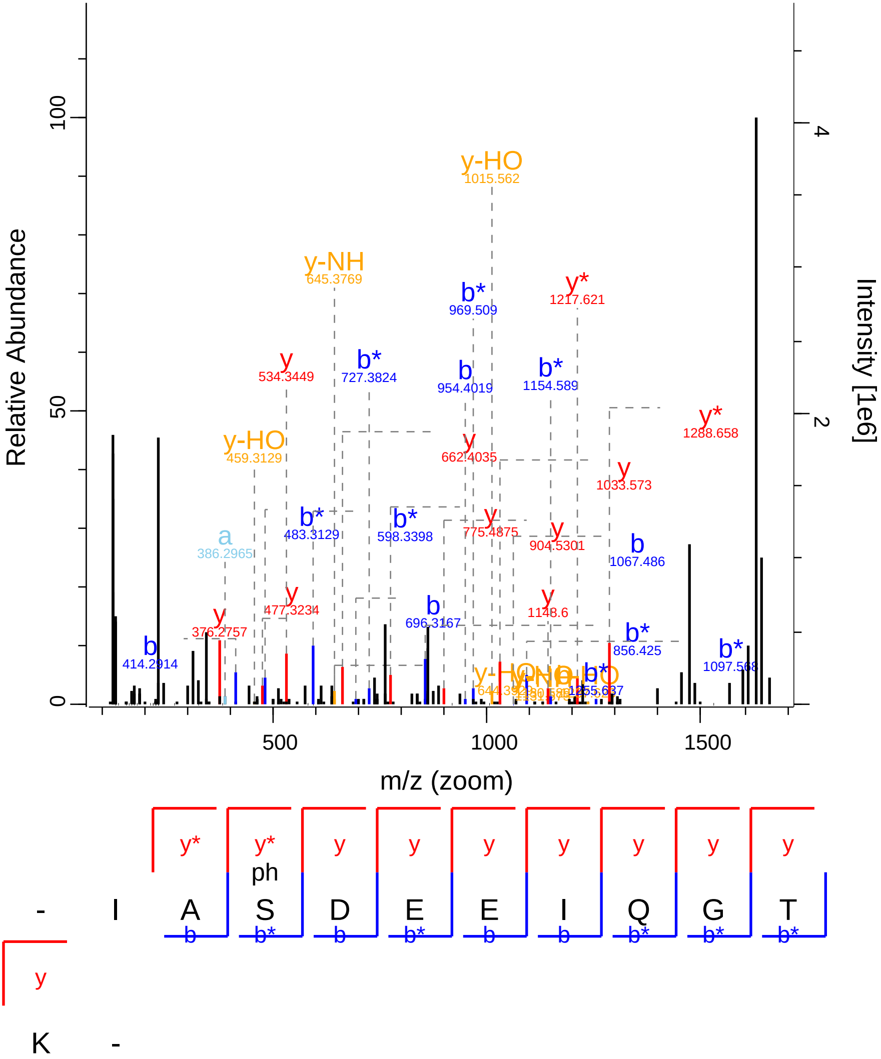

Raw File	Scan	Method	Score	m/z	Gene names
RO-QC-160121_ZackD_SS_Set_02_Fractior	19156	FTMS; HC	42.82	746.39	ZMYND8

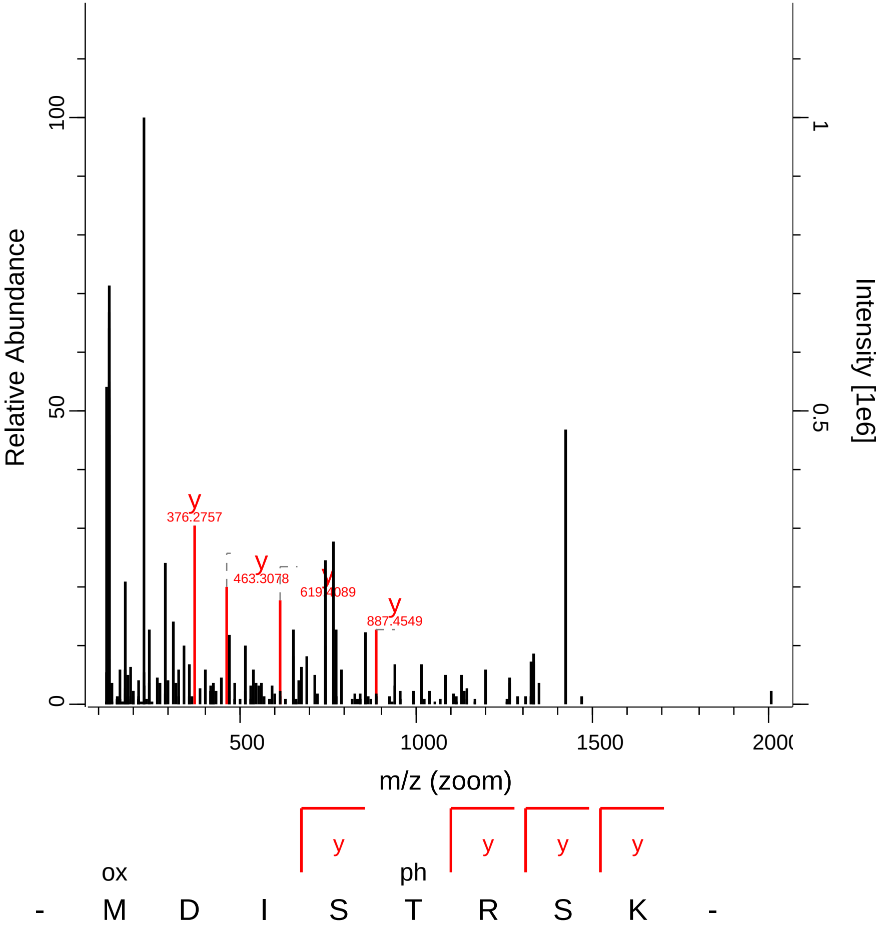

Raw File	Scan	Method	Score	m/z	Gene names
RO-QC-160121_ZackD_SS_Set_02_Fractior	19876	FTMS; HC	68.92	888.48	G3BP2

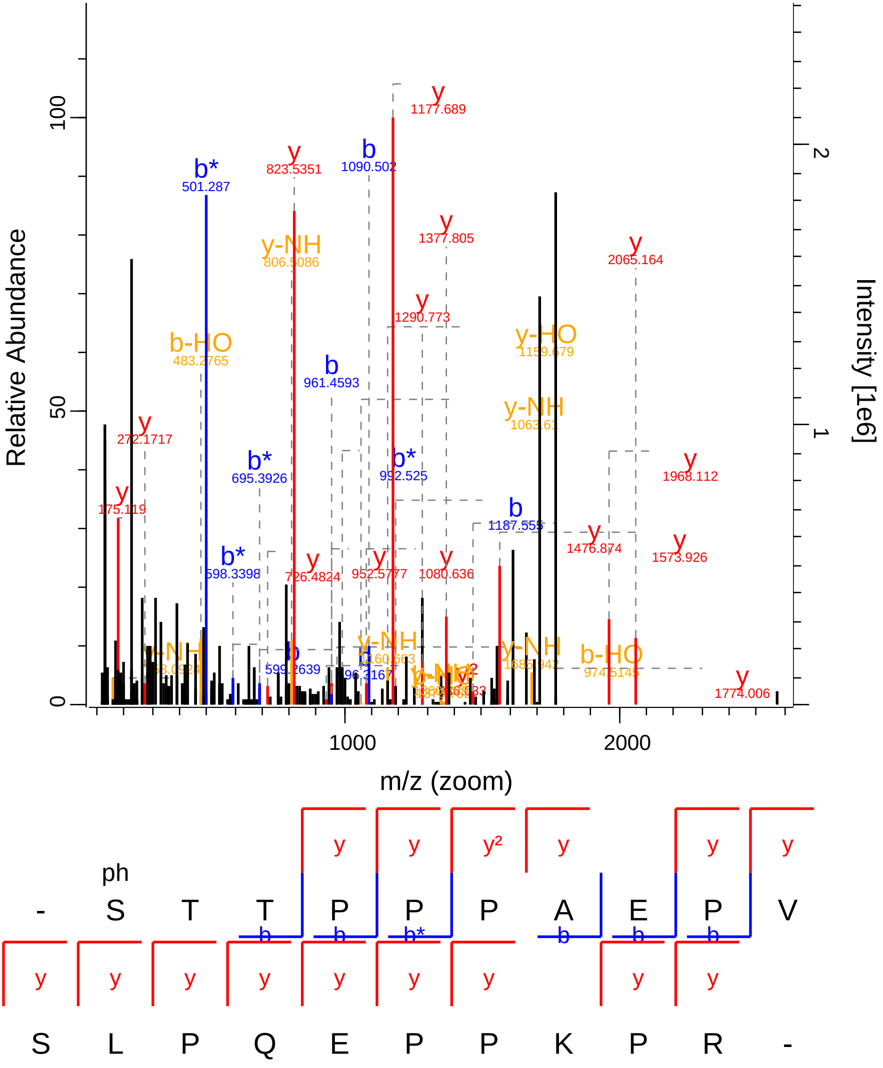

Raw File	Scan	Method	Score	m/z	Gene names
RO-QC-160121_ZackD_SS_Set_02_Fractior	20247	FTMS; HC	64.09	964.47	C3orf49

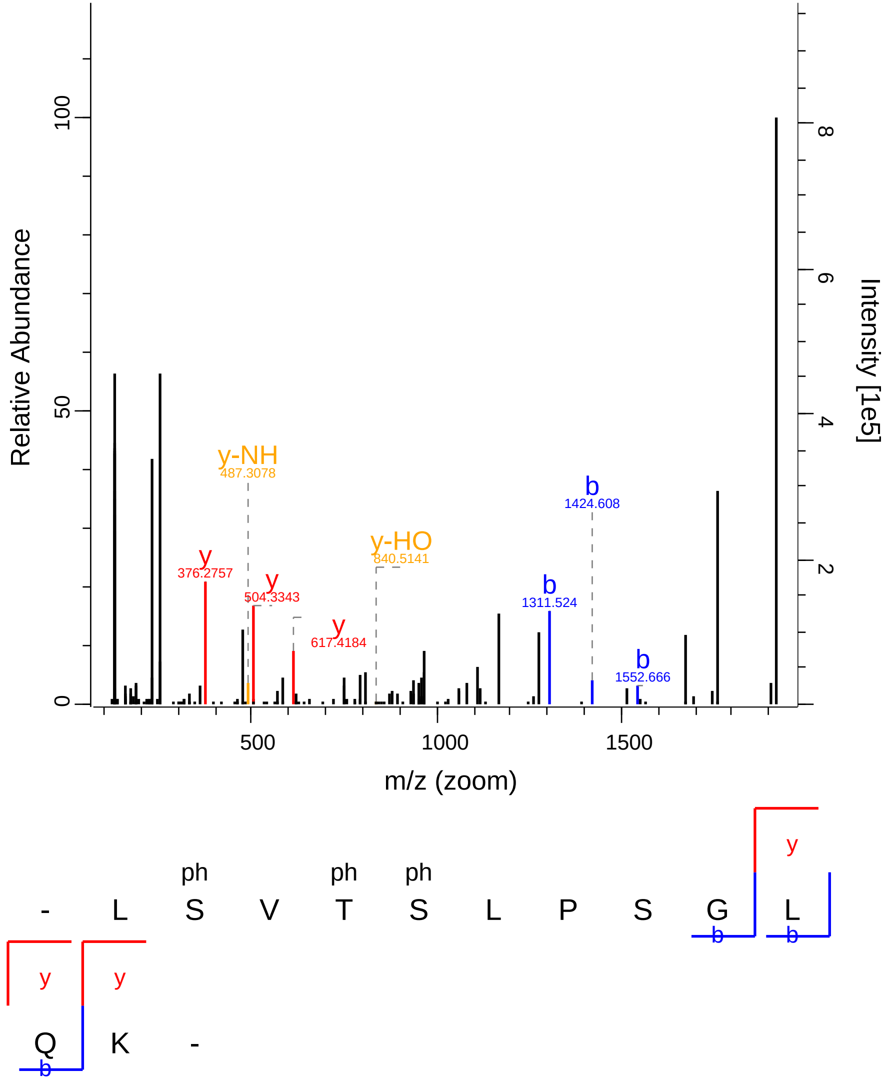

Raw File	Scan	Method	Score	m/z	Gene names
RO-QC-160121_ZackD_SS_Set_02_Fractior	20928	FTMS; HC	65.23	540.31	SAFB

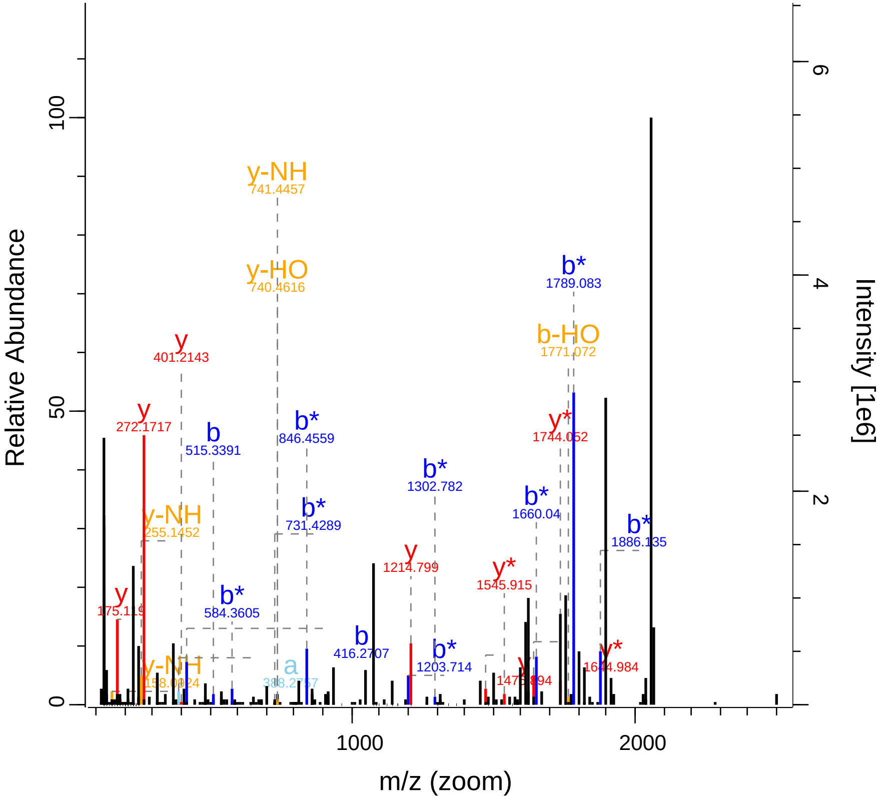

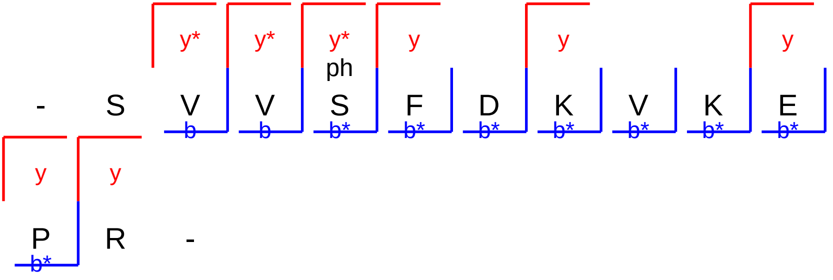

Raw File	Scan	Method	Score	m/z	Gene names
RO-QC-160121_ZackD_SS_Set_02_Fractioı	21682	FTMS; HC	236.61	955.46	NCAPG

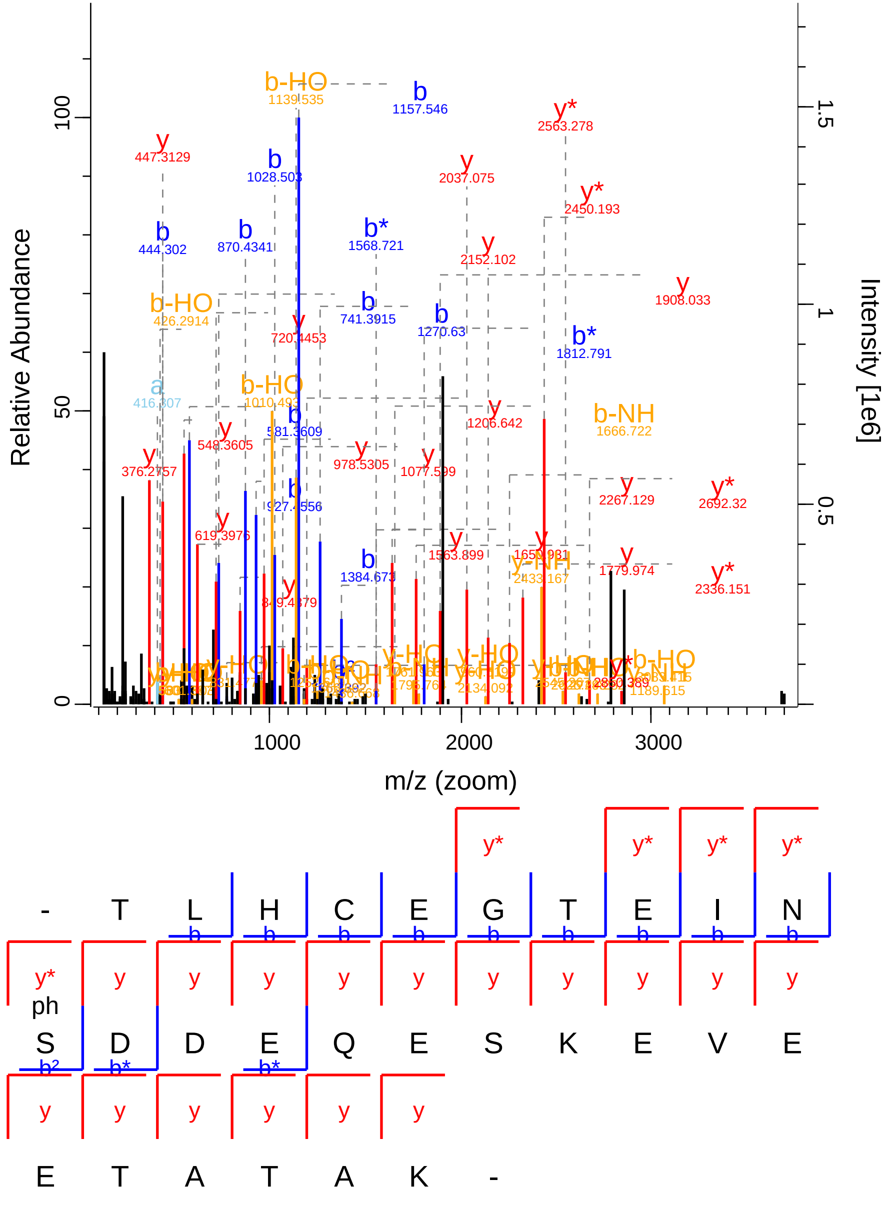

Raw File	Scan	Method	Score	m/z	Gene names
RO-QC-160121_ZackD_SS_Set_02_Fractioı	21725	FTMS; HC	220.55	907.94	ZRANB2

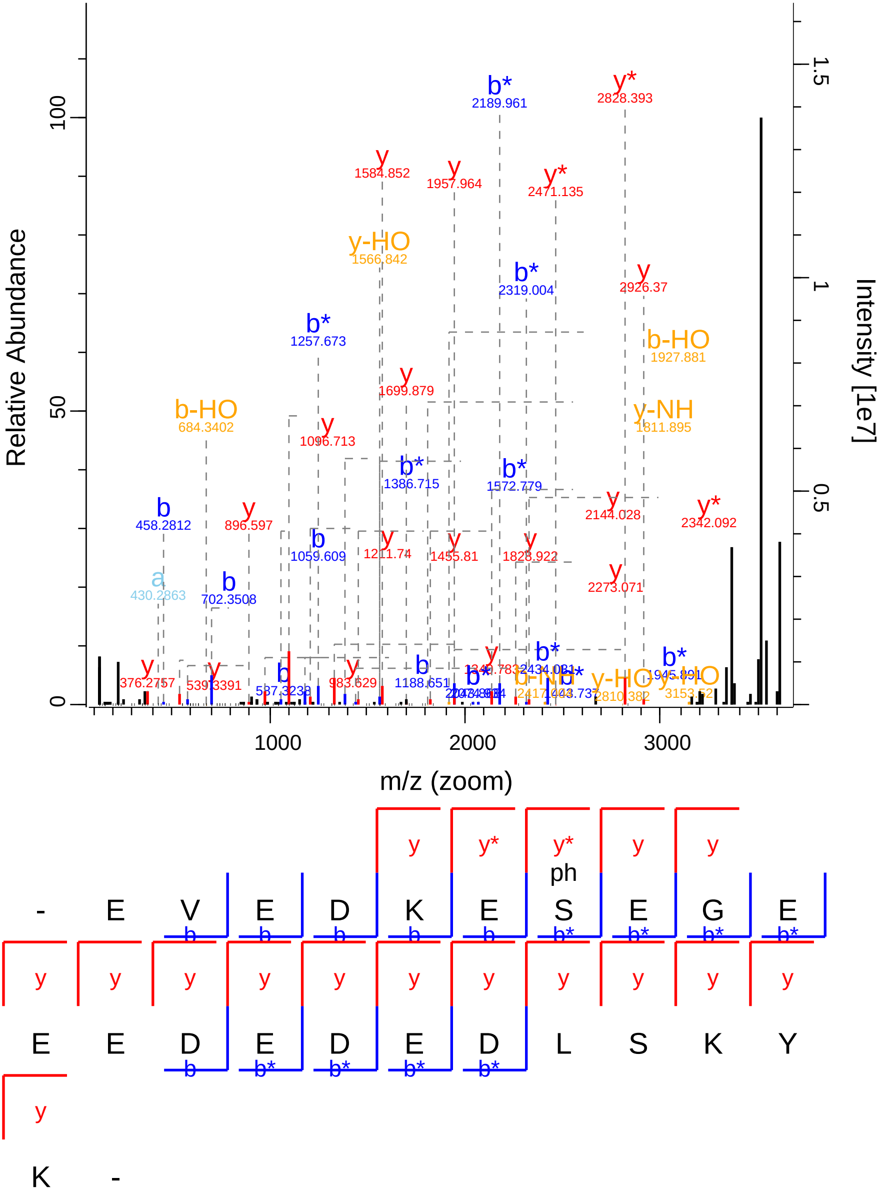

Raw File	Scan	Method	Score	m/z	Gene names
RO-QC-160121_ZackD_SS_Set_02_Fractior	21730	FTMS; HC	136.53	967.46	SRRM2

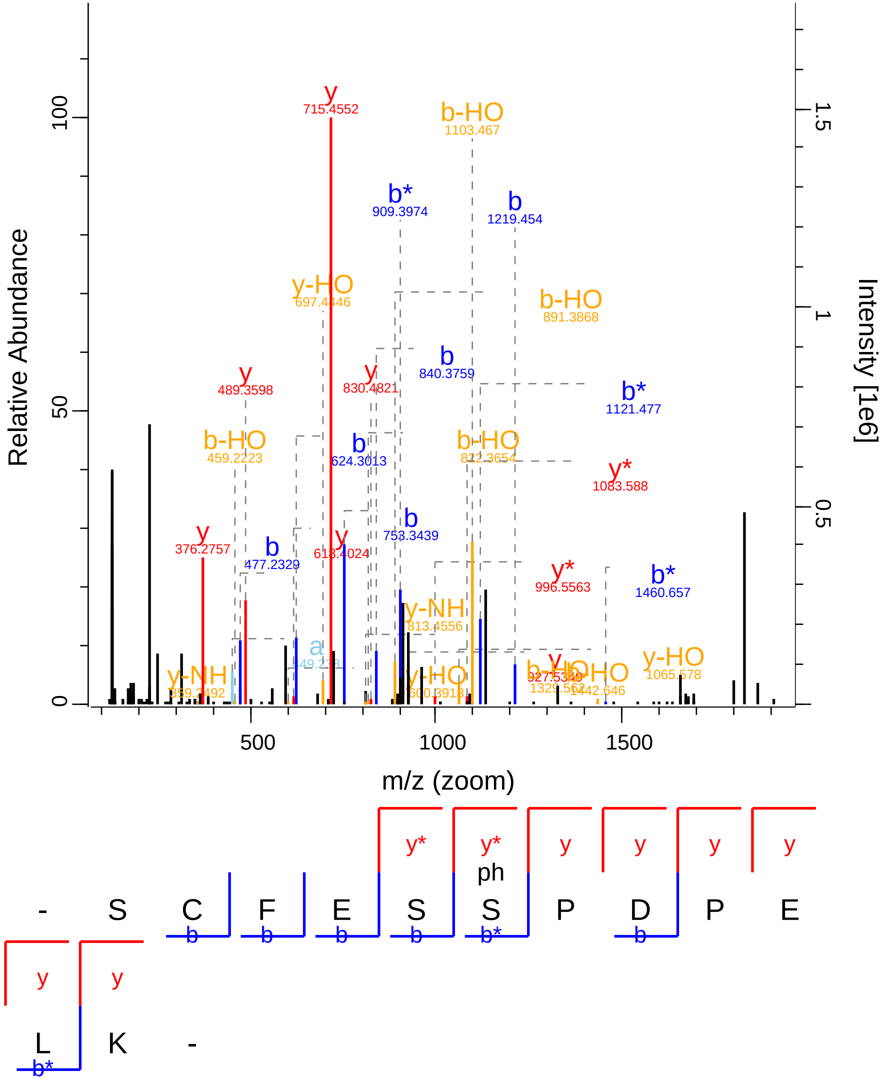

Raw File	Scan	Method	Score	m/z	Gene names
RO-QC-160121_ZackD_SS_Set_02_Fractio[illegible]	22379	FTMS; HC	103.63	651.16	CD44

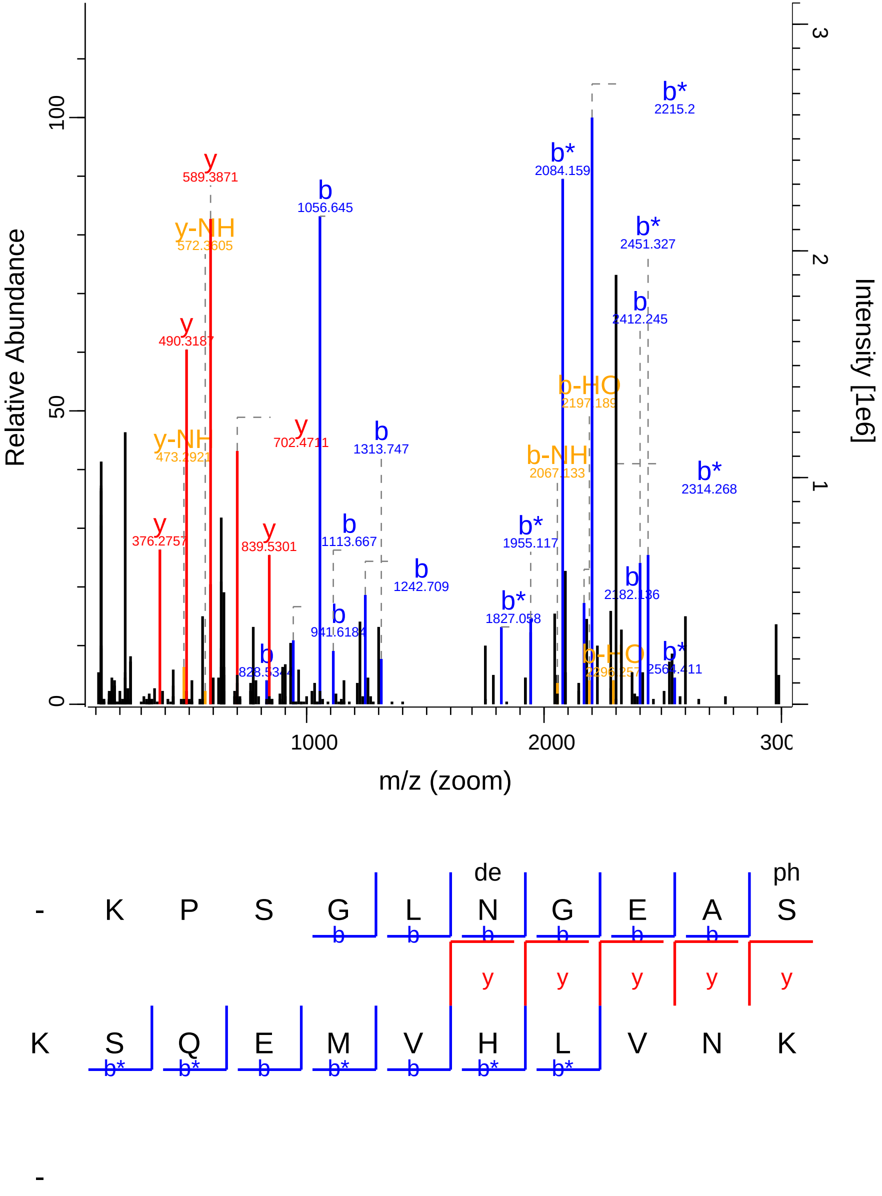

Raw File	Scan	Method	Score	m/z	Gene names
RO-QC-160121_ZackD_SS_Set_02_Fractior	22431	FTMS; HC	79.97	854.44	RABGAP1

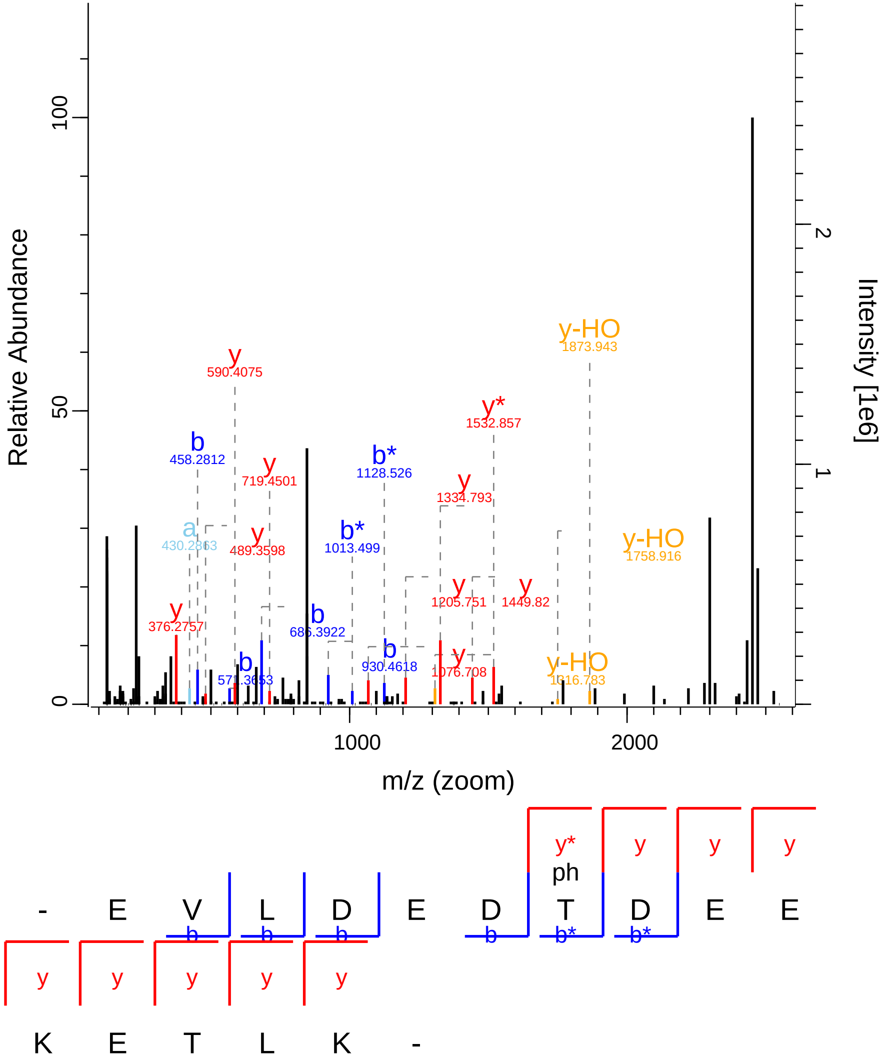

Raw File	Scan	Method	Score	m/z	Gene names
RO-QC-160121_ZackD_SS_Set_02_Fractior	22718	FTMS; HC	56.53	851.71	PRDX1

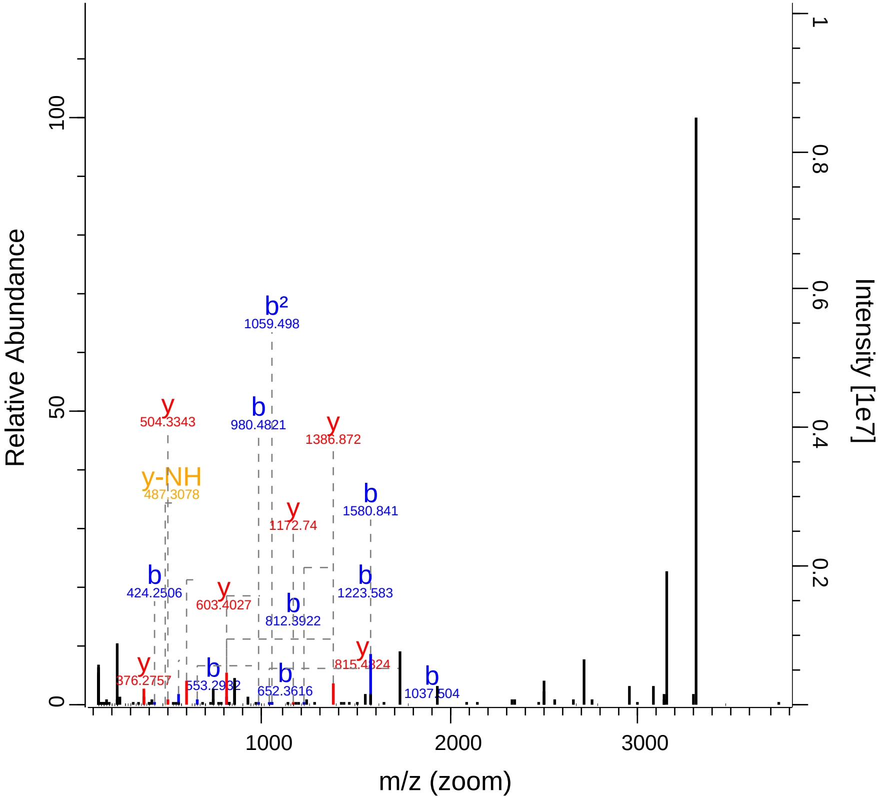

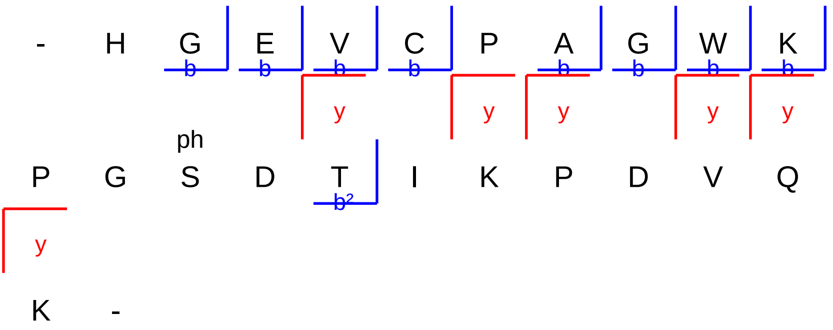

Raw File	Scan	Method	Score	m/z	Gene names
RO-QC-160121_ZackD_SS_Set_02_Fractior	23474	FTMS; HC	75.61	828.06	MAPK3

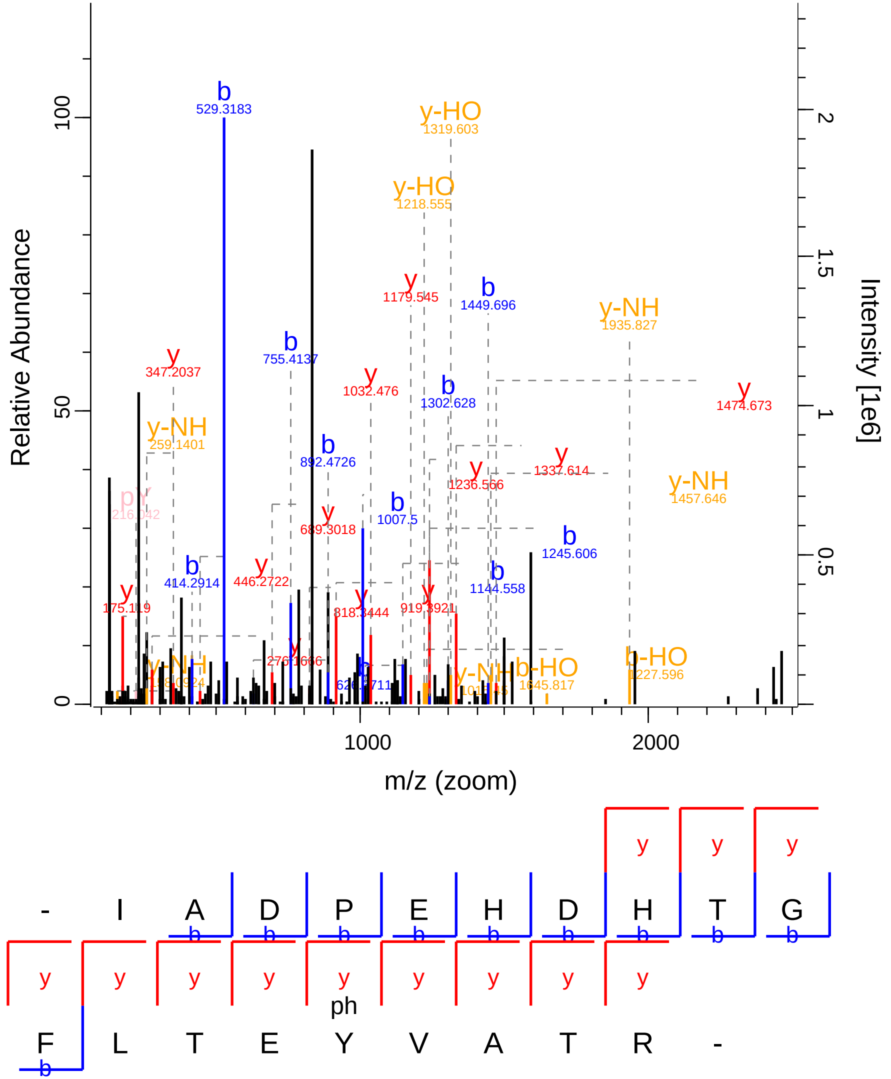

Raw File	Scan	Method	Score	m/z	Gene names
RO-QC-160121_ZackD_SS_Set_02_Fractior	23476	FTMS; HC	91.94	936.97	SAFB;SAFB2

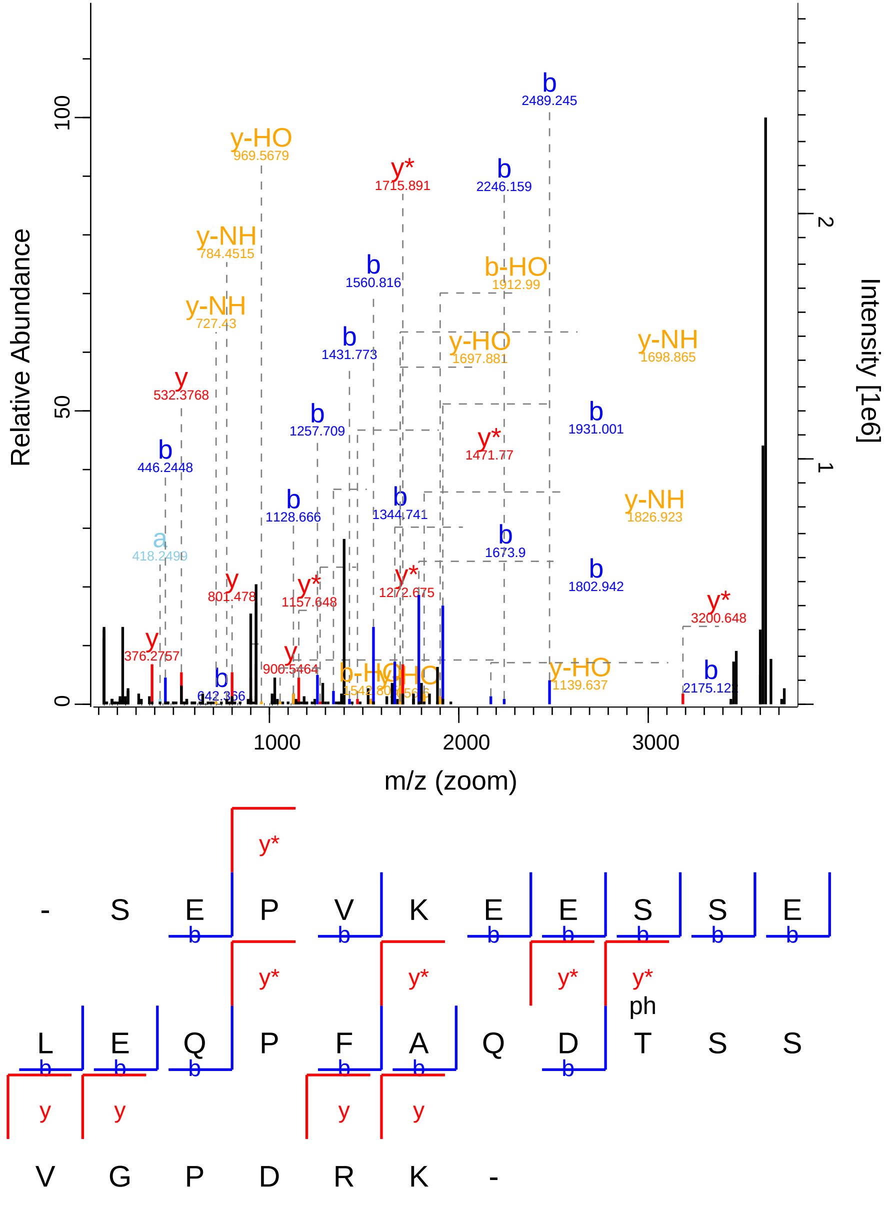

Raw File	Scan	Method	Score	m/z	Gene names
RO-QC-160121_ZackD_SS_Set_02_Fractior	23940	FTMS; HC	116.39	893.78	NEXN

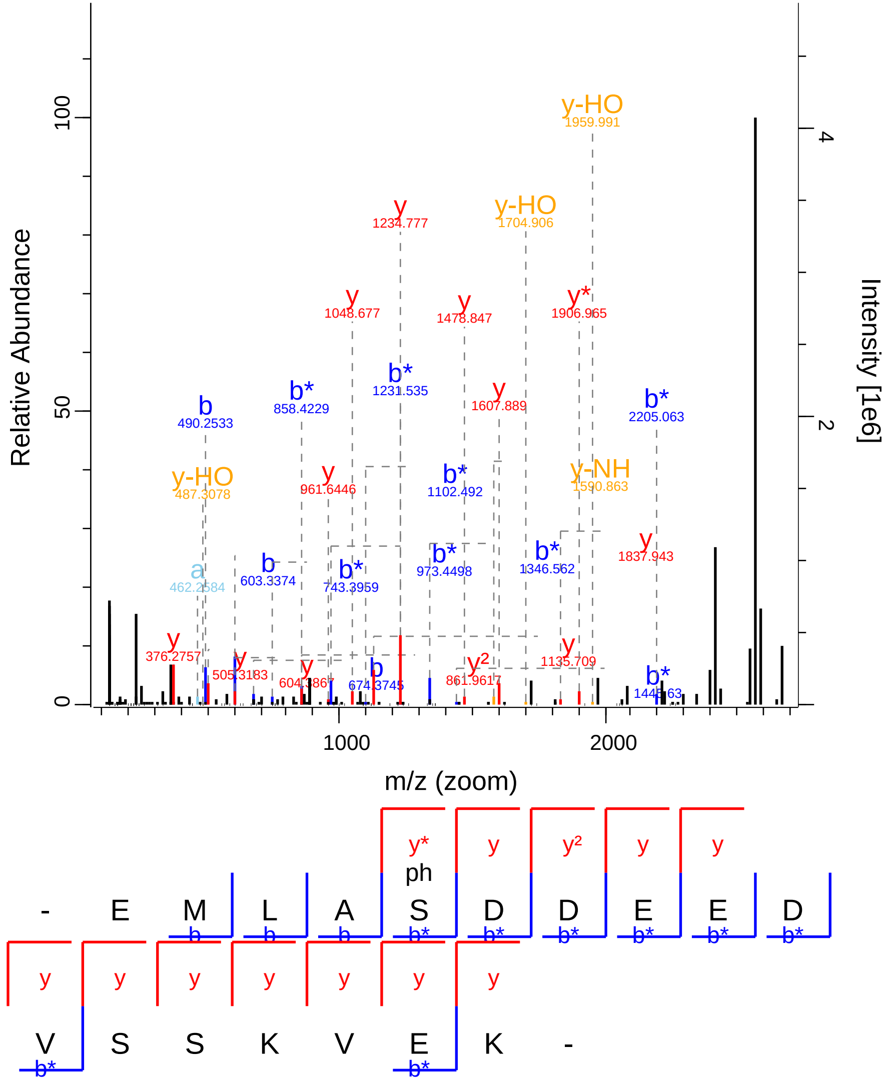

Raw File	Scan	Method	Score	m/z	Gene names
RO-QC-160121_ZackD_SS_Set_02_Fractio	24538	FTMS; HC	191.78	1057.51	CHCHD3

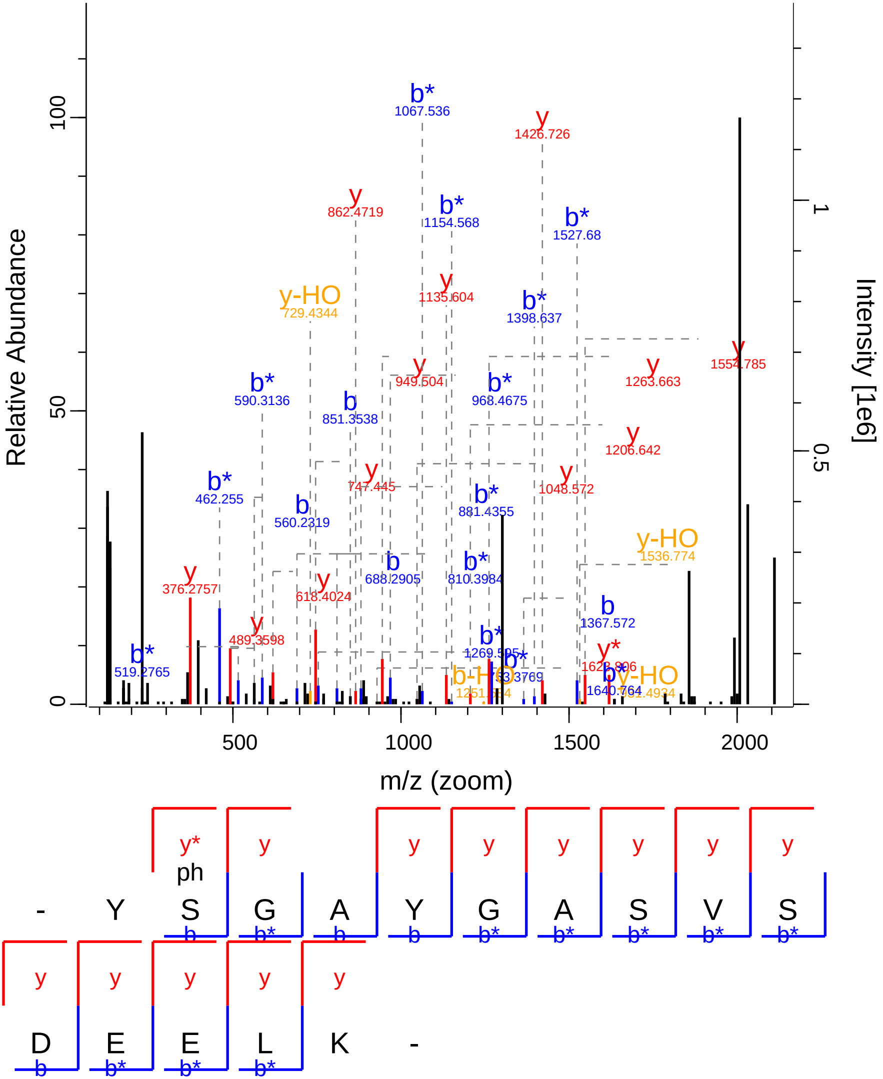

Raw File	Scan	Method	Score	m/z	Gene names
RO-QC-160121_ZackD_SS_Set_02_Fractio	24845	FTMS; HC	164.02	1157.56	AKAP2

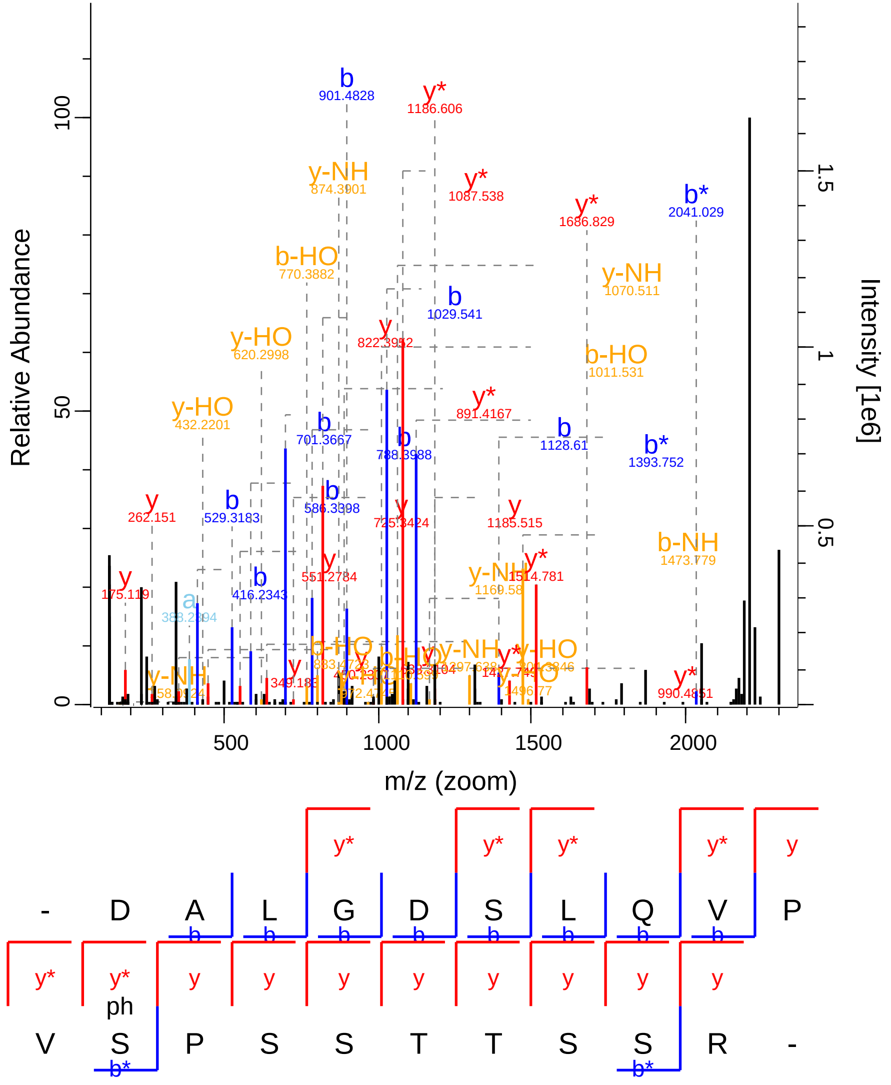

Raw File	Scan	Method	Score	m/z	Gene names
RO-QC-160121_ZackD_SS_Set_02_Fractio	24942	FTMS; HC	103.31	1006.79	CALU

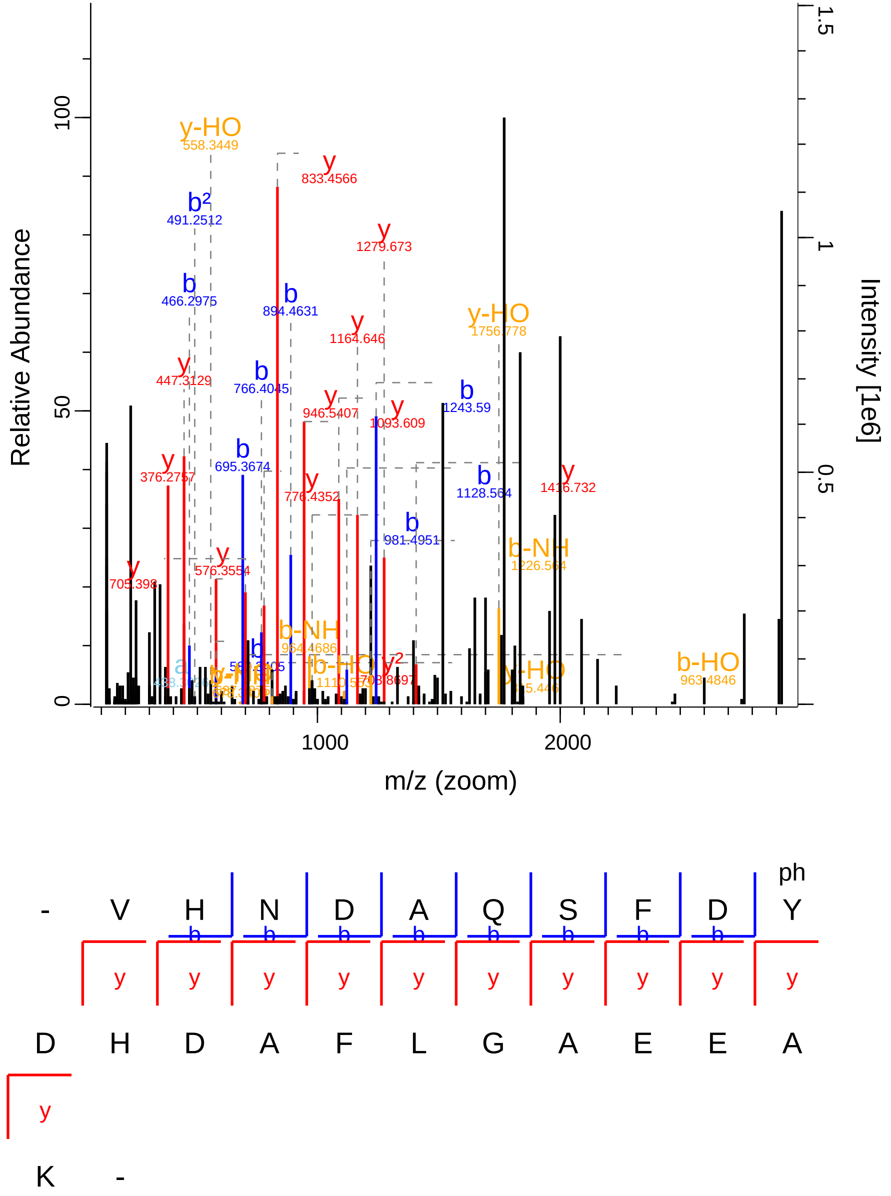

Raw File	Scan	Method	Score	m/z	Gene names
RO-QC-160121_ZackD_SS_Set_02_Fractio	25051	FTMS; HC	113.59	1509.69	CALU

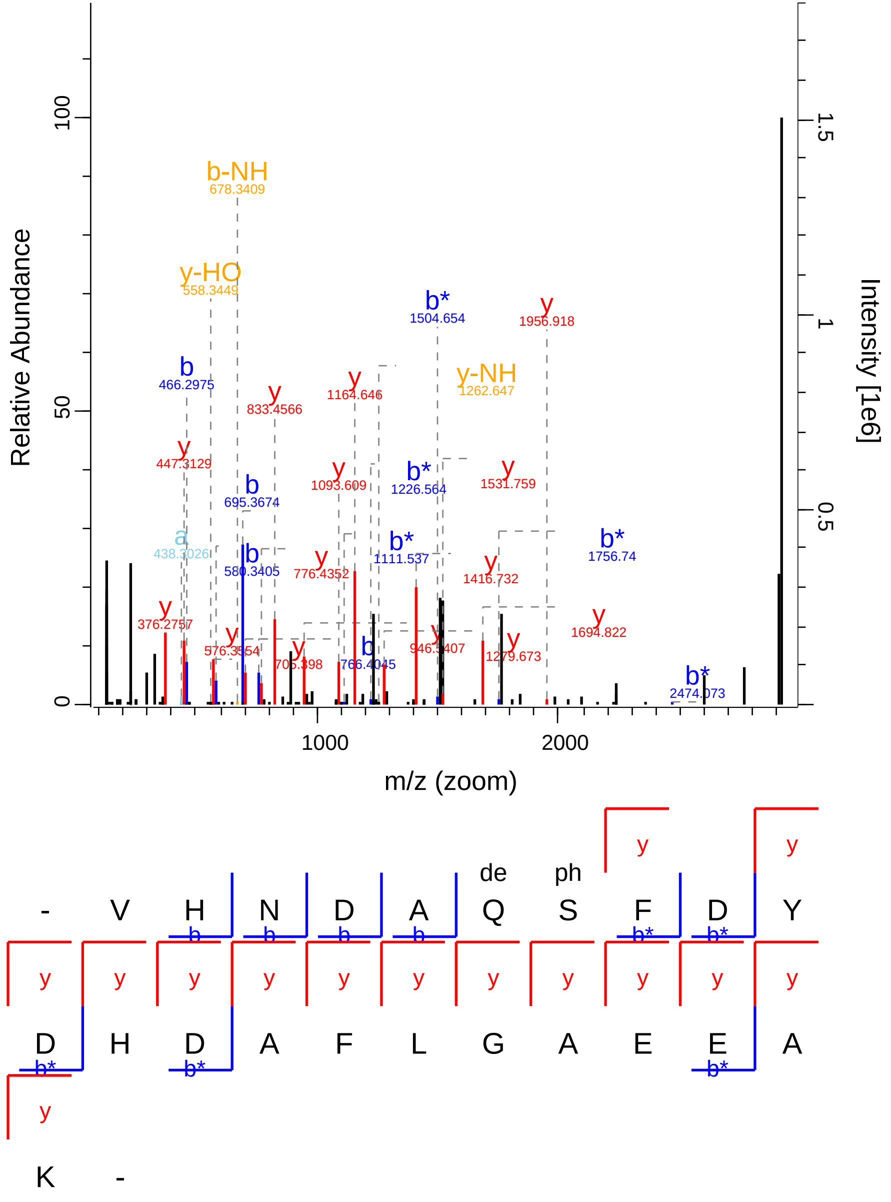

Raw File	Scan	Method	Score	m/z	Gene names
RO-QC-160121_ZackD_SS_Set_02_Fractio	25371	FTMS; HC	284.88	1274.02	NUCKS1

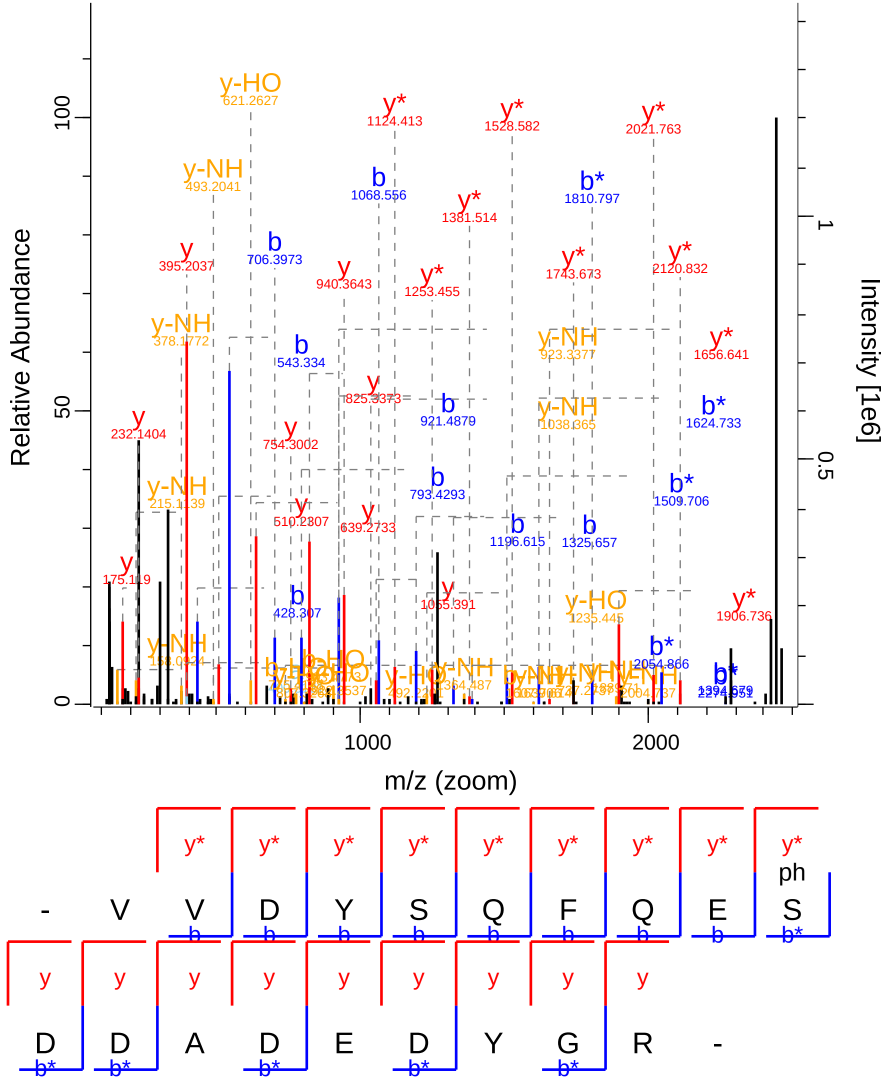

Raw File	Scan	Method	Score	m/z	Gene names
RO-QC-160121_ZackD_SS_Set_02_Fractioı	26114	FTMS; HC	105.49	1129.2	SF1

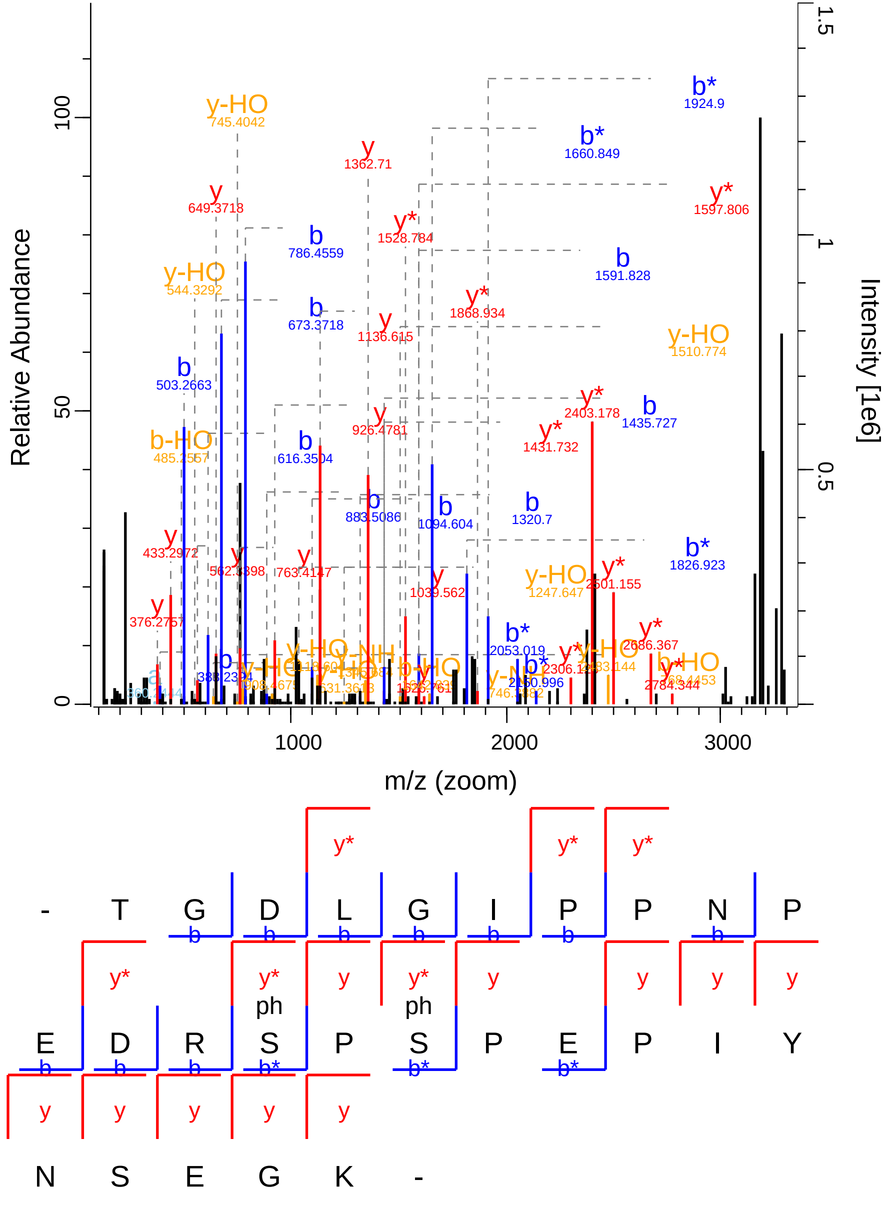

Raw File	Scan	Method	Score	m/z	Gene names
RO-QC-160121_ZackD_SS_Set_02_Fractio	26624	FTMS; HC	41.91	735.16	PLXNA4;PLXNA

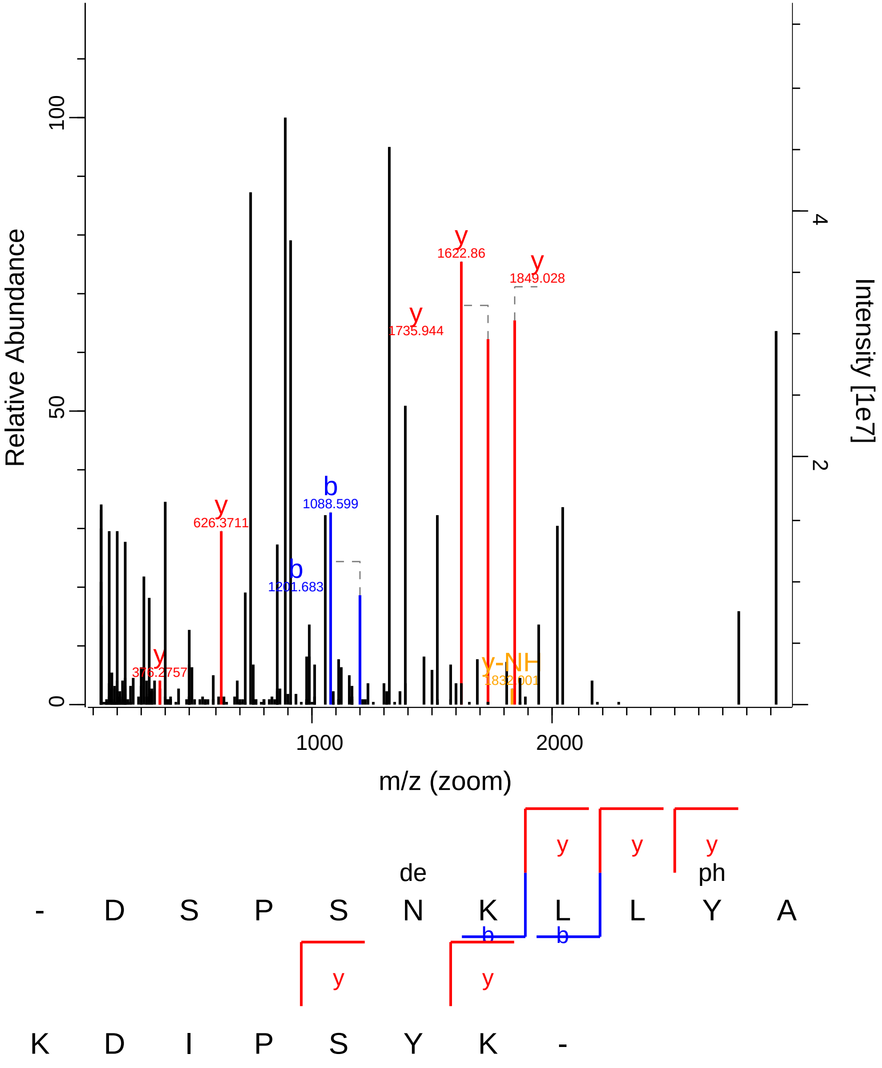

Raw File	Scan	Method	Score	m/z	Gene names
RO-QC-160121_ZackD_SS_Set_02_Fractio	27036	FTMS; HC	161.19	1116.53	SNTB1

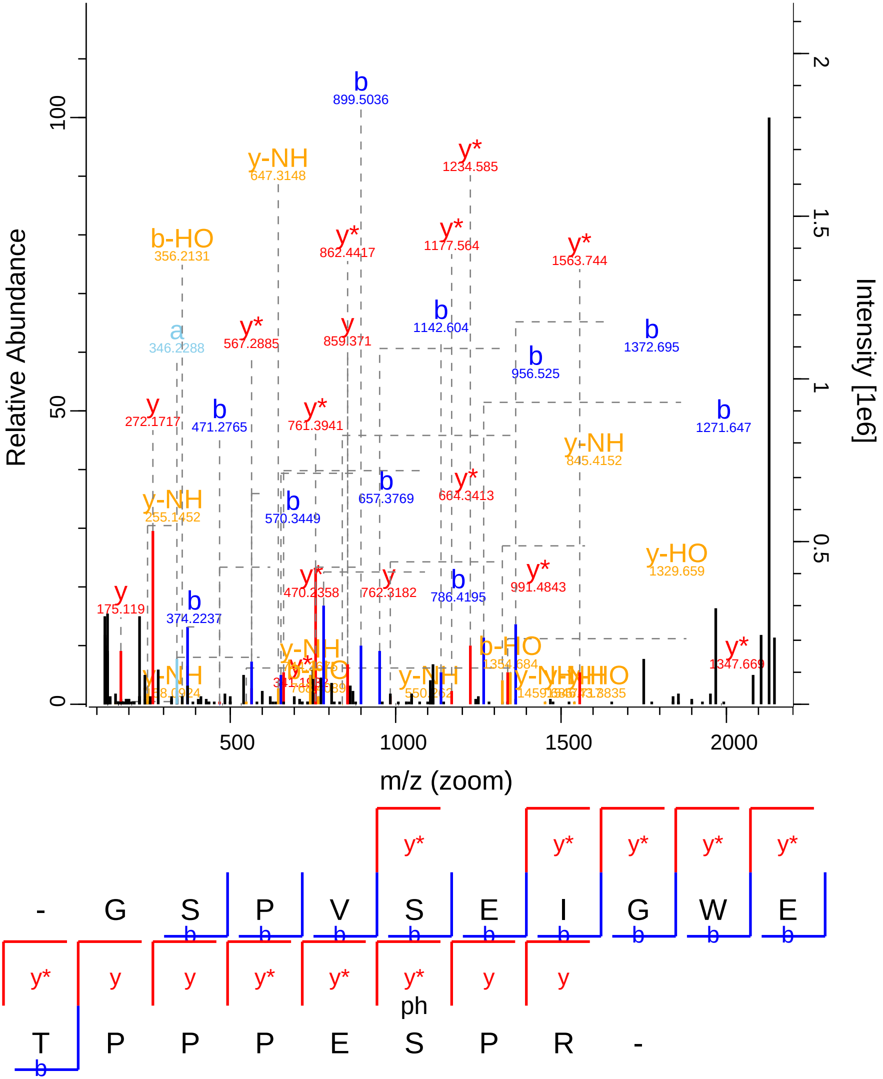

Raw File	Scan	Method	Score	m/z	Gene names
RO-QC-160121_ZackD_SS_Set_02_Fractior	27102	FTMS; HC	79.03	708.89	SLC6A15

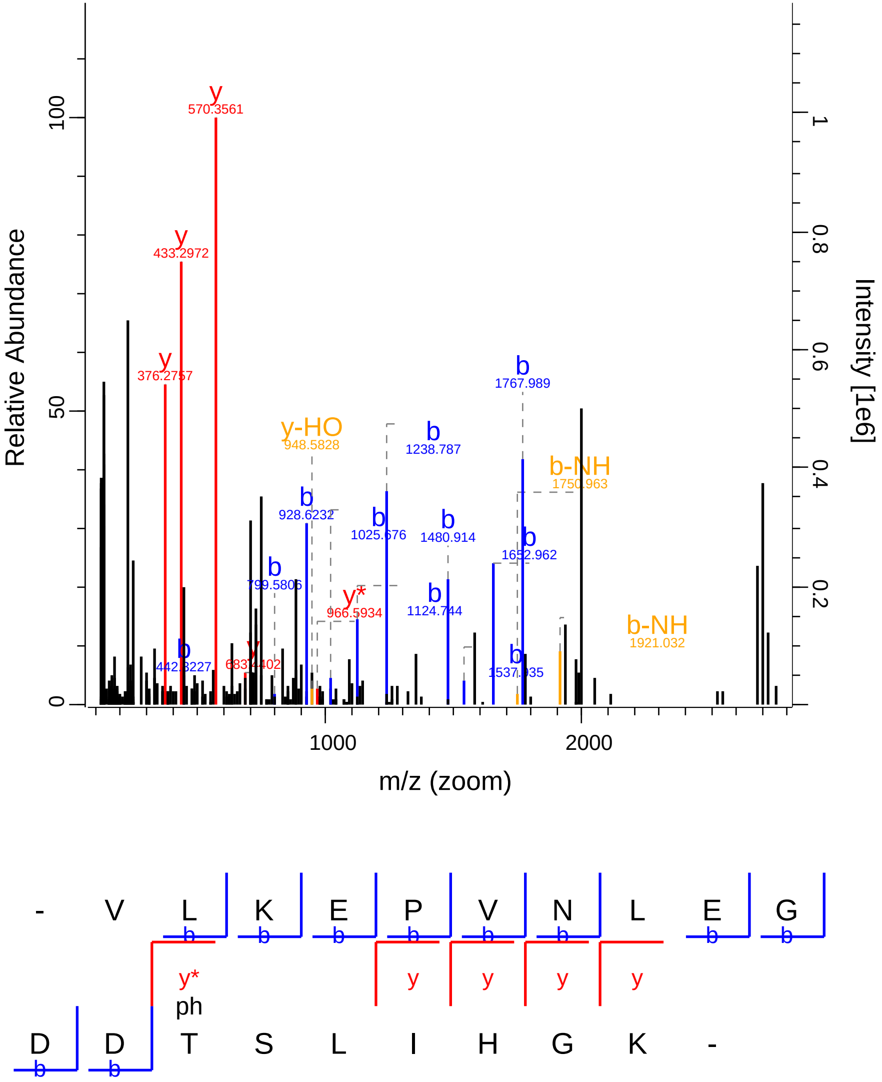

Raw File	Scan	Method	Score	m/z	Gene names
RO-QC-160121_ZackD_SS_Set_02_Fractior	27329	FTMS; HC	59.61	673.04	AKAP12

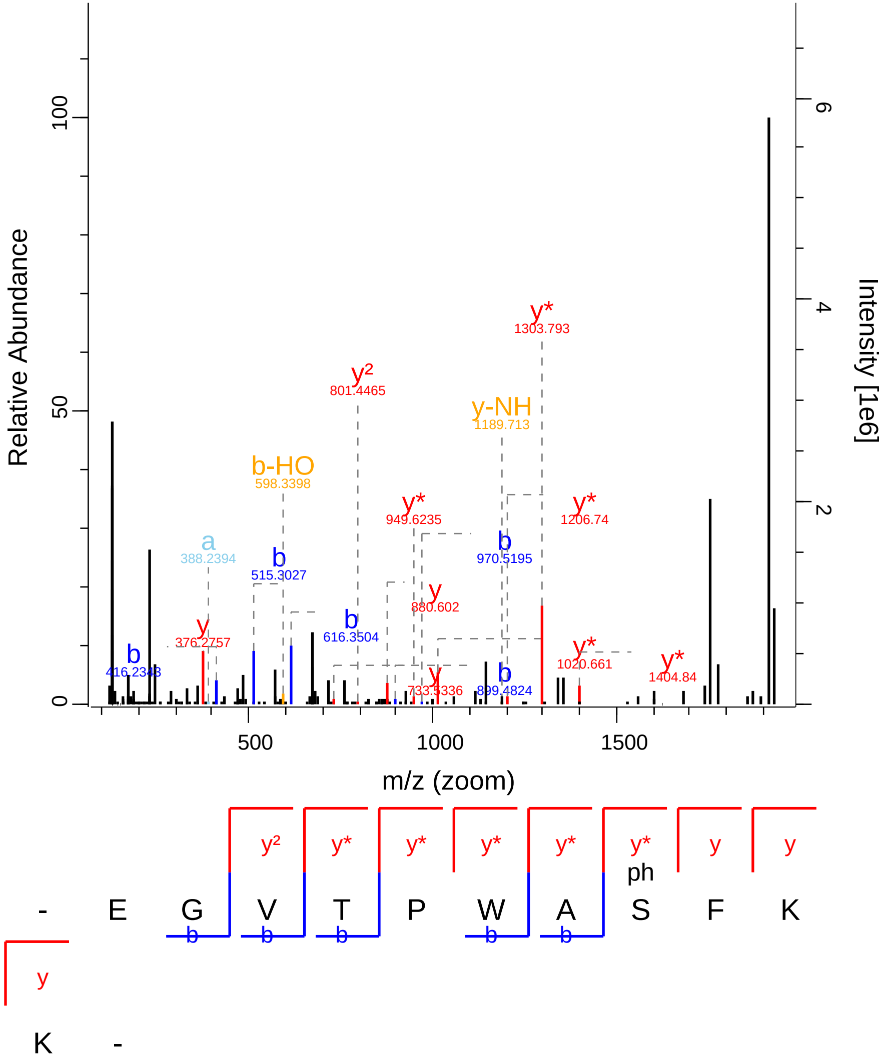

Raw File	Scan	Method	Score	m/z	Gene names
RO-QC-160121_ZackD_SS_Set_02_Fractior	27517	FTMS; HC	67.24	738.66	RSF1

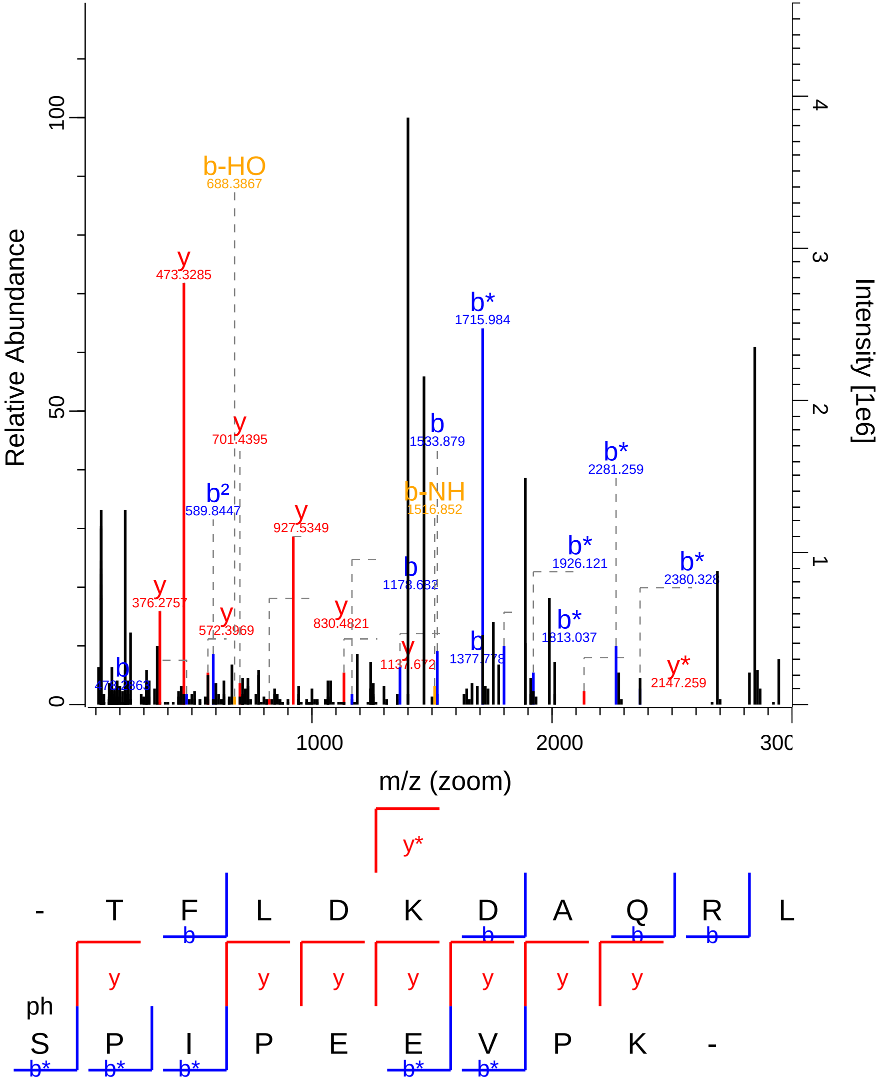

Raw File	Scan	Method	Score	m/z	Gene names
RO-QC-160121_ZackD_SS_Set_02_Fractior	27952	FTMS; HC	195.54	968.96	HTATSF1

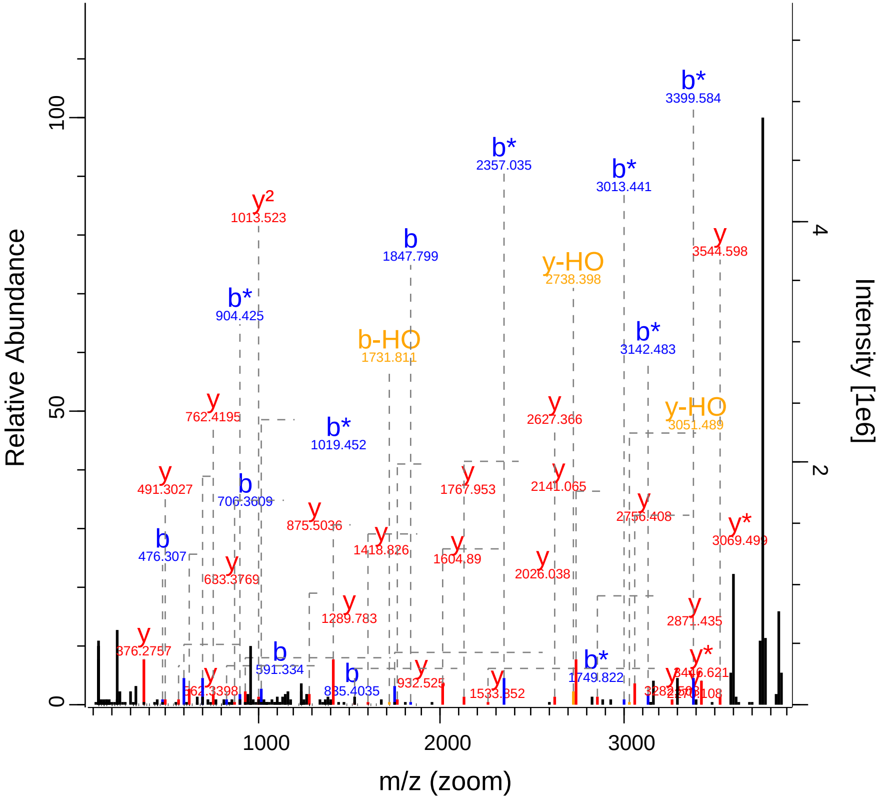

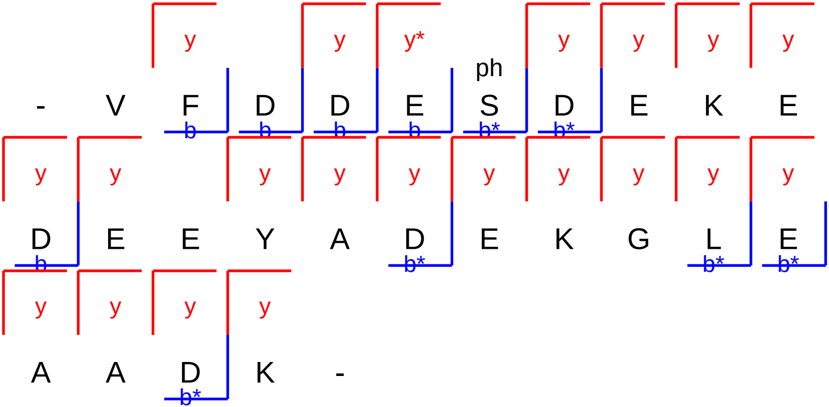

Raw File	Scan	Method	Score	m/z	Gene names
RO-QC-160121_ZackD_SS_Set_02_Fractio	28589	FTMS; HC	58.16	1020.52	LIAT1

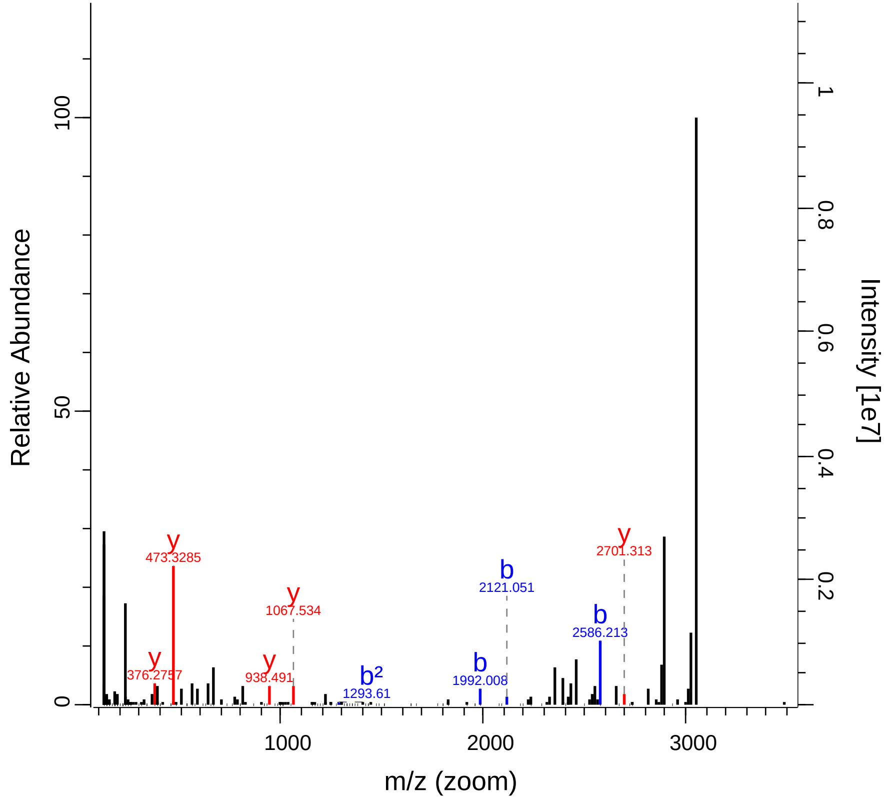

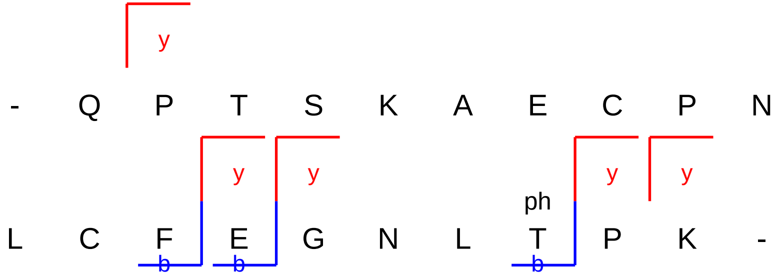

Raw File	Scan	Method	Score	m/z	Gene names
RO-QC-160121_ZackD_SS_Set_02_Fractio	29411	FTMS; HC	42.35	1085.25	NAP1L4

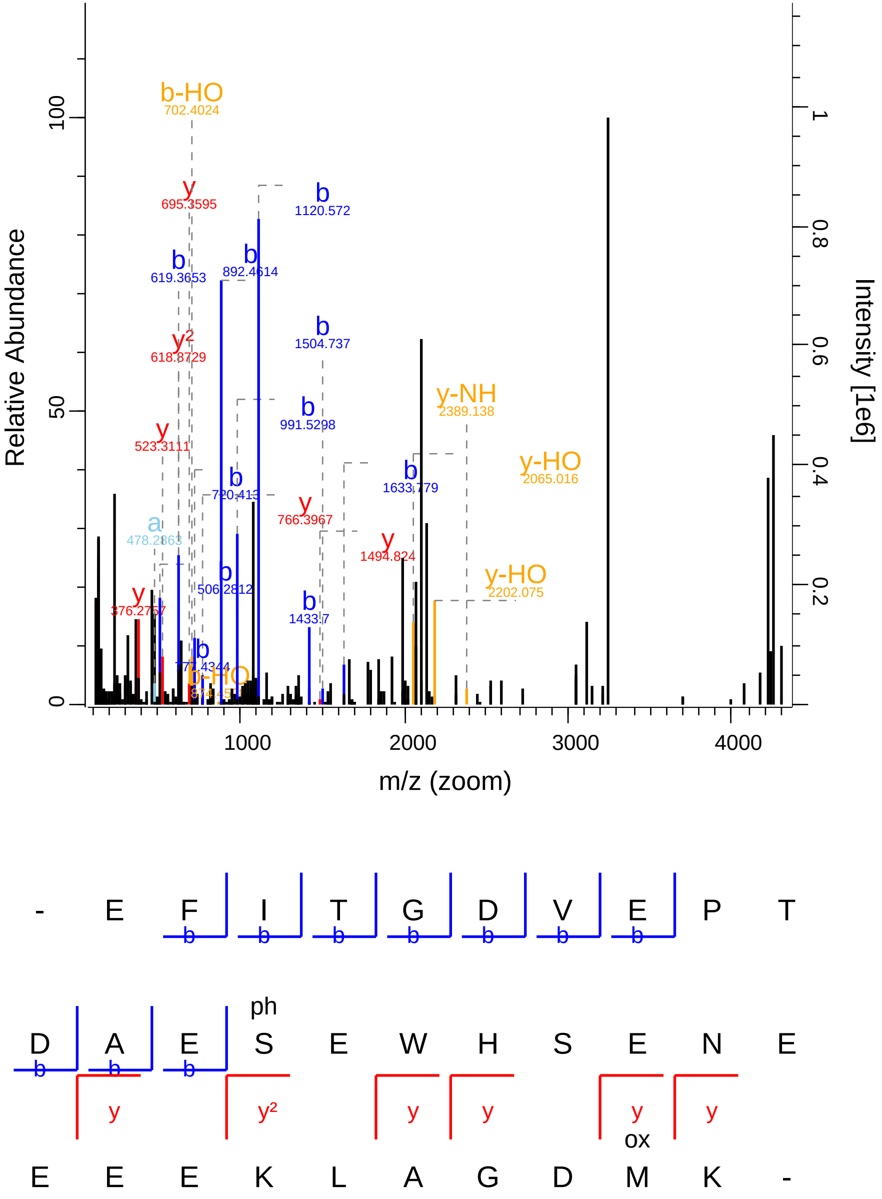

Raw File	Scan	Method	Score	m/z	Gene names
RO-QC-160121_ZackD_SS_Set_02_Fraction	30781	FTMS; HC	54.81	758.4	EFCAB10

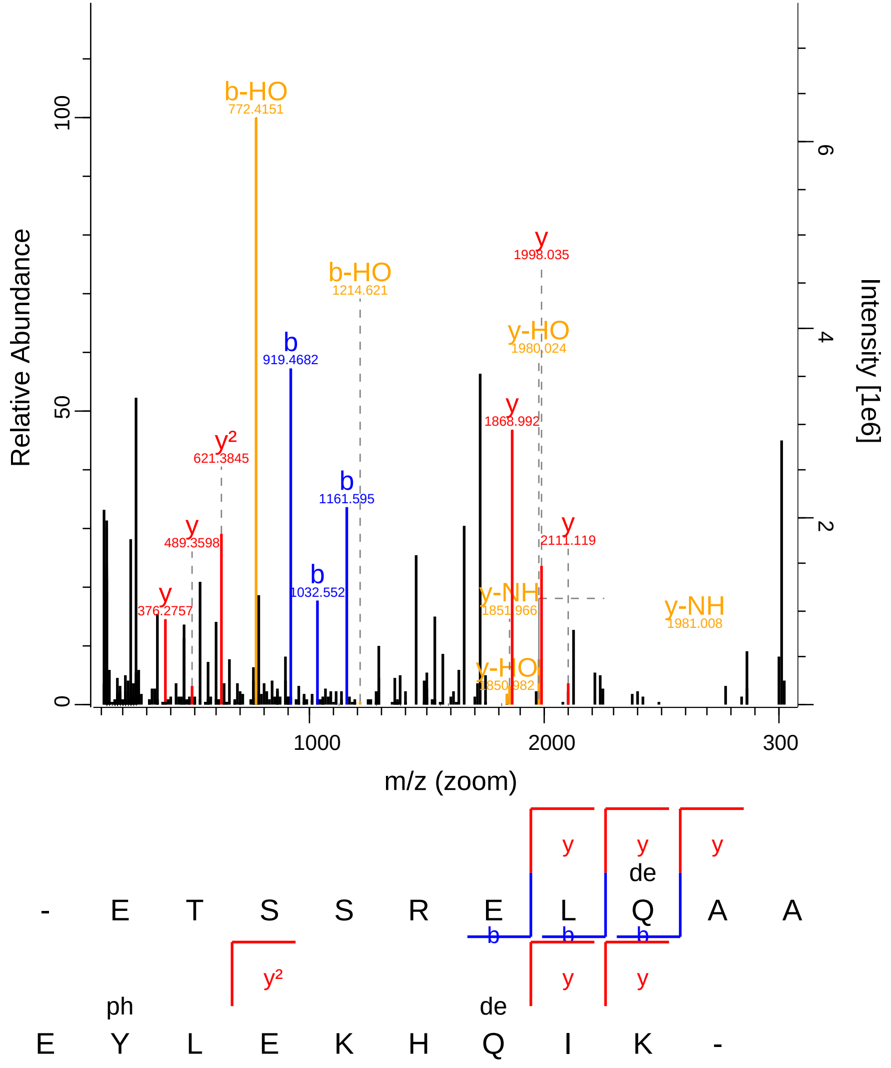

Raw File	Scan	Method	Score	m/z	Gene names
RO-QC-160121_ZackD_SS_Set_02_Fractio	30944	FTMS; HC	256.28	1136.06	AHNAK

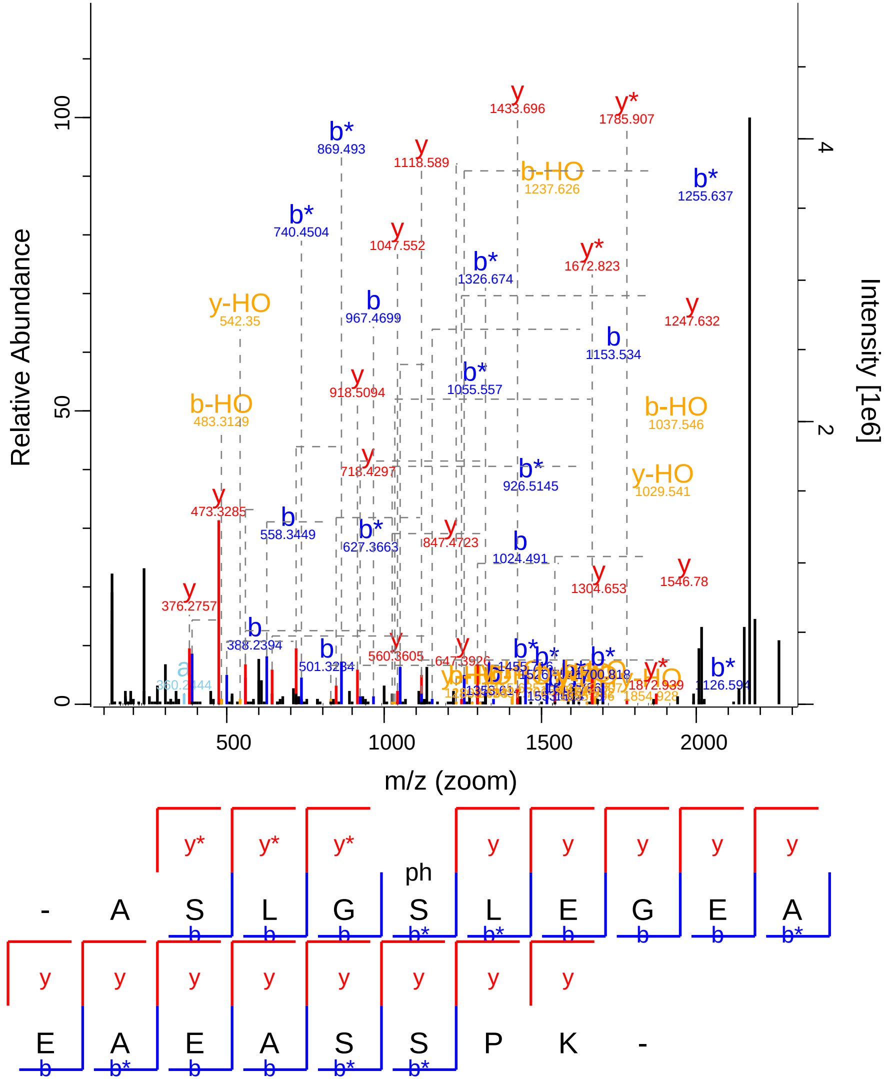

Raw File	Scan	Method	Score	m/z	Gene names
RO-QC-160121_ZackD_SS_Set_02_Fractio	31003	FTMS; HC	105.35	1175.64	MAP1B

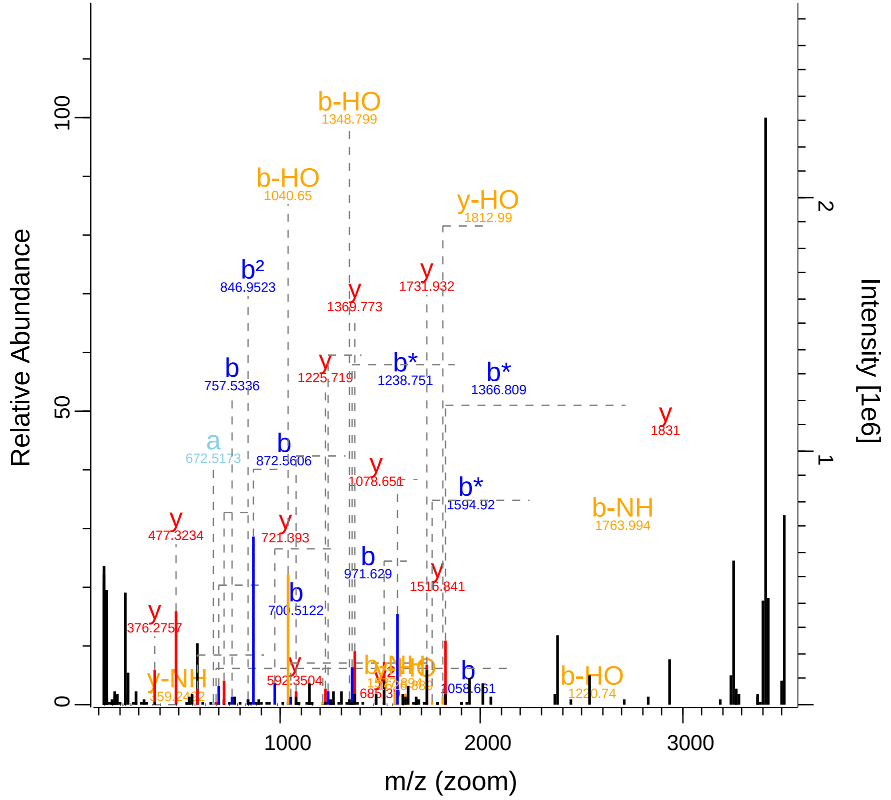

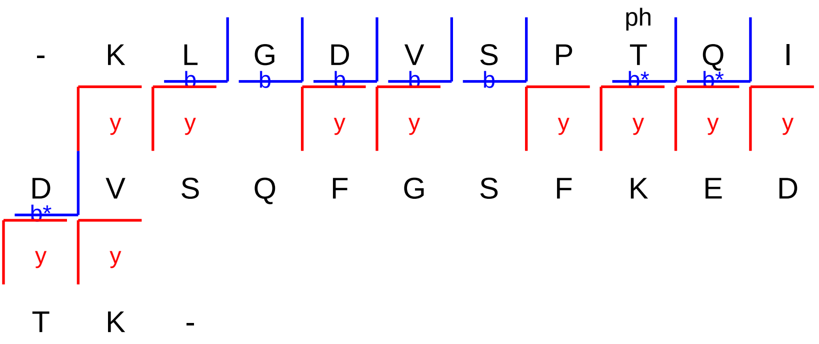

Raw File	Scan	Method	Score	m/z	Gene names
RO-QC-160121_ZackD_SS_Set_02_Fractioı	31549	FTMS; HC	125.12	1037.5	ARHGDIA

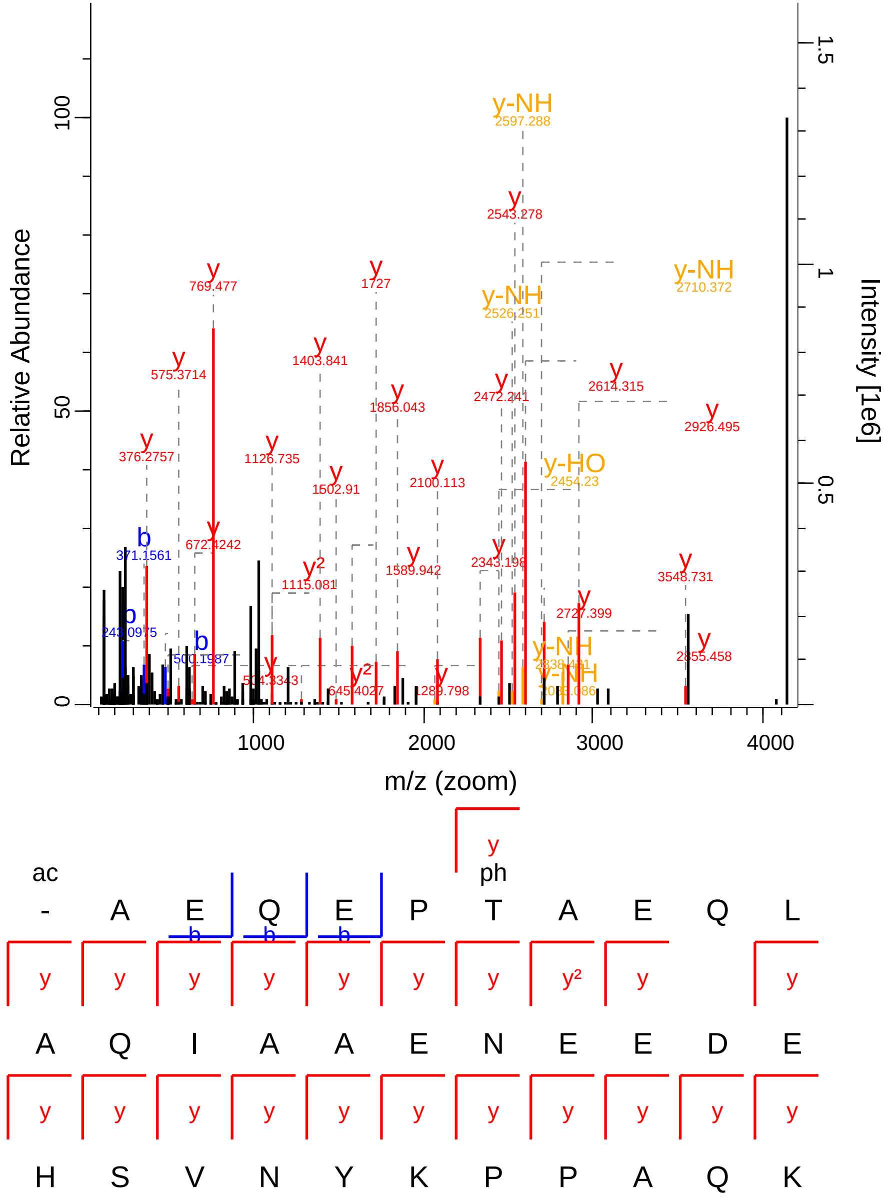

Raw File	Scan	Method	Score	m/z	Gene names
RO-QC-160121_ZackD_SS_Set_02_Fractior	32135	FTMS; HC	102.69	915.82	PGM1

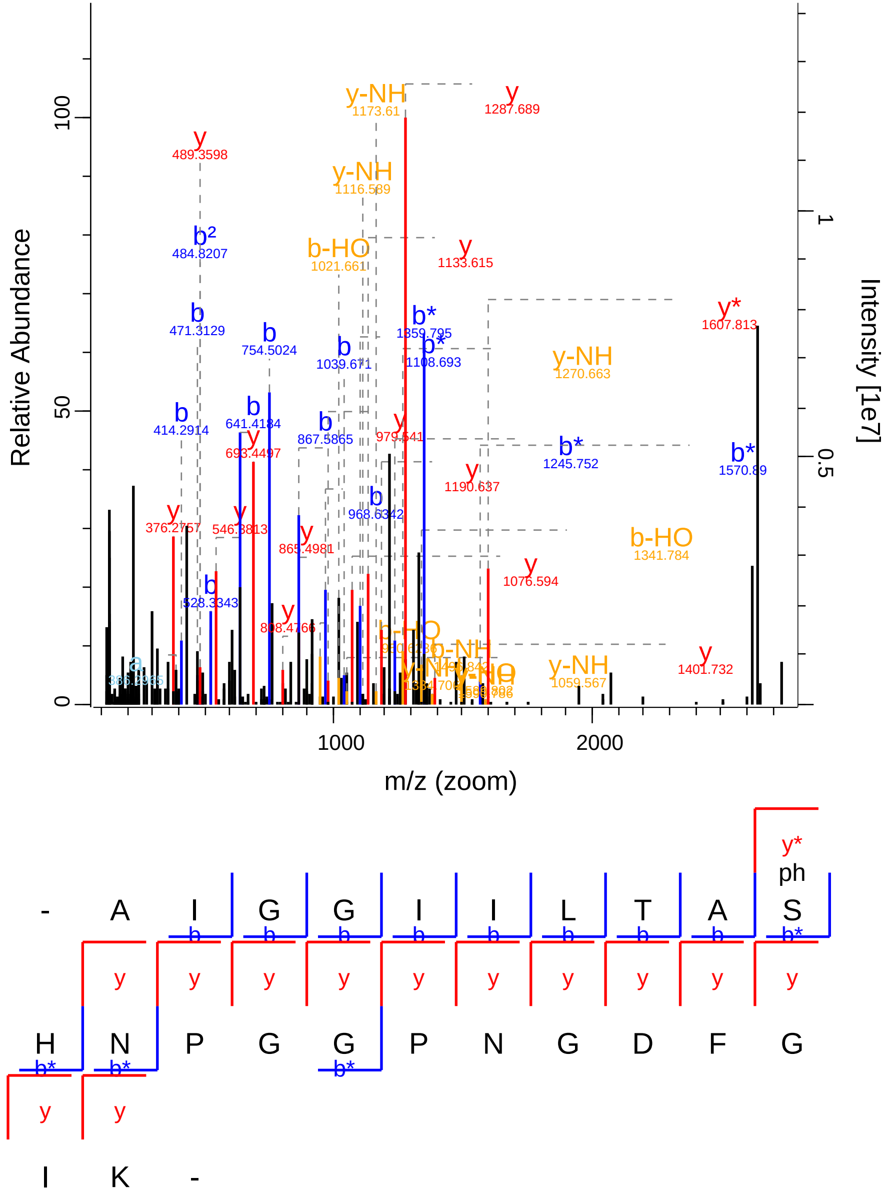

Raw File	Scan	Method	Score	m/z	Gene names
RO-QC-160121_ZackD_SS_Set_02_Fractior	32591	FTMS; HC	113.6	777.16	PAK2

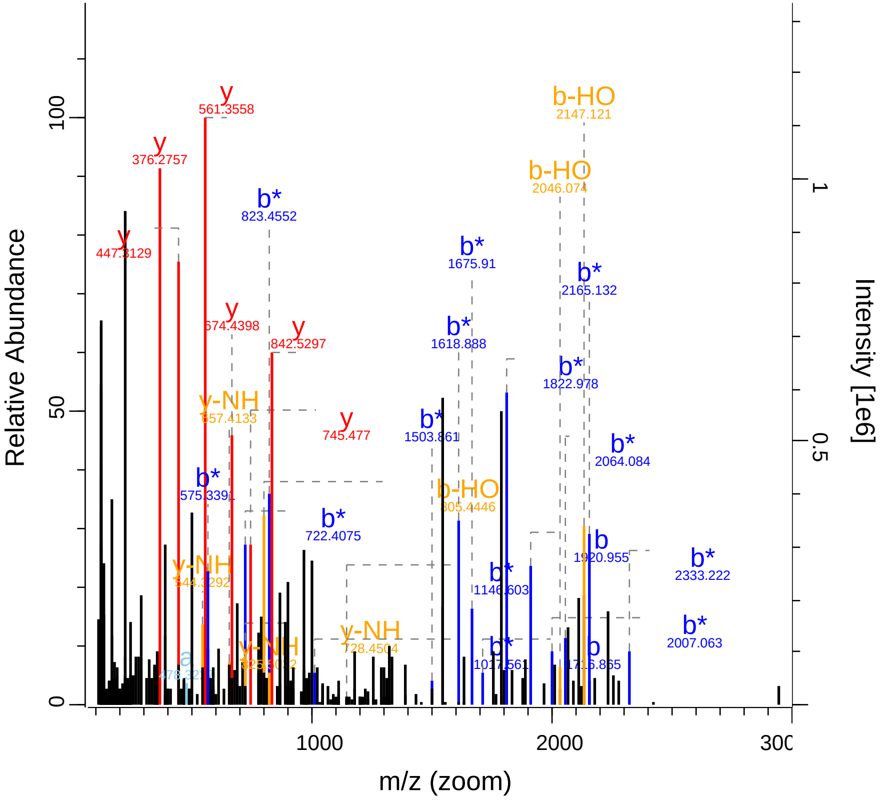

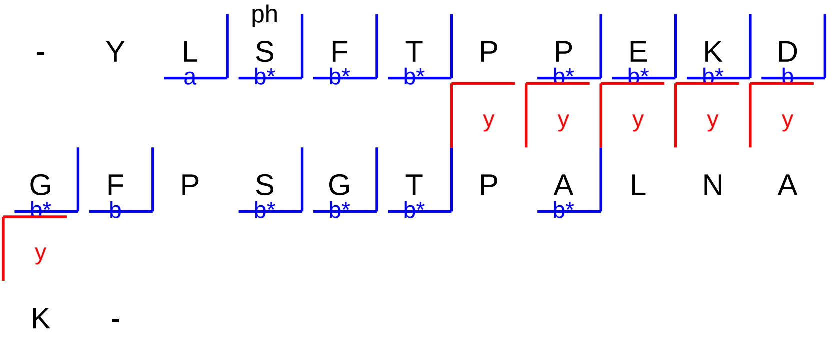

Raw File	Scan	Method	Score	m/z	Gene names
RO-QC-160121_ZackD_SS_Set_02_Fractio	33143	FTMS; HC	85.18	1017.83	AHNAK

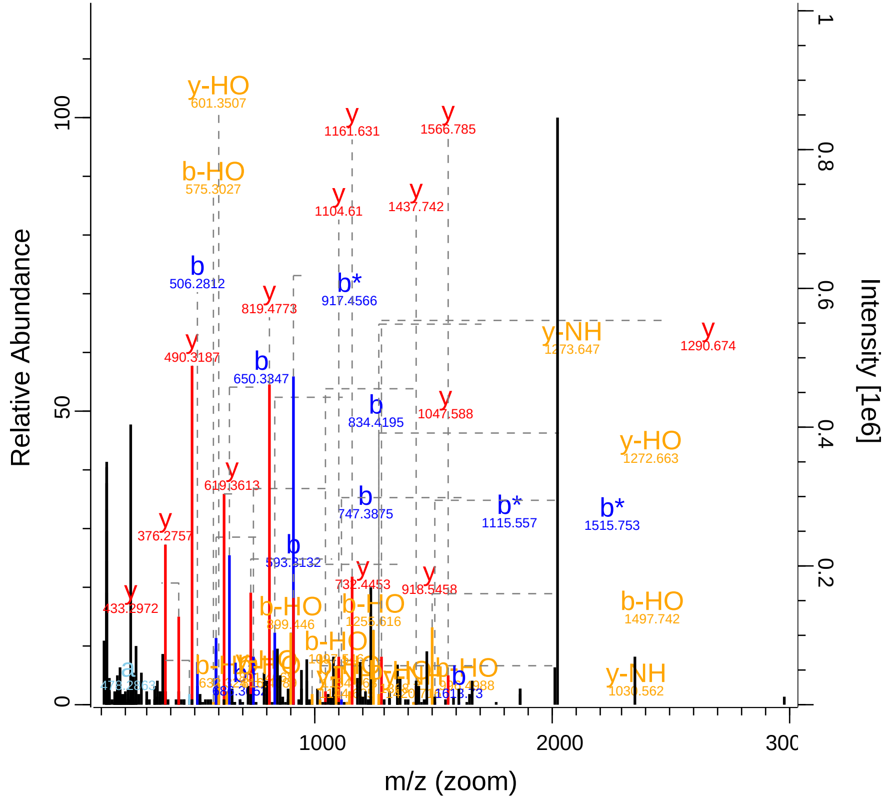

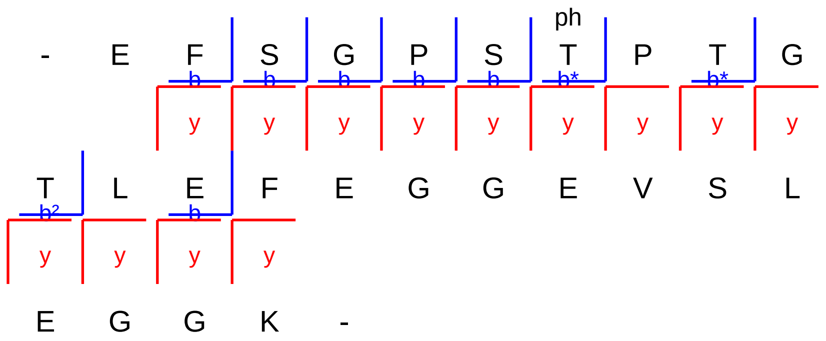

Raw File	Scan	Method	Score	m/z	Gene names
RO-QC-160121_ZackD_SS_Set_02_Fractior	33257	FTMS; HC	166.11	1339.6	PPP6R3

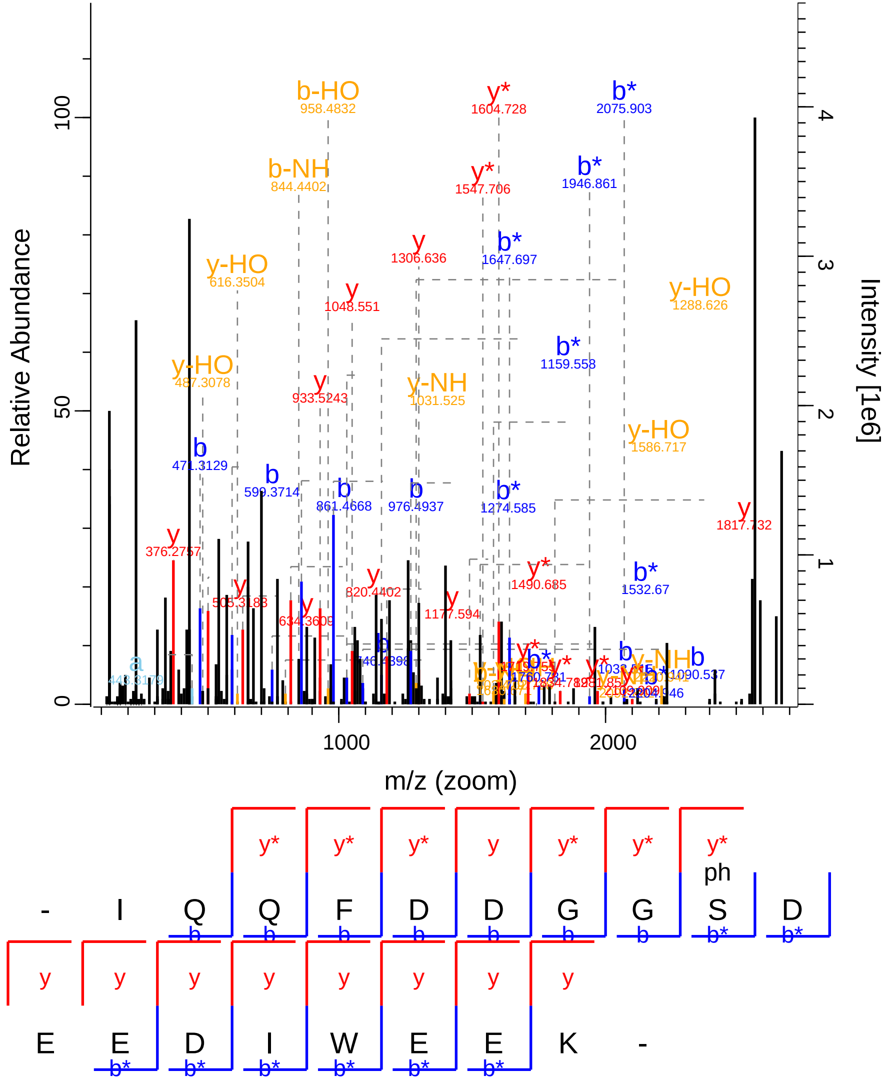

Raw File	Scan	Method	Score	m/z	Gene names
RO-QC-160121_ZackD_SS_Set_02_Fraction	33423	FTMS; HC	174.9	901.9	HACD3

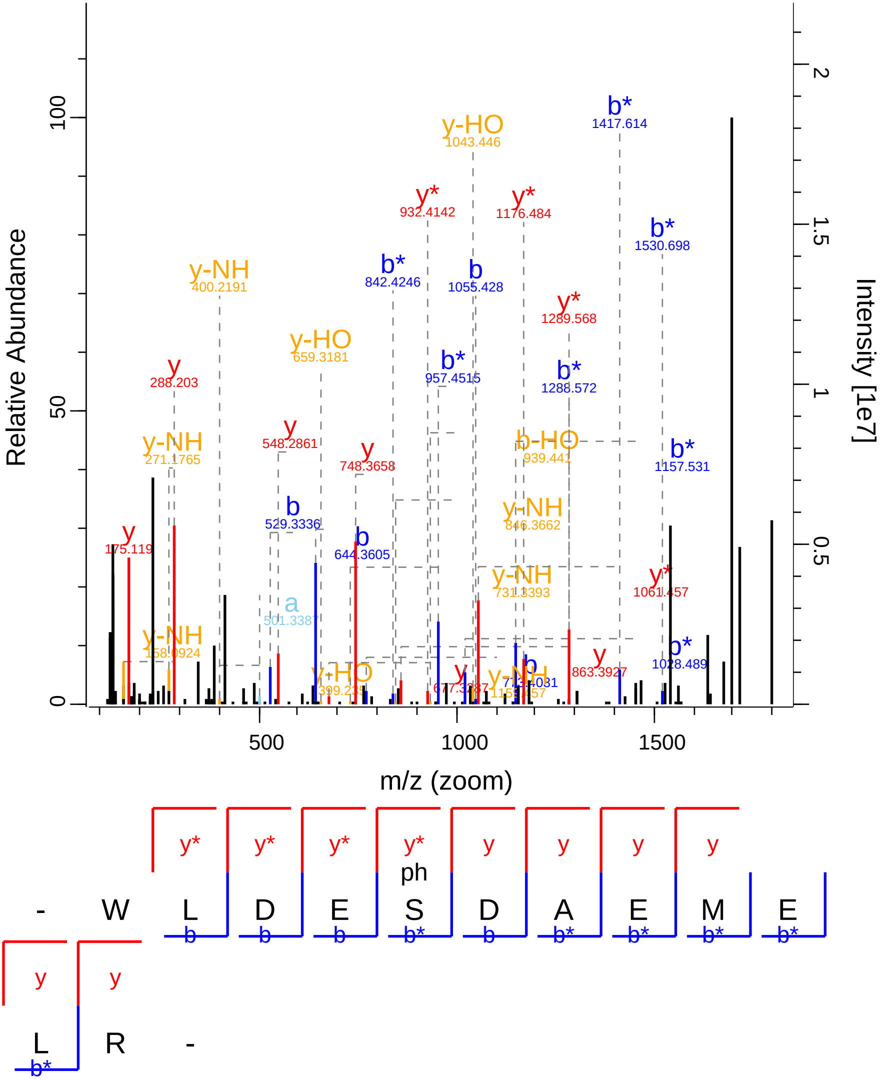

Raw File	Scan	Method	Score	m/z	Gene names
RO-QC-160121_ZackD_SS_Set_02_Fractio	33684	FTMS; HC	215.37	1029.04	SH3KBP1

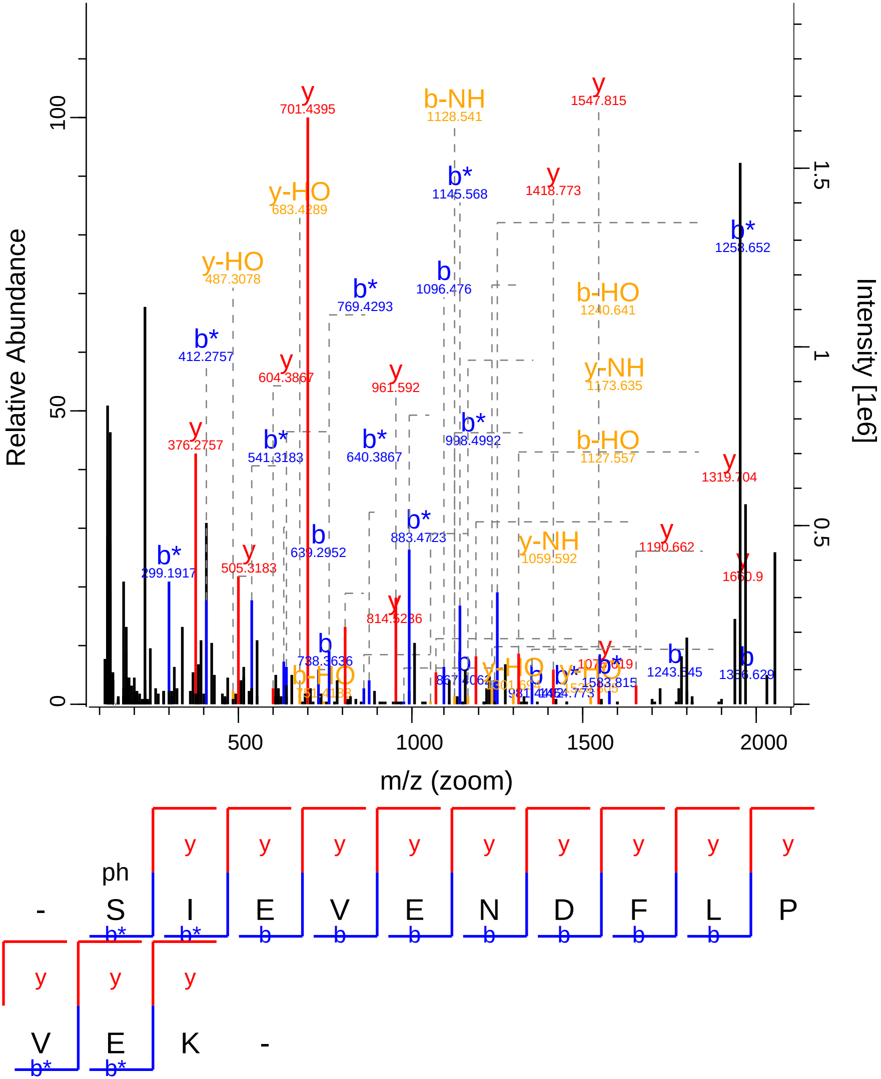

Raw File	Scan	Method	Score	m/z	Gene names
RO-QC-160121_ZackD_SS_Set_02_Fractio	34776	FTMS; HC	194.29	1377.33	RANBP2

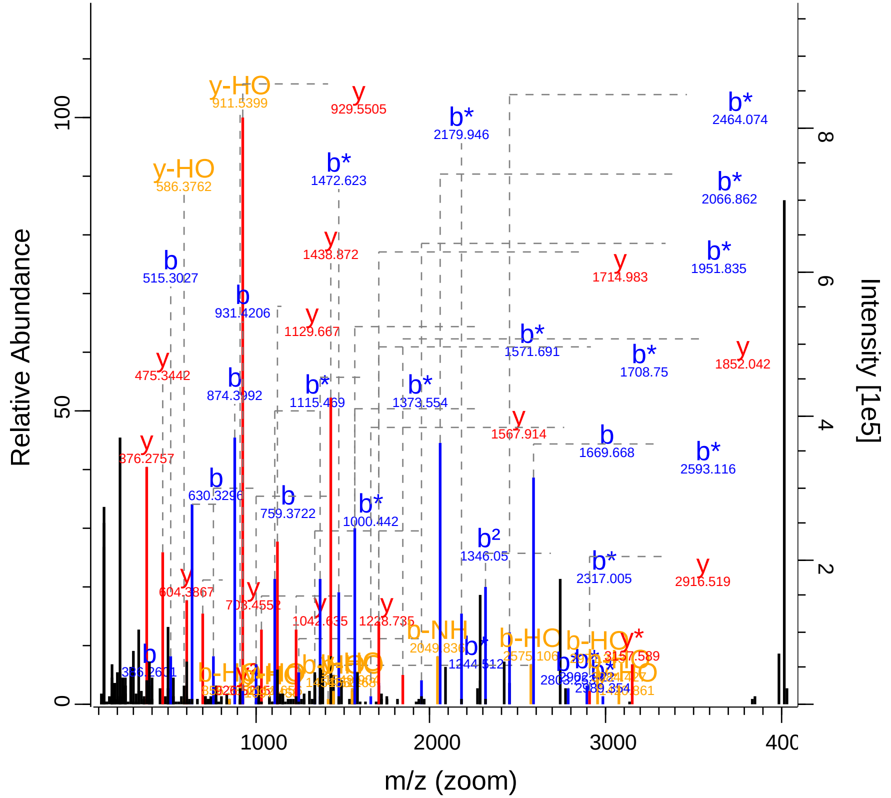

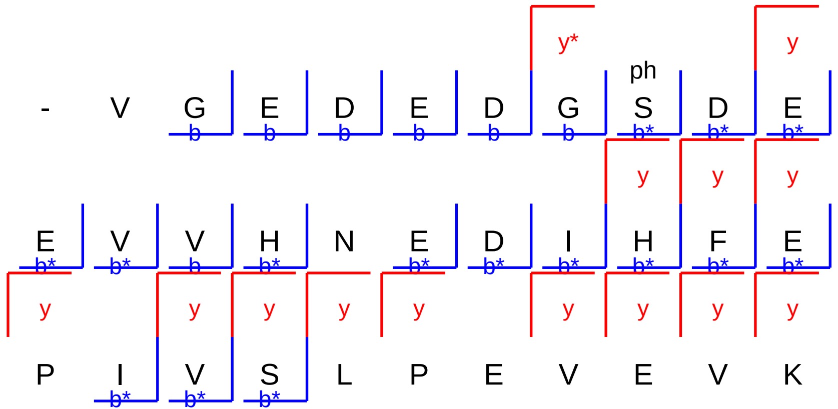

Raw File	Scan	Method	Score	m/z	Gene names
RO-QC-160121_ZackD_SS_Set_02_Fraction_	35330	FTMS; HC	108.46	559	CLU

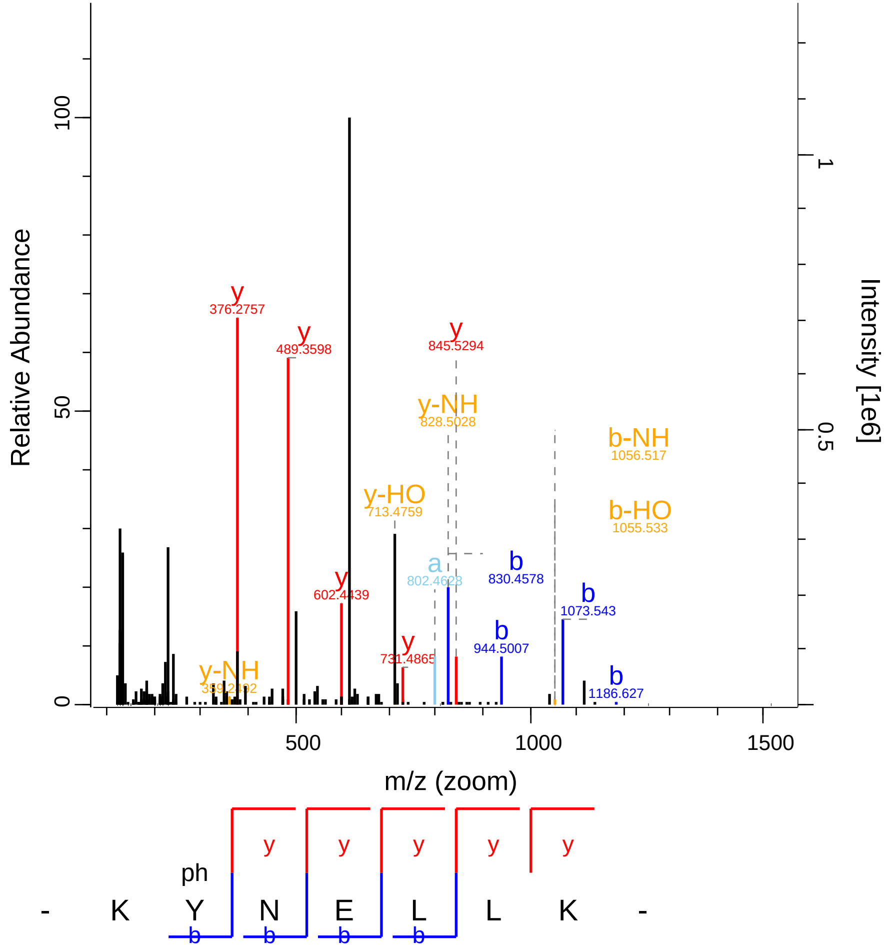

Raw File	Scan	Method	Score	m/z	Gene names
RO-QC-160121_ZackD_SS_Set_02_Fraction	35892	FTMS; HC	45.33	600.1	HMGXB3

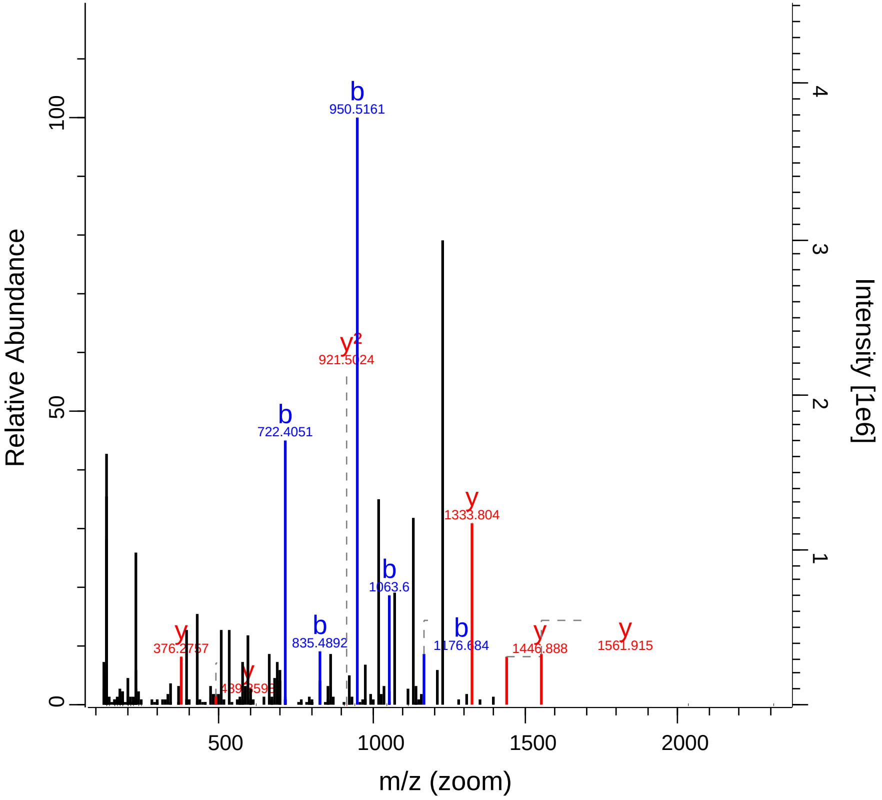

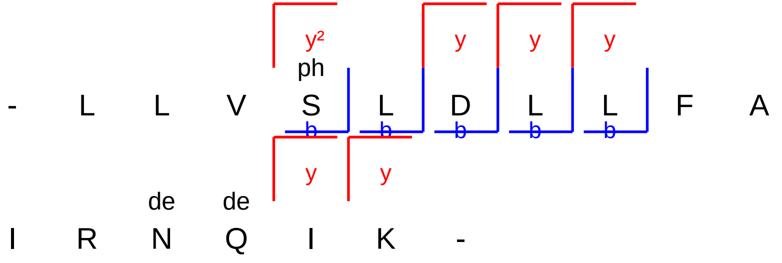

Raw File	Scan	Method	Score	m/z	Gene names
RO-QC-160121_ZackD_SS_Set_02_Fractio	36197	FTMS; HC	133.23	1029.51	RAB11FIP1

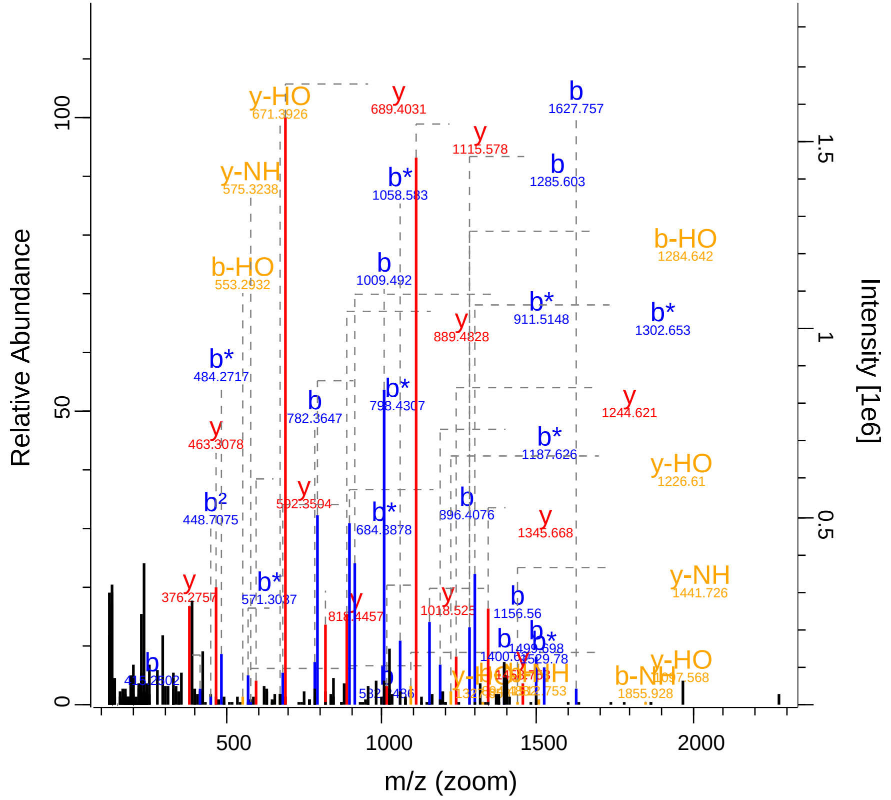

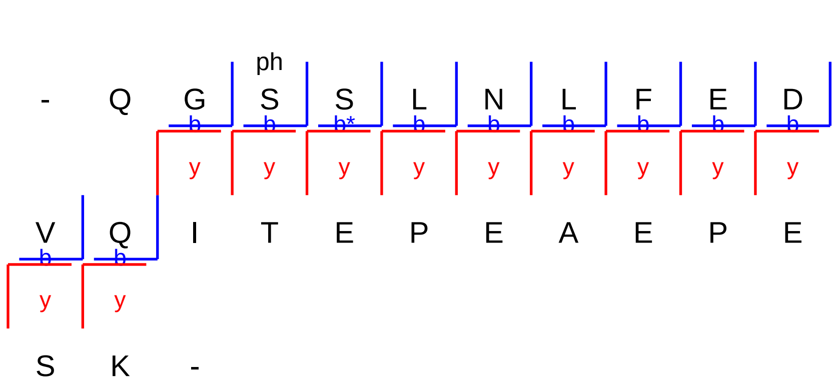

Raw File	Scan	Method	Score	m/z	Gene names
RO-QC-160121_ZackD_SS_Set_02_Fractio	36388	FTMS; HC	131.03	1071.25	SRRT

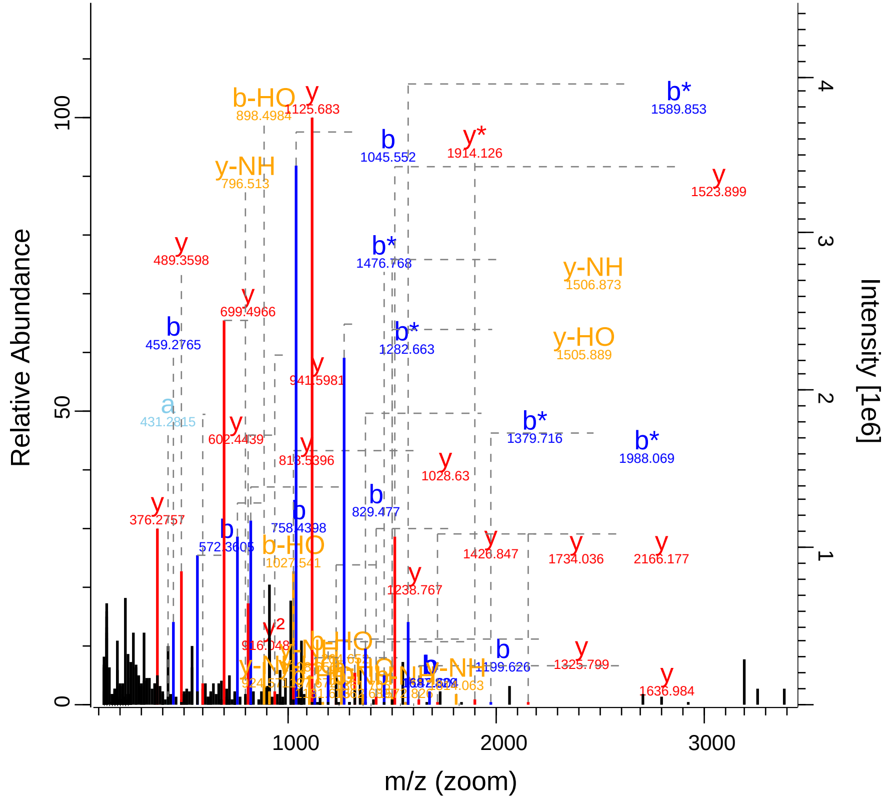

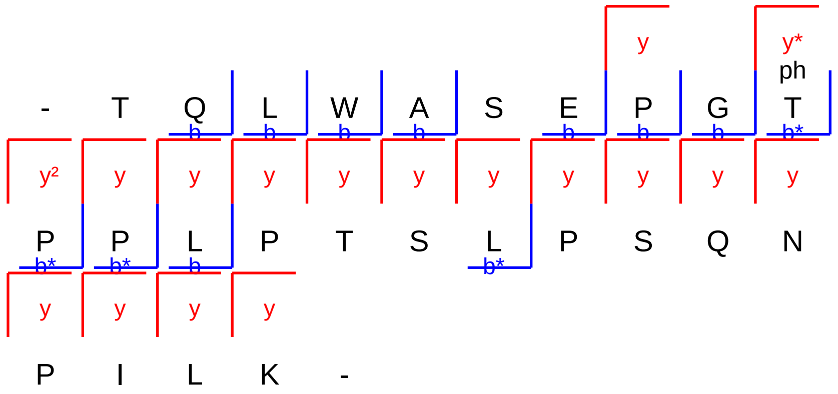

Raw File	Scan	Method	Score	m/z	Gene names
RO-QC-160121_ZackD_SS_Set_02_Fractio	37145	FTMS; HC	50.56	1565.81	CHRNA1

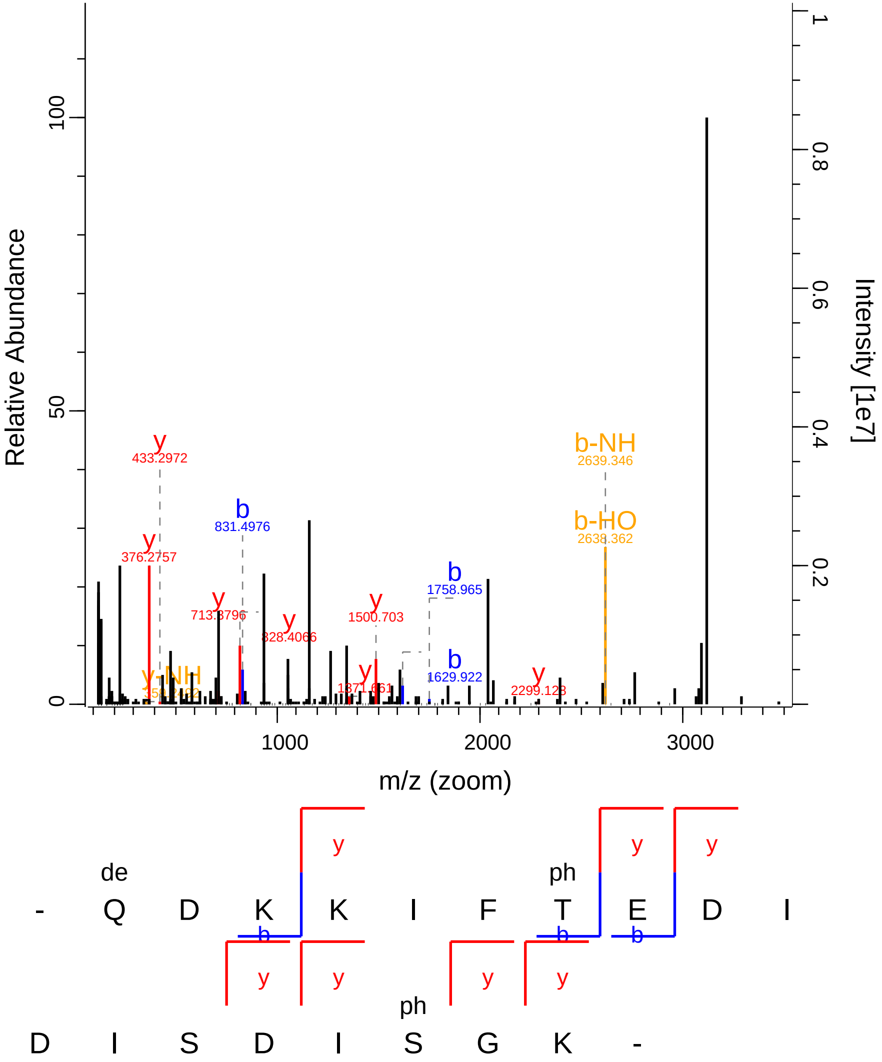

Raw File	Scan	Method	Score	m/z	Gene names
RO-QC-160121_ZackD_SS_Set_02_Fractio	37564	FTMS; HC	51.93	1337.16	CHD5

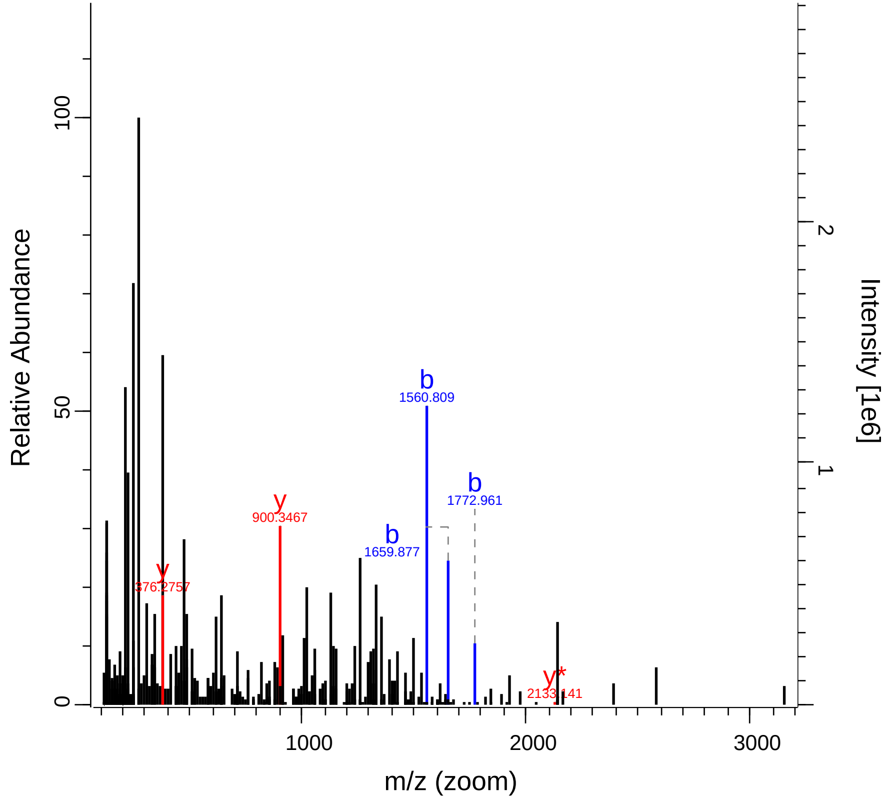

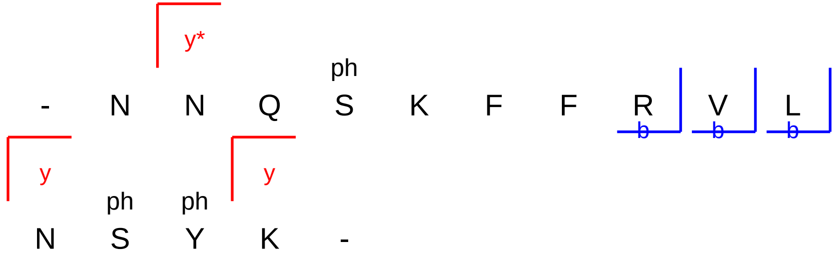

Raw File	Scan	Method	Score	m/z	Gene names
RO-QC-160121_ZackD_SS_Set_02_Fractior	37627	FTMS; HC	49.5	989.82	TMEM2

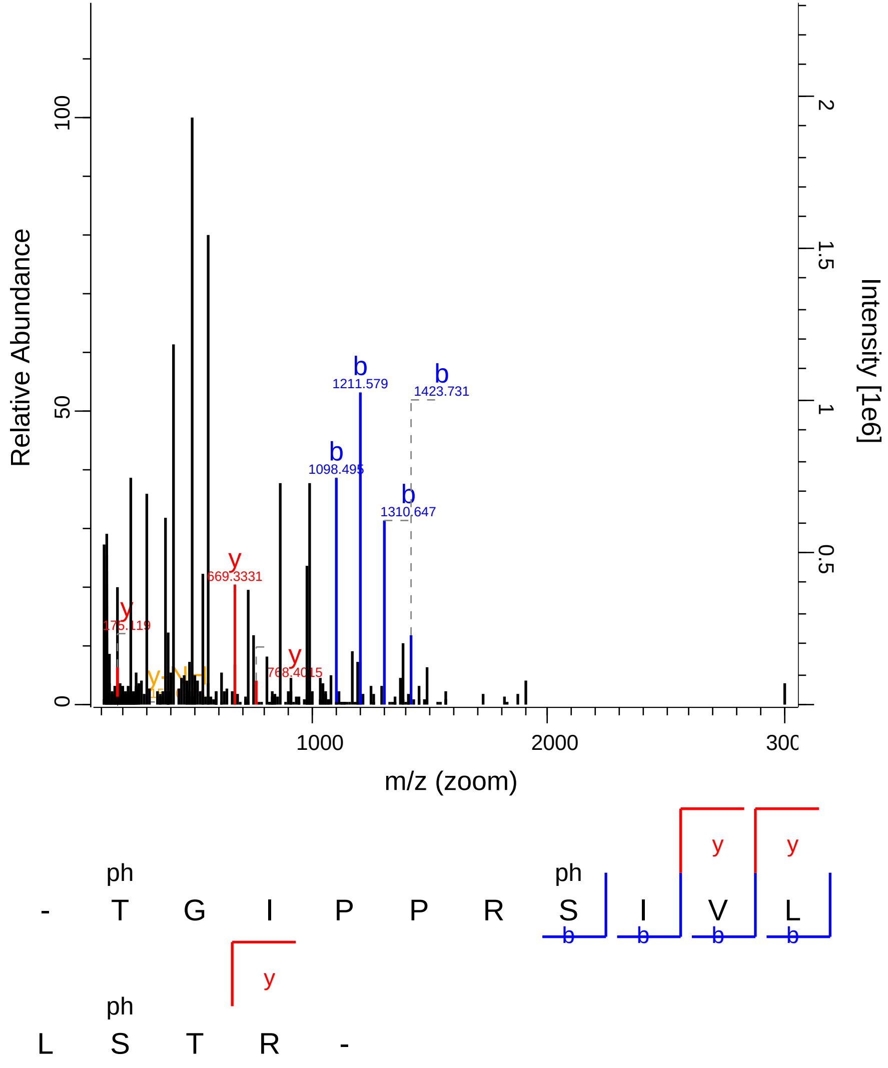

Raw File	Scan	Method	Score	m/z	Gene names
RO-QC-160121_ZackD_SS_Set_02_Fractio	37969	FTMS; HC	69.72	1132.13	FCHO1

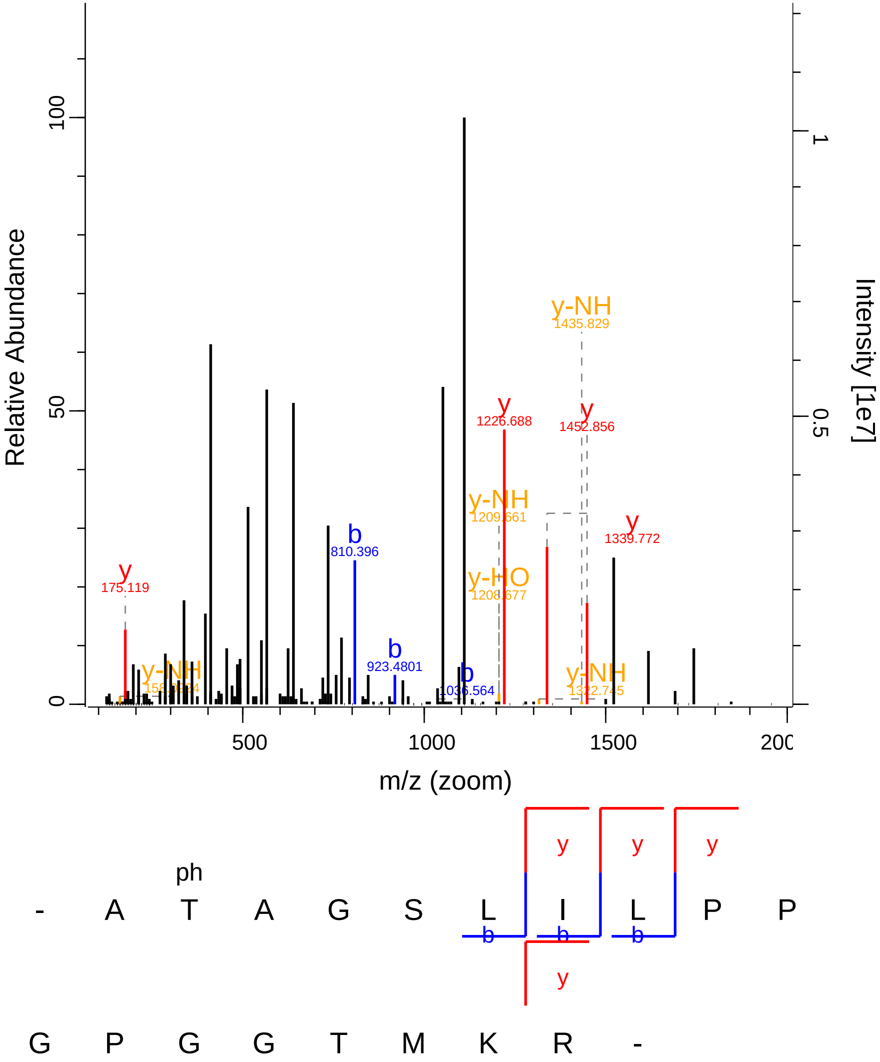

Supplement: Supplemental Figure S17 [file mmc17.pdf]
