# Supplementary material for: Proteome Landscape of Epithelial-to-Mesenchymal Transition (EMT) of Retinal Pigment Epithelium Shares Commonalities With Malignancy-Associated EMT
Source: Mol Cell Proteomics. 2021 Aug 27;20:100131. doi: 10.1016/j.mcpro.2021.100131 (PMC8482521; doi:10.1016/j.mcpro.2021.100131)
Supplement: Supplemental Figures S1–S15 [file mmc15.pdf]

Online Supplementary material

Epithelial to mesenchymal transition (EMT) of human stem cell-derived retinal pigment epithelium shares commonalities with malignancy-associated EMT: a proteomic analysis

Srinivasa R. Sripathi[1*], Ming-Wen Hu[1], Ravi Chakra Turaga[2], Joseph Mertz[1], Melissa M. Liu[1], Jun Wan[3], Julien Maruotti[4], Karl J. Wahlin[5], Cynthia A. Berlinicke,[1] Jiang Qian,[1] and Donald J. Zack[1,6,7*]

[1]Department of Ophthalmology, Stem Cell Ocular Regenerative Medicine Center, Wilmer Eye Institute, The Johns Hopkins University School of Medicine, Baltimore, MD 21205, USA.

[2]Caris Life Sciences, 350 W Washington St., Tempe, AZ 85281, USA.

[3]Department of Medical and Molecular Genetics, Indiana University School of Medicine, Indianapolis, IN 46202, USA.

[4]Phenocell, Grasse 06130, France.

[5]Shiley Eye Institute, University of California, San Diego, LA Jolla, CA 92093, USA.

[6]Solomon H. Snyder Department of Neuroscience, Department of Molecular Biology and Genetics, Department of Genetic Medicine, The Johns Hopkins University School of Medicine, Baltimore, MD 21205, USA.

[7]Institute for NanoBioTechnology, Johns Hopkins University, Whiting School of Engineering Baltimore, MD 21218, USA.

[*]For correspondence : Srinivasa R. Sripathi, ssripat1@jhmi.edu; Donald J. Zack, dzack@jhmi.edu

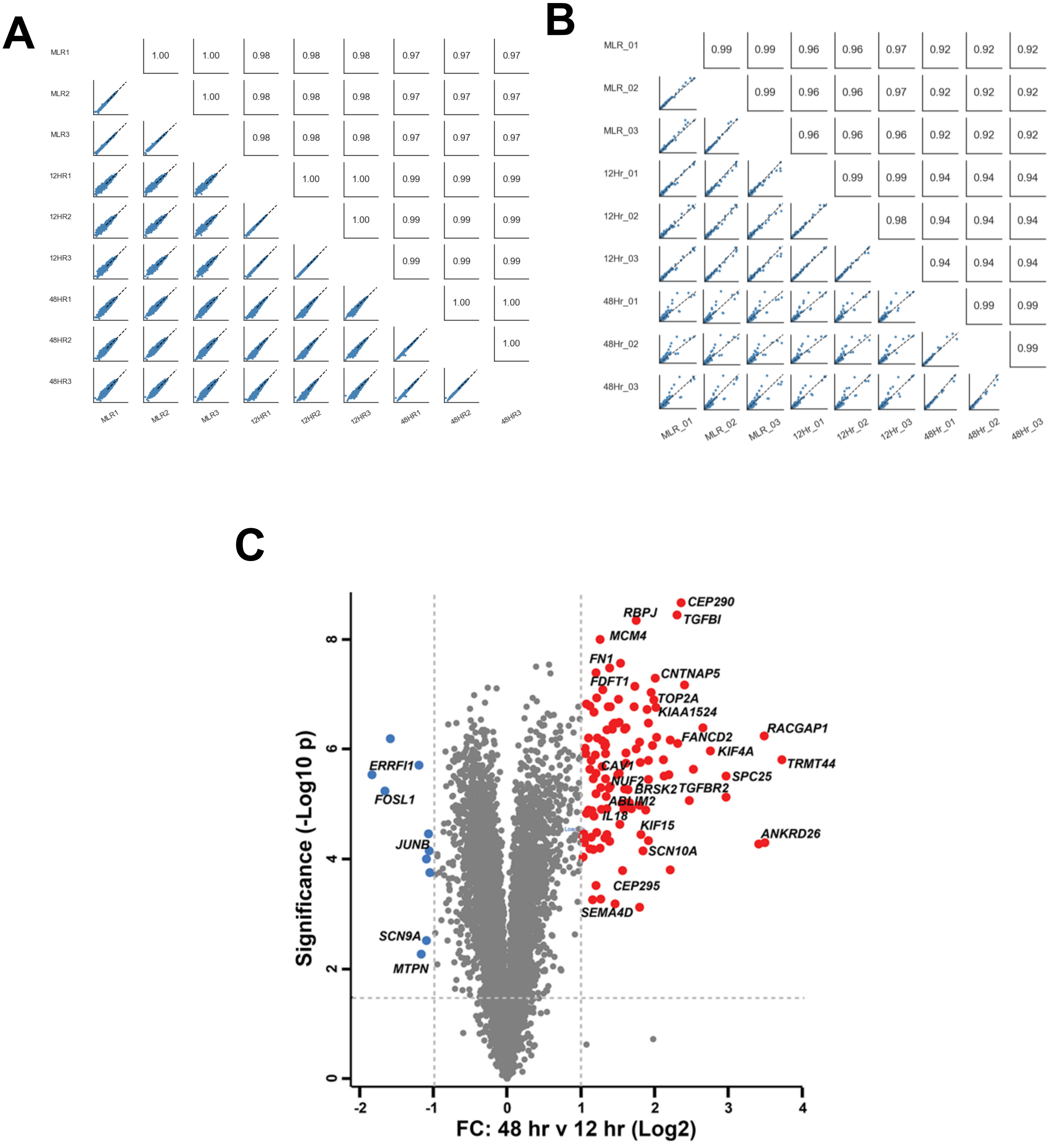

Supplemental Figure S1. Comparative analysis of RPE EMT proteome.

(A). The matrix of 36 correlation plots revealed high correlation between TMT intensities in triplicate samples (Pearson correlation coefficient 0.97–0.99 between biological replicates). (B) The matrix of 36 correlation plots revealed high correlation between triplicate samples from direct-DIA approach (Pearson correlation coefficient 0.92–0.99 between biological replicates). (C) Volcano plot illustration of TMT identified differentially expressed proteins during RPE EMT. Pairwise comparison between 12 hr and 48 hr time points. Expression fold changes (t-test difference, log2) were calculated and plotted against Benjamini-Hochberg corrected t-test P-value (log10).

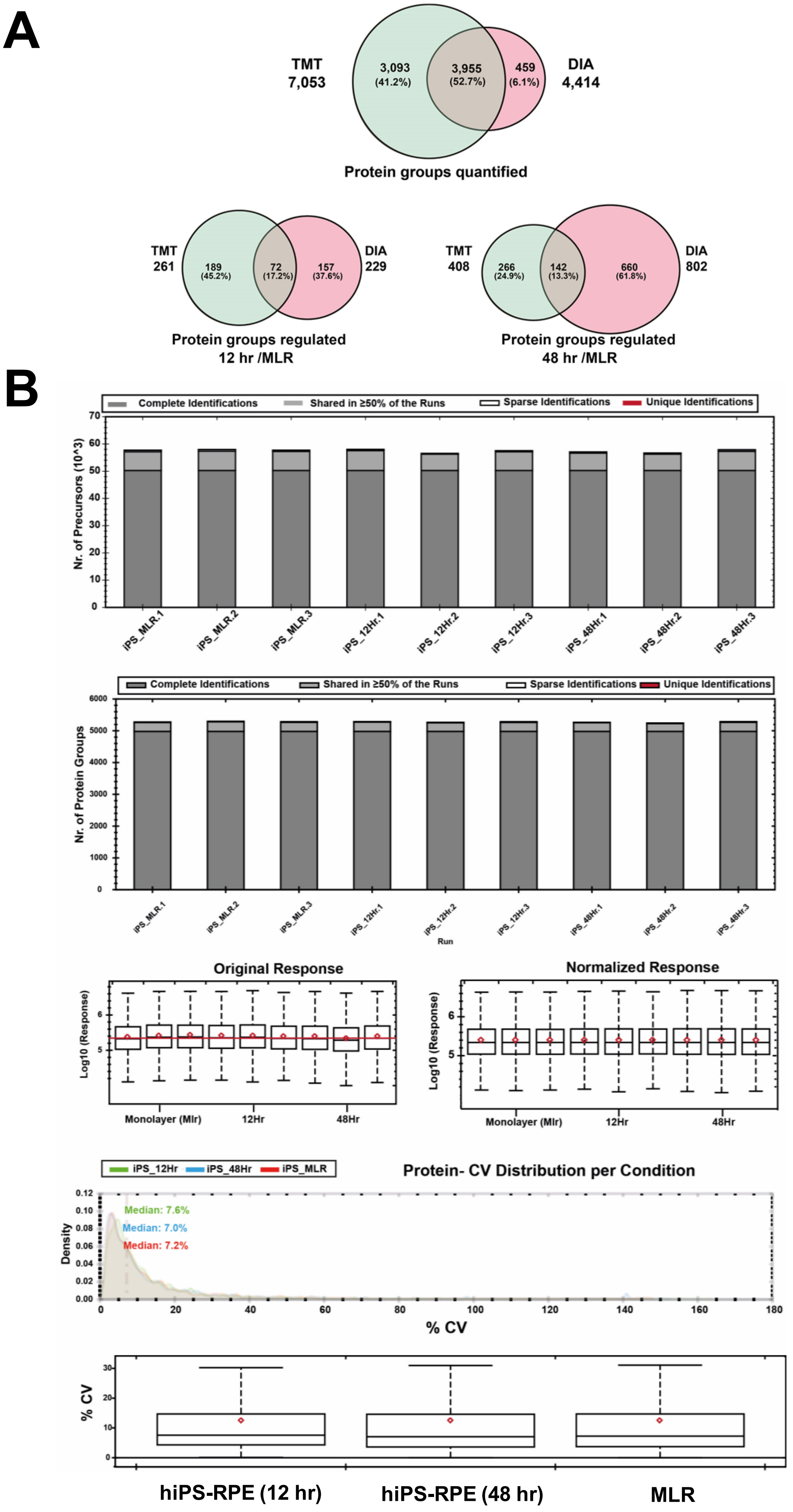

Supplemental Figure S2. (A) Venn diagram representation of comparative protein groups quantified and differentially regulated by TMT and direct-DIA approach. (B) Quantitative assessment of RPE-EMT conditions by direct-DIA approach.

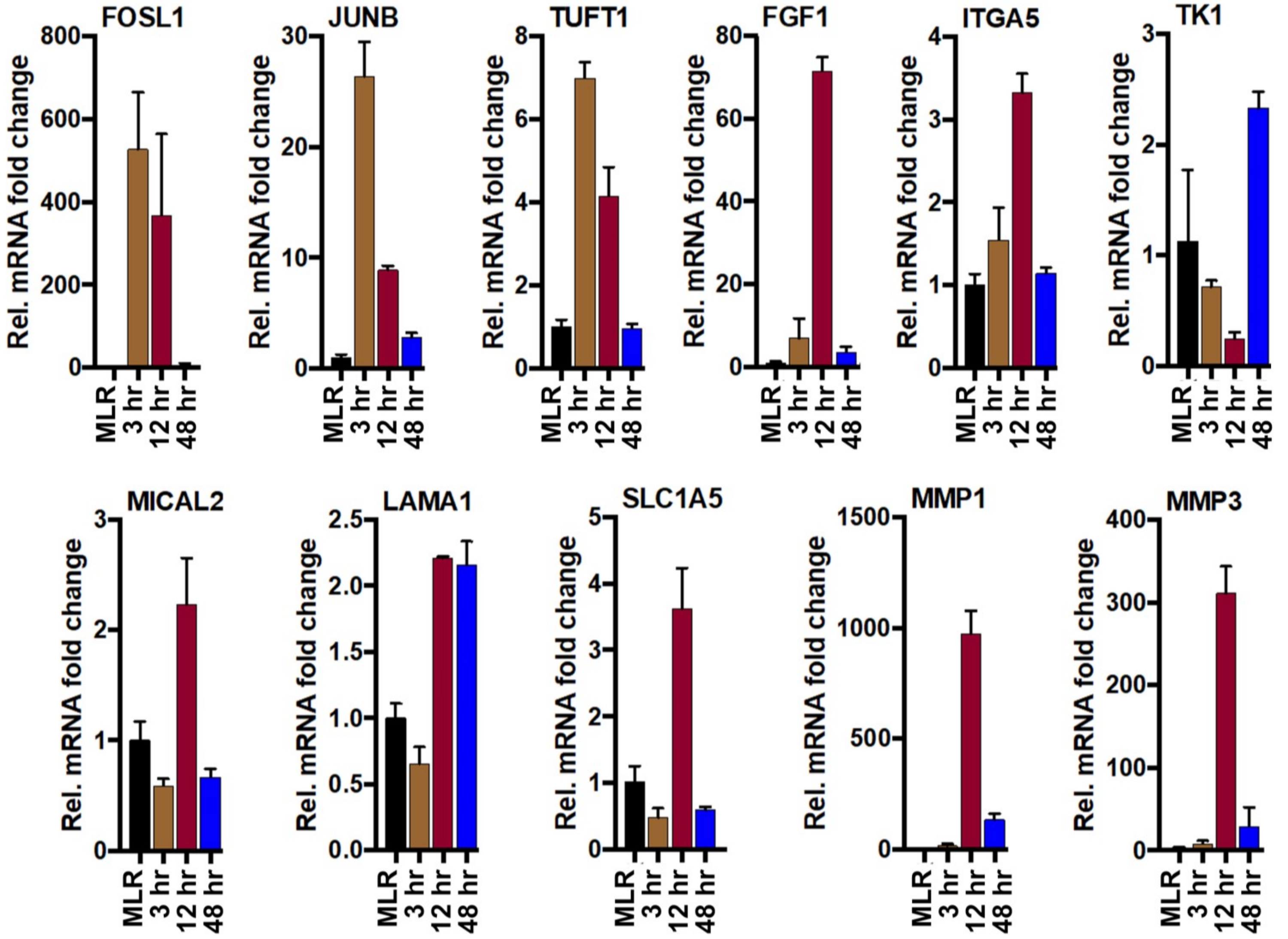

Supplemental Figure S3. qPCR validation of differentially regulated RPE-EMT factors in hiPS derived RPE (EP1). Differential expression of malignancy associated EMT associated factors identified from TMT labeling approach were measured by qRT-PCR after enzymatic dissociation of monolayers into single cells (3 hr, 12 hr and 48 hr). Data were normalized by the expression levels in undissociated monolayer control conditions.

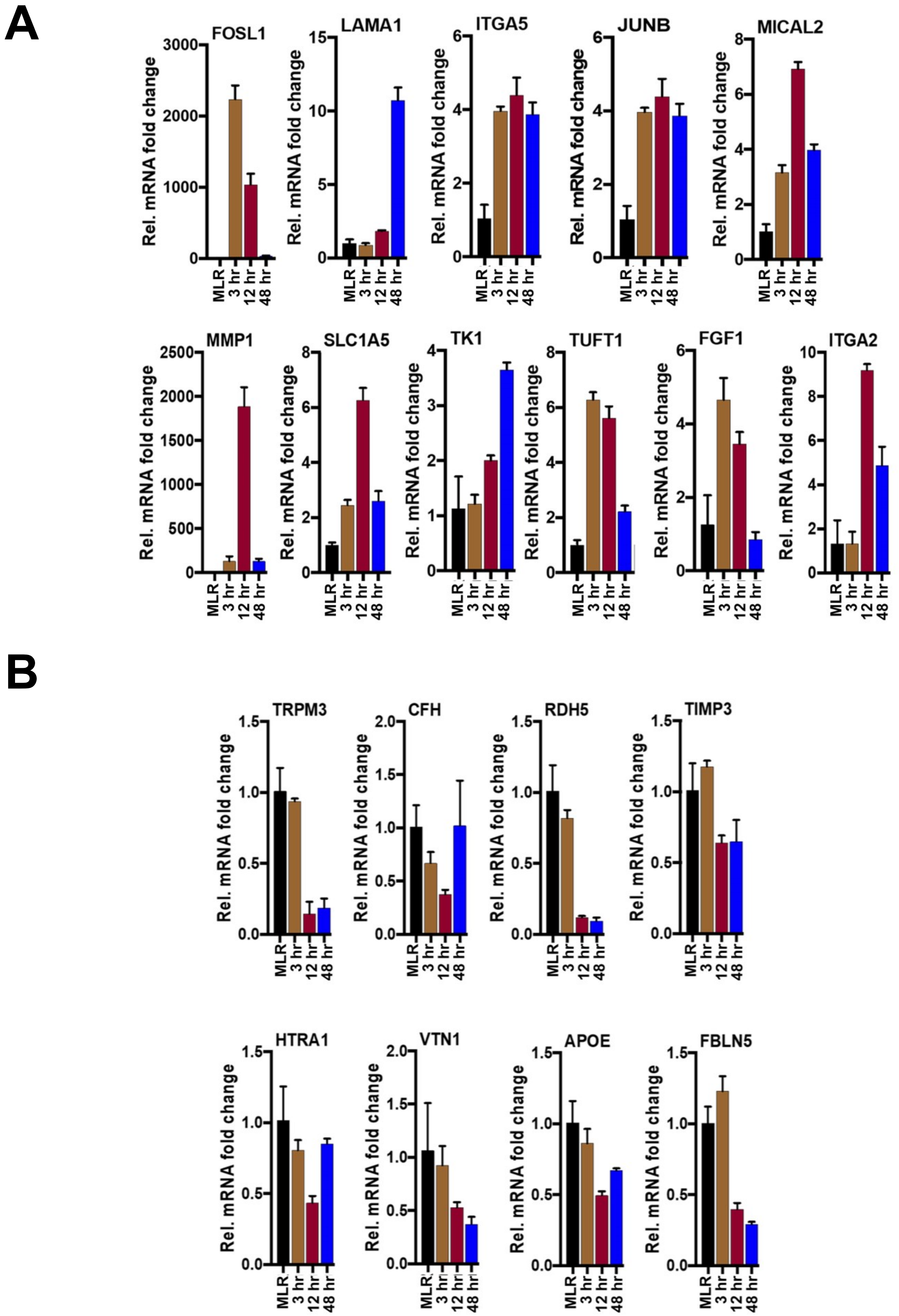

Supplemental Figure S4. qPCR validation of differentially regulated RPE-EMT factors in hiPSC derived RPE (IMR90.4).

Differential expression of (A) malignancy associated EMT and (B) AMD associated factors identified from TMT labeling approach were measured by qRT-PCR after enzymatic dissociation of monolayers into single cells (3 hr, 12 hr and 48 hr). Data were normalized by the expression levels in undissociated monolayer control conditions..

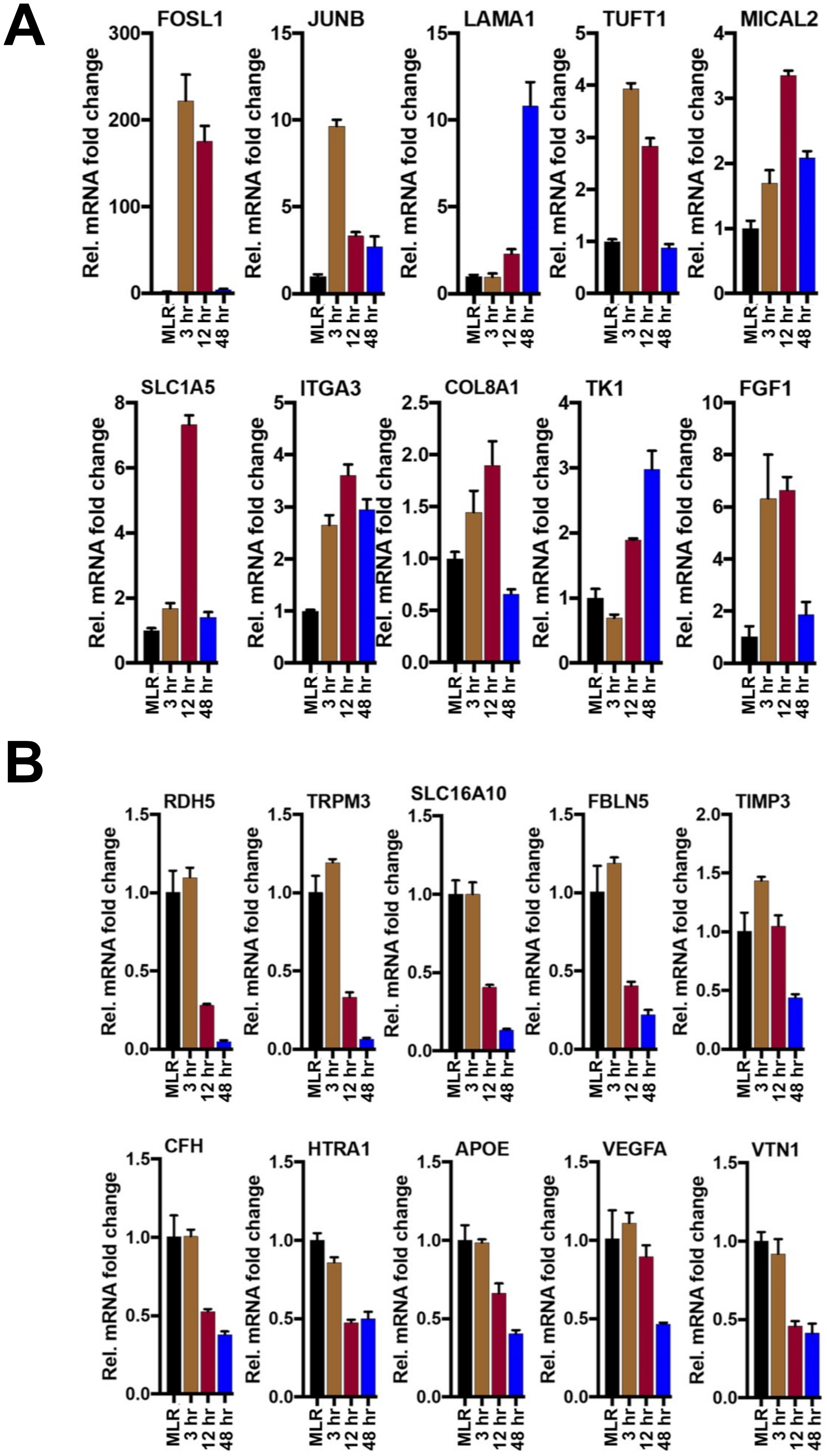

Supplemental Figure S5. qPCR validation of differentially regulated RPE-EMT factors in hESC derived RPE (H7). Differential expression of (A) malignancy associated EMT and (B) AMD associated factors identified from TMT labeling approach were measured by qRT-PCR after enzymatic dissociation of monolayers into single cells (3 hr, 12 hr and 48 hr). Data were normalized by the expression levels in undissociated monolayer control conditions.

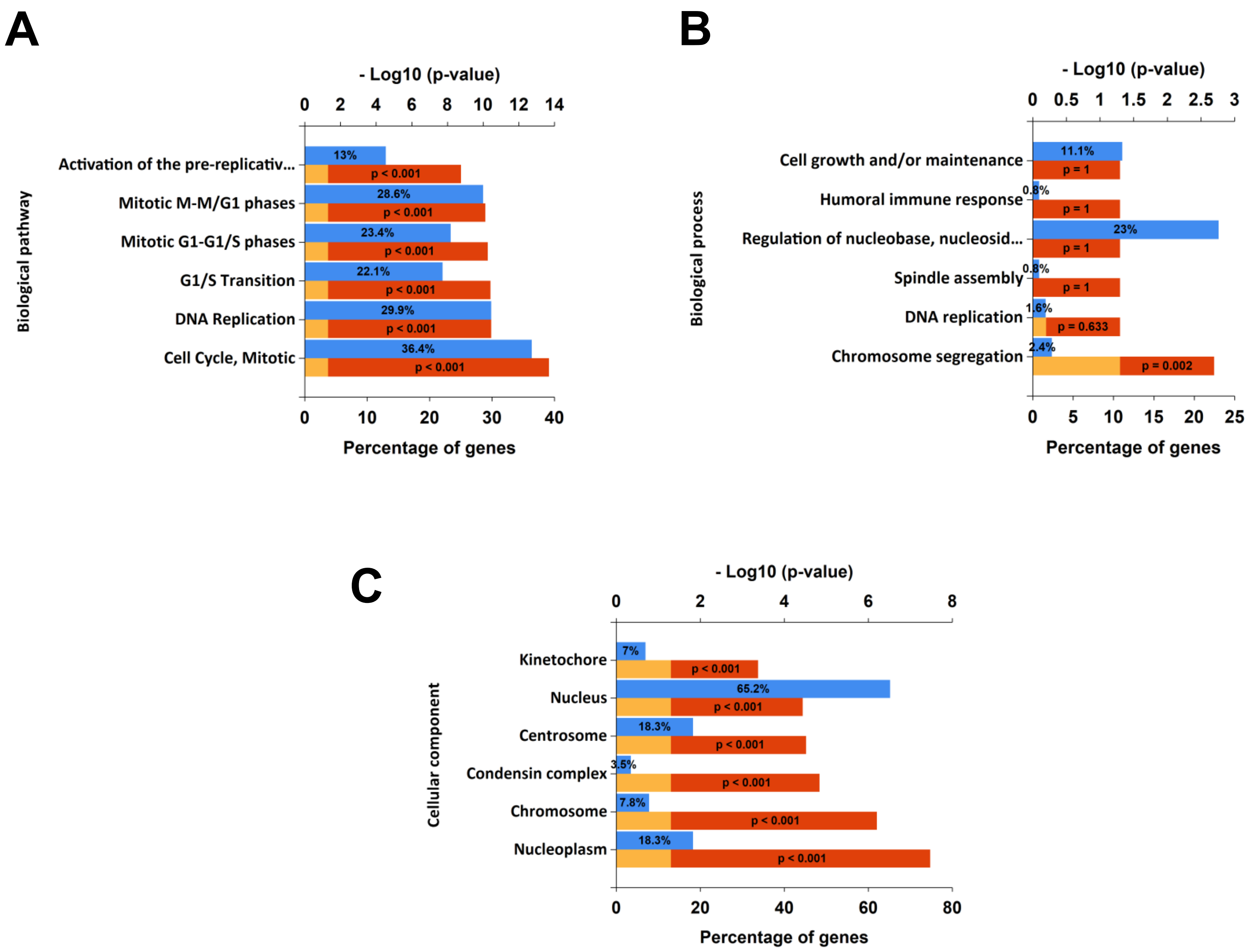

Supplemental Figure S6. Gene Ontology (GO) enrichment analysis of RPE EMT.
Functional enrichment of differentially regulated RPE-EMT proteins was analyzed using the FunRich tool. (A) Biological Pathways. (B) Molecular Function. (C) Cellular Components.

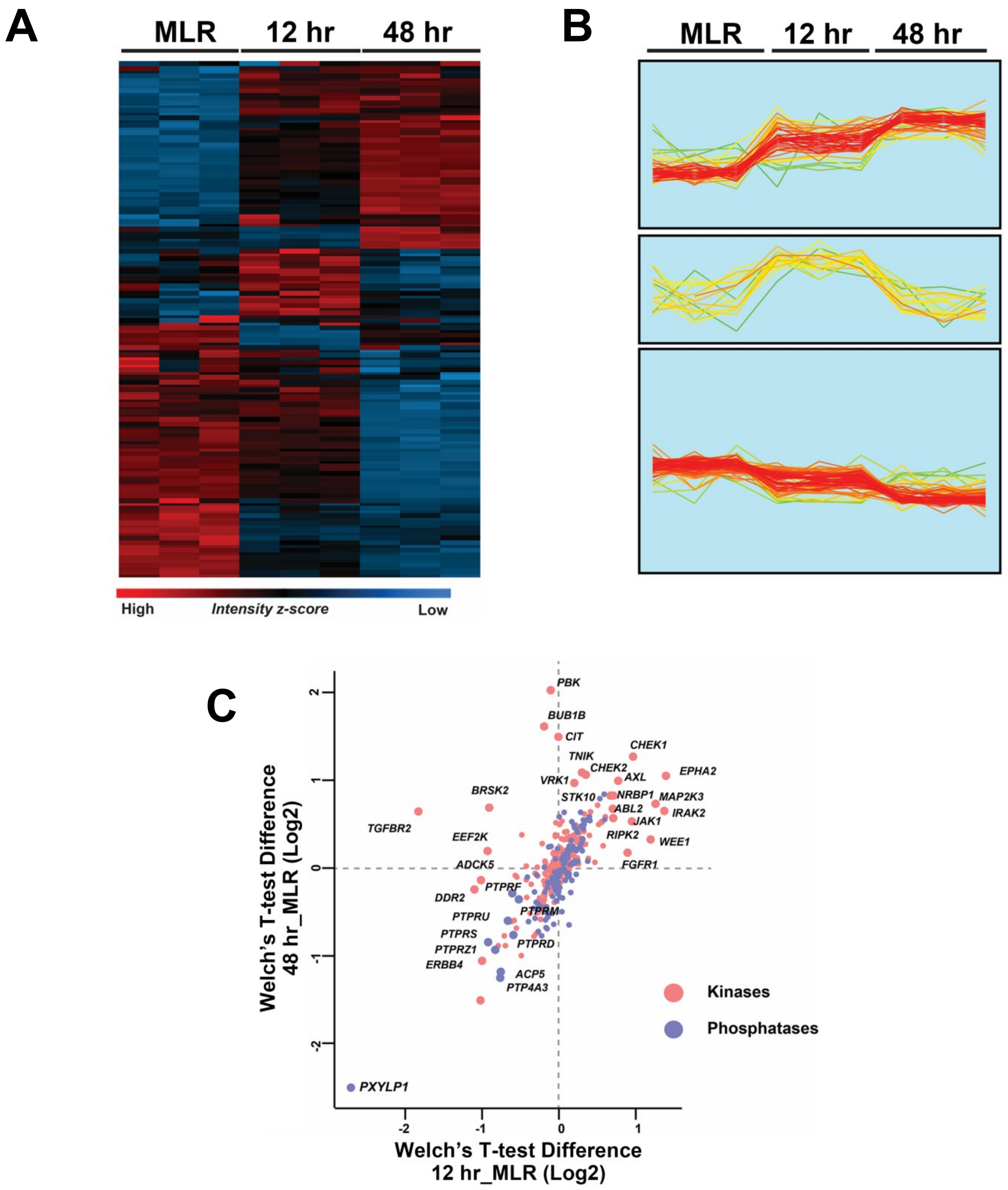

Supplemental Figure S7. RPE EMT induced changes in phosphatase expression.

(A) Heatmap representing altered phosphatase expression during dissociation induced EMT. (B) Profile plots of three selected clusters showing distinct behavior with respect to each time point: 1. strong increase at 12 hr and 48hr time points; 2. strong increase only at 12 hr; and 3. decrease at 12 hr and 48 hr time points. (C) Scatter plot of identified kinases and phosphatase changes across the 12h and 48h time points. Pink (Kinases), violet (phosphatases).

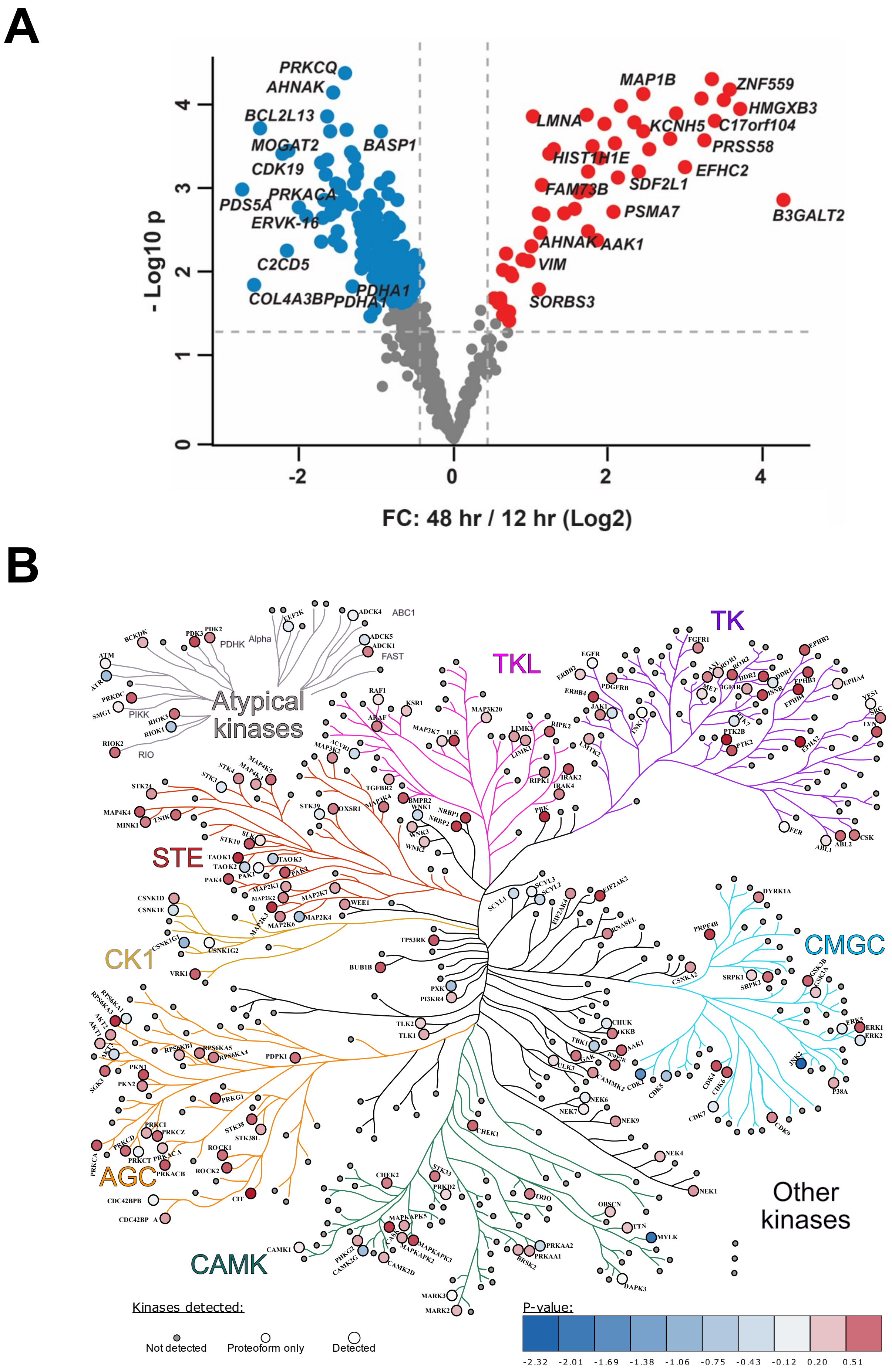

Supplemental Figure S8. (A) Volcano plot illustration of differentially expressed kinases during RPE EMT. Pairwise comparison between 12 hr and 48 hr time points. Expression fold changes (t-test difference, log2) were calculated and plotted against Benjamini-Hochberg corrected t-test P-value (log10). (B) Kinome tree representation of the altered kinase profiling in RPE-EMT.

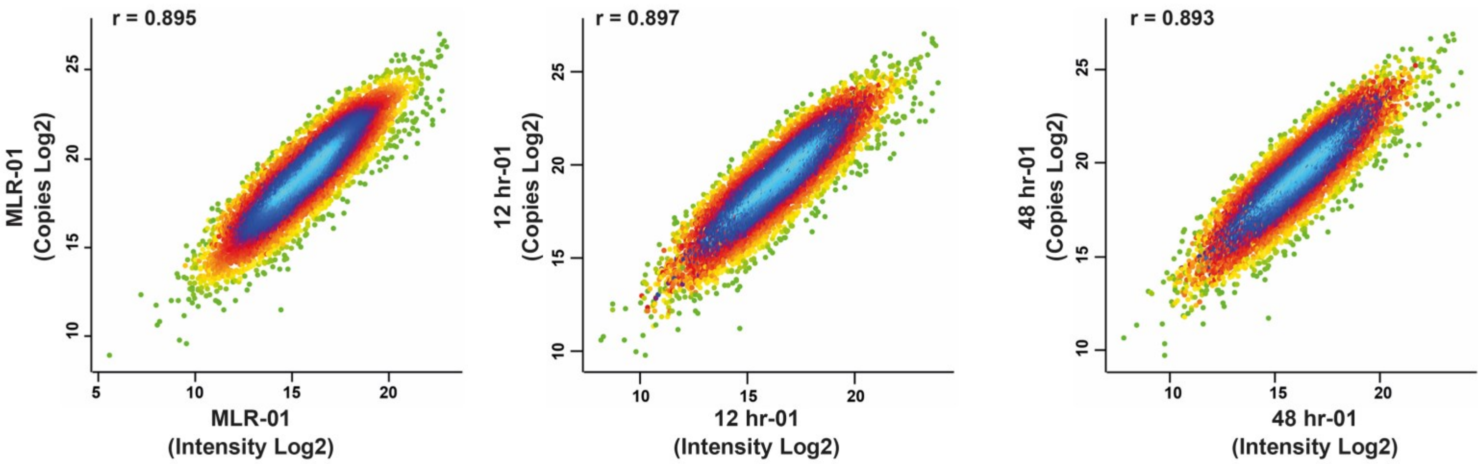

Supplemental Figure S9. Comparative analysis between copy numbers per cell and TMT intensities. Plots revealed high correlations between absolute protein copy numbers and TMT intensities in triplicate samples. (Pearson correlation coefficient 0.89 between biological replicates).

A

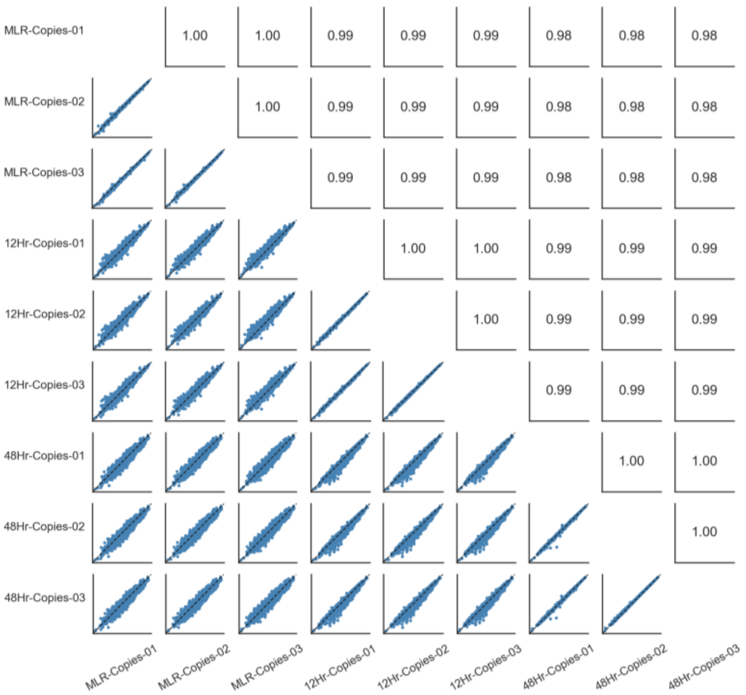

B

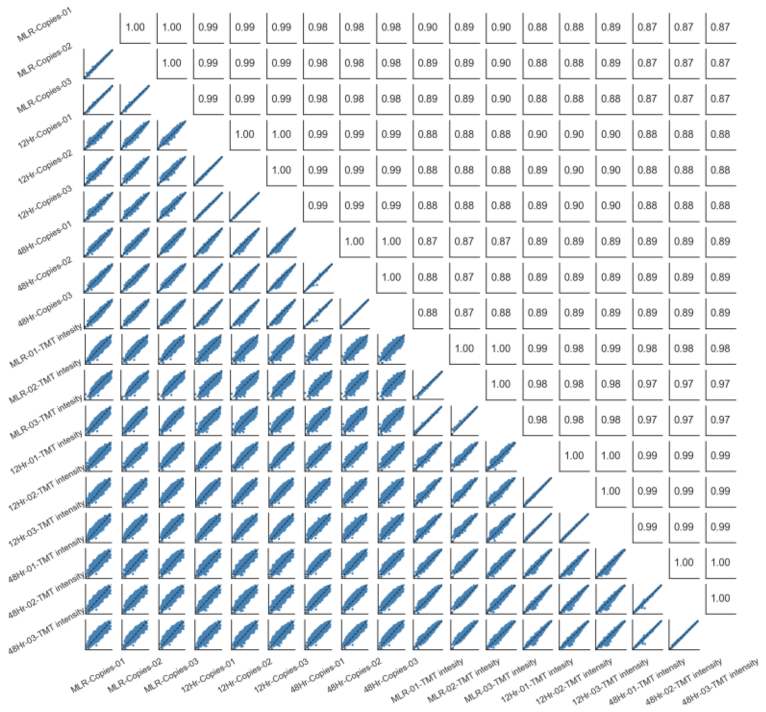

Supplemental Figure S10. Comparative analysis between copy numbers per cell and TMT intensities. (A) The matrix of 36 correlation plots revealed high correlations between absolute protein copy numbers and TMT intensities in triplicate samples (Pearson correlation coefficient 0.98 between biological replicates). (B) The matrix of 324 correlation plots revealed reasonable correlations between absolute protein copy numbers and TMT intensities in triplicate samples (Pearson correlation coefficient 0.87 between biological replicates).

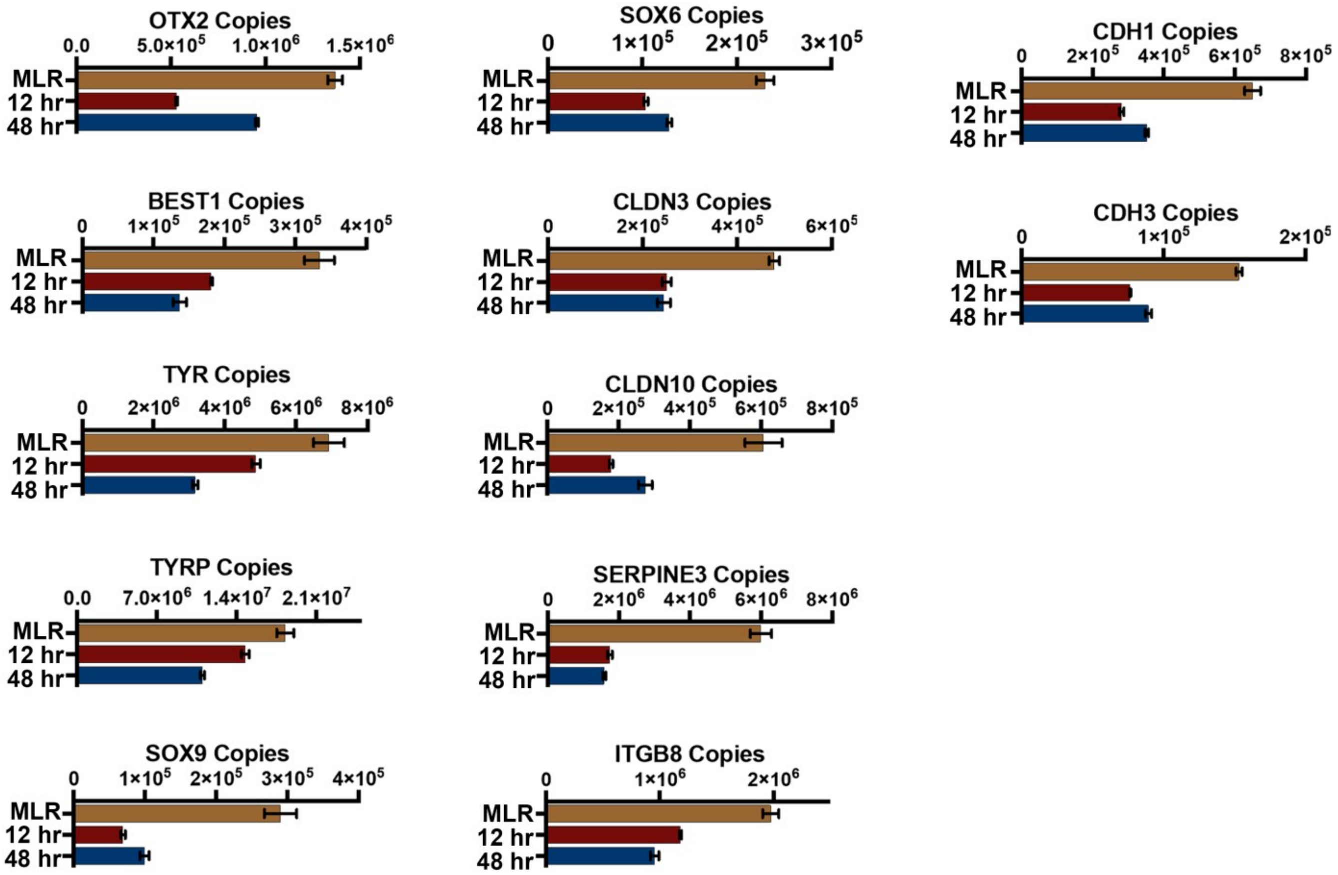

Supplemental Figure S11. Absolute quantification of RPE-EMT proteins. Bar graphs represent the absolute quantification (protein copy number per cell) of significantly altered malignancy-associated EMT factors.

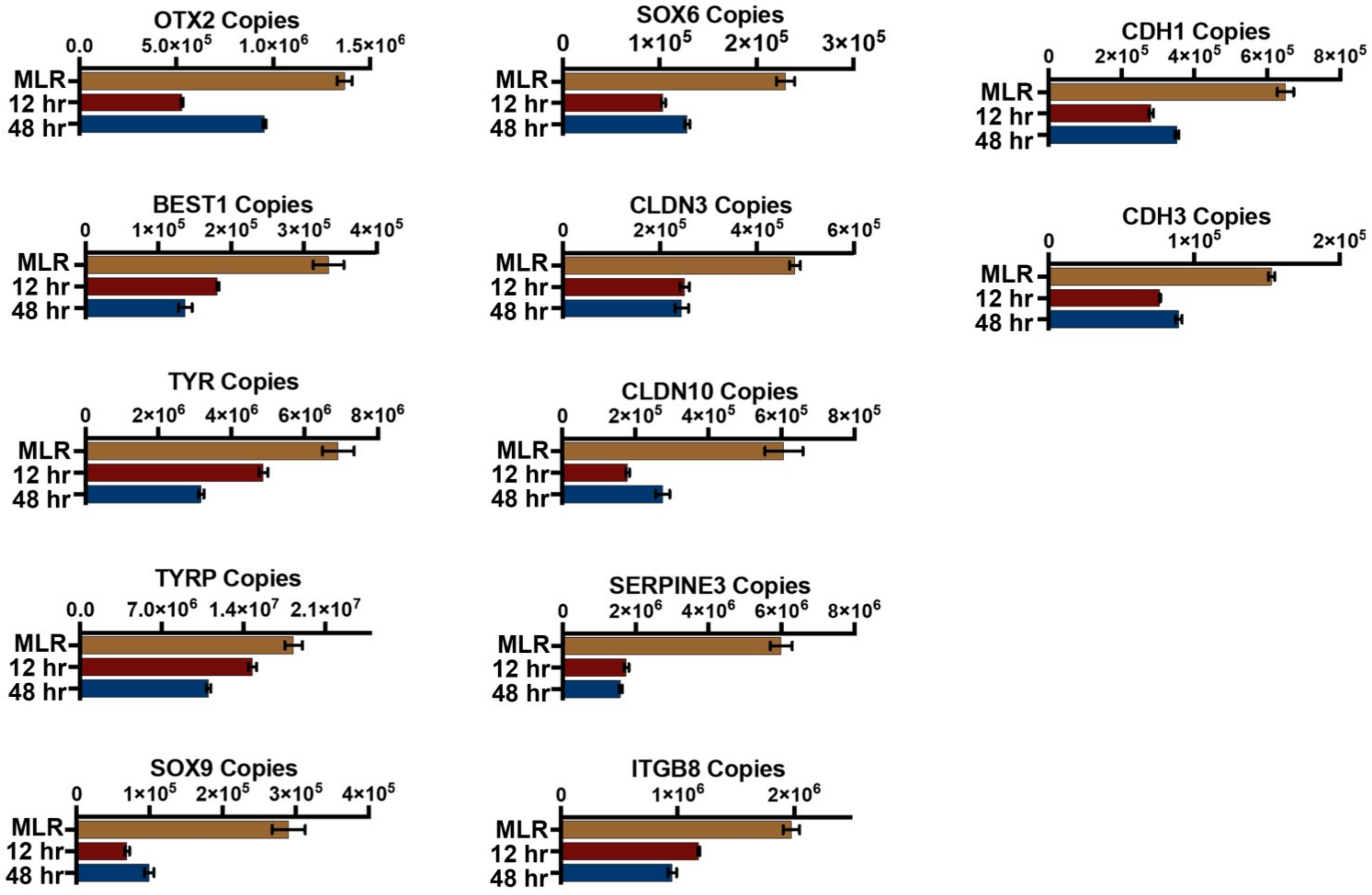

Supplemental Figure S12. Absolute quantification of RPE signature proteins. Bar graphs represent the absolute quantification (protein copy number per cell) of significantly altered RPE factors.

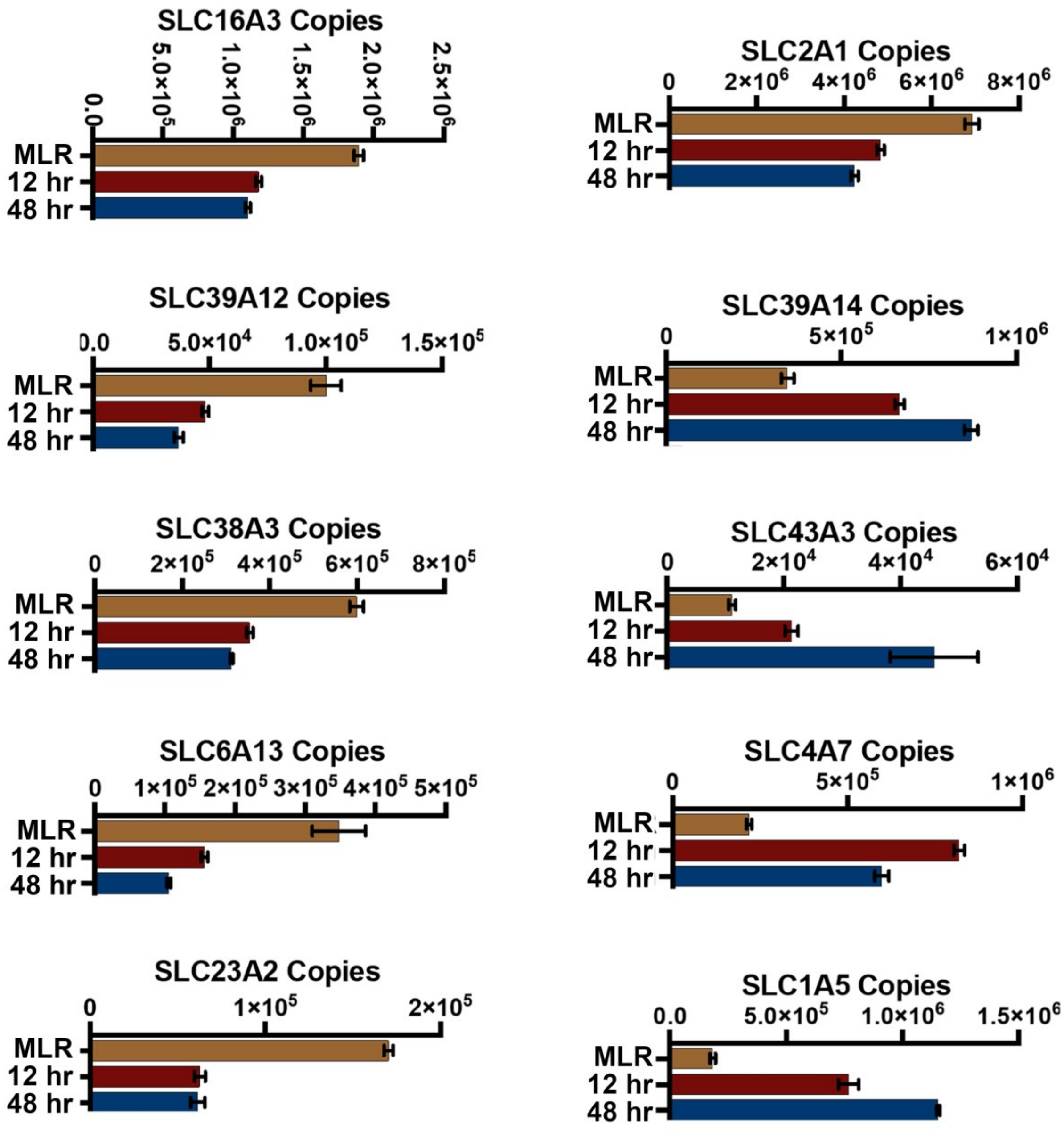

Supplemental Figure S13. Absolute quantification of solute carrier (SLC) group of membrane transport proteins. Bar graphs represent the absolute quantification (protein copy number per cell) of significantly altered SLC family-related proteins.

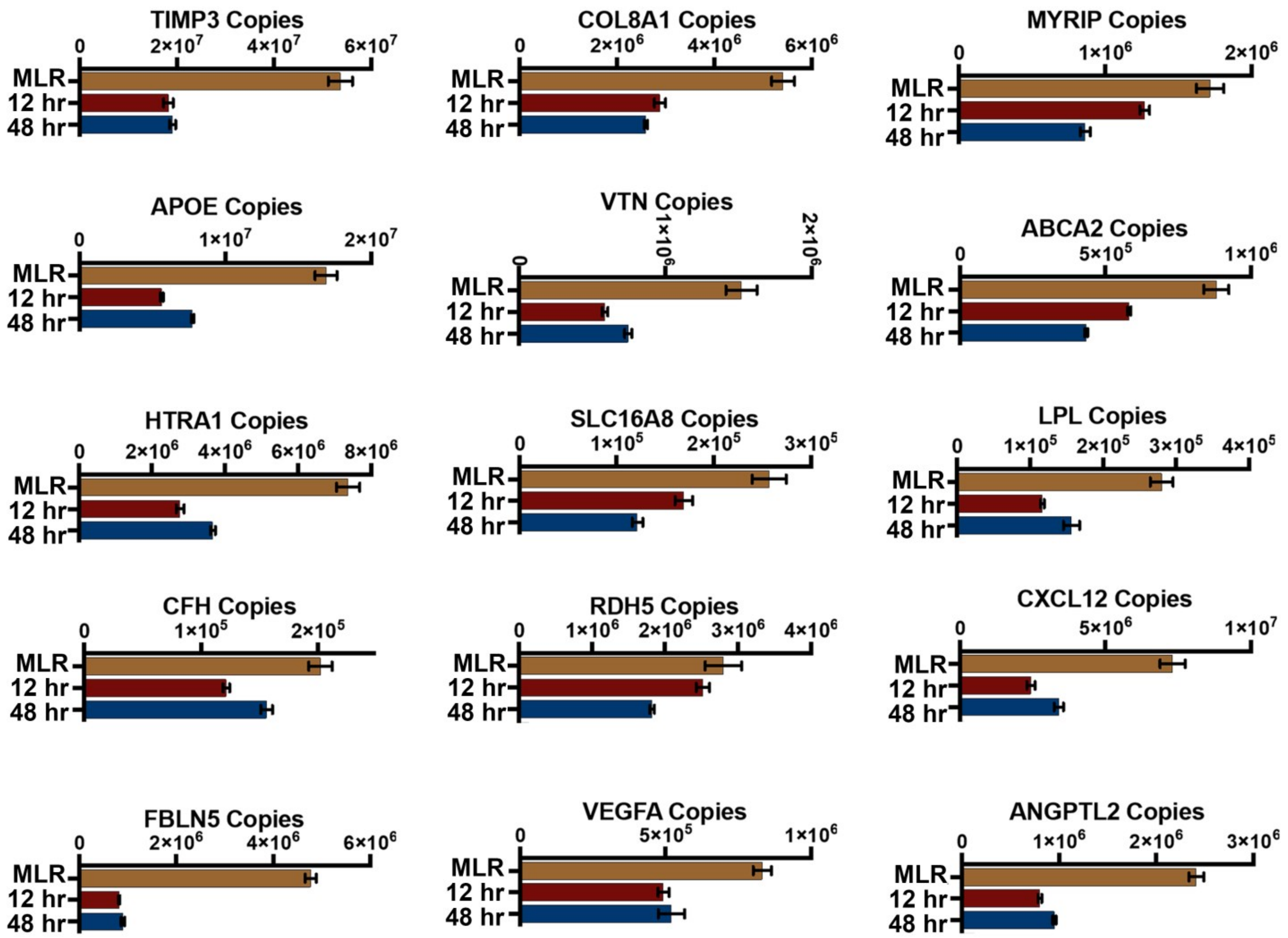

Supplemental Figure S14. Absolute quantification of RPE-EMT proteins. Bar graphs represent the absolute quantification (protein copy number per cell) of significantly altered AMD-associated factors.

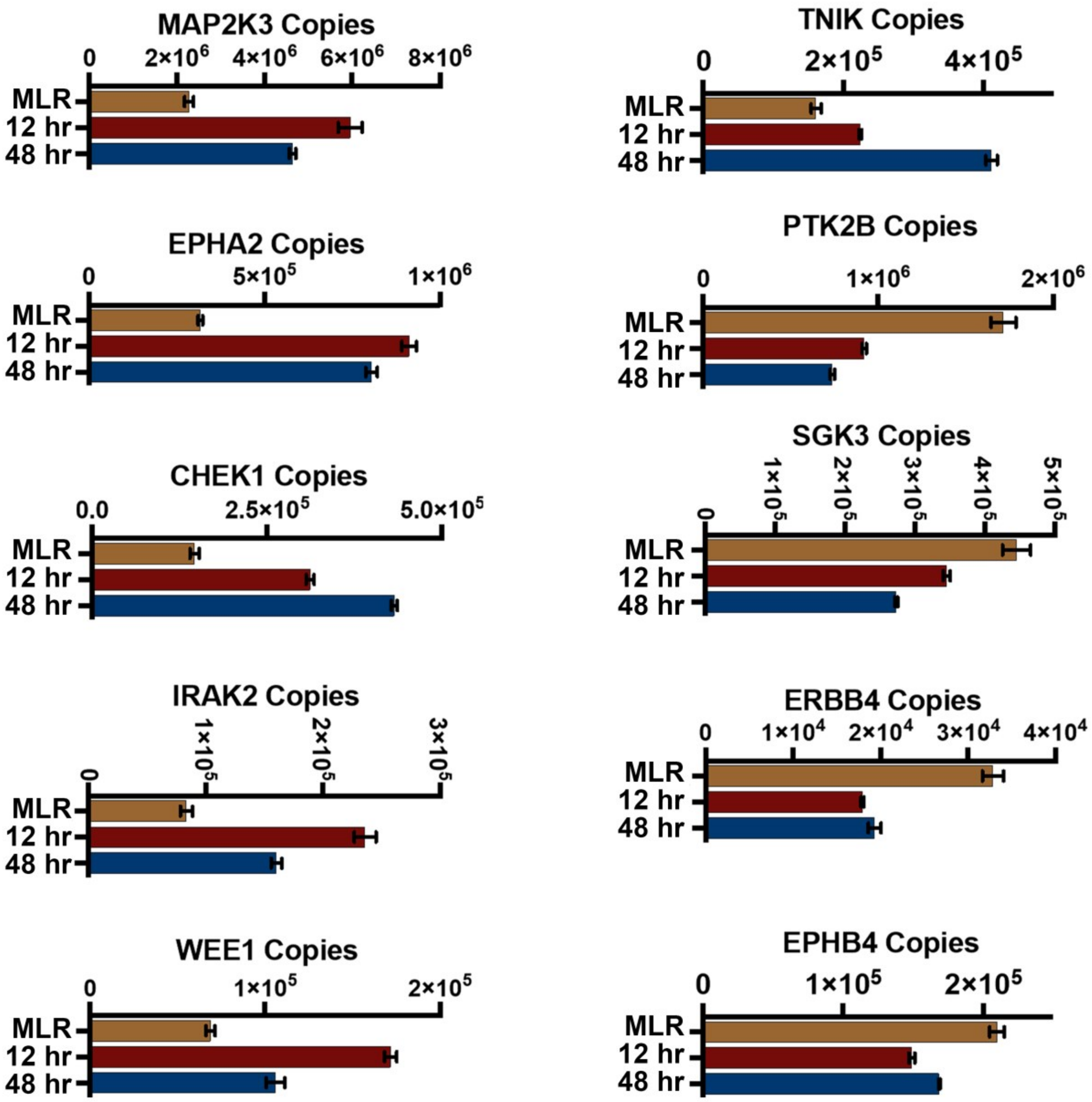

Supplemental Figure S15. Absolute quantification of RPE-EMT proteins.

Bar graphs represent the absolute quantification (protein copy number per cell) of significantly altered kinases during RPE-EMT factors.
